# Supplementary material for: Exploring online consumer behavior on fraudulent energy-saving products
Source: Sci Rep. 2024 Jun 21;14:14304. doi: 10.1038/s41598-024-65210-1 (PMC11192901; doi:10.1038/s41598-024-65210-1)
Supplement: Supplementary file 4 — Supplementary Table 4. [file 41598_2024_65210_MOESM4_ESM.pdf]

Jinfeng Hongyuan Flagship Store	Power Saving Expert 2	Logistics is super fast! Beautiful appearance! Looks like a great quality! I've never bought one before, so I'll try it first to see if it saves power. We'll see how much it saves.	Jingdong prefecture level city in Shandong
Jinfeng Hongyuan Flagship Store	Power Saving Expert 2	I saw online counterfeiting, energy saver is one of them, hahahahaha, jingdong also buy fake products	Jingdong prefecture level city in Shandong
Jinfeng Hongyuan Flagship Store	Power Saving Expert 2	Just received, installed, try a month to see how the effect and then follow up.	Jingdong prefecture level city in Shandong
Jinfeng Hongyuan Flagship Store	Power Saving Expert 2	Good for you! The goods are genuine! Good price for what you get!	Jingdong prefecture level city in Shandong
Jinfeng Hongyuan Flagship Store	Power Saving Expert 2	Things quickly received, immediately used to see if there is no effect, something river photo ⬆ the same, customer service is very professional	Jingdong prefecture level city in Shandong
Jinfeng Hongyuan Flagship Store	Power Saving Expert 2	The product is very convenient Looking forward to the subsequent use of the experience results	Jingdong prefecture level city in Shandong
Jinfeng Hongyuan Flagship Store	Power Saving Expert 2	I just received it and plugged it in, we'll see exactly when it's time to pay the electric bill	Jingdong prefecture level city in Shandong
Jinfeng Hongyuan Flagship Store	Power Saving Expert 2	Saver is good Packaging and looks good How effective it is to be shared after using it.	Jingdong prefecture level city in Shandong
Jinfeng Hongyuan Flagship Store	Power Saving Expert 2	There's no way to compare. I don't know if it's energy efficient.	Jingdong prefecture level city in Shandong
Jinfeng Hongyuan Flagship Store	Power Saving Expert 2	Just installed it, try it out and comment again	Jingdong prefecture level city in Shandong
Jinfeng Hongyuan Flagship Store	Power Saving Expert 2	Super save point also good to install, after a friend introduced then went to buy to, friends say it is very good to use, home I bought, try to use a period of time, to be honest, the economizer is not big or heavy, can be very convenient to put in a lot of places, the quality is also very good, power-saving function this time seems to be about 20% of the efficiency. The owner is also very warm contact, very	Jingdong prefecture level city in Shandong
Jinfeng Hongyuan Flagship Store	Power Saving Expert 2	The reviews online are mixed. Try it for a while, but I don't know if I can return it if it doesn't save any power after a couple months.	Jingdong prefecture level city in Shandong
Jinfeng Hongyuan Flagship Store	Power Saving Expert 2	The power consumption at home is high, bought a power saver, it looks very convenient to plug on the line, see if you can save some money next month, if good, recommend to friends down the road.	Jingdong prefecture level city in Shandong
Jinfeng Hongyuan Flagship Store	Power Saving Expert 2	It's beautiful, it's genuine, but I don't know the power-saving effect.	Jingdong prefecture level city in Shandong
Jinfeng Hongyuan Flagship Store	Power Saving Expert 2	This user thinks the product is good	Jingdong prefecture level city in Shandong

Jinfeng Hongyuan Flagship Store	Power Saving Expert 2	Nice exterior design with an at-a-glance display	Jingdong prefecture level city in Shandong
Jinfeng Hongyuan Flagship Store	Power Saving Expert 2	Home large and small things are purchased in the Jingdong Mall, have been accustomed to shopping in the Jingdong, many times can buy good quality and cheap products, and courier service attitude is particularly good, sometimes too many couriers are to help me to the home, so many years have been in the Jingdong Mall shopping, trust and support! Will also support the Jingdong as always!	Jingdong prefecture level city in Shandong
Jinfeng Hongyuan Flagship Store	Power Saving Expert 2	Good workmanship, mostly better than the original wall-plugged ones, and hopefully power factor compensation saves on electricity costs	Jingdong prefecture level city in Shandong
Jinfeng Hongyuan Flagship Store	Power Saving Expert 2	I don't feel anything, I said I won't know the effect for a month or two, let's see.	Jingdong prefecture level city in Shandong
Jinfeng Hongyuan Flagship Store	Power Saving Expert 2	Delivery was pretty fast, a little pricey, just not sure if it works or not	Jingdong prefecture level city in Shandong
Jinfeng Hongyuan Flagship Store	Power Saving Expert 2	The goods have been received, I do not know how effective.	Jingdong prefecture level city in Shandong
Jinfeng Hongyuan Flagship Store	Power Saving Expert 2	So far it doesn't seem to be too noticeable, so I'll comment later.	Jingdong prefecture level city in Shandong
Jinfeng Hongyuan Flagship Store	Power Saving Expert 2	I'll comment after I've used it	Jingdong prefecture level city in Shandong
Jinfeng Hongyuan Flagship Store	Power Saving Expert 2	Not bad. You'll save a lot on your electric bill in a year!	Jingdong prefecture level city in Shandong
Jinfeng Hongyuan Flagship Store	Power Saving Expert 2	I just got it. I haven't tried it yet. I don't know what it's like.	Jingdong prefecture level city in Shandong
Jinfeng Hongyuan Flagship Store	Power Saving Expert 2	Shopping at Jingdong has become a habit, and this disease will not be cured for a while.	Jingdong prefecture level city in Shandong
Jinfeng Hongyuan Flagship Store	Power Saving Expert 2	I've been using it for a while now. I don't know if I'm still saving power or not.	Jingdong prefecture level city in Shandong
Jinfeng Hongyuan Flagship Store	Power Saving Expert 2	Received the saver and it's great! There is also a digital voltage indication. Plugged in as instructed, easy to operate, hope you're our power saver!	Jingdong prefecture level city in Shandong
Jinfeng Hongyuan Flagship Store	Power Saving Expert 2	There is no power saving as the store says, using it is the same as not using it.	Jingdong prefecture level city in Shandong
Jinfeng Hongyuan Flagship Store	Power Saving Expert 2	Use it for a while and then comment again	Jingdong prefecture level city in Shandong
Jinfeng Hongyuan Flagship Store	Power Saving Expert 2	Feeling can, to use 1 to 2 months to see how the effect, there is a voltage display, can see the voltage fluctuations amplitude, quite good!	Jingdong prefecture level city in Shandong
Jinfeng Hongyuan Flagship Store	Power Saving Expert 2	I hope it works and saves me some electricity.	Jingdong prefecture level city in Shandong
Jinfeng Hongyuan Flagship Store	Power Saving Expert 2	Nice baby, happy with the after sales	Jingdong prefecture level city in Shandong
Jinfeng Hongyuan Flagship Store	Power Saving Expert 2	Use it and talk about it.	Jingdong prefecture level city in Shandong
Jinfeng Hongyuan Flagship Store	Power Saving Expert 2	Second purchase	Jingdong prefecture level city in Shandong
Jinfeng Hongyuan Flagship Store	Power Saving Expert 2	It works.	Jingdong prefecture level city in Shandong
Jinfeng Hongyuan Flagship Store	Power Saving Expert 2	It works.	Jingdong prefecture level city in Shandong
Jinfeng Hongyuan Flagship Store	Power Saving Expert 2	I don't know if it's a power saver until I start using it	Jingdong prefecture level city in Shandong
Jinfeng Hongyuan Flagship Store	Power Saving Expert 2	serviceable	Jingdong prefecture level city in Shandong
Jinfeng Hongyuan Flagship Store	Power Saving Expert 2	This user did not fill out the rating in time	Jingdong prefecture level city in Shandong
Jinfeng Hongyuan Flagship Store	Power Saving Expert 2	There is no power saving as the store says, using it is the same as not using it.	Jingdong prefecture level city in Shandong
Jinfeng Hongyuan Flagship Store	Power Saving Expert 2	This user did not fill out the rating in time	Jingdong prefecture level city in Shandong
Jinfeng Hongyuan Flagship Store	Power Saving Expert 2	Good...good.	Jingdong prefecture level city in Shandong
Jinfeng Hongyuan Flagship Store	Power Saving Expert 2	(of an unmarried couple) be close	Jingdong prefecture level city in Shandong
Jinfeng Hongyuan Flagship Store	Power Saving Expert 2	(of an unmarried couple) be close	Jingdong prefecture level city in Shandong
Jinfeng Hongyuan Flagship Store	Power Saving Expert 2	(of an unmarried couple) be close	Jingdong prefecture level city in Shandong
Jinfeng Hongyuan Flagship Store	Power Saving Expert 2	The goods are real, high value, high quality, worth every penny, material appearance and quality at a glance is very upscale, very like HAPPY100	Jingdong prefecture level city in Shandong
Jinfeng Hongyuan Flagship Store	Power Saving Expert 2	It is the ideal product, good quality, logistics is also very fast, worthy of praise 🎁 baby quality is very good, received the first time to open the first time to see, it is very good 👍, very satisfied 😊, customer service is also available to ask questions and answer, thank you 🙏	Jingdong prefecture level city in Shandong
Jinfeng Hongyuan Flagship Store	Power Saving Expert 2	I bought it and haven't used it yet, I'll use it again.	Jingdong prefecture level city in Shandong
Jinfeng Hongyuan Flagship Store	Power Saving Expert 2	It didn't work, I used it for a few days and it was the same as before, it didn't work, I should have to return it	Jingdong prefecture level city in Shandong
Jinfeng Hongyuan Flagship Store	Power Saving Expert 2	The goods have been received, but have not yet installed and use, feel good.	Jingdong prefecture level city in Shandong
Jinfeng Hongyuan Flagship Store	Power Saving Expert 2	Goods received, I forgot to take pictures, the goods look good, you can start, immediately start the school to see the effect then	Jingdong prefecture level city in Shandong
Jinfeng Hongyuan Flagship Store	Power Saving Expert 2	Looks good. I'll use it for a while.	Jingdong prefecture level city in Shandong
Jinfeng Hongyuan Flagship Store	Power Saving Expert 2	Great stuff, easy to operate, easy to use, good power saving, believe in the power of science!	Jingdong prefecture level city in Shandong
Jinfeng Hongyuan Flagship Store	Power Saving Expert 2	Especially great, comparison shopping before buying, the store owner is very good, answering questions very patiently and detailed, there is a need to come back!	Jingdong prefecture level city in Shandong
Jinfeng Hongyuan Flagship Store	Power Saving Expert 2	Trustworthy, feel free to use it boldly.	Jingdong prefecture level city in Shandong
Jinfeng Hongyuan Flagship Store	Power Saving Expert 2	Logistics is super fast! Beautiful appearance! Looks like a great quality! I've never bought one before, so I'll try it first to see if it saves power. We'll see how much it saves.	Jingdong prefecture level city in Shandong
Jinfeng Hongyuan Flagship Store	Power Saving Expert 2	I saw online counterfeiting, energy saver is one of them, hahahahaha, jingdong also buy fake products	Jingdong prefecture level city in Shandong
Jinfeng Hongyuan Flagship Store	Power Saving Expert 2	Just received, installed, try a month to see how the effect and then follow up.	Jingdong prefecture level city in Shandong
Jinfeng Hongyuan Flagship Store	Power Saving Expert 2	Good for you! The goods are genuine! Good price for what you get!	Jingdong prefecture level city in Shandong
Jinfeng Hongyuan Flagship Store	Power Saving Expert 2	Things quickly received, immediately used to see if there is no effect, something river photo 🔝 the same, customer service is very professional	Jingdong prefecture level city in Shandong
Jinfeng Hongyuan Flagship Store	Power Saving Expert 2	The product is very convenient Looking forward to the subsequent use of the experience results	Jingdong prefecture level city in Shandong
Jinfeng Hongyuan Flagship Store	Power Saving Expert 2	I just received it and plugged it in, we'll see exactly when it's time to pay the electric bill	Jingdong prefecture level city in Shandong
Jinfeng Hongyuan Flagship Store	Power Saving Expert 2	Saver is good Packaging and looks good How effective it is to be shared after using it.	Jingdong prefecture level city in Shandong
Jinfeng Hongyuan Flagship Store	Power Saving Expert 2	There's no way to compare. I don't know if it's energy efficient.	Jingdong prefecture level city in Shandong

Jinfeng Hongyuan Flagship Store	Power Saving Expert 2	Just installed it, try it out and comment again	Jingdong prefecture level city in Shandong
Jinfeng Hongyuan Flagship Store	Power Saving Expert 2	Super save point also good to install, after a friend introduced then went to buy to, friends say it is very good to use, home I bought, try to use a period of time, to be honest, the economizer is not big or heavy, can be very convenient to put in a lot of places, the quality is also very good, power-saving function this time seems to be about 20% of the efficiency. The owner is also very warm contact, very	Jingdong prefecture level city in Shandong
Jinfeng Hongyuan Flagship Store	Power Saving Expert 2	The reviews online are mixed. Try it for a while, but I don't know if I can return it if it doesn't save any power after a couple months.	Jingdong prefecture level city in Shandong
Jinfeng Hongyuan Flagship Store	Power Saving Expert 2	The power consumption at home is high, bought a power saver, it looks very convenient to plug on the line, see if you can save some money next month, if good, recommend to friends down the road.	Jingdong prefecture level city in Shandong
Jinfeng Hongyuan Flagship Store	Power Saving Expert 2	It's beautiful, it's genuine, but I don't know the power-saving effect.	Jingdong prefecture level city in Shandong
Jinfeng Hongyuan Flagship Store	Power Saving Expert 2	This user thinks the product is good	Jingdong prefecture level city in Shandong
Jinfeng Hongyuan Flagship Store	Power Saving Expert 2	Nice exterior design with an at-a-glance display	Jingdong prefecture level city in Shandong
Jinfeng Hongyuan Flagship Store	Power Saving Expert 2	Home large and small things are purchased in the Jingdong Mall, have been accustomed to shopping in the Jingdong, many times can buy good quality and cheap products, and courier service attitude is particularly good, sometimes too many couriers are to help me to the home, so many years have been in the Jingdong Mall shopping, trust and support! Will also support the Jingdong as always!	Jingdong prefecture level city in Shandong
Jinfeng Hongyuan Flagship Store	Power Saving Expert 2	Good workmanship, mostly better than the original wall-plugged ones, and hopefully power factor compensation saves on electricity costs	Jingdong prefecture level city in Shandong
Jinfeng Hongyuan Flagship Store	Power Saving Expert 2	I don't feel anything, I said I won't know the effect for a month or two, let's see.	Jingdong prefecture level city in Shandong
Jinfeng Hongyuan Flagship Store	Power Saving Expert 2	Delivery was pretty fast, a little pricey, just not sure if it works or not	Jingdong prefecture level city in Shandong
Jinfeng Hongyuan Flagship Store	Power Saving Expert 2	The goods have been received, I do not know how effective.	Jingdong prefecture level city in Shandong
Jinfeng Hongyuan Flagship Store	Power Saving Expert 2	So far it doesn't seem to be too noticeable, so I'll comment later.	Jingdong prefecture level city in Shandong
Jinfeng Hongyuan Flagship Store	Power Saving Expert 2	I'll comment after I've used it	Jingdong prefecture level city in Shandong
Jinfeng Hongyuan Flagship Store	Power Saving Expert 2	Not bad. You'll save a lot on your electric bill in a year!	Jingdong prefecture level city in Shandong
Jinfeng Hongyuan Flagship Store	Power Saving Expert 2	I just got it. I haven't tried it yet. I don't know what it's like.	Jingdong prefecture level city in Shandong
Jinfeng Hongyuan Flagship Store	Power Saving Expert 2	Shopping at Jingdong has become a habit, and this disease will not be cured for a while.	Jingdong prefecture level city in Shandong
Jinfeng Hongyuan Flagship Store	Power Saving Expert 2	I've been using it for a while now. I don't know if I'm still saving power or not.	Jingdong prefecture level city in Shandong
Jinfeng Hongyuan Flagship Store	Power Saving Expert 2	Received the saver and it's great! There is also a digital voltage indication. Plugged in as instructed, easy to operate, hope you're our power saver!	Jingdong prefecture level city in Shandong
Jinfeng Hongyuan Flagship Store	Power Saving Expert 2	There is no power saving as the store says, using it is the same as not using it.	Jingdong prefecture level city in Shandong
Jinfeng Hongyuan Flagship Store	Power Saving Expert 2	Use it for a while and then comment again	Jingdong prefecture level city in Shandong
Jinfeng Hongyuan Flagship Store	Power Saving Expert 2	Feeling can, to use 1 to 2 months to see how the effect, there is a voltage display, can see the voltage fluctuations amplitude, quite good!	Jingdong prefecture level city in Shandong
Jinfeng Hongyuan Flagship Store	Power Saving Expert 2	I hope it works and saves me some electricity.	Jingdong prefecture level city in Shandong
Jinfeng Hongyuan Flagship Store	Power Saving Expert 2	Nice baby, happy with the after sales	Jingdong prefecture level city in Shandong
Jinfeng Hongyuan Flagship Store	Power Saving Expert 2	Use it and talk about it.	Jingdong prefecture level city in Shandong
Jinfeng Hongyuan Flagship Store	Power Saving Expert 2	Second purchase	Jingdong prefecture level city in Shandong
Jinfeng Hongyuan Flagship Store	Power Saving Expert 2	It works.	Jingdong prefecture level city in Shandong
Jinfeng Hongyuan Flagship Store	Power Saving Expert 2	It works.	Jingdong prefecture level city in Shandong
Jinfeng Hongyuan Flagship Store	Power Saving Expert 2	I don't know if it's a power saver until I start using it	Jingdong prefecture level city in Shandong
Jinfeng Hongyuan Flagship Store	Power Saving Expert 2	serviceable	Jingdong prefecture level city in Shandong
Jinfeng Hongyuan Flagship Store	Power Saving Expert 2	This user did not fill out the rating in time	Jingdong prefecture level city in Shandong
Jinfeng Hongyuan Flagship Store	Power Saving Expert 2	There is no power saving as the store says, using it is the same as not using it.	Jingdong prefecture level city in Shandong
Jinfeng Hongyuan Flagship Store	Power Saving Expert 2	This user did not fill out the rating in time	Jingdong prefecture level city in Shandong
Jinfeng Hongyuan Flagship Store	Power Saving Expert 2	Good...good.	Jingdong prefecture level city in Shandong
Jinfeng Hongyuan Flagship Store	Power Saving Expert 2	(of an unmarried couple) be close	Jingdong prefecture level city in Shandong
Jinfeng Hongyuan Flagship Store	Power Saving Expert 2	(of an unmarried couple) be close	Jingdong prefecture level city in Shandong
Jinfeng Hongyuan Flagship Store	Power Saving Expert 2	(of an unmarried couple) be close	Jingdong prefecture level city in Shandong
Jinfeng Hongyuan Flagship Store	Power Saving Expert 2	The goods are real, high value, high quality, worth every penny, material appearance and quality at a glance is very upscale, very like HAPPY1 00	Jingdong prefecture level city in Shandong
Jinfeng Hongyuan Flagship Store	Power Saving Expert 2	It is the ideal product, good quality, logistics is also very fast, worthy of praise 🎁 baby quality is very good, received the first time to open the first time to see, it is very good 👍, very satisfied 😃, customer service is also available to ask questions and answer, thank you 🙏	Jingdong prefecture level city in Shandong
Jinfeng Hongyuan Flagship Store	Power Saving Expert 2	I bought it and haven't used it yet, I'll use it again.	Jingdong prefecture level city in Shandong
Jinfeng Hongyuan Flagship Store	Power Saving Expert 2	It didn't work, I used it for a few days and it was the same as before, it didn't work, I should have to return it	Jingdong prefecture level city in Shandong
Jinfeng Hongyuan Flagship Store	Power Saving Expert 2	The goods have been received, but have not yet installed and use, feel good.	Jingdong prefecture level city in Shandong
Jinfeng Hongyuan Flagship Store	Power Saving Expert 2	Goods received, I forgot to take pictures, the goods look good, you can start, immediately start the school to see the effect then	Jingdong prefecture level city in Shandong
Jinfeng Hongyuan Flagship Store	Power Saving Expert 2	Looks good. I'll use it for a while.	Jingdong prefecture level city in Shandong
Jinfeng Hongyuan Flagship Store	Power Saving Expert 2	Great stuff, easy to operate, easy to use, good power saving, believe in the power of science!	Jingdong prefecture level city in Shandong
Jinfeng Hongyuan Flagship Store	Power Saving Expert 2	Especially great, comparison shopping before buying, the store owner is very good, answering questions very patiently and detailed, there is a need to come back!	Jingdong prefecture level city in Shandong
Jinfeng Hongyuan Flagship Store	Power Saving Expert 2	Trustworthy, feel free to use it boldly.	Jingdong prefecture level city in Shandong
Jinfeng Hongyuan Flagship Store	Power Saving Expert 2	Logistics is super fast! Beautiful appearance! Looks like a great quality! I've never bought one before, so I'll try it first to see if it saves power. We'll see how much it saves.	Jingdong prefecture level city in Shandong
Jinfeng Hongyuan Flagship Store	Power Saving Expert 2	I saw online counterfeiting, energy saver is one of them, hahahahaha, jingdong also buy fake products	Jingdong prefecture level city in Shandong

Jinfeng Hongyuan Flagship Store	Power Saving Expert 2	Just received, installed, try a month to see how the effect and then follow up.	Jingdong prefecture level city in Shandong
Jinfeng Hongyuan Flagship Store	Power Saving Expert 2	Good for you! The goods are genuine! Good price for what you get!	Jingdong prefecture level city in Shandong
Jinfeng Hongyuan Flagship Store	Power Saving Expert 2	Things quickly received, immediately used to see if there is no effect, something river photo 🆙 the same, customer service is very professional	Jingdong prefecture level city in Shandong
Jinfeng Hongyuan Flagship Store	Power Saving Expert 2	The product is very convenient Looking forward to the subsequent use of the experience results	Jingdong prefecture level city in Shandong
Jinfeng Hongyuan Flagship Store	Power Saving Expert 2	I just received it and plugged it in, we'll see exactly when it's time to pay the electric bill	Jingdong prefecture level city in Shandong
Jinfeng Hongyuan Flagship Store	Power Saving Expert 2	Saver is good Packaging and looks good How effective it is to be shared after using it.	Jingdong prefecture level city in Shandong
Jinfeng Hongyuan Flagship Store	Power Saving Expert 2	There's no way to compare. I don't know if it's energy efficient.	Jingdong prefecture level city in Shandong
Jinfeng Hongyuan Flagship Store	Power Saving Expert 2	Just installed it, try it out and comment again	Jingdong prefecture level city in Shandong
Jinfeng Hongyuan Flagship Store	Power Saving Expert 2	Super save point also good to install, after a friend introduced then went to buy to, friends say it is very good to use, home I bought, try to use a period of time, to be honest, the economizer is not big or heavy, can be very convenient to put in a lot of places, the quality is also very good, power-saving function this time seems to be about 20% of the efficiency. The owner is also very warm contact, very	Jingdong prefecture level city in Shandong
Jinfeng Hongyuan Flagship Store	Power Saving Expert 2	The reviews online are mixed. Try it for a while, but I don't know if I can return it if it doesn't save any power after a couple months.	Jingdong prefecture level city in Shandong
Jinfeng Hongyuan Flagship Store	Power Saving Expert 2	The power consumption at home is high, bought a power saver, it looks very convenient to plug on the line, see if you can save some money next month, if good, recommend to friends down the road.	Jingdong prefecture level city in Shandong
Jinfeng Hongyuan Flagship Store	Power Saving Expert 2	It's beautiful, it's genuine, but I don't know the power-saving effect.	Jingdong prefecture level city in Shandong
Jinfeng Hongyuan Flagship Store	Power Saving Expert 2	This user thinks the product is good	Jingdong prefecture level city in Shandong
Jinfeng Hongyuan Flagship Store	Power Saving Expert 2	Nice exterior design with an at-a-glance display	Jingdong prefecture level city in Shandong
Jinfeng Hongyuan Flagship Store	Power Saving Expert 2	Home large and small things are purchased in the Jingdong Mall, have been accustomed to shopping in the Jingdong, many times can buy good quality and cheap products, and courier service attitude is particularly good, sometimes too many couriers are to help me to the home, so many years have been in the Jingdong Mall shopping, trust and support! Will also support the Jingdong as always!	Jingdong prefecture level city in Shandong
Jinfeng Hongyuan Flagship Store	Power Saving Expert 2	Good workmanship, mostly better than the original wall-plugged ones, and hopefully power factor compensation saves on electricity costs	Jingdong prefecture level city in Shandong
Jinfeng Hongyuan Flagship Store	Power Saving Expert 2	I don't feel anything, I said I won't know the effect for a month or two, let's see.	Jingdong prefecture level city in Shandong
Jinfeng Hongyuan Flagship Store	Power Saving Expert 2	Delivery was pretty fast, a little pricey, just not sure if it works or not	Jingdong prefecture level city in Shandong
Jinfeng Hongyuan Flagship Store	Power Saving Expert 2	The goods have been received, I do not know how effective.	Jingdong prefecture level city in Shandong
Jinfeng Hongyuan Flagship Store	Power Saving Expert 2	So far it doesn't seem to be too noticeable, so I'll comment later.	Jingdong prefecture level city in Shandong
Jinfeng Hongyuan Flagship Store	Power Saving Expert 2	I'll comment after I've used it	Jingdong prefecture level city in Shandong
Jinfeng Hongyuan Flagship Store	Power Saving Expert 2	Not bad. You'll save a lot on your electric bill in a year!	Jingdong prefecture level city in Shandong
Jinfeng Hongyuan Flagship Store	Power Saving Expert 2	I just got it. I haven't tried it yet. I don't know what it's like.	Jingdong prefecture level city in Shandong
Jinfeng Hongyuan Flagship Store	Power Saving Expert 2	Shopping at Jingdong has become a habit, and this disease will not be cured for a while.	Jingdong prefecture level city in Shandong
Jinfeng Hongyuan Flagship Store	Power Saving Expert 2	I've been using it for a while now. I don't know if I'm still saving power or not.	Jingdong prefecture level city in Shandong
Jinfeng Hongyuan Flagship Store	Power Saving Expert 2	Received the saver and it's great! There is also a digital voltage indication. Plugged in as instructed, easy to operate, hope you're our power saver!	Jingdong prefecture level city in Shandong
Jinfeng Hongyuan Flagship Store	Power Saving Expert 2	There is no power saving as the store says, using it is the same as not using it.	Jingdong prefecture level city in Shandong
Jinfeng Hongyuan Flagship Store	Power Saving Expert 2	Use it for a while and then comment again	Jingdong prefecture level city in Shandong
Jinfeng Hongyuan Flagship Store	Power Saving Expert 2	Feeling can, to use 1 to 2 months to see how the effect, there is a voltage display, can see the voltage fluctuations amplitude, quite good!	Jingdong prefecture level city in Shandong
Jinfeng Hongyuan Flagship Store	Power Saving Expert 2	I hope it works and saves me some electricity.	Jingdong prefecture level city in Shandong
Jinfeng Hongyuan Flagship Store	Power Saving Expert 2	Nice baby, happy with the after sales	Jingdong prefecture level city in Shandong
Jinfeng Hongyuan Flagship Store	Power Saving Expert 2	Use it and talk about it.	Jingdong prefecture level city in Shandong
Jinfeng Hongyuan Flagship Store	Power Saving Expert 2	Second purchase	Jingdong prefecture level city in Shandong
Jinfeng Hongyuan Flagship Store	Power Saving Expert 2	It works.	Jingdong prefecture level city in Shandong
Jinfeng Hongyuan Flagship Store	Power Saving Expert 2	It works.	Jingdong prefecture level city in Shandong
Jinfeng Hongyuan Flagship Store	Power Saving Expert 2	I don't know if it's a power saver until I start using it	Jingdong prefecture level city in Shandong
Jinfeng Hongyuan Flagship Store	Power Saving Expert 2	serviceable	Jingdong prefecture level city in Shandong
Jinfeng Hongyuan Flagship Store	Power Saving Expert 2	This user did not fill out the rating in time	Jingdong prefecture level city in Shandong
Jinfeng Hongyuan Flagship Store	Power Saving Expert 2	There is no power saving as the store says, using it is the same as not using it.	Jingdong prefecture level city in Shandong
Jinfeng Hongyuan Flagship Store	Power Saving Expert 2	This user did not fill out the rating in time	Jingdong prefecture level city in Shandong
Jinfeng Hongyuan Flagship Store	Power Saving Expert 2	Good...good.	Jingdong prefecture level city in Shandong
Jinfeng Hongyuan Flagship Store	Power Saving Expert 2	(of an unmarried couple) be close	Jingdong prefecture level city in Shandong
Jinfeng Hongyuan Flagship Store	Power Saving Expert 2	(of an unmarried couple) be close	Jingdong prefecture level city in Shandong
Jinfeng Hongyuan Flagship Store	Power Saving Expert 2	(of an unmarried couple) be close	Jingdong prefecture level city in Shandong
Jinfeng Hongyuan Flagship Store	Power Saving Expert 2	The goods are real, high value, high quality, worth every penny, material appearance and quality at a glance is very upscale, very like HAPPY1 00	Jingdong prefecture level city in Shandong
Jinfeng Hongyuan Flagship Store	Power Saving Expert 2	It is the ideal product, good quality, logistics is also very fast, worthy of praise 🎁 baby quality is very good, received the first time to open the first time to see, it is very good 👍, very satisfied 😃, customer service is also available to ask questions and answer, thank you 🙏	Jingdong prefecture level city in Shandong
Jinfeng Hongyuan Flagship Store	Power Saving Expert 2	I bought it and haven't used it yet, I'll use it again.	Jingdong prefecture level city in Shandong
Jinfeng Hongyuan Flagship Store	Power Saving Expert 2	It didn't work, I used it for a few days and it was the same as before, it didn't work, I should have to return it	Jingdong prefecture level city in Shandong
Jinfeng Hongyuan Flagship Store	Power Saving Expert 2	The goods have been received, but have not yet installed and use, feel good.	Jingdong prefecture level city in Shandong

Jinfeng Hongyuan Flagship Store	Power Saving Expert 2	Goods received, I forgot to take pictures, the goods look good, you can start, immediately start the school to see the effect then	Jingdong prefecture level city in Shandong
Jinfeng Hongyuan Flagship Store	Power Saving Expert 2	Looks good. I'll use it for a while.	Jingdong prefecture level city in Shandong
Jinfeng Hongyuan Flagship Store	Power Saving Expert 2	Great stuff, easy to operate, easy to use, good power saving, believe in the power of science!	Jingdong prefecture level city in Shandong
Jinfeng Hongyuan Flagship Store	Power Saving Expert 2	Especially great, comparison shopping before buying, the store owner is very good, answering questions very patiently and detailed, there is a need to come back!	Jingdong prefecture level city in Shandong
Jinfeng Hongyuan Flagship Store	Power Saving Expert 2	Trustworthy, feel free to use it boldly.	Jingdong prefecture level city in Shandong
Jinfeng Hongyuan Flagship Store	Power Saving Expert 2	Logistics is super fast! Beautiful appearance! Looks like a great quality! I've never bought one before, so I'll try it first to see if it saves power. We'll see how much it saves.	Jingdong prefecture level city in Shandong
Jinfeng Hongyuan Flagship Store	Power Saving Expert 2	I saw online counterfeiting, energy saver is one of them, hahahahaha, jingdong also buy fake products	Jingdong prefecture level city in Shandong
Jinfeng Hongyuan Flagship Store	Power Saving Expert 2	Just received, installed, try a month to see how the effect and then follow up.	Jingdong prefecture level city in Shandong
Jinfeng Hongyuan Flagship Store	Power Saving Expert 2	Good for you! The goods are genuine! Good price for what you get!	Jingdong prefecture level city in Shandong
Jinfeng Hongyuan Flagship Store	Power Saving Expert 2	Things quickly received, immediately used to see if there is no effect, something river photo ↑ the same, customer service is very professional	Jingdong prefecture level city in Shandong
Jinfeng Hongyuan Flagship Store	Power Saving Expert 2	The product is very convenient Looking forward to the subsequent use of the experience results	Jingdong prefecture level city in Shandong
Jinfeng Hongyuan Flagship Store	Power Saving Expert 2	I just received it and plugged it in, we'll see exactly when it's time to pay the electric bill	Jingdong prefecture level city in Shandong
Jinfeng Hongyuan Flagship Store	Power Saving Expert 2	Saver is good Packaging and looks good How effective it is to be shared after using it.	Jingdong prefecture level city in Shandong
Jinfeng Hongyuan Flagship Store	Power Saving Expert 2	There's no way to compare. I don't know if it's energy efficient.	Jingdong prefecture level city in Shandong
Jinfeng Hongyuan Flagship Store	Power Saving Expert 2	Just installed it, try it out and comment again	Jingdong prefecture level city in Shandong
Jinfeng Hongyuan Flagship Store	Power Saving Expert 2	Super save point also good to install, after a friend introduced then went to buy to, friends say it is very good to use, home I bought, try to use a period of time, to be honest, the economizer is not big or heavy, can be very convenient to put in a lot of places, the quality is also very good, power-saving function this time seems to be about 20% of the efficiency. The owner is also very warm contact, very	Jingdong prefecture level city in Shandong
Jinfeng Hongyuan Flagship Store	Power Saving Expert 2	The reviews online are mixed. Try it for a while, but I don't know if I can return it if it doesn't save any power after a couple months.	Jingdong prefecture level city in Shandong
Jinfeng Hongyuan Flagship Store	Power Saving Expert 2	The power consumption at home is high, bought a power saver, it looks very convenient to plug on the line, see if you can save some money next month, if good, recommend to friends down the road.	Jingdong prefecture level city in Shandong
Jinfeng Hongyuan Flagship Store	Power Saving Expert 2	It's beautiful, it's genuine, but I don't know the power-saving effect.	Jingdong prefecture level city in Shandong
Jinfeng Hongyuan Flagship Store	Power Saving Expert 2	This user thinks the product is good	Jingdong prefecture level city in Shandong
Jinfeng Hongyuan Flagship Store	Power Saving Expert 2	Nice exterior design with an at-a-glance display	Jingdong prefecture level city in Shandong
Jinfeng Hongyuan Flagship Store	Power Saving Expert 2	Home large and small things are purchased in the Jingdong Mall, have been accustomed to shopping in the Jingdong, many times can buy good quality and cheap products, and courier service attitude is particularly good, sometimes too many couriers are to help me to the home, so many years have been in the Jingdong Mall shopping, trust and support! Will also support the Jingdong as always!	Jingdong prefecture level city in Shandong
Jinfeng Hongyuan Flagship Store	Power Saving Expert 2	Good workmanship, mostly better than the original wall-plugged ones, and hopefully power factor compensation saves on electricity costs	Jingdong prefecture level city in Shandong
Jinfeng Hongyuan Flagship Store	Power Saving Expert 2	I don't feel anything, I said I won't know the effect for a month or two, let's see.	Jingdong prefecture level city in Shandong
Jinfeng Hongyuan Flagship Store	Power Saving Expert 2	Delivery was pretty fast, a little pricey, just not sure if it works or not	Jingdong prefecture level city in Shandong
Jinfeng Hongyuan Flagship Store	Power Saving Expert 2	The goods have been received, I do not know how effective.	Jingdong prefecture level city in Shandong
Jinfeng Hongyuan Flagship Store	Power Saving Expert 2	So far it doesn't seem to be too noticeable, so I'll comment later.	Jingdong prefecture level city in Shandong
Jinfeng Hongyuan Flagship Store	Power Saving Expert 2	I'll comment after I've used it	Jingdong prefecture level city in Shandong
Jinfeng Hongyuan Flagship Store	Power Saving Expert 2	Not bad. You'll save a lot on your electric bill in a year!	Jingdong prefecture level city in Shandong
Jinfeng Hongyuan Flagship Store	Power Saving Expert 2	I just got it. I haven't tried it yet. I don't know what it's like.	Jingdong prefecture level city in Shandong
Jinfeng Hongyuan Flagship Store	Power Saving Expert 2	Shopping at Jingdong has become a habit, and this disease will not be cured for a while.	Jingdong prefecture level city in Shandong
Jinfeng Hongyuan Flagship Store	Power Saving Expert 2	I've been using it for a while now. I don't know if I'm still saving power or not.	Jingdong prefecture level city in Shandong
Jinfeng Hongyuan Flagship Store	Power Saving Expert 2	Received the saver and it's great! There is also a digital voltage indication. Plugged in as instructed, easy to operate, hope you're our power saver!	Jingdong prefecture level city in Shandong
Jinfeng Hongyuan Flagship Store	Power Saving Expert 2	There is no power saving as the store says, using it is the same as not using it.	Jingdong prefecture level city in Shandong
Jinfeng Hongyuan Flagship Store	Power Saving Expert 2	Use it for a while and then comment again	Jingdong prefecture level city in Shandong
Jinfeng Hongyuan Flagship Store	Power Saving Expert 2	Feeling can, to use 1 to 2 months to see how the effect, there is a voltage display, can see the voltage fluctuations amplitude, quite good!	Jingdong prefecture level city in Shandong
Jinfeng Hongyuan Flagship Store	Power Saving Expert 2	I hope it works and saves me some electricity.	Jingdong prefecture level city in Shandong
Jinfeng Hongyuan Flagship Store	Power Saving Expert 2	Nice baby, happy with the after sales	Jingdong prefecture level city in Shandong
Jinfeng Hongyuan Flagship Store	Power Saving Expert 2	Use it and talk about it.	Jingdong prefecture level city in Shandong
Jinfeng Hongyuan Flagship Store	Power Saving Expert 2	Second purchase	Jingdong prefecture level city in Shandong
Jinfeng Hongyuan Flagship Store	Power Saving Expert 2	It works.	Jingdong prefecture level city in Shandong
Jinfeng Hongyuan Flagship Store	Power Saving Expert 2	It works.	Jingdong prefecture level city in Shandong
Jinfeng Hongyuan Flagship Store	Power Saving Expert 2	I don't know if it's a power saver until I start using it	Jingdong prefecture level city in Shandong
Jinfeng Hongyuan Flagship Store	Power Saving Expert 2	serviceable	Jingdong prefecture level city in Shandong
Jinfeng Hongyuan Flagship Store	Power Saving Expert 2	This user did not fill out the rating in time	Jingdong prefecture level city in Shandong
Jinfeng Hongyuan Flagship Store	Power Saving Expert 2	There is no power saving as the store says, using it is the same as not using it.	Jingdong prefecture level city in Shandong
Jinfeng Hongyuan Flagship Store	Power Saving Expert 2	This user did not fill out the rating in time	Jingdong prefecture level city in Shandong
Jinfeng Hongyuan Flagship Store	Power Saving Expert 2	Good...good.	Jingdong prefecture level city in Shandong
Jinfeng Hongyuan Flagship Store	Power Saving Expert 2	(of an unmarried couple) be close	Jingdong prefecture level city in Shandong

Jinfeng Hongyuan Flagship Store	Power Saving Expert 2	(of an unmarried couple) be close	Jingdong prefecture level city in Shandong
Jinfeng Hongyuan Flagship Store	Power Saving Expert 2	(of an unmarried couple) be close	Jingdong prefecture level city in Shandong
Jinfeng Hongyuan Flagship Store	Power Saving Expert 2	The goods are real, high value, high quality, worth every penny, material appearance and quality at a glance is very upscale, very like HAPPY1 00	Jingdong prefecture level city in Shandong
Jinfeng Hongyuan Flagship Store	Power Saving Expert 2	It is the ideal product, good quality, logistics is also very fast, worthy of praise 🎁 baby quality is very good, received the first time to open the first time to see, it is very good 👍, very satisfied 😊, customer service is also available to ask questions and answer, thank you 🙏	Jingdong prefecture level city in Shandong
Jinfeng Hongyuan Flagship Store	Power Saving Expert 2	I bought it and haven't used it yet, I'll use it again.	Jingdong prefecture level city in Shandong
Jinfeng Hongyuan Flagship Store	Power Saving Expert 2	It didn't work, I used it for a few days and it was the same as before, it didn't work, I should have to return it	Jingdong prefecture level city in Shandong
Jinfeng Hongyuan Flagship Store	Power Saving Expert 2	The goods have been received, but have not yet installed and use, feel good.	Jingdong prefecture level city in Shandong
Jinfeng Hongyuan Flagship Store	Power Saving Expert 2	Goods received, I forgot to take pictures, the goods look good, you can start, immediately start the school to see the effect then	Jingdong prefecture level city in Shandong
Jinfeng Hongyuan Flagship Store	Power Saving Expert 2	Looks good. I'll use it for a while.	Jingdong prefecture level city in Shandong
Jinfeng Hongyuan Flagship Store	Power Saving Expert 2	Great stuff, easy to operate, easy to use, good power saving, believe in the power of science!	Jingdong prefecture level city in Shandong
Jinfeng Hongyuan Flagship Store	Power Saving Expert 2	Especially great, comparison shopping before buying, the store owner is very good, answering questions very patiently and detailed, there is a need to come back!	Jingdong prefecture level city in Shandong
Jinfeng Hongyuan Flagship Store	Power Saving Expert 2	Trustworthy, feel free to use it boldly.	Jingdong prefecture level city in Shandong
Jinfeng Hongyuan Flagship Store	Power Saving Expert 2	Logistics is super fast! Beautiful appearance! Looks like a great quality! I've never bought one before, so I'll try it first to see if it saves power. We'll see how much it saves.	Jingdong prefecture level city in Shandong
Jinfeng Hongyuan Flagship Store	Power Saving Expert 2	I saw online counterfeiting, energy saver is one of them, hahahahaha, jingdong also buy fake products	Jingdong prefecture level city in Shandong
Jinfeng Hongyuan Flagship Store	Power Saving Expert 2	Just received, installed, try a month to see how the effect and then follow up.	Jingdong prefecture level city in Shandong
Jinfeng Hongyuan Flagship Store	Power Saving Expert 2	Good for you! The goods are genuine! Good price for what you get!	Jingdong prefecture level city in Shandong
Jinfeng Hongyuan Flagship Store	Power Saving Expert 2	Things quickly received, immediately used to see if there is no effect, something river photo ⬆ the same, customer service is very professional	Jingdong prefecture level city in Shandong
Jinfeng Hongyuan Flagship Store	Power Saving Expert 2	The product is very convenient Looking forward to the subsequent use of the experience results	Jingdong prefecture level city in Shandong
Jinfeng Hongyuan Flagship Store	Power Saving Expert 2	I just received it and plugged it in, we'll see exactly when it's time to pay the electric bill	Jingdong prefecture level city in Shandong
Jinfeng Hongyuan Flagship Store	Power Saving Expert 2	Saver is good Packaging and looks good How effective it is to be shared after using it.	Jingdong prefecture level city in Shandong
Jinfeng Hongyuan Flagship Store	Power Saving Expert 2	There's no way to compare. I don't know if it's energy efficient.	Jingdong prefecture level city in Shandong
Jinfeng Hongyuan Flagship Store	Power Saving Expert 2	Just installed it, try it out and comment again	Jingdong prefecture level city in Shandong
Jinfeng Hongyuan Flagship Store	Power Saving Expert 2	Super save point also good to install, after a friend introduced then went to buy to, friends say it is very good to use, home I bought, try to use a period of time, to be honest, the economizer is not big or heavy, can be very convenient to put in a lot of places, the quality is also very good, power-saving function this time seems to be about 20% of the efficiency. The owner is also very warm contact, very	Jingdong prefecture level city in Shandong
Jinfeng Hongyuan Flagship Store	Power Saving Expert 2	The reviews online are mixed. Try it for a while, but I don't know if I can return it if it doesn't save any power after a couple months.	Jingdong prefecture level city in Shandong
Jinfeng Hongyuan Flagship Store	Power Saving Expert 2	The power consumption at home is high, bought a power saver, it looks very convenient to plug on the line, see if you can save some money next month, if good, recommend to friends down the road.	Jingdong prefecture level city in Shandong
Jinfeng Hongyuan Flagship Store	Power Saving Expert 2	It's beautiful, it's genuine, but I don't know the power-saving effect.	Jingdong prefecture level city in Shandong
Jinfeng Hongyuan Flagship Store	Power Saving Expert 2	This user thinks the product is good	Jingdong prefecture level city in Shandong
Jinfeng Hongyuan Flagship Store	Power Saving Expert 2	Nice exterior design with an at-a-glance display	Jingdong prefecture level city in Shandong
Jinfeng Hongyuan Flagship Store	Power Saving Expert 2	Home large and small things are purchased in the Jingdong Mall, have been accustomed to shopping in the Jingdong, many times can buy good quality and cheap products, and courier service attitude is particularly good, sometimes too many couriers are to help me to the home, so many years have been in the Jingdong Mall shopping, trust and support! Will also support the Jingdong as always!	Jingdong prefecture level city in Shandong
Jinfeng Hongyuan Flagship Store	Power Saving Expert 2	Good workmanship, mostly better than the original wall-plugged ones, and hopefully power factor compensation saves on electricity costs	Jingdong prefecture level city in Shandong
Jinfeng Hongyuan Flagship Store	Power Saving Expert 2	I don't feel anything, I said I won't know the effect for a month or two, let's see.	Jingdong prefecture level city in Shandong
Jinfeng Hongyuan Flagship Store	Power Saving Expert 2	Delivery was pretty fast, a little pricey, just not sure if it works or not	Jingdong prefecture level city in Shandong
Jinfeng Hongyuan Flagship Store	Power Saving Expert 2	The goods have been received, I do not know how effective.	Jingdong prefecture level city in Shandong
Jinfeng Hongyuan Flagship Store	Power Saving Expert 2	So far it doesn't seem to be too noticeable, so I'll comment later.	Jingdong prefecture level city in Shandong
Jinfeng Hongyuan Flagship Store	Power Saving Expert 2	I'll comment after I've used it	Jingdong prefecture level city in Shandong
Jinfeng Hongyuan Flagship Store	Power Saving Expert 2	Not bad. You'll save a lot on your electric bill in a year!	Jingdong prefecture level city in Shandong
Jinfeng Hongyuan Flagship Store	Power Saving Expert 2	I just got it. I haven't tried it yet. I don't know what it's like.	Jingdong prefecture level city in Shandong
Jinfeng Hongyuan Flagship Store	Power Saving Expert 2	Shopping at Jingdong has become a habit, and this disease will not be cured for a while.	Jingdong prefecture level city in Shandong
Jinfeng Hongyuan Flagship Store	Power Saving Expert 2	I've been using it for a while now. I don't know if I'm still saving power or not.	Jingdong prefecture level city in Shandong
Jinfeng Hongyuan Flagship Store	Power Saving Expert 2	Received the saver and it's great! There is also a digital voltage indication. Plugged in as instructed, easy to operate, hope you're our power saver!	Jingdong prefecture level city in Shandong
Jinfeng Hongyuan Flagship Store	Power Saving Expert 2	There is no power saving as the store says, using it is the same as not using it.	Jingdong prefecture level city in Shandong
Jinfeng Hongyuan Flagship Store	Power Saving Expert 2	Use it for a while and then comment again	Jingdong prefecture level city in Shandong
Jinfeng Hongyuan Flagship Store	Power Saving Expert 2	Feeling can, to use 1 to 2 months to see how the effect, there is a voltage display, can see the voltage fluctuations amplitude, quite good!	Jingdong prefecture level city in Shandong
Jinfeng Hongyuan Flagship Store	Power Saving Expert 2	I hope it works and saves me some electricity.	Jingdong prefecture level city in Shandong
Jinfeng Hongyuan Flagship Store	Power Saving Expert 2	Nice baby, happy with the after sales	Jingdong prefecture level city in Shandong
Jinfeng Hongyuan Flagship Store	Power Saving Expert 2	Use it and talk about it.	Jingdong prefecture level city in Shandong
Jinfeng Hongyuan Flagship Store	Power Saving Expert 2	Second purchase	Jingdong prefecture level city in Shandong
Jinfeng Hongyuan Flagship Store	Power Saving Expert 2	It works.	Jingdong prefecture level city in Shandong
Jinfeng Hongyuan Flagship Store	Power Saving Expert 2	It works.	Jingdong prefecture level city in Shandong

Jinfeng Hongyuan Flagship Store	Power Saving Expert 2	I don't know if it's a power saver until I start using it	Jingdong prefecture level city in Shandong
Jinfeng Hongyuan Flagship Store	Power Saving Expert 2	serviceable	Jingdong prefecture level city in Shandong
Jinfeng Hongyuan Flagship Store	Power Saving Expert 2	This user did not fill out the rating in time	Jingdong prefecture level city in Shandong
Jinfeng Hongyuan Flagship Store	Power Saving Expert 2	There is no power saving as the store says, using it is the same as not using it.	Jingdong prefecture level city in Shandong
Jinfeng Hongyuan Flagship Store	Power Saving Expert 2	This user did not fill out the rating in time	Jingdong prefecture level city in Shandong
Jinfeng Hongyuan Flagship Store	Power Saving Expert 2	Good...good.	Jingdong prefecture level city in Shandong
Jinfeng Hongyuan Flagship Store	Power Saving Expert 2	(of an unmarried couple) be close	Jingdong prefecture level city in Shandong
Jinfeng Hongyuan Flagship Store	Power Saving Expert 2	(of an unmarried couple) be close	Jingdong prefecture level city in Shandong
Jinfeng Hongyuan Flagship Store	Power Saving Expert 2	(of an unmarried couple) be close	Jingdong prefecture level city in Shandong
Jinfeng Hongyuan Flagship Store	Power Saving Expert 2	The goods are real, high value, high quality, worth every penny, material appearance and quality at a glance is very upscale, very like HAPPY1 00	Jingdong prefecture level city in Shandong
Jinfeng Hongyuan Flagship Store	Power Saving Expert 2	It is the ideal product, good quality, logistics is also very fast, worthy of praise 🎁 baby quality is very good, received the first time to open the first time to see, it is very good 👍, very satisfied 😃, customer service is also available to ask questions and answer, thank you 🙏	Jingdong prefecture level city in Shandong
Jinfeng Hongyuan Flagship Store	Power Saving Expert 2	I bought it and haven't used it yet, I'll use it again.	Jingdong prefecture level city in Shandong
Jinfeng Hongyuan Flagship Store	Power Saving Expert 2	It didn't work, I used it for a few days and it was the same as before, it didn't work, I should have to return it	Jingdong prefecture level city in Shandong
Jinfeng Hongyuan Flagship Store	Power Saving Expert 2	The goods have been received, but have not yet installed and use, feel good.	Jingdong prefecture level city in Shandong
Jinfeng Hongyuan Flagship Store	Power Saving Expert 2	Goods received, I forgot to take pictures, the goods look good, you can start, immediately start the school to see the effect then	Jingdong prefecture level city in Shandong
Jinfeng Hongyuan Flagship Store	Power Saving Expert 2	Looks good. I'll use it for a while.	Jingdong prefecture level city in Shandong
Jinfeng Hongyuan Flagship Store	Power Saving Expert 2	Great stuff, easy to operate, easy to use, good power saving, believe in the power of science!	Jingdong prefecture level city in Shandong
Jinfeng Hongyuan Flagship Store	Power Saving Expert 2	Especially great, comparison shopping before buying, the store owner is very good, answering questions very patiently and detailed, there is a need to come back!	Jingdong prefecture level city in Shandong
Jinfeng Hongyuan Flagship Store	Power Saving Expert 2	Trustworthy, feel free to use it boldly.	Jingdong prefecture level city in Shandong
Jinfeng Hongyuan Flagship Store	Power Saving Expert 2	Logistics is super fast! Beautiful appearance! Looks like a great quality! I've never bought one before, so I'll try it first to see if it saves power. We'll see how much it saves.	Jingdong prefecture level city in Shandong
Jinfeng Hongyuan Flagship Store	Power Saving Expert 2	I saw online counterfeiting, energy saver is one of them, hahahahaha, jingdong also buy fake products	Jingdong prefecture level city in Shandong
Jinfeng Hongyuan Flagship Store	Power Saving Expert 2	Just received, installed, try a month to see how the effect and then follow up.	Jingdong prefecture level city in Shandong
Jinfeng Hongyuan Flagship Store	Power Saving Expert 2	Good for you! The goods are genuine! Good price for what you get!	Jingdong prefecture level city in Shandong
Jinfeng Hongyuan Flagship Store	Power Saving Expert 2	Things quickly received, immediately used to see if there is no effect, something river photo ⬆ the same, customer service is very professional	Jingdong prefecture level city in Shandong
Jinfeng Hongyuan Flagship Store	Power Saving Expert 2	The product is very convenient Looking forward to the subsequent use of the experience results	Jingdong prefecture level city in Shandong
Jinfeng Hongyuan Flagship Store	Power Saving Expert 2	I just received it and plugged it in, we'll see exactly when it's time to pay the electric bill	Jingdong prefecture level city in Shandong
Jinfeng Hongyuan Flagship Store	Power Saving Expert 2	Saver is good Packaging and looks good How effective it is to be shared after using it.	Jingdong prefecture level city in Shandong
Jinfeng Hongyuan Flagship Store	Power Saving Expert 2	There's no way to compare. I don't know if it's energy efficient.	Jingdong prefecture level city in Shandong
Jinfeng Hongyuan Flagship Store	Power Saving Expert 2	Just installed it, try it out and comment again	Jingdong prefecture level city in Shandong
Jinfeng Hongyuan Flagship Store	Power Saving Expert 2	Super save point also good to install, after a friend introduced then went to buy to, friends say it is very good to use, home I bought, try to use a period of time, to be honest, the economizer is not big or heavy, can be very convenient to put in a lot of places, the quality is also very good, power-saving function this time seems to be about 20% of the efficiency. The owner is also very warm contact, very	Jingdong prefecture level city in Shandong
Jinfeng Hongyuan Flagship Store	Power Saving Expert 2	The reviews online are mixed. Try it for a while, but I don't know if I can return it if it doesn't save any power after a couple months.	Jingdong prefecture level city in Shandong
Jinfeng Hongyuan Flagship Store	Power Saving Expert 2	The power consumption at home is high, bought a power saver, it looks very convenient to plug on the line, see if you can save some money next month, if good, recommend to friends down the road.	Jingdong prefecture level city in Shandong
Jinfeng Hongyuan Flagship Store	Power Saving Expert 2	It's beautiful, it's genuine, but I don't know the power-saving effect.	Jingdong prefecture level city in Shandong
Jinfeng Hongyuan Flagship Store	Power Saving Expert 2	This user thinks the product is good	Jingdong prefecture level city in Shandong
Jinfeng Hongyuan Flagship Store	Power Saving Expert 2	Nice exterior design with an at-a-glance display	Jingdong prefecture level city in Shandong
Jinfeng Hongyuan Flagship Store	Power Saving Expert 2	Home large and small things are purchased in the Jingdong Mall, have been accustomed to shopping in the Jingdong, many times can buy good quality and cheap products, and courier service attitude is particularly good, sometimes too many couriers are to help me to the home, so many years have been in the Jingdong Mall shopping, trust and support! Will also support the Jingdong as always!	Jingdong prefecture level city in Shandong
Jinfeng Hongyuan Flagship Store	Power Saving Expert 2	Good workmanship, mostly better than the original wall-plugged ones, and hopefully power factor compensation saves on electricity costs	Jingdong prefecture level city in Shandong
Jinfeng Hongyuan Flagship Store	Power Saving Expert 2	I don't feel anything, I said I won't know the effect for a month or two, let's see.	Jingdong prefecture level city in Shandong
Jinfeng Hongyuan Flagship Store	Power Saving Expert 2	Delivery was pretty fast, a little pricey, just not sure if it works or not	Jingdong prefecture level city in Shandong
Jinfeng Hongyuan Flagship Store	Power Saving Expert 2	The goods have been received, I do not know how effective.	Jingdong prefecture level city in Shandong
Jinfeng Hongyuan Flagship Store	Power Saving Expert 2	So far it doesn't seem to be too noticeable, so I'll comment later.	Jingdong prefecture level city in Shandong
Jinfeng Hongyuan Flagship Store	Power Saving Expert 2	I'll comment after I've used it	Jingdong prefecture level city in Shandong
Jinfeng Hongyuan Flagship Store	Power Saving Expert 2	Not bad. You'll save a lot on your electric bill in a year!	Jingdong prefecture level city in Shandong
Jinfeng Hongyuan Flagship Store	Power Saving Expert 2	I just got it. I haven't tried it yet. I don't know what it's like.	Jingdong prefecture level city in Shandong
Jinfeng Hongyuan Flagship Store	Power Saving Expert 2	Shopping at Jingdong has become a habit, and this disease will not be cured for a while.	Jingdong prefecture level city in Shandong
Jinfeng Hongyuan Flagship Store	Power Saving Expert 2	I've been using it for a while now. I don't know if I'm still saving power or not.	Jingdong prefecture level city in Shandong
Jinfeng Hongyuan Flagship Store	Power Saving Expert 2	Received the saver and it's great! There is also a digital voltage indication. Plugged in as instructed, easy to operate, hope you're our power saver!	Jingdong prefecture level city in Shandong
Jinfeng Hongyuan Flagship Store	Power Saving Expert 2	There is no power saving as the store says, using it is the same as not using it.	Jingdong prefecture level city in Shandong
Jinfeng Hongyuan Flagship Store	Power Saving Expert 2	Use it for a while and then comment again	Jingdong prefecture level city in Shandong

Jinfeng Hongyuan Flagship Store	Power Saving Expert 2	Feeling can, to use 1 to 2 months to see how the effect, there is a voltage display, can see the voltage fluctuations amplitude, quite good!	Jingdong prefecture level city in Shandong
Jinfeng Hongyuan Flagship Store	Power Saving Expert 2	I hope it works and saves me some electricity.	Jingdong prefecture level city in Shandong
Jinfeng Hongyuan Flagship Store	Power Saving Expert 2	Nice baby, happy with the after sales	Jingdong prefecture level city in Shandong
Jinfeng Hongyuan Flagship Store	Power Saving Expert 2	Use it and talk about it.	Jingdong prefecture level city in Shandong
Jinfeng Hongyuan Flagship Store	Power Saving Expert 2	Second purchase	Jingdong prefecture level city in Shandong
Jinfeng Hongyuan Flagship Store	Power Saving Expert 2	It works.	Jingdong prefecture level city in Shandong
Jinfeng Hongyuan Flagship Store	Power Saving Expert 2	It works.	Jingdong prefecture level city in Shandong
Jinfeng Hongyuan Flagship Store	Power Saving Expert 2	I don't know if it's a power saver until I start using it	Jingdong prefecture level city in Shandong
Jinfeng Hongyuan Flagship Store	Power Saving Expert 2	serviceable	Jingdong prefecture level city in Shandong
Jinfeng Hongyuan Flagship Store	Power Saving Expert 2	This user did not fill out the rating in time	Jingdong prefecture level city in Shandong
Jinfeng Hongyuan Flagship Store	Power Saving Expert 2	There is no power saving as the store says, using it is the same as not using it.	Jingdong prefecture level city in Shandong
Jinfeng Hongyuan Flagship Store	Power Saving Expert 2	This user did not fill out the rating in time	Jingdong prefecture level city in Shandong
Jinfeng Hongyuan Flagship Store	Power Saving Expert 2	Good...good.	Jingdong prefecture level city in Shandong
Jinfeng Hongyuan Flagship Store	Power Saving Expert 2	(of an unmarried couple) be close	Jingdong prefecture level city in Shandong
Jinfeng Hongyuan Flagship Store	Power Saving Expert 2	(of an unmarried couple) be close	Jingdong prefecture level city in Shandong
Jinfeng Hongyuan Flagship Store	Power Saving Expert 2	(of an unmarried couple) be close	Jingdong prefecture level city in Shandong
Jinfeng Hongyuan Flagship Store	Power Saving Expert 2	The goods are real, high value, high quality, worth every penny, material appearance and quality at a glance is very upscale, very like HAPPY1 00	Jingdong prefecture level city in Shandong
Jinfeng Hongyuan Flagship Store	Power Saving Expert 2	It is the ideal product, good quality, logistics is also very fast, worthy of praise 🎁 baby quality is very good, received the first time to open the first time to see, it is very good 👍, very satisfied 😃, customer service is also available to ask questions and answer, thank you 🙏	Jingdong prefecture level city in Shandong
Jinfeng Hongyuan Flagship Store	Power Saving Expert 2	I bought it and haven't used it yet, I'll use it again.	Jingdong prefecture level city in Shandong
Jinfeng Hongyuan Flagship Store	Power Saving Expert 2	It didn't work, I used it for a few days and it was the same as before, it didn't work, I should have to return it	Jingdong prefecture level city in Shandong
Jinfeng Hongyuan Flagship Store	Power Saving Expert 2	The goods have been received, but have not yet installed and use, feel good.	Jingdong prefecture level city in Shandong
Jinfeng Hongyuan Flagship Store	Power Saving Expert 2	Goods received, I forgot to take pictures, the goods look good, you can start, immediately start the school to see the effect then	Jingdong prefecture level city in Shandong
Jinfeng Hongyuan Flagship Store	Power Saving Expert 2	Looks good. I'll use it for a while.	Jingdong prefecture level city in Shandong
Jinfeng Hongyuan Flagship Store	Power Saving Expert 2	Great stuff, easy to operate, easy to use, good power saving, believe in the power of science!	Jingdong prefecture level city in Shandong
Jinfeng Hongyuan Flagship Store	Power Saving Expert 2	Especially great, comparison shopping before buying, the store owner is very good, answering questions very patiently and detailed, there is a need to come back!	Jingdong prefecture level city in Shandong
Jinfeng Hongyuan Flagship Store	Power Saving Expert 2	Trustworthy, feel free to use it boldly.	Jingdong prefecture level city in Shandong
Jinfeng Hongyuan Flagship Store	Power Saving Expert 2	Logistics is super fast! Beautiful appearance! Looks like a great quality! I've never bought one before, so I'll try it first to see if it saves power. We'll see how much it saves.	Jingdong prefecture level city in Shandong
Jinfeng Hongyuan Flagship Store	Power Saving Expert 2	I saw online counterfeiting, energy saver is one of them, hahahahaha, jingdong also buy fake products	Jingdong prefecture level city in Shandong
Jinfeng Hongyuan Flagship Store	Power Saving Expert 2	Just received, installed, try a month to see how the effect and then follow up.	Jingdong prefecture level city in Shandong
Jinfeng Hongyuan Flagship Store	Power Saving Expert 2	Good for you! The goods are genuine! Good price for what you get!	Jingdong prefecture level city in Shandong
Jinfeng Hongyuan Flagship Store	Power Saving Expert 2	Things quickly received, immediately used to see if there is no effect, something river photo ⬆ the same, customer service is very professional	Jingdong prefecture level city in Shandong
Jinfeng Hongyuan Flagship Store	Power Saving Expert 2	The product is very convenient Looking forward to the subsequent use of the experience results	Jingdong prefecture level city in Shandong
Jinfeng Hongyuan Flagship Store	Power Saving Expert 2	I just received it and plugged it in, we'll see exactly when it's time to pay the electric bill	Jingdong prefecture level city in Shandong
Jinfeng Hongyuan Flagship Store	Power Saving Expert 2	Saver is good Packaging and looks good How effective it is to be shared after using it.	Jingdong prefecture level city in Shandong
Jinfeng Hongyuan Flagship Store	Power Saving Expert 2	There's no way to compare. I don't know if it's energy efficient.	Jingdong prefecture level city in Shandong
Jinfeng Hongyuan Flagship Store	Power Saving Expert 2	Just installed it, try it out and comment again	Jingdong prefecture level city in Shandong
Jinfeng Hongyuan Flagship Store	Power Saving Expert 2	Super save point also good to install, after a friend introduced then went to buy to, friends say it is very good to use, home I bought, try to use a period of time, to be honest, the economizer is not big or heavy, can be very convenient to put in a lot of places, the quality is also very good, power-saving function this time seems to be about 20% of the efficiency. The owner is also very warm contact, very	Jingdong prefecture level city in Shandong
Jinfeng Hongyuan Flagship Store	Power Saving Expert 2	The reviews online are mixed. Try it for a while, but I don't know if I can return it if it doesn't save any power after a couple months.	Jingdong prefecture level city in Shandong
Jinfeng Hongyuan Flagship Store	Power Saving Expert 2	The power consumption at home is high, bought a power saver, it looks very convenient to plug on the line, see if you can save some money next month, if good, recommend to friends down the road.	Jingdong prefecture level city in Shandong
Jinfeng Hongyuan Flagship Store	Power Saving Expert 2	It's beautiful, it's genuine, but I don't know the power-saving effect.	Jingdong prefecture level city in Shandong
Jinfeng Hongyuan Flagship Store	Power Saving Expert 2	This user thinks the product is good	Jingdong prefecture level city in Shandong
Jinfeng Hongyuan Flagship Store	Power Saving Expert 2	Nice exterior design with an at-a-glance display	Jingdong prefecture level city in Shandong
Jinfeng Hongyuan Flagship Store	Power Saving Expert 2	Home large and small things are purchased in the Jingdong Mall, have been accustomed to shopping in the Jingdong, many times can buy good quality and cheap products, and courier service attitude is particularly good, sometimes too many couriers are to help me to the home, so many years have been in the Jingdong Mall shopping, trust and support! Will also support the Jingdong as always!	Jingdong prefecture level city in Shandong
Jinfeng Hongyuan Flagship Store	Power Saving Expert 2	Good workmanship, mostly better than the original wall-plugged ones, and hopefully power factor compensation saves on electricity costs	Jingdong prefecture level city in Shandong
Jinfeng Hongyuan Flagship Store	Power Saving Expert 2	I don't feel anything, I said I won't know the effect for a month or two, let's see.	Jingdong prefecture level city in Shandong
Jinfeng Hongyuan Flagship Store	Power Saving Expert 2	Delivery was pretty fast, a little pricey, just not sure if it works or not	Jingdong prefecture level city in Shandong
Jinfeng Hongyuan Flagship Store	Power Saving Expert 2	The goods have been received, I do not know how effective.	Jingdong prefecture level city in Shandong
Jinfeng Hongyuan Flagship Store	Power Saving Expert 2	So far it doesn't seem to be too noticeable, so I'll comment later.	Jingdong prefecture level city in Shandong
Jinfeng Hongyuan Flagship Store	Power Saving Expert 2	I'll comment after I've used it	Jingdong prefecture level city in Shandong

Jinfeng Hongyuan Flagship Store	Power Saving Expert 2	Not bad. You'll save a lot on your electric bill in a year!	Jingdong prefecture level city in Shandong
Jinfeng Hongyuan Flagship Store	Power Saving Expert 2	I just got it. I haven't tried it yet. I don't know what it's like.	Jingdong prefecture level city in Shandong
Jinfeng Hongyuan Flagship Store	Power Saving Expert 2	Shopping at Jingdong has become a habit, and this disease will not be cured for a while.	Jingdong prefecture level city in Shandong
Jinfeng Hongyuan Flagship Store	Power Saving Expert 2	I've been using it for a while now. I don't know if I'm still saving power or not.	Jingdong prefecture level city in Shandong
Jinfeng Hongyuan Flagship Store	Power Saving Expert 2	Received the saver and it's great! There is also a digital voltage indication. Plugged in as instructed, easy to operate, hope you're our power saver!	Jingdong prefecture level city in Shandong
Jinfeng Hongyuan Flagship Store	Power Saving Expert 2	There is no power saving as the store says, using it is the same as not using it.	Jingdong prefecture level city in Shandong
Jinfeng Hongyuan Flagship Store	Power Saving Expert 2	Use it for a while and then comment again	Jingdong prefecture level city in Shandong
Jinfeng Hongyuan Flagship Store	Power Saving Expert 2	Feeling can, to use 1 to 2 months to see how the effect, there is a voltage display, can see the voltage fluctuations amplitude, quite good!	Jingdong prefecture level city in Shandong
Jinfeng Hongyuan Flagship Store	Power Saving Expert 2	I hope it works and saves me some electricity.	Jingdong prefecture level city in Shandong
Jinfeng Hongyuan Flagship Store	Power Saving Expert 2	Nice baby, happy with the after sales	Jingdong prefecture level city in Shandong
Jinfeng Hongyuan Flagship Store	Power Saving Expert 2	Use it and talk about it.	Jingdong prefecture level city in Shandong
Jinfeng Hongyuan Flagship Store	Power Saving Expert 2	Second purchase	Jingdong prefecture level city in Shandong
Jinfeng Hongyuan Flagship Store	Power Saving Expert 2	It works.	Jingdong prefecture level city in Shandong
Jinfeng Hongyuan Flagship Store	Power Saving Expert 2	It works.	Jingdong prefecture level city in Shandong
Jinfeng Hongyuan Flagship Store	Power Saving Expert 2	I don't know if it's a power saver until I start using it	Jingdong prefecture level city in Shandong
Jinfeng Hongyuan Flagship Store	Power Saving Expert 2	serviceable	Jingdong prefecture level city in Shandong
Jinfeng Hongyuan Flagship Store	Power Saving Expert 2	This user did not fill out the rating in time	Jingdong prefecture level city in Shandong
Jinfeng Hongyuan Flagship Store	Power Saving Expert 2	There is no power saving as the store says, using it is the same as not using it.	Jingdong prefecture level city in Shandong
Jinfeng Hongyuan Flagship Store	Power Saving Expert 2	This user did not fill out the rating in time	Jingdong prefecture level city in Shandong
Jinfeng Hongyuan Flagship Store	Power Saving Expert 2	Good...good.	Jingdong prefecture level city in Shandong
Jinfeng Hongyuan Flagship Store	Power Saving Expert 2	(of an unmarried couple) be close	Jingdong prefecture level city in Shandong
Jinfeng Hongyuan Flagship Store	Power Saving Expert 2	(of an unmarried couple) be close	Jingdong prefecture level city in Shandong
Jinfeng Hongyuan Flagship Store	Power Saving Expert 2	(of an unmarried couple) be close	Jingdong prefecture level city in Shandong
Jinfeng Hongyuan Flagship Store	Power Saving Expert 2	The goods are real, high value, high quality, worth every penny, material appearance and quality at a glance is very upscale, very like HAPPY💯	Jingdong prefecture level city in Shandong
Jinfeng Hongyuan Flagship Store	Power Saving Expert 2	It is the ideal product, good quality, logistics is also very fast, worthy of praise 🎁 baby quality is very good, received the first time to open the first time to see, it is very good 👍, very satisfied 😃, customer service is also available to ask questions and answer, thank you 🙏	Jingdong prefecture level city in Shandong
Jinfeng Hongyuan Flagship Store	Power Saving Expert 2	I bought it and haven't used it yet, I'll use it again.	Jingdong prefecture level city in Shandong
Jinfeng Hongyuan Flagship Store	Power Saving Expert 2	It didn't work, I used it for a few days and it was the same as before, it didn't work, I should have to return it	Jingdong prefecture level city in Shandong
Jinfeng Hongyuan Flagship Store	Power Saving Expert 2	The goods have been received, but have not yet installed and use, feel good.	Jingdong prefecture level city in Shandong
Jinfeng Hongyuan Flagship Store	Power Saving Expert 2	Goods received, I forgot to take pictures, the goods look good, you can start, immediately start the school to see the effect then	Jingdong prefecture level city in Shandong
Jinfeng Hongyuan Flagship Store	Power Saving Expert 2	Looks good. I'll use it for a while.	Jingdong prefecture level city in Shandong
Jinfeng Hongyuan Flagship Store	Power Saving Expert 2	Great stuff, easy to operate, easy to use, good power saving, believe in the power of science!	Jingdong prefecture level city in Shandong
Jinfeng Hongyuan Flagship Store	Power Saving Expert 2	Especially great, comparison shopping before buying, the store owner is very good, answering questions very patiently and detailed, there is a need to come back!	Jingdong prefecture level city in Shandong
Jinfeng Hongyuan Flagship Store	Power Saving Expert 2	Trustworthy, feel free to use it boldly.	Jingdong prefecture level city in Shandong
Jinfeng Hongyuan Flagship Store	Power Saving Expert 2	Logistics is super fast! Beautiful appearance! Looks like a great quality! I've never bought one before, so I'll try it first to see if it saves power. We'll see how much it saves.	Jingdong prefecture level city in Shandong
Jinfeng Hongyuan Flagship Store	Power Saving Expert 2	I saw online counterfeiting, energy saver is one of them, hahahahaha, jingdong also buy fake products	Jingdong prefecture level city in Shandong
Jinfeng Hongyuan Flagship Store	Power Saving Expert 2	Just received, installed, try a month to see how the effect and then follow up.	Jingdong prefecture level city in Shandong
Jinfeng Hongyuan Flagship Store	Power Saving Expert 2	Good for you! The goods are genuine! Good price for what you get!	Jingdong prefecture level city in Shandong
Jinfeng Hongyuan Flagship Store	Power Saving Expert 2	Things quickly received, immediately used to see if there is no effect, something river photo ⬆ the same, customer service is very professional	Jingdong prefecture level city in Shandong
Jinfeng Hongyuan Flagship Store	Power Saving Expert 2	The product is very convenient Looking forward to the subsequent use of the experience results	Jingdong prefecture level city in Shandong
Jinfeng Hongyuan Flagship Store	Power Saving Expert 2	I just received it and plugged it in, we'll see exactly when it's time to pay the electric bill	Jingdong prefecture level city in Shandong
Jinfeng Hongyuan Flagship Store	Power Saving Expert 2	Saver is good Packaging and looks good How effective it is to be shared after using it.	Jingdong prefecture level city in Shandong
Jinfeng Hongyuan Flagship Store	Power Saving Expert 2	There's no way to compare. I don't know if it's energy efficient.	Jingdong prefecture level city in Shandong
Jinfeng Hongyuan Flagship Store	Power Saving Expert 2	Just installed it, try it out and comment again	Jingdong prefecture level city in Shandong
Jinfeng Hongyuan Flagship Store	Power Saving Expert 2	Super save point also good to install, after a friend introduced then went to buy to, friends say it is very good to use, home I bought, try to use a period of time, to be honest, the economizer is not big or heavy, can be very convenient to put in a lot of places, the quality is also very good, power-saving function this time seems to be about 20% of the efficiency. The owner is also very warm contact, very	Jingdong prefecture level city in Shandong
Jinfeng Hongyuan Flagship Store	Power Saving Expert 2	The reviews online are mixed. Try it for a while, but I don't know if I can return it if it doesn't save any power after a couple months.	Jingdong prefecture level city in Shandong
Jinfeng Hongyuan Flagship Store	Power Saving Expert 2	The power consumption at home is high, bought a power saver, it looks very convenient to plug on the line, see if you can save some money next month, if good, recommend to friends down the road.	Jingdong prefecture level city in Shandong
Jinfeng Hongyuan Flagship Store	Power Saving Expert 2	It's beautiful, it's genuine, but I don't know the power-saving effect.	Jingdong prefecture level city in Shandong
Jinfeng Hongyuan Flagship Store	Power Saving Expert 2	This user thinks the product is good	Jingdong prefecture level city in Shandong
Jinfeng Hongyuan Flagship Store	Power Saving Expert 2	Nice exterior design with an at-a-glance display	Jingdong prefecture level city in Shandong

Jinfeng Hongyuan Flagship Store	Power Saving Expert 2	Home large and small things are purchased in the Jingdong Mall, have been accustomed to shopping in the Jingdong, many times can buy good quality and cheap products, and courier service attitude is particularly good, sometimes too many couriers are to help me to the home, so many years have been in the Jingdong Mall shopping, trust and support! Will also support the Jingdong as always!	Jingdong prefecture level city in Shandong
Jinfeng Hongyuan Flagship Store	Power Saving Expert 2	Good workmanship, mostly better than the original wall-plugged ones, and hopefully power factor compensation saves on electricity costs	Jingdong prefecture level city in Shandong
Jinfeng Hongyuan Flagship Store	Power Saving Expert 2	I don't feel anything, I said I won't know the effect for a month or two, let's see.	Jingdong prefecture level city in Shandong
Jinfeng Hongyuan Flagship Store	Power Saving Expert 2	Delivery was pretty fast, a little pricey, just not sure if it works or not	Jingdong prefecture level city in Shandong
Jinfeng Hongyuan Flagship Store	Power Saving Expert 2	The goods have been received, I do not know how effective.	Jingdong prefecture level city in Shandong
Jinfeng Hongyuan Flagship Store	Power Saving Expert 2	So far it doesn't seem to be too noticeable, so I'll comment later.	Jingdong prefecture level city in Shandong
Jinfeng Hongyuan Flagship Store	Power Saving Expert 2	I'll comment after I've used it	Jingdong prefecture level city in Shandong
Jinfeng Hongyuan Flagship Store	Power Saving Expert 2	Not bad. You'll save a lot on your electric bill in a year!	Jingdong prefecture level city in Shandong
Jinfeng Hongyuan Flagship Store	Power Saving Expert 2	I just got it. I haven't tried it yet. I don't know what it's like.	Jingdong prefecture level city in Shandong
Jinfeng Hongyuan Flagship Store	Power Saving Expert 2	Shopping at Jingdong has become a habit, and this disease will not be cured for a while.	Jingdong prefecture level city in Shandong
Jinfeng Hongyuan Flagship Store	Power Saving Expert 2	I've been using it for a while now. I don't know if I'm still saving power or not.	Jingdong prefecture level city in Shandong
Jinfeng Hongyuan Flagship Store	Power Saving Expert 2	Received the saver and it's great! There is also a digital voltage indication. Plugged in as instructed, easy to operate, hope you're our power saver!	Jingdong prefecture level city in Shandong
Jinfeng Hongyuan Flagship Store	Power Saving Expert 2	There is no power saving as the store says, using it is the same as not using it.	Jingdong prefecture level city in Shandong
Jinfeng Hongyuan Flagship Store	Power Saving Expert 2	Use it for a while and then comment again	Jingdong prefecture level city in Shandong
Jinfeng Hongyuan Flagship Store	Power Saving Expert 2	Feeling can, to use 1 to 2 months to see how the effect, there is a voltage display, can see the voltage fluctuations amplitude, quite good!	Jingdong prefecture level city in Shandong
Jinfeng Hongyuan Flagship Store	Power Saving Expert 2	I hope it works and saves me some electricity.	Jingdong prefecture level city in Shandong
Jinfeng Hongyuan Flagship Store	Power Saving Expert 2	Nice baby, happy with the after sales	Jingdong prefecture level city in Shandong
Jinfeng Hongyuan Flagship Store	Power Saving Expert 2	Use it and talk about it.	Jingdong prefecture level city in Shandong
Jinfeng Hongyuan Flagship Store	Power Saving Expert 2	Second purchase	Jingdong prefecture level city in Shandong
Jinfeng Hongyuan Flagship Store	Power Saving Expert 2	It works.	Jingdong prefecture level city in Shandong
Jinfeng Hongyuan Flagship Store	Power Saving Expert 2	It works.	Jingdong prefecture level city in Shandong
Jinfeng Hongyuan Flagship Store	Power Saving Expert 2	I don't know if it's a power saver until I start using it	Jingdong prefecture level city in Shandong
Jinfeng Hongyuan Flagship Store	Power Saving Expert 2	serviceable	Jingdong prefecture level city in Shandong
Jinfeng Hongyuan Flagship Store	Power Saving Expert 2	This user did not fill out the rating in time	Jingdong prefecture level city in Shandong
Jinfeng Hongyuan Flagship Store	Power Saving Expert 2	There is no power saving as the store says, using it is the same as not using it.	Jingdong prefecture level city in Shandong
Jinfeng Hongyuan Flagship Store	Power Saving Expert 2	This user did not fill out the rating in time	Jingdong prefecture level city in Shandong
Jinfeng Hongyuan Flagship Store	Power Saving Expert 2	Good...good.	Jingdong prefecture level city in Shandong
Jinfeng Hongyuan Flagship Store	Power Saving Expert 2	(of an unmarried couple) be close	Jingdong prefecture level city in Shandong
Jinfeng Hongyuan Flagship Store	Power Saving Expert 2	(of an unmarried couple) be close	Jingdong prefecture level city in Shandong
Jinfeng Hongyuan Flagship Store	Power Saving Expert 2	(of an unmarried couple) be close	Jingdong prefecture level city in Shandong
Jinfeng Hongyuan Flagship Store	Power Saving Expert 2	The goods are real, high value, high quality, worth every penny, material appearance and quality at a glance is very upscale, very like HAPPY1 00	Jingdong prefecture level city in Shandong
Jinfeng Hongyuan Flagship Store	Power Saving Expert 2	It is the ideal product, good quality, logistics is also very fast, worthy of praise 🎁 baby quality is very good, received the first time to open the first time to see, it is very good 👍, very satisfied 😃, customer service is also available to ask questions and answer, thank you 🙏	Jingdong prefecture level city in Shandong
Jinfeng Hongyuan Flagship Store	Power Saving Expert 2	I bought it and haven't used it yet, I'll use it again.	Jingdong prefecture level city in Shandong
Jinfeng Hongyuan Flagship Store	Power Saving Expert 2	It didn't work, I used it for a few days and it was the same as before, it didn't work, I should have to return it	Jingdong prefecture level city in Shandong
Jinfeng Hongyuan Flagship Store	Power Saving Expert 2	The goods have been received, but have not yet installed and use, feel good.	Jingdong prefecture level city in Shandong
Jinfeng Hongyuan Flagship Store	Power Saving Expert 2	Goods received, I forgot to take pictures, the goods look good, you can start, immediately start the school to see the effect then	Jingdong prefecture level city in Shandong
Jinfeng Hongyuan Flagship Store	Power Saving Expert 2	Looks good. I'll use it for a while.	Jingdong prefecture level city in Shandong
Jinfeng Hongyuan Flagship Store	Power Saving Expert 2	Great stuff, easy to operate, easy to use, good power saving, believe in the power of science!	Jingdong prefecture level city in Shandong
Jinfeng Hongyuan Flagship Store	Power Saving Expert 2	Especially great, comparison shopping before buying, the store owner is very good, answering questions very patiently and detailed, there is a need to come back!	Jingdong prefecture level city in Shandong
Jinfeng Hongyuan Flagship Store	Power Saving Expert 2	Trustworthy, feel free to use it boldly.	Jingdong prefecture level city in Shandong
Jinfeng Hongyuan Flagship Store	Power Saving Expert 2	Logistics is super fast! Beautiful appearance! Looks like a great quality! I've never bought one before, so I'll try it first to see if it saves power. We'll see how much it saves.	Jingdong prefecture level city in Shandong
Jinfeng Hongyuan Flagship Store	Power Saving Expert 2	I saw online counterfeiting, energy saver is one of them, hahahahaha, jingdong also buy fake products	Jingdong prefecture level city in Shandong
Jinfeng Hongyuan Flagship Store	Power Saving Expert 2	Just received, installed, try a month to see how the effect and then follow up.	Jingdong prefecture level city in Shandong
Jinfeng Hongyuan Flagship Store	Power Saving Expert 2	Good for you! The goods are genuine! Good price for what you get!	Jingdong prefecture level city in Shandong
Jinfeng Hongyuan Flagship Store	Power Saving Expert 2	Things quickly received, immediately used to see if there is no effect, something river photo ⬆ the same, customer service is very professional	Jingdong prefecture level city in Shandong
Jinfeng Hongyuan Flagship Store	Power Saving Expert 2	The product is very convenient Looking forward to the subsequent use of the experience results	Jingdong prefecture level city in Shandong
Jinfeng Hongyuan Flagship Store	Power Saving Expert 2	I just received it and plugged it in, we'll see exactly when it's time to pay the electric bill	Jingdong prefecture level city in Shandong
Jinfeng Hongyuan Flagship Store	Power Saving Expert 2	Saver is good Packaging and looks good How effective it is to be shared after using it.	Jingdong prefecture level city in Shandong
Jinfeng Hongyuan Flagship Store	Power Saving Expert 2	There's no way to compare. I don't know if it's energy efficient.	Jingdong prefecture level city in Shandong
Jinfeng Hongyuan Flagship Store	Power Saving Expert 2	Just installed it, try it out and comment again	Jingdong prefecture level city in Shandong

Jinfeng Hongyuan Flagship Store	Power Saving Expert 2	Super save point also good to install, after a friend introduced then went to buy to, friends say it is very good to use, home I bought, try to use a period of time, to be honest, the economizer is not big or heavy, can be very convenient to put in a lot of places, the quality is also very good, power-saving function this time seems to be about 20% of the efficiency. The owner is also very warm contact, very	Jingdong prefecture level city in Shandong
Jinfeng Hongyuan Flagship Store	Power Saving Expert 2	The reviews online are mixed. Try it for a while, but I don't know if I can return it if it doesn't save any power after a couple months.	Jingdong prefecture level city in Shandong
Jinfeng Hongyuan Flagship Store	Power Saving Expert 2	The power consumption at home is high, bought a power saver, it looks very convenient to plug on the line, see if you can save some money next month, if good, recommend to friends down the road.	Jingdong prefecture level city in Shandong
Jinfeng Hongyuan Flagship Store	Power Saving Expert 2	It's beautiful, it's genuine, but I don't know the power-saving effect.	Jingdong prefecture level city in Shandong
Jinfeng Hongyuan Flagship Store	Power Saving Expert 2	This user thinks the product is good	Jingdong prefecture level city in Shandong
Jinfeng Hongyuan Flagship Store	Power Saving Expert 2	Nice exterior design with an at-a-glance display	Jingdong prefecture level city in Shandong
Jinfeng Hongyuan Flagship Store	Power Saving Expert 2	Home large and small things are purchased in the Jingdong Mall, have been accustomed to shopping in the Jingdong, many times can buy good quality and cheap products, and courier service attitude is particularly good, sometimes too many couriers are to help me to the home, so many years have been in the Jingdong Mall shopping, trust and support! Will also support the Jingdong as always!	Jingdong prefecture level city in Shandong
Jinfeng Hongyuan Flagship Store	Power Saving Expert 2	Good workmanship, mostly better than the original wall-plugged ones, and hopefully power factor compensation saves on electricity costs	Jingdong prefecture level city in Shandong
Jinfeng Hongyuan Flagship Store	Power Saving Expert 2	I don't feel anything, I said I won't know the effect for a month or two, let's see.	Jingdong prefecture level city in Shandong
Jinfeng Hongyuan Flagship Store	Power Saving Expert 2	Delivery was pretty fast, a little pricey, just not sure if it works or not	Jingdong prefecture level city in Shandong
Jinfeng Hongyuan Flagship Store	Power Saving Expert 2	The goods have been received, I do not know how effective.	Jingdong prefecture level city in Shandong
Jinfeng Hongyuan Flagship Store	Power Saving Expert 2	So far it doesn't seem to be too noticeable, so I'll comment later.	Jingdong prefecture level city in Shandong
Jinfeng Hongyuan Flagship Store	Power Saving Expert 2	I'll comment after I've used it	Jingdong prefecture level city in Shandong
Jinfeng Hongyuan Flagship Store	Power Saving Expert 2	Not bad. You'll save a lot on your electric bill in a year!	Jingdong prefecture level city in Shandong
Jinfeng Hongyuan Flagship Store	Power Saving Expert 2	I just got it. I haven't tried it yet. I don't know what it's like.	Jingdong prefecture level city in Shandong
Jinfeng Hongyuan Flagship Store	Power Saving Expert 2	Shopping at Jingdong has become a habit, and this disease will not be cured for a while.	Jingdong prefecture level city in Shandong
Jinfeng Hongyuan Flagship Store	Power Saving Expert 2	I've been using it for a while now. I don't know if I'm still saving power or not.	Jingdong prefecture level city in Shandong
Jinfeng Hongyuan Flagship Store	Power Saving Expert 2	Received the saver and it's great! There is also a digital voltage indication. Plugged in as instructed, easy to operate, hope you're our power saver!	Jingdong prefecture level city in Shandong
Jinfeng Hongyuan Flagship Store	Power Saving Expert 2	There is no power saving as the store says, using it is the same as not using it.	Jingdong prefecture level city in Shandong
Jinfeng Hongyuan Flagship Store	Power Saving Expert 2	Use it for a while and then comment again	Jingdong prefecture level city in Shandong
Jinfeng Hongyuan Flagship Store	Power Saving Expert 2	Feeling can, to use 1 to 2 months to see how the effect, there is a voltage display, can see the voltage fluctuations amplitude, quite good!	Jingdong prefecture level city in Shandong
Jinfeng Hongyuan Flagship Store	Power Saving Expert 2	I hope it works and saves me some electricity.	Jingdong prefecture level city in Shandong
Jinfeng Hongyuan Flagship Store	Power Saving Expert 2	Nice baby, happy with the after sales	Jingdong prefecture level city in Shandong
Jinfeng Hongyuan Flagship Store	Power Saving Expert 2	Use it and talk about it.	Jingdong prefecture level city in Shandong
Jinfeng Hongyuan Flagship Store	Power Saving Expert 2	Second purchase	Jingdong prefecture level city in Shandong
Jinfeng Hongyuan Flagship Store	Power Saving Expert 2	It works.	Jingdong prefecture level city in Shandong
Jinfeng Hongyuan Flagship Store	Power Saving Expert 2	It works.	Jingdong prefecture level city in Shandong
Jinfeng Hongyuan Flagship Store	Power Saving Expert 2	I don't know if it's a power saver until I start using it	Jingdong prefecture level city in Shandong
Jinfeng Hongyuan Flagship Store	Power Saving Expert 2	serviceable	Jingdong prefecture level city in Shandong
Jinfeng Hongyuan Flagship Store	Power Saving Expert 2	This user did not fill out the rating in time	Jingdong prefecture level city in Shandong
Jinfeng Hongyuan Flagship Store	Power Saving Expert 2	There is no power saving as the store says, using it is the same as not using it.	Jingdong prefecture level city in Shandong
Jinfeng Hongyuan Flagship Store	Power Saving Expert 2	This user did not fill out the rating in time	Jingdong prefecture level city in Shandong
Jinfeng Hongyuan Flagship Store	Power Saving Expert 2	Good...good.	Jingdong prefecture level city in Shandong
Jinfeng Hongyuan Flagship Store	Power Saving Expert 2	(of an unmarried couple) be close	Jingdong prefecture level city in Shandong
Jinfeng Hongyuan Flagship Store	Power Saving Expert 2	(of an unmarried couple) be close	Jingdong prefecture level city in Shandong
Jinfeng Hongyuan Flagship Store	Power Saving Expert 2	(of an unmarried couple) be close	Jingdong prefecture level city in Shandong
Jinfeng Hongyuan Flagship Store	Power Saving Expert 2	The goods are real, high value, high quality, worth every penny, material appearance and quality at a glance is very upscale, very like HAPPY1 00	Jingdong prefecture level city in Shandong
Jinfeng Hongyuan Flagship Store	Power Saving Expert 2	It is the ideal product, good quality, logistics is also very fast, worthy of praise 🎁 baby quality is very good, received the first time to open the first time to see, it is very good 👍, very satisfied 😀, customer service is also available to ask questions and answer, thank you 🙏	Jingdong prefecture level city in Shandong
Jinfeng Hongyuan Flagship Store	Power Saving Expert 2	I bought it and haven't used it yet, I'll use it again.	Jingdong prefecture level city in Shandong
Jinfeng Hongyuan Flagship Store	Power Saving Expert 2	It didn't work, I used it for a few days and it was the same as before, it didn't work, I should have to return it	Jingdong prefecture level city in Shandong
Jinfeng Hongyuan Flagship Store	Power Saving Expert 2	The goods have been received, but have not yet installed and use, feel good.	Jingdong prefecture level city in Shandong
Jinfeng Hongyuan Flagship Store	Power Saving Expert 2	Goods received, I forgot to take pictures, the goods look good, you can start, immediately start the school to see the effect then	Jingdong prefecture level city in Shandong
Jinfeng Hongyuan Flagship Store	Power Saving Expert 2	Looks good. I'll use it for a while.	Jingdong prefecture level city in Shandong
Jinfeng Hongyuan Flagship Store	Power Saving Expert 2	Great stuff, easy to operate, easy to use, good power saving, believe in the power of science!	Jingdong prefecture level city in Shandong
Jinfeng Hongyuan Flagship Store	Power Saving Expert 2	Especially great, comparison shopping before buying, the store owner is very good, answering questions very patiently and detailed, there is a need to come back!	Jingdong prefecture level city in Shandong
Jinfeng Hongyuan Flagship Store	Power Saving Expert 2	Trustworthy, feel free to use it boldly.	Jingdong prefecture level city in Shandong
Jinfeng Hongyuan Flagship Store	Power Saving Expert 2	Logistics is super fast! Beautiful appearance! Looks like a great quality! I've never bought one before, so I'll try it first to see if it saves power. We'll see how much it saves.	Jingdong prefecture level city in Shandong
Jinfeng Hongyuan Flagship Store	Power Saving Expert 2	I saw online counterfeiting, energy saver is one of them, hahahahaha, jingdong also buy fake products	Jingdong prefecture level city in Shandong
Jinfeng Hongyuan Flagship Store	Power Saving Expert 2	Just received, installed, try a month to see how the effect and then follow up.	Jingdong prefecture level city in Shandong

Jinfeng Hongyuan Flagship Store	Power Saving Expert 2	Good for you! The goods are genuine! Good price for what you get!	Jingdong prefecture level city in Shandong
Jinfeng Hongyuan Flagship Store	Power Saving Expert 2	Things quickly received, immediately used to see if there is no effect, something river photo ⬆ the same, customer service is very professional	Jingdong prefecture level city in Shandong
Jinfeng Hongyuan Flagship Store	Power Saving Expert 2	The product is very convenient Looking forward to the subsequent use of the experience results	Jingdong prefecture level city in Shandong
Jinfeng Hongyuan Flagship Store	Power Saving Expert 2	I just received it and plugged it in, we'll see exactly when it's time to pay the electric bill	Jingdong prefecture level city in Shandong
Jinfeng Hongyuan Flagship Store	Power Saving Expert 2	Saver is good Packaging and looks good How effective it is to be shared after using it.	Jingdong prefecture level city in Shandong
Jinfeng Hongyuan Flagship Store	Power Saving Expert 2	There's no way to compare. I don't know if it's energy efficient.	Jingdong prefecture level city in Shandong
Jinfeng Hongyuan Flagship Store	Power Saving Expert 2	Just installed it, try it out and comment again	Jingdong prefecture level city in Shandong
Jinfeng Hongyuan Flagship Store	Power Saving Expert 2	Super save point also good to install, after a friend introduced then went to buy to, friends say it is very good to use, home I bought, try to use a period of time, to be honest, the economizer is not big or heavy, can be very convenient to put in a lot of places, the quality is also very good, power-saving function this time seems to be about 20% of the efficiency. The owner is also very warm contact, very	Jingdong prefecture level city in Shandong
Jinfeng Hongyuan Flagship Store	Power Saving Expert 2	The reviews online are mixed. Try it for a while, but I don't know if I can return it if it doesn't save any power after a couple months.	Jingdong prefecture level city in Shandong
Jinfeng Hongyuan Flagship Store	Power Saving Expert 2	The power consumption at home is high, bought a power saver, it looks very convenient to plug on the line, see if you can save some money next month, if good, recommend to friends down the road.	Jingdong prefecture level city in Shandong
Jinfeng Hongyuan Flagship Store	Power Saving Expert 2	It's beautiful, it's genuine, but I don't know the power-saving effect.	Jingdong prefecture level city in Shandong
Jinfeng Hongyuan Flagship Store	Power Saving Expert 2	This user thinks the product is good	Jingdong prefecture level city in Shandong
Jinfeng Hongyuan Flagship Store	Power Saving Expert 2	Nice exterior design with an at-a-glance display	Jingdong prefecture level city in Shandong
Jinfeng Hongyuan Flagship Store	Power Saving Expert 2	Home large and small things are purchased in the Jingdong Mall, have been accustomed to shopping in the Jingdong, many times can buy good quality and cheap products, and courier service attitude is particularly good, sometimes too many couriers are to help me to the home, so many years have been in the Jingdong Mall shopping, trust and support! Will also support the Jingdong as always!	Jingdong prefecture level city in Shandong
Jinfeng Hongyuan Flagship Store	Power Saving Expert 2	Good workmanship, mostly better than the original wall-plugged ones, and hopefully power factor compensation saves on electricity costs	Jingdong prefecture level city in Shandong
Jinfeng Hongyuan Flagship Store	Power Saving Expert 2	I don't feel anything, I said I won't know the effect for a month or two, let's see.	Jingdong prefecture level city in Shandong
Jinfeng Hongyuan Flagship Store	Power Saving Expert 2	Delivery was pretty fast, a little pricey, just not sure if it works or not	Jingdong prefecture level city in Shandong
Jinfeng Hongyuan Flagship Store	Power Saving Expert 2	The goods have been received, I do not know how effective.	Jingdong prefecture level city in Shandong
Jinfeng Hongyuan Flagship Store	Power Saving Expert 2	So far it doesn't seem to be too noticeable, so I'll comment later.	Jingdong prefecture level city in Shandong
Jinfeng Hongyuan Flagship Store	Power Saving Expert 2	I'll comment after I've used it	Jingdong prefecture level city in Shandong
Jinfeng Hongyuan Flagship Store	Power Saving Expert 2	Not bad. You'll save a lot on your electric bill in a year!	Jingdong prefecture level city in Shandong
Jinfeng Hongyuan Flagship Store	Power Saving Expert 2	I just got it. I haven't tried it yet. I don't know what it's like.	Jingdong prefecture level city in Shandong
Jinfeng Hongyuan Flagship Store	Power Saving Expert 2	Shopping at Jingdong has become a habit, and this disease will not be cured for a while.	Jingdong prefecture level city in Shandong
Jinfeng Hongyuan Flagship Store	Power Saving Expert 2	I've been using it for a while now. I don't know if I'm still saving power or not.	Jingdong prefecture level city in Shandong
Jinfeng Hongyuan Flagship Store	Power Saving Expert 2	Received the saver and it's great! There is also a digital voltage indication. Plugged in as instructed, easy to operate, hope you're our power saver!	Jingdong prefecture level city in Shandong
Jinfeng Hongyuan Flagship Store	Power Saving Expert 2	There is no power saving as the store says, using it is the same as not using it.	Jingdong prefecture level city in Shandong
Jinfeng Hongyuan Flagship Store	Power Saving Expert 2	Use it for a while and then comment again	Jingdong prefecture level city in Shandong
Jinfeng Hongyuan Flagship Store	Power Saving Expert 2	Feeling can, to use 1 to 2 months to see how the effect, there is a voltage display, can see the voltage fluctuations amplitude, quite good!	Jingdong prefecture level city in Shandong
Jinfeng Hongyuan Flagship Store	Power Saving Expert 2	I hope it works and saves me some electricity.	Jingdong prefecture level city in Shandong
Jinfeng Hongyuan Flagship Store	Power Saving Expert 2	Nice baby, happy with the after sales	Jingdong prefecture level city in Shandong
Jinfeng Hongyuan Flagship Store	Power Saving Expert 2	Use it and talk about it.	Jingdong prefecture level city in Shandong
Jinfeng Hongyuan Flagship Store	Power Saving Expert 2	Second purchase	Jingdong prefecture level city in Shandong
Jinfeng Hongyuan Flagship Store	Power Saving Expert 2	It works.	Jingdong prefecture level city in Shandong
Jinfeng Hongyuan Flagship Store	Power Saving Expert 2	It works.	Jingdong prefecture level city in Shandong
Jinfeng Hongyuan Flagship Store	Power Saving Expert 2	I don't know if it's a power saver until I start using it	Jingdong prefecture level city in Shandong
Jinfeng Hongyuan Flagship Store	Power Saving Expert 2	serviceable	Jingdong prefecture level city in Shandong
Jinfeng Hongyuan Flagship Store	Power Saving Expert 2	This user did not fill out the rating in time	Jingdong prefecture level city in Shandong
Jinfeng Hongyuan Flagship Store	Power Saving Expert 2	There is no power saving as the store says, using it is the same as not using it.	Jingdong prefecture level city in Shandong
Jinfeng Hongyuan Flagship Store	Power Saving Expert 2	This user did not fill out the rating in time	Jingdong prefecture level city in Shandong
Jinfeng Hongyuan Flagship Store	Power Saving Expert 2	Good...good.	Jingdong prefecture level city in Shandong
Jinfeng Hongyuan Flagship Store	Power Saving Expert 2	(of an unmarried couple) be close	Jingdong prefecture level city in Shandong
Jinfeng Hongyuan Flagship Store	Power Saving Expert 2	(of an unmarried couple) be close	Jingdong prefecture level city in Shandong
Jinfeng Hongyuan Flagship Store	Power Saving Expert 2	(of an unmarried couple) be close	Jingdong prefecture level city in Shandong
Jinfeng Hongyuan Flagship Store	Power Saving Expert 2	The goods are real, high value, high quality, worth every penny, material appearance and quality at a glance is very upscale, very like HAPPY1 00	Jingdong prefecture level city in Shandong
Jinfeng Hongyuan Flagship Store	Power Saving Expert 2	It is the ideal product, good quality, logistics is also very fast, worthy of praise 🎁 baby quality is very good, received the first time to open the first time to see, it is very good 👍, very satisfied 😃, customer service is also available to ask questions and answer, thank you 🙏	Jingdong prefecture level city in Shandong
Jinfeng Hongyuan Flagship Store	Power Saving Expert 2	I bought it and haven't used it yet, I'll use it again.	Jingdong prefecture level city in Shandong
Jinfeng Hongyuan Flagship Store	Power Saving Expert 2	It didn't work, I used it for a few days and it was the same as before, it didn't work, I should have to return it	Jingdong prefecture level city in Shandong
Jinfeng Hongyuan Flagship Store	Power Saving Expert 2	The goods have been received, but have not yet installed and use, feel good.	Jingdong prefecture level city in Shandong
Jinfeng Hongyuan Flagship Store	Power Saving Expert 2	Goods received, I forgot to take pictures, the goods look good, you can start, immediately start the school to see the effect then	Jingdong prefecture level city in Shandong

Jinfeng Hongyuan Flagship Store	Power Saving Expert 2	Looks good. I'll use it for a while.	Jingdong prefecture level city in Shandong
Jinfeng Hongyuan Flagship Store	Power Saving Expert 2	Great stuff, easy to operate, easy to use, good power saving, believe in the power of science!	Jingdong prefecture level city in Shandong
Jinfeng Hongyuan Flagship Store	Power Saving Expert 2	Especially great, comparison shopping before buying, the store owner is very good, answering questions very patiently and detailed, there is a need to come back!	Jingdong prefecture level city in Shandong
Jinfeng Hongyuan Flagship Store	Power Saving Expert 2	Trustworthy, feel free to use it boldly.	Jingdong prefecture level city in Shandong
Jinfeng Hongyuan Flagship Store	Power Saving Expert 2	Logistics is super fast! Beautiful appearance! Looks like a great quality! I've never bought one before, so I'll try it first to see if it saves power. We'll see how much it saves.	Jingdong prefecture level city in Shandong
Jinfeng Hongyuan Flagship Store	Power Saving Expert 2	I saw online counterfeiting, energy saver is one of them, hahahahaha, jingdong also buy fake products	Jingdong prefecture level city in Shandong
Jinfeng Hongyuan Flagship Store	Power Saving Expert 2	Just received, installed, try a month to see how the effect and then follow up.	Jingdong prefecture level city in Shandong
Jinfeng Hongyuan Flagship Store	Power Saving Expert 2	Good for you! The goods are genuine! Good price for what you get!	Jingdong prefecture level city in Shandong
Jinfeng Hongyuan Flagship Store	Power Saving Expert 2	Things quickly received, immediately used to see if there is no effect, something river photo ⍐ the same, customer service is very professional	Jingdong prefecture level city in Shandong
Jinfeng Hongyuan Flagship Store	Power Saving Expert 2	The product is very convenient Looking forward to the subsequent use of the experience results	Jingdong prefecture level city in Shandong
Jinfeng Hongyuan Flagship Store	Power Saving Expert 2	I just received it and plugged it in, we'll see exactly when it's time to pay the electric bill	Jingdong prefecture level city in Shandong
Jinfeng Hongyuan Flagship Store	Power Saving Expert 2	Saver is good Packaging and looks good How effective it is to be shared after using it.	Jingdong prefecture level city in Shandong
Jinfeng Hongyuan Flagship Store	Power Saving Expert 2	There's no way to compare. I don't know if it's energy efficient.	Jingdong prefecture level city in Shandong
Jinfeng Hongyuan Flagship Store	Power Saving Expert 2	Just installed it, try it out and comment again	Jingdong prefecture level city in Shandong
Jinfeng Hongyuan Flagship Store	Power Saving Expert 2	Super save point also good to install, after a friend introduced then went to buy to, friends say it is very good to use, home I bought, try to use a period of time, to be honest, the economizer is not big or heavy, can be very convenient to put in a lot of places, the quality is also very good, power-saving function this time seems to be about 20% of the efficiency. The owner is also very warm contact, very	Jingdong prefecture level city in Shandong
Jinfeng Hongyuan Flagship Store	Power Saving Expert 2	The reviews online are mixed. Try it for a while, but I don't know if I can return it if it doesn't save any power after a couple months.	Jingdong prefecture level city in Shandong
Jinfeng Hongyuan Flagship Store	Power Saving Expert 2	The power consumption at home is high, bought a power saver, it looks very convenient to plug on the line, see if you can save some money next month, if good, recommend to friends down the road.	Jingdong prefecture level city in Shandong
Jinfeng Hongyuan Flagship Store	Power Saving Expert 2	It's beautiful, it's genuine, but I don't know the power-saving effect.	Jingdong prefecture level city in Shandong
Jinfeng Hongyuan Flagship Store	Power Saving Expert 2	This user thinks the product is good	Jingdong prefecture level city in Shandong
Jinfeng Hongyuan Flagship Store	Power Saving Expert 2	Nice exterior design with an at-a-glance display	Jingdong prefecture level city in Shandong
Jinfeng Hongyuan Flagship Store	Power Saving Expert 2	Home large and small things are purchased in the Jingdong Mall, have been accustomed to shopping in the Jingdong, many times can buy good quality and cheap products, and courier service attitude is particularly good, sometimes too many couriers are to help me to the home, so many years have been in the Jingdong Mall shopping, trust and support! Will also support the Jingdong as always!	Jingdong prefecture level city in Shandong
Jinfeng Hongyuan Flagship Store	Power Saving Expert 2	Good workmanship, mostly better than the original wall-plugged ones, and hopefully power factor compensation saves on electricity costs	Jingdong prefecture level city in Shandong
Jinfeng Hongyuan Flagship Store	Power Saving Expert 2	I don't feel anything, I said I won't know the effect for a month or two, let's see.	Jingdong prefecture level city in Shandong
Jinfeng Hongyuan Flagship Store	Power Saving Expert 2	Delivery was pretty fast, a little pricey, just not sure if it works or not	Jingdong prefecture level city in Shandong
Jinfeng Hongyuan Flagship Store	Power Saving Expert 2	The goods have been received, I do not know how effective.	Jingdong prefecture level city in Shandong
Jinfeng Hongyuan Flagship Store	Power Saving Expert 2	So far it doesn't seem to be too noticeable, so I'll comment later.	Jingdong prefecture level city in Shandong
Jinfeng Hongyuan Flagship Store	Power Saving Expert 2	I'll comment after I've used it	Jingdong prefecture level city in Shandong
Jinfeng Hongyuan Flagship Store	Power Saving Expert 2	Not bad. You'll save a lot on your electric bill in a year!	Jingdong prefecture level city in Shandong
Jinfeng Hongyuan Flagship Store	Power Saving Expert 2	I just got it. I haven't tried it yet. I don't know what it's like.	Jingdong prefecture level city in Shandong
Jinfeng Hongyuan Flagship Store	Power Saving Expert 2	Shopping at Jingdong has become a habit, and this disease will not be cured for a while.	Jingdong prefecture level city in Shandong
Jinfeng Hongyuan Flagship Store	Power Saving Expert 2	I've been using it for a while now. I don't know if I'm still saving power or not.	Jingdong prefecture level city in Shandong
Jinfeng Hongyuan Flagship Store	Power Saving Expert 2	Received the saver and it's great! There is also a digital voltage indication. Plugged in as instructed, easy to operate, hope you're our power saver!	Jingdong prefecture level city in Shandong
Jinfeng Hongyuan Flagship Store	Power Saving Expert 2	There is no power saving as the store says, using it is the same as not using it.	Jingdong prefecture level city in Shandong
Jinfeng Hongyuan Flagship Store	Power Saving Expert 2	Use it for a while and then comment again	Jingdong prefecture level city in Shandong
Jinfeng Hongyuan Flagship Store	Power Saving Expert 2	Feeling can, to use 1 to 2 months to see how the effect, there is a voltage display, can see the voltage fluctuations amplitude, quite good!	Jingdong prefecture level city in Shandong
Jinfeng Hongyuan Flagship Store	Power Saving Expert 2	I hope it works and saves me some electricity.	Jingdong prefecture level city in Shandong
Jinfeng Hongyuan Flagship Store	Power Saving Expert 2	Nice baby, happy with the after sales	Jingdong prefecture level city in Shandong
Jinfeng Hongyuan Flagship Store	Power Saving Expert 2	Use it and talk about it.	Jingdong prefecture level city in Shandong
Jinfeng Hongyuan Flagship Store	Power Saving Expert 2	Second purchase	Jingdong prefecture level city in Shandong
Jinfeng Hongyuan Flagship Store	Power Saving Expert 2	It works.	Jingdong prefecture level city in Shandong
Jinfeng Hongyuan Flagship Store	Power Saving Expert 2	It works.	Jingdong prefecture level city in Shandong
Jinfeng Hongyuan Flagship Store	Power Saving Expert 2	I don't know if it's a power saver until I start using it	Jingdong prefecture level city in Shandong
Jinfeng Hongyuan Flagship Store	Power Saving Expert 2	serviceable	Jingdong prefecture level city in Shandong
Jinfeng Hongyuan Flagship Store	Power Saving Expert 2	This user did not fill out the rating in time	Jingdong prefecture level city in Shandong
Jinfeng Hongyuan Flagship Store	Power Saving Expert 2	There is no power saving as the store says, using it is the same as not using it.	Jingdong prefecture level city in Shandong
Jinfeng Hongyuan Flagship Store	Power Saving Expert 2	This user did not fill out the rating in time	Jingdong prefecture level city in Shandong
Jinfeng Hongyuan Flagship Store	Power Saving Expert 2	Good...good.	Jingdong prefecture level city in Shandong
Jinfeng Hongyuan Flagship Store	Power Saving Expert 2	(of an unmarried couple) be close	Jingdong prefecture level city in Shandong
Jinfeng Hongyuan Flagship Store	Power Saving Expert 2	(of an unmarried couple) be close	Jingdong prefecture level city in Shandong

Jinfeng Hongyuan Flagship Store	Power Saving Expert 2	(of an unmarried couple) be close	Jingdong prefecture level city in Shandong
Jinfeng Hongyuan Flagship Store	Power Saving Expert 2	The goods are real, high value, high quality, worth every penny, material appearance and quality at a glance is very upscale, very like HAPPY💯	Jingdong prefecture level city in Shandong
Jinfeng Hongyuan Flagship Store	Power Saving Expert 2	It is the ideal product, good quality, logistics is also very fast, worthy of praise 🎁 baby quality is very good, received the first time to open the first time to see, it is very good 👍, very satisfied 😃, customer service is also available to ask questions and answer, thank you 🙏	Jingdong prefecture level city in Shandong
Jinfeng Hongyuan Flagship Store	Power Saving Expert 2	I bought it and haven't used it yet, I'll use it again.	Jingdong prefecture level city in Shandong
Jinfeng Hongyuan Flagship Store	Power Saving Expert 2	It didn't work, I used it for a few days and it was the same as before, it didn't work, I should have to return it	Jingdong prefecture level city in Shandong
Jinfeng Hongyuan Flagship Store	Power Saving Expert 2	The goods have been received, but have not yet installed and use, feel good.	Jingdong prefecture level city in Shandong
Jinfeng Hongyuan Flagship Store	Power Saving Expert 2	Goods received, I forgot to take pictures, the goods look good, you can start, immediately start the school to see the effect then	Jingdong prefecture level city in Shandong
Jinfeng Hongyuan Flagship Store	Power Saving Expert 2	Looks good. I'll use it for a while.	Jingdong prefecture level city in Shandong
Jinfeng Hongyuan Flagship Store	Power Saving Expert 2	Great stuff, easy to operate, easy to use, good power saving, believe in the power of science!	Jingdong prefecture level city in Shandong
Jinfeng Hongyuan Flagship Store	Power Saving Expert 2	Especially great, comparison shopping before buying, the store owner is very good, answering questions very patiently and detailed, there is a need to come back!	Jingdong prefecture level city in Shandong
Jinfeng Hongyuan Flagship Store	Power Saving Expert 2	Trustworthy, feel free to use it boldly.	Jingdong prefecture level city in Shandong
Jinfeng Hongyuan Flagship Store	Power Saving Expert 2	Logistics is super fast! Beautiful appearance! Looks like a great quality! I've never bought one before, so I'll try it first to see if it saves power. We'll see how much it saves.	Jingdong prefecture level city in Shandong
Jinfeng Hongyuan Flagship Store	Power Saving Expert 2	I saw online counterfeiting, energy saver is one of them, hahahahaha, jingdong also buy fake products	Jingdong prefecture level city in Shandong
Jinfeng Hongyuan Flagship Store	Power Saving Expert 2	Just received, installed, try a month to see how the effect and then follow up.	Jingdong prefecture level city in Shandong
Jinfeng Hongyuan Flagship Store	Power Saving Expert 2	Good for you! The goods are genuine! Good price for what you get!	Jingdong prefecture level city in Shandong
Jinfeng Hongyuan Flagship Store	Power Saving Expert 2	Things quickly received, immediately used to see if there is no effect, something river photo ⬆ the same, customer service is very professional	Jingdong prefecture level city in Shandong
Jinfeng Hongyuan Flagship Store	Power Saving Expert 2	The product is very convenient Looking forward to the subsequent use of the experience results	Jingdong prefecture level city in Shandong
Jinfeng Hongyuan Flagship Store	Power Saving Expert 2	I just received it and plugged it in, we'll see exactly when it's time to pay the electric bill	Jingdong prefecture level city in Shandong
Jinfeng Hongyuan Flagship Store	Power Saving Expert 2	Saver is good Packaging and looks good How effective it is to be shared after using it.	Jingdong prefecture level city in Shandong
Jinfeng Hongyuan Flagship Store	Power Saving Expert 2	There's no way to compare. I don't know if it's energy efficient.	Jingdong prefecture level city in Shandong
Jinfeng Hongyuan Flagship Store	Power Saving Expert 2	Just installed it, try it out and comment again	Jingdong prefecture level city in Shandong
Jinfeng Hongyuan Flagship Store	Power Saving Expert 2	Super save point also good to install, after a friend introduced then went to buy to, friends say it is very good to use, home I bought, try to use a period of time, to be honest, the economizer is not big or heavy, can be very convenient to put in a lot of places, the quality is also very good, power-saving function this time seems to be about 20% of the efficiency. The owner is also very warm contact, very	Jingdong prefecture level city in Shandong
Jinfeng Hongyuan Flagship Store	Power Saving Expert 2	The reviews online are mixed. Try it for a while, but I don't know if I can return it if it doesn't save any power after a couple months.	Jingdong prefecture level city in Shandong
Jinfeng Hongyuan Flagship Store	Power Saving Expert 2	The power consumption at home is high, bought a power saver, it looks very convenient to plug on the line, see if you can save some money next month, if good, recommend to friends down the road.	Jingdong prefecture level city in Shandong
Jinfeng Hongyuan Flagship Store	Power Saving Expert 2	It's beautiful, it's genuine, but I don't know the power-saving effect.	Jingdong prefecture level city in Shandong
Jinfeng Hongyuan Flagship Store	Power Saving Expert 2	This user thinks the product is good	Jingdong prefecture level city in Shandong
Jinfeng Hongyuan Flagship Store	Power Saving Expert 2	Nice exterior design with an at-a-glance display	Jingdong prefecture level city in Shandong
Jinfeng Hongyuan Flagship Store	Power Saving Expert 2	Home large and small things are purchased in the Jingdong Mall, have been accustomed to shopping in the Jingdong, many times can buy good quality and cheap products, and courier service attitude is particularly good, sometimes too many couriers are to help me to the home, so many years have been in the Jingdong Mall shopping, trust and support! Will also support the Jingdong as always!	Jingdong prefecture level city in Shandong
Jinfeng Hongyuan Flagship Store	Power Saving Expert 2	Good workmanship, mostly better than the original wall-plugged ones, and hopefully power factor compensation saves on electricity costs	Jingdong prefecture level city in Shandong
Jinfeng Hongyuan Flagship Store	Power Saving Expert 2	I don't feel anything, I said I won't know the effect for a month or two, let's see.	Jingdong prefecture level city in Shandong
Jinfeng Hongyuan Flagship Store	Power Saving Expert 2	Delivery was pretty fast, a little pricey, just not sure if it works or not	Jingdong prefecture level city in Shandong
Jinfeng Hongyuan Flagship Store	Power Saving Expert 2	The goods have been received, I do not know how effective.	Jingdong prefecture level city in Shandong
Jinfeng Hongyuan Flagship Store	Power Saving Expert 2	So far it doesn't seem to be too noticeable, so I'll comment later.	Jingdong prefecture level city in Shandong
Jinfeng Hongyuan Flagship Store	Power Saving Expert 2	I'll comment after I've used it	Jingdong prefecture level city in Shandong
Jinfeng Hongyuan Flagship Store	Power Saving Expert 2	Not bad. You'll save a lot on your electric bill in a year!	Jingdong prefecture level city in Shandong
Jinfeng Hongyuan Flagship Store	Power Saving Expert 2	I just got it. I haven't tried it yet. I don't know what it's like.	Jingdong prefecture level city in Shandong
Jinfeng Hongyuan Flagship Store	Power Saving Expert 2	Shopping at Jingdong has become a habit, and this disease will not be cured for a while.	Jingdong prefecture level city in Shandong
Jinfeng Hongyuan Flagship Store	Power Saving Expert 2	I've been using it for a while now. I don't know if I'm still saving power or not.	Jingdong prefecture level city in Shandong
Jinfeng Hongyuan Flagship Store	Power Saving Expert 2	Received the saver and it's great! There is also a digital voltage indication. Plugged in as instructed, easy to operate, hope you're our power saver!	Jingdong prefecture level city in Shandong
Jinfeng Hongyuan Flagship Store	Power Saving Expert 2	There is no power saving as the store says, using it is the same as not using it.	Jingdong prefecture level city in Shandong
Jinfeng Hongyuan Flagship Store	Power Saving Expert 2	Use it for a while and then comment again	Jingdong prefecture level city in Shandong
Jinfeng Hongyuan Flagship Store	Power Saving Expert 2	Feeling can, to use 1 to 2 months to see how the effect, there is a voltage display, can see the voltage fluctuations amplitude, quite good!	Jingdong prefecture level city in Shandong
Jinfeng Hongyuan Flagship Store	Power Saving Expert 2	I hope it works and saves me some electricity.	Jingdong prefecture level city in Shandong
Jinfeng Hongyuan Flagship Store	Power Saving Expert 2	Nice baby, happy with the after sales	Jingdong prefecture level city in Shandong
Jinfeng Hongyuan Flagship Store	Power Saving Expert 2	Use it and talk about it.	Jingdong prefecture level city in Shandong
Jinfeng Hongyuan Flagship Store	Power Saving Expert 2	Second purchase	Jingdong prefecture level city in Shandong
Jinfeng Hongyuan Flagship Store	Power Saving Expert 2	It works.	Jingdong prefecture level city in Shandong
Jinfeng Hongyuan Flagship Store	Power Saving Expert 2	It works.	Jingdong prefecture level city in Shandong
Jinfeng Hongyuan Flagship Store	Power Saving Expert 2	I don't know if it's a power saver until I start using it	Jingdong prefecture level city in Shandong

Jinfeng Hongyuan Flagship Store	Power Saving Expert 2	serviceable	Jingdong prefecture level city in Shandong
Jinfeng Hongyuan Flagship Store	Power Saving Expert 2	This user did not fill out the rating in time	Jingdong prefecture level city in Shandong
Jinfeng Hongyuan Flagship Store	Power Saving Expert 2	There is no power saving as the store says, using it is the same as not using it.	Jingdong prefecture level city in Shandong
Jinfeng Hongyuan Flagship Store	Power Saving Expert 2	This user did not fill out the rating in time	Jingdong prefecture level city in Shandong
Jinfeng Hongyuan Flagship Store	Power Saving Expert 2	Good...good.	Jingdong prefecture level city in Shandong
Jinfeng Hongyuan Flagship Store	Power Saving Expert 2	(of an unmarried couple) be close	Jingdong prefecture level city in Shandong
Jinfeng Hongyuan Flagship Store	Power Saving Expert 2	(of an unmarried couple) be close	Jingdong prefecture level city in Shandong
Jinfeng Hongyuan Flagship Store	Power Saving Expert 2	(of an unmarried couple) be close	Jingdong prefecture level city in Shandong
Jinfeng Hongyuan Flagship Store	Power Saving Expert 2	The goods are real, high value, high quality, worth every penny, material appearance and quality at a glance is very upscale, very like HAPPY100	Jingdong prefecture level city in Shandong
Jinfeng Hongyuan Flagship Store	Power Saving Expert 2	It is the ideal product, good quality, logistics is also very fast, worthy of praise 🎁 baby quality is very good, received the first time to open the first time to see, it is very good 👍, very satisfied 😃, customer service is also available to ask questions and answer, thank you 🙏	Jingdong prefecture level city in Shandong
Jinfeng Hongyuan Flagship Store	Power Saving Expert 2	I bought it and haven't used it yet, I'll use it again.	Jingdong prefecture level city in Shandong
Jinfeng Hongyuan Flagship Store	Power Saving Expert 2	It didn't work, I used it for a few days and it was the same as before, it didn't work, I should have to return it	Jingdong prefecture level city in Shandong
Jinfeng Hongyuan Flagship Store	Power Saving Expert 2	The goods have been received, but have not yet installed and use, feel good.	Jingdong prefecture level city in Shandong
Jinfeng Hongyuan Flagship Store	Power Saving Expert 2	Goods received, I forgot to take pictures, the goods look good, you can start, immediately start the school to see the effect then	Jingdong prefecture level city in Shandong
Jinfeng Hongyuan Flagship Store	Power Saving Expert 2	Looks good. I'll use it for a while.	Jingdong prefecture level city in Shandong
Jinfeng Hongyuan Flagship Store	Power Saving Expert 2	Great stuff, easy to operate, easy to use, good power saving, believe in the power of science!	Jingdong prefecture level city in Shandong
Jinfeng Hongyuan Flagship Store	Power Saving Expert 2	Especially great, comparison shopping before buying, the store owner is very good, answering questions very patiently and detailed, there is a need to come back!	Jingdong prefecture level city in Shandong
Jinfeng Hongyuan Flagship Store	Power Saving Expert 2	Trustworthy, feel free to use it boldly.	Jingdong prefecture level city in Shandong
Jinfeng Hongyuan Flagship Store	Power Saving Expert 2	Logistics is super fast! Beautiful appearance! Looks like a great quality! I've never bought one before, so I'll try it first to see if it saves power. We'll see how much it saves.	Jingdong prefecture level city in Shandong
Jinfeng Hongyuan Flagship Store	Power Saving Expert 2	I saw online counterfeiting, energy saver is one of them, hahahahaha, jingdong also buy fake products	Jingdong prefecture level city in Shandong
Jinfeng Hongyuan Flagship Store	Power Saving Expert 2	Just received, installed, try a month to see how the effect and then follow up.	Jingdong prefecture level city in Shandong
Jinfeng Hongyuan Flagship Store	Power Saving Expert 2	Good for you! The goods are genuine! Good price for what you get!	Jingdong prefecture level city in Shandong
Jinfeng Hongyuan Flagship Store	Power Saving Expert 2	Things quickly received, immediately used to see if there is no effect, something river photo ⬆ the same, customer service is very professional	Jingdong prefecture level city in Shandong
Jinfeng Hongyuan Flagship Store	Power Saving Expert 2	The product is very convenient Looking forward to the subsequent use of the experience results	Jingdong prefecture level city in Shandong
Jinfeng Hongyuan Flagship Store	Power Saving Expert 2	I just received it and plugged it in, we'll see exactly when it's time to pay the electric bill	Jingdong prefecture level city in Shandong
Jinfeng Hongyuan Flagship Store	Power Saving Expert 2	Saver is good Packaging and looks good How effective it is to be shared after using it.	Jingdong prefecture level city in Shandong
Jinfeng Hongyuan Flagship Store	Power Saving Expert 2	There's no way to compare. I don't know if it's energy efficient.	Jingdong prefecture level city in Shandong
Jinfeng Hongyuan Flagship Store	Power Saving Expert 2	Just installed it, try it out and comment again	Jingdong prefecture level city in Shandong
Jinfeng Hongyuan Flagship Store	Power Saving Expert 2	Super save point also good to install, after a friend introduced then went to buy to, friends say it is very good to use, home I bought, try to use a period of time, to be honest, the economizer is not big or heavy, can be very convenient to put in a lot of places, the quality is also very good, power-saving function this time seems to be about 20% of the efficiency. The owner is also very warm contact, very	Jingdong prefecture level city in Shandong
Jinfeng Hongyuan Flagship Store	Power Saving Expert 2	The reviews online are mixed. Try it for a while, but I don't know if I can return it if it doesn't save any power after a couple months.	Jingdong prefecture level city in Shandong
Jinfeng Hongyuan Flagship Store	Power Saving Expert 2	The power consumption at home is high, bought a power saver, it looks very convenient to plug on the line, see if you can save some money next month, if good, recommend to friends down the road.	Jingdong prefecture level city in Shandong
Jinfeng Hongyuan Flagship Store	Power Saving Expert 2	It's beautiful, it's genuine, but I don't know the power-saving effect.	Jingdong prefecture level city in Shandong
Jinfeng Hongyuan Flagship Store	Power Saving Expert 2	This user thinks the product is good	Jingdong prefecture level city in Shandong
Jinfeng Hongyuan Flagship Store	Power Saving Expert 2	Nice exterior design with an at-a-glance display	Jingdong prefecture level city in Shandong
Jinfeng Hongyuan Flagship Store	Power Saving Expert 2	Home large and small things are purchased in the Jingdong Mall, have been accustomed to shopping in the Jingdong, many times can buy good quality and cheap products, and courier service attitude is particularly good, sometimes too many couriers are to help me to the home, so many years have been in the Jingdong Mall shopping, trust and support! Will also support the Jingdong as always!	Jingdong prefecture level city in Shandong
Jinfeng Hongyuan Flagship Store	Power Saving Expert 2	Good workmanship, mostly better than the original wall-plugged ones, and hopefully power factor compensation saves on electricity costs	Jingdong prefecture level city in Shandong
Jinfeng Hongyuan Flagship Store	Power Saving Expert 2	I don't feel anything, I said I won't know the effect for a month or two, let's see.	Jingdong prefecture level city in Shandong
Jinfeng Hongyuan Flagship Store	Power Saving Expert 2	Delivery was pretty fast, a little pricey, just not sure if it works or not	Jingdong prefecture level city in Shandong
Jinfeng Hongyuan Flagship Store	Power Saving Expert 2	The goods have been received, I do not know how effective.	Jingdong prefecture level city in Shandong
Jinfeng Hongyuan Flagship Store	Power Saving Expert 2	So far it doesn't seem to be too noticeable, so I'll comment later.	Jingdong prefecture level city in Shandong
Jinfeng Hongyuan Flagship Store	Power Saving Expert 2	I'll comment after I've used it	Jingdong prefecture level city in Shandong
Jinfeng Hongyuan Flagship Store	Power Saving Expert 2	Not bad. You'll save a lot on your electric bill in a year!	Jingdong prefecture level city in Shandong
Jinfeng Hongyuan Flagship Store	Power Saving Expert 2	I just got it. I haven't tried it yet. I don't know what it's like.	Jingdong prefecture level city in Shandong
Jinfeng Hongyuan Flagship Store	Power Saving Expert 2	Shopping at Jingdong has become a habit, and this disease will not be cured for a while.	Jingdong prefecture level city in Shandong
Jinfeng Hongyuan Flagship Store	Power Saving Expert 2	I've been using it for a while now. I don't know if I'm still saving power or not.	Jingdong prefecture level city in Shandong
Jinfeng Hongyuan Flagship Store	Power Saving Expert 2	Received the saver and it's great! There is also a digital voltage indication. Plugged in as instructed, easy to operate, hope you're our power saver!	Jingdong prefecture level city in Shandong
Jinfeng Hongyuan Flagship Store	Power Saving Expert 2	There is no power saving as the store says, using it is the same as not using it.	Jingdong prefecture level city in Shandong
Jinfeng Hongyuan Flagship Store	Power Saving Expert 2	Use it for a while and then comment again	Jingdong prefecture level city in Shandong
Jinfeng Hongyuan Flagship Store	Power Saving Expert 2	Feeling can, to use 1 to 2 months to see how the effect, there is a voltage display, can see the voltage fluctuations amplitude, quite good!	Jingdong prefecture level city in Shandong

Jinfeng Hongyuan Flagship Store	Power Saving Expert 2	I hope it works and saves me some electricity.	Jingdong prefecture level city in Shandong
Jinfeng Hongyuan Flagship Store	Power Saving Expert 2	Nice baby, happy with the after sales	Jingdong prefecture level city in Shandong
Jinfeng Hongyuan Flagship Store	Power Saving Expert 2	Use it and talk about it.	Jingdong prefecture level city in Shandong
Jinfeng Hongyuan Flagship Store	Power Saving Expert 2	Second purchase	Jingdong prefecture level city in Shandong
Jinfeng Hongyuan Flagship Store	Power Saving Expert 2	It works.	Jingdong prefecture level city in Shandong
Jinfeng Hongyuan Flagship Store	Power Saving Expert 2	It works.	Jingdong prefecture level city in Shandong
Jinfeng Hongyuan Flagship Store	Power Saving Expert 2	I don't know if it's a power saver until I start using it	Jingdong prefecture level city in Shandong
Jinfeng Hongyuan Flagship Store	Power Saving Expert 2	serviceable	Jingdong prefecture level city in Shandong
Jinfeng Hongyuan Flagship Store	Power Saving Expert 2	This user did not fill out the rating in time	Jingdong prefecture level city in Shandong
Jinfeng Hongyuan Flagship Store	Power Saving Expert 2	There is no power saving as the store says, using it is the same as not using it.	Jingdong prefecture level city in Shandong
Jinfeng Hongyuan Flagship Store	Power Saving Expert 2	This user did not fill out the rating in time	Jingdong prefecture level city in Shandong
Jinfeng Hongyuan Flagship Store	Power Saving Expert 2	Good...good.	Jingdong prefecture level city in Shandong
Jinfeng Hongyuan Flagship Store	Power Saving Expert 2	(of an unmarried couple) be close	Jingdong prefecture level city in Shandong
Jinfeng Hongyuan Flagship Store	Power Saving Expert 2	(of an unmarried couple) be close	Jingdong prefecture level city in Shandong
Jinfeng Hongyuan Flagship Store	Power Saving Expert 2	(of an unmarried couple) be close	Jingdong prefecture level city in Shandong
Jinfeng Hongyuan Flagship Store	Power Saving Expert 2	The goods are real, high value, high quality, worth every penny, material appearance and quality at a glance is very upscale, very like HAPPY1 00	Jingdong prefecture level city in Shandong
Jinfeng Hongyuan Flagship Store	Power Saving Expert 2	It is the ideal product, good quality, logistics is also very fast, worthy of praise 🎁 baby quality is very good, received the first time to open the first time to see, it is very good 👍, very satisfied 😃, customer service is also available to ask questions and answer, thank you 🙏	Jingdong prefecture level city in Shandong
Jinfeng Hongyuan Flagship Store	Power Saving Expert 2	I bought it and haven't used it yet, I'll use it again.	Jingdong prefecture level city in Shandong
Jinfeng Hongyuan Flagship Store	Power Saving Expert 2	It didn't work, I used it for a few days and it was the same as before, it didn't work, I should have to return it	Jingdong prefecture level city in Shandong
Jinfeng Hongyuan Flagship Store	Power Saving Expert 2	The goods have been received, but have not yet installed and use, feel good.	Jingdong prefecture level city in Shandong
Jinfeng Hongyuan Flagship Store	Power Saving Expert 2	Goods received, I forgot to take pictures, the goods look good, you can start, immediately start the school to see the effect then	Jingdong prefecture level city in Shandong
Jinfeng Hongyuan Flagship Store	Power Saving Expert 2	Looks good. I'll use it for a while.	Jingdong prefecture level city in Shandong
Jinfeng Hongyuan Flagship Store	Power Saving Expert 2	Great stuff, easy to operate, easy to use, good power saving, believe in the power of science!	Jingdong prefecture level city in Shandong
Jinfeng Hongyuan Flagship Store	Power Saving Expert 2	Especially great, comparison shopping before buying, the store owner is very good, answering questions very patiently and detailed, there is a need to come back!	Jingdong prefecture level city in Shandong
Jinfeng Hongyuan Flagship Store	Power Saving Expert 2	Trustworthy, feel free to use it boldly.	Jingdong prefecture level city in Shandong
Jinfeng Hongyuan Flagship Store	Power Saving Expert 2	Logistics is super fast! Beautiful appearance! Looks like a great quality! I've never bought one before, so I'll try it first to see if it saves power. We'll see how much it saves.	Jingdong prefecture level city in Shandong
Jinfeng Hongyuan Flagship Store	Power Saving Expert 2	I saw online counterfeiting, energy saver is one of them, hahahahaha, jingdong also buy fake products	Jingdong prefecture level city in Shandong
Jinfeng Hongyuan Flagship Store	Power Saving Expert 2	Just received, installed, try a month to see how the effect and then follow up.	Jingdong prefecture level city in Shandong
Jinfeng Hongyuan Flagship Store	Power Saving Expert 2	Good for you! The goods are genuine! Good price for what you get!	Jingdong prefecture level city in Shandong
Jinfeng Hongyuan Flagship Store	Power Saving Expert 2	Things quickly received, immediately used to see if there is no effect, something river photo ⬆ the same, customer service is very professional	Jingdong prefecture level city in Shandong
Jinfeng Hongyuan Flagship Store	Power Saving Expert 2	The product is very convenient Looking forward to the subsequent use of the experience results	Jingdong prefecture level city in Shandong
Jinfeng Hongyuan Flagship Store	Power Saving Expert 2	I just received it and plugged it in, we'll see exactly when it's time to pay the electric bill	Jingdong prefecture level city in Shandong
Jinfeng Hongyuan Flagship Store	Power Saving Expert 2	Saver is good Packaging and looks good How effective it is to be shared after using it.	Jingdong prefecture level city in Shandong
Jinfeng Hongyuan Flagship Store	Power Saving Expert 2	There's no way to compare. I don't know if it's energy efficient.	Jingdong prefecture level city in Shandong
Jinfeng Hongyuan Flagship Store	Power Saving Expert 2	Just installed it, try it out and comment again	Jingdong prefecture level city in Shandong
Jinfeng Hongyuan Flagship Store	Power Saving Expert 2	Super save point also good to install, after a friend introduced then went to buy to, friends say it is very good to use, home I bought, try to use a period of time, to be honest, the economizer is not big or heavy, can be very convenient to put in a lot of places, the quality is also very good, power-saving function this time seems to be about 20% of the efficiency. The owner is also very warm contact, very	Jingdong prefecture level city in Shandong
Jinfeng Hongyuan Flagship Store	Power Saving Expert 2	The reviews online are mixed. Try it for a while, but I don't know if I can return it if it doesn't save any power after a couple months.	Jingdong prefecture level city in Shandong
Jinfeng Hongyuan Flagship Store	Power Saving Expert 2	The power consumption at home is high, bought a power saver, it looks very convenient to plug on the line, see if you can save some money next month, if good, recommend to friends down the road.	Jingdong prefecture level city in Shandong
Jinfeng Hongyuan Flagship Store	Power Saving Expert 2	It's beautiful, it's genuine, but I don't know the power-saving effect.	Jingdong prefecture level city in Shandong
Jinfeng Hongyuan Flagship Store	Power Saving Expert 2	This user thinks the product is good	Jingdong prefecture level city in Shandong
Jinfeng Hongyuan Flagship Store	Power Saving Expert 2	Nice exterior design with an at-a-glance display	Jingdong prefecture level city in Shandong
Jinfeng Hongyuan Flagship Store	Power Saving Expert 2	Home large and small things are purchased in the Jingdong Mall, have been accustomed to shopping in the Jingdong, many times can buy good quality and cheap products, and courier service attitude is particularly good, sometimes too many couriers are to help me to the home, so many years have been in the Jingdong Mall shopping, trust and support! Will also support the Jingdong as always!	Jingdong prefecture level city in Shandong
Jinfeng Hongyuan Flagship Store	Power Saving Expert 2	Good workmanship, mostly better than the original wall-plugged ones, and hopefully power factor compensation saves on electricity costs	Jingdong prefecture level city in Shandong
Jinfeng Hongyuan Flagship Store	Power Saving Expert 2	I don't feel anything, I said I won't know the effect for a month or two, let's see.	Jingdong prefecture level city in Shandong
Jinfeng Hongyuan Flagship Store	Power Saving Expert 2	Delivery was pretty fast, a little pricey, just not sure if it works or not	Jingdong prefecture level city in Shandong
Jinfeng Hongyuan Flagship Store	Power Saving Expert 2	The goods have been received, I do not know how effective.	Jingdong prefecture level city in Shandong
Jinfeng Hongyuan Flagship Store	Power Saving Expert 2	So far it doesn't seem to be too noticeable, so I'll comment later.	Jingdong prefecture level city in Shandong
Jinfeng Hongyuan Flagship Store	Power Saving Expert 2	I'll comment after I've used it	Jingdong prefecture level city in Shandong
Jinfeng Hongyuan Flagship Store	Power Saving Expert 2	Not bad. You'll save a lot on your electric bill in a year!	Jingdong prefecture level city in Shandong

Jinfeng Hongyuan Flagship Store	Power Saving Expert 2	I just got it. I haven't tried it yet. I don't know what it's like.	Jingdong prefecture level city in Shandong
Jinfeng Hongyuan Flagship Store	Power Saving Expert 2	Shopping at Jingdong has become a habit, and this disease will not be cured for a while.	Jingdong prefecture level city in Shandong
Jinfeng Hongyuan Flagship Store	Power Saving Expert 2	I've been using it for a while now. I don't know if I'm still saving power or not.	Jingdong prefecture level city in Shandong
Jinfeng Hongyuan Flagship Store	Power Saving Expert 2	Received the saver and it's great! There is also a digital voltage indication. Plugged in as instructed, easy to operate, hope you're our power saver!	Jingdong prefecture level city in Shandong
Jinfeng Hongyuan Flagship Store	Power Saving Expert 2	There is no power saving as the store says, using it is the same as not using it.	Jingdong prefecture level city in Shandong
Jinfeng Hongyuan Flagship Store	Power Saving Expert 2	Use it for a while and then comment again	Jingdong prefecture level city in Shandong
Jinfeng Hongyuan Flagship Store	Power Saving Expert 2	Feeling can, to use 1 to 2 months to see how the effect, there is a voltage display, can see the voltage fluctuations amplitude, quite good!	Jingdong prefecture level city in Shandong
Jinfeng Hongyuan Flagship Store	Power Saving Expert 2	I hope it works and saves me some electricity.	Jingdong prefecture level city in Shandong
Jinfeng Hongyuan Flagship Store	Power Saving Expert 2	Nice baby, happy with the after sales	Jingdong prefecture level city in Shandong
Jinfeng Hongyuan Flagship Store	Power Saving Expert 2	Use it and talk about it.	Jingdong prefecture level city in Shandong
Jinfeng Hongyuan Flagship Store	Power Saving Expert 2	Second purchase	Jingdong prefecture level city in Shandong
Jinfeng Hongyuan Flagship Store	Power Saving Expert 2	It works.	Jingdong prefecture level city in Shandong
Jinfeng Hongyuan Flagship Store	Power Saving Expert 2	It works.	Jingdong prefecture level city in Shandong
Jinfeng Hongyuan Flagship Store	Power Saving Expert 2	I don't know if it's a power saver until I start using it	Jingdong prefecture level city in Shandong
Jinfeng Hongyuan Flagship Store	Power Saving Expert 2	serviceable	Jingdong prefecture level city in Shandong
Jinfeng Hongyuan Flagship Store	Power Saving Expert 2	This user did not fill out the rating in time	Jingdong prefecture level city in Shandong
Jinfeng Hongyuan Flagship Store	Power Saving Expert 2	There is no power saving as the store says, using it is the same as not using it.	Jingdong prefecture level city in Shandong
Jinfeng Hongyuan Flagship Store	Power Saving Expert 2	This user did not fill out the rating in time	Jingdong prefecture level city in Shandong
Jinfeng Hongyuan Flagship Store	Power Saving Expert 2	Good...good.	Jingdong prefecture level city in Shandong
Jinfeng Hongyuan Flagship Store	Power Saving Expert 2	(of an unmarried couple) be close	Jingdong prefecture level city in Shandong
Jinfeng Hongyuan Flagship Store	Power Saving Expert 2	(of an unmarried couple) be close	Jingdong prefecture level city in Shandong
Jinfeng Hongyuan Flagship Store	Power Saving Expert 2	(of an unmarried couple) be close	Jingdong prefecture level city in Shandong
Jinfeng Hongyuan Flagship Store	Power Saving Expert 2	The goods are real, high value, high quality, worth every penny, material appearance and quality at a glance is very upscale, very like HAPPY1 00	Jingdong prefecture level city in Shandong
Jinfeng Hongyuan Flagship Store	Power Saving Expert 2	It is the ideal product, good quality, logistics is also very fast, worthy of praise 🎁 baby quality is very good, received the first time to open the first time to see, it is very good 👍, very satisfied 😊, customer service is also available to ask questions and answer, thank you 🙏	Jingdong prefecture level city in Shandong
Jinfeng Hongyuan Flagship Store	Power Saving Expert 2	I bought it and haven't used it yet, I'll use it again.	Jingdong prefecture level city in Shandong
Jinfeng Hongyuan Flagship Store	Power Saving Expert 2	It didn't work, I used it for a few days and it was the same as before, it didn't work, I should have to return it	Jingdong prefecture level city in Shandong
Jinfeng Hongyuan Flagship Store	Power Saving Expert 2	The goods have been received, but have not yet installed and use, feel good.	Jingdong prefecture level city in Shandong
Jinfeng Hongyuan Flagship Store	Power Saving Expert 2	Goods received, I forgot to take pictures, the goods look good, you can start, immediately start the school to see the effect then	Jingdong prefecture level city in Shandong
Jinfeng Hongyuan Flagship Store	Power Saving Expert 2	Looks good. I'll use it for a while.	Jingdong prefecture level city in Shandong
Jinfeng Hongyuan Flagship Store	Power Saving Expert 2	Great stuff, easy to operate, easy to use, good power saving, believe in the power of science!	Jingdong prefecture level city in Shandong
Jinfeng Hongyuan Flagship Store	Power Saving Expert 2	Especially great, comparison shopping before buying, the store owner is very good, answering questions very patiently and detailed, there is a need to come back!	Jingdong prefecture level city in Shandong
Jinfeng Hongyuan Flagship Store	Power Saving Expert 2	Trustworthy, feel free to use it boldly.	Jingdong prefecture level city in Shandong
Jinfeng Hongyuan Flagship Store	Power Saving Expert 2	Logistics is super fast! Beautiful appearance! Looks like a great quality! I've never bought one before, so I'll try it first to see if it saves power. We'll see how much it saves.	Jingdong prefecture level city in Shandong
Jinfeng Hongyuan Flagship Store	Power Saving Expert 2	I saw online counterfeiting, energy saver is one of them, hahahahaha, jingdong also buy fake products	Jingdong prefecture level city in Shandong
Jinfeng Hongyuan Flagship Store	Power Saving Expert 2	Just received, installed, try a month to see how the effect and then follow up.	Jingdong prefecture level city in Shandong
Jinfeng Hongyuan Flagship Store	Power Saving Expert 2	Good for you! The goods are genuine! Good price for what you get!	Jingdong prefecture level city in Shandong
Jinfeng Hongyuan Flagship Store	Power Saving Expert 2	Things quickly received, immediately used to see if there is no effect, something river photo ↑ the same, customer service is very professional	Jingdong prefecture level city in Shandong
Jinfeng Hongyuan Flagship Store	Power Saving Expert 2	The product is very convenient Looking forward to the subsequent use of the experience results	Jingdong prefecture level city in Shandong
Jinfeng Hongyuan Flagship Store	Power Saving Expert 2	I just received it and plugged it in, we'll see exactly when it's time to pay the electric bill	Jingdong prefecture level city in Shandong
Jinfeng Hongyuan Flagship Store	Power Saving Expert 2	Saver is good Packaging and looks good How effective it is to be shared after using it.	Jingdong prefecture level city in Shandong
Jinfeng Hongyuan Flagship Store	Power Saving Expert 2	There's no way to compare. I don't know if it's energy efficient.	Jingdong prefecture level city in Shandong
Jinfeng Hongyuan Flagship Store	Power Saving Expert 2	Just installed it, try it out and comment again	Jingdong prefecture level city in Shandong
Jinfeng Hongyuan Flagship Store	Power Saving Expert 2	Super save point also good to install, after a friend introduced then went to buy to, friends say it is very good to use, home I bought, try to use a period of time, to be honest, the economizer is not big or heavy, can be very convenient to put in a lot of places, the quality is also very good, power-saving function this time seems to be about 20% of the efficiency. The owner is also very warm contact, very	Jingdong prefecture level city in Shandong
Jinfeng Hongyuan Flagship Store	Power Saving Expert 2	The reviews online are mixed. Try it for a while, but I don't know if I can return it if it doesn't save any power after a couple months.	Jingdong prefecture level city in Shandong
Jinfeng Hongyuan Flagship Store	Power Saving Expert 2	The power consumption at home is high, bought a power saver, it looks very convenient to plug on the line, see if you can save some money next month, if good, recommend to friends down the road.	Jingdong prefecture level city in Shandong
Jinfeng Hongyuan Flagship Store	Power Saving Expert 2	It's beautiful, it's genuine, but I don't know the power-saving effect.	Jingdong prefecture level city in Shandong
Jinfeng Hongyuan Flagship Store	Power Saving Expert 2	This user thinks the product is good	Jingdong prefecture level city in Shandong
Jinfeng Hongyuan Flagship Store	Power Saving Expert 2	Nice exterior design with an at-a-glance display	Jingdong prefecture level city in Shandong
Jinfeng Hongyuan Flagship Store	Power Saving Expert 2	Home large and small things are purchased in the Jingdong Mall, have been accustomed to shopping in the Jingdong, many times can buy good quality and cheap products, and courier service attitude is particularly good, sometimes too many couriers are to help me to the home, so many years have been in the Jingdong Mall shopping, trust and support! Will also support the Jingdong as always!	Jingdong prefecture level city in Shandong

Jinfeng Hongyuan Flagship Store	Power Saving Expert 2	Good workmanship, mostly better than the original wall-plugged ones, and hopefully power factor compensation saves on electricity costs	Jingdong prefecture level city in Shandong
Jinfeng Hongyuan Flagship Store	Power Saving Expert 2	I don't feel anything, I said I won't know the effect for a month or two, let's see.	Jingdong prefecture level city in Shandong
Jinfeng Hongyuan Flagship Store	Power Saving Expert 2	Delivery was pretty fast, a little pricey, just not sure if it works or not	Jingdong prefecture level city in Shandong
Jinfeng Hongyuan Flagship Store	Power Saving Expert 2	The goods have been received, I do not know how effective.	Jingdong prefecture level city in Shandong
Jinfeng Hongyuan Flagship Store	Power Saving Expert 2	So far it doesn't seem to be too noticeable, so I'll comment later.	Jingdong prefecture level city in Shandong
Jinfeng Hongyuan Flagship Store	Power Saving Expert 2	I'll comment after I've used it	Jingdong prefecture level city in Shandong
Jinfeng Hongyuan Flagship Store	Power Saving Expert 2	Not bad. You'll save a lot on your electric bill in a year!	Jingdong prefecture level city in Shandong
Jinfeng Hongyuan Flagship Store	Power Saving Expert 2	I just got it. I haven't tried it yet. I don't know what it's like.	Jingdong prefecture level city in Shandong
Jinfeng Hongyuan Flagship Store	Power Saving Expert 2	Shopping at Jingdong has become a habit, and this disease will not be cured for a while.	Jingdong prefecture level city in Shandong
Jinfeng Hongyuan Flagship Store	Power Saving Expert 2	I've been using it for a while now. I don't know if I'm still saving power or not.	Jingdong prefecture level city in Shandong
Jinfeng Hongyuan Flagship Store	Power Saving Expert 2	Received the saver and it's great! There is also a digital voltage indication. Plugged in as instructed, easy to operate, hope you're our power saver!	Jingdong prefecture level city in Shandong
Jinfeng Hongyuan Flagship Store	Power Saving Expert 2	There is no power saving as the store says, using it is the same as not using it.	Jingdong prefecture level city in Shandong
Jinfeng Hongyuan Flagship Store	Power Saving Expert 2	Use it for a while and then comment again	Jingdong prefecture level city in Shandong
Jinfeng Hongyuan Flagship Store	Power Saving Expert 2	Feeling can, to use 1 to 2 months to see how the effect, there is a voltage display, can see the voltage fluctuations amplitude, quite good!	Jingdong prefecture level city in Shandong
Jinfeng Hongyuan Flagship Store	Power Saving Expert 2	I hope it works and saves me some electricity.	Jingdong prefecture level city in Shandong
Jinfeng Hongyuan Flagship Store	Power Saving Expert 2	Nice baby, happy with the after sales	Jingdong prefecture level city in Shandong
Jinfeng Hongyuan Flagship Store	Power Saving Expert 2	Use it and talk about it.	Jingdong prefecture level city in Shandong
Jinfeng Hongyuan Flagship Store	Power Saving Expert 2	Second purchase	Jingdong prefecture level city in Shandong
Jinfeng Hongyuan Flagship Store	Power Saving Expert 2	It works.	Jingdong prefecture level city in Shandong
Jinfeng Hongyuan Flagship Store	Power Saving Expert 2	It works.	Jingdong prefecture level city in Shandong
Jinfeng Hongyuan Flagship Store	Power Saving Expert 2	I don't know if it's a power saver until I start using it	Jingdong prefecture level city in Shandong
Jinfeng Hongyuan Flagship Store	Power Saving Expert 2	serviceable	Jingdong prefecture level city in Shandong
Jinfeng Hongyuan Flagship Store	Power Saving Expert 2	This user did not fill out the rating in time	Jingdong prefecture level city in Shandong
Jinfeng Hongyuan Flagship Store	Power Saving Expert 2	There is no power saving as the store says, using it is the same as not using it.	Jingdong prefecture level city in Shandong
Jinfeng Hongyuan Flagship Store	Power Saving Expert 2	This user did not fill out the rating in time	Jingdong prefecture level city in Shandong
Jinfeng Hongyuan Flagship Store	Power Saving Expert 2	Good...good.	Jingdong prefecture level city in Shandong
Jinfeng Hongyuan Flagship Store	Power Saving Expert 2	(of an unmarried couple) be close	Jingdong prefecture level city in Shandong
Jinfeng Hongyuan Flagship Store	Power Saving Expert 2	(of an unmarried couple) be close	Jingdong prefecture level city in Shandong
Jinfeng Hongyuan Flagship Store	Power Saving Expert 2	(of an unmarried couple) be close	Jingdong prefecture level city in Shandong
Jinfeng Hongyuan Flagship Store	Power Saving Expert 2	The goods are real, high value, high quality, worth every penny, material appearance and quality at a glance is very upscale, very like HAPPY100	Jingdong prefecture level city in Shandong
Jinfeng Hongyuan Flagship Store	Power Saving Expert 2	It is the ideal product, good quality, logistics is also very fast, worthy of praise 🎁 baby quality is very good, received the first time to open the first time to see, it is very good 👍, very satisfied 😃, customer service is also available to ask questions and answer, thank you 🙏	Jingdong prefecture level city in Shandong
Jinfeng Hongyuan Flagship Store	Power Saving Expert 2	I bought it and haven't used it yet, I'll use it again.	Jingdong prefecture level city in Shandong
Jinfeng Hongyuan Flagship Store	Power Saving Expert 2	It didn't work, I used it for a few days and it was the same as before, it didn't work, I should have to return it	Jingdong prefecture level city in Shandong
Jinfeng Hongyuan Flagship Store	Power Saving Expert 2	The goods have been received, but have not yet installed and use, feel good.	Jingdong prefecture level city in Shandong
Jinfeng Hongyuan Flagship Store	Power Saving Expert 2	Goods received, I forgot to take pictures, the goods look good, you can start, immediately start the school to see the effect then	Jingdong prefecture level city in Shandong
Jinfeng Hongyuan Flagship Store	Power Saving Expert 2	Looks good. I'll use it for a while.	Jingdong prefecture level city in Shandong
Jinfeng Hongyuan Flagship Store	Power Saving Expert 2	Great stuff, easy to operate, easy to use, good power saving, believe in the power of science!	Jingdong prefecture level city in Shandong
Jinfeng Hongyuan Flagship Store	Power Saving Expert 2	Especially great, comparison shopping before buying, the store owner is very good, answering questions very patiently and detailed, there is a need to come back!	Jingdong prefecture level city in Shandong
Jinfeng Hongyuan Flagship Store	Power Saving Expert 2	Trustworthy, feel free to use it boldly.	Jingdong prefecture level city in Shandong
Jinfeng Hongyuan Flagship Store	Power Saving Expert 2	Logistics is super fast! Beautiful appearance! Looks like a great quality! I've never bought one before, so I'll try it first to see if it saves power. We'll see how much it saves.	Jingdong prefecture level city in Shandong
Jinfeng Hongyuan Flagship Store	Power Saving Expert 2	I saw online counterfeiting, energy saver is one of them, hahahahaha, jingdong also buy fake products	Jingdong prefecture level city in Shandong
Jinfeng Hongyuan Flagship Store	Power Saving Expert 2	Just received, installed, try a month to see how the effect and then follow up.	Jingdong prefecture level city in Shandong
Jinfeng Hongyuan Flagship Store	Power Saving Expert 2	Good for you! The goods are genuine! Good price for what you get!	Jingdong prefecture level city in Shandong
Jinfeng Hongyuan Flagship Store	Power Saving Expert 2	Things quickly received, immediately used to see if there is no effect, something river photo ⬆ the same, customer service is very professional	Jingdong prefecture level city in Shandong
Jinfeng Hongyuan Flagship Store	Power Saving Expert 2	The product is very convenient Looking forward to the subsequent use of the experience results	Jingdong prefecture level city in Shandong
Jinfeng Hongyuan Flagship Store	Power Saving Expert 2	I just received it and plugged it in, we'll see exactly when it's time to pay the electric bill	Jingdong prefecture level city in Shandong
Jinfeng Hongyuan Flagship Store	Power Saving Expert 2	Saver is good Packaging and looks good How effective it is to be shared after using it.	Jingdong prefecture level city in Shandong
Jinfeng Hongyuan Flagship Store	Power Saving Expert 2	There's no way to compare. I don't know if it's energy efficient.	Jingdong prefecture level city in Shandong
Jinfeng Hongyuan Flagship Store	Power Saving Expert 2	Just installed it, try it out and comment again	Jingdong prefecture level city in Shandong
Jinfeng Hongyuan Flagship Store	Power Saving Expert 2	Super save point also good to install, after a friend introduced then went to buy to, friends say it is very good to use, home I bought, try to use a period of time, to be honest, the economizer is not big or heavy, can be very convenient to put in a lot of places, the quality is also very good, power-saving function this time seems to be about 20% of the efficiency. The owner is also very warm contact, very	Jingdong prefecture level city in Shandong

Jinfeng Hongyuan Flagship Store	Power Saving Expert 2	The reviews online are mixed. Try it for a while, but I don't know if I can return it if it doesn't save any power after a couple months.	Jingdong prefecture level city in Shandong
Jinfeng Hongyuan Flagship Store	Power Saving Expert 2	The power consumption at home is high, bought a power saver, it looks very convenient to plug on the line, see if you can save some money next month, if good, recommend to friends down the road.	Jingdong prefecture level city in Shandong
Jinfeng Hongyuan Flagship Store	Power Saving Expert 2	It's beautiful, it's genuine, but I don't know the power-saving effect.	Jingdong prefecture level city in Shandong
Jinfeng Hongyuan Flagship Store	Power Saving Expert 2	This user thinks the product is good	Jingdong prefecture level city in Shandong
Jinfeng Hongyuan Flagship Store	Power Saving Expert 2	Nice exterior design with an at-a-glance display	Jingdong prefecture level city in Shandong
Jinfeng Hongyuan Flagship Store	Power Saving Expert 2	Home large and small things are purchased in the Jingdong Mall, have been accustomed to shopping in the Jingdong, many times can buy good quality and cheap products, and courier service attitude is particularly good, sometimes too many couriers are to help me to the home, so many years have been in the Jingdong Mall shopping, trust and support! Will also support the Jingdong as always!	Jingdong prefecture level city in Shandong
Jinfeng Hongyuan Flagship Store	Power Saving Expert 2	Good workmanship, mostly better than the original wall-plugged ones, and hopefully power factor compensation saves on electricity costs	Jingdong prefecture level city in Shandong
Jinfeng Hongyuan Flagship Store	Power Saving Expert 2	I don't feel anything, I said I won't know the effect for a month or two, let's see.	Jingdong prefecture level city in Shandong
Jinfeng Hongyuan Flagship Store	Power Saving Expert 2	Delivery was pretty fast, a little pricey, just not sure if it works or not	Jingdong prefecture level city in Shandong
Jinfeng Hongyuan Flagship Store	Power Saving Expert 2	The goods have been received, I do not know how effective.	Jingdong prefecture level city in Shandong
Jinfeng Hongyuan Flagship Store	Power Saving Expert 2	So far it doesn't seem to be too noticeable, so I'll comment later.	Jingdong prefecture level city in Shandong
Jinfeng Hongyuan Flagship Store	Power Saving Expert 2	I'll comment after I've used it	Jingdong prefecture level city in Shandong
Jinfeng Hongyuan Flagship Store	Power Saving Expert 2	Not bad. You'll save a lot on your electric bill in a year!	Jingdong prefecture level city in Shandong
Jinfeng Hongyuan Flagship Store	Power Saving Expert 2	I just got it. I haven't tried it yet. I don't know what it's like.	Jingdong prefecture level city in Shandong
Jinfeng Hongyuan Flagship Store	Power Saving Expert 2	Shopping at Jingdong has become a habit, and this disease will not be cured for a while.	Jingdong prefecture level city in Shandong
Jinfeng Hongyuan Flagship Store	Power Saving Expert 2	I've been using it for a while now. I don't know if I'm still saving power or not.	Jingdong prefecture level city in Shandong
Jinfeng Hongyuan Flagship Store	Power Saving Expert 2	Received the saver and it's great! There is also a digital voltage indication. Plugged in as instructed, easy to operate, hope you're our power saver!	Jingdong prefecture level city in Shandong
Jinfeng Hongyuan Flagship Store	Power Saving Expert 2	There is no power saving as the store says, using it is the same as not using it.	Jingdong prefecture level city in Shandong
Jinfeng Hongyuan Flagship Store	Power Saving Expert 2	Use it for a while and then comment again	Jingdong prefecture level city in Shandong
Jinfeng Hongyuan Flagship Store	Power Saving Expert 2	Feeling can, to use 1 to 2 months to see how the effect, there is a voltage display, can see the voltage fluctuations amplitude, quite good!	Jingdong prefecture level city in Shandong
Jinfeng Hongyuan Flagship Store	Power Saving Expert 2	I hope it works and saves me some electricity.	Jingdong prefecture level city in Shandong
Jinfeng Hongyuan Flagship Store	Power Saving Expert 2	Nice baby, happy with the after sales	Jingdong prefecture level city in Shandong
Jinfeng Hongyuan Flagship Store	Power Saving Expert 2	Use it and talk about it.	Jingdong prefecture level city in Shandong
Jinfeng Hongyuan Flagship Store	Power Saving Expert 2	Second purchase	Jingdong prefecture level city in Shandong
Jinfeng Hongyuan Flagship Store	Power Saving Expert 2	It works.	Jingdong prefecture level city in Shandong
Jinfeng Hongyuan Flagship Store	Power Saving Expert 2	It works.	Jingdong prefecture level city in Shandong
Jinfeng Hongyuan Flagship Store	Power Saving Expert 2	I don't know if it's a power saver until I start using it	Jingdong prefecture level city in Shandong
Jinfeng Hongyuan Flagship Store	Power Saving Expert 2	serviceable	Jingdong prefecture level city in Shandong
Jinfeng Hongyuan Flagship Store	Power Saving Expert 2	This user did not fill out the rating in time	Jingdong prefecture level city in Shandong
Jinfeng Hongyuan Flagship Store	Power Saving Expert 2	There is no power saving as the store says, using it is the same as not using it.	Jingdong prefecture level city in Shandong
Jinfeng Hongyuan Flagship Store	Power Saving Expert 2	This user did not fill out the rating in time	Jingdong prefecture level city in Shandong
Jinfeng Hongyuan Flagship Store	Power Saving Expert 2	Good...good.	Jingdong prefecture level city in Shandong
Jinfeng Hongyuan Flagship Store	Power Saving Expert 2	(of an unmarried couple) be close	Jingdong prefecture level city in Shandong
Jinfeng Hongyuan Flagship Store	Power Saving Expert 2	(of an unmarried couple) be close	Jingdong prefecture level city in Shandong
Jinfeng Hongyuan Flagship Store	Power Saving Expert 2	(of an unmarried couple) be close	Jingdong prefecture level city in Shandong
Jinfeng Hongyuan Flagship Store	Power Saving Expert 2	The goods are real, high value, high quality, worth every penny, material appearance and quality at a glance is very upscale, very like HAPPY1 00	Jingdong prefecture level city in Shandong
Jinfeng Hongyuan Flagship Store	Power Saving Expert 2	It is the ideal product, good quality, logistics is also very fast, worthy of praise 🎁 baby quality is very good, received the first time to open the first time to see, it is very good 👍, very satisfied 😃, customer service is also available to ask questions and answer, thank you 🙏	Jingdong prefecture level city in Shandong
Jinfeng Hongyuan Flagship Store	Power Saving Expert 2	I bought it and haven't used it yet, I'll use it again.	Jingdong prefecture level city in Shandong
Jinfeng Hongyuan Flagship Store	Power Saving Expert 2	It didn't work, I used it for a few days and it was the same as before, it didn't work, I should have to return it	Jingdong prefecture level city in Shandong
Jinfeng Hongyuan Flagship Store	Power Saving Expert 2	The goods have been received, but have not yet installed and use, feel good.	Jingdong prefecture level city in Shandong
Jinfeng Hongyuan Flagship Store	Power Saving Expert 2	Goods received, I forgot to take pictures, the goods look good, you can start, immediately start the school to see the effect then	Jingdong prefecture level city in Shandong
Jinfeng Hongyuan Flagship Store	Power Saving Expert 2	Looks good. I'll use it for a while.	Jingdong prefecture level city in Shandong
Jinfeng Hongyuan Flagship Store	Power Saving Expert 2	Great stuff, easy to operate, easy to use, good power saving, believe in the power of science!	Jingdong prefecture level city in Shandong
Jinfeng Hongyuan Flagship Store	Power Saving Expert 2	Especially great, comparison shopping before buying, the store owner is very good, answering questions very patiently and detailed, there is a need to come back!	Jingdong prefecture level city in Shandong
Jinfeng Hongyuan Flagship Store	Power Saving Expert 2	Trustworthy, feel free to use it boldly.	Jingdong prefecture level city in Shandong
Jinfeng Hongyuan Flagship Store	Power Saving Expert 2	Logistics is super fast! Beautiful appearance! Looks like a great quality! I've never bought one before, so I'll try it first to see if it saves power. We'll see how much it saves.	Jingdong prefecture level city in Shandong
Jinfeng Hongyuan Flagship Store	Power Saving Expert 2	I saw online counterfeiting, energy saver is one of them, hahahahaha, jingdong also buy fake products	Jingdong prefecture level city in Shandong
Jinfeng Hongyuan Flagship Store	Power Saving Expert 2	Just received, installed, try a month to see how the effect and then follow up.	Jingdong prefecture level city in Shandong
Jinfeng Hongyuan Flagship Store	Power Saving Expert 2	Good for you! The goods are genuine! Good price for what you get!	Jingdong prefecture level city in Shandong
Jinfeng Hongyuan Flagship Store	Power Saving Expert 2	Things quickly received, immediately used to see if there is no effect, something river photo 🆙 the same, customer service is very professional	Jingdong prefecture level city in Shandong

Jinfeng Hongyuan Flagship Store	Power Saving Expert 2	The product is very convenient Looking forward to the subsequent use of the experience results	Jingdong prefecture level city in Shandong
Jinfeng Hongyuan Flagship Store	Power Saving Expert 2	I just received it and plugged it in, we'll see exactly when it's time to pay the electric bill	Jingdong prefecture level city in Shandong
Jinfeng Hongyuan Flagship Store	Power Saving Expert 2	Saver is good Packaging and looks good How effective it is to be shared after using it.	Jingdong prefecture level city in Shandong
Jinfeng Hongyuan Flagship Store	Power Saving Expert 2	There's no way to compare. I don't know if it's energy efficient.	Jingdong prefecture level city in Shandong
Jinfeng Hongyuan Flagship Store	Power Saving Expert 2	Just installed it, try it out and comment again	Jingdong prefecture level city in Shandong
Jinfeng Hongyuan Flagship Store	Power Saving Expert 2	Super save point also good to install, after a friend introduced then went to buy to, friends say it is very good to use, home I bought, try to use a period of time, to be honest, the economizer is not big or heavy, can be very convenient to put in a lot of places, the quality is also very good, power-saving function this time seems to be about 20% of the efficiency. The owner is also very warm contact, very	Jingdong prefecture level city in Shandong
Jinfeng Hongyuan Flagship Store	Power Saving Expert 2	The reviews online are mixed. Try it for a while, but I don't know if I can return it if it doesn't save any power after a couple months.	Jingdong prefecture level city in Shandong
Jinfeng Hongyuan Flagship Store	Power Saving Expert 2	The power consumption at home is high, bought a power saver, it looks very convenient to plug on the line, see if you can save some money next month, if good, recommend to friends down the road.	Jingdong prefecture level city in Shandong
Jinfeng Hongyuan Flagship Store	Power Saving Expert 2	It's beautiful, it's genuine, but I don't know the power-saving effect.	Jingdong prefecture level city in Shandong
Jinfeng Hongyuan Flagship Store	Power Saving Expert 2	This user thinks the product is good	Jingdong prefecture level city in Shandong
Jinfeng Hongyuan Flagship Store	Power Saving Expert 2	Nice exterior design with an at-a-glance display	Jingdong prefecture level city in Shandong
Jinfeng Hongyuan Flagship Store	Power Saving Expert 2	Home large and small things are purchased in the Jingdong Mall, have been accustomed to shopping in the Jingdong, many times can buy good quality and cheap products, and courier service attitude is particularly good, sometimes too many couriers are to help me to the home, so many years have been in the Jingdong Mall shopping, trust and support! Will also support the Jingdong as always!	Jingdong prefecture level city in Shandong
Jinfeng Hongyuan Flagship Store	Power Saving Expert 2	Good workmanship, mostly better than the original wall-plugged ones, and hopefully power factor compensation saves on electricity costs	Jingdong prefecture level city in Shandong
Jinfeng Hongyuan Flagship Store	Power Saving Expert 2	I don't feel anything, I said I won't know the effect for a month or two, let's see.	Jingdong prefecture level city in Shandong
Jinfeng Hongyuan Flagship Store	Power Saving Expert 2	Delivery was pretty fast, a little pricey, just not sure if it works or not	Jingdong prefecture level city in Shandong
Jinfeng Hongyuan Flagship Store	Power Saving Expert 2	The goods have been received, I do not know how effective.	Jingdong prefecture level city in Shandong
Jinfeng Hongyuan Flagship Store	Power Saving Expert 2	So far it doesn't seem to be too noticeable, so I'll comment later.	Jingdong prefecture level city in Shandong
Jinfeng Hongyuan Flagship Store	Power Saving Expert 2	I'll comment after I've used it	Jingdong prefecture level city in Shandong
Jinfeng Hongyuan Flagship Store	Power Saving Expert 2	Not bad. You'll save a lot on your electric bill in a year!	Jingdong prefecture level city in Shandong
Jinfeng Hongyuan Flagship Store	Power Saving Expert 2	I just got it. I haven't tried it yet. I don't know what it's like.	Jingdong prefecture level city in Shandong
Jinfeng Hongyuan Flagship Store	Power Saving Expert 2	Shopping at Jingdong has become a habit, and this disease will not be cured for a while.	Jingdong prefecture level city in Shandong
Jinfeng Hongyuan Flagship Store	Power Saving Expert 2	I've been using it for a while now. I don't know if I'm still saving power or not.	Jingdong prefecture level city in Shandong
Jinfeng Hongyuan Flagship Store	Power Saving Expert 2	Received the saver and it's great! There is also a digital voltage indication. Plugged in as instructed, easy to operate, hope you're our power saver!	Jingdong prefecture level city in Shandong
Jinfeng Hongyuan Flagship Store	Power Saving Expert 2	There is no power saving as the store says, using it is the same as not using it.	Jingdong prefecture level city in Shandong
Jinfeng Hongyuan Flagship Store	Power Saving Expert 2	Use it for a while and then comment again	Jingdong prefecture level city in Shandong
Jinfeng Hongyuan Flagship Store	Power Saving Expert 2	Feeling can, to use 1 to 2 months to see how the effect, there is a voltage display, can see the voltage fluctuations amplitude, quite good!	Jingdong prefecture level city in Shandong
Jinfeng Hongyuan Flagship Store	Power Saving Expert 2	I hope it works and saves me some electricity.	Jingdong prefecture level city in Shandong
Jinfeng Hongyuan Flagship Store	Power Saving Expert 2	Nice baby, happy with the after sales	Jingdong prefecture level city in Shandong
Jinfeng Hongyuan Flagship Store	Power Saving Expert 2	Use it and talk about it.	Jingdong prefecture level city in Shandong
Jinfeng Hongyuan Flagship Store	Power Saving Expert 2	Second purchase	Jingdong prefecture level city in Shandong
Jinfeng Hongyuan Flagship Store	Power Saving Expert 2	It works.	Jingdong prefecture level city in Shandong
Jinfeng Hongyuan Flagship Store	Power Saving Expert 2	It works.	Jingdong prefecture level city in Shandong
Jinfeng Hongyuan Flagship Store	Power Saving Expert 2	I don't know if it's a power saver until I start using it	Jingdong prefecture level city in Shandong
Jinfeng Hongyuan Flagship Store	Power Saving Expert 2	serviceable	Jingdong prefecture level city in Shandong
Jinfeng Hongyuan Flagship Store	Power Saving Expert 2	This user did not fill out the rating in time	Jingdong prefecture level city in Shandong
Jinfeng Hongyuan Flagship Store	Power Saving Expert 2	There is no power saving as the store says, using it is the same as not using it.	Jingdong prefecture level city in Shandong
Jinfeng Hongyuan Flagship Store	Power Saving Expert 2	This user did not fill out the rating in time	Jingdong prefecture level city in Shandong
Jinfeng Hongyuan Flagship Store	Power Saving Expert 2	Good...good.	Jingdong prefecture level city in Shandong
Jinfeng Hongyuan Flagship Store	Power Saving Expert 2	(of an unmarried couple) be close	Jingdong prefecture level city in Shandong
Jinfeng Hongyuan Flagship Store	Power Saving Expert 2	(of an unmarried couple) be close	Jingdong prefecture level city in Shandong
Jinfeng Hongyuan Flagship Store	Power Saving Expert 2	(of an unmarried couple) be close	Jingdong prefecture level city in Shandong
Jinfeng Hongyuan Flagship Store	Power Saving Expert 2	The goods are real, high value, high quality, worth every penny, material appearance and quality at a glance is very upscale, very like HAPPY1 00	Jingdong prefecture level city in Shandong
Jinfeng Hongyuan Flagship Store	Power Saving Expert 2	It is the ideal product, good quality, logistics is also very fast, worthy of praise 🎁 baby quality is very good, received the first time to open the first time to see, it is very good 👍, very satisfied 😃, customer service is also available to ask questions and answer, thank you 🙏	Jingdong prefecture level city in Shandong
Jinfeng Hongyuan Flagship Store	Power Saving Expert 2	I bought it and haven't used it yet, I'll use it again.	Jingdong prefecture level city in Shandong
Jinfeng Hongyuan Flagship Store	Power Saving Expert 2	It didn't work, I used it for a few days and it was the same as before, it didn't work, I should have to return it	Jingdong prefecture level city in Shandong
Jinfeng Hongyuan Flagship Store	Power Saving Expert 2	The goods have been received, but have not yet installed and use, feel good.	Jingdong prefecture level city in Shandong
Jinfeng Hongyuan Flagship Store	Power Saving Expert 2	Goods received, I forgot to take pictures, the goods look good, you can start, immediately start the school to see the effect then	Jingdong prefecture level city in Shandong
Jinfeng Hongyuan Flagship Store	Power Saving Expert 2	Looks good. I'll use it for a while.	Jingdong prefecture level city in Shandong
Jinfeng Hongyuan Flagship Store	Power Saving Expert 2	Great stuff, easy to operate, easy to use, good power saving, believe in the power of science!	Jingdong prefecture level city in Shandong

Jinfeng Hongyuan Flagship Store	Power Saving Expert 2	Especially great, comparison shopping before buying, the store owner is very good, answering questions very patiently and detailed, there is a need to come back!	Jingdong prefecture level city in Shandong
Jinfeng Hongyuan Flagship Store	Power Saving Expert 2	Trustworthy, feel free to use it boldly.	Jingdong prefecture level city in Shandong
Jinfeng Hongyuan Flagship Store	Power Saving Expert 2	Logistics is super fast! Beautiful appearance! Looks like a great quality! I've never bought one before, so I'll try it first to see if it saves power. We'll see how much it saves.	Jingdong prefecture level city in Shandong
Jinfeng Hongyuan Flagship Store	Power Saving Expert 2	I saw online counterfeiting, energy saver is one of them, hahahahaha, jingdong also buy fake products	Jingdong prefecture level city in Shandong
Jinfeng Hongyuan Flagship Store	Power Saving Expert 2	Just received, installed, try a month to see how the effect and then follow up.	Jingdong prefecture level city in Shandong
Jinfeng Hongyuan Flagship Store	Power Saving Expert 2	Good for you! The goods are genuine! Good price for what you get!	Jingdong prefecture level city in Shandong
Jinfeng Hongyuan Flagship Store	Power Saving Expert 2	Things quickly received, immediately used to see if there is no effect, something river photo ⇡ the same, customer service is very professional	Jingdong prefecture level city in Shandong
Jinfeng Hongyuan Flagship Store	Power Saving Expert 2	The product is very convenient Looking forward to the subsequent use of the experience results	Jingdong prefecture level city in Shandong
Jinfeng Hongyuan Flagship Store	Power Saving Expert 2	I just received it and plugged it in, we'll see exactly when it's time to pay the electric bill	Jingdong prefecture level city in Shandong
Jinfeng Hongyuan Flagship Store	Power Saving Expert 2	Saver is good Packaging and looks good How effective it is to be shared after using it.	Jingdong prefecture level city in Shandong
Jinfeng Hongyuan Flagship Store	Power Saving Expert 2	There's no way to compare. I don't know if it's energy efficient.	Jingdong prefecture level city in Shandong
Jinfeng Hongyuan Flagship Store	Power Saving Expert 2	Just installed it, try it out and comment again	Jingdong prefecture level city in Shandong
Jinfeng Hongyuan Flagship Store	Power Saving Expert 2	Super save point also good to install, after a friend introduced then went to buy to, friends say it is very good to use, home I bought, try to use a period of time, to be honest, the economizer is not big or heavy, can be very convenient to put in a lot of places, the quality is also very good, power-saving function this time seems to be about 20% of the efficiency. The owner is also very warm contact, very	Jingdong prefecture level city in Shandong
Jinfeng Hongyuan Flagship Store	Power Saving Expert 2	The reviews online are mixed. Try it for a while, but I don't know if I can return it if it doesn't save any power after a couple months.	Jingdong prefecture level city in Shandong
Jinfeng Hongyuan Flagship Store	Power Saving Expert 2	The power consumption at home is high, bought a power saver, it looks very convenient to plug on the line, see if you can save some money next month, if good, recommend to friends down the road.	Jingdong prefecture level city in Shandong
Jinfeng Hongyuan Flagship Store	Power Saving Expert 2	It's beautiful, it's genuine, but I don't know the power-saving effect.	Jingdong prefecture level city in Shandong
Jinfeng Hongyuan Flagship Store	Power Saving Expert 2	This user thinks the product is good	Jingdong prefecture level city in Shandong
Jinfeng Hongyuan Flagship Store	Power Saving Expert 2	Nice exterior design with an at-a-glance display	Jingdong prefecture level city in Shandong
Jinfeng Hongyuan Flagship Store	Power Saving Expert 2	Home large and small things are purchased in the Jingdong Mall, have been accustomed to shopping in the Jingdong, many times can buy good quality and cheap products, and courier service attitude is particularly good, sometimes too many couriers are to help me to the home, so many years have been in the Jingdong Mall shopping, trust and support! Will also support the Jingdong as always!	Jingdong prefecture level city in Shandong
Jinfeng Hongyuan Flagship Store	Power Saving Expert 2	Good workmanship, mostly better than the original wall-plugged ones, and hopefully power factor compensation saves on electricity costs	Jingdong prefecture level city in Shandong
Jinfeng Hongyuan Flagship Store	Power Saving Expert 2	I don't feel anything, I said I won't know the effect for a month or two, let's see.	Jingdong prefecture level city in Shandong
Jinfeng Hongyuan Flagship Store	Power Saving Expert 2	Delivery was pretty fast, a little pricey, just not sure if it works or not	Jingdong prefecture level city in Shandong
Jinfeng Hongyuan Flagship Store	Power Saving Expert 2	The goods have been received, I do not know how effective.	Jingdong prefecture level city in Shandong
Jinfeng Hongyuan Flagship Store	Power Saving Expert 2	So far it doesn't seem to be too noticeable, so I'll comment later.	Jingdong prefecture level city in Shandong
Jinfeng Hongyuan Flagship Store	Power Saving Expert 2	I'll comment after I've used it	Jingdong prefecture level city in Shandong
Jinfeng Hongyuan Flagship Store	Power Saving Expert 2	Not bad. You'll save a lot on your electric bill in a year!	Jingdong prefecture level city in Shandong
Jinfeng Hongyuan Flagship Store	Power Saving Expert 2	I just got it. I haven't tried it yet. I don't know what it's like.	Jingdong prefecture level city in Shandong
Jinfeng Hongyuan Flagship Store	Power Saving Expert 2	Shopping at Jingdong has become a habit, and this disease will not be cured for a while.	Jingdong prefecture level city in Shandong
Jinfeng Hongyuan Flagship Store	Power Saving Expert 2	I've been using it for a while now. I don't know if I'm still saving power or not.	Jingdong prefecture level city in Shandong
Jinfeng Hongyuan Flagship Store	Power Saving Expert 2	Received the saver and it's great! There is also a digital voltage indication. Plugged in as instructed, easy to operate, hope you're our power saver!	Jingdong prefecture level city in Shandong
Jinfeng Hongyuan Flagship Store	Power Saving Expert 2	There is no power saving as the store says, using it is the same as not using it.	Jingdong prefecture level city in Shandong
Jinfeng Hongyuan Flagship Store	Power Saving Expert 2	Use it for a while and then comment again	Jingdong prefecture level city in Shandong
Jinfeng Hongyuan Flagship Store	Power Saving Expert 2	Feeling can, to use 1 to 2 months to see how the effect, there is a voltage display, can see the voltage fluctuations amplitude, quite good!	Jingdong prefecture level city in Shandong
Jinfeng Hongyuan Flagship Store	Power Saving Expert 2	I hope it works and saves me some electricity.	Jingdong prefecture level city in Shandong
Jinfeng Hongyuan Flagship Store	Power Saving Expert 2	Nice baby, happy with the after sales	Jingdong prefecture level city in Shandong
Jinfeng Hongyuan Flagship Store	Power Saving Expert 2	Use it and talk about it.	Jingdong prefecture level city in Shandong
Jinfeng Hongyuan Flagship Store	Power Saving Expert 2	Second purchase	Jingdong prefecture level city in Shandong
Jinfeng Hongyuan Flagship Store	Power Saving Expert 2	It works.	Jingdong prefecture level city in Shandong
Jinfeng Hongyuan Flagship Store	Power Saving Expert 2	It works.	Jingdong prefecture level city in Shandong
Jinfeng Hongyuan Flagship Store	Power Saving Expert 2	I don't know if it's a power saver until I start using it	Jingdong prefecture level city in Shandong
Jinfeng Hongyuan Flagship Store	Power Saving Expert 2	serviceable	Jingdong prefecture level city in Shandong
Jinfeng Hongyuan Flagship Store	Power Saving Expert 2	This user did not fill out the rating in time	Jingdong prefecture level city in Shandong
Jinfeng Hongyuan Flagship Store	Power Saving Expert 2	There is no power saving as the store says, using it is the same as not using it.	Jingdong prefecture level city in Shandong
Jinfeng Hongyuan Flagship Store	Power Saving Expert 2	This user did not fill out the rating in time	Jingdong prefecture level city in Shandong
Jinfeng Hongyuan Flagship Store	Power Saving Expert 2	Good...good.	Jingdong prefecture level city in Shandong
Jinfeng Hongyuan Flagship Store	Power Saving Expert 2	(of an unmarried couple) be close	Jingdong prefecture level city in Shandong
Jinfeng Hongyuan Flagship Store	Power Saving Expert 2	(of an unmarried couple) be close	Jingdong prefecture level city in Shandong
Jinfeng Hongyuan Flagship Store	Power Saving Expert 2	(of an unmarried couple) be close	Jingdong prefecture level city in Shandong
Jinfeng Hongyuan Flagship Store	Power Saving Expert 2	The goods are real, high value, high quality, worth every penny, material appearance and quality at a glance is very upscale, very like HAPPY1 00	Jingdong prefecture level city in Shandong

Jinfeng Hongyuan Flagship Store	Power Saving Expert 2	It is the ideal product, good quality, logistics is also very fast, worthy of praise 🎁 baby quality is very good, received the first time to open the first time to see, it is very good 👍, very satisfied 😊, customer service is also available to ask questions and answer, thank you 🙏	Jingdong prefecture level city in Shandong
Jinfeng Hongyuan Flagship Store	Power Saving Expert 2	I bought it and haven't used it yet, I'll use it again.	Jingdong prefecture level city in Shandong
Jinfeng Hongyuan Flagship Store	Power Saving Expert 2	It didn't work, I used it for a few days and it was the same as before, it didn't work, I should have to return it	Jingdong prefecture level city in Shandong
Jinfeng Hongyuan Flagship Store	Power Saving Expert 2	The goods have been received, but have not yet installed and use, feel good.	Jingdong prefecture level city in Shandong
Jinfeng Hongyuan Flagship Store	Power Saving Expert 2	Goods received, I forgot to take pictures, the goods look good, you can start, immediately start the school to see the effect then	Jingdong prefecture level city in Shandong
Jinfeng Hongyuan Flagship Store	Power Saving Expert 2	Looks good. I'll use it for a while.	Jingdong prefecture level city in Shandong
Jinfeng Hongyuan Flagship Store	Power Saving Expert 2	Great stuff, easy to operate, easy to use, good power saving, believe in the power of science!	Jingdong prefecture level city in Shandong
Jinfeng Hongyuan Flagship Store	Power Saving Expert 2	Especially great, comparison shopping before buying, the store owner is very good, answering questions very patiently and detailed, there is a need to come back!	Jingdong prefecture level city in Shandong
Jinfeng Hongyuan Flagship Store	Power Saving Expert 2	Trustworthy, feel free to use it boldly.	Jingdong prefecture level city in Shandong
Jinfeng Hongyuan Flagship Store	Power Saving Expert 2	Logistics is super fast! Beautiful appearance! Looks like a great quality! I've never bought one before, so I'll try it first to see if it saves power. We'll see how much it saves.	Jingdong prefecture level city in Shandong
Jinfeng Hongyuan Flagship Store	Power Saving Expert 2	I saw online counterfeiting, energy saver is one of them, hahahahaha, jingdong also buy fake products	Jingdong prefecture level city in Shandong
Jinfeng Hongyuan Flagship Store	Power Saving Expert 2	Just received, installed, try a month to see how the effect and then follow up.	Jingdong prefecture level city in Shandong
Jinfeng Hongyuan Flagship Store	Power Saving Expert 2	Good for you! The goods are genuine! Good price for what you get!	Jingdong prefecture level city in Shandong
Jinfeng Hongyuan Flagship Store	Power Saving Expert 2	Things quickly received, immediately used to see if there is no effect, something river photo ⬆ the same, customer service is very professional	Jingdong prefecture level city in Shandong
Jinfeng Hongyuan Flagship Store	Power Saving Expert 2	The product is very convenient Looking forward to the subsequent use of the experience results	Jingdong prefecture level city in Shandong
Jinfeng Hongyuan Flagship Store	Power Saving Expert 2	I just received it and plugged it in, we'll see exactly when it's time to pay the electric bill	Jingdong prefecture level city in Shandong
Jinfeng Hongyuan Flagship Store	Power Saving Expert 2	Saver is good Packaging and looks good How effective it is to be shared after using it.	Jingdong prefecture level city in Shandong
Jinfeng Hongyuan Flagship Store	Power Saving Expert 2	There's no way to compare. I don't know if it's energy efficient.	Jingdong prefecture level city in Shandong
Jinfeng Hongyuan Flagship Store	Power Saving Expert 2	Just installed it, try it out and comment again	Jingdong prefecture level city in Shandong
Jinfeng Hongyuan Flagship Store	Power Saving Expert 2	Super save point also good to install, after a friend introduced then went to buy to, friends say it is very good to use, home I bought, try to use a period of time, to be honest, the economizer is not big or heavy, can be very convenient to put in a lot of places, the quality is also very good, power-saving function this time seems to be about 20% of the efficiency. The owner is also very warm contact, very	Jingdong prefecture level city in Shandong
Jinfeng Hongyuan Flagship Store	Power Saving Expert 2	The reviews online are mixed. Try it for a while, but I don't know if I can return it if it doesn't save any power after a couple months.	Jingdong prefecture level city in Shandong
Jinfeng Hongyuan Flagship Store	Power Saving Expert 2	The power consumption at home is high, bought a power saver, it looks very convenient to plug on the line, see if you can save some money next month, if good, recommend to friends down the road.	Jingdong prefecture level city in Shandong
Jinfeng Hongyuan Flagship Store	Power Saving Expert 2	It's beautiful, it's genuine, but I don't know the power-saving effect.	Jingdong prefecture level city in Shandong
Jinfeng Hongyuan Flagship Store	Power Saving Expert 2	This user thinks the product is good	Jingdong prefecture level city in Shandong
Jinfeng Hongyuan Flagship Store	Power Saving Expert 2	Nice exterior design with an at-a-glance display	Jingdong prefecture level city in Shandong
Jinfeng Hongyuan Flagship Store	Power Saving Expert 2	Home large and small things are purchased in the Jingdong Mall, have been accustomed to shopping in the Jingdong, many times can buy good quality and cheap products, and courier service attitude is particularly good, sometimes too many couriers are to help me to the home, so many years have been in the Jingdong Mall shopping, trust and support! Will also support the Jingdong as always!	Jingdong prefecture level city in Shandong
Jinfeng Hongyuan Flagship Store	Power Saving Expert 2	Good workmanship, mostly better than the original wall-plugged ones, and hopefully power factor compensation saves on electricity costs	Jingdong prefecture level city in Shandong
Jinfeng Hongyuan Flagship Store	Power Saving Expert 2	I don't feel anything, I said I won't know the effect for a month or two, let's see.	Jingdong prefecture level city in Shandong
Jinfeng Hongyuan Flagship Store	Power Saving Expert 2	Delivery was pretty fast, a little pricey, just not sure if it works or not	Jingdong prefecture level city in Shandong
Jinfeng Hongyuan Flagship Store	Power Saving Expert 2	The goods have been received, I do not know how effective.	Jingdong prefecture level city in Shandong
Jinfeng Hongyuan Flagship Store	Power Saving Expert 2	So far it doesn't seem to be too noticeable, so I'll comment later.	Jingdong prefecture level city in Shandong
Jinfeng Hongyuan Flagship Store	Power Saving Expert 2	I'll comment after I've used it	Jingdong prefecture level city in Shandong
Jinfeng Hongyuan Flagship Store	Power Saving Expert 2	Not bad. You'll save a lot on your electric bill in a year!	Jingdong prefecture level city in Shandong
Jinfeng Hongyuan Flagship Store	Power Saving Expert 2	I just got it. I haven't tried it yet. I don't know what it's like.	Jingdong prefecture level city in Shandong
Jinfeng Hongyuan Flagship Store	Power Saving Expert 2	Shopping at Jingdong has become a habit, and this disease will not be cured for a while.	Jingdong prefecture level city in Shandong
Jinfeng Hongyuan Flagship Store	Power Saving Expert 2	I've been using it for a while now. I don't know if I'm still saving power or not.	Jingdong prefecture level city in Shandong
Jinfeng Hongyuan Flagship Store	Power Saving Expert 2	Received the saver and it's great! There is also a digital voltage indication. Plugged in as instructed, easy to operate, hope you're our power saver!	Jingdong prefecture level city in Shandong
Jinfeng Hongyuan Flagship Store	Power Saving Expert 2	There is no power saving as the store says, using it is the same as not using it.	Jingdong prefecture level city in Shandong
Jinfeng Hongyuan Flagship Store	Power Saving Expert 2	Use it for a while and then comment again	Jingdong prefecture level city in Shandong
Jinfeng Hongyuan Flagship Store	Power Saving Expert 2	Feeling can, to use 1 to 2 months to see how the effect, there is a voltage display, can see the voltage fluctuations amplitude, quite good!	Jingdong prefecture level city in Shandong
Jinfeng Hongyuan Flagship Store	Power Saving Expert 2	I hope it works and saves me some electricity.	Jingdong prefecture level city in Shandong
Jinfeng Hongyuan Flagship Store	Power Saving Expert 2	Nice baby, happy with the after sales	Jingdong prefecture level city in Shandong
Jinfeng Hongyuan Flagship Store	Power Saving Expert 2	Use it and talk about it.	Jingdong prefecture level city in Shandong
Jinfeng Hongyuan Flagship Store	Power Saving Expert 2	Second purchase	Jingdong prefecture level city in Shandong
Jinfeng Hongyuan Flagship Store	Power Saving Expert 2	It works.	Jingdong prefecture level city in Shandong
Jinfeng Hongyuan Flagship Store	Power Saving Expert 2	It works.	Jingdong prefecture level city in Shandong
Jinfeng Hongyuan Flagship Store	Power Saving Expert 2	I don't know if it's a power saver until I start using it	Jingdong prefecture level city in Shandong
Jinfeng Hongyuan Flagship Store	Power Saving Expert 2	serviceable	Jingdong prefecture level city in Shandong
Jinfeng Hongyuan Flagship Store	Power Saving Expert 2	This user did not fill out the rating in time	Jingdong prefecture level city in Shandong

Jinfeng Hongyuan Flagship Store	Power Saving Expert 2	There is no power saving as the store says, using it is the same as not using it.	Jingdong prefecture level city in Shandong
Jinfeng Hongyuan Flagship Store	Power Saving Expert 2	This user did not fill out the rating in time	Jingdong prefecture level city in Shandong
Jinfeng Hongyuan Flagship Store	Power Saving Expert 2	Good...good.	Jingdong prefecture level city in Shandong
Jinfeng Hongyuan Flagship Store	Power Saving Expert 2	(of an unmarried couple) be close	Jingdong prefecture level city in Shandong
Jinfeng Hongyuan Flagship Store	Power Saving Expert 2	(of an unmarried couple) be close	Jingdong prefecture level city in Shandong
Jinfeng Hongyuan Flagship Store	Power Saving Expert 2	(of an unmarried couple) be close	Jingdong prefecture level city in Shandong
Jinfeng Hongyuan Flagship Store	Power Saving Expert 2	The goods are real, high value, high quality, worth every penny, material appearance and quality at a glance is very upscale, very like HAPPY1 00	Jingdong prefecture level city in Shandong
Jinfeng Hongyuan Flagship Store	Power Saving Expert 2	It is the ideal product, good quality, logistics is also very fast, worthy of praise 🎁 baby quality is very good, received the first time to open the first time to see, it is very good 👍, very satisfied 😃, customer service is also available to ask questions and answer, thank you 🙏	Jingdong prefecture level city in Shandong
Jinfeng Hongyuan Flagship Store	Power Saving Expert 2	I bought it and haven't used it yet, I'll use it again.	Jingdong prefecture level city in Shandong
Jinfeng Hongyuan Flagship Store	Power Saving Expert 2	It didn't work, I used it for a few days and it was the same as before, it didn't work, I should have to return it	Jingdong prefecture level city in Shandong
Jinfeng Hongyuan Flagship Store	Power Saving Expert 2	The goods have been received, but have not yet installed and use, feel good.	Jingdong prefecture level city in Shandong
Jinfeng Hongyuan Flagship Store	Power Saving Expert 2	Goods received, I forgot to take pictures, the goods look good, you can start, immediately start the school to see the effect then	Jingdong prefecture level city in Shandong
Jinfeng Hongyuan Flagship Store	Power Saving Expert 2	Looks good. I'll use it for a while.	Jingdong prefecture level city in Shandong
Jinfeng Hongyuan Flagship Store	Power Saving Expert 2	Great stuff, easy to operate, easy to use, good power saving, believe in the power of science!	Jingdong prefecture level city in Shandong
Jinfeng Hongyuan Flagship Store	Power Saving Expert 2	Especially great, comparison shopping before buying, the store owner is very good, answering questions very patiently and detailed, there is a need to come back!	Jingdong prefecture level city in Shandong
Jinfeng Hongyuan Flagship Store	Power Saving Expert 2	Trustworthy, feel free to use it boldly.	Jingdong prefecture level city in Shandong
Jinfeng Hongyuan Flagship Store	Power Saving Expert 2	Logistics is super fast! Beautiful appearance! Looks like a great quality! I've never bought one before, so I'll try it first to see if it saves power. We'll see how much it saves.	Jingdong prefecture level city in Shandong
Jinfeng Hongyuan Flagship Store	Power Saving Expert 2	I saw online counterfeiting, energy saver is one of them, hahahahaha, jingdong also buy fake products	Jingdong prefecture level city in Shandong
Jinfeng Hongyuan Flagship Store	Power Saving Expert 2	Just received, installed, try a month to see how the effect and then follow up.	Jingdong prefecture level city in Shandong
Jinfeng Hongyuan Flagship Store	Power Saving Expert 2	Good for you! The goods are genuine! Good price for what you get!	Jingdong prefecture level city in Shandong
Jinfeng Hongyuan Flagship Store	Power Saving Expert 2	Things quickly received, immediately used to see if there is no effect, something river photo ⬆ the same, customer service is very professional	Jingdong prefecture level city in Shandong
Jinfeng Hongyuan Flagship Store	Power Saving Expert 2	The product is very convenient Looking forward to the subsequent use of the experience results	Jingdong prefecture level city in Shandong
Jinfeng Hongyuan Flagship Store	Power Saving Expert 2	I just received it and plugged it in, we'll see exactly when it's time to pay the electric bill	Jingdong prefecture level city in Shandong
Jinfeng Hongyuan Flagship Store	Power Saving Expert 2	Saver is good Packaging and looks good How effective it is to be shared after using it.	Jingdong prefecture level city in Shandong
Jinfeng Hongyuan Flagship Store	Power Saving Expert 2	There's no way to compare. I don't know if it's energy efficient.	Jingdong prefecture level city in Shandong
Jinfeng Hongyuan Flagship Store	Power Saving Expert 2	Just installed it, try it out and comment again	Jingdong prefecture level city in Shandong
Jinfeng Hongyuan Flagship Store	Power Saving Expert 2	Super save point also good to install, after a friend introduced then went to buy to, friends say it is very good to use, home I bought, try to use a period of time, to be honest, the economizer is not big or heavy, can be very convenient to put in a lot of places, the quality is also very good, power-saving function this time seems to be about 20% of the efficiency. The owner is also very warm contact, very	Jingdong prefecture level city in Shandong
Jinfeng Hongyuan Flagship Store	Power Saving Expert 2	The reviews online are mixed. Try it for a while, but I don't know if I can return it if it doesn't save any power after a couple months.	Jingdong prefecture level city in Shandong
Jinfeng Hongyuan Flagship Store	Power Saving Expert 2	The power consumption at home is high, bought a power saver, it looks very convenient to plug on the line, see if you can save some money next month, if good, recommend to friends down the road.	Jingdong prefecture level city in Shandong
Jinfeng Hongyuan Flagship Store	Power Saving Expert 2	It's beautiful, it's genuine, but I don't know the power-saving effect.	Jingdong prefecture level city in Shandong
Jinfeng Hongyuan Flagship Store	Power Saving Expert 2	This user thinks the product is good	Jingdong prefecture level city in Shandong
Jinfeng Hongyuan Flagship Store	Power Saving Expert 2	Nice exterior design with an at-a-glance display	Jingdong prefecture level city in Shandong
Jinfeng Hongyuan Flagship Store	Power Saving Expert 2	Home large and small things are purchased in the Jingdong Mall, have been accustomed to shopping in the Jingdong, many times can buy good quality and cheap products, and courier service attitude is particularly good, sometimes too many couriers are to help me to the home, so many years have been in the Jingdong Mall shopping, trust and support! Will also support the Jingdong as always!	Jingdong prefecture level city in Shandong
Jinfeng Hongyuan Flagship Store	Power Saving Expert 2	Good workmanship, mostly better than the original wall-plugged ones, and hopefully power factor compensation saves on electricity costs	Jingdong prefecture level city in Shandong
Jinfeng Hongyuan Flagship Store	Power Saving Expert 2	I don't feel anything, I said I won't know the effect for a month or two, let's see.	Jingdong prefecture level city in Shandong
Jinfeng Hongyuan Flagship Store	Power Saving Expert 2	Delivery was pretty fast, a little pricey, just not sure if it works or not	Jingdong prefecture level city in Shandong
Jinfeng Hongyuan Flagship Store	Power Saving Expert 2	The goods have been received, I do not know how effective.	Jingdong prefecture level city in Shandong
Jinfeng Hongyuan Flagship Store	Power Saving Expert 2	So far it doesn't seem to be too noticeable, so I'll comment later.	Jingdong prefecture level city in Shandong
Jinfeng Hongyuan Flagship Store	Power Saving Expert 2	I'll comment after I've used it	Jingdong prefecture level city in Shandong
Jinfeng Hongyuan Flagship Store	Power Saving Expert 2	Not bad. You'll save a lot on your electric bill in a year!	Jingdong prefecture level city in Shandong
Jinfeng Hongyuan Flagship Store	Power Saving Expert 2	I just got it. I haven't tried it yet. I don't know what it's like.	Jingdong prefecture level city in Shandong
Jinfeng Hongyuan Flagship Store	Power Saving Expert 2	Shopping at Jingdong has become a habit, and this disease will not be cured for a while.	Jingdong prefecture level city in Shandong
Jinfeng Hongyuan Flagship Store	Power Saving Expert 2	I've been using it for a while now. I don't know if I'm still saving power or not.	Jingdong prefecture level city in Shandong
Jinfeng Hongyuan Flagship Store	Power Saving Expert 2	Received the saver and it's great! There is also a digital voltage indication. Plugged in as instructed, easy to operate, hope you're our power saver!	Jingdong prefecture level city in Shandong
Jinfeng Hongyuan Flagship Store	Power Saving Expert 2	There is no power saving as the store says, using it is the same as not using it.	Jingdong prefecture level city in Shandong
Jinfeng Hongyuan Flagship Store	Power Saving Expert 2	Use it for a while and then comment again	Jingdong prefecture level city in Shandong
Jinfeng Hongyuan Flagship Store	Power Saving Expert 2	Feeling can, to use 1 to 2 months to see how the effect, there is a voltage display, can see the voltage fluctuations amplitude, quite good!	Jingdong prefecture level city in Shandong
Jinfeng Hongyuan Flagship Store	Power Saving Expert 2	I hope it works and saves me some electricity.	Jingdong prefecture level city in Shandong
Jinfeng Hongyuan Flagship Store	Power Saving Expert 2	Nice baby, happy with the after sales	Jingdong prefecture level city in Shandong

Jinfeng Hongyuan Flagship Store	Power Saving Expert 2	Use it and talk about it.	Jingdong prefecture level city in Shandong
Jinfeng Hongyuan Flagship Store	Power Saving Expert 2	Second purchase	Jingdong prefecture level city in Shandong
Jinfeng Hongyuan Flagship Store	Power Saving Expert 2	It works.	Jingdong prefecture level city in Shandong
Jinfeng Hongyuan Flagship Store	Power Saving Expert 2	It works.	Jingdong prefecture level city in Shandong
Jinfeng Hongyuan Flagship Store	Power Saving Expert 2	I don't know if it's a power saver until I start using it	Jingdong prefecture level city in Shandong
Jinfeng Hongyuan Flagship Store	Power Saving Expert 2	serviceable	Jingdong prefecture level city in Shandong
Jinfeng Hongyuan Flagship Store	Power Saving Expert 2	This user did not fill out the rating in time	Jingdong prefecture level city in Shandong
Jinfeng Hongyuan Flagship Store	Power Saving Expert 2	There is no power saving as the store says, using it is the same as not using it.	Jingdong prefecture level city in Shandong
Jinfeng Hongyuan Flagship Store	Power Saving Expert 2	This user did not fill out the rating in time	Jingdong prefecture level city in Shandong
Jinfeng Hongyuan Flagship Store	Power Saving Expert 2	Good...good.	Jingdong prefecture level city in Shandong
Jinfeng Hongyuan Flagship Store	Power Saving Expert 2	(of an unmarried couple) be close	Jingdong prefecture level city in Shandong
Jinfeng Hongyuan Flagship Store	Power Saving Expert 2	(of an unmarried couple) be close	Jingdong prefecture level city in Shandong
Jinfeng Hongyuan Flagship Store	Power Saving Expert 2	(of an unmarried couple) be close	Jingdong prefecture level city in Shandong
Jinfeng Hongyuan Flagship Store	Power Saving Expert 2	The goods are real, high value, high quality, worth every penny, material appearance and quality at a glance is very upscale, very like HAPPY1 00	Jingdong prefecture level city in Shandong
Jinfeng Hongyuan Flagship Store	Power Saving Expert 2	It is the ideal product, good quality, logistics is also very fast, worthy of praise 🎁 baby quality is very good, received the first time to open the first time to see, it is very good 👍, very satisfied 😃, customer service is also available to ask questions and answer, thank you 🙏	Jingdong prefecture level city in Shandong
Jinfeng Hongyuan Flagship Store	Power Saving Expert 2	I bought it and haven't used it yet, I'll use it again.	Jingdong prefecture level city in Shandong
Jinfeng Hongyuan Flagship Store	Power Saving Expert 2	It didn't work, I used it for a few days and it was the same as before, it didn't work, I should have to return it	Jingdong prefecture level city in Shandong
Jinfeng Hongyuan Flagship Store	Power Saving Expert 2	The goods have been received, but have not yet installed and use, feel good.	Jingdong prefecture level city in Shandong
Jinfeng Hongyuan Flagship Store	Power Saving Expert 2	Goods received, I forgot to take pictures, the goods look good, you can start, immediately start the school to see the effect then	Jingdong prefecture level city in Shandong
Jinfeng Hongyuan Flagship Store	Power Saving Expert 2	Looks good. I'll use it for a while.	Jingdong prefecture level city in Shandong
Jinfeng Hongyuan Flagship Store	Power Saving Expert 2	Great stuff, easy to operate, easy to use, good power saving, believe in the power of science!	Jingdong prefecture level city in Shandong
Jinfeng Hongyuan Flagship Store	Power Saving Expert 2	Especially great, comparison shopping before buying, the store owner is very good, answering questions very patiently and detailed, there is a need to come back!	Jingdong prefecture level city in Shandong
Jinfeng Hongyuan Flagship Store	Power Saving Expert 2	Trustworthy, feel free to use it boldly.	Jingdong prefecture level city in Shandong
Jinfeng Hongyuan Flagship Store	Power Saving Expert 2	Logistics is super fast! Beautiful appearance! Looks like a great quality! I've never bought one before, so I'll try it first to see if it saves power. We'll see how much it saves.	Jingdong prefecture level city in Shandong
Jinfeng Hongyuan Flagship Store	Power Saving Expert 2	I saw online counterfeiting, energy saver is one of them, hahahahaha, jingdong also buy fake products	Jingdong prefecture level city in Shandong
Jinfeng Hongyuan Flagship Store	Power Saving Expert 2	Just received, installed, try a month to see how the effect and then follow up.	Jingdong prefecture level city in Shandong
Jinfeng Hongyuan Flagship Store	Power Saving Expert 2	Good for you! The goods are genuine! Good price for what you get!	Jingdong prefecture level city in Shandong
Jinfeng Hongyuan Flagship Store	Power Saving Expert 2	Things quickly received, immediately used to see if there is no effect, something river photo 🔝 the same, customer service is very professional	Jingdong prefecture level city in Shandong
Jinfeng Hongyuan Flagship Store	Power Saving Expert 2	The product is very convenient Looking forward to the subsequent use of the experience results	Jingdong prefecture level city in Shandong
Jinfeng Hongyuan Flagship Store	Power Saving Expert 2	I just received it and plugged it in, we'll see exactly when it's time to pay the electric bill	Jingdong prefecture level city in Shandong
Jinfeng Hongyuan Flagship Store	Power Saving Expert 2	Saver is good Packaging and looks good How effective it is to be shared after using it.	Jingdong prefecture level city in Shandong
Jinfeng Hongyuan Flagship Store	Power Saving Expert 2	There's no way to compare. I don't know if it's energy efficient.	Jingdong prefecture level city in Shandong
Jinfeng Hongyuan Flagship Store	Power Saving Expert 2	Just installed it, try it out and comment again	Jingdong prefecture level city in Shandong
Jinfeng Hongyuan Flagship Store	Power Saving Expert 2	Super save point also good to install, after a friend introduced then went to buy to, friends say it is very good to use, home I bought, try to use a period of time, to be honest, the economizer is not big or heavy, can be very convenient to put in a lot of places, the quality is also very good, power-saving function this time seems to be about 20% of the efficiency. The owner is also very warm contact, very	Jingdong prefecture level city in Shandong
Jinfeng Hongyuan Flagship Store	Power Saving Expert 2	The reviews online are mixed. Try it for a while, but I don't know if I can return it if it doesn't save any power after a couple months.	Jingdong prefecture level city in Shandong
Jinfeng Hongyuan Flagship Store	Power Saving Expert 2	The power consumption at home is high, bought a power saver, it looks very convenient to plug on the line, see if you can save some money next month, if good, recommend to friends down the road.	Jingdong prefecture level city in Shandong
Jinfeng Hongyuan Flagship Store	Power Saving Expert 2	It's beautiful, it's genuine, but I don't know the power-saving effect.	Jingdong prefecture level city in Shandong
Jinfeng Hongyuan Flagship Store	Power Saving Expert 2	This user thinks the product is good	Jingdong prefecture level city in Shandong
Jinfeng Hongyuan Flagship Store	Power Saving Expert 2	Nice exterior design with an at-a-glance display	Jingdong prefecture level city in Shandong
Jinfeng Hongyuan Flagship Store	Power Saving Expert 2	Home large and small things are purchased in the Jingdong Mall, have been accustomed to shopping in the Jingdong, many times can buy good quality and cheap products, and courier service attitude is particularly good, sometimes too many couriers are to help me to the home, so many years have been in the Jingdong Mall shopping, trust and support! Will also support the Jingdong as always!	Jingdong prefecture level city in Shandong
Jinfeng Hongyuan Flagship Store	Power Saving Expert 2	Good workmanship, mostly better than the original wall-plugged ones, and hopefully power factor compensation saves on electricity costs	Jingdong prefecture level city in Shandong
Jinfeng Hongyuan Flagship Store	Power Saving Expert 2	I don't feel anything, I said I won't know the effect for a month or two, let's see.	Jingdong prefecture level city in Shandong
Jinfeng Hongyuan Flagship Store	Power Saving Expert 2	Delivery was pretty fast, a little pricey, just not sure if it works or not	Jingdong prefecture level city in Shandong
Jinfeng Hongyuan Flagship Store	Power Saving Expert 2	The goods have been received, I do not know how effective.	Jingdong prefecture level city in Shandong
Jinfeng Hongyuan Flagship Store	Power Saving Expert 2	So far it doesn't seem to be too noticeable, so I'll comment later.	Jingdong prefecture level city in Shandong
Jinfeng Hongyuan Flagship Store	Power Saving Expert 2	I'll comment after I've used it	Jingdong prefecture level city in Shandong
Jinfeng Hongyuan Flagship Store	Power Saving Expert 2	Not bad. You'll save a lot on your electric bill in a year!	Jingdong prefecture level city in Shandong
Jinfeng Hongyuan Flagship Store	Power Saving Expert 2	I just got it. I haven't tried it yet. I don't know what it's like.	Jingdong prefecture level city in Shandong
Jinfeng Hongyuan Flagship Store	Power Saving Expert 2	Shopping at Jingdong has become a habit, and this disease will not be cured for a while.	Jingdong prefecture level city in Shandong

Jinfeng Hongyuan Flagship Store	Power Saving Expert 2	I've been using it for a while now. I don't know if I'm still saving power or not.	Jingdong prefecture level city in Shandong
Jinfeng Hongyuan Flagship Store	Power Saving Expert 2	Received the saver and it's great! There is also a digital voltage indication. Plugged in as instructed, easy to operate, hope you're our power saver!	Jingdong prefecture level city in Shandong
Jinfeng Hongyuan Flagship Store	Power Saving Expert 2	There is no power saving as the store says, using it is the same as not using it.	Jingdong prefecture level city in Shandong
Jinfeng Hongyuan Flagship Store	Power Saving Expert 2	Use it for a while and then comment again	Jingdong prefecture level city in Shandong
Jinfeng Hongyuan Flagship Store	Power Saving Expert 2	Feeling can, to use 1 to 2 months to see how the effect, there is a voltage display, can see the voltage fluctuations amplitude, quite good!	Jingdong prefecture level city in Shandong
Jinfeng Hongyuan Flagship Store	Power Saving Expert 2	I hope it works and saves me some electricity.	Jingdong prefecture level city in Shandong
Jinfeng Hongyuan Flagship Store	Power Saving Expert 2	Nice baby, happy with the after sales	Jingdong prefecture level city in Shandong
Jinfeng Hongyuan Flagship Store	Power Saving Expert 2	Use it and talk about it.	Jingdong prefecture level city in Shandong
Jinfeng Hongyuan Flagship Store	Power Saving Expert 2	Second purchase	Jingdong prefecture level city in Shandong
Jinfeng Hongyuan Flagship Store	Power Saving Expert 2	It works.	Jingdong prefecture level city in Shandong
Jinfeng Hongyuan Flagship Store	Power Saving Expert 2	It works.	Jingdong prefecture level city in Shandong
Jinfeng Hongyuan Flagship Store	Power Saving Expert 2	I don't know if it's a power saver until I start using it	Jingdong prefecture level city in Shandong
Jinfeng Hongyuan Flagship Store	Power Saving Expert 2	serviceable	Jingdong prefecture level city in Shandong
Jinfeng Hongyuan Flagship Store	Power Saving Expert 2	This user did not fill out the rating in time	Jingdong prefecture level city in Shandong
Jinfeng Hongyuan Flagship Store	Power Saving Expert 2	There is no power saving as the store says, using it is the same as not using it.	Jingdong prefecture level city in Shandong
Jinfeng Hongyuan Flagship Store	Power Saving Expert 2	This user did not fill out the rating in time	Jingdong prefecture level city in Shandong
Jinfeng Hongyuan Flagship Store	Power Saving Expert 2	Good...good.	Jingdong prefecture level city in Shandong
Jinfeng Hongyuan Flagship Store	Power Saving Expert 2	(of an unmarried couple) be close	Jingdong prefecture level city in Shandong
Jinfeng Hongyuan Flagship Store	Power Saving Expert 2	(of an unmarried couple) be close	Jingdong prefecture level city in Shandong
Jinfeng Hongyuan Flagship Store	Power Saving Expert 2	(of an unmarried couple) be close	Jingdong prefecture level city in Shandong
Jinfeng Hongyuan Flagship Store	Power Saving Expert 2	The goods are real, high value, high quality, worth every penny, material appearance and quality at a glance is very upscale, very like HAPPY100	Jingdong prefecture level city in Shandong
Jinfeng Hongyuan Flagship Store	Power Saving Expert 2	It is the ideal product, good quality, logistics is also very fast, worthy of praise 🎁 baby quality is very good, received the first time to open the first time to see, it is very good 👍, very satisfied 🙂, customer service is also available to ask questions and answer, thank you 🙏	Jingdong prefecture level city in Shandong
Jinfeng Hongyuan Flagship Store	Power Saving Expert 2	I bought it and haven't used it yet, I'll use it again.	Jingdong prefecture level city in Shandong
Jinfeng Hongyuan Flagship Store	Power Saving Expert 2	It didn't work, I used it for a few days and it was the same as before, it didn't work, I should have to return it	Jingdong prefecture level city in Shandong
Jinfeng Hongyuan Flagship Store	Power Saving Expert 2	The goods have been received, but have not yet installed and use, feel good.	Jingdong prefecture level city in Shandong
Jinfeng Hongyuan Flagship Store	Power Saving Expert 2	Goods received, I forgot to take pictures, the goods look good, you can start, immediately start the school to see the effect then	Jingdong prefecture level city in Shandong
Jinfeng Hongyuan Flagship Store	Power Saving Expert 2	Looks good. I'll use it for a while.	Jingdong prefecture level city in Shandong
Jinfeng Hongyuan Flagship Store	Power Saving Expert 2	Great stuff, easy to operate, easy to use, good power saving, believe in the power of science!	Jingdong prefecture level city in Shandong
Jinfeng Hongyuan Flagship Store	Power Saving Expert 2	Especially great, comparison shopping before buying, the store owner is very good, answering questions very patiently and detailed, there is a need to come back!	Jingdong prefecture level city in Shandong
Jinfeng Hongyuan Flagship Store	Power Saving Expert 2	Trustworthy, feel free to use it boldly.	Jingdong prefecture level city in Shandong
Jinfeng Hongyuan Flagship Store	Power Saving Expert 2	Logistics is super fast! Beautiful appearance! Looks like a great quality! I've never bought one before, so I'll try it first to see if it saves power. We'll see how much it saves.	Jingdong prefecture level city in Shandong
Jinfeng Hongyuan Flagship Store	Power Saving Expert 2	I saw online counterfeiting, energy saver is one of them, hahahahaha, jingdong also buy fake products	Jingdong prefecture level city in Shandong
Jinfeng Hongyuan Flagship Store	Power Saving Expert 2	Just received, installed, try a month to see how the effect and then follow up.	Jingdong prefecture level city in Shandong
Jinfeng Hongyuan Flagship Store	Power Saving Expert 2	Good for you! The goods are genuine! Good price for what you get!	Jingdong prefecture level city in Shandong
Jinfeng Hongyuan Flagship Store	Power Saving Expert 2	Things quickly received, immediately used to see if there is no effect, something river photo 🔝 the same, customer service is very professional	Jingdong prefecture level city in Shandong
Jinfeng Hongyuan Flagship Store	Power Saving Expert 2	The product is very convenient Looking forward to the subsequent use of the experience results	Jingdong prefecture level city in Shandong
Jinfeng Hongyuan Flagship Store	Power Saving Expert 2	I just received it and plugged it in, we'll see exactly when it's time to pay the electric bill	Jingdong prefecture level city in Shandong
Jinfeng Hongyuan Flagship Store	Power Saving Expert 2	Saver is good Packaging and looks good How effective it is to be shared after using it.	Jingdong prefecture level city in Shandong
Jinfeng Hongyuan Flagship Store	Power Saving Expert 2	There's no way to compare. I don't know if it's energy efficient.	Jingdong prefecture level city in Shandong
Jinfeng Hongyuan Flagship Store	Power Saving Expert 2	Just installed it, try it out and comment again	Jingdong prefecture level city in Shandong
Jinfeng Hongyuan Flagship Store	Power Saving Expert 2	Super save point also good to install, after a friend introduced then went to buy to, friends say it is very good to use, home I bought, try to use a period of time, to be honest, the economizer is not big or heavy, can be very convenient to put in a lot of places, the quality is also very good, power-saving function this time seems to be about 20% of the efficiency. The owner is also very warm contact, very	Jingdong prefecture level city in Shandong
Jinfeng Hongyuan Flagship Store	Power Saving Expert 2	The reviews online are mixed. Try it for a while, but I don't know if I can return it if it doesn't save any power after a couple months.	Jingdong prefecture level city in Shandong
Jinfeng Hongyuan Flagship Store	Power Saving Expert 2	The power consumption at home is high, bought a power saver, it looks very convenient to plug on the line, see if you can save some money next month, if good, recommend to friends down the road.	Jingdong prefecture level city in Shandong
Jinfeng Hongyuan Flagship Store	Power Saving Expert 2	It's beautiful, it's genuine, but I don't know the power-saving effect.	Jingdong prefecture level city in Shandong
Jinfeng Hongyuan Flagship Store	Power Saving Expert 2	This user thinks the product is good	Jingdong prefecture level city in Shandong
Jinfeng Hongyuan Flagship Store	Power Saving Expert 2	Nice exterior design with an at-a-glance display	Jingdong prefecture level city in Shandong
Jinfeng Hongyuan Flagship Store	Power Saving Expert 2	Home large and small things are purchased in the Jingdong Mall, have been accustomed to shopping in the Jingdong, many times can buy good quality and cheap products, and courier service attitude is particularly good, sometimes too many couriers are to help me to the home, so many years have been in the Jingdong Mall shopping, trust and support! Will also support the Jingdong as always!	Jingdong prefecture level city in Shandong
Jinfeng Hongyuan Flagship Store	Power Saving Expert 2	Good workmanship, mostly better than the original wall-plugged ones, and hopefully power factor compensation saves on electricity costs	Jingdong prefecture level city in Shandong
Jinfeng Hongyuan Flagship Store	Power Saving Expert 2	I don't feel anything, I said I won't know the effect for a month or two, let's see.	Jingdong prefecture level city in Shandong

Jinfeng Hongyuan Flagship Store	Power Saving Expert 2	Delivery was pretty fast, a little pricey, just not sure if it works or not	Jingdong prefecture level city in Shandong
Jinfeng Hongyuan Flagship Store	Power Saving Expert 2	The goods have been received, I do not know how effective.	Jingdong prefecture level city in Shandong
Jinfeng Hongyuan Flagship Store	Power Saving Expert 2	So far it doesn't seem to be too noticeable, so I'll comment later.	Jingdong prefecture level city in Shandong
Jinfeng Hongyuan Flagship Store	Power Saving Expert 2	I'll comment after I've used it	Jingdong prefecture level city in Shandong
Jinfeng Hongyuan Flagship Store	Power Saving Expert 2	Not bad. You'll save a lot on your electric bill in a year!	Jingdong prefecture level city in Shandong
Jinfeng Hongyuan Flagship Store	Power Saving Expert 2	I just got it. I haven't tried it yet. I don't know what it's like.	Jingdong prefecture level city in Shandong
Jinfeng Hongyuan Flagship Store	Power Saving Expert 2	Shopping at Jingdong has become a habit, and this disease will not be cured for a while.	Jingdong prefecture level city in Shandong
Jinfeng Hongyuan Flagship Store	Power Saving Expert 2	I've been using it for a while now. I don't know if I'm still saving power or not.	Jingdong prefecture level city in Shandong
Jinfeng Hongyuan Flagship Store	Power Saving Expert 2	Received the saver and it's great! There is also a digital voltage indication. Plugged in as instructed, easy to operate, hope you're our power saver!	Jingdong prefecture level city in Shandong
Jinfeng Hongyuan Flagship Store	Power Saving Expert 2	There is no power saving as the store says, using it is the same as not using it.	Jingdong prefecture level city in Shandong
Jinfeng Hongyuan Flagship Store	Power Saving Expert 2	Use it for a while and then comment again	Jingdong prefecture level city in Shandong
Jinfeng Hongyuan Flagship Store	Power Saving Expert 2	Feeling can, to use 1 to 2 months to see how the effect, there is a voltage display, can see the voltage fluctuations amplitude, quite good!	Jingdong prefecture level city in Shandong
Jinfeng Hongyuan Flagship Store	Power Saving Expert 2	I hope it works and saves me some electricity.	Jingdong prefecture level city in Shandong
Jinfeng Hongyuan Flagship Store	Power Saving Expert 2	Nice baby, happy with the after sales	Jingdong prefecture level city in Shandong
Jinfeng Hongyuan Flagship Store	Power Saving Expert 2	Use it and talk about it.	Jingdong prefecture level city in Shandong
Jinfeng Hongyuan Flagship Store	Power Saving Expert 2	Second purchase	Jingdong prefecture level city in Shandong
Jinfeng Hongyuan Flagship Store	Power Saving Expert 2	It works.	Jingdong prefecture level city in Shandong
Jinfeng Hongyuan Flagship Store	Power Saving Expert 2	It works.	Jingdong prefecture level city in Shandong
Jinfeng Hongyuan Flagship Store	Power Saving Expert 2	I don't know if it's a power saver until I start using it	Jingdong prefecture level city in Shandong
Jinfeng Hongyuan Flagship Store	Power Saving Expert 2	serviceable	Jingdong prefecture level city in Shandong
Jinfeng Hongyuan Flagship Store	Power Saving Expert 2	This user did not fill out the rating in time	Jingdong prefecture level city in Shandong
Jinfeng Hongyuan Flagship Store	Power Saving Expert 2	There is no power saving as the store says, using it is the same as not using it.	Jingdong prefecture level city in Shandong
Jinfeng Hongyuan Flagship Store	Power Saving Expert 2	This user did not fill out the rating in time	Jingdong prefecture level city in Shandong
Jinfeng Hongyuan Flagship Store	Power Saving Expert 2	Good...good.	Jingdong prefecture level city in Shandong
Jinfeng Hongyuan Flagship Store	Power Saving Expert 2	(of an unmarried couple) be close	Jingdong prefecture level city in Shandong
Jinfeng Hongyuan Flagship Store	Power Saving Expert 2	(of an unmarried couple) be close	Jingdong prefecture level city in Shandong
Jinfeng Hongyuan Flagship Store	Power Saving Expert 2	(of an unmarried couple) be close	Jingdong prefecture level city in Shandong
Jinfeng Hongyuan Flagship Store	Power saving voltage stabilizing compensator	Fine workmanship, solid materials, looking forward to the late power-saving effect. With leakage protection switch. As well as the voltage display and power indicator, the packaging is also very attentive, there is a separate box inside the outer box.	Jingdong prefecture level city in Shandong
Jinfeng Hongyuan Flagship Store	Power saving voltage stabilizing compensator	Just received, help a friend to buy, has not been installed to use, use and then add comment, the service is very good five-star praise!	Jingdong prefecture level city in Shandong
Jinfeng Hongyuan Flagship Store	Power saving voltage stabilizing compensator	Delivery was quick and good, I'll review the stuff after I use it.	Jingdong prefecture level city in Shandong
Jinfeng Hongyuan Flagship Store	Power saving voltage stabilizing compensator	It's not very effective. It says it comes with 5 insurance policies, but I don't see any.	Jingdong prefecture level city in Shandong
Jinfeng Hongyuan Flagship Store	Power saving voltage stabilizing compensator	In use, don't know the results, I'll follow up with a review	Jingdong prefecture level city in Shandong
Jinfeng Hongyuan Flagship Store	Power saving voltage stabilizing compensator	Received goods in the morning. Have started using it. Waiting for 1 to 2 months to see the effect. Follow-up review.	Jingdong prefecture level city in Shandong
Jinfeng Hongyuan Flagship Store	Power saving voltage stabilizing compensator	It doesn't seem to be working.	Jingdong prefecture level city in Shandong
Jinfeng Hongyuan Flagship Store	Power saving voltage stabilizing compensator	Received it, use it for a month and see the results!	Jingdong prefecture level city in Shandong
Jinfeng Hongyuan Flagship Store	Power saving voltage stabilizing compensator	It works well.	Jingdong prefecture level city in Shandong
Jinfeng Hongyuan Flagship Store	Three-phase voltage stabilizer and economizer	Logistics is very fast, the power saver received a few days without installation, today there is time to install a good, try two months to see how the effect will be, the quality is good, the business is very attentive, very satisfied with an online shopping!	Jingdong prefecture level city in Shandong
Jinfeng Hongyuan Flagship Store	Three-phase voltage stabilizer and economizer	Has been installed and completed, waiting for the effect, the business commitment of 2 months unlimited dissatisfaction can be returned, give a praise!	Jingdong prefecture level city in Shandong
Jinfeng Hongyuan Flagship Store	Three-phase voltage stabilizer and economizer	Good quality, business is very attentive, very satisfied	Jingdong prefecture level city in Shandong
Doraemon Hyakubo Shop	King of Power Saving	Home electricity consumption, bought a power saver, it looks very convenient a plug on the line, see if you can save some electricity next month, if good, recommend to friends under the next	Jingdong prefecture level city in Shandong
Doraemon Hyakubo Shop	King of Power Saving	Power saving effect can be, cost-effective, good appearance, good workmanship ! It is convenient to use, after using continue to observe, good live and then purchase.	Jingdong prefecture level city in Shandong
Doraemon Hyakubo Shop	King of Power Saving	Very good product, the effect is not yet known ah, with a few days, a month to summarize, with the convenience of the product quality is really good, which is used with peace of mind down to earth with safety, has been in the Jingdong shopping on the shopping! Trustworthy. Next time continue to support the	Jingdong prefecture level city in Shandong
Doraemon Hyakubo Shop	King of Power Saving	In line with the physical object, fast logistics, good service attitude of the deliveryman! The quality is very good! It will take up to 2 months to see if it can save power! Hope it's as good as advertised!	Jingdong prefecture level city in Shandong
Doraemon Hyakubo Shop	King of Power Saving	It's really good Save over 90 a month! Now the power consumption is not big customer service said 2 units will be enough, ready to buy another one!	Jingdong prefecture level city in Shandong
Doraemon Hyakubo Shop	King of Power Saving	Exquisite workmanship, affordable price, the electricity bill is indeed less, plug it in and use it, this day is too hot, the air conditioning and too much electricity, with this effect is good, recommended to	Jingdong prefecture level city in Shandong
Doraemon Hyakubo Shop	King of Power Saving	Very good to use, every day can save a little electricity, really good under the sample down a month to save dozens of dollars in electricity, good before I did not know that there is this? Haha, the price is	Jingdong prefecture level city in Shandong
Doraemon Hyakubo Shop	King of Power Saving	Looked and looked and finally chose this one, the quality of the hands does not look like the quality of shoddy goods. At first held the mind to try to buy after using the feeling can buy a second!	Jingdong prefecture level city in Shandong
Doraemon Hyakubo Shop	King of Power Saving	It rained a couple days ago and I just went to get the item back today , not bad, hey I looked at the meter and that red display light takes several seconds to jump, did not make a mistake buying it. After a period of time to evaluate.	Jingdong prefecture level city in Shandong
Doraemon Hyakubo Shop	King of Power Saving	Used more than 10 days to check the power, there is indeed some decline, customer service said, long-term use will save more points!	Jingdong prefecture level city in Shandong

Doraemon Hyakubo Shop	King of Power Saving	It's a proper IQ tax, and it costs electricity.	Jingdong prefecture level city in Shandong
Doraemon Hyakubo Shop	King of Power Saving	I've been using it for 17 days now, it really saves electricity Shipping is fast, you can save a lot in a year	Jingdong prefecture level city in Shandong
Doraemon Hyakubo Shop	King of Power Saving	I've been using it for 17 days and it really saves power Shipping is fast and you don't have to worry about the quality, free returns within 5 years	Jingdong prefecture level city in Shandong
Doraemon Hyakubo Shop	King of Power Saving	It's more regular, scanned the code and it's authentic, and the main thing is that you can try it for 90 days! Plugged in more than ten days, is less than before a few degrees per day. When the time to use a month to see Additional. Good to buy a few more	Jingdong prefecture level city in Shandong
Doraemon Hyakubo Shop	King of Power Saving	Logistics is very fast, fine workmanship, use it and then come back to the comments	Jingdong prefecture level city in Shandong
Doraemon Hyakubo Shop	King of Power Saving	The quality of workmanship is very good and the power saving effect is quite good, I regret not getting it earlier.	Jingdong prefecture level city in Shandong
Doraemon Hyakubo Shop	King of Power Saving	Received the goods, according to the instructions on the operation is very simple, to wait and see the effect, looks good!	Jingdong prefecture level city in Shandong
Doraemon Hyakubo Shop	King of Power Saving	It's really as safe and power-saving as the owner says, and it works great!	Jingdong prefecture level city in Shandong
Doraemon Hyakubo Shop	King of Power Saving	Received the goods, immediately used, looks good, to wait and see how the effect.	Jingdong prefecture level city in Shandong
Doraemon Hyakubo Shop	King of Power Saving	Afraid to buy back did not work, the store said the commitment to 90 days invalid refund, warranty 5 years. Saver also complimentary on the Pacific insurance, it is a lot of peace of mind, there are effects will continue to buy back drops!	Jingdong prefecture level city in Shandong
Doraemon Hyakubo Shop	King of Power Saving	Received the goods and used it, can save electricity a month later to see the effect!	Jingdong prefecture level city in Shandong
Doraemon Hyakubo Shop	King of Power Saving	Fancy 90-day trial, 5-year warranty before the order, the goods received, has been experiencing a week, can really feel the savings, in the introduction of friends also buy this!	Jingdong prefecture level city in Shandong
Doraemon Hyakubo Shop	King of Power Saving	Clothing stores use, neighbors recommended to buy, said a month can save more than 70 yuan, first plugged in to see the effect of the	Jingdong prefecture level city in Shandong
Doraemon Hyakubo Shop	King of Power Saving	A friend's home two months to save more than 80 dollars of electricity, recommended that I buy two, decisive purchase, looking forward to the effect of power saving, waiting for the next month effect.	Jingdong prefecture level city in Shandong
Doraemon Hyakubo Shop	King of Power Saving	The goods have been received, consistent with the description of the advertisement, quite good, I hope that in terms of power saving and purifying the air is really as the merchant said that would be better!	Jingdong prefecture level city in Shandong
Doraemon Hyakubo Shop	King of Power Saving	Power Saving King is really good to use the quality is very good than the physical store a lot cheaper we can rest assured that the purchase recommended to buy!	Jingdong prefecture level city in Shandong
Doraemon Hyakubo Shop	King of Power Saving	Energy saver has been used on the rental house inside the use of the meter is always running fast, with this can really save electricity, easy to use, plugged in can be, very satisfied!	Jingdong prefecture level city in Shandong
Doraemon Hyakubo Shop	King of Power Saving	Just use can not see what effect to,, wait for a period of time in the evaluation	Jingdong prefecture level city in Shandong
Doraemon Hyakubo Shop	King of Power Saving	Live in the 18th floor is too far away from the meter, afraid of no effect, look at the trial 90 days before buying. Currently used for a month, there is an effect, saving 27 dollars in electricity, next month should be able to save more!	Jingdong prefecture level city in Shandong
Doraemon Hyakubo Shop	King of Power Saving	I've been using it for almost a month now and it feels good, so I'll use it again.	Jingdong prefecture level city in Shandong
Doraemon Hyakubo Shop	King of Power Saving	It's ok, the electric bill is really less, originally I remember charging $100 and using it for 10 days. Now it lasts about 15 days. It's still noticeable.	Jingdong prefecture level city in Shandong
Doraemon Hyakubo Shop	King of Power Saving	Fast logistics, good power saving effect, very satisfied	Jingdong prefecture level city in Shandong
Doraemon Hyakubo Shop	King of Power Saving	Has been the second time back to buy, energy saving effect, worth recommending, you can try to see, power saving effect: very good Experience: can save about 30%!	Jingdong prefecture level city in Shandong
Doraemon Hyakubo Shop	King of Power Saving	I don't know how well it works for now	Jingdong prefecture level city in Shandong
Doraemon Hyakubo Shop	King of Power Saving	Factory direct is really good, plugged in and used for half a month did less probably almost 50 degrees of electricity! And the manufacturer's warranty is 5 years.	Jingdong prefecture level city in Shandong
Doraemon Hyakubo Shop	King of Power Saving	It's really effective. The previous month's electricity was $286, but after using the power saver for a month, I have $195 left this month, which is not bad.	Jingdong prefecture level city in Shandong
Doraemon Hyakubo Shop	King of Power Saving	I'll give you a good review and see how it goes.	Jingdong prefecture level city in Shandong
Doraemon Hyakubo Shop	King of Power Saving	Customer service is very professional and patient to guide the installation, the quality of workmanship is very fine. Durable, second repurchase. Really can save oh!	Jingdong prefecture level city in Shandong
Doraemon Hyakubo Shop	King of Power Saving	I've been using it for a month now and my electric bill is over $30 less than last month. I feel it's quite worth buying.	Jingdong prefecture level city in Shandong
Doraemon Hyakubo Shop	King of Power Saving	Small size, does not take up space. It's very good, it does work very effectively, the tool room has a lot of work with a lot of electricity. After installing it, it really reduces a lot of electricity bills. Before the day 30 degrees of electricity left stone, now work down really less a few degrees.	Jingdong prefecture level city in Shandong
Doraemon Hyakubo Shop	King of Power Saving	It does work for saving electricity, I've bought the kind from elsewhere before that was too simple not **all**. This time to buy this store, but also for the safety. Air conditioning is too much electricity, this use to save a lot of	Jingdong prefecture level city in Shandong
Doraemon Hyakubo Shop	King of Power Saving	Two air conditioners and a refrigerator at home, a month down the electricity bill is almost two hundred dollars, with this after the average savings of more than thirty dollars to spend less money to enjoy more, it's really very good!	Jingdong prefecture level city in Shandong
Doraemon Hyakubo Shop	King of Power Saving	Good. Very good.	Jingdong prefecture level city in Shandong
Doraemon Hyakubo Shop	King of Power Saving	Works great, easy to operate, great, very happy with it.	Jingdong prefecture level city in Shandong
Doraemon Hyakubo Shop	King of Power Saving	Received it and tried it out, it's great, great quality and worth having! Five stars praise!	Jingdong prefecture level city in Shandong
Doraemon Hyakubo Shop	King of Power Saving	Very trustworthy brand, quality is guaranteed, the price is still affordable, quite satisfied!	Jingdong prefecture level city in Shandong
Doraemon Hyakubo Shop	King of Power Saving	Use it before you speak.	Jingdong prefecture level city in Shandong
Doraemon Hyakubo Shop	King of Power Saving	The quality of workmanship is very good and the power saving effect is quite good, I regret not getting it earlier.	Jingdong prefecture level city in Shandong
Doraemon Hyakubo Shop	King of Power Saving	Merchants shipping speed, and customer service introduced the same very good, cost-effective	Jingdong prefecture level city in Shandong
Doraemon Hyakubo Shop	King of Power Saving	Feel a little effect Oh, where the power consumption where to plug, convenient and convenient	Jingdong prefecture level city in Shandong
Doraemon Hyakubo Shop	King of Power Saving	The quality of the imported saver is very good, practical and satisfactory. Cost-effective	Jingdong prefecture level city in Shandong
Doraemon Hyakubo Shop	King of Power Saving	Power Saver is especially good, good and inexpensive, very good	Jingdong prefecture level city in Shandong
Doraemon Hyakubo Shop	King of Power Saving	Fast shipping, awesome quality, affordable price, satisfied.	Jingdong prefecture level city in Shandong
Doraemon Hyakubo Shop	King of Power Saving	Energy saver received, the effect is consistent with the description, recommended to buy!	Jingdong prefecture level city in Shandong
Doraemon Hyakubo Shop	King of Power Saving	It's really good, the quality is great, I recommend it!	Jingdong prefecture level city in Shandong
Doraemon Hyakubo Shop	King of Power Saving	Works well. Inexpensive. Shipping was fast.	Jingdong prefecture level city in Shandong
Doraemon Hyakubo Shop	King of Power Saving	Logistics is fast, works well, energy saving is good	Jingdong prefecture level city in Shandong
Doraemon Hyakubo Shop	King of Power Saving	very happy	Jingdong prefecture level city in Shandong

Doraemon Hyakubo Shop	King of Power Saving	an excellent	Jingdong prefecture level city in Shandong
Doraemon Hyakubo Shop	King of Power Saving	It rained a couple days ago and I just went to get the item back today , not bad, hey I looked at the meter and that red display light takes several seconds to jump, did not make a mistake buying it. After a period of time to evaluate.	Jingdong prefecture level city in Shandong
Doraemon Hyakubo Shop	King of Power Saving	Used more than 10 days to check the power, there is indeed some decline, customer service said, long-term use will save more points!	Jingdong prefecture level city in Shandong
Doraemon Hyakubo Shop	King of Power Saving	It's a proper IQ tax, and it costs electricity.	Jingdong prefecture level city in Shandong
Doraemon Hyakubo Shop	King of Power Saving	I've been using it for 17 days now, it really saves electricity Shipping is fast, you can save a lot in a year	Jingdong prefecture level city in Shandong
Doraemon Hyakubo Shop	King of Power Saving	I've been using it for 17 days and it really saves power Shipping is fast and you don't have to worry about the quality, free returns within 5 years	Jingdong prefecture level city in Shandong
Doraemon Hyakubo Shop	King of Power Saving	It's more regular, scanned the code and it's authentic, and the main thing is that you can try it for 90 days! Plugged in more than ten days, is less than before a few degrees per day. When the time to use a month to see Additional. Good to buy a few more	Jingdong prefecture level city in Shandong
Doraemon Hyakubo Shop	King of Power Saving	Logistics is very fast, fine workmanship, use it and then come back to the comments	Jingdong prefecture level city in Shandong
Doraemon Hyakubo Shop	King of Power Saving	The quality of workmanship is very good and the power saving effect is quite good, I regret not getting it earlier.	Jingdong prefecture level city in Shandong
Doraemon Hyakubo Shop	King of Power Saving	Received the goods, according to the instructions on the operation is very simple, to wait and see the effect, looks good!	Jingdong prefecture level city in Shandong
Doraemon Hyakubo Shop	King of Power Saving	It's really as safe and power-saving as the owner says, and it works great!	Jingdong prefecture level city in Shandong
Doraemon Hyakubo Shop	King of Power Saving	Received the goods, immediately used, looks good, to wait and see how the effect.	Jingdong prefecture level city in Shandong
Doraemon Hyakubo Shop	King of Power Saving	Afraid to buy back did not work, the store said the commitment to 90 days invalid refund, warranty 5 years. Saver also complimentary on the Pacific insurance, it is a lot of peace of mind, there are effects will continue to buy back drops!	Jingdong prefecture level city in Shandong
Doraemon Hyakubo Shop	King of Power Saving	Received the goods and used it, can save electricity a month later to see the effect!	Jingdong prefecture level city in Shandong
Doraemon Hyakubo Shop	King of Power Saving	Fancy 90-day trial, 5-year warranty before the order, the goods received, has been experiencing a week, can really feel the savings, in the introduction of friends also buy this!	Jingdong prefecture level city in Shandong
Doraemon Hyakubo Shop	King of Power Saving	Clothing stores use, neighbors recommended to buy, said a month can save more than 70 yuan, first plugged in to see the effect of the	Jingdong prefecture level city in Shandong
Doraemon Hyakubo Shop	King of Power Saving	A friend's home two months to save more than 80 dollars of electricity, recommended that I buy two, decisive purchase, looking forward to the effect of power saving, waiting for the next month effect.	Jingdong prefecture level city in Shandong
Doraemon Hyakubo Shop	King of Power Saving	The goods have been received, consistent with the description of the advertisement, quite good, I hope that in terms of power saving and purifying the air is really as the merchant said that would be better!	Jingdong prefecture level city in Shandong
Doraemon Hyakubo Shop	King of Power Saving	Power Saving King is really good to use the quality is very good than the physical store a lot cheaper we can rest assured that the purchase recommended to buy!	Jingdong prefecture level city in Shandong
Doraemon Hyakubo Shop	King of Power Saving	Energy saver has been used on the rental house inside the use of the meter is always running fast, with this can really save electricity, easy to use, plugged in can be, very satisfied!	Jingdong prefecture level city in Shandong
Doraemon Hyakubo Shop	King of Power Saving	Just use can not see what effect to,, wait for a period of time in the evaluation	Jingdong prefecture level city in Shandong
Doraemon Hyakubo Shop	King of Power Saving	Live in the 18th floor is too far away from the meter, afraid of no effect, look at the trial 90 days before buying. Currently used for a month, there is an effect, saving 27 dollars in electricity, next month should be able to save more!	Jingdong prefecture level city in Shandong
Doraemon Hyakubo Shop	King of Power Saving	I've been using it for almost a month now and it feels good, so I'll use it again.	Jingdong prefecture level city in Shandong
Doraemon Hyakubo Shop	King of Power Saving	It's ok, the electric bill is really less, originally I remember charging $100 and using it for 10 days. Now it lasts about 15 days. It's still noticeable.	Jingdong prefecture level city in Shandong
Doraemon Hyakubo Shop	King of Power Saving	Fast logistics, good power saving effect, very satisfied	Jingdong prefecture level city in Shandong
Doraemon Hyakubo Shop	King of Power Saving	Has been the second time back to buy, energy saving effect, worth recommending, you can try to see, power saving effect: very good Experience: can save about 30%!	Jingdong prefecture level city in Shandong
Doraemon Hyakubo Shop	King of Power Saving	I don't know how well it works for now	Jingdong prefecture level city in Shandong
Doraemon Hyakubo Shop	King of Power Saving	Factory direct is really good, plugged in and used for half a month did less probably almost 50 degrees of electricity! And the manufacturer's warranty is 5 years.	Jingdong prefecture level city in Shandong
Doraemon Hyakubo Shop	King of Power Saving	It's really effective. The previous month's electricity was $286, but after using the power saver for a month, I have $195 left this month, which is not bad.	Jingdong prefecture level city in Shandong
Doraemon Hyakubo Shop	King of Power Saving	I'll give you a good review and see how it goes.	Jingdong prefecture level city in Shandong
Doraemon Hyakubo Shop	King of Power Saving	Customer service is very professional and patient to guide the installation, the quality of workmanship is very fine. Durable, second repurchase. Really can save oh!	Jingdong prefecture level city in Shandong
Doraemon Hyakubo Shop	King of Power Saving	I've been using it for a month now and my electric bill is over $30 less than last month. I feel it's quite worth buying.	Jingdong prefecture level city in Shandong
Doraemon Hyakubo Shop	King of Power Saving	Small size, does not take up space. It's very good, it does work very effectively, the tool room has a lot of work with a lot of electricity. After installing it, it really reduces a lot of electricity bills. Before the day 30 degrees of electricity left stone, now work down really less a few degrees.	Jingdong prefecture level city in Shandong
Doraemon Hyakubo Shop	King of Power Saving	It does work for saving electricity, I've bought the kind from elsewhere before that was too simple not **all**. This time to buy this store, but also for the safety. Air conditioning is too much electricity, this use to save a lot of	Jingdong prefecture level city in Shandong
Doraemon Hyakubo Shop	King of Power Saving	Two air conditioners and a refrigerator at home, a month down the electricity bill is almost two hundred dollars, with this after the average savings of more than thirty dollars to spend less money to enjoy more, it's really very good!	Jingdong prefecture level city in Shandong
Doraemon Hyakubo Shop	King of Power Saving	Good. Very good.	Jingdong prefecture level city in Shandong
Doraemon Hyakubo Shop	King of Power Saving	Works great, easy to operate, great, very happy with it.	Jingdong prefecture level city in Shandong
Doraemon Hyakubo Shop	King of Power Saving	Received it and tried it out, it's great, great quality and worth having! Five stars praise!	Jingdong prefecture level city in Shandong
Doraemon Hyakubo Shop	King of Power Saving	Very trustworthy brand, quality is guaranteed, the price is still affordable, quite satisfied!	Jingdong prefecture level city in Shandong
Doraemon Hyakubo Shop	King of Power Saving	Use it before you speak.	Jingdong prefecture level city in Shandong
Doraemon Hyakubo Shop	King of Power Saving	The quality of workmanship is very good and the power saving effect is quite good, I regret not getting it earlier.	Jingdong prefecture level city in Shandong
Doraemon Hyakubo Shop	King of Power Saving	Merchants shipping speed, and customer service introduced the same very good, cost-effective	Jingdong prefecture level city in Shandong
Doraemon Hyakubo Shop	King of Power Saving	Feel a little effect Oh, where the power consumption where to plug, convenient and convenient	Jingdong prefecture level city in Shandong
Doraemon Hyakubo Shop	King of Power Saving	The quality of the imported saver is very good, practical and satisfactory. Cost-effective	Jingdong prefecture level city in Shandong
Doraemon Hyakubo Shop	King of Power Saving	Power Saver is especially good, good and inexpensive, very good	Jingdong prefecture level city in Shandong
Doraemon Hyakubo Shop	King of Power Saving	Fast shipping, awesome quality, affordable price, satisfied.	Jingdong prefecture level city in Shandong
Doraemon Hyakubo Shop	King of Power Saving	Energy saver received, the effect is consistent with the description, recommended to buy!	Jingdong prefecture level city in Shandong
Doraemon Hyakubo Shop	King of Power Saving	It's really good, the quality is great, I recommend it!	Jingdong prefecture level city in Shandong

Doraemon Hyakubo Shop	King of Power Saving	Works well. Inexpensive. Shipping was fast.	Jingdong prefecture level city in Shandong
Doraemon Hyakubo Shop	King of Power Saving	Logistics is fast, works well, energy saving is good	Jingdong prefecture level city in Shandong
Doraemon Hyakubo Shop	King of Power Saving	very happy	Jingdong prefecture level city in Shandong
Doraemon Hyakubo Shop	King of Power Saving	an excellent	Jingdong prefecture level city in Shandong
Doraemon Hyakubo Shop	King of Power Saving	It rained a couple days ago and I just went to get the item back today , not bad, hey I looked at the meter and that red display light takes several seconds to jump, did not make a mistake buying it. After a period of time to evaluate.	Jingdong prefecture level city in Shandong
Doraemon Hyakubo Shop	King of Power Saving	Used more than 10 days to check the power, there is indeed some decline, customer service said, long-term use will save more points!	Jingdong prefecture level city in Shandong
Doraemon Hyakubo Shop	King of Power Saving	It's a proper IQ tax, and it costs electricity.	Jingdong prefecture level city in Shandong
Doraemon Hyakubo Shop	King of Power Saving	I've been using it for 17 days now, it really saves electricity Shipping is fast, you can save a lot in a year	Jingdong prefecture level city in Shandong
Doraemon Hyakubo Shop	King of Power Saving	I've been using it for 17 days and it really saves power Shipping is fast and you don't have to worry about the quality, free returns within 5 years	Jingdong prefecture level city in Shandong
Doraemon Hyakubo Shop	King of Power Saving	It's more regular, scanned the code and it's authentic, and the main thing is that you can try it for 90 days! Plugged in more than ten days, is less than before a few degrees per day. When the time to use a month to see Additional. Good to buy a few more	Jingdong prefecture level city in Shandong
Doraemon Hyakubo Shop	King of Power Saving	Logistics is very fast, fine workmanship, use it and then come back to the comments	Jingdong prefecture level city in Shandong
Doraemon Hyakubo Shop	King of Power Saving	The quality of workmanship is very good and the power saving effect is quite good, I regret not getting it earlier.	Jingdong prefecture level city in Shandong
Doraemon Hyakubo Shop	King of Power Saving	Received the goods, according to the instructions on the operation is very simple, to wait and see the effect, looks good!	Jingdong prefecture level city in Shandong
Doraemon Hyakubo Shop	King of Power Saving	It's really as safe and power-saving as the owner says, and it works great!	Jingdong prefecture level city in Shandong
Doraemon Hyakubo Shop	King of Power Saving	Received the goods, immediately used, looks good, to wait and see how the effect.	Jingdong prefecture level city in Shandong
Doraemon Hyakubo Shop	King of Power Saving	Afraid to buy back did not work, the store said the commitment to 90 days invalid refund, warranty 5 years. Saver also complimentary on the Pacific insurance, it is a lot of peace of mind, there are effects will continue to buy back drops!	Jingdong prefecture level city in Shandong
Doraemon Hyakubo Shop	King of Power Saving	Received the goods and used it, can save electricity a month later to see the effect!	Jingdong prefecture level city in Shandong
Doraemon Hyakubo Shop	King of Power Saving	Fancy 90-day trial, 5-year warranty before the order, the goods received, has been experiencing a week, can really feel the savings, in the introduction of friends also buy this!	Jingdong prefecture level city in Shandong
Doraemon Hyakubo Shop	King of Power Saving	Clothing stores use, neighbors recommended to buy, said a month can save more than 70 yuan, first plugged in to see the effect of the	Jingdong prefecture level city in Shandong
Doraemon Hyakubo Shop	King of Power Saving	A friend's home two months to save more than 80 dollars of electricity, recommended that I buy two, decisive purchase, looking forward to the effect of power saving, waiting for the next month effect.	Jingdong prefecture level city in Shandong
Doraemon Hyakubo Shop	King of Power Saving	The goods have been received, consistent with the description of the advertisement, quite good, I hope that in terms of power saving and purifying the air is really as the merchant said that would be better!	Jingdong prefecture level city in Shandong
Doraemon Hyakubo Shop	King of Power Saving	Power Saving King is really good to use the quality is very good than the physical store a lot cheaper we can rest assured that the purchase recommended to buy!	Jingdong prefecture level city in Shandong
Doraemon Hyakubo Shop	King of Power Saving	Energy saver has been used on the rental house inside the use of the meter is always running fast, with this can really save electricity, easy to use, plugged in can be, very satisfied!	Jingdong prefecture level city in Shandong
Doraemon Hyakubo Shop	King of Power Saving	Just use can not see what effect to,, wait for a period of time in the evaluation	Jingdong prefecture level city in Shandong
Doraemon Hyakubo Shop	King of Power Saving	Live in the 18th floor is too far away from the meter, afraid of no effect, look at the trial 90 days before buying. Currently used for a month, there is an effect, saving 27 dollars in electricity, next month should be able to save more!	Jingdong prefecture level city in Shandong
Doraemon Hyakubo Shop	King of Power Saving	I've been using it for almost a month now and it feels good, so I'll use it again.	Jingdong prefecture level city in Shandong
Doraemon Hyakubo Shop	King of Power Saving	It's ok, the electric bill is really less, originally I remember charging $100 and using it for 10 days. Now it lasts about 15 days. It's still noticeable.	Jingdong prefecture level city in Shandong
Doraemon Hyakubo Shop	King of Power Saving	Fast logistics, good power saving effect, very satisfied	Jingdong prefecture level city in Shandong
Doraemon Hyakubo Shop	King of Power Saving	Has been the second time back to buy, energy saving effect, worth recommending, you can try to see, power saving effect: very good Experience: can save about 30%!	Jingdong prefecture level city in Shandong
Doraemon Hyakubo Shop	King of Power Saving	I don't know how well it works for now	Jingdong prefecture level city in Shandong
Doraemon Hyakubo Shop	King of Power Saving	Factory direct is really good, plugged in and used for half a month did less probably almost 50 degrees of electricity! And the manufacturer's warranty is 5 years.	Jingdong prefecture level city in Shandong
Doraemon Hyakubo Shop	King of Power Saving	It's really effective. The previous month's electricity was $286, but after using the power saver for a month, I have $195 left this month, which is not bad.	Jingdong prefecture level city in Shandong
Doraemon Hyakubo Shop	King of Power Saving	I'll give you a good review and see how it goes.	Jingdong prefecture level city in Shandong
Doraemon Hyakubo Shop	King of Power Saving	Customer service is very professional and patient to guide the installation, the quality of workmanship is very fine. Durable, second repurchase. Really can save oh!	Jingdong prefecture level city in Shandong
Doraemon Hyakubo Shop	King of Power Saving	I've been using it for a month now and my electric bill is over $30 less than last month. I feel it's quite worth buying.	Jingdong prefecture level city in Shandong
Doraemon Hyakubo Shop	King of Power Saving	Small size, does not take up space. It's very good, it does work very effectively, the tool room has a lot of work with a lot of electricity. After installing it, it really reduces a lot of electricity bills. Before the day 30 degrees of electricity left stone, now work down really less a few degrees.	Jingdong prefecture level city in Shandong
Doraemon Hyakubo Shop	King of Power Saving	It does work for saving electricity, I've bought the kind from elsewhere before that was too simple not **all**. This time to buy this store, but also for the safety. Air conditioning is too much electricity, this use to save a lot of	Jingdong prefecture level city in Shandong
Doraemon Hyakubo Shop	King of Power Saving	Two air conditioners and a refrigerator at home, a month down the electricity bill is almost two hundred dollars, with this after the average savings of more than thirty dollars to spend less money to enjoy more, it's really very good!	Jingdong prefecture level city in Shandong
Doraemon Hyakubo Shop	King of Power Saving	Good. Very good.	Jingdong prefecture level city in Shandong
Doraemon Hyakubo Shop	King of Power Saving	Works great, easy to operate, great, very happy with it.	Jingdong prefecture level city in Shandong
Doraemon Hyakubo Shop	King of Power Saving	Received it and tried it out, it's great, great quality and worth having! Five stars praise!	Jingdong prefecture level city in Shandong
Doraemon Hyakubo Shop	King of Power Saving	Very trustworthy brand, quality is guaranteed, the price is still affordable, quite satisfied!	Jingdong prefecture level city in Shandong
Doraemon Hyakubo Shop	King of Power Saving	Use it before you speak.	Jingdong prefecture level city in Shandong
Doraemon Hyakubo Shop	King of Power Saving	The quality of workmanship is very good and the power saving effect is quite good, I regret not getting it earlier.	Jingdong prefecture level city in Shandong
Doraemon Hyakubo Shop	King of Power Saving	Merchants shipping speed, and customer service introduced the same very good, cost-effective	Jingdong prefecture level city in Shandong
Doraemon Hyakubo Shop	King of Power Saving	The quality of the imported saver is very good, practical and satisfactory. Cost-effective	Jingdong prefecture level city in Shandong
Doraemon Hyakubo Shop	King of Power Saving	Feel a little effect Oh, where the power consumption where to plug, convenient and convenient	Jingdong prefecture level city in Shandong
Doraemon Hyakubo Shop	King of Power Saving	Power Saver is especially good, good and inexpensive, very good	Jingdong prefecture level city in Shandong

Doraemon Hyakubo Shop	King of Power Saving	Fast shipping, awesome quality, affordable price, satisfied.	Jingdong prefecture level city in Shandong
Doraemon Hyakubo Shop	King of Power Saving	Energy saver received, the effect is consistent with the description, recommended to buy!	Jingdong prefecture level city in Shandong
Doraemon Hyakubo Shop	King of Power Saving	It's really good, the quality is great, I recommend it!	Jingdong prefecture level city in Shandong
Doraemon Hyakubo Shop	King of Power Saving	Works well. Inexpensive. Shipping was fast.	Jingdong prefecture level city in Shandong
Doraemon Hyakubo Shop	King of Power Saving	Logistics is fast, works well, energy saving is good	Jingdong prefecture level city in Shandong
Doraemon Hyakubo Shop	King of Power Saving	very happy	Jingdong prefecture level city in Shandong
Doraemon Hyakubo Shop	King of Power Saving	an excellent	Jingdong prefecture level city in Shandong
Doraemon Hyakubo Shop	King of Power Saving	It rained a couple days ago and I just went to get the item back today , not bad, hey I looked at the meter and that red display light takes several seconds to jump, did not make a mistake buying it. After a period of time to evaluate.	Jingdong prefecture level city in Shandong
Doraemon Hyakubo Shop	King of Power Saving	Used more than 10 days to check the power, there is indeed some decline, customer service said, long-term use will save more points!	Jingdong prefecture level city in Shandong
Doraemon Hyakubo Shop	King of Power Saving	It's a proper IQ tax, and it costs electricity.	Jingdong prefecture level city in Shandong
Doraemon Hyakubo Shop	King of Power Saving	I've been using it for 17 days now, it really saves electricity Shipping is fast, you can save a lot in a year	Jingdong prefecture level city in Shandong
Doraemon Hyakubo Shop	King of Power Saving	I've been using it for 17 days and it really saves power Shipping is fast and you don't have to worry about the quality, free returns within 5 years	Jingdong prefecture level city in Shandong
Doraemon Hyakubo Shop	King of Power Saving	It's more regular, scanned the code and it's authentic, and the main thing is that you can try it for 90 days! Plugged in more than ten days, is less than before a few degrees per day. When the time to use a month to see Additional. Good to buy a few more	Jingdong prefecture level city in Shandong
Doraemon Hyakubo Shop	King of Power Saving	Logistics is very fast, fine workmanship, use it and then come back to the comments	Jingdong prefecture level city in Shandong
Doraemon Hyakubo Shop	King of Power Saving	The quality of workmanship is very good and the power saving effect is quite good, I regret not getting it earlier.	Jingdong prefecture level city in Shandong
Doraemon Hyakubo Shop	King of Power Saving	Received the goods, according to the instructions on the operation is very simple, to wait and see the effect, looks good!	Jingdong prefecture level city in Shandong
Doraemon Hyakubo Shop	King of Power Saving	It's really as safe and power-saving as the owner says, and it works great!	Jingdong prefecture level city in Shandong
Doraemon Hyakubo Shop	King of Power Saving	Received the goods, immediately used, looks good, to wait and see how the effect.	Jingdong prefecture level city in Shandong
Doraemon Hyakubo Shop	King of Power Saving	Afraid to buy back did not work, the store said the commitment to 90 days invalid refund, warranty 5 years. Saver also complimentary on the Pacific insurance, it is a lot of peace of mind, there are effects will continue to buy back drops!	Jingdong prefecture level city in Shandong
Doraemon Hyakubo Shop	King of Power Saving	Received the goods and used it, can save electricity a month later to see the effect!	Jingdong prefecture level city in Shandong
Doraemon Hyakubo Shop	King of Power Saving	Fancy 90-day trial, 5-year warranty before the order, the goods received, has been experiencing a week, can really feel the savings, in the introduction of friends also buy this!	Jingdong prefecture level city in Shandong
Doraemon Hyakubo Shop	King of Power Saving	Clothing stores use, neighbors recommended to buy, said a month can save more than 70 yuan, first plugged in to see the effect of the	Jingdong prefecture level city in Shandong
Doraemon Hyakubo Shop	King of Power Saving	A friend's home two months to save more than 80 dollars of electricity, recommended that I buy two, decisive purchase, looking forward to the effect of power saving, waiting for the next month effect.	Jingdong prefecture level city in Shandong
Doraemon Hyakubo Shop	King of Power Saving	The goods have been received, consistent with the description of the advertisement, quite good, I hope that in terms of power saving and purifying the air is really as the merchant said that would be better!	Jingdong prefecture level city in Shandong
Doraemon Hyakubo Shop	King of Power Saving	Power Saving King is really good to use the quality is very good than the physical store a lot cheaper we can rest assured that the purchase recommended to buy!	Jingdong prefecture level city in Shandong
Doraemon Hyakubo Shop	King of Power Saving	Energy saver has been used on the rental house inside the use of the meter is always running fast, with this can really save electricity, easy to use, plugged in can be, very satisfied!	Jingdong prefecture level city in Shandong
Doraemon Hyakubo Shop	King of Power Saving	Just use can not see what effect to,, wait for a period of time in the evaluation	Jingdong prefecture level city in Shandong
Doraemon Hyakubo Shop	King of Power Saving	Live in the 18th floor is too far away from the meter, afraid of no effect, look at the trial 90 days before buying. Currently used for a month, there is an effect, saving 27 dollars in electricity, next month should be able to save more!	Jingdong prefecture level city in Shandong
Doraemon Hyakubo Shop	King of Power Saving	I've been using it for almost a month now and it feels good, so I'll use it again.	Jingdong prefecture level city in Shandong
Doraemon Hyakubo Shop	King of Power Saving	It's ok, the electric bill is really less, originally I remember charging $100 and using it for 10 days. Now it lasts about 15 days. It's still noticeable.	Jingdong prefecture level city in Shandong
Doraemon Hyakubo Shop	King of Power Saving	Fast logistics, good power saving effect, very satisfied	Jingdong prefecture level city in Shandong
Doraemon Hyakubo Shop	King of Power Saving	Has been the second time back to buy, energy saving effect, worth recommending, you can try to see, power saving effect: very good Experience: can save about 30%!	Jingdong prefecture level city in Shandong
Doraemon Hyakubo Shop	King of Power Saving	I don't know how well it works for now	Jingdong prefecture level city in Shandong
Doraemon Hyakubo Shop	King of Power Saving	Factory direct is really good, plugged in and used for half a month did less probably almost 50 degrees of electricity! And the manufacturer's warranty is 5 years.	Jingdong prefecture level city in Shandong
Doraemon Hyakubo Shop	King of Power Saving	It's really effective. The previous month's electricity was $286, but after using the power saver for a month, I have $195 left this month, which is not bad.	Jingdong prefecture level city in Shandong
Doraemon Hyakubo Shop	King of Power Saving	I'll give you a good review and see how it goes.	Jingdong prefecture level city in Shandong
Doraemon Hyakubo Shop	King of Power Saving	Customer service is very professional and patient to guide the installation, the quality of workmanship is very fine. Durable, second repurchase. Really can save oh!	Jingdong prefecture level city in Shandong
Doraemon Hyakubo Shop	King of Power Saving	I've been using it for a month now and my electric bill is over $30 less than last month. I feel it's quite worth buying.	Jingdong prefecture level city in Shandong
Doraemon Hyakubo Shop	King of Power Saving	Small size, does not take up space. It's very good, it does work very effectively, the tool room has a lot of work with a lot of electricity. After installing it, it really reduces a lot of electricity bills. Before the day 30 degrees of electricity left stone, now work down really less a few degrees.	Jingdong prefecture level city in Shandong
Doraemon Hyakubo Shop	King of Power Saving	It does work for saving electricity, I've bought the kind from elsewhere before that was too simple not **all**. This time to buy this store, but also for the safety. Air conditioning is too much electricity, this use to save a lot of	Jingdong prefecture level city in Shandong
Doraemon Hyakubo Shop	King of Power Saving	Two air conditioners and a refrigerator at home, a month down the electricity bill is almost two hundred dollars, with this after the average savings of more than thirty dollars to spend less money to enjoy more, it's really very good!	Jingdong prefecture level city in Shandong
Doraemon Hyakubo Shop	King of Power Saving	Good. Very good.	Jingdong prefecture level city in Shandong
Doraemon Hyakubo Shop	King of Power Saving	Works great, easy to operate, great, very happy with it.	Jingdong prefecture level city in Shandong
Doraemon Hyakubo Shop	King of Power Saving	Received it and tried it out, it's great, great quality and worth having! Five stars praise!	Jingdong prefecture level city in Shandong
Doraemon Hyakubo Shop	King of Power Saving	Very trustworthy brand, quality is guaranteed, the price is still affordable, quite satisfied!	Jingdong prefecture level city in Shandong
Doraemon Hyakubo Shop	King of Power Saving	Use it before you speak.	Jingdong prefecture level city in Shandong
Doraemon Hyakubo Shop	King of Power Saving	The quality of workmanship is very good and the power saving effect is quite good, I regret not getting it earlier.	Jingdong prefecture level city in Shandong
Doraemon Hyakubo Shop	King of Power Saving	Merchants shipping speed, and customer service introduced the same very good, cost-effective	Jingdong prefecture level city in Shandong

Doraemon Hyakubo Shop	King of Power Saving	Feel a little effect Oh, where the power consumption where to plug, convenient and convenient	Jingdong prefecture level city in Shandong
Doraemon Hyakubo Shop	King of Power Saving	The quality of the imported saver is very good, practical and satisfactory. Cost-effective	Jingdong prefecture level city in Shandong
Doraemon Hyakubo Shop	King of Power Saving	Power Saver is especially good, good and inexpensive, very good	Jingdong prefecture level city in Shandong
Doraemon Hyakubo Shop	King of Power Saving	Fast shipping, awesome quality, affordable price, satisfied.	Jingdong prefecture level city in Shandong
Doraemon Hyakubo Shop	King of Power Saving	Energy saver received, the effect is consistent with the description, recommended to buy!	Jingdong prefecture level city in Shandong
Doraemon Hyakubo Shop	King of Power Saving	It's really good, the quality is great, I recommend it!	Jingdong prefecture level city in Shandong
Doraemon Hyakubo Shop	King of Power Saving	Works well. Inexpensive. Shipping was fast.	Jingdong prefecture level city in Shandong
Doraemon Hyakubo Shop	King of Power Saving	Logistics is fast, works well, energy saving is good	Jingdong prefecture level city in Shandong
Doraemon Hyakubo Shop	King of Power Saving	very happy	Jingdong prefecture level city in Shandong
Doraemon Hyakubo Shop	King of Power Saving	an excellent	Jingdong prefecture level city in Shandong
Doraemon Hyakubo Shop	King of Power Saving	It rained a couple days ago and I just went to get the item back today , not bad, hey I looked at the meter and that red display light takes several seconds to jump, did not make a mistake buying it. After a period of time to evaluate.	Jingdong prefecture level city in Shandong
Doraemon Hyakubo Shop	King of Power Saving	Used more than 10 days to check the power, there is indeed some decline, customer service said, long-term use will save more points!	Jingdong prefecture level city in Shandong
Doraemon Hyakubo Shop	King of Power Saving	It's a proper IQ tax, and it costs electricity.	Jingdong prefecture level city in Shandong
Doraemon Hyakubo Shop	King of Power Saving	I've been using it for 17 days now, it really saves electricity Shipping is fast, you can save a lot in a year	Jingdong prefecture level city in Shandong
Doraemon Hyakubo Shop	King of Power Saving	I've been using it for 17 days and it really saves power Shipping is fast and you don't have to worry about the quality, free returns within 5 years	Jingdong prefecture level city in Shandong
Doraemon Hyakubo Shop	King of Power Saving	It's more regular, scanned the code and it's authentic, and the main thing is that you can try it for 90 days! Plugged in more than ten days, is less than before a few degrees per day. When the time to use a month to see Additional. Good to buy a few more	Jingdong prefecture level city in Shandong
Doraemon Hyakubo Shop	King of Power Saving	Logistics is very fast, fine workmanship, use it and then come back to the comments	Jingdong prefecture level city in Shandong
Doraemon Hyakubo Shop	King of Power Saving	The quality of workmanship is very good and the power saving effect is quite good, I regret not getting it earlier.	Jingdong prefecture level city in Shandong
Doraemon Hyakubo Shop	King of Power Saving	Received the goods, according to the instructions on the operation is very simple, to wait and see the effect, looks good!	Jingdong prefecture level city in Shandong
Doraemon Hyakubo Shop	King of Power Saving	It's really as safe and power-saving as the owner says, and it works great!	Jingdong prefecture level city in Shandong
Doraemon Hyakubo Shop	King of Power Saving	Received the goods, immediately used, looks good, to wait and see how the effect.	Jingdong prefecture level city in Shandong
Doraemon Hyakubo Shop	King of Power Saving	Afraid to buy back did not work, the store said the commitment to 90 days invalid refund, warranty 5 years. Saver also complimentary on the Pacific insurance, it is a lot of peace of mind, there are effects will continue to buy back drops!	Jingdong prefecture level city in Shandong
Doraemon Hyakubo Shop	King of Power Saving	Received the goods and used it, can save electricity a month later to see the effect!	Jingdong prefecture level city in Shandong
Doraemon Hyakubo Shop	King of Power Saving	Fancy 90-day trial, 5-year warranty before the order, the goods received, has been experiencing a week, can really feel the savings, in the introduction of friends also buy this!	Jingdong prefecture level city in Shandong
Doraemon Hyakubo Shop	King of Power Saving	Clothing stores use, neighbors recommended to buy, said a month can save more than 70 yuan, first plugged in to see the effect of the	Jingdong prefecture level city in Shandong
Doraemon Hyakubo Shop	King of Power Saving	A friend's home two months to save more than 80 dollars of electricity, recommended that I buy two, decisive purchase, looking forward to the effect of power saving, waiting for the next month effect.	Jingdong prefecture level city in Shandong
Doraemon Hyakubo Shop	King of Power Saving	The goods have been received, consistent with the description of the advertisement, quite good, I hope that in terms of power saving and purifying the air is really as the merchant said that would be better!	Jingdong prefecture level city in Shandong
Doraemon Hyakubo Shop	King of Power Saving	Power Saving King is really good to use the quality is very good than the physical store a lot cheaper we can rest assured that the purchase recommended to buy!	Jingdong prefecture level city in Shandong
Doraemon Hyakubo Shop	King of Power Saving	Energy saver has been used on the rental house inside the use of the meter is always running fast, with this can really save electricity, easy to use, plugged in can be, very satisfied!	Jingdong prefecture level city in Shandong
Doraemon Hyakubo Shop	King of Power Saving	Just use can not see what effect to,, wait for a period of time in the evaluation	Jingdong prefecture level city in Shandong
Doraemon Hyakubo Shop	King of Power Saving	Live in the 18th floor is too far away from the meter, afraid of no effect, look at the trial 90 days before buying. Currently used for a month, there is an effect, saving 27 dollars in electricity, next month should be able to save more!	Jingdong prefecture level city in Shandong
Doraemon Hyakubo Shop	King of Power Saving	I've been using it for almost a month now and it feels good, so I'll use it again.	Jingdong prefecture level city in Shandong
Doraemon Hyakubo Shop	King of Power Saving	It's ok, the electric bill is really less, originally I remember charging $100 and using it for 10 days. Now it lasts about 15 days. It's still noticeable.	Jingdong prefecture level city in Shandong
Doraemon Hyakubo Shop	King of Power Saving	Fast logistics, good power saving effect, very satisfied	Jingdong prefecture level city in Shandong
Doraemon Hyakubo Shop	King of Power Saving	Has been the second time back to buy, energy saving effect, worth recommending, you can try to see, power saving effect: very good Experience: can save about 30%!	Jingdong prefecture level city in Shandong
Doraemon Hyakubo Shop	King of Power Saving	I don't know how well it works for now	Jingdong prefecture level city in Shandong
Doraemon Hyakubo Shop	King of Power Saving	Factory direct is really good, plugged in and used for half a month did less probably almost 50 degrees of electricity! And the manufacturer's warranty is 5 years.	Jingdong prefecture level city in Shandong
Doraemon Hyakubo Shop	King of Power Saving	It's really effective. The previous month's electricity was $286, but after using the power saver for a month, I have $195 left this month, which is not bad.	Jingdong prefecture level city in Shandong
Doraemon Hyakubo Shop	King of Power Saving	I'll give you a good review and see how it goes.	Jingdong prefecture level city in Shandong
Doraemon Hyakubo Shop	King of Power Saving	Customer service is very professional and patient to guide the installation, the quality of workmanship is very fine. Durable, second repurchase. Really can save oh!	Jingdong prefecture level city in Shandong
Doraemon Hyakubo Shop	King of Power Saving	I've been using it for a month now and my electric bill is over $30 less than last month. I feel it's quite worth buying.	Jingdong prefecture level city in Shandong
Doraemon Hyakubo Shop	King of Power Saving	Small size, does not take up space. It's very good, it does work very effectively, the tool room has a lot of work with a lot of electricity. After installing it, it really reduces a lot of electricity bills. Before the day 30 degrees of electricity left stone, now work down really less a few degrees.	Jingdong prefecture level city in Shandong
Doraemon Hyakubo Shop	King of Power Saving	It does work for saving electricity, I've bought the kind from elsewhere before that was too simple not **all**. This time to buy this store, but also for the safety. Air conditioning is too much electricity, this use to save a lot of	Jingdong prefecture level city in Shandong
Doraemon Hyakubo Shop	King of Power Saving	Two air conditioners and a refrigerator at home, a month down the electricity bill is almost two hundred dollars, with this after the average savings of more than thirty dollars to spend less money to enjoy more, it's really very good!	Jingdong prefecture level city in Shandong
Doraemon Hyakubo Shop	King of Power Saving	Good. Very good.	Jingdong prefecture level city in Shandong
Doraemon Hyakubo Shop	King of Power Saving	Works great, easy to operate, great, very happy with it.	Jingdong prefecture level city in Shandong
Doraemon Hyakubo Shop	King of Power Saving	Received it and tried it out, it's great, great quality and worth having! Five stars praise!	Jingdong prefecture level city in Shandong
Doraemon Hyakubo Shop	King of Power Saving	Very trustworthy brand, quality is guaranteed, the price is still affordable, quite satisfied!	Jingdong prefecture level city in Shandong

Doraemon Hyakubo Shop	King of Power Saving	Use it before you speak.	Jingdong prefecture level city in Shandong
Doraemon Hyakubo Shop	King of Power Saving	The quality of workmanship is very good and the power saving effect is quite good, I regret not getting it earlier.	Jingdong prefecture level city in Shandong
Doraemon Hyakubo Shop	King of Power Saving	Merchants shipping speed, and customer service introduced the same very good, cost-effective	Jingdong prefecture level city in Shandong
Doraemon Hyakubo Shop	King of Power Saving	Feel a little effect Oh, where the power consumption where to plug, convenient and convenient	Jingdong prefecture level city in Shandong
Doraemon Hyakubo Shop	King of Power Saving	The quality of the imported saver is very good, practical and satisfactory. Cost-effective	Jingdong prefecture level city in Shandong
Doraemon Hyakubo Shop	King of Power Saving	Power Saver is especially good, good and inexpensive, very good	Jingdong prefecture level city in Shandong
Doraemon Hyakubo Shop	King of Power Saving	Fast shipping, awesome quality, affordable price, satisfied.	Jingdong prefecture level city in Shandong
Doraemon Hyakubo Shop	King of Power Saving	Energy saver received, the effect is consistent with the description, recommended to buy!	Jingdong prefecture level city in Shandong
Doraemon Hyakubo Shop	King of Power Saving	It's really good, the quality is great, I recommend it!	Jingdong prefecture level city in Shandong
Doraemon Hyakubo Shop	King of Power Saving	Works well. Inexpensive. Shipping was fast.	Jingdong prefecture level city in Shandong
Doraemon Hyakubo Shop	King of Power Saving	Logistics is fast, works well, energy saving is good	Jingdong prefecture level city in Shandong
Doraemon Hyakubo Shop	King of Power Saving	very happy	Jingdong prefecture level city in Shandong
Doraemon Hyakubo Shop	King of Power Saving	an excellent	Jingdong prefecture level city in Shandong
Doraemon Hyakubo Shop	King of Power Saving	It rained a couple days ago and I just went to get the item back today , not bad, hey I looked at the meter and that red display light takes several seconds to jump, did not make a mistake buying it. After a period of time to evaluate.	Jingdong prefecture level city in Shandong
Doraemon Hyakubo Shop	King of Power Saving	Used more than 10 days to check the power, there is indeed some decline, customer service said, long-term use will save more points!	Jingdong prefecture level city in Shandong
Doraemon Hyakubo Shop	King of Power Saving	It's a proper IQ tax, and it costs electricity.	Jingdong prefecture level city in Shandong
Doraemon Hyakubo Shop	King of Power Saving	I've been using it for 17 days now, it really saves electricity Shipping is fast, you can save a lot in a year	Jingdong prefecture level city in Shandong
Doraemon Hyakubo Shop	King of Power Saving	I've been using it for 17 days and it really saves power Shipping is fast and you don't have to worry about the quality, free returns within 5 years	Jingdong prefecture level city in Shandong
Doraemon Hyakubo Shop	King of Power Saving	It's more regular, scanned the code and it's authentic, and the main thing is that you can try it for 90 days! Plugged in more than ten days, is less than before a few degrees per day. When the time to use a month to see Additional. Good to buy a few more	Jingdong prefecture level city in Shandong
Doraemon Hyakubo Shop	King of Power Saving	Logistics is very fast, fine workmanship, use it and then come back to the comments	Jingdong prefecture level city in Shandong
Doraemon Hyakubo Shop	King of Power Saving	The quality of workmanship is very good and the power saving effect is quite good, I regret not getting it earlier.	Jingdong prefecture level city in Shandong
Doraemon Hyakubo Shop	King of Power Saving	Received the goods, according to the instructions on the operation is very simple, to wait and see the effect, looks good!	Jingdong prefecture level city in Shandong
Doraemon Hyakubo Shop	King of Power Saving	It's really as safe and power-saving as the owner says, and it works great!	Jingdong prefecture level city in Shandong
Doraemon Hyakubo Shop	King of Power Saving	Received the goods, immediately used, looks good, to wait and see how the effect.	Jingdong prefecture level city in Shandong
Doraemon Hyakubo Shop	King of Power Saving	Afraid to buy back did not work, the store said the commitment to 90 days invalid refund, warranty 5 years. Saver also complimentary on the Pacific insurance, it is a lot of peace of mind, there are effects will continue to buy back drops!	Jingdong prefecture level city in Shandong
Doraemon Hyakubo Shop	King of Power Saving	Received the goods and used it, can save electricity a month later to see the effect!	Jingdong prefecture level city in Shandong
Doraemon Hyakubo Shop	King of Power Saving	Fancy 90-day trial, 5-year warranty before the order, the goods received, has been experiencing a week, can really feel the savings, in the introduction of friends also buy this!	Jingdong prefecture level city in Shandong
Doraemon Hyakubo Shop	King of Power Saving	Clothing stores use, neighbors recommended to buy, said a month can save more than 70 yuan, first plugged in to see the effect of the	Jingdong prefecture level city in Shandong
Doraemon Hyakubo Shop	King of Power Saving	A friend's home two months to save more than 80 dollars of electricity, recommended that I buy two, decisive purchase, looking forward to the effect of power saving, waiting for the next month effect.	Jingdong prefecture level city in Shandong
Doraemon Hyakubo Shop	King of Power Saving	The goods have been received, consistent with the description of the advertisement, quite good, I hope that in terms of power saving and purifying the air is really as the merchant said that would be better!	Jingdong prefecture level city in Shandong
Doraemon Hyakubo Shop	King of Power Saving	Power Saving King is really good to use the quality is very good than the physical store a lot cheaper we can rest assured that the purchase recommended to buy!	Jingdong prefecture level city in Shandong
Doraemon Hyakubo Shop	King of Power Saving	Energy saver has been used on the rental house inside the use of the meter is always running fast, with this can really save electricity, easy to use, plugged in can be, very satisfied!	Jingdong prefecture level city in Shandong
Doraemon Hyakubo Shop	King of Power Saving	Just use can not see what effect to,, wait for a period of time in the evaluation	Jingdong prefecture level city in Shandong
Doraemon Hyakubo Shop	King of Power Saving	Live in the 18th floor is too far away from the meter, afraid of no effect, look at the trial 90 days before buying. Currently used for a month, there is an effect, saving 27 dollars in electricity, next month should be able to save more!	Jingdong prefecture level city in Shandong
Doraemon Hyakubo Shop	King of Power Saving	I've been using it for almost a month now and it feels good, so I'll use it again.	Jingdong prefecture level city in Shandong
Doraemon Hyakubo Shop	King of Power Saving	It's ok, the electric bill is really less, originally I remember charging $100 and using it for 10 days. Now it lasts about 15 days. It's still noticeable.	Jingdong prefecture level city in Shandong
Doraemon Hyakubo Shop	King of Power Saving	Fast logistics, good power saving effect, very satisfied	Jingdong prefecture level city in Shandong
Doraemon Hyakubo Shop	King of Power Saving	Has been the second time back to buy, energy saving effect, worth recommending, you can try to see, power saving effect: very good Experience: can save about 30%!	Jingdong prefecture level city in Shandong
Doraemon Hyakubo Shop	King of Power Saving	I don't know how well it works for now	Jingdong prefecture level city in Shandong
Doraemon Hyakubo Shop	King of Power Saving	Factory direct is really good, plugged in and used for half a month did less probably almost 50 degrees of electricity! And the manufacturer's warranty is 5 years.	Jingdong prefecture level city in Shandong
Doraemon Hyakubo Shop	King of Power Saving	It's really effective. The previous month's electricity was $286, but after using the power saver for a month, I have $195 left this month, which is not bad.	Jingdong prefecture level city in Shandong
Doraemon Hyakubo Shop	King of Power Saving	I'll give you a good review and see how it goes.	Jingdong prefecture level city in Shandong
Doraemon Hyakubo Shop	King of Power Saving	Customer service is very professional and patient to guide the installation, the quality of workmanship is very fine. Durable, second repurchase. Really can save oh!	Jingdong prefecture level city in Shandong
Doraemon Hyakubo Shop	King of Power Saving	I've been using it for a month now and my electric bill is over $30 less than last month. I feel it's quite worth buying.	Jingdong prefecture level city in Shandong
Doraemon Hyakubo Shop	King of Power Saving	Small size, does not take up space. It's very good, it does work very effectively, the tool room has a lot of work with a lot of electricity. After installing it, it really reduces a lot of electricity bills. Before the day 30 degrees of electricity left stone, now work down really less a few degrees.	Jingdong prefecture level city in Shandong
Doraemon Hyakubo Shop	King of Power Saving	It does work for saving electricity, I've bought the kind from elsewhere before that was too simple not **all**. This time to buy this store, but also for the safety. Air conditioning is too much electricity, this use to save a lot of	Jingdong prefecture level city in Shandong
Doraemon Hyakubo Shop	King of Power Saving	Two air conditioners and a refrigerator at home, a month down the electricity bill is almost two hundred dollars, with this after the average savings of more than thirty dollars to spend less money to enjoy more, it's really very good!	Jingdong prefecture level city in Shandong
Doraemon Hyakubo Shop	King of Power Saving	Good. Very good.	Jingdong prefecture level city in Shandong

Doraemon Hyakubo Shop	King of Power Saving	Works great, easy to operate, great, very happy with it.	Jingdong prefecture level city in Shandong
Doraemon Hyakubo Shop	King of Power Saving	Received it and tried it out, it's great, great quality and worth having! Five stars praise!	Jingdong prefecture level city in Shandong
Doraemon Hyakubo Shop	King of Power Saving	Very trustworthy brand, quality is guaranteed, the price is still affordable, quite satisfied!	Jingdong prefecture level city in Shandong
Doraemon Hyakubo Shop	King of Power Saving	Use it before you speak.	Jingdong prefecture level city in Shandong
Doraemon Hyakubo Shop	King of Power Saving	The quality of workmanship is very good and the power saving effect is quite good, I regret not getting it earlier.	Jingdong prefecture level city in Shandong
Doraemon Hyakubo Shop	King of Power Saving	Merchants shipping speed, and customer service introduced the same very good, cost-effective	Jingdong prefecture level city in Shandong
Doraemon Hyakubo Shop	King of Power Saving	Feel a little effect Oh, where the power consumption where to plug, convenient and convenient	Jingdong prefecture level city in Shandong
Doraemon Hyakubo Shop	King of Power Saving	The quality of the imported saver is very good, practical and satisfactory. Cost-effective	Jingdong prefecture level city in Shandong
Doraemon Hyakubo Shop	King of Power Saving	Power Saver is especially good, good and inexpensive, very good	Jingdong prefecture level city in Shandong
Doraemon Hyakubo Shop	King of Power Saving	Fast shipping, awesome quality, affordable price, satisfied.	Jingdong prefecture level city in Shandong
Doraemon Hyakubo Shop	King of Power Saving	Energy saver received, the effect is consistent with the description, recommended to buy!	Jingdong prefecture level city in Shandong
Doraemon Hyakubo Shop	King of Power Saving	It's really good, the quality is great, I recommend it!	Jingdong prefecture level city in Shandong
Doraemon Hyakubo Shop	King of Power Saving	Works well. Inexpensive. Shipping was fast.	Jingdong prefecture level city in Shandong
Doraemon Hyakubo Shop	King of Power Saving	Logistics is fast, works well, energy saving is good	Jingdong prefecture level city in Shandong
Doraemon Hyakubo Shop	King of Power Saving	very happy	Jingdong prefecture level city in Shandong
Doraemon Hyakubo Shop	King of Power Saving	an excellent	Jingdong prefecture level city in Shandong
Doraemon Hyakubo Shop	King of Power Saving	It rained a couple days ago and I just went to get the item back today , not bad, hey I looked at the meter and that red display light takes several seconds to jump, did not make a mistake buying it. After a period of time to evaluate.	Jingdong prefecture level city in Shandong
Doraemon Hyakubo Shop	King of Power Saving	Used more than 10 days to check the power, there is indeed some decline, customer service said, long-term use will save more points!	Jingdong prefecture level city in Shandong
Doraemon Hyakubo Shop	King of Power Saving	It's a proper IQ tax, and it costs electricity.	Jingdong prefecture level city in Shandong
Doraemon Hyakubo Shop	King of Power Saving	I've been using it for 17 days now, it really saves electricity Shipping is fast, you can save a lot in a year	Jingdong prefecture level city in Shandong
Doraemon Hyakubo Shop	King of Power Saving	I've been using it for 17 days and it really saves power Shipping is fast and you don't have to worry about the quality, free returns within 5 years	Jingdong prefecture level city in Shandong
Doraemon Hyakubo Shop	King of Power Saving	It's more regular, scanned the code and it's authentic, and the main thing is that you can try it for 90 days! Plugged in more than ten days, is less than before a few degrees per day. When the time to use a month to see Additional. Good to buy a few more	Jingdong prefecture level city in Shandong
Doraemon Hyakubo Shop	King of Power Saving	Logistics is very fast, fine workmanship, use it and then come back to the comments	Jingdong prefecture level city in Shandong
Doraemon Hyakubo Shop	King of Power Saving	The quality of workmanship is very good and the power saving effect is quite good, I regret not getting it earlier.	Jingdong prefecture level city in Shandong
Doraemon Hyakubo Shop	King of Power Saving	Received the goods, according to the instructions on the operation is very simple, to wait and see the effect, looks good!	Jingdong prefecture level city in Shandong
Doraemon Hyakubo Shop	King of Power Saving	It's really as safe and power-saving as the owner says, and it works great!	Jingdong prefecture level city in Shandong
Doraemon Hyakubo Shop	King of Power Saving	Received the goods, immediately used, looks good, to wait and see how the effect.	Jingdong prefecture level city in Shandong
Doraemon Hyakubo Shop	King of Power Saving	Afraid to buy back did not work, the store said the commitment to 90 days invalid refund, warranty 5 years. Saver also complimentary on the Pacific insurance, it is a lot of peace of mind, there are effects will continue to buy back drops!	Jingdong prefecture level city in Shandong
Doraemon Hyakubo Shop	King of Power Saving	Received the goods and used it, can save electricity a month later to see the effect!	Jingdong prefecture level city in Shandong
Doraemon Hyakubo Shop	King of Power Saving	Fancy 90-day trial, 5-year warranty before the order, the goods received, has been experiencing a week, can really feel the savings, in the introduction of friends also buy this!	Jingdong prefecture level city in Shandong
Doraemon Hyakubo Shop	King of Power Saving	Clothing stores use, neighbors recommended to buy, said a month can save more than 70 yuan, first plugged in to see the effect of the	Jingdong prefecture level city in Shandong
Doraemon Hyakubo Shop	King of Power Saving	A friend's home two months to save more than 80 dollars of electricity, recommended that I buy two, decisive purchase, looking forward to the effect of power saving, waiting for the next month effect.	Jingdong prefecture level city in Shandong
Doraemon Hyakubo Shop	King of Power Saving	The goods have been received, consistent with the description of the advertisement, quite good, I hope that in terms of power saving and purifying the air is really as the merchant said that would be better!	Jingdong prefecture level city in Shandong
Doraemon Hyakubo Shop	King of Power Saving	Power Saving King is really good to use the quality is very good than the physical store a lot cheaper we can rest assured that the purchase recommended to buy!	Jingdong prefecture level city in Shandong
Doraemon Hyakubo Shop	King of Power Saving	Energy saver has been used on the rental house inside the use of the meter is always running fast, with this can really save electricity, easy to use, plugged in can be, very satisfied!	Jingdong prefecture level city in Shandong
Doraemon Hyakubo Shop	King of Power Saving	Just use can not see what effect to,, wait for a period of time in the evaluation	Jingdong prefecture level city in Shandong
Doraemon Hyakubo Shop	King of Power Saving	Live in the 18th floor is too far away from the meter, afraid of no effect, look at the trial 90 days before buying. Currently used for a month, there is an effect, saving 27 dollars in electricity, next month should be able to save more!	Jingdong prefecture level city in Shandong
Doraemon Hyakubo Shop	King of Power Saving	I've been using it for almost a month now and it feels good, so I'll use it again.	Jingdong prefecture level city in Shandong
Doraemon Hyakubo Shop	King of Power Saving	It's ok, the electric bill is really less, originally I remember charging $100 and using it for 10 days. Now it lasts about 15 days. It's still noticeable.	Jingdong prefecture level city in Shandong
Doraemon Hyakubo Shop	King of Power Saving	Fast logistics, good power saving effect, very satisfied	Jingdong prefecture level city in Shandong
Doraemon Hyakubo Shop	King of Power Saving	Has been the second time back to buy, energy saving effect, worth recommending, you can try to see, power saving effect: very good Experience: can save about 30%!	Jingdong prefecture level city in Shandong
Doraemon Hyakubo Shop	King of Power Saving	I don't know how well it works for now	Jingdong prefecture level city in Shandong
Doraemon Hyakubo Shop	King of Power Saving	Factory direct is really good, plugged in and used for half a month did less probably almost 50 degrees of electricity! And the manufacturer's warranty is 5 years.	Jingdong prefecture level city in Shandong
Doraemon Hyakubo Shop	King of Power Saving	It's really effective. The previous month's electricity was $286, but after using the power saver for a month, I have $195 left this month, which is not bad.	Jingdong prefecture level city in Shandong
Doraemon Hyakubo Shop	King of Power Saving	I'll give you a good review and see how it goes.	Jingdong prefecture level city in Shandong
Doraemon Hyakubo Shop	King of Power Saving	Customer service is very professional and patient to guide the installation, the quality of workmanship is very fine. Durable, second repurchase. Really can save oh!	Jingdong prefecture level city in Shandong
Doraemon Hyakubo Shop	King of Power Saving	I've been using it for a month now and my electric bill is over $30 less than last month. I feel it's quite worth buying.	Jingdong prefecture level city in Shandong
Doraemon Hyakubo Shop	King of Power Saving	Small size, does not take up space. It's very good, it does work very effectively, the tool room has a lot of work with a lot of electricity. After installing it, it really reduces a lot of electricity bills. Before the day 30 degrees of electricity left stone, now work down really less a few degrees.	Jingdong prefecture level city in Shandong

Doraemon Hyakubo Shop	King of Power Saving	It does work for saving electricity, I've bought the kind from elsewhere before that was too simple not **all**. This time to buy this store, but also for the safety. Air conditioning is too much electricity, this use to save a lot of	Jingdong prefecture level city in Shandong
Doraemon Hyakubo Shop	King of Power Saving	Two air conditioners and a refrigerator at home, a month down the electricity bill is almost two hundred dollars, with this after the average savings of more than thirty dollars to spend less money to enjoy more, it's really very good!	Jingdong prefecture level city in Shandong
Doraemon Hyakubo Shop	King of Power Saving	Good. Very good.	Jingdong prefecture level city in Shandong
Doraemon Hyakubo Shop	King of Power Saving	Works great, easy to operate, great, very happy with it.	Jingdong prefecture level city in Shandong
Doraemon Hyakubo Shop	King of Power Saving	Received it and tried it out, it's great, great quality and worth having! Five stars praise!	Jingdong prefecture level city in Shandong
Doraemon Hyakubo Shop	King of Power Saving	Very trustworthy brand, quality is guaranteed, the price is still affordable, quite satisfied!	Jingdong prefecture level city in Shandong
Doraemon Hyakubo Shop	King of Power Saving	Use it before you speak.	Jingdong prefecture level city in Shandong
Doraemon Hyakubo Shop	King of Power Saving	The quality of workmanship is very good and the power saving effect is quite good, I regret not getting it earlier.	Jingdong prefecture level city in Shandong
Doraemon Hyakubo Shop	King of Power Saving	Merchants shipping speed, and customer service introduced the same very good, cost-effective	Jingdong prefecture level city in Shandong
Doraemon Hyakubo Shop	King of Power Saving	Feel a little effect Oh, where the power consumption where to plug, convenient and convenient	Jingdong prefecture level city in Shandong
Doraemon Hyakubo Shop	King of Power Saving	The quality of the imported saver is very good, practical and satisfactory. Cost-effective	Jingdong prefecture level city in Shandong
Doraemon Hyakubo Shop	King of Power Saving	Power Saver is especially good, good and inexpensive, very good	Jingdong prefecture level city in Shandong
Doraemon Hyakubo Shop	King of Power Saving	Fast shipping, awesome quality, affordable price, satisfied.	Jingdong prefecture level city in Shandong
Doraemon Hyakubo Shop	King of Power Saving	Energy saver received, the effect is consistent with the description, recommended to buy!	Jingdong prefecture level city in Shandong
Doraemon Hyakubo Shop	King of Power Saving	It's really good, the quality is great, I recommend it!	Jingdong prefecture level city in Shandong
Doraemon Hyakubo Shop	King of Power Saving	Works well. Inexpensive. Shipping was fast.	Jingdong prefecture level city in Shandong
Doraemon Hyakubo Shop	King of Power Saving	Logistics is fast, works well, energy saving is good	Jingdong prefecture level city in Shandong
Doraemon Hyakubo Shop	King of Power Saving	very happy	Jingdong prefecture level city in Shandong
Doraemon Hyakubo Shop	King of Power Saving	an excellent	Jingdong prefecture level city in Shandong
Doraemon Hyakubo Shop	King of Power Saving	It rained a couple days ago and I just went to get the item back today , not bad, hey I looked at the meter and that red display light takes several seconds to jump, did not make a mistake buying it. After a period of time to evaluate.	Jingdong prefecture level city in Shandong
Doraemon Hyakubo Shop	King of Power Saving	Used more than 10 days to check the power, there is indeed some decline, customer service said, long-term use will save more points!	Jingdong prefecture level city in Shandong
Doraemon Hyakubo Shop	King of Power Saving	It's a proper IQ tax, and it costs electricity.	Jingdong prefecture level city in Shandong
Doraemon Hyakubo Shop	King of Power Saving	I've been using it for 17 days now, it really saves electricity Shipping is fast, you can save a lot in a year	Jingdong prefecture level city in Shandong
Doraemon Hyakubo Shop	King of Power Saving	I've been using it for 17 days and it really saves power Shipping is fast and you don't have to worry about the quality, free returns within 5 years	Jingdong prefecture level city in Shandong
Doraemon Hyakubo Shop	King of Power Saving	It's more regular, scanned the code and it's authentic, and the main thing is that you can try it for 90 days! Plugged in more than ten days, is less than before a few degrees per day. When the time to use a month to see Additional. Good to buy a few more	Jingdong prefecture level city in Shandong
Doraemon Hyakubo Shop	King of Power Saving	Logistics is very fast, fine workmanship, use it and then come back to the comments	Jingdong prefecture level city in Shandong
Doraemon Hyakubo Shop	King of Power Saving	The quality of workmanship is very good and the power saving effect is quite good, I regret not getting it earlier.	Jingdong prefecture level city in Shandong
Doraemon Hyakubo Shop	King of Power Saving	Received the goods, according to the instructions on the operation is very simple, to wait and see the effect, looks good!	Jingdong prefecture level city in Shandong
Doraemon Hyakubo Shop	King of Power Saving	It's really as safe and power-saving as the owner says, and it works great!	Jingdong prefecture level city in Shandong
Doraemon Hyakubo Shop	King of Power Saving	Received the goods, immediately used, looks good, to wait and see how the effect.	Jingdong prefecture level city in Shandong
Doraemon Hyakubo Shop	King of Power Saving	Afraid to buy back did not work, the store said the commitment to 90 days invalid refund, warranty 5 years. Saver also complimentary on the Pacific insurance, it is a lot of peace of mind, there are effects will continue to buy back drops!	Jingdong prefecture level city in Shandong
Doraemon Hyakubo Shop	King of Power Saving	Received the goods and used it, can save electricity a month later to see the effect!	Jingdong prefecture level city in Shandong
Doraemon Hyakubo Shop	King of Power Saving	Fancy 90-day trial, 5-year warranty before the order, the goods received, has been experiencing a week, can really feel the savings, in the introduction of friends also buy this!	Jingdong prefecture level city in Shandong
Doraemon Hyakubo Shop	King of Power Saving	Clothing stores use, neighbors recommended to buy, said a month can save more than 70 yuan, first plugged in to see the effect of the	Jingdong prefecture level city in Shandong
Doraemon Hyakubo Shop	King of Power Saving	A friend's home two months to save more than 80 dollars of electricity, recommended that I buy two, decisive purchase, looking forward to the effect of power saving, waiting for the next month effect.	Jingdong prefecture level city in Shandong
Doraemon Hyakubo Shop	King of Power Saving	The goods have been received, consistent with the description of the advertisement, quite good, I hope that in terms of power saving and purifying the air is really as the merchant said that would be better!	Jingdong prefecture level city in Shandong
Doraemon Hyakubo Shop	King of Power Saving	Power Saving King is really good to use the quality is very good than the physical store a lot cheaper we can rest assured that the purchase recommended to buy!	Jingdong prefecture level city in Shandong
Doraemon Hyakubo Shop	King of Power Saving	Energy saver has been used on the rental house inside the use of the meter is always running fast, with this can really save electricity, easy to use, plugged in can be, very satisfied!	Jingdong prefecture level city in Shandong
Doraemon Hyakubo Shop	King of Power Saving	Just use can not see what effect to,, wait for a period of time in the evaluation	Jingdong prefecture level city in Shandong
Doraemon Hyakubo Shop	King of Power Saving	Live in the 18th floor is too far away from the meter, afraid of no effect, look at the trial 90 days before buying. Currently used for a month, there is an effect, saving 27 dollars in electricity, next month should be able to save more!	Jingdong prefecture level city in Shandong
Doraemon Hyakubo Shop	King of Power Saving	I've been using it for almost a month now and it feels good, so I'll use it again.	Jingdong prefecture level city in Shandong
Doraemon Hyakubo Shop	King of Power Saving	It's ok, the electric bill is really less, originally I remember charging $100 and using it for 10 days. Now it lasts about 15 days. It's still noticeable.	Jingdong prefecture level city in Shandong
Doraemon Hyakubo Shop	King of Power Saving	Fast logistics, good power saving effect, very satisfied	Jingdong prefecture level city in Shandong
Doraemon Hyakubo Shop	King of Power Saving	Has been the second time back to buy, energy saving effect, worth recommending, you can try to see, power saving effect: very good Experience: can save about 30%!	Jingdong prefecture level city in Shandong
Doraemon Hyakubo Shop	King of Power Saving	I don't know how well it works for now	Jingdong prefecture level city in Shandong
Doraemon Hyakubo Shop	King of Power Saving	Factory direct is really good, plugged in and used for half a month did less probably almost 50 degrees of electricity! And the manufacturer's warranty is 5 years.	Jingdong prefecture level city in Shandong
Doraemon Hyakubo Shop	King of Power Saving	It's really effective. The previous month's electricity was $286, but after using the power saver for a month, I have $195 left this month, which is not bad.	Jingdong prefecture level city in Shandong
Doraemon Hyakubo Shop	King of Power Saving	I'll give you a good review and see how it goes.	Jingdong prefecture level city in Shandong

Doraemon Hyakubo Shop	King of Power Saving	Customer service is very professional and patient to guide the installation, the quality of workmanship is very fine. Durable, second repurchase. Really can save oh!	Jingdong prefecture level city in Shandong
Doraemon Hyakubo Shop	King of Power Saving	I've been using it for a month now and my electric bill is over $30 less than last month. I feel it's quite worth buying.	Jingdong prefecture level city in Shandong
Doraemon Hyakubo Shop	King of Power Saving	Small size, does not take up space. It's very good, it does work very effectively, the tool room has a lot of work with a lot of electricity. After installing it, it really reduces a lot of electricity bills. Before the day 30 degrees of electricity left stone, now work down really less a few degrees.	Jingdong prefecture level city in Shandong
Doraemon Hyakubo Shop	King of Power Saving	It does work for saving electricity, I've bought the kind from elsewhere before that was too simple not **all**. This time to buy this store, but also for the safety. Air conditioning is too much electricity, this use to save a lot of	Jingdong prefecture level city in Shandong
Doraemon Hyakubo Shop	King of Power Saving	Two air conditioners and a refrigerator at home, a month down the electricity bill is almost two hundred dollars, with this after the average savings of more than thirty dollars to spend less money to enjoy more, it's really very good!	Jingdong prefecture level city in Shandong
Doraemon Hyakubo Shop	King of Power Saving	Good. Very good.	Jingdong prefecture level city in Shandong
Doraemon Hyakubo Shop	King of Power Saving	Works great, easy to operate, great, very happy with it.	Jingdong prefecture level city in Shandong
Doraemon Hyakubo Shop	King of Power Saving	Received it and tried it out, it's great, great quality and worth having! Five stars praise!	Jingdong prefecture level city in Shandong
Doraemon Hyakubo Shop	King of Power Saving	Very trustworthy brand, quality is guaranteed, the price is still affordable, quite satisfied!	Jingdong prefecture level city in Shandong
Doraemon Hyakubo Shop	King of Power Saving	Use it before you speak.	Jingdong prefecture level city in Shandong
Doraemon Hyakubo Shop	King of Power Saving	The quality of workmanship is very good and the power saving effect is quite good, I regret not getting it earlier.	Jingdong prefecture level city in Shandong
Doraemon Hyakubo Shop	King of Power Saving	Merchants shipping speed, and customer service introduced the same very good, cost-effective	Jingdong prefecture level city in Shandong
Doraemon Hyakubo Shop	King of Power Saving	Feel a little effect Oh, where the power consumption where to plug, convenient and convenient	Jingdong prefecture level city in Shandong
Doraemon Hyakubo Shop	King of Power Saving	The quality of the imported saver is very good, practical and satisfactory. Cost-effective	Jingdong prefecture level city in Shandong
Doraemon Hyakubo Shop	King of Power Saving	Power Saver is especially good, good and inexpensive, very good	Jingdong prefecture level city in Shandong
Doraemon Hyakubo Shop	King of Power Saving	Fast shipping, awesome quality, affordable price, satisfied.	Jingdong prefecture level city in Shandong
Doraemon Hyakubo Shop	King of Power Saving	Energy saver received, the effect is consistent with the description, recommended to buy!	Jingdong prefecture level city in Shandong
Doraemon Hyakubo Shop	King of Power Saving	It's really good, the quality is great, I recommend it!	Jingdong prefecture level city in Shandong
Doraemon Hyakubo Shop	King of Power Saving	Works well. Inexpensive. Shipping was fast.	Jingdong prefecture level city in Shandong
Doraemon Hyakubo Shop	King of Power Saving	Logistics is fast, works well, energy saving is good	Jingdong prefecture level city in Shandong
Doraemon Hyakubo Shop	King of Power Saving	very happy	Jingdong prefecture level city in Shandong
Doraemon Hyakubo Shop	King of Power Saving	an excellent	Jingdong prefecture level city in Shandong
Doraemon Hyakubo Shop	King of Power Saving	It rained a couple days ago and I just went to get the item back today , not bad, hey I looked at the meter and that red display light takes several seconds to jump, did not make a mistake buying it. After a period of time to evaluate.	Jingdong prefecture level city in Shandong
Doraemon Hyakubo Shop	King of Power Saving	Used more than 10 days to check the power, there is indeed some decline, customer service said, long-term use will save more points!	Jingdong prefecture level city in Shandong
Doraemon Hyakubo Shop	King of Power Saving	It's a proper IQ tax, and it costs electricity.	Jingdong prefecture level city in Shandong
Doraemon Hyakubo Shop	King of Power Saving	I've been using it for 17 days now, it really saves electricity Shipping is fast, you can save a lot in a year	Jingdong prefecture level city in Shandong
Doraemon Hyakubo Shop	King of Power Saving	I've been using it for 17 days and it really saves power Shipping is fast and you don't have to worry about the quality, free returns within 5 years	Jingdong prefecture level city in Shandong
Doraemon Hyakubo Shop	King of Power Saving	It's more regular, scanned the code and it's authentic, and the main thing is that you can try it for 90 days! Plugged in more than ten days, is less than before a few degrees per day. When the time to use a month to see Additional. Good to buy a few more	Jingdong prefecture level city in Shandong
Doraemon Hyakubo Shop	King of Power Saving	Logistics is very fast, fine workmanship, use it and then come back to the comments	Jingdong prefecture level city in Shandong
Doraemon Hyakubo Shop	King of Power Saving	The quality of workmanship is very good and the power saving effect is quite good, I regret not getting it earlier.	Jingdong prefecture level city in Shandong
Doraemon Hyakubo Shop	King of Power Saving	Received the goods, according to the instructions on the operation is very simple, to wait and see the effect, looks good!	Jingdong prefecture level city in Shandong
Doraemon Hyakubo Shop	King of Power Saving	It's really as safe and power-saving as the owner says, and it works great!	Jingdong prefecture level city in Shandong
Doraemon Hyakubo Shop	King of Power Saving	Received the goods, immediately used, looks good, to wait and see how the effect.	Jingdong prefecture level city in Shandong
Doraemon Hyakubo Shop	King of Power Saving	Afraid to buy back did not work, the store said the commitment to 90 days invalid refund, warranty 5 years. Saver also complimentary on the Pacific insurance, it is a lot of peace of mind, there are effects will continue to buy back drops!	Jingdong prefecture level city in Shandong
Doraemon Hyakubo Shop	King of Power Saving	Received the goods and used it, can save electricity a month later to see the effect!	Jingdong prefecture level city in Shandong
Doraemon Hyakubo Shop	King of Power Saving	Fancy 90-day trial, 5-year warranty before the order, the goods received, has been experiencing a week, can really feel the savings, in the introduction of friends also buy this!	Jingdong prefecture level city in Shandong
Doraemon Hyakubo Shop	King of Power Saving	Clothing stores use, neighbors recommended to buy, said a month can save more than 70 yuan, first plugged in to see the effect of the	Jingdong prefecture level city in Shandong
Doraemon Hyakubo Shop	King of Power Saving	A friend's home two months to save more than 80 dollars of electricity, recommended that I buy two, decisive purchase, looking forward to the effect of power saving, waiting for the next month effect.	Jingdong prefecture level city in Shandong
Doraemon Hyakubo Shop	King of Power Saving	The goods have been received, consistent with the description of the advertisement, quite good, I hope that in terms of power saving and purifying the air is really as the merchant said that would be better!	Jingdong prefecture level city in Shandong
Doraemon Hyakubo Shop	King of Power Saving	Power Saving King is really good to use the quality is very good than the physical store a lot cheaper we can rest assured that the purchase recommended to buy!	Jingdong prefecture level city in Shandong
Doraemon Hyakubo Shop	King of Power Saving	Energy saver has been used on the rental house inside the use of the meter is always running fast, with this can really save electricity, easy to use, plugged in can be, very satisfied!	Jingdong prefecture level city in Shandong
Doraemon Hyakubo Shop	King of Power Saving	Just use can not see what effect to,, wait for a period of time in the evaluation	Jingdong prefecture level city in Shandong
Doraemon Hyakubo Shop	King of Power Saving	Live in the 18th floor is too far away from the meter, afraid of no effect, look at the trial 90 days before buying. Currently used for a month, there is an effect, saving 27 dollars in electricity, next month should be able to save more!	Jingdong prefecture level city in Shandong
Doraemon Hyakubo Shop	King of Power Saving	I've been using it for almost a month now and it feels good, so I'll use it again.	Jingdong prefecture level city in Shandong
Doraemon Hyakubo Shop	King of Power Saving	It's ok, the electric bill is really less, originally I remember charging $100 and using it for 10 days. Now it lasts about 15 days. It's still noticeable.	Jingdong prefecture level city in Shandong
Doraemon Hyakubo Shop	King of Power Saving	Fast logistics, good power saving effect, very satisfied	Jingdong prefecture level city in Shandong
Doraemon Hyakubo Shop	King of Power Saving	Has been the second time back to buy, energy saving effect, worth recommending, you can try to see, power saving effect: very good Experience: can save about 30%!	Jingdong prefecture level city in Shandong
Doraemon Hyakubo Shop	King of Power Saving	I don't know how well it works for now	Jingdong prefecture level city in Shandong

Doraemon Hyakubo Shop	King of Power Saving	Factory direct is really good, plugged in and used for half a month did less probably almost 50 degrees of electricity! And the manufacturer's warranty is 5 years.	Jingdong prefecture level city in Shandong
Doraemon Hyakubo Shop	King of Power Saving	It's really effective. The previous month's electricity was $286, but after using the power saver for a month, I have $195 left this month, which is not bad.	Jingdong prefecture level city in Shandong
Doraemon Hyakubo Shop	King of Power Saving	I'll give you a good review and see how it goes.	Jingdong prefecture level city in Shandong
Doraemon Hyakubo Shop	King of Power Saving	Customer service is very professional and patient to guide the installation, the quality of workmanship is very fine. Durable, second repurchase. Really can save oh!	Jingdong prefecture level city in Shandong
Doraemon Hyakubo Shop	King of Power Saving	I've been using it for a month now and my electric bill is over $30 less than last month. I feel it's quite worth buying.	Jingdong prefecture level city in Shandong
Doraemon Hyakubo Shop	King of Power Saving	Small size, does not take up space. It's very good, it does work very effectively, the tool room has a lot of work with a lot of electricity. After installing it, it really reduces a lot of electricity bills. Before the day 30 degrees of electricity left stone, now work down really less a few degrees.	Jingdong prefecture level city in Shandong
Doraemon Hyakubo Shop	King of Power Saving	It does work for saving electricity, I've bought the kind from elsewhere before that was too simple not **all**. This time to buy this store, but also for the safety. Air conditioning is too much electricity, this use to save a lot of	Jingdong prefecture level city in Shandong
Doraemon Hyakubo Shop	King of Power Saving	Two air conditioners and a refrigerator at home, a month down the electricity bill is almost two hundred dollars, with this after the average savings of more than thirty dollars to spend less money to enjoy more, it's really very good!	Jingdong prefecture level city in Shandong
Doraemon Hyakubo Shop	King of Power Saving	Good. Very good.	Jingdong prefecture level city in Shandong
Doraemon Hyakubo Shop	King of Power Saving	Works great, easy to operate, great, very happy with it.	Jingdong prefecture level city in Shandong
Doraemon Hyakubo Shop	King of Power Saving	Received it and tried it out, it's great, great quality and worth having! Five stars praise!	Jingdong prefecture level city in Shandong
Doraemon Hyakubo Shop	King of Power Saving	Very trustworthy brand, quality is guaranteed, the price is still affordable, quite satisfied!	Jingdong prefecture level city in Shandong
Doraemon Hyakubo Shop	King of Power Saving	Use it before you speak.	Jingdong prefecture level city in Shandong
Doraemon Hyakubo Shop	King of Power Saving	The quality of workmanship is very good and the power saving effect is quite good, I regret not getting it earlier.	Jingdong prefecture level city in Shandong
Doraemon Hyakubo Shop	King of Power Saving	Merchants shipping speed, and customer service introduced the same very good, cost-effective	Jingdong prefecture level city in Shandong
Doraemon Hyakubo Shop	King of Power Saving	Feel a little effect Oh, where the power consumption where to plug, convenient and convenient	Jingdong prefecture level city in Shandong
Doraemon Hyakubo Shop	King of Power Saving	The quality of the imported saver is very good, practical and satisfactory. Cost-effective	Jingdong prefecture level city in Shandong
Doraemon Hyakubo Shop	King of Power Saving	Power Saver is especially good, good and inexpensive, very good	Jingdong prefecture level city in Shandong
Doraemon Hyakubo Shop	King of Power Saving	Fast shipping, awesome quality, affordable price, satisfied.	Jingdong prefecture level city in Shandong
Doraemon Hyakubo Shop	King of Power Saving	Energy saver received, the effect is consistent with the description, recommended to buy!	Jingdong prefecture level city in Shandong
Doraemon Hyakubo Shop	King of Power Saving	It's really good, the quality is great, I recommend it!	Jingdong prefecture level city in Shandong
Doraemon Hyakubo Shop	King of Power Saving	Works well. Inexpensive. Shipping was fast.	Jingdong prefecture level city in Shandong
Doraemon Hyakubo Shop	King of Power Saving	Logistics is fast, works well, energy saving is good	Jingdong prefecture level city in Shandong
Doraemon Hyakubo Shop	King of Power Saving	very happy	Jingdong prefecture level city in Shandong
Doraemon Hyakubo Shop	King of Power Saving	an excellent	Jingdong prefecture level city in Shandong
Doraemon Hyakubo Shop	King of Power Saving	It rained a couple days ago and I just went to get the item back today , not bad, hey I looked at the meter and that red display light takes several seconds to jump, did not make a mistake buying it. After a period of time to evaluate.	Jingdong prefecture level city in Shandong
Doraemon Hyakubo Shop	King of Power Saving	Used more than 10 days to check the power, there is indeed some decline, customer service said, long-term use will save more points!	Jingdong prefecture level city in Shandong
Doraemon Hyakubo Shop	King of Power Saving	It's a proper IQ tax, and it costs electricity.	Jingdong prefecture level city in Shandong
Doraemon Hyakubo Shop	King of Power Saving	I've been using it for 17 days now, it really saves electricity Shipping is fast, you can save a lot in a year	Jingdong prefecture level city in Shandong
Doraemon Hyakubo Shop	King of Power Saving	I've been using it for 17 days and it really saves power Shipping is fast and you don't have to worry about the quality, free returns within 5 years	Jingdong prefecture level city in Shandong
Doraemon Hyakubo Shop	King of Power Saving	It's more regular, scanned the code and it's authentic, and the main thing is that you can try it for 90 days! Plugged in more than ten days, is less than before a few degrees per day. When the time to use a month to see Additional. Good to buy a few more	Jingdong prefecture level city in Shandong
Doraemon Hyakubo Shop	King of Power Saving	Logistics is very fast, fine workmanship, use it and then come back to the comments	Jingdong prefecture level city in Shandong
Doraemon Hyakubo Shop	King of Power Saving	The quality of workmanship is very good and the power saving effect is quite good, I regret not getting it earlier.	Jingdong prefecture level city in Shandong
Doraemon Hyakubo Shop	King of Power Saving	Received the goods, according to the instructions on the operation is very simple, to wait and see the effect, looks good!	Jingdong prefecture level city in Shandong
Doraemon Hyakubo Shop	King of Power Saving	It's really as safe and power-saving as the owner says, and it works great!	Jingdong prefecture level city in Shandong
Doraemon Hyakubo Shop	King of Power Saving	Received the goods, immediately used, looks good, to wait and see how the effect.	Jingdong prefecture level city in Shandong
Doraemon Hyakubo Shop	King of Power Saving	Afraid to buy back did not work, the store said the commitment to 90 days invalid refund, warranty 5 years. Saver also complimentary on the Pacific insurance, it is a lot of peace of mind, there are effects will continue to buy back drops!	Jingdong prefecture level city in Shandong
Doraemon Hyakubo Shop	King of Power Saving	Received the goods and used it, can save electricity a month later to see the effect!	Jingdong prefecture level city in Shandong
Doraemon Hyakubo Shop	King of Power Saving	Fancy 90-day trial, 5-year warranty before the order, the goods received, has been experiencing a week, can really feel the savings, in the introduction of friends also buy this!	Jingdong prefecture level city in Shandong
Doraemon Hyakubo Shop	King of Power Saving	Clothing stores use, neighbors recommended to buy, said a month can save more than 70 yuan, first plugged in to see the effect of the	Jingdong prefecture level city in Shandong
Doraemon Hyakubo Shop	King of Power Saving	A friend's home two months to save more than 80 dollars of electricity, recommended that I buy two, decisive purchase, looking forward to the effect of power saving, waiting for the next month effect.	Jingdong prefecture level city in Shandong
Doraemon Hyakubo Shop	King of Power Saving	The goods have been received, consistent with the description of the advertisement, quite good, I hope that in terms of power saving and purifying the air is really as the merchant said that would be better!	Jingdong prefecture level city in Shandong
Doraemon Hyakubo Shop	King of Power Saving	Power Saving King is really good to use the quality is very good than the physical store a lot cheaper we can rest assured that the purchase recommended to buy!	Jingdong prefecture level city in Shandong
Doraemon Hyakubo Shop	King of Power Saving	Energy saver has been used on the rental house inside the use of the meter is always running fast, with this can really save electricity, easy to use, plugged in can be, very satisfied!	Jingdong prefecture level city in Shandong
Doraemon Hyakubo Shop	King of Power Saving	Just use can not see what effect to,, wait for a period of time in the evaluation	Jingdong prefecture level city in Shandong
Doraemon Hyakubo Shop	King of Power Saving	Live in the 18th floor is too far away from the meter, afraid of no effect, look at the trial 90 days before buying. Currently used for a month, there is an effect, saving 27 dollars in electricity, next month should be able to save more!	Jingdong prefecture level city in Shandong
Doraemon Hyakubo Shop	King of Power Saving	I've been using it for almost a month now and it feels good, so I'll use it again.	Jingdong prefecture level city in Shandong
Doraemon Hyakubo Shop	King of Power Saving	It's ok, the electric bill is really less, originally I remember charging $100 and using it for 10 days. Now it lasts about 15 days. It's still noticeable.	Jingdong prefecture level city in Shandong

Doraemon Hyakubo Shop	King of Power Saving	Fast logistics, good power saving effect, very satisfied	Jingdong prefecture level city in Shandong
Doraemon Hyakubo Shop	King of Power Saving	Has been the second time back to buy, energy saving effect, worth recommending, you can try to see, power saving effect: very good Experience: can save about 30%!	Jingdong prefecture level city in Shandong
Doraemon Hyakubo Shop	King of Power Saving	I don't know how well it works for now	Jingdong prefecture level city in Shandong
Doraemon Hyakubo Shop	King of Power Saving	Factory direct is really good, plugged in and used for half a month did less probably almost 50 degrees of electricity! And the manufacturer's warranty is 5 years.	Jingdong prefecture level city in Shandong
Doraemon Hyakubo Shop	King of Power Saving	It's really effective. The previous month's electricity was $286, but after using the power saver for a month, I have $195 left this month, which is not bad.	Jingdong prefecture level city in Shandong
Doraemon Hyakubo Shop	King of Power Saving	I'll give you a good review and see how it goes.	Jingdong prefecture level city in Shandong
Doraemon Hyakubo Shop	King of Power Saving	Customer service is very professional and patient to guide the installation, the quality of workmanship is very fine. Durable, second repurchase. Really can save oh!	Jingdong prefecture level city in Shandong
Doraemon Hyakubo Shop	King of Power Saving	I've been using it for a month now and my electric bill is over $30 less than last month. I feel it's quite worth buying.	Jingdong prefecture level city in Shandong
Doraemon Hyakubo Shop	King of Power Saving	Small size, does not take up space. It's very good, it does work very effectively, the tool room has a lot of work with a lot of electricity. After installing it, it really reduces a lot of electricity bills. Before the day 30 degrees of electricity left stone, now work down really less a few degrees.	Jingdong prefecture level city in Shandong
Doraemon Hyakubo Shop	King of Power Saving	It does work for saving electricity, I've bought the kind from elsewhere before that was too simple not **all**. This time to buy this store, but also for the safety. Air conditioning is too much electricity, this use to save a lot of	Jingdong prefecture level city in Shandong
Doraemon Hyakubo Shop	King of Power Saving	Two air conditioners and a refrigerator at home, a month down the electricity bill is almost two hundred dollars, with this after the average savings of more than thirty dollars to spend less money to enjoy more, it's really very good!	Jingdong prefecture level city in Shandong
Doraemon Hyakubo Shop	King of Power Saving	Good. Very good.	Jingdong prefecture level city in Shandong
Doraemon Hyakubo Shop	King of Power Saving	Works great, easy to operate, great, very happy with it.	Jingdong prefecture level city in Shandong
Doraemon Hyakubo Shop	King of Power Saving	Received it and tried it out, it's great, great quality and worth having! Five stars praise!	Jingdong prefecture level city in Shandong
Doraemon Hyakubo Shop	King of Power Saving	Very trustworthy brand, quality is guaranteed, the price is still affordable, quite satisfied!	Jingdong prefecture level city in Shandong
Doraemon Hyakubo Shop	King of Power Saving	Use it before you speak.	Jingdong prefecture level city in Shandong
Doraemon Hyakubo Shop	King of Power Saving	The quality of workmanship is very good and the power saving effect is quite good, I regret not getting it earlier.	Jingdong prefecture level city in Shandong
Doraemon Hyakubo Shop	King of Power Saving	Merchants shipping speed, and customer service introduced the same very good, cost-effective	Jingdong prefecture level city in Shandong
Doraemon Hyakubo Shop	King of Power Saving	Feel a little effect Oh, where the power consumption where to plug, convenient and convenient	Jingdong prefecture level city in Shandong
Doraemon Hyakubo Shop	King of Power Saving	The quality of the imported saver is very good, practical and satisfactory. Cost-effective	Jingdong prefecture level city in Shandong
Doraemon Hyakubo Shop	King of Power Saving	Power Saver is especially good, good and inexpensive, very good	Jingdong prefecture level city in Shandong
Doraemon Hyakubo Shop	King of Power Saving	Fast shipping, awesome quality, affordable price, satisfied.	Jingdong prefecture level city in Shandong
Doraemon Hyakubo Shop	King of Power Saving	Energy saver received, the effect is consistent with the description, recommended to buy!	Jingdong prefecture level city in Shandong
Doraemon Hyakubo Shop	King of Power Saving	It's really good, the quality is great, I recommend it!	Jingdong prefecture level city in Shandong
Doraemon Hyakubo Shop	King of Power Saving	Works well. Inexpensive. Shipping was fast.	Jingdong prefecture level city in Shandong
Doraemon Hyakubo Shop	King of Power Saving	Logistics is fast, works well, energy saving is good	Jingdong prefecture level city in Shandong
Doraemon Hyakubo Shop	King of Power Saving	very happy	Jingdong prefecture level city in Shandong
Doraemon Hyakubo Shop	King of Power Saving	an excellent	Jingdong prefecture level city in Shandong
Doraemon Hyakubo Shop	King of Power Saving	It rained a couple days ago and I just went to get the item back today , not bad, hey I looked at the meter and that red display light takes several seconds to jump, did not make a mistake buying it. After a period of time to evaluate.	Jingdong prefecture level city in Shandong
Doraemon Hyakubo Shop	King of Power Saving	Used more than 10 days to check the power, there is indeed some decline, customer service said, long-term use will save more points!	Jingdong prefecture level city in Shandong
Doraemon Hyakubo Shop	King of Power Saving	It's a proper IQ tax, and it costs electricity.	Jingdong prefecture level city in Shandong
Doraemon Hyakubo Shop	King of Power Saving	I've been using it for 17 days now, it really saves electricity Shipping is fast, you can save a lot in a year	Jingdong prefecture level city in Shandong
Doraemon Hyakubo Shop	King of Power Saving	I've been using it for 17 days and it really saves power Shipping is fast and you don't have to worry about the quality, free returns within 5 years	Jingdong prefecture level city in Shandong
Doraemon Hyakubo Shop	King of Power Saving	It's more regular, scanned the code and it's authentic, and the main thing is that you can try it for 90 days! Plugged in more than ten days, is less than before a few degrees per day. When the time to use a month to see Additional. Good to buy a few more	Jingdong prefecture level city in Shandong
Doraemon Hyakubo Shop	King of Power Saving	Logistics is very fast, fine workmanship, use it and then come back to the comments	Jingdong prefecture level city in Shandong
Doraemon Hyakubo Shop	King of Power Saving	The quality of workmanship is very good and the power saving effect is quite good, I regret not getting it earlier.	Jingdong prefecture level city in Shandong
Doraemon Hyakubo Shop	King of Power Saving	Received the goods, according to the instructions on the operation is very simple, to wait and see the effect, looks good!	Jingdong prefecture level city in Shandong
Doraemon Hyakubo Shop	King of Power Saving	It's really as safe and power-saving as the owner says, and it works great!	Jingdong prefecture level city in Shandong
Doraemon Hyakubo Shop	King of Power Saving	Received the goods, immediately used, looks good, to wait and see how the effect.	Jingdong prefecture level city in Shandong
Doraemon Hyakubo Shop	King of Power Saving	Afraid to buy back did not work, the store said the commitment to 90 days invalid refund, warranty 5 years. Saver also complimentary on the Pacific insurance, it is a lot of peace of mind, there are effects will continue to buy back drops!	Jingdong prefecture level city in Shandong
Doraemon Hyakubo Shop	King of Power Saving	Received the goods and used it, can save electricity a month later to see the effect!	Jingdong prefecture level city in Shandong
Doraemon Hyakubo Shop	King of Power Saving	Fancy 90-day trial, 5-year warranty before the order, the goods received, has been experiencing a week, can really feel the savings, in the introduction of friends also buy this!	Jingdong prefecture level city in Shandong
Doraemon Hyakubo Shop	King of Power Saving	Clothing stores use, neighbors recommended to buy, said a month can save more than 70 yuan, first plugged in to see the effect of the	Jingdong prefecture level city in Shandong
Doraemon Hyakubo Shop	King of Power Saving	A friend's home two months to save more than 80 dollars of electricity, recommended that I buy two, decisive purchase, looking forward to the effect of power saving, waiting for the next month effect.	Jingdong prefecture level city in Shandong
Doraemon Hyakubo Shop	King of Power Saving	The goods have been received, consistent with the description of the advertisement, quite good, I hope that in terms of power saving and purifying the air is really as the merchant said that would be better!	Jingdong prefecture level city in Shandong
Doraemon Hyakubo Shop	King of Power Saving	Power Saving King is really good to use the quality is very good than the physical store a lot cheaper we can rest assured that the purchase recommended to buy!	Jingdong prefecture level city in Shandong
Doraemon Hyakubo Shop	King of Power Saving	Energy saver has been used on the rental house inside the use of the meter is always running fast, with this can really save electricity, easy to use, plugged in can be, very satisfied!	Jingdong prefecture level city in Shandong
Doraemon Hyakubo Shop	King of Power Saving	Just use can not see what effect to,, wait for a period of time in the evaluation	Jingdong prefecture level city in Shandong

Doraemon Hyakubo Shop	King of Power Saving	Live in the 18th floor is too far away from the meter, afraid of no effect, look at the trial 90 days before buying. Currently used for a month, there is an effect, saving 27 dollars in electricity, next month should be able to save more!	Jingdong prefecture level city in Shandong
Doraemon Hyakubo Shop	King of Power Saving	I've been using it for almost a month now and it feels good, so I'll use it again.	Jingdong prefecture level city in Shandong
Doraemon Hyakubo Shop	King of Power Saving	It's ok, the electric bill is really less, originally I remember charging $100 and using it for 10 days. Now it lasts about 15 days. It's still noticeable.	Jingdong prefecture level city in Shandong
Doraemon Hyakubo Shop	King of Power Saving	Fast logistics, good power saving effect, very satisfied	Jingdong prefecture level city in Shandong
Doraemon Hyakubo Shop	King of Power Saving	Has been the second time back to buy, energy saving effect, worth recommending, you can try to see, power saving effect: very good Experience: can save about 30%!	Jingdong prefecture level city in Shandong
Doraemon Hyakubo Shop	King of Power Saving	I don't know how well it works for now	Jingdong prefecture level city in Shandong
Doraemon Hyakubo Shop	King of Power Saving	Factory direct is really good, plugged in and used for half a month did less probably almost 50 degrees of electricity! And the manufacturer's warranty is 5 years.	Jingdong prefecture level city in Shandong
Doraemon Hyakubo Shop	King of Power Saving	It's really effective. The previous month's electricity was $286, but after using the power saver for a month, I have $195 left this month, which is not bad.	Jingdong prefecture level city in Shandong
Doraemon Hyakubo Shop	King of Power Saving	I'll give you a good review and see how it goes.	Jingdong prefecture level city in Shandong
Doraemon Hyakubo Shop	King of Power Saving	Customer service is very professional and patient to guide the installation, the quality of workmanship is very fine. Durable, second repurchase. Really can save oh!	Jingdong prefecture level city in Shandong
Doraemon Hyakubo Shop	King of Power Saving	I've been using it for a month now and my electric bill is over $30 less than last month. I feel it's quite worth buying.	Jingdong prefecture level city in Shandong
Doraemon Hyakubo Shop	King of Power Saving	Small size, does not take up space. It's very good, it does work very effectively, the tool room has a lot of work with a lot of electricity. After installing it, it really reduces a lot of electricity bills. Before the day 30 degrees of electricity left stone, now work down really less a few degrees.	Jingdong prefecture level city in Shandong
Doraemon Hyakubo Shop	King of Power Saving	It does work for saving electricity, I've bought the kind from elsewhere before that was too simple not **all**. This time to buy this store, but also for the safety. Air conditioning is too much electricity, this use to save a lot of	Jingdong prefecture level city in Shandong
Doraemon Hyakubo Shop	King of Power Saving	Two air conditioners and a refrigerator at home, a month down the electricity bill is almost two hundred dollars, with this after the average savings of more than thirty dollars to spend less money to enjoy more, it's really very good!	Jingdong prefecture level city in Shandong
Doraemon Hyakubo Shop	King of Power Saving	Good. Very good.	Jingdong prefecture level city in Shandong
Doraemon Hyakubo Shop	King of Power Saving	Works great, easy to operate, great, very happy with it.	Jingdong prefecture level city in Shandong
Doraemon Hyakubo Shop	King of Power Saving	Received it and tried it out, it's great, great quality and worth having! Five stars praise!	Jingdong prefecture level city in Shandong
Doraemon Hyakubo Shop	King of Power Saving	Very trustworthy brand, quality is guaranteed, the price is still affordable, quite satisfied!	Jingdong prefecture level city in Shandong
Doraemon Hyakubo Shop	King of Power Saving	Use it before you speak.	Jingdong prefecture level city in Shandong
Doraemon Hyakubo Shop	King of Power Saving	The quality of workmanship is very good and the power saving effect is quite good, I regret not getting it earlier.	Jingdong prefecture level city in Shandong
Doraemon Hyakubo Shop	King of Power Saving	Merchants shipping speed, and customer service introduced the same very good, cost-effective	Jingdong prefecture level city in Shandong
Doraemon Hyakubo Shop	King of Power Saving	Feel a little effect Oh, where the power consumption where to plug, convenient and convenient	Jingdong prefecture level city in Shandong
Doraemon Hyakubo Shop	King of Power Saving	The quality of the imported saver is very good, practical and satisfactory. Cost-effective	Jingdong prefecture level city in Shandong
Doraemon Hyakubo Shop	King of Power Saving	Power Saver is especially good, good and inexpensive, very good	Jingdong prefecture level city in Shandong
Doraemon Hyakubo Shop	King of Power Saving	Fast shipping, awesome quality, affordable price, satisfied.	Jingdong prefecture level city in Shandong
Doraemon Hyakubo Shop	King of Power Saving	Energy saver received, the effect is consistent with the description, recommended to buy!	Jingdong prefecture level city in Shandong
Doraemon Hyakubo Shop	King of Power Saving	It's really good, the quality is great, I recommend it!	Jingdong prefecture level city in Shandong
Doraemon Hyakubo Shop	King of Power Saving	Works well. Inexpensive. Shipping was fast.	Jingdong prefecture level city in Shandong
Doraemon Hyakubo Shop	King of Power Saving	Logistics is fast, works well, energy saving is good	Jingdong prefecture level city in Shandong
Doraemon Hyakubo Shop	King of Power Saving	very happy	Jingdong prefecture level city in Shandong
Doraemon Hyakubo Shop	King of Power Saving	an excellent	Jingdong prefecture level city in Shandong
Doraemon Hyakubo Shop	King of Power Saving	It rained a couple days ago and I just went to get the item back today , not bad, hey I looked at the meter and that red display light takes several seconds to jump, did not make a mistake buying it. After a period of time to evaluate.	Jingdong prefecture level city in Shandong
Doraemon Hyakubo Shop	King of Power Saving	Used more than 10 days to check the power, there is indeed some decline, customer service said, long-term use will save more points!	Jingdong prefecture level city in Shandong
Doraemon Hyakubo Shop	King of Power Saving	It's a proper IQ tax, and it costs electricity.	Jingdong prefecture level city in Shandong
Doraemon Hyakubo Shop	King of Power Saving	I've been using it for 17 days now, it really saves electricity Shipping is fast, you can save a lot in a year	Jingdong prefecture level city in Shandong
Doraemon Hyakubo Shop	King of Power Saving	I've been using it for 17 days and it really saves power Shipping is fast and you don't have to worry about the quality, free returns within 5 years	Jingdong prefecture level city in Shandong
Doraemon Hyakubo Shop	King of Power Saving	It's more regular, scanned the code and it's authentic, and the main thing is that you can try it for 90 days! Plugged in more than ten days, is less than before a few degrees per day. When the time to use a month to see Additional. Good to buy a few more	Jingdong prefecture level city in Shandong
Doraemon Hyakubo Shop	King of Power Saving	Logistics is very fast, fine workmanship, use it and then come back to the comments	Jingdong prefecture level city in Shandong
Doraemon Hyakubo Shop	King of Power Saving	The quality of workmanship is very good and the power saving effect is quite good, I regret not getting it earlier.	Jingdong prefecture level city in Shandong
Doraemon Hyakubo Shop	King of Power Saving	Received the goods, according to the instructions on the operation is very simple, to wait and see the effect, looks good!	Jingdong prefecture level city in Shandong
Doraemon Hyakubo Shop	King of Power Saving	It's really as safe and power-saving as the owner says, and it works great!	Jingdong prefecture level city in Shandong
Doraemon Hyakubo Shop	King of Power Saving	Received the goods, immediately used, looks good, to wait and see how the effect.	Jingdong prefecture level city in Shandong
Doraemon Hyakubo Shop	King of Power Saving	Afraid to buy back did not work, the store said the commitment to 90 days invalid refund, warranty 5 years. Saver also complimentary on the Pacific insurance, it is a lot of peace of mind, there are effects will continue to buy back drops!	Jingdong prefecture level city in Shandong
Doraemon Hyakubo Shop	King of Power Saving	Received the goods and used it, can save electricity a month later to see the effect!	Jingdong prefecture level city in Shandong
Doraemon Hyakubo Shop	King of Power Saving	Fancy 90-day trial, 5-year warranty before the order, the goods received, has been experiencing a week, can really feel the savings, in the introduction of friends also buy this!	Jingdong prefecture level city in Shandong
Doraemon Hyakubo Shop	King of Power Saving	Clothing stores use, neighbors recommended to buy, said a month can save more than 70 yuan, first plugged in to see the effect of the	Jingdong prefecture level city in Shandong
Doraemon Hyakubo Shop	King of Power Saving	A friend's home two months to save more than 80 dollars of electricity, recommended that I buy two, decisive purchase, looking forward to the effect of power saving, waiting for the next month effect.	Jingdong prefecture level city in Shandong
Doraemon Hyakubo Shop	King of Power Saving	The goods have been received, consistent with the description of the advertisement, quite good, I hope that in terms of power saving and purifying the air is really as the merchant said that would be better!	Jingdong prefecture level city in Shandong

Doraemon Hyakubo Shop	King of Power Saving	Power Saving King is really good to use the quality is very good than the physical store a lot cheaper we can rest assured that the purchase recommended to buy!	Jingdong prefecture level city in Shandong
Doraemon Hyakubo Shop	King of Power Saving	Energy saver has been used on the rental house inside the use of the meter is always running fast, with this can really save electricity, easy to use, plugged in can be, very satisfied!	Jingdong prefecture level city in Shandong
Doraemon Hyakubo Shop	King of Power Saving	Just use can not see what effect to,, wait for a period of time in the evaluation	Jingdong prefecture level city in Shandong
Doraemon Hyakubo Shop	King of Power Saving	Live in the 18th floor is too far away from the meter, afraid of no effect, look at the trial 90 days before buying. Currently used for a month, there is an effect, saving 27 dollars in electricity, next month should be able to save more!	Jingdong prefecture level city in Shandong
Doraemon Hyakubo Shop	King of Power Saving	I've been using it for almost a month now and it feels good, so I'll use it again.	Jingdong prefecture level city in Shandong
Doraemon Hyakubo Shop	King of Power Saving	It's ok, the electric bill is really less, originally I remember charging $100 and using it for 10 days. Now it lasts about 15 days. It's still noticeable.	Jingdong prefecture level city in Shandong
Doraemon Hyakubo Shop	King of Power Saving	Fast logistics, good power saving effect, very satisfied	Jingdong prefecture level city in Shandong
Doraemon Hyakubo Shop	King of Power Saving	Has been the second time back to buy, energy saving effect, worth recommending, you can try to see, power saving effect: very good Experience: can save about 30%!	Jingdong prefecture level city in Shandong
Doraemon Hyakubo Shop	King of Power Saving	I don't know how well it works for now	Jingdong prefecture level city in Shandong
Doraemon Hyakubo Shop	King of Power Saving	Factory direct is really good, plugged in and used for half a month did less probably almost 50 degrees of electricity! And the manufacturer's warranty is 5 years.	Jingdong prefecture level city in Shandong
Doraemon Hyakubo Shop	King of Power Saving	It's really effective. The previous month's electricity was $286, but after using the power saver for a month, I have $195 left this month, which is not bad.	Jingdong prefecture level city in Shandong
Doraemon Hyakubo Shop	King of Power Saving	I'll give you a good review and see how it goes.	Jingdong prefecture level city in Shandong
Doraemon Hyakubo Shop	King of Power Saving	Customer service is very professional and patient to guide the installation, the quality of workmanship is very fine. Durable, second repurchase. Really can save oh!	Jingdong prefecture level city in Shandong
Doraemon Hyakubo Shop	King of Power Saving	I've been using it for a month now and my electric bill is over $30 less than last month. I feel it's quite worth buying.	Jingdong prefecture level city in Shandong
Doraemon Hyakubo Shop	King of Power Saving	Small size, does not take up space. It's very good, it does work very effectively, the tool room has a lot of work with a lot of electricity. After installing it, it really reduces a lot of electricity bills. Before the day 30 degrees of electricity left stone, now work down really less a few degrees.	Jingdong prefecture level city in Shandong
Doraemon Hyakubo Shop	King of Power Saving	It does work for saving electricity, I've bought the kind from elsewhere before that was too simple not **all**. This time to buy this store, but also for the safety. Air conditioning is too much electricity, this use to save a lot of	Jingdong prefecture level city in Shandong
Doraemon Hyakubo Shop	King of Power Saving	Two air conditioners and a refrigerator at home, a month down the electricity bill is almost two hundred dollars, with this after the average savings of more than thirty dollars to spend less money to enjoy more, it's really very good!	Jingdong prefecture level city in Shandong
Doraemon Hyakubo Shop	King of Power Saving	Good. Very good.	Jingdong prefecture level city in Shandong
Doraemon Hyakubo Shop	King of Power Saving	Works great, easy to operate, great, very happy with it.	Jingdong prefecture level city in Shandong
Doraemon Hyakubo Shop	King of Power Saving	Received it and tried it out, it's great, great quality and worth having! Five stars praise!	Jingdong prefecture level city in Shandong
Doraemon Hyakubo Shop	King of Power Saving	Very trustworthy brand, quality is guaranteed, the price is still affordable, quite satisfied!	Jingdong prefecture level city in Shandong
Doraemon Hyakubo Shop	King of Power Saving	Use it before you speak.	Jingdong prefecture level city in Shandong
Doraemon Hyakubo Shop	King of Power Saving	The quality of workmanship is very good and the power saving effect is quite good, I regret not getting it earlier.	Jingdong prefecture level city in Shandong
Doraemon Hyakubo Shop	King of Power Saving	Merchants shipping speed, and customer service introduced the same very good, cost-effective	Jingdong prefecture level city in Shandong
Doraemon Hyakubo Shop	King of Power Saving	Feel a little effect Oh, where the power consumption where to plug, convenient and convenient	Jingdong prefecture level city in Shandong
Doraemon Hyakubo Shop	King of Power Saving	The quality of the imported saver is very good, practical and satisfactory. Cost-effective	Jingdong prefecture level city in Shandong
Doraemon Hyakubo Shop	King of Power Saving	Power Saver is especially good, good and inexpensive, very good	Jingdong prefecture level city in Shandong
Doraemon Hyakubo Shop	King of Power Saving	Fast shipping, awesome quality, affordable price, satisfied.	Jingdong prefecture level city in Shandong
Doraemon Hyakubo Shop	King of Power Saving	Energy saver received, the effect is consistent with the description, recommended to buy!	Jingdong prefecture level city in Shandong
Doraemon Hyakubo Shop	King of Power Saving	It's really good, the quality is great, I recommend it!	Jingdong prefecture level city in Shandong
Doraemon Hyakubo Shop	King of Power Saving	Works well. Inexpensive. Shipping was fast.	Jingdong prefecture level city in Shandong
Doraemon Hyakubo Shop	King of Power Saving	Logistics is fast, works well, energy saving is good	Jingdong prefecture level city in Shandong
Doraemon Hyakubo Shop	King of Power Saving	very happy	Jingdong prefecture level city in Shandong
Doraemon Hyakubo Shop	King of Power Saving	an excellent	Jingdong prefecture level city in Shandong
Doraemon Hyakubo Shop	King of Power Saving	It rained a couple days ago and I just went to get the item back today , not bad, hey I looked at the meter and that red display light takes several seconds to jump, did not make a mistake buying it. After a period of time to evaluate.	Jingdong prefecture level city in Shandong
Doraemon Hyakubo Shop	King of Power Saving	Used more than 10 days to check the power, there is indeed some decline, customer service said, long-term use will save more points!	Jingdong prefecture level city in Shandong
Doraemon Hyakubo Shop	King of Power Saving	It's a proper IQ tax, and it costs electricity.	Jingdong prefecture level city in Shandong
Doraemon Hyakubo Shop	King of Power Saving	I've been using it for 17 days now, it really saves electricity Shipping is fast, you can save a lot in a year	Jingdong prefecture level city in Shandong
Doraemon Hyakubo Shop	King of Power Saving	I've been using it for 17 days and it really saves power Shipping is fast and you don't have to worry about the quality, free returns within 5 years	Jingdong prefecture level city in Shandong
Doraemon Hyakubo Shop	King of Power Saving	It's more regular, scanned the code and it's authentic, and the main thing is that you can try it for 90 days! Plugged in more than ten days, is less than before a few degrees per day. When the time to use a month to see Additional. Good to buy a few more	Jingdong prefecture level city in Shandong
Doraemon Hyakubo Shop	King of Power Saving	Logistics is very fast, fine workmanship, use it and then come back to the comments	Jingdong prefecture level city in Shandong
Doraemon Hyakubo Shop	King of Power Saving	The quality of workmanship is very good and the power saving effect is quite good, I regret not getting it earlier.	Jingdong prefecture level city in Shandong
Doraemon Hyakubo Shop	King of Power Saving	Received the goods, according to the instructions on the operation is very simple, to wait and see the effect, looks good!	Jingdong prefecture level city in Shandong
Doraemon Hyakubo Shop	King of Power Saving	It's really as safe and power-saving as the owner says, and it works great!	Jingdong prefecture level city in Shandong
Doraemon Hyakubo Shop	King of Power Saving	Received the goods, immediately used, looks good, to wait and see how the effect.	Jingdong prefecture level city in Shandong
Doraemon Hyakubo Shop	King of Power Saving	Afraid to buy back did not work, the store said the commitment to 90 days invalid refund, warranty 5 years. Saver also complimentary on the Pacific insurance, it is a lot of peace of mind, there are effects will continue to buy back drops!	Jingdong prefecture level city in Shandong
Doraemon Hyakubo Shop	King of Power Saving	Received the goods and used it, can save electricity a month later to see the effect!	Jingdong prefecture level city in Shandong
Doraemon Hyakubo Shop	King of Power Saving	Fancy 90-day trial, 5-year warranty before the order, the goods received, has been experiencing a week, can really feel the savings, in the introduction of friends also buy this!	Jingdong prefecture level city in Shandong

Doraemon Hyakubo Shop	King of Power Saving	Clothing stores use, neighbors recommended to buy, said a month can save more than 70 yuan, first plugged in to see the effect of the	Jingdong prefecture level city in Shandong
Doraemon Hyakubo Shop	King of Power Saving	A friend's home two months to save more than 80 dollars of electricity, recommended that I buy two, decisive purchase, looking forward to the effect of power saving, waiting for the next month effect.	Jingdong prefecture level city in Shandong
Doraemon Hyakubo Shop	King of Power Saving	The goods have been received, consistent with the description of the advertisement, quite good, I hope that in terms of power saving and purifying the air is really as the merchant said that would be better!	Jingdong prefecture level city in Shandong
Doraemon Hyakubo Shop	King of Power Saving	Power Saving King is really good to use the quality is very good than the physical store a lot cheaper we can rest assured that the purchase recommended to buy!	Jingdong prefecture level city in Shandong
Doraemon Hyakubo Shop	King of Power Saving	Energy saver has been used on the rental house inside the use of the meter is always running fast, with this can really save electricity, easy to use, plugged in can be, very satisfied!	Jingdong prefecture level city in Shandong
Doraemon Hyakubo Shop	King of Power Saving	Just use can not see what effect to,, wait for a period of time in the evaluation	Jingdong prefecture level city in Shandong
Doraemon Hyakubo Shop	King of Power Saving	Live in the 18th floor is too far away from the meter, afraid of no effect, look at the trial 90 days before buying. Currently used for a month, there is an effect, saving 27 dollars in electricity, next month should be able to save more!	Jingdong prefecture level city in Shandong
Doraemon Hyakubo Shop	King of Power Saving	I've been using it for almost a month now and it feels good, so I'll use it again.	Jingdong prefecture level city in Shandong
Doraemon Hyakubo Shop	King of Power Saving	It's ok, the electric bill is really less, originally I remember charging $100 and using it for 10 days. Now it lasts about 15 days. It's still noticeable.	Jingdong prefecture level city in Shandong
Doraemon Hyakubo Shop	King of Power Saving	Fast logistics, good power saving effect, very satisfied	Jingdong prefecture level city in Shandong
Doraemon Hyakubo Shop	King of Power Saving	Has been the second time back to buy, energy saving effect, worth recommending, you can try to see, power saving effect: very good Experience: can save about 30%!	Jingdong prefecture level city in Shandong
Doraemon Hyakubo Shop	King of Power Saving	I don't know how well it works for now	Jingdong prefecture level city in Shandong
Doraemon Hyakubo Shop	King of Power Saving	Factory direct is really good, plugged in and used for half a month did less probably almost 50 degrees of electricity! And the manufacturer's warranty is 5 years.	Jingdong prefecture level city in Shandong
Doraemon Hyakubo Shop	King of Power Saving	It's really effective. The previous month's electricity was $286, but after using the power saver for a month, I have $195 left this month, which is not bad.	Jingdong prefecture level city in Shandong
Doraemon Hyakubo Shop	King of Power Saving	I'll give you a good review and see how it goes.	Jingdong prefecture level city in Shandong
Doraemon Hyakubo Shop	King of Power Saving	Customer service is very professional and patient to guide the installation, the quality of workmanship is very fine. Durable, second repurchase. Really can save oh!	Jingdong prefecture level city in Shandong
Doraemon Hyakubo Shop	King of Power Saving	I've been using it for a month now and my electric bill is over $30 less than last month. I feel it's quite worth buying.	Jingdong prefecture level city in Shandong
Doraemon Hyakubo Shop	King of Power Saving	Small size, does not take up space. It's very good, it does work very effectively, the tool room has a lot of work with a lot of electricity. After installing it, it really reduces a lot of electricity bills. Before the day 30 degrees of electricity left stone, now work down really less a few degrees.	Jingdong prefecture level city in Shandong
Doraemon Hyakubo Shop	King of Power Saving	It does work for saving electricity, I've bought the kind from elsewhere before that was too simple not **all**. This time to buy this store, but also for the safety. Air conditioning is too much electricity, this use to save a lot of	Jingdong prefecture level city in Shandong
Doraemon Hyakubo Shop	King of Power Saving	Two air conditioners and a refrigerator at home, a month down the electricity bill is almost two hundred dollars, with this after the average savings of more than thirty dollars to spend less money to enjoy more, it's really very good!	Jingdong prefecture level city in Shandong
Doraemon Hyakubo Shop	King of Power Saving	Good. Very good.	Jingdong prefecture level city in Shandong
Doraemon Hyakubo Shop	King of Power Saving	Works great, easy to operate, great, very happy with it.	Jingdong prefecture level city in Shandong
Doraemon Hyakubo Shop	King of Power Saving	Received it and tried it out, it's great, great quality and worth having! Five stars praise!	Jingdong prefecture level city in Shandong
Doraemon Hyakubo Shop	King of Power Saving	Very trustworthy brand, quality is guaranteed, the price is still affordable, quite satisfied!	Jingdong prefecture level city in Shandong
Doraemon Hyakubo Shop	King of Power Saving	Use it before you speak.	Jingdong prefecture level city in Shandong
Doraemon Hyakubo Shop	King of Power Saving	The quality of workmanship is very good and the power saving effect is quite good, I regret not getting it earlier.	Jingdong prefecture level city in Shandong
Doraemon Hyakubo Shop	King of Power Saving	Merchants shipping speed, and customer service introduced the same very good, cost-effective	Jingdong prefecture level city in Shandong
Doraemon Hyakubo Shop	King of Power Saving	Feel a little effect Oh, where the power consumption where to plug, convenient and convenient	Jingdong prefecture level city in Shandong
Doraemon Hyakubo Shop	King of Power Saving	The quality of the imported saver is very good, practical and satisfactory. Cost-effective	Jingdong prefecture level city in Shandong
Doraemon Hyakubo Shop	King of Power Saving	Power Saver is especially good, good and inexpensive, very good	Jingdong prefecture level city in Shandong
Doraemon Hyakubo Shop	King of Power Saving	Fast shipping, awesome quality, affordable price, satisfied.	Jingdong prefecture level city in Shandong
Doraemon Hyakubo Shop	King of Power Saving	Energy saver received, the effect is consistent with the description, recommended to buy!	Jingdong prefecture level city in Shandong
Doraemon Hyakubo Shop	King of Power Saving	It's really good, the quality is great, I recommend it!	Jingdong prefecture level city in Shandong
Doraemon Hyakubo Shop	King of Power Saving	Works well. Inexpensive. Shipping was fast.	Jingdong prefecture level city in Shandong
Doraemon Hyakubo Shop	King of Power Saving	Logistics is fast, works well, energy saving is good	Jingdong prefecture level city in Shandong
Doraemon Hyakubo Shop	King of Power Saving	very happy	Jingdong prefecture level city in Shandong
Doraemon Hyakubo Shop	King of Power Saving	an excellent	Jingdong prefecture level city in Shandong
Doraemon Hyakubo Shop	King of Power Saving	It rained a couple days ago and I just went to get the item back today , not bad, hey I looked at the meter and that red display light takes several seconds to jump, did not make a mistake buying it. After a period of time to evaluate.	Jingdong prefecture level city in Shandong
Doraemon Hyakubo Shop	King of Power Saving	Used more than 10 days to check the power, there is indeed some decline, customer service said, long-term use will save more points!	Jingdong prefecture level city in Shandong
Doraemon Hyakubo Shop	King of Power Saving	It's a proper IQ tax, and it costs electricity.	Jingdong prefecture level city in Shandong
Doraemon Hyakubo Shop	King of Power Saving	I've been using it for 17 days now, it really saves electricity Shipping is fast, you can save a lot in a year	Jingdong prefecture level city in Shandong
Doraemon Hyakubo Shop	King of Power Saving	I've been using it for 17 days and it really saves power Shipping is fast and you don't have to worry about the quality, free returns within 5 years	Jingdong prefecture level city in Shandong
Doraemon Hyakubo Shop	King of Power Saving	It's more regular, scanned the code and it's authentic, and the main thing is that you can try it for 90 days! Plugged in more than ten days, is less than before a few degrees per day. When the time to use a month to see Additional. Good to buy a few more	Jingdong prefecture level city in Shandong
Doraemon Hyakubo Shop	King of Power Saving	Logistics is very fast, fine workmanship, use it and then come back to the comments	Jingdong prefecture level city in Shandong
Doraemon Hyakubo Shop	King of Power Saving	The quality of workmanship is very good and the power saving effect is quite good, I regret not getting it earlier.	Jingdong prefecture level city in Shandong
Doraemon Hyakubo Shop	King of Power Saving	Received the goods, according to the instructions on the operation is very simple, to wait and see the effect, looks good!	Jingdong prefecture level city in Shandong
Doraemon Hyakubo Shop	King of Power Saving	It's really as safe and power-saving as the owner says, and it works great!	Jingdong prefecture level city in Shandong
Doraemon Hyakubo Shop	King of Power Saving	Received the goods, immediately used, looks good, to wait and see how the effect.	Jingdong prefecture level city in Shandong

Doraemon Hyakubo Shop	King of Power Saving	Afraid to buy back did not work, the store said the commitment to 90 days invalid refund, warranty 5 years. Saver also complimentary on the Pacific insurance, it is a lot of peace of mind, there are effects will continue to buy back drops!	Jingdong prefecture level city in Shandong
Doraemon Hyakubo Shop	King of Power Saving	Received the goods and used it, can save electricity a month later to see the effect!	Jingdong prefecture level city in Shandong
Doraemon Hyakubo Shop	King of Power Saving	Fancy 90-day trial, 5-year warranty before the order, the goods received, has been experiencing a week, can really feel the savings, in the introduction of friends also buy this!	Jingdong prefecture level city in Shandong
Doraemon Hyakubo Shop	King of Power Saving	Clothing stores use, neighbors recommended to buy, said a month can save more than 70 yuan, first plugged in to see the effect of the	Jingdong prefecture level city in Shandong
Doraemon Hyakubo Shop	King of Power Saving	A friend's home two months to save more than 80 dollars of electricity, recommended that I buy two, decisive purchase, looking forward to the effect of power saving, waiting for the next month effect.	Jingdong prefecture level city in Shandong
Doraemon Hyakubo Shop	King of Power Saving	The goods have been received, consistent with the description of the advertisement, quite good, I hope that in terms of power saving and purifying the air is really as the merchant said that would be better!	Jingdong prefecture level city in Shandong
Doraemon Hyakubo Shop	King of Power Saving	Power Saving King is really good to use the quality is very good than the physical store a lot cheaper we can rest assured that the purchase recommended to buy!	Jingdong prefecture level city in Shandong
Doraemon Hyakubo Shop	King of Power Saving	Energy saver has been used on the rental house inside the use of the meter is always running fast, with this can really save electricity, easy to use, plugged in can be, very satisfied!	Jingdong prefecture level city in Shandong
Doraemon Hyakubo Shop	King of Power Saving	Just use can not see what effect to,, wait for a period of time in the evaluation	Jingdong prefecture level city in Shandong
Doraemon Hyakubo Shop	King of Power Saving	Live in the 18th floor is too far away from the meter, afraid of no effect, look at the trial 90 days before buying. Currently used for a month, there is an effect, saving 27 dollars in electricity, next month should be able to save more!	Jingdong prefecture level city in Shandong
Doraemon Hyakubo Shop	King of Power Saving	I've been using it for almost a month now and it feels good, so I'll use it again.	Jingdong prefecture level city in Shandong
Doraemon Hyakubo Shop	King of Power Saving	It's ok, the electric bill is really less, originally I remember charging $100 and using it for 10 days. Now it lasts about 15 days. It's still noticeable.	Jingdong prefecture level city in Shandong
Doraemon Hyakubo Shop	King of Power Saving	Fast logistics, good power saving effect, very satisfied	Jingdong prefecture level city in Shandong
Doraemon Hyakubo Shop	King of Power Saving	Has been the second time back to buy, energy saving effect, worth recommending, you can try to see, power saving effect: very good Experience: can save about 30%!	Jingdong prefecture level city in Shandong
Doraemon Hyakubo Shop	King of Power Saving	I don't know how well it works for now	Jingdong prefecture level city in Shandong
Doraemon Hyakubo Shop	King of Power Saving	Factory direct is really good, plugged in and used for half a month did less probably almost 50 degrees of electricity! And the manufacturer's warranty is 5 years.	Jingdong prefecture level city in Shandong
Doraemon Hyakubo Shop	King of Power Saving	It's really effective. The previous month's electricity was $286, but after using the power saver for a month, I have $195 left this month, which is not bad.	Jingdong prefecture level city in Shandong
Doraemon Hyakubo Shop	King of Power Saving	I'll give you a good review and see how it goes.	Jingdong prefecture level city in Shandong
Doraemon Hyakubo Shop	King of Power Saving	Customer service is very professional and patient to guide the installation, the quality of workmanship is very fine. Durable, second repurchase. Really can save oh!	Jingdong prefecture level city in Shandong
Doraemon Hyakubo Shop	King of Power Saving	I've been using it for a month now and my electric bill is over $30 less than last month. I feel it's quite worth buying.	Jingdong prefecture level city in Shandong
Doraemon Hyakubo Shop	King of Power Saving	Small size, does not take up space. It's very good, it does work very effectively, the tool room has a lot of work with a lot of electricity. After installing it, it really reduces a lot of electricity bills. Before the day 30 degrees of electricity left stone, now work down really less a few degrees.	Jingdong prefecture level city in Shandong
Doraemon Hyakubo Shop	King of Power Saving	It does work for saving electricity, I've bought the kind from elsewhere before that was too simple not **all**. This time to buy this store, but also for the safety. Air conditioning is too much electricity, this use to save a lot of	Jingdong prefecture level city in Shandong
Doraemon Hyakubo Shop	King of Power Saving	Two air conditioners and a refrigerator at home, a month down the electricity bill is almost two hundred dollars, with this after the average savings of more than thirty dollars to spend less money to enjoy more, it's really very good!	Jingdong prefecture level city in Shandong
Doraemon Hyakubo Shop	King of Power Saving	Good. Very good.	Jingdong prefecture level city in Shandong
Doraemon Hyakubo Shop	King of Power Saving	Works great, easy to operate, great, very happy with it.	Jingdong prefecture level city in Shandong
Doraemon Hyakubo Shop	King of Power Saving	Received it and tried it out, it's great, great quality and worth having! Five stars praise!	Jingdong prefecture level city in Shandong
Doraemon Hyakubo Shop	King of Power Saving	Very trustworthy brand, quality is guaranteed, the price is still affordable, quite satisfied!	Jingdong prefecture level city in Shandong
Doraemon Hyakubo Shop	King of Power Saving	Use it before you speak.	Jingdong prefecture level city in Shandong
Doraemon Hyakubo Shop	King of Power Saving	The quality of workmanship is very good and the power saving effect is quite good, I regret not getting it earlier.	Jingdong prefecture level city in Shandong
Doraemon Hyakubo Shop	King of Power Saving	Merchants shipping speed, and customer service introduced the same very good, cost-effective	Jingdong prefecture level city in Shandong
Doraemon Hyakubo Shop	King of Power Saving	Feel a little effect Oh, where the power consumption where to plug, convenient and convenient	Jingdong prefecture level city in Shandong
Doraemon Hyakubo Shop	King of Power Saving	The quality of the imported saver is very good, practical and satisfactory. Cost-effective	Jingdong prefecture level city in Shandong
Doraemon Hyakubo Shop	King of Power Saving	Power Saver is especially good, good and inexpensive, very good	Jingdong prefecture level city in Shandong
Doraemon Hyakubo Shop	King of Power Saving	Fast shipping, awesome quality, affordable price, satisfied.	Jingdong prefecture level city in Shandong
Doraemon Hyakubo Shop	King of Power Saving	Energy saver received, the effect is consistent with the description, recommended to buy!	Jingdong prefecture level city in Shandong
Doraemon Hyakubo Shop	King of Power Saving	It's really good, the quality is great, I recommend it!	Jingdong prefecture level city in Shandong
Doraemon Hyakubo Shop	King of Power Saving	Works well. Inexpensive. Shipping was fast.	Jingdong prefecture level city in Shandong
Doraemon Hyakubo Shop	King of Power Saving	Logistics is fast, works well, energy saving is good	Jingdong prefecture level city in Shandong
Doraemon Hyakubo Shop	King of Power Saving	very happy	Jingdong prefecture level city in Shandong
Doraemon Hyakubo Shop	King of Power Saving	an excellent	Jingdong prefecture level city in Shandong
Doraemon Hyakubo Shop	King of Power Saving	It rained a couple days ago and I just went to get the item back today , not bad, hey I looked at the meter and that red display light takes several seconds to jump, did not make a mistake buying it. After a period of time to evaluate.	Jingdong prefecture level city in Shandong
Doraemon Hyakubo Shop	King of Power Saving	Used more than 10 days to check the power, there is indeed some decline, customer service said, long-term use will save more points!	Jingdong prefecture level city in Shandong
Doraemon Hyakubo Shop	King of Power Saving	It's a proper IQ tax, and it costs electricity.	Jingdong prefecture level city in Shandong
Doraemon Hyakubo Shop	King of Power Saving	I've been using it for 17 days now, it really saves electricity Shipping is fast, you can save a lot in a year	Jingdong prefecture level city in Shandong
Doraemon Hyakubo Shop	King of Power Saving	I've been using it for 17 days and it really saves power Shipping is fast and you don't have to worry about the quality, free returns within 5 years	Jingdong prefecture level city in Shandong
Doraemon Hyakubo Shop	King of Power Saving	It's more regular, scanned the code and it's authentic, and the main thing is that you can try it for 90 days! Plugged in more than ten days, is less than before a few degrees per day. When the time to use a month to see Additional. Good to buy a few more	Jingdong prefecture level city in Shandong
Doraemon Hyakubo Shop	King of Power Saving	Logistics is very fast, fine workmanship, use it and then come back to the comments	Jingdong prefecture level city in Shandong
Doraemon Hyakubo Shop	King of Power Saving	The quality of workmanship is very good and the power saving effect is quite good, I regret not getting it earlier.	Jingdong prefecture level city in Shandong

Doraemon Hyakubo Shop	King of Power Saving	Received the goods, according to the instructions on the operation is very simple, to wait and see the effect, looks good!	Jingdong prefecture level city in Shandong
Doraemon Hyakubo Shop	King of Power Saving	It's really as safe and power-saving as the owner says, and it works great!	Jingdong prefecture level city in Shandong
Doraemon Hyakubo Shop	King of Power Saving	Received the goods, immediately used, looks good, to wait and see how the effect.	Jingdong prefecture level city in Shandong
Doraemon Hyakubo Shop	King of Power Saving	Afraid to buy back did not work, the store said the commitment to 90 days invalid refund, warranty 5 years. Saver also complimentary on the Pacific insurance, it is a lot of peace of mind, there are effects will continue to buy back drops!	Jingdong prefecture level city in Shandong
Doraemon Hyakubo Shop	King of Power Saving	Received the goods and used it, can save electricity a month later to see the effect!	Jingdong prefecture level city in Shandong
Doraemon Hyakubo Shop	King of Power Saving	Fancy 90-day trial, 5-year warranty before the order, the goods received, has been experiencing a week, can really feel the savings, in the introduction of friends also buy this!	Jingdong prefecture level city in Shandong
Doraemon Hyakubo Shop	King of Power Saving	Clothing stores use, neighbors recommended to buy, said a month can save more than 70 yuan, first plugged in to see the effect of the	Jingdong prefecture level city in Shandong
Doraemon Hyakubo Shop	King of Power Saving	A friend's home two months to save more than 80 dollars of electricity, recommended that I buy two, decisive purchase, looking forward to the effect of power saving, waiting for the next month effect.	Jingdong prefecture level city in Shandong
Doraemon Hyakubo Shop	King of Power Saving	The goods have been received, consistent with the description of the advertisement, quite good, I hope that in terms of power saving and purifying the air is really as the merchant said that would be better!	Jingdong prefecture level city in Shandong
Doraemon Hyakubo Shop	King of Power Saving	Power Saving King is really good to use the quality is very good than the physical store a lot cheaper we can rest assured that the purchase recommended to buy!	Jingdong prefecture level city in Shandong
Doraemon Hyakubo Shop	King of Power Saving	Energy saver has been used on the rental house inside the use of the meter is always running fast, with this can really save electricity, easy to use, plugged in can be, very satisfied!	Jingdong prefecture level city in Shandong
Doraemon Hyakubo Shop	King of Power Saving	Just use can not see what effect to,, wait for a period of time in the evaluation	Jingdong prefecture level city in Shandong
Doraemon Hyakubo Shop	King of Power Saving	Live in the 18th floor is too far away from the meter, afraid of no effect, look at the trial 90 days before buying. Currently used for a month, there is an effect, saving 27 dollars in electricity, next month should be able to save more!	Jingdong prefecture level city in Shandong
Doraemon Hyakubo Shop	King of Power Saving	I've been using it for almost a month now and it feels good, so I'll use it again.	Jingdong prefecture level city in Shandong
Doraemon Hyakubo Shop	King of Power Saving	It's ok, the electric bill is really less, originally I remember charging $100 and using it for 10 days. Now it lasts about 15 days. It's still noticeable.	Jingdong prefecture level city in Shandong
Doraemon Hyakubo Shop	King of Power Saving	Fast logistics, good power saving effect, very satisfied	Jingdong prefecture level city in Shandong
Doraemon Hyakubo Shop	King of Power Saving	Has been the second time back to buy, energy saving effect, worth recommending, you can try to see, power saving effect: very good Experience: can save about 30%!	Jingdong prefecture level city in Shandong
Doraemon Hyakubo Shop	King of Power Saving	I don't know how well it works for now	Jingdong prefecture level city in Shandong
Doraemon Hyakubo Shop	King of Power Saving	Factory direct is really good, plugged in and used for half a month did less probably almost 50 degrees of electricity! And the manufacturer's warranty is 5 years.	Jingdong prefecture level city in Shandong
Doraemon Hyakubo Shop	King of Power Saving	It's really effective. The previous month's electricity was $286, but after using the power saver for a month, I have $195 left this month, which is not bad.	Jingdong prefecture level city in Shandong
Doraemon Hyakubo Shop	King of Power Saving	I'll give you a good review and see how it goes.	Jingdong prefecture level city in Shandong
Doraemon Hyakubo Shop	King of Power Saving	Customer service is very professional and patient to guide the installation, the quality of workmanship is very fine. Durable, second repurchase. Really can save oh!	Jingdong prefecture level city in Shandong
Doraemon Hyakubo Shop	King of Power Saving	I've been using it for a month now and my electric bill is over $30 less than last month. I feel it's quite worth buying.	Jingdong prefecture level city in Shandong
Doraemon Hyakubo Shop	King of Power Saving	Small size, does not take up space. It's very good, it does work very effectively, the tool room has a lot of work with a lot of electricity. After installing it, it really reduces a lot of electricity bills. Before the day 30 degrees of electricity left stone, now work down really less a few degrees.	Jingdong prefecture level city in Shandong
Doraemon Hyakubo Shop	King of Power Saving	It does work for saving electricity, I've bought the kind from elsewhere before that was too simple not **all**. This time to buy this store, but also for the safety. Air conditioning is too much electricity, this use to save a lot of	Jingdong prefecture level city in Shandong
Doraemon Hyakubo Shop	King of Power Saving	Two air conditioners and a refrigerator at home, a month down the electricity bill is almost two hundred dollars, with this after the average savings of more than thirty dollars to spend less money to enjoy more, it's really very good!	Jingdong prefecture level city in Shandong
Doraemon Hyakubo Shop	King of Power Saving	Good. Very good.	Jingdong prefecture level city in Shandong
Doraemon Hyakubo Shop	King of Power Saving	Works great, easy to operate, great, very happy with it.	Jingdong prefecture level city in Shandong
Doraemon Hyakubo Shop	King of Power Saving	Received it and tried it out, it's great, great quality and worth having! Five stars praise!	Jingdong prefecture level city in Shandong
Doraemon Hyakubo Shop	King of Power Saving	Very trustworthy brand, quality is guaranteed, the price is still affordable, quite satisfied!	Jingdong prefecture level city in Shandong
Doraemon Hyakubo Shop	King of Power Saving	Use it before you speak.	Jingdong prefecture level city in Shandong
Doraemon Hyakubo Shop	King of Power Saving	The quality of workmanship is very good and the power saving effect is quite good, I regret not getting it earlier.	Jingdong prefecture level city in Shandong
Doraemon Hyakubo Shop	King of Power Saving	Merchants shipping speed, and customer service introduced the same very good, cost-effective	Jingdong prefecture level city in Shandong
Doraemon Hyakubo Shop	King of Power Saving	Feel a little effect Oh, where the power consumption where to plug, convenient and convenient	Jingdong prefecture level city in Shandong
Doraemon Hyakubo Shop	King of Power Saving	The quality of the imported saver is very good, practical and satisfactory. Cost-effective	Jingdong prefecture level city in Shandong
Doraemon Hyakubo Shop	King of Power Saving	Power Saver is especially good, good and inexpensive, very good	Jingdong prefecture level city in Shandong
Doraemon Hyakubo Shop	King of Power Saving	Fast shipping, awesome quality, affordable price, satisfied.	Jingdong prefecture level city in Shandong
Doraemon Hyakubo Shop	King of Power Saving	Energy saver received, the effect is consistent with the description, recommended to buy!	Jingdong prefecture level city in Shandong
Doraemon Hyakubo Shop	King of Power Saving	It's really good, the quality is great, I recommend it!	Jingdong prefecture level city in Shandong
Doraemon Hyakubo Shop	King of Power Saving	Works well. Inexpensive. Shipping was fast.	Jingdong prefecture level city in Shandong
Doraemon Hyakubo Shop	King of Power Saving	Logistics is fast, works well, energy saving is good	Jingdong prefecture level city in Shandong
Doraemon Hyakubo Shop	King of Power Saving	very happy	Jingdong prefecture level city in Shandong
Doraemon Hyakubo Shop	King of Power Saving	an excellent	Jingdong prefecture level city in Shandong
Doraemon Hyakubo Shop	King of Power Saving	It rained a couple days ago and I just went to get the item back today , not bad, hey I looked at the meter and that red display light takes several seconds to jump, did not make a mistake buying it. After a period of time to evaluate.	Jingdong prefecture level city in Shandong
Doraemon Hyakubo Shop	King of Power Saving	Used more than 10 days to check the power, there is indeed some decline, customer service said, long-term use will save more points!	Jingdong prefecture level city in Shandong
Doraemon Hyakubo Shop	King of Power Saving	It's a proper IQ tax, and it costs electricity.	Jingdong prefecture level city in Shandong
Doraemon Hyakubo Shop	King of Power Saving	I've been using it for 17 days now, it really saves electricity Shipping is fast, you can save a lot in a year	Jingdong prefecture level city in Shandong
Doraemon Hyakubo Shop	King of Power Saving	I've been using it for 17 days and it really saves power Shipping is fast and you don't have to worry about the quality, free returns within 5 years	Jingdong prefecture level city in Shandong

Doraemon Hyakubo Shop	King of Power Saving	It's more regular, scanned the code and it's authentic, and the main thing is that you can try it for 90 days! Plugged in more than ten days, is less than before a few degrees per day. When the time to use a month to see Additional. Good to buy a few more	Jingdong prefecture level city in Shandong
Doraemon Hyakubo Shop	King of Power Saving	Logistics is very fast, fine workmanship, use it and then come back to the comments	Jingdong prefecture level city in Shandong
Doraemon Hyakubo Shop	King of Power Saving	The quality of workmanship is very good and the power saving effect is quite good, I regret not getting it earlier.	Jingdong prefecture level city in Shandong
Doraemon Hyakubo Shop	King of Power Saving	Received the goods, according to the instructions on the operation is very simple, to wait and see the effect, looks good!	Jingdong prefecture level city in Shandong
Doraemon Hyakubo Shop	King of Power Saving	It's really as safe and power-saving as the owner says, and it works great!	Jingdong prefecture level city in Shandong
Doraemon Hyakubo Shop	King of Power Saving	Received the goods, immediately used, looks good, to wait and see how the effect.	Jingdong prefecture level city in Shandong
Doraemon Hyakubo Shop	King of Power Saving	Afraid to buy back did not work, the store said the commitment to 90 days invalid refund, warranty 5 years. Saver also complimentary on the Pacific insurance, it is a lot of peace of mind, there are effects will continue to buy back drops!	Jingdong prefecture level city in Shandong
Doraemon Hyakubo Shop	King of Power Saving	Received the goods and used it, can save electricity a month later to see the effect!	Jingdong prefecture level city in Shandong
Doraemon Hyakubo Shop	King of Power Saving	Fancy 90-day trial, 5-year warranty before the order, the goods received, has been experiencing a week, can really feel the savings, in the introduction of friends also buy this!	Jingdong prefecture level city in Shandong
Doraemon Hyakubo Shop	King of Power Saving	Clothing stores use, neighbors recommended to buy, said a month can save more than 70 yuan, first plugged in to see the effect of the	Jingdong prefecture level city in Shandong
Doraemon Hyakubo Shop	King of Power Saving	A friend's home two months to save more than 80 dollars of electricity, recommended that I buy two, decisive purchase, looking forward to the effect of power saving, waiting for the next month effect.	Jingdong prefecture level city in Shandong
Doraemon Hyakubo Shop	King of Power Saving	The goods have been received, consistent with the description of the advertisement, quite good, I hope that in terms of power saving and purifying the air is really as the merchant said that would be better!	Jingdong prefecture level city in Shandong
Doraemon Hyakubo Shop	King of Power Saving	Power Saving King is really good to use the quality is very good than the physical store a lot cheaper we can rest assured that the purchase recommended to buy!	Jingdong prefecture level city in Shandong
Doraemon Hyakubo Shop	King of Power Saving	Energy saver has been used on the rental house inside the use of the meter is always running fast, with this can really save electricity, easy to use, plugged in can be, very satisfied!	Jingdong prefecture level city in Shandong
Doraemon Hyakubo Shop	King of Power Saving	Just use can not see what effect to,, wait for a period of time in the evaluation	Jingdong prefecture level city in Shandong
Doraemon Hyakubo Shop	King of Power Saving	Live in the 18th floor is too far away from the meter, afraid of no effect, look at the trial 90 days before buying. Currently used for a month, there is an effect, saving 27 dollars in electricity, next month should be able to save more!	Jingdong prefecture level city in Shandong
Doraemon Hyakubo Shop	King of Power Saving	I've been using it for almost a month now and it feels good, so I'll use it again.	Jingdong prefecture level city in Shandong
Doraemon Hyakubo Shop	King of Power Saving	It's ok, the electric bill is really less, originally I remember charging $100 and using it for 10 days. Now it lasts about 15 days. It's still noticeable.	Jingdong prefecture level city in Shandong
Doraemon Hyakubo Shop	King of Power Saving	Fast logistics, good power saving effect, very satisfied	Jingdong prefecture level city in Shandong
Doraemon Hyakubo Shop	King of Power Saving	Has been the second time back to buy, energy saving effect, worth recommending, you can try to see, power saving effect: very good Experience: can save about 30%!	Jingdong prefecture level city in Shandong
Doraemon Hyakubo Shop	King of Power Saving	I don't know how well it works for now	Jingdong prefecture level city in Shandong
Doraemon Hyakubo Shop	King of Power Saving	Factory direct is really good, plugged in and used for half a month did less probably almost 50 degrees of electricity! And the manufacturer's warranty is 5 years.	Jingdong prefecture level city in Shandong
Doraemon Hyakubo Shop	King of Power Saving	It's really effective. The previous month's electricity was $286, but after using the power saver for a month, I have $195 left this month, which is not bad.	Jingdong prefecture level city in Shandong
Doraemon Hyakubo Shop	King of Power Saving	I'll give you a good review and see how it goes.	Jingdong prefecture level city in Shandong
Doraemon Hyakubo Shop	King of Power Saving	Customer service is very professional and patient to guide the installation, the quality of workmanship is very fine. Durable, second repurchase. Really can save oh!	Jingdong prefecture level city in Shandong
Doraemon Hyakubo Shop	King of Power Saving	I've been using it for a month now and my electric bill is over $30 less than last month. I feel it's quite worth buying.	Jingdong prefecture level city in Shandong
Doraemon Hyakubo Shop	King of Power Saving	Small size, does not take up space. It's very good, it does work very effectively, the tool room has a lot of work with a lot of electricity. After installing it, it really reduces a lot of electricity bills. Before the day 30 degrees of electricity left stone, now work down really less a few degrees.	Jingdong prefecture level city in Shandong
Doraemon Hyakubo Shop	King of Power Saving	It does work for saving electricity, I've bought the kind from elsewhere before that was too simple not **all**. This time to buy this store, but also for the safety. Air conditioning is too much electricity, this use to save a lot of	Jingdong prefecture level city in Shandong
Doraemon Hyakubo Shop	King of Power Saving	Two air conditioners and a refrigerator at home, a month down the electricity bill is almost two hundred dollars, with this after the average savings of more than thirty dollars to spend less money to enjoy more, it's really very good!	Jingdong prefecture level city in Shandong
Doraemon Hyakubo Shop	King of Power Saving	Good. Very good.	Jingdong prefecture level city in Shandong
Doraemon Hyakubo Shop	King of Power Saving	Works great, easy to operate, great, very happy with it.	Jingdong prefecture level city in Shandong
Doraemon Hyakubo Shop	King of Power Saving	Received it and tried it out, it's great, great quality and worth having! Five stars praise!	Jingdong prefecture level city in Shandong
Doraemon Hyakubo Shop	King of Power Saving	Very trustworthy brand, quality is guaranteed, the price is still affordable, quite satisfied!	Jingdong prefecture level city in Shandong
Doraemon Hyakubo Shop	King of Power Saving	Use it before you speak.	Jingdong prefecture level city in Shandong
Doraemon Hyakubo Shop	King of Power Saving	The quality of workmanship is very good and the power saving effect is quite good, I regret not getting it earlier.	Jingdong prefecture level city in Shandong
Doraemon Hyakubo Shop	King of Power Saving	Merchants shipping speed, and customer service introduced the same very good, cost-effective	Jingdong prefecture level city in Shandong
Doraemon Hyakubo Shop	King of Power Saving	Feel a little effect Oh, where the power consumption where to plug, convenient and convenient	Jingdong prefecture level city in Shandong
Doraemon Hyakubo Shop	King of Power Saving	The quality of the imported saver is very good, practical and satisfactory. Cost-effective	Jingdong prefecture level city in Shandong
Doraemon Hyakubo Shop	King of Power Saving	Power Saver is especially good, good and inexpensive, very good	Jingdong prefecture level city in Shandong
Doraemon Hyakubo Shop	King of Power Saving	Fast shipping, awesome quality, affordable price, satisfied.	Jingdong prefecture level city in Shandong
Doraemon Hyakubo Shop	King of Power Saving	Energy saver received, the effect is consistent with the description, recommended to buy!	Jingdong prefecture level city in Shandong
Doraemon Hyakubo Shop	King of Power Saving	It's really good, the quality is great, I recommend it!	Jingdong prefecture level city in Shandong
Doraemon Hyakubo Shop	King of Power Saving	Works well. Inexpensive. Shipping was fast.	Jingdong prefecture level city in Shandong
Doraemon Hyakubo Shop	King of Power Saving	Logistics is fast, works well, energy saving is good	Jingdong prefecture level city in Shandong
Doraemon Hyakubo Shop	King of Power Saving	very happy	Jingdong prefecture level city in Shandong
Doraemon Hyakubo Shop	King of Power Saving	an excellent	Jingdong prefecture level city in Shandong
Doraemon Hyakubo Shop	King of Power Saving	It rained a couple days ago and I just went to get the item back today , not bad, hey I looked at the meter and that red display light takes several seconds to jump, did not make a mistake buying it. After a period of time to evaluate.	Jingdong prefecture level city in Shandong
Doraemon Hyakubo Shop	King of Power Saving	Used more than 10 days to check the power, there is indeed some decline, customer service said, long-term use will save more points!	Jingdong prefecture level city in Shandong

Doraemon Hyakubo Shop	King of Power Saving	It's a proper IQ tax, and it costs electricity.	Jingdong prefecture level city in Shandong
Doraemon Hyakubo Shop	King of Power Saving	I've been using it for 17 days now, it really saves electricity Shipping is fast, you can save a lot in a year	Jingdong prefecture level city in Shandong
Doraemon Hyakubo Shop	King of Power Saving	I've been using it for 17 days and it really saves power Shipping is fast and you don't have to worry about the quality, free returns within 5 years	Jingdong prefecture level city in Shandong
Doraemon Hyakubo Shop	King of Power Saving	It's more regular, scanned the code and it's authentic, and the main thing is that you can try it for 90 days! Plugged in more than ten days, is less than before a few degrees per day. When the time to use a month to see Additional. Good to buy a few more	Jingdong prefecture level city in Shandong
Doraemon Hyakubo Shop	King of Power Saving	Logistics is very fast, fine workmanship, use it and then come back to the comments	Jingdong prefecture level city in Shandong
Doraemon Hyakubo Shop	King of Power Saving	The quality of workmanship is very good and the power saving effect is quite good, I regret not getting it earlier.	Jingdong prefecture level city in Shandong
Doraemon Hyakubo Shop	King of Power Saving	Received the goods, according to the instructions on the operation is very simple, to wait and see the effect, looks good!	Jingdong prefecture level city in Shandong
Doraemon Hyakubo Shop	King of Power Saving	It's really as safe and power-saving as the owner says, and it works great!	Jingdong prefecture level city in Shandong
Doraemon Hyakubo Shop	King of Power Saving	Received the goods, immediately used, looks good, to wait and see how the effect.	Jingdong prefecture level city in Shandong
Doraemon Hyakubo Shop	King of Power Saving	Afraid to buy back did not work, the store said the commitment to 90 days invalid refund, warranty 5 years. Saver also complimentary on the Pacific insurance, it is a lot of peace of mind, there are effects will continue to buy back drops!	Jingdong prefecture level city in Shandong
Doraemon Hyakubo Shop	King of Power Saving	Received the goods and used it, can save electricity a month later to see the effect!	Jingdong prefecture level city in Shandong
Doraemon Hyakubo Shop	King of Power Saving	Fancy 90-day trial, 5-year warranty before the order, the goods received, has been experiencing a week, can really feel the savings, in the introduction of friends also buy this!	Jingdong prefecture level city in Shandong
Doraemon Hyakubo Shop	King of Power Saving	Clothing stores use, neighbors recommended to buy, said a month can save more than 70 yuan, first plugged in to see the effect of the	Jingdong prefecture level city in Shandong
Doraemon Hyakubo Shop	King of Power Saving	A friend's home two months to save more than 80 dollars of electricity, recommended that I buy two, decisive purchase, looking forward to the effect of power saving, waiting for the next month effect.	Jingdong prefecture level city in Shandong
Doraemon Hyakubo Shop	King of Power Saving	The goods have been received, consistent with the description of the advertisement, quite good, I hope that in terms of power saving and purifying the air is really as the merchant said that would be better!	Jingdong prefecture level city in Shandong
Doraemon Hyakubo Shop	King of Power Saving	Power Saving King is really good to use the quality is very good than the physical store a lot cheaper we can rest assured that the purchase recommended to buy!	Jingdong prefecture level city in Shandong
Doraemon Hyakubo Shop	King of Power Saving	Energy saver has been used on the rental house inside the use of the meter is always running fast, with this can really save electricity, easy to use, plugged in can be, very satisfied!	Jingdong prefecture level city in Shandong
Doraemon Hyakubo Shop	King of Power Saving	Just use can not see what effect to,, wait for a period of time in the evaluation	Jingdong prefecture level city in Shandong
Doraemon Hyakubo Shop	King of Power Saving	Live in the 18th floor is too far away from the meter, afraid of no effect, look at the trial 90 days before buying. Currently used for a month, there is an effect, saving 27 dollars in electricity, next month should be able to save more!	Jingdong prefecture level city in Shandong
Doraemon Hyakubo Shop	King of Power Saving	I've been using it for almost a month now and it feels good, so I'll use it again.	Jingdong prefecture level city in Shandong
Doraemon Hyakubo Shop	King of Power Saving	It's ok, the electric bill is really less, originally I remember charging $100 and using it for 10 days. Now it lasts about 15 days. It's still noticeable.	Jingdong prefecture level city in Shandong
Doraemon Hyakubo Shop	King of Power Saving	Fast logistics, good power saving effect, very satisfied	Jingdong prefecture level city in Shandong
Doraemon Hyakubo Shop	King of Power Saving	Has been the second time back to buy, energy saving effect, worth recommending, you can try to see, power saving effect: very good Experience: can save about 30%!	Jingdong prefecture level city in Shandong
Doraemon Hyakubo Shop	King of Power Saving	I don't know how well it works for now	Jingdong prefecture level city in Shandong
Doraemon Hyakubo Shop	King of Power Saving	Factory direct is really good, plugged in and used for half a month did less probably almost 50 degrees of electricity! And the manufacturer's warranty is 5 years.	Jingdong prefecture level city in Shandong
Doraemon Hyakubo Shop	King of Power Saving	It's really effective. The previous month's electricity was $286, but after using the power saver for a month, I have $195 left this month, which is not bad.	Jingdong prefecture level city in Shandong
Doraemon Hyakubo Shop	King of Power Saving	I'll give you a good review and see how it goes.	Jingdong prefecture level city in Shandong
Doraemon Hyakubo Shop	King of Power Saving	Customer service is very professional and patient to guide the installation, the quality of workmanship is very fine. Durable, second repurchase. Really can save oh!	Jingdong prefecture level city in Shandong
Doraemon Hyakubo Shop	King of Power Saving	I've been using it for a month now and my electric bill is over $30 less than last month. I feel it's quite worth buying.	Jingdong prefecture level city in Shandong
Doraemon Hyakubo Shop	King of Power Saving	Small size, does not take up space. It's very good, it does work very effectively, the tool room has a lot of work with a lot of electricity. After installing it, it really reduces a lot of electricity bills. Before the day 30 degrees of electricity left stone, now work down really less a few degrees.	Jingdong prefecture level city in Shandong
Doraemon Hyakubo Shop	King of Power Saving	It does work for saving electricity, I've bought the kind from elsewhere before that was too simple not **all**. This time to buy this store, but also for the safety. Air conditioning is too much electricity, this use to save a lot of	Jingdong prefecture level city in Shandong
Doraemon Hyakubo Shop	King of Power Saving	Two air conditioners and a refrigerator at home, a month down the electricity bill is almost two hundred dollars, with this after the average savings of more than thirty dollars to spend less money to enjoy more, it's really very good!	Jingdong prefecture level city in Shandong
Doraemon Hyakubo Shop	King of Power Saving	Good. Very good.	Jingdong prefecture level city in Shandong
Doraemon Hyakubo Shop	King of Power Saving	Works great, easy to operate, great, very happy with it.	Jingdong prefecture level city in Shandong
Doraemon Hyakubo Shop	King of Power Saving	Received it and tried it out, it's great, great quality and worth having! Five stars praise!	Jingdong prefecture level city in Shandong
Doraemon Hyakubo Shop	King of Power Saving	Very trustworthy brand, quality is guaranteed, the price is still affordable, quite satisfied!	Jingdong prefecture level city in Shandong
Doraemon Hyakubo Shop	King of Power Saving	Use it before you speak.	Jingdong prefecture level city in Shandong
Doraemon Hyakubo Shop	King of Power Saving	The quality of workmanship is very good and the power saving effect is quite good, I regret not getting it earlier.	Jingdong prefecture level city in Shandong
Doraemon Hyakubo Shop	King of Power Saving	Merchants shipping speed, and customer service introduced the same very good, cost-effective	Jingdong prefecture level city in Shandong
Doraemon Hyakubo Shop	King of Power Saving	Feel a little effect Oh, where the power consumption where to plug, convenient and convenient	Jingdong prefecture level city in Shandong
Doraemon Hyakubo Shop	King of Power Saving	The quality of the imported saver is very good, practical and satisfactory. Cost-effective	Jingdong prefecture level city in Shandong
Doraemon Hyakubo Shop	King of Power Saving	Power Saver is especially good, good and inexpensive, very good	Jingdong prefecture level city in Shandong
Doraemon Hyakubo Shop	King of Power Saving	Fast shipping, awesome quality, affordable price, satisfied.	Jingdong prefecture level city in Shandong
Doraemon Hyakubo Shop	King of Power Saving	Energy saver received, the effect is consistent with the description, recommended to buy!	Jingdong prefecture level city in Shandong
Doraemon Hyakubo Shop	King of Power Saving	It's really good, the quality is great, I recommend it!	Jingdong prefecture level city in Shandong
Doraemon Hyakubo Shop	King of Power Saving	Works well. Inexpensive. Shipping was fast.	Jingdong prefecture level city in Shandong
Doraemon Hyakubo Shop	King of Power Saving	Logistics is fast, works well, energy saving is good	Jingdong prefecture level city in Shandong
Doraemon Hyakubo Shop	King of Power Saving	very happy	Jingdong prefecture level city in Shandong

Doraemon Hyakubo Shop	King of Power Saving	an excellent	Jingdong prefecture level city in Shandong
Doraemon Hyakubo Shop	King of Power Saving	It rained a couple days ago and I just went to get the item back today , not bad, hey I looked at the meter and that red display light takes several seconds to jump, did not make a mistake buying it. After a period of time to evaluate.	Jingdong prefecture level city in Shandong
Doraemon Hyakubo Shop	King of Power Saving	Used more than 10 days to check the power, there is indeed some decline, customer service said, long-term use will save more points!	Jingdong prefecture level city in Shandong
Doraemon Hyakubo Shop	King of Power Saving	It's a proper IQ tax, and it costs electricity.	Jingdong prefecture level city in Shandong
Doraemon Hyakubo Shop	King of Power Saving	I've been using it for 17 days now, it really saves electricity Shipping is fast, you can save a lot in a year	Jingdong prefecture level city in Shandong
Doraemon Hyakubo Shop	King of Power Saving	I've been using it for 17 days and it really saves power Shipping is fast and you don't have to worry about the quality, free returns within 5 years	Jingdong prefecture level city in Shandong
Doraemon Hyakubo Shop	King of Power Saving	It's more regular, scanned the code and it's authentic, and the main thing is that you can try it for 90 days! Plugged in more than ten days, is less than before a few degrees per day. When the time to use a month to see Additional. Good to buy a few more	Jingdong prefecture level city in Shandong
Doraemon Hyakubo Shop	King of Power Saving	Logistics is very fast, fine workmanship, use it and then come back to the comments	Jingdong prefecture level city in Shandong
Doraemon Hyakubo Shop	King of Power Saving	The quality of workmanship is very good and the power saving effect is quite good, I regret not getting it earlier.	Jingdong prefecture level city in Shandong
Doraemon Hyakubo Shop	King of Power Saving	Received the goods, according to the instructions on the operation is very simple, to wait and see the effect, looks good!	Jingdong prefecture level city in Shandong
Doraemon Hyakubo Shop	King of Power Saving	It's really as safe and power-saving as the owner says, and it works great!	Jingdong prefecture level city in Shandong
Doraemon Hyakubo Shop	King of Power Saving	Received the goods, immediately used, looks good, to wait and see how the effect.	Jingdong prefecture level city in Shandong
Doraemon Hyakubo Shop	King of Power Saving	Afraid to buy back did not work, the store said the commitment to 90 days invalid refund, warranty 5 years. Saver also complimentary on the Pacific insurance, it is a lot of peace of mind, there are effects will continue to buy back drops!	Jingdong prefecture level city in Shandong
Doraemon Hyakubo Shop	King of Power Saving	Received the goods and used it, can save electricity a month later to see the effect!	Jingdong prefecture level city in Shandong
Doraemon Hyakubo Shop	King of Power Saving	Fancy 90-day trial, 5-year warranty before the order, the goods received, has been experiencing a week, can really feel the savings, in the introduction of friends also buy this!	Jingdong prefecture level city in Shandong
Doraemon Hyakubo Shop	King of Power Saving	Clothing stores use, neighbors recommended to buy, said a month can save more than 70 yuan, first plugged in to see the effect of the	Jingdong prefecture level city in Shandong
Doraemon Hyakubo Shop	King of Power Saving	A friend's home two months to save more than 80 dollars of electricity, recommended that I buy two, decisive purchase, looking forward to the effect of power saving, waiting for the next month effect.	Jingdong prefecture level city in Shandong
Doraemon Hyakubo Shop	King of Power Saving	The goods have been received, consistent with the description of the advertisement, quite good, I hope that in terms of power saving and purifying the air is really as the merchant said that would be better!	Jingdong prefecture level city in Shandong
Doraemon Hyakubo Shop	King of Power Saving	Power Saving King is really good to use the quality is very good than the physical store a lot cheaper we can rest assured that the purchase recommended to buy!	Jingdong prefecture level city in Shandong
Doraemon Hyakubo Shop	King of Power Saving	Energy saver has been used on the rental house inside the use of the meter is always running fast, with this can really save electricity, easy to use, plugged in can be, very satisfied!	Jingdong prefecture level city in Shandong
Doraemon Hyakubo Shop	King of Power Saving	Just use can not see what effect to,, wait for a period of time in the evaluation	Jingdong prefecture level city in Shandong
Doraemon Hyakubo Shop	King of Power Saving	Live in the 18th floor is too far away from the meter, afraid of no effect, look at the trial 90 days before buying. Currently used for a month, there is an effect, saving 27 dollars in electricity, next month should be able to save more!	Jingdong prefecture level city in Shandong
Doraemon Hyakubo Shop	King of Power Saving	I've been using it for almost a month now and it feels good, so I'll use it again.	Jingdong prefecture level city in Shandong
Doraemon Hyakubo Shop	King of Power Saving	It's ok, the electric bill is really less, originally I remember charging $100 and using it for 10 days. Now it lasts about 15 days. It's still noticeable.	Jingdong prefecture level city in Shandong
Doraemon Hyakubo Shop	King of Power Saving	Fast logistics, good power saving effect, very satisfied	Jingdong prefecture level city in Shandong
Doraemon Hyakubo Shop	King of Power Saving	Has been the second time back to buy, energy saving effect, worth recommending, you can try to see, power saving effect: very good Experience: can save about 30%!	Jingdong prefecture level city in Shandong
Doraemon Hyakubo Shop	King of Power Saving	I don't know how well it works for now	Jingdong prefecture level city in Shandong
Doraemon Hyakubo Shop	King of Power Saving	Factory direct is really good, plugged in and used for half a month did less probably almost 50 degrees of electricity! And the manufacturer's warranty is 5 years.	Jingdong prefecture level city in Shandong
Doraemon Hyakubo Shop	King of Power Saving	It's really effective. The previous month's electricity was $286, but after using the power saver for a month, I have $195 left this month, which is not bad.	Jingdong prefecture level city in Shandong
Doraemon Hyakubo Shop	King of Power Saving	I'll give you a good review and see how it goes.	Jingdong prefecture level city in Shandong
Doraemon Hyakubo Shop	King of Power Saving	Customer service is very professional and patient to guide the installation, the quality of workmanship is very fine. Durable, second repurchase. Really can save oh!	Jingdong prefecture level city in Shandong
Doraemon Hyakubo Shop	King of Power Saving	I've been using it for a month now and my electric bill is over $30 less than last month. I feel it's quite worth buying.	Jingdong prefecture level city in Shandong
Doraemon Hyakubo Shop	King of Power Saving	Small size, does not take up space. It's very good, it does work very effectively, the tool room has a lot of work with a lot of electricity. After installing it, it really reduces a lot of electricity bills. Before the day 30 degrees of electricity left stone, now work down really less a few degrees.	Jingdong prefecture level city in Shandong
Doraemon Hyakubo Shop	King of Power Saving	It does work for saving electricity, I've bought the kind from elsewhere before that was too simple not **all**. This time to buy this store, but also for the safety. Air conditioning is too much electricity, this use to save a lot of	Jingdong prefecture level city in Shandong
Doraemon Hyakubo Shop	King of Power Saving	Two air conditioners and a refrigerator at home, a month down the electricity bill is almost two hundred dollars, with this after the average savings of more than thirty dollars to spend less money to enjoy more, it's really very good!	Jingdong prefecture level city in Shandong
Doraemon Hyakubo Shop	King of Power Saving	Good. Very good.	Jingdong prefecture level city in Shandong
Doraemon Hyakubo Shop	King of Power Saving	Works great, easy to operate, great, very happy with it.	Jingdong prefecture level city in Shandong
Doraemon Hyakubo Shop	King of Power Saving	Received it and tried it out, it's great, great quality and worth having! Five stars praise!	Jingdong prefecture level city in Shandong
Doraemon Hyakubo Shop	King of Power Saving	Very trustworthy brand, quality is guaranteed, the price is still affordable, quite satisfied!	Jingdong prefecture level city in Shandong
Doraemon Hyakubo Shop	King of Power Saving	Use it before you speak.	Jingdong prefecture level city in Shandong
Doraemon Hyakubo Shop	King of Power Saving	The quality of workmanship is very good and the power saving effect is quite good, I regret not getting it earlier.	Jingdong prefecture level city in Shandong
Doraemon Hyakubo Shop	King of Power Saving	Merchants shipping speed, and customer service introduced the same very good, cost-effective	Jingdong prefecture level city in Shandong
Doraemon Hyakubo Shop	King of Power Saving	Feel a little effect Oh, where the power consumption where to plug, convenient and convenient	Jingdong prefecture level city in Shandong
Doraemon Hyakubo Shop	King of Power Saving	The quality of the imported saver is very good, practical and satisfactory. Cost-effective	Jingdong prefecture level city in Shandong
Doraemon Hyakubo Shop	King of Power Saving	Power Saver is especially good, good and inexpensive, very good	Jingdong prefecture level city in Shandong
Doraemon Hyakubo Shop	King of Power Saving	Fast shipping, awesome quality, affordable price, satisfied.	Jingdong prefecture level city in Shandong
Doraemon Hyakubo Shop	King of Power Saving	Energy saver received, the effect is consistent with the description, recommended to buy!	Jingdong prefecture level city in Shandong
Doraemon Hyakubo Shop	King of Power Saving	It's really good, the quality is great, I recommend it!	Jingdong prefecture level city in Shandong

Doraemon Hyakubo Shop	King of Power Saving	Works well. Inexpensive. Shipping was fast.	Jingdong prefecture level city in Shandong
Doraemon Hyakubo Shop	King of Power Saving	Logistics is fast, works well, energy saving is good	Jingdong prefecture level city in Shandong
Doraemon Hyakubo Shop	King of Power Saving	very happy	Jingdong prefecture level city in Shandong
Doraemon Hyakubo Shop	King of Power Saving	an excellent	Jingdong prefecture level city in Shandong
Doraemon Hyakubo Shop	King of Power Saving	It rained a couple days ago and I just went to get the item back today , not bad, hey I looked at the meter and that red display light takes several seconds to jump, did not make a mistake buying it. After a period of time to evaluate.	Jingdong prefecture level city in Shandong
Doraemon Hyakubo Shop	King of Power Saving	Used more than 10 days to check the power, there is indeed some decline, customer service said, long-term use will save more points!	Jingdong prefecture level city in Shandong
Doraemon Hyakubo Shop	King of Power Saving	It's a proper IQ tax, and it costs electricity.	Jingdong prefecture level city in Shandong
Doraemon Hyakubo Shop	King of Power Saving	I've been using it for 17 days now, it really saves electricity Shipping is fast, you can save a lot in a year	Jingdong prefecture level city in Shandong
Doraemon Hyakubo Shop	King of Power Saving	I've been using it for 17 days and it really saves power Shipping is fast and you don't have to worry about the quality, free returns within 5 years	Jingdong prefecture level city in Shandong
Doraemon Hyakubo Shop	King of Power Saving	It's more regular, scanned the code and it's authentic, and the main thing is that you can try it for 90 days! Plugged in more than ten days, is less than before a few degrees per day. When the time to use a month to see Additional. Good to buy a few more	Jingdong prefecture level city in Shandong
Doraemon Hyakubo Shop	King of Power Saving	Logistics is very fast, fine workmanship, use it and then come back to the comments	Jingdong prefecture level city in Shandong
Doraemon Hyakubo Shop	King of Power Saving	The quality of workmanship is very good and the power saving effect is quite good, I regret not getting it earlier.	Jingdong prefecture level city in Shandong
Doraemon Hyakubo Shop	King of Power Saving	Received the goods, according to the instructions on the operation is very simple, to wait and see the effect, looks good!	Jingdong prefecture level city in Shandong
Doraemon Hyakubo Shop	King of Power Saving	It's really as safe and power-saving as the owner says, and it works great!	Jingdong prefecture level city in Shandong
Doraemon Hyakubo Shop	King of Power Saving	Received the goods, immediately used, looks good, to wait and see how the effect.	Jingdong prefecture level city in Shandong
Doraemon Hyakubo Shop	King of Power Saving	Afraid to buy back did not work, the store said the commitment to 90 days invalid refund, warranty 5 years. Saver also complimentary on the Pacific insurance, it is a lot of peace of mind, there are effects will continue to buy back drops!	Jingdong prefecture level city in Shandong
Doraemon Hyakubo Shop	King of Power Saving	Received the goods and used it, can save electricity a month later to see the effect!	Jingdong prefecture level city in Shandong
Doraemon Hyakubo Shop	King of Power Saving	Fancy 90-day trial, 5-year warranty before the order, the goods received, has been experiencing a week, can really feel the savings, in the introduction of friends also buy this!	Jingdong prefecture level city in Shandong
Doraemon Hyakubo Shop	King of Power Saving	Clothing stores use, neighbors recommended to buy, said a month can save more than 70 yuan, first plugged in to see the effect of the	Jingdong prefecture level city in Shandong
Doraemon Hyakubo Shop	King of Power Saving	A friend's home two months to save more than 80 dollars of electricity, recommended that I buy two, decisive purchase, looking forward to the effect of power saving, waiting for the next month effect.	Jingdong prefecture level city in Shandong
Doraemon Hyakubo Shop	King of Power Saving	The goods have been received, consistent with the description of the advertisement, quite good, I hope that in terms of power saving and purifying the air is really as the merchant said that would be better!	Jingdong prefecture level city in Shandong
Doraemon Hyakubo Shop	King of Power Saving	Power Saving King is really good to use the quality is very good than the physical store a lot cheaper we can rest assured that the purchase recommended to buy!	Jingdong prefecture level city in Shandong
Doraemon Hyakubo Shop	King of Power Saving	Energy saver has been used on the rental house inside the use of the meter is always running fast, with this can really save electricity, easy to use, plugged in can be, very satisfied!	Jingdong prefecture level city in Shandong
Doraemon Hyakubo Shop	King of Power Saving	Just use can not see what effect to,, wait for a period of time in the evaluation	Jingdong prefecture level city in Shandong
Doraemon Hyakubo Shop	King of Power Saving	Live in the 18th floor is too far away from the meter, afraid of no effect, look at the trial 90 days before buying. Currently used for a month, there is an effect, saving 27 dollars in electricity, next month should be able to save more!	Jingdong prefecture level city in Shandong
Doraemon Hyakubo Shop	King of Power Saving	I've been using it for almost a month now and it feels good, so I'll use it again.	Jingdong prefecture level city in Shandong
Doraemon Hyakubo Shop	King of Power Saving	It's ok, the electric bill is really less, originally I remember charging $100 and using it for 10 days. Now it lasts about 15 days. It's still noticeable.	Jingdong prefecture level city in Shandong
Doraemon Hyakubo Shop	King of Power Saving	Fast logistics, good power saving effect, very satisfied	Jingdong prefecture level city in Shandong
Doraemon Hyakubo Shop	King of Power Saving	I don't know how well it works for now	Jingdong prefecture level city in Shandong
Doraemon Hyakubo Shop	King of Power Saving	Has been the second time back to buy, energy saving effect, worth recommending, you can try to see, power saving effect: very good Experience: can save about 30%!	Jingdong prefecture level city in Shandong
Doraemon Hyakubo Shop	King of Power Saving	Factory direct is really good, plugged in and used for half a month did less probably almost 50 degrees of electricity! And the manufacturer's warranty is 5 years.	Jingdong prefecture level city in Shandong
Doraemon Hyakubo Shop	King of Power Saving	It's really effective. The previous month's electricity was $286, but after using the power saver for a month, I have $195 left this month, which is not bad.	Jingdong prefecture level city in Shandong
Doraemon Hyakubo Shop	King of Power Saving	I'll give you a good review and see how it goes.	Jingdong prefecture level city in Shandong
Doraemon Hyakubo Shop	King of Power Saving	Customer service is very professional and patient to guide the installation, the quality of workmanship is very fine. Durable, second repurchase. Really can save oh!	Jingdong prefecture level city in Shandong
Doraemon Hyakubo Shop	King of Power Saving	I've been using it for a month now and my electric bill is over $30 less than last month. I feel it's quite worth buying.	Jingdong prefecture level city in Shandong
Doraemon Hyakubo Shop	King of Power Saving	Small size, does not take up space. It's very good, it does work very effectively, the tool room has a lot of work with a lot of electricity. After installing it, it really reduces a lot of electricity bills. Before the day 30 degrees of electricity left stone, now work down really less a few degrees.	Jingdong prefecture level city in Shandong
Doraemon Hyakubo Shop	King of Power Saving	It does work for saving electricity, I've bought the kind from elsewhere before that was too simple not **all**. This time to buy this store, but also for the safety. Air conditioning is too much electricity, this use to save a lot of	Jingdong prefecture level city in Shandong
Doraemon Hyakubo Shop	King of Power Saving	Two air conditioners and a refrigerator at home, a month down the electricity bill is almost two hundred dollars, with this after the average savings of more than thirty dollars to spend less money to enjoy more, it's really very good!	Jingdong prefecture level city in Shandong
Doraemon Hyakubo Shop	King of Power Saving	Good. Very good.	Jingdong prefecture level city in Shandong
Doraemon Hyakubo Shop	King of Power Saving	Works great, easy to operate, great, very happy with it.	Jingdong prefecture level city in Shandong
Doraemon Hyakubo Shop	King of Power Saving	Received it and tried it out, it's great, great quality and worth having! Five stars praise!	Jingdong prefecture level city in Shandong
Doraemon Hyakubo Shop	King of Power Saving	Very trustworthy brand, quality is guaranteed, the price is still affordable, quite satisfied!	Jingdong prefecture level city in Shandong
Doraemon Hyakubo Shop	King of Power Saving	Use it before you speak.	Jingdong prefecture level city in Shandong
Doraemon Hyakubo Shop	King of Power Saving	The quality of workmanship is very good and the power saving effect is quite good, I regret not getting it earlier.	Jingdong prefecture level city in Shandong
Doraemon Hyakubo Shop	King of Power Saving	Merchants shipping speed, and customer service introduced the same very good, cost-effective	Jingdong prefecture level city in Shandong
Doraemon Hyakubo Shop	King of Power Saving	Feel a little effect Oh, where the power consumption where to plug, convenient and convenient	Jingdong prefecture level city in Shandong
Doraemon Hyakubo Shop	King of Power Saving	The quality of the imported saver is very good, practical and satisfactory. Cost-effective	Jingdong prefecture level city in Shandong
Doraemon Hyakubo Shop	King of Power Saving	Power Saver is especially good, good and inexpensive, very good	Jingdong prefecture level city in Shandong

Doraemon Hyakubo Shop	King of Power Saving	Fast shipping, awesome quality, affordable price, satisfied.	Jingdong prefecture level city in Shandong
Doraemon Hyakubo Shop	King of Power Saving	Energy saver received, the effect is consistent with the description, recommended to buy!	Jingdong prefecture level city in Shandong
Doraemon Hyakubo Shop	King of Power Saving	It's really good, the quality is great, I recommend it!	Jingdong prefecture level city in Shandong
Doraemon Hyakubo Shop	King of Power Saving	Works well. Inexpensive. Shipping was fast.	Jingdong prefecture level city in Shandong
Doraemon Hyakubo Shop	King of Power Saving	Logistics is fast, works well, energy saving is good	Jingdong prefecture level city in Shandong
Doraemon Hyakubo Shop	King of Power Saving	very happy	Jingdong prefecture level city in Shandong
Doraemon Hyakubo Shop	King of Power Saving	an excellent	Jingdong prefecture level city in Shandong
Doraemon Hyakubo Shop	King of Power Saving	It rained a couple days ago and I just went to get the item back today , not bad, hey I looked at the meter and that red display light takes several seconds to jump, did not make a mistake buying it. After a period of time to evaluate.	Jingdong prefecture level city in Shandong
Doraemon Hyakubo Shop	King of Power Saving	Used more than 10 days to check the power, there is indeed some decline, customer service said, long-term use will save more points!	Jingdong prefecture level city in Shandong
Doraemon Hyakubo Shop	King of Power Saving	It's a proper IQ tax, and it costs electricity.	Jingdong prefecture level city in Shandong
Doraemon Hyakubo Shop	King of Power Saving	I've been using it for 17 days now, it really saves electricity Shipping is fast, you can save a lot in a year	Jingdong prefecture level city in Shandong
Doraemon Hyakubo Shop	King of Power Saving	I've been using it for 17 days and it really saves power Shipping is fast and you don't have to worry about the quality, free returns within 5 years	Jingdong prefecture level city in Shandong
Doraemon Hyakubo Shop	King of Power Saving	It's more regular, scanned the code and it's authentic, and the main thing is that you can try it for 90 days! Plugged in more than ten days, is less than before a few degrees per day. When the time to use a month to see Additional. Good to buy a few more	Jingdong prefecture level city in Shandong
Doraemon Hyakubo Shop	King of Power Saving	Logistics is very fast, fine workmanship, use it and then come back to the comments	Jingdong prefecture level city in Shandong
Doraemon Hyakubo Shop	King of Power Saving	The quality of workmanship is very good and the power saving effect is quite good, I regret not getting it earlier.	Jingdong prefecture level city in Shandong
Doraemon Hyakubo Shop	King of Power Saving	Received the goods, according to the instructions on the operation is very simple, to wait and see the effect, looks good!	Jingdong prefecture level city in Shandong
Doraemon Hyakubo Shop	King of Power Saving	It's really as safe and power-saving as the owner says, and it works great!	Jingdong prefecture level city in Shandong
Doraemon Hyakubo Shop	King of Power Saving	Received the goods, immediately used, looks good, to wait and see how the effect.	Jingdong prefecture level city in Shandong
Doraemon Hyakubo Shop	King of Power Saving	Afraid to buy back did not work, the store said the commitment to 90 days invalid refund, warranty 5 years. Saver also complimentary on the Pacific insurance, it is a lot of peace of mind, there are effects will continue to buy back drops!	Jingdong prefecture level city in Shandong
Doraemon Hyakubo Shop	King of Power Saving	Received the goods and used it, can save electricity a month later to see the effect!	Jingdong prefecture level city in Shandong
Doraemon Hyakubo Shop	King of Power Saving	Fancy 90-day trial, 5-year warranty before the order, the goods received, has been experiencing a week, can really feel the savings, in the introduction of friends also buy this!	Jingdong prefecture level city in Shandong
Doraemon Hyakubo Shop	King of Power Saving	Clothing stores use, neighbors recommended to buy, said a month can save more than 70 yuan, first plugged in to see the effect of the	Jingdong prefecture level city in Shandong
Doraemon Hyakubo Shop	King of Power Saving	A friend's home two months to save more than 80 dollars of electricity, recommended that I buy two, decisive purchase, looking forward to the effect of power saving, waiting for the next month effect.	Jingdong prefecture level city in Shandong
Doraemon Hyakubo Shop	King of Power Saving	The goods have been received, consistent with the description of the advertisement, quite good, I hope that in terms of power saving and purifying the air is really as the merchant said that would be better!	Jingdong prefecture level city in Shandong
Doraemon Hyakubo Shop	King of Power Saving	Power Saving King is really good to use the quality is very good than the physical store a lot cheaper we can rest assured that the purchase recommended to buy!	Jingdong prefecture level city in Shandong
Doraemon Hyakubo Shop	King of Power Saving	Energy saver has been used on the rental house inside the use of the meter is always running fast, with this can really save electricity, easy to use, plugged in can be, very satisfied!	Jingdong prefecture level city in Shandong
Doraemon Hyakubo Shop	King of Power Saving	Just use can not see what effect to,, wait for a period of time in the evaluation	Jingdong prefecture level city in Shandong
Doraemon Hyakubo Shop	King of Power Saving	Live in the 18th floor is too far away from the meter, afraid of no effect, look at the trial 90 days before buying. Currently used for a month, there is an effect, saving 27 dollars in electricity, next month should be able to save more!	Jingdong prefecture level city in Shandong
Doraemon Hyakubo Shop	King of Power Saving	I've been using it for almost a month now and it feels good, so I'll use it again.	Jingdong prefecture level city in Shandong
Doraemon Hyakubo Shop	King of Power Saving	It's ok, the electric bill is really less, originally I remember charging $100 and using it for 10 days. Now it lasts about 15 days. It's still noticeable.	Jingdong prefecture level city in Shandong
Doraemon Hyakubo Shop	King of Power Saving	Fast logistics, good power saving effect, very satisfied	Jingdong prefecture level city in Shandong
Doraemon Hyakubo Shop	King of Power Saving	Has been the second time back to buy, energy saving effect, worth recommending, you can try to see, power saving effect: very good Experience: can save about 30%!	Jingdong prefecture level city in Shandong
Doraemon Hyakubo Shop	King of Power Saving	I don't know how well it works for now	Jingdong prefecture level city in Shandong
Doraemon Hyakubo Shop	King of Power Saving	Factory direct is really good, plugged in and used for half a month did less probably almost 50 degrees of electricity! And the manufacturer's warranty is 5 years.	Jingdong prefecture level city in Shandong
Doraemon Hyakubo Shop	King of Power Saving	It's really effective. The previous month's electricity was $286, but after using the power saver for a month, I have $195 left this month, which is not bad.	Jingdong prefecture level city in Shandong
Doraemon Hyakubo Shop	King of Power Saving	I'll give you a good review and see how it goes.	Jingdong prefecture level city in Shandong
Doraemon Hyakubo Shop	King of Power Saving	Customer service is very professional and patient to guide the installation, the quality of workmanship is very fine. Durable, second repurchase. Really can save oh!	Jingdong prefecture level city in Shandong
Doraemon Hyakubo Shop	King of Power Saving	I've been using it for a month now and my electric bill is over $30 less than last month. I feel it's quite worth buying.	Jingdong prefecture level city in Shandong
Doraemon Hyakubo Shop	King of Power Saving	Small size, does not take up space. It's very good, it does work very effectively, the tool room has a lot of work with a lot of electricity. After installing it, it really reduces a lot of electricity bills. Before the day 30 degrees of electricity left stone, now work down really less a few degrees.	Jingdong prefecture level city in Shandong
Doraemon Hyakubo Shop	King of Power Saving	It does work for saving electricity, I've bought the kind from elsewhere before that was too simple not **all**. This time to buy this store, but also for the safety. Air conditioning is too much electricity, this use to save a lot of	Jingdong prefecture level city in Shandong
Doraemon Hyakubo Shop	King of Power Saving	Two air conditioners and a refrigerator at home, a month down the electricity bill is almost two hundred dollars, with this after the average savings of more than thirty dollars to spend less money to enjoy more, it's really very good!	Jingdong prefecture level city in Shandong
Doraemon Hyakubo Shop	King of Power Saving	Good. Very good.	Jingdong prefecture level city in Shandong
Doraemon Hyakubo Shop	King of Power Saving	Works great, easy to operate, great, very happy with it.	Jingdong prefecture level city in Shandong
Doraemon Hyakubo Shop	King of Power Saving	Received it and tried it out, it's great, great quality and worth having! Five stars praise!	Jingdong prefecture level city in Shandong
Doraemon Hyakubo Shop	King of Power Saving	Very trustworthy brand, quality is guaranteed, the price is still affordable, quite satisfied!	Jingdong prefecture level city in Shandong
Doraemon Hyakubo Shop	King of Power Saving	Use it before you speak.	Jingdong prefecture level city in Shandong
Doraemon Hyakubo Shop	King of Power Saving	The quality of workmanship is very good and the power saving effect is quite good, I regret not getting it earlier.	Jingdong prefecture level city in Shandong
Doraemon Hyakubo Shop	King of Power Saving	Merchants shipping speed, and customer service introduced the same very good, cost-effective	Jingdong prefecture level city in Shandong

Doraemon Hyakubo Shop	King of Power Saving	Feel a little effect Oh, where the power consumption where to plug, convenient and convenient	Jingdong prefecture level city in Shandong
Doraemon Hyakubo Shop	King of Power Saving	The quality of the imported saver is very good, practical and satisfactory. Cost-effective	Jingdong prefecture level city in Shandong
Doraemon Hyakubo Shop	King of Power Saving	Power Saver is especially good, good and inexpensive, very good	Jingdong prefecture level city in Shandong
Doraemon Hyakubo Shop	King of Power Saving	Fast shipping, awesome quality, affordable price, satisfied.	Jingdong prefecture level city in Shandong
Doraemon Hyakubo Shop	King of Power Saving	Energy saver received, the effect is consistent with the description, recommended to buy!	Jingdong prefecture level city in Shandong
Doraemon Hyakubo Shop	King of Power Saving	It's really good, the quality is great, I recommend it!	Jingdong prefecture level city in Shandong
Doraemon Hyakubo Shop	King of Power Saving	Works well. Inexpensive. Shipping was fast.	Jingdong prefecture level city in Shandong
Doraemon Hyakubo Shop	King of Power Saving	Logistics is fast, works well, energy saving is good	Jingdong prefecture level city in Shandong
Doraemon Hyakubo Shop	King of Power Saving	very happy	Jingdong prefecture level city in Shandong
Doraemon Hyakubo Shop	King of Power Saving	an excellent	Jingdong prefecture level city in Shandong
Doraemon Hyakubo Shop	King of Power Saving	It rained a couple days ago and I just went to get the item back today , not bad, hey I looked at the meter and that red display light takes several seconds to jump, did not make a mistake buying it. After a period of time to evaluate.	Jingdong prefecture level city in Shandong
Doraemon Hyakubo Shop	King of Power Saving	Used more than 10 days to check the power, there is indeed some decline, customer service said, long-term use will save more points!	Jingdong prefecture level city in Shandong
Doraemon Hyakubo Shop	King of Power Saving	It's a proper IQ tax, and it costs electricity.	Jingdong prefecture level city in Shandong
Doraemon Hyakubo Shop	King of Power Saving	I've been using it for 17 days now, it really saves electricity Shipping is fast, you can save a lot in a year	Jingdong prefecture level city in Shandong
Doraemon Hyakubo Shop	King of Power Saving	I've been using it for 17 days and it really saves power Shipping is fast and you don't have to worry about the quality, free returns within 5 years	Jingdong prefecture level city in Shandong
Doraemon Hyakubo Shop	King of Power Saving	It's more regular, scanned the code and it's authentic, and the main thing is that you can try it for 90 days! Plugged in more than ten days, is less than before a few degrees per day. When the time to use a month to see Additional. Good to buy a few more	Jingdong prefecture level city in Shandong
Doraemon Hyakubo Shop	King of Power Saving	Logistics is very fast, fine workmanship, use it and then come back to the comments	Jingdong prefecture level city in Shandong
Doraemon Hyakubo Shop	King of Power Saving	The quality of workmanship is very good and the power saving effect is quite good, I regret not getting it earlier.	Jingdong prefecture level city in Shandong
Doraemon Hyakubo Shop	King of Power Saving	Received the goods, according to the instructions on the operation is very simple, to wait and see the effect, looks good!	Jingdong prefecture level city in Shandong
Doraemon Hyakubo Shop	King of Power Saving	It's really as safe and power-saving as the owner says, and it works great!	Jingdong prefecture level city in Shandong
Doraemon Hyakubo Shop	King of Power Saving	Received the goods, immediately used, looks good, to wait and see how the effect.	Jingdong prefecture level city in Shandong
Doraemon Hyakubo Shop	King of Power Saving	Afraid to buy back did not work, the store said the commitment to 90 days invalid refund, warranty 5 years. Saver also complimentary on the Pacific insurance, it is a lot of peace of mind, there are effects will continue to buy back drops!	Jingdong prefecture level city in Shandong
Doraemon Hyakubo Shop	King of Power Saving	Received the goods and used it, can save electricity a month later to see the effect!	Jingdong prefecture level city in Shandong
Doraemon Hyakubo Shop	King of Power Saving	Fancy 90-day trial, 5-year warranty before the order, the goods received, has been experiencing a week, can really feel the savings, in the introduction of friends also buy this!	Jingdong prefecture level city in Shandong
Doraemon Hyakubo Shop	King of Power Saving	Clothing stores use, neighbors recommended to buy, said a month can save more than 70 yuan, first plugged in to see the effect of the	Jingdong prefecture level city in Shandong
Doraemon Hyakubo Shop	King of Power Saving	A friend's home two months to save more than 80 dollars of electricity, recommended that I buy two, decisive purchase, looking forward to the effect of power saving, waiting for the next month effect.	Jingdong prefecture level city in Shandong
Doraemon Hyakubo Shop	King of Power Saving	The goods have been received, consistent with the description of the advertisement, quite good, I hope that in terms of power saving and purifying the air is really as the merchant said that would be better!	Jingdong prefecture level city in Shandong
Doraemon Hyakubo Shop	King of Power Saving	Power Saving King is really good to use the quality is very good than the physical store a lot cheaper we can rest assured that the purchase recommended to buy!	Jingdong prefecture level city in Shandong
Doraemon Hyakubo Shop	King of Power Saving	Energy saver has been used on the rental house inside the use of the meter is always running fast, with this can really save electricity, easy to use, plugged in can be, very satisfied!	Jingdong prefecture level city in Shandong
Doraemon Hyakubo Shop	King of Power Saving	Just use can not see what effect to,, wait for a period of time in the evaluation	Jingdong prefecture level city in Shandong
Doraemon Hyakubo Shop	King of Power Saving	Live in the 18th floor is too far away from the meter, afraid of no effect, look at the trial 90 days before buying. Currently used for a month, there is an effect, saving 27 dollars in electricity, next month should be able to save more!	Jingdong prefecture level city in Shandong
Doraemon Hyakubo Shop	King of Power Saving	I've been using it for almost a month now and it feels good, so I'll use it again.	Jingdong prefecture level city in Shandong
Doraemon Hyakubo Shop	King of Power Saving	It's ok, the electric bill is really less, originally I remember charging $100 and using it for 10 days. Now it lasts about 15 days. It's still noticeable.	Jingdong prefecture level city in Shandong
Doraemon Hyakubo Shop	King of Power Saving	Fast logistics, good power saving effect, very satisfied	Jingdong prefecture level city in Shandong
Doraemon Hyakubo Shop	King of Power Saving	Has been the second time back to buy, energy saving effect, worth recommending, you can try to see, power saving effect: very good Experience: can save about 30%!	Jingdong prefecture level city in Shandong
Doraemon Hyakubo Shop	King of Power Saving	I don't know how well it works for now	Jingdong prefecture level city in Shandong
Doraemon Hyakubo Shop	King of Power Saving	Factory direct is really good, plugged in and used for half a month did less probably almost 50 degrees of electricity! And the manufacturer's warranty is 5 years.	Jingdong prefecture level city in Shandong
Doraemon Hyakubo Shop	King of Power Saving	It's really effective. The previous month's electricity was $286, but after using the power saver for a month, I have $195 left this month, which is not bad.	Jingdong prefecture level city in Shandong
Doraemon Hyakubo Shop	King of Power Saving	I'll give you a good review and see how it goes.	Jingdong prefecture level city in Shandong
Doraemon Hyakubo Shop	King of Power Saving	Customer service is very professional and patient to guide the installation, the quality of workmanship is very fine. Durable, second repurchase. Really can save oh!	Jingdong prefecture level city in Shandong
Doraemon Hyakubo Shop	King of Power Saving	I've been using it for a month now and my electric bill is over $30 less than last month. I feel it's quite worth buying.	Jingdong prefecture level city in Shandong
Doraemon Hyakubo Shop	King of Power Saving	Small size, does not take up space. It's very good, it does work very effectively, the tool room has a lot of work with a lot of electricity. After installing it, it really reduces a lot of electricity bills. Before the day 30 degrees of electricity left stone, now work down really less a few degrees.	Jingdong prefecture level city in Shandong
Doraemon Hyakubo Shop	King of Power Saving	It does work for saving electricity, I've bought the kind from elsewhere before that was too simple not **all**. This time to buy this store, but also for the safety. Air conditioning is too much electricity, this use to save a lot of	Jingdong prefecture level city in Shandong
Doraemon Hyakubo Shop	King of Power Saving	Two air conditioners and a refrigerator at home, a month down the electricity bill is almost two hundred dollars, with this after the average savings of more than thirty dollars to spend less money to enjoy more, it's really very good!	Jingdong prefecture level city in Shandong
Doraemon Hyakubo Shop	King of Power Saving	Good. Very good.	Jingdong prefecture level city in Shandong
Doraemon Hyakubo Shop	King of Power Saving	Works great, easy to operate, great, very happy with it.	Jingdong prefecture level city in Shandong
Doraemon Hyakubo Shop	King of Power Saving	Received it and tried it out, it's great, great quality and worth having! Five stars praise!	Jingdong prefecture level city in Shandong
Doraemon Hyakubo Shop	King of Power Saving	Very trustworthy brand, quality is guaranteed, the price is still affordable, quite satisfied!	Jingdong prefecture level city in Shandong

Doraemon Hyakubo Shop	King of Power Saving	Use it before you speak.	Jingdong prefecture level city in Shandong
Doraemon Hyakubo Shop	King of Power Saving	The quality of workmanship is very good and the power saving effect is quite good, I regret not getting it earlier.	Jingdong prefecture level city in Shandong
Doraemon Hyakubo Shop	King of Power Saving	Merchants shipping speed, and customer service introduced the same very good, cost-effective	Jingdong prefecture level city in Shandong
Doraemon Hyakubo Shop	King of Power Saving	Feel a little effect Oh, where the power consumption where to plug, convenient and convenient	Jingdong prefecture level city in Shandong
Doraemon Hyakubo Shop	King of Power Saving	The quality of the imported saver is very good, practical and satisfactory. Cost-effective	Jingdong prefecture level city in Shandong
Doraemon Hyakubo Shop	King of Power Saving	Power Saver is especially good, good and inexpensive, very good	Jingdong prefecture level city in Shandong
Doraemon Hyakubo Shop	King of Power Saving	Fast shipping, awesome quality, affordable price, satisfied.	Jingdong prefecture level city in Shandong
Doraemon Hyakubo Shop	King of Power Saving	Energy saver received, the effect is consistent with the description, recommended to buy!	Jingdong prefecture level city in Shandong
Doraemon Hyakubo Shop	King of Power Saving	It's really good, the quality is great, I recommend it!	Jingdong prefecture level city in Shandong
Doraemon Hyakubo Shop	King of Power Saving	Works well. Inexpensive. Shipping was fast.	Jingdong prefecture level city in Shandong
Doraemon Hyakubo Shop	King of Power Saving	Logistics is fast, works well, energy saving is good	Jingdong prefecture level city in Shandong
Doraemon Hyakubo Shop	King of Power Saving	very happy	Jingdong prefecture level city in Shandong
Doraemon Hyakubo Shop	King of Power Saving	an excellent	Jingdong prefecture level city in Shandong
Doraemon Hyakubo Shop	King of Power Saving	It rained a couple days ago and I just went to get the item back today , not bad, hey I looked at the meter and that red display light takes several seconds to jump, did not make a mistake buying it. After a period of time to evaluate.	Jingdong prefecture level city in Shandong
Doraemon Hyakubo Shop	King of Power Saving	Used more than 10 days to check the power, there is indeed some decline, customer service said, long-term use will save more points!	Jingdong prefecture level city in Shandong
Doraemon Hyakubo Shop	King of Power Saving	It's a proper IQ tax, and it costs electricity.	Jingdong prefecture level city in Shandong
Doraemon Hyakubo Shop	King of Power Saving	I've been using it for 17 days now, it really saves electricity Shipping is fast, you can save a lot in a year	Jingdong prefecture level city in Shandong
Doraemon Hyakubo Shop	King of Power Saving	I've been using it for 17 days and it really saves power Shipping is fast and you don't have to worry about the quality, free returns within 5 years	Jingdong prefecture level city in Shandong
Doraemon Hyakubo Shop	King of Power Saving	It's more regular, scanned the code and it's authentic, and the main thing is that you can try it for 90 days! Plugged in more than ten days, is less than before a few degrees per day. When the time to use a month to see Additional. Good to buy a few more	Jingdong prefecture level city in Shandong
Doraemon Hyakubo Shop	King of Power Saving	Logistics is very fast, fine workmanship, use it and then come back to the comments	Jingdong prefecture level city in Shandong
Doraemon Hyakubo Shop	King of Power Saving	The quality of workmanship is very good and the power saving effect is quite good, I regret not getting it earlier.	Jingdong prefecture level city in Shandong
Doraemon Hyakubo Shop	King of Power Saving	Received the goods, according to the instructions on the operation is very simple, to wait and see the effect, looks good!	Jingdong prefecture level city in Shandong
Doraemon Hyakubo Shop	King of Power Saving	It's really as safe and power-saving as the owner says, and it works great!	Jingdong prefecture level city in Shandong
Doraemon Hyakubo Shop	King of Power Saving	Received the goods, immediately used, looks good, to wait and see how the effect.	Jingdong prefecture level city in Shandong
Doraemon Hyakubo Shop	King of Power Saving	Afraid to buy back did not work, the store said the commitment to 90 days invalid refund, warranty 5 years. Saver also complimentary on the Pacific insurance, it is a lot of peace of mind, there are effects will continue to buy back drops!	Jingdong prefecture level city in Shandong
Doraemon Hyakubo Shop	King of Power Saving	Received the goods and used it, can save electricity a month later to see the effect!	Jingdong prefecture level city in Shandong
Doraemon Hyakubo Shop	King of Power Saving	Fancy 90-day trial, 5-year warranty before the order, the goods received, has been experiencing a week, can really feel the savings, in the introduction of friends also buy this!	Jingdong prefecture level city in Shandong
Doraemon Hyakubo Shop	King of Power Saving	Clothing stores use, neighbors recommended to buy, said a month can save more than 70 yuan, first plugged in to see the effect of the	Jingdong prefecture level city in Shandong
Doraemon Hyakubo Shop	King of Power Saving	A friend's home two months to save more than 80 dollars of electricity, recommended that I buy two, decisive purchase, looking forward to the effect of power saving, waiting for the next month effect.	Jingdong prefecture level city in Shandong
Doraemon Hyakubo Shop	King of Power Saving	The goods have been received, consistent with the description of the advertisement, quite good, I hope that in terms of power saving and purifying the air is really as the merchant said that would be better!	Jingdong prefecture level city in Shandong
Doraemon Hyakubo Shop	King of Power Saving	Power Saving King is really good to use the quality is very good than the physical store a lot cheaper we can rest assured that the purchase recommended to buy!	Jingdong prefecture level city in Shandong
Doraemon Hyakubo Shop	King of Power Saving	Energy saver has been used on the rental house inside the use of the meter is always running fast, with this can really save electricity, easy to use, plugged in can be, very satisfied!	Jingdong prefecture level city in Shandong
Doraemon Hyakubo Shop	King of Power Saving	Just use can not see what effect to,, wait for a period of time in the evaluation	Jingdong prefecture level city in Shandong
Doraemon Hyakubo Shop	King of Power Saving	Live in the 18th floor is too far away from the meter, afraid of no effect, look at the trial 90 days before buying. Currently used for a month, there is an effect, saving 27 dollars in electricity, next month should be able to save more!	Jingdong prefecture level city in Shandong
Doraemon Hyakubo Shop	King of Power Saving	I've been using it for almost a month now and it feels good, so I'll use it again.	Jingdong prefecture level city in Shandong
Doraemon Hyakubo Shop	King of Power Saving	It's ok, the electric bill is really less, originally I remember charging $100 and using it for 10 days. Now it lasts about 15 days. It's still noticeable.	Jingdong prefecture level city in Shandong
Doraemon Hyakubo Shop	King of Power Saving	Fast logistics, good power saving effect, very satisfied	Jingdong prefecture level city in Shandong
Doraemon Hyakubo Shop	King of Power Saving	Has been the second time back to buy, energy saving effect, worth recommending, you can try to see, power saving effect: very good Experience: can save about 30%!	Jingdong prefecture level city in Shandong
Doraemon Hyakubo Shop	King of Power Saving	I don't know how well it works for now	Jingdong prefecture level city in Shandong
Doraemon Hyakubo Shop	King of Power Saving	Factory direct is really good, plugged in and used for half a month did less probably almost 50 degrees of electricity! And the manufacturer's warranty is 5 years.	Jingdong prefecture level city in Shandong
Doraemon Hyakubo Shop	King of Power Saving	It's really effective. The previous month's electricity was $286, but after using the power saver for a month, I have $195 left this month, which is not bad.	Jingdong prefecture level city in Shandong
Doraemon Hyakubo Shop	King of Power Saving	I'll give you a good review and see how it goes.	Jingdong prefecture level city in Shandong
Doraemon Hyakubo Shop	King of Power Saving	Customer service is very professional and patient to guide the installation, the quality of workmanship is very fine. Durable, second repurchase. Really can save oh!	Jingdong prefecture level city in Shandong
Doraemon Hyakubo Shop	King of Power Saving	I've been using it for a month now and my electric bill is over $30 less than last month. I feel it's quite worth buying.	Jingdong prefecture level city in Shandong
Doraemon Hyakubo Shop	King of Power Saving	Small size, does not take up space. It's very good, it does work very effectively, the tool room has a lot of work with a lot of electricity. After installing it, it really reduces a lot of electricity bills. Before the day 30 degrees of electricity left stone, now work down really less a few degrees.	Jingdong prefecture level city in Shandong
Doraemon Hyakubo Shop	King of Power Saving	It does work for saving electricity, I've bought the kind from elsewhere before that was too simple not **all**. This time to buy this store, but also for the safety. Air conditioning is too much electricity, this use to save a lot of	Jingdong prefecture level city in Shandong
Doraemon Hyakubo Shop	King of Power Saving	Two air conditioners and a refrigerator at home, a month down the electricity bill is almost two hundred dollars, with this after the average savings of more than thirty dollars to spend less money to enjoy more, it's really very good!	Jingdong prefecture level city in Shandong
Doraemon Hyakubo Shop	King of Power Saving	Good. Very good.	Jingdong prefecture level city in Shandong

Doraemon Hyakubo Shop	King of Power Saving	Works great, easy to operate, great, very happy with it.	Jingdong prefecture level city in Shandong
Doraemon Hyakubo Shop	King of Power Saving	Received it and tried it out, it's great, great quality and worth having! Five stars praise!	Jingdong prefecture level city in Shandong
Doraemon Hyakubo Shop	King of Power Saving	Very trustworthy brand, quality is guaranteed, the price is still affordable, quite satisfied!	Jingdong prefecture level city in Shandong
Doraemon Hyakubo Shop	King of Power Saving	Use it before you speak.	Jingdong prefecture level city in Shandong
Doraemon Hyakubo Shop	King of Power Saving	The quality of workmanship is very good and the power saving effect is quite good, I regret not getting it earlier.	Jingdong prefecture level city in Shandong
Doraemon Hyakubo Shop	King of Power Saving	Merchants shipping speed, and customer service introduced the same very good, cost-effective	Jingdong prefecture level city in Shandong
Doraemon Hyakubo Shop	King of Power Saving	Feel a little effect Oh, where the power consumption where to plug, convenient and convenient	Jingdong prefecture level city in Shandong
Doraemon Hyakubo Shop	King of Power Saving	The quality of the imported saver is very good, practical and satisfactory. Cost-effective	Jingdong prefecture level city in Shandong
Doraemon Hyakubo Shop	King of Power Saving	Power Saver is especially good, good and inexpensive, very good	Jingdong prefecture level city in Shandong
Doraemon Hyakubo Shop	King of Power Saving	Fast shipping, awesome quality, affordable price, satisfied.	Jingdong prefecture level city in Shandong
Doraemon Hyakubo Shop	King of Power Saving	Energy saver received, the effect is consistent with the description, recommended to buy!	Jingdong prefecture level city in Shandong
Doraemon Hyakubo Shop	King of Power Saving	It's really good, the quality is great, I recommend it!	Jingdong prefecture level city in Shandong
Doraemon Hyakubo Shop	King of Power Saving	Works well. Inexpensive. Shipping was fast.	Jingdong prefecture level city in Shandong
Doraemon Hyakubo Shop	King of Power Saving	Logistics is fast, works well, energy saving is good	Jingdong prefecture level city in Shandong
Doraemon Hyakubo Shop	King of Power Saving	very happy	Jingdong prefecture level city in Shandong
Doraemon Hyakubo Shop	King of Power Saving	an excellent	Jingdong prefecture level city in Shandong
Doraemon Hyakubo Shop	King of Power Saving	It rained a couple days ago and I just went to get the item back today , not bad, hey I looked at the meter and that red display light takes several seconds to jump, did not make a mistake buying it. After a period of time to evaluate.	Jingdong prefecture level city in Shandong
Doraemon Hyakubo Shop	King of Power Saving	Used more than 10 days to check the power, there is indeed some decline, customer service said, long-term use will save more points!	Jingdong prefecture level city in Shandong
Doraemon Hyakubo Shop	King of Power Saving	It's a proper IQ tax, and it costs electricity.	Jingdong prefecture level city in Shandong
Doraemon Hyakubo Shop	King of Power Saving	I've been using it for 17 days now, it really saves electricity Shipping is fast, you can save a lot in a year	Jingdong prefecture level city in Shandong
Doraemon Hyakubo Shop	King of Power Saving	I've been using it for 17 days and it really saves power Shipping is fast and you don't have to worry about the quality, free returns within 5 years	Jingdong prefecture level city in Shandong
Doraemon Hyakubo Shop	King of Power Saving	It's more regular, scanned the code and it's authentic, and the main thing is that you can try it for 90 days! Plugged in more than ten days, is less than before a few degrees per day. When the time to use a month to see Additional. Good to buy a few more	Jingdong prefecture level city in Shandong
Doraemon Hyakubo Shop	King of Power Saving	Logistics is very fast, fine workmanship, use it and then come back to the comments	Jingdong prefecture level city in Shandong
Doraemon Hyakubo Shop	King of Power Saving	The quality of workmanship is very good and the power saving effect is quite good, I regret not getting it earlier.	Jingdong prefecture level city in Shandong
Doraemon Hyakubo Shop	King of Power Saving	Received the goods, according to the instructions on the operation is very simple, to wait and see the effect, looks good!	Jingdong prefecture level city in Shandong
Doraemon Hyakubo Shop	King of Power Saving	It's really as safe and power-saving as the owner says, and it works great!	Jingdong prefecture level city in Shandong
Doraemon Hyakubo Shop	King of Power Saving	Received the goods, immediately used, looks good, to wait and see how the effect.	Jingdong prefecture level city in Shandong
Doraemon Hyakubo Shop	King of Power Saving	Afraid to buy back did not work, the store said the commitment to 90 days invalid refund, warranty 5 years. Saver also complimentary on the Pacific insurance, it is a lot of peace of mind, there are effects will continue to buy back drops!	Jingdong prefecture level city in Shandong
Doraemon Hyakubo Shop	King of Power Saving	Received the goods and used it, can save electricity a month later to see the effect!	Jingdong prefecture level city in Shandong
Doraemon Hyakubo Shop	King of Power Saving	Fancy 90-day trial, 5-year warranty before the order, the goods received, has been experiencing a week, can really feel the savings, in the introduction of friends also buy this!	Jingdong prefecture level city in Shandong
Doraemon Hyakubo Shop	King of Power Saving	Clothing stores use, neighbors recommended to buy, said a month can save more than 70 yuan, first plugged in to see the effect of the	Jingdong prefecture level city in Shandong
Doraemon Hyakubo Shop	King of Power Saving	A friend's home two months to save more than 80 dollars of electricity, recommended that I buy two, decisive purchase, looking forward to the effect of power saving, waiting for the next month effect.	Jingdong prefecture level city in Shandong
Doraemon Hyakubo Shop	King of Power Saving	The goods have been received, consistent with the description of the advertisement, quite good, I hope that in terms of power saving and purifying the air is really as the merchant said that would be better!	Jingdong prefecture level city in Shandong
Doraemon Hyakubo Shop	King of Power Saving	Power Saving King is really good to use the quality is very good than the physical store a lot cheaper we can rest assured that the purchase recommended to buy!	Jingdong prefecture level city in Shandong
Doraemon Hyakubo Shop	King of Power Saving	Energy saver has been used on the rental house inside the use of the meter is always running fast, with this can really save electricity, easy to use, plugged in can be, very satisfied!	Jingdong prefecture level city in Shandong
Doraemon Hyakubo Shop	King of Power Saving	Just use can not see what effect to,, wait for a period of time in the evaluation	Jingdong prefecture level city in Shandong
Doraemon Hyakubo Shop	King of Power Saving	Live in the 18th floor is too far away from the meter, afraid of no effect, look at the trial 90 days before buying. Currently used for a month, there is an effect, saving 27 dollars in electricity, next month should be able to save more!	Jingdong prefecture level city in Shandong
Doraemon Hyakubo Shop	King of Power Saving	I've been using it for almost a month now and it feels good, so I'll use it again.	Jingdong prefecture level city in Shandong
Doraemon Hyakubo Shop	King of Power Saving	It's ok, the electric bill is really less, originally I remember charging $100 and using it for 10 days. Now it lasts about 15 days. It's still noticeable.	Jingdong prefecture level city in Shandong
Doraemon Hyakubo Shop	King of Power Saving	Fast logistics, good power saving effect, very satisfied	Jingdong prefecture level city in Shandong
Doraemon Hyakubo Shop	King of Power Saving	Has been the second time back to buy, energy saving effect, worth recommending, you can try to see, power saving effect: very good Experience: can save about 30%!	Jingdong prefecture level city in Shandong
Doraemon Hyakubo Shop	King of Power Saving	I don't know how well it works for now	Jingdong prefecture level city in Shandong
Doraemon Hyakubo Shop	King of Power Saving	Factory direct is really good, plugged in and used for half a month did less probably almost 50 degrees of electricity! And the manufacturer's warranty is 5 years.	Jingdong prefecture level city in Shandong
Doraemon Hyakubo Shop	King of Power Saving	It's really effective. The previous month's electricity was $286, but after using the power saver for a month, I have $195 left this month, which is not bad.	Jingdong prefecture level city in Shandong
Doraemon Hyakubo Shop	King of Power Saving	I'll give you a good review and see how it goes.	Jingdong prefecture level city in Shandong
Doraemon Hyakubo Shop	King of Power Saving	Customer service is very professional and patient to guide the installation, the quality of workmanship is very fine. Durable, second repurchase. Really can save oh!	Jingdong prefecture level city in Shandong
Doraemon Hyakubo Shop	King of Power Saving	I've been using it for a month now and my electric bill is over $30 less than last month. I feel it's quite worth buying.	Jingdong prefecture level city in Shandong
Doraemon Hyakubo Shop	King of Power Saving	Small size, does not take up space. It's very good, it does work very effectively, the tool room has a lot of work with a lot of electricity. After installing it, it really reduces a lot of electricity bills. Before the day 30 degrees of electricity left stone, now work down really less a few degrees.	Jingdong prefecture level city in Shandong

Doraemon Hyakubo Shop	King of Power Saving	It does work for saving electricity, I've bought the kind from elsewhere before that was too simple not **all**. This time to buy this store, but also for the safety. Air conditioning is too much electricity, this use to save a lot of	Jingdong prefecture level city in Shandong
Doraemon Hyakubo Shop	King of Power Saving	Two air conditioners and a refrigerator at home, a month down the electricity bill is almost two hundred dollars, with this after the average savings of more than thirty dollars to spend less money to enjoy more, it's really very good!	Jingdong prefecture level city in Shandong
Doraemon Hyakubo Shop	King of Power Saving	Good. Very good.	Jingdong prefecture level city in Shandong
Doraemon Hyakubo Shop	King of Power Saving	Works great, easy to operate, great, very happy with it.	Jingdong prefecture level city in Shandong
Doraemon Hyakubo Shop	King of Power Saving	Received it and tried it out, it's great, great quality and worth having! Five stars praise!	Jingdong prefecture level city in Shandong
Doraemon Hyakubo Shop	King of Power Saving	Very trustworthy brand, quality is guaranteed, the price is still affordable, quite satisfied!	Jingdong prefecture level city in Shandong
Doraemon Hyakubo Shop	King of Power Saving	Use it before you speak.	Jingdong prefecture level city in Shandong
Doraemon Hyakubo Shop	King of Power Saving	The quality of workmanship is very good and the power saving effect is quite good, I regret not getting it earlier.	Jingdong prefecture level city in Shandong
Doraemon Hyakubo Shop	King of Power Saving	Merchants shipping speed, and customer service introduced the same very good, cost-effective	Jingdong prefecture level city in Shandong
Doraemon Hyakubo Shop	King of Power Saving	Feel a little effect Oh, where the power consumption where to plug, convenient and convenient	Jingdong prefecture level city in Shandong
Doraemon Hyakubo Shop	King of Power Saving	The quality of the imported saver is very good, practical and satisfactory. Cost-effective	Jingdong prefecture level city in Shandong
Doraemon Hyakubo Shop	King of Power Saving	Power Saver is especially good, good and inexpensive, very good	Jingdong prefecture level city in Shandong
Doraemon Hyakubo Shop	King of Power Saving	Fast shipping, awesome quality, affordable price, satisfied.	Jingdong prefecture level city in Shandong
Doraemon Hyakubo Shop	King of Power Saving	Energy saver received, the effect is consistent with the description, recommended to buy!	Jingdong prefecture level city in Shandong
Doraemon Hyakubo Shop	King of Power Saving	It's really good, the quality is great, I recommend it!	Jingdong prefecture level city in Shandong
Doraemon Hyakubo Shop	King of Power Saving	Works well. Inexpensive. Shipping was fast.	Jingdong prefecture level city in Shandong
Doraemon Hyakubo Shop	King of Power Saving	Logistics is fast, works well, energy saving is good	Jingdong prefecture level city in Shandong
Doraemon Hyakubo Shop	King of Power Saving	very happy	Jingdong prefecture level city in Shandong
Doraemon Hyakubo Shop	King of Power Saving	an excellent	Jingdong prefecture level city in Shandong
Doraemon Hyakubo Shop	King of Power Saving	It rained a couple days ago and I just went to get the item back today , not bad, hey I looked at the meter and that red display light takes several seconds to jump, did not make a mistake buying it. After a period of time to evaluate.	Jingdong prefecture level city in Shandong
Doraemon Hyakubo Shop	King of Power Saving	Used more than 10 days to check the power, there is indeed some decline, customer service said, long-term use will save more points!	Jingdong prefecture level city in Shandong
Doraemon Hyakubo Shop	King of Power Saving	It's a proper IQ tax, and it costs electricity.	Jingdong prefecture level city in Shandong
Doraemon Hyakubo Shop	King of Power Saving	I've been using it for 17 days now, it really saves electricity Shipping is fast, you can save a lot in a year	Jingdong prefecture level city in Shandong
Doraemon Hyakubo Shop	King of Power Saving	I've been using it for 17 days and it really saves power Shipping is fast and you don't have to worry about the quality, free returns within 5 years	Jingdong prefecture level city in Shandong
Doraemon Hyakubo Shop	King of Power Saving	It's more regular, scanned the code and it's authentic, and the main thing is that you can try it for 90 days! Plugged in more than ten days, is less than before a few degrees per day. When the time to use a month to see Additional. Good to buy a few more	Jingdong prefecture level city in Shandong
Doraemon Hyakubo Shop	King of Power Saving	Logistics is very fast, fine workmanship, use it and then come back to the comments	Jingdong prefecture level city in Shandong
Doraemon Hyakubo Shop	King of Power Saving	The quality of workmanship is very good and the power saving effect is quite good, I regret not getting it earlier.	Jingdong prefecture level city in Shandong
Doraemon Hyakubo Shop	King of Power Saving	Received the goods, according to the instructions on the operation is very simple, to wait and see the effect, looks good!	Jingdong prefecture level city in Shandong
Doraemon Hyakubo Shop	King of Power Saving	It's really as safe and power-saving as the owner says, and it works great!	Jingdong prefecture level city in Shandong
Doraemon Hyakubo Shop	King of Power Saving	Received the goods, immediately used, looks good, to wait and see how the effect.	Jingdong prefecture level city in Shandong
Doraemon Hyakubo Shop	King of Power Saving	Afraid to buy back did not work, the store said the commitment to 90 days invalid refund, warranty 5 years. Saver also complimentary on the Pacific insurance, it is a lot of peace of mind, there are effects will continue to buy back drops!	Jingdong prefecture level city in Shandong
Doraemon Hyakubo Shop	King of Power Saving	Received the goods and used it, can save electricity a month later to see the effect!	Jingdong prefecture level city in Shandong
Doraemon Hyakubo Shop	King of Power Saving	Fancy 90-day trial, 5-year warranty before the order, the goods received, has been experiencing a week, can really feel the savings, in the introduction of friends also buy this!	Jingdong prefecture level city in Shandong
Doraemon Hyakubo Shop	King of Power Saving	Clothing stores use, neighbors recommended to buy, said a month can save more than 70 yuan, first plugged in to see the effect of the	Jingdong prefecture level city in Shandong
Doraemon Hyakubo Shop	King of Power Saving	A friend's home two months to save more than 80 dollars of electricity, recommended that I buy two, decisive purchase, looking forward to the effect of power saving, waiting for the next month effect.	Jingdong prefecture level city in Shandong
Doraemon Hyakubo Shop	King of Power Saving	The goods have been received, consistent with the description of the advertisement, quite good, I hope that in terms of power saving and purifying the air is really as the merchant said that would be better!	Jingdong prefecture level city in Shandong
Doraemon Hyakubo Shop	King of Power Saving	Power Saving King is really good to use the quality is very good than the physical store a lot cheaper we can rest assured that the purchase recommended to buy!	Jingdong prefecture level city in Shandong
Doraemon Hyakubo Shop	King of Power Saving	Energy saver has been used on the rental house inside the use of the meter is always running fast, with this can really save electricity, easy to use, plugged in can be, very satisfied!	Jingdong prefecture level city in Shandong
Doraemon Hyakubo Shop	King of Power Saving	Just use can not see what effect to,, wait for a period of time in the evaluation	Jingdong prefecture level city in Shandong
Doraemon Hyakubo Shop	King of Power Saving	Live in the 18th floor is too far away from the meter, afraid of no effect, look at the trial 90 days before buying. Currently used for a month, there is an effect, saving 27 dollars in electricity, next month should be able to save more!	Jingdong prefecture level city in Shandong
Doraemon Hyakubo Shop	King of Power Saving	I've been using it for almost a month now and it feels good, so I'll use it again.	Jingdong prefecture level city in Shandong
Doraemon Hyakubo Shop	King of Power Saving	It's ok, the electric bill is really less, originally I remember charging $100 and using it for 10 days. Now it lasts about 15 days. It's still noticeable.	Jingdong prefecture level city in Shandong
Doraemon Hyakubo Shop	King of Power Saving	Fast logistics, good power saving effect, very satisfied	Jingdong prefecture level city in Shandong
Doraemon Hyakubo Shop	King of Power Saving	Has been the second time back to buy, energy saving effect, worth recommending, you can try to see, power saving effect: very good Experience: can save about 30%!	Jingdong prefecture level city in Shandong
Doraemon Hyakubo Shop	King of Power Saving	I don't know how well it works for now	Jingdong prefecture level city in Shandong
Doraemon Hyakubo Shop	King of Power Saving	Factory direct is really good, plugged in and used for half a month did less probably almost 50 degrees of electricity! And the manufacturer's warranty is 5 years.	Jingdong prefecture level city in Shandong
Doraemon Hyakubo Shop	King of Power Saving	It's really effective. The previous month's electricity was $286, but after using the power saver for a month, I have $195 left this month, which is not bad.	Jingdong prefecture level city in Shandong
Doraemon Hyakubo Shop	King of Power Saving	I'll give you a good review and see how it goes.	Jingdong prefecture level city in Shandong

Doraemon Hyakubo Shop	King of Power Saving	Customer service is very professional and patient to guide the installation, the quality of workmanship is very fine. Durable, second repurchase. Really can save oh!	Jingdong prefecture level city in Shandong
Doraemon Hyakubo Shop	King of Power Saving	I've been using it for a month now and my electric bill is over $30 less than last month. I feel it's quite worth buying.	Jingdong prefecture level city in Shandong
Doraemon Hyakubo Shop	King of Power Saving	Small size, does not take up space. It's very good, it does work very effectively, the tool room has a lot of work with a lot of electricity. After installing it, it really reduces a lot of electricity bills. Before the day 30 degrees of electricity left stone, now work down really less a few degrees.	Jingdong prefecture level city in Shandong
Doraemon Hyakubo Shop	King of Power Saving	It does work for saving electricity, I've bought the kind from elsewhere before that was too simple not **all**. This time to buy this store, but also for the safety. Air conditioning is too much electricity, this use to save a lot of	Jingdong prefecture level city in Shandong
Doraemon Hyakubo Shop	King of Power Saving	Two air conditioners and a refrigerator at home, a month down the electricity bill is almost two hundred dollars, with this after the average savings of more than thirty dollars to spend less money to enjoy more, it's really very good!	Jingdong prefecture level city in Shandong
Doraemon Hyakubo Shop	King of Power Saving	Good. Very good.	Jingdong prefecture level city in Shandong
Doraemon Hyakubo Shop	King of Power Saving	Works great, easy to operate, great, very happy with it.	Jingdong prefecture level city in Shandong
Doraemon Hyakubo Shop	King of Power Saving	Received it and tried it out, it's great, great quality and worth having! Five stars praise!	Jingdong prefecture level city in Shandong
Doraemon Hyakubo Shop	King of Power Saving	Very trustworthy brand, quality is guaranteed, the price is still affordable, quite satisfied!	Jingdong prefecture level city in Shandong
Doraemon Hyakubo Shop	King of Power Saving	Use it before you speak.	Jingdong prefecture level city in Shandong
Doraemon Hyakubo Shop	King of Power Saving	The quality of workmanship is very good and the power saving effect is quite good, I regret not getting it earlier.	Jingdong prefecture level city in Shandong
Doraemon Hyakubo Shop	King of Power Saving	Merchants shipping speed, and customer service introduced the same very good, cost-effective	Jingdong prefecture level city in Shandong
Doraemon Hyakubo Shop	King of Power Saving	Feel a little effect Oh, where the power consumption where to plug, convenient and convenient	Jingdong prefecture level city in Shandong
Doraemon Hyakubo Shop	King of Power Saving	The quality of the imported saver is very good, practical and satisfactory. Cost-effective	Jingdong prefecture level city in Shandong
Doraemon Hyakubo Shop	King of Power Saving	Power Saver is especially good, good and inexpensive, very good	Jingdong prefecture level city in Shandong
Doraemon Hyakubo Shop	King of Power Saving	Fast shipping, awesome quality, affordable price, satisfied.	Jingdong prefecture level city in Shandong
Doraemon Hyakubo Shop	King of Power Saving	Energy saver received, the effect is consistent with the description, recommended to buy!	Jingdong prefecture level city in Shandong
Doraemon Hyakubo Shop	King of Power Saving	It's really good, the quality is great, I recommend it!	Jingdong prefecture level city in Shandong
Doraemon Hyakubo Shop	King of Power Saving	Works well. Inexpensive. Shipping was fast.	Jingdong prefecture level city in Shandong
Doraemon Hyakubo Shop	King of Power Saving	Logistics is fast, works well, energy saving is good	Jingdong prefecture level city in Shandong
Doraemon Hyakubo Shop	King of Power Saving	very happy	Jingdong prefecture level city in Shandong
Doraemon Hyakubo Shop	King of Power Saving	an excellent	Jingdong prefecture level city in Shandong
Doraemon Hyakubo Shop	King of Power Saving	It rained a couple days ago and I just went to get the item back today , not bad, hey I looked at the meter and that red display light takes several seconds to jump, did not make a mistake buying it. After a period of time to evaluate.	Jingdong prefecture level city in Shandong
Doraemon Hyakubo Shop	King of Power Saving	Used more than 10 days to check the power, there is indeed some decline, customer service said, long-term use will save more points!	Jingdong prefecture level city in Shandong
Doraemon Hyakubo Shop	King of Power Saving	It's a proper IQ tax, and it costs electricity.	Jingdong prefecture level city in Shandong
Doraemon Hyakubo Shop	King of Power Saving	I've been using it for 17 days now, it really saves electricity Shipping is fast, you can save a lot in a year	Jingdong prefecture level city in Shandong
Doraemon Hyakubo Shop	King of Power Saving	I've been using it for 17 days and it really saves power Shipping is fast and you don't have to worry about the quality, free returns within 5 years	Jingdong prefecture level city in Shandong
Doraemon Hyakubo Shop	King of Power Saving	It's more regular, scanned the code and it's authentic, and the main thing is that you can try it for 90 days! Plugged in more than ten days, is less than before a few degrees per day. When the time to use a month to see Additional. Good to buy a few more	Jingdong prefecture level city in Shandong
Doraemon Hyakubo Shop	King of Power Saving	Logistics is very fast, fine workmanship, use it and then come back to the comments	Jingdong prefecture level city in Shandong
Doraemon Hyakubo Shop	King of Power Saving	The quality of workmanship is very good and the power saving effect is quite good, I regret not getting it earlier.	Jingdong prefecture level city in Shandong
Doraemon Hyakubo Shop	King of Power Saving	Received the goods, according to the instructions on the operation is very simple, to wait and see the effect, looks good!	Jingdong prefecture level city in Shandong
Doraemon Hyakubo Shop	King of Power Saving	It's really as safe and power-saving as the owner says, and it works great!	Jingdong prefecture level city in Shandong
Doraemon Hyakubo Shop	King of Power Saving	Received the goods, immediately used, looks good, to wait and see how the effect.	Jingdong prefecture level city in Shandong
Doraemon Hyakubo Shop	King of Power Saving	Afraid to buy back did not work, the store said the commitment to 90 days invalid refund, warranty 5 years. Saver also complimentary on the Pacific insurance, it is a lot of peace of mind, there are effects will continue to buy back drops!	Jingdong prefecture level city in Shandong
Doraemon Hyakubo Shop	King of Power Saving	Received the goods and used it, can save electricity a month later to see the effect!	Jingdong prefecture level city in Shandong
Doraemon Hyakubo Shop	King of Power Saving	Fancy 90-day trial, 5-year warranty before the order, the goods received, has been experiencing a week, can really feel the savings, in the introduction of friends also buy this!	Jingdong prefecture level city in Shandong
Doraemon Hyakubo Shop	King of Power Saving	Clothing stores use, neighbors recommended to buy, said a month can save more than 70 yuan, first plugged in to see the effect of the	Jingdong prefecture level city in Shandong
Doraemon Hyakubo Shop	King of Power Saving	A friend's home two months to save more than 80 dollars of electricity, recommended that I buy two, decisive purchase, looking forward to the effect of power saving, waiting for the next month effect.	Jingdong prefecture level city in Shandong
Doraemon Hyakubo Shop	King of Power Saving	The goods have been received, consistent with the description of the advertisement, quite good, I hope that in terms of power saving and purifying the air is really as the merchant said that would be better!	Jingdong prefecture level city in Shandong
Doraemon Hyakubo Shop	King of Power Saving	Power Saving King is really good to use the quality is very good than the physical store a lot cheaper we can rest assured that the purchase recommended to buy!	Jingdong prefecture level city in Shandong
Doraemon Hyakubo Shop	King of Power Saving	Energy saver has been used on the rental house inside the use of the meter is always running fast, with this can really save electricity, easy to use, plugged in can be, very satisfied!	Jingdong prefecture level city in Shandong
Doraemon Hyakubo Shop	King of Power Saving	Just use can not see what effect to,, wait for a period of time in the evaluation	Jingdong prefecture level city in Shandong
Doraemon Hyakubo Shop	King of Power Saving	Live in the 18th floor is too far away from the meter, afraid of no effect, look at the trial 90 days before buying. Currently used for a month, there is an effect, saving 27 dollars in electricity, next month should be able to save more!	Jingdong prefecture level city in Shandong
Doraemon Hyakubo Shop	King of Power Saving	I've been using it for almost a month now and it feels good, so I'll use it again.	Jingdong prefecture level city in Shandong
Doraemon Hyakubo Shop	King of Power Saving	It's ok, the electric bill is really less, originally I remember charging $100 and using it for 10 days. Now it lasts about 15 days. It's still noticeable.	Jingdong prefecture level city in Shandong
Doraemon Hyakubo Shop	King of Power Saving	Fast logistics, good power saving effect, very satisfied	Jingdong prefecture level city in Shandong
Doraemon Hyakubo Shop	King of Power Saving	Has been the second time back to buy, energy saving effect, worth recommending, you can try to see, power saving effect: very good Experience: can save about 30%!	Jingdong prefecture level city in Shandong
Doraemon Hyakubo Shop	King of Power Saving	I don't know how well it works for now	Jingdong prefecture level city in Shandong

Doraemon Hyakubo Shop	King of Power Saving	Factory direct is really good, plugged in and used for half a month did less probably almost 50 degrees of electricity! And the manufacturer's warranty is 5 years.	Jingdong prefecture level city in Shandong
Doraemon Hyakubo Shop	King of Power Saving	It's really effective. The previous month's electricity was $286, but after using the power saver for a month, I have $195 left this month, which is not bad.	Jingdong prefecture level city in Shandong
Doraemon Hyakubo Shop	King of Power Saving	I'll give you a good review and see how it goes.	Jingdong prefecture level city in Shandong
Doraemon Hyakubo Shop	King of Power Saving	Customer service is very professional and patient to guide the installation, the quality of workmanship is very fine. Durable, second repurchase. Really can save oh!	Jingdong prefecture level city in Shandong
Doraemon Hyakubo Shop	King of Power Saving	I've been using it for a month now and my electric bill is over $30 less than last month. I feel it's quite worth buying.	Jingdong prefecture level city in Shandong
Doraemon Hyakubo Shop	King of Power Saving	Small size, does not take up space. It's very good, it does work very effectively, the tool room has a lot of work with a lot of electricity. After installing it, it really reduces a lot of electricity bills. Before the day 30 degrees of electricity left stone, now work down really less a few degrees.	Jingdong prefecture level city in Shandong
Doraemon Hyakubo Shop	King of Power Saving	It does work for saving electricity, I've bought the kind from elsewhere before that was too simple not **all**. This time to buy this store, but also for the safety. Air conditioning is too much electricity, this use to save a lot of	Jingdong prefecture level city in Shandong
Doraemon Hyakubo Shop	King of Power Saving	Two air conditioners and a refrigerator at home, a month down the electricity bill is almost two hundred dollars, with this after the average savings of more than thirty dollars to spend less money to enjoy more, it's really very good!	Jingdong prefecture level city in Shandong
Doraemon Hyakubo Shop	King of Power Saving	Good. Very good.	Jingdong prefecture level city in Shandong
Doraemon Hyakubo Shop	King of Power Saving	Works great, easy to operate, great, very happy with it.	Jingdong prefecture level city in Shandong
Doraemon Hyakubo Shop	King of Power Saving	Received it and tried it out, it's great, great quality and worth having! Five stars praise!	Jingdong prefecture level city in Shandong
Doraemon Hyakubo Shop	King of Power Saving	Very trustworthy brand, quality is guaranteed, the price is still affordable, quite satisfied!	Jingdong prefecture level city in Shandong
Doraemon Hyakubo Shop	King of Power Saving	Use it before you speak.	Jingdong prefecture level city in Shandong
Doraemon Hyakubo Shop	King of Power Saving	The quality of workmanship is very good and the power saving effect is quite good, I regret not getting it earlier.	Jingdong prefecture level city in Shandong
Doraemon Hyakubo Shop	King of Power Saving	Merchants shipping speed, and customer service introduced the same very good, cost-effective	Jingdong prefecture level city in Shandong
Doraemon Hyakubo Shop	King of Power Saving	Feel a little effect Oh, where the power consumption where to plug, convenient and convenient	Jingdong prefecture level city in Shandong
Doraemon Hyakubo Shop	King of Power Saving	The quality of the imported saver is very good, practical and satisfactory. Cost-effective	Jingdong prefecture level city in Shandong
Doraemon Hyakubo Shop	King of Power Saving	Power Saver is especially good, good and inexpensive, very good	Jingdong prefecture level city in Shandong
Doraemon Hyakubo Shop	King of Power Saving	Fast shipping, awesome quality, affordable price, satisfied.	Jingdong prefecture level city in Shandong
Doraemon Hyakubo Shop	King of Power Saving	Energy saver received, the effect is consistent with the description, recommended to buy!	Jingdong prefecture level city in Shandong
Doraemon Hyakubo Shop	King of Power Saving	It's really good, the quality is great, I recommend it!	Jingdong prefecture level city in Shandong
Doraemon Hyakubo Shop	King of Power Saving	Works well. Inexpensive. Shipping was fast.	Jingdong prefecture level city in Shandong
Doraemon Hyakubo Shop	King of Power Saving	Logistics is fast, works well, energy saving is good	Jingdong prefecture level city in Shandong
Doraemon Hyakubo Shop	King of Power Saving	very happy	Jingdong prefecture level city in Shandong
Doraemon Hyakubo Shop	King of Power Saving	an excellent	Jingdong prefecture level city in Shandong
Doraemon Hyakubo Shop	King of Power Saving	It rained a couple days ago and I just went to get the item back today , not bad, hey I looked at the meter and that red display light takes several seconds to jump, did not make a mistake buying it. After a period of time to evaluate.	Jingdong prefecture level city in Shandong
Doraemon Hyakubo Shop	King of Power Saving	Used more than 10 days to check the power, there is indeed some decline, customer service said, long-term use will save more points!	Jingdong prefecture level city in Shandong
Doraemon Hyakubo Shop	King of Power Saving	It's a proper IQ tax, and it costs electricity.	Jingdong prefecture level city in Shandong
Doraemon Hyakubo Shop	King of Power Saving	I've been using it for 17 days now, it really saves electricity Shipping is fast, you can save a lot in a year	Jingdong prefecture level city in Shandong
Doraemon Hyakubo Shop	King of Power Saving	I've been using it for 17 days and it really saves power Shipping is fast and you don't have to worry about the quality, free returns within 5 years	Jingdong prefecture level city in Shandong
Doraemon Hyakubo Shop	King of Power Saving	It's more regular, scanned the code and it's authentic, and the main thing is that you can try it for 90 days! Plugged in more than ten days, is less than before a few degrees per day. When the time to use a month to see Additional. Good to buy a few more	Jingdong prefecture level city in Shandong
Doraemon Hyakubo Shop	King of Power Saving	Logistics is very fast, fine workmanship, use it and then come back to the comments	Jingdong prefecture level city in Shandong
Doraemon Hyakubo Shop	King of Power Saving	The quality of workmanship is very good and the power saving effect is quite good, I regret not getting it earlier.	Jingdong prefecture level city in Shandong
Doraemon Hyakubo Shop	King of Power Saving	Received the goods, according to the instructions on the operation is very simple, to wait and see the effect, looks good!	Jingdong prefecture level city in Shandong
Doraemon Hyakubo Shop	King of Power Saving	It's really as safe and power-saving as the owner says, and it works great!	Jingdong prefecture level city in Shandong
Doraemon Hyakubo Shop	King of Power Saving	Received the goods, immediately used, looks good, to wait and see how the effect.	Jingdong prefecture level city in Shandong
Doraemon Hyakubo Shop	King of Power Saving	Afraid to buy back did not work, the store said the commitment to 90 days invalid refund, warranty 5 years. Saver also complimentary on the Pacific insurance, it is a lot of peace of mind, there are effects will continue to buy back drops!	Jingdong prefecture level city in Shandong
Doraemon Hyakubo Shop	King of Power Saving	Received the goods and used it, can save electricity a month later to see the effect!	Jingdong prefecture level city in Shandong
Doraemon Hyakubo Shop	King of Power Saving	Fancy 90-day trial, 5-year warranty before the order, the goods received, has been experiencing a week, can really feel the savings, in the introduction of friends also buy this!	Jingdong prefecture level city in Shandong
Doraemon Hyakubo Shop	King of Power Saving	Clothing stores use, neighbors recommended to buy, said a month can save more than 70 yuan, first plugged in to see the effect of the	Jingdong prefecture level city in Shandong
Doraemon Hyakubo Shop	King of Power Saving	A friend's home two months to save more than 80 dollars of electricity, recommended that I buy two, decisive purchase, looking forward to the effect of power saving, waiting for the next month effect.	Jingdong prefecture level city in Shandong
Doraemon Hyakubo Shop	King of Power Saving	The goods have been received, consistent with the description of the advertisement, quite good, I hope that in terms of power saving and purifying the air is really as the merchant said that would be better!	Jingdong prefecture level city in Shandong
Doraemon Hyakubo Shop	King of Power Saving	Power Saving King is really good to use the quality is very good than the physical store a lot cheaper we can rest assured that the purchase recommended to buy!	Jingdong prefecture level city in Shandong
Doraemon Hyakubo Shop	King of Power Saving	Energy saver has been used on the rental house inside the use of the meter is always running fast, with this can really save electricity, easy to use, plugged in can be, very satisfied!	Jingdong prefecture level city in Shandong
Doraemon Hyakubo Shop	King of Power Saving	Just use can not see what effect to,, wait for a period of time in the evaluation	Jingdong prefecture level city in Shandong
Doraemon Hyakubo Shop	King of Power Saving	Live in the 18th floor is too far away from the meter, afraid of no effect, look at the trial 90 days before buying. Currently used for a month, there is an effect, saving 27 dollars in electricity, next month should be able to save more!	Jingdong prefecture level city in Shandong
Doraemon Hyakubo Shop	King of Power Saving	I've been using it for almost a month now and it feels good, so I'll use it again.	Jingdong prefecture level city in Shandong
Doraemon Hyakubo Shop	King of Power Saving	It's ok, the electric bill is really less, originally I remember charging $100 and using it for 10 days. Now it lasts about 15 days. It's still noticeable.	Jingdong prefecture level city in Shandong

Doraemon Hyakubo Shop	King of Power Saving	Fast logistics, good power saving effect, very satisfied	Jingdong prefecture level city in Shandong
Doraemon Hyakubo Shop	King of Power Saving	Has been the second time back to buy, energy saving effect, worth recommending, you can try to see, power saving effect: very good Experience: can save about 30%!	Jingdong prefecture level city in Shandong
Doraemon Hyakubo Shop	King of Power Saving	I don't know how well it works for now	Jingdong prefecture level city in Shandong
Doraemon Hyakubo Shop	King of Power Saving	Factory direct is really good, plugged in and used for half a month did less probably almost 50 degrees of electricity! And the manufacturer's warranty is 5 years.	Jingdong prefecture level city in Shandong
Doraemon Hyakubo Shop	King of Power Saving	It's really effective. The previous month's electricity was $286, but after using the power saver for a month, I have $195 left this month, which is not bad.	Jingdong prefecture level city in Shandong
Doraemon Hyakubo Shop	King of Power Saving	I'll give you a good review and see how it goes.	Jingdong prefecture level city in Shandong
Doraemon Hyakubo Shop	King of Power Saving	Customer service is very professional and patient to guide the installation, the quality of workmanship is very fine. Durable, second repurchase. Really can save oh!	Jingdong prefecture level city in Shandong
Doraemon Hyakubo Shop	King of Power Saving	I've been using it for a month now and my electric bill is over $30 less than last month. I feel it's quite worth buying.	Jingdong prefecture level city in Shandong
Doraemon Hyakubo Shop	King of Power Saving	Small size, does not take up space. It's very good, it does work very effectively, the tool room has a lot of work with a lot of electricity. After installing it, it really reduces a lot of electricity bills. Before the day 30 degrees of electricity left stone, now work down really less a few degrees.	Jingdong prefecture level city in Shandong
Doraemon Hyakubo Shop	King of Power Saving	It does work for saving electricity, I've bought the kind from elsewhere before that was too simple not **all**. This time to buy this store, but also for the safety. Air conditioning is too much electricity, this use to save a lot of	Jingdong prefecture level city in Shandong
Doraemon Hyakubo Shop	King of Power Saving	Two air conditioners and a refrigerator at home, a month down the electricity bill is almost two hundred dollars, with this after the average savings of more than thirty dollars to spend less money to enjoy more, it's really very good!	Jingdong prefecture level city in Shandong
Doraemon Hyakubo Shop	King of Power Saving	Good. Very good.	Jingdong prefecture level city in Shandong
Doraemon Hyakubo Shop	King of Power Saving	Works great, easy to operate, great, very happy with it.	Jingdong prefecture level city in Shandong
Doraemon Hyakubo Shop	King of Power Saving	Received it and tried it out, it's great, great quality and worth having! Five stars praise!	Jingdong prefecture level city in Shandong
Doraemon Hyakubo Shop	King of Power Saving	Very trustworthy brand, quality is guaranteed, the price is still affordable, quite satisfied!	Jingdong prefecture level city in Shandong
Doraemon Hyakubo Shop	King of Power Saving	Use it before you speak.	Jingdong prefecture level city in Shandong
Doraemon Hyakubo Shop	King of Power Saving	The quality of workmanship is very good and the power saving effect is quite good, I regret not getting it earlier.	Jingdong prefecture level city in Shandong
Doraemon Hyakubo Shop	King of Power Saving	Merchants shipping speed, and customer service introduced the same very good, cost-effective	Jingdong prefecture level city in Shandong
Doraemon Hyakubo Shop	King of Power Saving	Feel a little effect Oh, where the power consumption where to plug, convenient and convenient	Jingdong prefecture level city in Shandong
Doraemon Hyakubo Shop	King of Power Saving	The quality of the imported saver is very good, practical and satisfactory. Cost-effective	Jingdong prefecture level city in Shandong
Doraemon Hyakubo Shop	King of Power Saving	Power Saver is especially good, good and inexpensive, very good	Jingdong prefecture level city in Shandong
Doraemon Hyakubo Shop	King of Power Saving	Fast shipping, awesome quality, affordable price, satisfied.	Jingdong prefecture level city in Shandong
Doraemon Hyakubo Shop	King of Power Saving	Energy saver received, the effect is consistent with the description, recommended to buy!	Jingdong prefecture level city in Shandong
Doraemon Hyakubo Shop	King of Power Saving	It's really good, the quality is great, I recommend it!	Jingdong prefecture level city in Shandong
Doraemon Hyakubo Shop	King of Power Saving	Works well. Inexpensive. Shipping was fast.	Jingdong prefecture level city in Shandong
Doraemon Hyakubo Shop	King of Power Saving	Logistics is fast, works well, energy saving is good	Jingdong prefecture level city in Shandong
Doraemon Hyakubo Shop	King of Power Saving	very happy	Jingdong prefecture level city in Shandong
Doraemon Hyakubo Shop	King of Power Saving	an excellent	Jingdong prefecture level city in Shandong
Doraemon Hyakubo Shop	King of Power Saving	It rained a couple days ago and I just went to get the item back today , not bad, hey I looked at the meter and that red display light takes several seconds to jump, did not make a mistake buying it. After a period of time to evaluate.	Jingdong prefecture level city in Shandong
Doraemon Hyakubo Shop	King of Power Saving	Used more than 10 days to check the power, there is indeed some decline, customer service said, long-term use will save more points!	Jingdong prefecture level city in Shandong
Doraemon Hyakubo Shop	King of Power Saving	It's a proper IQ tax, and it costs electricity.	Jingdong prefecture level city in Shandong
Doraemon Hyakubo Shop	King of Power Saving	I've been using it for 17 days now, it really saves electricity Shipping is fast, you can save a lot in a year	Jingdong prefecture level city in Shandong
Doraemon Hyakubo Shop	King of Power Saving	I've been using it for 17 days and it really saves power Shipping is fast and you don't have to worry about the quality, free returns within 5 years	Jingdong prefecture level city in Shandong
Doraemon Hyakubo Shop	King of Power Saving	It's more regular, scanned the code and it's authentic, and the main thing is that you can try it for 90 days! Plugged in more than ten days, is less than before a few degrees per day. When the time to use a month to see Additional. Good to buy a few more	Jingdong prefecture level city in Shandong
Doraemon Hyakubo Shop	King of Power Saving	Logistics is very fast, fine workmanship, use it and then come back to the comments	Jingdong prefecture level city in Shandong
Doraemon Hyakubo Shop	King of Power Saving	The quality of workmanship is very good and the power saving effect is quite good, I regret not getting it earlier.	Jingdong prefecture level city in Shandong
Doraemon Hyakubo Shop	King of Power Saving	Received the goods, according to the instructions on the operation is very simple, to wait and see the effect, looks good!	Jingdong prefecture level city in Shandong
Doraemon Hyakubo Shop	King of Power Saving	It's really as safe and power-saving as the owner says, and it works great!	Jingdong prefecture level city in Shandong
Doraemon Hyakubo Shop	King of Power Saving	Received the goods, immediately used, looks good, to wait and see how the effect.	Jingdong prefecture level city in Shandong
Doraemon Hyakubo Shop	King of Power Saving	Afraid to buy back did not work, the store said the commitment to 90 days invalid refund, warranty 5 years. Saver also complimentary on the Pacific insurance, it is a lot of peace of mind, there are effects will continue to buy back drops!	Jingdong prefecture level city in Shandong
Doraemon Hyakubo Shop	King of Power Saving	Received the goods and used it, can save electricity a month later to see the effect!	Jingdong prefecture level city in Shandong
Doraemon Hyakubo Shop	King of Power Saving	Fancy 90-day trial, 5-year warranty before the order, the goods received, has been experiencing a week, can really feel the savings, in the introduction of friends also buy this!	Jingdong prefecture level city in Shandong
Doraemon Hyakubo Shop	King of Power Saving	Clothing stores use, neighbors recommended to buy, said a month can save more than 70 yuan, first plugged in to see the effect of the	Jingdong prefecture level city in Shandong
Doraemon Hyakubo Shop	King of Power Saving	A friend's home two months to save more than 80 dollars of electricity, recommended that I buy two, decisive purchase, looking forward to the effect of power saving, waiting for the next month effect.	Jingdong prefecture level city in Shandong
Doraemon Hyakubo Shop	King of Power Saving	The goods have been received, consistent with the description of the advertisement, quite good, I hope that in terms of power saving and purifying the air is really as the merchant said that would be better!	Jingdong prefecture level city in Shandong
Doraemon Hyakubo Shop	King of Power Saving	Power Saving King is really good to use the quality is very good than the physical store a lot cheaper we can rest assured that the purchase recommended to buy!	Jingdong prefecture level city in Shandong
Doraemon Hyakubo Shop	King of Power Saving	Energy saver has been used on the rental house inside the use of the meter is always running fast, with this can really save electricity, easy to use, plugged in can be, very satisfied!	Jingdong prefecture level city in Shandong
Doraemon Hyakubo Shop	King of Power Saving	Just use can not see what effect to,, wait for a period of time in the evaluation	Jingdong prefecture level city in Shandong

Doraemon Hyakubo Shop	King of Power Saving	Live in the 18th floor is too far away from the meter, afraid of no effect, look at the trial 90 days before buying. Currently used for a month, there is an effect, saving 27 dollars in electricity, next month should be able to save more!	Jingdong prefecture level city in Shandong
Doraemon Hyakubo Shop	King of Power Saving	I've been using it for almost a month now and it feels good, so I'll use it again.	Jingdong prefecture level city in Shandong
Doraemon Hyakubo Shop	King of Power Saving	It's ok, the electric bill is really less, originally I remember charging $100 and using it for 10 days. Now it lasts about 15 days. It's still noticeable.	Jingdong prefecture level city in Shandong
Doraemon Hyakubo Shop	King of Power Saving	Fast logistics, good power saving effect, very satisfied	Jingdong prefecture level city in Shandong
Doraemon Hyakubo Shop	King of Power Saving	Has been the second time back to buy, energy saving effect, worth recommending, you can try to see, power saving effect: very good Experience: can save about 30%!	Jingdong prefecture level city in Shandong
Doraemon Hyakubo Shop	King of Power Saving	I don't know how well it works for now	Jingdong prefecture level city in Shandong
Doraemon Hyakubo Shop	King of Power Saving	Factory direct is really good, plugged in and used for half a month did less probably almost 50 degrees of electricity! And the manufacturer's warranty is 5 years.	Jingdong prefecture level city in Shandong
Doraemon Hyakubo Shop	King of Power Saving	It's really effective. The previous month's electricity was $286, but after using the power saver for a month, I have $195 left this month, which is not bad.	Jingdong prefecture level city in Shandong
Doraemon Hyakubo Shop	King of Power Saving	I'll give you a good review and see how it goes.	Jingdong prefecture level city in Shandong
Doraemon Hyakubo Shop	King of Power Saving	Customer service is very professional and patient to guide the installation, the quality of workmanship is very fine. Durable, second repurchase. Really can save oh!	Jingdong prefecture level city in Shandong
Doraemon Hyakubo Shop	King of Power Saving	I've been using it for a month now and my electric bill is over $30 less than last month. I feel it's quite worth buying.	Jingdong prefecture level city in Shandong
Doraemon Hyakubo Shop	King of Power Saving	Small size, does not take up space. It's very good, it does work very effectively, the tool room has a lot of work with a lot of electricity. After installing it, it really reduces a lot of electricity bills. Before the day 30 degrees of electricity left stone, now work down really less a few degrees.	Jingdong prefecture level city in Shandong
Doraemon Hyakubo Shop	King of Power Saving	It does work for saving electricity, I've bought the kind from elsewhere before that was too simple not **all**. This time to buy this store, but also for the safety. Air conditioning is too much electricity, this use to save a lot of	Jingdong prefecture level city in Shandong
Doraemon Hyakubo Shop	King of Power Saving	Two air conditioners and a refrigerator at home, a month down the electricity bill is almost two hundred dollars, with this after the average savings of more than thirty dollars to spend less money to enjoy more, it's really very good!	Jingdong prefecture level city in Shandong
Doraemon Hyakubo Shop	King of Power Saving	Good. Very good.	Jingdong prefecture level city in Shandong
Doraemon Hyakubo Shop	King of Power Saving	Works great, easy to operate, great, very happy with it.	Jingdong prefecture level city in Shandong
Doraemon Hyakubo Shop	King of Power Saving	Received it and tried it out, it's great, great quality and worth having! Five stars praise!	Jingdong prefecture level city in Shandong
Doraemon Hyakubo Shop	King of Power Saving	Very trustworthy brand, quality is guaranteed, the price is still affordable, quite satisfied!	Jingdong prefecture level city in Shandong
Doraemon Hyakubo Shop	King of Power Saving	Use it before you speak.	Jingdong prefecture level city in Shandong
Doraemon Hyakubo Shop	King of Power Saving	The quality of workmanship is very good and the power saving effect is quite good, I regret not getting it earlier.	Jingdong prefecture level city in Shandong
Doraemon Hyakubo Shop	King of Power Saving	Merchants shipping speed, and customer service introduced the same very good, cost-effective	Jingdong prefecture level city in Shandong
Doraemon Hyakubo Shop	King of Power Saving	Feel a little effect Oh, where the power consumption where to plug, convenient and convenient	Jingdong prefecture level city in Shandong
Doraemon Hyakubo Shop	King of Power Saving	The quality of the imported saver is very good, practical and satisfactory. Cost-effective	Jingdong prefecture level city in Shandong
Doraemon Hyakubo Shop	King of Power Saving	Power Saver is especially good, good and inexpensive, very good	Jingdong prefecture level city in Shandong
Doraemon Hyakubo Shop	King of Power Saving	Fast shipping, awesome quality, affordable price, satisfied.	Jingdong prefecture level city in Shandong
Doraemon Hyakubo Shop	King of Power Saving	Energy saver received, the effect is consistent with the description, recommended to buy!	Jingdong prefecture level city in Shandong
Doraemon Hyakubo Shop	King of Power Saving	It's really good, the quality is great, I recommend it!	Jingdong prefecture level city in Shandong
Doraemon Hyakubo Shop	King of Power Saving	Works well. Inexpensive. Shipping was fast.	Jingdong prefecture level city in Shandong
Doraemon Hyakubo Shop	King of Power Saving	Logistics is fast, works well, energy saving is good	Jingdong prefecture level city in Shandong
Doraemon Hyakubo Shop	King of Power Saving	very happy	Jingdong prefecture level city in Shandong
Doraemon Hyakubo Shop	King of Power Saving	an excellent	Jingdong prefecture level city in Shandong
Doraemon Hyakubo Shop	King of Power Saving	It rained a couple days ago and I just went to get the item back today , not bad, hey I looked at the meter and that red display light takes several seconds to jump, did not make a mistake buying it. After a period of time to evaluate.	Jingdong prefecture level city in Shandong
Doraemon Hyakubo Shop	King of Power Saving	Used more than 10 days to check the power, there is indeed some decline, customer service said, long-term use will save more points!	Jingdong prefecture level city in Shandong
Doraemon Hyakubo Shop	King of Power Saving	It's a proper IQ tax, and it costs electricity.	Jingdong prefecture level city in Shandong
Doraemon Hyakubo Shop	King of Power Saving	I've been using it for 17 days now, it really saves electricity Shipping is fast, you can save a lot in a year	Jingdong prefecture level city in Shandong
Doraemon Hyakubo Shop	King of Power Saving	I've been using it for 17 days and it really saves power Shipping is fast and you don't have to worry about the quality, free returns within 5 years	Jingdong prefecture level city in Shandong
Doraemon Hyakubo Shop	King of Power Saving	It's more regular, scanned the code and it's authentic, and the main thing is that you can try it for 90 days! Plugged in more than ten days, is less than before a few degrees per day. When the time to use a month to see Additional. Good to buy a few more	Jingdong prefecture level city in Shandong
Doraemon Hyakubo Shop	King of Power Saving	Logistics is very fast, fine workmanship, use it and then come back to the comments	Jingdong prefecture level city in Shandong
Doraemon Hyakubo Shop	King of Power Saving	The quality of workmanship is very good and the power saving effect is quite good, I regret not getting it earlier.	Jingdong prefecture level city in Shandong
Doraemon Hyakubo Shop	King of Power Saving	Received the goods, according to the instructions on the operation is very simple, to wait and see the effect, looks good!	Jingdong prefecture level city in Shandong
Doraemon Hyakubo Shop	King of Power Saving	It's really as safe and power-saving as the owner says, and it works great!	Jingdong prefecture level city in Shandong
Doraemon Hyakubo Shop	King of Power Saving	Received the goods, immediately used, looks good, to wait and see how the effect.	Jingdong prefecture level city in Shandong
Doraemon Hyakubo Shop	King of Power Saving	Afraid to buy back did not work, the store said the commitment to 90 days invalid refund, warranty 5 years. Saver also complimentary on the Pacific insurance, it is a lot of peace of mind, there are effects will continue to buy back drops!	Jingdong prefecture level city in Shandong
Doraemon Hyakubo Shop	King of Power Saving	Received the goods and used it, can save electricity a month later to see the effect!	Jingdong prefecture level city in Shandong
Doraemon Hyakubo Shop	King of Power Saving	Fancy 90-day trial, 5-year warranty before the order, the goods received, has been experiencing a week, can really feel the savings, in the introduction of friends also buy this!	Jingdong prefecture level city in Shandong
Doraemon Hyakubo Shop	King of Power Saving	Clothing stores use, neighbors recommended to buy, said a month can save more than 70 yuan, first plugged in to see the effect of the	Jingdong prefecture level city in Shandong
Doraemon Hyakubo Shop	King of Power Saving	A friend's home two months to save more than 80 dollars of electricity, recommended that I buy two, decisive purchase, looking forward to the effect of power saving, waiting for the next month effect.	Jingdong prefecture level city in Shandong
Doraemon Hyakubo Shop	King of Power Saving	The goods have been received, consistent with the description of the advertisement, quite good, I hope that in terms of power saving and purifying the air is really as the merchant said that would be better!	Jingdong prefecture level city in Shandong

Doraemon Hyakubo Shop	King of Power Saving	Power Saving King is really good to use the quality is very good than the physical store a lot cheaper we can rest assured that the purchase recommended to buy!	Jingdong prefecture level city in Shandong
Doraemon Hyakubo Shop	King of Power Saving	Energy saver has been used on the rental house inside the use of the meter is always running fast, with this can really save electricity, easy to use, plugged in can be, very satisfied!	Jingdong prefecture level city in Shandong
Doraemon Hyakubo Shop	King of Power Saving	Just use can not see what effect to,, wait for a period of time in the evaluation	Jingdong prefecture level city in Shandong
Doraemon Hyakubo Shop	King of Power Saving	Live in the 18th floor is too far away from the meter, afraid of no effect, look at the trial 90 days before buying. Currently used for a month, there is an effect, saving 27 dollars in electricity, next month should be able to save more!	Jingdong prefecture level city in Shandong
Doraemon Hyakubo Shop	King of Power Saving	I've been using it for almost a month now and it feels good, so I'll use it again.	Jingdong prefecture level city in Shandong
Doraemon Hyakubo Shop	King of Power Saving	It's ok, the electric bill is really less, originally I remember charging $100 and using it for 10 days. Now it lasts about 15 days. It's still noticeable.	Jingdong prefecture level city in Shandong
Doraemon Hyakubo Shop	King of Power Saving	Fast logistics, good power saving effect, very satisfied	Jingdong prefecture level city in Shandong
Doraemon Hyakubo Shop	King of Power Saving	Has been the second time back to buy, energy saving effect, worth recommending, you can try to see, power saving effect: very good Experience: can save about 30%!	Jingdong prefecture level city in Shandong
Doraemon Hyakubo Shop	King of Power Saving	I don't know how well it works for now	Jingdong prefecture level city in Shandong
Doraemon Hyakubo Shop	King of Power Saving	Factory direct is really good, plugged in and used for half a month did less probably almost 50 degrees of electricity! And the manufacturer's warranty is 5 years.	Jingdong prefecture level city in Shandong
Doraemon Hyakubo Shop	King of Power Saving	It's really effective. The previous month's electricity was $286, but after using the power saver for a month, I have $195 left this month, which is not bad.	Jingdong prefecture level city in Shandong
Doraemon Hyakubo Shop	King of Power Saving	I'll give you a good review and see how it goes.	Jingdong prefecture level city in Shandong
Doraemon Hyakubo Shop	King of Power Saving	Customer service is very professional and patient to guide the installation, the quality of workmanship is very fine. Durable, second repurchase. Really can save oh!	Jingdong prefecture level city in Shandong
Doraemon Hyakubo Shop	King of Power Saving	I've been using it for a month now and my electric bill is over $30 less than last month. I feel it's quite worth buying.	Jingdong prefecture level city in Shandong
Doraemon Hyakubo Shop	King of Power Saving	Small size, does not take up space. It's very good, it does work very effectively, the tool room has a lot of work with a lot of electricity. After installing it, it really reduces a lot of electricity bills. Before the day 30 degrees of electricity left stone, now work down really less a few degrees.	Jingdong prefecture level city in Shandong
Doraemon Hyakubo Shop	King of Power Saving	It does work for saving electricity, I've bought the kind from elsewhere before that was too simple not **all**. This time to buy this store, but also for the safety. Air conditioning is too much electricity, this use to save a lot of	Jingdong prefecture level city in Shandong
Doraemon Hyakubo Shop	King of Power Saving	Two air conditioners and a refrigerator at home, a month down the electricity bill is almost two hundred dollars, with this after the average savings of more than thirty dollars to spend less money to enjoy more, it's really very good!	Jingdong prefecture level city in Shandong
Doraemon Hyakubo Shop	King of Power Saving	Good. Very good.	Jingdong prefecture level city in Shandong
Doraemon Hyakubo Shop	King of Power Saving	Works great, easy to operate, great, very happy with it.	Jingdong prefecture level city in Shandong
Doraemon Hyakubo Shop	King of Power Saving	Received it and tried it out, it's great, great quality and worth having! Five stars praise!	Jingdong prefecture level city in Shandong
Doraemon Hyakubo Shop	King of Power Saving	Very trustworthy brand, quality is guaranteed, the price is still affordable, quite satisfied!	Jingdong prefecture level city in Shandong
Doraemon Hyakubo Shop	King of Power Saving	Use it before you speak.	Jingdong prefecture level city in Shandong
Doraemon Hyakubo Shop	King of Power Saving	The quality of workmanship is very good and the power saving effect is quite good, I regret not getting it earlier.	Jingdong prefecture level city in Shandong
Doraemon Hyakubo Shop	King of Power Saving	Merchants shipping speed, and customer service introduced the same very good, cost-effective	Jingdong prefecture level city in Shandong
Doraemon Hyakubo Shop	King of Power Saving	Feel a little effect Oh, where the power consumption where to plug, convenient and convenient	Jingdong prefecture level city in Shandong
Doraemon Hyakubo Shop	King of Power Saving	The quality of the imported saver is very good, practical and satisfactory. Cost-effective	Jingdong prefecture level city in Shandong
Doraemon Hyakubo Shop	King of Power Saving	Power Saver is especially good, good and inexpensive, very good	Jingdong prefecture level city in Shandong
Doraemon Hyakubo Shop	King of Power Saving	Fast shipping, awesome quality, affordable price, satisfied.	Jingdong prefecture level city in Shandong
Doraemon Hyakubo Shop	King of Power Saving	Energy saver received, the effect is consistent with the description, recommended to buy!	Jingdong prefecture level city in Shandong
Doraemon Hyakubo Shop	King of Power Saving	It's really good, the quality is great, I recommend it!	Jingdong prefecture level city in Shandong
Doraemon Hyakubo Shop	King of Power Saving	Works well. Inexpensive. Shipping was fast.	Jingdong prefecture level city in Shandong
Doraemon Hyakubo Shop	King of Power Saving	Logistics is fast, works well, energy saving is good	Jingdong prefecture level city in Shandong
Doraemon Hyakubo Shop	King of Power Saving	very happy	Jingdong prefecture level city in Shandong
Doraemon Hyakubo Shop	King of Power Saving	an excellent	Jingdong prefecture level city in Shandong
Doraemon Hyakubo Shop	King of Power Saving	It rained a couple days ago and I just went to get the item back today , not bad, hey I looked at the meter and that red display light takes several seconds to jump, did not make a mistake buying it. After a period of time to evaluate.	Jingdong prefecture level city in Shandong
Doraemon Hyakubo Shop	King of Power Saving	Used more than 10 days to check the power, there is indeed some decline, customer service said, long-term use will save more points!	Jingdong prefecture level city in Shandong
Doraemon Hyakubo Shop	King of Power Saving	It's a proper IQ tax, and it costs electricity.	Jingdong prefecture level city in Shandong
Doraemon Hyakubo Shop	King of Power Saving	I've been using it for 17 days now, it really saves electricity Shipping is fast, you can save a lot in a year	Jingdong prefecture level city in Shandong
Doraemon Hyakubo Shop	King of Power Saving	I've been using it for 17 days and it really saves power Shipping is fast and you don't have to worry about the quality, free returns within 5 years	Jingdong prefecture level city in Shandong
Doraemon Hyakubo Shop	King of Power Saving	It's more regular, scanned the code and it's authentic, and the main thing is that you can try it for 90 days! Plugged in more than ten days, is less than before a few degrees per day. When the time to use a month to see Additional. Good to buy a few more	Jingdong prefecture level city in Shandong
Doraemon Hyakubo Shop	King of Power Saving	Logistics is very fast, fine workmanship, use it and then come back to the comments	Jingdong prefecture level city in Shandong
Doraemon Hyakubo Shop	King of Power Saving	The quality of workmanship is very good and the power saving effect is quite good, I regret not getting it earlier.	Jingdong prefecture level city in Shandong
Doraemon Hyakubo Shop	King of Power Saving	Received the goods, according to the instructions on the operation is very simple, to wait and see the effect, looks good!	Jingdong prefecture level city in Shandong
Doraemon Hyakubo Shop	King of Power Saving	It's really as safe and power-saving as the owner says, and it works great!	Jingdong prefecture level city in Shandong
Doraemon Hyakubo Shop	King of Power Saving	Received the goods, immediately used, looks good, to wait and see how the effect.	Jingdong prefecture level city in Shandong
Doraemon Hyakubo Shop	King of Power Saving	Afraid to buy back did not work, the store said the commitment to 90 days invalid refund, warranty 5 years. Saver also complimentary on the Pacific insurance, it is a lot of peace of mind, there are effects will continue to buy back drops!	Jingdong prefecture level city in Shandong
Doraemon Hyakubo Shop	King of Power Saving	Received the goods and used it, can save electricity a month later to see the effect!	Jingdong prefecture level city in Shandong
Doraemon Hyakubo Shop	King of Power Saving	Fancy 90-day trial, 5-year warranty before the order, the goods received, has been experiencing a week, can really feel the savings, in the introduction of friends also buy this!	Jingdong prefecture level city in Shandong

Doraemon Hyakubo Shop	King of Power Saving	Clothing stores use, neighbors recommended to buy, said a month can save more than 70 yuan, first plugged in to see the effect of the	Jingdong prefecture level city in Shandong
Doraemon Hyakubo Shop	King of Power Saving	A friend's home two months to save more than 80 dollars of electricity, recommended that I buy two, decisive purchase, looking forward to the effect of power saving, waiting for the next month effect.	Jingdong prefecture level city in Shandong
Doraemon Hyakubo Shop	King of Power Saving	The goods have been received, consistent with the description of the advertisement, quite good, I hope that in terms of power saving and purifying the air is really as the merchant said that would be better!	Jingdong prefecture level city in Shandong
Doraemon Hyakubo Shop	King of Power Saving	Power Saving King is really good to use the quality is very good than the physical store a lot cheaper we can rest assured that the purchase recommended to buy!	Jingdong prefecture level city in Shandong
Doraemon Hyakubo Shop	King of Power Saving	Energy saver has been used on the rental house inside the use of the meter is always running fast, with this can really save electricity, easy to use, plugged in can be, very satisfied!	Jingdong prefecture level city in Shandong
Doraemon Hyakubo Shop	King of Power Saving	Just use can not see what effect to,, wait for a period of time in the evaluation	Jingdong prefecture level city in Shandong
Doraemon Hyakubo Shop	King of Power Saving	Live in the 18th floor is too far away from the meter, afraid of no effect, look at the trial 90 days before buying. Currently used for a month, there is an effect, saving 27 dollars in electricity, next month should be able to save more!	Jingdong prefecture level city in Shandong
Doraemon Hyakubo Shop	King of Power Saving	I've been using it for almost a month now and it feels good, so I'll use it again.	Jingdong prefecture level city in Shandong
Doraemon Hyakubo Shop	King of Power Saving	It's ok, the electric bill is really less, originally I remember charging $100 and using it for 10 days. Now it lasts about 15 days. It's still noticeable.	Jingdong prefecture level city in Shandong
Doraemon Hyakubo Shop	King of Power Saving	Fast logistics, good power saving effect, very satisfied	Jingdong prefecture level city in Shandong
Doraemon Hyakubo Shop	King of Power Saving	Has been the second time back to buy, energy saving effect, worth recommending, you can try to see, power saving effect: very good Experience: can save about 30%!	Jingdong prefecture level city in Shandong
Doraemon Hyakubo Shop	King of Power Saving	I don't know how well it works for now	Jingdong prefecture level city in Shandong
Doraemon Hyakubo Shop	King of Power Saving	Factory direct is really good, plugged in and used for half a month did less probably almost 50 degrees of electricity! And the manufacturer's warranty is 5 years.	Jingdong prefecture level city in Shandong
Doraemon Hyakubo Shop	King of Power Saving	It's really effective. The previous month's electricity was $286, but after using the power saver for a month, I have $195 left this month, which is not bad.	Jingdong prefecture level city in Shandong
Doraemon Hyakubo Shop	King of Power Saving	I'll give you a good review and see how it goes.	Jingdong prefecture level city in Shandong
Doraemon Hyakubo Shop	King of Power Saving	Customer service is very professional and patient to guide the installation, the quality of workmanship is very fine. Durable, second repurchase. Really can save oh!	Jingdong prefecture level city in Shandong
Doraemon Hyakubo Shop	King of Power Saving	I've been using it for a month now and my electric bill is over $30 less than last month. I feel it's quite worth buying.	Jingdong prefecture level city in Shandong
Doraemon Hyakubo Shop	King of Power Saving	Small size, does not take up space. It's very good, it does work very effectively, the tool room has a lot of work with a lot of electricity. After installing it, it really reduces a lot of electricity bills. Before the day 30 degrees of electricity left stone, now work down really less a few degrees.	Jingdong prefecture level city in Shandong
Doraemon Hyakubo Shop	King of Power Saving	It does work for saving electricity, I've bought the kind from elsewhere before that was too simple not **all**. This time to buy this store, but also for the safety. Air conditioning is too much electricity, this use to save a lot of	Jingdong prefecture level city in Shandong
Doraemon Hyakubo Shop	King of Power Saving	Two air conditioners and a refrigerator at home, a month down the electricity bill is almost two hundred dollars, with this after the average savings of more than thirty dollars to spend less money to enjoy more, it's really very good!	Jingdong prefecture level city in Shandong
Doraemon Hyakubo Shop	King of Power Saving	Good. Very good.	Jingdong prefecture level city in Shandong
Doraemon Hyakubo Shop	King of Power Saving	Works great, easy to operate, great, very happy with it.	Jingdong prefecture level city in Shandong
Doraemon Hyakubo Shop	King of Power Saving	Received it and tried it out, it's great, great quality and worth having! Five stars praise!	Jingdong prefecture level city in Shandong
Doraemon Hyakubo Shop	King of Power Saving	Very trustworthy brand, quality is guaranteed, the price is still affordable, quite satisfied!	Jingdong prefecture level city in Shandong
Doraemon Hyakubo Shop	King of Power Saving	Use it before you speak.	Jingdong prefecture level city in Shandong
Doraemon Hyakubo Shop	King of Power Saving	The quality of workmanship is very good and the power saving effect is quite good, I regret not getting it earlier.	Jingdong prefecture level city in Shandong
Doraemon Hyakubo Shop	King of Power Saving	Merchants shipping speed, and customer service introduced the same very good, cost-effective	Jingdong prefecture level city in Shandong
Doraemon Hyakubo Shop	King of Power Saving	Feel a little effect Oh, where the power consumption where to plug, convenient and convenient	Jingdong prefecture level city in Shandong
Doraemon Hyakubo Shop	King of Power Saving	The quality of the imported saver is very good, practical and satisfactory. Cost-effective	Jingdong prefecture level city in Shandong
Doraemon Hyakubo Shop	King of Power Saving	Power Saver is especially good, good and inexpensive, very good	Jingdong prefecture level city in Shandong
Doraemon Hyakubo Shop	King of Power Saving	Fast shipping, awesome quality, affordable price, satisfied.	Jingdong prefecture level city in Shandong
Doraemon Hyakubo Shop	King of Power Saving	Energy saver received, the effect is consistent with the description, recommended to buy!	Jingdong prefecture level city in Shandong
Doraemon Hyakubo Shop	King of Power Saving	It's really good, the quality is great, I recommend it!	Jingdong prefecture level city in Shandong
Doraemon Hyakubo Shop	King of Power Saving	Works well. Inexpensive. Shipping was fast.	Jingdong prefecture level city in Shandong
Doraemon Hyakubo Shop	King of Power Saving	Logistics is fast, works well, energy saving is good	Jingdong prefecture level city in Shandong
Doraemon Hyakubo Shop	King of Power Saving	very happy	Jingdong prefecture level city in Shandong
Doraemon Hyakubo Shop	King of Power Saving	an excellent	Jingdong prefecture level city in Shandong
Doraemon Hyakubo Shop	King of Power Saving	It rained a couple days ago and I just went to get the item back today , not bad, hey I looked at the meter and that red display light takes several seconds to jump, did not make a mistake buying it. After a period of time to evaluate.	Jingdong prefecture level city in Shandong
Doraemon Hyakubo Shop	King of Power Saving	Used more than 10 days to check the power, there is indeed some decline, customer service said, long-term use will save more points!	Jingdong prefecture level city in Shandong
Doraemon Hyakubo Shop	King of Power Saving	It's a proper IQ tax, and it costs electricity.	Jingdong prefecture level city in Shandong
Doraemon Hyakubo Shop	King of Power Saving	I've been using it for 17 days now, it really saves electricity Shipping is fast, you can save a lot in a year	Jingdong prefecture level city in Shandong
Doraemon Hyakubo Shop	King of Power Saving	I've been using it for 17 days and it really saves power Shipping is fast and you don't have to worry about the quality, free returns within 5 years	Jingdong prefecture level city in Shandong
Doraemon Hyakubo Shop	King of Power Saving	It's more regular, scanned the code and it's authentic, and the main thing is that you can try it for 90 days! Plugged in more than ten days, is less than before a few degrees per day. When the time to use a month to see Additional. Good to buy a few more	Jingdong prefecture level city in Shandong
Doraemon Hyakubo Shop	King of Power Saving	Logistics is very fast, fine workmanship, use it and then come back to the comments	Jingdong prefecture level city in Shandong
Doraemon Hyakubo Shop	King of Power Saving	The quality of workmanship is very good and the power saving effect is quite good, I regret not getting it earlier.	Jingdong prefecture level city in Shandong
Doraemon Hyakubo Shop	King of Power Saving	Received the goods, according to the instructions on the operation is very simple, to wait and see the effect, looks good!	Jingdong prefecture level city in Shandong
Doraemon Hyakubo Shop	King of Power Saving	It's really as safe and power-saving as the owner says, and it works great!	Jingdong prefecture level city in Shandong
Doraemon Hyakubo Shop	King of Power Saving	Received the goods, immediately used, looks good, to wait and see how the effect.	Jingdong prefecture level city in Shandong

Doraemon Hyakubo Shop	King of Power Saving	Afraid to buy back did not work, the store said the commitment to 90 days invalid refund, warranty 5 years. Saver also complimentary on the Pacific insurance, it is a lot of peace of mind, there are effects will continue to buy back drops!	Jingdong prefecture level city in Shandong
Doraemon Hyakubo Shop	King of Power Saving	Received the goods and used it, can save electricity a month later to see the effect!	Jingdong prefecture level city in Shandong
Doraemon Hyakubo Shop	King of Power Saving	Fancy 90-day trial, 5-year warranty before the order, the goods received, has been experiencing a week, can really feel the savings, in the introduction of friends also buy this!	Jingdong prefecture level city in Shandong
Doraemon Hyakubo Shop	King of Power Saving	Clothing stores use, neighbors recommended to buy, said a month can save more than 70 yuan, first plugged in to see the effect of the	Jingdong prefecture level city in Shandong
Doraemon Hyakubo Shop	King of Power Saving	A friend's home two months to save more than 80 dollars of electricity, recommended that I buy two, decisive purchase, looking forward to the effect of power saving, waiting for the next month effect.	Jingdong prefecture level city in Shandong
Doraemon Hyakubo Shop	King of Power Saving	The goods have been received, consistent with the description of the advertisement, quite good, I hope that in terms of power saving and purifying the air is really as the merchant said that would be better!	Jingdong prefecture level city in Shandong
Doraemon Hyakubo Shop	King of Power Saving	Power Saving King is really good to use the quality is very good than the physical store a lot cheaper we can rest assured that the purchase recommended to buy!	Jingdong prefecture level city in Shandong
Doraemon Hyakubo Shop	King of Power Saving	Energy saver has been used on the rental house inside the use of the meter is always running fast, with this can really save electricity, easy to use, plugged in can be, very satisfied!	Jingdong prefecture level city in Shandong
Doraemon Hyakubo Shop	King of Power Saving	Just use can not see what effect to,, wait for a period of time in the evaluation	Jingdong prefecture level city in Shandong
Doraemon Hyakubo Shop	King of Power Saving	Live in the 18th floor is too far away from the meter, afraid of no effect, look at the trial 90 days before buying. Currently used for a month, there is an effect, saving 27 dollars in electricity, next month should be able to save more!	Jingdong prefecture level city in Shandong
Doraemon Hyakubo Shop	King of Power Saving	I've been using it for almost a month now and it feels good, so I'll use it again.	Jingdong prefecture level city in Shandong
Doraemon Hyakubo Shop	King of Power Saving	It's ok, the electric bill is really less, originally I remember charging $100 and using it for 10 days. Now it lasts about 15 days. It's still noticeable.	Jingdong prefecture level city in Shandong
Doraemon Hyakubo Shop	King of Power Saving	Fast logistics, good power saving effect, very satisfied	Jingdong prefecture level city in Shandong
Doraemon Hyakubo Shop	King of Power Saving	Has been the second time back to buy, energy saving effect, worth recommending, you can try to see, power saving effect: very good Experience: can save about 30%!	Jingdong prefecture level city in Shandong
Doraemon Hyakubo Shop	King of Power Saving	I don't know how well it works for now	Jingdong prefecture level city in Shandong
Doraemon Hyakubo Shop	King of Power Saving	Factory direct is really good, plugged in and used for half a month did less probably almost 50 degrees of electricity! And the manufacturer's warranty is 5 years.	Jingdong prefecture level city in Shandong
Doraemon Hyakubo Shop	King of Power Saving	It's really effective. The previous month's electricity was $286, but after using the power saver for a month, I have $195 left this month, which is not bad.	Jingdong prefecture level city in Shandong
Doraemon Hyakubo Shop	King of Power Saving	I'll give you a good review and see how it goes.	Jingdong prefecture level city in Shandong
Doraemon Hyakubo Shop	King of Power Saving	Customer service is very professional and patient to guide the installation, the quality of workmanship is very fine. Durable, second repurchase. Really can save oh!	Jingdong prefecture level city in Shandong
Doraemon Hyakubo Shop	King of Power Saving	I've been using it for a month now and my electric bill is over $30 less than last month. I feel it's quite worth buying.	Jingdong prefecture level city in Shandong
Doraemon Hyakubo Shop	King of Power Saving	Small size, does not take up space. It's very good, it does work very effectively, the tool room has a lot of work with a lot of electricity. After installing it, it really reduces a lot of electricity bills. Before the day 30 degrees of electricity left stone, now work down really less a few degrees.	Jingdong prefecture level city in Shandong
Doraemon Hyakubo Shop	King of Power Saving	It does work for saving electricity, I've bought the kind from elsewhere before that was too simple not **all**. This time to buy this store, but also for the safety. Air conditioning is too much electricity, this use to save a lot of	Jingdong prefecture level city in Shandong
Doraemon Hyakubo Shop	King of Power Saving	Two air conditioners and a refrigerator at home, a month down the electricity bill is almost two hundred dollars, with this after the average savings of more than thirty dollars to spend less money to enjoy more, it's really very good!	Jingdong prefecture level city in Shandong
Doraemon Hyakubo Shop	King of Power Saving	Good. Very good.	Jingdong prefecture level city in Shandong
Doraemon Hyakubo Shop	King of Power Saving	Works great, easy to operate, great, very happy with it.	Jingdong prefecture level city in Shandong
Doraemon Hyakubo Shop	King of Power Saving	Received it and tried it out, it's great, great quality and worth having! Five stars praise!	Jingdong prefecture level city in Shandong
Doraemon Hyakubo Shop	King of Power Saving	Very trustworthy brand, quality is guaranteed, the price is still affordable, quite satisfied!	Jingdong prefecture level city in Shandong
Doraemon Hyakubo Shop	King of Power Saving	Use it before you speak.	Jingdong prefecture level city in Shandong
Doraemon Hyakubo Shop	King of Power Saving	The quality of workmanship is very good and the power saving effect is quite good, I regret not getting it earlier.	Jingdong prefecture level city in Shandong
Doraemon Hyakubo Shop	King of Power Saving	Merchants shipping speed, and customer service introduced the same very good, cost-effective	Jingdong prefecture level city in Shandong
Doraemon Hyakubo Shop	King of Power Saving	Feel a little effect Oh, where the power consumption where to plug, convenient and convenient	Jingdong prefecture level city in Shandong
Doraemon Hyakubo Shop	King of Power Saving	The quality of the imported saver is very good, practical and satisfactory. Cost-effective	Jingdong prefecture level city in Shandong
Doraemon Hyakubo Shop	King of Power Saving	Power Saver is especially good, good and inexpensive, very good	Jingdong prefecture level city in Shandong
Doraemon Hyakubo Shop	King of Power Saving	Fast shipping, awesome quality, affordable price, satisfied.	Jingdong prefecture level city in Shandong
Doraemon Hyakubo Shop	King of Power Saving	Energy saver received, the effect is consistent with the description, recommended to buy!	Jingdong prefecture level city in Shandong
Doraemon Hyakubo Shop	King of Power Saving	It's really good, the quality is great, I recommend it!	Jingdong prefecture level city in Shandong
Doraemon Hyakubo Shop	King of Power Saving	Works well. Inexpensive. Shipping was fast.	Jingdong prefecture level city in Shandong
Doraemon Hyakubo Shop	King of Power Saving	Logistics is fast, works well, energy saving is good	Jingdong prefecture level city in Shandong
Doraemon Hyakubo Shop	King of Power Saving	very happy	Jingdong prefecture level city in Shandong
Doraemon Hyakubo Shop	King of Power Saving	an excellent	Jingdong prefecture level city in Shandong
Doraemon Hyakubo Shop	King of Power Saving	It rained a couple days ago and I just went to get the item back today , not bad, hey I looked at the meter and that red display light takes several seconds to jump, did not make a mistake buying it. After a period of time to evaluate.	Jingdong prefecture level city in Shandong
Doraemon Hyakubo Shop	King of Power Saving	Used more than 10 days to check the power, there is indeed some decline, customer service said, long-term use will save more points!	Jingdong prefecture level city in Shandong
Doraemon Hyakubo Shop	King of Power Saving	It's a proper IQ tax, and it costs electricity.	Jingdong prefecture level city in Shandong
Doraemon Hyakubo Shop	King of Power Saving	I've been using it for 17 days now, it really saves electricity Shipping is fast, you can save a lot in a year	Jingdong prefecture level city in Shandong
Doraemon Hyakubo Shop	King of Power Saving	I've been using it for 17 days and it really saves power Shipping is fast and you don't have to worry about the quality, free returns within 5 years	Jingdong prefecture level city in Shandong
Doraemon Hyakubo Shop	King of Power Saving	It's more regular, scanned the code and it's authentic, and the main thing is that you can try it for 90 days! Plugged in more than ten days, is less than before a few degrees per day. When the time to use a month to see Additional. Good to buy a few more	Jingdong prefecture level city in Shandong
Doraemon Hyakubo Shop	King of Power Saving	Logistics is very fast, fine workmanship, use it and then come back to the comments	Jingdong prefecture level city in Shandong
Doraemon Hyakubo Shop	King of Power Saving	The quality of workmanship is very good and the power saving effect is quite good, I regret not getting it earlier.	Jingdong prefecture level city in Shandong

Doraemon Hyakubo Shop	King of Power Saving	Received the goods, according to the instructions on the operation is very simple, to wait and see the effect, looks good!	Jingdong prefecture level city in Shandong
Doraemon Hyakubo Shop	King of Power Saving	It's really as safe and power-saving as the owner says, and it works great!	Jingdong prefecture level city in Shandong
Doraemon Hyakubo Shop	King of Power Saving	Received the goods, immediately used, looks good, to wait and see how the effect.	Jingdong prefecture level city in Shandong
Doraemon Hyakubo Shop	King of Power Saving	Afraid to buy back did not work, the store said the commitment to 90 days invalid refund, warranty 5 years. Saver also complimentary on the Pacific insurance, it is a lot of peace of mind, there are effects will continue to buy back drops!	Jingdong prefecture level city in Shandong
Doraemon Hyakubo Shop	King of Power Saving	Received the goods and used it, can save electricity a month later to see the effect!	Jingdong prefecture level city in Shandong
Doraemon Hyakubo Shop	King of Power Saving	Fancy 90-day trial, 5-year warranty before the order, the goods received, has been experiencing a week, can really feel the savings, in the introduction of friends also buy this!	Jingdong prefecture level city in Shandong
Doraemon Hyakubo Shop	King of Power Saving	Clothing stores use, neighbors recommended to buy, said a month can save more than 70 yuan, first plugged in to see the effect of the	Jingdong prefecture level city in Shandong
Doraemon Hyakubo Shop	King of Power Saving	A friend's home two months to save more than 80 dollars of electricity, recommended that I buy two, decisive purchase, looking forward to the effect of power saving, waiting for the next month effect.	Jingdong prefecture level city in Shandong
Doraemon Hyakubo Shop	King of Power Saving	The goods have been received, consistent with the description of the advertisement, quite good, I hope that in terms of power saving and purifying the air is really as the merchant said that would be better!	Jingdong prefecture level city in Shandong
Doraemon Hyakubo Shop	King of Power Saving	Power Saving King is really good to use the quality is very good than the physical store a lot cheaper we can rest assured that the purchase recommended to buy!	Jingdong prefecture level city in Shandong
Doraemon Hyakubo Shop	King of Power Saving	Energy saver has been used on the rental house inside the use of the meter is always running fast, with this can really save electricity, easy to use, plugged in can be, very satisfied!	Jingdong prefecture level city in Shandong
Doraemon Hyakubo Shop	King of Power Saving	Just use can not see what effect to,, wait for a period of time in the evaluation	Jingdong prefecture level city in Shandong
Doraemon Hyakubo Shop	King of Power Saving	Live in the 18th floor is too far away from the meter, afraid of no effect, look at the trial 90 days before buying. Currently used for a month, there is an effect, saving 27 dollars in electricity, next month should be able to save more!	Jingdong prefecture level city in Shandong
Doraemon Hyakubo Shop	King of Power Saving	I've been using it for almost a month now and it feels good, so I'll use it again.	Jingdong prefecture level city in Shandong
Doraemon Hyakubo Shop	King of Power Saving	It's ok, the electric bill is really less, originally I remember charging $100 and using it for 10 days. Now it lasts about 15 days. It's still noticeable.	Jingdong prefecture level city in Shandong
Doraemon Hyakubo Shop	King of Power Saving	Fast logistics, good power saving effect, very satisfied	Jingdong prefecture level city in Shandong
Doraemon Hyakubo Shop	King of Power Saving	Has been the second time back to buy, energy saving effect, worth recommending, you can try to see, power saving effect: very good Experience: can save about 30%!	Jingdong prefecture level city in Shandong
Doraemon Hyakubo Shop	King of Power Saving	I don't know how well it works for now	Jingdong prefecture level city in Shandong
Doraemon Hyakubo Shop	King of Power Saving	Factory direct is really good, plugged in and used for half a month did less probably almost 50 degrees of electricity! And the manufacturer's warranty is 5 years.	Jingdong prefecture level city in Shandong
Doraemon Hyakubo Shop	King of Power Saving	It's really effective. The previous month's electricity was $286, but after using the power saver for a month, I have $195 left this month, which is not bad.	Jingdong prefecture level city in Shandong
Doraemon Hyakubo Shop	King of Power Saving	I'll give you a good review and see how it goes.	Jingdong prefecture level city in Shandong
Doraemon Hyakubo Shop	King of Power Saving	Customer service is very professional and patient to guide the installation, the quality of workmanship is very fine. Durable, second repurchase. Really can save oh!	Jingdong prefecture level city in Shandong
Doraemon Hyakubo Shop	King of Power Saving	I've been using it for a month now and my electric bill is over $30 less than last month. I feel it's quite worth buying.	Jingdong prefecture level city in Shandong
Doraemon Hyakubo Shop	King of Power Saving	Small size, does not take up space. It's very good, it does work very effectively, the tool room has a lot of work with a lot of electricity. After installing it, it really reduces a lot of electricity bills. Before the day 30 degrees of electricity left stone, now work down really less a few degrees.	Jingdong prefecture level city in Shandong
Doraemon Hyakubo Shop	King of Power Saving	It does work for saving electricity, I've bought the kind from elsewhere before that was too simple not **all**. This time to buy this store, but also for the safety. Air conditioning is too much electricity, this use to save a lot of	Jingdong prefecture level city in Shandong
Doraemon Hyakubo Shop	King of Power Saving	Two air conditioners and a refrigerator at home, a month down the electricity bill is almost two hundred dollars, with this after the average savings of more than thirty dollars to spend less money to enjoy more, it's really very good!	Jingdong prefecture level city in Shandong
Doraemon Hyakubo Shop	King of Power Saving	Good. Very good.	Jingdong prefecture level city in Shandong
Doraemon Hyakubo Shop	King of Power Saving	Works great, easy to operate, great, very happy with it.	Jingdong prefecture level city in Shandong
Doraemon Hyakubo Shop	King of Power Saving	Received it and tried it out, it's great, great quality and worth having! Five stars praise!	Jingdong prefecture level city in Shandong
Doraemon Hyakubo Shop	King of Power Saving	Very trustworthy brand, quality is guaranteed, the price is still affordable, quite satisfied!	Jingdong prefecture level city in Shandong
Doraemon Hyakubo Shop	King of Power Saving	Use it before you speak.	Jingdong prefecture level city in Shandong
Doraemon Hyakubo Shop	King of Power Saving	The quality of workmanship is very good and the power saving effect is quite good, I regret not getting it earlier.	Jingdong prefecture level city in Shandong
Doraemon Hyakubo Shop	King of Power Saving	Merchants shipping speed, and customer service introduced the same very good, cost-effective	Jingdong prefecture level city in Shandong
Doraemon Hyakubo Shop	King of Power Saving	Feel a little effect Oh, where the power consumption where to plug, convenient and convenient	Jingdong prefecture level city in Shandong
Doraemon Hyakubo Shop	King of Power Saving	The quality of the imported saver is very good, practical and satisfactory. Cost-effective	Jingdong prefecture level city in Shandong
Doraemon Hyakubo Shop	King of Power Saving	Power Saver is especially good, good and inexpensive, very good	Jingdong prefecture level city in Shandong
Doraemon Hyakubo Shop	King of Power Saving	Fast shipping, awesome quality, affordable price, satisfied.	Jingdong prefecture level city in Shandong
Doraemon Hyakubo Shop	King of Power Saving	Energy saver received, the effect is consistent with the description, recommended to buy!	Jingdong prefecture level city in Shandong
Doraemon Hyakubo Shop	King of Power Saving	It's really good, the quality is great, I recommend it!	Jingdong prefecture level city in Shandong
Doraemon Hyakubo Shop	King of Power Saving	Works well. Inexpensive. Shipping was fast.	Jingdong prefecture level city in Shandong
Doraemon Hyakubo Shop	King of Power Saving	Logistics is fast, works well, energy saving is good	Jingdong prefecture level city in Shandong
Doraemon Hyakubo Shop	King of Power Saving	very happy	Jingdong prefecture level city in Shandong
Doraemon Hyakubo Shop	King of Power Saving	an excellent	Jingdong prefecture level city in Shandong
Doraemon Hyakubo Shop	King of Power Saving	It rained a couple days ago and I just went to get the item back today , not bad, hey I looked at the meter and that red display light takes several seconds to jump, did not make a mistake buying it. After a period of time to evaluate.	Jingdong prefecture level city in Shandong
Doraemon Hyakubo Shop	King of Power Saving	Used more than 10 days to check the power, there is indeed some decline, customer service said, long-term use will save more points!	Jingdong prefecture level city in Shandong
Doraemon Hyakubo Shop	King of Power Saving	It's a proper IQ tax, and it costs electricity.	Jingdong prefecture level city in Shandong
Doraemon Hyakubo Shop	King of Power Saving	I've been using it for 17 days now, it really saves electricity Shipping is fast, you can save a lot in a year	Jingdong prefecture level city in Shandong
Doraemon Hyakubo Shop	King of Power Saving	I've been using it for 17 days and it really saves power Shipping is fast and you don't have to worry about the quality, free returns within 5 years	Jingdong prefecture level city in Shandong

Doraemon Hyakubo Shop	King of Power Saving	It's more regular, scanned the code and it's authentic, and the main thing is that you can try it for 90 days! Plugged in more than ten days, is less than before a few degrees per day. When the time to use a month to see Additional. Good to buy a few more	Jingdong prefecture level city in Shandong
Doraemon Hyakubo Shop	King of Power Saving	Logistics is very fast, fine workmanship, use it and then come back to the comments	Jingdong prefecture level city in Shandong
Doraemon Hyakubo Shop	King of Power Saving	The quality of workmanship is very good and the power saving effect is quite good, I regret not getting it earlier.	Jingdong prefecture level city in Shandong
Doraemon Hyakubo Shop	King of Power Saving	Received the goods, according to the instructions on the operation is very simple, to wait and see the effect, looks good!	Jingdong prefecture level city in Shandong
Doraemon Hyakubo Shop	King of Power Saving	It's really as safe and power-saving as the owner says, and it works great!	Jingdong prefecture level city in Shandong
Doraemon Hyakubo Shop	King of Power Saving	Received the goods, immediately used, looks good, to wait and see how the effect.	Jingdong prefecture level city in Shandong
Doraemon Hyakubo Shop	King of Power Saving	Afraid to buy back did not work, the store said the commitment to 90 days invalid refund, warranty 5 years. Saver also complimentary on the Pacific insurance, it is a lot of peace of mind, there are effects will continue to buy back drops!	Jingdong prefecture level city in Shandong
Doraemon Hyakubo Shop	King of Power Saving	Received the goods and used it, can save electricity a month later to see the effect!	Jingdong prefecture level city in Shandong
Doraemon Hyakubo Shop	King of Power Saving	Fancy 90-day trial, 5-year warranty before the order, the goods received, has been experiencing a week, can really feel the savings, in the introduction of friends also buy this!	Jingdong prefecture level city in Shandong
Doraemon Hyakubo Shop	King of Power Saving	Clothing stores use, neighbors recommended to buy, said a month can save more than 70 yuan, first plugged in to see the effect of the	Jingdong prefecture level city in Shandong
Doraemon Hyakubo Shop	King of Power Saving	A friend's home two months to save more than 80 dollars of electricity, recommended that I buy two, decisive purchase, looking forward to the effect of power saving, waiting for the next month effect.	Jingdong prefecture level city in Shandong
Doraemon Hyakubo Shop	King of Power Saving	The goods have been received, consistent with the description of the advertisement, quite good, I hope that in terms of power saving and purifying the air is really as the merchant said that would be better!	Jingdong prefecture level city in Shandong
Doraemon Hyakubo Shop	King of Power Saving	Power Saving King is really good to use the quality is very good than the physical store a lot cheaper we can rest assured that the purchase recommended to buy!	Jingdong prefecture level city in Shandong
Doraemon Hyakubo Shop	King of Power Saving	Energy saver has been used on the rental house inside the use of the meter is always running fast, with this can really save electricity, easy to use, plugged in can be, very satisfied!	Jingdong prefecture level city in Shandong
Doraemon Hyakubo Shop	King of Power Saving	Just use can not see what effect to,, wait for a period of time in the evaluation	Jingdong prefecture level city in Shandong
Doraemon Hyakubo Shop	King of Power Saving	Live in the 18th floor is too far away from the meter, afraid of no effect, look at the trial 90 days before buying. Currently used for a month, there is an effect, saving 27 dollars in electricity, next month should be able to save more!	Jingdong prefecture level city in Shandong
Doraemon Hyakubo Shop	King of Power Saving	I've been using it for almost a month now and it feels good, so I'll use it again.	Jingdong prefecture level city in Shandong
Doraemon Hyakubo Shop	King of Power Saving	It's ok, the electric bill is really less, originally I remember charging $100 and using it for 10 days. Now it lasts about 15 days. It's still noticeable.	Jingdong prefecture level city in Shandong
Doraemon Hyakubo Shop	King of Power Saving	Fast logistics, good power saving effect, very satisfied	Jingdong prefecture level city in Shandong
Doraemon Hyakubo Shop	King of Power Saving	Has been the second time back to buy, energy saving effect, worth recommending, you can try to see, power saving effect: very good Experience: can save about 30%!	Jingdong prefecture level city in Shandong
Doraemon Hyakubo Shop	King of Power Saving	I don't know how well it works for now	Jingdong prefecture level city in Shandong
Doraemon Hyakubo Shop	King of Power Saving	Factory direct is really good, plugged in and used for half a month did less probably almost 50 degrees of electricity! And the manufacturer's warranty is 5 years.	Jingdong prefecture level city in Shandong
Doraemon Hyakubo Shop	King of Power Saving	It's really effective. The previous month's electricity was $286, but after using the power saver for a month, I have $195 left this month, which is not bad.	Jingdong prefecture level city in Shandong
Doraemon Hyakubo Shop	King of Power Saving	I'll give you a good review and see how it goes.	Jingdong prefecture level city in Shandong
Doraemon Hyakubo Shop	King of Power Saving	Customer service is very professional and patient to guide the installation, the quality of workmanship is very fine. Durable, second repurchase. Really can save oh!	Jingdong prefecture level city in Shandong
Doraemon Hyakubo Shop	King of Power Saving	I've been using it for a month now and my electric bill is over $30 less than last month. I feel it's quite worth buying.	Jingdong prefecture level city in Shandong
Doraemon Hyakubo Shop	King of Power Saving	Small size, does not take up space. It's very good, it does work very effectively, the tool room has a lot of work with a lot of electricity. After installing it, it really reduces a lot of electricity bills. Before the day 30 degrees of electricity left stone, now work down really less a few degrees.	Jingdong prefecture level city in Shandong
Doraemon Hyakubo Shop	King of Power Saving	It does work for saving electricity, I've bought the kind from elsewhere before that was too simple not **all**. This time to buy this store, but also for the safety. Air conditioning is too much electricity, this use to save a lot of	Jingdong prefecture level city in Shandong
Doraemon Hyakubo Shop	King of Power Saving	Two air conditioners and a refrigerator at home, a month down the electricity bill is almost two hundred dollars, with this after the average savings of more than thirty dollars to spend less money to enjoy more, it's really very good!	Jingdong prefecture level city in Shandong
Doraemon Hyakubo Shop	King of Power Saving	Good. Very good.	Jingdong prefecture level city in Shandong
Doraemon Hyakubo Shop	King of Power Saving	Works great, easy to operate, great, very happy with it.	Jingdong prefecture level city in Shandong
Doraemon Hyakubo Shop	King of Power Saving	Received it and tried it out, it's great, great quality and worth having! Five stars praise!	Jingdong prefecture level city in Shandong
Doraemon Hyakubo Shop	King of Power Saving	Very trustworthy brand, quality is guaranteed, the price is still affordable, quite satisfied!	Jingdong prefecture level city in Shandong
Doraemon Hyakubo Shop	King of Power Saving	Use it before you speak.	Jingdong prefecture level city in Shandong
Doraemon Hyakubo Shop	King of Power Saving	The quality of workmanship is very good and the power saving effect is quite good, I regret not getting it earlier.	Jingdong prefecture level city in Shandong
Doraemon Hyakubo Shop	King of Power Saving	Merchants shipping speed, and customer service introduced the same very good, cost-effective	Jingdong prefecture level city in Shandong
Doraemon Hyakubo Shop	King of Power Saving	Feel a little effect Oh, where the power consumption where to plug, convenient and convenient	Jingdong prefecture level city in Shandong
Doraemon Hyakubo Shop	King of Power Saving	The quality of the imported saver is very good, practical and satisfactory. Cost-effective	Jingdong prefecture level city in Shandong
Doraemon Hyakubo Shop	King of Power Saving	Power Saver is especially good, good and inexpensive, very good	Jingdong prefecture level city in Shandong
Doraemon Hyakubo Shop	King of Power Saving	Fast shipping, awesome quality, affordable price, satisfied.	Jingdong prefecture level city in Shandong
Doraemon Hyakubo Shop	King of Power Saving	Energy saver received, the effect is consistent with the description, recommended to buy!	Jingdong prefecture level city in Shandong
Doraemon Hyakubo Shop	King of Power Saving	It's really good, the quality is great, I recommend it!	Jingdong prefecture level city in Shandong
Doraemon Hyakubo Shop	King of Power Saving	Works well. Inexpensive. Shipping was fast.	Jingdong prefecture level city in Shandong
Doraemon Hyakubo Shop	King of Power Saving	Logistics is fast, works well, energy saving is good	Jingdong prefecture level city in Shandong
Doraemon Hyakubo Shop	King of Power Saving	very happy	Jingdong prefecture level city in Shandong
Doraemon Hyakubo Shop	King of Power Saving	an excellent	Jingdong prefecture level city in Shandong
Doraemon Hyakubo Shop	King of Power Saving	It rained a couple days ago and I just went to get the item back today , not bad, hey I looked at the meter and that red display light takes several seconds to jump, did not make a mistake buying it. After a period of time to evaluate.	Jingdong prefecture level city in Shandong
Doraemon Hyakubo Shop	King of Power Saving	Used more than 10 days to check the power, there is indeed some decline, customer service said, long-term use will save more points!	Jingdong prefecture level city in Shandong

Doraemon Hyakubo Shop	King of Power Saving	It's a proper IQ tax, and it costs electricity.	Jingdong prefecture level city in Shandong
Doraemon Hyakubo Shop	King of Power Saving	I've been using it for 17 days now, it really saves electricity Shipping is fast, you can save a lot in a year	Jingdong prefecture level city in Shandong
Doraemon Hyakubo Shop	King of Power Saving	I've been using it for 17 days and it really saves power Shipping is fast and you don't have to worry about the quality, free returns within 5 years	Jingdong prefecture level city in Shandong
Doraemon Hyakubo Shop	King of Power Saving	It's more regular, scanned the code and it's authentic, and the main thing is that you can try it for 90 days! Plugged in more than ten days, is less than before a few degrees per day. When the time to use a month to see Additional. Good to buy a few more	Jingdong prefecture level city in Shandong
Doraemon Hyakubo Shop	King of Power Saving	Logistics is very fast, fine workmanship, use it and then come back to the comments	Jingdong prefecture level city in Shandong
Doraemon Hyakubo Shop	King of Power Saving	The quality of workmanship is very good and the power saving effect is quite good, I regret not getting it earlier.	Jingdong prefecture level city in Shandong
Doraemon Hyakubo Shop	King of Power Saving	Received the goods, according to the instructions on the operation is very simple, to wait and see the effect, looks good!	Jingdong prefecture level city in Shandong
Doraemon Hyakubo Shop	King of Power Saving	It's really as safe and power-saving as the owner says, and it works great!	Jingdong prefecture level city in Shandong
Doraemon Hyakubo Shop	King of Power Saving	Received the goods, immediately used, looks good, to wait and see how the effect.	Jingdong prefecture level city in Shandong
Doraemon Hyakubo Shop	King of Power Saving	Afraid to buy back did not work, the store said the commitment to 90 days invalid refund, warranty 5 years. Saver also complimentary on the Pacific insurance, it is a lot of peace of mind, there are effects will continue to buy back drops!	Jingdong prefecture level city in Shandong
Doraemon Hyakubo Shop	King of Power Saving	Received the goods and used it, can save electricity a month later to see the effect!	Jingdong prefecture level city in Shandong
Doraemon Hyakubo Shop	King of Power Saving	Fancy 90-day trial, 5-year warranty before the order, the goods received, has been experiencing a week, can really feel the savings, in the introduction of friends also buy this!	Jingdong prefecture level city in Shandong
Doraemon Hyakubo Shop	King of Power Saving	Clothing stores use, neighbors recommended to buy, said a month can save more than 70 yuan, first plugged in to see the effect of the	Jingdong prefecture level city in Shandong
Doraemon Hyakubo Shop	King of Power Saving	A friend's home two months to save more than 80 dollars of electricity, recommended that I buy two, decisive purchase, looking forward to the effect of power saving, waiting for the next month effect.	Jingdong prefecture level city in Shandong
Doraemon Hyakubo Shop	King of Power Saving	The goods have been received, consistent with the description of the advertisement, quite good, I hope that in terms of power saving and purifying the air is really as the merchant said that would be better!	Jingdong prefecture level city in Shandong
Doraemon Hyakubo Shop	King of Power Saving	Power Saving King is really good to use the quality is very good than the physical store a lot cheaper we can rest assured that the purchase recommended to buy!	Jingdong prefecture level city in Shandong
Doraemon Hyakubo Shop	King of Power Saving	Energy saver has been used on the rental house inside the use of the meter is always running fast, with this can really save electricity, easy to use, plugged in can be, very satisfied!	Jingdong prefecture level city in Shandong
Doraemon Hyakubo Shop	King of Power Saving	Just use can not see what effect to,, wait for a period of time in the evaluation	Jingdong prefecture level city in Shandong
Doraemon Hyakubo Shop	King of Power Saving	Live in the 18th floor is too far away from the meter, afraid of no effect, look at the trial 90 days before buying. Currently used for a month, there is an effect, saving 27 dollars in electricity, next month should be able to save more!	Jingdong prefecture level city in Shandong
Doraemon Hyakubo Shop	King of Power Saving	I've been using it for almost a month now and it feels good, so I'll use it again.	Jingdong prefecture level city in Shandong
Doraemon Hyakubo Shop	King of Power Saving	It's ok, the electric bill is really less, originally I remember charging $100 and using it for 10 days. Now it lasts about 15 days. It's still noticeable.	Jingdong prefecture level city in Shandong
Doraemon Hyakubo Shop	King of Power Saving	Fast logistics, good power saving effect, very satisfied	Jingdong prefecture level city in Shandong
Doraemon Hyakubo Shop	King of Power Saving	Has been the second time back to buy, energy saving effect, worth recommending, you can try to see, power saving effect: very good Experience: can save about 30%!	Jingdong prefecture level city in Shandong
Doraemon Hyakubo Shop	King of Power Saving	I don't know how well it works for now	Jingdong prefecture level city in Shandong
Doraemon Hyakubo Shop	King of Power Saving	Factory direct is really good, plugged in and used for half a month did less probably almost 50 degrees of electricity! And the manufacturer's warranty is 5 years.	Jingdong prefecture level city in Shandong
Doraemon Hyakubo Shop	King of Power Saving	It's really effective. The previous month's electricity was $286, but after using the power saver for a month, I have $195 left this month, which is not bad.	Jingdong prefecture level city in Shandong
Doraemon Hyakubo Shop	King of Power Saving	I'll give you a good review and see how it goes.	Jingdong prefecture level city in Shandong
Doraemon Hyakubo Shop	King of Power Saving	Customer service is very professional and patient to guide the installation, the quality of workmanship is very fine. Durable, second repurchase. Really can save oh!	Jingdong prefecture level city in Shandong
Doraemon Hyakubo Shop	King of Power Saving	I've been using it for a month now and my electric bill is over $30 less than last month. I feel it's quite worth buying.	Jingdong prefecture level city in Shandong
Doraemon Hyakubo Shop	King of Power Saving	Small size, does not take up space. It's very good, it does work very effectively, the tool room has a lot of work with a lot of electricity. After installing it, it really reduces a lot of electricity bills. Before the day 30 degrees of electricity left stone, now work down really less a few degrees.	Jingdong prefecture level city in Shandong
Doraemon Hyakubo Shop	King of Power Saving	It does work for saving electricity, I've bought the kind from elsewhere before that was too simple not **all**. This time to buy this store, but also for the safety. Air conditioning is too much electricity, this use to save a lot of	Jingdong prefecture level city in Shandong
Doraemon Hyakubo Shop	King of Power Saving	Two air conditioners and a refrigerator at home, a month down the electricity bill is almost two hundred dollars, with this after the average savings of more than thirty dollars to spend less money to enjoy more, it's really very good!	Jingdong prefecture level city in Shandong
Doraemon Hyakubo Shop	King of Power Saving	Good. Very good.	Jingdong prefecture level city in Shandong
Doraemon Hyakubo Shop	King of Power Saving	Works great, easy to operate, great, very happy with it.	Jingdong prefecture level city in Shandong
Doraemon Hyakubo Shop	King of Power Saving	Received it and tried it out, it's great, great quality and worth having! Five stars praise!	Jingdong prefecture level city in Shandong
Doraemon Hyakubo Shop	King of Power Saving	Very trustworthy brand, quality is guaranteed, the price is still affordable, quite satisfied!	Jingdong prefecture level city in Shandong
Doraemon Hyakubo Shop	King of Power Saving	Use it before you speak.	Jingdong prefecture level city in Shandong
Doraemon Hyakubo Shop	King of Power Saving	The quality of workmanship is very good and the power saving effect is quite good, I regret not getting it earlier.	Jingdong prefecture level city in Shandong
Doraemon Hyakubo Shop	King of Power Saving	Merchants shipping speed, and customer service introduced the same very good, cost-effective	Jingdong prefecture level city in Shandong
Doraemon Hyakubo Shop	King of Power Saving	Feel a little effect Oh, where the power consumption where to plug, convenient and convenient	Jingdong prefecture level city in Shandong
Doraemon Hyakubo Shop	King of Power Saving	The quality of the imported saver is very good, practical and satisfactory. Cost-effective	Jingdong prefecture level city in Shandong
Doraemon Hyakubo Shop	King of Power Saving	Power Saver is especially good, good and inexpensive, very good	Jingdong prefecture level city in Shandong
Doraemon Hyakubo Shop	King of Power Saving	Fast shipping, awesome quality, affordable price, satisfied.	Jingdong prefecture level city in Shandong
Doraemon Hyakubo Shop	King of Power Saving	Energy saver received, the effect is consistent with the description, recommended to buy!	Jingdong prefecture level city in Shandong
Doraemon Hyakubo Shop	King of Power Saving	It's really good, the quality is great, I recommend it!	Jingdong prefecture level city in Shandong
Doraemon Hyakubo Shop	King of Power Saving	Works well. Inexpensive. Shipping was fast.	Jingdong prefecture level city in Shandong
Doraemon Hyakubo Shop	King of Power Saving	Logistics is fast, works well, energy saving is good	Jingdong prefecture level city in Shandong
Doraemon Hyakubo Shop	King of Power Saving	very happy	Jingdong prefecture level city in Shandong

Doraemon Hyakubo Shop	King of Power Saving	an excellent	Jingdong prefecture level city in Shandong
Doraemon Hyakubo Shop	King of Power Saving	It rained a couple days ago and I just went to get the item back today , not bad, hey I looked at the meter and that red display light takes several seconds to jump, did not make a mistake buying it. After a period of time to evaluate.	Jingdong prefecture level city in Shandong
Doraemon Hyakubo Shop	King of Power Saving	Used more than 10 days to check the power, there is indeed some decline, customer service said, long-term use will save more points!	Jingdong prefecture level city in Shandong
Doraemon Hyakubo Shop	King of Power Saving	It's a proper IQ tax, and it costs electricity.	Jingdong prefecture level city in Shandong
Doraemon Hyakubo Shop	King of Power Saving	I've been using it for 17 days now, it really saves electricity Shipping is fast, you can save a lot in a year	Jingdong prefecture level city in Shandong
Doraemon Hyakubo Shop	King of Power Saving	I've been using it for 17 days and it really saves power Shipping is fast and you don't have to worry about the quality, free returns within 5 years	Jingdong prefecture level city in Shandong
Doraemon Hyakubo Shop	King of Power Saving	It's more regular, scanned the code and it's authentic, and the main thing is that you can try it for 90 days! Plugged in more than ten days, is less than before a few degrees per day. When the time to use a month to see Additional. Good to buy a few more	Jingdong prefecture level city in Shandong
Doraemon Hyakubo Shop	King of Power Saving	Logistics is very fast, fine workmanship, use it and then come back to the comments	Jingdong prefecture level city in Shandong
Doraemon Hyakubo Shop	King of Power Saving	The quality of workmanship is very good and the power saving effect is quite good, I regret not getting it earlier.	Jingdong prefecture level city in Shandong
Doraemon Hyakubo Shop	King of Power Saving	Received the goods, according to the instructions on the operation is very simple, to wait and see the effect, looks good!	Jingdong prefecture level city in Shandong
Doraemon Hyakubo Shop	King of Power Saving	It's really as safe and power-saving as the owner says, and it works great!	Jingdong prefecture level city in Shandong
Doraemon Hyakubo Shop	King of Power Saving	Received the goods, immediately used, looks good, to wait and see how the effect.	Jingdong prefecture level city in Shandong
Doraemon Hyakubo Shop	King of Power Saving	Afraid to buy back did not work, the store said the commitment to 90 days invalid refund, warranty 5 years. Saver also complimentary on the Pacific insurance, it is a lot of peace of mind, there are effects will continue to buy back drops!	Jingdong prefecture level city in Shandong
Doraemon Hyakubo Shop	King of Power Saving	Received the goods and used it, can save electricity a month later to see the effect!	Jingdong prefecture level city in Shandong
Doraemon Hyakubo Shop	King of Power Saving	Fancy 90-day trial, 5-year warranty before the order, the goods received, has been experiencing a week, can really feel the savings, in the introduction of friends also buy this!	Jingdong prefecture level city in Shandong
Doraemon Hyakubo Shop	King of Power Saving	Clothing stores use, neighbors recommended to buy, said a month can save more than 70 yuan, first plugged in to see the effect of the	Jingdong prefecture level city in Shandong
Doraemon Hyakubo Shop	King of Power Saving	A friend's home two months to save more than 80 dollars of electricity, recommended that I buy two, decisive purchase, looking forward to the effect of power saving, waiting for the next month effect.	Jingdong prefecture level city in Shandong
Doraemon Hyakubo Shop	King of Power Saving	The goods have been received, consistent with the description of the advertisement, quite good, I hope that in terms of power saving and purifying the air is really as the merchant said that would be better!	Jingdong prefecture level city in Shandong
Doraemon Hyakubo Shop	King of Power Saving	Power Saving King is really good to use the quality is very good than the physical store a lot cheaper we can rest assured that the purchase recommended to buy!	Jingdong prefecture level city in Shandong
Doraemon Hyakubo Shop	King of Power Saving	Energy saver has been used on the rental house inside the use of the meter is always running fast, with this can really save electricity, easy to use, plugged in can be, very satisfied!	Jingdong prefecture level city in Shandong
Doraemon Hyakubo Shop	King of Power Saving	Just use can not see what effect to,, wait for a period of time in the evaluation	Jingdong prefecture level city in Shandong
Doraemon Hyakubo Shop	King of Power Saving	Live in the 18th floor is too far away from the meter, afraid of no effect, look at the trial 90 days before buying. Currently used for a month, there is an effect, saving 27 dollars in electricity, next month should be able to save more!	Jingdong prefecture level city in Shandong
Doraemon Hyakubo Shop	King of Power Saving	I've been using it for almost a month now and it feels good, so I'll use it again.	Jingdong prefecture level city in Shandong
Doraemon Hyakubo Shop	King of Power Saving	It's ok, the electric bill is really less, originally I remember charging $100 and using it for 10 days. Now it lasts about 15 days. It's still noticeable.	Jingdong prefecture level city in Shandong
Doraemon Hyakubo Shop	King of Power Saving	Fast logistics, good power saving effect, very satisfied	Jingdong prefecture level city in Shandong
Doraemon Hyakubo Shop	King of Power Saving	Has been the second time back to buy, energy saving effect, worth recommending, you can try to see, power saving effect: very good Experience: can save about 30%!	Jingdong prefecture level city in Shandong
Doraemon Hyakubo Shop	King of Power Saving	I don't know how well it works for now	Jingdong prefecture level city in Shandong
Doraemon Hyakubo Shop	King of Power Saving	Factory direct is really good, plugged in and used for half a month did less probably almost 50 degrees of electricity! And the manufacturer's warranty is 5 years.	Jingdong prefecture level city in Shandong
Doraemon Hyakubo Shop	King of Power Saving	It's really effective. The previous month's electricity was $286, but after using the power saver for a month, I have $195 left this month, which is not bad.	Jingdong prefecture level city in Shandong
Doraemon Hyakubo Shop	King of Power Saving	I'll give you a good review and see how it goes.	Jingdong prefecture level city in Shandong
Doraemon Hyakubo Shop	King of Power Saving	Customer service is very professional and patient to guide the installation, the quality of workmanship is very fine. Durable, second repurchase. Really can save oh!	Jingdong prefecture level city in Shandong
Doraemon Hyakubo Shop	King of Power Saving	I've been using it for a month now and my electric bill is over $30 less than last month. I feel it's quite worth buying.	Jingdong prefecture level city in Shandong
Doraemon Hyakubo Shop	King of Power Saving	Small size, does not take up space. It's very good, it does work very effectively, the tool room has a lot of work with a lot of electricity. After installing it, it really reduces a lot of electricity bills. Before the day 30 degrees of electricity left stone, now work down really less a few degrees.	Jingdong prefecture level city in Shandong
Doraemon Hyakubo Shop	King of Power Saving	It does work for saving electricity, I've bought the kind from elsewhere before that was too simple not **all**. This time to buy this store, but also for the safety. Air conditioning is too much electricity, this use to save a lot of	Jingdong prefecture level city in Shandong
Doraemon Hyakubo Shop	King of Power Saving	Two air conditioners and a refrigerator at home, a month down the electricity bill is almost two hundred dollars, with this after the average savings of more than thirty dollars to spend less money to enjoy more, it's really very good!	Jingdong prefecture level city in Shandong
Doraemon Hyakubo Shop	King of Power Saving	Good. Very good.	Jingdong prefecture level city in Shandong
Doraemon Hyakubo Shop	King of Power Saving	Works great, easy to operate, great, very happy with it.	Jingdong prefecture level city in Shandong
Doraemon Hyakubo Shop	King of Power Saving	Received it and tried it out, it's great, great quality and worth having! Five stars praise!	Jingdong prefecture level city in Shandong
Doraemon Hyakubo Shop	King of Power Saving	Very trustworthy brand, quality is guaranteed, the price is still affordable, quite satisfied!	Jingdong prefecture level city in Shandong
Doraemon Hyakubo Shop	King of Power Saving	Use it before you speak.	Jingdong prefecture level city in Shandong
Doraemon Hyakubo Shop	King of Power Saving	The quality of workmanship is very good and the power saving effect is quite good, I regret not getting it earlier.	Jingdong prefecture level city in Shandong
Doraemon Hyakubo Shop	King of Power Saving	Merchants shipping speed, and customer service introduced the same very good, cost-effective	Jingdong prefecture level city in Shandong
Doraemon Hyakubo Shop	King of Power Saving	Feel a little effect Oh, where the power consumption where to plug, convenient and convenient	Jingdong prefecture level city in Shandong
Doraemon Hyakubo Shop	King of Power Saving	The quality of the imported saver is very good, practical and satisfactory. Cost-effective	Jingdong prefecture level city in Shandong
Doraemon Hyakubo Shop	King of Power Saving	Power Saver is especially good, good and inexpensive, very good	Jingdong prefecture level city in Shandong
Doraemon Hyakubo Shop	King of Power Saving	Fast shipping, awesome quality, affordable price, satisfied.	Jingdong prefecture level city in Shandong
Doraemon Hyakubo Shop	King of Power Saving	Energy saver received, the effect is consistent with the description, recommended to buy!	Jingdong prefecture level city in Shandong
Doraemon Hyakubo Shop	King of Power Saving	It's really good, the quality is great, I recommend it!	Jingdong prefecture level city in Shandong

Doraemon Hyakubo Shop	King of Power Saving	Works well. Inexpensive. Shipping was fast.	Jingdong prefecture level city in Shandong
Doraemon Hyakubo Shop	King of Power Saving	Logistics is fast, works well, energy saving is good	Jingdong prefecture level city in Shandong
Doraemon Hyakubo Shop	King of Power Saving	very happy	Jingdong prefecture level city in Shandong
Doraemon Hyakubo Shop	King of Power Saving	an excellent	Jingdong prefecture level city in Shandong
Doraemon Hyakubo Shop	King of Power Saving	It rained a couple days ago and I just went to get the item back today , not bad, hey I looked at the meter and that red display light takes several seconds to jump, did not make a mistake buying it. After a period of time to evaluate.	Jingdong prefecture level city in Shandong
Doraemon Hyakubo Shop	King of Power Saving	Used more than 10 days to check the power, there is indeed some decline, customer service said, long-term use will save more points!	Jingdong prefecture level city in Shandong
Doraemon Hyakubo Shop	King of Power Saving	It's a proper IQ tax, and it costs electricity.	Jingdong prefecture level city in Shandong
Doraemon Hyakubo Shop	King of Power Saving	I've been using it for 17 days now, it really saves electricity Shipping is fast, you can save a lot in a year	Jingdong prefecture level city in Shandong
Doraemon Hyakubo Shop	King of Power Saving	I've been using it for 17 days and it really saves power Shipping is fast and you don't have to worry about the quality, free returns within 5 years	Jingdong prefecture level city in Shandong
Doraemon Hyakubo Shop	King of Power Saving	It's more regular, scanned the code and it's authentic, and the main thing is that you can try it for 90 days! Plugged in more than ten days, is less than before a few degrees per day. When the time to use a month to see Additional. Good to buy a few more	Jingdong prefecture level city in Shandong
Doraemon Hyakubo Shop	King of Power Saving	Logistics is very fast, fine workmanship, use it and then come back to the comments	Jingdong prefecture level city in Shandong
Doraemon Hyakubo Shop	King of Power Saving	The quality of workmanship is very good and the power saving effect is quite good, I regret not getting it earlier.	Jingdong prefecture level city in Shandong
Doraemon Hyakubo Shop	King of Power Saving	Received the goods, according to the instructions on the operation is very simple, to wait and see the effect, looks good!	Jingdong prefecture level city in Shandong
Doraemon Hyakubo Shop	King of Power Saving	It's really as safe and power-saving as the owner says, and it works great!	Jingdong prefecture level city in Shandong
Doraemon Hyakubo Shop	King of Power Saving	Received the goods, immediately used, looks good, to wait and see how the effect.	Jingdong prefecture level city in Shandong
Doraemon Hyakubo Shop	King of Power Saving	Afraid to buy back did not work, the store said the commitment to 90 days invalid refund, warranty 5 years. Saver also complimentary on the Pacific insurance, it is a lot of peace of mind, there are effects will continue to buy back drops!	Jingdong prefecture level city in Shandong
Doraemon Hyakubo Shop	King of Power Saving	Received the goods and used it, can save electricity a month later to see the effect!	Jingdong prefecture level city in Shandong
Doraemon Hyakubo Shop	King of Power Saving	Fancy 90-day trial, 5-year warranty before the order, the goods received, has been experiencing a week, can really feel the savings, in the introduction of friends also buy this!	Jingdong prefecture level city in Shandong
Doraemon Hyakubo Shop	King of Power Saving	Clothing stores use, neighbors recommended to buy, said a month can save more than 70 yuan, first plugged in to see the effect of the	Jingdong prefecture level city in Shandong
Doraemon Hyakubo Shop	King of Power Saving	A friend's home two months to save more than 80 dollars of electricity, recommended that I buy two, decisive purchase, looking forward to the effect of power saving, waiting for the next month effect.	Jingdong prefecture level city in Shandong
Doraemon Hyakubo Shop	King of Power Saving	The goods have been received, consistent with the description of the advertisement, quite good, I hope that in terms of power saving and purifying the air is really as the merchant said that would be better!	Jingdong prefecture level city in Shandong
Doraemon Hyakubo Shop	King of Power Saving	Power Saving King is really good to use the quality is very good than the physical store a lot cheaper we can rest assured that the purchase recommended to buy!	Jingdong prefecture level city in Shandong
Doraemon Hyakubo Shop	King of Power Saving	Energy saver has been used on the rental house inside the use of the meter is always running fast, with this can really save electricity, easy to use, plugged in can be, very satisfied!	Jingdong prefecture level city in Shandong
Doraemon Hyakubo Shop	King of Power Saving	Just use can not see what effect to,, wait for a period of time in the evaluation	Jingdong prefecture level city in Shandong
Doraemon Hyakubo Shop	King of Power Saving	Live in the 18th floor is too far away from the meter, afraid of no effect, look at the trial 90 days before buying. Currently used for a month, there is an effect, saving 27 dollars in electricity, next month should be able to save more!	Jingdong prefecture level city in Shandong
Doraemon Hyakubo Shop	King of Power Saving	I've been using it for almost a month now and it feels good, so I'll use it again.	Jingdong prefecture level city in Shandong
Doraemon Hyakubo Shop	King of Power Saving	It's ok, the electric bill is really less, originally I remember charging $100 and using it for 10 days. Now it lasts about 15 days. It's still noticeable.	Jingdong prefecture level city in Shandong
Doraemon Hyakubo Shop	King of Power Saving	Fast logistics, good power saving effect, very satisfied	Jingdong prefecture level city in Shandong
Doraemon Hyakubo Shop	King of Power Saving	Has been the second time back to buy, energy saving effect, worth recommending, you can try to see, power saving effect: very good Experience: can save about 30%!	Jingdong prefecture level city in Shandong
Doraemon Hyakubo Shop	King of Power Saving	I don't know how well it works for now	Jingdong prefecture level city in Shandong
Doraemon Hyakubo Shop	King of Power Saving	Factory direct is really good, plugged in and used for half a month did less probably almost 50 degrees of electricity! And the manufacturer's warranty is 5 years.	Jingdong prefecture level city in Shandong
Doraemon Hyakubo Shop	King of Power Saving	It's really effective. The previous month's electricity was $286, but after using the power saver for a month, I have $195 left this month, which is not bad.	Jingdong prefecture level city in Shandong
Doraemon Hyakubo Shop	King of Power Saving	I'll give you a good review and see how it goes.	Jingdong prefecture level city in Shandong
Doraemon Hyakubo Shop	King of Power Saving	Customer service is very professional and patient to guide the installation, the quality of workmanship is very fine. Durable, second repurchase. Really can save oh!	Jingdong prefecture level city in Shandong
Doraemon Hyakubo Shop	King of Power Saving	I've been using it for a month now and my electric bill is over $30 less than last month. I feel it's quite worth buying.	Jingdong prefecture level city in Shandong
Doraemon Hyakubo Shop	King of Power Saving	Small size, does not take up space. It's very good, it does work very effectively, the tool room has a lot of work with a lot of electricity. After installing it, it really reduces a lot of electricity bills. Before the day 30 degrees of electricity left stone, now work down really less a few degrees.	Jingdong prefecture level city in Shandong
Doraemon Hyakubo Shop	King of Power Saving	It does work for saving electricity, I've bought the kind from elsewhere before that was too simple not **all**. This time to buy this store, but also for the safety. Air conditioning is too much electricity, this use to save a lot of	Jingdong prefecture level city in Shandong
Doraemon Hyakubo Shop	King of Power Saving	Two air conditioners and a refrigerator at home, a month down the electricity bill is almost two hundred dollars, with this after the average savings of more than thirty dollars to spend less money to enjoy more, it's really very good!	Jingdong prefecture level city in Shandong
Doraemon Hyakubo Shop	King of Power Saving	Good. Very good.	Jingdong prefecture level city in Shandong
Doraemon Hyakubo Shop	King of Power Saving	Works great, easy to operate, great, very happy with it.	Jingdong prefecture level city in Shandong
Doraemon Hyakubo Shop	King of Power Saving	Received it and tried it out, it's great, great quality and worth having! Five stars praise!	Jingdong prefecture level city in Shandong
Doraemon Hyakubo Shop	King of Power Saving	Very trustworthy brand, quality is guaranteed, the price is still affordable, quite satisfied!	Jingdong prefecture level city in Shandong
Doraemon Hyakubo Shop	King of Power Saving	Use it before you speak.	Jingdong prefecture level city in Shandong
Doraemon Hyakubo Shop	King of Power Saving	The quality of workmanship is very good and the power saving effect is quite good, I regret not getting it earlier.	Jingdong prefecture level city in Shandong
Doraemon Hyakubo Shop	King of Power Saving	Merchants shipping speed, and customer service introduced the same very good, cost-effective	Jingdong prefecture level city in Shandong
Doraemon Hyakubo Shop	King of Power Saving	Feel a little effect Oh, where the power consumption where to plug, convenient and convenient	Jingdong prefecture level city in Shandong
Doraemon Hyakubo Shop	King of Power Saving	The quality of the imported saver is very good, practical and satisfactory. Cost-effective	Jingdong prefecture level city in Shandong
Doraemon Hyakubo Shop	King of Power Saving	Power Saver is especially good, good and inexpensive, very good	Jingdong prefecture level city in Shandong

Doraemon Hyakubo Shop	King of Power Saving	Fast shipping, awesome quality, affordable price, satisfied.	Jingdong prefecture level city in Shandong
Doraemon Hyakubo Shop	King of Power Saving	Energy saver received, the effect is consistent with the description, recommended to buy!	Jingdong prefecture level city in Shandong
Doraemon Hyakubo Shop	King of Power Saving	It's really good, the quality is great, I recommend it!	Jingdong prefecture level city in Shandong
Doraemon Hyakubo Shop	King of Power Saving	Works well. Inexpensive. Shipping was fast.	Jingdong prefecture level city in Shandong
Doraemon Hyakubo Shop	King of Power Saving	Logistics is fast, works well, energy saving is good	Jingdong prefecture level city in Shandong
Doraemon Hyakubo Shop	King of Power Saving	very happy	Jingdong prefecture level city in Shandong
Doraemon Hyakubo Shop	King of Power Saving	an excellent	Jingdong prefecture level city in Shandong
Doraemon Hyakubo Shop	King of Power Saving	It rained a couple days ago and I just went to get the item back today , not bad, hey I looked at the meter and that red display light takes several seconds to jump, did not make a mistake buying it. After a period of time to evaluate.	Jingdong prefecture level city in Shandong
Doraemon Hyakubo Shop	King of Power Saving	Used more than 10 days to check the power, there is indeed some decline, customer service said, long-term use will save more points!	Jingdong prefecture level city in Shandong
Doraemon Hyakubo Shop	King of Power Saving	It's a proper IQ tax, and it costs electricity.	Jingdong prefecture level city in Shandong
Doraemon Hyakubo Shop	King of Power Saving	I've been using it for 17 days now, it really saves electricity Shipping is fast, you can save a lot in a year	Jingdong prefecture level city in Shandong
Doraemon Hyakubo Shop	King of Power Saving	I've been using it for 17 days and it really saves power Shipping is fast and you don't have to worry about the quality, free returns within 5 years	Jingdong prefecture level city in Shandong
Doraemon Hyakubo Shop	King of Power Saving	It's more regular, scanned the code and it's authentic, and the main thing is that you can try it for 90 days! Plugged in more than ten days, is less than before a few degrees per day. When the time to use a month to see Additional. Good to buy a few more	Jingdong prefecture level city in Shandong
Doraemon Hyakubo Shop	King of Power Saving	Logistics is very fast, fine workmanship, use it and then come back to the comments	Jingdong prefecture level city in Shandong
Doraemon Hyakubo Shop	King of Power Saving	The quality of workmanship is very good and the power saving effect is quite good, I regret not getting it earlier.	Jingdong prefecture level city in Shandong
Doraemon Hyakubo Shop	King of Power Saving	Received the goods, according to the instructions on the operation is very simple, to wait and see the effect, looks good!	Jingdong prefecture level city in Shandong
Doraemon Hyakubo Shop	King of Power Saving	It's really as safe and power-saving as the owner says, and it works great!	Jingdong prefecture level city in Shandong
Doraemon Hyakubo Shop	King of Power Saving	Received the goods, immediately used, looks good, to wait and see how the effect.	Jingdong prefecture level city in Shandong
Doraemon Hyakubo Shop	King of Power Saving	Afraid to buy back did not work, the store said the commitment to 90 days invalid refund, warranty 5 years. Saver also complimentary on the Pacific insurance, it is a lot of peace of mind, there are effects will continue to buy back drops!	Jingdong prefecture level city in Shandong
Doraemon Hyakubo Shop	King of Power Saving	Received the goods and used it, can save electricity a month later to see the effect!	Jingdong prefecture level city in Shandong
Doraemon Hyakubo Shop	King of Power Saving	Fancy 90-day trial, 5-year warranty before the order, the goods received, has been experiencing a week, can really feel the savings, in the introduction of friends also buy this!	Jingdong prefecture level city in Shandong
Doraemon Hyakubo Shop	King of Power Saving	Clothing stores use, neighbors recommended to buy, said a month can save more than 70 yuan, first plugged in to see the effect of the	Jingdong prefecture level city in Shandong
Doraemon Hyakubo Shop	King of Power Saving	A friend's home two months to save more than 80 dollars of electricity, recommended that I buy two, decisive purchase, looking forward to the effect of power saving, waiting for the next month effect.	Jingdong prefecture level city in Shandong
Doraemon Hyakubo Shop	King of Power Saving	The goods have been received, consistent with the description of the advertisement, quite good, I hope that in terms of power saving and purifying the air is really as the merchant said that would be better!	Jingdong prefecture level city in Shandong
Doraemon Hyakubo Shop	King of Power Saving	Power Saving King is really good to use the quality is very good than the physical store a lot cheaper we can rest assured that the purchase recommended to buy!	Jingdong prefecture level city in Shandong
Doraemon Hyakubo Shop	King of Power Saving	Energy saver has been used on the rental house inside the use of the meter is always running fast, with this can really save electricity, easy to use, plugged in can be, very satisfied!	Jingdong prefecture level city in Shandong
Doraemon Hyakubo Shop	King of Power Saving	Just use can not see what effect to,, wait for a period of time in the evaluation	Jingdong prefecture level city in Shandong
Doraemon Hyakubo Shop	King of Power Saving	Live in the 18th floor is too far away from the meter, afraid of no effect, look at the trial 90 days before buying. Currently used for a month, there is an effect, saving 27 dollars in electricity, next month should be able to save more!	Jingdong prefecture level city in Shandong
Doraemon Hyakubo Shop	King of Power Saving	I've been using it for almost a month now and it feels good, so I'll use it again.	Jingdong prefecture level city in Shandong
Doraemon Hyakubo Shop	King of Power Saving	It's ok, the electric bill is really less, originally I remember charging $100 and using it for 10 days. Now it lasts about 15 days. It's still noticeable.	Jingdong prefecture level city in Shandong
Doraemon Hyakubo Shop	King of Power Saving	Fast logistics, good power saving effect, very satisfied	Jingdong prefecture level city in Shandong
Doraemon Hyakubo Shop	King of Power Saving	Has been the second time back to buy, energy saving effect, worth recommending, you can try to see, power saving effect: very good Experience: can save about 30%!	Jingdong prefecture level city in Shandong
Doraemon Hyakubo Shop	King of Power Saving	I don't know how well it works for now	Jingdong prefecture level city in Shandong
Doraemon Hyakubo Shop	King of Power Saving	Factory direct is really good, plugged in and used for half a month did less probably almost 50 degrees of electricity! And the manufacturer's warranty is 5 years.	Jingdong prefecture level city in Shandong
Doraemon Hyakubo Shop	King of Power Saving	It's really effective. The previous month's electricity was $286, but after using the power saver for a month, I have $195 left this month, which is not bad.	Jingdong prefecture level city in Shandong
Doraemon Hyakubo Shop	King of Power Saving	I'll give you a good review and see how it goes.	Jingdong prefecture level city in Shandong
Doraemon Hyakubo Shop	King of Power Saving	Customer service is very professional and patient to guide the installation, the quality of workmanship is very fine. Durable, second repurchase. Really can save oh!	Jingdong prefecture level city in Shandong
Doraemon Hyakubo Shop	King of Power Saving	I've been using it for a month now and my electric bill is over $30 less than last month. I feel it's quite worth buying.	Jingdong prefecture level city in Shandong
Doraemon Hyakubo Shop	King of Power Saving	Small size, does not take up space. It's very good, it does work very effectively, the tool room has a lot of work with a lot of electricity. After installing it, it really reduces a lot of electricity bills. Before the day 30 degrees of electricity left stone, now work down really less a few degrees.	Jingdong prefecture level city in Shandong
Doraemon Hyakubo Shop	King of Power Saving	It does work for saving electricity, I've bought the kind from elsewhere before that was too simple not **all**. This time to buy this store, but also for the safety. Air conditioning is too much electricity, this use to save a lot of	Jingdong prefecture level city in Shandong
Doraemon Hyakubo Shop	King of Power Saving	Two air conditioners and a refrigerator at home, a month down the electricity bill is almost two hundred dollars, with this after the average savings of more than thirty dollars to spend less money to enjoy more, it's really very good!	Jingdong prefecture level city in Shandong
Doraemon Hyakubo Shop	King of Power Saving	Good. Very good.	Jingdong prefecture level city in Shandong
Doraemon Hyakubo Shop	King of Power Saving	Works great, easy to operate, great, very happy with it.	Jingdong prefecture level city in Shandong
Doraemon Hyakubo Shop	King of Power Saving	Received it and tried it out, it's great, great quality and worth having! Five stars praise!	Jingdong prefecture level city in Shandong
Doraemon Hyakubo Shop	King of Power Saving	Very trustworthy brand, quality is guaranteed, the price is still affordable, quite satisfied!	Jingdong prefecture level city in Shandong
Doraemon Hyakubo Shop	King of Power Saving	Use it before you speak.	Jingdong prefecture level city in Shandong
Doraemon Hyakubo Shop	King of Power Saving	The quality of workmanship is very good and the power saving effect is quite good, I regret not getting it earlier.	Jingdong prefecture level city in Shandong
Doraemon Hyakubo Shop	King of Power Saving	Merchants shipping speed, and customer service introduced the same very good, cost-effective	Jingdong prefecture level city in Shandong

Doraemon Hyakubo Shop	King of Power Saving	Feel a little effect Oh, where the power consumption where to plug, convenient and convenient	Jingdong prefecture level city in Shandong
Doraemon Hyakubo Shop	King of Power Saving	The quality of the imported saver is very good, practical and satisfactory. Cost-effective	Jingdong prefecture level city in Shandong
Doraemon Hyakubo Shop	King of Power Saving	Power Saver is especially good, good and inexpensive, very good	Jingdong prefecture level city in Shandong
Doraemon Hyakubo Shop	King of Power Saving	Fast shipping, awesome quality, affordable price, satisfied.	Jingdong prefecture level city in Shandong
Doraemon Hyakubo Shop	King of Power Saving	Energy saver received, the effect is consistent with the description, recommended to buy!	Jingdong prefecture level city in Shandong
Doraemon Hyakubo Shop	King of Power Saving	It's really good, the quality is great, I recommend it!	Jingdong prefecture level city in Shandong
Doraemon Hyakubo Shop	King of Power Saving	Works well. Inexpensive. Shipping was fast.	Jingdong prefecture level city in Shandong
Doraemon Hyakubo Shop	King of Power Saving	Logistics is fast, works well, energy saving is good	Jingdong prefecture level city in Shandong
Doraemon Hyakubo Shop	King of Power Saving	very happy	Jingdong prefecture level city in Shandong
Doraemon Hyakubo Shop	King of Power Saving	an excellent	Jingdong prefecture level city in Shandong
Doraemon Hyakubo Shop	King of Power Saving	It rained a couple days ago and I just went to get the item back today , not bad, hey I looked at the meter and that red display light takes several seconds to jump, did not make a mistake buying it. After a period of time to evaluate.	Jingdong prefecture level city in Shandong
Doraemon Hyakubo Shop	King of Power Saving	Used more than 10 days to check the power, there is indeed some decline, customer service said, long-term use will save more points!	Jingdong prefecture level city in Shandong
Doraemon Hyakubo Shop	King of Power Saving	It's a proper IQ tax, and it costs electricity.	Jingdong prefecture level city in Shandong
Doraemon Hyakubo Shop	King of Power Saving	I've been using it for 17 days now, it really saves electricity Shipping is fast, you can save a lot in a year	Jingdong prefecture level city in Shandong
Doraemon Hyakubo Shop	King of Power Saving	I've been using it for 17 days and it really saves power Shipping is fast and you don't have to worry about the quality, free returns within 5 years	Jingdong prefecture level city in Shandong
Doraemon Hyakubo Shop	King of Power Saving	It's more regular, scanned the code and it's authentic, and the main thing is that you can try it for 90 days! Plugged in more than ten days, is less than before a few degrees per day. When the time to use a month to see Additional. Good to buy a few more	Jingdong prefecture level city in Shandong
Doraemon Hyakubo Shop	King of Power Saving	Logistics is very fast, fine workmanship, use it and then come back to the comments	Jingdong prefecture level city in Shandong
Doraemon Hyakubo Shop	King of Power Saving	The quality of workmanship is very good and the power saving effect is quite good, I regret not getting it earlier.	Jingdong prefecture level city in Shandong
Doraemon Hyakubo Shop	King of Power Saving	Received the goods, according to the instructions on the operation is very simple, to wait and see the effect, looks good!	Jingdong prefecture level city in Shandong
Doraemon Hyakubo Shop	King of Power Saving	It's really as safe and power-saving as the owner says, and it works great!	Jingdong prefecture level city in Shandong
Doraemon Hyakubo Shop	King of Power Saving	Received the goods, immediately used, looks good, to wait and see how the effect.	Jingdong prefecture level city in Shandong
Doraemon Hyakubo Shop	King of Power Saving	Afraid to buy back did not work, the store said the commitment to 90 days invalid refund, warranty 5 years. Saver also complimentary on the Pacific insurance, it is a lot of peace of mind, there are effects will continue to buy back drops!	Jingdong prefecture level city in Shandong
Doraemon Hyakubo Shop	King of Power Saving	Received the goods and used it, can save electricity a month later to see the effect!	Jingdong prefecture level city in Shandong
Doraemon Hyakubo Shop	King of Power Saving	Fancy 90-day trial, 5-year warranty before the order, the goods received, has been experiencing a week, can really feel the savings, in the introduction of friends also buy this!	Jingdong prefecture level city in Shandong
Doraemon Hyakubo Shop	King of Power Saving	Clothing stores use, neighbors recommended to buy, said a month can save more than 70 yuan, first plugged in to see the effect of the	Jingdong prefecture level city in Shandong
Doraemon Hyakubo Shop	King of Power Saving	A friend's home two months to save more than 80 dollars of electricity, recommended that I buy two, decisive purchase, looking forward to the effect of power saving, waiting for the next month effect.	Jingdong prefecture level city in Shandong
Doraemon Hyakubo Shop	King of Power Saving	The goods have been received, consistent with the description of the advertisement, quite good, I hope that in terms of power saving and purifying the air is really as the merchant said that would be better!	Jingdong prefecture level city in Shandong
Doraemon Hyakubo Shop	King of Power Saving	Power Saving King is really good to use the quality is very good than the physical store a lot cheaper we can rest assured that the purchase recommended to buy!	Jingdong prefecture level city in Shandong
Doraemon Hyakubo Shop	King of Power Saving	Energy saver has been used on the rental house inside the use of the meter is always running fast, with this can really save electricity, easy to use, plugged in can be, very satisfied!	Jingdong prefecture level city in Shandong
Doraemon Hyakubo Shop	King of Power Saving	Just use can not see what effect to,, wait for a period of time in the evaluation	Jingdong prefecture level city in Shandong
Doraemon Hyakubo Shop	King of Power Saving	Live in the 18th floor is too far away from the meter, afraid of no effect, look at the trial 90 days before buying. Currently used for a month, there is an effect, saving 27 dollars in electricity, next month should be able to save more!	Jingdong prefecture level city in Shandong
Doraemon Hyakubo Shop	King of Power Saving	I've been using it for almost a month now and it feels good, so I'll use it again.	Jingdong prefecture level city in Shandong
Doraemon Hyakubo Shop	King of Power Saving	It's ok, the electric bill is really less, originally I remember charging $100 and using it for 10 days. Now it lasts about 15 days. It's still noticeable.	Jingdong prefecture level city in Shandong
Doraemon Hyakubo Shop	King of Power Saving	Fast logistics, good power saving effect, very satisfied	Jingdong prefecture level city in Shandong
Doraemon Hyakubo Shop	King of Power Saving	Has been the second time back to buy, energy saving effect, worth recommending, you can try to see, power saving effect: very good Experience: can save about 30%!	Jingdong prefecture level city in Shandong
Doraemon Hyakubo Shop	King of Power Saving	I don't know how well it works for now	Jingdong prefecture level city in Shandong
Doraemon Hyakubo Shop	King of Power Saving	Factory direct is really good, plugged in and used for half a month did less probably almost 50 degrees of electricity! And the manufacturer's warranty is 5 years.	Jingdong prefecture level city in Shandong
Doraemon Hyakubo Shop	King of Power Saving	It's really effective. The previous month's electricity was $286, but after using the power saver for a month, I have $195 left this month, which is not bad.	Jingdong prefecture level city in Shandong
Doraemon Hyakubo Shop	King of Power Saving	I'll give you a good review and see how it goes.	Jingdong prefecture level city in Shandong
Doraemon Hyakubo Shop	King of Power Saving	Customer service is very professional and patient to guide the installation, the quality of workmanship is very fine. Durable, second repurchase. Really can save oh!	Jingdong prefecture level city in Shandong
Doraemon Hyakubo Shop	King of Power Saving	I've been using it for a month now and my electric bill is over $30 less than last month. I feel it's quite worth buying.	Jingdong prefecture level city in Shandong
Doraemon Hyakubo Shop	King of Power Saving	Small size, does not take up space. It's very good, it does work very effectively, the tool room has a lot of work with a lot of electricity. After installing it, it really reduces a lot of electricity bills. Before the day 30 degrees of electricity left stone, now work down really less a few degrees.	Jingdong prefecture level city in Shandong
Doraemon Hyakubo Shop	King of Power Saving	It does work for saving electricity, I've bought the kind from elsewhere before that was too simple not **all**. This time to buy this store, but also for the safety. Air conditioning is too much electricity, this use to save a lot of	Jingdong prefecture level city in Shandong
Doraemon Hyakubo Shop	King of Power Saving	Two air conditioners and a refrigerator at home, a month down the electricity bill is almost two hundred dollars, with this after the average savings of more than thirty dollars to spend less money to enjoy more, it's really very good!	Jingdong prefecture level city in Shandong
Doraemon Hyakubo Shop	King of Power Saving	Good. Very good.	Jingdong prefecture level city in Shandong
Doraemon Hyakubo Shop	King of Power Saving	Works great, easy to operate, great, very happy with it.	Jingdong prefecture level city in Shandong
Doraemon Hyakubo Shop	King of Power Saving	Received it and tried it out, it's great, great quality and worth having! Five stars praise!	Jingdong prefecture level city in Shandong
Doraemon Hyakubo Shop	King of Power Saving	Very trustworthy brand, quality is guaranteed, the price is still affordable, quite satisfied!	Jingdong prefecture level city in Shandong

Doraemon Hyakubo Shop	King of Power Saving	Use it before you speak.	Jingdong prefecture level city in Shandong
Doraemon Hyakubo Shop	King of Power Saving	The quality of workmanship is very good and the power saving effect is quite good, I regret not getting it earlier.	Jingdong prefecture level city in Shandong
Doraemon Hyakubo Shop	King of Power Saving	Merchants shipping speed, and customer service introduced the same very good, cost-effective	Jingdong prefecture level city in Shandong
Doraemon Hyakubo Shop	King of Power Saving	Feel a little effect Oh, where the power consumption where to plug, convenient and convenient	Jingdong prefecture level city in Shandong
Doraemon Hyakubo Shop	King of Power Saving	The quality of the imported saver is very good, practical and satisfactory. Cost-effective	Jingdong prefecture level city in Shandong
Doraemon Hyakubo Shop	King of Power Saving	Power Saver is especially good, good and inexpensive, very good	Jingdong prefecture level city in Shandong
Doraemon Hyakubo Shop	King of Power Saving	Fast shipping, awesome quality, affordable price, satisfied.	Jingdong prefecture level city in Shandong
Doraemon Hyakubo Shop	King of Power Saving	Energy saver received, the effect is consistent with the description, recommended to buy!	Jingdong prefecture level city in Shandong
Doraemon Hyakubo Shop	King of Power Saving	It's really good, the quality is great, I recommend it!	Jingdong prefecture level city in Shandong
Doraemon Hyakubo Shop	King of Power Saving	Works well. Inexpensive. Shipping was fast.	Jingdong prefecture level city in Shandong
Doraemon Hyakubo Shop	King of Power Saving	Logistics is fast, works well, energy saving is good	Jingdong prefecture level city in Shandong
Doraemon Hyakubo Shop	King of Power Saving	very happy	Jingdong prefecture level city in Shandong
Doraemon Hyakubo Shop	King of Power Saving	an excellent	Jingdong prefecture level city in Shandong
Doraemon Hyakubo Shop	King of Power Saving	It rained a couple days ago and I just went to get the item back today , not bad, hey I looked at the meter and that red display light takes several seconds to jump, did not make a mistake buying it. After a period of time to evaluate.	Jingdong prefecture level city in Shandong
Doraemon Hyakubo Shop	King of Power Saving	Used more than 10 days to check the power, there is indeed some decline, customer service said, long-term use will save more points!	Jingdong prefecture level city in Shandong
Doraemon Hyakubo Shop	King of Power Saving	It's a proper IQ tax, and it costs electricity.	Jingdong prefecture level city in Shandong
Doraemon Hyakubo Shop	King of Power Saving	I've been using it for 17 days now, it really saves electricity Shipping is fast, you can save a lot in a year	Jingdong prefecture level city in Shandong
Doraemon Hyakubo Shop	King of Power Saving	I've been using it for 17 days and it really saves power Shipping is fast and you don't have to worry about the quality, free returns within 5 years	Jingdong prefecture level city in Shandong
Doraemon Hyakubo Shop	King of Power Saving	It's more regular, scanned the code and it's authentic, and the main thing is that you can try it for 90 days! Plugged in more than ten days, is less than before a few degrees per day. When the time to use a month to see Additional. Good to buy a few more	Jingdong prefecture level city in Shandong
Doraemon Hyakubo Shop	King of Power Saving	Logistics is very fast, fine workmanship, use it and then come back to the comments	Jingdong prefecture level city in Shandong
Doraemon Hyakubo Shop	King of Power Saving	The quality of workmanship is very good and the power saving effect is quite good, I regret not getting it earlier.	Jingdong prefecture level city in Shandong
Doraemon Hyakubo Shop	King of Power Saving	Received the goods, according to the instructions on the operation is very simple, to wait and see the effect, looks good!	Jingdong prefecture level city in Shandong
Doraemon Hyakubo Shop	King of Power Saving	It's really as safe and power-saving as the owner says, and it works great!	Jingdong prefecture level city in Shandong
Doraemon Hyakubo Shop	King of Power Saving	Received the goods, immediately used, looks good, to wait and see how the effect.	Jingdong prefecture level city in Shandong
Doraemon Hyakubo Shop	King of Power Saving	Afraid to buy back did not work, the store said the commitment to 90 days invalid refund, warranty 5 years. Saver also complimentary on the Pacific insurance, it is a lot of peace of mind, there are effects will continue to buy back drops!	Jingdong prefecture level city in Shandong
Doraemon Hyakubo Shop	King of Power Saving	Received the goods and used it, can save electricity a month later to see the effect!	Jingdong prefecture level city in Shandong
Doraemon Hyakubo Shop	King of Power Saving	Fancy 90-day trial, 5-year warranty before the order, the goods received, has been experiencing a week, can really feel the savings, in the introduction of friends also buy this!	Jingdong prefecture level city in Shandong
Doraemon Hyakubo Shop	King of Power Saving	Clothing stores use, neighbors recommended to buy, said a month can save more than 70 yuan, first plugged in to see the effect of the	Jingdong prefecture level city in Shandong
Doraemon Hyakubo Shop	King of Power Saving	A friend's home two months to save more than 80 dollars of electricity, recommended that I buy two, decisive purchase, looking forward to the effect of power saving, waiting for the next month effect.	Jingdong prefecture level city in Shandong
Doraemon Hyakubo Shop	King of Power Saving	The goods have been received, consistent with the description of the advertisement, quite good, I hope that in terms of power saving and purifying the air is really as the merchant said that would be better!	Jingdong prefecture level city in Shandong
Doraemon Hyakubo Shop	King of Power Saving	Power Saving King is really good to use the quality is very good than the physical store a lot cheaper we can rest assured that the purchase recommended to buy!	Jingdong prefecture level city in Shandong
Doraemon Hyakubo Shop	King of Power Saving	Energy saver has been used on the rental house inside the use of the meter is always running fast, with this can really save electricity, easy to use, plugged in can be, very satisfied!	Jingdong prefecture level city in Shandong
Doraemon Hyakubo Shop	King of Power Saving	Just use can not see what effect to,, wait for a period of time in the evaluation	Jingdong prefecture level city in Shandong
Doraemon Hyakubo Shop	King of Power Saving	Live in the 18th floor is too far away from the meter, afraid of no effect, look at the trial 90 days before buying. Currently used for a month, there is an effect, saving 27 dollars in electricity, next month should be able to save more!	Jingdong prefecture level city in Shandong
Doraemon Hyakubo Shop	King of Power Saving	I've been using it for almost a month now and it feels good, so I'll use it again.	Jingdong prefecture level city in Shandong
Doraemon Hyakubo Shop	King of Power Saving	It's ok, the electric bill is really less, originally I remember charging $100 and using it for 10 days. Now it lasts about 15 days. It's still noticeable.	Jingdong prefecture level city in Shandong
Doraemon Hyakubo Shop	King of Power Saving	Fast logistics, good power saving effect, very satisfied	Jingdong prefecture level city in Shandong
Doraemon Hyakubo Shop	King of Power Saving	Has been the second time back to buy, energy saving effect, worth recommending, you can try to see, power saving effect: very good Experience: can save about 30%!	Jingdong prefecture level city in Shandong
Doraemon Hyakubo Shop	King of Power Saving	I don't know how well it works for now	Jingdong prefecture level city in Shandong
Doraemon Hyakubo Shop	King of Power Saving	Factory direct is really good, plugged in and used for half a month did less probably almost 50 degrees of electricity! And the manufacturer's warranty is 5 years.	Jingdong prefecture level city in Shandong
Doraemon Hyakubo Shop	King of Power Saving	It's really effective. The previous month's electricity was $286, but after using the power saver for a month, I have $195 left this month, which is not bad.	Jingdong prefecture level city in Shandong
Doraemon Hyakubo Shop	King of Power Saving	I'll give you a good review and see how it goes.	Jingdong prefecture level city in Shandong
Doraemon Hyakubo Shop	King of Power Saving	Customer service is very professional and patient to guide the installation, the quality of workmanship is very fine. Durable, second repurchase. Really can save oh!	Jingdong prefecture level city in Shandong
Doraemon Hyakubo Shop	King of Power Saving	I've been using it for a month now and my electric bill is over $30 less than last month. I feel it's quite worth buying.	Jingdong prefecture level city in Shandong
Doraemon Hyakubo Shop	King of Power Saving	Small size, does not take up space. It's very good, it does work very effectively, the tool room has a lot of work with a lot of electricity. After installing it, it really reduces a lot of electricity bills. Before the day 30 degrees of electricity left stone, now work down really less a few degrees.	Jingdong prefecture level city in Shandong
Doraemon Hyakubo Shop	King of Power Saving	It does work for saving electricity, I've bought the kind from elsewhere before that was too simple not **all**. This time to buy this store, but also for the safety. Air conditioning is too much electricity, this use to save a lot of	Jingdong prefecture level city in Shandong
Doraemon Hyakubo Shop	King of Power Saving	Two air conditioners and a refrigerator at home, a month down the electricity bill is almost two hundred dollars, with this after the average savings of more than thirty dollars to spend less money to enjoy more, it's really very good!	Jingdong prefecture level city in Shandong
Doraemon Hyakubo Shop	King of Power Saving	Good. Very good.	Jingdong prefecture level city in Shandong

Doraemon Hyakubo Shop	King of Power Saving	Works great, easy to operate, great, very happy with it.	Jingdong prefecture level city in Shandong
Doraemon Hyakubo Shop	King of Power Saving	Received it and tried it out, it's great, great quality and worth having! Five stars praise!	Jingdong prefecture level city in Shandong
Doraemon Hyakubo Shop	King of Power Saving	Very trustworthy brand, quality is guaranteed, the price is still affordable, quite satisfied!	Jingdong prefecture level city in Shandong
Doraemon Hyakubo Shop	King of Power Saving	Use it before you speak.	Jingdong prefecture level city in Shandong
Doraemon Hyakubo Shop	King of Power Saving	The quality of workmanship is very good and the power saving effect is quite good, I regret not getting it earlier.	Jingdong prefecture level city in Shandong
Doraemon Hyakubo Shop	King of Power Saving	Merchants shipping speed, and customer service introduced the same very good, cost-effective	Jingdong prefecture level city in Shandong
Doraemon Hyakubo Shop	King of Power Saving	Feel a little effect Oh, where the power consumption where to plug, convenient and convenient	Jingdong prefecture level city in Shandong
Doraemon Hyakubo Shop	King of Power Saving	The quality of the imported saver is very good, practical and satisfactory. Cost-effective	Jingdong prefecture level city in Shandong
Doraemon Hyakubo Shop	King of Power Saving	Power Saver is especially good, good and inexpensive, very good	Jingdong prefecture level city in Shandong
Doraemon Hyakubo Shop	King of Power Saving	Fast shipping, awesome quality, affordable price, satisfied.	Jingdong prefecture level city in Shandong
Doraemon Hyakubo Shop	King of Power Saving	Energy saver received, the effect is consistent with the description, recommended to buy!	Jingdong prefecture level city in Shandong
Doraemon Hyakubo Shop	King of Power Saving	It's really good, the quality is great, I recommend it!	Jingdong prefecture level city in Shandong
Doraemon Hyakubo Shop	King of Power Saving	Works well. Inexpensive. Shipping was fast.	Jingdong prefecture level city in Shandong
Doraemon Hyakubo Shop	King of Power Saving	Logistics is fast, works well, energy saving is good	Jingdong prefecture level city in Shandong
Doraemon Hyakubo Shop	King of Power Saving	very happy	Jingdong prefecture level city in Shandong
Doraemon Hyakubo Shop	King of Power Saving	an excellent	Jingdong prefecture level city in Shandong
Doraemon Hyakubo Shop	King of Power Saving	It rained a couple days ago and I just went to get the item back today , not bad, hey I looked at the meter and that red display light takes several seconds to jump, did not make a mistake buying it. After a period of time to evaluate.	Jingdong prefecture level city in Shandong
Doraemon Hyakubo Shop	King of Power Saving	Used more than 10 days to check the power, there is indeed some decline, customer service said, long-term use will save more points!	Jingdong prefecture level city in Shandong
Doraemon Hyakubo Shop	King of Power Saving	It's a proper IQ tax, and it costs electricity.	Jingdong prefecture level city in Shandong
Doraemon Hyakubo Shop	King of Power Saving	I've been using it for 17 days now, it really saves electricity Shipping is fast, you can save a lot in a year	Jingdong prefecture level city in Shandong
Doraemon Hyakubo Shop	King of Power Saving	I've been using it for 17 days and it really saves power Shipping is fast and you don't have to worry about the quality, free returns within 5 years	Jingdong prefecture level city in Shandong
Doraemon Hyakubo Shop	King of Power Saving	It's more regular, scanned the code and it's authentic, and the main thing is that you can try it for 90 days! Plugged in more than ten days, is less than before a few degrees per day. When the time to use a month to see Additional. Good to buy a few more	Jingdong prefecture level city in Shandong
Doraemon Hyakubo Shop	King of Power Saving	Logistics is very fast, fine workmanship, use it and then come back to the comments	Jingdong prefecture level city in Shandong
Doraemon Hyakubo Shop	King of Power Saving	The quality of workmanship is very good and the power saving effect is quite good, I regret not getting it earlier.	Jingdong prefecture level city in Shandong
Doraemon Hyakubo Shop	King of Power Saving	Received the goods, according to the instructions on the operation is very simple, to wait and see the effect, looks good!	Jingdong prefecture level city in Shandong
Doraemon Hyakubo Shop	King of Power Saving	It's really as safe and power-saving as the owner says, and it works great!	Jingdong prefecture level city in Shandong
Doraemon Hyakubo Shop	King of Power Saving	Received the goods, immediately used, looks good, to wait and see how the effect.	Jingdong prefecture level city in Shandong
Doraemon Hyakubo Shop	King of Power Saving	Afraid to buy back did not work, the store said the commitment to 90 days invalid refund, warranty 5 years. Saver also complimentary on the Pacific insurance, it is a lot of peace of mind, there are effects will continue to buy back drops!	Jingdong prefecture level city in Shandong
Doraemon Hyakubo Shop	King of Power Saving	Received the goods and used it, can save electricity a month later to see the effect!	Jingdong prefecture level city in Shandong
Doraemon Hyakubo Shop	King of Power Saving	Fancy 90-day trial, 5-year warranty before the order, the goods received, has been experiencing a week, can really feel the savings, in the introduction of friends also buy this!	Jingdong prefecture level city in Shandong
Doraemon Hyakubo Shop	King of Power Saving	Clothing stores use, neighbors recommended to buy, said a month can save more than 70 yuan, first plugged in to see the effect of the	Jingdong prefecture level city in Shandong
Doraemon Hyakubo Shop	King of Power Saving	A friend's home two months to save more than 80 dollars of electricity, recommended that I buy two, decisive purchase, looking forward to the effect of power saving, waiting for the next month effect.	Jingdong prefecture level city in Shandong
Doraemon Hyakubo Shop	King of Power Saving	The goods have been received, consistent with the description of the advertisement, quite good, I hope that in terms of power saving and purifying the air is really as the merchant said that would be better!	Jingdong prefecture level city in Shandong
Doraemon Hyakubo Shop	King of Power Saving	Power Saving King is really good to use the quality is very good than the physical store a lot cheaper we can rest assured that the purchase recommended to buy!	Jingdong prefecture level city in Shandong
Doraemon Hyakubo Shop	King of Power Saving	Energy saver has been used on the rental house inside the use of the meter is always running fast, with this can really save electricity, easy to use, plugged in can be, very satisfied!	Jingdong prefecture level city in Shandong
Doraemon Hyakubo Shop	King of Power Saving	Just use can not see what effect to,, wait for a period of time in the evaluation	Jingdong prefecture level city in Shandong
Doraemon Hyakubo Shop	King of Power Saving	Live in the 18th floor is too far away from the meter, afraid of no effect, look at the trial 90 days before buying. Currently used for a month, there is an effect, saving 27 dollars in electricity, next month should be able to save more!	Jingdong prefecture level city in Shandong
Doraemon Hyakubo Shop	King of Power Saving	I've been using it for almost a month now and it feels good, so I'll use it again.	Jingdong prefecture level city in Shandong
Doraemon Hyakubo Shop	King of Power Saving	It's ok, the electric bill is really less, originally I remember charging $100 and using it for 10 days. Now it lasts about 15 days. It's still noticeable.	Jingdong prefecture level city in Shandong
Doraemon Hyakubo Shop	King of Power Saving	Fast logistics, good power saving effect, very satisfied	Jingdong prefecture level city in Shandong
Doraemon Hyakubo Shop	King of Power Saving	Has been the second time back to buy, energy saving effect, worth recommending, you can try to see, power saving effect: very good Experience: can save about 30%!	Jingdong prefecture level city in Shandong
Doraemon Hyakubo Shop	King of Power Saving	I don't know how well it works for now	Jingdong prefecture level city in Shandong
Doraemon Hyakubo Shop	King of Power Saving	Factory direct is really good, plugged in and used for half a month did less probably almost 50 degrees of electricity! And the manufacturer's warranty is 5 years.	Jingdong prefecture level city in Shandong
Doraemon Hyakubo Shop	King of Power Saving	It's really effective. The previous month's electricity was $286, but after using the power saver for a month, I have $195 left this month, which is not bad.	Jingdong prefecture level city in Shandong
Doraemon Hyakubo Shop	King of Power Saving	I'll give you a good review and see how it goes.	Jingdong prefecture level city in Shandong
Doraemon Hyakubo Shop	King of Power Saving	Customer service is very professional and patient to guide the installation, the quality of workmanship is very fine. Durable, second repurchase. Really can save oh!	Jingdong prefecture level city in Shandong
Doraemon Hyakubo Shop	King of Power Saving	I've been using it for a month now and my electric bill is over $30 less than last month. I feel it's quite worth buying.	Jingdong prefecture level city in Shandong
Doraemon Hyakubo Shop	King of Power Saving	Small size, does not take up space. It's very good, it does work very effectively, the tool room has a lot of work with a lot of electricity. After installing it, it really reduces a lot of electricity bills. Before the day 30 degrees of electricity left stone, now work down really less a few degrees.	Jingdong prefecture level city in Shandong

Doraemon Hyakubo Shop	King of Power Saving	It does work for saving electricity, I've bought the kind from elsewhere before that was too simple not **all**. This time to buy this store, but also for the safety. Air conditioning is too much electricity, this use to save a lot of	Jingdong prefecture level city in Shandong
Doraemon Hyakubo Shop	King of Power Saving	Two air conditioners and a refrigerator at home, a month down the electricity bill is almost two hundred dollars, with this after the average savings of more than thirty dollars to spend less money to enjoy more, it's really very good!	Jingdong prefecture level city in Shandong
Doraemon Hyakubo Shop	King of Power Saving	Good. Very good.	Jingdong prefecture level city in Shandong
Doraemon Hyakubo Shop	King of Power Saving	Works great, easy to operate, great, very happy with it.	Jingdong prefecture level city in Shandong
Doraemon Hyakubo Shop	King of Power Saving	Received it and tried it out, it's great, great quality and worth having! Five stars praise!	Jingdong prefecture level city in Shandong
Doraemon Hyakubo Shop	King of Power Saving	Very trustworthy brand, quality is guaranteed, the price is still affordable, quite satisfied!	Jingdong prefecture level city in Shandong
Doraemon Hyakubo Shop	King of Power Saving	Use it before you speak.	Jingdong prefecture level city in Shandong
Doraemon Hyakubo Shop	King of Power Saving	The quality of workmanship is very good and the power saving effect is quite good, I regret not getting it earlier.	Jingdong prefecture level city in Shandong
Doraemon Hyakubo Shop	King of Power Saving	Merchants shipping speed, and customer service introduced the same very good, cost-effective	Jingdong prefecture level city in Shandong
Doraemon Hyakubo Shop	King of Power Saving	Feel a little effect Oh, where the power consumption where to plug, convenient and convenient	Jingdong prefecture level city in Shandong
Doraemon Hyakubo Shop	King of Power Saving	The quality of the imported saver is very good, practical and satisfactory. Cost-effective	Jingdong prefecture level city in Shandong
Doraemon Hyakubo Shop	King of Power Saving	Power Saver is especially good, good and inexpensive, very good	Jingdong prefecture level city in Shandong
Doraemon Hyakubo Shop	King of Power Saving	Fast shipping, awesome quality, affordable price, satisfied.	Jingdong prefecture level city in Shandong
Doraemon Hyakubo Shop	King of Power Saving	Energy saver received, the effect is consistent with the description, recommended to buy!	Jingdong prefecture level city in Shandong
Doraemon Hyakubo Shop	King of Power Saving	It's really good, the quality is great, I recommend it!	Jingdong prefecture level city in Shandong
Doraemon Hyakubo Shop	King of Power Saving	Works well. Inexpensive. Shipping was fast.	Jingdong prefecture level city in Shandong
Doraemon Hyakubo Shop	King of Power Saving	Logistics is fast, works well, energy saving is good	Jingdong prefecture level city in Shandong
Doraemon Hyakubo Shop	King of Power Saving	very happy	Jingdong prefecture level city in Shandong
Doraemon Hyakubo Shop	King of Power Saving	an excellent	Jingdong prefecture level city in Shandong
Doraemon Hyakubo Shop	King of Power Saving	It rained a couple days ago and I just went to get the item back today , not bad, hey I looked at the meter and that red display light takes several seconds to jump, did not make a mistake buying it. After a period of time to evaluate.	Jingdong prefecture level city in Shandong
Doraemon Hyakubo Shop	King of Power Saving	Used more than 10 days to check the power, there is indeed some decline, customer service said, long-term use will save more points!	Jingdong prefecture level city in Shandong
Doraemon Hyakubo Shop	King of Power Saving	It's a proper IQ tax, and it costs electricity.	Jingdong prefecture level city in Shandong
Doraemon Hyakubo Shop	King of Power Saving	I've been using it for 17 days now, it really saves electricity Shipping is fast, you can save a lot in a year	Jingdong prefecture level city in Shandong
Doraemon Hyakubo Shop	King of Power Saving	I've been using it for 17 days and it really saves power Shipping is fast and you don't have to worry about the quality, free returns within 5 years	Jingdong prefecture level city in Shandong
Doraemon Hyakubo Shop	King of Power Saving	It's more regular, scanned the code and it's authentic, and the main thing is that you can try it for 90 days! Plugged in more than ten days, is less than before a few degrees per day. When the time to use a month to see Additional. Good to buy a few more	Jingdong prefecture level city in Shandong
Doraemon Hyakubo Shop	King of Power Saving	Logistics is very fast, fine workmanship, use it and then come back to the comments	Jingdong prefecture level city in Shandong
Doraemon Hyakubo Shop	King of Power Saving	The quality of workmanship is very good and the power saving effect is quite good, I regret not getting it earlier.	Jingdong prefecture level city in Shandong
Doraemon Hyakubo Shop	King of Power Saving	Received the goods, according to the instructions on the operation is very simple, to wait and see the effect, looks good!	Jingdong prefecture level city in Shandong
Doraemon Hyakubo Shop	King of Power Saving	It's really as safe and power-saving as the owner says, and it works great!	Jingdong prefecture level city in Shandong
Doraemon Hyakubo Shop	King of Power Saving	Received the goods, immediately used, looks good, to wait and see how the effect.	Jingdong prefecture level city in Shandong
Doraemon Hyakubo Shop	King of Power Saving	Afraid to buy back did not work, the store said the commitment to 90 days invalid refund, warranty 5 years. Saver also complimentary on the Pacific insurance, it is a lot of peace of mind, there are effects will continue to buy back drops!	Jingdong prefecture level city in Shandong
Doraemon Hyakubo Shop	King of Power Saving	Received the goods and used it, can save electricity a month later to see the effect!	Jingdong prefecture level city in Shandong
Doraemon Hyakubo Shop	King of Power Saving	Fancy 90-day trial, 5-year warranty before the order, the goods received, has been experiencing a week, can really feel the savings, in the introduction of friends also buy this!	Jingdong prefecture level city in Shandong
Doraemon Hyakubo Shop	King of Power Saving	Clothing stores use, neighbors recommended to buy, said a month can save more than 70 yuan, first plugged in to see the effect of the	Jingdong prefecture level city in Shandong
Doraemon Hyakubo Shop	King of Power Saving	A friend's home two months to save more than 80 dollars of electricity, recommended that I buy two, decisive purchase, looking forward to the effect of power saving, waiting for the next month effect.	Jingdong prefecture level city in Shandong
Doraemon Hyakubo Shop	King of Power Saving	The goods have been received, consistent with the description of the advertisement, quite good, I hope that in terms of power saving and purifying the air is really as the merchant said that would be better!	Jingdong prefecture level city in Shandong
Doraemon Hyakubo Shop	King of Power Saving	Power Saving King is really good to use the quality is very good than the physical store a lot cheaper we can rest assured that the purchase recommended to buy!	Jingdong prefecture level city in Shandong
Doraemon Hyakubo Shop	King of Power Saving	Energy saver has been used on the rental house inside the use of the meter is always running fast, with this can really save electricity, easy to use, plugged in can be, very satisfied!	Jingdong prefecture level city in Shandong
Doraemon Hyakubo Shop	King of Power Saving	Just use can not see what effect to,, wait for a period of time in the evaluation	Jingdong prefecture level city in Shandong
Doraemon Hyakubo Shop	King of Power Saving	Live in the 18th floor is too far away from the meter, afraid of no effect, look at the trial 90 days before buying. Currently used for a month, there is an effect, saving 27 dollars in electricity, next month should be able to save more!	Jingdong prefecture level city in Shandong
Doraemon Hyakubo Shop	King of Power Saving	I've been using it for almost a month now and it feels good, so I'll use it again.	Jingdong prefecture level city in Shandong
Doraemon Hyakubo Shop	King of Power Saving	It's ok, the electric bill is really less, originally I remember charging $100 and using it for 10 days. Now it lasts about 15 days. It's still noticeable.	Jingdong prefecture level city in Shandong
Doraemon Hyakubo Shop	King of Power Saving	Fast logistics, good power saving effect, very satisfied	Jingdong prefecture level city in Shandong
Doraemon Hyakubo Shop	King of Power Saving	Has been the second time back to buy, energy saving effect, worth recommending, you can try to see, power saving effect: very good Experience: can save about 30%!	Jingdong prefecture level city in Shandong
Doraemon Hyakubo Shop	King of Power Saving	I don't know how well it works for now	Jingdong prefecture level city in Shandong
Doraemon Hyakubo Shop	King of Power Saving	Factory direct is really good, plugged in and used for half a month did less probably almost 50 degrees of electricity! And the manufacturer's warranty is 5 years.	Jingdong prefecture level city in Shandong
Doraemon Hyakubo Shop	King of Power Saving	It's really effective. The previous month's electricity was $286, but after using the power saver for a month, I have $195 left this month, which is not bad.	Jingdong prefecture level city in Shandong
Doraemon Hyakubo Shop	King of Power Saving	I'll give you a good review and see how it goes.	Jingdong prefecture level city in Shandong

Doraemon Hyakubo Shop	King of Power Saving	Customer service is very professional and patient to guide the installation, the quality of workmanship is very fine. Durable, second repurchase. Really can save oh!	Jingdong prefecture level city in Shandong
Doraemon Hyakubo Shop	King of Power Saving	I've been using it for a month now and my electric bill is over $30 less than last month. I feel it's quite worth buying.	Jingdong prefecture level city in Shandong
Doraemon Hyakubo Shop	King of Power Saving	Small size, does not take up space. It's very good, it does work very effectively, the tool room has a lot of work with a lot of electricity. After installing it, it really reduces a lot of electricity bills. Before the day 30 degrees of electricity left stone, now work down really less a few degrees.	Jingdong prefecture level city in Shandong
Doraemon Hyakubo Shop	King of Power Saving	It does work for saving electricity, I've bought the kind from elsewhere before that was too simple not **all**. This time to buy this store, but also for the safety. Air conditioning is too much electricity, this use to save a lot of	Jingdong prefecture level city in Shandong
Doraemon Hyakubo Shop	King of Power Saving	Two air conditioners and a refrigerator at home, a month down the electricity bill is almost two hundred dollars, with this after the average savings of more than thirty dollars to spend less money to enjoy more, it's really very good!	Jingdong prefecture level city in Shandong
Doraemon Hyakubo Shop	King of Power Saving	Good. Very good.	Jingdong prefecture level city in Shandong
Doraemon Hyakubo Shop	King of Power Saving	Works great, easy to operate, great, very happy with it.	Jingdong prefecture level city in Shandong
Doraemon Hyakubo Shop	King of Power Saving	Received it and tried it out, it's great, great quality and worth having! Five stars praise!	Jingdong prefecture level city in Shandong
Doraemon Hyakubo Shop	King of Power Saving	Very trustworthy brand, quality is guaranteed, the price is still affordable, quite satisfied!	Jingdong prefecture level city in Shandong
Doraemon Hyakubo Shop	King of Power Saving	Use it before you speak.	Jingdong prefecture level city in Shandong
Doraemon Hyakubo Shop	King of Power Saving	The quality of workmanship is very good and the power saving effect is quite good, I regret not getting it earlier.	Jingdong prefecture level city in Shandong
Doraemon Hyakubo Shop	King of Power Saving	Merchants shipping speed, and customer service introduced the same very good, cost-effective	Jingdong prefecture level city in Shandong
Doraemon Hyakubo Shop	King of Power Saving	Feel a little effect Oh, where the power consumption where to plug, convenient and convenient	Jingdong prefecture level city in Shandong
Doraemon Hyakubo Shop	King of Power Saving	The quality of the imported saver is very good, practical and satisfactory. Cost-effective	Jingdong prefecture level city in Shandong
Doraemon Hyakubo Shop	King of Power Saving	Power Saver is especially good, good and inexpensive, very good	Jingdong prefecture level city in Shandong
Doraemon Hyakubo Shop	King of Power Saving	Fast shipping, awesome quality, affordable price, satisfied.	Jingdong prefecture level city in Shandong
Doraemon Hyakubo Shop	King of Power Saving	Energy saver received, the effect is consistent with the description, recommended to buy!	Jingdong prefecture level city in Shandong
Doraemon Hyakubo Shop	King of Power Saving	It's really good, the quality is great, I recommend it!	Jingdong prefecture level city in Shandong
Doraemon Hyakubo Shop	King of Power Saving	Works well. Inexpensive. Shipping was fast.	Jingdong prefecture level city in Shandong
Doraemon Hyakubo Shop	King of Power Saving	Logistics is fast, works well, energy saving is good	Jingdong prefecture level city in Shandong
Doraemon Hyakubo Shop	King of Power Saving	very happy	Jingdong prefecture level city in Shandong
Doraemon Hyakubo Shop	King of Power Saving	an excellent	Jingdong prefecture level city in Shandong
Doraemon Hyakubo Shop	King of Power Saving	It rained a couple days ago and I just went to get the item back today , not bad, hey I looked at the meter and that red display light takes several seconds to jump, did not make a mistake buying it. After a period of time to evaluate.	Jingdong prefecture level city in Shandong
Doraemon Hyakubo Shop	King of Power Saving	Used more than 10 days to check the power, there is indeed some decline, customer service said, long-term use will save more points!	Jingdong prefecture level city in Shandong
Doraemon Hyakubo Shop	King of Power Saving	It's a proper IQ tax, and it costs electricity.	Jingdong prefecture level city in Shandong
Doraemon Hyakubo Shop	King of Power Saving	I've been using it for 17 days now, it really saves electricity Shipping is fast, you can save a lot in a year	Jingdong prefecture level city in Shandong
Doraemon Hyakubo Shop	King of Power Saving	I've been using it for 17 days and it really saves power Shipping is fast and you don't have to worry about the quality, free returns within 5 years	Jingdong prefecture level city in Shandong
Doraemon Hyakubo Shop	King of Power Saving	It's more regular, scanned the code and it's authentic, and the main thing is that you can try it for 90 days! Plugged in more than ten days, is less than before a few degrees per day. When the time to use a month to see Additional. Good to buy a few more	Jingdong prefecture level city in Shandong
Doraemon Hyakubo Shop	King of Power Saving	Logistics is very fast, fine workmanship, use it and then come back to the comments	Jingdong prefecture level city in Shandong
Doraemon Hyakubo Shop	King of Power Saving	The quality of workmanship is very good and the power saving effect is quite good, I regret not getting it earlier.	Jingdong prefecture level city in Shandong
Doraemon Hyakubo Shop	King of Power Saving	Received the goods, according to the instructions on the operation is very simple, to wait and see the effect, looks good!	Jingdong prefecture level city in Shandong
Doraemon Hyakubo Shop	King of Power Saving	It's really as safe and power-saving as the owner says, and it works great!	Jingdong prefecture level city in Shandong
Doraemon Hyakubo Shop	King of Power Saving	Received the goods, immediately used, looks good, to wait and see how the effect.	Jingdong prefecture level city in Shandong
Doraemon Hyakubo Shop	King of Power Saving	Afraid to buy back did not work, the store said the commitment to 90 days invalid refund, warranty 5 years. Saver also complimentary on the Pacific insurance, it is a lot of peace of mind, there are effects will continue to buy back drops!	Jingdong prefecture level city in Shandong
Doraemon Hyakubo Shop	King of Power Saving	Received the goods and used it, can save electricity a month later to see the effect!	Jingdong prefecture level city in Shandong
Doraemon Hyakubo Shop	King of Power Saving	Fancy 90-day trial, 5-year warranty before the order, the goods received, has been experiencing a week, can really feel the savings, in the introduction of friends also buy this!	Jingdong prefecture level city in Shandong
Doraemon Hyakubo Shop	King of Power Saving	Clothing stores use, neighbors recommended to buy, said a month can save more than 70 yuan, first plugged in to see the effect of the	Jingdong prefecture level city in Shandong
Doraemon Hyakubo Shop	King of Power Saving	A friend's home two months to save more than 80 dollars of electricity, recommended that I buy two, decisive purchase, looking forward to the effect of power saving, waiting for the next month effect.	Jingdong prefecture level city in Shandong
Doraemon Hyakubo Shop	King of Power Saving	The goods have been received, consistent with the description of the advertisement, quite good, I hope that in terms of power saving and purifying the air is really as the merchant said that would be better!	Jingdong prefecture level city in Shandong
Doraemon Hyakubo Shop	King of Power Saving	Power Saving King is really good to use the quality is very good than the physical store a lot cheaper we can rest assured that the purchase recommended to buy!	Jingdong prefecture level city in Shandong
Doraemon Hyakubo Shop	King of Power Saving	Energy saver has been used on the rental house inside the use of the meter is always running fast, with this can really save electricity, easy to use, plugged in can be, very satisfied!	Jingdong prefecture level city in Shandong
Doraemon Hyakubo Shop	King of Power Saving	Just use can not see what effect to,, wait for a period of time in the evaluation	Jingdong prefecture level city in Shandong
Doraemon Hyakubo Shop	King of Power Saving	Live in the 18th floor is too far away from the meter, afraid of no effect, look at the trial 90 days before buying. Currently used for a month, there is an effect, saving 27 dollars in electricity, next month should be able to save more!	Jingdong prefecture level city in Shandong
Doraemon Hyakubo Shop	King of Power Saving	I've been using it for almost a month now and it feels good, so I'll use it again.	Jingdong prefecture level city in Shandong
Doraemon Hyakubo Shop	King of Power Saving	It's ok, the electric bill is really less, originally I remember charging $100 and using it for 10 days. Now it lasts about 15 days. It's still noticeable.	Jingdong prefecture level city in Shandong
Doraemon Hyakubo Shop	King of Power Saving	Fast logistics, good power saving effect, very satisfied	Jingdong prefecture level city in Shandong
Doraemon Hyakubo Shop	King of Power Saving	Has been the second time back to buy, energy saving effect, worth recommending, you can try to see, power saving effect: very good Experience: can save about 30%!	Jingdong prefecture level city in Shandong
Doraemon Hyakubo Shop	King of Power Saving	I don't know how well it works for now	Jingdong prefecture level city in Shandong

Doraemon Hyakubo Shop	King of Power Saving	Factory direct is really good, plugged in and used for half a month did less probably almost 50 degrees of electricity! And the manufacturer's warranty is 5 years.	Jingdong prefecture level city in Shandong
Doraemon Hyakubo Shop	King of Power Saving	It's really effective. The previous month's electricity was $286, but after using the power saver for a month, I have $195 left this month, which is not bad.	Jingdong prefecture level city in Shandong
Doraemon Hyakubo Shop	King of Power Saving	I'll give you a good review and see how it goes.	Jingdong prefecture level city in Shandong
Doraemon Hyakubo Shop	King of Power Saving	Customer service is very professional and patient to guide the installation, the quality of workmanship is very fine. Durable, second repurchase. Really can save oh!	Jingdong prefecture level city in Shandong
Doraemon Hyakubo Shop	King of Power Saving	I've been using it for a month now and my electric bill is over $30 less than last month. I feel it's quite worth buying.	Jingdong prefecture level city in Shandong
Doraemon Hyakubo Shop	King of Power Saving	Small size, does not take up space. It's very good, it does work very effectively, the tool room has a lot of work with a lot of electricity. After installing it, it really reduces a lot of electricity bills. Before the day 30 degrees of electricity left stone, now work down really less a few degrees.	Jingdong prefecture level city in Shandong
Doraemon Hyakubo Shop	King of Power Saving	It does work for saving electricity, I've bought the kind from elsewhere before that was too simple not **all**. This time to buy this store, but also for the safety. Air conditioning is too much electricity, this use to save a lot of	Jingdong prefecture level city in Shandong
Doraemon Hyakubo Shop	King of Power Saving	Two air conditioners and a refrigerator at home, a month down the electricity bill is almost two hundred dollars, with this after the average savings of more than thirty dollars to spend less money to enjoy more, it's really very good!	Jingdong prefecture level city in Shandong
Doraemon Hyakubo Shop	King of Power Saving	Good. Very good.	Jingdong prefecture level city in Shandong
Doraemon Hyakubo Shop	King of Power Saving	Works great, easy to operate, great, very happy with it.	Jingdong prefecture level city in Shandong
Doraemon Hyakubo Shop	King of Power Saving	Received it and tried it out, it's great, great quality and worth having! Five stars praise!	Jingdong prefecture level city in Shandong
Doraemon Hyakubo Shop	King of Power Saving	Very trustworthy brand, quality is guaranteed, the price is still affordable, quite satisfied!	Jingdong prefecture level city in Shandong
Doraemon Hyakubo Shop	King of Power Saving	Use it before you speak.	Jingdong prefecture level city in Shandong
Doraemon Hyakubo Shop	King of Power Saving	The quality of workmanship is very good and the power saving effect is quite good, I regret not getting it earlier.	Jingdong prefecture level city in Shandong
Doraemon Hyakubo Shop	King of Power Saving	Merchants shipping speed, and customer service introduced the same very good, cost-effective	Jingdong prefecture level city in Shandong
Doraemon Hyakubo Shop	King of Power Saving	Feel a little effect Oh, where the power consumption where to plug, convenient and convenient	Jingdong prefecture level city in Shandong
Doraemon Hyakubo Shop	King of Power Saving	The quality of the imported saver is very good, practical and satisfactory. Cost-effective	Jingdong prefecture level city in Shandong
Doraemon Hyakubo Shop	King of Power Saving	Power Saver is especially good, good and inexpensive, very good	Jingdong prefecture level city in Shandong
Doraemon Hyakubo Shop	King of Power Saving	Fast shipping, awesome quality, affordable price, satisfied.	Jingdong prefecture level city in Shandong
Doraemon Hyakubo Shop	King of Power Saving	Energy saver received, the effect is consistent with the description, recommended to buy!	Jingdong prefecture level city in Shandong
Doraemon Hyakubo Shop	King of Power Saving	It's really good, the quality is great, I recommend it!	Jingdong prefecture level city in Shandong
Doraemon Hyakubo Shop	King of Power Saving	Works well. Inexpensive. Shipping was fast.	Jingdong prefecture level city in Shandong
Doraemon Hyakubo Shop	King of Power Saving	Logistics is fast, works well, energy saving is good	Jingdong prefecture level city in Shandong
Doraemon Hyakubo Shop	King of Power Saving	very happy	Jingdong prefecture level city in Shandong
Doraemon Hyakubo Shop	King of Power Saving	an excellent	Jingdong prefecture level city in Shandong
Doraemon Hyakubo Shop	King of Power Saving	It rained a couple days ago and I just went to get the item back today , not bad, hey I looked at the meter and that red display light takes several seconds to jump, did not make a mistake buying it. After a period of time to evaluate.	Jingdong prefecture level city in Shandong
Doraemon Hyakubo Shop	King of Power Saving	Used more than 10 days to check the power, there is indeed some decline, customer service said, long-term use will save more points!	Jingdong prefecture level city in Shandong
Doraemon Hyakubo Shop	King of Power Saving	It's a proper IQ tax, and it costs electricity.	Jingdong prefecture level city in Shandong
Doraemon Hyakubo Shop	King of Power Saving	I've been using it for 17 days now, it really saves electricity Shipping is fast, you can save a lot in a year	Jingdong prefecture level city in Shandong
Doraemon Hyakubo Shop	King of Power Saving	I've been using it for 17 days and it really saves power Shipping is fast and you don't have to worry about the quality, free returns within 5 years	Jingdong prefecture level city in Shandong
Doraemon Hyakubo Shop	King of Power Saving	It's more regular, scanned the code and it's authentic, and the main thing is that you can try it for 90 days! Plugged in more than ten days, is less than before a few degrees per day. When the time to use a month to see Additional. Good to buy a few more	Jingdong prefecture level city in Shandong
Doraemon Hyakubo Shop	King of Power Saving	Logistics is very fast, fine workmanship, use it and then come back to the comments	Jingdong prefecture level city in Shandong
Doraemon Hyakubo Shop	King of Power Saving	The quality of workmanship is very good and the power saving effect is quite good, I regret not getting it earlier.	Jingdong prefecture level city in Shandong
Doraemon Hyakubo Shop	King of Power Saving	Received the goods, according to the instructions on the operation is very simple, to wait and see the effect, looks good!	Jingdong prefecture level city in Shandong
Doraemon Hyakubo Shop	King of Power Saving	It's really as safe and power-saving as the owner says, and it works great!	Jingdong prefecture level city in Shandong
Doraemon Hyakubo Shop	King of Power Saving	Received the goods, immediately used, looks good, to wait and see how the effect.	Jingdong prefecture level city in Shandong
Doraemon Hyakubo Shop	King of Power Saving	Afraid to buy back did not work, the store said the commitment to 90 days invalid refund, warranty 5 years. Saver also complimentary on the Pacific insurance, it is a lot of peace of mind, there are effects will continue to buy back drops!	Jingdong prefecture level city in Shandong
Doraemon Hyakubo Shop	King of Power Saving	Received the goods and used it, can save electricity a month later to see the effect!	Jingdong prefecture level city in Shandong
Doraemon Hyakubo Shop	King of Power Saving	Fancy 90-day trial, 5-year warranty before the order, the goods received, has been experiencing a week, can really feel the savings, in the introduction of friends also buy this!	Jingdong prefecture level city in Shandong
Doraemon Hyakubo Shop	King of Power Saving	Clothing stores use, neighbors recommended to buy, said a month can save more than 70 yuan, first plugged in to see the effect of the	Jingdong prefecture level city in Shandong
Doraemon Hyakubo Shop	King of Power Saving	A friend's home two months to save more than 80 dollars of electricity, recommended that I buy two, decisive purchase, looking forward to the effect of power saving, waiting for the next month effect.	Jingdong prefecture level city in Shandong
Doraemon Hyakubo Shop	King of Power Saving	The goods have been received, consistent with the description of the advertisement, quite good, I hope that in terms of power saving and purifying the air is really as the merchant said that would be better!	Jingdong prefecture level city in Shandong
Doraemon Hyakubo Shop	King of Power Saving	Power Saving King is really good to use the quality is very good than the physical store a lot cheaper we can rest assured that the purchase recommended to buy!	Jingdong prefecture level city in Shandong
Doraemon Hyakubo Shop	King of Power Saving	Energy saver has been used on the rental house inside the use of the meter is always running fast, with this can really save electricity, easy to use, plugged in can be, very satisfied!	Jingdong prefecture level city in Shandong
Doraemon Hyakubo Shop	King of Power Saving	Just use can not see what effect to,, wait for a period of time in the evaluation	Jingdong prefecture level city in Shandong
Doraemon Hyakubo Shop	King of Power Saving	Live in the 18th floor is too far away from the meter, afraid of no effect, look at the trial 90 days before buying. Currently used for a month, there is an effect, saving 27 dollars in electricity, next month should be able to save more!	Jingdong prefecture level city in Shandong
Doraemon Hyakubo Shop	King of Power Saving	I've been using it for almost a month now and it feels good, so I'll use it again.	Jingdong prefecture level city in Shandong
Doraemon Hyakubo Shop	King of Power Saving	It's ok, the electric bill is really less, originally I remember charging $100 and using it for 10 days. Now it lasts about 15 days. It's still noticeable.	Jingdong prefecture level city in Shandong

Doraemon Hyakubo Shop	King of Power Saving	Fast logistics, good power saving effect, very satisfied	Jingdong prefecture level city in Shandong
Doraemon Hyakubo Shop	King of Power Saving	Has been the second time back to buy, energy saving effect, worth recommending, you can try to see, power saving effect: very good Experience: can save about 30%!	Jingdong prefecture level city in Shandong
Doraemon Hyakubo Shop	King of Power Saving	I don't know how well it works for now	Jingdong prefecture level city in Shandong
Doraemon Hyakubo Shop	King of Power Saving	Factory direct is really good, plugged in and used for half a month did less probably almost 50 degrees of electricity! And the manufacturer's warranty is 5 years.	Jingdong prefecture level city in Shandong
Doraemon Hyakubo Shop	King of Power Saving	It's really effective. The previous month's electricity was $286, but after using the power saver for a month, I have $195 left this month, which is not bad.	Jingdong prefecture level city in Shandong
Doraemon Hyakubo Shop	King of Power Saving	I'll give you a good review and see how it goes.	Jingdong prefecture level city in Shandong
Doraemon Hyakubo Shop	King of Power Saving	Customer service is very professional and patient to guide the installation, the quality of workmanship is very fine. Durable, second repurchase. Really can save oh!	Jingdong prefecture level city in Shandong
Doraemon Hyakubo Shop	King of Power Saving	I've been using it for a month now and my electric bill is over $30 less than last month. I feel it's quite worth buying.	Jingdong prefecture level city in Shandong
Doraemon Hyakubo Shop	King of Power Saving	Small size, does not take up space. It's very good, it does work very effectively, the tool room has a lot of work with a lot of electricity. After installing it, it really reduces a lot of electricity bills. Before the day 30 degrees of electricity left stone, now work down really less a few degrees.	Jingdong prefecture level city in Shandong
Doraemon Hyakubo Shop	King of Power Saving	It does work for saving electricity, I've bought the kind from elsewhere before that was too simple not **all**. This time to buy this store, but also for the safety. Air conditioning is too much electricity, this use to save a lot of	Jingdong prefecture level city in Shandong
Doraemon Hyakubo Shop	King of Power Saving	Two air conditioners and a refrigerator at home, a month down the electricity bill is almost two hundred dollars, with this after the average savings of more than thirty dollars to spend less money to enjoy more, it's really very good!	Jingdong prefecture level city in Shandong
Doraemon Hyakubo Shop	King of Power Saving	Good. Very good.	Jingdong prefecture level city in Shandong
Doraemon Hyakubo Shop	King of Power Saving	Works great, easy to operate, great, very happy with it.	Jingdong prefecture level city in Shandong
Doraemon Hyakubo Shop	King of Power Saving	Received it and tried it out, it's great, great quality and worth having! Five stars praise!	Jingdong prefecture level city in Shandong
Doraemon Hyakubo Shop	King of Power Saving	Very trustworthy brand, quality is guaranteed, the price is still affordable, quite satisfied!	Jingdong prefecture level city in Shandong
Doraemon Hyakubo Shop	King of Power Saving	Use it before you speak.	Jingdong prefecture level city in Shandong
Doraemon Hyakubo Shop	King of Power Saving	The quality of workmanship is very good and the power saving effect is quite good, I regret not getting it earlier.	Jingdong prefecture level city in Shandong
Doraemon Hyakubo Shop	King of Power Saving	Merchants shipping speed, and customer service introduced the same very good, cost-effective	Jingdong prefecture level city in Shandong
Doraemon Hyakubo Shop	King of Power Saving	Feel a little effect Oh, where the power consumption where to plug, convenient and convenient	Jingdong prefecture level city in Shandong
Doraemon Hyakubo Shop	King of Power Saving	The quality of the imported saver is very good, practical and satisfactory. Cost-effective	Jingdong prefecture level city in Shandong
Doraemon Hyakubo Shop	King of Power Saving	Power Saver is especially good, good and inexpensive, very good	Jingdong prefecture level city in Shandong
Doraemon Hyakubo Shop	King of Power Saving	Fast shipping, awesome quality, affordable price, satisfied.	Jingdong prefecture level city in Shandong
Doraemon Hyakubo Shop	King of Power Saving	Energy saver received, the effect is consistent with the description, recommended to buy!	Jingdong prefecture level city in Shandong
Doraemon Hyakubo Shop	King of Power Saving	It's really good, the quality is great, I recommend it!	Jingdong prefecture level city in Shandong
Doraemon Hyakubo Shop	King of Power Saving	Works well. Inexpensive. Shipping was fast.	Jingdong prefecture level city in Shandong
Doraemon Hyakubo Shop	King of Power Saving	Logistics is fast, works well, energy saving is good	Jingdong prefecture level city in Shandong
Doraemon Hyakubo Shop	King of Power Saving	very happy	Jingdong prefecture level city in Shandong
Doraemon Hyakubo Shop	King of Power Saving	an excellent	Jingdong prefecture level city in Shandong
Doraemon Hyakubo Shop	King of Power Saving	It rained a couple days ago and I just went to get the item back today , not bad, hey I looked at the meter and that red display light takes several seconds to jump, did not make a mistake buying it. After a period of time to evaluate.	Jingdong prefecture level city in Shandong
Doraemon Hyakubo Shop	King of Power Saving	Used more than 10 days to check the power, there is indeed some decline, customer service said, long-term use will save more points!	Jingdong prefecture level city in Shandong
Doraemon Hyakubo Shop	King of Power Saving	It's a proper IQ tax, and it costs electricity.	Jingdong prefecture level city in Shandong
Doraemon Hyakubo Shop	King of Power Saving	I've been using it for 17 days now, it really saves electricity Shipping is fast, you can save a lot in a year	Jingdong prefecture level city in Shandong
Doraemon Hyakubo Shop	King of Power Saving	I've been using it for 17 days and it really saves power Shipping is fast and you don't have to worry about the quality, free returns within 5 years	Jingdong prefecture level city in Shandong
Doraemon Hyakubo Shop	King of Power Saving	It's more regular, scanned the code and it's authentic, and the main thing is that you can try it for 90 days! Plugged in more than ten days, is less than before a few degrees per day. When the time to use a month to see Additional. Good to buy a few more	Jingdong prefecture level city in Shandong
Doraemon Hyakubo Shop	King of Power Saving	Logistics is very fast, fine workmanship, use it and then come back to the comments	Jingdong prefecture level city in Shandong
Doraemon Hyakubo Shop	King of Power Saving	The quality of workmanship is very good and the power saving effect is quite good, I regret not getting it earlier.	Jingdong prefecture level city in Shandong
Doraemon Hyakubo Shop	King of Power Saving	Received the goods, according to the instructions on the operation is very simple, to wait and see the effect, looks good!	Jingdong prefecture level city in Shandong
Doraemon Hyakubo Shop	King of Power Saving	It's really as safe and power-saving as the owner says, and it works great!	Jingdong prefecture level city in Shandong
Doraemon Hyakubo Shop	King of Power Saving	Received the goods, immediately used, looks good, to wait and see how the effect.	Jingdong prefecture level city in Shandong
Doraemon Hyakubo Shop	King of Power Saving	Afraid to buy back did not work, the store said the commitment to 90 days invalid refund, warranty 5 years. Saver also complimentary on the Pacific insurance, it is a lot of peace of mind, there are effects will continue to buy back drops!	Jingdong prefecture level city in Shandong
Doraemon Hyakubo Shop	King of Power Saving	Received the goods and used it, can save electricity a month later to see the effect!	Jingdong prefecture level city in Shandong
Doraemon Hyakubo Shop	King of Power Saving	Fancy 90-day trial, 5-year warranty before the order, the goods received, has been experiencing a week, can really feel the savings, in the introduction of friends also buy this!	Jingdong prefecture level city in Shandong
Doraemon Hyakubo Shop	King of Power Saving	Clothing stores use, neighbors recommended to buy, said a month can save more than 70 yuan, first plugged in to see the effect of the	Jingdong prefecture level city in Shandong
Doraemon Hyakubo Shop	King of Power Saving	A friend's home two months to save more than 80 dollars of electricity, recommended that I buy two, decisive purchase, looking forward to the effect of power saving, waiting for the next month effect.	Jingdong prefecture level city in Shandong
Doraemon Hyakubo Shop	King of Power Saving	The goods have been received, consistent with the description of the advertisement, quite good, I hope that in terms of power saving and purifying the air is really as the merchant said that would be better!	Jingdong prefecture level city in Shandong
Doraemon Hyakubo Shop	King of Power Saving	Power Saving King is really good to use the quality is very good than the physical store a lot cheaper we can rest assured that the purchase recommended to buy!	Jingdong prefecture level city in Shandong
Doraemon Hyakubo Shop	King of Power Saving	Energy saver has been used on the rental house inside the use of the meter is always running fast, with this can really save electricity, easy to use, plugged in can be, very satisfied!	Jingdong prefecture level city in Shandong
Doraemon Hyakubo Shop	King of Power Saving	Just use can not see what effect to,, wait for a period of time in the evaluation	Jingdong prefecture level city in Shandong

Doraemon Hyakubo Shop	King of Power Saving	Live in the 18th floor is too far away from the meter, afraid of no effect, look at the trial 90 days before buying. Currently used for a month, there is an effect, saving 27 dollars in electricity, next month should be able to save more!	Jingdong prefecture level city in Shandong
Doraemon Hyakubo Shop	King of Power Saving	I've been using it for almost a month now and it feels good, so I'll use it again.	Jingdong prefecture level city in Shandong
Doraemon Hyakubo Shop	King of Power Saving	It's ok, the electric bill is really less, originally I remember charging $100 and using it for 10 days. Now it lasts about 15 days. It's still noticeable.	Jingdong prefecture level city in Shandong
Doraemon Hyakubo Shop	King of Power Saving	Fast logistics, good power saving effect, very satisfied	Jingdong prefecture level city in Shandong
Doraemon Hyakubo Shop	King of Power Saving	Has been the second time back to buy, energy saving effect, worth recommending, you can try to see, power saving effect: very good Experience: can save about 30%!	Jingdong prefecture level city in Shandong
Doraemon Hyakubo Shop	King of Power Saving	I don't know how well it works for now	Jingdong prefecture level city in Shandong
Doraemon Hyakubo Shop	King of Power Saving	Factory direct is really good, plugged in and used for half a month did less probably almost 50 degrees of electricity! And the manufacturer's warranty is 5 years.	Jingdong prefecture level city in Shandong
Doraemon Hyakubo Shop	King of Power Saving	It's really effective. The previous month's electricity was $286, but after using the power saver for a month, I have $195 left this month, which is not bad.	Jingdong prefecture level city in Shandong
Doraemon Hyakubo Shop	King of Power Saving	I'll give you a good review and see how it goes.	Jingdong prefecture level city in Shandong
Doraemon Hyakubo Shop	King of Power Saving	Customer service is very professional and patient to guide the installation, the quality of workmanship is very fine. Durable, second repurchase. Really can save oh!	Jingdong prefecture level city in Shandong
Doraemon Hyakubo Shop	King of Power Saving	I've been using it for a month now and my electric bill is over $30 less than last month. I feel it's quite worth buying.	Jingdong prefecture level city in Shandong
Doraemon Hyakubo Shop	King of Power Saving	Small size, does not take up space. It's very good, it does work very effectively, the tool room has a lot of work with a lot of electricity. After installing it, it really reduces a lot of electricity bills. Before the day 30 degrees of electricity left stone, now work down really less a few degrees.	Jingdong prefecture level city in Shandong
Doraemon Hyakubo Shop	King of Power Saving	It does work for saving electricity, I've bought the kind from elsewhere before that was too simple not **all**. This time to buy this store, but also for the safety. Air conditioning is too much electricity, this use to save a lot of	Jingdong prefecture level city in Shandong
Doraemon Hyakubo Shop	King of Power Saving	Two air conditioners and a refrigerator at home, a month down the electricity bill is almost two hundred dollars, with this after the average savings of more than thirty dollars to spend less money to enjoy more, it's really very good!	Jingdong prefecture level city in Shandong
Doraemon Hyakubo Shop	King of Power Saving	Good. Very good.	Jingdong prefecture level city in Shandong
Doraemon Hyakubo Shop	King of Power Saving	Works great, easy to operate, great, very happy with it.	Jingdong prefecture level city in Shandong
Doraemon Hyakubo Shop	King of Power Saving	Received it and tried it out, it's great, great quality and worth having! Five stars praise!	Jingdong prefecture level city in Shandong
Doraemon Hyakubo Shop	King of Power Saving	Very trustworthy brand, quality is guaranteed, the price is still affordable, quite satisfied!	Jingdong prefecture level city in Shandong
Doraemon Hyakubo Shop	King of Power Saving	Use it before you speak.	Jingdong prefecture level city in Shandong
Doraemon Hyakubo Shop	King of Power Saving	The quality of workmanship is very good and the power saving effect is quite good, I regret not getting it earlier.	Jingdong prefecture level city in Shandong
Doraemon Hyakubo Shop	King of Power Saving	Merchants shipping speed, and customer service introduced the same very good, cost-effective	Jingdong prefecture level city in Shandong
Doraemon Hyakubo Shop	King of Power Saving	Feel a little effect Oh, where the power consumption where to plug, convenient and convenient	Jingdong prefecture level city in Shandong
Doraemon Hyakubo Shop	King of Power Saving	The quality of the imported saver is very good, practical and satisfactory. Cost-effective	Jingdong prefecture level city in Shandong
Doraemon Hyakubo Shop	King of Power Saving	Power Saver is especially good, good and inexpensive, very good	Jingdong prefecture level city in Shandong
Doraemon Hyakubo Shop	King of Power Saving	Fast shipping, awesome quality, affordable price, satisfied.	Jingdong prefecture level city in Shandong
Doraemon Hyakubo Shop	King of Power Saving	Energy saver received, the effect is consistent with the description, recommended to buy!	Jingdong prefecture level city in Shandong
Doraemon Hyakubo Shop	King of Power Saving	It's really good, the quality is great, I recommend it!	Jingdong prefecture level city in Shandong
Doraemon Hyakubo Shop	King of Power Saving	Works well. Inexpensive. Shipping was fast.	Jingdong prefecture level city in Shandong
Doraemon Hyakubo Shop	King of Power Saving	Logistics is fast, works well, energy saving is good	Jingdong prefecture level city in Shandong
Doraemon Hyakubo Shop	King of Power Saving	very happy	Jingdong prefecture level city in Shandong
Doraemon Hyakubo Shop	King of Power Saving	an excellent	Jingdong prefecture level city in Shandong
Doraemon Hyakubo Shop	King of Power Saving	It rained a couple days ago and I just went to get the item back today , not bad, hey I looked at the meter and that red display light takes several seconds to jump, did not make a mistake buying it. After a period of time to evaluate.	Jingdong prefecture level city in Shandong
Doraemon Hyakubo Shop	King of Power Saving	Used more than 10 days to check the power, there is indeed some decline, customer service said, long-term use will save more points!	Jingdong prefecture level city in Shandong
Doraemon Hyakubo Shop	King of Power Saving	It's a proper IQ tax, and it costs electricity.	Jingdong prefecture level city in Shandong
Doraemon Hyakubo Shop	King of Power Saving	I've been using it for 17 days now, it really saves electricity Shipping is fast, you can save a lot in a year	Jingdong prefecture level city in Shandong
Doraemon Hyakubo Shop	King of Power Saving	I've been using it for 17 days and it really saves power Shipping is fast and you don't have to worry about the quality, free returns within 5 years	Jingdong prefecture level city in Shandong
Doraemon Hyakubo Shop	King of Power Saving	It's more regular, scanned the code and it's authentic, and the main thing is that you can try it for 90 days! Plugged in more than ten days, is less than before a few degrees per day. When the time to use a month to see Additional. Good to buy a few more	Jingdong prefecture level city in Shandong
Doraemon Hyakubo Shop	King of Power Saving	Logistics is very fast, fine workmanship, use it and then come back to the comments	Jingdong prefecture level city in Shandong
Doraemon Hyakubo Shop	King of Power Saving	The quality of workmanship is very good and the power saving effect is quite good, I regret not getting it earlier.	Jingdong prefecture level city in Shandong
Doraemon Hyakubo Shop	King of Power Saving	Received the goods, according to the instructions on the operation is very simple, to wait and see the effect, looks good!	Jingdong prefecture level city in Shandong
Doraemon Hyakubo Shop	King of Power Saving	It's really as safe and power-saving as the owner says, and it works great!	Jingdong prefecture level city in Shandong
Doraemon Hyakubo Shop	King of Power Saving	Received the goods, immediately used, looks good, to wait and see how the effect.	Jingdong prefecture level city in Shandong
Doraemon Hyakubo Shop	King of Power Saving	Afraid to buy back did not work, the store said the commitment to 90 days invalid refund, warranty 5 years. Saver also complimentary on the Pacific insurance, it is a lot of peace of mind, there are effects will continue to buy back drops!	Jingdong prefecture level city in Shandong
Doraemon Hyakubo Shop	King of Power Saving	Received the goods and used it, can save electricity a month later to see the effect!	Jingdong prefecture level city in Shandong
Doraemon Hyakubo Shop	King of Power Saving	Fancy 90-day trial, 5-year warranty before the order, the goods received, has been experiencing a week, can really feel the savings, in the introduction of friends also buy this!	Jingdong prefecture level city in Shandong
Doraemon Hyakubo Shop	King of Power Saving	Clothing stores use, neighbors recommended to buy, said a month can save more than 70 yuan, first plugged in to see the effect of the	Jingdong prefecture level city in Shandong
Doraemon Hyakubo Shop	King of Power Saving	A friend's home two months to save more than 80 dollars of electricity, recommended that I buy two, decisive purchase, looking forward to the effect of power saving, waiting for the next month effect.	Jingdong prefecture level city in Shandong
Doraemon Hyakubo Shop	King of Power Saving	The goods have been received, consistent with the description of the advertisement, quite good, I hope that in terms of power saving and purifying the air is really as the merchant said that would be better!	Jingdong prefecture level city in Shandong

Doraemon Hyakubo Shop	King of Power Saving	Power Saving King is really good to use the quality is very good than the physical store a lot cheaper we can rest assured that the purchase recommended to buy!	Jingdong prefecture level city in Shandong
Doraemon Hyakubo Shop	King of Power Saving	Energy saver has been used on the rental house inside the use of the meter is always running fast, with this can really save electricity, easy to use, plugged in can be, very satisfied!	Jingdong prefecture level city in Shandong
Doraemon Hyakubo Shop	King of Power Saving	Just use can not see what effect to,, wait for a period of time in the evaluation	Jingdong prefecture level city in Shandong
Doraemon Hyakubo Shop	King of Power Saving	Live in the 18th floor is too far away from the meter, afraid of no effect, look at the trial 90 days before buying. Currently used for a month, there is an effect, saving 27 dollars in electricity, next month should be able to save more!	Jingdong prefecture level city in Shandong
Doraemon Hyakubo Shop	King of Power Saving	I've been using it for almost a month now and it feels good, so I'll use it again.	Jingdong prefecture level city in Shandong
Doraemon Hyakubo Shop	King of Power Saving	It's ok, the electric bill is really less, originally I remember charging $100 and using it for 10 days. Now it lasts about 15 days. It's still noticeable.	Jingdong prefecture level city in Shandong
Doraemon Hyakubo Shop	King of Power Saving	Fast logistics, good power saving effect, very satisfied	Jingdong prefecture level city in Shandong
Doraemon Hyakubo Shop	King of Power Saving	Has been the second time back to buy, energy saving effect, worth recommending, you can try to see, power saving effect: very good Experience: can save about 30%!	Jingdong prefecture level city in Shandong
Doraemon Hyakubo Shop	King of Power Saving	I don't know how well it works for now	Jingdong prefecture level city in Shandong
Doraemon Hyakubo Shop	King of Power Saving	Factory direct is really good, plugged in and used for half a month did less probably almost 50 degrees of electricity! And the manufacturer's warranty is 5 years.	Jingdong prefecture level city in Shandong
Doraemon Hyakubo Shop	King of Power Saving	It's really effective. The previous month's electricity was $286, but after using the power saver for a month, I have $195 left this month, which is not bad.	Jingdong prefecture level city in Shandong
Doraemon Hyakubo Shop	King of Power Saving	I'll give you a good review and see how it goes.	Jingdong prefecture level city in Shandong
Doraemon Hyakubo Shop	King of Power Saving	Customer service is very professional and patient to guide the installation, the quality of workmanship is very fine. Durable, second repurchase. Really can save oh!	Jingdong prefecture level city in Shandong
Doraemon Hyakubo Shop	King of Power Saving	I've been using it for a month now and my electric bill is over $30 less than last month. I feel it's quite worth buying.	Jingdong prefecture level city in Shandong
Doraemon Hyakubo Shop	King of Power Saving	Small size, does not take up space. It's very good, it does work very effectively, the tool room has a lot of work with a lot of electricity. After installing it, it really reduces a lot of electricity bills. Before the day 30 degrees of electricity left stone, now work down really less a few degrees.	Jingdong prefecture level city in Shandong
Doraemon Hyakubo Shop	King of Power Saving	It does work for saving electricity, I've bought the kind from elsewhere before that was too simple not **all**. This time to buy this store, but also for the safety. Air conditioning is too much electricity, this use to save a lot of	Jingdong prefecture level city in Shandong
Doraemon Hyakubo Shop	King of Power Saving	Two air conditioners and a refrigerator at home, a month down the electricity bill is almost two hundred dollars, with this after the average savings of more than thirty dollars to spend less money to enjoy more, it's really very good!	Jingdong prefecture level city in Shandong
Doraemon Hyakubo Shop	King of Power Saving	Good. Very good.	Jingdong prefecture level city in Shandong
Doraemon Hyakubo Shop	King of Power Saving	Works great, easy to operate, great, very happy with it.	Jingdong prefecture level city in Shandong
Doraemon Hyakubo Shop	King of Power Saving	Received it and tried it out, it's great, great quality and worth having! Five stars praise!	Jingdong prefecture level city in Shandong
Doraemon Hyakubo Shop	King of Power Saving	Very trustworthy brand, quality is guaranteed, the price is still affordable, quite satisfied!	Jingdong prefecture level city in Shandong
Doraemon Hyakubo Shop	King of Power Saving	Use it before you speak.	Jingdong prefecture level city in Shandong
Doraemon Hyakubo Shop	King of Power Saving	The quality of workmanship is very good and the power saving effect is quite good, I regret not getting it earlier.	Jingdong prefecture level city in Shandong
Doraemon Hyakubo Shop	King of Power Saving	Merchants shipping speed, and customer service introduced the same very good, cost-effective	Jingdong prefecture level city in Shandong
Doraemon Hyakubo Shop	King of Power Saving	Feel a little effect Oh, where the power consumption where to plug, convenient and convenient	Jingdong prefecture level city in Shandong
Doraemon Hyakubo Shop	King of Power Saving	The quality of the imported saver is very good, practical and satisfactory. Cost-effective	Jingdong prefecture level city in Shandong
Doraemon Hyakubo Shop	King of Power Saving	Power Saver is especially good, good and inexpensive, very good	Jingdong prefecture level city in Shandong
Doraemon Hyakubo Shop	King of Power Saving	Fast shipping, awesome quality, affordable price, satisfied.	Jingdong prefecture level city in Shandong
Doraemon Hyakubo Shop	King of Power Saving	Energy saver received, the effect is consistent with the description, recommended to buy!	Jingdong prefecture level city in Shandong
Doraemon Hyakubo Shop	King of Power Saving	It's really good, the quality is great, I recommend it!	Jingdong prefecture level city in Shandong
Doraemon Hyakubo Shop	King of Power Saving	Works well. Inexpensive. Shipping was fast.	Jingdong prefecture level city in Shandong
Doraemon Hyakubo Shop	King of Power Saving	Logistics is fast, works well, energy saving is good	Jingdong prefecture level city in Shandong
Doraemon Hyakubo Shop	King of Power Saving	very happy	Jingdong prefecture level city in Shandong
Doraemon Hyakubo Shop	King of Power Saving	an excellent	Jingdong prefecture level city in Shandong
Doraemon Hyakubo Shop	King of Power Saving	It rained a couple days ago and I just went to get the item back today , not bad, hey I looked at the meter and that red display light takes several seconds to jump, did not make a mistake buying it. After a period of time to evaluate.	Jingdong prefecture level city in Shandong
Doraemon Hyakubo Shop	King of Power Saving	Used more than 10 days to check the power, there is indeed some decline, customer service said, long-term use will save more points!	Jingdong prefecture level city in Shandong
Doraemon Hyakubo Shop	King of Power Saving	It's a proper IQ tax, and it costs electricity.	Jingdong prefecture level city in Shandong
Doraemon Hyakubo Shop	King of Power Saving	I've been using it for 17 days now, it really saves electricity Shipping is fast, you can save a lot in a year	Jingdong prefecture level city in Shandong
Doraemon Hyakubo Shop	King of Power Saving	I've been using it for 17 days and it really saves power Shipping is fast and you don't have to worry about the quality, free returns within 5 years	Jingdong prefecture level city in Shandong
Doraemon Hyakubo Shop	King of Power Saving	It's more regular, scanned the code and it's authentic, and the main thing is that you can try it for 90 days! Plugged in more than ten days, is less than before a few degrees per day. When the time to use a month to see Additional. Good to buy a few more	Jingdong prefecture level city in Shandong
Doraemon Hyakubo Shop	King of Power Saving	Logistics is very fast, fine workmanship, use it and then come back to the comments	Jingdong prefecture level city in Shandong
Doraemon Hyakubo Shop	King of Power Saving	The quality of workmanship is very good and the power saving effect is quite good, I regret not getting it earlier.	Jingdong prefecture level city in Shandong
Doraemon Hyakubo Shop	King of Power Saving	Received the goods, according to the instructions on the operation is very simple, to wait and see the effect, looks good!	Jingdong prefecture level city in Shandong
Doraemon Hyakubo Shop	King of Power Saving	It's really as safe and power-saving as the owner says, and it works great!	Jingdong prefecture level city in Shandong
Doraemon Hyakubo Shop	King of Power Saving	Received the goods, immediately used, looks good, to wait and see how the effect.	Jingdong prefecture level city in Shandong
Doraemon Hyakubo Shop	King of Power Saving	Afraid to buy back did not work, the store said the commitment to 90 days invalid refund, warranty 5 years. Saver also complimentary on the Pacific insurance, it is a lot of peace of mind, there are effects will continue to buy back drops!	Jingdong prefecture level city in Shandong
Doraemon Hyakubo Shop	King of Power Saving	Received the goods and used it, can save electricity a month later to see the effect!	Jingdong prefecture level city in Shandong
Doraemon Hyakubo Shop	King of Power Saving	Fancy 90-day trial, 5-year warranty before the order, the goods received, has been experiencing a week, can really feel the savings, in the introduction of friends also buy this!	Jingdong prefecture level city in Shandong

Doraemon Hyakubo Shop	King of Power Saving	Clothing stores use, neighbors recommended to buy, said a month can save more than 70 yuan, first plugged in to see the effect of the	Jingdong prefecture level city in Shandong
Doraemon Hyakubo Shop	King of Power Saving	A friend's home two months to save more than 80 dollars of electricity, recommended that I buy two, decisive purchase, looking forward to the effect of power saving, waiting for the next month effect.	Jingdong prefecture level city in Shandong
Doraemon Hyakubo Shop	King of Power Saving	The goods have been received, consistent with the description of the advertisement, quite good, I hope that in terms of power saving and purifying the air is really as the merchant said that would be better!	Jingdong prefecture level city in Shandong
Doraemon Hyakubo Shop	King of Power Saving	Power Saving King is really good to use the quality is very good than the physical store a lot cheaper we can rest assured that the purchase recommended to buy!	Jingdong prefecture level city in Shandong
Doraemon Hyakubo Shop	King of Power Saving	Energy saver has been used on the rental house inside the use of the meter is always running fast, with this can really save electricity, easy to use, plugged in can be, very satisfied!	Jingdong prefecture level city in Shandong
Doraemon Hyakubo Shop	King of Power Saving	Just use can not see what effect to,, wait for a period of time in the evaluation	Jingdong prefecture level city in Shandong
Doraemon Hyakubo Shop	King of Power Saving	Live in the 18th floor is too far away from the meter, afraid of no effect, look at the trial 90 days before buying. Currently used for a month, there is an effect, saving 27 dollars in electricity, next month should be able to save more!	Jingdong prefecture level city in Shandong
Doraemon Hyakubo Shop	King of Power Saving	I've been using it for almost a month now and it feels good, so I'll use it again.	Jingdong prefecture level city in Shandong
Doraemon Hyakubo Shop	King of Power Saving	It's ok, the electric bill is really less, originally I remember charging $100 and using it for 10 days. Now it lasts about 15 days. It's still noticeable.	Jingdong prefecture level city in Shandong
Doraemon Hyakubo Shop	King of Power Saving	Fast logistics, good power saving effect, very satisfied	Jingdong prefecture level city in Shandong
Doraemon Hyakubo Shop	King of Power Saving	Has been the second time back to buy, energy saving effect, worth recommending, you can try to see, power saving effect: very good Experience: can save about 30%!	Jingdong prefecture level city in Shandong
Doraemon Hyakubo Shop	King of Power Saving	I don't know how well it works for now	Jingdong prefecture level city in Shandong
Doraemon Hyakubo Shop	King of Power Saving	Factory direct is really good, plugged in and used for half a month did less probably almost 50 degrees of electricity! And the manufacturer's warranty is 5 years.	Jingdong prefecture level city in Shandong
Doraemon Hyakubo Shop	King of Power Saving	It's really effective. The previous month's electricity was $286, but after using the power saver for a month, I have $195 left this month, which is not bad.	Jingdong prefecture level city in Shandong
Doraemon Hyakubo Shop	King of Power Saving	I'll give you a good review and see how it goes.	Jingdong prefecture level city in Shandong
Doraemon Hyakubo Shop	King of Power Saving	Customer service is very professional and patient to guide the installation, the quality of workmanship is very fine. Durable, second repurchase. Really can save oh!	Jingdong prefecture level city in Shandong
Doraemon Hyakubo Shop	King of Power Saving	I've been using it for a month now and my electric bill is over $30 less than last month. I feel it's quite worth buying.	Jingdong prefecture level city in Shandong
Doraemon Hyakubo Shop	King of Power Saving	Small size, does not take up space. It's very good, it does work very effectively, the tool room has a lot of work with a lot of electricity. After installing it, it really reduces a lot of electricity bills. Before the day 30 degrees of electricity left stone, now work down really less a few degrees.	Jingdong prefecture level city in Shandong
Doraemon Hyakubo Shop	King of Power Saving	It does work for saving electricity, I've bought the kind from elsewhere before that was too simple not **all**. This time to buy this store, but also for the safety. Air conditioning is too much electricity, this use to save a lot of	Jingdong prefecture level city in Shandong
Doraemon Hyakubo Shop	King of Power Saving	Two air conditioners and a refrigerator at home, a month down the electricity bill is almost two hundred dollars, with this after the average savings of more than thirty dollars to spend less money to enjoy more, it's really very good!	Jingdong prefecture level city in Shandong
Doraemon Hyakubo Shop	King of Power Saving	Good. Very good.	Jingdong prefecture level city in Shandong
Doraemon Hyakubo Shop	King of Power Saving	Works great, easy to operate, great, very happy with it.	Jingdong prefecture level city in Shandong
Doraemon Hyakubo Shop	King of Power Saving	Received it and tried it out, it's great, great quality and worth having! Five stars praise!	Jingdong prefecture level city in Shandong
Doraemon Hyakubo Shop	King of Power Saving	Very trustworthy brand, quality is guaranteed, the price is still affordable, quite satisfied!	Jingdong prefecture level city in Shandong
Doraemon Hyakubo Shop	King of Power Saving	Use it before you speak.	Jingdong prefecture level city in Shandong
Doraemon Hyakubo Shop	King of Power Saving	The quality of workmanship is very good and the power saving effect is quite good, I regret not getting it earlier.	Jingdong prefecture level city in Shandong
Doraemon Hyakubo Shop	King of Power Saving	Merchants shipping speed, and customer service introduced the same very good, cost-effective	Jingdong prefecture level city in Shandong
Doraemon Hyakubo Shop	King of Power Saving	Feel a little effect Oh, where the power consumption where to plug, convenient and convenient	Jingdong prefecture level city in Shandong
Doraemon Hyakubo Shop	King of Power Saving	The quality of the imported saver is very good, practical and satisfactory. Cost-effective	Jingdong prefecture level city in Shandong
Doraemon Hyakubo Shop	King of Power Saving	Power Saver is especially good, good and inexpensive, very good	Jingdong prefecture level city in Shandong
Doraemon Hyakubo Shop	King of Power Saving	Fast shipping, awesome quality, affordable price, satisfied.	Jingdong prefecture level city in Shandong
Doraemon Hyakubo Shop	King of Power Saving	Energy saver received, the effect is consistent with the description, recommended to buy!	Jingdong prefecture level city in Shandong
Doraemon Hyakubo Shop	King of Power Saving	It's really good, the quality is great, I recommend it!	Jingdong prefecture level city in Shandong
Doraemon Hyakubo Shop	King of Power Saving	Works well. Inexpensive. Shipping was fast.	Jingdong prefecture level city in Shandong
Doraemon Hyakubo Shop	King of Power Saving	Logistics is fast, works well, energy saving is good	Jingdong prefecture level city in Shandong
Doraemon Hyakubo Shop	King of Power Saving	very happy	Jingdong prefecture level city in Shandong
Doraemon Hyakubo Shop	King of Power Saving	an excellent	Jingdong prefecture level city in Shandong
Doraemon Hyakubo Shop	King of Power Saving	It rained a couple days ago and I just went to get the item back today , not bad, hey I looked at the meter and that red display light takes several seconds to jump, did not make a mistake buying it. After a period of time to evaluate.	Jingdong prefecture level city in Shandong
Doraemon Hyakubo Shop	King of Power Saving	Used more than 10 days to check the power, there is indeed some decline, customer service said, long-term use will save more points!	Jingdong prefecture level city in Shandong
Doraemon Hyakubo Shop	King of Power Saving	It's a proper IQ tax, and it costs electricity.	Jingdong prefecture level city in Shandong
Doraemon Hyakubo Shop	King of Power Saving	I've been using it for 17 days now, it really saves electricity Shipping is fast, you can save a lot in a year	Jingdong prefecture level city in Shandong
Doraemon Hyakubo Shop	King of Power Saving	I've been using it for 17 days and it really saves power Shipping is fast and you don't have to worry about the quality, free returns within 5 years	Jingdong prefecture level city in Shandong
Doraemon Hyakubo Shop	King of Power Saving	It's more regular, scanned the code and it's authentic, and the main thing is that you can try it for 90 days! Plugged in more than ten days, is less than before a few degrees per day. When the time to use a month to see Additional. Good to buy a few more	Jingdong prefecture level city in Shandong
Doraemon Hyakubo Shop	King of Power Saving	Logistics is very fast, fine workmanship, use it and then come back to the comments	Jingdong prefecture level city in Shandong
Doraemon Hyakubo Shop	King of Power Saving	The quality of workmanship is very good and the power saving effect is quite good, I regret not getting it earlier.	Jingdong prefecture level city in Shandong
Doraemon Hyakubo Shop	King of Power Saving	Received the goods, according to the instructions on the operation is very simple, to wait and see the effect, looks good!	Jingdong prefecture level city in Shandong
Doraemon Hyakubo Shop	King of Power Saving	It's really as safe and power-saving as the owner says, and it works great!	Jingdong prefecture level city in Shandong
Doraemon Hyakubo Shop	King of Power Saving	Received the goods, immediately used, looks good, to wait and see how the effect.	Jingdong prefecture level city in Shandong

Doraemon Hyakubo Shop	King of Power Saving	Afraid to buy back did not work, the store said the commitment to 90 days invalid refund, warranty 5 years. Saver also complimentary on the Pacific insurance, it is a lot of peace of mind, there are effects will continue to buy back drops!	Jingdong prefecture level city in Shandong
Doraemon Hyakubo Shop	King of Power Saving	Received the goods and used it, can save electricity a month later to see the effect!	Jingdong prefecture level city in Shandong
Doraemon Hyakubo Shop	King of Power Saving	Fancy 90-day trial, 5-year warranty before the order, the goods received, has been experiencing a week, can really feel the savings, in the introduction of friends also buy this!	Jingdong prefecture level city in Shandong
Doraemon Hyakubo Shop	King of Power Saving	Clothing stores use, neighbors recommended to buy, said a month can save more than 70 yuan, first plugged in to see the effect of the	Jingdong prefecture level city in Shandong
Doraemon Hyakubo Shop	King of Power Saving	A friend's home two months to save more than 80 dollars of electricity, recommended that I buy two, decisive purchase, looking forward to the effect of power saving, waiting for the next month effect.	Jingdong prefecture level city in Shandong
Doraemon Hyakubo Shop	King of Power Saving	The goods have been received, consistent with the description of the advertisement, quite good, I hope that in terms of power saving and purifying the air is really as the merchant said that would be better!	Jingdong prefecture level city in Shandong
Doraemon Hyakubo Shop	King of Power Saving	Power Saving King is really good to use the quality is very good than the physical store a lot cheaper we can rest assured that the purchase recommended to buy!	Jingdong prefecture level city in Shandong
Doraemon Hyakubo Shop	King of Power Saving	Energy saver has been used on the rental house inside the use of the meter is always running fast, with this can really save electricity, easy to use, plugged in can be, very satisfied!	Jingdong prefecture level city in Shandong
Doraemon Hyakubo Shop	King of Power Saving	Just use can not see what effect to,, wait for a period of time in the evaluation	Jingdong prefecture level city in Shandong
Doraemon Hyakubo Shop	King of Power Saving	Live in the 18th floor is too far away from the meter, afraid of no effect, look at the trial 90 days before buying. Currently used for a month, there is an effect, saving 27 dollars in electricity, next month should be able to save more!	Jingdong prefecture level city in Shandong
Doraemon Hyakubo Shop	King of Power Saving	I've been using it for almost a month now and it feels good, so I'll use it again.	Jingdong prefecture level city in Shandong
Doraemon Hyakubo Shop	King of Power Saving	It's ok, the electric bill is really less, originally I remember charging $100 and using it for 10 days. Now it lasts about 15 days. It's still noticeable.	Jingdong prefecture level city in Shandong
Doraemon Hyakubo Shop	King of Power Saving	Fast logistics, good power saving effect, very satisfied	Jingdong prefecture level city in Shandong
Doraemon Hyakubo Shop	King of Power Saving	Has been the second time back to buy, energy saving effect, worth recommending, you can try to see, power saving effect: very good Experience: can save about 30%!	Jingdong prefecture level city in Shandong
Doraemon Hyakubo Shop	King of Power Saving	I don't know how well it works for now	Jingdong prefecture level city in Shandong
Doraemon Hyakubo Shop	King of Power Saving	Factory direct is really good, plugged in and used for half a month did less probably almost 50 degrees of electricity! And the manufacturer's warranty is 5 years.	Jingdong prefecture level city in Shandong
Doraemon Hyakubo Shop	King of Power Saving	It's really effective. The previous month's electricity was $286, but after using the power saver for a month, I have $195 left this month, which is not bad.	Jingdong prefecture level city in Shandong
Doraemon Hyakubo Shop	King of Power Saving	I'll give you a good review and see how it goes.	Jingdong prefecture level city in Shandong
Doraemon Hyakubo Shop	King of Power Saving	Customer service is very professional and patient to guide the installation, the quality of workmanship is very fine. Durable, second repurchase. Really can save oh!	Jingdong prefecture level city in Shandong
Doraemon Hyakubo Shop	King of Power Saving	I've been using it for a month now and my electric bill is over $30 less than last month. I feel it's quite worth buying.	Jingdong prefecture level city in Shandong
Doraemon Hyakubo Shop	King of Power Saving	Small size, does not take up space. It's very good, it does work very effectively, the tool room has a lot of work with a lot of electricity. After installing it, it really reduces a lot of electricity bills. Before the day 30 degrees of electricity left stone, now work down really less a few degrees.	Jingdong prefecture level city in Shandong
Doraemon Hyakubo Shop	King of Power Saving	It does work for saving electricity, I've bought the kind from elsewhere before that was too simple not **all**. This time to buy this store, but also for the safety. Air conditioning is too much electricity, this use to save a lot of	Jingdong prefecture level city in Shandong
Doraemon Hyakubo Shop	King of Power Saving	Two air conditioners and a refrigerator at home, a month down the electricity bill is almost two hundred dollars, with this after the average savings of more than thirty dollars to spend less money to enjoy more, it's really very good!	Jingdong prefecture level city in Shandong
Doraemon Hyakubo Shop	King of Power Saving	Good. Very good.	Jingdong prefecture level city in Shandong
Doraemon Hyakubo Shop	King of Power Saving	Works great, easy to operate, great, very happy with it.	Jingdong prefecture level city in Shandong
Doraemon Hyakubo Shop	King of Power Saving	Received it and tried it out, it's great, great quality and worth having! Five stars praise!	Jingdong prefecture level city in Shandong
Doraemon Hyakubo Shop	King of Power Saving	Very trustworthy brand, quality is guaranteed, the price is still affordable, quite satisfied!	Jingdong prefecture level city in Shandong
Doraemon Hyakubo Shop	King of Power Saving	Use it before you speak.	Jingdong prefecture level city in Shandong
Doraemon Hyakubo Shop	King of Power Saving	The quality of workmanship is very good and the power saving effect is quite good, I regret not getting it earlier.	Jingdong prefecture level city in Shandong
Doraemon Hyakubo Shop	King of Power Saving	Merchants shipping speed, and customer service introduced the same very good, cost-effective	Jingdong prefecture level city in Shandong
Doraemon Hyakubo Shop	King of Power Saving	Feel a little effect Oh, where the power consumption where to plug, convenient and convenient	Jingdong prefecture level city in Shandong
Doraemon Hyakubo Shop	King of Power Saving	The quality of the imported saver is very good, practical and satisfactory. Cost-effective	Jingdong prefecture level city in Shandong
Doraemon Hyakubo Shop	King of Power Saving	Power Saver is especially good, good and inexpensive, very good	Jingdong prefecture level city in Shandong
Doraemon Hyakubo Shop	King of Power Saving	Fast shipping, awesome quality, affordable price, satisfied.	Jingdong prefecture level city in Shandong
Doraemon Hyakubo Shop	King of Power Saving	Energy saver received, the effect is consistent with the description, recommended to buy!	Jingdong prefecture level city in Shandong
Doraemon Hyakubo Shop	King of Power Saving	It's really good, the quality is great, I recommend it!	Jingdong prefecture level city in Shandong
Doraemon Hyakubo Shop	King of Power Saving	Works well. Inexpensive. Shipping was fast.	Jingdong prefecture level city in Shandong
Doraemon Hyakubo Shop	King of Power Saving	Logistics is fast, works well, energy saving is good	Jingdong prefecture level city in Shandong
Doraemon Hyakubo Shop	King of Power Saving	very happy	Jingdong prefecture level city in Shandong
Doraemon Hyakubo Shop	King of Power Saving	an excellent	Jingdong prefecture level city in Shandong
Doraemon Hyakubo Shop	King of Power Saving	It rained a couple days ago and I just went to get the item back today , not bad, hey I looked at the meter and that red display light takes several seconds to jump, did not make a mistake buying it. After a period of time to evaluate.	Jingdong prefecture level city in Shandong
Doraemon Hyakubo Shop	King of Power Saving	Used more than 10 days to check the power, there is indeed some decline, customer service said, long-term use will save more points!	Jingdong prefecture level city in Shandong
Doraemon Hyakubo Shop	King of Power Saving	It's a proper IQ tax, and it costs electricity.	Jingdong prefecture level city in Shandong
Doraemon Hyakubo Shop	King of Power Saving	I've been using it for 17 days now, it really saves electricity Shipping is fast, you can save a lot in a year	Jingdong prefecture level city in Shandong
Doraemon Hyakubo Shop	King of Power Saving	I've been using it for 17 days and it really saves power Shipping is fast and you don't have to worry about the quality, free returns within 5 years	Jingdong prefecture level city in Shandong
Doraemon Hyakubo Shop	King of Power Saving	It's more regular, scanned the code and it's authentic, and the main thing is that you can try it for 90 days! Plugged in more than ten days, is less than before a few degrees per day. When the time to use a month to see Additional. Good to buy a few more	Jingdong prefecture level city in Shandong
Doraemon Hyakubo Shop	King of Power Saving	Logistics is very fast, fine workmanship, use it and then come back to the comments	Jingdong prefecture level city in Shandong
Doraemon Hyakubo Shop	King of Power Saving	The quality of workmanship is very good and the power saving effect is quite good, I regret not getting it earlier.	Jingdong prefecture level city in Shandong

Doraemon Hyakubo Shop	King of Power Saving	Received the goods, according to the instructions on the operation is very simple, to wait and see the effect, looks good!	Jingdong prefecture level city in Shandong
Doraemon Hyakubo Shop	King of Power Saving	It's really as safe and power-saving as the owner says, and it works great!	Jingdong prefecture level city in Shandong
Doraemon Hyakubo Shop	King of Power Saving	Received the goods, immediately used, looks good, to wait and see how the effect.	Jingdong prefecture level city in Shandong
Doraemon Hyakubo Shop	King of Power Saving	Afraid to buy back did not work, the store said the commitment to 90 days invalid refund, warranty 5 years. Saver also complimentary on the Pacific insurance, it is a lot of peace of mind, there are effects will continue to buy back drops!	Jingdong prefecture level city in Shandong
Doraemon Hyakubo Shop	King of Power Saving	Received the goods and used it, can save electricity a month later to see the effect!	Jingdong prefecture level city in Shandong
Doraemon Hyakubo Shop	King of Power Saving	Fancy 90-day trial, 5-year warranty before the order, the goods received, has been experiencing a week, can really feel the savings, in the introduction of friends also buy this!	Jingdong prefecture level city in Shandong
Doraemon Hyakubo Shop	King of Power Saving	Clothing stores use, neighbors recommended to buy, said a month can save more than 70 yuan, first plugged in to see the effect of the	Jingdong prefecture level city in Shandong
Doraemon Hyakubo Shop	King of Power Saving	A friend's home two months to save more than 80 dollars of electricity, recommended that I buy two, decisive purchase, looking forward to the effect of power saving, waiting for the next month effect.	Jingdong prefecture level city in Shandong
Doraemon Hyakubo Shop	King of Power Saving	The goods have been received, consistent with the description of the advertisement, quite good, I hope that in terms of power saving and purifying the air is really as the merchant said that would be better!	Jingdong prefecture level city in Shandong
Doraemon Hyakubo Shop	King of Power Saving	Power Saving King is really good to use the quality is very good than the physical store a lot cheaper we can rest assured that the purchase recommended to buy!	Jingdong prefecture level city in Shandong
Doraemon Hyakubo Shop	King of Power Saving	Energy saver has been used on the rental house inside the use of the meter is always running fast, with this can really save electricity, easy to use, plugged in can be, very satisfied!	Jingdong prefecture level city in Shandong
Doraemon Hyakubo Shop	King of Power Saving	Just use can not see what effect to,, wait for a period of time in the evaluation	Jingdong prefecture level city in Shandong
Doraemon Hyakubo Shop	King of Power Saving	Live in the 18th floor is too far away from the meter, afraid of no effect, look at the trial 90 days before buying. Currently used for a month, there is an effect, saving 27 dollars in electricity, next month should be able to save more!	Jingdong prefecture level city in Shandong
Doraemon Hyakubo Shop	King of Power Saving	I've been using it for almost a month now and it feels good, so I'll use it again.	Jingdong prefecture level city in Shandong
Doraemon Hyakubo Shop	King of Power Saving	It's ok, the electric bill is really less, originally I remember charging $100 and using it for 10 days. Now it lasts about 15 days. It's still noticeable.	Jingdong prefecture level city in Shandong
Doraemon Hyakubo Shop	King of Power Saving	Fast logistics, good power saving effect, very satisfied	Jingdong prefecture level city in Shandong
Doraemon Hyakubo Shop	King of Power Saving	Has been the second time back to buy, energy saving effect, worth recommending, you can try to see, power saving effect: very good Experience: can save about 30%!	Jingdong prefecture level city in Shandong
Doraemon Hyakubo Shop	King of Power Saving	I don't know how well it works for now	Jingdong prefecture level city in Shandong
Doraemon Hyakubo Shop	King of Power Saving	Factory direct is really good, plugged in and used for half a month did less probably almost 50 degrees of electricity! And the manufacturer's warranty is 5 years.	Jingdong prefecture level city in Shandong
Doraemon Hyakubo Shop	King of Power Saving	It's really effective. The previous month's electricity was $286, but after using the power saver for a month, I have $195 left this month, which is not bad.	Jingdong prefecture level city in Shandong
Doraemon Hyakubo Shop	King of Power Saving	I'll give you a good review and see how it goes.	Jingdong prefecture level city in Shandong
Doraemon Hyakubo Shop	King of Power Saving	Customer service is very professional and patient to guide the installation, the quality of workmanship is very fine. Durable, second repurchase. Really can save oh!	Jingdong prefecture level city in Shandong
Doraemon Hyakubo Shop	King of Power Saving	I've been using it for a month now and my electric bill is over $30 less than last month. I feel it's quite worth buying.	Jingdong prefecture level city in Shandong
Doraemon Hyakubo Shop	King of Power Saving	Small size, does not take up space. It's very good, it does work very effectively, the tool room has a lot of work with a lot of electricity. After installing it, it really reduces a lot of electricity bills. Before the day 30 degrees of electricity left stone, now work down really less a few degrees.	Jingdong prefecture level city in Shandong
Doraemon Hyakubo Shop	King of Power Saving	It does work for saving electricity, I've bought the kind from elsewhere before that was too simple not **all**. This time to buy this store, but also for the safety. Air conditioning is too much electricity, this use to save a lot of	Jingdong prefecture level city in Shandong
Doraemon Hyakubo Shop	King of Power Saving	Two air conditioners and a refrigerator at home, a month down the electricity bill is almost two hundred dollars, with this after the average savings of more than thirty dollars to spend less money to enjoy more, it's really very good!	Jingdong prefecture level city in Shandong
Doraemon Hyakubo Shop	King of Power Saving	Good. Very good.	Jingdong prefecture level city in Shandong
Doraemon Hyakubo Shop	King of Power Saving	Works great, easy to operate, great, very happy with it.	Jingdong prefecture level city in Shandong
Doraemon Hyakubo Shop	King of Power Saving	Received it and tried it out, it's great, great quality and worth having! Five stars praise!	Jingdong prefecture level city in Shandong
Doraemon Hyakubo Shop	King of Power Saving	Very trustworthy brand, quality is guaranteed, the price is still affordable, quite satisfied!	Jingdong prefecture level city in Shandong
Doraemon Hyakubo Shop	King of Power Saving	Use it before you speak.	Jingdong prefecture level city in Shandong
Doraemon Hyakubo Shop	King of Power Saving	The quality of workmanship is very good and the power saving effect is quite good, I regret not getting it earlier.	Jingdong prefecture level city in Shandong
Doraemon Hyakubo Shop	King of Power Saving	Merchants shipping speed, and customer service introduced the same very good, cost-effective	Jingdong prefecture level city in Shandong
Doraemon Hyakubo Shop	King of Power Saving	Feel a little effect Oh, where the power consumption where to plug, convenient and convenient	Jingdong prefecture level city in Shandong
Doraemon Hyakubo Shop	King of Power Saving	The quality of the imported saver is very good, practical and satisfactory. Cost-effective	Jingdong prefecture level city in Shandong
Doraemon Hyakubo Shop	King of Power Saving	Power Saver is especially good, good and inexpensive, very good	Jingdong prefecture level city in Shandong
Doraemon Hyakubo Shop	King of Power Saving	Fast shipping, awesome quality, affordable price, satisfied.	Jingdong prefecture level city in Shandong
Doraemon Hyakubo Shop	King of Power Saving	Energy saver received, the effect is consistent with the description, recommended to buy!	Jingdong prefecture level city in Shandong
Doraemon Hyakubo Shop	King of Power Saving	It's really good, the quality is great, I recommend it!	Jingdong prefecture level city in Shandong
Doraemon Hyakubo Shop	King of Power Saving	Works well. Inexpensive. Shipping was fast.	Jingdong prefecture level city in Shandong
Doraemon Hyakubo Shop	King of Power Saving	Logistics is fast, works well, energy saving is good	Jingdong prefecture level city in Shandong
Doraemon Hyakubo Shop	King of Power Saving	very happy	Jingdong prefecture level city in Shandong
Doraemon Hyakubo Shop	King of Power Saving	an excellent	Jingdong prefecture level city in Shandong
Doraemon Hyakubo Shop	King of Power Saving	It rained a couple days ago and I just went to get the item back today , not bad, hey I looked at the meter and that red display light takes several seconds to jump, did not make a mistake buying it. After a period of time to evaluate.	Jingdong prefecture level city in Shandong
Doraemon Hyakubo Shop	King of Power Saving	Used more than 10 days to check the power, there is indeed some decline, customer service said, long-term use will save more points!	Jingdong prefecture level city in Shandong
Doraemon Hyakubo Shop	King of Power Saving	It's a proper IQ tax, and it costs electricity.	Jingdong prefecture level city in Shandong
Doraemon Hyakubo Shop	King of Power Saving	I've been using it for 17 days now, it really saves electricity Shipping is fast, you can save a lot in a year	Jingdong prefecture level city in Shandong
Doraemon Hyakubo Shop	King of Power Saving	I've been using it for 17 days and it really saves power Shipping is fast and you don't have to worry about the quality, free returns within 5 years	Jingdong prefecture level city in Shandong

Doraemon Hyakubo Shop	King of Power Saving	It's more regular, scanned the code and it's authentic, and the main thing is that you can try it for 90 days! Plugged in more than ten days, is less than before a few degrees per day. When the time to use a month to see Additional. Good to buy a few more	Jingdong prefecture level city in Shandong
Doraemon Hyakubo Shop	King of Power Saving	Logistics is very fast, fine workmanship, use it and then come back to the comments	Jingdong prefecture level city in Shandong
Doraemon Hyakubo Shop	King of Power Saving	The quality of workmanship is very good and the power saving effect is quite good, I regret not getting it earlier.	Jingdong prefecture level city in Shandong
Doraemon Hyakubo Shop	King of Power Saving	Received the goods, according to the instructions on the operation is very simple, to wait and see the effect, looks good!	Jingdong prefecture level city in Shandong
Doraemon Hyakubo Shop	King of Power Saving	It's really as safe and power-saving as the owner says, and it works great!	Jingdong prefecture level city in Shandong
Doraemon Hyakubo Shop	King of Power Saving	Received the goods, immediately used, looks good, to wait and see how the effect.	Jingdong prefecture level city in Shandong
Doraemon Hyakubo Shop	King of Power Saving	Afraid to buy back did not work, the store said the commitment to 90 days invalid refund, warranty 5 years. Saver also complimentary on the Pacific insurance, it is a lot of peace of mind, there are effects will continue to buy back drops!	Jingdong prefecture level city in Shandong
Doraemon Hyakubo Shop	King of Power Saving	Received the goods and used it, can save electricity a month later to see the effect!	Jingdong prefecture level city in Shandong
Doraemon Hyakubo Shop	King of Power Saving	Fancy 90-day trial, 5-year warranty before the order, the goods received, has been experiencing a week, can really feel the savings, in the introduction of friends also buy this!	Jingdong prefecture level city in Shandong
Doraemon Hyakubo Shop	King of Power Saving	Clothing stores use, neighbors recommended to buy, said a month can save more than 70 yuan, first plugged in to see the effect of the	Jingdong prefecture level city in Shandong
Doraemon Hyakubo Shop	King of Power Saving	A friend's home two months to save more than 80 dollars of electricity, recommended that I buy two, decisive purchase, looking forward to the effect of power saving, waiting for the next month effect.	Jingdong prefecture level city in Shandong
Doraemon Hyakubo Shop	King of Power Saving	The goods have been received, consistent with the description of the advertisement, quite good, I hope that in terms of power saving and purifying the air is really as the merchant said that would be better!	Jingdong prefecture level city in Shandong
Doraemon Hyakubo Shop	King of Power Saving	Power Saving King is really good to use the quality is very good than the physical store a lot cheaper we can rest assured that the purchase recommended to buy!	Jingdong prefecture level city in Shandong
Doraemon Hyakubo Shop	King of Power Saving	Energy saver has been used on the rental house inside the use of the meter is always running fast, with this can really save electricity, easy to use, plugged in can be, very satisfied!	Jingdong prefecture level city in Shandong
Doraemon Hyakubo Shop	King of Power Saving	Just use can not see what effect to,, wait for a period of time in the evaluation	Jingdong prefecture level city in Shandong
Doraemon Hyakubo Shop	King of Power Saving	Live in the 18th floor is too far away from the meter, afraid of no effect, look at the trial 90 days before buying. Currently used for a month, there is an effect, saving 27 dollars in electricity, next month should be able to save more!	Jingdong prefecture level city in Shandong
Doraemon Hyakubo Shop	King of Power Saving	I've been using it for almost a month now and it feels good, so I'll use it again.	Jingdong prefecture level city in Shandong
Doraemon Hyakubo Shop	King of Power Saving	It's ok, the electric bill is really less, originally I remember charging $100 and using it for 10 days. Now it lasts about 15 days. It's still noticeable.	Jingdong prefecture level city in Shandong
Doraemon Hyakubo Shop	King of Power Saving	Fast logistics, good power saving effect, very satisfied	Jingdong prefecture level city in Shandong
Doraemon Hyakubo Shop	King of Power Saving	Has been the second time back to buy, energy saving effect, worth recommending, you can try to see, power saving effect: very good Experience: can save about 30%!	Jingdong prefecture level city in Shandong
Doraemon Hyakubo Shop	King of Power Saving	I don't know how well it works for now	Jingdong prefecture level city in Shandong
Doraemon Hyakubo Shop	King of Power Saving	Factory direct is really good, plugged in and used for half a month did less probably almost 50 degrees of electricity! And the manufacturer's warranty is 5 years.	Jingdong prefecture level city in Shandong
Doraemon Hyakubo Shop	King of Power Saving	It's really effective. The previous month's electricity was $286, but after using the power saver for a month, I have $195 left this month, which is not bad.	Jingdong prefecture level city in Shandong
Doraemon Hyakubo Shop	King of Power Saving	I'll give you a good review and see how it goes.	Jingdong prefecture level city in Shandong
Doraemon Hyakubo Shop	King of Power Saving	Customer service is very professional and patient to guide the installation, the quality of workmanship is very fine. Durable, second repurchase. Really can save oh!	Jingdong prefecture level city in Shandong
Doraemon Hyakubo Shop	King of Power Saving	I've been using it for a month now and my electric bill is over $30 less than last month. I feel it's quite worth buying.	Jingdong prefecture level city in Shandong
Doraemon Hyakubo Shop	King of Power Saving	Small size, does not take up space. It's very good, it does work very effectively, the tool room has a lot of work with a lot of electricity. After installing it, it really reduces a lot of electricity bills. Before the day 30 degrees of electricity left stone, now work down really less a few degrees.	Jingdong prefecture level city in Shandong
Doraemon Hyakubo Shop	King of Power Saving	It does work for saving electricity, I've bought the kind from elsewhere before that was too simple not **all**. This time to buy this store, but also for the safety. Air conditioning is too much electricity, this use to save a lot of	Jingdong prefecture level city in Shandong
Doraemon Hyakubo Shop	King of Power Saving	Two air conditioners and a refrigerator at home, a month down the electricity bill is almost two hundred dollars, with this after the average savings of more than thirty dollars to spend less money to enjoy more, it's really very good!	Jingdong prefecture level city in Shandong
Doraemon Hyakubo Shop	King of Power Saving	Good. Very good.	Jingdong prefecture level city in Shandong
Doraemon Hyakubo Shop	King of Power Saving	Works great, easy to operate, great, very happy with it.	Jingdong prefecture level city in Shandong
Doraemon Hyakubo Shop	King of Power Saving	Received it and tried it out, it's great, great quality and worth having! Five stars praise!	Jingdong prefecture level city in Shandong
Doraemon Hyakubo Shop	King of Power Saving	Very trustworthy brand, quality is guaranteed, the price is still affordable, quite satisfied!	Jingdong prefecture level city in Shandong
Doraemon Hyakubo Shop	King of Power Saving	Use it before you speak.	Jingdong prefecture level city in Shandong
Doraemon Hyakubo Shop	King of Power Saving	The quality of workmanship is very good and the power saving effect is quite good, I regret not getting it earlier.	Jingdong prefecture level city in Shandong
Doraemon Hyakubo Shop	King of Power Saving	Merchants shipping speed, and customer service introduced the same very good, cost-effective	Jingdong prefecture level city in Shandong
Doraemon Hyakubo Shop	King of Power Saving	Feel a little effect Oh, where the power consumption where to plug, convenient and convenient	Jingdong prefecture level city in Shandong
Doraemon Hyakubo Shop	King of Power Saving	The quality of the imported saver is very good, practical and satisfactory. Cost-effective	Jingdong prefecture level city in Shandong
Doraemon Hyakubo Shop	King of Power Saving	Power Saver is especially good, good and inexpensive, very good	Jingdong prefecture level city in Shandong
Doraemon Hyakubo Shop	King of Power Saving	Fast shipping, awesome quality, affordable price, satisfied.	Jingdong prefecture level city in Shandong
Doraemon Hyakubo Shop	King of Power Saving	Energy saver received, the effect is consistent with the description, recommended to buy!	Jingdong prefecture level city in Shandong
Doraemon Hyakubo Shop	King of Power Saving	It's really good, the quality is great, I recommend it!	Jingdong prefecture level city in Shandong
Doraemon Hyakubo Shop	King of Power Saving	Works well. Inexpensive. Shipping was fast.	Jingdong prefecture level city in Shandong
Doraemon Hyakubo Shop	King of Power Saving	Logistics is fast, works well, energy saving is good	Jingdong prefecture level city in Shandong
Doraemon Hyakubo Shop	King of Power Saving	very happy	Jingdong prefecture level city in Shandong
Doraemon Hyakubo Shop	King of Power Saving	an excellent	Jingdong prefecture level city in Shandong
Doraemon Hyakubo Shop	King of Power Saving	It rained a couple days ago and I just went to get the item back today , not bad, hey I looked at the meter and that red display light takes several seconds to jump, did not make a mistake buying it. After a period of time to evaluate.	Jingdong prefecture level city in Shandong
Doraemon Hyakubo Shop	King of Power Saving	Used more than 10 days to check the power, there is indeed some decline, customer service said, long-term use will save more points!	Jingdong prefecture level city in Shandong

Doraemon Hyakubo Shop	King of Power Saving	It's a proper IQ tax, and it costs electricity.	Jingdong prefecture level city in Shandong
Doraemon Hyakubo Shop	King of Power Saving	I've been using it for 17 days now, it really saves electricity Shipping is fast, you can save a lot in a year	Jingdong prefecture level city in Shandong
Doraemon Hyakubo Shop	King of Power Saving	I've been using it for 17 days and it really saves power Shipping is fast and you don't have to worry about the quality, free returns within 5 years	Jingdong prefecture level city in Shandong
Doraemon Hyakubo Shop	King of Power Saving	It's more regular, scanned the code and it's authentic, and the main thing is that you can try it for 90 days! Plugged in more than ten days, is less than before a few degrees per day. When the time to use a month to see Additional. Good to buy a few more	Jingdong prefecture level city in Shandong
Doraemon Hyakubo Shop	King of Power Saving	Logistics is very fast, fine workmanship, use it and then come back to the comments	Jingdong prefecture level city in Shandong
Doraemon Hyakubo Shop	King of Power Saving	The quality of workmanship is very good and the power saving effect is quite good, I regret not getting it earlier.	Jingdong prefecture level city in Shandong
Doraemon Hyakubo Shop	King of Power Saving	Received the goods, according to the instructions on the operation is very simple, to wait and see the effect, looks good!	Jingdong prefecture level city in Shandong
Doraemon Hyakubo Shop	King of Power Saving	It's really as safe and power-saving as the owner says, and it works great!	Jingdong prefecture level city in Shandong
Doraemon Hyakubo Shop	King of Power Saving	Received the goods, immediately used, looks good, to wait and see how the effect.	Jingdong prefecture level city in Shandong
Doraemon Hyakubo Shop	King of Power Saving	Afraid to buy back did not work, the store said the commitment to 90 days invalid refund, warranty 5 years. Saver also complimentary on the Pacific insurance, it is a lot of peace of mind, there are effects will continue to buy back drops!	Jingdong prefecture level city in Shandong
Doraemon Hyakubo Shop	King of Power Saving	Received the goods and used it, can save electricity a month later to see the effect!	Jingdong prefecture level city in Shandong
Doraemon Hyakubo Shop	King of Power Saving	Fancy 90-day trial, 5-year warranty before the order, the goods received, has been experiencing a week, can really feel the savings, in the introduction of friends also buy this!	Jingdong prefecture level city in Shandong
Doraemon Hyakubo Shop	King of Power Saving	Clothing stores use, neighbors recommended to buy, said a month can save more than 70 yuan, first plugged in to see the effect of the	Jingdong prefecture level city in Shandong
Doraemon Hyakubo Shop	King of Power Saving	A friend's home two months to save more than 80 dollars of electricity, recommended that I buy two, decisive purchase, looking forward to the effect of power saving, waiting for the next month effect.	Jingdong prefecture level city in Shandong
Doraemon Hyakubo Shop	King of Power Saving	The goods have been received, consistent with the description of the advertisement, quite good, I hope that in terms of power saving and purifying the air is really as the merchant said that would be better!	Jingdong prefecture level city in Shandong
Doraemon Hyakubo Shop	King of Power Saving	Power Saving King is really good to use the quality is very good than the physical store a lot cheaper we can rest assured that the purchase recommended to buy!	Jingdong prefecture level city in Shandong
Doraemon Hyakubo Shop	King of Power Saving	Energy saver has been used on the rental house inside the use of the meter is always running fast, with this can really save electricity, easy to use, plugged in can be, very satisfied!	Jingdong prefecture level city in Shandong
Doraemon Hyakubo Shop	King of Power Saving	Just use can not see what effect to,, wait for a period of time in the evaluation	Jingdong prefecture level city in Shandong
Doraemon Hyakubo Shop	King of Power Saving	Live in the 18th floor is too far away from the meter, afraid of no effect, look at the trial 90 days before buying. Currently used for a month, there is an effect, saving 27 dollars in electricity, next month should be able to save more!	Jingdong prefecture level city in Shandong
Doraemon Hyakubo Shop	King of Power Saving	I've been using it for almost a month now and it feels good, so I'll use it again.	Jingdong prefecture level city in Shandong
Doraemon Hyakubo Shop	King of Power Saving	It's ok, the electric bill is really less, originally I remember charging $100 and using it for 10 days. Now it lasts about 15 days. It's still noticeable.	Jingdong prefecture level city in Shandong
Doraemon Hyakubo Shop	King of Power Saving	Fast logistics, good power saving effect, very satisfied	Jingdong prefecture level city in Shandong
Doraemon Hyakubo Shop	King of Power Saving	Has been the second time back to buy, energy saving effect, worth recommending, you can try to see, power saving effect: very good Experience: can save about 30%!	Jingdong prefecture level city in Shandong
Doraemon Hyakubo Shop	King of Power Saving	I don't know how well it works for now	Jingdong prefecture level city in Shandong
Doraemon Hyakubo Shop	King of Power Saving	Factory direct is really good, plugged in and used for half a month did less probably almost 50 degrees of electricity! And the manufacturer's warranty is 5 years.	Jingdong prefecture level city in Shandong
Doraemon Hyakubo Shop	King of Power Saving	It's really effective. The previous month's electricity was $286, but after using the power saver for a month, I have $195 left this month, which is not bad.	Jingdong prefecture level city in Shandong
Doraemon Hyakubo Shop	King of Power Saving	I'll give you a good review and see how it goes.	Jingdong prefecture level city in Shandong
Doraemon Hyakubo Shop	King of Power Saving	Customer service is very professional and patient to guide the installation, the quality of workmanship is very fine. Durable, second repurchase. Really can save oh!	Jingdong prefecture level city in Shandong
Doraemon Hyakubo Shop	King of Power Saving	I've been using it for a month now and my electric bill is over $30 less than last month. I feel it's quite worth buying.	Jingdong prefecture level city in Shandong
Doraemon Hyakubo Shop	King of Power Saving	Small size, does not take up space. It's very good, it does work very effectively, the tool room has a lot of work with a lot of electricity. After installing it, it really reduces a lot of electricity bills. Before the day 30 degrees of electricity left stone, now work down really less a few degrees.	Jingdong prefecture level city in Shandong
Doraemon Hyakubo Shop	King of Power Saving	It does work for saving electricity, I've bought the kind from elsewhere before that was too simple not **all**. This time to buy this store, but also for the safety. Air conditioning is too much electricity, this use to save a lot of	Jingdong prefecture level city in Shandong
Doraemon Hyakubo Shop	King of Power Saving	Two air conditioners and a refrigerator at home, a month down the electricity bill is almost two hundred dollars, with this after the average savings of more than thirty dollars to spend less money to enjoy more, it's really very good!	Jingdong prefecture level city in Shandong
Doraemon Hyakubo Shop	King of Power Saving	Good. Very good.	Jingdong prefecture level city in Shandong
Doraemon Hyakubo Shop	King of Power Saving	Works great, easy to operate, great, very happy with it.	Jingdong prefecture level city in Shandong
Doraemon Hyakubo Shop	King of Power Saving	Received it and tried it out, it's great, great quality and worth having! Five stars praise!	Jingdong prefecture level city in Shandong
Doraemon Hyakubo Shop	King of Power Saving	Very trustworthy brand, quality is guaranteed, the price is still affordable, quite satisfied!	Jingdong prefecture level city in Shandong
Doraemon Hyakubo Shop	King of Power Saving	Use it before you speak.	Jingdong prefecture level city in Shandong
Doraemon Hyakubo Shop	King of Power Saving	The quality of workmanship is very good and the power saving effect is quite good, I regret not getting it earlier.	Jingdong prefecture level city in Shandong
Doraemon Hyakubo Shop	King of Power Saving	Merchants shipping speed, and customer service introduced the same very good, cost-effective	Jingdong prefecture level city in Shandong
Doraemon Hyakubo Shop	King of Power Saving	Feel a little effect Oh, where the power consumption where to plug, convenient and convenient	Jingdong prefecture level city in Shandong
Doraemon Hyakubo Shop	King of Power Saving	The quality of the imported saver is very good, practical and satisfactory. Cost-effective	Jingdong prefecture level city in Shandong
Doraemon Hyakubo Shop	King of Power Saving	Power Saver is especially good, good and inexpensive, very good	Jingdong prefecture level city in Shandong
Doraemon Hyakubo Shop	King of Power Saving	Fast shipping, awesome quality, affordable price, satisfied.	Jingdong prefecture level city in Shandong
Doraemon Hyakubo Shop	King of Power Saving	Energy saver received, the effect is consistent with the description, recommended to buy!	Jingdong prefecture level city in Shandong
Doraemon Hyakubo Shop	King of Power Saving	It's really good, the quality is great, I recommend it!	Jingdong prefecture level city in Shandong
Doraemon Hyakubo Shop	King of Power Saving	Works well. Inexpensive. Shipping was fast.	Jingdong prefecture level city in Shandong
Doraemon Hyakubo Shop	King of Power Saving	Logistics is fast, works well, energy saving is good	Jingdong prefecture level city in Shandong
Doraemon Hyakubo Shop	King of Power Saving	very happy	Jingdong prefecture level city in Shandong

Doraemon Hyakubo Shop	King of Power Saving	an excellent	Jingdong prefecture level city in Shandong
Doraemon Hyakubo Shop	King of Power Saving	It rained a couple days ago and I just went to get the item back today , not bad, hey I looked at the meter and that red display light takes several seconds to jump, did not make a mistake buying it. After a period of time to evaluate.	Jingdong prefecture level city in Shandong
Doraemon Hyakubo Shop	King of Power Saving	Used more than 10 days to check the power, there is indeed some decline, customer service said, long-term use will save more points!	Jingdong prefecture level city in Shandong
Doraemon Hyakubo Shop	King of Power Saving	It's a proper IQ tax, and it costs electricity.	Jingdong prefecture level city in Shandong
Doraemon Hyakubo Shop	King of Power Saving	I've been using it for 17 days now, it really saves electricity Shipping is fast, you can save a lot in a year	Jingdong prefecture level city in Shandong
Doraemon Hyakubo Shop	King of Power Saving	I've been using it for 17 days and it really saves power Shipping is fast and you don't have to worry about the quality, free returns within 5 years	Jingdong prefecture level city in Shandong
Doraemon Hyakubo Shop	King of Power Saving	It's more regular, scanned the code and it's authentic, and the main thing is that you can try it for 90 days! Plugged in more than ten days, is less than before a few degrees per day. When the time to use a month to see Additional. Good to buy a few more	Jingdong prefecture level city in Shandong
Doraemon Hyakubo Shop	King of Power Saving	Logistics is very fast, fine workmanship, use it and then come back to the comments	Jingdong prefecture level city in Shandong
Doraemon Hyakubo Shop	King of Power Saving	The quality of workmanship is very good and the power saving effect is quite good, I regret not getting it earlier.	Jingdong prefecture level city in Shandong
Doraemon Hyakubo Shop	King of Power Saving	Received the goods, according to the instructions on the operation is very simple, to wait and see the effect, looks good!	Jingdong prefecture level city in Shandong
Doraemon Hyakubo Shop	King of Power Saving	It's really as safe and power-saving as the owner says, and it works great!	Jingdong prefecture level city in Shandong
Doraemon Hyakubo Shop	King of Power Saving	Received the goods, immediately used, looks good, to wait and see how the effect.	Jingdong prefecture level city in Shandong
Doraemon Hyakubo Shop	King of Power Saving	Afraid to buy back did not work, the store said the commitment to 90 days invalid refund, warranty 5 years. Saver also complimentary on the Pacific insurance, it is a lot of peace of mind, there are effects will continue to buy back drops!	Jingdong prefecture level city in Shandong
Doraemon Hyakubo Shop	King of Power Saving	Received the goods and used it, can save electricity a month later to see the effect!	Jingdong prefecture level city in Shandong
Doraemon Hyakubo Shop	King of Power Saving	Fancy 90-day trial, 5-year warranty before the order, the goods received, has been experiencing a week, can really feel the savings, in the introduction of friends also buy this!	Jingdong prefecture level city in Shandong
Doraemon Hyakubo Shop	King of Power Saving	Clothing stores use, neighbors recommended to buy, said a month can save more than 70 yuan, first plugged in to see the effect of the	Jingdong prefecture level city in Shandong
Doraemon Hyakubo Shop	King of Power Saving	A friend's home two months to save more than 80 dollars of electricity, recommended that I buy two, decisive purchase, looking forward to the effect of power saving, waiting for the next month effect.	Jingdong prefecture level city in Shandong
Doraemon Hyakubo Shop	King of Power Saving	The goods have been received, consistent with the description of the advertisement, quite good, I hope that in terms of power saving and purifying the air is really as the merchant said that would be better!	Jingdong prefecture level city in Shandong
Doraemon Hyakubo Shop	King of Power Saving	Power Saving King is really good to use the quality is very good than the physical store a lot cheaper we can rest assured that the purchase recommended to buy!	Jingdong prefecture level city in Shandong
Doraemon Hyakubo Shop	King of Power Saving	Energy saver has been used on the rental house inside the use of the meter is always running fast, with this can really save electricity, easy to use, plugged in can be, very satisfied!	Jingdong prefecture level city in Shandong
Doraemon Hyakubo Shop	King of Power Saving	Just use can not see what effect to,, wait for a period of time in the evaluation	Jingdong prefecture level city in Shandong
Doraemon Hyakubo Shop	King of Power Saving	Live in the 18th floor is too far away from the meter, afraid of no effect, look at the trial 90 days before buying. Currently used for a month, there is an effect, saving 27 dollars in electricity, next month should be able to save more!	Jingdong prefecture level city in Shandong
Doraemon Hyakubo Shop	King of Power Saving	I've been using it for almost a month now and it feels good, so I'll use it again.	Jingdong prefecture level city in Shandong
Doraemon Hyakubo Shop	King of Power Saving	It's ok, the electric bill is really less, originally I remember charging $100 and using it for 10 days. Now it lasts about 15 days. It's still noticeable.	Jingdong prefecture level city in Shandong
Doraemon Hyakubo Shop	King of Power Saving	Fast logistics, good power saving effect, very satisfied	Jingdong prefecture level city in Shandong
Doraemon Hyakubo Shop	King of Power Saving	Has been the second time back to buy, energy saving effect, worth recommending, you can try to see, power saving effect: very good Experience: can save about 30%!	Jingdong prefecture level city in Shandong
Doraemon Hyakubo Shop	King of Power Saving	I don't know how well it works for now	Jingdong prefecture level city in Shandong
Doraemon Hyakubo Shop	King of Power Saving	Factory direct is really good, plugged in and used for half a month did less probably almost 50 degrees of electricity! And the manufacturer's warranty is 5 years.	Jingdong prefecture level city in Shandong
Doraemon Hyakubo Shop	King of Power Saving	It's really effective. The previous month's electricity was $286, but after using the power saver for a month, I have $195 left this month, which is not bad.	Jingdong prefecture level city in Shandong
Doraemon Hyakubo Shop	King of Power Saving	I'll give you a good review and see how it goes.	Jingdong prefecture level city in Shandong
Doraemon Hyakubo Shop	King of Power Saving	Customer service is very professional and patient to guide the installation, the quality of workmanship is very fine. Durable, second repurchase. Really can save oh!	Jingdong prefecture level city in Shandong
Doraemon Hyakubo Shop	King of Power Saving	I've been using it for a month now and my electric bill is over $30 less than last month. I feel it's quite worth buying.	Jingdong prefecture level city in Shandong
Doraemon Hyakubo Shop	King of Power Saving	Small size, does not take up space. It's very good, it does work very effectively, the tool room has a lot of work with a lot of electricity. After installing it, it really reduces a lot of electricity bills. Before the day 30 degrees of electricity left stone, now work down really less a few degrees.	Jingdong prefecture level city in Shandong
Doraemon Hyakubo Shop	King of Power Saving	It does work for saving electricity, I've bought the kind from elsewhere before that was too simple not **all**. This time to buy this store, but also for the safety. Air conditioning is too much electricity, this use to save a lot of	Jingdong prefecture level city in Shandong
Doraemon Hyakubo Shop	King of Power Saving	Two air conditioners and a refrigerator at home, a month down the electricity bill is almost two hundred dollars, with this after the average savings of more than thirty dollars to spend less money to enjoy more, it's really very good!	Jingdong prefecture level city in Shandong
Doraemon Hyakubo Shop	King of Power Saving	Good. Very good.	Jingdong prefecture level city in Shandong
Doraemon Hyakubo Shop	King of Power Saving	Works great, easy to operate, great, very happy with it.	Jingdong prefecture level city in Shandong
Doraemon Hyakubo Shop	King of Power Saving	Received it and tried it out, it's great, great quality and worth having! Five stars praise!	Jingdong prefecture level city in Shandong
Doraemon Hyakubo Shop	King of Power Saving	Very trustworthy brand, quality is guaranteed, the price is still affordable, quite satisfied!	Jingdong prefecture level city in Shandong
Doraemon Hyakubo Shop	King of Power Saving	Use it before you speak.	Jingdong prefecture level city in Shandong
Doraemon Hyakubo Shop	King of Power Saving	The quality of workmanship is very good and the power saving effect is quite good, I regret not getting it earlier.	Jingdong prefecture level city in Shandong
Doraemon Hyakubo Shop	King of Power Saving	Merchants shipping speed, and customer service introduced the same very good, cost-effective	Jingdong prefecture level city in Shandong
Doraemon Hyakubo Shop	King of Power Saving	Feel a little effect Oh, where the power consumption where to plug, convenient and convenient	Jingdong prefecture level city in Shandong
Doraemon Hyakubo Shop	King of Power Saving	The quality of the imported saver is very good, practical and satisfactory. Cost-effective	Jingdong prefecture level city in Shandong
Doraemon Hyakubo Shop	King of Power Saving	Power Saver is especially good, good and inexpensive, very good	Jingdong prefecture level city in Shandong
Doraemon Hyakubo Shop	King of Power Saving	Fast shipping, awesome quality, affordable price, satisfied.	Jingdong prefecture level city in Shandong
Doraemon Hyakubo Shop	King of Power Saving	Energy saver received, the effect is consistent with the description, recommended to buy!	Jingdong prefecture level city in Shandong
Doraemon Hyakubo Shop	King of Power Saving	It's really good, the quality is great, I recommend it!	Jingdong prefecture level city in Shandong

Doraemon Hyakubo Shop	King of Power Saving	Works well. Inexpensive. Shipping was fast.	Jingdong prefecture level city in Shandong
Doraemon Hyakubo Shop	King of Power Saving	Logistics is fast, works well, energy saving is good	Jingdong prefecture level city in Shandong
Doraemon Hyakubo Shop	King of Power Saving	very happy	Jingdong prefecture level city in Shandong
Doraemon Hyakubo Shop	King of Power Saving	an excellent	Jingdong prefecture level city in Shandong
Doraemon Hyakubo Shop	King of Power Saving	It rained a couple days ago and I just went to get the item back today , not bad, hey I looked at the meter and that red display light takes several seconds to jump, did not make a mistake buying it. After a period of time to evaluate.	Jingdong prefecture level city in Shandong
Doraemon Hyakubo Shop	King of Power Saving	Used more than 10 days to check the power, there is indeed some decline, customer service said, long-term use will save more points!	Jingdong prefecture level city in Shandong
Doraemon Hyakubo Shop	King of Power Saving	It's a proper IQ tax, and it costs electricity.	Jingdong prefecture level city in Shandong
Doraemon Hyakubo Shop	King of Power Saving	I've been using it for 17 days now, it really saves electricity Shipping is fast, you can save a lot in a year	Jingdong prefecture level city in Shandong
Doraemon Hyakubo Shop	King of Power Saving	I've been using it for 17 days and it really saves power Shipping is fast and you don't have to worry about the quality, free returns within 5 years	Jingdong prefecture level city in Shandong
Doraemon Hyakubo Shop	King of Power Saving	It's more regular, scanned the code and it's authentic, and the main thing is that you can try it for 90 days! Plugged in more than ten days, is less than before a few degrees per day. When the time to use a month to see Additional. Good to buy a few more	Jingdong prefecture level city in Shandong
Doraemon Hyakubo Shop	King of Power Saving	Logistics is very fast, fine workmanship, use it and then come back to the comments	Jingdong prefecture level city in Shandong
Doraemon Hyakubo Shop	King of Power Saving	The quality of workmanship is very good and the power saving effect is quite good, I regret not getting it earlier.	Jingdong prefecture level city in Shandong
Doraemon Hyakubo Shop	King of Power Saving	Received the goods, according to the instructions on the operation is very simple, to wait and see the effect, looks good!	Jingdong prefecture level city in Shandong
Doraemon Hyakubo Shop	King of Power Saving	It's really as safe and power-saving as the owner says, and it works great!	Jingdong prefecture level city in Shandong
Doraemon Hyakubo Shop	King of Power Saving	Received the goods, immediately used, looks good, to wait and see how the effect.	Jingdong prefecture level city in Shandong
Doraemon Hyakubo Shop	King of Power Saving	Afraid to buy back did not work, the store said the commitment to 90 days invalid refund, warranty 5 years. Saver also complimentary on the Pacific insurance, it is a lot of peace of mind, there are effects will continue to buy back drops!	Jingdong prefecture level city in Shandong
Doraemon Hyakubo Shop	King of Power Saving	Received the goods and used it, can save electricity a month later to see the effect!	Jingdong prefecture level city in Shandong
Doraemon Hyakubo Shop	King of Power Saving	Fancy 90-day trial, 5-year warranty before the order, the goods received, has been experiencing a week, can really feel the savings, in the introduction of friends also buy this!	Jingdong prefecture level city in Shandong
Doraemon Hyakubo Shop	King of Power Saving	Clothing stores use, neighbors recommended to buy, said a month can save more than 70 yuan, first plugged in to see the effect of the	Jingdong prefecture level city in Shandong
Doraemon Hyakubo Shop	King of Power Saving	A friend's home two months to save more than 80 dollars of electricity, recommended that I buy two, decisive purchase, looking forward to the effect of power saving, waiting for the next month effect.	Jingdong prefecture level city in Shandong
Doraemon Hyakubo Shop	King of Power Saving	The goods have been received, consistent with the description of the advertisement, quite good, I hope that in terms of power saving and purifying the air is really as the merchant said that would be better!	Jingdong prefecture level city in Shandong
Doraemon Hyakubo Shop	King of Power Saving	Power Saving King is really good to use the quality is very good than the physical store a lot cheaper we can rest assured that the purchase recommended to buy!	Jingdong prefecture level city in Shandong
Doraemon Hyakubo Shop	King of Power Saving	Energy saver has been used on the rental house inside the use of the meter is always running fast, with this can really save electricity, easy to use, plugged in can be, very satisfied!	Jingdong prefecture level city in Shandong
Doraemon Hyakubo Shop	King of Power Saving	Power Saver Power Saver King is good quality and affordable, very satisfied, worth buying.	Jingdong prefecture level city in Shandong
Doraemon Hyakubo Shop	King of Power Saving	Just use can not see what effect to,, wait for a period of time in the evaluation	Jingdong prefecture level city in Shandong
Doraemon Hyakubo Shop	King of Power Saving	Live in the 18th floor is too far away from the meter, afraid of no effect, look at the trial 90 days before buying. Currently used for a month, there is an effect, saving 27 dollars in electricity, next month should be able to save more!	Jingdong prefecture level city in Shandong
Doraemon Hyakubo Shop	King of Power Saving	I've been using it for almost a month now and it feels good, so I'll use it again.	Jingdong prefecture level city in Shandong
Doraemon Hyakubo Shop	King of Power Saving	It's ok, the electric bill is really less, originally I remember charging $100 and using it for 10 days. Now it lasts about 15 days. It's still noticeable.	Jingdong prefecture level city in Shandong
Doraemon Hyakubo Shop	King of Power Saving	Fast logistics, good power saving effect, very satisfied	Jingdong prefecture level city in Shandong
Doraemon Hyakubo Shop	King of Power Saving	Has been the second time back to buy, energy saving effect, worth recommending, you can try to see, power saving effect: very good Experience: can save about 30%!	Jingdong prefecture level city in Shandong
Doraemon Hyakubo Shop	King of Power Saving	I don't know how well it works for now	Jingdong prefecture level city in Shandong
Doraemon Hyakubo Shop	King of Power Saving	Factory direct is really good, plugged in and used for half a month did less probably almost 50 degrees of electricity! And the manufacturer's warranty is 5 years.	Jingdong prefecture level city in Shandong
Doraemon Hyakubo Shop	King of Power Saving	It's really effective. The previous month's electricity was $286, but after using the power saver for a month, I have $195 left this month, which is not bad.	Jingdong prefecture level city in Shandong
Doraemon Hyakubo Shop	King of Power Saving	I'll give you a good review and see how it goes.	Jingdong prefecture level city in Shandong
Doraemon Hyakubo Shop	King of Power Saving	Customer service is very professional and patient to guide the installation, the quality of workmanship is very fine. Durable, second repurchase. Really can save oh!	Jingdong prefecture level city in Shandong
Doraemon Hyakubo Shop	King of Power Saving	I've been using it for a month now and my electric bill is over $30 less than last month. I feel it's quite worth buying.	Jingdong prefecture level city in Shandong
Doraemon Hyakubo Shop	King of Power Saving	Small size, does not take up space. It's very good, it does work very effectively, the tool room has a lot of work with a lot of electricity. After installing it, it really reduces a lot of electricity bills. Before the day 30 degrees of electricity left stone, now work down really less a few degrees.	Jingdong prefecture level city in Shandong
Doraemon Hyakubo Shop	King of Power Saving	It does work for saving electricity, I've bought the kind from elsewhere before that was too simple not **all**. This time to buy this store, but also for the safety. Air conditioning is too much electricity, this use to save a lot of	Jingdong prefecture level city in Shandong
Doraemon Hyakubo Shop	King of Power Saving	Two air conditioners and a refrigerator at home, a month down the electricity bill is almost two hundred dollars, with this after the average savings of more than thirty dollars to spend less money to enjoy more, it's really very good!	Jingdong prefecture level city in Shandong
Doraemon Hyakubo Shop	King of Power Saving	Good. Very good.	Jingdong prefecture level city in Shandong
Doraemon Hyakubo Shop	King of Power Saving	Works great, easy to operate, great, very happy with it.	Jingdong prefecture level city in Shandong
Doraemon Hyakubo Shop	King of Power Saving	Received it and tried it out, it's great, great quality and worth having! Five stars praise!	Jingdong prefecture level city in Shandong
Doraemon Hyakubo Shop	King of Power Saving	Very trustworthy brand, quality is guaranteed, the price is still affordable, quite satisfied!	Jingdong prefecture level city in Shandong
Doraemon Hyakubo Shop	King of Power Saving	Use it before you speak.	Jingdong prefecture level city in Shandong
Doraemon Hyakubo Shop	King of Power Saving	The quality of workmanship is very good and the power saving effect is quite good, I regret not getting it earlier.	Jingdong prefecture level city in Shandong
Doraemon Hyakubo Shop	King of Power Saving	Merchants shipping speed, and customer service introduced the same very good, cost-effective	Jingdong prefecture level city in Shandong
Doraemon Hyakubo Shop	King of Power Saving	Feel a little effect Oh, where the power consumption where to plug, convenient and convenient	Jingdong prefecture level city in Shandong
Doraemon Hyakubo Shop	King of Power Saving	The quality of the imported saver is very good, practical and satisfactory. Cost-effective	Jingdong prefecture level city in Shandong

Doraemon Hyakubo Shop	King of Power Saving	Power Saver is especially good, good and inexpensive, very good	Jingdong prefecture level city in Shandong
Doraemon Hyakubo Shop	King of Power Saving	Fast shipping, awesome quality, affordable price, satisfied.	Jingdong prefecture level city in Shandong
Doraemon Hyakubo Shop	King of Power Saving	Energy saver received, the effect is consistent with the description, recommended to buy!	Jingdong prefecture level city in Shandong
Doraemon Hyakubo Shop	King of Power Saving	It's really good, the quality is great, I recommend it!	Jingdong prefecture level city in Shandong
Doraemon Hyakubo Shop	King of Power Saving	Works well. Inexpensive. Shipping was fast.	Jingdong prefecture level city in Shandong
Doraemon Hyakubo Shop	King of Power Saving	Logistics is fast, works well, energy saving is good	Jingdong prefecture level city in Shandong
Doraemon Hyakubo Shop	King of Power Saving	very happy	Jingdong prefecture level city in Shandong
Doraemon Hyakubo Shop	King of Power Saving	an excellent	Jingdong prefecture level city in Shandong
Doraemon Hyakubo Shop	King of Power Saving	It rained a couple days ago and I just went to get the item back today , not bad, hey I looked at the meter and that red display light takes several seconds to jump, did not make a mistake buying it. After a period of time to evaluate.	Jingdong prefecture level city in Shandong
Doraemon Hyakubo Shop	King of Power Saving	Used more than 10 days to check the power, there is indeed some decline, customer service said, long-term use will save more points!	Jingdong prefecture level city in Shandong
Doraemon Hyakubo Shop	King of Power Saving	It's a proper IQ tax, and it costs electricity.	Jingdong prefecture level city in Shandong
Doraemon Hyakubo Shop	King of Power Saving	I've been using it for 17 days now, it really saves electricity Shipping is fast, you can save a lot in a year	Jingdong prefecture level city in Shandong
Doraemon Hyakubo Shop	King of Power Saving	I've been using it for 17 days and it really saves power Shipping is fast and you don't have to worry about the quality, free returns within 5 years	Jingdong prefecture level city in Shandong
Doraemon Hyakubo Shop	King of Power Saving	It's more regular, scanned the code and it's authentic, and the main thing is that you can try it for 90 days! Plugged in more than ten days, is less than before a few degrees per day. When the time to use a month to see Additional. Good to buy a few more	Jingdong prefecture level city in Shandong
Doraemon Hyakubo Shop	King of Power Saving	Logistics is very fast, fine workmanship, use it and then come back to the comments	Jingdong prefecture level city in Shandong
Doraemon Hyakubo Shop	King of Power Saving	The quality of workmanship is very good and the power saving effect is quite good, I regret not getting it earlier.	Jingdong prefecture level city in Shandong
Doraemon Hyakubo Shop	King of Power Saving	Received the goods, according to the instructions on the operation is very simple, to wait and see the effect, looks good!	Jingdong prefecture level city in Shandong
Doraemon Hyakubo Shop	King of Power Saving	It's really as safe and power-saving as the owner says, and it works great!	Jingdong prefecture level city in Shandong
Doraemon Hyakubo Shop	King of Power Saving	Received the goods, immediately used, looks good, to wait and see how the effect.	Jingdong prefecture level city in Shandong
Doraemon Hyakubo Shop	King of Power Saving	Afraid to buy back did not work, the store said the commitment to 90 days invalid refund, warranty 5 years. Saver also complimentary on the Pacific insurance, it is a lot of peace of mind, there are effects will continue to buy back drops!	Jingdong prefecture level city in Shandong
Doraemon Hyakubo Shop	King of Power Saving	Received the goods and used it, can save electricity a month later to see the effect!	Jingdong prefecture level city in Shandong
Doraemon Hyakubo Shop	King of Power Saving	Fancy 90-day trial, 5-year warranty before the order, the goods received, has been experiencing a week, can really feel the savings, in the introduction of friends also buy this!	Jingdong prefecture level city in Shandong
Doraemon Hyakubo Shop	King of Power Saving	Clothing stores use, neighbors recommended to buy, said a month can save more than 70 yuan, first plugged in to see the effect of the	Jingdong prefecture level city in Shandong
Doraemon Hyakubo Shop	King of Power Saving	A friend's home two months to save more than 80 dollars of electricity, recommended that I buy two, decisive purchase, looking forward to the effect of power saving, waiting for the next month effect.	Jingdong prefecture level city in Shandong
Doraemon Hyakubo Shop	King of Power Saving	The goods have been received, consistent with the description of the advertisement, quite good, I hope that in terms of power saving and purifying the air is really as the merchant said that would be better!	Jingdong prefecture level city in Shandong
Doraemon Hyakubo Shop	King of Power Saving	Power Saving King is really good to use the quality is very good than the physical store a lot cheaper we can rest assured that the purchase recommended to buy!	Jingdong prefecture level city in Shandong
Doraemon Hyakubo Shop	King of Power Saving	Energy saver has been used on the rental house inside the use of the meter is always running fast, with this can really save electricity, easy to use, plugged in can be, very satisfied!	Jingdong prefecture level city in Shandong
Doraemon Hyakubo Shop	King of Power Saving	Power Saver Power Saver King is good quality and affordable, very satisfied, worth buying.	Jingdong prefecture level city in Shandong
Doraemon Hyakubo Shop	King of Power Saving	Just use can not see what effect to,, wait for a period of time in the evaluation	Jingdong prefecture level city in Shandong
Doraemon Hyakubo Shop	King of Power Saving	Live in the 18th floor is too far away from the meter, afraid of no effect, look at the trial 90 days before buying. Currently used for a month, there is an effect, saving 27 dollars in electricity, next month should be able to save more!	Jingdong prefecture level city in Shandong
Doraemon Hyakubo Shop	King of Power Saving	I've been using it for almost a month now and it feels good, so I'll use it again.	Jingdong prefecture level city in Shandong
Doraemon Hyakubo Shop	King of Power Saving	It's ok, the electric bill is really less, originally I remember charging $100 and using it for 10 days. Now it lasts about 15 days. It's still noticeable.	Jingdong prefecture level city in Shandong
Doraemon Hyakubo Shop	King of Power Saving	Fast logistics, good power saving effect, very satisfied	Jingdong prefecture level city in Shandong
Doraemon Hyakubo Shop	King of Power Saving	Has been the second time back to buy, energy saving effect, worth recommending, you can try to see, power saving effect: very good Experience: can save about 30%!	Jingdong prefecture level city in Shandong
Doraemon Hyakubo Shop	King of Power Saving	I don't know how well it works for now	Jingdong prefecture level city in Shandong
Doraemon Hyakubo Shop	King of Power Saving	Factory direct is really good, plugged in and used for half a month did less probably almost 50 degrees of electricity! And the manufacturer's warranty is 5 years.	Jingdong prefecture level city in Shandong
Doraemon Hyakubo Shop	King of Power Saving	It's really effective. The previous month's electricity was $286, but after using the power saver for a month, I have $195 left this month, which is not bad.	Jingdong prefecture level city in Shandong
Doraemon Hyakubo Shop	King of Power Saving	I'll give you a good review and see how it goes.	Jingdong prefecture level city in Shandong
Doraemon Hyakubo Shop	King of Power Saving	Customer service is very professional and patient to guide the installation, the quality of workmanship is very fine. Durable, second repurchase. Really can save oh!	Jingdong prefecture level city in Shandong
Doraemon Hyakubo Shop	King of Power Saving	I've been using it for a month now and my electric bill is over $30 less than last month. I feel it's quite worth buying.	Jingdong prefecture level city in Shandong
Doraemon Hyakubo Shop	King of Power Saving	Small size, does not take up space. It's very good, it does work very effectively, the tool room has a lot of work with a lot of electricity. After installing it, it really reduces a lot of electricity bills. Before the day 30 degrees of electricity left stone, now work down really less a few degrees.	Jingdong prefecture level city in Shandong
Doraemon Hyakubo Shop	King of Power Saving	It does work for saving electricity, I've bought the kind from elsewhere before that was too simple not **all**. This time to buy this store, but also for the safety. Air conditioning is too much electricity, this use to save a lot of	Jingdong prefecture level city in Shandong
Doraemon Hyakubo Shop	King of Power Saving	Two air conditioners and a refrigerator at home, a month down the electricity bill is almost two hundred dollars, with this after the average savings of more than thirty dollars to spend less money to enjoy more, it's really very good!	Jingdong prefecture level city in Shandong
Doraemon Hyakubo Shop	King of Power Saving	Good. Very good.	Jingdong prefecture level city in Shandong
Doraemon Hyakubo Shop	King of Power Saving	Works great, easy to operate, great, very happy with it.	Jingdong prefecture level city in Shandong
Doraemon Hyakubo Shop	King of Power Saving	Received it and tried it out, it's great, great quality and worth having! Five stars praise!	Jingdong prefecture level city in Shandong
Doraemon Hyakubo Shop	King of Power Saving	Very trustworthy brand, quality is guaranteed, the price is still affordable, quite satisfied!	Jingdong prefecture level city in Shandong
Doraemon Hyakubo Shop	King of Power Saving	Use it before you speak.	Jingdong prefecture level city in Shandong

Doraemon Hyakubo Shop	King of Power Saving	The quality of workmanship is very good and the power saving effect is quite good, I regret not getting it earlier.	Jingdong prefecture level city in Shandong
Doraemon Hyakubo Shop	King of Power Saving	Merchants shipping speed, and customer service introduced the same very good, cost-effective	Jingdong prefecture level city in Shandong
Doraemon Hyakubo Shop	King of Power Saving	Feel a little effect Oh, where the power consumption where to plug, convenient and convenient	Jingdong prefecture level city in Shandong
Doraemon Hyakubo Shop	King of Power Saving	The quality of the imported saver is very good, practical and satisfactory. Cost-effective	Jingdong prefecture level city in Shandong
Doraemon Hyakubo Shop	King of Power Saving	Power Saver is especially good, good and inexpensive, very good	Jingdong prefecture level city in Shandong
Doraemon Hyakubo Shop	King of Power Saving	Fast shipping, awesome quality, affordable price, satisfied.	Jingdong prefecture level city in Shandong
Doraemon Hyakubo Shop	King of Power Saving	Energy saver received, the effect is consistent with the description, recommended to buy!	Jingdong prefecture level city in Shandong
Doraemon Hyakubo Shop	King of Power Saving	It's really good, the quality is great, I recommend it!	Jingdong prefecture level city in Shandong
Doraemon Hyakubo Shop	King of Power Saving	Works well. Inexpensive. Shipping was fast.	Jingdong prefecture level city in Shandong
Doraemon Hyakubo Shop	King of Power Saving	Logistics is fast, works well, energy saving is good	Jingdong prefecture level city in Shandong
Doraemon Hyakubo Shop	King of Power Saving	very happy	Jingdong prefecture level city in Shandong
Doraemon Hyakubo Shop	King of Power Saving	an excellent	Jingdong prefecture level city in Shandong
Doraemon Hyakubo Shop	King of Power Saving	It rained a couple days ago and I just went to get the item back today , not bad, hey I looked at the meter and that red display light takes several seconds to jump, did not make a mistake buying it. After a period of time to evaluate.	Jingdong prefecture level city in Shandong
Doraemon Hyakubo Shop	King of Power Saving	Used more than 10 days to check the power, there is indeed some decline, customer service said, long-term use will save more points!	Jingdong prefecture level city in Shandong
Doraemon Hyakubo Shop	King of Power Saving	It's a proper IQ tax, and it costs electricity.	Jingdong prefecture level city in Shandong
Doraemon Hyakubo Shop	King of Power Saving	I've been using it for 17 days now, it really saves electricity Shipping is fast, you can save a lot in a year	Jingdong prefecture level city in Shandong
Doraemon Hyakubo Shop	King of Power Saving	I've been using it for 17 days and it really saves power Shipping is fast and you don't have to worry about the quality, free returns within 5 years	Jingdong prefecture level city in Shandong
Doraemon Hyakubo Shop	King of Power Saving	It's more regular, scanned the code and it's authentic, and the main thing is that you can try it for 90 days! Plugged in more than ten days, is less than before a few degrees per day. When the time to use a month to see Additional. Good to buy a few more	Jingdong prefecture level city in Shandong
Doraemon Hyakubo Shop	King of Power Saving	Logistics is very fast, fine workmanship, use it and then come back to the comments	Jingdong prefecture level city in Shandong
Doraemon Hyakubo Shop	King of Power Saving	The quality of workmanship is very good and the power saving effect is quite good, I regret not getting it earlier.	Jingdong prefecture level city in Shandong
Doraemon Hyakubo Shop	King of Power Saving	Received the goods, according to the instructions on the operation is very simple, to wait and see the effect, looks good!	Jingdong prefecture level city in Shandong
Doraemon Hyakubo Shop	King of Power Saving	It's really as safe and power-saving as the owner says, and it works great!	Jingdong prefecture level city in Shandong
Doraemon Hyakubo Shop	King of Power Saving	Received the goods, immediately used, looks good, to wait and see how the effect.	Jingdong prefecture level city in Shandong
Doraemon Hyakubo Shop	King of Power Saving	Afraid to buy back did not work, the store said the commitment to 90 days invalid refund, warranty 5 years. Saver also complimentary on the Pacific insurance, it is a lot of peace of mind, there are effects will continue to buy back drops!	Jingdong prefecture level city in Shandong
Doraemon Hyakubo Shop	King of Power Saving	Received the goods and used it, can save electricity a month later to see the effect!	Jingdong prefecture level city in Shandong
Doraemon Hyakubo Shop	King of Power Saving	Fancy 90-day trial, 5-year warranty before the order, the goods received, has been experiencing a week, can really feel the savings, in the introduction of friends also buy this!	Jingdong prefecture level city in Shandong
Doraemon Hyakubo Shop	King of Power Saving	Clothing stores use, neighbors recommended to buy, said a month can save more than 70 yuan, first plugged in to see the effect of the	Jingdong prefecture level city in Shandong
Doraemon Hyakubo Shop	King of Power Saving	A friend's home two months to save more than 80 dollars of electricity, recommended that I buy two, decisive purchase, looking forward to the effect of power saving, waiting for the next month effect.	Jingdong prefecture level city in Shandong
Doraemon Hyakubo Shop	King of Power Saving	The goods have been received, consistent with the description of the advertisement, quite good, I hope that in terms of power saving and purifying the air is really as the merchant said that would be better!	Jingdong prefecture level city in Shandong
Doraemon Hyakubo Shop	King of Power Saving	Power Saving King is really good to use the quality is very good than the physical store a lot cheaper we can rest assured that the purchase recommended to buy!	Jingdong prefecture level city in Shandong
Doraemon Hyakubo Shop	King of Power Saving	Energy saver has been used on the rental house inside the use of the meter is always running fast, with this can really save electricity, easy to use, plugged in can be, very satisfied!	Jingdong prefecture level city in Shandong
Doraemon Hyakubo Shop	King of Power Saving	Power Saver Power Saver King is good quality and affordable, very satisfied, worth buying.	Jingdong prefecture level city in Shandong
Doraemon Hyakubo Shop	King of Power Saving	Just use can not see what effect to,, wait for a period of time in the evaluation	Jingdong prefecture level city in Shandong
Doraemon Hyakubo Shop	King of Power Saving	Live in the 18th floor is too far away from the meter, afraid of no effect, look at the trial 90 days before buying. Currently used for a month, there is an effect, saving 27 dollars in electricity, next month should be able to save more!	Jingdong prefecture level city in Shandong
Doraemon Hyakubo Shop	King of Power Saving	I've been using it for almost a month now and it feels good, so I'll use it again.	Jingdong prefecture level city in Shandong
Doraemon Hyakubo Shop	King of Power Saving	It's ok, the electric bill is really less, originally I remember charging $100 and using it for 10 days. Now it lasts about 15 days. It's still noticeable.	Jingdong prefecture level city in Shandong
Doraemon Hyakubo Shop	King of Power Saving	Fast logistics, good power saving effect, very satisfied	Jingdong prefecture level city in Shandong
Doraemon Hyakubo Shop	King of Power Saving	Has been the second time back to buy, energy saving effect, worth recommending, you can try to see, power saving effect: very good Experience: can save about 30%!	Jingdong prefecture level city in Shandong
Doraemon Hyakubo Shop	King of Power Saving	I don't know how well it works for now	Jingdong prefecture level city in Shandong
Doraemon Hyakubo Shop	King of Power Saving	Factory direct is really good, plugged in and used for half a month did less probably almost 50 degrees of electricity! And the manufacturer's warranty is 5 years.	Jingdong prefecture level city in Shandong
Doraemon Hyakubo Shop	King of Power Saving	It's really effective. The previous month's electricity was $286, but after using the power saver for a month, I have $195 left this month, which is not bad.	Jingdong prefecture level city in Shandong
Doraemon Hyakubo Shop	King of Power Saving	I'll give you a good review and see how it goes.	Jingdong prefecture level city in Shandong
Doraemon Hyakubo Shop	King of Power Saving	Customer service is very professional and patient to guide the installation, the quality of workmanship is very fine. Durable, second repurchase. Really can save oh!	Jingdong prefecture level city in Shandong
Doraemon Hyakubo Shop	King of Power Saving	I've been using it for a month now and my electric bill is over $30 less than last month. I feel it's quite worth buying.	Jingdong prefecture level city in Shandong
Doraemon Hyakubo Shop	King of Power Saving	Small size, does not take up space. It's very good, it does work very effectively, the tool room has a lot of work with a lot of electricity. After installing it, it really reduces a lot of electricity bills. Before the day 30 degrees of electricity left stone, now work down really less a few degrees.	Jingdong prefecture level city in Shandong
Doraemon Hyakubo Shop	King of Power Saving	It does work for saving electricity, I've bought the kind from elsewhere before that was too simple not **all**. This time to buy this store, but also for the safety. Air conditioning is too much electricity, this use to save a lot of	Jingdong prefecture level city in Shandong
Doraemon Hyakubo Shop	King of Power Saving	Two air conditioners and a refrigerator at home, a month down the electricity bill is almost two hundred dollars, with this after the average savings of more than thirty dollars to spend less money to enjoy more, it's really very good!	Jingdong prefecture level city in Shandong
Doraemon Hyakubo Shop	King of Power Saving	Good. Very good.	Jingdong prefecture level city in Shandong

Doraemon Hyakubo Shop	King of Power Saving	Works great, easy to operate, great, very happy with it.	Jingdong prefecture level city in Shandong
Doraemon Hyakubo Shop	King of Power Saving	Received it and tried it out, it's great, great quality and worth having! Five stars praise!	Jingdong prefecture level city in Shandong
Doraemon Hyakubo Shop	King of Power Saving	Very trustworthy brand, quality is guaranteed, the price is still affordable, quite satisfied!	Jingdong prefecture level city in Shandong
Doraemon Hyakubo Shop	King of Power Saving	Use it before you speak.	Jingdong prefecture level city in Shandong
Doraemon Hyakubo Shop	King of Power Saving	The quality of workmanship is very good and the power saving effect is quite good, I regret not getting it earlier.	Jingdong prefecture level city in Shandong
Doraemon Hyakubo Shop	King of Power Saving	Merchants shipping speed, and customer service introduced the same very good, cost-effective	Jingdong prefecture level city in Shandong
Doraemon Hyakubo Shop	King of Power Saving	Feel a little effect Oh, where the power consumption where to plug, convenient and convenient	Jingdong prefecture level city in Shandong
Doraemon Hyakubo Shop	King of Power Saving	The quality of the imported saver is very good, practical and satisfactory. Cost-effective	Jingdong prefecture level city in Shandong
Doraemon Hyakubo Shop	King of Power Saving	Power Saver is especially good, good and inexpensive, very good	Jingdong prefecture level city in Shandong
Doraemon Hyakubo Shop	King of Power Saving	Fast shipping, awesome quality, affordable price, satisfied.	Jingdong prefecture level city in Shandong
Doraemon Hyakubo Shop	King of Power Saving	Energy saver received, the effect is consistent with the description, recommended to buy!	Jingdong prefecture level city in Shandong
Doraemon Hyakubo Shop	King of Power Saving	It's really good, the quality is great, I recommend it!	Jingdong prefecture level city in Shandong
Doraemon Hyakubo Shop	King of Power Saving	Works well. Inexpensive. Shipping was fast.	Jingdong prefecture level city in Shandong
Doraemon Hyakubo Shop	King of Power Saving	Logistics is fast, works well, energy saving is good	Jingdong prefecture level city in Shandong
Doraemon Hyakubo Shop	King of Power Saving	very happy	Jingdong prefecture level city in Shandong
Doraemon Hyakubo Shop	King of Power Saving	an excellent	Jingdong prefecture level city in Shandong
Doraemon Hyakubo Shop	King of Power Saving	It rained a couple days ago and I just went to get the item back today , not bad, hey I looked at the meter and that red display light takes several seconds to jump, did not make a mistake buying it. After a period of time to evaluate.	Jingdong prefecture level city in Shandong
Doraemon Hyakubo Shop	King of Power Saving	Used more than 10 days to check the power, there is indeed some decline, customer service said, long-term use will save more points!	Jingdong prefecture level city in Shandong
Doraemon Hyakubo Shop	King of Power Saving	It's a proper IQ tax, and it costs electricity.	Jingdong prefecture level city in Shandong
Doraemon Hyakubo Shop	King of Power Saving	I've been using it for 17 days now, it really saves electricity Shipping is fast, you can save a lot in a year	Jingdong prefecture level city in Shandong
Doraemon Hyakubo Shop	King of Power Saving	I've been using it for 17 days and it really saves power Shipping is fast and you don't have to worry about the quality, free returns within 5 years	Jingdong prefecture level city in Shandong
Doraemon Hyakubo Shop	King of Power Saving	It's more regular, scanned the code and it's authentic, and the main thing is that you can try it for 90 days! Plugged in more than ten days, is less than before a few degrees per day. When the time to use a month to see Additional. Good to buy a few more	Jingdong prefecture level city in Shandong
Doraemon Hyakubo Shop	King of Power Saving	Logistics is very fast, fine workmanship, use it and then come back to the comments	Jingdong prefecture level city in Shandong
Doraemon Hyakubo Shop	King of Power Saving	The quality of workmanship is very good and the power saving effect is quite good, I regret not getting it earlier.	Jingdong prefecture level city in Shandong
Doraemon Hyakubo Shop	King of Power Saving	Received the goods, according to the instructions on the operation is very simple, to wait and see the effect, looks good!	Jingdong prefecture level city in Shandong
Doraemon Hyakubo Shop	King of Power Saving	It's really as safe and power-saving as the owner says, and it works great!	Jingdong prefecture level city in Shandong
Doraemon Hyakubo Shop	King of Power Saving	Received the goods, immediately used, looks good, to wait and see how the effect.	Jingdong prefecture level city in Shandong
Doraemon Hyakubo Shop	King of Power Saving	Afraid to buy back did not work, the store said the commitment to 90 days invalid refund, warranty 5 years. Saver also complimentary on the Pacific insurance, it is a lot of peace of mind, there are effects will continue to buy back drops!	Jingdong prefecture level city in Shandong
Doraemon Hyakubo Shop	King of Power Saving	Received the goods and used it, can save electricity a month later to see the effect!	Jingdong prefecture level city in Shandong
Doraemon Hyakubo Shop	King of Power Saving	Fancy 90-day trial, 5-year warranty before the order, the goods received, has been experiencing a week, can really feel the savings, in the introduction of friends also buy this!	Jingdong prefecture level city in Shandong
Doraemon Hyakubo Shop	King of Power Saving	Clothing stores use, neighbors recommended to buy, said a month can save more than 70 yuan, first plugged in to see the effect of the	Jingdong prefecture level city in Shandong
Doraemon Hyakubo Shop	King of Power Saving	A friend's home two months to save more than 80 dollars of electricity, recommended that I buy two, decisive purchase, looking forward to the effect of power saving, waiting for the next month effect.	Jingdong prefecture level city in Shandong
Doraemon Hyakubo Shop	King of Power Saving	The goods have been received, consistent with the description of the advertisement, quite good, I hope that in terms of power saving and purifying the air is really as the merchant said that would be better!	Jingdong prefecture level city in Shandong
Doraemon Hyakubo Shop	King of Power Saving	Power Saving King is really good to use the quality is very good than the physical store a lot cheaper we can rest assured that the purchase recommended to buy!	Jingdong prefecture level city in Shandong
Doraemon Hyakubo Shop	King of Power Saving	Energy saver has been used on the rental house inside the use of the meter is always running fast, with this can really save electricity, easy to use, plugged in can be, very satisfied!	Jingdong prefecture level city in Shandong
Doraemon Hyakubo Shop	King of Power Saving	Power Saver Power Saver King is good quality and affordable, very satisfied, worth buying.	Jingdong prefecture level city in Shandong
Doraemon Hyakubo Shop	King of Power Saving	Just use can not see what effect to,, wait for a period of time in the evaluation	Jingdong prefecture level city in Shandong
Doraemon Hyakubo Shop	King of Power Saving	Live in the 18th floor is too far away from the meter, afraid of no effect, look at the trial 90 days before buying. Currently used for a month, there is an effect, saving 27 dollars in electricity, next month should be able to save more!	Jingdong prefecture level city in Shandong
Doraemon Hyakubo Shop	King of Power Saving	I've been using it for almost a month now and it feels good, so I'll use it again.	Jingdong prefecture level city in Shandong
Doraemon Hyakubo Shop	King of Power Saving	It's ok, the electric bill is really less, originally I remember charging $100 and using it for 10 days. Now it lasts about 15 days. It's still noticeable.	Jingdong prefecture level city in Shandong
Doraemon Hyakubo Shop	King of Power Saving	Fast logistics, good power saving effect, very satisfied	Jingdong prefecture level city in Shandong
Doraemon Hyakubo Shop	King of Power Saving	Has been the second time back to buy, energy saving effect, worth recommending, you can try to see, power saving effect: very good Experience: can save about 30%!	Jingdong prefecture level city in Shandong
Doraemon Hyakubo Shop	King of Power Saving	I don't know how well it works for now	Jingdong prefecture level city in Shandong
Doraemon Hyakubo Shop	King of Power Saving	Factory direct is really good, plugged in and used for half a month did less probably almost 50 degrees of electricity! And the manufacturer's warranty is 5 years.	Jingdong prefecture level city in Shandong
Doraemon Hyakubo Shop	King of Power Saving	It's really effective. The previous month's electricity was $286, but after using the power saver for a month, I have $195 left this month, which is not bad.	Jingdong prefecture level city in Shandong
Doraemon Hyakubo Shop	King of Power Saving	I'll give you a good review and see how it goes.	Jingdong prefecture level city in Shandong
Doraemon Hyakubo Shop	King of Power Saving	Customer service is very professional and patient to guide the installation, the quality of workmanship is very fine. Durable, second repurchase. Really can save oh!	Jingdong prefecture level city in Shandong
Doraemon Hyakubo Shop	King of Power Saving	I've been using it for a month now and my electric bill is over $30 less than last month. I feel it's quite worth buying.	Jingdong prefecture level city in Shandong
Doraemon Hyakubo Shop	King of Power Saving	Small size, does not take up space. It's very good, it does work very effectively, the tool room has a lot of work with a lot of electricity. After installing it, it really reduces a lot of electricity bills. Before the day 30 degrees of electricity left stone, now work down really less a few degrees.	Jingdong prefecture level city in Shandong

Doraemon Hyakubo Shop	King of Power Saving	It does work for saving electricity, I've bought the kind from elsewhere before that was too simple not **all**. This time to buy this store, but also for the safety. Air conditioning is too much electricity, this use to save a lot of	Jingdong prefecture level city in Shandong
Doraemon Hyakubo Shop	King of Power Saving	Two air conditioners and a refrigerator at home, a month down the electricity bill is almost two hundred dollars, with this after the average savings of more than thirty dollars to spend less money to enjoy more, it's really very good!	Jingdong prefecture level city in Shandong
Doraemon Hyakubo Shop	King of Power Saving	Good. Very good.	Jingdong prefecture level city in Shandong
Doraemon Hyakubo Shop	King of Power Saving	Works great, easy to operate, great, very happy with it.	Jingdong prefecture level city in Shandong
Doraemon Hyakubo Shop	King of Power Saving	Received it and tried it out, it's great, great quality and worth having! Five stars praise!	Jingdong prefecture level city in Shandong
Doraemon Hyakubo Shop	King of Power Saving	Very trustworthy brand, quality is guaranteed, the price is still affordable, quite satisfied!	Jingdong prefecture level city in Shandong
Doraemon Hyakubo Shop	King of Power Saving	Use it before you speak.	Jingdong prefecture level city in Shandong
Doraemon Hyakubo Shop	King of Power Saving	The quality of workmanship is very good and the power saving effect is quite good, I regret not getting it earlier.	Jingdong prefecture level city in Shandong
Doraemon Hyakubo Shop	King of Power Saving	Merchants shipping speed, and customer service introduced the same very good, cost-effective	Jingdong prefecture level city in Shandong
Doraemon Hyakubo Shop	King of Power Saving	Feel a little effect Oh, where the power consumption where to plug, convenient and convenient	Jingdong prefecture level city in Shandong
Doraemon Hyakubo Shop	King of Power Saving	The quality of the imported saver is very good, practical and satisfactory. Cost-effective	Jingdong prefecture level city in Shandong
Doraemon Hyakubo Shop	King of Power Saving	Power Saver is especially good, good and inexpensive, very good	Jingdong prefecture level city in Shandong
Doraemon Hyakubo Shop	King of Power Saving	Fast shipping, awesome quality, affordable price, satisfied.	Jingdong prefecture level city in Shandong
Doraemon Hyakubo Shop	King of Power Saving	Energy saver received, the effect is consistent with the description, recommended to buy!	Jingdong prefecture level city in Shandong
Doraemon Hyakubo Shop	King of Power Saving	It's really good, the quality is great, I recommend it!	Jingdong prefecture level city in Shandong
Doraemon Hyakubo Shop	King of Power Saving	Works well. Inexpensive. Shipping was fast.	Jingdong prefecture level city in Shandong
Doraemon Hyakubo Shop	King of Power Saving	Logistics is fast, works well, energy saving is good	Jingdong prefecture level city in Shandong
Doraemon Hyakubo Shop	King of Power Saving	very happy	Jingdong prefecture level city in Shandong
Doraemon Hyakubo Shop	King of Power Saving	an excellent	Jingdong prefecture level city in Shandong
Doraemon Hyakubo Shop	King of Power Saving	It rained a couple days ago and I just went to get the item back today , not bad, hey I looked at the meter and that red display light takes several seconds to jump, did not make a mistake buying it. After a period of time to evaluate.	Jingdong prefecture level city in Shandong
Doraemon Hyakubo Shop	King of Power Saving	Used more than 10 days to check the power, there is indeed some decline, customer service said, long-term use will save more points!	Jingdong prefecture level city in Shandong
Doraemon Hyakubo Shop	King of Power Saving	It's a proper IQ tax, and it costs electricity.	Jingdong prefecture level city in Shandong
Doraemon Hyakubo Shop	King of Power Saving	I've been using it for 17 days now, it really saves electricity Shipping is fast, you can save a lot in a year	Jingdong prefecture level city in Shandong
Doraemon Hyakubo Shop	King of Power Saving	I've been using it for 17 days and it really saves power Shipping is fast and you don't have to worry about the quality, free returns within 5 years	Jingdong prefecture level city in Shandong
Doraemon Hyakubo Shop	King of Power Saving	It's more regular, scanned the code and it's authentic, and the main thing is that you can try it for 90 days! Plugged in more than ten days, is less than before a few degrees per day. When the time to use a month to see Additional. Good to buy a few more	Jingdong prefecture level city in Shandong
Doraemon Hyakubo Shop	King of Power Saving	Logistics is very fast, fine workmanship, use it and then come back to the comments	Jingdong prefecture level city in Shandong
Doraemon Hyakubo Shop	King of Power Saving	The quality of workmanship is very good and the power saving effect is quite good, I regret not getting it earlier.	Jingdong prefecture level city in Shandong
Doraemon Hyakubo Shop	King of Power Saving	Received the goods, according to the instructions on the operation is very simple, to wait and see the effect, looks good!	Jingdong prefecture level city in Shandong
Doraemon Hyakubo Shop	King of Power Saving	It's really as safe and power-saving as the owner says, and it works great!	Jingdong prefecture level city in Shandong
Doraemon Hyakubo Shop	King of Power Saving	Received the goods, immediately used, looks good, to wait and see how the effect.	Jingdong prefecture level city in Shandong
Doraemon Hyakubo Shop	King of Power Saving	Afraid to buy back did not work, the store said the commitment to 90 days invalid refund, warranty 5 years. Saver also complimentary on the Pacific insurance, it is a lot of peace of mind, there are effects will continue to buy back drops!	Jingdong prefecture level city in Shandong
Doraemon Hyakubo Shop	King of Power Saving	Received the goods and used it, can save electricity a month later to see the effect!	Jingdong prefecture level city in Shandong
Doraemon Hyakubo Shop	King of Power Saving	Fancy 90-day trial, 5-year warranty before the order, the goods received, has been experiencing a week, can really feel the savings, in the introduction of friends also buy this!	Jingdong prefecture level city in Shandong
Doraemon Hyakubo Shop	King of Power Saving	Clothing stores use, neighbors recommended to buy, said a month can save more than 70 yuan, first plugged in to see the effect of the	Jingdong prefecture level city in Shandong
Doraemon Hyakubo Shop	King of Power Saving	A friend's home two months to save more than 80 dollars of electricity, recommended that I buy two, decisive purchase, looking forward to the effect of power saving, waiting for the next month effect.	Jingdong prefecture level city in Shandong
Doraemon Hyakubo Shop	King of Power Saving	The goods have been received, consistent with the description of the advertisement, quite good, I hope that in terms of power saving and purifying the air is really as the merchant said that would be better!	Jingdong prefecture level city in Shandong
Doraemon Hyakubo Shop	King of Power Saving	Power Saving King is really good to use the quality is very good than the physical store a lot cheaper we can rest assured that the purchase recommended to buy!	Jingdong prefecture level city in Shandong
Doraemon Hyakubo Shop	King of Power Saving	Energy saver has been used on the rental house inside the use of the meter is always running fast, with this can really save electricity, easy to use, plugged in can be, very satisfied!	Jingdong prefecture level city in Shandong
Doraemon Hyakubo Shop	King of Power Saving	Power Saver Power Saver King is good quality and affordable, very satisfied, worth buying.	Jingdong prefecture level city in Shandong
Doraemon Hyakubo Shop	King of Power Saving	Just use can not see what effect to,, wait for a period of time in the evaluation	Jingdong prefecture level city in Shandong
Doraemon Hyakubo Shop	King of Power Saving	Live in the 18th floor is too far away from the meter, afraid of no effect, look at the trial 90 days before buying. Currently used for a month, there is an effect, saving 27 dollars in electricity, next month should be able to save more!	Jingdong prefecture level city in Shandong
Doraemon Hyakubo Shop	King of Power Saving	I've been using it for almost a month now and it feels good, so I'll use it again.	Jingdong prefecture level city in Shandong
Doraemon Hyakubo Shop	King of Power Saving	It's ok, the electric bill is really less, originally I remember charging $100 and using it for 10 days. Now it lasts about 15 days. It's still noticeable.	Jingdong prefecture level city in Shandong
Doraemon Hyakubo Shop	King of Power Saving	Fast logistics, good power saving effect, very satisfied	Jingdong prefecture level city in Shandong
Doraemon Hyakubo Shop	King of Power Saving	Has been the second time back to buy, energy saving effect, worth recommending, you can try to see, power saving effect: very good Experience: can save about 30%!	Jingdong prefecture level city in Shandong
Doraemon Hyakubo Shop	King of Power Saving	I don't know how well it works for now	Jingdong prefecture level city in Shandong
Doraemon Hyakubo Shop	King of Power Saving	Factory direct is really good, plugged in and used for half a month did less probably almost 50 degrees of electricity! And the manufacturer's warranty is 5 years.	Jingdong prefecture level city in Shandong
Doraemon Hyakubo Shop	King of Power Saving	It's really effective. The previous month's electricity was $286, but after using the power saver for a month, I have $195 left this month, which is not bad.	Jingdong prefecture level city in Shandong

Doraemon Hyakubo Shop	King of Power Saving	I'll give you a good review and see how it goes.	Jingdong prefecture level city in Shandong
Doraemon Hyakubo Shop	King of Power Saving	Customer service is very professional and patient to guide the installation, the quality of workmanship is very fine. Durable, second repurchase. Really can save oh!	Jingdong prefecture level city in Shandong
Doraemon Hyakubo Shop	King of Power Saving	I've been using it for a month now and my electric bill is over $30 less than last month. I feel it's quite worth buying.	Jingdong prefecture level city in Shandong
Doraemon Hyakubo Shop	King of Power Saving	Small size, does not take up space. It's very good, it does work very effectively, the tool room has a lot of work with a lot of electricity. After installing it, it really reduces a lot of electricity bills. Before the day 30 degrees of electricity left stone, now work down really less a few degrees.	Jingdong prefecture level city in Shandong
Doraemon Hyakubo Shop	King of Power Saving	It does work for saving electricity, I've bought the kind from elsewhere before that was too simple not **all**. This time to buy this store, but also for the safety. Air conditioning is too much electricity, this use to save a lot of	Jingdong prefecture level city in Shandong
Doraemon Hyakubo Shop	King of Power Saving	Two air conditioners and a refrigerator at home, a month down the electricity bill is almost two hundred dollars, with this after the average savings of more than thirty dollars to spend less money to enjoy more, it's really very good!	Jingdong prefecture level city in Shandong
Doraemon Hyakubo Shop	King of Power Saving	Good. Very good.	Jingdong prefecture level city in Shandong
Doraemon Hyakubo Shop	King of Power Saving	Works great, easy to operate, great, very happy with it.	Jingdong prefecture level city in Shandong
Doraemon Hyakubo Shop	King of Power Saving	Received it and tried it out, it's great, great quality and worth having! Five stars praise!	Jingdong prefecture level city in Shandong
Doraemon Hyakubo Shop	King of Power Saving	Very trustworthy brand, quality is guaranteed, the price is still affordable, quite satisfied!	Jingdong prefecture level city in Shandong
Doraemon Hyakubo Shop	King of Power Saving	Use it before you speak.	Jingdong prefecture level city in Shandong
Doraemon Hyakubo Shop	King of Power Saving	The quality of workmanship is very good and the power saving effect is quite good, I regret not getting it earlier.	Jingdong prefecture level city in Shandong
Doraemon Hyakubo Shop	King of Power Saving	Merchants shipping speed, and customer service introduced the same very good, cost-effective	Jingdong prefecture level city in Shandong
Doraemon Hyakubo Shop	King of Power Saving	Feel a little effect Oh, where the power consumption where to plug, convenient and convenient	Jingdong prefecture level city in Shandong
Doraemon Hyakubo Shop	King of Power Saving	The quality of the imported saver is very good, practical and satisfactory. Cost-effective	Jingdong prefecture level city in Shandong
Doraemon Hyakubo Shop	King of Power Saving	Power Saver is especially good, good and inexpensive, very good	Jingdong prefecture level city in Shandong
Doraemon Hyakubo Shop	King of Power Saving	Fast shipping, awesome quality, affordable price, satisfied.	Jingdong prefecture level city in Shandong
Doraemon Hyakubo Shop	King of Power Saving	Energy saver received, the effect is consistent with the description, recommended to buy!	Jingdong prefecture level city in Shandong
Doraemon Hyakubo Shop	King of Power Saving	It's really good, the quality is great, I recommend it!	Jingdong prefecture level city in Shandong
Doraemon Hyakubo Shop	King of Power Saving	Works well. Inexpensive. Shipping was fast.	Jingdong prefecture level city in Shandong
Doraemon Hyakubo Shop	King of Power Saving	Logistics is fast, works well, energy saving is good	Jingdong prefecture level city in Shandong
Doraemon Hyakubo Shop	King of Power Saving	very happy	Jingdong prefecture level city in Shandong
Doraemon Hyakubo Shop	King of Power Saving	an excellent	Jingdong prefecture level city in Shandong
Doraemon Hyakubo Shop	King of Power Saving	It rained a couple days ago and I just went to get the item back today , not bad, hey I looked at the meter and that red display light takes several seconds to jump, did not make a mistake buying it. After a period of time to evaluate.	Jingdong prefecture level city in Shandong
Doraemon Hyakubo Shop	King of Power Saving	Used more than 10 days to check the power, there is indeed some decline, customer service said, long-term use will save more points!	Jingdong prefecture level city in Shandong
Doraemon Hyakubo Shop	King of Power Saving	It's a proper IQ tax, and it costs electricity.	Jingdong prefecture level city in Shandong
Doraemon Hyakubo Shop	King of Power Saving	I've been using it for 17 days now, it really saves electricity Shipping is fast, you can save a lot in a year	Jingdong prefecture level city in Shandong
Doraemon Hyakubo Shop	King of Power Saving	I've been using it for 17 days and it really saves power Shipping is fast and you don't have to worry about the quality, free returns within 5 years	Jingdong prefecture level city in Shandong
Doraemon Hyakubo Shop	King of Power Saving	It's more regular, scanned the code and it's authentic, and the main thing is that you can try it for 90 days! Plugged in more than ten days, is less than before a few degrees per day. When the time to use a month to see Additional. Good to buy a few more	Jingdong prefecture level city in Shandong
Doraemon Hyakubo Shop	King of Power Saving	Logistics is very fast, fine workmanship, use it and then come back to the comments	Jingdong prefecture level city in Shandong
Doraemon Hyakubo Shop	King of Power Saving	The quality of workmanship is very good and the power saving effect is quite good, I regret not getting it earlier.	Jingdong prefecture level city in Shandong
Doraemon Hyakubo Shop	King of Power Saving	Received the goods, according to the instructions on the operation is very simple, to wait and see the effect, looks good!	Jingdong prefecture level city in Shandong
Doraemon Hyakubo Shop	King of Power Saving	It's really as safe and power-saving as the owner says, and it works great!	Jingdong prefecture level city in Shandong
Doraemon Hyakubo Shop	King of Power Saving	Received the goods, immediately used, looks good, to wait and see how the effect.	Jingdong prefecture level city in Shandong
Doraemon Hyakubo Shop	King of Power Saving	Afraid to buy back did not work, the store said the commitment to 90 days invalid refund, warranty 5 years. Saver also complimentary on the Pacific insurance, it is a lot of peace of mind, there are effects will continue to buy back drops!	Jingdong prefecture level city in Shandong
Doraemon Hyakubo Shop	King of Power Saving	Received the goods and used it, can save electricity a month later to see the effect!	Jingdong prefecture level city in Shandong
Doraemon Hyakubo Shop	King of Power Saving	Fancy 90-day trial, 5-year warranty before the order, the goods received, has been experiencing a week, can really feel the savings, in the introduction of friends also buy this!	Jingdong prefecture level city in Shandong
Doraemon Hyakubo Shop	King of Power Saving	Clothing stores use, neighbors recommended to buy, said a month can save more than 70 yuan, first plugged in to see the effect of the	Jingdong prefecture level city in Shandong
Doraemon Hyakubo Shop	King of Power Saving	A friend's home two months to save more than 80 dollars of electricity, recommended that I buy two, decisive purchase, looking forward to the effect of power saving, waiting for the next month effect.	Jingdong prefecture level city in Shandong
Doraemon Hyakubo Shop	King of Power Saving	The goods have been received, consistent with the description of the advertisement, quite good, I hope that in terms of power saving and purifying the air is really as the merchant said that would be better!	Jingdong prefecture level city in Shandong
Doraemon Hyakubo Shop	King of Power Saving	Power Saving King is really good to use the quality is very good than the physical store a lot cheaper we can rest assured that the purchase recommended to buy!	Jingdong prefecture level city in Shandong
Doraemon Hyakubo Shop	King of Power Saving	Energy saver has been used on the rental house inside the use of the meter is always running fast, with this can really save electricity, easy to use, plugged in can be, very satisfied!	Jingdong prefecture level city in Shandong
Doraemon Hyakubo Shop	King of Power Saving	Power Saver Power Saver King is good quality and affordable, very satisfied, worth buying.	Jingdong prefecture level city in Shandong
Doraemon Hyakubo Shop	King of Power Saving	Just use can not see what effect to,, wait for a period of time in the evaluation	Jingdong prefecture level city in Shandong
Doraemon Hyakubo Shop	King of Power Saving	Live in the 18th floor is too far away from the meter, afraid of no effect, look at the trial 90 days before buying. Currently used for a month, there is an effect, saving 27 dollars in electricity, next month should be able to save more!	Jingdong prefecture level city in Shandong
Doraemon Hyakubo Shop	King of Power Saving	I've been using it for almost a month now and it feels good, so I'll use it again.	Jingdong prefecture level city in Shandong
Doraemon Hyakubo Shop	King of Power Saving	It's ok, the electric bill is really less, originally I remember charging $100 and using it for 10 days. Now it lasts about 15 days. It's still noticeable.	Jingdong prefecture level city in Shandong
Doraemon Hyakubo Shop	King of Power Saving	Fast logistics, good power saving effect, very satisfied	Jingdong prefecture level city in Shandong

Doraemon Hyakubo Shop	King of Power Saving	Has been the second time back to buy, energy saving effect, worth recommending, you can try to see, power saving effect: very good Experience: can save about 30%!	Jingdong prefecture level city in Shandong
Doraemon Hyakubo Shop	King of Power Saving	I don't know how well it works for now	Jingdong prefecture level city in Shandong
Doraemon Hyakubo Shop	King of Power Saving	Factory direct is really good, plugged in and used for half a month did less probably almost 50 degrees of electricity! And the manufacturer's warranty is 5 years.	Jingdong prefecture level city in Shandong
Doraemon Hyakubo Shop	King of Power Saving	It's really effective. The previous month's electricity was $286, but after using the power saver for a month, I have $195 left this month, which is not bad.	Jingdong prefecture level city in Shandong
Doraemon Hyakubo Shop	King of Power Saving	I'll give you a good review and see how it goes.	Jingdong prefecture level city in Shandong
Doraemon Hyakubo Shop	King of Power Saving	Customer service is very professional and patient to guide the installation, the quality of workmanship is very fine. Durable, second repurchase. Really can save oh!	Jingdong prefecture level city in Shandong
Doraemon Hyakubo Shop	King of Power Saving	I've been using it for a month now and my electric bill is over $30 less than last month. I feel it's quite worth buying.	Jingdong prefecture level city in Shandong
Doraemon Hyakubo Shop	King of Power Saving	Small size, does not take up space. It's very good, it does work very effectively, the tool room has a lot of work with a lot of electricity. After installing it, it really reduces a lot of electricity bills. Before the day 30 degrees of electricity left stone, now work down really less a few degrees.	Jingdong prefecture level city in Shandong
Doraemon Hyakubo Shop	King of Power Saving	It does work for saving electricity, I've bought the kind from elsewhere before that was too simple not **all**. This time to buy this store, but also for the safety. Air conditioning is too much electricity, this use to save a lot of	Jingdong prefecture level city in Shandong
Doraemon Hyakubo Shop	King of Power Saving	Two air conditioners and a refrigerator at home, a month down the electricity bill is almost two hundred dollars, with this after the average savings of more than thirty dollars to spend less money to enjoy more, it's really very good!	Jingdong prefecture level city in Shandong
Doraemon Hyakubo Shop	King of Power Saving	Good. Very good.	Jingdong prefecture level city in Shandong
Doraemon Hyakubo Shop	King of Power Saving	Works great, easy to operate, great, very happy with it.	Jingdong prefecture level city in Shandong
Doraemon Hyakubo Shop	King of Power Saving	Received it and tried it out, it's great, great quality and worth having! Five stars praise!	Jingdong prefecture level city in Shandong
Doraemon Hyakubo Shop	King of Power Saving	Very trustworthy brand, quality is guaranteed, the price is still affordable, quite satisfied!	Jingdong prefecture level city in Shandong
Doraemon Hyakubo Shop	King of Power Saving	Use it before you speak.	Jingdong prefecture level city in Shandong
Doraemon Hyakubo Shop	King of Power Saving	The quality of workmanship is very good and the power saving effect is quite good, I regret not getting it earlier.	Jingdong prefecture level city in Shandong
Doraemon Hyakubo Shop	King of Power Saving	Merchants shipping speed, and customer service introduced the same very good, cost-effective	Jingdong prefecture level city in Shandong
Doraemon Hyakubo Shop	King of Power Saving	Feel a little effect Oh, where the power consumption where to plug, convenient and convenient	Jingdong prefecture level city in Shandong
Doraemon Hyakubo Shop	King of Power Saving	The quality of the imported saver is very good, practical and satisfactory. Cost-effective	Jingdong prefecture level city in Shandong
Doraemon Hyakubo Shop	King of Power Saving	Power Saver is especially good, good and inexpensive, very good	Jingdong prefecture level city in Shandong
Doraemon Hyakubo Shop	King of Power Saving	Fast shipping, awesome quality, affordable price, satisfied.	Jingdong prefecture level city in Shandong
Doraemon Hyakubo Shop	King of Power Saving	Energy saver received, the effect is consistent with the description, recommended to buy!	Jingdong prefecture level city in Shandong
Doraemon Hyakubo Shop	King of Power Saving	It's really good, the quality is great, I recommend it!	Jingdong prefecture level city in Shandong
Doraemon Hyakubo Shop	King of Power Saving	Works well. Inexpensive. Shipping was fast.	Jingdong prefecture level city in Shandong
Doraemon Hyakubo Shop	King of Power Saving	Logistics is fast, works well, energy saving is good	Jingdong prefecture level city in Shandong
Doraemon Hyakubo Shop	King of Power Saving	very happy	Jingdong prefecture level city in Shandong
Doraemon Hyakubo Shop	King of Power Saving	an excellent	Jingdong prefecture level city in Shandong
Doraemon Hyakubo Shop	King of Power Saving	It rained a couple days ago and I just went to get the item back today , not bad, hey I looked at the meter and that red display light takes several seconds to jump, did not make a mistake buying it. After a period of time to evaluate.	Jingdong prefecture level city in Shandong
Doraemon Hyakubo Shop	King of Power Saving	Used more than 10 days to check the power, there is indeed some decline, customer service said, long-term use will save more points!	Jingdong prefecture level city in Shandong
Doraemon Hyakubo Shop	King of Power Saving	It's a proper IQ tax, and it costs electricity.	Jingdong prefecture level city in Shandong
Doraemon Hyakubo Shop	King of Power Saving	I've been using it for 17 days now, it really saves electricity Shipping is fast, you can save a lot in a year	Jingdong prefecture level city in Shandong
Doraemon Hyakubo Shop	King of Power Saving	I've been using it for 17 days and it really saves power Shipping is fast and you don't have to worry about the quality, free returns within 5 years	Jingdong prefecture level city in Shandong
Doraemon Hyakubo Shop	King of Power Saving	It's more regular, scanned the code and it's authentic, and the main thing is that you can try it for 90 days! Plugged in more than ten days, is less than before a few degrees per day. When the time to use a month to see Additional. Good to buy a few more	Jingdong prefecture level city in Shandong
Doraemon Hyakubo Shop	King of Power Saving	Logistics is very fast, fine workmanship, use it and then come back to the comments	Jingdong prefecture level city in Shandong
Doraemon Hyakubo Shop	King of Power Saving	The quality of workmanship is very good and the power saving effect is quite good, I regret not getting it earlier.	Jingdong prefecture level city in Shandong
Doraemon Hyakubo Shop	King of Power Saving	Received the goods, according to the instructions on the operation is very simple, to wait and see the effect, looks good!	Jingdong prefecture level city in Shandong
Doraemon Hyakubo Shop	King of Power Saving	It's really as safe and power-saving as the owner says, and it works great!	Jingdong prefecture level city in Shandong
Doraemon Hyakubo Shop	King of Power Saving	Received the goods, immediately used, looks good, to wait and see how the effect.	Jingdong prefecture level city in Shandong
Doraemon Hyakubo Shop	King of Power Saving	Afraid to buy back did not work, the store said the commitment to 90 days invalid refund, warranty 5 years. Saver also complimentary on the Pacific insurance, it is a lot of peace of mind, there are effects will continue to buy back drops!	Jingdong prefecture level city in Shandong
Doraemon Hyakubo Shop	King of Power Saving	Received the goods and used it, can save electricity a month later to see the effect!	Jingdong prefecture level city in Shandong
Doraemon Hyakubo Shop	King of Power Saving	Fancy 90-day trial, 5-year warranty before the order, the goods received, has been experiencing a week, can really feel the savings, in the introduction of friends also buy this!	Jingdong prefecture level city in Shandong
Doraemon Hyakubo Shop	King of Power Saving	Clothing stores use, neighbors recommended to buy, said a month can save more than 70 yuan, first plugged in to see the effect of the	Jingdong prefecture level city in Shandong
Doraemon Hyakubo Shop	King of Power Saving	A friend's home two months to save more than 80 dollars of electricity, recommended that I buy two, decisive purchase, looking forward to the effect of power saving, waiting for the next month effect.	Jingdong prefecture level city in Shandong
Doraemon Hyakubo Shop	King of Power Saving	The goods have been received, consistent with the description of the advertisement, quite good, I hope that in terms of power saving and purifying the air is really as the merchant said that would be better!	Jingdong prefecture level city in Shandong
Doraemon Hyakubo Shop	King of Power Saving	Power Saving King is really good to use the quality is very good than the physical store a lot cheaper we can rest assured that the purchase recommended to buy!	Jingdong prefecture level city in Shandong
Doraemon Hyakubo Shop	King of Power Saving	Energy saver has been used on the rental house inside the use of the meter is always running fast, with this can really save electricity, easy to use, plugged in can be, very satisfied!	Jingdong prefecture level city in Shandong
Doraemon Hyakubo Shop	King of Power Saving	Power Saver Power Saver King is good quality and affordable, very satisfied, worth buying.	Jingdong prefecture level city in Shandong
Doraemon Hyakubo Shop	King of Power Saving	Just use can not see what effect to,, wait for a period of time in the evaluation	Jingdong prefecture level city in Shandong

Doraemon Hyakubo Shop	King of Power Saving	Live in the 18th floor is too far away from the meter, afraid of no effect, look at the trial 90 days before buying. Currently used for a month, there is an effect, saving 27 dollars in electricity, next month should be able to save more!	Jingdong prefecture level city in Shandong
Doraemon Hyakubo Shop	King of Power Saving	I've been using it for almost a month now and it feels good, so I'll use it again.	Jingdong prefecture level city in Shandong
Doraemon Hyakubo Shop	King of Power Saving	It's ok, the electric bill is really less, originally I remember charging $100 and using it for 10 days. Now it lasts about 15 days. It's still noticeable.	Jingdong prefecture level city in Shandong
Doraemon Hyakubo Shop	King of Power Saving	Fast logistics, good power saving effect, very satisfied	Jingdong prefecture level city in Shandong
Doraemon Hyakubo Shop	King of Power Saving	Has been the second time back to buy, energy saving effect, worth recommending, you can try to see, power saving effect: very good Experience: can save about 30%!	Jingdong prefecture level city in Shandong
Doraemon Hyakubo Shop	King of Power Saving	I don't know how well it works for now	Jingdong prefecture level city in Shandong
Doraemon Hyakubo Shop	King of Power Saving	Factory direct is really good, plugged in and used for half a month did less probably almost 50 degrees of electricity! And the manufacturer's warranty is 5 years.	Jingdong prefecture level city in Shandong
Doraemon Hyakubo Shop	King of Power Saving	It's really effective. The previous month's electricity was $286, but after using the power saver for a month, I have $195 left this month, which is not bad.	Jingdong prefecture level city in Shandong
Doraemon Hyakubo Shop	King of Power Saving	I'll give you a good review and see how it goes.	Jingdong prefecture level city in Shandong
Doraemon Hyakubo Shop	King of Power Saving	Customer service is very professional and patient to guide the installation, the quality of workmanship is very fine. Durable, second repurchase. Really can save oh!	Jingdong prefecture level city in Shandong
Doraemon Hyakubo Shop	King of Power Saving	I've been using it for a month now and my electric bill is over $30 less than last month. I feel it's quite worth buying.	Jingdong prefecture level city in Shandong
Doraemon Hyakubo Shop	King of Power Saving	Small size, does not take up space. It's very good, it does work very effectively, the tool room has a lot of work with a lot of electricity. After installing it, it really reduces a lot of electricity bills. Before the day 30 degrees of electricity left stone, now work down really less a few degrees.	Jingdong prefecture level city in Shandong
Doraemon Hyakubo Shop	King of Power Saving	It does work for saving electricity, I've bought the kind from elsewhere before that was too simple not **all**. This time to buy this store, but also for the safety. Air conditioning is too much electricity, this use to save a lot of	Jingdong prefecture level city in Shandong
Doraemon Hyakubo Shop	King of Power Saving	Two air conditioners and a refrigerator at home, a month down the electricity bill is almost two hundred dollars, with this after the average savings of more than thirty dollars to spend less money to enjoy more, it's really very good!	Jingdong prefecture level city in Shandong
Doraemon Hyakubo Shop	King of Power Saving	Good. Very good.	Jingdong prefecture level city in Shandong
Doraemon Hyakubo Shop	King of Power Saving	Works great, easy to operate, great, very happy with it.	Jingdong prefecture level city in Shandong
Doraemon Hyakubo Shop	King of Power Saving	Received it and tried it out, it's great, great quality and worth having! Five stars praise!	Jingdong prefecture level city in Shandong
Doraemon Hyakubo Shop	King of Power Saving	Very trustworthy brand, quality is guaranteed, the price is still affordable, quite satisfied!	Jingdong prefecture level city in Shandong
Doraemon Hyakubo Shop	King of Power Saving	Use it before you speak.	Jingdong prefecture level city in Shandong
Doraemon Hyakubo Shop	King of Power Saving	The quality of workmanship is very good and the power saving effect is quite good, I regret not getting it earlier.	Jingdong prefecture level city in Shandong
Doraemon Hyakubo Shop	King of Power Saving	Merchants shipping speed, and customer service introduced the same very good, cost-effective	Jingdong prefecture level city in Shandong
Doraemon Hyakubo Shop	King of Power Saving	Feel a little effect Oh, where the power consumption where to plug, convenient and convenient	Jingdong prefecture level city in Shandong
Doraemon Hyakubo Shop	King of Power Saving	The quality of the imported saver is very good, practical and satisfactory. Cost-effective	Jingdong prefecture level city in Shandong
Doraemon Hyakubo Shop	King of Power Saving	Power Saver is especially good, good and inexpensive, very good	Jingdong prefecture level city in Shandong
Doraemon Hyakubo Shop	King of Power Saving	Fast shipping, awesome quality, affordable price, satisfied.	Jingdong prefecture level city in Shandong
Doraemon Hyakubo Shop	King of Power Saving	Energy saver received, the effect is consistent with the description, recommended to buy!	Jingdong prefecture level city in Shandong
Doraemon Hyakubo Shop	King of Power Saving	It's really good, the quality is great, I recommend it!	Jingdong prefecture level city in Shandong
Doraemon Hyakubo Shop	King of Power Saving	Works well. Inexpensive. Shipping was fast.	Jingdong prefecture level city in Shandong
Doraemon Hyakubo Shop	King of Power Saving	Logistics is fast, works well, energy saving is good	Jingdong prefecture level city in Shandong
Doraemon Hyakubo Shop	King of Power Saving	very happy	Jingdong prefecture level city in Shandong
Doraemon Hyakubo Shop	King of Power Saving	an excellent	Jingdong prefecture level city in Shandong
Doraemon Hyakubo Shop	King of Power Saving	It rained a couple days ago and I just went to get the item back today , not bad, hey I looked at the meter and that red display light takes several seconds to jump, did not make a mistake buying it. After a period of time to evaluate.	Jingdong prefecture level city in Shandong
Doraemon Hyakubo Shop	King of Power Saving	Used more than 10 days to check the power, there is indeed some decline, customer service said, long-term use will save more points!	Jingdong prefecture level city in Shandong
Doraemon Hyakubo Shop	King of Power Saving	It's a proper IQ tax, and it costs electricity.	Jingdong prefecture level city in Shandong
Doraemon Hyakubo Shop	King of Power Saving	I've been using it for 17 days now, it really saves electricity Shipping is fast, you can save a lot in a year	Jingdong prefecture level city in Shandong
Doraemon Hyakubo Shop	King of Power Saving	I've been using it for 17 days and it really saves power Shipping is fast and you don't have to worry about the quality, free returns within 5 years	Jingdong prefecture level city in Shandong
Doraemon Hyakubo Shop	King of Power Saving	It's more regular, scanned the code and it's authentic, and the main thing is that you can try it for 90 days! Plugged in more than ten days, is less than before a few degrees per day. When the time to use a month to see Additional. Good to buy a few more	Jingdong prefecture level city in Shandong
Doraemon Hyakubo Shop	King of Power Saving	Logistics is very fast, fine workmanship, use it and then come back to the comments	Jingdong prefecture level city in Shandong
Doraemon Hyakubo Shop	King of Power Saving	The quality of workmanship is very good and the power saving effect is quite good, I regret not getting it earlier.	Jingdong prefecture level city in Shandong
Doraemon Hyakubo Shop	King of Power Saving	Received the goods, according to the instructions on the operation is very simple, to wait and see the effect, looks good!	Jingdong prefecture level city in Shandong
Doraemon Hyakubo Shop	King of Power Saving	It's really as safe and power-saving as the owner says, and it works great!	Jingdong prefecture level city in Shandong
Doraemon Hyakubo Shop	King of Power Saving	Received the goods, immediately used, looks good, to wait and see how the effect.	Jingdong prefecture level city in Shandong
Doraemon Hyakubo Shop	King of Power Saving	Afraid to buy back did not work, the store said the commitment to 90 days invalid refund, warranty 5 years. Saver also complimentary on the Pacific insurance, it is a lot of peace of mind, there are effects will continue to buy back drops!	Jingdong prefecture level city in Shandong
Doraemon Hyakubo Shop	King of Power Saving	Received the goods and used it, can save electricity a month later to see the effect!	Jingdong prefecture level city in Shandong
Doraemon Hyakubo Shop	King of Power Saving	Fancy 90-day trial, 5-year warranty before the order, the goods received, has been experiencing a week, can really feel the savings, in the introduction of friends also buy this!	Jingdong prefecture level city in Shandong
Doraemon Hyakubo Shop	King of Power Saving	Clothing stores use, neighbors recommended to buy, said a month can save more than 70 yuan, first plugged in to see the effect of the	Jingdong prefecture level city in Shandong
Doraemon Hyakubo Shop	King of Power Saving	A friend's home two months to save more than 80 dollars of electricity, recommended that I buy two, decisive purchase, looking forward to the effect of power saving, waiting for the next month effect.	Jingdong prefecture level city in Shandong
Doraemon Hyakubo Shop	King of Power Saving	The goods have been received, consistent with the description of the advertisement, quite good, I hope that in terms of power saving and purifying the air is really as the merchant said that would be better!	Jingdong prefecture level city in Shandong

Doraemon Hyakubo Shop	King of Power Saving	Power Saving King is really good to use the quality is very good than the physical store a lot cheaper we can rest assured that the purchase recommended to buy!	Jingdong prefecture level city in Shandong
Doraemon Hyakubo Shop	King of Power Saving	Energy saver has been used on the rental house inside the use of the meter is always running fast, with this can really save electricity, easy to use, plugged in can be, very satisfied!	Jingdong prefecture level city in Shandong
Doraemon Hyakubo Shop	King of Power Saving	Power Saver Power Saver King is good quality and affordable, very satisfied, worth buying.	Jingdong prefecture level city in Shandong
Doraemon Hyakubo Shop	King of Power Saving	Just use can not see what effect to,, wait for a period of time in the evaluation	Jingdong prefecture level city in Shandong
Doraemon Hyakubo Shop	King of Power Saving	Live in the 18th floor is too far away from the meter, afraid of no effect, look at the trial 90 days before buying. Currently used for a month, there is an effect, saving 27 dollars in electricity, next month should be able to save more!	Jingdong prefecture level city in Shandong
Doraemon Hyakubo Shop	King of Power Saving	I've been using it for almost a month now and it feels good, so I'll use it again.	Jingdong prefecture level city in Shandong
Doraemon Hyakubo Shop	King of Power Saving	It's ok, the electric bill is really less, originally I remember charging $100 and using it for 10 days. Now it lasts about 15 days. It's still noticeable.	Jingdong prefecture level city in Shandong
Doraemon Hyakubo Shop	King of Power Saving	Fast logistics, good power saving effect, very satisfied	Jingdong prefecture level city in Shandong
Doraemon Hyakubo Shop	King of Power Saving	Has been the second time back to buy, energy saving effect, worth recommending, you can try to see, power saving effect: very good Experience: can save about 30%!	Jingdong prefecture level city in Shandong
Doraemon Hyakubo Shop	King of Power Saving	I don't know how well it works for now	Jingdong prefecture level city in Shandong
Doraemon Hyakubo Shop	King of Power Saving	Factory direct is really good, plugged in and used for half a month did less probably almost 50 degrees of electricity! And the manufacturer's warranty is 5 years.	Jingdong prefecture level city in Shandong
Doraemon Hyakubo Shop	King of Power Saving	It's really effective. The previous month's electricity was $286, but after using the power saver for a month, I have $195 left this month, which is not bad.	Jingdong prefecture level city in Shandong
Doraemon Hyakubo Shop	King of Power Saving	I'll give you a good review and see how it goes.	Jingdong prefecture level city in Shandong
Doraemon Hyakubo Shop	King of Power Saving	Customer service is very professional and patient to guide the installation, the quality of workmanship is very fine. Durable, second repurchase. Really can save oh!	Jingdong prefecture level city in Shandong
Doraemon Hyakubo Shop	King of Power Saving	I've been using it for a month now and my electric bill is over $30 less than last month. I feel it's quite worth buying.	Jingdong prefecture level city in Shandong
Doraemon Hyakubo Shop	King of Power Saving	Small size, does not take up space. It's very good, it does work very effectively, the tool room has a lot of work with a lot of electricity. After installing it, it really reduces a lot of electricity bills. Before the day 30 degrees of electricity left stone, now work down really less a few degrees.	Jingdong prefecture level city in Shandong
Doraemon Hyakubo Shop	King of Power Saving	It does work for saving electricity, I've bought the kind from elsewhere before that was too simple not **all**. This time to buy this store, but also for the safety. Air conditioning is too much electricity, this use to save a lot of	Jingdong prefecture level city in Shandong
Doraemon Hyakubo Shop	King of Power Saving	Two air conditioners and a refrigerator at home, a month down the electricity bill is almost two hundred dollars, with this after the average savings of more than thirty dollars to spend less money to enjoy more, it's really very good!	Jingdong prefecture level city in Shandong
Doraemon Hyakubo Shop	King of Power Saving	Good. Very good.	Jingdong prefecture level city in Shandong
Doraemon Hyakubo Shop	King of Power Saving	Works great, easy to operate, great, very happy with it.	Jingdong prefecture level city in Shandong
Doraemon Hyakubo Shop	King of Power Saving	Received it and tried it out, it's great, great quality and worth having! Five stars praise!	Jingdong prefecture level city in Shandong
Doraemon Hyakubo Shop	King of Power Saving	Very trustworthy brand, quality is guaranteed, the price is still affordable, quite satisfied!	Jingdong prefecture level city in Shandong
Doraemon Hyakubo Shop	King of Power Saving	Use it before you speak.	Jingdong prefecture level city in Shandong
Doraemon Hyakubo Shop	King of Power Saving	The quality of workmanship is very good and the power saving effect is quite good, I regret not getting it earlier.	Jingdong prefecture level city in Shandong
Doraemon Hyakubo Shop	King of Power Saving	Merchants shipping speed, and customer service introduced the same very good, cost-effective	Jingdong prefecture level city in Shandong
Doraemon Hyakubo Shop	King of Power Saving	Feel a little effect Oh, where the power consumption where to plug, convenient and convenient	Jingdong prefecture level city in Shandong
Doraemon Hyakubo Shop	King of Power Saving	The quality of the imported saver is very good, practical and satisfactory. Cost-effective	Jingdong prefecture level city in Shandong
Doraemon Hyakubo Shop	King of Power Saving	Power Saver is especially good, good and inexpensive, very good	Jingdong prefecture level city in Shandong
Doraemon Hyakubo Shop	King of Power Saving	Fast shipping, awesome quality, affordable price, satisfied.	Jingdong prefecture level city in Shandong
Doraemon Hyakubo Shop	King of Power Saving	Energy saver received, the effect is consistent with the description, recommended to buy!	Jingdong prefecture level city in Shandong
Doraemon Hyakubo Shop	King of Power Saving	It's really good, the quality is great, I recommend it!	Jingdong prefecture level city in Shandong
Doraemon Hyakubo Shop	King of Power Saving	Works well. Inexpensive. Shipping was fast.	Jingdong prefecture level city in Shandong
Doraemon Hyakubo Shop	King of Power Saving	Logistics is fast, works well, energy saving is good	Jingdong prefecture level city in Shandong
Doraemon Hyakubo Shop	King of Power Saving	very happy	Jingdong prefecture level city in Shandong
Doraemon Hyakubo Shop	King of Power Saving	an excellent	Jingdong prefecture level city in Shandong
Doraemon Hyakubo Shop	King of Power Saving	It rained a couple days ago and I just went to get the item back today , not bad, hey I looked at the meter and that red display light takes several seconds to jump, did not make a mistake buying it. After a period of time to evaluate.	Jingdong prefecture level city in Shandong
Doraemon Hyakubo Shop	King of Power Saving	Used more than 10 days to check the power, there is indeed some decline, customer service said, long-term use will save more points!	Jingdong prefecture level city in Shandong
Doraemon Hyakubo Shop	King of Power Saving	It's a proper IQ tax, and it costs electricity.	Jingdong prefecture level city in Shandong
Doraemon Hyakubo Shop	King of Power Saving	I've been using it for 17 days now, it really saves electricity Shipping is fast, you can save a lot in a year	Jingdong prefecture level city in Shandong
Doraemon Hyakubo Shop	King of Power Saving	I've been using it for 17 days and it really saves power Shipping is fast and you don't have to worry about the quality, free returns within 5 years	Jingdong prefecture level city in Shandong
Doraemon Hyakubo Shop	King of Power Saving	It's more regular, scanned the code and it's authentic, and the main thing is that you can try it for 90 days! Plugged in more than ten days, is less than before a few degrees per day. When the time to use a month to see Additional. Good to buy a few more	Jingdong prefecture level city in Shandong
Doraemon Hyakubo Shop	King of Power Saving	Logistics is very fast, fine workmanship, use it and then come back to the comments	Jingdong prefecture level city in Shandong
Doraemon Hyakubo Shop	King of Power Saving	The quality of workmanship is very good and the power saving effect is quite good, I regret not getting it earlier.	Jingdong prefecture level city in Shandong
Doraemon Hyakubo Shop	King of Power Saving	Received the goods, according to the instructions on the operation is very simple, to wait and see the effect, looks good!	Jingdong prefecture level city in Shandong
Doraemon Hyakubo Shop	King of Power Saving	It's really as safe and power-saving as the owner says, and it works great!	Jingdong prefecture level city in Shandong
Doraemon Hyakubo Shop	King of Power Saving	Received the goods, immediately used, looks good, to wait and see how the effect.	Jingdong prefecture level city in Shandong
Doraemon Hyakubo Shop	King of Power Saving	Afraid to buy back did not work, the store said the commitment to 90 days invalid refund, warranty 5 years. Saver also complimentary on the Pacific insurance, it is a lot of peace of mind, there are effects will continue to buy back drops!	Jingdong prefecture level city in Shandong
Doraemon Hyakubo Shop	King of Power Saving	Received the goods and used it, can save electricity a month later to see the effect!	Jingdong prefecture level city in Shandong

Doraemon Hyakubo Shop	King of Power Saving	Fancy 90-day trial, 5-year warranty before the order, the goods received, has been experiencing a week, can really feel the savings, in the introduction of friends also buy this!	Jingdong prefecture level city in Shandong
Doraemon Hyakubo Shop	King of Power Saving	Clothing stores use, neighbors recommended to buy, said a month can save more than 70 yuan, first plugged in to see the effect of the	Jingdong prefecture level city in Shandong
Doraemon Hyakubo Shop	King of Power Saving	A friend's home two months to save more than 80 dollars of electricity, recommended that I buy two, decisive purchase, looking forward to the effect of power saving, waiting for the next month effect.	Jingdong prefecture level city in Shandong
Doraemon Hyakubo Shop	King of Power Saving	The goods have been received, consistent with the description of the advertisement, quite good, I hope that in terms of power saving and purifying the air is really as the merchant said that would be better!	Jingdong prefecture level city in Shandong
Doraemon Hyakubo Shop	King of Power Saving	Power Saving King is really good to use the quality is very good than the physical store a lot cheaper we can rest assured that the purchase recommended to buy!	Jingdong prefecture level city in Shandong
Doraemon Hyakubo Shop	King of Power Saving	Energy saver has been used on the rental house inside the use of the meter is always running fast, with this can really save electricity, easy to use, plugged in can be, very satisfied!	Jingdong prefecture level city in Shandong
Doraemon Hyakubo Shop	King of Power Saving	Power Saver Power Saver King is good quality and affordable, very satisfied, worth buying.	Jingdong prefecture level city in Shandong
Doraemon Hyakubo Shop	King of Power Saving	Just use can not see what effect to,, wait for a period of time in the evaluation	Jingdong prefecture level city in Shandong
Doraemon Hyakubo Shop	King of Power Saving	Live in the 18th floor is too far away from the meter, afraid of no effect, look at the trial 90 days before buying. Currently used for a month, there is an effect, saving 27 dollars in electricity, next month should be able to save more!	Jingdong prefecture level city in Shandong
Doraemon Hyakubo Shop	King of Power Saving	I've been using it for almost a month now and it feels good, so I'll use it again.	Jingdong prefecture level city in Shandong
Doraemon Hyakubo Shop	King of Power Saving	It's ok, the electric bill is really less, originally I remember charging $100 and using it for 10 days. Now it lasts about 15 days. It's still noticeable.	Jingdong prefecture level city in Shandong
Doraemon Hyakubo Shop	King of Power Saving	Fast logistics, good power saving effect, very satisfied	Jingdong prefecture level city in Shandong
Doraemon Hyakubo Shop	King of Power Saving	Has been the second time back to buy, energy saving effect, worth recommending, you can try to see, power saving effect: very good Experience: can save about 30%!	Jingdong prefecture level city in Shandong
Doraemon Hyakubo Shop	King of Power Saving	I don't know how well it works for now	Jingdong prefecture level city in Shandong
Doraemon Hyakubo Shop	King of Power Saving	Factory direct is really good, plugged in and used for half a month did less probably almost 50 degrees of electricity! And the manufacturer's warranty is 5 years.	Jingdong prefecture level city in Shandong
Doraemon Hyakubo Shop	King of Power Saving	It's really effective. The previous month's electricity was $286, but after using the power saver for a month, I have $195 left this month, which is not bad.	Jingdong prefecture level city in Shandong
Doraemon Hyakubo Shop	King of Power Saving	I'll give you a good review and see how it goes.	Jingdong prefecture level city in Shandong
Doraemon Hyakubo Shop	King of Power Saving	Customer service is very professional and patient to guide the installation, the quality of workmanship is very fine. Durable, second repurchase. Really can save oh!	Jingdong prefecture level city in Shandong
Doraemon Hyakubo Shop	King of Power Saving	I've been using it for a month now and my electric bill is over $30 less than last month. I feel it's quite worth buying.	Jingdong prefecture level city in Shandong
Doraemon Hyakubo Shop	King of Power Saving	Small size, does not take up space. It's very good, it does work very effectively, the tool room has a lot of work with a lot of electricity. After installing it, it really reduces a lot of electricity bills. Before the day 30 degrees of electricity left stone, now work down really less a few degrees.	Jingdong prefecture level city in Shandong
Doraemon Hyakubo Shop	King of Power Saving	It does work for saving electricity, I've bought the kind from elsewhere before that was too simple not **all**. This time to buy this store, but also for the safety. Air conditioning is too much electricity, this use to save a lot of	Jingdong prefecture level city in Shandong
Doraemon Hyakubo Shop	King of Power Saving	Two air conditioners and a refrigerator at home, a month down the electricity bill is almost two hundred dollars, with this after the average savings of more than thirty dollars to spend less money to enjoy more, it's really very good!	Jingdong prefecture level city in Shandong
Doraemon Hyakubo Shop	King of Power Saving	Good. Very good.	Jingdong prefecture level city in Shandong
Doraemon Hyakubo Shop	King of Power Saving	Works great, easy to operate, great, very happy with it.	Jingdong prefecture level city in Shandong
Doraemon Hyakubo Shop	King of Power Saving	Received it and tried it out, it's great, great quality and worth having! Five stars praise!	Jingdong prefecture level city in Shandong
Doraemon Hyakubo Shop	King of Power Saving	Very trustworthy brand, quality is guaranteed, the price is still affordable, quite satisfied!	Jingdong prefecture level city in Shandong
Doraemon Hyakubo Shop	King of Power Saving	Use it before you speak.	Jingdong prefecture level city in Shandong
Doraemon Hyakubo Shop	King of Power Saving	The quality of workmanship is very good and the power saving effect is quite good, I regret not getting it earlier.	Jingdong prefecture level city in Shandong
Doraemon Hyakubo Shop	King of Power Saving	Merchants shipping speed, and customer service introduced the same very good, cost-effective	Jingdong prefecture level city in Shandong
Doraemon Hyakubo Shop	King of Power Saving	Feel a little effect Oh, where the power consumption where to plug, convenient and convenient	Jingdong prefecture level city in Shandong
Doraemon Hyakubo Shop	King of Power Saving	The quality of the imported saver is very good, practical and satisfactory. Cost-effective	Jingdong prefecture level city in Shandong
Doraemon Hyakubo Shop	King of Power Saving	Power Saver is especially good, good and inexpensive, very good	Jingdong prefecture level city in Shandong
Doraemon Hyakubo Shop	King of Power Saving	Fast shipping, awesome quality, affordable price, satisfied.	Jingdong prefecture level city in Shandong
Doraemon Hyakubo Shop	King of Power Saving	Energy saver received, the effect is consistent with the description, recommended to buy!	Jingdong prefecture level city in Shandong
Doraemon Hyakubo Shop	King of Power Saving	It's really good, the quality is great, I recommend it!	Jingdong prefecture level city in Shandong
Doraemon Hyakubo Shop	King of Power Saving	Works well. Inexpensive. Shipping was fast.	Jingdong prefecture level city in Shandong
Doraemon Hyakubo Shop	King of Power Saving	Logistics is fast, works well, energy saving is good	Jingdong prefecture level city in Shandong
Doraemon Hyakubo Shop	King of Power Saving	very happy	Jingdong prefecture level city in Shandong
Doraemon Hyakubo Shop	King of Power Saving	an excellent	Jingdong prefecture level city in Shandong
Doraemon Hyakubo Shop	King of Power Saving	It rained a couple days ago and I just went to get the item back today , not bad, hey I looked at the meter and that red display light takes several seconds to jump, did not make a mistake buying it. After a period of time to evaluate.	Jingdong prefecture level city in Shandong
Doraemon Hyakubo Shop	King of Power Saving	Used more than 10 days to check the power, there is indeed some decline, customer service said, long-term use will save more points!	Jingdong prefecture level city in Shandong
Doraemon Hyakubo Shop	King of Power Saving	It's a proper IQ tax, and it costs electricity.	Jingdong prefecture level city in Shandong
Doraemon Hyakubo Shop	King of Power Saving	I've been using it for 17 days now, it really saves electricity Shipping is fast, you can save a lot in a year	Jingdong prefecture level city in Shandong
Doraemon Hyakubo Shop	King of Power Saving	I've been using it for 17 days and it really saves power Shipping is fast and you don't have to worry about the quality, free returns within 5 years	Jingdong prefecture level city in Shandong
Doraemon Hyakubo Shop	King of Power Saving	It's more regular, scanned the code and it's authentic, and the main thing is that you can try it for 90 days! Plugged in more than ten days, is less than before a few degrees per day. When the time to use a month to see Additional. Good to buy a few more	Jingdong prefecture level city in Shandong
Doraemon Hyakubo Shop	King of Power Saving	Logistics is very fast, fine workmanship, use it and then come back to the comments	Jingdong prefecture level city in Shandong
Doraemon Hyakubo Shop	King of Power Saving	The quality of workmanship is very good and the power saving effect is quite good, I regret not getting it earlier.	Jingdong prefecture level city in Shandong
Doraemon Hyakubo Shop	King of Power Saving	Received the goods, according to the instructions on the operation is very simple, to wait and see the effect, looks good!	Jingdong prefecture level city in Shandong

Doraemon Hyakubo Shop	King of Power Saving	It's really as safe and power-saving as the owner says, and it works great!	Jingdong prefecture level city in Shandong
Doraemon Hyakubo Shop	King of Power Saving	Received the goods, immediately used, looks good, to wait and see how the effect.	Jingdong prefecture level city in Shandong
Doraemon Hyakubo Shop	King of Power Saving	Afraid to buy back did not work, the store said the commitment to 90 days invalid refund, warranty 5 years. Saver also complimentary on the Pacific insurance, it is a lot of peace of mind, there are effects will continue to buy back drops!	Jingdong prefecture level city in Shandong
Doraemon Hyakubo Shop	King of Power Saving	Received the goods and used it, can save electricity a month later to see the effect!	Jingdong prefecture level city in Shandong
Doraemon Hyakubo Shop	King of Power Saving	Fancy 90-day trial, 5-year warranty before the order, the goods received, has been experiencing a week, can really feel the savings, in the introduction of friends also buy this!	Jingdong prefecture level city in Shandong
Doraemon Hyakubo Shop	King of Power Saving	Clothing stores use, neighbors recommended to buy, said a month can save more than 70 yuan, first plugged in to see the effect of the	Jingdong prefecture level city in Shandong
Doraemon Hyakubo Shop	King of Power Saving	A friend's home two months to save more than 80 dollars of electricity, recommended that I buy two, decisive purchase, looking forward to the effect of power saving, waiting for the next month effect.	Jingdong prefecture level city in Shandong
Doraemon Hyakubo Shop	King of Power Saving	The goods have been received, consistent with the description of the advertisement, quite good, I hope that in terms of power saving and purifying the air is really as the merchant said that would be better!	Jingdong prefecture level city in Shandong
Doraemon Hyakubo Shop	King of Power Saving	Power Saving King is really good to use the quality is very good than the physical store a lot cheaper we can rest assured that the purchase recommended to buy!	Jingdong prefecture level city in Shandong
Doraemon Hyakubo Shop	King of Power Saving	Energy saver has been used on the rental house inside the use of the meter is always running fast, with this can really save electricity, easy to use, plugged in can be, very satisfied!	Jingdong prefecture level city in Shandong
Doraemon Hyakubo Shop	King of Power Saving	Power Saver Power Saver King is good quality and affordable, very satisfied, worth buying.	Jingdong prefecture level city in Shandong
Doraemon Hyakubo Shop	King of Power Saving	Just use can not see what effect to,, wait for a period of time in the evaluation	Jingdong prefecture level city in Shandong
Doraemon Hyakubo Shop	King of Power Saving	Live in the 18th floor is too far away from the meter, afraid of no effect, look at the trial 90 days before buying. Currently used for a month, there is an effect, saving 27 dollars in electricity, next month should be able to save more!	Jingdong prefecture level city in Shandong
Doraemon Hyakubo Shop	King of Power Saving	I've been using it for almost a month now and it feels good, so I'll use it again.	Jingdong prefecture level city in Shandong
Doraemon Hyakubo Shop	King of Power Saving	It's ok, the electric bill is really less, originally I remember charging $100 and using it for 10 days. Now it lasts about 15 days. It's still noticeable.	Jingdong prefecture level city in Shandong
Doraemon Hyakubo Shop	King of Power Saving	Fast logistics, good power saving effect, very satisfied	Jingdong prefecture level city in Shandong
Doraemon Hyakubo Shop	King of Power Saving	Has been the second time back to buy, energy saving effect, worth recommending, you can try to see, power saving effect: very good Experience: can save about 30%!	Jingdong prefecture level city in Shandong
Doraemon Hyakubo Shop	King of Power Saving	I don't know how well it works for now	Jingdong prefecture level city in Shandong
Doraemon Hyakubo Shop	King of Power Saving	Factory direct is really good, plugged in and used for half a month did less probably almost 50 degrees of electricity! And the manufacturer's warranty is 5 years.	Jingdong prefecture level city in Shandong
Doraemon Hyakubo Shop	King of Power Saving	It's really effective. The previous month's electricity was $286, but after using the power saver for a month, I have $195 left this month, which is not bad.	Jingdong prefecture level city in Shandong
Doraemon Hyakubo Shop	King of Power Saving	I'll give you a good review and see how it goes.	Jingdong prefecture level city in Shandong
Doraemon Hyakubo Shop	King of Power Saving	Customer service is very professional and patient to guide the installation, the quality of workmanship is very fine. Durable, second repurchase. Really can save oh!	Jingdong prefecture level city in Shandong
Doraemon Hyakubo Shop	King of Power Saving	I've been using it for a month now and my electric bill is over $30 less than last month. I feel it's quite worth buying.	Jingdong prefecture level city in Shandong
Doraemon Hyakubo Shop	King of Power Saving	Small size, does not take up space. It's very good, it does work very effectively, the tool room has a lot of work with a lot of electricity. After installing it, it really reduces a lot of electricity bills. Before the day 30 degrees of electricity left stone, now work down really less a few degrees.	Jingdong prefecture level city in Shandong
Doraemon Hyakubo Shop	King of Power Saving	It does work for saving electricity, I've bought the kind from elsewhere before that was too simple not **all**. This time to buy this store, but also for the safety. Air conditioning is too much electricity, this use to save a lot of	Jingdong prefecture level city in Shandong
Doraemon Hyakubo Shop	King of Power Saving	Two air conditioners and a refrigerator at home, a month down the electricity bill is almost two hundred dollars, with this after the average savings of more than thirty dollars to spend less money to enjoy more, it's really very good!	Jingdong prefecture level city in Shandong
Doraemon Hyakubo Shop	King of Power Saving	Good. Very good.	Jingdong prefecture level city in Shandong
Doraemon Hyakubo Shop	King of Power Saving	Works great, easy to operate, great, very happy with it.	Jingdong prefecture level city in Shandong
Doraemon Hyakubo Shop	King of Power Saving	Received it and tried it out, it's great, great quality and worth having! Five stars praise!	Jingdong prefecture level city in Shandong
Doraemon Hyakubo Shop	King of Power Saving	Very trustworthy brand, quality is guaranteed, the price is still affordable, quite satisfied!	Jingdong prefecture level city in Shandong
Doraemon Hyakubo Shop	King of Power Saving	Use it before you speak.	Jingdong prefecture level city in Shandong
Doraemon Hyakubo Shop	King of Power Saving	The quality of workmanship is very good and the power saving effect is quite good, I regret not getting it earlier.	Jingdong prefecture level city in Shandong
Doraemon Hyakubo Shop	King of Power Saving	Merchants shipping speed, and customer service introduced the same very good, cost-effective	Jingdong prefecture level city in Shandong
Doraemon Hyakubo Shop	King of Power Saving	Feel a little effect Oh, where the power consumption where to plug, convenient and convenient	Jingdong prefecture level city in Shandong
Doraemon Hyakubo Shop	King of Power Saving	The quality of the imported saver is very good, practical and satisfactory. Cost-effective	Jingdong prefecture level city in Shandong
Doraemon Hyakubo Shop	King of Power Saving	Power Saver is especially good, good and inexpensive, very good	Jingdong prefecture level city in Shandong
Doraemon Hyakubo Shop	King of Power Saving	Fast shipping, awesome quality, affordable price, satisfied.	Jingdong prefecture level city in Shandong
Doraemon Hyakubo Shop	King of Power Saving	Energy saver received, the effect is consistent with the description, recommended to buy!	Jingdong prefecture level city in Shandong
Doraemon Hyakubo Shop	King of Power Saving	It's really good, the quality is great, I recommend it!	Jingdong prefecture level city in Shandong
Doraemon Hyakubo Shop	King of Power Saving	Works well. Inexpensive. Shipping was fast.	Jingdong prefecture level city in Shandong
Doraemon Hyakubo Shop	King of Power Saving	Logistics is fast, works well, energy saving is good	Jingdong prefecture level city in Shandong
Doraemon Hyakubo Shop	King of Power Saving	very happy	Jingdong prefecture level city in Shandong
Doraemon Hyakubo Shop	King of Power Saving	an excellent	Jingdong prefecture level city in Shandong
Doraemon Hyakubo Shop	King of Power Saving	It rained a couple days ago and I just went to get the item back today , not bad, hey I looked at the meter and that red display light takes several seconds to jump, did not make a mistake buying it. After a period of time to evaluate.	Jingdong prefecture level city in Shandong
Doraemon Hyakubo Shop	King of Power Saving	Used more than 10 days to check the power, there is indeed some decline, customer service said, long-term use will save more points!	Jingdong prefecture level city in Shandong
Doraemon Hyakubo Shop	King of Power Saving	It's a proper IQ tax, and it costs electricity.	Jingdong prefecture level city in Shandong
Doraemon Hyakubo Shop	King of Power Saving	I've been using it for 17 days now, it really saves electricity Shipping is fast, you can save a lot in a year	Jingdong prefecture level city in Shandong
Doraemon Hyakubo Shop	King of Power Saving	I've been using it for 17 days and it really saves power Shipping is fast and you don't have to worry about the quality, free returns within 5 years	Jingdong prefecture level city in Shandong

Doraemon Hyakubo Shop	King of Power Saving	It's more regular, scanned the code and it's authentic, and the main thing is that you can try it for 90 days! Plugged in more than ten days, is less than before a few degrees per day. When the time to use a month to see Additional. Good to buy a few more	Jingdong prefecture level city in Shandong
Doraemon Hyakubo Shop	King of Power Saving	Logistics is very fast, fine workmanship, use it and then come back to the comments	Jingdong prefecture level city in Shandong
Doraemon Hyakubo Shop	King of Power Saving	The quality of workmanship is very good and the power saving effect is quite good, I regret not getting it earlier.	Jingdong prefecture level city in Shandong
Doraemon Hyakubo Shop	King of Power Saving	Received the goods, according to the instructions on the operation is very simple, to wait and see the effect, looks good!	Jingdong prefecture level city in Shandong
Doraemon Hyakubo Shop	King of Power Saving	It's really as safe and power-saving as the owner says, and it works great!	Jingdong prefecture level city in Shandong
Doraemon Hyakubo Shop	King of Power Saving	Received the goods, immediately used, looks good, to wait and see how the effect.	Jingdong prefecture level city in Shandong
Doraemon Hyakubo Shop	King of Power Saving	Afraid to buy back did not work, the store said the commitment to 90 days invalid refund, warranty 5 years. Saver also complimentary on the Pacific insurance, it is a lot of peace of mind, there are effects will continue to buy back drops!	Jingdong prefecture level city in Shandong
Doraemon Hyakubo Shop	King of Power Saving	Received the goods and used it, can save electricity a month later to see the effect!	Jingdong prefecture level city in Shandong
Doraemon Hyakubo Shop	King of Power Saving	Fancy 90-day trial, 5-year warranty before the order, the goods received, has been experiencing a week, can really feel the savings, in the introduction of friends also buy this!	Jingdong prefecture level city in Shandong
Doraemon Hyakubo Shop	King of Power Saving	Clothing stores use, neighbors recommended to buy, said a month can save more than 70 yuan, first plugged in to see the effect of the	Jingdong prefecture level city in Shandong
Doraemon Hyakubo Shop	King of Power Saving	A friend's home two months to save more than 80 dollars of electricity, recommended that I buy two, decisive purchase, looking forward to the effect of power saving, waiting for the next month effect.	Jingdong prefecture level city in Shandong
Doraemon Hyakubo Shop	King of Power Saving	The goods have been received, consistent with the description of the advertisement, quite good, I hope that in terms of power saving and purifying the air is really as the merchant said that would be better!	Jingdong prefecture level city in Shandong
Doraemon Hyakubo Shop	King of Power Saving	Power Saving King is really good to use the quality is very good than the physical store a lot cheaper we can rest assured that the purchase recommended to buy!	Jingdong prefecture level city in Shandong
Doraemon Hyakubo Shop	King of Power Saving	Energy saver has been used on the rental house inside the use of the meter is always running fast, with this can really save electricity, easy to use, plugged in can be, very satisfied!	Jingdong prefecture level city in Shandong
Doraemon Hyakubo Shop	King of Power Saving	Power Saver Power Saver King is good quality and affordable, very satisfied, worth buying.	Jingdong prefecture level city in Shandong
Doraemon Hyakubo Shop	King of Power Saving	Just use can not see what effect to,, wait for a period of time in the evaluation	Jingdong prefecture level city in Shandong
Doraemon Hyakubo Shop	King of Power Saving	Live in the 18th floor is too far away from the meter, afraid of no effect, look at the trial 90 days before buying. Currently used for a month, there is an effect, saving 27 dollars in electricity, next month should be able to save more!	Jingdong prefecture level city in Shandong
Doraemon Hyakubo Shop	King of Power Saving	I've been using it for almost a month now and it feels good, so I'll use it again.	Jingdong prefecture level city in Shandong
Doraemon Hyakubo Shop	King of Power Saving	It's ok, the electric bill is really less, originally I remember charging $100 and using it for 10 days. Now it lasts about 15 days. It's still noticeable.	Jingdong prefecture level city in Shandong
Doraemon Hyakubo Shop	King of Power Saving	Fast logistics, good power saving effect, very satisfied	Jingdong prefecture level city in Shandong
Doraemon Hyakubo Shop	King of Power Saving	Has been the second time back to buy, energy saving effect, worth recommending, you can try to see, power saving effect: very good Experience: can save about 30%!	Jingdong prefecture level city in Shandong
Doraemon Hyakubo Shop	King of Power Saving	I don't know how well it works for now	Jingdong prefecture level city in Shandong
Doraemon Hyakubo Shop	King of Power Saving	Factory direct is really good, plugged in and used for half a month did less probably almost 50 degrees of electricity! And the manufacturer's warranty is 5 years.	Jingdong prefecture level city in Shandong
Doraemon Hyakubo Shop	King of Power Saving	It's really effective. The previous month's electricity was $286, but after using the power saver for a month, I have $195 left this month, which is not bad.	Jingdong prefecture level city in Shandong
Doraemon Hyakubo Shop	King of Power Saving	I'll give you a good review and see how it goes.	Jingdong prefecture level city in Shandong
Doraemon Hyakubo Shop	King of Power Saving	Customer service is very professional and patient to guide the installation, the quality of workmanship is very fine. Durable, second repurchase. Really can save oh!	Jingdong prefecture level city in Shandong
Doraemon Hyakubo Shop	King of Power Saving	I've been using it for a month now and my electric bill is over $30 less than last month. I feel it's quite worth buying.	Jingdong prefecture level city in Shandong
Doraemon Hyakubo Shop	King of Power Saving	Small size, does not take up space. It's very good, it does work very effectively, the tool room has a lot of work with a lot of electricity. After installing it, it really reduces a lot of electricity bills. Before the day 30 degrees of electricity left stone, now work down really less a few degrees.	Jingdong prefecture level city in Shandong
Doraemon Hyakubo Shop	King of Power Saving	It does work for saving electricity, I've bought the kind from elsewhere before that was too simple not **all**. This time to buy this store, but also for the safety. Air conditioning is too much electricity, this use to save a lot of	Jingdong prefecture level city in Shandong
Doraemon Hyakubo Shop	King of Power Saving	Two air conditioners and a refrigerator at home, a month down the electricity bill is almost two hundred dollars, with this after the average savings of more than thirty dollars to spend less money to enjoy more, it's really very good!	Jingdong prefecture level city in Shandong
Doraemon Hyakubo Shop	King of Power Saving	Good. Very good.	Jingdong prefecture level city in Shandong
Doraemon Hyakubo Shop	King of Power Saving	Works great, easy to operate, great, very happy with it.	Jingdong prefecture level city in Shandong
Doraemon Hyakubo Shop	King of Power Saving	Received it and tried it out, it's great, great quality and worth having! Five stars praise!	Jingdong prefecture level city in Shandong
Doraemon Hyakubo Shop	King of Power Saving	Very trustworthy brand, quality is guaranteed, the price is still affordable, quite satisfied!	Jingdong prefecture level city in Shandong
Doraemon Hyakubo Shop	King of Power Saving	Use it before you speak.	Jingdong prefecture level city in Shandong
Doraemon Hyakubo Shop	King of Power Saving	The quality of workmanship is very good and the power saving effect is quite good, I regret not getting it earlier.	Jingdong prefecture level city in Shandong
Doraemon Hyakubo Shop	King of Power Saving	Merchants shipping speed, and customer service introduced the same very good, cost-effective	Jingdong prefecture level city in Shandong
Doraemon Hyakubo Shop	King of Power Saving	Feel a little effect Oh, where the power consumption where to plug, convenient and convenient	Jingdong prefecture level city in Shandong
Doraemon Hyakubo Shop	King of Power Saving	The quality of the imported saver is very good, practical and satisfactory. Cost-effective	Jingdong prefecture level city in Shandong
Doraemon Hyakubo Shop	King of Power Saving	Power Saver is especially good, good and inexpensive, very good	Jingdong prefecture level city in Shandong
Doraemon Hyakubo Shop	King of Power Saving	Fast shipping, awesome quality, affordable price, satisfied.	Jingdong prefecture level city in Shandong
Doraemon Hyakubo Shop	King of Power Saving	Energy saver received, the effect is consistent with the description, recommended to buy!	Jingdong prefecture level city in Shandong
Doraemon Hyakubo Shop	King of Power Saving	It's really good, the quality is great, I recommend it!	Jingdong prefecture level city in Shandong
Doraemon Hyakubo Shop	King of Power Saving	Works well. Inexpensive. Shipping was fast.	Jingdong prefecture level city in Shandong
Doraemon Hyakubo Shop	King of Power Saving	Logistics is fast, works well, energy saving is good	Jingdong prefecture level city in Shandong
Doraemon Hyakubo Shop	King of Power Saving	very happy	Jingdong prefecture level city in Shandong
Doraemon Hyakubo Shop	King of Power Saving	an excellent	Jingdong prefecture level city in Shandong
Doraemon Hyakubo Shop	King of Power Saving	It rained a couple days ago and I just went to get the item back today , not bad, hey I looked at the meter and that red display light takes several seconds to jump, did not make a mistake buying it. After a period of time to evaluate.	Jingdong prefecture level city in Shandong

Doraemon Hyakubo Shop	King of Power Saving	Used more than 10 days to check the power, there is indeed some decline, customer service said, long-term use will save more points!	Jingdong prefecture level city in Shandong
Doraemon Hyakubo Shop	King of Power Saving	It's a proper IQ tax, and it costs electricity.	Jingdong prefecture level city in Shandong
Doraemon Hyakubo Shop	King of Power Saving	I've been using it for 17 days now, it really saves electricity Shipping is fast, you can save a lot in a year	Jingdong prefecture level city in Shandong
Doraemon Hyakubo Shop	King of Power Saving	I've been using it for 17 days and it really saves power Shipping is fast and you don't have to worry about the quality, free returns within 5 years	Jingdong prefecture level city in Shandong
Doraemon Hyakubo Shop	King of Power Saving	It's more regular, scanned the code and it's authentic, and the main thing is that you can try it for 90 days! Plugged in more than ten days, is less than before a few degrees per day. When the time to use a month to see Additional. Good to buy a few more	Jingdong prefecture level city in Shandong
Doraemon Hyakubo Shop	King of Power Saving	Logistics is very fast, fine workmanship, use it and then come back to the comments	Jingdong prefecture level city in Shandong
Doraemon Hyakubo Shop	King of Power Saving	The quality of workmanship is very good and the power saving effect is quite good, I regret not getting it earlier.	Jingdong prefecture level city in Shandong
Doraemon Hyakubo Shop	King of Power Saving	Received the goods, according to the instructions on the operation is very simple, to wait and see the effect, looks good!	Jingdong prefecture level city in Shandong
Doraemon Hyakubo Shop	King of Power Saving	It's really as safe and power-saving as the owner says, and it works great!	Jingdong prefecture level city in Shandong
Doraemon Hyakubo Shop	King of Power Saving	Received the goods, immediately used, looks good, to wait and see how the effect.	Jingdong prefecture level city in Shandong
Doraemon Hyakubo Shop	King of Power Saving	Afraid to buy back did not work, the store said the commitment to 90 days invalid refund, warranty 5 years. Saver also complimentary on the Pacific insurance, it is a lot of peace of mind, there are effects will continue to buy back drops!	Jingdong prefecture level city in Shandong
Doraemon Hyakubo Shop	King of Power Saving	Received the goods and used it, can save electricity a month later to see the effect!	Jingdong prefecture level city in Shandong
Doraemon Hyakubo Shop	King of Power Saving	Fancy 90-day trial, 5-year warranty before the order, the goods received, has been experiencing a week, can really feel the savings, in the introduction of friends also buy this!	Jingdong prefecture level city in Shandong
Doraemon Hyakubo Shop	King of Power Saving	Clothing stores use, neighbors recommended to buy, said a month can save more than 70 yuan, first plugged in to see the effect of the	Jingdong prefecture level city in Shandong
Doraemon Hyakubo Shop	King of Power Saving	A friend's home two months to save more than 80 dollars of electricity, recommended that I buy two, decisive purchase, looking forward to the effect of power saving, waiting for the next month effect.	Jingdong prefecture level city in Shandong
Doraemon Hyakubo Shop	King of Power Saving	The goods have been received, consistent with the description of the advertisement, quite good, I hope that in terms of power saving and purifying the air is really as the merchant said that would be better!	Jingdong prefecture level city in Shandong
Doraemon Hyakubo Shop	King of Power Saving	Power Saving King is really good to use the quality is very good than the physical store a lot cheaper we can rest assured that the purchase recommended to buy!	Jingdong prefecture level city in Shandong
Doraemon Hyakubo Shop	King of Power Saving	Energy saver has been used on the rental house inside the use of the meter is always running fast, with this can really save electricity, easy to use, plugged in can be, very satisfied!	Jingdong prefecture level city in Shandong
Doraemon Hyakubo Shop	King of Power Saving	Power Saver Power Saver King is good quality and affordable, very satisfied, worth buying.	Jingdong prefecture level city in Shandong
Doraemon Hyakubo Shop	King of Power Saving	Just use can not see what effect to,, wait for a period of time in the evaluation	Jingdong prefecture level city in Shandong
Doraemon Hyakubo Shop	King of Power Saving	Live in the 18th floor is too far away from the meter, afraid of no effect, look at the trial 90 days before buying. Currently used for a month, there is an effect, saving 27 dollars in electricity, next month should be able to save more!	Jingdong prefecture level city in Shandong
Doraemon Hyakubo Shop	King of Power Saving	I've been using it for almost a month now and it feels good, so I'll use it again.	Jingdong prefecture level city in Shandong
Doraemon Hyakubo Shop	King of Power Saving	It's ok, the electric bill is really less, originally I remember charging $100 and using it for 10 days. Now it lasts about 15 days. It's still noticeable.	Jingdong prefecture level city in Shandong
Doraemon Hyakubo Shop	King of Power Saving	Fast logistics, good power saving effect, very satisfied	Jingdong prefecture level city in Shandong
Doraemon Hyakubo Shop	King of Power Saving	Has been the second time back to buy, energy saving effect, worth recommending, you can try to see, power saving effect: very good Experience: can save about 30%!	Jingdong prefecture level city in Shandong
Doraemon Hyakubo Shop	King of Power Saving	I don't know how well it works for now	Jingdong prefecture level city in Shandong
Doraemon Hyakubo Shop	King of Power Saving	Factory direct is really good, plugged in and used for half a month did less probably almost 50 degrees of electricity! And the manufacturer's warranty is 5 years.	Jingdong prefecture level city in Shandong
Doraemon Hyakubo Shop	King of Power Saving	It's really effective. The previous month's electricity was $286, but after using the power saver for a month, I have $195 left this month, which is not bad.	Jingdong prefecture level city in Shandong
Doraemon Hyakubo Shop	King of Power Saving	I'll give you a good review and see how it goes.	Jingdong prefecture level city in Shandong
Doraemon Hyakubo Shop	King of Power Saving	Customer service is very professional and patient to guide the installation, the quality of workmanship is very fine. Durable, second repurchase. Really can save oh!	Jingdong prefecture level city in Shandong
Doraemon Hyakubo Shop	King of Power Saving	I've been using it for a month now and my electric bill is over $30 less than last month. I feel it's quite worth buying.	Jingdong prefecture level city in Shandong
Doraemon Hyakubo Shop	King of Power Saving	Small size, does not take up space. It's very good, it does work very effectively, the tool room has a lot of work with a lot of electricity. After installing it, it really reduces a lot of electricity bills. Before the day 30 degrees of electricity left stone, now work down really less a few degrees.	Jingdong prefecture level city in Shandong
Doraemon Hyakubo Shop	King of Power Saving	It does work for saving electricity, I've bought the kind from elsewhere before that was too simple not **all**. This time to buy this store, but also for the safety. Air conditioning is too much electricity, this use to save a lot of	Jingdong prefecture level city in Shandong
Doraemon Hyakubo Shop	King of Power Saving	Two air conditioners and a refrigerator at home, a month down the electricity bill is almost two hundred dollars, with this after the average savings of more than thirty dollars to spend less money to enjoy more, it's really very good!	Jingdong prefecture level city in Shandong
Doraemon Hyakubo Shop	King of Power Saving	Good. Very good.	Jingdong prefecture level city in Shandong
Doraemon Hyakubo Shop	King of Power Saving	Works great, easy to operate, great, very happy with it.	Jingdong prefecture level city in Shandong
Doraemon Hyakubo Shop	King of Power Saving	Received it and tried it out, it's great, great quality and worth having! Five stars praise!	Jingdong prefecture level city in Shandong
Doraemon Hyakubo Shop	King of Power Saving	Very trustworthy brand, quality is guaranteed, the price is still affordable, quite satisfied!	Jingdong prefecture level city in Shandong
Doraemon Hyakubo Shop	King of Power Saving	Use it before you speak.	Jingdong prefecture level city in Shandong
Doraemon Hyakubo Shop	King of Power Saving	The quality of workmanship is very good and the power saving effect is quite good, I regret not getting it earlier.	Jingdong prefecture level city in Shandong
Doraemon Hyakubo Shop	King of Power Saving	Merchants shipping speed, and customer service introduced the same very good, cost-effective	Jingdong prefecture level city in Shandong
Doraemon Hyakubo Shop	King of Power Saving	Feel a little effect Oh, where the power consumption where to plug, convenient and convenient	Jingdong prefecture level city in Shandong
Doraemon Hyakubo Shop	King of Power Saving	The quality of the imported saver is very good, practical and satisfactory. Cost-effective	Jingdong prefecture level city in Shandong
Doraemon Hyakubo Shop	King of Power Saving	Power Saver is especially good, good and inexpensive, very good	Jingdong prefecture level city in Shandong
Doraemon Hyakubo Shop	King of Power Saving	Fast shipping, awesome quality, affordable price, satisfied.	Jingdong prefecture level city in Shandong
Doraemon Hyakubo Shop	King of Power Saving	Energy saver received, the effect is consistent with the description, recommended to buy!	Jingdong prefecture level city in Shandong
Doraemon Hyakubo Shop	King of Power Saving	It's really good, the quality is great, I recommend it!	Jingdong prefecture level city in Shandong
Doraemon Hyakubo Shop	King of Power Saving	Works well. Inexpensive. Shipping was fast.	Jingdong prefecture level city in Shandong

Doraemon Hyakubo Shop	King of Power Saving	Logistics is fast, works well, energy saving is good	Jingdong prefecture level city in Shandong
Doraemon Hyakubo Shop	King of Power Saving	very happy	Jingdong prefecture level city in Shandong
Doraemon Hyakubo Shop	King of Power Saving	an excellent	Jingdong prefecture level city in Shandong
Doraemon Hyakubo Shop	King of Power Saving	It rained a couple days ago and I just went to get the item back today , not bad, hey I looked at the meter and that red display light takes several seconds to jump, did not make a mistake buying it. After a period of time to evaluate.	Jingdong prefecture level city in Shandong
Doraemon Hyakubo Shop	King of Power Saving	Used more than 10 days to check the power, there is indeed some decline, customer service said, long-term use will save more points!	Jingdong prefecture level city in Shandong
Doraemon Hyakubo Shop	King of Power Saving	It's a proper IQ tax, and it costs electricity.	Jingdong prefecture level city in Shandong
Doraemon Hyakubo Shop	King of Power Saving	I've been using it for 17 days now, it really saves electricity Shipping is fast, you can save a lot in a year	Jingdong prefecture level city in Shandong
Doraemon Hyakubo Shop	King of Power Saving	I've been using it for 17 days and it really saves power Shipping is fast and you don't have to worry about the quality, free returns within 5 years	Jingdong prefecture level city in Shandong
Doraemon Hyakubo Shop	King of Power Saving	It's more regular, scanned the code and it's authentic, and the main thing is that you can try it for 90 days! Plugged in more than ten days, is less than before a few degrees per day. When the time to use a month to see Additional. Good to buy a few more	Jingdong prefecture level city in Shandong
Doraemon Hyakubo Shop	King of Power Saving	Logistics is very fast, fine workmanship, use it and then come back to the comments	Jingdong prefecture level city in Shandong
Doraemon Hyakubo Shop	King of Power Saving	The quality of workmanship is very good and the power saving effect is quite good, I regret not getting it earlier.	Jingdong prefecture level city in Shandong
Doraemon Hyakubo Shop	King of Power Saving	Received the goods, according to the instructions on the operation is very simple, to wait and see the effect, looks good!	Jingdong prefecture level city in Shandong
Doraemon Hyakubo Shop	King of Power Saving	It's really as safe and power-saving as the owner says, and it works great!	Jingdong prefecture level city in Shandong
Doraemon Hyakubo Shop	King of Power Saving	Received the goods, immediately used, looks good, to wait and see how the effect.	Jingdong prefecture level city in Shandong
Doraemon Hyakubo Shop	King of Power Saving	Afraid to buy back did not work, the store said the commitment to 90 days invalid refund, warranty 5 years. Saver also complimentary on the Pacific insurance, it is a lot of peace of mind, there are effects will continue to buy back drops!	Jingdong prefecture level city in Shandong
Doraemon Hyakubo Shop	King of Power Saving	Received the goods and used it, can save electricity a month later to see the effect!	Jingdong prefecture level city in Shandong
Doraemon Hyakubo Shop	King of Power Saving	Fancy 90-day trial, 5-year warranty before the order, the goods received, has been experiencing a week, can really feel the savings, in the introduction of friends also buy this!	Jingdong prefecture level city in Shandong
Doraemon Hyakubo Shop	King of Power Saving	Clothing stores use, neighbors recommended to buy, said a month can save more than 70 yuan, first plugged in to see the effect of the	Jingdong prefecture level city in Shandong
Doraemon Hyakubo Shop	King of Power Saving	A friend's home two months to save more than 80 dollars of electricity, recommended that I buy two, decisive purchase, looking forward to the effect of power saving, waiting for the next month effect.	Jingdong prefecture level city in Shandong
Doraemon Hyakubo Shop	King of Power Saving	The goods have been received, consistent with the description of the advertisement, quite good, I hope that in terms of power saving and purifying the air is really as the merchant said that would be better!	Jingdong prefecture level city in Shandong
Doraemon Hyakubo Shop	King of Power Saving	Power Saving King is really good to use the quality is very good than the physical store a lot cheaper we can rest assured that the purchase recommended to buy!	Jingdong prefecture level city in Shandong
Doraemon Hyakubo Shop	King of Power Saving	Energy saver has been used on the rental house inside the use of the meter is always running fast, with this can really save electricity, easy to use, plugged in can be, very satisfied!	Jingdong prefecture level city in Shandong
Doraemon Hyakubo Shop	King of Power Saving	Power Saver Power Saver King is good quality and affordable, very satisfied, worth buying.	Jingdong prefecture level city in Shandong
Doraemon Hyakubo Shop	King of Power Saving	Just use can not see what effect to,, wait for a period of time in the evaluation	Jingdong prefecture level city in Shandong
Doraemon Hyakubo Shop	King of Power Saving	Live in the 18th floor is too far away from the meter, afraid of no effect, look at the trial 90 days before buying. Currently used for a month, there is an effect, saving 27 dollars in electricity, next month should be able to save more!	Jingdong prefecture level city in Shandong
Doraemon Hyakubo Shop	King of Power Saving	I've been using it for almost a month now and it feels good, so I'll use it again.	Jingdong prefecture level city in Shandong
Doraemon Hyakubo Shop	King of Power Saving	It's ok, the electric bill is really less, originally I remember charging $100 and using it for 10 days. Now it lasts about 15 days. It's still noticeable.	Jingdong prefecture level city in Shandong
Doraemon Hyakubo Shop	King of Power Saving	Fast logistics, good power saving effect, very satisfied	Jingdong prefecture level city in Shandong
Doraemon Hyakubo Shop	King of Power Saving	Has been the second time back to buy, energy saving effect, worth recommending, you can try to see, power saving effect: very good Experience: can save about 30%!	Jingdong prefecture level city in Shandong
Doraemon Hyakubo Shop	King of Power Saving	I don't know how well it works for now	Jingdong prefecture level city in Shandong
Doraemon Hyakubo Shop	King of Power Saving	Factory direct is really good, plugged in and used for half a month did less probably almost 50 degrees of electricity! And the manufacturer's warranty is 5 years.	Jingdong prefecture level city in Shandong
Doraemon Hyakubo Shop	King of Power Saving	It's really effective. The previous month's electricity was $286, but after using the power saver for a month, I have $195 left this month, which is not bad.	Jingdong prefecture level city in Shandong
Doraemon Hyakubo Shop	King of Power Saving	I'll give you a good review and see how it goes.	Jingdong prefecture level city in Shandong
Doraemon Hyakubo Shop	King of Power Saving	Customer service is very professional and patient to guide the installation, the quality of workmanship is very fine. Durable, second repurchase. Really can save oh!	Jingdong prefecture level city in Shandong
Doraemon Hyakubo Shop	King of Power Saving	I've been using it for a month now and my electric bill is over $30 less than last month. I feel it's quite worth buying.	Jingdong prefecture level city in Shandong
Doraemon Hyakubo Shop	King of Power Saving	Small size, does not take up space. It's very good, it does work very effectively, the tool room has a lot of work with a lot of electricity. After installing it, it really reduces a lot of electricity bills. Before the day 30 degrees of electricity left stone, now work down really less a few degrees.	Jingdong prefecture level city in Shandong
Doraemon Hyakubo Shop	King of Power Saving	It does work for saving electricity, I've bought the kind from elsewhere before that was too simple not **all**. This time to buy this store, but also for the safety. Air conditioning is too much electricity, this use to save a lot of	Jingdong prefecture level city in Shandong
Doraemon Hyakubo Shop	King of Power Saving	Two air conditioners and a refrigerator at home, a month down the electricity bill is almost two hundred dollars, with this after the average savings of more than thirty dollars to spend less money to enjoy more, it's really very good!	Jingdong prefecture level city in Shandong
Doraemon Hyakubo Shop	King of Power Saving	Good. Very good.	Jingdong prefecture level city in Shandong
Doraemon Hyakubo Shop	King of Power Saving	Works great, easy to operate, great, very happy with it.	Jingdong prefecture level city in Shandong
Doraemon Hyakubo Shop	King of Power Saving	Received it and tried it out, it's great, great quality and worth having! Five stars praise!	Jingdong prefecture level city in Shandong
Doraemon Hyakubo Shop	King of Power Saving	Very trustworthy brand, quality is guaranteed, the price is still affordable, quite satisfied!	Jingdong prefecture level city in Shandong
Doraemon Hyakubo Shop	King of Power Saving	Use it before you speak.	Jingdong prefecture level city in Shandong
Doraemon Hyakubo Shop	King of Power Saving	The quality of workmanship is very good and the power saving effect is quite good, I regret not getting it earlier.	Jingdong prefecture level city in Shandong
Doraemon Hyakubo Shop	King of Power Saving	Merchants shipping speed, and customer service introduced the same very good, cost-effective	Jingdong prefecture level city in Shandong
Doraemon Hyakubo Shop	King of Power Saving	Feel a little effect Oh, where the power consumption where to plug, convenient and convenient	Jingdong prefecture level city in Shandong
Doraemon Hyakubo Shop	King of Power Saving	The quality of the imported saver is very good, practical and satisfactory. Cost-effective	Jingdong prefecture level city in Shandong
Doraemon Hyakubo Shop	King of Power Saving	Power Saver is especially good, good and inexpensive, very good	Jingdong prefecture level city in Shandong

Doraemon Hyakubo Shop	King of Power Saving	Fast shipping, awesome quality, affordable price, satisfied.	Jingdong prefecture level city in Shandong
Doraemon Hyakubo Shop	King of Power Saving	Energy saver received, the effect is consistent with the description, recommended to buy!	Jingdong prefecture level city in Shandong
Doraemon Hyakubo Shop	King of Power Saving	It's really good, the quality is great, I recommend it!	Jingdong prefecture level city in Shandong
Doraemon Hyakubo Shop	King of Power Saving	Works well. Inexpensive. Shipping was fast.	Jingdong prefecture level city in Shandong
Doraemon Hyakubo Shop	King of Power Saving	Logistics is fast, works well, energy saving is good	Jingdong prefecture level city in Shandong
Doraemon Hyakubo Shop	King of Power Saving	very happy	Jingdong prefecture level city in Shandong
Doraemon Hyakubo Shop	King of Power Saving	an excellent	Jingdong prefecture level city in Shandong
Doraemon Hyakubo Shop	King of Power Saving	It rained a couple days ago and I just went to get the item back today , not bad, hey I looked at the meter and that red display light takes several seconds to jump, did not make a mistake buying it. After a period of time to evaluate.	Jingdong prefecture level city in Shandong
Doraemon Hyakubo Shop	King of Power Saving	Used more than 10 days to check the power, there is indeed some decline, customer service said, long-term use will save more points!	Jingdong prefecture level city in Shandong
Doraemon Hyakubo Shop	King of Power Saving	It's a proper IQ tax, and it costs electricity.	Jingdong prefecture level city in Shandong
Doraemon Hyakubo Shop	King of Power Saving	I've been using it for 17 days now, it really saves electricity Shipping is fast, you can save a lot in a year	Jingdong prefecture level city in Shandong
Doraemon Hyakubo Shop	King of Power Saving	I've been using it for 17 days and it really saves power Shipping is fast and you don't have to worry about the quality, free returns within 5 years	Jingdong prefecture level city in Shandong
Doraemon Hyakubo Shop	King of Power Saving	It's more regular, scanned the code and it's authentic, and the main thing is that you can try it for 90 days! Plugged in more than ten days, is less than before a few degrees per day. When the time to use a month to see Additional. Good to buy a few more	Jingdong prefecture level city in Shandong
Doraemon Hyakubo Shop	King of Power Saving	Logistics is very fast, fine workmanship, use it and then come back to the comments	Jingdong prefecture level city in Shandong
Doraemon Hyakubo Shop	King of Power Saving	The quality of workmanship is very good and the power saving effect is quite good, I regret not getting it earlier.	Jingdong prefecture level city in Shandong
Doraemon Hyakubo Shop	King of Power Saving	Received the goods, according to the instructions on the operation is very simple, to wait and see the effect, looks good!	Jingdong prefecture level city in Shandong
Doraemon Hyakubo Shop	King of Power Saving	It's really as safe and power-saving as the owner says, and it works great!	Jingdong prefecture level city in Shandong
Doraemon Hyakubo Shop	King of Power Saving	Received the goods, immediately used, looks good, to wait and see how the effect.	Jingdong prefecture level city in Shandong
Doraemon Hyakubo Shop	King of Power Saving	Afraid to buy back did not work, the store said the commitment to 90 days invalid refund, warranty 5 years. Saver also complimentary on the Pacific insurance, it is a lot of peace of mind, there are effects will continue to buy back drops!	Jingdong prefecture level city in Shandong
Doraemon Hyakubo Shop	King of Power Saving	Received the goods and used it, can save electricity a month later to see the effect!	Jingdong prefecture level city in Shandong
Doraemon Hyakubo Shop	King of Power Saving	Fancy 90-day trial, 5-year warranty before the order, the goods received, has been experiencing a week, can really feel the savings, in the introduction of friends also buy this!	Jingdong prefecture level city in Shandong
Doraemon Hyakubo Shop	King of Power Saving	Clothing stores use, neighbors recommended to buy, said a month can save more than 70 yuan, first plugged in to see the effect of the	Jingdong prefecture level city in Shandong
Doraemon Hyakubo Shop	King of Power Saving	A friend's home two months to save more than 80 dollars of electricity, recommended that I buy two, decisive purchase, looking forward to the effect of power saving, waiting for the next month effect.	Jingdong prefecture level city in Shandong
Doraemon Hyakubo Shop	King of Power Saving	The goods have been received, consistent with the description of the advertisement, quite good, I hope that in terms of power saving and purifying the air is really as the merchant said that would be better!	Jingdong prefecture level city in Shandong
Doraemon Hyakubo Shop	King of Power Saving	Power Saving King is really good to use the quality is very good than the physical store a lot cheaper we can rest assured that the purchase recommended to buy!	Jingdong prefecture level city in Shandong
Doraemon Hyakubo Shop	King of Power Saving	Energy saver has been used on the rental house inside the use of the meter is always running fast, with this can really save electricity, easy to use, plugged in can be, very satisfied!	Jingdong prefecture level city in Shandong
Doraemon Hyakubo Shop	King of Power Saving	Power Saver Power Saver King is good quality and affordable, very satisfied, worth buying.	Jingdong prefecture level city in Shandong
Doraemon Hyakubo Shop	King of Power Saving	Just use can not see what effect to,, wait for a period of time in the evaluation	Jingdong prefecture level city in Shandong
Doraemon Hyakubo Shop	King of Power Saving	Live in the 18th floor is too far away from the meter, afraid of no effect, look at the trial 90 days before buying. Currently used for a month, there is an effect, saving 27 dollars in electricity, next month should be able to save more!	Jingdong prefecture level city in Shandong
Doraemon Hyakubo Shop	King of Power Saving	I've been using it for almost a month now and it feels good, so I'll use it again.	Jingdong prefecture level city in Shandong
Doraemon Hyakubo Shop	King of Power Saving	It's ok, the electric bill is really less, originally I remember charging $100 and using it for 10 days. Now it lasts about 15 days. It's still noticeable.	Jingdong prefecture level city in Shandong
Doraemon Hyakubo Shop	King of Power Saving	Fast logistics, good power saving effect, very satisfied	Jingdong prefecture level city in Shandong
Doraemon Hyakubo Shop	King of Power Saving	Has been the second time back to buy, energy saving effect, worth recommending, you can try to see, power saving effect: very good Experience: can save about 30%!	Jingdong prefecture level city in Shandong
Doraemon Hyakubo Shop	King of Power Saving	I don't know how well it works for now	Jingdong prefecture level city in Shandong
Doraemon Hyakubo Shop	King of Power Saving	Factory direct is really good, plugged in and used for half a month did less probably almost 50 degrees of electricity! And the manufacturer's warranty is 5 years.	Jingdong prefecture level city in Shandong
Doraemon Hyakubo Shop	King of Power Saving	It's really effective. The previous month's electricity was $286, but after using the power saver for a month, I have $195 left this month, which is not bad.	Jingdong prefecture level city in Shandong
Doraemon Hyakubo Shop	King of Power Saving	I'll give you a good review and see how it goes.	Jingdong prefecture level city in Shandong
Doraemon Hyakubo Shop	King of Power Saving	Customer service is very professional and patient to guide the installation, the quality of workmanship is very fine. Durable, second repurchase. Really can save oh!	Jingdong prefecture level city in Shandong
Doraemon Hyakubo Shop	King of Power Saving	I've been using it for a month now and my electric bill is over $30 less than last month. I feel it's quite worth buying.	Jingdong prefecture level city in Shandong
Doraemon Hyakubo Shop	King of Power Saving	Small size, does not take up space. It's very good, it does work very effectively, the tool room has a lot of work with a lot of electricity. After installing it, it really reduces a lot of electricity bills. Before the day 30 degrees of electricity left stone, now work down really less a few degrees.	Jingdong prefecture level city in Shandong
Doraemon Hyakubo Shop	King of Power Saving	It does work for saving electricity, I've bought the kind from elsewhere before that was too simple not **all**. This time to buy this store, but also for the safety. Air conditioning is too much electricity, this use to save a lot of	Jingdong prefecture level city in Shandong
Doraemon Hyakubo Shop	King of Power Saving	Two air conditioners and a refrigerator at home, a month down the electricity bill is almost two hundred dollars, with this after the average savings of more than thirty dollars to spend less money to enjoy more, it's really very good!	Jingdong prefecture level city in Shandong
Doraemon Hyakubo Shop	King of Power Saving	Good. Very good.	Jingdong prefecture level city in Shandong
Doraemon Hyakubo Shop	King of Power Saving	Works great, easy to operate, great, very happy with it.	Jingdong prefecture level city in Shandong
Doraemon Hyakubo Shop	King of Power Saving	Received it and tried it out, it's great, great quality and worth having! Five stars praise!	Jingdong prefecture level city in Shandong
Doraemon Hyakubo Shop	King of Power Saving	Very trustworthy brand, quality is guaranteed, the price is still affordable, quite satisfied!	Jingdong prefecture level city in Shandong
Doraemon Hyakubo Shop	King of Power Saving	Use it before you speak.	Jingdong prefecture level city in Shandong
Doraemon Hyakubo Shop	King of Power Saving	The quality of workmanship is very good and the power saving effect is quite good, I regret not getting it earlier.	Jingdong prefecture level city in Shandong

Doraemon Hyakubo Shop	King of Power Saving	Merchants shipping speed, and customer service introduced the same very good, cost-effective	Jingdong prefecture level city in Shandong
Doraemon Hyakubo Shop	King of Power Saving	Feel a little effect Oh, where the power consumption where to plug, convenient and convenient	Jingdong prefecture level city in Shandong
Doraemon Hyakubo Shop	King of Power Saving	The quality of the imported saver is very good, practical and satisfactory. Cost-effective	Jingdong prefecture level city in Shandong
Doraemon Hyakubo Shop	King of Power Saving	Power Saver is especially good, good and inexpensive, very good	Jingdong prefecture level city in Shandong
Doraemon Hyakubo Shop	King of Power Saving	Fast shipping, awesome quality, affordable price, satisfied.	Jingdong prefecture level city in Shandong
Doraemon Hyakubo Shop	King of Power Saving	Energy saver received, the effect is consistent with the description, recommended to buy!	Jingdong prefecture level city in Shandong
Doraemon Hyakubo Shop	King of Power Saving	It's really good, the quality is great, I recommend it!	Jingdong prefecture level city in Shandong
Doraemon Hyakubo Shop	King of Power Saving	Works well. Inexpensive. Shipping was fast.	Jingdong prefecture level city in Shandong
Doraemon Hyakubo Shop	King of Power Saving	Logistics is fast, works well, energy saving is good	Jingdong prefecture level city in Shandong
Doraemon Hyakubo Shop	King of Power Saving	very happy	Jingdong prefecture level city in Shandong
Doraemon Hyakubo Shop	King of Power Saving	an excellent	Jingdong prefecture level city in Shandong
Doraemon Hyakubo Shop	King of Power Saving	It rained a couple days ago and I just went to get the item back today , not bad, hey I looked at the meter and that red display light takes several seconds to jump, did not make a mistake buying it. After a period of time to evaluate.	Jingdong prefecture level city in Shandong
Doraemon Hyakubo Shop	King of Power Saving	Used more than 10 days to check the power, there is indeed some decline, customer service said, long-term use will save more points!	Jingdong prefecture level city in Shandong
Doraemon Hyakubo Shop	King of Power Saving	It's a proper IQ tax, and it costs electricity.	Jingdong prefecture level city in Shandong
Doraemon Hyakubo Shop	King of Power Saving	I've been using it for 17 days now, it really saves electricity Shipping is fast, you can save a lot in a year	Jingdong prefecture level city in Shandong
Doraemon Hyakubo Shop	King of Power Saving	I've been using it for 17 days and it really saves power Shipping is fast and you don't have to worry about the quality, free returns within 5 years	Jingdong prefecture level city in Shandong
Doraemon Hyakubo Shop	King of Power Saving	It's more regular, scanned the code and it's authentic, and the main thing is that you can try it for 90 days! Plugged in more than ten days, is less than before a few degrees per day. When the time to use a month to see Additional. Good to buy a few more	Jingdong prefecture level city in Shandong
Doraemon Hyakubo Shop	King of Power Saving	Logistics is very fast, fine workmanship, use it and then come back to the comments	Jingdong prefecture level city in Shandong
Doraemon Hyakubo Shop	King of Power Saving	The quality of workmanship is very good and the power saving effect is quite good, I regret not getting it earlier.	Jingdong prefecture level city in Shandong
Doraemon Hyakubo Shop	King of Power Saving	Received the goods, according to the instructions on the operation is very simple, to wait and see the effect, looks good!	Jingdong prefecture level city in Shandong
Doraemon Hyakubo Shop	King of Power Saving	It's really as safe and power-saving as the owner says, and it works great!	Jingdong prefecture level city in Shandong
Doraemon Hyakubo Shop	King of Power Saving	Received the goods, immediately used, looks good, to wait and see how the effect.	Jingdong prefecture level city in Shandong
Doraemon Hyakubo Shop	King of Power Saving	Afraid to buy back did not work, the store said the commitment to 90 days invalid refund, warranty 5 years. Saver also complimentary on the Pacific insurance, it is a lot of peace of mind, there are effects will continue to buy back drops!	Jingdong prefecture level city in Shandong
Doraemon Hyakubo Shop	King of Power Saving	Received the goods and used it, can save electricity a month later to see the effect!	Jingdong prefecture level city in Shandong
Doraemon Hyakubo Shop	King of Power Saving	Fancy 90-day trial, 5-year warranty before the order, the goods received, has been experiencing a week, can really feel the savings, in the introduction of friends also buy this!	Jingdong prefecture level city in Shandong
Doraemon Hyakubo Shop	King of Power Saving	Clothing stores use, neighbors recommended to buy, said a month can save more than 70 yuan, first plugged in to see the effect of the	Jingdong prefecture level city in Shandong
Doraemon Hyakubo Shop	King of Power Saving	A friend's home two months to save more than 80 dollars of electricity, recommended that I buy two, decisive purchase, looking forward to the effect of power saving, waiting for the next month effect.	Jingdong prefecture level city in Shandong
Doraemon Hyakubo Shop	King of Power Saving	The goods have been received, consistent with the description of the advertisement, quite good, I hope that in terms of power saving and purifying the air is really as the merchant said that would be better!	Jingdong prefecture level city in Shandong
Doraemon Hyakubo Shop	King of Power Saving	Power Saving King is really good to use the quality is very good than the physical store a lot cheaper we can rest assured that the purchase recommended to buy!	Jingdong prefecture level city in Shandong
Doraemon Hyakubo Shop	King of Power Saving	Energy saver has been used on the rental house inside the use of the meter is always running fast, with this can really save electricity, easy to use, plugged in can be, very satisfied!	Jingdong prefecture level city in Shandong
Doraemon Hyakubo Shop	King of Power Saving	Power Saver Power Saver King is good quality and affordable, very satisfied, worth buying.	Jingdong prefecture level city in Shandong
Doraemon Hyakubo Shop	King of Power Saving	Just use can not see what effect to,, wait for a period of time in the evaluation	Jingdong prefecture level city in Shandong
Doraemon Hyakubo Shop	King of Power Saving	Live in the 18th floor is too far away from the meter, afraid of no effect, look at the trial 90 days before buying. Currently used for a month, there is an effect, saving 27 dollars in electricity, next month should be able to save more!	Jingdong prefecture level city in Shandong
Doraemon Hyakubo Shop	King of Power Saving	I've been using it for almost a month now and it feels good, so I'll use it again.	Jingdong prefecture level city in Shandong
Doraemon Hyakubo Shop	King of Power Saving	It's ok, the electric bill is really less, originally I remember charging $100 and using it for 10 days. Now it lasts about 15 days. It's still noticeable.	Jingdong prefecture level city in Shandong
Doraemon Hyakubo Shop	King of Power Saving	Fast logistics, good power saving effect, very satisfied	Jingdong prefecture level city in Shandong
Doraemon Hyakubo Shop	King of Power Saving	Has been the second time back to buy, energy saving effect, worth recommending, you can try to see, power saving effect: very good Experience: can save about 30%!	Jingdong prefecture level city in Shandong
Doraemon Hyakubo Shop	King of Power Saving	I don't know how well it works for now	Jingdong prefecture level city in Shandong
Doraemon Hyakubo Shop	King of Power Saving	Factory direct is really good, plugged in and used for half a month did less probably almost 50 degrees of electricity! And the manufacturer's warranty is 5 years.	Jingdong prefecture level city in Shandong
Doraemon Hyakubo Shop	King of Power Saving	It's really effective. The previous month's electricity was $286, but after using the power saver for a month, I have $195 left this month, which is not bad.	Jingdong prefecture level city in Shandong
Doraemon Hyakubo Shop	King of Power Saving	I'll give you a good review and see how it goes.	Jingdong prefecture level city in Shandong
Doraemon Hyakubo Shop	King of Power Saving	Customer service is very professional and patient to guide the installation, the quality of workmanship is very fine. Durable, second repurchase. Really can save oh!	Jingdong prefecture level city in Shandong
Doraemon Hyakubo Shop	King of Power Saving	I've been using it for a month now and my electric bill is over $30 less than last month. I feel it's quite worth buying.	Jingdong prefecture level city in Shandong
Doraemon Hyakubo Shop	King of Power Saving	Small size, does not take up space. It's very good, it does work very effectively, the tool room has a lot of work with a lot of electricity. After installing it, it really reduces a lot of electricity bills. Before the day 30 degrees of electricity left stone, now work down really less a few degrees.	Jingdong prefecture level city in Shandong
Doraemon Hyakubo Shop	King of Power Saving	It does work for saving electricity, I've bought the kind from elsewhere before that was too simple not **all**. This time to buy this store, but also for the safety. Air conditioning is too much electricity, this use to save a lot of	Jingdong prefecture level city in Shandong
Doraemon Hyakubo Shop	King of Power Saving	Two air conditioners and a refrigerator at home, a month down the electricity bill is almost two hundred dollars, with this after the average savings of more than thirty dollars to spend less money to enjoy more, it's really very good!	Jingdong prefecture level city in Shandong
Doraemon Hyakubo Shop	King of Power Saving	Good. Very good.	Jingdong prefecture level city in Shandong
Doraemon Hyakubo Shop	King of Power Saving	Works great, easy to operate, great, very happy with it.	Jingdong prefecture level city in Shandong

Doraemon Hyakubo Shop	King of Power Saving	Received it and tried it out, it's great, great quality and worth having! Five stars praise!	Jingdong prefecture level city in Shandong
Doraemon Hyakubo Shop	King of Power Saving	Very trustworthy brand, quality is guaranteed, the price is still affordable, quite satisfied!	Jingdong prefecture level city in Shandong
Doraemon Hyakubo Shop	King of Power Saving	Use it before you speak.	Jingdong prefecture level city in Shandong
Doraemon Hyakubo Shop	King of Power Saving	The quality of workmanship is very good and the power saving effect is quite good, I regret not getting it earlier.	Jingdong prefecture level city in Shandong
Doraemon Hyakubo Shop	King of Power Saving	Merchants shipping speed, and customer service introduced the same very good, cost-effective	Jingdong prefecture level city in Shandong
Doraemon Hyakubo Shop	King of Power Saving	Feel a little effect Oh, where the power consumption where to plug, convenient and convenient	Jingdong prefecture level city in Shandong
Doraemon Hyakubo Shop	King of Power Saving	The quality of the imported saver is very good, practical and satisfactory. Cost-effective	Jingdong prefecture level city in Shandong
Doraemon Hyakubo Shop	King of Power Saving	Power Saver is especially good, good and inexpensive, very good	Jingdong prefecture level city in Shandong
Doraemon Hyakubo Shop	King of Power Saving	Fast shipping, awesome quality, affordable price, satisfied.	Jingdong prefecture level city in Shandong
Doraemon Hyakubo Shop	King of Power Saving	Energy saver received, the effect is consistent with the description, recommended to buy!	Jingdong prefecture level city in Shandong
Doraemon Hyakubo Shop	King of Power Saving	It's really good, the quality is great, I recommend it!	Jingdong prefecture level city in Shandong
Doraemon Hyakubo Shop	King of Power Saving	Works well. Inexpensive. Shipping was fast.	Jingdong prefecture level city in Shandong
Doraemon Hyakubo Shop	King of Power Saving	Logistics is fast, works well, energy saving is good	Jingdong prefecture level city in Shandong
Doraemon Hyakubo Shop	King of Power Saving	very happy	Jingdong prefecture level city in Shandong
Doraemon Hyakubo Shop	King of Power Saving	an excellent	Jingdong prefecture level city in Shandong
Doraemon Hyakubo Shop	King of Power Saving	It rained a couple days ago and I just went to get the item back today , not bad, hey I looked at the meter and that red display light takes several seconds to jump, did not make a mistake buying it. After a period of time to evaluate.	Jingdong prefecture level city in Shandong
Doraemon Hyakubo Shop	King of Power Saving	Used more than 10 days to check the power, there is indeed some decline, customer service said, long-term use will save more points!	Jingdong prefecture level city in Shandong
Doraemon Hyakubo Shop	King of Power Saving	It's a proper IQ tax, and it costs electricity.	Jingdong prefecture level city in Shandong
Doraemon Hyakubo Shop	King of Power Saving	I've been using it for 17 days now, it really saves electricity Shipping is fast, you can save a lot in a year	Jingdong prefecture level city in Shandong
Doraemon Hyakubo Shop	King of Power Saving	I've been using it for 17 days and it really saves power Shipping is fast and you don't have to worry about the quality, free returns within 5 years	Jingdong prefecture level city in Shandong
Doraemon Hyakubo Shop	King of Power Saving	It's more regular, scanned the code and it's authentic, and the main thing is that you can try it for 90 days! Plugged in more than ten days, is less than before a few degrees per day. When the time to use a month to see Additional. Good to buy a few more	Jingdong prefecture level city in Shandong
Doraemon Hyakubo Shop	King of Power Saving	Logistics is very fast, fine workmanship, use it and then come back to the comments	Jingdong prefecture level city in Shandong
Doraemon Hyakubo Shop	King of Power Saving	The quality of workmanship is very good and the power saving effect is quite good, I regret not getting it earlier.	Jingdong prefecture level city in Shandong
Doraemon Hyakubo Shop	King of Power Saving	Received the goods, according to the instructions on the operation is very simple, to wait and see the effect, looks good!	Jingdong prefecture level city in Shandong
Doraemon Hyakubo Shop	King of Power Saving	It's really as safe and power-saving as the owner says, and it works great!	Jingdong prefecture level city in Shandong
Doraemon Hyakubo Shop	King of Power Saving	Received the goods, immediately used, looks good, to wait and see how the effect.	Jingdong prefecture level city in Shandong
Doraemon Hyakubo Shop	King of Power Saving	Afraid to buy back did not work, the store said the commitment to 90 days invalid refund, warranty 5 years. Saver also complimentary on the Pacific insurance, it is a lot of peace of mind, there are effects will continue to buy back drops!	Jingdong prefecture level city in Shandong
Doraemon Hyakubo Shop	King of Power Saving	Received the goods and used it, can save electricity a month later to see the effect!	Jingdong prefecture level city in Shandong
Doraemon Hyakubo Shop	King of Power Saving	Fancy 90-day trial, 5-year warranty before the order, the goods received, has been experiencing a week, can really feel the savings, in the introduction of friends also buy this!	Jingdong prefecture level city in Shandong
Doraemon Hyakubo Shop	King of Power Saving	Clothing stores use, neighbors recommended to buy, said a month can save more than 70 yuan, first plugged in to see the effect of the	Jingdong prefecture level city in Shandong
Doraemon Hyakubo Shop	King of Power Saving	A friend's home two months to save more than 80 dollars of electricity, recommended that I buy two, decisive purchase, looking forward to the effect of power saving, waiting for the next month effect.	Jingdong prefecture level city in Shandong
Doraemon Hyakubo Shop	King of Power Saving	The goods have been received, consistent with the description of the advertisement, quite good, I hope that in terms of power saving and purifying the air is really as the merchant said that would be better!	Jingdong prefecture level city in Shandong
Doraemon Hyakubo Shop	King of Power Saving	Power Saving King is really good to use the quality is very good than the physical store a lot cheaper we can rest assured that the purchase recommended to buy!	Jingdong prefecture level city in Shandong
Doraemon Hyakubo Shop	King of Power Saving	Energy saver has been used on the rental house inside the use of the meter is always running fast, with this can really save electricity, easy to use, plugged in can be, very satisfied!	Jingdong prefecture level city in Shandong
Doraemon Hyakubo Shop	King of Power Saving	Power Saver Power Saver King is good quality and affordable, very satisfied, worth buying.	Jingdong prefecture level city in Shandong
Doraemon Hyakubo Shop	King of Power Saving	Just use can not see what effect to,, wait for a period of time in the evaluation	Jingdong prefecture level city in Shandong
Doraemon Hyakubo Shop	King of Power Saving	Live in the 18th floor is too far away from the meter, afraid of no effect, look at the trial 90 days before buying. Currently used for a month, there is an effect, saving 27 dollars in electricity, next month should be able to save more!	Jingdong prefecture level city in Shandong
Doraemon Hyakubo Shop	King of Power Saving	I've been using it for almost a month now and it feels good, so I'll use it again.	Jingdong prefecture level city in Shandong
Doraemon Hyakubo Shop	King of Power Saving	It's ok, the electric bill is really less, originally I remember charging $100 and using it for 10 days. Now it lasts about 15 days. It's still noticeable.	Jingdong prefecture level city in Shandong
Doraemon Hyakubo Shop	King of Power Saving	Fast logistics, good power saving effect, very satisfied	Jingdong prefecture level city in Shandong
Doraemon Hyakubo Shop	King of Power Saving	Has been the second time back to buy, energy saving effect, worth recommending, you can try to see, power saving effect: very good Experience: can save about 30%!	Jingdong prefecture level city in Shandong
Doraemon Hyakubo Shop	King of Power Saving	I don't know how well it works for now	Jingdong prefecture level city in Shandong
Doraemon Hyakubo Shop	King of Power Saving	Factory direct is really good, plugged in and used for half a month did less probably almost 50 degrees of electricity! And the manufacturer's warranty is 5 years.	Jingdong prefecture level city in Shandong
Doraemon Hyakubo Shop	King of Power Saving	It's really effective. The previous month's electricity was $286, but after using the power saver for a month, I have $195 left this month, which is not bad.	Jingdong prefecture level city in Shandong
Doraemon Hyakubo Shop	King of Power Saving	I'll give you a good review and see how it goes.	Jingdong prefecture level city in Shandong
Doraemon Hyakubo Shop	King of Power Saving	Customer service is very professional and patient to guide the installation, the quality of workmanship is very fine. Durable, second repurchase. Really can save oh!	Jingdong prefecture level city in Shandong
Doraemon Hyakubo Shop	King of Power Saving	I've been using it for a month now and my electric bill is over $30 less than last month. I feel it's quite worth buying.	Jingdong prefecture level city in Shandong
Doraemon Hyakubo Shop	King of Power Saving	Small size, does not take up space. It's very good, it does work very effectively, the tool room has a lot of work with a lot of electricity. After installing it, it really reduces a lot of electricity bills. Before the day 30 degrees of electricity left stone, now work down really less a few degrees.	Jingdong prefecture level city in Shandong

Doraemon Hyakubo Shop	King of Power Saving	It does work for saving electricity, I've bought the kind from elsewhere before that was too simple not **all**. This time to buy this store, but also for the safety. Air conditioning is too much electricity, this use to save a lot of	Jingdong prefecture level city in Shandong
Doraemon Hyakubo Shop	King of Power Saving	Two air conditioners and a refrigerator at home, a month down the electricity bill is almost two hundred dollars, with this after the average savings of more than thirty dollars to spend less money to enjoy more, it's really very good!	Jingdong prefecture level city in Shandong
Doraemon Hyakubo Shop	King of Power Saving	Good. Very good.	Jingdong prefecture level city in Shandong
Doraemon Hyakubo Shop	King of Power Saving	Works great, easy to operate, great, very happy with it.	Jingdong prefecture level city in Shandong
Doraemon Hyakubo Shop	King of Power Saving	Received it and tried it out, it's great, great quality and worth having! Five stars praise!	Jingdong prefecture level city in Shandong
Doraemon Hyakubo Shop	King of Power Saving	Very trustworthy brand, quality is guaranteed, the price is still affordable, quite satisfied!	Jingdong prefecture level city in Shandong
Doraemon Hyakubo Shop	King of Power Saving	Use it before you speak.	Jingdong prefecture level city in Shandong
Doraemon Hyakubo Shop	King of Power Saving	The quality of workmanship is very good and the power saving effect is quite good, I regret not getting it earlier.	Jingdong prefecture level city in Shandong
Doraemon Hyakubo Shop	King of Power Saving	Merchants shipping speed, and customer service introduced the same very good, cost-effective	Jingdong prefecture level city in Shandong
Doraemon Hyakubo Shop	King of Power Saving	Feel a little effect Oh, where the power consumption where to plug, convenient and convenient	Jingdong prefecture level city in Shandong
Doraemon Hyakubo Shop	King of Power Saving	The quality of the imported saver is very good, practical and satisfactory. Cost-effective	Jingdong prefecture level city in Shandong
Doraemon Hyakubo Shop	King of Power Saving	Power Saver is especially good, good and inexpensive, very good	Jingdong prefecture level city in Shandong
Doraemon Hyakubo Shop	King of Power Saving	Fast shipping, awesome quality, affordable price, satisfied.	Jingdong prefecture level city in Shandong
Doraemon Hyakubo Shop	King of Power Saving	Energy saver received, the effect is consistent with the description, recommended to buy!	Jingdong prefecture level city in Shandong
Doraemon Hyakubo Shop	King of Power Saving	It's really good, the quality is great, I recommend it!	Jingdong prefecture level city in Shandong
Doraemon Hyakubo Shop	King of Power Saving	Works well. Inexpensive. Shipping was fast.	Jingdong prefecture level city in Shandong
Doraemon Hyakubo Shop	King of Power Saving	Logistics is fast, works well, energy saving is good	Jingdong prefecture level city in Shandong
Doraemon Hyakubo Shop	King of Power Saving	very happy	Jingdong prefecture level city in Shandong
Doraemon Hyakubo Shop	King of Power Saving	an excellent	Jingdong prefecture level city in Shandong
Doraemon Hyakubo Shop	King of Power Saving	It rained a couple days ago and I just went to get the item back today , not bad, hey I looked at the meter and that red display light takes several seconds to jump, did not make a mistake buying it. After a period of time to evaluate.	Jingdong prefecture level city in Shandong
Doraemon Hyakubo Shop	King of Power Saving	Used more than 10 days to check the power, there is indeed some decline, customer service said, long-term use will save more points!	Jingdong prefecture level city in Shandong
Doraemon Hyakubo Shop	King of Power Saving	It's a proper IQ tax, and it costs electricity.	Jingdong prefecture level city in Shandong
Doraemon Hyakubo Shop	King of Power Saving	I've been using it for 17 days now, it really saves electricity Shipping is fast, you can save a lot in a year	Jingdong prefecture level city in Shandong
Doraemon Hyakubo Shop	King of Power Saving	I've been using it for 17 days and it really saves power Shipping is fast and you don't have to worry about the quality, free returns within 5 years	Jingdong prefecture level city in Shandong
Doraemon Hyakubo Shop	King of Power Saving	It's more regular, scanned the code and it's authentic, and the main thing is that you can try it for 90 days! Plugged in more than ten days, is less than before a few degrees per day. When the time to use a month to see Additional. Good to buy a few more	Jingdong prefecture level city in Shandong
Doraemon Hyakubo Shop	King of Power Saving	Logistics is very fast, fine workmanship, use it and then come back to the comments	Jingdong prefecture level city in Shandong
Doraemon Hyakubo Shop	King of Power Saving	The quality of workmanship is very good and the power saving effect is quite good, I regret not getting it earlier.	Jingdong prefecture level city in Shandong
Doraemon Hyakubo Shop	King of Power Saving	Received the goods, according to the instructions on the operation is very simple, to wait and see the effect, looks good!	Jingdong prefecture level city in Shandong
Doraemon Hyakubo Shop	King of Power Saving	It's really as safe and power-saving as the owner says, and it works great!	Jingdong prefecture level city in Shandong
Doraemon Hyakubo Shop	King of Power Saving	Received the goods, immediately used, looks good, to wait and see how the effect.	Jingdong prefecture level city in Shandong
Doraemon Hyakubo Shop	King of Power Saving	Afraid to buy back did not work, the store said the commitment to 90 days invalid refund, warranty 5 years. Saver also complimentary on the Pacific insurance, it is a lot of peace of mind, there are effects will continue to buy back drops!	Jingdong prefecture level city in Shandong
Doraemon Hyakubo Shop	King of Power Saving	Received the goods and used it, can save electricity a month later to see the effect!	Jingdong prefecture level city in Shandong
Doraemon Hyakubo Shop	King of Power Saving	Fancy 90-day trial, 5-year warranty before the order, the goods received, has been experiencing a week, can really feel the savings, in the introduction of friends also buy this!	Jingdong prefecture level city in Shandong
Doraemon Hyakubo Shop	King of Power Saving	Clothing stores use, neighbors recommended to buy, said a month can save more than 70 yuan, first plugged in to see the effect of the	Jingdong prefecture level city in Shandong
Doraemon Hyakubo Shop	King of Power Saving	A friend's home two months to save more than 80 dollars of electricity, recommended that I buy two, decisive purchase, looking forward to the effect of power saving, waiting for the next month effect.	Jingdong prefecture level city in Shandong
Doraemon Hyakubo Shop	King of Power Saving	The goods have been received, consistent with the description of the advertisement, quite good, I hope that in terms of power saving and purifying the air is really as the merchant said that would be better!	Jingdong prefecture level city in Shandong
Doraemon Hyakubo Shop	King of Power Saving	Power Saving King is really good to use the quality is very good than the physical store a lot cheaper we can rest assured that the purchase recommended to buy!	Jingdong prefecture level city in Shandong
Doraemon Hyakubo Shop	King of Power Saving	Energy saver has been used on the rental house inside the use of the meter is always running fast, with this can really save electricity, easy to use, plugged in can be, very satisfied!	Jingdong prefecture level city in Shandong
Doraemon Hyakubo Shop	King of Power Saving	Power Saver Power Saver King is good quality and affordable, very satisfied, worth buying.	Jingdong prefecture level city in Shandong
Doraemon Hyakubo Shop	King of Power Saving	Just use can not see what effect to,, wait for a period of time in the evaluation	Jingdong prefecture level city in Shandong
Doraemon Hyakubo Shop	King of Power Saving	Live in the 18th floor is too far away from the meter, afraid of no effect, look at the trial 90 days before buying. Currently used for a month, there is an effect, saving 27 dollars in electricity, next month should be able to save more!	Jingdong prefecture level city in Shandong
Doraemon Hyakubo Shop	King of Power Saving	I've been using it for almost a month now and it feels good, so I'll use it again.	Jingdong prefecture level city in Shandong
Doraemon Hyakubo Shop	King of Power Saving	It's ok, the electric bill is really less, originally I remember charging $100 and using it for 10 days. Now it lasts about 15 days. It's still noticeable.	Jingdong prefecture level city in Shandong
Doraemon Hyakubo Shop	King of Power Saving	Fast logistics, good power saving effect, very satisfied	Jingdong prefecture level city in Shandong
Doraemon Hyakubo Shop	King of Power Saving	Has been the second time back to buy, energy saving effect, worth recommending, you can try to see, power saving effect: very good Experience: can save about 30%!	Jingdong prefecture level city in Shandong
Doraemon Hyakubo Shop	King of Power Saving	I don't know how well it works for now	Jingdong prefecture level city in Shandong
Doraemon Hyakubo Shop	King of Power Saving	Factory direct is really good, plugged in and used for half a month did less probably almost 50 degrees of electricity! And the manufacturer's warranty is 5 years.	Jingdong prefecture level city in Shandong
Doraemon Hyakubo Shop	King of Power Saving	It's really effective. The previous month's electricity was $286, but after using the power saver for a month, I have $195 left this month, which is not bad.	Jingdong prefecture level city in Shandong

Doraemon Hyakubo Shop	King of Power Saving	I'll give you a good review and see how it goes.	Jingdong prefecture level city in Shandong
Doraemon Hyakubo Shop	King of Power Saving	Customer service is very professional and patient to guide the installation, the quality of workmanship is very fine. Durable, second repurchase. Really can save oh!	Jingdong prefecture level city in Shandong
Doraemon Hyakubo Shop	King of Power Saving	I've been using it for a month now and my electric bill is over $30 less than last month. I feel it's quite worth buying.	Jingdong prefecture level city in Shandong
Doraemon Hyakubo Shop	King of Power Saving	Small size, does not take up space. It's very good, it does work very effectively, the tool room has a lot of work with a lot of electricity. After installing it, it really reduces a lot of electricity bills. Before the day 30 degrees of electricity left stone, now work down really less a few degrees.	Jingdong prefecture level city in Shandong
Doraemon Hyakubo Shop	King of Power Saving	It does work for saving electricity, I've bought the kind from elsewhere before that was too simple not **all**. This time to buy this store, but also for the safety. Air conditioning is too much electricity, this use to save a lot of	Jingdong prefecture level city in Shandong
Doraemon Hyakubo Shop	King of Power Saving	Two air conditioners and a refrigerator at home, a month down the electricity bill is almost two hundred dollars, with this after the average savings of more than thirty dollars to spend less money to enjoy more, it's really very good!	Jingdong prefecture level city in Shandong
Doraemon Hyakubo Shop	King of Power Saving	Good. Very good.	Jingdong prefecture level city in Shandong
Doraemon Hyakubo Shop	King of Power Saving	Works great, easy to operate, great, very happy with it.	Jingdong prefecture level city in Shandong
Doraemon Hyakubo Shop	King of Power Saving	Received it and tried it out, it's great, great quality and worth having! Five stars praise!	Jingdong prefecture level city in Shandong
Doraemon Hyakubo Shop	King of Power Saving	Very trustworthy brand, quality is guaranteed, the price is still affordable, quite satisfied!	Jingdong prefecture level city in Shandong
Doraemon Hyakubo Shop	King of Power Saving	Use it before you speak.	Jingdong prefecture level city in Shandong
Doraemon Hyakubo Shop	King of Power Saving	The quality of workmanship is very good and the power saving effect is quite good, I regret not getting it earlier.	Jingdong prefecture level city in Shandong
Doraemon Hyakubo Shop	King of Power Saving	Merchants shipping speed, and customer service introduced the same very good, cost-effective	Jingdong prefecture level city in Shandong
Doraemon Hyakubo Shop	King of Power Saving	Feel a little effect Oh, where the power consumption where to plug, convenient and convenient	Jingdong prefecture level city in Shandong
Doraemon Hyakubo Shop	King of Power Saving	The quality of the imported saver is very good, practical and satisfactory. Cost-effective	Jingdong prefecture level city in Shandong
Doraemon Hyakubo Shop	King of Power Saving	Power Saver is especially good, good and inexpensive, very good	Jingdong prefecture level city in Shandong
Doraemon Hyakubo Shop	King of Power Saving	Fast shipping, awesome quality, affordable price, satisfied.	Jingdong prefecture level city in Shandong
Doraemon Hyakubo Shop	King of Power Saving	Energy saver received, the effect is consistent with the description, recommended to buy!	Jingdong prefecture level city in Shandong
Doraemon Hyakubo Shop	King of Power Saving	It's really good, the quality is great, I recommend it!	Jingdong prefecture level city in Shandong
Doraemon Hyakubo Shop	King of Power Saving	Works well. Inexpensive. Shipping was fast.	Jingdong prefecture level city in Shandong
Doraemon Hyakubo Shop	King of Power Saving	Logistics is fast, works well, energy saving is good	Jingdong prefecture level city in Shandong
Doraemon Hyakubo Shop	King of Power Saving	very happy	Jingdong prefecture level city in Shandong
Doraemon Hyakubo Shop	King of Power Saving	an excellent	Jingdong prefecture level city in Shandong
Doraemon Hyakubo Shop	King of Power Saving	It rained a couple days ago and I just went to get the item back today , not bad, hey I looked at the meter and that red display light takes several seconds to jump, did not make a mistake buying it. After a period of time to evaluate.	Jingdong prefecture level city in Shandong
Doraemon Hyakubo Shop	King of Power Saving	Used more than 10 days to check the power, there is indeed some decline, customer service said, long-term use will save more points!	Jingdong prefecture level city in Shandong
Doraemon Hyakubo Shop	King of Power Saving	It's a proper IQ tax, and it costs electricity.	Jingdong prefecture level city in Shandong
Doraemon Hyakubo Shop	King of Power Saving	I've been using it for 17 days now, it really saves electricity Shipping is fast, you can save a lot in a year	Jingdong prefecture level city in Shandong
Doraemon Hyakubo Shop	King of Power Saving	I've been using it for 17 days and it really saves power Shipping is fast and you don't have to worry about the quality, free returns within 5 years	Jingdong prefecture level city in Shandong
Doraemon Hyakubo Shop	King of Power Saving	It's more regular, scanned the code and it's authentic, and the main thing is that you can try it for 90 days! Plugged in more than ten days, is less than before a few degrees per day. When the time to use a month to see Additional. Good to buy a few more	Jingdong prefecture level city in Shandong
Doraemon Hyakubo Shop	King of Power Saving	Logistics is very fast, fine workmanship, use it and then come back to the comments	Jingdong prefecture level city in Shandong
Doraemon Hyakubo Shop	King of Power Saving	The quality of workmanship is very good and the power saving effect is quite good, I regret not getting it earlier.	Jingdong prefecture level city in Shandong
Doraemon Hyakubo Shop	King of Power Saving	Received the goods, according to the instructions on the operation is very simple, to wait and see the effect, looks good!	Jingdong prefecture level city in Shandong
Doraemon Hyakubo Shop	King of Power Saving	It's really as safe and power-saving as the owner says, and it works great!	Jingdong prefecture level city in Shandong
Doraemon Hyakubo Shop	King of Power Saving	Received the goods, immediately used, looks good, to wait and see how the effect.	Jingdong prefecture level city in Shandong
Doraemon Hyakubo Shop	King of Power Saving	Afraid to buy back did not work, the store said the commitment to 90 days invalid refund, warranty 5 years. Saver also complimentary on the Pacific insurance, it is a lot of peace of mind, there are effects will continue to buy back drops!	Jingdong prefecture level city in Shandong
Doraemon Hyakubo Shop	King of Power Saving	Received the goods and used it, can save electricity a month later to see the effect!	Jingdong prefecture level city in Shandong
Doraemon Hyakubo Shop	King of Power Saving	Fancy 90-day trial, 5-year warranty before the order, the goods received, has been experiencing a week, can really feel the savings, in the introduction of friends also buy this!	Jingdong prefecture level city in Shandong
Doraemon Hyakubo Shop	King of Power Saving	Clothing stores use, neighbors recommended to buy, said a month can save more than 70 yuan, first plugged in to see the effect of the	Jingdong prefecture level city in Shandong
Doraemon Hyakubo Shop	King of Power Saving	A friend's home two months to save more than 80 dollars of electricity, recommended that I buy two, decisive purchase, looking forward to the effect of power saving, waiting for the next month effect.	Jingdong prefecture level city in Shandong
Doraemon Hyakubo Shop	King of Power Saving	The goods have been received, consistent with the description of the advertisement, quite good, I hope that in terms of power saving and purifying the air is really as the merchant said that would be better!	Jingdong prefecture level city in Shandong
Doraemon Hyakubo Shop	King of Power Saving	Power Saving King is really good to use the quality is very good than the physical store a lot cheaper we can rest assured that the purchase recommended to buy!	Jingdong prefecture level city in Shandong
Doraemon Hyakubo Shop	King of Power Saving	Energy saver has been used on the rental house inside the use of the meter is always running fast, with this can really save electricity, easy to use, plugged in can be, very satisfied!	Jingdong prefecture level city in Shandong
Doraemon Hyakubo Shop	King of Power Saving	Power Saver Power Saver King is good quality and affordable, very satisfied, worth buying.	Jingdong prefecture level city in Shandong
Doraemon Hyakubo Shop	King of Power Saving	Just use can not see what effect to,, wait for a period of time in the evaluation	Jingdong prefecture level city in Shandong
Doraemon Hyakubo Shop	King of Power Saving	Live in the 18th floor is too far away from the meter, afraid of no effect, look at the trial 90 days before buying. Currently used for a month, there is an effect, saving 27 dollars in electricity, next month should be able to save more!	Jingdong prefecture level city in Shandong
Doraemon Hyakubo Shop	King of Power Saving	I've been using it for almost a month now and it feels good, so I'll use it again.	Jingdong prefecture level city in Shandong
Doraemon Hyakubo Shop	King of Power Saving	It's ok, the electric bill is really less, originally I remember charging $100 and using it for 10 days. Now it lasts about 15 days. It's still noticeable.	Jingdong prefecture level city in Shandong
Doraemon Hyakubo Shop	King of Power Saving	Fast logistics, good power saving effect, very satisfied	Jingdong prefecture level city in Shandong

Doraemon Hyakubo Shop	King of Power Saving	Has been the second time back to buy, energy saving effect, worth recommending, you can try to see, power saving effect: very good Experience: can save about 30%!	Jingdong prefecture level city in Shandong
Doraemon Hyakubo Shop	King of Power Saving	I don't know how well it works for now	Jingdong prefecture level city in Shandong
Doraemon Hyakubo Shop	King of Power Saving	Factory direct is really good, plugged in and used for half a month did less probably almost 50 degrees of electricity! And the manufacturer's warranty is 5 years.	Jingdong prefecture level city in Shandong
Doraemon Hyakubo Shop	King of Power Saving	It's really effective. The previous month's electricity was $286, but after using the power saver for a month, I have $195 left this month, which is not bad.	Jingdong prefecture level city in Shandong
Doraemon Hyakubo Shop	King of Power Saving	I'll give you a good review and see how it goes.	Jingdong prefecture level city in Shandong
Doraemon Hyakubo Shop	King of Power Saving	Customer service is very professional and patient to guide the installation, the quality of workmanship is very fine. Durable, second repurchase. Really can save oh!	Jingdong prefecture level city in Shandong
Doraemon Hyakubo Shop	King of Power Saving	I've been using it for a month now and my electric bill is over $30 less than last month. I feel it's quite worth buying.	Jingdong prefecture level city in Shandong
Doraemon Hyakubo Shop	King of Power Saving	Small size, does not take up space. It's very good, it does work very effectively, the tool room has a lot of work with a lot of electricity. After installing it, it really reduces a lot of electricity bills. Before the day 30 degrees of electricity left stone, now work down really less a few degrees.	Jingdong prefecture level city in Shandong
Doraemon Hyakubo Shop	King of Power Saving	It does work for saving electricity, I've bought the kind from elsewhere before that was too simple not **all**. This time to buy this store, but also for the safety. Air conditioning is too much electricity, this use to save a lot of	Jingdong prefecture level city in Shandong
Doraemon Hyakubo Shop	King of Power Saving	Two air conditioners and a refrigerator at home, a month down the electricity bill is almost two hundred dollars, with this after the average savings of more than thirty dollars to spend less money to enjoy more, it's really very good!	Jingdong prefecture level city in Shandong
Doraemon Hyakubo Shop	King of Power Saving	Good. Very good.	Jingdong prefecture level city in Shandong
Doraemon Hyakubo Shop	King of Power Saving	Works great, easy to operate, great, very happy with it.	Jingdong prefecture level city in Shandong
Doraemon Hyakubo Shop	King of Power Saving	Received it and tried it out, it's great, great quality and worth having! Five stars praise!	Jingdong prefecture level city in Shandong
Doraemon Hyakubo Shop	King of Power Saving	Very trustworthy brand, quality is guaranteed, the price is still affordable, quite satisfied!	Jingdong prefecture level city in Shandong
Doraemon Hyakubo Shop	King of Power Saving	Use it before you speak.	Jingdong prefecture level city in Shandong
Doraemon Hyakubo Shop	King of Power Saving	The quality of workmanship is very good and the power saving effect is quite good, I regret not getting it earlier.	Jingdong prefecture level city in Shandong
Doraemon Hyakubo Shop	King of Power Saving	Merchants shipping speed, and customer service introduced the same very good, cost-effective	Jingdong prefecture level city in Shandong
Doraemon Hyakubo Shop	King of Power Saving	Feel a little effect Oh, where the power consumption where to plug, convenient and convenient	Jingdong prefecture level city in Shandong
Doraemon Hyakubo Shop	King of Power Saving	The quality of the imported saver is very good, practical and satisfactory. Cost-effective	Jingdong prefecture level city in Shandong
Doraemon Hyakubo Shop	King of Power Saving	Power Saver is especially good, good and inexpensive, very good	Jingdong prefecture level city in Shandong
Doraemon Hyakubo Shop	King of Power Saving	Fast shipping, awesome quality, affordable price, satisfied.	Jingdong prefecture level city in Shandong
Doraemon Hyakubo Shop	King of Power Saving	Energy saver received, the effect is consistent with the description, recommended to buy!	Jingdong prefecture level city in Shandong
Doraemon Hyakubo Shop	King of Power Saving	It's really good, the quality is great, I recommend it!	Jingdong prefecture level city in Shandong
Doraemon Hyakubo Shop	King of Power Saving	Works well. Inexpensive. Shipping was fast.	Jingdong prefecture level city in Shandong
Doraemon Hyakubo Shop	King of Power Saving	Logistics is fast, works well, energy saving is good	Jingdong prefecture level city in Shandong
Doraemon Hyakubo Shop	King of Power Saving	very happy	Jingdong prefecture level city in Shandong
Doraemon Hyakubo Shop	King of Power Saving	an excellent	Jingdong prefecture level city in Shandong
Doraemon Hyakubo Shop	King of Power Saving	It rained a couple days ago and I just went to get the item back today , not bad, hey I looked at the meter and that red display light takes several seconds to jump, did not make a mistake buying it. After a period of time to evaluate.	Jingdong prefecture level city in Shandong
Doraemon Hyakubo Shop	King of Power Saving	Used more than 10 days to check the power, there is indeed some decline, customer service said, long-term use will save more points!	Jingdong prefecture level city in Shandong
Doraemon Hyakubo Shop	King of Power Saving	It's a proper IQ tax, and it costs electricity.	Jingdong prefecture level city in Shandong
Doraemon Hyakubo Shop	King of Power Saving	I've been using it for 17 days now, it really saves electricity Shipping is fast, you can save a lot in a year	Jingdong prefecture level city in Shandong
Doraemon Hyakubo Shop	King of Power Saving	I've been using it for 17 days and it really saves power Shipping is fast and you don't have to worry about the quality, free returns within 5 years	Jingdong prefecture level city in Shandong
Doraemon Hyakubo Shop	King of Power Saving	It's more regular, scanned the code and it's authentic, and the main thing is that you can try it for 90 days! Plugged in more than ten days, is less than before a few degrees per day. When the time to use a month to see Additional. Good to buy a few more	Jingdong prefecture level city in Shandong
Doraemon Hyakubo Shop	King of Power Saving	Logistics is very fast, fine workmanship, use it and then come back to the comments	Jingdong prefecture level city in Shandong
Doraemon Hyakubo Shop	King of Power Saving	The quality of workmanship is very good and the power saving effect is quite good, I regret not getting it earlier.	Jingdong prefecture level city in Shandong
Doraemon Hyakubo Shop	King of Power Saving	Received the goods, according to the instructions on the operation is very simple, to wait and see the effect, looks good!	Jingdong prefecture level city in Shandong
Doraemon Hyakubo Shop	King of Power Saving	It's really as safe and power-saving as the owner says, and it works great!	Jingdong prefecture level city in Shandong
Doraemon Hyakubo Shop	King of Power Saving	Received the goods, immediately used, looks good, to wait and see how the effect.	Jingdong prefecture level city in Shandong
Doraemon Hyakubo Shop	King of Power Saving	Afraid to buy back did not work, the store said the commitment to 90 days invalid refund, warranty 5 years. Saver also complimentary on the Pacific insurance, it is a lot of peace of mind, there are effects will continue to buy back drops!	Jingdong prefecture level city in Shandong
Doraemon Hyakubo Shop	King of Power Saving	Received the goods and used it, can save electricity a month later to see the effect!	Jingdong prefecture level city in Shandong
Doraemon Hyakubo Shop	King of Power Saving	Fancy 90-day trial, 5-year warranty before the order, the goods received, has been experiencing a week, can really feel the savings, in the introduction of friends also buy this!	Jingdong prefecture level city in Shandong
Doraemon Hyakubo Shop	King of Power Saving	Clothing stores use, neighbors recommended to buy, said a month can save more than 70 yuan, first plugged in to see the effect of the	Jingdong prefecture level city in Shandong
Doraemon Hyakubo Shop	King of Power Saving	A friend's home two months to save more than 80 dollars of electricity, recommended that I buy two, decisive purchase, looking forward to the effect of power saving, waiting for the next month effect.	Jingdong prefecture level city in Shandong
Doraemon Hyakubo Shop	King of Power Saving	The goods have been received, consistent with the description of the advertisement, quite good, I hope that in terms of power saving and purifying the air is really as the merchant said that would be better!	Jingdong prefecture level city in Shandong
Doraemon Hyakubo Shop	King of Power Saving	Power Saving King is really good to use the quality is very good than the physical store a lot cheaper we can rest assured that the purchase recommended to buy!	Jingdong prefecture level city in Shandong
Doraemon Hyakubo Shop	King of Power Saving	Energy saver has been used on the rental house inside the use of the meter is always running fast, with this can really save electricity, easy to use, plugged in can be, very satisfied!	Jingdong prefecture level city in Shandong
Doraemon Hyakubo Shop	King of Power Saving	Power Saver Power Saver King is good quality and affordable, very satisfied, worth buying.	Jingdong prefecture level city in Shandong
Doraemon Hyakubo Shop	King of Power Saving	Just use can not see what effect to,, wait for a period of time in the evaluation	Jingdong prefecture level city in Shandong

Doraemon Hyakubo Shop	King of Power Saving	Live in the 18th floor is too far away from the meter, afraid of no effect, look at the trial 90 days before buying. Currently used for a month, there is an effect, saving 27 dollars in electricity, next month should be able to save more!	Jingdong prefecture level city in Shandong
Doraemon Hyakubo Shop	King of Power Saving	I've been using it for almost a month now and it feels good, so I'll use it again.	Jingdong prefecture level city in Shandong
Doraemon Hyakubo Shop	King of Power Saving	It's ok, the electric bill is really less, originally I remember charging $100 and using it for 10 days. Now it lasts about 15 days. It's still noticeable.	Jingdong prefecture level city in Shandong
Doraemon Hyakubo Shop	King of Power Saving	Fast logistics, good power saving effect, very satisfied	Jingdong prefecture level city in Shandong
Doraemon Hyakubo Shop	King of Power Saving	Has been the second time back to buy, energy saving effect, worth recommending, you can try to see, power saving effect: very good Experience: can save about 30%!	Jingdong prefecture level city in Shandong
Doraemon Hyakubo Shop	King of Power Saving	I don't know how well it works for now	Jingdong prefecture level city in Shandong
Doraemon Hyakubo Shop	King of Power Saving	Factory direct is really good, plugged in and used for half a month did less probably almost 50 degrees of electricity! And the manufacturer's warranty is 5 years.	Jingdong prefecture level city in Shandong
Doraemon Hyakubo Shop	King of Power Saving	It's really effective. The previous month's electricity was $286, but after using the power saver for a month, I have $195 left this month, which is not bad.	Jingdong prefecture level city in Shandong
Doraemon Hyakubo Shop	King of Power Saving	I'll give you a good review and see how it goes.	Jingdong prefecture level city in Shandong
Doraemon Hyakubo Shop	King of Power Saving	Customer service is very professional and patient to guide the installation, the quality of workmanship is very fine. Durable, second repurchase. Really can save oh!	Jingdong prefecture level city in Shandong
Doraemon Hyakubo Shop	King of Power Saving	I've been using it for a month now and my electric bill is over $30 less than last month. I feel it's quite worth buying.	Jingdong prefecture level city in Shandong
Doraemon Hyakubo Shop	King of Power Saving	Small size, does not take up space. It's very good, it does work very effectively, the tool room has a lot of work with a lot of electricity. After installing it, it really reduces a lot of electricity bills. Before the day 30 degrees of electricity left stone, now work down really less a few degrees.	Jingdong prefecture level city in Shandong
Doraemon Hyakubo Shop	King of Power Saving	It does work for saving electricity, I've bought the kind from elsewhere before that was too simple not **all**. This time to buy this store, but also for the safety. Air conditioning is too much electricity, this use to save a lot of	Jingdong prefecture level city in Shandong
Doraemon Hyakubo Shop	King of Power Saving	Two air conditioners and a refrigerator at home, a month down the electricity bill is almost two hundred dollars, with this after the average savings of more than thirty dollars to spend less money to enjoy more, it's really very good!	Jingdong prefecture level city in Shandong
Doraemon Hyakubo Shop	King of Power Saving	Good. Very good.	Jingdong prefecture level city in Shandong
Doraemon Hyakubo Shop	King of Power Saving	Works great, easy to operate, great, very happy with it.	Jingdong prefecture level city in Shandong
Doraemon Hyakubo Shop	King of Power Saving	Received it and tried it out, it's great, great quality and worth having! Five stars praise!	Jingdong prefecture level city in Shandong
Doraemon Hyakubo Shop	King of Power Saving	Very trustworthy brand, quality is guaranteed, the price is still affordable, quite satisfied!	Jingdong prefecture level city in Shandong
Doraemon Hyakubo Shop	King of Power Saving	Use it before you speak.	Jingdong prefecture level city in Shandong
Doraemon Hyakubo Shop	King of Power Saving	The quality of workmanship is very good and the power saving effect is quite good, I regret not getting it earlier.	Jingdong prefecture level city in Shandong
Doraemon Hyakubo Shop	King of Power Saving	Merchants shipping speed, and customer service introduced the same very good, cost-effective	Jingdong prefecture level city in Shandong
Doraemon Hyakubo Shop	King of Power Saving	Feel a little effect Oh, where the power consumption where to plug, convenient and convenient	Jingdong prefecture level city in Shandong
Doraemon Hyakubo Shop	King of Power Saving	The quality of the imported saver is very good, practical and satisfactory. Cost-effective	Jingdong prefecture level city in Shandong
Doraemon Hyakubo Shop	King of Power Saving	Power Saver is especially good, good and inexpensive, very good	Jingdong prefecture level city in Shandong
Doraemon Hyakubo Shop	King of Power Saving	Fast shipping, awesome quality, affordable price, satisfied.	Jingdong prefecture level city in Shandong
Doraemon Hyakubo Shop	King of Power Saving	Energy saver received, the effect is consistent with the description, recommended to buy!	Jingdong prefecture level city in Shandong
Doraemon Hyakubo Shop	King of Power Saving	It's really good, the quality is great, I recommend it!	Jingdong prefecture level city in Shandong
Doraemon Hyakubo Shop	King of Power Saving	Works well. Inexpensive. Shipping was fast.	Jingdong prefecture level city in Shandong
Doraemon Hyakubo Shop	King of Power Saving	Logistics is fast, works well, energy saving is good	Jingdong prefecture level city in Shandong
Doraemon Hyakubo Shop	King of Power Saving	very happy	Jingdong prefecture level city in Shandong
Doraemon Hyakubo Shop	King of Power Saving	an excellent	Jingdong prefecture level city in Shandong
Doraemon Hyakubo Shop	King of Power Saving	It rained a couple days ago and I just went to get the item back today , not bad, hey I looked at the meter and that red display light takes several seconds to jump, did not make a mistake buying it. After a period of time to evaluate.	Jingdong prefecture level city in Shandong
Doraemon Hyakubo Shop	King of Power Saving	Used more than 10 days to check the power, there is indeed some decline, customer service said, long-term use will save more points!	Jingdong prefecture level city in Shandong
Doraemon Hyakubo Shop	King of Power Saving	It's a proper IQ tax, and it costs electricity.	Jingdong prefecture level city in Shandong
Doraemon Hyakubo Shop	King of Power Saving	I've been using it for 17 days now, it really saves electricity Shipping is fast, you can save a lot in a year	Jingdong prefecture level city in Shandong
Doraemon Hyakubo Shop	King of Power Saving	I've been using it for 17 days and it really saves power Shipping is fast and you don't have to worry about the quality, free returns within 5 years	Jingdong prefecture level city in Shandong
Doraemon Hyakubo Shop	King of Power Saving	It's more regular, scanned the code and it's authentic, and the main thing is that you can try it for 90 days! Plugged in more than ten days, is less than before a few degrees per day. When the time to use a month to see Additional. Good to buy a few more	Jingdong prefecture level city in Shandong
Doraemon Hyakubo Shop	King of Power Saving	Logistics is very fast, fine workmanship, use it and then come back to the comments	Jingdong prefecture level city in Shandong
Doraemon Hyakubo Shop	King of Power Saving	The quality of workmanship is very good and the power saving effect is quite good, I regret not getting it earlier.	Jingdong prefecture level city in Shandong
Doraemon Hyakubo Shop	King of Power Saving	Received the goods, according to the instructions on the operation is very simple, to wait and see the effect, looks good!	Jingdong prefecture level city in Shandong
Doraemon Hyakubo Shop	King of Power Saving	It's really as safe and power-saving as the owner says, and it works great!	Jingdong prefecture level city in Shandong
Doraemon Hyakubo Shop	King of Power Saving	Received the goods, immediately used, looks good, to wait and see how the effect.	Jingdong prefecture level city in Shandong
Doraemon Hyakubo Shop	King of Power Saving	Afraid to buy back did not work, the store said the commitment to 90 days invalid refund, warranty 5 years. Saver also complimentary on the Pacific insurance, it is a lot of peace of mind, there are effects will continue to buy back drops!	Jingdong prefecture level city in Shandong
Doraemon Hyakubo Shop	King of Power Saving	Received the goods and used it, can save electricity a month later to see the effect!	Jingdong prefecture level city in Shandong
Doraemon Hyakubo Shop	King of Power Saving	Fancy 90-day trial, 5-year warranty before the order, the goods received, has been experiencing a week, can really feel the savings, in the introduction of friends also buy this!	Jingdong prefecture level city in Shandong
Doraemon Hyakubo Shop	King of Power Saving	Clothing stores use, neighbors recommended to buy, said a month can save more than 70 yuan, first plugged in to see the effect of the	Jingdong prefecture level city in Shandong
Doraemon Hyakubo Shop	King of Power Saving	A friend's home two months to save more than 80 dollars of electricity, recommended that I buy two, decisive purchase, looking forward to the effect of power saving, waiting for the next month effect.	Jingdong prefecture level city in Shandong
Doraemon Hyakubo Shop	King of Power Saving	The goods have been received, consistent with the description of the advertisement, quite good, I hope that in terms of power saving and purifying the air is really as the merchant said that would be better!	Jingdong prefecture level city in Shandong

Doraemon Hyakubo Shop	King of Power Saving	Power Saving King is really good to use the quality is very good than the physical store a lot cheaper we can rest assured that the purchase recommended to buy!	Jingdong prefecture level city in Shandong
Doraemon Hyakubo Shop	King of Power Saving	Energy saver has been used on the rental house inside the use of the meter is always running fast, with this can really save electricity, easy to use, plugged in can be, very satisfied!	Jingdong prefecture level city in Shandong
Doraemon Hyakubo Shop	King of Power Saving	Power Saver Power Saver King is good quality and affordable, very satisfied, worth buying.	Jingdong prefecture level city in Shandong
Doraemon Hyakubo Shop	King of Power Saving	Just use can not see what effect to,, wait for a period of time in the evaluation	Jingdong prefecture level city in Shandong
Doraemon Hyakubo Shop	King of Power Saving	Live in the 18th floor is too far away from the meter, afraid of no effect, look at the trial 90 days before buying. Currently used for a month, there is an effect, saving 27 dollars in electricity, next month should be able to save more!	Jingdong prefecture level city in Shandong
Doraemon Hyakubo Shop	King of Power Saving	I've been using it for almost a month now and it feels good, so I'll use it again.	Jingdong prefecture level city in Shandong
Doraemon Hyakubo Shop	King of Power Saving	It's ok, the electric bill is really less, originally I remember charging $100 and using it for 10 days. Now it lasts about 15 days. It's still noticeable.	Jingdong prefecture level city in Shandong
Doraemon Hyakubo Shop	King of Power Saving	Fast logistics, good power saving effect, very satisfied	Jingdong prefecture level city in Shandong
Doraemon Hyakubo Shop	King of Power Saving	Has been the second time back to buy, energy saving effect, worth recommending, you can try to see, power saving effect: very good Experience: can save about 30%!	Jingdong prefecture level city in Shandong
Doraemon Hyakubo Shop	King of Power Saving	I don't know how well it works for now	Jingdong prefecture level city in Shandong
Doraemon Hyakubo Shop	King of Power Saving	Factory direct is really good, plugged in and used for half a month did less probably almost 50 degrees of electricity! And the manufacturer's warranty is 5 years.	Jingdong prefecture level city in Shandong
Doraemon Hyakubo Shop	King of Power Saving	It's really effective. The previous month's electricity was $286, but after using the power saver for a month, I have $195 left this month, which is not bad.	Jingdong prefecture level city in Shandong
Doraemon Hyakubo Shop	King of Power Saving	I'll give you a good review and see how it goes.	Jingdong prefecture level city in Shandong
Doraemon Hyakubo Shop	King of Power Saving	Customer service is very professional and patient to guide the installation, the quality of workmanship is very fine. Durable, second repurchase. Really can save oh!	Jingdong prefecture level city in Shandong
Doraemon Hyakubo Shop	King of Power Saving	I've been using it for a month now and my electric bill is over $30 less than last month. I feel it's quite worth buying.	Jingdong prefecture level city in Shandong
Doraemon Hyakubo Shop	King of Power Saving	Small size, does not take up space. It's very good, it does work very effectively, the tool room has a lot of work with a lot of electricity. After installing it, it really reduces a lot of electricity bills. Before the day 30 degrees of electricity left stone, now work down really less a few degrees.	Jingdong prefecture level city in Shandong
Doraemon Hyakubo Shop	King of Power Saving	It does work for saving electricity, I've bought the kind from elsewhere before that was too simple not **all**. This time to buy this store, but also for the safety. Air conditioning is too much electricity, this use to save a lot of	Jingdong prefecture level city in Shandong
Doraemon Hyakubo Shop	King of Power Saving	Two air conditioners and a refrigerator at home, a month down the electricity bill is almost two hundred dollars, with this after the average savings of more than thirty dollars to spend less money to enjoy more, it's really very good!	Jingdong prefecture level city in Shandong
Doraemon Hyakubo Shop	King of Power Saving	Good. Very good.	Jingdong prefecture level city in Shandong
Doraemon Hyakubo Shop	King of Power Saving	Works great, easy to operate, great, very happy with it.	Jingdong prefecture level city in Shandong
Doraemon Hyakubo Shop	King of Power Saving	Received it and tried it out, it's great, great quality and worth having! Five stars praise!	Jingdong prefecture level city in Shandong
Doraemon Hyakubo Shop	King of Power Saving	Very trustworthy brand, quality is guaranteed, the price is still affordable, quite satisfied!	Jingdong prefecture level city in Shandong
Doraemon Hyakubo Shop	King of Power Saving	Use it before you speak.	Jingdong prefecture level city in Shandong
Doraemon Hyakubo Shop	King of Power Saving	The quality of workmanship is very good and the power saving effect is quite good, I regret not getting it earlier.	Jingdong prefecture level city in Shandong
Doraemon Hyakubo Shop	King of Power Saving	Merchants shipping speed, and customer service introduced the same very good, cost-effective	Jingdong prefecture level city in Shandong
Doraemon Hyakubo Shop	King of Power Saving	Feel a little effect Oh, where the power consumption where to plug, convenient and convenient	Jingdong prefecture level city in Shandong
Doraemon Hyakubo Shop	King of Power Saving	The quality of the imported saver is very good, practical and satisfactory. Cost-effective	Jingdong prefecture level city in Shandong
Doraemon Hyakubo Shop	King of Power Saving	Power Saver is especially good, good and inexpensive, very good	Jingdong prefecture level city in Shandong
Doraemon Hyakubo Shop	King of Power Saving	Fast shipping, awesome quality, affordable price, satisfied.	Jingdong prefecture level city in Shandong
Doraemon Hyakubo Shop	King of Power Saving	Energy saver received, the effect is consistent with the description, recommended to buy!	Jingdong prefecture level city in Shandong
Doraemon Hyakubo Shop	King of Power Saving	It's really good, the quality is great, I recommend it!	Jingdong prefecture level city in Shandong
Doraemon Hyakubo Shop	King of Power Saving	Works well. Inexpensive. Shipping was fast.	Jingdong prefecture level city in Shandong
Doraemon Hyakubo Shop	King of Power Saving	Logistics is fast, works well, energy saving is good	Jingdong prefecture level city in Shandong
Doraemon Hyakubo Shop	King of Power Saving	very happy	Jingdong prefecture level city in Shandong
Doraemon Hyakubo Shop	King of Power Saving	an excellent	Jingdong prefecture level city in Shandong
Doraemon Hyakubo Shop	King of Power Saving	It rained a couple days ago and I just went to get the item back today , not bad, hey I looked at the meter and that red display light takes several seconds to jump, did not make a mistake buying it. After a period of time to evaluate.	Jingdong prefecture level city in Shandong
Doraemon Hyakubo Shop	King of Power Saving	Used more than 10 days to check the power, there is indeed some decline, customer service said, long-term use will save more points!	Jingdong prefecture level city in Shandong
Doraemon Hyakubo Shop	King of Power Saving	It's a proper IQ tax, and it costs electricity.	Jingdong prefecture level city in Shandong
Doraemon Hyakubo Shop	King of Power Saving	I've been using it for 17 days now, it really saves electricity Shipping is fast, you can save a lot in a year	Jingdong prefecture level city in Shandong
Doraemon Hyakubo Shop	King of Power Saving	I've been using it for 17 days and it really saves power Shipping is fast and you don't have to worry about the quality, free returns within 5 years	Jingdong prefecture level city in Shandong
Doraemon Hyakubo Shop	King of Power Saving	It's more regular, scanned the code and it's authentic, and the main thing is that you can try it for 90 days! Plugged in more than ten days, is less than before a few degrees per day. When the time to use a month to see Additional. Good to buy a few more	Jingdong prefecture level city in Shandong
Doraemon Hyakubo Shop	King of Power Saving	Logistics is very fast, fine workmanship, use it and then come back to the comments	Jingdong prefecture level city in Shandong
Doraemon Hyakubo Shop	King of Power Saving	The quality of workmanship is very good and the power saving effect is quite good, I regret not getting it earlier.	Jingdong prefecture level city in Shandong
Doraemon Hyakubo Shop	King of Power Saving	Received the goods, according to the instructions on the operation is very simple, to wait and see the effect, looks good!	Jingdong prefecture level city in Shandong
Doraemon Hyakubo Shop	King of Power Saving	It's really as safe and power-saving as the owner says, and it works great!	Jingdong prefecture level city in Shandong
Doraemon Hyakubo Shop	King of Power Saving	Received the goods, immediately used, looks good, to wait and see how the effect.	Jingdong prefecture level city in Shandong
Doraemon Hyakubo Shop	King of Power Saving	Afraid to buy back did not work, the store said the commitment to 90 days invalid refund, warranty 5 years. Saver also complimentary on the Pacific insurance, it is a lot of peace of mind, there are effects will continue to buy back drops!	Jingdong prefecture level city in Shandong
Doraemon Hyakubo Shop	King of Power Saving	Received the goods and used it, can save electricity a month later to see the effect!	Jingdong prefecture level city in Shandong

Doraemon Hyakubo Shop	King of Power Saving	Fancy 90-day trial, 5-year warranty before the order, the goods received, has been experiencing a week, can really feel the savings, in the introduction of friends also buy this!	Jingdong prefecture level city in Shandong
Doraemon Hyakubo Shop	King of Power Saving	Clothing stores use, neighbors recommended to buy, said a month can save more than 70 yuan, first plugged in to see the effect of the	Jingdong prefecture level city in Shandong
Doraemon Hyakubo Shop	King of Power Saving	A friend's home two months to save more than 80 dollars of electricity, recommended that I buy two, decisive purchase, looking forward to the effect of power saving, waiting for the next month effect.	Jingdong prefecture level city in Shandong
Doraemon Hyakubo Shop	King of Power Saving	The goods have been received, consistent with the description of the advertisement, quite good, I hope that in terms of power saving and purifying the air is really as the merchant said that would be better!	Jingdong prefecture level city in Shandong
Doraemon Hyakubo Shop	King of Power Saving	Power Saving King is really good to use the quality is very good than the physical store a lot cheaper we can rest assured that the purchase recommended to buy!	Jingdong prefecture level city in Shandong
Doraemon Hyakubo Shop	King of Power Saving	Energy saver has been used on the rental house inside the use of the meter is always running fast, with this can really save electricity, easy to use, plugged in can be, very satisfied!	Jingdong prefecture level city in Shandong
Doraemon Hyakubo Shop	King of Power Saving	Power Saver Power Saver King is good quality and affordable, very satisfied, worth buying.	Jingdong prefecture level city in Shandong
Doraemon Hyakubo Shop	King of Power Saving	Just use can not see what effect to,, wait for a period of time in the evaluation	Jingdong prefecture level city in Shandong
Doraemon Hyakubo Shop	King of Power Saving	Live in the 18th floor is too far away from the meter, afraid of no effect, look at the trial 90 days before buying. Currently used for a month, there is an effect, saving 27 dollars in electricity, next month should be able to save more!	Jingdong prefecture level city in Shandong
Doraemon Hyakubo Shop	King of Power Saving	I've been using it for almost a month now and it feels good, so I'll use it again.	Jingdong prefecture level city in Shandong
Doraemon Hyakubo Shop	King of Power Saving	It's ok, the electric bill is really less, originally I remember charging $100 and using it for 10 days. Now it lasts about 15 days. It's still noticeable.	Jingdong prefecture level city in Shandong
Doraemon Hyakubo Shop	King of Power Saving	Fast logistics, good power saving effect, very satisfied	Jingdong prefecture level city in Shandong
Doraemon Hyakubo Shop	King of Power Saving	Has been the second time back to buy, energy saving effect, worth recommending, you can try to see, power saving effect: very good Experience: can save about 30%!	Jingdong prefecture level city in Shandong
Doraemon Hyakubo Shop	King of Power Saving	I don't know how well it works for now	Jingdong prefecture level city in Shandong
Doraemon Hyakubo Shop	King of Power Saving	Factory direct is really good, plugged in and used for half a month did less probably almost 50 degrees of electricity! And the manufacturer's warranty is 5 years.	Jingdong prefecture level city in Shandong
Doraemon Hyakubo Shop	King of Power Saving	It's really effective. The previous month's electricity was $286, but after using the power saver for a month, I have $195 left this month, which is not bad.	Jingdong prefecture level city in Shandong
Doraemon Hyakubo Shop	King of Power Saving	I'll give you a good review and see how it goes.	Jingdong prefecture level city in Shandong
Doraemon Hyakubo Shop	King of Power Saving	Customer service is very professional and patient to guide the installation, the quality of workmanship is very fine. Durable, second repurchase. Really can save oh!	Jingdong prefecture level city in Shandong
Doraemon Hyakubo Shop	King of Power Saving	I've been using it for a month now and my electric bill is over $30 less than last month. I feel it's quite worth buying.	Jingdong prefecture level city in Shandong
Doraemon Hyakubo Shop	King of Power Saving	Small size, does not take up space. It's very good, it does work very effectively, the tool room has a lot of work with a lot of electricity. After installing it, it really reduces a lot of electricity bills. Before the day 30 degrees of electricity left stone, now work down really less a few degrees.	Jingdong prefecture level city in Shandong
Doraemon Hyakubo Shop	King of Power Saving	It does work for saving electricity, I've bought the kind from elsewhere before that was too simple not **all**. This time to buy this store, but also for the safety. Air conditioning is too much electricity, this use to save a lot of	Jingdong prefecture level city in Shandong
Doraemon Hyakubo Shop	King of Power Saving	Two air conditioners and a refrigerator at home, a month down the electricity bill is almost two hundred dollars, with this after the average savings of more than thirty dollars to spend less money to enjoy more, it's really very good!	Jingdong prefecture level city in Shandong
Doraemon Hyakubo Shop	King of Power Saving	Good. Very good.	Jingdong prefecture level city in Shandong
Doraemon Hyakubo Shop	King of Power Saving	Works great, easy to operate, great, very happy with it.	Jingdong prefecture level city in Shandong
Doraemon Hyakubo Shop	King of Power Saving	Received it and tried it out, it's great, great quality and worth having! Five stars praise!	Jingdong prefecture level city in Shandong
Doraemon Hyakubo Shop	King of Power Saving	Very trustworthy brand, quality is guaranteed, the price is still affordable, quite satisfied!	Jingdong prefecture level city in Shandong
Doraemon Hyakubo Shop	King of Power Saving	Use it before you speak.	Jingdong prefecture level city in Shandong
Doraemon Hyakubo Shop	King of Power Saving	The quality of workmanship is very good and the power saving effect is quite good, I regret not getting it earlier.	Jingdong prefecture level city in Shandong
Doraemon Hyakubo Shop	King of Power Saving	Merchants shipping speed, and customer service introduced the same very good, cost-effective	Jingdong prefecture level city in Shandong
Doraemon Hyakubo Shop	King of Power Saving	Feel a little effect Oh, where the power consumption where to plug, convenient and convenient	Jingdong prefecture level city in Shandong
Doraemon Hyakubo Shop	King of Power Saving	The quality of the imported saver is very good, practical and satisfactory. Cost-effective	Jingdong prefecture level city in Shandong
Doraemon Hyakubo Shop	King of Power Saving	Power Saver is especially good, good and inexpensive, very good	Jingdong prefecture level city in Shandong
Doraemon Hyakubo Shop	King of Power Saving	Fast shipping, awesome quality, affordable price, satisfied.	Jingdong prefecture level city in Shandong
Doraemon Hyakubo Shop	King of Power Saving	Energy saver received, the effect is consistent with the description, recommended to buy!	Jingdong prefecture level city in Shandong
Doraemon Hyakubo Shop	King of Power Saving	It's really good, the quality is great, I recommend it!	Jingdong prefecture level city in Shandong
Doraemon Hyakubo Shop	King of Power Saving	Works well. Inexpensive. Shipping was fast.	Jingdong prefecture level city in Shandong
Doraemon Hyakubo Shop	King of Power Saving	Logistics is fast, works well, energy saving is good	Jingdong prefecture level city in Shandong
Doraemon Hyakubo Shop	King of Power Saving	very happy	Jingdong prefecture level city in Shandong
Doraemon Hyakubo Shop	King of Power Saving	an excellent	Jingdong prefecture level city in Shandong
Doraemon Hyakubo Shop	King of Power Saving	It rained a couple days ago and I just went to get the item back today , not bad, hey I looked at the meter and that red display light takes several seconds to jump, did not make a mistake buying it. After a period of time to evaluate.	Jingdong prefecture level city in Shandong
Doraemon Hyakubo Shop	King of Power Saving	Used more than 10 days to check the power, there is indeed some decline, customer service said, long-term use will save more points!	Jingdong prefecture level city in Shandong
Doraemon Hyakubo Shop	King of Power Saving	It's a proper IQ tax, and it costs electricity.	Jingdong prefecture level city in Shandong
Doraemon Hyakubo Shop	King of Power Saving	I've been using it for 17 days now, it really saves electricity Shipping is fast, you can save a lot in a year	Jingdong prefecture level city in Shandong
Doraemon Hyakubo Shop	King of Power Saving	I've been using it for 17 days and it really saves power Shipping is fast and you don't have to worry about the quality, free returns within 5 years	Jingdong prefecture level city in Shandong
Doraemon Hyakubo Shop	King of Power Saving	It's more regular, scanned the code and it's authentic, and the main thing is that you can try it for 90 days! Plugged in more than ten days, is less than before a few degrees per day. When the time to use a month to see Additional. Good to buy a few more	Jingdong prefecture level city in Shandong
Doraemon Hyakubo Shop	King of Power Saving	Logistics is very fast, fine workmanship, use it and then come back to the comments	Jingdong prefecture level city in Shandong
Doraemon Hyakubo Shop	King of Power Saving	The quality of workmanship is very good and the power saving effect is quite good, I regret not getting it earlier.	Jingdong prefecture level city in Shandong
Doraemon Hyakubo Shop	King of Power Saving	Received the goods, according to the instructions on the operation is very simple, to wait and see the effect, looks good!	Jingdong prefecture level city in Shandong

Doraemon Hyakubo Shop	King of Power Saving	It's really as safe and power-saving as the owner says, and it works great!	Jingdong prefecture level city in Shandong
Doraemon Hyakubo Shop	King of Power Saving	Received the goods, immediately used, looks good, to wait and see how the effect.	Jingdong prefecture level city in Shandong
Doraemon Hyakubo Shop	King of Power Saving	Afraid to buy back did not work, the store said the commitment to 90 days invalid refund, warranty 5 years. Saver also complimentary on the Pacific insurance, it is a lot of peace of mind, there are effects will continue to buy back drops!	Jingdong prefecture level city in Shandong
Doraemon Hyakubo Shop	King of Power Saving	Received the goods and used it, can save electricity a month later to see the effect!	Jingdong prefecture level city in Shandong
Doraemon Hyakubo Shop	King of Power Saving	Fancy 90-day trial, 5-year warranty before the order, the goods received, has been experiencing a week, can really feel the savings, in the introduction of friends also buy this!	Jingdong prefecture level city in Shandong
Doraemon Hyakubo Shop	King of Power Saving	Clothing stores use, neighbors recommended to buy, said a month can save more than 70 yuan, first plugged in to see the effect of the	Jingdong prefecture level city in Shandong
Doraemon Hyakubo Shop	King of Power Saving	A friend's home two months to save more than 80 dollars of electricity, recommended that I buy two, decisive purchase, looking forward to the effect of power saving, waiting for the next month effect.	Jingdong prefecture level city in Shandong
Doraemon Hyakubo Shop	King of Power Saving	The goods have been received, consistent with the description of the advertisement, quite good, I hope that in terms of power saving and purifying the air is really as the merchant said that would be better!	Jingdong prefecture level city in Shandong
Doraemon Hyakubo Shop	King of Power Saving	Power Saving King is really good to use the quality is very good than the physical store a lot cheaper we can rest assured that the purchase recommended to buy!	Jingdong prefecture level city in Shandong
Doraemon Hyakubo Shop	King of Power Saving	Energy saver has been used on the rental house inside the use of the meter is always running fast, with this can really save electricity, easy to use, plugged in can be, very satisfied!	Jingdong prefecture level city in Shandong
Doraemon Hyakubo Shop	King of Power Saving	Power Saver Power Saver King is good quality and affordable, very satisfied, worth buying.	Jingdong prefecture level city in Shandong
Doraemon Hyakubo Shop	King of Power Saving	Just use can not see what effect to,, wait for a period of time in the evaluation	Jingdong prefecture level city in Shandong
Doraemon Hyakubo Shop	King of Power Saving	Live in the 18th floor is too far away from the meter, afraid of no effect, look at the trial 90 days before buying. Currently used for a month, there is an effect, saving 27 dollars in electricity, next month should be able to save more!	Jingdong prefecture level city in Shandong
Doraemon Hyakubo Shop	King of Power Saving	I've been using it for almost a month now and it feels good, so I'll use it again.	Jingdong prefecture level city in Shandong
Doraemon Hyakubo Shop	King of Power Saving	It's ok, the electric bill is really less, originally I remember charging $100 and using it for 10 days. Now it lasts about 15 days. It's still noticeable.	Jingdong prefecture level city in Shandong
Doraemon Hyakubo Shop	King of Power Saving	Fast logistics, good power saving effect, very satisfied	Jingdong prefecture level city in Shandong
Doraemon Hyakubo Shop	King of Power Saving	Has been the second time back to buy, energy saving effect, worth recommending, you can try to see, power saving effect: very good Experience: can save about 30%!	Jingdong prefecture level city in Shandong
Doraemon Hyakubo Shop	King of Power Saving	I don't know how well it works for now	Jingdong prefecture level city in Shandong
Doraemon Hyakubo Shop	King of Power Saving	Factory direct is really good, plugged in and used for half a month did less probably almost 50 degrees of electricity! And the manufacturer's warranty is 5 years.	Jingdong prefecture level city in Shandong
Doraemon Hyakubo Shop	King of Power Saving	It's really effective. The previous month's electricity was $286, but after using the power saver for a month, I have $195 left this month, which is not bad.	Jingdong prefecture level city in Shandong
Doraemon Hyakubo Shop	King of Power Saving	I'll give you a good review and see how it goes.	Jingdong prefecture level city in Shandong
Doraemon Hyakubo Shop	King of Power Saving	Customer service is very professional and patient to guide the installation, the quality of workmanship is very fine. Durable, second repurchase. Really can save oh!	Jingdong prefecture level city in Shandong
Doraemon Hyakubo Shop	King of Power Saving	I've been using it for a month now and my electric bill is over $30 less than last month. I feel it's quite worth buying.	Jingdong prefecture level city in Shandong
Doraemon Hyakubo Shop	King of Power Saving	Small size, does not take up space. It's very good, it does work very effectively, the tool room has a lot of work with a lot of electricity. After installing it, it really reduces a lot of electricity bills. Before the day 30 degrees of electricity left stone, now work down really less a few degrees.	Jingdong prefecture level city in Shandong
Doraemon Hyakubo Shop	King of Power Saving	It does work for saving electricity, I've bought the kind from elsewhere before that was too simple not **all**. This time to buy this store, but also for the safety. Air conditioning is too much electricity, this use to save a lot of	Jingdong prefecture level city in Shandong
Doraemon Hyakubo Shop	King of Power Saving	Two air conditioners and a refrigerator at home, a month down the electricity bill is almost two hundred dollars, with this after the average savings of more than thirty dollars to spend less money to enjoy more, it's really very good!	Jingdong prefecture level city in Shandong
Doraemon Hyakubo Shop	King of Power Saving	Good. Very good.	Jingdong prefecture level city in Shandong
Doraemon Hyakubo Shop	King of Power Saving	Works great, easy to operate, great, very happy with it.	Jingdong prefecture level city in Shandong
Doraemon Hyakubo Shop	King of Power Saving	Received it and tried it out, it's great, great quality and worth having! Five stars praise!	Jingdong prefecture level city in Shandong
Doraemon Hyakubo Shop	King of Power Saving	Very trustworthy brand, quality is guaranteed, the price is still affordable, quite satisfied!	Jingdong prefecture level city in Shandong
Doraemon Hyakubo Shop	King of Power Saving	Use it before you speak.	Jingdong prefecture level city in Shandong
Doraemon Hyakubo Shop	King of Power Saving	The quality of workmanship is very good and the power saving effect is quite good, I regret not getting it earlier.	Jingdong prefecture level city in Shandong
Doraemon Hyakubo Shop	King of Power Saving	Merchants shipping speed, and customer service introduced the same very good, cost-effective	Jingdong prefecture level city in Shandong
Doraemon Hyakubo Shop	King of Power Saving	Feel a little effect Oh, where the power consumption where to plug, convenient and convenient	Jingdong prefecture level city in Shandong
Doraemon Hyakubo Shop	King of Power Saving	The quality of the imported saver is very good, practical and satisfactory. Cost-effective	Jingdong prefecture level city in Shandong
Doraemon Hyakubo Shop	King of Power Saving	Power Saver is especially good, good and inexpensive, very good	Jingdong prefecture level city in Shandong
Doraemon Hyakubo Shop	King of Power Saving	Fast shipping, awesome quality, affordable price, satisfied.	Jingdong prefecture level city in Shandong
Doraemon Hyakubo Shop	King of Power Saving	Energy saver received, the effect is consistent with the description, recommended to buy!	Jingdong prefecture level city in Shandong
Doraemon Hyakubo Shop	King of Power Saving	It's really good, the quality is great, I recommend it!	Jingdong prefecture level city in Shandong
Doraemon Hyakubo Shop	King of Power Saving	Works well. Inexpensive. Shipping was fast.	Jingdong prefecture level city in Shandong
Doraemon Hyakubo Shop	King of Power Saving	Logistics is fast, works well, energy saving is good	Jingdong prefecture level city in Shandong
Doraemon Hyakubo Shop	King of Power Saving	very happy	Jingdong prefecture level city in Shandong
Doraemon Hyakubo Shop	King of Power Saving	an excellent	Jingdong prefecture level city in Shandong
Doraemon Hyakubo Shop	King of Power Saving	It rained a couple days ago and I just went to get the item back today , not bad, hey I looked at the meter and that red display light takes several seconds to jump, did not make a mistake buying it. After a period of time to evaluate.	Jingdong prefecture level city in Shandong
Doraemon Hyakubo Shop	King of Power Saving	Used more than 10 days to check the power, there is indeed some decline, customer service said, long-term use will save more points!	Jingdong prefecture level city in Shandong
Doraemon Hyakubo Shop	King of Power Saving	It's a proper IQ tax, and it costs electricity.	Jingdong prefecture level city in Shandong
Doraemon Hyakubo Shop	King of Power Saving	I've been using it for 17 days now, it really saves electricity Shipping is fast, you can save a lot in a year	Jingdong prefecture level city in Shandong
Doraemon Hyakubo Shop	King of Power Saving	I've been using it for 17 days and it really saves power Shipping is fast and you don't have to worry about the quality, free returns within 5 years	Jingdong prefecture level city in Shandong

Doraemon Hyakubo Shop	King of Power Saving	It's more regular, scanned the code and it's authentic, and the main thing is that you can try it for 90 days! Plugged in more than ten days, is less than before a few degrees per day. When the time to use a month to see Additional. Good to buy a few more	Jingdong prefecture level city in Shandong
Doraemon Hyakubo Shop	King of Power Saving	Logistics is very fast, fine workmanship, use it and then come back to the comments	Jingdong prefecture level city in Shandong
Doraemon Hyakubo Shop	King of Power Saving	The quality of workmanship is very good and the power saving effect is quite good, I regret not getting it earlier.	Jingdong prefecture level city in Shandong
Doraemon Hyakubo Shop	King of Power Saving	Received the goods, according to the instructions on the operation is very simple, to wait and see the effect, looks good!	Jingdong prefecture level city in Shandong
Doraemon Hyakubo Shop	King of Power Saving	It's really as safe and power-saving as the owner says, and it works great!	Jingdong prefecture level city in Shandong
Doraemon Hyakubo Shop	King of Power Saving	Received the goods, immediately used, looks good, to wait and see how the effect.	Jingdong prefecture level city in Shandong
Doraemon Hyakubo Shop	King of Power Saving	Afraid to buy back did not work, the store said the commitment to 90 days invalid refund, warranty 5 years. Saver also complimentary on the Pacific insurance, it is a lot of peace of mind, there are effects will continue to buy back drops!	Jingdong prefecture level city in Shandong
Doraemon Hyakubo Shop	King of Power Saving	Received the goods and used it, can save electricity a month later to see the effect!	Jingdong prefecture level city in Shandong
Doraemon Hyakubo Shop	King of Power Saving	Fancy 90-day trial, 5-year warranty before the order, the goods received, has been experiencing a week, can really feel the savings, in the introduction of friends also buy this!	Jingdong prefecture level city in Shandong
Doraemon Hyakubo Shop	King of Power Saving	Clothing stores use, neighbors recommended to buy, said a month can save more than 70 yuan, first plugged in to see the effect of the	Jingdong prefecture level city in Shandong
Doraemon Hyakubo Shop	King of Power Saving	A friend's home two months to save more than 80 dollars of electricity, recommended that I buy two, decisive purchase, looking forward to the effect of power saving, waiting for the next month effect.	Jingdong prefecture level city in Shandong
Doraemon Hyakubo Shop	King of Power Saving	The goods have been received, consistent with the description of the advertisement, quite good, I hope that in terms of power saving and purifying the air is really as the merchant said that would be better!	Jingdong prefecture level city in Shandong
Doraemon Hyakubo Shop	King of Power Saving	Power Saving King is really good to use the quality is very good than the physical store a lot cheaper we can rest assured that the purchase recommended to buy!	Jingdong prefecture level city in Shandong
Doraemon Hyakubo Shop	King of Power Saving	Energy saver has been used on the rental house inside the use of the meter is always running fast, with this can really save electricity, easy to use, plugged in can be, very satisfied!	Jingdong prefecture level city in Shandong
Doraemon Hyakubo Shop	King of Power Saving	Power Saver Power Saver King is good quality and affordable, very satisfied, worth buying.	Jingdong prefecture level city in Shandong
Doraemon Hyakubo Shop	King of Power Saving	Just use can not see what effect to,, wait for a period of time in the evaluation	Jingdong prefecture level city in Shandong
Doraemon Hyakubo Shop	King of Power Saving	Live in the 18th floor is too far away from the meter, afraid of no effect, look at the trial 90 days before buying. Currently used for a month, there is an effect, saving 27 dollars in electricity, next month should be able to save more!	Jingdong prefecture level city in Shandong
Doraemon Hyakubo Shop	King of Power Saving	I've been using it for almost a month now and it feels good, so I'll use it again.	Jingdong prefecture level city in Shandong
Doraemon Hyakubo Shop	King of Power Saving	It's ok, the electric bill is really less, originally I remember charging $100 and using it for 10 days. Now it lasts about 15 days. It's still noticeable.	Jingdong prefecture level city in Shandong
Doraemon Hyakubo Shop	King of Power Saving	Fast logistics, good power saving effect, very satisfied	Jingdong prefecture level city in Shandong
Doraemon Hyakubo Shop	King of Power Saving	Has been the second time back to buy, energy saving effect, worth recommending, you can try to see, power saving effect: very good Experience: can save about 30%!	Jingdong prefecture level city in Shandong
Doraemon Hyakubo Shop	King of Power Saving	I don't know how well it works for now	Jingdong prefecture level city in Shandong
Doraemon Hyakubo Shop	King of Power Saving	Factory direct is really good, plugged in and used for half a month did less probably almost 50 degrees of electricity! And the manufacturer's warranty is 5 years.	Jingdong prefecture level city in Shandong
Doraemon Hyakubo Shop	King of Power Saving	It's really effective. The previous month's electricity was $286, but after using the power saver for a month, I have $195 left this month, which is not bad.	Jingdong prefecture level city in Shandong
Doraemon Hyakubo Shop	King of Power Saving	I'll give you a good review and see how it goes.	Jingdong prefecture level city in Shandong
Doraemon Hyakubo Shop	King of Power Saving	Customer service is very professional and patient to guide the installation, the quality of workmanship is very fine. Durable, second repurchase. Really can save oh!	Jingdong prefecture level city in Shandong
Doraemon Hyakubo Shop	King of Power Saving	I've been using it for a month now and my electric bill is over $30 less than last month. I feel it's quite worth buying.	Jingdong prefecture level city in Shandong
Doraemon Hyakubo Shop	King of Power Saving	Small size, does not take up space. It's very good, it does work very effectively, the tool room has a lot of work with a lot of electricity. After installing it, it really reduces a lot of electricity bills. Before the day 30 degrees of electricity left stone, now work down really less a few degrees.	Jingdong prefecture level city in Shandong
Doraemon Hyakubo Shop	King of Power Saving	It does work for saving electricity, I've bought the kind from elsewhere before that was too simple not **all**. This time to buy this store, but also for the safety. Air conditioning is too much electricity, this use to save a lot of	Jingdong prefecture level city in Shandong
Doraemon Hyakubo Shop	King of Power Saving	Two air conditioners and a refrigerator at home, a month down the electricity bill is almost two hundred dollars, with this after the average savings of more than thirty dollars to spend less money to enjoy more, it's really very good!	Jingdong prefecture level city in Shandong
Doraemon Hyakubo Shop	King of Power Saving	Good. Very good.	Jingdong prefecture level city in Shandong
Doraemon Hyakubo Shop	King of Power Saving	Works great, easy to operate, great, very happy with it.	Jingdong prefecture level city in Shandong
Doraemon Hyakubo Shop	King of Power Saving	Received it and tried it out, it's great, great quality and worth having! Five stars praise!	Jingdong prefecture level city in Shandong
Doraemon Hyakubo Shop	King of Power Saving	Very trustworthy brand, quality is guaranteed, the price is still affordable, quite satisfied!	Jingdong prefecture level city in Shandong
Doraemon Hyakubo Shop	King of Power Saving	Use it before you speak.	Jingdong prefecture level city in Shandong
Doraemon Hyakubo Shop	King of Power Saving	The quality of workmanship is very good and the power saving effect is quite good, I regret not getting it earlier.	Jingdong prefecture level city in Shandong
Doraemon Hyakubo Shop	King of Power Saving	Merchants shipping speed, and customer service introduced the same very good, cost-effective	Jingdong prefecture level city in Shandong
Doraemon Hyakubo Shop	King of Power Saving	Feel a little effect Oh, where the power consumption where to plug, convenient and convenient	Jingdong prefecture level city in Shandong
Doraemon Hyakubo Shop	King of Power Saving	The quality of the imported saver is very good, practical and satisfactory. Cost-effective	Jingdong prefecture level city in Shandong
Doraemon Hyakubo Shop	King of Power Saving	Power Saver is especially good, good and inexpensive, very good	Jingdong prefecture level city in Shandong
Doraemon Hyakubo Shop	King of Power Saving	Fast shipping, awesome quality, affordable price, satisfied.	Jingdong prefecture level city in Shandong
Doraemon Hyakubo Shop	King of Power Saving	Energy saver received, the effect is consistent with the description, recommended to buy!	Jingdong prefecture level city in Shandong
Doraemon Hyakubo Shop	King of Power Saving	It's really good, the quality is great, I recommend it!	Jingdong prefecture level city in Shandong
Doraemon Hyakubo Shop	King of Power Saving	Works well. Inexpensive. Shipping was fast.	Jingdong prefecture level city in Shandong
Doraemon Hyakubo Shop	King of Power Saving	Logistics is fast, works well, energy saving is good	Jingdong prefecture level city in Shandong
Doraemon Hyakubo Shop	King of Power Saving	very happy	Jingdong prefecture level city in Shandong
Doraemon Hyakubo Shop	King of Power Saving	an excellent	Jingdong prefecture level city in Shandong
Doraemon Hyakubo Shop	King of Power Saving	It rained a couple days ago and I just went to get the item back today , not bad, hey I looked at the meter and that red display light takes several seconds to jump, did not make a mistake buying it. After a period of time to evaluate.	Jingdong prefecture level city in Shandong

Doraemon Hyakubo Shop	King of Power Saving	Used more than 10 days to check the power, there is indeed some decline, customer service said, long-term use will save more points!	Jingdong prefecture level city in Shandong
Doraemon Hyakubo Shop	King of Power Saving	It's a proper IQ tax, and it costs electricity.	Jingdong prefecture level city in Shandong
Doraemon Hyakubo Shop	King of Power Saving	I've been using it for 17 days now, it really saves electricity Shipping is fast, you can save a lot in a year	Jingdong prefecture level city in Shandong
Doraemon Hyakubo Shop	King of Power Saving	I've been using it for 17 days and it really saves power Shipping is fast and you don't have to worry about the quality, free returns within 5 years	Jingdong prefecture level city in Shandong
Doraemon Hyakubo Shop	King of Power Saving	It's more regular, scanned the code and it's authentic, and the main thing is that you can try it for 90 days! Plugged in more than ten days, is less than before a few degrees per day. When the time to use a month to see Additional. Good to buy a few more	Jingdong prefecture level city in Shandong
Doraemon Hyakubo Shop	King of Power Saving	Logistics is very fast, fine workmanship, use it and then come back to the comments	Jingdong prefecture level city in Shandong
Doraemon Hyakubo Shop	King of Power Saving	The quality of workmanship is very good and the power saving effect is quite good, I regret not getting it earlier.	Jingdong prefecture level city in Shandong
Doraemon Hyakubo Shop	King of Power Saving	Received the goods, according to the instructions on the operation is very simple, to wait and see the effect, looks good!	Jingdong prefecture level city in Shandong
Doraemon Hyakubo Shop	King of Power Saving	It's really as safe and power-saving as the owner says, and it works great!	Jingdong prefecture level city in Shandong
Doraemon Hyakubo Shop	King of Power Saving	Received the goods, immediately used, looks good, to wait and see how the effect.	Jingdong prefecture level city in Shandong
Doraemon Hyakubo Shop	King of Power Saving	Afraid to buy back did not work, the store said the commitment to 90 days invalid refund, warranty 5 years. Saver also complimentary on the Pacific insurance, it is a lot of peace of mind, there are effects will continue to buy back drops!	Jingdong prefecture level city in Shandong
Doraemon Hyakubo Shop	King of Power Saving	Received the goods and used it, can save electricity a month later to see the effect!	Jingdong prefecture level city in Shandong
Doraemon Hyakubo Shop	King of Power Saving	Fancy 90-day trial, 5-year warranty before the order, the goods received, has been experiencing a week, can really feel the savings, in the introduction of friends also buy this!	Jingdong prefecture level city in Shandong
Doraemon Hyakubo Shop	King of Power Saving	Clothing stores use, neighbors recommended to buy, said a month can save more than 70 yuan, first plugged in to see the effect of the	Jingdong prefecture level city in Shandong
Doraemon Hyakubo Shop	King of Power Saving	A friend's home two months to save more than 80 dollars of electricity, recommended that I buy two, decisive purchase, looking forward to the effect of power saving, waiting for the next month effect.	Jingdong prefecture level city in Shandong
Doraemon Hyakubo Shop	King of Power Saving	The goods have been received, consistent with the description of the advertisement, quite good, I hope that in terms of power saving and purifying the air is really as the merchant said that would be better!	Jingdong prefecture level city in Shandong
Doraemon Hyakubo Shop	King of Power Saving	Power Saving King is really good to use the quality is very good than the physical store a lot cheaper we can rest assured that the purchase recommended to buy!	Jingdong prefecture level city in Shandong
Doraemon Hyakubo Shop	King of Power Saving	Energy saver has been used on the rental house inside the use of the meter is always running fast, with this can really save electricity, easy to use, plugged in can be, very satisfied!	Jingdong prefecture level city in Shandong
Doraemon Hyakubo Shop	King of Power Saving	Power Saver Power Saver King is good quality and affordable, very satisfied, worth buying.	Jingdong prefecture level city in Shandong
Doraemon Hyakubo Shop	King of Power Saving	Just use can not see what effect to,, wait for a period of time in the evaluation	Jingdong prefecture level city in Shandong
Doraemon Hyakubo Shop	King of Power Saving	Live in the 18th floor is too far away from the meter, afraid of no effect, look at the trial 90 days before buying. Currently used for a month, there is an effect, saving 27 dollars in electricity, next month should be able to save more!	Jingdong prefecture level city in Shandong
Doraemon Hyakubo Shop	King of Power Saving	I've been using it for almost a month now and it feels good, so I'll use it again.	Jingdong prefecture level city in Shandong
Doraemon Hyakubo Shop	King of Power Saving	It's ok, the electric bill is really less, originally I remember charging $100 and using it for 10 days. Now it lasts about 15 days. It's still noticeable.	Jingdong prefecture level city in Shandong
Doraemon Hyakubo Shop	King of Power Saving	Fast logistics, good power saving effect, very satisfied	Jingdong prefecture level city in Shandong
Doraemon Hyakubo Shop	King of Power Saving	Has been the second time back to buy, energy saving effect, worth recommending, you can try to see, power saving effect: very good Experience: can save about 30%!	Jingdong prefecture level city in Shandong
Doraemon Hyakubo Shop	King of Power Saving	I don't know how well it works for now	Jingdong prefecture level city in Shandong
Doraemon Hyakubo Shop	King of Power Saving	Factory direct is really good, plugged in and used for half a month did less probably almost 50 degrees of electricity! And the manufacturer's warranty is 5 years.	Jingdong prefecture level city in Shandong
Doraemon Hyakubo Shop	King of Power Saving	It's really effective. The previous month's electricity was $286, but after using the power saver for a month, I have $195 left this month, which is not bad.	Jingdong prefecture level city in Shandong
Doraemon Hyakubo Shop	King of Power Saving	I'll give you a good review and see how it goes.	Jingdong prefecture level city in Shandong
Doraemon Hyakubo Shop	King of Power Saving	Customer service is very professional and patient to guide the installation, the quality of workmanship is very fine. Durable, second repurchase. Really can save oh!	Jingdong prefecture level city in Shandong
Doraemon Hyakubo Shop	King of Power Saving	I've been using it for a month now and my electric bill is over $30 less than last month. I feel it's quite worth buying.	Jingdong prefecture level city in Shandong
Doraemon Hyakubo Shop	King of Power Saving	Small size, does not take up space. It's very good, it does work very effectively, the tool room has a lot of work with a lot of electricity. After installing it, it really reduces a lot of electricity bills. Before the day 30 degrees of electricity left stone, now work down really less a few degrees.	Jingdong prefecture level city in Shandong
Doraemon Hyakubo Shop	King of Power Saving	It does work for saving electricity, I've bought the kind from elsewhere before that was too simple not **all**. This time to buy this store, but also for the safety. Air conditioning is too much electricity, this use to save a lot of	Jingdong prefecture level city in Shandong
Doraemon Hyakubo Shop	King of Power Saving	Two air conditioners and a refrigerator at home, a month down the electricity bill is almost two hundred dollars, with this after the average savings of more than thirty dollars to spend less money to enjoy more, it's really very good!	Jingdong prefecture level city in Shandong
Doraemon Hyakubo Shop	King of Power Saving	Good. Very good.	Jingdong prefecture level city in Shandong
Doraemon Hyakubo Shop	King of Power Saving	Works great, easy to operate, great, very happy with it.	Jingdong prefecture level city in Shandong
Doraemon Hyakubo Shop	King of Power Saving	Received it and tried it out, it's great, great quality and worth having! Five stars praise!	Jingdong prefecture level city in Shandong
Doraemon Hyakubo Shop	King of Power Saving	Very trustworthy brand, quality is guaranteed, the price is still affordable, quite satisfied!	Jingdong prefecture level city in Shandong
Doraemon Hyakubo Shop	King of Power Saving	Use it before you speak.	Jingdong prefecture level city in Shandong
Doraemon Hyakubo Shop	King of Power Saving	The quality of workmanship is very good and the power saving effect is quite good, I regret not getting it earlier.	Jingdong prefecture level city in Shandong
Doraemon Hyakubo Shop	King of Power Saving	Merchants shipping speed, and customer service introduced the same very good, cost-effective	Jingdong prefecture level city in Shandong
Doraemon Hyakubo Shop	King of Power Saving	Feel a little effect Oh, where the power consumption where to plug, convenient and convenient	Jingdong prefecture level city in Shandong
Doraemon Hyakubo Shop	King of Power Saving	The quality of the imported saver is very good, practical and satisfactory. Cost-effective	Jingdong prefecture level city in Shandong
Doraemon Hyakubo Shop	King of Power Saving	Power Saver is especially good, good and inexpensive, very good	Jingdong prefecture level city in Shandong
Doraemon Hyakubo Shop	King of Power Saving	Fast shipping, awesome quality, affordable price, satisfied.	Jingdong prefecture level city in Shandong
Doraemon Hyakubo Shop	King of Power Saving	Energy saver received, the effect is consistent with the description, recommended to buy!	Jingdong prefecture level city in Shandong
Doraemon Hyakubo Shop	King of Power Saving	It's really good, the quality is great, I recommend it!	Jingdong prefecture level city in Shandong
Doraemon Hyakubo Shop	King of Power Saving	Works well. Inexpensive. Shipping was fast.	Jingdong prefecture level city in Shandong

Doraemon Hyakubo Shop	King of Power Saving	Logistics is fast, works well, energy saving is good	Jingdong prefecture level city in Shandong
Doraemon Hyakubo Shop	King of Power Saving	very happy	Jingdong prefecture level city in Shandong
Doraemon Hyakubo Shop	King of Power Saving	an excellent	Jingdong prefecture level city in Shandong
Doraemon Hyakubo Shop	King of Power Saving	It rained a couple days ago and I just went to get the item back today , not bad, hey I looked at the meter and that red display light takes several seconds to jump, did not make a mistake buying it. After a period of time to evaluate.	Jingdong prefecture level city in Shandong
Doraemon Hyakubo Shop	King of Power Saving	Used more than 10 days to check the power, there is indeed some decline, customer service said, long-term use will save more points!	Jingdong prefecture level city in Shandong
Doraemon Hyakubo Shop	King of Power Saving	It's a proper IQ tax, and it costs electricity.	Jingdong prefecture level city in Shandong
Doraemon Hyakubo Shop	King of Power Saving	I've been using it for 17 days now, it really saves electricity Shipping is fast, you can save a lot in a year	Jingdong prefecture level city in Shandong
Doraemon Hyakubo Shop	King of Power Saving	I've been using it for 17 days and it really saves power Shipping is fast and you don't have to worry about the quality, free returns within 5 years	Jingdong prefecture level city in Shandong
Doraemon Hyakubo Shop	King of Power Saving	It's more regular, scanned the code and it's authentic, and the main thing is that you can try it for 90 days! Plugged in more than ten days, is less than before a few degrees per day. When the time to use a month to see Additional. Good to buy a few more	Jingdong prefecture level city in Shandong
Doraemon Hyakubo Shop	King of Power Saving	Logistics is very fast, fine workmanship, use it and then come back to the comments	Jingdong prefecture level city in Shandong
Doraemon Hyakubo Shop	King of Power Saving	The quality of workmanship is very good and the power saving effect is quite good, I regret not getting it earlier.	Jingdong prefecture level city in Shandong
Doraemon Hyakubo Shop	King of Power Saving	Received the goods, according to the instructions on the operation is very simple, to wait and see the effect, looks good!	Jingdong prefecture level city in Shandong
Doraemon Hyakubo Shop	King of Power Saving	It's really as safe and power-saving as the owner says, and it works great!	Jingdong prefecture level city in Shandong
Doraemon Hyakubo Shop	King of Power Saving	Received the goods, immediately used, looks good, to wait and see how the effect.	Jingdong prefecture level city in Shandong
Doraemon Hyakubo Shop	King of Power Saving	Afraid to buy back did not work, the store said the commitment to 90 days invalid refund, warranty 5 years. Saver also complimentary on the Pacific insurance, it is a lot of peace of mind, there are effects will continue to buy back drops!	Jingdong prefecture level city in Shandong
Doraemon Hyakubo Shop	King of Power Saving	Received the goods and used it, can save electricity a month later to see the effect!	Jingdong prefecture level city in Shandong
Doraemon Hyakubo Shop	King of Power Saving	Fancy 90-day trial, 5-year warranty before the order, the goods received, has been experiencing a week, can really feel the savings, in the introduction of friends also buy this!	Jingdong prefecture level city in Shandong
Doraemon Hyakubo Shop	King of Power Saving	Clothing stores use, neighbors recommended to buy, said a month can save more than 70 yuan, first plugged in to see the effect of the	Jingdong prefecture level city in Shandong
Doraemon Hyakubo Shop	King of Power Saving	A friend's home two months to save more than 80 dollars of electricity, recommended that I buy two, decisive purchase, looking forward to the effect of power saving, waiting for the next month effect.	Jingdong prefecture level city in Shandong
Doraemon Hyakubo Shop	King of Power Saving	The goods have been received, consistent with the description of the advertisement, quite good, I hope that in terms of power saving and purifying the air is really as the merchant said that would be better!	Jingdong prefecture level city in Shandong
Doraemon Hyakubo Shop	King of Power Saving	Power Saving King is really good to use the quality is very good than the physical store a lot cheaper we can rest assured that the purchase recommended to buy!	Jingdong prefecture level city in Shandong
Doraemon Hyakubo Shop	King of Power Saving	Energy saver has been used on the rental house inside the use of the meter is always running fast, with this can really save electricity, easy to use, plugged in can be, very satisfied!	Jingdong prefecture level city in Shandong
Doraemon Hyakubo Shop	King of Power Saving	Power Saver Power Saver King is good quality and affordable, very satisfied, worth buying.	Jingdong prefecture level city in Shandong
Doraemon Hyakubo Shop	King of Power Saving	Just use can not see what effect to,, wait for a period of time in the evaluation	Jingdong prefecture level city in Shandong
Doraemon Hyakubo Shop	King of Power Saving	Live in the 18th floor is too far away from the meter, afraid of no effect, look at the trial 90 days before buying. Currently used for a month, there is an effect, saving 27 dollars in electricity, next month should be able to save more!	Jingdong prefecture level city in Shandong
Doraemon Hyakubo Shop	King of Power Saving	I've been using it for almost a month now and it feels good, so I'll use it again.	Jingdong prefecture level city in Shandong
Doraemon Hyakubo Shop	King of Power Saving	It's ok, the electric bill is really less, originally I remember charging $100 and using it for 10 days. Now it lasts about 15 days. It's still noticeable.	Jingdong prefecture level city in Shandong
Doraemon Hyakubo Shop	King of Power Saving	Fast logistics, good power saving effect, very satisfied	Jingdong prefecture level city in Shandong
Doraemon Hyakubo Shop	King of Power Saving	Has been the second time back to buy, energy saving effect, worth recommending, you can try to see, power saving effect: very good Experience: can save about 30%!	Jingdong prefecture level city in Shandong
Doraemon Hyakubo Shop	King of Power Saving	I don't know how well it works for now	Jingdong prefecture level city in Shandong
Doraemon Hyakubo Shop	King of Power Saving	Factory direct is really good, plugged in and used for half a month did less probably almost 50 degrees of electricity! And the manufacturer's warranty is 5 years.	Jingdong prefecture level city in Shandong
Doraemon Hyakubo Shop	King of Power Saving	It's really effective. The previous month's electricity was $286, but after using the power saver for a month, I have $195 left this month, which is not bad.	Jingdong prefecture level city in Shandong
Doraemon Hyakubo Shop	King of Power Saving	I'll give you a good review and see how it goes.	Jingdong prefecture level city in Shandong
Doraemon Hyakubo Shop	King of Power Saving	Customer service is very professional and patient to guide the installation, the quality of workmanship is very fine. Durable, second repurchase. Really can save oh!	Jingdong prefecture level city in Shandong
Doraemon Hyakubo Shop	King of Power Saving	I've been using it for a month now and my electric bill is over $30 less than last month. I feel it's quite worth buying.	Jingdong prefecture level city in Shandong
Doraemon Hyakubo Shop	King of Power Saving	Small size, does not take up space. It's very good, it does work very effectively, the tool room has a lot of work with a lot of electricity. After installing it, it really reduces a lot of electricity bills. Before the day 30 degrees of electricity left stone, now work down really less a few degrees.	Jingdong prefecture level city in Shandong
Doraemon Hyakubo Shop	King of Power Saving	It does work for saving electricity, I've bought the kind from elsewhere before that was too simple not **all**. This time to buy this store, but also for the safety. Air conditioning is too much electricity, this use to save a lot of	Jingdong prefecture level city in Shandong
Doraemon Hyakubo Shop	King of Power Saving	Two air conditioners and a refrigerator at home, a month down the electricity bill is almost two hundred dollars, with this after the average savings of more than thirty dollars to spend less money to enjoy more, it's really very good!	Jingdong prefecture level city in Shandong
Doraemon Hyakubo Shop	King of Power Saving	Good. Very good.	Jingdong prefecture level city in Shandong
Doraemon Hyakubo Shop	King of Power Saving	Works great, easy to operate, great, very happy with it.	Jingdong prefecture level city in Shandong
Doraemon Hyakubo Shop	King of Power Saving	Received it and tried it out, it's great, great quality and worth having! Five stars praise!	Jingdong prefecture level city in Shandong
Doraemon Hyakubo Shop	King of Power Saving	Very trustworthy brand, quality is guaranteed, the price is still affordable, quite satisfied!	Jingdong prefecture level city in Shandong
Doraemon Hyakubo Shop	King of Power Saving	Use it before you speak.	Jingdong prefecture level city in Shandong
Doraemon Hyakubo Shop	King of Power Saving	The quality of workmanship is very good and the power saving effect is quite good, I regret not getting it earlier.	Jingdong prefecture level city in Shandong
Doraemon Hyakubo Shop	King of Power Saving	Merchants shipping speed, and customer service introduced the same very good, cost-effective	Jingdong prefecture level city in Shandong
Doraemon Hyakubo Shop	King of Power Saving	Feel a little effect Oh, where the power consumption where to plug, convenient and convenient	Jingdong prefecture level city in Shandong
Doraemon Hyakubo Shop	King of Power Saving	The quality of the imported saver is very good, practical and satisfactory. Cost-effective	Jingdong prefecture level city in Shandong
Doraemon Hyakubo Shop	King of Power Saving	Power Saver is especially good, good and inexpensive, very good	Jingdong prefecture level city in Shandong

Doraemon Hyakubo Shop	King of Power Saving	Fast shipping, awesome quality, affordable price, satisfied.	Jingdong prefecture level city in Shandong
Doraemon Hyakubo Shop	King of Power Saving	Energy saver received, the effect is consistent with the description, recommended to buy!	Jingdong prefecture level city in Shandong
Doraemon Hyakubo Shop	King of Power Saving	It's really good, the quality is great, I recommend it!	Jingdong prefecture level city in Shandong
Doraemon Hyakubo Shop	King of Power Saving	Works well. Inexpensive. Shipping was fast.	Jingdong prefecture level city in Shandong
Doraemon Hyakubo Shop	King of Power Saving	Logistics is fast, works well, energy saving is good	Jingdong prefecture level city in Shandong
Doraemon Hyakubo Shop	King of Power Saving	very happy	Jingdong prefecture level city in Shandong
Doraemon Hyakubo Shop	King of Power Saving	an excellent	Jingdong prefecture level city in Shandong
Doraemon Hyakubo Shop	King of Power Saving	It rained a couple days ago and I just went to get the item back today , not bad, hey I looked at the meter and that red display light takes several seconds to jump, did not make a mistake buying it. After a period of time to evaluate.	Jingdong prefecture level city in Shandong
Doraemon Hyakubo Shop	King of Power Saving	Used more than 10 days to check the power, there is indeed some decline, customer service said, long-term use will save more points!	Jingdong prefecture level city in Shandong
Doraemon Hyakubo Shop	King of Power Saving	It's a proper IQ tax, and it costs electricity.	Jingdong prefecture level city in Shandong
Doraemon Hyakubo Shop	King of Power Saving	I've been using it for 17 days now, it really saves electricity Shipping is fast, you can save a lot in a year	Jingdong prefecture level city in Shandong
Doraemon Hyakubo Shop	King of Power Saving	I've been using it for 17 days and it really saves power Shipping is fast and you don't have to worry about the quality, free returns within 5 years	Jingdong prefecture level city in Shandong
Doraemon Hyakubo Shop	King of Power Saving	It's more regular, scanned the code and it's authentic, and the main thing is that you can try it for 90 days! Plugged in more than ten days, is less than before a few degrees per day. When the time to use a month to see Additional. Good to buy a few more	Jingdong prefecture level city in Shandong
Doraemon Hyakubo Shop	King of Power Saving	Logistics is very fast, fine workmanship, use it and then come back to the comments	Jingdong prefecture level city in Shandong
Doraemon Hyakubo Shop	King of Power Saving	The quality of workmanship is very good and the power saving effect is quite good, I regret not getting it earlier.	Jingdong prefecture level city in Shandong
Doraemon Hyakubo Shop	King of Power Saving	Received the goods, according to the instructions on the operation is very simple, to wait and see the effect, looks good!	Jingdong prefecture level city in Shandong
Doraemon Hyakubo Shop	King of Power Saving	It's really as safe and power-saving as the owner says, and it works great!	Jingdong prefecture level city in Shandong
Doraemon Hyakubo Shop	King of Power Saving	Received the goods, immediately used, looks good, to wait and see how the effect.	Jingdong prefecture level city in Shandong
Doraemon Hyakubo Shop	King of Power Saving	Afraid to buy back did not work, the store said the commitment to 90 days invalid refund, warranty 5 years. Saver also complimentary on the Pacific insurance, it is a lot of peace of mind, there are effects will continue to buy back drops!	Jingdong prefecture level city in Shandong
Doraemon Hyakubo Shop	King of Power Saving	Received the goods and used it, can save electricity a month later to see the effect!	Jingdong prefecture level city in Shandong
Doraemon Hyakubo Shop	King of Power Saving	Fancy 90-day trial, 5-year warranty before the order, the goods received, has been experiencing a week, can really feel the savings, in the introduction of friends also buy this!	Jingdong prefecture level city in Shandong
Doraemon Hyakubo Shop	King of Power Saving	Clothing stores use, neighbors recommended to buy, said a month can save more than 70 yuan, first plugged in to see the effect of the	Jingdong prefecture level city in Shandong
Doraemon Hyakubo Shop	King of Power Saving	A friend's home two months to save more than 80 dollars of electricity, recommended that I buy two, decisive purchase, looking forward to the effect of power saving, waiting for the next month effect.	Jingdong prefecture level city in Shandong
Doraemon Hyakubo Shop	King of Power Saving	The goods have been received, consistent with the description of the advertisement, quite good, I hope that in terms of power saving and purifying the air is really as the merchant said that would be better!	Jingdong prefecture level city in Shandong
Doraemon Hyakubo Shop	King of Power Saving	Power Saving King is really good to use the quality is very good than the physical store a lot cheaper we can rest assured that the purchase recommended to buy!	Jingdong prefecture level city in Shandong
Doraemon Hyakubo Shop	King of Power Saving	Energy saver has been used on the rental house inside the use of the meter is always running fast, with this can really save electricity, easy to use, plugged in can be, very satisfied!	Jingdong prefecture level city in Shandong
Doraemon Hyakubo Shop	King of Power Saving	Power Saver Power Saver King is good quality and affordable, very satisfied, worth buying.	Jingdong prefecture level city in Shandong
Doraemon Hyakubo Shop	King of Power Saving	Just use can not see what effect to,, wait for a period of time in the evaluation	Jingdong prefecture level city in Shandong
Doraemon Hyakubo Shop	King of Power Saving	Live in the 18th floor is too far away from the meter, afraid of no effect, look at the trial 90 days before buying. Currently used for a month, there is an effect, saving 27 dollars in electricity, next month should be able to save more!	Jingdong prefecture level city in Shandong
Doraemon Hyakubo Shop	King of Power Saving	I've been using it for almost a month now and it feels good, so I'll use it again.	Jingdong prefecture level city in Shandong
Doraemon Hyakubo Shop	King of Power Saving	It's ok, the electric bill is really less, originally I remember charging $100 and using it for 10 days. Now it lasts about 15 days. It's still noticeable.	Jingdong prefecture level city in Shandong
Doraemon Hyakubo Shop	King of Power Saving	Fast logistics, good power saving effect, very satisfied	Jingdong prefecture level city in Shandong
Doraemon Hyakubo Shop	King of Power Saving	Has been the second time back to buy, energy saving effect, worth recommending, you can try to see, power saving effect: very good Experience: can save about 30%!	Jingdong prefecture level city in Shandong
Doraemon Hyakubo Shop	King of Power Saving	I don't know how well it works for now	Jingdong prefecture level city in Shandong
Doraemon Hyakubo Shop	King of Power Saving	Factory direct is really good, plugged in and used for half a month did less probably almost 50 degrees of electricity! And the manufacturer's warranty is 5 years.	Jingdong prefecture level city in Shandong
Doraemon Hyakubo Shop	King of Power Saving	It's really effective. The previous month's electricity was $286, but after using the power saver for a month, I have $195 left this month, which is not bad.	Jingdong prefecture level city in Shandong
Doraemon Hyakubo Shop	King of Power Saving	I'll give you a good review and see how it goes.	Jingdong prefecture level city in Shandong
Doraemon Hyakubo Shop	King of Power Saving	Customer service is very professional and patient to guide the installation, the quality of workmanship is very fine. Durable, second repurchase. Really can save oh!	Jingdong prefecture level city in Shandong
Doraemon Hyakubo Shop	King of Power Saving	I've been using it for a month now and my electric bill is over $30 less than last month. I feel it's quite worth buying.	Jingdong prefecture level city in Shandong
Doraemon Hyakubo Shop	King of Power Saving	Small size, does not take up space. It's very good, it does work very effectively, the tool room has a lot of work with a lot of electricity. After installing it, it really reduces a lot of electricity bills. Before the day 30 degrees of electricity left stone, now work down really less a few degrees.	Jingdong prefecture level city in Shandong
Doraemon Hyakubo Shop	King of Power Saving	It does work for saving electricity, I've bought the kind from elsewhere before that was too simple not **all**. This time to buy this store, but also for the safety. Air conditioning is too much electricity, this use to save a lot of	Jingdong prefecture level city in Shandong
Doraemon Hyakubo Shop	King of Power Saving	Two air conditioners and a refrigerator at home, a month down the electricity bill is almost two hundred dollars, with this after the average savings of more than thirty dollars to spend less money to enjoy more, it's really very good!	Jingdong prefecture level city in Shandong
Doraemon Hyakubo Shop	King of Power Saving	Good. Very good.	Jingdong prefecture level city in Shandong
Doraemon Hyakubo Shop	King of Power Saving	Works great, easy to operate, great, very happy with it.	Jingdong prefecture level city in Shandong
Doraemon Hyakubo Shop	King of Power Saving	Received it and tried it out, it's great, great quality and worth having! Five stars praise!	Jingdong prefecture level city in Shandong
Doraemon Hyakubo Shop	King of Power Saving	Very trustworthy brand, quality is guaranteed, the price is still affordable, quite satisfied!	Jingdong prefecture level city in Shandong
Doraemon Hyakubo Shop	King of Power Saving	Use it before you speak.	Jingdong prefecture level city in Shandong
Doraemon Hyakubo Shop	King of Power Saving	The quality of workmanship is very good and the power saving effect is quite good, I regret not getting it earlier.	Jingdong prefecture level city in Shandong

Doraemon Hyakubo Shop	King of Power Saving	Merchants shipping speed, and customer service introduced the same very good, cost-effective	Jingdong prefecture level city in Shandong
Doraemon Hyakubo Shop	King of Power Saving	Feel a little effect Oh, where the power consumption where to plug, convenient and convenient	Jingdong prefecture level city in Shandong
Doraemon Hyakubo Shop	King of Power Saving	The quality of the imported saver is very good, practical and satisfactory. Cost-effective	Jingdong prefecture level city in Shandong
Doraemon Hyakubo Shop	King of Power Saving	Power Saver is especially good, good and inexpensive, very good	Jingdong prefecture level city in Shandong
Doraemon Hyakubo Shop	King of Power Saving	Fast shipping, awesome quality, affordable price, satisfied.	Jingdong prefecture level city in Shandong
Doraemon Hyakubo Shop	King of Power Saving	Energy saver received, the effect is consistent with the description, recommended to buy!	Jingdong prefecture level city in Shandong
Doraemon Hyakubo Shop	King of Power Saving	It's really good, the quality is great, I recommend it!	Jingdong prefecture level city in Shandong
Doraemon Hyakubo Shop	King of Power Saving	Works well. Inexpensive. Shipping was fast.	Jingdong prefecture level city in Shandong
Doraemon Hyakubo Shop	King of Power Saving	Logistics is fast, works well, energy saving is good	Jingdong prefecture level city in Shandong
Doraemon Hyakubo Shop	King of Power Saving	very happy	Jingdong prefecture level city in Shandong
Doraemon Hyakubo Shop	King of Power Saving	an excellent	Jingdong prefecture level city in Shandong
Doraemon Hyakubo Shop	King of Power Saving	It rained a couple days ago and I just went to get the item back today , not bad, hey I looked at the meter and that red display light takes several seconds to jump, did not make a mistake buying it. After a period of time to evaluate.	Jingdong prefecture level city in Shandong
Doraemon Hyakubo Shop	King of Power Saving	Used more than 10 days to check the power, there is indeed some decline, customer service said, long-term use will save more points!	Jingdong prefecture level city in Shandong
Doraemon Hyakubo Shop	King of Power Saving	It's a proper IQ tax, and it costs electricity.	Jingdong prefecture level city in Shandong
Doraemon Hyakubo Shop	King of Power Saving	I've been using it for 17 days now, it really saves electricity Shipping is fast, you can save a lot in a year	Jingdong prefecture level city in Shandong
Doraemon Hyakubo Shop	King of Power Saving	I've been using it for 17 days and it really saves power Shipping is fast and you don't have to worry about the quality, free returns within 5 years	Jingdong prefecture level city in Shandong
Doraemon Hyakubo Shop	King of Power Saving	It's more regular, scanned the code and it's authentic, and the main thing is that you can try it for 90 days! Plugged in more than ten days, is less than before a few degrees per day. When the time to use a month to see Additional. Good to buy a few more	Jingdong prefecture level city in Shandong
Doraemon Hyakubo Shop	King of Power Saving	Logistics is very fast, fine workmanship, use it and then come back to the comments	Jingdong prefecture level city in Shandong
Doraemon Hyakubo Shop	King of Power Saving	The quality of workmanship is very good and the power saving effect is quite good, I regret not getting it earlier.	Jingdong prefecture level city in Shandong
Doraemon Hyakubo Shop	King of Power Saving	Received the goods, according to the instructions on the operation is very simple, to wait and see the effect, looks good!	Jingdong prefecture level city in Shandong
Doraemon Hyakubo Shop	King of Power Saving	It's really as safe and power-saving as the owner says, and it works great!	Jingdong prefecture level city in Shandong
Doraemon Hyakubo Shop	King of Power Saving	Received the goods, immediately used, looks good, to wait and see how the effect.	Jingdong prefecture level city in Shandong
Doraemon Hyakubo Shop	King of Power Saving	Afraid to buy back did not work, the store said the commitment to 90 days invalid refund, warranty 5 years. Saver also complimentary on the Pacific insurance, it is a lot of peace of mind, there are effects will continue to buy back drops!	Jingdong prefecture level city in Shandong
Doraemon Hyakubo Shop	King of Power Saving	Received the goods and used it, can save electricity a month later to see the effect!	Jingdong prefecture level city in Shandong
Doraemon Hyakubo Shop	King of Power Saving	Fancy 90-day trial, 5-year warranty before the order, the goods received, has been experiencing a week, can really feel the savings, in the introduction of friends also buy this!	Jingdong prefecture level city in Shandong
Doraemon Hyakubo Shop	King of Power Saving	Clothing stores use, neighbors recommended to buy, said a month can save more than 70 yuan, first plugged in to see the effect of the	Jingdong prefecture level city in Shandong
Doraemon Hyakubo Shop	King of Power Saving	A friend's home two months to save more than 80 dollars of electricity, recommended that I buy two, decisive purchase, looking forward to the effect of power saving, waiting for the next month effect.	Jingdong prefecture level city in Shandong
Doraemon Hyakubo Shop	King of Power Saving	The goods have been received, consistent with the description of the advertisement, quite good, I hope that in terms of power saving and purifying the air is really as the merchant said that would be better!	Jingdong prefecture level city in Shandong
Doraemon Hyakubo Shop	King of Power Saving	Power Saving King is really good to use the quality is very good than the physical store a lot cheaper we can rest assured that the purchase recommended to buy!	Jingdong prefecture level city in Shandong
Doraemon Hyakubo Shop	King of Power Saving	Energy saver has been used on the rental house inside the use of the meter is always running fast, with this can really save electricity, easy to use, plugged in can be, very satisfied!	Jingdong prefecture level city in Shandong
Doraemon Hyakubo Shop	King of Power Saving	Power Saver Power Saver King is good quality and affordable, very satisfied, worth buying.	Jingdong prefecture level city in Shandong
Doraemon Hyakubo Shop	King of Power Saving	Just use can not see what effect to,, wait for a period of time in the evaluation	Jingdong prefecture level city in Shandong
Doraemon Hyakubo Shop	King of Power Saving	Live in the 18th floor is too far away from the meter, afraid of no effect, look at the trial 90 days before buying. Currently used for a month, there is an effect, saving 27 dollars in electricity, next month should be able to save more!	Jingdong prefecture level city in Shandong
Doraemon Hyakubo Shop	King of Power Saving	I've been using it for almost a month now and it feels good, so I'll use it again.	Jingdong prefecture level city in Shandong
Doraemon Hyakubo Shop	King of Power Saving	It's ok, the electric bill is really less, originally I remember charging $100 and using it for 10 days. Now it lasts about 15 days. It's still noticeable.	Jingdong prefecture level city in Shandong
Doraemon Hyakubo Shop	King of Power Saving	Fast logistics, good power saving effect, very satisfied	Jingdong prefecture level city in Shandong
Doraemon Hyakubo Shop	King of Power Saving	Has been the second time back to buy, energy saving effect, worth recommending, you can try to see, power saving effect: very good Experience: can save about 30%!	Jingdong prefecture level city in Shandong
Doraemon Hyakubo Shop	King of Power Saving	I don't know how well it works for now	Jingdong prefecture level city in Shandong
Doraemon Hyakubo Shop	King of Power Saving	Factory direct is really good, plugged in and used for half a month did less probably almost 50 degrees of electricity! And the manufacturer's warranty is 5 years.	Jingdong prefecture level city in Shandong
Doraemon Hyakubo Shop	King of Power Saving	It's really effective. The previous month's electricity was $286, but after using the power saver for a month, I have $195 left this month, which is not bad.	Jingdong prefecture level city in Shandong
Doraemon Hyakubo Shop	King of Power Saving	I'll give you a good review and see how it goes.	Jingdong prefecture level city in Shandong
Doraemon Hyakubo Shop	King of Power Saving	Customer service is very professional and patient to guide the installation, the quality of workmanship is very fine. Durable, second repurchase. Really can save oh!	Jingdong prefecture level city in Shandong
Doraemon Hyakubo Shop	King of Power Saving	I've been using it for a month now and my electric bill is over $30 less than last month. I feel it's quite worth buying.	Jingdong prefecture level city in Shandong
Doraemon Hyakubo Shop	King of Power Saving	Small size, does not take up space. It's very good, it does work very effectively, the tool room has a lot of work with a lot of electricity. After installing it, it really reduces a lot of electricity bills. Before the day 30 degrees of electricity left stone, now work down really less a few degrees.	Jingdong prefecture level city in Shandong
Doraemon Hyakubo Shop	King of Power Saving	It does work for saving electricity, I've bought the kind from elsewhere before that was too simple not **all**. This time to buy this store, but also for the safety. Air conditioning is too much electricity, this use to save a lot of	Jingdong prefecture level city in Shandong
Doraemon Hyakubo Shop	King of Power Saving	Two air conditioners and a refrigerator at home, a month down the electricity bill is almost two hundred dollars, with this after the average savings of more than thirty dollars to spend less money to enjoy more, it's really very good!	Jingdong prefecture level city in Shandong
Doraemon Hyakubo Shop	King of Power Saving	Good. Very good.	Jingdong prefecture level city in Shandong
Doraemon Hyakubo Shop	King of Power Saving	Works great, easy to operate, great, very happy with it.	Jingdong prefecture level city in Shandong

Doraemon Hyakubo Shop	King of Power Saving	Received it and tried it out, it's great, great quality and worth having! Five stars praise!	Jingdong prefecture level city in Shandong
Doraemon Hyakubo Shop	King of Power Saving	Very trustworthy brand, quality is guaranteed, the price is still affordable, quite satisfied!	Jingdong prefecture level city in Shandong
Doraemon Hyakubo Shop	King of Power Saving	Use it before you speak.	Jingdong prefecture level city in Shandong
Doraemon Hyakubo Shop	King of Power Saving	The quality of workmanship is very good and the power saving effect is quite good, I regret not getting it earlier.	Jingdong prefecture level city in Shandong
Doraemon Hyakubo Shop	King of Power Saving	Merchants shipping speed, and customer service introduced the same very good, cost-effective	Jingdong prefecture level city in Shandong
Doraemon Hyakubo Shop	King of Power Saving	Feel a little effect Oh, where the power consumption where to plug, convenient and convenient	Jingdong prefecture level city in Shandong
Doraemon Hyakubo Shop	King of Power Saving	The quality of the imported saver is very good, practical and satisfactory. Cost-effective	Jingdong prefecture level city in Shandong
Doraemon Hyakubo Shop	King of Power Saving	Power Saver is especially good, good and inexpensive, very good	Jingdong prefecture level city in Shandong
Doraemon Hyakubo Shop	King of Power Saving	Fast shipping, awesome quality, affordable price, satisfied.	Jingdong prefecture level city in Shandong
Doraemon Hyakubo Shop	King of Power Saving	Energy saver received, the effect is consistent with the description, recommended to buy!	Jingdong prefecture level city in Shandong
Doraemon Hyakubo Shop	King of Power Saving	It's really good, the quality is great, I recommend it!	Jingdong prefecture level city in Shandong
Doraemon Hyakubo Shop	King of Power Saving	Works well. Inexpensive. Shipping was fast.	Jingdong prefecture level city in Shandong
Doraemon Hyakubo Shop	King of Power Saving	Logistics is fast, works well, energy saving is good	Jingdong prefecture level city in Shandong
Doraemon Hyakubo Shop	King of Power Saving	very happy	Jingdong prefecture level city in Shandong
Doraemon Hyakubo Shop	King of Power Saving	an excellent	Jingdong prefecture level city in Shandong
Doraemon Hyakubo Shop	King of Power Saving	It rained a couple days ago and I just went to get the item back today , not bad, hey I looked at the meter and that red display light takes several seconds to jump, did not make a mistake buying it. After a period of time to evaluate.	Jingdong prefecture level city in Shandong
Doraemon Hyakubo Shop	King of Power Saving	Used more than 10 days to check the power, there is indeed some decline, customer service said, long-term use will save more points!	Jingdong prefecture level city in Shandong
Doraemon Hyakubo Shop	King of Power Saving	It's a proper IQ tax, and it costs electricity.	Jingdong prefecture level city in Shandong
Doraemon Hyakubo Shop	King of Power Saving	I've been using it for 17 days now, it really saves electricity Shipping is fast, you can save a lot in a year	Jingdong prefecture level city in Shandong
Doraemon Hyakubo Shop	King of Power Saving	I've been using it for 17 days and it really saves power Shipping is fast and you don't have to worry about the quality, free returns within 5 years	Jingdong prefecture level city in Shandong
Doraemon Hyakubo Shop	King of Power Saving	It's more regular, scanned the code and it's authentic, and the main thing is that you can try it for 90 days! Plugged in more than ten days, is less than before a few degrees per day. When the time to use a month to see Additional. Good to buy a few more	Jingdong prefecture level city in Shandong
Doraemon Hyakubo Shop	King of Power Saving	Logistics is very fast, fine workmanship, use it and then come back to the comments	Jingdong prefecture level city in Shandong
Doraemon Hyakubo Shop	King of Power Saving	The quality of workmanship is very good and the power saving effect is quite good, I regret not getting it earlier.	Jingdong prefecture level city in Shandong
Doraemon Hyakubo Shop	King of Power Saving	Received the goods, according to the instructions on the operation is very simple, to wait and see the effect, looks good!	Jingdong prefecture level city in Shandong
Doraemon Hyakubo Shop	King of Power Saving	It's really as safe and power-saving as the owner says, and it works great!	Jingdong prefecture level city in Shandong
Doraemon Hyakubo Shop	King of Power Saving	Received the goods, immediately used, looks good, to wait and see how the effect.	Jingdong prefecture level city in Shandong
Doraemon Hyakubo Shop	King of Power Saving	Afraid to buy back did not work, the store said the commitment to 90 days invalid refund, warranty 5 years. Saver also complimentary on the Pacific insurance, it is a lot of peace of mind, there are effects will continue to buy back drops!	Jingdong prefecture level city in Shandong
Doraemon Hyakubo Shop	King of Power Saving	Received the goods and used it, can save electricity a month later to see the effect!	Jingdong prefecture level city in Shandong
Doraemon Hyakubo Shop	King of Power Saving	Fancy 90-day trial, 5-year warranty before the order, the goods received, has been experiencing a week, can really feel the savings, in the introduction of friends also buy this!	Jingdong prefecture level city in Shandong
Doraemon Hyakubo Shop	King of Power Saving	Clothing stores use, neighbors recommended to buy, said a month can save more than 70 yuan, first plugged in to see the effect of the	Jingdong prefecture level city in Shandong
Doraemon Hyakubo Shop	King of Power Saving	A friend's home two months to save more than 80 dollars of electricity, recommended that I buy two, decisive purchase, looking forward to the effect of power saving, waiting for the next month effect.	Jingdong prefecture level city in Shandong
Doraemon Hyakubo Shop	King of Power Saving	The goods have been received, consistent with the description of the advertisement, quite good, I hope that in terms of power saving and purifying the air is really as the merchant said that would be better!	Jingdong prefecture level city in Shandong
Doraemon Hyakubo Shop	King of Power Saving	Power Saving King is really good to use the quality is very good than the physical store a lot cheaper we can rest assured that the purchase recommended to buy!	Jingdong prefecture level city in Shandong
Doraemon Hyakubo Shop	King of Power Saving	Energy saver has been used on the rental house inside the use of the meter is always running fast, with this can really save electricity, easy to use, plugged in can be, very satisfied!	Jingdong prefecture level city in Shandong
Doraemon Hyakubo Shop	King of Power Saving	Power Saver Power Saver King is good quality and affordable, very satisfied, worth buying.	Jingdong prefecture level city in Shandong
Doraemon Hyakubo Shop	King of Power Saving	Just use can not see what effect to,, wait for a period of time in the evaluation	Jingdong prefecture level city in Shandong
Doraemon Hyakubo Shop	King of Power Saving	Live in the 18th floor is too far away from the meter, afraid of no effect, look at the trial 90 days before buying. Currently used for a month, there is an effect, saving 27 dollars in electricity, next month should be able to save more!	Jingdong prefecture level city in Shandong
Doraemon Hyakubo Shop	King of Power Saving	I've been using it for almost a month now and it feels good, so I'll use it again.	Jingdong prefecture level city in Shandong
Doraemon Hyakubo Shop	King of Power Saving	It's ok, the electric bill is really less, originally I remember charging $100 and using it for 10 days. Now it lasts about 15 days. It's still noticeable.	Jingdong prefecture level city in Shandong
Doraemon Hyakubo Shop	King of Power Saving	Fast logistics, good power saving effect, very satisfied	Jingdong prefecture level city in Shandong
Doraemon Hyakubo Shop	King of Power Saving	Has been the second time back to buy, energy saving effect, worth recommending, you can try to see, power saving effect: very good Experience: can save about 30%!	Jingdong prefecture level city in Shandong
Doraemon Hyakubo Shop	King of Power Saving	I don't know how well it works for now	Jingdong prefecture level city in Shandong
Doraemon Hyakubo Shop	King of Power Saving	Factory direct is really good, plugged in and used for half a month did less probably almost 50 degrees of electricity! And the manufacturer's warranty is 5 years.	Jingdong prefecture level city in Shandong
Doraemon Hyakubo Shop	King of Power Saving	It's really effective. The previous month's electricity was $286, but after using the power saver for a month, I have $195 left this month, which is not bad.	Jingdong prefecture level city in Shandong
Doraemon Hyakubo Shop	King of Power Saving	I'll give you a good review and see how it goes.	Jingdong prefecture level city in Shandong
Doraemon Hyakubo Shop	King of Power Saving	Customer service is very professional and patient to guide the installation, the quality of workmanship is very fine. Durable, second repurchase. Really can save oh!	Jingdong prefecture level city in Shandong
Doraemon Hyakubo Shop	King of Power Saving	I've been using it for a month now and my electric bill is over $30 less than last month. I feel it's quite worth buying.	Jingdong prefecture level city in Shandong
Doraemon Hyakubo Shop	King of Power Saving	Small size, does not take up space. It's very good, it does work very effectively, the tool room has a lot of work with a lot of electricity. After installing it, it really reduces a lot of electricity bills. Before the day 30 degrees of electricity left stone, now work down really less a few degrees.	Jingdong prefecture level city in Shandong

Doraemon Hyakubo Shop	King of Power Saving	It does work for saving electricity, I've bought the kind from elsewhere before that was too simple not **all**. This time to buy this store, but also for the safety. Air conditioning is too much electricity, this use to save a lot of	Jingdong prefecture level city in Shandong
Doraemon Hyakubo Shop	King of Power Saving	Two air conditioners and a refrigerator at home, a month down the electricity bill is almost two hundred dollars, with this after the average savings of more than thirty dollars to spend less money to enjoy more, it's really very good!	Jingdong prefecture level city in Shandong
Doraemon Hyakubo Shop	King of Power Saving	Good. Very good.	Jingdong prefecture level city in Shandong
Doraemon Hyakubo Shop	King of Power Saving	Works great, easy to operate, great, very happy with it.	Jingdong prefecture level city in Shandong
Doraemon Hyakubo Shop	King of Power Saving	Received it and tried it out, it's great, great quality and worth having! Five stars praise!	Jingdong prefecture level city in Shandong
Doraemon Hyakubo Shop	King of Power Saving	Very trustworthy brand, quality is guaranteed, the price is still affordable, quite satisfied!	Jingdong prefecture level city in Shandong
Doraemon Hyakubo Shop	King of Power Saving	Use it before you speak.	Jingdong prefecture level city in Shandong
Doraemon Hyakubo Shop	King of Power Saving	The quality of workmanship is very good and the power saving effect is quite good, I regret not getting it earlier.	Jingdong prefecture level city in Shandong
Doraemon Hyakubo Shop	King of Power Saving	Merchants shipping speed, and customer service introduced the same very good, cost-effective	Jingdong prefecture level city in Shandong
Doraemon Hyakubo Shop	King of Power Saving	Feel a little effect Oh, where the power consumption where to plug, convenient and convenient	Jingdong prefecture level city in Shandong
Doraemon Hyakubo Shop	King of Power Saving	The quality of the imported saver is very good, practical and satisfactory. Cost-effective	Jingdong prefecture level city in Shandong
Doraemon Hyakubo Shop	King of Power Saving	Power Saver is especially good, good and inexpensive, very good	Jingdong prefecture level city in Shandong
Doraemon Hyakubo Shop	King of Power Saving	Fast shipping, awesome quality, affordable price, satisfied.	Jingdong prefecture level city in Shandong
Doraemon Hyakubo Shop	King of Power Saving	Energy saver received, the effect is consistent with the description, recommended to buy!	Jingdong prefecture level city in Shandong
Doraemon Hyakubo Shop	King of Power Saving	It's really good, the quality is great, I recommend it!	Jingdong prefecture level city in Shandong
Doraemon Hyakubo Shop	King of Power Saving	Works well. Inexpensive. Shipping was fast.	Jingdong prefecture level city in Shandong
Doraemon Hyakubo Shop	King of Power Saving	Logistics is fast, works well, energy saving is good	Jingdong prefecture level city in Shandong
Doraemon Hyakubo Shop	King of Power Saving	very happy	Jingdong prefecture level city in Shandong
Doraemon Hyakubo Shop	King of Power Saving	an excellent	Jingdong prefecture level city in Shandong
Doraemon Hyakubo Shop	King of Power Saving	It rained a couple days ago and I just went to get the item back today , not bad, hey I looked at the meter and that red display light takes several seconds to jump, did not make a mistake buying it. After a period of time to evaluate.	Jingdong prefecture level city in Shandong
Doraemon Hyakubo Shop	King of Power Saving	Used more than 10 days to check the power, there is indeed some decline, customer service said, long-term use will save more points!	Jingdong prefecture level city in Shandong
Doraemon Hyakubo Shop	King of Power Saving	It's a proper IQ tax, and it costs electricity.	Jingdong prefecture level city in Shandong
Doraemon Hyakubo Shop	King of Power Saving	I've been using it for 17 days now, it really saves electricity Shipping is fast, you can save a lot in a year	Jingdong prefecture level city in Shandong
Doraemon Hyakubo Shop	King of Power Saving	I've been using it for 17 days and it really saves power Shipping is fast and you don't have to worry about the quality, free returns within 5 years	Jingdong prefecture level city in Shandong
Doraemon Hyakubo Shop	King of Power Saving	It's more regular, scanned the code and it's authentic, and the main thing is that you can try it for 90 days! Plugged in more than ten days, is less than before a few degrees per day. When the time to use a month to see Additional. Good to buy a few more	Jingdong prefecture level city in Shandong
Doraemon Hyakubo Shop	King of Power Saving	Logistics is very fast, fine workmanship, use it and then come back to the comments	Jingdong prefecture level city in Shandong
Doraemon Hyakubo Shop	King of Power Saving	The quality of workmanship is very good and the power saving effect is quite good, I regret not getting it earlier.	Jingdong prefecture level city in Shandong
Doraemon Hyakubo Shop	King of Power Saving	Received the goods, according to the instructions on the operation is very simple, to wait and see the effect, looks good!	Jingdong prefecture level city in Shandong
Doraemon Hyakubo Shop	King of Power Saving	It's really as safe and power-saving as the owner says, and it works great!	Jingdong prefecture level city in Shandong
Doraemon Hyakubo Shop	King of Power Saving	Received the goods, immediately used, looks good, to wait and see how the effect.	Jingdong prefecture level city in Shandong
Doraemon Hyakubo Shop	King of Power Saving	Afraid to buy back did not work, the store said the commitment to 90 days invalid refund, warranty 5 years. Saver also complimentary on the Pacific insurance, it is a lot of peace of mind, there are effects will continue to buy back drops!	Jingdong prefecture level city in Shandong
Doraemon Hyakubo Shop	King of Power Saving	Received the goods and used it, can save electricity a month later to see the effect!	Jingdong prefecture level city in Shandong
Doraemon Hyakubo Shop	King of Power Saving	Fancy 90-day trial, 5-year warranty before the order, the goods received, has been experiencing a week, can really feel the savings, in the introduction of friends also buy this!	Jingdong prefecture level city in Shandong
Doraemon Hyakubo Shop	King of Power Saving	Clothing stores use, neighbors recommended to buy, said a month can save more than 70 yuan, first plugged in to see the effect of the	Jingdong prefecture level city in Shandong
Doraemon Hyakubo Shop	King of Power Saving	A friend's home two months to save more than 80 dollars of electricity, recommended that I buy two, decisive purchase, looking forward to the effect of power saving, waiting for the next month effect.	Jingdong prefecture level city in Shandong
Doraemon Hyakubo Shop	King of Power Saving	The goods have been received, consistent with the description of the advertisement, quite good, I hope that in terms of power saving and purifying the air is really as the merchant said that would be better!	Jingdong prefecture level city in Shandong
Doraemon Hyakubo Shop	King of Power Saving	Power Saving King is really good to use the quality is very good than the physical store a lot cheaper we can rest assured that the purchase recommended to buy!	Jingdong prefecture level city in Shandong
Doraemon Hyakubo Shop	King of Power Saving	Energy saver has been used on the rental house inside the use of the meter is always running fast, with this can really save electricity, easy to use, plugged in can be, very satisfied!	Jingdong prefecture level city in Shandong
Doraemon Hyakubo Shop	King of Power Saving	Power Saver Power Saver King is good quality and affordable, very satisfied, worth buying.	Jingdong prefecture level city in Shandong
Doraemon Hyakubo Shop	King of Power Saving	Just use can not see what effect to,, wait for a period of time in the evaluation	Jingdong prefecture level city in Shandong
Doraemon Hyakubo Shop	King of Power Saving	Live in the 18th floor is too far away from the meter, afraid of no effect, look at the trial 90 days before buying. Currently used for a month, there is an effect, saving 27 dollars in electricity, next month should be able to save more!	Jingdong prefecture level city in Shandong
Doraemon Hyakubo Shop	King of Power Saving	I've been using it for almost a month now and it feels good, so I'll use it again.	Jingdong prefecture level city in Shandong
Doraemon Hyakubo Shop	King of Power Saving	It's ok, the electric bill is really less, originally I remember charging $100 and using it for 10 days. Now it lasts about 15 days. It's still noticeable.	Jingdong prefecture level city in Shandong
Doraemon Hyakubo Shop	King of Power Saving	Fast logistics, good power saving effect, very satisfied	Jingdong prefecture level city in Shandong
Doraemon Hyakubo Shop	King of Power Saving	Has been the second time back to buy, energy saving effect, worth recommending, you can try to see, power saving effect: very good Experience: can save about 30%!	Jingdong prefecture level city in Shandong
Doraemon Hyakubo Shop	King of Power Saving	I don't know how well it works for now	Jingdong prefecture level city in Shandong
Doraemon Hyakubo Shop	King of Power Saving	Factory direct is really good, plugged in and used for half a month did less probably almost 50 degrees of electricity! And the manufacturer's warranty is 5 years.	Jingdong prefecture level city in Shandong
Doraemon Hyakubo Shop	King of Power Saving	It's really effective. The previous month's electricity was $286, but after using the power saver for a month, I have $195 left this month, which is not bad.	Jingdong prefecture level city in Shandong

Doraemon Hyakubo Shop	King of Power Saving	I'll give you a good review and see how it goes.	Jingdong prefecture level city in Shandong
Doraemon Hyakubo Shop	King of Power Saving	Customer service is very professional and patient to guide the installation, the quality of workmanship is very fine. Durable, second repurchase. Really can save oh!	Jingdong prefecture level city in Shandong
Doraemon Hyakubo Shop	King of Power Saving	I've been using it for a month now and my electric bill is over $30 less than last month. I feel it's quite worth buying.	Jingdong prefecture level city in Shandong
Doraemon Hyakubo Shop	King of Power Saving	Small size, does not take up space. It's very good, it does work very effectively, the tool room has a lot of work with a lot of electricity. After installing it, it really reduces a lot of electricity bills. Before the day 30 degrees of electricity left stone, now work down really less a few degrees.	Jingdong prefecture level city in Shandong
Doraemon Hyakubo Shop	King of Power Saving	It does work for saving electricity, I've bought the kind from elsewhere before that was too simple not **all**. This time to buy this store, but also for the safety. Air conditioning is too much electricity, this use to save a lot of	Jingdong prefecture level city in Shandong
Doraemon Hyakubo Shop	King of Power Saving	Two air conditioners and a refrigerator at home, a month down the electricity bill is almost two hundred dollars, with this after the average savings of more than thirty dollars to spend less money to enjoy more, it's really very good!	Jingdong prefecture level city in Shandong
Doraemon Hyakubo Shop	King of Power Saving	Good. Very good.	Jingdong prefecture level city in Shandong
Doraemon Hyakubo Shop	King of Power Saving	Works great, easy to operate, great, very happy with it.	Jingdong prefecture level city in Shandong
Doraemon Hyakubo Shop	King of Power Saving	Received it and tried it out, it's great, great quality and worth having! Five stars praise!	Jingdong prefecture level city in Shandong
Doraemon Hyakubo Shop	King of Power Saving	Very trustworthy brand, quality is guaranteed, the price is still affordable, quite satisfied!	Jingdong prefecture level city in Shandong
Doraemon Hyakubo Shop	King of Power Saving	Use it before you speak.	Jingdong prefecture level city in Shandong
Doraemon Hyakubo Shop	King of Power Saving	The quality of workmanship is very good and the power saving effect is quite good, I regret not getting it earlier.	Jingdong prefecture level city in Shandong
Doraemon Hyakubo Shop	King of Power Saving	Merchants shipping speed, and customer service introduced the same very good, cost-effective	Jingdong prefecture level city in Shandong
Doraemon Hyakubo Shop	King of Power Saving	Feel a little effect Oh, where the power consumption where to plug, convenient and convenient	Jingdong prefecture level city in Shandong
Doraemon Hyakubo Shop	King of Power Saving	The quality of the imported saver is very good, practical and satisfactory. Cost-effective	Jingdong prefecture level city in Shandong
Doraemon Hyakubo Shop	King of Power Saving	Power Saver is especially good, good and inexpensive, very good	Jingdong prefecture level city in Shandong
Doraemon Hyakubo Shop	King of Power Saving	Fast shipping, awesome quality, affordable price, satisfied.	Jingdong prefecture level city in Shandong
Doraemon Hyakubo Shop	King of Power Saving	Energy saver received, the effect is consistent with the description, recommended to buy!	Jingdong prefecture level city in Shandong
Doraemon Hyakubo Shop	King of Power Saving	It's really good, the quality is great, I recommend it!	Jingdong prefecture level city in Shandong
Doraemon Hyakubo Shop	King of Power Saving	Works well. Inexpensive. Shipping was fast.	Jingdong prefecture level city in Shandong
Doraemon Hyakubo Shop	King of Power Saving	Logistics is fast, works well, energy saving is good	Jingdong prefecture level city in Shandong
Doraemon Hyakubo Shop	King of Power Saving	very happy	Jingdong prefecture level city in Shandong
Doraemon Hyakubo Shop	King of Power Saving	an excellent	Jingdong prefecture level city in Shandong
Doraemon Hyakubo Shop	King of Power Saving	It rained a couple days ago and I just went to get the item back today , not bad, hey I looked at the meter and that red display light takes several seconds to jump, did not make a mistake buying it. After a period of time to evaluate.	Jingdong prefecture level city in Shandong
Doraemon Hyakubo Shop	King of Power Saving	Used more than 10 days to check the power, there is indeed some decline, customer service said, long-term use will save more points!	Jingdong prefecture level city in Shandong
Doraemon Hyakubo Shop	King of Power Saving	It's a proper IQ tax, and it costs electricity.	Jingdong prefecture level city in Shandong
Doraemon Hyakubo Shop	King of Power Saving	I've been using it for 17 days now, it really saves electricity Shipping is fast, you can save a lot in a year	Jingdong prefecture level city in Shandong
Doraemon Hyakubo Shop	King of Power Saving	I've been using it for 17 days and it really saves power Shipping is fast and you don't have to worry about the quality, free returns within 5 years	Jingdong prefecture level city in Shandong
Doraemon Hyakubo Shop	King of Power Saving	It's more regular, scanned the code and it's authentic, and the main thing is that you can try it for 90 days! Plugged in more than ten days, is less than before a few degrees per day. When the time to use a month to see Additional. Good to buy a few more	Jingdong prefecture level city in Shandong
Doraemon Hyakubo Shop	King of Power Saving	Logistics is very fast, fine workmanship, use it and then come back to the comments	Jingdong prefecture level city in Shandong
Doraemon Hyakubo Shop	King of Power Saving	The quality of workmanship is very good and the power saving effect is quite good, I regret not getting it earlier.	Jingdong prefecture level city in Shandong
Doraemon Hyakubo Shop	King of Power Saving	Received the goods, according to the instructions on the operation is very simple, to wait and see the effect, looks good!	Jingdong prefecture level city in Shandong
Doraemon Hyakubo Shop	King of Power Saving	It's really as safe and power-saving as the owner says, and it works great!	Jingdong prefecture level city in Shandong
Doraemon Hyakubo Shop	King of Power Saving	Received the goods, immediately used, looks good, to wait and see how the effect.	Jingdong prefecture level city in Shandong
Doraemon Hyakubo Shop	King of Power Saving	Afraid to buy back did not work, the store said the commitment to 90 days invalid refund, warranty 5 years. Saver also complimentary on the Pacific insurance, it is a lot of peace of mind, there are effects will continue to buy back drops!	Jingdong prefecture level city in Shandong
Doraemon Hyakubo Shop	King of Power Saving	Received the goods and used it, can save electricity a month later to see the effect!	Jingdong prefecture level city in Shandong
Doraemon Hyakubo Shop	King of Power Saving	Fancy 90-day trial, 5-year warranty before the order, the goods received, has been experiencing a week, can really feel the savings, in the introduction of friends also buy this!	Jingdong prefecture level city in Shandong
Doraemon Hyakubo Shop	King of Power Saving	Clothing stores use, neighbors recommended to buy, said a month can save more than 70 yuan, first plugged in to see the effect of the	Jingdong prefecture level city in Shandong
Doraemon Hyakubo Shop	King of Power Saving	A friend's home two months to save more than 80 dollars of electricity, recommended that I buy two, decisive purchase, looking forward to the effect of power saving, waiting for the next month effect.	Jingdong prefecture level city in Shandong
Doraemon Hyakubo Shop	King of Power Saving	The goods have been received, consistent with the description of the advertisement, quite good, I hope that in terms of power saving and purifying the air is really as the merchant said that would be better!	Jingdong prefecture level city in Shandong
Doraemon Hyakubo Shop	King of Power Saving	Power Saving King is really good to use the quality is very good than the physical store a lot cheaper we can rest assured that the purchase recommended to buy!	Jingdong prefecture level city in Shandong
Doraemon Hyakubo Shop	King of Power Saving	Energy saver has been used on the rental house inside the use of the meter is always running fast, with this can really save electricity, easy to use, plugged in can be, very satisfied!	Jingdong prefecture level city in Shandong
Doraemon Hyakubo Shop	King of Power Saving	Power Saver Power Saver King is good quality and affordable, very satisfied, worth buying.	Jingdong prefecture level city in Shandong
Doraemon Hyakubo Shop	King of Power Saving	Just use can not see what effect to,, wait for a period of time in the evaluation	Jingdong prefecture level city in Shandong
Doraemon Hyakubo Shop	King of Power Saving	Live in the 18th floor is too far away from the meter, afraid of no effect, look at the trial 90 days before buying. Currently used for a month, there is an effect, saving 27 dollars in electricity, next month should be able to save more!	Jingdong prefecture level city in Shandong
Doraemon Hyakubo Shop	King of Power Saving	I've been using it for almost a month now and it feels good, so I'll use it again.	Jingdong prefecture level city in Shandong
Doraemon Hyakubo Shop	King of Power Saving	It's ok, the electric bill is really less, originally I remember charging $100 and using it for 10 days. Now it lasts about 15 days. It's still noticeable.	Jingdong prefecture level city in Shandong
Doraemon Hyakubo Shop	King of Power Saving	Fast logistics, good power saving effect, very satisfied	Jingdong prefecture level city in Shandong

Doraemon Hyakubo Shop	King of Power Saving	Has been the second time back to buy, energy saving effect, worth recommending, you can try to see, power saving effect: very good Experience: can save about 30%!	Jingdong prefecture level city in Shandong
Doraemon Hyakubo Shop	King of Power Saving	I don't know how well it works for now	Jingdong prefecture level city in Shandong
Doraemon Hyakubo Shop	King of Power Saving	Factory direct is really good, plugged in and used for half a month did less probably almost 50 degrees of electricity! And the manufacturer's warranty is 5 years.	Jingdong prefecture level city in Shandong
Doraemon Hyakubo Shop	King of Power Saving	It's really effective. The previous month's electricity was $286, but after using the power saver for a month, I have $195 left this month, which is not bad.	Jingdong prefecture level city in Shandong
Doraemon Hyakubo Shop	King of Power Saving	I'll give you a good review and see how it goes.	Jingdong prefecture level city in Shandong
Doraemon Hyakubo Shop	King of Power Saving	Customer service is very professional and patient to guide the installation, the quality of workmanship is very fine. Durable, second repurchase. Really can save oh!	Jingdong prefecture level city in Shandong
Doraemon Hyakubo Shop	King of Power Saving	I've been using it for a month now and my electric bill is over $30 less than last month. I feel it's quite worth buying.	Jingdong prefecture level city in Shandong
Doraemon Hyakubo Shop	King of Power Saving	Small size, does not take up space. It's very good, it does work very effectively, the tool room has a lot of work with a lot of electricity. After installing it, it really reduces a lot of electricity bills. Before the day 30 degrees of electricity left stone, now work down really less a few degrees.	Jingdong prefecture level city in Shandong
Doraemon Hyakubo Shop	King of Power Saving	It does work for saving electricity, I've bought the kind from elsewhere before that was too simple not **all**. This time to buy this store, but also for the safety. Air conditioning is too much electricity, this use to save a lot of	Jingdong prefecture level city in Shandong
Doraemon Hyakubo Shop	King of Power Saving	Two air conditioners and a refrigerator at home, a month down the electricity bill is almost two hundred dollars, with this after the average savings of more than thirty dollars to spend less money to enjoy more, it's really very good!	Jingdong prefecture level city in Shandong
Doraemon Hyakubo Shop	King of Power Saving	Good. Very good.	Jingdong prefecture level city in Shandong
Doraemon Hyakubo Shop	King of Power Saving	Works great, easy to operate, great, very happy with it.	Jingdong prefecture level city in Shandong
Doraemon Hyakubo Shop	King of Power Saving	Received it and tried it out, it's great, great quality and worth having! Five stars praise!	Jingdong prefecture level city in Shandong
Doraemon Hyakubo Shop	King of Power Saving	Very trustworthy brand, quality is guaranteed, the price is still affordable, quite satisfied!	Jingdong prefecture level city in Shandong
Doraemon Hyakubo Shop	King of Power Saving	Use it before you speak.	Jingdong prefecture level city in Shandong
Doraemon Hyakubo Shop	King of Power Saving	The quality of workmanship is very good and the power saving effect is quite good, I regret not getting it earlier.	Jingdong prefecture level city in Shandong
Doraemon Hyakubo Shop	King of Power Saving	Merchants shipping speed, and customer service introduced the same very good, cost-effective	Jingdong prefecture level city in Shandong
Doraemon Hyakubo Shop	King of Power Saving	Feel a little effect Oh, where the power consumption where to plug, convenient and convenient	Jingdong prefecture level city in Shandong
Doraemon Hyakubo Shop	King of Power Saving	The quality of the imported saver is very good, practical and satisfactory. Cost-effective	Jingdong prefecture level city in Shandong
Doraemon Hyakubo Shop	King of Power Saving	Power Saver is especially good, good and inexpensive, very good	Jingdong prefecture level city in Shandong
Doraemon Hyakubo Shop	King of Power Saving	Fast shipping, awesome quality, affordable price, satisfied.	Jingdong prefecture level city in Shandong
Doraemon Hyakubo Shop	King of Power Saving	Energy saver received, the effect is consistent with the description, recommended to buy!	Jingdong prefecture level city in Shandong
Doraemon Hyakubo Shop	King of Power Saving	It's really good, the quality is great, I recommend it!	Jingdong prefecture level city in Shandong
Doraemon Hyakubo Shop	King of Power Saving	Works well. Inexpensive. Shipping was fast.	Jingdong prefecture level city in Shandong
Doraemon Hyakubo Shop	King of Power Saving	Logistics is fast, works well, energy saving is good	Jingdong prefecture level city in Shandong
Doraemon Hyakubo Shop	King of Power Saving	very happy	Jingdong prefecture level city in Shandong
Doraemon Hyakubo Shop	King of Power Saving	an excellent	Jingdong prefecture level city in Shandong
Doraemon Hyakubo Shop	King of Power Saving	It rained a couple days ago and I just went to get the item back today , not bad, hey I looked at the meter and that red display light takes several seconds to jump, did not make a mistake buying it. After a period of time to evaluate.	Jingdong prefecture level city in Shandong
Doraemon Hyakubo Shop	King of Power Saving	Used more than 10 days to check the power, there is indeed some decline, customer service said, long-term use will save more points!	Jingdong prefecture level city in Shandong
Doraemon Hyakubo Shop	King of Power Saving	It's a proper IQ tax, and it costs electricity.	Jingdong prefecture level city in Shandong
Doraemon Hyakubo Shop	King of Power Saving	I've been using it for 17 days now, it really saves electricity Shipping is fast, you can save a lot in a year	Jingdong prefecture level city in Shandong
Doraemon Hyakubo Shop	King of Power Saving	I've been using it for 17 days and it really saves power Shipping is fast and you don't have to worry about the quality, free returns within 5 years	Jingdong prefecture level city in Shandong
Doraemon Hyakubo Shop	King of Power Saving	It's more regular, scanned the code and it's authentic, and the main thing is that you can try it for 90 days! Plugged in more than ten days, is less than before a few degrees per day. When the time to use a month to see Additional. Good to buy a few more	Jingdong prefecture level city in Shandong
Doraemon Hyakubo Shop	King of Power Saving	Logistics is very fast, fine workmanship, use it and then come back to the comments	Jingdong prefecture level city in Shandong
Doraemon Hyakubo Shop	King of Power Saving	The quality of workmanship is very good and the power saving effect is quite good, I regret not getting it earlier.	Jingdong prefecture level city in Shandong
Doraemon Hyakubo Shop	King of Power Saving	Received the goods, according to the instructions on the operation is very simple, to wait and see the effect, looks good!	Jingdong prefecture level city in Shandong
Doraemon Hyakubo Shop	King of Power Saving	It's really as safe and power-saving as the owner says, and it works great!	Jingdong prefecture level city in Shandong
Doraemon Hyakubo Shop	King of Power Saving	Received the goods, immediately used, looks good, to wait and see how the effect.	Jingdong prefecture level city in Shandong
Doraemon Hyakubo Shop	King of Power Saving	Afraid to buy back did not work, the store said the commitment to 90 days invalid refund, warranty 5 years. Saver also complimentary on the Pacific insurance, it is a lot of peace of mind, there are effects will continue to buy back drops!	Jingdong prefecture level city in Shandong
Doraemon Hyakubo Shop	King of Power Saving	Received the goods and used it, can save electricity a month later to see the effect!	Jingdong prefecture level city in Shandong
Doraemon Hyakubo Shop	King of Power Saving	Fancy 90-day trial, 5-year warranty before the order, the goods received, has been experiencing a week, can really feel the savings, in the introduction of friends also buy this!	Jingdong prefecture level city in Shandong
Doraemon Hyakubo Shop	King of Power Saving	Clothing stores use, neighbors recommended to buy, said a month can save more than 70 yuan, first plugged in to see the effect of the	Jingdong prefecture level city in Shandong
Doraemon Hyakubo Shop	King of Power Saving	A friend's home two months to save more than 80 dollars of electricity, recommended that I buy two, decisive purchase, looking forward to the effect of power saving, waiting for the next month effect.	Jingdong prefecture level city in Shandong
Doraemon Hyakubo Shop	King of Power Saving	The goods have been received, consistent with the description of the advertisement, quite good, I hope that in terms of power saving and purifying the air is really as the merchant said that would be better!	Jingdong prefecture level city in Shandong
Doraemon Hyakubo Shop	King of Power Saving	Power Saving King is really good to use the quality is very good than the physical store a lot cheaper we can rest assured that the purchase recommended to buy!	Jingdong prefecture level city in Shandong
Doraemon Hyakubo Shop	King of Power Saving	Energy saver has been used on the rental house inside the use of the meter is always running fast, with this can really save electricity, easy to use, plugged in can be, very satisfied!	Jingdong prefecture level city in Shandong
Doraemon Hyakubo Shop	King of Power Saving	Power Saver Power Saver King is good quality and affordable, very satisfied, worth buying.	Jingdong prefecture level city in Shandong
Doraemon Hyakubo Shop	King of Power Saving	Just use can not see what effect to,, wait for a period of time in the evaluation	Jingdong prefecture level city in Shandong

Doraemon Hyakubo Shop	King of Power Saving	Live in the 18th floor is too far away from the meter, afraid of no effect, look at the trial 90 days before buying. Currently used for a month, there is an effect, saving 27 dollars in electricity, next month should be able to save more!	Jingdong prefecture level city in Shandong
Doraemon Hyakubo Shop	King of Power Saving	I've been using it for almost a month now and it feels good, so I'll use it again.	Jingdong prefecture level city in Shandong
Doraemon Hyakubo Shop	King of Power Saving	It's ok, the electric bill is really less, originally I remember charging $100 and using it for 10 days. Now it lasts about 15 days. It's still noticeable.	Jingdong prefecture level city in Shandong
Doraemon Hyakubo Shop	King of Power Saving	Fast logistics, good power saving effect, very satisfied	Jingdong prefecture level city in Shandong
Doraemon Hyakubo Shop	King of Power Saving	Has been the second time back to buy, energy saving effect, worth recommending, you can try to see, power saving effect: very good Experience: can save about 30%!	Jingdong prefecture level city in Shandong
Doraemon Hyakubo Shop	King of Power Saving	I don't know how well it works for now	Jingdong prefecture level city in Shandong
Doraemon Hyakubo Shop	King of Power Saving	Factory direct is really good, plugged in and used for half a month did less probably almost 50 degrees of electricity! And the manufacturer's warranty is 5 years.	Jingdong prefecture level city in Shandong
Doraemon Hyakubo Shop	King of Power Saving	It's really effective. The previous month's electricity was $286, but after using the power saver for a month, I have $195 left this month, which is not bad.	Jingdong prefecture level city in Shandong
Doraemon Hyakubo Shop	King of Power Saving	I'll give you a good review and see how it goes.	Jingdong prefecture level city in Shandong
Doraemon Hyakubo Shop	King of Power Saving	Customer service is very professional and patient to guide the installation, the quality of workmanship is very fine. Durable, second repurchase. Really can save oh!	Jingdong prefecture level city in Shandong
Doraemon Hyakubo Shop	King of Power Saving	I've been using it for a month now and my electric bill is over $30 less than last month. I feel it's quite worth buying.	Jingdong prefecture level city in Shandong
Doraemon Hyakubo Shop	King of Power Saving	Small size, does not take up space. It's very good, it does work very effectively, the tool room has a lot of work with a lot of electricity. After installing it, it really reduces a lot of electricity bills. Before the day 30 degrees of electricity left stone, now work down really less a few degrees.	Jingdong prefecture level city in Shandong
Doraemon Hyakubo Shop	King of Power Saving	It does work for saving electricity, I've bought the kind from elsewhere before that was too simple not **all**. This time to buy this store, but also for the safety. Air conditioning is too much electricity, this use to save a lot of	Jingdong prefecture level city in Shandong
Doraemon Hyakubo Shop	King of Power Saving	Two air conditioners and a refrigerator at home, a month down the electricity bill is almost two hundred dollars, with this after the average savings of more than thirty dollars to spend less money to enjoy more, it's really very good!	Jingdong prefecture level city in Shandong
Doraemon Hyakubo Shop	King of Power Saving	Good. Very good.	Jingdong prefecture level city in Shandong
Doraemon Hyakubo Shop	King of Power Saving	Works great, easy to operate, great, very happy with it.	Jingdong prefecture level city in Shandong
Doraemon Hyakubo Shop	King of Power Saving	Received it and tried it out, it's great, great quality and worth having! Five stars praise!	Jingdong prefecture level city in Shandong
Doraemon Hyakubo Shop	King of Power Saving	Very trustworthy brand, quality is guaranteed, the price is still affordable, quite satisfied!	Jingdong prefecture level city in Shandong
Doraemon Hyakubo Shop	King of Power Saving	Use it before you speak.	Jingdong prefecture level city in Shandong
Doraemon Hyakubo Shop	King of Power Saving	The quality of workmanship is very good and the power saving effect is quite good, I regret not getting it earlier.	Jingdong prefecture level city in Shandong
Doraemon Hyakubo Shop	King of Power Saving	Merchants shipping speed, and customer service introduced the same very good, cost-effective	Jingdong prefecture level city in Shandong
Doraemon Hyakubo Shop	King of Power Saving	Feel a little effect Oh, where the power consumption where to plug, convenient and convenient	Jingdong prefecture level city in Shandong
Doraemon Hyakubo Shop	King of Power Saving	The quality of the imported saver is very good, practical and satisfactory. Cost-effective	Jingdong prefecture level city in Shandong
Doraemon Hyakubo Shop	King of Power Saving	Power Saver is especially good, good and inexpensive, very good	Jingdong prefecture level city in Shandong
Doraemon Hyakubo Shop	King of Power Saving	Fast shipping, awesome quality, affordable price, satisfied.	Jingdong prefecture level city in Shandong
Doraemon Hyakubo Shop	King of Power Saving	Energy saver received, the effect is consistent with the description, recommended to buy!	Jingdong prefecture level city in Shandong
Doraemon Hyakubo Shop	King of Power Saving	It's really good, the quality is great, I recommend it!	Jingdong prefecture level city in Shandong
Doraemon Hyakubo Shop	King of Power Saving	Works well. Inexpensive. Shipping was fast.	Jingdong prefecture level city in Shandong
Doraemon Hyakubo Shop	King of Power Saving	Logistics is fast, works well, energy saving is good	Jingdong prefecture level city in Shandong
Doraemon Hyakubo Shop	King of Power Saving	very happy	Jingdong prefecture level city in Shandong
Doraemon Hyakubo Shop	King of Power Saving	an excellent	Jingdong prefecture level city in Shandong
Doraemon Hyakubo Shop	King of Power Saving	It rained a couple days ago and I just went to get the item back today , not bad, hey I looked at the meter and that red display light takes several seconds to jump, did not make a mistake buying it. After a period of time to evaluate.	Jingdong prefecture level city in Shandong
Doraemon Hyakubo Shop	King of Power Saving	Used more than 10 days to check the power, there is indeed some decline, customer service said, long-term use will save more points!	Jingdong prefecture level city in Shandong
Doraemon Hyakubo Shop	King of Power Saving	It's a proper IQ tax, and it costs electricity.	Jingdong prefecture level city in Shandong
Doraemon Hyakubo Shop	King of Power Saving	I've been using it for 17 days now, it really saves electricity Shipping is fast, you can save a lot in a year	Jingdong prefecture level city in Shandong
Doraemon Hyakubo Shop	King of Power Saving	I've been using it for 17 days and it really saves power Shipping is fast and you don't have to worry about the quality, free returns within 5 years	Jingdong prefecture level city in Shandong
Doraemon Hyakubo Shop	King of Power Saving	It's more regular, scanned the code and it's authentic, and the main thing is that you can try it for 90 days! Plugged in more than ten days, is less than before a few degrees per day. When the time to use a month to see Additional. Good to buy a few more	Jingdong prefecture level city in Shandong
Doraemon Hyakubo Shop	King of Power Saving	Logistics is very fast, fine workmanship, use it and then come back to the comments	Jingdong prefecture level city in Shandong
Doraemon Hyakubo Shop	King of Power Saving	The quality of workmanship is very good and the power saving effect is quite good, I regret not getting it earlier.	Jingdong prefecture level city in Shandong
Doraemon Hyakubo Shop	King of Power Saving	Received the goods, according to the instructions on the operation is very simple, to wait and see the effect, looks good!	Jingdong prefecture level city in Shandong
Doraemon Hyakubo Shop	King of Power Saving	It's really as safe and power-saving as the owner says, and it works great!	Jingdong prefecture level city in Shandong
Doraemon Hyakubo Shop	King of Power Saving	Received the goods, immediately used, looks good, to wait and see how the effect.	Jingdong prefecture level city in Shandong
Doraemon Hyakubo Shop	King of Power Saving	Afraid to buy back did not work, the store said the commitment to 90 days invalid refund, warranty 5 years. Saver also complimentary on the Pacific insurance, it is a lot of peace of mind, there are effects will continue to buy back drops!	Jingdong prefecture level city in Shandong
Doraemon Hyakubo Shop	King of Power Saving	Received the goods and used it, can save electricity a month later to see the effect!	Jingdong prefecture level city in Shandong
Doraemon Hyakubo Shop	King of Power Saving	Fancy 90-day trial, 5-year warranty before the order, the goods received, has been experiencing a week, can really feel the savings, in the introduction of friends also buy this!	Jingdong prefecture level city in Shandong
Doraemon Hyakubo Shop	King of Power Saving	Clothing stores use, neighbors recommended to buy, said a month can save more than 70 yuan, first plugged in to see the effect of the	Jingdong prefecture level city in Shandong
Doraemon Hyakubo Shop	King of Power Saving	A friend's home two months to save more than 80 dollars of electricity, recommended that I buy two, decisive purchase, looking forward to the effect of power saving, waiting for the next month effect.	Jingdong prefecture level city in Shandong
Doraemon Hyakubo Shop	King of Power Saving	The goods have been received, consistent with the description of the advertisement, quite good, I hope that in terms of power saving and purifying the air is really as the merchant said that would be better!	Jingdong prefecture level city in Shandong

Doraemon Hyakubo Shop	King of Power Saving	Power Saving King is really good to use the quality is very good than the physical store a lot cheaper we can rest assured that the purchase recommended to buy!	Jingdong prefecture level city in Shandong
Doraemon Hyakubo Shop	King of Power Saving	Energy saver has been used on the rental house inside the use of the meter is always running fast, with this can really save electricity, easy to use, plugged in can be, very satisfied!	Jingdong prefecture level city in Shandong
Doraemon Hyakubo Shop	King of Power Saving	Power Saver Power Saver King is good quality and affordable, very satisfied, worth buying.	Jingdong prefecture level city in Shandong
Doraemon Hyakubo Shop	King of Power Saving	Just use can not see what effect to,, wait for a period of time in the evaluation	Jingdong prefecture level city in Shandong
Doraemon Hyakubo Shop	King of Power Saving	Live in the 18th floor is too far away from the meter, afraid of no effect, look at the trial 90 days before buying. Currently used for a month, there is an effect, saving 27 dollars in electricity, next month should be able to save more!	Jingdong prefecture level city in Shandong
Doraemon Hyakubo Shop	King of Power Saving	I've been using it for almost a month now and it feels good, so I'll use it again.	Jingdong prefecture level city in Shandong
Doraemon Hyakubo Shop	King of Power Saving	It's ok, the electric bill is really less, originally I remember charging $100 and using it for 10 days. Now it lasts about 15 days. It's still noticeable.	Jingdong prefecture level city in Shandong
Doraemon Hyakubo Shop	King of Power Saving	Fast logistics, good power saving effect, very satisfied	Jingdong prefecture level city in Shandong
Doraemon Hyakubo Shop	King of Power Saving	Has been the second time back to buy, energy saving effect, worth recommending, you can try to see, power saving effect: very good Experience: can save about 30%!	Jingdong prefecture level city in Shandong
Doraemon Hyakubo Shop	King of Power Saving	I don't know how well it works for now	Jingdong prefecture level city in Shandong
Doraemon Hyakubo Shop	King of Power Saving	Factory direct is really good, plugged in and used for half a month did less probably almost 50 degrees of electricity! And the manufacturer's warranty is 5 years.	Jingdong prefecture level city in Shandong
Doraemon Hyakubo Shop	King of Power Saving	It's really effective. The previous month's electricity was $286, but after using the power saver for a month, I have $195 left this month, which is not bad.	Jingdong prefecture level city in Shandong
Doraemon Hyakubo Shop	King of Power Saving	I'll give you a good review and see how it goes.	Jingdong prefecture level city in Shandong
Doraemon Hyakubo Shop	King of Power Saving	Customer service is very professional and patient to guide the installation, the quality of workmanship is very fine. Durable, second repurchase. Really can save oh!	Jingdong prefecture level city in Shandong
Doraemon Hyakubo Shop	King of Power Saving	I've been using it for a month now and my electric bill is over $30 less than last month. I feel it's quite worth buying.	Jingdong prefecture level city in Shandong
Doraemon Hyakubo Shop	King of Power Saving	Small size, does not take up space. It's very good, it does work very effectively, the tool room has a lot of work with a lot of electricity. After installing it, it really reduces a lot of electricity bills. Before the day 30 degrees of electricity left stone, now work down really less a few degrees.	Jingdong prefecture level city in Shandong
Doraemon Hyakubo Shop	King of Power Saving	It does work for saving electricity, I've bought the kind from elsewhere before that was too simple not **all**. This time to buy this store, but also for the safety. Air conditioning is too much electricity, this use to save a lot of	Jingdong prefecture level city in Shandong
Doraemon Hyakubo Shop	King of Power Saving	Two air conditioners and a refrigerator at home, a month down the electricity bill is almost two hundred dollars, with this after the average savings of more than thirty dollars to spend less money to enjoy more, it's really very good!	Jingdong prefecture level city in Shandong
Doraemon Hyakubo Shop	King of Power Saving	Good. Very good.	Jingdong prefecture level city in Shandong
Doraemon Hyakubo Shop	King of Power Saving	Works great, easy to operate, great, very happy with it.	Jingdong prefecture level city in Shandong
Doraemon Hyakubo Shop	King of Power Saving	Received it and tried it out, it's great, great quality and worth having! Five stars praise!	Jingdong prefecture level city in Shandong
Doraemon Hyakubo Shop	King of Power Saving	Very trustworthy brand, quality is guaranteed, the price is still affordable, quite satisfied!	Jingdong prefecture level city in Shandong
Doraemon Hyakubo Shop	King of Power Saving	Use it before you speak.	Jingdong prefecture level city in Shandong
Doraemon Hyakubo Shop	King of Power Saving	The quality of workmanship is very good and the power saving effect is quite good, I regret not getting it earlier.	Jingdong prefecture level city in Shandong
Doraemon Hyakubo Shop	King of Power Saving	Merchants shipping speed, and customer service introduced the same very good, cost-effective	Jingdong prefecture level city in Shandong
Doraemon Hyakubo Shop	King of Power Saving	Feel a little effect Oh, where the power consumption where to plug, convenient and convenient	Jingdong prefecture level city in Shandong
Doraemon Hyakubo Shop	King of Power Saving	The quality of the imported saver is very good, practical and satisfactory. Cost-effective	Jingdong prefecture level city in Shandong
Doraemon Hyakubo Shop	King of Power Saving	Power Saver is especially good, good and inexpensive, very good	Jingdong prefecture level city in Shandong
Doraemon Hyakubo Shop	King of Power Saving	Fast shipping, awesome quality, affordable price, satisfied.	Jingdong prefecture level city in Shandong
Doraemon Hyakubo Shop	King of Power Saving	Energy saver received, the effect is consistent with the description, recommended to buy!	Jingdong prefecture level city in Shandong
Doraemon Hyakubo Shop	King of Power Saving	It's really good, the quality is great, I recommend it!	Jingdong prefecture level city in Shandong
Doraemon Hyakubo Shop	King of Power Saving	Works well. Inexpensive. Shipping was fast.	Jingdong prefecture level city in Shandong
Doraemon Hyakubo Shop	King of Power Saving	Logistics is fast, works well, energy saving is good	Jingdong prefecture level city in Shandong
Doraemon Hyakubo Shop	King of Power Saving	very happy	Jingdong prefecture level city in Shandong
Doraemon Hyakubo Shop	King of Power Saving	an excellent	Jingdong prefecture level city in Shandong
Doraemon Hyakubo Shop	King of Power Saving	It rained a couple days ago and I just went to get the item back today , not bad, hey I looked at the meter and that red display light takes several seconds to jump, did not make a mistake buying it. After a period of time to evaluate.	Jingdong prefecture level city in Shandong
Doraemon Hyakubo Shop	King of Power Saving	Used more than 10 days to check the power, there is indeed some decline, customer service said, long-term use will save more points!	Jingdong prefecture level city in Shandong
Doraemon Hyakubo Shop	King of Power Saving	It's a proper IQ tax, and it costs electricity.	Jingdong prefecture level city in Shandong
Doraemon Hyakubo Shop	King of Power Saving	I've been using it for 17 days now, it really saves electricity Shipping is fast, you can save a lot in a year	Jingdong prefecture level city in Shandong
Doraemon Hyakubo Shop	King of Power Saving	I've been using it for 17 days and it really saves power Shipping is fast and you don't have to worry about the quality, free returns within 5 years	Jingdong prefecture level city in Shandong
Doraemon Hyakubo Shop	King of Power Saving	It's more regular, scanned the code and it's authentic, and the main thing is that you can try it for 90 days! Plugged in more than ten days, is less than before a few degrees per day. When the time to use a month to see Additional. Good to buy a few more	Jingdong prefecture level city in Shandong
Doraemon Hyakubo Shop	King of Power Saving	Logistics is very fast, fine workmanship, use it and then come back to the comments	Jingdong prefecture level city in Shandong
Doraemon Hyakubo Shop	King of Power Saving	The quality of workmanship is very good and the power saving effect is quite good, I regret not getting it earlier.	Jingdong prefecture level city in Shandong
Doraemon Hyakubo Shop	King of Power Saving	Received the goods, according to the instructions on the operation is very simple, to wait and see the effect, looks good!	Jingdong prefecture level city in Shandong
Doraemon Hyakubo Shop	King of Power Saving	It's really as safe and power-saving as the owner says, and it works great!	Jingdong prefecture level city in Shandong
Doraemon Hyakubo Shop	King of Power Saving	Received the goods, immediately used, looks good, to wait and see how the effect.	Jingdong prefecture level city in Shandong
Doraemon Hyakubo Shop	King of Power Saving	Afraid to buy back did not work, the store said the commitment to 90 days invalid refund, warranty 5 years. Saver also complimentary on the Pacific insurance, it is a lot of peace of mind, there are effects will continue to buy back drops!	Jingdong prefecture level city in Shandong
Doraemon Hyakubo Shop	King of Power Saving	Received the goods and used it, can save electricity a month later to see the effect!	Jingdong prefecture level city in Shandong

Doraemon Hyakubo Shop	King of Power Saving	Fancy 90-day trial, 5-year warranty before the order, the goods received, has been experiencing a week, can really feel the savings, in the introduction of friends also buy this!	Jingdong prefecture level city in Shandong
Doraemon Hyakubo Shop	King of Power Saving	Clothing stores use, neighbors recommended to buy, said a month can save more than 70 yuan, first plugged in to see the effect of the	Jingdong prefecture level city in Shandong
Doraemon Hyakubo Shop	King of Power Saving	A friend's home two months to save more than 80 dollars of electricity, recommended that I buy two, decisive purchase, looking forward to the effect of power saving, waiting for the next month effect.	Jingdong prefecture level city in Shandong
Doraemon Hyakubo Shop	King of Power Saving	The goods have been received, consistent with the description of the advertisement, quite good, I hope that in terms of power saving and purifying the air is really as the merchant said that would be better!	Jingdong prefecture level city in Shandong
Doraemon Hyakubo Shop	King of Power Saving	Power Saving King is really good to use the quality is very good than the physical store a lot cheaper we can rest assured that the purchase recommended to buy!	Jingdong prefecture level city in Shandong
Doraemon Hyakubo Shop	King of Power Saving	Energy saver has been used on the rental house inside the use of the meter is always running fast, with this can really save electricity, easy to use, plugged in can be, very satisfied!	Jingdong prefecture level city in Shandong
Doraemon Hyakubo Shop	King of Power Saving	Power Saver Power Saver King is good quality and affordable, very satisfied, worth buying.	Jingdong prefecture level city in Shandong
Doraemon Hyakubo Shop	King of Power Saving	Just use can not see what effect to,, wait for a period of time in the evaluation	Jingdong prefecture level city in Shandong
Doraemon Hyakubo Shop	King of Power Saving	Live in the 18th floor is too far away from the meter, afraid of no effect, look at the trial 90 days before buying. Currently used for a month, there is an effect, saving 27 dollars in electricity, next month should be able to save more!	Jingdong prefecture level city in Shandong
Doraemon Hyakubo Shop	King of Power Saving	I've been using it for almost a month now and it feels good, so I'll use it again.	Jingdong prefecture level city in Shandong
Doraemon Hyakubo Shop	King of Power Saving	It's ok, the electric bill is really less, originally I remember charging $100 and using it for 10 days. Now it lasts about 15 days. It's still noticeable.	Jingdong prefecture level city in Shandong
Doraemon Hyakubo Shop	King of Power Saving	Fast logistics, good power saving effect, very satisfied	Jingdong prefecture level city in Shandong
Doraemon Hyakubo Shop	King of Power Saving	Has been the second time back to buy, energy saving effect, worth recommending, you can try to see, power saving effect: very good Experience: can save about 30%!	Jingdong prefecture level city in Shandong
Doraemon Hyakubo Shop	King of Power Saving	I don't know how well it works for now	Jingdong prefecture level city in Shandong
Doraemon Hyakubo Shop	King of Power Saving	Factory direct is really good, plugged in and used for half a month did less probably almost 50 degrees of electricity! And the manufacturer's warranty is 5 years.	Jingdong prefecture level city in Shandong
Doraemon Hyakubo Shop	King of Power Saving	It's really effective. The previous month's electricity was $286, but after using the power saver for a month, I have $195 left this month, which is not bad.	Jingdong prefecture level city in Shandong
Doraemon Hyakubo Shop	King of Power Saving	I'll give you a good review and see how it goes.	Jingdong prefecture level city in Shandong
Doraemon Hyakubo Shop	King of Power Saving	Customer service is very professional and patient to guide the installation, the quality of workmanship is very fine. Durable, second repurchase. Really can save oh!	Jingdong prefecture level city in Shandong
Doraemon Hyakubo Shop	King of Power Saving	I've been using it for a month now and my electric bill is over $30 less than last month. I feel it's quite worth buying.	Jingdong prefecture level city in Shandong
Doraemon Hyakubo Shop	King of Power Saving	Small size, does not take up space. It's very good, it does work very effectively, the tool room has a lot of work with a lot of electricity. After installing it, it really reduces a lot of electricity bills. Before the day 30 degrees of electricity left stone, now work down really less a few degrees.	Jingdong prefecture level city in Shandong
Doraemon Hyakubo Shop	King of Power Saving	It does work for saving electricity, I've bought the kind from elsewhere before that was too simple not **all**. This time to buy this store, but also for the safety. Air conditioning is too much electricity, this use to save a lot of	Jingdong prefecture level city in Shandong
Doraemon Hyakubo Shop	King of Power Saving	Two air conditioners and a refrigerator at home, a month down the electricity bill is almost two hundred dollars, with this after the average savings of more than thirty dollars to spend less money to enjoy more, it's really very good!	Jingdong prefecture level city in Shandong
Doraemon Hyakubo Shop	King of Power Saving	Good. Very good.	Jingdong prefecture level city in Shandong
Doraemon Hyakubo Shop	King of Power Saving	Works great, easy to operate, great, very happy with it.	Jingdong prefecture level city in Shandong
Doraemon Hyakubo Shop	King of Power Saving	Received it and tried it out, it's great, great quality and worth having! Five stars praise!	Jingdong prefecture level city in Shandong
Doraemon Hyakubo Shop	King of Power Saving	Very trustworthy brand, quality is guaranteed, the price is still affordable, quite satisfied!	Jingdong prefecture level city in Shandong
Doraemon Hyakubo Shop	King of Power Saving	Use it before you speak.	Jingdong prefecture level city in Shandong
Doraemon Hyakubo Shop	King of Power Saving	The quality of workmanship is very good and the power saving effect is quite good, I regret not getting it earlier.	Jingdong prefecture level city in Shandong
Doraemon Hyakubo Shop	King of Power Saving	Merchants shipping speed, and customer service introduced the same very good, cost-effective	Jingdong prefecture level city in Shandong
Doraemon Hyakubo Shop	King of Power Saving	Feel a little effect Oh, where the power consumption where to plug, convenient and convenient	Jingdong prefecture level city in Shandong
Doraemon Hyakubo Shop	King of Power Saving	The quality of the imported saver is very good, practical and satisfactory. Cost-effective	Jingdong prefecture level city in Shandong
Doraemon Hyakubo Shop	King of Power Saving	Power Saver is especially good, good and inexpensive, very good	Jingdong prefecture level city in Shandong
Doraemon Hyakubo Shop	King of Power Saving	Fast shipping, awesome quality, affordable price, satisfied.	Jingdong prefecture level city in Shandong
Doraemon Hyakubo Shop	King of Power Saving	Energy saver received, the effect is consistent with the description, recommended to buy!	Jingdong prefecture level city in Shandong
Doraemon Hyakubo Shop	King of Power Saving	It's really good, the quality is great, I recommend it!	Jingdong prefecture level city in Shandong
Doraemon Hyakubo Shop	King of Power Saving	Works well. Inexpensive. Shipping was fast.	Jingdong prefecture level city in Shandong
Doraemon Hyakubo Shop	King of Power Saving	Logistics is fast, works well, energy saving is good	Jingdong prefecture level city in Shandong
Doraemon Hyakubo Shop	King of Power Saving	very happy	Jingdong prefecture level city in Shandong
Doraemon Hyakubo Shop	King of Power Saving	an excellent	Jingdong prefecture level city in Shandong
Doraemon Hyakubo Shop	King of Power Saving	It rained a couple days ago and I just went to get the item back today , not bad, hey I looked at the meter and that red display light takes several seconds to jump, did not make a mistake buying it. After a period of time to evaluate.	Jingdong prefecture level city in Shandong
Doraemon Hyakubo Shop	King of Power Saving	Used more than 10 days to check the power, there is indeed some decline, customer service said, long-term use will save more points!	Jingdong prefecture level city in Shandong
Doraemon Hyakubo Shop	King of Power Saving	It's a proper IQ tax, and it costs electricity.	Jingdong prefecture level city in Shandong
Doraemon Hyakubo Shop	King of Power Saving	I've been using it for 17 days now, it really saves electricity Shipping is fast, you can save a lot in a year	Jingdong prefecture level city in Shandong
Doraemon Hyakubo Shop	King of Power Saving	I've been using it for 17 days and it really saves power Shipping is fast and you don't have to worry about the quality, free returns within 5 years	Jingdong prefecture level city in Shandong
Doraemon Hyakubo Shop	King of Power Saving	It's more regular, scanned the code and it's authentic, and the main thing is that you can try it for 90 days! Plugged in more than ten days, is less than before a few degrees per day. When the time to use a month to see Additional. Good to buy a few more	Jingdong prefecture level city in Shandong
Doraemon Hyakubo Shop	King of Power Saving	Logistics is very fast, fine workmanship, use it and then come back to the comments	Jingdong prefecture level city in Shandong
Doraemon Hyakubo Shop	King of Power Saving	The quality of workmanship is very good and the power saving effect is quite good, I regret not getting it earlier.	Jingdong prefecture level city in Shandong
Doraemon Hyakubo Shop	King of Power Saving	Received the goods, according to the instructions on the operation is very simple, to wait and see the effect, looks good!	Jingdong prefecture level city in Shandong

Doraemon Hyakubo Shop	King of Power Saving	It's really as safe and power-saving as the owner says, and it works great!	Jingdong prefecture level city in Shandong
Doraemon Hyakubo Shop	King of Power Saving	Received the goods, immediately used, looks good, to wait and see how the effect.	Jingdong prefecture level city in Shandong
Doraemon Hyakubo Shop	King of Power Saving	Afraid to buy back did not work, the store said the commitment to 90 days invalid refund, warranty 5 years. Saver also complimentary on the Pacific insurance, it is a lot of peace of mind, there are effects will continue to buy back drops!	Jingdong prefecture level city in Shandong
Doraemon Hyakubo Shop	King of Power Saving	Received the goods and used it, can save electricity a month later to see the effect!	Jingdong prefecture level city in Shandong
Doraemon Hyakubo Shop	King of Power Saving	Fancy 90-day trial, 5-year warranty before the order, the goods received, has been experiencing a week, can really feel the savings, in the introduction of friends also buy this!	Jingdong prefecture level city in Shandong
Doraemon Hyakubo Shop	King of Power Saving	Clothing stores use, neighbors recommended to buy, said a month can save more than 70 yuan, first plugged in to see the effect of the	Jingdong prefecture level city in Shandong
Doraemon Hyakubo Shop	King of Power Saving	A friend's home two months to save more than 80 dollars of electricity, recommended that I buy two, decisive purchase, looking forward to the effect of power saving, waiting for the next month effect.	Jingdong prefecture level city in Shandong
Doraemon Hyakubo Shop	King of Power Saving	The goods have been received, consistent with the description of the advertisement, quite good, I hope that in terms of power saving and purifying the air is really as the merchant said that would be better!	Jingdong prefecture level city in Shandong
Doraemon Hyakubo Shop	King of Power Saving	Power Saving King is really good to use the quality is very good than the physical store a lot cheaper we can rest assured that the purchase recommended to buy!	Jingdong prefecture level city in Shandong
Doraemon Hyakubo Shop	King of Power Saving	Energy saver has been used on the rental house inside the use of the meter is always running fast, with this can really save electricity, easy to use, plugged in can be, very satisfied!	Jingdong prefecture level city in Shandong
Doraemon Hyakubo Shop	King of Power Saving	Power Saver Power Saver King is good quality and affordable, very satisfied, worth buying.	Jingdong prefecture level city in Shandong
Doraemon Hyakubo Shop	King of Power Saving	Just use can not see what effect to,, wait for a period of time in the evaluation	Jingdong prefecture level city in Shandong
Doraemon Hyakubo Shop	King of Power Saving	Live in the 18th floor is too far away from the meter, afraid of no effect, look at the trial 90 days before buying. Currently used for a month, there is an effect, saving 27 dollars in electricity, next month should be able to save more!	Jingdong prefecture level city in Shandong
Doraemon Hyakubo Shop	King of Power Saving	I've been using it for almost a month now and it feels good, so I'll use it again.	Jingdong prefecture level city in Shandong
Doraemon Hyakubo Shop	King of Power Saving	It's ok, the electric bill is really less, originally I remember charging $100 and using it for 10 days. Now it lasts about 15 days. It's still noticeable.	Jingdong prefecture level city in Shandong
Doraemon Hyakubo Shop	King of Power Saving	Fast logistics, good power saving effect, very satisfied	Jingdong prefecture level city in Shandong
Doraemon Hyakubo Shop	King of Power Saving	Has been the second time back to buy, energy saving effect, worth recommending, you can try to see, power saving effect: very good Experience: can save about 30%!	Jingdong prefecture level city in Shandong
Doraemon Hyakubo Shop	King of Power Saving	I don't know how well it works for now	Jingdong prefecture level city in Shandong
Doraemon Hyakubo Shop	King of Power Saving	Factory direct is really good, plugged in and used for half a month did less probably almost 50 degrees of electricity! And the manufacturer's warranty is 5 years.	Jingdong prefecture level city in Shandong
Doraemon Hyakubo Shop	King of Power Saving	It's really effective. The previous month's electricity was $286, but after using the power saver for a month, I have $195 left this month, which is not bad.	Jingdong prefecture level city in Shandong
Doraemon Hyakubo Shop	King of Power Saving	I'll give you a good review and see how it goes.	Jingdong prefecture level city in Shandong
Doraemon Hyakubo Shop	King of Power Saving	Customer service is very professional and patient to guide the installation, the quality of workmanship is very fine. Durable, second repurchase. Really can save oh!	Jingdong prefecture level city in Shandong
Doraemon Hyakubo Shop	King of Power Saving	I've been using it for a month now and my electric bill is over $30 less than last month. I feel it's quite worth buying.	Jingdong prefecture level city in Shandong
Doraemon Hyakubo Shop	King of Power Saving	Small size, does not take up space. It's very good, it does work very effectively, the tool room has a lot of work with a lot of electricity. After installing it, it really reduces a lot of electricity bills. Before the day 30 degrees of electricity left stone, now work down really less a few degrees.	Jingdong prefecture level city in Shandong
Doraemon Hyakubo Shop	King of Power Saving	It does work for saving electricity, I've bought the kind from elsewhere before that was too simple not **all**. This time to buy this store, but also for the safety. Air conditioning is too much electricity, this use to save a lot of	Jingdong prefecture level city in Shandong
Doraemon Hyakubo Shop	King of Power Saving	Two air conditioners and a refrigerator at home, a month down the electricity bill is almost two hundred dollars, with this after the average savings of more than thirty dollars to spend less money to enjoy more, it's really very good!	Jingdong prefecture level city in Shandong
Doraemon Hyakubo Shop	King of Power Saving	Good. Very good.	Jingdong prefecture level city in Shandong
Doraemon Hyakubo Shop	King of Power Saving	Works great, easy to operate, great, very happy with it.	Jingdong prefecture level city in Shandong
Doraemon Hyakubo Shop	King of Power Saving	Received it and tried it out, it's great, great quality and worth having! Five stars praise!	Jingdong prefecture level city in Shandong
Doraemon Hyakubo Shop	King of Power Saving	Very trustworthy brand, quality is guaranteed, the price is still affordable, quite satisfied!	Jingdong prefecture level city in Shandong
Doraemon Hyakubo Shop	King of Power Saving	Use it before you speak.	Jingdong prefecture level city in Shandong
Doraemon Hyakubo Shop	King of Power Saving	The quality of workmanship is very good and the power saving effect is quite good, I regret not getting it earlier.	Jingdong prefecture level city in Shandong
Doraemon Hyakubo Shop	King of Power Saving	Merchants shipping speed, and customer service introduced the same very good, cost-effective	Jingdong prefecture level city in Shandong
Doraemon Hyakubo Shop	King of Power Saving	Feel a little effect Oh, where the power consumption where to plug, convenient and convenient	Jingdong prefecture level city in Shandong
Doraemon Hyakubo Shop	King of Power Saving	The quality of the imported saver is very good, practical and satisfactory. Cost-effective	Jingdong prefecture level city in Shandong
Doraemon Hyakubo Shop	King of Power Saving	Power Saver is especially good, good and inexpensive, very good	Jingdong prefecture level city in Shandong
Doraemon Hyakubo Shop	King of Power Saving	Fast shipping, awesome quality, affordable price, satisfied.	Jingdong prefecture level city in Shandong
Doraemon Hyakubo Shop	King of Power Saving	Energy saver received, the effect is consistent with the description, recommended to buy!	Jingdong prefecture level city in Shandong
Doraemon Hyakubo Shop	King of Power Saving	It's really good, the quality is great, I recommend it!	Jingdong prefecture level city in Shandong
Doraemon Hyakubo Shop	King of Power Saving	Works well. Inexpensive. Shipping was fast.	Jingdong prefecture level city in Shandong
Doraemon Hyakubo Shop	King of Power Saving	Logistics is fast, works well, energy saving is good	Jingdong prefecture level city in Shandong
Doraemon Hyakubo Shop	King of Power Saving	very happy	Jingdong prefecture level city in Shandong
Doraemon Hyakubo Shop	King of Power Saving	an excellent	Jingdong prefecture level city in Shandong
Doraemon Hyakubo Shop	King of Power Saving	It rained a couple days ago and I just went to get the item back today , not bad, hey I looked at the meter and that red display light takes several seconds to jump, did not make a mistake buying it. After a period of time to evaluate.	Jingdong prefecture level city in Shandong
Doraemon Hyakubo Shop	King of Power Saving	Used more than 10 days to check the power, there is indeed some decline, customer service said, long-term use will save more points!	Jingdong prefecture level city in Shandong
Doraemon Hyakubo Shop	King of Power Saving	It's a proper IQ tax, and it costs electricity.	Jingdong prefecture level city in Shandong
Doraemon Hyakubo Shop	King of Power Saving	I've been using it for 17 days now, it really saves electricity Shipping is fast, you can save a lot in a year	Jingdong prefecture level city in Shandong
Doraemon Hyakubo Shop	King of Power Saving	I've been using it for 17 days and it really saves power Shipping is fast and you don't have to worry about the quality, free returns within 5 years	Jingdong prefecture level city in Shandong

Doraemon Hyakubo Shop	King of Power Saving	It's more regular, scanned the code and it's authentic, and the main thing is that you can try it for 90 days! Plugged in more than ten days, is less than before a few degrees per day. When the time to use a month to see Additional. Good to buy a few more	Jingdong prefecture level city in Shandong
Doraemon Hyakubo Shop	King of Power Saving	Logistics is very fast, fine workmanship, use it and then come back to the comments	Jingdong prefecture level city in Shandong
Doraemon Hyakubo Shop	King of Power Saving	The quality of workmanship is very good and the power saving effect is quite good, I regret not getting it earlier.	Jingdong prefecture level city in Shandong
Doraemon Hyakubo Shop	King of Power Saving	Received the goods, according to the instructions on the operation is very simple, to wait and see the effect, looks good!	Jingdong prefecture level city in Shandong
Doraemon Hyakubo Shop	King of Power Saving	It's really as safe and power-saving as the owner says, and it works great!	Jingdong prefecture level city in Shandong
Doraemon Hyakubo Shop	King of Power Saving	Received the goods, immediately used, looks good, to wait and see how the effect.	Jingdong prefecture level city in Shandong
Doraemon Hyakubo Shop	King of Power Saving	Afraid to buy back did not work, the store said the commitment to 90 days invalid refund, warranty 5 years. Saver also complimentary on the Pacific insurance, it is a lot of peace of mind, there are effects will continue to buy back drops!	Jingdong prefecture level city in Shandong
Doraemon Hyakubo Shop	King of Power Saving	Received the goods and used it, can save electricity a month later to see the effect!	Jingdong prefecture level city in Shandong
Doraemon Hyakubo Shop	King of Power Saving	Fancy 90-day trial, 5-year warranty before the order, the goods received, has been experiencing a week, can really feel the savings, in the introduction of friends also buy this!	Jingdong prefecture level city in Shandong
Doraemon Hyakubo Shop	King of Power Saving	Clothing stores use, neighbors recommended to buy, said a month can save more than 70 yuan, first plugged in to see the effect of the	Jingdong prefecture level city in Shandong
Doraemon Hyakubo Shop	King of Power Saving	A friend's home two months to save more than 80 dollars of electricity, recommended that I buy two, decisive purchase, looking forward to the effect of power saving, waiting for the next month effect.	Jingdong prefecture level city in Shandong
Doraemon Hyakubo Shop	King of Power Saving	The goods have been received, consistent with the description of the advertisement, quite good, I hope that in terms of power saving and purifying the air is really as the merchant said that would be better!	Jingdong prefecture level city in Shandong
Doraemon Hyakubo Shop	King of Power Saving	Power Saving King is really good to use the quality is very good than the physical store a lot cheaper we can rest assured that the purchase recommended to buy!	Jingdong prefecture level city in Shandong
Doraemon Hyakubo Shop	King of Power Saving	Energy saver has been used on the rental house inside the use of the meter is always running fast, with this can really save electricity, easy to use, plugged in can be, very satisfied!	Jingdong prefecture level city in Shandong
Doraemon Hyakubo Shop	King of Power Saving	Power Saver Power Saver King is good quality and affordable, very satisfied, worth buying.	Jingdong prefecture level city in Shandong
Doraemon Hyakubo Shop	King of Power Saving	Just use can not see what effect to,, wait for a period of time in the evaluation	Jingdong prefecture level city in Shandong
Doraemon Hyakubo Shop	King of Power Saving	Live in the 18th floor is too far away from the meter, afraid of no effect, look at the trial 90 days before buying. Currently used for a month, there is an effect, saving 27 dollars in electricity, next month should be able to save more!	Jingdong prefecture level city in Shandong
Doraemon Hyakubo Shop	King of Power Saving	I've been using it for almost a month now and it feels good, so I'll use it again.	Jingdong prefecture level city in Shandong
Doraemon Hyakubo Shop	King of Power Saving	It's ok, the electric bill is really less, originally I remember charging $100 and using it for 10 days. Now it lasts about 15 days. It's still noticeable.	Jingdong prefecture level city in Shandong
Doraemon Hyakubo Shop	King of Power Saving	Fast logistics, good power saving effect, very satisfied	Jingdong prefecture level city in Shandong
Doraemon Hyakubo Shop	King of Power Saving	Has been the second time back to buy, energy saving effect, worth recommending, you can try to see, power saving effect: very good Experience: can save about 30%!	Jingdong prefecture level city in Shandong
Doraemon Hyakubo Shop	King of Power Saving	I don't know how well it works for now	Jingdong prefecture level city in Shandong
Doraemon Hyakubo Shop	King of Power Saving	Factory direct is really good, plugged in and used for half a month did less probably almost 50 degrees of electricity! And the manufacturer's warranty is 5 years.	Jingdong prefecture level city in Shandong
Doraemon Hyakubo Shop	King of Power Saving	It's really effective. The previous month's electricity was $286, but after using the power saver for a month, I have $195 left this month, which is not bad.	Jingdong prefecture level city in Shandong
Doraemon Hyakubo Shop	King of Power Saving	I'll give you a good review and see how it goes.	Jingdong prefecture level city in Shandong
Doraemon Hyakubo Shop	King of Power Saving	Customer service is very professional and patient to guide the installation, the quality of workmanship is very fine. Durable, second repurchase. Really can save oh!	Jingdong prefecture level city in Shandong
Doraemon Hyakubo Shop	King of Power Saving	I've been using it for a month now and my electric bill is over $30 less than last month. I feel it's quite worth buying.	Jingdong prefecture level city in Shandong
Doraemon Hyakubo Shop	King of Power Saving	Small size, does not take up space. It's very good, it does work very effectively, the tool room has a lot of work with a lot of electricity. After installing it, it really reduces a lot of electricity bills. Before the day 30 degrees of electricity left stone, now work down really less a few degrees.	Jingdong prefecture level city in Shandong
Doraemon Hyakubo Shop	King of Power Saving	It does work for saving electricity, I've bought the kind from elsewhere before that was too simple not **all**. This time to buy this store, but also for the safety. Air conditioning is too much electricity, this use to save a lot of	Jingdong prefecture level city in Shandong
Doraemon Hyakubo Shop	King of Power Saving	Two air conditioners and a refrigerator at home, a month down the electricity bill is almost two hundred dollars, with this after the average savings of more than thirty dollars to spend less money to enjoy more, it's really very good!	Jingdong prefecture level city in Shandong
Doraemon Hyakubo Shop	King of Power Saving	Good. Very good.	Jingdong prefecture level city in Shandong
Doraemon Hyakubo Shop	King of Power Saving	Works great, easy to operate, great, very happy with it.	Jingdong prefecture level city in Shandong
Doraemon Hyakubo Shop	King of Power Saving	Received it and tried it out, it's great, great quality and worth having! Five stars praise!	Jingdong prefecture level city in Shandong
Doraemon Hyakubo Shop	King of Power Saving	Very trustworthy brand, quality is guaranteed, the price is still affordable, quite satisfied!	Jingdong prefecture level city in Shandong
Doraemon Hyakubo Shop	King of Power Saving	Use it before you speak.	Jingdong prefecture level city in Shandong
Doraemon Hyakubo Shop	King of Power Saving	The quality of workmanship is very good and the power saving effect is quite good, I regret not getting it earlier.	Jingdong prefecture level city in Shandong
Doraemon Hyakubo Shop	King of Power Saving	Merchants shipping speed, and customer service introduced the same very good, cost-effective	Jingdong prefecture level city in Shandong
Doraemon Hyakubo Shop	King of Power Saving	Feel a little effect Oh, where the power consumption where to plug, convenient and convenient	Jingdong prefecture level city in Shandong
Doraemon Hyakubo Shop	King of Power Saving	The quality of the imported saver is very good, practical and satisfactory. Cost-effective	Jingdong prefecture level city in Shandong
Doraemon Hyakubo Shop	King of Power Saving	Power Saver is especially good, good and inexpensive, very good	Jingdong prefecture level city in Shandong
Doraemon Hyakubo Shop	King of Power Saving	Fast shipping, awesome quality, affordable price, satisfied.	Jingdong prefecture level city in Shandong
Doraemon Hyakubo Shop	King of Power Saving	Energy saver received, the effect is consistent with the description, recommended to buy!	Jingdong prefecture level city in Shandong
Doraemon Hyakubo Shop	King of Power Saving	It's really good, the quality is great, I recommend it!	Jingdong prefecture level city in Shandong
Doraemon Hyakubo Shop	King of Power Saving	Works well. Inexpensive. Shipping was fast.	Jingdong prefecture level city in Shandong
Doraemon Hyakubo Shop	King of Power Saving	Logistics is fast, works well, energy saving is good	Jingdong prefecture level city in Shandong
Doraemon Hyakubo Shop	King of Power Saving	very happy	Jingdong prefecture level city in Shandong
Doraemon Hyakubo Shop	King of Power Saving	an excellent	Jingdong prefecture level city in Shandong
Doraemon Hyakubo Shop	King of Power Saving	It rained a couple days ago and I just went to get the item back today , not bad, hey I looked at the meter and that red display light takes several seconds to jump, did not make a mistake buying it. After a period of time to evaluate.	Jingdong prefecture level city in Shandong

Doraemon Hyakubo Shop	King of Power Saving	Used more than 10 days to check the power, there is indeed some decline, customer service said, long-term use will save more points!	Jingdong prefecture level city in Shandong
Doraemon Hyakubo Shop	King of Power Saving	It's a proper IQ tax, and it costs electricity.	Jingdong prefecture level city in Shandong
Doraemon Hyakubo Shop	King of Power Saving	I've been using it for 17 days now, it really saves electricity Shipping is fast, you can save a lot in a year	Jingdong prefecture level city in Shandong
Doraemon Hyakubo Shop	King of Power Saving	I've been using it for 17 days and it really saves power Shipping is fast and you don't have to worry about the quality, free returns within 5 years	Jingdong prefecture level city in Shandong
Doraemon Hyakubo Shop	King of Power Saving	It's more regular, scanned the code and it's authentic, and the main thing is that you can try it for 90 days! Plugged in more than ten days, is less than before a few degrees per day. When the time to use a month to see Additional. Good to buy a few more	Jingdong prefecture level city in Shandong
Doraemon Hyakubo Shop	King of Power Saving	Logistics is very fast, fine workmanship, use it and then come back to the comments	Jingdong prefecture level city in Shandong
Doraemon Hyakubo Shop	King of Power Saving	The quality of workmanship is very good and the power saving effect is quite good, I regret not getting it earlier.	Jingdong prefecture level city in Shandong
Doraemon Hyakubo Shop	King of Power Saving	Received the goods, according to the instructions on the operation is very simple, to wait and see the effect, looks good!	Jingdong prefecture level city in Shandong
Doraemon Hyakubo Shop	King of Power Saving	It's really as safe and power-saving as the owner says, and it works great!	Jingdong prefecture level city in Shandong
Doraemon Hyakubo Shop	King of Power Saving	Received the goods, immediately used, looks good, to wait and see how the effect.	Jingdong prefecture level city in Shandong
Doraemon Hyakubo Shop	King of Power Saving	Afraid to buy back did not work, the store said the commitment to 90 days invalid refund, warranty 5 years. Saver also complimentary on the Pacific insurance, it is a lot of peace of mind, there are effects will continue to buy back drops!	Jingdong prefecture level city in Shandong
Doraemon Hyakubo Shop	King of Power Saving	Received the goods and used it, can save electricity a month later to see the effect!	Jingdong prefecture level city in Shandong
Doraemon Hyakubo Shop	King of Power Saving	Fancy 90-day trial, 5-year warranty before the order, the goods received, has been experiencing a week, can really feel the savings, in the introduction of friends also buy this!	Jingdong prefecture level city in Shandong
Doraemon Hyakubo Shop	King of Power Saving	Clothing stores use, neighbors recommended to buy, said a month can save more than 70 yuan, first plugged in to see the effect of the	Jingdong prefecture level city in Shandong
Doraemon Hyakubo Shop	King of Power Saving	A friend's home two months to save more than 80 dollars of electricity, recommended that I buy two, decisive purchase, looking forward to the effect of power saving, waiting for the next month effect.	Jingdong prefecture level city in Shandong
Doraemon Hyakubo Shop	King of Power Saving	The goods have been received, consistent with the description of the advertisement, quite good, I hope that in terms of power saving and purifying the air is really as the merchant said that would be better!	Jingdong prefecture level city in Shandong
Doraemon Hyakubo Shop	King of Power Saving	Power Saving King is really good to use the quality is very good than the physical store a lot cheaper we can rest assured that the purchase recommended to buy!	Jingdong prefecture level city in Shandong
Doraemon Hyakubo Shop	King of Power Saving	Energy saver has been used on the rental house inside the use of the meter is always running fast, with this can really save electricity, easy to use, plugged in can be, very satisfied!	Jingdong prefecture level city in Shandong
Doraemon Hyakubo Shop	King of Power Saving	Power Saver Power Saver King is good quality and affordable, very satisfied, worth buying.	Jingdong prefecture level city in Shandong
Doraemon Hyakubo Shop	King of Power Saving	Just use can not see what effect to,, wait for a period of time in the evaluation	Jingdong prefecture level city in Shandong
Doraemon Hyakubo Shop	King of Power Saving	Live in the 18th floor is too far away from the meter, afraid of no effect, look at the trial 90 days before buying. Currently used for a month, there is an effect, saving 27 dollars in electricity, next month should be able to save more!	Jingdong prefecture level city in Shandong
Doraemon Hyakubo Shop	King of Power Saving	I've been using it for almost a month now and it feels good, so I'll use it again.	Jingdong prefecture level city in Shandong
Doraemon Hyakubo Shop	King of Power Saving	It's ok, the electric bill is really less, originally I remember charging $100 and using it for 10 days. Now it lasts about 15 days. It's still noticeable.	Jingdong prefecture level city in Shandong
Doraemon Hyakubo Shop	King of Power Saving	Fast logistics, good power saving effect, very satisfied	Jingdong prefecture level city in Shandong
Doraemon Hyakubo Shop	King of Power Saving	Has been the second time back to buy, energy saving effect, worth recommending, you can try to see, power saving effect: very good Experience: can save about 30%!	Jingdong prefecture level city in Shandong
Doraemon Hyakubo Shop	King of Power Saving	I don't know how well it works for now	Jingdong prefecture level city in Shandong
Doraemon Hyakubo Shop	King of Power Saving	Factory direct is really good, plugged in and used for half a month did less probably almost 50 degrees of electricity! And the manufacturer's warranty is 5 years.	Jingdong prefecture level city in Shandong
Doraemon Hyakubo Shop	King of Power Saving	It's really effective. The previous month's electricity was $286, but after using the power saver for a month, I have $195 left this month, which is not bad.	Jingdong prefecture level city in Shandong
Doraemon Hyakubo Shop	King of Power Saving	I'll give you a good review and see how it goes.	Jingdong prefecture level city in Shandong
Doraemon Hyakubo Shop	King of Power Saving	Customer service is very professional and patient to guide the installation, the quality of workmanship is very fine. Durable, second repurchase. Really can save oh!	Jingdong prefecture level city in Shandong
Doraemon Hyakubo Shop	King of Power Saving	I've been using it for a month now and my electric bill is over $30 less than last month. I feel it's quite worth buying.	Jingdong prefecture level city in Shandong
Doraemon Hyakubo Shop	King of Power Saving	Small size, does not take up space. It's very good, it does work very effectively, the tool room has a lot of work with a lot of electricity. After installing it, it really reduces a lot of electricity bills. Before the day 30 degrees of electricity left stone, now work down really less a few degrees.	Jingdong prefecture level city in Shandong
Doraemon Hyakubo Shop	King of Power Saving	It does work for saving electricity, I've bought the kind from elsewhere before that was too simple not **all**. This time to buy this store, but also for the safety. Air conditioning is too much electricity, this use to save a lot of	Jingdong prefecture level city in Shandong
Doraemon Hyakubo Shop	King of Power Saving	Two air conditioners and a refrigerator at home, a month down the electricity bill is almost two hundred dollars, with this after the average savings of more than thirty dollars to spend less money to enjoy more, it's really very good!	Jingdong prefecture level city in Shandong
Doraemon Hyakubo Shop	King of Power Saving	Good. Very good.	Jingdong prefecture level city in Shandong
Doraemon Hyakubo Shop	King of Power Saving	Works great, easy to operate, great, very happy with it.	Jingdong prefecture level city in Shandong
Doraemon Hyakubo Shop	King of Power Saving	Received it and tried it out, it's great, great quality and worth having! Five stars praise!	Jingdong prefecture level city in Shandong
Doraemon Hyakubo Shop	King of Power Saving	Very trustworthy brand, quality is guaranteed, the price is still affordable, quite satisfied!	Jingdong prefecture level city in Shandong
Doraemon Hyakubo Shop	King of Power Saving	Use it before you speak.	Jingdong prefecture level city in Shandong
Doraemon Hyakubo Shop	King of Power Saving	The quality of workmanship is very good and the power saving effect is quite good, I regret not getting it earlier.	Jingdong prefecture level city in Shandong
Doraemon Hyakubo Shop	King of Power Saving	Merchants shipping speed, and customer service introduced the same very good, cost-effective	Jingdong prefecture level city in Shandong
Doraemon Hyakubo Shop	King of Power Saving	Feel a little effect Oh, where the power consumption where to plug, convenient and convenient	Jingdong prefecture level city in Shandong
Doraemon Hyakubo Shop	King of Power Saving	The quality of the imported saver is very good, practical and satisfactory. Cost-effective	Jingdong prefecture level city in Shandong
Doraemon Hyakubo Shop	King of Power Saving	Power Saver is especially good, good and inexpensive, very good	Jingdong prefecture level city in Shandong
Doraemon Hyakubo Shop	King of Power Saving	Fast shipping, awesome quality, affordable price, satisfied.	Jingdong prefecture level city in Shandong
Doraemon Hyakubo Shop	King of Power Saving	Energy saver received, the effect is consistent with the description, recommended to buy!	Jingdong prefecture level city in Shandong
Doraemon Hyakubo Shop	King of Power Saving	It's really good, the quality is great, I recommend it!	Jingdong prefecture level city in Shandong
Doraemon Hyakubo Shop	King of Power Saving	Works well. Inexpensive. Shipping was fast.	Jingdong prefecture level city in Shandong

Doraemon Hyakubo Shop	King of Power Saving	Logistics is fast, works well, energy saving is good	Jingdong prefecture level city in Shandong
Doraemon Hyakubo Shop	King of Power Saving	very happy	Jingdong prefecture level city in Shandong
Doraemon Hyakubo Shop	King of Power Saving	an excellent	Jingdong prefecture level city in Shandong
Doraemon Hyakubo Shop	King of Power Saving	It rained a couple days ago and I just went to get the item back today , not bad, hey I looked at the meter and that red display light takes several seconds to jump, did not make a mistake buying it. After a period of time to evaluate.	Jingdong prefecture level city in Shandong
Doraemon Hyakubo Shop	King of Power Saving	Used more than 10 days to check the power, there is indeed some decline, customer service said, long-term use will save more points!	Jingdong prefecture level city in Shandong
Doraemon Hyakubo Shop	King of Power Saving	It's a proper IQ tax, and it costs electricity.	Jingdong prefecture level city in Shandong
Doraemon Hyakubo Shop	King of Power Saving	I've been using it for 17 days now, it really saves electricity Shipping is fast, you can save a lot in a year	Jingdong prefecture level city in Shandong
Doraemon Hyakubo Shop	King of Power Saving	I've been using it for 17 days and it really saves power Shipping is fast and you don't have to worry about the quality, free returns within 5 years	Jingdong prefecture level city in Shandong
Doraemon Hyakubo Shop	King of Power Saving	It's more regular, scanned the code and it's authentic, and the main thing is that you can try it for 90 days! Plugged in more than ten days, is less than before a few degrees per day. When the time to use a month to see Additional. Good to buy a few more	Jingdong prefecture level city in Shandong
Doraemon Hyakubo Shop	King of Power Saving	Logistics is very fast, fine workmanship, use it and then come back to the comments	Jingdong prefecture level city in Shandong
Doraemon Hyakubo Shop	King of Power Saving	The quality of workmanship is very good and the power saving effect is quite good, I regret not getting it earlier.	Jingdong prefecture level city in Shandong
Doraemon Hyakubo Shop	King of Power Saving	Received the goods, according to the instructions on the operation is very simple, to wait and see the effect, looks good!	Jingdong prefecture level city in Shandong
Doraemon Hyakubo Shop	King of Power Saving	It's really as safe and power-saving as the owner says, and it works great!	Jingdong prefecture level city in Shandong
Doraemon Hyakubo Shop	King of Power Saving	Received the goods, immediately used, looks good, to wait and see how the effect.	Jingdong prefecture level city in Shandong
Doraemon Hyakubo Shop	King of Power Saving	Afraid to buy back did not work, the store said the commitment to 90 days invalid refund, warranty 5 years. Saver also complimentary on the Pacific insurance, it is a lot of peace of mind, there are effects will continue to buy back drops!	Jingdong prefecture level city in Shandong
Doraemon Hyakubo Shop	King of Power Saving	Received the goods and used it, can save electricity a month later to see the effect!	Jingdong prefecture level city in Shandong
Doraemon Hyakubo Shop	King of Power Saving	Fancy 90-day trial, 5-year warranty before the order, the goods received, has been experiencing a week, can really feel the savings, in the introduction of friends also buy this!	Jingdong prefecture level city in Shandong
Doraemon Hyakubo Shop	King of Power Saving	Clothing stores use, neighbors recommended to buy, said a month can save more than 70 yuan, first plugged in to see the effect of the	Jingdong prefecture level city in Shandong
Doraemon Hyakubo Shop	King of Power Saving	A friend's home two months to save more than 80 dollars of electricity, recommended that I buy two, decisive purchase, looking forward to the effect of power saving, waiting for the next month effect.	Jingdong prefecture level city in Shandong
Doraemon Hyakubo Shop	King of Power Saving	The goods have been received, consistent with the description of the advertisement, quite good, I hope that in terms of power saving and purifying the air is really as the merchant said that would be better!	Jingdong prefecture level city in Shandong
Doraemon Hyakubo Shop	King of Power Saving	Power Saving King is really good to use the quality is very good than the physical store a lot cheaper we can rest assured that the purchase recommended to buy!	Jingdong prefecture level city in Shandong
Doraemon Hyakubo Shop	King of Power Saving	Energy saver has been used on the rental house inside the use of the meter is always running fast, with this can really save electricity, easy to use, plugged in can be, very satisfied!	Jingdong prefecture level city in Shandong
Doraemon Hyakubo Shop	King of Power Saving	Power Saver Power Saver King is good quality and affordable, very satisfied, worth buying.	Jingdong prefecture level city in Shandong
Doraemon Hyakubo Shop	King of Power Saving	Just use can not see what effect to,, wait for a period of time in the evaluation	Jingdong prefecture level city in Shandong
Doraemon Hyakubo Shop	King of Power Saving	Live in the 18th floor is too far away from the meter, afraid of no effect, look at the trial 90 days before buying. Currently used for a month, there is an effect, saving 27 dollars in electricity, next month should be able to save more!	Jingdong prefecture level city in Shandong
Doraemon Hyakubo Shop	King of Power Saving	I've been using it for almost a month now and it feels good, so I'll use it again.	Jingdong prefecture level city in Shandong
Doraemon Hyakubo Shop	King of Power Saving	It's ok, the electric bill is really less, originally I remember charging $100 and using it for 10 days. Now it lasts about 15 days. It's still noticeable.	Jingdong prefecture level city in Shandong
Doraemon Hyakubo Shop	King of Power Saving	Fast logistics, good power saving effect, very satisfied	Jingdong prefecture level city in Shandong
Doraemon Hyakubo Shop	King of Power Saving	Has been the second time back to buy, energy saving effect, worth recommending, you can try to see, power saving effect: very good Experience: can save about 30%!	Jingdong prefecture level city in Shandong
Doraemon Hyakubo Shop	King of Power Saving	I don't know how well it works for now	Jingdong prefecture level city in Shandong
Doraemon Hyakubo Shop	King of Power Saving	Factory direct is really good, plugged in and used for half a month did less probably almost 50 degrees of electricity! And the manufacturer's warranty is 5 years.	Jingdong prefecture level city in Shandong
Doraemon Hyakubo Shop	King of Power Saving	It's really effective. The previous month's electricity was $286, but after using the power saver for a month, I have $195 left this month, which is not bad.	Jingdong prefecture level city in Shandong
Doraemon Hyakubo Shop	King of Power Saving	I'll give you a good review and see how it goes.	Jingdong prefecture level city in Shandong
Doraemon Hyakubo Shop	King of Power Saving	Customer service is very professional and patient to guide the installation, the quality of workmanship is very fine. Durable, second repurchase. Really can save oh!	Jingdong prefecture level city in Shandong
Doraemon Hyakubo Shop	King of Power Saving	I've been using it for a month now and my electric bill is over $30 less than last month. I feel it's quite worth buying.	Jingdong prefecture level city in Shandong
Doraemon Hyakubo Shop	King of Power Saving	Small size, does not take up space. It's very good, it does work very effectively, the tool room has a lot of work with a lot of electricity. After installing it, it really reduces a lot of electricity bills. Before the day 30 degrees of electricity left stone, now work down really less a few degrees.	Jingdong prefecture level city in Shandong
Doraemon Hyakubo Shop	King of Power Saving	It does work for saving electricity, I've bought the kind from elsewhere before that was too simple not **all**. This time to buy this store, but also for the safety. Air conditioning is too much electricity, this use to save a lot of	Jingdong prefecture level city in Shandong
Doraemon Hyakubo Shop	King of Power Saving	Two air conditioners and a refrigerator at home, a month down the electricity bill is almost two hundred dollars, with this after the average savings of more than thirty dollars to spend less money to enjoy more, it's really very good!	Jingdong prefecture level city in Shandong
Doraemon Hyakubo Shop	King of Power Saving	Good. Very good.	Jingdong prefecture level city in Shandong
Doraemon Hyakubo Shop	King of Power Saving	Works great, easy to operate, great, very happy with it.	Jingdong prefecture level city in Shandong
Doraemon Hyakubo Shop	King of Power Saving	Received it and tried it out, it's great, great quality and worth having! Five stars praise!	Jingdong prefecture level city in Shandong
Doraemon Hyakubo Shop	King of Power Saving	Very trustworthy brand, quality is guaranteed, the price is still affordable, quite satisfied!	Jingdong prefecture level city in Shandong
Doraemon Hyakubo Shop	King of Power Saving	Use it before you speak.	Jingdong prefecture level city in Shandong
Doraemon Hyakubo Shop	King of Power Saving	The quality of workmanship is very good and the power saving effect is quite good, I regret not getting it earlier.	Jingdong prefecture level city in Shandong
Doraemon Hyakubo Shop	King of Power Saving	Merchants shipping speed, and customer service introduced the same very good, cost-effective	Jingdong prefecture level city in Shandong
Doraemon Hyakubo Shop	King of Power Saving	Feel a little effect Oh, where the power consumption where to plug, convenient and convenient	Jingdong prefecture level city in Shandong
Doraemon Hyakubo Shop	King of Power Saving	The quality of the imported saver is very good, practical and satisfactory. Cost-effective	Jingdong prefecture level city in Shandong
Doraemon Hyakubo Shop	King of Power Saving	Power Saver is especially good, good and inexpensive, very good	Jingdong prefecture level city in Shandong

Doraemon Hyakubo Shop	King of Power Saving	Fast shipping, awesome quality, affordable price, satisfied.	Jingdong prefecture level city in Shandong
Doraemon Hyakubo Shop	King of Power Saving	Energy saver received, the effect is consistent with the description, recommended to buy!	Jingdong prefecture level city in Shandong
Doraemon Hyakubo Shop	King of Power Saving	It's really good, the quality is great, I recommend it!	Jingdong prefecture level city in Shandong
Doraemon Hyakubo Shop	King of Power Saving	Works well. Inexpensive. Shipping was fast.	Jingdong prefecture level city in Shandong
Doraemon Hyakubo Shop	King of Power Saving	Logistics is fast, works well, energy saving is good	Jingdong prefecture level city in Shandong
Doraemon Hyakubo Shop	King of Power Saving	very happy	Jingdong prefecture level city in Shandong
Doraemon Hyakubo Shop	King of Power Saving	an excellent	Jingdong prefecture level city in Shandong
Doraemon Hyakubo Shop	King of Power Saving	It rained a couple days ago and I just went to get the item back today , not bad, hey I looked at the meter and that red display light takes several seconds to jump, did not make a mistake buying it. After a period of time to evaluate.	Jingdong prefecture level city in Shandong
Doraemon Hyakubo Shop	King of Power Saving	Used more than 10 days to check the power, there is indeed some decline, customer service said, long-term use will save more points!	Jingdong prefecture level city in Shandong
Doraemon Hyakubo Shop	King of Power Saving	It's a proper IQ tax, and it costs electricity.	Jingdong prefecture level city in Shandong
Doraemon Hyakubo Shop	King of Power Saving	I've been using it for 17 days now, it really saves electricity Shipping is fast, you can save a lot in a year	Jingdong prefecture level city in Shandong
Doraemon Hyakubo Shop	King of Power Saving	I've been using it for 17 days and it really saves power Shipping is fast and you don't have to worry about the quality, free returns within 5 years	Jingdong prefecture level city in Shandong
Doraemon Hyakubo Shop	King of Power Saving	It's more regular, scanned the code and it's authentic, and the main thing is that you can try it for 90 days! Plugged in more than ten days, is less than before a few degrees per day. When the time to use a month to see Additional. Good to buy a few more	Jingdong prefecture level city in Shandong
Doraemon Hyakubo Shop	King of Power Saving	Logistics is very fast, fine workmanship, use it and then come back to the comments	Jingdong prefecture level city in Shandong
Doraemon Hyakubo Shop	King of Power Saving	The quality of workmanship is very good and the power saving effect is quite good, I regret not getting it earlier.	Jingdong prefecture level city in Shandong
Doraemon Hyakubo Shop	King of Power Saving	Received the goods, according to the instructions on the operation is very simple, to wait and see the effect, looks good!	Jingdong prefecture level city in Shandong
Doraemon Hyakubo Shop	King of Power Saving	It's really as safe and power-saving as the owner says, and it works great!	Jingdong prefecture level city in Shandong
Doraemon Hyakubo Shop	King of Power Saving	Received the goods, immediately used, looks good, to wait and see how the effect.	Jingdong prefecture level city in Shandong
Doraemon Hyakubo Shop	King of Power Saving	Afraid to buy back did not work, the store said the commitment to 90 days invalid refund, warranty 5 years. Saver also complimentary on the Pacific insurance, it is a lot of peace of mind, there are effects will continue to buy back drops!	Jingdong prefecture level city in Shandong
Doraemon Hyakubo Shop	King of Power Saving	Received the goods and used it, can save electricity a month later to see the effect!	Jingdong prefecture level city in Shandong
Doraemon Hyakubo Shop	King of Power Saving	Fancy 90-day trial, 5-year warranty before the order, the goods received, has been experiencing a week, can really feel the savings, in the introduction of friends also buy this!	Jingdong prefecture level city in Shandong
Doraemon Hyakubo Shop	King of Power Saving	Clothing stores use, neighbors recommended to buy, said a month can save more than 70 yuan, first plugged in to see the effect of the	Jingdong prefecture level city in Shandong
Doraemon Hyakubo Shop	King of Power Saving	A friend's home two months to save more than 80 dollars of electricity, recommended that I buy two, decisive purchase, looking forward to the effect of power saving, waiting for the next month effect.	Jingdong prefecture level city in Shandong
Doraemon Hyakubo Shop	King of Power Saving	The goods have been received, consistent with the description of the advertisement, quite good, I hope that in terms of power saving and purifying the air is really as the merchant said that would be better!	Jingdong prefecture level city in Shandong
Doraemon Hyakubo Shop	King of Power Saving	Power Saving King is really good to use the quality is very good than the physical store a lot cheaper we can rest assured that the purchase recommended to buy!	Jingdong prefecture level city in Shandong
Doraemon Hyakubo Shop	King of Power Saving	Energy saver has been used on the rental house inside the use of the meter is always running fast, with this can really save electricity, easy to use, plugged in can be, very satisfied!	Jingdong prefecture level city in Shandong
Doraemon Hyakubo Shop	King of Power Saving	Power Saver Power Saver King is good quality and affordable, very satisfied, worth buying.	Jingdong prefecture level city in Shandong
Doraemon Hyakubo Shop	King of Power Saving	Just use can not see what effect to,, wait for a period of time in the evaluation	Jingdong prefecture level city in Shandong
Doraemon Hyakubo Shop	King of Power Saving	Live in the 18th floor is too far away from the meter, afraid of no effect, look at the trial 90 days before buying. Currently used for a month, there is an effect, saving 27 dollars in electricity, next month should be able to save more!	Jingdong prefecture level city in Shandong
Doraemon Hyakubo Shop	King of Power Saving	I've been using it for almost a month now and it feels good, so I'll use it again.	Jingdong prefecture level city in Shandong
Doraemon Hyakubo Shop	King of Power Saving	It's ok, the electric bill is really less, originally I remember charging $100 and using it for 10 days. Now it lasts about 15 days. It's still noticeable.	Jingdong prefecture level city in Shandong
Doraemon Hyakubo Shop	King of Power Saving	Fast logistics, good power saving effect, very satisfied	Jingdong prefecture level city in Shandong
Doraemon Hyakubo Shop	King of Power Saving	Has been the second time back to buy, energy saving effect, worth recommending, you can try to see, power saving effect: very good Experience: can save about 30%!	Jingdong prefecture level city in Shandong
Doraemon Hyakubo Shop	King of Power Saving	I don't know how well it works for now	Jingdong prefecture level city in Shandong
Doraemon Hyakubo Shop	King of Power Saving	Factory direct is really good, plugged in and used for half a month did less probably almost 50 degrees of electricity! And the manufacturer's warranty is 5 years.	Jingdong prefecture level city in Shandong
Doraemon Hyakubo Shop	King of Power Saving	It's really effective. The previous month's electricity was $286, but after using the power saver for a month, I have $195 left this month, which is not bad.	Jingdong prefecture level city in Shandong
Doraemon Hyakubo Shop	King of Power Saving	I'll give you a good review and see how it goes.	Jingdong prefecture level city in Shandong
Doraemon Hyakubo Shop	King of Power Saving	Customer service is very professional and patient to guide the installation, the quality of workmanship is very fine. Durable, second repurchase. Really can save oh!	Jingdong prefecture level city in Shandong
Doraemon Hyakubo Shop	King of Power Saving	I've been using it for a month now and my electric bill is over $30 less than last month. I feel it's quite worth buying.	Jingdong prefecture level city in Shandong
Doraemon Hyakubo Shop	King of Power Saving	Small size, does not take up space. It's very good, it does work very effectively, the tool room has a lot of work with a lot of electricity. After installing it, it really reduces a lot of electricity bills. Before the day 30 degrees of electricity left stone, now work down really less a few degrees.	Jingdong prefecture level city in Shandong
Doraemon Hyakubo Shop	King of Power Saving	It does work for saving electricity, I've bought the kind from elsewhere before that was too simple not **all**. This time to buy this store, but also for the safety. Air conditioning is too much electricity, this use to save a lot of	Jingdong prefecture level city in Shandong
Doraemon Hyakubo Shop	King of Power Saving	Two air conditioners and a refrigerator at home, a month down the electricity bill is almost two hundred dollars, with this after the average savings of more than thirty dollars to spend less money to enjoy more, it's really very good!	Jingdong prefecture level city in Shandong
Doraemon Hyakubo Shop	King of Power Saving	Good. Very good.	Jingdong prefecture level city in Shandong
Doraemon Hyakubo Shop	King of Power Saving	Works great, easy to operate, great, very happy with it.	Jingdong prefecture level city in Shandong
Doraemon Hyakubo Shop	King of Power Saving	Received it and tried it out, it's great, great quality and worth having! Five stars praise!	Jingdong prefecture level city in Shandong
Doraemon Hyakubo Shop	King of Power Saving	Very trustworthy brand, quality is guaranteed, the price is still affordable, quite satisfied!	Jingdong prefecture level city in Shandong
Doraemon Hyakubo Shop	King of Power Saving	Use it before you speak.	Jingdong prefecture level city in Shandong
Doraemon Hyakubo Shop	King of Power Saving	The quality of workmanship is very good and the power saving effect is quite good, I regret not getting it earlier.	Jingdong prefecture level city in Shandong

Doraemon Hyakubo Shop	King of Power Saving	Merchants shipping speed, and customer service introduced the same very good, cost-effective	Jingdong prefecture level city in Shandong
Doraemon Hyakubo Shop	King of Power Saving	Feel a little effect Oh, where the power consumption where to plug, convenient and convenient	Jingdong prefecture level city in Shandong
Doraemon Hyakubo Shop	King of Power Saving	The quality of the imported saver is very good, practical and satisfactory. Cost-effective	Jingdong prefecture level city in Shandong
Doraemon Hyakubo Shop	King of Power Saving	Power Saver is especially good, good and inexpensive, very good	Jingdong prefecture level city in Shandong
Doraemon Hyakubo Shop	King of Power Saving	Fast shipping, awesome quality, affordable price, satisfied.	Jingdong prefecture level city in Shandong
Doraemon Hyakubo Shop	King of Power Saving	Energy saver received, the effect is consistent with the description, recommended to buy!	Jingdong prefecture level city in Shandong
Doraemon Hyakubo Shop	King of Power Saving	It's really good, the quality is great, I recommend it!	Jingdong prefecture level city in Shandong
Doraemon Hyakubo Shop	King of Power Saving	Works well. Inexpensive. Shipping was fast.	Jingdong prefecture level city in Shandong
Doraemon Hyakubo Shop	King of Power Saving	Logistics is fast, works well, energy saving is good	Jingdong prefecture level city in Shandong
Doraemon Hyakubo Shop	King of Power Saving	very happy	Jingdong prefecture level city in Shandong
Doraemon Hyakubo Shop	King of Power Saving	an excellent	Jingdong prefecture level city in Shandong
Doraemon Hyakubo Shop	King of Power Saving	It rained a couple days ago and I just went to get the item back today , not bad, hey I looked at the meter and that red display light takes several seconds to jump, did not make a mistake buying it. After a period of time to evaluate.	Jingdong prefecture level city in Shandong
Doraemon Hyakubo Shop	King of Power Saving	Used more than 10 days to check the power, there is indeed some decline, customer service said, long-term use will save more points!	Jingdong prefecture level city in Shandong
Doraemon Hyakubo Shop	King of Power Saving	It's a proper IQ tax, and it costs electricity.	Jingdong prefecture level city in Shandong
Doraemon Hyakubo Shop	King of Power Saving	I've been using it for 17 days now, it really saves electricity Shipping is fast, you can save a lot in a year	Jingdong prefecture level city in Shandong
Doraemon Hyakubo Shop	King of Power Saving	I've been using it for 17 days and it really saves power Shipping is fast and you don't have to worry about the quality, free returns within 5 years	Jingdong prefecture level city in Shandong
Doraemon Hyakubo Shop	King of Power Saving	It's more regular, scanned the code and it's authentic, and the main thing is that you can try it for 90 days! Plugged in more than ten days, is less than before a few degrees per day. When the time to use a month to see Additional. Good to buy a few more	Jingdong prefecture level city in Shandong
Doraemon Hyakubo Shop	King of Power Saving	Logistics is very fast, fine workmanship, use it and then come back to the comments	Jingdong prefecture level city in Shandong
Doraemon Hyakubo Shop	King of Power Saving	The quality of workmanship is very good and the power saving effect is quite good, I regret not getting it earlier.	Jingdong prefecture level city in Shandong
Doraemon Hyakubo Shop	King of Power Saving	Received the goods, according to the instructions on the operation is very simple, to wait and see the effect, looks good!	Jingdong prefecture level city in Shandong
Doraemon Hyakubo Shop	King of Power Saving	It's really as safe and power-saving as the owner says, and it works great!	Jingdong prefecture level city in Shandong
Doraemon Hyakubo Shop	King of Power Saving	Received the goods, immediately used, looks good, to wait and see how the effect.	Jingdong prefecture level city in Shandong
Doraemon Hyakubo Shop	King of Power Saving	Afraid to buy back did not work, the store said the commitment to 90 days invalid refund, warranty 5 years. Saver also complimentary on the Pacific insurance, it is a lot of peace of mind, there are effects will continue to buy back drops!	Jingdong prefecture level city in Shandong
Doraemon Hyakubo Shop	King of Power Saving	Received the goods and used it, can save electricity a month later to see the effect!	Jingdong prefecture level city in Shandong
Doraemon Hyakubo Shop	King of Power Saving	Fancy 90-day trial, 5-year warranty before the order, the goods received, has been experiencing a week, can really feel the savings, in the introduction of friends also buy this!	Jingdong prefecture level city in Shandong
Doraemon Hyakubo Shop	King of Power Saving	Clothing stores use, neighbors recommended to buy, said a month can save more than 70 yuan, first plugged in to see the effect of the	Jingdong prefecture level city in Shandong
Doraemon Hyakubo Shop	King of Power Saving	A friend's home two months to save more than 80 dollars of electricity, recommended that I buy two, decisive purchase, looking forward to the effect of power saving, waiting for the next month effect.	Jingdong prefecture level city in Shandong
Doraemon Hyakubo Shop	King of Power Saving	The goods have been received, consistent with the description of the advertisement, quite good, I hope that in terms of power saving and purifying the air is really as the merchant said that would be better!	Jingdong prefecture level city in Shandong
Doraemon Hyakubo Shop	King of Power Saving	Power Saving King is really good to use the quality is very good than the physical store a lot cheaper we can rest assured that the purchase recommended to buy!	Jingdong prefecture level city in Shandong
Doraemon Hyakubo Shop	King of Power Saving	Energy saver has been used on the rental house inside the use of the meter is always running fast, with this can really save electricity, easy to use, plugged in can be, very satisfied!	Jingdong prefecture level city in Shandong
Doraemon Hyakubo Shop	King of Power Saving	Power Saver Power Saver King is good quality and affordable, very satisfied, worth buying.	Jingdong prefecture level city in Shandong
Doraemon Hyakubo Shop	King of Power Saving	Just use can not see what effect to,, wait for a period of time in the evaluation	Jingdong prefecture level city in Shandong
Doraemon Hyakubo Shop	King of Power Saving	Live in the 18th floor is too far away from the meter, afraid of no effect, look at the trial 90 days before buying. Currently used for a month, there is an effect, saving 27 dollars in electricity, next month should be able to save more!	Jingdong prefecture level city in Shandong
Doraemon Hyakubo Shop	King of Power Saving	I've been using it for almost a month now and it feels good, so I'll use it again.	Jingdong prefecture level city in Shandong
Doraemon Hyakubo Shop	King of Power Saving	It's ok, the electric bill is really less, originally I remember charging $100 and using it for 10 days. Now it lasts about 15 days. It's still noticeable.	Jingdong prefecture level city in Shandong
Doraemon Hyakubo Shop	King of Power Saving	Fast logistics, good power saving effect, very satisfied	Jingdong prefecture level city in Shandong
Doraemon Hyakubo Shop	King of Power Saving	Has been the second time back to buy, energy saving effect, worth recommending, you can try to see, power saving effect: very good Experience: can save about 30%!	Jingdong prefecture level city in Shandong
Doraemon Hyakubo Shop	King of Power Saving	I don't know how well it works for now	Jingdong prefecture level city in Shandong
Doraemon Hyakubo Shop	King of Power Saving	Factory direct is really good, plugged in and used for half a month did less probably almost 50 degrees of electricity! And the manufacturer's warranty is 5 years.	Jingdong prefecture level city in Shandong
Doraemon Hyakubo Shop	King of Power Saving	It's really effective. The previous month's electricity was $286, but after using the power saver for a month, I have $195 left this month, which is not bad.	Jingdong prefecture level city in Shandong
Doraemon Hyakubo Shop	King of Power Saving	I'll give you a good review and see how it goes.	Jingdong prefecture level city in Shandong
Doraemon Hyakubo Shop	King of Power Saving	Customer service is very professional and patient to guide the installation, the quality of workmanship is very fine. Durable, second repurchase. Really can save oh!	Jingdong prefecture level city in Shandong
Doraemon Hyakubo Shop	King of Power Saving	I've been using it for a month now and my electric bill is over $30 less than last month. I feel it's quite worth buying.	Jingdong prefecture level city in Shandong
Doraemon Hyakubo Shop	King of Power Saving	Small size, does not take up space. It's very good, it does work very effectively, the tool room has a lot of work with a lot of electricity. After installing it, it really reduces a lot of electricity bills. Before the day 30 degrees of electricity left stone, now work down really less a few degrees.	Jingdong prefecture level city in Shandong
Doraemon Hyakubo Shop	King of Power Saving	It does work for saving electricity, I've bought the kind from elsewhere before that was too simple not **all**. This time to buy this store, but also for the safety. Air conditioning is too much electricity, this use to save a lot of	Jingdong prefecture level city in Shandong
Doraemon Hyakubo Shop	King of Power Saving	Two air conditioners and a refrigerator at home, a month down the electricity bill is almost two hundred dollars, with this after the average savings of more than thirty dollars to spend less money to enjoy more, it's really very good!	Jingdong prefecture level city in Shandong
Doraemon Hyakubo Shop	King of Power Saving	Good. Very good.	Jingdong prefecture level city in Shandong
Doraemon Hyakubo Shop	King of Power Saving	Works great, easy to operate, great, very happy with it.	Jingdong prefecture level city in Shandong

Doraemon Hyakubo Shop	King of Power Saving	Received it and tried it out, it's great, great quality and worth having! Five stars praise!	Jingdong prefecture level city in Shandong
Doraemon Hyakubo Shop	King of Power Saving	Very trustworthy brand, quality is guaranteed, the price is still affordable, quite satisfied!	Jingdong prefecture level city in Shandong
Doraemon Hyakubo Shop	King of Power Saving	Use it before you speak.	Jingdong prefecture level city in Shandong
Doraemon Hyakubo Shop	King of Power Saving	The quality of workmanship is very good and the power saving effect is quite good, I regret not getting it earlier.	Jingdong prefecture level city in Shandong
Doraemon Hyakubo Shop	King of Power Saving	Merchants shipping speed, and customer service introduced the same very good, cost-effective	Jingdong prefecture level city in Shandong
Doraemon Hyakubo Shop	King of Power Saving	Feel a little effect Oh, where the power consumption where to plug, convenient and convenient	Jingdong prefecture level city in Shandong
Doraemon Hyakubo Shop	King of Power Saving	The quality of the imported saver is very good, practical and satisfactory. Cost-effective	Jingdong prefecture level city in Shandong
Doraemon Hyakubo Shop	King of Power Saving	Power Saver is especially good, good and inexpensive, very good	Jingdong prefecture level city in Shandong
Doraemon Hyakubo Shop	King of Power Saving	Fast shipping, awesome quality, affordable price, satisfied.	Jingdong prefecture level city in Shandong
Doraemon Hyakubo Shop	King of Power Saving	Energy saver received, the effect is consistent with the description, recommended to buy!	Jingdong prefecture level city in Shandong
Doraemon Hyakubo Shop	King of Power Saving	It's really good, the quality is great, I recommend it!	Jingdong prefecture level city in Shandong
Doraemon Hyakubo Shop	King of Power Saving	Works well. Inexpensive. Shipping was fast.	Jingdong prefecture level city in Shandong
Doraemon Hyakubo Shop	King of Power Saving	Logistics is fast, works well, energy saving is good	Jingdong prefecture level city in Shandong
Doraemon Hyakubo Shop	King of Power Saving	very happy	Jingdong prefecture level city in Shandong
Doraemon Hyakubo Shop	King of Power Saving	an excellent	Jingdong prefecture level city in Shandong
Doraemon Hyakubo Shop	King of Power Saving	It rained a couple days ago and I just went to get the item back today , not bad, hey I looked at the meter and that red display light takes several seconds to jump, did not make a mistake buying it. After a period of time to evaluate.	Jingdong prefecture level city in Shandong
Doraemon Hyakubo Shop	King of Power Saving	Used more than 10 days to check the power, there is indeed some decline, customer service said, long-term use will save more points!	Jingdong prefecture level city in Shandong
Doraemon Hyakubo Shop	King of Power Saving	It's a proper IQ tax, and it costs electricity.	Jingdong prefecture level city in Shandong
Doraemon Hyakubo Shop	King of Power Saving	I've been using it for 17 days now, it really saves electricity Shipping is fast, you can save a lot in a year	Jingdong prefecture level city in Shandong
Doraemon Hyakubo Shop	King of Power Saving	I've been using it for 17 days and it really saves power Shipping is fast and you don't have to worry about the quality, free returns within 5 years	Jingdong prefecture level city in Shandong
Doraemon Hyakubo Shop	King of Power Saving	It's more regular, scanned the code and it's authentic, and the main thing is that you can try it for 90 days! Plugged in more than ten days, is less than before a few degrees per day. When the time to use a month to see Additional. Good to buy a few more	Jingdong prefecture level city in Shandong
Doraemon Hyakubo Shop	King of Power Saving	Logistics is very fast, fine workmanship, use it and then come back to the comments	Jingdong prefecture level city in Shandong
Doraemon Hyakubo Shop	King of Power Saving	The quality of workmanship is very good and the power saving effect is quite good, I regret not getting it earlier.	Jingdong prefecture level city in Shandong
Doraemon Hyakubo Shop	King of Power Saving	Received the goods, according to the instructions on the operation is very simple, to wait and see the effect, looks good!	Jingdong prefecture level city in Shandong
Doraemon Hyakubo Shop	King of Power Saving	It's really as safe and power-saving as the owner says, and it works great!	Jingdong prefecture level city in Shandong
Doraemon Hyakubo Shop	King of Power Saving	Received the goods, immediately used, looks good, to wait and see how the effect.	Jingdong prefecture level city in Shandong
Doraemon Hyakubo Shop	King of Power Saving	Afraid to buy back did not work, the store said the commitment to 90 days invalid refund, warranty 5 years. Saver also complimentary on the Pacific insurance, it is a lot of peace of mind, there are effects will continue to buy back drops!	Jingdong prefecture level city in Shandong
Doraemon Hyakubo Shop	King of Power Saving	Received the goods and used it, can save electricity a month later to see the effect!	Jingdong prefecture level city in Shandong
Doraemon Hyakubo Shop	King of Power Saving	Fancy 90-day trial, 5-year warranty before the order, the goods received, has been experiencing a week, can really feel the savings, in the introduction of friends also buy this!	Jingdong prefecture level city in Shandong
Doraemon Hyakubo Shop	King of Power Saving	Clothing stores use, neighbors recommended to buy, said a month can save more than 70 yuan, first plugged in to see the effect of the	Jingdong prefecture level city in Shandong
Doraemon Hyakubo Shop	King of Power Saving	A friend's home two months to save more than 80 dollars of electricity, recommended that I buy two, decisive purchase, looking forward to the effect of power saving, waiting for the next month effect.	Jingdong prefecture level city in Shandong
Doraemon Hyakubo Shop	King of Power Saving	The goods have been received, consistent with the description of the advertisement, quite good, I hope that in terms of power saving and purifying the air is really as the merchant said that would be better!	Jingdong prefecture level city in Shandong
Doraemon Hyakubo Shop	King of Power Saving	Power Saving King is really good to use the quality is very good than the physical store a lot cheaper we can rest assured that the purchase recommended to buy!	Jingdong prefecture level city in Shandong
Doraemon Hyakubo Shop	King of Power Saving	Energy saver has been used on the rental house inside the use of the meter is always running fast, with this can really save electricity, easy to use, plugged in can be, very satisfied!	Jingdong prefecture level city in Shandong
Doraemon Hyakubo Shop	King of Power Saving	Power Saver Power Saver King is good quality and affordable, very satisfied, worth buying.	Jingdong prefecture level city in Shandong
Doraemon Hyakubo Shop	King of Power Saving	Just use can not see what effect to,, wait for a period of time in the evaluation	Jingdong prefecture level city in Shandong
Doraemon Hyakubo Shop	King of Power Saving	Live in the 18th floor is too far away from the meter, afraid of no effect, look at the trial 90 days before buying. Currently used for a month, there is an effect, saving 27 dollars in electricity, next month should be able to save more!	Jingdong prefecture level city in Shandong
Doraemon Hyakubo Shop	King of Power Saving	I've been using it for almost a month now and it feels good, so I'll use it again.	Jingdong prefecture level city in Shandong
Doraemon Hyakubo Shop	King of Power Saving	It's ok, the electric bill is really less, originally I remember charging $100 and using it for 10 days. Now it lasts about 15 days. It's still noticeable.	Jingdong prefecture level city in Shandong
Doraemon Hyakubo Shop	King of Power Saving	Fast logistics, good power saving effect, very satisfied	Jingdong prefecture level city in Shandong
Doraemon Hyakubo Shop	King of Power Saving	Has been the second time back to buy, energy saving effect, worth recommending, you can try to see, power saving effect: very good Experience: can save about 30%!	Jingdong prefecture level city in Shandong
Doraemon Hyakubo Shop	King of Power Saving	I don't know how well it works for now	Jingdong prefecture level city in Shandong
Doraemon Hyakubo Shop	King of Power Saving	Factory direct is really good, plugged in and used for half a month did less probably almost 50 degrees of electricity! And the manufacturer's warranty is 5 years.	Jingdong prefecture level city in Shandong
Doraemon Hyakubo Shop	King of Power Saving	It's really effective. The previous month's electricity was $286, but after using the power saver for a month, I have $195 left this month, which is not bad.	Jingdong prefecture level city in Shandong
Doraemon Hyakubo Shop	King of Power Saving	I'll give you a good review and see how it goes.	Jingdong prefecture level city in Shandong
Doraemon Hyakubo Shop	King of Power Saving	Customer service is very professional and patient to guide the installation, the quality of workmanship is very fine. Durable, second repurchase. Really can save oh!	Jingdong prefecture level city in Shandong
Doraemon Hyakubo Shop	King of Power Saving	I've been using it for a month now and my electric bill is over $30 less than last month. I feel it's quite worth buying.	Jingdong prefecture level city in Shandong
Doraemon Hyakubo Shop	King of Power Saving	Small size, does not take up space. It's very good, it does work very effectively, the tool room has a lot of work with a lot of electricity. After installing it, it really reduces a lot of electricity bills. Before the day 30 degrees of electricity left stone, now work down really less a few degrees.	Jingdong prefecture level city in Shandong

Doraemon Hyakubo Shop	King of Power Saving	It does work for saving electricity, I've bought the kind from elsewhere before that was too simple not **all**. This time to buy this store, but also for the safety. Air conditioning is too much electricity, this use to save a lot of	Jingdong prefecture level city in Shandong
Doraemon Hyakubo Shop	King of Power Saving	Two air conditioners and a refrigerator at home, a month down the electricity bill is almost two hundred dollars, with this after the average savings of more than thirty dollars to spend less money to enjoy more, it's really very good!	Jingdong prefecture level city in Shandong
Doraemon Hyakubo Shop	King of Power Saving	Good. Very good.	Jingdong prefecture level city in Shandong
Doraemon Hyakubo Shop	King of Power Saving	Works great, easy to operate, great, very happy with it.	Jingdong prefecture level city in Shandong
Doraemon Hyakubo Shop	King of Power Saving	Received it and tried it out, it's great, great quality and worth having! Five stars praise!	Jingdong prefecture level city in Shandong
Doraemon Hyakubo Shop	King of Power Saving	Very trustworthy brand, quality is guaranteed, the price is still affordable, quite satisfied!	Jingdong prefecture level city in Shandong
Doraemon Hyakubo Shop	King of Power Saving	Use it before you speak.	Jingdong prefecture level city in Shandong
Doraemon Hyakubo Shop	King of Power Saving	The quality of workmanship is very good and the power saving effect is quite good, I regret not getting it earlier.	Jingdong prefecture level city in Shandong
Doraemon Hyakubo Shop	King of Power Saving	Merchants shipping speed, and customer service introduced the same very good, cost-effective	Jingdong prefecture level city in Shandong
Doraemon Hyakubo Shop	King of Power Saving	Feel a little effect Oh, where the power consumption where to plug, convenient and convenient	Jingdong prefecture level city in Shandong
Doraemon Hyakubo Shop	King of Power Saving	The quality of the imported saver is very good, practical and satisfactory. Cost-effective	Jingdong prefecture level city in Shandong
Doraemon Hyakubo Shop	King of Power Saving	Power Saver is especially good, good and inexpensive, very good	Jingdong prefecture level city in Shandong
Doraemon Hyakubo Shop	King of Power Saving	Fast shipping, awesome quality, affordable price, satisfied.	Jingdong prefecture level city in Shandong
Doraemon Hyakubo Shop	King of Power Saving	Energy saver received, the effect is consistent with the description, recommended to buy!	Jingdong prefecture level city in Shandong
Doraemon Hyakubo Shop	King of Power Saving	It's really good, the quality is great, I recommend it!	Jingdong prefecture level city in Shandong
Doraemon Hyakubo Shop	King of Power Saving	Works well. Inexpensive. Shipping was fast.	Jingdong prefecture level city in Shandong
Doraemon Hyakubo Shop	King of Power Saving	Logistics is fast, works well, energy saving is good	Jingdong prefecture level city in Shandong
Doraemon Hyakubo Shop	King of Power Saving	very happy	Jingdong prefecture level city in Shandong
Doraemon Hyakubo Shop	King of Power Saving	an excellent	Jingdong prefecture level city in Shandong
Doraemon Hyakubo Shop	King of Power Saving	It rained a couple days ago and I just went to get the item back today , not bad, hey I looked at the meter and that red display light takes several seconds to jump, did not make a mistake buying it. After a period of time to evaluate.	Jingdong prefecture level city in Shandong
Doraemon Hyakubo Shop	King of Power Saving	Used more than 10 days to check the power, there is indeed some decline, customer service said, long-term use will save more points!	Jingdong prefecture level city in Shandong
Doraemon Hyakubo Shop	King of Power Saving	It's a proper IQ tax, and it costs electricity.	Jingdong prefecture level city in Shandong
Doraemon Hyakubo Shop	King of Power Saving	I've been using it for 17 days now, it really saves electricity Shipping is fast, you can save a lot in a year	Jingdong prefecture level city in Shandong
Doraemon Hyakubo Shop	King of Power Saving	I've been using it for 17 days and it really saves power Shipping is fast and you don't have to worry about the quality, free returns within 5 years	Jingdong prefecture level city in Shandong
Doraemon Hyakubo Shop	King of Power Saving	It's more regular, scanned the code and it's authentic, and the main thing is that you can try it for 90 days! Plugged in more than ten days, is less than before a few degrees per day. When the time to use a month to see Additional. Good to buy a few more	Jingdong prefecture level city in Shandong
Doraemon Hyakubo Shop	King of Power Saving	Logistics is very fast, fine workmanship, use it and then come back to the comments	Jingdong prefecture level city in Shandong
Doraemon Hyakubo Shop	King of Power Saving	The quality of workmanship is very good and the power saving effect is quite good, I regret not getting it earlier.	Jingdong prefecture level city in Shandong
Doraemon Hyakubo Shop	King of Power Saving	Received the goods, according to the instructions on the operation is very simple, to wait and see the effect, looks good!	Jingdong prefecture level city in Shandong
Doraemon Hyakubo Shop	King of Power Saving	It's really as safe and power-saving as the owner says, and it works great!	Jingdong prefecture level city in Shandong
Doraemon Hyakubo Shop	King of Power Saving	Received the goods, immediately used, looks good, to wait and see how the effect.	Jingdong prefecture level city in Shandong
Doraemon Hyakubo Shop	King of Power Saving	Afraid to buy back did not work, the store said the commitment to 90 days invalid refund, warranty 5 years. Saver also complimentary on the Pacific insurance, it is a lot of peace of mind, there are effects will continue to buy back drops!	Jingdong prefecture level city in Shandong
Doraemon Hyakubo Shop	King of Power Saving	Received the goods and used it, can save electricity a month later to see the effect!	Jingdong prefecture level city in Shandong
Doraemon Hyakubo Shop	King of Power Saving	Fancy 90-day trial, 5-year warranty before the order, the goods received, has been experiencing a week, can really feel the savings, in the introduction of friends also buy this!	Jingdong prefecture level city in Shandong
Doraemon Hyakubo Shop	King of Power Saving	Clothing stores use, neighbors recommended to buy, said a month can save more than 70 yuan, first plugged in to see the effect of the	Jingdong prefecture level city in Shandong
Doraemon Hyakubo Shop	King of Power Saving	A friend's home two months to save more than 80 dollars of electricity, recommended that I buy two, decisive purchase, looking forward to the effect of power saving, waiting for the next month effect.	Jingdong prefecture level city in Shandong
Doraemon Hyakubo Shop	King of Power Saving	The goods have been received, consistent with the description of the advertisement, quite good, I hope that in terms of power saving and purifying the air is really as the merchant said that would be better!	Jingdong prefecture level city in Shandong
Doraemon Hyakubo Shop	King of Power Saving	Power Saving King is really good to use the quality is very good than the physical store a lot cheaper we can rest assured that the purchase recommended to buy!	Jingdong prefecture level city in Shandong
Doraemon Hyakubo Shop	King of Power Saving	Energy saver has been used on the rental house inside the use of the meter is always running fast, with this can really save electricity, easy to use, plugged in can be, very satisfied!	Jingdong prefecture level city in Shandong
Doraemon Hyakubo Shop	King of Power Saving	Power Saver Power Saver King is good quality and affordable, very satisfied, worth buying.	Jingdong prefecture level city in Shandong
Doraemon Hyakubo Shop	King of Power Saving	Just use can not see what effect to,, wait for a period of time in the evaluation	Jingdong prefecture level city in Shandong
Doraemon Hyakubo Shop	King of Power Saving	Live in the 18th floor is too far away from the meter, afraid of no effect, look at the trial 90 days before buying. Currently used for a month, there is an effect, saving 27 dollars in electricity, next month should be able to save more!	Jingdong prefecture level city in Shandong
Doraemon Hyakubo Shop	King of Power Saving	I've been using it for almost a month now and it feels good, so I'll use it again.	Jingdong prefecture level city in Shandong
Doraemon Hyakubo Shop	King of Power Saving	It's ok, the electric bill is really less, originally I remember charging $100 and using it for 10 days. Now it lasts about 15 days. It's still noticeable.	Jingdong prefecture level city in Shandong
Doraemon Hyakubo Shop	King of Power Saving	Fast logistics, good power saving effect, very satisfied	Jingdong prefecture level city in Shandong
Doraemon Hyakubo Shop	King of Power Saving	Has been the second time back to buy, energy saving effect, worth recommending, you can try to see, power saving effect: very good Experience: can save about 30%!	Jingdong prefecture level city in Shandong
Doraemon Hyakubo Shop	King of Power Saving	I don't know how well it works for now	Jingdong prefecture level city in Shandong
Doraemon Hyakubo Shop	King of Power Saving	Factory direct is really good, plugged in and used for half a month did less probably almost 50 degrees of electricity! And the manufacturer's warranty is 5 years.	Jingdong prefecture level city in Shandong
Doraemon Hyakubo Shop	King of Power Saving	It's really effective. The previous month's electricity was $286, but after using the power saver for a month, I have $195 left this month, which is not bad.	Jingdong prefecture level city in Shandong

Doraemon Hyakubo Shop	King of Power Saving	I'll give you a good review and see how it goes.	Jingdong prefecture level city in Shandong
Doraemon Hyakubo Shop	King of Power Saving	Customer service is very professional and patient to guide the installation, the quality of workmanship is very fine. Durable, second repurchase. Really can save oh!	Jingdong prefecture level city in Shandong
Doraemon Hyakubo Shop	King of Power Saving	I've been using it for a month now and my electric bill is over $30 less than last month. I feel it's quite worth buying.	Jingdong prefecture level city in Shandong
Doraemon Hyakubo Shop	King of Power Saving	Small size, does not take up space. It's very good, it does work very effectively, the tool room has a lot of work with a lot of electricity. After installing it, it really reduces a lot of electricity bills. Before the day 30 degrees of electricity left stone, now work down really less a few degrees.	Jingdong prefecture level city in Shandong
Doraemon Hyakubo Shop	King of Power Saving	It does work for saving electricity, I've bought the kind from elsewhere before that was too simple not **all**. This time to buy this store, but also for the safety. Air conditioning is too much electricity, this use to save a lot of	Jingdong prefecture level city in Shandong
Doraemon Hyakubo Shop	King of Power Saving	Two air conditioners and a refrigerator at home, a month down the electricity bill is almost two hundred dollars, with this after the average savings of more than thirty dollars to spend less money to enjoy more, it's really very good!	Jingdong prefecture level city in Shandong
Doraemon Hyakubo Shop	King of Power Saving	Good. Very good.	Jingdong prefecture level city in Shandong
Doraemon Hyakubo Shop	King of Power Saving	Works great, easy to operate, great, very happy with it.	Jingdong prefecture level city in Shandong
Doraemon Hyakubo Shop	King of Power Saving	Received it and tried it out, it's great, great quality and worth having! Five stars praise!	Jingdong prefecture level city in Shandong
Doraemon Hyakubo Shop	King of Power Saving	Very trustworthy brand, quality is guaranteed, the price is still affordable, quite satisfied!	Jingdong prefecture level city in Shandong
Doraemon Hyakubo Shop	King of Power Saving	Use it before you speak.	Jingdong prefecture level city in Shandong
Doraemon Hyakubo Shop	King of Power Saving	The quality of workmanship is very good and the power saving effect is quite good, I regret not getting it earlier.	Jingdong prefecture level city in Shandong
Doraemon Hyakubo Shop	King of Power Saving	Merchants shipping speed, and customer service introduced the same very good, cost-effective	Jingdong prefecture level city in Shandong
Doraemon Hyakubo Shop	King of Power Saving	Feel a little effect Oh, where the power consumption where to plug, convenient and convenient	Jingdong prefecture level city in Shandong
Doraemon Hyakubo Shop	King of Power Saving	The quality of the imported saver is very good, practical and satisfactory. Cost-effective	Jingdong prefecture level city in Shandong
Doraemon Hyakubo Shop	King of Power Saving	Power Saver is especially good, good and inexpensive, very good	Jingdong prefecture level city in Shandong
Doraemon Hyakubo Shop	King of Power Saving	Fast shipping, awesome quality, affordable price, satisfied.	Jingdong prefecture level city in Shandong
Doraemon Hyakubo Shop	King of Power Saving	Energy saver received, the effect is consistent with the description, recommended to buy!	Jingdong prefecture level city in Shandong
Doraemon Hyakubo Shop	King of Power Saving	It's really good, the quality is great, I recommend it!	Jingdong prefecture level city in Shandong
Doraemon Hyakubo Shop	King of Power Saving	Works well. Inexpensive. Shipping was fast.	Jingdong prefecture level city in Shandong
Doraemon Hyakubo Shop	King of Power Saving	Logistics is fast, works well, energy saving is good	Jingdong prefecture level city in Shandong
Doraemon Hyakubo Shop	King of Power Saving	very happy	Jingdong prefecture level city in Shandong
Doraemon Hyakubo Shop	King of Power Saving	an excellent	Jingdong prefecture level city in Shandong
Doraemon Hyakubo Shop	King of Power Saving	It rained a couple days ago and I just went to get the item back today , not bad, hey I looked at the meter and that red display light takes several seconds to jump, did not make a mistake buying it. After a period of time to evaluate.	Jingdong prefecture level city in Shandong
Doraemon Hyakubo Shop	King of Power Saving	Used more than 10 days to check the power, there is indeed some decline, customer service said, long-term use will save more points!	Jingdong prefecture level city in Shandong
Doraemon Hyakubo Shop	King of Power Saving	It's a proper IQ tax, and it costs electricity.	Jingdong prefecture level city in Shandong
Doraemon Hyakubo Shop	King of Power Saving	I've been using it for 17 days now, it really saves electricity Shipping is fast, you can save a lot in a year	Jingdong prefecture level city in Shandong
Doraemon Hyakubo Shop	King of Power Saving	I've been using it for 17 days and it really saves power Shipping is fast and you don't have to worry about the quality, free returns within 5 years	Jingdong prefecture level city in Shandong
Doraemon Hyakubo Shop	King of Power Saving	It's more regular, scanned the code and it's authentic, and the main thing is that you can try it for 90 days! Plugged in more than ten days, is less than before a few degrees per day. When the time to use a month to see Additional. Good to buy a few more	Jingdong prefecture level city in Shandong
Doraemon Hyakubo Shop	King of Power Saving	Logistics is very fast, fine workmanship, use it and then come back to the comments	Jingdong prefecture level city in Shandong
Doraemon Hyakubo Shop	King of Power Saving	The quality of workmanship is very good and the power saving effect is quite good, I regret not getting it earlier.	Jingdong prefecture level city in Shandong
Doraemon Hyakubo Shop	King of Power Saving	Received the goods, according to the instructions on the operation is very simple, to wait and see the effect, looks good!	Jingdong prefecture level city in Shandong
Doraemon Hyakubo Shop	King of Power Saving	It's really as safe and power-saving as the owner says, and it works great!	Jingdong prefecture level city in Shandong
Doraemon Hyakubo Shop	King of Power Saving	Received the goods, immediately used, looks good, to wait and see how the effect.	Jingdong prefecture level city in Shandong
Doraemon Hyakubo Shop	King of Power Saving	Afraid to buy back did not work, the store said the commitment to 90 days invalid refund, warranty 5 years. Saver also complimentary on the Pacific insurance, it is a lot of peace of mind, there are effects will continue to buy back drops!	Jingdong prefecture level city in Shandong
Doraemon Hyakubo Shop	King of Power Saving	Received the goods and used it, can save electricity a month later to see the effect!	Jingdong prefecture level city in Shandong
Doraemon Hyakubo Shop	King of Power Saving	Fancy 90-day trial, 5-year warranty before the order, the goods received, has been experiencing a week, can really feel the savings, in the introduction of friends also buy this!	Jingdong prefecture level city in Shandong
Doraemon Hyakubo Shop	King of Power Saving	Clothing stores use, neighbors recommended to buy, said a month can save more than 70 yuan, first plugged in to see the effect of the	Jingdong prefecture level city in Shandong
Doraemon Hyakubo Shop	King of Power Saving	A friend's home two months to save more than 80 dollars of electricity, recommended that I buy two, decisive purchase, looking forward to the effect of power saving, waiting for the next month effect.	Jingdong prefecture level city in Shandong
Doraemon Hyakubo Shop	King of Power Saving	The goods have been received, consistent with the description of the advertisement, quite good, I hope that in terms of power saving and purifying the air is really as the merchant said that would be better!	Jingdong prefecture level city in Shandong
Doraemon Hyakubo Shop	King of Power Saving	Power Saving King is really good to use the quality is very good than the physical store a lot cheaper we can rest assured that the purchase recommended to buy!	Jingdong prefecture level city in Shandong
Doraemon Hyakubo Shop	King of Power Saving	Energy saver has been used on the rental house inside the use of the meter is always running fast, with this can really save electricity, easy to use, plugged in can be, very satisfied!	Jingdong prefecture level city in Shandong
Doraemon Hyakubo Shop	King of Power Saving	Power Saver Power Saver King is good quality and affordable, very satisfied, worth buying.	Jingdong prefecture level city in Shandong
Doraemon Hyakubo Shop	King of Power Saving	Just use can not see what effect to,, wait for a period of time in the evaluation	Jingdong prefecture level city in Shandong
Doraemon Hyakubo Shop	King of Power Saving	Live in the 18th floor is too far away from the meter, afraid of no effect, look at the trial 90 days before buying. Currently used for a month, there is an effect, saving 27 dollars in electricity, next month should be able to save more!	Jingdong prefecture level city in Shandong
Doraemon Hyakubo Shop	King of Power Saving	I've been using it for almost a month now and it feels good, so I'll use it again.	Jingdong prefecture level city in Shandong
Doraemon Hyakubo Shop	King of Power Saving	It's ok, the electric bill is really less, originally I remember charging $100 and using it for 10 days. Now it lasts about 15 days. It's still noticeable.	Jingdong prefecture level city in Shandong
Doraemon Hyakubo Shop	King of Power Saving	Fast logistics, good power saving effect, very satisfied	Jingdong prefecture level city in Shandong

Doraemon Hyakubo Shop	King of Power Saving	Has been the second time back to buy, energy saving effect, worth recommending, you can try to see, power saving effect: very good Experience: can save about 30%!	Jingdong prefecture level city in Shandong
Doraemon Hyakubo Shop	King of Power Saving	I don't know how well it works for now	Jingdong prefecture level city in Shandong
Doraemon Hyakubo Shop	King of Power Saving	Factory direct is really good, plugged in and used for half a month did less probably almost 50 degrees of electricity! And the manufacturer's warranty is 5 years.	Jingdong prefecture level city in Shandong
Doraemon Hyakubo Shop	King of Power Saving	It's really effective. The previous month's electricity was $286, but after using the power saver for a month, I have $195 left this month, which is not bad.	Jingdong prefecture level city in Shandong
Doraemon Hyakubo Shop	King of Power Saving	I'll give you a good review and see how it goes.	Jingdong prefecture level city in Shandong
Doraemon Hyakubo Shop	King of Power Saving	Customer service is very professional and patient to guide the installation, the quality of workmanship is very fine. Durable, second repurchase. Really can save oh!	Jingdong prefecture level city in Shandong
Doraemon Hyakubo Shop	King of Power Saving	I've been using it for a month now and my electric bill is over $30 less than last month. I feel it's quite worth buying.	Jingdong prefecture level city in Shandong
Doraemon Hyakubo Shop	King of Power Saving	Small size, does not take up space. It's very good, it does work very effectively, the tool room has a lot of work with a lot of electricity. After installing it, it really reduces a lot of electricity bills. Before the day 30 degrees of electricity left stone, now work down really less a few degrees.	Jingdong prefecture level city in Shandong
Doraemon Hyakubo Shop	King of Power Saving	It does work for saving electricity, I've bought the kind from elsewhere before that was too simple not **all**. This time to buy this store, but also for the safety. Air conditioning is too much electricity, this use to save a lot of	Jingdong prefecture level city in Shandong
Doraemon Hyakubo Shop	King of Power Saving	Two air conditioners and a refrigerator at home, a month down the electricity bill is almost two hundred dollars, with this after the average savings of more than thirty dollars to spend less money to enjoy more, it's really very good!	Jingdong prefecture level city in Shandong
Doraemon Hyakubo Shop	King of Power Saving	Good. Very good.	Jingdong prefecture level city in Shandong
Doraemon Hyakubo Shop	King of Power Saving	Works great, easy to operate, great, very happy with it.	Jingdong prefecture level city in Shandong
Doraemon Hyakubo Shop	King of Power Saving	Received it and tried it out, it's great, great quality and worth having! Five stars praise!	Jingdong prefecture level city in Shandong
Doraemon Hyakubo Shop	King of Power Saving	Very trustworthy brand, quality is guaranteed, the price is still affordable, quite satisfied!	Jingdong prefecture level city in Shandong
Doraemon Hyakubo Shop	King of Power Saving	Use it before you speak.	Jingdong prefecture level city in Shandong
Doraemon Hyakubo Shop	King of Power Saving	The quality of workmanship is very good and the power saving effect is quite good, I regret not getting it earlier.	Jingdong prefecture level city in Shandong
Doraemon Hyakubo Shop	King of Power Saving	Merchants shipping speed, and customer service introduced the same very good, cost-effective	Jingdong prefecture level city in Shandong
Doraemon Hyakubo Shop	King of Power Saving	Feel a little effect Oh, where the power consumption where to plug, convenient and convenient	Jingdong prefecture level city in Shandong
Doraemon Hyakubo Shop	King of Power Saving	The quality of the imported saver is very good, practical and satisfactory. Cost-effective	Jingdong prefecture level city in Shandong
Doraemon Hyakubo Shop	King of Power Saving	Power Saver is especially good, good and inexpensive, very good	Jingdong prefecture level city in Shandong
Doraemon Hyakubo Shop	King of Power Saving	Fast shipping, awesome quality, affordable price, satisfied.	Jingdong prefecture level city in Shandong
Doraemon Hyakubo Shop	King of Power Saving	Energy saver received, the effect is consistent with the description, recommended to buy!	Jingdong prefecture level city in Shandong
Doraemon Hyakubo Shop	King of Power Saving	It's really good, the quality is great, I recommend it!	Jingdong prefecture level city in Shandong
Doraemon Hyakubo Shop	King of Power Saving	Works well. Inexpensive. Shipping was fast.	Jingdong prefecture level city in Shandong
Doraemon Hyakubo Shop	King of Power Saving	Logistics is fast, works well, energy saving is good	Jingdong prefecture level city in Shandong
Doraemon Hyakubo Shop	King of Power Saving	very happy	Jingdong prefecture level city in Shandong
Doraemon Hyakubo Shop	King of Power Saving	an excellent	Jingdong prefecture level city in Shandong
Doraemon Hyakubo Shop	King of Power Saving	It rained a couple days ago and I just went to get the item back today , not bad, hey I looked at the meter and that red display light takes several seconds to jump, did not make a mistake buying it. After a period of time to evaluate.	Jingdong prefecture level city in Shandong
Doraemon Hyakubo Shop	King of Power Saving	Used more than 10 days to check the power, there is indeed some decline, customer service said, long-term use will save more points!	Jingdong prefecture level city in Shandong
Doraemon Hyakubo Shop	King of Power Saving	It's a proper IQ tax, and it costs electricity.	Jingdong prefecture level city in Shandong
Doraemon Hyakubo Shop	King of Power Saving	I've been using it for 17 days now, it really saves electricity Shipping is fast, you can save a lot in a year	Jingdong prefecture level city in Shandong
Doraemon Hyakubo Shop	King of Power Saving	I've been using it for 17 days and it really saves power Shipping is fast and you don't have to worry about the quality, free returns within 5 years	Jingdong prefecture level city in Shandong
Doraemon Hyakubo Shop	King of Power Saving	It's more regular, scanned the code and it's authentic, and the main thing is that you can try it for 90 days! Plugged in more than ten days, is less than before a few degrees per day. When the time to use a month to see Additional. Good to buy a few more	Jingdong prefecture level city in Shandong
Doraemon Hyakubo Shop	King of Power Saving	Logistics is very fast, fine workmanship, use it and then come back to the comments	Jingdong prefecture level city in Shandong
Doraemon Hyakubo Shop	King of Power Saving	The quality of workmanship is very good and the power saving effect is quite good, I regret not getting it earlier.	Jingdong prefecture level city in Shandong
Doraemon Hyakubo Shop	King of Power Saving	Received the goods, according to the instructions on the operation is very simple, to wait and see the effect, looks good!	Jingdong prefecture level city in Shandong
Doraemon Hyakubo Shop	King of Power Saving	It's really as safe and power-saving as the owner says, and it works great!	Jingdong prefecture level city in Shandong
Doraemon Hyakubo Shop	King of Power Saving	Received the goods, immediately used, looks good, to wait and see how the effect.	Jingdong prefecture level city in Shandong
Doraemon Hyakubo Shop	King of Power Saving	Afraid to buy back did not work, the store said the commitment to 90 days invalid refund, warranty 5 years. Saver also complimentary on the Pacific insurance, it is a lot of peace of mind, there are effects will continue to buy back drops!	Jingdong prefecture level city in Shandong
Doraemon Hyakubo Shop	King of Power Saving	Received the goods and used it, can save electricity a month later to see the effect!	Jingdong prefecture level city in Shandong
Doraemon Hyakubo Shop	King of Power Saving	Fancy 90-day trial, 5-year warranty before the order, the goods received, has been experiencing a week, can really feel the savings, in the introduction of friends also buy this!	Jingdong prefecture level city in Shandong
Doraemon Hyakubo Shop	King of Power Saving	Clothing stores use, neighbors recommended to buy, said a month can save more than 70 yuan, first plugged in to see the effect of the	Jingdong prefecture level city in Shandong
Doraemon Hyakubo Shop	King of Power Saving	A friend's home two months to save more than 80 dollars of electricity, recommended that I buy two, decisive purchase, looking forward to the effect of power saving, waiting for the next month effect.	Jingdong prefecture level city in Shandong
Doraemon Hyakubo Shop	King of Power Saving	The goods have been received, consistent with the description of the advertisement, quite good, I hope that in terms of power saving and purifying the air is really as the merchant said that would be better!	Jingdong prefecture level city in Shandong
Doraemon Hyakubo Shop	King of Power Saving	Power Saving King is really good to use the quality is very good than the physical store a lot cheaper we can rest assured that the purchase recommended to buy!	Jingdong prefecture level city in Shandong
Doraemon Hyakubo Shop	King of Power Saving	Energy saver has been used on the rental house inside the use of the meter is always running fast, with this can really save electricity, easy to use, plugged in can be, very satisfied!	Jingdong prefecture level city in Shandong
Doraemon Hyakubo Shop	King of Power Saving	Power Saver Power Saver King is good quality and affordable, very satisfied, worth buying.	Jingdong prefecture level city in Shandong
Doraemon Hyakubo Shop	King of Power Saving	Just use can not see what effect to,, wait for a period of time in the evaluation	Jingdong prefecture level city in Shandong

Doraemon Hyakubo Shop	King of Power Saving	Live in the 18th floor is too far away from the meter, afraid of no effect, look at the trial 90 days before buying. Currently used for a month, there is an effect, saving 27 dollars in electricity, next month should be able to save more!	Jingdong prefecture level city in Shandong
Doraemon Hyakubo Shop	King of Power Saving	I've been using it for almost a month now and it feels good, so I'll use it again.	Jingdong prefecture level city in Shandong
Doraemon Hyakubo Shop	King of Power Saving	It's ok, the electric bill is really less, originally I remember charging $100 and using it for 10 days. Now it lasts about 15 days. It's still noticeable.	Jingdong prefecture level city in Shandong
Doraemon Hyakubo Shop	King of Power Saving	Fast logistics, good power saving effect, very satisfied	Jingdong prefecture level city in Shandong
Doraemon Hyakubo Shop	King of Power Saving	Has been the second time back to buy, energy saving effect, worth recommending, you can try to see, power saving effect: very good Experience: can save about 30%!	Jingdong prefecture level city in Shandong
Doraemon Hyakubo Shop	King of Power Saving	I don't know how well it works for now	Jingdong prefecture level city in Shandong
Doraemon Hyakubo Shop	King of Power Saving	Factory direct is really good, plugged in and used for half a month did less probably almost 50 degrees of electricity! And the manufacturer's warranty is 5 years.	Jingdong prefecture level city in Shandong
Doraemon Hyakubo Shop	King of Power Saving	It's really effective. The previous month's electricity was $286, but after using the power saver for a month, I have $195 left this month, which is not bad.	Jingdong prefecture level city in Shandong
Doraemon Hyakubo Shop	King of Power Saving	I'll give you a good review and see how it goes.	Jingdong prefecture level city in Shandong
Doraemon Hyakubo Shop	King of Power Saving	Customer service is very professional and patient to guide the installation, the quality of workmanship is very fine. Durable, second repurchase. Really can save oh!	Jingdong prefecture level city in Shandong
Doraemon Hyakubo Shop	King of Power Saving	I've been using it for a month now and my electric bill is over $30 less than last month. I feel it's quite worth buying.	Jingdong prefecture level city in Shandong
Doraemon Hyakubo Shop	King of Power Saving	Small size, does not take up space. It's very good, it does work very effectively, the tool room has a lot of work with a lot of electricity. After installing it, it really reduces a lot of electricity bills. Before the day 30 degrees of electricity left stone, now work down really less a few degrees.	Jingdong prefecture level city in Shandong
Doraemon Hyakubo Shop	King of Power Saving	It does work for saving electricity, I've bought the kind from elsewhere before that was too simple not **all**. This time to buy this store, but also for the safety. Air conditioning is too much electricity, this use to save a lot of	Jingdong prefecture level city in Shandong
Doraemon Hyakubo Shop	King of Power Saving	Two air conditioners and a refrigerator at home, a month down the electricity bill is almost two hundred dollars, with this after the average savings of more than thirty dollars to spend less money to enjoy more, it's really very good!	Jingdong prefecture level city in Shandong
Doraemon Hyakubo Shop	King of Power Saving	Good. Very good.	Jingdong prefecture level city in Shandong
Doraemon Hyakubo Shop	King of Power Saving	Works great, easy to operate, great, very happy with it.	Jingdong prefecture level city in Shandong
Doraemon Hyakubo Shop	King of Power Saving	Received it and tried it out, it's great, great quality and worth having! Five stars praise!	Jingdong prefecture level city in Shandong
Doraemon Hyakubo Shop	King of Power Saving	Very trustworthy brand, quality is guaranteed, the price is still affordable, quite satisfied!	Jingdong prefecture level city in Shandong
Doraemon Hyakubo Shop	King of Power Saving	Use it before you speak.	Jingdong prefecture level city in Shandong
Doraemon Hyakubo Shop	King of Power Saving	The quality of workmanship is very good and the power saving effect is quite good, I regret not getting it earlier.	Jingdong prefecture level city in Shandong
Doraemon Hyakubo Shop	King of Power Saving	Merchants shipping speed, and customer service introduced the same very good, cost-effective	Jingdong prefecture level city in Shandong
Doraemon Hyakubo Shop	King of Power Saving	Feel a little effect Oh, where the power consumption where to plug, convenient and convenient	Jingdong prefecture level city in Shandong
Doraemon Hyakubo Shop	King of Power Saving	The quality of the imported saver is very good, practical and satisfactory. Cost-effective	Jingdong prefecture level city in Shandong
Doraemon Hyakubo Shop	King of Power Saving	Power Saver is especially good, good and inexpensive, very good	Jingdong prefecture level city in Shandong
Doraemon Hyakubo Shop	King of Power Saving	Fast shipping, awesome quality, affordable price, satisfied.	Jingdong prefecture level city in Shandong
Doraemon Hyakubo Shop	King of Power Saving	Energy saver received, the effect is consistent with the description, recommended to buy!	Jingdong prefecture level city in Shandong
Doraemon Hyakubo Shop	King of Power Saving	It's really good, the quality is great, I recommend it!	Jingdong prefecture level city in Shandong
Doraemon Hyakubo Shop	King of Power Saving	Works well. Inexpensive. Shipping was fast.	Jingdong prefecture level city in Shandong
Doraemon Hyakubo Shop	King of Power Saving	Logistics is fast, works well, energy saving is good	Jingdong prefecture level city in Shandong
Doraemon Hyakubo Shop	King of Power Saving	very happy	Jingdong prefecture level city in Shandong
Doraemon Hyakubo Shop	King of Power Saving	an excellent	Jingdong prefecture level city in Shandong
Doraemon Hyakubo Shop	King of Power Saving	It rained a couple days ago and I just went to get the item back today , not bad, hey I looked at the meter and that red display light takes several seconds to jump, did not make a mistake buying it. After a period of time to evaluate.	Jingdong prefecture level city in Shandong
Doraemon Hyakubo Shop	King of Power Saving	Used more than 10 days to check the power, there is indeed some decline, customer service said, long-term use will save more points!	Jingdong prefecture level city in Shandong
Doraemon Hyakubo Shop	King of Power Saving	It's a proper IQ tax, and it costs electricity.	Jingdong prefecture level city in Shandong
Doraemon Hyakubo Shop	King of Power Saving	I've been using it for 17 days now, it really saves electricity Shipping is fast, you can save a lot in a year	Jingdong prefecture level city in Shandong
Doraemon Hyakubo Shop	King of Power Saving	I've been using it for 17 days and it really saves power Shipping is fast and you don't have to worry about the quality, free returns within 5 years	Jingdong prefecture level city in Shandong
Doraemon Hyakubo Shop	King of Power Saving	It's more regular, scanned the code and it's authentic, and the main thing is that you can try it for 90 days! Plugged in more than ten days, is less than before a few degrees per day. When the time to use a month to see Additional. Good to buy a few more	Jingdong prefecture level city in Shandong
Doraemon Hyakubo Shop	King of Power Saving	Logistics is very fast, fine workmanship, use it and then come back to the comments	Jingdong prefecture level city in Shandong
Doraemon Hyakubo Shop	King of Power Saving	The quality of workmanship is very good and the power saving effect is quite good, I regret not getting it earlier.	Jingdong prefecture level city in Shandong
Doraemon Hyakubo Shop	King of Power Saving	Received the goods, according to the instructions on the operation is very simple, to wait and see the effect, looks good!	Jingdong prefecture level city in Shandong
Doraemon Hyakubo Shop	King of Power Saving	It's really as safe and power-saving as the owner says, and it works great!	Jingdong prefecture level city in Shandong
Doraemon Hyakubo Shop	King of Power Saving	Received the goods, immediately used, looks good, to wait and see how the effect.	Jingdong prefecture level city in Shandong
Doraemon Hyakubo Shop	King of Power Saving	Afraid to buy back did not work, the store said the commitment to 90 days invalid refund, warranty 5 years. Saver also complimentary on the Pacific insurance, it is a lot of peace of mind, there are effects will continue to buy back drops!	Jingdong prefecture level city in Shandong
Doraemon Hyakubo Shop	King of Power Saving	Received the goods and used it, can save electricity a month later to see the effect!	Jingdong prefecture level city in Shandong
Doraemon Hyakubo Shop	King of Power Saving	Fancy 90-day trial, 5-year warranty before the order, the goods received, has been experiencing a week, can really feel the savings, in the introduction of friends also buy this!	Jingdong prefecture level city in Shandong
Doraemon Hyakubo Shop	King of Power Saving	Clothing stores use, neighbors recommended to buy, said a month can save more than 70 yuan, first plugged in to see the effect of the	Jingdong prefecture level city in Shandong
Doraemon Hyakubo Shop	King of Power Saving	A friend's home two months to save more than 80 dollars of electricity, recommended that I buy two, decisive purchase, looking forward to the effect of power saving, waiting for the next month effect.	Jingdong prefecture level city in Shandong
Doraemon Hyakubo Shop	King of Power Saving	The goods have been received, consistent with the description of the advertisement, quite good, I hope that in terms of power saving and purifying the air is really as the merchant said that would be better!	Jingdong prefecture level city in Shandong

Doraemon Hyakubo Shop	King of Power Saving	Power Saving King is really good to use the quality is very good than the physical store a lot cheaper we can rest assured that the purchase recommended to buy!	Jingdong prefecture level city in Shandong
Doraemon Hyakubo Shop	King of Power Saving	Energy saver has been used on the rental house inside the use of the meter is always running fast, with this can really save electricity, easy to use, plugged in can be, very satisfied!	Jingdong prefecture level city in Shandong
Doraemon Hyakubo Shop	King of Power Saving	Power Saver Power Saver King is good quality and affordable, very satisfied, worth buying.	Jingdong prefecture level city in Shandong
Doraemon Hyakubo Shop	King of Power Saving	Just use can not see what effect to,, wait for a period of time in the evaluation	Jingdong prefecture level city in Shandong
Doraemon Hyakubo Shop	King of Power Saving	Live in the 18th floor is too far away from the meter, afraid of no effect, look at the trial 90 days before buying. Currently used for a month, there is an effect, saving 27 dollars in electricity, next month should be able to save more!	Jingdong prefecture level city in Shandong
Doraemon Hyakubo Shop	King of Power Saving	I've been using it for almost a month now and it feels good, so I'll use it again.	Jingdong prefecture level city in Shandong
Doraemon Hyakubo Shop	King of Power Saving	It's ok, the electric bill is really less, originally I remember charging $100 and using it for 10 days. Now it lasts about 15 days. It's still noticeable.	Jingdong prefecture level city in Shandong
Doraemon Hyakubo Shop	King of Power Saving	Fast logistics, good power saving effect, very satisfied	Jingdong prefecture level city in Shandong
Doraemon Hyakubo Shop	King of Power Saving	Has been the second time back to buy, energy saving effect, worth recommending, you can try to see, power saving effect: very good Experience: can save about 30%!	Jingdong prefecture level city in Shandong
Doraemon Hyakubo Shop	King of Power Saving	I don't know how well it works for now	Jingdong prefecture level city in Shandong
Doraemon Hyakubo Shop	King of Power Saving	Factory direct is really good, plugged in and used for half a month did less probably almost 50 degrees of electricity! And the manufacturer's warranty is 5 years.	Jingdong prefecture level city in Shandong
Doraemon Hyakubo Shop	King of Power Saving	It's really effective. The previous month's electricity was $286, but after using the power saver for a month, I have $195 left this month, which is not bad.	Jingdong prefecture level city in Shandong
Doraemon Hyakubo Shop	King of Power Saving	I'll give you a good review and see how it goes.	Jingdong prefecture level city in Shandong
Doraemon Hyakubo Shop	King of Power Saving	Customer service is very professional and patient to guide the installation, the quality of workmanship is very fine. Durable, second repurchase. Really can save oh!	Jingdong prefecture level city in Shandong
Doraemon Hyakubo Shop	King of Power Saving	I've been using it for a month now and my electric bill is over $30 less than last month. I feel it's quite worth buying.	Jingdong prefecture level city in Shandong
Doraemon Hyakubo Shop	King of Power Saving	Small size, does not take up space. It's very good, it does work very effectively, the tool room has a lot of work with a lot of electricity. After installing it, it really reduces a lot of electricity bills. Before the day 30 degrees of electricity left stone, now work down really less a few degrees.	Jingdong prefecture level city in Shandong
Doraemon Hyakubo Shop	King of Power Saving	It does work for saving electricity, I've bought the kind from elsewhere before that was too simple not **all**. This time to buy this store, but also for the safety. Air conditioning is too much electricity, this use to save a lot of	Jingdong prefecture level city in Shandong
Doraemon Hyakubo Shop	King of Power Saving	Two air conditioners and a refrigerator at home, a month down the electricity bill is almost two hundred dollars, with this after the average savings of more than thirty dollars to spend less money to enjoy more, it's really very good!	Jingdong prefecture level city in Shandong
Doraemon Hyakubo Shop	King of Power Saving	Good. Very good.	Jingdong prefecture level city in Shandong
Doraemon Hyakubo Shop	King of Power Saving	Works great, easy to operate, great, very happy with it.	Jingdong prefecture level city in Shandong
Doraemon Hyakubo Shop	King of Power Saving	Received it and tried it out, it's great, great quality and worth having! Five stars praise!	Jingdong prefecture level city in Shandong
Doraemon Hyakubo Shop	King of Power Saving	Very trustworthy brand, quality is guaranteed, the price is still affordable, quite satisfied!	Jingdong prefecture level city in Shandong
Doraemon Hyakubo Shop	King of Power Saving	Use it before you speak.	Jingdong prefecture level city in Shandong
Doraemon Hyakubo Shop	King of Power Saving	The quality of workmanship is very good and the power saving effect is quite good, I regret not getting it earlier.	Jingdong prefecture level city in Shandong
Doraemon Hyakubo Shop	King of Power Saving	Merchants shipping speed, and customer service introduced the same very good, cost-effective	Jingdong prefecture level city in Shandong
Doraemon Hyakubo Shop	King of Power Saving	Feel a little effect Oh, where the power consumption where to plug, convenient and convenient	Jingdong prefecture level city in Shandong
Doraemon Hyakubo Shop	King of Power Saving	The quality of the imported saver is very good, practical and satisfactory. Cost-effective	Jingdong prefecture level city in Shandong
Doraemon Hyakubo Shop	King of Power Saving	Power Saver is especially good, good and inexpensive, very good	Jingdong prefecture level city in Shandong
Doraemon Hyakubo Shop	King of Power Saving	Fast shipping, awesome quality, affordable price, satisfied.	Jingdong prefecture level city in Shandong
Doraemon Hyakubo Shop	King of Power Saving	Energy saver received, the effect is consistent with the description, recommended to buy!	Jingdong prefecture level city in Shandong
Doraemon Hyakubo Shop	King of Power Saving	It's really good, the quality is great, I recommend it!	Jingdong prefecture level city in Shandong
Doraemon Hyakubo Shop	King of Power Saving	Works well. Inexpensive. Shipping was fast.	Jingdong prefecture level city in Shandong
Doraemon Hyakubo Shop	King of Power Saving	Logistics is fast, works well, energy saving is good	Jingdong prefecture level city in Shandong
Doraemon Hyakubo Shop	King of Power Saving	very happy	Jingdong prefecture level city in Shandong
Doraemon Hyakubo Shop	King of Power Saving	an excellent	Jingdong prefecture level city in Shandong
Doraemon Hyakubo Shop	King of Power Saving	It rained a couple days ago and I just went to get the item back today , not bad, hey I looked at the meter and that red display light takes several seconds to jump, did not make a mistake buying it. After a period of time to evaluate.	Jingdong prefecture level city in Shandong
Doraemon Hyakubo Shop	King of Power Saving	Used more than 10 days to check the power, there is indeed some decline, customer service said, long-term use will save more points!	Jingdong prefecture level city in Shandong
Doraemon Hyakubo Shop	King of Power Saving	It's a proper IQ tax, and it costs electricity.	Jingdong prefecture level city in Shandong
Doraemon Hyakubo Shop	King of Power Saving	I've been using it for 17 days now, it really saves electricity Shipping is fast, you can save a lot in a year	Jingdong prefecture level city in Shandong
Doraemon Hyakubo Shop	King of Power Saving	I've been using it for 17 days and it really saves power Shipping is fast and you don't have to worry about the quality, free returns within 5 years	Jingdong prefecture level city in Shandong
Doraemon Hyakubo Shop	King of Power Saving	It's more regular, scanned the code and it's authentic, and the main thing is that you can try it for 90 days! Plugged in more than ten days, is less than before a few degrees per day. When the time to use a month to see Additional. Good to buy a few more	Jingdong prefecture level city in Shandong
Doraemon Hyakubo Shop	King of Power Saving	Logistics is very fast, fine workmanship, use it and then come back to the comments	Jingdong prefecture level city in Shandong
Doraemon Hyakubo Shop	King of Power Saving	The quality of workmanship is very good and the power saving effect is quite good, I regret not getting it earlier.	Jingdong prefecture level city in Shandong
Doraemon Hyakubo Shop	King of Power Saving	Received the goods, according to the instructions on the operation is very simple, to wait and see the effect, looks good!	Jingdong prefecture level city in Shandong
Doraemon Hyakubo Shop	King of Power Saving	It's really as safe and power-saving as the owner says, and it works great!	Jingdong prefecture level city in Shandong
Doraemon Hyakubo Shop	King of Power Saving	Received the goods, immediately used, looks good, to wait and see how the effect.	Jingdong prefecture level city in Shandong
Doraemon Hyakubo Shop	King of Power Saving	Afraid to buy back did not work, the store said the commitment to 90 days invalid refund, warranty 5 years. Saver also complimentary on the Pacific insurance, it is a lot of peace of mind, there are effects will continue to buy back drops!	Jingdong prefecture level city in Shandong
Doraemon Hyakubo Shop	King of Power Saving	Received the goods and used it, can save electricity a month later to see the effect!	Jingdong prefecture level city in Shandong

Doraemon Hyakubo Shop	King of Power Saving	Fancy 90-day trial, 5-year warranty before the order, the goods received, has been experiencing a week, can really feel the savings, in the introduction of friends also buy this!	Jingdong prefecture level city in Shandong
Doraemon Hyakubo Shop	King of Power Saving	Clothing stores use, neighbors recommended to buy, said a month can save more than 70 yuan, first plugged in to see the effect of the	Jingdong prefecture level city in Shandong
Doraemon Hyakubo Shop	King of Power Saving	A friend's home two months to save more than 80 dollars of electricity, recommended that I buy two, decisive purchase, looking forward to the effect of power saving, waiting for the next month effect.	Jingdong prefecture level city in Shandong
Doraemon Hyakubo Shop	King of Power Saving	The goods have been received, consistent with the description of the advertisement, quite good, I hope that in terms of power saving and purifying the air is really as the merchant said that would be better!	Jingdong prefecture level city in Shandong
Doraemon Hyakubo Shop	King of Power Saving	Power Saving King is really good to use the quality is very good than the physical store a lot cheaper we can rest assured that the purchase recommended to buy!	Jingdong prefecture level city in Shandong
Doraemon Hyakubo Shop	King of Power Saving	Energy saver has been used on the rental house inside the use of the meter is always running fast, with this can really save electricity, easy to use, plugged in can be, very satisfied!	Jingdong prefecture level city in Shandong
Doraemon Hyakubo Shop	King of Power Saving	Power Saver Power Saver King is good quality and affordable, very satisfied, worth buying.	Jingdong prefecture level city in Shandong
Doraemon Hyakubo Shop	King of Power Saving	Just use can not see what effect to,, wait for a period of time in the evaluation	Jingdong prefecture level city in Shandong
Doraemon Hyakubo Shop	King of Power Saving	Live in the 18th floor is too far away from the meter, afraid of no effect, look at the trial 90 days before buying. Currently used for a month, there is an effect, saving 27 dollars in electricity, next month should be able to save more!	Jingdong prefecture level city in Shandong
Doraemon Hyakubo Shop	King of Power Saving	I've been using it for almost a month now and it feels good, so I'll use it again.	Jingdong prefecture level city in Shandong
Doraemon Hyakubo Shop	King of Power Saving	It's ok, the electric bill is really less, originally I remember charging $100 and using it for 10 days. Now it lasts about 15 days. It's still noticeable.	Jingdong prefecture level city in Shandong
Doraemon Hyakubo Shop	King of Power Saving	Fast logistics, good power saving effect, very satisfied	Jingdong prefecture level city in Shandong
Doraemon Hyakubo Shop	King of Power Saving	Has been the second time back to buy, energy saving effect, worth recommending, you can try to see, power saving effect: very good Experience: can save about 30%!	Jingdong prefecture level city in Shandong
Doraemon Hyakubo Shop	King of Power Saving	I don't know how well it works for now	Jingdong prefecture level city in Shandong
Doraemon Hyakubo Shop	King of Power Saving	Factory direct is really good, plugged in and used for half a month did less probably almost 50 degrees of electricity! And the manufacturer's warranty is 5 years.	Jingdong prefecture level city in Shandong
Doraemon Hyakubo Shop	King of Power Saving	It's really effective. The previous month's electricity was $286, but after using the power saver for a month, I have $195 left this month, which is not bad.	Jingdong prefecture level city in Shandong
Doraemon Hyakubo Shop	King of Power Saving	I'll give you a good review and see how it goes.	Jingdong prefecture level city in Shandong
Doraemon Hyakubo Shop	King of Power Saving	Customer service is very professional and patient to guide the installation, the quality of workmanship is very fine. Durable, second repurchase. Really can save oh!	Jingdong prefecture level city in Shandong
Doraemon Hyakubo Shop	King of Power Saving	I've been using it for a month now and my electric bill is over $30 less than last month. I feel it's quite worth buying.	Jingdong prefecture level city in Shandong
Doraemon Hyakubo Shop	King of Power Saving	Small size, does not take up space. It's very good, it does work very effectively, the tool room has a lot of work with a lot of electricity. After installing it, it really reduces a lot of electricity bills. Before the day 30 degrees of electricity left stone, now work down really less a few degrees.	Jingdong prefecture level city in Shandong
Doraemon Hyakubo Shop	King of Power Saving	It does work for saving electricity, I've bought the kind from elsewhere before that was too simple not **all**. This time to buy this store, but also for the safety. Air conditioning is too much electricity, this use to save a lot of	Jingdong prefecture level city in Shandong
Doraemon Hyakubo Shop	King of Power Saving	Two air conditioners and a refrigerator at home, a month down the electricity bill is almost two hundred dollars, with this after the average savings of more than thirty dollars to spend less money to enjoy more, it's really very good!	Jingdong prefecture level city in Shandong
Doraemon Hyakubo Shop	King of Power Saving	Good. Very good.	Jingdong prefecture level city in Shandong
Doraemon Hyakubo Shop	King of Power Saving	Works great, easy to operate, great, very happy with it.	Jingdong prefecture level city in Shandong
Doraemon Hyakubo Shop	King of Power Saving	Received it and tried it out, it's great, great quality and worth having! Five stars praise!	Jingdong prefecture level city in Shandong
Doraemon Hyakubo Shop	King of Power Saving	Very trustworthy brand, quality is guaranteed, the price is still affordable, quite satisfied!	Jingdong prefecture level city in Shandong
Doraemon Hyakubo Shop	King of Power Saving	Use it before you speak.	Jingdong prefecture level city in Shandong
Doraemon Hyakubo Shop	King of Power Saving	The quality of workmanship is very good and the power saving effect is quite good, I regret not getting it earlier.	Jingdong prefecture level city in Shandong
Doraemon Hyakubo Shop	King of Power Saving	Merchants shipping speed, and customer service introduced the same very good, cost-effective	Jingdong prefecture level city in Shandong
Doraemon Hyakubo Shop	King of Power Saving	Feel a little effect Oh, where the power consumption where to plug, convenient and convenient	Jingdong prefecture level city in Shandong
Doraemon Hyakubo Shop	King of Power Saving	The quality of the imported saver is very good, practical and satisfactory. Cost-effective	Jingdong prefecture level city in Shandong
Doraemon Hyakubo Shop	King of Power Saving	Power Saver is especially good, good and inexpensive, very good	Jingdong prefecture level city in Shandong
Doraemon Hyakubo Shop	King of Power Saving	Fast shipping, awesome quality, affordable price, satisfied.	Jingdong prefecture level city in Shandong
Doraemon Hyakubo Shop	King of Power Saving	Energy saver received, the effect is consistent with the description, recommended to buy!	Jingdong prefecture level city in Shandong
Doraemon Hyakubo Shop	King of Power Saving	It's really good, the quality is great, I recommend it!	Jingdong prefecture level city in Shandong
Doraemon Hyakubo Shop	King of Power Saving	Works well. Inexpensive. Shipping was fast.	Jingdong prefecture level city in Shandong
Doraemon Hyakubo Shop	King of Power Saving	Logistics is fast, works well, energy saving is good	Jingdong prefecture level city in Shandong
Doraemon Hyakubo Shop	King of Power Saving	very happy	Jingdong prefecture level city in Shandong
Doraemon Hyakubo Shop	King of Power Saving	an excellent	Jingdong prefecture level city in Shandong
Doraemon Hyakubo Shop	King of Power Saving	It rained a couple days ago and I just went to get the item back today , not bad, hey I looked at the meter and that red display light takes several seconds to jump, did not make a mistake buying it. After a period of time to evaluate.	Jingdong prefecture level city in Shandong
Doraemon Hyakubo Shop	King of Power Saving	Used more than 10 days to check the power, there is indeed some decline, customer service said, long-term use will save more points!	Jingdong prefecture level city in Shandong
Doraemon Hyakubo Shop	King of Power Saving	It's a proper IQ tax, and it costs electricity.	Jingdong prefecture level city in Shandong
Doraemon Hyakubo Shop	King of Power Saving	I've been using it for 17 days now, it really saves electricity Shipping is fast, you can save a lot in a year	Jingdong prefecture level city in Shandong
Doraemon Hyakubo Shop	King of Power Saving	I've been using it for 17 days and it really saves power Shipping is fast and you don't have to worry about the quality, free returns within 5 years	Jingdong prefecture level city in Shandong
Doraemon Hyakubo Shop	King of Power Saving	It's more regular, scanned the code and it's authentic, and the main thing is that you can try it for 90 days! Plugged in more than ten days, is less than before a few degrees per day. When the time to use a month to see Additional. Good to buy a few more	Jingdong prefecture level city in Shandong
Doraemon Hyakubo Shop	King of Power Saving	Logistics is very fast, fine workmanship, use it and then come back to the comments	Jingdong prefecture level city in Shandong
Doraemon Hyakubo Shop	King of Power Saving	The quality of workmanship is very good and the power saving effect is quite good, I regret not getting it earlier.	Jingdong prefecture level city in Shandong
Doraemon Hyakubo Shop	King of Power Saving	Received the goods, according to the instructions on the operation is very simple, to wait and see the effect, looks good!	Jingdong prefecture level city in Shandong

Doraemon Hyakubo Shop	King of Power Saving	It's really as safe and power-saving as the owner says, and it works great!	Jingdong prefecture level city in Shandong
Doraemon Hyakubo Shop	King of Power Saving	Received the goods, immediately used, looks good, to wait and see how the effect.	Jingdong prefecture level city in Shandong
Doraemon Hyakubo Shop	King of Power Saving	Afraid to buy back did not work, the store said the commitment to 90 days invalid refund, warranty 5 years. Saver also complimentary on the Pacific insurance, it is a lot of peace of mind, there are effects will continue to buy back drops!	Jingdong prefecture level city in Shandong
Doraemon Hyakubo Shop	King of Power Saving	Received the goods and used it, can save electricity a month later to see the effect!	Jingdong prefecture level city in Shandong
Doraemon Hyakubo Shop	King of Power Saving	Fancy 90-day trial, 5-year warranty before the order, the goods received, has been experiencing a week, can really feel the savings, in the introduction of friends also buy this!	Jingdong prefecture level city in Shandong
Doraemon Hyakubo Shop	King of Power Saving	Clothing stores use, neighbors recommended to buy, said a month can save more than 70 yuan, first plugged in to see the effect of the	Jingdong prefecture level city in Shandong
Doraemon Hyakubo Shop	King of Power Saving	A friend's home two months to save more than 80 dollars of electricity, recommended that I buy two, decisive purchase, looking forward to the effect of power saving, waiting for the next month effect.	Jingdong prefecture level city in Shandong
Doraemon Hyakubo Shop	King of Power Saving	The goods have been received, consistent with the description of the advertisement, quite good, I hope that in terms of power saving and purifying the air is really as the merchant said that would be better!	Jingdong prefecture level city in Shandong
Doraemon Hyakubo Shop	King of Power Saving	Power Saving King is really good to use the quality is very good than the physical store a lot cheaper we can rest assured that the purchase recommended to buy!	Jingdong prefecture level city in Shandong
Doraemon Hyakubo Shop	King of Power Saving	Energy saver has been used on the rental house inside the use of the meter is always running fast, with this can really save electricity, easy to use, plugged in can be, very satisfied!	Jingdong prefecture level city in Shandong
Doraemon Hyakubo Shop	King of Power Saving	Power Saver Power Saver King is good quality and affordable, very satisfied, worth buying.	Jingdong prefecture level city in Shandong
Doraemon Hyakubo Shop	King of Power Saving	Just use can not see what effect to,, wait for a period of time in the evaluation	Jingdong prefecture level city in Shandong
Doraemon Hyakubo Shop	King of Power Saving	Live in the 18th floor is too far away from the meter, afraid of no effect, look at the trial 90 days before buying. Currently used for a month, there is an effect, saving 27 dollars in electricity, next month should be able to save more!	Jingdong prefecture level city in Shandong
Doraemon Hyakubo Shop	King of Power Saving	I've been using it for almost a month now and it feels good, so I'll use it again.	Jingdong prefecture level city in Shandong
Doraemon Hyakubo Shop	King of Power Saving	It's ok, the electric bill is really less, originally I remember charging $100 and using it for 10 days. Now it lasts about 15 days. It's still noticeable.	Jingdong prefecture level city in Shandong
Doraemon Hyakubo Shop	King of Power Saving	Fast logistics, good power saving effect, very satisfied	Jingdong prefecture level city in Shandong
Doraemon Hyakubo Shop	King of Power Saving	Has been the second time back to buy, energy saving effect, worth recommending, you can try to see, power saving effect: very good Experience: can save about 30%!	Jingdong prefecture level city in Shandong
Doraemon Hyakubo Shop	King of Power Saving	I don't know how well it works for now	Jingdong prefecture level city in Shandong
Doraemon Hyakubo Shop	King of Power Saving	Factory direct is really good, plugged in and used for half a month did less probably almost 50 degrees of electricity! And the manufacturer's warranty is 5 years.	Jingdong prefecture level city in Shandong
Doraemon Hyakubo Shop	King of Power Saving	It's really effective. The previous month's electricity was $286, but after using the power saver for a month, I have $195 left this month, which is not bad.	Jingdong prefecture level city in Shandong
Doraemon Hyakubo Shop	King of Power Saving	I'll give you a good review and see how it goes.	Jingdong prefecture level city in Shandong
Doraemon Hyakubo Shop	King of Power Saving	Customer service is very professional and patient to guide the installation, the quality of workmanship is very fine. Durable, second repurchase. Really can save oh!	Jingdong prefecture level city in Shandong
Doraemon Hyakubo Shop	King of Power Saving	I've been using it for a month now and my electric bill is over $30 less than last month. I feel it's quite worth buying.	Jingdong prefecture level city in Shandong
Doraemon Hyakubo Shop	King of Power Saving	Small size, does not take up space. It's very good, it does work very effectively, the tool room has a lot of work with a lot of electricity. After installing it, it really reduces a lot of electricity bills. Before the day 30 degrees of electricity left stone, now work down really less a few degrees.	Jingdong prefecture level city in Shandong
Doraemon Hyakubo Shop	King of Power Saving	It does work for saving electricity, I've bought the kind from elsewhere before that was too simple not **all**. This time to buy this store, but also for the safety. Air conditioning is too much electricity, this use to save a lot of	Jingdong prefecture level city in Shandong
Doraemon Hyakubo Shop	King of Power Saving	Two air conditioners and a refrigerator at home, a month down the electricity bill is almost two hundred dollars, with this after the average savings of more than thirty dollars to spend less money to enjoy more, it's really very good!	Jingdong prefecture level city in Shandong
Doraemon Hyakubo Shop	King of Power Saving	Good. Very good.	Jingdong prefecture level city in Shandong
Doraemon Hyakubo Shop	King of Power Saving	Works great, easy to operate, great, very happy with it.	Jingdong prefecture level city in Shandong
Doraemon Hyakubo Shop	King of Power Saving	Received it and tried it out, it's great, great quality and worth having! Five stars praise!	Jingdong prefecture level city in Shandong
Doraemon Hyakubo Shop	King of Power Saving	Very trustworthy brand, quality is guaranteed, the price is still affordable, quite satisfied!	Jingdong prefecture level city in Shandong
Doraemon Hyakubo Shop	King of Power Saving	Use it before you speak.	Jingdong prefecture level city in Shandong
Doraemon Hyakubo Shop	King of Power Saving	The quality of workmanship is very good and the power saving effect is quite good, I regret not getting it earlier.	Jingdong prefecture level city in Shandong
Doraemon Hyakubo Shop	King of Power Saving	Merchants shipping speed, and customer service introduced the same very good, cost-effective	Jingdong prefecture level city in Shandong
Doraemon Hyakubo Shop	King of Power Saving	Feel a little effect Oh, where the power consumption where to plug, convenient and convenient	Jingdong prefecture level city in Shandong
Doraemon Hyakubo Shop	King of Power Saving	The quality of the imported saver is very good, practical and satisfactory. Cost-effective	Jingdong prefecture level city in Shandong
Doraemon Hyakubo Shop	King of Power Saving	Power Saver is especially good, good and inexpensive, very good	Jingdong prefecture level city in Shandong
Doraemon Hyakubo Shop	King of Power Saving	Fast shipping, awesome quality, affordable price, satisfied.	Jingdong prefecture level city in Shandong
Doraemon Hyakubo Shop	King of Power Saving	Energy saver received, the effect is consistent with the description, recommended to buy!	Jingdong prefecture level city in Shandong
Doraemon Hyakubo Shop	King of Power Saving	It's really good, the quality is great, I recommend it!	Jingdong prefecture level city in Shandong
Doraemon Hyakubo Shop	King of Power Saving	Works well. Inexpensive. Shipping was fast.	Jingdong prefecture level city in Shandong
Doraemon Hyakubo Shop	King of Power Saving	Logistics is fast, works well, energy saving is good	Jingdong prefecture level city in Shandong
Doraemon Hyakubo Shop	King of Power Saving	very happy	Jingdong prefecture level city in Shandong
Doraemon Hyakubo Shop	King of Power Saving	an excellent	Jingdong prefecture level city in Shandong
Doraemon Hyakubo Shop	King of Power Saving	It rained a couple days ago and I just went to get the item back today , not bad, hey I looked at the meter and that red display light takes several seconds to jump, did not make a mistake buying it. After a period of time to evaluate.	Jingdong prefecture level city in Shandong
Doraemon Hyakubo Shop	King of Power Saving	Used more than 10 days to check the power, there is indeed some decline, customer service said, long-term use will save more points!	Jingdong prefecture level city in Shandong
Doraemon Hyakubo Shop	King of Power Saving	It's a proper IQ tax, and it costs electricity.	Jingdong prefecture level city in Shandong
Doraemon Hyakubo Shop	King of Power Saving	I've been using it for 17 days now, it really saves electricity Shipping is fast, you can save a lot in a year	Jingdong prefecture level city in Shandong
Doraemon Hyakubo Shop	King of Power Saving	I've been using it for 17 days and it really saves power Shipping is fast and you don't have to worry about the quality, free returns within 5 years	Jingdong prefecture level city in Shandong

Doraemon Hyakubo Shop	King of Power Saving	It's more regular, scanned the code and it's authentic, and the main thing is that you can try it for 90 days! Plugged in more than ten days, is less than before a few degrees per day. When the time to use a month to see Additional. Good to buy a few more	Jingdong prefecture level city in Shandong
Doraemon Hyakubo Shop	King of Power Saving	Logistics is very fast, fine workmanship, use it and then come back to the comments	Jingdong prefecture level city in Shandong
Doraemon Hyakubo Shop	King of Power Saving	The quality of workmanship is very good and the power saving effect is quite good, I regret not getting it earlier.	Jingdong prefecture level city in Shandong
Doraemon Hyakubo Shop	King of Power Saving	Received the goods, according to the instructions on the operation is very simple, to wait and see the effect, looks good!	Jingdong prefecture level city in Shandong
Doraemon Hyakubo Shop	King of Power Saving	It's really as safe and power-saving as the owner says, and it works great!	Jingdong prefecture level city in Shandong
Doraemon Hyakubo Shop	King of Power Saving	Received the goods, immediately used, looks good, to wait and see how the effect.	Jingdong prefecture level city in Shandong
Doraemon Hyakubo Shop	King of Power Saving	Afraid to buy back did not work, the store said the commitment to 90 days invalid refund, warranty 5 years. Saver also complimentary on the Pacific insurance, it is a lot of peace of mind, there are effects will continue to buy back drops!	Jingdong prefecture level city in Shandong
Doraemon Hyakubo Shop	King of Power Saving	Received the goods and used it, can save electricity a month later to see the effect!	Jingdong prefecture level city in Shandong
Doraemon Hyakubo Shop	King of Power Saving	Fancy 90-day trial, 5-year warranty before the order, the goods received, has been experiencing a week, can really feel the savings, in the introduction of friends also buy this!	Jingdong prefecture level city in Shandong
Doraemon Hyakubo Shop	King of Power Saving	Clothing stores use, neighbors recommended to buy, said a month can save more than 70 yuan, first plugged in to see the effect of the	Jingdong prefecture level city in Shandong
Doraemon Hyakubo Shop	King of Power Saving	A friend's home two months to save more than 80 dollars of electricity, recommended that I buy two, decisive purchase, looking forward to the effect of power saving, waiting for the next month effect.	Jingdong prefecture level city in Shandong
Doraemon Hyakubo Shop	King of Power Saving	The goods have been received, consistent with the description of the advertisement, quite good, I hope that in terms of power saving and purifying the air is really as the merchant said that would be better!	Jingdong prefecture level city in Shandong
Doraemon Hyakubo Shop	King of Power Saving	Power Saving King is really good to use the quality is very good than the physical store a lot cheaper we can rest assured that the purchase recommended to buy!	Jingdong prefecture level city in Shandong
Doraemon Hyakubo Shop	King of Power Saving	Energy saver has been used on the rental house inside the use of the meter is always running fast, with this can really save electricity, easy to use, plugged in can be, very satisfied!	Jingdong prefecture level city in Shandong
Doraemon Hyakubo Shop	King of Power Saving	Power Saver Power Saver King is good quality and affordable, very satisfied, worth buying.	Jingdong prefecture level city in Shandong
Doraemon Hyakubo Shop	King of Power Saving	Just use can not see what effect to,, wait for a period of time in the evaluation	Jingdong prefecture level city in Shandong
Doraemon Hyakubo Shop	King of Power Saving	Live in the 18th floor is too far away from the meter, afraid of no effect, look at the trial 90 days before buying. Currently used for a month, there is an effect, saving 27 dollars in electricity, next month should be able to save more!	Jingdong prefecture level city in Shandong
Doraemon Hyakubo Shop	King of Power Saving	I've been using it for almost a month now and it feels good, so I'll use it again.	Jingdong prefecture level city in Shandong
Doraemon Hyakubo Shop	King of Power Saving	It's ok, the electric bill is really less, originally I remember charging $100 and using it for 10 days. Now it lasts about 15 days. It's still noticeable.	Jingdong prefecture level city in Shandong
Doraemon Hyakubo Shop	King of Power Saving	Fast logistics, good power saving effect, very satisfied	Jingdong prefecture level city in Shandong
Doraemon Hyakubo Shop	King of Power Saving	Has been the second time back to buy, energy saving effect, worth recommending, you can try to see, power saving effect: very good Experience: can save about 30%!	Jingdong prefecture level city in Shandong
Doraemon Hyakubo Shop	King of Power Saving	I don't know how well it works for now	Jingdong prefecture level city in Shandong
Doraemon Hyakubo Shop	King of Power Saving	Factory direct is really good, plugged in and used for half a month did less probably almost 50 degrees of electricity! And the manufacturer's warranty is 5 years.	Jingdong prefecture level city in Shandong
Doraemon Hyakubo Shop	King of Power Saving	It's really effective. The previous month's electricity was $286, but after using the power saver for a month, I have $195 left this month, which is not bad.	Jingdong prefecture level city in Shandong
Doraemon Hyakubo Shop	King of Power Saving	I'll give you a good review and see how it goes.	Jingdong prefecture level city in Shandong
Doraemon Hyakubo Shop	King of Power Saving	Customer service is very professional and patient to guide the installation, the quality of workmanship is very fine. Durable, second repurchase. Really can save oh!	Jingdong prefecture level city in Shandong
Doraemon Hyakubo Shop	King of Power Saving	I've been using it for a month now and my electric bill is over $30 less than last month. I feel it's quite worth buying.	Jingdong prefecture level city in Shandong
Doraemon Hyakubo Shop	King of Power Saving	Small size, does not take up space. It's very good, it does work very effectively, the tool room has a lot of work with a lot of electricity. After installing it, it really reduces a lot of electricity bills. Before the day 30 degrees of electricity left stone, now work down really less a few degrees.	Jingdong prefecture level city in Shandong
Doraemon Hyakubo Shop	King of Power Saving	It does work for saving electricity, I've bought the kind from elsewhere before that was too simple not **all**. This time to buy this store, but also for the safety. Air conditioning is too much electricity, this use to save a lot of	Jingdong prefecture level city in Shandong
Doraemon Hyakubo Shop	King of Power Saving	Two air conditioners and a refrigerator at home, a month down the electricity bill is almost two hundred dollars, with this after the average savings of more than thirty dollars to spend less money to enjoy more, it's really very good!	Jingdong prefecture level city in Shandong
Doraemon Hyakubo Shop	King of Power Saving	Good. Very good.	Jingdong prefecture level city in Shandong
Doraemon Hyakubo Shop	King of Power Saving	Works great, easy to operate, great, very happy with it.	Jingdong prefecture level city in Shandong
Doraemon Hyakubo Shop	King of Power Saving	Received it and tried it out, it's great, great quality and worth having! Five stars praise!	Jingdong prefecture level city in Shandong
Doraemon Hyakubo Shop	King of Power Saving	Very trustworthy brand, quality is guaranteed, the price is still affordable, quite satisfied!	Jingdong prefecture level city in Shandong
Doraemon Hyakubo Shop	King of Power Saving	Use it before you speak.	Jingdong prefecture level city in Shandong
Doraemon Hyakubo Shop	King of Power Saving	The quality of workmanship is very good and the power saving effect is quite good, I regret not getting it earlier.	Jingdong prefecture level city in Shandong
Doraemon Hyakubo Shop	King of Power Saving	Merchants shipping speed, and customer service introduced the same very good, cost-effective	Jingdong prefecture level city in Shandong
Doraemon Hyakubo Shop	King of Power Saving	Feel a little effect Oh, where the power consumption where to plug, convenient and convenient	Jingdong prefecture level city in Shandong
Doraemon Hyakubo Shop	King of Power Saving	The quality of the imported saver is very good, practical and satisfactory. Cost-effective	Jingdong prefecture level city in Shandong
Doraemon Hyakubo Shop	King of Power Saving	Power Saver is especially good, good and inexpensive, very good	Jingdong prefecture level city in Shandong
Doraemon Hyakubo Shop	King of Power Saving	Fast shipping, awesome quality, affordable price, satisfied.	Jingdong prefecture level city in Shandong
Doraemon Hyakubo Shop	King of Power Saving	Energy saver received, the effect is consistent with the description, recommended to buy!	Jingdong prefecture level city in Shandong
Doraemon Hyakubo Shop	King of Power Saving	It's really good, the quality is great, I recommend it!	Jingdong prefecture level city in Shandong
Doraemon Hyakubo Shop	King of Power Saving	Works well. Inexpensive. Shipping was fast.	Jingdong prefecture level city in Shandong
Doraemon Hyakubo Shop	King of Power Saving	Logistics is fast, works well, energy saving is good	Jingdong prefecture level city in Shandong
Doraemon Hyakubo Shop	King of Power Saving	very happy	Jingdong prefecture level city in Shandong
Doraemon Hyakubo Shop	King of Power Saving	an excellent	Jingdong prefecture level city in Shandong
Doraemon Hyakubo Shop	King of Power Saving	It rained a couple days ago and I just went to get the item back today , not bad, hey I looked at the meter and that red display light takes several seconds to jump, did not make a mistake buying it. After a period of time to evaluate.	Jingdong prefecture level city in Shandong

Doraemon Hyakubo Shop	King of Power Saving	Used more than 10 days to check the power, there is indeed some decline, customer service said, long-term use will save more points!	Jingdong prefecture level city in Shandong
Doraemon Hyakubo Shop	King of Power Saving	It's a proper IQ tax, and it costs electricity.	Jingdong prefecture level city in Shandong
Doraemon Hyakubo Shop	King of Power Saving	I've been using it for 17 days now, it really saves electricity Shipping is fast, you can save a lot in a year	Jingdong prefecture level city in Shandong
Doraemon Hyakubo Shop	King of Power Saving	I've been using it for 17 days and it really saves power Shipping is fast and you don't have to worry about the quality, free returns within 5 years	Jingdong prefecture level city in Shandong
Doraemon Hyakubo Shop	King of Power Saving	It's more regular, scanned the code and it's authentic, and the main thing is that you can try it for 90 days! Plugged in more than ten days, is less than before a few degrees per day. When the time to use a month to see Additional. Good to buy a few more	Jingdong prefecture level city in Shandong
Doraemon Hyakubo Shop	King of Power Saving	Logistics is very fast, fine workmanship, use it and then come back to the comments	Jingdong prefecture level city in Shandong
Doraemon Hyakubo Shop	King of Power Saving	The quality of workmanship is very good and the power saving effect is quite good, I regret not getting it earlier.	Jingdong prefecture level city in Shandong
Doraemon Hyakubo Shop	King of Power Saving	Received the goods, according to the instructions on the operation is very simple, to wait and see the effect, looks good!	Jingdong prefecture level city in Shandong
Doraemon Hyakubo Shop	King of Power Saving	It's really as safe and power-saving as the owner says, and it works great!	Jingdong prefecture level city in Shandong
Doraemon Hyakubo Shop	King of Power Saving	Received the goods, immediately used, looks good, to wait and see how the effect.	Jingdong prefecture level city in Shandong
Doraemon Hyakubo Shop	King of Power Saving	Afraid to buy back did not work, the store said the commitment to 90 days invalid refund, warranty 5 years. Saver also complimentary on the Pacific insurance, it is a lot of peace of mind, there are effects will continue to buy back drops!	Jingdong prefecture level city in Shandong
Doraemon Hyakubo Shop	King of Power Saving	Received the goods and used it, can save electricity a month later to see the effect!	Jingdong prefecture level city in Shandong
Doraemon Hyakubo Shop	King of Power Saving	Fancy 90-day trial, 5-year warranty before the order, the goods received, has been experiencing a week, can really feel the savings, in the introduction of friends also buy this!	Jingdong prefecture level city in Shandong
Doraemon Hyakubo Shop	King of Power Saving	Clothing stores use, neighbors recommended to buy, said a month can save more than 70 yuan, first plugged in to see the effect of the	Jingdong prefecture level city in Shandong
Doraemon Hyakubo Shop	King of Power Saving	A friend's home two months to save more than 80 dollars of electricity, recommended that I buy two, decisive purchase, looking forward to the effect of power saving, waiting for the next month effect.	Jingdong prefecture level city in Shandong
Doraemon Hyakubo Shop	King of Power Saving	The goods have been received, consistent with the description of the advertisement, quite good, I hope that in terms of power saving and purifying the air is really as the merchant said that would be better!	Jingdong prefecture level city in Shandong
Doraemon Hyakubo Shop	King of Power Saving	Power Saving King is really good to use the quality is very good than the physical store a lot cheaper we can rest assured that the purchase recommended to buy!	Jingdong prefecture level city in Shandong
Doraemon Hyakubo Shop	King of Power Saving	Energy saver has been used on the rental house inside the use of the meter is always running fast, with this can really save electricity, easy to use, plugged in can be, very satisfied!	Jingdong prefecture level city in Shandong
Doraemon Hyakubo Shop	King of Power Saving	Power Saver Power Saver King is good quality and affordable, very satisfied, worth buying.	Jingdong prefecture level city in Shandong
Doraemon Hyakubo Shop	King of Power Saving	Just use can not see what effect to,, wait for a period of time in the evaluation	Jingdong prefecture level city in Shandong
Doraemon Hyakubo Shop	King of Power Saving	Live in the 18th floor is too far away from the meter, afraid of no effect, look at the trial 90 days before buying. Currently used for a month, there is an effect, saving 27 dollars in electricity, next month should be able to save more!	Jingdong prefecture level city in Shandong
Doraemon Hyakubo Shop	King of Power Saving	I've been using it for almost a month now and it feels good, so I'll use it again.	Jingdong prefecture level city in Shandong
Doraemon Hyakubo Shop	King of Power Saving	It's ok, the electric bill is really less, originally I remember charging $100 and using it for 10 days. Now it lasts about 15 days. It's still noticeable.	Jingdong prefecture level city in Shandong
Doraemon Hyakubo Shop	King of Power Saving	Fast logistics, good power saving effect, very satisfied	Jingdong prefecture level city in Shandong
Doraemon Hyakubo Shop	King of Power Saving	Has been the second time back to buy, energy saving effect, worth recommending, you can try to see, power saving effect: very good Experience: can save about 30%!	Jingdong prefecture level city in Shandong
Doraemon Hyakubo Shop	King of Power Saving	I don't know how well it works for now	Jingdong prefecture level city in Shandong
Doraemon Hyakubo Shop	King of Power Saving	Factory direct is really good, plugged in and used for half a month did less probably almost 50 degrees of electricity! And the manufacturer's warranty is 5 years.	Jingdong prefecture level city in Shandong
Doraemon Hyakubo Shop	King of Power Saving	It's really effective. The previous month's electricity was $286, but after using the power saver for a month, I have $195 left this month, which is not bad.	Jingdong prefecture level city in Shandong
Doraemon Hyakubo Shop	King of Power Saving	I'll give you a good review and see how it goes.	Jingdong prefecture level city in Shandong
Doraemon Hyakubo Shop	King of Power Saving	Customer service is very professional and patient to guide the installation, the quality of workmanship is very fine. Durable, second repurchase. Really can save oh!	Jingdong prefecture level city in Shandong
Doraemon Hyakubo Shop	King of Power Saving	I've been using it for a month now and my electric bill is over $30 less than last month. I feel it's quite worth buying.	Jingdong prefecture level city in Shandong
Doraemon Hyakubo Shop	King of Power Saving	Small size, does not take up space. It's very good, it does work very effectively, the tool room has a lot of work with a lot of electricity. After installing it, it really reduces a lot of electricity bills. Before the day 30 degrees of electricity left stone, now work down really less a few degrees.	Jingdong prefecture level city in Shandong
Doraemon Hyakubo Shop	King of Power Saving	It does work for saving electricity, I've bought the kind from elsewhere before that was too simple not **all**. This time to buy this store, but also for the safety. Air conditioning is too much electricity, this use to save a lot of	Jingdong prefecture level city in Shandong
Doraemon Hyakubo Shop	King of Power Saving	Two air conditioners and a refrigerator at home, a month down the electricity bill is almost two hundred dollars, with this after the average savings of more than thirty dollars to spend less money to enjoy more, it's really very good!	Jingdong prefecture level city in Shandong
Doraemon Hyakubo Shop	King of Power Saving	Good. Very good.	Jingdong prefecture level city in Shandong
Doraemon Hyakubo Shop	King of Power Saving	Works great, easy to operate, great, very happy with it.	Jingdong prefecture level city in Shandong
Doraemon Hyakubo Shop	King of Power Saving	Received it and tried it out, it's great, great quality and worth having! Five stars praise!	Jingdong prefecture level city in Shandong
Doraemon Hyakubo Shop	King of Power Saving	Very trustworthy brand, quality is guaranteed, the price is still affordable, quite satisfied!	Jingdong prefecture level city in Shandong
Doraemon Hyakubo Shop	King of Power Saving	Use it before you speak.	Jingdong prefecture level city in Shandong
Doraemon Hyakubo Shop	King of Power Saving	The quality of workmanship is very good and the power saving effect is quite good, I regret not getting it earlier.	Jingdong prefecture level city in Shandong
Doraemon Hyakubo Shop	King of Power Saving	Merchants shipping speed, and customer service introduced the same very good, cost-effective	Jingdong prefecture level city in Shandong
Doraemon Hyakubo Shop	King of Power Saving	Feel a little effect Oh, where the power consumption where to plug, convenient and convenient	Jingdong prefecture level city in Shandong
Doraemon Hyakubo Shop	King of Power Saving	The quality of the imported saver is very good, practical and satisfactory. Cost-effective	Jingdong prefecture level city in Shandong
Doraemon Hyakubo Shop	King of Power Saving	Power Saver is especially good, good and inexpensive, very good	Jingdong prefecture level city in Shandong
Doraemon Hyakubo Shop	King of Power Saving	Fast shipping, awesome quality, affordable price, satisfied.	Jingdong prefecture level city in Shandong
Doraemon Hyakubo Shop	King of Power Saving	Energy saver received, the effect is consistent with the description, recommended to buy!	Jingdong prefecture level city in Shandong
Doraemon Hyakubo Shop	King of Power Saving	It's really good, the quality is great, I recommend it!	Jingdong prefecture level city in Shandong
Doraemon Hyakubo Shop	King of Power Saving	Works well. Inexpensive. Shipping was fast.	Jingdong prefecture level city in Shandong

Doraemon Hyakubo Shop	King of Power Saving	Logistics is fast, works well, energy saving is good	Jingdong prefecture level city in Shandong
Doraemon Hyakubo Shop	King of Power Saving	very happy	Jingdong prefecture level city in Shandong
Doraemon Hyakubo Shop	King of Power Saving	an excellent	Jingdong prefecture level city in Shandong
Doraemon Hyakubo Shop	King of Power Saving	It rained a couple days ago and I just went to get the item back today , not bad, hey I looked at the meter and that red display light takes several seconds to jump, did not make a mistake buying it. After a period of time to evaluate.	Jingdong prefecture level city in Shandong
Doraemon Hyakubo Shop	King of Power Saving	Used more than 10 days to check the power, there is indeed some decline, customer service said, long-term use will save more points!	Jingdong prefecture level city in Shandong
Doraemon Hyakubo Shop	King of Power Saving	It's a proper IQ tax, and it costs electricity.	Jingdong prefecture level city in Shandong
Doraemon Hyakubo Shop	King of Power Saving	I've been using it for 17 days now, it really saves electricity Shipping is fast, you can save a lot in a year	Jingdong prefecture level city in Shandong
Doraemon Hyakubo Shop	King of Power Saving	I've been using it for 17 days and it really saves power Shipping is fast and you don't have to worry about the quality, free returns within 5 years	Jingdong prefecture level city in Shandong
Doraemon Hyakubo Shop	King of Power Saving	It's more regular, scanned the code and it's authentic, and the main thing is that you can try it for 90 days! Plugged in more than ten days, is less than before a few degrees per day. When the time to use a month to see Additional. Good to buy a few more	Jingdong prefecture level city in Shandong
Doraemon Hyakubo Shop	King of Power Saving	Logistics is very fast, fine workmanship, use it and then come back to the comments	Jingdong prefecture level city in Shandong
Doraemon Hyakubo Shop	King of Power Saving	The quality of workmanship is very good and the power saving effect is quite good, I regret not getting it earlier.	Jingdong prefecture level city in Shandong
Doraemon Hyakubo Shop	King of Power Saving	Received the goods, according to the instructions on the operation is very simple, to wait and see the effect, looks good!	Jingdong prefecture level city in Shandong
Doraemon Hyakubo Shop	King of Power Saving	It's really as safe and power-saving as the owner says, and it works great!	Jingdong prefecture level city in Shandong
Doraemon Hyakubo Shop	King of Power Saving	Received the goods, immediately used, looks good, to wait and see how the effect.	Jingdong prefecture level city in Shandong
Doraemon Hyakubo Shop	King of Power Saving	Afraid to buy back did not work, the store said the commitment to 90 days invalid refund, warranty 5 years. Saver also complimentary on the Pacific insurance, it is a lot of peace of mind, there are effects will continue to buy back drops!	Jingdong prefecture level city in Shandong
Doraemon Hyakubo Shop	King of Power Saving	Received the goods and used it, can save electricity a month later to see the effect!	Jingdong prefecture level city in Shandong
Doraemon Hyakubo Shop	King of Power Saving	Fancy 90-day trial, 5-year warranty before the order, the goods received, has been experiencing a week, can really feel the savings, in the introduction of friends also buy this!	Jingdong prefecture level city in Shandong
Doraemon Hyakubo Shop	King of Power Saving	Clothing stores use, neighbors recommended to buy, said a month can save more than 70 yuan, first plugged in to see the effect of the	Jingdong prefecture level city in Shandong
Doraemon Hyakubo Shop	King of Power Saving	A friend's home two months to save more than 80 dollars of electricity, recommended that I buy two, decisive purchase, looking forward to the effect of power saving, waiting for the next month effect.	Jingdong prefecture level city in Shandong
Doraemon Hyakubo Shop	King of Power Saving	The goods have been received, consistent with the description of the advertisement, quite good, I hope that in terms of power saving and purifying the air is really as the merchant said that would be better!	Jingdong prefecture level city in Shandong
Doraemon Hyakubo Shop	King of Power Saving	Power Saving King is really good to use the quality is very good than the physical store a lot cheaper we can rest assured that the purchase recommended to buy!	Jingdong prefecture level city in Shandong
Doraemon Hyakubo Shop	King of Power Saving	Energy saver has been used on the rental house inside the use of the meter is always running fast, with this can really save electricity, easy to use, plugged in can be, very satisfied!	Jingdong prefecture level city in Shandong
Doraemon Hyakubo Shop	King of Power Saving	Power Saver Power Saver King is good quality and affordable, very satisfied, worth buying.	Jingdong prefecture level city in Shandong
Doraemon Hyakubo Shop	King of Power Saving	Just use can not see what effect to,, wait for a period of time in the evaluation	Jingdong prefecture level city in Shandong
Doraemon Hyakubo Shop	King of Power Saving	Live in the 18th floor is too far away from the meter, afraid of no effect, look at the trial 90 days before buying. Currently used for a month, there is an effect, saving 27 dollars in electricity, next month should be able to save more!	Jingdong prefecture level city in Shandong
Doraemon Hyakubo Shop	King of Power Saving	I've been using it for almost a month now and it feels good, so I'll use it again.	Jingdong prefecture level city in Shandong
Doraemon Hyakubo Shop	King of Power Saving	It's ok, the electric bill is really less, originally I remember charging $100 and using it for 10 days. Now it lasts about 15 days. It's still noticeable.	Jingdong prefecture level city in Shandong
Doraemon Hyakubo Shop	King of Power Saving	Fast logistics, good power saving effect, very satisfied	Jingdong prefecture level city in Shandong
Doraemon Hyakubo Shop	King of Power Saving	Has been the second time back to buy, energy saving effect, worth recommending, you can try to see, power saving effect: very good Experience: can save about 30%!	Jingdong prefecture level city in Shandong
Doraemon Hyakubo Shop	King of Power Saving	I don't know how well it works for now	Jingdong prefecture level city in Shandong
Doraemon Hyakubo Shop	King of Power Saving	Factory direct is really good, plugged in and used for half a month did less probably almost 50 degrees of electricity! And the manufacturer's warranty is 5 years.	Jingdong prefecture level city in Shandong
Doraemon Hyakubo Shop	King of Power Saving	It's really effective. The previous month's electricity was $286, but after using the power saver for a month, I have $195 left this month, which is not bad.	Jingdong prefecture level city in Shandong
Doraemon Hyakubo Shop	King of Power Saving	I'll give you a good review and see how it goes.	Jingdong prefecture level city in Shandong
Doraemon Hyakubo Shop	King of Power Saving	Customer service is very professional and patient to guide the installation, the quality of workmanship is very fine. Durable, second repurchase. Really can save oh!	Jingdong prefecture level city in Shandong
Doraemon Hyakubo Shop	King of Power Saving	I've been using it for a month now and my electric bill is over $30 less than last month. I feel it's quite worth buying.	Jingdong prefecture level city in Shandong
Doraemon Hyakubo Shop	King of Power Saving	Small size, does not take up space. It's very good, it does work very effectively, the tool room has a lot of work with a lot of electricity. After installing it, it really reduces a lot of electricity bills. Before the day 30 degrees of electricity left stone, now work down really less a few degrees.	Jingdong prefecture level city in Shandong
Doraemon Hyakubo Shop	King of Power Saving	It does work for saving electricity, I've bought the kind from elsewhere before that was too simple not **all**. This time to buy this store, but also for the safety. Air conditioning is too much electricity, this use to save a lot of	Jingdong prefecture level city in Shandong
Doraemon Hyakubo Shop	King of Power Saving	Two air conditioners and a refrigerator at home, a month down the electricity bill is almost two hundred dollars, with this after the average savings of more than thirty dollars to spend less money to enjoy more, it's really very good!	Jingdong prefecture level city in Shandong
Doraemon Hyakubo Shop	King of Power Saving	Good. Very good.	Jingdong prefecture level city in Shandong
Doraemon Hyakubo Shop	King of Power Saving	Works great, easy to operate, great, very happy with it.	Jingdong prefecture level city in Shandong
Doraemon Hyakubo Shop	King of Power Saving	Received it and tried it out, it's great, great quality and worth having! Five stars praise!	Jingdong prefecture level city in Shandong
Doraemon Hyakubo Shop	King of Power Saving	Very trustworthy brand, quality is guaranteed, the price is still affordable, quite satisfied!	Jingdong prefecture level city in Shandong
Doraemon Hyakubo Shop	King of Power Saving	Use it before you speak.	Jingdong prefecture level city in Shandong
Doraemon Hyakubo Shop	King of Power Saving	The quality of workmanship is very good and the power saving effect is quite good, I regret not getting it earlier.	Jingdong prefecture level city in Shandong
Doraemon Hyakubo Shop	King of Power Saving	Merchants shipping speed, and customer service introduced the same very good, cost-effective	Jingdong prefecture level city in Shandong
Doraemon Hyakubo Shop	King of Power Saving	Feel a little effect Oh, where the power consumption where to plug, convenient and convenient	Jingdong prefecture level city in Shandong
Doraemon Hyakubo Shop	King of Power Saving	The quality of the imported saver is very good, practical and satisfactory. Cost-effective	Jingdong prefecture level city in Shandong
Doraemon Hyakubo Shop	King of Power Saving	Power Saver is especially good, good and inexpensive, very good	Jingdong prefecture level city in Shandong

Doraemon Hyakubo Shop	King of Power Saving	Fast shipping, awesome quality, affordable price, satisfied.	Jingdong prefecture level city in Shandong
Doraemon Hyakubo Shop	King of Power Saving	Energy saver received, the effect is consistent with the description, recommended to buy!	Jingdong prefecture level city in Shandong
Doraemon Hyakubo Shop	King of Power Saving	It's really good, the quality is great, I recommend it!	Jingdong prefecture level city in Shandong
Doraemon Hyakubo Shop	King of Power Saving	Works well. Inexpensive. Shipping was fast.	Jingdong prefecture level city in Shandong
Doraemon Hyakubo Shop	King of Power Saving	Logistics is fast, works well, energy saving is good	Jingdong prefecture level city in Shandong
Doraemon Hyakubo Shop	King of Power Saving	very happy	Jingdong prefecture level city in Shandong
Doraemon Hyakubo Shop	King of Power Saving	an excellent	Jingdong prefecture level city in Shandong
IXCHSQ Flagship Store	Green Intelligent Power Saver	A month to save more than 300, the saver is really too good to use, at once bought 3, commercial electricity, electricity high daily power consumption, before a month of more than 600, would have just wanted to try, did not want to use more after a month to save so much, happy!	Jingdong prefecture level city in Shandong
IXCHSQ Flagship Store	Green Intelligent Power Saver	Good use, this month only used 93.75, last month 345 dollars, very save electricity, very powerful power saver, plug into the socket can be used, save a lot of electricity, recommend everyone to use!	Jingdong prefecture level city in Shandong
IXCHSQ Flagship Store	Green Intelligent Power Saver	Section intelligent power saver received, with the back to see how the effect, logistics is very fast, thanks to the courier brother worked hard to send home!	Jingdong prefecture level city in Shandong
IXCHSQ Flagship Store	Green Intelligent Power Saver	Intelligent power saver received, logistics is very fast, thanks to the courier brother hard work to the doorstep!	Jingdong prefecture level city in Shandong
IXCHSQ Flagship Store	Green Intelligent Power Saver	Regular customer, third purchase, really powerful, bought a fourth looking forward to next month's electricity bill	Jingdong prefecture level city in Shandong
IXCHSQ Flagship Store	Green Intelligent Power Saver	I installed the product as soon as I received it, and I just used it for 2 days, so I can't see the effect yet. To be used for a period of time to evaluate	Jingdong prefecture level city in Shandong
IXCHSQ Flagship Store	Green Intelligent Power Saver	IXCHSQ power saver intelligent home appliance power saving i king received, the packaging is very good, the quality is very good, the style is new, it is very convenient to use.	Jingdong prefecture level city in Shandong
IXCHSQ Flagship Store	Green Intelligent Power Saver	Same as last time, watching the payment history saves electricity, buy a few more	Jingdong prefecture level city in Shandong
IXCHSQ Flagship Store	Green Intelligent Power Saver	It's not useful at all, plug it in and the electricity bill is as much as it is, don't buy this if it's a scam!	Jingdong prefecture level city in Shandong
IXCHSQ Flagship Store	Green Intelligent Power Saver	The quality is ok, the power is still trying for a month	Jingdong prefecture level city in Shandong
IXCHSQ Flagship Store	Green Intelligent Power Saver	Reserve your opinion and review after the research.	Jingdong prefecture level city in Shandong
IXCHSQ Flagship Store	Green Intelligent Power Saver	I'll review the results when they come out	Jingdong prefecture level city in Shandong
IXCHSQ Flagship Store	Green Intelligent Power Saver	Cheat nothing, no change in power at all.	Jingdong prefecture level city in Shandong
IXCHSQ Flagship Store	Green Intelligent Power Saver	It's not useful at all, plug it in and the electricity bill is as much as it is, don't buy this if it's a scam!	Jingdong prefecture level city in Shandong
IXCHSQ Flagship Store	Green Intelligent Power Saver	The quality is ok, the power is still trying for a month	Jingdong prefecture level city in Shandong
IXCHSQ Flagship Store	Green Intelligent Power Saver	Reserve your opinion and review after the research.	Jingdong prefecture level city in Shandong
IXCHSQ Flagship Store	Green Intelligent Power Saver	I'll review the results when they come out	Jingdong prefecture level city in Shandong
IXCHSQ Flagship Store	Green Intelligent Power Saver	Cheat nothing, no change in power at all.	Jingdong prefecture level city in Shandong
IXCHSQ Flagship Store	Green Intelligent Power Saver	It's not useful at all, plug it in and the electricity bill is as much as it is, don't buy this if it's a scam!	Jingdong prefecture level city in Shandong
IXCHSQ Flagship Store	Green Intelligent Power Saver	The quality is ok, the power is still trying for a month	Jingdong prefecture level city in Shandong
IXCHSQ Flagship Store	Green Intelligent Power Saver	Reserve your opinion and review after the research.	Jingdong prefecture level city in Shandong
IXCHSQ Flagship Store	Green Intelligent Power Saver	I'll review the results when they come out	Jingdong prefecture level city in Shandong
IXCHSQ Flagship Store	Green Intelligent Power Saver	Cheat nothing, no change in power at all.	Jingdong prefecture level city in Shandong
IXCHSQ Flagship Store	Green Intelligent Power Saver	It's not useful at all, plug it in and the electricity bill is as much as it is, don't buy this if it's a scam!	Jingdong prefecture level city in Shandong
IXCHSQ Flagship Store	Green Intelligent Power Saver	The quality is ok, the power is still trying for a month	Jingdong prefecture level city in Shandong
IXCHSQ Flagship Store	Green Intelligent Power Saver	Reserve your opinion and review after the research.	Jingdong prefecture level city in Shandong
IXCHSQ Flagship Store	Green Intelligent Power Saver	I'll review the results when they come out	Jingdong prefecture level city in Shandong
IXCHSQ Flagship Store	Green Intelligent Power Saver	Cheat nothing, no change in power at all.	Jingdong prefecture level city in Shandong
IXCHSQ Flagship Store	Green Intelligent Power Saver	It's not useful at all, plug it in and the electricity bill is as much as it is, don't buy this if it's a scam!	Jingdong prefecture level city in Shandong
IXCHSQ Flagship Store	Green Intelligent Power Saver	The quality is ok, the power is still trying for a month	Jingdong prefecture level city in Shandong
IXCHSQ Flagship Store	Green Intelligent Power Saver	Reserve your opinion and review after the research.	Jingdong prefecture level city in Shandong
IXCHSQ Flagship Store	Green Intelligent Power Saver	I'll review the results when they come out	Jingdong prefecture level city in Shandong
IXCHSQ Flagship Store	Green Intelligent Power Saver	Cheat nothing, no change in power at all.	Jingdong prefecture level city in Shandong
IXCHSQ Flagship Store	Green Intelligent Power Saver	It's not useful at all, plug it in and the electricity bill is as much as it is, don't buy this if it's a scam!	Jingdong prefecture level city in Shandong
IXCHSQ Flagship Store	Green Intelligent Power Saver	The quality is ok, the power is still trying for a month	Jingdong prefecture level city in Shandong
IXCHSQ Flagship Store	Green Intelligent Power Saver	Reserve your opinion and review after the research.	Jingdong prefecture level city in Shandong
IXCHSQ Flagship Store	Green Intelligent Power Saver	I'll review the results when they come out	Jingdong prefecture level city in Shandong
IXCHSQ Flagship Store	Green Intelligent Power Saver	Cheat nothing, no change in power at all.	Jingdong prefecture level city in Shandong
IXCHSQ Flagship Store	Green Intelligent Power Saver	It's not useful at all, plug it in and the electricity bill is as much as it is, don't buy this if it's a scam!	Jingdong prefecture level city in Shandong

IXCHSQ Flagship Store	Green Intelligent Power Saver	The quality is ok, the power is still trying for a month	Jingdong prefecture level city in Shandong
IXCHSQ Flagship Store	Green Intelligent Power Saver	Reserve your opinion and review after the research.	Jingdong prefecture level city in Shandong
IXCHSQ Flagship Store	Green Intelligent Power Saver	I’ll review the results when they come out	Jingdong prefecture level city in Shandong
IXCHSQ Flagship Store	Green Intelligent Power Saver	Cheat nothing, no change in power at all.	Jingdong prefecture level city in Shandong
IXCHSQ Flagship Store	Green Intelligent Power Saver	It’s not useful at all, plug it in and the electricity bill is as much as it is, don’t buy this if it’s a scam!	Jingdong prefecture level city in Shandong
IXCHSQ Flagship Store	Green Intelligent Power Saver	The quality is ok, the power is still trying for a month	Jingdong prefecture level city in Shandong
IXCHSQ Flagship Store	Green Intelligent Power Saver	Reserve your opinion and review after the research.	Jingdong prefecture level city in Shandong
IXCHSQ Flagship Store	Green Intelligent Power Saver	I’ll review the results when they come out	Jingdong prefecture level city in Shandong
IXCHSQ Flagship Store	Green Intelligent Power Saver	Cheat nothing, no change in power at all.	Jingdong prefecture level city in Shandong
IXCHSQ Flagship Store	Green Intelligent Power Saver	It’s not useful at all, plug it in and the electricity bill is as much as it is, don’t buy this if it’s a scam!	Jingdong prefecture level city in Shandong
IXCHSQ Flagship Store	Green Intelligent Power Saver	The quality is ok, the power is still trying for a month	Jingdong prefecture level city in Shandong
IXCHSQ Flagship Store	Green Intelligent Power Saver	Reserve your opinion and review after the research.	Jingdong prefecture level city in Shandong
IXCHSQ Flagship Store	Green Intelligent Power Saver	I’ll review the results when they come out	Jingdong prefecture level city in Shandong
IXCHSQ Flagship Store	Green Intelligent Power Saver	Cheat nothing, no change in power at all.	Jingdong prefecture level city in Shandong
IXCHSQ Flagship Store	Green Intelligent Power Saver	It’s not useful at all, plug it in and the electricity bill is as much as it is, don’t buy this if it’s a scam!	Jingdong prefecture level city in Shandong
IXCHSQ Flagship Store	Green Intelligent Power Saver	The quality is ok, the power is still trying for a month	Jingdong prefecture level city in Shandong
IXCHSQ Flagship Store	Green Intelligent Power Saver	Reserve your opinion and review after the research.	Jingdong prefecture level city in Shandong
IXCHSQ Flagship Store	Green Intelligent Power Saver	I’ll review the results when they come out	Jingdong prefecture level city in Shandong
IXCHSQ Flagship Store	Green Intelligent Power Saver	Cheat nothing, no change in power at all.	Jingdong prefecture level city in Shandong
IXCHSQ Flagship Store	Green Intelligent Power Saver	It’s not useful at all, plug it in and the electricity bill is as much as it is, don’t buy this if it’s a scam!	Jingdong prefecture level city in Shandong
IXCHSQ Flagship Store	Green Intelligent Power Saver	The quality is ok, the power is still trying for a month	Jingdong prefecture level city in Shandong
IXCHSQ Flagship Store	Green Intelligent Power Saver	Reserve your opinion and review after the research.	Jingdong prefecture level city in Shandong
IXCHSQ Flagship Store	Green Intelligent Power Saver	I’ll review the results when they come out	Jingdong prefecture level city in Shandong
IXCHSQ Flagship Store	Green Intelligent Power Saver	Cheat nothing, no change in power at all.	Jingdong prefecture level city in Shandong
IXCHSQ Flagship Store	Green Intelligent Power Saver	It’s not useful at all, plug it in and the electricity bill is as much as it is, don’t buy this if it’s a scam!	Jingdong prefecture level city in Shandong
IXCHSQ Flagship Store	Green Intelligent Power Saver	The quality is ok, the power is still trying for a month	Jingdong prefecture level city in Shandong
IXCHSQ Flagship Store	Green Intelligent Power Saver	Reserve your opinion and review after the research.	Jingdong prefecture level city in Shandong
IXCHSQ Flagship Store	Green Intelligent Power Saver	I’ll review the results when they come out	Jingdong prefecture level city in Shandong
IXCHSQ Flagship Store	Green Intelligent Power Saver	Cheat nothing, no change in power at all.	Jingdong prefecture level city in Shandong
IXCHSQ Flagship Store	Green Intelligent Power Saver	It’s not useful at all, plug it in and the electricity bill is as much as it is, don’t buy this if it’s a scam!	Jingdong prefecture level city in Shandong
IXCHSQ Flagship Store	Green Intelligent Power Saver	The quality is ok, the power is still trying for a month	Jingdong prefecture level city in Shandong
IXCHSQ Flagship Store	Green Intelligent Power Saver	Reserve your opinion and review after the research.	Jingdong prefecture level city in Shandong
IXCHSQ Flagship Store	Green Intelligent Power Saver	I’ll review the results when they come out	Jingdong prefecture level city in Shandong
IXCHSQ Flagship Store	Green Intelligent Power Saver	Cheat nothing, no change in power at all.	Jingdong prefecture level city in Shandong
IXCHSQ Flagship Store	Green Intelligent Power Saver	It’s not useful at all, plug it in and the electricity bill is as much as it is, don’t buy this if it’s a scam!	Jingdong prefecture level city in Shandong
IXCHSQ Flagship Store	Green Intelligent Power Saver	The quality is ok, the power is still trying for a month	Jingdong prefecture level city in Shandong
IXCHSQ Flagship Store	Green Intelligent Power Saver	Reserve your opinion and review after the research.	Jingdong prefecture level city in Shandong
IXCHSQ Flagship Store	Green Intelligent Power Saver	I’ll review the results when they come out	Jingdong prefecture level city in Shandong
IXCHSQ Flagship Store	Green Intelligent Power Saver	Cheat nothing, no change in power at all.	Jingdong prefecture level city in Shandong
IXCHSQ Flagship Store	Green Intelligent Power Saver	It’s not useful at all, plug it in and the electricity bill is as much as it is, don’t buy this if it’s a scam!	Jingdong prefecture level city in Shandong
IXCHSQ Flagship Store	Green Intelligent Power Saver	The quality is ok, the power is still trying for a month	Jingdong prefecture level city in Shandong
IXCHSQ Flagship Store	Green Intelligent Power Saver	Reserve your opinion and review after the research.	Jingdong prefecture level city in Shandong
IXCHSQ Flagship Store	Green Intelligent Power Saver	I’ll review the results when they come out	Jingdong prefecture level city in Shandong
IXCHSQ Flagship Store	Green Intelligent Power Saver	Cheat nothing, no change in power at all.	Jingdong prefecture level city in Shandong
IXCHSQ Flagship Store	Green Intelligent Power Saver	It’s not useful at all, plug it in and the electricity bill is as much as it is, don’t buy this if it’s a scam!	Jingdong prefecture level city in Shandong
IXCHSQ Flagship Store	Green Intelligent Power Saver	The quality is ok, the power is still trying for a month	Jingdong prefecture level city in Shandong
IXCHSQ Flagship Store	Green Intelligent Power Saver	Reserve your opinion and review after the research.	Jingdong prefecture level city in Shandong
IXCHSQ Flagship Store	Green Intelligent Power Saver	I’ll review the results when they come out	Jingdong prefecture level city in Shandong
IXCHSQ Flagship Store	Green Intelligent Power Saver	Cheat nothing, no change in power at all.	Jingdong prefecture level city in Shandong
IXCHSQ Flagship Store	Green Intelligent Power Saver	It’s not useful at all, plug it in and the electricity bill is as much as it is, don’t buy this if it’s a scam!	Jingdong prefecture level city in Shandong
IXCHSQ Flagship Store	Green Intelligent Power Saver	The quality is ok, the power is still trying for a month	Jingdong prefecture level city in Shandong

IXCHSQ Flagship Store	Green Intelligent Power Saver	Reserve your opinion and review after the research.	Jingdong prefecture level city in Shandong
IXCHSQ Flagship Store	Green Intelligent Power Saver	I’ll review the results when they come out	Jingdong prefecture level city in Shandong
IXCHSQ Flagship Store	Green Intelligent Power Saver	Cheat nothing, no change in power at all.	Jingdong prefecture level city in Shandong
IXCHSQ Flagship Store	Green Intelligent Power Saver	It’s not useful at all, plug it in and the electricity bill is as much as it is, don’t buy this if it’s a scam!	Jingdong prefecture level city in Shandong
IXCHSQ Flagship Store	Green Intelligent Power Saver	The quality is ok, the power is still trying for a month	Jingdong prefecture level city in Shandong
IXCHSQ Flagship Store	Green Intelligent Power Saver	Reserve your opinion and review after the research.	Jingdong prefecture level city in Shandong
IXCHSQ Flagship Store	Green Intelligent Power Saver	I’ll review the results when they come out	Jingdong prefecture level city in Shandong
IXCHSQ Flagship Store	Green Intelligent Power Saver	Cheat nothing, no change in power at all.	Jingdong prefecture level city in Shandong
IXCHSQ Flagship Store	Green Intelligent Power Saver	It’s not useful at all, plug it in and the electricity bill is as much as it is, don’t buy this if it’s a scam!	Jingdong prefecture level city in Shandong
IXCHSQ Flagship Store	Green Intelligent Power Saver	The quality is ok, the power is still trying for a month	Jingdong prefecture level city in Shandong
IXCHSQ Flagship Store	Green Intelligent Power Saver	Reserve your opinion and review after the research.	Jingdong prefecture level city in Shandong
IXCHSQ Flagship Store	Green Intelligent Power Saver	I’ll review the results when they come out	Jingdong prefecture level city in Shandong
IXCHSQ Flagship Store	Green Intelligent Power Saver	Cheat nothing, no change in power at all.	Jingdong prefecture level city in Shandong
IXCHSQ Flagship Store	Green Intelligent Power Saver	It’s not useful at all, plug it in and the electricity bill is as much as it is, don’t buy this if it’s a scam!	Jingdong prefecture level city in Shandong
IXCHSQ Flagship Store	Green Intelligent Power Saver	The quality is ok, the power is still trying for a month	Jingdong prefecture level city in Shandong
IXCHSQ Flagship Store	Green Intelligent Power Saver	Reserve your opinion and review after the research.	Jingdong prefecture level city in Shandong
IXCHSQ Flagship Store	Green Intelligent Power Saver	I’ll review the results when they come out	Jingdong prefecture level city in Shandong
IXCHSQ Flagship Store	Green Intelligent Power Saver	Cheat nothing, no change in power at all.	Jingdong prefecture level city in Shandong
IXCHSQ Flagship Store	Green Intelligent Power Saver	It’s not useful at all, plug it in and the electricity bill is as much as it is, don’t buy this if it’s a scam!	Jingdong prefecture level city in Shandong
IXCHSQ Flagship Store	Green Intelligent Power Saver	The quality is ok, the power is still trying for a month	Jingdong prefecture level city in Shandong
IXCHSQ Flagship Store	Green Intelligent Power Saver	Reserve your opinion and review after the research.	Jingdong prefecture level city in Shandong
IXCHSQ Flagship Store	Green Intelligent Power Saver	I’ll review the results when they come out	Jingdong prefecture level city in Shandong
IXCHSQ Flagship Store	Green Intelligent Power Saver	Cheat nothing, no change in power at all.	Jingdong prefecture level city in Shandong
IXCHSQ Flagship Store	Green Intelligent Power Saver	It’s not useful at all, plug it in and the electricity bill is as much as it is, don’t buy this if it’s a scam!	Jingdong prefecture level city in Shandong
IXCHSQ Flagship Store	Green Intelligent Power Saver	The quality is ok, the power is still trying for a month	Jingdong prefecture level city in Shandong
IXCHSQ Flagship Store	Green Intelligent Power Saver	Reserve your opinion and review after the research.	Jingdong prefecture level city in Shandong
IXCHSQ Flagship Store	Green Intelligent Power Saver	I’ll review the results when they come out	Jingdong prefecture level city in Shandong
IXCHSQ Flagship Store	Green Intelligent Power Saver	Cheat nothing, no change in power at all.	Jingdong prefecture level city in Shandong
IXCHSQ Flagship Store	Green Intelligent Power Saver	It’s not useful at all, plug it in and the electricity bill is as much as it is, don’t buy this if it’s a scam!	Jingdong prefecture level city in Shandong
IXCHSQ Flagship Store	Green Intelligent Power Saver	The quality is ok, the power is still trying for a month	Jingdong prefecture level city in Shandong
IXCHSQ Flagship Store	Green Intelligent Power Saver	Reserve your opinion and review after the research.	Jingdong prefecture level city in Shandong
IXCHSQ Flagship Store	Green Intelligent Power Saver	I’ll review the results when they come out	Jingdong prefecture level city in Shandong
IXCHSQ Flagship Store	Green Intelligent Power Saver	Cheat nothing, no change in power at all.	Jingdong prefecture level city in Shandong
IXCHSQ Flagship Store	Green Intelligent Power Saver	It’s not useful at all, plug it in and the electricity bill is as much as it is, don’t buy this if it’s a scam!	Jingdong prefecture level city in Shandong
IXCHSQ Flagship Store	Green Intelligent Power Saver	The quality is ok, the power is still trying for a month	Jingdong prefecture level city in Shandong
IXCHSQ Flagship Store	Green Intelligent Power Saver	Reserve your opinion and review after the research.	Jingdong prefecture level city in Shandong
IXCHSQ Flagship Store	Green Intelligent Power Saver	I’ll review the results when they come out	Jingdong prefecture level city in Shandong
IXCHSQ Flagship Store	Green Intelligent Power Saver	Cheat nothing, no change in power at all.	Jingdong prefecture level city in Shandong
IXCHSQ Flagship Store	Green Intelligent Power Saver	It’s not useful at all, plug it in and the electricity bill is as much as it is, don’t buy this if it’s a scam!	Jingdong prefecture level city in Shandong
IXCHSQ Flagship Store	Green Intelligent Power Saver	The quality is ok, the power is still trying for a month	Jingdong prefecture level city in Shandong
IXCHSQ Flagship Store	Green Intelligent Power Saver	Reserve your opinion and review after the research.	Jingdong prefecture level city in Shandong
IXCHSQ Flagship Store	Green Intelligent Power Saver	I’ll review the results when they come out	Jingdong prefecture level city in Shandong
IXCHSQ Flagship Store	Green Intelligent Power Saver	Cheat nothing, no change in power at all.	Jingdong prefecture level city in Shandong
IXCHSQ Flagship Store	Green Intelligent Power Saver	It’s not useful at all, plug it in and the electricity bill is as much as it is, don’t buy this if it’s a scam!	Jingdong prefecture level city in Shandong
IXCHSQ Flagship Store	Green Intelligent Power Saver	The quality is ok, the power is still trying for a month	Jingdong prefecture level city in Shandong
IXCHSQ Flagship Store	Green Intelligent Power Saver	Reserve your opinion and review after the research.	Jingdong prefecture level city in Shandong
IXCHSQ Flagship Store	Green Intelligent Power Saver	I’ll review the results when they come out	Jingdong prefecture level city in Shandong
IXCHSQ Flagship Store	Green Intelligent Power Saver	Cheat nothing, no change in power at all.	Jingdong prefecture level city in Shandong
IXCHSQ Flagship Store	Green Intelligent Power Saver	It’s not useful at all, plug it in and the electricity bill is as much as it is, don’t buy this if it’s a scam!	Jingdong prefecture level city in Shandong
IXCHSQ Flagship Store	Green Intelligent Power Saver	The quality is ok, the power is still trying for a month	Jingdong prefecture level city in Shandong
IXCHSQ Flagship Store	Green Intelligent Power Saver	Reserve your opinion and review after the research.	Jingdong prefecture level city in Shandong

IXCHSQ Flagship Store	Green Intelligent Power Saver	I’ll review the results when they come out	Jingdong prefecture level city in Shandong
IXCHSQ Flagship Store	Green Intelligent Power Saver	Cheat nothing, no change in power at all.	Jingdong prefecture level city in Shandong
IXCHSQ Flagship Store	Green Intelligent Power Saver	It’s not useful at all, plug it in and the electricity bill is as much as it is, don’t buy this if it's a scam!	Jingdong prefecture level city in Shandong
IXCHSQ Flagship Store	Green Intelligent Power Saver	The quality is ok, the power is still trying for a month	Jingdong prefecture level city in Shandong
IXCHSQ Flagship Store	Green Intelligent Power Saver	Reserve your opinion and review after the research.	Jingdong prefecture level city in Shandong
IXCHSQ Flagship Store	Green Intelligent Power Saver	I’ll review the results when they come out	Jingdong prefecture level city in Shandong
IXCHSQ Flagship Store	Green Intelligent Power Saver	Cheat nothing, no change in power at all.	Jingdong prefecture level city in Shandong
IXCHSQ Flagship Store	Green Intelligent Power Saver	It’s not useful at all, plug it in and the electricity bill is as much as it is, don’t buy this if it's a scam!	Jingdong prefecture level city in Shandong
IXCHSQ Flagship Store	Green Intelligent Power Saver	The quality is ok, the power is still trying for a month	Jingdong prefecture level city in Shandong
IXCHSQ Flagship Store	Green Intelligent Power Saver	Reserve your opinion and review after the research.	Jingdong prefecture level city in Shandong
IXCHSQ Flagship Store	Green Intelligent Power Saver	I’ll review the results when they come out	Jingdong prefecture level city in Shandong
IXCHSQ Flagship Store	Green Intelligent Power Saver	Cheat nothing, no change in power at all.	Jingdong prefecture level city in Shandong
IXCHSQ Flagship Store	Green Intelligent Power Saver	It’s not useful at all, plug it in and the electricity bill is as much as it is, don’t buy this if it's a scam!	Jingdong prefecture level city in Shandong
IXCHSQ Flagship Store	Green Intelligent Power Saver	The quality is ok, the power is still trying for a month	Jingdong prefecture level city in Shandong
IXCHSQ Flagship Store	Green Intelligent Power Saver	Reserve your opinion and review after the research.	Jingdong prefecture level city in Shandong
IXCHSQ Flagship Store	Green Intelligent Power Saver	I’ll review the results when they come out	Jingdong prefecture level city in Shandong
IXCHSQ Flagship Store	Green Intelligent Power Saver	Cheat nothing, no change in power at all.	Jingdong prefecture level city in Shandong
IXCHSQ Flagship Store	Green Intelligent Power Saver	It’s not useful at all, plug it in and the electricity bill is as much as it is, don’t buy this if it's a scam!	Jingdong prefecture level city in Shandong
IXCHSQ Flagship Store	Green Intelligent Power Saver	The quality is ok, the power is still trying for a month	Jingdong prefecture level city in Shandong
IXCHSQ Flagship Store	Green Intelligent Power Saver	Reserve your opinion and review after the research.	Jingdong prefecture level city in Shandong
IXCHSQ Flagship Store	Green Intelligent Power Saver	I’ll review the results when they come out	Jingdong prefecture level city in Shandong
IXCHSQ Flagship Store	Green Intelligent Power Saver	Cheat nothing, no change in power at all.	Jingdong prefecture level city in Shandong
IXCHSQ Flagship Store	Green Intelligent Power Saver	It’s not useful at all, plug it in and the electricity bill is as much as it is, don’t buy this if it's a scam!	Jingdong prefecture level city in Shandong
IXCHSQ Flagship Store	Green Intelligent Power Saver	The quality is ok, the power is still trying for a month	Jingdong prefecture level city in Shandong
IXCHSQ Flagship Store	Green Intelligent Power Saver	Reserve your opinion and review after the research.	Jingdong prefecture level city in Shandong
IXCHSQ Flagship Store	Green Intelligent Power Saver	I’ll review the results when they come out	Jingdong prefecture level city in Shandong
IXCHSQ Flagship Store	Green Intelligent Power Saver	Cheat nothing, no change in power at all.	Jingdong prefecture level city in Shandong
IXCHSQ Flagship Store	Green Intelligent Power Saver	It’s not useful at all, plug it in and the electricity bill is as much as it is, don’t buy this if it's a scam!	Jingdong prefecture level city in Shandong
IXCHSQ Flagship Store	Green Intelligent Power Saver	The quality is ok, the power is still trying for a month	Jingdong prefecture level city in Shandong
IXCHSQ Flagship Store	Green Intelligent Power Saver	Reserve your opinion and review after the research.	Jingdong prefecture level city in Shandong
IXCHSQ Flagship Store	Green Intelligent Power Saver	I’ll review the results when they come out	Jingdong prefecture level city in Shandong
IXCHSQ Flagship Store	Green Intelligent Power Saver	Cheat nothing, no change in power at all.	Jingdong prefecture level city in Shandong
IXCHSQ Flagship Store	Green Intelligent Power Saver	It’s not useful at all, plug it in and the electricity bill is as much as it is, don’t buy this if it's a scam!	Jingdong prefecture level city in Shandong
IXCHSQ Flagship Store	Green Intelligent Power Saver	The quality is ok, the power is still trying for a month	Jingdong prefecture level city in Shandong
IXCHSQ Flagship Store	Green Intelligent Power Saver	Reserve your opinion and review after the research.	Jingdong prefecture level city in Shandong
IXCHSQ Flagship Store	Green Intelligent Power Saver	I’ll review the results when they come out	Jingdong prefecture level city in Shandong
IXCHSQ Flagship Store	Green Intelligent Power Saver	Cheat nothing, no change in power at all.	Jingdong prefecture level city in Shandong
IXCHSQ Flagship Store	Green Intelligent Power Saver	It’s not useful at all, plug it in and the electricity bill is as much as it is, don’t buy this if it's a scam!	Jingdong prefecture level city in Shandong
IXCHSQ Flagship Store	Green Intelligent Power Saver	The quality is ok, the power is still trying for a month	Jingdong prefecture level city in Shandong
IXCHSQ Flagship Store	Green Intelligent Power Saver	Reserve your opinion and review after the research.	Jingdong prefecture level city in Shandong
IXCHSQ Flagship Store	Green Intelligent Power Saver	I’ll review the results when they come out	Jingdong prefecture level city in Shandong
IXCHSQ Flagship Store	Green Intelligent Power Saver	Cheat nothing, no change in power at all.	Jingdong prefecture level city in Shandong
IXCHSQ Flagship Store	Green Intelligent Power Saver	It’s not useful at all, plug it in and the electricity bill is as much as it is, don’t buy this if it's a scam!	Jingdong prefecture level city in Shandong
IXCHSQ Flagship Store	Green Intelligent Power Saver	The quality is ok, the power is still trying for a month	Jingdong prefecture level city in Shandong
IXCHSQ Flagship Store	Green Intelligent Power Saver	Reserve your opinion and review after the research.	Jingdong prefecture level city in Shandong
IXCHSQ Flagship Store	Green Intelligent Power Saver	I’ll review the results when they come out	Jingdong prefecture level city in Shandong
IXCHSQ Flagship Store	Green Intelligent Power Saver	Cheat nothing, no change in power at all.	Jingdong prefecture level city in Shandong
IXCHSQ Flagship Store	Green Intelligent Power Saver	It’s not useful at all, plug it in and the electricity bill is as much as it is, don’t buy this if it's a scam!	Jingdong prefecture level city in Shandong
IXCHSQ Flagship Store	Green Intelligent Power Saver	The quality is ok, the power is still trying for a month	Jingdong prefecture level city in Shandong
IXCHSQ Flagship Store	Green Intelligent Power Saver	Reserve your opinion and review after the research.	Jingdong prefecture level city in Shandong
IXCHSQ Flagship Store	Green Intelligent Power Saver	I’ll review the results when they come out	Jingdong prefecture level city in Shandong

IXCHSQ Flagship Store	Green Intelligent Power Saver	Cheat nothing, no change in power at all.	Jingdong prefecture level city in Shandong
IXCHSQ Flagship Store	Green Intelligent Power Saver	It’s not useful at all, plug it in and the electricity bill is as much as it is, don’t buy this if it’s a scam!	Jingdong prefecture level city in Shandong
IXCHSQ Flagship Store	Green Intelligent Power Saver	The quality is ok, the power is still trying for a month	Jingdong prefecture level city in Shandong
IXCHSQ Flagship Store	Green Intelligent Power Saver	Reserve your opinion and review after the research.	Jingdong prefecture level city in Shandong
IXCHSQ Flagship Store	Green Intelligent Power Saver	I’ll review the results when they come out	Jingdong prefecture level city in Shandong
IXCHSQ Flagship Store	Green Intelligent Power Saver	Cheat nothing, no change in power at all.	Jingdong prefecture level city in Shandong
IXCHSQ Flagship Store	Green Intelligent Power Saver	It’s not useful at all, plug it in and the electricity bill is as much as it is, don’t buy this if it’s a scam!	Jingdong prefecture level city in Shandong
IXCHSQ Flagship Store	Green Intelligent Power Saver	The quality is ok, the power is still trying for a month	Jingdong prefecture level city in Shandong
IXCHSQ Flagship Store	Green Intelligent Power Saver	Reserve your opinion and review after the research.	Jingdong prefecture level city in Shandong
IXCHSQ Flagship Store	Green Intelligent Power Saver	I’ll review the results when they come out	Jingdong prefecture level city in Shandong
IXCHSQ Flagship Store	Green Intelligent Power Saver	Cheat nothing, no change in power at all.	Jingdong prefecture level city in Shandong
IXCHSQ Flagship Store	Green Intelligent Power Saver	It’s not useful at all, plug it in and the electricity bill is as much as it is, don’t buy this if it’s a scam!	Jingdong prefecture level city in Shandong
IXCHSQ Flagship Store	Green Intelligent Power Saver	The quality is ok, the power is still trying for a month	Jingdong prefecture level city in Shandong
IXCHSQ Flagship Store	Green Intelligent Power Saver	Reserve your opinion and review after the research.	Jingdong prefecture level city in Shandong
IXCHSQ Flagship Store	Green Intelligent Power Saver	I’ll review the results when they come out	Jingdong prefecture level city in Shandong
IXCHSQ Flagship Store	Green Intelligent Power Saver	Cheat nothing, no change in power at all.	Jingdong prefecture level city in Shandong
IXCHSQ Flagship Store	Green Intelligent Power Saver	It’s not useful at all, plug it in and the electricity bill is as much as it is, don’t buy this if it’s a scam!	Jingdong prefecture level city in Shandong
IXCHSQ Flagship Store	Green Intelligent Power Saver	The quality is ok, the power is still trying for a month	Jingdong prefecture level city in Shandong
IXCHSQ Flagship Store	Green Intelligent Power Saver	Reserve your opinion and review after the research.	Jingdong prefecture level city in Shandong
IXCHSQ Flagship Store	Green Intelligent Power Saver	I’ll review the results when they come out	Jingdong prefecture level city in Shandong
IXCHSQ Flagship Store	Green Intelligent Power Saver	Cheat nothing, no change in power at all.	Jingdong prefecture level city in Shandong
IXCHSQ Flagship Store	Green Intelligent Power Saver	It’s not useful at all, plug it in and the electricity bill is as much as it is, don’t buy this if it’s a scam!	Jingdong prefecture level city in Shandong
IXCHSQ Flagship Store	Green Intelligent Power Saver	The quality is ok, the power is still trying for a month	Jingdong prefecture level city in Shandong
IXCHSQ Flagship Store	Green Intelligent Power Saver	Reserve your opinion and review after the research.	Jingdong prefecture level city in Shandong
IXCHSQ Flagship Store	Green Intelligent Power Saver	I’ll review the results when they come out	Jingdong prefecture level city in Shandong
IXCHSQ Flagship Store	Green Intelligent Power Saver	Cheat nothing, no change in power at all.	Jingdong prefecture level city in Shandong
IXCHSQ Flagship Store	Green Intelligent Power Saver	It’s not useful at all, plug it in and the electricity bill is as much as it is, don’t buy this if it’s a scam!	Jingdong prefecture level city in Shandong
IXCHSQ Flagship Store	Green Intelligent Power Saver	The quality is ok, the power is still trying for a month	Jingdong prefecture level city in Shandong
IXCHSQ Flagship Store	Green Intelligent Power Saver	Reserve your opinion and review after the research.	Jingdong prefecture level city in Shandong
IXCHSQ Flagship Store	Green Intelligent Power Saver	I’ll review the results when they come out	Jingdong prefecture level city in Shandong
IXCHSQ Flagship Store	Green Intelligent Power Saver	Cheat nothing, no change in power at all.	Jingdong prefecture level city in Shandong
IXCHSQ Flagship Store	Green Intelligent Power Saver	It’s not useful at all, plug it in and the electricity bill is as much as it is, don’t buy this if it’s a scam!	Jingdong prefecture level city in Shandong
IXCHSQ Flagship Store	Green Intelligent Power Saver	The quality is ok, the power is still trying for a month	Jingdong prefecture level city in Shandong
IXCHSQ Flagship Store	Green Intelligent Power Saver	Reserve your opinion and review after the research.	Jingdong prefecture level city in Shandong
IXCHSQ Flagship Store	Green Intelligent Power Saver	I’ll review the results when they come out	Jingdong prefecture level city in Shandong
IXCHSQ Flagship Store	Green Intelligent Power Saver	Cheat nothing, no change in power at all.	Jingdong prefecture level city in Shandong
IXCHSQ Flagship Store	Green Intelligent Power Saver	It’s not useful at all, plug it in and the electricity bill is as much as it is, don’t buy this if it’s a scam!	Jingdong prefecture level city in Shandong
IXCHSQ Flagship Store	Green Intelligent Power Saver	The quality is ok, the power is still trying for a month	Jingdong prefecture level city in Shandong
IXCHSQ Flagship Store	Green Intelligent Power Saver	Reserve your opinion and review after the research.	Jingdong prefecture level city in Shandong
IXCHSQ Flagship Store	Green Intelligent Power Saver	I’ll review the results when they come out	Jingdong prefecture level city in Shandong
IXCHSQ Flagship Store	Green Intelligent Power Saver	Cheat nothing, no change in power at all.	Jingdong prefecture level city in Shandong
IXCHSQ Flagship Store	Green Intelligent Power Saver	It’s not useful at all, plug it in and the electricity bill is as much as it is, don’t buy this if it’s a scam!	Jingdong prefecture level city in Shandong
IXCHSQ Flagship Store	Green Intelligent Power Saver	The quality is ok, the power is still trying for a month	Jingdong prefecture level city in Shandong
IXCHSQ Flagship Store	Green Intelligent Power Saver	Reserve your opinion and review after the research.	Jingdong prefecture level city in Shandong
IXCHSQ Flagship Store	Green Intelligent Power Saver	I’ll review the results when they come out	Jingdong prefecture level city in Shandong
IXCHSQ Flagship Store	Green Intelligent Power Saver	Cheat nothing, no change in power at all.	Jingdong prefecture level city in Shandong
IXCHSQ Flagship Store	Green Intelligent Power Saver	It’s not useful at all, plug it in and the electricity bill is as much as it is, don’t buy this if it’s a scam!	Jingdong prefecture level city in Shandong
IXCHSQ Flagship Store	Green Intelligent Power Saver	The quality is ok, the power is still trying for a month	Jingdong prefecture level city in Shandong
IXCHSQ Flagship Store	Green Intelligent Power Saver	Reserve your opinion and review after the research.	Jingdong prefecture level city in Shandong
IXCHSQ Flagship Store	Green Intelligent Power Saver	I’ll review the results when they come out	Jingdong prefecture level city in Shandong
IXCHSQ Flagship Store	Green Intelligent Power Saver	Cheat nothing, no change in power at all.	Jingdong prefecture level city in Shandong

IXCHSQ Flagship Store	Green Intelligent Power Saver	It’s not useful at all, plug it in and the electricity bill is as much as it is, don’t buy this if it’s a scam!	Jingdong prefecture level city in Shandong
IXCHSQ Flagship Store	Green Intelligent Power Saver	The quality is ok, the power is still trying for a month	Jingdong prefecture level city in Shandong
IXCHSQ Flagship Store	Green Intelligent Power Saver	Reserve your opinion and review after the research.	Jingdong prefecture level city in Shandong
IXCHSQ Flagship Store	Green Intelligent Power Saver	I’ll review the results when they come out	Jingdong prefecture level city in Shandong
IXCHSQ Flagship Store	Green Intelligent Power Saver	Cheat nothing, no change in power at all.	Jingdong prefecture level city in Shandong
IXCHSQ Flagship Store	Green Intelligent Power Saver	It’s not useful at all, plug it in and the electricity bill is as much as it is, don’t buy this if it’s a scam!	Jingdong prefecture level city in Shandong
IXCHSQ Flagship Store	Green Intelligent Power Saver	The quality is ok, the power is still trying for a month	Jingdong prefecture level city in Shandong
IXCHSQ Flagship Store	Green Intelligent Power Saver	Reserve your opinion and review after the research.	Jingdong prefecture level city in Shandong
IXCHSQ Flagship Store	Green Intelligent Power Saver	I’ll review the results when they come out	Jingdong prefecture level city in Shandong
IXCHSQ Flagship Store	Green Intelligent Power Saver	Cheat nothing, no change in power at all.	Jingdong prefecture level city in Shandong
IXCHSQ Flagship Store	Green Intelligent Power Saver	It’s not useful at all, plug it in and the electricity bill is as much as it is, don’t buy this if it’s a scam!	Jingdong prefecture level city in Shandong
IXCHSQ Flagship Store	Green Intelligent Power Saver	The quality is ok, the power is still trying for a month	Jingdong prefecture level city in Shandong
IXCHSQ Flagship Store	Green Intelligent Power Saver	Reserve your opinion and review after the research.	Jingdong prefecture level city in Shandong
IXCHSQ Flagship Store	Green Intelligent Power Saver	I’ll review the results when they come out	Jingdong prefecture level city in Shandong
IXCHSQ Flagship Store	Green Intelligent Power Saver	Cheat nothing, no change in power at all.	Jingdong prefecture level city in Shandong
IXCHSQ Flagship Store	Green Intelligent Power Saver	It’s not useful at all, plug it in and the electricity bill is as much as it is, don’t buy this if it’s a scam!	Jingdong prefecture level city in Shandong
IXCHSQ Flagship Store	Green Intelligent Power Saver	The quality is ok, the power is still trying for a month	Jingdong prefecture level city in Shandong
IXCHSQ Flagship Store	Green Intelligent Power Saver	Reserve your opinion and review after the research.	Jingdong prefecture level city in Shandong
IXCHSQ Flagship Store	Green Intelligent Power Saver	I’ll review the results when they come out	Jingdong prefecture level city in Shandong
IXCHSQ Flagship Store	Green Intelligent Power Saver	Cheat nothing, no change in power at all.	Jingdong prefecture level city in Shandong
IXCHSQ Flagship Store	Green Intelligent Power Saver	It’s not useful at all, plug it in and the electricity bill is as much as it is, don’t buy this if it’s a scam!	Jingdong prefecture level city in Shandong
IXCHSQ Flagship Store	Green Intelligent Power Saver	The quality is ok, the power is still trying for a month	Jingdong prefecture level city in Shandong
IXCHSQ Flagship Store	Green Intelligent Power Saver	Reserve your opinion and review after the research.	Jingdong prefecture level city in Shandong
IXCHSQ Flagship Store	Green Intelligent Power Saver	I’ll review the results when they come out	Jingdong prefecture level city in Shandong
IXCHSQ Flagship Store	Green Intelligent Power Saver	Cheat nothing, no change in power at all.	Jingdong prefecture level city in Shandong
IXCHSQ Flagship Store	Green Intelligent Power Saver	It’s not useful at all, plug it in and the electricity bill is as much as it is, don’t buy this if it’s a scam!	Jingdong prefecture level city in Shandong
IXCHSQ Flagship Store	Green Intelligent Power Saver	The quality is ok, the power is still trying for a month	Jingdong prefecture level city in Shandong
IXCHSQ Flagship Store	Green Intelligent Power Saver	Reserve your opinion and review after the research.	Jingdong prefecture level city in Shandong
IXCHSQ Flagship Store	Green Intelligent Power Saver	I’ll review the results when they come out	Jingdong prefecture level city in Shandong
IXCHSQ Flagship Store	Green Intelligent Power Saver	Cheat nothing, no change in power at all.	Jingdong prefecture level city in Shandong
IXCHSQ Flagship Store	Green Intelligent Power Saver	It’s not useful at all, plug it in and the electricity bill is as much as it is, don’t buy this if it’s a scam!	Jingdong prefecture level city in Shandong
IXCHSQ Flagship Store	Green Intelligent Power Saver	The quality is ok, the power is still trying for a month	Jingdong prefecture level city in Shandong
IXCHSQ Flagship Store	Green Intelligent Power Saver	Reserve your opinion and review after the research.	Jingdong prefecture level city in Shandong
IXCHSQ Flagship Store	Green Intelligent Power Saver	I’ll review the results when they come out	Jingdong prefecture level city in Shandong
IXCHSQ Flagship Store	Green Intelligent Power Saver	Cheat nothing, no change in power at all.	Jingdong prefecture level city in Shandong
IXCHSQ Flagship Store	Green Intelligent Power Saver	It’s not useful at all, plug it in and the electricity bill is as much as it is, don’t buy this if it’s a scam!	Jingdong prefecture level city in Shandong
IXCHSQ Flagship Store	Green Intelligent Power Saver	The quality is ok, the power is still trying for a month	Jingdong prefecture level city in Shandong
IXCHSQ Flagship Store	Green Intelligent Power Saver	Reserve your opinion and review after the research.	Jingdong prefecture level city in Shandong
IXCHSQ Flagship Store	Green Intelligent Power Saver	I’ll review the results when they come out	Jingdong prefecture level city in Shandong
IXCHSQ Flagship Store	Green Intelligent Power Saver	Cheat nothing, no change in power at all.	Jingdong prefecture level city in Shandong
IXCHSQ Flagship Store	Green Intelligent Power Saver	It’s not useful at all, plug it in and the electricity bill is as much as it is, don’t buy this if it’s a scam!	Jingdong prefecture level city in Shandong
IXCHSQ Flagship Store	Green Intelligent Power Saver	The quality is ok, the power is still trying for a month	Jingdong prefecture level city in Shandong
IXCHSQ Flagship Store	Green Intelligent Power Saver	Reserve your opinion and review after the research.	Jingdong prefecture level city in Shandong
IXCHSQ Flagship Store	Green Intelligent Power Saver	I’ll review the results when they come out	Jingdong prefecture level city in Shandong
IXCHSQ Flagship Store	Green Intelligent Power Saver	Cheat nothing, no change in power at all.	Jingdong prefecture level city in Shandong
IXCHSQ Flagship Store	Green Intelligent Power Saver	It’s not useful at all, plug it in and the electricity bill is as much as it is, don’t buy this if it’s a scam!	Jingdong prefecture level city in Shandong
IXCHSQ Flagship Store	Green Intelligent Power Saver	The quality is ok, the power is still trying for a month	Jingdong prefecture level city in Shandong
IXCHSQ Flagship Store	Green Intelligent Power Saver	Reserve your opinion and review after the research.	Jingdong prefecture level city in Shandong
IXCHSQ Flagship Store	Green Intelligent Power Saver	I’ll review the results when they come out	Jingdong prefecture level city in Shandong
IXCHSQ Flagship Store	Green Intelligent Power Saver	Cheat nothing, no change in power at all.	Jingdong prefecture level city in Shandong
IXCHSQ Flagship Store	Green Intelligent Power Saver	It’s not useful at all, plug it in and the electricity bill is as much as it is, don’t buy this if it’s a scam!	Jingdong prefecture level city in Shandong

IXCHSQ Flagship Store	Green Intelligent Power Saver	The quality is ok, the power is still trying for a month	Jingdong prefecture level city in Shandong
IXCHSQ Flagship Store	Green Intelligent Power Saver	Reserve your opinion and review after the research.	Jingdong prefecture level city in Shandong
IXCHSQ Flagship Store	Green Intelligent Power Saver	I’ll review the results when they come out	Jingdong prefecture level city in Shandong
IXCHSQ Flagship Store	Green Intelligent Power Saver	Cheat nothing, no change in power at all.	Jingdong prefecture level city in Shandong
IXCHSQ Flagship Store	Green Intelligent Power Saver	It’s not useful at all, plug it in and the electricity bill is as much as it is, don’t buy this if it’s a scam!	Jingdong prefecture level city in Shandong
IXCHSQ Flagship Store	Green Intelligent Power Saver	The quality is ok, the power is still trying for a month	Jingdong prefecture level city in Shandong
IXCHSQ Flagship Store	Green Intelligent Power Saver	Reserve your opinion and review after the research.	Jingdong prefecture level city in Shandong
IXCHSQ Flagship Store	Green Intelligent Power Saver	I’ll review the results when they come out	Jingdong prefecture level city in Shandong
IXCHSQ Flagship Store	Green Intelligent Power Saver	Cheat nothing, no change in power at all.	Jingdong prefecture level city in Shandong
IXCHSQ Flagship Store	Green Intelligent Power Saver	It’s not useful at all, plug it in and the electricity bill is as much as it is, don’t buy this if it’s a scam!	Jingdong prefecture level city in Shandong
IXCHSQ Flagship Store	Green Intelligent Power Saver	The quality is ok, the power is still trying for a month	Jingdong prefecture level city in Shandong
IXCHSQ Flagship Store	Green Intelligent Power Saver	Reserve your opinion and review after the research.	Jingdong prefecture level city in Shandong
IXCHSQ Flagship Store	Green Intelligent Power Saver	I’ll review the results when they come out	Jingdong prefecture level city in Shandong
IXCHSQ Flagship Store	Green Intelligent Power Saver	Cheat nothing, no change in power at all.	Jingdong prefecture level city in Shandong
IXCHSQ Flagship Store	Green Intelligent Power Saver	It’s not useful at all, plug it in and the electricity bill is as much as it is, don’t buy this if it’s a scam!	Jingdong prefecture level city in Shandong
IXCHSQ Flagship Store	Green Intelligent Power Saver	The quality is ok, the power is still trying for a month	Jingdong prefecture level city in Shandong
IXCHSQ Flagship Store	Green Intelligent Power Saver	Reserve your opinion and review after the research.	Jingdong prefecture level city in Shandong
IXCHSQ Flagship Store	Green Intelligent Power Saver	I’ll review the results when they come out	Jingdong prefecture level city in Shandong
IXCHSQ Flagship Store	Green Intelligent Power Saver	Cheat nothing, no change in power at all.	Jingdong prefecture level city in Shandong
IXCHSQ Flagship Store	Green Intelligent Power Saver	It’s not useful at all, plug it in and the electricity bill is as much as it is, don’t buy this if it’s a scam!	Jingdong prefecture level city in Shandong
IXCHSQ Flagship Store	Green Intelligent Power Saver	The quality is ok, the power is still trying for a month	Jingdong prefecture level city in Shandong
IXCHSQ Flagship Store	Green Intelligent Power Saver	Reserve your opinion and review after the research.	Jingdong prefecture level city in Shandong
IXCHSQ Flagship Store	Green Intelligent Power Saver	I’ll review the results when they come out	Jingdong prefecture level city in Shandong
IXCHSQ Flagship Store	Green Intelligent Power Saver	Cheat nothing, no change in power at all.	Jingdong prefecture level city in Shandong
IXCHSQ Flagship Store	Green Intelligent Power Saver	It’s not useful at all, plug it in and the electricity bill is as much as it is, don’t buy this if it’s a scam!	Jingdong prefecture level city in Shandong
IXCHSQ Flagship Store	Green Intelligent Power Saver	The quality is ok, the power is still trying for a month	Jingdong prefecture level city in Shandong
IXCHSQ Flagship Store	Green Intelligent Power Saver	Reserve your opinion and review after the research.	Jingdong prefecture level city in Shandong
IXCHSQ Flagship Store	Green Intelligent Power Saver	I’ll review the results when they come out	Jingdong prefecture level city in Shandong
IXCHSQ Flagship Store	Green Intelligent Power Saver	Cheat nothing, no change in power at all.	Jingdong prefecture level city in Shandong
IXCHSQ Flagship Store	Green Intelligent Power Saver	It’s not useful at all, plug it in and the electricity bill is as much as it is, don’t buy this if it’s a scam!	Jingdong prefecture level city in Shandong
IXCHSQ Flagship Store	Green Intelligent Power Saver	The quality is ok, the power is still trying for a month	Jingdong prefecture level city in Shandong
IXCHSQ Flagship Store	Green Intelligent Power Saver	Reserve your opinion and review after the research.	Jingdong prefecture level city in Shandong
IXCHSQ Flagship Store	Green Intelligent Power Saver	I’ll review the results when they come out	Jingdong prefecture level city in Shandong
IXCHSQ Flagship Store	Green Intelligent Power Saver	Cheat nothing, no change in power at all.	Jingdong prefecture level city in Shandong
IXCHSQ Flagship Store	Green Intelligent Power Saver	It’s not useful at all, plug it in and the electricity bill is as much as it is, don’t buy this if it’s a scam!	Jingdong prefecture level city in Shandong
IXCHSQ Flagship Store	Green Intelligent Power Saver	The quality is ok, the power is still trying for a month	Jingdong prefecture level city in Shandong
IXCHSQ Flagship Store	Green Intelligent Power Saver	Reserve your opinion and review after the research.	Jingdong prefecture level city in Shandong
IXCHSQ Flagship Store	Green Intelligent Power Saver	I’ll review the results when they come out	Jingdong prefecture level city in Shandong
IXCHSQ Flagship Store	Green Intelligent Power Saver	Cheat nothing, no change in power at all.	Jingdong prefecture level city in Shandong
IXCHSQ Flagship Store	Green Intelligent Power Saver	It’s not useful at all, plug it in and the electricity bill is as much as it is, don’t buy this if it’s a scam!	Jingdong prefecture level city in Shandong
IXCHSQ Flagship Store	Green Intelligent Power Saver	The quality is ok, the power is still trying for a month	Jingdong prefecture level city in Shandong
IXCHSQ Flagship Store	Green Intelligent Power Saver	Reserve your opinion and review after the research.	Jingdong prefecture level city in Shandong
IXCHSQ Flagship Store	Green Intelligent Power Saver	I’ll review the results when they come out	Jingdong prefecture level city in Shandong
IXCHSQ Flagship Store	Green Intelligent Power Saver	Cheat nothing, no change in power at all.	Jingdong prefecture level city in Shandong
IXCHSQ Flagship Store	Green Intelligent Power Saver	It’s not useful at all, plug it in and the electricity bill is as much as it is, don’t buy this if it’s a scam!	Jingdong prefecture level city in Shandong
IXCHSQ Flagship Store	Green Intelligent Power Saver	The quality is ok, the power is still trying for a month	Jingdong prefecture level city in Shandong
IXCHSQ Flagship Store	Green Intelligent Power Saver	Reserve your opinion and review after the research.	Jingdong prefecture level city in Shandong
IXCHSQ Flagship Store	Green Intelligent Power Saver	I’ll review the results when they come out	Jingdong prefecture level city in Shandong
IXCHSQ Flagship Store	Green Intelligent Power Saver	Cheat nothing, no change in power at all.	Jingdong prefecture level city in Shandong
IXCHSQ Flagship Store	Green Intelligent Power Saver	It’s not useful at all, plug it in and the electricity bill is as much as it is, don’t buy this if it’s a scam!	Jingdong prefecture level city in Shandong
IXCHSQ Flagship Store	Green Intelligent Power Saver	The quality is ok, the power is still trying for a month	Jingdong prefecture level city in Shandong

IXCHSQ Flagship Store	Green Intelligent Power Saver	Reserve your opinion and review after the research.	Jingdong prefecture level city in Shandong
IXCHSQ Flagship Store	Green Intelligent Power Saver	I’ll review the results when they come out	Jingdong prefecture level city in Shandong
IXCHSQ Flagship Store	Green Intelligent Power Saver	Cheat nothing, no change in power at all.	Jingdong prefecture level city in Shandong
IXCHSQ Flagship Store	Green Intelligent Power Saver	It’s not useful at all, plug it in and the electricity bill is as much as it is, don’t buy this if it’s a scam!	Jingdong prefecture level city in Shandong
IXCHSQ Flagship Store	Green Intelligent Power Saver	The quality is ok, the power is still trying for a month	Jingdong prefecture level city in Shandong
IXCHSQ Flagship Store	Green Intelligent Power Saver	Reserve your opinion and review after the research.	Jingdong prefecture level city in Shandong
IXCHSQ Flagship Store	Green Intelligent Power Saver	I’ll review the results when they come out	Jingdong prefecture level city in Shandong
IXCHSQ Flagship Store	Green Intelligent Power Saver	Cheat nothing, no change in power at all.	Jingdong prefecture level city in Shandong
IXCHSQ Flagship Store	Green Intelligent Power Saver	It’s not useful at all, plug it in and the electricity bill is as much as it is, don’t buy this if it’s a scam!	Jingdong prefecture level city in Shandong
IXCHSQ Flagship Store	Green Intelligent Power Saver	The quality is ok, the power is still trying for a month	Jingdong prefecture level city in Shandong
IXCHSQ Flagship Store	Green Intelligent Power Saver	Reserve your opinion and review after the research.	Jingdong prefecture level city in Shandong
IXCHSQ Flagship Store	Green Intelligent Power Saver	I’ll review the results when they come out	Jingdong prefecture level city in Shandong
IXCHSQ Flagship Store	Green Intelligent Power Saver	Cheat nothing, no change in power at all.	Jingdong prefecture level city in Shandong
IXCHSQ Flagship Store	Green Intelligent Power Saver	It’s not useful at all, plug it in and the electricity bill is as much as it is, don’t buy this if it’s a scam!	Jingdong prefecture level city in Shandong
IXCHSQ Flagship Store	Green Intelligent Power Saver	The quality is ok, the power is still trying for a month	Jingdong prefecture level city in Shandong
IXCHSQ Flagship Store	Green Intelligent Power Saver	Reserve your opinion and review after the research.	Jingdong prefecture level city in Shandong
IXCHSQ Flagship Store	Green Intelligent Power Saver	I’ll review the results when they come out	Jingdong prefecture level city in Shandong
IXCHSQ Flagship Store	Green Intelligent Power Saver	Cheat nothing, no change in power at all.	Jingdong prefecture level city in Shandong
IXCHSQ Flagship Store	Green Intelligent Power Saver	It’s not useful at all, plug it in and the electricity bill is as much as it is, don’t buy this if it’s a scam!	Jingdong prefecture level city in Shandong
IXCHSQ Flagship Store	Green Intelligent Power Saver	The quality is ok, the power is still trying for a month	Jingdong prefecture level city in Shandong
IXCHSQ Flagship Store	Green Intelligent Power Saver	Reserve your opinion and review after the research.	Jingdong prefecture level city in Shandong
IXCHSQ Flagship Store	Green Intelligent Power Saver	I’ll review the results when they come out	Jingdong prefecture level city in Shandong
IXCHSQ Flagship Store	Green Intelligent Power Saver	Cheat nothing, no change in power at all.	Jingdong prefecture level city in Shandong
IXCHSQ Flagship Store	Green Intelligent Power Saver	It’s not useful at all, plug it in and the electricity bill is as much as it is, don’t buy this if it’s a scam!	Jingdong prefecture level city in Shandong
IXCHSQ Flagship Store	Green Intelligent Power Saver	The quality is ok, the power is still trying for a month	Jingdong prefecture level city in Shandong
IXCHSQ Flagship Store	Green Intelligent Power Saver	Reserve your opinion and review after the research.	Jingdong prefecture level city in Shandong
IXCHSQ Flagship Store	Green Intelligent Power Saver	I’ll review the results when they come out	Jingdong prefecture level city in Shandong
IXCHSQ Flagship Store	Green Intelligent Power Saver	Cheat nothing, no change in power at all.	Jingdong prefecture level city in Shandong
IXCHSQ Flagship Store	Green Intelligent Power Saver	It’s not useful at all, plug it in and the electricity bill is as much as it is, don’t buy this if it’s a scam!	Jingdong prefecture level city in Shandong
IXCHSQ Flagship Store	Green Intelligent Power Saver	The quality is ok, the power is still trying for a month	Jingdong prefecture level city in Shandong
IXCHSQ Flagship Store	Green Intelligent Power Saver	Reserve your opinion and review after the research.	Jingdong prefecture level city in Shandong
IXCHSQ Flagship Store	Green Intelligent Power Saver	I’ll review the results when they come out	Jingdong prefecture level city in Shandong
IXCHSQ Flagship Store	Green Intelligent Power Saver	Cheat nothing, no change in power at all.	Jingdong prefecture level city in Shandong
IXCHSQ Flagship Store	Green Intelligent Power Saver	It’s not useful at all, plug it in and the electricity bill is as much as it is, don’t buy this if it’s a scam!	Jingdong prefecture level city in Shandong
IXCHSQ Flagship Store	Green Intelligent Power Saver	The quality is ok, the power is still trying for a month	Jingdong prefecture level city in Shandong
IXCHSQ Flagship Store	Green Intelligent Power Saver	Reserve your opinion and review after the research.	Jingdong prefecture level city in Shandong
IXCHSQ Flagship Store	Green Intelligent Power Saver	I’ll review the results when they come out	Jingdong prefecture level city in Shandong
IXCHSQ Flagship Store	Green Intelligent Power Saver	Cheat nothing, no change in power at all.	Jingdong prefecture level city in Shandong
IXCHSQ Flagship Store	Green Intelligent Power Saver	It’s not useful at all, plug it in and the electricity bill is as much as it is, don’t buy this if it’s a scam!	Jingdong prefecture level city in Shandong
IXCHSQ Flagship Store	Green Intelligent Power Saver	The quality is ok, the power is still trying for a month	Jingdong prefecture level city in Shandong
IXCHSQ Flagship Store	Green Intelligent Power Saver	Reserve your opinion and review after the research.	Jingdong prefecture level city in Shandong
IXCHSQ Flagship Store	Green Intelligent Power Saver	I’ll review the results when they come out	Jingdong prefecture level city in Shandong
IXCHSQ Flagship Store	Green Intelligent Power Saver	Cheat nothing, no change in power at all.	Jingdong prefecture level city in Shandong
IXCHSQ Flagship Store	Green Intelligent Power Saver	It’s not useful at all, plug it in and the electricity bill is as much as it is, don’t buy this if it’s a scam!	Jingdong prefecture level city in Shandong
IXCHSQ Flagship Store	Green Intelligent Power Saver	The quality is ok, the power is still trying for a month	Jingdong prefecture level city in Shandong
IXCHSQ Flagship Store	Green Intelligent Power Saver	Reserve your opinion and review after the research.	Jingdong prefecture level city in Shandong
IXCHSQ Flagship Store	Green Intelligent Power Saver	I’ll review the results when they come out	Jingdong prefecture level city in Shandong
IXCHSQ Flagship Store	Green Intelligent Power Saver	Cheat nothing, no change in power at all.	Jingdong prefecture level city in Shandong
IXCHSQ Flagship Store	Green Intelligent Power Saver	It’s not useful at all, plug it in and the electricity bill is as much as it is, don’t buy this if it’s a scam!	Jingdong prefecture level city in Shandong
IXCHSQ Flagship Store	Green Intelligent Power Saver	The quality is ok, the power is still trying for a month	Jingdong prefecture level city in Shandong
IXCHSQ Flagship Store	Green Intelligent Power Saver	Reserve your opinion and review after the research.	Jingdong prefecture level city in Shandong

IXCHSQ Flagship Store	Green Intelligent Power Saver	I’ll review the results when they come out	Jingdong prefecture level city in Shandong
IXCHSQ Flagship Store	Green Intelligent Power Saver	Cheat nothing, no change in power at all.	Jingdong prefecture level city in Shandong
IXCHSQ Flagship Store	Green Intelligent Power Saver	It’s not useful at all, plug it in and the electricity bill is as much as it is, don’t buy this if it’s a scam!	Jingdong prefecture level city in Shandong
IXCHSQ Flagship Store	Green Intelligent Power Saver	The quality is ok, the power is still trying for a month	Jingdong prefecture level city in Shandong
IXCHSQ Flagship Store	Green Intelligent Power Saver	Reserve your opinion and review after the research.	Jingdong prefecture level city in Shandong
IXCHSQ Flagship Store	Green Intelligent Power Saver	I’ll review the results when they come out	Jingdong prefecture level city in Shandong
IXCHSQ Flagship Store	Green Intelligent Power Saver	Cheat nothing, no change in power at all.	Jingdong prefecture level city in Shandong
IXCHSQ Flagship Store	Green Intelligent Power Saver	It’s not useful at all, plug it in and the electricity bill is as much as it is, don’t buy this if it’s a scam!	Jingdong prefecture level city in Shandong
IXCHSQ Flagship Store	Green Intelligent Power Saver	Reserve your opinion and review after the research.	Jingdong prefecture level city in Shandong
IXCHSQ Flagship Store	Green Intelligent Power Saver	The quality is ok, the power is still trying for a month	Jingdong prefecture level city in Shandong
IXCHSQ Flagship Store	Green Intelligent Power Saver	I’ll review the results when they come out	Jingdong prefecture level city in Shandong
IXCHSQ Flagship Store	Green Intelligent Power Saver	Cheat nothing, no change in power at all.	Jingdong prefecture level city in Shandong
IXCHSQ Flagship Store	Green Intelligent Power Saver	It’s not useful at all, plug it in and the electricity bill is as much as it is, don’t buy this if it’s a scam!	Jingdong prefecture level city in Shandong
IXCHSQ Flagship Store	Green Intelligent Power Saver	The quality is ok, the power is still trying for a month	Jingdong prefecture level city in Shandong
IXCHSQ Flagship Store	Green Intelligent Power Saver	Reserve your opinion and review after the research.	Jingdong prefecture level city in Shandong
IXCHSQ Flagship Store	Green Intelligent Power Saver	I’ll review the results when they come out	Jingdong prefecture level city in Shandong
IXCHSQ Flagship Store	Green Intelligent Power Saver	Cheat nothing, no change in power at all.	Jingdong prefecture level city in Shandong
IXCHSQ Flagship Store	Green Intelligent Power Saver	It’s not useful at all, plug it in and the electricity bill is as much as it is, don’t buy this if it’s a scam!	Jingdong prefecture level city in Shandong
IXCHSQ Flagship Store	Green Intelligent Power Saver	The quality is ok, the power is still trying for a month	Jingdong prefecture level city in Shandong
IXCHSQ Flagship Store	Green Intelligent Power Saver	Reserve your opinion and review after the research.	Jingdong prefecture level city in Shandong
IXCHSQ Flagship Store	Green Intelligent Power Saver	I’ll review the results when they come out	Jingdong prefecture level city in Shandong
IXCHSQ Flagship Store	Green Intelligent Power Saver	Cheat nothing, no change in power at all.	Jingdong prefecture level city in Shandong
IXCHSQ Flagship Store	Green Intelligent Power Saver	It’s not useful at all, plug it in and the electricity bill is as much as it is, don’t buy this if it’s a scam!	Jingdong prefecture level city in Shandong
IXCHSQ Flagship Store	Green Intelligent Power Saver	The quality is ok, the power is still trying for a month	Jingdong prefecture level city in Shandong
IXCHSQ Flagship Store	Green Intelligent Power Saver	Reserve your opinion and review after the research.	Jingdong prefecture level city in Shandong
IXCHSQ Flagship Store	Green Intelligent Power Saver	I’ll review the results when they come out	Jingdong prefecture level city in Shandong
IXCHSQ Flagship Store	Green Intelligent Power Saver	Cheat nothing, no change in power at all.	Jingdong prefecture level city in Shandong
IXCHSQ Flagship Store	Green Intelligent Power Saver	It’s not useful at all, plug it in and the electricity bill is as much as it is, don’t buy this if it’s a scam!	Jingdong prefecture level city in Shandong
IXCHSQ Flagship Store	Green Intelligent Power Saver	The quality is ok, the power is still trying for a month	Jingdong prefecture level city in Shandong
IXCHSQ Flagship Store	Green Intelligent Power Saver	Reserve your opinion and review after the research.	Jingdong prefecture level city in Shandong
IXCHSQ Flagship Store	Green Intelligent Power Saver	I’ll review the results when they come out	Jingdong prefecture level city in Shandong
IXCHSQ Flagship Store	Green Intelligent Power Saver	Cheat nothing, no change in power at all.	Jingdong prefecture level city in Shandong
IXCHSQ Flagship Store	Green Intelligent Power Saver	It’s not useful at all, plug it in and the electricity bill is as much as it is, don’t buy this if it’s a scam!	Jingdong prefecture level city in Shandong
IXCHSQ Flagship Store	Green Intelligent Power Saver	The quality is ok, the power is still trying for a month	Jingdong prefecture level city in Shandong
IXCHSQ Flagship Store	Green Intelligent Power Saver	Reserve your opinion and review after the research.	Jingdong prefecture level city in Shandong
IXCHSQ Flagship Store	Green Intelligent Power Saver	I’ll review the results when they come out	Jingdong prefecture level city in Shandong
IXCHSQ Flagship Store	Green Intelligent Power Saver	Cheat nothing, no change in power at all.	Jingdong prefecture level city in Shandong
IXCHSQ Flagship Store	Green Intelligent Power Saver	It’s not useful at all, plug it in and the electricity bill is as much as it is, don’t buy this if it’s a scam!	Jingdong prefecture level city in Shandong
IXCHSQ Flagship Store	Green Intelligent Power Saver	The quality is ok, the power is still trying for a month	Jingdong prefecture level city in Shandong
IXCHSQ Flagship Store	Green Intelligent Power Saver	Reserve your opinion and review after the research.	Jingdong prefecture level city in Shandong
IXCHSQ Flagship Store	Green Intelligent Power Saver	I’ll review the results when they come out	Jingdong prefecture level city in Shandong
IXCHSQ Flagship Store	Green Intelligent Power Saver	Cheat nothing, no change in power at all.	Jingdong prefecture level city in Shandong
IXCHSQ Flagship Store	Green Intelligent Power Saver	It’s not useful at all, plug it in and the electricity bill is as much as it is, don’t buy this if it’s a scam!	Jingdong prefecture level city in Shandong
IXCHSQ Flagship Store	Green Intelligent Power Saver	The quality is ok, the power is still trying for a month	Jingdong prefecture level city in Shandong
IXCHSQ Flagship Store	Green Intelligent Power Saver	I’ll review the results when they come out	Jingdong prefecture level city in Shandong
IXCHSQ Flagship Store	Green Intelligent Power Saver	Reserve your opinion and review after the research.	Jingdong prefecture level city in Shandong
IXCHSQ Flagship Store	Green Intelligent Power Saver	Cheat nothing, no change in power at all.	Jingdong prefecture level city in Shandong
IXCHSQ Flagship Store	Green Intelligent Power Saver	It’s not useful at all, plug it in and the electricity bill is as much as it is, don’t buy this if it’s a scam!	Jingdong prefecture level city in Shandong
IXCHSQ Flagship Store	Green Intelligent Power Saver	The quality is ok, the power is still trying for a month	Jingdong prefecture level city in Shandong
IXCHSQ Flagship Store	Green Intelligent Power Saver	Reserve your opinion and review after the research.	Jingdong prefecture level city in Shandong
IXCHSQ Flagship Store	Green Intelligent Power Saver	I’ll review the results when they come out	Jingdong prefecture level city in Shandong

IXCHSQ Flagship Store	Green Intelligent Power Saver	Cheat nothing, no change in power at all.	Jingdong prefecture level city in Shandong
IXCHSQ Flagship Store	Green Intelligent Power Saver	It’s not useful at all, plug it in and the electricity bill is as much as it is, don’t buy this if it’s a scam!	Jingdong prefecture level city in Shandong
IXCHSQ Flagship Store	Green Intelligent Power Saver	The quality is ok, the power is still trying for a month	Jingdong prefecture level city in Shandong
IXCHSQ Flagship Store	Green Intelligent Power Saver	Reserve your opinion and review after the research.	Jingdong prefecture level city in Shandong
IXCHSQ Flagship Store	Green Intelligent Power Saver	I’ll review the results when they come out	Jingdong prefecture level city in Shandong
IXCHSQ Flagship Store	Green Intelligent Power Saver	Cheat nothing, no change in power at all.	Jingdong prefecture level city in Shandong
IXCHSQ Flagship Store	Green Intelligent Power Saver	It’s not useful at all, plug it in and the electricity bill is as much as it is, don’t buy this if it’s a scam!	Jingdong prefecture level city in Shandong
IXCHSQ Flagship Store	Green Intelligent Power Saver	The quality is ok, the power is still trying for a month	Jingdong prefecture level city in Shandong
IXCHSQ Flagship Store	Green Intelligent Power Saver	Reserve your opinion and review after the research.	Jingdong prefecture level city in Shandong
IXCHSQ Flagship Store	Green Intelligent Power Saver	I’ll review the results when they come out	Jingdong prefecture level city in Shandong
IXCHSQ Flagship Store	Green Intelligent Power Saver	Cheat nothing, no change in power at all.	Jingdong prefecture level city in Shandong
IXCHSQ Flagship Store	Green Intelligent Power Saver	It’s not useful at all, plug it in and the electricity bill is as much as it is, don’t buy this if it’s a scam!	Jingdong prefecture level city in Shandong
IXCHSQ Flagship Store	Green Intelligent Power Saver	The quality is ok, the power is still trying for a month	Jingdong prefecture level city in Shandong
IXCHSQ Flagship Store	Green Intelligent Power Saver	Reserve your opinion and review after the research.	Jingdong prefecture level city in Shandong
IXCHSQ Flagship Store	Green Intelligent Power Saver	I’ll review the results when they come out	Jingdong prefecture level city in Shandong
IXCHSQ Flagship Store	Green Intelligent Power Saver	Cheat nothing, no change in power at all.	Jingdong prefecture level city in Shandong
IXCHSQ Flagship Store	Green Intelligent Power Saver	It’s not useful at all, plug it in and the electricity bill is as much as it is, don’t buy this if it’s a scam!	Jingdong prefecture level city in Shandong
IXCHSQ Flagship Store	Green Intelligent Power Saver	The quality is ok, the power is still trying for a month	Jingdong prefecture level city in Shandong
IXCHSQ Flagship Store	Green Intelligent Power Saver	Reserve your opinion and review after the research.	Jingdong prefecture level city in Shandong
IXCHSQ Flagship Store	Green Intelligent Power Saver	I’ll review the results when they come out	Jingdong prefecture level city in Shandong
IXCHSQ Flagship Store	Green Intelligent Power Saver	Cheat nothing, no change in power at all.	Jingdong prefecture level city in Shandong
IXCHSQ Flagship Store	Green Intelligent Power Saver	It’s not useful at all, plug it in and the electricity bill is as much as it is, don’t buy this if it’s a scam!	Jingdong prefecture level city in Shandong
IXCHSQ Flagship Store	Green Intelligent Power Saver	The quality is ok, the power is still trying for a month	Jingdong prefecture level city in Shandong
IXCHSQ Flagship Store	Green Intelligent Power Saver	Reserve your opinion and review after the research.	Jingdong prefecture level city in Shandong
IXCHSQ Flagship Store	Green Intelligent Power Saver	I’ll review the results when they come out	Jingdong prefecture level city in Shandong
IXCHSQ Flagship Store	Green Intelligent Power Saver	Cheat nothing, no change in power at all.	Jingdong prefecture level city in Shandong
IXCHSQ Flagship Store	Green Intelligent Power Saver	It’s not useful at all, plug it in and the electricity bill is as much as it is, don’t buy this if it’s a scam!	Jingdong prefecture level city in Shandong
IXCHSQ Flagship Store	Green Intelligent Power Saver	The quality is ok, the power is still trying for a month	Jingdong prefecture level city in Shandong
IXCHSQ Flagship Store	Green Intelligent Power Saver	Reserve your opinion and review after the research.	Jingdong prefecture level city in Shandong
IXCHSQ Flagship Store	Green Intelligent Power Saver	I’ll review the results when they come out	Jingdong prefecture level city in Shandong
IXCHSQ Flagship Store	Green Intelligent Power Saver	Cheat nothing, no change in power at all.	Jingdong prefecture level city in Shandong
IXCHSQ Flagship Store	Green Intelligent Power Saver	It’s not useful at all, plug it in and the electricity bill is as much as it is, don’t buy this if it’s a scam!	Jingdong prefecture level city in Shandong
IXCHSQ Flagship Store	Green Intelligent Power Saver	The quality is ok, the power is still trying for a month	Jingdong prefecture level city in Shandong
IXCHSQ Flagship Store	Green Intelligent Power Saver	Reserve your opinion and review after the research.	Jingdong prefecture level city in Shandong
IXCHSQ Flagship Store	Green Intelligent Power Saver	I’ll review the results when they come out	Jingdong prefecture level city in Shandong
IXCHSQ Flagship Store	Green Intelligent Power Saver	Cheat nothing, no change in power at all.	Jingdong prefecture level city in Shandong
IXCHSQ Flagship Store	Green Intelligent Power Saver	It’s not useful at all, plug it in and the electricity bill is as much as it is, don’t buy this if it’s a scam!	Jingdong prefecture level city in Shandong
IXCHSQ Flagship Store	Green Intelligent Power Saver	The quality is ok, the power is still trying for a month	Jingdong prefecture level city in Shandong
IXCHSQ Flagship Store	Green Intelligent Power Saver	Reserve your opinion and review after the research.	Jingdong prefecture level city in Shandong
IXCHSQ Flagship Store	Green Intelligent Power Saver	I’ll review the results when they come out	Jingdong prefecture level city in Shandong
IXCHSQ Flagship Store	Green Intelligent Power Saver	Cheat nothing, no change in power at all.	Jingdong prefecture level city in Shandong
IXCHSQ Flagship Store	Green Intelligent Power Saver	It’s not useful at all, plug it in and the electricity bill is as much as it is, don’t buy this if it’s a scam!	Jingdong prefecture level city in Shandong
IXCHSQ Flagship Store	Green Intelligent Power Saver	The quality is ok, the power is still trying for a month	Jingdong prefecture level city in Shandong
IXCHSQ Flagship Store	Green Intelligent Power Saver	Reserve your opinion and review after the research.	Jingdong prefecture level city in Shandong
IXCHSQ Flagship Store	Green Intelligent Power Saver	I’ll review the results when they come out	Jingdong prefecture level city in Shandong
IXCHSQ Flagship Store	Green Intelligent Power Saver	Cheat nothing, no change in power at all.	Jingdong prefecture level city in Shandong
IXCHSQ Flagship Store	Green Intelligent Power Saver	It’s not useful at all, plug it in and the electricity bill is as much as it is, don’t buy this if it’s a scam!	Jingdong prefecture level city in Shandong
IXCHSQ Flagship Store	Green Intelligent Power Saver	The quality is ok, the power is still trying for a month	Jingdong prefecture level city in Shandong
IXCHSQ Flagship Store	Green Intelligent Power Saver	Reserve your opinion and review after the research.	Jingdong prefecture level city in Shandong
IXCHSQ Flagship Store	Green Intelligent Power Saver	I’ll review the results when they come out	Jingdong prefecture level city in Shandong
IXCHSQ Flagship Store	Green Intelligent Power Saver	Cheat nothing, no change in power at all.	Jingdong prefecture level city in Shandong

IXCHSQ Flagship Store	Green Intelligent Power Saver	Definitely a great product, it's especially easy to use and plugs right into the plug board at home. In the past few days to turn on the air conditioning to see the meter turn a little slower than before. But the electricity consumption is a little less than before. This product is trustworthy, like	Jingdong prefecture level city in Shandong
IXCHSQ Flagship Store	Green Intelligent Power Saver	Indeed can save some, itself I also use electricity not too much, about a month to save more than 100 or so, very can, really good. Colleagues recommended to me, inadvertently told me she bought this with a period of time evaluation, found that quite good, let me also buy a back to try, of course, listen to her words to buy a back to try and found that it is still quite good!	Jingdong prefecture level city in Shandong
IXCHSQ Flagship Store	White Intelligent Power Saver	Baby received immediately broadcast on the electricity used, looks very beautiful and generous, and the number of voltage, delivery and logistics is also very fast, and the same as described, business service attitude is also very good.	Jingdong prefecture level city in Shandong
IXCHSQ Flagship Store	White Intelligent Power Saver	Received intelligent power-saving products, power-saving effect is good, good packaging, the product is intact, the quality is very good, is genuine, with power saving, power saving is real.	Jingdong prefecture level city in Shandong
IXCHSQ Flagship Store	White Intelligent Power Saver	This high-power special edition power-saving equipment, from the appearance of the production process to the rigorous workmanship, belonging to the genuine manufacturing process, saving electricity and money, genuine, I like the service attitude is very enthusiastic and thoughtful.	Jingdong prefecture level city in Shandong
IXCHSQ Flagship Store	a power saver (electronics)	After using a month after a look at the savings of more than 40 dollars, there is a display of electricity savings, this high-power intelligent inverter power saver, affordable and cheap, logistics is very fast, the service attitude is very good, very satisfied with a shopping.	Jingdong prefecture level city in Shandong
IXCHSQ Flagship Store	a power saver (electronics)	Energy saver is really good, really effective, have tried a few days, the home two air conditioners open after the electricity meter dawdling run very fast, after plugging in this energy saver, immediately slowed down a lot, this is a good home appliances energy-saving appliances, good!	Jingdong prefecture level city in Shandong
IXCHSQ Flagship Store	a power saver (electronics)	High-power super saver received immediately used, the price is not expensive quality is very good, save electricity and money, is very convenient, conscientious business, must be praised, specially recommended	Jingdong prefecture level city in Shandong
IXCHSQ Flagship Store	a power saver (electronics)	Good quality and good, as described by customer service, logistics is very fast.	Jingdong prefecture level city in Shandong
IXCHSQ Flagship Store	a power saver (electronics)	Recommended to me by a friend, it does have power-saving effect.	Jingdong prefecture level city in Shandong
IXCHSQ Flagship Store	a power saver (electronics)	The power saver received, tested really save electricity, power saving effect is very good, product manual, after-sales commitment, maintenance warranty card, genuine logo should be complete, is genuine.	Jingdong prefecture level city in Shandong
IXCHSQ Flagship Store	a power saver (electronics)	With this you can turn on the air conditioner as you like. I tried it and it saves energy.	Jingdong prefecture level city in Shandong
IXCHSQ Flagship Store	a power saver (electronics)	The quality is very good, the delivery speed is very timely, praise!	Jingdong prefecture level city in Shandong
IXCHSQ Flagship Store	a power saver (electronics)	The product did not work at all.	Jingdong prefecture level city in Shandong
IXCHSQ Flagship Store	King of Power Saving	High-power power saver has been received, well-packed, fast logistics, the quality of the power saver is very good, texture, one machine, one code, anti-counterfeiting marking. Has been used, looked at the meter, it does turn slower, work all normal.	Jingdong prefecture level city in Shandong
IXCHSQ Flagship Store	King of Power Saving	Good use! This month only 93.75 yuan, last month 345 yuan, very power saving, very powerful saver, plugged into the outlet directly can be used, save a lot of electricity ah, recommend that everyone get!	Jingdong prefecture level city in Shandong
IXCHSQ Flagship Store	King of Power Saving	Use the pipe thing to save thirty percent, the quality of the economizer is very good, good-looking, plug and play is very convenient, the price is not expensive but also can save a lot of electricity, cost-	Jingdong prefecture level city in Shandong
IXCHSQ Flagship Store	King of Power Saving	Baby received, thanks! I wish the store a prosperous business and a lot of money!	Jingdong prefecture level city in Shandong
IXCHSQ Flagship Store	King of Power Saving	The first time to buy this energy saver, do not know that there is this thing, hold a try to buy the mentality, the business commitment to no effect full refund, after receiving the product there is a manual, it is very convenient, the saver is compact and does not take up space, my family's power consumption is very large, the purchase of 6, the effect of the open after the chase comment!	Jingdong prefecture level city in Shandong
IXCHSQ Flagship Store	King of Power Saving	Home two-story residential building, listening to the seller of a layer inserted a, used more than 40 days, the effect is still relatively good, save more than 100 points. It is worth buying, the service after sale is also very thoughtful, customer service explained in detail, very professional!	Jingdong prefecture level city in Shandong
IXCHSQ Flagship Store	King of Power Saving	Purposefully try the electricity, air conditioning on the electricity quickly go slower, my family light monthly electricity bill of more than 7 hundred, can save a lot. Electrical appliances more very costly, since the use of this after saving a lot of electricity, ready to get two more, very good. Looking forward to more surprises next month	Jingdong prefecture level city in Shandong
IXCHSQ Flagship Store	King of Power Saving	After using a month after a look at the savings of more than 40 dollars, there is a display of electricity savings, this high-power intelligent inverter power saver, affordable and cheap, logistics is very fast, the service attitude is very good, very satisfied with a shopping.	Jingdong prefecture level city in Shandong
IXCHSQ Flagship Store	King of Power Saving	There are a lot of appliances in the house, the summer air conditioning is very costly, I bought this in front of the air conditioning effect is very good, I explain that I bought three in one breath, I use more electricity a month down to 200 or so, very satisfied.	Jingdong prefecture level city in Shandong
IXCHSQ Flagship Store	King of Power Saving	The voltage is very stable has been maintained at 220v this value, high power super power saving, trustworthy, reasonable design, shape bright, logistics is quite fast, business warranty period, excellent service, logistics fast!	Jingdong prefecture level city in Shandong
IXCHSQ Flagship Store	King of Power Saving	Use a month after a look at the savings of more than 40 dollars, there is a display of electricity savings, this high-power intelligent frequency converter economizer, affordable and cheap, logistics is very fast, the service attitude is very good, very satisfied with a shopping.	Jingdong prefecture level city in Shandong
IXCHSQ Flagship Store	King of Power Saving	Used for a period of time, it seems that every day can really can save a few degrees of electricity, workmanship quite can, with a period of time and then continue to look at it, anyway, the price is not	Jingdong prefecture level city in Shandong
IXCHSQ Flagship Store	King of Power Saving	Used for a period of time, it seems that every day can indeed save two several degrees of electricity, workmanship quite can, with a period of time and then continue to look at it, anyway, the price is not	Jingdong prefecture level city in Shandong
IXCHSQ Flagship Store	King of Power Saving	High-power super saver received immediately used, the price is not expensive quality is very good, save electricity and money, very convenient, conscientious business, must be praised, recommended to everyone!	Jingdong prefecture level city in Shandong
IXCHSQ Flagship Store	King of Power Saving	Home enhancement version of the intelligent power saver, is in use, I think the price is cheap, value for money, efficacy, power, power saving effect is very good.	Jingdong prefecture level city in Shandong
IXCHSQ Flagship Store	King of Power Saving	Bought three at once, one for each room, summer electricity consumption is very high, despite the three air conditioners on the electricity bill is still down a lot, too Nessie!	Jingdong prefecture level city in Shandong
IXCHSQ Flagship Store	King of Power Saving	Without further ado, the electric bill came down, $102 less this month, personal satisfaction, it counts okay, give you a good review	Jingdong prefecture level city in Shandong
IXCHSQ Flagship Store	King of Power Saving	The power saver has arrived, the installation is really convenient, plugged in on the line operation is quite simple, power saving effect is also a strong!	Jingdong prefecture level city in Shandong
IXCHSQ Flagship Store	King of Power Saving	Saver received, good quality, beautiful appearance, fine workmanship, simple installation	Jingdong prefecture level city in Shandong
IXCHSQ Flagship Store	King of Power Saving	Product design style modern, worth buying	Jingdong prefecture level city in Shandong
IXCHSQ Flagship Store	King of Power Saving	Energy saving, good power saving effect, practical, great value	Jingdong prefecture level city in Shandong
IXCHSQ Flagship Store	King of Power Saving	It's good quality, it's good, it's energy efficient, and I'll buy it again.	Jingdong prefecture level city in Shandong
IXCHSQ Flagship Store	King of Power Saving	Installation saves a lot of electricity, fast logistics, real energy-saving appliances	Jingdong prefecture level city in Shandong
IXCHSQ Flagship Store	King of Power Saving	The effect is good and the power saving is obvious after using it for a month and saving tens of dollars, the product is trustworthy, I now buy another 4 colors to prepare for their siblings' family to pay for	Jingdong prefecture level city in Shandong
IXCHSQ Flagship Store	King of Power Saving	The quality is particularly good workmanship is no problem, the arrival of the very fast oh, especially power-saving	Jingdong prefecture level city in Shandong
IXCHSQ Flagship Store	King of Power Saving	The quality is very good! In use, observed for a day, not as used before, so much power consumption.	Jingdong prefecture level city in Shandong
IXCHSQ Flagship Store	King of Power Saving	Goods have been received, the effect is good is not good, power saving is not yet know, wait for me to use it and then evaluate it.	Jingdong prefecture level city in Shandong
IXCHSQ Flagship Store	King of Power Saving	In-kind and the same as described, the quality is quite good, the seller's attitude is also good, ask questions and answer them, the speed of delivery bar, worth buying Oh!	Jingdong prefecture level city in Shandong
IXCHSQ Flagship Store	King of Power Saving	Nice stuff, came to comment after using it, it does save power.	Jingdong prefecture level city in Shandong

IXCHSQ Flagship Store	King of Power Saving	Fast charging, but also save power, the effect is very good, satisfied, praise!	Jingdong prefecture level city in Shandong
IXCHSQ Flagship Store	King of Power Saving	The goods have been received, I do not know how effective, after using the flat price again!	Jingdong prefecture level city in Shandong
IXCHSQ Flagship Store	King of Power Saving	Merchant service is very good Reply quickly The key is the quality and that quality is good	Jingdong prefecture level city in Shandong
IXCHSQ Flagship Store	King of Power Saving	It's working well now, I'll do a follow up review after I actually use it for a while	Jingdong prefecture level city in Shandong
IXCHSQ Flagship Store	King of Power Saving	The quality is good. After installing it, I feel like I've saved a lot of money on my electricity bill.	Jingdong prefecture level city in Shandong
IXCHSQ Flagship Store	King of Power Saving	Make up a review after use.	Jingdong prefecture level city in Shandong
IXCHSQ Flagship Store	King of Power Saving	review after use	Jingdong prefecture level city in Shandong
IXCHSQ Flagship Store	King of Power Saving	It's a small capacitor. It's not energy efficient.	Jingdong prefecture level city in Shandong
IXCHSQ Flagship Store	King of Power Saving	I'll follow up with a review after I've used it and had results.	Jingdong prefecture level city in Shandong
IXCHSQ Flagship Store	King of Power Saving	No power saving? Not good!	Jingdong prefecture level city in Shandong
IXCHSQ Flagship Store	King of Power Saving	Been using the same appliances and running a 1.5 air conditioner at night for what feels like no time, and after half a month of use it feels like the power is only increasing. Doesn't show any power saving	Jingdong prefecture level city in Shandong
IXCHSQ Flagship Store	King of Power Saving	serviceable	Jingdong prefecture level city in Shandong
IXCHSQ Flagship Store	King of Power Saving	It's a piece of junk. It doesn't save any power.	Jingdong prefecture level city in Shandong
IXCHSQ Flagship Store	King of Power Saving	Use the pipe thing to save thirty percent, the quality of the economizer is very good, good-looking, plug and play is very convenient, the price is not expensive but also can save a lot of electricity, cost-	Jingdong prefecture level city in Shandong
IXCHSQ Flagship Store	King of Power Saving	There are a lot of appliances in the house, the summer air conditioning is very costly, I bought this in front of the air conditioning effect is very good, I explain that I bought three in one breath, I use more electricity a month down to 200 or so, very satisfied.	Jingdong prefecture level city in Shandong
IXCHSQ Flagship Store	King of Power Saving	The voltage is very stable has been maintained at 220v this value, high power super power saving, trustworthy, reasonable design, shape bright, logistics is quite fast, business warranty period, excellent service, logistics fast!	Jingdong prefecture level city in Shandong
IXCHSQ Flagship Store	King of Power Saving	Use a month after a look at the savings of more than 40 dollars, there is a display of electricity savings, this high-power intelligent frequency converter economizer, affordable and cheap, logistics is very fast, the service attitude is very good, very satisfied with a shopping.	Jingdong prefecture level city in Shandong
IXCHSQ Flagship Store	King of Power Saving	Used for a period of time, it seems that every day can really can save a few degrees of electricity, workmanship quite can, with a period of time and then continue to look at it, anyway, the price is not	Jingdong prefecture level city in Shandong
IXCHSQ Flagship Store	King of Power Saving	Used for a period of time, it seems that every day can indeed save two several degrees of electricity, workmanship quite can, with a period of time and then continue to look at it, anyway, the price is not	Jingdong prefecture level city in Shandong
IXCHSQ Flagship Store	King of Power Saving	High-power super saver received immediately used, the price is not expensive quality is very good, save electricity and money, very convenient, conscientious business, must be praised, recommended to everyone!	Jingdong prefecture level city in Shandong
IXCHSQ Flagship Store	King of Power Saving	Home enhancement version of the intelligent power saver, is in use, I think the price is cheap, value for money, efficacy, power, power saving effect is very good.	Jingdong prefecture level city in Shandong
IXCHSQ Flagship Store	King of Power Saving	Bought three at once, one for each room, summer electricity consumption is very high, despite the three air conditioners on the electricity bill is still down a lot, too Nessie!	Jingdong prefecture level city in Shandong
IXCHSQ Flagship Store	King of Power Saving	Without further ado, the electric bill came down, $102 less this month, personal satisfaction, it counts okay, give you a good review	Jingdong prefecture level city in Shandong
IXCHSQ Flagship Store	King of Power Saving	The power saver has arrived, the installation is really convenient, plugged in on the line operation is quite simple, power saving effect is also a strong!	Jingdong prefecture level city in Shandong
IXCHSQ Flagship Store	King of Power Saving	Saver received, good quality, beautiful appearance, fine workmanship, simple installation	Jingdong prefecture level city in Shandong
IXCHSQ Flagship Store	King of Power Saving	Product design style modern, worth buying	Jingdong prefecture level city in Shandong
IXCHSQ Flagship Store	King of Power Saving	Energy saving, good power saving effect, practical, great value	Jingdong prefecture level city in Shandong
IXCHSQ Flagship Store	King of Power Saving	It's good quality, it's good, it's energy efficient, and I'll buy it again.	Jingdong prefecture level city in Shandong
IXCHSQ Flagship Store	King of Power Saving	Installation saves a lot of electricity, fast logistics, real energy-saving appliances	Jingdong prefecture level city in Shandong
IXCHSQ Flagship Store	King of Power Saving	The effect is good and the power saving is obvious after using it for a month and saving tens of dollars, the product is trustworthy, I now buy another 4 colors to prepare for their siblings' family to pay for	Jingdong prefecture level city in Shandong
IXCHSQ Flagship Store	King of Power Saving	The quality is particularly good workmanship is no problem, the arrival of the very fast oh, especially power-saving	Jingdong prefecture level city in Shandong
IXCHSQ Flagship Store	King of Power Saving	The quality is very good! In use, observed for a day, not as used before, so much power consumption.	Jingdong prefecture level city in Shandong
IXCHSQ Flagship Store	King of Power Saving	Goods have been received, the effect is good is not good, power saving is not yet know, wait for me to use it and then evaluate it.	Jingdong prefecture level city in Shandong
IXCHSQ Flagship Store	King of Power Saving	In-kind and the same as described, the quality is quite good, the seller's attitude is also good, ask questions and answer them, the speed of delivery bar, worth buying Oh!	Jingdong prefecture level city in Shandong
IXCHSQ Flagship Store	King of Power Saving	Nice stuff, came to comment after using it, it does save power.	Jingdong prefecture level city in Shandong
IXCHSQ Flagship Store	King of Power Saving	Fast charging, but also save power, the effect is very good, satisfied, praise!	Jingdong prefecture level city in Shandong
IXCHSQ Flagship Store	King of Power Saving	The goods have been received, I do not know how effective, after using the flat price again!	Jingdong prefecture level city in Shandong
IXCHSQ Flagship Store	King of Power Saving	Merchant service is very good Reply quickly The key is the quality and that quality is good	Jingdong prefecture level city in Shandong
IXCHSQ Flagship Store	King of Power Saving	It's working well now, I'll do a follow up review after I actually use it for a while	Jingdong prefecture level city in Shandong
IXCHSQ Flagship Store	King of Power Saving	The quality is good. After installing it, I feel like I've saved a lot of money on my electricity bill.	Jingdong prefecture level city in Shandong
IXCHSQ Flagship Store	King of Power Saving	Make up a review after use.	Jingdong prefecture level city in Shandong
IXCHSQ Flagship Store	King of Power Saving	review after use	Jingdong prefecture level city in Shandong
IXCHSQ Flagship Store	King of Power Saving	It's a small capacitor. It's not energy efficient.	Jingdong prefecture level city in Shandong
IXCHSQ Flagship Store	King of Power Saving	I'll follow up with a review after I've used it and had results.	Jingdong prefecture level city in Shandong
IXCHSQ Flagship Store	King of Power Saving	No power saving? Not good!	Jingdong prefecture level city in Shandong
IXCHSQ Flagship Store	King of Power Saving	Been using the same appliances and running a 1.5 air conditioner at night for what feels like no time, and after half a month of use it feels like the power is only increasing. Doesn't show any power saving	Jingdong prefecture level city in Shandong
IXCHSQ Flagship Store	King of Power Saving	serviceable	Jingdong prefecture level city in Shandong
IXCHSQ Flagship Store	King of Power Saving	It's a piece of junk. It doesn't save any power.	Jingdong prefecture level city in Shandong
IXCHSQ Flagship Store	King of Power Saving	Use the pipe thing to save thirty percent, the quality of the economizer is very good, good-looking, plug and play is very convenient, the price is not expensive but also can save a lot of electricity, cost-	Jingdong prefecture level city in Shandong

IXCHSQ Flagship Store	King of Power Saving	There are a lot of appliances in the house, the summer air conditioning is very costly, I bought this in front of the air conditioning effect is very good, I explain that I bought three in one breath, I use more electricity a month down to 200 or so, very satisfied.	Jingdong prefecture level city in Shandong
IXCHSQ Flagship Store	King of Power Saving	The voltage is very stable has been maintained at 220v this value, high power super power saving, trustworthy, reasonable design, shape bright, logistics is quite fast, business warranty period, excellent service, logistics fast!	Jingdong prefecture level city in Shandong
IXCHSQ Flagship Store	King of Power Saving	Use a month after a look at the savings of more than 40 dollars, there is a display of electricity savings, this high-power intelligent frequency converter economizer, affordable and cheap, logistics is very fast, the service attitude is very good, very satisfied with a shopping.	Jingdong prefecture level city in Shandong
IXCHSQ Flagship Store	King of Power Saving	Used for a period of time, it seems that every day can really can save a few degrees of electricity, workmanship quite can, with a period of time and then continue to look at it, anyway, the price is not	Jingdong prefecture level city in Shandong
IXCHSQ Flagship Store	King of Power Saving	Used for a period of time, it seems that every day can indeed save two several degrees of electricity, workmanship quite can, with a period of time and then continue to look at it, anyway, the price is not	Jingdong prefecture level city in Shandong
IXCHSQ Flagship Store	King of Power Saving	High-power super saver received immediately used, the price is not expensive quality is very good, save electricity and money, very convenient, conscientious business, must be praised, recommended to everyone!	Jingdong prefecture level city in Shandong
IXCHSQ Flagship Store	King of Power Saving	Home enhancement version of the intelligent power saver, is in use, I think the price is cheap, value for money, efficacy, power, power saving effect is very good.	Jingdong prefecture level city in Shandong
IXCHSQ Flagship Store	King of Power Saving	Bought three at once, one for each room, summer electricity consumption is very high, despite the three air conditioners on the electricity bill is still down a lot, too Nessie!	Jingdong prefecture level city in Shandong
IXCHSQ Flagship Store	King of Power Saving	Without further ado, the electric bill came down, $102 less this month, personal satisfaction, it counts okay, give you a good review	Jingdong prefecture level city in Shandong
IXCHSQ Flagship Store	King of Power Saving	The power saver has arrived, the installation is really convenient, plugged in on the line operation is quite simple, power saving effect is also a strong!	Jingdong prefecture level city in Shandong
IXCHSQ Flagship Store	King of Power Saving	Saver received, good quality, beautiful appearance, fine workmanship, simple installation	Jingdong prefecture level city in Shandong
IXCHSQ Flagship Store	King of Power Saving	Product design style modern, worth buying	Jingdong prefecture level city in Shandong
IXCHSQ Flagship Store	King of Power Saving	Energy saving, good power saving effect, practical, great value	Jingdong prefecture level city in Shandong
IXCHSQ Flagship Store	King of Power Saving	It's good quality, it's good, it's energy efficient, and I'll buy it again.	Jingdong prefecture level city in Shandong
IXCHSQ Flagship Store	King of Power Saving	Installation saves a lot of electricity, fast logistics, real energy-saving appliances	Jingdong prefecture level city in Shandong
IXCHSQ Flagship Store	King of Power Saving	The effect is good and the power saving is obvious after using it for a month and saving tens of dollars, the product is trustworthy, I now buy another 4 colors to prepare for their siblings' family to pay for	Jingdong prefecture level city in Shandong
IXCHSQ Flagship Store	King of Power Saving	The quality is particularly good workmanship is no problem, the arrival of the very fast oh, especially power-saving	Jingdong prefecture level city in Shandong
IXCHSQ Flagship Store	King of Power Saving	The quality is very good! In use, observed for a day, not as used before, so much power consumption.	Jingdong prefecture level city in Shandong
IXCHSQ Flagship Store	King of Power Saving	Goods have been received, the effect is good is not good, power saving is not yet know, wait for me to use it and then evaluate it.	Jingdong prefecture level city in Shandong
IXCHSQ Flagship Store	King of Power Saving	In-kind and the same as described, the quality is quite good, the seller's attitude is also good, ask questions and answer them, the speed of delivery bar, worth buying Oh!	Jingdong prefecture level city in Shandong
IXCHSQ Flagship Store	King of Power Saving	Nice stuff, came to comment after using it, it does save power.	Jingdong prefecture level city in Shandong
IXCHSQ Flagship Store	King of Power Saving	Fast charging, but also save power, the effect is very good, satisfied, praise!	Jingdong prefecture level city in Shandong
IXCHSQ Flagship Store	King of Power Saving	The goods have been received, I do not know how effective, after using the flat price again!	Jingdong prefecture level city in Shandong
IXCHSQ Flagship Store	King of Power Saving	Merchant service is very good Reply quickly The key is the quality and that quality is good	Jingdong prefecture level city in Shandong
IXCHSQ Flagship Store	King of Power Saving	It's working well now, I'll do a follow up review after I actually use it for a while	Jingdong prefecture level city in Shandong
IXCHSQ Flagship Store	King of Power Saving	The quality is good. After installing it, I feel like I've saved a lot of money on my electricity bill.	Jingdong prefecture level city in Shandong
IXCHSQ Flagship Store	King of Power Saving	Make up a review after use.	Jingdong prefecture level city in Shandong
IXCHSQ Flagship Store	King of Power Saving	review after use	Jingdong prefecture level city in Shandong
IXCHSQ Flagship Store	King of Power Saving	It's a small capacitor. It's not energy efficient.	Jingdong prefecture level city in Shandong
IXCHSQ Flagship Store	King of Power Saving	I'll follow up with a review after I've used it and had results.	Jingdong prefecture level city in Shandong
IXCHSQ Flagship Store	King of Power Saving	No power saving? Not good!	Jingdong prefecture level city in Shandong
IXCHSQ Flagship Store	King of Power Saving	Been using the same appliances and running a 1.5 air conditioner at night for what feels like no time, and after half a month of use it feels like the power is only increasing. Doesn't show any power saving	Jingdong prefecture level city in Shandong
IXCHSQ Flagship Store	King of Power Saving	serviceable	Jingdong prefecture level city in Shandong
IXCHSQ Flagship Store	King of Power Saving	It's a piece of junk. It doesn't save any power.	Jingdong prefecture level city in Shandong
IXCHSQ Flagship Store	King of Power Saving	Use the pipe thing to save thirty percent, the quality of the economizer is very good, good-looking, plug and play is very convenient, the price is not expensive but also can save a lot of electricity, cost-	Jingdong prefecture level city in Shandong
IXCHSQ Flagship Store	King of Power Saving	There are a lot of appliances in the house, the summer air conditioning is very costly, I bought this in front of the air conditioning effect is very good, I explain that I bought three in one breath, I use more electricity a month down to 200 or so, very satisfied.	Jingdong prefecture level city in Shandong
IXCHSQ Flagship Store	King of Power Saving	The voltage is very stable has been maintained at 220v this value, high power super power saving, trustworthy, reasonable design, shape bright, logistics is quite fast, business warranty period, excellent service, logistics fast!	Jingdong prefecture level city in Shandong
IXCHSQ Flagship Store	King of Power Saving	Use a month after a look at the savings of more than 40 dollars, there is a display of electricity savings, this high-power intelligent frequency converter economizer, affordable and cheap, logistics is very fast, the service attitude is very good, very satisfied with a shopping.	Jingdong prefecture level city in Shandong
IXCHSQ Flagship Store	King of Power Saving	Used for a period of time, it seems that every day can really can save a few degrees of electricity, workmanship quite can, with a period of time and then continue to look at it, anyway, the price is not	Jingdong prefecture level city in Shandong
IXCHSQ Flagship Store	King of Power Saving	Used for a period of time, it seems that every day can indeed save two several degrees of electricity, workmanship quite can, with a period of time and then continue to look at it, anyway, the price is not	Jingdong prefecture level city in Shandong
IXCHSQ Flagship Store	King of Power Saving	High-power super saver received immediately used, the price is not expensive quality is very good, save electricity and money, very convenient, conscientious business, must be praised, recommended to everyone!	Jingdong prefecture level city in Shandong
IXCHSQ Flagship Store	King of Power Saving	Home enhancement version of the intelligent power saver, is in use, I think the price is cheap, value for money, efficacy, power, power saving effect is very good.	Jingdong prefecture level city in Shandong
IXCHSQ Flagship Store	King of Power Saving	Bought three at once, one for each room, summer electricity consumption is very high, despite the three air conditioners on the electricity bill is still down a lot, too Nessie!	Jingdong prefecture level city in Shandong
IXCHSQ Flagship Store	King of Power Saving	Without further ado, the electric bill came down, $102 less this month, personal satisfaction, it counts okay, give you a good review	Jingdong prefecture level city in Shandong
IXCHSQ Flagship Store	King of Power Saving	The power saver has arrived, the installation is really convenient, plugged in on the line operation is quite simple, power saving effect is also a strong!	Jingdong prefecture level city in Shandong
IXCHSQ Flagship Store	King of Power Saving	Saver received, good quality, beautiful appearance, fine workmanship, simple installation	Jingdong prefecture level city in Shandong
IXCHSQ Flagship Store	King of Power Saving	Product design style modern, worth buying	Jingdong prefecture level city in Shandong
IXCHSQ Flagship Store	King of Power Saving	Energy saving, good power saving effect, practical, great value	Jingdong prefecture level city in Shandong

IXCHSQ Flagship Store	King of Power Saving	It's good quality, it's good, it's energy efficient, and I'll buy it again.	Jingdong prefecture level city in Shandong
IXCHSQ Flagship Store	King of Power Saving	Installation saves a lot of electricity, fast logistics, real energy-saving appliances	Jingdong prefecture level city in Shandong
IXCHSQ Flagship Store	King of Power Saving	The effect is good and the power saving is obvious after using it for a month and saving tens of dollars, the product is trustworthy, I now buy another 4 colors to prepare for their siblings' family to pay for	Jingdong prefecture level city in Shandong
IXCHSQ Flagship Store	King of Power Saving	The quality is particularly good workmanship is no problem, the arrival of the very fast oh, especially power-saving	Jingdong prefecture level city in Shandong
IXCHSQ Flagship Store	King of Power Saving	The quality is very good! In use, observed for a day, not as used before, so much power consumption.	Jingdong prefecture level city in Shandong
IXCHSQ Flagship Store	King of Power Saving	Goods have been received, the effect is good is not good, power saving is not yet know, wait for me to use it and then evaluate it.	Jingdong prefecture level city in Shandong
IXCHSQ Flagship Store	King of Power Saving	In-kind and the same as described, the quality is quite good, the seller's attitude is also good, ask questions and answer them, the speed of delivery bar, worth buying Oh!	Jingdong prefecture level city in Shandong
IXCHSQ Flagship Store	King of Power Saving	Nice stuff, came to comment after using it, it does save power.	Jingdong prefecture level city in Shandong
IXCHSQ Flagship Store	King of Power Saving	Fast charging, but also save power, the effect is very good, satisfied, praise!	Jingdong prefecture level city in Shandong
IXCHSQ Flagship Store	King of Power Saving	The goods have been received, I do not know how effective, after using the flat price again!	Jingdong prefecture level city in Shandong
IXCHSQ Flagship Store	King of Power Saving	Merchant service is very good Reply quickly The key is the quality and that quality is good	Jingdong prefecture level city in Shandong
IXCHSQ Flagship Store	King of Power Saving	It's working well now, I'll do a follow up review after I actually use it for a while	Jingdong prefecture level city in Shandong
IXCHSQ Flagship Store	King of Power Saving	The quality is good. After installing it, I feel like I've saved a lot of money on my electricity bill.	Jingdong prefecture level city in Shandong
IXCHSQ Flagship Store	King of Power Saving	Make up a review after use.	Jingdong prefecture level city in Shandong
IXCHSQ Flagship Store	King of Power Saving	review after use	Jingdong prefecture level city in Shandong
IXCHSQ Flagship Store	King of Power Saving	It's a small capacitor. It's not energy efficient.	Jingdong prefecture level city in Shandong
IXCHSQ Flagship Store	King of Power Saving	I'll follow up with a review after I've used it and had results.	Jingdong prefecture level city in Shandong
IXCHSQ Flagship Store	King of Power Saving	No power saving? Not good!	Jingdong prefecture level city in Shandong
IXCHSQ Flagship Store	King of Power Saving	Been using the same appliances and running a 1.5 air conditioner at night for what feels like no time, and after half a month of use it feels like the power is only increasing. Doesn't show any power saving	Jingdong prefecture level city in Shandong
IXCHSQ Flagship Store	King of Power Saving	serviceable	Jingdong prefecture level city in Shandong
IXCHSQ Flagship Store	King of Power Saving	It's a piece of junk. It doesn't save any power.	Jingdong prefecture level city in Shandong
IXCHSQ Flagship Store	King of Power Saving	Use the pipe thing to save thirty percent, the quality of the economizer is very good, good-looking, plug and play is very convenient, the price is not expensive but also can save a lot of electricity, cost-	Jingdong prefecture level city in Shandong
IXCHSQ Flagship Store	King of Power Saving	There are a lot of appliances in the house, the summer air conditioning is very costly, I bought this in front of the air conditioning effect is very good, I explain that I bought three in one breath, I use more electricity a month down to 200 or so, very satisfied.	Jingdong prefecture level city in Shandong
IXCHSQ Flagship Store	King of Power Saving	The voltage is very stable has been maintained at 220v this value, high power super power saving, trustworthy, reasonable design, shape bright, logistics is quite fast, business warranty period, excellent service, logistics fast!	Jingdong prefecture level city in Shandong
IXCHSQ Flagship Store	King of Power Saving	Use a month after a look at the savings of more than 40 dollars, there is a display of electricity savings, this high-power intelligent frequency converter economizer, affordable and cheap, logistics is very fast, the service attitude is very good, very satisfied with a shopping.	Jingdong prefecture level city in Shandong
IXCHSQ Flagship Store	King of Power Saving	Used for a period of time, it seems that every day can really can save a few degrees of electricity, workmanship quite can, with a period of time and then continue to look at it, anyway, the price is not	Jingdong prefecture level city in Shandong
IXCHSQ Flagship Store	King of Power Saving	Used for a period of time, it seems that every day can indeed save two several degrees of electricity, workmanship quite can, with a period of time and then continue to look at it, anyway, the price is not	Jingdong prefecture level city in Shandong
IXCHSQ Flagship Store	King of Power Saving	High-power super saver received immediately used, the price is not expensive quality is very good, save electricity and money, very convenient, conscientious business, must be praised, recommended to everyone!	Jingdong prefecture level city in Shandong
IXCHSQ Flagship Store	King of Power Saving	Home enhancement version of the intelligent power saver, is in use, I think the price is cheap, value for money, efficacy, power, power saving effect is very good.	Jingdong prefecture level city in Shandong
IXCHSQ Flagship Store	King of Power Saving	Bought three at once, one for each room, summer electricity consumption is very high, despite the three air conditioners on the electricity bill is still down a lot, too Nessie!	Jingdong prefecture level city in Shandong
IXCHSQ Flagship Store	King of Power Saving	Without further ado, the electric bill came down, $102 less this month, personal satisfaction, it counts okay, give you a good review	Jingdong prefecture level city in Shandong
IXCHSQ Flagship Store	King of Power Saving	The power saver has arrived, the installation is really convenient, plugged in on the line operation is quite simple, power saving effect is also a strong!	Jingdong prefecture level city in Shandong
IXCHSQ Flagship Store	King of Power Saving	Saver received, good quality, beautiful appearance, fine workmanship, simple installation	Jingdong prefecture level city in Shandong
IXCHSQ Flagship Store	King of Power Saving	Product design style modern, worth buying	Jingdong prefecture level city in Shandong
IXCHSQ Flagship Store	King of Power Saving	Energy saving, good power saving effect, practical, great value	Jingdong prefecture level city in Shandong
IXCHSQ Flagship Store	King of Power Saving	It's good quality, it's good, it's energy efficient, and I'll buy it again.	Jingdong prefecture level city in Shandong
IXCHSQ Flagship Store	King of Power Saving	Installation saves a lot of electricity, fast logistics, real energy-saving appliances	Jingdong prefecture level city in Shandong
IXCHSQ Flagship Store	King of Power Saving	The effect is good and the power saving is obvious after using it for a month and saving tens of dollars, the product is trustworthy, I now buy another 4 colors to prepare for their siblings' family to pay for	Jingdong prefecture level city in Shandong
IXCHSQ Flagship Store	King of Power Saving	The quality is particularly good workmanship is no problem, the arrival of the very fast oh, especially power-saving	Jingdong prefecture level city in Shandong
IXCHSQ Flagship Store	King of Power Saving	The quality is very good! In use, observed for a day, not as used before, so much power consumption.	Jingdong prefecture level city in Shandong
IXCHSQ Flagship Store	King of Power Saving	Goods have been received, the effect is good is not good, power saving is not yet know, wait for me to use it and then evaluate it.	Jingdong prefecture level city in Shandong
IXCHSQ Flagship Store	King of Power Saving	In-kind and the same as described, the quality is quite good, the seller's attitude is also good, ask questions and answer them, the speed of delivery bar, worth buying Oh!	Jingdong prefecture level city in Shandong
IXCHSQ Flagship Store	King of Power Saving	Nice stuff, came to comment after using it, it does save power.	Jingdong prefecture level city in Shandong
IXCHSQ Flagship Store	King of Power Saving	Fast charging, but also save power, the effect is very good, satisfied, praise!	Jingdong prefecture level city in Shandong
IXCHSQ Flagship Store	King of Power Saving	The goods have been received, I do not know how effective, after using the flat price again!	Jingdong prefecture level city in Shandong
IXCHSQ Flagship Store	King of Power Saving	Merchant service is very good Reply quickly The key is the quality and that quality is good	Jingdong prefecture level city in Shandong
IXCHSQ Flagship Store	King of Power Saving	It's working well now, I'll do a follow up review after I actually use it for a while	Jingdong prefecture level city in Shandong
IXCHSQ Flagship Store	King of Power Saving	The quality is good. After installing it, I feel like I've saved a lot of money on my electricity bill.	Jingdong prefecture level city in Shandong
IXCHSQ Flagship Store	King of Power Saving	Make up a review after use.	Jingdong prefecture level city in Shandong
IXCHSQ Flagship Store	King of Power Saving	review after use	Jingdong prefecture level city in Shandong

IXCHSQ Flagship Store	King of Power Saving	It's a small capacitor. It's not energy efficient.	Jingdong prefecture level city in Shandong
IXCHSQ Flagship Store	King of Power Saving	I'll follow up with a review after I've used it and had results.	Jingdong prefecture level city in Shandong
IXCHSQ Flagship Store	King of Power Saving	No power saving? Not good!	Jingdong prefecture level city in Shandong
IXCHSQ Flagship Store	King of Power Saving	Been using the same appliances and running a 1.5 air conditioner at night for what feels like no time, and after half a month of use it feels like the power is only increasing. Doesn't show any power saving	Jingdong prefecture level city in Shandong
IXCHSQ Flagship Store	King of Power Saving	serviceable	Jingdong prefecture level city in Shandong
IXCHSQ Flagship Store	King of Power Saving	It's a piece of junk. It doesn't save any power.	Jingdong prefecture level city in Shandong
IXCHSQ Flagship Store	King of Power Saving	Use the pipe thing to save thirty percent, the quality of the economizer is very good, good-looking, plug and play is very convenient, the price is not expensive but also can save a lot of electricity, cost-	Jingdong prefecture level city in Shandong
IXCHSQ Flagship Store	King of Power Saving	There are a lot of appliances in the house, the summer air conditioning is very costly, I bought this in front of the air conditioning effect is very good, I explain that I bought three in one breath, I use more electricity a month down to 200 or so, very satisfied.	Jingdong prefecture level city in Shandong
IXCHSQ Flagship Store	King of Power Saving	The voltage is very stable has been maintained at 220v this value, high power super power saving, trustworthy, reasonable design, shape bright, logistics is quite fast, business warranty period, excellent service, logistics fast!	Jingdong prefecture level city in Shandong
IXCHSQ Flagship Store	King of Power Saving	Use a month after a look at the savings of more than 40 dollars, there is a display of electricity savings, this high-power intelligent frequency converter economizer, affordable and cheap, logistics is very fast, the service attitude is very good, very satisfied with a shopping.	Jingdong prefecture level city in Shandong
IXCHSQ Flagship Store	King of Power Saving	Used for a period of time, it seems that every day can really can save a few degrees of electricity, workmanship quite can, with a period of time and then continue to look at it, anyway, the price is not	Jingdong prefecture level city in Shandong
IXCHSQ Flagship Store	King of Power Saving	Used for a period of time, it seems that every day can indeed save two several degrees of electricity, workmanship quite can, with a period of time and then continue to look at it, anyway, the price is not	Jingdong prefecture level city in Shandong
IXCHSQ Flagship Store	King of Power Saving	High-power super saver received immediately used, the price is not expensive quality is very good, save electricity and money, very convenient, conscientious business, must be praised, recommended to everyone!	Jingdong prefecture level city in Shandong
IXCHSQ Flagship Store	King of Power Saving	Home enhancement version of the intelligent power saver, is in use, I think the price is cheap, value for money, efficacy, power, power saving effect is very good.	Jingdong prefecture level city in Shandong
IXCHSQ Flagship Store	King of Power Saving	Bought three at once, one for each room, summer electricity consumption is very high, despite the three air conditioners on the electricity bill is still down a lot, too Nessie!	Jingdong prefecture level city in Shandong
IXCHSQ Flagship Store	King of Power Saving	Without further ado, the electric bill came down, $102 less this month, personal satisfaction, it counts okay, give you a good review	Jingdong prefecture level city in Shandong
IXCHSQ Flagship Store	King of Power Saving	The power saver has arrived, the installation is really convenient, plugged in on the line operation is quite simple, power saving effect is also a strong!	Jingdong prefecture level city in Shandong
IXCHSQ Flagship Store	King of Power Saving	Saver received, good quality, beautiful appearance, fine workmanship, simple installation	Jingdong prefecture level city in Shandong
IXCHSQ Flagship Store	King of Power Saving	Product design style modern, worth buying	Jingdong prefecture level city in Shandong
IXCHSQ Flagship Store	King of Power Saving	Energy saving, good power saving effect, practical, great value	Jingdong prefecture level city in Shandong
IXCHSQ Flagship Store	King of Power Saving	It's good quality, it's good, it's energy efficient, and I'll buy it again.	Jingdong prefecture level city in Shandong
IXCHSQ Flagship Store	King of Power Saving	Installation saves a lot of electricity, fast logistics, real energy-saving appliances	Jingdong prefecture level city in Shandong
IXCHSQ Flagship Store	King of Power Saving	The effect is good and the power saving is obvious after using it for a month and saving tens of dollars, the product is trustworthy, I now buy another 4 colors to prepare for their siblings' family to pay for	Jingdong prefecture level city in Shandong
IXCHSQ Flagship Store	King of Power Saving	The quality is particularly good workmanship is no problem, the arrival of the very fast oh, especially power-saving	Jingdong prefecture level city in Shandong
IXCHSQ Flagship Store	King of Power Saving	The quality is very good! In use, observed for a day, not as used before, so much power consumption.	Jingdong prefecture level city in Shandong
IXCHSQ Flagship Store	King of Power Saving	Goods have been received, the effect is good is not good, power saving is not yet know, wait for me to use it and then evaluate it.	Jingdong prefecture level city in Shandong
IXCHSQ Flagship Store	King of Power Saving	In-kind and the same as described, the quality is quite good, the seller's attitude is also good, ask questions and answer them, the speed of delivery bar, worth buying Oh!	Jingdong prefecture level city in Shandong
IXCHSQ Flagship Store	King of Power Saving	Nice stuff, came to comment after using it, it does save power.	Jingdong prefecture level city in Shandong
IXCHSQ Flagship Store	King of Power Saving	Fast charging, but also save power, the effect is very good, satisfied, praise!	Jingdong prefecture level city in Shandong
IXCHSQ Flagship Store	King of Power Saving	The goods have been received, I do not know how effective, after using the flat price again!	Jingdong prefecture level city in Shandong
IXCHSQ Flagship Store	King of Power Saving	Merchant service is very good Reply quickly The key is the quality and that quality is good	Jingdong prefecture level city in Shandong
IXCHSQ Flagship Store	King of Power Saving	It's working well now, I'll do a follow up review after I actually use it for a while	Jingdong prefecture level city in Shandong
IXCHSQ Flagship Store	King of Power Saving	The quality is good. After installing it, I feel like I've saved a lot of money on my electricity bill.	Jingdong prefecture level city in Shandong
IXCHSQ Flagship Store	King of Power Saving	Make up a review after use.	Jingdong prefecture level city in Shandong
IXCHSQ Flagship Store	King of Power Saving	review after use	Jingdong prefecture level city in Shandong
IXCHSQ Flagship Store	King of Power Saving	It's a small capacitor. It's not energy efficient.	Jingdong prefecture level city in Shandong
IXCHSQ Flagship Store	King of Power Saving	I'll follow up with a review after I've used it and had results.	Jingdong prefecture level city in Shandong
IXCHSQ Flagship Store	King of Power Saving	No power saving? Not good!	Jingdong prefecture level city in Shandong
IXCHSQ Flagship Store	King of Power Saving	Been using the same appliances and running a 1.5 air conditioner at night for what feels like no time, and after half a month of use it feels like the power is only increasing. Doesn't show any power saving	Jingdong prefecture level city in Shandong
IXCHSQ Flagship Store	King of Power Saving	serviceable	Jingdong prefecture level city in Shandong
IXCHSQ Flagship Store	King of Power Saving	It's a piece of junk. It doesn't save any power.	Jingdong prefecture level city in Shandong
IXCHSQ Flagship Store	King of Power Saving	Use the pipe thing to save thirty percent, the quality of the economizer is very good, good-looking, plug and play is very convenient, the price is not expensive but also can save a lot of electricity, cost-	Jingdong prefecture level city in Shandong
IXCHSQ Flagship Store	King of Power Saving	There are a lot of appliances in the house, the summer air conditioning is very costly, I bought this in front of the air conditioning effect is very good, I explain that I bought three in one breath, I use more electricity a month down to 200 or so, very satisfied.	Jingdong prefecture level city in Shandong
IXCHSQ Flagship Store	King of Power Saving	The voltage is very stable has been maintained at 220v this value, high power super power saving, trustworthy, reasonable design, shape bright, logistics is quite fast, business warranty period, excellent service, logistics fast!	Jingdong prefecture level city in Shandong
IXCHSQ Flagship Store	King of Power Saving	Use a month after a look at the savings of more than 40 dollars, there is a display of electricity savings, this high-power intelligent frequency converter economizer, affordable and cheap, logistics is very fast, the service attitude is very good, very satisfied with a shopping.	Jingdong prefecture level city in Shandong
IXCHSQ Flagship Store	King of Power Saving	Used for a period of time, it seems that every day can really can save a few degrees of electricity, workmanship quite can, with a period of time and then continue to look at it, anyway, the price is not	Jingdong prefecture level city in Shandong
IXCHSQ Flagship Store	King of Power Saving	Used for a period of time, it seems that every day can indeed save two several degrees of electricity, workmanship quite can, with a period of time and then continue to look at it, anyway, the price is not	Jingdong prefecture level city in Shandong
IXCHSQ Flagship Store	King of Power Saving	High-power super saver received immediately used, the price is not expensive quality is very good, save electricity and money, very convenient, conscientious business, must be praised, recommended to everyone!	Jingdong prefecture level city in Shandong

IXCHSQ Flagship Store	King of Power Saving	Home enhancement version of the intelligent power saver, is in use, I think the price is cheap, value for money, efficacy, power, power saving effect is very good.	Jingdong prefecture level city in Shandong
IXCHSQ Flagship Store	King of Power Saving	Bought three at once, one for each room, summer electricity consumption is very high, despite the three air conditioners on the electricity bill is still down a lot, too Nessie!	Jingdong prefecture level city in Shandong
IXCHSQ Flagship Store	King of Power Saving	Without further ado, the electric bill came down, $102 less this month, personal satisfaction, it counts okay, give you a good review	Jingdong prefecture level city in Shandong
IXCHSQ Flagship Store	King of Power Saving	The power saver has arrived, the installation is really convenient, plugged in on the line operation is quite simple, power saving effect is also a strong!	Jingdong prefecture level city in Shandong
IXCHSQ Flagship Store	King of Power Saving	Saver received, good quality, beautiful appearance, fine workmanship, simple installation	Jingdong prefecture level city in Shandong
IXCHSQ Flagship Store	King of Power Saving	Product design style modern, worth buying	Jingdong prefecture level city in Shandong
IXCHSQ Flagship Store	King of Power Saving	Energy saving, good power saving effect, practical, great value	Jingdong prefecture level city in Shandong
IXCHSQ Flagship Store	King of Power Saving	It's good quality, it's good, it's energy efficient, and I'll buy it again.	Jingdong prefecture level city in Shandong
IXCHSQ Flagship Store	King of Power Saving	Installation saves a lot of electricity, fast logistics, real energy-saving appliances	Jingdong prefecture level city in Shandong
IXCHSQ Flagship Store	King of Power Saving	The effect is good and the power saving is obvious after using it for a month and saving tens of dollars, the product is trustworthy, I now buy another 4 colors to prepare for their siblings' family to pay for	Jingdong prefecture level city in Shandong
IXCHSQ Flagship Store	King of Power Saving	The quality is particularly good workmanship is no problem, the arrival of the very fast oh, especially power-saving	Jingdong prefecture level city in Shandong
IXCHSQ Flagship Store	King of Power Saving	The quality is very good! In use, observed for a day, not as used before, so much power consumption.	Jingdong prefecture level city in Shandong
IXCHSQ Flagship Store	King of Power Saving	Goods have been received, the effect is good is not good, power saving is not yet know, wait for me to use it and then evaluate it.	Jingdong prefecture level city in Shandong
IXCHSQ Flagship Store	King of Power Saving	In-kind and the same as described, the quality is quite good, the seller's attitude is also good, ask questions and answer them, the speed of delivery bar, worth buying Oh!	Jingdong prefecture level city in Shandong
IXCHSQ Flagship Store	King of Power Saving	Nice stuff, came to comment after using it, it does save power.	Jingdong prefecture level city in Shandong
IXCHSQ Flagship Store	King of Power Saving	Fast charging, but also save power, the effect is very good, satisfied, praise!	Jingdong prefecture level city in Shandong
IXCHSQ Flagship Store	King of Power Saving	The goods have been received, I do not know how effective, after using the flat price again!	Jingdong prefecture level city in Shandong
IXCHSQ Flagship Store	King of Power Saving	Merchant service is very good Reply quickly The key is the quality and that quality is good	Jingdong prefecture level city in Shandong
IXCHSQ Flagship Store	King of Power Saving	It's working well now, I'll do a follow up review after I actually use it for a while	Jingdong prefecture level city in Shandong
IXCHSQ Flagship Store	King of Power Saving	The quality is good. After installing it, I feel like I've saved a lot of money on my electricity bill.	Jingdong prefecture level city in Shandong
IXCHSQ Flagship Store	King of Power Saving	Make up a review after use.	Jingdong prefecture level city in Shandong
IXCHSQ Flagship Store	King of Power Saving	review after use	Jingdong prefecture level city in Shandong
IXCHSQ Flagship Store	King of Power Saving	It's a small capacitor. It's not energy efficient.	Jingdong prefecture level city in Shandong
IXCHSQ Flagship Store	King of Power Saving	I'll follow up with a review after I've used it and had results.	Jingdong prefecture level city in Shandong
IXCHSQ Flagship Store	King of Power Saving	No power saving? Not good!	Jingdong prefecture level city in Shandong
IXCHSQ Flagship Store	King of Power Saving	Been using the same appliances and running a 1.5 air conditioner at night for what feels like no time, and after half a month of use it feels like the power is only increasing. Doesn't show any power saving	Jingdong prefecture level city in Shandong
IXCHSQ Flagship Store	King of Power Saving	serviceable	Jingdong prefecture level city in Shandong
IXCHSQ Flagship Store	King of Power Saving	It's a piece of junk. It doesn't save any power.	Jingdong prefecture level city in Shandong
IXCHSQ Flagship Store	King of Power Saving	Use the pipe thing to save thirty percent, the quality of the economizer is very good, good-looking, plug and play is very convenient, the price is not expensive but also can save a lot of electricity, cost-	Jingdong prefecture level city in Shandong
IXCHSQ Flagship Store	King of Power Saving	There are a lot of appliances in the house, the summer air conditioning is very costly, I bought this in front of the air conditioning effect is very good, I explain that I bought three in one breath, I use more electricity a month down to 200 or so, very satisfied.	Jingdong prefecture level city in Shandong
IXCHSQ Flagship Store	King of Power Saving	The voltage is very stable has been maintained at 220v this value, high power super power saving, trustworthy, reasonable design, shape bright, logistics is quite fast, business warranty period, excellent service, logistics fast!	Jingdong prefecture level city in Shandong
IXCHSQ Flagship Store	King of Power Saving	Use a month after a look at the savings of more than 40 dollars, there is a display of electricity savings, this high-power intelligent frequency converter economizer, affordable and cheap, logistics is very fast, the service attitude is very good, very satisfied with a shopping.	Jingdong prefecture level city in Shandong
IXCHSQ Flagship Store	King of Power Saving	Used for a period of time, it seems that every day can really can save a few degrees of electricity, workmanship quite can, with a period of time and then continue to look at it, anyway, the price is not	Jingdong prefecture level city in Shandong
IXCHSQ Flagship Store	King of Power Saving	Used for a period of time, it seems that every day can indeed save two several degrees of electricity, workmanship quite can, with a period of time and then continue to look at it, anyway, the price is not	Jingdong prefecture level city in Shandong
IXCHSQ Flagship Store	King of Power Saving	High-power super saver received immediately used, the price is not expensive quality is very good, save electricity and money, very convenient, conscientious business, must be praised, recommended to everyone!	Jingdong prefecture level city in Shandong
IXCHSQ Flagship Store	King of Power Saving	Home enhancement version of the intelligent power saver, is in use, I think the price is cheap, value for money, efficacy, power, power saving effect is very good.	Jingdong prefecture level city in Shandong
IXCHSQ Flagship Store	King of Power Saving	Bought three at once, one for each room, summer electricity consumption is very high, despite the three air conditioners on the electricity bill is still down a lot, too Nessie!	Jingdong prefecture level city in Shandong
IXCHSQ Flagship Store	King of Power Saving	Without further ado, the electric bill came down, $102 less this month, personal satisfaction, it counts okay, give you a good review	Jingdong prefecture level city in Shandong
IXCHSQ Flagship Store	King of Power Saving	The power saver has arrived, the installation is really convenient, plugged in on the line operation is quite simple, power saving effect is also a strong!	Jingdong prefecture level city in Shandong
IXCHSQ Flagship Store	King of Power Saving	Saver received, good quality, beautiful appearance, fine workmanship, simple installation	Jingdong prefecture level city in Shandong
IXCHSQ Flagship Store	King of Power Saving	Product design style modern, worth buying	Jingdong prefecture level city in Shandong
IXCHSQ Flagship Store	King of Power Saving	Energy saving, good power saving effect, practical, great value	Jingdong prefecture level city in Shandong
IXCHSQ Flagship Store	King of Power Saving	It's good quality, it's good, it's energy efficient, and I'll buy it again.	Jingdong prefecture level city in Shandong
IXCHSQ Flagship Store	King of Power Saving	Installation saves a lot of electricity, fast logistics, real energy-saving appliances	Jingdong prefecture level city in Shandong
IXCHSQ Flagship Store	King of Power Saving	The effect is good and the power saving is obvious after using it for a month and saving tens of dollars, the product is trustworthy, I now buy another 4 colors to prepare for their siblings' family to pay for	Jingdong prefecture level city in Shandong
IXCHSQ Flagship Store	King of Power Saving	The quality is particularly good workmanship is no problem, the arrival of the very fast oh, especially power-saving	Jingdong prefecture level city in Shandong
IXCHSQ Flagship Store	King of Power Saving	The quality is very good! In use, observed for a day, not as used before, so much power consumption.	Jingdong prefecture level city in Shandong
IXCHSQ Flagship Store	King of Power Saving	Goods have been received, the effect is good is not good, power saving is not yet know, wait for me to use it and then evaluate it.	Jingdong prefecture level city in Shandong
IXCHSQ Flagship Store	King of Power Saving	In-kind and the same as described, the quality is quite good, the seller's attitude is also good, ask questions and answer them, the speed of delivery bar, worth buying Oh!	Jingdong prefecture level city in Shandong
IXCHSQ Flagship Store	King of Power Saving	Nice stuff, came to comment after using it, it does save power.	Jingdong prefecture level city in Shandong

IXCHSQ Flagship Store	King of Power Saving	Fast charging, but also save power, the effect is very good, satisfied, praise!	Jingdong prefecture level city in Shandong
IXCHSQ Flagship Store	King of Power Saving	The goods have been received, I do not know how effective, after using the flat price again!	Jingdong prefecture level city in Shandong
IXCHSQ Flagship Store	King of Power Saving	Merchant service is very good Reply quickly The key is the quality and that quality is good	Jingdong prefecture level city in Shandong
IXCHSQ Flagship Store	King of Power Saving	It's working well now, I'll do a follow up review after I actually use it for a while	Jingdong prefecture level city in Shandong
IXCHSQ Flagship Store	King of Power Saving	The quality is good. After installing it, I feel like I've saved a lot of money on my electricity bill.	Jingdong prefecture level city in Shandong
IXCHSQ Flagship Store	King of Power Saving	Make up a review after use.	Jingdong prefecture level city in Shandong
IXCHSQ Flagship Store	King of Power Saving	review after use	Jingdong prefecture level city in Shandong
IXCHSQ Flagship Store	King of Power Saving	It's a small capacitor. It's not energy efficient.	Jingdong prefecture level city in Shandong
IXCHSQ Flagship Store	King of Power Saving	I'll follow up with a review after I've used it and had results.	Jingdong prefecture level city in Shandong
IXCHSQ Flagship Store	King of Power Saving	No power saving? Not good!	Jingdong prefecture level city in Shandong
IXCHSQ Flagship Store	King of Power Saving	Been using the same appliances and running a 1.5 air conditioner at night for what feels like no time, and after half a month of use it feels like the power is only increasing. Doesn't show any power saving	Jingdong prefecture level city in Shandong
IXCHSQ Flagship Store	King of Power Saving	serviceable	Jingdong prefecture level city in Shandong
IXCHSQ Flagship Store	King of Power Saving	It's a piece of junk. It doesn't save any power.	Jingdong prefecture level city in Shandong
IXCHSQ Flagship Store	King of Power Saving	Use the pipe thing to save thirty percent, the quality of the economizer is very good, good-looking, plug and play is very convenient, the price is not expensive but also can save a lot of electricity, cost-	Jingdong prefecture level city in Shandong
IXCHSQ Flagship Store	King of Power Saving	There are a lot of appliances in the house, the summer air conditioning is very costly, I bought this in front of the air conditioning effect is very good, I explain that I bought three in one breath, I use more electricity a month down to 200 or so, very satisfied.	Jingdong prefecture level city in Shandong
IXCHSQ Flagship Store	King of Power Saving	The voltage is very stable has been maintained at 220v this value, high power super power saving, trustworthy, reasonable design, shape bright, logistics is quite fast, business warranty period, excellent service, logistics fast!	Jingdong prefecture level city in Shandong
IXCHSQ Flagship Store	King of Power Saving	Use a month after a look at the savings of more than 40 dollars, there is a display of electricity savings, this high-power intelligent frequency converter economizer, affordable and cheap, logistics is very fast, the service attitude is very good, very satisfied with a shopping.	Jingdong prefecture level city in Shandong
IXCHSQ Flagship Store	King of Power Saving	Used for a period of time, it seems that every day can really can save a few degrees of electricity, workmanship quite can, with a period of time and then continue to look at it, anyway, the price is not	Jingdong prefecture level city in Shandong
IXCHSQ Flagship Store	King of Power Saving	Used for a period of time, it seems that every day can indeed save two several degrees of electricity, workmanship quite can, with a period of time and then continue to look at it, anyway, the price is not	Jingdong prefecture level city in Shandong
IXCHSQ Flagship Store	King of Power Saving	High-power super saver received immediately used, the price is not expensive quality is very good, save electricity and money, very convenient, conscientious business, must be praised, recommended to everyone!	Jingdong prefecture level city in Shandong
IXCHSQ Flagship Store	King of Power Saving	Home enhancement version of the intelligent power saver, is in use, I think the price is cheap, value for money, efficacy, power, power saving effect is very good.	Jingdong prefecture level city in Shandong
IXCHSQ Flagship Store	King of Power Saving	Bought three at once, one for each room, summer electricity consumption is very high, despite the three air conditioners on the electricity bill is still down a lot, too Nessie!	Jingdong prefecture level city in Shandong
IXCHSQ Flagship Store	King of Power Saving	Without further ado, the electric bill came down, $102 less this month, personal satisfaction, it counts okay, give you a good review	Jingdong prefecture level city in Shandong
IXCHSQ Flagship Store	King of Power Saving	The power saver has arrived, the installation is really convenient, plugged in on the line operation is quite simple, power saving effect is also a strong!	Jingdong prefecture level city in Shandong
IXCHSQ Flagship Store	King of Power Saving	Saver received, good quality, beautiful appearance, fine workmanship, simple installation	Jingdong prefecture level city in Shandong
IXCHSQ Flagship Store	King of Power Saving	Product design style modern, worth buying	Jingdong prefecture level city in Shandong
IXCHSQ Flagship Store	King of Power Saving	Energy saving, good power saving effect, practical, great value	Jingdong prefecture level city in Shandong
IXCHSQ Flagship Store	King of Power Saving	It's good quality, it's good, it's energy efficient, and I'll buy it again.	Jingdong prefecture level city in Shandong
IXCHSQ Flagship Store	King of Power Saving	Installation saves a lot of electricity, fast logistics, real energy-saving appliances	Jingdong prefecture level city in Shandong
IXCHSQ Flagship Store	King of Power Saving	The effect is good and the power saving is obvious after using it for a month and saving tens of dollars, the product is trustworthy, I now buy another 4 colors to prepare for their siblings' family to pay for	Jingdong prefecture level city in Shandong
IXCHSQ Flagship Store	King of Power Saving	The quality is particularly good workmanship is no problem, the arrival of the very fast oh, especially power-saving	Jingdong prefecture level city in Shandong
IXCHSQ Flagship Store	King of Power Saving	The quality is very good! In use, observed for a day, not as used before, so much power consumption.	Jingdong prefecture level city in Shandong
IXCHSQ Flagship Store	King of Power Saving	Goods have been received, the effect is good is not good, power saving is not yet know, wait for me to use it and then evaluate it.	Jingdong prefecture level city in Shandong
IXCHSQ Flagship Store	King of Power Saving	In-kind and the same as described, the quality is quite good, the seller's attitude is also good, ask questions and answer them, the speed of delivery bar, worth buying Oh!	Jingdong prefecture level city in Shandong
IXCHSQ Flagship Store	King of Power Saving	Nice stuff, came to comment after using it, it does save power.	Jingdong prefecture level city in Shandong
IXCHSQ Flagship Store	King of Power Saving	Fast charging, but also save power, the effect is very good, satisfied, praise!	Jingdong prefecture level city in Shandong
IXCHSQ Flagship Store	King of Power Saving	The goods have been received, I do not know how effective, after using the flat price again!	Jingdong prefecture level city in Shandong
IXCHSQ Flagship Store	King of Power Saving	Merchant service is very good Reply quickly The key is the quality and that quality is good	Jingdong prefecture level city in Shandong
IXCHSQ Flagship Store	King of Power Saving	It's working well now, I'll do a follow up review after I actually use it for a while	Jingdong prefecture level city in Shandong
IXCHSQ Flagship Store	King of Power Saving	The quality is good. After installing it, I feel like I've saved a lot of money on my electricity bill.	Jingdong prefecture level city in Shandong
IXCHSQ Flagship Store	King of Power Saving	Make up a review after use.	Jingdong prefecture level city in Shandong
IXCHSQ Flagship Store	King of Power Saving	review after use	Jingdong prefecture level city in Shandong
IXCHSQ Flagship Store	King of Power Saving	It's a small capacitor. It's not energy efficient.	Jingdong prefecture level city in Shandong
IXCHSQ Flagship Store	King of Power Saving	I'll follow up with a review after I've used it and had results.	Jingdong prefecture level city in Shandong
IXCHSQ Flagship Store	King of Power Saving	No power saving? Not good!	Jingdong prefecture level city in Shandong
IXCHSQ Flagship Store	King of Power Saving	Been using the same appliances and running a 1.5 air conditioner at night for what feels like no time, and after half a month of use it feels like the power is only increasing. Doesn't show any power saving	Jingdong prefecture level city in Shandong
IXCHSQ Flagship Store	King of Power Saving	serviceable	Jingdong prefecture level city in Shandong
IXCHSQ Flagship Store	King of Power Saving	It's a piece of junk. It doesn't save any power.	Jingdong prefecture level city in Shandong
IXCHSQ Flagship Store	King of Power Saving	Use the pipe thing to save thirty percent, the quality of the economizer is very good, good-looking, plug and play is very convenient, the price is not expensive but also can save a lot of electricity, cost-	Jingdong prefecture level city in Shandong

IXCHSQ Flagship Store	King of Power Saving	There are a lot of appliances in the house, the summer air conditioning is very costly, I bought this in front of the air conditioning effect is very good, I explain that I bought three in one breath, I use more electricity a month down to 200 or so, very satisfied.	Jingdong prefecture level city in Shandong
IXCHSQ Flagship Store	King of Power Saving	The voltage is very stable has been maintained at 220v this value, high power super power saving, trustworthy, reasonable design, shape bright, logistics is quite fast, business warranty period, excellent service, logistics fast!	Jingdong prefecture level city in Shandong
IXCHSQ Flagship Store	King of Power Saving	Use a month after a look at the savings of more than 40 dollars, there is a display of electricity savings, this high-power intelligent frequency converter economizer, affordable and cheap, logistics is very fast, the service attitude is very good, very satisfied with a shopping.	Jingdong prefecture level city in Shandong
IXCHSQ Flagship Store	King of Power Saving	Used for a period of time, it seems that every day can really can save a few degrees of electricity, workmanship quite can, with a period of time and then continue to look at it, anyway, the price is not	Jingdong prefecture level city in Shandong
IXCHSQ Flagship Store	King of Power Saving	Used for a period of time, it seems that every day can indeed save two several degrees of electricity, workmanship quite can, with a period of time and then continue to look at it, anyway, the price is not	Jingdong prefecture level city in Shandong
IXCHSQ Flagship Store	King of Power Saving	High-power super saver received immediately used, the price is not expensive quality is very good, save electricity and money, very convenient, conscientious business, must be praised, recommended to everyone!	Jingdong prefecture level city in Shandong
IXCHSQ Flagship Store	King of Power Saving	Home enhancement version of the intelligent power saver, is in use, I think the price is cheap, value for money, efficacy, power, power saving effect is very good.	Jingdong prefecture level city in Shandong
IXCHSQ Flagship Store	King of Power Saving	Bought three at once, one for each room, summer electricity consumption is very high, despite the three air conditioners on the electricity bill is still down a lot, too Nessie!	Jingdong prefecture level city in Shandong
IXCHSQ Flagship Store	King of Power Saving	Without further ado, the electric bill came down, $102 less this month, personal satisfaction, it counts okay, give you a good review	Jingdong prefecture level city in Shandong
IXCHSQ Flagship Store	King of Power Saving	The power saver has arrived, the installation is really convenient, plugged in on the line operation is quite simple, power saving effect is also a strong!	Jingdong prefecture level city in Shandong
IXCHSQ Flagship Store	King of Power Saving	Saver received, good quality, beautiful appearance, fine workmanship, simple installation	Jingdong prefecture level city in Shandong
IXCHSQ Flagship Store	King of Power Saving	Product design style modern, worth buying	Jingdong prefecture level city in Shandong
IXCHSQ Flagship Store	King of Power Saving	Energy saving, good power saving effect, practical, great value	Jingdong prefecture level city in Shandong
IXCHSQ Flagship Store	King of Power Saving	It's good quality, it's good, it's energy efficient, and I'll buy it again.	Jingdong prefecture level city in Shandong
IXCHSQ Flagship Store	King of Power Saving	Installation saves a lot of electricity, fast logistics, real energy-saving appliances	Jingdong prefecture level city in Shandong
IXCHSQ Flagship Store	King of Power Saving	The effect is good and the power saving is obvious after using it for a month and saving tens of dollars, the product is trustworthy, I now buy another 4 colors to prepare for their siblings' family to pay for	Jingdong prefecture level city in Shandong
IXCHSQ Flagship Store	King of Power Saving	The quality is particularly good workmanship is no problem, the arrival of the very fast oh, especially power-saving	Jingdong prefecture level city in Shandong
IXCHSQ Flagship Store	King of Power Saving	The quality is very good! In use, observed for a day, not as used before, so much power consumption.	Jingdong prefecture level city in Shandong
IXCHSQ Flagship Store	King of Power Saving	Goods have been received, the effect is good is not good, power saving is not yet know, wait for me to use it and then evaluate it.	Jingdong prefecture level city in Shandong
IXCHSQ Flagship Store	King of Power Saving	In-kind and the same as described, the quality is quite good, the seller's attitude is also good, ask questions and answer them, the speed of delivery bar, worth buying Oh!	Jingdong prefecture level city in Shandong
IXCHSQ Flagship Store	King of Power Saving	Nice stuff, came to comment after using it, it does save power.	Jingdong prefecture level city in Shandong
IXCHSQ Flagship Store	King of Power Saving	Fast charging, but also save power, the effect is very good, satisfied, praise!	Jingdong prefecture level city in Shandong
IXCHSQ Flagship Store	King of Power Saving	The goods have been received, I do not know how effective, after using the flat price again!	Jingdong prefecture level city in Shandong
IXCHSQ Flagship Store	King of Power Saving	Merchant service is very good Reply quickly The key is the quality and that quality is good	Jingdong prefecture level city in Shandong
IXCHSQ Flagship Store	King of Power Saving	It's working well now, I'll do a follow up review after I actually use it for a while	Jingdong prefecture level city in Shandong
IXCHSQ Flagship Store	King of Power Saving	The quality is good. After installing it, I feel like I've saved a lot of money on my electricity bill.	Jingdong prefecture level city in Shandong
IXCHSQ Flagship Store	King of Power Saving	Make up a review after use.	Jingdong prefecture level city in Shandong
IXCHSQ Flagship Store	King of Power Saving	review after use	Jingdong prefecture level city in Shandong
IXCHSQ Flagship Store	King of Power Saving	It's a small capacitor. It's not energy efficient.	Jingdong prefecture level city in Shandong
IXCHSQ Flagship Store	King of Power Saving	I'll follow up with a review after I've used it and had results.	Jingdong prefecture level city in Shandong
IXCHSQ Flagship Store	King of Power Saving	No power saving? Not good!	Jingdong prefecture level city in Shandong
IXCHSQ Flagship Store	King of Power Saving	Been using the same appliances and running a 1.5 air conditioner at night for what feels like no time, and after half a month of use it feels like the power is only increasing. Doesn't show any power saving	Jingdong prefecture level city in Shandong
IXCHSQ Flagship Store	King of Power Saving	serviceable	Jingdong prefecture level city in Shandong
IXCHSQ Flagship Store	King of Power Saving	It's a piece of junk. It doesn't save any power.	Jingdong prefecture level city in Shandong
IXCHSQ Flagship Store	King of Power Saving	Use the pipe thing to save thirty percent, the quality of the economizer is very good, good-looking, plug and play is very convenient, the price is not expensive but also can save a lot of electricity, cost-	Jingdong prefecture level city in Shandong
IXCHSQ Flagship Store	King of Power Saving	There are a lot of appliances in the house, the summer air conditioning is very costly, I bought this in front of the air conditioning effect is very good, I explain that I bought three in one breath, I use more electricity a month down to 200 or so, very satisfied.	Jingdong prefecture level city in Shandong
IXCHSQ Flagship Store	King of Power Saving	The voltage is very stable has been maintained at 220v this value, high power super power saving, trustworthy, reasonable design, shape bright, logistics is quite fast, business warranty period, excellent service, logistics fast!	Jingdong prefecture level city in Shandong
IXCHSQ Flagship Store	King of Power Saving	Use a month after a look at the savings of more than 40 dollars, there is a display of electricity savings, this high-power intelligent frequency converter economizer, affordable and cheap, logistics is very fast, the service attitude is very good, very satisfied with a shopping.	Jingdong prefecture level city in Shandong
IXCHSQ Flagship Store	King of Power Saving	Used for a period of time, it seems that every day can really can save a few degrees of electricity, workmanship quite can, with a period of time and then continue to look at it, anyway, the price is not	Jingdong prefecture level city in Shandong
IXCHSQ Flagship Store	King of Power Saving	Used for a period of time, it seems that every day can indeed save two several degrees of electricity, workmanship quite can, with a period of time and then continue to look at it, anyway, the price is not	Jingdong prefecture level city in Shandong
IXCHSQ Flagship Store	King of Power Saving	High-power super saver received immediately used, the price is not expensive quality is very good, save electricity and money, very convenient, conscientious business, must be praised, recommended to everyone!	Jingdong prefecture level city in Shandong
IXCHSQ Flagship Store	King of Power Saving	Home enhancement version of the intelligent power saver, is in use, I think the price is cheap, value for money, efficacy, power, power saving effect is very good.	Jingdong prefecture level city in Shandong
IXCHSQ Flagship Store	King of Power Saving	Bought three at once, one for each room, summer electricity consumption is very high, despite the three air conditioners on the electricity bill is still down a lot, too Nessie!	Jingdong prefecture level city in Shandong
IXCHSQ Flagship Store	King of Power Saving	Without further ado, the electric bill came down, $102 less this month, personal satisfaction, it counts okay, give you a good review	Jingdong prefecture level city in Shandong
IXCHSQ Flagship Store	King of Power Saving	The power saver has arrived, the installation is really convenient, plugged in on the line operation is quite simple, power saving effect is also a strong!	Jingdong prefecture level city in Shandong
IXCHSQ Flagship Store	King of Power Saving	Saver received, good quality, beautiful appearance, fine workmanship, simple installation	Jingdong prefecture level city in Shandong
IXCHSQ Flagship Store	King of Power Saving	Product design style modern, worth buying	Jingdong prefecture level city in Shandong
IXCHSQ Flagship Store	King of Power Saving	Energy saving, good power saving effect, practical, great value	Jingdong prefecture level city in Shandong

IXCHSQ Flagship Store	King of Power Saving	It's good quality, it's good, it's energy efficient, and I'll buy it again.	Jingdong prefecture level city in Shandong
IXCHSQ Flagship Store	King of Power Saving	Installation saves a lot of electricity, fast logistics, real energy-saving appliances	Jingdong prefecture level city in Shandong
IXCHSQ Flagship Store	King of Power Saving	The effect is good and the power saving is obvious after using it for a month and saving tens of dollars, the product is trustworthy, I now buy another 4 colors to prepare for their siblings' family to pay for	Jingdong prefecture level city in Shandong
IXCHSQ Flagship Store	King of Power Saving	The quality is particularly good workmanship is no problem, the arrival of the very fast oh, especially power-saving	Jingdong prefecture level city in Shandong
IXCHSQ Flagship Store	King of Power Saving	The quality is very good! In use, observed for a day, not as used before, so much power consumption.	Jingdong prefecture level city in Shandong
IXCHSQ Flagship Store	King of Power Saving	Goods have been received, the effect is good is not good, power saving is not yet know, wait for me to use it and then evaluate it.	Jingdong prefecture level city in Shandong
IXCHSQ Flagship Store	King of Power Saving	In-kind and the same as described, the quality is quite good, the seller's attitude is also good, ask questions and answer them, the speed of delivery bar, worth buying Oh!	Jingdong prefecture level city in Shandong
IXCHSQ Flagship Store	King of Power Saving	Nice stuff, came to comment after using it, it does save power.	Jingdong prefecture level city in Shandong
IXCHSQ Flagship Store	King of Power Saving	Fast charging, but also save power, the effect is very good, satisfied, praise!	Jingdong prefecture level city in Shandong
IXCHSQ Flagship Store	King of Power Saving	The goods have been received, I do not know how effective, after using the flat price again!	Jingdong prefecture level city in Shandong
IXCHSQ Flagship Store	King of Power Saving	Merchant service is very good Reply quickly The key is the quality and that quality is good	Jingdong prefecture level city in Shandong
IXCHSQ Flagship Store	King of Power Saving	It's working well now, I'll do a follow up review after I actually use it for a while	Jingdong prefecture level city in Shandong
IXCHSQ Flagship Store	King of Power Saving	The quality is good. After installing it, I feel like I've saved a lot of money on my electricity bill.	Jingdong prefecture level city in Shandong
IXCHSQ Flagship Store	King of Power Saving	Make up a review after use.	Jingdong prefecture level city in Shandong
IXCHSQ Flagship Store	King of Power Saving	review after use	Jingdong prefecture level city in Shandong
IXCHSQ Flagship Store	King of Power Saving	It's a small capacitor. It's not energy efficient.	Jingdong prefecture level city in Shandong
IXCHSQ Flagship Store	King of Power Saving	I'll follow up with a review after I've used it and had results.	Jingdong prefecture level city in Shandong
IXCHSQ Flagship Store	King of Power Saving	No power saving? Not good!	Jingdong prefecture level city in Shandong
IXCHSQ Flagship Store	King of Power Saving	Been using the same appliances and running a 1.5 air conditioner at night for what feels like no time, and after half a month of use it feels like the power is only increasing. Doesn't show any power saving	Jingdong prefecture level city in Shandong
IXCHSQ Flagship Store	King of Power Saving	serviceable	Jingdong prefecture level city in Shandong
IXCHSQ Flagship Store	King of Power Saving	It's a piece of junk. It doesn't save any power.	Jingdong prefecture level city in Shandong
IXCHSQ Flagship Store	King of Power Saving	Use the pipe thing to save thirty percent, the quality of the economizer is very good, good-looking, plug and play is very convenient, the price is not expensive but also can save a lot of electricity, cost-	Jingdong prefecture level city in Shandong
IXCHSQ Flagship Store	King of Power Saving	There are a lot of appliances in the house, the summer air conditioning is very costly, I bought this in front of the air conditioning effect is very good, I explain that I bought three in one breath, I use more electricity a month down to 200 or so, very satisfied.	Jingdong prefecture level city in Shandong
IXCHSQ Flagship Store	King of Power Saving	The voltage is very stable has been maintained at 220v this value, high power super power saving, trustworthy, reasonable design, shape bright, logistics is quite fast, business warranty period, excellent service, logistics fast!	Jingdong prefecture level city in Shandong
IXCHSQ Flagship Store	King of Power Saving	Use a month after a look at the savings of more than 40 dollars, there is a display of electricity savings, this high-power intelligent frequency converter economizer, affordable and cheap, logistics is very fast, the service attitude is very good, very satisfied with a shopping.	Jingdong prefecture level city in Shandong
IXCHSQ Flagship Store	King of Power Saving	Used for a period of time, it seems that every day can really can save a few degrees of electricity, workmanship quite can, with a period of time and then continue to look at it, anyway, the price is not	Jingdong prefecture level city in Shandong
IXCHSQ Flagship Store	King of Power Saving	Used for a period of time, it seems that every day can indeed save two several degrees of electricity, workmanship quite can, with a period of time and then continue to look at it, anyway, the price is not	Jingdong prefecture level city in Shandong
IXCHSQ Flagship Store	King of Power Saving	High-power super saver received immediately used, the price is not expensive quality is very good, save electricity and money, very convenient, conscientious business, must be praised, recommended to everyone!	Jingdong prefecture level city in Shandong
IXCHSQ Flagship Store	King of Power Saving	Home enhancement version of the intelligent power saver, is in use, I think the price is cheap, value for money, efficacy, power, power saving effect is very good.	Jingdong prefecture level city in Shandong
IXCHSQ Flagship Store	King of Power Saving	Bought three at once, one for each room, summer electricity consumption is very high, despite the three air conditioners on the electricity bill is still down a lot, too Nessie!	Jingdong prefecture level city in Shandong
IXCHSQ Flagship Store	King of Power Saving	Without further ado, the electric bill came down, $102 less this month, personal satisfaction, it counts okay, give you a good review	Jingdong prefecture level city in Shandong
IXCHSQ Flagship Store	King of Power Saving	The power saver has arrived, the installation is really convenient, plugged in on the line operation is quite simple, power saving effect is also a strong!	Jingdong prefecture level city in Shandong
IXCHSQ Flagship Store	King of Power Saving	Saver received, good quality, beautiful appearance, fine workmanship, simple installation	Jingdong prefecture level city in Shandong
IXCHSQ Flagship Store	King of Power Saving	Product design style modern, worth buying	Jingdong prefecture level city in Shandong
IXCHSQ Flagship Store	King of Power Saving	Energy saving, good power saving effect, practical, great value	Jingdong prefecture level city in Shandong
IXCHSQ Flagship Store	King of Power Saving	It's good quality, it's good, it's energy efficient, and I'll buy it again.	Jingdong prefecture level city in Shandong
IXCHSQ Flagship Store	King of Power Saving	Installation saves a lot of electricity, fast logistics, real energy-saving appliances	Jingdong prefecture level city in Shandong
IXCHSQ Flagship Store	King of Power Saving	The effect is good and the power saving is obvious after using it for a month and saving tens of dollars, the product is trustworthy, I now buy another 4 colors to prepare for their siblings' family to pay for	Jingdong prefecture level city in Shandong
IXCHSQ Flagship Store	King of Power Saving	The quality is particularly good workmanship is no problem, the arrival of the very fast oh, especially power-saving	Jingdong prefecture level city in Shandong
IXCHSQ Flagship Store	King of Power Saving	The quality is very good! In use, observed for a day, not as used before, so much power consumption.	Jingdong prefecture level city in Shandong
IXCHSQ Flagship Store	King of Power Saving	In-kind and the same as described, the quality is quite good, the seller's attitude is also good, ask questions and answer them, the speed of delivery bar, worth buying Oh!	Jingdong prefecture level city in Shandong
IXCHSQ Flagship Store	King of Power Saving	Goods have been received, the effect is good is not good, power saving is not yet know, wait for me to use it and then evaluate it.	Jingdong prefecture level city in Shandong
IXCHSQ Flagship Store	King of Power Saving	Nice stuff, came to comment after using it, it does save power.	Jingdong prefecture level city in Shandong
IXCHSQ Flagship Store	King of Power Saving	Fast charging, but also save power, the effect is very good, satisfied, praise!	Jingdong prefecture level city in Shandong
IXCHSQ Flagship Store	King of Power Saving	Merchant service is very good Reply quickly The key is the quality and that quality is good	Jingdong prefecture level city in Shandong
IXCHSQ Flagship Store	King of Power Saving	The goods have been received, I do not know how effective, after using the flat price again!	Jingdong prefecture level city in Shandong
IXCHSQ Flagship Store	King of Power Saving	It's working well now, I'll do a follow up review after I actually use it for a while	Jingdong prefecture level city in Shandong
IXCHSQ Flagship Store	King of Power Saving	The quality is good. After installing it, I feel like I've saved a lot of money on my electricity bill.	Jingdong prefecture level city in Shandong
IXCHSQ Flagship Store	King of Power Saving	It's a small capacitor. It's not energy efficient.	Jingdong prefecture level city in Shandong
IXCHSQ Flagship Store	King of Power Saving	I'll follow up with a review after I've used it and had results.	Jingdong prefecture level city in Shandong

IXCHSQ Flagship Store	King of Power Saving	Make up a review after use.	Jingdong prefecture level city in Shandong
IXCHSQ Flagship Store	King of Power Saving	review after use	Jingdong prefecture level city in Shandong
IXCHSQ Flagship Store	King of Power Saving	No power saving? Not good!	Jingdong prefecture level city in Shandong
IXCHSQ Flagship Store	King of Power Saving	Been using the same appliances and running a 1.5 air conditioner at night for what feels like no time, and after half a month of use it feels like the power is only increasing. Doesn't show any power saving	Jingdong prefecture level city in Shandong
IXCHSQ Flagship Store	King of Power Saving	serviceable	Jingdong prefecture level city in Shandong
IXCHSQ Flagship Store	King of Power Saving	It's a piece of junk. It doesn't save any power.	Jingdong prefecture level city in Shandong
IXCHSQ Flagship Store	King of Power Saving	Use the pipe thing to save thirty percent, the quality of the economizer is very good, good-looking, plug and play is very convenient, the price is not expensive but also can save a lot of electricity, cost-	Jingdong prefecture level city in Shandong
IXCHSQ Flagship Store	King of Power Saving	There are a lot of appliances in the house, the summer air conditioning is very costly, I bought this in front of the air conditioning effect is very good, I explain that I bought three in one breath, I use more electricity a month down to 200 or so, very satisfied.	Jingdong prefecture level city in Shandong
IXCHSQ Flagship Store	King of Power Saving	The voltage is very stable has been maintained at 220v this value, high power super power saving, trustworthy, reasonable design, shape bright, logistics is quite fast, business warranty period, excellent service, logistics fast!	Jingdong prefecture level city in Shandong
IXCHSQ Flagship Store	King of Power Saving	Use a month after a look at the savings of more than 40 dollars, there is a display of electricity savings, this high-power intelligent frequency converter economizer, affordable and cheap, logistics is very fast, the service attitude is very good, very satisfied with a shopping.	Jingdong prefecture level city in Shandong
IXCHSQ Flagship Store	King of Power Saving	Used for a period of time, it seems that every day can really can save a few degrees of electricity, workmanship quite can, with a period of time and then continue to look at it, anyway, the price is not	Jingdong prefecture level city in Shandong
IXCHSQ Flagship Store	King of Power Saving	Used for a period of time, it seems that every day can indeed save two several degrees of electricity, workmanship quite can, with a period of time and then continue to look at it, anyway, the price is not	Jingdong prefecture level city in Shandong
IXCHSQ Flagship Store	King of Power Saving	High-power super saver received immediately used, the price is not expensive quality is very good, save electricity and money, very convenient, conscientious business, must be praised, recommended to everyone!	Jingdong prefecture level city in Shandong
IXCHSQ Flagship Store	King of Power Saving	Home enhancement version of the intelligent power saver, is in use, I think the price is cheap, value for money, efficacy, power, power saving effect is very good.	Jingdong prefecture level city in Shandong
IXCHSQ Flagship Store	King of Power Saving	Bought three at once, one for each room, summer electricity consumption is very high, despite the three air conditioners on the electricity bill is still down a lot, too Nessie!	Jingdong prefecture level city in Shandong
IXCHSQ Flagship Store	King of Power Saving	Without further ado, the electric bill came down, $102 less this month, personal satisfaction, it counts okay, give you a good review	Jingdong prefecture level city in Shandong
IXCHSQ Flagship Store	King of Power Saving	The power saver has arrived, the installation is really convenient, plugged in on the line operation is quite simple, power saving effect is also a strong!	Jingdong prefecture level city in Shandong
IXCHSQ Flagship Store	King of Power Saving	Saver received, good quality, beautiful appearance, fine workmanship, simple installation	Jingdong prefecture level city in Shandong
IXCHSQ Flagship Store	King of Power Saving	Product design style modern, worth buying	Jingdong prefecture level city in Shandong
IXCHSQ Flagship Store	King of Power Saving	Energy saving, good power saving effect, practical, great value	Jingdong prefecture level city in Shandong
IXCHSQ Flagship Store	King of Power Saving	It's good quality, it's good, it's energy efficient, and I'll buy it again.	Jingdong prefecture level city in Shandong
IXCHSQ Flagship Store	King of Power Saving	Installation saves a lot of electricity, fast logistics, real energy-saving appliances	Jingdong prefecture level city in Shandong
IXCHSQ Flagship Store	King of Power Saving	The effect is good and the power saving is obvious after using it for a month and saving tens of dollars, the product is trustworthy, I now buy another 4 colors to prepare for their siblings' family to pay for	Jingdong prefecture level city in Shandong
IXCHSQ Flagship Store	King of Power Saving	The quality is particularly good workmanship is no problem, the arrival of the very fast oh, especially power-saving	Jingdong prefecture level city in Shandong
IXCHSQ Flagship Store	King of Power Saving	The quality is very good! In use, observed for a day, not as used before, so much power consumption.	Jingdong prefecture level city in Shandong
IXCHSQ Flagship Store	King of Power Saving	Goods have been received, the effect is good is not good, power saving is not yet know, wait for me to use it and then evaluate it.	Jingdong prefecture level city in Shandong
IXCHSQ Flagship Store	King of Power Saving	In-kind and the same as described, the quality is quite good, the seller's attitude is also good, ask questions and answer them, the speed of delivery bar, worth buying Oh!	Jingdong prefecture level city in Shandong
IXCHSQ Flagship Store	King of Power Saving	Nice stuff, came to comment after using it, it does save power.	Jingdong prefecture level city in Shandong
IXCHSQ Flagship Store	King of Power Saving	Fast charging, but also save power, the effect is very good, satisfied, praise!	Jingdong prefecture level city in Shandong
IXCHSQ Flagship Store	King of Power Saving	The goods have been received, I do not know how effective, after using the flat price again!	Jingdong prefecture level city in Shandong
IXCHSQ Flagship Store	King of Power Saving	Merchant service is very good Reply quickly The key is the quality and that quality is good	Jingdong prefecture level city in Shandong
IXCHSQ Flagship Store	King of Power Saving	It's working well now, I'll do a follow up review after I actually use it for a while	Jingdong prefecture level city in Shandong
IXCHSQ Flagship Store	King of Power Saving	The quality is good. After installing it, I feel like I've saved a lot of money on my electricity bill.	Jingdong prefecture level city in Shandong
IXCHSQ Flagship Store	King of Power Saving	Make up a review after use.	Jingdong prefecture level city in Shandong
IXCHSQ Flagship Store	King of Power Saving	review after use	Jingdong prefecture level city in Shandong
IXCHSQ Flagship Store	King of Power Saving	It's a small capacitor. It's not energy efficient.	Jingdong prefecture level city in Shandong
IXCHSQ Flagship Store	King of Power Saving	I'll follow up with a review after I've used it and had results.	Jingdong prefecture level city in Shandong
IXCHSQ Flagship Store	King of Power Saving	No power saving? Not good!	Jingdong prefecture level city in Shandong
IXCHSQ Flagship Store	King of Power Saving	Been using the same appliances and running a 1.5 air conditioner at night for what feels like no time, and after half a month of use it feels like the power is only increasing. Doesn't show any power saving	Jingdong prefecture level city in Shandong
IXCHSQ Flagship Store	King of Power Saving	serviceable	Jingdong prefecture level city in Shandong
IXCHSQ Flagship Store	King of Power Saving	It's a piece of junk. It doesn't save any power.	Jingdong prefecture level city in Shandong
IXCHSQ Flagship Store	King of Power Saving	Use the pipe thing to save thirty percent, the quality of the economizer is very good, good-looking, plug and play is very convenient, the price is not expensive but also can save a lot of electricity, cost-	Jingdong prefecture level city in Shandong
IXCHSQ Flagship Store	King of Power Saving	There are a lot of appliances in the house, the summer air conditioning is very costly, I bought this in front of the air conditioning effect is very good, I explain that I bought three in one breath, I use more electricity a month down to 200 or so, very satisfied.	Jingdong prefecture level city in Shandong
IXCHSQ Flagship Store	King of Power Saving	The voltage is very stable has been maintained at 220v this value, high power super power saving, trustworthy, reasonable design, shape bright, logistics is quite fast, business warranty period, excellent service, logistics fast!	Jingdong prefecture level city in Shandong
IXCHSQ Flagship Store	King of Power Saving	Use a month after a look at the savings of more than 40 dollars, there is a display of electricity savings, this high-power intelligent frequency converter economizer, affordable and cheap, logistics is very fast, the service attitude is very good, very satisfied with a shopping.	Jingdong prefecture level city in Shandong
IXCHSQ Flagship Store	King of Power Saving	Used for a period of time, it seems that every day can really can save a few degrees of electricity, workmanship quite can, with a period of time and then continue to look at it, anyway, the price is not	Jingdong prefecture level city in Shandong
IXCHSQ Flagship Store	King of Power Saving	Used for a period of time, it seems that every day can indeed save two several degrees of electricity, workmanship quite can, with a period of time and then continue to look at it, anyway, the price is not	Jingdong prefecture level city in Shandong
IXCHSQ Flagship Store	King of Power Saving	High-power super saver received immediately used, the price is not expensive quality is very good, save electricity and money, very convenient, conscientious business, must be praised, recommended to everyone!	Jingdong prefecture level city in Shandong

IXCHSQ Flagship Store	King of Power Saving	Home enhancement version of the intelligent power saver, is in use, I think the price is cheap, value for money, efficacy, power, power saving effect is very good.	Jingdong prefecture level city in Shandong
IXCHSQ Flagship Store	King of Power Saving	Bought three at once, one for each room, summer electricity consumption is very high, despite the three air conditioners on the electricity bill is still down a lot, too Nessie!	Jingdong prefecture level city in Shandong
IXCHSQ Flagship Store	King of Power Saving	Without further ado, the electric bill came down, $102 less this month, personal satisfaction, it counts okay, give you a good review	Jingdong prefecture level city in Shandong
IXCHSQ Flagship Store	King of Power Saving	The power saver has arrived, the installation is really convenient, plugged in on the line operation is quite simple, power saving effect is also a strong!	Jingdong prefecture level city in Shandong
IXCHSQ Flagship Store	King of Power Saving	Saver received, good quality, beautiful appearance, fine workmanship, simple installation	Jingdong prefecture level city in Shandong
IXCHSQ Flagship Store	King of Power Saving	Product design style modern, worth buying	Jingdong prefecture level city in Shandong
IXCHSQ Flagship Store	King of Power Saving	Energy saving, good power saving effect, practical, great value	Jingdong prefecture level city in Shandong
IXCHSQ Flagship Store	King of Power Saving	It's good quality, it's good, it's energy efficient, and I'll buy it again.	Jingdong prefecture level city in Shandong
IXCHSQ Flagship Store	King of Power Saving	Installation saves a lot of electricity, fast logistics, real energy-saving appliances	Jingdong prefecture level city in Shandong
IXCHSQ Flagship Store	King of Power Saving	The effect is good and the power saving is obvious after using it for a month and saving tens of dollars, the product is trustworthy, I now buy another 4 colors to prepare for their siblings' family to pay for	Jingdong prefecture level city in Shandong
IXCHSQ Flagship Store	King of Power Saving	The quality is particularly good workmanship is no problem, the arrival of the very fast oh, especially power-saving	Jingdong prefecture level city in Shandong
IXCHSQ Flagship Store	King of Power Saving	The quality is very good! In use, observed for a day, not as used before, so much power consumption.	Jingdong prefecture level city in Shandong
IXCHSQ Flagship Store	King of Power Saving	Goods have been received, the effect is good is not good, power saving is not yet know, wait for me to use it and then evaluate it.	Jingdong prefecture level city in Shandong
IXCHSQ Flagship Store	King of Power Saving	In-kind and the same as described, the quality is quite good, the seller's attitude is also good, ask questions and answer them, the speed of delivery bar, worth buying Oh!	Jingdong prefecture level city in Shandong
IXCHSQ Flagship Store	King of Power Saving	Nice stuff, came to comment after using it, it does save power.	Jingdong prefecture level city in Shandong
IXCHSQ Flagship Store	King of Power Saving	Fast charging, but also save power, the effect is very good, satisfied, praise!	Jingdong prefecture level city in Shandong
IXCHSQ Flagship Store	King of Power Saving	The goods have been received, I do not know how effective, after using the flat price again!	Jingdong prefecture level city in Shandong
IXCHSQ Flagship Store	King of Power Saving	Merchant service is very good Reply quickly The key is the quality and that quality is good	Jingdong prefecture level city in Shandong
IXCHSQ Flagship Store	King of Power Saving	It's working well now, I'll do a follow up review after I actually use it for a while	Jingdong prefecture level city in Shandong
IXCHSQ Flagship Store	King of Power Saving	The quality is good. After installing it, I feel like I've saved a lot of money on my electricity bill.	Jingdong prefecture level city in Shandong
IXCHSQ Flagship Store	King of Power Saving	Make up a review after use.	Jingdong prefecture level city in Shandong
IXCHSQ Flagship Store	King of Power Saving	review after use	Jingdong prefecture level city in Shandong
IXCHSQ Flagship Store	King of Power Saving	It's a small capacitor. It's not energy efficient.	Jingdong prefecture level city in Shandong
IXCHSQ Flagship Store	King of Power Saving	I'll follow up with a review after I've used it and had results.	Jingdong prefecture level city in Shandong
IXCHSQ Flagship Store	King of Power Saving	No power saving? Not good!	Jingdong prefecture level city in Shandong
IXCHSQ Flagship Store	King of Power Saving	Been using the same appliances and running a 1.5 air conditioner at night for what feels like no time, and after half a month of use it feels like the power is only increasing. Doesn't show any power saving	Jingdong prefecture level city in Shandong
IXCHSQ Flagship Store	King of Power Saving	serviceable	Jingdong prefecture level city in Shandong
IXCHSQ Flagship Store	King of Power Saving	It's a piece of junk. It doesn't save any power.	Jingdong prefecture level city in Shandong
IXCHSQ Flagship Store	King of Power Saving	Use the pipe thing to save thirty percent, the quality of the economizer is very good, good-looking, plug and play is very convenient, the price is not expensive but also can save a lot of electricity, cost-	Jingdong prefecture level city in Shandong
IXCHSQ Flagship Store	King of Power Saving	There are a lot of appliances in the house, the summer air conditioning is very costly, I bought this in front of the air conditioning effect is very good, I explain that I bought three in one breath, I use more electricity a month down to 200 or so, very satisfied.	Jingdong prefecture level city in Shandong
IXCHSQ Flagship Store	King of Power Saving	The voltage is very stable has been maintained at 220v this value, high power super power saving, trustworthy, reasonable design, shape bright, logistics is quite fast, business warranty period, excellent service, logistics fast!	Jingdong prefecture level city in Shandong
IXCHSQ Flagship Store	King of Power Saving	Use a month after a look at the savings of more than 40 dollars, there is a display of electricity savings, this high-power intelligent frequency converter economizer, affordable and cheap, logistics is very fast, the service attitude is very good, very satisfied with a shopping.	Jingdong prefecture level city in Shandong
IXCHSQ Flagship Store	King of Power Saving	Used for a period of time, it seems that every day can really can save a few degrees of electricity, workmanship quite can, with a period of time and then continue to look at it, anyway, the price is not	Jingdong prefecture level city in Shandong
IXCHSQ Flagship Store	King of Power Saving	Used for a period of time, it seems that every day can indeed save two several degrees of electricity, workmanship quite can, with a period of time and then continue to look at it, anyway, the price is not	Jingdong prefecture level city in Shandong
IXCHSQ Flagship Store	King of Power Saving	High-power super saver received immediately used, the price is not expensive quality is very good, save electricity and money, very convenient, conscientious business, must be praised, recommended to everyone!	Jingdong prefecture level city in Shandong
IXCHSQ Flagship Store	King of Power Saving	Home enhancement version of the intelligent power saver, is in use, I think the price is cheap, value for money, efficacy, power, power saving effect is very good.	Jingdong prefecture level city in Shandong
IXCHSQ Flagship Store	King of Power Saving	Bought three at once, one for each room, summer electricity consumption is very high, despite the three air conditioners on the electricity bill is still down a lot, too Nessie!	Jingdong prefecture level city in Shandong
IXCHSQ Flagship Store	King of Power Saving	Without further ado, the electric bill came down, $102 less this month, personal satisfaction, it counts okay, give you a good review	Jingdong prefecture level city in Shandong
IXCHSQ Flagship Store	King of Power Saving	The power saver has arrived, the installation is really convenient, plugged in on the line operation is quite simple, power saving effect is also a strong!	Jingdong prefecture level city in Shandong
IXCHSQ Flagship Store	King of Power Saving	Saver received, good quality, beautiful appearance, fine workmanship, simple installation	Jingdong prefecture level city in Shandong
IXCHSQ Flagship Store	King of Power Saving	Product design style modern, worth buying	Jingdong prefecture level city in Shandong
IXCHSQ Flagship Store	King of Power Saving	Energy saving, good power saving effect, practical, great value	Jingdong prefecture level city in Shandong
IXCHSQ Flagship Store	King of Power Saving	It's good quality, it's good, it's energy efficient, and I'll buy it again.	Jingdong prefecture level city in Shandong
IXCHSQ Flagship Store	King of Power Saving	Installation saves a lot of electricity, fast logistics, real energy-saving appliances	Jingdong prefecture level city in Shandong
IXCHSQ Flagship Store	King of Power Saving	The effect is good and the power saving is obvious after using it for a month and saving tens of dollars, the product is trustworthy, I now buy another 4 colors to prepare for their siblings' family to pay for	Jingdong prefecture level city in Shandong
IXCHSQ Flagship Store	King of Power Saving	The quality is particularly good workmanship is no problem, the arrival of the very fast oh, especially power-saving	Jingdong prefecture level city in Shandong
IXCHSQ Flagship Store	King of Power Saving	The quality is very good! In use, observed for a day, not as used before, so much power consumption.	Jingdong prefecture level city in Shandong
IXCHSQ Flagship Store	King of Power Saving	Goods have been received, the effect is good is not good, power saving is not yet know, wait for me to use it and then evaluate it.	Jingdong prefecture level city in Shandong
IXCHSQ Flagship Store	King of Power Saving	In-kind and the same as described, the quality is quite good, the seller's attitude is also good, ask questions and answer them, the speed of delivery bar, worth buying Oh!	Jingdong prefecture level city in Shandong
IXCHSQ Flagship Store	King of Power Saving	Nice stuff, came to comment after using it, it does save power.	Jingdong prefecture level city in Shandong

IXCHSQ Flagship Store	King of Power Saving	Fast charging, but also save power, the effect is very good, satisfied, praise!	Jingdong prefecture level city in Shandong
IXCHSQ Flagship Store	King of Power Saving	The goods have been received, I do not know how effective, after using the flat price again!	Jingdong prefecture level city in Shandong
IXCHSQ Flagship Store	King of Power Saving	Merchant service is very good Reply quickly The key is the quality and that quality is good	Jingdong prefecture level city in Shandong
IXCHSQ Flagship Store	King of Power Saving	It's working well now, I'll do a follow up review after I actually use it for a while	Jingdong prefecture level city in Shandong
IXCHSQ Flagship Store	King of Power Saving	The quality is good. After installing it, I feel like I've saved a lot of money on my electricity bill.	Jingdong prefecture level city in Shandong
IXCHSQ Flagship Store	King of Power Saving	Make up a review after use.	Jingdong prefecture level city in Shandong
IXCHSQ Flagship Store	King of Power Saving	review after use	Jingdong prefecture level city in Shandong
IXCHSQ Flagship Store	King of Power Saving	It's a small capacitor. It's not energy efficient.	Jingdong prefecture level city in Shandong
IXCHSQ Flagship Store	King of Power Saving	I'll follow up with a review after I've used it and had results.	Jingdong prefecture level city in Shandong
IXCHSQ Flagship Store	King of Power Saving	No power saving? Not good!	Jingdong prefecture level city in Shandong
IXCHSQ Flagship Store	King of Power Saving	Been using the same appliances and running a 1.5 air conditioner at night for what feels like no time, and after half a month of use it feels like the power is only increasing. Doesn't show any power saving	Jingdong prefecture level city in Shandong
IXCHSQ Flagship Store	King of Power Saving	serviceable	Jingdong prefecture level city in Shandong
IXCHSQ Flagship Store	King of Power Saving	It's a piece of junk. It doesn't save any power.	Jingdong prefecture level city in Shandong
IXCHSQ Flagship Store	King of Power Saving	Use the pipe thing to save thirty percent, the quality of the economizer is very good, good-looking, plug and play is very convenient, the price is not expensive but also can save a lot of electricity, cost-	Jingdong prefecture level city in Shandong
IXCHSQ Flagship Store	King of Power Saving	There are a lot of appliances in the house, the summer air conditioning is very costly, I bought this in front of the air conditioning effect is very good, I explain that I bought three in one breath, I use more electricity a month down to 200 or so, very satisfied.	Jingdong prefecture level city in Shandong
IXCHSQ Flagship Store	King of Power Saving	The voltage is very stable has been maintained at 220v this value, high power super power saving, trustworthy, reasonable design, shape bright, logistics is quite fast, business warranty period, excellent service, logistics fast!	Jingdong prefecture level city in Shandong
IXCHSQ Flagship Store	King of Power Saving	Use a month after a look at the savings of more than 40 dollars, there is a display of electricity savings, this high-power intelligent frequency converter economizer, affordable and cheap, logistics is very fast, the service attitude is very good, very satisfied with a shopping.	Jingdong prefecture level city in Shandong
IXCHSQ Flagship Store	King of Power Saving	Used for a period of time, it seems that every day can really can save a few degrees of electricity, workmanship quite can, with a period of time and then continue to look at it, anyway, the price is not	Jingdong prefecture level city in Shandong
IXCHSQ Flagship Store	King of Power Saving	Used for a period of time, it seems that every day can indeed save two several degrees of electricity, workmanship quite can, with a period of time and then continue to look at it, anyway, the price is not	Jingdong prefecture level city in Shandong
IXCHSQ Flagship Store	King of Power Saving	High-power super saver received immediately used, the price is not expensive quality is very good, save electricity and money, very convenient, conscientious business, must be praised, recommended to everyone!	Jingdong prefecture level city in Shandong
IXCHSQ Flagship Store	King of Power Saving	Home enhancement version of the intelligent power saver, is in use, I think the price is cheap, value for money, efficacy, power, power saving effect is very good.	Jingdong prefecture level city in Shandong
IXCHSQ Flagship Store	King of Power Saving	Bought three at once, one for each room, summer electricity consumption is very high, despite the three air conditioners on the electricity bill is still down a lot, too Nessie!	Jingdong prefecture level city in Shandong
IXCHSQ Flagship Store	King of Power Saving	Without further ado, the electric bill came down, $102 less this month, personal satisfaction, it counts okay, give you a good review	Jingdong prefecture level city in Shandong
IXCHSQ Flagship Store	King of Power Saving	The power saver has arrived, the installation is really convenient, plugged in on the line operation is quite simple, power saving effect is also a strong!	Jingdong prefecture level city in Shandong
IXCHSQ Flagship Store	King of Power Saving	Saver received, good quality, beautiful appearance, fine workmanship, simple installation	Jingdong prefecture level city in Shandong
IXCHSQ Flagship Store	King of Power Saving	Product design style modern, worth buying	Jingdong prefecture level city in Shandong
IXCHSQ Flagship Store	King of Power Saving	Energy saving, good power saving effect, practical, great value	Jingdong prefecture level city in Shandong
IXCHSQ Flagship Store	King of Power Saving	It's good quality, it's good, it's energy efficient, and I'll buy it again.	Jingdong prefecture level city in Shandong
IXCHSQ Flagship Store	King of Power Saving	Installation saves a lot of electricity, fast logistics, real energy-saving appliances	Jingdong prefecture level city in Shandong
IXCHSQ Flagship Store	King of Power Saving	The effect is good and the power saving is obvious after using it for a month and saving tens of dollars, the product is trustworthy, I now buy another 4 colors to prepare for their siblings' family to pay for	Jingdong prefecture level city in Shandong
IXCHSQ Flagship Store	King of Power Saving	The quality is particularly good workmanship is no problem, the arrival of the very fast oh, especially power-saving	Jingdong prefecture level city in Shandong
IXCHSQ Flagship Store	King of Power Saving	The quality is very good! In use, observed for a day, not as used before, so much power consumption.	Jingdong prefecture level city in Shandong
IXCHSQ Flagship Store	King of Power Saving	Goods have been received, the effect is good is not good, power saving is not yet know, wait for me to use it and then evaluate it.	Jingdong prefecture level city in Shandong
IXCHSQ Flagship Store	King of Power Saving	In-kind and the same as described, the quality is quite good, the seller's attitude is also good, ask questions and answer them, the speed of delivery bar, worth buying Oh!	Jingdong prefecture level city in Shandong
IXCHSQ Flagship Store	King of Power Saving	Nice stuff, came to comment after using it, it does save power.	Jingdong prefecture level city in Shandong
IXCHSQ Flagship Store	King of Power Saving	Fast charging, but also save power, the effect is very good, satisfied, praise!	Jingdong prefecture level city in Shandong
IXCHSQ Flagship Store	King of Power Saving	The goods have been received, I do not know how effective, after using the flat price again!	Jingdong prefecture level city in Shandong
IXCHSQ Flagship Store	King of Power Saving	Merchant service is very good Reply quickly The key is the quality and that quality is good	Jingdong prefecture level city in Shandong
IXCHSQ Flagship Store	King of Power Saving	It's working well now, I'll do a follow up review after I actually use it for a while	Jingdong prefecture level city in Shandong
IXCHSQ Flagship Store	King of Power Saving	The quality is good. After installing it, I feel like I've saved a lot of money on my electricity bill.	Jingdong prefecture level city in Shandong
IXCHSQ Flagship Store	King of Power Saving	Make up a review after use.	Jingdong prefecture level city in Shandong
IXCHSQ Flagship Store	King of Power Saving	review after use	Jingdong prefecture level city in Shandong
IXCHSQ Flagship Store	King of Power Saving	It's a small capacitor. It's not energy efficient.	Jingdong prefecture level city in Shandong
IXCHSQ Flagship Store	King of Power Saving	I'll follow up with a review after I've used it and had results.	Jingdong prefecture level city in Shandong
IXCHSQ Flagship Store	King of Power Saving	No power saving? Not good!	Jingdong prefecture level city in Shandong
IXCHSQ Flagship Store	King of Power Saving	Been using the same appliances and running a 1.5 air conditioner at night for what feels like no time, and after half a month of use it feels like the power is only increasing. Doesn't show any power saving	Jingdong prefecture level city in Shandong
IXCHSQ Flagship Store	King of Power Saving	serviceable	Jingdong prefecture level city in Shandong
IXCHSQ Flagship Store	King of Power Saving	It's a piece of junk. It doesn't save any power.	Jingdong prefecture level city in Shandong
IXCHSQ Flagship Store	King of Power Saving	Use the pipe thing to save thirty percent, the quality of the economizer is very good, good-looking, plug and play is very convenient, the price is not expensive but also can save a lot of electricity, cost-	Jingdong prefecture level city in Shandong

IXCHSQ Flagship Store	King of Power Saving	There are a lot of appliances in the house, the summer air conditioning is very costly, I bought this in front of the air conditioning effect is very good, I explain that I bought three in one breath, I use more electricity a month down to 200 or so, very satisfied.	Jingdong prefecture level city in Shandong
IXCHSQ Flagship Store	King of Power Saving	The voltage is very stable has been maintained at 220v this value, high power super power saving, trustworthy, reasonable design, shape bright, logistics is quite fast, business warranty period, excellent service, logistics fast!	Jingdong prefecture level city in Shandong
IXCHSQ Flagship Store	King of Power Saving	Use a month after a look at the savings of more than 40 dollars, there is a display of electricity savings, this high-power intelligent frequency converter economizer, affordable and cheap, logistics is very fast, the service attitude is very good, very satisfied with a shopping.	Jingdong prefecture level city in Shandong
IXCHSQ Flagship Store	King of Power Saving	Used for a period of time, it seems that every day can really can save a few degrees of electricity, workmanship quite can, with a period of time and then continue to look at it, anyway, the price is not	Jingdong prefecture level city in Shandong
IXCHSQ Flagship Store	King of Power Saving	Used for a period of time, it seems that every day can indeed save two several degrees of electricity, workmanship quite can, with a period of time and then continue to look at it, anyway, the price is not	Jingdong prefecture level city in Shandong
IXCHSQ Flagship Store	King of Power Saving	High-power super saver received immediately used, the price is not expensive quality is very good, save electricity and money, very convenient, conscientious business, must be praised, recommended to everyone!	Jingdong prefecture level city in Shandong
IXCHSQ Flagship Store	King of Power Saving	Home enhancement version of the intelligent power saver, is in use, I think the price is cheap, value for money, efficacy, power, power saving effect is very good.	Jingdong prefecture level city in Shandong
IXCHSQ Flagship Store	King of Power Saving	Bought three at once, one for each room, summer electricity consumption is very high, despite the three air conditioners on the electricity bill is still down a lot, too Nessie!	Jingdong prefecture level city in Shandong
IXCHSQ Flagship Store	King of Power Saving	Without further ado, the electric bill came down, $102 less this month, personal satisfaction, it counts okay, give you a good review	Jingdong prefecture level city in Shandong
IXCHSQ Flagship Store	King of Power Saving	The power saver has arrived, the installation is really convenient, plugged in on the line operation is quite simple, power saving effect is also a strong!	Jingdong prefecture level city in Shandong
IXCHSQ Flagship Store	King of Power Saving	Saver received, good quality, beautiful appearance, fine workmanship, simple installation	Jingdong prefecture level city in Shandong
IXCHSQ Flagship Store	King of Power Saving	Product design style modern, worth buying	Jingdong prefecture level city in Shandong
IXCHSQ Flagship Store	King of Power Saving	Energy saving, good power saving effect, practical, great value	Jingdong prefecture level city in Shandong
IXCHSQ Flagship Store	King of Power Saving	It's good quality, it's good, it's energy efficient, and I'll buy it again.	Jingdong prefecture level city in Shandong
IXCHSQ Flagship Store	King of Power Saving	Installation saves a lot of electricity, fast logistics, real energy-saving appliances	Jingdong prefecture level city in Shandong
IXCHSQ Flagship Store	King of Power Saving	The effect is good and the power saving is obvious after using it for a month and saving tens of dollars, the product is trustworthy, I now buy another 4 colors to prepare for their siblings' family to pay for	Jingdong prefecture level city in Shandong
IXCHSQ Flagship Store	King of Power Saving	The quality is particularly good workmanship is no problem, the arrival of the very fast oh, especially power-saving	Jingdong prefecture level city in Shandong
IXCHSQ Flagship Store	King of Power Saving	The quality is very good! In use, observed for a day, not as used before, so much power consumption.	Jingdong prefecture level city in Shandong
IXCHSQ Flagship Store	King of Power Saving	Goods have been received, the effect is good is not good, power saving is not yet know, wait for me to use it and then evaluate it.	Jingdong prefecture level city in Shandong
IXCHSQ Flagship Store	King of Power Saving	In-kind and the same as described, the quality is quite good, the seller's attitude is also good, ask questions and answer them, the speed of delivery bar, worth buying Oh!	Jingdong prefecture level city in Shandong
IXCHSQ Flagship Store	King of Power Saving	Nice stuff, came to comment after using it, it does save power.	Jingdong prefecture level city in Shandong
IXCHSQ Flagship Store	King of Power Saving	Fast charging, but also save power, the effect is very good, satisfied, praise!	Jingdong prefecture level city in Shandong
IXCHSQ Flagship Store	King of Power Saving	The goods have been received, I do not know how effective, after using the flat price again!	Jingdong prefecture level city in Shandong
IXCHSQ Flagship Store	King of Power Saving	Merchant service is very good Reply quickly The key is the quality and that quality is good	Jingdong prefecture level city in Shandong
IXCHSQ Flagship Store	King of Power Saving	It's working well now, I'll do a follow up review after I actually use it for a while	Jingdong prefecture level city in Shandong
IXCHSQ Flagship Store	King of Power Saving	The quality is good. After installing it, I feel like I've saved a lot of money on my electricity bill.	Jingdong prefecture level city in Shandong
IXCHSQ Flagship Store	King of Power Saving	Make up a review after use.	Jingdong prefecture level city in Shandong
IXCHSQ Flagship Store	King of Power Saving	review after use	Jingdong prefecture level city in Shandong
IXCHSQ Flagship Store	King of Power Saving	It's a small capacitor. It's not energy efficient.	Jingdong prefecture level city in Shandong
IXCHSQ Flagship Store	King of Power Saving	I'll follow up with a review after I've used it and had results.	Jingdong prefecture level city in Shandong
IXCHSQ Flagship Store	King of Power Saving	No power saving? Not good!	Jingdong prefecture level city in Shandong
IXCHSQ Flagship Store	King of Power Saving	Been using the same appliances and running a 1.5 air conditioner at night for what feels like no time, and after half a month of use it feels like the power is only increasing. Doesn't show any power saving	Jingdong prefecture level city in Shandong
IXCHSQ Flagship Store	King of Power Saving	serviceable	Jingdong prefecture level city in Shandong
IXCHSQ Flagship Store	King of Power Saving	It's a piece of junk. It doesn't save any power.	Jingdong prefecture level city in Shandong
IXCHSQ Flagship Store	King of Power Saving	Use the pipe thing to save thirty percent, the quality of the economizer is very good, good-looking, plug and play is very convenient, the price is not expensive but also can save a lot of electricity, cost-	Jingdong prefecture level city in Shandong
IXCHSQ Flagship Store	King of Power Saving	There are a lot of appliances in the house, the summer air conditioning is very costly, I bought this in front of the air conditioning effect is very good, I explain that I bought three in one breath, I use more electricity a month down to 200 or so, very satisfied.	Jingdong prefecture level city in Shandong
IXCHSQ Flagship Store	King of Power Saving	The voltage is very stable has been maintained at 220v this value, high power super power saving, trustworthy, reasonable design, shape bright, logistics is quite fast, business warranty period, excellent service, logistics fast!	Jingdong prefecture level city in Shandong
IXCHSQ Flagship Store	King of Power Saving	Use a month after a look at the savings of more than 40 dollars, there is a display of electricity savings, this high-power intelligent frequency converter economizer, affordable and cheap, logistics is very fast, the service attitude is very good, very satisfied with a shopping.	Jingdong prefecture level city in Shandong
IXCHSQ Flagship Store	King of Power Saving	Used for a period of time, it seems that every day can really can save a few degrees of electricity, workmanship quite can, with a period of time and then continue to look at it, anyway, the price is not	Jingdong prefecture level city in Shandong
IXCHSQ Flagship Store	King of Power Saving	Used for a period of time, it seems that every day can indeed save two several degrees of electricity, workmanship quite can, with a period of time and then continue to look at it, anyway, the price is not	Jingdong prefecture level city in Shandong
IXCHSQ Flagship Store	King of Power Saving	High-power super saver received immediately used, the price is not expensive quality is very good, save electricity and money, very convenient, conscientious business, must be praised, recommended to everyone!	Jingdong prefecture level city in Shandong
IXCHSQ Flagship Store	King of Power Saving	Home enhancement version of the intelligent power saver, is in use, I think the price is cheap, value for money, efficacy, power, power saving effect is very good.	Jingdong prefecture level city in Shandong
IXCHSQ Flagship Store	King of Power Saving	Bought three at once, one for each room, summer electricity consumption is very high, despite the three air conditioners on the electricity bill is still down a lot, too Nessie!	Jingdong prefecture level city in Shandong
IXCHSQ Flagship Store	King of Power Saving	Without further ado, the electric bill came down, $102 less this month, personal satisfaction, it counts okay, give you a good review	Jingdong prefecture level city in Shandong
IXCHSQ Flagship Store	King of Power Saving	The power saver has arrived, the installation is really convenient, plugged in on the line operation is quite simple, power saving effect is also a strong!	Jingdong prefecture level city in Shandong
IXCHSQ Flagship Store	King of Power Saving	Saver received, good quality, beautiful appearance, fine workmanship, simple installation	Jingdong prefecture level city in Shandong
IXCHSQ Flagship Store	King of Power Saving	Product design style modern, worth buying	Jingdong prefecture level city in Shandong
IXCHSQ Flagship Store	King of Power Saving	Energy saving, good power saving effect, practical, great value	Jingdong prefecture level city in Shandong

IXCHSQ Flagship Store	King of Power Saving	It's good quality, it's good, it's energy efficient, and I'll buy it again.	Jingdong prefecture level city in Shandong
IXCHSQ Flagship Store	King of Power Saving	Installation saves a lot of electricity, fast logistics, real energy-saving appliances	Jingdong prefecture level city in Shandong
IXCHSQ Flagship Store	King of Power Saving	The effect is good and the power saving is obvious after using it for a month and saving tens of dollars, the product is trustworthy, I now buy another 4 colors to prepare for their siblings' family to pay for	Jingdong prefecture level city in Shandong
IXCHSQ Flagship Store	King of Power Saving	The quality is particularly good workmanship is no problem, the arrival of the very fast oh, especially power-saving	Jingdong prefecture level city in Shandong
IXCHSQ Flagship Store	King of Power Saving	The quality is very good! In use, observed for a day, not as used before, so much power consumption.	Jingdong prefecture level city in Shandong
IXCHSQ Flagship Store	King of Power Saving	Goods have been received, the effect is good is not good, power saving is not yet know, wait for me to use it and then evaluate it.	Jingdong prefecture level city in Shandong
IXCHSQ Flagship Store	King of Power Saving	In-kind and the same as described, the quality is quite good, the seller's attitude is also good, ask questions and answer them, the speed of delivery bar, worth buying Oh!	Jingdong prefecture level city in Shandong
IXCHSQ Flagship Store	King of Power Saving	Nice stuff, came to comment after using it, it does save power.	Jingdong prefecture level city in Shandong
IXCHSQ Flagship Store	King of Power Saving	Fast charging, but also save power, the effect is very good, satisfied, praise!	Jingdong prefecture level city in Shandong
IXCHSQ Flagship Store	King of Power Saving	The goods have been received, I do not know how effective, after using the flat price again!	Jingdong prefecture level city in Shandong
IXCHSQ Flagship Store	King of Power Saving	Merchant service is very good Reply quickly The key is the quality and that quality is good	Jingdong prefecture level city in Shandong
IXCHSQ Flagship Store	King of Power Saving	It's working well now, I'll do a follow up review after I actually use it for a while	Jingdong prefecture level city in Shandong
IXCHSQ Flagship Store	King of Power Saving	The quality is good. After installing it, I feel like I've saved a lot of money on my electricity bill.	Jingdong prefecture level city in Shandong
IXCHSQ Flagship Store	King of Power Saving	Make up a review after use.	Jingdong prefecture level city in Shandong
IXCHSQ Flagship Store	King of Power Saving	review after use	Jingdong prefecture level city in Shandong
IXCHSQ Flagship Store	King of Power Saving	It's a small capacitor. It's not energy efficient.	Jingdong prefecture level city in Shandong
IXCHSQ Flagship Store	King of Power Saving	I'll follow up with a review after I've used it and had results.	Jingdong prefecture level city in Shandong
IXCHSQ Flagship Store	King of Power Saving	No power saving? Not good!	Jingdong prefecture level city in Shandong
IXCHSQ Flagship Store	King of Power Saving	Been using the same appliances and running a 1.5 air conditioner at night for what feels like no time, and after half a month of use it feels like the power is only increasing. Doesn't show any power saving	Jingdong prefecture level city in Shandong
IXCHSQ Flagship Store	King of Power Saving	serviceable	Jingdong prefecture level city in Shandong
IXCHSQ Flagship Store	King of Power Saving	It's a piece of junk. It doesn't save any power.	Jingdong prefecture level city in Shandong
IXCHSQ Flagship Store	King of Power Saving	Use the pipe thing to save thirty percent, the quality of the economizer is very good, good-looking, plug and play is very convenient, the price is not expensive but also can save a lot of electricity, cost-	Jingdong prefecture level city in Shandong
IXCHSQ Flagship Store	King of Power Saving	There are a lot of appliances in the house, the summer air conditioning is very costly, I bought this in front of the air conditioning effect is very good, I explain that I bought three in one breath, I use more electricity a month down to 200 or so, very satisfied.	Jingdong prefecture level city in Shandong
IXCHSQ Flagship Store	King of Power Saving	The voltage is very stable has been maintained at 220v this value, high power super power saving, trustworthy, reasonable design, shape bright, logistics is quite fast, business warranty period, excellent service, logistics fast!	Jingdong prefecture level city in Shandong
IXCHSQ Flagship Store	King of Power Saving	Use a month after a look at the savings of more than 40 dollars, there is a display of electricity savings, this high-power intelligent frequency converter economizer, affordable and cheap, logistics is very fast, the service attitude is very good, very satisfied with a shopping.	Jingdong prefecture level city in Shandong
IXCHSQ Flagship Store	King of Power Saving	Used for a period of time, it seems that every day can indeed save two several degrees of electricity, workmanship quite can, with a period of time and then continue to look at it, anyway, the price is not	Jingdong prefecture level city in Shandong
IXCHSQ Flagship Store	King of Power Saving	Used for a period of time, it seems that every day can really can save a few degrees of electricity, workmanship quite can, with a period of time and then continue to look at it, anyway, the price is not	Jingdong prefecture level city in Shandong
IXCHSQ Flagship Store	King of Power Saving	High-power super saver received immediately used, the price is not expensive quality is very good, save electricity and money, very convenient, conscientious business, must be praised, recommended to everyone!	Jingdong prefecture level city in Shandong
IXCHSQ Flagship Store	King of Power Saving	Home enhancement version of the intelligent power saver, is in use, I think the price is cheap, value for money, efficacy, power, power saving effect is very good.	Jingdong prefecture level city in Shandong
IXCHSQ Flagship Store	King of Power Saving	Without further ado, the electric bill came down, $102 less this month, personal satisfaction, it counts okay, give you a good review	Jingdong prefecture level city in Shandong
IXCHSQ Flagship Store	King of Power Saving	Bought three at once, one for each room, summer electricity consumption is very high, despite the three air conditioners on the electricity bill is still down a lot, too Nessie!	Jingdong prefecture level city in Shandong
IXCHSQ Flagship Store	King of Power Saving	The power saver has arrived, the installation is really convenient, plugged in on the line operation is quite simple, power saving effect is also a strong!	Jingdong prefecture level city in Shandong
IXCHSQ Flagship Store	King of Power Saving	Saver received, good quality, beautiful appearance, fine workmanship, simple installation	Jingdong prefecture level city in Shandong
IXCHSQ Flagship Store	King of Power Saving	It's good quality, it's good, it's energy efficient, and I'll buy it again.	Jingdong prefecture level city in Shandong
IXCHSQ Flagship Store	King of Power Saving	Product design style modern, worth buying	Jingdong prefecture level city in Shandong
IXCHSQ Flagship Store	King of Power Saving	Energy saving, good power saving effect, practical, great value	Jingdong prefecture level city in Shandong
IXCHSQ Flagship Store	King of Power Saving	Installation saves a lot of electricity, fast logistics, real energy-saving appliances	Jingdong prefecture level city in Shandong
IXCHSQ Flagship Store	King of Power Saving	The quality is particularly good workmanship is no problem, the arrival of the very fast oh, especially power-saving	Jingdong prefecture level city in Shandong
IXCHSQ Flagship Store	King of Power Saving	The quality is very good! In use, observed for a day, not as used before, so much power consumption.	Jingdong prefecture level city in Shandong
IXCHSQ Flagship Store	King of Power Saving	The effect is good and the power saving is obvious after using it for a month and saving tens of dollars, the product is trustworthy, I now buy another 4 colors to prepare for their siblings' family to pay for	Jingdong prefecture level city in Shandong
IXCHSQ Flagship Store	King of Power Saving	Goods have been received, the effect is good is not good, power saving is not yet know, wait for me to use it and then evaluate it.	Jingdong prefecture level city in Shandong
IXCHSQ Flagship Store	King of Power Saving	In-kind and the same as described, the quality is quite good, the seller's attitude is also good, ask questions and answer them, the speed of delivery bar, worth buying Oh!	Jingdong prefecture level city in Shandong
IXCHSQ Flagship Store	King of Power Saving	Fast charging, but also save power, the effect is very good, satisfied, praise!	Jingdong prefecture level city in Shandong
IXCHSQ Flagship Store	King of Power Saving	Nice stuff, came to comment after using it, it does save power.	Jingdong prefecture level city in Shandong
IXCHSQ Flagship Store	King of Power Saving	The goods have been received, I do not know how effective, after using the flat price again!	Jingdong prefecture level city in Shandong
IXCHSQ Flagship Store	King of Power Saving	Merchant service is very good Reply quickly The key is the quality and that quality is good	Jingdong prefecture level city in Shandong
IXCHSQ Flagship Store	King of Power Saving	The quality is good. After installing it, I feel like I've saved a lot of money on my electricity bill.	Jingdong prefecture level city in Shandong
IXCHSQ Flagship Store	King of Power Saving	It's working well now, I'll do a follow up review after I actually use it for a while	Jingdong prefecture level city in Shandong
IXCHSQ Flagship Store	King of Power Saving	Make up a review after use.	Jingdong prefecture level city in Shandong
IXCHSQ Flagship Store	King of Power Saving	I'll follow up with a review after I've used it and had results.	Jingdong prefecture level city in Shandong

IXCHSQ Flagship Store	King of Power Saving	No power saving? Not good!	Jingdong prefecture level city in Shandong
IXCHSQ Flagship Store	King of Power Saving	Been using the same appliances and running a 1.5 air conditioner at night for what feels like no time, and after half a month of use it feels like the power is only increasing. Doesn't show any power saving	Jingdong prefecture level city in Shandong
IXCHSQ Flagship Store	King of Power Saving	It's a small capacitor. It's not energy efficient.	Jingdong prefecture level city in Shandong
IXCHSQ Flagship Store	King of Power Saving	review after use	Jingdong prefecture level city in Shandong
IXCHSQ Flagship Store	King of Power Saving	serviceable	Jingdong prefecture level city in Shandong
IXCHSQ Flagship Store	King of Power Saving	It's a piece of junk. It doesn't save any power.	Jingdong prefecture level city in Shandong
IXCHSQ Flagship Store	King of Power Saving	Use the pipe thing to save thirty percent, the quality of the economizer is very good, good-looking, plug and play is very convenient, the price is not expensive but also can save a lot of electricity, cost-	Jingdong prefecture level city in Shandong
IXCHSQ Flagship Store	King of Power Saving	There are a lot of appliances in the house, the summer air conditioning is very costly, I bought this in front of the air conditioning effect is very good, I explain that I bought three in one breath, I use more electricity a month down to 200 or so, very satisfied.	Jingdong prefecture level city in Shandong
IXCHSQ Flagship Store	King of Power Saving	The voltage is very stable has been maintained at 220v this value, high power super power saving, trustworthy, reasonable design, shape bright, logistics is quite fast, business warranty period, excellent service, logistics fast!	Jingdong prefecture level city in Shandong
IXCHSQ Flagship Store	King of Power Saving	Use a month after a look at the savings of more than 40 dollars, there is a display of electricity savings, this high-power intelligent frequency converter economizer, affordable and cheap, logistics is very fast, the service attitude is very good, very satisfied with a shopping.	Jingdong prefecture level city in Shandong
IXCHSQ Flagship Store	King of Power Saving	Used for a period of time, it seems that every day can really can save a few degrees of electricity, workmanship quite can, with a period of time and then continue to look at it, anyway, the price is not	Jingdong prefecture level city in Shandong
IXCHSQ Flagship Store	King of Power Saving	Used for a period of time, it seems that every day can indeed save two several degrees of electricity, workmanship quite can, with a period of time and then continue to look at it, anyway, the price is not	Jingdong prefecture level city in Shandong
IXCHSQ Flagship Store	King of Power Saving	High-power super saver received immediately used, the price is not expensive quality is very good, save electricity and money, very convenient, conscientious business, must be praised, recommended to everyone!	Jingdong prefecture level city in Shandong
IXCHSQ Flagship Store	King of Power Saving	Home enhancement version of the intelligent power saver, is in use, I think the price is cheap, value for money, efficacy, power, power saving effect is very good.	Jingdong prefecture level city in Shandong
IXCHSQ Flagship Store	King of Power Saving	Bought three at once, one for each room, summer electricity consumption is very high, despite the three air conditioners on the electricity bill is still down a lot, too Nessie!	Jingdong prefecture level city in Shandong
IXCHSQ Flagship Store	King of Power Saving	Without further ado, the electric bill came down, $102 less this month, personal satisfaction, it counts okay, give you a good review	Jingdong prefecture level city in Shandong
IXCHSQ Flagship Store	King of Power Saving	The power saver has arrived, the installation is really convenient, plugged in on the line operation is quite simple, power saving effect is also a strong!	Jingdong prefecture level city in Shandong
IXCHSQ Flagship Store	King of Power Saving	Saver received, good quality, beautiful appearance, fine workmanship, simple installation	Jingdong prefecture level city in Shandong
IXCHSQ Flagship Store	King of Power Saving	Product design style modern, worth buying	Jingdong prefecture level city in Shandong
IXCHSQ Flagship Store	King of Power Saving	Energy saving, good power saving effect, practical, great value	Jingdong prefecture level city in Shandong
IXCHSQ Flagship Store	King of Power Saving	It's good quality, it's good, it's energy efficient, and I'll buy it again.	Jingdong prefecture level city in Shandong
IXCHSQ Flagship Store	King of Power Saving	Installation saves a lot of electricity, fast logistics, real energy-saving appliances	Jingdong prefecture level city in Shandong
IXCHSQ Flagship Store	King of Power Saving	The effect is good and the power saving is obvious after using it for a month and saving tens of dollars, the product is trustworthy, I now buy another 4 colors to prepare for their siblings' family to pay for	Jingdong prefecture level city in Shandong
IXCHSQ Flagship Store	King of Power Saving	The quality is particularly good workmanship is no problem, the arrival of the very fast oh, especially power-saving	Jingdong prefecture level city in Shandong
IXCHSQ Flagship Store	King of Power Saving	The quality is very good! In use, observed for a day, not as used before, so much power consumption.	Jingdong prefecture level city in Shandong
IXCHSQ Flagship Store	King of Power Saving	Goods have been received, the effect is good is not good, power saving is not yet know, wait for me to use it and then evaluate it.	Jingdong prefecture level city in Shandong
IXCHSQ Flagship Store	King of Power Saving	In-kind and the same as described, the quality is quite good, the seller's attitude is also good, ask questions and answer them, the speed of delivery bar, worth buying Oh!	Jingdong prefecture level city in Shandong
IXCHSQ Flagship Store	King of Power Saving	Nice stuff, came to comment after using it, it does save power.	Jingdong prefecture level city in Shandong
IXCHSQ Flagship Store	King of Power Saving	Fast charging, but also save power, the effect is very good, satisfied, praise!	Jingdong prefecture level city in Shandong
IXCHSQ Flagship Store	King of Power Saving	The goods have been received, I do not know how effective, after using the flat price again!	Jingdong prefecture level city in Shandong
IXCHSQ Flagship Store	King of Power Saving	Merchant service is very good Reply quickly The key is the quality and that quality is good	Jingdong prefecture level city in Shandong
IXCHSQ Flagship Store	King of Power Saving	It's working well now, I'll do a follow up review after I actually use it for a while	Jingdong prefecture level city in Shandong
IXCHSQ Flagship Store	King of Power Saving	The quality is good. After installing it, I feel like I've saved a lot of money on my electricity bill.	Jingdong prefecture level city in Shandong
IXCHSQ Flagship Store	King of Power Saving	Make up a review after use.	Jingdong prefecture level city in Shandong
IXCHSQ Flagship Store	King of Power Saving	review after use	Jingdong prefecture level city in Shandong
IXCHSQ Flagship Store	King of Power Saving	It's a small capacitor. It's not energy efficient.	Jingdong prefecture level city in Shandong
IXCHSQ Flagship Store	King of Power Saving	I'll follow up with a review after I've used it and had results.	Jingdong prefecture level city in Shandong
IXCHSQ Flagship Store	King of Power Saving	No power saving? Not good!	Jingdong prefecture level city in Shandong
IXCHSQ Flagship Store	King of Power Saving	Been using the same appliances and running a 1.5 air conditioner at night for what feels like no time, and after half a month of use it feels like the power is only increasing. Doesn't show any power saving	Jingdong prefecture level city in Shandong
IXCHSQ Flagship Store	King of Power Saving	serviceable	Jingdong prefecture level city in Shandong
IXCHSQ Flagship Store	King of Power Saving	It's a piece of junk. It doesn't save any power.	Jingdong prefecture level city in Shandong
IXCHSQ Flagship Store	King of Power Saving	Use the pipe thing to save thirty percent, the quality of the economizer is very good, good-looking, plug and play is very convenient, the price is not expensive but also can save a lot of electricity, cost-	Jingdong prefecture level city in Shandong
IXCHSQ Flagship Store	King of Power Saving	There are a lot of appliances in the house, the summer air conditioning is very costly, I bought this in front of the air conditioning effect is very good, I explain that I bought three in one breath, I use more electricity a month down to 200 or so, very satisfied.	Jingdong prefecture level city in Shandong
IXCHSQ Flagship Store	King of Power Saving	The voltage is very stable has been maintained at 220v this value, high power super power saving, trustworthy, reasonable design, shape bright, logistics is quite fast, business warranty period, excellent service, logistics fast!	Jingdong prefecture level city in Shandong
IXCHSQ Flagship Store	King of Power Saving	Use a month after a look at the savings of more than 40 dollars, there is a display of electricity savings, this high-power intelligent frequency converter economizer, affordable and cheap, logistics is very fast, the service attitude is very good, very satisfied with a shopping.	Jingdong prefecture level city in Shandong
IXCHSQ Flagship Store	King of Power Saving	Used for a period of time, it seems that every day can really can save a few degrees of electricity, workmanship quite can, with a period of time and then continue to look at it, anyway, the price is not	Jingdong prefecture level city in Shandong
IXCHSQ Flagship Store	King of Power Saving	Used for a period of time, it seems that every day can indeed save two several degrees of electricity, workmanship quite can, with a period of time and then continue to look at it, anyway, the price is not	Jingdong prefecture level city in Shandong
IXCHSQ Flagship Store	King of Power Saving	High-power super saver received immediately used, the price is not expensive quality is very good, save electricity and money, very convenient, conscientious business, must be praised, recommended to everyone!	Jingdong prefecture level city in Shandong

IXCHSQ Flagship Store	King of Power Saving	Home enhancement version of the intelligent power saver, is in use, I think the price is cheap, value for money, efficacy, power, power saving effect is very good.	Jingdong prefecture level city in Shandong
IXCHSQ Flagship Store	King of Power Saving	Bought three at once, one for each room, summer electricity consumption is very high, despite the three air conditioners on the electricity bill is still down a lot, too Nessie!	Jingdong prefecture level city in Shandong
IXCHSQ Flagship Store	King of Power Saving	Without further ado, the electric bill came down, $102 less this month, personal satisfaction, it counts okay, give you a good review	Jingdong prefecture level city in Shandong
IXCHSQ Flagship Store	King of Power Saving	The power saver has arrived, the installation is really convenient, plugged in on the line operation is quite simple, power saving effect is also a strong!	Jingdong prefecture level city in Shandong
IXCHSQ Flagship Store	King of Power Saving	Saver received, good quality, beautiful appearance, fine workmanship, simple installation	Jingdong prefecture level city in Shandong
IXCHSQ Flagship Store	King of Power Saving	Product design style modern, worth buying	Jingdong prefecture level city in Shandong
IXCHSQ Flagship Store	King of Power Saving	Energy saving, good power saving effect, practical, great value	Jingdong prefecture level city in Shandong
IXCHSQ Flagship Store	King of Power Saving	It's good quality, it's good, it's energy efficient, and I'll buy it again.	Jingdong prefecture level city in Shandong
IXCHSQ Flagship Store	King of Power Saving	Installation saves a lot of electricity, fast logistics, real energy-saving appliances	Jingdong prefecture level city in Shandong
IXCHSQ Flagship Store	King of Power Saving	The effect is good and the power saving is obvious after using it for a month and saving tens of dollars, the product is trustworthy, I now buy another 4 colors to prepare for their siblings' family to pay for	Jingdong prefecture level city in Shandong
IXCHSQ Flagship Store	King of Power Saving	The quality is particularly good workmanship is no problem, the arrival of the very fast oh, especially power-saving	Jingdong prefecture level city in Shandong
IXCHSQ Flagship Store	King of Power Saving	The quality is very good! In use, observed for a day, not as used before, so much power consumption.	Jingdong prefecture level city in Shandong
IXCHSQ Flagship Store	King of Power Saving	Goods have been received, the effect is good is not good, power saving is not yet know, wait for me to use it and then evaluate it.	Jingdong prefecture level city in Shandong
IXCHSQ Flagship Store	King of Power Saving	In-kind and the same as described, the quality is quite good, the seller's attitude is also good, ask questions and answer them, the speed of delivery bar, worth buying Oh!	Jingdong prefecture level city in Shandong
IXCHSQ Flagship Store	King of Power Saving	Nice stuff, came to comment after using it, it does save power.	Jingdong prefecture level city in Shandong
IXCHSQ Flagship Store	King of Power Saving	Fast charging, but also save power, the effect is very good, satisfied, praise!	Jingdong prefecture level city in Shandong
IXCHSQ Flagship Store	King of Power Saving	The goods have been received, I do not know how effective, after using the flat price again!	Jingdong prefecture level city in Shandong
IXCHSQ Flagship Store	King of Power Saving	Merchant service is very good Reply quickly The key is the quality and that quality is good	Jingdong prefecture level city in Shandong
IXCHSQ Flagship Store	King of Power Saving	It's working well now, I'll do a follow up review after I actually use it for a while	Jingdong prefecture level city in Shandong
IXCHSQ Flagship Store	King of Power Saving	The quality is good. After installing it, I feel like I've saved a lot of money on my electricity bill.	Jingdong prefecture level city in Shandong
IXCHSQ Flagship Store	King of Power Saving	Make up a review after use.	Jingdong prefecture level city in Shandong
IXCHSQ Flagship Store	King of Power Saving	review after use	Jingdong prefecture level city in Shandong
IXCHSQ Flagship Store	King of Power Saving	It's a small capacitor. It's not energy efficient.	Jingdong prefecture level city in Shandong
IXCHSQ Flagship Store	King of Power Saving	I'll follow up with a review after I've used it and had results.	Jingdong prefecture level city in Shandong
IXCHSQ Flagship Store	King of Power Saving	No power saving? Not good!	Jingdong prefecture level city in Shandong
IXCHSQ Flagship Store	King of Power Saving	Been using the same appliances and running a 1.5 air conditioner at night for what feels like no time, and after half a month of use it feels like the power is only increasing. Doesn't show any power saving	Jingdong prefecture level city in Shandong
IXCHSQ Flagship Store	King of Power Saving	serviceable	Jingdong prefecture level city in Shandong
IXCHSQ Flagship Store	King of Power Saving	It's a piece of junk. It doesn't save any power.	Jingdong prefecture level city in Shandong
IXCHSQ Flagship Store	King of Power Saving	Use the pipe thing to save thirty percent, the quality of the economizer is very good, good-looking, plug and play is very convenient, the price is not expensive but also can save a lot of electricity, cost-	Jingdong prefecture level city in Shandong
IXCHSQ Flagship Store	King of Power Saving	There are a lot of appliances in the house, the summer air conditioning is very costly, I bought this in front of the air conditioning effect is very good, I explain that I bought three in one breath, I use more electricity a month down to 200 or so, very satisfied.	Jingdong prefecture level city in Shandong
IXCHSQ Flagship Store	King of Power Saving	The voltage is very stable has been maintained at 220v this value, high power super power saving, trustworthy, reasonable design, shape bright, logistics is quite fast, business warranty period, excellent service, logistics fast!	Jingdong prefecture level city in Shandong
IXCHSQ Flagship Store	King of Power Saving	Use a month after a look at the savings of more than 40 dollars, there is a display of electricity savings, this high-power intelligent frequency converter economizer, affordable and cheap, logistics is very fast, the service attitude is very good, very satisfied with a shopping.	Jingdong prefecture level city in Shandong
IXCHSQ Flagship Store	King of Power Saving	Used for a period of time, it seems that every day can really can save a few degrees of electricity, workmanship quite can, with a period of time and then continue to look at it, anyway, the price is not	Jingdong prefecture level city in Shandong
IXCHSQ Flagship Store	King of Power Saving	Used for a period of time, it seems that every day can indeed save two several degrees of electricity, workmanship quite can, with a period of time and then continue to look at it, anyway, the price is not	Jingdong prefecture level city in Shandong
IXCHSQ Flagship Store	King of Power Saving	High-power super saver received immediately used, the price is not expensive quality is very good, save electricity and money, very convenient, conscientious business, must be praised, recommended to everyone!	Jingdong prefecture level city in Shandong
IXCHSQ Flagship Store	King of Power Saving	Home enhancement version of the intelligent power saver, is in use, I think the price is cheap, value for money, efficacy, power, power saving effect is very good.	Jingdong prefecture level city in Shandong
IXCHSQ Flagship Store	King of Power Saving	Bought three at once, one for each room, summer electricity consumption is very high, despite the three air conditioners on the electricity bill is still down a lot, too Nessie!	Jingdong prefecture level city in Shandong
IXCHSQ Flagship Store	King of Power Saving	Without further ado, the electric bill came down, $102 less this month, personal satisfaction, it counts okay, give you a good review	Jingdong prefecture level city in Shandong
IXCHSQ Flagship Store	King of Power Saving	The power saver has arrived, the installation is really convenient, plugged in on the line operation is quite simple, power saving effect is also a strong!	Jingdong prefecture level city in Shandong
IXCHSQ Flagship Store	King of Power Saving	Saver received, good quality, beautiful appearance, fine workmanship, simple installation	Jingdong prefecture level city in Shandong
IXCHSQ Flagship Store	King of Power Saving	Product design style modern, worth buying	Jingdong prefecture level city in Shandong
IXCHSQ Flagship Store	King of Power Saving	Energy saving, good power saving effect, practical, great value	Jingdong prefecture level city in Shandong
IXCHSQ Flagship Store	King of Power Saving	It's good quality, it's good, it's energy efficient, and I'll buy it again.	Jingdong prefecture level city in Shandong
IXCHSQ Flagship Store	King of Power Saving	Installation saves a lot of electricity, fast logistics, real energy-saving appliances	Jingdong prefecture level city in Shandong
IXCHSQ Flagship Store	King of Power Saving	The effect is good and the power saving is obvious after using it for a month and saving tens of dollars, the product is trustworthy, I now buy another 4 colors to prepare for their siblings' family to pay for	Jingdong prefecture level city in Shandong
IXCHSQ Flagship Store	King of Power Saving	The quality is particularly good workmanship is no problem, the arrival of the very fast oh, especially power-saving	Jingdong prefecture level city in Shandong
IXCHSQ Flagship Store	King of Power Saving	The quality is very good! In use, observed for a day, not as used before, so much power consumption.	Jingdong prefecture level city in Shandong
IXCHSQ Flagship Store	King of Power Saving	Goods have been received, the effect is good is not good, power saving is not yet know, wait for me to use it and then evaluate it.	Jingdong prefecture level city in Shandong
IXCHSQ Flagship Store	King of Power Saving	In-kind and the same as described, the quality is quite good, the seller's attitude is also good, ask questions and answer them, the speed of delivery bar, worth buying Oh!	Jingdong prefecture level city in Shandong
IXCHSQ Flagship Store	King of Power Saving	Nice stuff, came to comment after using it, it does save power.	Jingdong prefecture level city in Shandong

IXCHSQ Flagship Store	King of Power Saving	Fast charging, but also save power, the effect is very good, satisfied, praise!	Jingdong prefecture level city in Shandong
IXCHSQ Flagship Store	King of Power Saving	The goods have been received, I do not know how effective, after using the flat price again!	Jingdong prefecture level city in Shandong
IXCHSQ Flagship Store	King of Power Saving	Merchant service is very good Reply quickly The key is the quality and that quality is good	Jingdong prefecture level city in Shandong
IXCHSQ Flagship Store	King of Power Saving	It's working well now, I'll do a follow up review after I actually use it for a while	Jingdong prefecture level city in Shandong
IXCHSQ Flagship Store	King of Power Saving	The quality is good. After installing it, I feel like I've saved a lot of money on my electricity bill.	Jingdong prefecture level city in Shandong
IXCHSQ Flagship Store	King of Power Saving	Make up a review after use.	Jingdong prefecture level city in Shandong
IXCHSQ Flagship Store	King of Power Saving	review after use	Jingdong prefecture level city in Shandong
IXCHSQ Flagship Store	King of Power Saving	It's a small capacitor. It's not energy efficient.	Jingdong prefecture level city in Shandong
IXCHSQ Flagship Store	King of Power Saving	I'll follow up with a review after I've used it and had results.	Jingdong prefecture level city in Shandong
IXCHSQ Flagship Store	King of Power Saving	No power saving? Not good!	Jingdong prefecture level city in Shandong
IXCHSQ Flagship Store	King of Power Saving	Been using the same appliances and running a 1.5 air conditioner at night for what feels like no time, and after half a month of use it feels like the power is only increasing. Doesn't show any power saving	Jingdong prefecture level city in Shandong
IXCHSQ Flagship Store	King of Power Saving	serviceable	Jingdong prefecture level city in Shandong
IXCHSQ Flagship Store	King of Power Saving	It's a piece of junk. It doesn't save any power.	Jingdong prefecture level city in Shandong
IXCHSQ Flagship Store	King of Power Saving	Use the pipe thing to save thirty percent, the quality of the economizer is very good, good-looking, plug and play is very convenient, the price is not expensive but also can save a lot of electricity, cost-	Jingdong prefecture level city in Shandong
IXCHSQ Flagship Store	King of Power Saving	There are a lot of appliances in the house, the summer air conditioning is very costly, I bought this in front of the air conditioning effect is very good, I explain that I bought three in one breath, I use more electricity a month down to 200 or so, very satisfied.	Jingdong prefecture level city in Shandong
IXCHSQ Flagship Store	King of Power Saving	The voltage is very stable has been maintained at 220v this value, high power super power saving, trustworthy, reasonable design, shape bright, logistics is quite fast, business warranty period, excellent service, logistics fast!	Jingdong prefecture level city in Shandong
IXCHSQ Flagship Store	King of Power Saving	Use a month after a look at the savings of more than 40 dollars, there is a display of electricity savings, this high-power intelligent frequency converter economizer, affordable and cheap, logistics is very fast, the service attitude is very good, very satisfied with a shopping.	Jingdong prefecture level city in Shandong
IXCHSQ Flagship Store	King of Power Saving	Used for a period of time, it seems that every day can really can save a few degrees of electricity, workmanship quite can, with a period of time and then continue to look at it, anyway, the price is not	Jingdong prefecture level city in Shandong
IXCHSQ Flagship Store	King of Power Saving	Used for a period of time, it seems that every day can indeed save two several degrees of electricity, workmanship quite can, with a period of time and then continue to look at it, anyway, the price is not	Jingdong prefecture level city in Shandong
IXCHSQ Flagship Store	King of Power Saving	High-power super saver received immediately used, the price is not expensive quality is very good, save electricity and money, very convenient, conscientious business, must be praised, recommended to everyone!	Jingdong prefecture level city in Shandong
IXCHSQ Flagship Store	King of Power Saving	Home enhancement version of the intelligent power saver, is in use, I think the price is cheap, value for money, efficacy, power, power saving effect is very good.	Jingdong prefecture level city in Shandong
IXCHSQ Flagship Store	King of Power Saving	Bought three at once, one for each room, summer electricity consumption is very high, despite the three air conditioners on the electricity bill is still down a lot, too Nessie!	Jingdong prefecture level city in Shandong
IXCHSQ Flagship Store	King of Power Saving	Without further ado, the electric bill came down, $102 less this month, personal satisfaction, it counts okay, give you a good review	Jingdong prefecture level city in Shandong
IXCHSQ Flagship Store	King of Power Saving	The power saver has arrived, the installation is really convenient, plugged in on the line operation is quite simple, power saving effect is also a strong!	Jingdong prefecture level city in Shandong
IXCHSQ Flagship Store	King of Power Saving	Saver received, good quality, beautiful appearance, fine workmanship, simple installation	Jingdong prefecture level city in Shandong
IXCHSQ Flagship Store	King of Power Saving	Product design style modern, worth buying	Jingdong prefecture level city in Shandong
IXCHSQ Flagship Store	King of Power Saving	Energy saving, good power saving effect, practical, great value	Jingdong prefecture level city in Shandong
IXCHSQ Flagship Store	King of Power Saving	It's good quality, it's good, it's energy efficient, and I'll buy it again.	Jingdong prefecture level city in Shandong
IXCHSQ Flagship Store	King of Power Saving	Installation saves a lot of electricity, fast logistics, real energy-saving appliances	Jingdong prefecture level city in Shandong
IXCHSQ Flagship Store	King of Power Saving	The effect is good and the power saving is obvious after using it for a month and saving tens of dollars, the product is trustworthy, I now buy another 4 colors to prepare for their siblings' family to pay for	Jingdong prefecture level city in Shandong
IXCHSQ Flagship Store	King of Power Saving	The quality is particularly good workmanship is no problem, the arrival of the very fast oh, especially power-saving	Jingdong prefecture level city in Shandong
IXCHSQ Flagship Store	King of Power Saving	The quality is very good! In use, observed for a day, not as used before, so much power consumption.	Jingdong prefecture level city in Shandong
IXCHSQ Flagship Store	King of Power Saving	Goods have been received, the effect is good is not good, power saving is not yet know, wait for me to use it and then evaluate it.	Jingdong prefecture level city in Shandong
IXCHSQ Flagship Store	King of Power Saving	In-kind and the same as described, the quality is quite good, the seller's attitude is also good, ask questions and answer them, the speed of delivery bar, worth buying Oh!	Jingdong prefecture level city in Shandong
IXCHSQ Flagship Store	King of Power Saving	Nice stuff, came to comment after using it, it does save power.	Jingdong prefecture level city in Shandong
IXCHSQ Flagship Store	King of Power Saving	Fast charging, but also save power, the effect is very good, satisfied, praise!	Jingdong prefecture level city in Shandong
IXCHSQ Flagship Store	King of Power Saving	The goods have been received, I do not know how effective, after using the flat price again!	Jingdong prefecture level city in Shandong
IXCHSQ Flagship Store	King of Power Saving	Merchant service is very good Reply quickly The key is the quality and that quality is good	Jingdong prefecture level city in Shandong
IXCHSQ Flagship Store	King of Power Saving	It's working well now, I'll do a follow up review after I actually use it for a while	Jingdong prefecture level city in Shandong
IXCHSQ Flagship Store	King of Power Saving	The quality is good. After installing it, I feel like I've saved a lot of money on my electricity bill.	Jingdong prefecture level city in Shandong
IXCHSQ Flagship Store	King of Power Saving	Make up a review after use.	Jingdong prefecture level city in Shandong
IXCHSQ Flagship Store	King of Power Saving	review after use	Jingdong prefecture level city in Shandong
IXCHSQ Flagship Store	King of Power Saving	It's a small capacitor. It's not energy efficient.	Jingdong prefecture level city in Shandong
IXCHSQ Flagship Store	King of Power Saving	I'll follow up with a review after I've used it and had results.	Jingdong prefecture level city in Shandong
IXCHSQ Flagship Store	King of Power Saving	No power saving? Not good!	Jingdong prefecture level city in Shandong
IXCHSQ Flagship Store	King of Power Saving	Been using the same appliances and running a 1.5 air conditioner at night for what feels like no time, and after half a month of use it feels like the power is only increasing. Doesn't show any power saving	Jingdong prefecture level city in Shandong
IXCHSQ Flagship Store	King of Power Saving	serviceable	Jingdong prefecture level city in Shandong
IXCHSQ Flagship Store	King of Power Saving	It's a piece of junk. It doesn't save any power.	Jingdong prefecture level city in Shandong
IXCHSQ Flagship Store	King of Power Saving	Use the pipe thing to save thirty percent, the quality of the economizer is very good, good-looking, plug and play is very convenient, the price is not expensive but also can save a lot of electricity, cost-	Jingdong prefecture level city in Shandong

IXCHSQ Flagship Store	King of Power Saving	There are a lot of appliances in the house, the summer air conditioning is very costly, I bought this in front of the air conditioning effect is very good, I explain that I bought three in one breath, I use more electricity a month down to 200 or so, very satisfied.	Jingdong prefecture level city in Shandong
IXCHSQ Flagship Store	King of Power Saving	The voltage is very stable has been maintained at 220v this value, high power super power saving, trustworthy, reasonable design, shape bright, logistics is quite fast, business warranty period, excellent service, logistics fast!	Jingdong prefecture level city in Shandong
IXCHSQ Flagship Store	King of Power Saving	Use a month after a look at the savings of more than 40 dollars, there is a display of electricity savings, this high-power intelligent frequency converter economizer, affordable and cheap, logistics is very fast, the service attitude is very good, very satisfied with a shopping.	Jingdong prefecture level city in Shandong
IXCHSQ Flagship Store	King of Power Saving	Used for a period of time, it seems that every day can really can save a few degrees of electricity, workmanship quite can, with a period of time and then continue to look at it, anyway, the price is not	Jingdong prefecture level city in Shandong
IXCHSQ Flagship Store	King of Power Saving	Used for a period of time, it seems that every day can indeed save two several degrees of electricity, workmanship quite can, with a period of time and then continue to look at it, anyway, the price is not	Jingdong prefecture level city in Shandong
IXCHSQ Flagship Store	King of Power Saving	High-power super saver received immediately used, the price is not expensive quality is very good, save electricity and money, very convenient, conscientious business, must be praised, recommended to everyone!	Jingdong prefecture level city in Shandong
IXCHSQ Flagship Store	King of Power Saving	Home enhancement version of the intelligent power saver, is in use, I think the price is cheap, value for money, efficacy, power, power saving effect is very good.	Jingdong prefecture level city in Shandong
IXCHSQ Flagship Store	King of Power Saving	Bought three at once, one for each room, summer electricity consumption is very high, despite the three air conditioners on the electricity bill is still down a lot, too Nessie!	Jingdong prefecture level city in Shandong
IXCHSQ Flagship Store	King of Power Saving	Without further ado, the electric bill came down, $102 less this month, personal satisfaction, it counts okay, give you a good review	Jingdong prefecture level city in Shandong
IXCHSQ Flagship Store	King of Power Saving	The power saver has arrived, the installation is really convenient, plugged in on the line operation is quite simple, power saving effect is also a strong!	Jingdong prefecture level city in Shandong
IXCHSQ Flagship Store	King of Power Saving	Saver received, good quality, beautiful appearance, fine workmanship, simple installation	Jingdong prefecture level city in Shandong
IXCHSQ Flagship Store	King of Power Saving	Product design style modern, worth buying	Jingdong prefecture level city in Shandong
IXCHSQ Flagship Store	King of Power Saving	Energy saving, good power saving effect, practical, great value	Jingdong prefecture level city in Shandong
IXCHSQ Flagship Store	King of Power Saving	It's good quality, it's good, it's energy efficient, and I'll buy it again.	Jingdong prefecture level city in Shandong
IXCHSQ Flagship Store	King of Power Saving	Installation saves a lot of electricity, fast logistics, real energy-saving appliances	Jingdong prefecture level city in Shandong
IXCHSQ Flagship Store	King of Power Saving	The effect is good and the power saving is obvious after using it for a month and saving tens of dollars, the product is trustworthy, I now buy another 4 colors to prepare for their siblings' family to pay for	Jingdong prefecture level city in Shandong
IXCHSQ Flagship Store	King of Power Saving	The quality is particularly good workmanship is no problem, the arrival of the very fast oh, especially power-saving	Jingdong prefecture level city in Shandong
IXCHSQ Flagship Store	King of Power Saving	The quality is very good! In use, observed for a day, not as used before, so much power consumption.	Jingdong prefecture level city in Shandong
IXCHSQ Flagship Store	King of Power Saving	Goods have been received, the effect is good is not good, power saving is not yet know, wait for me to use it and then evaluate it.	Jingdong prefecture level city in Shandong
IXCHSQ Flagship Store	King of Power Saving	In-kind and the same as described, the quality is quite good, the seller's attitude is also good, ask questions and answer them, the speed of delivery bar, worth buying Oh!	Jingdong prefecture level city in Shandong
IXCHSQ Flagship Store	King of Power Saving	Nice stuff, came to comment after using it, it does save power.	Jingdong prefecture level city in Shandong
IXCHSQ Flagship Store	King of Power Saving	Fast charging, but also save power, the effect is very good, satisfied, praise!	Jingdong prefecture level city in Shandong
IXCHSQ Flagship Store	King of Power Saving	The goods have been received, I do not know how effective, after using the flat price again!	Jingdong prefecture level city in Shandong
IXCHSQ Flagship Store	King of Power Saving	Merchant service is very good Reply quickly The key is the quality and that quality is good	Jingdong prefecture level city in Shandong
IXCHSQ Flagship Store	King of Power Saving	It's working well now, I'll do a follow up review after I actually use it for a while	Jingdong prefecture level city in Shandong
IXCHSQ Flagship Store	King of Power Saving	The quality is good. After installing it, I feel like I've saved a lot of money on my electricity bill.	Jingdong prefecture level city in Shandong
IXCHSQ Flagship Store	King of Power Saving	Make up a review after use.	Jingdong prefecture level city in Shandong
IXCHSQ Flagship Store	King of Power Saving	review after use	Jingdong prefecture level city in Shandong
IXCHSQ Flagship Store	King of Power Saving	It's a small capacitor. It's not energy efficient.	Jingdong prefecture level city in Shandong
IXCHSQ Flagship Store	King of Power Saving	I'll follow up with a review after I've used it and had results.	Jingdong prefecture level city in Shandong
IXCHSQ Flagship Store	King of Power Saving	No power saving? Not good!	Jingdong prefecture level city in Shandong
IXCHSQ Flagship Store	King of Power Saving	Been using the same appliances and running a 1.5 air conditioner at night for what feels like no time, and after half a month of use it feels like the power is only increasing. Doesn't show any power saving	Jingdong prefecture level city in Shandong
IXCHSQ Flagship Store	King of Power Saving	serviceable	Jingdong prefecture level city in Shandong
IXCHSQ Flagship Store	King of Power Saving	It's a piece of junk. It doesn't save any power.	Jingdong prefecture level city in Shandong
IXCHSQ Flagship Store	King of Power Saving	Use the pipe thing to save thirty percent, the quality of the economizer is very good, good-looking, plug and play is very convenient, the price is not expensive but also can save a lot of electricity, cost-	Jingdong prefecture level city in Shandong
IXCHSQ Flagship Store	King of Power Saving	There are a lot of appliances in the house, the summer air conditioning is very costly, I bought this in front of the air conditioning effect is very good, I explain that I bought three in one breath, I use more electricity a month down to 200 or so, very satisfied.	Jingdong prefecture level city in Shandong
IXCHSQ Flagship Store	King of Power Saving	The voltage is very stable has been maintained at 220v this value, high power super power saving, trustworthy, reasonable design, shape bright, logistics is quite fast, business warranty period, excellent service, logistics fast!	Jingdong prefecture level city in Shandong
IXCHSQ Flagship Store	King of Power Saving	Use a month after a look at the savings of more than 40 dollars, there is a display of electricity savings, this high-power intelligent frequency converter economizer, affordable and cheap, logistics is very fast, the service attitude is very good, very satisfied with a shopping.	Jingdong prefecture level city in Shandong
IXCHSQ Flagship Store	King of Power Saving	Used for a period of time, it seems that every day can really can save a few degrees of electricity, workmanship quite can, with a period of time and then continue to look at it, anyway, the price is not	Jingdong prefecture level city in Shandong
IXCHSQ Flagship Store	King of Power Saving	Used for a period of time, it seems that every day can indeed save two several degrees of electricity, workmanship quite can, with a period of time and then continue to look at it, anyway, the price is not	Jingdong prefecture level city in Shandong
IXCHSQ Flagship Store	King of Power Saving	High-power super saver received immediately used, the price is not expensive quality is very good, save electricity and money, very convenient, conscientious business, must be praised, recommended to everyone!	Jingdong prefecture level city in Shandong
IXCHSQ Flagship Store	King of Power Saving	Home enhancement version of the intelligent power saver, is in use, I think the price is cheap, value for money, efficacy, power, power saving effect is very good.	Jingdong prefecture level city in Shandong
IXCHSQ Flagship Store	King of Power Saving	Bought three at once, one for each room, summer electricity consumption is very high, despite the three air conditioners on the electricity bill is still down a lot, too Nessie!	Jingdong prefecture level city in Shandong
IXCHSQ Flagship Store	King of Power Saving	Without further ado, the electric bill came down, $102 less this month, personal satisfaction, it counts okay, give you a good review	Jingdong prefecture level city in Shandong
IXCHSQ Flagship Store	King of Power Saving	The power saver has arrived, the installation is really convenient, plugged in on the line operation is quite simple, power saving effect is also a strong!	Jingdong prefecture level city in Shandong
IXCHSQ Flagship Store	King of Power Saving	Saver received, good quality, beautiful appearance, fine workmanship, simple installation	Jingdong prefecture level city in Shandong
IXCHSQ Flagship Store	King of Power Saving	Product design style modern, worth buying	Jingdong prefecture level city in Shandong
IXCHSQ Flagship Store	King of Power Saving	Energy saving, good power saving effect, practical, great value	Jingdong prefecture level city in Shandong

IXCHSQ Flagship Store	King of Power Saving	It's good quality, it's good, it's energy efficient, and I'll buy it again.	Jingdong prefecture level city in Shandong
IXCHSQ Flagship Store	King of Power Saving	Installation saves a lot of electricity, fast logistics, real energy-saving appliances	Jingdong prefecture level city in Shandong
IXCHSQ Flagship Store	King of Power Saving	The effect is good and the power saving is obvious after using it for a month and saving tens of dollars, the product is trustworthy, I now buy another 4 colors to prepare for their siblings' family to pay for	Jingdong prefecture level city in Shandong
IXCHSQ Flagship Store	King of Power Saving	The quality is particularly good workmanship is no problem, the arrival of the very fast oh, especially power-saving	Jingdong prefecture level city in Shandong
IXCHSQ Flagship Store	King of Power Saving	The quality is very good! In use, observed for a day, not as used before, so much power consumption.	Jingdong prefecture level city in Shandong
IXCHSQ Flagship Store	King of Power Saving	Goods have been received, the effect is good is not good, power saving is not yet know, wait for me to use it and then evaluate it.	Jingdong prefecture level city in Shandong
IXCHSQ Flagship Store	King of Power Saving	In-kind and the same as described, the quality is quite good, the seller's attitude is also good, ask questions and answer them, the speed of delivery bar, worth buying Oh!	Jingdong prefecture level city in Shandong
IXCHSQ Flagship Store	King of Power Saving	Nice stuff, came to comment after using it, it does save power.	Jingdong prefecture level city in Shandong
IXCHSQ Flagship Store	King of Power Saving	Fast charging, but also save power, the effect is very good, satisfied, praise!	Jingdong prefecture level city in Shandong
IXCHSQ Flagship Store	King of Power Saving	The goods have been received, I do not know how effective, after using the flat price again!	Jingdong prefecture level city in Shandong
IXCHSQ Flagship Store	King of Power Saving	Merchant service is very good Reply quickly The key is the quality and that quality is good	Jingdong prefecture level city in Shandong
IXCHSQ Flagship Store	King of Power Saving	It's working well now, I'll do a follow up review after I actually use it for a while	Jingdong prefecture level city in Shandong
IXCHSQ Flagship Store	King of Power Saving	The quality is good. After installing it, I feel like I've saved a lot of money on my electricity bill.	Jingdong prefecture level city in Shandong
IXCHSQ Flagship Store	King of Power Saving	Make up a review after use.	Jingdong prefecture level city in Shandong
IXCHSQ Flagship Store	King of Power Saving	review after use	Jingdong prefecture level city in Shandong
IXCHSQ Flagship Store	King of Power Saving	It's a small capacitor. It's not energy efficient.	Jingdong prefecture level city in Shandong
IXCHSQ Flagship Store	King of Power Saving	I'll follow up with a review after I've used it and had results.	Jingdong prefecture level city in Shandong
IXCHSQ Flagship Store	King of Power Saving	No power saving? Not good!	Jingdong prefecture level city in Shandong
IXCHSQ Flagship Store	King of Power Saving	Been using the same appliances and running a 1.5 air conditioner at night for what feels like no time, and after half a month of use it feels like the power is only increasing. Doesn't show any power saving	Jingdong prefecture level city in Shandong
IXCHSQ Flagship Store	King of Power Saving	serviceable	Jingdong prefecture level city in Shandong
IXCHSQ Flagship Store	King of Power Saving	It's a piece of junk. It doesn't save any power.	Jingdong prefecture level city in Shandong
IXCHSQ Flagship Store	King of Power Saving	Use the pipe thing to save thirty percent, the quality of the economizer is very good, good-looking, plug and play is very convenient, the price is not expensive but also can save a lot of electricity, cost-	Jingdong prefecture level city in Shandong
IXCHSQ Flagship Store	King of Power Saving	There are a lot of appliances in the house, the summer air conditioning is very costly, I bought this in front of the air conditioning effect is very good, I explain that I bought three in one breath, I use more electricity a month down to 200 or so, very satisfied.	Jingdong prefecture level city in Shandong
IXCHSQ Flagship Store	King of Power Saving	The voltage is very stable has been maintained at 220v this value, high power super power saving, trustworthy, reasonable design, shape bright, logistics is quite fast, business warranty period, excellent service, logistics fast!	Jingdong prefecture level city in Shandong
IXCHSQ Flagship Store	King of Power Saving	Use a month after a look at the savings of more than 40 dollars, there is a display of electricity savings, this high-power intelligent frequency converter economizer, affordable and cheap, logistics is very fast, the service attitude is very good, very satisfied with a shopping.	Jingdong prefecture level city in Shandong
IXCHSQ Flagship Store	King of Power Saving	Used for a period of time, it seems that every day can really can save a few degrees of electricity, workmanship quite can, with a period of time and then continue to look at it, anyway, the price is not	Jingdong prefecture level city in Shandong
IXCHSQ Flagship Store	King of Power Saving	Used for a period of time, it seems that every day can indeed save two several degrees of electricity, workmanship quite can, with a period of time and then continue to look at it, anyway, the price is not	Jingdong prefecture level city in Shandong
IXCHSQ Flagship Store	King of Power Saving	High-power super saver received immediately used, the price is not expensive quality is very good, save electricity and money, very convenient, conscientious business, must be praised, recommended to everyone!	Jingdong prefecture level city in Shandong
IXCHSQ Flagship Store	King of Power Saving	Home enhancement version of the intelligent power saver, is in use, I think the price is cheap, value for money, efficacy, power, power saving effect is very good.	Jingdong prefecture level city in Shandong
IXCHSQ Flagship Store	King of Power Saving	Bought three at once, one for each room, summer electricity consumption is very high, despite the three air conditioners on the electricity bill is still down a lot, too Nessie!	Jingdong prefecture level city in Shandong
IXCHSQ Flagship Store	King of Power Saving	Without further ado, the electric bill came down, $102 less this month, personal satisfaction, it counts okay, give you a good review	Jingdong prefecture level city in Shandong
IXCHSQ Flagship Store	King of Power Saving	The power saver has arrived, the installation is really convenient, plugged in on the line operation is quite simple, power saving effect is also a strong!	Jingdong prefecture level city in Shandong
IXCHSQ Flagship Store	King of Power Saving	Saver received, good quality, beautiful appearance, fine workmanship, simple installation	Jingdong prefecture level city in Shandong
IXCHSQ Flagship Store	King of Power Saving	Product design style modern, worth buying	Jingdong prefecture level city in Shandong
IXCHSQ Flagship Store	King of Power Saving	Energy saving, good power saving effect, practical, great value	Jingdong prefecture level city in Shandong
IXCHSQ Flagship Store	King of Power Saving	It's good quality, it's good, it's energy efficient, and I'll buy it again.	Jingdong prefecture level city in Shandong
IXCHSQ Flagship Store	King of Power Saving	Installation saves a lot of electricity, fast logistics, real energy-saving appliances	Jingdong prefecture level city in Shandong
IXCHSQ Flagship Store	King of Power Saving	The effect is good and the power saving is obvious after using it for a month and saving tens of dollars, the product is trustworthy, I now buy another 4 colors to prepare for their siblings' family to pay for	Jingdong prefecture level city in Shandong
IXCHSQ Flagship Store	King of Power Saving	The quality is particularly good workmanship is no problem, the arrival of the very fast oh, especially power-saving	Jingdong prefecture level city in Shandong
IXCHSQ Flagship Store	King of Power Saving	The quality is very good! In use, observed for a day, not as used before, so much power consumption.	Jingdong prefecture level city in Shandong
IXCHSQ Flagship Store	King of Power Saving	Goods have been received, the effect is good is not good, power saving is not yet know, wait for me to use it and then evaluate it.	Jingdong prefecture level city in Shandong
IXCHSQ Flagship Store	King of Power Saving	In-kind and the same as described, the quality is quite good, the seller's attitude is also good, ask questions and answer them, the speed of delivery bar, worth buying Oh!	Jingdong prefecture level city in Shandong
IXCHSQ Flagship Store	King of Power Saving	Nice stuff, came to comment after using it, it does save power.	Jingdong prefecture level city in Shandong
IXCHSQ Flagship Store	King of Power Saving	Fast charging, but also save power, the effect is very good, satisfied, praise!	Jingdong prefecture level city in Shandong
IXCHSQ Flagship Store	King of Power Saving	The goods have been received, I do not know how effective, after using the flat price again!	Jingdong prefecture level city in Shandong
IXCHSQ Flagship Store	King of Power Saving	Merchant service is very good Reply quickly The key is the quality and that quality is good	Jingdong prefecture level city in Shandong
IXCHSQ Flagship Store	King of Power Saving	It's working well now, I'll do a follow up review after I actually use it for a while	Jingdong prefecture level city in Shandong
IXCHSQ Flagship Store	King of Power Saving	The quality is good. After installing it, I feel like I've saved a lot of money on my electricity bill.	Jingdong prefecture level city in Shandong
IXCHSQ Flagship Store	King of Power Saving	Make up a review after use.	Jingdong prefecture level city in Shandong
IXCHSQ Flagship Store	King of Power Saving	review after use	Jingdong prefecture level city in Shandong

IXCHSQ Flagship Store	King of Power Saving	It's a small capacitor. It's not energy efficient.	Jingdong prefecture level city in Shandong
IXCHSQ Flagship Store	King of Power Saving	I'll follow up with a review after I've used it and had results.	Jingdong prefecture level city in Shandong
IXCHSQ Flagship Store	King of Power Saving	No power saving? Not good!	Jingdong prefecture level city in Shandong
IXCHSQ Flagship Store	King of Power Saving	Been using the same appliances and running a 1.5 air conditioner at night for what feels like no time, and after half a month of use it feels like the power is only increasing. Doesn't show any power saving	Jingdong prefecture level city in Shandong
IXCHSQ Flagship Store	King of Power Saving	serviceable	Jingdong prefecture level city in Shandong
IXCHSQ Flagship Store	King of Power Saving	It's a piece of junk. It doesn't save any power.	Jingdong prefecture level city in Shandong
IXCHSQ Flagship Store	King of Power Saving	Use the pipe thing to save thirty percent, the quality of the economizer is very good, good-looking, plug and play is very convenient, the price is not expensive but also can save a lot of electricity, cost-	Jingdong prefecture level city in Shandong
IXCHSQ Flagship Store	King of Power Saving	There are a lot of appliances in the house, the summer air conditioning is very costly, I bought this in front of the air conditioning effect is very good, I explain that I bought three in one breath, I use more electricity a month down to 200 or so, very satisfied.	Jingdong prefecture level city in Shandong
IXCHSQ Flagship Store	King of Power Saving	The voltage is very stable has been maintained at 220v this value, high power super power saving, trustworthy, reasonable design, shape bright, logistics is quite fast, business warranty period, excellent service, logistics fast!	Jingdong prefecture level city in Shandong
IXCHSQ Flagship Store	King of Power Saving	Use a month after a look at the savings of more than 40 dollars, there is a display of electricity savings, this high-power intelligent frequency converter economizer, affordable and cheap, logistics is very fast, the service attitude is very good, very satisfied with a shopping.	Jingdong prefecture level city in Shandong
IXCHSQ Flagship Store	King of Power Saving	Used for a period of time, it seems that every day can really can save a few degrees of electricity, workmanship quite can, with a period of time and then continue to look at it, anyway, the price is not	Jingdong prefecture level city in Shandong
IXCHSQ Flagship Store	King of Power Saving	Used for a period of time, it seems that every day can indeed save two several degrees of electricity, workmanship quite can, with a period of time and then continue to look at it, anyway, the price is not	Jingdong prefecture level city in Shandong
IXCHSQ Flagship Store	King of Power Saving	High-power super saver received immediately used, the price is not expensive quality is very good, save electricity and money, very convenient, conscientious business, must be praised, recommended to everyone!	Jingdong prefecture level city in Shandong
IXCHSQ Flagship Store	King of Power Saving	Home enhancement version of the intelligent power saver, is in use, I think the price is cheap, value for money, efficacy, power, power saving effect is very good.	Jingdong prefecture level city in Shandong
IXCHSQ Flagship Store	King of Power Saving	Bought three at once, one for each room, summer electricity consumption is very high, despite the three air conditioners on the electricity bill is still down a lot, too Nessie!	Jingdong prefecture level city in Shandong
IXCHSQ Flagship Store	King of Power Saving	Without further ado, the electric bill came down, $102 less this month, personal satisfaction, it counts okay, give you a good review	Jingdong prefecture level city in Shandong
IXCHSQ Flagship Store	King of Power Saving	The power saver has arrived, the installation is really convenient, plugged in on the line operation is quite simple, power saving effect is also a strong!	Jingdong prefecture level city in Shandong
IXCHSQ Flagship Store	King of Power Saving	Saver received, good quality, beautiful appearance, fine workmanship, simple installation	Jingdong prefecture level city in Shandong
IXCHSQ Flagship Store	King of Power Saving	Product design style modern, worth buying	Jingdong prefecture level city in Shandong
IXCHSQ Flagship Store	King of Power Saving	Energy saving, good power saving effect, practical, great value	Jingdong prefecture level city in Shandong
IXCHSQ Flagship Store	King of Power Saving	It's good quality, it's good, it's energy efficient, and I'll buy it again.	Jingdong prefecture level city in Shandong
IXCHSQ Flagship Store	King of Power Saving	Installation saves a lot of electricity, fast logistics, real energy-saving appliances	Jingdong prefecture level city in Shandong
IXCHSQ Flagship Store	King of Power Saving	The effect is good and the power saving is obvious after using it for a month and saving tens of dollars, the product is trustworthy, I now buy another 4 colors to prepare for their siblings' family to pay for	Jingdong prefecture level city in Shandong
IXCHSQ Flagship Store	King of Power Saving	The quality is particularly good workmanship is no problem, the arrival of the very fast oh, especially power-saving	Jingdong prefecture level city in Shandong
IXCHSQ Flagship Store	King of Power Saving	The quality is very good! In use, observed for a day, not as used before, so much power consumption.	Jingdong prefecture level city in Shandong
IXCHSQ Flagship Store	King of Power Saving	Goods have been received, the effect is good is not good, power saving is not yet know, wait for me to use it and then evaluate it.	Jingdong prefecture level city in Shandong
IXCHSQ Flagship Store	King of Power Saving	In-kind and the same as described, the quality is quite good, the seller's attitude is also good, ask questions and answer them, the speed of delivery bar, worth buying Oh!	Jingdong prefecture level city in Shandong
IXCHSQ Flagship Store	King of Power Saving	Nice stuff, came to comment after using it, it does save power.	Jingdong prefecture level city in Shandong
IXCHSQ Flagship Store	King of Power Saving	Fast charging, but also save power, the effect is very good, satisfied, praise!	Jingdong prefecture level city in Shandong
IXCHSQ Flagship Store	King of Power Saving	The goods have been received, I do not know how effective, after using the flat price again!	Jingdong prefecture level city in Shandong
IXCHSQ Flagship Store	King of Power Saving	Merchant service is very good Reply quickly The key is the quality and that quality is good	Jingdong prefecture level city in Shandong
IXCHSQ Flagship Store	King of Power Saving	It's working well now, I'll do a follow up review after I actually use it for a while	Jingdong prefecture level city in Shandong
IXCHSQ Flagship Store	King of Power Saving	The quality is good. After installing it, I feel like I've saved a lot of money on my electricity bill.	Jingdong prefecture level city in Shandong
IXCHSQ Flagship Store	King of Power Saving	Make up a review after use.	Jingdong prefecture level city in Shandong
IXCHSQ Flagship Store	King of Power Saving	review after use	Jingdong prefecture level city in Shandong
IXCHSQ Flagship Store	King of Power Saving	It's a small capacitor. It's not energy efficient.	Jingdong prefecture level city in Shandong
IXCHSQ Flagship Store	King of Power Saving	I'll follow up with a review after I've used it and had results.	Jingdong prefecture level city in Shandong
IXCHSQ Flagship Store	King of Power Saving	No power saving? Not good!	Jingdong prefecture level city in Shandong
IXCHSQ Flagship Store	King of Power Saving	Been using the same appliances and running a 1.5 air conditioner at night for what feels like no time, and after half a month of use it feels like the power is only increasing. Doesn't show any power saving	Jingdong prefecture level city in Shandong
IXCHSQ Flagship Store	King of Power Saving	serviceable	Jingdong prefecture level city in Shandong
IXCHSQ Flagship Store	King of Power Saving	It's a piece of junk. It doesn't save any power.	Jingdong prefecture level city in Shandong
IXCHSQ Flagship Store	King of Power Saving	Use the pipe thing to save thirty percent, the quality of the economizer is very good, good-looking, plug and play is very convenient, the price is not expensive but also can save a lot of electricity, cost-	Jingdong prefecture level city in Shandong
IXCHSQ Flagship Store	King of Power Saving	There are a lot of appliances in the house, the summer air conditioning is very costly, I bought this in front of the air conditioning effect is very good, I explain that I bought three in one breath, I use more electricity a month down to 200 or so, very satisfied.	Jingdong prefecture level city in Shandong
IXCHSQ Flagship Store	King of Power Saving	The voltage is very stable has been maintained at 220v this value, high power super power saving, trustworthy, reasonable design, shape bright, logistics is quite fast, business warranty period, excellent service, logistics fast!	Jingdong prefecture level city in Shandong
IXCHSQ Flagship Store	King of Power Saving	Use a month after a look at the savings of more than 40 dollars, there is a display of electricity savings, this high-power intelligent frequency converter economizer, affordable and cheap, logistics is very fast, the service attitude is very good, very satisfied with a shopping.	Jingdong prefecture level city in Shandong
IXCHSQ Flagship Store	King of Power Saving	Used for a period of time, it seems that every day can really can save a few degrees of electricity, workmanship quite can, with a period of time and then continue to look at it, anyway, the price is not	Jingdong prefecture level city in Shandong
IXCHSQ Flagship Store	King of Power Saving	Used for a period of time, it seems that every day can indeed save two several degrees of electricity, workmanship quite can, with a period of time and then continue to look at it, anyway, the price is not	Jingdong prefecture level city in Shandong
IXCHSQ Flagship Store	King of Power Saving	High-power super saver received immediately used, the price is not expensive quality is very good, save electricity and money, very convenient, conscientious business, must be praised, recommended to everyone!	Jingdong prefecture level city in Shandong

IXCHSQ Flagship Store	King of Power Saving	Home enhancement version of the intelligent power saver, is in use, I think the price is cheap, value for money, efficacy, power, power saving effect is very good.	Jingdong prefecture level city in Shandong
IXCHSQ Flagship Store	King of Power Saving	Bought three at once, one for each room, summer electricity consumption is very high, despite the three air conditioners on the electricity bill is still down a lot, too Nessie!	Jingdong prefecture level city in Shandong
IXCHSQ Flagship Store	King of Power Saving	Without further ado, the electric bill came down, $102 less this month, personal satisfaction, it counts okay, give you a good review	Jingdong prefecture level city in Shandong
IXCHSQ Flagship Store	King of Power Saving	The power saver has arrived, the installation is really convenient, plugged in on the line operation is quite simple, power saving effect is also a strong!	Jingdong prefecture level city in Shandong
IXCHSQ Flagship Store	King of Power Saving	Saver received, good quality, beautiful appearance, fine workmanship, simple installation	Jingdong prefecture level city in Shandong
IXCHSQ Flagship Store	King of Power Saving	Product design style modern, worth buying	Jingdong prefecture level city in Shandong
IXCHSQ Flagship Store	King of Power Saving	Energy saving, good power saving effect, practical, great value	Jingdong prefecture level city in Shandong
IXCHSQ Flagship Store	King of Power Saving	It's good quality, it's good, it's energy efficient, and I'll buy it again.	Jingdong prefecture level city in Shandong
IXCHSQ Flagship Store	King of Power Saving	Installation saves a lot of electricity, fast logistics, real energy-saving appliances	Jingdong prefecture level city in Shandong
IXCHSQ Flagship Store	King of Power Saving	The effect is good and the power saving is obvious after using it for a month and saving tens of dollars, the product is trustworthy, I now buy another 4 colors to prepare for their siblings' family to pay for	Jingdong prefecture level city in Shandong
IXCHSQ Flagship Store	King of Power Saving	The quality is particularly good workmanship is no problem, the arrival of the very fast oh, especially power-saving	Jingdong prefecture level city in Shandong
IXCHSQ Flagship Store	King of Power Saving	The quality is very good! In use, observed for a day, not as used before, so much power consumption.	Jingdong prefecture level city in Shandong
IXCHSQ Flagship Store	King of Power Saving	Goods have been received, the effect is good is not good, power saving is not yet know, wait for me to use it and then evaluate it.	Jingdong prefecture level city in Shandong
IXCHSQ Flagship Store	King of Power Saving	In-kind and the same as described, the quality is quite good, the seller's attitude is also good, ask questions and answer them, the speed of delivery bar, worth buying Oh!	Jingdong prefecture level city in Shandong
IXCHSQ Flagship Store	King of Power Saving	Nice stuff, came to comment after using it, it does save power.	Jingdong prefecture level city in Shandong
IXCHSQ Flagship Store	King of Power Saving	Fast charging, but also save power, the effect is very good, satisfied, praise!	Jingdong prefecture level city in Shandong
IXCHSQ Flagship Store	King of Power Saving	The goods have been received, I do not know how effective, after using the flat price again!	Jingdong prefecture level city in Shandong
IXCHSQ Flagship Store	King of Power Saving	Merchant service is very good Reply quickly The key is the quality and that quality is good	Jingdong prefecture level city in Shandong
IXCHSQ Flagship Store	King of Power Saving	It's working well now, I'll do a follow up review after I actually use it for a while	Jingdong prefecture level city in Shandong
IXCHSQ Flagship Store	King of Power Saving	The quality is good. After installing it, I feel like I've saved a lot of money on my electricity bill.	Jingdong prefecture level city in Shandong
IXCHSQ Flagship Store	King of Power Saving	Make up a review after use.	Jingdong prefecture level city in Shandong
IXCHSQ Flagship Store	King of Power Saving	review after use	Jingdong prefecture level city in Shandong
IXCHSQ Flagship Store	King of Power Saving	It's a small capacitor. It's not energy efficient.	Jingdong prefecture level city in Shandong
IXCHSQ Flagship Store	King of Power Saving	I'll follow up with a review after I've used it and had results.	Jingdong prefecture level city in Shandong
IXCHSQ Flagship Store	King of Power Saving	No power saving? Not good!	Jingdong prefecture level city in Shandong
IXCHSQ Flagship Store	King of Power Saving	Been using the same appliances and running a 1.5 air conditioner at night for what feels like no time, and after half a month of use it feels like the power is only increasing. Doesn't show any power saving	Jingdong prefecture level city in Shandong
IXCHSQ Flagship Store	King of Power Saving	serviceable	Jingdong prefecture level city in Shandong
IXCHSQ Flagship Store	King of Power Saving	It's a piece of junk. It doesn't save any power.	Jingdong prefecture level city in Shandong
IXCHSQ Flagship Store	King of Power Saving	Use the pipe thing to save thirty percent, the quality of the economizer is very good, good-looking, plug and play is very convenient, the price is not expensive but also can save a lot of electricity, cost-	Jingdong prefecture level city in Shandong
IXCHSQ Flagship Store	King of Power Saving	There are a lot of appliances in the house, the summer air conditioning is very costly, I bought this in front of the air conditioning effect is very good, I explain that I bought three in one breath, I use more electricity a month down to 200 or so, very satisfied.	Jingdong prefecture level city in Shandong
IXCHSQ Flagship Store	King of Power Saving	The voltage is very stable has been maintained at 220v this value, high power super power saving, trustworthy, reasonable design, shape bright, logistics is quite fast, business warranty period, excellent service, logistics fast!	Jingdong prefecture level city in Shandong
IXCHSQ Flagship Store	King of Power Saving	Use a month after a look at the savings of more than 40 dollars, there is a display of electricity savings, this high-power intelligent frequency converter economizer, affordable and cheap, logistics is very fast, the service attitude is very good, very satisfied with a shopping.	Jingdong prefecture level city in Shandong
IXCHSQ Flagship Store	King of Power Saving	Used for a period of time, it seems that every day can really can save a few degrees of electricity, workmanship quite can, with a period of time and then continue to look at it, anyway, the price is not	Jingdong prefecture level city in Shandong
IXCHSQ Flagship Store	King of Power Saving	Used for a period of time, it seems that every day can indeed save two several degrees of electricity, workmanship quite can, with a period of time and then continue to look at it, anyway, the price is not	Jingdong prefecture level city in Shandong
IXCHSQ Flagship Store	King of Power Saving	High-power super saver received immediately used, the price is not expensive quality is very good, save electricity and money, very convenient, conscientious business, must be praised, recommended to everyone!	Jingdong prefecture level city in Shandong
IXCHSQ Flagship Store	King of Power Saving	Home enhancement version of the intelligent power saver, is in use, I think the price is cheap, value for money, efficacy, power, power saving effect is very good.	Jingdong prefecture level city in Shandong
IXCHSQ Flagship Store	King of Power Saving	Bought three at once, one for each room, summer electricity consumption is very high, despite the three air conditioners on the electricity bill is still down a lot, too Nessie!	Jingdong prefecture level city in Shandong
IXCHSQ Flagship Store	King of Power Saving	Without further ado, the electric bill came down, $102 less this month, personal satisfaction, it counts okay, give you a good review	Jingdong prefecture level city in Shandong
IXCHSQ Flagship Store	King of Power Saving	The power saver has arrived, the installation is really convenient, plugged in on the line operation is quite simple, power saving effect is also a strong!	Jingdong prefecture level city in Shandong
IXCHSQ Flagship Store	King of Power Saving	Saver received, good quality, beautiful appearance, fine workmanship, simple installation	Jingdong prefecture level city in Shandong
IXCHSQ Flagship Store	King of Power Saving	Product design style modern, worth buying	Jingdong prefecture level city in Shandong
IXCHSQ Flagship Store	King of Power Saving	Energy saving, good power saving effect, practical, great value	Jingdong prefecture level city in Shandong
IXCHSQ Flagship Store	King of Power Saving	It's good quality, it's good, it's energy efficient, and I'll buy it again.	Jingdong prefecture level city in Shandong
IXCHSQ Flagship Store	King of Power Saving	Installation saves a lot of electricity, fast logistics, real energy-saving appliances	Jingdong prefecture level city in Shandong
IXCHSQ Flagship Store	King of Power Saving	The effect is good and the power saving is obvious after using it for a month and saving tens of dollars, the product is trustworthy, I now buy another 4 colors to prepare for their siblings' family to pay for	Jingdong prefecture level city in Shandong
IXCHSQ Flagship Store	King of Power Saving	The quality is particularly good workmanship is no problem, the arrival of the very fast oh, especially power-saving	Jingdong prefecture level city in Shandong
IXCHSQ Flagship Store	King of Power Saving	The quality is very good! In use, observed for a day, not as used before, so much power consumption.	Jingdong prefecture level city in Shandong
IXCHSQ Flagship Store	King of Power Saving	Goods have been received, the effect is good is not good, power saving is not yet know, wait for me to use it and then evaluate it.	Jingdong prefecture level city in Shandong
IXCHSQ Flagship Store	King of Power Saving	In-kind and the same as described, the quality is quite good, the seller's attitude is also good, ask questions and answer them, the speed of delivery bar, worth buying Oh!	Jingdong prefecture level city in Shandong
IXCHSQ Flagship Store	King of Power Saving	Nice stuff, came to comment after using it, it does save power.	Jingdong prefecture level city in Shandong

IXCHSQ Flagship Store	King of Power Saving	Fast charging, but also save power, the effect is very good, satisfied, praise!	Jingdong prefecture level city in Shandong
IXCHSQ Flagship Store	King of Power Saving	The goods have been received, I do not know how effective, after using the flat price again!	Jingdong prefecture level city in Shandong
IXCHSQ Flagship Store	King of Power Saving	Merchant service is very good Reply quickly The key is the quality and that quality is good	Jingdong prefecture level city in Shandong
IXCHSQ Flagship Store	King of Power Saving	It's working well now, I'll do a follow up review after I actually use it for a while	Jingdong prefecture level city in Shandong
IXCHSQ Flagship Store	King of Power Saving	The quality is good. After installing it, I feel like I've saved a lot of money on my electricity bill.	Jingdong prefecture level city in Shandong
IXCHSQ Flagship Store	King of Power Saving	Make up a review after use.	Jingdong prefecture level city in Shandong
IXCHSQ Flagship Store	King of Power Saving	review after use	Jingdong prefecture level city in Shandong
IXCHSQ Flagship Store	King of Power Saving	It's a small capacitor. It's not energy efficient.	Jingdong prefecture level city in Shandong
IXCHSQ Flagship Store	King of Power Saving	I'll follow up with a review after I've used it and had results.	Jingdong prefecture level city in Shandong
IXCHSQ Flagship Store	King of Power Saving	No power saving? Not good!	Jingdong prefecture level city in Shandong
IXCHSQ Flagship Store	King of Power Saving	Been using the same appliances and running a 1.5 air conditioner at night for what feels like no time, and after half a month of use it feels like the power is only increasing. Doesn't show any power saving	Jingdong prefecture level city in Shandong
IXCHSQ Flagship Store	King of Power Saving	serviceable	Jingdong prefecture level city in Shandong
IXCHSQ Flagship Store	King of Power Saving	It's a piece of junk. It doesn't save any power.	Jingdong prefecture level city in Shandong
IXCHSQ Flagship Store	King of Power Saving	Use the pipe thing to save thirty percent, the quality of the economizer is very good, good-looking, plug and play is very convenient, the price is not expensive but also can save a lot of electricity, cost-	Jingdong prefecture level city in Shandong
IXCHSQ Flagship Store	King of Power Saving	There are a lot of appliances in the house, the summer air conditioning is very costly, I bought this in front of the air conditioning effect is very good, I explain that I bought three in one breath, I use more electricity a month down to 200 or so, very satisfied.	Jingdong prefecture level city in Shandong
IXCHSQ Flagship Store	King of Power Saving	The voltage is very stable has been maintained at 220v this value, high power super power saving, trustworthy, reasonable design, shape bright, logistics is quite fast, business warranty period, excellent service, logistics fast!	Jingdong prefecture level city in Shandong
IXCHSQ Flagship Store	King of Power Saving	Use a month after a look at the savings of more than 40 dollars, there is a display of electricity savings, this high-power intelligent frequency converter economizer, affordable and cheap, logistics is very fast, the service attitude is very good, very satisfied with a shopping.	Jingdong prefecture level city in Shandong
IXCHSQ Flagship Store	King of Power Saving	Used for a period of time, it seems that every day can really can save a few degrees of electricity, workmanship quite can, with a period of time and then continue to look at it, anyway, the price is not	Jingdong prefecture level city in Shandong
IXCHSQ Flagship Store	King of Power Saving	Used for a period of time, it seems that every day can indeed save two several degrees of electricity, workmanship quite can, with a period of time and then continue to look at it, anyway, the price is not	Jingdong prefecture level city in Shandong
IXCHSQ Flagship Store	King of Power Saving	High-power super saver received immediately used, the price is not expensive quality is very good, save electricity and money, very convenient, conscientious business, must be praised, recommended to everyone!	Jingdong prefecture level city in Shandong
IXCHSQ Flagship Store	King of Power Saving	Home enhancement version of the intelligent power saver, is in use, I think the price is cheap, value for money, efficacy, power, power saving effect is very good.	Jingdong prefecture level city in Shandong
IXCHSQ Flagship Store	King of Power Saving	Bought three at once, one for each room, summer electricity consumption is very high, despite the three air conditioners on the electricity bill is still down a lot, too Nessie!	Jingdong prefecture level city in Shandong
IXCHSQ Flagship Store	King of Power Saving	Without further ado, the electric bill came down, $102 less this month, personal satisfaction, it counts okay, give you a good review	Jingdong prefecture level city in Shandong
IXCHSQ Flagship Store	King of Power Saving	The power saver has arrived, the installation is really convenient, plugged in on the line operation is quite simple, power saving effect is also a strong!	Jingdong prefecture level city in Shandong
IXCHSQ Flagship Store	King of Power Saving	Saver received, good quality, beautiful appearance, fine workmanship, simple installation	Jingdong prefecture level city in Shandong
IXCHSQ Flagship Store	King of Power Saving	Product design style modern, worth buying	Jingdong prefecture level city in Shandong
IXCHSQ Flagship Store	King of Power Saving	Energy saving, good power saving effect, practical, great value	Jingdong prefecture level city in Shandong
IXCHSQ Flagship Store	King of Power Saving	It's good quality, it's good, it's energy efficient, and I'll buy it again.	Jingdong prefecture level city in Shandong
IXCHSQ Flagship Store	King of Power Saving	Installation saves a lot of electricity, fast logistics, real energy-saving appliances	Jingdong prefecture level city in Shandong
IXCHSQ Flagship Store	King of Power Saving	The effect is good and the power saving is obvious after using it for a month and saving tens of dollars, the product is trustworthy, I now buy another 4 colors to prepare for their siblings' family to pay for	Jingdong prefecture level city in Shandong
IXCHSQ Flagship Store	King of Power Saving	The quality is particularly good workmanship is no problem, the arrival of the very fast oh, especially power-saving	Jingdong prefecture level city in Shandong
IXCHSQ Flagship Store	King of Power Saving	The quality is very good! In use, observed for a day, not as used before, so much power consumption.	Jingdong prefecture level city in Shandong
IXCHSQ Flagship Store	King of Power Saving	Goods have been received, the effect is good is not good, power saving is not yet know, wait for me to use it and then evaluate it.	Jingdong prefecture level city in Shandong
IXCHSQ Flagship Store	King of Power Saving	In-kind and the same as described, the quality is quite good, the seller's attitude is also good, ask questions and answer them, the speed of delivery bar, worth buying Oh!	Jingdong prefecture level city in Shandong
IXCHSQ Flagship Store	King of Power Saving	Nice stuff, came to comment after using it, it does save power.	Jingdong prefecture level city in Shandong
IXCHSQ Flagship Store	King of Power Saving	Fast charging, but also save power, the effect is very good, satisfied, praise!	Jingdong prefecture level city in Shandong
IXCHSQ Flagship Store	King of Power Saving	The goods have been received, I do not know how effective, after using the flat price again!	Jingdong prefecture level city in Shandong
IXCHSQ Flagship Store	King of Power Saving	Merchant service is very good Reply quickly The key is the quality and that quality is good	Jingdong prefecture level city in Shandong
IXCHSQ Flagship Store	King of Power Saving	It's working well now, I'll do a follow up review after I actually use it for a while	Jingdong prefecture level city in Shandong
IXCHSQ Flagship Store	King of Power Saving	The quality is good. After installing it, I feel like I've saved a lot of money on my electricity bill.	Jingdong prefecture level city in Shandong
IXCHSQ Flagship Store	King of Power Saving	Make up a review after use.	Jingdong prefecture level city in Shandong
IXCHSQ Flagship Store	King of Power Saving	review after use	Jingdong prefecture level city in Shandong
IXCHSQ Flagship Store	King of Power Saving	It's a small capacitor. It's not energy efficient.	Jingdong prefecture level city in Shandong
IXCHSQ Flagship Store	King of Power Saving	I'll follow up with a review after I've used it and had results.	Jingdong prefecture level city in Shandong
IXCHSQ Flagship Store	King of Power Saving	No power saving? Not good!	Jingdong prefecture level city in Shandong
IXCHSQ Flagship Store	King of Power Saving	Been using the same appliances and running a 1.5 air conditioner at night for what feels like no time, and after half a month of use it feels like the power is only increasing. Doesn't show any power saving	Jingdong prefecture level city in Shandong
IXCHSQ Flagship Store	King of Power Saving	serviceable	Jingdong prefecture level city in Shandong
IXCHSQ Flagship Store	King of Power Saving	It's a piece of junk. It doesn't save any power.	Jingdong prefecture level city in Shandong
IXCHSQ Flagship Store	King of Power Saving	Use the pipe thing to save thirty percent, the quality of the economizer is very good, good-looking, plug and play is very convenient, the price is not expensive but also can save a lot of electricity, cost-	Jingdong prefecture level city in Shandong

IXCHSQ Flagship Store	King of Power Saving	There are a lot of appliances in the house, the summer air conditioning is very costly, I bought this in front of the air conditioning effect is very good, I explain that I bought three in one breath, I use more electricity a month down to 200 or so, very satisfied.	Jingdong prefecture level city in Shandong
IXCHSQ Flagship Store	King of Power Saving	The voltage is very stable has been maintained at 220v this value, high power super power saving, trustworthy, reasonable design, shape bright, logistics is quite fast, business warranty period, excellent service, logistics fast!	Jingdong prefecture level city in Shandong
IXCHSQ Flagship Store	King of Power Saving	Use a month after a look at the savings of more than 40 dollars, there is a display of electricity savings, this high-power intelligent frequency converter economizer, affordable and cheap, logistics is very fast, the service attitude is very good, very satisfied with a shopping.	Jingdong prefecture level city in Shandong
IXCHSQ Flagship Store	King of Power Saving	Used for a period of time, it seems that every day can really can save a few degrees of electricity, workmanship quite can, with a period of time and then continue to look at it, anyway, the price is not	Jingdong prefecture level city in Shandong
IXCHSQ Flagship Store	King of Power Saving	Used for a period of time, it seems that every day can indeed save two several degrees of electricity, workmanship quite can, with a period of time and then continue to look at it, anyway, the price is not	Jingdong prefecture level city in Shandong
IXCHSQ Flagship Store	King of Power Saving	High-power super saver received immediately used, the price is not expensive quality is very good, save electricity and money, very convenient, conscientious business, must be praised, recommended to everyone!	Jingdong prefecture level city in Shandong
IXCHSQ Flagship Store	King of Power Saving	Home enhancement version of the intelligent power saver, is in use, I think the price is cheap, value for money, efficacy, power, power saving effect is very good.	Jingdong prefecture level city in Shandong
IXCHSQ Flagship Store	King of Power Saving	Bought three at once, one for each room, summer electricity consumption is very high, despite the three air conditioners on the electricity bill is still down a lot, too Nessie!	Jingdong prefecture level city in Shandong
IXCHSQ Flagship Store	King of Power Saving	Without further ado, the electric bill came down, $102 less this month, personal satisfaction, it counts okay, give you a good review	Jingdong prefecture level city in Shandong
IXCHSQ Flagship Store	King of Power Saving	The power saver has arrived, the installation is really convenient, plugged in on the line operation is quite simple, power saving effect is also a strong!	Jingdong prefecture level city in Shandong
IXCHSQ Flagship Store	King of Power Saving	Saver received, good quality, beautiful appearance, fine workmanship, simple installation	Jingdong prefecture level city in Shandong
IXCHSQ Flagship Store	King of Power Saving	Product design style modern, worth buying	Jingdong prefecture level city in Shandong
IXCHSQ Flagship Store	King of Power Saving	Energy saving, good power saving effect, practical, great value	Jingdong prefecture level city in Shandong
IXCHSQ Flagship Store	King of Power Saving	It's good quality, it's good, it's energy efficient, and I'll buy it again.	Jingdong prefecture level city in Shandong
IXCHSQ Flagship Store	King of Power Saving	Installation saves a lot of electricity, fast logistics, real energy-saving appliances	Jingdong prefecture level city in Shandong
IXCHSQ Flagship Store	King of Power Saving	The effect is good and the power saving is obvious after using it for a month and saving tens of dollars, the product is trustworthy, I now buy another 4 colors to prepare for their siblings' family to pay for	Jingdong prefecture level city in Shandong
IXCHSQ Flagship Store	King of Power Saving	The quality is particularly good workmanship is no problem, the arrival of the very fast oh, especially power-saving	Jingdong prefecture level city in Shandong
IXCHSQ Flagship Store	King of Power Saving	The quality is very good! In use, observed for a day, not as used before, so much power consumption.	Jingdong prefecture level city in Shandong
IXCHSQ Flagship Store	King of Power Saving	Goods have been received, the effect is good is not good, power saving is not yet know, wait for me to use it and then evaluate it.	Jingdong prefecture level city in Shandong
IXCHSQ Flagship Store	King of Power Saving	In-kind and the same as described, the quality is quite good, the seller's attitude is also good, ask questions and answer them, the speed of delivery bar, worth buying Oh!	Jingdong prefecture level city in Shandong
IXCHSQ Flagship Store	King of Power Saving	Nice stuff, came to comment after using it, it does save power.	Jingdong prefecture level city in Shandong
IXCHSQ Flagship Store	King of Power Saving	Fast charging, but also save power, the effect is very good, satisfied, praise!	Jingdong prefecture level city in Shandong
IXCHSQ Flagship Store	King of Power Saving	The goods have been received, I do not know how effective, after using the flat price again!	Jingdong prefecture level city in Shandong
IXCHSQ Flagship Store	King of Power Saving	Merchant service is very good Reply quickly The key is the quality and that quality is good	Jingdong prefecture level city in Shandong
IXCHSQ Flagship Store	King of Power Saving	It's working well now, I'll do a follow up review after I actually use it for a while	Jingdong prefecture level city in Shandong
IXCHSQ Flagship Store	King of Power Saving	The quality is good. After installing it, I feel like I've saved a lot of money on my electricity bill.	Jingdong prefecture level city in Shandong
IXCHSQ Flagship Store	King of Power Saving	Make up a review after use.	Jingdong prefecture level city in Shandong
IXCHSQ Flagship Store	King of Power Saving	review after use	Jingdong prefecture level city in Shandong
IXCHSQ Flagship Store	King of Power Saving	It's a small capacitor. It's not energy efficient.	Jingdong prefecture level city in Shandong
IXCHSQ Flagship Store	King of Power Saving	I'll follow up with a review after I've used it and had results.	Jingdong prefecture level city in Shandong
IXCHSQ Flagship Store	King of Power Saving	No power saving? Not good!	Jingdong prefecture level city in Shandong
IXCHSQ Flagship Store	King of Power Saving	Been using the same appliances and running a 1.5 air conditioner at night for what feels like no time, and after half a month of use it feels like the power is only increasing. Doesn't show any power saving	Jingdong prefecture level city in Shandong
IXCHSQ Flagship Store	King of Power Saving	serviceable	Jingdong prefecture level city in Shandong
IXCHSQ Flagship Store	King of Power Saving	It's a piece of junk. It doesn't save any power.	Jingdong prefecture level city in Shandong
IXCHSQ Flagship Store	King of Power Saving	Use the pipe thing to save thirty percent, the quality of the economizer is very good, good-looking, plug and play is very convenient, the price is not expensive but also can save a lot of electricity, cost-	Jingdong prefecture level city in Shandong
IXCHSQ Flagship Store	King of Power Saving	There are a lot of appliances in the house, the summer air conditioning is very costly, I bought this in front of the air conditioning effect is very good, I explain that I bought three in one breath, I use more electricity a month down to 200 or so, very satisfied.	Jingdong prefecture level city in Shandong
IXCHSQ Flagship Store	King of Power Saving	The voltage is very stable has been maintained at 220v this value, high power super power saving, trustworthy, reasonable design, shape bright, logistics is quite fast, business warranty period, excellent service, logistics fast!	Jingdong prefecture level city in Shandong
IXCHSQ Flagship Store	King of Power Saving	Use a month after a look at the savings of more than 40 dollars, there is a display of electricity savings, this high-power intelligent frequency converter economizer, affordable and cheap, logistics is very fast, the service attitude is very good, very satisfied with a shopping.	Jingdong prefecture level city in Shandong
IXCHSQ Flagship Store	King of Power Saving	Used for a period of time, it seems that every day can really can save a few degrees of electricity, workmanship quite can, with a period of time and then continue to look at it, anyway, the price is not	Jingdong prefecture level city in Shandong
IXCHSQ Flagship Store	King of Power Saving	Used for a period of time, it seems that every day can indeed save two several degrees of electricity, workmanship quite can, with a period of time and then continue to look at it, anyway, the price is not	Jingdong prefecture level city in Shandong
IXCHSQ Flagship Store	King of Power Saving	High-power super saver received immediately used, the price is not expensive quality is very good, save electricity and money, very convenient, conscientious business, must be praised, recommended to everyone!	Jingdong prefecture level city in Shandong
IXCHSQ Flagship Store	King of Power Saving	Home enhancement version of the intelligent power saver, is in use, I think the price is cheap, value for money, efficacy, power, power saving effect is very good.	Jingdong prefecture level city in Shandong
IXCHSQ Flagship Store	King of Power Saving	Bought three at once, one for each room, summer electricity consumption is very high, despite the three air conditioners on the electricity bill is still down a lot, too Nessie!	Jingdong prefecture level city in Shandong
IXCHSQ Flagship Store	King of Power Saving	Without further ado, the electric bill came down, $102 less this month, personal satisfaction, it counts okay, give you a good review	Jingdong prefecture level city in Shandong
IXCHSQ Flagship Store	King of Power Saving	The power saver has arrived, the installation is really convenient, plugged in on the line operation is quite simple, power saving effect is also a strong!	Jingdong prefecture level city in Shandong
IXCHSQ Flagship Store	King of Power Saving	Saver received, good quality, beautiful appearance, fine workmanship, simple installation	Jingdong prefecture level city in Shandong
IXCHSQ Flagship Store	King of Power Saving	Product design style modern, worth buying	Jingdong prefecture level city in Shandong
IXCHSQ Flagship Store	King of Power Saving	Energy saving, good power saving effect, practical, great value	Jingdong prefecture level city in Shandong

IXCHSQ Flagship Store	King of Power Saving	It's good quality, it's good, it's energy efficient, and I'll buy it again.	Jingdong prefecture level city in Shandong
IXCHSQ Flagship Store	King of Power Saving	Installation saves a lot of electricity, fast logistics, real energy-saving appliances	Jingdong prefecture level city in Shandong
IXCHSQ Flagship Store	King of Power Saving	The effect is good and the power saving is obvious after using it for a month and saving tens of dollars, the product is trustworthy, I now buy another 4 colors to prepare for their siblings' family to pay for	Jingdong prefecture level city in Shandong
IXCHSQ Flagship Store	King of Power Saving	The quality is particularly good workmanship is no problem, the arrival of the very fast oh, especially power-saving	Jingdong prefecture level city in Shandong
IXCHSQ Flagship Store	King of Power Saving	The quality is very good! In use, observed for a day, not as used before, so much power consumption.	Jingdong prefecture level city in Shandong
IXCHSQ Flagship Store	King of Power Saving	Goods have been received, the effect is good is not good, power saving is not yet know, wait for me to use it and then evaluate it.	Jingdong prefecture level city in Shandong
IXCHSQ Flagship Store	King of Power Saving	In-kind and the same as described, the quality is quite good, the seller's attitude is also good, ask questions and answer them, the speed of delivery bar, worth buying Oh!	Jingdong prefecture level city in Shandong
IXCHSQ Flagship Store	King of Power Saving	Nice stuff, came to comment after using it, it does save power.	Jingdong prefecture level city in Shandong
IXCHSQ Flagship Store	King of Power Saving	Fast charging, but also save power, the effect is very good, satisfied, praise!	Jingdong prefecture level city in Shandong
IXCHSQ Flagship Store	King of Power Saving	The goods have been received, I do not know how effective, after using the flat price again!	Jingdong prefecture level city in Shandong
IXCHSQ Flagship Store	King of Power Saving	Merchant service is very good Reply quickly The key is the quality and that quality is good	Jingdong prefecture level city in Shandong
IXCHSQ Flagship Store	King of Power Saving	It's working well now, I'll do a follow up review after I actually use it for a while	Jingdong prefecture level city in Shandong
IXCHSQ Flagship Store	King of Power Saving	The quality is good. After installing it, I feel like I've saved a lot of money on my electricity bill.	Jingdong prefecture level city in Shandong
IXCHSQ Flagship Store	King of Power Saving	Make up a review after use.	Jingdong prefecture level city in Shandong
IXCHSQ Flagship Store	King of Power Saving	review after use	Jingdong prefecture level city in Shandong
IXCHSQ Flagship Store	King of Power Saving	It's a small capacitor. It's not energy efficient.	Jingdong prefecture level city in Shandong
IXCHSQ Flagship Store	King of Power Saving	I'll follow up with a review after I've used it and had results.	Jingdong prefecture level city in Shandong
IXCHSQ Flagship Store	King of Power Saving	No power saving? Not good!	Jingdong prefecture level city in Shandong
IXCHSQ Flagship Store	King of Power Saving	Been using the same appliances and running a 1.5 air conditioner at night for what feels like no time, and after half a month of use it feels like the power is only increasing. Doesn't show any power saving	Jingdong prefecture level city in Shandong
IXCHSQ Flagship Store	King of Power Saving	serviceable	Jingdong prefecture level city in Shandong
IXCHSQ Flagship Store	King of Power Saving	It's a piece of junk. It doesn't save any power.	Jingdong prefecture level city in Shandong
IXCHSQ Flagship Store	King of Power Saving	Use the pipe thing to save thirty percent, the quality of the economizer is very good, good-looking, plug and play is very convenient, the price is not expensive but also can save a lot of electricity, cost-	Jingdong prefecture level city in Shandong
IXCHSQ Flagship Store	King of Power Saving	There are a lot of appliances in the house, the summer air conditioning is very costly, I bought this in front of the air conditioning effect is very good, I explain that I bought three in one breath, I use more electricity a month down to 200 or so, very satisfied.	Jingdong prefecture level city in Shandong
IXCHSQ Flagship Store	King of Power Saving	The voltage is very stable has been maintained at 220v this value, high power super power saving, trustworthy, reasonable design, shape bright, logistics is quite fast, business warranty period, excellent service, logistics fast!	Jingdong prefecture level city in Shandong
IXCHSQ Flagship Store	King of Power Saving	Use a month after a look at the savings of more than 40 dollars, there is a display of electricity savings, this high-power intelligent frequency converter economizer, affordable and cheap, logistics is very fast, the service attitude is very good, very satisfied with a shopping.	Jingdong prefecture level city in Shandong
IXCHSQ Flagship Store	King of Power Saving	Used for a period of time, it seems that every day can really can save a few degrees of electricity, workmanship quite can, with a period of time and then continue to look at it, anyway, the price is not	Jingdong prefecture level city in Shandong
IXCHSQ Flagship Store	King of Power Saving	Used for a period of time, it seems that every day can indeed save two several degrees of electricity, workmanship quite can, with a period of time and then continue to look at it, anyway, the price is not	Jingdong prefecture level city in Shandong
IXCHSQ Flagship Store	King of Power Saving	High-power super saver received immediately used, the price is not expensive quality is very good, save electricity and money, very convenient, conscientious business, must be praised, recommended to everyone!	Jingdong prefecture level city in Shandong
IXCHSQ Flagship Store	King of Power Saving	Home enhancement version of the intelligent power saver, is in use, I think the price is cheap, value for money, efficacy, power, power saving effect is very good.	Jingdong prefecture level city in Shandong
IXCHSQ Flagship Store	King of Power Saving	Bought three at once, one for each room, summer electricity consumption is very high, despite the three air conditioners on the electricity bill is still down a lot, too Nessie!	Jingdong prefecture level city in Shandong
IXCHSQ Flagship Store	King of Power Saving	Without further ado, the electric bill came down, $102 less this month, personal satisfaction, it counts okay, give you a good review	Jingdong prefecture level city in Shandong
IXCHSQ Flagship Store	King of Power Saving	The power saver has arrived, the installation is really convenient, plugged in on the line operation is quite simple, power saving effect is also a strong!	Jingdong prefecture level city in Shandong
IXCHSQ Flagship Store	King of Power Saving	Saver received, good quality, beautiful appearance, fine workmanship, simple installation	Jingdong prefecture level city in Shandong
IXCHSQ Flagship Store	King of Power Saving	Product design style modern, worth buying	Jingdong prefecture level city in Shandong
IXCHSQ Flagship Store	King of Power Saving	Energy saving, good power saving effect, practical, great value	Jingdong prefecture level city in Shandong
IXCHSQ Flagship Store	King of Power Saving	It's good quality, it's good, it's energy efficient, and I'll buy it again.	Jingdong prefecture level city in Shandong
IXCHSQ Flagship Store	King of Power Saving	Installation saves a lot of electricity, fast logistics, real energy-saving appliances	Jingdong prefecture level city in Shandong
IXCHSQ Flagship Store	King of Power Saving	The effect is good and the power saving is obvious after using it for a month and saving tens of dollars, the product is trustworthy, I now buy another 4 colors to prepare for their siblings' family to pay for	Jingdong prefecture level city in Shandong
IXCHSQ Flagship Store	King of Power Saving	The quality is particularly good workmanship is no problem, the arrival of the very fast oh, especially power-saving	Jingdong prefecture level city in Shandong
IXCHSQ Flagship Store	King of Power Saving	The quality is very good! In use, observed for a day, not as used before, so much power consumption.	Jingdong prefecture level city in Shandong
IXCHSQ Flagship Store	King of Power Saving	Goods have been received, the effect is good is not good, power saving is not yet know, wait for me to use it and then evaluate it.	Jingdong prefecture level city in Shandong
IXCHSQ Flagship Store	King of Power Saving	In-kind and the same as described, the quality is quite good, the seller's attitude is also good, ask questions and answer them, the speed of delivery bar, worth buying Oh!	Jingdong prefecture level city in Shandong
IXCHSQ Flagship Store	King of Power Saving	Nice stuff, came to comment after using it, it does save power.	Jingdong prefecture level city in Shandong
IXCHSQ Flagship Store	King of Power Saving	Fast charging, but also save power, the effect is very good, satisfied, praise!	Jingdong prefecture level city in Shandong
IXCHSQ Flagship Store	King of Power Saving	The goods have been received, I do not know how effective, after using the flat price again!	Jingdong prefecture level city in Shandong
IXCHSQ Flagship Store	King of Power Saving	Merchant service is very good Reply quickly The key is the quality and that quality is good	Jingdong prefecture level city in Shandong
IXCHSQ Flagship Store	King of Power Saving	It's working well now, I'll do a follow up review after I actually use it for a while	Jingdong prefecture level city in Shandong
IXCHSQ Flagship Store	King of Power Saving	The quality is good. After installing it, I feel like I've saved a lot of money on my electricity bill.	Jingdong prefecture level city in Shandong
IXCHSQ Flagship Store	King of Power Saving	Make up a review after use.	Jingdong prefecture level city in Shandong
IXCHSQ Flagship Store	King of Power Saving	review after use	Jingdong prefecture level city in Shandong

IXCHSQ Flagship Store	King of Power Saving	It's a small capacitor. It's not energy efficient.	Jingdong prefecture level city in Shandong
IXCHSQ Flagship Store	King of Power Saving	I'll follow up with a review after I've used it and had results.	Jingdong prefecture level city in Shandong
IXCHSQ Flagship Store	King of Power Saving	No power saving? Not good!	Jingdong prefecture level city in Shandong
IXCHSQ Flagship Store	King of Power Saving	Been using the same appliances and running a 1.5 air conditioner at night for what feels like no time, and after half a month of use it feels like the power is only increasing. Doesn't show any power saving	Jingdong prefecture level city in Shandong
IXCHSQ Flagship Store	King of Power Saving	serviceable	Jingdong prefecture level city in Shandong
IXCHSQ Flagship Store	King of Power Saving	It's a piece of junk. It doesn't save any power.	Jingdong prefecture level city in Shandong
IXCHSQ Flagship Store	King of Power Saving	Use the pipe thing to save thirty percent, the quality of the economizer is very good, good-looking, plug and play is very convenient, the price is not expensive but also can save a lot of electricity, cost-	Jingdong prefecture level city in Shandong
IXCHSQ Flagship Store	King of Power Saving	There are a lot of appliances in the house, the summer air conditioning is very costly, I bought this in front of the air conditioning effect is very good, I explain that I bought three in one breath, I use more electricity a month down to 200 or so, very satisfied.	Jingdong prefecture level city in Shandong
IXCHSQ Flagship Store	King of Power Saving	The voltage is very stable has been maintained at 220v this value, high power super power saving, trustworthy, reasonable design, shape bright, logistics is quite fast, business warranty period, excellent service, logistics fast!	Jingdong prefecture level city in Shandong
IXCHSQ Flagship Store	King of Power Saving	Use a month after a look at the savings of more than 40 dollars, there is a display of electricity savings, this high-power intelligent frequency converter economizer, affordable and cheap, logistics is very fast, the service attitude is very good, very satisfied with a shopping.	Jingdong prefecture level city in Shandong
IXCHSQ Flagship Store	King of Power Saving	Used for a period of time, it seems that every day can really can save a few degrees of electricity, workmanship quite can, with a period of time and then continue to look at it, anyway, the price is not	Jingdong prefecture level city in Shandong
IXCHSQ Flagship Store	King of Power Saving	Used for a period of time, it seems that every day can indeed save two several degrees of electricity, workmanship quite can, with a period of time and then continue to look at it, anyway, the price is not	Jingdong prefecture level city in Shandong
IXCHSQ Flagship Store	King of Power Saving	High-power super saver received immediately used, the price is not expensive quality is very good, save electricity and money, very convenient, conscientious business, must be praised, recommended to everyone!	Jingdong prefecture level city in Shandong
IXCHSQ Flagship Store	King of Power Saving	Home enhancement version of the intelligent power saver, is in use, I think the price is cheap, value for money, efficacy, power, power saving effect is very good.	Jingdong prefecture level city in Shandong
IXCHSQ Flagship Store	King of Power Saving	Bought three at once, one for each room, summer electricity consumption is very high, despite the three air conditioners on the electricity bill is still down a lot, too Nessie!	Jingdong prefecture level city in Shandong
IXCHSQ Flagship Store	King of Power Saving	Without further ado, the electric bill came down, $102 less this month, personal satisfaction, it counts okay, give you a good review	Jingdong prefecture level city in Shandong
IXCHSQ Flagship Store	King of Power Saving	The power saver has arrived, the installation is really convenient, plugged in on the line operation is quite simple, power saving effect is also a strong!	Jingdong prefecture level city in Shandong
IXCHSQ Flagship Store	King of Power Saving	Saver received, good quality, beautiful appearance, fine workmanship, simple installation	Jingdong prefecture level city in Shandong
IXCHSQ Flagship Store	King of Power Saving	Product design style modern, worth buying	Jingdong prefecture level city in Shandong
IXCHSQ Flagship Store	King of Power Saving	Energy saving, good power saving effect, practical, great value	Jingdong prefecture level city in Shandong
IXCHSQ Flagship Store	King of Power Saving	It's good quality, it's good, it's energy efficient, and I'll buy it again.	Jingdong prefecture level city in Shandong
IXCHSQ Flagship Store	King of Power Saving	Installation saves a lot of electricity, fast logistics, real energy-saving appliances	Jingdong prefecture level city in Shandong
IXCHSQ Flagship Store	King of Power Saving	The effect is good and the power saving is obvious after using it for a month and saving tens of dollars, the product is trustworthy, I now buy another 4 colors to prepare for their siblings' family to pay for	Jingdong prefecture level city in Shandong
IXCHSQ Flagship Store	King of Power Saving	The quality is particularly good workmanship is no problem, the arrival of the very fast oh, especially power-saving	Jingdong prefecture level city in Shandong
IXCHSQ Flagship Store	King of Power Saving	The quality is very good! In use, observed for a day, not as used before, so much power consumption.	Jingdong prefecture level city in Shandong
IXCHSQ Flagship Store	King of Power Saving	Goods have been received, the effect is good is not good, power saving is not yet know, wait for me to use it and then evaluate it.	Jingdong prefecture level city in Shandong
IXCHSQ Flagship Store	King of Power Saving	In-kind and the same as described, the quality is quite good, the seller's attitude is also good, ask questions and answer them, the speed of delivery bar, worth buying Oh!	Jingdong prefecture level city in Shandong
IXCHSQ Flagship Store	King of Power Saving	Nice stuff, came to comment after using it, it does save power.	Jingdong prefecture level city in Shandong
IXCHSQ Flagship Store	King of Power Saving	Fast charging, but also save power, the effect is very good, satisfied, praise!	Jingdong prefecture level city in Shandong
IXCHSQ Flagship Store	King of Power Saving	The goods have been received, I do not know how effective, after using the flat price again!	Jingdong prefecture level city in Shandong
IXCHSQ Flagship Store	King of Power Saving	Merchant service is very good Reply quickly The key is the quality and that quality is good	Jingdong prefecture level city in Shandong
IXCHSQ Flagship Store	King of Power Saving	It's working well now, I'll do a follow up review after I actually use it for a while	Jingdong prefecture level city in Shandong
IXCHSQ Flagship Store	King of Power Saving	The quality is good. After installing it, I feel like I've saved a lot of money on my electricity bill.	Jingdong prefecture level city in Shandong
IXCHSQ Flagship Store	King of Power Saving	Make up a review after use.	Jingdong prefecture level city in Shandong
IXCHSQ Flagship Store	King of Power Saving	review after use	Jingdong prefecture level city in Shandong
IXCHSQ Flagship Store	King of Power Saving	It's a small capacitor. It's not energy efficient.	Jingdong prefecture level city in Shandong
IXCHSQ Flagship Store	King of Power Saving	I'll follow up with a review after I've used it and had results.	Jingdong prefecture level city in Shandong
IXCHSQ Flagship Store	King of Power Saving	No power saving? Not good!	Jingdong prefecture level city in Shandong
IXCHSQ Flagship Store	King of Power Saving	Been using the same appliances and running a 1.5 air conditioner at night for what feels like no time, and after half a month of use it feels like the power is only increasing. Doesn't show any power saving	Jingdong prefecture level city in Shandong
IXCHSQ Flagship Store	King of Power Saving	serviceable	Jingdong prefecture level city in Shandong
IXCHSQ Flagship Store	King of Power Saving	It's a piece of junk. It doesn't save any power.	Jingdong prefecture level city in Shandong
IXCHSQ Flagship Store	King of Power Saving	Use the pipe thing to save thirty percent, the quality of the economizer is very good, good-looking, plug and play is very convenient, the price is not expensive but also can save a lot of electricity, cost-	Jingdong prefecture level city in Shandong
IXCHSQ Flagship Store	King of Power Saving	There are a lot of appliances in the house, the summer air conditioning is very costly, I bought this in front of the air conditioning effect is very good, I explain that I bought three in one breath, I use more electricity a month down to 200 or so, very satisfied.	Jingdong prefecture level city in Shandong
IXCHSQ Flagship Store	King of Power Saving	The voltage is very stable has been maintained at 220v this value, high power super power saving, trustworthy, reasonable design, shape bright, logistics is quite fast, business warranty period, excellent service, logistics fast!	Jingdong prefecture level city in Shandong
IXCHSQ Flagship Store	King of Power Saving	Use a month after a look at the savings of more than 40 dollars, there is a display of electricity savings, this high-power intelligent frequency converter economizer, affordable and cheap, logistics is very fast, the service attitude is very good, very satisfied with a shopping.	Jingdong prefecture level city in Shandong
IXCHSQ Flagship Store	King of Power Saving	Used for a period of time, it seems that every day can really can save a few degrees of electricity, workmanship quite can, with a period of time and then continue to look at it, anyway, the price is not	Jingdong prefecture level city in Shandong
IXCHSQ Flagship Store	King of Power Saving	Used for a period of time, it seems that every day can indeed save two several degrees of electricity, workmanship quite can, with a period of time and then continue to look at it, anyway, the price is not	Jingdong prefecture level city in Shandong
IXCHSQ Flagship Store	King of Power Saving	High-power super saver received immediately used, the price is not expensive quality is very good, save electricity and money, very convenient, conscientious business, must be praised, recommended to everyone!	Jingdong prefecture level city in Shandong

IXCHSQ Flagship Store	King of Power Saving	Home enhancement version of the intelligent power saver, is in use, I think the price is cheap, value for money, efficacy, power, power saving effect is very good.	Jingdong prefecture level city in Shandong
IXCHSQ Flagship Store	King of Power Saving	Bought three at once, one for each room, summer electricity consumption is very high, despite the three air conditioners on the electricity bill is still down a lot, too Nessie!	Jingdong prefecture level city in Shandong
IXCHSQ Flagship Store	King of Power Saving	Without further ado, the electric bill came down, $102 less this month, personal satisfaction, it counts okay, give you a good review	Jingdong prefecture level city in Shandong
IXCHSQ Flagship Store	King of Power Saving	The power saver has arrived, the installation is really convenient, plugged in on the line operation is quite simple, power saving effect is also a strong!	Jingdong prefecture level city in Shandong
IXCHSQ Flagship Store	King of Power Saving	Saver received, good quality, beautiful appearance, fine workmanship, simple installation	Jingdong prefecture level city in Shandong
IXCHSQ Flagship Store	King of Power Saving	Product design style modern, worth buying	Jingdong prefecture level city in Shandong
IXCHSQ Flagship Store	King of Power Saving	Energy saving, good power saving effect, practical, great value	Jingdong prefecture level city in Shandong
IXCHSQ Flagship Store	King of Power Saving	It's good quality, it's good, it's energy efficient, and I'll buy it again.	Jingdong prefecture level city in Shandong
IXCHSQ Flagship Store	King of Power Saving	Installation saves a lot of electricity, fast logistics, real energy-saving appliances	Jingdong prefecture level city in Shandong
IXCHSQ Flagship Store	King of Power Saving	The effect is good and the power saving is obvious after using it for a month and saving tens of dollars, the product is trustworthy, I now buy another 4 colors to prepare for their siblings' family to pay for	Jingdong prefecture level city in Shandong
IXCHSQ Flagship Store	King of Power Saving	The quality is particularly good workmanship is no problem, the arrival of the very fast oh, especially power-saving	Jingdong prefecture level city in Shandong
IXCHSQ Flagship Store	King of Power Saving	The quality is very good! In use, observed for a day, not as used before, so much power consumption.	Jingdong prefecture level city in Shandong
IXCHSQ Flagship Store	King of Power Saving	Goods have been received, the effect is good is not good, power saving is not yet know, wait for me to use it and then evaluate it.	Jingdong prefecture level city in Shandong
IXCHSQ Flagship Store	King of Power Saving	In-kind and the same as described, the quality is quite good, the seller's attitude is also good, ask questions and answer them, the speed of delivery bar, worth buying Oh!	Jingdong prefecture level city in Shandong
IXCHSQ Flagship Store	King of Power Saving	Nice stuff, came to comment after using it, it does save power.	Jingdong prefecture level city in Shandong
IXCHSQ Flagship Store	King of Power Saving	Fast charging, but also save power, the effect is very good, satisfied, praise!	Jingdong prefecture level city in Shandong
IXCHSQ Flagship Store	King of Power Saving	The goods have been received, I do not know how effective, after using the flat price again!	Jingdong prefecture level city in Shandong
IXCHSQ Flagship Store	King of Power Saving	Merchant service is very good Reply quickly The key is the quality and that quality is good	Jingdong prefecture level city in Shandong
IXCHSQ Flagship Store	King of Power Saving	It's working well now, I'll do a follow up review after I actually use it for a while	Jingdong prefecture level city in Shandong
IXCHSQ Flagship Store	King of Power Saving	The quality is good. After installing it, I feel like I've saved a lot of money on my electricity bill.	Jingdong prefecture level city in Shandong
IXCHSQ Flagship Store	King of Power Saving	Make up a review after use.	Jingdong prefecture level city in Shandong
IXCHSQ Flagship Store	King of Power Saving	review after use	Jingdong prefecture level city in Shandong
IXCHSQ Flagship Store	King of Power Saving	It's a small capacitor. It's not energy efficient.	Jingdong prefecture level city in Shandong
IXCHSQ Flagship Store	King of Power Saving	I'll follow up with a review after I've used it and had results.	Jingdong prefecture level city in Shandong
IXCHSQ Flagship Store	King of Power Saving	No power saving? Not good!	Jingdong prefecture level city in Shandong
IXCHSQ Flagship Store	King of Power Saving	Been using the same appliances and running a 1.5 air conditioner at night for what feels like no time, and after half a month of use it feels like the power is only increasing. Doesn't show any power saving	Jingdong prefecture level city in Shandong
IXCHSQ Flagship Store	King of Power Saving	serviceable	Jingdong prefecture level city in Shandong
IXCHSQ Flagship Store	King of Power Saving	It's a piece of junk. It doesn't save any power.	Jingdong prefecture level city in Shandong
IXCHSQ Flagship Store	King of Power Saving	Use the pipe thing to save thirty percent, the quality of the economizer is very good, good-looking, plug and play is very convenient, the price is not expensive but also can save a lot of electricity, cost-	Jingdong prefecture level city in Shandong
IXCHSQ Flagship Store	King of Power Saving	There are a lot of appliances in the house, the summer air conditioning is very costly, I bought this in front of the air conditioning effect is very good, I explain that I bought three in one breath, I use more electricity a month down to 200 or so, very satisfied.	Jingdong prefecture level city in Shandong
IXCHSQ Flagship Store	King of Power Saving	The voltage is very stable has been maintained at 220v this value, high power super power saving, trustworthy, reasonable design, shape bright, logistics is quite fast, business warranty period, excellent service, logistics fast!	Jingdong prefecture level city in Shandong
IXCHSQ Flagship Store	King of Power Saving	Use a month after a look at the savings of more than 40 dollars, there is a display of electricity savings, this high-power intelligent frequency converter economizer, affordable and cheap, logistics is very fast, the service attitude is very good, very satisfied with a shopping.	Jingdong prefecture level city in Shandong
IXCHSQ Flagship Store	King of Power Saving	Used for a period of time, it seems that every day can really can save a few degrees of electricity, workmanship quite can, with a period of time and then continue to look at it, anyway, the price is not	Jingdong prefecture level city in Shandong
IXCHSQ Flagship Store	King of Power Saving	Used for a period of time, it seems that every day can indeed save two several degrees of electricity, workmanship quite can, with a period of time and then continue to look at it, anyway, the price is not	Jingdong prefecture level city in Shandong
IXCHSQ Flagship Store	King of Power Saving	High-power super saver received immediately used, the price is not expensive quality is very good, save electricity and money, very convenient, conscientious business, must be praised, recommended to everyone!	Jingdong prefecture level city in Shandong
IXCHSQ Flagship Store	King of Power Saving	Home enhancement version of the intelligent power saver, is in use, I think the price is cheap, value for money, efficacy, power, power saving effect is very good.	Jingdong prefecture level city in Shandong
IXCHSQ Flagship Store	King of Power Saving	Bought three at once, one for each room, summer electricity consumption is very high, despite the three air conditioners on the electricity bill is still down a lot, too Nessie!	Jingdong prefecture level city in Shandong
IXCHSQ Flagship Store	King of Power Saving	Without further ado, the electric bill came down, $102 less this month, personal satisfaction, it counts okay, give you a good review	Jingdong prefecture level city in Shandong
IXCHSQ Flagship Store	King of Power Saving	The power saver has arrived, the installation is really convenient, plugged in on the line operation is quite simple, power saving effect is also a strong!	Jingdong prefecture level city in Shandong
IXCHSQ Flagship Store	King of Power Saving	Saver received, good quality, beautiful appearance, fine workmanship, simple installation	Jingdong prefecture level city in Shandong
IXCHSQ Flagship Store	King of Power Saving	Product design style modern, worth buying	Jingdong prefecture level city in Shandong
IXCHSQ Flagship Store	King of Power Saving	Energy saving, good power saving effect, practical, great value	Jingdong prefecture level city in Shandong
IXCHSQ Flagship Store	King of Power Saving	It's good quality, it's good, it's energy efficient, and I'll buy it again.	Jingdong prefecture level city in Shandong
IXCHSQ Flagship Store	King of Power Saving	Installation saves a lot of electricity, fast logistics, real energy-saving appliances	Jingdong prefecture level city in Shandong
IXCHSQ Flagship Store	King of Power Saving	The effect is good and the power saving is obvious after using it for a month and saving tens of dollars, the product is trustworthy, I now buy another 4 colors to prepare for their siblings' family to pay for	Jingdong prefecture level city in Shandong
IXCHSQ Flagship Store	King of Power Saving	The quality is particularly good workmanship is no problem, the arrival of the very fast oh, especially power-saving	Jingdong prefecture level city in Shandong
IXCHSQ Flagship Store	King of Power Saving	The quality is very good! In use, observed for a day, not as used before, so much power consumption.	Jingdong prefecture level city in Shandong
IXCHSQ Flagship Store	King of Power Saving	Goods have been received, the effect is good is not good, power saving is not yet know, wait for me to use it and then evaluate it.	Jingdong prefecture level city in Shandong
IXCHSQ Flagship Store	King of Power Saving	In-kind and the same as described, the quality is quite good, the seller's attitude is also good, ask questions and answer them, the speed of delivery bar, worth buying Oh!	Jingdong prefecture level city in Shandong
IXCHSQ Flagship Store	King of Power Saving	Nice stuff, came to comment after using it, it does save power.	Jingdong prefecture level city in Shandong

IXCHSQ Flagship Store	King of Power Saving	Fast charging, but also save power, the effect is very good, satisfied, praise!	Jingdong prefecture level city in Shandong
IXCHSQ Flagship Store	King of Power Saving	The goods have been received, I do not know how effective, after using the flat price again!	Jingdong prefecture level city in Shandong
IXCHSQ Flagship Store	King of Power Saving	Merchant service is very good Reply quickly The key is the quality and that quality is good	Jingdong prefecture level city in Shandong
IXCHSQ Flagship Store	King of Power Saving	It's working well now, I'll do a follow up review after I actually use it for a while	Jingdong prefecture level city in Shandong
IXCHSQ Flagship Store	King of Power Saving	The quality is good. After installing it, I feel like I've saved a lot of money on my electricity bill.	Jingdong prefecture level city in Shandong
IXCHSQ Flagship Store	King of Power Saving	Make up a review after use.	Jingdong prefecture level city in Shandong
IXCHSQ Flagship Store	King of Power Saving	review after use	Jingdong prefecture level city in Shandong
IXCHSQ Flagship Store	King of Power Saving	It's a small capacitor. It's not energy efficient.	Jingdong prefecture level city in Shandong
IXCHSQ Flagship Store	King of Power Saving	I'll follow up with a review after I've used it and had results.	Jingdong prefecture level city in Shandong
IXCHSQ Flagship Store	King of Power Saving	No power saving? Not good!	Jingdong prefecture level city in Shandong
IXCHSQ Flagship Store	King of Power Saving	Been using the same appliances and running a 1.5 air conditioner at night for what feels like no time, and after half a month of use it feels like the power is only increasing. Doesn't show any power saving	Jingdong prefecture level city in Shandong
IXCHSQ Flagship Store	King of Power Saving	serviceable	Jingdong prefecture level city in Shandong
IXCHSQ Flagship Store	King of Power Saving	It's a piece of junk. It doesn't save any power.	Jingdong prefecture level city in Shandong
IXCHSQ Flagship Store	King of Power Saving	Use the pipe thing to save thirty percent, the quality of the economizer is very good, good-looking, plug and play is very convenient, the price is not expensive but also can save a lot of electricity, cost-	Jingdong prefecture level city in Shandong
IXCHSQ Flagship Store	King of Power Saving	There are a lot of appliances in the house, the summer air conditioning is very costly, I bought this in front of the air conditioning effect is very good, I explain that I bought three in one breath, I use more electricity a month down to 200 or so, very satisfied.	Jingdong prefecture level city in Shandong
IXCHSQ Flagship Store	King of Power Saving	The voltage is very stable has been maintained at 220v this value, high power super power saving, trustworthy, reasonable design, shape bright, logistics is quite fast, business warranty period, excellent service, logistics fast!	Jingdong prefecture level city in Shandong
IXCHSQ Flagship Store	King of Power Saving	Use a month after a look at the savings of more than 40 dollars, there is a display of electricity savings, this high-power intelligent frequency converter economizer, affordable and cheap, logistics is very fast, the service attitude is very good, very satisfied with a shopping.	Jingdong prefecture level city in Shandong
IXCHSQ Flagship Store	King of Power Saving	Used for a period of time, it seems that every day can really can save a few degrees of electricity, workmanship quite can, with a period of time and then continue to look at it, anyway, the price is not	Jingdong prefecture level city in Shandong
IXCHSQ Flagship Store	King of Power Saving	Used for a period of time, it seems that every day can indeed save two several degrees of electricity, workmanship quite can, with a period of time and then continue to look at it, anyway, the price is not	Jingdong prefecture level city in Shandong
IXCHSQ Flagship Store	King of Power Saving	High-power super saver received immediately used, the price is not expensive quality is very good, save electricity and money, very convenient, conscientious business, must be praised, recommended to everyone!	Jingdong prefecture level city in Shandong
IXCHSQ Flagship Store	King of Power Saving	Home enhancement version of the intelligent power saver, is in use, I think the price is cheap, value for money, efficacy, power, power saving effect is very good.	Jingdong prefecture level city in Shandong
IXCHSQ Flagship Store	King of Power Saving	Bought three at once, one for each room, summer electricity consumption is very high, despite the three air conditioners on the electricity bill is still down a lot, too Nessie!	Jingdong prefecture level city in Shandong
IXCHSQ Flagship Store	King of Power Saving	Without further ado, the electric bill came down, $102 less this month, personal satisfaction, it counts okay, give you a good review	Jingdong prefecture level city in Shandong
IXCHSQ Flagship Store	King of Power Saving	The power saver has arrived, the installation is really convenient, plugged in on the line operation is quite simple, power saving effect is also a strong!	Jingdong prefecture level city in Shandong
IXCHSQ Flagship Store	King of Power Saving	Saver received, good quality, beautiful appearance, fine workmanship, simple installation	Jingdong prefecture level city in Shandong
IXCHSQ Flagship Store	King of Power Saving	Product design style modern, worth buying	Jingdong prefecture level city in Shandong
IXCHSQ Flagship Store	King of Power Saving	Energy saving, good power saving effect, practical, great value	Jingdong prefecture level city in Shandong
IXCHSQ Flagship Store	King of Power Saving	It's good quality, it's good, it's energy efficient, and I'll buy it again.	Jingdong prefecture level city in Shandong
IXCHSQ Flagship Store	King of Power Saving	Installation saves a lot of electricity, fast logistics, real energy-saving appliances	Jingdong prefecture level city in Shandong
IXCHSQ Flagship Store	King of Power Saving	The effect is good and the power saving is obvious after using it for a month and saving tens of dollars, the product is trustworthy, I now buy another 4 colors to prepare for their siblings' family to pay for	Jingdong prefecture level city in Shandong
IXCHSQ Flagship Store	King of Power Saving	The quality is particularly good workmanship is no problem, the arrival of the very fast oh, especially power-saving	Jingdong prefecture level city in Shandong
IXCHSQ Flagship Store	King of Power Saving	The quality is very good! In use, observed for a day, not as used before, so much power consumption.	Jingdong prefecture level city in Shandong
IXCHSQ Flagship Store	King of Power Saving	Goods have been received, the effect is good is not good, power saving is not yet know, wait for me to use it and then evaluate it.	Jingdong prefecture level city in Shandong
IXCHSQ Flagship Store	King of Power Saving	In-kind and the same as described, the quality is quite good, the seller's attitude is also good, ask questions and answer them, the speed of delivery bar, worth buying Oh!	Jingdong prefecture level city in Shandong
IXCHSQ Flagship Store	King of Power Saving	Nice stuff, came to comment after using it, it does save power.	Jingdong prefecture level city in Shandong
IXCHSQ Flagship Store	King of Power Saving	Fast charging, but also save power, the effect is very good, satisfied, praise!	Jingdong prefecture level city in Shandong
IXCHSQ Flagship Store	King of Power Saving	The goods have been received, I do not know how effective, after using the flat price again!	Jingdong prefecture level city in Shandong
IXCHSQ Flagship Store	King of Power Saving	Merchant service is very good Reply quickly The key is the quality and that quality is good	Jingdong prefecture level city in Shandong
IXCHSQ Flagship Store	King of Power Saving	It's working well now, I'll do a follow up review after I actually use it for a while	Jingdong prefecture level city in Shandong
IXCHSQ Flagship Store	King of Power Saving	The quality is good. After installing it, I feel like I've saved a lot of money on my electricity bill.	Jingdong prefecture level city in Shandong
IXCHSQ Flagship Store	King of Power Saving	Make up a review after use.	Jingdong prefecture level city in Shandong
IXCHSQ Flagship Store	King of Power Saving	review after use	Jingdong prefecture level city in Shandong
IXCHSQ Flagship Store	King of Power Saving	It's a small capacitor. It's not energy efficient.	Jingdong prefecture level city in Shandong
IXCHSQ Flagship Store	King of Power Saving	I'll follow up with a review after I've used it and had results.	Jingdong prefecture level city in Shandong
IXCHSQ Flagship Store	King of Power Saving	No power saving? Not good!	Jingdong prefecture level city in Shandong
IXCHSQ Flagship Store	King of Power Saving	Been using the same appliances and running a 1.5 air conditioner at night for what feels like no time, and after half a month of use it feels like the power is only increasing. Doesn't show any power saving	Jingdong prefecture level city in Shandong
IXCHSQ Flagship Store	King of Power Saving	serviceable	Jingdong prefecture level city in Shandong
IXCHSQ Flagship Store	King of Power Saving	It's a piece of junk. It doesn't save any power.	Jingdong prefecture level city in Shandong
IXCHSQ Flagship Store	King of Power Saving	Use the pipe thing to save thirty percent, the quality of the economizer is very good, good-looking, plug and play is very convenient, the price is not expensive but also can save a lot of electricity, cost-	Jingdong prefecture level city in Shandong

IXCHSQ Flagship Store	King of Power Saving	There are a lot of appliances in the house, the summer air conditioning is very costly, I bought this in front of the air conditioning effect is very good, I explain that I bought three in one breath, I use more electricity a month down to 200 or so, very satisfied.	Jingdong prefecture level city in Shandong
IXCHSQ Flagship Store	King of Power Saving	The voltage is very stable has been maintained at 220v this value, high power super power saving, trustworthy, reasonable design, shape bright, logistics is quite fast, business warranty period, excellent service, logistics fast!	Jingdong prefecture level city in Shandong
IXCHSQ Flagship Store	King of Power Saving	Use a month after a look at the savings of more than 40 dollars, there is a display of electricity savings, this high-power intelligent frequency converter economizer, affordable and cheap, logistics is very fast, the service attitude is very good, very satisfied with a shopping.	Jingdong prefecture level city in Shandong
IXCHSQ Flagship Store	King of Power Saving	Used for a period of time, it seems that every day can really can save a few degrees of electricity, workmanship quite can, with a period of time and then continue to look at it, anyway, the price is not	Jingdong prefecture level city in Shandong
IXCHSQ Flagship Store	King of Power Saving	Used for a period of time, it seems that every day can indeed save two several degrees of electricity, workmanship quite can, with a period of time and then continue to look at it, anyway, the price is not	Jingdong prefecture level city in Shandong
IXCHSQ Flagship Store	King of Power Saving	High-power super saver received immediately used, the price is not expensive quality is very good, save electricity and money, very convenient, conscientious business, must be praised, recommended to everyone!	Jingdong prefecture level city in Shandong
IXCHSQ Flagship Store	King of Power Saving	Home enhancement version of the intelligent power saver, is in use, I think the price is cheap, value for money, efficacy, power, power saving effect is very good.	Jingdong prefecture level city in Shandong
IXCHSQ Flagship Store	King of Power Saving	Bought three at once, one for each room, summer electricity consumption is very high, despite the three air conditioners on the electricity bill is still down a lot, too Nessie!	Jingdong prefecture level city in Shandong
IXCHSQ Flagship Store	King of Power Saving	Without further ado, the electric bill came down, $102 less this month, personal satisfaction, it counts okay, give you a good review	Jingdong prefecture level city in Shandong
IXCHSQ Flagship Store	King of Power Saving	The power saver has arrived, the installation is really convenient, plugged in on the line operation is quite simple, power saving effect is also a strong!	Jingdong prefecture level city in Shandong
IXCHSQ Flagship Store	King of Power Saving	Saver received, good quality, beautiful appearance, fine workmanship, simple installation	Jingdong prefecture level city in Shandong
IXCHSQ Flagship Store	King of Power Saving	Product design style modern, worth buying	Jingdong prefecture level city in Shandong
IXCHSQ Flagship Store	King of Power Saving	Energy saving, good power saving effect, practical, great value	Jingdong prefecture level city in Shandong
IXCHSQ Flagship Store	King of Power Saving	It's good quality, it's good, it's energy efficient, and I'll buy it again.	Jingdong prefecture level city in Shandong
IXCHSQ Flagship Store	King of Power Saving	Installation saves a lot of electricity, fast logistics, real energy-saving appliances	Jingdong prefecture level city in Shandong
IXCHSQ Flagship Store	King of Power Saving	The effect is good and the power saving is obvious after using it for a month and saving tens of dollars, the product is trustworthy, I now buy another 4 colors to prepare for their siblings' family to pay for	Jingdong prefecture level city in Shandong
IXCHSQ Flagship Store	King of Power Saving	The quality is particularly good workmanship is no problem, the arrival of the very fast oh, especially power-saving	Jingdong prefecture level city in Shandong
IXCHSQ Flagship Store	King of Power Saving	The quality is very good! In use, observed for a day, not as used before, so much power consumption.	Jingdong prefecture level city in Shandong
IXCHSQ Flagship Store	King of Power Saving	Goods have been received, the effect is good is not good, power saving is not yet know, wait for me to use it and then evaluate it.	Jingdong prefecture level city in Shandong
IXCHSQ Flagship Store	King of Power Saving	In-kind and the same as described, the quality is quite good, the seller's attitude is also good, ask questions and answer them, the speed of delivery bar, worth buying Oh!	Jingdong prefecture level city in Shandong
IXCHSQ Flagship Store	King of Power Saving	Nice stuff, came to comment after using it, it does save power.	Jingdong prefecture level city in Shandong
IXCHSQ Flagship Store	King of Power Saving	Fast charging, but also save power, the effect is very good, satisfied, praise!	Jingdong prefecture level city in Shandong
IXCHSQ Flagship Store	King of Power Saving	The goods have been received, I do not know how effective, after using the flat price again!	Jingdong prefecture level city in Shandong
IXCHSQ Flagship Store	King of Power Saving	Merchant service is very good Reply quickly The key is the quality and that quality is good	Jingdong prefecture level city in Shandong
IXCHSQ Flagship Store	King of Power Saving	It's working well now, I'll do a follow up review after I actually use it for a while	Jingdong prefecture level city in Shandong
IXCHSQ Flagship Store	King of Power Saving	The quality is good. After installing it, I feel like I've saved a lot of money on my electricity bill.	Jingdong prefecture level city in Shandong
IXCHSQ Flagship Store	King of Power Saving	Make up a review after use.	Jingdong prefecture level city in Shandong
IXCHSQ Flagship Store	King of Power Saving	review after use	Jingdong prefecture level city in Shandong
IXCHSQ Flagship Store	King of Power Saving	It's a small capacitor. It's not energy efficient.	Jingdong prefecture level city in Shandong
IXCHSQ Flagship Store	King of Power Saving	I'll follow up with a review after I've used it and had results.	Jingdong prefecture level city in Shandong
IXCHSQ Flagship Store	King of Power Saving	No power saving? Not good!	Jingdong prefecture level city in Shandong
IXCHSQ Flagship Store	King of Power Saving	Been using the same appliances and running a 1.5 air conditioner at night for what feels like no time, and after half a month of use it feels like the power is only increasing. Doesn't show any power saving	Jingdong prefecture level city in Shandong
IXCHSQ Flagship Store	King of Power Saving	serviceable	Jingdong prefecture level city in Shandong
IXCHSQ Flagship Store	King of Power Saving	It's a piece of junk. It doesn't save any power.	Jingdong prefecture level city in Shandong
IXCHSQ Flagship Store	King of Power Saving	Use the pipe thing to save thirty percent, the quality of the economizer is very good, good-looking, plug and play is very convenient, the price is not expensive but also can save a lot of electricity, cost-	Jingdong prefecture level city in Shandong
IXCHSQ Flagship Store	King of Power Saving	There are a lot of appliances in the house, the summer air conditioning is very costly, I bought this in front of the air conditioning effect is very good, I explain that I bought three in one breath, I use more electricity a month down to 200 or so, very satisfied.	Jingdong prefecture level city in Shandong
IXCHSQ Flagship Store	King of Power Saving	The voltage is very stable has been maintained at 220v this value, high power super power saving, trustworthy, reasonable design, shape bright, logistics is quite fast, business warranty period, excellent service, logistics fast!	Jingdong prefecture level city in Shandong
IXCHSQ Flagship Store	King of Power Saving	Use a month after a look at the savings of more than 40 dollars, there is a display of electricity savings, this high-power intelligent frequency converter economizer, affordable and cheap, logistics is very fast, the service attitude is very good, very satisfied with a shopping.	Jingdong prefecture level city in Shandong
IXCHSQ Flagship Store	King of Power Saving	Used for a period of time, it seems that every day can really can save a few degrees of electricity, workmanship quite can, with a period of time and then continue to look at it, anyway, the price is not	Jingdong prefecture level city in Shandong
IXCHSQ Flagship Store	King of Power Saving	Used for a period of time, it seems that every day can indeed save two several degrees of electricity, workmanship quite can, with a period of time and then continue to look at it, anyway, the price is not	Jingdong prefecture level city in Shandong
IXCHSQ Flagship Store	King of Power Saving	High-power super saver received immediately used, the price is not expensive quality is very good, save electricity and money, very convenient, conscientious business, must be praised, recommended to everyone!	Jingdong prefecture level city in Shandong
IXCHSQ Flagship Store	King of Power Saving	Home enhancement version of the intelligent power saver, is in use, I think the price is cheap, value for money, efficacy, power, power saving effect is very good.	Jingdong prefecture level city in Shandong
IXCHSQ Flagship Store	King of Power Saving	Bought three at once, one for each room, summer electricity consumption is very high, despite the three air conditioners on the electricity bill is still down a lot, too Nessie!	Jingdong prefecture level city in Shandong
IXCHSQ Flagship Store	King of Power Saving	Without further ado, the electric bill came down, $102 less this month, personal satisfaction, it counts okay, give you a good review	Jingdong prefecture level city in Shandong
IXCHSQ Flagship Store	King of Power Saving	The power saver has arrived, the installation is really convenient, plugged in on the line operation is quite simple, power saving effect is also a strong!	Jingdong prefecture level city in Shandong
IXCHSQ Flagship Store	King of Power Saving	Saver received, good quality, beautiful appearance, fine workmanship, simple installation	Jingdong prefecture level city in Shandong
IXCHSQ Flagship Store	King of Power Saving	Product design style modern, worth buying	Jingdong prefecture level city in Shandong
IXCHSQ Flagship Store	King of Power Saving	Energy saving, good power saving effect, practical, great value	Jingdong prefecture level city in Shandong

IXCHSQ Flagship Store	King of Power Saving	It's good quality, it's good, it's energy efficient, and I'll buy it again.	Jingdong prefecture level city in Shandong
IXCHSQ Flagship Store	King of Power Saving	Installation saves a lot of electricity, fast logistics, real energy-saving appliances	Jingdong prefecture level city in Shandong
IXCHSQ Flagship Store	King of Power Saving	The effect is good and the power saving is obvious after using it for a month and saving tens of dollars, the product is trustworthy, I now buy another 4 colors to prepare for their siblings' family to pay for	Jingdong prefecture level city in Shandong
IXCHSQ Flagship Store	King of Power Saving	The quality is particularly good workmanship is no problem, the arrival of the very fast oh, especially power-saving	Jingdong prefecture level city in Shandong
IXCHSQ Flagship Store	King of Power Saving	The quality is very good! In use, observed for a day, not as used before, so much power consumption.	Jingdong prefecture level city in Shandong
IXCHSQ Flagship Store	King of Power Saving	Goods have been received, the effect is good is not good, power saving is not yet know, wait for me to use it and then evaluate it.	Jingdong prefecture level city in Shandong
IXCHSQ Flagship Store	King of Power Saving	In-kind and the same as described, the quality is quite good, the seller's attitude is also good, ask questions and answer them, the speed of delivery bar, worth buying Oh!	Jingdong prefecture level city in Shandong
IXCHSQ Flagship Store	King of Power Saving	Nice stuff, came to comment after using it, it does save power.	Jingdong prefecture level city in Shandong
IXCHSQ Flagship Store	King of Power Saving	Fast charging, but also save power, the effect is very good, satisfied, praise!	Jingdong prefecture level city in Shandong
IXCHSQ Flagship Store	King of Power Saving	The goods have been received, I do not know how effective, after using the flat price again!	Jingdong prefecture level city in Shandong
IXCHSQ Flagship Store	King of Power Saving	Merchant service is very good Reply quickly The key is the quality and that quality is good	Jingdong prefecture level city in Shandong
IXCHSQ Flagship Store	King of Power Saving	It's working well now, I'll do a follow up review after I actually use it for a while	Jingdong prefecture level city in Shandong
IXCHSQ Flagship Store	King of Power Saving	The quality is good. After installing it, I feel like I've saved a lot of money on my electricity bill.	Jingdong prefecture level city in Shandong
IXCHSQ Flagship Store	King of Power Saving	Make up a review after use.	Jingdong prefecture level city in Shandong
IXCHSQ Flagship Store	King of Power Saving	review after use	Jingdong prefecture level city in Shandong
IXCHSQ Flagship Store	King of Power Saving	It's a small capacitor. It's not energy efficient.	Jingdong prefecture level city in Shandong
IXCHSQ Flagship Store	King of Power Saving	I'll follow up with a review after I've used it and had results.	Jingdong prefecture level city in Shandong
IXCHSQ Flagship Store	King of Power Saving	No power saving? Not good!	Jingdong prefecture level city in Shandong
IXCHSQ Flagship Store	King of Power Saving	Been using the same appliances and running a 1.5 air conditioner at night for what feels like no time, and after half a month of use it feels like the power is only increasing. Doesn't show any power saving	Jingdong prefecture level city in Shandong
IXCHSQ Flagship Store	King of Power Saving	serviceable	Jingdong prefecture level city in Shandong
IXCHSQ Flagship Store	King of Power Saving	It's a piece of junk. It doesn't save any power.	Jingdong prefecture level city in Shandong
IXCHSQ Flagship Store	King of Power Saving	There are a lot of appliances in the house, the summer air conditioning is very costly, I bought this in front of the air conditioning effect is very good, I explain that I bought three in one breath, I use more electricity a month down to 200 or so, very satisfied.	Jingdong prefecture level city in Shandong
IXCHSQ Flagship Store	King of Power Saving	The voltage is very stable has been maintained at 220v this value, high power super power saving, trustworthy, reasonable design, shape bright, logistics is quite fast, business warranty period, excellent service, logistics fast!	Jingdong prefecture level city in Shandong
IXCHSQ Flagship Store	King of Power Saving	Use a month after a look at the savings of more than 40 dollars, there is a display of electricity savings, this high-power intelligent frequency converter economizer, affordable and cheap, logistics is very fast, the service attitude is very good, very satisfied with a shopping.	Jingdong prefecture level city in Shandong
IXCHSQ Flagship Store	King of Power Saving	Used for a period of time, it seems that every day can really can save a few degrees of electricity, workmanship quite can, with a period of time and then continue to look at it, anyway, the price is not	Jingdong prefecture level city in Shandong
IXCHSQ Flagship Store	King of Power Saving	Used for a period of time, it seems that every day can indeed save two several degrees of electricity, workmanship quite can, with a period of time and then continue to look at it, anyway, the price is not	Jingdong prefecture level city in Shandong
IXCHSQ Flagship Store	King of Power Saving	High-power super saver received immediately used, the price is not expensive quality is very good, save electricity and money, very convenient, conscientious business, must be praised, recommended to everyone!	Jingdong prefecture level city in Shandong
IXCHSQ Flagship Store	King of Power Saving	Home enhancement version of the intelligent power saver, is in use, I think the price is cheap, value for money, efficacy, power, power saving effect is very good.	Jingdong prefecture level city in Shandong
IXCHSQ Flagship Store	King of Power Saving	Bought three at once, one for each room, summer electricity consumption is very high, despite the three air conditioners on the electricity bill is still down a lot, too Nessie!	Jingdong prefecture level city in Shandong
IXCHSQ Flagship Store	King of Power Saving	Without further ado, the electric bill came down, $102 less this month, personal satisfaction, it counts okay, give you a good review	Jingdong prefecture level city in Shandong
IXCHSQ Flagship Store	King of Power Saving	The power saver has arrived, the installation is really convenient, plugged in on the line operation is quite simple, power saving effect is also a strong!	Jingdong prefecture level city in Shandong
IXCHSQ Flagship Store	King of Power Saving	Saver received, good quality, beautiful appearance, fine workmanship, simple installation	Jingdong prefecture level city in Shandong
IXCHSQ Flagship Store	King of Power Saving	Product design style modern, worth buying	Jingdong prefecture level city in Shandong
IXCHSQ Flagship Store	King of Power Saving	Energy saving, good power saving effect, practical, great value	Jingdong prefecture level city in Shandong
IXCHSQ Flagship Store	King of Power Saving	It's good quality, it's good, it's energy efficient, and I'll buy it again.	Jingdong prefecture level city in Shandong
IXCHSQ Flagship Store	King of Power Saving	Installation saves a lot of electricity, fast logistics, real energy-saving appliances	Jingdong prefecture level city in Shandong
IXCHSQ Flagship Store	King of Power Saving	The effect is good and the power saving is obvious after using it for a month and saving tens of dollars, the product is trustworthy, I now buy another 4 colors to prepare for their siblings' family to pay for	Jingdong prefecture level city in Shandong
IXCHSQ Flagship Store	King of Power Saving	The quality is particularly good workmanship is no problem, the arrival of the very fast oh, especially power-saving	Jingdong prefecture level city in Shandong
IXCHSQ Flagship Store	King of Power Saving	The quality is very good! In use, observed for a day, not as used before, so much power consumption.	Jingdong prefecture level city in Shandong
IXCHSQ Flagship Store	King of Power Saving	Goods have been received, the effect is good is not good, power saving is not yet know, wait for me to use it and then evaluate it.	Jingdong prefecture level city in Shandong
IXCHSQ Flagship Store	King of Power Saving	In-kind and the same as described, the quality is quite good, the seller's attitude is also good, ask questions and answer them, the speed of delivery bar, worth buying Oh!	Jingdong prefecture level city in Shandong
IXCHSQ Flagship Store	King of Power Saving	Nice stuff, came to comment after using it, it does save power.	Jingdong prefecture level city in Shandong
IXCHSQ Flagship Store	King of Power Saving	Fast charging, but also save power, the effect is very good, satisfied, praise!	Jingdong prefecture level city in Shandong
IXCHSQ Flagship Store	King of Power Saving	The goods have been received, I do not know how effective, after using the flat price again!	Jingdong prefecture level city in Shandong
IXCHSQ Flagship Store	King of Power Saving	Merchant service is very good Reply quickly The key is the quality and that quality is good	Jingdong prefecture level city in Shandong
IXCHSQ Flagship Store	King of Power Saving	It's working well now, I'll do a follow up review after I actually use it for a while	Jingdong prefecture level city in Shandong
IXCHSQ Flagship Store	King of Power Saving	The quality is good. After installing it, I feel like I've saved a lot of money on my electricity bill.	Jingdong prefecture level city in Shandong
IXCHSQ Flagship Store	King of Power Saving	Make up a review after use.	Jingdong prefecture level city in Shandong
IXCHSQ Flagship Store	King of Power Saving	review after use	Jingdong prefecture level city in Shandong
IXCHSQ Flagship Store	King of Power Saving	It's a small capacitor. It's not energy efficient.	Jingdong prefecture level city in Shandong

IXCHSQ Flagship Store	King of Power Saving	I'll follow up with a review after I've used it and had results.	Jingdong prefecture level city in Shandong
IXCHSQ Flagship Store	King of Power Saving	No power saving? Not good!	Jingdong prefecture level city in Shandong
IXCHSQ Flagship Store	King of Power Saving	Been using the same appliances and running a 1.5 air conditioner at night for what feels like no time, and after half a month of use it feels like the power is only increasing. Doesn't show any power saving	Jingdong prefecture level city in Shandong
IXCHSQ Flagship Store	King of Power Saving	serviceable	Jingdong prefecture level city in Shandong
IXCHSQ Flagship Store	King of Power Saving	It's a piece of junk. It doesn't save any power.	Jingdong prefecture level city in Shandong
IXCHSQ Flagship Store	King of Power Saving	There are a lot of appliances in the house, the summer air conditioning is very costly, I bought this in front of the air conditioning effect is very good, I explain that I bought three in one breath, I use more electricity a month down to 200 or so, very satisfied.	Jingdong prefecture level city in Shandong
IXCHSQ Flagship Store	King of Power Saving	The voltage is very stable has been maintained at 220v this value, high power super power saving, trustworthy, reasonable design, shape bright, logistics is quite fast, business warranty period, excellent service, logistics fast!	Jingdong prefecture level city in Shandong
IXCHSQ Flagship Store	King of Power Saving	Use a month after a look at the savings of more than 40 dollars, there is a display of electricity savings, this high-power intelligent frequency converter economizer, affordable and cheap, logistics is very fast, the service attitude is very good, very satisfied with a shopping.	Jingdong prefecture level city in Shandong
IXCHSQ Flagship Store	King of Power Saving	Used for a period of time, it seems that every day can really can save a few degrees of electricity, workmanship quite can, with a period of time and then continue to look at it, anyway, the price is not	Jingdong prefecture level city in Shandong
IXCHSQ Flagship Store	King of Power Saving	Used for a period of time, it seems that every day can indeed save two several degrees of electricity, workmanship quite can, with a period of time and then continue to look at it, anyway, the price is not	Jingdong prefecture level city in Shandong
IXCHSQ Flagship Store	King of Power Saving	High-power super saver received immediately used, the price is not expensive quality is very good, save electricity and money, very convenient, conscientious business, must be praised, recommended to everyone!	Jingdong prefecture level city in Shandong
IXCHSQ Flagship Store	King of Power Saving	Home enhancement version of the intelligent power saver, is in use, I think the price is cheap, value for money, efficacy, power, power saving effect is very good.	Jingdong prefecture level city in Shandong
IXCHSQ Flagship Store	King of Power Saving	Bought three at once, one for each room, summer electricity consumption is very high, despite the three air conditioners on the electricity bill is still down a lot, too Nessie!	Jingdong prefecture level city in Shandong
IXCHSQ Flagship Store	King of Power Saving	Without further ado, the electric bill came down, $102 less this month, personal satisfaction, it counts okay, give you a good review	Jingdong prefecture level city in Shandong
IXCHSQ Flagship Store	King of Power Saving	The power saver has arrived, the installation is really convenient, plugged in on the line operation is quite simple, power saving effect is also a strong!	Jingdong prefecture level city in Shandong
IXCHSQ Flagship Store	King of Power Saving	Saver received, good quality, beautiful appearance, fine workmanship, simple installation	Jingdong prefecture level city in Shandong
IXCHSQ Flagship Store	King of Power Saving	Product design style modern, worth buying	Jingdong prefecture level city in Shandong
IXCHSQ Flagship Store	King of Power Saving	Energy saving, good power saving effect, practical, great value	Jingdong prefecture level city in Shandong
IXCHSQ Flagship Store	King of Power Saving	It's good quality, it's good, it's energy efficient, and I'll buy it again.	Jingdong prefecture level city in Shandong
IXCHSQ Flagship Store	King of Power Saving	Installation saves a lot of electricity, fast logistics, real energy-saving appliances	Jingdong prefecture level city in Shandong
IXCHSQ Flagship Store	King of Power Saving	The effect is good and the power saving is obvious after using it for a month and saving tens of dollars, the product is trustworthy, I now buy another 4 colors to prepare for their siblings' family to pay for	Jingdong prefecture level city in Shandong
IXCHSQ Flagship Store	King of Power Saving	The quality is particularly good workmanship is no problem, the arrival of the very fast oh, especially power-saving	Jingdong prefecture level city in Shandong
IXCHSQ Flagship Store	King of Power Saving	The quality is very good! In use, observed for a day, not as used before, so much power consumption.	Jingdong prefecture level city in Shandong
IXCHSQ Flagship Store	King of Power Saving	Goods have been received, the effect is good is not good, power saving is not yet know, wait for me to use it and then evaluate it.	Jingdong prefecture level city in Shandong
IXCHSQ Flagship Store	King of Power Saving	In-kind and the same as described, the quality is quite good, the seller's attitude is also good, ask questions and answer them, the speed of delivery bar, worth buying Oh!	Jingdong prefecture level city in Shandong
IXCHSQ Flagship Store	King of Power Saving	Nice stuff, came to comment after using it, it does save power.	Jingdong prefecture level city in Shandong
IXCHSQ Flagship Store	King of Power Saving	Fast charging, but also save power, the effect is very good, satisfied, praise!	Jingdong prefecture level city in Shandong
IXCHSQ Flagship Store	King of Power Saving	The goods have been received, I do not know how effective, after using the flat price again!	Jingdong prefecture level city in Shandong
IXCHSQ Flagship Store	King of Power Saving	Merchant service is very good Reply quickly The key is the quality and that quality is good	Jingdong prefecture level city in Shandong
IXCHSQ Flagship Store	King of Power Saving	It's working well now, I'll do a follow up review after I actually use it for a while	Jingdong prefecture level city in Shandong
IXCHSQ Flagship Store	King of Power Saving	The quality is good. After installing it, I feel like I've saved a lot of money on my electricity bill.	Jingdong prefecture level city in Shandong
IXCHSQ Flagship Store	King of Power Saving	Make up a review after use.	Jingdong prefecture level city in Shandong
IXCHSQ Flagship Store	King of Power Saving	review after use	Jingdong prefecture level city in Shandong
IXCHSQ Flagship Store	King of Power Saving	It's a small capacitor. It's not energy efficient.	Jingdong prefecture level city in Shandong
IXCHSQ Flagship Store	King of Power Saving	I'll follow up with a review after I've used it and had results.	Jingdong prefecture level city in Shandong
IXCHSQ Flagship Store	King of Power Saving	No power saving? Not good!	Jingdong prefecture level city in Shandong
IXCHSQ Flagship Store	King of Power Saving	Been using the same appliances and running a 1.5 air conditioner at night for what feels like no time, and after half a month of use it feels like the power is only increasing. Doesn't show any power saving	Jingdong prefecture level city in Shandong
IXCHSQ Flagship Store	King of Power Saving	serviceable	Jingdong prefecture level city in Shandong
IXCHSQ Flagship Store	King of Power Saving	It's a piece of junk. It doesn't save any power.	Jingdong prefecture level city in Shandong
IXCHSQ Flagship Store	King of Power Saving	There are a lot of appliances in the house, the summer air conditioning is very costly, I bought this in front of the air conditioning effect is very good, I explain that I bought three in one breath, I use more electricity a month down to 200 or so, very satisfied.	Jingdong prefecture level city in Shandong
IXCHSQ Flagship Store	King of Power Saving	The voltage is very stable has been maintained at 220v this value, high power super power saving, trustworthy, reasonable design, shape bright, logistics is quite fast, business warranty period, excellent service, logistics fast!	Jingdong prefecture level city in Shandong
IXCHSQ Flagship Store	King of Power Saving	Use a month after a look at the savings of more than 40 dollars, there is a display of electricity savings, this high-power intelligent frequency converter economizer, affordable and cheap, logistics is very fast, the service attitude is very good, very satisfied with a shopping.	Jingdong prefecture level city in Shandong
IXCHSQ Flagship Store	King of Power Saving	Used for a period of time, it seems that every day can really can save a few degrees of electricity, workmanship quite can, with a period of time and then continue to look at it, anyway, the price is not	Jingdong prefecture level city in Shandong
IXCHSQ Flagship Store	King of Power Saving	Used for a period of time, it seems that every day can indeed save two several degrees of electricity, workmanship quite can, with a period of time and then continue to look at it, anyway, the price is not	Jingdong prefecture level city in Shandong
IXCHSQ Flagship Store	King of Power Saving	High-power super saver received immediately used, the price is not expensive quality is very good, save electricity and money, very convenient, conscientious business, must be praised, recommended to everyone!	Jingdong prefecture level city in Shandong
IXCHSQ Flagship Store	King of Power Saving	Home enhancement version of the intelligent power saver, is in use, I think the price is cheap, value for money, efficacy, power, power saving effect is very good.	Jingdong prefecture level city in Shandong
IXCHSQ Flagship Store	King of Power Saving	Bought three at once, one for each room, summer electricity consumption is very high, despite the three air conditioners on the electricity bill is still down a lot, too Nessie!	Jingdong prefecture level city in Shandong
IXCHSQ Flagship Store	King of Power Saving	Without further ado, the electric bill came down, $102 less this month, personal satisfaction, it counts okay, give you a good review	Jingdong prefecture level city in Shandong

IXCHSQ Flagship Store	King of Power Saving	The power saver has arrived, the installation is really convenient, plugged in on the line operation is quite simple, power saving effect is also a strong!	Jingdong prefecture level city in Shandong
IXCHSQ Flagship Store	King of Power Saving	Saver received, good quality, beautiful appearance, fine workmanship, simple installation	Jingdong prefecture level city in Shandong
IXCHSQ Flagship Store	King of Power Saving	Product design style modern, worth buying	Jingdong prefecture level city in Shandong
IXCHSQ Flagship Store	King of Power Saving	Energy saving, good power saving effect, practical, great value	Jingdong prefecture level city in Shandong
IXCHSQ Flagship Store	King of Power Saving	It's good quality, it's good, it's energy efficient, and I'll buy it again.	Jingdong prefecture level city in Shandong
IXCHSQ Flagship Store	King of Power Saving	Installation saves a lot of electricity, fast logistics, real energy-saving appliances	Jingdong prefecture level city in Shandong
IXCHSQ Flagship Store	King of Power Saving	The effect is good and the power saving is obvious after using it for a month and saving tens of dollars, the product is trustworthy, I now buy another 4 colors to prepare for their siblings' family to pay for	Jingdong prefecture level city in Shandong
IXCHSQ Flagship Store	King of Power Saving	The quality is particularly good workmanship is no problem, the arrival of the very fast oh, especially power-saving	Jingdong prefecture level city in Shandong
IXCHSQ Flagship Store	King of Power Saving	The quality is very good! In use, observed for a day, not as used before, so much power consumption.	Jingdong prefecture level city in Shandong
IXCHSQ Flagship Store	King of Power Saving	Goods have been received, the effect is good is not good, power saving is not yet know, wait for me to use it and then evaluate it.	Jingdong prefecture level city in Shandong
IXCHSQ Flagship Store	King of Power Saving	In-kind and the same as described, the quality is quite good, the seller's attitude is also good, ask questions and answer them, the speed of delivery bar, worth buying Oh!	Jingdong prefecture level city in Shandong
IXCHSQ Flagship Store	King of Power Saving	Nice stuff, came to comment after using it, it does save power.	Jingdong prefecture level city in Shandong
IXCHSQ Flagship Store	King of Power Saving	Fast charging, but also save power, the effect is very good, satisfied, praise!	Jingdong prefecture level city in Shandong
IXCHSQ Flagship Store	King of Power Saving	The goods have been received, I do not know how effective, after using the flat price again!	Jingdong prefecture level city in Shandong
IXCHSQ Flagship Store	King of Power Saving	Merchant service is very good Reply quickly The key is the quality and that quality is good	Jingdong prefecture level city in Shandong
IXCHSQ Flagship Store	King of Power Saving	It's working well now, I'll do a follow up review after I actually use it for a while	Jingdong prefecture level city in Shandong
IXCHSQ Flagship Store	King of Power Saving	The quality is good. After installing it, I feel like I've saved a lot of money on my electricity bill.	Jingdong prefecture level city in Shandong
IXCHSQ Flagship Store	King of Power Saving	Make up a review after use.	Jingdong prefecture level city in Shandong
IXCHSQ Flagship Store	King of Power Saving	review after use	Jingdong prefecture level city in Shandong
IXCHSQ Flagship Store	King of Power Saving	It's a small capacitor. It's not energy efficient.	Jingdong prefecture level city in Shandong
IXCHSQ Flagship Store	King of Power Saving	I'll follow up with a review after I've used it and had results.	Jingdong prefecture level city in Shandong
IXCHSQ Flagship Store	King of Power Saving	No power saving? Not good!	Jingdong prefecture level city in Shandong
IXCHSQ Flagship Store	King of Power Saving	Been using the same appliances and running a 1.5 air conditioner at night for what feels like no time, and after half a month of use it feels like the power is only increasing. Doesn't show any power saving	Jingdong prefecture level city in Shandong
IXCHSQ Flagship Store	King of Power Saving	serviceable	Jingdong prefecture level city in Shandong
IXCHSQ Flagship Store	King of Power Saving	It's a piece of junk. It doesn't save any power.	Jingdong prefecture level city in Shandong
IXCHSQ Flagship Store	King of Power Saving	There are a lot of appliances in the house, the summer air conditioning is very costly, I bought this in front of the air conditioning effect is very good, I explain that I bought three in one breath, I use more electricity a month down to 200 or so, very satisfied.	Jingdong prefecture level city in Shandong
IXCHSQ Flagship Store	King of Power Saving	The voltage is very stable has been maintained at 220v this value, high power super power saving, trustworthy, reasonable design, shape bright, logistics is quite fast, business warranty period, excellent service, logistics fast!	Jingdong prefecture level city in Shandong
IXCHSQ Flagship Store	King of Power Saving	Use a month after a look at the savings of more than 40 dollars, there is a display of electricity savings, this high-power intelligent frequency converter economizer, affordable and cheap, logistics is very fast, the service attitude is very good, very satisfied with a shopping.	Jingdong prefecture level city in Shandong
IXCHSQ Flagship Store	King of Power Saving	Used for a period of time, it seems that every day can really can save a few degrees of electricity, workmanship quite can, with a period of time and then continue to look at it, anyway, the price is not	Jingdong prefecture level city in Shandong
IXCHSQ Flagship Store	King of Power Saving	Used for a period of time, it seems that every day can indeed save two several degrees of electricity, workmanship quite can, with a period of time and then continue to look at it, anyway, the price is not	Jingdong prefecture level city in Shandong
IXCHSQ Flagship Store	King of Power Saving	High-power super saver received immediately used, the price is not expensive quality is very good, save electricity and money, very convenient, conscientious business, must be praised, recommended to everyone!	Jingdong prefecture level city in Shandong
IXCHSQ Flagship Store	King of Power Saving	Home enhancement version of the intelligent power saver, is in use, I think the price is cheap, value for money, efficacy, power, power saving effect is very good.	Jingdong prefecture level city in Shandong
IXCHSQ Flagship Store	King of Power Saving	Bought three at once, one for each room, summer electricity consumption is very high, despite the three air conditioners on the electricity bill is still down a lot, too Nessie!	Jingdong prefecture level city in Shandong
IXCHSQ Flagship Store	King of Power Saving	Without further ado, the electric bill came down, $102 less this month, personal satisfaction, it counts okay, give you a good review	Jingdong prefecture level city in Shandong
IXCHSQ Flagship Store	King of Power Saving	The power saver has arrived, the installation is really convenient, plugged in on the line operation is quite simple, power saving effect is also a strong!	Jingdong prefecture level city in Shandong
IXCHSQ Flagship Store	King of Power Saving	Saver received, good quality, beautiful appearance, fine workmanship, simple installation	Jingdong prefecture level city in Shandong
IXCHSQ Flagship Store	King of Power Saving	Product design style modern, worth buying	Jingdong prefecture level city in Shandong
IXCHSQ Flagship Store	King of Power Saving	Energy saving, good power saving effect, practical, great value	Jingdong prefecture level city in Shandong
IXCHSQ Flagship Store	King of Power Saving	It's good quality, it's good, it's energy efficient, and I'll buy it again.	Jingdong prefecture level city in Shandong
IXCHSQ Flagship Store	King of Power Saving	Installation saves a lot of electricity, fast logistics, real energy-saving appliances	Jingdong prefecture level city in Shandong
IXCHSQ Flagship Store	King of Power Saving	The effect is good and the power saving is obvious after using it for a month and saving tens of dollars, the product is trustworthy, I now buy another 4 colors to prepare for their siblings' family to pay for	Jingdong prefecture level city in Shandong
IXCHSQ Flagship Store	King of Power Saving	The quality is particularly good workmanship is no problem, the arrival of the very fast oh, especially power-saving	Jingdong prefecture level city in Shandong
IXCHSQ Flagship Store	King of Power Saving	The quality is very good! In use, observed for a day, not as used before, so much power consumption.	Jingdong prefecture level city in Shandong
IXCHSQ Flagship Store	King of Power Saving	Goods have been received, the effect is good is not good, power saving is not yet know, wait for me to use it and then evaluate it.	Jingdong prefecture level city in Shandong
IXCHSQ Flagship Store	King of Power Saving	In-kind and the same as described, the quality is quite good, the seller's attitude is also good, ask questions and answer them, the speed of delivery bar, worth buying Oh!	Jingdong prefecture level city in Shandong
IXCHSQ Flagship Store	King of Power Saving	Nice stuff, came to comment after using it, it does save power.	Jingdong prefecture level city in Shandong
IXCHSQ Flagship Store	King of Power Saving	Fast charging, but also save power, the effect is very good, satisfied, praise!	Jingdong prefecture level city in Shandong
IXCHSQ Flagship Store	King of Power Saving	The goods have been received, I do not know how effective, after using the flat price again!	Jingdong prefecture level city in Shandong
IXCHSQ Flagship Store	King of Power Saving	Merchant service is very good Reply quickly The key is the quality and that quality is good	Jingdong prefecture level city in Shandong
IXCHSQ Flagship Store	King of Power Saving	It's working well now, I'll do a follow up review after I actually use it for a while	Jingdong prefecture level city in Shandong

IXCHSQ Flagship Store	King of Power Saving	The quality is good. After installing it, I feel like I’ve saved a lot of money on my electricity bill.	Jingdong prefecture level city in Shandong
IXCHSQ Flagship Store	King of Power Saving	Make up a review after use.	Jingdong prefecture level city in Shandong
IXCHSQ Flagship Store	King of Power Saving	review after use	Jingdong prefecture level city in Shandong
IXCHSQ Flagship Store	King of Power Saving	It’s a small capacitor. It’s not energy efficient.	Jingdong prefecture level city in Shandong
IXCHSQ Flagship Store	King of Power Saving	I’ll follow up with a review after I’ve used it and had results.	Jingdong prefecture level city in Shandong
IXCHSQ Flagship Store	King of Power Saving	No power saving? Not good!	Jingdong prefecture level city in Shandong
IXCHSQ Flagship Store	King of Power Saving	Been using the same appliances and running a 1.5 air conditioner at night for what feels like no time, and after half a month of use it feels like the power is only increasing. Doesn’t show any power saving	Jingdong prefecture level city in Shandong
IXCHSQ Flagship Store	King of Power Saving	serviceable	Jingdong prefecture level city in Shandong
IXCHSQ Flagship Store	King of Power Saving	It’s a piece of junk. It doesn’t save any power.	Jingdong prefecture level city in Shandong
IXCHSQ Flagship Store	King of Power Saving	There are a lot of appliances in the house, the summer air conditioning is very costly, I bought this in front of the air conditioning effect is very good, I explain that I bought three in one breath, I use more electricity a month down to 200 or so, very satisfied.	Jingdong prefecture level city in Shandong
IXCHSQ Flagship Store	King of Power Saving	The voltage is very stable has been maintained at 220v this value, high power super power saving, trustworthy, reasonable design, shape bright, logistics is quite fast, business warranty period, excellent service, logistics fast!	Jingdong prefecture level city in Shandong
IXCHSQ Flagship Store	King of Power Saving	Use a month after a look at the savings of more than 40 dollars, there is a display of electricity savings, this high-power intelligent frequency converter economizer, affordable and cheap, logistics is very fast, the service attitude is very good, very satisfied with a shopping.	Jingdong prefecture level city in Shandong
IXCHSQ Flagship Store	King of Power Saving	Used for a period of time, it seems that every day can really can save a few degrees of electricity, workmanship quite can, with a period of time and then continue to look at it, anyway, the price is not	Jingdong prefecture level city in Shandong
IXCHSQ Flagship Store	King of Power Saving	Used for a period of time, it seems that every day can indeed save two several degrees of electricity, workmanship quite can, with a period of time and then continue to look at it, anyway, the price is not	Jingdong prefecture level city in Shandong
IXCHSQ Flagship Store	King of Power Saving	High-power super saver received immediately used, the price is not expensive quality is very good, save electricity and money, very convenient, conscientious business, must be praised, recommended to everyone!	Jingdong prefecture level city in Shandong
IXCHSQ Flagship Store	King of Power Saving	Home enhancement version of the intelligent power saver, is in use, I think the price is cheap, value for money, efficacy, power, power saving effect is very good.	Jingdong prefecture level city in Shandong
IXCHSQ Flagship Store	King of Power Saving	Bought three at once, one for each room, summer electricity consumption is very high, despite the three air conditioners on the electricity bill is still down a lot, too Nessie!	Jingdong prefecture level city in Shandong
IXCHSQ Flagship Store	King of Power Saving	Without further ado, the electric bill came down, $102 less this month, personal satisfaction, it counts okay, give you a good review	Jingdong prefecture level city in Shandong
IXCHSQ Flagship Store	King of Power Saving	The power saver has arrived, the installation is really convenient, plugged in on the line operation is quite simple, power saving effect is also a strong!	Jingdong prefecture level city in Shandong
IXCHSQ Flagship Store	King of Power Saving	Saver received, good quality, beautiful appearance, fine workmanship, simple installation	Jingdong prefecture level city in Shandong
IXCHSQ Flagship Store	King of Power Saving	Product design style modern, worth buying	Jingdong prefecture level city in Shandong
IXCHSQ Flagship Store	King of Power Saving	Energy saving, good power saving effect, practical, great value	Jingdong prefecture level city in Shandong
IXCHSQ Flagship Store	King of Power Saving	It’s good quality, it’s good, it’s energy efficient, and I’ll buy it again.	Jingdong prefecture level city in Shandong
IXCHSQ Flagship Store	King of Power Saving	Installation saves a lot of electricity, fast logistics, real energy-saving appliances	Jingdong prefecture level city in Shandong
IXCHSQ Flagship Store	King of Power Saving	The effect is good and the power saving is obvious after using it for a month and saving tens of dollars, the product is trustworthy, I now buy another 4 colors to prepare for their siblings’ family to pay for	Jingdong prefecture level city in Shandong
IXCHSQ Flagship Store	King of Power Saving	The quality is particularly good workmanship is no problem, the arrival of the very fast oh, especially power-saving	Jingdong prefecture level city in Shandong
IXCHSQ Flagship Store	King of Power Saving	The quality is very good! In use, observed for a day, not as used before, so much power consumption.	Jingdong prefecture level city in Shandong
IXCHSQ Flagship Store	King of Power Saving	Goods have been received, the effect is good is not good, power saving is not yet know, wait for me to use it and then evaluate it.	Jingdong prefecture level city in Shandong
IXCHSQ Flagship Store	King of Power Saving	In-kind and the same as described, the quality is quite good, the seller's attitude is also good, ask questions and answer them, the speed of delivery bar, worth buying Oh!	Jingdong prefecture level city in Shandong
IXCHSQ Flagship Store	King of Power Saving	Nice stuff, came to comment after using it, it does save power.	Jingdong prefecture level city in Shandong
IXCHSQ Flagship Store	King of Power Saving	Fast charging, but also save power, the effect is very good, satisfied, praise!	Jingdong prefecture level city in Shandong
IXCHSQ Flagship Store	King of Power Saving	The goods have been received, I do not know how effective, after using the flat price again!	Jingdong prefecture level city in Shandong
IXCHSQ Flagship Store	King of Power Saving	Merchant service is very good Reply quickly The key is the quality and that quality is good	Jingdong prefecture level city in Shandong
IXCHSQ Flagship Store	King of Power Saving	It’s working well now, I’ll do a follow up review after I actually use it for a while	Jingdong prefecture level city in Shandong
IXCHSQ Flagship Store	King of Power Saving	The quality is good. After installing it, I feel like I’ve saved a lot of money on my electricity bill.	Jingdong prefecture level city in Shandong
IXCHSQ Flagship Store	King of Power Saving	Make up a review after use.	Jingdong prefecture level city in Shandong
IXCHSQ Flagship Store	King of Power Saving	review after use	Jingdong prefecture level city in Shandong
IXCHSQ Flagship Store	King of Power Saving	It’s a small capacitor. It’s not energy efficient.	Jingdong prefecture level city in Shandong
IXCHSQ Flagship Store	King of Power Saving	I’ll follow up with a review after I’ve used it and had results.	Jingdong prefecture level city in Shandong
IXCHSQ Flagship Store	King of Power Saving	No power saving? Not good!	Jingdong prefecture level city in Shandong
IXCHSQ Flagship Store	King of Power Saving	Been using the same appliances and running a 1.5 air conditioner at night for what feels like no time, and after half a month of use it feels like the power is only increasing. Doesn’t show any power saving	Jingdong prefecture level city in Shandong
IXCHSQ Flagship Store	King of Power Saving	serviceable	Jingdong prefecture level city in Shandong
IXCHSQ Flagship Store	King of Power Saving	It’s a piece of junk. It doesn’t save any power.	Jingdong prefecture level city in Shandong
IXCHSQ Flagship Store	King of Power Saving	There are a lot of appliances in the house, the summer air conditioning is very costly, I bought this in front of the air conditioning effect is very good, I explain that I bought three in one breath, I use more electricity a month down to 200 or so, very satisfied.	Jingdong prefecture level city in Shandong
IXCHSQ Flagship Store	King of Power Saving	The voltage is very stable has been maintained at 220v this value, high power super power saving, trustworthy, reasonable design, shape bright, logistics is quite fast, business warranty period, excellent service, logistics fast!	Jingdong prefecture level city in Shandong
IXCHSQ Flagship Store	King of Power Saving	Use a month after a look at the savings of more than 40 dollars, there is a display of electricity savings, this high-power intelligent frequency converter economizer, affordable and cheap, logistics is very fast, the service attitude is very good, very satisfied with a shopping.	Jingdong prefecture level city in Shandong
IXCHSQ Flagship Store	King of Power Saving	Used for a period of time, it seems that every day can really can save a few degrees of electricity, workmanship quite can, with a period of time and then continue to look at it, anyway, the price is not	Jingdong prefecture level city in Shandong
IXCHSQ Flagship Store	King of Power Saving	Used for a period of time, it seems that every day can indeed save two several degrees of electricity, workmanship quite can, with a period of time and then continue to look at it, anyway, the price is not	Jingdong prefecture level city in Shandong

IXCHSQ Flagship Store	King of Power Saving	High-power super saver received immediately used, the price is not expensive quality is very good, save electricity and money, very convenient, conscientious business, must be praised, recommended to everyone!	Jingdong prefecture level city in Shandong
IXCHSQ Flagship Store	King of Power Saving	Home enhancement version of the intelligent power saver, is in use, I think the price is cheap, value for money, efficacy, power, power saving effect is very good.	Jingdong prefecture level city in Shandong
IXCHSQ Flagship Store	King of Power Saving	Bought three at once, one for each room, summer electricity consumption is very high, despite the three air conditioners on the electricity bill is still down a lot, too Nessie!	Jingdong prefecture level city in Shandong
IXCHSQ Flagship Store	King of Power Saving	Without further ado, the electric bill came down, $102 less this month, personal satisfaction, it counts okay, give you a good review	Jingdong prefecture level city in Shandong
IXCHSQ Flagship Store	King of Power Saving	The power saver has arrived, the installation is really convenient, plugged in on the line operation is quite simple, power saving effect is also a strong!	Jingdong prefecture level city in Shandong
IXCHSQ Flagship Store	King of Power Saving	Saver received, good quality, beautiful appearance, fine workmanship, simple installation	Jingdong prefecture level city in Shandong
IXCHSQ Flagship Store	King of Power Saving	Product design style modern, worth buying	Jingdong prefecture level city in Shandong
IXCHSQ Flagship Store	King of Power Saving	Energy saving, good power saving effect, practical, great value	Jingdong prefecture level city in Shandong
IXCHSQ Flagship Store	King of Power Saving	It's good quality, it's good, it's energy efficient, and I'll buy it again.	Jingdong prefecture level city in Shandong
IXCHSQ Flagship Store	King of Power Saving	Installation saves a lot of electricity, fast logistics, real energy-saving appliances	Jingdong prefecture level city in Shandong
IXCHSQ Flagship Store	King of Power Saving	The effect is good and the power saving is obvious after using it for a month and saving tens of dollars, the product is trustworthy, I now buy another 4 colors to prepare for their siblings' family to pay for	Jingdong prefecture level city in Shandong
IXCHSQ Flagship Store	King of Power Saving	The quality is particularly good workmanship is no problem, the arrival of the very fast oh, especially power-saving	Jingdong prefecture level city in Shandong
IXCHSQ Flagship Store	King of Power Saving	The quality is very good! In use, observed for a day, not as used before, so much power consumption.	Jingdong prefecture level city in Shandong
IXCHSQ Flagship Store	King of Power Saving	Goods have been received, the effect is good is not good, power saving is not yet know, wait for me to use it and then evaluate it.	Jingdong prefecture level city in Shandong
IXCHSQ Flagship Store	King of Power Saving	In-kind and the same as described, the quality is quite good, the seller's attitude is also good, ask questions and answer them, the speed of delivery bar, worth buying Oh!	Jingdong prefecture level city in Shandong
IXCHSQ Flagship Store	King of Power Saving	Nice stuff, came to comment after using it, it does save power.	Jingdong prefecture level city in Shandong
IXCHSQ Flagship Store	King of Power Saving	Fast charging, but also save power, the effect is very good, satisfied, praise!	Jingdong prefecture level city in Shandong
IXCHSQ Flagship Store	King of Power Saving	The goods have been received, I do not know how effective, after using the flat price again!	Jingdong prefecture level city in Shandong
IXCHSQ Flagship Store	King of Power Saving	Merchant service is very good Reply quickly The key is the quality and that quality is good	Jingdong prefecture level city in Shandong
IXCHSQ Flagship Store	King of Power Saving	It's working well now, I'll do a follow up review after I actually use it for a while	Jingdong prefecture level city in Shandong
IXCHSQ Flagship Store	King of Power Saving	The quality is good. After installing it, I feel like I've saved a lot of money on my electricity bill.	Jingdong prefecture level city in Shandong
IXCHSQ Flagship Store	King of Power Saving	Make up a review after use.	Jingdong prefecture level city in Shandong
IXCHSQ Flagship Store	King of Power Saving	review after use	Jingdong prefecture level city in Shandong
IXCHSQ Flagship Store	King of Power Saving	It's a small capacitor. It's not energy efficient.	Jingdong prefecture level city in Shandong
IXCHSQ Flagship Store	King of Power Saving	I'll follow up with a review after I've used it and had results.	Jingdong prefecture level city in Shandong
IXCHSQ Flagship Store	King of Power Saving	No power saving? Not good!	Jingdong prefecture level city in Shandong
IXCHSQ Flagship Store	King of Power Saving	Been using the same appliances and running a 1.5 air conditioner at night for what feels like no time, and after half a month of use it feels like the power is only increasing. Doesn't show any power saving	Jingdong prefecture level city in Shandong
IXCHSQ Flagship Store	King of Power Saving	serviceable	Jingdong prefecture level city in Shandong
IXCHSQ Flagship Store	King of Power Saving	It's a piece of junk. It doesn't save any power.	Jingdong prefecture level city in Shandong
IXCHSQ Flagship Store	King of Power Saving	There are a lot of appliances in the house, the summer air conditioning is very costly, I bought this in front of the air conditioning effect is very good, I explain that I bought three in one breath, I use more electricity a month down to 200 or so, very satisfied.	Jingdong prefecture level city in Shandong
IXCHSQ Flagship Store	King of Power Saving	The voltage is very stable has been maintained at 220v this value, high power super power saving, trustworthy, reasonable design, shape bright, logistics is quite fast, business warranty period, excellent service, logistics fast!	Jingdong prefecture level city in Shandong
IXCHSQ Flagship Store	King of Power Saving	Use a month after a look at the savings of more than 40 dollars, there is a display of electricity savings, this high-power intelligent frequency converter economizer, affordable and cheap, logistics is very fast, the service attitude is very good, very satisfied with a shopping.	Jingdong prefecture level city in Shandong
IXCHSQ Flagship Store	King of Power Saving	Used for a period of time, it seems that every day can really can save a few degrees of electricity, workmanship quite can, with a period of time and then continue to look at it, anyway, the price is not	Jingdong prefecture level city in Shandong
IXCHSQ Flagship Store	King of Power Saving	Used for a period of time, it seems that every day can indeed save two several degrees of electricity, workmanship quite can, with a period of time and then continue to look at it, anyway, the price is not	Jingdong prefecture level city in Shandong
IXCHSQ Flagship Store	King of Power Saving	High-power super saver received immediately used, the price is not expensive quality is very good, save electricity and money, very convenient, conscientious business, must be praised, recommended to everyone!	Jingdong prefecture level city in Shandong
IXCHSQ Flagship Store	King of Power Saving	Home enhancement version of the intelligent power saver, is in use, I think the price is cheap, value for money, efficacy, power, power saving effect is very good.	Jingdong prefecture level city in Shandong
IXCHSQ Flagship Store	King of Power Saving	Bought three at once, one for each room, summer electricity consumption is very high, despite the three air conditioners on the electricity bill is still down a lot, too Nessie!	Jingdong prefecture level city in Shandong
IXCHSQ Flagship Store	King of Power Saving	Without further ado, the electric bill came down, $102 less this month, personal satisfaction, it counts okay, give you a good review	Jingdong prefecture level city in Shandong
IXCHSQ Flagship Store	King of Power Saving	The power saver has arrived, the installation is really convenient, plugged in on the line operation is quite simple, power saving effect is also a strong!	Jingdong prefecture level city in Shandong
IXCHSQ Flagship Store	King of Power Saving	Saver received, good quality, beautiful appearance, fine workmanship, simple installation	Jingdong prefecture level city in Shandong
IXCHSQ Flagship Store	King of Power Saving	Product design style modern, worth buying	Jingdong prefecture level city in Shandong
IXCHSQ Flagship Store	King of Power Saving	Energy saving, good power saving effect, practical, great value	Jingdong prefecture level city in Shandong
IXCHSQ Flagship Store	King of Power Saving	It's good quality, it's good, it's energy efficient, and I'll buy it again.	Jingdong prefecture level city in Shandong
IXCHSQ Flagship Store	King of Power Saving	Installation saves a lot of electricity, fast logistics, real energy-saving appliances	Jingdong prefecture level city in Shandong
IXCHSQ Flagship Store	King of Power Saving	The effect is good and the power saving is obvious after using it for a month and saving tens of dollars, the product is trustworthy, I now buy another 4 colors to prepare for their siblings' family to pay for	Jingdong prefecture level city in Shandong
IXCHSQ Flagship Store	King of Power Saving	The quality is particularly good workmanship is no problem, the arrival of the very fast oh, especially power-saving	Jingdong prefecture level city in Shandong
IXCHSQ Flagship Store	King of Power Saving	The quality is very good! In use, observed for a day, not as used before, so much power consumption.	Jingdong prefecture level city in Shandong
IXCHSQ Flagship Store	King of Power Saving	Goods have been received, the effect is good is not good, power saving is not yet know, wait for me to use it and then evaluate it.	Jingdong prefecture level city in Shandong
IXCHSQ Flagship Store	King of Power Saving	In-kind and the same as described, the quality is quite good, the seller's attitude is also good, ask questions and answer them, the speed of delivery bar, worth buying Oh!	Jingdong prefecture level city in Shandong
IXCHSQ Flagship Store	King of Power Saving	Nice stuff, came to comment after using it, it does save power.	Jingdong prefecture level city in Shandong

IXCHSQ Flagship Store	King of Power Saving	Fast charging, but also save power, the effect is very good, satisfied, praise!	Jingdong prefecture level city in Shandong
IXCHSQ Flagship Store	King of Power Saving	The goods have been received, I do not know how effective, after using the flat price again!	Jingdong prefecture level city in Shandong
IXCHSQ Flagship Store	King of Power Saving	Merchant service is very good Reply quickly The key is the quality and that quality is good	Jingdong prefecture level city in Shandong
IXCHSQ Flagship Store	King of Power Saving	It's working well now, I'll do a follow up review after I actually use it for a while	Jingdong prefecture level city in Shandong
IXCHSQ Flagship Store	King of Power Saving	The quality is good. After installing it, I feel like I've saved a lot of money on my electricity bill.	Jingdong prefecture level city in Shandong
IXCHSQ Flagship Store	King of Power Saving	Make up a review after use.	Jingdong prefecture level city in Shandong
IXCHSQ Flagship Store	King of Power Saving	review after use	Jingdong prefecture level city in Shandong
IXCHSQ Flagship Store	King of Power Saving	It's a small capacitor. It's not energy efficient.	Jingdong prefecture level city in Shandong
IXCHSQ Flagship Store	King of Power Saving	I'll follow up with a review after I've used it and had results.	Jingdong prefecture level city in Shandong
IXCHSQ Flagship Store	King of Power Saving	No power saving? Not good!	Jingdong prefecture level city in Shandong
IXCHSQ Flagship Store	King of Power Saving	Been using the same appliances and running a 1.5 air conditioner at night for what feels like no time, and after half a month of use it feels like the power is only increasing. Doesn't show any power saving	Jingdong prefecture level city in Shandong
IXCHSQ Flagship Store	King of Power Saving	serviceable	Jingdong prefecture level city in Shandong
IXCHSQ Flagship Store	King of Power Saving	It's a piece of junk. It doesn't save any power.	Jingdong prefecture level city in Shandong
IXCHSQ Flagship Store	King of Power Saving	There are a lot of appliances in the house, the summer air conditioning is very costly, I bought this in front of the air conditioning effect is very good, I explain that I bought three in one breath, I use more electricity a month down to 200 or so, very satisfied.	Jingdong prefecture level city in Shandong
IXCHSQ Flagship Store	King of Power Saving	The voltage is very stable has been maintained at 220v this value, high power super power saving, trustworthy, reasonable design, shape bright, logistics is quite fast, business warranty period, excellent service, logistics fast!	Jingdong prefecture level city in Shandong
IXCHSQ Flagship Store	King of Power Saving	Use a month after a look at the savings of more than 40 dollars, there is a display of electricity savings, this high-power intelligent frequency converter economizer, affordable and cheap, logistics is very fast, the service attitude is very good, very satisfied with a shopping.	Jingdong prefecture level city in Shandong
IXCHSQ Flagship Store	King of Power Saving	Used for a period of time, it seems that every day can really can save a few degrees of electricity, workmanship quite can, with a period of time and then continue to look at it, anyway, the price is not	Jingdong prefecture level city in Shandong
IXCHSQ Flagship Store	King of Power Saving	Used for a period of time, it seems that every day can indeed save two several degrees of electricity, workmanship quite can, with a period of time and then continue to look at it, anyway, the price is not	Jingdong prefecture level city in Shandong
IXCHSQ Flagship Store	King of Power Saving	High-power super saver received immediately used, the price is not expensive quality is very good, save electricity and money, very convenient, conscientious business, must be praised, recommended to everyone!	Jingdong prefecture level city in Shandong
IXCHSQ Flagship Store	King of Power Saving	Home enhancement version of the intelligent power saver, is in use, I think the price is cheap, value for money, efficacy, power, power saving effect is very good.	Jingdong prefecture level city in Shandong
IXCHSQ Flagship Store	King of Power Saving	Bought three at once, one for each room, summer electricity consumption is very high, despite the three air conditioners on the electricity bill is still down a lot, too Nessie!	Jingdong prefecture level city in Shandong
IXCHSQ Flagship Store	King of Power Saving	Without further ado, the electric bill came down, $102 less this month, personal satisfaction, it counts okay, give you a good review	Jingdong prefecture level city in Shandong
IXCHSQ Flagship Store	King of Power Saving	The power saver has arrived, the installation is really convenient, plugged in on the line operation is quite simple, power saving effect is also a strong!	Jingdong prefecture level city in Shandong
IXCHSQ Flagship Store	King of Power Saving	Saver received, good quality, beautiful appearance, fine workmanship, simple installation	Jingdong prefecture level city in Shandong
IXCHSQ Flagship Store	King of Power Saving	Product design style modern, worth buying	Jingdong prefecture level city in Shandong
IXCHSQ Flagship Store	King of Power Saving	Energy saving, good power saving effect, practical, great value	Jingdong prefecture level city in Shandong
IXCHSQ Flagship Store	King of Power Saving	It's good quality, it's good, it's energy efficient, and I'll buy it again.	Jingdong prefecture level city in Shandong
IXCHSQ Flagship Store	King of Power Saving	Installation saves a lot of electricity, fast logistics, real energy-saving appliances	Jingdong prefecture level city in Shandong
IXCHSQ Flagship Store	King of Power Saving	The effect is good and the power saving is obvious after using it for a month and saving tens of dollars, the product is trustworthy, I now buy another 4 colors to prepare for their siblings' family to pay for	Jingdong prefecture level city in Shandong
IXCHSQ Flagship Store	King of Power Saving	The quality is particularly good workmanship is no problem, the arrival of the very fast oh, especially power-saving	Jingdong prefecture level city in Shandong
IXCHSQ Flagship Store	King of Power Saving	The quality is very good! In use, observed for a day, not as used before, so much power consumption.	Jingdong prefecture level city in Shandong
IXCHSQ Flagship Store	King of Power Saving	Goods have been received, the effect is good is not good, power saving is not yet know, wait for me to use it and then evaluate it.	Jingdong prefecture level city in Shandong
IXCHSQ Flagship Store	King of Power Saving	In-kind and the same as described, the quality is quite good, the seller's attitude is also good, ask questions and answer them, the speed of delivery bar, worth buying Oh!	Jingdong prefecture level city in Shandong
IXCHSQ Flagship Store	King of Power Saving	Nice stuff, came to comment after using it, it does save power.	Jingdong prefecture level city in Shandong
IXCHSQ Flagship Store	King of Power Saving	Fast charging, but also save power, the effect is very good, satisfied, praise!	Jingdong prefecture level city in Shandong
IXCHSQ Flagship Store	King of Power Saving	The goods have been received, I do not know how effective, after using the flat price again!	Jingdong prefecture level city in Shandong
IXCHSQ Flagship Store	King of Power Saving	Merchant service is very good Reply quickly The key is the quality and that quality is good	Jingdong prefecture level city in Shandong
IXCHSQ Flagship Store	King of Power Saving	It's working well now, I'll do a follow up review after I actually use it for a while	Jingdong prefecture level city in Shandong
IXCHSQ Flagship Store	King of Power Saving	The quality is good. After installing it, I feel like I've saved a lot of money on my electricity bill.	Jingdong prefecture level city in Shandong
IXCHSQ Flagship Store	King of Power Saving	Make up a review after use.	Jingdong prefecture level city in Shandong
IXCHSQ Flagship Store	King of Power Saving	review after use	Jingdong prefecture level city in Shandong
IXCHSQ Flagship Store	King of Power Saving	It's a small capacitor. It's not energy efficient.	Jingdong prefecture level city in Shandong
IXCHSQ Flagship Store	King of Power Saving	I'll follow up with a review after I've used it and had results.	Jingdong prefecture level city in Shandong
IXCHSQ Flagship Store	King of Power Saving	No power saving? Not good!	Jingdong prefecture level city in Shandong
IXCHSQ Flagship Store	King of Power Saving	Been using the same appliances and running a 1.5 air conditioner at night for what feels like no time, and after half a month of use it feels like the power is only increasing. Doesn't show any power saving	Jingdong prefecture level city in Shandong
IXCHSQ Flagship Store	King of Power Saving	serviceable	Jingdong prefecture level city in Shandong
IXCHSQ Flagship Store	King of Power Saving	It's a piece of junk. It doesn't save any power.	Jingdong prefecture level city in Shandong
IXCHSQ Flagship Store	King of Power Saving	There are a lot of appliances in the house, the summer air conditioning is very costly, I bought this in front of the air conditioning effect is very good, I explain that I bought three in one breath, I use more electricity a month down to 200 or so, very satisfied.	Jingdong prefecture level city in Shandong
IXCHSQ Flagship Store	King of Power Saving	The voltage is very stable has been maintained at 220v this value, high power super power saving, trustworthy, reasonable design, shape bright, logistics is quite fast, business warranty period, excellent service, logistics fast!	Jingdong prefecture level city in Shandong

IXCHSQ Flagship Store	King of Power Saving	Use a month after a look at the savings of more than 40 dollars, there is a display of electricity savings, this high-power intelligent frequency converter economizer, affordable and cheap, logistics is very fast, the service attitude is very good, very satisfied with a shopping.	Jingdong prefecture level city in Shandong
IXCHSQ Flagship Store	King of Power Saving	Used for a period of time, it seems that every day can really can save a few degrees of electricity, workmanship quite can, with a period of time and then continue to look at it, anyway, the price is not	Jingdong prefecture level city in Shandong
IXCHSQ Flagship Store	King of Power Saving	Used for a period of time, it seems that every day can indeed save two several degrees of electricity, workmanship quite can, with a period of time and then continue to look at it, anyway, the price is not	Jingdong prefecture level city in Shandong
IXCHSQ Flagship Store	King of Power Saving	High-power super saver received immediately used, the price is not expensive quality is very good, save electricity and money, very convenient, conscientious business, must be praised, recommended to everyone!	Jingdong prefecture level city in Shandong
IXCHSQ Flagship Store	King of Power Saving	Home enhancement version of the intelligent power saver, is in use, I think the price is cheap, value for money, efficacy, power, power saving effect is very good.	Jingdong prefecture level city in Shandong
IXCHSQ Flagship Store	King of Power Saving	Bought three at once, one for each room, summer electricity consumption is very high, despite the three air conditioners on the electricity bill is still down a lot, too Nessie!	Jingdong prefecture level city in Shandong
IXCHSQ Flagship Store	King of Power Saving	Without further ado, the electric bill came down, $102 less this month, personal satisfaction, it counts okay, give you a good review	Jingdong prefecture level city in Shandong
IXCHSQ Flagship Store	King of Power Saving	The power saver has arrived, the installation is really convenient, plugged in on the line operation is quite simple, power saving effect is also a strong!	Jingdong prefecture level city in Shandong
IXCHSQ Flagship Store	King of Power Saving	Saver received, good quality, beautiful appearance, fine workmanship, simple installation	Jingdong prefecture level city in Shandong
IXCHSQ Flagship Store	King of Power Saving	Product design style modern, worth buying	Jingdong prefecture level city in Shandong
IXCHSQ Flagship Store	King of Power Saving	Energy saving, good power saving effect, practical, great value	Jingdong prefecture level city in Shandong
IXCHSQ Flagship Store	King of Power Saving	It's good quality, it's good, it's energy efficient, and I'll buy it again.	Jingdong prefecture level city in Shandong
IXCHSQ Flagship Store	King of Power Saving	Installation saves a lot of electricity, fast logistics, real energy-saving appliances	Jingdong prefecture level city in Shandong
IXCHSQ Flagship Store	King of Power Saving	The effect is good and the power saving is obvious after using it for a month and saving tens of dollars, the product is trustworthy, I now buy another 4 colors to prepare for their siblings' family to pay for	Jingdong prefecture level city in Shandong
IXCHSQ Flagship Store	King of Power Saving	The quality is particularly good workmanship is no problem, the arrival of the very fast oh, especially power-saving	Jingdong prefecture level city in Shandong
IXCHSQ Flagship Store	King of Power Saving	The quality is very good! In use, observed for a day, not as used before, so much power consumption.	Jingdong prefecture level city in Shandong
IXCHSQ Flagship Store	King of Power Saving	Goods have been received, the effect is good is not good, power saving is not yet know, wait for me to use it and then evaluate it.	Jingdong prefecture level city in Shandong
IXCHSQ Flagship Store	King of Power Saving	In-kind and the same as described, the quality is quite good, the seller's attitude is also good, ask questions and answer them, the speed of delivery bar, worth buying Oh!	Jingdong prefecture level city in Shandong
IXCHSQ Flagship Store	King of Power Saving	Nice stuff, came to comment after using it, it does save power.	Jingdong prefecture level city in Shandong
IXCHSQ Flagship Store	King of Power Saving	Fast charging, but also save power, the effect is very good, satisfied, praise!	Jingdong prefecture level city in Shandong
IXCHSQ Flagship Store	King of Power Saving	The goods have been received, I do not know how effective, after using the flat price again!	Jingdong prefecture level city in Shandong
IXCHSQ Flagship Store	King of Power Saving	Merchant service is very good Reply quickly The key is the quality and that quality is good	Jingdong prefecture level city in Shandong
IXCHSQ Flagship Store	King of Power Saving	It's working well now, I'll do a follow up review after I actually use it for a while	Jingdong prefecture level city in Shandong
IXCHSQ Flagship Store	King of Power Saving	The quality is good. After installing it, I feel like I've saved a lot of money on my electricity bill.	Jingdong prefecture level city in Shandong
IXCHSQ Flagship Store	King of Power Saving	Make up a review after use.	Jingdong prefecture level city in Shandong
IXCHSQ Flagship Store	King of Power Saving	review after use	Jingdong prefecture level city in Shandong
IXCHSQ Flagship Store	King of Power Saving	It's a small capacitor. It's not energy efficient.	Jingdong prefecture level city in Shandong
IXCHSQ Flagship Store	King of Power Saving	I'll follow up with a review after I've used it and had results.	Jingdong prefecture level city in Shandong
IXCHSQ Flagship Store	King of Power Saving	No power saving? Not good!	Jingdong prefecture level city in Shandong
IXCHSQ Flagship Store	King of Power Saving	Been using the same appliances and running a 1.5 air conditioner at night for what feels like no time, and after half a month of use it feels like the power is only increasing. Doesn't show any power saving	Jingdong prefecture level city in Shandong
IXCHSQ Flagship Store	King of Power Saving	serviceable	Jingdong prefecture level city in Shandong
IXCHSQ Flagship Store	King of Power Saving	It's a piece of junk. It doesn't save any power.	Jingdong prefecture level city in Shandong
IXCHSQ Flagship Store	King of Power Saving	There are a lot of appliances in the house, the summer air conditioning is very costly, I bought this in front of the air conditioning effect is very good, I explain that I bought three in one breath, I use more electricity a month down to 200 or so, very satisfied.	Jingdong prefecture level city in Shandong
IXCHSQ Flagship Store	King of Power Saving	The voltage is very stable has been maintained at 220v this value, high power super power saving, trustworthy, reasonable design, shape bright, logistics is quite fast, business warranty period, excellent service, logistics fast!	Jingdong prefecture level city in Shandong
IXCHSQ Flagship Store	King of Power Saving	Use a month after a look at the savings of more than 40 dollars, there is a display of electricity savings, this high-power intelligent frequency converter economizer, affordable and cheap, logistics is very fast, the service attitude is very good, very satisfied with a shopping.	Jingdong prefecture level city in Shandong
IXCHSQ Flagship Store	King of Power Saving	Used for a period of time, it seems that every day can really can save a few degrees of electricity, workmanship quite can, with a period of time and then continue to look at it, anyway, the price is not	Jingdong prefecture level city in Shandong
IXCHSQ Flagship Store	King of Power Saving	Used for a period of time, it seems that every day can indeed save two several degrees of electricity, workmanship quite can, with a period of time and then continue to look at it, anyway, the price is not	Jingdong prefecture level city in Shandong
IXCHSQ Flagship Store	King of Power Saving	High-power super saver received immediately used, the price is not expensive quality is very good, save electricity and money, very convenient, conscientious business, must be praised, recommended to everyone!	Jingdong prefecture level city in Shandong
IXCHSQ Flagship Store	King of Power Saving	Home enhancement version of the intelligent power saver, is in use, I think the price is cheap, value for money, efficacy, power, power saving effect is very good.	Jingdong prefecture level city in Shandong
IXCHSQ Flagship Store	King of Power Saving	Bought three at once, one for each room, summer electricity consumption is very high, despite the three air conditioners on the electricity bill is still down a lot, too Nessie!	Jingdong prefecture level city in Shandong
IXCHSQ Flagship Store	King of Power Saving	Without further ado, the electric bill came down, $102 less this month, personal satisfaction, it counts okay, give you a good review	Jingdong prefecture level city in Shandong
IXCHSQ Flagship Store	King of Power Saving	The power saver has arrived, the installation is really convenient, plugged in on the line operation is quite simple, power saving effect is also a strong!	Jingdong prefecture level city in Shandong
IXCHSQ Flagship Store	King of Power Saving	Saver received, good quality, beautiful appearance, fine workmanship, simple installation	Jingdong prefecture level city in Shandong
IXCHSQ Flagship Store	King of Power Saving	Product design style modern, worth buying	Jingdong prefecture level city in Shandong
IXCHSQ Flagship Store	King of Power Saving	Energy saving, good power saving effect, practical, great value	Jingdong prefecture level city in Shandong
IXCHSQ Flagship Store	King of Power Saving	It's good quality, it's good, it's energy efficient, and I'll buy it again.	Jingdong prefecture level city in Shandong
IXCHSQ Flagship Store	King of Power Saving	Installation saves a lot of electricity, fast logistics, real energy-saving appliances	Jingdong prefecture level city in Shandong
IXCHSQ Flagship Store	King of Power Saving	The effect is good and the power saving is obvious after using it for a month and saving tens of dollars, the product is trustworthy, I now buy another 4 colors to prepare for their siblings' family to pay for	Jingdong prefecture level city in Shandong
IXCHSQ Flagship Store	King of Power Saving	The quality is particularly good workmanship is no problem, the arrival of the very fast oh, especially power-saving	Jingdong prefecture level city in Shandong

IXCHSQ Flagship Store	King of Power Saving	The quality is very good! In use, observed for a day, not as used before, so much power consumption.	Jingdong prefecture level city in Shandong
IXCHSQ Flagship Store	King of Power Saving	Goods have been received, the effect is good is not good, power saving is not yet know, wait for me to use it and then evaluate it.	Jingdong prefecture level city in Shandong
IXCHSQ Flagship Store	King of Power Saving	In-kind and the same as described, the quality is quite good, the seller's attitude is also good, ask questions and answer them, the speed of delivery bar, worth buying Oh!	Jingdong prefecture level city in Shandong
IXCHSQ Flagship Store	King of Power Saving	Nice stuff, came to comment after using it, it does save power.	Jingdong prefecture level city in Shandong
IXCHSQ Flagship Store	King of Power Saving	Fast charging, but also save power, the effect is very good, satisfied, praise!	Jingdong prefecture level city in Shandong
IXCHSQ Flagship Store	King of Power Saving	The goods have been received, I do not know how effective, after using the flat price again!	Jingdong prefecture level city in Shandong
IXCHSQ Flagship Store	King of Power Saving	Merchant service is very good Reply quickly The key is the quality and that quality is good	Jingdong prefecture level city in Shandong
IXCHSQ Flagship Store	King of Power Saving	It's working well now, I'll do a follow up review after I actually use it for a while	Jingdong prefecture level city in Shandong
IXCHSQ Flagship Store	King of Power Saving	The quality is good. After installing it, I feel like I've saved a lot of money on my electricity bill.	Jingdong prefecture level city in Shandong
IXCHSQ Flagship Store	King of Power Saving	Make up a review after use.	Jingdong prefecture level city in Shandong
IXCHSQ Flagship Store	King of Power Saving	review after use	Jingdong prefecture level city in Shandong
IXCHSQ Flagship Store	King of Power Saving	It's a small capacitor. It's not energy efficient.	Jingdong prefecture level city in Shandong
IXCHSQ Flagship Store	King of Power Saving	I'll follow up with a review after I've used it and had results.	Jingdong prefecture level city in Shandong
IXCHSQ Flagship Store	King of Power Saving	No power saving? Not good!	Jingdong prefecture level city in Shandong
IXCHSQ Flagship Store	King of Power Saving	Been using the same appliances and running a 1.5 air conditioner at night for what feels like no time, and after half a month of use it feels like the power is only increasing. Doesn't show any power saving	Jingdong prefecture level city in Shandong
IXCHSQ Flagship Store	King of Power Saving	serviceable	Jingdong prefecture level city in Shandong
IXCHSQ Flagship Store	King of Power Saving	It's a piece of junk. It doesn't save any power.	Jingdong prefecture level city in Shandong
IXCHSQ Flagship Store	King of Power Saving	There are a lot of appliances in the house, the summer air conditioning is very costly, I bought this in front of the air conditioning effect is very good, I explain that I bought three in one breath, I use more electricity a month down to 200 or so, very satisfied.	Jingdong prefecture level city in Shandong
IXCHSQ Flagship Store	King of Power Saving	The voltage is very stable has been maintained at 220v this value, high power super power saving, trustworthy, reasonable design, shape bright, logistics is quite fast, business warranty period, excellent service, logistics fast!	Jingdong prefecture level city in Shandong
IXCHSQ Flagship Store	King of Power Saving	Use a month after a look at the savings of more than 40 dollars, there is a display of electricity savings, this high-power intelligent frequency converter economizer, affordable and cheap, logistics is very fast, the service attitude is very good, very satisfied with a shopping.	Jingdong prefecture level city in Shandong
IXCHSQ Flagship Store	King of Power Saving	Used for a period of time, it seems that every day can really can save a few degrees of electricity, workmanship quite can, with a period of time and then continue to look at it, anyway, the price is not	Jingdong prefecture level city in Shandong
IXCHSQ Flagship Store	King of Power Saving	Used for a period of time, it seems that every day can indeed save two several degrees of electricity, workmanship quite can, with a period of time and then continue to look at it, anyway, the price is not	Jingdong prefecture level city in Shandong
IXCHSQ Flagship Store	King of Power Saving	High-power super saver received immediately used, the price is not expensive quality is very good, save electricity and money, very convenient, conscientious business, must be praised, recommended to everyone!	Jingdong prefecture level city in Shandong
IXCHSQ Flagship Store	King of Power Saving	Home enhancement version of the intelligent power saver, is in use, I think the price is cheap, value for money, efficacy, power, power saving effect is very good.	Jingdong prefecture level city in Shandong
IXCHSQ Flagship Store	King of Power Saving	Bought three at once, one for each room, summer electricity consumption is very high, despite the three air conditioners on the electricity bill is still down a lot, too Nessie!	Jingdong prefecture level city in Shandong
IXCHSQ Flagship Store	King of Power Saving	Without further ado, the electric bill came down, $102 less this month, personal satisfaction, it counts okay, give you a good review	Jingdong prefecture level city in Shandong
IXCHSQ Flagship Store	King of Power Saving	The power saver has arrived, the installation is really convenient, plugged in on the line operation is quite simple, power saving effect is also a strong!	Jingdong prefecture level city in Shandong
IXCHSQ Flagship Store	King of Power Saving	Saver received, good quality, beautiful appearance, fine workmanship, simple installation	Jingdong prefecture level city in Shandong
IXCHSQ Flagship Store	King of Power Saving	Product design style modern, worth buying	Jingdong prefecture level city in Shandong
IXCHSQ Flagship Store	King of Power Saving	Energy saving, good power saving effect, practical, great value	Jingdong prefecture level city in Shandong
IXCHSQ Flagship Store	King of Power Saving	It's good quality, it's good, it's energy efficient, and I'll buy it again.	Jingdong prefecture level city in Shandong
IXCHSQ Flagship Store	King of Power Saving	Installation saves a lot of electricity, fast logistics, real energy-saving appliances	Jingdong prefecture level city in Shandong
IXCHSQ Flagship Store	King of Power Saving	The effect is good and the power saving is obvious after using it for a month and saving tens of dollars, the product is trustworthy, I now buy another 4 colors to prepare for their siblings' family to pay for	Jingdong prefecture level city in Shandong
IXCHSQ Flagship Store	King of Power Saving	The quality is particularly good workmanship is no problem, the arrival of the very fast oh, especially power-saving	Jingdong prefecture level city in Shandong
IXCHSQ Flagship Store	King of Power Saving	The quality is very good! In use, observed for a day, not as used before, so much power consumption.	Jingdong prefecture level city in Shandong
IXCHSQ Flagship Store	King of Power Saving	Goods have been received, the effect is good is not good, power saving is not yet know, wait for me to use it and then evaluate it.	Jingdong prefecture level city in Shandong
IXCHSQ Flagship Store	King of Power Saving	In-kind and the same as described, the quality is quite good, the seller's attitude is also good, ask questions and answer them, the speed of delivery bar, worth buying Oh!	Jingdong prefecture level city in Shandong
IXCHSQ Flagship Store	King of Power Saving	Nice stuff, came to comment after using it, it does save power.	Jingdong prefecture level city in Shandong
IXCHSQ Flagship Store	King of Power Saving	Fast charging, but also save power, the effect is very good, satisfied, praise!	Jingdong prefecture level city in Shandong
IXCHSQ Flagship Store	King of Power Saving	The goods have been received, I do not know how effective, after using the flat price again!	Jingdong prefecture level city in Shandong
IXCHSQ Flagship Store	King of Power Saving	Merchant service is very good Reply quickly The key is the quality and that quality is good	Jingdong prefecture level city in Shandong
IXCHSQ Flagship Store	King of Power Saving	It's working well now, I'll do a follow up review after I actually use it for a while	Jingdong prefecture level city in Shandong
IXCHSQ Flagship Store	King of Power Saving	The quality is good. After installing it, I feel like I've saved a lot of money on my electricity bill.	Jingdong prefecture level city in Shandong
IXCHSQ Flagship Store	King of Power Saving	Make up a review after use.	Jingdong prefecture level city in Shandong
IXCHSQ Flagship Store	King of Power Saving	review after use	Jingdong prefecture level city in Shandong
IXCHSQ Flagship Store	King of Power Saving	It's a small capacitor. It's not energy efficient.	Jingdong prefecture level city in Shandong
IXCHSQ Flagship Store	King of Power Saving	I'll follow up with a review after I've used it and had results.	Jingdong prefecture level city in Shandong
IXCHSQ Flagship Store	King of Power Saving	No power saving? Not good!	Jingdong prefecture level city in Shandong
IXCHSQ Flagship Store	King of Power Saving	Been using the same appliances and running a 1.5 air conditioner at night for what feels like no time, and after half a month of use it feels like the power is only increasing. Doesn't show any power saving	Jingdong prefecture level city in Shandong
IXCHSQ Flagship Store	King of Power Saving	serviceable	Jingdong prefecture level city in Shandong

IXCHSQ Flagship Store	King of Power Saving	It's a piece of junk. It doesn't save any power.	Jingdong prefecture level city in Shandong
IXCHSQ Flagship Store	King of Power Saving	There are a lot of appliances in the house, the summer air conditioning is very costly, I bought this in front of the air conditioning effect is very good, I explain that I bought three in one breath, I use more electricity a month down to 200 or so, very satisfied.	Jingdong prefecture level city in Shandong
IXCHSQ Flagship Store	King of Power Saving	The voltage is very stable has been maintained at 220v this value, high power super power saving, trustworthy, reasonable design, shape bright, logistics is quite fast, business warranty period, excellent service, logistics fast!	Jingdong prefecture level city in Shandong
IXCHSQ Flagship Store	King of Power Saving	Use a month after a look at the savings of more than 40 dollars, there is a display of electricity savings, this high-power intelligent frequency converter economizer, affordable and cheap, logistics is very fast, the service attitude is very good, very satisfied with a shopping.	Jingdong prefecture level city in Shandong
IXCHSQ Flagship Store	King of Power Saving	Used for a period of time, it seems that every day can really can save a few degrees of electricity, workmanship quite can, with a period of time and then continue to look at it, anyway, the price is not	Jingdong prefecture level city in Shandong
IXCHSQ Flagship Store	King of Power Saving	Used for a period of time, it seems that every day can indeed save two several degrees of electricity, workmanship quite can, with a period of time and then continue to look at it, anyway, the price is not	Jingdong prefecture level city in Shandong
IXCHSQ Flagship Store	King of Power Saving	High-power super saver received immediately used, the price is not expensive quality is very good, save electricity and money, very convenient, conscientious business, must be praised, recommended to everyone!	Jingdong prefecture level city in Shandong
IXCHSQ Flagship Store	King of Power Saving	Home enhancement version of the intelligent power saver, is in use, I think the price is cheap, value for money, efficacy, power, power saving effect is very good.	Jingdong prefecture level city in Shandong
IXCHSQ Flagship Store	King of Power Saving	Bought three at once, one for each room, summer electricity consumption is very high, despite the three air conditioners on the electricity bill is still down a lot, too Nessie!	Jingdong prefecture level city in Shandong
IXCHSQ Flagship Store	King of Power Saving	Without further ado, the electric bill came down, $102 less this month, personal satisfaction, it counts okay, give you a good review	Jingdong prefecture level city in Shandong
IXCHSQ Flagship Store	King of Power Saving	The power saver has arrived, the installation is really convenient, plugged in on the line operation is quite simple, power saving effect is also a strong!	Jingdong prefecture level city in Shandong
IXCHSQ Flagship Store	King of Power Saving	Saver received, good quality, beautiful appearance, fine workmanship, simple installation	Jingdong prefecture level city in Shandong
IXCHSQ Flagship Store	King of Power Saving	Product design style modern, worth buying	Jingdong prefecture level city in Shandong
IXCHSQ Flagship Store	King of Power Saving	Energy saving, good power saving effect, practical, great value	Jingdong prefecture level city in Shandong
IXCHSQ Flagship Store	King of Power Saving	It's good quality, it's good, it's energy efficient, and I'll buy it again.	Jingdong prefecture level city in Shandong
IXCHSQ Flagship Store	King of Power Saving	Installation saves a lot of electricity, fast logistics, real energy-saving appliances	Jingdong prefecture level city in Shandong
IXCHSQ Flagship Store	King of Power Saving	The effect is good and the power saving is obvious after using it for a month and saving tens of dollars, the product is trustworthy, I now buy another 4 colors to prepare for their siblings' family to pay for	Jingdong prefecture level city in Shandong
IXCHSQ Flagship Store	King of Power Saving	The quality is particularly good workmanship is no problem, the arrival of the very fast oh, especially power-saving	Jingdong prefecture level city in Shandong
IXCHSQ Flagship Store	King of Power Saving	The quality is very good! In use, observed for a day, not as used before, so much power consumption.	Jingdong prefecture level city in Shandong
IXCHSQ Flagship Store	King of Power Saving	Goods have been received, the effect is good is not good, power saving is not yet know, wait for me to use it and then evaluate it.	Jingdong prefecture level city in Shandong
IXCHSQ Flagship Store	King of Power Saving	In-kind and the same as described, the quality is quite good, the seller's attitude is also good, ask questions and answer them, the speed of delivery bar, worth buying Oh!	Jingdong prefecture level city in Shandong
IXCHSQ Flagship Store	King of Power Saving	Nice stuff, came to comment after using it, it does save power.	Jingdong prefecture level city in Shandong
IXCHSQ Flagship Store	King of Power Saving	Fast charging, but also save power, the effect is very good, satisfied, praise!	Jingdong prefecture level city in Shandong
IXCHSQ Flagship Store	King of Power Saving	The goods have been received, I do not know how effective, after using the flat price again!	Jingdong prefecture level city in Shandong
IXCHSQ Flagship Store	King of Power Saving	Merchant service is very good Reply quickly The key is the quality and that quality is good	Jingdong prefecture level city in Shandong
IXCHSQ Flagship Store	King of Power Saving	It's working well now, I'll do a follow up review after I actually use it for a while	Jingdong prefecture level city in Shandong
IXCHSQ Flagship Store	King of Power Saving	The quality is good. After installing it, I feel like I've saved a lot of money on my electricity bill.	Jingdong prefecture level city in Shandong
IXCHSQ Flagship Store	King of Power Saving	Make up a review after use.	Jingdong prefecture level city in Shandong
IXCHSQ Flagship Store	King of Power Saving	review after use	Jingdong prefecture level city in Shandong
IXCHSQ Flagship Store	King of Power Saving	It's a small capacitor. It's not energy efficient.	Jingdong prefecture level city in Shandong
IXCHSQ Flagship Store	King of Power Saving	I'll follow up with a review after I've used it and had results.	Jingdong prefecture level city in Shandong
IXCHSQ Flagship Store	King of Power Saving	No power saving? Not good!	Jingdong prefecture level city in Shandong
IXCHSQ Flagship Store	King of Power Saving	Been using the same appliances and running a 1.5 air conditioner at night for what feels like no time, and after half a month of use it feels like the power is only increasing. Doesn't show any power saving	Jingdong prefecture level city in Shandong
IXCHSQ Flagship Store	King of Power Saving	serviceable	Jingdong prefecture level city in Shandong
IXCHSQ Flagship Store	King of Power Saving	It's a piece of junk. It doesn't save any power.	Jingdong prefecture level city in Shandong
IXCHSQ Flagship Store	King of Power Saving	There are a lot of appliances in the house, the summer air conditioning is very costly, I bought this in front of the air conditioning effect is very good, I explain that I bought three in one breath, I use more electricity a month down to 200 or so, very satisfied.	Jingdong prefecture level city in Shandong
IXCHSQ Flagship Store	King of Power Saving	The voltage is very stable has been maintained at 220v this value, high power super power saving, trustworthy, reasonable design, shape bright, logistics is quite fast, business warranty period, excellent service, logistics fast!	Jingdong prefecture level city in Shandong
IXCHSQ Flagship Store	King of Power Saving	Use a month after a look at the savings of more than 40 dollars, there is a display of electricity savings, this high-power intelligent frequency converter economizer, affordable and cheap, logistics is very fast, the service attitude is very good, very satisfied with a shopping.	Jingdong prefecture level city in Shandong
IXCHSQ Flagship Store	King of Power Saving	Used for a period of time, it seems that every day can really can save a few degrees of electricity, workmanship quite can, with a period of time and then continue to look at it, anyway, the price is not	Jingdong prefecture level city in Shandong
IXCHSQ Flagship Store	King of Power Saving	Used for a period of time, it seems that every day can indeed save two several degrees of electricity, workmanship quite can, with a period of time and then continue to look at it, anyway, the price is not	Jingdong prefecture level city in Shandong
IXCHSQ Flagship Store	King of Power Saving	High-power super saver received immediately used, the price is not expensive quality is very good, save electricity and money, very convenient, conscientious business, must be praised, recommended to everyone!	Jingdong prefecture level city in Shandong
IXCHSQ Flagship Store	King of Power Saving	Home enhancement version of the intelligent power saver, is in use, I think the price is cheap, value for money, efficacy, power, power saving effect is very good.	Jingdong prefecture level city in Shandong
IXCHSQ Flagship Store	King of Power Saving	Bought three at once, one for each room, summer electricity consumption is very high, despite the three air conditioners on the electricity bill is still down a lot, too Nessie!	Jingdong prefecture level city in Shandong
IXCHSQ Flagship Store	King of Power Saving	Without further ado, the electric bill came down, $102 less this month, personal satisfaction, it counts okay, give you a good review	Jingdong prefecture level city in Shandong
IXCHSQ Flagship Store	King of Power Saving	The power saver has arrived, the installation is really convenient, plugged in on the line operation is quite simple, power saving effect is also a strong!	Jingdong prefecture level city in Shandong
IXCHSQ Flagship Store	King of Power Saving	Saver received, good quality, beautiful appearance, fine workmanship, simple installation	Jingdong prefecture level city in Shandong
IXCHSQ Flagship Store	King of Power Saving	Product design style modern, worth buying	Jingdong prefecture level city in Shandong
IXCHSQ Flagship Store	King of Power Saving	Energy saving, good power saving effect, practical, great value	Jingdong prefecture level city in Shandong

IXCHSQ Flagship Store	King of Power Saving	It's good quality, it's good, it's energy efficient, and I'll buy it again.	Jingdong prefecture level city in Shandong
IXCHSQ Flagship Store	King of Power Saving	Installation saves a lot of electricity, fast logistics, real energy-saving appliances	Jingdong prefecture level city in Shandong
IXCHSQ Flagship Store	King of Power Saving	The effect is good and the power saving is obvious after using it for a month and saving tens of dollars, the product is trustworthy, I now buy another 4 colors to prepare for their siblings' family to pay for	Jingdong prefecture level city in Shandong
IXCHSQ Flagship Store	King of Power Saving	The quality is particularly good workmanship is no problem, the arrival of the very fast oh, especially power-saving	Jingdong prefecture level city in Shandong
IXCHSQ Flagship Store	King of Power Saving	The quality is very good! In use, observed for a day, not as used before, so much power consumption.	Jingdong prefecture level city in Shandong
IXCHSQ Flagship Store	King of Power Saving	Goods have been received, the effect is good is not good, power saving is not yet know, wait for me to use it and then evaluate it.	Jingdong prefecture level city in Shandong
IXCHSQ Flagship Store	King of Power Saving	In-kind and the same as described, the quality is quite good, the seller's attitude is also good, ask questions and answer them, the speed of delivery bar, worth buying Oh!	Jingdong prefecture level city in Shandong
IXCHSQ Flagship Store	King of Power Saving	Nice stuff, came to comment after using it, it does save power.	Jingdong prefecture level city in Shandong
IXCHSQ Flagship Store	King of Power Saving	Fast charging, but also save power, the effect is very good, satisfied, praise!	Jingdong prefecture level city in Shandong
IXCHSQ Flagship Store	King of Power Saving	The goods have been received, I do not know how effective, after using the flat price again!	Jingdong prefecture level city in Shandong
IXCHSQ Flagship Store	King of Power Saving	Merchant service is very good Reply quickly The key is the quality and that quality is good	Jingdong prefecture level city in Shandong
IXCHSQ Flagship Store	King of Power Saving	It's working well now, I'll do a follow up review after I actually use it for a while	Jingdong prefecture level city in Shandong
IXCHSQ Flagship Store	King of Power Saving	The quality is good. After installing it, I feel like I've saved a lot of money on my electricity bill.	Jingdong prefecture level city in Shandong
IXCHSQ Flagship Store	King of Power Saving	Make up a review after use.	Jingdong prefecture level city in Shandong
IXCHSQ Flagship Store	King of Power Saving	review after use	Jingdong prefecture level city in Shandong
IXCHSQ Flagship Store	King of Power Saving	It's a small capacitor. It's not energy efficient.	Jingdong prefecture level city in Shandong
IXCHSQ Flagship Store	King of Power Saving	I'll follow up with a review after I've used it and had results.	Jingdong prefecture level city in Shandong
IXCHSQ Flagship Store	King of Power Saving	No power saving? Not good!	Jingdong prefecture level city in Shandong
IXCHSQ Flagship Store	King of Power Saving	Been using the same appliances and running a 1.5 air conditioner at night for what feels like no time, and after half a month of use it feels like the power is only increasing. Doesn't show any power saving	Jingdong prefecture level city in Shandong
IXCHSQ Flagship Store	King of Power Saving	serviceable	Jingdong prefecture level city in Shandong
IXCHSQ Flagship Store	King of Power Saving	It's a piece of junk. It doesn't save any power.	Jingdong prefecture level city in Shandong
IXCHSQ Flagship Store	King of Power Saving	There are a lot of appliances in the house, the summer air conditioning is very costly, I bought this in front of the air conditioning effect is very good, I explain that I bought three in one breath, I use more electricity a month down to 200 or so, very satisfied.	Jingdong prefecture level city in Shandong
IXCHSQ Flagship Store	King of Power Saving	The voltage is very stable has been maintained at 220v this value, high power super power saving, trustworthy, reasonable design, shape bright, logistics is quite fast, business warranty period, excellent service, logistics fast!	Jingdong prefecture level city in Shandong
IXCHSQ Flagship Store	King of Power Saving	Use a month after a look at the savings of more than 40 dollars, there is a display of electricity savings, this high-power intelligent frequency converter economizer, affordable and cheap, logistics is very fast, the service attitude is very good, very satisfied with a shopping.	Jingdong prefecture level city in Shandong
IXCHSQ Flagship Store	King of Power Saving	Used for a period of time, it seems that every day can really can save a few degrees of electricity, workmanship quite can, with a period of time and then continue to look at it, anyway, the price is not	Jingdong prefecture level city in Shandong
IXCHSQ Flagship Store	King of Power Saving	Used for a period of time, it seems that every day can indeed save two several degrees of electricity, workmanship quite can, with a period of time and then continue to look at it, anyway, the price is not	Jingdong prefecture level city in Shandong
IXCHSQ Flagship Store	King of Power Saving	High-power super saver received immediately used, the price is not expensive quality is very good, save electricity and money, very convenient, conscientious business, must be praised, recommended to everyone!	Jingdong prefecture level city in Shandong
IXCHSQ Flagship Store	King of Power Saving	Home enhancement version of the intelligent power saver, is in use, I think the price is cheap, value for money, efficacy, power, power saving effect is very good.	Jingdong prefecture level city in Shandong
IXCHSQ Flagship Store	King of Power Saving	Bought three at once, one for each room, summer electricity consumption is very high, despite the three air conditioners on the electricity bill is still down a lot, too Nessie!	Jingdong prefecture level city in Shandong
IXCHSQ Flagship Store	King of Power Saving	Without further ado, the electric bill came down, $102 less this month, personal satisfaction, it counts okay, give you a good review	Jingdong prefecture level city in Shandong
IXCHSQ Flagship Store	King of Power Saving	The power saver has arrived, the installation is really convenient, plugged in on the line operation is quite simple, power saving effect is also a strong!	Jingdong prefecture level city in Shandong
IXCHSQ Flagship Store	King of Power Saving	Saver received, good quality, beautiful appearance, fine workmanship, simple installation	Jingdong prefecture level city in Shandong
IXCHSQ Flagship Store	King of Power Saving	Product design style modern, worth buying	Jingdong prefecture level city in Shandong
IXCHSQ Flagship Store	King of Power Saving	Energy saving, good power saving effect, practical, great value	Jingdong prefecture level city in Shandong
IXCHSQ Flagship Store	King of Power Saving	It's good quality, it's good, it's energy efficient, and I'll buy it again.	Jingdong prefecture level city in Shandong
IXCHSQ Flagship Store	King of Power Saving	Installation saves a lot of electricity, fast logistics, real energy-saving appliances	Jingdong prefecture level city in Shandong
IXCHSQ Flagship Store	King of Power Saving	The effect is good and the power saving is obvious after using it for a month and saving tens of dollars, the product is trustworthy, I now buy another 4 colors to prepare for their siblings' family to pay for	Jingdong prefecture level city in Shandong
IXCHSQ Flagship Store	King of Power Saving	The quality is particularly good workmanship is no problem, the arrival of the very fast oh, especially power-saving	Jingdong prefecture level city in Shandong
IXCHSQ Flagship Store	King of Power Saving	The quality is very good! In use, observed for a day, not as used before, so much power consumption.	Jingdong prefecture level city in Shandong
IXCHSQ Flagship Store	King of Power Saving	Goods have been received, the effect is good is not good, power saving is not yet know, wait for me to use it and then evaluate it.	Jingdong prefecture level city in Shandong
IXCHSQ Flagship Store	King of Power Saving	In-kind and the same as described, the quality is quite good, the seller's attitude is also good, ask questions and answer them, the speed of delivery bar, worth buying Oh!	Jingdong prefecture level city in Shandong
IXCHSQ Flagship Store	King of Power Saving	Nice stuff, came to comment after using it, it does save power.	Jingdong prefecture level city in Shandong
IXCHSQ Flagship Store	King of Power Saving	Fast charging, but also save power, the effect is very good, satisfied, praise!	Jingdong prefecture level city in Shandong
IXCHSQ Flagship Store	King of Power Saving	The goods have been received, I do not know how effective, after using the flat price again!	Jingdong prefecture level city in Shandong
IXCHSQ Flagship Store	King of Power Saving	Merchant service is very good Reply quickly The key is the quality and that quality is good	Jingdong prefecture level city in Shandong
IXCHSQ Flagship Store	King of Power Saving	It's working well now, I'll do a follow up review after I actually use it for a while	Jingdong prefecture level city in Shandong
IXCHSQ Flagship Store	King of Power Saving	The quality is good. After installing it, I feel like I've saved a lot of money on my electricity bill.	Jingdong prefecture level city in Shandong
IXCHSQ Flagship Store	King of Power Saving	Make up a review after use.	Jingdong prefecture level city in Shandong
IXCHSQ Flagship Store	King of Power Saving	review after use	Jingdong prefecture level city in Shandong
IXCHSQ Flagship Store	King of Power Saving	It's a small capacitor. It's not energy efficient.	Jingdong prefecture level city in Shandong

IXCHSQ Flagship Store	King of Power Saving	I'll follow up with a review after I've used it and had results.	Jingdong prefecture level city in Shandong
IXCHSQ Flagship Store	King of Power Saving	No power saving? Not good!	Jingdong prefecture level city in Shandong
IXCHSQ Flagship Store	King of Power Saving	Been using the same appliances and running a 1.5 air conditioner at night for what feels like no time, and after half a month of use it feels like the power is only increasing. Doesn't show any power saving	Jingdong prefecture level city in Shandong
IXCHSQ Flagship Store	King of Power Saving	serviceable	Jingdong prefecture level city in Shandong
IXCHSQ Flagship Store	King of Power Saving	It's a piece of junk. It doesn't save any power.	Jingdong prefecture level city in Shandong
IXCHSQ Flagship Store	King of Power Saving	There are a lot of appliances in the house, the summer air conditioning is very costly, I bought this in front of the air conditioning effect is very good, I explain that I bought three in one breath, I use more electricity a month down to 200 or so, very satisfied.	Jingdong prefecture level city in Shandong
IXCHSQ Flagship Store	King of Power Saving	The voltage is very stable has been maintained at 220v this value, high power super power saving, trustworthy, reasonable design, shape bright, logistics is quite fast, business warranty period, excellent service, logistics fast!	Jingdong prefecture level city in Shandong
IXCHSQ Flagship Store	King of Power Saving	Use a month after a look at the savings of more than 40 dollars, there is a display of electricity savings, this high-power intelligent frequency converter economizer, affordable and cheap, logistics is very fast, the service attitude is very good, very satisfied with a shopping.	Jingdong prefecture level city in Shandong
IXCHSQ Flagship Store	King of Power Saving	Used for a period of time, it seems that every day can really can save a few degrees of electricity, workmanship quite can, with a period of time and then continue to look at it, anyway, the price is not	Jingdong prefecture level city in Shandong
IXCHSQ Flagship Store	King of Power Saving	Used for a period of time, it seems that every day can indeed save two several degrees of electricity, workmanship quite can, with a period of time and then continue to look at it, anyway, the price is not	Jingdong prefecture level city in Shandong
IXCHSQ Flagship Store	King of Power Saving	High-power super saver received immediately used, the price is not expensive quality is very good, save electricity and money, very convenient, conscientious business, must be praised, recommended to everyone!	Jingdong prefecture level city in Shandong
IXCHSQ Flagship Store	King of Power Saving	Home enhancement version of the intelligent power saver, is in use, I think the price is cheap, value for money, efficacy, power, power saving effect is very good.	Jingdong prefecture level city in Shandong
IXCHSQ Flagship Store	King of Power Saving	Bought three at once, one for each room, summer electricity consumption is very high, despite the three air conditioners on the electricity bill is still down a lot, too Nessie!	Jingdong prefecture level city in Shandong
IXCHSQ Flagship Store	King of Power Saving	Without further ado, the electric bill came down, $102 less this month, personal satisfaction, it counts okay, give you a good review	Jingdong prefecture level city in Shandong
IXCHSQ Flagship Store	King of Power Saving	The power saver has arrived, the installation is really convenient, plugged in on the line operation is quite simple, power saving effect is also a strong!	Jingdong prefecture level city in Shandong
IXCHSQ Flagship Store	King of Power Saving	Saver received, good quality, beautiful appearance, fine workmanship, simple installation	Jingdong prefecture level city in Shandong
IXCHSQ Flagship Store	King of Power Saving	Product design style modern, worth buying	Jingdong prefecture level city in Shandong
IXCHSQ Flagship Store	King of Power Saving	Energy saving, good power saving effect, practical, great value	Jingdong prefecture level city in Shandong
IXCHSQ Flagship Store	King of Power Saving	It's good quality, it's good, it's energy efficient, and I'll buy it again.	Jingdong prefecture level city in Shandong
IXCHSQ Flagship Store	King of Power Saving	Installation saves a lot of electricity, fast logistics, real energy-saving appliances	Jingdong prefecture level city in Shandong
IXCHSQ Flagship Store	King of Power Saving	The effect is good and the power saving is obvious after using it for a month and saving tens of dollars, the product is trustworthy, I now buy another 4 colors to prepare for their siblings' family to pay for	Jingdong prefecture level city in Shandong
IXCHSQ Flagship Store	King of Power Saving	The quality is particularly good workmanship is no problem, the arrival of the very fast oh, especially power-saving	Jingdong prefecture level city in Shandong
IXCHSQ Flagship Store	King of Power Saving	The quality is very good! In use, observed for a day, not as used before, so much power consumption.	Jingdong prefecture level city in Shandong
IXCHSQ Flagship Store	King of Power Saving	Goods have been received, the effect is good is not good, power saving is not yet know, wait for me to use it and then evaluate it.	Jingdong prefecture level city in Shandong
IXCHSQ Flagship Store	King of Power Saving	In-kind and the same as described, the quality is quite good, the seller's attitude is also good, ask questions and answer them, the speed of delivery bar, worth buying Oh!	Jingdong prefecture level city in Shandong
IXCHSQ Flagship Store	King of Power Saving	Nice stuff, came to comment after using it, it does save power.	Jingdong prefecture level city in Shandong
IXCHSQ Flagship Store	King of Power Saving	Fast charging, but also save power, the effect is very good, satisfied, praise!	Jingdong prefecture level city in Shandong
IXCHSQ Flagship Store	King of Power Saving	The goods have been received, I do not know how effective, after using the flat price again!	Jingdong prefecture level city in Shandong
IXCHSQ Flagship Store	King of Power Saving	Merchant service is very good Reply quickly The key is the quality and that quality is good	Jingdong prefecture level city in Shandong
IXCHSQ Flagship Store	King of Power Saving	It's working well now, I'll do a follow up review after I actually use it for a while	Jingdong prefecture level city in Shandong
IXCHSQ Flagship Store	King of Power Saving	The quality is good. After installing it, I feel like I've saved a lot of money on my electricity bill.	Jingdong prefecture level city in Shandong
IXCHSQ Flagship Store	King of Power Saving	Make up a review after use.	Jingdong prefecture level city in Shandong
IXCHSQ Flagship Store	King of Power Saving	review after use	Jingdong prefecture level city in Shandong
IXCHSQ Flagship Store	King of Power Saving	It's a small capacitor. It's not energy efficient.	Jingdong prefecture level city in Shandong
IXCHSQ Flagship Store	King of Power Saving	I'll follow up with a review after I've used it and had results.	Jingdong prefecture level city in Shandong
IXCHSQ Flagship Store	King of Power Saving	No power saving? Not good!	Jingdong prefecture level city in Shandong
IXCHSQ Flagship Store	King of Power Saving	Been using the same appliances and running a 1.5 air conditioner at night for what feels like no time, and after half a month of use it feels like the power is only increasing. Doesn't show any power saving	Jingdong prefecture level city in Shandong
IXCHSQ Flagship Store	King of Power Saving	serviceable	Jingdong prefecture level city in Shandong
IXCHSQ Flagship Store	King of Power Saving	It's a piece of junk. It doesn't save any power.	Jingdong prefecture level city in Shandong
IXCHSQ Flagship Store	King of Power Saving	There are a lot of appliances in the house, the summer air conditioning is very costly, I bought this in front of the air conditioning effect is very good, I explain that I bought three in one breath, I use more electricity a month down to 200 or so, very satisfied.	Jingdong prefecture level city in Shandong
IXCHSQ Flagship Store	King of Power Saving	The voltage is very stable has been maintained at 220v this value, high power super power saving, trustworthy, reasonable design, shape bright, logistics is quite fast, business warranty period, excellent service, logistics fast!	Jingdong prefecture level city in Shandong
IXCHSQ Flagship Store	King of Power Saving	Use a month after a look at the savings of more than 40 dollars, there is a display of electricity savings, this high-power intelligent frequency converter economizer, affordable and cheap, logistics is very fast, the service attitude is very good, very satisfied with a shopping.	Jingdong prefecture level city in Shandong
IXCHSQ Flagship Store	King of Power Saving	Used for a period of time, it seems that every day can really can save a few degrees of electricity, workmanship quite can, with a period of time and then continue to look at it, anyway, the price is not	Jingdong prefecture level city in Shandong
IXCHSQ Flagship Store	King of Power Saving	Used for a period of time, it seems that every day can indeed save two several degrees of electricity, workmanship quite can, with a period of time and then continue to look at it, anyway, the price is not	Jingdong prefecture level city in Shandong
IXCHSQ Flagship Store	King of Power Saving	High-power super saver received immediately used, the price is not expensive quality is very good, save electricity and money, very convenient, conscientious business, must be praised, recommended to everyone!	Jingdong prefecture level city in Shandong
IXCHSQ Flagship Store	King of Power Saving	Home enhancement version of the intelligent power saver, is in use, I think the price is cheap, value for money, efficacy, power, power saving effect is very good.	Jingdong prefecture level city in Shandong
IXCHSQ Flagship Store	King of Power Saving	Bought three at once, one for each room, summer electricity consumption is very high, despite the three air conditioners on the electricity bill is still down a lot, too Nessie!	Jingdong prefecture level city in Shandong
IXCHSQ Flagship Store	King of Power Saving	Without further ado, the electric bill came down, $102 less this month, personal satisfaction, it counts okay, give you a good review	Jingdong prefecture level city in Shandong

IXCHSQ Flagship Store	King of Power Saving	The power saver has arrived, the installation is really convenient, plugged in on the line operation is quite simple, power saving effect is also a strong!	Jingdong prefecture level city in Shandong
IXCHSQ Flagship Store	King of Power Saving	Saver received, good quality, beautiful appearance, fine workmanship, simple installation	Jingdong prefecture level city in Shandong
IXCHSQ Flagship Store	King of Power Saving	Product design style modern, worth buying	Jingdong prefecture level city in Shandong
IXCHSQ Flagship Store	King of Power Saving	Energy saving, good power saving effect, practical, great value	Jingdong prefecture level city in Shandong
IXCHSQ Flagship Store	King of Power Saving	It's good quality, it's good, it's energy efficient, and I'll buy it again.	Jingdong prefecture level city in Shandong
IXCHSQ Flagship Store	King of Power Saving	Installation saves a lot of electricity, fast logistics, real energy-saving appliances	Jingdong prefecture level city in Shandong
IXCHSQ Flagship Store	King of Power Saving	The effect is good and the power saving is obvious after using it for a month and saving tens of dollars, the product is trustworthy, I now buy another 4 colors to prepare for their siblings' family to pay for	Jingdong prefecture level city in Shandong
IXCHSQ Flagship Store	King of Power Saving	The quality is particularly good workmanship is no problem, the arrival of the very fast oh, especially power-saving	Jingdong prefecture level city in Shandong
IXCHSQ Flagship Store	King of Power Saving	The quality is very good! In use, observed for a day, not as used before, so much power consumption.	Jingdong prefecture level city in Shandong
IXCHSQ Flagship Store	King of Power Saving	Goods have been received, the effect is good is not good, power saving is not yet know, wait for me to use it and then evaluate it.	Jingdong prefecture level city in Shandong
IXCHSQ Flagship Store	King of Power Saving	In-kind and the same as described, the quality is quite good, the seller's attitude is also good, ask questions and answer them, the speed of delivery bar, worth buying Oh!	Jingdong prefecture level city in Shandong
IXCHSQ Flagship Store	King of Power Saving	Nice stuff, came to comment after using it, it does save power.	Jingdong prefecture level city in Shandong
IXCHSQ Flagship Store	King of Power Saving	Fast charging, but also save power, the effect is very good, satisfied, praise!	Jingdong prefecture level city in Shandong
IXCHSQ Flagship Store	King of Power Saving	The goods have been received, I do not know how effective, after using the flat price again!	Jingdong prefecture level city in Shandong
IXCHSQ Flagship Store	King of Power Saving	Merchant service is very good Reply quickly The key is the quality and that quality is good	Jingdong prefecture level city in Shandong
IXCHSQ Flagship Store	King of Power Saving	It's working well now, I'll do a follow up review after I actually use it for a while	Jingdong prefecture level city in Shandong
IXCHSQ Flagship Store	King of Power Saving	The quality is good. After installing it, I feel like I've saved a lot of money on my electricity bill.	Jingdong prefecture level city in Shandong
IXCHSQ Flagship Store	King of Power Saving	Make up a review after use.	Jingdong prefecture level city in Shandong
IXCHSQ Flagship Store	King of Power Saving	review after use	Jingdong prefecture level city in Shandong
IXCHSQ Flagship Store	King of Power Saving	It's a small capacitor. It's not energy efficient.	Jingdong prefecture level city in Shandong
IXCHSQ Flagship Store	King of Power Saving	I'll follow up with a review after I've used it and had results.	Jingdong prefecture level city in Shandong
IXCHSQ Flagship Store	King of Power Saving	No power saving? Not good!	Jingdong prefecture level city in Shandong
IXCHSQ Flagship Store	King of Power Saving	Been using the same appliances and running a 1.5 air conditioner at night for what feels like no time, and after half a month of use it feels like the power is only increasing. Doesn't show any power saving	Jingdong prefecture level city in Shandong
IXCHSQ Flagship Store	King of Power Saving	serviceable	Jingdong prefecture level city in Shandong
IXCHSQ Flagship Store	King of Power Saving	It's a piece of junk. It doesn't save any power.	Jingdong prefecture level city in Shandong
IXCHSQ Flagship Store	King of Power Saving	There are a lot of appliances in the house, the summer air conditioning is very costly, I bought this in front of the air conditioning effect is very good, I explain that I bought three in one breath, I use more electricity a month down to 200 or so, very satisfied.	Jingdong prefecture level city in Shandong
IXCHSQ Flagship Store	King of Power Saving	The voltage is very stable has been maintained at 220v this value, high power super power saving, trustworthy, reasonable design, shape bright, logistics is quite fast, business warranty period, excellent service, logistics fast!	Jingdong prefecture level city in Shandong
IXCHSQ Flagship Store	King of Power Saving	Use a month after a look at the savings of more than 40 dollars, there is a display of electricity savings, this high-power intelligent frequency converter economizer, affordable and cheap, logistics is very fast, the service attitude is very good, very satisfied with a shopping.	Jingdong prefecture level city in Shandong
IXCHSQ Flagship Store	King of Power Saving	Used for a period of time, it seems that every day can really can save a few degrees of electricity, workmanship quite can, with a period of time and then continue to look at it, anyway, the price is not	Jingdong prefecture level city in Shandong
IXCHSQ Flagship Store	King of Power Saving	Used for a period of time, it seems that every day can indeed save two several degrees of electricity, workmanship quite can, with a period of time and then continue to look at it, anyway, the price is not	Jingdong prefecture level city in Shandong
IXCHSQ Flagship Store	King of Power Saving	High-power super saver received immediately used, the price is not expensive quality is very good, save electricity and money, very convenient, conscientious business, must be praised, recommended to everyone!	Jingdong prefecture level city in Shandong
IXCHSQ Flagship Store	King of Power Saving	Home enhancement version of the intelligent power saver, is in use, I think the price is cheap, value for money, efficacy, power, power saving effect is very good.	Jingdong prefecture level city in Shandong
IXCHSQ Flagship Store	King of Power Saving	Bought three at once, one for each room, summer electricity consumption is very high, despite the three air conditioners on the electricity bill is still down a lot, too Nessie!	Jingdong prefecture level city in Shandong
IXCHSQ Flagship Store	King of Power Saving	Without further ado, the electric bill came down, $102 less this month, personal satisfaction, it counts okay, give you a good review	Jingdong prefecture level city in Shandong
IXCHSQ Flagship Store	King of Power Saving	The power saver has arrived, the installation is really convenient, plugged in on the line operation is quite simple, power saving effect is also a strong!	Jingdong prefecture level city in Shandong
IXCHSQ Flagship Store	King of Power Saving	Saver received, good quality, beautiful appearance, fine workmanship, simple installation	Jingdong prefecture level city in Shandong
IXCHSQ Flagship Store	King of Power Saving	Product design style modern, worth buying	Jingdong prefecture level city in Shandong
IXCHSQ Flagship Store	King of Power Saving	Energy saving, good power saving effect, practical, great value	Jingdong prefecture level city in Shandong
IXCHSQ Flagship Store	King of Power Saving	It's good quality, it's good, it's energy efficient, and I'll buy it again.	Jingdong prefecture level city in Shandong
IXCHSQ Flagship Store	King of Power Saving	Installation saves a lot of electricity, fast logistics, real energy-saving appliances	Jingdong prefecture level city in Shandong
IXCHSQ Flagship Store	King of Power Saving	The effect is good and the power saving is obvious after using it for a month and saving tens of dollars, the product is trustworthy, I now buy another 4 colors to prepare for their siblings' family to pay for	Jingdong prefecture level city in Shandong
IXCHSQ Flagship Store	King of Power Saving	The quality is particularly good workmanship is no problem, the arrival of the very fast oh, especially power-saving	Jingdong prefecture level city in Shandong
IXCHSQ Flagship Store	King of Power Saving	The quality is very good! In use, observed for a day, not as used before, so much power consumption.	Jingdong prefecture level city in Shandong
IXCHSQ Flagship Store	King of Power Saving	Goods have been received, the effect is good is not good, power saving is not yet know, wait for me to use it and then evaluate it.	Jingdong prefecture level city in Shandong
IXCHSQ Flagship Store	King of Power Saving	In-kind and the same as described, the quality is quite good, the seller's attitude is also good, ask questions and answer them, the speed of delivery bar, worth buying Oh!	Jingdong prefecture level city in Shandong
IXCHSQ Flagship Store	King of Power Saving	Nice stuff, came to comment after using it, it does save power.	Jingdong prefecture level city in Shandong
IXCHSQ Flagship Store	King of Power Saving	Fast charging, but also save power, the effect is very good, satisfied, praise!	Jingdong prefecture level city in Shandong
IXCHSQ Flagship Store	King of Power Saving	The goods have been received, I do not know how effective, after using the flat price again!	Jingdong prefecture level city in Shandong
IXCHSQ Flagship Store	King of Power Saving	Merchant service is very good Reply quickly The key is the quality and that quality is good	Jingdong prefecture level city in Shandong
IXCHSQ Flagship Store	King of Power Saving	It's working well now, I'll do a follow up review after I actually use it for a while	Jingdong prefecture level city in Shandong

IXCHSQ Flagship Store	King of Power Saving	The quality is good. After installing it, I feel like I've saved a lot of money on my electricity bill.	Jingdong prefecture level city in Shandong
IXCHSQ Flagship Store	King of Power Saving	Make up a review after use.	Jingdong prefecture level city in Shandong
IXCHSQ Flagship Store	King of Power Saving	review after use	Jingdong prefecture level city in Shandong
IXCHSQ Flagship Store	King of Power Saving	It's a small capacitor. It's not energy efficient.	Jingdong prefecture level city in Shandong
IXCHSQ Flagship Store	King of Power Saving	I'll follow up with a review after I've used it and had results.	Jingdong prefecture level city in Shandong
IXCHSQ Flagship Store	King of Power Saving	No power saving? Not good!	Jingdong prefecture level city in Shandong
IXCHSQ Flagship Store	King of Power Saving	Been using the same appliances and running a 1.5 air conditioner at night for what feels like no time, and after half a month of use it feels like the power is only increasing. Doesn't show any power saving	Jingdong prefecture level city in Shandong
IXCHSQ Flagship Store	King of Power Saving	serviceable	Jingdong prefecture level city in Shandong
IXCHSQ Flagship Store	King of Power Saving	It's a piece of junk. It doesn't save any power.	Jingdong prefecture level city in Shandong
IXCHSQ Flagship Store	King of Power Saving	There are a lot of appliances in the house, the summer air conditioning is very costly, I bought this in front of the air conditioning effect is very good, I explain that I bought three in one breath, I use more electricity a month down to 200 or so, very satisfied.	Jingdong prefecture level city in Shandong
IXCHSQ Flagship Store	King of Power Saving	The voltage is very stable has been maintained at 220v this value, high power super power saving, trustworthy, reasonable design, shape bright, logistics is quite fast, business warranty period, excellent service, logistics fast!	Jingdong prefecture level city in Shandong
IXCHSQ Flagship Store	King of Power Saving	Use a month after a look at the savings of more than 40 dollars, there is a display of electricity savings, this high-power intelligent frequency converter economizer, affordable and cheap, logistics is very fast, the service attitude is very good, very satisfied with a shopping.	Jingdong prefecture level city in Shandong
IXCHSQ Flagship Store	King of Power Saving	Used for a period of time, it seems that every day can really can save a few degrees of electricity, workmanship quite can, with a period of time and then continue to look at it, anyway, the price is not	Jingdong prefecture level city in Shandong
IXCHSQ Flagship Store	King of Power Saving	Used for a period of time, it seems that every day can indeed save two several degrees of electricity, workmanship quite can, with a period of time and then continue to look at it, anyway, the price is not	Jingdong prefecture level city in Shandong
IXCHSQ Flagship Store	King of Power Saving	High-power super saver received immediately used, the price is not expensive quality is very good, save electricity and money, very convenient, conscientious business, must be praised, recommended to everyone!	Jingdong prefecture level city in Shandong
IXCHSQ Flagship Store	King of Power Saving	Home enhancement version of the intelligent power saver, is in use, I think the price is cheap, value for money, efficacy, power, power saving effect is very good.	Jingdong prefecture level city in Shandong
IXCHSQ Flagship Store	King of Power Saving	Bought three at once, one for each room, summer electricity consumption is very high, despite the three air conditioners on the electricity bill is still down a lot, too Nessie!	Jingdong prefecture level city in Shandong
IXCHSQ Flagship Store	King of Power Saving	Without further ado, the electric bill came down, $102 less this month, personal satisfaction, it counts okay, give you a good review	Jingdong prefecture level city in Shandong
IXCHSQ Flagship Store	King of Power Saving	The power saver has arrived, the installation is really convenient, plugged in on the line operation is quite simple, power saving effect is also a strong!	Jingdong prefecture level city in Shandong
IXCHSQ Flagship Store	King of Power Saving	Saver received, good quality, beautiful appearance, fine workmanship, simple installation	Jingdong prefecture level city in Shandong
IXCHSQ Flagship Store	King of Power Saving	Product design style modern, worth buying	Jingdong prefecture level city in Shandong
IXCHSQ Flagship Store	King of Power Saving	Energy saving, good power saving effect, practical, great value	Jingdong prefecture level city in Shandong
IXCHSQ Flagship Store	King of Power Saving	It's good quality, it's good, it's energy efficient, and I'll buy it again.	Jingdong prefecture level city in Shandong
IXCHSQ Flagship Store	King of Power Saving	Installation saves a lot of electricity, fast logistics, real energy-saving appliances	Jingdong prefecture level city in Shandong
IXCHSQ Flagship Store	King of Power Saving	The effect is good and the power saving is obvious after using it for a month and saving tens of dollars, the product is trustworthy, I now buy another 4 colors to prepare for their siblings' family to pay for	Jingdong prefecture level city in Shandong
IXCHSQ Flagship Store	King of Power Saving	The quality is particularly good workmanship is no problem, the arrival of the very fast oh, especially power-saving	Jingdong prefecture level city in Shandong
IXCHSQ Flagship Store	King of Power Saving	The quality is very good! In use, observed for a day, not as used before, so much power consumption.	Jingdong prefecture level city in Shandong
IXCHSQ Flagship Store	King of Power Saving	Goods have been received, the effect is good is not good, power saving is not yet know, wait for me to use it and then evaluate it.	Jingdong prefecture level city in Shandong
IXCHSQ Flagship Store	King of Power Saving	In-kind and the same as described, the quality is quite good, the seller's attitude is also good, ask questions and answer them, the speed of delivery bar, worth buying Oh!	Jingdong prefecture level city in Shandong
IXCHSQ Flagship Store	King of Power Saving	Nice stuff, came to comment after using it, it does save power.	Jingdong prefecture level city in Shandong
IXCHSQ Flagship Store	King of Power Saving	Fast charging, but also save power, the effect is very good, satisfied, praise!	Jingdong prefecture level city in Shandong
IXCHSQ Flagship Store	King of Power Saving	The goods have been received, I do not know how effective, after using the flat price again!	Jingdong prefecture level city in Shandong
IXCHSQ Flagship Store	King of Power Saving	Merchant service is very good Reply quickly The key is the quality and that quality is good	Jingdong prefecture level city in Shandong
IXCHSQ Flagship Store	King of Power Saving	It's working well now, I'll do a follow up review after I actually use it for a while	Jingdong prefecture level city in Shandong
IXCHSQ Flagship Store	King of Power Saving	The quality is good. After installing it, I feel like I've saved a lot of money on my electricity bill.	Jingdong prefecture level city in Shandong
IXCHSQ Flagship Store	King of Power Saving	Make up a review after use.	Jingdong prefecture level city in Shandong
IXCHSQ Flagship Store	King of Power Saving	review after use	Jingdong prefecture level city in Shandong
IXCHSQ Flagship Store	King of Power Saving	It's a small capacitor. It's not energy efficient.	Jingdong prefecture level city in Shandong
IXCHSQ Flagship Store	King of Power Saving	I'll follow up with a review after I've used it and had results.	Jingdong prefecture level city in Shandong
IXCHSQ Flagship Store	King of Power Saving	No power saving? Not good!	Jingdong prefecture level city in Shandong
IXCHSQ Flagship Store	King of Power Saving	Been using the same appliances and running a 1.5 air conditioner at night for what feels like no time, and after half a month of use it feels like the power is only increasing. Doesn't show any power saving	Jingdong prefecture level city in Shandong
IXCHSQ Flagship Store	King of Power Saving	serviceable	Jingdong prefecture level city in Shandong
IXCHSQ Flagship Store	King of Power Saving	It's a piece of junk. It doesn't save any power.	Jingdong prefecture level city in Shandong
IXCHSQ Flagship Store	King of Power Saving	There are a lot of appliances in the house, the summer air conditioning is very costly, I bought this in front of the air conditioning effect is very good, I explain that I bought three in one breath, I use more electricity a month down to 200 or so, very satisfied.	Jingdong prefecture level city in Shandong
IXCHSQ Flagship Store	King of Power Saving	The voltage is very stable has been maintained at 220v this value, high power super power saving, trustworthy, reasonable design, shape bright, logistics is quite fast, business warranty period, excellent service, logistics fast!	Jingdong prefecture level city in Shandong
IXCHSQ Flagship Store	King of Power Saving	Use a month after a look at the savings of more than 40 dollars, there is a display of electricity savings, this high-power intelligent frequency converter economizer, affordable and cheap, logistics is very fast, the service attitude is very good, very satisfied with a shopping.	Jingdong prefecture level city in Shandong
IXCHSQ Flagship Store	King of Power Saving	Used for a period of time, it seems that every day can really can save a few degrees of electricity, workmanship quite can, with a period of time and then continue to look at it, anyway, the price is not	Jingdong prefecture level city in Shandong
IXCHSQ Flagship Store	King of Power Saving	Used for a period of time, it seems that every day can indeed save two several degrees of electricity, workmanship quite can, with a period of time and then continue to look at it, anyway, the price is not	Jingdong prefecture level city in Shandong

IXCHSQ Flagship Store	King of Power Saving	High-power super saver received immediately used, the price is not expensive quality is very good, save electricity and money, very convenient, conscientious business, must be praised, recommended to everyone!	Jingdong prefecture level city in Shandong
IXCHSQ Flagship Store	King of Power Saving	Home enhancement version of the intelligent power saver, is in use, I think the price is cheap, value for money, efficacy, power, power saving effect is very good.	Jingdong prefecture level city in Shandong
IXCHSQ Flagship Store	King of Power Saving	Bought three at once, one for each room, summer electricity consumption is very high, despite the three air conditioners on the electricity bill is still down a lot, too Nessie!	Jingdong prefecture level city in Shandong
IXCHSQ Flagship Store	King of Power Saving	Without further ado, the electric bill came down, $102 less this month, personal satisfaction, it counts okay, give you a good review	Jingdong prefecture level city in Shandong
IXCHSQ Flagship Store	King of Power Saving	The power saver has arrived, the installation is really convenient, plugged in on the line operation is quite simple, power saving effect is also a strong!	Jingdong prefecture level city in Shandong
IXCHSQ Flagship Store	King of Power Saving	Saver received, good quality, beautiful appearance, fine workmanship, simple installation	Jingdong prefecture level city in Shandong
IXCHSQ Flagship Store	King of Power Saving	Product design style modern, worth buying	Jingdong prefecture level city in Shandong
IXCHSQ Flagship Store	King of Power Saving	Energy saving, good power saving effect, practical, great value	Jingdong prefecture level city in Shandong
IXCHSQ Flagship Store	King of Power Saving	It's good quality, it's good, it's energy efficient, and I'll buy it again.	Jingdong prefecture level city in Shandong
IXCHSQ Flagship Store	King of Power Saving	Installation saves a lot of electricity, fast logistics, real energy-saving appliances	Jingdong prefecture level city in Shandong
IXCHSQ Flagship Store	King of Power Saving	The effect is good and the power saving is obvious after using it for a month and saving tens of dollars, the product is trustworthy, I now buy another 4 colors to prepare for their siblings' family to pay for	Jingdong prefecture level city in Shandong
IXCHSQ Flagship Store	King of Power Saving	The quality is particularly good workmanship is no problem, the arrival of the very fast oh, especially power-saving	Jingdong prefecture level city in Shandong
IXCHSQ Flagship Store	King of Power Saving	The quality is very good! In use, observed for a day, not as used before, so much power consumption.	Jingdong prefecture level city in Shandong
IXCHSQ Flagship Store	King of Power Saving	Goods have been received, the effect is good is not good, power saving is not yet know, wait for me to use it and then evaluate it.	Jingdong prefecture level city in Shandong
IXCHSQ Flagship Store	King of Power Saving	In-kind and the same as described, the quality is quite good, the seller's attitude is also good, ask questions and answer them, the speed of delivery bar, worth buying Oh!	Jingdong prefecture level city in Shandong
IXCHSQ Flagship Store	King of Power Saving	Nice stuff, came to comment after using it, it does save power.	Jingdong prefecture level city in Shandong
IXCHSQ Flagship Store	King of Power Saving	Fast charging, but also save power, the effect is very good, satisfied, praise!	Jingdong prefecture level city in Shandong
IXCHSQ Flagship Store	King of Power Saving	The goods have been received, I do not know how effective, after using the flat price again!	Jingdong prefecture level city in Shandong
IXCHSQ Flagship Store	King of Power Saving	Merchant service is very good Reply quickly The key is the quality and that quality is good	Jingdong prefecture level city in Shandong
IXCHSQ Flagship Store	King of Power Saving	It's working well now, I'll do a follow up review after I actually use it for a while	Jingdong prefecture level city in Shandong
IXCHSQ Flagship Store	King of Power Saving	The quality is good. After installing it, I feel like I've saved a lot of money on my electricity bill.	Jingdong prefecture level city in Shandong
IXCHSQ Flagship Store	King of Power Saving	Make up a review after use.	Jingdong prefecture level city in Shandong
IXCHSQ Flagship Store	King of Power Saving	review after use	Jingdong prefecture level city in Shandong
IXCHSQ Flagship Store	King of Power Saving	It's a small capacitor. It's not energy efficient.	Jingdong prefecture level city in Shandong
IXCHSQ Flagship Store	King of Power Saving	I'll follow up with a review after I've used it and had results.	Jingdong prefecture level city in Shandong
IXCHSQ Flagship Store	King of Power Saving	No power saving? Not good!	Jingdong prefecture level city in Shandong
IXCHSQ Flagship Store	King of Power Saving	Been using the same appliances and running a 1.5 air conditioner at night for what feels like no time, and after half a month of use it feels like the power is only increasing. Doesn't show any power saving	Jingdong prefecture level city in Shandong
IXCHSQ Flagship Store	King of Power Saving	serviceable	Jingdong prefecture level city in Shandong
IXCHSQ Flagship Store	King of Power Saving	It's a piece of junk. It doesn't save any power.	Jingdong prefecture level city in Shandong
IXCHSQ Flagship Store	King of Power Saving	There are a lot of appliances in the house, the summer air conditioning is very costly, I bought this in front of the air conditioning effect is very good, I explain that I bought three in one breath, I use more electricity a month down to 200 or so, very satisfied.	Jingdong prefecture level city in Shandong
IXCHSQ Flagship Store	King of Power Saving	The voltage is very stable has been maintained at 220v this value, high power super power saving, trustworthy, reasonable design, shape bright, logistics is quite fast, business warranty period, excellent service, logistics fast!	Jingdong prefecture level city in Shandong
IXCHSQ Flagship Store	King of Power Saving	Use a month after a look at the savings of more than 40 dollars, there is a display of electricity savings, this high-power intelligent frequency converter economizer, affordable and cheap, logistics is very fast, the service attitude is very good, very satisfied with a shopping.	Jingdong prefecture level city in Shandong
IXCHSQ Flagship Store	King of Power Saving	Used for a period of time, it seems that every day can really can save a few degrees of electricity, workmanship quite can, with a period of time and then continue to look at it, anyway, the price is not	Jingdong prefecture level city in Shandong
IXCHSQ Flagship Store	King of Power Saving	Used for a period of time, it seems that every day can indeed save two several degrees of electricity, workmanship quite can, with a period of time and then continue to look at it, anyway, the price is not	Jingdong prefecture level city in Shandong
IXCHSQ Flagship Store	King of Power Saving	High-power super saver received immediately used, the price is not expensive quality is very good, save electricity and money, very convenient, conscientious business, must be praised, recommended to everyone!	Jingdong prefecture level city in Shandong
IXCHSQ Flagship Store	King of Power Saving	Home enhancement version of the intelligent power saver, is in use, I think the price is cheap, value for money, efficacy, power, power saving effect is very good.	Jingdong prefecture level city in Shandong
IXCHSQ Flagship Store	King of Power Saving	Bought three at once, one for each room, summer electricity consumption is very high, despite the three air conditioners on the electricity bill is still down a lot, too Nessie!	Jingdong prefecture level city in Shandong
IXCHSQ Flagship Store	King of Power Saving	Without further ado, the electric bill came down, $102 less this month, personal satisfaction, it counts okay, give you a good review	Jingdong prefecture level city in Shandong
IXCHSQ Flagship Store	King of Power Saving	The power saver has arrived, the installation is really convenient, plugged in on the line operation is quite simple, power saving effect is also a strong!	Jingdong prefecture level city in Shandong
IXCHSQ Flagship Store	King of Power Saving	Saver received, good quality, beautiful appearance, fine workmanship, simple installation	Jingdong prefecture level city in Shandong
IXCHSQ Flagship Store	King of Power Saving	Product design style modern, worth buying	Jingdong prefecture level city in Shandong
IXCHSQ Flagship Store	King of Power Saving	Energy saving, good power saving effect, practical, great value	Jingdong prefecture level city in Shandong
IXCHSQ Flagship Store	King of Power Saving	It's good quality, it's good, it's energy efficient, and I'll buy it again.	Jingdong prefecture level city in Shandong
IXCHSQ Flagship Store	King of Power Saving	Installation saves a lot of electricity, fast logistics, real energy-saving appliances	Jingdong prefecture level city in Shandong
IXCHSQ Flagship Store	King of Power Saving	The effect is good and the power saving is obvious after using it for a month and saving tens of dollars, the product is trustworthy, I now buy another 4 colors to prepare for their siblings' family to pay for	Jingdong prefecture level city in Shandong
IXCHSQ Flagship Store	King of Power Saving	The quality is particularly good workmanship is no problem, the arrival of the very fast oh, especially power-saving	Jingdong prefecture level city in Shandong
IXCHSQ Flagship Store	King of Power Saving	The quality is very good! In use, observed for a day, not as used before, so much power consumption.	Jingdong prefecture level city in Shandong
IXCHSQ Flagship Store	King of Power Saving	Goods have been received, the effect is good is not good, power saving is not yet know, wait for me to use it and then evaluate it.	Jingdong prefecture level city in Shandong
IXCHSQ Flagship Store	King of Power Saving	In-kind and the same as described, the quality is quite good, the seller's attitude is also good, ask questions and answer them, the speed of delivery bar, worth buying Oh!	Jingdong prefecture level city in Shandong
IXCHSQ Flagship Store	King of Power Saving	Nice stuff, came to comment after using it, it does save power.	Jingdong prefecture level city in Shandong

IXCHSQ Flagship Store	King of Power Saving	Fast charging, but also save power, the effect is very good, satisfied, praise!	Jingdong prefecture level city in Shandong
IXCHSQ Flagship Store	King of Power Saving	The goods have been received, I do not know how effective, after using the flat price again!	Jingdong prefecture level city in Shandong
IXCHSQ Flagship Store	King of Power Saving	Merchant service is very good Reply quickly The key is the quality and that quality is good	Jingdong prefecture level city in Shandong
IXCHSQ Flagship Store	King of Power Saving	It's working well now, I'll do a follow up review after I actually use it for a while	Jingdong prefecture level city in Shandong
IXCHSQ Flagship Store	King of Power Saving	The quality is good. After installing it, I feel like I've saved a lot of money on my electricity bill.	Jingdong prefecture level city in Shandong
IXCHSQ Flagship Store	King of Power Saving	Make up a review after use.	Jingdong prefecture level city in Shandong
IXCHSQ Flagship Store	King of Power Saving	review after use	Jingdong prefecture level city in Shandong
IXCHSQ Flagship Store	King of Power Saving	It's a small capacitor. It's not energy efficient.	Jingdong prefecture level city in Shandong
IXCHSQ Flagship Store	King of Power Saving	I'll follow up with a review after I've used it and had results.	Jingdong prefecture level city in Shandong
IXCHSQ Flagship Store	King of Power Saving	No power saving? Not good!	Jingdong prefecture level city in Shandong
IXCHSQ Flagship Store	King of Power Saving	Been using the same appliances and running a 1.5 air conditioner at night for what feels like no time, and after half a month of use it feels like the power is only increasing. Doesn't show any power saving	Jingdong prefecture level city in Shandong
IXCHSQ Flagship Store	King of Power Saving	serviceable	Jingdong prefecture level city in Shandong
IXCHSQ Flagship Store	King of Power Saving	It's a piece of junk. It doesn't save any power.	Jingdong prefecture level city in Shandong
IXCHSQ Flagship Store	King of Power Saving	There are a lot of appliances in the house, the summer air conditioning is very costly, I bought this in front of the air conditioning effect is very good, I explain that I bought three in one breath, I use more electricity a month down to 200 or so, very satisfied.	Jingdong prefecture level city in Shandong
IXCHSQ Flagship Store	King of Power Saving	The voltage is very stable has been maintained at 220v this value, high power super power saving, trustworthy, reasonable design, shape bright, logistics is quite fast, business warranty period, excellent service, logistics fast!	Jingdong prefecture level city in Shandong
IXCHSQ Flagship Store	King of Power Saving	Use a month after a look at the savings of more than 40 dollars, there is a display of electricity savings, this high-power intelligent frequency converter economizer, affordable and cheap, logistics is very fast, the service attitude is very good, very satisfied with a shopping.	Jingdong prefecture level city in Shandong
IXCHSQ Flagship Store	King of Power Saving	Used for a period of time, it seems that every day can really can save a few degrees of electricity, workmanship quite can, with a period of time and then continue to look at it, anyway, the price is not	Jingdong prefecture level city in Shandong
IXCHSQ Flagship Store	King of Power Saving	Used for a period of time, it seems that every day can indeed save two several degrees of electricity, workmanship quite can, with a period of time and then continue to look at it, anyway, the price is not	Jingdong prefecture level city in Shandong
IXCHSQ Flagship Store	King of Power Saving	High-power super saver received immediately used, the price is not expensive quality is very good, save electricity and money, very convenient, conscientious business, must be praised, recommended to everyone!	Jingdong prefecture level city in Shandong
IXCHSQ Flagship Store	King of Power Saving	Home enhancement version of the intelligent power saver, is in use, I think the price is cheap, value for money, efficacy, power, power saving effect is very good.	Jingdong prefecture level city in Shandong
IXCHSQ Flagship Store	King of Power Saving	Bought three at once, one for each room, summer electricity consumption is very high, despite the three air conditioners on the electricity bill is still down a lot, too Nessie!	Jingdong prefecture level city in Shandong
IXCHSQ Flagship Store	King of Power Saving	Without further ado, the electric bill came down, $102 less this month, personal satisfaction, it counts okay, give you a good review	Jingdong prefecture level city in Shandong
IXCHSQ Flagship Store	King of Power Saving	The power saver has arrived, the installation is really convenient, plugged in on the line operation is quite simple, power saving effect is also a strong!	Jingdong prefecture level city in Shandong
IXCHSQ Flagship Store	King of Power Saving	Saver received, good quality, beautiful appearance, fine workmanship, simple installation	Jingdong prefecture level city in Shandong
IXCHSQ Flagship Store	King of Power Saving	Product design style modern, worth buying	Jingdong prefecture level city in Shandong
IXCHSQ Flagship Store	King of Power Saving	Energy saving, good power saving effect, practical, great value	Jingdong prefecture level city in Shandong
IXCHSQ Flagship Store	King of Power Saving	It's good quality, it's good, it's energy efficient, and I'll buy it again.	Jingdong prefecture level city in Shandong
IXCHSQ Flagship Store	King of Power Saving	Installation saves a lot of electricity, fast logistics, real energy-saving appliances	Jingdong prefecture level city in Shandong
IXCHSQ Flagship Store	King of Power Saving	The effect is good and the power saving is obvious after using it for a month and saving tens of dollars, the product is trustworthy, I now buy another 4 colors to prepare for their siblings' family to pay for	Jingdong prefecture level city in Shandong
IXCHSQ Flagship Store	King of Power Saving	The quality is particularly good workmanship is no problem, the arrival of the very fast oh, especially power-saving	Jingdong prefecture level city in Shandong
IXCHSQ Flagship Store	King of Power Saving	The quality is very good! In use, observed for a day, not as used before, so much power consumption.	Jingdong prefecture level city in Shandong
IXCHSQ Flagship Store	King of Power Saving	Goods have been received, the effect is good is not good, power saving is not yet know, wait for me to use it and then evaluate it.	Jingdong prefecture level city in Shandong
IXCHSQ Flagship Store	King of Power Saving	In-kind and the same as described, the quality is quite good, the seller's attitude is also good, ask questions and answer them, the speed of delivery bar, worth buying Oh!	Jingdong prefecture level city in Shandong
IXCHSQ Flagship Store	King of Power Saving	Nice stuff, came to comment after using it, it does save power.	Jingdong prefecture level city in Shandong
IXCHSQ Flagship Store	King of Power Saving	Fast charging, but also save power, the effect is very good, satisfied, praise!	Jingdong prefecture level city in Shandong
IXCHSQ Flagship Store	King of Power Saving	The goods have been received, I do not know how effective, after using the flat price again!	Jingdong prefecture level city in Shandong
IXCHSQ Flagship Store	King of Power Saving	Merchant service is very good Reply quickly The key is the quality and that quality is good	Jingdong prefecture level city in Shandong
IXCHSQ Flagship Store	King of Power Saving	It's working well now, I'll do a follow up review after I actually use it for a while	Jingdong prefecture level city in Shandong
IXCHSQ Flagship Store	King of Power Saving	The quality is good. After installing it, I feel like I've saved a lot of money on my electricity bill.	Jingdong prefecture level city in Shandong
IXCHSQ Flagship Store	King of Power Saving	Make up a review after use.	Jingdong prefecture level city in Shandong
IXCHSQ Flagship Store	King of Power Saving	review after use	Jingdong prefecture level city in Shandong
IXCHSQ Flagship Store	King of Power Saving	It's a small capacitor. It's not energy efficient.	Jingdong prefecture level city in Shandong
IXCHSQ Flagship Store	King of Power Saving	I'll follow up with a review after I've used it and had results.	Jingdong prefecture level city in Shandong
IXCHSQ Flagship Store	King of Power Saving	No power saving? Not good!	Jingdong prefecture level city in Shandong
IXCHSQ Flagship Store	King of Power Saving	Been using the same appliances and running a 1.5 air conditioner at night for what feels like no time, and after half a month of use it feels like the power is only increasing. Doesn't show any power saving	Jingdong prefecture level city in Shandong
IXCHSQ Flagship Store	King of Power Saving	serviceable	Jingdong prefecture level city in Shandong
IXCHSQ Flagship Store	King of Power Saving	It's a piece of junk. It doesn't save any power.	Jingdong prefecture level city in Shandong
IXCHSQ Flagship Store	King of Power Saving	There are a lot of appliances in the house, the summer air conditioning is very costly, I bought this in front of the air conditioning effect is very good, I explain that I bought three in one breath, I use more electricity a month down to 200 or so, very satisfied.	Jingdong prefecture level city in Shandong
IXCHSQ Flagship Store	King of Power Saving	The voltage is very stable has been maintained at 220v this value, high power super power saving, trustworthy, reasonable design, shape bright, logistics is quite fast, business warranty period, excellent service, logistics fast!	Jingdong prefecture level city in Shandong

IXCHSQ Flagship Store	King of Power Saving	Use a month after a look at the savings of more than 40 dollars, there is a display of electricity savings, this high-power intelligent frequency converter economizer, affordable and cheap, logistics is very fast, the service attitude is very good, very satisfied with a shopping.	Jingdong prefecture level city in Shandong
IXCHSQ Flagship Store	King of Power Saving	Used for a period of time, it seems that every day can really can save a few degrees of electricity, workmanship quite can, with a period of time and then continue to look at it, anyway, the price is not	Jingdong prefecture level city in Shandong
IXCHSQ Flagship Store	King of Power Saving	Used for a period of time, it seems that every day can indeed save two several degrees of electricity, workmanship quite can, with a period of time and then continue to look at it, anyway, the price is not	Jingdong prefecture level city in Shandong
IXCHSQ Flagship Store	King of Power Saving	High-power super saver received immediately used, the price is not expensive quality is very good, save electricity and money, very convenient, conscientious business, must be praised, recommended to everyone!	Jingdong prefecture level city in Shandong
IXCHSQ Flagship Store	King of Power Saving	Home enhancement version of the intelligent power saver, is in use, I think the price is cheap, value for money, efficacy, power, power saving effect is very good.	Jingdong prefecture level city in Shandong
IXCHSQ Flagship Store	King of Power Saving	Bought three at once, one for each room, summer electricity consumption is very high, despite the three air conditioners on the electricity bill is still down a lot, too Nessie!	Jingdong prefecture level city in Shandong
IXCHSQ Flagship Store	King of Power Saving	Without further ado, the electric bill came down, $102 less this month, personal satisfaction, it counts okay, give you a good review	Jingdong prefecture level city in Shandong
IXCHSQ Flagship Store	King of Power Saving	The power saver has arrived, the installation is really convenient, plugged in on the line operation is quite simple, power saving effect is also a strong!	Jingdong prefecture level city in Shandong
IXCHSQ Flagship Store	King of Power Saving	Saver received, good quality, beautiful appearance, fine workmanship, simple installation	Jingdong prefecture level city in Shandong
IXCHSQ Flagship Store	King of Power Saving	Product design style modern, worth buying	Jingdong prefecture level city in Shandong
IXCHSQ Flagship Store	King of Power Saving	Energy saving, good power saving effect, practical, great value	Jingdong prefecture level city in Shandong
IXCHSQ Flagship Store	King of Power Saving	It's good quality, it's good, it's energy efficient, and I'll buy it again.	Jingdong prefecture level city in Shandong
IXCHSQ Flagship Store	King of Power Saving	Installation saves a lot of electricity, fast logistics, real energy-saving appliances	Jingdong prefecture level city in Shandong
IXCHSQ Flagship Store	King of Power Saving	The effect is good and the power saving is obvious after using it for a month and saving tens of dollars, the product is trustworthy, I now buy another 4 colors to prepare for their siblings' family to pay for	Jingdong prefecture level city in Shandong
IXCHSQ Flagship Store	King of Power Saving	The quality is particularly good workmanship is no problem, the arrival of the very fast oh, especially power-saving	Jingdong prefecture level city in Shandong
IXCHSQ Flagship Store	King of Power Saving	The quality is very good! In use, observed for a day, not as used before, so much power consumption.	Jingdong prefecture level city in Shandong
IXCHSQ Flagship Store	King of Power Saving	Goods have been received, the effect is good is not good, power saving is not yet know, wait for me to use it and then evaluate it.	Jingdong prefecture level city in Shandong
IXCHSQ Flagship Store	King of Power Saving	In-kind and the same as described, the quality is quite good, the seller's attitude is also good, ask questions and answer them, the speed of delivery bar, worth buying Oh!	Jingdong prefecture level city in Shandong
IXCHSQ Flagship Store	King of Power Saving	Nice stuff, came to comment after using it, it does save power.	Jingdong prefecture level city in Shandong
IXCHSQ Flagship Store	King of Power Saving	Fast charging, but also save power, the effect is very good, satisfied, praise!	Jingdong prefecture level city in Shandong
IXCHSQ Flagship Store	King of Power Saving	The goods have been received, I do not know how effective, after using the flat price again!	Jingdong prefecture level city in Shandong
IXCHSQ Flagship Store	King of Power Saving	Merchant service is very good Reply quickly The key is the quality and that quality is good	Jingdong prefecture level city in Shandong
IXCHSQ Flagship Store	King of Power Saving	It's working well now, I'll do a follow up review after I actually use it for a while	Jingdong prefecture level city in Shandong
IXCHSQ Flagship Store	King of Power Saving	The quality is good. After installing it, I feel like I've saved a lot of money on my electricity bill.	Jingdong prefecture level city in Shandong
IXCHSQ Flagship Store	King of Power Saving	Make up a review after use.	Jingdong prefecture level city in Shandong
IXCHSQ Flagship Store	King of Power Saving	review after use	Jingdong prefecture level city in Shandong
IXCHSQ Flagship Store	King of Power Saving	It's a small capacitor. It's not energy efficient.	Jingdong prefecture level city in Shandong
IXCHSQ Flagship Store	King of Power Saving	I'll follow up with a review after I've used it and had results.	Jingdong prefecture level city in Shandong
IXCHSQ Flagship Store	King of Power Saving	No power saving? Not good!	Jingdong prefecture level city in Shandong
IXCHSQ Flagship Store	King of Power Saving	Been using the same appliances and running a 1.5 air conditioner at night for what feels like no time, and after half a month of use it feels like the power is only increasing. Doesn't show any power saving	Jingdong prefecture level city in Shandong
IXCHSQ Flagship Store	King of Power Saving	serviceable	Jingdong prefecture level city in Shandong
IXCHSQ Flagship Store	King of Power Saving	It's a piece of junk. It doesn't save any power.	Jingdong prefecture level city in Shandong
IXCHSQ Flagship Store	King of Power Saving	There are a lot of appliances in the house, the summer air conditioning is very costly, I bought this in front of the air conditioning effect is very good, I explain that I bought three in one breath, I use more electricity a month down to 200 or so, very satisfied.	Jingdong prefecture level city in Shandong
IXCHSQ Flagship Store	King of Power Saving	The voltage is very stable has been maintained at 220v this value, high power super power saving, trustworthy, reasonable design, shape bright, logistics is quite fast, business warranty period, excellent service, logistics fast!	Jingdong prefecture level city in Shandong
IXCHSQ Flagship Store	King of Power Saving	Use a month after a look at the savings of more than 40 dollars, there is a display of electricity savings, this high-power intelligent frequency converter economizer, affordable and cheap, logistics is very fast, the service attitude is very good, very satisfied with a shopping.	Jingdong prefecture level city in Shandong
IXCHSQ Flagship Store	King of Power Saving	Used for a period of time, it seems that every day can really can save a few degrees of electricity, workmanship quite can, with a period of time and then continue to look at it, anyway, the price is not	Jingdong prefecture level city in Shandong
IXCHSQ Flagship Store	King of Power Saving	Used for a period of time, it seems that every day can indeed save two several degrees of electricity, workmanship quite can, with a period of time and then continue to look at it, anyway, the price is not	Jingdong prefecture level city in Shandong
IXCHSQ Flagship Store	King of Power Saving	High-power super saver received immediately used, the price is not expensive quality is very good, save electricity and money, very convenient, conscientious business, must be praised, recommended to everyone!	Jingdong prefecture level city in Shandong
IXCHSQ Flagship Store	King of Power Saving	Home enhancement version of the intelligent power saver, is in use, I think the price is cheap, value for money, efficacy, power, power saving effect is very good.	Jingdong prefecture level city in Shandong
IXCHSQ Flagship Store	King of Power Saving	Bought three at once, one for each room, summer electricity consumption is very high, despite the three air conditioners on the electricity bill is still down a lot, too Nessie!	Jingdong prefecture level city in Shandong
IXCHSQ Flagship Store	King of Power Saving	Without further ado, the electric bill came down, $102 less this month, personal satisfaction, it counts okay, give you a good review	Jingdong prefecture level city in Shandong
IXCHSQ Flagship Store	King of Power Saving	The power saver has arrived, the installation is really convenient, plugged in on the line operation is quite simple, power saving effect is also a strong!	Jingdong prefecture level city in Shandong
IXCHSQ Flagship Store	King of Power Saving	Saver received, good quality, beautiful appearance, fine workmanship, simple installation	Jingdong prefecture level city in Shandong
IXCHSQ Flagship Store	King of Power Saving	Product design style modern, worth buying	Jingdong prefecture level city in Shandong
IXCHSQ Flagship Store	King of Power Saving	Energy saving, good power saving effect, practical, great value	Jingdong prefecture level city in Shandong
IXCHSQ Flagship Store	King of Power Saving	It's good quality, it's good, it's energy efficient, and I'll buy it again.	Jingdong prefecture level city in Shandong
IXCHSQ Flagship Store	King of Power Saving	Installation saves a lot of electricity, fast logistics, real energy-saving appliances	Jingdong prefecture level city in Shandong
IXCHSQ Flagship Store	King of Power Saving	The effect is good and the power saving is obvious after using it for a month and saving tens of dollars, the product is trustworthy, I now buy another 4 colors to prepare for their siblings' family to pay for	Jingdong prefecture level city in Shandong
IXCHSQ Flagship Store	King of Power Saving	The quality is particularly good workmanship is no problem, the arrival of the very fast oh, especially power-saving	Jingdong prefecture level city in Shandong

IXCHSQ Flagship Store	King of Power Saving	The quality is very good! In use, observed for a day, not as used before, so much power consumption.	Jingdong prefecture level city in Shandong
IXCHSQ Flagship Store	King of Power Saving	Goods have been received, the effect is good is not good, power saving is not yet know, wait for me to use it and then evaluate it.	Jingdong prefecture level city in Shandong
IXCHSQ Flagship Store	King of Power Saving	In-kind and the same as described, the quality is quite good, the seller's attitude is also good, ask questions and answer them, the speed of delivery bar, worth buying Oh!	Jingdong prefecture level city in Shandong
IXCHSQ Flagship Store	King of Power Saving	Nice stuff, came to comment after using it, it does save power.	Jingdong prefecture level city in Shandong
IXCHSQ Flagship Store	King of Power Saving	Fast charging, but also save power, the effect is very good, satisfied, praise!	Jingdong prefecture level city in Shandong
IXCHSQ Flagship Store	King of Power Saving	The goods have been received, I do not know how effective, after using the flat price again!	Jingdong prefecture level city in Shandong
IXCHSQ Flagship Store	King of Power Saving	Merchant service is very good Reply quickly The key is the quality and that quality is good	Jingdong prefecture level city in Shandong
IXCHSQ Flagship Store	King of Power Saving	It's working well now, I'll do a follow up review after I actually use it for a while	Jingdong prefecture level city in Shandong
IXCHSQ Flagship Store	King of Power Saving	The quality is good. After installing it, I feel like I've saved a lot of money on my electricity bill.	Jingdong prefecture level city in Shandong
IXCHSQ Flagship Store	King of Power Saving	Make up a review after use.	Jingdong prefecture level city in Shandong
IXCHSQ Flagship Store	King of Power Saving	review after use	Jingdong prefecture level city in Shandong
IXCHSQ Flagship Store	King of Power Saving	It's a small capacitor. It's not energy efficient.	Jingdong prefecture level city in Shandong
IXCHSQ Flagship Store	King of Power Saving	I'll follow up with a review after I've used it and had results.	Jingdong prefecture level city in Shandong
IXCHSQ Flagship Store	King of Power Saving	No power saving? Not good!	Jingdong prefecture level city in Shandong
IXCHSQ Flagship Store	King of Power Saving	Been using the same appliances and running a 1.5 air conditioner at night for what feels like no time, and after half a month of use it feels like the power is only increasing. Doesn't show any power saving	Jingdong prefecture level city in Shandong
IXCHSQ Flagship Store	King of Power Saving	serviceable	Jingdong prefecture level city in Shandong
IXCHSQ Flagship Store	King of Power Saving	It's a piece of junk. It doesn't save any power.	Jingdong prefecture level city in Shandong
IXCHSQ Flagship Store	King of Power Saving	There are a lot of appliances in the house, the summer air conditioning is very costly, I bought this in front of the air conditioning effect is very good, I explain that I bought three in one breath, I use more electricity a month down to 200 or so, very satisfied.	Jingdong prefecture level city in Shandong
IXCHSQ Flagship Store	King of Power Saving	The voltage is very stable has been maintained at 220v this value, high power super power saving, trustworthy, reasonable design, shape bright, logistics is quite fast, business warranty period, excellent service, logistics fast!	Jingdong prefecture level city in Shandong
IXCHSQ Flagship Store	King of Power Saving	Use a month after a look at the savings of more than 40 dollars, there is a display of electricity savings, this high-power intelligent frequency converter economizer, affordable and cheap, logistics is very fast, the service attitude is very good, very satisfied with a shopping.	Jingdong prefecture level city in Shandong
IXCHSQ Flagship Store	King of Power Saving	Used for a period of time, it seems that every day can really can save a few degrees of electricity, workmanship quite can, with a period of time and then continue to look at it, anyway, the price is not	Jingdong prefecture level city in Shandong
IXCHSQ Flagship Store	King of Power Saving	Used for a period of time, it seems that every day can indeed save two several degrees of electricity, workmanship quite can, with a period of time and then continue to look at it, anyway, the price is not	Jingdong prefecture level city in Shandong
IXCHSQ Flagship Store	King of Power Saving	High-power super saver received immediately used, the price is not expensive quality is very good, save electricity and money, very convenient, conscientious business, must be praised, recommended to everyone!	Jingdong prefecture level city in Shandong
IXCHSQ Flagship Store	King of Power Saving	Home enhancement version of the intelligent power saver, is in use, I think the price is cheap, value for money, efficacy, power, power saving effect is very good.	Jingdong prefecture level city in Shandong
IXCHSQ Flagship Store	King of Power Saving	Bought three at once, one for each room, summer electricity consumption is very high, despite the three air conditioners on the electricity bill is still down a lot, too Nessie!	Jingdong prefecture level city in Shandong
IXCHSQ Flagship Store	King of Power Saving	Without further ado, the electric bill came down, $102 less this month, personal satisfaction, it counts okay, give you a good review	Jingdong prefecture level city in Shandong
IXCHSQ Flagship Store	King of Power Saving	The power saver has arrived, the installation is really convenient, plugged in on the line operation is quite simple, power saving effect is also a strong!	Jingdong prefecture level city in Shandong
IXCHSQ Flagship Store	King of Power Saving	Saver received, good quality, beautiful appearance, fine workmanship, simple installation	Jingdong prefecture level city in Shandong
IXCHSQ Flagship Store	King of Power Saving	Product design style modern, worth buying	Jingdong prefecture level city in Shandong
IXCHSQ Flagship Store	King of Power Saving	Energy saving, good power saving effect, practical, great value	Jingdong prefecture level city in Shandong
IXCHSQ Flagship Store	King of Power Saving	It's good quality, it's good, it's energy efficient, and I'll buy it again.	Jingdong prefecture level city in Shandong
IXCHSQ Flagship Store	King of Power Saving	Installation saves a lot of electricity, fast logistics, real energy-saving appliances	Jingdong prefecture level city in Shandong
IXCHSQ Flagship Store	King of Power Saving	The effect is good and the power saving is obvious after using it for a month and saving tens of dollars, the product is trustworthy, I now buy another 4 colors to prepare for their siblings' family to pay for	Jingdong prefecture level city in Shandong
IXCHSQ Flagship Store	King of Power Saving	The quality is particularly good workmanship is no problem, the arrival of the very fast oh, especially power-saving	Jingdong prefecture level city in Shandong
IXCHSQ Flagship Store	King of Power Saving	The quality is very good! In use, observed for a day, not as used before, so much power consumption.	Jingdong prefecture level city in Shandong
IXCHSQ Flagship Store	King of Power Saving	Goods have been received, the effect is good is not good, power saving is not yet know, wait for me to use it and then evaluate it.	Jingdong prefecture level city in Shandong
IXCHSQ Flagship Store	King of Power Saving	In-kind and the same as described, the quality is quite good, the seller's attitude is also good, ask questions and answer them, the speed of delivery bar, worth buying Oh!	Jingdong prefecture level city in Shandong
IXCHSQ Flagship Store	King of Power Saving	Nice stuff, came to comment after using it, it does save power.	Jingdong prefecture level city in Shandong
IXCHSQ Flagship Store	King of Power Saving	Fast charging, but also save power, the effect is very good, satisfied, praise!	Jingdong prefecture level city in Shandong
IXCHSQ Flagship Store	King of Power Saving	The goods have been received, I do not know how effective, after using the flat price again!	Jingdong prefecture level city in Shandong
IXCHSQ Flagship Store	King of Power Saving	Merchant service is very good Reply quickly The key is the quality and that quality is good	Jingdong prefecture level city in Shandong
IXCHSQ Flagship Store	King of Power Saving	It's working well now, I'll do a follow up review after I actually use it for a while	Jingdong prefecture level city in Shandong
IXCHSQ Flagship Store	King of Power Saving	The quality is good. After installing it, I feel like I've saved a lot of money on my electricity bill.	Jingdong prefecture level city in Shandong
IXCHSQ Flagship Store	King of Power Saving	Make up a review after use.	Jingdong prefecture level city in Shandong
IXCHSQ Flagship Store	King of Power Saving	review after use	Jingdong prefecture level city in Shandong
IXCHSQ Flagship Store	King of Power Saving	It's a small capacitor. It's not energy efficient.	Jingdong prefecture level city in Shandong
IXCHSQ Flagship Store	King of Power Saving	I'll follow up with a review after I've used it and had results.	Jingdong prefecture level city in Shandong
IXCHSQ Flagship Store	King of Power Saving	No power saving? Not good!	Jingdong prefecture level city in Shandong
IXCHSQ Flagship Store	King of Power Saving	Been using the same appliances and running a 1.5 air conditioner at night for what feels like no time, and after half a month of use it feels like the power is only increasing. Doesn't show any power saving	Jingdong prefecture level city in Shandong
IXCHSQ Flagship Store	King of Power Saving	serviceable	Jingdong prefecture level city in Shandong

IXCHSQ Flagship Store	King of Power Saving	It's a piece of junk. It doesn't save any power.	Jingdong prefecture level city in Shandong
IXCHSQ Flagship Store	King of Power Saving	There are a lot of appliances in the house, the summer air conditioning is very costly, I bought this in front of the air conditioning effect is very good, I explain that I bought three in one breath, I use more electricity a month down to 200 or so, very satisfied.	Jingdong prefecture level city in Shandong
IXCHSQ Flagship Store	King of Power Saving	The voltage is very stable has been maintained at 220v this value, high power super power saving, trustworthy, reasonable design, shape bright, logistics is quite fast, business warranty period, excellent service, logistics fast!	Jingdong prefecture level city in Shandong
IXCHSQ Flagship Store	King of Power Saving	Use a month after a look at the savings of more than 40 dollars, there is a display of electricity savings, this high-power intelligent frequency converter economizer, affordable and cheap, logistics is very fast, the service attitude is very good, very satisfied with a shopping.	Jingdong prefecture level city in Shandong
IXCHSQ Flagship Store	King of Power Saving	Used for a period of time, it seems that every day can really can save a few degrees of electricity, workmanship quite can, with a period of time and then continue to look at it, anyway, the price is not	Jingdong prefecture level city in Shandong
IXCHSQ Flagship Store	King of Power Saving	Used for a period of time, it seems that every day can indeed save two several degrees of electricity, workmanship quite can, with a period of time and then continue to look at it, anyway, the price is not	Jingdong prefecture level city in Shandong
IXCHSQ Flagship Store	King of Power Saving	High-power super saver received immediately used, the price is not expensive quality is very good, save electricity and money, very convenient, conscientious business, must be praised, recommended to everyone!	Jingdong prefecture level city in Shandong
IXCHSQ Flagship Store	King of Power Saving	Home enhancement version of the intelligent power saver, is in use, I think the price is cheap, value for money, efficacy, power, power saving effect is very good.	Jingdong prefecture level city in Shandong
IXCHSQ Flagship Store	King of Power Saving	Bought three at once, one for each room, summer electricity consumption is very high, despite the three air conditioners on the electricity bill is still down a lot, too Nessie!	Jingdong prefecture level city in Shandong
IXCHSQ Flagship Store	King of Power Saving	Without further ado, the electric bill came down, $102 less this month, personal satisfaction, it counts okay, give you a good review	Jingdong prefecture level city in Shandong
IXCHSQ Flagship Store	King of Power Saving	The power saver has arrived, the installation is really convenient, plugged in on the line operation is quite simple, power saving effect is also a strong!	Jingdong prefecture level city in Shandong
IXCHSQ Flagship Store	King of Power Saving	Saver received, good quality, beautiful appearance, fine workmanship, simple installation	Jingdong prefecture level city in Shandong
IXCHSQ Flagship Store	King of Power Saving	Product design style modern, worth buying	Jingdong prefecture level city in Shandong
IXCHSQ Flagship Store	King of Power Saving	Energy saving, good power saving effect, practical, great value	Jingdong prefecture level city in Shandong
IXCHSQ Flagship Store	King of Power Saving	It's good quality, it's good, it's energy efficient, and I'll buy it again.	Jingdong prefecture level city in Shandong
IXCHSQ Flagship Store	King of Power Saving	Installation saves a lot of electricity, fast logistics, real energy-saving appliances	Jingdong prefecture level city in Shandong
IXCHSQ Flagship Store	King of Power Saving	The effect is good and the power saving is obvious after using it for a month and saving tens of dollars, the product is trustworthy, I now buy another 4 colors to prepare for their siblings' family to pay for	Jingdong prefecture level city in Shandong
IXCHSQ Flagship Store	King of Power Saving	The quality is particularly good workmanship is no problem, the arrival of the very fast oh, especially power-saving	Jingdong prefecture level city in Shandong
IXCHSQ Flagship Store	King of Power Saving	The quality is very good! In use, observed for a day, not as used before, so much power consumption.	Jingdong prefecture level city in Shandong
IXCHSQ Flagship Store	King of Power Saving	Goods have been received, the effect is good is not good, power saving is not yet know, wait for me to use it and then evaluate it.	Jingdong prefecture level city in Shandong
IXCHSQ Flagship Store	King of Power Saving	In-kind and the same as described, the quality is quite good, the seller's attitude is also good, ask questions and answer them, the speed of delivery bar, worth buying Oh!	Jingdong prefecture level city in Shandong
IXCHSQ Flagship Store	King of Power Saving	Nice stuff, came to comment after using it, it does save power.	Jingdong prefecture level city in Shandong
IXCHSQ Flagship Store	King of Power Saving	Fast charging, but also save power, the effect is very good, satisfied, praise!	Jingdong prefecture level city in Shandong
IXCHSQ Flagship Store	King of Power Saving	The goods have been received, I do not know how effective, after using the flat price again!	Jingdong prefecture level city in Shandong
IXCHSQ Flagship Store	King of Power Saving	Merchant service is very good Reply quickly The key is the quality and that quality is good	Jingdong prefecture level city in Shandong
IXCHSQ Flagship Store	King of Power Saving	It's working well now, I'll do a follow up review after I actually use it for a while	Jingdong prefecture level city in Shandong
IXCHSQ Flagship Store	King of Power Saving	The quality is good. After installing it, I feel like I've saved a lot of money on my electricity bill.	Jingdong prefecture level city in Shandong
IXCHSQ Flagship Store	King of Power Saving	Make up a review after use.	Jingdong prefecture level city in Shandong
IXCHSQ Flagship Store	King of Power Saving	review after use	Jingdong prefecture level city in Shandong
IXCHSQ Flagship Store	King of Power Saving	It's a small capacitor. It's not energy efficient.	Jingdong prefecture level city in Shandong
IXCHSQ Flagship Store	King of Power Saving	I'll follow up with a review after I've used it and had results.	Jingdong prefecture level city in Shandong
IXCHSQ Flagship Store	King of Power Saving	No power saving? Not good!	Jingdong prefecture level city in Shandong
IXCHSQ Flagship Store	King of Power Saving	Been using the same appliances and running a 1.5 air conditioner at night for what feels like no time, and after half a month of use it feels like the power is only increasing. Doesn't show any power saving	Jingdong prefecture level city in Shandong
IXCHSQ Flagship Store	King of Power Saving	serviceable	Jingdong prefecture level city in Shandong
IXCHSQ Flagship Store	King of Power Saving	It's a piece of junk. It doesn't save any power.	Jingdong prefecture level city in Shandong
IXCHSQ Flagship Store	King of Power Saving	There are a lot of appliances in the house, the summer air conditioning is very costly, I bought this in front of the air conditioning effect is very good, I explain that I bought three in one breath, I use more electricity a month down to 200 or so, very satisfied.	Jingdong prefecture level city in Shandong
IXCHSQ Flagship Store	King of Power Saving	The voltage is very stable has been maintained at 220v this value, high power super power saving, trustworthy, reasonable design, shape bright, logistics is quite fast, business warranty period, excellent service, logistics fast!	Jingdong prefecture level city in Shandong
IXCHSQ Flagship Store	King of Power Saving	Use a month after a look at the savings of more than 40 dollars, there is a display of electricity savings, this high-power intelligent frequency converter economizer, affordable and cheap, logistics is very fast, the service attitude is very good, very satisfied with a shopping.	Jingdong prefecture level city in Shandong
IXCHSQ Flagship Store	King of Power Saving	Used for a period of time, it seems that every day can really can save a few degrees of electricity, workmanship quite can, with a period of time and then continue to look at it, anyway, the price is not	Jingdong prefecture level city in Shandong
IXCHSQ Flagship Store	King of Power Saving	Used for a period of time, it seems that every day can indeed save two several degrees of electricity, workmanship quite can, with a period of time and then continue to look at it, anyway, the price is not	Jingdong prefecture level city in Shandong
IXCHSQ Flagship Store	King of Power Saving	High-power super saver received immediately used, the price is not expensive quality is very good, save electricity and money, very convenient, conscientious business, must be praised, recommended to everyone!	Jingdong prefecture level city in Shandong
IXCHSQ Flagship Store	King of Power Saving	Home enhancement version of the intelligent power saver, is in use, I think the price is cheap, value for money, efficacy, power, power saving effect is very good.	Jingdong prefecture level city in Shandong
IXCHSQ Flagship Store	King of Power Saving	Bought three at once, one for each room, summer electricity consumption is very high, despite the three air conditioners on the electricity bill is still down a lot, too Nessie!	Jingdong prefecture level city in Shandong
IXCHSQ Flagship Store	King of Power Saving	Without further ado, the electric bill came down, $102 less this month, personal satisfaction, it counts okay, give you a good review	Jingdong prefecture level city in Shandong
IXCHSQ Flagship Store	King of Power Saving	The power saver has arrived, the installation is really convenient, plugged in on the line operation is quite simple, power saving effect is also a strong!	Jingdong prefecture level city in Shandong
IXCHSQ Flagship Store	King of Power Saving	Saver received, good quality, beautiful appearance, fine workmanship, simple installation	Jingdong prefecture level city in Shandong
IXCHSQ Flagship Store	King of Power Saving	Product design style modern, worth buying	Jingdong prefecture level city in Shandong
IXCHSQ Flagship Store	King of Power Saving	Energy saving, good power saving effect, practical, great value	Jingdong prefecture level city in Shandong

IXCHSQ Flagship Store	King of Power Saving	It's good quality, it's good, it's energy efficient, and I'll buy it again.	Jingdong prefecture level city in Shandong
IXCHSQ Flagship Store	King of Power Saving	Installation saves a lot of electricity, fast logistics, real energy-saving appliances	Jingdong prefecture level city in Shandong
IXCHSQ Flagship Store	King of Power Saving	The effect is good and the power saving is obvious after using it for a month and saving tens of dollars, the product is trustworthy, I now buy another 4 colors to prepare for their siblings' family to pay for	Jingdong prefecture level city in Shandong
IXCHSQ Flagship Store	King of Power Saving	The quality is particularly good workmanship is no problem, the arrival of the very fast oh, especially power-saving	Jingdong prefecture level city in Shandong
IXCHSQ Flagship Store	King of Power Saving	The quality is very good! In use, observed for a day, not as used before, so much power consumption.	Jingdong prefecture level city in Shandong
IXCHSQ Flagship Store	King of Power Saving	Goods have been received, the effect is good is not good, power saving is not yet know, wait for me to use it and then evaluate it.	Jingdong prefecture level city in Shandong
IXCHSQ Flagship Store	King of Power Saving	In-kind and the same as described, the quality is quite good, the seller's attitude is also good, ask questions and answer them, the speed of delivery bar, worth buying Oh!	Jingdong prefecture level city in Shandong
IXCHSQ Flagship Store	King of Power Saving	Nice stuff, came to comment after using it, it does save power.	Jingdong prefecture level city in Shandong
IXCHSQ Flagship Store	King of Power Saving	Fast charging, but also save power, the effect is very good, satisfied, praise!	Jingdong prefecture level city in Shandong
IXCHSQ Flagship Store	King of Power Saving	The goods have been received, I do not know how effective, after using the flat price again!	Jingdong prefecture level city in Shandong
IXCHSQ Flagship Store	King of Power Saving	Merchant service is very good Reply quickly The key is the quality and that quality is good	Jingdong prefecture level city in Shandong
IXCHSQ Flagship Store	King of Power Saving	It's working well now, I'll do a follow up review after I actually use it for a while	Jingdong prefecture level city in Shandong
IXCHSQ Flagship Store	King of Power Saving	The quality is good. After installing it, I feel like I've saved a lot of money on my electricity bill.	Jingdong prefecture level city in Shandong
IXCHSQ Flagship Store	King of Power Saving	Make up a review after use.	Jingdong prefecture level city in Shandong
IXCHSQ Flagship Store	King of Power Saving	review after use	Jingdong prefecture level city in Shandong
IXCHSQ Flagship Store	King of Power Saving	It's a small capacitor. It's not energy efficient.	Jingdong prefecture level city in Shandong
IXCHSQ Flagship Store	King of Power Saving	I'll follow up with a review after I've used it and had results.	Jingdong prefecture level city in Shandong
IXCHSQ Flagship Store	King of Power Saving	No power saving? Not good!	Jingdong prefecture level city in Shandong
IXCHSQ Flagship Store	King of Power Saving	Been using the same appliances and running a 1.5 air conditioner at night for what feels like no time, and after half a month of use it feels like the power is only increasing. Doesn't show any power saving	Jingdong prefecture level city in Shandong
IXCHSQ Flagship Store	King of Power Saving	serviceable	Jingdong prefecture level city in Shandong
IXCHSQ Flagship Store	King of Power Saving	It's a piece of junk. It doesn't save any power.	Jingdong prefecture level city in Shandong
IXCHSQ Flagship Store	King of Power Saving	There are a lot of appliances in the house, the summer air conditioning is very costly, I bought this in front of the air conditioning effect is very good, I explain that I bought three in one breath, I use more electricity a month down to 200 or so, very satisfied.	Jingdong prefecture level city in Shandong
IXCHSQ Flagship Store	King of Power Saving	The voltage is very stable has been maintained at 220v this value, high power super power saving, trustworthy, reasonable design, shape bright, logistics is quite fast, business warranty period, excellent service, logistics fast!	Jingdong prefecture level city in Shandong
IXCHSQ Flagship Store	King of Power Saving	Use a month after a look at the savings of more than 40 dollars, there is a display of electricity savings, this high-power intelligent frequency converter economizer, affordable and cheap, logistics is very fast, the service attitude is very good, very satisfied with a shopping.	Jingdong prefecture level city in Shandong
IXCHSQ Flagship Store	King of Power Saving	Used for a period of time, it seems that every day can really can save a few degrees of electricity, workmanship quite can, with a period of time and then continue to look at it, anyway, the price is not	Jingdong prefecture level city in Shandong
IXCHSQ Flagship Store	King of Power Saving	Used for a period of time, it seems that every day can indeed save two several degrees of electricity, workmanship quite can, with a period of time and then continue to look at it, anyway, the price is not	Jingdong prefecture level city in Shandong
IXCHSQ Flagship Store	King of Power Saving	High-power super saver received immediately used, the price is not expensive quality is very good, save electricity and money, very convenient, conscientious business, must be praised, recommended to everyone!	Jingdong prefecture level city in Shandong
IXCHSQ Flagship Store	King of Power Saving	Home enhancement version of the intelligent power saver, is in use, I think the price is cheap, value for money, efficacy, power, power saving effect is very good.	Jingdong prefecture level city in Shandong
IXCHSQ Flagship Store	King of Power Saving	Bought three at once, one for each room, summer electricity consumption is very high, despite the three air conditioners on the electricity bill is still down a lot, too Nessie!	Jingdong prefecture level city in Shandong
IXCHSQ Flagship Store	King of Power Saving	Without further ado, the electric bill came down, $102 less this month, personal satisfaction, it counts okay, give you a good review	Jingdong prefecture level city in Shandong
IXCHSQ Flagship Store	King of Power Saving	The power saver has arrived, the installation is really convenient, plugged in on the line operation is quite simple, power saving effect is also a strong!	Jingdong prefecture level city in Shandong
IXCHSQ Flagship Store	King of Power Saving	Saver received, good quality, beautiful appearance, fine workmanship, simple installation	Jingdong prefecture level city in Shandong
IXCHSQ Flagship Store	King of Power Saving	Product design style modern, worth buying	Jingdong prefecture level city in Shandong
IXCHSQ Flagship Store	King of Power Saving	Energy saving, good power saving effect, practical, great value	Jingdong prefecture level city in Shandong
IXCHSQ Flagship Store	King of Power Saving	It's good quality, it's good, it's energy efficient, and I'll buy it again.	Jingdong prefecture level city in Shandong
IXCHSQ Flagship Store	King of Power Saving	Installation saves a lot of electricity, fast logistics, real energy-saving appliances	Jingdong prefecture level city in Shandong
IXCHSQ Flagship Store	King of Power Saving	The effect is good and the power saving is obvious after using it for a month and saving tens of dollars, the product is trustworthy, I now buy another 4 colors to prepare for their siblings' family to pay for	Jingdong prefecture level city in Shandong
IXCHSQ Flagship Store	King of Power Saving	The quality is particularly good workmanship is no problem, the arrival of the very fast oh, especially power-saving	Jingdong prefecture level city in Shandong
IXCHSQ Flagship Store	King of Power Saving	The quality is very good! In use, observed for a day, not as used before, so much power consumption.	Jingdong prefecture level city in Shandong
IXCHSQ Flagship Store	King of Power Saving	Goods have been received, the effect is good is not good, power saving is not yet know, wait for me to use it and then evaluate it.	Jingdong prefecture level city in Shandong
IXCHSQ Flagship Store	King of Power Saving	In-kind and the same as described, the quality is quite good, the seller's attitude is also good, ask questions and answer them, the speed of delivery bar, worth buying Oh!	Jingdong prefecture level city in Shandong
IXCHSQ Flagship Store	King of Power Saving	Nice stuff, came to comment after using it, it does save power.	Jingdong prefecture level city in Shandong
IXCHSQ Flagship Store	King of Power Saving	Fast charging, but also save power, the effect is very good, satisfied, praise!	Jingdong prefecture level city in Shandong
IXCHSQ Flagship Store	King of Power Saving	The goods have been received, I do not know how effective, after using the flat price again!	Jingdong prefecture level city in Shandong
IXCHSQ Flagship Store	King of Power Saving	Merchant service is very good Reply quickly The key is the quality and that quality is good	Jingdong prefecture level city in Shandong
IXCHSQ Flagship Store	King of Power Saving	It's working well now, I'll do a follow up review after I actually use it for a while	Jingdong prefecture level city in Shandong
IXCHSQ Flagship Store	King of Power Saving	The quality is good. After installing it, I feel like I've saved a lot of money on my electricity bill.	Jingdong prefecture level city in Shandong
IXCHSQ Flagship Store	King of Power Saving	Make up a review after use.	Jingdong prefecture level city in Shandong
IXCHSQ Flagship Store	King of Power Saving	review after use	Jingdong prefecture level city in Shandong
IXCHSQ Flagship Store	King of Power Saving	It's a small capacitor. It's not energy efficient.	Jingdong prefecture level city in Shandong

IXCHSQ Flagship Store	King of Power Saving	I'll follow up with a review after I've used it and had results.	Jingdong prefecture level city in Shandong
IXCHSQ Flagship Store	King of Power Saving	No power saving? Not good!	Jingdong prefecture level city in Shandong
IXCHSQ Flagship Store	King of Power Saving	Been using the same appliances and running a 1.5 air conditioner at night for what feels like no time, and after half a month of use it feels like the power is only increasing. Doesn't show any power saving	Jingdong prefecture level city in Shandong
IXCHSQ Flagship Store	King of Power Saving	serviceable	Jingdong prefecture level city in Shandong
IXCHSQ Flagship Store	King of Power Saving	It's a piece of junk. It doesn't save any power.	Jingdong prefecture level city in Shandong
IXCHSQ Flagship Store	King of Power Saving	There are a lot of appliances in the house, the summer air conditioning is very costly, I bought this in front of the air conditioning effect is very good, I explain that I bought three in one breath, I use more electricity a month down to 200 or so, very satisfied.	Jingdong prefecture level city in Shandong
IXCHSQ Flagship Store	King of Power Saving	The voltage is very stable has been maintained at 220v this value, high power super power saving, trustworthy, reasonable design, shape bright, logistics is quite fast, business warranty period, excellent service, logistics fast!	Jingdong prefecture level city in Shandong
IXCHSQ Flagship Store	King of Power Saving	Use a month after a look at the savings of more than 40 dollars, there is a display of electricity savings, this high-power intelligent frequency converter economizer, affordable and cheap, logistics is very fast, the service attitude is very good, very satisfied with a shopping.	Jingdong prefecture level city in Shandong
IXCHSQ Flagship Store	King of Power Saving	Used for a period of time, it seems that every day can really can save a few degrees of electricity, workmanship quite can, with a period of time and then continue to look at it, anyway, the price is not	Jingdong prefecture level city in Shandong
IXCHSQ Flagship Store	King of Power Saving	Used for a period of time, it seems that every day can indeed save two several degrees of electricity, workmanship quite can, with a period of time and then continue to look at it, anyway, the price is not	Jingdong prefecture level city in Shandong
IXCHSQ Flagship Store	King of Power Saving	High-power super saver received immediately used, the price is not expensive quality is very good, save electricity and money, very convenient, conscientious business, must be praised, recommended to everyone!	Jingdong prefecture level city in Shandong
IXCHSQ Flagship Store	King of Power Saving	Home enhancement version of the intelligent power saver, is in use, I think the price is cheap, value for money, efficacy, power, power saving effect is very good.	Jingdong prefecture level city in Shandong
IXCHSQ Flagship Store	King of Power Saving	Bought three at once, one for each room, summer electricity consumption is very high, despite the three air conditioners on the electricity bill is still down a lot, too Nessie!	Jingdong prefecture level city in Shandong
IXCHSQ Flagship Store	King of Power Saving	Without further ado, the electric bill came down, $102 less this month, personal satisfaction, it counts okay, give you a good review	Jingdong prefecture level city in Shandong
IXCHSQ Flagship Store	King of Power Saving	The power saver has arrived, the installation is really convenient, plugged in on the line operation is quite simple, power saving effect is also a strong!	Jingdong prefecture level city in Shandong
IXCHSQ Flagship Store	King of Power Saving	Saver received, good quality, beautiful appearance, fine workmanship, simple installation	Jingdong prefecture level city in Shandong
IXCHSQ Flagship Store	King of Power Saving	Product design style modern, worth buying	Jingdong prefecture level city in Shandong
IXCHSQ Flagship Store	King of Power Saving	Energy saving, good power saving effect, practical, great value	Jingdong prefecture level city in Shandong
IXCHSQ Flagship Store	King of Power Saving	It's good quality, it's good, it's energy efficient, and I'll buy it again.	Jingdong prefecture level city in Shandong
IXCHSQ Flagship Store	King of Power Saving	Installation saves a lot of electricity, fast logistics, real energy-saving appliances	Jingdong prefecture level city in Shandong
IXCHSQ Flagship Store	King of Power Saving	The effect is good and the power saving is obvious after using it for a month and saving tens of dollars, the product is trustworthy, I now buy another 4 colors to prepare for their siblings' family to pay for	Jingdong prefecture level city in Shandong
IXCHSQ Flagship Store	King of Power Saving	The quality is particularly good workmanship is no problem, the arrival of the very fast oh, especially power-saving	Jingdong prefecture level city in Shandong
IXCHSQ Flagship Store	King of Power Saving	The quality is very good! In use, observed for a day, not as used before, so much power consumption.	Jingdong prefecture level city in Shandong
IXCHSQ Flagship Store	King of Power Saving	Goods have been received, the effect is good is not good, power saving is not yet know, wait for me to use it and then evaluate it.	Jingdong prefecture level city in Shandong
IXCHSQ Flagship Store	King of Power Saving	In-kind and the same as described, the quality is quite good, the seller's attitude is also good, ask questions and answer them, the speed of delivery bar, worth buying Oh!	Jingdong prefecture level city in Shandong
IXCHSQ Flagship Store	King of Power Saving	Nice stuff, came to comment after using it, it does save power.	Jingdong prefecture level city in Shandong
IXCHSQ Flagship Store	King of Power Saving	Fast charging, but also save power, the effect is very good, satisfied, praise!	Jingdong prefecture level city in Shandong
IXCHSQ Flagship Store	King of Power Saving	The goods have been received, I do not know how effective, after using the flat price again!	Jingdong prefecture level city in Shandong
IXCHSQ Flagship Store	King of Power Saving	Merchant service is very good Reply quickly The key is the quality and that quality is good	Jingdong prefecture level city in Shandong
IXCHSQ Flagship Store	King of Power Saving	It's working well now, I'll do a follow up review after I actually use it for a while	Jingdong prefecture level city in Shandong
IXCHSQ Flagship Store	King of Power Saving	The quality is good. After installing it, I feel like I've saved a lot of money on my electricity bill.	Jingdong prefecture level city in Shandong
IXCHSQ Flagship Store	King of Power Saving	Make up a review after use.	Jingdong prefecture level city in Shandong
IXCHSQ Flagship Store	King of Power Saving	review after use	Jingdong prefecture level city in Shandong
IXCHSQ Flagship Store	King of Power Saving	It's a small capacitor. It's not energy efficient.	Jingdong prefecture level city in Shandong
IXCHSQ Flagship Store	King of Power Saving	I'll follow up with a review after I've used it and had results.	Jingdong prefecture level city in Shandong
IXCHSQ Flagship Store	King of Power Saving	No power saving? Not good!	Jingdong prefecture level city in Shandong
IXCHSQ Flagship Store	King of Power Saving	Been using the same appliances and running a 1.5 air conditioner at night for what feels like no time, and after half a month of use it feels like the power is only increasing. Doesn't show any power saving	Jingdong prefecture level city in Shandong
IXCHSQ Flagship Store	King of Power Saving	serviceable	Jingdong prefecture level city in Shandong
IXCHSQ Flagship Store	King of Power Saving	It's a piece of junk. It doesn't save any power.	Jingdong prefecture level city in Shandong
IXCHSQ Flagship Store	King of Power Saving	There are a lot of appliances in the house, the summer air conditioning is very costly, I bought this in front of the air conditioning effect is very good, I explain that I bought three in one breath, I use more electricity a month down to 200 or so, very satisfied.	Jingdong prefecture level city in Shandong
IXCHSQ Flagship Store	King of Power Saving	The voltage is very stable has been maintained at 220v this value, high power super power saving, trustworthy, reasonable design, shape bright, logistics is quite fast, business warranty period, excellent service, logistics fast!	Jingdong prefecture level city in Shandong
IXCHSQ Flagship Store	King of Power Saving	Use a month after a look at the savings of more than 40 dollars, there is a display of electricity savings, this high-power intelligent frequency converter economizer, affordable and cheap, logistics is very fast, the service attitude is very good, very satisfied with a shopping.	Jingdong prefecture level city in Shandong
IXCHSQ Flagship Store	King of Power Saving	Used for a period of time, it seems that every day can really can save a few degrees of electricity, workmanship quite can, with a period of time and then continue to look at it, anyway, the price is not	Jingdong prefecture level city in Shandong
IXCHSQ Flagship Store	King of Power Saving	Used for a period of time, it seems that every day can indeed save two several degrees of electricity, workmanship quite can, with a period of time and then continue to look at it, anyway, the price is not	Jingdong prefecture level city in Shandong
IXCHSQ Flagship Store	King of Power Saving	High-power super saver received immediately used, the price is not expensive quality is very good, save electricity and money, very convenient, conscientious business, must be praised, recommended to everyone!	Jingdong prefecture level city in Shandong
IXCHSQ Flagship Store	King of Power Saving	Home enhancement version of the intelligent power saver, is in use, I think the price is cheap, value for money, efficacy, power, power saving effect is very good.	Jingdong prefecture level city in Shandong
IXCHSQ Flagship Store	King of Power Saving	Bought three at once, one for each room, summer electricity consumption is very high, despite the three air conditioners on the electricity bill is still down a lot, too Nessie!	Jingdong prefecture level city in Shandong
IXCHSQ Flagship Store	King of Power Saving	Without further ado, the electric bill came down, $102 less this month, personal satisfaction, it counts okay, give you a good review	Jingdong prefecture level city in Shandong

IXCHSQ Flagship Store	King of Power Saving	The power saver has arrived, the installation is really convenient, plugged in on the line operation is quite simple, power saving effect is also a strong!	Jingdong prefecture level city in Shandong
IXCHSQ Flagship Store	King of Power Saving	Saver received, good quality, beautiful appearance, fine workmanship, simple installation	Jingdong prefecture level city in Shandong
IXCHSQ Flagship Store	King of Power Saving	Product design style modern, worth buying	Jingdong prefecture level city in Shandong
IXCHSQ Flagship Store	King of Power Saving	Energy saving, good power saving effect, practical, great value	Jingdong prefecture level city in Shandong
IXCHSQ Flagship Store	King of Power Saving	It's good quality, it's good, it's energy efficient, and I'll buy it again.	Jingdong prefecture level city in Shandong
IXCHSQ Flagship Store	King of Power Saving	Installation saves a lot of electricity, fast logistics, real energy-saving appliances	Jingdong prefecture level city in Shandong
IXCHSQ Flagship Store	King of Power Saving	The effect is good and the power saving is obvious after using it for a month and saving tens of dollars, the product is trustworthy, I now buy another 4 colors to prepare for their siblings' family to pay for	Jingdong prefecture level city in Shandong
IXCHSQ Flagship Store	King of Power Saving	The quality is particularly good workmanship is no problem, the arrival of the very fast oh, especially power-saving	Jingdong prefecture level city in Shandong
IXCHSQ Flagship Store	King of Power Saving	The quality is very good! In use, observed for a day, not as used before, so much power consumption.	Jingdong prefecture level city in Shandong
IXCHSQ Flagship Store	King of Power Saving	Goods have been received, the effect is good is not good, power saving is not yet know, wait for me to use it and then evaluate it.	Jingdong prefecture level city in Shandong
IXCHSQ Flagship Store	King of Power Saving	In-kind and the same as described, the quality is quite good, the seller's attitude is also good, ask questions and answer them, the speed of delivery bar, worth buying Oh!	Jingdong prefecture level city in Shandong
IXCHSQ Flagship Store	King of Power Saving	Nice stuff, came to comment after using it, it does save power.	Jingdong prefecture level city in Shandong
IXCHSQ Flagship Store	King of Power Saving	Fast charging, but also save power, the effect is very good, satisfied, praise!	Jingdong prefecture level city in Shandong
IXCHSQ Flagship Store	King of Power Saving	The goods have been received, I do not know how effective, after using the flat price again!	Jingdong prefecture level city in Shandong
IXCHSQ Flagship Store	King of Power Saving	Merchant service is very good Reply quickly The key is the quality and that quality is good	Jingdong prefecture level city in Shandong
IXCHSQ Flagship Store	King of Power Saving	It's working well now, I'll do a follow up review after I actually use it for a while	Jingdong prefecture level city in Shandong
IXCHSQ Flagship Store	King of Power Saving	The quality is good. After installing it, I feel like I've saved a lot of money on my electricity bill.	Jingdong prefecture level city in Shandong
IXCHSQ Flagship Store	King of Power Saving	Make up a review after use.	Jingdong prefecture level city in Shandong
IXCHSQ Flagship Store	King of Power Saving	review after use	Jingdong prefecture level city in Shandong
IXCHSQ Flagship Store	King of Power Saving	It's a small capacitor. It's not energy efficient.	Jingdong prefecture level city in Shandong
IXCHSQ Flagship Store	King of Power Saving	I'll follow up with a review after I've used it and had results.	Jingdong prefecture level city in Shandong
IXCHSQ Flagship Store	King of Power Saving	No power saving? Not good!	Jingdong prefecture level city in Shandong
IXCHSQ Flagship Store	King of Power Saving	Been using the same appliances and running a 1.5 air conditioner at night for what feels like no time, and after half a month of use it feels like the power is only increasing. Doesn't show any power saving	Jingdong prefecture level city in Shandong
IXCHSQ Flagship Store	King of Power Saving	serviceable	Jingdong prefecture level city in Shandong
IXCHSQ Flagship Store	King of Power Saving	It's a piece of junk. It doesn't save any power.	Jingdong prefecture level city in Shandong
IXCHSQ Flagship Store	King of Power Saving	There are a lot of appliances in the house, the summer air conditioning is very costly, I bought this in front of the air conditioning effect is very good, I explain that I bought three in one breath, I use more electricity a month down to 200 or so, very satisfied.	Jingdong prefecture level city in Shandong
IXCHSQ Flagship Store	King of Power Saving	The voltage is very stable has been maintained at 220v this value, high power super power saving, trustworthy, reasonable design, shape bright, logistics is quite fast, business warranty period, excellent service, logistics fast!	Jingdong prefecture level city in Shandong
IXCHSQ Flagship Store	King of Power Saving	Use a month after a look at the savings of more than 40 dollars, there is a display of electricity savings, this high-power intelligent frequency converter economizer, affordable and cheap, logistics is very fast, the service attitude is very good, very satisfied with a shopping.	Jingdong prefecture level city in Shandong
IXCHSQ Flagship Store	King of Power Saving	Used for a period of time, it seems that every day can really can save a few degrees of electricity, workmanship quite can, with a period of time and then continue to look at it, anyway, the price is not	Jingdong prefecture level city in Shandong
IXCHSQ Flagship Store	King of Power Saving	Used for a period of time, it seems that every day can indeed save two several degrees of electricity, workmanship quite can, with a period of time and then continue to look at it, anyway, the price is not	Jingdong prefecture level city in Shandong
IXCHSQ Flagship Store	King of Power Saving	High-power super saver received immediately used, the price is not expensive quality is very good, save electricity and money, very convenient, conscientious business, must be praised, recommended to everyone!	Jingdong prefecture level city in Shandong
IXCHSQ Flagship Store	King of Power Saving	Home enhancement version of the intelligent power saver, is in use, I think the price is cheap, value for money, efficacy, power, power saving effect is very good.	Jingdong prefecture level city in Shandong
IXCHSQ Flagship Store	King of Power Saving	Bought three at once, one for each room, summer electricity consumption is very high, despite the three air conditioners on the electricity bill is still down a lot, too Nessie!	Jingdong prefecture level city in Shandong
IXCHSQ Flagship Store	King of Power Saving	Without further ado, the electric bill came down, $102 less this month, personal satisfaction, it counts okay, give you a good review	Jingdong prefecture level city in Shandong
IXCHSQ Flagship Store	King of Power Saving	The power saver has arrived, the installation is really convenient, plugged in on the line operation is quite simple, power saving effect is also a strong!	Jingdong prefecture level city in Shandong
IXCHSQ Flagship Store	King of Power Saving	Saver received, good quality, beautiful appearance, fine workmanship, simple installation	Jingdong prefecture level city in Shandong
IXCHSQ Flagship Store	King of Power Saving	Product design style modern, worth buying	Jingdong prefecture level city in Shandong
IXCHSQ Flagship Store	King of Power Saving	Energy saving, good power saving effect, practical, great value	Jingdong prefecture level city in Shandong
IXCHSQ Flagship Store	King of Power Saving	It's good quality, it's good, it's energy efficient, and I'll buy it again.	Jingdong prefecture level city in Shandong
IXCHSQ Flagship Store	King of Power Saving	Installation saves a lot of electricity, fast logistics, real energy-saving appliances	Jingdong prefecture level city in Shandong
IXCHSQ Flagship Store	King of Power Saving	The effect is good and the power saving is obvious after using it for a month and saving tens of dollars, the product is trustworthy, I now buy another 4 colors to prepare for their siblings' family to pay for	Jingdong prefecture level city in Shandong
IXCHSQ Flagship Store	King of Power Saving	The quality is particularly good workmanship is no problem, the arrival of the very fast oh, especially power-saving	Jingdong prefecture level city in Shandong
IXCHSQ Flagship Store	King of Power Saving	The quality is very good! In use, observed for a day, not as used before, so much power consumption.	Jingdong prefecture level city in Shandong
IXCHSQ Flagship Store	King of Power Saving	Goods have been received, the effect is good is not good, power saving is not yet know, wait for me to use it and then evaluate it.	Jingdong prefecture level city in Shandong
IXCHSQ Flagship Store	King of Power Saving	In-kind and the same as described, the quality is quite good, the seller's attitude is also good, ask questions and answer them, the speed of delivery bar, worth buying Oh!	Jingdong prefecture level city in Shandong
IXCHSQ Flagship Store	King of Power Saving	Nice stuff, came to comment after using it, it does save power.	Jingdong prefecture level city in Shandong
IXCHSQ Flagship Store	King of Power Saving	Fast charging, but also save power, the effect is very good, satisfied, praise!	Jingdong prefecture level city in Shandong
IXCHSQ Flagship Store	King of Power Saving	The goods have been received, I do not know how effective, after using the flat price again!	Jingdong prefecture level city in Shandong
IXCHSQ Flagship Store	King of Power Saving	Merchant service is very good Reply quickly The key is the quality and that quality is good	Jingdong prefecture level city in Shandong
IXCHSQ Flagship Store	King of Power Saving	It's working well now, I'll do a follow up review after I actually use it for a while	Jingdong prefecture level city in Shandong

IXCHSQ Flagship Store	King of Power Saving	The quality is good. After installing it, I feel like I've saved a lot of money on my electricity bill.	Jingdong prefecture level city in Shandong
IXCHSQ Flagship Store	King of Power Saving	Make up a review after use.	Jingdong prefecture level city in Shandong
IXCHSQ Flagship Store	King of Power Saving	review after use	Jingdong prefecture level city in Shandong
IXCHSQ Flagship Store	King of Power Saving	It's a small capacitor. It's not energy efficient.	Jingdong prefecture level city in Shandong
IXCHSQ Flagship Store	King of Power Saving	I'll follow up with a review after I've used it and had results.	Jingdong prefecture level city in Shandong
IXCHSQ Flagship Store	King of Power Saving	No power saving? Not good!	Jingdong prefecture level city in Shandong
IXCHSQ Flagship Store	King of Power Saving	Been using the same appliances and running a 1.5 air conditioner at night for what feels like no time, and after half a month of use it feels like the power is only increasing. Doesn't show any power saving	Jingdong prefecture level city in Shandong
IXCHSQ Flagship Store	King of Power Saving	serviceable	Jingdong prefecture level city in Shandong
IXCHSQ Flagship Store	King of Power Saving	It's a piece of junk. It doesn't save any power.	Jingdong prefecture level city in Shandong
IXCHSQ Flagship Store	King of Power Saving	There are a lot of appliances in the house, the summer air conditioning is very costly, I bought this in front of the air conditioning effect is very good, I explain that I bought three in one breath, I use more electricity a month down to 200 or so, very satisfied.	Jingdong prefecture level city in Shandong
IXCHSQ Flagship Store	King of Power Saving	The voltage is very stable has been maintained at 220v this value, high power super power saving, trustworthy, reasonable design, shape bright, logistics is quite fast, business warranty period, excellent service, logistics fast!	Jingdong prefecture level city in Shandong
IXCHSQ Flagship Store	King of Power Saving	Use a month after a look at the savings of more than 40 dollars, there is a display of electricity savings, this high-power intelligent frequency converter economizer, affordable and cheap, logistics is very fast, the service attitude is very good, very satisfied with a shopping.	Jingdong prefecture level city in Shandong
IXCHSQ Flagship Store	King of Power Saving	Used for a period of time, it seems that every day can really can save a few degrees of electricity, workmanship quite can, with a period of time and then continue to look at it, anyway, the price is not	Jingdong prefecture level city in Shandong
IXCHSQ Flagship Store	King of Power Saving	Used for a period of time, it seems that every day can indeed save two several degrees of electricity, workmanship quite can, with a period of time and then continue to look at it, anyway, the price is not	Jingdong prefecture level city in Shandong
IXCHSQ Flagship Store	King of Power Saving	High-power super saver received immediately used, the price is not expensive quality is very good, save electricity and money, very convenient, conscientious business, must be praised, recommended to everyone!	Jingdong prefecture level city in Shandong
IXCHSQ Flagship Store	King of Power Saving	Home enhancement version of the intelligent power saver, is in use, I think the price is cheap, value for money, efficacy, power, power saving effect is very good.	Jingdong prefecture level city in Shandong
IXCHSQ Flagship Store	King of Power Saving	Bought three at once, one for each room, summer electricity consumption is very high, despite the three air conditioners on the electricity bill is still down a lot, too Nessie!	Jingdong prefecture level city in Shandong
IXCHSQ Flagship Store	King of Power Saving	Without further ado, the electric bill came down, $102 less this month, personal satisfaction, it counts okay, give you a good review	Jingdong prefecture level city in Shandong
IXCHSQ Flagship Store	King of Power Saving	The power saver has arrived, the installation is really convenient, plugged in on the line operation is quite simple, power saving effect is also a strong!	Jingdong prefecture level city in Shandong
IXCHSQ Flagship Store	King of Power Saving	Saver received, good quality, beautiful appearance, fine workmanship, simple installation	Jingdong prefecture level city in Shandong
IXCHSQ Flagship Store	King of Power Saving	Product design style modern, worth buying	Jingdong prefecture level city in Shandong
IXCHSQ Flagship Store	King of Power Saving	Energy saving, good power saving effect, practical, great value	Jingdong prefecture level city in Shandong
IXCHSQ Flagship Store	King of Power Saving	It's good quality, it's good, it's energy efficient, and I'll buy it again.	Jingdong prefecture level city in Shandong
IXCHSQ Flagship Store	King of Power Saving	Installation saves a lot of electricity, fast logistics, real energy-saving appliances	Jingdong prefecture level city in Shandong
IXCHSQ Flagship Store	King of Power Saving	The effect is good and the power saving is obvious after using it for a month and saving tens of dollars, the product is trustworthy, I now buy another 4 colors to prepare for their siblings' family to pay for	Jingdong prefecture level city in Shandong
IXCHSQ Flagship Store	King of Power Saving	The quality is particularly good workmanship is no problem, the arrival of the very fast oh, especially power-saving	Jingdong prefecture level city in Shandong
IXCHSQ Flagship Store	King of Power Saving	The quality is very good! In use, observed for a day, not as used before, so much power consumption.	Jingdong prefecture level city in Shandong
IXCHSQ Flagship Store	King of Power Saving	Goods have been received, the effect is good is not good, power saving is not yet know, wait for me to use it and then evaluate it.	Jingdong prefecture level city in Shandong
IXCHSQ Flagship Store	King of Power Saving	In-kind and the same as described, the quality is quite good, the seller's attitude is also good, ask questions and answer them, the speed of delivery bar, worth buying Oh!	Jingdong prefecture level city in Shandong
IXCHSQ Flagship Store	King of Power Saving	Nice stuff, came to comment after using it, it does save power.	Jingdong prefecture level city in Shandong
IXCHSQ Flagship Store	King of Power Saving	Fast charging, but also save power, the effect is very good, satisfied, praise!	Jingdong prefecture level city in Shandong
IXCHSQ Flagship Store	King of Power Saving	The goods have been received, I do not know how effective, after using the flat price again!	Jingdong prefecture level city in Shandong
IXCHSQ Flagship Store	King of Power Saving	Merchant service is very good Reply quickly The key is the quality and that quality is good	Jingdong prefecture level city in Shandong
IXCHSQ Flagship Store	King of Power Saving	It's working well now, I'll do a follow up review after I actually use it for a while	Jingdong prefecture level city in Shandong
IXCHSQ Flagship Store	King of Power Saving	The quality is good. After installing it, I feel like I've saved a lot of money on my electricity bill.	Jingdong prefecture level city in Shandong
IXCHSQ Flagship Store	King of Power Saving	Make up a review after use.	Jingdong prefecture level city in Shandong
IXCHSQ Flagship Store	King of Power Saving	review after use	Jingdong prefecture level city in Shandong
IXCHSQ Flagship Store	King of Power Saving	It's a small capacitor. It's not energy efficient.	Jingdong prefecture level city in Shandong
IXCHSQ Flagship Store	King of Power Saving	I'll follow up with a review after I've used it and had results.	Jingdong prefecture level city in Shandong
IXCHSQ Flagship Store	King of Power Saving	No power saving? Not good!	Jingdong prefecture level city in Shandong
IXCHSQ Flagship Store	King of Power Saving	Been using the same appliances and running a 1.5 air conditioner at night for what feels like no time, and after half a month of use it feels like the power is only increasing. Doesn't show any power saving	Jingdong prefecture level city in Shandong
IXCHSQ Flagship Store	King of Power Saving	serviceable	Jingdong prefecture level city in Shandong
IXCHSQ Flagship Store	King of Power Saving	It's a piece of junk. It doesn't save any power.	Jingdong prefecture level city in Shandong
IXCHSQ Flagship Store	King of Power Saving	There are a lot of appliances in the house, the summer air conditioning is very costly, I bought this in front of the air conditioning effect is very good, I explain that I bought three in one breath, I use more electricity a month down to 200 or so, very satisfied.	Jingdong prefecture level city in Shandong
IXCHSQ Flagship Store	King of Power Saving	The voltage is very stable has been maintained at 220v this value, high power super power saving, trustworthy, reasonable design, shape bright, logistics is quite fast, business warranty period, excellent service, logistics fast!	Jingdong prefecture level city in Shandong
IXCHSQ Flagship Store	King of Power Saving	Use a month after a look at the savings of more than 40 dollars, there is a display of electricity savings, this high-power intelligent frequency converter economizer, affordable and cheap, logistics is very fast, the service attitude is very good, very satisfied with a shopping.	Jingdong prefecture level city in Shandong
IXCHSQ Flagship Store	King of Power Saving	Used for a period of time, it seems that every day can really can save a few degrees of electricity, workmanship quite can, with a period of time and then continue to look at it, anyway, the price is not	Jingdong prefecture level city in Shandong
IXCHSQ Flagship Store	King of Power Saving	Used for a period of time, it seems that every day can indeed save two several degrees of electricity, workmanship quite can, with a period of time and then continue to look at it, anyway, the price is not	Jingdong prefecture level city in Shandong

IXCHSQ Flagship Store	King of Power Saving	High-power super saver received immediately used, the price is not expensive quality is very good, save electricity and money, very convenient, conscientious business, must be praised, recommended to everyone!	Jingdong prefecture level city in Shandong
IXCHSQ Flagship Store	King of Power Saving	Home enhancement version of the intelligent power saver, is in use, I think the price is cheap, value for money, efficacy, power, power saving effect is very good.	Jingdong prefecture level city in Shandong
IXCHSQ Flagship Store	King of Power Saving	Bought three at once, one for each room, summer electricity consumption is very high, despite the three air conditioners on the electricity bill is still down a lot, too Nessie!	Jingdong prefecture level city in Shandong
IXCHSQ Flagship Store	King of Power Saving	Without further ado, the electric bill came down, $102 less this month, personal satisfaction, it counts okay, give you a good review	Jingdong prefecture level city in Shandong
IXCHSQ Flagship Store	King of Power Saving	The power saver has arrived, the installation is really convenient, plugged in on the line operation is quite simple, power saving effect is also a strong!	Jingdong prefecture level city in Shandong
IXCHSQ Flagship Store	King of Power Saving	Saver received, good quality, beautiful appearance, fine workmanship, simple installation	Jingdong prefecture level city in Shandong
IXCHSQ Flagship Store	King of Power Saving	Product design style modern, worth buying	Jingdong prefecture level city in Shandong
IXCHSQ Flagship Store	King of Power Saving	Energy saving, good power saving effect, practical, great value	Jingdong prefecture level city in Shandong
IXCHSQ Flagship Store	King of Power Saving	It’s good quality, it’s good, it’s energy efficient, and I’ll buy it again.	Jingdong prefecture level city in Shandong
IXCHSQ Flagship Store	King of Power Saving	Installation saves a lot of electricity, fast logistics, real energy-saving appliances	Jingdong prefecture level city in Shandong
IXCHSQ Flagship Store	King of Power Saving	The effect is good and the power saving is obvious after using it for a month and saving tens of dollars, the product is trustworthy, I now buy another 4 colors to prepare for their siblings’ family to pay for	Jingdong prefecture level city in Shandong
IXCHSQ Flagship Store	King of Power Saving	The quality is particularly good workmanship is no problem, the arrival of the very fast oh, especially power-saving	Jingdong prefecture level city in Shandong
IXCHSQ Flagship Store	King of Power Saving	The quality is very good! In use, observed for a day, not as used before, so much power consumption.	Jingdong prefecture level city in Shandong
IXCHSQ Flagship Store	King of Power Saving	Goods have been received, the effect is good is not good, power saving is not yet know, wait for me to use it and then evaluate it.	Jingdong prefecture level city in Shandong
IXCHSQ Flagship Store	King of Power Saving	In-kind and the same as described, the quality is quite good, the seller’s attitude is also good, ask questions and answer them, the speed of delivery bar, worth buying Oh!	Jingdong prefecture level city in Shandong
IXCHSQ Flagship Store	King of Power Saving	Nice stuff, came to comment after using it, it does save power.	Jingdong prefecture level city in Shandong
IXCHSQ Flagship Store	King of Power Saving	Fast charging, but also save power, the effect is very good, satisfied, praise!	Jingdong prefecture level city in Shandong
IXCHSQ Flagship Store	King of Power Saving	The goods have been received, I do not know how effective, after using the flat price again!	Jingdong prefecture level city in Shandong
IXCHSQ Flagship Store	King of Power Saving	Merchant service is very good Reply quickly The key is the quality and that quality is good	Jingdong prefecture level city in Shandong
IXCHSQ Flagship Store	King of Power Saving	It’s working well now, I’ll do a follow up review after I actually use it for a while	Jingdong prefecture level city in Shandong
IXCHSQ Flagship Store	King of Power Saving	The quality is good. After installing it, I feel like I’ve saved a lot of money on my electricity bill.	Jingdong prefecture level city in Shandong
IXCHSQ Flagship Store	King of Power Saving	Make up a review after use.	Jingdong prefecture level city in Shandong
IXCHSQ Flagship Store	King of Power Saving	review after use	Jingdong prefecture level city in Shandong
IXCHSQ Flagship Store	King of Power Saving	It’s a small capacitor. It’s not energy efficient.	Jingdong prefecture level city in Shandong
IXCHSQ Flagship Store	King of Power Saving	I’ll follow up with a review after I’ve used it and had results.	Jingdong prefecture level city in Shandong
IXCHSQ Flagship Store	King of Power Saving	No power saving? Not good!	Jingdong prefecture level city in Shandong
IXCHSQ Flagship Store	King of Power Saving	Been using the same appliances and running a 1.5 air conditioner at night for what feels like no time, and after half a month of use it feels like the power is only increasing. Doesn't show any power saving	Jingdong prefecture level city in Shandong
IXCHSQ Flagship Store	King of Power Saving	serviceable	Jingdong prefecture level city in Shandong
IXCHSQ Flagship Store	King of Power Saving	It’s a piece of junk. It doesn't save any power.	Jingdong prefecture level city in Shandong
IXCHSQ Flagship Store	King of Power Saving	There are a lot of appliances in the house, the summer air conditioning is very costly, I bought this in front of the air conditioning effect is very good, I explain that I bought three in one breath, I use more electricity a month down to 200 or so, very satisfied.	Jingdong prefecture level city in Shandong
IXCHSQ Flagship Store	King of Power Saving	The voltage is very stable has been maintained at 220v this value, high power super power saving, trustworthy, reasonable design, shape bright, logistics is quite fast, business warranty period, excellent service, logistics fast!	Jingdong prefecture level city in Shandong
IXCHSQ Flagship Store	King of Power Saving	Use a month after a look at the savings of more than 40 dollars, there is a display of electricity savings, this high-power intelligent frequency converter economizer, affordable and cheap, logistics is very fast, the service attitude is very good, very satisfied with a shopping.	Jingdong prefecture level city in Shandong
IXCHSQ Flagship Store	King of Power Saving	Used for a period of time, it seems that every day can really can save a few degrees of electricity, workmanship quite can, with a period of time and then continue to look at it, anyway, the price is not	Jingdong prefecture level city in Shandong
IXCHSQ Flagship Store	King of Power Saving	Used for a period of time, it seems that every day can indeed save two several degrees of electricity, workmanship quite can, with a period of time and then continue to look at it, anyway, the price is not	Jingdong prefecture level city in Shandong
IXCHSQ Flagship Store	King of Power Saving	High-power super saver received immediately used, the price is not expensive quality is very good, save electricity and money, very convenient, conscientious business, must be praised, recommended to everyone!	Jingdong prefecture level city in Shandong
IXCHSQ Flagship Store	King of Power Saving	Home enhancement version of the intelligent power saver, is in use, I think the price is cheap, value for money, efficacy, power, power saving effect is very good.	Jingdong prefecture level city in Shandong
IXCHSQ Flagship Store	King of Power Saving	Bought three at once, one for each room, summer electricity consumption is very high, despite the three air conditioners on the electricity bill is still down a lot, too Nessie!	Jingdong prefecture level city in Shandong
IXCHSQ Flagship Store	King of Power Saving	Without further ado, the electric bill came down, $102 less this month, personal satisfaction, it counts okay, give you a good review	Jingdong prefecture level city in Shandong
IXCHSQ Flagship Store	King of Power Saving	The power saver has arrived, the installation is really convenient, plugged in on the line operation is quite simple, power saving effect is also a strong!	Jingdong prefecture level city in Shandong
IXCHSQ Flagship Store	King of Power Saving	Saver received, good quality, beautiful appearance, fine workmanship, simple installation	Jingdong prefecture level city in Shandong
IXCHSQ Flagship Store	King of Power Saving	Product design style modern, worth buying	Jingdong prefecture level city in Shandong
IXCHSQ Flagship Store	King of Power Saving	Energy saving, good power saving effect, practical, great value	Jingdong prefecture level city in Shandong
IXCHSQ Flagship Store	King of Power Saving	It’s good quality, it’s good, it’s energy efficient, and I’ll buy it again.	Jingdong prefecture level city in Shandong
IXCHSQ Flagship Store	King of Power Saving	Installation saves a lot of electricity, fast logistics, real energy-saving appliances	Jingdong prefecture level city in Shandong
IXCHSQ Flagship Store	King of Power Saving	The effect is good and the power saving is obvious after using it for a month and saving tens of dollars, the product is trustworthy, I now buy another 4 colors to prepare for their siblings’ family to pay for	Jingdong prefecture level city in Shandong
IXCHSQ Flagship Store	King of Power Saving	The quality is particularly good workmanship is no problem, the arrival of the very fast oh, especially power-saving	Jingdong prefecture level city in Shandong
IXCHSQ Flagship Store	King of Power Saving	The quality is very good! In use, observed for a day, not as used before, so much power consumption.	Jingdong prefecture level city in Shandong
IXCHSQ Flagship Store	King of Power Saving	Goods have been received, the effect is good is not good, power saving is not yet know, wait for me to use it and then evaluate it.	Jingdong prefecture level city in Shandong
IXCHSQ Flagship Store	King of Power Saving	In-kind and the same as described, the quality is quite good, the seller’s attitude is also good, ask questions and answer them, the speed of delivery bar, worth buying Oh!	Jingdong prefecture level city in Shandong
IXCHSQ Flagship Store	King of Power Saving	Nice stuff, came to comment after using it, it does save power.	Jingdong prefecture level city in Shandong

IXCHSQ Flagship Store	King of Power Saving	Fast charging, but also save power, the effect is very good, satisfied, praise!	Jingdong prefecture level city in Shandong
IXCHSQ Flagship Store	King of Power Saving	The goods have been received, I do not know how effective, after using the flat price again!	Jingdong prefecture level city in Shandong
IXCHSQ Flagship Store	King of Power Saving	Merchant service is very good Reply quickly The key is the quality and that quality is good	Jingdong prefecture level city in Shandong
IXCHSQ Flagship Store	King of Power Saving	It's working well now, I'll do a follow up review after I actually use it for a while	Jingdong prefecture level city in Shandong
IXCHSQ Flagship Store	King of Power Saving	The quality is good. After installing it, I feel like I've saved a lot of money on my electricity bill.	Jingdong prefecture level city in Shandong
IXCHSQ Flagship Store	King of Power Saving	Make up a review after use.	Jingdong prefecture level city in Shandong
IXCHSQ Flagship Store	King of Power Saving	review after use	Jingdong prefecture level city in Shandong
IXCHSQ Flagship Store	King of Power Saving	It's a small capacitor. It's not energy efficient.	Jingdong prefecture level city in Shandong
IXCHSQ Flagship Store	King of Power Saving	I'll follow up with a review after I've used it and had results.	Jingdong prefecture level city in Shandong
IXCHSQ Flagship Store	King of Power Saving	No power saving? Not good!	Jingdong prefecture level city in Shandong
IXCHSQ Flagship Store	King of Power Saving	Been using the same appliances and running a 1.5 air conditioner at night for what feels like no time, and after half a month of use it feels like the power is only increasing. Doesn't show any power saving	Jingdong prefecture level city in Shandong
IXCHSQ Flagship Store	King of Power Saving	serviceable	Jingdong prefecture level city in Shandong
IXCHSQ Flagship Store	King of Power Saving	It's a piece of junk. It doesn't save any power.	Jingdong prefecture level city in Shandong
IXCHSQ Flagship Store	King of Power Saving	There are a lot of appliances in the house, the summer air conditioning is very costly, I bought this in front of the air conditioning effect is very good, I explain that I bought three in one breath, I use more electricity a month down to 200 or so, very satisfied.	Jingdong prefecture level city in Shandong
IXCHSQ Flagship Store	King of Power Saving	The voltage is very stable has been maintained at 220v this value, high power super power saving, trustworthy, reasonable design, shape bright, logistics is quite fast, business warranty period, excellent service, logistics fast!	Jingdong prefecture level city in Shandong
IXCHSQ Flagship Store	King of Power Saving	Use a month after a look at the savings of more than 40 dollars, there is a display of electricity savings, this high-power intelligent frequency converter economizer, affordable and cheap, logistics is very fast, the service attitude is very good, very satisfied with a shopping.	Jingdong prefecture level city in Shandong
IXCHSQ Flagship Store	King of Power Saving	Used for a period of time, it seems that every day can really can save a few degrees of electricity, workmanship quite can, with a period of time and then continue to look at it, anyway, the price is not	Jingdong prefecture level city in Shandong
IXCHSQ Flagship Store	King of Power Saving	Used for a period of time, it seems that every day can indeed save two several degrees of electricity, workmanship quite can, with a period of time and then continue to look at it, anyway, the price is not	Jingdong prefecture level city in Shandong
IXCHSQ Flagship Store	King of Power Saving	High-power super saver received immediately used, the price is not expensive quality is very good, save electricity and money, very convenient, conscientious business, must be praised, recommended to everyone!	Jingdong prefecture level city in Shandong
IXCHSQ Flagship Store	King of Power Saving	Home enhancement version of the intelligent power saver, is in use, I think the price is cheap, value for money, efficacy, power, power saving effect is very good.	Jingdong prefecture level city in Shandong
IXCHSQ Flagship Store	King of Power Saving	Bought three at once, one for each room, summer electricity consumption is very high, despite the three air conditioners on the electricity bill is still down a lot, too Nessie!	Jingdong prefecture level city in Shandong
IXCHSQ Flagship Store	King of Power Saving	Without further ado, the electric bill came down, $102 less this month, personal satisfaction, it counts okay, give you a good review	Jingdong prefecture level city in Shandong
IXCHSQ Flagship Store	King of Power Saving	The power saver has arrived, the installation is really convenient, plugged in on the line operation is quite simple, power saving effect is also a strong!	Jingdong prefecture level city in Shandong
IXCHSQ Flagship Store	King of Power Saving	Saver received, good quality, beautiful appearance, fine workmanship, simple installation	Jingdong prefecture level city in Shandong
IXCHSQ Flagship Store	King of Power Saving	Product design style modern, worth buying	Jingdong prefecture level city in Shandong
IXCHSQ Flagship Store	King of Power Saving	Energy saving, good power saving effect, practical, great value	Jingdong prefecture level city in Shandong
IXCHSQ Flagship Store	King of Power Saving	It's good quality, it's good, it's energy efficient, and I'll buy it again.	Jingdong prefecture level city in Shandong
IXCHSQ Flagship Store	King of Power Saving	Installation saves a lot of electricity, fast logistics, real energy-saving appliances	Jingdong prefecture level city in Shandong
IXCHSQ Flagship Store	King of Power Saving	The effect is good and the power saving is obvious after using it for a month and saving tens of dollars, the product is trustworthy, I now buy another 4 colors to prepare for their siblings' family to pay for	Jingdong prefecture level city in Shandong
IXCHSQ Flagship Store	King of Power Saving	The quality is particularly good workmanship is no problem, the arrival of the very fast oh, especially power-saving	Jingdong prefecture level city in Shandong
IXCHSQ Flagship Store	King of Power Saving	The quality is very good! In use, observed for a day, not as used before, so much power consumption.	Jingdong prefecture level city in Shandong
IXCHSQ Flagship Store	King of Power Saving	Goods have been received, the effect is good is not good, power saving is not yet know, wait for me to use it and then evaluate it.	Jingdong prefecture level city in Shandong
IXCHSQ Flagship Store	King of Power Saving	In-kind and the same as described, the quality is quite good, the seller's attitude is also good, ask questions and answer them, the speed of delivery bar, worth buying Oh!	Jingdong prefecture level city in Shandong
IXCHSQ Flagship Store	King of Power Saving	Nice stuff, came to comment after using it, it does save power.	Jingdong prefecture level city in Shandong
IXCHSQ Flagship Store	King of Power Saving	Fast charging, but also save power, the effect is very good, satisfied, praise!	Jingdong prefecture level city in Shandong
IXCHSQ Flagship Store	King of Power Saving	The goods have been received, I do not know how effective, after using the flat price again!	Jingdong prefecture level city in Shandong
IXCHSQ Flagship Store	King of Power Saving	Merchant service is very good Reply quickly The key is the quality and that quality is good	Jingdong prefecture level city in Shandong
IXCHSQ Flagship Store	King of Power Saving	It's working well now, I'll do a follow up review after I actually use it for a while	Jingdong prefecture level city in Shandong
IXCHSQ Flagship Store	King of Power Saving	The quality is good. After installing it, I feel like I've saved a lot of money on my electricity bill.	Jingdong prefecture level city in Shandong
IXCHSQ Flagship Store	King of Power Saving	Make up a review after use.	Jingdong prefecture level city in Shandong
IXCHSQ Flagship Store	King of Power Saving	review after use	Jingdong prefecture level city in Shandong
IXCHSQ Flagship Store	King of Power Saving	It's a small capacitor. It's not energy efficient.	Jingdong prefecture level city in Shandong
IXCHSQ Flagship Store	King of Power Saving	I'll follow up with a review after I've used it and had results.	Jingdong prefecture level city in Shandong
IXCHSQ Flagship Store	King of Power Saving	No power saving? Not good!	Jingdong prefecture level city in Shandong
IXCHSQ Flagship Store	King of Power Saving	Been using the same appliances and running a 1.5 air conditioner at night for what feels like no time, and after half a month of use it feels like the power is only increasing. Doesn't show any power saving	Jingdong prefecture level city in Shandong
IXCHSQ Flagship Store	King of Power Saving	serviceable	Jingdong prefecture level city in Shandong
IXCHSQ Flagship Store	King of Power Saving	It's a piece of junk. It doesn't save any power.	Jingdong prefecture level city in Shandong
IXCHSQ Flagship Store	King of Power Saving	There are a lot of appliances in the house, the summer air conditioning is very costly, I bought this in front of the air conditioning effect is very good, I explain that I bought three in one breath, I use more electricity a month down to 200 or so, very satisfied.	Jingdong prefecture level city in Shandong
IXCHSQ Flagship Store	King of Power Saving	The voltage is very stable has been maintained at 220v this value, high power super power saving, trustworthy, reasonable design, shape bright, logistics is quite fast, business warranty period, excellent service, logistics fast!	Jingdong prefecture level city in Shandong

IXCHSQ Flagship Store	King of Power Saving	Use a month after a look at the savings of more than 40 dollars, there is a display of electricity savings, this high-power intelligent frequency converter economizer, affordable and cheap, logistics is very fast, the service attitude is very good, very satisfied with a shopping.	Jingdong prefecture level city in Shandong
IXCHSQ Flagship Store	King of Power Saving	Used for a period of time, it seems that every day can really can save a few degrees of electricity, workmanship quite can, with a period of time and then continue to look at it, anyway, the price is not	Jingdong prefecture level city in Shandong
IXCHSQ Flagship Store	King of Power Saving	Used for a period of time, it seems that every day can indeed save two several degrees of electricity, workmanship quite can, with a period of time and then continue to look at it, anyway, the price is not	Jingdong prefecture level city in Shandong
IXCHSQ Flagship Store	King of Power Saving	High-power super saver received immediately used, the price is not expensive quality is very good, save electricity and money, very convenient, conscientious business, must be praised, recommended to everyone!	Jingdong prefecture level city in Shandong
IXCHSQ Flagship Store	King of Power Saving	Home enhancement version of the intelligent power saver, is in use, I think the price is cheap, value for money, efficacy, power, power saving effect is very good.	Jingdong prefecture level city in Shandong
IXCHSQ Flagship Store	King of Power Saving	Bought three at once, one for each room, summer electricity consumption is very high, despite the three air conditioners on the electricity bill is still down a lot, too Nessie!	Jingdong prefecture level city in Shandong
IXCHSQ Flagship Store	King of Power Saving	Without further ado, the electric bill came down, $102 less this month, personal satisfaction, it counts okay, give you a good review	Jingdong prefecture level city in Shandong
IXCHSQ Flagship Store	King of Power Saving	The power saver has arrived, the installation is really convenient, plugged in on the line operation is quite simple, power saving effect is also a strong!	Jingdong prefecture level city in Shandong
IXCHSQ Flagship Store	King of Power Saving	Saver received, good quality, beautiful appearance, fine workmanship, simple installation	Jingdong prefecture level city in Shandong
IXCHSQ Flagship Store	King of Power Saving	Product design style modern, worth buying	Jingdong prefecture level city in Shandong
IXCHSQ Flagship Store	King of Power Saving	Energy saving, good power saving effect, practical, great value	Jingdong prefecture level city in Shandong
IXCHSQ Flagship Store	King of Power Saving	It's good quality, it's good, it's energy efficient, and I'll buy it again.	Jingdong prefecture level city in Shandong
IXCHSQ Flagship Store	King of Power Saving	Installation saves a lot of electricity, fast logistics, real energy-saving appliances	Jingdong prefecture level city in Shandong
IXCHSQ Flagship Store	King of Power Saving	The effect is good and the power saving is obvious after using it for a month and saving tens of dollars, the product is trustworthy, I now buy another 4 colors to prepare for their siblings' family to pay for	Jingdong prefecture level city in Shandong
IXCHSQ Flagship Store	King of Power Saving	The quality is particularly good workmanship is no problem, the arrival of the very fast oh, especially power-saving	Jingdong prefecture level city in Shandong
IXCHSQ Flagship Store	King of Power Saving	The quality is very good! In use, observed for a day, not as used before, so much power consumption.	Jingdong prefecture level city in Shandong
IXCHSQ Flagship Store	King of Power Saving	Goods have been received, the effect is good is not good, power saving is not yet know, wait for me to use it and then evaluate it.	Jingdong prefecture level city in Shandong
IXCHSQ Flagship Store	King of Power Saving	In-kind and the same as described, the quality is quite good, the seller's attitude is also good, ask questions and answer them, the speed of delivery bar, worth buying Oh!	Jingdong prefecture level city in Shandong
IXCHSQ Flagship Store	King of Power Saving	Nice stuff, came to comment after using it, it does save power.	Jingdong prefecture level city in Shandong
IXCHSQ Flagship Store	King of Power Saving	Fast charging, but also save power, the effect is very good, satisfied, praise!	Jingdong prefecture level city in Shandong
IXCHSQ Flagship Store	King of Power Saving	The goods have been received, I do not know how effective, after using the flat price again!	Jingdong prefecture level city in Shandong
IXCHSQ Flagship Store	King of Power Saving	Merchant service is very good Reply quickly The key is the quality and that quality is good	Jingdong prefecture level city in Shandong
IXCHSQ Flagship Store	King of Power Saving	It's working well now, I'll do a follow up review after I actually use it for a while	Jingdong prefecture level city in Shandong
IXCHSQ Flagship Store	King of Power Saving	The quality is good. After installing it, I feel like I've saved a lot of money on my electricity bill.	Jingdong prefecture level city in Shandong
IXCHSQ Flagship Store	King of Power Saving	Make up a review after use.	Jingdong prefecture level city in Shandong
IXCHSQ Flagship Store	King of Power Saving	review after use	Jingdong prefecture level city in Shandong
IXCHSQ Flagship Store	King of Power Saving	It's a small capacitor. It's not energy efficient.	Jingdong prefecture level city in Shandong
IXCHSQ Flagship Store	King of Power Saving	I'll follow up with a review after I've used it and had results.	Jingdong prefecture level city in Shandong
IXCHSQ Flagship Store	King of Power Saving	No power saving? Not good!	Jingdong prefecture level city in Shandong
IXCHSQ Flagship Store	King of Power Saving	Been using the same appliances and running a 1.5 air conditioner at night for what feels like no time, and after half a month of use it feels like the power is only increasing. Doesn't show any power saving	Jingdong prefecture level city in Shandong
IXCHSQ Flagship Store	King of Power Saving	serviceable	Jingdong prefecture level city in Shandong
IXCHSQ Flagship Store	King of Power Saving	It's a piece of junk. It doesn't save any power.	Jingdong prefecture level city in Shandong
IXCHSQ Flagship Store	King of Power Saving	There are a lot of appliances in the house, the summer air conditioning is very costly, I bought this in front of the air conditioning effect is very good, I explain that I bought three in one breath, I use more electricity a month down to 200 or so, very satisfied.	Jingdong prefecture level city in Shandong
IXCHSQ Flagship Store	King of Power Saving	The voltage is very stable has been maintained at 220v this value, high power super power saving, trustworthy, reasonable design, shape bright, logistics is quite fast, business warranty period, excellent service, logistics fast!	Jingdong prefecture level city in Shandong
IXCHSQ Flagship Store	King of Power Saving	Use a month after a look at the savings of more than 40 dollars, there is a display of electricity savings, this high-power intelligent frequency converter economizer, affordable and cheap, logistics is very fast, the service attitude is very good, very satisfied with a shopping.	Jingdong prefecture level city in Shandong
IXCHSQ Flagship Store	King of Power Saving	Used for a period of time, it seems that every day can really can save a few degrees of electricity, workmanship quite can, with a period of time and then continue to look at it, anyway, the price is not	Jingdong prefecture level city in Shandong
IXCHSQ Flagship Store	King of Power Saving	Used for a period of time, it seems that every day can indeed save two several degrees of electricity, workmanship quite can, with a period of time and then continue to look at it, anyway, the price is not	Jingdong prefecture level city in Shandong
IXCHSQ Flagship Store	King of Power Saving	High-power super saver received immediately used, the price is not expensive quality is very good, save electricity and money, very convenient, conscientious business, must be praised, recommended to everyone!	Jingdong prefecture level city in Shandong
IXCHSQ Flagship Store	King of Power Saving	Home enhancement version of the intelligent power saver, is in use, I think the price is cheap, value for money, efficacy, power, power saving effect is very good.	Jingdong prefecture level city in Shandong
IXCHSQ Flagship Store	King of Power Saving	Bought three at once, one for each room, summer electricity consumption is very high, despite the three air conditioners on the electricity bill is still down a lot, too Nessie!	Jingdong prefecture level city in Shandong
IXCHSQ Flagship Store	King of Power Saving	Without further ado, the electric bill came down, $102 less this month, personal satisfaction, it counts okay, give you a good review	Jingdong prefecture level city in Shandong
IXCHSQ Flagship Store	King of Power Saving	The power saver has arrived, the installation is really convenient, plugged in on the line operation is quite simple, power saving effect is also a strong!	Jingdong prefecture level city in Shandong
IXCHSQ Flagship Store	King of Power Saving	Saver received, good quality, beautiful appearance, fine workmanship, simple installation	Jingdong prefecture level city in Shandong
IXCHSQ Flagship Store	King of Power Saving	Product design style modern, worth buying	Jingdong prefecture level city in Shandong
IXCHSQ Flagship Store	King of Power Saving	Energy saving, good power saving effect, practical, great value	Jingdong prefecture level city in Shandong
IXCHSQ Flagship Store	King of Power Saving	It's good quality, it's good, it's energy efficient, and I'll buy it again.	Jingdong prefecture level city in Shandong
IXCHSQ Flagship Store	King of Power Saving	Installation saves a lot of electricity, fast logistics, real energy-saving appliances	Jingdong prefecture level city in Shandong
IXCHSQ Flagship Store	King of Power Saving	The effect is good and the power saving is obvious after using it for a month and saving tens of dollars, the product is trustworthy, I now buy another 4 colors to prepare for their siblings' family to pay for	Jingdong prefecture level city in Shandong
IXCHSQ Flagship Store	King of Power Saving	The quality is particularly good workmanship is no problem, the arrival of the very fast oh, especially power-saving	Jingdong prefecture level city in Shandong

IXCHSQ Flagship Store	King of Power Saving	The quality is very good! In use, observed for a day, not as used before, so much power consumption.	Jingdong prefecture level city in Shandong
IXCHSQ Flagship Store	King of Power Saving	Goods have been received, the effect is good is not good, power saving is not yet know, wait for me to use it and then evaluate it.	Jingdong prefecture level city in Shandong
IXCHSQ Flagship Store	King of Power Saving	In-kind and the same as described, the quality is quite good, the seller's attitude is also good, ask questions and answer them, the speed of delivery bar, worth buying Oh!	Jingdong prefecture level city in Shandong
IXCHSQ Flagship Store	King of Power Saving	Nice stuff, came to comment after using it, it does save power.	Jingdong prefecture level city in Shandong
IXCHSQ Flagship Store	King of Power Saving	Fast charging, but also save power, the effect is very good, satisfied, praise!	Jingdong prefecture level city in Shandong
IXCHSQ Flagship Store	King of Power Saving	The goods have been received, I do not know how effective, after using the flat price again!	Jingdong prefecture level city in Shandong
IXCHSQ Flagship Store	King of Power Saving	Merchant service is very good Reply quickly The key is the quality and that quality is good	Jingdong prefecture level city in Shandong
IXCHSQ Flagship Store	King of Power Saving	It's working well now, I'll do a follow up review after I actually use it for a while	Jingdong prefecture level city in Shandong
IXCHSQ Flagship Store	King of Power Saving	The quality is good. After installing it, I feel like I've saved a lot of money on my electricity bill.	Jingdong prefecture level city in Shandong
IXCHSQ Flagship Store	King of Power Saving	Make up a review after use.	Jingdong prefecture level city in Shandong
IXCHSQ Flagship Store	King of Power Saving	review after use	Jingdong prefecture level city in Shandong
IXCHSQ Flagship Store	King of Power Saving	It's a small capacitor. It's not energy efficient.	Jingdong prefecture level city in Shandong
IXCHSQ Flagship Store	King of Power Saving	I'll follow up with a review after I've used it and had results.	Jingdong prefecture level city in Shandong
IXCHSQ Flagship Store	King of Power Saving	No power saving? Not good!	Jingdong prefecture level city in Shandong
IXCHSQ Flagship Store	King of Power Saving	Been using the same appliances and running a 1.5 air conditioner at night for what feels like no time, and after half a month of use it feels like the power is only increasing. Doesn't show any power saving	Jingdong prefecture level city in Shandong
IXCHSQ Flagship Store	King of Power Saving	serviceable	Jingdong prefecture level city in Shandong
IXCHSQ Flagship Store	King of Power Saving	It's a piece of junk. It doesn't save any power.	Jingdong prefecture level city in Shandong
IXCHSQ Flagship Store	King of Power Saving	There are a lot of appliances in the house, the summer air conditioning is very costly, I bought this in front of the air conditioning effect is very good, I explain that I bought three in one breath, I use more electricity a month down to 200 or so, very satisfied.	Jingdong prefecture level city in Shandong
IXCHSQ Flagship Store	King of Power Saving	The voltage is very stable has been maintained at 220v this value, high power super power saving, trustworthy, reasonable design, shape bright, logistics is quite fast, business warranty period, excellent service, logistics fast!	Jingdong prefecture level city in Shandong
IXCHSQ Flagship Store	King of Power Saving	Use a month after a look at the savings of more than 40 dollars, there is a display of electricity savings, this high-power intelligent frequency converter economizer, affordable and cheap, logistics is very fast, the service attitude is very good, very satisfied with a shopping.	Jingdong prefecture level city in Shandong
IXCHSQ Flagship Store	King of Power Saving	Used for a period of time, it seems that every day can really can save a few degrees of electricity, workmanship quite can, with a period of time and then continue to look at it, anyway, the price is not	Jingdong prefecture level city in Shandong
IXCHSQ Flagship Store	King of Power Saving	Used for a period of time, it seems that every day can indeed save two several degrees of electricity, workmanship quite can, with a period of time and then continue to look at it, anyway, the price is not	Jingdong prefecture level city in Shandong
IXCHSQ Flagship Store	King of Power Saving	High-power super saver received immediately used, the price is not expensive quality is very good, save electricity and money, very convenient, conscientious business, must be praised, recommended to everyone!	Jingdong prefecture level city in Shandong
IXCHSQ Flagship Store	King of Power Saving	Home enhancement version of the intelligent power saver, is in use, I think the price is cheap, value for money, efficacy, power, power saving effect is very good.	Jingdong prefecture level city in Shandong
IXCHSQ Flagship Store	King of Power Saving	Bought three at once, one for each room, summer electricity consumption is very high, despite the three air conditioners on the electricity bill is still down a lot, too Nessie!	Jingdong prefecture level city in Shandong
IXCHSQ Flagship Store	King of Power Saving	Without further ado, the electric bill came down, $102 less this month, personal satisfaction, it counts okay, give you a good review	Jingdong prefecture level city in Shandong
IXCHSQ Flagship Store	King of Power Saving	The power saver has arrived, the installation is really convenient, plugged in on the line operation is quite simple, power saving effect is also a strong!	Jingdong prefecture level city in Shandong
IXCHSQ Flagship Store	King of Power Saving	Saver received, good quality, beautiful appearance, fine workmanship, simple installation	Jingdong prefecture level city in Shandong
IXCHSQ Flagship Store	King of Power Saving	Product design style modern, worth buying	Jingdong prefecture level city in Shandong
IXCHSQ Flagship Store	King of Power Saving	Energy saving, good power saving effect, practical, great value	Jingdong prefecture level city in Shandong
IXCHSQ Flagship Store	King of Power Saving	It's good quality, it's good, it's energy efficient, and I'll buy it again.	Jingdong prefecture level city in Shandong
IXCHSQ Flagship Store	King of Power Saving	Installation saves a lot of electricity, fast logistics, real energy-saving appliances	Jingdong prefecture level city in Shandong
IXCHSQ Flagship Store	King of Power Saving	The effect is good and the power saving is obvious after using it for a month and saving tens of dollars, the product is trustworthy, I now buy another 4 colors to prepare for their siblings' family to pay for	Jingdong prefecture level city in Shandong
IXCHSQ Flagship Store	King of Power Saving	The quality is particularly good workmanship is no problem, the arrival of the very fast oh, especially power-saving	Jingdong prefecture level city in Shandong
IXCHSQ Flagship Store	King of Power Saving	The quality is very good! In use, observed for a day, not as used before, so much power consumption.	Jingdong prefecture level city in Shandong
IXCHSQ Flagship Store	King of Power Saving	Goods have been received, the effect is good is not good, power saving is not yet know, wait for me to use it and then evaluate it.	Jingdong prefecture level city in Shandong
IXCHSQ Flagship Store	King of Power Saving	In-kind and the same as described, the quality is quite good, the seller's attitude is also good, ask questions and answer them, the speed of delivery bar, worth buying Oh!	Jingdong prefecture level city in Shandong
IXCHSQ Flagship Store	King of Power Saving	Nice stuff, came to comment after using it, it does save power.	Jingdong prefecture level city in Shandong
IXCHSQ Flagship Store	King of Power Saving	Fast charging, but also save power, the effect is very good, satisfied, praise!	Jingdong prefecture level city in Shandong
IXCHSQ Flagship Store	King of Power Saving	The goods have been received, I do not know how effective, after using the flat price again!	Jingdong prefecture level city in Shandong
IXCHSQ Flagship Store	King of Power Saving	Merchant service is very good Reply quickly The key is the quality and that quality is good	Jingdong prefecture level city in Shandong
IXCHSQ Flagship Store	King of Power Saving	It's working well now, I'll do a follow up review after I actually use it for a while	Jingdong prefecture level city in Shandong
IXCHSQ Flagship Store	King of Power Saving	The quality is good. After installing it, I feel like I've saved a lot of money on my electricity bill.	Jingdong prefecture level city in Shandong
IXCHSQ Flagship Store	King of Power Saving	Make up a review after use.	Jingdong prefecture level city in Shandong
IXCHSQ Flagship Store	King of Power Saving	review after use	Jingdong prefecture level city in Shandong
IXCHSQ Flagship Store	King of Power Saving	It's a small capacitor. It's not energy efficient.	Jingdong prefecture level city in Shandong
IXCHSQ Flagship Store	King of Power Saving	I'll follow up with a review after I've used it and had results.	Jingdong prefecture level city in Shandong
IXCHSQ Flagship Store	King of Power Saving	No power saving? Not good!	Jingdong prefecture level city in Shandong
IXCHSQ Flagship Store	King of Power Saving	Been using the same appliances and running a 1.5 air conditioner at night for what feels like no time, and after half a month of use it feels like the power is only increasing. Doesn't show any power saving	Jingdong prefecture level city in Shandong
IXCHSQ Flagship Store	King of Power Saving	serviceable	Jingdong prefecture level city in Shandong

IXCHSQ Flagship Store	King of Power Saving	It’s a piece of junk. It doesn’t save any power.	Jingdong prefecture level city in Shandong
IXCHSQ Flagship Store	King of Power Saving	There are a lot of appliances in the house, the summer air conditioning is very costly, I bought this in front of the air conditioning effect is very good, I explain that I bought three in one breath, I use more electricity a month down to 200 or so, very satisfied.	Jingdong prefecture level city in Shandong
IXCHSQ Flagship Store	King of Power Saving	The voltage is very stable has been maintained at 220v this value, high power super power saving, trustworthy, reasonable design, shape bright, logistics is quite fast, business warranty period, excellent service, logistics fast!	Jingdong prefecture level city in Shandong
IXCHSQ Flagship Store	King of Power Saving	Use a month after a look at the savings of more than 40 dollars, there is a display of electricity savings, this high-power intelligent frequency converter economizer, affordable and cheap, logistics is very fast, the service attitude is very good, very satisfied with a shopping.	Jingdong prefecture level city in Shandong
IXCHSQ Flagship Store	King of Power Saving	Used for a period of time, it seems that every day can really can save a few degrees of electricity, workmanship quite can, with a period of time and then continue to look at it, anyway, the price is not	Jingdong prefecture level city in Shandong
IXCHSQ Flagship Store	King of Power Saving	Used for a period of time, it seems that every day can indeed save two several degrees of electricity, workmanship quite can, with a period of time and then continue to look at it, anyway, the price is not	Jingdong prefecture level city in Shandong
IXCHSQ Flagship Store	King of Power Saving	High-power super saver received immediately used, the price is not expensive quality is very good, save electricity and money, very convenient, conscientious business, must be praised, recommended to everyone!	Jingdong prefecture level city in Shandong
IXCHSQ Flagship Store	King of Power Saving	Home enhancement version of the intelligent power saver, is in use, I think the price is cheap, value for money, efficacy, power, power saving effect is very good.	Jingdong prefecture level city in Shandong
IXCHSQ Flagship Store	King of Power Saving	Bought three at once, one for each room, summer electricity consumption is very high, despite the three air conditioners on the electricity bill is still down a lot, too Nessie!	Jingdong prefecture level city in Shandong
IXCHSQ Flagship Store	King of Power Saving	Without further ado, the electric bill came down, $102 less this month, personal satisfaction, it counts okay, give you a good review	Jingdong prefecture level city in Shandong
IXCHSQ Flagship Store	King of Power Saving	The power saver has arrived, the installation is really convenient, plugged in on the line operation is quite simple, power saving effect is also a strong!	Jingdong prefecture level city in Shandong
IXCHSQ Flagship Store	King of Power Saving	Saver received, good quality, beautiful appearance, fine workmanship, simple installation	Jingdong prefecture level city in Shandong
IXCHSQ Flagship Store	King of Power Saving	Product design style modern, worth buying	Jingdong prefecture level city in Shandong
IXCHSQ Flagship Store	King of Power Saving	Energy saving, good power saving effect, practical, great value	Jingdong prefecture level city in Shandong
IXCHSQ Flagship Store	King of Power Saving	It’s good quality, it’s good, it’s energy efficient, and I’ll buy it again.	Jingdong prefecture level city in Shandong
IXCHSQ Flagship Store	King of Power Saving	Installation saves a lot of electricity, fast logistics, real energy-saving appliances	Jingdong prefecture level city in Shandong
IXCHSQ Flagship Store	King of Power Saving	The effect is good and the power saving is obvious after using it for a month and saving tens of dollars, the product is trustworthy, I now buy another 4 colors to prepare for their siblings' family to pay for	Jingdong prefecture level city in Shandong
IXCHSQ Flagship Store	King of Power Saving	The quality is particularly good workmanship is no problem, the arrival of the very fast oh, especially power-saving	Jingdong prefecture level city in Shandong
IXCHSQ Flagship Store	King of Power Saving	The quality is very good! In use, observed for a day, not as used before, so much power consumption.	Jingdong prefecture level city in Shandong
IXCHSQ Flagship Store	King of Power Saving	Goods have been received, the effect is good is not good, power saving is not yet know, wait for me to use it and then evaluate it.	Jingdong prefecture level city in Shandong
IXCHSQ Flagship Store	King of Power Saving	In-kind and the same as described, the quality is quite good, the seller's attitude is also good, ask questions and answer them, the speed of delivery bar, worth buying Oh!	Jingdong prefecture level city in Shandong
IXCHSQ Flagship Store	King of Power Saving	Nice stuff, came to comment after using it, it does save power.	Jingdong prefecture level city in Shandong
IXCHSQ Flagship Store	King of Power Saving	Fast charging, but also save power, the effect is very good, satisfied, praise!	Jingdong prefecture level city in Shandong
IXCHSQ Flagship Store	King of Power Saving	The goods have been received, I do not know how effective, after using the flat price again!	Jingdong prefecture level city in Shandong
IXCHSQ Flagship Store	King of Power Saving	Merchant service is very good Reply quickly The key is the quality and that quality is good	Jingdong prefecture level city in Shandong
IXCHSQ Flagship Store	King of Power Saving	It’s working well now, I’ll do a follow up review after I actually use it for a while	Jingdong prefecture level city in Shandong
IXCHSQ Flagship Store	King of Power Saving	The quality is good. After installing it, I feel like I’ve saved a lot of money on my electricity bill.	Jingdong prefecture level city in Shandong
IXCHSQ Flagship Store	King of Power Saving	Make up a review after use.	Jingdong prefecture level city in Shandong
IXCHSQ Flagship Store	King of Power Saving	review after use	Jingdong prefecture level city in Shandong
IXCHSQ Flagship Store	King of Power Saving	It’s a small capacitor. It’s not energy efficient.	Jingdong prefecture level city in Shandong
IXCHSQ Flagship Store	King of Power Saving	I’ll follow up with a review after I’ve used it and had results.	Jingdong prefecture level city in Shandong
IXCHSQ Flagship Store	King of Power Saving	No power saving? Not good!	Jingdong prefecture level city in Shandong
IXCHSQ Flagship Store	King of Power Saving	Been using the same appliances and running a 1.5 air conditioner at night for what feels like no time, and after half a month of use it feels like the power is only increasing. Doesn't show any power saving	Jingdong prefecture level city in Shandong
IXCHSQ Flagship Store	King of Power Saving	serviceable	Jingdong prefecture level city in Shandong
IXCHSQ Flagship Store	King of Power Saving	It’s a piece of junk. It doesn’t save any power.	Jingdong prefecture level city in Shandong
IXCHSQ Flagship Store	King of Power Saving	There are a lot of appliances in the house, the summer air conditioning is very costly, I bought this in front of the air conditioning effect is very good, I explain that I bought three in one breath, I use more electricity a month down to 200 or so, very satisfied.	Jingdong prefecture level city in Shandong
IXCHSQ Flagship Store	King of Power Saving	The voltage is very stable has been maintained at 220v this value, high power super power saving, trustworthy, reasonable design, shape bright, logistics is quite fast, business warranty period, excellent service, logistics fast!	Jingdong prefecture level city in Shandong
IXCHSQ Flagship Store	King of Power Saving	Use a month after a look at the savings of more than 40 dollars, there is a display of electricity savings, this high-power intelligent frequency converter economizer, affordable and cheap, logistics is very fast, the service attitude is very good, very satisfied with a shopping.	Jingdong prefecture level city in Shandong
IXCHSQ Flagship Store	King of Power Saving	Used for a period of time, it seems that every day can really can save a few degrees of electricity, workmanship quite can, with a period of time and then continue to look at it, anyway, the price is not	Jingdong prefecture level city in Shandong
IXCHSQ Flagship Store	King of Power Saving	Used for a period of time, it seems that every day can indeed save two several degrees of electricity, workmanship quite can, with a period of time and then continue to look at it, anyway, the price is not	Jingdong prefecture level city in Shandong
IXCHSQ Flagship Store	King of Power Saving	High-power super saver received immediately used, the price is not expensive quality is very good, save electricity and money, very convenient, conscientious business, must be praised, recommended to everyone!	Jingdong prefecture level city in Shandong
IXCHSQ Flagship Store	King of Power Saving	Home enhancement version of the intelligent power saver, is in use, I think the price is cheap, value for money, efficacy, power, power saving effect is very good.	Jingdong prefecture level city in Shandong
IXCHSQ Flagship Store	King of Power Saving	Bought three at once, one for each room, summer electricity consumption is very high, despite the three air conditioners on the electricity bill is still down a lot, too Nessie!	Jingdong prefecture level city in Shandong
IXCHSQ Flagship Store	King of Power Saving	Without further ado, the electric bill came down, $102 less this month, personal satisfaction, it counts okay, give you a good review	Jingdong prefecture level city in Shandong
IXCHSQ Flagship Store	King of Power Saving	The power saver has arrived, the installation is really convenient, plugged in on the line operation is quite simple, power saving effect is also a strong!	Jingdong prefecture level city in Shandong
IXCHSQ Flagship Store	King of Power Saving	Saver received, good quality, beautiful appearance, fine workmanship, simple installation	Jingdong prefecture level city in Shandong
IXCHSQ Flagship Store	King of Power Saving	Product design style modern, worth buying	Jingdong prefecture level city in Shandong
IXCHSQ Flagship Store	King of Power Saving	Energy saving, good power saving effect, practical, great value	Jingdong prefecture level city in Shandong

IXCHSQ Flagship Store	King of Power Saving	It's good quality, it's good, it's energy efficient, and I'll buy it again.	Jingdong prefecture level city in Shandong
IXCHSQ Flagship Store	King of Power Saving	Installation saves a lot of electricity, fast logistics, real energy-saving appliances	Jingdong prefecture level city in Shandong
IXCHSQ Flagship Store	King of Power Saving	The effect is good and the power saving is obvious after using it for a month and saving tens of dollars, the product is trustworthy, I now buy another 4 colors to prepare for their siblings' family to pay for	Jingdong prefecture level city in Shandong
IXCHSQ Flagship Store	King of Power Saving	The quality is particularly good workmanship is no problem, the arrival of the very fast oh, especially power-saving	Jingdong prefecture level city in Shandong
IXCHSQ Flagship Store	King of Power Saving	The quality is very good! In use, observed for a day, not as used before, so much power consumption.	Jingdong prefecture level city in Shandong
IXCHSQ Flagship Store	King of Power Saving	Goods have been received, the effect is good is not good, power saving is not yet know, wait for me to use it and then evaluate it.	Jingdong prefecture level city in Shandong
IXCHSQ Flagship Store	King of Power Saving	In-kind and the same as described, the quality is quite good, the seller's attitude is also good, ask questions and answer them, the speed of delivery bar, worth buying Oh!	Jingdong prefecture level city in Shandong
IXCHSQ Flagship Store	King of Power Saving	Nice stuff, came to comment after using it, it does save power.	Jingdong prefecture level city in Shandong
IXCHSQ Flagship Store	King of Power Saving	Fast charging, but also save power, the effect is very good, satisfied, praise!	Jingdong prefecture level city in Shandong
IXCHSQ Flagship Store	King of Power Saving	The goods have been received, I do not know how effective, after using the flat price again!	Jingdong prefecture level city in Shandong
IXCHSQ Flagship Store	King of Power Saving	Merchant service is very good Reply quickly The key is the quality and that quality is good	Jingdong prefecture level city in Shandong
IXCHSQ Flagship Store	King of Power Saving	It's working well now, I'll do a follow up review after I actually use it for a while	Jingdong prefecture level city in Shandong
IXCHSQ Flagship Store	King of Power Saving	The quality is good. After installing it, I feel like I've saved a lot of money on my electricity bill.	Jingdong prefecture level city in Shandong
IXCHSQ Flagship Store	King of Power Saving	Make up a review after use.	Jingdong prefecture level city in Shandong
IXCHSQ Flagship Store	King of Power Saving	review after use	Jingdong prefecture level city in Shandong
IXCHSQ Flagship Store	King of Power Saving	It's a small capacitor. It's not energy efficient.	Jingdong prefecture level city in Shandong
IXCHSQ Flagship Store	King of Power Saving	I'll follow up with a review after I've used it and had results.	Jingdong prefecture level city in Shandong
IXCHSQ Flagship Store	King of Power Saving	No power saving? Not good!	Jingdong prefecture level city in Shandong
IXCHSQ Flagship Store	King of Power Saving	Been using the same appliances and running a 1.5 air conditioner at night for what feels like no time, and after half a month of use it feels like the power is only increasing. Doesn't show any power saving	Jingdong prefecture level city in Shandong
IXCHSQ Flagship Store	King of Power Saving	serviceable	Jingdong prefecture level city in Shandong
IXCHSQ Flagship Store	King of Power Saving	It's a piece of junk. It doesn't save any power.	Jingdong prefecture level city in Shandong
IXCHSQ Flagship Store	King of Power Saving	There are a lot of appliances in the house, the summer air conditioning is very costly, I bought this in front of the air conditioning effect is very good, I explain that I bought three in one breath, I use more electricity a month down to 200 or so, very satisfied.	Jingdong prefecture level city in Shandong
IXCHSQ Flagship Store	King of Power Saving	The voltage is very stable has been maintained at 220v this value, high power super power saving, trustworthy, reasonable design, shape bright, logistics is quite fast, business warranty period, excellent service, logistics fast!	Jingdong prefecture level city in Shandong
IXCHSQ Flagship Store	King of Power Saving	Use a month after a look at the savings of more than 40 dollars, there is a display of electricity savings, this high-power intelligent frequency converter economizer, affordable and cheap, logistics is very fast, the service attitude is very good, very satisfied with a shopping.	Jingdong prefecture level city in Shandong
IXCHSQ Flagship Store	King of Power Saving	Used for a period of time, it seems that every day can really can save a few degrees of electricity, workmanship quite can, with a period of time and then continue to look at it, anyway, the price is not	Jingdong prefecture level city in Shandong
IXCHSQ Flagship Store	King of Power Saving	Used for a period of time, it seems that every day can indeed save two several degrees of electricity, workmanship quite can, with a period of time and then continue to look at it, anyway, the price is not	Jingdong prefecture level city in Shandong
IXCHSQ Flagship Store	King of Power Saving	High-power super saver received immediately used, the price is not expensive quality is very good, save electricity and money, very convenient, conscientious business, must be praised, recommended to everyone!	Jingdong prefecture level city in Shandong
IXCHSQ Flagship Store	King of Power Saving	Home enhancement version of the intelligent power saver, is in use, I think the price is cheap, value for money, efficacy, power, power saving effect is very good.	Jingdong prefecture level city in Shandong
IXCHSQ Flagship Store	King of Power Saving	Bought three at once, one for each room, summer electricity consumption is very high, despite the three air conditioners on the electricity bill is still down a lot, too Nessie!	Jingdong prefecture level city in Shandong
IXCHSQ Flagship Store	King of Power Saving	Without further ado, the electric bill came down, $102 less this month, personal satisfaction, it counts okay, give you a good review	Jingdong prefecture level city in Shandong
IXCHSQ Flagship Store	King of Power Saving	The power saver has arrived, the installation is really convenient, plugged in on the line operation is quite simple, power saving effect is also a strong!	Jingdong prefecture level city in Shandong
IXCHSQ Flagship Store	King of Power Saving	Saver received, good quality, beautiful appearance, fine workmanship, simple installation	Jingdong prefecture level city in Shandong
IXCHSQ Flagship Store	King of Power Saving	Product design style modern, worth buying	Jingdong prefecture level city in Shandong
IXCHSQ Flagship Store	King of Power Saving	Energy saving, good power saving effect, practical, great value	Jingdong prefecture level city in Shandong
IXCHSQ Flagship Store	King of Power Saving	It's good quality, it's good, it's energy efficient, and I'll buy it again.	Jingdong prefecture level city in Shandong
IXCHSQ Flagship Store	King of Power Saving	Installation saves a lot of electricity, fast logistics, real energy-saving appliances	Jingdong prefecture level city in Shandong
IXCHSQ Flagship Store	King of Power Saving	The effect is good and the power saving is obvious after using it for a month and saving tens of dollars, the product is trustworthy, I now buy another 4 colors to prepare for their siblings' family to pay for	Jingdong prefecture level city in Shandong
IXCHSQ Flagship Store	King of Power Saving	The quality is particularly good workmanship is no problem, the arrival of the very fast oh, especially power-saving	Jingdong prefecture level city in Shandong
IXCHSQ Flagship Store	King of Power Saving	The quality is very good! In use, observed for a day, not as used before, so much power consumption.	Jingdong prefecture level city in Shandong
IXCHSQ Flagship Store	King of Power Saving	Goods have been received, the effect is good is not good, power saving is not yet know, wait for me to use it and then evaluate it.	Jingdong prefecture level city in Shandong
IXCHSQ Flagship Store	King of Power Saving	In-kind and the same as described, the quality is quite good, the seller's attitude is also good, ask questions and answer them, the speed of delivery bar, worth buying Oh!	Jingdong prefecture level city in Shandong
IXCHSQ Flagship Store	King of Power Saving	Nice stuff, came to comment after using it, it does save power.	Jingdong prefecture level city in Shandong
IXCHSQ Flagship Store	King of Power Saving	Fast charging, but also save power, the effect is very good, satisfied, praise!	Jingdong prefecture level city in Shandong
IXCHSQ Flagship Store	King of Power Saving	The goods have been received, I do not know how effective, after using the flat price again!	Jingdong prefecture level city in Shandong
IXCHSQ Flagship Store	King of Power Saving	Merchant service is very good Reply quickly The key is the quality and that quality is good	Jingdong prefecture level city in Shandong
IXCHSQ Flagship Store	King of Power Saving	It's working well now, I'll do a follow up review after I actually use it for a while	Jingdong prefecture level city in Shandong
IXCHSQ Flagship Store	King of Power Saving	The quality is good. After installing it, I feel like I've saved a lot of money on my electricity bill.	Jingdong prefecture level city in Shandong
IXCHSQ Flagship Store	King of Power Saving	Make up a review after use.	Jingdong prefecture level city in Shandong
IXCHSQ Flagship Store	King of Power Saving	review after use	Jingdong prefecture level city in Shandong
IXCHSQ Flagship Store	King of Power Saving	It's a small capacitor. It's not energy efficient.	Jingdong prefecture level city in Shandong

IXCHSQ Flagship Store	King of Power Saving	I’ll follow up with a review after I’ve used it and had results.	Jingdong prefecture level city in Shandong
IXCHSQ Flagship Store	King of Power Saving	No power saving? Not good!	Jingdong prefecture level city in Shandong
IXCHSQ Flagship Store	King of Power Saving	Been using the same appliances and running a 1.5 air conditioner at night for what feels like no time, and after half a month of use it feels like the power is only increasing. Doesn’t show any power saving	Jingdong prefecture level city in Shandong
IXCHSQ Flagship Store	King of Power Saving	serviceable	Jingdong prefecture level city in Shandong
IXCHSQ Flagship Store	King of Power Saving	It’s a piece of junk. It doesn’t save any power.	Jingdong prefecture level city in Shandong
IXCHSQ Flagship Store	King of Power Saving	There are a lot of appliances in the house, the summer air conditioning is very costly, I bought this in front of the air conditioning effect is very good, I explain that I bought three in one breath, I use more electricity a month down to 200 or so, very satisfied.	Jingdong prefecture level city in Shandong
IXCHSQ Flagship Store	King of Power Saving	The voltage is very stable has been maintained at 220v this value, high power super power saving, trustworthy, reasonable design, shape bright, logistics is quite fast, business warranty period, excellent service, logistics fast!	Jingdong prefecture level city in Shandong
IXCHSQ Flagship Store	King of Power Saving	Use a month after a look at the savings of more than 40 dollars, there is a display of electricity savings, this high-power intelligent frequency converter economizer, affordable and cheap, logistics is very fast, the service attitude is very good, very satisfied with a shopping.	Jingdong prefecture level city in Shandong
IXCHSQ Flagship Store	King of Power Saving	Used for a period of time, it seems that every day can really can save a few degrees of electricity, workmanship quite can, with a period of time and then continue to look at it, anyway, the price is not	Jingdong prefecture level city in Shandong
IXCHSQ Flagship Store	King of Power Saving	Used for a period of time, it seems that every day can indeed save two several degrees of electricity, workmanship quite can, with a period of time and then continue to look at it, anyway, the price is not	Jingdong prefecture level city in Shandong
IXCHSQ Flagship Store	King of Power Saving	High-power super saver received immediately used, the price is not expensive quality is very good, save electricity and money, very convenient, conscientious business, must be praised, recommended to everyone!	Jingdong prefecture level city in Shandong
IXCHSQ Flagship Store	King of Power Saving	Home enhancement version of the intelligent power saver, is in use, I think the price is cheap, value for money, efficacy, power, power saving effect is very good.	Jingdong prefecture level city in Shandong
IXCHSQ Flagship Store	King of Power Saving	Bought three at once, one for each room, summer electricity consumption is very high, despite the three air conditioners on the electricity bill is still down a lot, too Nessie!	Jingdong prefecture level city in Shandong
IXCHSQ Flagship Store	King of Power Saving	Without further ado, the electric bill came down, $102 less this month, personal satisfaction, it counts okay, give you a good review	Jingdong prefecture level city in Shandong
IXCHSQ Flagship Store	King of Power Saving	The power saver has arrived, the installation is really convenient, plugged in on the line operation is quite simple, power saving effect is also a strong!	Jingdong prefecture level city in Shandong
IXCHSQ Flagship Store	King of Power Saving	Saver received, good quality, beautiful appearance, fine workmanship, simple installation	Jingdong prefecture level city in Shandong
IXCHSQ Flagship Store	King of Power Saving	Product design style modern, worth buying	Jingdong prefecture level city in Shandong
IXCHSQ Flagship Store	King of Power Saving	Energy saving, good power saving effect, practical, great value	Jingdong prefecture level city in Shandong
IXCHSQ Flagship Store	King of Power Saving	It’s good quality, it’s good, it’s energy efficient, and I’ll buy it again.	Jingdong prefecture level city in Shandong
IXCHSQ Flagship Store	King of Power Saving	Installation saves a lot of electricity, fast logistics, real energy-saving appliances	Jingdong prefecture level city in Shandong
IXCHSQ Flagship Store	King of Power Saving	The effect is good and the power saving is obvious after using it for a month and saving tens of dollars, the product is trustworthy, I now buy another 4 colors to prepare for their siblings' family to pay for	Jingdong prefecture level city in Shandong
IXCHSQ Flagship Store	King of Power Saving	The quality is particularly good workmanship is no problem, the arrival of the very fast oh, especially power-saving	Jingdong prefecture level city in Shandong
IXCHSQ Flagship Store	King of Power Saving	The quality is very good! In use, observed for a day, not as used before, so much power consumption.	Jingdong prefecture level city in Shandong
IXCHSQ Flagship Store	King of Power Saving	Goods have been received, the effect is good is not good, power saving is not yet know, wait for me to use it and then evaluate it.	Jingdong prefecture level city in Shandong
IXCHSQ Flagship Store	King of Power Saving	In-kind and the same as described, the quality is quite good, the seller's attitude is also good, ask questions and answer them, the speed of delivery bar, worth buying Oh!	Jingdong prefecture level city in Shandong
IXCHSQ Flagship Store	King of Power Saving	Nice stuff, came to comment after using it, it does save power.	Jingdong prefecture level city in Shandong
IXCHSQ Flagship Store	King of Power Saving	Fast charging, but also save power, the effect is very good, satisfied, praise!	Jingdong prefecture level city in Shandong
IXCHSQ Flagship Store	King of Power Saving	The goods have been received, I do not know how effective, after using the flat price again!	Jingdong prefecture level city in Shandong
IXCHSQ Flagship Store	King of Power Saving	Merchant service is very good Reply quickly The key is the quality and that quality is good	Jingdong prefecture level city in Shandong
IXCHSQ Flagship Store	King of Power Saving	It’s working well now, I’ll do a follow up review after I actually use it for a while	Jingdong prefecture level city in Shandong
IXCHSQ Flagship Store	King of Power Saving	The quality is good. After installing it, I feel like I’ve saved a lot of money on my electricity bill.	Jingdong prefecture level city in Shandong
IXCHSQ Flagship Store	King of Power Saving	Make up a review after use.	Jingdong prefecture level city in Shandong
IXCHSQ Flagship Store	King of Power Saving	review after use	Jingdong prefecture level city in Shandong
IXCHSQ Flagship Store	King of Power Saving	It’s a small capacitor. It’s not energy efficient.	Jingdong prefecture level city in Shandong
IXCHSQ Flagship Store	King of Power Saving	I’ll follow up with a review after I’ve used it and had results.	Jingdong prefecture level city in Shandong
IXCHSQ Flagship Store	King of Power Saving	No power saving? Not good!	Jingdong prefecture level city in Shandong
IXCHSQ Flagship Store	King of Power Saving	Been using the same appliances and running a 1.5 air conditioner at night for what feels like no time, and after half a month of use it feels like the power is only increasing. Doesn’t show any power saving	Jingdong prefecture level city in Shandong
IXCHSQ Flagship Store	King of Power Saving	serviceable	Jingdong prefecture level city in Shandong
IXCHSQ Flagship Store	King of Power Saving	It’s a piece of junk. It doesn’t save any power.	Jingdong prefecture level city in Shandong
IXCHSQ Flagship Store	King of Power Saving	There are a lot of appliances in the house, the summer air conditioning is very costly, I bought this in front of the air conditioning effect is very good, I explain that I bought three in one breath, I use more electricity a month down to 200 or so, very satisfied.	Jingdong prefecture level city in Shandong
IXCHSQ Flagship Store	King of Power Saving	The voltage is very stable has been maintained at 220v this value, high power super power saving, trustworthy, reasonable design, shape bright, logistics is quite fast, business warranty period, excellent service, logistics fast!	Jingdong prefecture level city in Shandong
IXCHSQ Flagship Store	King of Power Saving	Use a month after a look at the savings of more than 40 dollars, there is a display of electricity savings, this high-power intelligent frequency converter economizer, affordable and cheap, logistics is very fast, the service attitude is very good, very satisfied with a shopping.	Jingdong prefecture level city in Shandong
IXCHSQ Flagship Store	King of Power Saving	Used for a period of time, it seems that every day can really can save a few degrees of electricity, workmanship quite can, with a period of time and then continue to look at it, anyway, the price is not	Jingdong prefecture level city in Shandong
IXCHSQ Flagship Store	King of Power Saving	Used for a period of time, it seems that every day can indeed save two several degrees of electricity, workmanship quite can, with a period of time and then continue to look at it, anyway, the price is not	Jingdong prefecture level city in Shandong
IXCHSQ Flagship Store	King of Power Saving	High-power super saver received immediately used, the price is not expensive quality is very good, save electricity and money, very convenient, conscientious business, must be praised, recommended to everyone!	Jingdong prefecture level city in Shandong
IXCHSQ Flagship Store	King of Power Saving	Home enhancement version of the intelligent power saver, is in use, I think the price is cheap, value for money, efficacy, power, power saving effect is very good.	Jingdong prefecture level city in Shandong
IXCHSQ Flagship Store	King of Power Saving	Bought three at once, one for each room, summer electricity consumption is very high, despite the three air conditioners on the electricity bill is still down a lot, too Nessie!	Jingdong prefecture level city in Shandong
IXCHSQ Flagship Store	King of Power Saving	Without further ado, the electric bill came down, $102 less this month, personal satisfaction, it counts okay, give you a good review	Jingdong prefecture level city in Shandong

IXCHSQ Flagship Store	King of Power Saving	The power saver has arrived, the installation is really convenient, plugged in on the line operation is quite simple, power saving effect is also a strong!	Jingdong prefecture level city in Shandong
IXCHSQ Flagship Store	King of Power Saving	Saver received, good quality, beautiful appearance, fine workmanship, simple installation	Jingdong prefecture level city in Shandong
IXCHSQ Flagship Store	King of Power Saving	Product design style modern, worth buying	Jingdong prefecture level city in Shandong
IXCHSQ Flagship Store	King of Power Saving	Energy saving, good power saving effect, practical, great value	Jingdong prefecture level city in Shandong
IXCHSQ Flagship Store	King of Power Saving	It's good quality, it's good, it's energy efficient, and I'll buy it again.	Jingdong prefecture level city in Shandong
IXCHSQ Flagship Store	King of Power Saving	Installation saves a lot of electricity, fast logistics, real energy-saving appliances	Jingdong prefecture level city in Shandong
IXCHSQ Flagship Store	King of Power Saving	The effect is good and the power saving is obvious after using it for a month and saving tens of dollars, the product is trustworthy, I now buy another 4 colors to prepare for their siblings' family to pay for	Jingdong prefecture level city in Shandong
IXCHSQ Flagship Store	King of Power Saving	The quality is particularly good workmanship is no problem, the arrival of the very fast oh, especially power-saving	Jingdong prefecture level city in Shandong
IXCHSQ Flagship Store	King of Power Saving	The quality is very good! In use, observed for a day, not as used before, so much power consumption.	Jingdong prefecture level city in Shandong
IXCHSQ Flagship Store	King of Power Saving	Goods have been received, the effect is good is not good, power saving is not yet know, wait for me to use it and then evaluate it.	Jingdong prefecture level city in Shandong
IXCHSQ Flagship Store	King of Power Saving	In-kind and the same as described, the quality is quite good, the seller's attitude is also good, ask questions and answer them, the speed of delivery bar, worth buying Oh!	Jingdong prefecture level city in Shandong
IXCHSQ Flagship Store	King of Power Saving	Nice stuff, came to comment after using it, it does save power.	Jingdong prefecture level city in Shandong
IXCHSQ Flagship Store	King of Power Saving	Fast charging, but also save power, the effect is very good, satisfied, praise!	Jingdong prefecture level city in Shandong
IXCHSQ Flagship Store	King of Power Saving	The goods have been received, I do not know how effective, after using the flat price again!	Jingdong prefecture level city in Shandong
IXCHSQ Flagship Store	King of Power Saving	Merchant service is very good Reply quickly The key is the quality and that quality is good	Jingdong prefecture level city in Shandong
IXCHSQ Flagship Store	King of Power Saving	It's working well now, I'll do a follow up review after I actually use it for a while	Jingdong prefecture level city in Shandong
IXCHSQ Flagship Store	King of Power Saving	The quality is good. After installing it, I feel like I've saved a lot of money on my electricity bill.	Jingdong prefecture level city in Shandong
IXCHSQ Flagship Store	King of Power Saving	Make up a review after use.	Jingdong prefecture level city in Shandong
IXCHSQ Flagship Store	King of Power Saving	review after use	Jingdong prefecture level city in Shandong
IXCHSQ Flagship Store	King of Power Saving	It's a small capacitor. It's not energy efficient.	Jingdong prefecture level city in Shandong
IXCHSQ Flagship Store	King of Power Saving	I'll follow up with a review after I've used it and had results.	Jingdong prefecture level city in Shandong
IXCHSQ Flagship Store	King of Power Saving	No power saving? Not good!	Jingdong prefecture level city in Shandong
IXCHSQ Flagship Store	King of Power Saving	Been using the same appliances and running a 1.5 air conditioner at night for what feels like no time, and after half a month of use it feels like the power is only increasing. Doesn't show any power saving	Jingdong prefecture level city in Shandong
IXCHSQ Flagship Store	King of Power Saving	serviceable	Jingdong prefecture level city in Shandong
IXCHSQ Flagship Store	King of Power Saving	It's a piece of junk. It doesn't save any power.	Jingdong prefecture level city in Shandong
IXCHSQ Flagship Store	King of Power Saving	There are a lot of appliances in the house, the summer air conditioning is very costly, I bought this in front of the air conditioning effect is very good, I explain that I bought three in one breath, I use more electricity a month down to 200 or so, very satisfied.	Jingdong prefecture level city in Shandong
IXCHSQ Flagship Store	King of Power Saving	The voltage is very stable has been maintained at 220v this value, high power super power saving, trustworthy, reasonable design, shape bright, logistics is quite fast, business warranty period, excellent service, logistics fast!	Jingdong prefecture level city in Shandong
IXCHSQ Flagship Store	King of Power Saving	Use a month after a look at the savings of more than 40 dollars, there is a display of electricity savings, this high-power intelligent frequency converter economizer, affordable and cheap, logistics is very fast, the service attitude is very good, very satisfied with a shopping.	Jingdong prefecture level city in Shandong
IXCHSQ Flagship Store	King of Power Saving	Used for a period of time, it seems that every day can really can save a few degrees of electricity, workmanship quite can, with a period of time and then continue to look at it, anyway, the price is not	Jingdong prefecture level city in Shandong
IXCHSQ Flagship Store	King of Power Saving	Used for a period of time, it seems that every day can indeed save two several degrees of electricity, workmanship quite can, with a period of time and then continue to look at it, anyway, the price is not	Jingdong prefecture level city in Shandong
IXCHSQ Flagship Store	King of Power Saving	High-power super saver received immediately used, the price is not expensive quality is very good, save electricity and money, very convenient, conscientious business, must be praised, recommended to everyone!	Jingdong prefecture level city in Shandong
IXCHSQ Flagship Store	King of Power Saving	Home enhancement version of the intelligent power saver, is in use, I think the price is cheap, value for money, efficacy, power, power saving effect is very good.	Jingdong prefecture level city in Shandong
IXCHSQ Flagship Store	King of Power Saving	Bought three at once, one for each room, summer electricity consumption is very high, despite the three air conditioners on the electricity bill is still down a lot, too Nessie!	Jingdong prefecture level city in Shandong
IXCHSQ Flagship Store	King of Power Saving	Without further ado, the electric bill came down, $102 less this month, personal satisfaction, it counts okay, give you a good review	Jingdong prefecture level city in Shandong
IXCHSQ Flagship Store	King of Power Saving	The power saver has arrived, the installation is really convenient, plugged in on the line operation is quite simple, power saving effect is also a strong!	Jingdong prefecture level city in Shandong
IXCHSQ Flagship Store	King of Power Saving	Saver received, good quality, beautiful appearance, fine workmanship, simple installation	Jingdong prefecture level city in Shandong
IXCHSQ Flagship Store	King of Power Saving	Product design style modern, worth buying	Jingdong prefecture level city in Shandong
IXCHSQ Flagship Store	King of Power Saving	Energy saving, good power saving effect, practical, great value	Jingdong prefecture level city in Shandong
IXCHSQ Flagship Store	King of Power Saving	It's good quality, it's good, it's energy efficient, and I'll buy it again.	Jingdong prefecture level city in Shandong
IXCHSQ Flagship Store	King of Power Saving	Installation saves a lot of electricity, fast logistics, real energy-saving appliances	Jingdong prefecture level city in Shandong
IXCHSQ Flagship Store	King of Power Saving	The effect is good and the power saving is obvious after using it for a month and saving tens of dollars, the product is trustworthy, I now buy another 4 colors to prepare for their siblings' family to pay for	Jingdong prefecture level city in Shandong
IXCHSQ Flagship Store	King of Power Saving	The quality is particularly good workmanship is no problem, the arrival of the very fast oh, especially power-saving	Jingdong prefecture level city in Shandong
IXCHSQ Flagship Store	King of Power Saving	The quality is very good! In use, observed for a day, not as used before, so much power consumption.	Jingdong prefecture level city in Shandong
IXCHSQ Flagship Store	King of Power Saving	Goods have been received, the effect is good is not good, power saving is not yet know, wait for me to use it and then evaluate it.	Jingdong prefecture level city in Shandong
IXCHSQ Flagship Store	King of Power Saving	In-kind and the same as described, the quality is quite good, the seller's attitude is also good, ask questions and answer them, the speed of delivery bar, worth buying Oh!	Jingdong prefecture level city in Shandong
IXCHSQ Flagship Store	King of Power Saving	Nice stuff, came to comment after using it, it does save power.	Jingdong prefecture level city in Shandong
IXCHSQ Flagship Store	King of Power Saving	Fast charging, but also save power, the effect is very good, satisfied, praise!	Jingdong prefecture level city in Shandong
IXCHSQ Flagship Store	King of Power Saving	The goods have been received, I do not know how effective, after using the flat price again!	Jingdong prefecture level city in Shandong
IXCHSQ Flagship Store	King of Power Saving	Merchant service is very good Reply quickly The key is the quality and that quality is good	Jingdong prefecture level city in Shandong
IXCHSQ Flagship Store	King of Power Saving	It's working well now, I'll do a follow up review after I actually use it for a while	Jingdong prefecture level city in Shandong

IXCHSQ Flagship Store	King of Power Saving	The quality is good. After installing it, I feel like I've saved a lot of money on my electricity bill.	Jingdong prefecture level city in Shandong
IXCHSQ Flagship Store	King of Power Saving	Make up a review after use.	Jingdong prefecture level city in Shandong
IXCHSQ Flagship Store	King of Power Saving	review after use	Jingdong prefecture level city in Shandong
IXCHSQ Flagship Store	King of Power Saving	It's a small capacitor. It's not energy efficient.	Jingdong prefecture level city in Shandong
IXCHSQ Flagship Store	King of Power Saving	I'll follow up with a review after I've used it and had results.	Jingdong prefecture level city in Shandong
IXCHSQ Flagship Store	King of Power Saving	No power saving? Not good!	Jingdong prefecture level city in Shandong
IXCHSQ Flagship Store	King of Power Saving	Been using the same appliances and running a 1.5 air conditioner at night for what feels like no time, and after half a month of use it feels like the power is only increasing. Doesn't show any power saving	Jingdong prefecture level city in Shandong
IXCHSQ Flagship Store	King of Power Saving	serviceable	Jingdong prefecture level city in Shandong
IXCHSQ Flagship Store	King of Power Saving	It's a piece of junk. It doesn't save any power.	Jingdong prefecture level city in Shandong
IXCHSQ Flagship Store	King of Power Saving	There are a lot of appliances in the house, the summer air conditioning is very costly, I bought this in front of the air conditioning effect is very good, I explain that I bought three in one breath, I use more electricity a month down to 200 or so, very satisfied.	Jingdong prefecture level city in Shandong
IXCHSQ Flagship Store	King of Power Saving	The voltage is very stable has been maintained at 220v this value, high power super power saving, trustworthy, reasonable design, shape bright, logistics is quite fast, business warranty period, excellent service, logistics fast!	Jingdong prefecture level city in Shandong
IXCHSQ Flagship Store	King of Power Saving	Use a month after a look at the savings of more than 40 dollars, there is a display of electricity savings, this high-power intelligent frequency converter economizer, affordable and cheap, logistics is very fast, the service attitude is very good, very satisfied with a shopping.	Jingdong prefecture level city in Shandong
IXCHSQ Flagship Store	King of Power Saving	Used for a period of time, it seems that every day can really can save a few degrees of electricity, workmanship quite can, with a period of time and then continue to look at it, anyway, the price is not	Jingdong prefecture level city in Shandong
IXCHSQ Flagship Store	King of Power Saving	Used for a period of time, it seems that every day can indeed save two several degrees of electricity, workmanship quite can, with a period of time and then continue to look at it, anyway, the price is not	Jingdong prefecture level city in Shandong
IXCHSQ Flagship Store	King of Power Saving	High-power super saver received immediately used, the price is not expensive quality is very good, save electricity and money, very convenient, conscientious business, must be praised, recommended to everyone!	Jingdong prefecture level city in Shandong
IXCHSQ Flagship Store	King of Power Saving	Home enhancement version of the intelligent power saver, is in use, I think the price is cheap, value for money, efficacy, power, power saving effect is very good.	Jingdong prefecture level city in Shandong
IXCHSQ Flagship Store	King of Power Saving	Bought three at once, one for each room, summer electricity consumption is very high, despite the three air conditioners on the electricity bill is still down a lot, too Nessie!	Jingdong prefecture level city in Shandong
IXCHSQ Flagship Store	King of Power Saving	Without further ado, the electric bill came down, $102 less this month, personal satisfaction, it counts okay, give you a good review	Jingdong prefecture level city in Shandong
IXCHSQ Flagship Store	King of Power Saving	The power saver has arrived, the installation is really convenient, plugged in on the line operation is quite simple, power saving effect is also a strong!	Jingdong prefecture level city in Shandong
IXCHSQ Flagship Store	King of Power Saving	Saver received, good quality, beautiful appearance, fine workmanship, simple installation	Jingdong prefecture level city in Shandong
IXCHSQ Flagship Store	King of Power Saving	Product design style modern, worth buying	Jingdong prefecture level city in Shandong
IXCHSQ Flagship Store	King of Power Saving	Energy saving, good power saving effect, practical, great value	Jingdong prefecture level city in Shandong
IXCHSQ Flagship Store	King of Power Saving	It's good quality, it's good, it's energy efficient, and I'll buy it again.	Jingdong prefecture level city in Shandong
IXCHSQ Flagship Store	King of Power Saving	Installation saves a lot of electricity, fast logistics, real energy-saving appliances	Jingdong prefecture level city in Shandong
IXCHSQ Flagship Store	King of Power Saving	The effect is good and the power saving is obvious after using it for a month and saving tens of dollars, the product is trustworthy, I now buy another 4 colors to prepare for their siblings' family to pay for	Jingdong prefecture level city in Shandong
IXCHSQ Flagship Store	King of Power Saving	The quality is particularly good workmanship is no problem, the arrival of the very fast oh, especially power-saving	Jingdong prefecture level city in Shandong
IXCHSQ Flagship Store	King of Power Saving	The quality is very good! In use, observed for a day, not as used before, so much power consumption.	Jingdong prefecture level city in Shandong
IXCHSQ Flagship Store	King of Power Saving	Goods have been received, the effect is good is not good, power saving is not yet know, wait for me to use it and then evaluate it.	Jingdong prefecture level city in Shandong
IXCHSQ Flagship Store	King of Power Saving	In-kind and the same as described, the quality is quite good, the seller's attitude is also good, ask questions and answer them, the speed of delivery bar, worth buying Oh!	Jingdong prefecture level city in Shandong
IXCHSQ Flagship Store	King of Power Saving	Nice stuff, came to comment after using it, it does save power.	Jingdong prefecture level city in Shandong
IXCHSQ Flagship Store	King of Power Saving	Fast charging, but also save power, the effect is very good, satisfied, praise!	Jingdong prefecture level city in Shandong
IXCHSQ Flagship Store	King of Power Saving	The goods have been received, I do not know how effective, after using the flat price again!	Jingdong prefecture level city in Shandong
IXCHSQ Flagship Store	King of Power Saving	Merchant service is very good Reply quickly The key is the quality and that quality is good	Jingdong prefecture level city in Shandong
IXCHSQ Flagship Store	King of Power Saving	It's working well now, I'll do a follow up review after I actually use it for a while	Jingdong prefecture level city in Shandong
IXCHSQ Flagship Store	King of Power Saving	The quality is good. After installing it, I feel like I've saved a lot of money on my electricity bill.	Jingdong prefecture level city in Shandong
IXCHSQ Flagship Store	King of Power Saving	Make up a review after use.	Jingdong prefecture level city in Shandong
IXCHSQ Flagship Store	King of Power Saving	review after use	Jingdong prefecture level city in Shandong
IXCHSQ Flagship Store	King of Power Saving	It's a small capacitor. It's not energy efficient.	Jingdong prefecture level city in Shandong
IXCHSQ Flagship Store	King of Power Saving	I'll follow up with a review after I've used it and had results.	Jingdong prefecture level city in Shandong
IXCHSQ Flagship Store	King of Power Saving	No power saving? Not good!	Jingdong prefecture level city in Shandong
IXCHSQ Flagship Store	King of Power Saving	Been using the same appliances and running a 1.5 air conditioner at night for what feels like no time, and after half a month of use it feels like the power is only increasing. Doesn't show any power saving	Jingdong prefecture level city in Shandong
IXCHSQ Flagship Store	King of Power Saving	serviceable	Jingdong prefecture level city in Shandong
IXCHSQ Flagship Store	King of Power Saving	It's a piece of junk. It doesn't save any power.	Jingdong prefecture level city in Shandong
IXCHSQ Flagship Store	King of Power Saving	There are a lot of appliances in the house, the summer air conditioning is very costly, I bought this in front of the air conditioning effect is very good, I explain that I bought three in one breath, I use more electricity a month down to 200 or so, very satisfied.	Jingdong prefecture level city in Shandong
IXCHSQ Flagship Store	King of Power Saving	The voltage is very stable has been maintained at 220v this value, high power super power saving, trustworthy, reasonable design, shape bright, logistics is quite fast, business warranty period, excellent service, logistics fast!	Jingdong prefecture level city in Shandong
IXCHSQ Flagship Store	King of Power Saving	Use a month after a look at the savings of more than 40 dollars, there is a display of electricity savings, this high-power intelligent frequency converter economizer, affordable and cheap, logistics is very fast, the service attitude is very good, very satisfied with a shopping.	Jingdong prefecture level city in Shandong
IXCHSQ Flagship Store	King of Power Saving	Used for a period of time, it seems that every day can really can save a few degrees of electricity, workmanship quite can, with a period of time and then continue to look at it, anyway, the price is not	Jingdong prefecture level city in Shandong
IXCHSQ Flagship Store	King of Power Saving	Used for a period of time, it seems that every day can indeed save two several degrees of electricity, workmanship quite can, with a period of time and then continue to look at it, anyway, the price is not	Jingdong prefecture level city in Shandong

IXCHSQ Flagship Store	King of Power Saving	High-power super saver received immediately used, the price is not expensive quality is very good, save electricity and money, very convenient, conscientious business, must be praised, recommended to everyone!	Jingdong prefecture level city in Shandong
IXCHSQ Flagship Store	King of Power Saving	Home enhancement version of the intelligent power saver, is in use, I think the price is cheap, value for money, efficacy, power, power saving effect is very good.	Jingdong prefecture level city in Shandong
IXCHSQ Flagship Store	King of Power Saving	Bought three at once, one for each room, summer electricity consumption is very high, despite the three air conditioners on the electricity bill is still down a lot, too Nessie!	Jingdong prefecture level city in Shandong
IXCHSQ Flagship Store	King of Power Saving	Without further ado, the electric bill came down, $102 less this month, personal satisfaction, it counts okay, give you a good review	Jingdong prefecture level city in Shandong
IXCHSQ Flagship Store	King of Power Saving	The power saver has arrived, the installation is really convenient, plugged in on the line operation is quite simple, power saving effect is also a strong!	Jingdong prefecture level city in Shandong
IXCHSQ Flagship Store	King of Power Saving	Saver received, good quality, beautiful appearance, fine workmanship, simple installation	Jingdong prefecture level city in Shandong
IXCHSQ Flagship Store	King of Power Saving	Product design style modern, worth buying	Jingdong prefecture level city in Shandong
IXCHSQ Flagship Store	King of Power Saving	Energy saving, good power saving effect, practical, great value	Jingdong prefecture level city in Shandong
IXCHSQ Flagship Store	King of Power Saving	It's good quality, it's good, it's energy efficient, and I'll buy it again.	Jingdong prefecture level city in Shandong
IXCHSQ Flagship Store	King of Power Saving	Installation saves a lot of electricity, fast logistics, real energy-saving appliances	Jingdong prefecture level city in Shandong
IXCHSQ Flagship Store	King of Power Saving	The effect is good and the power saving is obvious after using it for a month and saving tens of dollars, the product is trustworthy, I now buy another 4 colors to prepare for their siblings' family to pay for	Jingdong prefecture level city in Shandong
IXCHSQ Flagship Store	King of Power Saving	The quality is particularly good workmanship is no problem, the arrival of the very fast oh, especially power-saving	Jingdong prefecture level city in Shandong
IXCHSQ Flagship Store	King of Power Saving	The quality is very good! In use, observed for a day, not as used before, so much power consumption.	Jingdong prefecture level city in Shandong
IXCHSQ Flagship Store	King of Power Saving	Goods have been received, the effect is good is not good, power saving is not yet know, wait for me to use it and then evaluate it.	Jingdong prefecture level city in Shandong
IXCHSQ Flagship Store	King of Power Saving	In-kind and the same as described, the quality is quite good, the seller's attitude is also good, ask questions and answer them, the speed of delivery bar, worth buying Oh!	Jingdong prefecture level city in Shandong
IXCHSQ Flagship Store	King of Power Saving	Nice stuff, came to comment after using it, it does save power.	Jingdong prefecture level city in Shandong
IXCHSQ Flagship Store	King of Power Saving	Fast charging, but also save power, the effect is very good, satisfied, praise!	Jingdong prefecture level city in Shandong
IXCHSQ Flagship Store	King of Power Saving	The goods have been received, I do not know how effective, after using the flat price again!	Jingdong prefecture level city in Shandong
IXCHSQ Flagship Store	King of Power Saving	Merchant service is very good Reply quickly The key is the quality and that quality is good	Jingdong prefecture level city in Shandong
IXCHSQ Flagship Store	King of Power Saving	It's working well now, I'll do a follow up review after I actually use it for a while	Jingdong prefecture level city in Shandong
IXCHSQ Flagship Store	King of Power Saving	The quality is good. After installing it, I feel like I've saved a lot of money on my electricity bill.	Jingdong prefecture level city in Shandong
IXCHSQ Flagship Store	King of Power Saving	Make up a review after use.	Jingdong prefecture level city in Shandong
IXCHSQ Flagship Store	King of Power Saving	review after use	Jingdong prefecture level city in Shandong
IXCHSQ Flagship Store	King of Power Saving	It's a small capacitor. It's not energy efficient.	Jingdong prefecture level city in Shandong
IXCHSQ Flagship Store	King of Power Saving	I'll follow up with a review after I've used it and had results.	Jingdong prefecture level city in Shandong
IXCHSQ Flagship Store	King of Power Saving	No power saving? Not good!	Jingdong prefecture level city in Shandong
IXCHSQ Flagship Store	King of Power Saving	Been using the same appliances and running a 1.5 air conditioner at night for what feels like no time, and after half a month of use it feels like the power is only increasing. Doesn't show any power saving	Jingdong prefecture level city in Shandong
IXCHSQ Flagship Store	King of Power Saving	serviceable	Jingdong prefecture level city in Shandong
IXCHSQ Flagship Store	King of Power Saving	It's a piece of junk. It doesn't save any power.	Jingdong prefecture level city in Shandong
IXCHSQ Flagship Store	King of Power Saving	There are a lot of appliances in the house, the summer air conditioning is very costly, I bought this in front of the air conditioning effect is very good, I explain that I bought three in one breath, I use more electricity a month down to 200 or so, very satisfied.	Jingdong prefecture level city in Shandong
IXCHSQ Flagship Store	King of Power Saving	The voltage is very stable has been maintained at 220v this value, high power super power saving, trustworthy, reasonable design, shape bright, logistics is quite fast, business warranty period, excellent service, logistics fast!	Jingdong prefecture level city in Shandong
IXCHSQ Flagship Store	King of Power Saving	Use a month after a look at the savings of more than 40 dollars, there is a display of electricity savings, this high-power intelligent frequency converter economizer, affordable and cheap, logistics is very fast, the service attitude is very good, very satisfied with a shopping.	Jingdong prefecture level city in Shandong
IXCHSQ Flagship Store	King of Power Saving	Used for a period of time, it seems that every day can really can save a few degrees of electricity, workmanship quite can, with a period of time and then continue to look at it, anyway, the price is not	Jingdong prefecture level city in Shandong
IXCHSQ Flagship Store	King of Power Saving	Used for a period of time, it seems that every day can indeed save two several degrees of electricity, workmanship quite can, with a period of time and then continue to look at it, anyway, the price is not	Jingdong prefecture level city in Shandong
IXCHSQ Flagship Store	King of Power Saving	High-power super saver received immediately used, the price is not expensive quality is very good, save electricity and money, very convenient, conscientious business, must be praised, recommended to everyone!	Jingdong prefecture level city in Shandong
IXCHSQ Flagship Store	King of Power Saving	Home enhancement version of the intelligent power saver, is in use, I think the price is cheap, value for money, efficacy, power, power saving effect is very good.	Jingdong prefecture level city in Shandong
IXCHSQ Flagship Store	King of Power Saving	Bought three at once, one for each room, summer electricity consumption is very high, despite the three air conditioners on the electricity bill is still down a lot, too Nessie!	Jingdong prefecture level city in Shandong
IXCHSQ Flagship Store	King of Power Saving	Without further ado, the electric bill came down, $102 less this month, personal satisfaction, it counts okay, give you a good review	Jingdong prefecture level city in Shandong
IXCHSQ Flagship Store	King of Power Saving	The power saver has arrived, the installation is really convenient, plugged in on the line operation is quite simple, power saving effect is also a strong!	Jingdong prefecture level city in Shandong
IXCHSQ Flagship Store	King of Power Saving	Saver received, good quality, beautiful appearance, fine workmanship, simple installation	Jingdong prefecture level city in Shandong
IXCHSQ Flagship Store	King of Power Saving	Product design style modern, worth buying	Jingdong prefecture level city in Shandong
IXCHSQ Flagship Store	King of Power Saving	Energy saving, good power saving effect, practical, great value	Jingdong prefecture level city in Shandong
IXCHSQ Flagship Store	King of Power Saving	It's good quality, it's good, it's energy efficient, and I'll buy it again.	Jingdong prefecture level city in Shandong
IXCHSQ Flagship Store	King of Power Saving	Installation saves a lot of electricity, fast logistics, real energy-saving appliances	Jingdong prefecture level city in Shandong
IXCHSQ Flagship Store	King of Power Saving	The effect is good and the power saving is obvious after using it for a month and saving tens of dollars, the product is trustworthy, I now buy another 4 colors to prepare for their siblings' family to pay for	Jingdong prefecture level city in Shandong
IXCHSQ Flagship Store	King of Power Saving	The quality is particularly good workmanship is no problem, the arrival of the very fast oh, especially power-saving	Jingdong prefecture level city in Shandong
IXCHSQ Flagship Store	King of Power Saving	The quality is very good! In use, observed for a day, not as used before, so much power consumption.	Jingdong prefecture level city in Shandong
IXCHSQ Flagship Store	King of Power Saving	Goods have been received, the effect is good is not good, power saving is not yet know, wait for me to use it and then evaluate it.	Jingdong prefecture level city in Shandong
IXCHSQ Flagship Store	King of Power Saving	In-kind and the same as described, the quality is quite good, the seller's attitude is also good, ask questions and answer them, the speed of delivery bar, worth buying Oh!	Jingdong prefecture level city in Shandong
IXCHSQ Flagship Store	King of Power Saving	Nice stuff, came to comment after using it, it does save power.	Jingdong prefecture level city in Shandong

IXCHSQ Flagship Store	King of Power Saving	Fast charging, but also save power, the effect is very good, satisfied, praise!	Jingdong prefecture level city in Shandong
IXCHSQ Flagship Store	King of Power Saving	The goods have been received, I do not know how effective, after using the flat price again!	Jingdong prefecture level city in Shandong
IXCHSQ Flagship Store	King of Power Saving	Merchant service is very good Reply quickly The key is the quality and that quality is good	Jingdong prefecture level city in Shandong
IXCHSQ Flagship Store	King of Power Saving	It's working well now, I'll do a follow up review after I actually use it for a while	Jingdong prefecture level city in Shandong
IXCHSQ Flagship Store	King of Power Saving	The quality is good. After installing it, I feel like I've saved a lot of money on my electricity bill.	Jingdong prefecture level city in Shandong
IXCHSQ Flagship Store	King of Power Saving	Make up a review after use.	Jingdong prefecture level city in Shandong
IXCHSQ Flagship Store	King of Power Saving	review after use	Jingdong prefecture level city in Shandong
IXCHSQ Flagship Store	King of Power Saving	It's a small capacitor. It's not energy efficient.	Jingdong prefecture level city in Shandong
IXCHSQ Flagship Store	King of Power Saving	I'll follow up with a review after I've used it and had results.	Jingdong prefecture level city in Shandong
IXCHSQ Flagship Store	King of Power Saving	No power saving? Not good!	Jingdong prefecture level city in Shandong
IXCHSQ Flagship Store	King of Power Saving	Been using the same appliances and running a 1.5 air conditioner at night for what feels like no time, and after half a month of use it feels like the power is only increasing. Doesn't show any power saving	Jingdong prefecture level city in Shandong
IXCHSQ Flagship Store	King of Power Saving	serviceable	Jingdong prefecture level city in Shandong
IXCHSQ Flagship Store	King of Power Saving	It's a piece of junk. It doesn't save any power.	Jingdong prefecture level city in Shandong
IXCHSQ Flagship Store	King of Power Saving	There are a lot of appliances in the house, the summer air conditioning is very costly, I bought this in front of the air conditioning effect is very good, I explain that I bought three in one breath, I use more electricity a month down to 200 or so, very satisfied.	Jingdong prefecture level city in Shandong
IXCHSQ Flagship Store	King of Power Saving	The voltage is very stable has been maintained at 220v this value, high power super power saving, trustworthy, reasonable design, shape bright, logistics is quite fast, business warranty period, excellent service, logistics fast!	Jingdong prefecture level city in Shandong
IXCHSQ Flagship Store	King of Power Saving	Use a month after a look at the savings of more than 40 dollars, there is a display of electricity savings, this high-power intelligent frequency converter economizer, affordable and cheap, logistics is very fast, the service attitude is very good, very satisfied with a shopping.	Jingdong prefecture level city in Shandong
IXCHSQ Flagship Store	King of Power Saving	Used for a period of time, it seems that every day can really can save a few degrees of electricity, workmanship quite can, with a period of time and then continue to look at it, anyway, the price is not	Jingdong prefecture level city in Shandong
IXCHSQ Flagship Store	King of Power Saving	Used for a period of time, it seems that every day can indeed save two several degrees of electricity, workmanship quite can, with a period of time and then continue to look at it, anyway, the price is not	Jingdong prefecture level city in Shandong
IXCHSQ Flagship Store	King of Power Saving	High-power super saver received immediately used, the price is not expensive quality is very good, save electricity and money, very convenient, conscientious business, must be praised, recommended to everyone!	Jingdong prefecture level city in Shandong
IXCHSQ Flagship Store	King of Power Saving	Home enhancement version of the intelligent power saver, is in use, I think the price is cheap, value for money, efficacy, power, power saving effect is very good.	Jingdong prefecture level city in Shandong
IXCHSQ Flagship Store	King of Power Saving	Bought three at once, one for each room, summer electricity consumption is very high, despite the three air conditioners on the electricity bill is still down a lot, too Nessie!	Jingdong prefecture level city in Shandong
IXCHSQ Flagship Store	King of Power Saving	Without further ado, the electric bill came down, $102 less this month, personal satisfaction, it counts okay, give you a good review	Jingdong prefecture level city in Shandong
IXCHSQ Flagship Store	King of Power Saving	The power saver has arrived, the installation is really convenient, plugged in on the line operation is quite simple, power saving effect is also a strong!	Jingdong prefecture level city in Shandong
IXCHSQ Flagship Store	King of Power Saving	Saver received, good quality, beautiful appearance, fine workmanship, simple installation	Jingdong prefecture level city in Shandong
IXCHSQ Flagship Store	King of Power Saving	Product design style modern, worth buying	Jingdong prefecture level city in Shandong
IXCHSQ Flagship Store	King of Power Saving	Energy saving, good power saving effect, practical, great value	Jingdong prefecture level city in Shandong
IXCHSQ Flagship Store	King of Power Saving	It's good quality, it's good, it's energy efficient, and I'll buy it again.	Jingdong prefecture level city in Shandong
IXCHSQ Flagship Store	King of Power Saving	Installation saves a lot of electricity, fast logistics, real energy-saving appliances	Jingdong prefecture level city in Shandong
IXCHSQ Flagship Store	King of Power Saving	The effect is good and the power saving is obvious after using it for a month and saving tens of dollars, the product is trustworthy, I now buy another 4 colors to prepare for their siblings' family to pay for	Jingdong prefecture level city in Shandong
IXCHSQ Flagship Store	King of Power Saving	The quality is particularly good workmanship is no problem, the arrival of the very fast oh, especially power-saving	Jingdong prefecture level city in Shandong
IXCHSQ Flagship Store	King of Power Saving	The quality is very good! In use, observed for a day, not as used before, so much power consumption.	Jingdong prefecture level city in Shandong
IXCHSQ Flagship Store	King of Power Saving	Goods have been received, the effect is good is not good, power saving is not yet know, wait for me to use it and then evaluate it.	Jingdong prefecture level city in Shandong
IXCHSQ Flagship Store	King of Power Saving	In-kind and the same as described, the quality is quite good, the seller's attitude is also good, ask questions and answer them, the speed of delivery bar, worth buying Oh!	Jingdong prefecture level city in Shandong
IXCHSQ Flagship Store	King of Power Saving	Nice stuff, came to comment after using it, it does save power.	Jingdong prefecture level city in Shandong
IXCHSQ Flagship Store	King of Power Saving	Fast charging, but also save power, the effect is very good, satisfied, praise!	Jingdong prefecture level city in Shandong
IXCHSQ Flagship Store	King of Power Saving	The goods have been received, I do not know how effective, after using the flat price again!	Jingdong prefecture level city in Shandong
IXCHSQ Flagship Store	King of Power Saving	Merchant service is very good Reply quickly The key is the quality and that quality is good	Jingdong prefecture level city in Shandong
IXCHSQ Flagship Store	King of Power Saving	It's working well now, I'll do a follow up review after I actually use it for a while	Jingdong prefecture level city in Shandong
IXCHSQ Flagship Store	King of Power Saving	The quality is good. After installing it, I feel like I've saved a lot of money on my electricity bill.	Jingdong prefecture level city in Shandong
IXCHSQ Flagship Store	King of Power Saving	Make up a review after use.	Jingdong prefecture level city in Shandong
IXCHSQ Flagship Store	King of Power Saving	review after use	Jingdong prefecture level city in Shandong
IXCHSQ Flagship Store	King of Power Saving	It's a small capacitor. It's not energy efficient.	Jingdong prefecture level city in Shandong
IXCHSQ Flagship Store	King of Power Saving	I'll follow up with a review after I've used it and had results.	Jingdong prefecture level city in Shandong
IXCHSQ Flagship Store	King of Power Saving	No power saving? Not good!	Jingdong prefecture level city in Shandong
IXCHSQ Flagship Store	King of Power Saving	Been using the same appliances and running a 1.5 air conditioner at night for what feels like no time, and after half a month of use it feels like the power is only increasing. Doesn't show any power saving	Jingdong prefecture level city in Shandong
IXCHSQ Flagship Store	King of Power Saving	serviceable	Jingdong prefecture level city in Shandong
IXCHSQ Flagship Store	King of Power Saving	It's a piece of junk. It doesn't save any power.	Jingdong prefecture level city in Shandong
IXCHSQ Flagship Store	King of Power Saving	There are a lot of appliances in the house, the summer air conditioning is very costly, I bought this in front of the air conditioning effect is very good, I explain that I bought three in one breath, I use more electricity a month down to 200 or so, very satisfied.	Jingdong prefecture level city in Shandong
IXCHSQ Flagship Store	King of Power Saving	The voltage is very stable has been maintained at 220v this value, high power super power saving, trustworthy, reasonable design, shape bright, logistics is quite fast, business warranty period, excellent service, logistics fast!	Jingdong prefecture level city in Shandong

IXCHSQ Flagship Store	King of Power Saving	Use a month after a look at the savings of more than 40 dollars, there is a display of electricity savings, this high-power intelligent frequency converter economizer, affordable and cheap, logistics is very fast, the service attitude is very good, very satisfied with a shopping.	Jingdong prefecture level city in Shandong
IXCHSQ Flagship Store	King of Power Saving	Used for a period of time, it seems that every day can really can save a few degrees of electricity, workmanship quite can, with a period of time and then continue to look at it, anyway, the price is not	Jingdong prefecture level city in Shandong
IXCHSQ Flagship Store	King of Power Saving	Used for a period of time, it seems that every day can indeed save two several degrees of electricity, workmanship quite can, with a period of time and then continue to look at it, anyway, the price is not	Jingdong prefecture level city in Shandong
IXCHSQ Flagship Store	King of Power Saving	High-power super saver received immediately used, the price is not expensive quality is very good, save electricity and money, very convenient, conscientious business, must be praised, recommended to everyone!	Jingdong prefecture level city in Shandong
IXCHSQ Flagship Store	King of Power Saving	Home enhancement version of the intelligent power saver, is in use, I think the price is cheap, value for money, efficacy, power, power saving effect is very good.	Jingdong prefecture level city in Shandong
IXCHSQ Flagship Store	King of Power Saving	Bought three at once, one for each room, summer electricity consumption is very high, despite the three air conditioners on the electricity bill is still down a lot, too Nessie!	Jingdong prefecture level city in Shandong
IXCHSQ Flagship Store	King of Power Saving	Without further ado, the electric bill came down, $102 less this month, personal satisfaction, it counts okay, give you a good review	Jingdong prefecture level city in Shandong
IXCHSQ Flagship Store	King of Power Saving	The power saver has arrived, the installation is really convenient, plugged in on the line operation is quite simple, power saving effect is also a strong!	Jingdong prefecture level city in Shandong
IXCHSQ Flagship Store	King of Power Saving	Saver received, good quality, beautiful appearance, fine workmanship, simple installation	Jingdong prefecture level city in Shandong
IXCHSQ Flagship Store	King of Power Saving	Product design style modern, worth buying	Jingdong prefecture level city in Shandong
IXCHSQ Flagship Store	King of Power Saving	Energy saving, good power saving effect, practical, great value	Jingdong prefecture level city in Shandong
IXCHSQ Flagship Store	King of Power Saving	It's good quality, it's good, it's energy efficient, and I'll buy it again.	Jingdong prefecture level city in Shandong
IXCHSQ Flagship Store	King of Power Saving	Installation saves a lot of electricity, fast logistics, real energy-saving appliances	Jingdong prefecture level city in Shandong
IXCHSQ Flagship Store	King of Power Saving	The effect is good and the power saving is obvious after using it for a month and saving tens of dollars, the product is trustworthy, I now buy another 4 colors to prepare for their siblings' family to pay for	Jingdong prefecture level city in Shandong
IXCHSQ Flagship Store	King of Power Saving	The quality is particularly good workmanship is no problem, the arrival of the very fast oh, especially power-saving	Jingdong prefecture level city in Shandong
IXCHSQ Flagship Store	King of Power Saving	The quality is very good! In use, observed for a day, not as used before, so much power consumption.	Jingdong prefecture level city in Shandong
IXCHSQ Flagship Store	King of Power Saving	Goods have been received, the effect is good is not good, power saving is not yet know, wait for me to use it and then evaluate it.	Jingdong prefecture level city in Shandong
IXCHSQ Flagship Store	King of Power Saving	In-kind and the same as described, the quality is quite good, the seller's attitude is also good, ask questions and answer them, the speed of delivery bar, worth buying Oh!	Jingdong prefecture level city in Shandong
IXCHSQ Flagship Store	King of Power Saving	Nice stuff, came to comment after using it, it does save power.	Jingdong prefecture level city in Shandong
IXCHSQ Flagship Store	King of Power Saving	Fast charging, but also save power, the effect is very good, satisfied, praise!	Jingdong prefecture level city in Shandong
IXCHSQ Flagship Store	King of Power Saving	The goods have been received, I do not know how effective, after using the flat price again!	Jingdong prefecture level city in Shandong
IXCHSQ Flagship Store	King of Power Saving	Merchant service is very good Reply quickly The key is the quality and that quality is good	Jingdong prefecture level city in Shandong
IXCHSQ Flagship Store	King of Power Saving	It's working well now, I'll do a follow up review after I actually use it for a while	Jingdong prefecture level city in Shandong
IXCHSQ Flagship Store	King of Power Saving	The quality is good. After installing it, I feel like I've saved a lot of money on my electricity bill.	Jingdong prefecture level city in Shandong
IXCHSQ Flagship Store	King of Power Saving	Make up a review after use.	Jingdong prefecture level city in Shandong
IXCHSQ Flagship Store	King of Power Saving	review after use	Jingdong prefecture level city in Shandong
IXCHSQ Flagship Store	King of Power Saving	It's a small capacitor. It's not energy efficient.	Jingdong prefecture level city in Shandong
IXCHSQ Flagship Store	King of Power Saving	I'll follow up with a review after I've used it and had results.	Jingdong prefecture level city in Shandong
IXCHSQ Flagship Store	King of Power Saving	No power saving? Not good!	Jingdong prefecture level city in Shandong
IXCHSQ Flagship Store	King of Power Saving	Been using the same appliances and running a 1.5 air conditioner at night for what feels like no time, and after half a month of use it feels like the power is only increasing. Doesn't show any power saving	Jingdong prefecture level city in Shandong
IXCHSQ Flagship Store	King of Power Saving	serviceable	Jingdong prefecture level city in Shandong
IXCHSQ Flagship Store	King of Power Saving	It's a piece of junk. It doesn't save any power.	Jingdong prefecture level city in Shandong
IXCHSQ Flagship Store	King of Power Saving	There are a lot of appliances in the house, the summer air conditioning is very costly, I bought this in front of the air conditioning effect is very good, I explain that I bought three in one breath, I use more electricity a month down to 200 or so, very satisfied.	Jingdong prefecture level city in Shandong
IXCHSQ Flagship Store	King of Power Saving	The voltage is very stable has been maintained at 220v this value, high power super power saving, trustworthy, reasonable design, shape bright, logistics is quite fast, business warranty period, excellent service, logistics fast!	Jingdong prefecture level city in Shandong
IXCHSQ Flagship Store	King of Power Saving	Use a month after a look at the savings of more than 40 dollars, there is a display of electricity savings, this high-power intelligent frequency converter economizer, affordable and cheap, logistics is very fast, the service attitude is very good, very satisfied with a shopping.	Jingdong prefecture level city in Shandong
IXCHSQ Flagship Store	King of Power Saving	Used for a period of time, it seems that every day can really can save a few degrees of electricity, workmanship quite can, with a period of time and then continue to look at it, anyway, the price is not	Jingdong prefecture level city in Shandong
IXCHSQ Flagship Store	King of Power Saving	Used for a period of time, it seems that every day can indeed save two several degrees of electricity, workmanship quite can, with a period of time and then continue to look at it, anyway, the price is not	Jingdong prefecture level city in Shandong
IXCHSQ Flagship Store	King of Power Saving	High-power super saver received immediately used, the price is not expensive quality is very good, save electricity and money, very convenient, conscientious business, must be praised, recommended to everyone!	Jingdong prefecture level city in Shandong
IXCHSQ Flagship Store	King of Power Saving	Home enhancement version of the intelligent power saver, is in use, I think the price is cheap, value for money, efficacy, power, power saving effect is very good.	Jingdong prefecture level city in Shandong
IXCHSQ Flagship Store	King of Power Saving	Bought three at once, one for each room, summer electricity consumption is very high, despite the three air conditioners on the electricity bill is still down a lot, too Nessie!	Jingdong prefecture level city in Shandong
IXCHSQ Flagship Store	King of Power Saving	Without further ado, the electric bill came down, $102 less this month, personal satisfaction, it counts okay, give you a good review	Jingdong prefecture level city in Shandong
IXCHSQ Flagship Store	King of Power Saving	The power saver has arrived, the installation is really convenient, plugged in on the line operation is quite simple, power saving effect is also a strong!	Jingdong prefecture level city in Shandong
IXCHSQ Flagship Store	King of Power Saving	Saver received, good quality, beautiful appearance, fine workmanship, simple installation	Jingdong prefecture level city in Shandong
IXCHSQ Flagship Store	King of Power Saving	Product design style modern, worth buying	Jingdong prefecture level city in Shandong
IXCHSQ Flagship Store	King of Power Saving	Energy saving, good power saving effect, practical, great value	Jingdong prefecture level city in Shandong
IXCHSQ Flagship Store	King of Power Saving	It's good quality, it's good, it's energy efficient, and I'll buy it again.	Jingdong prefecture level city in Shandong
IXCHSQ Flagship Store	King of Power Saving	Installation saves a lot of electricity, fast logistics, real energy-saving appliances	Jingdong prefecture level city in Shandong
IXCHSQ Flagship Store	King of Power Saving	The effect is good and the power saving is obvious after using it for a month and saving tens of dollars, the product is trustworthy, I now buy another 4 colors to prepare for their siblings' family to pay for	Jingdong prefecture level city in Shandong
IXCHSQ Flagship Store	King of Power Saving	The quality is particularly good workmanship is no problem, the arrival of the very fast oh, especially power-saving	Jingdong prefecture level city in Shandong

IXCHSQ Flagship Store	King of Power Saving	The quality is very good! In use, observed for a day, not as used before, so much power consumption.	Jingdong prefecture level city in Shandong
IXCHSQ Flagship Store	King of Power Saving	Goods have been received, the effect is good is not good, power saving is not yet know, wait for me to use it and then evaluate it.	Jingdong prefecture level city in Shandong
IXCHSQ Flagship Store	King of Power Saving	In-kind and the same as described, the quality is quite good, the seller's attitude is also good, ask questions and answer them, the speed of delivery bar, worth buying Oh!	Jingdong prefecture level city in Shandong
IXCHSQ Flagship Store	King of Power Saving	Nice stuff, came to comment after using it, it does save power.	Jingdong prefecture level city in Shandong
IXCHSQ Flagship Store	King of Power Saving	Fast charging, but also save power, the effect is very good, satisfied, praise!	Jingdong prefecture level city in Shandong
IXCHSQ Flagship Store	King of Power Saving	The goods have been received, I do not know how effective, after using the flat price again!	Jingdong prefecture level city in Shandong
IXCHSQ Flagship Store	King of Power Saving	Merchant service is very good Reply quickly The key is the quality and that quality is good	Jingdong prefecture level city in Shandong
IXCHSQ Flagship Store	King of Power Saving	It's working well now, I'll do a follow up review after I actually use it for a while	Jingdong prefecture level city in Shandong
IXCHSQ Flagship Store	King of Power Saving	The quality is good. After installing it, I feel like I've saved a lot of money on my electricity bill.	Jingdong prefecture level city in Shandong
IXCHSQ Flagship Store	King of Power Saving	Make up a review after use.	Jingdong prefecture level city in Shandong
IXCHSQ Flagship Store	King of Power Saving	review after use	Jingdong prefecture level city in Shandong
IXCHSQ Flagship Store	King of Power Saving	It's a small capacitor. It's not energy efficient.	Jingdong prefecture level city in Shandong
IXCHSQ Flagship Store	King of Power Saving	I'll follow up with a review after I've used it and had results.	Jingdong prefecture level city in Shandong
IXCHSQ Flagship Store	King of Power Saving	No power saving? Not good!	Jingdong prefecture level city in Shandong
IXCHSQ Flagship Store	King of Power Saving	Been using the same appliances and running a 1.5 air conditioner at night for what feels like no time, and after half a month of use it feels like the power is only increasing. Doesn't show any power saving	Jingdong prefecture level city in Shandong
IXCHSQ Flagship Store	King of Power Saving	serviceable	Jingdong prefecture level city in Shandong
IXCHSQ Flagship Store	King of Power Saving	It's a piece of junk. It doesn't save any power.	Jingdong prefecture level city in Shandong
IXCHSQ Flagship Store	King of Power Saving	There are a lot of appliances in the house, the summer air conditioning is very costly, I bought this in front of the air conditioning effect is very good, I explain that I bought three in one breath, I use more electricity a month down to 200 or so, very satisfied.	Jingdong prefecture level city in Shandong
IXCHSQ Flagship Store	King of Power Saving	The voltage is very stable has been maintained at 220v this value, high power super power saving, trustworthy, reasonable design, shape bright, logistics is quite fast, business warranty period, excellent service, logistics fast!	Jingdong prefecture level city in Shandong
IXCHSQ Flagship Store	King of Power Saving	Use a month after a look at the savings of more than 40 dollars, there is a display of electricity savings, this high-power intelligent frequency converter economizer, affordable and cheap, logistics is very fast, the service attitude is very good, very satisfied with a shopping.	Jingdong prefecture level city in Shandong
IXCHSQ Flagship Store	King of Power Saving	Used for a period of time, it seems that every day can really can save a few degrees of electricity, workmanship quite can, with a period of time and then continue to look at it, anyway, the price is not	Jingdong prefecture level city in Shandong
IXCHSQ Flagship Store	King of Power Saving	Used for a period of time, it seems that every day can indeed save two several degrees of electricity, workmanship quite can, with a period of time and then continue to look at it, anyway, the price is not	Jingdong prefecture level city in Shandong
IXCHSQ Flagship Store	King of Power Saving	High-power super saver received immediately used, the price is not expensive quality is very good, save electricity and money, very convenient, conscientious business, must be praised, recommended to everyone!	Jingdong prefecture level city in Shandong
IXCHSQ Flagship Store	King of Power Saving	Home enhancement version of the intelligent power saver, is in use, I think the price is cheap, value for money, efficacy, power, power saving effect is very good.	Jingdong prefecture level city in Shandong
IXCHSQ Flagship Store	King of Power Saving	Bought three at once, one for each room, summer electricity consumption is very high, despite the three air conditioners on the electricity bill is still down a lot, too Nessie!	Jingdong prefecture level city in Shandong
IXCHSQ Flagship Store	King of Power Saving	Without further ado, the electric bill came down, $102 less this month, personal satisfaction, it counts okay, give you a good review	Jingdong prefecture level city in Shandong
IXCHSQ Flagship Store	King of Power Saving	The power saver has arrived, the installation is really convenient, plugged in on the line operation is quite simple, power saving effect is also a strong!	Jingdong prefecture level city in Shandong
IXCHSQ Flagship Store	King of Power Saving	Saver received, good quality, beautiful appearance, fine workmanship, simple installation	Jingdong prefecture level city in Shandong
IXCHSQ Flagship Store	King of Power Saving	Product design style modern, worth buying	Jingdong prefecture level city in Shandong
IXCHSQ Flagship Store	King of Power Saving	Energy saving, good power saving effect, practical, great value	Jingdong prefecture level city in Shandong
IXCHSQ Flagship Store	King of Power Saving	It's good quality, it's good, it's energy efficient, and I'll buy it again.	Jingdong prefecture level city in Shandong
IXCHSQ Flagship Store	King of Power Saving	Installation saves a lot of electricity, fast logistics, real energy-saving appliances	Jingdong prefecture level city in Shandong
IXCHSQ Flagship Store	King of Power Saving	The effect is good and the power saving is obvious after using it for a month and saving tens of dollars, the product is trustworthy, I now buy another 4 colors to prepare for their siblings' family to pay for	Jingdong prefecture level city in Shandong
IXCHSQ Flagship Store	King of Power Saving	The quality is particularly good workmanship is no problem, the arrival of the very fast oh, especially power-saving	Jingdong prefecture level city in Shandong
IXCHSQ Flagship Store	King of Power Saving	The quality is very good! In use, observed for a day, not as used before, so much power consumption.	Jingdong prefecture level city in Shandong
IXCHSQ Flagship Store	King of Power Saving	Goods have been received, the effect is good is not good, power saving is not yet know, wait for me to use it and then evaluate it.	Jingdong prefecture level city in Shandong
IXCHSQ Flagship Store	King of Power Saving	In-kind and the same as described, the quality is quite good, the seller's attitude is also good, ask questions and answer them, the speed of delivery bar, worth buying Oh!	Jingdong prefecture level city in Shandong
IXCHSQ Flagship Store	King of Power Saving	Nice stuff, came to comment after using it, it does save power.	Jingdong prefecture level city in Shandong
IXCHSQ Flagship Store	King of Power Saving	Fast charging, but also save power, the effect is very good, satisfied, praise!	Jingdong prefecture level city in Shandong
IXCHSQ Flagship Store	King of Power Saving	The goods have been received, I do not know how effective, after using the flat price again!	Jingdong prefecture level city in Shandong
IXCHSQ Flagship Store	King of Power Saving	Merchant service is very good Reply quickly The key is the quality and that quality is good	Jingdong prefecture level city in Shandong
IXCHSQ Flagship Store	King of Power Saving	It's working well now, I'll do a follow up review after I actually use it for a while	Jingdong prefecture level city in Shandong
IXCHSQ Flagship Store	King of Power Saving	The quality is good. After installing it, I feel like I've saved a lot of money on my electricity bill.	Jingdong prefecture level city in Shandong
IXCHSQ Flagship Store	King of Power Saving	Make up a review after use.	Jingdong prefecture level city in Shandong
IXCHSQ Flagship Store	King of Power Saving	review after use	Jingdong prefecture level city in Shandong
IXCHSQ Flagship Store	King of Power Saving	It's a small capacitor. It's not energy efficient.	Jingdong prefecture level city in Shandong
IXCHSQ Flagship Store	King of Power Saving	I'll follow up with a review after I've used it and had results.	Jingdong prefecture level city in Shandong
IXCHSQ Flagship Store	King of Power Saving	No power saving? Not good!	Jingdong prefecture level city in Shandong
IXCHSQ Flagship Store	King of Power Saving	Been using the same appliances and running a 1.5 air conditioner at night for what feels like no time, and after half a month of use it feels like the power is only increasing. Doesn't show any power saving	Jingdong prefecture level city in Shandong
IXCHSQ Flagship Store	King of Power Saving	serviceable	Jingdong prefecture level city in Shandong

IXCHSQ Flagship Store	King of Power Saving	It's a piece of junk. It doesn't save any power.	Jingdong prefecture level city in Shandong
IXCHSQ Flagship Store	King of Power Saving	There are a lot of appliances in the house, the summer air conditioning is very costly, I bought this in front of the air conditioning effect is very good, I explain that I bought three in one breath, I use more electricity a month down to 200 or so, very satisfied.	Jingdong prefecture level city in Shandong
IXCHSQ Flagship Store	King of Power Saving	The voltage is very stable has been maintained at 220v this value, high power super power saving, trustworthy, reasonable design, shape bright, logistics is quite fast, business warranty period, excellent service, logistics fast!	Jingdong prefecture level city in Shandong
IXCHSQ Flagship Store	King of Power Saving	Use a month after a look at the savings of more than 40 dollars, there is a display of electricity savings, this high-power intelligent frequency converter economizer, affordable and cheap, logistics is very fast, the service attitude is very good, very satisfied with a shopping.	Jingdong prefecture level city in Shandong
IXCHSQ Flagship Store	King of Power Saving	Used for a period of time, it seems that every day can really can save a few degrees of electricity, workmanship quite can, with a period of time and then continue to look at it, anyway, the price is not	Jingdong prefecture level city in Shandong
IXCHSQ Flagship Store	King of Power Saving	Used for a period of time, it seems that every day can indeed save two several degrees of electricity, workmanship quite can, with a period of time and then continue to look at it, anyway, the price is not	Jingdong prefecture level city in Shandong
IXCHSQ Flagship Store	King of Power Saving	High-power super saver received immediately used, the price is not expensive quality is very good, save electricity and money, very convenient, conscientious business, must be praised, recommended to everyone!	Jingdong prefecture level city in Shandong
IXCHSQ Flagship Store	King of Power Saving	Home enhancement version of the intelligent power saver, is in use, I think the price is cheap, value for money, efficacy, power, power saving effect is very good.	Jingdong prefecture level city in Shandong
IXCHSQ Flagship Store	King of Power Saving	Bought three at once, one for each room, summer electricity consumption is very high, despite the three air conditioners on the electricity bill is still down a lot, too Nessie!	Jingdong prefecture level city in Shandong
IXCHSQ Flagship Store	King of Power Saving	Without further ado, the electric bill came down, $102 less this month, personal satisfaction, it counts okay, give you a good review	Jingdong prefecture level city in Shandong
IXCHSQ Flagship Store	King of Power Saving	The power saver has arrived, the installation is really convenient, plugged in on the line operation is quite simple, power saving effect is also a strong!	Jingdong prefecture level city in Shandong
IXCHSQ Flagship Store	King of Power Saving	Saver received, good quality, beautiful appearance, fine workmanship, simple installation	Jingdong prefecture level city in Shandong
IXCHSQ Flagship Store	King of Power Saving	Product design style modern, worth buying	Jingdong prefecture level city in Shandong
IXCHSQ Flagship Store	King of Power Saving	Energy saving, good power saving effect, practical, great value	Jingdong prefecture level city in Shandong
IXCHSQ Flagship Store	King of Power Saving	It's good quality, it's good, it's energy efficient, and I'll buy it again.	Jingdong prefecture level city in Shandong
IXCHSQ Flagship Store	King of Power Saving	Installation saves a lot of electricity, fast logistics, real energy-saving appliances	Jingdong prefecture level city in Shandong
IXCHSQ Flagship Store	King of Power Saving	The effect is good and the power saving is obvious after using it for a month and saving tens of dollars, the product is trustworthy, I now buy another 4 colors to prepare for their siblings' family to pay for	Jingdong prefecture level city in Shandong
IXCHSQ Flagship Store	King of Power Saving	The quality is particularly good workmanship is no problem, the arrival of the very fast oh, especially power-saving	Jingdong prefecture level city in Shandong
IXCHSQ Flagship Store	King of Power Saving	The quality is very good! In use, observed for a day, not as used before, so much power consumption.	Jingdong prefecture level city in Shandong
IXCHSQ Flagship Store	King of Power Saving	Goods have been received, the effect is good is not good, power saving is not yet know, wait for me to use it and then evaluate it.	Jingdong prefecture level city in Shandong
IXCHSQ Flagship Store	King of Power Saving	In-kind and the same as described, the quality is quite good, the seller's attitude is also good, ask questions and answer them, the speed of delivery bar, worth buying Oh!	Jingdong prefecture level city in Shandong
IXCHSQ Flagship Store	King of Power Saving	Nice stuff, came to comment after using it, it does save power.	Jingdong prefecture level city in Shandong
IXCHSQ Flagship Store	King of Power Saving	Fast charging, but also save power, the effect is very good, satisfied, praise!	Jingdong prefecture level city in Shandong
IXCHSQ Flagship Store	King of Power Saving	The goods have been received, I do not know how effective, after using the flat price again!	Jingdong prefecture level city in Shandong
IXCHSQ Flagship Store	King of Power Saving	Merchant service is very good Reply quickly The key is the quality and that quality is good	Jingdong prefecture level city in Shandong
IXCHSQ Flagship Store	King of Power Saving	It's working well now, I'll do a follow up review after I actually use it for a while	Jingdong prefecture level city in Shandong
IXCHSQ Flagship Store	King of Power Saving	The quality is good. After installing it, I feel like I've saved a lot of money on my electricity bill.	Jingdong prefecture level city in Shandong
IXCHSQ Flagship Store	King of Power Saving	Make up a review after use.	Jingdong prefecture level city in Shandong
IXCHSQ Flagship Store	King of Power Saving	review after use	Jingdong prefecture level city in Shandong
IXCHSQ Flagship Store	King of Power Saving	It's a small capacitor. It's not energy efficient.	Jingdong prefecture level city in Shandong
IXCHSQ Flagship Store	King of Power Saving	I'll follow up with a review after I've used it and had results.	Jingdong prefecture level city in Shandong
IXCHSQ Flagship Store	King of Power Saving	No power saving? Not good!	Jingdong prefecture level city in Shandong
IXCHSQ Flagship Store	King of Power Saving	Been using the same appliances and running a 1.5 air conditioner at night for what feels like no time, and after half a month of use it feels like the power is only increasing. Doesn't show any power saving	Jingdong prefecture level city in Shandong
IXCHSQ Flagship Store	King of Power Saving	serviceable	Jingdong prefecture level city in Shandong
IXCHSQ Flagship Store	King of Power Saving	It's a piece of junk. It doesn't save any power.	Jingdong prefecture level city in Shandong
IXCHSQ Flagship Store	King of Power Saving	There are a lot of appliances in the house, the summer air conditioning is very costly, I bought this in front of the air conditioning effect is very good, I explain that I bought three in one breath, I use more electricity a month down to 200 or so, very satisfied.	Jingdong prefecture level city in Shandong
IXCHSQ Flagship Store	King of Power Saving	The voltage is very stable has been maintained at 220v this value, high power super power saving, trustworthy, reasonable design, shape bright, logistics is quite fast, business warranty period, excellent service, logistics fast!	Jingdong prefecture level city in Shandong
IXCHSQ Flagship Store	King of Power Saving	Use a month after a look at the savings of more than 40 dollars, there is a display of electricity savings, this high-power intelligent frequency converter economizer, affordable and cheap, logistics is very fast, the service attitude is very good, very satisfied with a shopping.	Jingdong prefecture level city in Shandong
IXCHSQ Flagship Store	King of Power Saving	Used for a period of time, it seems that every day can really can save a few degrees of electricity, workmanship quite can, with a period of time and then continue to look at it, anyway, the price is not	Jingdong prefecture level city in Shandong
IXCHSQ Flagship Store	King of Power Saving	Used for a period of time, it seems that every day can indeed save two several degrees of electricity, workmanship quite can, with a period of time and then continue to look at it, anyway, the price is not	Jingdong prefecture level city in Shandong
IXCHSQ Flagship Store	King of Power Saving	High-power super saver received immediately used, the price is not expensive quality is very good, save electricity and money, very convenient, conscientious business, must be praised, recommended to everyone!	Jingdong prefecture level city in Shandong
IXCHSQ Flagship Store	King of Power Saving	Home enhancement version of the intelligent power saver, is in use, I think the price is cheap, value for money, efficacy, power, power saving effect is very good.	Jingdong prefecture level city in Shandong
IXCHSQ Flagship Store	King of Power Saving	Bought three at once, one for each room, summer electricity consumption is very high, despite the three air conditioners on the electricity bill is still down a lot, too Nessie!	Jingdong prefecture level city in Shandong
IXCHSQ Flagship Store	King of Power Saving	Without further ado, the electric bill came down, $102 less this month, personal satisfaction, it counts okay, give you a good review	Jingdong prefecture level city in Shandong
IXCHSQ Flagship Store	King of Power Saving	The power saver has arrived, the installation is really convenient, plugged in on the line operation is quite simple, power saving effect is also a strong!	Jingdong prefecture level city in Shandong
IXCHSQ Flagship Store	King of Power Saving	Saver received, good quality, beautiful appearance, fine workmanship, simple installation	Jingdong prefecture level city in Shandong
IXCHSQ Flagship Store	King of Power Saving	Product design style modern, worth buying	Jingdong prefecture level city in Shandong
IXCHSQ Flagship Store	King of Power Saving	Energy saving, good power saving effect, practical, great value	Jingdong prefecture level city in Shandong

IXCHSQ Flagship Store	King of Power Saving	It’s good quality, it’s good, it’s energy efficient, and I’ll buy it again.	Jingdong prefecture level city in Shandong
IXCHSQ Flagship Store	King of Power Saving	Installation saves a lot of electricity, fast logistics, real energy-saving appliances	Jingdong prefecture level city in Shandong
IXCHSQ Flagship Store	King of Power Saving	The effect is good and the power saving is obvious after using it for a month and saving tens of dollars, the product is trustworthy, I now buy another 4 colors to prepare for their siblings’ family to pay for	Jingdong prefecture level city in Shandong
IXCHSQ Flagship Store	King of Power Saving	The quality is particularly good workmanship is no problem, the arrival of the very fast oh, especially power-saving	Jingdong prefecture level city in Shandong
IXCHSQ Flagship Store	King of Power Saving	The quality is very good! In use, observed for a day, not as used before, so much power consumption.	Jingdong prefecture level city in Shandong
IXCHSQ Flagship Store	King of Power Saving	Goods have been received, the effect is good is not good, power saving is not yet know, wait for me to use it and then evaluate it.	Jingdong prefecture level city in Shandong
IXCHSQ Flagship Store	King of Power Saving	In-kind and the same as described, the quality is quite good, the seller’s attitude is also good, ask questions and answer them, the speed of delivery bar, worth buying Oh!	Jingdong prefecture level city in Shandong
IXCHSQ Flagship Store	King of Power Saving	Nice stuff, came to comment after using it, it does save power.	Jingdong prefecture level city in Shandong
IXCHSQ Flagship Store	King of Power Saving	Fast charging, but also save power, the effect is very good, satisfied, praise!	Jingdong prefecture level city in Shandong
IXCHSQ Flagship Store	King of Power Saving	The goods have been received, I do not know how effective, after using the flat price again!	Jingdong prefecture level city in Shandong
IXCHSQ Flagship Store	King of Power Saving	Merchant service is very good Reply quickly The key is the quality and that quality is good	Jingdong prefecture level city in Shandong
IXCHSQ Flagship Store	King of Power Saving	It’s working well now, I’ll do a follow up review after I actually use it for a while	Jingdong prefecture level city in Shandong
IXCHSQ Flagship Store	King of Power Saving	The quality is good. After installing it, I feel like I’ve saved a lot of money on my electricity bill.	Jingdong prefecture level city in Shandong
IXCHSQ Flagship Store	King of Power Saving	Make up a review after use.	Jingdong prefecture level city in Shandong
IXCHSQ Flagship Store	King of Power Saving	review after use	Jingdong prefecture level city in Shandong
IXCHSQ Flagship Store	King of Power Saving	It’s a small capacitor. It’s not energy efficient.	Jingdong prefecture level city in Shandong
IXCHSQ Flagship Store	King of Power Saving	I’ll follow up with a review after I’ve used it and had results.	Jingdong prefecture level city in Shandong
IXCHSQ Flagship Store	King of Power Saving	No power saving? Not good!	Jingdong prefecture level city in Shandong
IXCHSQ Flagship Store	King of Power Saving	Been using the same appliances and running a 1.5 air conditioner at night for what feels like no time, and after half a month of use it feels like the power is only increasing. Doesn’t show any power saving	Jingdong prefecture level city in Shandong
IXCHSQ Flagship Store	King of Power Saving	serviceable	Jingdong prefecture level city in Shandong
IXCHSQ Flagship Store	King of Power Saving	It’s a piece of junk. It doesn’t save any power.	Jingdong prefecture level city in Shandong
IXCHSQ Flagship Store	King of Power Saving	There are a lot of appliances in the house, the summer air conditioning is very costly, I bought this in front of the air conditioning effect is very good, I explain that I bought three in one breath, I use more electricity a month down to 200 or so, very satisfied.	Jingdong prefecture level city in Shandong
IXCHSQ Flagship Store	King of Power Saving	The voltage is very stable has been maintained at 220v this value, high power super power saving, trustworthy, reasonable design, shape bright, logistics is quite fast, business warranty period, excellent service, logistics fast!	Jingdong prefecture level city in Shandong
IXCHSQ Flagship Store	King of Power Saving	Use a month after a look at the savings of more than 40 dollars, there is a display of electricity savings, this high-power intelligent frequency converter economizer, affordable and cheap, logistics is very fast, the service attitude is very good, very satisfied with a shopping.	Jingdong prefecture level city in Shandong
IXCHSQ Flagship Store	King of Power Saving	Used for a period of time, it seems that every day can really can save a few degrees of electricity, workmanship quite can, with a period of time and then continue to look at it, anyway, the price is not	Jingdong prefecture level city in Shandong
IXCHSQ Flagship Store	King of Power Saving	Used for a period of time, it seems that every day can indeed save two several degrees of electricity, workmanship quite can, with a period of time and then continue to look at it, anyway, the price is not	Jingdong prefecture level city in Shandong
IXCHSQ Flagship Store	King of Power Saving	High-power super saver received immediately used, the price is not expensive quality is very good, save electricity and money, very convenient, conscientious business, must be praised, recommended to everyone!	Jingdong prefecture level city in Shandong
IXCHSQ Flagship Store	King of Power Saving	Home enhancement version of the intelligent power saver, is in use, I think the price is cheap, value for money, efficacy, power, power saving effect is very good.	Jingdong prefecture level city in Shandong
IXCHSQ Flagship Store	King of Power Saving	Bought three at once, one for each room, summer electricity consumption is very high, despite the three air conditioners on the electricity bill is still down a lot, too Nessie!	Jingdong prefecture level city in Shandong
IXCHSQ Flagship Store	King of Power Saving	Without further ado, the electric bill came down, $102 less this month, personal satisfaction, it counts okay, give you a good review	Jingdong prefecture level city in Shandong
IXCHSQ Flagship Store	King of Power Saving	The power saver has arrived, the installation is really convenient, plugged in on the line operation is quite simple, power saving effect is also a strong!	Jingdong prefecture level city in Shandong
IXCHSQ Flagship Store	King of Power Saving	Saver received, good quality, beautiful appearance, fine workmanship, simple installation	Jingdong prefecture level city in Shandong
IXCHSQ Flagship Store	King of Power Saving	Product design style modern, worth buying	Jingdong prefecture level city in Shandong
IXCHSQ Flagship Store	King of Power Saving	Energy saving, good power saving effect, practical, great value	Jingdong prefecture level city in Shandong
IXCHSQ Flagship Store	King of Power Saving	It’s good quality, it’s good, it’s energy efficient, and I’ll buy it again.	Jingdong prefecture level city in Shandong
IXCHSQ Flagship Store	King of Power Saving	Installation saves a lot of electricity, fast logistics, real energy-saving appliances	Jingdong prefecture level city in Shandong
IXCHSQ Flagship Store	King of Power Saving	The effect is good and the power saving is obvious after using it for a month and saving tens of dollars, the product is trustworthy, I now buy another 4 colors to prepare for their siblings’ family to pay for	Jingdong prefecture level city in Shandong
IXCHSQ Flagship Store	King of Power Saving	The quality is particularly good workmanship is no problem, the arrival of the very fast oh, especially power-saving	Jingdong prefecture level city in Shandong
IXCHSQ Flagship Store	King of Power Saving	The quality is very good! In use, observed for a day, not as used before, so much power consumption.	Jingdong prefecture level city in Shandong
IXCHSQ Flagship Store	King of Power Saving	Goods have been received, the effect is good is not good, power saving is not yet know, wait for me to use it and then evaluate it.	Jingdong prefecture level city in Shandong
IXCHSQ Flagship Store	King of Power Saving	In-kind and the same as described, the quality is quite good, the seller’s attitude is also good, ask questions and answer them, the speed of delivery bar, worth buying Oh!	Jingdong prefecture level city in Shandong
IXCHSQ Flagship Store	King of Power Saving	Nice stuff, came to comment after using it, it does save power.	Jingdong prefecture level city in Shandong
IXCHSQ Flagship Store	King of Power Saving	Fast charging, but also save power, the effect is very good, satisfied, praise!	Jingdong prefecture level city in Shandong
IXCHSQ Flagship Store	King of Power Saving	The goods have been received, I do not know how effective, after using the flat price again!	Jingdong prefecture level city in Shandong
IXCHSQ Flagship Store	King of Power Saving	Merchant service is very good Reply quickly The key is the quality and that quality is good	Jingdong prefecture level city in Shandong
IXCHSQ Flagship Store	King of Power Saving	It’s working well now, I’ll do a follow up review after I actually use it for a while	Jingdong prefecture level city in Shandong
IXCHSQ Flagship Store	King of Power Saving	The quality is good. After installing it, I feel like I’ve saved a lot of money on my electricity bill.	Jingdong prefecture level city in Shandong
IXCHSQ Flagship Store	King of Power Saving	Make up a review after use.	Jingdong prefecture level city in Shandong
IXCHSQ Flagship Store	King of Power Saving	review after use	Jingdong prefecture level city in Shandong
IXCHSQ Flagship Store	King of Power Saving	It’s a small capacitor. It’s not energy efficient.	Jingdong prefecture level city in Shandong

IXCHSQ Flagship Store	King of Power Saving	I'll follow up with a review after I've used it and had results.	Jingdong prefecture level city in Shandong
IXCHSQ Flagship Store	King of Power Saving	No power saving? Not good!	Jingdong prefecture level city in Shandong
IXCHSQ Flagship Store	King of Power Saving	Been using the same appliances and running a 1.5 air conditioner at night for what feels like no time, and after half a month of use it feels like the power is only increasing. Doesn't show any power saving	Jingdong prefecture level city in Shandong
IXCHSQ Flagship Store	King of Power Saving	serviceable	Jingdong prefecture level city in Shandong
IXCHSQ Flagship Store	King of Power Saving	It's a piece of junk. It doesn't save any power.	Jingdong prefecture level city in Shandong
IXCHSQ Flagship Store	King of Power Saving	There are a lot of appliances in the house, the summer air conditioning is very costly, I bought this in front of the air conditioning effect is very good, I explain that I bought three in one breath, I use more electricity a month down to 200 or so, very satisfied.	Jingdong prefecture level city in Shandong
IXCHSQ Flagship Store	King of Power Saving	The voltage is very stable has been maintained at 220v this value, high power super power saving, trustworthy, reasonable design, shape bright, logistics is quite fast, business warranty period, excellent service, logistics fast!	Jingdong prefecture level city in Shandong
IXCHSQ Flagship Store	King of Power Saving	Use a month after a look at the savings of more than 40 dollars, there is a display of electricity savings, this high-power intelligent frequency converter economizer, affordable and cheap, logistics is very fast, the service attitude is very good, very satisfied with a shopping.	Jingdong prefecture level city in Shandong
IXCHSQ Flagship Store	King of Power Saving	Used for a period of time, it seems that every day can really can save a few degrees of electricity, workmanship quite can, with a period of time and then continue to look at it, anyway, the price is not	Jingdong prefecture level city in Shandong
IXCHSQ Flagship Store	King of Power Saving	Used for a period of time, it seems that every day can indeed save two several degrees of electricity, workmanship quite can, with a period of time and then continue to look at it, anyway, the price is not	Jingdong prefecture level city in Shandong
IXCHSQ Flagship Store	King of Power Saving	High-power super saver received immediately used, the price is not expensive quality is very good, save electricity and money, very convenient, conscientious business, must be praised, recommended to everyone!	Jingdong prefecture level city in Shandong
IXCHSQ Flagship Store	King of Power Saving	Home enhancement version of the intelligent power saver, is in use, I think the price is cheap, value for money, efficacy, power, power saving effect is very good.	Jingdong prefecture level city in Shandong
IXCHSQ Flagship Store	King of Power Saving	Bought three at once, one for each room, summer electricity consumption is very high, despite the three air conditioners on the electricity bill is still down a lot, too Nessie!	Jingdong prefecture level city in Shandong
IXCHSQ Flagship Store	King of Power Saving	Without further ado, the electric bill came down, $102 less this month, personal satisfaction, it counts okay, give you a good review	Jingdong prefecture level city in Shandong
IXCHSQ Flagship Store	King of Power Saving	The power saver has arrived, the installation is really convenient, plugged in on the line operation is quite simple, power saving effect is also a strong!	Jingdong prefecture level city in Shandong
IXCHSQ Flagship Store	King of Power Saving	Saver received, good quality, beautiful appearance, fine workmanship, simple installation	Jingdong prefecture level city in Shandong
IXCHSQ Flagship Store	King of Power Saving	Product design style modern, worth buying	Jingdong prefecture level city in Shandong
IXCHSQ Flagship Store	King of Power Saving	Energy saving, good power saving effect, practical, great value	Jingdong prefecture level city in Shandong
IXCHSQ Flagship Store	King of Power Saving	It's good quality, it's good, it's energy efficient, and I'll buy it again.	Jingdong prefecture level city in Shandong
IXCHSQ Flagship Store	King of Power Saving	Installation saves a lot of electricity, fast logistics, real energy-saving appliances	Jingdong prefecture level city in Shandong
IXCHSQ Flagship Store	King of Power Saving	The effect is good and the power saving is obvious after using it for a month and saving tens of dollars, the product is trustworthy, I now buy another 4 colors to prepare for their siblings' family to pay for	Jingdong prefecture level city in Shandong
IXCHSQ Flagship Store	King of Power Saving	The quality is particularly good workmanship is no problem, the arrival of the very fast oh, especially power-saving	Jingdong prefecture level city in Shandong
IXCHSQ Flagship Store	King of Power Saving	The quality is very good! In use, observed for a day, not as used before, so much power consumption.	Jingdong prefecture level city in Shandong
IXCHSQ Flagship Store	King of Power Saving	Goods have been received, the effect is good is not good, power saving is not yet know, wait for me to use it and then evaluate it.	Jingdong prefecture level city in Shandong
IXCHSQ Flagship Store	King of Power Saving	In-kind and the same as described, the quality is quite good, the seller's attitude is also good, ask questions and answer them, the speed of delivery bar, worth buying Oh!	Jingdong prefecture level city in Shandong
IXCHSQ Flagship Store	King of Power Saving	Nice stuff, came to comment after using it, it does save power.	Jingdong prefecture level city in Shandong
IXCHSQ Flagship Store	King of Power Saving	Fast charging, but also save power, the effect is very good, satisfied, praise!	Jingdong prefecture level city in Shandong
IXCHSQ Flagship Store	King of Power Saving	The goods have been received, I do not know how effective, after using the flat price again!	Jingdong prefecture level city in Shandong
IXCHSQ Flagship Store	King of Power Saving	Merchant service is very good Reply quickly The key is the quality and that quality is good	Jingdong prefecture level city in Shandong
IXCHSQ Flagship Store	King of Power Saving	It's working well now, I'll do a follow up review after I actually use it for a while	Jingdong prefecture level city in Shandong
IXCHSQ Flagship Store	King of Power Saving	The quality is good. After installing it, I feel like I've saved a lot of money on my electricity bill.	Jingdong prefecture level city in Shandong
IXCHSQ Flagship Store	King of Power Saving	Make up a review after use.	Jingdong prefecture level city in Shandong
IXCHSQ Flagship Store	King of Power Saving	review after use	Jingdong prefecture level city in Shandong
IXCHSQ Flagship Store	King of Power Saving	It's a small capacitor. It's not energy efficient.	Jingdong prefecture level city in Shandong
IXCHSQ Flagship Store	King of Power Saving	I'll follow up with a review after I've used it and had results.	Jingdong prefecture level city in Shandong
IXCHSQ Flagship Store	King of Power Saving	No power saving? Not good!	Jingdong prefecture level city in Shandong
IXCHSQ Flagship Store	King of Power Saving	Been using the same appliances and running a 1.5 air conditioner at night for what feels like no time, and after half a month of use it feels like the power is only increasing. Doesn't show any power saving	Jingdong prefecture level city in Shandong
IXCHSQ Flagship Store	King of Power Saving	serviceable	Jingdong prefecture level city in Shandong
IXCHSQ Flagship Store	King of Power Saving	It's a piece of junk. It doesn't save any power.	Jingdong prefecture level city in Shandong
IXCHSQ Flagship Store	King of Power Saving	There are a lot of appliances in the house, the summer air conditioning is very costly, I bought this in front of the air conditioning effect is very good, I explain that I bought three in one breath, I use more electricity a month down to 200 or so, very satisfied.	Jingdong prefecture level city in Shandong
IXCHSQ Flagship Store	King of Power Saving	The voltage is very stable has been maintained at 220v this value, high power super power saving, trustworthy, reasonable design, shape bright, logistics is quite fast, business warranty period, excellent service, logistics fast!	Jingdong prefecture level city in Shandong
IXCHSQ Flagship Store	King of Power Saving	Use a month after a look at the savings of more than 40 dollars, there is a display of electricity savings, this high-power intelligent frequency converter economizer, affordable and cheap, logistics is very fast, the service attitude is very good, very satisfied with a shopping.	Jingdong prefecture level city in Shandong
IXCHSQ Flagship Store	King of Power Saving	Used for a period of time, it seems that every day can really can save a few degrees of electricity, workmanship quite can, with a period of time and then continue to look at it, anyway, the price is not	Jingdong prefecture level city in Shandong
IXCHSQ Flagship Store	King of Power Saving	Used for a period of time, it seems that every day can indeed save two several degrees of electricity, workmanship quite can, with a period of time and then continue to look at it, anyway, the price is not	Jingdong prefecture level city in Shandong
IXCHSQ Flagship Store	King of Power Saving	High-power super saver received immediately used, the price is not expensive quality is very good, save electricity and money, very convenient, conscientious business, must be praised, recommended to everyone!	Jingdong prefecture level city in Shandong
IXCHSQ Flagship Store	King of Power Saving	Home enhancement version of the intelligent power saver, is in use, I think the price is cheap, value for money, efficacy, power, power saving effect is very good.	Jingdong prefecture level city in Shandong
IXCHSQ Flagship Store	King of Power Saving	Bought three at once, one for each room, summer electricity consumption is very high, despite the three air conditioners on the electricity bill is still down a lot, too Nessie!	Jingdong prefecture level city in Shandong
IXCHSQ Flagship Store	King of Power Saving	Without further ado, the electric bill came down, $102 less this month, personal satisfaction, it counts okay, give you a good review	Jingdong prefecture level city in Shandong

IXCHSQ Flagship Store	King of Power Saving	The power saver has arrived, the installation is really convenient, plugged in on the line operation is quite simple, power saving effect is also a strong!	Jingdong prefecture level city in Shandong
IXCHSQ Flagship Store	King of Power Saving	Saver received, good quality, beautiful appearance, fine workmanship, simple installation	Jingdong prefecture level city in Shandong
IXCHSQ Flagship Store	King of Power Saving	Product design style modern, worth buying	Jingdong prefecture level city in Shandong
IXCHSQ Flagship Store	King of Power Saving	Energy saving, good power saving effect, practical, great value	Jingdong prefecture level city in Shandong
IXCHSQ Flagship Store	King of Power Saving	It's good quality, it's good, it's energy efficient, and I'll buy it again.	Jingdong prefecture level city in Shandong
IXCHSQ Flagship Store	King of Power Saving	Installation saves a lot of electricity, fast logistics, real energy-saving appliances	Jingdong prefecture level city in Shandong
IXCHSQ Flagship Store	King of Power Saving	The effect is good and the power saving is obvious after using it for a month and saving tens of dollars, the product is trustworthy, I now buy another 4 colors to prepare for their siblings' family to pay for	Jingdong prefecture level city in Shandong
IXCHSQ Flagship Store	King of Power Saving	The quality is particularly good workmanship is no problem, the arrival of the very fast oh, especially power-saving	Jingdong prefecture level city in Shandong
IXCHSQ Flagship Store	King of Power Saving	The quality is very good! In use, observed for a day, not as used before, so much power consumption.	Jingdong prefecture level city in Shandong
IXCHSQ Flagship Store	King of Power Saving	Goods have been received, the effect is good is not good, power saving is not yet know, wait for me to use it and then evaluate it.	Jingdong prefecture level city in Shandong
IXCHSQ Flagship Store	King of Power Saving	In-kind and the same as described, the quality is quite good, the seller's attitude is also good, ask questions and answer them, the speed of delivery bar, worth buying Oh!	Jingdong prefecture level city in Shandong
IXCHSQ Flagship Store	King of Power Saving	Nice stuff, came to comment after using it, it does save power.	Jingdong prefecture level city in Shandong
IXCHSQ Flagship Store	King of Power Saving	Fast charging, but also save power, the effect is very good, satisfied, praise!	Jingdong prefecture level city in Shandong
IXCHSQ Flagship Store	King of Power Saving	The goods have been received, I do not know how effective, after using the flat price again!	Jingdong prefecture level city in Shandong
IXCHSQ Flagship Store	King of Power Saving	Merchant service is very good Reply quickly The key is the quality and that quality is good	Jingdong prefecture level city in Shandong
IXCHSQ Flagship Store	King of Power Saving	It's working well now, I'll do a follow up review after I actually use it for a while	Jingdong prefecture level city in Shandong
IXCHSQ Flagship Store	King of Power Saving	The quality is good. After installing it, I feel like I've saved a lot of money on my electricity bill.	Jingdong prefecture level city in Shandong
IXCHSQ Flagship Store	King of Power Saving	Make up a review after use.	Jingdong prefecture level city in Shandong
IXCHSQ Flagship Store	King of Power Saving	review after use	Jingdong prefecture level city in Shandong
IXCHSQ Flagship Store	King of Power Saving	It's a small capacitor. It's not energy efficient.	Jingdong prefecture level city in Shandong
IXCHSQ Flagship Store	King of Power Saving	I'll follow up with a review after I've used it and had results.	Jingdong prefecture level city in Shandong
IXCHSQ Flagship Store	King of Power Saving	No power saving? Not good!	Jingdong prefecture level city in Shandong
IXCHSQ Flagship Store	King of Power Saving	Been using the same appliances and running a 1.5 air conditioner at night for what feels like no time, and after half a month of use it feels like the power is only increasing. Doesn't show any power saving	Jingdong prefecture level city in Shandong
IXCHSQ Flagship Store	King of Power Saving	serviceable	Jingdong prefecture level city in Shandong
IXCHSQ Flagship Store	King of Power Saving	It's a piece of junk. It doesn't save any power.	Jingdong prefecture level city in Shandong
IXCHSQ Flagship Store	King of Power Saving	There are a lot of appliances in the house, the summer air conditioning is very costly, I bought this in front of the air conditioning effect is very good, I explain that I bought three in one breath, I use more electricity a month down to 200 or so, very satisfied.	Jingdong prefecture level city in Shandong
IXCHSQ Flagship Store	King of Power Saving	The voltage is very stable has been maintained at 220v this value, high power super power saving, trustworthy, reasonable design, shape bright, logistics is quite fast, business warranty period, excellent service, logistics fast!	Jingdong prefecture level city in Shandong
IXCHSQ Flagship Store	King of Power Saving	Use a month after a look at the savings of more than 40 dollars, there is a display of electricity savings, this high-power intelligent frequency converter economizer, affordable and cheap, logistics is very fast, the service attitude is very good, very satisfied with a shopping.	Jingdong prefecture level city in Shandong
IXCHSQ Flagship Store	King of Power Saving	Used for a period of time, it seems that every day can really can save a few degrees of electricity, workmanship quite can, with a period of time and then continue to look at it, anyway, the price is not	Jingdong prefecture level city in Shandong
IXCHSQ Flagship Store	King of Power Saving	Used for a period of time, it seems that every day can indeed save two several degrees of electricity, workmanship quite can, with a period of time and then continue to look at it, anyway, the price is not	Jingdong prefecture level city in Shandong
IXCHSQ Flagship Store	King of Power Saving	High-power super saver received immediately used, the price is not expensive quality is very good, save electricity and money, very convenient, conscientious business, must be praised, recommended to everyone!	Jingdong prefecture level city in Shandong
IXCHSQ Flagship Store	King of Power Saving	Home enhancement version of the intelligent power saver, is in use, I think the price is cheap, value for money, efficacy, power, power saving effect is very good.	Jingdong prefecture level city in Shandong
IXCHSQ Flagship Store	King of Power Saving	Bought three at once, one for each room, summer electricity consumption is very high, despite the three air conditioners on the electricity bill is still down a lot, too Nessie!	Jingdong prefecture level city in Shandong
IXCHSQ Flagship Store	King of Power Saving	Without further ado, the electric bill came down, $102 less this month, personal satisfaction, it counts okay, give you a good review	Jingdong prefecture level city in Shandong
IXCHSQ Flagship Store	King of Power Saving	The power saver has arrived, the installation is really convenient, plugged in on the line operation is quite simple, power saving effect is also a strong!	Jingdong prefecture level city in Shandong
IXCHSQ Flagship Store	King of Power Saving	Saver received, good quality, beautiful appearance, fine workmanship, simple installation	Jingdong prefecture level city in Shandong
IXCHSQ Flagship Store	King of Power Saving	Product design style modern, worth buying	Jingdong prefecture level city in Shandong
IXCHSQ Flagship Store	King of Power Saving	Energy saving, good power saving effect, practical, great value	Jingdong prefecture level city in Shandong
IXCHSQ Flagship Store	King of Power Saving	It's good quality, it's good, it's energy efficient, and I'll buy it again.	Jingdong prefecture level city in Shandong
IXCHSQ Flagship Store	King of Power Saving	Installation saves a lot of electricity, fast logistics, real energy-saving appliances	Jingdong prefecture level city in Shandong
IXCHSQ Flagship Store	King of Power Saving	The effect is good and the power saving is obvious after using it for a month and saving tens of dollars, the product is trustworthy, I now buy another 4 colors to prepare for their siblings' family to pay for	Jingdong prefecture level city in Shandong
IXCHSQ Flagship Store	King of Power Saving	The quality is particularly good workmanship is no problem, the arrival of the very fast oh, especially power-saving	Jingdong prefecture level city in Shandong
IXCHSQ Flagship Store	King of Power Saving	The quality is very good! In use, observed for a day, not as used before, so much power consumption.	Jingdong prefecture level city in Shandong
IXCHSQ Flagship Store	King of Power Saving	Goods have been received, the effect is good is not good, power saving is not yet know, wait for me to use it and then evaluate it.	Jingdong prefecture level city in Shandong
IXCHSQ Flagship Store	King of Power Saving	In-kind and the same as described, the quality is quite good, the seller's attitude is also good, ask questions and answer them, the speed of delivery bar, worth buying Oh!	Jingdong prefecture level city in Shandong
IXCHSQ Flagship Store	King of Power Saving	Nice stuff, came to comment after using it, it does save power.	Jingdong prefecture level city in Shandong
IXCHSQ Flagship Store	King of Power Saving	Fast charging, but also save power, the effect is very good, satisfied, praise!	Jingdong prefecture level city in Shandong
IXCHSQ Flagship Store	King of Power Saving	The goods have been received, I do not know how effective, after using the flat price again!	Jingdong prefecture level city in Shandong
IXCHSQ Flagship Store	King of Power Saving	Merchant service is very good Reply quickly The key is the quality and that quality is good	Jingdong prefecture level city in Shandong
IXCHSQ Flagship Store	King of Power Saving	It's working well now, I'll do a follow up review after I actually use it for a while	Jingdong prefecture level city in Shandong

IXCHSQ Flagship Store	King of Power Saving	The quality is good. After installing it, I feel like I've saved a lot of money on my electricity bill.	Jingdong prefecture level city in Shandong
IXCHSQ Flagship Store	King of Power Saving	Make up a review after use.	Jingdong prefecture level city in Shandong
IXCHSQ Flagship Store	King of Power Saving	review after use	Jingdong prefecture level city in Shandong
IXCHSQ Flagship Store	King of Power Saving	It's a small capacitor. It's not energy efficient.	Jingdong prefecture level city in Shandong
IXCHSQ Flagship Store	King of Power Saving	I'll follow up with a review after I've used it and had results.	Jingdong prefecture level city in Shandong
IXCHSQ Flagship Store	King of Power Saving	No power saving? Not good!	Jingdong prefecture level city in Shandong
IXCHSQ Flagship Store	King of Power Saving	Been using the same appliances and running a 1.5 air conditioner at night for what feels like no time, and after half a month of use it feels like the power is only increasing. Doesn't show any power saving	Jingdong prefecture level city in Shandong
IXCHSQ Flagship Store	King of Power Saving	serviceable	Jingdong prefecture level city in Shandong
IXCHSQ Flagship Store	King of Power Saving	It's a piece of junk. It doesn't save any power.	Jingdong prefecture level city in Shandong
IXCHSQ Flagship Store	King of Power Saving	There are a lot of appliances in the house, the summer air conditioning is very costly, I bought this in front of the air conditioning effect is very good, I explain that I bought three in one breath, I use more electricity a month down to 200 or so, very satisfied.	Jingdong prefecture level city in Shandong
IXCHSQ Flagship Store	King of Power Saving	The voltage is very stable has been maintained at 220v this value, high power super power saving, trustworthy, reasonable design, shape bright, logistics is quite fast, business warranty period, excellent service, logistics fast!	Jingdong prefecture level city in Shandong
IXCHSQ Flagship Store	King of Power Saving	Use a month after a look at the savings of more than 40 dollars, there is a display of electricity savings, this high-power intelligent frequency converter economizer, affordable and cheap, logistics is very fast, the service attitude is very good, very satisfied with a shopping.	Jingdong prefecture level city in Shandong
IXCHSQ Flagship Store	King of Power Saving	Used for a period of time, it seems that every day can really can save a few degrees of electricity, workmanship quite can, with a period of time and then continue to look at it, anyway, the price is not	Jingdong prefecture level city in Shandong
IXCHSQ Flagship Store	King of Power Saving	Used for a period of time, it seems that every day can indeed save two several degrees of electricity, workmanship quite can, with a period of time and then continue to look at it, anyway, the price is not	Jingdong prefecture level city in Shandong
IXCHSQ Flagship Store	King of Power Saving	High-power super saver received immediately used, the price is not expensive quality is very good, save electricity and money, very convenient, conscientious business, must be praised, recommended to everyone!	Jingdong prefecture level city in Shandong
IXCHSQ Flagship Store	King of Power Saving	Home enhancement version of the intelligent power saver, is in use, I think the price is cheap, value for money, efficacy, power, power saving effect is very good.	Jingdong prefecture level city in Shandong
IXCHSQ Flagship Store	King of Power Saving	Bought three at once, one for each room, summer electricity consumption is very high, despite the three air conditioners on the electricity bill is still down a lot, too Nessie!	Jingdong prefecture level city in Shandong
IXCHSQ Flagship Store	King of Power Saving	Without further ado, the electric bill came down, $102 less this month, personal satisfaction, it counts okay, give you a good review	Jingdong prefecture level city in Shandong
IXCHSQ Flagship Store	King of Power Saving	The power saver has arrived, the installation is really convenient, plugged in on the line operation is quite simple, power saving effect is also a strong!	Jingdong prefecture level city in Shandong
IXCHSQ Flagship Store	King of Power Saving	Saver received, good quality, beautiful appearance, fine workmanship, simple installation	Jingdong prefecture level city in Shandong
IXCHSQ Flagship Store	King of Power Saving	Product design style modern, worth buying	Jingdong prefecture level city in Shandong
IXCHSQ Flagship Store	King of Power Saving	Energy saving, good power saving effect, practical, great value	Jingdong prefecture level city in Shandong
IXCHSQ Flagship Store	King of Power Saving	It's good quality, it's good, it's energy efficient, and I'll buy it again.	Jingdong prefecture level city in Shandong
IXCHSQ Flagship Store	King of Power Saving	Installation saves a lot of electricity, fast logistics, real energy-saving appliances	Jingdong prefecture level city in Shandong
IXCHSQ Flagship Store	King of Power Saving	The effect is good and the power saving is obvious after using it for a month and saving tens of dollars, the product is trustworthy, I now buy another 4 colors to prepare for their siblings' family to pay for	Jingdong prefecture level city in Shandong
IXCHSQ Flagship Store	King of Power Saving	The quality is particularly good workmanship is no problem, the arrival of the very fast oh, especially power-saving	Jingdong prefecture level city in Shandong
IXCHSQ Flagship Store	King of Power Saving	The quality is very good! In use, observed for a day, not as used before, so much power consumption.	Jingdong prefecture level city in Shandong
IXCHSQ Flagship Store	King of Power Saving	Goods have been received, the effect is good is not good, power saving is not yet know, wait for me to use it and then evaluate it.	Jingdong prefecture level city in Shandong
IXCHSQ Flagship Store	King of Power Saving	In-kind and the same as described, the quality is quite good, the seller's attitude is also good, ask questions and answer them, the speed of delivery bar, worth buying Oh!	Jingdong prefecture level city in Shandong
IXCHSQ Flagship Store	King of Power Saving	Nice stuff, came to comment after using it, it does save power.	Jingdong prefecture level city in Shandong
IXCHSQ Flagship Store	King of Power Saving	Fast charging, but also save power, the effect is very good, satisfied, praise!	Jingdong prefecture level city in Shandong
IXCHSQ Flagship Store	King of Power Saving	The goods have been received, I do not know how effective, after using the flat price again!	Jingdong prefecture level city in Shandong
IXCHSQ Flagship Store	King of Power Saving	Merchant service is very good Reply quickly The key is the quality and that quality is good	Jingdong prefecture level city in Shandong
IXCHSQ Flagship Store	King of Power Saving	It's working well now, I'll do a follow up review after I actually use it for a while	Jingdong prefecture level city in Shandong
IXCHSQ Flagship Store	King of Power Saving	The quality is good. After installing it, I feel like I've saved a lot of money on my electricity bill.	Jingdong prefecture level city in Shandong
IXCHSQ Flagship Store	King of Power Saving	Make up a review after use.	Jingdong prefecture level city in Shandong
IXCHSQ Flagship Store	King of Power Saving	review after use	Jingdong prefecture level city in Shandong
IXCHSQ Flagship Store	King of Power Saving	It's a small capacitor. It's not energy efficient.	Jingdong prefecture level city in Shandong
IXCHSQ Flagship Store	King of Power Saving	I'll follow up with a review after I've used it and had results.	Jingdong prefecture level city in Shandong
IXCHSQ Flagship Store	King of Power Saving	No power saving? Not good!	Jingdong prefecture level city in Shandong
IXCHSQ Flagship Store	King of Power Saving	Been using the same appliances and running a 1.5 air conditioner at night for what feels like no time, and after half a month of use it feels like the power is only increasing. Doesn't show any power saving	Jingdong prefecture level city in Shandong
IXCHSQ Flagship Store	King of Power Saving	serviceable	Jingdong prefecture level city in Shandong
IXCHSQ Flagship Store	King of Power Saving	It's a piece of junk. It doesn't save any power.	Jingdong prefecture level city in Shandong
IXCHSQ Flagship Store	King of Power Saving	There are a lot of appliances in the house, the summer air conditioning is very costly, I bought this in front of the air conditioning effect is very good, I explain that I bought three in one breath, I use more electricity a month down to 200 or so, very satisfied.	Jingdong prefecture level city in Shandong
IXCHSQ Flagship Store	King of Power Saving	The voltage is very stable has been maintained at 220v this value, high power super power saving, trustworthy, reasonable design, shape bright, logistics is quite fast, business warranty period, excellent service, logistics fast!	Jingdong prefecture level city in Shandong
IXCHSQ Flagship Store	King of Power Saving	Use a month after a look at the savings of more than 40 dollars, there is a display of electricity savings, this high-power intelligent frequency converter economizer, affordable and cheap, logistics is very fast, the service attitude is very good, very satisfied with a shopping.	Jingdong prefecture level city in Shandong
IXCHSQ Flagship Store	King of Power Saving	Used for a period of time, it seems that every day can really can save a few degrees of electricity, workmanship quite can, with a period of time and then continue to look at it, anyway, the price is not	Jingdong prefecture level city in Shandong
IXCHSQ Flagship Store	King of Power Saving	Used for a period of time, it seems that every day can indeed save two several degrees of electricity, workmanship quite can, with a period of time and then continue to look at it, anyway, the price is not	Jingdong prefecture level city in Shandong

IXCHSQ Flagship Store	King of Power Saving	High-power super saver received immediately used, the price is not expensive quality is very good, save electricity and money, very convenient, conscientious business, must be praised, recommended to everyone!	Jingdong prefecture level city in Shandong
IXCHSQ Flagship Store	King of Power Saving	Home enhancement version of the intelligent power saver, is in use, I think the price is cheap, value for money, efficacy, power, power saving effect is very good.	Jingdong prefecture level city in Shandong
IXCHSQ Flagship Store	King of Power Saving	Bought three at once, one for each room, summer electricity consumption is very high, despite the three air conditioners on the electricity bill is still down a lot, too Nessie!	Jingdong prefecture level city in Shandong
IXCHSQ Flagship Store	King of Power Saving	Without further ado, the electric bill came down, $102 less this month, personal satisfaction, it counts okay, give you a good review	Jingdong prefecture level city in Shandong
IXCHSQ Flagship Store	King of Power Saving	The power saver has arrived, the installation is really convenient, plugged in on the line operation is quite simple, power saving effect is also a strong!	Jingdong prefecture level city in Shandong
IXCHSQ Flagship Store	King of Power Saving	Saver received, good quality, beautiful appearance, fine workmanship, simple installation	Jingdong prefecture level city in Shandong
IXCHSQ Flagship Store	King of Power Saving	Product design style modern, worth buying	Jingdong prefecture level city in Shandong
IXCHSQ Flagship Store	King of Power Saving	Energy saving, good power saving effect, practical, great value	Jingdong prefecture level city in Shandong
IXCHSQ Flagship Store	King of Power Saving	It's good quality, it's good, it's energy efficient, and I'll buy it again.	Jingdong prefecture level city in Shandong
IXCHSQ Flagship Store	King of Power Saving	Installation saves a lot of electricity, fast logistics, real energy-saving appliances	Jingdong prefecture level city in Shandong
IXCHSQ Flagship Store	King of Power Saving	The effect is good and the power saving is obvious after using it for a month and saving tens of dollars, the product is trustworthy, I now buy another 4 colors to prepare for their siblings' family to pay for	Jingdong prefecture level city in Shandong
IXCHSQ Flagship Store	King of Power Saving	The quality is particularly good workmanship is no problem, the arrival of the very fast oh, especially power-saving	Jingdong prefecture level city in Shandong
IXCHSQ Flagship Store	King of Power Saving	The quality is very good! In use, observed for a day, not as used before, so much power consumption.	Jingdong prefecture level city in Shandong
IXCHSQ Flagship Store	King of Power Saving	Goods have been received, the effect is good is not good, power saving is not yet know, wait for me to use it and then evaluate it.	Jingdong prefecture level city in Shandong
IXCHSQ Flagship Store	King of Power Saving	In-kind and the same as described, the quality is quite good, the seller's attitude is also good, ask questions and answer them, the speed of delivery bar, worth buying Oh!	Jingdong prefecture level city in Shandong
IXCHSQ Flagship Store	King of Power Saving	Nice stuff, came to comment after using it, it does save power.	Jingdong prefecture level city in Shandong
IXCHSQ Flagship Store	King of Power Saving	Fast charging, but also save power, the effect is very good, satisfied, praise!	Jingdong prefecture level city in Shandong
IXCHSQ Flagship Store	King of Power Saving	The goods have been received, I do not know how effective, after using the flat price again!	Jingdong prefecture level city in Shandong
IXCHSQ Flagship Store	King of Power Saving	Merchant service is very good Reply quickly The key is the quality and that quality is good	Jingdong prefecture level city in Shandong
IXCHSQ Flagship Store	King of Power Saving	It's working well now, I'll do a follow up review after I actually use it for a while	Jingdong prefecture level city in Shandong
IXCHSQ Flagship Store	King of Power Saving	The quality is good. After installing it, I feel like I've saved a lot of money on my electricity bill.	Jingdong prefecture level city in Shandong
IXCHSQ Flagship Store	King of Power Saving	Make up a review after use.	Jingdong prefecture level city in Shandong
IXCHSQ Flagship Store	King of Power Saving	review after use	Jingdong prefecture level city in Shandong
IXCHSQ Flagship Store	King of Power Saving	It's a small capacitor. It's not energy efficient.	Jingdong prefecture level city in Shandong
IXCHSQ Flagship Store	King of Power Saving	I'll follow up with a review after I've used it and had results.	Jingdong prefecture level city in Shandong
IXCHSQ Flagship Store	King of Power Saving	No power saving? Not good!	Jingdong prefecture level city in Shandong
IXCHSQ Flagship Store	King of Power Saving	Been using the same appliances and running a 1.5 air conditioner at night for what feels like no time, and after half a month of use it feels like the power is only increasing. Doesn't show any power saving	Jingdong prefecture level city in Shandong
IXCHSQ Flagship Store	King of Power Saving	serviceable	Jingdong prefecture level city in Shandong
IXCHSQ Flagship Store	King of Power Saving	It's a piece of junk. It doesn't save any power.	Jingdong prefecture level city in Shandong
IXCHSQ Flagship Store	King of Power Saving	There are a lot of appliances in the house, the summer air conditioning is very costly, I bought this in front of the air conditioning effect is very good, I explain that I bought three in one breath, I use more electricity a month down to 200 or so, very satisfied.	Jingdong prefecture level city in Shandong
IXCHSQ Flagship Store	King of Power Saving	The voltage is very stable has been maintained at 220v this value, high power super power saving, trustworthy, reasonable design, shape bright, logistics is quite fast, business warranty period, excellent service, logistics fast!	Jingdong prefecture level city in Shandong
IXCHSQ Flagship Store	King of Power Saving	Use a month after a look at the savings of more than 40 dollars, there is a display of electricity savings, this high-power intelligent frequency converter economizer, affordable and cheap, logistics is very fast, the service attitude is very good, very satisfied with a shopping.	Jingdong prefecture level city in Shandong
IXCHSQ Flagship Store	King of Power Saving	Used for a period of time, it seems that every day can really can save a few degrees of electricity, workmanship quite can, with a period of time and then continue to look at it, anyway, the price is not	Jingdong prefecture level city in Shandong
IXCHSQ Flagship Store	King of Power Saving	Used for a period of time, it seems that every day can indeed save two several degrees of electricity, workmanship quite can, with a period of time and then continue to look at it, anyway, the price is not	Jingdong prefecture level city in Shandong
IXCHSQ Flagship Store	King of Power Saving	High-power super saver received immediately used, the price is not expensive quality is very good, save electricity and money, very convenient, conscientious business, must be praised, recommended to everyone!	Jingdong prefecture level city in Shandong
IXCHSQ Flagship Store	King of Power Saving	Home enhancement version of the intelligent power saver, is in use, I think the price is cheap, value for money, efficacy, power, power saving effect is very good.	Jingdong prefecture level city in Shandong
IXCHSQ Flagship Store	King of Power Saving	Bought three at once, one for each room, summer electricity consumption is very high, despite the three air conditioners on the electricity bill is still down a lot, too Nessie!	Jingdong prefecture level city in Shandong
IXCHSQ Flagship Store	King of Power Saving	Without further ado, the electric bill came down, $102 less this month, personal satisfaction, it counts okay, give you a good review	Jingdong prefecture level city in Shandong
IXCHSQ Flagship Store	King of Power Saving	The power saver has arrived, the installation is really convenient, plugged in on the line operation is quite simple, power saving effect is also a strong!	Jingdong prefecture level city in Shandong
IXCHSQ Flagship Store	King of Power Saving	Saver received, good quality, beautiful appearance, fine workmanship, simple installation	Jingdong prefecture level city in Shandong
IXCHSQ Flagship Store	King of Power Saving	Product design style modern, worth buying	Jingdong prefecture level city in Shandong
IXCHSQ Flagship Store	King of Power Saving	Energy saving, good power saving effect, practical, great value	Jingdong prefecture level city in Shandong
IXCHSQ Flagship Store	King of Power Saving	It's good quality, it's good, it's energy efficient, and I'll buy it again.	Jingdong prefecture level city in Shandong
IXCHSQ Flagship Store	King of Power Saving	Installation saves a lot of electricity, fast logistics, real energy-saving appliances	Jingdong prefecture level city in Shandong
IXCHSQ Flagship Store	King of Power Saving	The effect is good and the power saving is obvious after using it for a month and saving tens of dollars, the product is trustworthy, I now buy another 4 colors to prepare for their siblings' family to pay for	Jingdong prefecture level city in Shandong
IXCHSQ Flagship Store	King of Power Saving	The quality is particularly good workmanship is no problem, the arrival of the very fast oh, especially power-saving	Jingdong prefecture level city in Shandong
IXCHSQ Flagship Store	King of Power Saving	The quality is very good! In use, observed for a day, not as used before, so much power consumption.	Jingdong prefecture level city in Shandong
IXCHSQ Flagship Store	King of Power Saving	Goods have been received, the effect is good is not good, power saving is not yet know, wait for me to use it and then evaluate it.	Jingdong prefecture level city in Shandong
IXCHSQ Flagship Store	King of Power Saving	In-kind and the same as described, the quality is quite good, the seller's attitude is also good, ask questions and answer them, the speed of delivery bar, worth buying Oh!	Jingdong prefecture level city in Shandong
IXCHSQ Flagship Store	King of Power Saving	Nice stuff, came to comment after using it, it does save power.	Jingdong prefecture level city in Shandong

IXCHSQ Flagship Store	King of Power Saving	Fast charging, but also save power, the effect is very good, satisfied, praise!	Jingdong prefecture level city in Shandong
IXCHSQ Flagship Store	King of Power Saving	The goods have been received, I do not know how effective, after using the flat price again!	Jingdong prefecture level city in Shandong
IXCHSQ Flagship Store	King of Power Saving	Merchant service is very good Reply quickly The key is the quality and that quality is good	Jingdong prefecture level city in Shandong
IXCHSQ Flagship Store	King of Power Saving	It's working well now, I'll do a follow up review after I actually use it for a while	Jingdong prefecture level city in Shandong
IXCHSQ Flagship Store	King of Power Saving	The quality is good. After installing it, I feel like I've saved a lot of money on my electricity bill.	Jingdong prefecture level city in Shandong
IXCHSQ Flagship Store	King of Power Saving	Make up a review after use.	Jingdong prefecture level city in Shandong
IXCHSQ Flagship Store	King of Power Saving	review after use	Jingdong prefecture level city in Shandong
IXCHSQ Flagship Store	King of Power Saving	It's a small capacitor. It's not energy efficient.	Jingdong prefecture level city in Shandong
IXCHSQ Flagship Store	King of Power Saving	I'll follow up with a review after I've used it and had results.	Jingdong prefecture level city in Shandong
IXCHSQ Flagship Store	King of Power Saving	No power saving? Not good!	Jingdong prefecture level city in Shandong
IXCHSQ Flagship Store	King of Power Saving	Been using the same appliances and running a 1.5 air conditioner at night for what feels like no time, and after half a month of use it feels like the power is only increasing. Doesn't show any power saving	Jingdong prefecture level city in Shandong
IXCHSQ Flagship Store	King of Power Saving	serviceable	Jingdong prefecture level city in Shandong
IXCHSQ Flagship Store	King of Power Saving	It's a piece of junk. It doesn't save any power.	Jingdong prefecture level city in Shandong
IXCHSQ Flagship Store	King of Power Saving	There are a lot of appliances in the house, the summer air conditioning is very costly, I bought this in front of the air conditioning effect is very good, I explain that I bought three in one breath, I use more electricity a month down to 200 or so, very satisfied.	Jingdong prefecture level city in Shandong
IXCHSQ Flagship Store	King of Power Saving	The voltage is very stable has been maintained at 220v this value, high power super power saving, trustworthy, reasonable design, shape bright, logistics is quite fast, business warranty period, excellent service, logistics fast!	Jingdong prefecture level city in Shandong
IXCHSQ Flagship Store	King of Power Saving	Use a month after a look at the savings of more than 40 dollars, there is a display of electricity savings, this high-power intelligent frequency converter economizer, affordable and cheap, logistics is very fast, the service attitude is very good, very satisfied with a shopping.	Jingdong prefecture level city in Shandong
IXCHSQ Flagship Store	King of Power Saving	Used for a period of time, it seems that every day can really can save a few degrees of electricity, workmanship quite can, with a period of time and then continue to look at it, anyway, the price is not	Jingdong prefecture level city in Shandong
IXCHSQ Flagship Store	King of Power Saving	Used for a period of time, it seems that every day can indeed save two several degrees of electricity, workmanship quite can, with a period of time and then continue to look at it, anyway, the price is not	Jingdong prefecture level city in Shandong
IXCHSQ Flagship Store	King of Power Saving	High-power super saver received immediately used, the price is not expensive quality is very good, save electricity and money, very convenient, conscientious business, must be praised, recommended to everyone!	Jingdong prefecture level city in Shandong
IXCHSQ Flagship Store	King of Power Saving	Home enhancement version of the intelligent power saver, is in use, I think the price is cheap, value for money, efficacy, power, power saving effect is very good.	Jingdong prefecture level city in Shandong
IXCHSQ Flagship Store	King of Power Saving	Bought three at once, one for each room, summer electricity consumption is very high, despite the three air conditioners on the electricity bill is still down a lot, too Nessie!	Jingdong prefecture level city in Shandong
IXCHSQ Flagship Store	King of Power Saving	Without further ado, the electric bill came down, $102 less this month, personal satisfaction, it counts okay, give you a good review	Jingdong prefecture level city in Shandong
IXCHSQ Flagship Store	King of Power Saving	The power saver has arrived, the installation is really convenient, plugged in on the line operation is quite simple, power saving effect is also a strong!	Jingdong prefecture level city in Shandong
IXCHSQ Flagship Store	King of Power Saving	Saver received, good quality, beautiful appearance, fine workmanship, simple installation	Jingdong prefecture level city in Shandong
IXCHSQ Flagship Store	King of Power Saving	Product design style modern, worth buying	Jingdong prefecture level city in Shandong
IXCHSQ Flagship Store	King of Power Saving	Energy saving, good power saving effect, practical, great value	Jingdong prefecture level city in Shandong
IXCHSQ Flagship Store	King of Power Saving	It's good quality, it's good, it's energy efficient, and I'll buy it again.	Jingdong prefecture level city in Shandong
IXCHSQ Flagship Store	King of Power Saving	Installation saves a lot of electricity, fast logistics, real energy-saving appliances	Jingdong prefecture level city in Shandong
IXCHSQ Flagship Store	King of Power Saving	The effect is good and the power saving is obvious after using it for a month and saving tens of dollars, the product is trustworthy, I now buy another 4 colors to prepare for their siblings' family to pay for	Jingdong prefecture level city in Shandong
IXCHSQ Flagship Store	King of Power Saving	The quality is particularly good workmanship is no problem, the arrival of the very fast oh, especially power-saving	Jingdong prefecture level city in Shandong
IXCHSQ Flagship Store	King of Power Saving	The quality is very good! In use, observed for a day, not as used before, so much power consumption.	Jingdong prefecture level city in Shandong
IXCHSQ Flagship Store	King of Power Saving	Goods have been received, the effect is good is not good, power saving is not yet know, wait for me to use it and then evaluate it.	Jingdong prefecture level city in Shandong
IXCHSQ Flagship Store	King of Power Saving	In-kind and the same as described, the quality is quite good, the seller's attitude is also good, ask questions and answer them, the speed of delivery bar, worth buying Oh!	Jingdong prefecture level city in Shandong
IXCHSQ Flagship Store	King of Power Saving	Nice stuff, came to comment after using it, it does save power.	Jingdong prefecture level city in Shandong
IXCHSQ Flagship Store	King of Power Saving	Fast charging, but also save power, the effect is very good, satisfied, praise!	Jingdong prefecture level city in Shandong
IXCHSQ Flagship Store	King of Power Saving	The goods have been received, I do not know how effective, after using the flat price again!	Jingdong prefecture level city in Shandong
IXCHSQ Flagship Store	King of Power Saving	Merchant service is very good Reply quickly The key is the quality and that quality is good	Jingdong prefecture level city in Shandong
IXCHSQ Flagship Store	King of Power Saving	It's working well now, I'll do a follow up review after I actually use it for a while	Jingdong prefecture level city in Shandong
IXCHSQ Flagship Store	King of Power Saving	The quality is good. After installing it, I feel like I've saved a lot of money on my electricity bill.	Jingdong prefecture level city in Shandong
IXCHSQ Flagship Store	King of Power Saving	Make up a review after use.	Jingdong prefecture level city in Shandong
IXCHSQ Flagship Store	King of Power Saving	review after use	Jingdong prefecture level city in Shandong
IXCHSQ Flagship Store	King of Power Saving	It's a small capacitor. It's not energy efficient.	Jingdong prefecture level city in Shandong
IXCHSQ Flagship Store	King of Power Saving	I'll follow up with a review after I've used it and had results.	Jingdong prefecture level city in Shandong
IXCHSQ Flagship Store	King of Power Saving	No power saving? Not good!	Jingdong prefecture level city in Shandong
IXCHSQ Flagship Store	King of Power Saving	Been using the same appliances and running a 1.5 air conditioner at night for what feels like no time, and after half a month of use it feels like the power is only increasing. Doesn't show any power saving	Jingdong prefecture level city in Shandong
IXCHSQ Flagship Store	King of Power Saving	serviceable	Jingdong prefecture level city in Shandong
IXCHSQ Flagship Store	King of Power Saving	It's a piece of junk. It doesn't save any power.	Jingdong prefecture level city in Shandong
IXCHSQ Flagship Store	King of Power Saving	Received on the insertion of the meter under the, so far more sugar fixed, home electricity is not hot gate. I am an old house in the countryside! Home line is a bit chaotic at the same time open electric cozy from time to time tripping, listen to the seller said the product has a phase pressure effect, decisive order. Currently experiencing 2 weeks indeed voltage is very stable did not appear to have tripped the gate, the effect is very not also have a power-saving effect. Effect: very good Cost-effective: high Appearance style: good-looking Workmanship material: very good	Jingdong prefecture level city in Shandong

IXCHSQ Flagship Store	King of Power Saving	Received on the insertion of the meter down, currently more stable, home electricity is not tripped, I am the old house in the countryside! Home lines are a bit chaotic while opening electrical appliances from time to time tripping, listening to the seller said the product has a stabilized voltage, decisive order. Currently experiencing 2 weeks indeed voltage is very stable without tripping, the effect is very good, there is also a power-saving effect. Power-saving effect: very good Cost-effective: high Appearance style: good-looking Workmanship material: very good	Jingdong prefecture level city in Shandong
Dragon Quality Goods Shop	Green Intelligent Power Saver	Not worth the price at all it's so fake...working display...it meows just a couple of words	Jingdong prefecture level city in Shandong
Dragon Quality Goods Shop	Green Intelligent Power Saver	Intelligent compensation economizer received, looks very sturdy, like it very much. It is being used, very satisfied with a shopping, very easy to use. Buy home and plug it into the socket. Recommended to buy with confidence!	Jingdong prefecture level city in Shandong
Dragon Quality Goods Shop	Green Intelligent Power Saver	The quality of the saver is very good, good value for money, fine workmanship, the use of the effect is very good, the	Jingdong prefecture level city in Shandong
Dragon Quality Goods Shop	Green Intelligent Power Saver	Received specially used 3 months to evaluate, in fact, these days at home with electricity or growth, with a power saver instead of more power than before, the power saving rate is not bad, almost a month to save more than 200 bucks, measured effect!	Jingdong prefecture level city in Shandong
Dragon Quality Goods Shop	Green Intelligent Power Saver	. Used a period of time to look at the meter, check the price is indeed less, in the use of electricity when plugged into the meter is slow, this instrument is really good, I also want to buy two, bedroom and living room are installed Use experience, simple, convenient, fast!	Jingdong prefecture level city in Shandong
Dragon Quality Goods Shop	Green Intelligent Power Saver	Simple and convenient, just which plug a plug can save points, a month can save twenty or thirty dollars a year down to save a lot of good products cost-effective, everyone can afford to use, the use of experience: convenient for everyone, small objects of great use!	Jingdong prefecture level city in Shandong
Dragon Quality Goods Shop	Green Intelligent Power Saver	X at home is really good to save ah ! Received the goods can not wait to plug in, the meter I feel slowed down! Does it say good power saving good obvious it!	Jingdong prefecture level city in Shandong
Dragon Quality Goods Shop	Green Intelligent Power Saver	Fake scam no use, returns are not handled, don't fall for it friends.	Jingdong prefecture level city in Shandong
Dragon Quality Goods Shop	Green Intelligent Power Saver	Saver has received, but real can save electricity, the quality is very good, I times I buy a few more, too good.	Jingdong prefecture level city in Shandong
Dragon Quality Goods Shop	Green Intelligent Power Saver	Great quality as always, a very favorite product, great! o(*￣︶￣*)o	Jingdong prefecture level city in Shandong
Dragon Quality Goods Shop	Green Intelligent Power Saver	If we can reduce the electricity bill, for individuals can save money, for the country can protect the environment, now the weather is getting hotter and hotter, to protect the environment to reduce emissions is very important. Already use the product, whether to reduce the electricity bill depends on next month!	Jingdong prefecture level city in Shandong
Dragon Quality Goods Shop	Green Intelligent Power Saver	Purchased a power saver received on the put into use, rent a room with many electrical appliances, power consumption is very large, we have a piece of electricity or 1.2 dollars a time, with this power saver is much better, every day can save a few cents, very convenient and practical!	Jingdong prefecture level city in Shandong
Dragon Quality Goods Shop	Green Intelligent Power Saver	The product is better to use before not to use when a month 1500 yuan electricity bill, now using the 5 second month electricity bill directly down 470 dollars or very good. A save money to buy several power saver money ZanZanZanZan👍	Jingdong prefecture level city in Shandong
Dragon Quality Goods Shop	Green Intelligent Power Saver	The moment I received the goods really satisfied with this product, not only high-grade packaging, the body are base alloy, up to the price, I was also introduced by a friend, he used last month did save a lot of electricity, but also recommended to me!	Jingdong prefecture level city in Shandong
Dragon Quality Goods Shop	Green Intelligent Power Saver	I got it. Good review. I'm very satisfied. Reasonable price.	Jingdong prefecture level city in Shandong
Dragon Quality Goods Shop	Green Intelligent Power Saver	Intelligent power saver received and used, looking at the quality is good, the quality of the plug sent is also very good. Expect the effect of the economizer is obvious, first five-star praise, with a period of time to follow up!	Jingdong prefecture level city in Shandong
Dragon Quality Goods Shop	Green Intelligent Power Saver	The saver has been received. It was plugged into an outlet and used immediately after receipt. Whether it really saves electricity is not clear yet, I believe that this brand of energy saver really saves	Jingdong prefecture level city in Shandong
Dragon Quality Goods Shop	Green Intelligent Power Saver	Okay, okay, okay. Thank you so much. Fast is good. Fast. Item was as described, mainly because the event was cheap and you can save a lot of money.	Jingdong prefecture level city in Shandong
Dragon Quality Goods Shop	Green Intelligent Power Saver	Received it a couple days ago! Plugged it in the same day! Let's see what the electric bill is in a month! Hopefully it works well oh!	Jingdong prefecture level city in Shandong
Dragon Quality Goods Shop	Green Intelligent Power Saver	Looking at the reviews good, energy-saving small products, compact, the results after 2 months to see, because the electricity bill season payment	Jingdong prefecture level city in Shandong
Dragon Quality Goods Shop	Green Intelligent Power Saver	I just received it and will try it out to see if it works well. I'll comment later.	Jingdong prefecture level city in Shandong
Dragon Quality Goods Shop	Green Intelligent Power Saver	It looks a little small and not very aesthetically pleasing, I don't know how effective it will be until I use it	Jingdong prefecture level city in Shandong
Dragon Quality Goods Shop	Green Intelligent Power Saver	Not only to the point of being useless yet	Jingdong prefecture level city in Shandong
Dragon Quality Goods Shop	Green Intelligent Power Saver	Used it for a month, nothing works, the meter you turn very fast. This is not a scam, but also sold on Jingdong. To cheat a bunch of people?	Jingdong prefecture level city in Shandong
Dragon Quality Goods Shop	Green Intelligent Power Saver	Very good to use, every day can save a little electricity, really good under the sample down a month to save a few dozen dollars in electricity, good before I did not know that there is this 👆 haha, the price is not expensive praise.	Jingdong prefecture level city in Shandong
Dragon Quality Goods Shop	Green Intelligent Power Saver	Tried it for ten days and the change is almost nothing, can't see the power saving yet, use it some more days and see how it goes.	Jingdong prefecture level city in Shandong
Dragon Quality Goods Shop	Green Intelligent Power Saver	Received very quickly, the quality is very good, really save electricity, business service attitude is also very good, I hope you do not miss this opportunity, I continue to buy a few in	Jingdong prefecture level city in Shandong
Dragon Quality Goods Shop	Green Intelligent Power Saver	Quality is very good Oh, the delivery is also very fast, a very favorite store, I want to crazy recommend to friends!	Jingdong prefecture level city in Shandong
Dragon Quality Goods Shop	Green Intelligent Power Saver	The goods have just received, fine workmanship, and the same as described by the merchant, to use a period of time to see if it can achieve the purpose of power saving.	Jingdong prefecture level city in Shandong
Dragon Quality Goods Shop	Green Intelligent Power Saver	Received the goods first use a period of time to see if there is no effect, the business commitment to ninety days invalid refund, first praise, good service!	Jingdong prefecture level city in Shandong
Dragon Quality Goods Shop	Green Intelligent Power Saver	The product is used for a month without energy-saving and power-saving effect does not have any effect	Jingdong prefecture level city in Shandong
Dragon Quality Goods Shop	Green Intelligent Power Saver	The baby is really great, I like it very much, my friends also say good to come, recommended to buy!	Jingdong prefecture level city in Shandong
Dragon Quality Goods Shop	Green Intelligent Power Saver	Logistics is fast, courier boy service is good, after using to see how the fruit, and then follow up.	Jingdong prefecture level city in Shandong
Dragon Quality Goods Shop	Green Intelligent Power Saver	Baby has been received to try, I believe that as the business advertised energy saving. The courier is very responsible.	Jingdong prefecture level city in Shandong
Dragon Quality Goods Shop	Green Intelligent Power Saver	Save Electric Energy King received, good quality, beautiful appearance, very satisfied!	Jingdong prefecture level city in Shandong
Dragon Quality Goods Shop	Green Intelligent Power Saver	Three no products no manufacturers no phone pure fraud induced consumption cheat	Jingdong prefecture level city in Shandong
Dragon Quality Goods Shop	Green Intelligent Power Saver	I'm using it now, I don't know if it's as good as it says, I'll follow up after a month	Jingdong prefecture level city in Shandong
Dragon Quality Goods Shop	Green Intelligent Power Saver	Good quality logistics and fast. Fine workmanship. The description is consistent cost-effective	Jingdong prefecture level city in Shandong
Dragon Quality Goods Shop	Green Intelligent Power Saver	Received, whether it is good or not, to wait a few days and then evaluate	Jingdong prefecture level city in Shandong
Dragon Quality Goods Shop	Green Intelligent Power Saver	I bought it and started using it, I won't know if it works well until I pay my electric bill	Jingdong prefecture level city in Shandong
Dragon Quality Goods Shop	Green Intelligent Power Saver	Especially good, energy saving and power saving, especially good quality, good	Jingdong prefecture level city in Shandong
Dragon Quality Goods Shop	Green Intelligent Power Saver	Logistics is strong, the quality is very good, paste really save electricity, as described, will buy back!	Jingdong prefecture level city in Shandong
Dragon Quality Goods Shop	Green Intelligent Power Saver	Great stuff at a great price, worth the money!	Jingdong prefecture level city in Shandong

Dragon Quality Goods Shop	Green Intelligent Power Saver	Received the power saver, very good quality, very power saving!	Jingdong prefecture level city in Shandong
Dragon Quality Goods Shop	Green Intelligent Power Saver	There are no fruits, only samples...	Jingdong prefecture level city in Shandong
Dragon Quality Goods Shop	Green Intelligent Power Saver	Save a few dollars a month.	Jingdong prefecture level city in Shandong
Dragon Quality Goods Shop	Green Intelligent Power Saver	The quality is particularly good, it works well, and the seller has a good service attitude	Jingdong prefecture level city in Shandong
Dragon Quality Goods Shop	Green Intelligent Power Saver	I've compared them a couple of times and I don't feel any difference	Jingdong prefecture level city in Shandong
Dragon Quality Goods Shop	Green Intelligent Power Saver	I don't see the savings. It's a sham.	Jingdong prefecture level city in Shandong
Dragon Quality Goods Shop	Green Intelligent Power Saver	The power saving effect is very good, and the logistics is very fast. The seller's service attitude is very good	Jingdong prefecture level city in Shandong
Dragon Quality Goods Shop	Green Intelligent Power Saver	Received, well packaged, haven't tried it yet!	Jingdong prefecture level city in Shandong
Dragon Quality Goods Shop	Green Intelligent Power Saver	I don't know how it works, I'll comment after I use it.	Jingdong prefecture level city in Shandong
Dragon Quality Goods Shop	Green Intelligent Power Saver	The product doesn't work well enough to be of any use	Jingdong prefecture level city in Shandong
Dragon Quality Goods Shop	Green Intelligent Power Saver	On Trial 。。。。。。。	Jingdong prefecture level city in Shandong
Dragon Quality Goods Shop	Green Intelligent Power Saver	Being used with unknown results	Jingdong prefecture level city in Shandong
Dragon Quality Goods Shop	Green Intelligent Power Saver	Smart power saving and great quality	Jingdong prefecture level city in Shandong
Dragon Quality Goods Shop	Green Intelligent Power Saver	The saver was received, it works very well and the price is affordable!	Jingdong prefecture level city in Shandong
Dragon Quality Goods Shop	Green Intelligent Power Saver	How can I feel the power saving and how long does it last	Jingdong prefecture level city in Shandong
Dragon Quality Goods Shop	Green Intelligent Power Saver	And I know if it saves electricity or not.	Jingdong prefecture level city in Shandong
Dragon Quality Goods Shop	Green Intelligent Power Saver	It won't save any electricity. No.	Jingdong prefecture level city in Shandong
Dragon Quality Goods Shop	Green Intelligent Power Saver	Invalid. Fake.	Jingdong prefecture level city in Shandong
Dragon Quality Goods Shop	Green Intelligent Power Saver	It's good!	Jingdong prefecture level city in Shandong
Dragon Quality Goods Shop	Green Intelligent Power Saver	have no effect	Jingdong prefecture level city in Shandong
Dragon Quality Goods Shop	Green Intelligent Power Saver	Not used.	Jingdong prefecture level city in Shandong
Dragon Quality Goods Shop	Green Intelligent Power Saver	There is no load range, packaging thrift, ten days also did not develop invoices, received the goods with a receipt, hand-written receipt also has no financial stamp, communication for nearly a week a moment to say that the electronic invoice, a moment to say that the paper invoice, said it is mailed to me, ten days also did not see anything, the invoice invoice can not be found to say that did not stay on file, single single number can not be found, said that the warehouse more than one to a one to find out, in addition to the perfunctory or perfunctory. Products are no certificate of conformity, proper three	Jingdong prefecture level city in Shandong
Dragon Quality Goods Shop	Green Intelligent Power Saver	Satisfaction.	Jingdong prefecture level city in Shandong
Dragon Quality Goods Shop	Green Intelligent Power Saver	There is not a bit of power saving effect, and ringing up an increase in the electricity bill, because you have to add the cost of this machine's own electricity consumption and purchase, so I think it is better to use this machine instead of increasing the expenditure, uneconomical, rather than not use it.	Jingdong prefecture level city in Shandong
Dragon Quality Goods Shop	Green Intelligent Power Saver	I've been using it for two days now and I don't feel any effect.	Jingdong prefecture level city in Shandong
Dragon Quality Goods Shop	Green Intelligent Power Saver	What it looked like was nothing like what was sent.	Jingdong prefecture level city in Shandong
Dragon Quality Goods Shop	Green Intelligent Power Saver	It didn't work. It didn't work for a month or so.	Jingdong prefecture level city in Shandong
Dragon Quality Goods Shop	Green Intelligent Power Saver	Garbage products, physical and description is not the same, no effect at all, pit # no discussion, to buy the fine, garbage, garbage, garbage	Jingdong prefecture level city in Shandong
Dragon Quality Goods Shop	Green Intelligent Power Saver	general	Jingdong prefecture level city in Shandong
Dragon Quality Goods Shop	Green Intelligent Power Saver	IQ tax. Instead of saving electricity, it increases the power consumption of an appliance.	Jingdong prefecture level city in Shandong
Dragon Quality Goods Shop	Green Intelligent Power Saver	There's no trash at all.	Jingdong prefecture level city in Shandong
Dragon Quality Goods Shop	Green Intelligent Power Saver	It doesn't do anything.	Jingdong prefecture level city in Shandong
Dragon Quality Goods Shop	Green Intelligent Power Saver	(of an unmarried couple) be close	Jingdong prefecture level city in Shandong
Dragon Quality Goods Shop	Green Intelligent Power Saver	(of an unmarried couple) be close	Jingdong prefecture level city in Shandong
Dragon Quality Goods Shop	Green Intelligent Power Saver	no electricity saving	Jingdong prefecture level city in Shandong
Dragon Quality Goods Shop	Green Intelligent Power Saver	differ from	Jingdong prefecture level city in Shandong
Dragon Quality Goods Shop	Green Intelligent Power Saver	Saver has received, but real can save electricity, the quality is very good, I times I buy a few more, too good.	Jingdong prefecture level city in Shandong
Dragon Quality Goods Shop	Green Intelligent Power Saver	Great quality as always, a very favorite product, great! o(*￣︶￣*)o	Jingdong prefecture level city in Shandong
Dragon Quality Goods Shop	Green Intelligent Power Saver	If we can reduce the electricity bill, for individuals can save money, for the country can protect the environment, now the weather is getting hotter and hotter, to protect the environment to reduce emissions is very important. Already use the product, whether to reduce the electricity bill depends on next month!	Jingdong prefecture level city in Shandong
Dragon Quality Goods Shop	Green Intelligent Power Saver	Purchased a power saver received on the put into use, rent a room with many electrical appliances, power consumption is very large, we have a piece of electricity or 1.2 dollars a time, with this power saver is much better, every day can save a few cents, very convenient and practical!	Jingdong prefecture level city in Shandong
Dragon Quality Goods Shop	Green Intelligent Power Saver	The product is better to use before not to use when a month 1500 yuan electricity bill, now using the 5 second month electricity bill directly down 470 dollars or very good. A save money to buy several power saver money ZanZanZanZan👍	Jingdong prefecture level city in Shandong
Dragon Quality Goods Shop	Green Intelligent Power Saver	The moment I received the goods really satisfied with this product, not only high-grade packaging, the body are base alloy, up to the price, I was also introduced by a friend, he used last month did save a lot of electricity, but also recommended to me!	Jingdong prefecture level city in Shandong
Dragon Quality Goods Shop	Green Intelligent Power Saver	I got it. Good review. I'm very satisfied. Reasonable price.	Jingdong prefecture level city in Shandong
Dragon Quality Goods Shop	Green Intelligent Power Saver	Intelligent power saver received and used, looking at the quality is good, the quality of the plug sent is also very good. Expect the effect of the economizer is obvious, first five-star praise, with a period of time to follow up!	Jingdong prefecture level city in Shandong
Dragon Quality Goods Shop	Green Intelligent Power Saver	The saver has been received. It was plugged into an outlet and used immediately after receipt. Whether it really saves electricity is not clear yet, I believe that this brand of energy saver really saves	Jingdong prefecture level city in Shandong
Dragon Quality Goods Shop	Green Intelligent Power Saver	Okay, okay, okay. Thank you so much. Fast is good. Fast. Item was as described, mainly because the event was cheap and you can save a lot of money.	Jingdong prefecture level city in Shandong

Dragon Quality Goods Shop	Green Intelligent Power Saver	Received it a couple days ago! Plugged it in the same day! Let's see what the electric bill is in a month! Hopefully it works well oh!	Jingdong prefecture level city in Shandong
Dragon Quality Goods Shop	Green Intelligent Power Saver	Looking at the reviews good, energy-saving small products, compact, the results after 2 months to see, because the electricity bill season payment	Jingdong prefecture level city in Shandong
Dragon Quality Goods Shop	Green Intelligent Power Saver	I just received it and will try it out to see if it works well. I'll comment later.	Jingdong prefecture level city in Shandong
Dragon Quality Goods Shop	Green Intelligent Power Saver	It looks a little small and not very aesthetically pleasing, I don't know how effective it will be until I use it	Jingdong prefecture level city in Shandong
Dragon Quality Goods Shop	Green Intelligent Power Saver	Not only to the point of being useless yet	Jingdong prefecture level city in Shandong
Dragon Quality Goods Shop	Green Intelligent Power Saver	Used it for a month, nothing works, the meter you turn very fast. This is not a scam, but also sold on Jingdong. To cheat a bunch of people?	Jingdong prefecture level city in Shandong
Dragon Quality Goods Shop	Green Intelligent Power Saver	Very good to use, every day can save a little electricity, really good under the sample down a month to save a few dozen dollars in electricity, good before I did not know that there is this haha, the price is not expensive praise.	Jingdong prefecture level city in Shandong
Dragon Quality Goods Shop	Green Intelligent Power Saver	Tried it for ten days and the change is almost nothing, can't see the power saving yet, use it some more days and see how it goes.	Jingdong prefecture level city in Shandong
Dragon Quality Goods Shop	Green Intelligent Power Saver	Received very quickly, the quality is very good, really save electricity, business service attitude is also very good, I hope you do not miss this opportunity, I continue to buy a few in	Jingdong prefecture level city in Shandong
Dragon Quality Goods Shop	Green Intelligent Power Saver	Quality is very good Oh, the delivery is also very fast, a very favorite store, I want to crazy recommend to friends!	Jingdong prefecture level city in Shandong
Dragon Quality Goods Shop	Green Intelligent Power Saver	The goods have just received, fine workmanship, and the same as described by the merchant, to use a period of time to see if it can achieve the purpose of power saving.	Jingdong prefecture level city in Shandong
Dragon Quality Goods Shop	Green Intelligent Power Saver	Received the goods first use a period of time to see if there is no effect, the business commitment to ninety days invalid refund, first praise, good service!	Jingdong prefecture level city in Shandong
Dragon Quality Goods Shop	Green Intelligent Power Saver	The product is used for a month without energy-saving and power-saving effect does not have any effect	Jingdong prefecture level city in Shandong
Dragon Quality Goods Shop	Green Intelligent Power Saver	The baby is really great, I like it very much, my friends also say good to come, recommended to buy!	Jingdong prefecture level city in Shandong
Dragon Quality Goods Shop	Green Intelligent Power Saver	Logistics is fast, courier boy service is good, after using to see how the fruit, and then follow up.	Jingdong prefecture level city in Shandong
Dragon Quality Goods Shop	Green Intelligent Power Saver	Baby has been received to try, I believe that as the business advertised energy saving. The courier is very responsible.	Jingdong prefecture level city in Shandong
Dragon Quality Goods Shop	Green Intelligent Power Saver	Save Electric Energy King received, good quality, beautiful appearance, very satisfied!	Jingdong prefecture level city in Shandong
Dragon Quality Goods Shop	Green Intelligent Power Saver	Three no products no manufacturers no phone pure fraud induced consumption cheat	Jingdong prefecture level city in Shandong
Dragon Quality Goods Shop	Green Intelligent Power Saver	I'm using it now, I don't know if it's as good as it says, I'll follow up after a month	Jingdong prefecture level city in Shandong
Dragon Quality Goods Shop	Green Intelligent Power Saver	Good quality logistics and fast. Fine workmanship. The description is consistent cost-effective	Jingdong prefecture level city in Shandong
Dragon Quality Goods Shop	Green Intelligent Power Saver	Received, whether it is good or not, to wait a few days and then evaluate	Jingdong prefecture level city in Shandong
Dragon Quality Goods Shop	Green Intelligent Power Saver	I bought it and started using it, I won't know if it works well until I pay my electric bill	Jingdong prefecture level city in Shandong
Dragon Quality Goods Shop	Green Intelligent Power Saver	Especially good, energy saving and power saving, especially good quality, good	Jingdong prefecture level city in Shandong
Dragon Quality Goods Shop	Green Intelligent Power Saver	Logistics is strong, the quality is very good, paste really save electricity, as described, will buy back!	Jingdong prefecture level city in Shandong
Dragon Quality Goods Shop	Green Intelligent Power Saver	Great stuff at a great price, worth the money!	Jingdong prefecture level city in Shandong
Dragon Quality Goods Shop	Green Intelligent Power Saver	Received the power saver, very good quality, very power saving!	Jingdong prefecture level city in Shandong
Dragon Quality Goods Shop	Green Intelligent Power Saver	There are no fruits, only samples...	Jingdong prefecture level city in Shandong
Dragon Quality Goods Shop	Green Intelligent Power Saver	Save a few dollars a month.	Jingdong prefecture level city in Shandong
Dragon Quality Goods Shop	Green Intelligent Power Saver	The quality is particularly good, it works well, and the seller has a good service attitude	Jingdong prefecture level city in Shandong
Dragon Quality Goods Shop	Green Intelligent Power Saver	I've compared them a couple of times and I don't feel any difference	Jingdong prefecture level city in Shandong
Dragon Quality Goods Shop	Green Intelligent Power Saver	I don't see the savings. It's a sham.	Jingdong prefecture level city in Shandong
Dragon Quality Goods Shop	Green Intelligent Power Saver	The power saving effect is very good, and the logistics is very fast. The seller's service attitude is very good	Jingdong prefecture level city in Shandong
Dragon Quality Goods Shop	Green Intelligent Power Saver	Received, well packaged, haven't tried it yet!	Jingdong prefecture level city in Shandong
Dragon Quality Goods Shop	Green Intelligent Power Saver	I don't know how it works, I'll comment after I use it.	Jingdong prefecture level city in Shandong
Dragon Quality Goods Shop	Green Intelligent Power Saver	The product doesn't work well enough to be of any use	Jingdong prefecture level city in Shandong
Dragon Quality Goods Shop	Green Intelligent Power Saver	On Trial 。。。。。。。	Jingdong prefecture level city in Shandong
Dragon Quality Goods Shop	Green Intelligent Power Saver	Being used with unknown results	Jingdong prefecture level city in Shandong
Dragon Quality Goods Shop	Green Intelligent Power Saver	Smart power saving and great quality	Jingdong prefecture level city in Shandong
Dragon Quality Goods Shop	Green Intelligent Power Saver	The saver was received, it works very well and the price is affordable!	Jingdong prefecture level city in Shandong
Dragon Quality Goods Shop	Green Intelligent Power Saver	How can I feel the power saving and how long does it last	Jingdong prefecture level city in Shandong
Dragon Quality Goods Shop	Green Intelligent Power Saver	And I know if it saves electricity or not.	Jingdong prefecture level city in Shandong
Dragon Quality Goods Shop	Green Intelligent Power Saver	It won't save any electricity. No.	Jingdong prefecture level city in Shandong
Dragon Quality Goods Shop	Green Intelligent Power Saver	Invalid. Fake.	Jingdong prefecture level city in Shandong
Dragon Quality Goods Shop	Green Intelligent Power Saver	It's good!	Jingdong prefecture level city in Shandong
Dragon Quality Goods Shop	Green Intelligent Power Saver	have no effect	Jingdong prefecture level city in Shandong
Dragon Quality Goods Shop	Green Intelligent Power Saver	Not used.	Jingdong prefecture level city in Shandong
Dragon Quality Goods Shop	Green Intelligent Power Saver	There is no load range, packaging thrift, ten days also did not develop invoices, received the goods with a receipt, hand-written receipt also has no financial stamp, communication for nearly a week a moment to say that the electronic invoice, a moment to say that the paper invoice, said it is mailed to me, ten days also did not see anything, the invoice invoice can not be found to say that did not stay on file, single single number can not be found, said that the warehouse more than one to a one to find out, in addition to the perfunctory or perfunctory. Products are no certificate of conformity, proper three	Jingdong prefecture level city in Shandong
Dragon Quality Goods Shop	Green Intelligent Power Saver	Satisfaction.	Jingdong prefecture level city in Shandong

Dragon Quality Goods Shop	Green Intelligent Power Saver	There is not a bit of power saving effect, and ringing up an increase in the electricity bill, because you have to add the cost of this machine's own electricity consumption and purchase, so I think it is better to use this machine instead of increasing the expenditure, uneconomical, rather than not use it.	Jingdong prefecture level city in Shandong
Dragon Quality Goods Shop	Green Intelligent Power Saver	I've been using it for two days now and I don't feel any effect.	Jingdong prefecture level city in Shandong
Dragon Quality Goods Shop	Green Intelligent Power Saver	What it looked like was nothing like what was sent.	Jingdong prefecture level city in Shandong
Dragon Quality Goods Shop	Green Intelligent Power Saver	It didn't work. It didn't work for a month or so.	Jingdong prefecture level city in Shandong
Dragon Quality Goods Shop	Green Intelligent Power Saver	Garbage products, physical and description is not the same, no effect at all, pit # no discussion, to buy the fine, garbage, garbage, garbage	Jingdong prefecture level city in Shandong
Dragon Quality Goods Shop	Green Intelligent Power Saver	general	Jingdong prefecture level city in Shandong
Dragon Quality Goods Shop	Green Intelligent Power Saver	IQ tax. Instead of saving electricity, it increases the power consumption of an appliance.	Jingdong prefecture level city in Shandong
Dragon Quality Goods Shop	Green Intelligent Power Saver	There's no trash at all.	Jingdong prefecture level city in Shandong
Dragon Quality Goods Shop	Green Intelligent Power Saver	It doesn't do anything.	Jingdong prefecture level city in Shandong
Dragon Quality Goods Shop	Green Intelligent Power Saver	(of an unmarried couple) be close	Jingdong prefecture level city in Shandong
Dragon Quality Goods Shop	Green Intelligent Power Saver	(of an unmarried couple) be close	Jingdong prefecture level city in Shandong
Dragon Quality Goods Shop	Green Intelligent Power Saver	no electricity saving	Jingdong prefecture level city in Shandong
Dragon Quality Goods Shop	Green Intelligent Power Saver	differ from	Jingdong prefecture level city in Shandong
Dragon Quality Goods Shop	Green Intelligent Power Saver	Saver has received, but real can save electricity, the quality is very good, I times I buy a few more, too good.	Jingdong prefecture level city in Shandong
Dragon Quality Goods Shop	Green Intelligent Power Saver	Great quality as always, a very favorite product, great! o(*￣︶￣*)o	Jingdong prefecture level city in Shandong
Dragon Quality Goods Shop	Green Intelligent Power Saver	If we can reduce the electricity bill, for individuals can save money, for the country can protect the environment, now the weather is getting hotter and hotter, to protect the environment to reduce emissions is very important. Already use the product, whether to reduce the electricity bill depends on next month!	Jingdong prefecture level city in Shandong
Dragon Quality Goods Shop	Green Intelligent Power Saver	Purchased a power saver received on the put into use, rent a room with many electrical appliances, power consumption is very large, we have a piece of electricity or 1.2 dollars a time, with this power saver is much better, every day can save a few cents, very convenient and practical!	Jingdong prefecture level city in Shandong
Dragon Quality Goods Shop	Green Intelligent Power Saver	The product is better to use before not to use when a month 1500 yuan electricity bill, now using the 5 second month electricity bill directly down 470 dollars or very good. A save money to buy several power saver money ZanZanZanZan👍	Jingdong prefecture level city in Shandong
Dragon Quality Goods Shop	Green Intelligent Power Saver	The moment I received the goods really satisfied with this product, not only high-grade packaging, the body are base alloy, up to the price, I was also introduced by a friend, he used last month did save a lot of electricity, but also recommended to me!	Jingdong prefecture level city in Shandong
Dragon Quality Goods Shop	Green Intelligent Power Saver	I got it. Good review. I'm very satisfied. Reasonable price.	Jingdong prefecture level city in Shandong
Dragon Quality Goods Shop	Green Intelligent Power Saver	Intelligent power saver received and used, looking at the quality is good, the quality of the plug sent is also very good. Expect the effect of the economizer is obvious, first five-star praise, with a period of time to follow up!	Jingdong prefecture level city in Shandong
Dragon Quality Goods Shop	Green Intelligent Power Saver	The saver has been received. It was plugged into an outlet and used immediately after receipt. Whether it really saves electricity is not clear yet, I believe that this brand of energy saver really saves	Jingdong prefecture level city in Shandong
Dragon Quality Goods Shop	Green Intelligent Power Saver	Okay, okay, okay. Thank you so much. Fast is good. Fast. Item was as described, mainly because the event was cheap and you can save a lot of money.	Jingdong prefecture level city in Shandong
Dragon Quality Goods Shop	Green Intelligent Power Saver	Received it a couple days ago! Plugged it in the same day! Let's see what the electric bill is in a month! Hopefully it works well oh!	Jingdong prefecture level city in Shandong
Dragon Quality Goods Shop	Green Intelligent Power Saver	Looking at the reviews good, energy-saving small products, compact, the results after 2 months to see, because the electricity bill season payment	Jingdong prefecture level city in Shandong
Dragon Quality Goods Shop	Green Intelligent Power Saver	I just received it and will try it out to see if it works well. I'll comment later.	Jingdong prefecture level city in Shandong
Dragon Quality Goods Shop	Green Intelligent Power Saver	It looks a little small and not very aesthetically pleasing, I don't know how effective it will be until I use it	Jingdong prefecture level city in Shandong
Dragon Quality Goods Shop	Green Intelligent Power Saver	Not only to the point of being useless yet	Jingdong prefecture level city in Shandong
Dragon Quality Goods Shop	Green Intelligent Power Saver	Used it for a month, nothing works, the meter you turn very fast. This is not a scam, but also sold on Jingdong. To cheat a bunch of people?	Jingdong prefecture level city in Shandong
Dragon Quality Goods Shop	Green Intelligent Power Saver	Very good to use, every day can save a little electricity, really good under the sample down a month to save a few dozen dollars in electricity, good before I did not know that there is this 👆 haha, the price is not expensive praise.	Jingdong prefecture level city in Shandong
Dragon Quality Goods Shop	Green Intelligent Power Saver	Tried it for ten days and the change is almost nothing, can't see the power saving yet, use it some more days and see how it goes.	Jingdong prefecture level city in Shandong
Dragon Quality Goods Shop	Green Intelligent Power Saver	Received very quickly, the quality is very good, really save electricity, business service attitude is also very good, I hope you do not miss this opportunity, I continue to buy a few in	Jingdong prefecture level city in Shandong
Dragon Quality Goods Shop	Green Intelligent Power Saver	Quality is very good Oh, the delivery is also very fast, a very favorite store, I want to crazy recommend to friends!	Jingdong prefecture level city in Shandong
Dragon Quality Goods Shop	Green Intelligent Power Saver	The goods have just received, fine workmanship, and the same as described by the merchant, to use a period of time to see if it can achieve the purpose of power saving.	Jingdong prefecture level city in Shandong
Dragon Quality Goods Shop	Green Intelligent Power Saver	Received the goods first use a period of time to see if there is no effect, the business commitment to ninety days invalid refund, first praise, good service!	Jingdong prefecture level city in Shandong
Dragon Quality Goods Shop	Green Intelligent Power Saver	The product is used for a month without energy-saving and power-saving effect does not have any effect	Jingdong prefecture level city in Shandong
Dragon Quality Goods Shop	Green Intelligent Power Saver	The baby is really great, I like it very much, my friends also say good to come, recommended to buy!	Jingdong prefecture level city in Shandong
Dragon Quality Goods Shop	Green Intelligent Power Saver	Logistics is fast, courier boy service is good, after using to see how the fruit, and then follow up.	Jingdong prefecture level city in Shandong
Dragon Quality Goods Shop	Green Intelligent Power Saver	Baby has been received to try, I believe that as the business advertised energy saving. The courier is very responsible.	Jingdong prefecture level city in Shandong
Dragon Quality Goods Shop	Green Intelligent Power Saver	Save Electric Energy King received, good quality, beautiful appearance, very satisfied!	Jingdong prefecture level city in Shandong
Dragon Quality Goods Shop	Green Intelligent Power Saver	Three no products no manufacturers no phone pure fraud induced consumption cheat	Jingdong prefecture level city in Shandong
Dragon Quality Goods Shop	Green Intelligent Power Saver	I'm using it now, I don't know if it's as good as it says, I'll follow up after a month	Jingdong prefecture level city in Shandong
Dragon Quality Goods Shop	Green Intelligent Power Saver	Good quality logistics and fast. Fine workmanship. The description is consistent cost-effective	Jingdong prefecture level city in Shandong
Dragon Quality Goods Shop	Green Intelligent Power Saver	Received, whether it is good or not, to wait a few days and then evaluate	Jingdong prefecture level city in Shandong
Dragon Quality Goods Shop	Green Intelligent Power Saver	I bought it and started using it, I won't know if it works well until I pay my electric bill	Jingdong prefecture level city in Shandong
Dragon Quality Goods Shop	Green Intelligent Power Saver	Especially good, energy saving and power saving, especially good quality, good	Jingdong prefecture level city in Shandong
Dragon Quality Goods Shop	Green Intelligent Power Saver	Logistics is strong, the quality is very good, paste really save electricity, as described, will buy back!	Jingdong prefecture level city in Shandong
Dragon Quality Goods Shop	Green Intelligent Power Saver	Great stuff at a great price, worth the money!	Jingdong prefecture level city in Shandong

Dragon Quality Goods Shop	Green Intelligent Power Saver	Received the power saver, very good quality, very power saving!	Jingdong prefecture level city in Shandong
Dragon Quality Goods Shop	Green Intelligent Power Saver	There are no fruits, only samples...	Jingdong prefecture level city in Shandong
Dragon Quality Goods Shop	Green Intelligent Power Saver	Save a few dollars a month.	Jingdong prefecture level city in Shandong
Dragon Quality Goods Shop	Green Intelligent Power Saver	The quality is particularly good, it works well, and the seller has a good service attitude	Jingdong prefecture level city in Shandong
Dragon Quality Goods Shop	Green Intelligent Power Saver	I've compared them a couple of times and I don't feel any difference	Jingdong prefecture level city in Shandong
Dragon Quality Goods Shop	Green Intelligent Power Saver	I don't see the savings. It's a sham.	Jingdong prefecture level city in Shandong
Dragon Quality Goods Shop	Green Intelligent Power Saver	The power saving effect is very good, and the logistics is very fast. The seller's service attitude is very good	Jingdong prefecture level city in Shandong
Dragon Quality Goods Shop	Green Intelligent Power Saver	Received, well packaged, haven't tried it yet!	Jingdong prefecture level city in Shandong
Dragon Quality Goods Shop	Green Intelligent Power Saver	I don't know how it works, I'll comment after I use it.	Jingdong prefecture level city in Shandong
Dragon Quality Goods Shop	Green Intelligent Power Saver	The product doesn't work well enough to be of any use	Jingdong prefecture level city in Shandong
Dragon Quality Goods Shop	Green Intelligent Power Saver	On Trial 。。。。。。。	Jingdong prefecture level city in Shandong
Dragon Quality Goods Shop	Green Intelligent Power Saver	Being used with unknown results	Jingdong prefecture level city in Shandong
Dragon Quality Goods Shop	Green Intelligent Power Saver	Smart power saving and great quality	Jingdong prefecture level city in Shandong
Dragon Quality Goods Shop	Green Intelligent Power Saver	The saver was received, it works very well and the price is affordable!	Jingdong prefecture level city in Shandong
Dragon Quality Goods Shop	Green Intelligent Power Saver	How can I feel the power saving and how long does it last	Jingdong prefecture level city in Shandong
Dragon Quality Goods Shop	Green Intelligent Power Saver	And I know if it saves electricity or not.	Jingdong prefecture level city in Shandong
Dragon Quality Goods Shop	Green Intelligent Power Saver	It won't save any electricity. No.	Jingdong prefecture level city in Shandong
Dragon Quality Goods Shop	Green Intelligent Power Saver	Invalid. Fake.	Jingdong prefecture level city in Shandong
Dragon Quality Goods Shop	Green Intelligent Power Saver	It's good!	Jingdong prefecture level city in Shandong
Dragon Quality Goods Shop	Green Intelligent Power Saver	have no effect	Jingdong prefecture level city in Shandong
Dragon Quality Goods Shop	Green Intelligent Power Saver	Not used.	Jingdong prefecture level city in Shandong
Dragon Quality Goods Shop	Green Intelligent Power Saver	There is no load range, packaging thrift, ten days also did not develop invoices, received the goods with a receipt, hand-written receipt also has no financial stamp, communication for nearly a week a moment to say that the electronic invoice, a moment to say that the paper invoice, said it is mailed to me, ten days also did not see anything, the invoice invoice can not be found to say that did not stay on file, single single number can not be found, said that the warehouse more than one to a one to find out, in addition to the perfunctory or perfunctory. Products are no certificate of conformity, proper three	Jingdong prefecture level city in Shandong
Dragon Quality Goods Shop	Green Intelligent Power Saver	Satisfaction.	Jingdong prefecture level city in Shandong
Dragon Quality Goods Shop	Green Intelligent Power Saver	There is not a bit of power saving effect, and ringing up an increase in the electricity bill, because you have to add the cost of this machine's own electricity consumption and purchase, so I think it is better to use this machine instead of increasing the expenditure, uneconomical, rather than not use it.	Jingdong prefecture level city in Shandong
Dragon Quality Goods Shop	Green Intelligent Power Saver	I've been using it for two days now and I don't feel any effect.	Jingdong prefecture level city in Shandong
Dragon Quality Goods Shop	Green Intelligent Power Saver	What it looked like was nothing like what was sent.	Jingdong prefecture level city in Shandong
Dragon Quality Goods Shop	Green Intelligent Power Saver	It didn't work. It didn't work for a month or so.	Jingdong prefecture level city in Shandong
Dragon Quality Goods Shop	Green Intelligent Power Saver	Garbage products, physical and description is not the same, no effect at all, pit # no discussion, to buy the fine, garbage, garbage, garbage	Jingdong prefecture level city in Shandong
Dragon Quality Goods Shop	Green Intelligent Power Saver	general	Jingdong prefecture level city in Shandong
Dragon Quality Goods Shop	Green Intelligent Power Saver	IQ tax. Instead of saving electricity, it increases the power consumption of an appliance.	Jingdong prefecture level city in Shandong
Dragon Quality Goods Shop	Green Intelligent Power Saver	There's no trash at all.	Jingdong prefecture level city in Shandong
Dragon Quality Goods Shop	Green Intelligent Power Saver	It doesn't do anything.	Jingdong prefecture level city in Shandong
Dragon Quality Goods Shop	Green Intelligent Power Saver	(of an unmarried couple) be close	Jingdong prefecture level city in Shandong
Dragon Quality Goods Shop	Green Intelligent Power Saver	(of an unmarried couple) be close	Jingdong prefecture level city in Shandong
Dragon Quality Goods Shop	Green Intelligent Power Saver	no electricity saving	Jingdong prefecture level city in Shandong
Dragon Quality Goods Shop	Green Intelligent Power Saver	differ from	Jingdong prefecture level city in Shandong
Dragon Quality Goods Shop	Green Intelligent Power Saver	Saver has received, but real can save electricity, the quality is very good, I times I buy a few more, too good.	Jingdong prefecture level city in Shandong
Dragon Quality Goods Shop	Green Intelligent Power Saver	Great quality as always, a very favorite product, great! o(*￣︶￣*)o	Jingdong prefecture level city in Shandong
Dragon Quality Goods Shop	Green Intelligent Power Saver	If we can reduce the electricity bill, for individuals can save money, for the country can protect the environment, now the weather is getting hotter and hotter, to protect the environment to reduce emissions is very important. Already use the product, whether to reduce the electricity bill depends on next month!	Jingdong prefecture level city in Shandong
Dragon Quality Goods Shop	Green Intelligent Power Saver	Purchased a power saver received on the put into use, rent a room with many electrical appliances, power consumption is very large, we have a piece of electricity or 1.2 dollars a time, with this power saver is much better, every day can save a few cents, very convenient and practical!	Jingdong prefecture level city in Shandong
Dragon Quality Goods Shop	Green Intelligent Power Saver	The product is better to use before not to use when a month 1500 yuan electricity bill, now using the 5 second month electricity bill directly down 470 dollars or very good. A save money to buy several power saver money ZanZanZanZan👍	Jingdong prefecture level city in Shandong
Dragon Quality Goods Shop	Green Intelligent Power Saver	The moment I received the goods really satisfied with this product, not only high-grade packaging, the body are base alloy, up to the price, I was also introduced by a friend, he used last month did save a lot of electricity, but also recommended to me!	Jingdong prefecture level city in Shandong
Dragon Quality Goods Shop	Green Intelligent Power Saver	I got it. Good review. I'm very satisfied. Reasonable price.	Jingdong prefecture level city in Shandong
Dragon Quality Goods Shop	Green Intelligent Power Saver	Intelligent power saver received and used, looking at the quality is good, the quality of the plug sent is also very good. Expect the effect of the economizer is obvious, first five-star praise, with a period of time to follow up!	Jingdong prefecture level city in Shandong
Dragon Quality Goods Shop	Green Intelligent Power Saver	The saver has been received. It was plugged into an outlet and used immediately after receipt. Whether it really saves electricity is not clear yet, I believe that this brand of energy saver really saves	Jingdong prefecture level city in Shandong
Dragon Quality Goods Shop	Green Intelligent Power Saver	Okay, okay, okay. Thank you so much. Fast is good. Fast. Item was as described, mainly because the event was cheap and you can save a lot of money.	Jingdong prefecture level city in Shandong

Dragon Quality Goods Shop	Green Intelligent Power Saver	Received it a couple days ago! Plugged it in the same day! Let's see what the electric bill is in a month! Hopefully it works well oh!	Jingdong prefecture level city in Shandong
Dragon Quality Goods Shop	Green Intelligent Power Saver	Looking at the reviews good, energy-saving small products, compact, the results after 2 months to see, because the electricity bill season payment	Jingdong prefecture level city in Shandong
Dragon Quality Goods Shop	Green Intelligent Power Saver	I just received it and will try it out to see if it works well. I'll comment later.	Jingdong prefecture level city in Shandong
Dragon Quality Goods Shop	Green Intelligent Power Saver	It looks a little small and not very aesthetically pleasing, I don't know how effective it will be until I use it	Jingdong prefecture level city in Shandong
Dragon Quality Goods Shop	Green Intelligent Power Saver	Not only to the point of being useless yet	Jingdong prefecture level city in Shandong
Dragon Quality Goods Shop	Green Intelligent Power Saver	Used it for a month, nothing works, the meter you turn very fast. This is not a scam, but also sold on Jingdong. To cheat a bunch of people?	Jingdong prefecture level city in Shandong
Dragon Quality Goods Shop	Green Intelligent Power Saver	Very good to use, every day can save a little electricity, really good under the sample down a month to save a few dozen dollars in electricity, good before I did not know that there is this 👆 haha, the price is not expensive praise.	Jingdong prefecture level city in Shandong
Dragon Quality Goods Shop	Green Intelligent Power Saver	Tried it for ten days and the change is almost nothing, can't see the power saving yet, use it some more days and see how it goes.	Jingdong prefecture level city in Shandong
Dragon Quality Goods Shop	Green Intelligent Power Saver	Received very quickly, the quality is very good, really save electricity, business service attitude is also very good, I hope you do not miss this opportunity, I continue to buy a few in	Jingdong prefecture level city in Shandong
Dragon Quality Goods Shop	Green Intelligent Power Saver	Quality is very good Oh, the delivery is also very fast, a very favorite store, I want to crazy recommend to friends!	Jingdong prefecture level city in Shandong
Dragon Quality Goods Shop	Green Intelligent Power Saver	The goods have just received, fine workmanship, and the same as described by the merchant, to use a period of time to see if it can achieve the purpose of power saving.	Jingdong prefecture level city in Shandong
Dragon Quality Goods Shop	Green Intelligent Power Saver	Received the goods first use a period of time to see if there is no effect, the business commitment to ninety days invalid refund, first praise, good service!	Jingdong prefecture level city in Shandong
Dragon Quality Goods Shop	Green Intelligent Power Saver	The product is used for a month without energy-saving and power-saving effect does not have any effect	Jingdong prefecture level city in Shandong
Dragon Quality Goods Shop	Green Intelligent Power Saver	The baby is really great, I like it very much, my friends also say good to come, recommended to buy!	Jingdong prefecture level city in Shandong
Dragon Quality Goods Shop	Green Intelligent Power Saver	Logistics is fast, courier boy service is good, after using to see how the fruit, and then follow up.	Jingdong prefecture level city in Shandong
Dragon Quality Goods Shop	Green Intelligent Power Saver	Baby has been received to try, I believe that as the business advertised energy saving. The courier is very responsible.	Jingdong prefecture level city in Shandong
Dragon Quality Goods Shop	Green Intelligent Power Saver	Save Electric Energy King received, good quality, beautiful appearance, very satisfied!	Jingdong prefecture level city in Shandong
Dragon Quality Goods Shop	Green Intelligent Power Saver	Three no products no manufacturers no phone pure fraud induced consumption cheat	Jingdong prefecture level city in Shandong
Dragon Quality Goods Shop	Green Intelligent Power Saver	I'm using it now, I don't know if it's as good as it says, I'll follow up after a month	Jingdong prefecture level city in Shandong
Dragon Quality Goods Shop	Green Intelligent Power Saver	Good quality logistics and fast. Fine workmanship. The description is consistent cost-effective	Jingdong prefecture level city in Shandong
Dragon Quality Goods Shop	Green Intelligent Power Saver	Received, whether it is good or not, to wait a few days and then evaluate	Jingdong prefecture level city in Shandong
Dragon Quality Goods Shop	Green Intelligent Power Saver	I bought it and started using it, I won't know if it works well until I pay my electric bill	Jingdong prefecture level city in Shandong
Dragon Quality Goods Shop	Green Intelligent Power Saver	Especially good, energy saving and power saving, especially good quality, good	Jingdong prefecture level city in Shandong
Dragon Quality Goods Shop	Green Intelligent Power Saver	Logistics is strong, the quality is very good, paste really save electricity, as described, will buy back!	Jingdong prefecture level city in Shandong
Dragon Quality Goods Shop	Green Intelligent Power Saver	Great stuff at a great price, worth the money!	Jingdong prefecture level city in Shandong
Dragon Quality Goods Shop	Green Intelligent Power Saver	Received the power saver, very good quality, very power saving!	Jingdong prefecture level city in Shandong
Dragon Quality Goods Shop	Green Intelligent Power Saver	There are no fruits, only samples...	Jingdong prefecture level city in Shandong
Dragon Quality Goods Shop	Green Intelligent Power Saver	Save a few dollars a month.	Jingdong prefecture level city in Shandong
Dragon Quality Goods Shop	Green Intelligent Power Saver	The quality is particularly good, it works well, and the seller has a good service attitude	Jingdong prefecture level city in Shandong
Dragon Quality Goods Shop	Green Intelligent Power Saver	I've compared them a couple of times and I don't feel any difference	Jingdong prefecture level city in Shandong
Dragon Quality Goods Shop	Green Intelligent Power Saver	I don't see the savings. It's a sham.	Jingdong prefecture level city in Shandong
Dragon Quality Goods Shop	Green Intelligent Power Saver	The power saving effect is very good, and the logistics is very fast. The seller's service attitude is very good	Jingdong prefecture level city in Shandong
Dragon Quality Goods Shop	Green Intelligent Power Saver	I don't know how it works, I'll comment after I use it.	Jingdong prefecture level city in Shandong
Dragon Quality Goods Shop	Green Intelligent Power Saver	Received, well packaged, haven't tried it yet!	Jingdong prefecture level city in Shandong
Dragon Quality Goods Shop	Green Intelligent Power Saver	The product doesn't work well enough to be of any use	Jingdong prefecture level city in Shandong
Dragon Quality Goods Shop	Green Intelligent Power Saver	On Trial 。。。。。。。	Jingdong prefecture level city in Shandong
Dragon Quality Goods Shop	Green Intelligent Power Saver	Being used with unknown results	Jingdong prefecture level city in Shandong
Dragon Quality Goods Shop	Green Intelligent Power Saver	Smart power saving and great quality	Jingdong prefecture level city in Shandong
Dragon Quality Goods Shop	Green Intelligent Power Saver	The saver was received, it works very well and the price is affordable!	Jingdong prefecture level city in Shandong
Dragon Quality Goods Shop	Green Intelligent Power Saver	How can I feel the power saving and how long does it last	Jingdong prefecture level city in Shandong
Dragon Quality Goods Shop	Green Intelligent Power Saver	And I know if it saves electricity or not.	Jingdong prefecture level city in Shandong
Dragon Quality Goods Shop	Green Intelligent Power Saver	It won't save any electricity. No.	Jingdong prefecture level city in Shandong
Dragon Quality Goods Shop	Green Intelligent Power Saver	Invalid. Fake.	Jingdong prefecture level city in Shandong
Dragon Quality Goods Shop	Green Intelligent Power Saver	It's good!	Jingdong prefecture level city in Shandong
Dragon Quality Goods Shop	Green Intelligent Power Saver	have no effect	Jingdong prefecture level city in Shandong
Dragon Quality Goods Shop	Green Intelligent Power Saver	Not used.	Jingdong prefecture level city in Shandong
Dragon Quality Goods Shop	Green Intelligent Power Saver	There is no load range, packaging thrift, ten days also did not develop invoices, received the goods with a receipt, hand-written receipt also has no financial stamp, communication for nearly a week a moment to say that the electronic invoice, a moment to say that the paper invoice, said it is mailed to me, ten days also did not see anything, the invoice invoice can not be found to say that did not stay on file, single single number can not be found, said that the warehouse more than one to a one to find out, in addition to the perfunctory or perfunctory. Products are no certificate of conformity, proper three	Jingdong prefecture level city in Shandong
Dragon Quality Goods Shop	Green Intelligent Power Saver	Satisfaction.	Jingdong prefecture level city in Shandong

Dragon Quality Goods Shop	Green Intelligent Power Saver	There is not a bit of power saving effect, and ringing up an increase in the electricity bill, because you have to add the cost of this machine's own electricity consumption and purchase, so I think it is better to use this machine instead of increasing the expenditure, uneconomical, rather than not use it.	Jingdong prefecture level city in Shandong
Dragon Quality Goods Shop	Green Intelligent Power Saver	I've been using it for two days now and I don't feel any effect.	Jingdong prefecture level city in Shandong
Dragon Quality Goods Shop	Green Intelligent Power Saver	What it looked like was nothing like what was sent.	Jingdong prefecture level city in Shandong
Dragon Quality Goods Shop	Green Intelligent Power Saver	It didn't work. It didn't work for a month or so.	Jingdong prefecture level city in Shandong
Dragon Quality Goods Shop	Green Intelligent Power Saver	Garbage products, physical and description is not the same, no effect at all, pit # no discussion, to buy the fine, garbage, garbage, garbage	Jingdong prefecture level city in Shandong
Dragon Quality Goods Shop	Green Intelligent Power Saver	general	Jingdong prefecture level city in Shandong
Dragon Quality Goods Shop	Green Intelligent Power Saver	IQ tax. Instead of saving electricity, it increases the power consumption of an appliance.	Jingdong prefecture level city in Shandong
Dragon Quality Goods Shop	Green Intelligent Power Saver	There's no trash at all.	Jingdong prefecture level city in Shandong
Dragon Quality Goods Shop	Green Intelligent Power Saver	It doesn't do anything.	Jingdong prefecture level city in Shandong
Dragon Quality Goods Shop	Green Intelligent Power Saver	(of an unmarried couple) be close	Jingdong prefecture level city in Shandong
Dragon Quality Goods Shop	Green Intelligent Power Saver	(of an unmarried couple) be close	Jingdong prefecture level city in Shandong
Dragon Quality Goods Shop	Green Intelligent Power Saver	no electricity saving	Jingdong prefecture level city in Shandong
Dragon Quality Goods Shop	Green Intelligent Power Saver	differ from	Jingdong prefecture level city in Shandong
Dragon Quality Goods Shop	Green Intelligent Power Saver	Saver has received, but real can save electricity, the quality is very good, I times I buy a few more, too good.	Jingdong prefecture level city in Shandong
Dragon Quality Goods Shop	Green Intelligent Power Saver	Great quality as always, a very favorite product, great! o(*￣︶￣*)o	Jingdong prefecture level city in Shandong
Dragon Quality Goods Shop	Green Intelligent Power Saver	If we can reduce the electricity bill, for individuals can save money, for the country can protect the environment, now the weather is getting hotter and hotter, to protect the environment to reduce emissions is very important. Already use the product, whether to reduce the electricity bill depends on next month!	Jingdong prefecture level city in Shandong
Dragon Quality Goods Shop	Green Intelligent Power Saver	Purchased a power saver received on the put into use, rent a room with many electrical appliances, power consumption is very large, we have a piece of electricity or 1.2 dollars a time, with this power saver is much better, every day can save a few cents, very convenient and practical!	Jingdong prefecture level city in Shandong
Dragon Quality Goods Shop	Green Intelligent Power Saver	The product is better to use before not to use when a month 1500 yuan electricity bill, now using the 5 second month electricity bill directly down 470 dollars or very good. A save money to buy several power saver money ZanZanZanZan👍	Jingdong prefecture level city in Shandong
Dragon Quality Goods Shop	Green Intelligent Power Saver	The moment I received the goods really satisfied with this product, not only high-grade packaging, the body are base alloy, up to the price, I was also introduced by a friend, he used last month did save a lot of electricity, but also recommended to me!	Jingdong prefecture level city in Shandong
Dragon Quality Goods Shop	Green Intelligent Power Saver	I got it. Good review. I'm very satisfied. Reasonable price.	Jingdong prefecture level city in Shandong
Dragon Quality Goods Shop	Green Intelligent Power Saver	Intelligent power saver received and used, looking at the quality is good, the quality of the plug sent is also very good. Expect the effect of the economizer is obvious, first five-star praise, with a period of time to follow up!	Jingdong prefecture level city in Shandong
Dragon Quality Goods Shop	Green Intelligent Power Saver	The saver has been received. It was plugged into an outlet and used immediately after receipt. Whether it really saves electricity is not clear yet, I believe that this brand of energy saver really saves	Jingdong prefecture level city in Shandong
Dragon Quality Goods Shop	Green Intelligent Power Saver	Okay, okay, okay. Thank you so much. Fast is good. Fast. Item was as described, mainly because the event was cheap and you can save a lot of money.	Jingdong prefecture level city in Shandong
Dragon Quality Goods Shop	Green Intelligent Power Saver	Received it a couple days ago! Plugged it in the same day! Let's see what the electric bill is in a month! Hopefully it works well oh!	Jingdong prefecture level city in Shandong
Dragon Quality Goods Shop	Green Intelligent Power Saver	Looking at the reviews good, energy-saving small products, compact, the results after 2 months to see, because the electricity bill season payment	Jingdong prefecture level city in Shandong
Dragon Quality Goods Shop	Green Intelligent Power Saver	I just received it and will try it out to see if it works well. I'll comment later.	Jingdong prefecture level city in Shandong
Dragon Quality Goods Shop	Green Intelligent Power Saver	It looks a little small and not very aesthetically pleasing, I don't know how effective it will be until I use it	Jingdong prefecture level city in Shandong
Dragon Quality Goods Shop	Green Intelligent Power Saver	Not only to the point of being useless yet	Jingdong prefecture level city in Shandong
Dragon Quality Goods Shop	Green Intelligent Power Saver	Used it for a month, nothing works, the meter you turn very fast. This is not a scam, but also sold on Jingdong. To cheat a bunch of people?	Jingdong prefecture level city in Shandong
Dragon Quality Goods Shop	Green Intelligent Power Saver	Very good to use, every day can save a little electricity, really good under the sample down a month to save a few dozen dollars in electricity, good before I did not know that there is this 👆 haha, the price is not expensive praise.	Jingdong prefecture level city in Shandong
Dragon Quality Goods Shop	Green Intelligent Power Saver	Tried it for ten days and the change is almost nothing, can't see the power saving yet, use it some more days and see how it goes.	Jingdong prefecture level city in Shandong
Dragon Quality Goods Shop	Green Intelligent Power Saver	Received very quickly, the quality is very good, really save electricity, business service attitude is also very good, I hope you do not miss this opportunity, I continue to buy a few in	Jingdong prefecture level city in Shandong
Dragon Quality Goods Shop	Green Intelligent Power Saver	Quality is very good Oh, the delivery is also very fast, a very favorite store, I want to crazy recommend to friends!	Jingdong prefecture level city in Shandong
Dragon Quality Goods Shop	Green Intelligent Power Saver	The goods have just received, fine workmanship, and the same as described by the merchant, to use a period of time to see if it can achieve the purpose of power saving.	Jingdong prefecture level city in Shandong
Dragon Quality Goods Shop	Green Intelligent Power Saver	Received the goods first use a period of time to see if there is no effect, the business commitment to ninety days invalid refund, first praise, good service!	Jingdong prefecture level city in Shandong
Dragon Quality Goods Shop	Green Intelligent Power Saver	The product is used for a month without energy-saving and power-saving effect does not have any effect	Jingdong prefecture level city in Shandong
Dragon Quality Goods Shop	Green Intelligent Power Saver	The baby is really great, I like it very much, my friends also say good to come, recommended to buy!	Jingdong prefecture level city in Shandong
Dragon Quality Goods Shop	Green Intelligent Power Saver	Logistics is fast, courier boy service is good, after using to see how the fruit, and then follow up.	Jingdong prefecture level city in Shandong
Dragon Quality Goods Shop	Green Intelligent Power Saver	Baby has been received to try, I believe that as the business advertised energy saving. The courier is very responsible.	Jingdong prefecture level city in Shandong
Dragon Quality Goods Shop	Green Intelligent Power Saver	Save Electric Energy King received, good quality, beautiful appearance, very satisfied!	Jingdong prefecture level city in Shandong
Dragon Quality Goods Shop	Green Intelligent Power Saver	Three no products no manufacturers no phone pure fraud induced consumption cheat	Jingdong prefecture level city in Shandong
Dragon Quality Goods Shop	Green Intelligent Power Saver	I'm using it now, I don't know if it's as good as it says, I'll follow up after a month	Jingdong prefecture level city in Shandong
Dragon Quality Goods Shop	Green Intelligent Power Saver	Good quality logistics and fast. Fine workmanship. The description is consistent cost-effective	Jingdong prefecture level city in Shandong
Dragon Quality Goods Shop	Green Intelligent Power Saver	Received, whether it is good or not, to wait a few days and then evaluate	Jingdong prefecture level city in Shandong
Dragon Quality Goods Shop	Green Intelligent Power Saver	I bought it and started using it, I won't know if it works well until I pay my electric bill	Jingdong prefecture level city in Shandong
Dragon Quality Goods Shop	Green Intelligent Power Saver	Especially good, energy saving and power saving, especially good quality, good	Jingdong prefecture level city in Shandong
Dragon Quality Goods Shop	Green Intelligent Power Saver	Logistics is strong, the quality is very good, paste really save electricity, as described, will buy back!	Jingdong prefecture level city in Shandong
Dragon Quality Goods Shop	Green Intelligent Power Saver	Great stuff at a great price, worth the money!	Jingdong prefecture level city in Shandong

Dragon Quality Goods Shop	Green Intelligent Power Saver	Received the power saver, very good quality, very power saving!	Jingdong prefecture level city in Shandong
Dragon Quality Goods Shop	Green Intelligent Power Saver	There are no fruits, only samples...	Jingdong prefecture level city in Shandong
Dragon Quality Goods Shop	Green Intelligent Power Saver	Save a few dollars a month.	Jingdong prefecture level city in Shandong
Dragon Quality Goods Shop	Green Intelligent Power Saver	The quality is particularly good, it works well, and the seller has a good service attitude	Jingdong prefecture level city in Shandong
Dragon Quality Goods Shop	Green Intelligent Power Saver	I've compared them a couple of times and I don't feel any difference	Jingdong prefecture level city in Shandong
Dragon Quality Goods Shop	Green Intelligent Power Saver	I don't see the savings. It's a sham.	Jingdong prefecture level city in Shandong
Dragon Quality Goods Shop	Green Intelligent Power Saver	The power saving effect is very good, and the logistics is very fast. The seller's service attitude is very good	Jingdong prefecture level city in Shandong
Dragon Quality Goods Shop	Green Intelligent Power Saver	Received, well packaged, haven't tried it yet!	Jingdong prefecture level city in Shandong
Dragon Quality Goods Shop	Green Intelligent Power Saver	I don't know how it works, I'll comment after I use it.	Jingdong prefecture level city in Shandong
Dragon Quality Goods Shop	Green Intelligent Power Saver	The product doesn't work well enough to be of any use	Jingdong prefecture level city in Shandong
Dragon Quality Goods Shop	Green Intelligent Power Saver	On Trial 。。。。。。。	Jingdong prefecture level city in Shandong
Dragon Quality Goods Shop	Green Intelligent Power Saver	Being used with unknown results	Jingdong prefecture level city in Shandong
Dragon Quality Goods Shop	Green Intelligent Power Saver	Smart power saving and great quality	Jingdong prefecture level city in Shandong
Dragon Quality Goods Shop	Green Intelligent Power Saver	The saver was received, it works very well and the price is affordable!	Jingdong prefecture level city in Shandong
Dragon Quality Goods Shop	Green Intelligent Power Saver	How can I feel the power saving and how long does it last	Jingdong prefecture level city in Shandong
Dragon Quality Goods Shop	Green Intelligent Power Saver	And I know if it saves electricity or not.	Jingdong prefecture level city in Shandong
Dragon Quality Goods Shop	Green Intelligent Power Saver	It won't save any electricity. No.	Jingdong prefecture level city in Shandong
Dragon Quality Goods Shop	Green Intelligent Power Saver	Invalid. Fake.	Jingdong prefecture level city in Shandong
Dragon Quality Goods Shop	Green Intelligent Power Saver	It's good!	Jingdong prefecture level city in Shandong
Dragon Quality Goods Shop	Green Intelligent Power Saver	have no effect	Jingdong prefecture level city in Shandong
Dragon Quality Goods Shop	Green Intelligent Power Saver	Not used.	Jingdong prefecture level city in Shandong
Dragon Quality Goods Shop	Green Intelligent Power Saver	There is no load range, packaging thrift, ten days also did not develop invoices, received the goods with a receipt, hand-written receipt also has no financial stamp, communication for nearly a week a moment to say that the electronic invoice, a moment to say that the paper invoice, said it is mailed to me, ten days also did not see anything, the invoice invoice can not be found to say that did not stay on file, single single number can not be found, said that the warehouse more than one to a one to find out, in addition to the perfunctory or perfunctory. Products are no certificate of conformity, proper three	Jingdong prefecture level city in Shandong
Dragon Quality Goods Shop	Green Intelligent Power Saver	Satisfaction.	Jingdong prefecture level city in Shandong
Dragon Quality Goods Shop	Green Intelligent Power Saver	There is not a bit of power saving effect, and ringing up an increase in the electricity bill, because you have to add the cost of this machine's own electricity consumption and purchase, so I think it is better to use this machine instead of increasing the expenditure, uneconomical, rather than not use it.	Jingdong prefecture level city in Shandong
Dragon Quality Goods Shop	Green Intelligent Power Saver	I've been using it for two days now and I don't feel any effect.	Jingdong prefecture level city in Shandong
Dragon Quality Goods Shop	Green Intelligent Power Saver	What it looked like was nothing like what was sent.	Jingdong prefecture level city in Shandong
Dragon Quality Goods Shop	Green Intelligent Power Saver	It didn't work. It didn't work for a month or so.	Jingdong prefecture level city in Shandong
Dragon Quality Goods Shop	Green Intelligent Power Saver	Garbage products, physical and description is not the same, no effect at all, pit # no discussion, to buy the fine, garbage, garbage, garbage	Jingdong prefecture level city in Shandong
Dragon Quality Goods Shop	Green Intelligent Power Saver	general	Jingdong prefecture level city in Shandong
Dragon Quality Goods Shop	Green Intelligent Power Saver	IQ tax. Instead of saving electricity, it increases the power consumption of an appliance.	Jingdong prefecture level city in Shandong
Dragon Quality Goods Shop	Green Intelligent Power Saver	There's no trash at all.	Jingdong prefecture level city in Shandong
Dragon Quality Goods Shop	Green Intelligent Power Saver	It doesn't do anything.	Jingdong prefecture level city in Shandong
Dragon Quality Goods Shop	Green Intelligent Power Saver	(of an unmarried couple) be close	Jingdong prefecture level city in Shandong
Dragon Quality Goods Shop	Green Intelligent Power Saver	(of an unmarried couple) be close	Jingdong prefecture level city in Shandong
Dragon Quality Goods Shop	Green Intelligent Power Saver	no electricity saving	Jingdong prefecture level city in Shandong
Dragon Quality Goods Shop	Green Intelligent Power Saver	differ from	Jingdong prefecture level city in Shandong
Dragon Quality Goods Shop	Green Intelligent Power Saver	Saver has received, but real can save electricity, the quality is very good, I times I buy a few more, too good.	Jingdong prefecture level city in Shandong
Dragon Quality Goods Shop	Green Intelligent Power Saver	Great quality as always, a very favorite product, great! o(*￣︶￣*)o	Jingdong prefecture level city in Shandong
Dragon Quality Goods Shop	Green Intelligent Power Saver	If we can reduce the electricity bill, for individuals can save money, for the country can protect the environment, now the weather is getting hotter and hotter, to protect the environment to reduce emissions is very important. Already use the product, whether to reduce the electricity bill depends on next month!	Jingdong prefecture level city in Shandong
Dragon Quality Goods Shop	Green Intelligent Power Saver	Purchased a power saver received on the put into use, rent a room with many electrical appliances, power consumption is very large, we have a piece of electricity or 1.2 dollars a time, with this power saver is much better, every day can save a few cents, very convenient and practical!	Jingdong prefecture level city in Shandong
Dragon Quality Goods Shop	Green Intelligent Power Saver	The product is better to use before not to use when a month 1500 yuan electricity bill, now using the 5 second month electricity bill directly down 470 dollars or very good. A save money to buy several power saver money ZanZanZanZan👍	Jingdong prefecture level city in Shandong
Dragon Quality Goods Shop	Green Intelligent Power Saver	The moment I received the goods really satisfied with this product, not only high-grade packaging, the body are base alloy, up to the price, I was also introduced by a friend, he used last month did save a lot of electricity, but also recommended to me!	Jingdong prefecture level city in Shandong
Dragon Quality Goods Shop	Green Intelligent Power Saver	I got it. Good review. I'm very satisfied. Reasonable price.	Jingdong prefecture level city in Shandong
Dragon Quality Goods Shop	Green Intelligent Power Saver	Intelligent power saver received and used, looking at the quality is good, the quality of the plug sent is also very good. Expect the effect of the economizer is obvious, first five-star praise, with a period of time to follow up!	Jingdong prefecture level city in Shandong
Dragon Quality Goods Shop	Green Intelligent Power Saver	The saver has been received. It was plugged into an outlet and used immediately after receipt. Whether it really saves electricity is not clear yet, I believe that this brand of energy saver really saves	Jingdong prefecture level city in Shandong
Dragon Quality Goods Shop	Green Intelligent Power Saver	Okay, okay, okay. Thank you so much. Fast is good. Fast. Item was as described, mainly because the event was cheap and you can save a lot of money.	Jingdong prefecture level city in Shandong

Dragon Quality Goods Shop	Green Intelligent Power Saver	Received it a couple days ago! Plugged it in the same day! Let's see what the electric bill is in a month! Hopefully it works well oh!	Jingdong prefecture level city in Shandong
Dragon Quality Goods Shop	Green Intelligent Power Saver	Looking at the reviews good, energy-saving small products, compact, the results after 2 months to see, because the electricity bill season payment	Jingdong prefecture level city in Shandong
Dragon Quality Goods Shop	Green Intelligent Power Saver	I just received it and will try it out to see if it works well. I'll comment later.	Jingdong prefecture level city in Shandong
Dragon Quality Goods Shop	Green Intelligent Power Saver	It looks a little small and not very aesthetically pleasing, I don't know how effective it will be until I use it	Jingdong prefecture level city in Shandong
Dragon Quality Goods Shop	Green Intelligent Power Saver	Not only to the point of being useless yet	Jingdong prefecture level city in Shandong
Dragon Quality Goods Shop	Green Intelligent Power Saver	Used it for a month, nothing works, the meter you turn very fast. This is not a scam, but also sold on Jingdong. To cheat a bunch of people?	Jingdong prefecture level city in Shandong
Dragon Quality Goods Shop	Green Intelligent Power Saver	Very good to use, every day can save a little electricity, really good under the sample down a month to save a few dozen dollars in electricity, good before I did not know that there is this ☝ haha, the price is not expensive praise.	Jingdong prefecture level city in Shandong
Dragon Quality Goods Shop	Green Intelligent Power Saver	Tried it for ten days and the change is almost nothing, can't see the power saving yet, use it some more days and see how it goes.	Jingdong prefecture level city in Shandong
Dragon Quality Goods Shop	Green Intelligent Power Saver	Received very quickly, the quality is very good, really save electricity, business service attitude is also very good, I hope you do not miss this opportunity, I continue to buy a few in	Jingdong prefecture level city in Shandong
Dragon Quality Goods Shop	Green Intelligent Power Saver	Quality is very good Oh, the delivery is also very fast, a very favorite store, I want to crazy recommend to friends!	Jingdong prefecture level city in Shandong
Dragon Quality Goods Shop	Green Intelligent Power Saver	The goods have just received, fine workmanship, and the same as described by the merchant, to use a period of time to see if it can achieve the purpose of power saving.	Jingdong prefecture level city in Shandong
Dragon Quality Goods Shop	Green Intelligent Power Saver	Received the goods first use a period of time to see if there is no effect, the business commitment to ninety days invalid refund, first praise, good service!	Jingdong prefecture level city in Shandong
Dragon Quality Goods Shop	Green Intelligent Power Saver	The product is used for a month without energy-saving and power-saving effect does not have any effect	Jingdong prefecture level city in Shandong
Dragon Quality Goods Shop	Green Intelligent Power Saver	The baby is really great, I like it very much, my friends also say good to come, recommended to buy!	Jingdong prefecture level city in Shandong
Dragon Quality Goods Shop	Green Intelligent Power Saver	Logistics is fast, courier boy service is good, after using to see how the fruit, and then follow up.	Jingdong prefecture level city in Shandong
Dragon Quality Goods Shop	Green Intelligent Power Saver	Baby has been received to try, I believe that as the business advertised energy saving. The courier is very responsible.	Jingdong prefecture level city in Shandong
Dragon Quality Goods Shop	Green Intelligent Power Saver	Save Electric Energy King received, good quality, beautiful appearance, very satisfied!	Jingdong prefecture level city in Shandong
Dragon Quality Goods Shop	Green Intelligent Power Saver	Three no products no manufacturers no phone pure fraud induced consumption cheat	Jingdong prefecture level city in Shandong
Dragon Quality Goods Shop	Green Intelligent Power Saver	I'm using it now, I don't know if it's as good as it says, I'll follow up after a month	Jingdong prefecture level city in Shandong
Dragon Quality Goods Shop	Green Intelligent Power Saver	Good quality logistics and fast. Fine workmanship. The description is consistent cost-effective	Jingdong prefecture level city in Shandong
Dragon Quality Goods Shop	Green Intelligent Power Saver	Received, whether it is good or not, to wait a few days and then evaluate	Jingdong prefecture level city in Shandong
Dragon Quality Goods Shop	Green Intelligent Power Saver	I bought it and started using it, I won't know if it works well until I pay my electric bill	Jingdong prefecture level city in Shandong
Dragon Quality Goods Shop	Green Intelligent Power Saver	Especially good, energy saving and power saving, especially good quality, good	Jingdong prefecture level city in Shandong
Dragon Quality Goods Shop	Green Intelligent Power Saver	Logistics is strong, the quality is very good, paste really save electricity, as described, will buy back!	Jingdong prefecture level city in Shandong
Dragon Quality Goods Shop	Green Intelligent Power Saver	Great stuff at a great price, worth the money!	Jingdong prefecture level city in Shandong
Dragon Quality Goods Shop	Green Intelligent Power Saver	Received the power saver, very good quality, very power saving!	Jingdong prefecture level city in Shandong
Dragon Quality Goods Shop	Green Intelligent Power Saver	There are no fruits, only samples...	Jingdong prefecture level city in Shandong
Dragon Quality Goods Shop	Green Intelligent Power Saver	Save a few dollars a month.	Jingdong prefecture level city in Shandong
Dragon Quality Goods Shop	Green Intelligent Power Saver	The quality is particularly good, it works well, and the seller has a good service attitude	Jingdong prefecture level city in Shandong
Dragon Quality Goods Shop	Green Intelligent Power Saver	I've compared them a couple of times and I don't feel any difference	Jingdong prefecture level city in Shandong
Dragon Quality Goods Shop	Green Intelligent Power Saver	I don't see the savings. It's a sham.	Jingdong prefecture level city in Shandong
Dragon Quality Goods Shop	Green Intelligent Power Saver	The power saving effect is very good, and the logistics is very fast. The seller's service attitude is very good	Jingdong prefecture level city in Shandong
Dragon Quality Goods Shop	Green Intelligent Power Saver	Received, well packaged, haven't tried it yet!	Jingdong prefecture level city in Shandong
Dragon Quality Goods Shop	Green Intelligent Power Saver	I don't know how it works, I'll comment after I use it.	Jingdong prefecture level city in Shandong
Dragon Quality Goods Shop	Green Intelligent Power Saver	The product doesn't work well enough to be of any use	Jingdong prefecture level city in Shandong
Dragon Quality Goods Shop	Green Intelligent Power Saver	On Trial 。。。。。。。	Jingdong prefecture level city in Shandong
Dragon Quality Goods Shop	Green Intelligent Power Saver	Being used with unknown results	Jingdong prefecture level city in Shandong
Dragon Quality Goods Shop	Green Intelligent Power Saver	Smart power saving and great quality	Jingdong prefecture level city in Shandong
Dragon Quality Goods Shop	Green Intelligent Power Saver	The saver was received, it works very well and the price is affordable!	Jingdong prefecture level city in Shandong
Dragon Quality Goods Shop	Green Intelligent Power Saver	How can I feel the power saving and how long does it last	Jingdong prefecture level city in Shandong
Dragon Quality Goods Shop	Green Intelligent Power Saver	And I know if it saves electricity or not.	Jingdong prefecture level city in Shandong
Dragon Quality Goods Shop	Green Intelligent Power Saver	It won't save any electricity. No.	Jingdong prefecture level city in Shandong
Dragon Quality Goods Shop	Green Intelligent Power Saver	Invalid. Fake.	Jingdong prefecture level city in Shandong
Dragon Quality Goods Shop	Green Intelligent Power Saver	It's good!	Jingdong prefecture level city in Shandong
Dragon Quality Goods Shop	Green Intelligent Power Saver	have no effect	Jingdong prefecture level city in Shandong
Dragon Quality Goods Shop	Green Intelligent Power Saver	Not used.	Jingdong prefecture level city in Shandong
Dragon Quality Goods Shop	Green Intelligent Power Saver	There is no load range, packaging thrift, ten days also did not develop invoices, received the goods with a receipt, hand-written receipt also has no financial stamp, communication for nearly a week a moment to say that the electronic invoice, a moment to say that the paper invoice, said it is mailed to me, ten days also did not see anything, the invoice invoice can not be found to say that did not stay on file, single single number can not be found, said that the warehouse more than one to a one to find out, in addition to the perfunctory or perfunctory. Products are no certificate of conformity, proper three	Jingdong prefecture level city in Shandong
Dragon Quality Goods Shop	Green Intelligent Power Saver	Satisfaction.	Jingdong prefecture level city in Shandong

Dragon Quality Goods Shop	Green Intelligent Power Saver	There is not a bit of power saving effect, and ringing up an increase in the electricity bill, because you have to add the cost of this machine's own electricity consumption and purchase, so I think it is better to use this machine instead of increasing the expenditure, uneconomical, rather than not use it.	Jingdong prefecture level city in Shandong
Dragon Quality Goods Shop	Green Intelligent Power Saver	I've been using it for two days now and I don't feel any effect.	Jingdong prefecture level city in Shandong
Dragon Quality Goods Shop	Green Intelligent Power Saver	What it looked like was nothing like what was sent.	Jingdong prefecture level city in Shandong
Dragon Quality Goods Shop	Green Intelligent Power Saver	It didn't work. It didn't work for a month or so.	Jingdong prefecture level city in Shandong
Dragon Quality Goods Shop	Green Intelligent Power Saver	Garbage products, physical and description is not the same, no effect at all, pit # no discussion, to buy the fine, garbage, garbage, garbage	Jingdong prefecture level city in Shandong
Dragon Quality Goods Shop	Green Intelligent Power Saver	general	Jingdong prefecture level city in Shandong
Dragon Quality Goods Shop	Green Intelligent Power Saver	IQ tax. Instead of saving electricity, it increases the power consumption of an appliance.	Jingdong prefecture level city in Shandong
Dragon Quality Goods Shop	Green Intelligent Power Saver	There's no trash at all.	Jingdong prefecture level city in Shandong
Dragon Quality Goods Shop	Green Intelligent Power Saver	It doesn't do anything.	Jingdong prefecture level city in Shandong
Dragon Quality Goods Shop	Green Intelligent Power Saver	(of an unmarried couple) be close	Jingdong prefecture level city in Shandong
Dragon Quality Goods Shop	Green Intelligent Power Saver	(of an unmarried couple) be close	Jingdong prefecture level city in Shandong
Dragon Quality Goods Shop	Green Intelligent Power Saver	no electricity saving	Jingdong prefecture level city in Shandong
Dragon Quality Goods Shop	Green Intelligent Power Saver	differ from	Jingdong prefecture level city in Shandong
Dragon Quality Goods Shop	Green Intelligent Power Saver	Saver has received, but real can save electricity, the quality is very good, I times I buy a few more, too good.	Jingdong prefecture level city in Shandong
Dragon Quality Goods Shop	Green Intelligent Power Saver	Great quality as always, a very favorite product, great! o(*￣︶￣*)o	Jingdong prefecture level city in Shandong
Dragon Quality Goods Shop	Green Intelligent Power Saver	If we can reduce the electricity bill, for individuals can save money, for the country can protect the environment, now the weather is getting hotter and hotter, to protect the environment to reduce emissions is very important. Already use the product, whether to reduce the electricity bill depends on next month!	Jingdong prefecture level city in Shandong
Dragon Quality Goods Shop	Green Intelligent Power Saver	Purchased a power saver received on the put into use, rent a room with many electrical appliances, power consumption is very large, we have a piece of electricity or 1.2 dollars a time, with this power saver is much better, every day can save a few cents, very convenient and practical!	Jingdong prefecture level city in Shandong
Dragon Quality Goods Shop	Green Intelligent Power Saver	The product is better to use before not to use when a month 1500 yuan electricity bill, now using the 5 second month electricity bill directly down 470 dollars or very good. A save money to buy several power saver money ZanZanZanZan👍	Jingdong prefecture level city in Shandong
Dragon Quality Goods Shop	Green Intelligent Power Saver	The moment I received the goods really satisfied with this product, not only high-grade packaging, the body are base alloy, up to the price, I was also introduced by a friend, he used last month did save a lot of electricity, but also recommended to me!	Jingdong prefecture level city in Shandong
Dragon Quality Goods Shop	Green Intelligent Power Saver	I got it. Good review. I'm very satisfied. Reasonable price.	Jingdong prefecture level city in Shandong
Dragon Quality Goods Shop	Green Intelligent Power Saver	Intelligent power saver received and used, looking at the quality is good, the quality of the plug sent is also very good. Expect the effect of the economizer is obvious, first five-star praise, with a period of time to follow up!	Jingdong prefecture level city in Shandong
Dragon Quality Goods Shop	Green Intelligent Power Saver	The saver has been received. It was plugged into an outlet and used immediately after receipt. Whether it really saves electricity is not clear yet, I believe that this brand of energy saver really saves	Jingdong prefecture level city in Shandong
Dragon Quality Goods Shop	Green Intelligent Power Saver	Okay, okay, okay. Thank you so much. Fast is good. Fast. Item was as described, mainly because the event was cheap and you can save a lot of money.	Jingdong prefecture level city in Shandong
Dragon Quality Goods Shop	Green Intelligent Power Saver	Received it a couple days ago! Plugged it in the same day! Let's see what the electric bill is in a month! Hopefully it works well oh!	Jingdong prefecture level city in Shandong
Dragon Quality Goods Shop	Green Intelligent Power Saver	Looking at the reviews good, energy-saving small products, compact, the results after 2 months to see, because the electricity bill season payment	Jingdong prefecture level city in Shandong
Dragon Quality Goods Shop	Green Intelligent Power Saver	I just received it and will try it out to see if it works well. I'll comment later.	Jingdong prefecture level city in Shandong
Dragon Quality Goods Shop	Green Intelligent Power Saver	It looks a little small and not very aesthetically pleasing, I don't know how effective it will be until I use it	Jingdong prefecture level city in Shandong
Dragon Quality Goods Shop	Green Intelligent Power Saver	Not only to the point of being useless yet	Jingdong prefecture level city in Shandong
Dragon Quality Goods Shop	Green Intelligent Power Saver	Used it for a month, nothing works, the meter you turn very fast. This is not a scam, but also sold on Jingdong. To cheat a bunch of people?	Jingdong prefecture level city in Shandong
Dragon Quality Goods Shop	Green Intelligent Power Saver	Very good to use, every day can save a little electricity, really good under the sample down a month to save a few dozen dollars in electricity, good before I did not know that there is this 👆 haha, the price is not expensive praise.	Jingdong prefecture level city in Shandong
Dragon Quality Goods Shop	Green Intelligent Power Saver	Tried it for ten days and the change is almost nothing, can't see the power saving yet, use it some more days and see how it goes.	Jingdong prefecture level city in Shandong
Dragon Quality Goods Shop	Green Intelligent Power Saver	Received very quickly, the quality is very good, really save electricity, business service attitude is also very good, I hope you do not miss this opportunity, I continue to buy a few in	Jingdong prefecture level city in Shandong
Dragon Quality Goods Shop	Green Intelligent Power Saver	Quality is very good Oh, the delivery is also very fast, a very favorite store, I want to crazy recommend to friends!	Jingdong prefecture level city in Shandong
Dragon Quality Goods Shop	Green Intelligent Power Saver	The goods have just received, fine workmanship, and the same as described by the merchant, to use a period of time to see if it can achieve the purpose of power saving.	Jingdong prefecture level city in Shandong
Dragon Quality Goods Shop	Green Intelligent Power Saver	Received the goods first use a period of time to see if there is no effect, the business commitment to ninety days invalid refund, first praise, good service!	Jingdong prefecture level city in Shandong
Dragon Quality Goods Shop	Green Intelligent Power Saver	The product is used for a month without energy-saving and power-saving effect does not have any effect	Jingdong prefecture level city in Shandong
Dragon Quality Goods Shop	Green Intelligent Power Saver	The baby is really great, I like it very much, my friends also say good to come, recommended to buy!	Jingdong prefecture level city in Shandong
Dragon Quality Goods Shop	Green Intelligent Power Saver	Logistics is fast, courier boy service is good, after using to see how the fruit, and then follow up.	Jingdong prefecture level city in Shandong
Dragon Quality Goods Shop	Green Intelligent Power Saver	Baby has been received to try, I believe that as the business advertised energy saving. The courier is very responsible.	Jingdong prefecture level city in Shandong
Dragon Quality Goods Shop	Green Intelligent Power Saver	Save Electric Energy King received, good quality, beautiful appearance, very satisfied!	Jingdong prefecture level city in Shandong
Dragon Quality Goods Shop	Green Intelligent Power Saver	Three no products no manufacturers no phone pure fraud induced consumption cheat	Jingdong prefecture level city in Shandong
Dragon Quality Goods Shop	Green Intelligent Power Saver	I'm using it now, I don't know if it's as good as it says, I'll follow up after a month	Jingdong prefecture level city in Shandong
Dragon Quality Goods Shop	Green Intelligent Power Saver	Good quality logistics and fast. Fine workmanship. The description is consistent cost-effective	Jingdong prefecture level city in Shandong
Dragon Quality Goods Shop	Green Intelligent Power Saver	Received, whether it is good or not, to wait a few days and then evaluate	Jingdong prefecture level city in Shandong
Dragon Quality Goods Shop	Green Intelligent Power Saver	I bought it and started using it, I won't know if it works well until I pay my electric bill	Jingdong prefecture level city in Shandong
Dragon Quality Goods Shop	Green Intelligent Power Saver	Especially good, energy saving and power saving, especially good quality, good	Jingdong prefecture level city in Shandong
Dragon Quality Goods Shop	Green Intelligent Power Saver	Logistics is strong, the quality is very good, paste really save electricity, as described, will buy back!	Jingdong prefecture level city in Shandong
Dragon Quality Goods Shop	Green Intelligent Power Saver	Great stuff at a great price, worth the money!	Jingdong prefecture level city in Shandong

Dragon Quality Goods Shop	Green Intelligent Power Saver	Received the power saver, very good quality, very power saving!	Jingdong prefecture level city in Shandong
Dragon Quality Goods Shop	Green Intelligent Power Saver	There are no fruits, only samples...	Jingdong prefecture level city in Shandong
Dragon Quality Goods Shop	Green Intelligent Power Saver	Save a few dollars a month.	Jingdong prefecture level city in Shandong
Dragon Quality Goods Shop	Green Intelligent Power Saver	The quality is particularly good, it works well, and the seller has a good service attitude	Jingdong prefecture level city in Shandong
Dragon Quality Goods Shop	Green Intelligent Power Saver	I've compared them a couple of times and I don't feel any difference	Jingdong prefecture level city in Shandong
Dragon Quality Goods Shop	Green Intelligent Power Saver	I don't see the savings. It's a sham.	Jingdong prefecture level city in Shandong
Dragon Quality Goods Shop	Green Intelligent Power Saver	The power saving effect is very good, and the logistics is very fast. The seller's service attitude is very good	Jingdong prefecture level city in Shandong
Dragon Quality Goods Shop	Green Intelligent Power Saver	Received, well packaged, haven't tried it yet!	Jingdong prefecture level city in Shandong
Dragon Quality Goods Shop	Green Intelligent Power Saver	I don't know how it works, I'll comment after I use it.	Jingdong prefecture level city in Shandong
Dragon Quality Goods Shop	Green Intelligent Power Saver	The product doesn't work well enough to be of any use	Jingdong prefecture level city in Shandong
Dragon Quality Goods Shop	Green Intelligent Power Saver	On Trial 。。。。。。。	Jingdong prefecture level city in Shandong
Dragon Quality Goods Shop	Green Intelligent Power Saver	Being used with unknown results	Jingdong prefecture level city in Shandong
Dragon Quality Goods Shop	Green Intelligent Power Saver	Smart power saving and great quality	Jingdong prefecture level city in Shandong
Dragon Quality Goods Shop	Green Intelligent Power Saver	The saver was received, it works very well and the price is affordable!	Jingdong prefecture level city in Shandong
Dragon Quality Goods Shop	Green Intelligent Power Saver	How can I feel the power saving and how long does it last	Jingdong prefecture level city in Shandong
Dragon Quality Goods Shop	Green Intelligent Power Saver	And I know if it saves electricity or not.	Jingdong prefecture level city in Shandong
Dragon Quality Goods Shop	Green Intelligent Power Saver	It won't save any electricity. No.	Jingdong prefecture level city in Shandong
Dragon Quality Goods Shop	Green Intelligent Power Saver	Invalid. Fake.	Jingdong prefecture level city in Shandong
Dragon Quality Goods Shop	Green Intelligent Power Saver	It's good!	Jingdong prefecture level city in Shandong
Dragon Quality Goods Shop	Green Intelligent Power Saver	have no effect	Jingdong prefecture level city in Shandong
Dragon Quality Goods Shop	Green Intelligent Power Saver	Not used.	Jingdong prefecture level city in Shandong
Dragon Quality Goods Shop	Green Intelligent Power Saver	There is no load range, packaging thrift, ten days also did not develop invoices, received the goods with a receipt, hand-written receipt also has no financial stamp, communication for nearly a week a moment to say that the electronic invoice, a moment to say that the paper invoice, said it is mailed to me, ten days also did not see anything, the invoice invoice can not be found to say that did not stay on file, single single number can not be found, said that the warehouse more than one to a one to find out, in addition to the perfunctory or perfunctory. Products are no certificate of conformity, proper three	Jingdong prefecture level city in Shandong
Dragon Quality Goods Shop	Green Intelligent Power Saver	Satisfaction.	Jingdong prefecture level city in Shandong
Dragon Quality Goods Shop	Green Intelligent Power Saver	There is not a bit of power saving effect, and ringing up an increase in the electricity bill, because you have to add the cost of this machine's own electricity consumption and purchase, so I think it is better to use this machine instead of increasing the expenditure, uneconomical, rather than not use it.	Jingdong prefecture level city in Shandong
Dragon Quality Goods Shop	Green Intelligent Power Saver	I've been using it for two days now and I don't feel any effect.	Jingdong prefecture level city in Shandong
Dragon Quality Goods Shop	Green Intelligent Power Saver	What it looked like was nothing like what was sent.	Jingdong prefecture level city in Shandong
Dragon Quality Goods Shop	Green Intelligent Power Saver	It didn't work. It didn't work for a month or so.	Jingdong prefecture level city in Shandong
Dragon Quality Goods Shop	Green Intelligent Power Saver	Garbage products, physical and description is not the same, no effect at all, pit # no discussion, to buy the fine, garbage, garbage, garbage	Jingdong prefecture level city in Shandong
Dragon Quality Goods Shop	Green Intelligent Power Saver	general	Jingdong prefecture level city in Shandong
Dragon Quality Goods Shop	Green Intelligent Power Saver	IQ tax. Instead of saving electricity, it increases the power consumption of an appliance.	Jingdong prefecture level city in Shandong
Dragon Quality Goods Shop	Green Intelligent Power Saver	There's no trash at all.	Jingdong prefecture level city in Shandong
Dragon Quality Goods Shop	Green Intelligent Power Saver	It doesn't do anything.	Jingdong prefecture level city in Shandong
Dragon Quality Goods Shop	Green Intelligent Power Saver	(of an unmarried couple) be close	Jingdong prefecture level city in Shandong
Dragon Quality Goods Shop	Green Intelligent Power Saver	(of an unmarried couple) be close	Jingdong prefecture level city in Shandong
Dragon Quality Goods Shop	Green Intelligent Power Saver	no electricity saving	Jingdong prefecture level city in Shandong
Dragon Quality Goods Shop	Green Intelligent Power Saver	differ from	Jingdong prefecture level city in Shandong
Dragon Quality Goods Shop	Green Intelligent Power Saver	Saver has received, but real can save electricity, the quality is very good, I times I buy a few more, too good.	Jingdong prefecture level city in Shandong
Dragon Quality Goods Shop	Green Intelligent Power Saver	Great quality as always, a very favorite product, great! o(*￣︶￣*)o	Jingdong prefecture level city in Shandong
Dragon Quality Goods Shop	Green Intelligent Power Saver	If we can reduce the electricity bill, for individuals can save money, for the country can protect the environment, now the weather is getting hotter and hotter, to protect the environment to reduce emissions is very important. Already use the product, whether to reduce the electricity bill depends on next month!	Jingdong prefecture level city in Shandong
Dragon Quality Goods Shop	Green Intelligent Power Saver	Purchased a power saver received on the put into use, rent a room with many electrical appliances, power consumption is very large, we have a piece of electricity or 1.2 dollars a time, with this power saver is much better, every day can save a few cents, very convenient and practical!	Jingdong prefecture level city in Shandong
Dragon Quality Goods Shop	Green Intelligent Power Saver	The product is better to use before not to use when a month 1500 yuan electricity bill, now using the 5 second month electricity bill directly down 470 dollars or very good. A save money to buy several power saver money ZanZanZanZan👍	Jingdong prefecture level city in Shandong
Dragon Quality Goods Shop	Green Intelligent Power Saver	The moment I received the goods really satisfied with this product, not only high-grade packaging, the body are base alloy, up to the price, I was also introduced by a friend, he used last month did save a lot of electricity, but also recommended to me!	Jingdong prefecture level city in Shandong
Dragon Quality Goods Shop	Green Intelligent Power Saver	I got it. Good review. I'm very satisfied. Reasonable price.	Jingdong prefecture level city in Shandong
Dragon Quality Goods Shop	Green Intelligent Power Saver	Intelligent power saver received and used, looking at the quality is good, the quality of the plug sent is also very good. Expect the effect of the economizer is obvious, first five-star praise, with a period of time to follow up!	Jingdong prefecture level city in Shandong
Dragon Quality Goods Shop	Green Intelligent Power Saver	The saver has been received. It was plugged into an outlet and used immediately after receipt. Whether it really saves electricity is not clear yet, I believe that this brand of energy saver really saves	Jingdong prefecture level city in Shandong
Dragon Quality Goods Shop	Green Intelligent Power Saver	Okay, okay, okay. Thank you so much. Fast is good. Fast. Item was as described, mainly because the event was cheap and you can save a lot of money.	Jingdong prefecture level city in Shandong

Dragon Quality Goods Shop	Green Intelligent Power Saver	Received it a couple days ago! Plugged it in the same day! Let's see what the electric bill is in a month! Hopefully it works well oh!	Jingdong prefecture level city in Shandong
Dragon Quality Goods Shop	Green Intelligent Power Saver	Looking at the reviews good, energy-saving small products, compact, the results after 2 months to see, because the electricity bill season payment	Jingdong prefecture level city in Shandong
Dragon Quality Goods Shop	Green Intelligent Power Saver	I just received it and will try it out to see if it works well. I'll comment later.	Jingdong prefecture level city in Shandong
Dragon Quality Goods Shop	Green Intelligent Power Saver	It looks a little small and not very aesthetically pleasing, I don't know how effective it will be until I use it	Jingdong prefecture level city in Shandong
Dragon Quality Goods Shop	Green Intelligent Power Saver	Not only to the point of being useless yet	Jingdong prefecture level city in Shandong
Dragon Quality Goods Shop	Green Intelligent Power Saver	Used it for a month, nothing works, the meter you turn very fast. This is not a scam, but also sold on Jingdong. To cheat a bunch of people?	Jingdong prefecture level city in Shandong
Dragon Quality Goods Shop	Green Intelligent Power Saver	Very good to use, every day can save a little electricity, really good under the sample down a month to save a few dozen dollars in electricity, good before I did not know that there is this ☝ haha, the price is not expensive praise.	Jingdong prefecture level city in Shandong
Dragon Quality Goods Shop	Green Intelligent Power Saver	Tried it for ten days and the change is almost nothing, can't see the power saving yet, use it some more days and see how it goes.	Jingdong prefecture level city in Shandong
Dragon Quality Goods Shop	Green Intelligent Power Saver	Received very quickly, the quality is very good, really save electricity, business service attitude is also very good, I hope you do not miss this opportunity, I continue to buy a few in	Jingdong prefecture level city in Shandong
Dragon Quality Goods Shop	Green Intelligent Power Saver	Quality is very good Oh, the delivery is also very fast, a very favorite store, I want to crazy recommend to friends!	Jingdong prefecture level city in Shandong
Dragon Quality Goods Shop	Green Intelligent Power Saver	The goods have just received, fine workmanship, and the same as described by the merchant, to use a period of time to see if it can achieve the purpose of power saving.	Jingdong prefecture level city in Shandong
Dragon Quality Goods Shop	Green Intelligent Power Saver	Received the goods first use a period of time to see if there is no effect, the business commitment to ninety days invalid refund, first praise, good service!	Jingdong prefecture level city in Shandong
Dragon Quality Goods Shop	Green Intelligent Power Saver	The product is used for a month without energy-saving and power-saving effect does not have any effect	Jingdong prefecture level city in Shandong
Dragon Quality Goods Shop	Green Intelligent Power Saver	The baby is really great, I like it very much, my friends also say good to come, recommended to buy!	Jingdong prefecture level city in Shandong
Dragon Quality Goods Shop	Green Intelligent Power Saver	Logistics is fast, courier boy service is good, after using to see how the fruit, and then follow up.	Jingdong prefecture level city in Shandong
Dragon Quality Goods Shop	Green Intelligent Power Saver	Baby has been received to try, I believe that as the business advertised energy saving. The courier is very responsible.	Jingdong prefecture level city in Shandong
Dragon Quality Goods Shop	Green Intelligent Power Saver	Save Electric Energy King received, good quality, beautiful appearance, very satisfied!	Jingdong prefecture level city in Shandong
Dragon Quality Goods Shop	Green Intelligent Power Saver	Three no products no manufacturers no phone pure fraud induced consumption cheat	Jingdong prefecture level city in Shandong
Dragon Quality Goods Shop	Green Intelligent Power Saver	I'm using it now, I don't know if it's as good as it says, I'll follow up after a month	Jingdong prefecture level city in Shandong
Dragon Quality Goods Shop	Green Intelligent Power Saver	Good quality logistics and fast. Fine workmanship. The description is consistent cost-effective	Jingdong prefecture level city in Shandong
Dragon Quality Goods Shop	Green Intelligent Power Saver	Received, whether it is good or not, to wait a few days and then evaluate	Jingdong prefecture level city in Shandong
Dragon Quality Goods Shop	Green Intelligent Power Saver	I bought it and started using it, I won't know if it works well until I pay my electric bill	Jingdong prefecture level city in Shandong
Dragon Quality Goods Shop	Green Intelligent Power Saver	Especially good, energy saving and power saving, especially good quality, good	Jingdong prefecture level city in Shandong
Dragon Quality Goods Shop	Green Intelligent Power Saver	Logistics is strong, the quality is very good, paste really save electricity, as described, will buy back!	Jingdong prefecture level city in Shandong
Dragon Quality Goods Shop	Green Intelligent Power Saver	Great stuff at a great price, worth the money!	Jingdong prefecture level city in Shandong
Dragon Quality Goods Shop	Green Intelligent Power Saver	Received the power saver, very good quality, very power saving!	Jingdong prefecture level city in Shandong
Dragon Quality Goods Shop	Green Intelligent Power Saver	There are no fruits, only samples...	Jingdong prefecture level city in Shandong
Dragon Quality Goods Shop	Green Intelligent Power Saver	Save a few dollars a month.	Jingdong prefecture level city in Shandong
Dragon Quality Goods Shop	Green Intelligent Power Saver	The quality is particularly good, it works well, and the seller has a good service attitude	Jingdong prefecture level city in Shandong
Dragon Quality Goods Shop	Green Intelligent Power Saver	I've compared them a couple of times and I don't feel any difference	Jingdong prefecture level city in Shandong
Dragon Quality Goods Shop	Green Intelligent Power Saver	I don't see the savings. It's a sham.	Jingdong prefecture level city in Shandong
Dragon Quality Goods Shop	Green Intelligent Power Saver	The power saving effect is very good, and the logistics is very fast. The seller's service attitude is very good	Jingdong prefecture level city in Shandong
Dragon Quality Goods Shop	Green Intelligent Power Saver	Received, well packaged, haven't tried it yet!	Jingdong prefecture level city in Shandong
Dragon Quality Goods Shop	Green Intelligent Power Saver	I don't know how it works, I'll comment after I use it.	Jingdong prefecture level city in Shandong
Dragon Quality Goods Shop	Green Intelligent Power Saver	The product doesn't work well enough to be of any use	Jingdong prefecture level city in Shandong
Dragon Quality Goods Shop	Green Intelligent Power Saver	On Trial 。。。。。。。	Jingdong prefecture level city in Shandong
Dragon Quality Goods Shop	Green Intelligent Power Saver	Being used with unknown results	Jingdong prefecture level city in Shandong
Dragon Quality Goods Shop	Green Intelligent Power Saver	Smart power saving and great quality	Jingdong prefecture level city in Shandong
Dragon Quality Goods Shop	Green Intelligent Power Saver	The saver was received, it works very well and the price is affordable!	Jingdong prefecture level city in Shandong
Dragon Quality Goods Shop	Green Intelligent Power Saver	How can I feel the power saving and how long does it last	Jingdong prefecture level city in Shandong
Dragon Quality Goods Shop	Green Intelligent Power Saver	And I know if it saves electricity or not.	Jingdong prefecture level city in Shandong
Dragon Quality Goods Shop	Green Intelligent Power Saver	It won't save any electricity. No.	Jingdong prefecture level city in Shandong
Dragon Quality Goods Shop	Green Intelligent Power Saver	Invalid. Fake.	Jingdong prefecture level city in Shandong
Dragon Quality Goods Shop	Green Intelligent Power Saver	It's good!	Jingdong prefecture level city in Shandong
Dragon Quality Goods Shop	Green Intelligent Power Saver	have no effect	Jingdong prefecture level city in Shandong
Dragon Quality Goods Shop	Green Intelligent Power Saver	Not used.	Jingdong prefecture level city in Shandong
Dragon Quality Goods Shop	Green Intelligent Power Saver	There is no load range, packaging thrift, ten days also did not develop invoices, received the goods with a receipt, hand-written receipt also has no financial stamp, communication for nearly a week a moment to say that the electronic invoice, a moment to say that the paper invoice, said it is mailed to me, ten days also did not see anything, the invoice invoice can not be found to say that did not stay on file, single single number can not be found, said that the warehouse more than one to a one to find out, in addition to the perfunctory or perfunctory. Products are no certificate of conformity, proper three	Jingdong prefecture level city in Shandong
Dragon Quality Goods Shop	Green Intelligent Power Saver	Satisfaction.	Jingdong prefecture level city in Shandong

Dragon Quality Goods Shop	Green Intelligent Power Saver	There is not a bit of power saving effect, and ringing up an increase in the electricity bill, because you have to add the cost of this machine's own electricity consumption and purchase, so I think it is better to use this machine instead of increasing the expenditure, uneconomical, rather than not use it.	Jingdong prefecture level city in Shandong
Dragon Quality Goods Shop	Green Intelligent Power Saver	I've been using it for two days now and I don't feel any effect.	Jingdong prefecture level city in Shandong
Dragon Quality Goods Shop	Green Intelligent Power Saver	What it looked like was nothing like what was sent.	Jingdong prefecture level city in Shandong
Dragon Quality Goods Shop	Green Intelligent Power Saver	It didn't work. It didn't work for a month or so.	Jingdong prefecture level city in Shandong
Dragon Quality Goods Shop	Green Intelligent Power Saver	Garbage products, physical and description is not the same, no effect at all, pit # no discussion, to buy the fine, garbage, garbage, garbage	Jingdong prefecture level city in Shandong
Dragon Quality Goods Shop	Green Intelligent Power Saver	general	Jingdong prefecture level city in Shandong
Dragon Quality Goods Shop	Green Intelligent Power Saver	IQ tax. Instead of saving electricity, it increases the power consumption of an appliance.	Jingdong prefecture level city in Shandong
Dragon Quality Goods Shop	Green Intelligent Power Saver	There's no trash at all.	Jingdong prefecture level city in Shandong
Dragon Quality Goods Shop	Green Intelligent Power Saver	It doesn't do anything.	Jingdong prefecture level city in Shandong
Dragon Quality Goods Shop	Green Intelligent Power Saver	(of an unmarried couple) be close	Jingdong prefecture level city in Shandong
Dragon Quality Goods Shop	Green Intelligent Power Saver	(of an unmarried couple) be close	Jingdong prefecture level city in Shandong
Dragon Quality Goods Shop	Green Intelligent Power Saver	no electricity saving	Jingdong prefecture level city in Shandong
Dragon Quality Goods Shop	Green Intelligent Power Saver	differ from	Jingdong prefecture level city in Shandong
Dragon Quality Goods Shop	Green Intelligent Power Saver	Saver has received, but real can save electricity, the quality is very good, I times I buy a few more, too good.	Jingdong prefecture level city in Shandong
Dragon Quality Goods Shop	Green Intelligent Power Saver	Great quality as always, a very favorite product, great! o(*￣︶￣*)o	Jingdong prefecture level city in Shandong
Dragon Quality Goods Shop	Green Intelligent Power Saver	If we can reduce the electricity bill, for individuals can save money, for the country can protect the environment, now the weather is getting hotter and hotter, to protect the environment to reduce emissions is very important. Already use the product, whether to reduce the electricity bill depends on next month!	Jingdong prefecture level city in Shandong
Dragon Quality Goods Shop	Green Intelligent Power Saver	Purchased a power saver received on the put into use, rent a room with many electrical appliances, power consumption is very large, we have a piece of electricity or 1.2 dollars a time, with this power saver is much better, every day can save a few cents, very convenient and practical!	Jingdong prefecture level city in Shandong
Dragon Quality Goods Shop	Green Intelligent Power Saver	The product is better to use before not to use when a month 1500 yuan electricity bill, now using the 5 second month electricity bill directly down 470 dollars or very good. A save money to buy several power saver money ZanZanZanZan👍	Jingdong prefecture level city in Shandong
Dragon Quality Goods Shop	Green Intelligent Power Saver	The moment I received the goods really satisfied with this product, not only high-grade packaging, the body are base alloy, up to the price, I was also introduced by a friend, he used last month did save a lot of electricity, but also recommended to me!	Jingdong prefecture level city in Shandong
Dragon Quality Goods Shop	Green Intelligent Power Saver	I got it. Good review. I'm very satisfied. Reasonable price.	Jingdong prefecture level city in Shandong
Dragon Quality Goods Shop	Green Intelligent Power Saver	Intelligent power saver received and used, looking at the quality is good, the quality of the plug sent is also very good. Expect the effect of the economizer is obvious, first five-star praise, with a period of time to follow up!	Jingdong prefecture level city in Shandong
Dragon Quality Goods Shop	Green Intelligent Power Saver	The saver has been received. It was plugged into an outlet and used immediately after receipt. Whether it really saves electricity is not clear yet, I believe that this brand of energy saver really saves	Jingdong prefecture level city in Shandong
Dragon Quality Goods Shop	Green Intelligent Power Saver	Okay, okay, okay. Thank you so much. Fast is good. Fast. Item was as described, mainly because the event was cheap and you can save a lot of money.	Jingdong prefecture level city in Shandong
Dragon Quality Goods Shop	Green Intelligent Power Saver	Received it a couple days ago! Plugged it in the same day! Let's see what the electric bill is in a month! Hopefully it works well oh!	Jingdong prefecture level city in Shandong
Dragon Quality Goods Shop	Green Intelligent Power Saver	Looking at the reviews good, energy-saving small products, compact, the results after 2 months to see, because the electricity bill season payment	Jingdong prefecture level city in Shandong
Dragon Quality Goods Shop	Green Intelligent Power Saver	I just received it and will try it out to see if it works well. I'll comment later.	Jingdong prefecture level city in Shandong
Dragon Quality Goods Shop	Green Intelligent Power Saver	It looks a little small and not very aesthetically pleasing, I don't know how effective it will be until I use it	Jingdong prefecture level city in Shandong
Dragon Quality Goods Shop	Green Intelligent Power Saver	Not only to the point of being useless yet	Jingdong prefecture level city in Shandong
Dragon Quality Goods Shop	Green Intelligent Power Saver	Used it for a month, nothing works, the meter you turn very fast. This is not a scam, but also sold on Jingdong. To cheat a bunch of people?	Jingdong prefecture level city in Shandong
Dragon Quality Goods Shop	Green Intelligent Power Saver	Very good to use, every day can save a little electricity, really good under the sample down a month to save a few dozen dollars in electricity, good before I did not know that there is this 👆 haha, the price is not expensive praise.	Jingdong prefecture level city in Shandong
Dragon Quality Goods Shop	Green Intelligent Power Saver	Tried it for ten days and the change is almost nothing, can't see the power saving yet, use it some more days and see how it goes.	Jingdong prefecture level city in Shandong
Dragon Quality Goods Shop	Green Intelligent Power Saver	Received very quickly, the quality is very good, really save electricity, business service attitude is also very good, I hope you do not miss this opportunity, I continue to buy a few in	Jingdong prefecture level city in Shandong
Dragon Quality Goods Shop	Green Intelligent Power Saver	Quality is very good Oh, the delivery is also very fast, a very favorite store, I want to crazy recommend to friends!	Jingdong prefecture level city in Shandong
Dragon Quality Goods Shop	Green Intelligent Power Saver	The goods have just received, fine workmanship, and the same as described by the merchant, to use a period of time to see if it can achieve the purpose of power saving.	Jingdong prefecture level city in Shandong
Dragon Quality Goods Shop	Green Intelligent Power Saver	Received the goods first use a period of time to see if there is no effect, the business commitment to ninety days invalid refund, first praise, good service!	Jingdong prefecture level city in Shandong
Dragon Quality Goods Shop	Green Intelligent Power Saver	The product is used for a month without energy-saving and power-saving effect does not have any effect	Jingdong prefecture level city in Shandong
Dragon Quality Goods Shop	Green Intelligent Power Saver	The baby is really great, I like it very much, my friends also say good to come, recommended to buy!	Jingdong prefecture level city in Shandong
Dragon Quality Goods Shop	Green Intelligent Power Saver	Logistics is fast, courier boy service is good, after using to see how the fruit, and then follow up.	Jingdong prefecture level city in Shandong
Dragon Quality Goods Shop	Green Intelligent Power Saver	Baby has been received to try, I believe that as the business advertised energy saving. The courier is very responsible.	Jingdong prefecture level city in Shandong
Dragon Quality Goods Shop	Green Intelligent Power Saver	Save Electric Energy King received, good quality, beautiful appearance, very satisfied!	Jingdong prefecture level city in Shandong
Dragon Quality Goods Shop	Green Intelligent Power Saver	Three no products no manufacturers no phone pure fraud induced consumption cheat	Jingdong prefecture level city in Shandong
Dragon Quality Goods Shop	Green Intelligent Power Saver	I'm using it now, I don't know if it's as good as it says, I'll follow up after a month	Jingdong prefecture level city in Shandong
Dragon Quality Goods Shop	Green Intelligent Power Saver	Good quality logistics and fast. Fine workmanship. The description is consistent cost-effective	Jingdong prefecture level city in Shandong
Dragon Quality Goods Shop	Green Intelligent Power Saver	Received, whether it is good or not, to wait a few days and then evaluate	Jingdong prefecture level city in Shandong
Dragon Quality Goods Shop	Green Intelligent Power Saver	I bought it and started using it, I won't know if it works well until I pay my electric bill	Jingdong prefecture level city in Shandong
Dragon Quality Goods Shop	Green Intelligent Power Saver	Especially good, energy saving and power saving, especially good quality, good	Jingdong prefecture level city in Shandong
Dragon Quality Goods Shop	Green Intelligent Power Saver	Logistics is strong, the quality is very good, paste really save electricity, as described, will buy back!	Jingdong prefecture level city in Shandong
Dragon Quality Goods Shop	Green Intelligent Power Saver	Great stuff at a great price, worth the money!	Jingdong prefecture level city in Shandong

Dragon Quality Goods Shop	Green Intelligent Power Saver	Received the power saver, very good quality, very power saving!	Jingdong prefecture level city in Shandong
Dragon Quality Goods Shop	Green Intelligent Power Saver	There are no fruits, only samples...	Jingdong prefecture level city in Shandong
Dragon Quality Goods Shop	Green Intelligent Power Saver	Save a few dollars a month.	Jingdong prefecture level city in Shandong
Dragon Quality Goods Shop	Green Intelligent Power Saver	The quality is particularly good, it works well, and the seller has a good service attitude	Jingdong prefecture level city in Shandong
Dragon Quality Goods Shop	Green Intelligent Power Saver	I've compared them a couple of times and I don't feel any difference	Jingdong prefecture level city in Shandong
Dragon Quality Goods Shop	Green Intelligent Power Saver	I don't see the savings. It's a sham.	Jingdong prefecture level city in Shandong
Dragon Quality Goods Shop	Green Intelligent Power Saver	The power saving effect is very good, and the logistics is very fast. The seller's service attitude is very good	Jingdong prefecture level city in Shandong
Dragon Quality Goods Shop	Green Intelligent Power Saver	Received, well packaged, haven't tried it yet!	Jingdong prefecture level city in Shandong
Dragon Quality Goods Shop	Green Intelligent Power Saver	I don't know how it works, I'll comment after I use it.	Jingdong prefecture level city in Shandong
Dragon Quality Goods Shop	Green Intelligent Power Saver	The product doesn't work well enough to be of any use	Jingdong prefecture level city in Shandong
Dragon Quality Goods Shop	Green Intelligent Power Saver	On Trial 。。。。。。。	Jingdong prefecture level city in Shandong
Dragon Quality Goods Shop	Green Intelligent Power Saver	Being used with unknown results	Jingdong prefecture level city in Shandong
Dragon Quality Goods Shop	Green Intelligent Power Saver	Smart power saving and great quality	Jingdong prefecture level city in Shandong
Dragon Quality Goods Shop	Green Intelligent Power Saver	The saver was received, it works very well and the price is affordable!	Jingdong prefecture level city in Shandong
Dragon Quality Goods Shop	Green Intelligent Power Saver	How can I feel the power saving and how long does it last	Jingdong prefecture level city in Shandong
Dragon Quality Goods Shop	Green Intelligent Power Saver	And I know if it saves electricity or not.	Jingdong prefecture level city in Shandong
Dragon Quality Goods Shop	Green Intelligent Power Saver	It won't save any electricity. No.	Jingdong prefecture level city in Shandong
Dragon Quality Goods Shop	Green Intelligent Power Saver	Invalid. Fake.	Jingdong prefecture level city in Shandong
Dragon Quality Goods Shop	Green Intelligent Power Saver	It's good!	Jingdong prefecture level city in Shandong
Dragon Quality Goods Shop	Green Intelligent Power Saver	have no effect	Jingdong prefecture level city in Shandong
Dragon Quality Goods Shop	Green Intelligent Power Saver	Not used.	Jingdong prefecture level city in Shandong
Dragon Quality Goods Shop	Green Intelligent Power Saver	There is no load range, packaging thrift, ten days also did not develop invoices, received the goods with a receipt, hand-written receipt also has no financial stamp, communication for nearly a week a moment to say that the electronic invoice, a moment to say that the paper invoice, said it is mailed to me, ten days also did not see anything, the invoice invoice can not be found to say that did not stay on file, single single number can not be found, said that the warehouse more than one to a one to find out, in addition to the perfunctory or perfunctory. Products are no certificate of conformity, proper three	Jingdong prefecture level city in Shandong
Dragon Quality Goods Shop	Green Intelligent Power Saver	Satisfaction.	Jingdong prefecture level city in Shandong
Dragon Quality Goods Shop	Green Intelligent Power Saver	There is not a bit of power saving effect, and ringing up an increase in the electricity bill, because you have to add the cost of this machine's own electricity consumption and purchase, so I think it is better to use this machine instead of increasing the expenditure, uneconomical, rather than not use it.	Jingdong prefecture level city in Shandong
Dragon Quality Goods Shop	Green Intelligent Power Saver	I've been using it for two days now and I don't feel any effect.	Jingdong prefecture level city in Shandong
Dragon Quality Goods Shop	Green Intelligent Power Saver	What it looked like was nothing like what was sent.	Jingdong prefecture level city in Shandong
Dragon Quality Goods Shop	Green Intelligent Power Saver	It didn't work. It didn't work for a month or so.	Jingdong prefecture level city in Shandong
Dragon Quality Goods Shop	Green Intelligent Power Saver	Garbage products, physical and description is not the same, no effect at all, pit # no discussion, to buy the fine, garbage, garbage, garbage	Jingdong prefecture level city in Shandong
Dragon Quality Goods Shop	Green Intelligent Power Saver	general	Jingdong prefecture level city in Shandong
Dragon Quality Goods Shop	Green Intelligent Power Saver	IQ tax. Instead of saving electricity, it increases the power consumption of an appliance.	Jingdong prefecture level city in Shandong
Dragon Quality Goods Shop	Green Intelligent Power Saver	There's no trash at all.	Jingdong prefecture level city in Shandong
Dragon Quality Goods Shop	Green Intelligent Power Saver	It doesn't do anything.	Jingdong prefecture level city in Shandong
Dragon Quality Goods Shop	Green Intelligent Power Saver	(of an unmarried couple) be close	Jingdong prefecture level city in Shandong
Dragon Quality Goods Shop	Green Intelligent Power Saver	(of an unmarried couple) be close	Jingdong prefecture level city in Shandong
Dragon Quality Goods Shop	Green Intelligent Power Saver	no electricity saving	Jingdong prefecture level city in Shandong
Dragon Quality Goods Shop	Green Intelligent Power Saver	differ from	Jingdong prefecture level city in Shandong
Dragon Quality Goods Shop	Green Intelligent Power Saver	Saver has received, but real can save electricity, the quality is very good, I times I buy a few more, too good.	Jingdong prefecture level city in Shandong
Dragon Quality Goods Shop	Green Intelligent Power Saver	Great quality as always, a very favorite product, great! o(*￣︶￣*)o	Jingdong prefecture level city in Shandong
Dragon Quality Goods Shop	Green Intelligent Power Saver	If we can reduce the electricity bill, for individuals can save money, for the country can protect the environment, now the weather is getting hotter and hotter, to protect the environment to reduce emissions is very important. Already use the product, whether to reduce the electricity bill depends on next month!	Jingdong prefecture level city in Shandong
Dragon Quality Goods Shop	Green Intelligent Power Saver	Purchased a power saver received on the put into use, rent a room with many electrical appliances, power consumption is very large, we have a piece of electricity or 1.2 dollars a time, with this power saver is much better, every day can save a few cents, very convenient and practical!	Jingdong prefecture level city in Shandong
Dragon Quality Goods Shop	Green Intelligent Power Saver	The product is better to use before not to use when a month 1500 yuan electricity bill, now using the 5 second month electricity bill directly down 470 dollars or very good. A save money to buy several power saver money ZanZanZanZan👍	Jingdong prefecture level city in Shandong
Dragon Quality Goods Shop	Green Intelligent Power Saver	The moment I received the goods really satisfied with this product, not only high-grade packaging, the body are base alloy, up to the price, I was also introduced by a friend, he used last month did save a lot of electricity, but also recommended to me!	Jingdong prefecture level city in Shandong
Dragon Quality Goods Shop	Green Intelligent Power Saver	I got it. Good review. I'm very satisfied. Reasonable price.	Jingdong prefecture level city in Shandong
Dragon Quality Goods Shop	Green Intelligent Power Saver	Intelligent power saver received and used, looking at the quality is good, the quality of the plug sent is also very good. Expect the effect of the economizer is obvious, first five-star praise, with a period of time to follow up!	Jingdong prefecture level city in Shandong
Dragon Quality Goods Shop	Green Intelligent Power Saver	The saver has been received. It was plugged into an outlet and used immediately after receipt. Whether it really saves electricity is not clear yet, I believe that this brand of energy saver really saves	Jingdong prefecture level city in Shandong
Dragon Quality Goods Shop	Green Intelligent Power Saver	Okay, okay, okay. Thank you so much. Fast is good. Fast. Item was as described, mainly because the event was cheap and you can save a lot of money.	Jingdong prefecture level city in Shandong

Dragon Quality Goods Shop	Green Intelligent Power Saver	Received it a couple days ago! Plugged it in the same day! Let's see what the electric bill is in a month! Hopefully it works well oh!	Jingdong prefecture level city in Shandong
Dragon Quality Goods Shop	Green Intelligent Power Saver	Looking at the reviews good, energy-saving small products, compact, the results after 2 months to see, because the electricity bill season payment	Jingdong prefecture level city in Shandong
Dragon Quality Goods Shop	Green Intelligent Power Saver	I just received it and will try it out to see if it works well. I'll comment later.	Jingdong prefecture level city in Shandong
Dragon Quality Goods Shop	Green Intelligent Power Saver	It looks a little small and not very aesthetically pleasing, I don't know how effective it will be until I use it	Jingdong prefecture level city in Shandong
Dragon Quality Goods Shop	Green Intelligent Power Saver	Not only to the point of being useless yet	Jingdong prefecture level city in Shandong
Dragon Quality Goods Shop	Green Intelligent Power Saver	Used it for a month, nothing works, the meter you turn very fast. This is not a scam, but also sold on Jingdong. To cheat a bunch of people?	Jingdong prefecture level city in Shandong
Dragon Quality Goods Shop	Green Intelligent Power Saver	Very good to use, every day can save a little electricity, really good under the sample down a month to save a few dozen dollars in electricity, good before I did not know that there is this haha, the price is not expensive praise.	Jingdong prefecture level city in Shandong
Dragon Quality Goods Shop	Green Intelligent Power Saver	Tried it for ten days and the change is almost nothing, can't see the power saving yet, use it some more days and see how it goes.	Jingdong prefecture level city in Shandong
Dragon Quality Goods Shop	Green Intelligent Power Saver	Received very quickly, the quality is very good, really save electricity, business service attitude is also very good, I hope you do not miss this opportunity, I continue to buy a few in	Jingdong prefecture level city in Shandong
Dragon Quality Goods Shop	Green Intelligent Power Saver	Quality is very good Oh, the delivery is also very fast, a very favorite store, I want to crazy recommend to friends!	Jingdong prefecture level city in Shandong
Dragon Quality Goods Shop	Green Intelligent Power Saver	The goods have just received, fine workmanship, and the same as described by the merchant, to use a period of time to see if it can achieve the purpose of power saving.	Jingdong prefecture level city in Shandong
Dragon Quality Goods Shop	Green Intelligent Power Saver	Received the goods first use a period of time to see if there is no effect, the business commitment to ninety days invalid refund, first praise, good service!	Jingdong prefecture level city in Shandong
Dragon Quality Goods Shop	Green Intelligent Power Saver	The product is used for a month without energy-saving and power-saving effect does not have any effect	Jingdong prefecture level city in Shandong
Dragon Quality Goods Shop	Green Intelligent Power Saver	The baby is really great, I like it very much, my friends also say good to come, recommended to buy!	Jingdong prefecture level city in Shandong
Dragon Quality Goods Shop	Green Intelligent Power Saver	Logistics is fast, courier boy service is good, after using to see how the fruit, and then follow up.	Jingdong prefecture level city in Shandong
Dragon Quality Goods Shop	Green Intelligent Power Saver	Baby has been received to try, I believe that as the business advertised energy saving. The courier is very responsible.	Jingdong prefecture level city in Shandong
Dragon Quality Goods Shop	Green Intelligent Power Saver	Save Electric Energy King received, good quality, beautiful appearance, very satisfied!	Jingdong prefecture level city in Shandong
Dragon Quality Goods Shop	Green Intelligent Power Saver	Three no products no manufacturers no phone pure fraud induced consumption cheat	Jingdong prefecture level city in Shandong
Dragon Quality Goods Shop	Green Intelligent Power Saver	I'm using it now, I don't know if it's as good as it says, I'll follow up after a month	Jingdong prefecture level city in Shandong
Dragon Quality Goods Shop	Green Intelligent Power Saver	Good quality logistics and fast. Fine workmanship. The description is consistent cost-effective	Jingdong prefecture level city in Shandong
Dragon Quality Goods Shop	Green Intelligent Power Saver	Received, whether it is good or not, to wait a few days and then evaluate	Jingdong prefecture level city in Shandong
Dragon Quality Goods Shop	Green Intelligent Power Saver	I bought it and started using it, I won't know if it works well until I pay my electric bill	Jingdong prefecture level city in Shandong
Dragon Quality Goods Shop	Green Intelligent Power Saver	Especially good, energy saving and power saving, especially good quality, good	Jingdong prefecture level city in Shandong
Dragon Quality Goods Shop	Green Intelligent Power Saver	Logistics is strong, the quality is very good, paste really save electricity, as described, will buy back!	Jingdong prefecture level city in Shandong
Dragon Quality Goods Shop	Green Intelligent Power Saver	Great stuff at a great price, worth the money!	Jingdong prefecture level city in Shandong
Dragon Quality Goods Shop	Green Intelligent Power Saver	Received the power saver, very good quality, very power saving!	Jingdong prefecture level city in Shandong
Dragon Quality Goods Shop	Green Intelligent Power Saver	There are no fruits, only samples...	Jingdong prefecture level city in Shandong
Dragon Quality Goods Shop	Green Intelligent Power Saver	Save a few dollars a month.	Jingdong prefecture level city in Shandong
Dragon Quality Goods Shop	Green Intelligent Power Saver	The quality is particularly good, it works well, and the seller has a good service attitude	Jingdong prefecture level city in Shandong
Dragon Quality Goods Shop	Green Intelligent Power Saver	I've compared them a couple of times and I don't feel any difference	Jingdong prefecture level city in Shandong
Dragon Quality Goods Shop	Green Intelligent Power Saver	I don't see the savings. It's a sham.	Jingdong prefecture level city in Shandong
Dragon Quality Goods Shop	Green Intelligent Power Saver	The power saving effect is very good, and the logistics is very fast. The seller's service attitude is very good	Jingdong prefecture level city in Shandong
Dragon Quality Goods Shop	Green Intelligent Power Saver	Received, well packaged, haven't tried it yet!	Jingdong prefecture level city in Shandong
Dragon Quality Goods Shop	Green Intelligent Power Saver	I don't know how it works, I'll comment after I use it.	Jingdong prefecture level city in Shandong
Dragon Quality Goods Shop	Green Intelligent Power Saver	The product doesn't work well enough to be of any use	Jingdong prefecture level city in Shandong
Dragon Quality Goods Shop	Green Intelligent Power Saver	On Trial 。。。。。。。	Jingdong prefecture level city in Shandong
Dragon Quality Goods Shop	Green Intelligent Power Saver	Being used with unknown results	Jingdong prefecture level city in Shandong
Dragon Quality Goods Shop	Green Intelligent Power Saver	Smart power saving and great quality	Jingdong prefecture level city in Shandong
Dragon Quality Goods Shop	Green Intelligent Power Saver	The saver was received, it works very well and the price is affordable!	Jingdong prefecture level city in Shandong
Dragon Quality Goods Shop	Green Intelligent Power Saver	How can I feel the power saving and how long does it last	Jingdong prefecture level city in Shandong
Dragon Quality Goods Shop	Green Intelligent Power Saver	And I know if it saves electricity or not.	Jingdong prefecture level city in Shandong
Dragon Quality Goods Shop	Green Intelligent Power Saver	It won't save any electricity. No.	Jingdong prefecture level city in Shandong
Dragon Quality Goods Shop	Green Intelligent Power Saver	Invalid. Fake.	Jingdong prefecture level city in Shandong
Dragon Quality Goods Shop	Green Intelligent Power Saver	It's good!	Jingdong prefecture level city in Shandong
Dragon Quality Goods Shop	Green Intelligent Power Saver	have no effect	Jingdong prefecture level city in Shandong
Dragon Quality Goods Shop	Green Intelligent Power Saver	Not used.	Jingdong prefecture level city in Shandong
Dragon Quality Goods Shop	Green Intelligent Power Saver	There is no load range, packaging thrift, ten days also did not develop invoices, received the goods with a receipt, hand-written receipt also has no financial stamp, communication for nearly a week a moment to say that the electronic invoice, a moment to say that the paper invoice, said it is mailed to me, ten days also did not see anything, the invoice invoice can not be found to say that did not stay on file, single single number can not be found, said that the warehouse more than one to a one to find out, in addition to the perfunctory or perfunctory. Products are no certificate of conformity, proper three	Jingdong prefecture level city in Shandong
Dragon Quality Goods Shop	Green Intelligent Power Saver	Satisfaction.	Jingdong prefecture level city in Shandong

Dragon Quality Goods Shop	Green Intelligent Power Saver	There is not a bit of power saving effect, and ringing up an increase in the electricity bill, because you have to add the cost of this machine's own electricity consumption and purchase, so I think it is better to use this machine instead of increasing the expenditure, uneconomical, rather than not use it.	Jingdong prefecture level city in Shandong
Dragon Quality Goods Shop	Green Intelligent Power Saver	I've been using it for two days now and I don't feel any effect.	Jingdong prefecture level city in Shandong
Dragon Quality Goods Shop	Green Intelligent Power Saver	What it looked like was nothing like what was sent.	Jingdong prefecture level city in Shandong
Dragon Quality Goods Shop	Green Intelligent Power Saver	It didn't work. It didn't work for a month or so.	Jingdong prefecture level city in Shandong
Dragon Quality Goods Shop	Green Intelligent Power Saver	Garbage products, physical and description is not the same, no effect at all, pit # no discussion, to buy the fine, garbage, garbage, garbage	Jingdong prefecture level city in Shandong
Dragon Quality Goods Shop	Green Intelligent Power Saver	general	Jingdong prefecture level city in Shandong
Dragon Quality Goods Shop	Green Intelligent Power Saver	IQ tax. Instead of saving electricity, it increases the power consumption of an appliance.	Jingdong prefecture level city in Shandong
Dragon Quality Goods Shop	Green Intelligent Power Saver	There's no trash at all.	Jingdong prefecture level city in Shandong
Dragon Quality Goods Shop	Green Intelligent Power Saver	It doesn't do anything.	Jingdong prefecture level city in Shandong
Dragon Quality Goods Shop	Green Intelligent Power Saver	(of an unmarried couple) be close	Jingdong prefecture level city in Shandong
Dragon Quality Goods Shop	Green Intelligent Power Saver	(of an unmarried couple) be close	Jingdong prefecture level city in Shandong
Dragon Quality Goods Shop	Green Intelligent Power Saver	no electricity saving	Jingdong prefecture level city in Shandong
Dragon Quality Goods Shop	Green Intelligent Power Saver	differ from	Jingdong prefecture level city in Shandong
Dragon Quality Goods Shop	Green Intelligent Power Saver	Saver has received, but real can save electricity, the quality is very good, I times I buy a few more, too good.	Jingdong prefecture level city in Shandong
Dragon Quality Goods Shop	Green Intelligent Power Saver	Great quality as always, a very favorite product, great! o(*￣︶￣*)o	Jingdong prefecture level city in Shandong
Dragon Quality Goods Shop	Green Intelligent Power Saver	If we can reduce the electricity bill, for individuals can save money, for the country can protect the environment, now the weather is getting hotter and hotter, to protect the environment to reduce emissions is very important. Already use the product, whether to reduce the electricity bill depends on next month!	Jingdong prefecture level city in Shandong
Dragon Quality Goods Shop	Green Intelligent Power Saver	Purchased a power saver received on the put into use, rent a room with many electrical appliances, power consumption is very large, we have a piece of electricity or 1.2 dollars a time, with this power saver is much better, every day can save a few cents, very convenient and practical!	Jingdong prefecture level city in Shandong
Dragon Quality Goods Shop	Green Intelligent Power Saver	The product is better to use before not to use when a month 1500 yuan electricity bill, now using the 5 second month electricity bill directly down 470 dollars or very good. A save money to buy several power saver money ZanZanZanZan👍	Jingdong prefecture level city in Shandong
Dragon Quality Goods Shop	Green Intelligent Power Saver	The moment I received the goods really satisfied with this product, not only high-grade packaging, the body are base alloy, up to the price, I was also introduced by a friend, he used last month did save a lot of electricity, but also recommended to me!	Jingdong prefecture level city in Shandong
Dragon Quality Goods Shop	Green Intelligent Power Saver	I got it. Good review. I'm very satisfied. Reasonable price.	Jingdong prefecture level city in Shandong
Dragon Quality Goods Shop	Green Intelligent Power Saver	Intelligent power saver received and used, looking at the quality is good, the quality of the plug sent is also very good. Expect the effect of the economizer is obvious, first five-star praise, with a period of time to follow up!	Jingdong prefecture level city in Shandong
Dragon Quality Goods Shop	Green Intelligent Power Saver	The saver has been received. It was plugged into an outlet and used immediately after receipt. Whether it really saves electricity is not clear yet, I believe that this brand of energy saver really saves	Jingdong prefecture level city in Shandong
Dragon Quality Goods Shop	Green Intelligent Power Saver	Okay, okay, okay. Thank you so much. Fast is good. Fast. Item was as described, mainly because the event was cheap and you can save a lot of money.	Jingdong prefecture level city in Shandong
Dragon Quality Goods Shop	Green Intelligent Power Saver	Received it a couple days ago! Plugged it in the same day! Let's see what the electric bill is in a month! Hopefully it works well oh!	Jingdong prefecture level city in Shandong
Dragon Quality Goods Shop	Green Intelligent Power Saver	Looking at the reviews good, energy-saving small products, compact, the results after 2 months to see, because the electricity bill season payment	Jingdong prefecture level city in Shandong
Dragon Quality Goods Shop	Green Intelligent Power Saver	I just received it and will try it out to see if it works well. I'll comment later.	Jingdong prefecture level city in Shandong
Dragon Quality Goods Shop	Green Intelligent Power Saver	It looks a little small and not very aesthetically pleasing, I don't know how effective it will be until I use it	Jingdong prefecture level city in Shandong
Dragon Quality Goods Shop	Green Intelligent Power Saver	Not only to the point of being useless yet	Jingdong prefecture level city in Shandong
Dragon Quality Goods Shop	Green Intelligent Power Saver	Used it for a month, nothing works, the meter you turn very fast. This is not a scam, but also sold on Jingdong. To cheat a bunch of people?	Jingdong prefecture level city in Shandong
Dragon Quality Goods Shop	Green Intelligent Power Saver	Very good to use, every day can save a little electricity, really good under the sample down a month to save a few dozen dollars in electricity, good before I did not know that there is this 👆 haha, the price is not expensive praise.	Jingdong prefecture level city in Shandong
Dragon Quality Goods Shop	Green Intelligent Power Saver	Tried it for ten days and the change is almost nothing, can't see the power saving yet, use it some more days and see how it goes.	Jingdong prefecture level city in Shandong
Dragon Quality Goods Shop	Green Intelligent Power Saver	Received very quickly, the quality is very good, really save electricity, business service attitude is also very good, I hope you do not miss this opportunity, I continue to buy a few in	Jingdong prefecture level city in Shandong
Dragon Quality Goods Shop	Green Intelligent Power Saver	Quality is very good Oh, the delivery is also very fast, a very favorite store, I want to crazy recommend to friends!	Jingdong prefecture level city in Shandong
Dragon Quality Goods Shop	Green Intelligent Power Saver	The goods have just received, fine workmanship, and the same as described by the merchant, to use a period of time to see if it can achieve the purpose of power saving.	Jingdong prefecture level city in Shandong
Dragon Quality Goods Shop	Green Intelligent Power Saver	Received the goods first use a period of time to see if there is no effect, the business commitment to ninety days invalid refund, first praise, good service!	Jingdong prefecture level city in Shandong
Dragon Quality Goods Shop	Green Intelligent Power Saver	The product is used for a month without energy-saving and power-saving effect does not have any effect	Jingdong prefecture level city in Shandong
Dragon Quality Goods Shop	Green Intelligent Power Saver	The baby is really great, I like it very much, my friends also say good to come, recommended to buy!	Jingdong prefecture level city in Shandong
Dragon Quality Goods Shop	Green Intelligent Power Saver	Logistics is fast, courier boy service is good, after using to see how the fruit, and then follow up.	Jingdong prefecture level city in Shandong
Dragon Quality Goods Shop	Green Intelligent Power Saver	Baby has been received to try, I believe that as the business advertised energy saving. The courier is very responsible.	Jingdong prefecture level city in Shandong
Dragon Quality Goods Shop	Green Intelligent Power Saver	Save Electric Energy King received, good quality, beautiful appearance, very satisfied!	Jingdong prefecture level city in Shandong
Dragon Quality Goods Shop	Green Intelligent Power Saver	Three no products no manufacturers no phone pure fraud induced consumption cheat	Jingdong prefecture level city in Shandong
Dragon Quality Goods Shop	Green Intelligent Power Saver	I'm using it now, I don't know if it's as good as it says, I'll follow up after a month	Jingdong prefecture level city in Shandong
Dragon Quality Goods Shop	Green Intelligent Power Saver	Good quality logistics and fast. Fine workmanship. The description is consistent cost-effective	Jingdong prefecture level city in Shandong
Dragon Quality Goods Shop	Green Intelligent Power Saver	Received, whether it is good or not, to wait a few days and then evaluate	Jingdong prefecture level city in Shandong
Dragon Quality Goods Shop	Green Intelligent Power Saver	I bought it and started using it, I won't know if it works well until I pay my electric bill	Jingdong prefecture level city in Shandong
Dragon Quality Goods Shop	Green Intelligent Power Saver	Especially good, energy saving and power saving, especially good quality, good	Jingdong prefecture level city in Shandong
Dragon Quality Goods Shop	Green Intelligent Power Saver	Logistics is strong, the quality is very good, paste really save electricity, as described, will buy back!	Jingdong prefecture level city in Shandong
Dragon Quality Goods Shop	Green Intelligent Power Saver	Great stuff at a great price, worth the money!	Jingdong prefecture level city in Shandong

Dragon Quality Goods Shop	Green Intelligent Power Saver	Received the power saver, very good quality, very power saving!	Jingdong prefecture level city in Shandong
Dragon Quality Goods Shop	Green Intelligent Power Saver	There are no fruits, only samples...	Jingdong prefecture level city in Shandong
Dragon Quality Goods Shop	Green Intelligent Power Saver	Save a few dollars a month.	Jingdong prefecture level city in Shandong
Dragon Quality Goods Shop	Green Intelligent Power Saver	The quality is particularly good, it works well, and the seller has a good service attitude	Jingdong prefecture level city in Shandong
Dragon Quality Goods Shop	Green Intelligent Power Saver	I've compared them a couple of times and I don't feel any difference	Jingdong prefecture level city in Shandong
Dragon Quality Goods Shop	Green Intelligent Power Saver	I don't see the savings. It's a sham.	Jingdong prefecture level city in Shandong
Dragon Quality Goods Shop	Green Intelligent Power Saver	The power saving effect is very good, and the logistics is very fast. The seller's service attitude is very good	Jingdong prefecture level city in Shandong
Dragon Quality Goods Shop	Green Intelligent Power Saver	Received, well packaged, haven't tried it yet!	Jingdong prefecture level city in Shandong
Dragon Quality Goods Shop	Green Intelligent Power Saver	I don't know how it works, I'll comment after I use it.	Jingdong prefecture level city in Shandong
Dragon Quality Goods Shop	Green Intelligent Power Saver	The product doesn't work well enough to be of any use	Jingdong prefecture level city in Shandong
Dragon Quality Goods Shop	Green Intelligent Power Saver	On Trial 。。。。。。。	Jingdong prefecture level city in Shandong
Dragon Quality Goods Shop	Green Intelligent Power Saver	Being used with unknown results	Jingdong prefecture level city in Shandong
Dragon Quality Goods Shop	Green Intelligent Power Saver	Smart power saving and great quality	Jingdong prefecture level city in Shandong
Dragon Quality Goods Shop	Green Intelligent Power Saver	The saver was received, it works very well and the price is affordable!	Jingdong prefecture level city in Shandong
Dragon Quality Goods Shop	Green Intelligent Power Saver	How can I feel the power saving and how long does it last	Jingdong prefecture level city in Shandong
Dragon Quality Goods Shop	Green Intelligent Power Saver	And I know if it saves electricity or not.	Jingdong prefecture level city in Shandong
Dragon Quality Goods Shop	Green Intelligent Power Saver	It won't save any electricity. No.	Jingdong prefecture level city in Shandong
Dragon Quality Goods Shop	Green Intelligent Power Saver	Invalid. Fake.	Jingdong prefecture level city in Shandong
Dragon Quality Goods Shop	Green Intelligent Power Saver	It's good!	Jingdong prefecture level city in Shandong
Dragon Quality Goods Shop	Green Intelligent Power Saver	have no effect	Jingdong prefecture level city in Shandong
Dragon Quality Goods Shop	Green Intelligent Power Saver	Not used.	Jingdong prefecture level city in Shandong
Dragon Quality Goods Shop	Green Intelligent Power Saver	There is no load range, packaging thrift, ten days also did not develop invoices, received the goods with a receipt, hand-written receipt also has no financial stamp, communication for nearly a week a moment to say that the electronic invoice, a moment to say that the paper invoice, said it is mailed to me, ten days also did not see anything, the invoice invoice can not be found to say that did not stay on file, single single number can not be found, said that the warehouse more than one to a one to find out, in addition to the perfunctory or perfunctory. Products are no certificate of conformity, proper three	Jingdong prefecture level city in Shandong
Dragon Quality Goods Shop	Green Intelligent Power Saver	Satisfaction.	Jingdong prefecture level city in Shandong
Dragon Quality Goods Shop	Green Intelligent Power Saver	There is not a bit of power saving effect, and ringing up an increase in the electricity bill, because you have to add the cost of this machine's own electricity consumption and purchase, so I think it is better to use this machine instead of increasing the expenditure, uneconomical, rather than not use it.	Jingdong prefecture level city in Shandong
Dragon Quality Goods Shop	Green Intelligent Power Saver	I've been using it for two days now and I don't feel any effect.	Jingdong prefecture level city in Shandong
Dragon Quality Goods Shop	Green Intelligent Power Saver	What it looked like was nothing like what was sent.	Jingdong prefecture level city in Shandong
Dragon Quality Goods Shop	Green Intelligent Power Saver	It didn't work. It didn't work for a month or so.	Jingdong prefecture level city in Shandong
Dragon Quality Goods Shop	Green Intelligent Power Saver	Garbage products, physical and description is not the same, no effect at all, pit # no discussion, to buy the fine, garbage, garbage, garbage	Jingdong prefecture level city in Shandong
Dragon Quality Goods Shop	Green Intelligent Power Saver	general	Jingdong prefecture level city in Shandong
Dragon Quality Goods Shop	Green Intelligent Power Saver	IQ tax. Instead of saving electricity, it increases the power consumption of an appliance.	Jingdong prefecture level city in Shandong
Dragon Quality Goods Shop	Green Intelligent Power Saver	There's no trash at all.	Jingdong prefecture level city in Shandong
Dragon Quality Goods Shop	Green Intelligent Power Saver	It doesn't do anything.	Jingdong prefecture level city in Shandong
Dragon Quality Goods Shop	Green Intelligent Power Saver	(of an unmarried couple) be close	Jingdong prefecture level city in Shandong
Dragon Quality Goods Shop	Green Intelligent Power Saver	(of an unmarried couple) be close	Jingdong prefecture level city in Shandong
Dragon Quality Goods Shop	Green Intelligent Power Saver	no electricity saving	Jingdong prefecture level city in Shandong
Dragon Quality Goods Shop	Green Intelligent Power Saver	differ from	Jingdong prefecture level city in Shandong
Dragon Quality Goods Shop	Green Intelligent Power Saver	Saver has received, but real can save electricity, the quality is very good, I times I buy a few more, too good.	Jingdong prefecture level city in Shandong
Dragon Quality Goods Shop	Green Intelligent Power Saver	Great quality as always, a very favorite product, great! o(*￣︶￣*)o	Jingdong prefecture level city in Shandong
Dragon Quality Goods Shop	Green Intelligent Power Saver	If we can reduce the electricity bill, for individuals can save money, for the country can protect the environment, now the weather is getting hotter and hotter, to protect the environment to reduce emissions is very important. Already use the product, whether to reduce the electricity bill depends on next month!	Jingdong prefecture level city in Shandong
Dragon Quality Goods Shop	Green Intelligent Power Saver	Purchased a power saver received on the put into use, rent a room with many electrical appliances, power consumption is very large, we have a piece of electricity or 1.2 dollars a time, with this power saver is much better, every day can save a few cents, very convenient and practical!	Jingdong prefecture level city in Shandong
Dragon Quality Goods Shop	Green Intelligent Power Saver	The product is better to use before not to use when a month 1500 yuan electricity bill, now using the 5 second month electricity bill directly down 470 dollars or very good. A save money to buy several power saver money ZanZanZanZan👍	Jingdong prefecture level city in Shandong
Dragon Quality Goods Shop	Green Intelligent Power Saver	The moment I received the goods really satisfied with this product, not only high-grade packaging, the body are base alloy, up to the price, I was also introduced by a friend, he used last month did save a lot of electricity, but also recommended to me!	Jingdong prefecture level city in Shandong
Dragon Quality Goods Shop	Green Intelligent Power Saver	I got it. Good review. I'm very satisfied. Reasonable price.	Jingdong prefecture level city in Shandong
Dragon Quality Goods Shop	Green Intelligent Power Saver	Intelligent power saver received and used, looking at the quality is good, the quality of the plug sent is also very good. Expect the effect of the economizer is obvious, first five-star praise, with a period of time to follow up!	Jingdong prefecture level city in Shandong
Dragon Quality Goods Shop	Green Intelligent Power Saver	The saver has been received. It was plugged into an outlet and used immediately after receipt. Whether it really saves electricity is not clear yet, I believe that this brand of energy saver really saves	Jingdong prefecture level city in Shandong
Dragon Quality Goods Shop	Green Intelligent Power Saver	Okay, okay, okay. Thank you so much. Fast is good. Fast. Item was as described, mainly because the event was cheap and you can save a lot of money.	Jingdong prefecture level city in Shandong

Dragon Quality Goods Shop	Green Intelligent Power Saver	Received it a couple days ago! Plugged it in the same day! Let's see what the electric bill is in a month! Hopefully it works well oh!	Jingdong prefecture level city in Shandong
Dragon Quality Goods Shop	Green Intelligent Power Saver	Looking at the reviews good, energy-saving small products, compact, the results after 2 months to see, because the electricity bill season payment	Jingdong prefecture level city in Shandong
Dragon Quality Goods Shop	Green Intelligent Power Saver	I just received it and will try it out to see if it works well. I'll comment later.	Jingdong prefecture level city in Shandong
Dragon Quality Goods Shop	Green Intelligent Power Saver	It looks a little small and not very aesthetically pleasing, I don't know how effective it will be until I use it	Jingdong prefecture level city in Shandong
Dragon Quality Goods Shop	Green Intelligent Power Saver	Not only to the point of being useless yet	Jingdong prefecture level city in Shandong
Dragon Quality Goods Shop	Green Intelligent Power Saver	Used it for a month, nothing works, the meter you turn very fast. This is not a scam, but also sold on Jingdong. To cheat a bunch of people?	Jingdong prefecture level city in Shandong
Dragon Quality Goods Shop	Green Intelligent Power Saver	Very good to use, every day can save a little electricity, really good under the sample down a month to save a few dozen dollars in electricity, good before I did not know that there is this haha, the price is not expensive praise.	Jingdong prefecture level city in Shandong
Dragon Quality Goods Shop	Green Intelligent Power Saver	Tried it for ten days and the change is almost nothing, can't see the power saving yet, use it some more days and see how it goes.	Jingdong prefecture level city in Shandong
Dragon Quality Goods Shop	Green Intelligent Power Saver	Received very quickly, the quality is very good, really save electricity, business service attitude is also very good, I hope you do not miss this opportunity, I continue to buy a few in	Jingdong prefecture level city in Shandong
Dragon Quality Goods Shop	Green Intelligent Power Saver	Quality is very good Oh, the delivery is also very fast, a very favorite store, I want to crazy recommend to friends!	Jingdong prefecture level city in Shandong
Dragon Quality Goods Shop	Green Intelligent Power Saver	The goods have just received, fine workmanship, and the same as described by the merchant, to use a period of time to see if it can achieve the purpose of power saving.	Jingdong prefecture level city in Shandong
Dragon Quality Goods Shop	Green Intelligent Power Saver	Received the goods first use a period of time to see if there is no effect, the business commitment to ninety days invalid refund, first praise, good service!	Jingdong prefecture level city in Shandong
Dragon Quality Goods Shop	Green Intelligent Power Saver	The product is used for a month without energy-saving and power-saving effect does not have any effect	Jingdong prefecture level city in Shandong
Dragon Quality Goods Shop	Green Intelligent Power Saver	The baby is really great, I like it very much, my friends also say good to come, recommended to buy!	Jingdong prefecture level city in Shandong
Dragon Quality Goods Shop	Green Intelligent Power Saver	Logistics is fast, courier boy service is good, after using to see how the fruit, and then follow up.	Jingdong prefecture level city in Shandong
Dragon Quality Goods Shop	Green Intelligent Power Saver	Baby has been received to try, I believe that as the business advertised energy saving. The courier is very responsible.	Jingdong prefecture level city in Shandong
Dragon Quality Goods Shop	Green Intelligent Power Saver	Save Electric Energy King received, good quality, beautiful appearance, very satisfied!	Jingdong prefecture level city in Shandong
Dragon Quality Goods Shop	Green Intelligent Power Saver	Three no products no manufacturers no phone pure fraud induced consumption cheat	Jingdong prefecture level city in Shandong
Dragon Quality Goods Shop	Green Intelligent Power Saver	I'm using it now, I don't know if it's as good as it says, I'll follow up after a month	Jingdong prefecture level city in Shandong
Dragon Quality Goods Shop	Green Intelligent Power Saver	Good quality logistics and fast. Fine workmanship. The description is consistent cost-effective	Jingdong prefecture level city in Shandong
Dragon Quality Goods Shop	Green Intelligent Power Saver	Received, whether it is good or not, to wait a few days and then evaluate	Jingdong prefecture level city in Shandong
Dragon Quality Goods Shop	Green Intelligent Power Saver	I bought it and started using it, I won't know if it works well until I pay my electric bill	Jingdong prefecture level city in Shandong
Dragon Quality Goods Shop	Green Intelligent Power Saver	Especially good, energy saving and power saving, especially good quality, good	Jingdong prefecture level city in Shandong
Dragon Quality Goods Shop	Green Intelligent Power Saver	Logistics is strong, the quality is very good, paste really save electricity, as described, will buy back!	Jingdong prefecture level city in Shandong
Dragon Quality Goods Shop	Green Intelligent Power Saver	Great stuff at a great price, worth the money!	Jingdong prefecture level city in Shandong
Dragon Quality Goods Shop	Green Intelligent Power Saver	Received the power saver, very good quality, very power saving!	Jingdong prefecture level city in Shandong
Dragon Quality Goods Shop	Green Intelligent Power Saver	There are no fruits, only samples...	Jingdong prefecture level city in Shandong
Dragon Quality Goods Shop	Green Intelligent Power Saver	Save a few dollars a month.	Jingdong prefecture level city in Shandong
Dragon Quality Goods Shop	Green Intelligent Power Saver	The quality is particularly good, it works well, and the seller has a good service attitude	Jingdong prefecture level city in Shandong
Dragon Quality Goods Shop	Green Intelligent Power Saver	I've compared them a couple of times and I don't feel any difference	Jingdong prefecture level city in Shandong
Dragon Quality Goods Shop	Green Intelligent Power Saver	I don't see the savings. It's a sham.	Jingdong prefecture level city in Shandong
Dragon Quality Goods Shop	Green Intelligent Power Saver	The power saving effect is very good, and the logistics is very fast. The seller's service attitude is very good	Jingdong prefecture level city in Shandong
Dragon Quality Goods Shop	Green Intelligent Power Saver	Received, well packaged, haven't tried it yet!	Jingdong prefecture level city in Shandong
Dragon Quality Goods Shop	Green Intelligent Power Saver	I don't know how it works, I'll comment after I use it.	Jingdong prefecture level city in Shandong
Dragon Quality Goods Shop	Green Intelligent Power Saver	The product doesn't work well enough to be of any use	Jingdong prefecture level city in Shandong
Dragon Quality Goods Shop	Green Intelligent Power Saver	On Trial 。。。。。。。	Jingdong prefecture level city in Shandong
Dragon Quality Goods Shop	Green Intelligent Power Saver	Being used with unknown results	Jingdong prefecture level city in Shandong
Dragon Quality Goods Shop	Green Intelligent Power Saver	Smart power saving and great quality	Jingdong prefecture level city in Shandong
Dragon Quality Goods Shop	Green Intelligent Power Saver	The saver was received, it works very well and the price is affordable!	Jingdong prefecture level city in Shandong
Dragon Quality Goods Shop	Green Intelligent Power Saver	How can I feel the power saving and how long does it last	Jingdong prefecture level city in Shandong
Dragon Quality Goods Shop	Green Intelligent Power Saver	And I know if it saves electricity or not.	Jingdong prefecture level city in Shandong
Dragon Quality Goods Shop	Green Intelligent Power Saver	It won't save any electricity. No.	Jingdong prefecture level city in Shandong
Dragon Quality Goods Shop	Green Intelligent Power Saver	Invalid. Fake.	Jingdong prefecture level city in Shandong
Dragon Quality Goods Shop	Green Intelligent Power Saver	It's good!	Jingdong prefecture level city in Shandong
Dragon Quality Goods Shop	Green Intelligent Power Saver	have no effect	Jingdong prefecture level city in Shandong
Dragon Quality Goods Shop	Green Intelligent Power Saver	Not used.	Jingdong prefecture level city in Shandong
Dragon Quality Goods Shop	Green Intelligent Power Saver	There is no load range, packaging thrift, ten days also did not develop invoices, received the goods with a receipt, hand-written receipt also has no financial stamp, communication for nearly a week a moment to say that the electronic invoice, a moment to say that the paper invoice, said it is mailed to me, ten days also did not see anything, the invoice invoice can not be found to say that did not stay on file, single single number can not be found, said that the warehouse more than one to a one to find out, in addition to the perfunctory or perfunctory. Products are no certificate of conformity, proper three	Jingdong prefecture level city in Shandong
Dragon Quality Goods Shop	Green Intelligent Power Saver	Satisfaction.	Jingdong prefecture level city in Shandong

Dragon Quality Goods Shop	Green Intelligent Power Saver	There is not a bit of power saving effect, and ringing up an increase in the electricity bill, because you have to add the cost of this machine's own electricity consumption and purchase, so I think it is better to use this machine instead of increasing the expenditure, uneconomical, rather than not use it.	Jingdong prefecture level city in Shandong
Dragon Quality Goods Shop	Green Intelligent Power Saver	I've been using it for two days now and I don't feel any effect.	Jingdong prefecture level city in Shandong
Dragon Quality Goods Shop	Green Intelligent Power Saver	What it looked like was nothing like what was sent.	Jingdong prefecture level city in Shandong
Dragon Quality Goods Shop	Green Intelligent Power Saver	It didn't work. It didn't work for a month or so.	Jingdong prefecture level city in Shandong
Dragon Quality Goods Shop	Green Intelligent Power Saver	Garbage products, physical and description is not the same, no effect at all, pit # no discussion, to buy the fine, garbage, garbage, garbage	Jingdong prefecture level city in Shandong
Dragon Quality Goods Shop	Green Intelligent Power Saver	general	Jingdong prefecture level city in Shandong
Dragon Quality Goods Shop	Green Intelligent Power Saver	IQ tax. Instead of saving electricity, it increases the power consumption of an appliance.	Jingdong prefecture level city in Shandong
Dragon Quality Goods Shop	Green Intelligent Power Saver	There's no trash at all.	Jingdong prefecture level city in Shandong
Dragon Quality Goods Shop	Green Intelligent Power Saver	It doesn't do anything.	Jingdong prefecture level city in Shandong
Dragon Quality Goods Shop	Green Intelligent Power Saver	(of an unmarried couple) be close	Jingdong prefecture level city in Shandong
Dragon Quality Goods Shop	Green Intelligent Power Saver	(of an unmarried couple) be close	Jingdong prefecture level city in Shandong
Dragon Quality Goods Shop	Green Intelligent Power Saver	no electricity saving	Jingdong prefecture level city in Shandong
Dragon Quality Goods Shop	Green Intelligent Power Saver	differ from	Jingdong prefecture level city in Shandong
Dragon Quality Goods Shop	Green Intelligent Power Saver	Saver has received, but real can save electricity, the quality is very good, I times I buy a few more, too good.	Jingdong prefecture level city in Shandong
Dragon Quality Goods Shop	Green Intelligent Power Saver	Great quality as always, a very favorite product, great! o(*￣︶￣*)o	Jingdong prefecture level city in Shandong
Dragon Quality Goods Shop	Green Intelligent Power Saver	If we can reduce the electricity bill, for individuals can save money, for the country can protect the environment, now the weather is getting hotter and hotter, to protect the environment to reduce emissions is very important. Already use the product, whether to reduce the electricity bill depends on next month!	Jingdong prefecture level city in Shandong
Dragon Quality Goods Shop	Green Intelligent Power Saver	Purchased a power saver received on the put into use, rent a room with many electrical appliances, power consumption is very large, we have a piece of electricity or 1.2 dollars a time, with this power saver is much better, every day can save a few cents, very convenient and practical!	Jingdong prefecture level city in Shandong
Dragon Quality Goods Shop	Green Intelligent Power Saver	The product is better to use before not to use when a month 1500 yuan electricity bill, now using the 5 second month electricity bill directly down 470 dollars or very good. A save money to buy several power saver money ZanZanZanZan👍	Jingdong prefecture level city in Shandong
Dragon Quality Goods Shop	Green Intelligent Power Saver	The moment I received the goods really satisfied with this product, not only high-grade packaging, the body are base alloy, up to the price, I was also introduced by a friend, he used last month did save a lot of electricity, but also recommended to me!	Jingdong prefecture level city in Shandong
Dragon Quality Goods Shop	Green Intelligent Power Saver	I got it. Good review. I'm very satisfied. Reasonable price.	Jingdong prefecture level city in Shandong
Dragon Quality Goods Shop	Green Intelligent Power Saver	Intelligent power saver received and used, looking at the quality is good, the quality of the plug sent is also very good. Expect the effect of the economizer is obvious, first five-star praise, with a period of time to follow up!	Jingdong prefecture level city in Shandong
Dragon Quality Goods Shop	Green Intelligent Power Saver	The saver has been received. It was plugged into an outlet and used immediately after receipt. Whether it really saves electricity is not clear yet, I believe that this brand of energy saver really saves	Jingdong prefecture level city in Shandong
Dragon Quality Goods Shop	Green Intelligent Power Saver	Okay, okay, okay. Thank you so much. Fast is good. Fast. Item was as described, mainly because the event was cheap and you can save a lot of money.	Jingdong prefecture level city in Shandong
Dragon Quality Goods Shop	Green Intelligent Power Saver	Received it a couple days ago! Plugged it in the same day! Let's see what the electric bill is in a month! Hopefully it works well oh!	Jingdong prefecture level city in Shandong
Dragon Quality Goods Shop	Green Intelligent Power Saver	Looking at the reviews good, energy-saving small products, compact, the results after 2 months to see, because the electricity bill season payment	Jingdong prefecture level city in Shandong
Dragon Quality Goods Shop	Green Intelligent Power Saver	I just received it and will try it out to see if it works well. I'll comment later.	Jingdong prefecture level city in Shandong
Dragon Quality Goods Shop	Green Intelligent Power Saver	It looks a little small and not very aesthetically pleasing, I don't know how effective it will be until I use it	Jingdong prefecture level city in Shandong
Dragon Quality Goods Shop	Green Intelligent Power Saver	Not only to the point of being useless yet	Jingdong prefecture level city in Shandong
Dragon Quality Goods Shop	Green Intelligent Power Saver	Used it for a month, nothing works, the meter you turn very fast. This is not a scam, but also sold on Jingdong. To cheat a bunch of people?	Jingdong prefecture level city in Shandong
Dragon Quality Goods Shop	Green Intelligent Power Saver	Very good to use, every day can save a little electricity, really good under the sample down a month to save a few dozen dollars in electricity, good before I did not know that there is this 👆 haha, the price is not expensive praise.	Jingdong prefecture level city in Shandong
Dragon Quality Goods Shop	Green Intelligent Power Saver	Tried it for ten days and the change is almost nothing, can't see the power saving yet, use it some more days and see how it goes.	Jingdong prefecture level city in Shandong
Dragon Quality Goods Shop	Green Intelligent Power Saver	Received very quickly, the quality is very good, really save electricity, business service attitude is also very good, I hope you do not miss this opportunity, I continue to buy a few in	Jingdong prefecture level city in Shandong
Dragon Quality Goods Shop	Green Intelligent Power Saver	Quality is very good Oh, the delivery is also very fast, a very favorite store, I want to crazy recommend to friends!	Jingdong prefecture level city in Shandong
Dragon Quality Goods Shop	Green Intelligent Power Saver	The goods have just received, fine workmanship, and the same as described by the merchant, to use a period of time to see if it can achieve the purpose of power saving.	Jingdong prefecture level city in Shandong
Dragon Quality Goods Shop	Green Intelligent Power Saver	Received the goods first use a period of time to see if there is no effect, the business commitment to ninety days invalid refund, first praise, good service!	Jingdong prefecture level city in Shandong
Dragon Quality Goods Shop	Green Intelligent Power Saver	The product is used for a month without energy-saving and power-saving effect does not have any effect	Jingdong prefecture level city in Shandong
Dragon Quality Goods Shop	Green Intelligent Power Saver	The baby is really great, I like it very much, my friends also say good to come, recommended to buy!	Jingdong prefecture level city in Shandong
Dragon Quality Goods Shop	Green Intelligent Power Saver	Logistics is fast, courier boy service is good, after using to see how the fruit, and then follow up.	Jingdong prefecture level city in Shandong
Dragon Quality Goods Shop	Green Intelligent Power Saver	Baby has been received to try, I believe that as the business advertised energy saving. The courier is very responsible.	Jingdong prefecture level city in Shandong
Dragon Quality Goods Shop	Green Intelligent Power Saver	Save Electric Energy King received, good quality, beautiful appearance, very satisfied!	Jingdong prefecture level city in Shandong
Dragon Quality Goods Shop	Green Intelligent Power Saver	Three no products no manufacturers no phone pure fraud induced consumption cheat	Jingdong prefecture level city in Shandong
Dragon Quality Goods Shop	Green Intelligent Power Saver	I'm using it now, I don't know if it's as good as it says, I'll follow up after a month	Jingdong prefecture level city in Shandong
Dragon Quality Goods Shop	Green Intelligent Power Saver	Good quality logistics and fast. Fine workmanship. The description is consistent cost-effective	Jingdong prefecture level city in Shandong
Dragon Quality Goods Shop	Green Intelligent Power Saver	Received, whether it is good or not, to wait a few days and then evaluate	Jingdong prefecture level city in Shandong
Dragon Quality Goods Shop	Green Intelligent Power Saver	I bought it and started using it, I won't know if it works well until I pay my electric bill	Jingdong prefecture level city in Shandong
Dragon Quality Goods Shop	Green Intelligent Power Saver	Especially good, energy saving and power saving, especially good quality, good	Jingdong prefecture level city in Shandong
Dragon Quality Goods Shop	Green Intelligent Power Saver	Logistics is strong, the quality is very good, paste really save electricity, as described, will buy back!	Jingdong prefecture level city in Shandong
Dragon Quality Goods Shop	Green Intelligent Power Saver	Great stuff at a great price, worth the money!	Jingdong prefecture level city in Shandong

Dragon Quality Goods Shop	Green Intelligent Power Saver	Received the power saver, very good quality, very power saving!	Jingdong prefecture level city in Shandong
Dragon Quality Goods Shop	Green Intelligent Power Saver	There are no fruits, only samples...	Jingdong prefecture level city in Shandong
Dragon Quality Goods Shop	Green Intelligent Power Saver	Save a few dollars a month.	Jingdong prefecture level city in Shandong
Dragon Quality Goods Shop	Green Intelligent Power Saver	The quality is particularly good, it works well, and the seller has a good service attitude	Jingdong prefecture level city in Shandong
Dragon Quality Goods Shop	Green Intelligent Power Saver	I've compared them a couple of times and I don't feel any difference	Jingdong prefecture level city in Shandong
Dragon Quality Goods Shop	Green Intelligent Power Saver	I don't see the savings. It's a sham.	Jingdong prefecture level city in Shandong
Dragon Quality Goods Shop	Green Intelligent Power Saver	The power saving effect is very good, and the logistics is very fast. The seller's service attitude is very good	Jingdong prefecture level city in Shandong
Dragon Quality Goods Shop	Green Intelligent Power Saver	Received, well packaged, haven't tried it yet!	Jingdong prefecture level city in Shandong
Dragon Quality Goods Shop	Green Intelligent Power Saver	I don't know how it works, I'll comment after I use it.	Jingdong prefecture level city in Shandong
Dragon Quality Goods Shop	Green Intelligent Power Saver	The product doesn't work well enough to be of any use	Jingdong prefecture level city in Shandong
Dragon Quality Goods Shop	Green Intelligent Power Saver	On Trial 。。。。。。。	Jingdong prefecture level city in Shandong
Dragon Quality Goods Shop	Green Intelligent Power Saver	Being used with unknown results	Jingdong prefecture level city in Shandong
Dragon Quality Goods Shop	Green Intelligent Power Saver	Smart power saving and great quality	Jingdong prefecture level city in Shandong
Dragon Quality Goods Shop	Green Intelligent Power Saver	The saver was received, it works very well and the price is affordable!	Jingdong prefecture level city in Shandong
Dragon Quality Goods Shop	Green Intelligent Power Saver	How can I feel the power saving and how long does it last	Jingdong prefecture level city in Shandong
Dragon Quality Goods Shop	Green Intelligent Power Saver	And I know if it saves electricity or not.	Jingdong prefecture level city in Shandong
Dragon Quality Goods Shop	Green Intelligent Power Saver	It won't save any electricity. No.	Jingdong prefecture level city in Shandong
Dragon Quality Goods Shop	Green Intelligent Power Saver	Invalid. Fake.	Jingdong prefecture level city in Shandong
Dragon Quality Goods Shop	Green Intelligent Power Saver	It's good!	Jingdong prefecture level city in Shandong
Dragon Quality Goods Shop	Green Intelligent Power Saver	have no effect	Jingdong prefecture level city in Shandong
Dragon Quality Goods Shop	Green Intelligent Power Saver	Not used.	Jingdong prefecture level city in Shandong
Dragon Quality Goods Shop	Green Intelligent Power Saver	There is no load range, packaging thrift, ten days also did not develop invoices, received the goods with a receipt, hand-written receipt also has no financial stamp, communication for nearly a week a moment to say that the electronic invoice, a moment to say that the paper invoice, said it is mailed to me, ten days also did not see anything, the invoice invoice can not be found to say that did not stay on file, single single number can not be found, said that the warehouse more than one to a one to find out, in addition to the perfunctory or perfunctory. Products are no certificate of conformity, proper three	Jingdong prefecture level city in Shandong
Dragon Quality Goods Shop	Green Intelligent Power Saver	Satisfaction.	Jingdong prefecture level city in Shandong
Dragon Quality Goods Shop	Green Intelligent Power Saver	There is not a bit of power saving effect, and ringing up an increase in the electricity bill, because you have to add the cost of this machine's own electricity consumption and purchase, so I think it is better to use this machine instead of increasing the expenditure, uneconomical, rather than not use it.	Jingdong prefecture level city in Shandong
Dragon Quality Goods Shop	Green Intelligent Power Saver	I've been using it for two days now and I don't feel any effect.	Jingdong prefecture level city in Shandong
Dragon Quality Goods Shop	Green Intelligent Power Saver	What it looked like was nothing like what was sent.	Jingdong prefecture level city in Shandong
Dragon Quality Goods Shop	Green Intelligent Power Saver	It didn't work. It didn't work for a month or so.	Jingdong prefecture level city in Shandong
Dragon Quality Goods Shop	Green Intelligent Power Saver	Garbage products, physical and description is not the same, no effect at all, pit # no discussion, to buy the fine, garbage, garbage, garbage	Jingdong prefecture level city in Shandong
Dragon Quality Goods Shop	Green Intelligent Power Saver	general	Jingdong prefecture level city in Shandong
Dragon Quality Goods Shop	Green Intelligent Power Saver	IQ tax. Instead of saving electricity, it increases the power consumption of an appliance.	Jingdong prefecture level city in Shandong
Dragon Quality Goods Shop	Green Intelligent Power Saver	There's no trash at all.	Jingdong prefecture level city in Shandong
Dragon Quality Goods Shop	Green Intelligent Power Saver	It doesn't do anything.	Jingdong prefecture level city in Shandong
Dragon Quality Goods Shop	Green Intelligent Power Saver	(of an unmarried couple) be close	Jingdong prefecture level city in Shandong
Dragon Quality Goods Shop	Green Intelligent Power Saver	(of an unmarried couple) be close	Jingdong prefecture level city in Shandong
Dragon Quality Goods Shop	Green Intelligent Power Saver	no electricity saving	Jingdong prefecture level city in Shandong
Dragon Quality Goods Shop	Green Intelligent Power Saver	differ from	Jingdong prefecture level city in Shandong
Dragon Quality Goods Shop	Green Intelligent Power Saver	Saver has received, but real can save electricity, the quality is very good, I times I buy a few more, too good.	Jingdong prefecture level city in Shandong
Dragon Quality Goods Shop	Green Intelligent Power Saver	Great quality as always, a very favorite product, great! o(*￣︶￣*)o	Jingdong prefecture level city in Shandong
Dragon Quality Goods Shop	Green Intelligent Power Saver	If we can reduce the electricity bill, for individuals can save money, for the country can protect the environment, now the weather is getting hotter and hotter, to protect the environment to reduce emissions is very important. Already use the product, whether to reduce the electricity bill depends on next month!	Jingdong prefecture level city in Shandong
Dragon Quality Goods Shop	Green Intelligent Power Saver	Purchased a power saver received on the put into use, rent a room with many electrical appliances, power consumption is very large, we have a piece of electricity or 1.2 dollars a time, with this power saver is much better, every day can save a few cents, very convenient and practical!	Jingdong prefecture level city in Shandong
Dragon Quality Goods Shop	Green Intelligent Power Saver	The product is better to use before not to use when a month 1500 yuan electricity bill, now using the 5 second month electricity bill directly down 470 dollars or very good. A save money to buy several power saver money ZanZanZanZan👍	Jingdong prefecture level city in Shandong
Dragon Quality Goods Shop	Green Intelligent Power Saver	The moment I received the goods really satisfied with this product, not only high-grade packaging, the body are base alloy, up to the price, I was also introduced by a friend, he used last month did save a lot of electricity, but also recommended to me!	Jingdong prefecture level city in Shandong
Dragon Quality Goods Shop	Green Intelligent Power Saver	I got it. Good review. I'm very satisfied. Reasonable price.	Jingdong prefecture level city in Shandong
Dragon Quality Goods Shop	Green Intelligent Power Saver	Intelligent power saver received and used, looking at the quality is good, the quality of the plug sent is also very good. Expect the effect of the economizer is obvious, first five-star praise, with a period of time to follow up!	Jingdong prefecture level city in Shandong
Dragon Quality Goods Shop	Green Intelligent Power Saver	The saver has been received. It was plugged into an outlet and used immediately after receipt. Whether it really saves electricity is not clear yet, I believe that this brand of energy saver really saves	Jingdong prefecture level city in Shandong
Dragon Quality Goods Shop	Green Intelligent Power Saver	Okay, okay, okay. Thank you so much. Fast is good. Fast. Item was as described, mainly because the event was cheap and you can save a lot of money.	Jingdong prefecture level city in Shandong

Dragon Quality Goods Shop	Green Intelligent Power Saver	Received it a couple days ago! Plugged it in the same day! Let's see what the electric bill is in a month! Hopefully it works well oh!	Jingdong prefecture level city in Shandong
Dragon Quality Goods Shop	Green Intelligent Power Saver	Looking at the reviews good, energy-saving small products, compact, the results after 2 months to see, because the electricity bill season payment	Jingdong prefecture level city in Shandong
Dragon Quality Goods Shop	Green Intelligent Power Saver	I just received it and will try it out to see if it works well. I'll comment later.	Jingdong prefecture level city in Shandong
Dragon Quality Goods Shop	Green Intelligent Power Saver	It looks a little small and not very aesthetically pleasing, I don't know how effective it will be until I use it	Jingdong prefecture level city in Shandong
Dragon Quality Goods Shop	Green Intelligent Power Saver	Not only to the point of being useless yet	Jingdong prefecture level city in Shandong
Dragon Quality Goods Shop	Green Intelligent Power Saver	Used it for a month, nothing works, the meter you turn very fast. This is not a scam, but also sold on Jingdong. To cheat a bunch of people?	Jingdong prefecture level city in Shandong
Dragon Quality Goods Shop	Green Intelligent Power Saver	Very good to use, every day can save a little electricity, really good under the sample down a month to save a few dozen dollars in electricity, good before I did not know that there is this haha, the price is not expensive praise.	Jingdong prefecture level city in Shandong
Dragon Quality Goods Shop	Green Intelligent Power Saver	Tried it for ten days and the change is almost nothing, can't see the power saving yet, use it some more days and see how it goes.	Jingdong prefecture level city in Shandong
Dragon Quality Goods Shop	Green Intelligent Power Saver	Received very quickly, the quality is very good, really save electricity, business service attitude is also very good, I hope you do not miss this opportunity, I continue to buy a few in	Jingdong prefecture level city in Shandong
Dragon Quality Goods Shop	Green Intelligent Power Saver	Quality is very good Oh, the delivery is also very fast, a very favorite store, I want to crazy recommend to friends!	Jingdong prefecture level city in Shandong
Dragon Quality Goods Shop	Green Intelligent Power Saver	The goods have just received, fine workmanship, and the same as described by the merchant, to use a period of time to see if it can achieve the purpose of power saving.	Jingdong prefecture level city in Shandong
Dragon Quality Goods Shop	Green Intelligent Power Saver	Received the goods first use a period of time to see if there is no effect, the business commitment to ninety days invalid refund, first praise, good service!	Jingdong prefecture level city in Shandong
Dragon Quality Goods Shop	Green Intelligent Power Saver	The product is used for a month without energy-saving and power-saving effect does not have any effect	Jingdong prefecture level city in Shandong
Dragon Quality Goods Shop	Green Intelligent Power Saver	The baby is really great, I like it very much, my friends also say good to come, recommended to buy!	Jingdong prefecture level city in Shandong
Dragon Quality Goods Shop	Green Intelligent Power Saver	Logistics is fast, courier boy service is good, after using to see how the fruit, and then follow up.	Jingdong prefecture level city in Shandong
Dragon Quality Goods Shop	Green Intelligent Power Saver	Baby has been received to try, I believe that as the business advertised energy saving. The courier is very responsible.	Jingdong prefecture level city in Shandong
Dragon Quality Goods Shop	Green Intelligent Power Saver	Save Electric Energy King received, good quality, beautiful appearance, very satisfied!	Jingdong prefecture level city in Shandong
Dragon Quality Goods Shop	Green Intelligent Power Saver	Three no products no manufacturers no phone pure fraud induced consumption cheat	Jingdong prefecture level city in Shandong
Dragon Quality Goods Shop	Green Intelligent Power Saver	I'm using it now, I don't know if it's as good as it says, I'll follow up after a month	Jingdong prefecture level city in Shandong
Dragon Quality Goods Shop	Green Intelligent Power Saver	Good quality logistics and fast. Fine workmanship. The description is consistent cost-effective	Jingdong prefecture level city in Shandong
Dragon Quality Goods Shop	Green Intelligent Power Saver	Received, whether it is good or not, to wait a few days and then evaluate	Jingdong prefecture level city in Shandong
Dragon Quality Goods Shop	Green Intelligent Power Saver	I bought it and started using it, I won't know if it works well until I pay my electric bill	Jingdong prefecture level city in Shandong
Dragon Quality Goods Shop	Green Intelligent Power Saver	Especially good, energy saving and power saving, especially good quality, good	Jingdong prefecture level city in Shandong
Dragon Quality Goods Shop	Green Intelligent Power Saver	Logistics is strong, the quality is very good, paste really save electricity, as described, will buy back!	Jingdong prefecture level city in Shandong
Dragon Quality Goods Shop	Green Intelligent Power Saver	Great stuff at a great price, worth the money!	Jingdong prefecture level city in Shandong
Dragon Quality Goods Shop	Green Intelligent Power Saver	Received the power saver, very good quality, very power saving!	Jingdong prefecture level city in Shandong
Dragon Quality Goods Shop	Green Intelligent Power Saver	There are no fruits, only samples...	Jingdong prefecture level city in Shandong
Dragon Quality Goods Shop	Green Intelligent Power Saver	Save a few dollars a month.	Jingdong prefecture level city in Shandong
Dragon Quality Goods Shop	Green Intelligent Power Saver	The quality is particularly good, it works well, and the seller has a good service attitude	Jingdong prefecture level city in Shandong
Dragon Quality Goods Shop	Green Intelligent Power Saver	I've compared them a couple of times and I don't feel any difference	Jingdong prefecture level city in Shandong
Dragon Quality Goods Shop	Green Intelligent Power Saver	I don't see the savings. It's a sham.	Jingdong prefecture level city in Shandong
Dragon Quality Goods Shop	Green Intelligent Power Saver	The power saving effect is very good, and the logistics is very fast. The seller's service attitude is very good	Jingdong prefecture level city in Shandong
Dragon Quality Goods Shop	Green Intelligent Power Saver	Received, well packaged, haven't tried it yet!	Jingdong prefecture level city in Shandong
Dragon Quality Goods Shop	Green Intelligent Power Saver	I don't know how it works, I'll comment after I use it.	Jingdong prefecture level city in Shandong
Dragon Quality Goods Shop	Green Intelligent Power Saver	The product doesn't work well enough to be of any use	Jingdong prefecture level city in Shandong
Dragon Quality Goods Shop	Green Intelligent Power Saver	On Trial 。。。。。。。	Jingdong prefecture level city in Shandong
Dragon Quality Goods Shop	Green Intelligent Power Saver	Being used with unknown results	Jingdong prefecture level city in Shandong
Dragon Quality Goods Shop	Green Intelligent Power Saver	Smart power saving and great quality	Jingdong prefecture level city in Shandong
Dragon Quality Goods Shop	Green Intelligent Power Saver	The saver was received, it works very well and the price is affordable!	Jingdong prefecture level city in Shandong
Dragon Quality Goods Shop	Green Intelligent Power Saver	How can I feel the power saving and how long does it last	Jingdong prefecture level city in Shandong
Dragon Quality Goods Shop	Green Intelligent Power Saver	And I know if it saves electricity or not.	Jingdong prefecture level city in Shandong
Dragon Quality Goods Shop	Green Intelligent Power Saver	It won't save any electricity. No.	Jingdong prefecture level city in Shandong
Dragon Quality Goods Shop	Green Intelligent Power Saver	Invalid. Fake.	Jingdong prefecture level city in Shandong
Dragon Quality Goods Shop	Green Intelligent Power Saver	It's good!	Jingdong prefecture level city in Shandong
Dragon Quality Goods Shop	Green Intelligent Power Saver	have no effect	Jingdong prefecture level city in Shandong
Dragon Quality Goods Shop	Green Intelligent Power Saver	Not used.	Jingdong prefecture level city in Shandong
Dragon Quality Goods Shop	Green Intelligent Power Saver	There is no load range, packaging thrift, ten days also did not develop invoices, received the goods with a receipt, hand-written receipt also has no financial stamp, communication for nearly a week a moment to say that the electronic invoice, a moment to say that the paper invoice, said it is mailed to me, ten days also did not see anything, the invoice invoice can not be found to say that did not stay on file, single single number can not be found, said that the warehouse more than one to a one to find out, in addition to the perfunctory or perfunctory. Products are no certificate of conformity, proper three	Jingdong prefecture level city in Shandong
Dragon Quality Goods Shop	Green Intelligent Power Saver	Satisfaction.	Jingdong prefecture level city in Shandong

Dragon Quality Goods Shop	Green Intelligent Power Saver	There is not a bit of power saving effect, and ringing up an increase in the electricity bill, because you have to add the cost of this machine's own electricity consumption and purchase, so I think it is better to use this machine instead of increasing the expenditure, uneconomical, rather than not use it.	Jingdong prefecture level city in Shandong
Dragon Quality Goods Shop	Green Intelligent Power Saver	I've been using it for two days now and I don't feel any effect.	Jingdong prefecture level city in Shandong
Dragon Quality Goods Shop	Green Intelligent Power Saver	What it looked like was nothing like what was sent.	Jingdong prefecture level city in Shandong
Dragon Quality Goods Shop	Green Intelligent Power Saver	It didn't work. It didn't work for a month or so.	Jingdong prefecture level city in Shandong
Dragon Quality Goods Shop	Green Intelligent Power Saver	Garbage products, physical and description is not the same, no effect at all, pit # no discussion, to buy the fine, garbage, garbage, garbage	Jingdong prefecture level city in Shandong
Dragon Quality Goods Shop	Green Intelligent Power Saver	general	Jingdong prefecture level city in Shandong
Dragon Quality Goods Shop	Green Intelligent Power Saver	IQ tax. Instead of saving electricity, it increases the power consumption of an appliance.	Jingdong prefecture level city in Shandong
Dragon Quality Goods Shop	Green Intelligent Power Saver	There's no trash at all.	Jingdong prefecture level city in Shandong
Dragon Quality Goods Shop	Green Intelligent Power Saver	It doesn't do anything.	Jingdong prefecture level city in Shandong
Dragon Quality Goods Shop	Green Intelligent Power Saver	(of an unmarried couple) be close	Jingdong prefecture level city in Shandong
Dragon Quality Goods Shop	Green Intelligent Power Saver	(of an unmarried couple) be close	Jingdong prefecture level city in Shandong
Dragon Quality Goods Shop	Green Intelligent Power Saver	no electricity saving	Jingdong prefecture level city in Shandong
Dragon Quality Goods Shop	Green Intelligent Power Saver	differ from	Jingdong prefecture level city in Shandong
Dragon Quality Goods Shop	Green Intelligent Power Saver	Saver has received, but real can save electricity, the quality is very good, I times I buy a few more, too good.	Jingdong prefecture level city in Shandong
Dragon Quality Goods Shop	Green Intelligent Power Saver	Great quality as always, a very favorite product, great! o(*￣︶￣*)o	Jingdong prefecture level city in Shandong
Dragon Quality Goods Shop	Green Intelligent Power Saver	If we can reduce the electricity bill, for individuals can save money, for the country can protect the environment, now the weather is getting hotter and hotter, to protect the environment to reduce emissions is very important. Already use the product, whether to reduce the electricity bill depends on next month!	Jingdong prefecture level city in Shandong
Dragon Quality Goods Shop	Green Intelligent Power Saver	Purchased a power saver received on the put into use, rent a room with many electrical appliances, power consumption is very large, we have a piece of electricity or 1.2 dollars a time, with this power saver is much better, every day can save a few cents, very convenient and practical!	Jingdong prefecture level city in Shandong
Dragon Quality Goods Shop	Green Intelligent Power Saver	The product is better to use before not to use when a month 1500 yuan electricity bill, now using the 5 second month electricity bill directly down 470 dollars or very good. A save money to buy several power saver money ZanZanZanZan👍	Jingdong prefecture level city in Shandong
Dragon Quality Goods Shop	Green Intelligent Power Saver	The moment I received the goods really satisfied with this product, not only high-grade packaging, the body are base alloy, up to the price, I was also introduced by a friend, he used last month did save a lot of electricity, but also recommended to me!	Jingdong prefecture level city in Shandong
Dragon Quality Goods Shop	Green Intelligent Power Saver	I got it. Good review. I'm very satisfied. Reasonable price.	Jingdong prefecture level city in Shandong
Dragon Quality Goods Shop	Green Intelligent Power Saver	Intelligent power saver received and used, looking at the quality is good, the quality of the plug sent is also very good. Expect the effect of the economizer is obvious, first five-star praise, with a period of time to follow up!	Jingdong prefecture level city in Shandong
Dragon Quality Goods Shop	Green Intelligent Power Saver	The saver has been received. It was plugged into an outlet and used immediately after receipt. Whether it really saves electricity is not clear yet, I believe that this brand of energy saver really saves	Jingdong prefecture level city in Shandong
Dragon Quality Goods Shop	Green Intelligent Power Saver	Okay, okay, okay. Thank you so much. Fast is good. Fast. Item was as described, mainly because the event was cheap and you can save a lot of money.	Jingdong prefecture level city in Shandong
Dragon Quality Goods Shop	Green Intelligent Power Saver	Received it a couple days ago! Plugged it in the same day! Let's see what the electric bill is in a month! Hopefully it works well oh!	Jingdong prefecture level city in Shandong
Dragon Quality Goods Shop	Green Intelligent Power Saver	Looking at the reviews good, energy-saving small products, compact, the results after 2 months to see, because the electricity bill season payment	Jingdong prefecture level city in Shandong
Dragon Quality Goods Shop	Green Intelligent Power Saver	I just received it and will try it out to see if it works well. I'll comment later.	Jingdong prefecture level city in Shandong
Dragon Quality Goods Shop	Green Intelligent Power Saver	It looks a little small and not very aesthetically pleasing, I don't know how effective it will be until I use it	Jingdong prefecture level city in Shandong
Dragon Quality Goods Shop	Green Intelligent Power Saver	Not only to the point of being useless yet	Jingdong prefecture level city in Shandong
Dragon Quality Goods Shop	Green Intelligent Power Saver	Used it for a month, nothing works, the meter you turn very fast. This is not a scam, but also sold on Jingdong. To cheat a bunch of people?	Jingdong prefecture level city in Shandong
Dragon Quality Goods Shop	Green Intelligent Power Saver	Very good to use, every day can save a little electricity, really good under the sample down a month to save a few dozen dollars in electricity, good before I did not know that there is this 👆 haha, the price is not expensive praise.	Jingdong prefecture level city in Shandong
Dragon Quality Goods Shop	Green Intelligent Power Saver	Tried it for ten days and the change is almost nothing, can't see the power saving yet, use it some more days and see how it goes.	Jingdong prefecture level city in Shandong
Dragon Quality Goods Shop	Green Intelligent Power Saver	Received very quickly, the quality is very good, really save electricity, business service attitude is also very good, I hope you do not miss this opportunity, I continue to buy a few in	Jingdong prefecture level city in Shandong
Dragon Quality Goods Shop	Green Intelligent Power Saver	Quality is very good Oh, the delivery is also very fast, a very favorite store, I want to crazy recommend to friends!	Jingdong prefecture level city in Shandong
Dragon Quality Goods Shop	Green Intelligent Power Saver	The goods have just received, fine workmanship, and the same as described by the merchant, to use a period of time to see if it can achieve the purpose of power saving.	Jingdong prefecture level city in Shandong
Dragon Quality Goods Shop	Green Intelligent Power Saver	Received the goods first use a period of time to see if there is no effect, the business commitment to ninety days invalid refund, first praise, good service!	Jingdong prefecture level city in Shandong
Dragon Quality Goods Shop	Green Intelligent Power Saver	The product is used for a month without energy-saving and power-saving effect does not have any effect	Jingdong prefecture level city in Shandong
Dragon Quality Goods Shop	Green Intelligent Power Saver	The baby is really great, I like it very much, my friends also say good to come, recommended to buy!	Jingdong prefecture level city in Shandong
Dragon Quality Goods Shop	Green Intelligent Power Saver	Logistics is fast, courier boy service is good, after using to see how the fruit, and then follow up.	Jingdong prefecture level city in Shandong
Dragon Quality Goods Shop	Green Intelligent Power Saver	Baby has been received to try, I believe that as the business advertised energy saving. The courier is very responsible.	Jingdong prefecture level city in Shandong
Dragon Quality Goods Shop	Green Intelligent Power Saver	Save Electric Energy King received, good quality, beautiful appearance, very satisfied!	Jingdong prefecture level city in Shandong
Dragon Quality Goods Shop	Green Intelligent Power Saver	Three no products no manufacturers no phone pure fraud induced consumption cheat	Jingdong prefecture level city in Shandong
Dragon Quality Goods Shop	Green Intelligent Power Saver	I'm using it now, I don't know if it's as good as it says, I'll follow up after a month	Jingdong prefecture level city in Shandong
Dragon Quality Goods Shop	Green Intelligent Power Saver	Good quality logistics and fast. Fine workmanship. The description is consistent cost-effective	Jingdong prefecture level city in Shandong
Dragon Quality Goods Shop	Green Intelligent Power Saver	Received, whether it is good or not, to wait a few days and then evaluate	Jingdong prefecture level city in Shandong
Dragon Quality Goods Shop	Green Intelligent Power Saver	I bought it and started using it, I won't know if it works well until I pay my electric bill	Jingdong prefecture level city in Shandong
Dragon Quality Goods Shop	Green Intelligent Power Saver	Especially good, energy saving and power saving, especially good quality, good	Jingdong prefecture level city in Shandong
Dragon Quality Goods Shop	Green Intelligent Power Saver	Logistics is strong, the quality is very good, paste really save electricity, as described, will buy back!	Jingdong prefecture level city in Shandong
Dragon Quality Goods Shop	Green Intelligent Power Saver	Great stuff at a great price, worth the money!	Jingdong prefecture level city in Shandong

Dragon Quality Goods Shop	Green Intelligent Power Saver	Received the power saver, very good quality, very power saving!	Jingdong prefecture level city in Shandong
Dragon Quality Goods Shop	Green Intelligent Power Saver	There are no fruits, only samples...	Jingdong prefecture level city in Shandong
Dragon Quality Goods Shop	Green Intelligent Power Saver	Save a few dollars a month.	Jingdong prefecture level city in Shandong
Dragon Quality Goods Shop	Green Intelligent Power Saver	The quality is particularly good, it works well, and the seller has a good service attitude	Jingdong prefecture level city in Shandong
Dragon Quality Goods Shop	Green Intelligent Power Saver	I've compared them a couple of times and I don't feel any difference	Jingdong prefecture level city in Shandong
Dragon Quality Goods Shop	Green Intelligent Power Saver	I don't see the savings. It's a sham.	Jingdong prefecture level city in Shandong
Dragon Quality Goods Shop	Green Intelligent Power Saver	The power saving effect is very good, and the logistics is very fast. The seller's service attitude is very good	Jingdong prefecture level city in Shandong
Dragon Quality Goods Shop	Green Intelligent Power Saver	Received, well packaged, haven't tried it yet!	Jingdong prefecture level city in Shandong
Dragon Quality Goods Shop	Green Intelligent Power Saver	I don't know how it works, I'll comment after I use it.	Jingdong prefecture level city in Shandong
Dragon Quality Goods Shop	Green Intelligent Power Saver	The product doesn't work well enough to be of any use	Jingdong prefecture level city in Shandong
Dragon Quality Goods Shop	Green Intelligent Power Saver	On Trial 。。。。。。。	Jingdong prefecture level city in Shandong
Dragon Quality Goods Shop	Green Intelligent Power Saver	Being used with unknown results	Jingdong prefecture level city in Shandong
Dragon Quality Goods Shop	Green Intelligent Power Saver	Smart power saving and great quality	Jingdong prefecture level city in Shandong
Dragon Quality Goods Shop	Green Intelligent Power Saver	The saver was received, it works very well and the price is affordable!	Jingdong prefecture level city in Shandong
Dragon Quality Goods Shop	Green Intelligent Power Saver	How can I feel the power saving and how long does it last	Jingdong prefecture level city in Shandong
Dragon Quality Goods Shop	Green Intelligent Power Saver	And I know if it saves electricity or not.	Jingdong prefecture level city in Shandong
Dragon Quality Goods Shop	Green Intelligent Power Saver	It won't save any electricity. No.	Jingdong prefecture level city in Shandong
Dragon Quality Goods Shop	Green Intelligent Power Saver	Invalid. Fake.	Jingdong prefecture level city in Shandong
Dragon Quality Goods Shop	Green Intelligent Power Saver	It's good!	Jingdong prefecture level city in Shandong
Dragon Quality Goods Shop	Green Intelligent Power Saver	have no effect	Jingdong prefecture level city in Shandong
Dragon Quality Goods Shop	Green Intelligent Power Saver	Not used.	Jingdong prefecture level city in Shandong
Dragon Quality Goods Shop	Green Intelligent Power Saver	There is no load range, packaging thrift, ten days also did not develop invoices, received the goods with a receipt, hand-written receipt also has no financial stamp, communication for nearly a week a moment to say that the electronic invoice, a moment to say that the paper invoice, said it is mailed to me, ten days also did not see anything, the invoice invoice can not be found to say that did not stay on file, single single number can not be found, said that the warehouse more than one to a one to find out, in addition to the perfunctory or perfunctory. Products are no certificate of conformity, proper three	Jingdong prefecture level city in Shandong
Dragon Quality Goods Shop	Green Intelligent Power Saver	Satisfaction.	Jingdong prefecture level city in Shandong
Dragon Quality Goods Shop	Green Intelligent Power Saver	There is not a bit of power saving effect, and ringing up an increase in the electricity bill, because you have to add the cost of this machine's own electricity consumption and purchase, so I think it is better to use this machine instead of increasing the expenditure, uneconomical, rather than not use it.	Jingdong prefecture level city in Shandong
Dragon Quality Goods Shop	Green Intelligent Power Saver	I've been using it for two days now and I don't feel any effect.	Jingdong prefecture level city in Shandong
Dragon Quality Goods Shop	Green Intelligent Power Saver	What it looked like was nothing like what was sent.	Jingdong prefecture level city in Shandong
Dragon Quality Goods Shop	Green Intelligent Power Saver	It didn't work. It didn't work for a month or so.	Jingdong prefecture level city in Shandong
Dragon Quality Goods Shop	Green Intelligent Power Saver	Garbage products, physical and description is not the same, no effect at all, pit # no discussion, to buy the fine, garbage, garbage, garbage	Jingdong prefecture level city in Shandong
Dragon Quality Goods Shop	Green Intelligent Power Saver	general	Jingdong prefecture level city in Shandong
Dragon Quality Goods Shop	Green Intelligent Power Saver	IQ tax. Instead of saving electricity, it increases the power consumption of an appliance.	Jingdong prefecture level city in Shandong
Dragon Quality Goods Shop	Green Intelligent Power Saver	There's no trash at all.	Jingdong prefecture level city in Shandong
Dragon Quality Goods Shop	Green Intelligent Power Saver	It doesn't do anything.	Jingdong prefecture level city in Shandong
Dragon Quality Goods Shop	Green Intelligent Power Saver	(of an unmarried couple) be close	Jingdong prefecture level city in Shandong
Dragon Quality Goods Shop	Green Intelligent Power Saver	(of an unmarried couple) be close	Jingdong prefecture level city in Shandong
Dragon Quality Goods Shop	Green Intelligent Power Saver	no electricity saving	Jingdong prefecture level city in Shandong
Dragon Quality Goods Shop	Green Intelligent Power Saver	differ from	Jingdong prefecture level city in Shandong
Dragon Quality Goods Shop	Green Intelligent Power Saver	Saver has received, but real can save electricity, the quality is very good, I times I buy a few more, too good.	Jingdong prefecture level city in Shandong
Dragon Quality Goods Shop	Green Intelligent Power Saver	Great quality as always, a very favorite product, great! o(*￣︶￣*)o	Jingdong prefecture level city in Shandong
Dragon Quality Goods Shop	Green Intelligent Power Saver	If we can reduce the electricity bill, for individuals can save money, for the country can protect the environment, now the weather is getting hotter and hotter, to protect the environment to reduce emissions is very important. Already use the product, whether to reduce the electricity bill depends on next month!	Jingdong prefecture level city in Shandong
Dragon Quality Goods Shop	Green Intelligent Power Saver	Purchased a power saver received on the put into use, rent a room with many electrical appliances, power consumption is very large, we have a piece of electricity or 1.2 dollars a time, with this power saver is much better, every day can save a few cents, very convenient and practical!	Jingdong prefecture level city in Shandong
Dragon Quality Goods Shop	Green Intelligent Power Saver	The product is better to use before not to use when a month 1500 yuan electricity bill, now using the 5 second month electricity bill directly down 470 dollars or very good. A save money to buy several power saver money ZanZanZanZan👍	Jingdong prefecture level city in Shandong
Dragon Quality Goods Shop	Green Intelligent Power Saver	The moment I received the goods really satisfied with this product, not only high-grade packaging, the body are base alloy, up to the price, I was also introduced by a friend, he used last month did save a lot of electricity, but also recommended to me!	Jingdong prefecture level city in Shandong
Dragon Quality Goods Shop	Green Intelligent Power Saver	I got it. Good review. I'm very satisfied. Reasonable price.	Jingdong prefecture level city in Shandong
Dragon Quality Goods Shop	Green Intelligent Power Saver	Intelligent power saver received and used, looking at the quality is good, the quality of the plug sent is also very good. Expect the effect of the economizer is obvious, first five-star praise, with a period of time to follow up!	Jingdong prefecture level city in Shandong
Dragon Quality Goods Shop	Green Intelligent Power Saver	The saver has been received. It was plugged into an outlet and used immediately after receipt. Whether it really saves electricity is not clear yet, I believe that this brand of energy saver really saves	Jingdong prefecture level city in Shandong
Dragon Quality Goods Shop	Green Intelligent Power Saver	Okay, okay, okay. Thank you so much. Fast is good. Fast. Item was as described, mainly because the event was cheap and you can save a lot of money.	Jingdong prefecture level city in Shandong

Dragon Quality Goods Shop	Green Intelligent Power Saver	Received it a couple days ago! Plugged it in the same day! Let's see what the electric bill is in a month! Hopefully it works well oh!	Jingdong prefecture level city in Shandong
Dragon Quality Goods Shop	Green Intelligent Power Saver	Looking at the reviews good, energy-saving small products, compact, the results after 2 months to see, because the electricity bill season payment	Jingdong prefecture level city in Shandong
Dragon Quality Goods Shop	Green Intelligent Power Saver	I just received it and will try it out to see if it works well. I'll comment later.	Jingdong prefecture level city in Shandong
Dragon Quality Goods Shop	Green Intelligent Power Saver	It looks a little small and not very aesthetically pleasing, I don't know how effective it will be until I use it	Jingdong prefecture level city in Shandong
Dragon Quality Goods Shop	Green Intelligent Power Saver	Not only to the point of being useless yet	Jingdong prefecture level city in Shandong
Dragon Quality Goods Shop	Green Intelligent Power Saver	Used it for a month, nothing works, the meter you turn very fast. This is not a scam, but also sold on Jingdong. To cheat a bunch of people?	Jingdong prefecture level city in Shandong
Dragon Quality Goods Shop	Green Intelligent Power Saver	Very good to use, every day can save a little electricity, really good under the sample down a month to save a few dozen dollars in electricity, good before I did not know that there is this haha, the price is not expensive praise.	Jingdong prefecture level city in Shandong
Dragon Quality Goods Shop	Green Intelligent Power Saver	Tried it for ten days and the change is almost nothing, can't see the power saving yet, use it some more days and see how it goes.	Jingdong prefecture level city in Shandong
Dragon Quality Goods Shop	Green Intelligent Power Saver	Received very quickly, the quality is very good, really save electricity, business service attitude is also very good, I hope you do not miss this opportunity, I continue to buy a few in	Jingdong prefecture level city in Shandong
Dragon Quality Goods Shop	Green Intelligent Power Saver	Quality is very good Oh, the delivery is also very fast, a very favorite store, I want to crazy recommend to friends!	Jingdong prefecture level city in Shandong
Dragon Quality Goods Shop	Green Intelligent Power Saver	The goods have just received, fine workmanship, and the same as described by the merchant, to use a period of time to see if it can achieve the purpose of power saving.	Jingdong prefecture level city in Shandong
Dragon Quality Goods Shop	Green Intelligent Power Saver	Received the goods first use a period of time to see if there is no effect, the business commitment to ninety days invalid refund, first praise, good service!	Jingdong prefecture level city in Shandong
Dragon Quality Goods Shop	Green Intelligent Power Saver	The product is used for a month without energy-saving and power-saving effect does not have any effect	Jingdong prefecture level city in Shandong
Dragon Quality Goods Shop	Green Intelligent Power Saver	The baby is really great, I like it very much, my friends also say good to come, recommended to buy!	Jingdong prefecture level city in Shandong
Dragon Quality Goods Shop	Green Intelligent Power Saver	Logistics is fast, courier boy service is good, after using to see how the fruit, and then follow up.	Jingdong prefecture level city in Shandong
Dragon Quality Goods Shop	Green Intelligent Power Saver	Baby has been received to try, I believe that as the business advertised energy saving. The courier is very responsible.	Jingdong prefecture level city in Shandong
Dragon Quality Goods Shop	Green Intelligent Power Saver	Save Electric Energy King received, good quality, beautiful appearance, very satisfied!	Jingdong prefecture level city in Shandong
Dragon Quality Goods Shop	Green Intelligent Power Saver	Three no products no manufacturers no phone pure fraud induced consumption cheat	Jingdong prefecture level city in Shandong
Dragon Quality Goods Shop	Green Intelligent Power Saver	I'm using it now, I don't know if it's as good as it says, I'll follow up after a month	Jingdong prefecture level city in Shandong
Dragon Quality Goods Shop	Green Intelligent Power Saver	Good quality logistics and fast. Fine workmanship. The description is consistent cost-effective	Jingdong prefecture level city in Shandong
Dragon Quality Goods Shop	Green Intelligent Power Saver	Received, whether it is good or not, to wait a few days and then evaluate	Jingdong prefecture level city in Shandong
Dragon Quality Goods Shop	Green Intelligent Power Saver	I bought it and started using it, I won't know if it works well until I pay my electric bill	Jingdong prefecture level city in Shandong
Dragon Quality Goods Shop	Green Intelligent Power Saver	Especially good, energy saving and power saving, especially good quality, good	Jingdong prefecture level city in Shandong
Dragon Quality Goods Shop	Green Intelligent Power Saver	Logistics is strong, the quality is very good, paste really save electricity, as described, will buy back!	Jingdong prefecture level city in Shandong
Dragon Quality Goods Shop	Green Intelligent Power Saver	Great stuff at a great price, worth the money!	Jingdong prefecture level city in Shandong
Dragon Quality Goods Shop	Green Intelligent Power Saver	Received the power saver, very good quality, very power saving!	Jingdong prefecture level city in Shandong
Dragon Quality Goods Shop	Green Intelligent Power Saver	There are no fruits, only samples...	Jingdong prefecture level city in Shandong
Dragon Quality Goods Shop	Green Intelligent Power Saver	Save a few dollars a month.	Jingdong prefecture level city in Shandong
Dragon Quality Goods Shop	Green Intelligent Power Saver	The quality is particularly good, it works well, and the seller has a good service attitude	Jingdong prefecture level city in Shandong
Dragon Quality Goods Shop	Green Intelligent Power Saver	I've compared them a couple of times and I don't feel any difference	Jingdong prefecture level city in Shandong
Dragon Quality Goods Shop	Green Intelligent Power Saver	I don't see the savings. It's a sham.	Jingdong prefecture level city in Shandong
Dragon Quality Goods Shop	Green Intelligent Power Saver	The power saving effect is very good, and the logistics is very fast. The seller's service attitude is very good	Jingdong prefecture level city in Shandong
Dragon Quality Goods Shop	Green Intelligent Power Saver	Received, well packaged, haven't tried it yet!	Jingdong prefecture level city in Shandong
Dragon Quality Goods Shop	Green Intelligent Power Saver	I don't know how it works, I'll comment after I use it.	Jingdong prefecture level city in Shandong
Dragon Quality Goods Shop	Green Intelligent Power Saver	The product doesn't work well enough to be of any use	Jingdong prefecture level city in Shandong
Dragon Quality Goods Shop	Green Intelligent Power Saver	On Trial 。。。。。。。	Jingdong prefecture level city in Shandong
Dragon Quality Goods Shop	Green Intelligent Power Saver	Being used with unknown results	Jingdong prefecture level city in Shandong
Dragon Quality Goods Shop	Green Intelligent Power Saver	Smart power saving and great quality	Jingdong prefecture level city in Shandong
Dragon Quality Goods Shop	Green Intelligent Power Saver	The saver was received, it works very well and the price is affordable!	Jingdong prefecture level city in Shandong
Dragon Quality Goods Shop	Green Intelligent Power Saver	How can I feel the power saving and how long does it last	Jingdong prefecture level city in Shandong
Dragon Quality Goods Shop	Green Intelligent Power Saver	And I know if it saves electricity or not.	Jingdong prefecture level city in Shandong
Dragon Quality Goods Shop	Green Intelligent Power Saver	It won't save any electricity. No.	Jingdong prefecture level city in Shandong
Dragon Quality Goods Shop	Green Intelligent Power Saver	Invalid. Fake.	Jingdong prefecture level city in Shandong
Dragon Quality Goods Shop	Green Intelligent Power Saver	It's good!	Jingdong prefecture level city in Shandong
Dragon Quality Goods Shop	Green Intelligent Power Saver	have no effect	Jingdong prefecture level city in Shandong
Dragon Quality Goods Shop	Green Intelligent Power Saver	Not used.	Jingdong prefecture level city in Shandong
Dragon Quality Goods Shop	Green Intelligent Power Saver	There is no load range, packaging thrift, ten days also did not develop invoices, received the goods with a receipt, hand-written receipt also has no financial stamp, communication for nearly a week a moment to say that the electronic invoice, a moment to say that the paper invoice, said it is mailed to me, ten days also did not see anything, the invoice invoice can not be found to say that did not stay on file, single single number can not be found, said that the warehouse more than one to a one to find out, in addition to the perfunctory or perfunctory. Products are no certificate of conformity, proper three	Jingdong prefecture level city in Shandong
Dragon Quality Goods Shop	Green Intelligent Power Saver	Satisfaction.	Jingdong prefecture level city in Shandong

Dragon Quality Goods Shop	Green Intelligent Power Saver	There is not a bit of power saving effect, and ringing up an increase in the electricity bill, because you have to add the cost of this machine's own electricity consumption and purchase, so I think it is better to use this machine instead of increasing the expenditure, uneconomical, rather than not use it.	Jingdong prefecture level city in Shandong
Dragon Quality Goods Shop	Green Intelligent Power Saver	I've been using it for two days now and I don't feel any effect.	Jingdong prefecture level city in Shandong
Dragon Quality Goods Shop	Green Intelligent Power Saver	What it looked like was nothing like what was sent.	Jingdong prefecture level city in Shandong
Dragon Quality Goods Shop	Green Intelligent Power Saver	It didn't work. It didn't work for a month or so.	Jingdong prefecture level city in Shandong
Dragon Quality Goods Shop	Green Intelligent Power Saver	Garbage products, physical and description is not the same, no effect at all, pit # no discussion, to buy the fine, garbage, garbage, garbage	Jingdong prefecture level city in Shandong
Dragon Quality Goods Shop	Green Intelligent Power Saver	general	Jingdong prefecture level city in Shandong
Dragon Quality Goods Shop	Green Intelligent Power Saver	IQ tax. Instead of saving electricity, it increases the power consumption of an appliance.	Jingdong prefecture level city in Shandong
Dragon Quality Goods Shop	Green Intelligent Power Saver	There's no trash at all.	Jingdong prefecture level city in Shandong
Dragon Quality Goods Shop	Green Intelligent Power Saver	It doesn't do anything.	Jingdong prefecture level city in Shandong
Dragon Quality Goods Shop	Green Intelligent Power Saver	(of an unmarried couple) be close	Jingdong prefecture level city in Shandong
Dragon Quality Goods Shop	Green Intelligent Power Saver	(of an unmarried couple) be close	Jingdong prefecture level city in Shandong
Dragon Quality Goods Shop	Green Intelligent Power Saver	no electricity saving	Jingdong prefecture level city in Shandong
Dragon Quality Goods Shop	Green Intelligent Power Saver	differ from	Jingdong prefecture level city in Shandong
Dragon Quality Goods Shop	Green Intelligent Power Saver	Saver has received, but real can save electricity, the quality is very good, I times I buy a few more, too good.	Jingdong prefecture level city in Shandong
Dragon Quality Goods Shop	Green Intelligent Power Saver	Great quality as always, a very favorite product, great! o(*￣︶￣*)o	Jingdong prefecture level city in Shandong
Dragon Quality Goods Shop	Green Intelligent Power Saver	If we can reduce the electricity bill, for individuals can save money, for the country can protect the environment, now the weather is getting hotter and hotter, to protect the environment to reduce emissions is very important. Already use the product, whether to reduce the electricity bill depends on next month!	Jingdong prefecture level city in Shandong
Dragon Quality Goods Shop	Green Intelligent Power Saver	Purchased a power saver received on the put into use, rent a room with many electrical appliances, power consumption is very large, we have a piece of electricity or 1.2 dollars a time, with this power saver is much better, every day can save a few cents, very convenient and practical!	Jingdong prefecture level city in Shandong
Dragon Quality Goods Shop	Green Intelligent Power Saver	The product is better to use before not to use when a month 1500 yuan electricity bill, now using the 5 second month electricity bill directly down 470 dollars or very good. A save money to buy several power saver money ZanZanZanZan👍	Jingdong prefecture level city in Shandong
Dragon Quality Goods Shop	Green Intelligent Power Saver	The moment I received the goods really satisfied with this product, not only high-grade packaging, the body are base alloy, up to the price, I was also introduced by a friend, he used last month did save a lot of electricity, but also recommended to me!	Jingdong prefecture level city in Shandong
Dragon Quality Goods Shop	Green Intelligent Power Saver	I got it. Good review. I'm very satisfied. Reasonable price.	Jingdong prefecture level city in Shandong
Dragon Quality Goods Shop	Green Intelligent Power Saver	Intelligent power saver received and used, looking at the quality is good, the quality of the plug sent is also very good. Expect the effect of the economizer is obvious, first five-star praise, with a period of time to follow up!	Jingdong prefecture level city in Shandong
Dragon Quality Goods Shop	Green Intelligent Power Saver	The saver has been received. It was plugged into an outlet and used immediately after receipt. Whether it really saves electricity is not clear yet, I believe that this brand of energy saver really saves	Jingdong prefecture level city in Shandong
Dragon Quality Goods Shop	Green Intelligent Power Saver	Okay, okay, okay. Thank you so much. Fast is good. Fast. Item was as described, mainly because the event was cheap and you can save a lot of money.	Jingdong prefecture level city in Shandong
Dragon Quality Goods Shop	Green Intelligent Power Saver	Received it a couple days ago! Plugged it in the same day! Let's see what the electric bill is in a month! Hopefully it works well oh!	Jingdong prefecture level city in Shandong
Dragon Quality Goods Shop	Green Intelligent Power Saver	Looking at the reviews good, energy-saving small products, compact, the results after 2 months to see, because the electricity bill season payment	Jingdong prefecture level city in Shandong
Dragon Quality Goods Shop	Green Intelligent Power Saver	I just received it and will try it out to see if it works well. I'll comment later.	Jingdong prefecture level city in Shandong
Dragon Quality Goods Shop	Green Intelligent Power Saver	It looks a little small and not very aesthetically pleasing, I don't know how effective it will be until I use it	Jingdong prefecture level city in Shandong
Dragon Quality Goods Shop	Green Intelligent Power Saver	Not only to the point of being useless yet	Jingdong prefecture level city in Shandong
Dragon Quality Goods Shop	Green Intelligent Power Saver	Used it for a month, nothing works, the meter you turn very fast. This is not a scam, but also sold on Jingdong. To cheat a bunch of people?	Jingdong prefecture level city in Shandong
Dragon Quality Goods Shop	Green Intelligent Power Saver	Very good to use, every day can save a little electricity, really good under the sample down a month to save a few dozen dollars in electricity, good before I did not know that there is this 👆 haha, the price is not expensive praise.	Jingdong prefecture level city in Shandong
Dragon Quality Goods Shop	Green Intelligent Power Saver	Tried it for ten days and the change is almost nothing, can't see the power saving yet, use it some more days and see how it goes.	Jingdong prefecture level city in Shandong
Dragon Quality Goods Shop	Green Intelligent Power Saver	Received very quickly, the quality is very good, really save electricity, business service attitude is also very good, I hope you do not miss this opportunity, I continue to buy a few in	Jingdong prefecture level city in Shandong
Dragon Quality Goods Shop	Green Intelligent Power Saver	Quality is very good Oh, the delivery is also very fast, a very favorite store, I want to crazy recommend to friends!	Jingdong prefecture level city in Shandong
Dragon Quality Goods Shop	Green Intelligent Power Saver	The goods have just received, fine workmanship, and the same as described by the merchant, to use a period of time to see if it can achieve the purpose of power saving.	Jingdong prefecture level city in Shandong
Dragon Quality Goods Shop	Green Intelligent Power Saver	Received the goods first use a period of time to see if there is no effect, the business commitment to ninety days invalid refund, first praise, good service!	Jingdong prefecture level city in Shandong
Dragon Quality Goods Shop	Green Intelligent Power Saver	The product is used for a month without energy-saving and power-saving effect does not have any effect	Jingdong prefecture level city in Shandong
Dragon Quality Goods Shop	Green Intelligent Power Saver	The baby is really great, I like it very much, my friends also say good to come, recommended to buy!	Jingdong prefecture level city in Shandong
Dragon Quality Goods Shop	Green Intelligent Power Saver	Logistics is fast, courier boy service is good, after using to see how the fruit, and then follow up.	Jingdong prefecture level city in Shandong
Dragon Quality Goods Shop	Green Intelligent Power Saver	Baby has been received to try, I believe that as the business advertised energy saving. The courier is very responsible.	Jingdong prefecture level city in Shandong
Dragon Quality Goods Shop	Green Intelligent Power Saver	Save Electric Energy King received, good quality, beautiful appearance, very satisfied!	Jingdong prefecture level city in Shandong
Dragon Quality Goods Shop	Green Intelligent Power Saver	Three no products no manufacturers no phone pure fraud induced consumption cheat	Jingdong prefecture level city in Shandong
Dragon Quality Goods Shop	Green Intelligent Power Saver	I'm using it now, I don't know if it's as good as it says, I'll follow up after a month	Jingdong prefecture level city in Shandong
Dragon Quality Goods Shop	Green Intelligent Power Saver	Good quality logistics and fast. Fine workmanship. The description is consistent cost-effective	Jingdong prefecture level city in Shandong
Dragon Quality Goods Shop	Green Intelligent Power Saver	Received, whether it is good or not, to wait a few days and then evaluate	Jingdong prefecture level city in Shandong
Dragon Quality Goods Shop	Green Intelligent Power Saver	I bought it and started using it, I won't know if it works well until I pay my electric bill	Jingdong prefecture level city in Shandong
Dragon Quality Goods Shop	Green Intelligent Power Saver	Especially good, energy saving and power saving, especially good quality, good	Jingdong prefecture level city in Shandong
Dragon Quality Goods Shop	Green Intelligent Power Saver	Logistics is strong, the quality is very good, paste really save electricity, as described, will buy back!	Jingdong prefecture level city in Shandong
Dragon Quality Goods Shop	Green Intelligent Power Saver	Great stuff at a great price, worth the money!	Jingdong prefecture level city in Shandong

Dragon Quality Goods Shop	Green Intelligent Power Saver	Received the power saver, very good quality, very power saving!	Jingdong prefecture level city in Shandong
Dragon Quality Goods Shop	Green Intelligent Power Saver	There are no fruits, only samples...	Jingdong prefecture level city in Shandong
Dragon Quality Goods Shop	Green Intelligent Power Saver	Save a few dollars a month.	Jingdong prefecture level city in Shandong
Dragon Quality Goods Shop	Green Intelligent Power Saver	The quality is particularly good, it works well, and the seller has a good service attitude	Jingdong prefecture level city in Shandong
Dragon Quality Goods Shop	Green Intelligent Power Saver	I've compared them a couple of times and I don't feel any difference	Jingdong prefecture level city in Shandong
Dragon Quality Goods Shop	Green Intelligent Power Saver	I don't see the savings. It's a sham.	Jingdong prefecture level city in Shandong
Dragon Quality Goods Shop	Green Intelligent Power Saver	The power saving effect is very good, and the logistics is very fast. The seller's service attitude is very good	Jingdong prefecture level city in Shandong
Dragon Quality Goods Shop	Green Intelligent Power Saver	Received, well packaged, haven't tried it yet!	Jingdong prefecture level city in Shandong
Dragon Quality Goods Shop	Green Intelligent Power Saver	I don't know how it works, I'll comment after I use it.	Jingdong prefecture level city in Shandong
Dragon Quality Goods Shop	Green Intelligent Power Saver	The product doesn't work well enough to be of any use	Jingdong prefecture level city in Shandong
Dragon Quality Goods Shop	Green Intelligent Power Saver	On Trial 。。。。。。。	Jingdong prefecture level city in Shandong
Dragon Quality Goods Shop	Green Intelligent Power Saver	Being used with unknown results	Jingdong prefecture level city in Shandong
Dragon Quality Goods Shop	Green Intelligent Power Saver	Smart power saving and great quality	Jingdong prefecture level city in Shandong
Dragon Quality Goods Shop	Green Intelligent Power Saver	The saver was received, it works very well and the price is affordable!	Jingdong prefecture level city in Shandong
Dragon Quality Goods Shop	Green Intelligent Power Saver	How can I feel the power saving and how long does it last	Jingdong prefecture level city in Shandong
Dragon Quality Goods Shop	Green Intelligent Power Saver	And I know if it saves electricity or not.	Jingdong prefecture level city in Shandong
Dragon Quality Goods Shop	Green Intelligent Power Saver	It won't save any electricity. No.	Jingdong prefecture level city in Shandong
Dragon Quality Goods Shop	Green Intelligent Power Saver	Invalid. Fake.	Jingdong prefecture level city in Shandong
Dragon Quality Goods Shop	Green Intelligent Power Saver	It's good!	Jingdong prefecture level city in Shandong
Dragon Quality Goods Shop	Green Intelligent Power Saver	have no effect	Jingdong prefecture level city in Shandong
Dragon Quality Goods Shop	Green Intelligent Power Saver	Not used.	Jingdong prefecture level city in Shandong
Dragon Quality Goods Shop	Green Intelligent Power Saver	There is no load range, packaging thrift, ten days also did not develop invoices, received the goods with a receipt, hand-written receipt also has no financial stamp, communication for nearly a week a moment to say that the electronic invoice, a moment to say that the paper invoice, said it is mailed to me, ten days also did not see anything, the invoice invoice can not be found to say that did not stay on file, single single number can not be found, said that the warehouse more than one to a one to find out, in addition to the perfunctory or perfunctory. Products are no certificate of conformity, proper three	Jingdong prefecture level city in Shandong
Dragon Quality Goods Shop	Green Intelligent Power Saver	Satisfaction.	Jingdong prefecture level city in Shandong
Dragon Quality Goods Shop	Green Intelligent Power Saver	There is not a bit of power saving effect, and ringing up an increase in the electricity bill, because you have to add the cost of this machine's own electricity consumption and purchase, so I think it is better to use this machine instead of increasing the expenditure, uneconomical, rather than not use it.	Jingdong prefecture level city in Shandong
Dragon Quality Goods Shop	Green Intelligent Power Saver	I've been using it for two days now and I don't feel any effect.	Jingdong prefecture level city in Shandong
Dragon Quality Goods Shop	Green Intelligent Power Saver	What it looked like was nothing like what was sent.	Jingdong prefecture level city in Shandong
Dragon Quality Goods Shop	Green Intelligent Power Saver	It didn't work. It didn't work for a month or so.	Jingdong prefecture level city in Shandong
Dragon Quality Goods Shop	Green Intelligent Power Saver	Garbage products, physical and description is not the same, no effect at all, pit # no discussion, to buy the fine, garbage, garbage, garbage	Jingdong prefecture level city in Shandong
Dragon Quality Goods Shop	Green Intelligent Power Saver	general	Jingdong prefecture level city in Shandong
Dragon Quality Goods Shop	Green Intelligent Power Saver	IQ tax. Instead of saving electricity, it increases the power consumption of an appliance.	Jingdong prefecture level city in Shandong
Dragon Quality Goods Shop	Green Intelligent Power Saver	There's no trash at all.	Jingdong prefecture level city in Shandong
Dragon Quality Goods Shop	Green Intelligent Power Saver	It doesn't do anything.	Jingdong prefecture level city in Shandong
Dragon Quality Goods Shop	Green Intelligent Power Saver	(of an unmarried couple) be close	Jingdong prefecture level city in Shandong
Dragon Quality Goods Shop	Green Intelligent Power Saver	(of an unmarried couple) be close	Jingdong prefecture level city in Shandong
Dragon Quality Goods Shop	Green Intelligent Power Saver	no electricity saving	Jingdong prefecture level city in Shandong
Dragon Quality Goods Shop	Green Intelligent Power Saver	differ from	Jingdong prefecture level city in Shandong
Dragon Quality Goods Shop	Green Intelligent Power Saver	Saver has received, but real can save electricity, the quality is very good, I times I buy a few more, too good.	Jingdong prefecture level city in Shandong
Dragon Quality Goods Shop	Green Intelligent Power Saver	Great quality as always, a very favorite product, great! o(*￣︶￣*)o	Jingdong prefecture level city in Shandong
Dragon Quality Goods Shop	Green Intelligent Power Saver	If we can reduce the electricity bill, for individuals can save money, for the country can protect the environment, now the weather is getting hotter and hotter, to protect the environment to reduce emissions is very important. Already use the product, whether to reduce the electricity bill depends on next month!	Jingdong prefecture level city in Shandong
Dragon Quality Goods Shop	Green Intelligent Power Saver	Purchased a power saver received on the put into use, rent a room with many electrical appliances, power consumption is very large, we have a piece of electricity or 1.2 dollars a time, with this power saver is much better, every day can save a few cents, very convenient and practical!	Jingdong prefecture level city in Shandong
Dragon Quality Goods Shop	Green Intelligent Power Saver	The product is better to use before not to use when a month 1500 yuan electricity bill, now using the 5 second month electricity bill directly down 470 dollars or very good. A save money to buy several power saver money ZanZanZanZan👍	Jingdong prefecture level city in Shandong
Dragon Quality Goods Shop	Green Intelligent Power Saver	The moment I received the goods really satisfied with this product, not only high-grade packaging, the body are base alloy, up to the price, I was also introduced by a friend, he used last month did save a lot of electricity, but also recommended to me!	Jingdong prefecture level city in Shandong
Dragon Quality Goods Shop	Green Intelligent Power Saver	I got it. Good review. I'm very satisfied. Reasonable price.	Jingdong prefecture level city in Shandong
Dragon Quality Goods Shop	Green Intelligent Power Saver	Intelligent power saver received and used, looking at the quality is good, the quality of the plug sent is also very good. Expect the effect of the economizer is obvious, first five-star praise, with a period of time to follow up!	Jingdong prefecture level city in Shandong
Dragon Quality Goods Shop	Green Intelligent Power Saver	The saver has been received. It was plugged into an outlet and used immediately after receipt. Whether it really saves electricity is not clear yet, I believe that this brand of energy saver really saves	Jingdong prefecture level city in Shandong
Dragon Quality Goods Shop	Green Intelligent Power Saver	Okay, okay, okay. Thank you so much. Fast is good. Fast. Item was as described, mainly because the event was cheap and you can save a lot of money.	Jingdong prefecture level city in Shandong

Dragon Quality Goods Shop	Green Intelligent Power Saver	Received it a couple days ago! Plugged it in the same day! Let's see what the electric bill is in a month! Hopefully it works well oh!	Jingdong prefecture level city in Shandong
Dragon Quality Goods Shop	Green Intelligent Power Saver	Looking at the reviews good, energy-saving small products, compact, the results after 2 months to see, because the electricity bill season payment	Jingdong prefecture level city in Shandong
Dragon Quality Goods Shop	Green Intelligent Power Saver	I just received it and will try it out to see if it works well. I'll comment later.	Jingdong prefecture level city in Shandong
Dragon Quality Goods Shop	Green Intelligent Power Saver	It looks a little small and not very aesthetically pleasing, I don't know how effective it will be until I use it	Jingdong prefecture level city in Shandong
Dragon Quality Goods Shop	Green Intelligent Power Saver	Not only to the point of being useless yet	Jingdong prefecture level city in Shandong
Dragon Quality Goods Shop	Green Intelligent Power Saver	Used it for a month, nothing works, the meter you turn very fast. This is not a scam, but also sold on Jingdong. To cheat a bunch of people?	Jingdong prefecture level city in Shandong
Dragon Quality Goods Shop	Green Intelligent Power Saver	Very good to use, every day can save a little electricity, really good under the sample down a month to save a few dozen dollars in electricity, good before I did not know that there is this 👆 haha, the price is not expensive praise.	Jingdong prefecture level city in Shandong
Dragon Quality Goods Shop	Green Intelligent Power Saver	Tried it for ten days and the change is almost nothing, can't see the power saving yet, use it some more days and see how it goes.	Jingdong prefecture level city in Shandong
Dragon Quality Goods Shop	Green Intelligent Power Saver	Received very quickly, the quality is very good, really save electricity, business service attitude is also very good, I hope you do not miss this opportunity, I continue to buy a few in	Jingdong prefecture level city in Shandong
Dragon Quality Goods Shop	Green Intelligent Power Saver	Quality is very good Oh, the delivery is also very fast, a very favorite store, I want to crazy recommend to friends!	Jingdong prefecture level city in Shandong
Dragon Quality Goods Shop	Green Intelligent Power Saver	The goods have just received, fine workmanship, and the same as described by the merchant, to use a period of time to see if it can achieve the purpose of power saving.	Jingdong prefecture level city in Shandong
Dragon Quality Goods Shop	Green Intelligent Power Saver	Received the goods first use a period of time to see if there is no effect, the business commitment to ninety days invalid refund, first praise, good service!	Jingdong prefecture level city in Shandong
Dragon Quality Goods Shop	Green Intelligent Power Saver	The product is used for a month without energy-saving and power-saving effect does not have any effect	Jingdong prefecture level city in Shandong
Dragon Quality Goods Shop	Green Intelligent Power Saver	The baby is really great, I like it very much, my friends also say good to come, recommended to buy!	Jingdong prefecture level city in Shandong
Dragon Quality Goods Shop	Green Intelligent Power Saver	Logistics is fast, courier boy service is good, after using to see how the fruit, and then follow up.	Jingdong prefecture level city in Shandong
Dragon Quality Goods Shop	Green Intelligent Power Saver	Baby has been received to try, I believe that as the business advertised energy saving. The courier is very responsible.	Jingdong prefecture level city in Shandong
Dragon Quality Goods Shop	Green Intelligent Power Saver	Save Electric Energy King received, good quality, beautiful appearance, very satisfied!	Jingdong prefecture level city in Shandong
Dragon Quality Goods Shop	Green Intelligent Power Saver	Three no products no manufacturers no phone pure fraud induced consumption cheat	Jingdong prefecture level city in Shandong
Dragon Quality Goods Shop	Green Intelligent Power Saver	I'm using it now, I don't know if it's as good as it says, I'll follow up after a month	Jingdong prefecture level city in Shandong
Dragon Quality Goods Shop	Green Intelligent Power Saver	Good quality logistics and fast. Fine workmanship. The description is consistent cost-effective	Jingdong prefecture level city in Shandong
Dragon Quality Goods Shop	Green Intelligent Power Saver	Received, whether it is good or not, to wait a few days and then evaluate	Jingdong prefecture level city in Shandong
Dragon Quality Goods Shop	Green Intelligent Power Saver	I bought it and started using it, I won't know if it works well until I pay my electric bill	Jingdong prefecture level city in Shandong
Dragon Quality Goods Shop	Green Intelligent Power Saver	Especially good, energy saving and power saving, especially good quality, good	Jingdong prefecture level city in Shandong
Dragon Quality Goods Shop	Green Intelligent Power Saver	Logistics is strong, the quality is very good, paste really save electricity, as described, will buy back!	Jingdong prefecture level city in Shandong
Dragon Quality Goods Shop	Green Intelligent Power Saver	Great stuff at a great price, worth the money!	Jingdong prefecture level city in Shandong
Dragon Quality Goods Shop	Green Intelligent Power Saver	Received the power saver, very good quality, very power saving!	Jingdong prefecture level city in Shandong
Dragon Quality Goods Shop	Green Intelligent Power Saver	There are no fruits, only samples...	Jingdong prefecture level city in Shandong
Dragon Quality Goods Shop	Green Intelligent Power Saver	Save a few dollars a month.	Jingdong prefecture level city in Shandong
Dragon Quality Goods Shop	Green Intelligent Power Saver	The quality is particularly good, it works well, and the seller has a good service attitude	Jingdong prefecture level city in Shandong
Dragon Quality Goods Shop	Green Intelligent Power Saver	I've compared them a couple of times and I don't feel any difference	Jingdong prefecture level city in Shandong
Dragon Quality Goods Shop	Green Intelligent Power Saver	I don't see the savings. It's a sham.	Jingdong prefecture level city in Shandong
Dragon Quality Goods Shop	Green Intelligent Power Saver	The power saving effect is very good, and the logistics is very fast. The seller's service attitude is very good	Jingdong prefecture level city in Shandong
Dragon Quality Goods Shop	Green Intelligent Power Saver	Received, well packaged, haven't tried it yet!	Jingdong prefecture level city in Shandong
Dragon Quality Goods Shop	Green Intelligent Power Saver	I don't know how it works, I'll comment after I use it.	Jingdong prefecture level city in Shandong
Dragon Quality Goods Shop	Green Intelligent Power Saver	The product doesn't work well enough to be of any use	Jingdong prefecture level city in Shandong
Dragon Quality Goods Shop	Green Intelligent Power Saver	On Trial 。。。。。。。	Jingdong prefecture level city in Shandong
Dragon Quality Goods Shop	Green Intelligent Power Saver	Being used with unknown results	Jingdong prefecture level city in Shandong
Dragon Quality Goods Shop	Green Intelligent Power Saver	Smart power saving and great quality	Jingdong prefecture level city in Shandong
Dragon Quality Goods Shop	Green Intelligent Power Saver	The saver was received, it works very well and the price is affordable!	Jingdong prefecture level city in Shandong
Dragon Quality Goods Shop	Green Intelligent Power Saver	How can I feel the power saving and how long does it last	Jingdong prefecture level city in Shandong
Dragon Quality Goods Shop	Green Intelligent Power Saver	And I know if it saves electricity or not.	Jingdong prefecture level city in Shandong
Dragon Quality Goods Shop	Green Intelligent Power Saver	It won't save any electricity. No.	Jingdong prefecture level city in Shandong
Dragon Quality Goods Shop	Green Intelligent Power Saver	Invalid. Fake.	Jingdong prefecture level city in Shandong
Dragon Quality Goods Shop	Green Intelligent Power Saver	It's good!	Jingdong prefecture level city in Shandong
Dragon Quality Goods Shop	Green Intelligent Power Saver	have no effect	Jingdong prefecture level city in Shandong
Dragon Quality Goods Shop	Green Intelligent Power Saver	Not used.	Jingdong prefecture level city in Shandong
Dragon Quality Goods Shop	Green Intelligent Power Saver	There is no load range, packaging thrift, ten days also did not develop invoices, received the goods with a receipt, hand-written receipt also has no financial stamp, communication for nearly a week a moment to say that the electronic invoice, a moment to say that the paper invoice, said it is mailed to me, ten days also did not see anything, the invoice invoice can not be found to say that did not stay on file, single single number can not be found, said that the warehouse more than one to a one to find out, in addition to the perfunctory or perfunctory. Products are no certificate of conformity, proper three	Jingdong prefecture level city in Shandong
Dragon Quality Goods Shop	Green Intelligent Power Saver	Satisfaction.	Jingdong prefecture level city in Shandong

Dragon Quality Goods Shop	Green Intelligent Power Saver	There is not a bit of power saving effect, and ringing up an increase in the electricity bill, because you have to add the cost of this machine's own electricity consumption and purchase, so I think it is better to use this machine instead of increasing the expenditure, uneconomical, rather than not use it.	Jingdong prefecture level city in Shandong
Dragon Quality Goods Shop	Green Intelligent Power Saver	I've been using it for two days now and I don't feel any effect.	Jingdong prefecture level city in Shandong
Dragon Quality Goods Shop	Green Intelligent Power Saver	What it looked like was nothing like what was sent.	Jingdong prefecture level city in Shandong
Dragon Quality Goods Shop	Green Intelligent Power Saver	It didn't work. It didn't work for a month or so.	Jingdong prefecture level city in Shandong
Dragon Quality Goods Shop	Green Intelligent Power Saver	Garbage products, physical and description is not the same, no effect at all, pit # no discussion, to buy the fine, garbage, garbage, garbage	Jingdong prefecture level city in Shandong
Dragon Quality Goods Shop	Green Intelligent Power Saver	general	Jingdong prefecture level city in Shandong
Dragon Quality Goods Shop	Green Intelligent Power Saver	IQ tax. Instead of saving electricity, it increases the power consumption of an appliance.	Jingdong prefecture level city in Shandong
Dragon Quality Goods Shop	Green Intelligent Power Saver	There's no trash at all.	Jingdong prefecture level city in Shandong
Dragon Quality Goods Shop	Green Intelligent Power Saver	It doesn't do anything.	Jingdong prefecture level city in Shandong
Dragon Quality Goods Shop	Green Intelligent Power Saver	(of an unmarried couple) be close	Jingdong prefecture level city in Shandong
Dragon Quality Goods Shop	Green Intelligent Power Saver	(of an unmarried couple) be close	Jingdong prefecture level city in Shandong
Dragon Quality Goods Shop	Green Intelligent Power Saver	no electricity saving	Jingdong prefecture level city in Shandong
Dragon Quality Goods Shop	Green Intelligent Power Saver	differ from	Jingdong prefecture level city in Shandong
Dragon Quality Goods Shop	Green Intelligent Power Saver	Saver has received, but real can save electricity, the quality is very good, I times I buy a few more, too good.	Jingdong prefecture level city in Shandong
Dragon Quality Goods Shop	Green Intelligent Power Saver	Great quality as always, a very favorite product, great! o(*￣︶￣*)o	Jingdong prefecture level city in Shandong
Dragon Quality Goods Shop	Green Intelligent Power Saver	If we can reduce the electricity bill, for individuals can save money, for the country can protect the environment, now the weather is getting hotter and hotter, to protect the environment to reduce emissions is very important. Already use the product, whether to reduce the electricity bill depends on next month!	Jingdong prefecture level city in Shandong
Dragon Quality Goods Shop	Green Intelligent Power Saver	Purchased a power saver received on the put into use, rent a room with many electrical appliances, power consumption is very large, we have a piece of electricity or 1.2 dollars a time, with this power saver is much better, every day can save a few cents, very convenient and practical!	Jingdong prefecture level city in Shandong
Dragon Quality Goods Shop	Green Intelligent Power Saver	The product is better to use before not to use when a month 1500 yuan electricity bill, now using the 5 second month electricity bill directly down 470 dollars or very good. A save money to buy several power saver money ZanZanZanZan👍	Jingdong prefecture level city in Shandong
Dragon Quality Goods Shop	Green Intelligent Power Saver	The moment I received the goods really satisfied with this product, not only high-grade packaging, the body are base alloy, up to the price, I was also introduced by a friend, he used last month did save a lot of electricity, but also recommended to me!	Jingdong prefecture level city in Shandong
Dragon Quality Goods Shop	Green Intelligent Power Saver	I got it. Good review. I'm very satisfied. Reasonable price.	Jingdong prefecture level city in Shandong
Dragon Quality Goods Shop	Green Intelligent Power Saver	Intelligent power saver received and used, looking at the quality is good, the quality of the plug sent is also very good. Expect the effect of the economizer is obvious, first five-star praise, with a period of time to follow up!	Jingdong prefecture level city in Shandong
Dragon Quality Goods Shop	Green Intelligent Power Saver	The saver has been received. It was plugged into an outlet and used immediately after receipt. Whether it really saves electricity is not clear yet, I believe that this brand of energy saver really saves	Jingdong prefecture level city in Shandong
Dragon Quality Goods Shop	Green Intelligent Power Saver	Okay, okay, okay. Thank you so much. Fast is good. Fast. Item was as described, mainly because the event was cheap and you can save a lot of money.	Jingdong prefecture level city in Shandong
Dragon Quality Goods Shop	Green Intelligent Power Saver	Received it a couple days ago! Plugged it in the same day! Let's see what the electric bill is in a month! Hopefully it works well oh!	Jingdong prefecture level city in Shandong
Dragon Quality Goods Shop	Green Intelligent Power Saver	Looking at the reviews good, energy-saving small products, compact, the results after 2 months to see, because the electricity bill season payment	Jingdong prefecture level city in Shandong
Dragon Quality Goods Shop	Green Intelligent Power Saver	I just received it and will try it out to see if it works well. I'll comment later.	Jingdong prefecture level city in Shandong
Dragon Quality Goods Shop	Green Intelligent Power Saver	It looks a little small and not very aesthetically pleasing, I don't know how effective it will be until I use it	Jingdong prefecture level city in Shandong
Dragon Quality Goods Shop	Green Intelligent Power Saver	Not only to the point of being useless yet	Jingdong prefecture level city in Shandong
Dragon Quality Goods Shop	Green Intelligent Power Saver	Used it for a month, nothing works, the meter you turn very fast. This is not a scam, but also sold on Jingdong. To cheat a bunch of people?	Jingdong prefecture level city in Shandong
Dragon Quality Goods Shop	Green Intelligent Power Saver	Very good to use, every day can save a little electricity, really good under the sample down a month to save a few dozen dollars in electricity, good before I did not know that there is this 👆 haha, the price is not expensive praise.	Jingdong prefecture level city in Shandong
Dragon Quality Goods Shop	Green Intelligent Power Saver	Tried it for ten days and the change is almost nothing, can't see the power saving yet, use it some more days and see how it goes.	Jingdong prefecture level city in Shandong
Dragon Quality Goods Shop	Green Intelligent Power Saver	Received very quickly, the quality is very good, really save electricity, business service attitude is also very good, I hope you do not miss this opportunity, I continue to buy a few in	Jingdong prefecture level city in Shandong
Dragon Quality Goods Shop	Green Intelligent Power Saver	Quality is very good Oh, the delivery is also very fast, a very favorite store, I want to crazy recommend to friends!	Jingdong prefecture level city in Shandong
Dragon Quality Goods Shop	Green Intelligent Power Saver	The goods have just received, fine workmanship, and the same as described by the merchant, to use a period of time to see if it can achieve the purpose of power saving.	Jingdong prefecture level city in Shandong
Dragon Quality Goods Shop	Green Intelligent Power Saver	Received the goods first use a period of time to see if there is no effect, the business commitment to ninety days invalid refund, first praise, good service!	Jingdong prefecture level city in Shandong
Dragon Quality Goods Shop	Green Intelligent Power Saver	The product is used for a month without energy-saving and power-saving effect does not have any effect	Jingdong prefecture level city in Shandong
Dragon Quality Goods Shop	Green Intelligent Power Saver	The baby is really great, I like it very much, my friends also say good to come, recommended to buy!	Jingdong prefecture level city in Shandong
Dragon Quality Goods Shop	Green Intelligent Power Saver	Logistics is fast, courier boy service is good, after using to see how the fruit, and then follow up.	Jingdong prefecture level city in Shandong
Dragon Quality Goods Shop	Green Intelligent Power Saver	Baby has been received to try, I believe that as the business advertised energy saving. The courier is very responsible.	Jingdong prefecture level city in Shandong
Dragon Quality Goods Shop	Green Intelligent Power Saver	Save Electric Energy King received, good quality, beautiful appearance, very satisfied!	Jingdong prefecture level city in Shandong
Dragon Quality Goods Shop	Green Intelligent Power Saver	Three no products no manufacturers no phone pure fraud induced consumption cheat	Jingdong prefecture level city in Shandong
Dragon Quality Goods Shop	Green Intelligent Power Saver	I'm using it now, I don't know if it's as good as it says, I'll follow up after a month	Jingdong prefecture level city in Shandong
Dragon Quality Goods Shop	Green Intelligent Power Saver	Good quality logistics and fast. Fine workmanship. The description is consistent cost-effective	Jingdong prefecture level city in Shandong
Dragon Quality Goods Shop	Green Intelligent Power Saver	Received, whether it is good or not, to wait a few days and then evaluate	Jingdong prefecture level city in Shandong
Dragon Quality Goods Shop	Green Intelligent Power Saver	I bought it and started using it, I won't know if it works well until I pay my electric bill	Jingdong prefecture level city in Shandong
Dragon Quality Goods Shop	Green Intelligent Power Saver	Especially good, energy saving and power saving, especially good quality, good	Jingdong prefecture level city in Shandong
Dragon Quality Goods Shop	Green Intelligent Power Saver	Logistics is strong, the quality is very good, paste really save electricity, as described, will buy back!	Jingdong prefecture level city in Shandong
Dragon Quality Goods Shop	Green Intelligent Power Saver	Great stuff at a great price, worth the money!	Jingdong prefecture level city in Shandong

Dragon Quality Goods Shop	Green Intelligent Power Saver	Received the power saver, very good quality, very power saving!	Jingdong prefecture level city in Shandong
Dragon Quality Goods Shop	Green Intelligent Power Saver	There are no fruits, only samples...	Jingdong prefecture level city in Shandong
Dragon Quality Goods Shop	Green Intelligent Power Saver	Save a few dollars a month.	Jingdong prefecture level city in Shandong
Dragon Quality Goods Shop	Green Intelligent Power Saver	The quality is particularly good, it works well, and the seller has a good service attitude	Jingdong prefecture level city in Shandong
Dragon Quality Goods Shop	Green Intelligent Power Saver	I've compared them a couple of times and I don't feel any difference	Jingdong prefecture level city in Shandong
Dragon Quality Goods Shop	Green Intelligent Power Saver	I don't see the savings. It's a sham.	Jingdong prefecture level city in Shandong
Dragon Quality Goods Shop	Green Intelligent Power Saver	The power saving effect is very good, and the logistics is very fast. The seller's service attitude is very good	Jingdong prefecture level city in Shandong
Dragon Quality Goods Shop	Green Intelligent Power Saver	Received, well packaged, haven't tried it yet!	Jingdong prefecture level city in Shandong
Dragon Quality Goods Shop	Green Intelligent Power Saver	I don't know how it works, I'll comment after I use it.	Jingdong prefecture level city in Shandong
Dragon Quality Goods Shop	Green Intelligent Power Saver	The product doesn't work well enough to be of any use	Jingdong prefecture level city in Shandong
Dragon Quality Goods Shop	Green Intelligent Power Saver	On Trial 。。。。。。。	Jingdong prefecture level city in Shandong
Dragon Quality Goods Shop	Green Intelligent Power Saver	Being used with unknown results	Jingdong prefecture level city in Shandong
Dragon Quality Goods Shop	Green Intelligent Power Saver	Smart power saving and great quality	Jingdong prefecture level city in Shandong
Dragon Quality Goods Shop	Green Intelligent Power Saver	The saver was received, it works very well and the price is affordable!	Jingdong prefecture level city in Shandong
Dragon Quality Goods Shop	Green Intelligent Power Saver	How can I feel the power saving and how long does it last	Jingdong prefecture level city in Shandong
Dragon Quality Goods Shop	Green Intelligent Power Saver	And I know if it saves electricity or not.	Jingdong prefecture level city in Shandong
Dragon Quality Goods Shop	Green Intelligent Power Saver	It won't save any electricity. No.	Jingdong prefecture level city in Shandong
Dragon Quality Goods Shop	Green Intelligent Power Saver	Invalid. Fake.	Jingdong prefecture level city in Shandong
Dragon Quality Goods Shop	Green Intelligent Power Saver	It's good!	Jingdong prefecture level city in Shandong
Dragon Quality Goods Shop	Green Intelligent Power Saver	have no effect	Jingdong prefecture level city in Shandong
Dragon Quality Goods Shop	Green Intelligent Power Saver	Not used.	Jingdong prefecture level city in Shandong
Dragon Quality Goods Shop	Green Intelligent Power Saver	There is no load range, packaging thrift, ten days also did not develop invoices, received the goods with a receipt, hand-written receipt also has no financial stamp, communication for nearly a week a moment to say that the electronic invoice, a moment to say that the paper invoice, said it is mailed to me, ten days also did not see anything, the invoice invoice can not be found to say that did not stay on file, single single number can not be found, said that the warehouse more than one to a one to find out, in addition to the perfunctory or perfunctory. Products are no certificate of conformity, proper three	Jingdong prefecture level city in Shandong
Dragon Quality Goods Shop	Green Intelligent Power Saver	Satisfaction.	Jingdong prefecture level city in Shandong
Dragon Quality Goods Shop	Green Intelligent Power Saver	There is not a bit of power saving effect, and ringing up an increase in the electricity bill, because you have to add the cost of this machine's own electricity consumption and purchase, so I think it is better to use this machine instead of increasing the expenditure, uneconomical, rather than not use it.	Jingdong prefecture level city in Shandong
Dragon Quality Goods Shop	Green Intelligent Power Saver	I've been using it for two days now and I don't feel any effect.	Jingdong prefecture level city in Shandong
Dragon Quality Goods Shop	Green Intelligent Power Saver	What it looked like was nothing like what was sent.	Jingdong prefecture level city in Shandong
Dragon Quality Goods Shop	Green Intelligent Power Saver	It didn't work. It didn't work for a month or so.	Jingdong prefecture level city in Shandong
Dragon Quality Goods Shop	Green Intelligent Power Saver	Garbage products, physical and description is not the same, no effect at all, pit # no discussion, to buy the fine, garbage, garbage, garbage	Jingdong prefecture level city in Shandong
Dragon Quality Goods Shop	Green Intelligent Power Saver	general	Jingdong prefecture level city in Shandong
Dragon Quality Goods Shop	Green Intelligent Power Saver	IQ tax. Instead of saving electricity, it increases the power consumption of an appliance.	Jingdong prefecture level city in Shandong
Dragon Quality Goods Shop	Green Intelligent Power Saver	There's no trash at all.	Jingdong prefecture level city in Shandong
Dragon Quality Goods Shop	Green Intelligent Power Saver	It doesn't do anything.	Jingdong prefecture level city in Shandong
Dragon Quality Goods Shop	Green Intelligent Power Saver	(of an unmarried couple) be close	Jingdong prefecture level city in Shandong
Dragon Quality Goods Shop	Green Intelligent Power Saver	(of an unmarried couple) be close	Jingdong prefecture level city in Shandong
Dragon Quality Goods Shop	Green Intelligent Power Saver	no electricity saving	Jingdong prefecture level city in Shandong
Dragon Quality Goods Shop	Green Intelligent Power Saver	differ from	Jingdong prefecture level city in Shandong
Dragon Quality Goods Shop	Green Intelligent Power Saver	Saver has received, but real can save electricity, the quality is very good, I times I buy a few more, too good.	Jingdong prefecture level city in Shandong
Dragon Quality Goods Shop	Green Intelligent Power Saver	Great quality as always, a very favorite product, great! o(*￣︶￣*)o	Jingdong prefecture level city in Shandong
Dragon Quality Goods Shop	Green Intelligent Power Saver	If we can reduce the electricity bill, for individuals can save money, for the country can protect the environment, now the weather is getting hotter and hotter, to protect the environment to reduce emissions is very important. Already use the product, whether to reduce the electricity bill depends on next month!	Jingdong prefecture level city in Shandong
Dragon Quality Goods Shop	Green Intelligent Power Saver	Purchased a power saver received on the put into use, rent a room with many electrical appliances, power consumption is very large, we have a piece of electricity or 1.2 dollars a time, with this power saver is much better, every day can save a few cents, very convenient and practical!	Jingdong prefecture level city in Shandong
Dragon Quality Goods Shop	Green Intelligent Power Saver	The product is better to use before not to use when a month 1500 yuan electricity bill, now using the 5 second month electricity bill directly down 470 dollars or very good. A save money to buy several power saver money ZanZanZanZan👍	Jingdong prefecture level city in Shandong
Dragon Quality Goods Shop	Green Intelligent Power Saver	The moment I received the goods really satisfied with this product, not only high-grade packaging, the body are base alloy, up to the price, I was also introduced by a friend, he used last month did save a lot of electricity, but also recommended to me!	Jingdong prefecture level city in Shandong
Dragon Quality Goods Shop	Green Intelligent Power Saver	I got it. Good review. I'm very satisfied. Reasonable price.	Jingdong prefecture level city in Shandong
Dragon Quality Goods Shop	Green Intelligent Power Saver	Intelligent power saver received and used, looking at the quality is good, the quality of the plug sent is also very good. Expect the effect of the economizer is obvious, first five-star praise, with a period of time to follow up!	Jingdong prefecture level city in Shandong
Dragon Quality Goods Shop	Green Intelligent Power Saver	The saver has been received. It was plugged into an outlet and used immediately after receipt. Whether it really saves electricity is not clear yet, I believe that this brand of energy saver really saves	Jingdong prefecture level city in Shandong
Dragon Quality Goods Shop	Green Intelligent Power Saver	Okay, okay, okay. Thank you so much. Fast is good. Fast. Item was as described, mainly because the event was cheap and you can save a lot of money.	Jingdong prefecture level city in Shandong

Dragon Quality Goods Shop	Green Intelligent Power Saver	Received it a couple days ago! Plugged it in the same day! Let's see what the electric bill is in a month! Hopefully it works well oh!	Jingdong prefecture level city in Shandong
Dragon Quality Goods Shop	Green Intelligent Power Saver	Looking at the reviews good, energy-saving small products, compact, the results after 2 months to see, because the electricity bill season payment	Jingdong prefecture level city in Shandong
Dragon Quality Goods Shop	Green Intelligent Power Saver	I just received it and will try it out to see if it works well. I'll comment later.	Jingdong prefecture level city in Shandong
Dragon Quality Goods Shop	Green Intelligent Power Saver	It looks a little small and not very aesthetically pleasing, I don't know how effective it will be until I use it	Jingdong prefecture level city in Shandong
Dragon Quality Goods Shop	Green Intelligent Power Saver	Not only to the point of being useless yet	Jingdong prefecture level city in Shandong
Dragon Quality Goods Shop	Green Intelligent Power Saver	Used it for a month, nothing works, the meter you turn very fast. This is not a scam, but also sold on Jingdong. To cheat a bunch of people?	Jingdong prefecture level city in Shandong
Dragon Quality Goods Shop	Green Intelligent Power Saver	Very good to use, every day can save a little electricity, really good under the sample down a month to save a few dozen dollars in electricity, good before I did not know that there is this haha, the price is not expensive praise.	Jingdong prefecture level city in Shandong
Dragon Quality Goods Shop	Green Intelligent Power Saver	Tried it for ten days and the change is almost nothing, can't see the power saving yet, use it some more days and see how it goes.	Jingdong prefecture level city in Shandong
Dragon Quality Goods Shop	Green Intelligent Power Saver	Received very quickly, the quality is very good, really save electricity, business service attitude is also very good, I hope you do not miss this opportunity, I continue to buy a few in	Jingdong prefecture level city in Shandong
Dragon Quality Goods Shop	Green Intelligent Power Saver	Quality is very good Oh, the delivery is also very fast, a very favorite store, I want to crazy recommend to friends!	Jingdong prefecture level city in Shandong
Dragon Quality Goods Shop	Green Intelligent Power Saver	The goods have just received, fine workmanship, and the same as described by the merchant, to use a period of time to see if it can achieve the purpose of power saving.	Jingdong prefecture level city in Shandong
Dragon Quality Goods Shop	Green Intelligent Power Saver	Received the goods first use a period of time to see if there is no effect, the business commitment to ninety days invalid refund, first praise, good service!	Jingdong prefecture level city in Shandong
Dragon Quality Goods Shop	Green Intelligent Power Saver	The product is used for a month without energy-saving and power-saving effect does not have any effect	Jingdong prefecture level city in Shandong
Dragon Quality Goods Shop	Green Intelligent Power Saver	The baby is really great, I like it very much, my friends also say good to come, recommended to buy!	Jingdong prefecture level city in Shandong
Dragon Quality Goods Shop	Green Intelligent Power Saver	Logistics is fast, courier boy service is good, after using to see how the fruit, and then follow up.	Jingdong prefecture level city in Shandong
Dragon Quality Goods Shop	Green Intelligent Power Saver	Baby has been received to try, I believe that as the business advertised energy saving. The courier is very responsible.	Jingdong prefecture level city in Shandong
Dragon Quality Goods Shop	Green Intelligent Power Saver	Save Electric Energy King received, good quality, beautiful appearance, very satisfied!	Jingdong prefecture level city in Shandong
Dragon Quality Goods Shop	Green Intelligent Power Saver	Three no products no manufacturers no phone pure fraud induced consumption cheat	Jingdong prefecture level city in Shandong
Dragon Quality Goods Shop	Green Intelligent Power Saver	I'm using it now, I don't know if it's as good as it says, I'll follow up after a month	Jingdong prefecture level city in Shandong
Dragon Quality Goods Shop	Green Intelligent Power Saver	Good quality logistics and fast. Fine workmanship. The description is consistent cost-effective	Jingdong prefecture level city in Shandong
Dragon Quality Goods Shop	Green Intelligent Power Saver	Received, whether it is good or not, to wait a few days and then evaluate	Jingdong prefecture level city in Shandong
Dragon Quality Goods Shop	Green Intelligent Power Saver	I bought it and started using it, I won't know if it works well until I pay my electric bill	Jingdong prefecture level city in Shandong
Dragon Quality Goods Shop	Green Intelligent Power Saver	Especially good, energy saving and power saving, especially good quality, good	Jingdong prefecture level city in Shandong
Dragon Quality Goods Shop	Green Intelligent Power Saver	Logistics is strong, the quality is very good, paste really save electricity, as described, will buy back!	Jingdong prefecture level city in Shandong
Dragon Quality Goods Shop	Green Intelligent Power Saver	Great stuff at a great price, worth the money!	Jingdong prefecture level city in Shandong
Dragon Quality Goods Shop	Green Intelligent Power Saver	Received the power saver, very good quality, very power saving!	Jingdong prefecture level city in Shandong
Dragon Quality Goods Shop	Green Intelligent Power Saver	There are no fruits, only samples...	Jingdong prefecture level city in Shandong
Dragon Quality Goods Shop	Green Intelligent Power Saver	Save a few dollars a month.	Jingdong prefecture level city in Shandong
Dragon Quality Goods Shop	Green Intelligent Power Saver	The quality is particularly good, it works well, and the seller has a good service attitude	Jingdong prefecture level city in Shandong
Dragon Quality Goods Shop	Green Intelligent Power Saver	I've compared them a couple of times and I don't feel any difference	Jingdong prefecture level city in Shandong
Dragon Quality Goods Shop	Green Intelligent Power Saver	I don't see the savings. It's a sham.	Jingdong prefecture level city in Shandong
Dragon Quality Goods Shop	Green Intelligent Power Saver	The power saving effect is very good, and the logistics is very fast. The seller's service attitude is very good	Jingdong prefecture level city in Shandong
Dragon Quality Goods Shop	Green Intelligent Power Saver	Received, well packaged, haven't tried it yet!	Jingdong prefecture level city in Shandong
Dragon Quality Goods Shop	Green Intelligent Power Saver	I don't know how it works, I'll comment after I use it.	Jingdong prefecture level city in Shandong
Dragon Quality Goods Shop	Green Intelligent Power Saver	The product doesn't work well enough to be of any use	Jingdong prefecture level city in Shandong
Dragon Quality Goods Shop	Green Intelligent Power Saver	On Trial 。。。。。。。	Jingdong prefecture level city in Shandong
Dragon Quality Goods Shop	Green Intelligent Power Saver	Being used with unknown results	Jingdong prefecture level city in Shandong
Dragon Quality Goods Shop	Green Intelligent Power Saver	Smart power saving and great quality	Jingdong prefecture level city in Shandong
Dragon Quality Goods Shop	Green Intelligent Power Saver	The saver was received, it works very well and the price is affordable!	Jingdong prefecture level city in Shandong
Dragon Quality Goods Shop	Green Intelligent Power Saver	How can I feel the power saving and how long does it last	Jingdong prefecture level city in Shandong
Dragon Quality Goods Shop	Green Intelligent Power Saver	And I know if it saves electricity or not.	Jingdong prefecture level city in Shandong
Dragon Quality Goods Shop	Green Intelligent Power Saver	It won't save any electricity. No.	Jingdong prefecture level city in Shandong
Dragon Quality Goods Shop	Green Intelligent Power Saver	Invalid. Fake.	Jingdong prefecture level city in Shandong
Dragon Quality Goods Shop	Green Intelligent Power Saver	It's good!	Jingdong prefecture level city in Shandong
Dragon Quality Goods Shop	Green Intelligent Power Saver	have no effect	Jingdong prefecture level city in Shandong
Dragon Quality Goods Shop	Green Intelligent Power Saver	Not used.	Jingdong prefecture level city in Shandong
Dragon Quality Goods Shop	Green Intelligent Power Saver	There is no load range, packaging thrift, ten days also did not develop invoices, received the goods with a receipt, hand-written receipt also has no financial stamp, communication for nearly a week a moment to say that the electronic invoice, a moment to say that the paper invoice, said it is mailed to me, ten days also did not see anything, the invoice invoice can not be found to say that did not stay on file, single single number can not be found, said that the warehouse more than one to a one to find out, in addition to the perfunctory or perfunctory. Products are no certificate of conformity, proper three	Jingdong prefecture level city in Shandong
Dragon Quality Goods Shop	Green Intelligent Power Saver	Satisfaction.	Jingdong prefecture level city in Shandong

Dragon Quality Goods Shop	Green Intelligent Power Saver	There is not a bit of power saving effect, and ringing up an increase in the electricity bill, because you have to add the cost of this machine's own electricity consumption and purchase, so I think it is better to use this machine instead of increasing the expenditure, uneconomical, rather than not use it.	Jingdong prefecture level city in Shandong
Dragon Quality Goods Shop	Green Intelligent Power Saver	I've been using it for two days now and I don't feel any effect.	Jingdong prefecture level city in Shandong
Dragon Quality Goods Shop	Green Intelligent Power Saver	What it looked like was nothing like what was sent.	Jingdong prefecture level city in Shandong
Dragon Quality Goods Shop	Green Intelligent Power Saver	It didn't work. It didn't work for a month or so.	Jingdong prefecture level city in Shandong
Dragon Quality Goods Shop	Green Intelligent Power Saver	Garbage products, physical and description is not the same, no effect at all, pit # no discussion, to buy the fine, garbage, garbage, garbage	Jingdong prefecture level city in Shandong
Dragon Quality Goods Shop	Green Intelligent Power Saver	general	Jingdong prefecture level city in Shandong
Dragon Quality Goods Shop	Green Intelligent Power Saver	IQ tax. Instead of saving electricity, it increases the power consumption of an appliance.	Jingdong prefecture level city in Shandong
Dragon Quality Goods Shop	Green Intelligent Power Saver	There's no trash at all.	Jingdong prefecture level city in Shandong
Dragon Quality Goods Shop	Green Intelligent Power Saver	It doesn't do anything.	Jingdong prefecture level city in Shandong
Dragon Quality Goods Shop	Green Intelligent Power Saver	(of an unmarried couple) be close	Jingdong prefecture level city in Shandong
Dragon Quality Goods Shop	Green Intelligent Power Saver	(of an unmarried couple) be close	Jingdong prefecture level city in Shandong
Dragon Quality Goods Shop	Green Intelligent Power Saver	no electricity saving	Jingdong prefecture level city in Shandong
Dragon Quality Goods Shop	Green Intelligent Power Saver	differ from	Jingdong prefecture level city in Shandong
Dragon Quality Goods Shop	Green Intelligent Power Saver	Saver has received, but real can save electricity, the quality is very good, I times I buy a few more, too good.	Jingdong prefecture level city in Shandong
Dragon Quality Goods Shop	Green Intelligent Power Saver	Great quality as always, a very favorite product, great! o(*￣︶￣*)o	Jingdong prefecture level city in Shandong
Dragon Quality Goods Shop	Green Intelligent Power Saver	If we can reduce the electricity bill, for individuals can save money, for the country can protect the environment, now the weather is getting hotter and hotter, to protect the environment to reduce emissions is very important. Already use the product, whether to reduce the electricity bill depends on next month!	Jingdong prefecture level city in Shandong
Dragon Quality Goods Shop	Green Intelligent Power Saver	Purchased a power saver received on the put into use, rent a room with many electrical appliances, power consumption is very large, we have a piece of electricity or 1.2 dollars a time, with this power saver is much better, every day can save a few cents, very convenient and practical!	Jingdong prefecture level city in Shandong
Dragon Quality Goods Shop	Green Intelligent Power Saver	The product is better to use before not to use when a month 1500 yuan electricity bill, now using the 5 second month electricity bill directly down 470 dollars or very good. A save money to buy several power saver money ZanZanZanZan👍	Jingdong prefecture level city in Shandong
Dragon Quality Goods Shop	Green Intelligent Power Saver	The moment I received the goods really satisfied with this product, not only high-grade packaging, the body are base alloy, up to the price, I was also introduced by a friend, he used last month did save a lot of electricity, but also recommended to me!	Jingdong prefecture level city in Shandong
Dragon Quality Goods Shop	Green Intelligent Power Saver	I got it. Good review. I'm very satisfied. Reasonable price.	Jingdong prefecture level city in Shandong
Dragon Quality Goods Shop	Green Intelligent Power Saver	Intelligent power saver received and used, looking at the quality is good, the quality of the plug sent is also very good. Expect the effect of the economizer is obvious, first five-star praise, with a period of time to follow up!	Jingdong prefecture level city in Shandong
Dragon Quality Goods Shop	Green Intelligent Power Saver	The saver has been received. It was plugged into an outlet and used immediately after receipt. Whether it really saves electricity is not clear yet, I believe that this brand of energy saver really saves	Jingdong prefecture level city in Shandong
Dragon Quality Goods Shop	Green Intelligent Power Saver	Okay, okay, okay. Thank you so much. Fast is good. Fast. Item was as described, mainly because the event was cheap and you can save a lot of money.	Jingdong prefecture level city in Shandong
Dragon Quality Goods Shop	Green Intelligent Power Saver	Received it a couple days ago! Plugged it in the same day! Let's see what the electric bill is in a month! Hopefully it works well oh!	Jingdong prefecture level city in Shandong
Dragon Quality Goods Shop	Green Intelligent Power Saver	Looking at the reviews good, energy-saving small products, compact, the results after 2 months to see, because the electricity bill season payment	Jingdong prefecture level city in Shandong
Dragon Quality Goods Shop	Green Intelligent Power Saver	I just received it and will try it out to see if it works well. I'll comment later.	Jingdong prefecture level city in Shandong
Dragon Quality Goods Shop	Green Intelligent Power Saver	It looks a little small and not very aesthetically pleasing, I don't know how effective it will be until I use it	Jingdong prefecture level city in Shandong
Dragon Quality Goods Shop	Green Intelligent Power Saver	Not only to the point of being useless yet	Jingdong prefecture level city in Shandong
Dragon Quality Goods Shop	Green Intelligent Power Saver	Used it for a month, nothing works, the meter you turn very fast. This is not a scam, but also sold on Jingdong. To cheat a bunch of people?	Jingdong prefecture level city in Shandong
Dragon Quality Goods Shop	Green Intelligent Power Saver	Very good to use, every day can save a little electricity, really good under the sample down a month to save a few dozen dollars in electricity, good before I did not know that there is this 👆 haha, the price is not expensive praise.	Jingdong prefecture level city in Shandong
Dragon Quality Goods Shop	Green Intelligent Power Saver	Tried it for ten days and the change is almost nothing, can't see the power saving yet, use it some more days and see how it goes.	Jingdong prefecture level city in Shandong
Dragon Quality Goods Shop	Green Intelligent Power Saver	Received very quickly, the quality is very good, really save electricity, business service attitude is also very good, I hope you do not miss this opportunity, I continue to buy a few in	Jingdong prefecture level city in Shandong
Dragon Quality Goods Shop	Green Intelligent Power Saver	Quality is very good Oh, the delivery is also very fast, a very favorite store, I want to crazy recommend to friends!	Jingdong prefecture level city in Shandong
Dragon Quality Goods Shop	Green Intelligent Power Saver	The goods have just received, fine workmanship, and the same as described by the merchant, to use a period of time to see if it can achieve the purpose of power saving.	Jingdong prefecture level city in Shandong
Dragon Quality Goods Shop	Green Intelligent Power Saver	Received the goods first use a period of time to see if there is no effect, the business commitment to ninety days invalid refund, first praise, good service!	Jingdong prefecture level city in Shandong
Dragon Quality Goods Shop	Green Intelligent Power Saver	The product is used for a month without energy-saving and power-saving effect does not have any effect	Jingdong prefecture level city in Shandong
Dragon Quality Goods Shop	Green Intelligent Power Saver	The baby is really great, I like it very much, my friends also say good to come, recommended to buy!	Jingdong prefecture level city in Shandong
Dragon Quality Goods Shop	Green Intelligent Power Saver	Logistics is fast, courier boy service is good, after using to see how the fruit, and then follow up.	Jingdong prefecture level city in Shandong
Dragon Quality Goods Shop	Green Intelligent Power Saver	Baby has been received to try, I believe that as the business advertised energy saving. The courier is very responsible.	Jingdong prefecture level city in Shandong
Dragon Quality Goods Shop	Green Intelligent Power Saver	Save Electric Energy King received, good quality, beautiful appearance, very satisfied!	Jingdong prefecture level city in Shandong
Dragon Quality Goods Shop	Green Intelligent Power Saver	Three no products no manufacturers no phone pure fraud induced consumption cheat	Jingdong prefecture level city in Shandong
Dragon Quality Goods Shop	Green Intelligent Power Saver	I'm using it now, I don't know if it's as good as it says, I'll follow up after a month	Jingdong prefecture level city in Shandong
Dragon Quality Goods Shop	Green Intelligent Power Saver	Good quality logistics and fast. Fine workmanship. The description is consistent cost-effective	Jingdong prefecture level city in Shandong
Dragon Quality Goods Shop	Green Intelligent Power Saver	Received, whether it is good or not, to wait a few days and then evaluate	Jingdong prefecture level city in Shandong
Dragon Quality Goods Shop	Green Intelligent Power Saver	I bought it and started using it, I won't know if it works well until I pay my electric bill	Jingdong prefecture level city in Shandong
Dragon Quality Goods Shop	Green Intelligent Power Saver	Especially good, energy saving and power saving, especially good quality, good	Jingdong prefecture level city in Shandong
Dragon Quality Goods Shop	Green Intelligent Power Saver	Logistics is strong, the quality is very good, paste really save electricity, as described, will buy back!	Jingdong prefecture level city in Shandong
Dragon Quality Goods Shop	Green Intelligent Power Saver	Great stuff at a great price, worth the money!	Jingdong prefecture level city in Shandong

Dragon Quality Goods Shop	Green Intelligent Power Saver	Received the power saver, very good quality, very power saving!	Jingdong prefecture level city in Shandong
Dragon Quality Goods Shop	Green Intelligent Power Saver	There are no fruits, only samples...	Jingdong prefecture level city in Shandong
Dragon Quality Goods Shop	Green Intelligent Power Saver	Save a few dollars a month.	Jingdong prefecture level city in Shandong
Dragon Quality Goods Shop	Green Intelligent Power Saver	The quality is particularly good, it works well, and the seller has a good service attitude	Jingdong prefecture level city in Shandong
Dragon Quality Goods Shop	Green Intelligent Power Saver	I've compared them a couple of times and I don't feel any difference	Jingdong prefecture level city in Shandong
Dragon Quality Goods Shop	Green Intelligent Power Saver	I don't see the savings. It's a sham.	Jingdong prefecture level city in Shandong
Dragon Quality Goods Shop	Green Intelligent Power Saver	The power saving effect is very good, and the logistics is very fast. The seller's service attitude is very good	Jingdong prefecture level city in Shandong
Dragon Quality Goods Shop	Green Intelligent Power Saver	Received, well packaged, haven't tried it yet!	Jingdong prefecture level city in Shandong
Dragon Quality Goods Shop	Green Intelligent Power Saver	I don't know how it works, I'll comment after I use it.	Jingdong prefecture level city in Shandong
Dragon Quality Goods Shop	Green Intelligent Power Saver	The product doesn't work well enough to be of any use	Jingdong prefecture level city in Shandong
Dragon Quality Goods Shop	Green Intelligent Power Saver	On Trial 。。。。。。。	Jingdong prefecture level city in Shandong
Dragon Quality Goods Shop	Green Intelligent Power Saver	Being used with unknown results	Jingdong prefecture level city in Shandong
Dragon Quality Goods Shop	Green Intelligent Power Saver	Smart power saving and great quality	Jingdong prefecture level city in Shandong
Dragon Quality Goods Shop	Green Intelligent Power Saver	The saver was received, it works very well and the price is affordable!	Jingdong prefecture level city in Shandong
Dragon Quality Goods Shop	Green Intelligent Power Saver	How can I feel the power saving and how long does it last	Jingdong prefecture level city in Shandong
Dragon Quality Goods Shop	Green Intelligent Power Saver	And I know if it saves electricity or not.	Jingdong prefecture level city in Shandong
Dragon Quality Goods Shop	Green Intelligent Power Saver	It won't save any electricity. No.	Jingdong prefecture level city in Shandong
Dragon Quality Goods Shop	Green Intelligent Power Saver	Invalid. Fake.	Jingdong prefecture level city in Shandong
Dragon Quality Goods Shop	Green Intelligent Power Saver	It's good!	Jingdong prefecture level city in Shandong
Dragon Quality Goods Shop	Green Intelligent Power Saver	have no effect	Jingdong prefecture level city in Shandong
Dragon Quality Goods Shop	Green Intelligent Power Saver	Not used.	Jingdong prefecture level city in Shandong
Dragon Quality Goods Shop	Green Intelligent Power Saver	There is no load range, packaging thrift, ten days also did not develop invoices, received the goods with a receipt, hand-written receipt also has no financial stamp, communication for nearly a week a moment to say that the electronic invoice, a moment to say that the paper invoice, said it is mailed to me, ten days also did not see anything, the invoice invoice can not be found to say that did not stay on file, single single number can not be found, said that the warehouse more than one to a one to find out, in addition to the perfunctory or perfunctory. Products are no certificate of conformity, proper three	Jingdong prefecture level city in Shandong
Dragon Quality Goods Shop	Green Intelligent Power Saver	Satisfaction.	Jingdong prefecture level city in Shandong
Dragon Quality Goods Shop	Green Intelligent Power Saver	There is not a bit of power saving effect, and ringing up an increase in the electricity bill, because you have to add the cost of this machine's own electricity consumption and purchase, so I think it is better to use this machine instead of increasing the expenditure, uneconomical, rather than not use it.	Jingdong prefecture level city in Shandong
Dragon Quality Goods Shop	Green Intelligent Power Saver	I've been using it for two days now and I don't feel any effect.	Jingdong prefecture level city in Shandong
Dragon Quality Goods Shop	Green Intelligent Power Saver	What it looked like was nothing like what was sent.	Jingdong prefecture level city in Shandong
Dragon Quality Goods Shop	Green Intelligent Power Saver	It didn't work. It didn't work for a month or so.	Jingdong prefecture level city in Shandong
Dragon Quality Goods Shop	Green Intelligent Power Saver	Garbage products, physical and description is not the same, no effect at all, pit # no discussion, to buy the fine, garbage, garbage, garbage	Jingdong prefecture level city in Shandong
Dragon Quality Goods Shop	Green Intelligent Power Saver	general	Jingdong prefecture level city in Shandong
Dragon Quality Goods Shop	Green Intelligent Power Saver	IQ tax. Instead of saving electricity, it increases the power consumption of an appliance.	Jingdong prefecture level city in Shandong
Dragon Quality Goods Shop	Green Intelligent Power Saver	There's no trash at all.	Jingdong prefecture level city in Shandong
Dragon Quality Goods Shop	Green Intelligent Power Saver	It doesn't do anything.	Jingdong prefecture level city in Shandong
Dragon Quality Goods Shop	Green Intelligent Power Saver	(of an unmarried couple) be close	Jingdong prefecture level city in Shandong
Dragon Quality Goods Shop	Green Intelligent Power Saver	(of an unmarried couple) be close	Jingdong prefecture level city in Shandong
Dragon Quality Goods Shop	Green Intelligent Power Saver	no electricity saving	Jingdong prefecture level city in Shandong
Dragon Quality Goods Shop	Green Intelligent Power Saver	differ from	Jingdong prefecture level city in Shandong
Dragon Quality Goods Shop	Green Intelligent Power Saver	Saver has received, but real can save electricity, the quality is very good, I times I buy a few more, too good.	Jingdong prefecture level city in Shandong
Dragon Quality Goods Shop	Green Intelligent Power Saver	Great quality as always, a very favorite product, great! o(*￣︶￣*)o	Jingdong prefecture level city in Shandong
Dragon Quality Goods Shop	Green Intelligent Power Saver	If we can reduce the electricity bill, for individuals can save money, for the country can protect the environment, now the weather is getting hotter and hotter, to protect the environment to reduce emissions is very important. Already use the product, whether to reduce the electricity bill depends on next month!	Jingdong prefecture level city in Shandong
Dragon Quality Goods Shop	Green Intelligent Power Saver	Purchased a power saver received on the put into use, rent a room with many electrical appliances, power consumption is very large, we have a piece of electricity or 1.2 dollars a time, with this power saver is much better, every day can save a few cents, very convenient and practical!	Jingdong prefecture level city in Shandong
Dragon Quality Goods Shop	Green Intelligent Power Saver	The product is better to use before not to use when a month 1500 yuan electricity bill, now using the 5 second month electricity bill directly down 470 dollars or very good. A save money to buy several power saver money ZanZanZanZan👍	Jingdong prefecture level city in Shandong
Dragon Quality Goods Shop	Green Intelligent Power Saver	The moment I received the goods really satisfied with this product, not only high-grade packaging, the body are base alloy, up to the price, I was also introduced by a friend, he used last month did save a lot of electricity, but also recommended to me!	Jingdong prefecture level city in Shandong
Dragon Quality Goods Shop	Green Intelligent Power Saver	I got it. Good review. I'm very satisfied. Reasonable price.	Jingdong prefecture level city in Shandong
Dragon Quality Goods Shop	Green Intelligent Power Saver	Intelligent power saver received and used, looking at the quality is good, the quality of the plug sent is also very good. Expect the effect of the economizer is obvious, first five-star praise, with a period of time to follow up!	Jingdong prefecture level city in Shandong
Dragon Quality Goods Shop	Green Intelligent Power Saver	The saver has been received. It was plugged into an outlet and used immediately after receipt. Whether it really saves electricity is not clear yet, I believe that this brand of energy saver really saves	Jingdong prefecture level city in Shandong
Dragon Quality Goods Shop	Green Intelligent Power Saver	Okay, okay, okay. Thank you so much. Fast is good. Fast. Item was as described, mainly because the event was cheap and you can save a lot of money.	Jingdong prefecture level city in Shandong

Dragon Quality Goods Shop	Green Intelligent Power Saver	Received it a couple days ago! Plugged it in the same day! Let's see what the electric bill is in a month! Hopefully it works well oh!	Jingdong prefecture level city in Shandong
Dragon Quality Goods Shop	Green Intelligent Power Saver	Looking at the reviews good, energy-saving small products, compact, the results after 2 months to see, because the electricity bill season payment	Jingdong prefecture level city in Shandong
Dragon Quality Goods Shop	Green Intelligent Power Saver	I just received it and will try it out to see if it works well. I'll comment later.	Jingdong prefecture level city in Shandong
Dragon Quality Goods Shop	Green Intelligent Power Saver	It looks a little small and not very aesthetically pleasing, I don't know how effective it will be until I use it	Jingdong prefecture level city in Shandong
Dragon Quality Goods Shop	Green Intelligent Power Saver	Not only to the point of being useless yet	Jingdong prefecture level city in Shandong
Dragon Quality Goods Shop	Green Intelligent Power Saver	Used it for a month, nothing works, the meter you turn very fast. This is not a scam, but also sold on Jingdong. To cheat a bunch of people?	Jingdong prefecture level city in Shandong
Dragon Quality Goods Shop	Green Intelligent Power Saver	Very good to use, every day can save a little electricity, really good under the sample down a month to save a few dozen dollars in electricity, good before I did not know that there is this haha, the price is not expensive praise.	Jingdong prefecture level city in Shandong
Dragon Quality Goods Shop	Green Intelligent Power Saver	Tried it for ten days and the change is almost nothing, can't see the power saving yet, use it some more days and see how it goes.	Jingdong prefecture level city in Shandong
Dragon Quality Goods Shop	Green Intelligent Power Saver	Received very quickly, the quality is very good, really save electricity, business service attitude is also very good, I hope you do not miss this opportunity, I continue to buy a few in	Jingdong prefecture level city in Shandong
Dragon Quality Goods Shop	Green Intelligent Power Saver	Quality is very good Oh, the delivery is also very fast, a very favorite store, I want to crazy recommend to friends!	Jingdong prefecture level city in Shandong
Dragon Quality Goods Shop	Green Intelligent Power Saver	The goods have just received, fine workmanship, and the same as described by the merchant, to use a period of time to see if it can achieve the purpose of power saving.	Jingdong prefecture level city in Shandong
Dragon Quality Goods Shop	Green Intelligent Power Saver	Received the goods first use a period of time to see if there is no effect, the business commitment to ninety days invalid refund, first praise, good service!	Jingdong prefecture level city in Shandong
Dragon Quality Goods Shop	Green Intelligent Power Saver	The product is used for a month without energy-saving and power-saving effect does not have any effect	Jingdong prefecture level city in Shandong
Dragon Quality Goods Shop	Green Intelligent Power Saver	The baby is really great, I like it very much, my friends also say good to come, recommended to buy!	Jingdong prefecture level city in Shandong
Dragon Quality Goods Shop	Green Intelligent Power Saver	Logistics is fast, courier boy service is good, after using to see how the fruit, and then follow up.	Jingdong prefecture level city in Shandong
Dragon Quality Goods Shop	Green Intelligent Power Saver	Baby has been received to try, I believe that as the business advertised energy saving. The courier is very responsible.	Jingdong prefecture level city in Shandong
Dragon Quality Goods Shop	Green Intelligent Power Saver	Save Electric Energy King received, good quality, beautiful appearance, very satisfied!	Jingdong prefecture level city in Shandong
Dragon Quality Goods Shop	Green Intelligent Power Saver	Three no products no manufacturers no phone pure fraud induced consumption cheat	Jingdong prefecture level city in Shandong
Dragon Quality Goods Shop	Green Intelligent Power Saver	I'm using it now, I don't know if it's as good as it says, I'll follow up after a month	Jingdong prefecture level city in Shandong
Dragon Quality Goods Shop	Green Intelligent Power Saver	Good quality logistics and fast. Fine workmanship. The description is consistent cost-effective	Jingdong prefecture level city in Shandong
Dragon Quality Goods Shop	Green Intelligent Power Saver	Received, whether it is good or not, to wait a few days and then evaluate	Jingdong prefecture level city in Shandong
Dragon Quality Goods Shop	Green Intelligent Power Saver	I bought it and started using it, I won't know if it works well until I pay my electric bill	Jingdong prefecture level city in Shandong
Dragon Quality Goods Shop	Green Intelligent Power Saver	Especially good, energy saving and power saving, especially good quality, good	Jingdong prefecture level city in Shandong
Dragon Quality Goods Shop	Green Intelligent Power Saver	Logistics is strong, the quality is very good, paste really save electricity, as described, will buy back!	Jingdong prefecture level city in Shandong
Dragon Quality Goods Shop	Green Intelligent Power Saver	Great stuff at a great price, worth the money!	Jingdong prefecture level city in Shandong
Dragon Quality Goods Shop	Green Intelligent Power Saver	Received the power saver, very good quality, very power saving!	Jingdong prefecture level city in Shandong
Dragon Quality Goods Shop	Green Intelligent Power Saver	There are no fruits, only samples...	Jingdong prefecture level city in Shandong
Dragon Quality Goods Shop	Green Intelligent Power Saver	Save a few dollars a month.	Jingdong prefecture level city in Shandong
Dragon Quality Goods Shop	Green Intelligent Power Saver	The quality is particularly good, it works well, and the seller has a good service attitude	Jingdong prefecture level city in Shandong
Dragon Quality Goods Shop	Green Intelligent Power Saver	I've compared them a couple of times and I don't feel any difference	Jingdong prefecture level city in Shandong
Dragon Quality Goods Shop	Green Intelligent Power Saver	I don't see the savings. It's a sham.	Jingdong prefecture level city in Shandong
Dragon Quality Goods Shop	Green Intelligent Power Saver	The power saving effect is very good, and the logistics is very fast. The seller's service attitude is very good	Jingdong prefecture level city in Shandong
Dragon Quality Goods Shop	Green Intelligent Power Saver	Received, well packaged, haven't tried it yet!	Jingdong prefecture level city in Shandong
Dragon Quality Goods Shop	Green Intelligent Power Saver	I don't know how it works, I'll comment after I use it.	Jingdong prefecture level city in Shandong
Dragon Quality Goods Shop	Green Intelligent Power Saver	The product doesn't work well enough to be of any use	Jingdong prefecture level city in Shandong
Dragon Quality Goods Shop	Green Intelligent Power Saver	On Trial 。。。。。。。	Jingdong prefecture level city in Shandong
Dragon Quality Goods Shop	Green Intelligent Power Saver	Being used with unknown results	Jingdong prefecture level city in Shandong
Dragon Quality Goods Shop	Green Intelligent Power Saver	Smart power saving and great quality	Jingdong prefecture level city in Shandong
Dragon Quality Goods Shop	Green Intelligent Power Saver	The saver was received, it works very well and the price is affordable!	Jingdong prefecture level city in Shandong
Dragon Quality Goods Shop	Green Intelligent Power Saver	How can I feel the power saving and how long does it last	Jingdong prefecture level city in Shandong
Dragon Quality Goods Shop	Green Intelligent Power Saver	And I know if it saves electricity or not.	Jingdong prefecture level city in Shandong
Dragon Quality Goods Shop	Green Intelligent Power Saver	It won't save any electricity. No.	Jingdong prefecture level city in Shandong
Dragon Quality Goods Shop	Green Intelligent Power Saver	Invalid. Fake.	Jingdong prefecture level city in Shandong
Dragon Quality Goods Shop	Green Intelligent Power Saver	It's good!	Jingdong prefecture level city in Shandong
Dragon Quality Goods Shop	Green Intelligent Power Saver	have no effect	Jingdong prefecture level city in Shandong
Dragon Quality Goods Shop	Green Intelligent Power Saver	Not used.	Jingdong prefecture level city in Shandong
Dragon Quality Goods Shop	Green Intelligent Power Saver	There is no load range, packaging thrift, ten days also did not develop invoices, received the goods with a receipt, hand-written receipt also has no financial stamp, communication for nearly a week a moment to say that the electronic invoice, a moment to say that the paper invoice, said it is mailed to me, ten days also did not see anything, the invoice invoice can not be found to say that did not stay on file, single single number can not be found, said that the warehouse more than one to a one to find out, in addition to the perfunctory or perfunctory. Products are no certificate of conformity, proper three	Jingdong prefecture level city in Shandong
Dragon Quality Goods Shop	Green Intelligent Power Saver	Satisfaction.	Jingdong prefecture level city in Shandong

Dragon Quality Goods Shop	Green Intelligent Power Saver	There is not a bit of power saving effect, and ringing up an increase in the electricity bill, because you have to add the cost of this machine's own electricity consumption and purchase, so I think it is better to use this machine instead of increasing the expenditure, uneconomical, rather than not use it.	Jingdong prefecture level city in Shandong
Dragon Quality Goods Shop	Green Intelligent Power Saver	I've been using it for two days now and I don't feel any effect.	Jingdong prefecture level city in Shandong
Dragon Quality Goods Shop	Green Intelligent Power Saver	What it looked like was nothing like what was sent.	Jingdong prefecture level city in Shandong
Dragon Quality Goods Shop	Green Intelligent Power Saver	It didn't work. It didn't work for a month or so.	Jingdong prefecture level city in Shandong
Dragon Quality Goods Shop	Green Intelligent Power Saver	Garbage products, physical and description is not the same, no effect at all, pit # no discussion, to buy the fine, garbage, garbage, garbage	Jingdong prefecture level city in Shandong
Dragon Quality Goods Shop	Green Intelligent Power Saver	general	Jingdong prefecture level city in Shandong
Dragon Quality Goods Shop	Green Intelligent Power Saver	IQ tax. Instead of saving electricity, it increases the power consumption of an appliance.	Jingdong prefecture level city in Shandong
Dragon Quality Goods Shop	Green Intelligent Power Saver	There's no trash at all.	Jingdong prefecture level city in Shandong
Dragon Quality Goods Shop	Green Intelligent Power Saver	It doesn't do anything.	Jingdong prefecture level city in Shandong
Dragon Quality Goods Shop	Green Intelligent Power Saver	(of an unmarried couple) be close	Jingdong prefecture level city in Shandong
Dragon Quality Goods Shop	Green Intelligent Power Saver	(of an unmarried couple) be close	Jingdong prefecture level city in Shandong
Dragon Quality Goods Shop	Green Intelligent Power Saver	no electricity saving	Jingdong prefecture level city in Shandong
Dragon Quality Goods Shop	Green Intelligent Power Saver	differ from	Jingdong prefecture level city in Shandong
Dragon Quality Goods Shop	Green Intelligent Power Saver	Saver has received, but real can save electricity, the quality is very good, I times I buy a few more, too good.	Jingdong prefecture level city in Shandong
Dragon Quality Goods Shop	Green Intelligent Power Saver	Great quality as always, a very favorite product, great! o(*￣︶￣*)o	Jingdong prefecture level city in Shandong
Dragon Quality Goods Shop	Green Intelligent Power Saver	If we can reduce the electricity bill, for individuals can save money, for the country can protect the environment, now the weather is getting hotter and hotter, to protect the environment to reduce emissions is very important. Already use the product, whether to reduce the electricity bill depends on next month!	Jingdong prefecture level city in Shandong
Dragon Quality Goods Shop	Green Intelligent Power Saver	Purchased a power saver received on the put into use, rent a room with many electrical appliances, power consumption is very large, we have a piece of electricity or 1.2 dollars a time, with this power saver is much better, every day can save a few cents, very convenient and practical!	Jingdong prefecture level city in Shandong
Dragon Quality Goods Shop	Green Intelligent Power Saver	The product is better to use before not to use when a month 1500 yuan electricity bill, now using the 5 second month electricity bill directly down 470 dollars or very good. A save money to buy several power saver money ZanZanZanZan👍	Jingdong prefecture level city in Shandong
Dragon Quality Goods Shop	Green Intelligent Power Saver	The moment I received the goods really satisfied with this product, not only high-grade packaging, the body are base alloy, up to the price, I was also introduced by a friend, he used last month did save a lot of electricity, but also recommended to me!	Jingdong prefecture level city in Shandong
Dragon Quality Goods Shop	Green Intelligent Power Saver	I got it. Good review. I'm very satisfied. Reasonable price.	Jingdong prefecture level city in Shandong
Dragon Quality Goods Shop	Green Intelligent Power Saver	Intelligent power saver received and used, looking at the quality is good, the quality of the plug sent is also very good. Expect the effect of the economizer is obvious, first five-star praise, with a period of time to follow up!	Jingdong prefecture level city in Shandong
Dragon Quality Goods Shop	Green Intelligent Power Saver	The saver has been received. It was plugged into an outlet and used immediately after receipt. Whether it really saves electricity is not clear yet, I believe that this brand of energy saver really saves	Jingdong prefecture level city in Shandong
Dragon Quality Goods Shop	Green Intelligent Power Saver	Okay, okay, okay. Thank you so much. Fast is good. Fast. Item was as described, mainly because the event was cheap and you can save a lot of money.	Jingdong prefecture level city in Shandong
Dragon Quality Goods Shop	Green Intelligent Power Saver	Received it a couple days ago! Plugged it in the same day! Let's see what the electric bill is in a month! Hopefully it works well oh!	Jingdong prefecture level city in Shandong
Dragon Quality Goods Shop	Green Intelligent Power Saver	Looking at the reviews good, energy-saving small products, compact, the results after 2 months to see, because the electricity bill season payment	Jingdong prefecture level city in Shandong
Dragon Quality Goods Shop	Green Intelligent Power Saver	I just received it and will try it out to see if it works well. I'll comment later.	Jingdong prefecture level city in Shandong
Dragon Quality Goods Shop	Green Intelligent Power Saver	It looks a little small and not very aesthetically pleasing, I don't know how effective it will be until I use it	Jingdong prefecture level city in Shandong
Dragon Quality Goods Shop	Green Intelligent Power Saver	Not only to the point of being useless yet	Jingdong prefecture level city in Shandong
Dragon Quality Goods Shop	Green Intelligent Power Saver	Used it for a month, nothing works, the meter you turn very fast. This is not a scam, but also sold on Jingdong. To cheat a bunch of people?	Jingdong prefecture level city in Shandong
Dragon Quality Goods Shop	Green Intelligent Power Saver	Very good to use, every day can save a little electricity, really good under the sample down a month to save a few dozen dollars in electricity, good before I did not know that there is this 👆 haha, the price is not expensive praise.	Jingdong prefecture level city in Shandong
Dragon Quality Goods Shop	Green Intelligent Power Saver	Tried it for ten days and the change is almost nothing, can't see the power saving yet, use it some more days and see how it goes.	Jingdong prefecture level city in Shandong
Dragon Quality Goods Shop	Green Intelligent Power Saver	Received very quickly, the quality is very good, really save electricity, business service attitude is also very good, I hope you do not miss this opportunity, I continue to buy a few in	Jingdong prefecture level city in Shandong
Dragon Quality Goods Shop	Green Intelligent Power Saver	Quality is very good Oh, the delivery is also very fast, a very favorite store, I want to crazy recommend to friends!	Jingdong prefecture level city in Shandong
Dragon Quality Goods Shop	Green Intelligent Power Saver	The goods have just received, fine workmanship, and the same as described by the merchant, to use a period of time to see if it can achieve the purpose of power saving.	Jingdong prefecture level city in Shandong
Dragon Quality Goods Shop	Green Intelligent Power Saver	Received the goods first use a period of time to see if there is no effect, the business commitment to ninety days invalid refund, first praise, good service!	Jingdong prefecture level city in Shandong
Dragon Quality Goods Shop	Green Intelligent Power Saver	The product is used for a month without energy-saving and power-saving effect does not have any effect	Jingdong prefecture level city in Shandong
Dragon Quality Goods Shop	Green Intelligent Power Saver	The baby is really great, I like it very much, my friends also say good to come, recommended to buy!	Jingdong prefecture level city in Shandong
Dragon Quality Goods Shop	Green Intelligent Power Saver	Logistics is fast, courier boy service is good, after using to see how the fruit, and then follow up.	Jingdong prefecture level city in Shandong
Dragon Quality Goods Shop	Green Intelligent Power Saver	Baby has been received to try, I believe that as the business advertised energy saving. The courier is very responsible.	Jingdong prefecture level city in Shandong
Dragon Quality Goods Shop	Green Intelligent Power Saver	Save Electric Energy King received, good quality, beautiful appearance, very satisfied!	Jingdong prefecture level city in Shandong
Dragon Quality Goods Shop	Green Intelligent Power Saver	Three no products no manufacturers no phone pure fraud induced consumption cheat	Jingdong prefecture level city in Shandong
Dragon Quality Goods Shop	Green Intelligent Power Saver	I'm using it now, I don't know if it's as good as it says, I'll follow up after a month	Jingdong prefecture level city in Shandong
Dragon Quality Goods Shop	Green Intelligent Power Saver	Good quality logistics and fast. Fine workmanship. The description is consistent cost-effective	Jingdong prefecture level city in Shandong
Dragon Quality Goods Shop	Green Intelligent Power Saver	Received, whether it is good or not, to wait a few days and then evaluate	Jingdong prefecture level city in Shandong
Dragon Quality Goods Shop	Green Intelligent Power Saver	I bought it and started using it, I won't know if it works well until I pay my electric bill	Jingdong prefecture level city in Shandong
Dragon Quality Goods Shop	Green Intelligent Power Saver	Especially good, energy saving and power saving, especially good quality, good	Jingdong prefecture level city in Shandong
Dragon Quality Goods Shop	Green Intelligent Power Saver	Logistics is strong, the quality is very good, paste really save electricity, as described, will buy back!	Jingdong prefecture level city in Shandong
Dragon Quality Goods Shop	Green Intelligent Power Saver	Great stuff at a great price, worth the money!	Jingdong prefecture level city in Shandong

Dragon Quality Goods Shop	Green Intelligent Power Saver	Received the power saver, very good quality, very power saving!	Jingdong prefecture level city in Shandong
Dragon Quality Goods Shop	Green Intelligent Power Saver	There are no fruits, only samples...	Jingdong prefecture level city in Shandong
Dragon Quality Goods Shop	Green Intelligent Power Saver	Save a few dollars a month.	Jingdong prefecture level city in Shandong
Dragon Quality Goods Shop	Green Intelligent Power Saver	The quality is particularly good, it works well, and the seller has a good service attitude	Jingdong prefecture level city in Shandong
Dragon Quality Goods Shop	Green Intelligent Power Saver	I've compared them a couple of times and I don't feel any difference	Jingdong prefecture level city in Shandong
Dragon Quality Goods Shop	Green Intelligent Power Saver	I don't see the savings. It's a sham.	Jingdong prefecture level city in Shandong
Dragon Quality Goods Shop	Green Intelligent Power Saver	The power saving effect is very good, and the logistics is very fast. The seller's service attitude is very good	Jingdong prefecture level city in Shandong
Dragon Quality Goods Shop	Green Intelligent Power Saver	Received, well packaged, haven't tried it yet!	Jingdong prefecture level city in Shandong
Dragon Quality Goods Shop	Green Intelligent Power Saver	I don't know how it works, I'll comment after I use it.	Jingdong prefecture level city in Shandong
Dragon Quality Goods Shop	Green Intelligent Power Saver	The product doesn't work well enough to be of any use	Jingdong prefecture level city in Shandong
Dragon Quality Goods Shop	Green Intelligent Power Saver	On Trial 。。。。。。。	Jingdong prefecture level city in Shandong
Dragon Quality Goods Shop	Green Intelligent Power Saver	Being used with unknown results	Jingdong prefecture level city in Shandong
Dragon Quality Goods Shop	Green Intelligent Power Saver	Smart power saving and great quality	Jingdong prefecture level city in Shandong
Dragon Quality Goods Shop	Green Intelligent Power Saver	The saver was received, it works very well and the price is affordable!	Jingdong prefecture level city in Shandong
Dragon Quality Goods Shop	Green Intelligent Power Saver	How can I feel the power saving and how long does it last	Jingdong prefecture level city in Shandong
Dragon Quality Goods Shop	Green Intelligent Power Saver	And I know if it saves electricity or not.	Jingdong prefecture level city in Shandong
Dragon Quality Goods Shop	Green Intelligent Power Saver	It won't save any electricity. No.	Jingdong prefecture level city in Shandong
Dragon Quality Goods Shop	Green Intelligent Power Saver	Invalid. Fake.	Jingdong prefecture level city in Shandong
Dragon Quality Goods Shop	Green Intelligent Power Saver	It's good!	Jingdong prefecture level city in Shandong
Dragon Quality Goods Shop	Green Intelligent Power Saver	have no effect	Jingdong prefecture level city in Shandong
Dragon Quality Goods Shop	Green Intelligent Power Saver	Not used.	Jingdong prefecture level city in Shandong
Dragon Quality Goods Shop	Green Intelligent Power Saver	There is no load range, packaging thrift, ten days also did not develop invoices, received the goods with a receipt, hand-written receipt also has no financial stamp, communication for nearly a week a moment to say that the electronic invoice, a moment to say that the paper invoice, said it is mailed to me, ten days also did not see anything, the invoice invoice can not be found to say that did not stay on file, single single number can not be found, said that the warehouse more than one to a one to find out, in addition to the perfunctory or perfunctory. Products are no certificate of conformity, proper three	Jingdong prefecture level city in Shandong
Dragon Quality Goods Shop	Green Intelligent Power Saver	Satisfaction.	Jingdong prefecture level city in Shandong
Dragon Quality Goods Shop	Green Intelligent Power Saver	There is not a bit of power saving effect, and ringing up an increase in the electricity bill, because you have to add the cost of this machine's own electricity consumption and purchase, so I think it is better to use this machine instead of increasing the expenditure, uneconomical, rather than not use it.	Jingdong prefecture level city in Shandong
Dragon Quality Goods Shop	Green Intelligent Power Saver	I've been using it for two days now and I don't feel any effect.	Jingdong prefecture level city in Shandong
Dragon Quality Goods Shop	Green Intelligent Power Saver	What it looked like was nothing like what was sent.	Jingdong prefecture level city in Shandong
Dragon Quality Goods Shop	Green Intelligent Power Saver	It didn't work. It didn't work for a month or so.	Jingdong prefecture level city in Shandong
Dragon Quality Goods Shop	Green Intelligent Power Saver	Garbage products, physical and description is not the same, no effect at all, pit # no discussion, to buy the fine, garbage, garbage, garbage	Jingdong prefecture level city in Shandong
Dragon Quality Goods Shop	Green Intelligent Power Saver	general	Jingdong prefecture level city in Shandong
Dragon Quality Goods Shop	Green Intelligent Power Saver	IQ tax. Instead of saving electricity, it increases the power consumption of an appliance.	Jingdong prefecture level city in Shandong
Dragon Quality Goods Shop	Green Intelligent Power Saver	There's no trash at all.	Jingdong prefecture level city in Shandong
Dragon Quality Goods Shop	Green Intelligent Power Saver	It doesn't do anything.	Jingdong prefecture level city in Shandong
Dragon Quality Goods Shop	Green Intelligent Power Saver	(of an unmarried couple) be close	Jingdong prefecture level city in Shandong
Dragon Quality Goods Shop	Green Intelligent Power Saver	(of an unmarried couple) be close	Jingdong prefecture level city in Shandong
Dragon Quality Goods Shop	Green Intelligent Power Saver	no electricity saving	Jingdong prefecture level city in Shandong
Dragon Quality Goods Shop	Green Intelligent Power Saver	differ from	Jingdong prefecture level city in Shandong
Dragon Quality Goods Shop	Green Intelligent Power Saver	Saver has received, but real can save electricity, the quality is very good, I times I buy a few more, too good.	Jingdong prefecture level city in Shandong
Dragon Quality Goods Shop	Green Intelligent Power Saver	Great quality as always, a very favorite product, great! o(*￣︶￣*)o	Jingdong prefecture level city in Shandong
Dragon Quality Goods Shop	Green Intelligent Power Saver	If we can reduce the electricity bill, for individuals can save money, for the country can protect the environment, now the weather is getting hotter and hotter, to protect the environment to reduce emissions is very important. Already use the product, whether to reduce the electricity bill depends on next month!	Jingdong prefecture level city in Shandong
Dragon Quality Goods Shop	Green Intelligent Power Saver	Purchased a power saver received on the put into use, rent a room with many electrical appliances, power consumption is very large, we have a piece of electricity or 1.2 dollars a time, with this power saver is much better, every day can save a few cents, very convenient and practical!	Jingdong prefecture level city in Shandong
Dragon Quality Goods Shop	Green Intelligent Power Saver	The product is better to use before not to use when a month 1500 yuan electricity bill, now using the 5 second month electricity bill directly down 470 dollars or very good. A save money to buy several power saver money ZanZanZanZan👍	Jingdong prefecture level city in Shandong
Dragon Quality Goods Shop	Green Intelligent Power Saver	The moment I received the goods really satisfied with this product, not only high-grade packaging, the body are base alloy, up to the price, I was also introduced by a friend, he used last month did save a lot of electricity, but also recommended to me!	Jingdong prefecture level city in Shandong
Dragon Quality Goods Shop	Green Intelligent Power Saver	I got it. Good review. I'm very satisfied. Reasonable price.	Jingdong prefecture level city in Shandong
Dragon Quality Goods Shop	Green Intelligent Power Saver	Intelligent power saver received and used, looking at the quality is good, the quality of the plug sent is also very good. Expect the effect of the economizer is obvious, first five-star praise, with a period of time to follow up!	Jingdong prefecture level city in Shandong
Dragon Quality Goods Shop	Green Intelligent Power Saver	The saver has been received. It was plugged into an outlet and used immediately after receipt. Whether it really saves electricity is not clear yet, I believe that this brand of energy saver really saves	Jingdong prefecture level city in Shandong
Dragon Quality Goods Shop	Green Intelligent Power Saver	Okay, okay, okay. Thank you so much. Fast is good. Fast. Item was as described, mainly because the event was cheap and you can save a lot of money.	Jingdong prefecture level city in Shandong

Dragon Quality Goods Shop	Green Intelligent Power Saver	Received it a couple days ago! Plugged it in the same day! Let's see what the electric bill is in a month! Hopefully it works well oh!	Jingdong prefecture level city in Shandong
Dragon Quality Goods Shop	Green Intelligent Power Saver	Looking at the reviews good, energy-saving small products, compact, the results after 2 months to see, because the electricity bill season payment	Jingdong prefecture level city in Shandong
Dragon Quality Goods Shop	Green Intelligent Power Saver	I just received it and will try it out to see if it works well. I'll comment later.	Jingdong prefecture level city in Shandong
Dragon Quality Goods Shop	Green Intelligent Power Saver	It looks a little small and not very aesthetically pleasing, I don't know how effective it will be until I use it	Jingdong prefecture level city in Shandong
Dragon Quality Goods Shop	Green Intelligent Power Saver	Not only to the point of being useless yet	Jingdong prefecture level city in Shandong
Dragon Quality Goods Shop	Green Intelligent Power Saver	Used it for a month, nothing works, the meter you turn very fast. This is not a scam, but also sold on Jingdong. To cheat a bunch of people?	Jingdong prefecture level city in Shandong
Dragon Quality Goods Shop	Green Intelligent Power Saver	Very good to use, every day can save a little electricity, really good under the sample down a month to save a few dozen dollars in electricity, good before I did not know that there is this haha, the price is not expensive praise.	Jingdong prefecture level city in Shandong
Dragon Quality Goods Shop	Green Intelligent Power Saver	Tried it for ten days and the change is almost nothing, can't see the power saving yet, use it some more days and see how it goes.	Jingdong prefecture level city in Shandong
Dragon Quality Goods Shop	Green Intelligent Power Saver	Received very quickly, the quality is very good, really save electricity, business service attitude is also very good, I hope you do not miss this opportunity, I continue to buy a few in	Jingdong prefecture level city in Shandong
Dragon Quality Goods Shop	Green Intelligent Power Saver	Quality is very good Oh, the delivery is also very fast, a very favorite store, I want to crazy recommend to friends!	Jingdong prefecture level city in Shandong
Dragon Quality Goods Shop	Green Intelligent Power Saver	The goods have just received, fine workmanship, and the same as described by the merchant, to use a period of time to see if it can achieve the purpose of power saving.	Jingdong prefecture level city in Shandong
Dragon Quality Goods Shop	Green Intelligent Power Saver	Received the goods first use a period of time to see if there is no effect, the business commitment to ninety days invalid refund, first praise, good service!	Jingdong prefecture level city in Shandong
Dragon Quality Goods Shop	Green Intelligent Power Saver	The product is used for a month without energy-saving and power-saving effect does not have any effect	Jingdong prefecture level city in Shandong
Dragon Quality Goods Shop	Green Intelligent Power Saver	The baby is really great, I like it very much, my friends also say good to come, recommended to buy!	Jingdong prefecture level city in Shandong
Dragon Quality Goods Shop	Green Intelligent Power Saver	Logistics is fast, courier boy service is good, after using to see how the fruit, and then follow up.	Jingdong prefecture level city in Shandong
Dragon Quality Goods Shop	Green Intelligent Power Saver	Baby has been received to try, I believe that as the business advertised energy saving. The courier is very responsible.	Jingdong prefecture level city in Shandong
Dragon Quality Goods Shop	Green Intelligent Power Saver	Save Electric Energy King received, good quality, beautiful appearance, very satisfied!	Jingdong prefecture level city in Shandong
Dragon Quality Goods Shop	Green Intelligent Power Saver	Three no products no manufacturers no phone pure fraud induced consumption cheat	Jingdong prefecture level city in Shandong
Dragon Quality Goods Shop	Green Intelligent Power Saver	I'm using it now, I don't know if it's as good as it says, I'll follow up after a month	Jingdong prefecture level city in Shandong
Dragon Quality Goods Shop	Green Intelligent Power Saver	Good quality logistics and fast. Fine workmanship. The description is consistent cost-effective	Jingdong prefecture level city in Shandong
Dragon Quality Goods Shop	Green Intelligent Power Saver	Received, whether it is good or not, to wait a few days and then evaluate	Jingdong prefecture level city in Shandong
Dragon Quality Goods Shop	Green Intelligent Power Saver	I bought it and started using it, I won't know if it works well until I pay my electric bill	Jingdong prefecture level city in Shandong
Dragon Quality Goods Shop	Green Intelligent Power Saver	Especially good, energy saving and power saving, especially good quality, good	Jingdong prefecture level city in Shandong
Dragon Quality Goods Shop	Green Intelligent Power Saver	Logistics is strong, the quality is very good, paste really save electricity, as described, will buy back!	Jingdong prefecture level city in Shandong
Dragon Quality Goods Shop	Green Intelligent Power Saver	Great stuff at a great price, worth the money!	Jingdong prefecture level city in Shandong
Dragon Quality Goods Shop	Green Intelligent Power Saver	Received the power saver, very good quality, very power saving!	Jingdong prefecture level city in Shandong
Dragon Quality Goods Shop	Green Intelligent Power Saver	There are no fruits, only samples...	Jingdong prefecture level city in Shandong
Dragon Quality Goods Shop	Green Intelligent Power Saver	Save a few dollars a month.	Jingdong prefecture level city in Shandong
Dragon Quality Goods Shop	Green Intelligent Power Saver	The quality is particularly good, it works well, and the seller has a good service attitude	Jingdong prefecture level city in Shandong
Dragon Quality Goods Shop	Green Intelligent Power Saver	I've compared them a couple of times and I don't feel any difference	Jingdong prefecture level city in Shandong
Dragon Quality Goods Shop	Green Intelligent Power Saver	I don't see the savings. It's a sham.	Jingdong prefecture level city in Shandong
Dragon Quality Goods Shop	Green Intelligent Power Saver	The power saving effect is very good, and the logistics is very fast. The seller's service attitude is very good	Jingdong prefecture level city in Shandong
Dragon Quality Goods Shop	Green Intelligent Power Saver	Received, well packaged, haven't tried it yet!	Jingdong prefecture level city in Shandong
Dragon Quality Goods Shop	Green Intelligent Power Saver	I don't know how it works, I'll comment after I use it.	Jingdong prefecture level city in Shandong
Dragon Quality Goods Shop	Green Intelligent Power Saver	The product doesn't work well enough to be of any use	Jingdong prefecture level city in Shandong
Dragon Quality Goods Shop	Green Intelligent Power Saver	On Trial 。。。。。。。	Jingdong prefecture level city in Shandong
Dragon Quality Goods Shop	Green Intelligent Power Saver	Being used with unknown results	Jingdong prefecture level city in Shandong
Dragon Quality Goods Shop	Green Intelligent Power Saver	Smart power saving and great quality	Jingdong prefecture level city in Shandong
Dragon Quality Goods Shop	Green Intelligent Power Saver	The saver was received, it works very well and the price is affordable!	Jingdong prefecture level city in Shandong
Dragon Quality Goods Shop	Green Intelligent Power Saver	How can I feel the power saving and how long does it last	Jingdong prefecture level city in Shandong
Dragon Quality Goods Shop	Green Intelligent Power Saver	And I know if it saves electricity or not.	Jingdong prefecture level city in Shandong
Dragon Quality Goods Shop	Green Intelligent Power Saver	It won't save any electricity. No.	Jingdong prefecture level city in Shandong
Dragon Quality Goods Shop	Green Intelligent Power Saver	Invalid. Fake.	Jingdong prefecture level city in Shandong
Dragon Quality Goods Shop	Green Intelligent Power Saver	It's good!	Jingdong prefecture level city in Shandong
Dragon Quality Goods Shop	Green Intelligent Power Saver	have no effect	Jingdong prefecture level city in Shandong
Dragon Quality Goods Shop	Green Intelligent Power Saver	Not used.	Jingdong prefecture level city in Shandong
Dragon Quality Goods Shop	Green Intelligent Power Saver	There is no load range, packaging thrift, ten days also did not develop invoices, received the goods with a receipt, hand-written receipt also has no financial stamp, communication for nearly a week a moment to say that the electronic invoice, a moment to say that the paper invoice, said it is mailed to me, ten days also did not see anything, the invoice invoice can not be found to say that did not stay on file, single single number can not be found, said that the warehouse more than one to a one to find out, in addition to the perfunctory or perfunctory. Products are no certificate of conformity, proper three	Jingdong prefecture level city in Shandong
Dragon Quality Goods Shop	Green Intelligent Power Saver	Satisfaction.	Jingdong prefecture level city in Shandong

Dragon Quality Goods Shop	Green Intelligent Power Saver	There is not a bit of power saving effect, and ringing up an increase in the electricity bill, because you have to add the cost of this machine's own electricity consumption and purchase, so I think it is better to use this machine instead of increasing the expenditure, uneconomical, rather than not use it.	Jingdong prefecture level city in Shandong
Dragon Quality Goods Shop	Green Intelligent Power Saver	I've been using it for two days now and I don't feel any effect.	Jingdong prefecture level city in Shandong
Dragon Quality Goods Shop	Green Intelligent Power Saver	What it looked like was nothing like what was sent.	Jingdong prefecture level city in Shandong
Dragon Quality Goods Shop	Green Intelligent Power Saver	It didn't work. It didn't work for a month or so.	Jingdong prefecture level city in Shandong
Dragon Quality Goods Shop	Green Intelligent Power Saver	Garbage products, physical and description is not the same, no effect at all, pit # no discussion, to buy the fine, garbage, garbage, garbage	Jingdong prefecture level city in Shandong
Dragon Quality Goods Shop	Green Intelligent Power Saver	general	Jingdong prefecture level city in Shandong
Dragon Quality Goods Shop	Green Intelligent Power Saver	IQ tax. Instead of saving electricity, it increases the power consumption of an appliance.	Jingdong prefecture level city in Shandong
Dragon Quality Goods Shop	Green Intelligent Power Saver	There's no trash at all.	Jingdong prefecture level city in Shandong
Dragon Quality Goods Shop	Green Intelligent Power Saver	It doesn't do anything.	Jingdong prefecture level city in Shandong
Dragon Quality Goods Shop	Green Intelligent Power Saver	(of an unmarried couple) be close	Jingdong prefecture level city in Shandong
Dragon Quality Goods Shop	Green Intelligent Power Saver	(of an unmarried couple) be close	Jingdong prefecture level city in Shandong
Dragon Quality Goods Shop	Green Intelligent Power Saver	no electricity saving	Jingdong prefecture level city in Shandong
Dragon Quality Goods Shop	Green Intelligent Power Saver	differ from	Jingdong prefecture level city in Shandong
Dragon Quality Goods Shop	Green Intelligent Power Saver	Saver has received, but real can save electricity, the quality is very good, I times I buy a few more, too good.	Jingdong prefecture level city in Shandong
Dragon Quality Goods Shop	Green Intelligent Power Saver	Great quality as always, a very favorite product, great! o(*￣︶￣*)o	Jingdong prefecture level city in Shandong
Dragon Quality Goods Shop	Green Intelligent Power Saver	If we can reduce the electricity bill, for individuals can save money, for the country can protect the environment, now the weather is getting hotter and hotter, to protect the environment to reduce emissions is very important. Already use the product, whether to reduce the electricity bill depends on next month!	Jingdong prefecture level city in Shandong
Dragon Quality Goods Shop	Green Intelligent Power Saver	Purchased a power saver received on the put into use, rent a room with many electrical appliances, power consumption is very large, we have a piece of electricity or 1.2 dollars a time, with this power saver is much better, every day can save a few cents, very convenient and practical!	Jingdong prefecture level city in Shandong
Dragon Quality Goods Shop	Green Intelligent Power Saver	The product is better to use before not to use when a month 1500 yuan electricity bill, now using the 5 second month electricity bill directly down 470 dollars or very good. A save money to buy several power saver money ZanZanZanZan👍	Jingdong prefecture level city in Shandong
Dragon Quality Goods Shop	Green Intelligent Power Saver	The moment I received the goods really satisfied with this product, not only high-grade packaging, the body are base alloy, up to the price, I was also introduced by a friend, he used last month did save a lot of electricity, but also recommended to me!	Jingdong prefecture level city in Shandong
Dragon Quality Goods Shop	Green Intelligent Power Saver	I got it. Good review. I'm very satisfied. Reasonable price.	Jingdong prefecture level city in Shandong
Dragon Quality Goods Shop	Green Intelligent Power Saver	Intelligent power saver received and used, looking at the quality is good, the quality of the plug sent is also very good. Expect the effect of the economizer is obvious, first five-star praise, with a period of time to follow up!	Jingdong prefecture level city in Shandong
Dragon Quality Goods Shop	Green Intelligent Power Saver	The saver has been received. It was plugged into an outlet and used immediately after receipt. Whether it really saves electricity is not clear yet, I believe that this brand of energy saver really saves	Jingdong prefecture level city in Shandong
Dragon Quality Goods Shop	Green Intelligent Power Saver	Okay, okay, okay. Thank you so much. Fast is good. Fast. Item was as described, mainly because the event was cheap and you can save a lot of money.	Jingdong prefecture level city in Shandong
Dragon Quality Goods Shop	Green Intelligent Power Saver	Received it a couple days ago! Plugged it in the same day! Let's see what the electric bill is in a month! Hopefully it works well oh!	Jingdong prefecture level city in Shandong
Dragon Quality Goods Shop	Green Intelligent Power Saver	Looking at the reviews good, energy-saving small products, compact, the results after 2 months to see, because the electricity bill season payment	Jingdong prefecture level city in Shandong
Dragon Quality Goods Shop	Green Intelligent Power Saver	I just received it and will try it out to see if it works well. I'll comment later.	Jingdong prefecture level city in Shandong
Dragon Quality Goods Shop	Green Intelligent Power Saver	It looks a little small and not very aesthetically pleasing, I don't know how effective it will be until I use it	Jingdong prefecture level city in Shandong
Dragon Quality Goods Shop	Green Intelligent Power Saver	Not only to the point of being useless yet	Jingdong prefecture level city in Shandong
Dragon Quality Goods Shop	Green Intelligent Power Saver	Used it for a month, nothing works, the meter you turn very fast. This is not a scam, but also sold on Jingdong. To cheat a bunch of people?	Jingdong prefecture level city in Shandong
Dragon Quality Goods Shop	Green Intelligent Power Saver	Very good to use, every day can save a little electricity, really good under the sample down a month to save a few dozen dollars in electricity, good before I did not know that there is this 👆 haha, the price is not expensive praise.	Jingdong prefecture level city in Shandong
Dragon Quality Goods Shop	Green Intelligent Power Saver	Tried it for ten days and the change is almost nothing, can't see the power saving yet, use it some more days and see how it goes.	Jingdong prefecture level city in Shandong
Dragon Quality Goods Shop	Green Intelligent Power Saver	Received very quickly, the quality is very good, really save electricity, business service attitude is also very good, I hope you do not miss this opportunity, I continue to buy a few in	Jingdong prefecture level city in Shandong
Dragon Quality Goods Shop	Green Intelligent Power Saver	Quality is very good Oh, the delivery is also very fast, a very favorite store, I want to crazy recommend to friends!	Jingdong prefecture level city in Shandong
Dragon Quality Goods Shop	Green Intelligent Power Saver	The goods have just received, fine workmanship, and the same as described by the merchant, to use a period of time to see if it can achieve the purpose of power saving.	Jingdong prefecture level city in Shandong
Dragon Quality Goods Shop	Green Intelligent Power Saver	Received the goods first use a period of time to see if there is no effect, the business commitment to ninety days invalid refund, first praise, good service!	Jingdong prefecture level city in Shandong
Dragon Quality Goods Shop	Green Intelligent Power Saver	The product is used for a month without energy-saving and power-saving effect does not have any effect	Jingdong prefecture level city in Shandong
Dragon Quality Goods Shop	Green Intelligent Power Saver	The baby is really great, I like it very much, my friends also say good to come, recommended to buy!	Jingdong prefecture level city in Shandong
Dragon Quality Goods Shop	Green Intelligent Power Saver	Logistics is fast, courier boy service is good, after using to see how the fruit, and then follow up.	Jingdong prefecture level city in Shandong
Dragon Quality Goods Shop	Green Intelligent Power Saver	Baby has been received to try, I believe that as the business advertised energy saving. The courier is very responsible.	Jingdong prefecture level city in Shandong
Dragon Quality Goods Shop	Green Intelligent Power Saver	Save Electric Energy King received, good quality, beautiful appearance, very satisfied!	Jingdong prefecture level city in Shandong
Dragon Quality Goods Shop	Green Intelligent Power Saver	Three no products no manufacturers no phone pure fraud induced consumption cheat	Jingdong prefecture level city in Shandong
Dragon Quality Goods Shop	Green Intelligent Power Saver	I'm using it now, I don't know if it's as good as it says, I'll follow up after a month	Jingdong prefecture level city in Shandong
Dragon Quality Goods Shop	Green Intelligent Power Saver	Good quality logistics and fast. Fine workmanship. The description is consistent cost-effective	Jingdong prefecture level city in Shandong
Dragon Quality Goods Shop	Green Intelligent Power Saver	Received, whether it is good or not, to wait a few days and then evaluate	Jingdong prefecture level city in Shandong
Dragon Quality Goods Shop	Green Intelligent Power Saver	I bought it and started using it, I won't know if it works well until I pay my electric bill	Jingdong prefecture level city in Shandong
Dragon Quality Goods Shop	Green Intelligent Power Saver	Especially good, energy saving and power saving, especially good quality, good	Jingdong prefecture level city in Shandong
Dragon Quality Goods Shop	Green Intelligent Power Saver	Logistics is strong, the quality is very good, paste really save electricity, as described, will buy back!	Jingdong prefecture level city in Shandong
Dragon Quality Goods Shop	Green Intelligent Power Saver	Great stuff at a great price, worth the money!	Jingdong prefecture level city in Shandong

Dragon Quality Goods Shop	Green Intelligent Power Saver	Received the power saver, very good quality, very power saving!	Jingdong prefecture level city in Shandong
Dragon Quality Goods Shop	Green Intelligent Power Saver	There are no fruits, only samples...	Jingdong prefecture level city in Shandong
Dragon Quality Goods Shop	Green Intelligent Power Saver	Save a few dollars a month.	Jingdong prefecture level city in Shandong
Dragon Quality Goods Shop	Green Intelligent Power Saver	The quality is particularly good, it works well, and the seller has a good service attitude	Jingdong prefecture level city in Shandong
Dragon Quality Goods Shop	Green Intelligent Power Saver	I've compared them a couple of times and I don't feel any difference	Jingdong prefecture level city in Shandong
Dragon Quality Goods Shop	Green Intelligent Power Saver	I don't see the savings. It's a sham.	Jingdong prefecture level city in Shandong
Dragon Quality Goods Shop	Green Intelligent Power Saver	The power saving effect is very good, and the logistics is very fast. The seller's service attitude is very good	Jingdong prefecture level city in Shandong
Dragon Quality Goods Shop	Green Intelligent Power Saver	Received, well packaged, haven't tried it yet!	Jingdong prefecture level city in Shandong
Dragon Quality Goods Shop	Green Intelligent Power Saver	I don't know how it works, I'll comment after I use it.	Jingdong prefecture level city in Shandong
Dragon Quality Goods Shop	Green Intelligent Power Saver	The product doesn't work well enough to be of any use	Jingdong prefecture level city in Shandong
Dragon Quality Goods Shop	Green Intelligent Power Saver	On Trial 。。。。。。。	Jingdong prefecture level city in Shandong
Dragon Quality Goods Shop	Green Intelligent Power Saver	Being used with unknown results	Jingdong prefecture level city in Shandong
Dragon Quality Goods Shop	Green Intelligent Power Saver	Smart power saving and great quality	Jingdong prefecture level city in Shandong
Dragon Quality Goods Shop	Green Intelligent Power Saver	The saver was received, it works very well and the price is affordable!	Jingdong prefecture level city in Shandong
Dragon Quality Goods Shop	Green Intelligent Power Saver	How can I feel the power saving and how long does it last	Jingdong prefecture level city in Shandong
Dragon Quality Goods Shop	Green Intelligent Power Saver	And I know if it saves electricity or not.	Jingdong prefecture level city in Shandong
Dragon Quality Goods Shop	Green Intelligent Power Saver	It won't save any electricity. No.	Jingdong prefecture level city in Shandong
Dragon Quality Goods Shop	Green Intelligent Power Saver	Invalid. Fake.	Jingdong prefecture level city in Shandong
Dragon Quality Goods Shop	Green Intelligent Power Saver	It's good!	Jingdong prefecture level city in Shandong
Dragon Quality Goods Shop	Green Intelligent Power Saver	have no effect	Jingdong prefecture level city in Shandong
Dragon Quality Goods Shop	Green Intelligent Power Saver	Not used.	Jingdong prefecture level city in Shandong
Dragon Quality Goods Shop	Green Intelligent Power Saver	There is no load range, packaging thrift, ten days also did not develop invoices, received the goods with a receipt, hand-written receipt also has no financial stamp, communication for nearly a week a moment to say that the electronic invoice, a moment to say that the paper invoice, said it is mailed to me, ten days also did not see anything, the invoice invoice can not be found to say that did not stay on file, single single number can not be found, said that the warehouse more than one to a one to find out, in addition to the perfunctory or perfunctory. Products are no certificate of conformity, proper three	Jingdong prefecture level city in Shandong
Dragon Quality Goods Shop	Green Intelligent Power Saver	Satisfaction.	Jingdong prefecture level city in Shandong
Dragon Quality Goods Shop	Green Intelligent Power Saver	There is not a bit of power saving effect, and ringing up an increase in the electricity bill, because you have to add the cost of this machine's own electricity consumption and purchase, so I think it is better to use this machine instead of increasing the expenditure, uneconomical, rather than not use it.	Jingdong prefecture level city in Shandong
Dragon Quality Goods Shop	Green Intelligent Power Saver	I've been using it for two days now and I don't feel any effect.	Jingdong prefecture level city in Shandong
Dragon Quality Goods Shop	Green Intelligent Power Saver	What it looked like was nothing like what was sent.	Jingdong prefecture level city in Shandong
Dragon Quality Goods Shop	Green Intelligent Power Saver	It didn't work. It didn't work for a month or so.	Jingdong prefecture level city in Shandong
Dragon Quality Goods Shop	Green Intelligent Power Saver	Garbage products, physical and description is not the same, no effect at all, pit # no discussion, to buy the fine, garbage, garbage, garbage	Jingdong prefecture level city in Shandong
Dragon Quality Goods Shop	Green Intelligent Power Saver	general	Jingdong prefecture level city in Shandong
Dragon Quality Goods Shop	Green Intelligent Power Saver	IQ tax. Instead of saving electricity, it increases the power consumption of an appliance.	Jingdong prefecture level city in Shandong
Dragon Quality Goods Shop	Green Intelligent Power Saver	There's no trash at all.	Jingdong prefecture level city in Shandong
Dragon Quality Goods Shop	Green Intelligent Power Saver	It doesn't do anything.	Jingdong prefecture level city in Shandong
Dragon Quality Goods Shop	Green Intelligent Power Saver	(of an unmarried couple) be close	Jingdong prefecture level city in Shandong
Dragon Quality Goods Shop	Green Intelligent Power Saver	(of an unmarried couple) be close	Jingdong prefecture level city in Shandong
Dragon Quality Goods Shop	Green Intelligent Power Saver	no electricity saving	Jingdong prefecture level city in Shandong
Dragon Quality Goods Shop	Green Intelligent Power Saver	differ from	Jingdong prefecture level city in Shandong
Dragon Quality Goods Shop	Green Intelligent Power Saver	Saver has received, but real can save electricity, the quality is very good, I times I buy a few more, too good.	Jingdong prefecture level city in Shandong
Dragon Quality Goods Shop	Green Intelligent Power Saver	Great quality as always, a very favorite product, great! o(*￣︶￣*)o	Jingdong prefecture level city in Shandong
Dragon Quality Goods Shop	Green Intelligent Power Saver	If we can reduce the electricity bill, for individuals can save money, for the country can protect the environment, now the weather is getting hotter and hotter, to protect the environment to reduce emissions is very important. Already use the product, whether to reduce the electricity bill depends on next month!	Jingdong prefecture level city in Shandong
Dragon Quality Goods Shop	Green Intelligent Power Saver	Purchased a power saver received on the put into use, rent a room with many electrical appliances, power consumption is very large, we have a piece of electricity or 1.2 dollars a time, with this power saver is much better, every day can save a few cents, very convenient and practical!	Jingdong prefecture level city in Shandong
Dragon Quality Goods Shop	Green Intelligent Power Saver	The product is better to use before not to use when a month 1500 yuan electricity bill, now using the 5 second month electricity bill directly down 470 dollars or very good. A save money to buy several power saver money ZanZanZanZan👍	Jingdong prefecture level city in Shandong
Dragon Quality Goods Shop	Green Intelligent Power Saver	The moment I received the goods really satisfied with this product, not only high-grade packaging, the body are base alloy, up to the price, I was also introduced by a friend, he used last month did save a lot of electricity, but also recommended to me!	Jingdong prefecture level city in Shandong
Dragon Quality Goods Shop	Green Intelligent Power Saver	I got it. Good review. I'm very satisfied. Reasonable price.	Jingdong prefecture level city in Shandong
Dragon Quality Goods Shop	Green Intelligent Power Saver	Intelligent power saver received and used, looking at the quality is good, the quality of the plug sent is also very good. Expect the effect of the economizer is obvious, first five-star praise, with a period of time to follow up!	Jingdong prefecture level city in Shandong
Dragon Quality Goods Shop	Green Intelligent Power Saver	The saver has been received. It was plugged into an outlet and used immediately after receipt. Whether it really saves electricity is not clear yet, I believe that this brand of energy saver really saves	Jingdong prefecture level city in Shandong
Dragon Quality Goods Shop	Green Intelligent Power Saver	Okay, okay, okay. Thank you so much. Fast is good. Fast. Item was as described, mainly because the event was cheap and you can save a lot of money.	Jingdong prefecture level city in Shandong

Dragon Quality Goods Shop	Green Intelligent Power Saver	Received it a couple days ago! Plugged it in the same day! Let's see what the electric bill is in a month! Hopefully it works well oh!	Jingdong prefecture level city in Shandong
Dragon Quality Goods Shop	Green Intelligent Power Saver	Looking at the reviews good, energy-saving small products, compact, the results after 2 months to see, because the electricity bill season payment	Jingdong prefecture level city in Shandong
Dragon Quality Goods Shop	Green Intelligent Power Saver	I just received it and will try it out to see if it works well. I'll comment later.	Jingdong prefecture level city in Shandong
Dragon Quality Goods Shop	Green Intelligent Power Saver	It looks a little small and not very aesthetically pleasing, I don't know how effective it will be until I use it	Jingdong prefecture level city in Shandong
Dragon Quality Goods Shop	Green Intelligent Power Saver	Not only to the point of being useless yet	Jingdong prefecture level city in Shandong
Dragon Quality Goods Shop	Green Intelligent Power Saver	Used it for a month, nothing works, the meter you turn very fast. This is not a scam, but also sold on Jingdong. To cheat a bunch of people?	Jingdong prefecture level city in Shandong
Dragon Quality Goods Shop	Green Intelligent Power Saver	Very good to use, every day can save a little electricity, really good under the sample down a month to save a few dozen dollars in electricity, good before I did not know that there is this ☝ haha, the price is not expensive praise.	Jingdong prefecture level city in Shandong
Dragon Quality Goods Shop	Green Intelligent Power Saver	Tried it for ten days and the change is almost nothing, can't see the power saving yet, use it some more days and see how it goes.	Jingdong prefecture level city in Shandong
Dragon Quality Goods Shop	Green Intelligent Power Saver	Received very quickly, the quality is very good, really save electricity, business service attitude is also very good, I hope you do not miss this opportunity, I continue to buy a few in	Jingdong prefecture level city in Shandong
Dragon Quality Goods Shop	Green Intelligent Power Saver	Quality is very good Oh, the delivery is also very fast, a very favorite store, I want to crazy recommend to friends!	Jingdong prefecture level city in Shandong
Dragon Quality Goods Shop	Green Intelligent Power Saver	The goods have just received, fine workmanship, and the same as described by the merchant, to use a period of time to see if it can achieve the purpose of power saving.	Jingdong prefecture level city in Shandong
Dragon Quality Goods Shop	Green Intelligent Power Saver	Received the goods first use a period of time to see if there is no effect, the business commitment to ninety days invalid refund, first praise, good service!	Jingdong prefecture level city in Shandong
Dragon Quality Goods Shop	Green Intelligent Power Saver	The product is used for a month without energy-saving and power-saving effect does not have any effect	Jingdong prefecture level city in Shandong
Dragon Quality Goods Shop	Green Intelligent Power Saver	The baby is really great, I like it very much, my friends also say good to come, recommended to buy!	Jingdong prefecture level city in Shandong
Dragon Quality Goods Shop	Green Intelligent Power Saver	Logistics is fast, courier boy service is good, after using to see how the fruit, and then follow up.	Jingdong prefecture level city in Shandong
Dragon Quality Goods Shop	Green Intelligent Power Saver	Baby has been received to try, I believe that as the business advertised energy saving. The courier is very responsible.	Jingdong prefecture level city in Shandong
Dragon Quality Goods Shop	Green Intelligent Power Saver	Save Electric Energy King received, good quality, beautiful appearance, very satisfied!	Jingdong prefecture level city in Shandong
Dragon Quality Goods Shop	Green Intelligent Power Saver	Three no products no manufacturers no phone pure fraud induced consumption cheat	Jingdong prefecture level city in Shandong
Dragon Quality Goods Shop	Green Intelligent Power Saver	I'm using it now, I don't know if it's as good as it says, I'll follow up after a month	Jingdong prefecture level city in Shandong
Dragon Quality Goods Shop	Green Intelligent Power Saver	Good quality logistics and fast. Fine workmanship. The description is consistent cost-effective	Jingdong prefecture level city in Shandong
Dragon Quality Goods Shop	Green Intelligent Power Saver	Received, whether it is good or not, to wait a few days and then evaluate	Jingdong prefecture level city in Shandong
Dragon Quality Goods Shop	Green Intelligent Power Saver	I bought it and started using it, I won't know if it works well until I pay my electric bill	Jingdong prefecture level city in Shandong
Dragon Quality Goods Shop	Green Intelligent Power Saver	Especially good, energy saving and power saving, especially good quality, good	Jingdong prefecture level city in Shandong
Dragon Quality Goods Shop	Green Intelligent Power Saver	Logistics is strong, the quality is very good, paste really save electricity, as described, will buy back!	Jingdong prefecture level city in Shandong
Dragon Quality Goods Shop	Green Intelligent Power Saver	Great stuff at a great price, worth the money!	Jingdong prefecture level city in Shandong
Dragon Quality Goods Shop	Green Intelligent Power Saver	Received the power saver, very good quality, very power saving!	Jingdong prefecture level city in Shandong
Dragon Quality Goods Shop	Green Intelligent Power Saver	There are no fruits, only samples...	Jingdong prefecture level city in Shandong
Dragon Quality Goods Shop	Green Intelligent Power Saver	Save a few dollars a month.	Jingdong prefecture level city in Shandong
Dragon Quality Goods Shop	Green Intelligent Power Saver	The quality is particularly good, it works well, and the seller has a good service attitude	Jingdong prefecture level city in Shandong
Dragon Quality Goods Shop	Green Intelligent Power Saver	I've compared them a couple of times and I don't feel any difference	Jingdong prefecture level city in Shandong
Dragon Quality Goods Shop	Green Intelligent Power Saver	I don't see the savings. It's a sham.	Jingdong prefecture level city in Shandong
Dragon Quality Goods Shop	Green Intelligent Power Saver	The power saving effect is very good, and the logistics is very fast. The seller's service attitude is very good	Jingdong prefecture level city in Shandong
Dragon Quality Goods Shop	Green Intelligent Power Saver	Received, well packaged, haven't tried it yet!	Jingdong prefecture level city in Shandong
Dragon Quality Goods Shop	Green Intelligent Power Saver	I don't know how it works, I'll comment after I use it.	Jingdong prefecture level city in Shandong
Dragon Quality Goods Shop	Green Intelligent Power Saver	The product doesn't work well enough to be of any use	Jingdong prefecture level city in Shandong
Dragon Quality Goods Shop	Green Intelligent Power Saver	On Trial 。。。。。。。	Jingdong prefecture level city in Shandong
Dragon Quality Goods Shop	Green Intelligent Power Saver	Being used with unknown results	Jingdong prefecture level city in Shandong
Dragon Quality Goods Shop	Green Intelligent Power Saver	Smart power saving and great quality	Jingdong prefecture level city in Shandong
Dragon Quality Goods Shop	Green Intelligent Power Saver	The saver was received, it works very well and the price is affordable!	Jingdong prefecture level city in Shandong
Dragon Quality Goods Shop	Green Intelligent Power Saver	How can I feel the power saving and how long does it last	Jingdong prefecture level city in Shandong
Dragon Quality Goods Shop	Green Intelligent Power Saver	And I know if it saves electricity or not.	Jingdong prefecture level city in Shandong
Dragon Quality Goods Shop	Green Intelligent Power Saver	It won't save any electricity. No.	Jingdong prefecture level city in Shandong
Dragon Quality Goods Shop	Green Intelligent Power Saver	Invalid. Fake.	Jingdong prefecture level city in Shandong
Dragon Quality Goods Shop	Green Intelligent Power Saver	It's good!	Jingdong prefecture level city in Shandong
Dragon Quality Goods Shop	Green Intelligent Power Saver	have no effect	Jingdong prefecture level city in Shandong
Dragon Quality Goods Shop	Green Intelligent Power Saver	Not used.	Jingdong prefecture level city in Shandong
Dragon Quality Goods Shop	Green Intelligent Power Saver	There is no load range, packaging thrift, ten days also did not develop invoices, received the goods with a receipt, hand-written receipt also has no financial stamp, communication for nearly a week a moment to say that the electronic invoice, a moment to say that the paper invoice, said it is mailed to me, ten days also did not see anything, the invoice invoice can not be found to say that did not stay on file, single single number can not be found, said that the warehouse more than one to a one to find out, in addition to the perfunctory or perfunctory. Products are no certificate of conformity, proper three	Jingdong prefecture level city in Shandong
Dragon Quality Goods Shop	Green Intelligent Power Saver	Satisfaction.	Jingdong prefecture level city in Shandong

Dragon Quality Goods Shop	Green Intelligent Power Saver	There is not a bit of power saving effect, and ringing up an increase in the electricity bill, because you have to add the cost of this machine's own electricity consumption and purchase, so I think it is better to use this machine instead of increasing the expenditure, uneconomical, rather than not use it.	Jingdong prefecture level city in Shandong
Dragon Quality Goods Shop	Green Intelligent Power Saver	I've been using it for two days now and I don't feel any effect.	Jingdong prefecture level city in Shandong
Dragon Quality Goods Shop	Green Intelligent Power Saver	What it looked like was nothing like what was sent.	Jingdong prefecture level city in Shandong
Dragon Quality Goods Shop	Green Intelligent Power Saver	It didn't work. It didn't work for a month or so.	Jingdong prefecture level city in Shandong
Dragon Quality Goods Shop	Green Intelligent Power Saver	Garbage products, physical and description is not the same, no effect at all, pit # no discussion, to buy the fine, garbage, garbage, garbage	Jingdong prefecture level city in Shandong
Dragon Quality Goods Shop	Green Intelligent Power Saver	general	Jingdong prefecture level city in Shandong
Dragon Quality Goods Shop	Green Intelligent Power Saver	IQ tax. Instead of saving electricity, it increases the power consumption of an appliance.	Jingdong prefecture level city in Shandong
Dragon Quality Goods Shop	Green Intelligent Power Saver	There's no trash at all.	Jingdong prefecture level city in Shandong
Dragon Quality Goods Shop	Green Intelligent Power Saver	It doesn't do anything.	Jingdong prefecture level city in Shandong
Dragon Quality Goods Shop	Green Intelligent Power Saver	(of an unmarried couple) be close	Jingdong prefecture level city in Shandong
Dragon Quality Goods Shop	Green Intelligent Power Saver	(of an unmarried couple) be close	Jingdong prefecture level city in Shandong
Dragon Quality Goods Shop	Green Intelligent Power Saver	no electricity saving	Jingdong prefecture level city in Shandong
Dragon Quality Goods Shop	Green Intelligent Power Saver	differ from	Jingdong prefecture level city in Shandong
Dragon Quality Goods Shop	Green Intelligent Power Saver	Saver has received, but real can save electricity, the quality is very good, I times I buy a few more, too good.	Jingdong prefecture level city in Shandong
Dragon Quality Goods Shop	Green Intelligent Power Saver	Great quality as always, a very favorite product, great! o(*￣︶￣*)o	Jingdong prefecture level city in Shandong
Dragon Quality Goods Shop	Green Intelligent Power Saver	If we can reduce the electricity bill, for individuals can save money, for the country can protect the environment, now the weather is getting hotter and hotter, to protect the environment to reduce emissions is very important. Already use the product, whether to reduce the electricity bill depends on next month!	Jingdong prefecture level city in Shandong
Dragon Quality Goods Shop	Green Intelligent Power Saver	Purchased a power saver received on the put into use, rent a room with many electrical appliances, power consumption is very large, we have a piece of electricity or 1.2 dollars a time, with this power saver is much better, every day can save a few cents, very convenient and practical!	Jingdong prefecture level city in Shandong
Dragon Quality Goods Shop	Green Intelligent Power Saver	The product is better to use before not to use when a month 1500 yuan electricity bill, now using the 5 second month electricity bill directly down 470 dollars or very good. A save money to buy several power saver money ZanZanZanZan👍	Jingdong prefecture level city in Shandong
Dragon Quality Goods Shop	Green Intelligent Power Saver	The moment I received the goods really satisfied with this product, not only high-grade packaging, the body are base alloy, up to the price, I was also introduced by a friend, he used last month did save a lot of electricity, but also recommended to me!	Jingdong prefecture level city in Shandong
Dragon Quality Goods Shop	Green Intelligent Power Saver	I got it. Good review. I'm very satisfied. Reasonable price.	Jingdong prefecture level city in Shandong
Dragon Quality Goods Shop	Green Intelligent Power Saver	Intelligent power saver received and used, looking at the quality is good, the quality of the plug sent is also very good. Expect the effect of the economizer is obvious, first five-star praise, with a period of time to follow up!	Jingdong prefecture level city in Shandong
Dragon Quality Goods Shop	Green Intelligent Power Saver	The saver has been received. It was plugged into an outlet and used immediately after receipt. Whether it really saves electricity is not clear yet, I believe that this brand of energy saver really saves	Jingdong prefecture level city in Shandong
Dragon Quality Goods Shop	Green Intelligent Power Saver	Okay, okay, okay. Thank you so much. Fast is good. Fast. Item was as described, mainly because the event was cheap and you can save a lot of money.	Jingdong prefecture level city in Shandong
Dragon Quality Goods Shop	Green Intelligent Power Saver	Received it a couple days ago! Plugged it in the same day! Let's see what the electric bill is in a month! Hopefully it works well oh!	Jingdong prefecture level city in Shandong
Dragon Quality Goods Shop	Green Intelligent Power Saver	Looking at the reviews good, energy-saving small products, compact, the results after 2 months to see, because the electricity bill season payment	Jingdong prefecture level city in Shandong
Dragon Quality Goods Shop	Green Intelligent Power Saver	I just received it and will try it out to see if it works well. I'll comment later.	Jingdong prefecture level city in Shandong
Dragon Quality Goods Shop	Green Intelligent Power Saver	It looks a little small and not very aesthetically pleasing, I don't know how effective it will be until I use it	Jingdong prefecture level city in Shandong
Dragon Quality Goods Shop	Green Intelligent Power Saver	Not only to the point of being useless yet	Jingdong prefecture level city in Shandong
Dragon Quality Goods Shop	Green Intelligent Power Saver	Used it for a month, nothing works, the meter you turn very fast. This is not a scam, but also sold on Jingdong. To cheat a bunch of people?	Jingdong prefecture level city in Shandong
Dragon Quality Goods Shop	Green Intelligent Power Saver	Very good to use, every day can save a little electricity, really good under the sample down a month to save a few dozen dollars in electricity, good before I did not know that there is this 👆 haha, the price is not expensive praise.	Jingdong prefecture level city in Shandong
Dragon Quality Goods Shop	Green Intelligent Power Saver	Tried it for ten days and the change is almost nothing, can't see the power saving yet, use it some more days and see how it goes.	Jingdong prefecture level city in Shandong
Dragon Quality Goods Shop	Green Intelligent Power Saver	Received very quickly, the quality is very good, really save electricity, business service attitude is also very good, I hope you do not miss this opportunity, I continue to buy a few in	Jingdong prefecture level city in Shandong
Dragon Quality Goods Shop	Green Intelligent Power Saver	Quality is very good Oh, the delivery is also very fast, a very favorite store, I want to crazy recommend to friends!	Jingdong prefecture level city in Shandong
Dragon Quality Goods Shop	Green Intelligent Power Saver	The goods have just received, fine workmanship, and the same as described by the merchant, to use a period of time to see if it can achieve the purpose of power saving.	Jingdong prefecture level city in Shandong
Dragon Quality Goods Shop	Green Intelligent Power Saver	Received the goods first use a period of time to see if there is no effect, the business commitment to ninety days invalid refund, first praise, good service!	Jingdong prefecture level city in Shandong
Dragon Quality Goods Shop	Green Intelligent Power Saver	The product is used for a month without energy-saving and power-saving effect does not have any effect	Jingdong prefecture level city in Shandong
Dragon Quality Goods Shop	Green Intelligent Power Saver	The baby is really great, I like it very much, my friends also say good to come, recommended to buy!	Jingdong prefecture level city in Shandong
Dragon Quality Goods Shop	Green Intelligent Power Saver	Logistics is fast, courier boy service is good, after using to see how the fruit, and then follow up.	Jingdong prefecture level city in Shandong
Dragon Quality Goods Shop	Green Intelligent Power Saver	Baby has been received to try, I believe that as the business advertised energy saving. The courier is very responsible.	Jingdong prefecture level city in Shandong
Dragon Quality Goods Shop	Green Intelligent Power Saver	Save Electric Energy King received, good quality, beautiful appearance, very satisfied!	Jingdong prefecture level city in Shandong
Dragon Quality Goods Shop	Green Intelligent Power Saver	Three no products no manufacturers no phone pure fraud induced consumption cheat	Jingdong prefecture level city in Shandong
Dragon Quality Goods Shop	Green Intelligent Power Saver	I'm using it now, I don't know if it's as good as it says, I'll follow up after a month	Jingdong prefecture level city in Shandong
Dragon Quality Goods Shop	Green Intelligent Power Saver	Good quality logistics and fast. Fine workmanship. The description is consistent cost-effective	Jingdong prefecture level city in Shandong
Dragon Quality Goods Shop	Green Intelligent Power Saver	Received, whether it is good or not, to wait a few days and then evaluate	Jingdong prefecture level city in Shandong
Dragon Quality Goods Shop	Green Intelligent Power Saver	I bought it and started using it, I won't know if it works well until I pay my electric bill	Jingdong prefecture level city in Shandong
Dragon Quality Goods Shop	Green Intelligent Power Saver	Especially good, energy saving and power saving, especially good quality, good	Jingdong prefecture level city in Shandong
Dragon Quality Goods Shop	Green Intelligent Power Saver	Logistics is strong, the quality is very good, paste really save electricity, as described, will buy back!	Jingdong prefecture level city in Shandong
Dragon Quality Goods Shop	Green Intelligent Power Saver	Great stuff at a great price, worth the money!	Jingdong prefecture level city in Shandong

Dragon Quality Goods Shop	Green Intelligent Power Saver	Received the power saver, very good quality, very power saving!	Jingdong prefecture level city in Shandong
Dragon Quality Goods Shop	Green Intelligent Power Saver	There are no fruits, only samples...	Jingdong prefecture level city in Shandong
Dragon Quality Goods Shop	Green Intelligent Power Saver	Save a few dollars a month.	Jingdong prefecture level city in Shandong
Dragon Quality Goods Shop	Green Intelligent Power Saver	The quality is particularly good, it works well, and the seller has a good service attitude	Jingdong prefecture level city in Shandong
Dragon Quality Goods Shop	Green Intelligent Power Saver	I've compared them a couple of times and I don't feel any difference	Jingdong prefecture level city in Shandong
Dragon Quality Goods Shop	Green Intelligent Power Saver	I don't see the savings. It's a sham.	Jingdong prefecture level city in Shandong
Dragon Quality Goods Shop	Green Intelligent Power Saver	The power saving effect is very good, and the logistics is very fast. The seller's service attitude is very good	Jingdong prefecture level city in Shandong
Dragon Quality Goods Shop	Green Intelligent Power Saver	Received, well packaged, haven't tried it yet!	Jingdong prefecture level city in Shandong
Dragon Quality Goods Shop	Green Intelligent Power Saver	I don't know how it works, I'll comment after I use it.	Jingdong prefecture level city in Shandong
Dragon Quality Goods Shop	Green Intelligent Power Saver	The product doesn't work well enough to be of any use	Jingdong prefecture level city in Shandong
Dragon Quality Goods Shop	Green Intelligent Power Saver	On Trial 。。。。。。。	Jingdong prefecture level city in Shandong
Dragon Quality Goods Shop	Green Intelligent Power Saver	Being used with unknown results	Jingdong prefecture level city in Shandong
Dragon Quality Goods Shop	Green Intelligent Power Saver	Smart power saving and great quality	Jingdong prefecture level city in Shandong
Dragon Quality Goods Shop	Green Intelligent Power Saver	The saver was received, it works very well and the price is affordable!	Jingdong prefecture level city in Shandong
Dragon Quality Goods Shop	Green Intelligent Power Saver	How can I feel the power saving and how long does it last	Jingdong prefecture level city in Shandong
Dragon Quality Goods Shop	Green Intelligent Power Saver	And I know if it saves electricity or not.	Jingdong prefecture level city in Shandong
Dragon Quality Goods Shop	Green Intelligent Power Saver	It won't save any electricity. No.	Jingdong prefecture level city in Shandong
Dragon Quality Goods Shop	Green Intelligent Power Saver	Invalid. Fake.	Jingdong prefecture level city in Shandong
Dragon Quality Goods Shop	Green Intelligent Power Saver	It's good!	Jingdong prefecture level city in Shandong
Dragon Quality Goods Shop	Green Intelligent Power Saver	have no effect	Jingdong prefecture level city in Shandong
Dragon Quality Goods Shop	Green Intelligent Power Saver	Not used.	Jingdong prefecture level city in Shandong
Dragon Quality Goods Shop	Green Intelligent Power Saver	There is no load range, packaging thrift, ten days also did not develop invoices, received the goods with a receipt, hand-written receipt also has no financial stamp, communication for nearly a week a moment to say that the electronic invoice, a moment to say that the paper invoice, said it is mailed to me, ten days also did not see anything, the invoice invoice can not be found to say that did not stay on file, single single number can not be found, said that the warehouse more than one to a one to find out, in addition to the perfunctory or perfunctory. Products are no certificate of conformity, proper three	Jingdong prefecture level city in Shandong
Dragon Quality Goods Shop	Green Intelligent Power Saver	Satisfaction.	Jingdong prefecture level city in Shandong
Dragon Quality Goods Shop	Green Intelligent Power Saver	There is not a bit of power saving effect, and ringing up an increase in the electricity bill, because you have to add the cost of this machine's own electricity consumption and purchase, so I think it is better to use this machine instead of increasing the expenditure, uneconomical, rather than not use it.	Jingdong prefecture level city in Shandong
Dragon Quality Goods Shop	Green Intelligent Power Saver	I've been using it for two days now and I don't feel any effect.	Jingdong prefecture level city in Shandong
Dragon Quality Goods Shop	Green Intelligent Power Saver	What it looked like was nothing like what was sent.	Jingdong prefecture level city in Shandong
Dragon Quality Goods Shop	Green Intelligent Power Saver	It didn't work. It didn't work for a month or so.	Jingdong prefecture level city in Shandong
Dragon Quality Goods Shop	Green Intelligent Power Saver	Garbage products, physical and description is not the same, no effect at all, pit # no discussion, to buy the fine, garbage, garbage, garbage	Jingdong prefecture level city in Shandong
Dragon Quality Goods Shop	Green Intelligent Power Saver	general	Jingdong prefecture level city in Shandong
Dragon Quality Goods Shop	Green Intelligent Power Saver	IQ tax. Instead of saving electricity, it increases the power consumption of an appliance.	Jingdong prefecture level city in Shandong
Dragon Quality Goods Shop	Green Intelligent Power Saver	There's no trash at all.	Jingdong prefecture level city in Shandong
Dragon Quality Goods Shop	Green Intelligent Power Saver	It doesn't do anything.	Jingdong prefecture level city in Shandong
Dragon Quality Goods Shop	Green Intelligent Power Saver	(of an unmarried couple) be close	Jingdong prefecture level city in Shandong
Dragon Quality Goods Shop	Green Intelligent Power Saver	(of an unmarried couple) be close	Jingdong prefecture level city in Shandong
Dragon Quality Goods Shop	Green Intelligent Power Saver	no electricity saving	Jingdong prefecture level city in Shandong
Dragon Quality Goods Shop	Green Intelligent Power Saver	differ from	Jingdong prefecture level city in Shandong
Dragon Quality Goods Shop	Green Intelligent Power Saver	Saver has received, but real can save electricity, the quality is very good, I times I buy a few more, too good.	Jingdong prefecture level city in Shandong
Dragon Quality Goods Shop	Green Intelligent Power Saver	Great quality as always, a very favorite product, great! o(*￣︶￣*)o	Jingdong prefecture level city in Shandong
Dragon Quality Goods Shop	Green Intelligent Power Saver	If we can reduce the electricity bill, for individuals can save money, for the country can protect the environment, now the weather is getting hotter and hotter, to protect the environment to reduce emissions is very important. Already use the product, whether to reduce the electricity bill depends on next month!	Jingdong prefecture level city in Shandong
Dragon Quality Goods Shop	Green Intelligent Power Saver	Purchased a power saver received on the put into use, rent a room with many electrical appliances, power consumption is very large, we have a piece of electricity or 1.2 dollars a time, with this power saver is much better, every day can save a few cents, very convenient and practical!	Jingdong prefecture level city in Shandong
Dragon Quality Goods Shop	Green Intelligent Power Saver	The product is better to use before not to use when a month 1500 yuan electricity bill, now using the 5 second month electricity bill directly down 470 dollars or very good. A save money to buy several power saver money ZanZanZanZan👍	Jingdong prefecture level city in Shandong
Dragon Quality Goods Shop	Green Intelligent Power Saver	The moment I received the goods really satisfied with this product, not only high-grade packaging, the body are base alloy, up to the price, I was also introduced by a friend, he used last month did save a lot of electricity, but also recommended to me!	Jingdong prefecture level city in Shandong
Dragon Quality Goods Shop	Green Intelligent Power Saver	I got it. Good review. I'm very satisfied. Reasonable price.	Jingdong prefecture level city in Shandong
Dragon Quality Goods Shop	Green Intelligent Power Saver	Intelligent power saver received and used, looking at the quality is good, the quality of the plug sent is also very good. Expect the effect of the economizer is obvious, first five-star praise, with a period of time to follow up!	Jingdong prefecture level city in Shandong
Dragon Quality Goods Shop	Green Intelligent Power Saver	The saver has been received. It was plugged into an outlet and used immediately after receipt. Whether it really saves electricity is not clear yet, I believe that this brand of energy saver really saves	Jingdong prefecture level city in Shandong
Dragon Quality Goods Shop	Green Intelligent Power Saver	Okay, okay, okay. Thank you so much. Fast is good. Fast. Item was as described, mainly because the event was cheap and you can save a lot of money.	Jingdong prefecture level city in Shandong

Dragon Quality Goods Shop	Green Intelligent Power Saver	Received it a couple days ago! Plugged it in the same day! Let's see what the electric bill is in a month! Hopefully it works well oh!	Jingdong prefecture level city in Shandong
Dragon Quality Goods Shop	Green Intelligent Power Saver	Looking at the reviews good, energy-saving small products, compact, the results after 2 months to see, because the electricity bill season payment	Jingdong prefecture level city in Shandong
Dragon Quality Goods Shop	Green Intelligent Power Saver	I just received it and will try it out to see if it works well. I'll comment later.	Jingdong prefecture level city in Shandong
Dragon Quality Goods Shop	Green Intelligent Power Saver	It looks a little small and not very aesthetically pleasing, I don't know how effective it will be until I use it	Jingdong prefecture level city in Shandong
Dragon Quality Goods Shop	Green Intelligent Power Saver	Not only to the point of being useless yet	Jingdong prefecture level city in Shandong
Dragon Quality Goods Shop	Green Intelligent Power Saver	Used it for a month, nothing works, the meter you turn very fast. This is not a scam, but also sold on Jingdong. To cheat a bunch of people?	Jingdong prefecture level city in Shandong
Dragon Quality Goods Shop	Green Intelligent Power Saver	Very good to use, every day can save a little electricity, really good under the sample down a month to save a few dozen dollars in electricity, good before I did not know that there is this haha, the price is not expensive praise.	Jingdong prefecture level city in Shandong
Dragon Quality Goods Shop	Green Intelligent Power Saver	Tried it for ten days and the change is almost nothing, can't see the power saving yet, use it some more days and see how it goes.	Jingdong prefecture level city in Shandong
Dragon Quality Goods Shop	Green Intelligent Power Saver	Received very quickly, the quality is very good, really save electricity, business service attitude is also very good, I hope you do not miss this opportunity, I continue to buy a few in	Jingdong prefecture level city in Shandong
Dragon Quality Goods Shop	Green Intelligent Power Saver	Quality is very good Oh, the delivery is also very fast, a very favorite store, I want to crazy recommend to friends!	Jingdong prefecture level city in Shandong
Dragon Quality Goods Shop	Green Intelligent Power Saver	The goods have just received, fine workmanship, and the same as described by the merchant, to use a period of time to see if it can achieve the purpose of power saving.	Jingdong prefecture level city in Shandong
Dragon Quality Goods Shop	Green Intelligent Power Saver	Received the goods first use a period of time to see if there is no effect, the business commitment to ninety days invalid refund, first praise, good service!	Jingdong prefecture level city in Shandong
Dragon Quality Goods Shop	Green Intelligent Power Saver	The product is used for a month without energy-saving and power-saving effect does not have any effect	Jingdong prefecture level city in Shandong
Dragon Quality Goods Shop	Green Intelligent Power Saver	The baby is really great, I like it very much, my friends also say good to come, recommended to buy!	Jingdong prefecture level city in Shandong
Dragon Quality Goods Shop	Green Intelligent Power Saver	Logistics is fast, courier boy service is good, after using to see how the fruit, and then follow up.	Jingdong prefecture level city in Shandong
Dragon Quality Goods Shop	Green Intelligent Power Saver	Baby has been received to try, I believe that as the business advertised energy saving. The courier is very responsible.	Jingdong prefecture level city in Shandong
Dragon Quality Goods Shop	Green Intelligent Power Saver	Save Electric Energy King received, good quality, beautiful appearance, very satisfied!	Jingdong prefecture level city in Shandong
Dragon Quality Goods Shop	Green Intelligent Power Saver	Three no products no manufacturers no phone pure fraud induced consumption cheat	Jingdong prefecture level city in Shandong
Dragon Quality Goods Shop	Green Intelligent Power Saver	I'm using it now, I don't know if it's as good as it says, I'll follow up after a month	Jingdong prefecture level city in Shandong
Dragon Quality Goods Shop	Green Intelligent Power Saver	Good quality logistics and fast. Fine workmanship. The description is consistent cost-effective	Jingdong prefecture level city in Shandong
Dragon Quality Goods Shop	Green Intelligent Power Saver	Received, whether it is good or not, to wait a few days and then evaluate	Jingdong prefecture level city in Shandong
Dragon Quality Goods Shop	Green Intelligent Power Saver	I bought it and started using it, I won't know if it works well until I pay my electric bill	Jingdong prefecture level city in Shandong
Dragon Quality Goods Shop	Green Intelligent Power Saver	Especially good, energy saving and power saving, especially good quality, good	Jingdong prefecture level city in Shandong
Dragon Quality Goods Shop	Green Intelligent Power Saver	Logistics is strong, the quality is very good, paste really save electricity, as described, will buy back!	Jingdong prefecture level city in Shandong
Dragon Quality Goods Shop	Green Intelligent Power Saver	Great stuff at a great price, worth the money!	Jingdong prefecture level city in Shandong
Dragon Quality Goods Shop	Green Intelligent Power Saver	Received the power saver, very good quality, very power saving!	Jingdong prefecture level city in Shandong
Dragon Quality Goods Shop	Green Intelligent Power Saver	There are no fruits, only samples...	Jingdong prefecture level city in Shandong
Dragon Quality Goods Shop	Green Intelligent Power Saver	Save a few dollars a month.	Jingdong prefecture level city in Shandong
Dragon Quality Goods Shop	Green Intelligent Power Saver	The quality is particularly good, it works well, and the seller has a good service attitude	Jingdong prefecture level city in Shandong
Dragon Quality Goods Shop	Green Intelligent Power Saver	I've compared them a couple of times and I don't feel any difference	Jingdong prefecture level city in Shandong
Dragon Quality Goods Shop	Green Intelligent Power Saver	I don't see the savings. It's a sham.	Jingdong prefecture level city in Shandong
Dragon Quality Goods Shop	Green Intelligent Power Saver	The power saving effect is very good, and the logistics is very fast. The seller's service attitude is very good	Jingdong prefecture level city in Shandong
Dragon Quality Goods Shop	Green Intelligent Power Saver	Received, well packaged, haven't tried it yet!	Jingdong prefecture level city in Shandong
Dragon Quality Goods Shop	Green Intelligent Power Saver	I don't know how it works, I'll comment after I use it.	Jingdong prefecture level city in Shandong
Dragon Quality Goods Shop	Green Intelligent Power Saver	The product doesn't work well enough to be of any use	Jingdong prefecture level city in Shandong
Dragon Quality Goods Shop	Green Intelligent Power Saver	On Trial 。。。。。。。	Jingdong prefecture level city in Shandong
Dragon Quality Goods Shop	Green Intelligent Power Saver	Being used with unknown results	Jingdong prefecture level city in Shandong
Dragon Quality Goods Shop	Green Intelligent Power Saver	Smart power saving and great quality	Jingdong prefecture level city in Shandong
Dragon Quality Goods Shop	Green Intelligent Power Saver	The saver was received, it works very well and the price is affordable!	Jingdong prefecture level city in Shandong
Dragon Quality Goods Shop	Green Intelligent Power Saver	How can I feel the power saving and how long does it last	Jingdong prefecture level city in Shandong
Dragon Quality Goods Shop	Green Intelligent Power Saver	And I know if it saves electricity or not.	Jingdong prefecture level city in Shandong
Dragon Quality Goods Shop	Green Intelligent Power Saver	It won't save any electricity. No.	Jingdong prefecture level city in Shandong
Dragon Quality Goods Shop	Green Intelligent Power Saver	Invalid. Fake.	Jingdong prefecture level city in Shandong
Dragon Quality Goods Shop	Green Intelligent Power Saver	It's good!	Jingdong prefecture level city in Shandong
Dragon Quality Goods Shop	Green Intelligent Power Saver	have no effect	Jingdong prefecture level city in Shandong
Dragon Quality Goods Shop	Green Intelligent Power Saver	Not used.	Jingdong prefecture level city in Shandong
Dragon Quality Goods Shop	Green Intelligent Power Saver	There is no load range, packaging thrift, ten days also did not develop invoices, received the goods with a receipt, hand-written receipt also has no financial stamp, communication for nearly a week a moment to say that the electronic invoice, a moment to say that the paper invoice, said it is mailed to me, ten days also did not see anything, the invoice invoice can not be found to say that did not stay on file, single single number can not be found, said that the warehouse more than one to a one to find out, in addition to the perfunctory or perfunctory. Products are no certificate of conformity, proper three	Jingdong prefecture level city in Shandong
Dragon Quality Goods Shop	Green Intelligent Power Saver	Satisfaction.	Jingdong prefecture level city in Shandong

Dragon Quality Goods Shop	Green Intelligent Power Saver	There is not a bit of power saving effect, and ringing up an increase in the electricity bill, because you have to add the cost of this machine's own electricity consumption and purchase, so I think it is better to use this machine instead of increasing the expenditure, uneconomical, rather than not use it.	Jingdong prefecture level city in Shandong
Dragon Quality Goods Shop	Green Intelligent Power Saver	I've been using it for two days now and I don't feel any effect.	Jingdong prefecture level city in Shandong
Dragon Quality Goods Shop	Green Intelligent Power Saver	What it looked like was nothing like what was sent.	Jingdong prefecture level city in Shandong
Dragon Quality Goods Shop	Green Intelligent Power Saver	It didn't work. It didn't work for a month or so.	Jingdong prefecture level city in Shandong
Dragon Quality Goods Shop	Green Intelligent Power Saver	Garbage products, physical and description is not the same, no effect at all, pit # no discussion, to buy the fine, garbage, garbage, garbage	Jingdong prefecture level city in Shandong
Dragon Quality Goods Shop	Green Intelligent Power Saver	general	Jingdong prefecture level city in Shandong
Dragon Quality Goods Shop	Green Intelligent Power Saver	IQ tax. Instead of saving electricity, it increases the power consumption of an appliance.	Jingdong prefecture level city in Shandong
Dragon Quality Goods Shop	Green Intelligent Power Saver	There's no trash at all.	Jingdong prefecture level city in Shandong
Dragon Quality Goods Shop	Green Intelligent Power Saver	It doesn't do anything.	Jingdong prefecture level city in Shandong
Dragon Quality Goods Shop	Green Intelligent Power Saver	(of an unmarried couple) be close	Jingdong prefecture level city in Shandong
Dragon Quality Goods Shop	Green Intelligent Power Saver	(of an unmarried couple) be close	Jingdong prefecture level city in Shandong
Dragon Quality Goods Shop	Green Intelligent Power Saver	no electricity saving	Jingdong prefecture level city in Shandong
Dragon Quality Goods Shop	Green Intelligent Power Saver	differ from	Jingdong prefecture level city in Shandong
Dragon Quality Goods Shop	Green Intelligent Power Saver	Saver has received, but real can save electricity, the quality is very good, I times I buy a few more, too good.	Jingdong prefecture level city in Shandong
Dragon Quality Goods Shop	Green Intelligent Power Saver	Great quality as always, a very favorite product, great! o(*￣︶￣*)o	Jingdong prefecture level city in Shandong
Dragon Quality Goods Shop	Green Intelligent Power Saver	If we can reduce the electricity bill, for individuals can save money, for the country can protect the environment, now the weather is getting hotter and hotter, to protect the environment to reduce emissions is very important. Already use the product, whether to reduce the electricity bill depends on next month!	Jingdong prefecture level city in Shandong
Dragon Quality Goods Shop	Green Intelligent Power Saver	Purchased a power saver received on the put into use, rent a room with many electrical appliances, power consumption is very large, we have a piece of electricity or 1.2 dollars a time, with this power saver is much better, every day can save a few cents, very convenient and practical!	Jingdong prefecture level city in Shandong
Dragon Quality Goods Shop	Green Intelligent Power Saver	The product is better to use before not to use when a month 1500 yuan electricity bill, now using the 5 second month electricity bill directly down 470 dollars or very good. A save money to buy several power saver money ZanZanZanZan👍	Jingdong prefecture level city in Shandong
Dragon Quality Goods Shop	Green Intelligent Power Saver	The moment I received the goods really satisfied with this product, not only high-grade packaging, the body are base alloy, up to the price, I was also introduced by a friend, he used last month did save a lot of electricity, but also recommended to me!	Jingdong prefecture level city in Shandong
Dragon Quality Goods Shop	Green Intelligent Power Saver	I got it. Good review. I'm very satisfied. Reasonable price.	Jingdong prefecture level city in Shandong
Dragon Quality Goods Shop	Green Intelligent Power Saver	Intelligent power saver received and used, looking at the quality is good, the quality of the plug sent is also very good. Expect the effect of the economizer is obvious, first five-star praise, with a period of time to follow up!	Jingdong prefecture level city in Shandong
Dragon Quality Goods Shop	Green Intelligent Power Saver	The saver has been received. It was plugged into an outlet and used immediately after receipt. Whether it really saves electricity is not clear yet, I believe that this brand of energy saver really saves	Jingdong prefecture level city in Shandong
Dragon Quality Goods Shop	Green Intelligent Power Saver	Okay, okay, okay. Thank you so much. Fast is good. Fast. Item was as described, mainly because the event was cheap and you can save a lot of money.	Jingdong prefecture level city in Shandong
Dragon Quality Goods Shop	Green Intelligent Power Saver	Received it a couple days ago! Plugged it in the same day! Let's see what the electric bill is in a month! Hopefully it works well oh!	Jingdong prefecture level city in Shandong
Dragon Quality Goods Shop	Green Intelligent Power Saver	Looking at the reviews good, energy-saving small products, compact, the results after 2 months to see, because the electricity bill season payment	Jingdong prefecture level city in Shandong
Dragon Quality Goods Shop	Green Intelligent Power Saver	I just received it and will try it out to see if it works well. I'll comment later.	Jingdong prefecture level city in Shandong
Dragon Quality Goods Shop	Green Intelligent Power Saver	It looks a little small and not very aesthetically pleasing, I don't know how effective it will be until I use it	Jingdong prefecture level city in Shandong
Dragon Quality Goods Shop	Green Intelligent Power Saver	Not only to the point of being useless yet	Jingdong prefecture level city in Shandong
Dragon Quality Goods Shop	Green Intelligent Power Saver	Used it for a month, nothing works, the meter you turn very fast. This is not a scam, but also sold on Jingdong. To cheat a bunch of people?	Jingdong prefecture level city in Shandong
Dragon Quality Goods Shop	Green Intelligent Power Saver	Very good to use, every day can save a little electricity, really good under the sample down a month to save a few dozen dollars in electricity, good before I did not know that there is this 👆 haha, the price is not expensive praise.	Jingdong prefecture level city in Shandong
Dragon Quality Goods Shop	Green Intelligent Power Saver	Tried it for ten days and the change is almost nothing, can't see the power saving yet, use it some more days and see how it goes.	Jingdong prefecture level city in Shandong
Dragon Quality Goods Shop	Green Intelligent Power Saver	Received very quickly, the quality is very good, really save electricity, business service attitude is also very good, I hope you do not miss this opportunity, I continue to buy a few in	Jingdong prefecture level city in Shandong
Dragon Quality Goods Shop	Green Intelligent Power Saver	Quality is very good Oh, the delivery is also very fast, a very favorite store, I want to crazy recommend to friends!	Jingdong prefecture level city in Shandong
Dragon Quality Goods Shop	Green Intelligent Power Saver	The goods have just received, fine workmanship, and the same as described by the merchant, to use a period of time to see if it can achieve the purpose of power saving.	Jingdong prefecture level city in Shandong
Dragon Quality Goods Shop	Green Intelligent Power Saver	Received the goods first use a period of time to see if there is no effect, the business commitment to ninety days invalid refund, first praise, good service!	Jingdong prefecture level city in Shandong
Dragon Quality Goods Shop	Green Intelligent Power Saver	The product is used for a month without energy-saving and power-saving effect does not have any effect	Jingdong prefecture level city in Shandong
Dragon Quality Goods Shop	Green Intelligent Power Saver	The baby is really great, I like it very much, my friends also say good to come, recommended to buy!	Jingdong prefecture level city in Shandong
Dragon Quality Goods Shop	Green Intelligent Power Saver	Logistics is fast, courier boy service is good, after using to see how the fruit, and then follow up.	Jingdong prefecture level city in Shandong
Dragon Quality Goods Shop	Green Intelligent Power Saver	Baby has been received to try, I believe that as the business advertised energy saving. The courier is very responsible.	Jingdong prefecture level city in Shandong
Dragon Quality Goods Shop	Green Intelligent Power Saver	Save Electric Energy King received, good quality, beautiful appearance, very satisfied!	Jingdong prefecture level city in Shandong
Dragon Quality Goods Shop	Green Intelligent Power Saver	Three no products no manufacturers no phone pure fraud induced consumption cheat	Jingdong prefecture level city in Shandong
Dragon Quality Goods Shop	Green Intelligent Power Saver	I'm using it now, I don't know if it's as good as it says, I'll follow up after a month	Jingdong prefecture level city in Shandong
Dragon Quality Goods Shop	Green Intelligent Power Saver	Good quality logistics and fast. Fine workmanship. The description is consistent cost-effective	Jingdong prefecture level city in Shandong
Dragon Quality Goods Shop	Green Intelligent Power Saver	Received, whether it is good or not, to wait a few days and then evaluate	Jingdong prefecture level city in Shandong
Dragon Quality Goods Shop	Green Intelligent Power Saver	I bought it and started using it, I won't know if it works well until I pay my electric bill	Jingdong prefecture level city in Shandong
Dragon Quality Goods Shop	Green Intelligent Power Saver	Especially good, energy saving and power saving, especially good quality, good	Jingdong prefecture level city in Shandong
Dragon Quality Goods Shop	Green Intelligent Power Saver	Logistics is strong, the quality is very good, paste really save electricity, as described, will buy back!	Jingdong prefecture level city in Shandong
Dragon Quality Goods Shop	Green Intelligent Power Saver	Great stuff at a great price, worth the money!	Jingdong prefecture level city in Shandong

Dragon Quality Goods Shop	Green Intelligent Power Saver	Received the power saver, very good quality, very power saving!	Jingdong prefecture level city in Shandong
Dragon Quality Goods Shop	Green Intelligent Power Saver	There are no fruits, only samples...	Jingdong prefecture level city in Shandong
Dragon Quality Goods Shop	Green Intelligent Power Saver	Save a few dollars a month.	Jingdong prefecture level city in Shandong
Dragon Quality Goods Shop	Green Intelligent Power Saver	The quality is particularly good, it works well, and the seller has a good service attitude	Jingdong prefecture level city in Shandong
Dragon Quality Goods Shop	Green Intelligent Power Saver	I've compared them a couple of times and I don't feel any difference	Jingdong prefecture level city in Shandong
Dragon Quality Goods Shop	Green Intelligent Power Saver	I don't see the savings. It's a sham.	Jingdong prefecture level city in Shandong
Dragon Quality Goods Shop	Green Intelligent Power Saver	The power saving effect is very good, and the logistics is very fast. The seller's service attitude is very good	Jingdong prefecture level city in Shandong
Dragon Quality Goods Shop	Green Intelligent Power Saver	Received, well packaged, haven't tried it yet!	Jingdong prefecture level city in Shandong
Dragon Quality Goods Shop	Green Intelligent Power Saver	I don't know how it works, I'll comment after I use it.	Jingdong prefecture level city in Shandong
Dragon Quality Goods Shop	Green Intelligent Power Saver	The product doesn't work well enough to be of any use	Jingdong prefecture level city in Shandong
Dragon Quality Goods Shop	Green Intelligent Power Saver	On Trial 。。。。。。。	Jingdong prefecture level city in Shandong
Dragon Quality Goods Shop	Green Intelligent Power Saver	Being used with unknown results	Jingdong prefecture level city in Shandong
Dragon Quality Goods Shop	Green Intelligent Power Saver	Smart power saving and great quality	Jingdong prefecture level city in Shandong
Dragon Quality Goods Shop	Green Intelligent Power Saver	The saver was received, it works very well and the price is affordable!	Jingdong prefecture level city in Shandong
Dragon Quality Goods Shop	Green Intelligent Power Saver	How can I feel the power saving and how long does it last	Jingdong prefecture level city in Shandong
Dragon Quality Goods Shop	Green Intelligent Power Saver	And I know if it saves electricity or not.	Jingdong prefecture level city in Shandong
Dragon Quality Goods Shop	Green Intelligent Power Saver	It won't save any electricity. No.	Jingdong prefecture level city in Shandong
Dragon Quality Goods Shop	Green Intelligent Power Saver	Invalid. Fake.	Jingdong prefecture level city in Shandong
Dragon Quality Goods Shop	Green Intelligent Power Saver	It's good!	Jingdong prefecture level city in Shandong
Dragon Quality Goods Shop	Green Intelligent Power Saver	have no effect	Jingdong prefecture level city in Shandong
Dragon Quality Goods Shop	Green Intelligent Power Saver	Not used.	Jingdong prefecture level city in Shandong
Dragon Quality Goods Shop	Green Intelligent Power Saver	There is no load range, packaging thrift, ten days also did not develop invoices, received the goods with a receipt, hand-written receipt also has no financial stamp, communication for nearly a week a moment to say that the electronic invoice, a moment to say that the paper invoice, said it is mailed to me, ten days also did not see anything, the invoice invoice can not be found to say that did not stay on file, single single number can not be found, said that the warehouse more than one to a one to find out, in addition to the perfunctory or perfunctory. Products are no certificate of conformity, proper three	Jingdong prefecture level city in Shandong
Dragon Quality Goods Shop	Green Intelligent Power Saver	Satisfaction.	Jingdong prefecture level city in Shandong
Dragon Quality Goods Shop	Green Intelligent Power Saver	There is not a bit of power saving effect, and ringing up an increase in the electricity bill, because you have to add the cost of this machine's own electricity consumption and purchase, so I think it is better to use this machine instead of increasing the expenditure, uneconomical, rather than not use it.	Jingdong prefecture level city in Shandong
Dragon Quality Goods Shop	Green Intelligent Power Saver	I've been using it for two days now and I don't feel any effect.	Jingdong prefecture level city in Shandong
Dragon Quality Goods Shop	Green Intelligent Power Saver	What it looked like was nothing like what was sent.	Jingdong prefecture level city in Shandong
Dragon Quality Goods Shop	Green Intelligent Power Saver	It didn't work. It didn't work for a month or so.	Jingdong prefecture level city in Shandong
Dragon Quality Goods Shop	Green Intelligent Power Saver	Garbage products, physical and description is not the same, no effect at all, pit # no discussion, to buy the fine, garbage, garbage, garbage	Jingdong prefecture level city in Shandong
Dragon Quality Goods Shop	Green Intelligent Power Saver	general	Jingdong prefecture level city in Shandong
Dragon Quality Goods Shop	Green Intelligent Power Saver	IQ tax. Instead of saving electricity, it increases the power consumption of an appliance.	Jingdong prefecture level city in Shandong
Dragon Quality Goods Shop	Green Intelligent Power Saver	There's no trash at all.	Jingdong prefecture level city in Shandong
Dragon Quality Goods Shop	Green Intelligent Power Saver	It doesn't do anything.	Jingdong prefecture level city in Shandong
Dragon Quality Goods Shop	Green Intelligent Power Saver	(of an unmarried couple) be close	Jingdong prefecture level city in Shandong
Dragon Quality Goods Shop	Green Intelligent Power Saver	(of an unmarried couple) be close	Jingdong prefecture level city in Shandong
Dragon Quality Goods Shop	Green Intelligent Power Saver	no electricity saving	Jingdong prefecture level city in Shandong
Dragon Quality Goods Shop	Green Intelligent Power Saver	differ from	Jingdong prefecture level city in Shandong
Dragon Quality Goods Shop	Green Intelligent Power Saver	Saver has received, but real can save electricity, the quality is very good, I times I buy a few more, too good.	Jingdong prefecture level city in Shandong
Dragon Quality Goods Shop	Green Intelligent Power Saver	Great quality as always, a very favorite product, great! o(*￣︶￣*)o	Jingdong prefecture level city in Shandong
Dragon Quality Goods Shop	Green Intelligent Power Saver	If we can reduce the electricity bill, for individuals can save money, for the country can protect the environment, now the weather is getting hotter and hotter, to protect the environment to reduce emissions is very important. Already use the product, whether to reduce the electricity bill depends on next month!	Jingdong prefecture level city in Shandong
Dragon Quality Goods Shop	Green Intelligent Power Saver	Purchased a power saver received on the put into use, rent a room with many electrical appliances, power consumption is very large, we have a piece of electricity or 1.2 dollars a time, with this power saver is much better, every day can save a few cents, very convenient and practical!	Jingdong prefecture level city in Shandong
Dragon Quality Goods Shop	Green Intelligent Power Saver	The product is better to use before not to use when a month 1500 yuan electricity bill, now using the 5 second month electricity bill directly down 470 dollars or very good. A save money to buy several power saver money ZanZanZanZan👍	Jingdong prefecture level city in Shandong
Dragon Quality Goods Shop	Green Intelligent Power Saver	The moment I received the goods really satisfied with this product, not only high-grade packaging, the body are base alloy, up to the price, I was also introduced by a friend, he used last month did save a lot of electricity, but also recommended to me!	Jingdong prefecture level city in Shandong
Dragon Quality Goods Shop	Green Intelligent Power Saver	I got it. Good review. I'm very satisfied. Reasonable price.	Jingdong prefecture level city in Shandong
Dragon Quality Goods Shop	Green Intelligent Power Saver	Intelligent power saver received and used, looking at the quality is good, the quality of the plug sent is also very good. Expect the effect of the economizer is obvious, first five-star praise, with a period of time to follow up!	Jingdong prefecture level city in Shandong
Dragon Quality Goods Shop	Green Intelligent Power Saver	The saver has been received. It was plugged into an outlet and used immediately after receipt. Whether it really saves electricity is not clear yet, I believe that this brand of energy saver really saves	Jingdong prefecture level city in Shandong
Dragon Quality Goods Shop	Green Intelligent Power Saver	Okay, okay, okay. Thank you so much. Fast is good. Fast. Item was as described, mainly because the event was cheap and you can save a lot of money.	Jingdong prefecture level city in Shandong

Dragon Quality Goods Shop	Green Intelligent Power Saver	Received it a couple days ago! Plugged it in the same day! Let's see what the electric bill is in a month! Hopefully it works well oh!	Jingdong prefecture level city in Shandong
Dragon Quality Goods Shop	Green Intelligent Power Saver	Looking at the reviews good, energy-saving small products, compact, the results after 2 months to see, because the electricity bill season payment	Jingdong prefecture level city in Shandong
Dragon Quality Goods Shop	Green Intelligent Power Saver	I just received it and will try it out to see if it works well. I'll comment later.	Jingdong prefecture level city in Shandong
Dragon Quality Goods Shop	Green Intelligent Power Saver	It looks a little small and not very aesthetically pleasing, I don't know how effective it will be until I use it	Jingdong prefecture level city in Shandong
Dragon Quality Goods Shop	Green Intelligent Power Saver	Not only to the point of being useless yet	Jingdong prefecture level city in Shandong
Dragon Quality Goods Shop	Green Intelligent Power Saver	Used it for a month, nothing works, the meter you turn very fast. This is not a scam, but also sold on Jingdong. To cheat a bunch of people?	Jingdong prefecture level city in Shandong
Dragon Quality Goods Shop	Green Intelligent Power Saver	Very good to use, every day can save a little electricity, really good under the sample down a month to save a few dozen dollars in electricity, good before I did not know that there is this haha, the price is not expensive praise.	Jingdong prefecture level city in Shandong
Dragon Quality Goods Shop	Green Intelligent Power Saver	Tried it for ten days and the change is almost nothing, can't see the power saving yet, use it some more days and see how it goes.	Jingdong prefecture level city in Shandong
Dragon Quality Goods Shop	Green Intelligent Power Saver	Received very quickly, the quality is very good, really save electricity, business service attitude is also very good, I hope you do not miss this opportunity, I continue to buy a few in	Jingdong prefecture level city in Shandong
Dragon Quality Goods Shop	Green Intelligent Power Saver	Quality is very good Oh, the delivery is also very fast, a very favorite store, I want to crazy recommend to friends!	Jingdong prefecture level city in Shandong
Dragon Quality Goods Shop	Green Intelligent Power Saver	The goods have just received, fine workmanship, and the same as described by the merchant, to use a period of time to see if it can achieve the purpose of power saving.	Jingdong prefecture level city in Shandong
Dragon Quality Goods Shop	Green Intelligent Power Saver	Received the goods first use a period of time to see if there is no effect, the business commitment to ninety days invalid refund, first praise, good service!	Jingdong prefecture level city in Shandong
Dragon Quality Goods Shop	Green Intelligent Power Saver	The product is used for a month without energy-saving and power-saving effect does not have any effect	Jingdong prefecture level city in Shandong
Dragon Quality Goods Shop	Green Intelligent Power Saver	The baby is really great, I like it very much, my friends also say good to come, recommended to buy!	Jingdong prefecture level city in Shandong
Dragon Quality Goods Shop	Green Intelligent Power Saver	Logistics is fast, courier boy service is good, after using to see how the fruit, and then follow up.	Jingdong prefecture level city in Shandong
Dragon Quality Goods Shop	Green Intelligent Power Saver	Baby has been received to try, I believe that as the business advertised energy saving. The courier is very responsible.	Jingdong prefecture level city in Shandong
Dragon Quality Goods Shop	Green Intelligent Power Saver	Save Electric Energy King received, good quality, beautiful appearance, very satisfied!	Jingdong prefecture level city in Shandong
Dragon Quality Goods Shop	Green Intelligent Power Saver	Three no products no manufacturers no phone pure fraud induced consumption cheat	Jingdong prefecture level city in Shandong
Dragon Quality Goods Shop	Green Intelligent Power Saver	I'm using it now, I don't know if it's as good as it says, I'll follow up after a month	Jingdong prefecture level city in Shandong
Dragon Quality Goods Shop	Green Intelligent Power Saver	Good quality logistics and fast. Fine workmanship. The description is consistent cost-effective	Jingdong prefecture level city in Shandong
Dragon Quality Goods Shop	Green Intelligent Power Saver	Received, whether it is good or not, to wait a few days and then evaluate	Jingdong prefecture level city in Shandong
Dragon Quality Goods Shop	Green Intelligent Power Saver	I bought it and started using it, I won't know if it works well until I pay my electric bill	Jingdong prefecture level city in Shandong
Dragon Quality Goods Shop	Green Intelligent Power Saver	Especially good, energy saving and power saving, especially good quality, good	Jingdong prefecture level city in Shandong
Dragon Quality Goods Shop	Green Intelligent Power Saver	Logistics is strong, the quality is very good, paste really save electricity, as described, will buy back!	Jingdong prefecture level city in Shandong
Dragon Quality Goods Shop	Green Intelligent Power Saver	Great stuff at a great price, worth the money!	Jingdong prefecture level city in Shandong
Dragon Quality Goods Shop	Green Intelligent Power Saver	Received the power saver, very good quality, very power saving!	Jingdong prefecture level city in Shandong
Dragon Quality Goods Shop	Green Intelligent Power Saver	There are no fruits, only samples...	Jingdong prefecture level city in Shandong
Dragon Quality Goods Shop	Green Intelligent Power Saver	Save a few dollars a month.	Jingdong prefecture level city in Shandong
Dragon Quality Goods Shop	Green Intelligent Power Saver	The quality is particularly good, it works well, and the seller has a good service attitude	Jingdong prefecture level city in Shandong
Dragon Quality Goods Shop	Green Intelligent Power Saver	I've compared them a couple of times and I don't feel any difference	Jingdong prefecture level city in Shandong
Dragon Quality Goods Shop	Green Intelligent Power Saver	I don't see the savings. It's a sham.	Jingdong prefecture level city in Shandong
Dragon Quality Goods Shop	Green Intelligent Power Saver	The power saving effect is very good, and the logistics is very fast. The seller's service attitude is very good	Jingdong prefecture level city in Shandong
Dragon Quality Goods Shop	Green Intelligent Power Saver	Received, well packaged, haven't tried it yet!	Jingdong prefecture level city in Shandong
Dragon Quality Goods Shop	Green Intelligent Power Saver	I don't know how it works, I'll comment after I use it.	Jingdong prefecture level city in Shandong
Dragon Quality Goods Shop	Green Intelligent Power Saver	The product doesn't work well enough to be of any use	Jingdong prefecture level city in Shandong
Dragon Quality Goods Shop	Green Intelligent Power Saver	On Trial 。。。。。。。	Jingdong prefecture level city in Shandong
Dragon Quality Goods Shop	Green Intelligent Power Saver	Being used with unknown results	Jingdong prefecture level city in Shandong
Dragon Quality Goods Shop	Green Intelligent Power Saver	Smart power saving and great quality	Jingdong prefecture level city in Shandong
Dragon Quality Goods Shop	Green Intelligent Power Saver	The saver was received, it works very well and the price is affordable!	Jingdong prefecture level city in Shandong
Dragon Quality Goods Shop	Green Intelligent Power Saver	How can I feel the power saving and how long does it last	Jingdong prefecture level city in Shandong
Dragon Quality Goods Shop	Green Intelligent Power Saver	And I know if it saves electricity or not.	Jingdong prefecture level city in Shandong
Dragon Quality Goods Shop	Green Intelligent Power Saver	It won't save any electricity. No.	Jingdong prefecture level city in Shandong
Dragon Quality Goods Shop	Green Intelligent Power Saver	Invalid. Fake.	Jingdong prefecture level city in Shandong
Dragon Quality Goods Shop	Green Intelligent Power Saver	It's good!	Jingdong prefecture level city in Shandong
Dragon Quality Goods Shop	Green Intelligent Power Saver	have no effect	Jingdong prefecture level city in Shandong
Dragon Quality Goods Shop	Green Intelligent Power Saver	Not used.	Jingdong prefecture level city in Shandong
Dragon Quality Goods Shop	Green Intelligent Power Saver	There is no load range, packaging thrift, ten days also did not develop invoices, received the goods with a receipt, hand-written receipt also has no financial stamp, communication for nearly a week a moment to say that the electronic invoice, a moment to say that the paper invoice, said it is mailed to me, ten days also did not see anything, the invoice invoice can not be found to say that did not stay on file, single single number can not be found, said that the warehouse more than one to a one to find out, in addition to the perfunctory or perfunctory. Products are no certificate of conformity, proper three	Jingdong prefecture level city in Shandong
Dragon Quality Goods Shop	Green Intelligent Power Saver	Satisfaction.	Jingdong prefecture level city in Shandong

Dragon Quality Goods Shop	Green Intelligent Power Saver	There is not a bit of power saving effect, and ringing up an increase in the electricity bill, because you have to add the cost of this machine's own electricity consumption and purchase, so I think it is better to use this machine instead of increasing the expenditure, uneconomical, rather than not use it.	Jingdong prefecture level city in Shandong
Dragon Quality Goods Shop	Green Intelligent Power Saver	I've been using it for two days now and I don't feel any effect.	Jingdong prefecture level city in Shandong
Dragon Quality Goods Shop	Green Intelligent Power Saver	What it looked like was nothing like what was sent.	Jingdong prefecture level city in Shandong
Dragon Quality Goods Shop	Green Intelligent Power Saver	It didn't work. It didn't work for a month or so.	Jingdong prefecture level city in Shandong
Dragon Quality Goods Shop	Green Intelligent Power Saver	Garbage products, physical and description is not the same, no effect at all, pit # no discussion, to buy the fine, garbage, garbage, garbage	Jingdong prefecture level city in Shandong
Dragon Quality Goods Shop	Green Intelligent Power Saver	general	Jingdong prefecture level city in Shandong
Dragon Quality Goods Shop	Green Intelligent Power Saver	IQ tax. Instead of saving electricity, it increases the power consumption of an appliance.	Jingdong prefecture level city in Shandong
Dragon Quality Goods Shop	Green Intelligent Power Saver	There's no trash at all.	Jingdong prefecture level city in Shandong
Dragon Quality Goods Shop	Green Intelligent Power Saver	It doesn't do anything.	Jingdong prefecture level city in Shandong
Dragon Quality Goods Shop	Green Intelligent Power Saver	(of an unmarried couple) be close	Jingdong prefecture level city in Shandong
Dragon Quality Goods Shop	Green Intelligent Power Saver	(of an unmarried couple) be close	Jingdong prefecture level city in Shandong
Dragon Quality Goods Shop	Green Intelligent Power Saver	no electricity saving	Jingdong prefecture level city in Shandong
Dragon Quality Goods Shop	Green Intelligent Power Saver	differ from	Jingdong prefecture level city in Shandong
Dragon Quality Goods Shop	Green Intelligent Power Saver	Saver has received, but real can save electricity, the quality is very good, I times I buy a few more, too good.	Jingdong prefecture level city in Shandong
Dragon Quality Goods Shop	Green Intelligent Power Saver	Great quality as always, a very favorite product, great! o(*￣︶￣*)o	Jingdong prefecture level city in Shandong
Dragon Quality Goods Shop	Green Intelligent Power Saver	If we can reduce the electricity bill, for individuals can save money, for the country can protect the environment, now the weather is getting hotter and hotter, to protect the environment to reduce emissions is very important. Already use the product, whether to reduce the electricity bill depends on next month!	Jingdong prefecture level city in Shandong
Dragon Quality Goods Shop	Green Intelligent Power Saver	Purchased a power saver received on the put into use, rent a room with many electrical appliances, power consumption is very large, we have a piece of electricity or 1.2 dollars a time, with this power saver is much better, every day can save a few cents, very convenient and practical!	Jingdong prefecture level city in Shandong
Dragon Quality Goods Shop	Green Intelligent Power Saver	The product is better to use before not to use when a month 1500 yuan electricity bill, now using the 5 second month electricity bill directly down 470 dollars or very good. A save money to buy several power saver money ZanZanZanZan👍	Jingdong prefecture level city in Shandong
Dragon Quality Goods Shop	Green Intelligent Power Saver	The moment I received the goods really satisfied with this product, not only high-grade packaging, the body are base alloy, up to the price, I was also introduced by a friend, he used last month did save a lot of electricity, but also recommended to me!	Jingdong prefecture level city in Shandong
Dragon Quality Goods Shop	Green Intelligent Power Saver	I got it. Good review. I'm very satisfied. Reasonable price.	Jingdong prefecture level city in Shandong
Dragon Quality Goods Shop	Green Intelligent Power Saver	Intelligent power saver received and used, looking at the quality is good, the quality of the plug sent is also very good. Expect the effect of the economizer is obvious, first five-star praise, with a period of time to follow up!	Jingdong prefecture level city in Shandong
Dragon Quality Goods Shop	Green Intelligent Power Saver	The saver has been received. It was plugged into an outlet and used immediately after receipt. Whether it really saves electricity is not clear yet, I believe that this brand of energy saver really saves	Jingdong prefecture level city in Shandong
Dragon Quality Goods Shop	Green Intelligent Power Saver	Okay, okay, okay. Thank you so much. Fast is good. Fast. Item was as described, mainly because the event was cheap and you can save a lot of money.	Jingdong prefecture level city in Shandong
Dragon Quality Goods Shop	Green Intelligent Power Saver	Received it a couple days ago! Plugged it in the same day! Let's see what the electric bill is in a month! Hopefully it works well oh!	Jingdong prefecture level city in Shandong
Dragon Quality Goods Shop	Green Intelligent Power Saver	Looking at the reviews good, energy-saving small products, compact, the results after 2 months to see, because the electricity bill season payment	Jingdong prefecture level city in Shandong
Dragon Quality Goods Shop	Green Intelligent Power Saver	I just received it and will try it out to see if it works well. I'll comment later.	Jingdong prefecture level city in Shandong
Dragon Quality Goods Shop	Green Intelligent Power Saver	It looks a little small and not very aesthetically pleasing, I don't know how effective it will be until I use it	Jingdong prefecture level city in Shandong
Dragon Quality Goods Shop	Green Intelligent Power Saver	Not only to the point of being useless yet	Jingdong prefecture level city in Shandong
Dragon Quality Goods Shop	Green Intelligent Power Saver	Used it for a month, nothing works, the meter you turn very fast. This is not a scam, but also sold on Jingdong. To cheat a bunch of people?	Jingdong prefecture level city in Shandong
Dragon Quality Goods Shop	Green Intelligent Power Saver	Very good to use, every day can save a little electricity, really good under the sample down a month to save a few dozen dollars in electricity, good before I did not know that there is this 👍 haha, the price is not expensive praise.	Jingdong prefecture level city in Shandong
Dragon Quality Goods Shop	Green Intelligent Power Saver	Tried it for ten days and the change is almost nothing, can't see the power saving yet, use it some more days and see how it goes.	Jingdong prefecture level city in Shandong
Dragon Quality Goods Shop	Green Intelligent Power Saver	Received very quickly, the quality is very good, really save electricity, business service attitude is also very good, I hope you do not miss this opportunity, I continue to buy a few in	Jingdong prefecture level city in Shandong
Dragon Quality Goods Shop	Green Intelligent Power Saver	Quality is very good Oh, the delivery is also very fast, a very favorite store, I want to crazy recommend to friends!	Jingdong prefecture level city in Shandong
Dragon Quality Goods Shop	Green Intelligent Power Saver	The goods have just received, fine workmanship, and the same as described by the merchant, to use a period of time to see if it can achieve the purpose of power saving.	Jingdong prefecture level city in Shandong
Dragon Quality Goods Shop	Green Intelligent Power Saver	Received the goods first use a period of time to see if there is no effect, the business commitment to ninety days invalid refund, first praise, good service!	Jingdong prefecture level city in Shandong
Dragon Quality Goods Shop	Green Intelligent Power Saver	The product is used for a month without energy-saving and power-saving effect does not have any effect	Jingdong prefecture level city in Shandong
Dragon Quality Goods Shop	Green Intelligent Power Saver	The baby is really great, I like it very much, my friends also say good to come, recommended to buy!	Jingdong prefecture level city in Shandong
Dragon Quality Goods Shop	Green Intelligent Power Saver	Logistics is fast, courier boy service is good, after using to see how the fruit, and then follow up.	Jingdong prefecture level city in Shandong
Dragon Quality Goods Shop	Green Intelligent Power Saver	Baby has been received to try, I believe that as the business advertised energy saving. The courier is very responsible.	Jingdong prefecture level city in Shandong
Dragon Quality Goods Shop	Green Intelligent Power Saver	Save Electric Energy King received, good quality, beautiful appearance, very satisfied!	Jingdong prefecture level city in Shandong
Dragon Quality Goods Shop	Green Intelligent Power Saver	Three no products no manufacturers no phone pure fraud induced consumption cheat	Jingdong prefecture level city in Shandong
Dragon Quality Goods Shop	Green Intelligent Power Saver	I'm using it now, I don't know if it's as good as it says, I'll follow up after a month	Jingdong prefecture level city in Shandong
Dragon Quality Goods Shop	Green Intelligent Power Saver	Good quality logistics and fast. Fine workmanship. The description is consistent cost-effective	Jingdong prefecture level city in Shandong
Dragon Quality Goods Shop	Green Intelligent Power Saver	Received, whether it is good or not, to wait a few days and then evaluate	Jingdong prefecture level city in Shandong
Dragon Quality Goods Shop	Green Intelligent Power Saver	I bought it and started using it, I won't know if it works well until I pay my electric bill	Jingdong prefecture level city in Shandong
Dragon Quality Goods Shop	Green Intelligent Power Saver	Especially good, energy saving and power saving, especially good quality, good	Jingdong prefecture level city in Shandong
Dragon Quality Goods Shop	Green Intelligent Power Saver	Logistics is strong, the quality is very good, paste really save electricity, as described, will buy back!	Jingdong prefecture level city in Shandong
Dragon Quality Goods Shop	Green Intelligent Power Saver	Great stuff at a great price, worth the money!	Jingdong prefecture level city in Shandong

Dragon Quality Goods Shop	Green Intelligent Power Saver	Received the power saver, very good quality, very power saving!	Jingdong prefecture level city in Shandong
Dragon Quality Goods Shop	Green Intelligent Power Saver	There are no fruits, only samples...	Jingdong prefecture level city in Shandong
Dragon Quality Goods Shop	Green Intelligent Power Saver	Save a few dollars a month.	Jingdong prefecture level city in Shandong
Dragon Quality Goods Shop	Green Intelligent Power Saver	The quality is particularly good, it works well, and the seller has a good service attitude	Jingdong prefecture level city in Shandong
Dragon Quality Goods Shop	Green Intelligent Power Saver	I've compared them a couple of times and I don't feel any difference	Jingdong prefecture level city in Shandong
Dragon Quality Goods Shop	Green Intelligent Power Saver	I don't see the savings. It's a sham.	Jingdong prefecture level city in Shandong
Dragon Quality Goods Shop	Green Intelligent Power Saver	The power saving effect is very good, and the logistics is very fast. The seller's service attitude is very good	Jingdong prefecture level city in Shandong
Dragon Quality Goods Shop	Green Intelligent Power Saver	Received, well packaged, haven't tried it yet!	Jingdong prefecture level city in Shandong
Dragon Quality Goods Shop	Green Intelligent Power Saver	I don't know how it works, I'll comment after I use it.	Jingdong prefecture level city in Shandong
Dragon Quality Goods Shop	Green Intelligent Power Saver	The product doesn't work well enough to be of any use	Jingdong prefecture level city in Shandong
Dragon Quality Goods Shop	Green Intelligent Power Saver	On Trial 。。。。。。。	Jingdong prefecture level city in Shandong
Dragon Quality Goods Shop	Green Intelligent Power Saver	Being used with unknown results	Jingdong prefecture level city in Shandong
Dragon Quality Goods Shop	Green Intelligent Power Saver	Smart power saving and great quality	Jingdong prefecture level city in Shandong
Dragon Quality Goods Shop	Green Intelligent Power Saver	The saver was received, it works very well and the price is affordable!	Jingdong prefecture level city in Shandong
Dragon Quality Goods Shop	Green Intelligent Power Saver	How can I feel the power saving and how long does it last	Jingdong prefecture level city in Shandong
Dragon Quality Goods Shop	Green Intelligent Power Saver	And I know if it saves electricity or not.	Jingdong prefecture level city in Shandong
Dragon Quality Goods Shop	Green Intelligent Power Saver	It won't save any electricity. No.	Jingdong prefecture level city in Shandong
Dragon Quality Goods Shop	Green Intelligent Power Saver	Invalid. Fake.	Jingdong prefecture level city in Shandong
Dragon Quality Goods Shop	Green Intelligent Power Saver	It's good!	Jingdong prefecture level city in Shandong
Dragon Quality Goods Shop	Green Intelligent Power Saver	have no effect	Jingdong prefecture level city in Shandong
Dragon Quality Goods Shop	Green Intelligent Power Saver	Not used.	Jingdong prefecture level city in Shandong
Dragon Quality Goods Shop	Green Intelligent Power Saver	There is no load range, packaging thrift, ten days also did not develop invoices, received the goods with a receipt, hand-written receipt also has no financial stamp, communication for nearly a week a moment to say that the electronic invoice, a moment to say that the paper invoice, said it is mailed to me, ten days also did not see anything, the invoice invoice can not be found to say that did not stay on file, single single number can not be found, said that the warehouse more than one to a one to find out, in addition to the perfunctory or perfunctory. Products are no certificate of conformity, proper three	Jingdong prefecture level city in Shandong
Dragon Quality Goods Shop	Green Intelligent Power Saver	Satisfaction.	Jingdong prefecture level city in Shandong
Dragon Quality Goods Shop	Green Intelligent Power Saver	There is not a bit of power saving effect, and ringing up an increase in the electricity bill, because you have to add the cost of this machine's own electricity consumption and purchase, so I think it is better to use this machine instead of increasing the expenditure, uneconomical, rather than not use it.	Jingdong prefecture level city in Shandong
Dragon Quality Goods Shop	Green Intelligent Power Saver	I've been using it for two days now and I don't feel any effect.	Jingdong prefecture level city in Shandong
Dragon Quality Goods Shop	Green Intelligent Power Saver	What it looked like was nothing like what was sent.	Jingdong prefecture level city in Shandong
Dragon Quality Goods Shop	Green Intelligent Power Saver	It didn't work. It didn't work for a month or so.	Jingdong prefecture level city in Shandong
Dragon Quality Goods Shop	Green Intelligent Power Saver	Garbage products, physical and description is not the same, no effect at all, pit # no discussion, to buy the fine, garbage, garbage, garbage	Jingdong prefecture level city in Shandong
Dragon Quality Goods Shop	Green Intelligent Power Saver	general	Jingdong prefecture level city in Shandong
Dragon Quality Goods Shop	Green Intelligent Power Saver	IQ tax. Instead of saving electricity, it increases the power consumption of an appliance.	Jingdong prefecture level city in Shandong
Dragon Quality Goods Shop	Green Intelligent Power Saver	There's no trash at all.	Jingdong prefecture level city in Shandong
Dragon Quality Goods Shop	Green Intelligent Power Saver	It doesn't do anything.	Jingdong prefecture level city in Shandong
Dragon Quality Goods Shop	Green Intelligent Power Saver	(of an unmarried couple) be close	Jingdong prefecture level city in Shandong
Dragon Quality Goods Shop	Green Intelligent Power Saver	(of an unmarried couple) be close	Jingdong prefecture level city in Shandong
Dragon Quality Goods Shop	Green Intelligent Power Saver	no electricity saving	Jingdong prefecture level city in Shandong
Dragon Quality Goods Shop	Green Intelligent Power Saver	differ from	Jingdong prefecture level city in Shandong
Dragon Quality Goods Shop	Green Intelligent Power Saver	Saver has received, but real can save electricity, the quality is very good, I times I buy a few more, too good.	Jingdong prefecture level city in Shandong
Dragon Quality Goods Shop	Green Intelligent Power Saver	Great quality as always, a very favorite product, great! o(*￣︶￣*)o	Jingdong prefecture level city in Shandong
Dragon Quality Goods Shop	Green Intelligent Power Saver	If we can reduce the electricity bill, for individuals can save money, for the country can protect the environment, now the weather is getting hotter and hotter, to protect the environment to reduce emissions is very important. Already use the product, whether to reduce the electricity bill depends on next month!	Jingdong prefecture level city in Shandong
Dragon Quality Goods Shop	Green Intelligent Power Saver	Purchased a power saver received on the put into use, rent a room with many electrical appliances, power consumption is very large, we have a piece of electricity or 1.2 dollars a time, with this power saver is much better, every day can save a few cents, very convenient and practical!	Jingdong prefecture level city in Shandong
Dragon Quality Goods Shop	Green Intelligent Power Saver	The product is better to use before not to use when a month 1500 yuan electricity bill, now using the 5 second month electricity bill directly down 470 dollars or very good. A save money to buy several power saver money ZanZanZanZan👍	Jingdong prefecture level city in Shandong
Dragon Quality Goods Shop	Green Intelligent Power Saver	The moment I received the goods really satisfied with this product, not only high-grade packaging, the body are base alloy, up to the price, I was also introduced by a friend, he used last month did save a lot of electricity, but also recommended to me!	Jingdong prefecture level city in Shandong
Dragon Quality Goods Shop	Green Intelligent Power Saver	I got it. Good review. I'm very satisfied. Reasonable price.	Jingdong prefecture level city in Shandong
Dragon Quality Goods Shop	Green Intelligent Power Saver	Intelligent power saver received and used, looking at the quality is good, the quality of the plug sent is also very good. Expect the effect of the economizer is obvious, first five-star praise, with a period of time to follow up!	Jingdong prefecture level city in Shandong
Dragon Quality Goods Shop	Green Intelligent Power Saver	The saver has been received. It was plugged into an outlet and used immediately after receipt. Whether it really saves electricity is not clear yet, I believe that this brand of energy saver really saves	Jingdong prefecture level city in Shandong
Dragon Quality Goods Shop	Green Intelligent Power Saver	Okay, okay, okay. Thank you so much. Fast is good. Fast. Item was as described, mainly because the event was cheap and you can save a lot of money.	Jingdong prefecture level city in Shandong

Dragon Quality Goods Shop	Green Intelligent Power Saver	Received it a couple days ago! Plugged it in the same day! Let's see what the electric bill is in a month! Hopefully it works well oh!	Jingdong prefecture level city in Shandong
Dragon Quality Goods Shop	Green Intelligent Power Saver	Looking at the reviews good, energy-saving small products, compact, the results after 2 months to see, because the electricity bill season payment	Jingdong prefecture level city in Shandong
Dragon Quality Goods Shop	Green Intelligent Power Saver	I just received it and will try it out to see if it works well. I'll comment later.	Jingdong prefecture level city in Shandong
Dragon Quality Goods Shop	Green Intelligent Power Saver	It looks a little small and not very aesthetically pleasing, I don't know how effective it will be until I use it	Jingdong prefecture level city in Shandong
Dragon Quality Goods Shop	Green Intelligent Power Saver	Not only to the point of being useless yet	Jingdong prefecture level city in Shandong
Dragon Quality Goods Shop	Green Intelligent Power Saver	Used it for a month, nothing works, the meter you turn very fast. This is not a scam, but also sold on Jingdong. To cheat a bunch of people?	Jingdong prefecture level city in Shandong
Dragon Quality Goods Shop	Green Intelligent Power Saver	Very good to use, every day can save a little electricity, really good under the sample down a month to save a few dozen dollars in electricity, good before I did not know that there is this haha, the price is not expensive praise.	Jingdong prefecture level city in Shandong
Dragon Quality Goods Shop	Green Intelligent Power Saver	Tried it for ten days and the change is almost nothing, can't see the power saving yet, use it some more days and see how it goes.	Jingdong prefecture level city in Shandong
Dragon Quality Goods Shop	Green Intelligent Power Saver	Received very quickly, the quality is very good, really save electricity, business service attitude is also very good, I hope you do not miss this opportunity, I continue to buy a few in	Jingdong prefecture level city in Shandong
Dragon Quality Goods Shop	Green Intelligent Power Saver	Quality is very good Oh, the delivery is also very fast, a very favorite store, I want to crazy recommend to friends!	Jingdong prefecture level city in Shandong
Dragon Quality Goods Shop	Green Intelligent Power Saver	The goods have just received, fine workmanship, and the same as described by the merchant, to use a period of time to see if it can achieve the purpose of power saving.	Jingdong prefecture level city in Shandong
Dragon Quality Goods Shop	Green Intelligent Power Saver	Received the goods first use a period of time to see if there is no effect, the business commitment to ninety days invalid refund, first praise, good service!	Jingdong prefecture level city in Shandong
Dragon Quality Goods Shop	Green Intelligent Power Saver	The product is used for a month without energy-saving and power-saving effect does not have any effect	Jingdong prefecture level city in Shandong
Dragon Quality Goods Shop	Green Intelligent Power Saver	The baby is really great, I like it very much, my friends also say good to come, recommended to buy!	Jingdong prefecture level city in Shandong
Dragon Quality Goods Shop	Green Intelligent Power Saver	Logistics is fast, courier boy service is good, after using to see how the fruit, and then follow up.	Jingdong prefecture level city in Shandong
Dragon Quality Goods Shop	Green Intelligent Power Saver	Baby has been received to try, I believe that as the business advertised energy saving. The courier is very responsible.	Jingdong prefecture level city in Shandong
Dragon Quality Goods Shop	Green Intelligent Power Saver	Save Electric Energy King received, good quality, beautiful appearance, very satisfied!	Jingdong prefecture level city in Shandong
Dragon Quality Goods Shop	Green Intelligent Power Saver	Three no products no manufacturers no phone pure fraud induced consumption cheat	Jingdong prefecture level city in Shandong
Dragon Quality Goods Shop	Green Intelligent Power Saver	I'm using it now, I don't know if it's as good as it says, I'll follow up after a month	Jingdong prefecture level city in Shandong
Dragon Quality Goods Shop	Green Intelligent Power Saver	Good quality logistics and fast. Fine workmanship. The description is consistent cost-effective	Jingdong prefecture level city in Shandong
Dragon Quality Goods Shop	Green Intelligent Power Saver	Received, whether it is good or not, to wait a few days and then evaluate	Jingdong prefecture level city in Shandong
Dragon Quality Goods Shop	Green Intelligent Power Saver	I bought it and started using it, I won't know if it works well until I pay my electric bill	Jingdong prefecture level city in Shandong
Dragon Quality Goods Shop	Green Intelligent Power Saver	Especially good, energy saving and power saving, especially good quality, good	Jingdong prefecture level city in Shandong
Dragon Quality Goods Shop	Green Intelligent Power Saver	Logistics is strong, the quality is very good, paste really save electricity, as described, will buy back!	Jingdong prefecture level city in Shandong
Dragon Quality Goods Shop	Green Intelligent Power Saver	Great stuff at a great price, worth the money!	Jingdong prefecture level city in Shandong
Dragon Quality Goods Shop	Green Intelligent Power Saver	Received the power saver, very good quality, very power saving!	Jingdong prefecture level city in Shandong
Dragon Quality Goods Shop	Green Intelligent Power Saver	There are no fruits, only samples...	Jingdong prefecture level city in Shandong
Dragon Quality Goods Shop	Green Intelligent Power Saver	Save a few dollars a month.	Jingdong prefecture level city in Shandong
Dragon Quality Goods Shop	Green Intelligent Power Saver	The quality is particularly good, it works well, and the seller has a good service attitude	Jingdong prefecture level city in Shandong
Dragon Quality Goods Shop	Green Intelligent Power Saver	I've compared them a couple of times and I don't feel any difference	Jingdong prefecture level city in Shandong
Dragon Quality Goods Shop	Green Intelligent Power Saver	I don't see the savings. It's a sham.	Jingdong prefecture level city in Shandong
Dragon Quality Goods Shop	Green Intelligent Power Saver	The power saving effect is very good, and the logistics is very fast. The seller's service attitude is very good	Jingdong prefecture level city in Shandong
Dragon Quality Goods Shop	Green Intelligent Power Saver	Received, well packaged, haven't tried it yet!	Jingdong prefecture level city in Shandong
Dragon Quality Goods Shop	Green Intelligent Power Saver	I don't know how it works, I'll comment after I use it.	Jingdong prefecture level city in Shandong
Dragon Quality Goods Shop	Green Intelligent Power Saver	The product doesn't work well enough to be of any use	Jingdong prefecture level city in Shandong
Dragon Quality Goods Shop	Green Intelligent Power Saver	On Trial 。。。。。。。	Jingdong prefecture level city in Shandong
Dragon Quality Goods Shop	Green Intelligent Power Saver	Being used with unknown results	Jingdong prefecture level city in Shandong
Dragon Quality Goods Shop	Green Intelligent Power Saver	Smart power saving and great quality	Jingdong prefecture level city in Shandong
Dragon Quality Goods Shop	Green Intelligent Power Saver	The saver was received, it works very well and the price is affordable!	Jingdong prefecture level city in Shandong
Dragon Quality Goods Shop	Green Intelligent Power Saver	How can I feel the power saving and how long does it last	Jingdong prefecture level city in Shandong
Dragon Quality Goods Shop	Green Intelligent Power Saver	And I know if it saves electricity or not.	Jingdong prefecture level city in Shandong
Dragon Quality Goods Shop	Green Intelligent Power Saver	It won't save any electricity. No.	Jingdong prefecture level city in Shandong
Dragon Quality Goods Shop	Green Intelligent Power Saver	Invalid. Fake.	Jingdong prefecture level city in Shandong
Dragon Quality Goods Shop	Green Intelligent Power Saver	It's good!	Jingdong prefecture level city in Shandong
Dragon Quality Goods Shop	Green Intelligent Power Saver	have no effect	Jingdong prefecture level city in Shandong
Dragon Quality Goods Shop	Green Intelligent Power Saver	Not used.	Jingdong prefecture level city in Shandong
Dragon Quality Goods Shop	Green Intelligent Power Saver	There is no load range, packaging thrift, ten days also did not develop invoices, received the goods with a receipt, hand-written receipt also has no financial stamp, communication for nearly a week a moment to say that the electronic invoice, a moment to say that the paper invoice, said it is mailed to me, ten days also did not see anything, the invoice invoice can not be found to say that did not stay on file, single single number can not be found, said that the warehouse more than one to a one to find out, in addition to the perfunctory or perfunctory. Products are no certificate of conformity, proper three	Jingdong prefecture level city in Shandong
Dragon Quality Goods Shop	Green Intelligent Power Saver	Satisfaction.	Jingdong prefecture level city in Shandong

Dragon Quality Goods Shop	Green Intelligent Power Saver	There is not a bit of power saving effect, and ringing up an increase in the electricity bill, because you have to add the cost of this machine's own electricity consumption and purchase, so I think it is better to use this machine instead of increasing the expenditure, uneconomical, rather than not use it.	Jingdong prefecture level city in Shandong
Dragon Quality Goods Shop	Green Intelligent Power Saver	I've been using it for two days now and I don't feel any effect.	Jingdong prefecture level city in Shandong
Dragon Quality Goods Shop	Green Intelligent Power Saver	What it looked like was nothing like what was sent.	Jingdong prefecture level city in Shandong
Dragon Quality Goods Shop	Green Intelligent Power Saver	It didn't work. It didn't work for a month or so.	Jingdong prefecture level city in Shandong
Dragon Quality Goods Shop	Green Intelligent Power Saver	Garbage products, physical and description is not the same, no effect at all, pit # no discussion, to buy the fine, garbage, garbage, garbage	Jingdong prefecture level city in Shandong
Dragon Quality Goods Shop	Green Intelligent Power Saver	general	Jingdong prefecture level city in Shandong
Dragon Quality Goods Shop	Green Intelligent Power Saver	IQ tax. Instead of saving electricity, it increases the power consumption of an appliance.	Jingdong prefecture level city in Shandong
Dragon Quality Goods Shop	Green Intelligent Power Saver	There's no trash at all.	Jingdong prefecture level city in Shandong
Dragon Quality Goods Shop	Green Intelligent Power Saver	It doesn't do anything.	Jingdong prefecture level city in Shandong
Dragon Quality Goods Shop	Green Intelligent Power Saver	(of an unmarried couple) be close	Jingdong prefecture level city in Shandong
Dragon Quality Goods Shop	Green Intelligent Power Saver	(of an unmarried couple) be close	Jingdong prefecture level city in Shandong
Dragon Quality Goods Shop	Green Intelligent Power Saver	no electricity saving	Jingdong prefecture level city in Shandong
Dragon Quality Goods Shop	Green Intelligent Power Saver	differ from	Jingdong prefecture level city in Shandong
Dragon Quality Goods Shop	Green Intelligent Power Saver	Saver has received, but real can save electricity, the quality is very good, I times I buy a few more, too good.	Jingdong prefecture level city in Shandong
Dragon Quality Goods Shop	Green Intelligent Power Saver	Great quality as always, a very favorite product, great! o(*￣︶￣*)o	Jingdong prefecture level city in Shandong
Dragon Quality Goods Shop	Green Intelligent Power Saver	If we can reduce the electricity bill, for individuals can save money, for the country can protect the environment, now the weather is getting hotter and hotter, to protect the environment to reduce emissions is very important. Already use the product, whether to reduce the electricity bill depends on next month!	Jingdong prefecture level city in Shandong
Dragon Quality Goods Shop	Green Intelligent Power Saver	Purchased a power saver received on the put into use, rent a room with many electrical appliances, power consumption is very large, we have a piece of electricity or 1.2 dollars a time, with this power saver is much better, every day can save a few cents, very convenient and practical!	Jingdong prefecture level city in Shandong
Dragon Quality Goods Shop	Green Intelligent Power Saver	The product is better to use before not to use when a month 1500 yuan electricity bill, now using the 5 second month electricity bill directly down 470 dollars or very good. A save money to buy several power saver money ZanZanZanZan	Jingdong prefecture level city in Shandong
Dragon Quality Goods Shop	Green Intelligent Power Saver	The moment I received the goods really satisfied with this product, not only high-grade packaging, the body are base alloy, up to the price, I was also introduced by a friend, he used last month did save a lot of electricity, but also recommended to me!	Jingdong prefecture level city in Shandong
Dragon Quality Goods Shop	Green Intelligent Power Saver	I got it. Good review. I'm very satisfied. Reasonable price.	Jingdong prefecture level city in Shandong
Dragon Quality Goods Shop	Green Intelligent Power Saver	Intelligent power saver received and used, looking at the quality is good, the quality of the plug sent is also very good. Expect the effect of the economizer is obvious, first five-star praise, with a period of time to follow up!	Jingdong prefecture level city in Shandong
Dragon Quality Goods Shop	Green Intelligent Power Saver	The saver has been received. It was plugged into an outlet and used immediately after receipt. Whether it really saves electricity is not clear yet, I believe that this brand of energy saver really saves	Jingdong prefecture level city in Shandong
Dragon Quality Goods Shop	Green Intelligent Power Saver	Okay, okay, okay. Thank you so much. Fast is good. Fast. Item was as described, mainly because the event was cheap and you can save a lot of money.	Jingdong prefecture level city in Shandong
Dragon Quality Goods Shop	Green Intelligent Power Saver	Received it a couple days ago! Plugged it in the same day! Let's see what the electric bill is in a month! Hopefully it works well oh!	Jingdong prefecture level city in Shandong
Dragon Quality Goods Shop	Green Intelligent Power Saver	Looking at the reviews good, energy-saving small products, compact, the results after 2 months to see, because the electricity bill season payment	Jingdong prefecture level city in Shandong
Dragon Quality Goods Shop	Green Intelligent Power Saver	I just received it and will try it out to see if it works well. I'll comment later.	Jingdong prefecture level city in Shandong
Dragon Quality Goods Shop	Green Intelligent Power Saver	It looks a little small and not very aesthetically pleasing, I don't know how effective it will be until I use it	Jingdong prefecture level city in Shandong
Dragon Quality Goods Shop	Green Intelligent Power Saver	Not only to the point of being useless yet	Jingdong prefecture level city in Shandong
Dragon Quality Goods Shop	Green Intelligent Power Saver	Used it for a month, nothing works, the meter you turn very fast. This is not a scam, but also sold on Jingdong. To cheat a bunch of people?	Jingdong prefecture level city in Shandong
Dragon Quality Goods Shop	Green Intelligent Power Saver	Very good to use, every day can save a little electricity, really good under the sample down a month to save a few dozen dollars in electricity, good before I did not know that there is this haha, the price is not expensive praise.	Jingdong prefecture level city in Shandong
Dragon Quality Goods Shop	Green Intelligent Power Saver	Tried it for ten days and the change is almost nothing, can't see the power saving yet, use it some more days and see how it goes.	Jingdong prefecture level city in Shandong
Dragon Quality Goods Shop	Green Intelligent Power Saver	Received very quickly, the quality is very good, really save electricity, business service attitude is also very good, I hope you do not miss this opportunity, I continue to buy a few in	Jingdong prefecture level city in Shandong
Dragon Quality Goods Shop	Green Intelligent Power Saver	Quality is very good Oh, the delivery is also very fast, a very favorite store, I want to crazy recommend to friends!	Jingdong prefecture level city in Shandong
Dragon Quality Goods Shop	Green Intelligent Power Saver	The goods have just received, fine workmanship, and the same as described by the merchant, to use a period of time to see if it can achieve the purpose of power saving.	Jingdong prefecture level city in Shandong
Dragon Quality Goods Shop	Green Intelligent Power Saver	Received the goods first use a period of time to see if there is no effect, the business commitment to ninety days invalid refund, first praise, good service!	Jingdong prefecture level city in Shandong
Dragon Quality Goods Shop	Green Intelligent Power Saver	The product is used for a month without energy-saving and power-saving effect does not have any effect	Jingdong prefecture level city in Shandong
Dragon Quality Goods Shop	Green Intelligent Power Saver	The baby is really great, I like it very much, my friends also say good to come, recommended to buy!	Jingdong prefecture level city in Shandong
Dragon Quality Goods Shop	Green Intelligent Power Saver	Logistics is fast, courier boy service is good, after using to see how the fruit, and then follow up.	Jingdong prefecture level city in Shandong
Dragon Quality Goods Shop	Green Intelligent Power Saver	Baby has been received to try, I believe that as the business advertised energy saving. The courier is very responsible.	Jingdong prefecture level city in Shandong
Dragon Quality Goods Shop	Green Intelligent Power Saver	Save Electric Energy King received, good quality, beautiful appearance, very satisfied!	Jingdong prefecture level city in Shandong
Dragon Quality Goods Shop	Green Intelligent Power Saver	Three no products no manufacturers no phone pure fraud induced consumption cheat	Jingdong prefecture level city in Shandong
Dragon Quality Goods Shop	Green Intelligent Power Saver	I'm using it now, I don't know if it's as good as it says, I'll follow up after a month	Jingdong prefecture level city in Shandong
Dragon Quality Goods Shop	Green Intelligent Power Saver	Good quality logistics and fast. Fine workmanship. The description is consistent cost-effective	Jingdong prefecture level city in Shandong
Dragon Quality Goods Shop	Green Intelligent Power Saver	Received, whether it is good or not, to wait a few days and then evaluate	Jingdong prefecture level city in Shandong
Dragon Quality Goods Shop	Green Intelligent Power Saver	I bought it and started using it, I won't know if it works well until I pay my electric bill	Jingdong prefecture level city in Shandong
Dragon Quality Goods Shop	Green Intelligent Power Saver	Especially good, energy saving and power saving, especially good quality, good	Jingdong prefecture level city in Shandong
Dragon Quality Goods Shop	Green Intelligent Power Saver	Logistics is strong, the quality is very good, paste really save electricity, as described, will buy back!	Jingdong prefecture level city in Shandong
Dragon Quality Goods Shop	Green Intelligent Power Saver	Great stuff at a great price, worth the money!	Jingdong prefecture level city in Shandong

Dragon Quality Goods Shop	Green Intelligent Power Saver	Received the power saver, very good quality, very power saving!	Jingdong prefecture level city in Shandong
Dragon Quality Goods Shop	Green Intelligent Power Saver	There are no fruits, only samples...	Jingdong prefecture level city in Shandong
Dragon Quality Goods Shop	Green Intelligent Power Saver	Save a few dollars a month.	Jingdong prefecture level city in Shandong
Dragon Quality Goods Shop	Green Intelligent Power Saver	The quality is particularly good, it works well, and the seller has a good service attitude	Jingdong prefecture level city in Shandong
Dragon Quality Goods Shop	Green Intelligent Power Saver	I've compared them a couple of times and I don't feel any difference	Jingdong prefecture level city in Shandong
Dragon Quality Goods Shop	Green Intelligent Power Saver	I don't see the savings. It's a sham.	Jingdong prefecture level city in Shandong
Dragon Quality Goods Shop	Green Intelligent Power Saver	The power saving effect is very good, and the logistics is very fast. The seller's service attitude is very good	Jingdong prefecture level city in Shandong
Dragon Quality Goods Shop	Green Intelligent Power Saver	Received, well packaged, haven't tried it yet!	Jingdong prefecture level city in Shandong
Dragon Quality Goods Shop	Green Intelligent Power Saver	I don't know how it works, I'll comment after I use it.	Jingdong prefecture level city in Shandong
Dragon Quality Goods Shop	Green Intelligent Power Saver	The product doesn't work well enough to be of any use	Jingdong prefecture level city in Shandong
Dragon Quality Goods Shop	Green Intelligent Power Saver	On Trial 。。。。。。。	Jingdong prefecture level city in Shandong
Dragon Quality Goods Shop	Green Intelligent Power Saver	Being used with unknown results	Jingdong prefecture level city in Shandong
Dragon Quality Goods Shop	Green Intelligent Power Saver	Smart power saving and great quality	Jingdong prefecture level city in Shandong
Dragon Quality Goods Shop	Green Intelligent Power Saver	The saver was received, it works very well and the price is affordable!	Jingdong prefecture level city in Shandong
Dragon Quality Goods Shop	Green Intelligent Power Saver	How can I feel the power saving and how long does it last	Jingdong prefecture level city in Shandong
Dragon Quality Goods Shop	Green Intelligent Power Saver	And I know if it saves electricity or not.	Jingdong prefecture level city in Shandong
Dragon Quality Goods Shop	Green Intelligent Power Saver	It won't save any electricity. No.	Jingdong prefecture level city in Shandong
Dragon Quality Goods Shop	Green Intelligent Power Saver	Invalid. Fake.	Jingdong prefecture level city in Shandong
Dragon Quality Goods Shop	Green Intelligent Power Saver	It's good!	Jingdong prefecture level city in Shandong
Dragon Quality Goods Shop	Green Intelligent Power Saver	have no effect	Jingdong prefecture level city in Shandong
Dragon Quality Goods Shop	Green Intelligent Power Saver	Not used.	Jingdong prefecture level city in Shandong
Dragon Quality Goods Shop	Green Intelligent Power Saver	There is no load range, packaging thrift, ten days also did not develop invoices, received the goods with a receipt, hand-written receipt also has no financial stamp, communication for nearly a week a moment to say that the electronic invoice, a moment to say that the paper invoice, said it is mailed to me, ten days also did not see anything, the invoice invoice can not be found to say that did not stay on file, single single number can not be found, said that the warehouse more than one to a one to find out, in addition to the perfunctory or perfunctory. Products are no certificate of conformity, proper three	Jingdong prefecture level city in Shandong
Dragon Quality Goods Shop	Green Intelligent Power Saver	Satisfaction.	Jingdong prefecture level city in Shandong
Dragon Quality Goods Shop	Green Intelligent Power Saver	There is not a bit of power saving effect, and ringing up an increase in the electricity bill, because you have to add the cost of this machine's own electricity consumption and purchase, so I think it is better to use this machine instead of increasing the expenditure, uneconomical, rather than not use it.	Jingdong prefecture level city in Shandong
Dragon Quality Goods Shop	Green Intelligent Power Saver	I've been using it for two days now and I don't feel any effect.	Jingdong prefecture level city in Shandong
Dragon Quality Goods Shop	Green Intelligent Power Saver	What it looked like was nothing like what was sent.	Jingdong prefecture level city in Shandong
Dragon Quality Goods Shop	Green Intelligent Power Saver	It didn't work. It didn't work for a month or so.	Jingdong prefecture level city in Shandong
Dragon Quality Goods Shop	Green Intelligent Power Saver	Garbage products, physical and description is not the same, no effect at all, pit # no discussion, to buy the fine, garbage, garbage, garbage	Jingdong prefecture level city in Shandong
Dragon Quality Goods Shop	Green Intelligent Power Saver	general	Jingdong prefecture level city in Shandong
Dragon Quality Goods Shop	Green Intelligent Power Saver	IQ tax. Instead of saving electricity, it increases the power consumption of an appliance.	Jingdong prefecture level city in Shandong
Dragon Quality Goods Shop	Green Intelligent Power Saver	There's no trash at all.	Jingdong prefecture level city in Shandong
Dragon Quality Goods Shop	Green Intelligent Power Saver	It doesn't do anything.	Jingdong prefecture level city in Shandong
Dragon Quality Goods Shop	Green Intelligent Power Saver	(of an unmarried couple) be close	Jingdong prefecture level city in Shandong
Dragon Quality Goods Shop	Green Intelligent Power Saver	(of an unmarried couple) be close	Jingdong prefecture level city in Shandong
Dragon Quality Goods Shop	Green Intelligent Power Saver	no electricity saving	Jingdong prefecture level city in Shandong
Dragon Quality Goods Shop	Green Intelligent Power Saver	differ from	Jingdong prefecture level city in Shandong
Dragon Quality Goods Shop	Green Intelligent Power Saver	Saver has received, but real can save electricity, the quality is very good, I times I buy a few more, too good.	Jingdong prefecture level city in Shandong
Dragon Quality Goods Shop	Green Intelligent Power Saver	Great quality as always, a very favorite product, great! o(*￣︶￣*)o	Jingdong prefecture level city in Shandong
Dragon Quality Goods Shop	Green Intelligent Power Saver	If we can reduce the electricity bill, for individuals can save money, for the country can protect the environment, now the weather is getting hotter and hotter, to protect the environment to reduce emissions is very important. Already use the product, whether to reduce the electricity bill depends on next month!	Jingdong prefecture level city in Shandong
Dragon Quality Goods Shop	Green Intelligent Power Saver	Purchased a power saver received on the put into use, rent a room with many electrical appliances, power consumption is very large, we have a piece of electricity or 1.2 dollars a time, with this power saver is much better, every day can save a few cents, very convenient and practical!	Jingdong prefecture level city in Shandong
Dragon Quality Goods Shop	Green Intelligent Power Saver	The product is better to use before not to use when a month 1500 yuan electricity bill, now using the 5 second month electricity bill directly down 470 dollars or very good. A save money to buy several power saver money ZanZanZanZan👍	Jingdong prefecture level city in Shandong
Dragon Quality Goods Shop	Green Intelligent Power Saver	The moment I received the goods really satisfied with this product, not only high-grade packaging, the body are base alloy, up to the price, I was also introduced by a friend, he used last month did save a lot of electricity, but also recommended to me!	Jingdong prefecture level city in Shandong
Dragon Quality Goods Shop	Green Intelligent Power Saver	I got it. Good review. I'm very satisfied. Reasonable price.	Jingdong prefecture level city in Shandong
Dragon Quality Goods Shop	Green Intelligent Power Saver	Intelligent power saver received and used, looking at the quality is good, the quality of the plug sent is also very good. Expect the effect of the economizer is obvious, first five-star praise, with a period of time to follow up!	Jingdong prefecture level city in Shandong
Dragon Quality Goods Shop	Green Intelligent Power Saver	The saver has been received. It was plugged into an outlet and used immediately after receipt. Whether it really saves electricity is not clear yet, I believe that this brand of energy saver really saves	Jingdong prefecture level city in Shandong
Dragon Quality Goods Shop	Green Intelligent Power Saver	Okay, okay, okay. Thank you so much. Fast is good. Fast. Item was as described, mainly because the event was cheap and you can save a lot of money.	Jingdong prefecture level city in Shandong

Dragon Quality Goods Shop	Green Intelligent Power Saver	Received it a couple days ago! Plugged it in the same day! Let's see what the electric bill is in a month! Hopefully it works well oh!	Jingdong prefecture level city in Shandong
Dragon Quality Goods Shop	Green Intelligent Power Saver	Looking at the reviews good, energy-saving small products, compact, the results after 2 months to see, because the electricity bill season payment	Jingdong prefecture level city in Shandong
Dragon Quality Goods Shop	Green Intelligent Power Saver	Used it for a month, nothing works, the meter you turn very fast. This is not a scam, but also sold on Jingdong. To cheat a bunch of people?	Jingdong prefecture level city in Shandong
Dragon Quality Goods Shop	Green Intelligent Power Saver	It looks a little small and not very aesthetically pleasing, I don't know how effective it will be until I use it	Jingdong prefecture level city in Shandong
Dragon Quality Goods Shop	Green Intelligent Power Saver	Not only to the point of being useless yet	Jingdong prefecture level city in Shandong
Dragon Quality Goods Shop	Green Intelligent Power Saver	I just received it and will try it out to see if it works well. I'll comment later.	Jingdong prefecture level city in Shandong
Dragon Quality Goods Shop	Green Intelligent Power Saver	Very good to use, every day can save a little electricity, really good under the sample down a month to save a few dozen dollars in electricity, good before I did not know that there is this haha, the price is not expensive praise.	Jingdong prefecture level city in Shandong
Dragon Quality Goods Shop	Green Intelligent Power Saver	Tried it for ten days and the change is almost nothing, can't see the power saving yet, use it some more days and see how it goes.	Jingdong prefecture level city in Shandong
Dragon Quality Goods Shop	Green Intelligent Power Saver	The goods have just received, fine workmanship, and the same as described by the merchant, to use a period of time to see if it can achieve the purpose of power saving.	Jingdong prefecture level city in Shandong
Dragon Quality Goods Shop	Green Intelligent Power Saver	Quality is very good Oh, the delivery is also very fast, a very favorite store, I want to crazy recommend to friends!	Jingdong prefecture level city in Shandong
Dragon Quality Goods Shop	Green Intelligent Power Saver	Received very quickly, the quality is very good, really save electricity, business service attitude is also very good, I hope you do not miss this opportunity, I continue to buy a few in	Jingdong prefecture level city in Shandong
Dragon Quality Goods Shop	Green Intelligent Power Saver	Received the goods first use a period of time to see if there is no effect, the business commitment to ninety days invalid refund, first praise, good service!	Jingdong prefecture level city in Shandong
Dragon Quality Goods Shop	Green Intelligent Power Saver	The product is used for a month without energy-saving and power-saving effect does not have any effect	Jingdong prefecture level city in Shandong
Dragon Quality Goods Shop	Green Intelligent Power Saver	The baby is really great, I like it very much, my friends also say good to come, recommended to buy!	Jingdong prefecture level city in Shandong
Dragon Quality Goods Shop	Green Intelligent Power Saver	Logistics is fast, courier boy service is good, after using to see how the fruit, and then follow up.	Jingdong prefecture level city in Shandong
Dragon Quality Goods Shop	Green Intelligent Power Saver	Baby has been received to try, I believe that as the business advertised energy saving. The courier is very responsible.	Jingdong prefecture level city in Shandong
Dragon Quality Goods Shop	Green Intelligent Power Saver	Save Electric Energy King received, good quality, beautiful appearance, very satisfied!	Jingdong prefecture level city in Shandong
Dragon Quality Goods Shop	Green Intelligent Power Saver	Three no products no manufacturers no phone pure fraud induced consumption cheat	Jingdong prefecture level city in Shandong
Dragon Quality Goods Shop	Green Intelligent Power Saver	I'm using it now, I don't know if it's as good as it says, I'll follow up after a month	Jingdong prefecture level city in Shandong
Dragon Quality Goods Shop	Green Intelligent Power Saver	Good quality logistics and fast. Fine workmanship. The description is consistent cost-effective	Jingdong prefecture level city in Shandong
Dragon Quality Goods Shop	Green Intelligent Power Saver	Received, whether it is good or not, to wait a few days and then evaluate	Jingdong prefecture level city in Shandong
Dragon Quality Goods Shop	Green Intelligent Power Saver	I bought it and started using it, I won't know if it works well until I pay my electric bill	Jingdong prefecture level city in Shandong
Dragon Quality Goods Shop	Green Intelligent Power Saver	Especially good, energy saving and power saving, especially good quality, good	Jingdong prefecture level city in Shandong
Dragon Quality Goods Shop	Green Intelligent Power Saver	Logistics is strong, the quality is very good, paste really save electricity, as described, will buy back!	Jingdong prefecture level city in Shandong
Dragon Quality Goods Shop	Green Intelligent Power Saver	Great stuff at a great price, worth the money!	Jingdong prefecture level city in Shandong
Dragon Quality Goods Shop	Green Intelligent Power Saver	Received the power saver, very good quality, very power saving!	Jingdong prefecture level city in Shandong
Dragon Quality Goods Shop	Green Intelligent Power Saver	There are no fruits, only samples...	Jingdong prefecture level city in Shandong
Dragon Quality Goods Shop	Green Intelligent Power Saver	Save a few dollars a month.	Jingdong prefecture level city in Shandong
Dragon Quality Goods Shop	Green Intelligent Power Saver	The quality is particularly good, it works well, and the seller has a good service attitude	Jingdong prefecture level city in Shandong
Dragon Quality Goods Shop	Green Intelligent Power Saver	I've compared them a couple of times and I don't feel any difference	Jingdong prefecture level city in Shandong
Dragon Quality Goods Shop	Green Intelligent Power Saver	I don't see the savings. It's a sham.	Jingdong prefecture level city in Shandong
Dragon Quality Goods Shop	Green Intelligent Power Saver	The power saving effect is very good, and the logistics is very fast. The seller's service attitude is very good	Jingdong prefecture level city in Shandong
Dragon Quality Goods Shop	Green Intelligent Power Saver	Received, well packaged, haven't tried it yet!	Jingdong prefecture level city in Shandong
Dragon Quality Goods Shop	Green Intelligent Power Saver	I don't know how it works, I'll comment after I use it.	Jingdong prefecture level city in Shandong
Dragon Quality Goods Shop	Green Intelligent Power Saver	The product doesn't work well enough to be of any use	Jingdong prefecture level city in Shandong
Dragon Quality Goods Shop	Green Intelligent Power Saver	On Trial 。。。。。。。	Jingdong prefecture level city in Shandong
Dragon Quality Goods Shop	Green Intelligent Power Saver	Being used with unknown results	Jingdong prefecture level city in Shandong
Dragon Quality Goods Shop	Green Intelligent Power Saver	Smart power saving and great quality	Jingdong prefecture level city in Shandong
Dragon Quality Goods Shop	Green Intelligent Power Saver	The saver was received, it works very well and the price is affordable!	Jingdong prefecture level city in Shandong
Dragon Quality Goods Shop	Green Intelligent Power Saver	How can I feel the power saving and how long does it last	Jingdong prefecture level city in Shandong
Dragon Quality Goods Shop	Green Intelligent Power Saver	And I know if it saves electricity or not.	Jingdong prefecture level city in Shandong
Dragon Quality Goods Shop	Green Intelligent Power Saver	It won't save any electricity. No.	Jingdong prefecture level city in Shandong
Dragon Quality Goods Shop	Green Intelligent Power Saver	Invalid. Fake.	Jingdong prefecture level city in Shandong
Dragon Quality Goods Shop	Green Intelligent Power Saver	It's good!	Jingdong prefecture level city in Shandong
Dragon Quality Goods Shop	Green Intelligent Power Saver	have no effect	Jingdong prefecture level city in Shandong
Dragon Quality Goods Shop	Green Intelligent Power Saver	Not used.	Jingdong prefecture level city in Shandong
Dragon Quality Goods Shop	Green Intelligent Power Saver	There is no load range, packaging thrift, ten days also did not develop invoices, received the goods with a receipt, hand-written receipt also has no financial stamp, communication for nearly a week a moment to say that the electronic invoice, a moment to say that the paper invoice, said it is mailed to me, ten days also did not see anything, the invoice invoice can not be found to say that did not stay on file, single single number can not be found, said that the warehouse more than one to a one to find out, in addition to the perfunctory or perfunctory. Products are no certificate of conformity, proper three	Jingdong prefecture level city in Shandong
Dragon Quality Goods Shop	Green Intelligent Power Saver	Satisfaction.	Jingdong prefecture level city in Shandong

Dragon Quality Goods Shop	Green Intelligent Power Saver	There is not a bit of power saving effect, and ringing up an increase in the electricity bill, because you have to add the cost of this machine's own electricity consumption and purchase, so I think it is better to use this machine instead of increasing the expenditure, uneconomical, rather than not use it.	Jingdong prefecture level city in Shandong
Dragon Quality Goods Shop	Green Intelligent Power Saver	I've been using it for two days now and I don't feel any effect.	Jingdong prefecture level city in Shandong
Dragon Quality Goods Shop	Green Intelligent Power Saver	What it looked like was nothing like what was sent.	Jingdong prefecture level city in Shandong
Dragon Quality Goods Shop	Green Intelligent Power Saver	It didn't work. It didn't work for a month or so.	Jingdong prefecture level city in Shandong
Dragon Quality Goods Shop	Green Intelligent Power Saver	Garbage products, physical and description is not the same, no effect at all, pit # no discussion, to buy the fine, garbage, garbage, garbage	Jingdong prefecture level city in Shandong
Dragon Quality Goods Shop	Green Intelligent Power Saver	general	Jingdong prefecture level city in Shandong
Dragon Quality Goods Shop	Green Intelligent Power Saver	IQ tax. Instead of saving electricity, it increases the power consumption of an appliance.	Jingdong prefecture level city in Shandong
Dragon Quality Goods Shop	Green Intelligent Power Saver	There's no trash at all.	Jingdong prefecture level city in Shandong
Dragon Quality Goods Shop	Green Intelligent Power Saver	It doesn't do anything.	Jingdong prefecture level city in Shandong
Dragon Quality Goods Shop	Green Intelligent Power Saver	(of an unmarried couple) be close	Jingdong prefecture level city in Shandong
Dragon Quality Goods Shop	Green Intelligent Power Saver	(of an unmarried couple) be close	Jingdong prefecture level city in Shandong
Dragon Quality Goods Shop	Green Intelligent Power Saver	no electricity saving	Jingdong prefecture level city in Shandong
Dragon Quality Goods Shop	Green Intelligent Power Saver	differ from	Jingdong prefecture level city in Shandong
Dragon Quality Goods Shop	Green Intelligent Power Saver	Saver has received, but real can save electricity, the quality is very good, I times I buy a few more, too good.	Jingdong prefecture level city in Shandong
Dragon Quality Goods Shop	Green Intelligent Power Saver	Great quality as always, a very favorite product, great! o(*￣︶￣*)o	Jingdong prefecture level city in Shandong
Dragon Quality Goods Shop	Green Intelligent Power Saver	If we can reduce the electricity bill, for individuals can save money, for the country can protect the environment, now the weather is getting hotter and hotter, to protect the environment to reduce emissions is very important. Already use the product, whether to reduce the electricity bill depends on next month!	Jingdong prefecture level city in Shandong
Dragon Quality Goods Shop	Green Intelligent Power Saver	Purchased a power saver received on the put into use, rent a room with many electrical appliances, power consumption is very large, we have a piece of electricity or 1.2 dollars a time, with this power saver is much better, every day can save a few cents, very convenient and practical!	Jingdong prefecture level city in Shandong
Dragon Quality Goods Shop	Green Intelligent Power Saver	The product is better to use before not to use when a month 1500 yuan electricity bill, now using the 5 second month electricity bill directly down 470 dollars or very good. A save money to buy several power saver money ZanZanZanZan👍	Jingdong prefecture level city in Shandong
Dragon Quality Goods Shop	Green Intelligent Power Saver	The moment I received the goods really satisfied with this product, not only high-grade packaging, the body are base alloy, up to the price, I was also introduced by a friend, he used last month did save a lot of electricity, but also recommended to me!	Jingdong prefecture level city in Shandong
Dragon Quality Goods Shop	Green Intelligent Power Saver	I got it. Good review. I'm very satisfied. Reasonable price.	Jingdong prefecture level city in Shandong
Dragon Quality Goods Shop	Green Intelligent Power Saver	Intelligent power saver received and used, looking at the quality is good, the quality of the plug sent is also very good. Expect the effect of the economizer is obvious, first five-star praise, with a period of time to follow up!	Jingdong prefecture level city in Shandong
Dragon Quality Goods Shop	Green Intelligent Power Saver	The saver has been received. It was plugged into an outlet and used immediately after receipt. Whether it really saves electricity is not clear yet, I believe that this brand of energy saver really saves	Jingdong prefecture level city in Shandong
Dragon Quality Goods Shop	Green Intelligent Power Saver	Okay, okay, okay. Thank you so much. Fast is good. Fast. Item was as described, mainly because the event was cheap and you can save a lot of money.	Jingdong prefecture level city in Shandong
Dragon Quality Goods Shop	Green Intelligent Power Saver	Received it a couple days ago! Plugged it in the same day! Let's see what the electric bill is in a month! Hopefully it works well oh!	Jingdong prefecture level city in Shandong
Dragon Quality Goods Shop	Green Intelligent Power Saver	Looking at the reviews good, energy-saving small products, compact, the results after 2 months to see, because the electricity bill season payment	Jingdong prefecture level city in Shandong
Dragon Quality Goods Shop	Green Intelligent Power Saver	I just received it and will try it out to see if it works well. I'll comment later.	Jingdong prefecture level city in Shandong
Dragon Quality Goods Shop	Green Intelligent Power Saver	It looks a little small and not very aesthetically pleasing, I don't know how effective it will be until I use it	Jingdong prefecture level city in Shandong
Dragon Quality Goods Shop	Green Intelligent Power Saver	Not only to the point of being useless yet	Jingdong prefecture level city in Shandong
Dragon Quality Goods Shop	Green Intelligent Power Saver	Used it for a month, nothing works, the meter you turn very fast. This is not a scam, but also sold on Jingdong. To cheat a bunch of people?	Jingdong prefecture level city in Shandong
Dragon Quality Goods Shop	Green Intelligent Power Saver	Very good to use, every day can save a little electricity, really good under the sample down a month to save a few dozen dollars in electricity, good before I did not know that there is this 👆 haha, the price is not expensive praise.	Jingdong prefecture level city in Shandong
Dragon Quality Goods Shop	Green Intelligent Power Saver	Tried it for ten days and the change is almost nothing, can't see the power saving yet, use it some more days and see how it goes.	Jingdong prefecture level city in Shandong
Dragon Quality Goods Shop	Green Intelligent Power Saver	Received very quickly, the quality is very good, really save electricity, business service attitude is also very good, I hope you do not miss this opportunity, I continue to buy a few in	Jingdong prefecture level city in Shandong
Dragon Quality Goods Shop	Green Intelligent Power Saver	Quality is very good Oh, the delivery is also very fast, a very favorite store, I want to crazy recommend to friends!	Jingdong prefecture level city in Shandong
Dragon Quality Goods Shop	Green Intelligent Power Saver	The goods have just received, fine workmanship, and the same as described by the merchant, to use a period of time to see if it can achieve the purpose of power saving.	Jingdong prefecture level city in Shandong
Dragon Quality Goods Shop	Green Intelligent Power Saver	Received the goods first use a period of time to see if there is no effect, the business commitment to ninety days invalid refund, first praise, good service!	Jingdong prefecture level city in Shandong
Dragon Quality Goods Shop	Green Intelligent Power Saver	The product is used for a month without energy-saving and power-saving effect does not have any effect	Jingdong prefecture level city in Shandong
Dragon Quality Goods Shop	Green Intelligent Power Saver	The baby is really great, I like it very much, my friends also say good to come, recommended to buy!	Jingdong prefecture level city in Shandong
Dragon Quality Goods Shop	Green Intelligent Power Saver	Logistics is fast, courier boy service is good, after using to see how the fruit, and then follow up.	Jingdong prefecture level city in Shandong
Dragon Quality Goods Shop	Green Intelligent Power Saver	Baby has been received to try, I believe that as the business advertised energy saving. The courier is very responsible.	Jingdong prefecture level city in Shandong
Dragon Quality Goods Shop	Green Intelligent Power Saver	Save Electric Energy King received, good quality, beautiful appearance, very satisfied!	Jingdong prefecture level city in Shandong
Dragon Quality Goods Shop	Green Intelligent Power Saver	Three no products no manufacturers no phone pure fraud induced consumption cheat	Jingdong prefecture level city in Shandong
Dragon Quality Goods Shop	Green Intelligent Power Saver	I'm using it now, I don't know if it's as good as it says, I'll follow up after a month	Jingdong prefecture level city in Shandong
Dragon Quality Goods Shop	Green Intelligent Power Saver	Good quality logistics and fast. Fine workmanship. The description is consistent cost-effective	Jingdong prefecture level city in Shandong
Dragon Quality Goods Shop	Green Intelligent Power Saver	Received, whether it is good or not, to wait a few days and then evaluate	Jingdong prefecture level city in Shandong
Dragon Quality Goods Shop	Green Intelligent Power Saver	I bought it and started using it, I won't know if it works well until I pay my electric bill	Jingdong prefecture level city in Shandong
Dragon Quality Goods Shop	Green Intelligent Power Saver	Especially good, energy saving and power saving, especially good quality, good	Jingdong prefecture level city in Shandong
Dragon Quality Goods Shop	Green Intelligent Power Saver	Logistics is strong, the quality is very good, paste really save electricity, as described, will buy back!	Jingdong prefecture level city in Shandong
Dragon Quality Goods Shop	Green Intelligent Power Saver	Great stuff at a great price, worth the money!	Jingdong prefecture level city in Shandong

Dragon Quality Goods Shop	Green Intelligent Power Saver	Received the power saver, very good quality, very power saving!	Jingdong prefecture level city in Shandong
Dragon Quality Goods Shop	Green Intelligent Power Saver	There are no fruits, only samples...	Jingdong prefecture level city in Shandong
Dragon Quality Goods Shop	Green Intelligent Power Saver	Save a few dollars a month.	Jingdong prefecture level city in Shandong
Dragon Quality Goods Shop	Green Intelligent Power Saver	The quality is particularly good, it works well, and the seller has a good service attitude	Jingdong prefecture level city in Shandong
Dragon Quality Goods Shop	Green Intelligent Power Saver	I've compared them a couple of times and I don't feel any difference	Jingdong prefecture level city in Shandong
Dragon Quality Goods Shop	Green Intelligent Power Saver	I don't see the savings. It's a sham.	Jingdong prefecture level city in Shandong
Dragon Quality Goods Shop	Green Intelligent Power Saver	The power saving effect is very good, and the logistics is very fast. The seller's service attitude is very good	Jingdong prefecture level city in Shandong
Dragon Quality Goods Shop	Green Intelligent Power Saver	Received, well packaged, haven't tried it yet!	Jingdong prefecture level city in Shandong
Dragon Quality Goods Shop	Green Intelligent Power Saver	I don't know how it works, I'll comment after I use it.	Jingdong prefecture level city in Shandong
Dragon Quality Goods Shop	Green Intelligent Power Saver	The product doesn't work well enough to be of any use	Jingdong prefecture level city in Shandong
Dragon Quality Goods Shop	Green Intelligent Power Saver	On Trial 。。。。。。。	Jingdong prefecture level city in Shandong
Dragon Quality Goods Shop	Green Intelligent Power Saver	Being used with unknown results	Jingdong prefecture level city in Shandong
Dragon Quality Goods Shop	Green Intelligent Power Saver	Smart power saving and great quality	Jingdong prefecture level city in Shandong
Dragon Quality Goods Shop	Green Intelligent Power Saver	The saver was received, it works very well and the price is affordable!	Jingdong prefecture level city in Shandong
Dragon Quality Goods Shop	Green Intelligent Power Saver	How can I feel the power saving and how long does it last	Jingdong prefecture level city in Shandong
Dragon Quality Goods Shop	Green Intelligent Power Saver	And I know if it saves electricity or not.	Jingdong prefecture level city in Shandong
Dragon Quality Goods Shop	Green Intelligent Power Saver	It won't save any electricity. No.	Jingdong prefecture level city in Shandong
Dragon Quality Goods Shop	Green Intelligent Power Saver	Invalid. Fake.	Jingdong prefecture level city in Shandong
Dragon Quality Goods Shop	Green Intelligent Power Saver	It's good!	Jingdong prefecture level city in Shandong
Dragon Quality Goods Shop	Green Intelligent Power Saver	have no effect	Jingdong prefecture level city in Shandong
Dragon Quality Goods Shop	Green Intelligent Power Saver	Not used.	Jingdong prefecture level city in Shandong
Dragon Quality Goods Shop	Green Intelligent Power Saver	There is no load range, packaging thrift, ten days also did not develop invoices, received the goods with a receipt, hand-written receipt also has no financial stamp, communication for nearly a week a moment to say that the electronic invoice, a moment to say that the paper invoice, said it is mailed to me, ten days also did not see anything, the invoice invoice can not be found to say that did not stay on file, single single number can not be found, said that the warehouse more than one to a one to find out, in addition to the perfunctory or perfunctory. Products are no certificate of conformity, proper three	Jingdong prefecture level city in Shandong
Dragon Quality Goods Shop	Green Intelligent Power Saver	Satisfaction.	Jingdong prefecture level city in Shandong
Dragon Quality Goods Shop	Green Intelligent Power Saver	There is not a bit of power saving effect, and ringing up an increase in the electricity bill, because you have to add the cost of this machine's own electricity consumption and purchase, so I think it is better to use this machine instead of increasing the expenditure, uneconomical, rather than not use it.	Jingdong prefecture level city in Shandong
Dragon Quality Goods Shop	Green Intelligent Power Saver	I've been using it for two days now and I don't feel any effect.	Jingdong prefecture level city in Shandong
Dragon Quality Goods Shop	Green Intelligent Power Saver	What it looked like was nothing like what was sent.	Jingdong prefecture level city in Shandong
Dragon Quality Goods Shop	Green Intelligent Power Saver	It didn't work. It didn't work for a month or so.	Jingdong prefecture level city in Shandong
Dragon Quality Goods Shop	Green Intelligent Power Saver	Garbage products, physical and description is not the same, no effect at all, pit # no discussion, to buy the fine, garbage, garbage, garbage	Jingdong prefecture level city in Shandong
Dragon Quality Goods Shop	Green Intelligent Power Saver	general	Jingdong prefecture level city in Shandong
Dragon Quality Goods Shop	Green Intelligent Power Saver	IQ tax. Instead of saving electricity, it increases the power consumption of an appliance.	Jingdong prefecture level city in Shandong
Dragon Quality Goods Shop	Green Intelligent Power Saver	There's no trash at all.	Jingdong prefecture level city in Shandong
Dragon Quality Goods Shop	Green Intelligent Power Saver	It doesn't do anything.	Jingdong prefecture level city in Shandong
Dragon Quality Goods Shop	Green Intelligent Power Saver	(of an unmarried couple) be close	Jingdong prefecture level city in Shandong
Dragon Quality Goods Shop	Green Intelligent Power Saver	(of an unmarried couple) be close	Jingdong prefecture level city in Shandong
Dragon Quality Goods Shop	Green Intelligent Power Saver	no electricity saving	Jingdong prefecture level city in Shandong
Dragon Quality Goods Shop	Green Intelligent Power Saver	differ from	Jingdong prefecture level city in Shandong
Dragon Quality Goods Shop	Green Intelligent Power Saver	Saver has received, but real can save electricity, the quality is very good, I times I buy a few more, too good.	Jingdong prefecture level city in Shandong
Dragon Quality Goods Shop	Green Intelligent Power Saver	Great quality as always, a very favorite product, great! o(*￣︶￣*)o	Jingdong prefecture level city in Shandong
Dragon Quality Goods Shop	Green Intelligent Power Saver	If we can reduce the electricity bill, for individuals can save money, for the country can protect the environment, now the weather is getting hotter and hotter, to protect the environment to reduce emissions is very important. Already use the product, whether to reduce the electricity bill depends on next month!	Jingdong prefecture level city in Shandong
Dragon Quality Goods Shop	Green Intelligent Power Saver	Purchased a power saver received on the put into use, rent a room with many electrical appliances, power consumption is very large, we have a piece of electricity or 1.2 dollars a time, with this power saver is much better, every day can save a few cents, very convenient and practical!	Jingdong prefecture level city in Shandong
Dragon Quality Goods Shop	Green Intelligent Power Saver	The product is better to use before not to use when a month 1500 yuan electricity bill, now using the 5 second month electricity bill directly down 470 dollars or very good. A save money to buy several power saver money ZanZanZanZan👍	Jingdong prefecture level city in Shandong
Dragon Quality Goods Shop	Green Intelligent Power Saver	The moment I received the goods really satisfied with this product, not only high-grade packaging, the body are base alloy, up to the price, I was also introduced by a friend, he used last month did save a lot of electricity, but also recommended to me!	Jingdong prefecture level city in Shandong
Dragon Quality Goods Shop	Green Intelligent Power Saver	I got it. Good review. I'm very satisfied. Reasonable price.	Jingdong prefecture level city in Shandong
Dragon Quality Goods Shop	Green Intelligent Power Saver	Intelligent power saver received and used, looking at the quality is good, the quality of the plug sent is also very good. Expect the effect of the economizer is obvious, first five-star praise, with a period of time to follow up!	Jingdong prefecture level city in Shandong
Dragon Quality Goods Shop	Green Intelligent Power Saver	The saver has been received. It was plugged into an outlet and used immediately after receipt. Whether it really saves electricity is not clear yet, I believe that this brand of energy saver really saves	Jingdong prefecture level city in Shandong
Dragon Quality Goods Shop	Green Intelligent Power Saver	Okay, okay, okay. Thank you so much. Fast is good. Fast. Item was as described, mainly because the event was cheap and you can save a lot of money.	Jingdong prefecture level city in Shandong

Dragon Quality Goods Shop	Green Intelligent Power Saver	Received it a couple days ago! Plugged it in the same day! Let's see what the electric bill is in a month! Hopefully it works well oh!	Jingdong prefecture level city in Shandong
Dragon Quality Goods Shop	Green Intelligent Power Saver	Looking at the reviews good, energy-saving small products, compact, the results after 2 months to see, because the electricity bill season payment	Jingdong prefecture level city in Shandong
Dragon Quality Goods Shop	Green Intelligent Power Saver	I just received it and will try it out to see if it works well. I'll comment later.	Jingdong prefecture level city in Shandong
Dragon Quality Goods Shop	Green Intelligent Power Saver	It looks a little small and not very aesthetically pleasing, I don't know how effective it will be until I use it	Jingdong prefecture level city in Shandong
Dragon Quality Goods Shop	Green Intelligent Power Saver	Not only to the point of being useless yet	Jingdong prefecture level city in Shandong
Dragon Quality Goods Shop	Green Intelligent Power Saver	Used it for a month, nothing works, the meter you turn very fast. This is not a scam, but also sold on Jingdong. To cheat a bunch of people?	Jingdong prefecture level city in Shandong
Dragon Quality Goods Shop	Green Intelligent Power Saver	Very good to use, every day can save a little electricity, really good under the sample down a month to save a few dozen dollars in electricity, good before I did not know that there is this haha, the price is not expensive praise.	Jingdong prefecture level city in Shandong
Dragon Quality Goods Shop	Green Intelligent Power Saver	Tried it for ten days and the change is almost nothing, can't see the power saving yet, use it some more days and see how it goes.	Jingdong prefecture level city in Shandong
Dragon Quality Goods Shop	Green Intelligent Power Saver	Received very quickly, the quality is very good, really save electricity, business service attitude is also very good, I hope you do not miss this opportunity, I continue to buy a few in	Jingdong prefecture level city in Shandong
Dragon Quality Goods Shop	Green Intelligent Power Saver	Quality is very good Oh, the delivery is also very fast, a very favorite store, I want to crazy recommend to friends!	Jingdong prefecture level city in Shandong
Dragon Quality Goods Shop	Green Intelligent Power Saver	The goods have just received, fine workmanship, and the same as described by the merchant, to use a period of time to see if it can achieve the purpose of power saving.	Jingdong prefecture level city in Shandong
Dragon Quality Goods Shop	Green Intelligent Power Saver	Received the goods first use a period of time to see if there is no effect, the business commitment to ninety days invalid refund, first praise, good service!	Jingdong prefecture level city in Shandong
Dragon Quality Goods Shop	Green Intelligent Power Saver	The product is used for a month without energy-saving and power-saving effect does not have any effect	Jingdong prefecture level city in Shandong
Dragon Quality Goods Shop	Green Intelligent Power Saver	The baby is really great, I like it very much, my friends also say good to come, recommended to buy!	Jingdong prefecture level city in Shandong
Dragon Quality Goods Shop	Green Intelligent Power Saver	Logistics is fast, courier boy service is good, after using to see how the fruit, and then follow up.	Jingdong prefecture level city in Shandong
Dragon Quality Goods Shop	Green Intelligent Power Saver	Baby has been received to try, I believe that as the business advertised energy saving. The courier is very responsible.	Jingdong prefecture level city in Shandong
Dragon Quality Goods Shop	Green Intelligent Power Saver	Save Electric Energy King received, good quality, beautiful appearance, very satisfied!	Jingdong prefecture level city in Shandong
Dragon Quality Goods Shop	Green Intelligent Power Saver	Three no products no manufacturers no phone pure fraud induced consumption cheat	Jingdong prefecture level city in Shandong
Dragon Quality Goods Shop	Green Intelligent Power Saver	I'm using it now, I don't know if it's as good as it says, I'll follow up after a month	Jingdong prefecture level city in Shandong
Dragon Quality Goods Shop	Green Intelligent Power Saver	Good quality logistics and fast. Fine workmanship. The description is consistent cost-effective	Jingdong prefecture level city in Shandong
Dragon Quality Goods Shop	Green Intelligent Power Saver	Received, whether it is good or not, to wait a few days and then evaluate	Jingdong prefecture level city in Shandong
Dragon Quality Goods Shop	Green Intelligent Power Saver	I bought it and started using it, I won't know if it works well until I pay my electric bill	Jingdong prefecture level city in Shandong
Dragon Quality Goods Shop	Green Intelligent Power Saver	Especially good, energy saving and power saving, especially good quality, good	Jingdong prefecture level city in Shandong
Dragon Quality Goods Shop	Green Intelligent Power Saver	Logistics is strong, the quality is very good, paste really save electricity, as described, will buy back!	Jingdong prefecture level city in Shandong
Dragon Quality Goods Shop	Green Intelligent Power Saver	Great stuff at a great price, worth the money!	Jingdong prefecture level city in Shandong
Dragon Quality Goods Shop	Green Intelligent Power Saver	Received the power saver, very good quality, very power saving!	Jingdong prefecture level city in Shandong
Dragon Quality Goods Shop	Green Intelligent Power Saver	There are no fruits, only samples...	Jingdong prefecture level city in Shandong
Dragon Quality Goods Shop	Green Intelligent Power Saver	Save a few dollars a month.	Jingdong prefecture level city in Shandong
Dragon Quality Goods Shop	Green Intelligent Power Saver	The quality is particularly good, it works well, and the seller has a good service attitude	Jingdong prefecture level city in Shandong
Dragon Quality Goods Shop	Green Intelligent Power Saver	I've compared them a couple of times and I don't feel any difference	Jingdong prefecture level city in Shandong
Dragon Quality Goods Shop	Green Intelligent Power Saver	I don't see the savings. It's a sham.	Jingdong prefecture level city in Shandong
Dragon Quality Goods Shop	Green Intelligent Power Saver	The power saving effect is very good, and the logistics is very fast. The seller's service attitude is very good	Jingdong prefecture level city in Shandong
Dragon Quality Goods Shop	Green Intelligent Power Saver	Received, well packaged, haven't tried it yet!	Jingdong prefecture level city in Shandong
Dragon Quality Goods Shop	Green Intelligent Power Saver	I don't know how it works, I'll comment after I use it.	Jingdong prefecture level city in Shandong
Dragon Quality Goods Shop	Green Intelligent Power Saver	The product doesn't work well enough to be of any use	Jingdong prefecture level city in Shandong
Dragon Quality Goods Shop	Green Intelligent Power Saver	On Trial 。。。。。。。	Jingdong prefecture level city in Shandong
Dragon Quality Goods Shop	Green Intelligent Power Saver	Being used with unknown results	Jingdong prefecture level city in Shandong
Dragon Quality Goods Shop	Green Intelligent Power Saver	Smart power saving and great quality	Jingdong prefecture level city in Shandong
Dragon Quality Goods Shop	Green Intelligent Power Saver	The saver was received, it works very well and the price is affordable!	Jingdong prefecture level city in Shandong
Dragon Quality Goods Shop	Green Intelligent Power Saver	How can I feel the power saving and how long does it last	Jingdong prefecture level city in Shandong
Dragon Quality Goods Shop	Green Intelligent Power Saver	And I know if it saves electricity or not.	Jingdong prefecture level city in Shandong
Dragon Quality Goods Shop	Green Intelligent Power Saver	It won't save any electricity. No.	Jingdong prefecture level city in Shandong
Dragon Quality Goods Shop	Green Intelligent Power Saver	Invalid. Fake.	Jingdong prefecture level city in Shandong
Dragon Quality Goods Shop	Green Intelligent Power Saver	It's good!	Jingdong prefecture level city in Shandong
Dragon Quality Goods Shop	Green Intelligent Power Saver	have no effect	Jingdong prefecture level city in Shandong
Dragon Quality Goods Shop	Green Intelligent Power Saver	Not used.	Jingdong prefecture level city in Shandong
Dragon Quality Goods Shop	Green Intelligent Power Saver	There is no load range, packaging thrift, ten days also did not develop invoices, received the goods with a receipt, hand-written receipt also has no financial stamp, communication for nearly a week a moment to say that the electronic invoice, a moment to say that the paper invoice, said it is mailed to me, ten days also did not see anything, the invoice invoice can not be found to say that did not stay on file, single single number can not be found, said that the warehouse more than one to a one to find out, in addition to the perfunctory or perfunctory. Products are no certificate of conformity, proper three	Jingdong prefecture level city in Shandong
Dragon Quality Goods Shop	Green Intelligent Power Saver	Satisfaction.	Jingdong prefecture level city in Shandong

Dragon Quality Goods Shop	Green Intelligent Power Saver	There is not a bit of power saving effect, and ringing up an increase in the electricity bill, because you have to add the cost of this machine's own electricity consumption and purchase, so I think it is better to use this machine instead of increasing the expenditure, uneconomical, rather than not use it.	Jingdong prefecture level city in Shandong
Dragon Quality Goods Shop	Green Intelligent Power Saver	I've been using it for two days now and I don't feel any effect.	Jingdong prefecture level city in Shandong
Dragon Quality Goods Shop	Green Intelligent Power Saver	What it looked like was nothing like what was sent.	Jingdong prefecture level city in Shandong
Dragon Quality Goods Shop	Green Intelligent Power Saver	It didn't work. It didn't work for a month or so.	Jingdong prefecture level city in Shandong
Dragon Quality Goods Shop	Green Intelligent Power Saver	Garbage products, physical and description is not the same, no effect at all, pit # no discussion, to buy the fine, garbage, garbage, garbage	Jingdong prefecture level city in Shandong
Dragon Quality Goods Shop	Green Intelligent Power Saver	general	Jingdong prefecture level city in Shandong
Dragon Quality Goods Shop	Green Intelligent Power Saver	IQ tax. Instead of saving electricity, it increases the power consumption of an appliance.	Jingdong prefecture level city in Shandong
Dragon Quality Goods Shop	Green Intelligent Power Saver	There's no trash at all.	Jingdong prefecture level city in Shandong
Dragon Quality Goods Shop	Green Intelligent Power Saver	It doesn't do anything.	Jingdong prefecture level city in Shandong
Dragon Quality Goods Shop	Green Intelligent Power Saver	(of an unmarried couple) be close	Jingdong prefecture level city in Shandong
Dragon Quality Goods Shop	Green Intelligent Power Saver	(of an unmarried couple) be close	Jingdong prefecture level city in Shandong
Dragon Quality Goods Shop	Green Intelligent Power Saver	no electricity saving	Jingdong prefecture level city in Shandong
Dragon Quality Goods Shop	Green Intelligent Power Saver	differ from	Jingdong prefecture level city in Shandong
Dragon Quality Goods Shop	Green Intelligent Power Saver	Saver has received, but real can save electricity, the quality is very good, I times I buy a few more, too good.	Jingdong prefecture level city in Shandong
Dragon Quality Goods Shop	Green Intelligent Power Saver	Great quality as always, a very favorite product, great! o(*￣︶￣*)o	Jingdong prefecture level city in Shandong
Dragon Quality Goods Shop	Green Intelligent Power Saver	If we can reduce the electricity bill, for individuals can save money, for the country can protect the environment, now the weather is getting hotter and hotter, to protect the environment to reduce emissions is very important. Already use the product, whether to reduce the electricity bill depends on next month!	Jingdong prefecture level city in Shandong
Dragon Quality Goods Shop	Green Intelligent Power Saver	Purchased a power saver received on the put into use, rent a room with many electrical appliances, power consumption is very large, we have a piece of electricity or 1.2 dollars a time, with this power saver is much better, every day can save a few cents, very convenient and practical!	Jingdong prefecture level city in Shandong
Dragon Quality Goods Shop	Green Intelligent Power Saver	The product is better to use before not to use when a month 1500 yuan electricity bill, now using the 5 second month electricity bill directly down 470 dollars or very good. A save money to buy several power saver money ZanZanZanZan👍	Jingdong prefecture level city in Shandong
Dragon Quality Goods Shop	Green Intelligent Power Saver	The moment I received the goods really satisfied with this product, not only high-grade packaging, the body are base alloy, up to the price, I was also introduced by a friend, he used last month did save a lot of electricity, but also recommended to me!	Jingdong prefecture level city in Shandong
Dragon Quality Goods Shop	Green Intelligent Power Saver	I got it. Good review. I'm very satisfied. Reasonable price.	Jingdong prefecture level city in Shandong
Dragon Quality Goods Shop	Green Intelligent Power Saver	Intelligent power saver received and used, looking at the quality is good, the quality of the plug sent is also very good. Expect the effect of the economizer is obvious, first five-star praise, with a period of time to follow up!	Jingdong prefecture level city in Shandong
Dragon Quality Goods Shop	Green Intelligent Power Saver	The saver has been received. It was plugged into an outlet and used immediately after receipt. Whether it really saves electricity is not clear yet, I believe that this brand of energy saver really saves	Jingdong prefecture level city in Shandong
Dragon Quality Goods Shop	Green Intelligent Power Saver	Okay, okay, okay. Thank you so much. Fast is good. Fast. Item was as described, mainly because the event was cheap and you can save a lot of money.	Jingdong prefecture level city in Shandong
Dragon Quality Goods Shop	Green Intelligent Power Saver	Received it a couple days ago! Plugged it in the same day! Let's see what the electric bill is in a month! Hopefully it works well oh!	Jingdong prefecture level city in Shandong
Dragon Quality Goods Shop	Green Intelligent Power Saver	Looking at the reviews good, energy-saving small products, compact, the results after 2 months to see, because the electricity bill season payment	Jingdong prefecture level city in Shandong
Dragon Quality Goods Shop	Green Intelligent Power Saver	I just received it and will try it out to see if it works well. I'll comment later.	Jingdong prefecture level city in Shandong
Dragon Quality Goods Shop	Green Intelligent Power Saver	It looks a little small and not very aesthetically pleasing, I don't know how effective it will be until I use it	Jingdong prefecture level city in Shandong
Dragon Quality Goods Shop	Green Intelligent Power Saver	Not only to the point of being useless yet	Jingdong prefecture level city in Shandong
Dragon Quality Goods Shop	Green Intelligent Power Saver	Used it for a month, nothing works, the meter you turn very fast. This is not a scam, but also sold on Jingdong. To cheat a bunch of people?	Jingdong prefecture level city in Shandong
Dragon Quality Goods Shop	Green Intelligent Power Saver	Very good to use, every day can save a little electricity, really good under the sample down a month to save a few dozen dollars in electricity, good before I did not know that there is this 👆 haha, the price is not expensive praise.	Jingdong prefecture level city in Shandong
Dragon Quality Goods Shop	Green Intelligent Power Saver	Tried it for ten days and the change is almost nothing, can't see the power saving yet, use it some more days and see how it goes.	Jingdong prefecture level city in Shandong
Dragon Quality Goods Shop	Green Intelligent Power Saver	Received very quickly, the quality is very good, really save electricity, business service attitude is also very good, I hope you do not miss this opportunity, I continue to buy a few in	Jingdong prefecture level city in Shandong
Dragon Quality Goods Shop	Green Intelligent Power Saver	Quality is very good Oh, the delivery is also very fast, a very favorite store, I want to crazy recommend to friends!	Jingdong prefecture level city in Shandong
Dragon Quality Goods Shop	Green Intelligent Power Saver	The goods have just received, fine workmanship, and the same as described by the merchant, to use a period of time to see if it can achieve the purpose of power saving.	Jingdong prefecture level city in Shandong
Dragon Quality Goods Shop	Green Intelligent Power Saver	Received the goods first use a period of time to see if there is no effect, the business commitment to ninety days invalid refund, first praise, good service!	Jingdong prefecture level city in Shandong
Dragon Quality Goods Shop	Green Intelligent Power Saver	The product is used for a month without energy-saving and power-saving effect does not have any effect	Jingdong prefecture level city in Shandong
Dragon Quality Goods Shop	Green Intelligent Power Saver	The baby is really great, I like it very much, my friends also say good to come, recommended to buy!	Jingdong prefecture level city in Shandong
Dragon Quality Goods Shop	Green Intelligent Power Saver	Logistics is fast, courier boy service is good, after using to see how the fruit, and then follow up.	Jingdong prefecture level city in Shandong
Dragon Quality Goods Shop	Green Intelligent Power Saver	Baby has been received to try, I believe that as the business advertised energy saving. The courier is very responsible.	Jingdong prefecture level city in Shandong
Dragon Quality Goods Shop	Green Intelligent Power Saver	Save Electric Energy King received, good quality, beautiful appearance, very satisfied!	Jingdong prefecture level city in Shandong
Dragon Quality Goods Shop	Green Intelligent Power Saver	Three no products no manufacturers no phone pure fraud induced consumption cheat	Jingdong prefecture level city in Shandong
Dragon Quality Goods Shop	Green Intelligent Power Saver	I'm using it now, I don't know if it's as good as it says, I'll follow up after a month	Jingdong prefecture level city in Shandong
Dragon Quality Goods Shop	Green Intelligent Power Saver	Good quality logistics and fast. Fine workmanship. The description is consistent cost-effective	Jingdong prefecture level city in Shandong
Dragon Quality Goods Shop	Green Intelligent Power Saver	Received, whether it is good or not, to wait a few days and then evaluate	Jingdong prefecture level city in Shandong
Dragon Quality Goods Shop	Green Intelligent Power Saver	I bought it and started using it, I won't know if it works well until I pay my electric bill	Jingdong prefecture level city in Shandong
Dragon Quality Goods Shop	Green Intelligent Power Saver	Especially good, energy saving and power saving, especially good quality, good	Jingdong prefecture level city in Shandong
Dragon Quality Goods Shop	Green Intelligent Power Saver	Logistics is strong, the quality is very good, paste really save electricity, as described, will buy back!	Jingdong prefecture level city in Shandong
Dragon Quality Goods Shop	Green Intelligent Power Saver	Great stuff at a great price, worth the money!	Jingdong prefecture level city in Shandong

Dragon Quality Goods Shop	Green Intelligent Power Saver	Received the power saver, very good quality, very power saving!	Jingdong prefecture level city in Shandong
Dragon Quality Goods Shop	Green Intelligent Power Saver	There are no fruits, only samples...	Jingdong prefecture level city in Shandong
Dragon Quality Goods Shop	Green Intelligent Power Saver	Save a few dollars a month.	Jingdong prefecture level city in Shandong
Dragon Quality Goods Shop	Green Intelligent Power Saver	The quality is particularly good, it works well, and the seller has a good service attitude	Jingdong prefecture level city in Shandong
Dragon Quality Goods Shop	Green Intelligent Power Saver	I've compared them a couple of times and I don't feel any difference	Jingdong prefecture level city in Shandong
Dragon Quality Goods Shop	Green Intelligent Power Saver	I don't see the savings. It's a sham.	Jingdong prefecture level city in Shandong
Dragon Quality Goods Shop	Green Intelligent Power Saver	The power saving effect is very good, and the logistics is very fast. The seller's service attitude is very good	Jingdong prefecture level city in Shandong
Dragon Quality Goods Shop	Green Intelligent Power Saver	Received, well packaged, haven't tried it yet!	Jingdong prefecture level city in Shandong
Dragon Quality Goods Shop	Green Intelligent Power Saver	I don't know how it works, I'll comment after I use it.	Jingdong prefecture level city in Shandong
Dragon Quality Goods Shop	Green Intelligent Power Saver	The product doesn't work well enough to be of any use	Jingdong prefecture level city in Shandong
Dragon Quality Goods Shop	Green Intelligent Power Saver	On Trial 。。。。。。。	Jingdong prefecture level city in Shandong
Dragon Quality Goods Shop	Green Intelligent Power Saver	Being used with unknown results	Jingdong prefecture level city in Shandong
Dragon Quality Goods Shop	Green Intelligent Power Saver	Smart power saving and great quality	Jingdong prefecture level city in Shandong
Dragon Quality Goods Shop	Green Intelligent Power Saver	The saver was received, it works very well and the price is affordable!	Jingdong prefecture level city in Shandong
Dragon Quality Goods Shop	Green Intelligent Power Saver	How can I feel the power saving and how long does it last	Jingdong prefecture level city in Shandong
Dragon Quality Goods Shop	Green Intelligent Power Saver	And I know if it saves electricity or not.	Jingdong prefecture level city in Shandong
Dragon Quality Goods Shop	Green Intelligent Power Saver	It won't save any electricity. No.	Jingdong prefecture level city in Shandong
Dragon Quality Goods Shop	Green Intelligent Power Saver	Invalid. Fake.	Jingdong prefecture level city in Shandong
Dragon Quality Goods Shop	Green Intelligent Power Saver	It's good!	Jingdong prefecture level city in Shandong
Dragon Quality Goods Shop	Green Intelligent Power Saver	have no effect	Jingdong prefecture level city in Shandong
Dragon Quality Goods Shop	Green Intelligent Power Saver	Not used.	Jingdong prefecture level city in Shandong
Dragon Quality Goods Shop	Green Intelligent Power Saver	There is no load range, packaging thrift, ten days also did not develop invoices, received the goods with a receipt, hand-written receipt also has no financial stamp, communication for nearly a week a moment to say that the electronic invoice, a moment to say that the paper invoice, said it is mailed to me, ten days also did not see anything, the invoice invoice can not be found to say that did not stay on file, single single number can not be found, said that the warehouse more than one to a one to find out, in addition to the perfunctory or perfunctory. Products are no certificate of conformity, proper three	Jingdong prefecture level city in Shandong
Dragon Quality Goods Shop	Green Intelligent Power Saver	Satisfaction.	Jingdong prefecture level city in Shandong
Dragon Quality Goods Shop	Green Intelligent Power Saver	There is not a bit of power saving effect, and ringing up an increase in the electricity bill, because you have to add the cost of this machine's own electricity consumption and purchase, so I think it is better to use this machine instead of increasing the expenditure, uneconomical, rather than not use it.	Jingdong prefecture level city in Shandong
Dragon Quality Goods Shop	Green Intelligent Power Saver	I've been using it for two days now and I don't feel any effect.	Jingdong prefecture level city in Shandong
Dragon Quality Goods Shop	Green Intelligent Power Saver	What it looked like was nothing like what was sent.	Jingdong prefecture level city in Shandong
Dragon Quality Goods Shop	Green Intelligent Power Saver	It didn't work. It didn't work for a month or so.	Jingdong prefecture level city in Shandong
Dragon Quality Goods Shop	Green Intelligent Power Saver	Garbage products, physical and description is not the same, no effect at all, pit # no discussion, to buy the fine, garbage, garbage, garbage	Jingdong prefecture level city in Shandong
Dragon Quality Goods Shop	Green Intelligent Power Saver	general	Jingdong prefecture level city in Shandong
Dragon Quality Goods Shop	Green Intelligent Power Saver	IQ tax. Instead of saving electricity, it increases the power consumption of an appliance.	Jingdong prefecture level city in Shandong
Dragon Quality Goods Shop	Green Intelligent Power Saver	There's no trash at all.	Jingdong prefecture level city in Shandong
Dragon Quality Goods Shop	Green Intelligent Power Saver	It doesn't do anything.	Jingdong prefecture level city in Shandong
Dragon Quality Goods Shop	Green Intelligent Power Saver	(of an unmarried couple) be close	Jingdong prefecture level city in Shandong
Dragon Quality Goods Shop	Green Intelligent Power Saver	(of an unmarried couple) be close	Jingdong prefecture level city in Shandong
Dragon Quality Goods Shop	Green Intelligent Power Saver	no electricity saving	Jingdong prefecture level city in Shandong
Dragon Quality Goods Shop	Green Intelligent Power Saver	differ from	Jingdong prefecture level city in Shandong
Dragon Quality Goods Shop	Green Intelligent Power Saver	Saver has received, but real can save electricity, the quality is very good, I times I buy a few more, too good.	Jingdong prefecture level city in Shandong
Dragon Quality Goods Shop	Green Intelligent Power Saver	Great quality as always, a very favorite product, great! o(*￣︶￣*)o	Jingdong prefecture level city in Shandong
Dragon Quality Goods Shop	Green Intelligent Power Saver	If we can reduce the electricity bill, for individuals can save money, for the country can protect the environment, now the weather is getting hotter and hotter, to protect the environment to reduce emissions is very important. Already use the product, whether to reduce the electricity bill depends on next month!	Jingdong prefecture level city in Shandong
Dragon Quality Goods Shop	Green Intelligent Power Saver	Purchased a power saver received on the put into use, rent a room with many electrical appliances, power consumption is very large, we have a piece of electricity or 1.2 dollars a time, with this power saver is much better, every day can save a few cents, very convenient and practical!	Jingdong prefecture level city in Shandong
Dragon Quality Goods Shop	Green Intelligent Power Saver	The product is better to use before not to use when a month 1500 yuan electricity bill, now using the 5 second month electricity bill directly down 470 dollars or very good. A save money to buy several power saver money ZanZanZanZan👍	Jingdong prefecture level city in Shandong
Dragon Quality Goods Shop	Green Intelligent Power Saver	The moment I received the goods really satisfied with this product, not only high-grade packaging, the body are base alloy, up to the price, I was also introduced by a friend, he used last month did save a lot of electricity, but also recommended to me!	Jingdong prefecture level city in Shandong
Dragon Quality Goods Shop	Green Intelligent Power Saver	I got it. Good review. I'm very satisfied. Reasonable price.	Jingdong prefecture level city in Shandong
Dragon Quality Goods Shop	Green Intelligent Power Saver	Intelligent power saver received and used, looking at the quality is good, the quality of the plug sent is also very good. Expect the effect of the economizer is obvious, first five-star praise, with a period of time to follow up!	Jingdong prefecture level city in Shandong
Dragon Quality Goods Shop	Green Intelligent Power Saver	The saver has been received. It was plugged into an outlet and used immediately after receipt. Whether it really saves electricity is not clear yet, I believe that this brand of energy saver really saves	Jingdong prefecture level city in Shandong
Dragon Quality Goods Shop	Green Intelligent Power Saver	Okay, okay, okay. Thank you so much. Fast is good. Fast. Item was as described, mainly because the event was cheap and you can save a lot of money.	Jingdong prefecture level city in Shandong

Dragon Quality Goods Shop	Green Intelligent Power Saver	Received it a couple days ago! Plugged it in the same day! Let's see what the electric bill is in a month! Hopefully it works well oh!	Jingdong prefecture level city in Shandong
Dragon Quality Goods Shop	Green Intelligent Power Saver	Looking at the reviews good, energy-saving small products, compact, the results after 2 months to see, because the electricity bill season payment	Jingdong prefecture level city in Shandong
Dragon Quality Goods Shop	Green Intelligent Power Saver	I just received it and will try it out to see if it works well. I'll comment later.	Jingdong prefecture level city in Shandong
Dragon Quality Goods Shop	Green Intelligent Power Saver	It looks a little small and not very aesthetically pleasing, I don't know how effective it will be until I use it	Jingdong prefecture level city in Shandong
Dragon Quality Goods Shop	Green Intelligent Power Saver	Not only to the point of being useless yet	Jingdong prefecture level city in Shandong
Dragon Quality Goods Shop	Green Intelligent Power Saver	Used it for a month, nothing works, the meter you turn very fast. This is not a scam, but also sold on Jingdong. To cheat a bunch of people?	Jingdong prefecture level city in Shandong
Dragon Quality Goods Shop	Green Intelligent Power Saver	Very good to use, every day can save a little electricity, really good under the sample down a month to save a few dozen dollars in electricity, good before I did not know that there is this haha, the price is not expensive praise.	Jingdong prefecture level city in Shandong
Dragon Quality Goods Shop	Green Intelligent Power Saver	Tried it for ten days and the change is almost nothing, can't see the power saving yet, use it some more days and see how it goes.	Jingdong prefecture level city in Shandong
Dragon Quality Goods Shop	Green Intelligent Power Saver	Received very quickly, the quality is very good, really save electricity, business service attitude is also very good, I hope you do not miss this opportunity, I continue to buy a few in	Jingdong prefecture level city in Shandong
Dragon Quality Goods Shop	Green Intelligent Power Saver	Quality is very good Oh, the delivery is also very fast, a very favorite store, I want to crazy recommend to friends!	Jingdong prefecture level city in Shandong
Dragon Quality Goods Shop	Green Intelligent Power Saver	The goods have just received, fine workmanship, and the same as described by the merchant, to use a period of time to see if it can achieve the purpose of power saving.	Jingdong prefecture level city in Shandong
Dragon Quality Goods Shop	Green Intelligent Power Saver	Received the goods first use a period of time to see if there is no effect, the business commitment to ninety days invalid refund, first praise, good service!	Jingdong prefecture level city in Shandong
Dragon Quality Goods Shop	Green Intelligent Power Saver	The product is used for a month without energy-saving and power-saving effect does not have any effect	Jingdong prefecture level city in Shandong
Dragon Quality Goods Shop	Green Intelligent Power Saver	The baby is really great, I like it very much, my friends also say good to come, recommended to buy!	Jingdong prefecture level city in Shandong
Dragon Quality Goods Shop	Green Intelligent Power Saver	Logistics is fast, courier boy service is good, after using to see how the fruit, and then follow up.	Jingdong prefecture level city in Shandong
Dragon Quality Goods Shop	Green Intelligent Power Saver	Baby has been received to try, I believe that as the business advertised energy saving. The courier is very responsible.	Jingdong prefecture level city in Shandong
Dragon Quality Goods Shop	Green Intelligent Power Saver	Save Electric Energy King received, good quality, beautiful appearance, very satisfied!	Jingdong prefecture level city in Shandong
Dragon Quality Goods Shop	Green Intelligent Power Saver	Three no products no manufacturers no phone pure fraud induced consumption cheat	Jingdong prefecture level city in Shandong
Dragon Quality Goods Shop	Green Intelligent Power Saver	I'm using it now, I don't know if it's as good as it says, I'll follow up after a month	Jingdong prefecture level city in Shandong
Dragon Quality Goods Shop	Green Intelligent Power Saver	Good quality logistics and fast. Fine workmanship. The description is consistent cost-effective	Jingdong prefecture level city in Shandong
Dragon Quality Goods Shop	Green Intelligent Power Saver	Received, whether it is good or not, to wait a few days and then evaluate	Jingdong prefecture level city in Shandong
Dragon Quality Goods Shop	Green Intelligent Power Saver	I bought it and started using it, I won't know if it works well until I pay my electric bill	Jingdong prefecture level city in Shandong
Dragon Quality Goods Shop	Green Intelligent Power Saver	Especially good, energy saving and power saving, especially good quality, good	Jingdong prefecture level city in Shandong
Dragon Quality Goods Shop	Green Intelligent Power Saver	Logistics is strong, the quality is very good, paste really save electricity, as described, will buy back!	Jingdong prefecture level city in Shandong
Dragon Quality Goods Shop	Green Intelligent Power Saver	Great stuff at a great price, worth the money!	Jingdong prefecture level city in Shandong
Dragon Quality Goods Shop	Green Intelligent Power Saver	Received the power saver, very good quality, very power saving!	Jingdong prefecture level city in Shandong
Dragon Quality Goods Shop	Green Intelligent Power Saver	There are no fruits, only samples...	Jingdong prefecture level city in Shandong
Dragon Quality Goods Shop	Green Intelligent Power Saver	Save a few dollars a month.	Jingdong prefecture level city in Shandong
Dragon Quality Goods Shop	Green Intelligent Power Saver	The quality is particularly good, it works well, and the seller has a good service attitude	Jingdong prefecture level city in Shandong
Dragon Quality Goods Shop	Green Intelligent Power Saver	I've compared them a couple of times and I don't feel any difference	Jingdong prefecture level city in Shandong
Dragon Quality Goods Shop	Green Intelligent Power Saver	I don't see the savings. It's a sham.	Jingdong prefecture level city in Shandong
Dragon Quality Goods Shop	Green Intelligent Power Saver	The power saving effect is very good, and the logistics is very fast. The seller's service attitude is very good	Jingdong prefecture level city in Shandong
Dragon Quality Goods Shop	Green Intelligent Power Saver	Received, well packaged, haven't tried it yet!	Jingdong prefecture level city in Shandong
Dragon Quality Goods Shop	Green Intelligent Power Saver	I don't know how it works, I'll comment after I use it.	Jingdong prefecture level city in Shandong
Dragon Quality Goods Shop	Green Intelligent Power Saver	The product doesn't work well enough to be of any use	Jingdong prefecture level city in Shandong
Dragon Quality Goods Shop	Green Intelligent Power Saver	On Trial 。。。。。。。	Jingdong prefecture level city in Shandong
Dragon Quality Goods Shop	Green Intelligent Power Saver	Being used with unknown results	Jingdong prefecture level city in Shandong
Dragon Quality Goods Shop	Green Intelligent Power Saver	Smart power saving and great quality	Jingdong prefecture level city in Shandong
Dragon Quality Goods Shop	Green Intelligent Power Saver	The saver was received, it works very well and the price is affordable!	Jingdong prefecture level city in Shandong
Dragon Quality Goods Shop	Green Intelligent Power Saver	How can I feel the power saving and how long does it last	Jingdong prefecture level city in Shandong
Dragon Quality Goods Shop	Green Intelligent Power Saver	And I know if it saves electricity or not.	Jingdong prefecture level city in Shandong
Dragon Quality Goods Shop	Green Intelligent Power Saver	It won't save any electricity. No.	Jingdong prefecture level city in Shandong
Dragon Quality Goods Shop	Green Intelligent Power Saver	Invalid. Fake.	Jingdong prefecture level city in Shandong
Dragon Quality Goods Shop	Green Intelligent Power Saver	It's good!	Jingdong prefecture level city in Shandong
Dragon Quality Goods Shop	Green Intelligent Power Saver	have no effect	Jingdong prefecture level city in Shandong
Dragon Quality Goods Shop	Green Intelligent Power Saver	Not used.	Jingdong prefecture level city in Shandong
Dragon Quality Goods Shop	Green Intelligent Power Saver	There is no load range, packaging thrift, ten days also did not develop invoices, received the goods with a receipt, hand-written receipt also has no financial stamp, communication for nearly a week a moment to say that the electronic invoice, a moment to say that the paper invoice, said it is mailed to me, ten days also did not see anything, the invoice invoice can not be found to say that did not stay on file, single single number can not be found, said that the warehouse more than one to a one to find out, in addition to the perfunctory or perfunctory. Products are no certificate of conformity, proper three	Jingdong prefecture level city in Shandong
Dragon Quality Goods Shop	Green Intelligent Power Saver	Satisfaction.	Jingdong prefecture level city in Shandong

Dragon Quality Goods Shop	Green Intelligent Power Saver	There is not a bit of power saving effect, and ringing up an increase in the electricity bill, because you have to add the cost of this machine's own electricity consumption and purchase, so I think it is better to use this machine instead of increasing the expenditure, uneconomical, rather than not use it.	Jingdong prefecture level city in Shandong
Dragon Quality Goods Shop	Green Intelligent Power Saver	I've been using it for two days now and I don't feel any effect.	Jingdong prefecture level city in Shandong
Dragon Quality Goods Shop	Green Intelligent Power Saver	What it looked like was nothing like what was sent.	Jingdong prefecture level city in Shandong
Dragon Quality Goods Shop	Green Intelligent Power Saver	It didn't work. It didn't work for a month or so.	Jingdong prefecture level city in Shandong
Dragon Quality Goods Shop	Green Intelligent Power Saver	Garbage products, physical and description is not the same, no effect at all, pit # no discussion, to buy the fine, garbage, garbage, garbage	Jingdong prefecture level city in Shandong
Dragon Quality Goods Shop	Green Intelligent Power Saver	general	Jingdong prefecture level city in Shandong
Dragon Quality Goods Shop	Green Intelligent Power Saver	IQ tax. Instead of saving electricity, it increases the power consumption of an appliance.	Jingdong prefecture level city in Shandong
Dragon Quality Goods Shop	Green Intelligent Power Saver	There's no trash at all.	Jingdong prefecture level city in Shandong
Dragon Quality Goods Shop	Green Intelligent Power Saver	It doesn't do anything.	Jingdong prefecture level city in Shandong
Dragon Quality Goods Shop	Green Intelligent Power Saver	(of an unmarried couple) be close	Jingdong prefecture level city in Shandong
Dragon Quality Goods Shop	Green Intelligent Power Saver	(of an unmarried couple) be close	Jingdong prefecture level city in Shandong
Dragon Quality Goods Shop	Green Intelligent Power Saver	no electricity saving	Jingdong prefecture level city in Shandong
Dragon Quality Goods Shop	Green Intelligent Power Saver	differ from	Jingdong prefecture level city in Shandong
Dragon Quality Goods Shop	Green Intelligent Power Saver	Saver has received, but real can save electricity, the quality is very good, I times I buy a few more, too good.	Jingdong prefecture level city in Shandong
Dragon Quality Goods Shop	Green Intelligent Power Saver	Great quality as always, a very favorite product, great! o(*￣︶￣*)o	Jingdong prefecture level city in Shandong
Dragon Quality Goods Shop	Green Intelligent Power Saver	If we can reduce the electricity bill, for individuals can save money, for the country can protect the environment, now the weather is getting hotter and hotter, to protect the environment to reduce emissions is very important. Already use the product, whether to reduce the electricity bill depends on next month!	Jingdong prefecture level city in Shandong
Dragon Quality Goods Shop	Green Intelligent Power Saver	Purchased a power saver received on the put into use, rent a room with many electrical appliances, power consumption is very large, we have a piece of electricity or 1.2 dollars a time, with this power saver is much better, every day can save a few cents, very convenient and practical!	Jingdong prefecture level city in Shandong
Dragon Quality Goods Shop	Green Intelligent Power Saver	The product is better to use before not to use when a month 1500 yuan electricity bill, now using the 5 second month electricity bill directly down 470 dollars or very good. A save money to buy several power saver money ZanZanZanZan👍	Jingdong prefecture level city in Shandong
Dragon Quality Goods Shop	Green Intelligent Power Saver	The moment I received the goods really satisfied with this product, not only high-grade packaging, the body are base alloy, up to the price, I was also introduced by a friend, he used last month did save a lot of electricity, but also recommended to me!	Jingdong prefecture level city in Shandong
Dragon Quality Goods Shop	Green Intelligent Power Saver	I got it. Good review. I'm very satisfied. Reasonable price.	Jingdong prefecture level city in Shandong
Dragon Quality Goods Shop	Green Intelligent Power Saver	Intelligent power saver received and used, looking at the quality is good, the quality of the plug sent is also very good. Expect the effect of the economizer is obvious, first five-star praise, with a period of time to follow up!	Jingdong prefecture level city in Shandong
Dragon Quality Goods Shop	Green Intelligent Power Saver	The saver has been received. It was plugged into an outlet and used immediately after receipt. Whether it really saves electricity is not clear yet, I believe that this brand of energy saver really saves	Jingdong prefecture level city in Shandong
Dragon Quality Goods Shop	Green Intelligent Power Saver	Okay, okay, okay. Thank you so much. Fast is good. Fast. Item was as described, mainly because the event was cheap and you can save a lot of money.	Jingdong prefecture level city in Shandong
Dragon Quality Goods Shop	Green Intelligent Power Saver	Received it a couple days ago! Plugged it in the same day! Let's see what the electric bill is in a month! Hopefully it works well oh!	Jingdong prefecture level city in Shandong
Dragon Quality Goods Shop	Green Intelligent Power Saver	Looking at the reviews good, energy-saving small products, compact, the results after 2 months to see, because the electricity bill season payment	Jingdong prefecture level city in Shandong
Dragon Quality Goods Shop	Green Intelligent Power Saver	I just received it and will try it out to see if it works well. I'll comment later.	Jingdong prefecture level city in Shandong
Dragon Quality Goods Shop	Green Intelligent Power Saver	It looks a little small and not very aesthetically pleasing, I don't know how effective it will be until I use it	Jingdong prefecture level city in Shandong
Dragon Quality Goods Shop	Green Intelligent Power Saver	Not only to the point of being useless yet	Jingdong prefecture level city in Shandong
Dragon Quality Goods Shop	Green Intelligent Power Saver	Used it for a month, nothing works, the meter you turn very fast. This is not a scam, but also sold on Jingdong. To cheat a bunch of people?	Jingdong prefecture level city in Shandong
Dragon Quality Goods Shop	Green Intelligent Power Saver	Very good to use, every day can save a little electricity, really good under the sample down a month to save a few dozen dollars in electricity, good before I did not know that there is this 👆 haha, the price is not expensive praise.	Jingdong prefecture level city in Shandong
Dragon Quality Goods Shop	Green Intelligent Power Saver	Tried it for ten days and the change is almost nothing, can't see the power saving yet, use it some more days and see how it goes.	Jingdong prefecture level city in Shandong
Dragon Quality Goods Shop	Green Intelligent Power Saver	Received very quickly, the quality is very good, really save electricity, business service attitude is also very good, I hope you do not miss this opportunity, I continue to buy a few in	Jingdong prefecture level city in Shandong
Dragon Quality Goods Shop	Green Intelligent Power Saver	Quality is very good Oh, the delivery is also very fast, a very favorite store, I want to crazy recommend to friends!	Jingdong prefecture level city in Shandong
Dragon Quality Goods Shop	Green Intelligent Power Saver	The goods have just received, fine workmanship, and the same as described by the merchant, to use a period of time to see if it can achieve the purpose of power saving.	Jingdong prefecture level city in Shandong
Dragon Quality Goods Shop	Green Intelligent Power Saver	Received the goods first use a period of time to see if there is no effect, the business commitment to ninety days invalid refund, first praise, good service!	Jingdong prefecture level city in Shandong
Dragon Quality Goods Shop	Green Intelligent Power Saver	The product is used for a month without energy-saving and power-saving effect does not have any effect	Jingdong prefecture level city in Shandong
Dragon Quality Goods Shop	Green Intelligent Power Saver	The baby is really great, I like it very much, my friends also say good to come, recommended to buy!	Jingdong prefecture level city in Shandong
Dragon Quality Goods Shop	Green Intelligent Power Saver	Logistics is fast, courier boy service is good, after using to see how the fruit, and then follow up.	Jingdong prefecture level city in Shandong
Dragon Quality Goods Shop	Green Intelligent Power Saver	Baby has been received to try, I believe that as the business advertised energy saving. The courier is very responsible.	Jingdong prefecture level city in Shandong
Dragon Quality Goods Shop	Green Intelligent Power Saver	Save Electric Energy King received, good quality, beautiful appearance, very satisfied!	Jingdong prefecture level city in Shandong
Dragon Quality Goods Shop	Green Intelligent Power Saver	Three no products no manufacturers no phone pure fraud induced consumption cheat	Jingdong prefecture level city in Shandong
Dragon Quality Goods Shop	Green Intelligent Power Saver	I'm using it now, I don't know if it's as good as it says, I'll follow up after a month	Jingdong prefecture level city in Shandong
Dragon Quality Goods Shop	Green Intelligent Power Saver	Good quality logistics and fast. Fine workmanship. The description is consistent cost-effective	Jingdong prefecture level city in Shandong
Dragon Quality Goods Shop	Green Intelligent Power Saver	Received, whether it is good or not, to wait a few days and then evaluate	Jingdong prefecture level city in Shandong
Dragon Quality Goods Shop	Green Intelligent Power Saver	I bought it and started using it, I won't know if it works well until I pay my electric bill	Jingdong prefecture level city in Shandong
Dragon Quality Goods Shop	Green Intelligent Power Saver	Especially good, energy saving and power saving, especially good quality, good	Jingdong prefecture level city in Shandong
Dragon Quality Goods Shop	Green Intelligent Power Saver	Logistics is strong, the quality is very good, paste really save electricity, as described, will buy back!	Jingdong prefecture level city in Shandong
Dragon Quality Goods Shop	Green Intelligent Power Saver	Great stuff at a great price, worth the money!	Jingdong prefecture level city in Shandong

Dragon Quality Goods Shop	Green Intelligent Power Saver	Received the power saver, very good quality, very power saving!	Jingdong prefecture level city in Shandong
Dragon Quality Goods Shop	Green Intelligent Power Saver	There are no fruits, only samples...	Jingdong prefecture level city in Shandong
Dragon Quality Goods Shop	Green Intelligent Power Saver	Save a few dollars a month.	Jingdong prefecture level city in Shandong
Dragon Quality Goods Shop	Green Intelligent Power Saver	The quality is particularly good, it works well, and the seller has a good service attitude	Jingdong prefecture level city in Shandong
Dragon Quality Goods Shop	Green Intelligent Power Saver	I've compared them a couple of times and I don't feel any difference	Jingdong prefecture level city in Shandong
Dragon Quality Goods Shop	Green Intelligent Power Saver	I don't see the savings. It's a sham.	Jingdong prefecture level city in Shandong
Dragon Quality Goods Shop	Green Intelligent Power Saver	The power saving effect is very good, and the logistics is very fast. The seller's service attitude is very good	Jingdong prefecture level city in Shandong
Dragon Quality Goods Shop	Green Intelligent Power Saver	Received, well packaged, haven't tried it yet!	Jingdong prefecture level city in Shandong
Dragon Quality Goods Shop	Green Intelligent Power Saver	I don't know how it works, I'll comment after I use it.	Jingdong prefecture level city in Shandong
Dragon Quality Goods Shop	Green Intelligent Power Saver	The product doesn't work well enough to be of any use	Jingdong prefecture level city in Shandong
Dragon Quality Goods Shop	Green Intelligent Power Saver	On Trial 。。。。。。。	Jingdong prefecture level city in Shandong
Dragon Quality Goods Shop	Green Intelligent Power Saver	Being used with unknown results	Jingdong prefecture level city in Shandong
Dragon Quality Goods Shop	Green Intelligent Power Saver	Smart power saving and great quality	Jingdong prefecture level city in Shandong
Dragon Quality Goods Shop	Green Intelligent Power Saver	The saver was received, it works very well and the price is affordable!	Jingdong prefecture level city in Shandong
Dragon Quality Goods Shop	Green Intelligent Power Saver	How can I feel the power saving and how long does it last	Jingdong prefecture level city in Shandong
Dragon Quality Goods Shop	Green Intelligent Power Saver	And I know if it saves electricity or not.	Jingdong prefecture level city in Shandong
Dragon Quality Goods Shop	Green Intelligent Power Saver	It won't save any electricity. No.	Jingdong prefecture level city in Shandong
Dragon Quality Goods Shop	Green Intelligent Power Saver	Invalid. Fake.	Jingdong prefecture level city in Shandong
Dragon Quality Goods Shop	Green Intelligent Power Saver	It's good!	Jingdong prefecture level city in Shandong
Dragon Quality Goods Shop	Green Intelligent Power Saver	have no effect	Jingdong prefecture level city in Shandong
Dragon Quality Goods Shop	Green Intelligent Power Saver	Not used.	Jingdong prefecture level city in Shandong
Dragon Quality Goods Shop	Green Intelligent Power Saver	There is no load range, packaging thrift, ten days also did not develop invoices, received the goods with a receipt, hand-written receipt also has no financial stamp, communication for nearly a week a moment to say that the electronic invoice, a moment to say that the paper invoice, said it is mailed to me, ten days also did not see anything, the invoice invoice can not be found to say that did not stay on file, single single number can not be found, said that the warehouse more than one to a one to find out, in addition to the perfunctory or perfunctory. Products are no certificate of conformity, proper three	Jingdong prefecture level city in Shandong
Dragon Quality Goods Shop	Green Intelligent Power Saver	Satisfaction.	Jingdong prefecture level city in Shandong
Dragon Quality Goods Shop	Green Intelligent Power Saver	There is not a bit of power saving effect, and ringing up an increase in the electricity bill, because you have to add the cost of this machine's own electricity consumption and purchase, so I think it is better to use this machine instead of increasing the expenditure, uneconomical, rather than not use it.	Jingdong prefecture level city in Shandong
Dragon Quality Goods Shop	Green Intelligent Power Saver	I've been using it for two days now and I don't feel any effect.	Jingdong prefecture level city in Shandong
Dragon Quality Goods Shop	Green Intelligent Power Saver	What it looked like was nothing like what was sent.	Jingdong prefecture level city in Shandong
Dragon Quality Goods Shop	Green Intelligent Power Saver	It didn't work. It didn't work for a month or so.	Jingdong prefecture level city in Shandong
Dragon Quality Goods Shop	Green Intelligent Power Saver	Garbage products, physical and description is not the same, no effect at all, pit # no discussion, to buy the fine, garbage, garbage, garbage	Jingdong prefecture level city in Shandong
Dragon Quality Goods Shop	Green Intelligent Power Saver	general	Jingdong prefecture level city in Shandong
Dragon Quality Goods Shop	Green Intelligent Power Saver	IQ tax. Instead of saving electricity, it increases the power consumption of an appliance.	Jingdong prefecture level city in Shandong
Dragon Quality Goods Shop	Green Intelligent Power Saver	There's no trash at all.	Jingdong prefecture level city in Shandong
Dragon Quality Goods Shop	Green Intelligent Power Saver	It doesn't do anything.	Jingdong prefecture level city in Shandong
Dragon Quality Goods Shop	Green Intelligent Power Saver	(of an unmarried couple) be close	Jingdong prefecture level city in Shandong
Dragon Quality Goods Shop	Green Intelligent Power Saver	(of an unmarried couple) be close	Jingdong prefecture level city in Shandong
Dragon Quality Goods Shop	Green Intelligent Power Saver	no electricity saving	Jingdong prefecture level city in Shandong
Dragon Quality Goods Shop	Green Intelligent Power Saver	differ from	Jingdong prefecture level city in Shandong
Dragon Quality Goods Shop	Green Intelligent Power Saver	Saver has received, but real can save electricity, the quality is very good, I times I buy a few more, too good.	Jingdong prefecture level city in Shandong
Dragon Quality Goods Shop	Green Intelligent Power Saver	Great quality as always, a very favorite product, great! o(*￣︶￣*)o	Jingdong prefecture level city in Shandong
Dragon Quality Goods Shop	Green Intelligent Power Saver	If we can reduce the electricity bill, for individuals can save money, for the country can protect the environment, now the weather is getting hotter and hotter, to protect the environment to reduce emissions is very important. Already use the product, whether to reduce the electricity bill depends on next month!	Jingdong prefecture level city in Shandong
Dragon Quality Goods Shop	Green Intelligent Power Saver	Purchased a power saver received on the put into use, rent a room with many electrical appliances, power consumption is very large, we have a piece of electricity or 1.2 dollars a time, with this power saver is much better, every day can save a few cents, very convenient and practical!	Jingdong prefecture level city in Shandong
Dragon Quality Goods Shop	Green Intelligent Power Saver	The product is better to use before not to use when a month 1500 yuan electricity bill, now using the 5 second month electricity bill directly down 470 dollars or very good. A save money to buy several power saver money ZanZanZanZan👍	Jingdong prefecture level city in Shandong
Dragon Quality Goods Shop	Green Intelligent Power Saver	The moment I received the goods really satisfied with this product, not only high-grade packaging, the body are base alloy, up to the price, I was also introduced by a friend, he used last month did save a lot of electricity, but also recommended to me!	Jingdong prefecture level city in Shandong
Dragon Quality Goods Shop	Green Intelligent Power Saver	I got it. Good review. I'm very satisfied. Reasonable price.	Jingdong prefecture level city in Shandong
Dragon Quality Goods Shop	Green Intelligent Power Saver	Intelligent power saver received and used, looking at the quality is good, the quality of the plug sent is also very good. Expect the effect of the economizer is obvious, first five-star praise, with a period of time to follow up!	Jingdong prefecture level city in Shandong
Dragon Quality Goods Shop	Green Intelligent Power Saver	The saver has been received. It was plugged into an outlet and used immediately after receipt. Whether it really saves electricity is not clear yet, I believe that this brand of energy saver really saves	Jingdong prefecture level city in Shandong
Dragon Quality Goods Shop	Green Intelligent Power Saver	Okay, okay, okay. Thank you so much. Fast is good. Fast. Item was as described, mainly because the event was cheap and you can save a lot of money.	Jingdong prefecture level city in Shandong

Dragon Quality Goods Shop	Green Intelligent Power Saver	Received it a couple days ago! Plugged it in the same day! Let's see what the electric bill is in a month! Hopefully it works well oh!	Jingdong prefecture level city in Shandong
Dragon Quality Goods Shop	Green Intelligent Power Saver	Looking at the reviews good, energy-saving small products, compact, the results after 2 months to see, because the electricity bill season payment	Jingdong prefecture level city in Shandong
Dragon Quality Goods Shop	Green Intelligent Power Saver	I just received it and will try it out to see if it works well. I'll comment later.	Jingdong prefecture level city in Shandong
Dragon Quality Goods Shop	Green Intelligent Power Saver	It looks a little small and not very aesthetically pleasing, I don't know how effective it will be until I use it	Jingdong prefecture level city in Shandong
Dragon Quality Goods Shop	Green Intelligent Power Saver	Not only to the point of being useless yet	Jingdong prefecture level city in Shandong
Dragon Quality Goods Shop	Green Intelligent Power Saver	Used it for a month, nothing works, the meter you turn very fast. This is not a scam, but also sold on Jingdong. To cheat a bunch of people?	Jingdong prefecture level city in Shandong
Dragon Quality Goods Shop	Green Intelligent Power Saver	Very good to use, every day can save a little electricity, really good under the sample down a month to save a few dozen dollars in electricity, good before I did not know that there is this haha, the price is not expensive praise.	Jingdong prefecture level city in Shandong
Dragon Quality Goods Shop	Green Intelligent Power Saver	Tried it for ten days and the change is almost nothing, can't see the power saving yet, use it some more days and see how it goes.	Jingdong prefecture level city in Shandong
Dragon Quality Goods Shop	Green Intelligent Power Saver	Received very quickly, the quality is very good, really save electricity, business service attitude is also very good, I hope you do not miss this opportunity, I continue to buy a few in	Jingdong prefecture level city in Shandong
Dragon Quality Goods Shop	Green Intelligent Power Saver	Quality is very good Oh, the delivery is also very fast, a very favorite store, I want to crazy recommend to friends!	Jingdong prefecture level city in Shandong
Dragon Quality Goods Shop	Green Intelligent Power Saver	The goods have just received, fine workmanship, and the same as described by the merchant, to use a period of time to see if it can achieve the purpose of power saving.	Jingdong prefecture level city in Shandong
Dragon Quality Goods Shop	Green Intelligent Power Saver	Received the goods first use a period of time to see if there is no effect, the business commitment to ninety days invalid refund, first praise, good service!	Jingdong prefecture level city in Shandong
Dragon Quality Goods Shop	Green Intelligent Power Saver	The product is used for a month without energy-saving and power-saving effect does not have any effect	Jingdong prefecture level city in Shandong
Dragon Quality Goods Shop	Green Intelligent Power Saver	The baby is really great, I like it very much, my friends also say good to come, recommended to buy!	Jingdong prefecture level city in Shandong
Dragon Quality Goods Shop	Green Intelligent Power Saver	Logistics is fast, courier boy service is good, after using to see how the fruit, and then follow up.	Jingdong prefecture level city in Shandong
Dragon Quality Goods Shop	Green Intelligent Power Saver	Baby has been received to try, I believe that as the business advertised energy saving. The courier is very responsible.	Jingdong prefecture level city in Shandong
Dragon Quality Goods Shop	Green Intelligent Power Saver	Save Electric Energy King received, good quality, beautiful appearance, very satisfied!	Jingdong prefecture level city in Shandong
Dragon Quality Goods Shop	Green Intelligent Power Saver	Three no products no manufacturers no phone pure fraud induced consumption cheat	Jingdong prefecture level city in Shandong
Dragon Quality Goods Shop	Green Intelligent Power Saver	I'm using it now, I don't know if it's as good as it says, I'll follow up after a month	Jingdong prefecture level city in Shandong
Dragon Quality Goods Shop	Green Intelligent Power Saver	Good quality logistics and fast. Fine workmanship. The description is consistent cost-effective	Jingdong prefecture level city in Shandong
Dragon Quality Goods Shop	Green Intelligent Power Saver	Received, whether it is good or not, to wait a few days and then evaluate	Jingdong prefecture level city in Shandong
Dragon Quality Goods Shop	Green Intelligent Power Saver	I bought it and started using it, I won't know if it works well until I pay my electric bill	Jingdong prefecture level city in Shandong
Dragon Quality Goods Shop	Green Intelligent Power Saver	Especially good, energy saving and power saving, especially good quality, good	Jingdong prefecture level city in Shandong
Dragon Quality Goods Shop	Green Intelligent Power Saver	Logistics is strong, the quality is very good, paste really save electricity, as described, will buy back!	Jingdong prefecture level city in Shandong
Dragon Quality Goods Shop	Green Intelligent Power Saver	Great stuff at a great price, worth the money!	Jingdong prefecture level city in Shandong
Dragon Quality Goods Shop	Green Intelligent Power Saver	Received the power saver, very good quality, very power saving!	Jingdong prefecture level city in Shandong
Dragon Quality Goods Shop	Green Intelligent Power Saver	There are no fruits, only samples...	Jingdong prefecture level city in Shandong
Dragon Quality Goods Shop	Green Intelligent Power Saver	Save a few dollars a month.	Jingdong prefecture level city in Shandong
Dragon Quality Goods Shop	Green Intelligent Power Saver	The quality is particularly good, it works well, and the seller has a good service attitude	Jingdong prefecture level city in Shandong
Dragon Quality Goods Shop	Green Intelligent Power Saver	I've compared them a couple of times and I don't feel any difference	Jingdong prefecture level city in Shandong
Dragon Quality Goods Shop	Green Intelligent Power Saver	I don't see the savings. It's a sham.	Jingdong prefecture level city in Shandong
Dragon Quality Goods Shop	Green Intelligent Power Saver	The power saving effect is very good, and the logistics is very fast. The seller's service attitude is very good	Jingdong prefecture level city in Shandong
Dragon Quality Goods Shop	Green Intelligent Power Saver	Received, well packaged, haven't tried it yet!	Jingdong prefecture level city in Shandong
Dragon Quality Goods Shop	Green Intelligent Power Saver	I don't know how it works, I'll comment after I use it.	Jingdong prefecture level city in Shandong
Dragon Quality Goods Shop	Green Intelligent Power Saver	The product doesn't work well enough to be of any use	Jingdong prefecture level city in Shandong
Dragon Quality Goods Shop	Green Intelligent Power Saver	On Trial 。。。。。。。	Jingdong prefecture level city in Shandong
Dragon Quality Goods Shop	Green Intelligent Power Saver	Being used with unknown results	Jingdong prefecture level city in Shandong
Dragon Quality Goods Shop	Green Intelligent Power Saver	Smart power saving and great quality	Jingdong prefecture level city in Shandong
Dragon Quality Goods Shop	Green Intelligent Power Saver	The saver was received, it works very well and the price is affordable!	Jingdong prefecture level city in Shandong
Dragon Quality Goods Shop	Green Intelligent Power Saver	How can I feel the power saving and how long does it last	Jingdong prefecture level city in Shandong
Dragon Quality Goods Shop	Green Intelligent Power Saver	And I know if it saves electricity or not.	Jingdong prefecture level city in Shandong
Dragon Quality Goods Shop	Green Intelligent Power Saver	It won't save any electricity. No.	Jingdong prefecture level city in Shandong
Dragon Quality Goods Shop	Green Intelligent Power Saver	Invalid. Fake.	Jingdong prefecture level city in Shandong
Dragon Quality Goods Shop	Green Intelligent Power Saver	It's good!	Jingdong prefecture level city in Shandong
Dragon Quality Goods Shop	Green Intelligent Power Saver	have no effect	Jingdong prefecture level city in Shandong
Dragon Quality Goods Shop	Green Intelligent Power Saver	Not used.	Jingdong prefecture level city in Shandong
Dragon Quality Goods Shop	Green Intelligent Power Saver	There is no load range, packaging thrift, ten days also did not develop invoices, received the goods with a receipt, hand-written receipt also has no financial stamp, communication for nearly a week a moment to say that the electronic invoice, a moment to say that the paper invoice, said it is mailed to me, ten days also did not see anything, the invoice invoice can not be found to say that did not stay on file, single single number can not be found, said that the warehouse more than one to a one to find out, in addition to the perfunctory or perfunctory. Products are no certificate of conformity, proper three	Jingdong prefecture level city in Shandong
Dragon Quality Goods Shop	Green Intelligent Power Saver	Satisfaction.	Jingdong prefecture level city in Shandong

Dragon Quality Goods Shop	Green Intelligent Power Saver	There is not a bit of power saving effect, and ringing up an increase in the electricity bill, because you have to add the cost of this machine’s own electricity consumption and purchase, so I think it is better to use this machine instead of increasing the expenditure, uneconomical, rather than not use it.	Jingdong prefecture level city in Shandong
Dragon Quality Goods Shop	Green Intelligent Power Saver	I’ve been using it for two days now and I don’t feel any effect.	Jingdong prefecture level city in Shandong
Dragon Quality Goods Shop	Green Intelligent Power Saver	What it looked like was nothing like what was sent.	Jingdong prefecture level city in Shandong
Dragon Quality Goods Shop	Green Intelligent Power Saver	It didn’t work. It didn’t work for a month or so.	Jingdong prefecture level city in Shandong
Dragon Quality Goods Shop	Green Intelligent Power Saver	Garbage products, physical and description is not the same, no effect at all, pit # no discussion, to buy the fine, garbage, garbage, garbage	Jingdong prefecture level city in Shandong
Dragon Quality Goods Shop	Green Intelligent Power Saver	general	Jingdong prefecture level city in Shandong
Dragon Quality Goods Shop	Green Intelligent Power Saver	IQ tax. Instead of saving electricity, it increases the power consumption of an appliance.	Jingdong prefecture level city in Shandong
Dragon Quality Goods Shop	Green Intelligent Power Saver	There’s no trash at all.	Jingdong prefecture level city in Shandong
Dragon Quality Goods Shop	Green Intelligent Power Saver	It doesn’t do anything.	Jingdong prefecture level city in Shandong
Dragon Quality Goods Shop	Green Intelligent Power Saver	(of an unmarried couple) be close	Jingdong prefecture level city in Shandong
Dragon Quality Goods Shop	Green Intelligent Power Saver	(of an unmarried couple) be close	Jingdong prefecture level city in Shandong
Dragon Quality Goods Shop	Green Intelligent Power Saver	no electricity saving	Jingdong prefecture level city in Shandong
Dragon Quality Goods Shop	Green Intelligent Power Saver	differ from	Jingdong prefecture level city in Shandong
Dragon Quality Goods Shop	Green Intelligent Power Saver	Saver has received, but real can save electricity, the quality is very good, I times I buy a few more, too good.	Jingdong prefecture level city in Shandong
Dragon Quality Goods Shop	Green Intelligent Power Saver	Great quality as always, a very favorite product, great! o(*￣︶￣*)o	Jingdong prefecture level city in Shandong
Dragon Quality Goods Shop	Green Intelligent Power Saver	If we can reduce the electricity bill, for individuals can save money, for the country can protect the environment, now the weather is getting hotter and hotter, to protect the environment to reduce emissions is very important. Already use the product, whether to reduce the electricity bill depends on next month!	Jingdong prefecture level city in Shandong
Dragon Quality Goods Shop	Green Intelligent Power Saver	Purchased a power saver received on the put into use, rent a room with many electrical appliances, power consumption is very large, we have a piece of electricity or 1.2 dollars a time, with this power saver is much better, every day can save a few cents, very convenient and practical!	Jingdong prefecture level city in Shandong
Dragon Quality Goods Shop	Green Intelligent Power Saver	The product is better to use before not to use when a month 1500 yuan electricity bill, now using the 5 second month electricity bill directly down 470 dollars or very good. A save money to buy several power saver money ZanZanZanZan👍	Jingdong prefecture level city in Shandong
Dragon Quality Goods Shop	Green Intelligent Power Saver	The moment I received the goods really satisfied with this product, not only high-grade packaging, the body are base alloy, up to the price, I was also introduced by a friend, he used last month did save a lot of electricity, but also recommended to me!	Jingdong prefecture level city in Shandong
Dragon Quality Goods Shop	Green Intelligent Power Saver	I got it. Good review. I’m very satisfied. Reasonable price.	Jingdong prefecture level city in Shandong
Dragon Quality Goods Shop	Green Intelligent Power Saver	Intelligent power saver received and used, looking at the quality is good, the quality of the plug sent is also very good. Expect the effect of the economizer is obvious, first five-star praise, with a period of time to follow up!	Jingdong prefecture level city in Shandong
Dragon Quality Goods Shop	Green Intelligent Power Saver	The saver has been received. It was plugged into an outlet and used immediately after receipt. Whether it really saves electricity is not clear yet, I believe that this brand of energy saver really saves	Jingdong prefecture level city in Shandong
Dragon Quality Goods Shop	Green Intelligent Power Saver	Okay, okay, okay. Thank you so much. Fast is good. Fast. Item was as described, mainly because the event was cheap and you can save a lot of money.	Jingdong prefecture level city in Shandong
Dragon Quality Goods Shop	Green Intelligent Power Saver	Received it a couple days ago! Plugged it in the same day! Let’s see what the electric bill is in a month! Hopefully it works well oh!	Jingdong prefecture level city in Shandong
Dragon Quality Goods Shop	Green Intelligent Power Saver	Looking at the reviews good, energy-saving small products, compact, the results after 2 months to see, because the electricity bill season payment	Jingdong prefecture level city in Shandong
Dragon Quality Goods Shop	Green Intelligent Power Saver	I just received it and will try it out to see if it works well. I’ll comment later.	Jingdong prefecture level city in Shandong
Dragon Quality Goods Shop	Green Intelligent Power Saver	It looks a little small and not very aesthetically pleasing, I don’t know how effective it will be until I use it	Jingdong prefecture level city in Shandong
Dragon Quality Goods Shop	Green Intelligent Power Saver	Not only to the point of being useless yet	Jingdong prefecture level city in Shandong
Dragon Quality Goods Shop	Green Intelligent Power Saver	Used it for a month, nothing works, the meter you turn very fast. This is not a scam, but also sold on Jingdong. To cheat a bunch of people?	Jingdong prefecture level city in Shandong
Dragon Quality Goods Shop	Green Intelligent Power Saver	Very good to use, every day can save a little electricity, really good under the sample down a month to save a few dozen dollars in electricity, good before I did not know that there is this 👆 haha, the price is not expensive praise.	Jingdong prefecture level city in Shandong
Dragon Quality Goods Shop	Green Intelligent Power Saver	Tried it for ten days and the change is almost nothing, can’t see the power saving yet, use it some more days and see how it goes.	Jingdong prefecture level city in Shandong
Dragon Quality Goods Shop	Green Intelligent Power Saver	Received very quickly, the quality is very good, really save electricity, business service attitude is also very good, I hope you do not miss this opportunity, I continue to buy a few in	Jingdong prefecture level city in Shandong
Dragon Quality Goods Shop	Green Intelligent Power Saver	Quality is very good Oh, the delivery is also very fast, a very favorite store, I want to crazy recommend to friends!	Jingdong prefecture level city in Shandong
Dragon Quality Goods Shop	Green Intelligent Power Saver	The goods have just received, fine workmanship, and the same as described by the merchant, to use a period of time to see if it can achieve the purpose of power saving.	Jingdong prefecture level city in Shandong
Dragon Quality Goods Shop	Green Intelligent Power Saver	Received the goods first use a period of time to see if there is no effect, the business commitment to ninety days invalid refund, first praise, good service!	Jingdong prefecture level city in Shandong
Dragon Quality Goods Shop	Green Intelligent Power Saver	The product is used for a month without energy-saving and power-saving effect does not have any effect	Jingdong prefecture level city in Shandong
Dragon Quality Goods Shop	Green Intelligent Power Saver	The baby is really great, I like it very much, my friends also say good to come, recommended to buy!	Jingdong prefecture level city in Shandong
Dragon Quality Goods Shop	Green Intelligent Power Saver	Logistics is fast, courier boy service is good, after using to see how the fruit, and then follow up.	Jingdong prefecture level city in Shandong
Dragon Quality Goods Shop	Green Intelligent Power Saver	Baby has been received to try, I believe that as the business advertised energy saving. The courier is very responsible.	Jingdong prefecture level city in Shandong
Dragon Quality Goods Shop	Green Intelligent Power Saver	Save Electric Energy King received, good quality, beautiful appearance, very satisfied!	Jingdong prefecture level city in Shandong
Dragon Quality Goods Shop	Green Intelligent Power Saver	Three no products no manufacturers no phone pure fraud induced consumption cheat	Jingdong prefecture level city in Shandong
Dragon Quality Goods Shop	Green Intelligent Power Saver	I’m using it now, I don’t know if it’s as good as it says, I’ll follow up after a month	Jingdong prefecture level city in Shandong
Dragon Quality Goods Shop	Green Intelligent Power Saver	Good quality logistics and fast. Fine workmanship. The description is consistent cost-effective	Jingdong prefecture level city in Shandong
Dragon Quality Goods Shop	Green Intelligent Power Saver	Received, whether it is good or not, to wait a few days and then evaluate	Jingdong prefecture level city in Shandong
Dragon Quality Goods Shop	Green Intelligent Power Saver	I bought it and started using it, I won’t know if it works well until I pay my electric bill	Jingdong prefecture level city in Shandong
Dragon Quality Goods Shop	Green Intelligent Power Saver	Especially good, energy saving and power saving, especially good quality, good	Jingdong prefecture level city in Shandong
Dragon Quality Goods Shop	Green Intelligent Power Saver	Logistics is strong, the quality is very good, paste really save electricity, as described, will buy back!	Jingdong prefecture level city in Shandong
Dragon Quality Goods Shop	Green Intelligent Power Saver	Great stuff at a great price, worth the money!	Jingdong prefecture level city in Shandong

Dragon Quality Goods Shop	Green Intelligent Power Saver	Received the power saver, very good quality, very power saving!	Jingdong prefecture level city in Shandong
Dragon Quality Goods Shop	Green Intelligent Power Saver	There are no fruits, only samples...	Jingdong prefecture level city in Shandong
Dragon Quality Goods Shop	Green Intelligent Power Saver	Save a few dollars a month.	Jingdong prefecture level city in Shandong
Dragon Quality Goods Shop	Green Intelligent Power Saver	The quality is particularly good, it works well, and the seller has a good service attitude	Jingdong prefecture level city in Shandong
Dragon Quality Goods Shop	Green Intelligent Power Saver	I've compared them a couple of times and I don't feel any difference	Jingdong prefecture level city in Shandong
Dragon Quality Goods Shop	Green Intelligent Power Saver	I don't see the savings. It's a sham.	Jingdong prefecture level city in Shandong
Dragon Quality Goods Shop	Green Intelligent Power Saver	The power saving effect is very good, and the logistics is very fast. The seller's service attitude is very good	Jingdong prefecture level city in Shandong
Dragon Quality Goods Shop	Green Intelligent Power Saver	Received, well packaged, haven't tried it yet!	Jingdong prefecture level city in Shandong
Dragon Quality Goods Shop	Green Intelligent Power Saver	I don't know how it works, I'll comment after I use it.	Jingdong prefecture level city in Shandong
Dragon Quality Goods Shop	Green Intelligent Power Saver	The product doesn't work well enough to be of any use	Jingdong prefecture level city in Shandong
Dragon Quality Goods Shop	Green Intelligent Power Saver	On Trial 。。。。。。。	Jingdong prefecture level city in Shandong
Dragon Quality Goods Shop	Green Intelligent Power Saver	Being used with unknown results	Jingdong prefecture level city in Shandong
Dragon Quality Goods Shop	Green Intelligent Power Saver	Smart power saving and great quality	Jingdong prefecture level city in Shandong
Dragon Quality Goods Shop	Green Intelligent Power Saver	The saver was received, it works very well and the price is affordable!	Jingdong prefecture level city in Shandong
Dragon Quality Goods Shop	Green Intelligent Power Saver	How can I feel the power saving and how long does it last	Jingdong prefecture level city in Shandong
Dragon Quality Goods Shop	Green Intelligent Power Saver	And I know if it saves electricity or not.	Jingdong prefecture level city in Shandong
Dragon Quality Goods Shop	Green Intelligent Power Saver	It won't save any electricity. No.	Jingdong prefecture level city in Shandong
Dragon Quality Goods Shop	Green Intelligent Power Saver	Invalid. Fake.	Jingdong prefecture level city in Shandong
Dragon Quality Goods Shop	Green Intelligent Power Saver	It's good!	Jingdong prefecture level city in Shandong
Dragon Quality Goods Shop	Green Intelligent Power Saver	have no effect	Jingdong prefecture level city in Shandong
Dragon Quality Goods Shop	Green Intelligent Power Saver	Not used.	Jingdong prefecture level city in Shandong
Dragon Quality Goods Shop	Green Intelligent Power Saver	There is no load range, packaging thrift, ten days also did not develop invoices, received the goods with a receipt, hand-written receipt also has no financial stamp, communication for nearly a week a moment to say that the electronic invoice, a moment to say that the paper invoice, said it is mailed to me, ten days also did not see anything, the invoice invoice can not be found to say that did not stay on file, single single number can not be found, said that the warehouse more than one to a one to find out, in addition to the perfunctory or perfunctory. Products are no certificate of conformity, proper three	Jingdong prefecture level city in Shandong
Dragon Quality Goods Shop	Green Intelligent Power Saver	Satisfaction.	Jingdong prefecture level city in Shandong
Dragon Quality Goods Shop	Green Intelligent Power Saver	There is not a bit of power saving effect, and ringing up an increase in the electricity bill, because you have to add the cost of this machine's own electricity consumption and purchase, so I think it is better to use this machine instead of increasing the expenditure, uneconomical, rather than not use it.	Jingdong prefecture level city in Shandong
Dragon Quality Goods Shop	Green Intelligent Power Saver	I've been using it for two days now and I don't feel any effect.	Jingdong prefecture level city in Shandong
Dragon Quality Goods Shop	Green Intelligent Power Saver	What it looked like was nothing like what was sent.	Jingdong prefecture level city in Shandong
Dragon Quality Goods Shop	Green Intelligent Power Saver	It didn't work. It didn't work for a month or so.	Jingdong prefecture level city in Shandong
Dragon Quality Goods Shop	Green Intelligent Power Saver	Garbage products, physical and description is not the same, no effect at all, pit # no discussion, to buy the fine, garbage, garbage, garbage	Jingdong prefecture level city in Shandong
Dragon Quality Goods Shop	Green Intelligent Power Saver	general	Jingdong prefecture level city in Shandong
Dragon Quality Goods Shop	Green Intelligent Power Saver	IQ tax. Instead of saving electricity, it increases the power consumption of an appliance.	Jingdong prefecture level city in Shandong
Dragon Quality Goods Shop	Green Intelligent Power Saver	There's no trash at all.	Jingdong prefecture level city in Shandong
Dragon Quality Goods Shop	Green Intelligent Power Saver	It doesn't do anything.	Jingdong prefecture level city in Shandong
Dragon Quality Goods Shop	Green Intelligent Power Saver	(of an unmarried couple) be close	Jingdong prefecture level city in Shandong
Dragon Quality Goods Shop	Green Intelligent Power Saver	(of an unmarried couple) be close	Jingdong prefecture level city in Shandong
Dragon Quality Goods Shop	Green Intelligent Power Saver	no electricity saving	Jingdong prefecture level city in Shandong
Dragon Quality Goods Shop	Green Intelligent Power Saver	differ from	Jingdong prefecture level city in Shandong
Dragon Quality Goods Shop	Green Intelligent Power Saver	Saver has received, but real can save electricity, the quality is very good, I times I buy a few more, too good.	Jingdong prefecture level city in Shandong
Dragon Quality Goods Shop	Green Intelligent Power Saver	Great quality as always, a very favorite product, great! o(*￣︶￣*)o	Jingdong prefecture level city in Shandong
Dragon Quality Goods Shop	Green Intelligent Power Saver	If we can reduce the electricity bill, for individuals can save money, for the country can protect the environment, now the weather is getting hotter and hotter, to protect the environment to reduce emissions is very important. Already use the product, whether to reduce the electricity bill depends on next month!	Jingdong prefecture level city in Shandong
Dragon Quality Goods Shop	Green Intelligent Power Saver	Purchased a power saver received on the put into use, rent a room with many electrical appliances, power consumption is very large, we have a piece of electricity or 1.2 dollars a time, with this power saver is much better, every day can save a few cents, very convenient and practical!	Jingdong prefecture level city in Shandong
Dragon Quality Goods Shop	Green Intelligent Power Saver	The product is better to use before not to use when a month 1500 yuan electricity bill, now using the 5 second month electricity bill directly down 470 dollars or very good. A save money to buy several power saver money ZanZanZanZan👍	Jingdong prefecture level city in Shandong
Dragon Quality Goods Shop	Green Intelligent Power Saver	The moment I received the goods really satisfied with this product, not only high-grade packaging, the body are base alloy, up to the price, I was also introduced by a friend, he used last month did save a lot of electricity, but also recommended to me!	Jingdong prefecture level city in Shandong
Dragon Quality Goods Shop	Green Intelligent Power Saver	I got it. Good review. I'm very satisfied. Reasonable price.	Jingdong prefecture level city in Shandong
Dragon Quality Goods Shop	Green Intelligent Power Saver	Intelligent power saver received and used, looking at the quality is good, the quality of the plug sent is also very good. Expect the effect of the economizer is obvious, first five-star praise, with a period of time to follow up!	Jingdong prefecture level city in Shandong
Dragon Quality Goods Shop	Green Intelligent Power Saver	The saver has been received. It was plugged into an outlet and used immediately after receipt. Whether it really saves electricity is not clear yet, I believe that this brand of energy saver really saves	Jingdong prefecture level city in Shandong
Dragon Quality Goods Shop	Green Intelligent Power Saver	Okay, okay, okay. Thank you so much. Fast is good. Fast. Item was as described, mainly because the event was cheap and you can save a lot of money.	Jingdong prefecture level city in Shandong

Dragon Quality Goods Shop	Green Intelligent Power Saver	Received it a couple days ago! Plugged it in the same day! Let's see what the electric bill is in a month! Hopefully it works well oh!	Jingdong prefecture level city in Shandong
Dragon Quality Goods Shop	Green Intelligent Power Saver	Looking at the reviews good, energy-saving small products, compact, the results after 2 months to see, because the electricity bill season payment	Jingdong prefecture level city in Shandong
Dragon Quality Goods Shop	Green Intelligent Power Saver	I just received it and will try it out to see if it works well. I'll comment later.	Jingdong prefecture level city in Shandong
Dragon Quality Goods Shop	Green Intelligent Power Saver	It looks a little small and not very aesthetically pleasing, I don't know how effective it will be until I use it	Jingdong prefecture level city in Shandong
Dragon Quality Goods Shop	Green Intelligent Power Saver	Not only to the point of being useless yet	Jingdong prefecture level city in Shandong
Dragon Quality Goods Shop	Green Intelligent Power Saver	Used it for a month, nothing works, the meter you turn very fast. This is not a scam, but also sold on Jingdong. To cheat a bunch of people?	Jingdong prefecture level city in Shandong
Dragon Quality Goods Shop	Green Intelligent Power Saver	Very good to use, every day can save a little electricity, really good under the sample down a month to save a few dozen dollars in electricity, good before I did not know that there is this haha, the price is not expensive praise.	Jingdong prefecture level city in Shandong
Dragon Quality Goods Shop	Green Intelligent Power Saver	Tried it for ten days and the change is almost nothing, can't see the power saving yet, use it some more days and see how it goes.	Jingdong prefecture level city in Shandong
Dragon Quality Goods Shop	Green Intelligent Power Saver	Received very quickly, the quality is very good, really save electricity, business service attitude is also very good, I hope you do not miss this opportunity, I continue to buy a few in	Jingdong prefecture level city in Shandong
Dragon Quality Goods Shop	Green Intelligent Power Saver	Quality is very good Oh, the delivery is also very fast, a very favorite store, I want to crazy recommend to friends!	Jingdong prefecture level city in Shandong
Dragon Quality Goods Shop	Green Intelligent Power Saver	The goods have just received, fine workmanship, and the same as described by the merchant, to use a period of time to see if it can achieve the purpose of power saving.	Jingdong prefecture level city in Shandong
Dragon Quality Goods Shop	Green Intelligent Power Saver	Received the goods first use a period of time to see if there is no effect, the business commitment to ninety days invalid refund, first praise, good service!	Jingdong prefecture level city in Shandong
Dragon Quality Goods Shop	Green Intelligent Power Saver	The product is used for a month without energy-saving and power-saving effect does not have any effect	Jingdong prefecture level city in Shandong
Dragon Quality Goods Shop	Green Intelligent Power Saver	The baby is really great, I like it very much, my friends also say good to come, recommended to buy!	Jingdong prefecture level city in Shandong
Dragon Quality Goods Shop	Green Intelligent Power Saver	Logistics is fast, courier boy service is good, after using to see how the fruit, and then follow up.	Jingdong prefecture level city in Shandong
Dragon Quality Goods Shop	Green Intelligent Power Saver	Baby has been received to try, I believe that as the business advertised energy saving. The courier is very responsible.	Jingdong prefecture level city in Shandong
Dragon Quality Goods Shop	Green Intelligent Power Saver	Save Electric Energy King received, good quality, beautiful appearance, very satisfied!	Jingdong prefecture level city in Shandong
Dragon Quality Goods Shop	Green Intelligent Power Saver	Three no products no manufacturers no phone pure fraud induced consumption cheat	Jingdong prefecture level city in Shandong
Dragon Quality Goods Shop	Green Intelligent Power Saver	I'm using it now, I don't know if it's as good as it says, I'll follow up after a month	Jingdong prefecture level city in Shandong
Dragon Quality Goods Shop	Green Intelligent Power Saver	Good quality logistics and fast. Fine workmanship. The description is consistent cost-effective	Jingdong prefecture level city in Shandong
Dragon Quality Goods Shop	Green Intelligent Power Saver	Received, whether it is good or not, to wait a few days and then evaluate	Jingdong prefecture level city in Shandong
Dragon Quality Goods Shop	Green Intelligent Power Saver	I bought it and started using it, I won't know if it works well until I pay my electric bill	Jingdong prefecture level city in Shandong
Dragon Quality Goods Shop	Green Intelligent Power Saver	Especially good, energy saving and power saving, especially good quality, good	Jingdong prefecture level city in Shandong
Dragon Quality Goods Shop	Green Intelligent Power Saver	Logistics is strong, the quality is very good, paste really save electricity, as described, will buy back!	Jingdong prefecture level city in Shandong
Dragon Quality Goods Shop	Green Intelligent Power Saver	Great stuff at a great price, worth the money!	Jingdong prefecture level city in Shandong
Dragon Quality Goods Shop	Green Intelligent Power Saver	Received the power saver, very good quality, very power saving!	Jingdong prefecture level city in Shandong
Dragon Quality Goods Shop	Green Intelligent Power Saver	There are no fruits, only samples...	Jingdong prefecture level city in Shandong
Dragon Quality Goods Shop	Green Intelligent Power Saver	Save a few dollars a month.	Jingdong prefecture level city in Shandong
Dragon Quality Goods Shop	Green Intelligent Power Saver	The quality is particularly good, it works well, and the seller has a good service attitude	Jingdong prefecture level city in Shandong
Dragon Quality Goods Shop	Green Intelligent Power Saver	I've compared them a couple of times and I don't feel any difference	Jingdong prefecture level city in Shandong
Dragon Quality Goods Shop	Green Intelligent Power Saver	I don't see the savings. It's a sham.	Jingdong prefecture level city in Shandong
Dragon Quality Goods Shop	Green Intelligent Power Saver	The power saving effect is very good, and the logistics is very fast. The seller's service attitude is very good	Jingdong prefecture level city in Shandong
Dragon Quality Goods Shop	Green Intelligent Power Saver	Received, well packaged, haven't tried it yet!	Jingdong prefecture level city in Shandong
Dragon Quality Goods Shop	Green Intelligent Power Saver	I don't know how it works, I'll comment after I use it.	Jingdong prefecture level city in Shandong
Dragon Quality Goods Shop	Green Intelligent Power Saver	The product doesn't work well enough to be of any use	Jingdong prefecture level city in Shandong
Dragon Quality Goods Shop	Green Intelligent Power Saver	On Trial 。。。。。。。	Jingdong prefecture level city in Shandong
Dragon Quality Goods Shop	Green Intelligent Power Saver	Being used with unknown results	Jingdong prefecture level city in Shandong
Dragon Quality Goods Shop	Green Intelligent Power Saver	Smart power saving and great quality	Jingdong prefecture level city in Shandong
Dragon Quality Goods Shop	Green Intelligent Power Saver	The saver was received, it works very well and the price is affordable!	Jingdong prefecture level city in Shandong
Dragon Quality Goods Shop	Green Intelligent Power Saver	How can I feel the power saving and how long does it last	Jingdong prefecture level city in Shandong
Dragon Quality Goods Shop	Green Intelligent Power Saver	And I know if it saves electricity or not.	Jingdong prefecture level city in Shandong
Dragon Quality Goods Shop	Green Intelligent Power Saver	It won't save any electricity. No.	Jingdong prefecture level city in Shandong
Dragon Quality Goods Shop	Green Intelligent Power Saver	Invalid. Fake.	Jingdong prefecture level city in Shandong
Dragon Quality Goods Shop	Green Intelligent Power Saver	It's good!	Jingdong prefecture level city in Shandong
Dragon Quality Goods Shop	Green Intelligent Power Saver	have no effect	Jingdong prefecture level city in Shandong
Dragon Quality Goods Shop	Green Intelligent Power Saver	Not used.	Jingdong prefecture level city in Shandong
Dragon Quality Goods Shop	Green Intelligent Power Saver	There is no load range, packaging thrift, ten days also did not develop invoices, received the goods with a receipt, hand-written receipt also has no financial stamp, communication for nearly a week a moment to say that the electronic invoice, a moment to say that the paper invoice, said it is mailed to me, ten days also did not see anything, the invoice invoice can not be found to say that did not stay on file, single single number can not be found, said that the warehouse more than one to a one to find out, in addition to the perfunctory or perfunctory. Products are no certificate of conformity, proper three	Jingdong prefecture level city in Shandong
Dragon Quality Goods Shop	Green Intelligent Power Saver	Satisfaction.	Jingdong prefecture level city in Shandong

Dragon Quality Goods Shop	Green Intelligent Power Saver	There is not a bit of power saving effect, and ringing up an increase in the electricity bill, because you have to add the cost of this machine's own electricity consumption and purchase, so I think it is better to use this machine instead of increasing the expenditure, uneconomical, rather than not use it.	Jingdong prefecture level city in Shandong
Dragon Quality Goods Shop	Green Intelligent Power Saver	I've been using it for two days now and I don't feel any effect.	Jingdong prefecture level city in Shandong
Dragon Quality Goods Shop	Green Intelligent Power Saver	What it looked like was nothing like what was sent.	Jingdong prefecture level city in Shandong
Dragon Quality Goods Shop	Green Intelligent Power Saver	It didn't work. It didn't work for a month or so.	Jingdong prefecture level city in Shandong
Dragon Quality Goods Shop	Green Intelligent Power Saver	Garbage products, physical and description is not the same, no effect at all, pit # no discussion, to buy the fine, garbage, garbage, garbage	Jingdong prefecture level city in Shandong
Dragon Quality Goods Shop	Green Intelligent Power Saver	general	Jingdong prefecture level city in Shandong
Dragon Quality Goods Shop	Green Intelligent Power Saver	IQ tax. Instead of saving electricity, it increases the power consumption of an appliance.	Jingdong prefecture level city in Shandong
Dragon Quality Goods Shop	Green Intelligent Power Saver	There's no trash at all.	Jingdong prefecture level city in Shandong
Dragon Quality Goods Shop	Green Intelligent Power Saver	It doesn't do anything.	Jingdong prefecture level city in Shandong
Dragon Quality Goods Shop	Green Intelligent Power Saver	(of an unmarried couple) be close	Jingdong prefecture level city in Shandong
Dragon Quality Goods Shop	Green Intelligent Power Saver	(of an unmarried couple) be close	Jingdong prefecture level city in Shandong
Dragon Quality Goods Shop	Green Intelligent Power Saver	no electricity saving	Jingdong prefecture level city in Shandong
Dragon Quality Goods Shop	Green Intelligent Power Saver	differ from	Jingdong prefecture level city in Shandong
Dragon Quality Goods Shop	Green Intelligent Power Saver	Saver has received, but real can save electricity, the quality is very good, I times I buy a few more, too good.	Jingdong prefecture level city in Shandong
Dragon Quality Goods Shop	Green Intelligent Power Saver	Great quality as always, a very favorite product, great! o(*￣︶￣*)o	Jingdong prefecture level city in Shandong
Dragon Quality Goods Shop	Green Intelligent Power Saver	If we can reduce the electricity bill, for individuals can save money, for the country can protect the environment, now the weather is getting hotter and hotter, to protect the environment to reduce emissions is very important. Already use the product, whether to reduce the electricity bill depends on next month!	Jingdong prefecture level city in Shandong
Dragon Quality Goods Shop	Green Intelligent Power Saver	Purchased a power saver received on the put into use, rent a room with many electrical appliances, power consumption is very large, we have a piece of electricity or 1.2 dollars a time, with this power saver is much better, every day can save a few cents, very convenient and practical!	Jingdong prefecture level city in Shandong
Dragon Quality Goods Shop	Green Intelligent Power Saver	The product is better to use before not to use when a month 1500 yuan electricity bill, now using the 5 second month electricity bill directly down 470 dollars or very good. A save money to buy several power saver money ZanZanZanZan👍	Jingdong prefecture level city in Shandong
Dragon Quality Goods Shop	Green Intelligent Power Saver	The moment I received the goods really satisfied with this product, not only high-grade packaging, the body are base alloy, up to the price, I was also introduced by a friend, he used last month did save a lot of electricity, but also recommended to me!	Jingdong prefecture level city in Shandong
Dragon Quality Goods Shop	Green Intelligent Power Saver	I got it. Good review. I'm very satisfied. Reasonable price.	Jingdong prefecture level city in Shandong
Dragon Quality Goods Shop	Green Intelligent Power Saver	Intelligent power saver received and used, looking at the quality is good, the quality of the plug sent is also very good. Expect the effect of the economizer is obvious, first five-star praise, with a period of time to follow up!	Jingdong prefecture level city in Shandong
Dragon Quality Goods Shop	Green Intelligent Power Saver	The saver has been received. It was plugged into an outlet and used immediately after receipt. Whether it really saves electricity is not clear yet, I believe that this brand of energy saver really saves	Jingdong prefecture level city in Shandong
Dragon Quality Goods Shop	Green Intelligent Power Saver	Okay, okay, okay. Thank you so much. Fast is good. Fast. Item was as described, mainly because the event was cheap and you can save a lot of money.	Jingdong prefecture level city in Shandong
Dragon Quality Goods Shop	Green Intelligent Power Saver	Received it a couple days ago! Plugged it in the same day! Let's see what the electric bill is in a month! Hopefully it works well oh!	Jingdong prefecture level city in Shandong
Dragon Quality Goods Shop	Green Intelligent Power Saver	Looking at the reviews good, energy-saving small products, compact, the results after 2 months to see, because the electricity bill season payment	Jingdong prefecture level city in Shandong
Dragon Quality Goods Shop	Green Intelligent Power Saver	I just received it and will try it out to see if it works well. I'll comment later.	Jingdong prefecture level city in Shandong
Dragon Quality Goods Shop	Green Intelligent Power Saver	It looks a little small and not very aesthetically pleasing, I don't know how effective it will be until I use it	Jingdong prefecture level city in Shandong
Dragon Quality Goods Shop	Green Intelligent Power Saver	Not only to the point of being useless yet	Jingdong prefecture level city in Shandong
Dragon Quality Goods Shop	Green Intelligent Power Saver	Used it for a month, nothing works, the meter you turn very fast. This is not a scam, but also sold on Jingdong. To cheat a bunch of people?	Jingdong prefecture level city in Shandong
Dragon Quality Goods Shop	Green Intelligent Power Saver	Very good to use, every day can save a little electricity, really good under the sample down a month to save a few dozen dollars in electricity, good before I did not know that there is this 👆 haha, the price is not expensive praise.	Jingdong prefecture level city in Shandong
Dragon Quality Goods Shop	Green Intelligent Power Saver	Tried it for ten days and the change is almost nothing, can't see the power saving yet, use it some more days and see how it goes.	Jingdong prefecture level city in Shandong
Dragon Quality Goods Shop	Green Intelligent Power Saver	Received very quickly, the quality is very good, really save electricity, business service attitude is also very good, I hope you do not miss this opportunity, I continue to buy a few in	Jingdong prefecture level city in Shandong
Dragon Quality Goods Shop	Green Intelligent Power Saver	Quality is very good Oh, the delivery is also very fast, a very favorite store, I want to crazy recommend to friends!	Jingdong prefecture level city in Shandong
Dragon Quality Goods Shop	Green Intelligent Power Saver	The goods have just received, fine workmanship, and the same as described by the merchant, to use a period of time to see if it can achieve the purpose of power saving.	Jingdong prefecture level city in Shandong
Dragon Quality Goods Shop	Green Intelligent Power Saver	Received the goods first use a period of time to see if there is no effect, the business commitment to ninety days invalid refund, first praise, good service!	Jingdong prefecture level city in Shandong
Dragon Quality Goods Shop	Green Intelligent Power Saver	The product is used for a month without energy-saving and power-saving effect does not have any effect	Jingdong prefecture level city in Shandong
Dragon Quality Goods Shop	Green Intelligent Power Saver	The baby is really great, I like it very much, my friends also say good to come, recommended to buy!	Jingdong prefecture level city in Shandong
Dragon Quality Goods Shop	Green Intelligent Power Saver	Logistics is fast, courier boy service is good, after using to see how the fruit, and then follow up.	Jingdong prefecture level city in Shandong
Dragon Quality Goods Shop	Green Intelligent Power Saver	Baby has been received to try, I believe that as the business advertised energy saving. The courier is very responsible.	Jingdong prefecture level city in Shandong
Dragon Quality Goods Shop	Green Intelligent Power Saver	Save Electric Energy King received, good quality, beautiful appearance, very satisfied!	Jingdong prefecture level city in Shandong
Dragon Quality Goods Shop	Green Intelligent Power Saver	Three no products no manufacturers no phone pure fraud induced consumption cheat	Jingdong prefecture level city in Shandong
Dragon Quality Goods Shop	Green Intelligent Power Saver	I'm using it now, I don't know if it's as good as it says, I'll follow up after a month	Jingdong prefecture level city in Shandong
Dragon Quality Goods Shop	Green Intelligent Power Saver	Good quality logistics and fast. Fine workmanship. The description is consistent cost-effective	Jingdong prefecture level city in Shandong
Dragon Quality Goods Shop	Green Intelligent Power Saver	Received, whether it is good or not, to wait a few days and then evaluate	Jingdong prefecture level city in Shandong
Dragon Quality Goods Shop	Green Intelligent Power Saver	I bought it and started using it, I won't know if it works well until I pay my electric bill	Jingdong prefecture level city in Shandong
Dragon Quality Goods Shop	Green Intelligent Power Saver	Especially good, energy saving and power saving, especially good quality, good	Jingdong prefecture level city in Shandong
Dragon Quality Goods Shop	Green Intelligent Power Saver	Logistics is strong, the quality is very good, paste really save electricity, as described, will buy back!	Jingdong prefecture level city in Shandong
Dragon Quality Goods Shop	Green Intelligent Power Saver	Great stuff at a great price, worth the money!	Jingdong prefecture level city in Shandong

Dragon Quality Goods Shop	Green Intelligent Power Saver	Received the power saver, very good quality, very power saving!	Jingdong prefecture level city in Shandong
Dragon Quality Goods Shop	Green Intelligent Power Saver	There are no fruits, only samples...	Jingdong prefecture level city in Shandong
Dragon Quality Goods Shop	Green Intelligent Power Saver	Save a few dollars a month.	Jingdong prefecture level city in Shandong
Dragon Quality Goods Shop	Green Intelligent Power Saver	The quality is particularly good, it works well, and the seller has a good service attitude	Jingdong prefecture level city in Shandong
Dragon Quality Goods Shop	Green Intelligent Power Saver	I've compared them a couple of times and I don't feel any difference	Jingdong prefecture level city in Shandong
Dragon Quality Goods Shop	Green Intelligent Power Saver	I don't see the savings. It's a sham.	Jingdong prefecture level city in Shandong
Dragon Quality Goods Shop	Green Intelligent Power Saver	The power saving effect is very good, and the logistics is very fast. The seller's service attitude is very good	Jingdong prefecture level city in Shandong
Dragon Quality Goods Shop	Green Intelligent Power Saver	Received, well packaged, haven't tried it yet!	Jingdong prefecture level city in Shandong
Dragon Quality Goods Shop	Green Intelligent Power Saver	I don't know how it works, I'll comment after I use it.	Jingdong prefecture level city in Shandong
Dragon Quality Goods Shop	Green Intelligent Power Saver	The product doesn't work well enough to be of any use	Jingdong prefecture level city in Shandong
Dragon Quality Goods Shop	Green Intelligent Power Saver	On Trial 。。。。。。。	Jingdong prefecture level city in Shandong
Dragon Quality Goods Shop	Green Intelligent Power Saver	Being used with unknown results	Jingdong prefecture level city in Shandong
Dragon Quality Goods Shop	Green Intelligent Power Saver	Smart power saving and great quality	Jingdong prefecture level city in Shandong
Dragon Quality Goods Shop	Green Intelligent Power Saver	The saver was received, it works very well and the price is affordable!	Jingdong prefecture level city in Shandong
Dragon Quality Goods Shop	Green Intelligent Power Saver	How can I feel the power saving and how long does it last	Jingdong prefecture level city in Shandong
Dragon Quality Goods Shop	Green Intelligent Power Saver	And I know if it saves electricity or not.	Jingdong prefecture level city in Shandong
Dragon Quality Goods Shop	Green Intelligent Power Saver	It won't save any electricity. No.	Jingdong prefecture level city in Shandong
Dragon Quality Goods Shop	Green Intelligent Power Saver	Invalid. Fake.	Jingdong prefecture level city in Shandong
Dragon Quality Goods Shop	Green Intelligent Power Saver	It's good!	Jingdong prefecture level city in Shandong
Dragon Quality Goods Shop	Green Intelligent Power Saver	have no effect	Jingdong prefecture level city in Shandong
Dragon Quality Goods Shop	Green Intelligent Power Saver	Not used.	Jingdong prefecture level city in Shandong
Dragon Quality Goods Shop	Green Intelligent Power Saver	There is no load range, packaging thrift, ten days also did not develop invoices, received the goods with a receipt, hand-written receipt also has no financial stamp, communication for nearly a week a moment to say that the electronic invoice, a moment to say that the paper invoice, said it is mailed to me, ten days also did not see anything, the invoice invoice can not be found to say that did not stay on file, single single number can not be found, said that the warehouse more than one to a one to find out, in addition to the perfunctory or perfunctory. Products are no certificate of conformity, proper three	Jingdong prefecture level city in Shandong
Dragon Quality Goods Shop	Green Intelligent Power Saver	Satisfaction.	Jingdong prefecture level city in Shandong
Dragon Quality Goods Shop	Green Intelligent Power Saver	There is not a bit of power saving effect, and ringing up an increase in the electricity bill, because you have to add the cost of this machine's own electricity consumption and purchase, so I think it is better to use this machine instead of increasing the expenditure, uneconomical, rather than not use it.	Jingdong prefecture level city in Shandong
Dragon Quality Goods Shop	Green Intelligent Power Saver	I've been using it for two days now and I don't feel any effect.	Jingdong prefecture level city in Shandong
Dragon Quality Goods Shop	Green Intelligent Power Saver	What it looked like was nothing like what was sent.	Jingdong prefecture level city in Shandong
Dragon Quality Goods Shop	Green Intelligent Power Saver	It didn't work. It didn't work for a month or so.	Jingdong prefecture level city in Shandong
Dragon Quality Goods Shop	Green Intelligent Power Saver	Garbage products, physical and description is not the same, no effect at all, pit # no discussion, to buy the fine, garbage, garbage, garbage	Jingdong prefecture level city in Shandong
Dragon Quality Goods Shop	Green Intelligent Power Saver	general	Jingdong prefecture level city in Shandong
Dragon Quality Goods Shop	Green Intelligent Power Saver	IQ tax. Instead of saving electricity, it increases the power consumption of an appliance.	Jingdong prefecture level city in Shandong
Dragon Quality Goods Shop	Green Intelligent Power Saver	There's no trash at all.	Jingdong prefecture level city in Shandong
Dragon Quality Goods Shop	Green Intelligent Power Saver	It doesn't do anything.	Jingdong prefecture level city in Shandong
Dragon Quality Goods Shop	Green Intelligent Power Saver	(of an unmarried couple) be close	Jingdong prefecture level city in Shandong
Dragon Quality Goods Shop	Green Intelligent Power Saver	(of an unmarried couple) be close	Jingdong prefecture level city in Shandong
Dragon Quality Goods Shop	Green Intelligent Power Saver	no electricity saving	Jingdong prefecture level city in Shandong
Dragon Quality Goods Shop	Green Intelligent Power Saver	differ from	Jingdong prefecture level city in Shandong
Dragon Quality Goods Shop	Green Intelligent Power Saver	Saver has received, but real can save electricity, the quality is very good, I times I buy a few more, too good.	Jingdong prefecture level city in Shandong
Dragon Quality Goods Shop	Green Intelligent Power Saver	Great quality as always, a very favorite product, great! o(*￣︶￣*)o	Jingdong prefecture level city in Shandong
Dragon Quality Goods Shop	Green Intelligent Power Saver	If we can reduce the electricity bill, for individuals can save money, for the country can protect the environment, now the weather is getting hotter and hotter, to protect the environment to reduce emissions is very important. Already use the product, whether to reduce the electricity bill depends on next month!	Jingdong prefecture level city in Shandong
Dragon Quality Goods Shop	Green Intelligent Power Saver	Purchased a power saver received on the put into use, rent a room with many electrical appliances, power consumption is very large, we have a piece of electricity or 1.2 dollars a time, with this power saver is much better, every day can save a few cents, very convenient and practical!	Jingdong prefecture level city in Shandong
Dragon Quality Goods Shop	Green Intelligent Power Saver	The product is better to use before not to use when a month 1500 yuan electricity bill, now using the 5 second month electricity bill directly down 470 dollars or very good. A save money to buy several power saver money ZanZanZanZan👍	Jingdong prefecture level city in Shandong
Dragon Quality Goods Shop	Green Intelligent Power Saver	The moment I received the goods really satisfied with this product, not only high-grade packaging, the body are base alloy, up to the price, I was also introduced by a friend, he used last month did save a lot of electricity, but also recommended to me!	Jingdong prefecture level city in Shandong
Dragon Quality Goods Shop	Green Intelligent Power Saver	I got it. Good review. I'm very satisfied. Reasonable price.	Jingdong prefecture level city in Shandong
Dragon Quality Goods Shop	Green Intelligent Power Saver	Intelligent power saver received and used, looking at the quality is good, the quality of the plug sent is also very good. Expect the effect of the economizer is obvious, first five-star praise, with a period of time to follow up!	Jingdong prefecture level city in Shandong
Dragon Quality Goods Shop	Green Intelligent Power Saver	The saver has been received. It was plugged into an outlet and used immediately after receipt. Whether it really saves electricity is not clear yet, I believe that this brand of energy saver really saves	Jingdong prefecture level city in Shandong
Dragon Quality Goods Shop	Green Intelligent Power Saver	Okay, okay, okay. Thank you so much. Fast is good. Fast. Item was as described, mainly because the event was cheap and you can save a lot of money.	Jingdong prefecture level city in Shandong

Dragon Quality Goods Shop	Green Intelligent Power Saver	Received it a couple days ago! Plugged it in the same day! Let's see what the electric bill is in a month! Hopefully it works well oh!	Jingdong prefecture level city in Shandong
Dragon Quality Goods Shop	Green Intelligent Power Saver	Looking at the reviews good, energy-saving small products, compact, the results after 2 months to see, because the electricity bill season payment	Jingdong prefecture level city in Shandong
Dragon Quality Goods Shop	Green Intelligent Power Saver	I just received it and will try it out to see if it works well. I'll comment later.	Jingdong prefecture level city in Shandong
Dragon Quality Goods Shop	Green Intelligent Power Saver	It looks a little small and not very aesthetically pleasing, I don't know how effective it will be until I use it	Jingdong prefecture level city in Shandong
Dragon Quality Goods Shop	Green Intelligent Power Saver	Not only to the point of being useless yet	Jingdong prefecture level city in Shandong
Dragon Quality Goods Shop	Green Intelligent Power Saver	Used it for a month, nothing works, the meter you turn very fast. This is not a scam, but also sold on Jingdong. To cheat a bunch of people?	Jingdong prefecture level city in Shandong
Dragon Quality Goods Shop	Green Intelligent Power Saver	Very good to use, every day can save a little electricity, really good under the sample down a month to save a few dozen dollars in electricity, good before I did not know that there is this haha, the price is not expensive praise.	Jingdong prefecture level city in Shandong
Dragon Quality Goods Shop	Green Intelligent Power Saver	Tried it for ten days and the change is almost nothing, can't see the power saving yet, use it some more days and see how it goes.	Jingdong prefecture level city in Shandong
Dragon Quality Goods Shop	Green Intelligent Power Saver	Received very quickly, the quality is very good, really save electricity, business service attitude is also very good, I hope you do not miss this opportunity, I continue to buy a few in	Jingdong prefecture level city in Shandong
Dragon Quality Goods Shop	Green Intelligent Power Saver	Quality is very good Oh, the delivery is also very fast, a very favorite store, I want to crazy recommend to friends!	Jingdong prefecture level city in Shandong
Dragon Quality Goods Shop	Green Intelligent Power Saver	The goods have just received, fine workmanship, and the same as described by the merchant, to use a period of time to see if it can achieve the purpose of power saving.	Jingdong prefecture level city in Shandong
Dragon Quality Goods Shop	Green Intelligent Power Saver	Received the goods first use a period of time to see if there is no effect, the business commitment to ninety days invalid refund, first praise, good service!	Jingdong prefecture level city in Shandong
Dragon Quality Goods Shop	Green Intelligent Power Saver	The product is used for a month without energy-saving and power-saving effect does not have any effect	Jingdong prefecture level city in Shandong
Dragon Quality Goods Shop	Green Intelligent Power Saver	The baby is really great, I like it very much, my friends also say good to come, recommended to buy!	Jingdong prefecture level city in Shandong
Dragon Quality Goods Shop	Green Intelligent Power Saver	Logistics is fast, courier boy service is good, after using to see how the fruit, and then follow up.	Jingdong prefecture level city in Shandong
Dragon Quality Goods Shop	Green Intelligent Power Saver	Baby has been received to try, I believe that as the business advertised energy saving. The courier is very responsible.	Jingdong prefecture level city in Shandong
Dragon Quality Goods Shop	Green Intelligent Power Saver	Save Electric Energy King received, good quality, beautiful appearance, very satisfied!	Jingdong prefecture level city in Shandong
Dragon Quality Goods Shop	Green Intelligent Power Saver	Three no products no manufacturers no phone pure fraud induced consumption cheat	Jingdong prefecture level city in Shandong
Dragon Quality Goods Shop	Green Intelligent Power Saver	I'm using it now, I don't know if it's as good as it says, I'll follow up after a month	Jingdong prefecture level city in Shandong
Dragon Quality Goods Shop	Green Intelligent Power Saver	Good quality logistics and fast. Fine workmanship. The description is consistent cost-effective	Jingdong prefecture level city in Shandong
Dragon Quality Goods Shop	Green Intelligent Power Saver	Received, whether it is good or not, to wait a few days and then evaluate	Jingdong prefecture level city in Shandong
Dragon Quality Goods Shop	Green Intelligent Power Saver	I bought it and started using it, I won't know if it works well until I pay my electric bill	Jingdong prefecture level city in Shandong
Dragon Quality Goods Shop	Green Intelligent Power Saver	Especially good, energy saving and power saving, especially good quality, good	Jingdong prefecture level city in Shandong
Dragon Quality Goods Shop	Green Intelligent Power Saver	Logistics is strong, the quality is very good, paste really save electricity, as described, will buy back!	Jingdong prefecture level city in Shandong
Dragon Quality Goods Shop	Green Intelligent Power Saver	Great stuff at a great price, worth the money!	Jingdong prefecture level city in Shandong
Dragon Quality Goods Shop	Green Intelligent Power Saver	Received the power saver, very good quality, very power saving!	Jingdong prefecture level city in Shandong
Dragon Quality Goods Shop	Green Intelligent Power Saver	There are no fruits, only samples...	Jingdong prefecture level city in Shandong
Dragon Quality Goods Shop	Green Intelligent Power Saver	Save a few dollars a month.	Jingdong prefecture level city in Shandong
Dragon Quality Goods Shop	Green Intelligent Power Saver	The quality is particularly good, it works well, and the seller has a good service attitude	Jingdong prefecture level city in Shandong
Dragon Quality Goods Shop	Green Intelligent Power Saver	I've compared them a couple of times and I don't feel any difference	Jingdong prefecture level city in Shandong
Dragon Quality Goods Shop	Green Intelligent Power Saver	I don't see the savings. It's a sham.	Jingdong prefecture level city in Shandong
Dragon Quality Goods Shop	Green Intelligent Power Saver	The power saving effect is very good, and the logistics is very fast. The seller's service attitude is very good	Jingdong prefecture level city in Shandong
Dragon Quality Goods Shop	Green Intelligent Power Saver	Received, well packaged, haven't tried it yet!	Jingdong prefecture level city in Shandong
Dragon Quality Goods Shop	Green Intelligent Power Saver	I don't know how it works, I'll comment after I use it.	Jingdong prefecture level city in Shandong
Dragon Quality Goods Shop	Green Intelligent Power Saver	The product doesn't work well enough to be of any use	Jingdong prefecture level city in Shandong
Dragon Quality Goods Shop	Green Intelligent Power Saver	On Trial 。。。。。。。	Jingdong prefecture level city in Shandong
Dragon Quality Goods Shop	Green Intelligent Power Saver	Being used with unknown results	Jingdong prefecture level city in Shandong
Dragon Quality Goods Shop	Green Intelligent Power Saver	Smart power saving and great quality	Jingdong prefecture level city in Shandong
Dragon Quality Goods Shop	Green Intelligent Power Saver	The saver was received, it works very well and the price is affordable!	Jingdong prefecture level city in Shandong
Dragon Quality Goods Shop	Green Intelligent Power Saver	How can I feel the power saving and how long does it last	Jingdong prefecture level city in Shandong
Dragon Quality Goods Shop	Green Intelligent Power Saver	And I know if it saves electricity or not.	Jingdong prefecture level city in Shandong
Dragon Quality Goods Shop	Green Intelligent Power Saver	It won't save any electricity. No.	Jingdong prefecture level city in Shandong
Dragon Quality Goods Shop	Green Intelligent Power Saver	Invalid. Fake.	Jingdong prefecture level city in Shandong
Dragon Quality Goods Shop	Green Intelligent Power Saver	It's good!	Jingdong prefecture level city in Shandong
Dragon Quality Goods Shop	Green Intelligent Power Saver	have no effect	Jingdong prefecture level city in Shandong
Dragon Quality Goods Shop	Green Intelligent Power Saver	Not used.	Jingdong prefecture level city in Shandong
Dragon Quality Goods Shop	Green Intelligent Power Saver	There is no load range, packaging thrift, ten days also did not develop invoices, received the goods with a receipt, hand-written receipt also has no financial stamp, communication for nearly a week a moment to say that the electronic invoice, a moment to say that the paper invoice, said it is mailed to me, ten days also did not see anything, the invoice invoice can not be found to say that did not stay on file, single single number can not be found, said that the warehouse more than one to a one to find out, in addition to the perfunctory or perfunctory. Products are no certificate of conformity, proper three	Jingdong prefecture level city in Shandong
Dragon Quality Goods Shop	Green Intelligent Power Saver	Satisfaction.	Jingdong prefecture level city in Shandong

Dragon Quality Goods Shop	Green Intelligent Power Saver	There is not a bit of power saving effect, and ringing up an increase in the electricity bill, because you have to add the cost of this machine's own electricity consumption and purchase, so I think it is better to use this machine instead of increasing the expenditure, uneconomical, rather than not use it.	Jingdong prefecture level city in Shandong
Dragon Quality Goods Shop	Green Intelligent Power Saver	I've been using it for two days now and I don't feel any effect.	Jingdong prefecture level city in Shandong
Dragon Quality Goods Shop	Green Intelligent Power Saver	What it looked like was nothing like what was sent.	Jingdong prefecture level city in Shandong
Dragon Quality Goods Shop	Green Intelligent Power Saver	It didn't work. It didn't work for a month or so.	Jingdong prefecture level city in Shandong
Dragon Quality Goods Shop	Green Intelligent Power Saver	Garbage products, physical and description is not the same, no effect at all, pit # no discussion, to buy the fine, garbage, garbage, garbage	Jingdong prefecture level city in Shandong
Dragon Quality Goods Shop	Green Intelligent Power Saver	general	Jingdong prefecture level city in Shandong
Dragon Quality Goods Shop	Green Intelligent Power Saver	IQ tax. Instead of saving electricity, it increases the power consumption of an appliance.	Jingdong prefecture level city in Shandong
Dragon Quality Goods Shop	Green Intelligent Power Saver	There's no trash at all.	Jingdong prefecture level city in Shandong
Dragon Quality Goods Shop	Green Intelligent Power Saver	It doesn't do anything.	Jingdong prefecture level city in Shandong
Dragon Quality Goods Shop	Green Intelligent Power Saver	(of an unmarried couple) be close	Jingdong prefecture level city in Shandong
Dragon Quality Goods Shop	Green Intelligent Power Saver	(of an unmarried couple) be close	Jingdong prefecture level city in Shandong
Dragon Quality Goods Shop	Green Intelligent Power Saver	no electricity saving	Jingdong prefecture level city in Shandong
Dragon Quality Goods Shop	Green Intelligent Power Saver	differ from	Jingdong prefecture level city in Shandong
Dragon Quality Goods Shop	Green Intelligent Power Saver	Saver has received, but real can save electricity, the quality is very good, I times I buy a few more, too good.	Jingdong prefecture level city in Shandong
Dragon Quality Goods Shop	Green Intelligent Power Saver	Great quality as always, a very favorite product, great! o(*￣︶￣*)o	Jingdong prefecture level city in Shandong
Dragon Quality Goods Shop	Green Intelligent Power Saver	If we can reduce the electricity bill, for individuals can save money, for the country can protect the environment, now the weather is getting hotter and hotter, to protect the environment to reduce emissions is very important. Already use the product, whether to reduce the electricity bill depends on next month!	Jingdong prefecture level city in Shandong
Dragon Quality Goods Shop	Green Intelligent Power Saver	Purchased a power saver received on the put into use, rent a room with many electrical appliances, power consumption is very large, we have a piece of electricity or 1.2 dollars a time, with this power saver is much better, every day can save a few cents, very convenient and practical!	Jingdong prefecture level city in Shandong
Dragon Quality Goods Shop	Green Intelligent Power Saver	The product is better to use before not to use when a month 1500 yuan electricity bill, now using the 5 second month electricity bill directly down 470 dollars or very good. A save money to buy several power saver money ZanZanZanZan👍	Jingdong prefecture level city in Shandong
Dragon Quality Goods Shop	Green Intelligent Power Saver	The moment I received the goods really satisfied with this product, not only high-grade packaging, the body are base alloy, up to the price, I was also introduced by a friend, he used last month did save a lot of electricity, but also recommended to me!	Jingdong prefecture level city in Shandong
Dragon Quality Goods Shop	Green Intelligent Power Saver	I got it. Good review. I'm very satisfied. Reasonable price.	Jingdong prefecture level city in Shandong
Dragon Quality Goods Shop	Green Intelligent Power Saver	Intelligent power saver received and used, looking at the quality is good, the quality of the plug sent is also very good. Expect the effect of the economizer is obvious, first five-star praise, with a period of time to follow up!	Jingdong prefecture level city in Shandong
Dragon Quality Goods Shop	Green Intelligent Power Saver	The saver has been received. It was plugged into an outlet and used immediately after receipt. Whether it really saves electricity is not clear yet, I believe that this brand of energy saver really saves	Jingdong prefecture level city in Shandong
Dragon Quality Goods Shop	Green Intelligent Power Saver	Okay, okay, okay. Thank you so much. Fast is good. Fast. Item was as described, mainly because the event was cheap and you can save a lot of money.	Jingdong prefecture level city in Shandong
Dragon Quality Goods Shop	Green Intelligent Power Saver	Received it a couple days ago! Plugged it in the same day! Let's see what the electric bill is in a month! Hopefully it works well oh!	Jingdong prefecture level city in Shandong
Dragon Quality Goods Shop	Green Intelligent Power Saver	Looking at the reviews good, energy-saving small products, compact, the results after 2 months to see, because the electricity bill season payment	Jingdong prefecture level city in Shandong
Dragon Quality Goods Shop	Green Intelligent Power Saver	I just received it and will try it out to see if it works well. I'll comment later.	Jingdong prefecture level city in Shandong
Dragon Quality Goods Shop	Green Intelligent Power Saver	It looks a little small and not very aesthetically pleasing, I don't know how effective it will be until I use it	Jingdong prefecture level city in Shandong
Dragon Quality Goods Shop	Green Intelligent Power Saver	Not only to the point of being useless yet	Jingdong prefecture level city in Shandong
Dragon Quality Goods Shop	Green Intelligent Power Saver	Used it for a month, nothing works, the meter you turn very fast. This is not a scam, but also sold on Jingdong. To cheat a bunch of people?	Jingdong prefecture level city in Shandong
Dragon Quality Goods Shop	Green Intelligent Power Saver	Very good to use, every day can save a little electricity, really good under the sample down a month to save a few dozen dollars in electricity, good before I did not know that there is this 👍 haha, the price is not expensive praise.	Jingdong prefecture level city in Shandong
Dragon Quality Goods Shop	Green Intelligent Power Saver	Tried it for ten days and the change is almost nothing, can't see the power saving yet, use it some more days and see how it goes.	Jingdong prefecture level city in Shandong
Dragon Quality Goods Shop	Green Intelligent Power Saver	Received very quickly, the quality is very good, really save electricity, business service attitude is also very good, I hope you do not miss this opportunity, I continue to buy a few in	Jingdong prefecture level city in Shandong
Dragon Quality Goods Shop	Green Intelligent Power Saver	Quality is very good Oh, the delivery is also very fast, a very favorite store, I want to crazy recommend to friends!	Jingdong prefecture level city in Shandong
Dragon Quality Goods Shop	Green Intelligent Power Saver	The goods have just received, fine workmanship, and the same as described by the merchant, to use a period of time to see if it can achieve the purpose of power saving.	Jingdong prefecture level city in Shandong
Dragon Quality Goods Shop	Green Intelligent Power Saver	Received the goods first use a period of time to see if there is no effect, the business commitment to ninety days invalid refund, first praise, good service!	Jingdong prefecture level city in Shandong
Dragon Quality Goods Shop	Green Intelligent Power Saver	The product is used for a month without energy-saving and power-saving effect does not have any effect	Jingdong prefecture level city in Shandong
Dragon Quality Goods Shop	Green Intelligent Power Saver	The baby is really great, I like it very much, my friends also say good to come, recommended to buy!	Jingdong prefecture level city in Shandong
Dragon Quality Goods Shop	Green Intelligent Power Saver	Logistics is fast, courier boy service is good, after using to see how the fruit, and then follow up.	Jingdong prefecture level city in Shandong
Dragon Quality Goods Shop	Green Intelligent Power Saver	Baby has been received to try, I believe that as the business advertised energy saving. The courier is very responsible.	Jingdong prefecture level city in Shandong
Dragon Quality Goods Shop	Green Intelligent Power Saver	Save Electric Energy King received, good quality, beautiful appearance, very satisfied!	Jingdong prefecture level city in Shandong
Dragon Quality Goods Shop	Green Intelligent Power Saver	Three no products no manufacturers no phone pure fraud induced consumption cheat	Jingdong prefecture level city in Shandong
Dragon Quality Goods Shop	Green Intelligent Power Saver	I'm using it now, I don't know if it's as good as it says, I'll follow up after a month	Jingdong prefecture level city in Shandong
Dragon Quality Goods Shop	Green Intelligent Power Saver	Good quality logistics and fast. Fine workmanship. The description is consistent cost-effective	Jingdong prefecture level city in Shandong
Dragon Quality Goods Shop	Green Intelligent Power Saver	Received, whether it is good or not, to wait a few days and then evaluate	Jingdong prefecture level city in Shandong
Dragon Quality Goods Shop	Green Intelligent Power Saver	I bought it and started using it, I won't know if it works well until I pay my electric bill	Jingdong prefecture level city in Shandong
Dragon Quality Goods Shop	Green Intelligent Power Saver	Especially good, energy saving and power saving, especially good quality, good	Jingdong prefecture level city in Shandong
Dragon Quality Goods Shop	Green Intelligent Power Saver	Logistics is strong, the quality is very good, paste really save electricity, as described, will buy back!	Jingdong prefecture level city in Shandong
Dragon Quality Goods Shop	Green Intelligent Power Saver	Great stuff at a great price, worth the money!	Jingdong prefecture level city in Shandong

Dragon Quality Goods Shop	Green Intelligent Power Saver	Received the power saver, very good quality, very power saving!	Jingdong prefecture level city in Shandong
Dragon Quality Goods Shop	Green Intelligent Power Saver	There are no fruits, only samples...	Jingdong prefecture level city in Shandong
Dragon Quality Goods Shop	Green Intelligent Power Saver	Save a few dollars a month.	Jingdong prefecture level city in Shandong
Dragon Quality Goods Shop	Green Intelligent Power Saver	The quality is particularly good, it works well, and the seller has a good service attitude	Jingdong prefecture level city in Shandong
Dragon Quality Goods Shop	Green Intelligent Power Saver	I've compared them a couple of times and I don't feel any difference	Jingdong prefecture level city in Shandong
Dragon Quality Goods Shop	Green Intelligent Power Saver	I don't see the savings. It's a sham.	Jingdong prefecture level city in Shandong
Dragon Quality Goods Shop	Green Intelligent Power Saver	The power saving effect is very good, and the logistics is very fast. The seller's service attitude is very good	Jingdong prefecture level city in Shandong
Dragon Quality Goods Shop	Green Intelligent Power Saver	Received, well packaged, haven't tried it yet!	Jingdong prefecture level city in Shandong
Dragon Quality Goods Shop	Green Intelligent Power Saver	I don't know how it works, I'll comment after I use it.	Jingdong prefecture level city in Shandong
Dragon Quality Goods Shop	Green Intelligent Power Saver	The product doesn't work well enough to be of any use	Jingdong prefecture level city in Shandong
Dragon Quality Goods Shop	Green Intelligent Power Saver	On Trial 。。。。。。。	Jingdong prefecture level city in Shandong
Dragon Quality Goods Shop	Green Intelligent Power Saver	Being used with unknown results	Jingdong prefecture level city in Shandong
Dragon Quality Goods Shop	Green Intelligent Power Saver	Smart power saving and great quality	Jingdong prefecture level city in Shandong
Dragon Quality Goods Shop	Green Intelligent Power Saver	The saver was received, it works very well and the price is affordable!	Jingdong prefecture level city in Shandong
Dragon Quality Goods Shop	Green Intelligent Power Saver	How can I feel the power saving and how long does it last	Jingdong prefecture level city in Shandong
Dragon Quality Goods Shop	Green Intelligent Power Saver	And I know if it saves electricity or not.	Jingdong prefecture level city in Shandong
Dragon Quality Goods Shop	Green Intelligent Power Saver	It won't save any electricity. No.	Jingdong prefecture level city in Shandong
Dragon Quality Goods Shop	Green Intelligent Power Saver	Invalid. Fake.	Jingdong prefecture level city in Shandong
Dragon Quality Goods Shop	Green Intelligent Power Saver	It's good!	Jingdong prefecture level city in Shandong
Dragon Quality Goods Shop	Green Intelligent Power Saver	have no effect	Jingdong prefecture level city in Shandong
Dragon Quality Goods Shop	Green Intelligent Power Saver	Not used.	Jingdong prefecture level city in Shandong
Dragon Quality Goods Shop	Green Intelligent Power Saver	There is no load range, packaging thrift, ten days also did not develop invoices, received the goods with a receipt, hand-written receipt also has no financial stamp, communication for nearly a week a moment to say that the electronic invoice, a moment to say that the paper invoice, said it is mailed to me, ten days also did not see anything, the invoice invoice can not be found to say that did not stay on file, single single number can not be found, said that the warehouse more than one to a one to find out, in addition to the perfunctory or perfunctory. Products are no certificate of conformity, proper three	Jingdong prefecture level city in Shandong
Dragon Quality Goods Shop	Green Intelligent Power Saver	Satisfaction.	Jingdong prefecture level city in Shandong
Dragon Quality Goods Shop	Green Intelligent Power Saver	There is not a bit of power saving effect, and ringing up an increase in the electricity bill, because you have to add the cost of this machine's own electricity consumption and purchase, so I think it is better to use this machine instead of increasing the expenditure, uneconomical, rather than not use it.	Jingdong prefecture level city in Shandong
Dragon Quality Goods Shop	Green Intelligent Power Saver	I've been using it for two days now and I don't feel any effect.	Jingdong prefecture level city in Shandong
Dragon Quality Goods Shop	Green Intelligent Power Saver	What it looked like was nothing like what was sent.	Jingdong prefecture level city in Shandong
Dragon Quality Goods Shop	Green Intelligent Power Saver	It didn't work. It didn't work for a month or so.	Jingdong prefecture level city in Shandong
Dragon Quality Goods Shop	Green Intelligent Power Saver	Garbage products, physical and description is not the same, no effect at all, pit # no discussion, to buy the fine, garbage, garbage, garbage	Jingdong prefecture level city in Shandong
Dragon Quality Goods Shop	Green Intelligent Power Saver	general	Jingdong prefecture level city in Shandong
Dragon Quality Goods Shop	Green Intelligent Power Saver	IQ tax. Instead of saving electricity, it increases the power consumption of an appliance.	Jingdong prefecture level city in Shandong
Dragon Quality Goods Shop	Green Intelligent Power Saver	There's no trash at all.	Jingdong prefecture level city in Shandong
Dragon Quality Goods Shop	Green Intelligent Power Saver	It doesn't do anything.	Jingdong prefecture level city in Shandong
Dragon Quality Goods Shop	Green Intelligent Power Saver	(of an unmarried couple) be close	Jingdong prefecture level city in Shandong
Dragon Quality Goods Shop	Green Intelligent Power Saver	(of an unmarried couple) be close	Jingdong prefecture level city in Shandong
Dragon Quality Goods Shop	Green Intelligent Power Saver	no electricity saving	Jingdong prefecture level city in Shandong
Dragon Quality Goods Shop	Green Intelligent Power Saver	differ from	Jingdong prefecture level city in Shandong
Dragon Quality Goods Shop	Green Intelligent Power Saver	Saver has received, but real can save electricity, the quality is very good, I times I buy a few more, too good.	Jingdong prefecture level city in Shandong
Dragon Quality Goods Shop	Green Intelligent Power Saver	Great quality as always, a very favorite product, great! o(*￣︶￣*)o	Jingdong prefecture level city in Shandong
Dragon Quality Goods Shop	Green Intelligent Power Saver	If we can reduce the electricity bill, for individuals can save money, for the country can protect the environment, now the weather is getting hotter and hotter, to protect the environment to reduce emissions is very important. Already use the product, whether to reduce the electricity bill depends on next month!	Jingdong prefecture level city in Shandong
Dragon Quality Goods Shop	Green Intelligent Power Saver	Purchased a power saver received on the put into use, rent a room with many electrical appliances, power consumption is very large, we have a piece of electricity or 1.2 dollars a time, with this power saver is much better, every day can save a few cents, very convenient and practical!	Jingdong prefecture level city in Shandong
Dragon Quality Goods Shop	Green Intelligent Power Saver	The product is better to use before not to use when a month 1500 yuan electricity bill, now using the 5 second month electricity bill directly down 470 dollars or very good. A save money to buy several power saver money ZanZanZanZan👍	Jingdong prefecture level city in Shandong
Dragon Quality Goods Shop	Green Intelligent Power Saver	The moment I received the goods really satisfied with this product, not only high-grade packaging, the body are base alloy, up to the price, I was also introduced by a friend, he used last month did save a lot of electricity, but also recommended to me!	Jingdong prefecture level city in Shandong
Dragon Quality Goods Shop	Green Intelligent Power Saver	I got it. Good review. I'm very satisfied. Reasonable price.	Jingdong prefecture level city in Shandong
Dragon Quality Goods Shop	Green Intelligent Power Saver	Intelligent power saver received and used, looking at the quality is good, the quality of the plug sent is also very good. Expect the effect of the economizer is obvious, first five-star praise, with a period of time to follow up!	Jingdong prefecture level city in Shandong
Dragon Quality Goods Shop	Green Intelligent Power Saver	The saver has been received. It was plugged into an outlet and used immediately after receipt. Whether it really saves electricity is not clear yet, I believe that this brand of energy saver really saves	Jingdong prefecture level city in Shandong
Dragon Quality Goods Shop	Green Intelligent Power Saver	Okay, okay, okay. Thank you so much. Fast is good. Fast. Item was as described, mainly because the event was cheap and you can save a lot of money.	Jingdong prefecture level city in Shandong

Dragon Quality Goods Shop	Green Intelligent Power Saver	Received it a couple days ago! Plugged it in the same day! Let's see what the electric bill is in a month! Hopefully it works well oh!	Jingdong prefecture level city in Shandong
Dragon Quality Goods Shop	Green Intelligent Power Saver	Looking at the reviews good, energy-saving small products, compact, the results after 2 months to see, because the electricity bill season payment	Jingdong prefecture level city in Shandong
Dragon Quality Goods Shop	Green Intelligent Power Saver	I just received it and will try it out to see if it works well. I'll comment later.	Jingdong prefecture level city in Shandong
Dragon Quality Goods Shop	Green Intelligent Power Saver	It looks a little small and not very aesthetically pleasing, I don't know how effective it will be until I use it	Jingdong prefecture level city in Shandong
Dragon Quality Goods Shop	Green Intelligent Power Saver	Not only to the point of being useless yet	Jingdong prefecture level city in Shandong
Dragon Quality Goods Shop	Green Intelligent Power Saver	Used it for a month, nothing works, the meter you turn very fast. This is not a scam, but also sold on Jingdong. To cheat a bunch of people?	Jingdong prefecture level city in Shandong
Dragon Quality Goods Shop	Green Intelligent Power Saver	Very good to use, every day can save a little electricity, really good under the sample down a month to save a few dozen dollars in electricity, good before I did not know that there is this haha, the price is not expensive praise.	Jingdong prefecture level city in Shandong
Dragon Quality Goods Shop	Green Intelligent Power Saver	Tried it for ten days and the change is almost nothing, can't see the power saving yet, use it some more days and see how it goes.	Jingdong prefecture level city in Shandong
Dragon Quality Goods Shop	Green Intelligent Power Saver	Received very quickly, the quality is very good, really save electricity, business service attitude is also very good, I hope you do not miss this opportunity, I continue to buy a few in	Jingdong prefecture level city in Shandong
Dragon Quality Goods Shop	Green Intelligent Power Saver	Quality is very good Oh, the delivery is also very fast, a very favorite store, I want to crazy recommend to friends!	Jingdong prefecture level city in Shandong
Dragon Quality Goods Shop	Green Intelligent Power Saver	The goods have just received, fine workmanship, and the same as described by the merchant, to use a period of time to see if it can achieve the purpose of power saving.	Jingdong prefecture level city in Shandong
Dragon Quality Goods Shop	Green Intelligent Power Saver	Received the goods first use a period of time to see if there is no effect, the business commitment to ninety days invalid refund, first praise, good service!	Jingdong prefecture level city in Shandong
Dragon Quality Goods Shop	Green Intelligent Power Saver	The product is used for a month without energy-saving and power-saving effect does not have any effect	Jingdong prefecture level city in Shandong
Dragon Quality Goods Shop	Green Intelligent Power Saver	The baby is really great, I like it very much, my friends also say good to come, recommended to buy!	Jingdong prefecture level city in Shandong
Dragon Quality Goods Shop	Green Intelligent Power Saver	Logistics is fast, courier boy service is good, after using to see how the fruit, and then follow up.	Jingdong prefecture level city in Shandong
Dragon Quality Goods Shop	Green Intelligent Power Saver	Baby has been received to try, I believe that as the business advertised energy saving. The courier is very responsible.	Jingdong prefecture level city in Shandong
Dragon Quality Goods Shop	Green Intelligent Power Saver	Save Electric Energy King received, good quality, beautiful appearance, very satisfied!	Jingdong prefecture level city in Shandong
Dragon Quality Goods Shop	Green Intelligent Power Saver	Three no products no manufacturers no phone pure fraud induced consumption cheat	Jingdong prefecture level city in Shandong
Dragon Quality Goods Shop	Green Intelligent Power Saver	I'm using it now, I don't know if it's as good as it says, I'll follow up after a month	Jingdong prefecture level city in Shandong
Dragon Quality Goods Shop	Green Intelligent Power Saver	Good quality logistics and fast. Fine workmanship. The description is consistent cost-effective	Jingdong prefecture level city in Shandong
Dragon Quality Goods Shop	Green Intelligent Power Saver	Received, whether it is good or not, to wait a few days and then evaluate	Jingdong prefecture level city in Shandong
Dragon Quality Goods Shop	Green Intelligent Power Saver	I bought it and started using it, I won't know if it works well until I pay my electric bill	Jingdong prefecture level city in Shandong
Dragon Quality Goods Shop	Green Intelligent Power Saver	Especially good, energy saving and power saving, especially good quality, good	Jingdong prefecture level city in Shandong
Dragon Quality Goods Shop	Green Intelligent Power Saver	Logistics is strong, the quality is very good, paste really save electricity, as described, will buy back!	Jingdong prefecture level city in Shandong
Dragon Quality Goods Shop	Green Intelligent Power Saver	Great stuff at a great price, worth the money!	Jingdong prefecture level city in Shandong
Dragon Quality Goods Shop	Green Intelligent Power Saver	Received the power saver, very good quality, very power saving!	Jingdong prefecture level city in Shandong
Dragon Quality Goods Shop	Green Intelligent Power Saver	There are no fruits, only samples...	Jingdong prefecture level city in Shandong
Dragon Quality Goods Shop	Green Intelligent Power Saver	Save a few dollars a month.	Jingdong prefecture level city in Shandong
Dragon Quality Goods Shop	Green Intelligent Power Saver	The quality is particularly good, it works well, and the seller has a good service attitude	Jingdong prefecture level city in Shandong
Dragon Quality Goods Shop	Green Intelligent Power Saver	I've compared them a couple of times and I don't feel any difference	Jingdong prefecture level city in Shandong
Dragon Quality Goods Shop	Green Intelligent Power Saver	I don't see the savings. It's a sham.	Jingdong prefecture level city in Shandong
Dragon Quality Goods Shop	Green Intelligent Power Saver	The power saving effect is very good, and the logistics is very fast. The seller's service attitude is very good	Jingdong prefecture level city in Shandong
Dragon Quality Goods Shop	Green Intelligent Power Saver	Received, well packaged, haven't tried it yet!	Jingdong prefecture level city in Shandong
Dragon Quality Goods Shop	Green Intelligent Power Saver	I don't know how it works, I'll comment after I use it.	Jingdong prefecture level city in Shandong
Dragon Quality Goods Shop	Green Intelligent Power Saver	The product doesn't work well enough to be of any use	Jingdong prefecture level city in Shandong
Dragon Quality Goods Shop	Green Intelligent Power Saver	On Trial 。。。。。。。	Jingdong prefecture level city in Shandong
Dragon Quality Goods Shop	Green Intelligent Power Saver	Being used with unknown results	Jingdong prefecture level city in Shandong
Dragon Quality Goods Shop	Green Intelligent Power Saver	Smart power saving and great quality	Jingdong prefecture level city in Shandong
Dragon Quality Goods Shop	Green Intelligent Power Saver	The saver was received, it works very well and the price is affordable!	Jingdong prefecture level city in Shandong
Dragon Quality Goods Shop	Green Intelligent Power Saver	How can I feel the power saving and how long does it last	Jingdong prefecture level city in Shandong
Dragon Quality Goods Shop	Green Intelligent Power Saver	And I know if it saves electricity or not.	Jingdong prefecture level city in Shandong
Dragon Quality Goods Shop	Green Intelligent Power Saver	It won't save any electricity. No.	Jingdong prefecture level city in Shandong
Dragon Quality Goods Shop	Green Intelligent Power Saver	Invalid. Fake.	Jingdong prefecture level city in Shandong
Dragon Quality Goods Shop	Green Intelligent Power Saver	It's good!	Jingdong prefecture level city in Shandong
Dragon Quality Goods Shop	Green Intelligent Power Saver	have no effect	Jingdong prefecture level city in Shandong
Dragon Quality Goods Shop	Green Intelligent Power Saver	Not used.	Jingdong prefecture level city in Shandong
Dragon Quality Goods Shop	Green Intelligent Power Saver	There is no load range, packaging thrift, ten days also did not develop invoices, received the goods with a receipt, hand-written receipt also has no financial stamp, communication for nearly a week a moment to say that the electronic invoice, a moment to say that the paper invoice, said it is mailed to me, ten days also did not see anything, the invoice invoice can not be found to say that did not stay on file, single single number can not be found, said that the warehouse more than one to a one to find out, in addition to the perfunctory or perfunctory. Products are no certificate of conformity, proper three	Jingdong prefecture level city in Shandong
Dragon Quality Goods Shop	Green Intelligent Power Saver	Satisfaction.	Jingdong prefecture level city in Shandong

Dragon Quality Goods Shop	Green Intelligent Power Saver	There is not a bit of power saving effect, and ringing up an increase in the electricity bill, because you have to add the cost of this machine's own electricity consumption and purchase, so I think it is better to use this machine instead of increasing the expenditure, uneconomical, rather than not use it.	Jingdong prefecture level city in Shandong
Dragon Quality Goods Shop	Green Intelligent Power Saver	I've been using it for two days now and I don't feel any effect.	Jingdong prefecture level city in Shandong
Dragon Quality Goods Shop	Green Intelligent Power Saver	What it looked like was nothing like what was sent.	Jingdong prefecture level city in Shandong
Dragon Quality Goods Shop	Green Intelligent Power Saver	It didn't work. It didn't work for a month or so.	Jingdong prefecture level city in Shandong
Dragon Quality Goods Shop	Green Intelligent Power Saver	Garbage products, physical and description is not the same, no effect at all, pit # no discussion, to buy the fine, garbage, garbage, garbage	Jingdong prefecture level city in Shandong
Dragon Quality Goods Shop	Green Intelligent Power Saver	general	Jingdong prefecture level city in Shandong
Dragon Quality Goods Shop	Green Intelligent Power Saver	IQ tax. Instead of saving electricity, it increases the power consumption of an appliance.	Jingdong prefecture level city in Shandong
Dragon Quality Goods Shop	Green Intelligent Power Saver	There's no trash at all.	Jingdong prefecture level city in Shandong
Dragon Quality Goods Shop	Green Intelligent Power Saver	It doesn't do anything.	Jingdong prefecture level city in Shandong
Dragon Quality Goods Shop	Green Intelligent Power Saver	(of an unmarried couple) be close	Jingdong prefecture level city in Shandong
Dragon Quality Goods Shop	Green Intelligent Power Saver	(of an unmarried couple) be close	Jingdong prefecture level city in Shandong
Dragon Quality Goods Shop	Green Intelligent Power Saver	no electricity saving	Jingdong prefecture level city in Shandong
Dragon Quality Goods Shop	Green Intelligent Power Saver	differ from	Jingdong prefecture level city in Shandong
Dragon Quality Goods Shop	Green Intelligent Power Saver	Saver has received, but real can save electricity, the quality is very good, I times I buy a few more, too good.	Jingdong prefecture level city in Shandong
Dragon Quality Goods Shop	Green Intelligent Power Saver	Great quality as always, a very favorite product, great! o(*￣︶￣*)o	Jingdong prefecture level city in Shandong
Dragon Quality Goods Shop	Green Intelligent Power Saver	If we can reduce the electricity bill, for individuals can save money, for the country can protect the environment, now the weather is getting hotter and hotter, to protect the environment to reduce emissions is very important. Already use the product, whether to reduce the electricity bill depends on next month!	Jingdong prefecture level city in Shandong
Dragon Quality Goods Shop	Green Intelligent Power Saver	Purchased a power saver received on the put into use, rent a room with many electrical appliances, power consumption is very large, we have a piece of electricity or 1.2 dollars a time, with this power saver is much better, every day can save a few cents, very convenient and practical!	Jingdong prefecture level city in Shandong
Dragon Quality Goods Shop	Green Intelligent Power Saver	The product is better to use before not to use when a month 1500 yuan electricity bill, now using the 5 second month electricity bill directly down 470 dollars or very good. A save money to buy several power saver money ZanZanZanZan👍	Jingdong prefecture level city in Shandong
Dragon Quality Goods Shop	Green Intelligent Power Saver	The moment I received the goods really satisfied with this product, not only high-grade packaging, the body are base alloy, up to the price, I was also introduced by a friend, he used last month did save a lot of electricity, but also recommended to me!	Jingdong prefecture level city in Shandong
Dragon Quality Goods Shop	Green Intelligent Power Saver	I got it. Good review. I'm very satisfied. Reasonable price.	Jingdong prefecture level city in Shandong
Dragon Quality Goods Shop	Green Intelligent Power Saver	Intelligent power saver received and used, looking at the quality is good, the quality of the plug sent is also very good. Expect the effect of the economizer is obvious, first five-star praise, with a period of time to follow up!	Jingdong prefecture level city in Shandong
Dragon Quality Goods Shop	Green Intelligent Power Saver	The saver has been received. It was plugged into an outlet and used immediately after receipt. Whether it really saves electricity is not clear yet, I believe that this brand of energy saver really saves	Jingdong prefecture level city in Shandong
Dragon Quality Goods Shop	Green Intelligent Power Saver	Okay, okay, okay. Thank you so much. Fast is good. Fast. Item was as described, mainly because the event was cheap and you can save a lot of money.	Jingdong prefecture level city in Shandong
Dragon Quality Goods Shop	Green Intelligent Power Saver	Received it a couple days ago! Plugged it in the same day! Let's see what the electric bill is in a month! Hopefully it works well oh!	Jingdong prefecture level city in Shandong
Dragon Quality Goods Shop	Green Intelligent Power Saver	Looking at the reviews good, energy-saving small products, compact, the results after 2 months to see, because the electricity bill season payment	Jingdong prefecture level city in Shandong
Dragon Quality Goods Shop	Green Intelligent Power Saver	I just received it and will try it out to see if it works well. I'll comment later.	Jingdong prefecture level city in Shandong
Dragon Quality Goods Shop	Green Intelligent Power Saver	It looks a little small and not very aesthetically pleasing, I don't know how effective it will be until I use it	Jingdong prefecture level city in Shandong
Dragon Quality Goods Shop	Green Intelligent Power Saver	Not only to the point of being useless yet	Jingdong prefecture level city in Shandong
Dragon Quality Goods Shop	Green Intelligent Power Saver	Used it for a month, nothing works, the meter you turn very fast. This is not a scam, but also sold on Jingdong. To cheat a bunch of people?	Jingdong prefecture level city in Shandong
Dragon Quality Goods Shop	Green Intelligent Power Saver	Very good to use, every day can save a little electricity, really good under the sample down a month to save a few dozen dollars in electricity, good before I did not know that there is this 👆 haha, the price is not expensive praise.	Jingdong prefecture level city in Shandong
Dragon Quality Goods Shop	Green Intelligent Power Saver	Tried it for ten days and the change is almost nothing, can't see the power saving yet, use it some more days and see how it goes.	Jingdong prefecture level city in Shandong
Dragon Quality Goods Shop	Green Intelligent Power Saver	Received very quickly, the quality is very good, really save electricity, business service attitude is also very good, I hope you do not miss this opportunity, I continue to buy a few in	Jingdong prefecture level city in Shandong
Dragon Quality Goods Shop	Green Intelligent Power Saver	Quality is very good Oh, the delivery is also very fast, a very favorite store, I want to crazy recommend to friends!	Jingdong prefecture level city in Shandong
Dragon Quality Goods Shop	Green Intelligent Power Saver	The goods have just received, fine workmanship, and the same as described by the merchant, to use a period of time to see if it can achieve the purpose of power saving.	Jingdong prefecture level city in Shandong
Dragon Quality Goods Shop	Green Intelligent Power Saver	Received the goods first use a period of time to see if there is no effect, the business commitment to ninety days invalid refund, first praise, good service!	Jingdong prefecture level city in Shandong
Dragon Quality Goods Shop	Green Intelligent Power Saver	The product is used for a month without energy-saving and power-saving effect does not have any effect	Jingdong prefecture level city in Shandong
Dragon Quality Goods Shop	Green Intelligent Power Saver	The baby is really great, I like it very much, my friends also say good to come, recommended to buy!	Jingdong prefecture level city in Shandong
Dragon Quality Goods Shop	Green Intelligent Power Saver	Logistics is fast, courier boy service is good, after using to see how the fruit, and then follow up.	Jingdong prefecture level city in Shandong
Dragon Quality Goods Shop	Green Intelligent Power Saver	Baby has been received to try, I believe that as the business advertised energy saving. The courier is very responsible.	Jingdong prefecture level city in Shandong
Dragon Quality Goods Shop	Green Intelligent Power Saver	Save Electric Energy King received, good quality, beautiful appearance, very satisfied!	Jingdong prefecture level city in Shandong
Dragon Quality Goods Shop	Green Intelligent Power Saver	Three no products no manufacturers no phone pure fraud induced consumption cheat	Jingdong prefecture level city in Shandong
Dragon Quality Goods Shop	Green Intelligent Power Saver	I'm using it now, I don't know if it's as good as it says, I'll follow up after a month	Jingdong prefecture level city in Shandong
Dragon Quality Goods Shop	Green Intelligent Power Saver	Good quality logistics and fast. Fine workmanship. The description is consistent cost-effective	Jingdong prefecture level city in Shandong
Dragon Quality Goods Shop	Green Intelligent Power Saver	Received, whether it is good or not, to wait a few days and then evaluate	Jingdong prefecture level city in Shandong
Dragon Quality Goods Shop	Green Intelligent Power Saver	I bought it and started using it, I won't know if it works well until I pay my electric bill	Jingdong prefecture level city in Shandong
Dragon Quality Goods Shop	Green Intelligent Power Saver	Especially good, energy saving and power saving, especially good quality, good	Jingdong prefecture level city in Shandong
Dragon Quality Goods Shop	Green Intelligent Power Saver	Logistics is strong, the quality is very good, paste really save electricity, as described, will buy back!	Jingdong prefecture level city in Shandong
Dragon Quality Goods Shop	Green Intelligent Power Saver	Great stuff at a great price, worth the money!	Jingdong prefecture level city in Shandong

Dragon Quality Goods Shop	Green Intelligent Power Saver	Received the power saver, very good quality, very power saving!	Jingdong prefecture level city in Shandong
Dragon Quality Goods Shop	Green Intelligent Power Saver	There are no fruits, only samples...	Jingdong prefecture level city in Shandong
Dragon Quality Goods Shop	Green Intelligent Power Saver	Save a few dollars a month.	Jingdong prefecture level city in Shandong
Dragon Quality Goods Shop	Green Intelligent Power Saver	The quality is particularly good, it works well, and the seller has a good service attitude	Jingdong prefecture level city in Shandong
Dragon Quality Goods Shop	Green Intelligent Power Saver	I've compared them a couple of times and I don't feel any difference	Jingdong prefecture level city in Shandong
Dragon Quality Goods Shop	Green Intelligent Power Saver	I don't see the savings. It's a sham.	Jingdong prefecture level city in Shandong
Dragon Quality Goods Shop	Green Intelligent Power Saver	The power saving effect is very good, and the logistics is very fast. The seller's service attitude is very good	Jingdong prefecture level city in Shandong
Dragon Quality Goods Shop	Green Intelligent Power Saver	Received, well packaged, haven't tried it yet!	Jingdong prefecture level city in Shandong
Dragon Quality Goods Shop	Green Intelligent Power Saver	I don't know how it works, I'll comment after I use it.	Jingdong prefecture level city in Shandong
Dragon Quality Goods Shop	Green Intelligent Power Saver	The product doesn't work well enough to be of any use	Jingdong prefecture level city in Shandong
Dragon Quality Goods Shop	Green Intelligent Power Saver	On Trial 。。。。。。。	Jingdong prefecture level city in Shandong
Dragon Quality Goods Shop	Green Intelligent Power Saver	Being used with unknown results	Jingdong prefecture level city in Shandong
Dragon Quality Goods Shop	Green Intelligent Power Saver	Smart power saving and great quality	Jingdong prefecture level city in Shandong
Dragon Quality Goods Shop	Green Intelligent Power Saver	The saver was received, it works very well and the price is affordable!	Jingdong prefecture level city in Shandong
Dragon Quality Goods Shop	Green Intelligent Power Saver	How can I feel the power saving and how long does it last	Jingdong prefecture level city in Shandong
Dragon Quality Goods Shop	Green Intelligent Power Saver	And I know if it saves electricity or not.	Jingdong prefecture level city in Shandong
Dragon Quality Goods Shop	Green Intelligent Power Saver	It won't save any electricity. No.	Jingdong prefecture level city in Shandong
Dragon Quality Goods Shop	Green Intelligent Power Saver	Invalid. Fake.	Jingdong prefecture level city in Shandong
Dragon Quality Goods Shop	Green Intelligent Power Saver	It's good!	Jingdong prefecture level city in Shandong
Dragon Quality Goods Shop	Green Intelligent Power Saver	have no effect	Jingdong prefecture level city in Shandong
Dragon Quality Goods Shop	Green Intelligent Power Saver	Not used.	Jingdong prefecture level city in Shandong
Dragon Quality Goods Shop	Green Intelligent Power Saver	There is no load range, packaging thrift, ten days also did not develop invoices, received the goods with a receipt, hand-written receipt also has no financial stamp, communication for nearly a week a moment to say that the electronic invoice, a moment to say that the paper invoice, said it is mailed to me, ten days also did not see anything, the invoice invoice can not be found to say that did not stay on file, single single number can not be found, said that the warehouse more than one to a one to find out, in addition to the perfunctory or perfunctory. Products are no certificate of conformity, proper three	Jingdong prefecture level city in Shandong
Dragon Quality Goods Shop	Green Intelligent Power Saver	Satisfaction.	Jingdong prefecture level city in Shandong
Dragon Quality Goods Shop	Green Intelligent Power Saver	There is not a bit of power saving effect, and ringing up an increase in the electricity bill, because you have to add the cost of this machine's own electricity consumption and purchase, so I think it is better to use this machine instead of increasing the expenditure, uneconomical, rather than not use it.	Jingdong prefecture level city in Shandong
Dragon Quality Goods Shop	Green Intelligent Power Saver	I've been using it for two days now and I don't feel any effect.	Jingdong prefecture level city in Shandong
Dragon Quality Goods Shop	Green Intelligent Power Saver	What it looked like was nothing like what was sent.	Jingdong prefecture level city in Shandong
Dragon Quality Goods Shop	Green Intelligent Power Saver	It didn't work. It didn't work for a month or so.	Jingdong prefecture level city in Shandong
Dragon Quality Goods Shop	Green Intelligent Power Saver	Garbage products, physical and description is not the same, no effect at all, pit # no discussion, to buy the fine, garbage, garbage, garbage	Jingdong prefecture level city in Shandong
Dragon Quality Goods Shop	Green Intelligent Power Saver	general	Jingdong prefecture level city in Shandong
Dragon Quality Goods Shop	Green Intelligent Power Saver	IQ tax. Instead of saving electricity, it increases the power consumption of an appliance.	Jingdong prefecture level city in Shandong
Dragon Quality Goods Shop	Green Intelligent Power Saver	There's no trash at all.	Jingdong prefecture level city in Shandong
Dragon Quality Goods Shop	Green Intelligent Power Saver	It doesn't do anything.	Jingdong prefecture level city in Shandong
Dragon Quality Goods Shop	Green Intelligent Power Saver	(of an unmarried couple) be close	Jingdong prefecture level city in Shandong
Dragon Quality Goods Shop	Green Intelligent Power Saver	(of an unmarried couple) be close	Jingdong prefecture level city in Shandong
Dragon Quality Goods Shop	Green Intelligent Power Saver	no electricity saving	Jingdong prefecture level city in Shandong
Dragon Quality Goods Shop	Green Intelligent Power Saver	differ from	Jingdong prefecture level city in Shandong
Dragon Quality Goods Shop	Green Intelligent Power Saver	Saver has received, but real can save electricity, the quality is very good, I times I buy a few more, too good.	Jingdong prefecture level city in Shandong
Dragon Quality Goods Shop	Green Intelligent Power Saver	Great quality as always, a very favorite product, great! o(*￣︶￣*)o	Jingdong prefecture level city in Shandong
Dragon Quality Goods Shop	Green Intelligent Power Saver	If we can reduce the electricity bill, for individuals can save money, for the country can protect the environment, now the weather is getting hotter and hotter, to protect the environment to reduce emissions is very important. Already use the product, whether to reduce the electricity bill depends on next month!	Jingdong prefecture level city in Shandong
Dragon Quality Goods Shop	Green Intelligent Power Saver	Purchased a power saver received on the put into use, rent a room with many electrical appliances, power consumption is very large, we have a piece of electricity or 1.2 dollars a time, with this power saver is much better, every day can save a few cents, very convenient and practical!	Jingdong prefecture level city in Shandong
Dragon Quality Goods Shop	Green Intelligent Power Saver	The product is better to use before not to use when a month 1500 yuan electricity bill, now using the 5 second month electricity bill directly down 470 dollars or very good. A save money to buy several power saver money ZanZanZanZan👍	Jingdong prefecture level city in Shandong
Dragon Quality Goods Shop	Green Intelligent Power Saver	The moment I received the goods really satisfied with this product, not only high-grade packaging, the body are base alloy, up to the price, I was also introduced by a friend, he used last month did save a lot of electricity, but also recommended to me!	Jingdong prefecture level city in Shandong
Dragon Quality Goods Shop	Green Intelligent Power Saver	I got it. Good review. I'm very satisfied. Reasonable price.	Jingdong prefecture level city in Shandong
Dragon Quality Goods Shop	Green Intelligent Power Saver	Intelligent power saver received and used, looking at the quality is good, the quality of the plug sent is also very good. Expect the effect of the economizer is obvious, first five-star praise, with a period of time to follow up!	Jingdong prefecture level city in Shandong
Dragon Quality Goods Shop	Green Intelligent Power Saver	The saver has been received. It was plugged into an outlet and used immediately after receipt. Whether it really saves electricity is not clear yet, I believe that this brand of energy saver really saves	Jingdong prefecture level city in Shandong
Dragon Quality Goods Shop	Green Intelligent Power Saver	Okay, okay, okay. Thank you so much. Fast is good. Fast. Item was as described, mainly because the event was cheap and you can save a lot of money.	Jingdong prefecture level city in Shandong

Dragon Quality Goods Shop	Green Intelligent Power Saver	Received it a couple days ago! Plugged it in the same day! Let's see what the electric bill is in a month! Hopefully it works well oh!	Jingdong prefecture level city in Shandong
Dragon Quality Goods Shop	Green Intelligent Power Saver	Looking at the reviews good, energy-saving small products, compact, the results after 2 months to see, because the electricity bill season payment	Jingdong prefecture level city in Shandong
Dragon Quality Goods Shop	Green Intelligent Power Saver	I just received it and will try it out to see if it works well. I'll comment later.	Jingdong prefecture level city in Shandong
Dragon Quality Goods Shop	Green Intelligent Power Saver	It looks a little small and not very aesthetically pleasing, I don't know how effective it will be until I use it	Jingdong prefecture level city in Shandong
Dragon Quality Goods Shop	Green Intelligent Power Saver	Not only to the point of being useless yet	Jingdong prefecture level city in Shandong
Dragon Quality Goods Shop	Green Intelligent Power Saver	Used it for a month, nothing works, the meter you turn very fast. This is not a scam, but also sold on Jingdong. To cheat a bunch of people?	Jingdong prefecture level city in Shandong
Dragon Quality Goods Shop	Green Intelligent Power Saver	Very good to use, every day can save a little electricity, really good under the sample down a month to save a few dozen dollars in electricity, good before I did not know that there is this haha, the price is not expensive praise.	Jingdong prefecture level city in Shandong
Dragon Quality Goods Shop	Green Intelligent Power Saver	Tried it for ten days and the change is almost nothing, can't see the power saving yet, use it some more days and see how it goes.	Jingdong prefecture level city in Shandong
Dragon Quality Goods Shop	Green Intelligent Power Saver	Received very quickly, the quality is very good, really save electricity, business service attitude is also very good, I hope you do not miss this opportunity, I continue to buy a few in	Jingdong prefecture level city in Shandong
Dragon Quality Goods Shop	Green Intelligent Power Saver	Quality is very good Oh, the delivery is also very fast, a very favorite store, I want to crazy recommend to friends!	Jingdong prefecture level city in Shandong
Dragon Quality Goods Shop	Green Intelligent Power Saver	The goods have just received, fine workmanship, and the same as described by the merchant, to use a period of time to see if it can achieve the purpose of power saving.	Jingdong prefecture level city in Shandong
Dragon Quality Goods Shop	Green Intelligent Power Saver	Received the goods first use a period of time to see if there is no effect, the business commitment to ninety days invalid refund, first praise, good service!	Jingdong prefecture level city in Shandong
Dragon Quality Goods Shop	Green Intelligent Power Saver	The product is used for a month without energy-saving and power-saving effect does not have any effect	Jingdong prefecture level city in Shandong
Dragon Quality Goods Shop	Green Intelligent Power Saver	The baby is really great, I like it very much, my friends also say good to come, recommended to buy!	Jingdong prefecture level city in Shandong
Dragon Quality Goods Shop	Green Intelligent Power Saver	Logistics is fast, courier boy service is good, after using to see how the fruit, and then follow up.	Jingdong prefecture level city in Shandong
Dragon Quality Goods Shop	Green Intelligent Power Saver	Baby has been received to try, I believe that as the business advertised energy saving. The courier is very responsible.	Jingdong prefecture level city in Shandong
Dragon Quality Goods Shop	Green Intelligent Power Saver	Save Electric Energy King received, good quality, beautiful appearance, very satisfied!	Jingdong prefecture level city in Shandong
Dragon Quality Goods Shop	Green Intelligent Power Saver	Three no products no manufacturers no phone pure fraud induced consumption cheat	Jingdong prefecture level city in Shandong
Dragon Quality Goods Shop	Green Intelligent Power Saver	I'm using it now, I don't know if it's as good as it says, I'll follow up after a month	Jingdong prefecture level city in Shandong
Dragon Quality Goods Shop	Green Intelligent Power Saver	Good quality logistics and fast. Fine workmanship. The description is consistent cost-effective	Jingdong prefecture level city in Shandong
Dragon Quality Goods Shop	Green Intelligent Power Saver	Received, whether it is good or not, to wait a few days and then evaluate	Jingdong prefecture level city in Shandong
Dragon Quality Goods Shop	Green Intelligent Power Saver	I bought it and started using it, I won't know if it works well until I pay my electric bill	Jingdong prefecture level city in Shandong
Dragon Quality Goods Shop	Green Intelligent Power Saver	Especially good, energy saving and power saving, especially good quality, good	Jingdong prefecture level city in Shandong
Dragon Quality Goods Shop	Green Intelligent Power Saver	Logistics is strong, the quality is very good, paste really save electricity, as described, will buy back!	Jingdong prefecture level city in Shandong
Dragon Quality Goods Shop	Green Intelligent Power Saver	Great stuff at a great price, worth the money!	Jingdong prefecture level city in Shandong
Dragon Quality Goods Shop	Green Intelligent Power Saver	Received the power saver, very good quality, very power saving!	Jingdong prefecture level city in Shandong
Dragon Quality Goods Shop	Green Intelligent Power Saver	There are no fruits, only samples...	Jingdong prefecture level city in Shandong
Dragon Quality Goods Shop	Green Intelligent Power Saver	Save a few dollars a month.	Jingdong prefecture level city in Shandong
Dragon Quality Goods Shop	Green Intelligent Power Saver	The quality is particularly good, it works well, and the seller has a good service attitude	Jingdong prefecture level city in Shandong
Dragon Quality Goods Shop	Green Intelligent Power Saver	I've compared them a couple of times and I don't feel any difference	Jingdong prefecture level city in Shandong
Dragon Quality Goods Shop	Green Intelligent Power Saver	I don't see the savings. It's a sham.	Jingdong prefecture level city in Shandong
Dragon Quality Goods Shop	Green Intelligent Power Saver	The power saving effect is very good, and the logistics is very fast. The seller's service attitude is very good	Jingdong prefecture level city in Shandong
Dragon Quality Goods Shop	Green Intelligent Power Saver	Received, well packaged, haven't tried it yet!	Jingdong prefecture level city in Shandong
Dragon Quality Goods Shop	Green Intelligent Power Saver	I don't know how it works, I'll comment after I use it.	Jingdong prefecture level city in Shandong
Dragon Quality Goods Shop	Green Intelligent Power Saver	The product doesn't work well enough to be of any use	Jingdong prefecture level city in Shandong
Dragon Quality Goods Shop	Green Intelligent Power Saver	On Trial 。。。。。。。	Jingdong prefecture level city in Shandong
Dragon Quality Goods Shop	Green Intelligent Power Saver	Being used with unknown results	Jingdong prefecture level city in Shandong
Dragon Quality Goods Shop	Green Intelligent Power Saver	Smart power saving and great quality	Jingdong prefecture level city in Shandong
Dragon Quality Goods Shop	Green Intelligent Power Saver	The saver was received, it works very well and the price is affordable!	Jingdong prefecture level city in Shandong
Dragon Quality Goods Shop	Green Intelligent Power Saver	How can I feel the power saving and how long does it last	Jingdong prefecture level city in Shandong
Dragon Quality Goods Shop	Green Intelligent Power Saver	And I know if it saves electricity or not.	Jingdong prefecture level city in Shandong
Dragon Quality Goods Shop	Green Intelligent Power Saver	It won't save any electricity. No.	Jingdong prefecture level city in Shandong
Dragon Quality Goods Shop	Green Intelligent Power Saver	Invalid. Fake.	Jingdong prefecture level city in Shandong
Dragon Quality Goods Shop	Green Intelligent Power Saver	It's good!	Jingdong prefecture level city in Shandong
Dragon Quality Goods Shop	Green Intelligent Power Saver	have no effect	Jingdong prefecture level city in Shandong
Dragon Quality Goods Shop	Green Intelligent Power Saver	Not used.	Jingdong prefecture level city in Shandong
Dragon Quality Goods Shop	Green Intelligent Power Saver	There is no load range, packaging thrift, ten days also did not develop invoices, received the goods with a receipt, hand-written receipt also has no financial stamp, communication for nearly a week a moment to say that the electronic invoice, a moment to say that the paper invoice, said it is mailed to me, ten days also did not see anything, the invoice invoice can not be found to say that did not stay on file, single single number can not be found, said that the warehouse more than one to a one to find out, in addition to the perfunctory or perfunctory. Products are no certificate of conformity, proper three	Jingdong prefecture level city in Shandong
Dragon Quality Goods Shop	Green Intelligent Power Saver	Satisfaction.	Jingdong prefecture level city in Shandong

Dragon Quality Goods Shop	Green Intelligent Power Saver	There is not a bit of power saving effect, and ringing up an increase in the electricity bill, because you have to add the cost of this machine's own electricity consumption and purchase, so I think it is better to use this machine instead of increasing the expenditure, uneconomical, rather than not use it.	Jingdong prefecture level city in Shandong
Dragon Quality Goods Shop	Green Intelligent Power Saver	I've been using it for two days now and I don't feel any effect.	Jingdong prefecture level city in Shandong
Dragon Quality Goods Shop	Green Intelligent Power Saver	What it looked like was nothing like what was sent.	Jingdong prefecture level city in Shandong
Dragon Quality Goods Shop	Green Intelligent Power Saver	It didn't work. It didn't work for a month or so.	Jingdong prefecture level city in Shandong
Dragon Quality Goods Shop	Green Intelligent Power Saver	Garbage products, physical and description is not the same, no effect at all, pit # no discussion, to buy the fine, garbage, garbage, garbage	Jingdong prefecture level city in Shandong
Dragon Quality Goods Shop	Green Intelligent Power Saver	general	Jingdong prefecture level city in Shandong
Dragon Quality Goods Shop	Green Intelligent Power Saver	IQ tax. Instead of saving electricity, it increases the power consumption of an appliance.	Jingdong prefecture level city in Shandong
Dragon Quality Goods Shop	Green Intelligent Power Saver	There's no trash at all.	Jingdong prefecture level city in Shandong
Dragon Quality Goods Shop	Green Intelligent Power Saver	It doesn't do anything.	Jingdong prefecture level city in Shandong
Dragon Quality Goods Shop	Green Intelligent Power Saver	(of an unmarried couple) be close	Jingdong prefecture level city in Shandong
Dragon Quality Goods Shop	Green Intelligent Power Saver	(of an unmarried couple) be close	Jingdong prefecture level city in Shandong
Dragon Quality Goods Shop	Green Intelligent Power Saver	no electricity saving	Jingdong prefecture level city in Shandong
Dragon Quality Goods Shop	Green Intelligent Power Saver	differ from	Jingdong prefecture level city in Shandong
Dragon Quality Goods Shop	Green Intelligent Power Saver	Saver has received, but real can save electricity, the quality is very good, I times I buy a few more, too good.	Jingdong prefecture level city in Shandong
Dragon Quality Goods Shop	Green Intelligent Power Saver	Great quality as always, a very favorite product, great! o(*￣︶￣*)o	Jingdong prefecture level city in Shandong
Dragon Quality Goods Shop	Green Intelligent Power Saver	If we can reduce the electricity bill, for individuals can save money, for the country can protect the environment, now the weather is getting hotter and hotter, to protect the environment to reduce emissions is very important. Already use the product, whether to reduce the electricity bill depends on next month!	Jingdong prefecture level city in Shandong
Dragon Quality Goods Shop	Green Intelligent Power Saver	Purchased a power saver received on the put into use, rent a room with many electrical appliances, power consumption is very large, we have a piece of electricity or 1.2 dollars a time, with this power saver is much better, every day can save a few cents, very convenient and practical!	Jingdong prefecture level city in Shandong
Dragon Quality Goods Shop	Green Intelligent Power Saver	The product is better to use before not to use when a month 1500 yuan electricity bill, now using the 5 second month electricity bill directly down 470 dollars or very good. A save money to buy several power saver money ZanZanZanZan👍	Jingdong prefecture level city in Shandong
Dragon Quality Goods Shop	Green Intelligent Power Saver	The moment I received the goods really satisfied with this product, not only high-grade packaging, the body are base alloy, up to the price, I was also introduced by a friend, he used last month did save a lot of electricity, but also recommended to me!	Jingdong prefecture level city in Shandong
Dragon Quality Goods Shop	Green Intelligent Power Saver	I got it. Good review. I'm very satisfied. Reasonable price.	Jingdong prefecture level city in Shandong
Dragon Quality Goods Shop	Green Intelligent Power Saver	Intelligent power saver received and used, looking at the quality is good, the quality of the plug sent is also very good. Expect the effect of the economizer is obvious, first five-star praise, with a period of time to follow up!	Jingdong prefecture level city in Shandong
Dragon Quality Goods Shop	Green Intelligent Power Saver	The saver has been received. It was plugged into an outlet and used immediately after receipt. Whether it really saves electricity is not clear yet, I believe that this brand of energy saver really saves	Jingdong prefecture level city in Shandong
Dragon Quality Goods Shop	Green Intelligent Power Saver	Okay, okay, okay. Thank you so much. Fast is good. Fast. Item was as described, mainly because the event was cheap and you can save a lot of money.	Jingdong prefecture level city in Shandong
Dragon Quality Goods Shop	Green Intelligent Power Saver	Received it a couple days ago! Plugged it in the same day! Let's see what the electric bill is in a month! Hopefully it works well oh!	Jingdong prefecture level city in Shandong
Dragon Quality Goods Shop	Green Intelligent Power Saver	Looking at the reviews good, energy-saving small products, compact, the results after 2 months to see, because the electricity bill season payment	Jingdong prefecture level city in Shandong
Dragon Quality Goods Shop	Green Intelligent Power Saver	I just received it and will try it out to see if it works well. I'll comment later.	Jingdong prefecture level city in Shandong
Dragon Quality Goods Shop	Green Intelligent Power Saver	It looks a little small and not very aesthetically pleasing, I don't know how effective it will be until I use it	Jingdong prefecture level city in Shandong
Dragon Quality Goods Shop	Green Intelligent Power Saver	Not only to the point of being useless yet	Jingdong prefecture level city in Shandong
Dragon Quality Goods Shop	Green Intelligent Power Saver	Used it for a month, nothing works, the meter you turn very fast. This is not a scam, but also sold on Jingdong. To cheat a bunch of people?	Jingdong prefecture level city in Shandong
Dragon Quality Goods Shop	Green Intelligent Power Saver	Very good to use, every day can save a little electricity, really good under the sample down a month to save a few dozen dollars in electricity, good before I did not know that there is this 👆 haha, the price is not expensive praise.	Jingdong prefecture level city in Shandong
Dragon Quality Goods Shop	Green Intelligent Power Saver	Tried it for ten days and the change is almost nothing, can't see the power saving yet, use it some more days and see how it goes.	Jingdong prefecture level city in Shandong
Dragon Quality Goods Shop	Green Intelligent Power Saver	Received very quickly, the quality is very good, really save electricity, business service attitude is also very good, I hope you do not miss this opportunity, I continue to buy a few in	Jingdong prefecture level city in Shandong
Dragon Quality Goods Shop	Green Intelligent Power Saver	Quality is very good Oh, the delivery is also very fast, a very favorite store, I want to crazy recommend to friends!	Jingdong prefecture level city in Shandong
Dragon Quality Goods Shop	Green Intelligent Power Saver	The goods have just received, fine workmanship, and the same as described by the merchant, to use a period of time to see if it can achieve the purpose of power saving.	Jingdong prefecture level city in Shandong
Dragon Quality Goods Shop	Green Intelligent Power Saver	Received the goods first use a period of time to see if there is no effect, the business commitment to ninety days invalid refund, first praise, good service!	Jingdong prefecture level city in Shandong
Dragon Quality Goods Shop	Green Intelligent Power Saver	The product is used for a month without energy-saving and power-saving effect does not have any effect	Jingdong prefecture level city in Shandong
Dragon Quality Goods Shop	Green Intelligent Power Saver	The baby is really great, I like it very much, my friends also say good to come, recommended to buy!	Jingdong prefecture level city in Shandong
Dragon Quality Goods Shop	Green Intelligent Power Saver	Logistics is fast, courier boy service is good, after using to see how the fruit, and then follow up.	Jingdong prefecture level city in Shandong
Dragon Quality Goods Shop	Green Intelligent Power Saver	Baby has been received to try, I believe that as the business advertised energy saving. The courier is very responsible.	Jingdong prefecture level city in Shandong
Dragon Quality Goods Shop	Green Intelligent Power Saver	Save Electric Energy King received, good quality, beautiful appearance, very satisfied!	Jingdong prefecture level city in Shandong
Dragon Quality Goods Shop	Green Intelligent Power Saver	Three no products no manufacturers no phone pure fraud induced consumption cheat	Jingdong prefecture level city in Shandong
Dragon Quality Goods Shop	Green Intelligent Power Saver	I'm using it now, I don't know if it's as good as it says, I'll follow up after a month	Jingdong prefecture level city in Shandong
Dragon Quality Goods Shop	Green Intelligent Power Saver	Good quality logistics and fast. Fine workmanship. The description is consistent cost-effective	Jingdong prefecture level city in Shandong
Dragon Quality Goods Shop	Green Intelligent Power Saver	Received, whether it is good or not, to wait a few days and then evaluate	Jingdong prefecture level city in Shandong
Dragon Quality Goods Shop	Green Intelligent Power Saver	I bought it and started using it, I won't know if it works well until I pay my electric bill	Jingdong prefecture level city in Shandong
Dragon Quality Goods Shop	Green Intelligent Power Saver	Especially good, energy saving and power saving, especially good quality, good	Jingdong prefecture level city in Shandong
Dragon Quality Goods Shop	Green Intelligent Power Saver	Logistics is strong, the quality is very good, paste really save electricity, as described, will buy back!	Jingdong prefecture level city in Shandong
Dragon Quality Goods Shop	Green Intelligent Power Saver	Great stuff at a great price, worth the money!	Jingdong prefecture level city in Shandong

Dragon Quality Goods Shop	Green Intelligent Power Saver	Received the power saver, very good quality, very power saving!	Jingdong prefecture level city in Shandong
Dragon Quality Goods Shop	Green Intelligent Power Saver	There are no fruits, only samples...	Jingdong prefecture level city in Shandong
Dragon Quality Goods Shop	Green Intelligent Power Saver	Save a few dollars a month.	Jingdong prefecture level city in Shandong
Dragon Quality Goods Shop	Green Intelligent Power Saver	The quality is particularly good, it works well, and the seller has a good service attitude	Jingdong prefecture level city in Shandong
Dragon Quality Goods Shop	Green Intelligent Power Saver	I've compared them a couple of times and I don't feel any difference	Jingdong prefecture level city in Shandong
Dragon Quality Goods Shop	Green Intelligent Power Saver	I don't see the savings. It's a sham.	Jingdong prefecture level city in Shandong
Dragon Quality Goods Shop	Green Intelligent Power Saver	The power saving effect is very good, and the logistics is very fast. The seller's service attitude is very good	Jingdong prefecture level city in Shandong
Dragon Quality Goods Shop	Green Intelligent Power Saver	Received, well packaged, haven't tried it yet!	Jingdong prefecture level city in Shandong
Dragon Quality Goods Shop	Green Intelligent Power Saver	I don't know how it works, I'll comment after I use it.	Jingdong prefecture level city in Shandong
Dragon Quality Goods Shop	Green Intelligent Power Saver	The product doesn't work well enough to be of any use	Jingdong prefecture level city in Shandong
Dragon Quality Goods Shop	Green Intelligent Power Saver	On Trial 。。。。。。。	Jingdong prefecture level city in Shandong
Dragon Quality Goods Shop	Green Intelligent Power Saver	Being used with unknown results	Jingdong prefecture level city in Shandong
Dragon Quality Goods Shop	Green Intelligent Power Saver	Smart power saving and great quality	Jingdong prefecture level city in Shandong
Dragon Quality Goods Shop	Green Intelligent Power Saver	The saver was received, it works very well and the price is affordable!	Jingdong prefecture level city in Shandong
Dragon Quality Goods Shop	Green Intelligent Power Saver	How can I feel the power saving and how long does it last	Jingdong prefecture level city in Shandong
Dragon Quality Goods Shop	Green Intelligent Power Saver	And I know if it saves electricity or not.	Jingdong prefecture level city in Shandong
Dragon Quality Goods Shop	Green Intelligent Power Saver	It won't save any electricity. No.	Jingdong prefecture level city in Shandong
Dragon Quality Goods Shop	Green Intelligent Power Saver	Invalid. Fake.	Jingdong prefecture level city in Shandong
Dragon Quality Goods Shop	Green Intelligent Power Saver	It's good!	Jingdong prefecture level city in Shandong
Dragon Quality Goods Shop	Green Intelligent Power Saver	have no effect	Jingdong prefecture level city in Shandong
Dragon Quality Goods Shop	Green Intelligent Power Saver	Not used.	Jingdong prefecture level city in Shandong
Dragon Quality Goods Shop	Green Intelligent Power Saver	There is no load range, packaging thrift, ten days also did not develop invoices, received the goods with a receipt, hand-written receipt also has no financial stamp, communication for nearly a week a moment to say that the electronic invoice, a moment to say that the paper invoice, said it is mailed to me, ten days also did not see anything, the invoice invoice can not be found to say that did not stay on file, single single number can not be found, said that the warehouse more than one to a one to find out, in addition to the perfunctory or perfunctory. Products are no certificate of conformity, proper three	Jingdong prefecture level city in Shandong
Dragon Quality Goods Shop	Green Intelligent Power Saver	Satisfaction.	Jingdong prefecture level city in Shandong
Dragon Quality Goods Shop	Green Intelligent Power Saver	There is not a bit of power saving effect, and ringing up an increase in the electricity bill, because you have to add the cost of this machine's own electricity consumption and purchase, so I think it is better to use this machine instead of increasing the expenditure, uneconomical, rather than not use it.	Jingdong prefecture level city in Shandong
Dragon Quality Goods Shop	Green Intelligent Power Saver	I've been using it for two days now and I don't feel any effect.	Jingdong prefecture level city in Shandong
Dragon Quality Goods Shop	Green Intelligent Power Saver	What it looked like was nothing like what was sent.	Jingdong prefecture level city in Shandong
Dragon Quality Goods Shop	Green Intelligent Power Saver	It didn't work. It didn't work for a month or so.	Jingdong prefecture level city in Shandong
Dragon Quality Goods Shop	Green Intelligent Power Saver	Garbage products, physical and description is not the same, no effect at all, pit # no discussion, to buy the fine, garbage, garbage, garbage	Jingdong prefecture level city in Shandong
Dragon Quality Goods Shop	Green Intelligent Power Saver	general	Jingdong prefecture level city in Shandong
Dragon Quality Goods Shop	Green Intelligent Power Saver	IQ tax. Instead of saving electricity, it increases the power consumption of an appliance.	Jingdong prefecture level city in Shandong
Dragon Quality Goods Shop	Green Intelligent Power Saver	There's no trash at all.	Jingdong prefecture level city in Shandong
Dragon Quality Goods Shop	Green Intelligent Power Saver	It doesn't do anything.	Jingdong prefecture level city in Shandong
Dragon Quality Goods Shop	Green Intelligent Power Saver	(of an unmarried couple) be close	Jingdong prefecture level city in Shandong
Dragon Quality Goods Shop	Green Intelligent Power Saver	(of an unmarried couple) be close	Jingdong prefecture level city in Shandong
Dragon Quality Goods Shop	Green Intelligent Power Saver	no electricity saving	Jingdong prefecture level city in Shandong
Dragon Quality Goods Shop	Green Intelligent Power Saver	differ from	Jingdong prefecture level city in Shandong
Dragon Quality Goods Shop	Green Intelligent Power Saver	Saver has received, but real can save electricity, the quality is very good, I times I buy a few more, too good.	Jingdong prefecture level city in Shandong
Dragon Quality Goods Shop	Green Intelligent Power Saver	Great quality as always, a very favorite product, great! o(*￣︶￣*)o	Jingdong prefecture level city in Shandong
Dragon Quality Goods Shop	Green Intelligent Power Saver	If we can reduce the electricity bill, for individuals can save money, for the country can protect the environment, now the weather is getting hotter and hotter, to protect the environment to reduce emissions is very important. Already use the product, whether to reduce the electricity bill depends on next month!	Jingdong prefecture level city in Shandong
Dragon Quality Goods Shop	Green Intelligent Power Saver	Purchased a power saver received on the put into use, rent a room with many electrical appliances, power consumption is very large, we have a piece of electricity or 1.2 dollars a time, with this power saver is much better, every day can save a few cents, very convenient and practical!	Jingdong prefecture level city in Shandong
Dragon Quality Goods Shop	Green Intelligent Power Saver	The product is better to use before not to use when a month 1500 yuan electricity bill, now using the 5 second month electricity bill directly down 470 dollars or very good. A save money to buy several power saver money ZanZanZanZan👍	Jingdong prefecture level city in Shandong
Dragon Quality Goods Shop	Green Intelligent Power Saver	The moment I received the goods really satisfied with this product, not only high-grade packaging, the body are base alloy, up to the price, I was also introduced by a friend, he used last month did save a lot of electricity, but also recommended to me!	Jingdong prefecture level city in Shandong
Dragon Quality Goods Shop	Green Intelligent Power Saver	I got it. Good review. I'm very satisfied. Reasonable price.	Jingdong prefecture level city in Shandong
Dragon Quality Goods Shop	Green Intelligent Power Saver	Intelligent power saver received and used, looking at the quality is good, the quality of the plug sent is also very good. Expect the effect of the economizer is obvious, first five-star praise, with a period of time to follow up!	Jingdong prefecture level city in Shandong
Dragon Quality Goods Shop	Green Intelligent Power Saver	The saver has been received. It was plugged into an outlet and used immediately after receipt. Whether it really saves electricity is not clear yet, I believe that this brand of energy saver really saves	Jingdong prefecture level city in Shandong
Dragon Quality Goods Shop	Green Intelligent Power Saver	Okay, okay, okay. Thank you so much. Fast is good. Fast. Item was as described, mainly because the event was cheap and you can save a lot of money.	Jingdong prefecture level city in Shandong

Dragon Quality Goods Shop	Green Intelligent Power Saver	Received it a couple days ago! Plugged it in the same day! Let's see what the electric bill is in a month! Hopefully it works well oh!	Jingdong prefecture level city in Shandong
Dragon Quality Goods Shop	Green Intelligent Power Saver	Looking at the reviews good, energy-saving small products, compact, the results after 2 months to see, because the electricity bill season payment	Jingdong prefecture level city in Shandong
Dragon Quality Goods Shop	Green Intelligent Power Saver	I just received it and will try it out to see if it works well. I'll comment later.	Jingdong prefecture level city in Shandong
Dragon Quality Goods Shop	Green Intelligent Power Saver	It looks a little small and not very aesthetically pleasing, I don't know how effective it will be until I use it	Jingdong prefecture level city in Shandong
Dragon Quality Goods Shop	Green Intelligent Power Saver	Not only to the point of being useless yet	Jingdong prefecture level city in Shandong
Dragon Quality Goods Shop	Green Intelligent Power Saver	Used it for a month, nothing works, the meter you turn very fast. This is not a scam, but also sold on Jingdong. To cheat a bunch of people?	Jingdong prefecture level city in Shandong
Dragon Quality Goods Shop	Green Intelligent Power Saver	Very good to use, every day can save a little electricity, really good under the sample down a month to save a few dozen dollars in electricity, good before I did not know that there is this haha, the price is not expensive praise.	Jingdong prefecture level city in Shandong
Dragon Quality Goods Shop	Green Intelligent Power Saver	Tried it for ten days and the change is almost nothing, can't see the power saving yet, use it some more days and see how it goes.	Jingdong prefecture level city in Shandong
Dragon Quality Goods Shop	Green Intelligent Power Saver	Received very quickly, the quality is very good, really save electricity, business service attitude is also very good, I hope you do not miss this opportunity, I continue to buy a few in	Jingdong prefecture level city in Shandong
Dragon Quality Goods Shop	Green Intelligent Power Saver	Quality is very good Oh, the delivery is also very fast, a very favorite store, I want to crazy recommend to friends!	Jingdong prefecture level city in Shandong
Dragon Quality Goods Shop	Green Intelligent Power Saver	The goods have just received, fine workmanship, and the same as described by the merchant, to use a period of time to see if it can achieve the purpose of power saving.	Jingdong prefecture level city in Shandong
Dragon Quality Goods Shop	Green Intelligent Power Saver	Received the goods first use a period of time to see if there is no effect, the business commitment to ninety days invalid refund, first praise, good service!	Jingdong prefecture level city in Shandong
Dragon Quality Goods Shop	Green Intelligent Power Saver	The product is used for a month without energy-saving and power-saving effect does not have any effect	Jingdong prefecture level city in Shandong
Dragon Quality Goods Shop	Green Intelligent Power Saver	The baby is really great, I like it very much, my friends also say good to come, recommended to buy!	Jingdong prefecture level city in Shandong
Dragon Quality Goods Shop	Green Intelligent Power Saver	Logistics is fast, courier boy service is good, after using to see how the fruit, and then follow up.	Jingdong prefecture level city in Shandong
Dragon Quality Goods Shop	Green Intelligent Power Saver	Baby has been received to try, I believe that as the business advertised energy saving. The courier is very responsible.	Jingdong prefecture level city in Shandong
Dragon Quality Goods Shop	Green Intelligent Power Saver	Save Electric Energy King received, good quality, beautiful appearance, very satisfied!	Jingdong prefecture level city in Shandong
Dragon Quality Goods Shop	Green Intelligent Power Saver	Three no products no manufacturers no phone pure fraud induced consumption cheat	Jingdong prefecture level city in Shandong
Dragon Quality Goods Shop	Green Intelligent Power Saver	I'm using it now, I don't know if it's as good as it says, I'll follow up after a month	Jingdong prefecture level city in Shandong
Dragon Quality Goods Shop	Green Intelligent Power Saver	Good quality logistics and fast. Fine workmanship. The description is consistent cost-effective	Jingdong prefecture level city in Shandong
Dragon Quality Goods Shop	Green Intelligent Power Saver	Received, whether it is good or not, to wait a few days and then evaluate	Jingdong prefecture level city in Shandong
Dragon Quality Goods Shop	Green Intelligent Power Saver	I bought it and started using it, I won't know if it works well until I pay my electric bill	Jingdong prefecture level city in Shandong
Dragon Quality Goods Shop	Green Intelligent Power Saver	Especially good, energy saving and power saving, especially good quality, good	Jingdong prefecture level city in Shandong
Dragon Quality Goods Shop	Green Intelligent Power Saver	Logistics is strong, the quality is very good, paste really save electricity, as described, will buy back!	Jingdong prefecture level city in Shandong
Dragon Quality Goods Shop	Green Intelligent Power Saver	Great stuff at a great price, worth the money!	Jingdong prefecture level city in Shandong
Dragon Quality Goods Shop	Green Intelligent Power Saver	Received the power saver, very good quality, very power saving!	Jingdong prefecture level city in Shandong
Dragon Quality Goods Shop	Green Intelligent Power Saver	There are no fruits, only samples...	Jingdong prefecture level city in Shandong
Dragon Quality Goods Shop	Green Intelligent Power Saver	Save a few dollars a month.	Jingdong prefecture level city in Shandong
Dragon Quality Goods Shop	Green Intelligent Power Saver	The quality is particularly good, it works well, and the seller has a good service attitude	Jingdong prefecture level city in Shandong
Dragon Quality Goods Shop	Green Intelligent Power Saver	I've compared them a couple of times and I don't feel any difference	Jingdong prefecture level city in Shandong
Dragon Quality Goods Shop	Green Intelligent Power Saver	I don't see the savings. It's a sham.	Jingdong prefecture level city in Shandong
Dragon Quality Goods Shop	Green Intelligent Power Saver	The power saving effect is very good, and the logistics is very fast. The seller's service attitude is very good	Jingdong prefecture level city in Shandong
Dragon Quality Goods Shop	Green Intelligent Power Saver	Received, well packaged, haven't tried it yet!	Jingdong prefecture level city in Shandong
Dragon Quality Goods Shop	Green Intelligent Power Saver	I don't know how it works, I'll comment after I use it.	Jingdong prefecture level city in Shandong
Dragon Quality Goods Shop	Green Intelligent Power Saver	The product doesn't work well enough to be of any use	Jingdong prefecture level city in Shandong
Dragon Quality Goods Shop	Green Intelligent Power Saver	On Trial 。。。。。。。	Jingdong prefecture level city in Shandong
Dragon Quality Goods Shop	Green Intelligent Power Saver	Being used with unknown results	Jingdong prefecture level city in Shandong
Dragon Quality Goods Shop	Green Intelligent Power Saver	Smart power saving and great quality	Jingdong prefecture level city in Shandong
Dragon Quality Goods Shop	Green Intelligent Power Saver	The saver was received, it works very well and the price is affordable!	Jingdong prefecture level city in Shandong
Dragon Quality Goods Shop	Green Intelligent Power Saver	How can I feel the power saving and how long does it last	Jingdong prefecture level city in Shandong
Dragon Quality Goods Shop	Green Intelligent Power Saver	And I know if it saves electricity or not.	Jingdong prefecture level city in Shandong
Dragon Quality Goods Shop	Green Intelligent Power Saver	It won't save any electricity. No.	Jingdong prefecture level city in Shandong
Dragon Quality Goods Shop	Green Intelligent Power Saver	Invalid. Fake.	Jingdong prefecture level city in Shandong
Dragon Quality Goods Shop	Green Intelligent Power Saver	It's good!	Jingdong prefecture level city in Shandong
Dragon Quality Goods Shop	Green Intelligent Power Saver	have no effect	Jingdong prefecture level city in Shandong
Dragon Quality Goods Shop	Green Intelligent Power Saver	Not used.	Jingdong prefecture level city in Shandong
Dragon Quality Goods Shop	Green Intelligent Power Saver	There is no load range, packaging thrift, ten days also did not develop invoices, received the goods with a receipt, hand-written receipt also has no financial stamp, communication for nearly a week a moment to say that the electronic invoice, a moment to say that the paper invoice, said it is mailed to me, ten days also did not see anything, the invoice invoice can not be found to say that did not stay on file, single single number can not be found, said that the warehouse more than one to a one to find out, in addition to the perfunctory or perfunctory. Products are no certificate of conformity, proper three	Jingdong prefecture level city in Shandong
Dragon Quality Goods Shop	Green Intelligent Power Saver	Satisfaction.	Jingdong prefecture level city in Shandong

Dragon Quality Goods Shop	Green Intelligent Power Saver	There is not a bit of power saving effect, and ringing up an increase in the electricity bill, because you have to add the cost of this machine's own electricity consumption and purchase, so I think it is better to use this machine instead of increasing the expenditure, uneconomical, rather than not use it.	Jingdong prefecture level city in Shandong
Dragon Quality Goods Shop	Green Intelligent Power Saver	I've been using it for two days now and I don't feel any effect.	Jingdong prefecture level city in Shandong
Dragon Quality Goods Shop	Green Intelligent Power Saver	What it looked like was nothing like what was sent.	Jingdong prefecture level city in Shandong
Dragon Quality Goods Shop	Green Intelligent Power Saver	It didn't work. It didn't work for a month or so.	Jingdong prefecture level city in Shandong
Dragon Quality Goods Shop	Green Intelligent Power Saver	Garbage products, physical and description is not the same, no effect at all, pit # no discussion, to buy the fine, garbage, garbage, garbage	Jingdong prefecture level city in Shandong
Dragon Quality Goods Shop	Green Intelligent Power Saver	general	Jingdong prefecture level city in Shandong
Dragon Quality Goods Shop	Green Intelligent Power Saver	IQ tax. Instead of saving electricity, it increases the power consumption of an appliance.	Jingdong prefecture level city in Shandong
Dragon Quality Goods Shop	Green Intelligent Power Saver	There's no trash at all.	Jingdong prefecture level city in Shandong
Dragon Quality Goods Shop	Green Intelligent Power Saver	It doesn't do anything.	Jingdong prefecture level city in Shandong
Dragon Quality Goods Shop	Green Intelligent Power Saver	(of an unmarried couple) be close	Jingdong prefecture level city in Shandong
Dragon Quality Goods Shop	Green Intelligent Power Saver	(of an unmarried couple) be close	Jingdong prefecture level city in Shandong
Dragon Quality Goods Shop	Green Intelligent Power Saver	no electricity saving	Jingdong prefecture level city in Shandong
Dragon Quality Goods Shop	Green Intelligent Power Saver	differ from	Jingdong prefecture level city in Shandong
Dragon Quality Goods Shop	Green Intelligent Power Saver	Saver has received, but real can save electricity, the quality is very good, I times I buy a few more, too good.	Jingdong prefecture level city in Shandong
Dragon Quality Goods Shop	Green Intelligent Power Saver	Great quality as always, a very favorite product, great! o(*￣︶￣*)o	Jingdong prefecture level city in Shandong
Dragon Quality Goods Shop	Green Intelligent Power Saver	If we can reduce the electricity bill, for individuals can save money, for the country can protect the environment, now the weather is getting hotter and hotter, to protect the environment to reduce emissions is very important. Already use the product, whether to reduce the electricity bill depends on next month!	Jingdong prefecture level city in Shandong
Dragon Quality Goods Shop	Green Intelligent Power Saver	Purchased a power saver received on the put into use, rent a room with many electrical appliances, power consumption is very large, we have a piece of electricity or 1.2 dollars a time, with this power saver is much better, every day can save a few cents, very convenient and practical!	Jingdong prefecture level city in Shandong
Dragon Quality Goods Shop	Green Intelligent Power Saver	The product is better to use before not to use when a month 1500 yuan electricity bill, now using the 5 second month electricity bill directly down 470 dollars or very good. A save money to buy several power saver money ZanZanZanZan👍	Jingdong prefecture level city in Shandong
Dragon Quality Goods Shop	Green Intelligent Power Saver	The moment I received the goods really satisfied with this product, not only high-grade packaging, the body are base alloy, up to the price, I was also introduced by a friend, he used last month did save a lot of electricity, but also recommended to me!	Jingdong prefecture level city in Shandong
Dragon Quality Goods Shop	Green Intelligent Power Saver	I got it. Good review. I'm very satisfied. Reasonable price.	Jingdong prefecture level city in Shandong
Dragon Quality Goods Shop	Green Intelligent Power Saver	Intelligent power saver received and used, looking at the quality is good, the quality of the plug sent is also very good. Expect the effect of the economizer is obvious, first five-star praise, with a period of time to follow up!	Jingdong prefecture level city in Shandong
Dragon Quality Goods Shop	Green Intelligent Power Saver	The saver has been received. It was plugged into an outlet and used immediately after receipt. Whether it really saves electricity is not clear yet, I believe that this brand of energy saver really saves	Jingdong prefecture level city in Shandong
Dragon Quality Goods Shop	Green Intelligent Power Saver	Okay, okay, okay. Thank you so much. Fast is good. Fast. Item was as described, mainly because the event was cheap and you can save a lot of money.	Jingdong prefecture level city in Shandong
Dragon Quality Goods Shop	Green Intelligent Power Saver	Received it a couple days ago! Plugged it in the same day! Let's see what the electric bill is in a month! Hopefully it works well oh!	Jingdong prefecture level city in Shandong
Dragon Quality Goods Shop	Green Intelligent Power Saver	Looking at the reviews good, energy-saving small products, compact, the results after 2 months to see, because the electricity bill season payment	Jingdong prefecture level city in Shandong
Dragon Quality Goods Shop	Green Intelligent Power Saver	I just received it and will try it out to see if it works well. I'll comment later.	Jingdong prefecture level city in Shandong
Dragon Quality Goods Shop	Green Intelligent Power Saver	It looks a little small and not very aesthetically pleasing, I don't know how effective it will be until I use it	Jingdong prefecture level city in Shandong
Dragon Quality Goods Shop	Green Intelligent Power Saver	Not only to the point of being useless yet	Jingdong prefecture level city in Shandong
Dragon Quality Goods Shop	Green Intelligent Power Saver	Used it for a month, nothing works, the meter you turn very fast. This is not a scam, but also sold on Jingdong. To cheat a bunch of people?	Jingdong prefecture level city in Shandong
Dragon Quality Goods Shop	Green Intelligent Power Saver	Very good to use, every day can save a little electricity, really good under the sample down a month to save a few dozen dollars in electricity, good before I did not know that there is this 👆 haha, the price is not expensive praise.	Jingdong prefecture level city in Shandong
Dragon Quality Goods Shop	Green Intelligent Power Saver	Tried it for ten days and the change is almost nothing, can't see the power saving yet, use it some more days and see how it goes.	Jingdong prefecture level city in Shandong
Dragon Quality Goods Shop	Green Intelligent Power Saver	Received very quickly, the quality is very good, really save electricity, business service attitude is also very good, I hope you do not miss this opportunity, I continue to buy a few in	Jingdong prefecture level city in Shandong
Dragon Quality Goods Shop	Green Intelligent Power Saver	Quality is very good Oh, the delivery is also very fast, a very favorite store, I want to crazy recommend to friends!	Jingdong prefecture level city in Shandong
Dragon Quality Goods Shop	Green Intelligent Power Saver	The goods have just received, fine workmanship, and the same as described by the merchant, to use a period of time to see if it can achieve the purpose of power saving.	Jingdong prefecture level city in Shandong
Dragon Quality Goods Shop	Green Intelligent Power Saver	Received the goods first use a period of time to see if there is no effect, the business commitment to ninety days invalid refund, first praise, good service!	Jingdong prefecture level city in Shandong
Dragon Quality Goods Shop	Green Intelligent Power Saver	The product is used for a month without energy-saving and power-saving effect does not have any effect	Jingdong prefecture level city in Shandong
Dragon Quality Goods Shop	Green Intelligent Power Saver	The baby is really great, I like it very much, my friends also say good to come, recommended to buy!	Jingdong prefecture level city in Shandong
Dragon Quality Goods Shop	Green Intelligent Power Saver	Logistics is fast, courier boy service is good, after using to see how the fruit, and then follow up.	Jingdong prefecture level city in Shandong
Dragon Quality Goods Shop	Green Intelligent Power Saver	Baby has been received to try, I believe that as the business advertised energy saving. The courier is very responsible.	Jingdong prefecture level city in Shandong
Dragon Quality Goods Shop	Green Intelligent Power Saver	Save Electric Energy King received, good quality, beautiful appearance, very satisfied!	Jingdong prefecture level city in Shandong
Dragon Quality Goods Shop	Green Intelligent Power Saver	Three no products no manufacturers no phone pure fraud induced consumption cheat	Jingdong prefecture level city in Shandong
Dragon Quality Goods Shop	Green Intelligent Power Saver	I'm using it now, I don't know if it's as good as it says, I'll follow up after a month	Jingdong prefecture level city in Shandong
Dragon Quality Goods Shop	Green Intelligent Power Saver	Good quality logistics and fast. Fine workmanship. The description is consistent cost-effective	Jingdong prefecture level city in Shandong
Dragon Quality Goods Shop	Green Intelligent Power Saver	Received, whether it is good or not, to wait a few days and then evaluate	Jingdong prefecture level city in Shandong
Dragon Quality Goods Shop	Green Intelligent Power Saver	I bought it and started using it, I won't know if it works well until I pay my electric bill	Jingdong prefecture level city in Shandong
Dragon Quality Goods Shop	Green Intelligent Power Saver	Especially good, energy saving and power saving, especially good quality, good	Jingdong prefecture level city in Shandong
Dragon Quality Goods Shop	Green Intelligent Power Saver	Logistics is strong, the quality is very good, paste really save electricity, as described, will buy back!	Jingdong prefecture level city in Shandong
Dragon Quality Goods Shop	Green Intelligent Power Saver	Great stuff at a great price, worth the money!	Jingdong prefecture level city in Shandong

Dragon Quality Goods Shop	Green Intelligent Power Saver	Received the power saver, very good quality, very power saving!	Jingdong prefecture level city in Shandong
Dragon Quality Goods Shop	Green Intelligent Power Saver	There are no fruits, only samples...	Jingdong prefecture level city in Shandong
Dragon Quality Goods Shop	Green Intelligent Power Saver	Save a few dollars a month.	Jingdong prefecture level city in Shandong
Dragon Quality Goods Shop	Green Intelligent Power Saver	The quality is particularly good, it works well, and the seller has a good service attitude	Jingdong prefecture level city in Shandong
Dragon Quality Goods Shop	Green Intelligent Power Saver	I've compared them a couple of times and I don't feel any difference	Jingdong prefecture level city in Shandong
Dragon Quality Goods Shop	Green Intelligent Power Saver	I don't see the savings. It's a sham.	Jingdong prefecture level city in Shandong
Dragon Quality Goods Shop	Green Intelligent Power Saver	The power saving effect is very good, and the logistics is very fast. The seller's service attitude is very good	Jingdong prefecture level city in Shandong
Dragon Quality Goods Shop	Green Intelligent Power Saver	Received, well packaged, haven't tried it yet!	Jingdong prefecture level city in Shandong
Dragon Quality Goods Shop	Green Intelligent Power Saver	I don't know how it works, I'll comment after I use it.	Jingdong prefecture level city in Shandong
Dragon Quality Goods Shop	Green Intelligent Power Saver	The product doesn't work well enough to be of any use	Jingdong prefecture level city in Shandong
Dragon Quality Goods Shop	Green Intelligent Power Saver	On Trial 。。。。。。。	Jingdong prefecture level city in Shandong
Dragon Quality Goods Shop	Green Intelligent Power Saver	Being used with unknown results	Jingdong prefecture level city in Shandong
Dragon Quality Goods Shop	Green Intelligent Power Saver	Smart power saving and great quality	Jingdong prefecture level city in Shandong
Dragon Quality Goods Shop	Green Intelligent Power Saver	The saver was received, it works very well and the price is affordable!	Jingdong prefecture level city in Shandong
Dragon Quality Goods Shop	Green Intelligent Power Saver	How can I feel the power saving and how long does it last	Jingdong prefecture level city in Shandong
Dragon Quality Goods Shop	Green Intelligent Power Saver	And I know if it saves electricity or not.	Jingdong prefecture level city in Shandong
Dragon Quality Goods Shop	Green Intelligent Power Saver	It won't save any electricity. No.	Jingdong prefecture level city in Shandong
Dragon Quality Goods Shop	Green Intelligent Power Saver	Invalid. Fake.	Jingdong prefecture level city in Shandong
Dragon Quality Goods Shop	Green Intelligent Power Saver	It's good!	Jingdong prefecture level city in Shandong
Dragon Quality Goods Shop	Green Intelligent Power Saver	have no effect	Jingdong prefecture level city in Shandong
Dragon Quality Goods Shop	Green Intelligent Power Saver	Not used.	Jingdong prefecture level city in Shandong
Dragon Quality Goods Shop	Green Intelligent Power Saver	There is no load range, packaging thrift, ten days also did not develop invoices, received the goods with a receipt, hand-written receipt also has no financial stamp, communication for nearly a week a moment to say that the electronic invoice, a moment to say that the paper invoice, said it is mailed to me, ten days also did not see anything, the invoice invoice can not be found to say that did not stay on file, single single number can not be found, said that the warehouse more than one to a one to find out, in addition to the perfunctory or perfunctory. Products are no certificate of conformity, proper three	Jingdong prefecture level city in Shandong
Dragon Quality Goods Shop	Green Intelligent Power Saver	Satisfaction.	Jingdong prefecture level city in Shandong
Dragon Quality Goods Shop	Green Intelligent Power Saver	There is not a bit of power saving effect, and ringing up an increase in the electricity bill, because you have to add the cost of this machine's own electricity consumption and purchase, so I think it is better to use this machine instead of increasing the expenditure, uneconomical, rather than not use it.	Jingdong prefecture level city in Shandong
Dragon Quality Goods Shop	Green Intelligent Power Saver	I've been using it for two days now and I don't feel any effect.	Jingdong prefecture level city in Shandong
Dragon Quality Goods Shop	Green Intelligent Power Saver	What it looked like was nothing like what was sent.	Jingdong prefecture level city in Shandong
Dragon Quality Goods Shop	Green Intelligent Power Saver	It didn't work. It didn't work for a month or so.	Jingdong prefecture level city in Shandong
Dragon Quality Goods Shop	Green Intelligent Power Saver	Garbage products, physical and description is not the same, no effect at all, pit # no discussion, to buy the fine, garbage, garbage, garbage	Jingdong prefecture level city in Shandong
Dragon Quality Goods Shop	Green Intelligent Power Saver	general	Jingdong prefecture level city in Shandong
Dragon Quality Goods Shop	Green Intelligent Power Saver	IQ tax. Instead of saving electricity, it increases the power consumption of an appliance.	Jingdong prefecture level city in Shandong
Dragon Quality Goods Shop	Green Intelligent Power Saver	There's no trash at all.	Jingdong prefecture level city in Shandong
Dragon Quality Goods Shop	Green Intelligent Power Saver	It doesn't do anything.	Jingdong prefecture level city in Shandong
Dragon Quality Goods Shop	Green Intelligent Power Saver	(of an unmarried couple) be close	Jingdong prefecture level city in Shandong
Dragon Quality Goods Shop	Green Intelligent Power Saver	(of an unmarried couple) be close	Jingdong prefecture level city in Shandong
Dragon Quality Goods Shop	Green Intelligent Power Saver	no electricity saving	Jingdong prefecture level city in Shandong
Dragon Quality Goods Shop	Green Intelligent Power Saver	differ from	Jingdong prefecture level city in Shandong
Dragon Quality Goods Shop	Green Intelligent Power Saver	Saver has received, but real can save electricity, the quality is very good, I times I buy a few more, too good.	Jingdong prefecture level city in Shandong
Dragon Quality Goods Shop	Green Intelligent Power Saver	Great quality as always, a very favorite product, great! o(*￣︶￣*)o	Jingdong prefecture level city in Shandong
Dragon Quality Goods Shop	Green Intelligent Power Saver	If we can reduce the electricity bill, for individuals can save money, for the country can protect the environment, now the weather is getting hotter and hotter, to protect the environment to reduce emissions is very important. Already use the product, whether to reduce the electricity bill depends on next month!	Jingdong prefecture level city in Shandong
Dragon Quality Goods Shop	Green Intelligent Power Saver	Purchased a power saver received on the put into use, rent a room with many electrical appliances, power consumption is very large, we have a piece of electricity or 1.2 dollars a time, with this power saver is much better, every day can save a few cents, very convenient and practical!	Jingdong prefecture level city in Shandong
Dragon Quality Goods Shop	Green Intelligent Power Saver	The product is better to use before not to use when a month 1500 yuan electricity bill, now using the 5 second month electricity bill directly down 470 dollars or very good. A save money to buy several power saver money ZanZanZanZan👍	Jingdong prefecture level city in Shandong
Dragon Quality Goods Shop	Green Intelligent Power Saver	The moment I received the goods really satisfied with this product, not only high-grade packaging, the body are base alloy, up to the price, I was also introduced by a friend, he used last month did save a lot of electricity, but also recommended to me!	Jingdong prefecture level city in Shandong
Dragon Quality Goods Shop	Green Intelligent Power Saver	I got it. Good review. I'm very satisfied. Reasonable price.	Jingdong prefecture level city in Shandong
Dragon Quality Goods Shop	Green Intelligent Power Saver	Intelligent power saver received and used, looking at the quality is good, the quality of the plug sent is also very good. Expect the effect of the economizer is obvious, first five-star praise, with a period of time to follow up!	Jingdong prefecture level city in Shandong
Dragon Quality Goods Shop	Green Intelligent Power Saver	The saver has been received. It was plugged into an outlet and used immediately after receipt. Whether it really saves electricity is not clear yet, I believe that this brand of energy saver really saves	Jingdong prefecture level city in Shandong
Dragon Quality Goods Shop	Green Intelligent Power Saver	Okay, okay, okay. Thank you so much. Fast is good. Fast. Item was as described, mainly because the event was cheap and you can save a lot of money.	Jingdong prefecture level city in Shandong

Dragon Quality Goods Shop	Green Intelligent Power Saver	Received it a couple days ago! Plugged it in the same day! Let's see what the electric bill is in a month! Hopefully it works well oh!	Jingdong prefecture level city in Shandong
Dragon Quality Goods Shop	Green Intelligent Power Saver	Looking at the reviews good, energy-saving small products, compact, the results after 2 months to see, because the electricity bill season payment	Jingdong prefecture level city in Shandong
Dragon Quality Goods Shop	Green Intelligent Power Saver	I just received it and will try it out to see if it works well. I'll comment later.	Jingdong prefecture level city in Shandong
Dragon Quality Goods Shop	Green Intelligent Power Saver	It looks a little small and not very aesthetically pleasing, I don't know how effective it will be until I use it	Jingdong prefecture level city in Shandong
Dragon Quality Goods Shop	Green Intelligent Power Saver	Not only to the point of being useless yet	Jingdong prefecture level city in Shandong
Dragon Quality Goods Shop	Green Intelligent Power Saver	Used it for a month, nothing works, the meter you turn very fast. This is not a scam, but also sold on Jingdong. To cheat a bunch of people?	Jingdong prefecture level city in Shandong
Dragon Quality Goods Shop	Green Intelligent Power Saver	Very good to use, every day can save a little electricity, really good under the sample down a month to save a few dozen dollars in electricity, good before I did not know that there is this ☝ haha, the price is not expensive praise.	Jingdong prefecture level city in Shandong
Dragon Quality Goods Shop	Green Intelligent Power Saver	Tried it for ten days and the change is almost nothing, can't see the power saving yet, use it some more days and see how it goes.	Jingdong prefecture level city in Shandong
Dragon Quality Goods Shop	Green Intelligent Power Saver	Received very quickly, the quality is very good, really save electricity, business service attitude is also very good, I hope you do not miss this opportunity, I continue to buy a few in	Jingdong prefecture level city in Shandong
Dragon Quality Goods Shop	Green Intelligent Power Saver	Quality is very good Oh, the delivery is also very fast, a very favorite store, I want to crazy recommend to friends!	Jingdong prefecture level city in Shandong
Dragon Quality Goods Shop	Green Intelligent Power Saver	The goods have just received, fine workmanship, and the same as described by the merchant, to use a period of time to see if it can achieve the purpose of power saving.	Jingdong prefecture level city in Shandong
Dragon Quality Goods Shop	Green Intelligent Power Saver	Received the goods first use a period of time to see if there is no effect, the business commitment to ninety days invalid refund, first praise, good service!	Jingdong prefecture level city in Shandong
Dragon Quality Goods Shop	Green Intelligent Power Saver	The product is used for a month without energy-saving and power-saving effect does not have any effect	Jingdong prefecture level city in Shandong
Dragon Quality Goods Shop	Green Intelligent Power Saver	The baby is really great, I like it very much, my friends also say good to come, recommended to buy!	Jingdong prefecture level city in Shandong
Dragon Quality Goods Shop	Green Intelligent Power Saver	Logistics is fast, courier boy service is good, after using to see how the fruit, and then follow up.	Jingdong prefecture level city in Shandong
Dragon Quality Goods Shop	Green Intelligent Power Saver	Baby has been received to try, I believe that as the business advertised energy saving. The courier is very responsible.	Jingdong prefecture level city in Shandong
Dragon Quality Goods Shop	Green Intelligent Power Saver	Save Electric Energy King received, good quality, beautiful appearance, very satisfied!	Jingdong prefecture level city in Shandong
Dragon Quality Goods Shop	Green Intelligent Power Saver	Three no products no manufacturers no phone pure fraud induced consumption cheat	Jingdong prefecture level city in Shandong
Dragon Quality Goods Shop	Green Intelligent Power Saver	I'm using it now, I don't know if it's as good as it says, I'll follow up after a month	Jingdong prefecture level city in Shandong
Dragon Quality Goods Shop	Green Intelligent Power Saver	Good quality logistics and fast. Fine workmanship. The description is consistent cost-effective	Jingdong prefecture level city in Shandong
Dragon Quality Goods Shop	Green Intelligent Power Saver	Received, whether it is good or not, to wait a few days and then evaluate	Jingdong prefecture level city in Shandong
Dragon Quality Goods Shop	Green Intelligent Power Saver	I bought it and started using it, I won't know if it works well until I pay my electric bill	Jingdong prefecture level city in Shandong
Dragon Quality Goods Shop	Green Intelligent Power Saver	Especially good, energy saving and power saving, especially good quality, good	Jingdong prefecture level city in Shandong
Dragon Quality Goods Shop	Green Intelligent Power Saver	Logistics is strong, the quality is very good, paste really save electricity, as described, will buy back!	Jingdong prefecture level city in Shandong
Dragon Quality Goods Shop	Green Intelligent Power Saver	Great stuff at a great price, worth the money!	Jingdong prefecture level city in Shandong
Dragon Quality Goods Shop	Green Intelligent Power Saver	Received the power saver, very good quality, very power saving!	Jingdong prefecture level city in Shandong
Dragon Quality Goods Shop	Green Intelligent Power Saver	There are no fruits, only samples...	Jingdong prefecture level city in Shandong
Dragon Quality Goods Shop	Green Intelligent Power Saver	Save a few dollars a month.	Jingdong prefecture level city in Shandong
Dragon Quality Goods Shop	Green Intelligent Power Saver	The quality is particularly good, it works well, and the seller has a good service attitude	Jingdong prefecture level city in Shandong
Dragon Quality Goods Shop	Green Intelligent Power Saver	I've compared them a couple of times and I don't feel any difference	Jingdong prefecture level city in Shandong
Dragon Quality Goods Shop	Green Intelligent Power Saver	I don't see the savings. It's a sham.	Jingdong prefecture level city in Shandong
Dragon Quality Goods Shop	Green Intelligent Power Saver	The power saving effect is very good, and the logistics is very fast. The seller's service attitude is very good	Jingdong prefecture level city in Shandong
Dragon Quality Goods Shop	Green Intelligent Power Saver	Received, well packaged, haven't tried it yet!	Jingdong prefecture level city in Shandong
Dragon Quality Goods Shop	Green Intelligent Power Saver	I don't know how it works, I'll comment after I use it.	Jingdong prefecture level city in Shandong
Dragon Quality Goods Shop	Green Intelligent Power Saver	The product doesn't work well enough to be of any use	Jingdong prefecture level city in Shandong
Dragon Quality Goods Shop	Green Intelligent Power Saver	On Trial 。。。。。。。	Jingdong prefecture level city in Shandong
Dragon Quality Goods Shop	Green Intelligent Power Saver	Being used with unknown results	Jingdong prefecture level city in Shandong
Dragon Quality Goods Shop	Green Intelligent Power Saver	Smart power saving and great quality	Jingdong prefecture level city in Shandong
Dragon Quality Goods Shop	Green Intelligent Power Saver	The saver was received, it works very well and the price is affordable!	Jingdong prefecture level city in Shandong
Dragon Quality Goods Shop	Green Intelligent Power Saver	How can I feel the power saving and how long does it last	Jingdong prefecture level city in Shandong
Dragon Quality Goods Shop	Green Intelligent Power Saver	And I know if it saves electricity or not.	Jingdong prefecture level city in Shandong
Dragon Quality Goods Shop	Green Intelligent Power Saver	It won't save any electricity. No.	Jingdong prefecture level city in Shandong
Dragon Quality Goods Shop	Green Intelligent Power Saver	Invalid. Fake.	Jingdong prefecture level city in Shandong
Dragon Quality Goods Shop	Green Intelligent Power Saver	It's good!	Jingdong prefecture level city in Shandong
Dragon Quality Goods Shop	Green Intelligent Power Saver	have no effect	Jingdong prefecture level city in Shandong
Dragon Quality Goods Shop	Green Intelligent Power Saver	Not used.	Jingdong prefecture level city in Shandong
Dragon Quality Goods Shop	Green Intelligent Power Saver	There is no load range, packaging thrift, ten days also did not develop invoices, received the goods with a receipt, hand-written receipt also has no financial stamp, communication for nearly a week a moment to say that the electronic invoice, a moment to say that the paper invoice, said it is mailed to me, ten days also did not see anything, the invoice invoice can not be found to say that did not stay on file, single single number can not be found, said that the warehouse more than one to a one to find out, in addition to the perfunctory or perfunctory. Products are no certificate of conformity, proper three	Jingdong prefecture level city in Shandong
Dragon Quality Goods Shop	Green Intelligent Power Saver	Satisfaction.	Jingdong prefecture level city in Shandong

Dragon Quality Goods Shop	Green Intelligent Power Saver	There is not a bit of power saving effect, and ringing up an increase in the electricity bill, because you have to add the cost of this machine's own electricity consumption and purchase, so I think it is better to use this machine instead of increasing the expenditure, uneconomical, rather than not use it.	Jingdong prefecture level city in Shandong
Dragon Quality Goods Shop	Green Intelligent Power Saver	I've been using it for two days now and I don't feel any effect.	Jingdong prefecture level city in Shandong
Dragon Quality Goods Shop	Green Intelligent Power Saver	What it looked like was nothing like what was sent.	Jingdong prefecture level city in Shandong
Dragon Quality Goods Shop	Green Intelligent Power Saver	It didn't work. It didn't work for a month or so.	Jingdong prefecture level city in Shandong
Dragon Quality Goods Shop	Green Intelligent Power Saver	Garbage products, physical and description is not the same, no effect at all, pit # no discussion, to buy the fine, garbage, garbage, garbage	Jingdong prefecture level city in Shandong
Dragon Quality Goods Shop	Green Intelligent Power Saver	general	Jingdong prefecture level city in Shandong
Dragon Quality Goods Shop	Green Intelligent Power Saver	IQ tax. Instead of saving electricity, it increases the power consumption of an appliance.	Jingdong prefecture level city in Shandong
Dragon Quality Goods Shop	Green Intelligent Power Saver	There's no trash at all.	Jingdong prefecture level city in Shandong
Dragon Quality Goods Shop	Green Intelligent Power Saver	It doesn't do anything.	Jingdong prefecture level city in Shandong
Dragon Quality Goods Shop	Green Intelligent Power Saver	(of an unmarried couple) be close	Jingdong prefecture level city in Shandong
Dragon Quality Goods Shop	Green Intelligent Power Saver	(of an unmarried couple) be close	Jingdong prefecture level city in Shandong
Dragon Quality Goods Shop	Green Intelligent Power Saver	no electricity saving	Jingdong prefecture level city in Shandong
Dragon Quality Goods Shop	Green Intelligent Power Saver	differ from	Jingdong prefecture level city in Shandong
Dragon Quality Goods Shop	Green Intelligent Power Saver	Saver has received, but real can save electricity, the quality is very good, I times I buy a few more, too good.	Jingdong prefecture level city in Shandong
Dragon Quality Goods Shop	Green Intelligent Power Saver	Great quality as always, a very favorite product, great! o(*￣︶￣*)o	Jingdong prefecture level city in Shandong
Dragon Quality Goods Shop	Green Intelligent Power Saver	If we can reduce the electricity bill, for individuals can save money, for the country can protect the environment, now the weather is getting hotter and hotter, to protect the environment to reduce emissions is very important. Already use the product, whether to reduce the electricity bill depends on next month!	Jingdong prefecture level city in Shandong
Dragon Quality Goods Shop	Green Intelligent Power Saver	Purchased a power saver received on the put into use, rent a room with many electrical appliances, power consumption is very large, we have a piece of electricity or 1.2 dollars a time, with this power saver is much better, every day can save a few cents, very convenient and practical!	Jingdong prefecture level city in Shandong
Dragon Quality Goods Shop	Green Intelligent Power Saver	The product is better to use before not to use when a month 1500 yuan electricity bill, now using the 5 second month electricity bill directly down 470 dollars or very good. A save money to buy several power saver money ZanZanZanZan👍	Jingdong prefecture level city in Shandong
Dragon Quality Goods Shop	Green Intelligent Power Saver	The moment I received the goods really satisfied with this product, not only high-grade packaging, the body are base alloy, up to the price, I was also introduced by a friend, he used last month did save a lot of electricity, but also recommended to me!	Jingdong prefecture level city in Shandong
Dragon Quality Goods Shop	Green Intelligent Power Saver	I got it. Good review. I'm very satisfied. Reasonable price.	Jingdong prefecture level city in Shandong
Dragon Quality Goods Shop	Green Intelligent Power Saver	Intelligent power saver received and used, looking at the quality is good, the quality of the plug sent is also very good. Expect the effect of the economizer is obvious, first five-star praise, with a period of time to follow up!	Jingdong prefecture level city in Shandong
Dragon Quality Goods Shop	Green Intelligent Power Saver	The saver has been received. It was plugged into an outlet and used immediately after receipt. Whether it really saves electricity is not clear yet, I believe that this brand of energy saver really saves	Jingdong prefecture level city in Shandong
Dragon Quality Goods Shop	Green Intelligent Power Saver	Okay, okay, okay. Thank you so much. Fast is good. Fast. Item was as described, mainly because the event was cheap and you can save a lot of money.	Jingdong prefecture level city in Shandong
Dragon Quality Goods Shop	Green Intelligent Power Saver	Received it a couple days ago! Plugged it in the same day! Let's see what the electric bill is in a month! Hopefully it works well oh!	Jingdong prefecture level city in Shandong
Dragon Quality Goods Shop	Green Intelligent Power Saver	Looking at the reviews good, energy-saving small products, compact, the results after 2 months to see, because the electricity bill season payment	Jingdong prefecture level city in Shandong
Dragon Quality Goods Shop	Green Intelligent Power Saver	I just received it and will try it out to see if it works well. I'll comment later.	Jingdong prefecture level city in Shandong
Dragon Quality Goods Shop	Green Intelligent Power Saver	It looks a little small and not very aesthetically pleasing, I don't know how effective it will be until I use it	Jingdong prefecture level city in Shandong
Dragon Quality Goods Shop	Green Intelligent Power Saver	Not only to the point of being useless yet	Jingdong prefecture level city in Shandong
Dragon Quality Goods Shop	Green Intelligent Power Saver	Used it for a month, nothing works, the meter you turn very fast. This is not a scam, but also sold on Jingdong. To cheat a bunch of people?	Jingdong prefecture level city in Shandong
Dragon Quality Goods Shop	Green Intelligent Power Saver	Very good to use, every day can save a little electricity, really good under the sample down a month to save a few dozen dollars in electricity, good before I did not know that there is this 👍 haha, the price is not expensive praise.	Jingdong prefecture level city in Shandong
Dragon Quality Goods Shop	Green Intelligent Power Saver	Tried it for ten days and the change is almost nothing, can't see the power saving yet, use it some more days and see how it goes.	Jingdong prefecture level city in Shandong
Dragon Quality Goods Shop	Green Intelligent Power Saver	Received very quickly, the quality is very good, really save electricity, business service attitude is also very good, I hope you do not miss this opportunity, I continue to buy a few in	Jingdong prefecture level city in Shandong
Dragon Quality Goods Shop	Green Intelligent Power Saver	Quality is very good Oh, the delivery is also very fast, a very favorite store, I want to crazy recommend to friends!	Jingdong prefecture level city in Shandong
Dragon Quality Goods Shop	Green Intelligent Power Saver	The goods have just received, fine workmanship, and the same as described by the merchant, to use a period of time to see if it can achieve the purpose of power saving.	Jingdong prefecture level city in Shandong
Dragon Quality Goods Shop	Green Intelligent Power Saver	Received the goods first use a period of time to see if there is no effect, the business commitment to ninety days invalid refund, first praise, good service!	Jingdong prefecture level city in Shandong
Dragon Quality Goods Shop	Green Intelligent Power Saver	The product is used for a month without energy-saving and power-saving effect does not have any effect	Jingdong prefecture level city in Shandong
Dragon Quality Goods Shop	Green Intelligent Power Saver	The baby is really great, I like it very much, my friends also say good to come, recommended to buy!	Jingdong prefecture level city in Shandong
Dragon Quality Goods Shop	Green Intelligent Power Saver	Logistics is fast, courier boy service is good, after using to see how the fruit, and then follow up.	Jingdong prefecture level city in Shandong
Dragon Quality Goods Shop	Green Intelligent Power Saver	Baby has been received to try, I believe that as the business advertised energy saving. The courier is very responsible.	Jingdong prefecture level city in Shandong
Dragon Quality Goods Shop	Green Intelligent Power Saver	Save Electric Energy King received, good quality, beautiful appearance, very satisfied!	Jingdong prefecture level city in Shandong
Dragon Quality Goods Shop	Green Intelligent Power Saver	Three no products no manufacturers no phone pure fraud induced consumption cheat	Jingdong prefecture level city in Shandong
Dragon Quality Goods Shop	Green Intelligent Power Saver	I'm using it now, I don't know if it's as good as it says, I'll follow up after a month	Jingdong prefecture level city in Shandong
Dragon Quality Goods Shop	Green Intelligent Power Saver	Good quality logistics and fast. Fine workmanship. The description is consistent cost-effective	Jingdong prefecture level city in Shandong
Dragon Quality Goods Shop	Green Intelligent Power Saver	Received, whether it is good or not, to wait a few days and then evaluate	Jingdong prefecture level city in Shandong
Dragon Quality Goods Shop	Green Intelligent Power Saver	I bought it and started using it, I won't know if it works well until I pay my electric bill	Jingdong prefecture level city in Shandong
Dragon Quality Goods Shop	Green Intelligent Power Saver	Especially good, energy saving and power saving, especially good quality, good	Jingdong prefecture level city in Shandong
Dragon Quality Goods Shop	Green Intelligent Power Saver	Logistics is strong, the quality is very good, paste really save electricity, as described, will buy back!	Jingdong prefecture level city in Shandong
Dragon Quality Goods Shop	Green Intelligent Power Saver	Great stuff at a great price, worth the money!	Jingdong prefecture level city in Shandong

Dragon Quality Goods Shop	Green Intelligent Power Saver	Received the power saver, very good quality, very power saving!	Jingdong prefecture level city in Shandong
Dragon Quality Goods Shop	Green Intelligent Power Saver	There are no fruits, only samples...	Jingdong prefecture level city in Shandong
Dragon Quality Goods Shop	Green Intelligent Power Saver	Save a few dollars a month.	Jingdong prefecture level city in Shandong
Dragon Quality Goods Shop	Green Intelligent Power Saver	The quality is particularly good, it works well, and the seller has a good service attitude	Jingdong prefecture level city in Shandong
Dragon Quality Goods Shop	Green Intelligent Power Saver	I've compared them a couple of times and I don't feel any difference	Jingdong prefecture level city in Shandong
Dragon Quality Goods Shop	Green Intelligent Power Saver	I don't see the savings. It's a sham.	Jingdong prefecture level city in Shandong
Dragon Quality Goods Shop	Green Intelligent Power Saver	The power saving effect is very good, and the logistics is very fast. The seller's service attitude is very good	Jingdong prefecture level city in Shandong
Dragon Quality Goods Shop	Green Intelligent Power Saver	Received, well packaged, haven't tried it yet!	Jingdong prefecture level city in Shandong
Dragon Quality Goods Shop	Green Intelligent Power Saver	I don't know how it works, I'll comment after I use it.	Jingdong prefecture level city in Shandong
Dragon Quality Goods Shop	Green Intelligent Power Saver	The product doesn't work well enough to be of any use	Jingdong prefecture level city in Shandong
Dragon Quality Goods Shop	Green Intelligent Power Saver	On Trial 。。。。。。。	Jingdong prefecture level city in Shandong
Dragon Quality Goods Shop	Green Intelligent Power Saver	Being used with unknown results	Jingdong prefecture level city in Shandong
Dragon Quality Goods Shop	Green Intelligent Power Saver	Smart power saving and great quality	Jingdong prefecture level city in Shandong
Dragon Quality Goods Shop	Green Intelligent Power Saver	The saver was received, it works very well and the price is affordable!	Jingdong prefecture level city in Shandong
Dragon Quality Goods Shop	Green Intelligent Power Saver	How can I feel the power saving and how long does it last	Jingdong prefecture level city in Shandong
Dragon Quality Goods Shop	Green Intelligent Power Saver	And I know if it saves electricity or not.	Jingdong prefecture level city in Shandong
Dragon Quality Goods Shop	Green Intelligent Power Saver	It won't save any electricity. No.	Jingdong prefecture level city in Shandong
Dragon Quality Goods Shop	Green Intelligent Power Saver	Invalid. Fake.	Jingdong prefecture level city in Shandong
Dragon Quality Goods Shop	Green Intelligent Power Saver	It's good!	Jingdong prefecture level city in Shandong
Dragon Quality Goods Shop	Green Intelligent Power Saver	have no effect	Jingdong prefecture level city in Shandong
Dragon Quality Goods Shop	Green Intelligent Power Saver	Not used.	Jingdong prefecture level city in Shandong
Dragon Quality Goods Shop	Green Intelligent Power Saver	There is no load range, packaging thrift, ten days also did not develop invoices, received the goods with a receipt, hand-written receipt also has no financial stamp, communication for nearly a week a moment to say that the electronic invoice, a moment to say that the paper invoice, said it is mailed to me, ten days also did not see anything, the invoice invoice can not be found to say that did not stay on file, single single number can not be found, said that the warehouse more than one to a one to find out, in addition to the perfunctory or perfunctory. Products are no certificate of conformity, proper three	Jingdong prefecture level city in Shandong
Dragon Quality Goods Shop	Green Intelligent Power Saver	Satisfaction.	Jingdong prefecture level city in Shandong
Dragon Quality Goods Shop	Green Intelligent Power Saver	There is not a bit of power saving effect, and ringing up an increase in the electricity bill, because you have to add the cost of this machine's own electricity consumption and purchase, so I think it is better to use this machine instead of increasing the expenditure, uneconomical, rather than not use it.	Jingdong prefecture level city in Shandong
Dragon Quality Goods Shop	Green Intelligent Power Saver	I've been using it for two days now and I don't feel any effect.	Jingdong prefecture level city in Shandong
Dragon Quality Goods Shop	Green Intelligent Power Saver	What it looked like was nothing like what was sent.	Jingdong prefecture level city in Shandong
Dragon Quality Goods Shop	Green Intelligent Power Saver	It didn't work. It didn't work for a month or so.	Jingdong prefecture level city in Shandong
Dragon Quality Goods Shop	Green Intelligent Power Saver	Garbage products, physical and description is not the same, no effect at all, pit # no discussion, to buy the fine, garbage, garbage, garbage	Jingdong prefecture level city in Shandong
Dragon Quality Goods Shop	Green Intelligent Power Saver	general	Jingdong prefecture level city in Shandong
Dragon Quality Goods Shop	Green Intelligent Power Saver	IQ tax. Instead of saving electricity, it increases the power consumption of an appliance.	Jingdong prefecture level city in Shandong
Dragon Quality Goods Shop	Green Intelligent Power Saver	There's no trash at all.	Jingdong prefecture level city in Shandong
Dragon Quality Goods Shop	Green Intelligent Power Saver	It doesn't do anything.	Jingdong prefecture level city in Shandong
Dragon Quality Goods Shop	Green Intelligent Power Saver	(of an unmarried couple) be close	Jingdong prefecture level city in Shandong
Dragon Quality Goods Shop	Green Intelligent Power Saver	(of an unmarried couple) be close	Jingdong prefecture level city in Shandong
Dragon Quality Goods Shop	Green Intelligent Power Saver	no electricity saving	Jingdong prefecture level city in Shandong
Dragon Quality Goods Shop	Green Intelligent Power Saver	differ from	Jingdong prefecture level city in Shandong
Dragon Quality Goods Shop	Green Intelligent Power Saver	Saver has received, but real can save electricity, the quality is very good, I times I buy a few more, too good.	Jingdong prefecture level city in Shandong
Dragon Quality Goods Shop	Green Intelligent Power Saver	Great quality as always, a very favorite product, great! o(*￣︶￣*)o	Jingdong prefecture level city in Shandong
Dragon Quality Goods Shop	Green Intelligent Power Saver	If we can reduce the electricity bill, for individuals can save money, for the country can protect the environment, now the weather is getting hotter and hotter, to protect the environment to reduce emissions is very important. Already use the product, whether to reduce the electricity bill depends on next month!	Jingdong prefecture level city in Shandong
Dragon Quality Goods Shop	Green Intelligent Power Saver	Purchased a power saver received on the put into use, rent a room with many electrical appliances, power consumption is very large, we have a piece of electricity or 1.2 dollars a time, with this power saver is much better, every day can save a few cents, very convenient and practical!	Jingdong prefecture level city in Shandong
Dragon Quality Goods Shop	Green Intelligent Power Saver	The product is better to use before not to use when a month 1500 yuan electricity bill, now using the 5 second month electricity bill directly down 470 dollars or very good. A save money to buy several power saver money ZanZanZanZan👍	Jingdong prefecture level city in Shandong
Dragon Quality Goods Shop	Green Intelligent Power Saver	The moment I received the goods really satisfied with this product, not only high-grade packaging, the body are base alloy, up to the price, I was also introduced by a friend, he used last month did save a lot of electricity, but also recommended to me!	Jingdong prefecture level city in Shandong
Dragon Quality Goods Shop	Green Intelligent Power Saver	I got it. Good review. I'm very satisfied. Reasonable price.	Jingdong prefecture level city in Shandong
Dragon Quality Goods Shop	Green Intelligent Power Saver	Intelligent power saver received and used, looking at the quality is good, the quality of the plug sent is also very good. Expect the effect of the economizer is obvious, first five-star praise, with a period of time to follow up!	Jingdong prefecture level city in Shandong
Dragon Quality Goods Shop	Green Intelligent Power Saver	The saver has been received. It was plugged into an outlet and used immediately after receipt. Whether it really saves electricity is not clear yet, I believe that this brand of energy saver really saves	Jingdong prefecture level city in Shandong
Dragon Quality Goods Shop	Green Intelligent Power Saver	Okay, okay, okay. Thank you so much. Fast is good. Fast. Item was as described, mainly because the event was cheap and you can save a lot of money.	Jingdong prefecture level city in Shandong

Dragon Quality Goods Shop	Green Intelligent Power Saver	Received it a couple days ago! Plugged it in the same day! Let's see what the electric bill is in a month! Hopefully it works well oh!	Jingdong prefecture level city in Shandong
Dragon Quality Goods Shop	Green Intelligent Power Saver	Looking at the reviews good, energy-saving small products, compact, the results after 2 months to see, because the electricity bill season payment	Jingdong prefecture level city in Shandong
Dragon Quality Goods Shop	Green Intelligent Power Saver	I just received it and will try it out to see if it works well. I'll comment later.	Jingdong prefecture level city in Shandong
Dragon Quality Goods Shop	Green Intelligent Power Saver	It looks a little small and not very aesthetically pleasing, I don't know how effective it will be until I use it	Jingdong prefecture level city in Shandong
Dragon Quality Goods Shop	Green Intelligent Power Saver	Not only to the point of being useless yet	Jingdong prefecture level city in Shandong
Dragon Quality Goods Shop	Green Intelligent Power Saver	Used it for a month, nothing works, the meter you turn very fast. This is not a scam, but also sold on Jingdong. To cheat a bunch of people?	Jingdong prefecture level city in Shandong
Dragon Quality Goods Shop	Green Intelligent Power Saver	Very good to use, every day can save a little electricity, really good under the sample down a month to save a few dozen dollars in electricity, good before I did not know that there is this haha, the price is not expensive praise.	Jingdong prefecture level city in Shandong
Dragon Quality Goods Shop	Green Intelligent Power Saver	Tried it for ten days and the change is almost nothing, can't see the power saving yet, use it some more days and see how it goes.	Jingdong prefecture level city in Shandong
Dragon Quality Goods Shop	Green Intelligent Power Saver	Received very quickly, the quality is very good, really save electricity, business service attitude is also very good, I hope you do not miss this opportunity, I continue to buy a few in	Jingdong prefecture level city in Shandong
Dragon Quality Goods Shop	Green Intelligent Power Saver	Quality is very good Oh, the delivery is also very fast, a very favorite store, I want to crazy recommend to friends!	Jingdong prefecture level city in Shandong
Dragon Quality Goods Shop	Green Intelligent Power Saver	The goods have just received, fine workmanship, and the same as described by the merchant, to use a period of time to see if it can achieve the purpose of power saving.	Jingdong prefecture level city in Shandong
Dragon Quality Goods Shop	Green Intelligent Power Saver	Received the goods first use a period of time to see if there is no effect, the business commitment to ninety days invalid refund, first praise, good service!	Jingdong prefecture level city in Shandong
Dragon Quality Goods Shop	Green Intelligent Power Saver	The product is used for a month without energy-saving and power-saving effect does not have any effect	Jingdong prefecture level city in Shandong
Dragon Quality Goods Shop	Green Intelligent Power Saver	The baby is really great, I like it very much, my friends also say good to come, recommended to buy!	Jingdong prefecture level city in Shandong
Dragon Quality Goods Shop	Green Intelligent Power Saver	Logistics is fast, courier boy service is good, after using to see how the fruit, and then follow up.	Jingdong prefecture level city in Shandong
Dragon Quality Goods Shop	Green Intelligent Power Saver	Baby has been received to try, I believe that as the business advertised energy saving. The courier is very responsible.	Jingdong prefecture level city in Shandong
Dragon Quality Goods Shop	Green Intelligent Power Saver	Save Electric Energy King received, good quality, beautiful appearance, very satisfied!	Jingdong prefecture level city in Shandong
Dragon Quality Goods Shop	Green Intelligent Power Saver	Three no products no manufacturers no phone pure fraud induced consumption cheat	Jingdong prefecture level city in Shandong
Dragon Quality Goods Shop	Green Intelligent Power Saver	I'm using it now, I don't know if it's as good as it says, I'll follow up after a month	Jingdong prefecture level city in Shandong
Dragon Quality Goods Shop	Green Intelligent Power Saver	Good quality logistics and fast. Fine workmanship. The description is consistent cost-effective	Jingdong prefecture level city in Shandong
Dragon Quality Goods Shop	Green Intelligent Power Saver	Received, whether it is good or not, to wait a few days and then evaluate	Jingdong prefecture level city in Shandong
Dragon Quality Goods Shop	Green Intelligent Power Saver	I bought it and started using it, I won't know if it works well until I pay my electric bill	Jingdong prefecture level city in Shandong
Dragon Quality Goods Shop	Green Intelligent Power Saver	Especially good, energy saving and power saving, especially good quality, good	Jingdong prefecture level city in Shandong
Dragon Quality Goods Shop	Green Intelligent Power Saver	Logistics is strong, the quality is very good, paste really save electricity, as described, will buy back!	Jingdong prefecture level city in Shandong
Dragon Quality Goods Shop	Green Intelligent Power Saver	Great stuff at a great price, worth the money!	Jingdong prefecture level city in Shandong
Dragon Quality Goods Shop	Green Intelligent Power Saver	Received the power saver, very good quality, very power saving!	Jingdong prefecture level city in Shandong
Dragon Quality Goods Shop	Green Intelligent Power Saver	There are no fruits, only samples...	Jingdong prefecture level city in Shandong
Dragon Quality Goods Shop	Green Intelligent Power Saver	Save a few dollars a month.	Jingdong prefecture level city in Shandong
Dragon Quality Goods Shop	Green Intelligent Power Saver	The quality is particularly good, it works well, and the seller has a good service attitude	Jingdong prefecture level city in Shandong
Dragon Quality Goods Shop	Green Intelligent Power Saver	I've compared them a couple of times and I don't feel any difference	Jingdong prefecture level city in Shandong
Dragon Quality Goods Shop	Green Intelligent Power Saver	I don't see the savings. It's a sham.	Jingdong prefecture level city in Shandong
Dragon Quality Goods Shop	Green Intelligent Power Saver	The power saving effect is very good, and the logistics is very fast. The seller's service attitude is very good	Jingdong prefecture level city in Shandong
Dragon Quality Goods Shop	Green Intelligent Power Saver	Received, well packaged, haven't tried it yet!	Jingdong prefecture level city in Shandong
Dragon Quality Goods Shop	Green Intelligent Power Saver	I don't know how it works, I'll comment after I use it.	Jingdong prefecture level city in Shandong
Dragon Quality Goods Shop	Green Intelligent Power Saver	The product doesn't work well enough to be of any use	Jingdong prefecture level city in Shandong
Dragon Quality Goods Shop	Green Intelligent Power Saver	On Trial 。。。。。。。	Jingdong prefecture level city in Shandong
Dragon Quality Goods Shop	Green Intelligent Power Saver	Being used with unknown results	Jingdong prefecture level city in Shandong
Dragon Quality Goods Shop	Green Intelligent Power Saver	Smart power saving and great quality	Jingdong prefecture level city in Shandong
Dragon Quality Goods Shop	Green Intelligent Power Saver	The saver was received, it works very well and the price is affordable!	Jingdong prefecture level city in Shandong
Dragon Quality Goods Shop	Green Intelligent Power Saver	How can I feel the power saving and how long does it last	Jingdong prefecture level city in Shandong
Dragon Quality Goods Shop	Green Intelligent Power Saver	And I know if it saves electricity or not.	Jingdong prefecture level city in Shandong
Dragon Quality Goods Shop	Green Intelligent Power Saver	It won't save any electricity. No.	Jingdong prefecture level city in Shandong
Dragon Quality Goods Shop	Green Intelligent Power Saver	Invalid. Fake.	Jingdong prefecture level city in Shandong
Dragon Quality Goods Shop	Green Intelligent Power Saver	It's good!	Jingdong prefecture level city in Shandong
Dragon Quality Goods Shop	Green Intelligent Power Saver	have no effect	Jingdong prefecture level city in Shandong
Dragon Quality Goods Shop	Green Intelligent Power Saver	Not used.	Jingdong prefecture level city in Shandong
Dragon Quality Goods Shop	Green Intelligent Power Saver	There is no load range, packaging thrift, ten days also did not develop invoices, received the goods with a receipt, hand-written receipt also has no financial stamp, communication for nearly a week a moment to say that the electronic invoice, a moment to say that the paper invoice, said it is mailed to me, ten days also did not see anything, the invoice invoice can not be found to say that did not stay on file, single single number can not be found, said that the warehouse more than one to a one to find out, in addition to the perfunctory or perfunctory. Products are no certificate of conformity, proper three	Jingdong prefecture level city in Shandong
Dragon Quality Goods Shop	Green Intelligent Power Saver	Satisfaction.	Jingdong prefecture level city in Shandong

Dragon Quality Goods Shop	Green Intelligent Power Saver	There is not a bit of power saving effect, and ringing up an increase in the electricity bill, because you have to add the cost of this machine's own electricity consumption and purchase, so I think it is better to use this machine instead of increasing the expenditure, uneconomical, rather than not use it.	Jingdong prefecture level city in Shandong
Dragon Quality Goods Shop	Green Intelligent Power Saver	I've been using it for two days now and I don't feel any effect.	Jingdong prefecture level city in Shandong
Dragon Quality Goods Shop	Green Intelligent Power Saver	What it looked like was nothing like what was sent.	Jingdong prefecture level city in Shandong
Dragon Quality Goods Shop	Green Intelligent Power Saver	It didn't work. It didn't work for a month or so.	Jingdong prefecture level city in Shandong
Dragon Quality Goods Shop	Green Intelligent Power Saver	Garbage products, physical and description is not the same, no effect at all, pit # no discussion, to buy the fine, garbage, garbage, garbage	Jingdong prefecture level city in Shandong
Dragon Quality Goods Shop	Green Intelligent Power Saver	general	Jingdong prefecture level city in Shandong
Dragon Quality Goods Shop	Green Intelligent Power Saver	IQ tax. Instead of saving electricity, it increases the power consumption of an appliance.	Jingdong prefecture level city in Shandong
Dragon Quality Goods Shop	Green Intelligent Power Saver	There's no trash at all.	Jingdong prefecture level city in Shandong
Dragon Quality Goods Shop	Green Intelligent Power Saver	It doesn't do anything.	Jingdong prefecture level city in Shandong
Dragon Quality Goods Shop	Green Intelligent Power Saver	(of an unmarried couple) be close	Jingdong prefecture level city in Shandong
Dragon Quality Goods Shop	Green Intelligent Power Saver	(of an unmarried couple) be close	Jingdong prefecture level city in Shandong
Dragon Quality Goods Shop	Green Intelligent Power Saver	no electricity saving	Jingdong prefecture level city in Shandong
Dragon Quality Goods Shop	Green Intelligent Power Saver	differ from	Jingdong prefecture level city in Shandong
Dragon Quality Goods Shop	Green Intelligent Power Saver	Saver has received, but real can save electricity, the quality is very good, I times I buy a few more, too good.	Jingdong prefecture level city in Shandong
Dragon Quality Goods Shop	Green Intelligent Power Saver	Great quality as always, a very favorite product, great! o(*￣︶￣*)o	Jingdong prefecture level city in Shandong
Dragon Quality Goods Shop	Green Intelligent Power Saver	If we can reduce the electricity bill, for individuals can save money, for the country can protect the environment, now the weather is getting hotter and hotter, to protect the environment to reduce emissions is very important. Already use the product, whether to reduce the electricity bill depends on next month!	Jingdong prefecture level city in Shandong
Dragon Quality Goods Shop	Green Intelligent Power Saver	Purchased a power saver received on the put into use, rent a room with many electrical appliances, power consumption is very large, we have a piece of electricity or 1.2 dollars a time, with this power saver is much better, every day can save a few cents, very convenient and practical!	Jingdong prefecture level city in Shandong
Dragon Quality Goods Shop	Green Intelligent Power Saver	The product is better to use before not to use when a month 1500 yuan electricity bill, now using the 5 second month electricity bill directly down 470 dollars or very good. A save money to buy several power saver money ZanZanZanZan👍	Jingdong prefecture level city in Shandong
Dragon Quality Goods Shop	Green Intelligent Power Saver	The moment I received the goods really satisfied with this product, not only high-grade packaging, the body are base alloy, up to the price, I was also introduced by a friend, he used last month did save a lot of electricity, but also recommended to me!	Jingdong prefecture level city in Shandong
Dragon Quality Goods Shop	Green Intelligent Power Saver	I got it. Good review. I'm very satisfied. Reasonable price.	Jingdong prefecture level city in Shandong
Dragon Quality Goods Shop	Green Intelligent Power Saver	Intelligent power saver received and used, looking at the quality is good, the quality of the plug sent is also very good. Expect the effect of the economizer is obvious, first five-star praise, with a period of time to follow up!	Jingdong prefecture level city in Shandong
Dragon Quality Goods Shop	Green Intelligent Power Saver	The saver has been received. It was plugged into an outlet and used immediately after receipt. Whether it really saves electricity is not clear yet, I believe that this brand of energy saver really saves	Jingdong prefecture level city in Shandong
Dragon Quality Goods Shop	Green Intelligent Power Saver	Okay, okay, okay. Thank you so much. Fast is good. Fast. Item was as described, mainly because the event was cheap and you can save a lot of money.	Jingdong prefecture level city in Shandong
Dragon Quality Goods Shop	Green Intelligent Power Saver	Received it a couple days ago! Plugged it in the same day! Let's see what the electric bill is in a month! Hopefully it works well oh!	Jingdong prefecture level city in Shandong
Dragon Quality Goods Shop	Green Intelligent Power Saver	Looking at the reviews good, energy-saving small products, compact, the results after 2 months to see, because the electricity bill season payment	Jingdong prefecture level city in Shandong
Dragon Quality Goods Shop	Green Intelligent Power Saver	I just received it and will try it out to see if it works well. I'll comment later.	Jingdong prefecture level city in Shandong
Dragon Quality Goods Shop	Green Intelligent Power Saver	It looks a little small and not very aesthetically pleasing, I don't know how effective it will be until I use it	Jingdong prefecture level city in Shandong
Dragon Quality Goods Shop	Green Intelligent Power Saver	Not only to the point of being useless yet	Jingdong prefecture level city in Shandong
Dragon Quality Goods Shop	Green Intelligent Power Saver	Used it for a month, nothing works, the meter you turn very fast. This is not a scam, but also sold on Jingdong. To cheat a bunch of people?	Jingdong prefecture level city in Shandong
Dragon Quality Goods Shop	Green Intelligent Power Saver	Very good to use, every day can save a little electricity, really good under the sample down a month to save a few dozen dollars in electricity, good before I did not know that there is this 👆 haha, the price is not expensive praise.	Jingdong prefecture level city in Shandong
Dragon Quality Goods Shop	Green Intelligent Power Saver	Tried it for ten days and the change is almost nothing, can't see the power saving yet, use it some more days and see how it goes.	Jingdong prefecture level city in Shandong
Dragon Quality Goods Shop	Green Intelligent Power Saver	Received very quickly, the quality is very good, really save electricity, business service attitude is also very good, I hope you do not miss this opportunity, I continue to buy a few in	Jingdong prefecture level city in Shandong
Dragon Quality Goods Shop	Green Intelligent Power Saver	Quality is very good Oh, the delivery is also very fast, a very favorite store, I want to crazy recommend to friends!	Jingdong prefecture level city in Shandong
Dragon Quality Goods Shop	Green Intelligent Power Saver	The goods have just received, fine workmanship, and the same as described by the merchant, to use a period of time to see if it can achieve the purpose of power saving.	Jingdong prefecture level city in Shandong
Dragon Quality Goods Shop	Green Intelligent Power Saver	Received the goods first use a period of time to see if there is no effect, the business commitment to ninety days invalid refund, first praise, good service!	Jingdong prefecture level city in Shandong
Dragon Quality Goods Shop	Green Intelligent Power Saver	The product is used for a month without energy-saving and power-saving effect does not have any effect	Jingdong prefecture level city in Shandong
Dragon Quality Goods Shop	Green Intelligent Power Saver	The baby is really great, I like it very much, my friends also say good to come, recommended to buy!	Jingdong prefecture level city in Shandong
Dragon Quality Goods Shop	Green Intelligent Power Saver	Logistics is fast, courier boy service is good, after using to see how the fruit, and then follow up.	Jingdong prefecture level city in Shandong
Dragon Quality Goods Shop	Green Intelligent Power Saver	Baby has been received to try, I believe that as the business advertised energy saving. The courier is very responsible.	Jingdong prefecture level city in Shandong
Dragon Quality Goods Shop	Green Intelligent Power Saver	Save Electric Energy King received, good quality, beautiful appearance, very satisfied!	Jingdong prefecture level city in Shandong
Dragon Quality Goods Shop	Green Intelligent Power Saver	Three no products no manufacturers no phone pure fraud induced consumption cheat	Jingdong prefecture level city in Shandong
Dragon Quality Goods Shop	Green Intelligent Power Saver	I'm using it now, I don't know if it's as good as it says, I'll follow up after a month	Jingdong prefecture level city in Shandong
Dragon Quality Goods Shop	Green Intelligent Power Saver	Good quality logistics and fast. Fine workmanship. The description is consistent cost-effective	Jingdong prefecture level city in Shandong
Dragon Quality Goods Shop	Green Intelligent Power Saver	Received, whether it is good or not, to wait a few days and then evaluate	Jingdong prefecture level city in Shandong
Dragon Quality Goods Shop	Green Intelligent Power Saver	I bought it and started using it, I won't know if it works well until I pay my electric bill	Jingdong prefecture level city in Shandong
Dragon Quality Goods Shop	Green Intelligent Power Saver	Especially good, energy saving and power saving, especially good quality, good	Jingdong prefecture level city in Shandong
Dragon Quality Goods Shop	Green Intelligent Power Saver	Logistics is strong, the quality is very good, paste really save electricity, as described, will buy back!	Jingdong prefecture level city in Shandong
Dragon Quality Goods Shop	Green Intelligent Power Saver	Great stuff at a great price, worth the money!	Jingdong prefecture level city in Shandong

Dragon Quality Goods Shop	Green Intelligent Power Saver	Received the power saver, very good quality, very power saving!	Jingdong prefecture level city in Shandong
Dragon Quality Goods Shop	Green Intelligent Power Saver	There are no fruits, only samples...	Jingdong prefecture level city in Shandong
Dragon Quality Goods Shop	Green Intelligent Power Saver	Save a few dollars a month.	Jingdong prefecture level city in Shandong
Dragon Quality Goods Shop	Green Intelligent Power Saver	The quality is particularly good, it works well, and the seller has a good service attitude	Jingdong prefecture level city in Shandong
Dragon Quality Goods Shop	Green Intelligent Power Saver	I've compared them a couple of times and I don't feel any difference	Jingdong prefecture level city in Shandong
Dragon Quality Goods Shop	Green Intelligent Power Saver	I don't see the savings. It's a sham.	Jingdong prefecture level city in Shandong
Dragon Quality Goods Shop	Green Intelligent Power Saver	The power saving effect is very good, and the logistics is very fast. The seller's service attitude is very good	Jingdong prefecture level city in Shandong
Dragon Quality Goods Shop	Green Intelligent Power Saver	Received, well packaged, haven't tried it yet!	Jingdong prefecture level city in Shandong
Dragon Quality Goods Shop	Green Intelligent Power Saver	I don't know how it works, I'll comment after I use it.	Jingdong prefecture level city in Shandong
Dragon Quality Goods Shop	Green Intelligent Power Saver	The product doesn't work well enough to be of any use	Jingdong prefecture level city in Shandong
Dragon Quality Goods Shop	Green Intelligent Power Saver	On Trial 。。。。。。。	Jingdong prefecture level city in Shandong
Dragon Quality Goods Shop	Green Intelligent Power Saver	Being used with unknown results	Jingdong prefecture level city in Shandong
Dragon Quality Goods Shop	Green Intelligent Power Saver	Smart power saving and great quality	Jingdong prefecture level city in Shandong
Dragon Quality Goods Shop	Green Intelligent Power Saver	The saver was received, it works very well and the price is affordable!	Jingdong prefecture level city in Shandong
Dragon Quality Goods Shop	Green Intelligent Power Saver	How can I feel the power saving and how long does it last	Jingdong prefecture level city in Shandong
Dragon Quality Goods Shop	Green Intelligent Power Saver	And I know if it saves electricity or not.	Jingdong prefecture level city in Shandong
Dragon Quality Goods Shop	Green Intelligent Power Saver	It won't save any electricity. No.	Jingdong prefecture level city in Shandong
Dragon Quality Goods Shop	Green Intelligent Power Saver	Invalid. Fake.	Jingdong prefecture level city in Shandong
Dragon Quality Goods Shop	Green Intelligent Power Saver	It's good!	Jingdong prefecture level city in Shandong
Dragon Quality Goods Shop	Green Intelligent Power Saver	have no effect	Jingdong prefecture level city in Shandong
Dragon Quality Goods Shop	Green Intelligent Power Saver	Not used.	Jingdong prefecture level city in Shandong
Dragon Quality Goods Shop	Green Intelligent Power Saver	There is no load range, packaging thrift, ten days also did not develop invoices, received the goods with a receipt, hand-written receipt also has no financial stamp, communication for nearly a week a moment to say that the electronic invoice, a moment to say that the paper invoice, said it is mailed to me, ten days also did not see anything, the invoice invoice can not be found to say that did not stay on file, single single number can not be found, said that the warehouse more than one to a one to find out, in addition to the perfunctory or perfunctory. Products are no certificate of conformity, proper three	Jingdong prefecture level city in Shandong
Dragon Quality Goods Shop	Green Intelligent Power Saver	Satisfaction.	Jingdong prefecture level city in Shandong
Dragon Quality Goods Shop	Green Intelligent Power Saver	There is not a bit of power saving effect, and ringing up an increase in the electricity bill, because you have to add the cost of this machine's own electricity consumption and purchase, so I think it is better to use this machine instead of increasing the expenditure, uneconomical, rather than not use it.	Jingdong prefecture level city in Shandong
Dragon Quality Goods Shop	Green Intelligent Power Saver	I've been using it for two days now and I don't feel any effect.	Jingdong prefecture level city in Shandong
Dragon Quality Goods Shop	Green Intelligent Power Saver	What it looked like was nothing like what was sent.	Jingdong prefecture level city in Shandong
Dragon Quality Goods Shop	Green Intelligent Power Saver	It didn't work. It didn't work for a month or so.	Jingdong prefecture level city in Shandong
Dragon Quality Goods Shop	Green Intelligent Power Saver	Garbage products, physical and description is not the same, no effect at all, pit # no discussion, to buy the fine, garbage, garbage, garbage	Jingdong prefecture level city in Shandong
Dragon Quality Goods Shop	Green Intelligent Power Saver	general	Jingdong prefecture level city in Shandong
Dragon Quality Goods Shop	Green Intelligent Power Saver	IQ tax. Instead of saving electricity, it increases the power consumption of an appliance.	Jingdong prefecture level city in Shandong
Dragon Quality Goods Shop	Green Intelligent Power Saver	There's no trash at all.	Jingdong prefecture level city in Shandong
Dragon Quality Goods Shop	Green Intelligent Power Saver	It doesn't do anything.	Jingdong prefecture level city in Shandong
Dragon Quality Goods Shop	Green Intelligent Power Saver	(of an unmarried couple) be close	Jingdong prefecture level city in Shandong
Dragon Quality Goods Shop	Green Intelligent Power Saver	(of an unmarried couple) be close	Jingdong prefecture level city in Shandong
Dragon Quality Goods Shop	Green Intelligent Power Saver	no electricity saving	Jingdong prefecture level city in Shandong
Dragon Quality Goods Shop	Green Intelligent Power Saver	differ from	Jingdong prefecture level city in Shandong
Dragon Quality Goods Shop	Green Intelligent Power Saver	Saver has received, but real can save electricity, the quality is very good, I times I buy a few more, too good.	Jingdong prefecture level city in Shandong
Dragon Quality Goods Shop	Green Intelligent Power Saver	Great quality as always, a very favorite product, great! o(*￣︶￣*)o	Jingdong prefecture level city in Shandong
Dragon Quality Goods Shop	Green Intelligent Power Saver	If we can reduce the electricity bill, for individuals can save money, for the country can protect the environment, now the weather is getting hotter and hotter, to protect the environment to reduce emissions is very important. Already use the product, whether to reduce the electricity bill depends on next month!	Jingdong prefecture level city in Shandong
Dragon Quality Goods Shop	Green Intelligent Power Saver	Purchased a power saver received on the put into use, rent a room with many electrical appliances, power consumption is very large, we have a piece of electricity or 1.2 dollars a time, with this power saver is much better, every day can save a few cents, very convenient and practical!	Jingdong prefecture level city in Shandong
Dragon Quality Goods Shop	Green Intelligent Power Saver	The product is better to use before not to use when a month 1500 yuan electricity bill, now using the 5 second month electricity bill directly down 470 dollars or very good. A save money to buy several power saver money ZanZanZanZan👍	Jingdong prefecture level city in Shandong
Dragon Quality Goods Shop	Green Intelligent Power Saver	The moment I received the goods really satisfied with this product, not only high-grade packaging, the body are base alloy, up to the price, I was also introduced by a friend, he used last month did save a lot of electricity, but also recommended to me!	Jingdong prefecture level city in Shandong
Dragon Quality Goods Shop	Green Intelligent Power Saver	I got it. Good review. I'm very satisfied. Reasonable price.	Jingdong prefecture level city in Shandong
Dragon Quality Goods Shop	Green Intelligent Power Saver	Intelligent power saver received and used, looking at the quality is good, the quality of the plug sent is also very good. Expect the effect of the economizer is obvious, first five-star praise, with a period of time to follow up!	Jingdong prefecture level city in Shandong
Dragon Quality Goods Shop	Green Intelligent Power Saver	The saver has been received. It was plugged into an outlet and used immediately after receipt. Whether it really saves electricity is not clear yet, I believe that this brand of energy saver really saves	Jingdong prefecture level city in Shandong
Dragon Quality Goods Shop	Green Intelligent Power Saver	Okay, okay, okay. Thank you so much. Fast is good. Fast. Item was as described, mainly because the event was cheap and you can save a lot of money.	Jingdong prefecture level city in Shandong

Dragon Quality Goods Shop	Green Intelligent Power Saver	Received it a couple days ago! Plugged it in the same day! Let's see what the electric bill is in a month! Hopefully it works well oh!	Jingdong prefecture level city in Shandong
Dragon Quality Goods Shop	Green Intelligent Power Saver	Looking at the reviews good, energy-saving small products, compact, the results after 2 months to see, because the electricity bill season payment	Jingdong prefecture level city in Shandong
Dragon Quality Goods Shop	Green Intelligent Power Saver	I just received it and will try it out to see if it works well. I'll comment later.	Jingdong prefecture level city in Shandong
Dragon Quality Goods Shop	Green Intelligent Power Saver	It looks a little small and not very aesthetically pleasing, I don't know how effective it will be until I use it	Jingdong prefecture level city in Shandong
Dragon Quality Goods Shop	Green Intelligent Power Saver	Not only to the point of being useless yet	Jingdong prefecture level city in Shandong
Dragon Quality Goods Shop	Green Intelligent Power Saver	Used it for a month, nothing works, the meter you turn very fast. This is not a scam, but also sold on Jingdong. To cheat a bunch of people?	Jingdong prefecture level city in Shandong
Dragon Quality Goods Shop	Green Intelligent Power Saver	Very good to use, every day can save a little electricity, really good under the sample down a month to save a few dozen dollars in electricity, good before I did not know that there is this 👆 haha, the price is not expensive praise.	Jingdong prefecture level city in Shandong
Dragon Quality Goods Shop	Green Intelligent Power Saver	Tried it for ten days and the change is almost nothing, can't see the power saving yet, use it some more days and see how it goes.	Jingdong prefecture level city in Shandong
Dragon Quality Goods Shop	Green Intelligent Power Saver	Received very quickly, the quality is very good, really save electricity, business service attitude is also very good, I hope you do not miss this opportunity, I continue to buy a few in	Jingdong prefecture level city in Shandong
Dragon Quality Goods Shop	Green Intelligent Power Saver	Quality is very good Oh, the delivery is also very fast, a very favorite store, I want to crazy recommend to friends!	Jingdong prefecture level city in Shandong
Dragon Quality Goods Shop	Green Intelligent Power Saver	The goods have just received, fine workmanship, and the same as described by the merchant, to use a period of time to see if it can achieve the purpose of power saving.	Jingdong prefecture level city in Shandong
Dragon Quality Goods Shop	Green Intelligent Power Saver	Received the goods first use a period of time to see if there is no effect, the business commitment to ninety days invalid refund, first praise, good service!	Jingdong prefecture level city in Shandong
Dragon Quality Goods Shop	Green Intelligent Power Saver	The product is used for a month without energy-saving and power-saving effect does not have any effect	Jingdong prefecture level city in Shandong
Dragon Quality Goods Shop	Green Intelligent Power Saver	The baby is really great, I like it very much, my friends also say good to come, recommended to buy!	Jingdong prefecture level city in Shandong
Dragon Quality Goods Shop	Green Intelligent Power Saver	Logistics is fast, courier boy service is good, after using to see how the fruit, and then follow up.	Jingdong prefecture level city in Shandong
Dragon Quality Goods Shop	Green Intelligent Power Saver	Baby has been received to try, I believe that as the business advertised energy saving. The courier is very responsible.	Jingdong prefecture level city in Shandong
Dragon Quality Goods Shop	Green Intelligent Power Saver	Save Electric Energy King received, good quality, beautiful appearance, very satisfied!	Jingdong prefecture level city in Shandong
Dragon Quality Goods Shop	Green Intelligent Power Saver	Three no products no manufacturers no phone pure fraud induced consumption cheat	Jingdong prefecture level city in Shandong
Dragon Quality Goods Shop	Green Intelligent Power Saver	I'm using it now, I don't know if it's as good as it says, I'll follow up after a month	Jingdong prefecture level city in Shandong
Dragon Quality Goods Shop	Green Intelligent Power Saver	Good quality logistics and fast. Fine workmanship. The description is consistent cost-effective	Jingdong prefecture level city in Shandong
Dragon Quality Goods Shop	Green Intelligent Power Saver	Received, whether it is good or not, to wait a few days and then evaluate	Jingdong prefecture level city in Shandong
Dragon Quality Goods Shop	Green Intelligent Power Saver	I bought it and started using it, I won't know if it works well until I pay my electric bill	Jingdong prefecture level city in Shandong
Dragon Quality Goods Shop	Green Intelligent Power Saver	Especially good, energy saving and power saving, especially good quality, good	Jingdong prefecture level city in Shandong
Dragon Quality Goods Shop	Green Intelligent Power Saver	Logistics is strong, the quality is very good, paste really save electricity, as described, will buy back!	Jingdong prefecture level city in Shandong
Dragon Quality Goods Shop	Green Intelligent Power Saver	Great stuff at a great price, worth the money!	Jingdong prefecture level city in Shandong
Dragon Quality Goods Shop	Green Intelligent Power Saver	Received the power saver, very good quality, very power saving!	Jingdong prefecture level city in Shandong
Dragon Quality Goods Shop	Green Intelligent Power Saver	There are no fruits, only samples...	Jingdong prefecture level city in Shandong
Dragon Quality Goods Shop	Green Intelligent Power Saver	Save a few dollars a month.	Jingdong prefecture level city in Shandong
Dragon Quality Goods Shop	Green Intelligent Power Saver	The quality is particularly good, it works well, and the seller has a good service attitude	Jingdong prefecture level city in Shandong
Dragon Quality Goods Shop	Green Intelligent Power Saver	I've compared them a couple of times and I don't feel any difference	Jingdong prefecture level city in Shandong
Dragon Quality Goods Shop	Green Intelligent Power Saver	I don't see the savings. It's a sham.	Jingdong prefecture level city in Shandong
Dragon Quality Goods Shop	Green Intelligent Power Saver	The power saving effect is very good, and the logistics is very fast. The seller's service attitude is very good	Jingdong prefecture level city in Shandong
Dragon Quality Goods Shop	Green Intelligent Power Saver	Received, well packaged, haven't tried it yet!	Jingdong prefecture level city in Shandong
Dragon Quality Goods Shop	Green Intelligent Power Saver	I don't know how it works, I'll comment after I use it.	Jingdong prefecture level city in Shandong
Dragon Quality Goods Shop	Green Intelligent Power Saver	The product doesn't work well enough to be of any use	Jingdong prefecture level city in Shandong
Dragon Quality Goods Shop	Green Intelligent Power Saver	On Trial 。。。。。。。	Jingdong prefecture level city in Shandong
Dragon Quality Goods Shop	Green Intelligent Power Saver	Being used with unknown results	Jingdong prefecture level city in Shandong
Dragon Quality Goods Shop	Green Intelligent Power Saver	Smart power saving and great quality	Jingdong prefecture level city in Shandong
Dragon Quality Goods Shop	Green Intelligent Power Saver	The saver was received, it works very well and the price is affordable!	Jingdong prefecture level city in Shandong
Dragon Quality Goods Shop	Green Intelligent Power Saver	How can I feel the power saving and how long does it last	Jingdong prefecture level city in Shandong
Dragon Quality Goods Shop	Green Intelligent Power Saver	And I know if it saves electricity or not.	Jingdong prefecture level city in Shandong
Dragon Quality Goods Shop	Green Intelligent Power Saver	It won't save any electricity. No.	Jingdong prefecture level city in Shandong
Dragon Quality Goods Shop	Green Intelligent Power Saver	Invalid. Fake.	Jingdong prefecture level city in Shandong
Dragon Quality Goods Shop	Green Intelligent Power Saver	It's good!	Jingdong prefecture level city in Shandong
Dragon Quality Goods Shop	Green Intelligent Power Saver	have no effect	Jingdong prefecture level city in Shandong
Dragon Quality Goods Shop	Green Intelligent Power Saver	Not used.	Jingdong prefecture level city in Shandong
Dragon Quality Goods Shop	Green Intelligent Power Saver	There is no load range, packaging thrift, ten days also did not develop invoices, received the goods with a receipt, hand-written receipt also has no financial stamp, communication for nearly a week a moment to say that the electronic invoice, a moment to say that the paper invoice, said it is mailed to me, ten days also did not see anything, the invoice invoice can not be found to say that did not stay on file, single single number can not be found, said that the warehouse more than one to a one to find out, in addition to the perfunctory or perfunctory. Products are no certificate of conformity, proper three	Jingdong prefecture level city in Shandong
Dragon Quality Goods Shop	Green Intelligent Power Saver	Satisfaction.	Jingdong prefecture level city in Shandong

Dragon Quality Goods Shop	Green Intelligent Power Saver	There is not a bit of power saving effect, and ringing up an increase in the electricity bill, because you have to add the cost of this machine's own electricity consumption and purchase, so I think it is better to use this machine instead of increasing the expenditure, uneconomical, rather than not use it.	Jingdong prefecture level city in Shandong
Dragon Quality Goods Shop	Green Intelligent Power Saver	I've been using it for two days now and I don't feel any effect.	Jingdong prefecture level city in Shandong
Dragon Quality Goods Shop	Green Intelligent Power Saver	What it looked like was nothing like what was sent.	Jingdong prefecture level city in Shandong
Dragon Quality Goods Shop	Green Intelligent Power Saver	It didn't work. It didn't work for a month or so.	Jingdong prefecture level city in Shandong
Dragon Quality Goods Shop	Green Intelligent Power Saver	Garbage products, physical and description is not the same, no effect at all, pit # no discussion, to buy the fine, garbage, garbage, garbage	Jingdong prefecture level city in Shandong
Dragon Quality Goods Shop	Green Intelligent Power Saver	general	Jingdong prefecture level city in Shandong
Dragon Quality Goods Shop	Green Intelligent Power Saver	IQ tax. Instead of saving electricity, it increases the power consumption of an appliance.	Jingdong prefecture level city in Shandong
Dragon Quality Goods Shop	Green Intelligent Power Saver	There's no trash at all.	Jingdong prefecture level city in Shandong
Dragon Quality Goods Shop	Green Intelligent Power Saver	It doesn't do anything.	Jingdong prefecture level city in Shandong
Dragon Quality Goods Shop	Green Intelligent Power Saver	(of an unmarried couple) be close	Jingdong prefecture level city in Shandong
Dragon Quality Goods Shop	Green Intelligent Power Saver	(of an unmarried couple) be close	Jingdong prefecture level city in Shandong
Dragon Quality Goods Shop	Green Intelligent Power Saver	no electricity saving	Jingdong prefecture level city in Shandong
Dragon Quality Goods Shop	Green Intelligent Power Saver	differ from	Jingdong prefecture level city in Shandong
Dragon Quality Goods Shop	Green Intelligent Power Saver	Saver has received, but real can save electricity, the quality is very good, I times I buy a few more, too good.	Jingdong prefecture level city in Shandong
Dragon Quality Goods Shop	Green Intelligent Power Saver	Great quality as always, a very favorite product, great! o(*￣︶￣*)o	Jingdong prefecture level city in Shandong
Dragon Quality Goods Shop	Green Intelligent Power Saver	If we can reduce the electricity bill, for individuals can save money, for the country can protect the environment, now the weather is getting hotter and hotter, to protect the environment to reduce emissions is very important. Already use the product, whether to reduce the electricity bill depends on next month!	Jingdong prefecture level city in Shandong
Dragon Quality Goods Shop	Green Intelligent Power Saver	Purchased a power saver received on the put into use, rent a room with many electrical appliances, power consumption is very large, we have a piece of electricity or 1.2 dollars a time, with this power saver is much better, every day can save a few cents, very convenient and practical!	Jingdong prefecture level city in Shandong
Dragon Quality Goods Shop	Green Intelligent Power Saver	The product is better to use before not to use when a month 1500 yuan electricity bill, now using the 5 second month electricity bill directly down 470 dollars or very good. A save money to buy several power saver money ZanZanZanZan👍	Jingdong prefecture level city in Shandong
Dragon Quality Goods Shop	Green Intelligent Power Saver	The moment I received the goods really satisfied with this product, not only high-grade packaging, the body are base alloy, up to the price, I was also introduced by a friend, he used last month did save a lot of electricity, but also recommended to me!	Jingdong prefecture level city in Shandong
Dragon Quality Goods Shop	Green Intelligent Power Saver	I got it. Good review. I'm very satisfied. Reasonable price.	Jingdong prefecture level city in Shandong
Dragon Quality Goods Shop	Green Intelligent Power Saver	Intelligent power saver received and used, looking at the quality is good, the quality of the plug sent is also very good. Expect the effect of the economizer is obvious, first five-star praise, with a period of time to follow up!	Jingdong prefecture level city in Shandong
Dragon Quality Goods Shop	Green Intelligent Power Saver	The saver has been received. It was plugged into an outlet and used immediately after receipt. Whether it really saves electricity is not clear yet, I believe that this brand of energy saver really saves	Jingdong prefecture level city in Shandong
Dragon Quality Goods Shop	Green Intelligent Power Saver	Okay, okay, okay. Thank you so much. Fast is good. Fast. Item was as described, mainly because the event was cheap and you can save a lot of money.	Jingdong prefecture level city in Shandong
Dragon Quality Goods Shop	Green Intelligent Power Saver	Received it a couple days ago! Plugged it in the same day! Let's see what the electric bill is in a month! Hopefully it works well oh!	Jingdong prefecture level city in Shandong
Dragon Quality Goods Shop	Green Intelligent Power Saver	Looking at the reviews good, energy-saving small products, compact, the results after 2 months to see, because the electricity bill season payment	Jingdong prefecture level city in Shandong
Dragon Quality Goods Shop	Green Intelligent Power Saver	I just received it and will try it out to see if it works well. I'll comment later.	Jingdong prefecture level city in Shandong
Dragon Quality Goods Shop	Green Intelligent Power Saver	It looks a little small and not very aesthetically pleasing, I don't know how effective it will be until I use it	Jingdong prefecture level city in Shandong
Dragon Quality Goods Shop	Green Intelligent Power Saver	Not only to the point of being useless yet	Jingdong prefecture level city in Shandong
Dragon Quality Goods Shop	Green Intelligent Power Saver	Used it for a month, nothing works, the meter you turn very fast. This is not a scam, but also sold on Jingdong. To cheat a bunch of people?	Jingdong prefecture level city in Shandong
Dragon Quality Goods Shop	Green Intelligent Power Saver	Very good to use, every day can save a little electricity, really good under the sample down a month to save a few dozen dollars in electricity, good before I did not know that there is this 👆 haha, the price is not expensive praise.	Jingdong prefecture level city in Shandong
Dragon Quality Goods Shop	Green Intelligent Power Saver	Tried it for ten days and the change is almost nothing, can't see the power saving yet, use it some more days and see how it goes.	Jingdong prefecture level city in Shandong
Dragon Quality Goods Shop	Green Intelligent Power Saver	Received very quickly, the quality is very good, really save electricity, business service attitude is also very good, I hope you do not miss this opportunity, I continue to buy a few in	Jingdong prefecture level city in Shandong
Dragon Quality Goods Shop	Green Intelligent Power Saver	Quality is very good Oh, the delivery is also very fast, a very favorite store, I want to crazy recommend to friends!	Jingdong prefecture level city in Shandong
Dragon Quality Goods Shop	Green Intelligent Power Saver	The goods have just received, fine workmanship, and the same as described by the merchant, to use a period of time to see if it can achieve the purpose of power saving.	Jingdong prefecture level city in Shandong
Dragon Quality Goods Shop	Green Intelligent Power Saver	Received the goods first use a period of time to see if there is no effect, the business commitment to ninety days invalid refund, first praise, good service!	Jingdong prefecture level city in Shandong
Dragon Quality Goods Shop	Green Intelligent Power Saver	The product is used for a month without energy-saving and power-saving effect does not have any effect	Jingdong prefecture level city in Shandong
Dragon Quality Goods Shop	Green Intelligent Power Saver	The baby is really great, I like it very much, my friends also say good to come, recommended to buy!	Jingdong prefecture level city in Shandong
Dragon Quality Goods Shop	Green Intelligent Power Saver	Logistics is fast, courier boy service is good, after using to see how the fruit, and then follow up.	Jingdong prefecture level city in Shandong
Dragon Quality Goods Shop	Green Intelligent Power Saver	Baby has been received to try, I believe that as the business advertised energy saving. The courier is very responsible.	Jingdong prefecture level city in Shandong
Dragon Quality Goods Shop	Green Intelligent Power Saver	Save Electric Energy King received, good quality, beautiful appearance, very satisfied!	Jingdong prefecture level city in Shandong
Dragon Quality Goods Shop	Green Intelligent Power Saver	Three no products no manufacturers no phone pure fraud induced consumption cheat	Jingdong prefecture level city in Shandong
Dragon Quality Goods Shop	Green Intelligent Power Saver	I'm using it now, I don't know if it's as good as it says, I'll follow up after a month	Jingdong prefecture level city in Shandong
Dragon Quality Goods Shop	Green Intelligent Power Saver	Good quality logistics and fast. Fine workmanship. The description is consistent cost-effective	Jingdong prefecture level city in Shandong
Dragon Quality Goods Shop	Green Intelligent Power Saver	Received, whether it is good or not, to wait a few days and then evaluate	Jingdong prefecture level city in Shandong
Dragon Quality Goods Shop	Green Intelligent Power Saver	I bought it and started using it, I won't know if it works well until I pay my electric bill	Jingdong prefecture level city in Shandong
Dragon Quality Goods Shop	Green Intelligent Power Saver	Especially good, energy saving and power saving, especially good quality, good	Jingdong prefecture level city in Shandong
Dragon Quality Goods Shop	Green Intelligent Power Saver	Logistics is strong, the quality is very good, paste really save electricity, as described, will buy back!	Jingdong prefecture level city in Shandong
Dragon Quality Goods Shop	Green Intelligent Power Saver	Great stuff at a great price, worth the money!	Jingdong prefecture level city in Shandong

Dragon Quality Goods Shop	Green Intelligent Power Saver	Received the power saver, very good quality, very power saving!	Jingdong prefecture level city in Shandong
Dragon Quality Goods Shop	Green Intelligent Power Saver	There are no fruits, only samples...	Jingdong prefecture level city in Shandong
Dragon Quality Goods Shop	Green Intelligent Power Saver	Save a few dollars a month.	Jingdong prefecture level city in Shandong
Dragon Quality Goods Shop	Green Intelligent Power Saver	The quality is particularly good, it works well, and the seller has a good service attitude	Jingdong prefecture level city in Shandong
Dragon Quality Goods Shop	Green Intelligent Power Saver	I've compared them a couple of times and I don't feel any difference	Jingdong prefecture level city in Shandong
Dragon Quality Goods Shop	Green Intelligent Power Saver	I don't see the savings. It's a sham.	Jingdong prefecture level city in Shandong
Dragon Quality Goods Shop	Green Intelligent Power Saver	The power saving effect is very good, and the logistics is very fast. The seller's service attitude is very good	Jingdong prefecture level city in Shandong
Dragon Quality Goods Shop	Green Intelligent Power Saver	Received, well packaged, haven't tried it yet!	Jingdong prefecture level city in Shandong
Dragon Quality Goods Shop	Green Intelligent Power Saver	I don't know how it works, I'll comment after I use it.	Jingdong prefecture level city in Shandong
Dragon Quality Goods Shop	Green Intelligent Power Saver	The product doesn't work well enough to be of any use	Jingdong prefecture level city in Shandong
Dragon Quality Goods Shop	Green Intelligent Power Saver	On Trial 。。。。。。。	Jingdong prefecture level city in Shandong
Dragon Quality Goods Shop	Green Intelligent Power Saver	Being used with unknown results	Jingdong prefecture level city in Shandong
Dragon Quality Goods Shop	Green Intelligent Power Saver	Smart power saving and great quality	Jingdong prefecture level city in Shandong
Dragon Quality Goods Shop	Green Intelligent Power Saver	The saver was received, it works very well and the price is affordable!	Jingdong prefecture level city in Shandong
Dragon Quality Goods Shop	Green Intelligent Power Saver	How can I feel the power saving and how long does it last	Jingdong prefecture level city in Shandong
Dragon Quality Goods Shop	Green Intelligent Power Saver	And I know if it saves electricity or not.	Jingdong prefecture level city in Shandong
Dragon Quality Goods Shop	Green Intelligent Power Saver	It won't save any electricity. No.	Jingdong prefecture level city in Shandong
Dragon Quality Goods Shop	Green Intelligent Power Saver	Invalid. Fake.	Jingdong prefecture level city in Shandong
Dragon Quality Goods Shop	Green Intelligent Power Saver	It's good!	Jingdong prefecture level city in Shandong
Dragon Quality Goods Shop	Green Intelligent Power Saver	have no effect	Jingdong prefecture level city in Shandong
Dragon Quality Goods Shop	Green Intelligent Power Saver	Not used.	Jingdong prefecture level city in Shandong
Dragon Quality Goods Shop	Green Intelligent Power Saver	There is no load range, packaging thrift, ten days also did not develop invoices, received the goods with a receipt, hand-written receipt also has no financial stamp, communication for nearly a week a moment to say that the electronic invoice, a moment to say that the paper invoice, said it is mailed to me, ten days also did not see anything, the invoice invoice can not be found to say that did not stay on file, single single number can not be found, said that the warehouse more than one to a one to find out, in addition to the perfunctory or perfunctory. Products are no certificate of conformity, proper three	Jingdong prefecture level city in Shandong
Dragon Quality Goods Shop	Green Intelligent Power Saver	Satisfaction.	Jingdong prefecture level city in Shandong
Dragon Quality Goods Shop	Green Intelligent Power Saver	There is not a bit of power saving effect, and ringing up an increase in the electricity bill, because you have to add the cost of this machine's own electricity consumption and purchase, so I think it is better to use this machine instead of increasing the expenditure, uneconomical, rather than not use it.	Jingdong prefecture level city in Shandong
Dragon Quality Goods Shop	Green Intelligent Power Saver	I've been using it for two days now and I don't feel any effect.	Jingdong prefecture level city in Shandong
Dragon Quality Goods Shop	Green Intelligent Power Saver	What it looked like was nothing like what was sent.	Jingdong prefecture level city in Shandong
Dragon Quality Goods Shop	Green Intelligent Power Saver	It didn't work. It didn't work for a month or so.	Jingdong prefecture level city in Shandong
Dragon Quality Goods Shop	Green Intelligent Power Saver	Garbage products, physical and description is not the same, no effect at all, pit # no discussion, to buy the fine, garbage, garbage, garbage	Jingdong prefecture level city in Shandong
Dragon Quality Goods Shop	Green Intelligent Power Saver	general	Jingdong prefecture level city in Shandong
Dragon Quality Goods Shop	Green Intelligent Power Saver	IQ tax. Instead of saving electricity, it increases the power consumption of an appliance.	Jingdong prefecture level city in Shandong
Dragon Quality Goods Shop	Green Intelligent Power Saver	There's no trash at all.	Jingdong prefecture level city in Shandong
Dragon Quality Goods Shop	Green Intelligent Power Saver	It doesn't do anything.	Jingdong prefecture level city in Shandong
Dragon Quality Goods Shop	Green Intelligent Power Saver	(of an unmarried couple) be close	Jingdong prefecture level city in Shandong
Dragon Quality Goods Shop	Green Intelligent Power Saver	(of an unmarried couple) be close	Jingdong prefecture level city in Shandong
Dragon Quality Goods Shop	Green Intelligent Power Saver	no electricity saving	Jingdong prefecture level city in Shandong
Dragon Quality Goods Shop	Green Intelligent Power Saver	differ from	Jingdong prefecture level city in Shandong
Dragon Quality Goods Shop	Green Intelligent Power Saver	Saver has received, but real can save electricity, the quality is very good, I times I buy a few more, too good.	Jingdong prefecture level city in Shandong
Dragon Quality Goods Shop	Green Intelligent Power Saver	Great quality as always, a very favorite product, great! o(*￣︶￣*)o	Jingdong prefecture level city in Shandong
Dragon Quality Goods Shop	Green Intelligent Power Saver	If we can reduce the electricity bill, for individuals can save money, for the country can protect the environment, now the weather is getting hotter and hotter, to protect the environment to reduce emissions is very important. Already use the product, whether to reduce the electricity bill depends on next month!	Jingdong prefecture level city in Shandong
Dragon Quality Goods Shop	Green Intelligent Power Saver	Purchased a power saver received on the put into use, rent a room with many electrical appliances, power consumption is very large, we have a piece of electricity or 1.2 dollars a time, with this power saver is much better, every day can save a few cents, very convenient and practical!	Jingdong prefecture level city in Shandong
Dragon Quality Goods Shop	Green Intelligent Power Saver	The product is better to use before not to use when a month 1500 yuan electricity bill, now using the 5 second month electricity bill directly down 470 dollars or very good. A save money to buy several power saver money ZanZanZanZan👍	Jingdong prefecture level city in Shandong
Dragon Quality Goods Shop	Green Intelligent Power Saver	The moment I received the goods really satisfied with this product, not only high-grade packaging, the body are base alloy, up to the price, I was also introduced by a friend, he used last month did save a lot of electricity, but also recommended to me!	Jingdong prefecture level city in Shandong
Dragon Quality Goods Shop	Green Intelligent Power Saver	I got it. Good review. I'm very satisfied. Reasonable price.	Jingdong prefecture level city in Shandong
Dragon Quality Goods Shop	Green Intelligent Power Saver	Intelligent power saver received and used, looking at the quality is good, the quality of the plug sent is also very good. Expect the effect of the economizer is obvious, first five-star praise, with a period of time to follow up!	Jingdong prefecture level city in Shandong
Dragon Quality Goods Shop	Green Intelligent Power Saver	The saver has been received. It was plugged into an outlet and used immediately after receipt. Whether it really saves electricity is not clear yet, I believe that this brand of energy saver really saves	Jingdong prefecture level city in Shandong
Dragon Quality Goods Shop	Green Intelligent Power Saver	Okay, okay, okay. Thank you so much. Fast is good. Fast. Item was as described, mainly because the event was cheap and you can save a lot of money.	Jingdong prefecture level city in Shandong

Dragon Quality Goods Shop	Green Intelligent Power Saver	Received it a couple days ago! Plugged it in the same day! Let's see what the electric bill is in a month! Hopefully it works well oh!	Jingdong prefecture level city in Shandong
Dragon Quality Goods Shop	Green Intelligent Power Saver	Looking at the reviews good, energy-saving small products, compact, the results after 2 months to see, because the electricity bill season payment	Jingdong prefecture level city in Shandong
Dragon Quality Goods Shop	Green Intelligent Power Saver	I just received it and will try it out to see if it works well. I'll comment later.	Jingdong prefecture level city in Shandong
Dragon Quality Goods Shop	Green Intelligent Power Saver	It looks a little small and not very aesthetically pleasing, I don't know how effective it will be until I use it	Jingdong prefecture level city in Shandong
Dragon Quality Goods Shop	Green Intelligent Power Saver	Not only to the point of being useless yet	Jingdong prefecture level city in Shandong
Dragon Quality Goods Shop	Green Intelligent Power Saver	Used it for a month, nothing works, the meter you turn very fast. This is not a scam, but also sold on Jingdong. To cheat a bunch of people?	Jingdong prefecture level city in Shandong
Dragon Quality Goods Shop	Green Intelligent Power Saver	Very good to use, every day can save a little electricity, really good under the sample down a month to save a few dozen dollars in electricity, good before I did not know that there is this haha, the price is not expensive praise.	Jingdong prefecture level city in Shandong
Dragon Quality Goods Shop	Green Intelligent Power Saver	Tried it for ten days and the change is almost nothing, can't see the power saving yet, use it some more days and see how it goes.	Jingdong prefecture level city in Shandong
Dragon Quality Goods Shop	Green Intelligent Power Saver	Received very quickly, the quality is very good, really save electricity, business service attitude is also very good, I hope you do not miss this opportunity, I continue to buy a few in	Jingdong prefecture level city in Shandong
Dragon Quality Goods Shop	Green Intelligent Power Saver	Quality is very good Oh, the delivery is also very fast, a very favorite store, I want to crazy recommend to friends!	Jingdong prefecture level city in Shandong
Dragon Quality Goods Shop	Green Intelligent Power Saver	The goods have just received, fine workmanship, and the same as described by the merchant, to use a period of time to see if it can achieve the purpose of power saving.	Jingdong prefecture level city in Shandong
Dragon Quality Goods Shop	Green Intelligent Power Saver	Received the goods first use a period of time to see if there is no effect, the business commitment to ninety days invalid refund, first praise, good service!	Jingdong prefecture level city in Shandong
Dragon Quality Goods Shop	Green Intelligent Power Saver	The product is used for a month without energy-saving and power-saving effect does not have any effect	Jingdong prefecture level city in Shandong
Dragon Quality Goods Shop	Green Intelligent Power Saver	The baby is really great, I like it very much, my friends also say good to come, recommended to buy!	Jingdong prefecture level city in Shandong
Dragon Quality Goods Shop	Green Intelligent Power Saver	Logistics is fast, courier boy service is good, after using to see how the fruit, and then follow up.	Jingdong prefecture level city in Shandong
Dragon Quality Goods Shop	Green Intelligent Power Saver	Baby has been received to try, I believe that as the business advertised energy saving. The courier is very responsible.	Jingdong prefecture level city in Shandong
Dragon Quality Goods Shop	Green Intelligent Power Saver	Save Electric Energy King received, good quality, beautiful appearance, very satisfied!	Jingdong prefecture level city in Shandong
Dragon Quality Goods Shop	Green Intelligent Power Saver	Three no products no manufacturers no phone pure fraud induced consumption cheat	Jingdong prefecture level city in Shandong
Dragon Quality Goods Shop	Green Intelligent Power Saver	I'm using it now, I don't know if it's as good as it says, I'll follow up after a month	Jingdong prefecture level city in Shandong
Dragon Quality Goods Shop	Green Intelligent Power Saver	Good quality logistics and fast. Fine workmanship. The description is consistent cost-effective	Jingdong prefecture level city in Shandong
Dragon Quality Goods Shop	Green Intelligent Power Saver	Received, whether it is good or not, to wait a few days and then evaluate	Jingdong prefecture level city in Shandong
Dragon Quality Goods Shop	Green Intelligent Power Saver	I bought it and started using it, I won't know if it works well until I pay my electric bill	Jingdong prefecture level city in Shandong
Dragon Quality Goods Shop	Green Intelligent Power Saver	Especially good, energy saving and power saving, especially good quality, good	Jingdong prefecture level city in Shandong
Dragon Quality Goods Shop	Green Intelligent Power Saver	Logistics is strong, the quality is very good, paste really save electricity, as described, will buy back!	Jingdong prefecture level city in Shandong
Dragon Quality Goods Shop	Green Intelligent Power Saver	Great stuff at a great price, worth the money!	Jingdong prefecture level city in Shandong
Dragon Quality Goods Shop	Green Intelligent Power Saver	Received the power saver, very good quality, very power saving!	Jingdong prefecture level city in Shandong
Dragon Quality Goods Shop	Green Intelligent Power Saver	There are no fruits, only samples...	Jingdong prefecture level city in Shandong
Dragon Quality Goods Shop	Green Intelligent Power Saver	Save a few dollars a month.	Jingdong prefecture level city in Shandong
Dragon Quality Goods Shop	Green Intelligent Power Saver	The quality is particularly good, it works well, and the seller has a good service attitude	Jingdong prefecture level city in Shandong
Dragon Quality Goods Shop	Green Intelligent Power Saver	I've compared them a couple of times and I don't feel any difference	Jingdong prefecture level city in Shandong
Dragon Quality Goods Shop	Green Intelligent Power Saver	I don't see the savings. It's a sham.	Jingdong prefecture level city in Shandong
Dragon Quality Goods Shop	Green Intelligent Power Saver	The power saving effect is very good, and the logistics is very fast. The seller's service attitude is very good	Jingdong prefecture level city in Shandong
Dragon Quality Goods Shop	Green Intelligent Power Saver	Received, well packaged, haven't tried it yet!	Jingdong prefecture level city in Shandong
Dragon Quality Goods Shop	Green Intelligent Power Saver	I don't know how it works, I'll comment after I use it.	Jingdong prefecture level city in Shandong
Dragon Quality Goods Shop	Green Intelligent Power Saver	The product doesn't work well enough to be of any use	Jingdong prefecture level city in Shandong
Dragon Quality Goods Shop	Green Intelligent Power Saver	On Trial 。。。。。。。	Jingdong prefecture level city in Shandong
Dragon Quality Goods Shop	Green Intelligent Power Saver	Being used with unknown results	Jingdong prefecture level city in Shandong
Dragon Quality Goods Shop	Green Intelligent Power Saver	Smart power saving and great quality	Jingdong prefecture level city in Shandong
Dragon Quality Goods Shop	Green Intelligent Power Saver	The saver was received, it works very well and the price is affordable!	Jingdong prefecture level city in Shandong
Dragon Quality Goods Shop	Green Intelligent Power Saver	How can I feel the power saving and how long does it last	Jingdong prefecture level city in Shandong
Dragon Quality Goods Shop	Green Intelligent Power Saver	And I know if it saves electricity or not.	Jingdong prefecture level city in Shandong
Dragon Quality Goods Shop	Green Intelligent Power Saver	It won't save any electricity. No.	Jingdong prefecture level city in Shandong
Dragon Quality Goods Shop	Green Intelligent Power Saver	Invalid. Fake.	Jingdong prefecture level city in Shandong
Dragon Quality Goods Shop	Green Intelligent Power Saver	It's good!	Jingdong prefecture level city in Shandong
Dragon Quality Goods Shop	Green Intelligent Power Saver	have no effect	Jingdong prefecture level city in Shandong
Dragon Quality Goods Shop	Green Intelligent Power Saver	Not used.	Jingdong prefecture level city in Shandong
Dragon Quality Goods Shop	Green Intelligent Power Saver	There is no load range, packaging thrift, ten days also did not develop invoices, received the goods with a receipt, hand-written receipt also has no financial stamp, communication for nearly a week a moment to say that the electronic invoice, a moment to say that the paper invoice, said it is mailed to me, ten days also did not see anything, the invoice invoice can not be found to say that did not stay on file, single single number can not be found, said that the warehouse more than one to a one to find out, in addition to the perfunctory or perfunctory. Products are no certificate of conformity, proper three	Jingdong prefecture level city in Shandong
Dragon Quality Goods Shop	Green Intelligent Power Saver	Satisfaction.	Jingdong prefecture level city in Shandong

Dragon Quality Goods Shop	Green Intelligent Power Saver	There is not a bit of power saving effect, and ringing up an increase in the electricity bill, because you have to add the cost of this machine's own electricity consumption and purchase, so I think it is better to use this machine instead of increasing the expenditure, uneconomical, rather than not use it.	Jingdong prefecture level city in Shandong
Dragon Quality Goods Shop	Green Intelligent Power Saver	I've been using it for two days now and I don't feel any effect.	Jingdong prefecture level city in Shandong
Dragon Quality Goods Shop	Green Intelligent Power Saver	What it looked like was nothing like what was sent.	Jingdong prefecture level city in Shandong
Dragon Quality Goods Shop	Green Intelligent Power Saver	It didn't work. It didn't work for a month or so.	Jingdong prefecture level city in Shandong
Dragon Quality Goods Shop	Green Intelligent Power Saver	Garbage products, physical and description is not the same, no effect at all, pit # no discussion, to buy the fine, garbage, garbage, garbage	Jingdong prefecture level city in Shandong
Dragon Quality Goods Shop	Green Intelligent Power Saver	general	Jingdong prefecture level city in Shandong
Dragon Quality Goods Shop	Green Intelligent Power Saver	IQ tax. Instead of saving electricity, it increases the power consumption of an appliance.	Jingdong prefecture level city in Shandong
Dragon Quality Goods Shop	Green Intelligent Power Saver	There's no trash at all.	Jingdong prefecture level city in Shandong
Dragon Quality Goods Shop	Green Intelligent Power Saver	It doesn't do anything.	Jingdong prefecture level city in Shandong
Dragon Quality Goods Shop	Green Intelligent Power Saver	(of an unmarried couple) be close	Jingdong prefecture level city in Shandong
Dragon Quality Goods Shop	Green Intelligent Power Saver	(of an unmarried couple) be close	Jingdong prefecture level city in Shandong
Dragon Quality Goods Shop	Green Intelligent Power Saver	no electricity saving	Jingdong prefecture level city in Shandong
Dragon Quality Goods Shop	Green Intelligent Power Saver	differ from	Jingdong prefecture level city in Shandong
Dragon Quality Goods Shop	Green Intelligent Power Saver	Saver has received, but real can save electricity, the quality is very good, I times I buy a few more, too good.	Jingdong prefecture level city in Shandong
Dragon Quality Goods Shop	Green Intelligent Power Saver	Great quality as always, a very favorite product, great! o(*￣︶￣*)o	Jingdong prefecture level city in Shandong
Dragon Quality Goods Shop	Green Intelligent Power Saver	If we can reduce the electricity bill, for individuals can save money, for the country can protect the environment, now the weather is getting hotter and hotter, to protect the environment to reduce emissions is very important. Already use the product, whether to reduce the electricity bill depends on next month!	Jingdong prefecture level city in Shandong
Dragon Quality Goods Shop	Green Intelligent Power Saver	Purchased a power saver received on the put into use, rent a room with many electrical appliances, power consumption is very large, we have a piece of electricity or 1.2 dollars a time, with this power saver is much better, every day can save a few cents, very convenient and practical!	Jingdong prefecture level city in Shandong
Dragon Quality Goods Shop	Green Intelligent Power Saver	The product is better to use before not to use when a month 1500 yuan electricity bill, now using the 5 second month electricity bill directly down 470 dollars or very good. A save money to buy several power saver money ZanZanZanZan👍	Jingdong prefecture level city in Shandong
Dragon Quality Goods Shop	Green Intelligent Power Saver	The moment I received the goods really satisfied with this product, not only high-grade packaging, the body are base alloy, up to the price, I was also introduced by a friend, he used last month did save a lot of electricity, but also recommended to me!	Jingdong prefecture level city in Shandong
Dragon Quality Goods Shop	Green Intelligent Power Saver	I got it. Good review. I'm very satisfied. Reasonable price.	Jingdong prefecture level city in Shandong
Dragon Quality Goods Shop	Green Intelligent Power Saver	Intelligent power saver received and used, looking at the quality is good, the quality of the plug sent is also very good. Expect the effect of the economizer is obvious, first five-star praise, with a period of time to follow up!	Jingdong prefecture level city in Shandong
Dragon Quality Goods Shop	Green Intelligent Power Saver	The saver has been received. It was plugged into an outlet and used immediately after receipt. Whether it really saves electricity is not clear yet, I believe that this brand of energy saver really saves	Jingdong prefecture level city in Shandong
Dragon Quality Goods Shop	Green Intelligent Power Saver	Okay, okay, okay. Thank you so much. Fast is good. Fast. Item was as described, mainly because the event was cheap and you can save a lot of money.	Jingdong prefecture level city in Shandong
Dragon Quality Goods Shop	Green Intelligent Power Saver	Received it a couple days ago! Plugged it in the same day! Let's see what the electric bill is in a month! Hopefully it works well oh!	Jingdong prefecture level city in Shandong
Dragon Quality Goods Shop	Green Intelligent Power Saver	Looking at the reviews good, energy-saving small products, compact, the results after 2 months to see, because the electricity bill season payment	Jingdong prefecture level city in Shandong
Dragon Quality Goods Shop	Green Intelligent Power Saver	I just received it and will try it out to see if it works well. I'll comment later.	Jingdong prefecture level city in Shandong
Dragon Quality Goods Shop	Green Intelligent Power Saver	It looks a little small and not very aesthetically pleasing, I don't know how effective it will be until I use it	Jingdong prefecture level city in Shandong
Dragon Quality Goods Shop	Green Intelligent Power Saver	Not only to the point of being useless yet	Jingdong prefecture level city in Shandong
Dragon Quality Goods Shop	Green Intelligent Power Saver	Used it for a month, nothing works, the meter you turn very fast. This is not a scam, but also sold on Jingdong. To cheat a bunch of people?	Jingdong prefecture level city in Shandong
Dragon Quality Goods Shop	Green Intelligent Power Saver	Very good to use, every day can save a little electricity, really good under the sample down a month to save a few dozen dollars in electricity, good before I did not know that there is this 👍 haha, the price is not expensive praise.	Jingdong prefecture level city in Shandong
Dragon Quality Goods Shop	Green Intelligent Power Saver	Tried it for ten days and the change is almost nothing, can't see the power saving yet, use it some more days and see how it goes.	Jingdong prefecture level city in Shandong
Dragon Quality Goods Shop	Green Intelligent Power Saver	Received very quickly, the quality is very good, really save electricity, business service attitude is also very good, I hope you do not miss this opportunity, I continue to buy a few in	Jingdong prefecture level city in Shandong
Dragon Quality Goods Shop	Green Intelligent Power Saver	Quality is very good Oh, the delivery is also very fast, a very favorite store, I want to crazy recommend to friends!	Jingdong prefecture level city in Shandong
Dragon Quality Goods Shop	Green Intelligent Power Saver	The goods have just received, fine workmanship, and the same as described by the merchant, to use a period of time to see if it can achieve the purpose of power saving.	Jingdong prefecture level city in Shandong
Dragon Quality Goods Shop	Green Intelligent Power Saver	Received the goods first use a period of time to see if there is no effect, the business commitment to ninety days invalid refund, first praise, good service!	Jingdong prefecture level city in Shandong
Dragon Quality Goods Shop	Green Intelligent Power Saver	The product is used for a month without energy-saving and power-saving effect does not have any effect	Jingdong prefecture level city in Shandong
Dragon Quality Goods Shop	Green Intelligent Power Saver	The baby is really great, I like it very much, my friends also say good to come, recommended to buy!	Jingdong prefecture level city in Shandong
Dragon Quality Goods Shop	Green Intelligent Power Saver	Logistics is fast, courier boy service is good, after using to see how the fruit, and then follow up.	Jingdong prefecture level city in Shandong
Dragon Quality Goods Shop	Green Intelligent Power Saver	Baby has been received to try, I believe that as the business advertised energy saving. The courier is very responsible.	Jingdong prefecture level city in Shandong
Dragon Quality Goods Shop	Green Intelligent Power Saver	Save Electric Energy King received, good quality, beautiful appearance, very satisfied!	Jingdong prefecture level city in Shandong
Dragon Quality Goods Shop	Green Intelligent Power Saver	Three no products no manufacturers no phone pure fraud induced consumption cheat	Jingdong prefecture level city in Shandong
Dragon Quality Goods Shop	Green Intelligent Power Saver	I'm using it now, I don't know if it's as good as it says, I'll follow up after a month	Jingdong prefecture level city in Shandong
Dragon Quality Goods Shop	Green Intelligent Power Saver	Good quality logistics and fast. Fine workmanship. The description is consistent cost-effective	Jingdong prefecture level city in Shandong
Dragon Quality Goods Shop	Green Intelligent Power Saver	Received, whether it is good or not, to wait a few days and then evaluate	Jingdong prefecture level city in Shandong
Dragon Quality Goods Shop	Green Intelligent Power Saver	I bought it and started using it, I won't know if it works well until I pay my electric bill	Jingdong prefecture level city in Shandong
Dragon Quality Goods Shop	Green Intelligent Power Saver	Especially good, energy saving and power saving, especially good quality, good	Jingdong prefecture level city in Shandong
Dragon Quality Goods Shop	Green Intelligent Power Saver	Logistics is strong, the quality is very good, paste really save electricity, as described, will buy back!	Jingdong prefecture level city in Shandong
Dragon Quality Goods Shop	Green Intelligent Power Saver	Great stuff at a great price, worth the money!	Jingdong prefecture level city in Shandong

Dragon Quality Goods Shop	Green Intelligent Power Saver	Received the power saver, very good quality, very power saving!	Jingdong prefecture level city in Shandong
Dragon Quality Goods Shop	Green Intelligent Power Saver	There are no fruits, only samples...	Jingdong prefecture level city in Shandong
Dragon Quality Goods Shop	Green Intelligent Power Saver	Save a few dollars a month.	Jingdong prefecture level city in Shandong
Dragon Quality Goods Shop	Green Intelligent Power Saver	The quality is particularly good, it works well, and the seller has a good service attitude	Jingdong prefecture level city in Shandong
Dragon Quality Goods Shop	Green Intelligent Power Saver	I've compared them a couple of times and I don't feel any difference	Jingdong prefecture level city in Shandong
Dragon Quality Goods Shop	Green Intelligent Power Saver	I don't see the savings. It's a sham.	Jingdong prefecture level city in Shandong
Dragon Quality Goods Shop	Green Intelligent Power Saver	The power saving effect is very good, and the logistics is very fast. The seller's service attitude is very good	Jingdong prefecture level city in Shandong
Dragon Quality Goods Shop	Green Intelligent Power Saver	Received, well packaged, haven't tried it yet!	Jingdong prefecture level city in Shandong
Dragon Quality Goods Shop	Green Intelligent Power Saver	I don't know how it works, I'll comment after I use it.	Jingdong prefecture level city in Shandong
Dragon Quality Goods Shop	Green Intelligent Power Saver	The product doesn't work well enough to be of any use	Jingdong prefecture level city in Shandong
Dragon Quality Goods Shop	Green Intelligent Power Saver	On Trial 。。。。。。。	Jingdong prefecture level city in Shandong
Dragon Quality Goods Shop	Green Intelligent Power Saver	Being used with unknown results	Jingdong prefecture level city in Shandong
Dragon Quality Goods Shop	Green Intelligent Power Saver	Smart power saving and great quality	Jingdong prefecture level city in Shandong
Dragon Quality Goods Shop	Green Intelligent Power Saver	The saver was received, it works very well and the price is affordable!	Jingdong prefecture level city in Shandong
Dragon Quality Goods Shop	Green Intelligent Power Saver	How can I feel the power saving and how long does it last	Jingdong prefecture level city in Shandong
Dragon Quality Goods Shop	Green Intelligent Power Saver	And I know if it saves electricity or not.	Jingdong prefecture level city in Shandong
Dragon Quality Goods Shop	Green Intelligent Power Saver	It won't save any electricity. No.	Jingdong prefecture level city in Shandong
Dragon Quality Goods Shop	Green Intelligent Power Saver	Invalid. Fake.	Jingdong prefecture level city in Shandong
Dragon Quality Goods Shop	Green Intelligent Power Saver	It's good!	Jingdong prefecture level city in Shandong
Dragon Quality Goods Shop	Green Intelligent Power Saver	have no effect	Jingdong prefecture level city in Shandong
Dragon Quality Goods Shop	Green Intelligent Power Saver	Not used.	Jingdong prefecture level city in Shandong
Dragon Quality Goods Shop	Green Intelligent Power Saver	There is no load range, packaging thrift, ten days also did not develop invoices, received the goods with a receipt, hand-written receipt also has no financial stamp, communication for nearly a week a moment to say that the electronic invoice, a moment to say that the paper invoice, said it is mailed to me, ten days also did not see anything, the invoice invoice can not be found to say that did not stay on file, single single number can not be found, said that the warehouse more than one to a one to find out, in addition to the perfunctory or perfunctory. Products are no certificate of conformity, proper three	Jingdong prefecture level city in Shandong
Dragon Quality Goods Shop	Green Intelligent Power Saver	Satisfaction.	Jingdong prefecture level city in Shandong
Dragon Quality Goods Shop	Green Intelligent Power Saver	There is not a bit of power saving effect, and ringing up an increase in the electricity bill, because you have to add the cost of this machine's own electricity consumption and purchase, so I think it is better to use this machine instead of increasing the expenditure, uneconomical, rather than not use it.	Jingdong prefecture level city in Shandong
Dragon Quality Goods Shop	Green Intelligent Power Saver	I've been using it for two days now and I don't feel any effect.	Jingdong prefecture level city in Shandong
Dragon Quality Goods Shop	Green Intelligent Power Saver	What it looked like was nothing like what was sent.	Jingdong prefecture level city in Shandong
Dragon Quality Goods Shop	Green Intelligent Power Saver	It didn't work. It didn't work for a month or so.	Jingdong prefecture level city in Shandong
Dragon Quality Goods Shop	Green Intelligent Power Saver	Garbage products, physical and description is not the same, no effect at all, pit # no discussion, to buy the fine, garbage, garbage, garbage	Jingdong prefecture level city in Shandong
Dragon Quality Goods Shop	Green Intelligent Power Saver	general	Jingdong prefecture level city in Shandong
Dragon Quality Goods Shop	Green Intelligent Power Saver	IQ tax. Instead of saving electricity, it increases the power consumption of an appliance.	Jingdong prefecture level city in Shandong
Dragon Quality Goods Shop	Green Intelligent Power Saver	There's no trash at all.	Jingdong prefecture level city in Shandong
Dragon Quality Goods Shop	Green Intelligent Power Saver	It doesn't do anything.	Jingdong prefecture level city in Shandong
Dragon Quality Goods Shop	Green Intelligent Power Saver	(of an unmarried couple) be close	Jingdong prefecture level city in Shandong
Dragon Quality Goods Shop	Green Intelligent Power Saver	(of an unmarried couple) be close	Jingdong prefecture level city in Shandong
Dragon Quality Goods Shop	Green Intelligent Power Saver	no electricity saving	Jingdong prefecture level city in Shandong
Dragon Quality Goods Shop	Green Intelligent Power Saver	differ from	Jingdong prefecture level city in Shandong
Dragon Quality Goods Shop	Green Intelligent Power Saver	Saver has received, but real can save electricity, the quality is very good, I times I buy a few more, too good.	Jingdong prefecture level city in Shandong
Dragon Quality Goods Shop	Green Intelligent Power Saver	Great quality as always, a very favorite product, great! o(*￣︶￣*)o	Jingdong prefecture level city in Shandong
Dragon Quality Goods Shop	Green Intelligent Power Saver	If we can reduce the electricity bill, for individuals can save money, for the country can protect the environment, now the weather is getting hotter and hotter, to protect the environment to reduce emissions is very important. Already use the product, whether to reduce the electricity bill depends on next month!	Jingdong prefecture level city in Shandong
Dragon Quality Goods Shop	Green Intelligent Power Saver	Purchased a power saver received on the put into use, rent a room with many electrical appliances, power consumption is very large, we have a piece of electricity or 1.2 dollars a time, with this power saver is much better, every day can save a few cents, very convenient and practical!	Jingdong prefecture level city in Shandong
Dragon Quality Goods Shop	Green Intelligent Power Saver	The product is better to use before not to use when a month 1500 yuan electricity bill, now using the 5 second month electricity bill directly down 470 dollars or very good. A save money to buy several power saver money ZanZanZanZan👍	Jingdong prefecture level city in Shandong
Dragon Quality Goods Shop	Green Intelligent Power Saver	The moment I received the goods really satisfied with this product, not only high-grade packaging, the body are base alloy, up to the price, I was also introduced by a friend, he used last month did save a lot of electricity, but also recommended to me!	Jingdong prefecture level city in Shandong
Dragon Quality Goods Shop	Green Intelligent Power Saver	I got it. Good review. I'm very satisfied. Reasonable price.	Jingdong prefecture level city in Shandong
Dragon Quality Goods Shop	Green Intelligent Power Saver	Intelligent power saver received and used, looking at the quality is good, the quality of the plug sent is also very good. Expect the effect of the economizer is obvious, first five-star praise, with a period of time to follow up!	Jingdong prefecture level city in Shandong
Dragon Quality Goods Shop	Green Intelligent Power Saver	The saver has been received. It was plugged into an outlet and used immediately after receipt. Whether it really saves electricity is not clear yet, I believe that this brand of energy saver really saves	Jingdong prefecture level city in Shandong
Dragon Quality Goods Shop	Green Intelligent Power Saver	Okay, okay, okay. Thank you so much. Fast is good. Fast. Item was as described, mainly because the event was cheap and you can save a lot of money.	Jingdong prefecture level city in Shandong

Dragon Quality Goods Shop	Green Intelligent Power Saver	Received it a couple days ago! Plugged it in the same day! Let's see what the electric bill is in a month! Hopefully it works well oh!	Jingdong prefecture level city in Shandong
Dragon Quality Goods Shop	Green Intelligent Power Saver	Looking at the reviews good, energy-saving small products, compact, the results after 2 months to see, because the electricity bill season payment	Jingdong prefecture level city in Shandong
Dragon Quality Goods Shop	Green Intelligent Power Saver	I just received it and will try it out to see if it works well. I'll comment later.	Jingdong prefecture level city in Shandong
Dragon Quality Goods Shop	Green Intelligent Power Saver	It looks a little small and not very aesthetically pleasing, I don't know how effective it will be until I use it	Jingdong prefecture level city in Shandong
Dragon Quality Goods Shop	Green Intelligent Power Saver	Not only to the point of being useless yet	Jingdong prefecture level city in Shandong
Dragon Quality Goods Shop	Green Intelligent Power Saver	Used it for a month, nothing works, the meter you turn very fast. This is not a scam, but also sold on Jingdong. To cheat a bunch of people?	Jingdong prefecture level city in Shandong
Dragon Quality Goods Shop	Green Intelligent Power Saver	Very good to use, every day can save a little electricity, really good under the sample down a month to save a few dozen dollars in electricity, good before I did not know that there is this ☝ haha, the price is not expensive praise.	Jingdong prefecture level city in Shandong
Dragon Quality Goods Shop	Green Intelligent Power Saver	Tried it for ten days and the change is almost nothing, can't see the power saving yet, use it some more days and see how it goes.	Jingdong prefecture level city in Shandong
Dragon Quality Goods Shop	Green Intelligent Power Saver	Received very quickly, the quality is very good, really save electricity, business service attitude is also very good, I hope you do not miss this opportunity, I continue to buy a few in	Jingdong prefecture level city in Shandong
Dragon Quality Goods Shop	Green Intelligent Power Saver	Quality is very good Oh, the delivery is also very fast, a very favorite store, I want to crazy recommend to friends!	Jingdong prefecture level city in Shandong
Dragon Quality Goods Shop	Green Intelligent Power Saver	The goods have just received, fine workmanship, and the same as described by the merchant, to use a period of time to see if it can achieve the purpose of power saving.	Jingdong prefecture level city in Shandong
Dragon Quality Goods Shop	Green Intelligent Power Saver	Received the goods first use a period of time to see if there is no effect, the business commitment to ninety days invalid refund, first praise, good service!	Jingdong prefecture level city in Shandong
Dragon Quality Goods Shop	Green Intelligent Power Saver	The product is used for a month without energy-saving and power-saving effect does not have any effect	Jingdong prefecture level city in Shandong
Dragon Quality Goods Shop	Green Intelligent Power Saver	The baby is really great, I like it very much, my friends also say good to come, recommended to buy!	Jingdong prefecture level city in Shandong
Dragon Quality Goods Shop	Green Intelligent Power Saver	Logistics is fast, courier boy service is good, after using to see how the fruit, and then follow up.	Jingdong prefecture level city in Shandong
Dragon Quality Goods Shop	Green Intelligent Power Saver	Baby has been received to try, I believe that as the business advertised energy saving. The courier is very responsible.	Jingdong prefecture level city in Shandong
Dragon Quality Goods Shop	Green Intelligent Power Saver	Save Electric Energy King received, good quality, beautiful appearance, very satisfied!	Jingdong prefecture level city in Shandong
Dragon Quality Goods Shop	Green Intelligent Power Saver	Three no products no manufacturers no phone pure fraud induced consumption cheat	Jingdong prefecture level city in Shandong
Dragon Quality Goods Shop	Green Intelligent Power Saver	I'm using it now, I don't know if it's as good as it says, I'll follow up after a month	Jingdong prefecture level city in Shandong
Dragon Quality Goods Shop	Green Intelligent Power Saver	Good quality logistics and fast. Fine workmanship. The description is consistent cost-effective	Jingdong prefecture level city in Shandong
Dragon Quality Goods Shop	Green Intelligent Power Saver	Received, whether it is good or not, to wait a few days and then evaluate	Jingdong prefecture level city in Shandong
Dragon Quality Goods Shop	Green Intelligent Power Saver	I bought it and started using it, I won't know if it works well until I pay my electric bill	Jingdong prefecture level city in Shandong
Dragon Quality Goods Shop	Green Intelligent Power Saver	Especially good, energy saving and power saving, especially good quality, good	Jingdong prefecture level city in Shandong
Dragon Quality Goods Shop	Green Intelligent Power Saver	Logistics is strong, the quality is very good, paste really save electricity, as described, will buy back!	Jingdong prefecture level city in Shandong
Dragon Quality Goods Shop	Green Intelligent Power Saver	Great stuff at a great price, worth the money!	Jingdong prefecture level city in Shandong
Dragon Quality Goods Shop	Green Intelligent Power Saver	Received the power saver, very good quality, very power saving!	Jingdong prefecture level city in Shandong
Dragon Quality Goods Shop	Green Intelligent Power Saver	There are no fruits, only samples...	Jingdong prefecture level city in Shandong
Dragon Quality Goods Shop	Green Intelligent Power Saver	Save a few dollars a month.	Jingdong prefecture level city in Shandong
Dragon Quality Goods Shop	Green Intelligent Power Saver	The quality is particularly good, it works well, and the seller has a good service attitude	Jingdong prefecture level city in Shandong
Dragon Quality Goods Shop	Green Intelligent Power Saver	I've compared them a couple of times and I don't feel any difference	Jingdong prefecture level city in Shandong
Dragon Quality Goods Shop	Green Intelligent Power Saver	I don't see the savings. It's a sham.	Jingdong prefecture level city in Shandong
Dragon Quality Goods Shop	Green Intelligent Power Saver	The power saving effect is very good, and the logistics is very fast. The seller's service attitude is very good	Jingdong prefecture level city in Shandong
Dragon Quality Goods Shop	Green Intelligent Power Saver	Received, well packaged, haven't tried it yet!	Jingdong prefecture level city in Shandong
Dragon Quality Goods Shop	Green Intelligent Power Saver	I don't know how it works, I'll comment after I use it.	Jingdong prefecture level city in Shandong
Dragon Quality Goods Shop	Green Intelligent Power Saver	The product doesn't work well enough to be of any use	Jingdong prefecture level city in Shandong
Dragon Quality Goods Shop	Green Intelligent Power Saver	On Trial 。。。。。。。	Jingdong prefecture level city in Shandong
Dragon Quality Goods Shop	Green Intelligent Power Saver	Being used with unknown results	Jingdong prefecture level city in Shandong
Dragon Quality Goods Shop	Green Intelligent Power Saver	Smart power saving and great quality	Jingdong prefecture level city in Shandong
Dragon Quality Goods Shop	Green Intelligent Power Saver	The saver was received, it works very well and the price is affordable!	Jingdong prefecture level city in Shandong
Dragon Quality Goods Shop	Green Intelligent Power Saver	How can I feel the power saving and how long does it last	Jingdong prefecture level city in Shandong
Dragon Quality Goods Shop	Green Intelligent Power Saver	And I know if it saves electricity or not.	Jingdong prefecture level city in Shandong
Dragon Quality Goods Shop	Green Intelligent Power Saver	It won't save any electricity. No.	Jingdong prefecture level city in Shandong
Dragon Quality Goods Shop	Green Intelligent Power Saver	Invalid. Fake.	Jingdong prefecture level city in Shandong
Dragon Quality Goods Shop	Green Intelligent Power Saver	It's good!	Jingdong prefecture level city in Shandong
Dragon Quality Goods Shop	Green Intelligent Power Saver	have no effect	Jingdong prefecture level city in Shandong
Dragon Quality Goods Shop	Green Intelligent Power Saver	Not used.	Jingdong prefecture level city in Shandong
Dragon Quality Goods Shop	Green Intelligent Power Saver	There is no load range, packaging thrift, ten days also did not develop invoices, received the goods with a receipt, hand-written receipt also has no financial stamp, communication for nearly a week a moment to say that the electronic invoice, a moment to say that the paper invoice, said it is mailed to me, ten days also did not see anything, the invoice invoice can not be found to say that did not stay on file, single single number can not be found, said that the warehouse more than one to a one to find out, in addition to the perfunctory or perfunctory. Products are no certificate of conformity, proper three	Jingdong prefecture level city in Shandong
Dragon Quality Goods Shop	Green Intelligent Power Saver	Satisfaction.	Jingdong prefecture level city in Shandong

Dragon Quality Goods Shop	Green Intelligent Power Saver	There is not a bit of power saving effect, and ringing up an increase in the electricity bill, because you have to add the cost of this machine's own electricity consumption and purchase, so I think it is better to use this machine instead of increasing the expenditure, uneconomical, rather than not use it.	Jingdong prefecture level city in Shandong
Dragon Quality Goods Shop	Green Intelligent Power Saver	I've been using it for two days now and I don't feel any effect.	Jingdong prefecture level city in Shandong
Dragon Quality Goods Shop	Green Intelligent Power Saver	What it looked like was nothing like what was sent.	Jingdong prefecture level city in Shandong
Dragon Quality Goods Shop	Green Intelligent Power Saver	It didn't work. It didn't work for a month or so.	Jingdong prefecture level city in Shandong
Dragon Quality Goods Shop	Green Intelligent Power Saver	Garbage products, physical and description is not the same, no effect at all, pit # no discussion, to buy the fine, garbage, garbage, garbage	Jingdong prefecture level city in Shandong
Dragon Quality Goods Shop	Green Intelligent Power Saver	general	Jingdong prefecture level city in Shandong
Dragon Quality Goods Shop	Green Intelligent Power Saver	IQ tax. Instead of saving electricity, it increases the power consumption of an appliance.	Jingdong prefecture level city in Shandong
Dragon Quality Goods Shop	Green Intelligent Power Saver	There's no trash at all.	Jingdong prefecture level city in Shandong
Dragon Quality Goods Shop	Green Intelligent Power Saver	It doesn't do anything.	Jingdong prefecture level city in Shandong
Dragon Quality Goods Shop	Green Intelligent Power Saver	(of an unmarried couple) be close	Jingdong prefecture level city in Shandong
Dragon Quality Goods Shop	Green Intelligent Power Saver	(of an unmarried couple) be close	Jingdong prefecture level city in Shandong
Dragon Quality Goods Shop	Green Intelligent Power Saver	no electricity saving	Jingdong prefecture level city in Shandong
Dragon Quality Goods Shop	Green Intelligent Power Saver	differ from	Jingdong prefecture level city in Shandong
Dragon Quality Goods Shop	Green Intelligent Power Saver	Saver has received, but real can save electricity, the quality is very good, I times I buy a few more, too good.	Jingdong prefecture level city in Shandong
Dragon Quality Goods Shop	Green Intelligent Power Saver	Great quality as always, a very favorite product, great! o(*￣︶￣*)o	Jingdong prefecture level city in Shandong
Dragon Quality Goods Shop	Green Intelligent Power Saver	If we can reduce the electricity bill, for individuals can save money, for the country can protect the environment, now the weather is getting hotter and hotter, to protect the environment to reduce emissions is very important. Already use the product, whether to reduce the electricity bill depends on next month!	Jingdong prefecture level city in Shandong
Dragon Quality Goods Shop	Green Intelligent Power Saver	Purchased a power saver received on the put into use, rent a room with many electrical appliances, power consumption is very large, we have a piece of electricity or 1.2 dollars a time, with this power saver is much better, every day can save a few cents, very convenient and practical!	Jingdong prefecture level city in Shandong
Dragon Quality Goods Shop	Green Intelligent Power Saver	The product is better to use before not to use when a month 1500 yuan electricity bill, now using the 5 second month electricity bill directly down 470 dollars or very good. A save money to buy several power saver money ZanZanZanZan	Jingdong prefecture level city in Shandong
Dragon Quality Goods Shop	Green Intelligent Power Saver	The moment I received the goods really satisfied with this product, not only high-grade packaging, the body are base alloy, up to the price, I was also introduced by a friend, he used last month did save a lot of electricity, but also recommended to me!	Jingdong prefecture level city in Shandong
Dragon Quality Goods Shop	Green Intelligent Power Saver	I got it. Good review. I'm very satisfied. Reasonable price.	Jingdong prefecture level city in Shandong
Dragon Quality Goods Shop	Green Intelligent Power Saver	Intelligent power saver received and used, looking at the quality is good, the quality of the plug sent is also very good. Expect the effect of the economizer is obvious, first five-star praise, with a period of time to follow up!	Jingdong prefecture level city in Shandong
Dragon Quality Goods Shop	Green Intelligent Power Saver	The saver has been received. It was plugged into an outlet and used immediately after receipt. Whether it really saves electricity is not clear yet, I believe that this brand of energy saver really saves	Jingdong prefecture level city in Shandong
Dragon Quality Goods Shop	Green Intelligent Power Saver	Okay, okay, okay. Thank you so much. Fast is good. Fast. Item was as described, mainly because the event was cheap and you can save a lot of money.	Jingdong prefecture level city in Shandong
Dragon Quality Goods Shop	Green Intelligent Power Saver	Received it a couple days ago! Plugged it in the same day! Let's see what the electric bill is in a month! Hopefully it works well oh!	Jingdong prefecture level city in Shandong
Dragon Quality Goods Shop	Green Intelligent Power Saver	Looking at the reviews good, energy-saving small products, compact, the results after 2 months to see, because the electricity bill season payment	Jingdong prefecture level city in Shandong
Dragon Quality Goods Shop	Green Intelligent Power Saver	I just received it and will try it out to see if it works well. I'll comment later.	Jingdong prefecture level city in Shandong
Dragon Quality Goods Shop	Green Intelligent Power Saver	It looks a little small and not very aesthetically pleasing, I don't know how effective it will be until I use it	Jingdong prefecture level city in Shandong
Dragon Quality Goods Shop	Green Intelligent Power Saver	Not only to the point of being useless yet	Jingdong prefecture level city in Shandong
Dragon Quality Goods Shop	Green Intelligent Power Saver	Used it for a month, nothing works, the meter you turn very fast. This is not a scam, but also sold on Jingdong. To cheat a bunch of people?	Jingdong prefecture level city in Shandong
Dragon Quality Goods Shop	Green Intelligent Power Saver	Very good to use, every day can save a little electricity, really good under the sample down a month to save a few dozen dollars in electricity, good before I did not know that there is this haha, the price is not expensive praise.	Jingdong prefecture level city in Shandong
Dragon Quality Goods Shop	Green Intelligent Power Saver	Tried it for ten days and the change is almost nothing, can't see the power saving yet, use it some more days and see how it goes.	Jingdong prefecture level city in Shandong
Dragon Quality Goods Shop	Green Intelligent Power Saver	Received very quickly, the quality is very good, really save electricity, business service attitude is also very good, I hope you do not miss this opportunity, I continue to buy a few in	Jingdong prefecture level city in Shandong
Dragon Quality Goods Shop	Green Intelligent Power Saver	Quality is very good Oh, the delivery is also very fast, a very favorite store, I want to crazy recommend to friends!	Jingdong prefecture level city in Shandong
Dragon Quality Goods Shop	Green Intelligent Power Saver	The goods have just received, fine workmanship, and the same as described by the merchant, to use a period of time to see if it can achieve the purpose of power saving.	Jingdong prefecture level city in Shandong
Dragon Quality Goods Shop	Green Intelligent Power Saver	Received the goods first use a period of time to see if there is no effect, the business commitment to ninety days invalid refund, first praise, good service!	Jingdong prefecture level city in Shandong
Dragon Quality Goods Shop	Green Intelligent Power Saver	The product is used for a month without energy-saving and power-saving effect does not have any effect	Jingdong prefecture level city in Shandong
Dragon Quality Goods Shop	Green Intelligent Power Saver	The baby is really great, I like it very much, my friends also say good to come, recommended to buy!	Jingdong prefecture level city in Shandong
Dragon Quality Goods Shop	Green Intelligent Power Saver	Logistics is fast, courier boy service is good, after using to see how the fruit, and then follow up.	Jingdong prefecture level city in Shandong
Dragon Quality Goods Shop	Green Intelligent Power Saver	Baby has been received to try, I believe that as the business advertised energy saving. The courier is very responsible.	Jingdong prefecture level city in Shandong
Dragon Quality Goods Shop	Green Intelligent Power Saver	Save Electric Energy King received, good quality, beautiful appearance, very satisfied!	Jingdong prefecture level city in Shandong
Dragon Quality Goods Shop	Green Intelligent Power Saver	Three no products no manufacturers no phone pure fraud induced consumption cheat	Jingdong prefecture level city in Shandong
Dragon Quality Goods Shop	Green Intelligent Power Saver	I'm using it now, I don't know if it's as good as it says, I'll follow up after a month	Jingdong prefecture level city in Shandong
Dragon Quality Goods Shop	Green Intelligent Power Saver	Good quality logistics and fast. Fine workmanship. The description is consistent cost-effective	Jingdong prefecture level city in Shandong
Dragon Quality Goods Shop	Green Intelligent Power Saver	Received, whether it is good or not, to wait a few days and then evaluate	Jingdong prefecture level city in Shandong
Dragon Quality Goods Shop	Green Intelligent Power Saver	I bought it and started using it, I won't know if it works well until I pay my electric bill	Jingdong prefecture level city in Shandong
Dragon Quality Goods Shop	Green Intelligent Power Saver	Especially good, energy saving and power saving, especially good quality, good	Jingdong prefecture level city in Shandong
Dragon Quality Goods Shop	Green Intelligent Power Saver	Logistics is strong, the quality is very good, paste really save electricity, as described, will buy back!	Jingdong prefecture level city in Shandong
Dragon Quality Goods Shop	Green Intelligent Power Saver	Great stuff at a great price, worth the money!	Jingdong prefecture level city in Shandong

Dragon Quality Goods Shop	Green Intelligent Power Saver	Received the power saver, very good quality, very power saving!	Jingdong prefecture level city in Shandong
Dragon Quality Goods Shop	Green Intelligent Power Saver	There are no fruits, only samples...	Jingdong prefecture level city in Shandong
Dragon Quality Goods Shop	Green Intelligent Power Saver	Save a few dollars a month.	Jingdong prefecture level city in Shandong
Dragon Quality Goods Shop	Green Intelligent Power Saver	The quality is particularly good, it works well, and the seller has a good service attitude	Jingdong prefecture level city in Shandong
Dragon Quality Goods Shop	Green Intelligent Power Saver	I've compared them a couple of times and I don't feel any difference	Jingdong prefecture level city in Shandong
Dragon Quality Goods Shop	Green Intelligent Power Saver	I don't see the savings. It's a sham.	Jingdong prefecture level city in Shandong
Dragon Quality Goods Shop	Green Intelligent Power Saver	The power saving effect is very good, and the logistics is very fast. The seller's service attitude is very good	Jingdong prefecture level city in Shandong
Dragon Quality Goods Shop	Green Intelligent Power Saver	Received, well packaged, haven't tried it yet!	Jingdong prefecture level city in Shandong
Dragon Quality Goods Shop	Green Intelligent Power Saver	I don't know how it works, I'll comment after I use it.	Jingdong prefecture level city in Shandong
Dragon Quality Goods Shop	Green Intelligent Power Saver	The product doesn't work well enough to be of any use	Jingdong prefecture level city in Shandong
Dragon Quality Goods Shop	Green Intelligent Power Saver	On Trial 。。。。。。。	Jingdong prefecture level city in Shandong
Dragon Quality Goods Shop	Green Intelligent Power Saver	Being used with unknown results	Jingdong prefecture level city in Shandong
Dragon Quality Goods Shop	Green Intelligent Power Saver	Smart power saving and great quality	Jingdong prefecture level city in Shandong
Dragon Quality Goods Shop	Green Intelligent Power Saver	The saver was received, it works very well and the price is affordable!	Jingdong prefecture level city in Shandong
Dragon Quality Goods Shop	Green Intelligent Power Saver	How can I feel the power saving and how long does it last	Jingdong prefecture level city in Shandong
Dragon Quality Goods Shop	Green Intelligent Power Saver	And I know if it saves electricity or not.	Jingdong prefecture level city in Shandong
Dragon Quality Goods Shop	Green Intelligent Power Saver	It won't save any electricity. No.	Jingdong prefecture level city in Shandong
Dragon Quality Goods Shop	Green Intelligent Power Saver	Invalid. Fake.	Jingdong prefecture level city in Shandong
Dragon Quality Goods Shop	Green Intelligent Power Saver	It's good!	Jingdong prefecture level city in Shandong
Dragon Quality Goods Shop	Green Intelligent Power Saver	have no effect	Jingdong prefecture level city in Shandong
Dragon Quality Goods Shop	Green Intelligent Power Saver	Not used.	Jingdong prefecture level city in Shandong
Dragon Quality Goods Shop	Green Intelligent Power Saver	There is no load range, packaging thrift, ten days also did not develop invoices, received the goods with a receipt, hand-written receipt also has no financial stamp, communication for nearly a week a moment to say that the electronic invoice, a moment to say that the paper invoice, said it is mailed to me, ten days also did not see anything, the invoice invoice can not be found to say that did not stay on file, single single number can not be found, said that the warehouse more than one to a one to find out, in addition to the perfunctory or perfunctory. Products are no certificate of conformity, proper three	Jingdong prefecture level city in Shandong
Dragon Quality Goods Shop	Green Intelligent Power Saver	Satisfaction.	Jingdong prefecture level city in Shandong
Dragon Quality Goods Shop	Green Intelligent Power Saver	There is not a bit of power saving effect, and ringing up an increase in the electricity bill, because you have to add the cost of this machine's own electricity consumption and purchase, so I think it is better to use this machine instead of increasing the expenditure, uneconomical, rather than not use it.	Jingdong prefecture level city in Shandong
Dragon Quality Goods Shop	Green Intelligent Power Saver	I've been using it for two days now and I don't feel any effect.	Jingdong prefecture level city in Shandong
Dragon Quality Goods Shop	Green Intelligent Power Saver	What it looked like was nothing like what was sent.	Jingdong prefecture level city in Shandong
Dragon Quality Goods Shop	Green Intelligent Power Saver	It didn't work. It didn't work for a month or so.	Jingdong prefecture level city in Shandong
Dragon Quality Goods Shop	Green Intelligent Power Saver	Garbage products, physical and description is not the same, no effect at all, pit # no discussion, to buy the fine, garbage, garbage, garbage	Jingdong prefecture level city in Shandong
Dragon Quality Goods Shop	Green Intelligent Power Saver	general	Jingdong prefecture level city in Shandong
Dragon Quality Goods Shop	Green Intelligent Power Saver	IQ tax. Instead of saving electricity, it increases the power consumption of an appliance.	Jingdong prefecture level city in Shandong
Dragon Quality Goods Shop	Green Intelligent Power Saver	There's no trash at all.	Jingdong prefecture level city in Shandong
Dragon Quality Goods Shop	Green Intelligent Power Saver	It doesn't do anything.	Jingdong prefecture level city in Shandong
Dragon Quality Goods Shop	Green Intelligent Power Saver	(of an unmarried couple) be close	Jingdong prefecture level city in Shandong
Dragon Quality Goods Shop	Green Intelligent Power Saver	(of an unmarried couple) be close	Jingdong prefecture level city in Shandong
Dragon Quality Goods Shop	Green Intelligent Power Saver	no electricity saving	Jingdong prefecture level city in Shandong
Dragon Quality Goods Shop	Green Intelligent Power Saver	differ from	Jingdong prefecture level city in Shandong
Dragon Quality Goods Shop	Green Intelligent Power Saver	Saver has received, but real can save electricity, the quality is very good, I times I buy a few more, too good.	Jingdong prefecture level city in Shandong
Dragon Quality Goods Shop	Green Intelligent Power Saver	Great quality as always, a very favorite product, great! o(*￣︶￣*)o	Jingdong prefecture level city in Shandong
Dragon Quality Goods Shop	Green Intelligent Power Saver	If we can reduce the electricity bill, for individuals can save money, for the country can protect the environment, now the weather is getting hotter and hotter, to protect the environment to reduce emissions is very important. Already use the product, whether to reduce the electricity bill depends on next month!	Jingdong prefecture level city in Shandong
Dragon Quality Goods Shop	Green Intelligent Power Saver	Purchased a power saver received on the put into use, rent a room with many electrical appliances, power consumption is very large, we have a piece of electricity or 1.2 dollars a time, with this power saver is much better, every day can save a few cents, very convenient and practical!	Jingdong prefecture level city in Shandong
Dragon Quality Goods Shop	Green Intelligent Power Saver	The product is better to use before not to use when a month 1500 yuan electricity bill, now using the 5 second month electricity bill directly down 470 dollars or very good. A save money to buy several power saver money ZanZanZanZan👍	Jingdong prefecture level city in Shandong
Dragon Quality Goods Shop	Green Intelligent Power Saver	The moment I received the goods really satisfied with this product, not only high-grade packaging, the body are base alloy, up to the price, I was also introduced by a friend, he used last month did save a lot of electricity, but also recommended to me!	Jingdong prefecture level city in Shandong
Dragon Quality Goods Shop	Green Intelligent Power Saver	I got it. Good review. I'm very satisfied. Reasonable price.	Jingdong prefecture level city in Shandong
Dragon Quality Goods Shop	Green Intelligent Power Saver	Intelligent power saver received and used, looking at the quality is good, the quality of the plug sent is also very good. Expect the effect of the economizer is obvious, first five-star praise, with a period of time to follow up!	Jingdong prefecture level city in Shandong
Dragon Quality Goods Shop	Green Intelligent Power Saver	The saver has been received. It was plugged into an outlet and used immediately after receipt. Whether it really saves electricity is not clear yet, I believe that this brand of energy saver really saves	Jingdong prefecture level city in Shandong
Dragon Quality Goods Shop	Green Intelligent Power Saver	Okay, okay, okay. Thank you so much. Fast is good. Fast. Item was as described, mainly because the event was cheap and you can save a lot of money.	Jingdong prefecture level city in Shandong

Dragon Quality Goods Shop	Green Intelligent Power Saver	Received it a couple days ago! Plugged it in the same day! Let's see what the electric bill is in a month! Hopefully it works well oh!	Jingdong prefecture level city in Shandong
Dragon Quality Goods Shop	Green Intelligent Power Saver	Looking at the reviews good, energy-saving small products, compact, the results after 2 months to see, because the electricity bill season payment	Jingdong prefecture level city in Shandong
Dragon Quality Goods Shop	Green Intelligent Power Saver	I just received it and will try it out to see if it works well. I'll comment later.	Jingdong prefecture level city in Shandong
Dragon Quality Goods Shop	Green Intelligent Power Saver	It looks a little small and not very aesthetically pleasing, I don't know how effective it will be until I use it	Jingdong prefecture level city in Shandong
Dragon Quality Goods Shop	Green Intelligent Power Saver	Not only to the point of being useless yet	Jingdong prefecture level city in Shandong
Dragon Quality Goods Shop	Green Intelligent Power Saver	Used it for a month, nothing works, the meter you turn very fast. This is not a scam, but also sold on Jingdong. To cheat a bunch of people?	Jingdong prefecture level city in Shandong
Dragon Quality Goods Shop	Green Intelligent Power Saver	Very good to use, every day can save a little electricity, really good under the sample down a month to save a few dozen dollars in electricity, good before I did not know that there is this 👆 haha, the price is not expensive praise.	Jingdong prefecture level city in Shandong
Dragon Quality Goods Shop	Green Intelligent Power Saver	Tried it for ten days and the change is almost nothing, can't see the power saving yet, use it some more days and see how it goes.	Jingdong prefecture level city in Shandong
Dragon Quality Goods Shop	Green Intelligent Power Saver	Received very quickly, the quality is very good, really save electricity, business service attitude is also very good, I hope you do not miss this opportunity, I continue to buy a few in	Jingdong prefecture level city in Shandong
Dragon Quality Goods Shop	Green Intelligent Power Saver	Quality is very good Oh, the delivery is also very fast, a very favorite store, I want to crazy recommend to friends!	Jingdong prefecture level city in Shandong
Dragon Quality Goods Shop	Green Intelligent Power Saver	The goods have just received, fine workmanship, and the same as described by the merchant, to use a period of time to see if it can achieve the purpose of power saving.	Jingdong prefecture level city in Shandong
Dragon Quality Goods Shop	Green Intelligent Power Saver	Received the goods first use a period of time to see if there is no effect, the business commitment to ninety days invalid refund, first praise, good service!	Jingdong prefecture level city in Shandong
Dragon Quality Goods Shop	Green Intelligent Power Saver	The product is used for a month without energy-saving and power-saving effect does not have any effect	Jingdong prefecture level city in Shandong
Dragon Quality Goods Shop	Green Intelligent Power Saver	The baby is really great, I like it very much, my friends also say good to come, recommended to buy!	Jingdong prefecture level city in Shandong
Dragon Quality Goods Shop	Green Intelligent Power Saver	Logistics is fast, courier boy service is good, after using to see how the fruit, and then follow up.	Jingdong prefecture level city in Shandong
Dragon Quality Goods Shop	Green Intelligent Power Saver	Baby has been received to try, I believe that as the business advertised energy saving. The courier is very responsible.	Jingdong prefecture level city in Shandong
Dragon Quality Goods Shop	Green Intelligent Power Saver	Save Electric Energy King received, good quality, beautiful appearance, very satisfied!	Jingdong prefecture level city in Shandong
Dragon Quality Goods Shop	Green Intelligent Power Saver	Three no products no manufacturers no phone pure fraud induced consumption cheat	Jingdong prefecture level city in Shandong
Dragon Quality Goods Shop	Green Intelligent Power Saver	I'm using it now, I don't know if it's as good as it says, I'll follow up after a month	Jingdong prefecture level city in Shandong
Dragon Quality Goods Shop	Green Intelligent Power Saver	Good quality logistics and fast. Fine workmanship. The description is consistent cost-effective	Jingdong prefecture level city in Shandong
Dragon Quality Goods Shop	Green Intelligent Power Saver	Received, whether it is good or not, to wait a few days and then evaluate	Jingdong prefecture level city in Shandong
Dragon Quality Goods Shop	Green Intelligent Power Saver	I bought it and started using it, I won't know if it works well until I pay my electric bill	Jingdong prefecture level city in Shandong
Dragon Quality Goods Shop	Green Intelligent Power Saver	Especially good, energy saving and power saving, especially good quality, good	Jingdong prefecture level city in Shandong
Dragon Quality Goods Shop	Green Intelligent Power Saver	Logistics is strong, the quality is very good, paste really save electricity, as described, will buy back!	Jingdong prefecture level city in Shandong
Dragon Quality Goods Shop	Green Intelligent Power Saver	Great stuff at a great price, worth the money!	Jingdong prefecture level city in Shandong
Dragon Quality Goods Shop	Green Intelligent Power Saver	Received the power saver, very good quality, very power saving!	Jingdong prefecture level city in Shandong
Dragon Quality Goods Shop	Green Intelligent Power Saver	There are no fruits, only samples...	Jingdong prefecture level city in Shandong
Dragon Quality Goods Shop	Green Intelligent Power Saver	Save a few dollars a month.	Jingdong prefecture level city in Shandong
Dragon Quality Goods Shop	Green Intelligent Power Saver	The quality is particularly good, it works well, and the seller has a good service attitude	Jingdong prefecture level city in Shandong
Dragon Quality Goods Shop	Green Intelligent Power Saver	I've compared them a couple of times and I don't feel any difference	Jingdong prefecture level city in Shandong
Dragon Quality Goods Shop	Green Intelligent Power Saver	I don't see the savings. It's a sham.	Jingdong prefecture level city in Shandong
Dragon Quality Goods Shop	Green Intelligent Power Saver	The power saving effect is very good, and the logistics is very fast. The seller's service attitude is very good	Jingdong prefecture level city in Shandong
Dragon Quality Goods Shop	Green Intelligent Power Saver	Received, well packaged, haven't tried it yet!	Jingdong prefecture level city in Shandong
Dragon Quality Goods Shop	Green Intelligent Power Saver	I don't know how it works, I'll comment after I use it.	Jingdong prefecture level city in Shandong
Dragon Quality Goods Shop	Green Intelligent Power Saver	The product doesn't work well enough to be of any use	Jingdong prefecture level city in Shandong
Dragon Quality Goods Shop	Green Intelligent Power Saver	On Trial 。。。。。。。	Jingdong prefecture level city in Shandong
Dragon Quality Goods Shop	Green Intelligent Power Saver	Being used with unknown results	Jingdong prefecture level city in Shandong
Dragon Quality Goods Shop	Green Intelligent Power Saver	Smart power saving and great quality	Jingdong prefecture level city in Shandong
Dragon Quality Goods Shop	Green Intelligent Power Saver	The saver was received, it works very well and the price is affordable!	Jingdong prefecture level city in Shandong
Dragon Quality Goods Shop	Green Intelligent Power Saver	How can I feel the power saving and how long does it last	Jingdong prefecture level city in Shandong
Dragon Quality Goods Shop	Green Intelligent Power Saver	And I know if it saves electricity or not.	Jingdong prefecture level city in Shandong
Dragon Quality Goods Shop	Green Intelligent Power Saver	It won't save any electricity. No.	Jingdong prefecture level city in Shandong
Dragon Quality Goods Shop	Green Intelligent Power Saver	Invalid. Fake.	Jingdong prefecture level city in Shandong
Dragon Quality Goods Shop	Green Intelligent Power Saver	It's good!	Jingdong prefecture level city in Shandong
Dragon Quality Goods Shop	Green Intelligent Power Saver	have no effect	Jingdong prefecture level city in Shandong
Dragon Quality Goods Shop	Green Intelligent Power Saver	Not used.	Jingdong prefecture level city in Shandong
Dragon Quality Goods Shop	Green Intelligent Power Saver	There is no load range, packaging thrift, ten days also did not develop invoices, received the goods with a receipt, hand-written receipt also has no financial stamp, communication for nearly a week a moment to say that the electronic invoice, a moment to say that the paper invoice, said it is mailed to me, ten days also did not see anything, the invoice invoice can not be found to say that did not stay on file, single single number can not be found, said that the warehouse more than one to a one to find out, in addition to the perfunctory or perfunctory. Products are no certificate of conformity, proper three	Jingdong prefecture level city in Shandong
Dragon Quality Goods Shop	Green Intelligent Power Saver	Satisfaction.	Jingdong prefecture level city in Shandong

Dragon Quality Goods Shop	Green Intelligent Power Saver	There is not a bit of power saving effect, and ringing up an increase in the electricity bill, because you have to add the cost of this machine's own electricity consumption and purchase, so I think it is better to use this machine instead of increasing the expenditure, uneconomical, rather than not use it.	Jingdong prefecture level city in Shandong
Dragon Quality Goods Shop	Green Intelligent Power Saver	I've been using it for two days now and I don't feel any effect.	Jingdong prefecture level city in Shandong
Dragon Quality Goods Shop	Green Intelligent Power Saver	What it looked like was nothing like what was sent.	Jingdong prefecture level city in Shandong
Dragon Quality Goods Shop	Green Intelligent Power Saver	It didn't work. It didn't work for a month or so.	Jingdong prefecture level city in Shandong
Dragon Quality Goods Shop	Green Intelligent Power Saver	Garbage products, physical and description is not the same, no effect at all, pit # no discussion, to buy the fine, garbage, garbage, garbage	Jingdong prefecture level city in Shandong
Dragon Quality Goods Shop	Green Intelligent Power Saver	general	Jingdong prefecture level city in Shandong
Dragon Quality Goods Shop	Green Intelligent Power Saver	IQ tax. Instead of saving electricity, it increases the power consumption of an appliance.	Jingdong prefecture level city in Shandong
Dragon Quality Goods Shop	Green Intelligent Power Saver	There's no trash at all.	Jingdong prefecture level city in Shandong
Dragon Quality Goods Shop	Green Intelligent Power Saver	It doesn't do anything.	Jingdong prefecture level city in Shandong
Dragon Quality Goods Shop	Green Intelligent Power Saver	(of an unmarried couple) be close	Jingdong prefecture level city in Shandong
Dragon Quality Goods Shop	Green Intelligent Power Saver	(of an unmarried couple) be close	Jingdong prefecture level city in Shandong
Dragon Quality Goods Shop	Green Intelligent Power Saver	no electricity saving	Jingdong prefecture level city in Shandong
Dragon Quality Goods Shop	Green Intelligent Power Saver	differ from	Jingdong prefecture level city in Shandong
Dragon Quality Goods Shop	Green Intelligent Power Saver	Saver has received, but real can save electricity, the quality is very good, I times I buy a few more, too good.	Jingdong prefecture level city in Shandong
Dragon Quality Goods Shop	Green Intelligent Power Saver	Great quality as always, a very favorite product, great! o(*￣︶￣*)o	Jingdong prefecture level city in Shandong
Dragon Quality Goods Shop	Green Intelligent Power Saver	If we can reduce the electricity bill, for individuals can save money, for the country can protect the environment, now the weather is getting hotter and hotter, to protect the environment to reduce emissions is very important. Already use the product, whether to reduce the electricity bill depends on next month!	Jingdong prefecture level city in Shandong
Dragon Quality Goods Shop	Green Intelligent Power Saver	Purchased a power saver received on the put into use, rent a room with many electrical appliances, power consumption is very large, we have a piece of electricity or 1.2 dollars a time, with this power saver is much better, every day can save a few cents, very convenient and practical!	Jingdong prefecture level city in Shandong
Dragon Quality Goods Shop	Green Intelligent Power Saver	The product is better to use before not to use when a month 1500 yuan electricity bill, now using the 5 second month electricity bill directly down 470 dollars or very good. A save money to buy several power saver money ZanZanZanZan👍	Jingdong prefecture level city in Shandong
Dragon Quality Goods Shop	Green Intelligent Power Saver	The moment I received the goods really satisfied with this product, not only high-grade packaging, the body are base alloy, up to the price, I was also introduced by a friend, he used last month did save a lot of electricity, but also recommended to me!	Jingdong prefecture level city in Shandong
Dragon Quality Goods Shop	Green Intelligent Power Saver	I got it. Good review. I'm very satisfied. Reasonable price.	Jingdong prefecture level city in Shandong
Dragon Quality Goods Shop	Green Intelligent Power Saver	Intelligent power saver received and used, looking at the quality is good, the quality of the plug sent is also very good. Expect the effect of the economizer is obvious, first five-star praise, with a period of time to follow up!	Jingdong prefecture level city in Shandong
Dragon Quality Goods Shop	Green Intelligent Power Saver	The saver has been received. It was plugged into an outlet and used immediately after receipt. Whether it really saves electricity is not clear yet, I believe that this brand of energy saver really saves	Jingdong prefecture level city in Shandong
Dragon Quality Goods Shop	Green Intelligent Power Saver	Okay, okay, okay. Thank you so much. Fast is good. Fast. Item was as described, mainly because the event was cheap and you can save a lot of money.	Jingdong prefecture level city in Shandong
Dragon Quality Goods Shop	Green Intelligent Power Saver	Received it a couple days ago! Plugged it in the same day! Let's see what the electric bill is in a month! Hopefully it works well oh!	Jingdong prefecture level city in Shandong
Dragon Quality Goods Shop	Green Intelligent Power Saver	Looking at the reviews good, energy-saving small products, compact, the results after 2 months to see, because the electricity bill season payment	Jingdong prefecture level city in Shandong
Dragon Quality Goods Shop	Green Intelligent Power Saver	I just received it and will try it out to see if it works well. I'll comment later.	Jingdong prefecture level city in Shandong
Dragon Quality Goods Shop	Green Intelligent Power Saver	It looks a little small and not very aesthetically pleasing, I don't know how effective it will be until I use it	Jingdong prefecture level city in Shandong
Dragon Quality Goods Shop	Green Intelligent Power Saver	Not only to the point of being useless yet	Jingdong prefecture level city in Shandong
Dragon Quality Goods Shop	Green Intelligent Power Saver	Used it for a month, nothing works, the meter you turn very fast. This is not a scam, but also sold on Jingdong. To cheat a bunch of people?	Jingdong prefecture level city in Shandong
Dragon Quality Goods Shop	Green Intelligent Power Saver	Very good to use, every day can save a little electricity, really good under the sample down a month to save a few dozen dollars in electricity, good before I did not know that there is this 👆 haha, the price is not expensive praise.	Jingdong prefecture level city in Shandong
Dragon Quality Goods Shop	Green Intelligent Power Saver	Tried it for ten days and the change is almost nothing, can't see the power saving yet, use it some more days and see how it goes.	Jingdong prefecture level city in Shandong
Dragon Quality Goods Shop	Green Intelligent Power Saver	Received very quickly, the quality is very good, really save electricity, business service attitude is also very good, I hope you do not miss this opportunity, I continue to buy a few in	Jingdong prefecture level city in Shandong
Dragon Quality Goods Shop	Green Intelligent Power Saver	Quality is very good Oh, the delivery is also very fast, a very favorite store, I want to crazy recommend to friends!	Jingdong prefecture level city in Shandong
Dragon Quality Goods Shop	Green Intelligent Power Saver	The goods have just received, fine workmanship, and the same as described by the merchant, to use a period of time to see if it can achieve the purpose of power saving.	Jingdong prefecture level city in Shandong
Dragon Quality Goods Shop	Green Intelligent Power Saver	Received the goods first use a period of time to see if there is no effect, the business commitment to ninety days invalid refund, first praise, good service!	Jingdong prefecture level city in Shandong
Dragon Quality Goods Shop	Green Intelligent Power Saver	The product is used for a month without energy-saving and power-saving effect does not have any effect	Jingdong prefecture level city in Shandong
Dragon Quality Goods Shop	Green Intelligent Power Saver	The baby is really great, I like it very much, my friends also say good to come, recommended to buy!	Jingdong prefecture level city in Shandong
Dragon Quality Goods Shop	Green Intelligent Power Saver	Logistics is fast, courier boy service is good, after using to see how the fruit, and then follow up.	Jingdong prefecture level city in Shandong
Dragon Quality Goods Shop	Green Intelligent Power Saver	Baby has been received to try, I believe that as the business advertised energy saving. The courier is very responsible.	Jingdong prefecture level city in Shandong
Dragon Quality Goods Shop	Green Intelligent Power Saver	Save Electric Energy King received, good quality, beautiful appearance, very satisfied!	Jingdong prefecture level city in Shandong
Dragon Quality Goods Shop	Green Intelligent Power Saver	Three no products no manufacturers no phone pure fraud induced consumption cheat	Jingdong prefecture level city in Shandong
Dragon Quality Goods Shop	Green Intelligent Power Saver	I'm using it now, I don't know if it's as good as it says, I'll follow up after a month	Jingdong prefecture level city in Shandong
Dragon Quality Goods Shop	Green Intelligent Power Saver	Good quality logistics and fast. Fine workmanship. The description is consistent cost-effective	Jingdong prefecture level city in Shandong
Dragon Quality Goods Shop	Green Intelligent Power Saver	Received, whether it is good or not, to wait a few days and then evaluate	Jingdong prefecture level city in Shandong
Dragon Quality Goods Shop	Green Intelligent Power Saver	I bought it and started using it, I won't know if it works well until I pay my electric bill	Jingdong prefecture level city in Shandong
Dragon Quality Goods Shop	Green Intelligent Power Saver	Especially good, energy saving and power saving, especially good quality, good	Jingdong prefecture level city in Shandong
Dragon Quality Goods Shop	Green Intelligent Power Saver	Logistics is strong, the quality is very good, paste really save electricity, as described, will buy back!	Jingdong prefecture level city in Shandong
Dragon Quality Goods Shop	Green Intelligent Power Saver	Great stuff at a great price, worth the money!	Jingdong prefecture level city in Shandong

Dragon Quality Goods Shop	Green Intelligent Power Saver	Received the power saver, very good quality, very power saving!	Jingdong prefecture level city in Shandong
Dragon Quality Goods Shop	Green Intelligent Power Saver	There are no fruits, only samples...	Jingdong prefecture level city in Shandong
Dragon Quality Goods Shop	Green Intelligent Power Saver	Save a few dollars a month.	Jingdong prefecture level city in Shandong
Dragon Quality Goods Shop	Green Intelligent Power Saver	The quality is particularly good, it works well, and the seller has a good service attitude	Jingdong prefecture level city in Shandong
Dragon Quality Goods Shop	Green Intelligent Power Saver	I've compared them a couple of times and I don't feel any difference	Jingdong prefecture level city in Shandong
Dragon Quality Goods Shop	Green Intelligent Power Saver	I don't see the savings. It's a sham.	Jingdong prefecture level city in Shandong
Dragon Quality Goods Shop	Green Intelligent Power Saver	The power saving effect is very good, and the logistics is very fast. The seller's service attitude is very good	Jingdong prefecture level city in Shandong
Dragon Quality Goods Shop	Green Intelligent Power Saver	Received, well packaged, haven't tried it yet!	Jingdong prefecture level city in Shandong
Dragon Quality Goods Shop	Green Intelligent Power Saver	I don't know how it works, I'll comment after I use it.	Jingdong prefecture level city in Shandong
Dragon Quality Goods Shop	Green Intelligent Power Saver	The product doesn't work well enough to be of any use	Jingdong prefecture level city in Shandong
Dragon Quality Goods Shop	Green Intelligent Power Saver	On Trial 。。。。。。。	Jingdong prefecture level city in Shandong
Dragon Quality Goods Shop	Green Intelligent Power Saver	Being used with unknown results	Jingdong prefecture level city in Shandong
Dragon Quality Goods Shop	Green Intelligent Power Saver	Smart power saving and great quality	Jingdong prefecture level city in Shandong
Dragon Quality Goods Shop	Green Intelligent Power Saver	The saver was received, it works very well and the price is affordable!	Jingdong prefecture level city in Shandong
Dragon Quality Goods Shop	Green Intelligent Power Saver	How can I feel the power saving and how long does it last	Jingdong prefecture level city in Shandong
Dragon Quality Goods Shop	Green Intelligent Power Saver	And I know if it saves electricity or not.	Jingdong prefecture level city in Shandong
Dragon Quality Goods Shop	Green Intelligent Power Saver	It won't save any electricity. No.	Jingdong prefecture level city in Shandong
Dragon Quality Goods Shop	Green Intelligent Power Saver	Invalid. Fake.	Jingdong prefecture level city in Shandong
Dragon Quality Goods Shop	Green Intelligent Power Saver	It's good!	Jingdong prefecture level city in Shandong
Dragon Quality Goods Shop	Green Intelligent Power Saver	have no effect	Jingdong prefecture level city in Shandong
Dragon Quality Goods Shop	Green Intelligent Power Saver	Not used.	Jingdong prefecture level city in Shandong
Dragon Quality Goods Shop	Green Intelligent Power Saver	There is no load range, packaging thrift, ten days also did not develop invoices, received the goods with a receipt, hand-written receipt also has no financial stamp, communication for nearly a week a moment to say that the electronic invoice, a moment to say that the paper invoice, said it is mailed to me, ten days also did not see anything, the invoice invoice can not be found to say that did not stay on file, single single number can not be found, said that the warehouse more than one to a one to find out, in addition to the perfunctory or perfunctory. Products are no certificate of conformity, proper three	Jingdong prefecture level city in Shandong
Dragon Quality Goods Shop	Green Intelligent Power Saver	Satisfaction.	Jingdong prefecture level city in Shandong
Dragon Quality Goods Shop	Green Intelligent Power Saver	There is not a bit of power saving effect, and ringing up an increase in the electricity bill, because you have to add the cost of this machine's own electricity consumption and purchase, so I think it is better to use this machine instead of increasing the expenditure, uneconomical, rather than not use it.	Jingdong prefecture level city in Shandong
Dragon Quality Goods Shop	Green Intelligent Power Saver	I've been using it for two days now and I don't feel any effect.	Jingdong prefecture level city in Shandong
Dragon Quality Goods Shop	Green Intelligent Power Saver	What it looked like was nothing like what was sent.	Jingdong prefecture level city in Shandong
Dragon Quality Goods Shop	Green Intelligent Power Saver	It didn't work. It didn't work for a month or so.	Jingdong prefecture level city in Shandong
Dragon Quality Goods Shop	Green Intelligent Power Saver	Garbage products, physical and description is not the same, no effect at all, pit # no discussion, to buy the fine, garbage, garbage, garbage	Jingdong prefecture level city in Shandong
Dragon Quality Goods Shop	Green Intelligent Power Saver	general	Jingdong prefecture level city in Shandong
Dragon Quality Goods Shop	Green Intelligent Power Saver	IQ tax. Instead of saving electricity, it increases the power consumption of an appliance.	Jingdong prefecture level city in Shandong
Dragon Quality Goods Shop	Green Intelligent Power Saver	There's no trash at all.	Jingdong prefecture level city in Shandong
Dragon Quality Goods Shop	Green Intelligent Power Saver	It doesn't do anything.	Jingdong prefecture level city in Shandong
Dragon Quality Goods Shop	Green Intelligent Power Saver	(of an unmarried couple) be close	Jingdong prefecture level city in Shandong
Dragon Quality Goods Shop	Green Intelligent Power Saver	(of an unmarried couple) be close	Jingdong prefecture level city in Shandong
Dragon Quality Goods Shop	Green Intelligent Power Saver	no electricity saving	Jingdong prefecture level city in Shandong
Dragon Quality Goods Shop	Green Intelligent Power Saver	differ from	Jingdong prefecture level city in Shandong
Dragon Quality Goods Shop	Green Intelligent Power Saver	Saver has received, but real can save electricity, the quality is very good, I times I buy a few more, too good.	Jingdong prefecture level city in Shandong
Dragon Quality Goods Shop	Green Intelligent Power Saver	Great quality as always, a very favorite product, great! o(*￣︶￣*)o	Jingdong prefecture level city in Shandong
Dragon Quality Goods Shop	Green Intelligent Power Saver	If we can reduce the electricity bill, for individuals can save money, for the country can protect the environment, now the weather is getting hotter and hotter, to protect the environment to reduce emissions is very important. Already use the product, whether to reduce the electricity bill depends on next month!	Jingdong prefecture level city in Shandong
Dragon Quality Goods Shop	Green Intelligent Power Saver	Purchased a power saver received on the put into use, rent a room with many electrical appliances, power consumption is very large, we have a piece of electricity or 1.2 dollars a time, with this power saver is much better, every day can save a few cents, very convenient and practical!	Jingdong prefecture level city in Shandong
Dragon Quality Goods Shop	Green Intelligent Power Saver	The product is better to use before not to use when a month 1500 yuan electricity bill, now using the 5 second month electricity bill directly down 470 dollars or very good. A save money to buy several power saver money ZanZanZanZan👍	Jingdong prefecture level city in Shandong
Dragon Quality Goods Shop	Green Intelligent Power Saver	The moment I received the goods really satisfied with this product, not only high-grade packaging, the body are base alloy, up to the price, I was also introduced by a friend, he used last month did save a lot of electricity, but also recommended to me!	Jingdong prefecture level city in Shandong
Dragon Quality Goods Shop	Green Intelligent Power Saver	I got it. Good review. I'm very satisfied. Reasonable price.	Jingdong prefecture level city in Shandong
Dragon Quality Goods Shop	Green Intelligent Power Saver	Intelligent power saver received and used, looking at the quality is good, the quality of the plug sent is also very good. Expect the effect of the economizer is obvious, first five-star praise, with a period of time to follow up!	Jingdong prefecture level city in Shandong
Dragon Quality Goods Shop	Green Intelligent Power Saver	The saver has been received. It was plugged into an outlet and used immediately after receipt. Whether it really saves electricity is not clear yet, I believe that this brand of energy saver really saves	Jingdong prefecture level city in Shandong
Dragon Quality Goods Shop	Green Intelligent Power Saver	Okay, okay, okay. Thank you so much. Fast is good. Fast. Item was as described, mainly because the event was cheap and you can save a lot of money.	Jingdong prefecture level city in Shandong

Dragon Quality Goods Shop	Green Intelligent Power Saver	Received it a couple days ago! Plugged it in the same day! Let's see what the electric bill is in a month! Hopefully it works well oh!	Jingdong prefecture level city in Shandong
Dragon Quality Goods Shop	Green Intelligent Power Saver	Looking at the reviews good, energy-saving small products, compact, the results after 2 months to see, because the electricity bill season payment	Jingdong prefecture level city in Shandong
Dragon Quality Goods Shop	Green Intelligent Power Saver	I just received it and will try it out to see if it works well. I'll comment later.	Jingdong prefecture level city in Shandong
Dragon Quality Goods Shop	Green Intelligent Power Saver	It looks a little small and not very aesthetically pleasing, I don't know how effective it will be until I use it	Jingdong prefecture level city in Shandong
Dragon Quality Goods Shop	Green Intelligent Power Saver	Not only to the point of being useless yet	Jingdong prefecture level city in Shandong
Dragon Quality Goods Shop	Green Intelligent Power Saver	Used it for a month, nothing works, the meter you turn very fast. This is not a scam, but also sold on Jingdong. To cheat a bunch of people?	Jingdong prefecture level city in Shandong
Dragon Quality Goods Shop	Green Intelligent Power Saver	Very good to use, every day can save a little electricity, really good under the sample down a month to save a few dozen dollars in electricity, good before I did not know that there is this haha, the price is not expensive praise.	Jingdong prefecture level city in Shandong
Dragon Quality Goods Shop	Green Intelligent Power Saver	Tried it for ten days and the change is almost nothing, can't see the power saving yet, use it some more days and see how it goes.	Jingdong prefecture level city in Shandong
Dragon Quality Goods Shop	Green Intelligent Power Saver	Received very quickly, the quality is very good, really save electricity, business service attitude is also very good, I hope you do not miss this opportunity, I continue to buy a few in	Jingdong prefecture level city in Shandong
Dragon Quality Goods Shop	Green Intelligent Power Saver	Quality is very good Oh, the delivery is also very fast, a very favorite store, I want to crazy recommend to friends!	Jingdong prefecture level city in Shandong
Dragon Quality Goods Shop	Green Intelligent Power Saver	The goods have just received, fine workmanship, and the same as described by the merchant, to use a period of time to see if it can achieve the purpose of power saving.	Jingdong prefecture level city in Shandong
Dragon Quality Goods Shop	Green Intelligent Power Saver	Received the goods first use a period of time to see if there is no effect, the business commitment to ninety days invalid refund, first praise, good service!	Jingdong prefecture level city in Shandong
Dragon Quality Goods Shop	Green Intelligent Power Saver	The product is used for a month without energy-saving and power-saving effect does not have any effect	Jingdong prefecture level city in Shandong
Dragon Quality Goods Shop	Green Intelligent Power Saver	The baby is really great, I like it very much, my friends also say good to come, recommended to buy!	Jingdong prefecture level city in Shandong
Dragon Quality Goods Shop	Green Intelligent Power Saver	Logistics is fast, courier boy service is good, after using to see how the fruit, and then follow up.	Jingdong prefecture level city in Shandong
Dragon Quality Goods Shop	Green Intelligent Power Saver	Baby has been received to try, I believe that as the business advertised energy saving. The courier is very responsible.	Jingdong prefecture level city in Shandong
Dragon Quality Goods Shop	Green Intelligent Power Saver	Save Electric Energy King received, good quality, beautiful appearance, very satisfied!	Jingdong prefecture level city in Shandong
Dragon Quality Goods Shop	Green Intelligent Power Saver	Three no products no manufacturers no phone pure fraud induced consumption cheat	Jingdong prefecture level city in Shandong
Dragon Quality Goods Shop	Green Intelligent Power Saver	I'm using it now, I don't know if it's as good as it says, I'll follow up after a month	Jingdong prefecture level city in Shandong
Dragon Quality Goods Shop	Green Intelligent Power Saver	Good quality logistics and fast. Fine workmanship. The description is consistent cost-effective	Jingdong prefecture level city in Shandong
Dragon Quality Goods Shop	Green Intelligent Power Saver	Received, whether it is good or not, to wait a few days and then evaluate	Jingdong prefecture level city in Shandong
Dragon Quality Goods Shop	Green Intelligent Power Saver	I bought it and started using it, I won't know if it works well until I pay my electric bill	Jingdong prefecture level city in Shandong
Dragon Quality Goods Shop	Green Intelligent Power Saver	Especially good, energy saving and power saving, especially good quality, good	Jingdong prefecture level city in Shandong
Dragon Quality Goods Shop	Green Intelligent Power Saver	Logistics is strong, the quality is very good, paste really save electricity, as described, will buy back!	Jingdong prefecture level city in Shandong
Dragon Quality Goods Shop	Green Intelligent Power Saver	Great stuff at a great price, worth the money!	Jingdong prefecture level city in Shandong
Dragon Quality Goods Shop	Green Intelligent Power Saver	Received the power saver, very good quality, very power saving!	Jingdong prefecture level city in Shandong
Dragon Quality Goods Shop	Green Intelligent Power Saver	There are no fruits, only samples...	Jingdong prefecture level city in Shandong
Dragon Quality Goods Shop	Green Intelligent Power Saver	Save a few dollars a month.	Jingdong prefecture level city in Shandong
Dragon Quality Goods Shop	Green Intelligent Power Saver	The quality is particularly good, it works well, and the seller has a good service attitude	Jingdong prefecture level city in Shandong
Dragon Quality Goods Shop	Green Intelligent Power Saver	I've compared them a couple of times and I don't feel any difference	Jingdong prefecture level city in Shandong
Dragon Quality Goods Shop	Green Intelligent Power Saver	I don't see the savings. It's a sham.	Jingdong prefecture level city in Shandong
Dragon Quality Goods Shop	Green Intelligent Power Saver	The power saving effect is very good, and the logistics is very fast. The seller's service attitude is very good	Jingdong prefecture level city in Shandong
Dragon Quality Goods Shop	Green Intelligent Power Saver	Received, well packaged, haven't tried it yet!	Jingdong prefecture level city in Shandong
Dragon Quality Goods Shop	Green Intelligent Power Saver	I don't know how it works, I'll comment after I use it.	Jingdong prefecture level city in Shandong
Dragon Quality Goods Shop	Green Intelligent Power Saver	The product doesn't work well enough to be of any use	Jingdong prefecture level city in Shandong
Dragon Quality Goods Shop	Green Intelligent Power Saver	On Trial 。。。。。。。	Jingdong prefecture level city in Shandong
Dragon Quality Goods Shop	Green Intelligent Power Saver	Being used with unknown results	Jingdong prefecture level city in Shandong
Dragon Quality Goods Shop	Green Intelligent Power Saver	Smart power saving and great quality	Jingdong prefecture level city in Shandong
Dragon Quality Goods Shop	Green Intelligent Power Saver	The saver was received, it works very well and the price is affordable!	Jingdong prefecture level city in Shandong
Dragon Quality Goods Shop	Green Intelligent Power Saver	How can I feel the power saving and how long does it last	Jingdong prefecture level city in Shandong
Dragon Quality Goods Shop	Green Intelligent Power Saver	And I know if it saves electricity or not.	Jingdong prefecture level city in Shandong
Dragon Quality Goods Shop	Green Intelligent Power Saver	It won't save any electricity. No.	Jingdong prefecture level city in Shandong
Dragon Quality Goods Shop	Green Intelligent Power Saver	Invalid. Fake.	Jingdong prefecture level city in Shandong
Dragon Quality Goods Shop	Green Intelligent Power Saver	It's good!	Jingdong prefecture level city in Shandong
Dragon Quality Goods Shop	Green Intelligent Power Saver	have no effect	Jingdong prefecture level city in Shandong
Dragon Quality Goods Shop	Green Intelligent Power Saver	Not used.	Jingdong prefecture level city in Shandong
Dragon Quality Goods Shop	Green Intelligent Power Saver	There is no load range, packaging thrift, ten days also did not develop invoices, received the goods with a receipt, hand-written receipt also has no financial stamp, communication for nearly a week a moment to say that the electronic invoice, a moment to say that the paper invoice, said it is mailed to me, ten days also did not see anything, the invoice invoice can not be found to say that did not stay on file, single single number can not be found, said that the warehouse more than one to a one to find out, in addition to the perfunctory or perfunctory. Products are no certificate of conformity, proper three	Jingdong prefecture level city in Shandong
Dragon Quality Goods Shop	Green Intelligent Power Saver	Satisfaction.	Jingdong prefecture level city in Shandong

Dragon Quality Goods Shop	Green Intelligent Power Saver	There is not a bit of power saving effect, and ringing up an increase in the electricity bill, because you have to add the cost of this machine's own electricity consumption and purchase, so I think it is better to use this machine instead of increasing the expenditure, uneconomical, rather than not use it.	Jingdong prefecture level city in Shandong
Dragon Quality Goods Shop	Green Intelligent Power Saver	I've been using it for two days now and I don't feel any effect.	Jingdong prefecture level city in Shandong
Dragon Quality Goods Shop	Green Intelligent Power Saver	What it looked like was nothing like what was sent.	Jingdong prefecture level city in Shandong
Dragon Quality Goods Shop	Green Intelligent Power Saver	It didn't work. It didn't work for a month or so.	Jingdong prefecture level city in Shandong
Dragon Quality Goods Shop	Green Intelligent Power Saver	Garbage products, physical and description is not the same, no effect at all, pit # no discussion, to buy the fine, garbage, garbage, garbage	Jingdong prefecture level city in Shandong
Dragon Quality Goods Shop	Green Intelligent Power Saver	general	Jingdong prefecture level city in Shandong
Dragon Quality Goods Shop	Green Intelligent Power Saver	IQ tax. Instead of saving electricity, it increases the power consumption of an appliance.	Jingdong prefecture level city in Shandong
Dragon Quality Goods Shop	Green Intelligent Power Saver	There's no trash at all.	Jingdong prefecture level city in Shandong
Dragon Quality Goods Shop	Green Intelligent Power Saver	It doesn't do anything.	Jingdong prefecture level city in Shandong
Dragon Quality Goods Shop	Green Intelligent Power Saver	(of an unmarried couple) be close	Jingdong prefecture level city in Shandong
Dragon Quality Goods Shop	Green Intelligent Power Saver	(of an unmarried couple) be close	Jingdong prefecture level city in Shandong
Dragon Quality Goods Shop	Green Intelligent Power Saver	no electricity saving	Jingdong prefecture level city in Shandong
Dragon Quality Goods Shop	Green Intelligent Power Saver	differ from	Jingdong prefecture level city in Shandong
Dragon Quality Goods Shop	Green Intelligent Power Saver	Saver has received, but real can save electricity, the quality is very good, I times I buy a few more, too good.	Jingdong prefecture level city in Shandong
Dragon Quality Goods Shop	Green Intelligent Power Saver	Great quality as always, a very favorite product, great! o(*￣︶￣*)o	Jingdong prefecture level city in Shandong
Dragon Quality Goods Shop	Green Intelligent Power Saver	If we can reduce the electricity bill, for individuals can save money, for the country can protect the environment, now the weather is getting hotter and hotter, to protect the environment to reduce emissions is very important. Already use the product, whether to reduce the electricity bill depends on next month!	Jingdong prefecture level city in Shandong
Dragon Quality Goods Shop	Green Intelligent Power Saver	Purchased a power saver received on the put into use, rent a room with many electrical appliances, power consumption is very large, we have a piece of electricity or 1.2 dollars a time, with this power saver is much better, every day can save a few cents, very convenient and practical!	Jingdong prefecture level city in Shandong
Dragon Quality Goods Shop	Green Intelligent Power Saver	The product is better to use before not to use when a month 1500 yuan electricity bill, now using the 5 second month electricity bill directly down 470 dollars or very good. A save money to buy several power saver money ZanZanZanZan👍	Jingdong prefecture level city in Shandong
Dragon Quality Goods Shop	Green Intelligent Power Saver	The moment I received the goods really satisfied with this product, not only high-grade packaging, the body are base alloy, up to the price, I was also introduced by a friend, he used last month did save a lot of electricity, but also recommended to me!	Jingdong prefecture level city in Shandong
Dragon Quality Goods Shop	Green Intelligent Power Saver	I got it. Good review. I'm very satisfied. Reasonable price.	Jingdong prefecture level city in Shandong
Dragon Quality Goods Shop	Green Intelligent Power Saver	Intelligent power saver received and used, looking at the quality is good, the quality of the plug sent is also very good. Expect the effect of the economizer is obvious, first five-star praise, with a period of time to follow up!	Jingdong prefecture level city in Shandong
Dragon Quality Goods Shop	Green Intelligent Power Saver	The saver has been received. It was plugged into an outlet and used immediately after receipt. Whether it really saves electricity is not clear yet, I believe that this brand of energy saver really saves	Jingdong prefecture level city in Shandong
Dragon Quality Goods Shop	Green Intelligent Power Saver	Okay, okay, okay. Thank you so much. Fast is good. Fast. Item was as described, mainly because the event was cheap and you can save a lot of money.	Jingdong prefecture level city in Shandong
Dragon Quality Goods Shop	Green Intelligent Power Saver	Received it a couple days ago! Plugged it in the same day! Let's see what the electric bill is in a month! Hopefully it works well oh!	Jingdong prefecture level city in Shandong
Dragon Quality Goods Shop	Green Intelligent Power Saver	Looking at the reviews good, energy-saving small products, compact, the results after 2 months to see, because the electricity bill season payment	Jingdong prefecture level city in Shandong
Dragon Quality Goods Shop	Green Intelligent Power Saver	I just received it and will try it out to see if it works well. I'll comment later.	Jingdong prefecture level city in Shandong
Dragon Quality Goods Shop	Green Intelligent Power Saver	It looks a little small and not very aesthetically pleasing, I don't know how effective it will be until I use it	Jingdong prefecture level city in Shandong
Dragon Quality Goods Shop	Green Intelligent Power Saver	Not only to the point of being useless yet	Jingdong prefecture level city in Shandong
Dragon Quality Goods Shop	Green Intelligent Power Saver	Used it for a month, nothing works, the meter you turn very fast. This is not a scam, but also sold on Jingdong. To cheat a bunch of people?	Jingdong prefecture level city in Shandong
Dragon Quality Goods Shop	Green Intelligent Power Saver	Very good to use, every day can save a little electricity, really good under the sample down a month to save a few dozen dollars in electricity, good before I did not know that there is this 👆 haha, the price is not expensive praise.	Jingdong prefecture level city in Shandong
Dragon Quality Goods Shop	Green Intelligent Power Saver	Tried it for ten days and the change is almost nothing, can't see the power saving yet, use it some more days and see how it goes.	Jingdong prefecture level city in Shandong
Dragon Quality Goods Shop	Green Intelligent Power Saver	Received very quickly, the quality is very good, really save electricity, business service attitude is also very good, I hope you do not miss this opportunity, I continue to buy a few in	Jingdong prefecture level city in Shandong
Dragon Quality Goods Shop	Green Intelligent Power Saver	Quality is very good Oh, the delivery is also very fast, a very favorite store, I want to crazy recommend to friends!	Jingdong prefecture level city in Shandong
Dragon Quality Goods Shop	Green Intelligent Power Saver	The goods have just received, fine workmanship, and the same as described by the merchant, to use a period of time to see if it can achieve the purpose of power saving.	Jingdong prefecture level city in Shandong
Dragon Quality Goods Shop	Green Intelligent Power Saver	Received the goods first use a period of time to see if there is no effect, the business commitment to ninety days invalid refund, first praise, good service!	Jingdong prefecture level city in Shandong
Dragon Quality Goods Shop	Green Intelligent Power Saver	The product is used for a month without energy-saving and power-saving effect does not have any effect	Jingdong prefecture level city in Shandong
Dragon Quality Goods Shop	Green Intelligent Power Saver	The baby is really great, I like it very much, my friends also say good to come, recommended to buy!	Jingdong prefecture level city in Shandong
Dragon Quality Goods Shop	Green Intelligent Power Saver	Logistics is fast, courier boy service is good, after using to see how the fruit, and then follow up.	Jingdong prefecture level city in Shandong
Dragon Quality Goods Shop	Green Intelligent Power Saver	Baby has been received to try, I believe that as the business advertised energy saving. The courier is very responsible.	Jingdong prefecture level city in Shandong
Dragon Quality Goods Shop	Green Intelligent Power Saver	Save Electric Energy King received, good quality, beautiful appearance, very satisfied!	Jingdong prefecture level city in Shandong
Dragon Quality Goods Shop	Green Intelligent Power Saver	Three no products no manufacturers no phone pure fraud induced consumption cheat	Jingdong prefecture level city in Shandong
Dragon Quality Goods Shop	Green Intelligent Power Saver	I'm using it now, I don't know if it's as good as it says, I'll follow up after a month	Jingdong prefecture level city in Shandong
Dragon Quality Goods Shop	Green Intelligent Power Saver	Good quality logistics and fast. Fine workmanship. The description is consistent cost-effective	Jingdong prefecture level city in Shandong
Dragon Quality Goods Shop	Green Intelligent Power Saver	Received, whether it is good or not, to wait a few days and then evaluate	Jingdong prefecture level city in Shandong
Dragon Quality Goods Shop	Green Intelligent Power Saver	I bought it and started using it, I won't know if it works well until I pay my electric bill	Jingdong prefecture level city in Shandong
Dragon Quality Goods Shop	Green Intelligent Power Saver	Especially good, energy saving and power saving, especially good quality, good	Jingdong prefecture level city in Shandong
Dragon Quality Goods Shop	Green Intelligent Power Saver	Logistics is strong, the quality is very good, paste really save electricity, as described, will buy back!	Jingdong prefecture level city in Shandong
Dragon Quality Goods Shop	Green Intelligent Power Saver	Great stuff at a great price, worth the money!	Jingdong prefecture level city in Shandong

Dragon Quality Goods Shop	Green Intelligent Power Saver	Received the power saver, very good quality, very power saving!	Jingdong prefecture level city in Shandong
Dragon Quality Goods Shop	Green Intelligent Power Saver	There are no fruits, only samples...	Jingdong prefecture level city in Shandong
Dragon Quality Goods Shop	Green Intelligent Power Saver	Save a few dollars a month.	Jingdong prefecture level city in Shandong
Dragon Quality Goods Shop	Green Intelligent Power Saver	The quality is particularly good, it works well, and the seller has a good service attitude	Jingdong prefecture level city in Shandong
Dragon Quality Goods Shop	Green Intelligent Power Saver	I've compared them a couple of times and I don't feel any difference	Jingdong prefecture level city in Shandong
Dragon Quality Goods Shop	Green Intelligent Power Saver	I don't see the savings. It's a sham.	Jingdong prefecture level city in Shandong
Dragon Quality Goods Shop	Green Intelligent Power Saver	The power saving effect is very good, and the logistics is very fast. The seller's service attitude is very good	Jingdong prefecture level city in Shandong
Dragon Quality Goods Shop	Green Intelligent Power Saver	Received, well packaged, haven't tried it yet!	Jingdong prefecture level city in Shandong
Dragon Quality Goods Shop	Green Intelligent Power Saver	I don't know how it works, I'll comment after I use it.	Jingdong prefecture level city in Shandong
Dragon Quality Goods Shop	Green Intelligent Power Saver	The product doesn't work well enough to be of any use	Jingdong prefecture level city in Shandong
Dragon Quality Goods Shop	Green Intelligent Power Saver	On Trial 。。。。。。。	Jingdong prefecture level city in Shandong
Dragon Quality Goods Shop	Green Intelligent Power Saver	Being used with unknown results	Jingdong prefecture level city in Shandong
Dragon Quality Goods Shop	Green Intelligent Power Saver	Smart power saving and great quality	Jingdong prefecture level city in Shandong
Dragon Quality Goods Shop	Green Intelligent Power Saver	The saver was received, it works very well and the price is affordable!	Jingdong prefecture level city in Shandong
Dragon Quality Goods Shop	Green Intelligent Power Saver	How can I feel the power saving and how long does it last	Jingdong prefecture level city in Shandong
Dragon Quality Goods Shop	Green Intelligent Power Saver	And I know if it saves electricity or not.	Jingdong prefecture level city in Shandong
Dragon Quality Goods Shop	Green Intelligent Power Saver	It won't save any electricity. No.	Jingdong prefecture level city in Shandong
Dragon Quality Goods Shop	Green Intelligent Power Saver	Invalid. Fake.	Jingdong prefecture level city in Shandong
Dragon Quality Goods Shop	Green Intelligent Power Saver	It's good!	Jingdong prefecture level city in Shandong
Dragon Quality Goods Shop	Green Intelligent Power Saver	have no effect	Jingdong prefecture level city in Shandong
Dragon Quality Goods Shop	Green Intelligent Power Saver	Not used.	Jingdong prefecture level city in Shandong
Dragon Quality Goods Shop	Green Intelligent Power Saver	There is no load range, packaging thrift, ten days also did not develop invoices, received the goods with a receipt, hand-written receipt also has no financial stamp, communication for nearly a week a moment to say that the electronic invoice, a moment to say that the paper invoice, said it is mailed to me, ten days also did not see anything, the invoice invoice can not be found to say that did not stay on file, single single number can not be found, said that the warehouse more than one to a one to find out, in addition to the perfunctory or perfunctory. Products are no certificate of conformity, proper three	Jingdong prefecture level city in Shandong
Dragon Quality Goods Shop	Green Intelligent Power Saver	Satisfaction.	Jingdong prefecture level city in Shandong
Dragon Quality Goods Shop	Green Intelligent Power Saver	There is not a bit of power saving effect, and ringing up an increase in the electricity bill, because you have to add the cost of this machine's own electricity consumption and purchase, so I think it is better to use this machine instead of increasing the expenditure, uneconomical, rather than not use it.	Jingdong prefecture level city in Shandong
Dragon Quality Goods Shop	Green Intelligent Power Saver	I've been using it for two days now and I don't feel any effect.	Jingdong prefecture level city in Shandong
Dragon Quality Goods Shop	Green Intelligent Power Saver	What it looked like was nothing like what was sent.	Jingdong prefecture level city in Shandong
Dragon Quality Goods Shop	Green Intelligent Power Saver	It didn't work. It didn't work for a month or so.	Jingdong prefecture level city in Shandong
Dragon Quality Goods Shop	Green Intelligent Power Saver	Garbage products, physical and description is not the same, no effect at all, pit # no discussion, to buy the fine, garbage, garbage, garbage	Jingdong prefecture level city in Shandong
Dragon Quality Goods Shop	Green Intelligent Power Saver	general	Jingdong prefecture level city in Shandong
Dragon Quality Goods Shop	Green Intelligent Power Saver	IQ tax. Instead of saving electricity, it increases the power consumption of an appliance.	Jingdong prefecture level city in Shandong
Dragon Quality Goods Shop	Green Intelligent Power Saver	There's no trash at all.	Jingdong prefecture level city in Shandong
Dragon Quality Goods Shop	Green Intelligent Power Saver	It doesn't do anything.	Jingdong prefecture level city in Shandong
Dragon Quality Goods Shop	Green Intelligent Power Saver	(of an unmarried couple) be close	Jingdong prefecture level city in Shandong
Dragon Quality Goods Shop	Green Intelligent Power Saver	(of an unmarried couple) be close	Jingdong prefecture level city in Shandong
Dragon Quality Goods Shop	Green Intelligent Power Saver	no electricity saving	Jingdong prefecture level city in Shandong
Dragon Quality Goods Shop	Green Intelligent Power Saver	differ from	Jingdong prefecture level city in Shandong
Dragon Quality Goods Shop	Green Intelligent Power Saver	Saver has received, but real can save electricity, the quality is very good, I times I buy a few more, too good.	Jingdong prefecture level city in Shandong
Dragon Quality Goods Shop	Green Intelligent Power Saver	Great quality as always, a very favorite product, great! o(*￣︶￣*)o	Jingdong prefecture level city in Shandong
Dragon Quality Goods Shop	Green Intelligent Power Saver	If we can reduce the electricity bill, for individuals can save money, for the country can protect the environment, now the weather is getting hotter and hotter, to protect the environment to reduce emissions is very important. Already use the product, whether to reduce the electricity bill depends on next month!	Jingdong prefecture level city in Shandong
Dragon Quality Goods Shop	Green Intelligent Power Saver	Purchased a power saver received on the put into use, rent a room with many electrical appliances, power consumption is very large, we have a piece of electricity or 1.2 dollars a time, with this power saver is much better, every day can save a few cents, very convenient and practical!	Jingdong prefecture level city in Shandong
Dragon Quality Goods Shop	Green Intelligent Power Saver	The product is better to use before not to use when a month 1500 yuan electricity bill, now using the 5 second month electricity bill directly down 470 dollars or very good. A save money to buy several power saver money ZanZanZanZan👍	Jingdong prefecture level city in Shandong
Dragon Quality Goods Shop	Green Intelligent Power Saver	The moment I received the goods really satisfied with this product, not only high-grade packaging, the body are base alloy, up to the price, I was also introduced by a friend, he used last month did save a lot of electricity, but also recommended to me!	Jingdong prefecture level city in Shandong
Dragon Quality Goods Shop	Green Intelligent Power Saver	I got it. Good review. I'm very satisfied. Reasonable price.	Jingdong prefecture level city in Shandong
Dragon Quality Goods Shop	Green Intelligent Power Saver	Intelligent power saver received and used, looking at the quality is good, the quality of the plug sent is also very good. Expect the effect of the economizer is obvious, first five-star praise, with a period of time to follow up!	Jingdong prefecture level city in Shandong
Dragon Quality Goods Shop	Green Intelligent Power Saver	The saver has been received. It was plugged into an outlet and used immediately after receipt. Whether it really saves electricity is not clear yet, I believe that this brand of energy saver really saves	Jingdong prefecture level city in Shandong
Dragon Quality Goods Shop	Green Intelligent Power Saver	Okay, okay, okay. Thank you so much. Fast is good. Fast. Item was as described, mainly because the event was cheap and you can save a lot of money.	Jingdong prefecture level city in Shandong

Dragon Quality Goods Shop	Green Intelligent Power Saver	Received it a couple days ago! Plugged it in the same day! Let's see what the electric bill is in a month! Hopefully it works well oh!	Jingdong prefecture level city in Shandong
Dragon Quality Goods Shop	Green Intelligent Power Saver	Looking at the reviews good, energy-saving small products, compact, the results after 2 months to see, because the electricity bill season payment	Jingdong prefecture level city in Shandong
Dragon Quality Goods Shop	Green Intelligent Power Saver	I just received it and will try it out to see if it works well. I'll comment later.	Jingdong prefecture level city in Shandong
Dragon Quality Goods Shop	Green Intelligent Power Saver	It looks a little small and not very aesthetically pleasing, I don't know how effective it will be until I use it	Jingdong prefecture level city in Shandong
Dragon Quality Goods Shop	Green Intelligent Power Saver	Not only to the point of being useless yet	Jingdong prefecture level city in Shandong
Dragon Quality Goods Shop	Green Intelligent Power Saver	Used it for a month, nothing works, the meter you turn very fast. This is not a scam, but also sold on Jingdong. To cheat a bunch of people?	Jingdong prefecture level city in Shandong
Dragon Quality Goods Shop	Green Intelligent Power Saver	Very good to use, every day can save a little electricity, really good under the sample down a month to save a few dozen dollars in electricity, good before I did not know that there is this haha, the price is not expensive praise.	Jingdong prefecture level city in Shandong
Dragon Quality Goods Shop	Green Intelligent Power Saver	Tried it for ten days and the change is almost nothing, can't see the power saving yet, use it some more days and see how it goes.	Jingdong prefecture level city in Shandong
Dragon Quality Goods Shop	Green Intelligent Power Saver	Received very quickly, the quality is very good, really save electricity, business service attitude is also very good, I hope you do not miss this opportunity, I continue to buy a few in	Jingdong prefecture level city in Shandong
Dragon Quality Goods Shop	Green Intelligent Power Saver	Quality is very good Oh, the delivery is also very fast, a very favorite store, I want to crazy recommend to friends!	Jingdong prefecture level city in Shandong
Dragon Quality Goods Shop	Green Intelligent Power Saver	The goods have just received, fine workmanship, and the same as described by the merchant, to use a period of time to see if it can achieve the purpose of power saving.	Jingdong prefecture level city in Shandong
Dragon Quality Goods Shop	Green Intelligent Power Saver	Received the goods first use a period of time to see if there is no effect, the business commitment to ninety days invalid refund, first praise, good service!	Jingdong prefecture level city in Shandong
Dragon Quality Goods Shop	Green Intelligent Power Saver	The product is used for a month without energy-saving and power-saving effect does not have any effect	Jingdong prefecture level city in Shandong
Dragon Quality Goods Shop	Green Intelligent Power Saver	The baby is really great, I like it very much, my friends also say good to come, recommended to buy!	Jingdong prefecture level city in Shandong
Dragon Quality Goods Shop	Green Intelligent Power Saver	Logistics is fast, courier boy service is good, after using to see how the fruit, and then follow up.	Jingdong prefecture level city in Shandong
Dragon Quality Goods Shop	Green Intelligent Power Saver	Baby has been received to try, I believe that as the business advertised energy saving. The courier is very responsible.	Jingdong prefecture level city in Shandong
Dragon Quality Goods Shop	Green Intelligent Power Saver	Save Electric Energy King received, good quality, beautiful appearance, very satisfied!	Jingdong prefecture level city in Shandong
Dragon Quality Goods Shop	Green Intelligent Power Saver	Three no products no manufacturers no phone pure fraud induced consumption cheat	Jingdong prefecture level city in Shandong
Dragon Quality Goods Shop	Green Intelligent Power Saver	I'm using it now, I don't know if it's as good as it says, I'll follow up after a month	Jingdong prefecture level city in Shandong
Dragon Quality Goods Shop	Green Intelligent Power Saver	Good quality logistics and fast. Fine workmanship. The description is consistent cost-effective	Jingdong prefecture level city in Shandong
Dragon Quality Goods Shop	Green Intelligent Power Saver	Received, whether it is good or not, to wait a few days and then evaluate	Jingdong prefecture level city in Shandong
Dragon Quality Goods Shop	Green Intelligent Power Saver	I bought it and started using it, I won't know if it works well until I pay my electric bill	Jingdong prefecture level city in Shandong
Dragon Quality Goods Shop	Green Intelligent Power Saver	Especially good, energy saving and power saving, especially good quality, good	Jingdong prefecture level city in Shandong
Dragon Quality Goods Shop	Green Intelligent Power Saver	Logistics is strong, the quality is very good, paste really save electricity, as described, will buy back!	Jingdong prefecture level city in Shandong
Dragon Quality Goods Shop	Green Intelligent Power Saver	Great stuff at a great price, worth the money!	Jingdong prefecture level city in Shandong
Dragon Quality Goods Shop	Green Intelligent Power Saver	Received the power saver, very good quality, very power saving!	Jingdong prefecture level city in Shandong
Dragon Quality Goods Shop	Green Intelligent Power Saver	There are no fruits, only samples...	Jingdong prefecture level city in Shandong
Dragon Quality Goods Shop	Green Intelligent Power Saver	Save a few dollars a month.	Jingdong prefecture level city in Shandong
Dragon Quality Goods Shop	Green Intelligent Power Saver	The quality is particularly good, it works well, and the seller has a good service attitude	Jingdong prefecture level city in Shandong
Dragon Quality Goods Shop	Green Intelligent Power Saver	I've compared them a couple of times and I don't feel any difference	Jingdong prefecture level city in Shandong
Dragon Quality Goods Shop	Green Intelligent Power Saver	I don't see the savings. It's a sham.	Jingdong prefecture level city in Shandong
Dragon Quality Goods Shop	Green Intelligent Power Saver	The power saving effect is very good, and the logistics is very fast. The seller's service attitude is very good	Jingdong prefecture level city in Shandong
Dragon Quality Goods Shop	Green Intelligent Power Saver	Received, well packaged, haven't tried it yet!	Jingdong prefecture level city in Shandong
Dragon Quality Goods Shop	Green Intelligent Power Saver	I don't know how it works, I'll comment after I use it.	Jingdong prefecture level city in Shandong
Dragon Quality Goods Shop	Green Intelligent Power Saver	The product doesn't work well enough to be of any use	Jingdong prefecture level city in Shandong
Dragon Quality Goods Shop	Green Intelligent Power Saver	On Trial 。。。。。。。	Jingdong prefecture level city in Shandong
Dragon Quality Goods Shop	Green Intelligent Power Saver	Being used with unknown results	Jingdong prefecture level city in Shandong
Dragon Quality Goods Shop	Green Intelligent Power Saver	Smart power saving and great quality	Jingdong prefecture level city in Shandong
Dragon Quality Goods Shop	Green Intelligent Power Saver	The saver was received, it works very well and the price is affordable!	Jingdong prefecture level city in Shandong
Dragon Quality Goods Shop	Green Intelligent Power Saver	How can I feel the power saving and how long does it last	Jingdong prefecture level city in Shandong
Dragon Quality Goods Shop	Green Intelligent Power Saver	And I know if it saves electricity or not.	Jingdong prefecture level city in Shandong
Dragon Quality Goods Shop	Green Intelligent Power Saver	It won't save any electricity. No.	Jingdong prefecture level city in Shandong
Dragon Quality Goods Shop	Green Intelligent Power Saver	Invalid. Fake.	Jingdong prefecture level city in Shandong
Dragon Quality Goods Shop	Green Intelligent Power Saver	It's good!	Jingdong prefecture level city in Shandong
Dragon Quality Goods Shop	Green Intelligent Power Saver	have no effect	Jingdong prefecture level city in Shandong
Dragon Quality Goods Shop	Green Intelligent Power Saver	Not used.	Jingdong prefecture level city in Shandong
Dragon Quality Goods Shop	Green Intelligent Power Saver	There is no load range, packaging thrift, ten days also did not develop invoices, received the goods with a receipt, hand-written receipt also has no financial stamp, communication for nearly a week a moment to say that the electronic invoice, a moment to say that the paper invoice, said it is mailed to me, ten days also did not see anything, the invoice invoice can not be found to say that did not stay on file, single single number can not be found, said that the warehouse more than one to a one to find out, in addition to the perfunctory or perfunctory. Products are no certificate of conformity, proper three	Jingdong prefecture level city in Shandong
Dragon Quality Goods Shop	Green Intelligent Power Saver	Satisfaction.	Jingdong prefecture level city in Shandong

Dragon Quality Goods Shop	Green Intelligent Power Saver	There is not a bit of power saving effect, and ringing up an increase in the electricity bill, because you have to add the cost of this machine's own electricity consumption and purchase, so I think it is better to use this machine instead of increasing the expenditure, uneconomical, rather than not use it.	Jingdong prefecture level city in Shandong
Dragon Quality Goods Shop	Green Intelligent Power Saver	I've been using it for two days now and I don't feel any effect.	Jingdong prefecture level city in Shandong
Dragon Quality Goods Shop	Green Intelligent Power Saver	What it looked like was nothing like what was sent.	Jingdong prefecture level city in Shandong
Dragon Quality Goods Shop	Green Intelligent Power Saver	It didn't work. It didn't work for a month or so.	Jingdong prefecture level city in Shandong
Dragon Quality Goods Shop	Green Intelligent Power Saver	Garbage products, physical and description is not the same, no effect at all, pit # no discussion, to buy the fine, garbage, garbage, garbage	Jingdong prefecture level city in Shandong
Dragon Quality Goods Shop	Green Intelligent Power Saver	general	Jingdong prefecture level city in Shandong
Dragon Quality Goods Shop	Green Intelligent Power Saver	IQ tax. Instead of saving electricity, it increases the power consumption of an appliance.	Jingdong prefecture level city in Shandong
Dragon Quality Goods Shop	Green Intelligent Power Saver	There's no trash at all.	Jingdong prefecture level city in Shandong
Dragon Quality Goods Shop	Green Intelligent Power Saver	It doesn't do anything.	Jingdong prefecture level city in Shandong
Dragon Quality Goods Shop	Green Intelligent Power Saver	(of an unmarried couple) be close	Jingdong prefecture level city in Shandong
Dragon Quality Goods Shop	Green Intelligent Power Saver	(of an unmarried couple) be close	Jingdong prefecture level city in Shandong
Dragon Quality Goods Shop	Green Intelligent Power Saver	no electricity saving	Jingdong prefecture level city in Shandong
Dragon Quality Goods Shop	Green Intelligent Power Saver	differ from	Jingdong prefecture level city in Shandong
Dragon Quality Goods Shop	Green Intelligent Power Saver	Saver has received, but real can save electricity, the quality is very good, I times I buy a few more, too good.	Jingdong prefecture level city in Shandong
Dragon Quality Goods Shop	Green Intelligent Power Saver	Great quality as always, a very favorite product, great! o(*￣︶￣*)o	Jingdong prefecture level city in Shandong
Dragon Quality Goods Shop	Green Intelligent Power Saver	If we can reduce the electricity bill, for individuals can save money, for the country can protect the environment, now the weather is getting hotter and hotter, to protect the environment to reduce emissions is very important. Already use the product, whether to reduce the electricity bill depends on next month!	Jingdong prefecture level city in Shandong
Dragon Quality Goods Shop	Green Intelligent Power Saver	Purchased a power saver received on the put into use, rent a room with many electrical appliances, power consumption is very large, we have a piece of electricity or 1.2 dollars a time, with this power saver is much better, every day can save a few cents, very convenient and practical!	Jingdong prefecture level city in Shandong
Dragon Quality Goods Shop	Green Intelligent Power Saver	The product is better to use before not to use when a month 1500 yuan electricity bill, now using the 5 second month electricity bill directly down 470 dollars or very good. A save money to buy several power saver money ZanZanZanZan👍	Jingdong prefecture level city in Shandong
Dragon Quality Goods Shop	Green Intelligent Power Saver	The moment I received the goods really satisfied with this product, not only high-grade packaging, the body are base alloy, up to the price, I was also introduced by a friend, he used last month did save a lot of electricity, but also recommended to me!	Jingdong prefecture level city in Shandong
Dragon Quality Goods Shop	Green Intelligent Power Saver	I got it. Good review. I'm very satisfied. Reasonable price.	Jingdong prefecture level city in Shandong
Dragon Quality Goods Shop	Green Intelligent Power Saver	Intelligent power saver received and used, looking at the quality is good, the quality of the plug sent is also very good. Expect the effect of the economizer is obvious, first five-star praise, with a period of time to follow up!	Jingdong prefecture level city in Shandong
Dragon Quality Goods Shop	Green Intelligent Power Saver	The saver has been received. It was plugged into an outlet and used immediately after receipt. Whether it really saves electricity is not clear yet, I believe that this brand of energy saver really saves	Jingdong prefecture level city in Shandong
Dragon Quality Goods Shop	Green Intelligent Power Saver	Okay, okay, okay. Thank you so much. Fast is good. Fast. Item was as described, mainly because the event was cheap and you can save a lot of money.	Jingdong prefecture level city in Shandong
Dragon Quality Goods Shop	Green Intelligent Power Saver	Received it a couple days ago! Plugged it in the same day! Let's see what the electric bill is in a month! Hopefully it works well oh!	Jingdong prefecture level city in Shandong
Dragon Quality Goods Shop	Green Intelligent Power Saver	Looking at the reviews good, energy-saving small products, compact, the results after 2 months to see, because the electricity bill season payment	Jingdong prefecture level city in Shandong
Dragon Quality Goods Shop	Green Intelligent Power Saver	I just received it and will try it out to see if it works well. I'll comment later.	Jingdong prefecture level city in Shandong
Dragon Quality Goods Shop	Green Intelligent Power Saver	It looks a little small and not very aesthetically pleasing, I don't know how effective it will be until I use it	Jingdong prefecture level city in Shandong
Dragon Quality Goods Shop	Green Intelligent Power Saver	Not only to the point of being useless yet	Jingdong prefecture level city in Shandong
Dragon Quality Goods Shop	Green Intelligent Power Saver	Used it for a month, nothing works, the meter you turn very fast. This is not a scam, but also sold on Jingdong. To cheat a bunch of people?	Jingdong prefecture level city in Shandong
Dragon Quality Goods Shop	Green Intelligent Power Saver	Very good to use, every day can save a little electricity, really good under the sample down a month to save a few dozen dollars in electricity, good before I did not know that there is this 👆 haha, the price is not expensive praise.	Jingdong prefecture level city in Shandong
Dragon Quality Goods Shop	Green Intelligent Power Saver	Tried it for ten days and the change is almost nothing, can't see the power saving yet, use it some more days and see how it goes.	Jingdong prefecture level city in Shandong
Dragon Quality Goods Shop	Green Intelligent Power Saver	Received very quickly, the quality is very good, really save electricity, business service attitude is also very good, I hope you do not miss this opportunity, I continue to buy a few in	Jingdong prefecture level city in Shandong
Dragon Quality Goods Shop	Green Intelligent Power Saver	Quality is very good Oh, the delivery is also very fast, a very favorite store, I want to crazy recommend to friends!	Jingdong prefecture level city in Shandong
Dragon Quality Goods Shop	Green Intelligent Power Saver	The goods have just received, fine workmanship, and the same as described by the merchant, to use a period of time to see if it can achieve the purpose of power saving.	Jingdong prefecture level city in Shandong
Dragon Quality Goods Shop	Green Intelligent Power Saver	Received the goods first use a period of time to see if there is no effect, the business commitment to ninety days invalid refund, first praise, good service!	Jingdong prefecture level city in Shandong
Dragon Quality Goods Shop	Green Intelligent Power Saver	The product is used for a month without energy-saving and power-saving effect does not have any effect	Jingdong prefecture level city in Shandong
Dragon Quality Goods Shop	Green Intelligent Power Saver	The baby is really great, I like it very much, my friends also say good to come, recommended to buy!	Jingdong prefecture level city in Shandong
Dragon Quality Goods Shop	Green Intelligent Power Saver	Logistics is fast, courier boy service is good, after using to see how the fruit, and then follow up.	Jingdong prefecture level city in Shandong
Dragon Quality Goods Shop	Green Intelligent Power Saver	Baby has been received to try, I believe that as the business advertised energy saving. The courier is very responsible.	Jingdong prefecture level city in Shandong
Dragon Quality Goods Shop	Green Intelligent Power Saver	Save Electric Energy King received, good quality, beautiful appearance, very satisfied!	Jingdong prefecture level city in Shandong
Dragon Quality Goods Shop	Green Intelligent Power Saver	Three no products no manufacturers no phone pure fraud induced consumption cheat	Jingdong prefecture level city in Shandong
Dragon Quality Goods Shop	Green Intelligent Power Saver	I'm using it now, I don't know if it's as good as it says, I'll follow up after a month	Jingdong prefecture level city in Shandong
Dragon Quality Goods Shop	Green Intelligent Power Saver	Good quality logistics and fast. Fine workmanship. The description is consistent cost-effective	Jingdong prefecture level city in Shandong
Dragon Quality Goods Shop	Green Intelligent Power Saver	Received, whether it is good or not, to wait a few days and then evaluate	Jingdong prefecture level city in Shandong
Dragon Quality Goods Shop	Green Intelligent Power Saver	I bought it and started using it, I won't know if it works well until I pay my electric bill	Jingdong prefecture level city in Shandong
Dragon Quality Goods Shop	Green Intelligent Power Saver	Especially good, energy saving and power saving, especially good quality, good	Jingdong prefecture level city in Shandong
Dragon Quality Goods Shop	Green Intelligent Power Saver	Logistics is strong, the quality is very good, paste really save electricity, as described, will buy back!	Jingdong prefecture level city in Shandong
Dragon Quality Goods Shop	Green Intelligent Power Saver	Great stuff at a great price, worth the money!	Jingdong prefecture level city in Shandong

Dragon Quality Goods Shop	Green Intelligent Power Saver	Received the power saver, very good quality, very power saving!	Jingdong prefecture level city in Shandong
Dragon Quality Goods Shop	Green Intelligent Power Saver	There are no fruits, only samples...	Jingdong prefecture level city in Shandong
Dragon Quality Goods Shop	Green Intelligent Power Saver	Save a few dollars a month.	Jingdong prefecture level city in Shandong
Dragon Quality Goods Shop	Green Intelligent Power Saver	The quality is particularly good, it works well, and the seller has a good service attitude	Jingdong prefecture level city in Shandong
Dragon Quality Goods Shop	Green Intelligent Power Saver	I've compared them a couple of times and I don't feel any difference	Jingdong prefecture level city in Shandong
Dragon Quality Goods Shop	Green Intelligent Power Saver	I don't see the savings. It's a sham.	Jingdong prefecture level city in Shandong
Dragon Quality Goods Shop	Green Intelligent Power Saver	The power saving effect is very good, and the logistics is very fast. The seller's service attitude is very good	Jingdong prefecture level city in Shandong
Dragon Quality Goods Shop	Green Intelligent Power Saver	Received, well packaged, haven't tried it yet!	Jingdong prefecture level city in Shandong
Dragon Quality Goods Shop	Green Intelligent Power Saver	I don't know how it works, I'll comment after I use it.	Jingdong prefecture level city in Shandong
Dragon Quality Goods Shop	Green Intelligent Power Saver	The product doesn't work well enough to be of any use	Jingdong prefecture level city in Shandong
Dragon Quality Goods Shop	Green Intelligent Power Saver	On Trial 。。。。。。。	Jingdong prefecture level city in Shandong
Dragon Quality Goods Shop	Green Intelligent Power Saver	Being used with unknown results	Jingdong prefecture level city in Shandong
Dragon Quality Goods Shop	Green Intelligent Power Saver	Smart power saving and great quality	Jingdong prefecture level city in Shandong
Dragon Quality Goods Shop	Green Intelligent Power Saver	The saver was received, it works very well and the price is affordable!	Jingdong prefecture level city in Shandong
Dragon Quality Goods Shop	Green Intelligent Power Saver	How can I feel the power saving and how long does it last	Jingdong prefecture level city in Shandong
Dragon Quality Goods Shop	Green Intelligent Power Saver	And I know if it saves electricity or not.	Jingdong prefecture level city in Shandong
Dragon Quality Goods Shop	Green Intelligent Power Saver	It won't save any electricity. No.	Jingdong prefecture level city in Shandong
Dragon Quality Goods Shop	Green Intelligent Power Saver	Invalid. Fake.	Jingdong prefecture level city in Shandong
Dragon Quality Goods Shop	Green Intelligent Power Saver	It's good!	Jingdong prefecture level city in Shandong
Dragon Quality Goods Shop	Green Intelligent Power Saver	have no effect	Jingdong prefecture level city in Shandong
Dragon Quality Goods Shop	Green Intelligent Power Saver	Not used.	Jingdong prefecture level city in Shandong
Dragon Quality Goods Shop	Green Intelligent Power Saver	There is no load range, packaging thrift, ten days also did not develop invoices, received the goods with a receipt, hand-written receipt also has no financial stamp, communication for nearly a week a moment to say that the electronic invoice, a moment to say that the paper invoice, said it is mailed to me, ten days also did not see anything, the invoice invoice can not be found to say that did not stay on file, single single number can not be found, said that the warehouse more than one to a one to find out, in addition to the perfunctory or perfunctory. Products are no certificate of conformity, proper three	Jingdong prefecture level city in Shandong
Dragon Quality Goods Shop	Green Intelligent Power Saver	Satisfaction.	Jingdong prefecture level city in Shandong
Dragon Quality Goods Shop	Green Intelligent Power Saver	There is not a bit of power saving effect, and ringing up an increase in the electricity bill, because you have to add the cost of this machine's own electricity consumption and purchase, so I think it is better to use this machine instead of increasing the expenditure, uneconomical, rather than not use it.	Jingdong prefecture level city in Shandong
Dragon Quality Goods Shop	Green Intelligent Power Saver	I've been using it for two days now and I don't feel any effect.	Jingdong prefecture level city in Shandong
Dragon Quality Goods Shop	Green Intelligent Power Saver	What it looked like was nothing like what was sent.	Jingdong prefecture level city in Shandong
Dragon Quality Goods Shop	Green Intelligent Power Saver	It didn't work. It didn't work for a month or so.	Jingdong prefecture level city in Shandong
Dragon Quality Goods Shop	Green Intelligent Power Saver	Garbage products, physical and description is not the same, no effect at all, pit # no discussion, to buy the fine, garbage, garbage, garbage	Jingdong prefecture level city in Shandong
Dragon Quality Goods Shop	Green Intelligent Power Saver	general	Jingdong prefecture level city in Shandong
Dragon Quality Goods Shop	Green Intelligent Power Saver	IQ tax. Instead of saving electricity, it increases the power consumption of an appliance.	Jingdong prefecture level city in Shandong
Dragon Quality Goods Shop	Green Intelligent Power Saver	There's no trash at all.	Jingdong prefecture level city in Shandong
Dragon Quality Goods Shop	Green Intelligent Power Saver	It doesn't do anything.	Jingdong prefecture level city in Shandong
Dragon Quality Goods Shop	Green Intelligent Power Saver	(of an unmarried couple) be close	Jingdong prefecture level city in Shandong
Dragon Quality Goods Shop	Green Intelligent Power Saver	(of an unmarried couple) be close	Jingdong prefecture level city in Shandong
Dragon Quality Goods Shop	Green Intelligent Power Saver	no electricity saving	Jingdong prefecture level city in Shandong
Dragon Quality Goods Shop	Green Intelligent Power Saver	differ from	Jingdong prefecture level city in Shandong
Dragon Quality Goods Shop	Green Intelligent Power Saver	Saver has received, but real can save electricity, the quality is very good, I times I buy a few more, too good.	Jingdong prefecture level city in Shandong
Dragon Quality Goods Shop	Green Intelligent Power Saver	Great quality as always, a very favorite product, great! o(*￣︶￣*)o	Jingdong prefecture level city in Shandong
Dragon Quality Goods Shop	Green Intelligent Power Saver	If we can reduce the electricity bill, for individuals can save money, for the country can protect the environment, now the weather is getting hotter and hotter, to protect the environment to reduce emissions is very important. Already use the product, whether to reduce the electricity bill depends on next month!	Jingdong prefecture level city in Shandong
Dragon Quality Goods Shop	Green Intelligent Power Saver	Purchased a power saver received on the put into use, rent a room with many electrical appliances, power consumption is very large, we have a piece of electricity or 1.2 dollars a time, with this power saver is much better, every day can save a few cents, very convenient and practical!	Jingdong prefecture level city in Shandong
Dragon Quality Goods Shop	Green Intelligent Power Saver	The product is better to use before not to use when a month 1500 yuan electricity bill, now using the 5 second month electricity bill directly down 470 dollars or very good. A save money to buy several power saver money ZanZanZanZan👍	Jingdong prefecture level city in Shandong
Dragon Quality Goods Shop	Green Intelligent Power Saver	The moment I received the goods really satisfied with this product, not only high-grade packaging, the body are base alloy, up to the price, I was also introduced by a friend, he used last month did save a lot of electricity, but also recommended to me!	Jingdong prefecture level city in Shandong
Dragon Quality Goods Shop	Green Intelligent Power Saver	I got it. Good review. I'm very satisfied. Reasonable price.	Jingdong prefecture level city in Shandong
Dragon Quality Goods Shop	Green Intelligent Power Saver	Intelligent power saver received and used, looking at the quality is good, the quality of the plug sent is also very good. Expect the effect of the economizer is obvious, first five-star praise, with a period of time to follow up!	Jingdong prefecture level city in Shandong
Dragon Quality Goods Shop	Green Intelligent Power Saver	The saver has been received. It was plugged into an outlet and used immediately after receipt. Whether it really saves electricity is not clear yet, I believe that this brand of energy saver really saves	Jingdong prefecture level city in Shandong
Dragon Quality Goods Shop	Green Intelligent Power Saver	Okay, okay, okay. Thank you so much. Fast is good. Fast. Item was as described, mainly because the event was cheap and you can save a lot of money.	Jingdong prefecture level city in Shandong

Dragon Quality Goods Shop	Green Intelligent Power Saver	Received it a couple days ago! Plugged it in the same day! Let's see what the electric bill is in a month! Hopefully it works well oh!	Jingdong prefecture level city in Shandong
Dragon Quality Goods Shop	Green Intelligent Power Saver	Looking at the reviews good, energy-saving small products, compact, the results after 2 months to see, because the electricity bill season payment	Jingdong prefecture level city in Shandong
Dragon Quality Goods Shop	Green Intelligent Power Saver	I just received it and will try it out to see if it works well. I'll comment later.	Jingdong prefecture level city in Shandong
Dragon Quality Goods Shop	Green Intelligent Power Saver	It looks a little small and not very aesthetically pleasing, I don't know how effective it will be until I use it	Jingdong prefecture level city in Shandong
Dragon Quality Goods Shop	Green Intelligent Power Saver	Not only to the point of being useless yet	Jingdong prefecture level city in Shandong
Dragon Quality Goods Shop	Green Intelligent Power Saver	Used it for a month, nothing works, the meter you turn very fast. This is not a scam, but also sold on Jingdong. To cheat a bunch of people?	Jingdong prefecture level city in Shandong
Dragon Quality Goods Shop	Green Intelligent Power Saver	Very good to use, every day can save a little electricity, really good under the sample down a month to save a few dozen dollars in electricity, good before I did not know that there is this haha, the price is not expensive praise.	Jingdong prefecture level city in Shandong
Dragon Quality Goods Shop	Green Intelligent Power Saver	Tried it for ten days and the change is almost nothing, can't see the power saving yet, use it some more days and see how it goes.	Jingdong prefecture level city in Shandong
Dragon Quality Goods Shop	Green Intelligent Power Saver	Received very quickly, the quality is very good, really save electricity, business service attitude is also very good, I hope you do not miss this opportunity, I continue to buy a few in	Jingdong prefecture level city in Shandong
Dragon Quality Goods Shop	Green Intelligent Power Saver	Quality is very good Oh, the delivery is also very fast, a very favorite store, I want to crazy recommend to friends!	Jingdong prefecture level city in Shandong
Dragon Quality Goods Shop	Green Intelligent Power Saver	The goods have just received, fine workmanship, and the same as described by the merchant, to use a period of time to see if it can achieve the purpose of power saving.	Jingdong prefecture level city in Shandong
Dragon Quality Goods Shop	Green Intelligent Power Saver	Received the goods first use a period of time to see if there is no effect, the business commitment to ninety days invalid refund, first praise, good service!	Jingdong prefecture level city in Shandong
Dragon Quality Goods Shop	Green Intelligent Power Saver	The product is used for a month without energy-saving and power-saving effect does not have any effect	Jingdong prefecture level city in Shandong
Dragon Quality Goods Shop	Green Intelligent Power Saver	The baby is really great, I like it very much, my friends also say good to come, recommended to buy!	Jingdong prefecture level city in Shandong
Dragon Quality Goods Shop	Green Intelligent Power Saver	Logistics is fast, courier boy service is good, after using to see how the fruit, and then follow up.	Jingdong prefecture level city in Shandong
Dragon Quality Goods Shop	Green Intelligent Power Saver	Baby has been received to try, I believe that as the business advertised energy saving. The courier is very responsible.	Jingdong prefecture level city in Shandong
Dragon Quality Goods Shop	Green Intelligent Power Saver	Save Electric Energy King received, good quality, beautiful appearance, very satisfied!	Jingdong prefecture level city in Shandong
Dragon Quality Goods Shop	Green Intelligent Power Saver	Three no products no manufacturers no phone pure fraud induced consumption cheat	Jingdong prefecture level city in Shandong
Dragon Quality Goods Shop	Green Intelligent Power Saver	I'm using it now, I don't know if it's as good as it says, I'll follow up after a month	Jingdong prefecture level city in Shandong
Dragon Quality Goods Shop	Green Intelligent Power Saver	Good quality logistics and fast. Fine workmanship. The description is consistent cost-effective	Jingdong prefecture level city in Shandong
Dragon Quality Goods Shop	Green Intelligent Power Saver	Received, whether it is good or not, to wait a few days and then evaluate	Jingdong prefecture level city in Shandong
Dragon Quality Goods Shop	Green Intelligent Power Saver	I bought it and started using it, I won't know if it works well until I pay my electric bill	Jingdong prefecture level city in Shandong
Dragon Quality Goods Shop	Green Intelligent Power Saver	Especially good, energy saving and power saving, especially good quality, good	Jingdong prefecture level city in Shandong
Dragon Quality Goods Shop	Green Intelligent Power Saver	Logistics is strong, the quality is very good, paste really save electricity, as described, will buy back!	Jingdong prefecture level city in Shandong
Dragon Quality Goods Shop	Green Intelligent Power Saver	Great stuff at a great price, worth the money!	Jingdong prefecture level city in Shandong
Dragon Quality Goods Shop	Green Intelligent Power Saver	Received the power saver, very good quality, very power saving!	Jingdong prefecture level city in Shandong
Dragon Quality Goods Shop	Green Intelligent Power Saver	There are no fruits, only samples...	Jingdong prefecture level city in Shandong
Dragon Quality Goods Shop	Green Intelligent Power Saver	Save a few dollars a month.	Jingdong prefecture level city in Shandong
Dragon Quality Goods Shop	Green Intelligent Power Saver	The quality is particularly good, it works well, and the seller has a good service attitude	Jingdong prefecture level city in Shandong
Dragon Quality Goods Shop	Green Intelligent Power Saver	I've compared them a couple of times and I don't feel any difference	Jingdong prefecture level city in Shandong
Dragon Quality Goods Shop	Green Intelligent Power Saver	I don't see the savings. It's a sham.	Jingdong prefecture level city in Shandong
Dragon Quality Goods Shop	Green Intelligent Power Saver	The power saving effect is very good, and the logistics is very fast. The seller's service attitude is very good	Jingdong prefecture level city in Shandong
Dragon Quality Goods Shop	Green Intelligent Power Saver	Received, well packaged, haven't tried it yet!	Jingdong prefecture level city in Shandong
Dragon Quality Goods Shop	Green Intelligent Power Saver	I don't know how it works, I'll comment after I use it.	Jingdong prefecture level city in Shandong
Dragon Quality Goods Shop	Green Intelligent Power Saver	The product doesn't work well enough to be of any use	Jingdong prefecture level city in Shandong
Dragon Quality Goods Shop	Green Intelligent Power Saver	On Trial 。。。。。。。	Jingdong prefecture level city in Shandong
Dragon Quality Goods Shop	Green Intelligent Power Saver	Being used with unknown results	Jingdong prefecture level city in Shandong
Dragon Quality Goods Shop	Green Intelligent Power Saver	Smart power saving and great quality	Jingdong prefecture level city in Shandong
Dragon Quality Goods Shop	Green Intelligent Power Saver	The saver was received, it works very well and the price is affordable!	Jingdong prefecture level city in Shandong
Dragon Quality Goods Shop	Green Intelligent Power Saver	How can I feel the power saving and how long does it last	Jingdong prefecture level city in Shandong
Dragon Quality Goods Shop	Green Intelligent Power Saver	And I know if it saves electricity or not.	Jingdong prefecture level city in Shandong
Dragon Quality Goods Shop	Green Intelligent Power Saver	It won't save any electricity. No.	Jingdong prefecture level city in Shandong
Dragon Quality Goods Shop	Green Intelligent Power Saver	Invalid. Fake.	Jingdong prefecture level city in Shandong
Dragon Quality Goods Shop	Green Intelligent Power Saver	It's good!	Jingdong prefecture level city in Shandong
Dragon Quality Goods Shop	Green Intelligent Power Saver	have no effect	Jingdong prefecture level city in Shandong
Dragon Quality Goods Shop	Green Intelligent Power Saver	Not used.	Jingdong prefecture level city in Shandong
Dragon Quality Goods Shop	Green Intelligent Power Saver	There is no load range, packaging thrift, ten days also did not develop invoices, received the goods with a receipt, hand-written receipt also has no financial stamp, communication for nearly a week a moment to say that the electronic invoice, a moment to say that the paper invoice, said it is mailed to me, ten days also did not see anything, the invoice invoice can not be found to say that did not stay on file, single single number can not be found, said that the warehouse more than one to a one to find out, in addition to the perfunctory or perfunctory. Products are no certificate of conformity, proper three	Jingdong prefecture level city in Shandong
Dragon Quality Goods Shop	Green Intelligent Power Saver	Satisfaction.	Jingdong prefecture level city in Shandong

Dragon Quality Goods Shop	Green Intelligent Power Saver	There is not a bit of power saving effect, and ringing up an increase in the electricity bill, because you have to add the cost of this machine's own electricity consumption and purchase, so I think it is better to use this machine instead of increasing the expenditure, uneconomical, rather than not use it.	Jingdong prefecture level city in Shandong
Dragon Quality Goods Shop	Green Intelligent Power Saver	I've been using it for two days now and I don't feel any effect.	Jingdong prefecture level city in Shandong
Dragon Quality Goods Shop	Green Intelligent Power Saver	What it looked like was nothing like what was sent.	Jingdong prefecture level city in Shandong
Dragon Quality Goods Shop	Green Intelligent Power Saver	It didn't work. It didn't work for a month or so.	Jingdong prefecture level city in Shandong
Dragon Quality Goods Shop	Green Intelligent Power Saver	Garbage products, physical and description is not the same, no effect at all, pit # no discussion, to buy the fine, garbage, garbage, garbage	Jingdong prefecture level city in Shandong
Dragon Quality Goods Shop	Green Intelligent Power Saver	general	Jingdong prefecture level city in Shandong
Dragon Quality Goods Shop	Green Intelligent Power Saver	IQ tax. Instead of saving electricity, it increases the power consumption of an appliance.	Jingdong prefecture level city in Shandong
Dragon Quality Goods Shop	Green Intelligent Power Saver	There's no trash at all.	Jingdong prefecture level city in Shandong
Dragon Quality Goods Shop	Green Intelligent Power Saver	It doesn't do anything.	Jingdong prefecture level city in Shandong
Dragon Quality Goods Shop	Green Intelligent Power Saver	(of an unmarried couple) be close	Jingdong prefecture level city in Shandong
Dragon Quality Goods Shop	Green Intelligent Power Saver	(of an unmarried couple) be close	Jingdong prefecture level city in Shandong
Dragon Quality Goods Shop	Green Intelligent Power Saver	no electricity saving	Jingdong prefecture level city in Shandong
Dragon Quality Goods Shop	Green Intelligent Power Saver	differ from	Jingdong prefecture level city in Shandong
Dragon Quality Goods Shop	Green Intelligent Power Saver	Saver has received, but real can save electricity, the quality is very good, I times I buy a few more, too good.	Jingdong prefecture level city in Shandong
Dragon Quality Goods Shop	Green Intelligent Power Saver	Great quality as always, a very favorite product, great! o(*￣︶￣*)o	Jingdong prefecture level city in Shandong
Dragon Quality Goods Shop	Green Intelligent Power Saver	If we can reduce the electricity bill, for individuals can save money, for the country can protect the environment, now the weather is getting hotter and hotter, to protect the environment to reduce emissions is very important. Already use the product, whether to reduce the electricity bill depends on next month!	Jingdong prefecture level city in Shandong
Dragon Quality Goods Shop	Green Intelligent Power Saver	Purchased a power saver received on the put into use, rent a room with many electrical appliances, power consumption is very large, we have a piece of electricity or 1.2 dollars a time, with this power saver is much better, every day can save a few cents, very convenient and practical!	Jingdong prefecture level city in Shandong
Dragon Quality Goods Shop	Green Intelligent Power Saver	The product is better to use before not to use when a month 1500 yuan electricity bill, now using the 5 second month electricity bill directly down 470 dollars or very good. A save money to buy several power saver money ZanZanZanZan👍	Jingdong prefecture level city in Shandong
Dragon Quality Goods Shop	Green Intelligent Power Saver	The moment I received the goods really satisfied with this product, not only high-grade packaging, the body are base alloy, up to the price, I was also introduced by a friend, he used last month did save a lot of electricity, but also recommended to me!	Jingdong prefecture level city in Shandong
Dragon Quality Goods Shop	Green Intelligent Power Saver	I got it. Good review. I'm very satisfied. Reasonable price.	Jingdong prefecture level city in Shandong
Dragon Quality Goods Shop	Green Intelligent Power Saver	Intelligent power saver received and used, looking at the quality is good, the quality of the plug sent is also very good. Expect the effect of the economizer is obvious, first five-star praise, with a period of time to follow up!	Jingdong prefecture level city in Shandong
Dragon Quality Goods Shop	Green Intelligent Power Saver	The saver has been received. It was plugged into an outlet and used immediately after receipt. Whether it really saves electricity is not clear yet, I believe that this brand of energy saver really saves	Jingdong prefecture level city in Shandong
Dragon Quality Goods Shop	Green Intelligent Power Saver	Okay, okay, okay. Thank you so much. Fast is good. Fast. Item was as described, mainly because the event was cheap and you can save a lot of money.	Jingdong prefecture level city in Shandong
Dragon Quality Goods Shop	Green Intelligent Power Saver	Received it a couple days ago! Plugged it in the same day! Let's see what the electric bill is in a month! Hopefully it works well oh!	Jingdong prefecture level city in Shandong
Dragon Quality Goods Shop	Green Intelligent Power Saver	Looking at the reviews good, energy-saving small products, compact, the results after 2 months to see, because the electricity bill season payment	Jingdong prefecture level city in Shandong
Dragon Quality Goods Shop	Green Intelligent Power Saver	I just received it and will try it out to see if it works well. I'll comment later.	Jingdong prefecture level city in Shandong
Dragon Quality Goods Shop	Green Intelligent Power Saver	It looks a little small and not very aesthetically pleasing, I don't know how effective it will be until I use it	Jingdong prefecture level city in Shandong
Dragon Quality Goods Shop	Green Intelligent Power Saver	Not only to the point of being useless yet	Jingdong prefecture level city in Shandong
Dragon Quality Goods Shop	Green Intelligent Power Saver	Used it for a month, nothing works, the meter you turn very fast. This is not a scam, but also sold on Jingdong. To cheat a bunch of people?	Jingdong prefecture level city in Shandong
Dragon Quality Goods Shop	Green Intelligent Power Saver	Very good to use, every day can save a little electricity, really good under the sample down a month to save a few dozen dollars in electricity, good before I did not know that there is this 👆 haha, the price is not expensive praise.	Jingdong prefecture level city in Shandong
Dragon Quality Goods Shop	Green Intelligent Power Saver	Tried it for ten days and the change is almost nothing, can't see the power saving yet, use it some more days and see how it goes.	Jingdong prefecture level city in Shandong
Dragon Quality Goods Shop	Green Intelligent Power Saver	Received very quickly, the quality is very good, really save electricity, business service attitude is also very good, I hope you do not miss this opportunity, I continue to buy a few in	Jingdong prefecture level city in Shandong
Dragon Quality Goods Shop	Green Intelligent Power Saver	Quality is very good Oh, the delivery is also very fast, a very favorite store, I want to crazy recommend to friends!	Jingdong prefecture level city in Shandong
Dragon Quality Goods Shop	Green Intelligent Power Saver	The goods have just received, fine workmanship, and the same as described by the merchant, to use a period of time to see if it can achieve the purpose of power saving.	Jingdong prefecture level city in Shandong
Dragon Quality Goods Shop	Green Intelligent Power Saver	Received the goods first use a period of time to see if there is no effect, the business commitment to ninety days invalid refund, first praise, good service!	Jingdong prefecture level city in Shandong
Dragon Quality Goods Shop	Green Intelligent Power Saver	The product is used for a month without energy-saving and power-saving effect does not have any effect	Jingdong prefecture level city in Shandong
Dragon Quality Goods Shop	Green Intelligent Power Saver	The baby is really great, I like it very much, my friends also say good to come, recommended to buy!	Jingdong prefecture level city in Shandong
Dragon Quality Goods Shop	Green Intelligent Power Saver	Logistics is fast, courier boy service is good, after using to see how the fruit, and then follow up.	Jingdong prefecture level city in Shandong
Dragon Quality Goods Shop	Green Intelligent Power Saver	Baby has been received to try, I believe that as the business advertised energy saving. The courier is very responsible.	Jingdong prefecture level city in Shandong
Dragon Quality Goods Shop	Green Intelligent Power Saver	Save Electric Energy King received, good quality, beautiful appearance, very satisfied!	Jingdong prefecture level city in Shandong
Dragon Quality Goods Shop	Green Intelligent Power Saver	Three no products no manufacturers no phone pure fraud induced consumption cheat	Jingdong prefecture level city in Shandong
Dragon Quality Goods Shop	Green Intelligent Power Saver	I'm using it now, I don't know if it's as good as it says, I'll follow up after a month	Jingdong prefecture level city in Shandong
Dragon Quality Goods Shop	Green Intelligent Power Saver	Good quality logistics and fast. Fine workmanship. The description is consistent cost-effective	Jingdong prefecture level city in Shandong
Dragon Quality Goods Shop	Green Intelligent Power Saver	Received, whether it is good or not, to wait a few days and then evaluate	Jingdong prefecture level city in Shandong
Dragon Quality Goods Shop	Green Intelligent Power Saver	I bought it and started using it, I won't know if it works well until I pay my electric bill	Jingdong prefecture level city in Shandong
Dragon Quality Goods Shop	Green Intelligent Power Saver	Especially good, energy saving and power saving, especially good quality, good	Jingdong prefecture level city in Shandong
Dragon Quality Goods Shop	Green Intelligent Power Saver	Logistics is strong, the quality is very good, paste really save electricity, as described, will buy back!	Jingdong prefecture level city in Shandong
Dragon Quality Goods Shop	Green Intelligent Power Saver	Great stuff at a great price, worth the money!	Jingdong prefecture level city in Shandong

Dragon Quality Goods Shop	Green Intelligent Power Saver	Received the power saver, very good quality, very power saving!	Jingdong prefecture level city in Shandong
Dragon Quality Goods Shop	Green Intelligent Power Saver	There are no fruits, only samples...	Jingdong prefecture level city in Shandong
Dragon Quality Goods Shop	Green Intelligent Power Saver	Save a few dollars a month.	Jingdong prefecture level city in Shandong
Dragon Quality Goods Shop	Green Intelligent Power Saver	The quality is particularly good, it works well, and the seller has a good service attitude	Jingdong prefecture level city in Shandong
Dragon Quality Goods Shop	Green Intelligent Power Saver	I've compared them a couple of times and I don't feel any difference	Jingdong prefecture level city in Shandong
Dragon Quality Goods Shop	Green Intelligent Power Saver	I don't see the savings. It's a sham.	Jingdong prefecture level city in Shandong
Dragon Quality Goods Shop	Green Intelligent Power Saver	The power saving effect is very good, and the logistics is very fast. The seller's service attitude is very good	Jingdong prefecture level city in Shandong
Dragon Quality Goods Shop	Green Intelligent Power Saver	Received, well packaged, haven't tried it yet!	Jingdong prefecture level city in Shandong
Dragon Quality Goods Shop	Green Intelligent Power Saver	I don't know how it works, I'll comment after I use it.	Jingdong prefecture level city in Shandong
Dragon Quality Goods Shop	Green Intelligent Power Saver	The product doesn't work well enough to be of any use	Jingdong prefecture level city in Shandong
Dragon Quality Goods Shop	Green Intelligent Power Saver	On Trial 。。。。。。。	Jingdong prefecture level city in Shandong
Dragon Quality Goods Shop	Green Intelligent Power Saver	Being used with unknown results	Jingdong prefecture level city in Shandong
Dragon Quality Goods Shop	Green Intelligent Power Saver	Smart power saving and great quality	Jingdong prefecture level city in Shandong
Dragon Quality Goods Shop	Green Intelligent Power Saver	The saver was received, it works very well and the price is affordable!	Jingdong prefecture level city in Shandong
Dragon Quality Goods Shop	Green Intelligent Power Saver	How can I feel the power saving and how long does it last	Jingdong prefecture level city in Shandong
Dragon Quality Goods Shop	Green Intelligent Power Saver	And I know if it saves electricity or not.	Jingdong prefecture level city in Shandong
Dragon Quality Goods Shop	Green Intelligent Power Saver	It won't save any electricity. No.	Jingdong prefecture level city in Shandong
Dragon Quality Goods Shop	Green Intelligent Power Saver	Invalid. Fake.	Jingdong prefecture level city in Shandong
Dragon Quality Goods Shop	Green Intelligent Power Saver	It's good!	Jingdong prefecture level city in Shandong
Dragon Quality Goods Shop	Green Intelligent Power Saver	have no effect	Jingdong prefecture level city in Shandong
Dragon Quality Goods Shop	Green Intelligent Power Saver	Not used.	Jingdong prefecture level city in Shandong
Dragon Quality Goods Shop	Green Intelligent Power Saver	There is no load range, packaging thrift, ten days also did not develop invoices, received the goods with a receipt, hand-written receipt also has no financial stamp, communication for nearly a week a moment to say that the electronic invoice, a moment to say that the paper invoice, said it is mailed to me, ten days also did not see anything, the invoice invoice can not be found to say that did not stay on file, single single number can not be found, said that the warehouse more than one to a one to find out, in addition to the perfunctory or perfunctory. Products are no certificate of conformity, proper three	Jingdong prefecture level city in Shandong
Dragon Quality Goods Shop	Green Intelligent Power Saver	Satisfaction.	Jingdong prefecture level city in Shandong
Dragon Quality Goods Shop	Green Intelligent Power Saver	There is not a bit of power saving effect, and ringing up an increase in the electricity bill, because you have to add the cost of this machine's own electricity consumption and purchase, so I think it is better to use this machine instead of increasing the expenditure, uneconomical, rather than not use it.	Jingdong prefecture level city in Shandong
Dragon Quality Goods Shop	Green Intelligent Power Saver	I've been using it for two days now and I don't feel any effect.	Jingdong prefecture level city in Shandong
Dragon Quality Goods Shop	Green Intelligent Power Saver	What it looked like was nothing like what was sent.	Jingdong prefecture level city in Shandong
Dragon Quality Goods Shop	Green Intelligent Power Saver	It didn't work. It didn't work for a month or so.	Jingdong prefecture level city in Shandong
Dragon Quality Goods Shop	Green Intelligent Power Saver	Garbage products, physical and description is not the same, no effect at all, pit # no discussion, to buy the fine, garbage, garbage, garbage	Jingdong prefecture level city in Shandong
Dragon Quality Goods Shop	Green Intelligent Power Saver	general	Jingdong prefecture level city in Shandong
Dragon Quality Goods Shop	Green Intelligent Power Saver	IQ tax. Instead of saving electricity, it increases the power consumption of an appliance.	Jingdong prefecture level city in Shandong
Dragon Quality Goods Shop	Green Intelligent Power Saver	There's no trash at all.	Jingdong prefecture level city in Shandong
Dragon Quality Goods Shop	Green Intelligent Power Saver	It doesn't do anything.	Jingdong prefecture level city in Shandong
Dragon Quality Goods Shop	Green Intelligent Power Saver	(of an unmarried couple) be close	Jingdong prefecture level city in Shandong
Dragon Quality Goods Shop	Green Intelligent Power Saver	(of an unmarried couple) be close	Jingdong prefecture level city in Shandong
Dragon Quality Goods Shop	Green Intelligent Power Saver	no electricity saving	Jingdong prefecture level city in Shandong
Dragon Quality Goods Shop	Green Intelligent Power Saver	differ from	Jingdong prefecture level city in Shandong
Dragon Quality Goods Shop	Green Intelligent Power Saver	Saver has received, but real can save electricity, the quality is very good, I times I buy a few more, too good.	Jingdong prefecture level city in Shandong
Dragon Quality Goods Shop	Green Intelligent Power Saver	Great quality as always, a very favorite product, great! o(*￣︶￣*)o	Jingdong prefecture level city in Shandong
Dragon Quality Goods Shop	Green Intelligent Power Saver	If we can reduce the electricity bill, for individuals can save money, for the country can protect the environment, now the weather is getting hotter and hotter, to protect the environment to reduce emissions is very important. Already use the product, whether to reduce the electricity bill depends on next month!	Jingdong prefecture level city in Shandong
Dragon Quality Goods Shop	Green Intelligent Power Saver	Purchased a power saver received on the put into use, rent a room with many electrical appliances, power consumption is very large, we have a piece of electricity or 1.2 dollars a time, with this power saver is much better, every day can save a few cents, very convenient and practical!	Jingdong prefecture level city in Shandong
Dragon Quality Goods Shop	Green Intelligent Power Saver	The product is better to use before not to use when a month 1500 yuan electricity bill, now using the 5 second month electricity bill directly down 470 dollars or very good. A save money to buy several power saver money ZanZanZanZan👍	Jingdong prefecture level city in Shandong
Dragon Quality Goods Shop	Green Intelligent Power Saver	The moment I received the goods really satisfied with this product, not only high-grade packaging, the body are base alloy, up to the price, I was also introduced by a friend, he used last month did save a lot of electricity, but also recommended to me!	Jingdong prefecture level city in Shandong
Dragon Quality Goods Shop	Green Intelligent Power Saver	I got it. Good review. I'm very satisfied. Reasonable price.	Jingdong prefecture level city in Shandong
Dragon Quality Goods Shop	Green Intelligent Power Saver	Intelligent power saver received and used, looking at the quality is good, the quality of the plug sent is also very good. Expect the effect of the economizer is obvious, first five-star praise, with a period of time to follow up!	Jingdong prefecture level city in Shandong
Dragon Quality Goods Shop	Green Intelligent Power Saver	The saver has been received. It was plugged into an outlet and used immediately after receipt. Whether it really saves electricity is not clear yet, I believe that this brand of energy saver really saves	Jingdong prefecture level city in Shandong
Dragon Quality Goods Shop	Green Intelligent Power Saver	Okay, okay, okay. Thank you so much. Fast is good. Fast. Item was as described, mainly because the event was cheap and you can save a lot of money.	Jingdong prefecture level city in Shandong

Dragon Quality Goods Shop	Green Intelligent Power Saver	Received it a couple days ago! Plugged it in the same day! Let's see what the electric bill is in a month! Hopefully it works well oh!	Jingdong prefecture level city in Shandong
Dragon Quality Goods Shop	Green Intelligent Power Saver	Looking at the reviews good, energy-saving small products, compact, the results after 2 months to see, because the electricity bill season payment	Jingdong prefecture level city in Shandong
Dragon Quality Goods Shop	Green Intelligent Power Saver	I just received it and will try it out to see if it works well. I'll comment later.	Jingdong prefecture level city in Shandong
Dragon Quality Goods Shop	Green Intelligent Power Saver	It looks a little small and not very aesthetically pleasing, I don't know how effective it will be until I use it	Jingdong prefecture level city in Shandong
Dragon Quality Goods Shop	Green Intelligent Power Saver	Not only to the point of being useless yet	Jingdong prefecture level city in Shandong
Dragon Quality Goods Shop	Green Intelligent Power Saver	Used it for a month, nothing works, the meter you turn very fast. This is not a scam, but also sold on Jingdong. To cheat a bunch of people?	Jingdong prefecture level city in Shandong
Dragon Quality Goods Shop	Green Intelligent Power Saver	Very good to use, every day can save a little electricity, really good under the sample down a month to save a few dozen dollars in electricity, good before I did not know that there is this haha, the price is not expensive praise.	Jingdong prefecture level city in Shandong
Dragon Quality Goods Shop	Green Intelligent Power Saver	Tried it for ten days and the change is almost nothing, can't see the power saving yet, use it some more days and see how it goes.	Jingdong prefecture level city in Shandong
Dragon Quality Goods Shop	Green Intelligent Power Saver	Received very quickly, the quality is very good, really save electricity, business service attitude is also very good, I hope you do not miss this opportunity, I continue to buy a few in	Jingdong prefecture level city in Shandong
Dragon Quality Goods Shop	Green Intelligent Power Saver	Quality is very good Oh, the delivery is also very fast, a very favorite store, I want to crazy recommend to friends!	Jingdong prefecture level city in Shandong
Dragon Quality Goods Shop	Green Intelligent Power Saver	The goods have just received, fine workmanship, and the same as described by the merchant, to use a period of time to see if it can achieve the purpose of power saving.	Jingdong prefecture level city in Shandong
Dragon Quality Goods Shop	Green Intelligent Power Saver	Received the goods first use a period of time to see if there is no effect, the business commitment to ninety days invalid refund, first praise, good service!	Jingdong prefecture level city in Shandong
Dragon Quality Goods Shop	Green Intelligent Power Saver	The product is used for a month without energy-saving and power-saving effect does not have any effect	Jingdong prefecture level city in Shandong
Dragon Quality Goods Shop	Green Intelligent Power Saver	The baby is really great, I like it very much, my friends also say good to come, recommended to buy!	Jingdong prefecture level city in Shandong
Dragon Quality Goods Shop	Green Intelligent Power Saver	Logistics is fast, courier boy service is good, after using to see how the fruit, and then follow up.	Jingdong prefecture level city in Shandong
Dragon Quality Goods Shop	Green Intelligent Power Saver	Baby has been received to try, I believe that as the business advertised energy saving. The courier is very responsible.	Jingdong prefecture level city in Shandong
Dragon Quality Goods Shop	Green Intelligent Power Saver	Save Electric Energy King received, good quality, beautiful appearance, very satisfied!	Jingdong prefecture level city in Shandong
Dragon Quality Goods Shop	Green Intelligent Power Saver	Three no products no manufacturers no phone pure fraud induced consumption cheat	Jingdong prefecture level city in Shandong
Dragon Quality Goods Shop	Green Intelligent Power Saver	I'm using it now, I don't know if it's as good as it says, I'll follow up after a month	Jingdong prefecture level city in Shandong
Dragon Quality Goods Shop	Green Intelligent Power Saver	Good quality logistics and fast. Fine workmanship. The description is consistent cost-effective	Jingdong prefecture level city in Shandong
Dragon Quality Goods Shop	Green Intelligent Power Saver	Received, whether it is good or not, to wait a few days and then evaluate	Jingdong prefecture level city in Shandong
Dragon Quality Goods Shop	Green Intelligent Power Saver	I bought it and started using it, I won't know if it works well until I pay my electric bill	Jingdong prefecture level city in Shandong
Dragon Quality Goods Shop	Green Intelligent Power Saver	Especially good, energy saving and power saving, especially good quality, good	Jingdong prefecture level city in Shandong
Dragon Quality Goods Shop	Green Intelligent Power Saver	Logistics is strong, the quality is very good, paste really save electricity, as described, will buy back!	Jingdong prefecture level city in Shandong
Dragon Quality Goods Shop	Green Intelligent Power Saver	Great stuff at a great price, worth the money!	Jingdong prefecture level city in Shandong
Dragon Quality Goods Shop	Green Intelligent Power Saver	Received the power saver, very good quality, very power saving!	Jingdong prefecture level city in Shandong
Dragon Quality Goods Shop	Green Intelligent Power Saver	There are no fruits, only samples...	Jingdong prefecture level city in Shandong
Dragon Quality Goods Shop	Green Intelligent Power Saver	Save a few dollars a month.	Jingdong prefecture level city in Shandong
Dragon Quality Goods Shop	Green Intelligent Power Saver	The quality is particularly good, it works well, and the seller has a good service attitude	Jingdong prefecture level city in Shandong
Dragon Quality Goods Shop	Green Intelligent Power Saver	I've compared them a couple of times and I don't feel any difference	Jingdong prefecture level city in Shandong
Dragon Quality Goods Shop	Green Intelligent Power Saver	I don't see the savings. It's a sham.	Jingdong prefecture level city in Shandong
Dragon Quality Goods Shop	Green Intelligent Power Saver	The power saving effect is very good, and the logistics is very fast. The seller's service attitude is very good	Jingdong prefecture level city in Shandong
Dragon Quality Goods Shop	Green Intelligent Power Saver	Received, well packaged, haven't tried it yet!	Jingdong prefecture level city in Shandong
Dragon Quality Goods Shop	Green Intelligent Power Saver	I don't know how it works, I'll comment after I use it.	Jingdong prefecture level city in Shandong
Dragon Quality Goods Shop	Green Intelligent Power Saver	The product doesn't work well enough to be of any use	Jingdong prefecture level city in Shandong
Dragon Quality Goods Shop	Green Intelligent Power Saver	On Trial 。。。。。。。	Jingdong prefecture level city in Shandong
Dragon Quality Goods Shop	Green Intelligent Power Saver	Being used with unknown results	Jingdong prefecture level city in Shandong
Dragon Quality Goods Shop	Green Intelligent Power Saver	Smart power saving and great quality	Jingdong prefecture level city in Shandong
Dragon Quality Goods Shop	Green Intelligent Power Saver	The saver was received, it works very well and the price is affordable!	Jingdong prefecture level city in Shandong
Dragon Quality Goods Shop	Green Intelligent Power Saver	How can I feel the power saving and how long does it last	Jingdong prefecture level city in Shandong
Dragon Quality Goods Shop	Green Intelligent Power Saver	And I know if it saves electricity or not.	Jingdong prefecture level city in Shandong
Dragon Quality Goods Shop	Green Intelligent Power Saver	It won't save any electricity. No.	Jingdong prefecture level city in Shandong
Dragon Quality Goods Shop	Green Intelligent Power Saver	Invalid. Fake.	Jingdong prefecture level city in Shandong
Dragon Quality Goods Shop	Green Intelligent Power Saver	It's good!	Jingdong prefecture level city in Shandong
Dragon Quality Goods Shop	Green Intelligent Power Saver	have no effect	Jingdong prefecture level city in Shandong
Dragon Quality Goods Shop	Green Intelligent Power Saver	Not used.	Jingdong prefecture level city in Shandong
Dragon Quality Goods Shop	Green Intelligent Power Saver	There is no load range, packaging thrift, ten days also did not develop invoices, received the goods with a receipt, hand-written receipt also has no financial stamp, communication for nearly a week a moment to say that the electronic invoice, a moment to say that the paper invoice, said it is mailed to me, ten days also did not see anything, the invoice invoice can not be found to say that did not stay on file, single single number can not be found, said that the warehouse more than one to a one to find out, in addition to the perfunctory or perfunctory. Products are no certificate of conformity, proper three	Jingdong prefecture level city in Shandong
Dragon Quality Goods Shop	Green Intelligent Power Saver	Satisfaction.	Jingdong prefecture level city in Shandong

Dragon Quality Goods Shop	Green Intelligent Power Saver	There is not a bit of power saving effect, and ringing up an increase in the electricity bill, because you have to add the cost of this machine's own electricity consumption and purchase, so I think it is better to use this machine instead of increasing the expenditure, uneconomical, rather than not use it.	Jingdong prefecture level city in Shandong
Dragon Quality Goods Shop	Green Intelligent Power Saver	I've been using it for two days now and I don't feel any effect.	Jingdong prefecture level city in Shandong
Dragon Quality Goods Shop	Green Intelligent Power Saver	What it looked like was nothing like what was sent.	Jingdong prefecture level city in Shandong
Dragon Quality Goods Shop	Green Intelligent Power Saver	It didn't work. It didn't work for a month or so.	Jingdong prefecture level city in Shandong
Dragon Quality Goods Shop	Green Intelligent Power Saver	Garbage products, physical and description is not the same, no effect at all, pit # no discussion, to buy the fine, garbage, garbage, garbage	Jingdong prefecture level city in Shandong
Dragon Quality Goods Shop	Green Intelligent Power Saver	general	Jingdong prefecture level city in Shandong
Dragon Quality Goods Shop	Green Intelligent Power Saver	IQ tax. Instead of saving electricity, it increases the power consumption of an appliance.	Jingdong prefecture level city in Shandong
Dragon Quality Goods Shop	Green Intelligent Power Saver	There's no trash at all.	Jingdong prefecture level city in Shandong
Dragon Quality Goods Shop	Green Intelligent Power Saver	It doesn't do anything.	Jingdong prefecture level city in Shandong
Dragon Quality Goods Shop	Green Intelligent Power Saver	(of an unmarried couple) be close	Jingdong prefecture level city in Shandong
Dragon Quality Goods Shop	Green Intelligent Power Saver	(of an unmarried couple) be close	Jingdong prefecture level city in Shandong
Dragon Quality Goods Shop	Green Intelligent Power Saver	no electricity saving	Jingdong prefecture level city in Shandong
Dragon Quality Goods Shop	Green Intelligent Power Saver	differ from	Jingdong prefecture level city in Shandong
Dragon Quality Goods Shop	Green Intelligent Power Saver	Saver has received, but real can save electricity, the quality is very good, I times I buy a few more, too good.	Jingdong prefecture level city in Shandong
Dragon Quality Goods Shop	Green Intelligent Power Saver	Great quality as always, a very favorite product, great! o(*￣︶￣*)o	Jingdong prefecture level city in Shandong
Dragon Quality Goods Shop	Green Intelligent Power Saver	If we can reduce the electricity bill, for individuals can save money, for the country can protect the environment, now the weather is getting hotter and hotter, to protect the environment to reduce emissions is very important. Already use the product, whether to reduce the electricity bill depends on next month!	Jingdong prefecture level city in Shandong
Dragon Quality Goods Shop	Green Intelligent Power Saver	Purchased a power saver received on the put into use, rent a room with many electrical appliances, power consumption is very large, we have a piece of electricity or 1.2 dollars a time, with this power saver is much better, every day can save a few cents, very convenient and practical!	Jingdong prefecture level city in Shandong
Dragon Quality Goods Shop	Green Intelligent Power Saver	The product is better to use before not to use when a month 1500 yuan electricity bill, now using the 5 second month electricity bill directly down 470 dollars or very good. A save money to buy several power saver money ZanZanZanZan👍	Jingdong prefecture level city in Shandong
Dragon Quality Goods Shop	Green Intelligent Power Saver	The moment I received the goods really satisfied with this product, not only high-grade packaging, the body are base alloy, up to the price, I was also introduced by a friend, he used last month did save a lot of electricity, but also recommended to me!	Jingdong prefecture level city in Shandong
Dragon Quality Goods Shop	Green Intelligent Power Saver	I got it. Good review. I'm very satisfied. Reasonable price.	Jingdong prefecture level city in Shandong
Dragon Quality Goods Shop	Green Intelligent Power Saver	Intelligent power saver received and used, looking at the quality is good, the quality of the plug sent is also very good. Expect the effect of the economizer is obvious, first five-star praise, with a period of time to follow up!	Jingdong prefecture level city in Shandong
Dragon Quality Goods Shop	Green Intelligent Power Saver	The saver has been received. It was plugged into an outlet and used immediately after receipt. Whether it really saves electricity is not clear yet, I believe that this brand of energy saver really saves	Jingdong prefecture level city in Shandong
Dragon Quality Goods Shop	Green Intelligent Power Saver	Okay, okay, okay. Thank you so much. Fast is good. Fast. Item was as described, mainly because the event was cheap and you can save a lot of money.	Jingdong prefecture level city in Shandong
Dragon Quality Goods Shop	Green Intelligent Power Saver	Received it a couple days ago! Plugged it in the same day! Let's see what the electric bill is in a month! Hopefully it works well oh!	Jingdong prefecture level city in Shandong
Dragon Quality Goods Shop	Green Intelligent Power Saver	Looking at the reviews good, energy-saving small products, compact, the results after 2 months to see, because the electricity bill season payment	Jingdong prefecture level city in Shandong
Dragon Quality Goods Shop	Green Intelligent Power Saver	I just received it and will try it out to see if it works well. I'll comment later.	Jingdong prefecture level city in Shandong
Dragon Quality Goods Shop	Green Intelligent Power Saver	It looks a little small and not very aesthetically pleasing, I don't know how effective it will be until I use it	Jingdong prefecture level city in Shandong
Dragon Quality Goods Shop	Green Intelligent Power Saver	Not only to the point of being useless yet	Jingdong prefecture level city in Shandong
Dragon Quality Goods Shop	Green Intelligent Power Saver	Used it for a month, nothing works, the meter you turn very fast. This is not a scam, but also sold on Jingdong. To cheat a bunch of people?	Jingdong prefecture level city in Shandong
Dragon Quality Goods Shop	Green Intelligent Power Saver	Very good to use, every day can save a little electricity, really good under the sample down a month to save a few dozen dollars in electricity, good before I did not know that there is this 👆 haha, the price is not expensive praise.	Jingdong prefecture level city in Shandong
Dragon Quality Goods Shop	Green Intelligent Power Saver	Tried it for ten days and the change is almost nothing, can't see the power saving yet, use it some more days and see how it goes.	Jingdong prefecture level city in Shandong
Dragon Quality Goods Shop	Green Intelligent Power Saver	Received very quickly, the quality is very good, really save electricity, business service attitude is also very good, I hope you do not miss this opportunity, I continue to buy a few in	Jingdong prefecture level city in Shandong
Dragon Quality Goods Shop	Green Intelligent Power Saver	Quality is very good Oh, the delivery is also very fast, a very favorite store, I want to crazy recommend to friends!	Jingdong prefecture level city in Shandong
Dragon Quality Goods Shop	Green Intelligent Power Saver	The goods have just received, fine workmanship, and the same as described by the merchant, to use a period of time to see if it can achieve the purpose of power saving.	Jingdong prefecture level city in Shandong
Dragon Quality Goods Shop	Green Intelligent Power Saver	Received the goods first use a period of time to see if there is no effect, the business commitment to ninety days invalid refund, first praise, good service!	Jingdong prefecture level city in Shandong
Dragon Quality Goods Shop	Green Intelligent Power Saver	The product is used for a month without energy-saving and power-saving effect does not have any effect	Jingdong prefecture level city in Shandong
Dragon Quality Goods Shop	Green Intelligent Power Saver	The baby is really great, I like it very much, my friends also say good to come, recommended to buy!	Jingdong prefecture level city in Shandong
Dragon Quality Goods Shop	Green Intelligent Power Saver	Logistics is fast, courier boy service is good, after using to see how the fruit, and then follow up.	Jingdong prefecture level city in Shandong
Dragon Quality Goods Shop	Green Intelligent Power Saver	Baby has been received to try, I believe that as the business advertised energy saving. The courier is very responsible.	Jingdong prefecture level city in Shandong
Dragon Quality Goods Shop	Green Intelligent Power Saver	Save Electric Energy King received, good quality, beautiful appearance, very satisfied!	Jingdong prefecture level city in Shandong
Dragon Quality Goods Shop	Green Intelligent Power Saver	Three no products no manufacturers no phone pure fraud induced consumption cheat	Jingdong prefecture level city in Shandong
Dragon Quality Goods Shop	Green Intelligent Power Saver	I'm using it now, I don't know if it's as good as it says, I'll follow up after a month	Jingdong prefecture level city in Shandong
Dragon Quality Goods Shop	Green Intelligent Power Saver	Good quality logistics and fast. Fine workmanship. The description is consistent cost-effective	Jingdong prefecture level city in Shandong
Dragon Quality Goods Shop	Green Intelligent Power Saver	Received, whether it is good or not, to wait a few days and then evaluate	Jingdong prefecture level city in Shandong
Dragon Quality Goods Shop	Green Intelligent Power Saver	I bought it and started using it, I won't know if it works well until I pay my electric bill	Jingdong prefecture level city in Shandong
Dragon Quality Goods Shop	Green Intelligent Power Saver	Especially good, energy saving and power saving, especially good quality, good	Jingdong prefecture level city in Shandong
Dragon Quality Goods Shop	Green Intelligent Power Saver	Logistics is strong, the quality is very good, paste really save electricity, as described, will buy back!	Jingdong prefecture level city in Shandong
Dragon Quality Goods Shop	Green Intelligent Power Saver	Great stuff at a great price, worth the money!	Jingdong prefecture level city in Shandong

Dragon Quality Goods Shop	Green Intelligent Power Saver	Received the power saver, very good quality, very power saving!	Jingdong prefecture level city in Shandong
Dragon Quality Goods Shop	Green Intelligent Power Saver	There are no fruits, only samples...	Jingdong prefecture level city in Shandong
Dragon Quality Goods Shop	Green Intelligent Power Saver	Save a few dollars a month.	Jingdong prefecture level city in Shandong
Dragon Quality Goods Shop	Green Intelligent Power Saver	The quality is particularly good, it works well, and the seller has a good service attitude	Jingdong prefecture level city in Shandong
Dragon Quality Goods Shop	Green Intelligent Power Saver	I've compared them a couple of times and I don't feel any difference	Jingdong prefecture level city in Shandong
Dragon Quality Goods Shop	Green Intelligent Power Saver	I don't see the savings. It's a sham.	Jingdong prefecture level city in Shandong
Dragon Quality Goods Shop	Green Intelligent Power Saver	The power saving effect is very good, and the logistics is very fast. The seller's service attitude is very good	Jingdong prefecture level city in Shandong
Dragon Quality Goods Shop	Green Intelligent Power Saver	Received, well packaged, haven't tried it yet!	Jingdong prefecture level city in Shandong
Dragon Quality Goods Shop	Green Intelligent Power Saver	I don't know how it works, I'll comment after I use it.	Jingdong prefecture level city in Shandong
Dragon Quality Goods Shop	Green Intelligent Power Saver	The product doesn't work well enough to be of any use	Jingdong prefecture level city in Shandong
Dragon Quality Goods Shop	Green Intelligent Power Saver	On Trial 。。。。。。。	Jingdong prefecture level city in Shandong
Dragon Quality Goods Shop	Green Intelligent Power Saver	Being used with unknown results	Jingdong prefecture level city in Shandong
Dragon Quality Goods Shop	Green Intelligent Power Saver	Smart power saving and great quality	Jingdong prefecture level city in Shandong
Dragon Quality Goods Shop	Green Intelligent Power Saver	The saver was received, it works very well and the price is affordable!	Jingdong prefecture level city in Shandong
Dragon Quality Goods Shop	Green Intelligent Power Saver	How can I feel the power saving and how long does it last	Jingdong prefecture level city in Shandong
Dragon Quality Goods Shop	Green Intelligent Power Saver	And I know if it saves electricity or not.	Jingdong prefecture level city in Shandong
Dragon Quality Goods Shop	Green Intelligent Power Saver	It won't save any electricity. No.	Jingdong prefecture level city in Shandong
Dragon Quality Goods Shop	Green Intelligent Power Saver	Invalid. Fake.	Jingdong prefecture level city in Shandong
Dragon Quality Goods Shop	Green Intelligent Power Saver	It's good!	Jingdong prefecture level city in Shandong
Dragon Quality Goods Shop	Green Intelligent Power Saver	have no effect	Jingdong prefecture level city in Shandong
Dragon Quality Goods Shop	Green Intelligent Power Saver	Not used.	Jingdong prefecture level city in Shandong
Dragon Quality Goods Shop	Green Intelligent Power Saver	There is no load range, packaging thrift, ten days also did not develop invoices, received the goods with a receipt, hand-written receipt also has no financial stamp, communication for nearly a week a moment to say that the electronic invoice, a moment to say that the paper invoice, said it is mailed to me, ten days also did not see anything, the invoice invoice can not be found to say that did not stay on file, single single number can not be found, said that the warehouse more than one to a one to find out, in addition to the perfunctory or perfunctory. Products are no certificate of conformity, proper three	Jingdong prefecture level city in Shandong
Dragon Quality Goods Shop	Green Intelligent Power Saver	Satisfaction.	Jingdong prefecture level city in Shandong
Dragon Quality Goods Shop	Green Intelligent Power Saver	There is not a bit of power saving effect, and ringing up an increase in the electricity bill, because you have to add the cost of this machine's own electricity consumption and purchase, so I think it is better to use this machine instead of increasing the expenditure, uneconomical, rather than not use it.	Jingdong prefecture level city in Shandong
Dragon Quality Goods Shop	Green Intelligent Power Saver	I've been using it for two days now and I don't feel any effect.	Jingdong prefecture level city in Shandong
Dragon Quality Goods Shop	Green Intelligent Power Saver	What it looked like was nothing like what was sent.	Jingdong prefecture level city in Shandong
Dragon Quality Goods Shop	Green Intelligent Power Saver	It didn't work. It didn't work for a month or so.	Jingdong prefecture level city in Shandong
Dragon Quality Goods Shop	Green Intelligent Power Saver	Garbage products, physical and description is not the same, no effect at all, pit # no discussion, to buy the fine, garbage, garbage, garbage	Jingdong prefecture level city in Shandong
Dragon Quality Goods Shop	Green Intelligent Power Saver	general	Jingdong prefecture level city in Shandong
Dragon Quality Goods Shop	Green Intelligent Power Saver	IQ tax. Instead of saving electricity, it increases the power consumption of an appliance.	Jingdong prefecture level city in Shandong
Dragon Quality Goods Shop	Green Intelligent Power Saver	There's no trash at all.	Jingdong prefecture level city in Shandong
Dragon Quality Goods Shop	Green Intelligent Power Saver	It doesn't do anything.	Jingdong prefecture level city in Shandong
Dragon Quality Goods Shop	Green Intelligent Power Saver	(of an unmarried couple) be close	Jingdong prefecture level city in Shandong
Dragon Quality Goods Shop	Green Intelligent Power Saver	(of an unmarried couple) be close	Jingdong prefecture level city in Shandong
Dragon Quality Goods Shop	Green Intelligent Power Saver	no electricity saving	Jingdong prefecture level city in Shandong
Dragon Quality Goods Shop	Green Intelligent Power Saver	differ from	Jingdong prefecture level city in Shandong
Dragon Quality Goods Shop	Green Intelligent Power Saver	Saver has received, but real can save electricity, the quality is very good, I times I buy a few more, too good.	Jingdong prefecture level city in Shandong
Dragon Quality Goods Shop	Green Intelligent Power Saver	Great quality as always, a very favorite product, great! o(*￣︶￣*)o	Jingdong prefecture level city in Shandong
Dragon Quality Goods Shop	Green Intelligent Power Saver	If we can reduce the electricity bill, for individuals can save money, for the country can protect the environment, now the weather is getting hotter and hotter, to protect the environment to reduce emissions is very important. Already use the product, whether to reduce the electricity bill depends on next month!	Jingdong prefecture level city in Shandong
Dragon Quality Goods Shop	Green Intelligent Power Saver	Purchased a power saver received on the put into use, rent a room with many electrical appliances, power consumption is very large, we have a piece of electricity or 1.2 dollars a time, with this power saver is much better, every day can save a few cents, very convenient and practical!	Jingdong prefecture level city in Shandong
Dragon Quality Goods Shop	Green Intelligent Power Saver	The product is better to use before not to use when a month 1500 yuan electricity bill, now using the 5 second month electricity bill directly down 470 dollars or very good. A save money to buy several power saver money ZanZanZanZan👍	Jingdong prefecture level city in Shandong
Dragon Quality Goods Shop	Green Intelligent Power Saver	The moment I received the goods really satisfied with this product, not only high-grade packaging, the body are base alloy, up to the price, I was also introduced by a friend, he used last month did save a lot of electricity, but also recommended to me!	Jingdong prefecture level city in Shandong
Dragon Quality Goods Shop	Green Intelligent Power Saver	I got it. Good review. I'm very satisfied. Reasonable price.	Jingdong prefecture level city in Shandong
Dragon Quality Goods Shop	Green Intelligent Power Saver	Intelligent power saver received and used, looking at the quality is good, the quality of the plug sent is also very good. Expect the effect of the economizer is obvious, first five-star praise, with a period of time to follow up!	Jingdong prefecture level city in Shandong
Dragon Quality Goods Shop	Green Intelligent Power Saver	The saver has been received. It was plugged into an outlet and used immediately after receipt. Whether it really saves electricity is not clear yet, I believe that this brand of energy saver really saves	Jingdong prefecture level city in Shandong
Dragon Quality Goods Shop	Green Intelligent Power Saver	Okay, okay, okay. Thank you so much. Fast is good. Fast. Item was as described, mainly because the event was cheap and you can save a lot of money.	Jingdong prefecture level city in Shandong

Dragon Quality Goods Shop	Green Intelligent Power Saver	Received it a couple days ago! Plugged it in the same day! Let's see what the electric bill is in a month! Hopefully it works well oh!	Jingdong prefecture level city in Shandong
Dragon Quality Goods Shop	Green Intelligent Power Saver	Looking at the reviews good, energy-saving small products, compact, the results after 2 months to see, because the electricity bill season payment	Jingdong prefecture level city in Shandong
Dragon Quality Goods Shop	Green Intelligent Power Saver	I just received it and will try it out to see if it works well. I'll comment later.	Jingdong prefecture level city in Shandong
Dragon Quality Goods Shop	Green Intelligent Power Saver	It looks a little small and not very aesthetically pleasing, I don't know how effective it will be until I use it	Jingdong prefecture level city in Shandong
Dragon Quality Goods Shop	Green Intelligent Power Saver	Not only to the point of being useless yet	Jingdong prefecture level city in Shandong
Dragon Quality Goods Shop	Green Intelligent Power Saver	Used it for a month, nothing works, the meter you turn very fast. This is not a scam, but also sold on Jingdong. To cheat a bunch of people?	Jingdong prefecture level city in Shandong
Dragon Quality Goods Shop	Green Intelligent Power Saver	Very good to use, every day can save a little electricity, really good under the sample down a month to save a few dozen dollars in electricity, good before I did not know that there is this 👆 haha, the price is not expensive praise.	Jingdong prefecture level city in Shandong
Dragon Quality Goods Shop	Green Intelligent Power Saver	Tried it for ten days and the change is almost nothing, can't see the power saving yet, use it some more days and see how it goes.	Jingdong prefecture level city in Shandong
Dragon Quality Goods Shop	Green Intelligent Power Saver	Received very quickly, the quality is very good, really save electricity, business service attitude is also very good, I hope you do not miss this opportunity, I continue to buy a few in	Jingdong prefecture level city in Shandong
Dragon Quality Goods Shop	Green Intelligent Power Saver	Quality is very good Oh, the delivery is also very fast, a very favorite store, I want to crazy recommend to friends!	Jingdong prefecture level city in Shandong
Dragon Quality Goods Shop	Green Intelligent Power Saver	The goods have just received, fine workmanship, and the same as described by the merchant, to use a period of time to see if it can achieve the purpose of power saving.	Jingdong prefecture level city in Shandong
Dragon Quality Goods Shop	Green Intelligent Power Saver	Received the goods first use a period of time to see if there is no effect, the business commitment to ninety days invalid refund, first praise, good service!	Jingdong prefecture level city in Shandong
Dragon Quality Goods Shop	Green Intelligent Power Saver	The product is used for a month without energy-saving and power-saving effect does not have any effect	Jingdong prefecture level city in Shandong
Dragon Quality Goods Shop	Green Intelligent Power Saver	The baby is really great, I like it very much, my friends also say good to come, recommended to buy!	Jingdong prefecture level city in Shandong
Dragon Quality Goods Shop	Green Intelligent Power Saver	Logistics is fast, courier boy service is good, after using to see how the fruit, and then follow up.	Jingdong prefecture level city in Shandong
Dragon Quality Goods Shop	Green Intelligent Power Saver	Baby has been received to try, I believe that as the business advertised energy saving. The courier is very responsible.	Jingdong prefecture level city in Shandong
Dragon Quality Goods Shop	Green Intelligent Power Saver	Save Electric Energy King received, good quality, beautiful appearance, very satisfied!	Jingdong prefecture level city in Shandong
Dragon Quality Goods Shop	Green Intelligent Power Saver	Three no products no manufacturers no phone pure fraud induced consumption cheat	Jingdong prefecture level city in Shandong
Dragon Quality Goods Shop	Green Intelligent Power Saver	I'm using it now, I don't know if it's as good as it says, I'll follow up after a month	Jingdong prefecture level city in Shandong
Dragon Quality Goods Shop	Green Intelligent Power Saver	Good quality logistics and fast. Fine workmanship. The description is consistent cost-effective	Jingdong prefecture level city in Shandong
Dragon Quality Goods Shop	Green Intelligent Power Saver	Received, whether it is good or not, to wait a few days and then evaluate	Jingdong prefecture level city in Shandong
Dragon Quality Goods Shop	Green Intelligent Power Saver	I bought it and started using it, I won't know if it works well until I pay my electric bill	Jingdong prefecture level city in Shandong
Dragon Quality Goods Shop	Green Intelligent Power Saver	Especially good, energy saving and power saving, especially good quality, good	Jingdong prefecture level city in Shandong
Dragon Quality Goods Shop	Green Intelligent Power Saver	Logistics is strong, the quality is very good, paste really save electricity, as described, will buy back!	Jingdong prefecture level city in Shandong
Dragon Quality Goods Shop	Green Intelligent Power Saver	Great stuff at a great price, worth the money!	Jingdong prefecture level city in Shandong
Dragon Quality Goods Shop	Green Intelligent Power Saver	Received the power saver, very good quality, very power saving!	Jingdong prefecture level city in Shandong
Dragon Quality Goods Shop	Green Intelligent Power Saver	There are no fruits, only samples...	Jingdong prefecture level city in Shandong
Dragon Quality Goods Shop	Green Intelligent Power Saver	Save a few dollars a month.	Jingdong prefecture level city in Shandong
Dragon Quality Goods Shop	Green Intelligent Power Saver	The quality is particularly good, it works well, and the seller has a good service attitude	Jingdong prefecture level city in Shandong
Dragon Quality Goods Shop	Green Intelligent Power Saver	I've compared them a couple of times and I don't feel any difference	Jingdong prefecture level city in Shandong
Dragon Quality Goods Shop	Green Intelligent Power Saver	I don't see the savings. It's a sham.	Jingdong prefecture level city in Shandong
Dragon Quality Goods Shop	Green Intelligent Power Saver	The power saving effect is very good, and the logistics is very fast. The seller's service attitude is very good	Jingdong prefecture level city in Shandong
Dragon Quality Goods Shop	Green Intelligent Power Saver	Received, well packaged, haven't tried it yet!	Jingdong prefecture level city in Shandong
Dragon Quality Goods Shop	Green Intelligent Power Saver	I don't know how it works, I'll comment after I use it.	Jingdong prefecture level city in Shandong
Dragon Quality Goods Shop	Green Intelligent Power Saver	The product doesn't work well enough to be of any use	Jingdong prefecture level city in Shandong
Dragon Quality Goods Shop	Green Intelligent Power Saver	On Trial 。。。。。。。	Jingdong prefecture level city in Shandong
Dragon Quality Goods Shop	Green Intelligent Power Saver	Being used with unknown results	Jingdong prefecture level city in Shandong
Dragon Quality Goods Shop	Green Intelligent Power Saver	Smart power saving and great quality	Jingdong prefecture level city in Shandong
Dragon Quality Goods Shop	Green Intelligent Power Saver	The saver was received, it works very well and the price is affordable!	Jingdong prefecture level city in Shandong
Dragon Quality Goods Shop	Green Intelligent Power Saver	How can I feel the power saving and how long does it last	Jingdong prefecture level city in Shandong
Dragon Quality Goods Shop	Green Intelligent Power Saver	And I know if it saves electricity or not.	Jingdong prefecture level city in Shandong
Dragon Quality Goods Shop	Green Intelligent Power Saver	It won't save any electricity. No.	Jingdong prefecture level city in Shandong
Dragon Quality Goods Shop	Green Intelligent Power Saver	Invalid. Fake.	Jingdong prefecture level city in Shandong
Dragon Quality Goods Shop	Green Intelligent Power Saver	It's good!	Jingdong prefecture level city in Shandong
Dragon Quality Goods Shop	Green Intelligent Power Saver	have no effect	Jingdong prefecture level city in Shandong
Dragon Quality Goods Shop	Green Intelligent Power Saver	Not used.	Jingdong prefecture level city in Shandong
Dragon Quality Goods Shop	Green Intelligent Power Saver	There is no load range, packaging thrift, ten days also did not develop invoices, received the goods with a receipt, hand-written receipt also has no financial stamp, communication for nearly a week a moment to say that the electronic invoice, a moment to say that the paper invoice, said it is mailed to me, ten days also did not see anything, the invoice invoice can not be found to say that did not stay on file, single single number can not be found, said that the warehouse more than one to a one to find out, in addition to the perfunctory or perfunctory. Products are no certificate of conformity, proper three	Jingdong prefecture level city in Shandong
Dragon Quality Goods Shop	Green Intelligent Power Saver	Satisfaction.	Jingdong prefecture level city in Shandong

Dragon Quality Goods Shop	Green Intelligent Power Saver	There is not a bit of power saving effect, and ringing up an increase in the electricity bill, because you have to add the cost of this machine's own electricity consumption and purchase, so I think it is better to use this machine instead of increasing the expenditure, uneconomical, rather than not use it.	Jingdong prefecture level city in Shandong
Dragon Quality Goods Shop	Green Intelligent Power Saver	I've been using it for two days now and I don't feel any effect.	Jingdong prefecture level city in Shandong
Dragon Quality Goods Shop	Green Intelligent Power Saver	What it looked like was nothing like what was sent.	Jingdong prefecture level city in Shandong
Dragon Quality Goods Shop	Green Intelligent Power Saver	It didn't work. It didn't work for a month or so.	Jingdong prefecture level city in Shandong
Dragon Quality Goods Shop	Green Intelligent Power Saver	Garbage products, physical and description is not the same, no effect at all, pit # no discussion, to buy the fine, garbage, garbage, garbage	Jingdong prefecture level city in Shandong
Dragon Quality Goods Shop	Green Intelligent Power Saver	general	Jingdong prefecture level city in Shandong
Dragon Quality Goods Shop	Green Intelligent Power Saver	IQ tax. Instead of saving electricity, it increases the power consumption of an appliance.	Jingdong prefecture level city in Shandong
Dragon Quality Goods Shop	Green Intelligent Power Saver	There's no trash at all.	Jingdong prefecture level city in Shandong
Dragon Quality Goods Shop	Green Intelligent Power Saver	It doesn't do anything.	Jingdong prefecture level city in Shandong
Dragon Quality Goods Shop	Green Intelligent Power Saver	(of an unmarried couple) be close	Jingdong prefecture level city in Shandong
Dragon Quality Goods Shop	Green Intelligent Power Saver	(of an unmarried couple) be close	Jingdong prefecture level city in Shandong
Dragon Quality Goods Shop	Green Intelligent Power Saver	no electricity saving	Jingdong prefecture level city in Shandong
Dragon Quality Goods Shop	Green Intelligent Power Saver	differ from	Jingdong prefecture level city in Shandong
Dragon Quality Goods Shop	Green Intelligent Power Saver	Saver has received, but real can save electricity, the quality is very good, I times I buy a few more, too good.	Jingdong prefecture level city in Shandong
Dragon Quality Goods Shop	Green Intelligent Power Saver	Great quality as always, a very favorite product, great! o(*￣︶￣*)o	Jingdong prefecture level city in Shandong
Dragon Quality Goods Shop	Green Intelligent Power Saver	If we can reduce the electricity bill, for individuals can save money, for the country can protect the environment, now the weather is getting hotter and hotter, to protect the environment to reduce emissions is very important. Already use the product, whether to reduce the electricity bill depends on next month!	Jingdong prefecture level city in Shandong
Dragon Quality Goods Shop	Green Intelligent Power Saver	Purchased a power saver received on the put into use, rent a room with many electrical appliances, power consumption is very large, we have a piece of electricity or 1.2 dollars a time, with this power saver is much better, every day can save a few cents, very convenient and practical!	Jingdong prefecture level city in Shandong
Dragon Quality Goods Shop	Green Intelligent Power Saver	The product is better to use before not to use when a month 1500 yuan electricity bill, now using the 5 second month electricity bill directly down 470 dollars or very good. A save money to buy several power saver money ZanZanZanZan👍	Jingdong prefecture level city in Shandong
Dragon Quality Goods Shop	Green Intelligent Power Saver	The moment I received the goods really satisfied with this product, not only high-grade packaging, the body are base alloy, up to the price, I was also introduced by a friend, he used last month did save a lot of electricity, but also recommended to me!	Jingdong prefecture level city in Shandong
Dragon Quality Goods Shop	Green Intelligent Power Saver	I got it. Good review. I'm very satisfied. Reasonable price.	Jingdong prefecture level city in Shandong
Dragon Quality Goods Shop	Green Intelligent Power Saver	Intelligent power saver received and used, looking at the quality is good, the quality of the plug sent is also very good. Expect the effect of the economizer is obvious, first five-star praise, with a period of time to follow up!	Jingdong prefecture level city in Shandong
Dragon Quality Goods Shop	Green Intelligent Power Saver	The saver has been received. It was plugged into an outlet and used immediately after receipt. Whether it really saves electricity is not clear yet, I believe that this brand of energy saver really saves	Jingdong prefecture level city in Shandong
Dragon Quality Goods Shop	Green Intelligent Power Saver	Okay, okay, okay. Thank you so much. Fast is good. Fast. Item was as described, mainly because the event was cheap and you can save a lot of money.	Jingdong prefecture level city in Shandong
Dragon Quality Goods Shop	Green Intelligent Power Saver	Received it a couple days ago! Plugged it in the same day! Let's see what the electric bill is in a month! Hopefully it works well oh!	Jingdong prefecture level city in Shandong
Dragon Quality Goods Shop	Green Intelligent Power Saver	Looking at the reviews good, energy-saving small products, compact, the results after 2 months to see, because the electricity bill season payment	Jingdong prefecture level city in Shandong
Dragon Quality Goods Shop	Green Intelligent Power Saver	I just received it and will try it out to see if it works well. I'll comment later.	Jingdong prefecture level city in Shandong
Dragon Quality Goods Shop	Green Intelligent Power Saver	It looks a little small and not very aesthetically pleasing, I don't know how effective it will be until I use it	Jingdong prefecture level city in Shandong
Dragon Quality Goods Shop	Green Intelligent Power Saver	Not only to the point of being useless yet	Jingdong prefecture level city in Shandong
Dragon Quality Goods Shop	Green Intelligent Power Saver	Used it for a month, nothing works, the meter you turn very fast. This is not a scam, but also sold on Jingdong. To cheat a bunch of people?	Jingdong prefecture level city in Shandong
Dragon Quality Goods Shop	Green Intelligent Power Saver	Very good to use, every day can save a little electricity, really good under the sample down a month to save a few dozen dollars in electricity, good before I did not know that there is this 👆 haha, the price is not expensive praise.	Jingdong prefecture level city in Shandong
Dragon Quality Goods Shop	Green Intelligent Power Saver	Tried it for ten days and the change is almost nothing, can't see the power saving yet, use it some more days and see how it goes.	Jingdong prefecture level city in Shandong
Dragon Quality Goods Shop	Green Intelligent Power Saver	Received very quickly, the quality is very good, really save electricity, business service attitude is also very good, I hope you do not miss this opportunity, I continue to buy a few in	Jingdong prefecture level city in Shandong
Dragon Quality Goods Shop	Green Intelligent Power Saver	Quality is very good Oh, the delivery is also very fast, a very favorite store, I want to crazy recommend to friends!	Jingdong prefecture level city in Shandong
Dragon Quality Goods Shop	Green Intelligent Power Saver	The goods have just received, fine workmanship, and the same as described by the merchant, to use a period of time to see if it can achieve the purpose of power saving.	Jingdong prefecture level city in Shandong
Dragon Quality Goods Shop	Green Intelligent Power Saver	Received the goods first use a period of time to see if there is no effect, the business commitment to ninety days invalid refund, first praise, good service!	Jingdong prefecture level city in Shandong
Dragon Quality Goods Shop	Green Intelligent Power Saver	The product is used for a month without energy-saving and power-saving effect does not have any effect	Jingdong prefecture level city in Shandong
Dragon Quality Goods Shop	Green Intelligent Power Saver	The baby is really great, I like it very much, my friends also say good to come, recommended to buy!	Jingdong prefecture level city in Shandong
Dragon Quality Goods Shop	Green Intelligent Power Saver	Logistics is fast, courier boy service is good, after using to see how the fruit, and then follow up.	Jingdong prefecture level city in Shandong
Dragon Quality Goods Shop	Green Intelligent Power Saver	Baby has been received to try, I believe that as the business advertised energy saving. The courier is very responsible.	Jingdong prefecture level city in Shandong
Dragon Quality Goods Shop	Green Intelligent Power Saver	Save Electric Energy King received, good quality, beautiful appearance, very satisfied!	Jingdong prefecture level city in Shandong
Dragon Quality Goods Shop	Green Intelligent Power Saver	Three no products no manufacturers no phone pure fraud induced consumption cheat	Jingdong prefecture level city in Shandong
Dragon Quality Goods Shop	Green Intelligent Power Saver	I'm using it now, I don't know if it's as good as it says, I'll follow up after a month	Jingdong prefecture level city in Shandong
Dragon Quality Goods Shop	Green Intelligent Power Saver	Good quality logistics and fast. Fine workmanship. The description is consistent cost-effective	Jingdong prefecture level city in Shandong
Dragon Quality Goods Shop	Green Intelligent Power Saver	Received, whether it is good or not, to wait a few days and then evaluate	Jingdong prefecture level city in Shandong
Dragon Quality Goods Shop	Green Intelligent Power Saver	I bought it and started using it, I won't know if it works well until I pay my electric bill	Jingdong prefecture level city in Shandong
Dragon Quality Goods Shop	Green Intelligent Power Saver	Especially good, energy saving and power saving, especially good quality, good	Jingdong prefecture level city in Shandong
Dragon Quality Goods Shop	Green Intelligent Power Saver	Logistics is strong, the quality is very good, paste really save electricity, as described, will buy back!	Jingdong prefecture level city in Shandong
Dragon Quality Goods Shop	Green Intelligent Power Saver	Great stuff at a great price, worth the money!	Jingdong prefecture level city in Shandong

Dragon Quality Goods Shop	Green Intelligent Power Saver	Received the power saver, very good quality, very power saving!	Jingdong prefecture level city in Shandong
Dragon Quality Goods Shop	Green Intelligent Power Saver	There are no fruits, only samples...	Jingdong prefecture level city in Shandong
Dragon Quality Goods Shop	Green Intelligent Power Saver	Save a few dollars a month.	Jingdong prefecture level city in Shandong
Dragon Quality Goods Shop	Green Intelligent Power Saver	The quality is particularly good, it works well, and the seller has a good service attitude	Jingdong prefecture level city in Shandong
Dragon Quality Goods Shop	Green Intelligent Power Saver	I've compared them a couple of times and I don't feel any difference	Jingdong prefecture level city in Shandong
Dragon Quality Goods Shop	Green Intelligent Power Saver	I don't see the savings. It's a sham.	Jingdong prefecture level city in Shandong
Dragon Quality Goods Shop	Green Intelligent Power Saver	The power saving effect is very good, and the logistics is very fast. The seller's service attitude is very good	Jingdong prefecture level city in Shandong
Dragon Quality Goods Shop	Green Intelligent Power Saver	Received, well packaged, haven't tried it yet!	Jingdong prefecture level city in Shandong
Dragon Quality Goods Shop	Green Intelligent Power Saver	I don't know how it works, I'll comment after I use it.	Jingdong prefecture level city in Shandong
Dragon Quality Goods Shop	Green Intelligent Power Saver	The product doesn't work well enough to be of any use	Jingdong prefecture level city in Shandong
Dragon Quality Goods Shop	Green Intelligent Power Saver	On Trial 。。。。。。。	Jingdong prefecture level city in Shandong
Dragon Quality Goods Shop	Green Intelligent Power Saver	Being used with unknown results	Jingdong prefecture level city in Shandong
Dragon Quality Goods Shop	Green Intelligent Power Saver	Smart power saving and great quality	Jingdong prefecture level city in Shandong
Dragon Quality Goods Shop	Green Intelligent Power Saver	The saver was received, it works very well and the price is affordable!	Jingdong prefecture level city in Shandong
Dragon Quality Goods Shop	Green Intelligent Power Saver	How can I feel the power saving and how long does it last	Jingdong prefecture level city in Shandong
Dragon Quality Goods Shop	Green Intelligent Power Saver	And I know if it saves electricity or not.	Jingdong prefecture level city in Shandong
Dragon Quality Goods Shop	Green Intelligent Power Saver	It won't save any electricity. No.	Jingdong prefecture level city in Shandong
Dragon Quality Goods Shop	Green Intelligent Power Saver	Invalid. Fake.	Jingdong prefecture level city in Shandong
Dragon Quality Goods Shop	Green Intelligent Power Saver	It's good!	Jingdong prefecture level city in Shandong
Dragon Quality Goods Shop	Green Intelligent Power Saver	have no effect	Jingdong prefecture level city in Shandong
Dragon Quality Goods Shop	Green Intelligent Power Saver	Not used.	Jingdong prefecture level city in Shandong
Dragon Quality Goods Shop	Green Intelligent Power Saver	There is no load range, packaging thrift, ten days also did not develop invoices, received the goods with a receipt, hand-written receipt also has no financial stamp, communication for nearly a week a moment to say that the electronic invoice, a moment to say that the paper invoice, said it is mailed to me, ten days also did not see anything, the invoice invoice can not be found to say that did not stay on file, single single number can not be found, said that the warehouse more than one to a one to find out, in addition to the perfunctory or perfunctory. Products are no certificate of conformity, proper three	Jingdong prefecture level city in Shandong
Dragon Quality Goods Shop	Green Intelligent Power Saver	Satisfaction.	Jingdong prefecture level city in Shandong
Dragon Quality Goods Shop	Green Intelligent Power Saver	There is not a bit of power saving effect, and ringing up an increase in the electricity bill, because you have to add the cost of this machine's own electricity consumption and purchase, so I think it is better to use this machine instead of increasing the expenditure, uneconomical, rather than not use it.	Jingdong prefecture level city in Shandong
Dragon Quality Goods Shop	Green Intelligent Power Saver	I've been using it for two days now and I don't feel any effect.	Jingdong prefecture level city in Shandong
Dragon Quality Goods Shop	Green Intelligent Power Saver	What it looked like was nothing like what was sent.	Jingdong prefecture level city in Shandong
Dragon Quality Goods Shop	Green Intelligent Power Saver	It didn't work. It didn't work for a month or so.	Jingdong prefecture level city in Shandong
Dragon Quality Goods Shop	Green Intelligent Power Saver	Garbage products, physical and description is not the same, no effect at all, pit # no discussion, to buy the fine, garbage, garbage, garbage	Jingdong prefecture level city in Shandong
Dragon Quality Goods Shop	Green Intelligent Power Saver	general	Jingdong prefecture level city in Shandong
Dragon Quality Goods Shop	Green Intelligent Power Saver	IQ tax. Instead of saving electricity, it increases the power consumption of an appliance.	Jingdong prefecture level city in Shandong
Dragon Quality Goods Shop	Green Intelligent Power Saver	There's no trash at all.	Jingdong prefecture level city in Shandong
Dragon Quality Goods Shop	Green Intelligent Power Saver	It doesn't do anything.	Jingdong prefecture level city in Shandong
Dragon Quality Goods Shop	Green Intelligent Power Saver	(of an unmarried couple) be close	Jingdong prefecture level city in Shandong
Dragon Quality Goods Shop	Green Intelligent Power Saver	(of an unmarried couple) be close	Jingdong prefecture level city in Shandong
Dragon Quality Goods Shop	Green Intelligent Power Saver	no electricity saving	Jingdong prefecture level city in Shandong
Dragon Quality Goods Shop	Green Intelligent Power Saver	differ from	Jingdong prefecture level city in Shandong
Dragon Quality Goods Shop	Green Intelligent Power Saver	Saver has received, but real can save electricity, the quality is very good, I times I buy a few more, too good.	Jingdong prefecture level city in Shandong
Dragon Quality Goods Shop	Green Intelligent Power Saver	Great quality as always, a very favorite product, great! o(*￣︶￣*)o	Jingdong prefecture level city in Shandong
Dragon Quality Goods Shop	Green Intelligent Power Saver	If we can reduce the electricity bill, for individuals can save money, for the country can protect the environment, now the weather is getting hotter and hotter, to protect the environment to reduce emissions is very important. Already use the product, whether to reduce the electricity bill depends on next month!	Jingdong prefecture level city in Shandong
Dragon Quality Goods Shop	Green Intelligent Power Saver	Purchased a power saver received on the put into use, rent a room with many electrical appliances, power consumption is very large, we have a piece of electricity or 1.2 dollars a time, with this power saver is much better, every day can save a few cents, very convenient and practical!	Jingdong prefecture level city in Shandong
Dragon Quality Goods Shop	Green Intelligent Power Saver	The product is better to use before not to use when a month 1500 yuan electricity bill, now using the 5 second month electricity bill directly down 470 dollars or very good. A save money to buy several power saver money ZanZanZanZan👍	Jingdong prefecture level city in Shandong
Dragon Quality Goods Shop	Green Intelligent Power Saver	The moment I received the goods really satisfied with this product, not only high-grade packaging, the body are base alloy, up to the price, I was also introduced by a friend, he used last month did save a lot of electricity, but also recommended to me!	Jingdong prefecture level city in Shandong
Dragon Quality Goods Shop	Green Intelligent Power Saver	I got it. Good review. I'm very satisfied. Reasonable price.	Jingdong prefecture level city in Shandong
Dragon Quality Goods Shop	Green Intelligent Power Saver	Intelligent power saver received and used, looking at the quality is good, the quality of the plug sent is also very good. Expect the effect of the economizer is obvious, first five-star praise, with a period of time to follow up!	Jingdong prefecture level city in Shandong
Dragon Quality Goods Shop	Green Intelligent Power Saver	The saver has been received. It was plugged into an outlet and used immediately after receipt. Whether it really saves electricity is not clear yet, I believe that this brand of energy saver really saves	Jingdong prefecture level city in Shandong
Dragon Quality Goods Shop	Green Intelligent Power Saver	Okay, okay, okay. Thank you so much. Fast is good. Fast. Item was as described, mainly because the event was cheap and you can save a lot of money.	Jingdong prefecture level city in Shandong

Dragon Quality Goods Shop	Green Intelligent Power Saver	Received it a couple days ago! Plugged it in the same day! Let's see what the electric bill is in a month! Hopefully it works well oh!	Jingdong prefecture level city in Shandong
Dragon Quality Goods Shop	Green Intelligent Power Saver	Looking at the reviews good, energy-saving small products, compact, the results after 2 months to see, because the electricity bill season payment	Jingdong prefecture level city in Shandong
Dragon Quality Goods Shop	Green Intelligent Power Saver	I just received it and will try it out to see if it works well. I'll comment later.	Jingdong prefecture level city in Shandong
Dragon Quality Goods Shop	Green Intelligent Power Saver	It looks a little small and not very aesthetically pleasing, I don't know how effective it will be until I use it	Jingdong prefecture level city in Shandong
Dragon Quality Goods Shop	Green Intelligent Power Saver	Not only to the point of being useless yet	Jingdong prefecture level city in Shandong
Dragon Quality Goods Shop	Green Intelligent Power Saver	Used it for a month, nothing works, the meter you turn very fast. This is not a scam, but also sold on Jingdong. To cheat a bunch of people?	Jingdong prefecture level city in Shandong
Dragon Quality Goods Shop	Green Intelligent Power Saver	Very good to use, every day can save a little electricity, really good under the sample down a month to save a few dozen dollars in electricity, good before I did not know that there is this haha, the price is not expensive praise.	Jingdong prefecture level city in Shandong
Dragon Quality Goods Shop	Green Intelligent Power Saver	Tried it for ten days and the change is almost nothing, can't see the power saving yet, use it some more days and see how it goes.	Jingdong prefecture level city in Shandong
Dragon Quality Goods Shop	Green Intelligent Power Saver	Received very quickly, the quality is very good, really save electricity, business service attitude is also very good, I hope you do not miss this opportunity, I continue to buy a few in	Jingdong prefecture level city in Shandong
Dragon Quality Goods Shop	Green Intelligent Power Saver	Quality is very good Oh, the delivery is also very fast, a very favorite store, I want to crazy recommend to friends!	Jingdong prefecture level city in Shandong
Dragon Quality Goods Shop	Green Intelligent Power Saver	The goods have just received, fine workmanship, and the same as described by the merchant, to use a period of time to see if it can achieve the purpose of power saving.	Jingdong prefecture level city in Shandong
Dragon Quality Goods Shop	Green Intelligent Power Saver	Received the goods first use a period of time to see if there is no effect, the business commitment to ninety days invalid refund, first praise, good service!	Jingdong prefecture level city in Shandong
Dragon Quality Goods Shop	Green Intelligent Power Saver	The product is used for a month without energy-saving and power-saving effect does not have any effect	Jingdong prefecture level city in Shandong
Dragon Quality Goods Shop	Green Intelligent Power Saver	The baby is really great, I like it very much, my friends also say good to come, recommended to buy!	Jingdong prefecture level city in Shandong
Dragon Quality Goods Shop	Green Intelligent Power Saver	Logistics is fast, courier boy service is good, after using to see how the fruit, and then follow up.	Jingdong prefecture level city in Shandong
Dragon Quality Goods Shop	Green Intelligent Power Saver	Baby has been received to try, I believe that as the business advertised energy saving. The courier is very responsible.	Jingdong prefecture level city in Shandong
Dragon Quality Goods Shop	Green Intelligent Power Saver	Save Electric Energy King received, good quality, beautiful appearance, very satisfied!	Jingdong prefecture level city in Shandong
Dragon Quality Goods Shop	Green Intelligent Power Saver	Three no products no manufacturers no phone pure fraud induced consumption cheat	Jingdong prefecture level city in Shandong
Dragon Quality Goods Shop	Green Intelligent Power Saver	I'm using it now, I don't know if it's as good as it says, I'll follow up after a month	Jingdong prefecture level city in Shandong
Dragon Quality Goods Shop	Green Intelligent Power Saver	Good quality logistics and fast. Fine workmanship. The description is consistent cost-effective	Jingdong prefecture level city in Shandong
Dragon Quality Goods Shop	Green Intelligent Power Saver	Received, whether it is good or not, to wait a few days and then evaluate	Jingdong prefecture level city in Shandong
Dragon Quality Goods Shop	Green Intelligent Power Saver	I bought it and started using it, I won't know if it works well until I pay my electric bill	Jingdong prefecture level city in Shandong
Dragon Quality Goods Shop	Green Intelligent Power Saver	Especially good, energy saving and power saving, especially good quality, good	Jingdong prefecture level city in Shandong
Dragon Quality Goods Shop	Green Intelligent Power Saver	Logistics is strong, the quality is very good, paste really save electricity, as described, will buy back!	Jingdong prefecture level city in Shandong
Dragon Quality Goods Shop	Green Intelligent Power Saver	Great stuff at a great price, worth the money!	Jingdong prefecture level city in Shandong
Dragon Quality Goods Shop	Green Intelligent Power Saver	Received the power saver, very good quality, very power saving!	Jingdong prefecture level city in Shandong
Dragon Quality Goods Shop	Green Intelligent Power Saver	There are no fruits, only samples...	Jingdong prefecture level city in Shandong
Dragon Quality Goods Shop	Green Intelligent Power Saver	Save a few dollars a month.	Jingdong prefecture level city in Shandong
Dragon Quality Goods Shop	Green Intelligent Power Saver	The quality is particularly good, it works well, and the seller has a good service attitude	Jingdong prefecture level city in Shandong
Dragon Quality Goods Shop	Green Intelligent Power Saver	I've compared them a couple of times and I don't feel any difference	Jingdong prefecture level city in Shandong
Dragon Quality Goods Shop	Green Intelligent Power Saver	I don't see the savings. It's a sham.	Jingdong prefecture level city in Shandong
Dragon Quality Goods Shop	Green Intelligent Power Saver	The power saving effect is very good, and the logistics is very fast. The seller's service attitude is very good	Jingdong prefecture level city in Shandong
Dragon Quality Goods Shop	Green Intelligent Power Saver	Received, well packaged, haven't tried it yet!	Jingdong prefecture level city in Shandong
Dragon Quality Goods Shop	Green Intelligent Power Saver	I don't know how it works, I'll comment after I use it.	Jingdong prefecture level city in Shandong
Dragon Quality Goods Shop	Green Intelligent Power Saver	The product doesn't work well enough to be of any use	Jingdong prefecture level city in Shandong
Dragon Quality Goods Shop	Green Intelligent Power Saver	On Trial 。。。。。。。	Jingdong prefecture level city in Shandong
Dragon Quality Goods Shop	Green Intelligent Power Saver	Being used with unknown results	Jingdong prefecture level city in Shandong
Dragon Quality Goods Shop	Green Intelligent Power Saver	Smart power saving and great quality	Jingdong prefecture level city in Shandong
Dragon Quality Goods Shop	Green Intelligent Power Saver	The saver was received, it works very well and the price is affordable!	Jingdong prefecture level city in Shandong
Dragon Quality Goods Shop	Green Intelligent Power Saver	How can I feel the power saving and how long does it last	Jingdong prefecture level city in Shandong
Dragon Quality Goods Shop	Green Intelligent Power Saver	And I know if it saves electricity or not.	Jingdong prefecture level city in Shandong
Dragon Quality Goods Shop	Green Intelligent Power Saver	It won't save any electricity. No.	Jingdong prefecture level city in Shandong
Dragon Quality Goods Shop	Green Intelligent Power Saver	Invalid. Fake.	Jingdong prefecture level city in Shandong
Dragon Quality Goods Shop	Green Intelligent Power Saver	It's good!	Jingdong prefecture level city in Shandong
Dragon Quality Goods Shop	Green Intelligent Power Saver	have no effect	Jingdong prefecture level city in Shandong
Dragon Quality Goods Shop	Green Intelligent Power Saver	Not used.	Jingdong prefecture level city in Shandong
Dragon Quality Goods Shop	Green Intelligent Power Saver	There is no load range, packaging thrift, ten days also did not develop invoices, received the goods with a receipt, hand-written receipt also has no financial stamp, communication for nearly a week a moment to say that the electronic invoice, a moment to say that the paper invoice, said it is mailed to me, ten days also did not see anything, the invoice invoice can not be found to say that did not stay on file, single single number can not be found, said that the warehouse more than one to a one to find out, in addition to the perfunctory or perfunctory. Products are no certificate of conformity, proper three	Jingdong prefecture level city in Shandong
Dragon Quality Goods Shop	Green Intelligent Power Saver	Satisfaction.	Jingdong prefecture level city in Shandong

Dragon Quality Goods Shop	Green Intelligent Power Saver	There is not a bit of power saving effect, and ringing up an increase in the electricity bill, because you have to add the cost of this machine's own electricity consumption and purchase, so I think it is better to use this machine instead of increasing the expenditure, uneconomical, rather than not use it.	Jingdong prefecture level city in Shandong
Dragon Quality Goods Shop	Green Intelligent Power Saver	I've been using it for two days now and I don't feel any effect.	Jingdong prefecture level city in Shandong
Dragon Quality Goods Shop	Green Intelligent Power Saver	What it looked like was nothing like what was sent.	Jingdong prefecture level city in Shandong
Dragon Quality Goods Shop	Green Intelligent Power Saver	It didn't work. It didn't work for a month or so.	Jingdong prefecture level city in Shandong
Dragon Quality Goods Shop	Green Intelligent Power Saver	Garbage products, physical and description is not the same, no effect at all, pit # no discussion, to buy the fine, garbage, garbage, garbage	Jingdong prefecture level city in Shandong
Dragon Quality Goods Shop	Green Intelligent Power Saver	general	Jingdong prefecture level city in Shandong
Dragon Quality Goods Shop	Green Intelligent Power Saver	IQ tax. Instead of saving electricity, it increases the power consumption of an appliance.	Jingdong prefecture level city in Shandong
Dragon Quality Goods Shop	Green Intelligent Power Saver	There's no trash at all.	Jingdong prefecture level city in Shandong
Dragon Quality Goods Shop	Green Intelligent Power Saver	It doesn't do anything.	Jingdong prefecture level city in Shandong
Dragon Quality Goods Shop	Green Intelligent Power Saver	(of an unmarried couple) be close	Jingdong prefecture level city in Shandong
Dragon Quality Goods Shop	Green Intelligent Power Saver	(of an unmarried couple) be close	Jingdong prefecture level city in Shandong
Dragon Quality Goods Shop	Green Intelligent Power Saver	no electricity saving	Jingdong prefecture level city in Shandong
Dragon Quality Goods Shop	Green Intelligent Power Saver	differ from	Jingdong prefecture level city in Shandong
Dragon Quality Goods Shop	Green Intelligent Power Saver	Saver has received, but real can save electricity, the quality is very good, I times I buy a few more, too good.	Jingdong prefecture level city in Shandong
Dragon Quality Goods Shop	Green Intelligent Power Saver	Great quality as always, a very favorite product, great! o(*￣︶￣*)o	Jingdong prefecture level city in Shandong
Dragon Quality Goods Shop	Green Intelligent Power Saver	If we can reduce the electricity bill, for individuals can save money, for the country can protect the environment, now the weather is getting hotter and hotter, to protect the environment to reduce emissions is very important. Already use the product, whether to reduce the electricity bill depends on next month!	Jingdong prefecture level city in Shandong
Dragon Quality Goods Shop	Green Intelligent Power Saver	Purchased a power saver received on the put into use, rent a room with many electrical appliances, power consumption is very large, we have a piece of electricity or 1.2 dollars a time, with this power saver is much better, every day can save a few cents, very convenient and practical!	Jingdong prefecture level city in Shandong
Dragon Quality Goods Shop	Green Intelligent Power Saver	The product is better to use before not to use when a month 1500 yuan electricity bill, now using the 5 second month electricity bill directly down 470 dollars or very good. A save money to buy several power saver money ZanZanZanZan👍	Jingdong prefecture level city in Shandong
Dragon Quality Goods Shop	Green Intelligent Power Saver	The moment I received the goods really satisfied with this product, not only high-grade packaging, the body are base alloy, up to the price, I was also introduced by a friend, he used last month did save a lot of electricity, but also recommended to me!	Jingdong prefecture level city in Shandong
Dragon Quality Goods Shop	Green Intelligent Power Saver	I got it. Good review. I'm very satisfied. Reasonable price.	Jingdong prefecture level city in Shandong
Dragon Quality Goods Shop	Green Intelligent Power Saver	Intelligent power saver received and used, looking at the quality is good, the quality of the plug sent is also very good. Expect the effect of the economizer is obvious, first five-star praise, with a period of time to follow up!	Jingdong prefecture level city in Shandong
Dragon Quality Goods Shop	Green Intelligent Power Saver	The saver has been received. It was plugged into an outlet and used immediately after receipt. Whether it really saves electricity is not clear yet, I believe that this brand of energy saver really saves	Jingdong prefecture level city in Shandong
Dragon Quality Goods Shop	Green Intelligent Power Saver	Okay, okay, okay. Thank you so much. Fast is good. Fast. Item was as described, mainly because the event was cheap and you can save a lot of money.	Jingdong prefecture level city in Shandong
Dragon Quality Goods Shop	Green Intelligent Power Saver	Received it a couple days ago! Plugged it in the same day! Let's see what the electric bill is in a month! Hopefully it works well oh!	Jingdong prefecture level city in Shandong
Dragon Quality Goods Shop	Green Intelligent Power Saver	Looking at the reviews good, energy-saving small products, compact, the results after 2 months to see, because the electricity bill season payment	Jingdong prefecture level city in Shandong
Dragon Quality Goods Shop	Green Intelligent Power Saver	I just received it and will try it out to see if it works well. I'll comment later.	Jingdong prefecture level city in Shandong
Dragon Quality Goods Shop	Green Intelligent Power Saver	It looks a little small and not very aesthetically pleasing, I don't know how effective it will be until I use it	Jingdong prefecture level city in Shandong
Dragon Quality Goods Shop	Green Intelligent Power Saver	Not only to the point of being useless yet	Jingdong prefecture level city in Shandong
Dragon Quality Goods Shop	Green Intelligent Power Saver	Used it for a month, nothing works, the meter you turn very fast. This is not a scam, but also sold on Jingdong. To cheat a bunch of people?	Jingdong prefecture level city in Shandong
Dragon Quality Goods Shop	Green Intelligent Power Saver	Very good to use, every day can save a little electricity, really good under the sample down a month to save a few dozen dollars in electricity, good before I did not know that there is this 👆 haha, the price is not expensive praise.	Jingdong prefecture level city in Shandong
Dragon Quality Goods Shop	Green Intelligent Power Saver	Tried it for ten days and the change is almost nothing, can't see the power saving yet, use it some more days and see how it goes.	Jingdong prefecture level city in Shandong
Dragon Quality Goods Shop	Green Intelligent Power Saver	Received very quickly, the quality is very good, really save electricity, business service attitude is also very good, I hope you do not miss this opportunity, I continue to buy a few in	Jingdong prefecture level city in Shandong
Dragon Quality Goods Shop	Green Intelligent Power Saver	Quality is very good Oh, the delivery is also very fast, a very favorite store, I want to crazy recommend to friends!	Jingdong prefecture level city in Shandong
Dragon Quality Goods Shop	Green Intelligent Power Saver	The goods have just received, fine workmanship, and the same as described by the merchant, to use a period of time to see if it can achieve the purpose of power saving.	Jingdong prefecture level city in Shandong
Dragon Quality Goods Shop	Green Intelligent Power Saver	Received the goods first use a period of time to see if there is no effect, the business commitment to ninety days invalid refund, first praise, good service!	Jingdong prefecture level city in Shandong
Dragon Quality Goods Shop	Green Intelligent Power Saver	The product is used for a month without energy-saving and power-saving effect does not have any effect	Jingdong prefecture level city in Shandong
Dragon Quality Goods Shop	Green Intelligent Power Saver	The baby is really great, I like it very much, my friends also say good to come, recommended to buy!	Jingdong prefecture level city in Shandong
Dragon Quality Goods Shop	Green Intelligent Power Saver	Logistics is fast, courier boy service is good, after using to see how the fruit, and then follow up.	Jingdong prefecture level city in Shandong
Dragon Quality Goods Shop	Green Intelligent Power Saver	Baby has been received to try, I believe that as the business advertised energy saving. The courier is very responsible.	Jingdong prefecture level city in Shandong
Dragon Quality Goods Shop	Green Intelligent Power Saver	Save Electric Energy King received, good quality, beautiful appearance, very satisfied!	Jingdong prefecture level city in Shandong
Dragon Quality Goods Shop	Green Intelligent Power Saver	Three no products no manufacturers no phone pure fraud induced consumption cheat	Jingdong prefecture level city in Shandong
Dragon Quality Goods Shop	Green Intelligent Power Saver	I'm using it now, I don't know if it's as good as it says, I'll follow up after a month	Jingdong prefecture level city in Shandong
Dragon Quality Goods Shop	Green Intelligent Power Saver	Good quality logistics and fast. Fine workmanship. The description is consistent cost-effective	Jingdong prefecture level city in Shandong
Dragon Quality Goods Shop	Green Intelligent Power Saver	Received, whether it is good or not, to wait a few days and then evaluate	Jingdong prefecture level city in Shandong
Dragon Quality Goods Shop	Green Intelligent Power Saver	I bought it and started using it, I won't know if it works well until I pay my electric bill	Jingdong prefecture level city in Shandong
Dragon Quality Goods Shop	Green Intelligent Power Saver	Especially good, energy saving and power saving, especially good quality, good	Jingdong prefecture level city in Shandong
Dragon Quality Goods Shop	Green Intelligent Power Saver	Logistics is strong, the quality is very good, paste really save electricity, as described, will buy back!	Jingdong prefecture level city in Shandong
Dragon Quality Goods Shop	Green Intelligent Power Saver	Great stuff at a great price, worth the money!	Jingdong prefecture level city in Shandong

Dragon Quality Goods Shop	Green Intelligent Power Saver	Received the power saver, very good quality, very power saving!	Jingdong prefecture level city in Shandong
Dragon Quality Goods Shop	Green Intelligent Power Saver	There are no fruits, only samples...	Jingdong prefecture level city in Shandong
Dragon Quality Goods Shop	Green Intelligent Power Saver	Save a few dollars a month.	Jingdong prefecture level city in Shandong
Dragon Quality Goods Shop	Green Intelligent Power Saver	The quality is particularly good, it works well, and the seller has a good service attitude	Jingdong prefecture level city in Shandong
Dragon Quality Goods Shop	Green Intelligent Power Saver	I've compared them a couple of times and I don't feel any difference	Jingdong prefecture level city in Shandong
Dragon Quality Goods Shop	Green Intelligent Power Saver	I don't see the savings. It's a sham.	Jingdong prefecture level city in Shandong
Dragon Quality Goods Shop	Green Intelligent Power Saver	The power saving effect is very good, and the logistics is very fast. The seller's service attitude is very good	Jingdong prefecture level city in Shandong
Dragon Quality Goods Shop	Green Intelligent Power Saver	Received, well packaged, haven't tried it yet!	Jingdong prefecture level city in Shandong
Dragon Quality Goods Shop	Green Intelligent Power Saver	I don't know how it works, I'll comment after I use it.	Jingdong prefecture level city in Shandong
Dragon Quality Goods Shop	Green Intelligent Power Saver	The product doesn't work well enough to be of any use	Jingdong prefecture level city in Shandong
Dragon Quality Goods Shop	Green Intelligent Power Saver	On Trial 。。。。。。。	Jingdong prefecture level city in Shandong
Dragon Quality Goods Shop	Green Intelligent Power Saver	Being used with unknown results	Jingdong prefecture level city in Shandong
Dragon Quality Goods Shop	Green Intelligent Power Saver	Smart power saving and great quality	Jingdong prefecture level city in Shandong
Dragon Quality Goods Shop	Green Intelligent Power Saver	The saver was received, it works very well and the price is affordable!	Jingdong prefecture level city in Shandong
Dragon Quality Goods Shop	Green Intelligent Power Saver	How can I feel the power saving and how long does it last	Jingdong prefecture level city in Shandong
Dragon Quality Goods Shop	Green Intelligent Power Saver	And I know if it saves electricity or not.	Jingdong prefecture level city in Shandong
Dragon Quality Goods Shop	Green Intelligent Power Saver	It won't save any electricity. No.	Jingdong prefecture level city in Shandong
Dragon Quality Goods Shop	Green Intelligent Power Saver	Invalid. Fake.	Jingdong prefecture level city in Shandong
Dragon Quality Goods Shop	Green Intelligent Power Saver	It's good!	Jingdong prefecture level city in Shandong
Dragon Quality Goods Shop	Green Intelligent Power Saver	have no effect	Jingdong prefecture level city in Shandong
Dragon Quality Goods Shop	Green Intelligent Power Saver	Not used.	Jingdong prefecture level city in Shandong
Dragon Quality Goods Shop	Green Intelligent Power Saver	There is no load range, packaging thrift, ten days also did not develop invoices, received the goods with a receipt, hand-written receipt also has no financial stamp, communication for nearly a week a moment to say that the electronic invoice, a moment to say that the paper invoice, said it is mailed to me, ten days also did not see anything, the invoice invoice can not be found to say that did not stay on file, single single number can not be found, said that the warehouse more than one to a one to find out, in addition to the perfunctory or perfunctory. Products are no certificate of conformity, proper three	Jingdong prefecture level city in Shandong
Dragon Quality Goods Shop	Green Intelligent Power Saver	Satisfaction.	Jingdong prefecture level city in Shandong
Dragon Quality Goods Shop	Green Intelligent Power Saver	There is not a bit of power saving effect, and ringing up an increase in the electricity bill, because you have to add the cost of this machine's own electricity consumption and purchase, so I think it is better to use this machine instead of increasing the expenditure, uneconomical, rather than not use it.	Jingdong prefecture level city in Shandong
Dragon Quality Goods Shop	Green Intelligent Power Saver	I've been using it for two days now and I don't feel any effect.	Jingdong prefecture level city in Shandong
Dragon Quality Goods Shop	Green Intelligent Power Saver	What it looked like was nothing like what was sent.	Jingdong prefecture level city in Shandong
Dragon Quality Goods Shop	Green Intelligent Power Saver	It didn't work. It didn't work for a month or so.	Jingdong prefecture level city in Shandong
Dragon Quality Goods Shop	Green Intelligent Power Saver	Garbage products, physical and description is not the same, no effect at all, pit # no discussion, to buy the fine, garbage, garbage, garbage	Jingdong prefecture level city in Shandong
Dragon Quality Goods Shop	Green Intelligent Power Saver	general	Jingdong prefecture level city in Shandong
Dragon Quality Goods Shop	Green Intelligent Power Saver	IQ tax. Instead of saving electricity, it increases the power consumption of an appliance.	Jingdong prefecture level city in Shandong
Dragon Quality Goods Shop	Green Intelligent Power Saver	There's no trash at all.	Jingdong prefecture level city in Shandong
Dragon Quality Goods Shop	Green Intelligent Power Saver	It doesn't do anything.	Jingdong prefecture level city in Shandong
Dragon Quality Goods Shop	Green Intelligent Power Saver	(of an unmarried couple) be close	Jingdong prefecture level city in Shandong
Dragon Quality Goods Shop	Green Intelligent Power Saver	(of an unmarried couple) be close	Jingdong prefecture level city in Shandong
Dragon Quality Goods Shop	Green Intelligent Power Saver	no electricity saving	Jingdong prefecture level city in Shandong
Dragon Quality Goods Shop	Green Intelligent Power Saver	differ from	Jingdong prefecture level city in Shandong
Dragon Quality Goods Shop	Green Intelligent Power Saver	Saver has received, but real can save electricity, the quality is very good, I times I buy a few more, too good.	Jingdong prefecture level city in Shandong
Dragon Quality Goods Shop	Green Intelligent Power Saver	Great quality as always, a very favorite product, great! o(*￣︶￣*)o	Jingdong prefecture level city in Shandong
Dragon Quality Goods Shop	Green Intelligent Power Saver	If we can reduce the electricity bill, for individuals can save money, for the country can protect the environment, now the weather is getting hotter and hotter, to protect the environment to reduce emissions is very important. Already use the product, whether to reduce the electricity bill depends on next month!	Jingdong prefecture level city in Shandong
Dragon Quality Goods Shop	Green Intelligent Power Saver	Purchased a power saver received on the put into use, rent a room with many electrical appliances, power consumption is very large, we have a piece of electricity or 1.2 dollars a time, with this power saver is much better, every day can save a few cents, very convenient and practical!	Jingdong prefecture level city in Shandong
Dragon Quality Goods Shop	Green Intelligent Power Saver	The product is better to use before not to use when a month 1500 yuan electricity bill, now using the 5 second month electricity bill directly down 470 dollars or very good. A save money to buy several power saver money ZanZanZanZan👍	Jingdong prefecture level city in Shandong
Dragon Quality Goods Shop	Green Intelligent Power Saver	The moment I received the goods really satisfied with this product, not only high-grade packaging, the body are base alloy, up to the price, I was also introduced by a friend, he used last month did save a lot of electricity, but also recommended to me!	Jingdong prefecture level city in Shandong
Dragon Quality Goods Shop	Green Intelligent Power Saver	I got it. Good review. I'm very satisfied. Reasonable price.	Jingdong prefecture level city in Shandong
Dragon Quality Goods Shop	Green Intelligent Power Saver	Intelligent power saver received and used, looking at the quality is good, the quality of the plug sent is also very good. Expect the effect of the economizer is obvious, first five-star praise, with a period of time to follow up!	Jingdong prefecture level city in Shandong
Dragon Quality Goods Shop	Green Intelligent Power Saver	The saver has been received. It was plugged into an outlet and used immediately after receipt. Whether it really saves electricity is not clear yet, I believe that this brand of energy saver really saves	Jingdong prefecture level city in Shandong
Dragon Quality Goods Shop	Green Intelligent Power Saver	Okay, okay, okay. Thank you so much. Fast is good. Fast. Item was as described, mainly because the event was cheap and you can save a lot of money.	Jingdong prefecture level city in Shandong

Dragon Quality Goods Shop	Green Intelligent Power Saver	Received it a couple days ago! Plugged it in the same day! Let's see what the electric bill is in a month! Hopefully it works well oh!	Jingdong prefecture level city in Shandong
Dragon Quality Goods Shop	Green Intelligent Power Saver	Looking at the reviews good, energy-saving small products, compact, the results after 2 months to see, because the electricity bill season payment	Jingdong prefecture level city in Shandong
Dragon Quality Goods Shop	Green Intelligent Power Saver	I just received it and will try it out to see if it works well. I'll comment later.	Jingdong prefecture level city in Shandong
Dragon Quality Goods Shop	Green Intelligent Power Saver	It looks a little small and not very aesthetically pleasing, I don't know how effective it will be until I use it	Jingdong prefecture level city in Shandong
Dragon Quality Goods Shop	Green Intelligent Power Saver	Not only to the point of being useless yet	Jingdong prefecture level city in Shandong
Dragon Quality Goods Shop	Green Intelligent Power Saver	Used it for a month, nothing works, the meter you turn very fast. This is not a scam, but also sold on Jingdong. To cheat a bunch of people?	Jingdong prefecture level city in Shandong
Dragon Quality Goods Shop	Green Intelligent Power Saver	Very good to use, every day can save a little electricity, really good under the sample down a month to save a few dozen dollars in electricity, good before I did not know that there is this haha, the price is not expensive praise.	Jingdong prefecture level city in Shandong
Dragon Quality Goods Shop	Green Intelligent Power Saver	Tried it for ten days and the change is almost nothing, can't see the power saving yet, use it some more days and see how it goes.	Jingdong prefecture level city in Shandong
Dragon Quality Goods Shop	Green Intelligent Power Saver	Received very quickly, the quality is very good, really save electricity, business service attitude is also very good, I hope you do not miss this opportunity, I continue to buy a few in	Jingdong prefecture level city in Shandong
Dragon Quality Goods Shop	Green Intelligent Power Saver	Quality is very good Oh, the delivery is also very fast, a very favorite store, I want to crazy recommend to friends!	Jingdong prefecture level city in Shandong
Dragon Quality Goods Shop	Green Intelligent Power Saver	The goods have just received, fine workmanship, and the same as described by the merchant, to use a period of time to see if it can achieve the purpose of power saving.	Jingdong prefecture level city in Shandong
Dragon Quality Goods Shop	Green Intelligent Power Saver	Received the goods first use a period of time to see if there is no effect, the business commitment to ninety days invalid refund, first praise, good service!	Jingdong prefecture level city in Shandong
Dragon Quality Goods Shop	Green Intelligent Power Saver	The product is used for a month without energy-saving and power-saving effect does not have any effect	Jingdong prefecture level city in Shandong
Dragon Quality Goods Shop	Green Intelligent Power Saver	The baby is really great, I like it very much, my friends also say good to come, recommended to buy!	Jingdong prefecture level city in Shandong
Dragon Quality Goods Shop	Green Intelligent Power Saver	Logistics is fast, courier boy service is good, after using to see how the fruit, and then follow up.	Jingdong prefecture level city in Shandong
Dragon Quality Goods Shop	Green Intelligent Power Saver	Baby has been received to try, I believe that as the business advertised energy saving. The courier is very responsible.	Jingdong prefecture level city in Shandong
Dragon Quality Goods Shop	Green Intelligent Power Saver	Save Electric Energy King received, good quality, beautiful appearance, very satisfied!	Jingdong prefecture level city in Shandong
Dragon Quality Goods Shop	Green Intelligent Power Saver	Three no products no manufacturers no phone pure fraud induced consumption cheat	Jingdong prefecture level city in Shandong
Dragon Quality Goods Shop	Green Intelligent Power Saver	I'm using it now, I don't know if it's as good as it says, I'll follow up after a month	Jingdong prefecture level city in Shandong
Dragon Quality Goods Shop	Green Intelligent Power Saver	Good quality logistics and fast. Fine workmanship. The description is consistent cost-effective	Jingdong prefecture level city in Shandong
Dragon Quality Goods Shop	Green Intelligent Power Saver	Received, whether it is good or not, to wait a few days and then evaluate	Jingdong prefecture level city in Shandong
Dragon Quality Goods Shop	Green Intelligent Power Saver	I bought it and started using it, I won't know if it works well until I pay my electric bill	Jingdong prefecture level city in Shandong
Dragon Quality Goods Shop	Green Intelligent Power Saver	Especially good, energy saving and power saving, especially good quality, good	Jingdong prefecture level city in Shandong
Dragon Quality Goods Shop	Green Intelligent Power Saver	Logistics is strong, the quality is very good, paste really save electricity, as described, will buy back!	Jingdong prefecture level city in Shandong
Dragon Quality Goods Shop	Green Intelligent Power Saver	Great stuff at a great price, worth the money!	Jingdong prefecture level city in Shandong
Dragon Quality Goods Shop	Green Intelligent Power Saver	Received the power saver, very good quality, very power saving!	Jingdong prefecture level city in Shandong
Dragon Quality Goods Shop	Green Intelligent Power Saver	There are no fruits, only samples...	Jingdong prefecture level city in Shandong
Dragon Quality Goods Shop	Green Intelligent Power Saver	Save a few dollars a month.	Jingdong prefecture level city in Shandong
Dragon Quality Goods Shop	Green Intelligent Power Saver	The quality is particularly good, it works well, and the seller has a good service attitude	Jingdong prefecture level city in Shandong
Dragon Quality Goods Shop	Green Intelligent Power Saver	I've compared them a couple of times and I don't feel any difference	Jingdong prefecture level city in Shandong
Dragon Quality Goods Shop	Green Intelligent Power Saver	I don't see the savings. It's a sham.	Jingdong prefecture level city in Shandong
Dragon Quality Goods Shop	Green Intelligent Power Saver	The power saving effect is very good, and the logistics is very fast. The seller's service attitude is very good	Jingdong prefecture level city in Shandong
Dragon Quality Goods Shop	Green Intelligent Power Saver	Received, well packaged, haven't tried it yet!	Jingdong prefecture level city in Shandong
Dragon Quality Goods Shop	Green Intelligent Power Saver	I don't know how it works, I'll comment after I use it.	Jingdong prefecture level city in Shandong
Dragon Quality Goods Shop	Green Intelligent Power Saver	The product doesn't work well enough to be of any use	Jingdong prefecture level city in Shandong
Dragon Quality Goods Shop	Green Intelligent Power Saver	On Trial 。。。。。。。	Jingdong prefecture level city in Shandong
Dragon Quality Goods Shop	Green Intelligent Power Saver	Being used with unknown results	Jingdong prefecture level city in Shandong
Dragon Quality Goods Shop	Green Intelligent Power Saver	Smart power saving and great quality	Jingdong prefecture level city in Shandong
Dragon Quality Goods Shop	Green Intelligent Power Saver	The saver was received, it works very well and the price is affordable!	Jingdong prefecture level city in Shandong
Dragon Quality Goods Shop	Green Intelligent Power Saver	How can I feel the power saving and how long does it last	Jingdong prefecture level city in Shandong
Dragon Quality Goods Shop	Green Intelligent Power Saver	And I know if it saves electricity or not.	Jingdong prefecture level city in Shandong
Dragon Quality Goods Shop	Green Intelligent Power Saver	It won't save any electricity. No.	Jingdong prefecture level city in Shandong
Dragon Quality Goods Shop	Green Intelligent Power Saver	Invalid. Fake.	Jingdong prefecture level city in Shandong
Dragon Quality Goods Shop	Green Intelligent Power Saver	It's good!	Jingdong prefecture level city in Shandong
Dragon Quality Goods Shop	Green Intelligent Power Saver	have no effect	Jingdong prefecture level city in Shandong
Dragon Quality Goods Shop	Green Intelligent Power Saver	Not used.	Jingdong prefecture level city in Shandong
Dragon Quality Goods Shop	Green Intelligent Power Saver	There is no load range, packaging thrift, ten days also did not develop invoices, received the goods with a receipt, hand-written receipt also has no financial stamp, communication for nearly a week a moment to say that the electronic invoice, a moment to say that the paper invoice, said it is mailed to me, ten days also did not see anything, the invoice invoice can not be found to say that did not stay on file, single single number can not be found, said that the warehouse more than one to a one to find out, in addition to the perfunctory or perfunctory. Products are no certificate of conformity, proper three	Jingdong prefecture level city in Shandong
Dragon Quality Goods Shop	Green Intelligent Power Saver	Satisfaction.	Jingdong prefecture level city in Shandong

Dragon Quality Goods Shop	Green Intelligent Power Saver	There is not a bit of power saving effect, and ringing up an increase in the electricity bill, because you have to add the cost of this machine's own electricity consumption and purchase, so I think it is better to use this machine instead of increasing the expenditure, uneconomical, rather than not use it.	Jingdong prefecture level city in Shandong
Dragon Quality Goods Shop	Green Intelligent Power Saver	I've been using it for two days now and I don't feel any effect.	Jingdong prefecture level city in Shandong
Dragon Quality Goods Shop	Green Intelligent Power Saver	What it looked like was nothing like what was sent.	Jingdong prefecture level city in Shandong
Dragon Quality Goods Shop	Green Intelligent Power Saver	It didn't work. It didn't work for a month or so.	Jingdong prefecture level city in Shandong
Dragon Quality Goods Shop	Green Intelligent Power Saver	Garbage products, physical and description is not the same, no effect at all, pit # no discussion, to buy the fine, garbage, garbage, garbage	Jingdong prefecture level city in Shandong
Dragon Quality Goods Shop	Green Intelligent Power Saver	general	Jingdong prefecture level city in Shandong
Dragon Quality Goods Shop	Green Intelligent Power Saver	IQ tax. Instead of saving electricity, it increases the power consumption of an appliance.	Jingdong prefecture level city in Shandong
Dragon Quality Goods Shop	Green Intelligent Power Saver	There's no trash at all.	Jingdong prefecture level city in Shandong
Dragon Quality Goods Shop	Green Intelligent Power Saver	It doesn't do anything.	Jingdong prefecture level city in Shandong
Dragon Quality Goods Shop	Green Intelligent Power Saver	(of an unmarried couple) be close	Jingdong prefecture level city in Shandong
Dragon Quality Goods Shop	Green Intelligent Power Saver	(of an unmarried couple) be close	Jingdong prefecture level city in Shandong
Dragon Quality Goods Shop	Green Intelligent Power Saver	no electricity saving	Jingdong prefecture level city in Shandong
Dragon Quality Goods Shop	Green Intelligent Power Saver	differ from	Jingdong prefecture level city in Shandong
Dragon Quality Goods Shop	Green Intelligent Power Saver	Saver has received, but real can save electricity, the quality is very good, I times I buy a few more, too good.	Jingdong prefecture level city in Shandong
Dragon Quality Goods Shop	Green Intelligent Power Saver	Great quality as always, a very favorite product, great! o(*￣︶￣*)o	Jingdong prefecture level city in Shandong
Dragon Quality Goods Shop	Green Intelligent Power Saver	If we can reduce the electricity bill, for individuals can save money, for the country can protect the environment, now the weather is getting hotter and hotter, to protect the environment to reduce emissions is very important. Already use the product, whether to reduce the electricity bill depends on next month!	Jingdong prefecture level city in Shandong
Dragon Quality Goods Shop	Green Intelligent Power Saver	Purchased a power saver received on the put into use, rent a room with many electrical appliances, power consumption is very large, we have a piece of electricity or 1.2 dollars a time, with this power saver is much better, every day can save a few cents, very convenient and practical!	Jingdong prefecture level city in Shandong
Dragon Quality Goods Shop	Green Intelligent Power Saver	The product is better to use before not to use when a month 1500 yuan electricity bill, now using the 5 second month electricity bill directly down 470 dollars or very good. A save money to buy several power saver money ZanZanZanZan👍	Jingdong prefecture level city in Shandong
Dragon Quality Goods Shop	Green Intelligent Power Saver	The moment I received the goods really satisfied with this product, not only high-grade packaging, the body are base alloy, up to the price, I was also introduced by a friend, he used last month did save a lot of electricity, but also recommended to me!	Jingdong prefecture level city in Shandong
Dragon Quality Goods Shop	Green Intelligent Power Saver	I got it. Good review. I'm very satisfied. Reasonable price.	Jingdong prefecture level city in Shandong
Dragon Quality Goods Shop	Green Intelligent Power Saver	Intelligent power saver received and used, looking at the quality is good, the quality of the plug sent is also very good. Expect the effect of the economizer is obvious, first five-star praise, with a period of time to follow up!	Jingdong prefecture level city in Shandong
Dragon Quality Goods Shop	Green Intelligent Power Saver	The saver has been received. It was plugged into an outlet and used immediately after receipt. Whether it really saves electricity is not clear yet, I believe that this brand of energy saver really saves	Jingdong prefecture level city in Shandong
Dragon Quality Goods Shop	Green Intelligent Power Saver	Okay, okay, okay. Thank you so much. Fast is good. Fast. Item was as described, mainly because the event was cheap and you can save a lot of money.	Jingdong prefecture level city in Shandong
Dragon Quality Goods Shop	Green Intelligent Power Saver	Received it a couple days ago! Plugged it in the same day! Let's see what the electric bill is in a month! Hopefully it works well oh!	Jingdong prefecture level city in Shandong
Dragon Quality Goods Shop	Green Intelligent Power Saver	Looking at the reviews good, energy-saving small products, compact, the results after 2 months to see, because the electricity bill season payment	Jingdong prefecture level city in Shandong
Dragon Quality Goods Shop	Green Intelligent Power Saver	I just received it and will try it out to see if it works well. I'll comment later.	Jingdong prefecture level city in Shandong
Dragon Quality Goods Shop	Green Intelligent Power Saver	It looks a little small and not very aesthetically pleasing, I don't know how effective it will be until I use it	Jingdong prefecture level city in Shandong
Dragon Quality Goods Shop	Green Intelligent Power Saver	Not only to the point of being useless yet	Jingdong prefecture level city in Shandong
Dragon Quality Goods Shop	Green Intelligent Power Saver	Used it for a month, nothing works, the meter you turn very fast. This is not a scam, but also sold on Jingdong. To cheat a bunch of people?	Jingdong prefecture level city in Shandong
Dragon Quality Goods Shop	Green Intelligent Power Saver	Very good to use, every day can save a little electricity, really good under the sample down a month to save a few dozen dollars in electricity, good before I did not know that there is this 👆 haha, the price is not expensive praise.	Jingdong prefecture level city in Shandong
Dragon Quality Goods Shop	Green Intelligent Power Saver	Tried it for ten days and the change is almost nothing, can't see the power saving yet, use it some more days and see how it goes.	Jingdong prefecture level city in Shandong
Dragon Quality Goods Shop	Green Intelligent Power Saver	Received very quickly, the quality is very good, really save electricity, business service attitude is also very good, I hope you do not miss this opportunity, I continue to buy a few in	Jingdong prefecture level city in Shandong
Dragon Quality Goods Shop	Green Intelligent Power Saver	Quality is very good Oh, the delivery is also very fast, a very favorite store, I want to crazy recommend to friends!	Jingdong prefecture level city in Shandong
Dragon Quality Goods Shop	Green Intelligent Power Saver	The goods have just received, fine workmanship, and the same as described by the merchant, to use a period of time to see if it can achieve the purpose of power saving.	Jingdong prefecture level city in Shandong
Dragon Quality Goods Shop	Green Intelligent Power Saver	Received the goods first use a period of time to see if there is no effect, the business commitment to ninety days invalid refund, first praise, good service!	Jingdong prefecture level city in Shandong
Dragon Quality Goods Shop	Green Intelligent Power Saver	The product is used for a month without energy-saving and power-saving effect does not have any effect	Jingdong prefecture level city in Shandong
Dragon Quality Goods Shop	Green Intelligent Power Saver	The baby is really great, I like it very much, my friends also say good to come, recommended to buy!	Jingdong prefecture level city in Shandong
Dragon Quality Goods Shop	Green Intelligent Power Saver	Logistics is fast, courier boy service is good, after using to see how the fruit, and then follow up.	Jingdong prefecture level city in Shandong
Dragon Quality Goods Shop	Green Intelligent Power Saver	Baby has been received to try, I believe that as the business advertised energy saving. The courier is very responsible.	Jingdong prefecture level city in Shandong
Dragon Quality Goods Shop	Green Intelligent Power Saver	Save Electric Energy King received, good quality, beautiful appearance, very satisfied!	Jingdong prefecture level city in Shandong
Dragon Quality Goods Shop	Green Intelligent Power Saver	Three no products no manufacturers no phone pure fraud induced consumption cheat	Jingdong prefecture level city in Shandong
Dragon Quality Goods Shop	Green Intelligent Power Saver	I'm using it now, I don't know if it's as good as it says, I'll follow up after a month	Jingdong prefecture level city in Shandong
Dragon Quality Goods Shop	Green Intelligent Power Saver	Good quality logistics and fast. Fine workmanship. The description is consistent cost-effective	Jingdong prefecture level city in Shandong
Dragon Quality Goods Shop	Green Intelligent Power Saver	Received, whether it is good or not, to wait a few days and then evaluate	Jingdong prefecture level city in Shandong
Dragon Quality Goods Shop	Green Intelligent Power Saver	I bought it and started using it, I won't know if it works well until I pay my electric bill	Jingdong prefecture level city in Shandong
Dragon Quality Goods Shop	Green Intelligent Power Saver	Especially good, energy saving and power saving, especially good quality, good	Jingdong prefecture level city in Shandong
Dragon Quality Goods Shop	Green Intelligent Power Saver	Logistics is strong, the quality is very good, paste really save electricity, as described, will buy back!	Jingdong prefecture level city in Shandong
Dragon Quality Goods Shop	Green Intelligent Power Saver	Great stuff at a great price, worth the money!	Jingdong prefecture level city in Shandong

Dragon Quality Goods Shop	Green Intelligent Power Saver	Received the power saver, very good quality, very power saving!	Jingdong prefecture level city in Shandong
Dragon Quality Goods Shop	Green Intelligent Power Saver	There are no fruits, only samples...	Jingdong prefecture level city in Shandong
Dragon Quality Goods Shop	Green Intelligent Power Saver	Save a few dollars a month.	Jingdong prefecture level city in Shandong
Dragon Quality Goods Shop	Green Intelligent Power Saver	The quality is particularly good, it works well, and the seller has a good service attitude	Jingdong prefecture level city in Shandong
Dragon Quality Goods Shop	Green Intelligent Power Saver	I've compared them a couple of times and I don't feel any difference	Jingdong prefecture level city in Shandong
Dragon Quality Goods Shop	Green Intelligent Power Saver	I don't see the savings. It's a sham.	Jingdong prefecture level city in Shandong
Dragon Quality Goods Shop	Green Intelligent Power Saver	The power saving effect is very good, and the logistics is very fast. The seller's service attitude is very good	Jingdong prefecture level city in Shandong
Dragon Quality Goods Shop	Green Intelligent Power Saver	Received, well packaged, haven't tried it yet!	Jingdong prefecture level city in Shandong
Dragon Quality Goods Shop	Green Intelligent Power Saver	I don't know how it works, I'll comment after I use it.	Jingdong prefecture level city in Shandong
Dragon Quality Goods Shop	Green Intelligent Power Saver	The product doesn't work well enough to be of any use	Jingdong prefecture level city in Shandong
Dragon Quality Goods Shop	Green Intelligent Power Saver	On Trial 。。。。。。。	Jingdong prefecture level city in Shandong
Dragon Quality Goods Shop	Green Intelligent Power Saver	Being used with unknown results	Jingdong prefecture level city in Shandong
Dragon Quality Goods Shop	Green Intelligent Power Saver	Smart power saving and great quality	Jingdong prefecture level city in Shandong
Dragon Quality Goods Shop	Green Intelligent Power Saver	The saver was received, it works very well and the price is affordable!	Jingdong prefecture level city in Shandong
Dragon Quality Goods Shop	Green Intelligent Power Saver	How can I feel the power saving and how long does it last	Jingdong prefecture level city in Shandong
Dragon Quality Goods Shop	Green Intelligent Power Saver	And I know if it saves electricity or not.	Jingdong prefecture level city in Shandong
Dragon Quality Goods Shop	Green Intelligent Power Saver	It won't save any electricity. No.	Jingdong prefecture level city in Shandong
Dragon Quality Goods Shop	Green Intelligent Power Saver	Invalid. Fake.	Jingdong prefecture level city in Shandong
Dragon Quality Goods Shop	Green Intelligent Power Saver	It's good!	Jingdong prefecture level city in Shandong
Dragon Quality Goods Shop	Green Intelligent Power Saver	have no effect	Jingdong prefecture level city in Shandong
Dragon Quality Goods Shop	Green Intelligent Power Saver	Not used.	Jingdong prefecture level city in Shandong
Dragon Quality Goods Shop	Green Intelligent Power Saver	There is no load range, packaging thrift, ten days also did not develop invoices, received the goods with a receipt, hand-written receipt also has no financial stamp, communication for nearly a week a moment to say that the electronic invoice, a moment to say that the paper invoice, said it is mailed to me, ten days also did not see anything, the invoice invoice can not be found to say that did not stay on file, single single number can not be found, said that the warehouse more than one to a one to find out, in addition to the perfunctory or perfunctory. Products are no certificate of conformity, proper three	Jingdong prefecture level city in Shandong
Dragon Quality Goods Shop	Green Intelligent Power Saver	Satisfaction.	Jingdong prefecture level city in Shandong
Dragon Quality Goods Shop	Green Intelligent Power Saver	There is not a bit of power saving effect, and ringing up an increase in the electricity bill, because you have to add the cost of this machine's own electricity consumption and purchase, so I think it is better to use this machine instead of increasing the expenditure, uneconomical, rather than not use it.	Jingdong prefecture level city in Shandong
Dragon Quality Goods Shop	Green Intelligent Power Saver	I've been using it for two days now and I don't feel any effect.	Jingdong prefecture level city in Shandong
Dragon Quality Goods Shop	Green Intelligent Power Saver	What it looked like was nothing like what was sent.	Jingdong prefecture level city in Shandong
Dragon Quality Goods Shop	Green Intelligent Power Saver	It didn't work. It didn't work for a month or so.	Jingdong prefecture level city in Shandong
Dragon Quality Goods Shop	Green Intelligent Power Saver	Garbage products, physical and description is not the same, no effect at all, pit # no discussion, to buy the fine, garbage, garbage, garbage	Jingdong prefecture level city in Shandong
Dragon Quality Goods Shop	Green Intelligent Power Saver	general	Jingdong prefecture level city in Shandong
Dragon Quality Goods Shop	Green Intelligent Power Saver	IQ tax. Instead of saving electricity, it increases the power consumption of an appliance.	Jingdong prefecture level city in Shandong
Dragon Quality Goods Shop	Green Intelligent Power Saver	There's no trash at all.	Jingdong prefecture level city in Shandong
Dragon Quality Goods Shop	Green Intelligent Power Saver	It doesn't do anything.	Jingdong prefecture level city in Shandong
Dragon Quality Goods Shop	Green Intelligent Power Saver	(of an unmarried couple) be close	Jingdong prefecture level city in Shandong
Dragon Quality Goods Shop	Green Intelligent Power Saver	(of an unmarried couple) be close	Jingdong prefecture level city in Shandong
Dragon Quality Goods Shop	Green Intelligent Power Saver	no electricity saving	Jingdong prefecture level city in Shandong
Dragon Quality Goods Shop	Green Intelligent Power Saver	differ from	Jingdong prefecture level city in Shandong
Dragon Quality Goods Shop	Green Intelligent Power Saver	Saver has received, but real can save electricity, the quality is very good, I times I buy a few more, too good.	Jingdong prefecture level city in Shandong
Dragon Quality Goods Shop	Green Intelligent Power Saver	Great quality as always, a very favorite product, great! o(*￣︶￣*)o	Jingdong prefecture level city in Shandong
Dragon Quality Goods Shop	Green Intelligent Power Saver	If we can reduce the electricity bill, for individuals can save money, for the country can protect the environment, now the weather is getting hotter and hotter, to protect the environment to reduce emissions is very important. Already use the product, whether to reduce the electricity bill depends on next month!	Jingdong prefecture level city in Shandong
Dragon Quality Goods Shop	Green Intelligent Power Saver	Purchased a power saver received on the put into use, rent a room with many electrical appliances, power consumption is very large, we have a piece of electricity or 1.2 dollars a time, with this power saver is much better, every day can save a few cents, very convenient and practical!	Jingdong prefecture level city in Shandong
Dragon Quality Goods Shop	Green Intelligent Power Saver	The product is better to use before not to use when a month 1500 yuan electricity bill, now using the 5 second month electricity bill directly down 470 dollars or very good. A save money to buy several power saver money ZanZanZanZan👍	Jingdong prefecture level city in Shandong
Dragon Quality Goods Shop	Green Intelligent Power Saver	The moment I received the goods really satisfied with this product, not only high-grade packaging, the body are base alloy, up to the price, I was also introduced by a friend, he used last month did save a lot of electricity, but also recommended to me!	Jingdong prefecture level city in Shandong
Dragon Quality Goods Shop	Green Intelligent Power Saver	I got it. Good review. I'm very satisfied. Reasonable price.	Jingdong prefecture level city in Shandong
Dragon Quality Goods Shop	Green Intelligent Power Saver	Intelligent power saver received and used, looking at the quality is good, the quality of the plug sent is also very good. Expect the effect of the economizer is obvious, first five-star praise, with a period of time to follow up!	Jingdong prefecture level city in Shandong
Dragon Quality Goods Shop	Green Intelligent Power Saver	The saver has been received. It was plugged into an outlet and used immediately after receipt. Whether it really saves electricity is not clear yet, I believe that this brand of energy saver really saves	Jingdong prefecture level city in Shandong
Dragon Quality Goods Shop	Green Intelligent Power Saver	Okay, okay, okay. Thank you so much. Fast is good. Fast. Item was as described, mainly because the event was cheap and you can save a lot of money.	Jingdong prefecture level city in Shandong

Dragon Quality Goods Shop	Green Intelligent Power Saver	Received it a couple days ago! Plugged it in the same day! Let's see what the electric bill is in a month! Hopefully it works well oh!	Jingdong prefecture level city in Shandong
Dragon Quality Goods Shop	Green Intelligent Power Saver	Looking at the reviews good, energy-saving small products, compact, the results after 2 months to see, because the electricity bill season payment	Jingdong prefecture level city in Shandong
Dragon Quality Goods Shop	Green Intelligent Power Saver	I just received it and will try it out to see if it works well. I'll comment later.	Jingdong prefecture level city in Shandong
Dragon Quality Goods Shop	Green Intelligent Power Saver	It looks a little small and not very aesthetically pleasing, I don't know how effective it will be until I use it	Jingdong prefecture level city in Shandong
Dragon Quality Goods Shop	Green Intelligent Power Saver	Not only to the point of being useless yet	Jingdong prefecture level city in Shandong
Dragon Quality Goods Shop	Green Intelligent Power Saver	Used it for a month, nothing works, the meter you turn very fast. This is not a scam, but also sold on Jingdong. To cheat a bunch of people?	Jingdong prefecture level city in Shandong
Dragon Quality Goods Shop	Green Intelligent Power Saver	Very good to use, every day can save a little electricity, really good under the sample down a month to save a few dozen dollars in electricity, good before I did not know that there is this haha, the price is not expensive praise.	Jingdong prefecture level city in Shandong
Dragon Quality Goods Shop	Green Intelligent Power Saver	Tried it for ten days and the change is almost nothing, can't see the power saving yet, use it some more days and see how it goes.	Jingdong prefecture level city in Shandong
Dragon Quality Goods Shop	Green Intelligent Power Saver	Received very quickly, the quality is very good, really save electricity, business service attitude is also very good, I hope you do not miss this opportunity, I continue to buy a few in	Jingdong prefecture level city in Shandong
Dragon Quality Goods Shop	Green Intelligent Power Saver	Quality is very good Oh, the delivery is also very fast, a very favorite store, I want to crazy recommend to friends!	Jingdong prefecture level city in Shandong
Dragon Quality Goods Shop	Green Intelligent Power Saver	The goods have just received, fine workmanship, and the same as described by the merchant, to use a period of time to see if it can achieve the purpose of power saving.	Jingdong prefecture level city in Shandong
Dragon Quality Goods Shop	Green Intelligent Power Saver	Received the goods first use a period of time to see if there is no effect, the business commitment to ninety days invalid refund, first praise, good service!	Jingdong prefecture level city in Shandong
Dragon Quality Goods Shop	Green Intelligent Power Saver	The product is used for a month without energy-saving and power-saving effect does not have any effect	Jingdong prefecture level city in Shandong
Dragon Quality Goods Shop	Green Intelligent Power Saver	The baby is really great, I like it very much, my friends also say good to come, recommended to buy!	Jingdong prefecture level city in Shandong
Dragon Quality Goods Shop	Green Intelligent Power Saver	Logistics is fast, courier boy service is good, after using to see how the fruit, and then follow up.	Jingdong prefecture level city in Shandong
Dragon Quality Goods Shop	Green Intelligent Power Saver	Baby has been received to try, I believe that as the business advertised energy saving. The courier is very responsible.	Jingdong prefecture level city in Shandong
Dragon Quality Goods Shop	Green Intelligent Power Saver	Save Electric Energy King received, good quality, beautiful appearance, very satisfied!	Jingdong prefecture level city in Shandong
Dragon Quality Goods Shop	Green Intelligent Power Saver	Three no products no manufacturers no phone pure fraud induced consumption cheat	Jingdong prefecture level city in Shandong
Dragon Quality Goods Shop	Green Intelligent Power Saver	I'm using it now, I don't know if it's as good as it says, I'll follow up after a month	Jingdong prefecture level city in Shandong
Dragon Quality Goods Shop	Green Intelligent Power Saver	Good quality logistics and fast. Fine workmanship. The description is consistent cost-effective	Jingdong prefecture level city in Shandong
Dragon Quality Goods Shop	Green Intelligent Power Saver	Received, whether it is good or not, to wait a few days and then evaluate	Jingdong prefecture level city in Shandong
Dragon Quality Goods Shop	Green Intelligent Power Saver	I bought it and started using it, I won't know if it works well until I pay my electric bill	Jingdong prefecture level city in Shandong
Dragon Quality Goods Shop	Green Intelligent Power Saver	Especially good, energy saving and power saving, especially good quality, good	Jingdong prefecture level city in Shandong
Dragon Quality Goods Shop	Green Intelligent Power Saver	Logistics is strong, the quality is very good, paste really save electricity, as described, will buy back!	Jingdong prefecture level city in Shandong
Dragon Quality Goods Shop	Green Intelligent Power Saver	Great stuff at a great price, worth the money!	Jingdong prefecture level city in Shandong
Dragon Quality Goods Shop	Green Intelligent Power Saver	Received the power saver, very good quality, very power saving!	Jingdong prefecture level city in Shandong
Dragon Quality Goods Shop	Green Intelligent Power Saver	There are no fruits, only samples...	Jingdong prefecture level city in Shandong
Dragon Quality Goods Shop	Green Intelligent Power Saver	Save a few dollars a month.	Jingdong prefecture level city in Shandong
Dragon Quality Goods Shop	Green Intelligent Power Saver	The quality is particularly good, it works well, and the seller has a good service attitude	Jingdong prefecture level city in Shandong
Dragon Quality Goods Shop	Green Intelligent Power Saver	I've compared them a couple of times and I don't feel any difference	Jingdong prefecture level city in Shandong
Dragon Quality Goods Shop	Green Intelligent Power Saver	I don't see the savings. It's a sham.	Jingdong prefecture level city in Shandong
Dragon Quality Goods Shop	Green Intelligent Power Saver	The power saving effect is very good, and the logistics is very fast. The seller's service attitude is very good	Jingdong prefecture level city in Shandong
Dragon Quality Goods Shop	Green Intelligent Power Saver	Received, well packaged, haven't tried it yet!	Jingdong prefecture level city in Shandong
Dragon Quality Goods Shop	Green Intelligent Power Saver	I don't know how it works, I'll comment after I use it.	Jingdong prefecture level city in Shandong
Dragon Quality Goods Shop	Green Intelligent Power Saver	The product doesn't work well enough to be of any use	Jingdong prefecture level city in Shandong
Dragon Quality Goods Shop	Green Intelligent Power Saver	On Trial 。。。。。。。	Jingdong prefecture level city in Shandong
Dragon Quality Goods Shop	Green Intelligent Power Saver	Being used with unknown results	Jingdong prefecture level city in Shandong
Dragon Quality Goods Shop	Green Intelligent Power Saver	Smart power saving and great quality	Jingdong prefecture level city in Shandong
Dragon Quality Goods Shop	Green Intelligent Power Saver	The saver was received, it works very well and the price is affordable!	Jingdong prefecture level city in Shandong
Dragon Quality Goods Shop	Green Intelligent Power Saver	How can I feel the power saving and how long does it last	Jingdong prefecture level city in Shandong
Dragon Quality Goods Shop	Green Intelligent Power Saver	And I know if it saves electricity or not.	Jingdong prefecture level city in Shandong
Dragon Quality Goods Shop	Green Intelligent Power Saver	It won't save any electricity. No.	Jingdong prefecture level city in Shandong
Dragon Quality Goods Shop	Green Intelligent Power Saver	Invalid. Fake.	Jingdong prefecture level city in Shandong
Dragon Quality Goods Shop	Green Intelligent Power Saver	It's good!	Jingdong prefecture level city in Shandong
Dragon Quality Goods Shop	Green Intelligent Power Saver	have no effect	Jingdong prefecture level city in Shandong
Dragon Quality Goods Shop	Green Intelligent Power Saver	Not used.	Jingdong prefecture level city in Shandong
Dragon Quality Goods Shop	Green Intelligent Power Saver	There is no load range, packaging thrift, ten days also did not develop invoices, received the goods with a receipt, hand-written receipt also has no financial stamp, communication for nearly a week a moment to say that the electronic invoice, a moment to say that the paper invoice, said it is mailed to me, ten days also did not see anything, the invoice invoice can not be found to say that did not stay on file, single single number can not be found, said that the warehouse more than one to a one to find out, in addition to the perfunctory or perfunctory. Products are no certificate of conformity, proper three	Jingdong prefecture level city in Shandong
Dragon Quality Goods Shop	Green Intelligent Power Saver	Satisfaction.	Jingdong prefecture level city in Shandong

Dragon Quality Goods Shop	Green Intelligent Power Saver	There is not a bit of power saving effect, and ringing up an increase in the electricity bill, because you have to add the cost of this machine's own electricity consumption and purchase, so I think it is better to use this machine instead of increasing the expenditure, uneconomical, rather than not use it.	Jingdong prefecture level city in Shandong
Dragon Quality Goods Shop	Green Intelligent Power Saver	I've been using it for two days now and I don't feel any effect.	Jingdong prefecture level city in Shandong
Dragon Quality Goods Shop	Green Intelligent Power Saver	What it looked like was nothing like what was sent.	Jingdong prefecture level city in Shandong
Dragon Quality Goods Shop	Green Intelligent Power Saver	It didn't work. It didn't work for a month or so.	Jingdong prefecture level city in Shandong
Dragon Quality Goods Shop	Green Intelligent Power Saver	Garbage products, physical and description is not the same, no effect at all, pit # no discussion, to buy the fine, garbage, garbage, garbage	Jingdong prefecture level city in Shandong
Dragon Quality Goods Shop	Green Intelligent Power Saver	general	Jingdong prefecture level city in Shandong
Dragon Quality Goods Shop	Green Intelligent Power Saver	IQ tax. Instead of saving electricity, it increases the power consumption of an appliance.	Jingdong prefecture level city in Shandong
Dragon Quality Goods Shop	Green Intelligent Power Saver	There's no trash at all.	Jingdong prefecture level city in Shandong
Dragon Quality Goods Shop	Green Intelligent Power Saver	It doesn't do anything.	Jingdong prefecture level city in Shandong
Dragon Quality Goods Shop	Green Intelligent Power Saver	(of an unmarried couple) be close	Jingdong prefecture level city in Shandong
Dragon Quality Goods Shop	Green Intelligent Power Saver	(of an unmarried couple) be close	Jingdong prefecture level city in Shandong
Dragon Quality Goods Shop	Green Intelligent Power Saver	no electricity saving	Jingdong prefecture level city in Shandong
Dragon Quality Goods Shop	Green Intelligent Power Saver	differ from	Jingdong prefecture level city in Shandong
Dragon Quality Goods Shop	Green Intelligent Power Saver	Saver has received, but real can save electricity, the quality is very good, I times I buy a few more, too good.	Jingdong prefecture level city in Shandong
Dragon Quality Goods Shop	Green Intelligent Power Saver	Great quality as always, a very favorite product, great! o(*￣︶￣*)o	Jingdong prefecture level city in Shandong
Dragon Quality Goods Shop	Green Intelligent Power Saver	If we can reduce the electricity bill, for individuals can save money, for the country can protect the environment, now the weather is getting hotter and hotter, to protect the environment to reduce emissions is very important. Already use the product, whether to reduce the electricity bill depends on next month!	Jingdong prefecture level city in Shandong
Dragon Quality Goods Shop	Green Intelligent Power Saver	Purchased a power saver received on the put into use, rent a room with many electrical appliances, power consumption is very large, we have a piece of electricity or 1.2 dollars a time, with this power saver is much better, every day can save a few cents, very convenient and practical!	Jingdong prefecture level city in Shandong
Dragon Quality Goods Shop	Green Intelligent Power Saver	The product is better to use before not to use when a month 1500 yuan electricity bill, now using the 5 second month electricity bill directly down 470 dollars or very good. A save money to buy several power saver money ZanZanZanZan👍	Jingdong prefecture level city in Shandong
Dragon Quality Goods Shop	Green Intelligent Power Saver	The moment I received the goods really satisfied with this product, not only high-grade packaging, the body are base alloy, up to the price, I was also introduced by a friend, he used last month did save a lot of electricity, but also recommended to me!	Jingdong prefecture level city in Shandong
Dragon Quality Goods Shop	Green Intelligent Power Saver	I got it. Good review. I'm very satisfied. Reasonable price.	Jingdong prefecture level city in Shandong
Dragon Quality Goods Shop	Green Intelligent Power Saver	Intelligent power saver received and used, looking at the quality is good, the quality of the plug sent is also very good. Expect the effect of the economizer is obvious, first five-star praise, with a period of time to follow up!	Jingdong prefecture level city in Shandong
Dragon Quality Goods Shop	Green Intelligent Power Saver	The saver has been received. It was plugged into an outlet and used immediately after receipt. Whether it really saves electricity is not clear yet, I believe that this brand of energy saver really saves	Jingdong prefecture level city in Shandong
Dragon Quality Goods Shop	Green Intelligent Power Saver	Okay, okay, okay. Thank you so much. Fast is good. Fast. Item was as described, mainly because the event was cheap and you can save a lot of money.	Jingdong prefecture level city in Shandong
Dragon Quality Goods Shop	Green Intelligent Power Saver	Received it a couple days ago! Plugged it in the same day! Let's see what the electric bill is in a month! Hopefully it works well oh!	Jingdong prefecture level city in Shandong
Dragon Quality Goods Shop	Green Intelligent Power Saver	Looking at the reviews good, energy-saving small products, compact, the results after 2 months to see, because the electricity bill season payment	Jingdong prefecture level city in Shandong
Dragon Quality Goods Shop	Green Intelligent Power Saver	I just received it and will try it out to see if it works well. I'll comment later.	Jingdong prefecture level city in Shandong
Dragon Quality Goods Shop	Green Intelligent Power Saver	It looks a little small and not very aesthetically pleasing, I don't know how effective it will be until I use it	Jingdong prefecture level city in Shandong
Dragon Quality Goods Shop	Green Intelligent Power Saver	Not only to the point of being useless yet	Jingdong prefecture level city in Shandong
Dragon Quality Goods Shop	Green Intelligent Power Saver	Used it for a month, nothing works, the meter you turn very fast. This is not a scam, but also sold on Jingdong. To cheat a bunch of people?	Jingdong prefecture level city in Shandong
Dragon Quality Goods Shop	Green Intelligent Power Saver	Very good to use, every day can save a little electricity, really good under the sample down a month to save a few dozen dollars in electricity, good before I did not know that there is this 👆 haha, the price is not expensive praise.	Jingdong prefecture level city in Shandong
Dragon Quality Goods Shop	Green Intelligent Power Saver	Tried it for ten days and the change is almost nothing, can't see the power saving yet, use it some more days and see how it goes.	Jingdong prefecture level city in Shandong
Dragon Quality Goods Shop	Green Intelligent Power Saver	Received very quickly, the quality is very good, really save electricity, business service attitude is also very good, I hope you do not miss this opportunity, I continue to buy a few in	Jingdong prefecture level city in Shandong
Dragon Quality Goods Shop	Green Intelligent Power Saver	Quality is very good Oh, the delivery is also very fast, a very favorite store, I want to crazy recommend to friends!	Jingdong prefecture level city in Shandong
Dragon Quality Goods Shop	Green Intelligent Power Saver	The goods have just received, fine workmanship, and the same as described by the merchant, to use a period of time to see if it can achieve the purpose of power saving.	Jingdong prefecture level city in Shandong
Dragon Quality Goods Shop	Green Intelligent Power Saver	Received the goods first use a period of time to see if there is no effect, the business commitment to ninety days invalid refund, first praise, good service!	Jingdong prefecture level city in Shandong
Dragon Quality Goods Shop	Green Intelligent Power Saver	The product is used for a month without energy-saving and power-saving effect does not have any effect	Jingdong prefecture level city in Shandong
Dragon Quality Goods Shop	Green Intelligent Power Saver	The baby is really great, I like it very much, my friends also say good to come, recommended to buy!	Jingdong prefecture level city in Shandong
Dragon Quality Goods Shop	Green Intelligent Power Saver	Logistics is fast, courier boy service is good, after using to see how the fruit, and then follow up.	Jingdong prefecture level city in Shandong
Dragon Quality Goods Shop	Green Intelligent Power Saver	Baby has been received to try, I believe that as the business advertised energy saving. The courier is very responsible.	Jingdong prefecture level city in Shandong
Dragon Quality Goods Shop	Green Intelligent Power Saver	Save Electric Energy King received, good quality, beautiful appearance, very satisfied!	Jingdong prefecture level city in Shandong
Dragon Quality Goods Shop	Green Intelligent Power Saver	Three no products no manufacturers no phone pure fraud induced consumption cheat	Jingdong prefecture level city in Shandong
Dragon Quality Goods Shop	Green Intelligent Power Saver	I'm using it now, I don't know if it's as good as it says, I'll follow up after a month	Jingdong prefecture level city in Shandong
Dragon Quality Goods Shop	Green Intelligent Power Saver	Good quality logistics and fast. Fine workmanship. The description is consistent cost-effective	Jingdong prefecture level city in Shandong
Dragon Quality Goods Shop	Green Intelligent Power Saver	Received, whether it is good or not, to wait a few days and then evaluate	Jingdong prefecture level city in Shandong
Dragon Quality Goods Shop	Green Intelligent Power Saver	I bought it and started using it, I won't know if it works well until I pay my electric bill	Jingdong prefecture level city in Shandong
Dragon Quality Goods Shop	Green Intelligent Power Saver	Especially good, energy saving and power saving, especially good quality, good	Jingdong prefecture level city in Shandong
Dragon Quality Goods Shop	Green Intelligent Power Saver	Logistics is strong, the quality is very good, paste really save electricity, as described, will buy back!	Jingdong prefecture level city in Shandong
Dragon Quality Goods Shop	Green Intelligent Power Saver	Great stuff at a great price, worth the money!	Jingdong prefecture level city in Shandong

Dragon Quality Goods Shop	Green Intelligent Power Saver	Received the power saver, very good quality, very power saving!	Jingdong prefecture level city in Shandong
Dragon Quality Goods Shop	Green Intelligent Power Saver	There are no fruits, only samples...	Jingdong prefecture level city in Shandong
Dragon Quality Goods Shop	Green Intelligent Power Saver	Save a few dollars a month.	Jingdong prefecture level city in Shandong
Dragon Quality Goods Shop	Green Intelligent Power Saver	The quality is particularly good, it works well, and the seller has a good service attitude	Jingdong prefecture level city in Shandong
Dragon Quality Goods Shop	Green Intelligent Power Saver	I've compared them a couple of times and I don't feel any difference	Jingdong prefecture level city in Shandong
Dragon Quality Goods Shop	Green Intelligent Power Saver	I don't see the savings. It's a sham.	Jingdong prefecture level city in Shandong
Dragon Quality Goods Shop	Green Intelligent Power Saver	The power saving effect is very good, and the logistics is very fast. The seller's service attitude is very good	Jingdong prefecture level city in Shandong
Dragon Quality Goods Shop	Green Intelligent Power Saver	Received, well packaged, haven't tried it yet!	Jingdong prefecture level city in Shandong
Dragon Quality Goods Shop	Green Intelligent Power Saver	I don't know how it works, I'll comment after I use it.	Jingdong prefecture level city in Shandong
Dragon Quality Goods Shop	Green Intelligent Power Saver	The product doesn't work well enough to be of any use	Jingdong prefecture level city in Shandong
Dragon Quality Goods Shop	Green Intelligent Power Saver	On Trial 。。。。。。。	Jingdong prefecture level city in Shandong
Dragon Quality Goods Shop	Green Intelligent Power Saver	Being used with unknown results	Jingdong prefecture level city in Shandong
Dragon Quality Goods Shop	Green Intelligent Power Saver	Smart power saving and great quality	Jingdong prefecture level city in Shandong
Dragon Quality Goods Shop	Green Intelligent Power Saver	The saver was received, it works very well and the price is affordable!	Jingdong prefecture level city in Shandong
Dragon Quality Goods Shop	Green Intelligent Power Saver	How can I feel the power saving and how long does it last	Jingdong prefecture level city in Shandong
Dragon Quality Goods Shop	Green Intelligent Power Saver	And I know if it saves electricity or not.	Jingdong prefecture level city in Shandong
Dragon Quality Goods Shop	Green Intelligent Power Saver	It won't save any electricity. No.	Jingdong prefecture level city in Shandong
Dragon Quality Goods Shop	Green Intelligent Power Saver	Invalid. Fake.	Jingdong prefecture level city in Shandong
Dragon Quality Goods Shop	Green Intelligent Power Saver	It's good!	Jingdong prefecture level city in Shandong
Dragon Quality Goods Shop	Green Intelligent Power Saver	have no effect	Jingdong prefecture level city in Shandong
Dragon Quality Goods Shop	Green Intelligent Power Saver	Not used.	Jingdong prefecture level city in Shandong
Dragon Quality Goods Shop	Green Intelligent Power Saver	There is no load range, packaging thrift, ten days also did not develop invoices, received the goods with a receipt, hand-written receipt also has no financial stamp, communication for nearly a week a moment to say that the electronic invoice, a moment to say that the paper invoice, said it is mailed to me, ten days also did not see anything, the invoice invoice can not be found to say that did not stay on file, single single number can not be found, said that the warehouse more than one to a one to find out, in addition to the perfunctory or perfunctory. Products are no certificate of conformity, proper three	Jingdong prefecture level city in Shandong
Dragon Quality Goods Shop	Green Intelligent Power Saver	Satisfaction.	Jingdong prefecture level city in Shandong
Dragon Quality Goods Shop	Green Intelligent Power Saver	There is not a bit of power saving effect, and ringing up an increase in the electricity bill, because you have to add the cost of this machine's own electricity consumption and purchase, so I think it is better to use this machine instead of increasing the expenditure, uneconomical, rather than not use it.	Jingdong prefecture level city in Shandong
Dragon Quality Goods Shop	Green Intelligent Power Saver	I've been using it for two days now and I don't feel any effect.	Jingdong prefecture level city in Shandong
Dragon Quality Goods Shop	Green Intelligent Power Saver	What it looked like was nothing like what was sent.	Jingdong prefecture level city in Shandong
Dragon Quality Goods Shop	Green Intelligent Power Saver	It didn't work. It didn't work for a month or so.	Jingdong prefecture level city in Shandong
Dragon Quality Goods Shop	Green Intelligent Power Saver	Garbage products, physical and description is not the same, no effect at all, pit # no discussion, to buy the fine, garbage, garbage, garbage	Jingdong prefecture level city in Shandong
Dragon Quality Goods Shop	Green Intelligent Power Saver	general	Jingdong prefecture level city in Shandong
Dragon Quality Goods Shop	Green Intelligent Power Saver	IQ tax. Instead of saving electricity, it increases the power consumption of an appliance.	Jingdong prefecture level city in Shandong
Dragon Quality Goods Shop	Green Intelligent Power Saver	There's no trash at all.	Jingdong prefecture level city in Shandong
Dragon Quality Goods Shop	Green Intelligent Power Saver	It doesn't do anything.	Jingdong prefecture level city in Shandong
Dragon Quality Goods Shop	Green Intelligent Power Saver	(of an unmarried couple) be close	Jingdong prefecture level city in Shandong
Dragon Quality Goods Shop	Green Intelligent Power Saver	(of an unmarried couple) be close	Jingdong prefecture level city in Shandong
Dragon Quality Goods Shop	Green Intelligent Power Saver	no electricity saving	Jingdong prefecture level city in Shandong
Dragon Quality Goods Shop	Green Intelligent Power Saver	differ from	Jingdong prefecture level city in Shandong
Dragon Quality Goods Shop	Green Intelligent Power Saver	Saver has received, but real can save electricity, the quality is very good, I times I buy a few more, too good.	Jingdong prefecture level city in Shandong
Dragon Quality Goods Shop	Green Intelligent Power Saver	Great quality as always, a very favorite product, great! o(*￣︶￣*)o	Jingdong prefecture level city in Shandong
Dragon Quality Goods Shop	Green Intelligent Power Saver	If we can reduce the electricity bill, for individuals can save money, for the country can protect the environment, now the weather is getting hotter and hotter, to protect the environment to reduce emissions is very important. Already use the product, whether to reduce the electricity bill depends on next month!	Jingdong prefecture level city in Shandong
Dragon Quality Goods Shop	Green Intelligent Power Saver	Purchased a power saver received on the put into use, rent a room with many electrical appliances, power consumption is very large, we have a piece of electricity or 1.2 dollars a time, with this power saver is much better, every day can save a few cents, very convenient and practical!	Jingdong prefecture level city in Shandong
Dragon Quality Goods Shop	Green Intelligent Power Saver	The product is better to use before not to use when a month 1500 yuan electricity bill, now using the 5 second month electricity bill directly down 470 dollars or very good. A save money to buy several power saver money ZanZanZanZan👍	Jingdong prefecture level city in Shandong
Dragon Quality Goods Shop	Green Intelligent Power Saver	The moment I received the goods really satisfied with this product, not only high-grade packaging, the body are base alloy, up to the price, I was also introduced by a friend, he used last month did save a lot of electricity, but also recommended to me!	Jingdong prefecture level city in Shandong
Dragon Quality Goods Shop	Green Intelligent Power Saver	I got it. Good review. I'm very satisfied. Reasonable price.	Jingdong prefecture level city in Shandong
Dragon Quality Goods Shop	Green Intelligent Power Saver	Intelligent power saver received and used, looking at the quality is good, the quality of the plug sent is also very good. Expect the effect of the economizer is obvious, first five-star praise, with a period of time to follow up!	Jingdong prefecture level city in Shandong
Dragon Quality Goods Shop	Green Intelligent Power Saver	The saver has been received. It was plugged into an outlet and used immediately after receipt. Whether it really saves electricity is not clear yet, I believe that this brand of energy saver really saves	Jingdong prefecture level city in Shandong
Dragon Quality Goods Shop	Green Intelligent Power Saver	Okay, okay, okay. Thank you so much. Fast is good. Fast. Item was as described, mainly because the event was cheap and you can save a lot of money.	Jingdong prefecture level city in Shandong

Dragon Quality Goods Shop	Green Intelligent Power Saver	Received it a couple days ago! Plugged it in the same day! Let's see what the electric bill is in a month! Hopefully it works well oh!	Jingdong prefecture level city in Shandong
Dragon Quality Goods Shop	Green Intelligent Power Saver	Looking at the reviews good, energy-saving small products, compact, the results after 2 months to see, because the electricity bill season payment	Jingdong prefecture level city in Shandong
Dragon Quality Goods Shop	Green Intelligent Power Saver	I just received it and will try it out to see if it works well. I'll comment later.	Jingdong prefecture level city in Shandong
Dragon Quality Goods Shop	Green Intelligent Power Saver	It looks a little small and not very aesthetically pleasing, I don't know how effective it will be until I use it	Jingdong prefecture level city in Shandong
Dragon Quality Goods Shop	Green Intelligent Power Saver	Not only to the point of being useless yet	Jingdong prefecture level city in Shandong
Dragon Quality Goods Shop	Green Intelligent Power Saver	Used it for a month, nothing works, the meter you turn very fast. This is not a scam, but also sold on Jingdong. To cheat a bunch of people?	Jingdong prefecture level city in Shandong
Dragon Quality Goods Shop	Green Intelligent Power Saver	Very good to use, every day can save a little electricity, really good under the sample down a month to save a few dozen dollars in electricity, good before I did not know that there is this haha, the price is not expensive praise.	Jingdong prefecture level city in Shandong
Dragon Quality Goods Shop	Green Intelligent Power Saver	Tried it for ten days and the change is almost nothing, can't see the power saving yet, use it some more days and see how it goes.	Jingdong prefecture level city in Shandong
Dragon Quality Goods Shop	Green Intelligent Power Saver	Received very quickly, the quality is very good, really save electricity, business service attitude is also very good, I hope you do not miss this opportunity, I continue to buy a few in	Jingdong prefecture level city in Shandong
Dragon Quality Goods Shop	Green Intelligent Power Saver	Quality is very good Oh, the delivery is also very fast, a very favorite store, I want to crazy recommend to friends!	Jingdong prefecture level city in Shandong
Dragon Quality Goods Shop	Green Intelligent Power Saver	The goods have just received, fine workmanship, and the same as described by the merchant, to use a period of time to see if it can achieve the purpose of power saving.	Jingdong prefecture level city in Shandong
Dragon Quality Goods Shop	Green Intelligent Power Saver	Received the goods first use a period of time to see if there is no effect, the business commitment to ninety days invalid refund, first praise, good service!	Jingdong prefecture level city in Shandong
Dragon Quality Goods Shop	Green Intelligent Power Saver	The product is used for a month without energy-saving and power-saving effect does not have any effect	Jingdong prefecture level city in Shandong
Dragon Quality Goods Shop	Green Intelligent Power Saver	The baby is really great, I like it very much, my friends also say good to come, recommended to buy!	Jingdong prefecture level city in Shandong
Dragon Quality Goods Shop	Green Intelligent Power Saver	Logistics is fast, courier boy service is good, after using to see how the fruit, and then follow up.	Jingdong prefecture level city in Shandong
Dragon Quality Goods Shop	Green Intelligent Power Saver	Baby has been received to try, I believe that as the business advertised energy saving. The courier is very responsible.	Jingdong prefecture level city in Shandong
Dragon Quality Goods Shop	Green Intelligent Power Saver	Save Electric Energy King received, good quality, beautiful appearance, very satisfied!	Jingdong prefecture level city in Shandong
Dragon Quality Goods Shop	Green Intelligent Power Saver	Three no products no manufacturers no phone pure fraud induced consumption cheat	Jingdong prefecture level city in Shandong
Dragon Quality Goods Shop	Green Intelligent Power Saver	I'm using it now, I don't know if it's as good as it says, I'll follow up after a month	Jingdong prefecture level city in Shandong
Dragon Quality Goods Shop	Green Intelligent Power Saver	Good quality logistics and fast. Fine workmanship. The description is consistent cost-effective	Jingdong prefecture level city in Shandong
Dragon Quality Goods Shop	Green Intelligent Power Saver	Received, whether it is good or not, to wait a few days and then evaluate	Jingdong prefecture level city in Shandong
Dragon Quality Goods Shop	Green Intelligent Power Saver	I bought it and started using it, I won't know if it works well until I pay my electric bill	Jingdong prefecture level city in Shandong
Dragon Quality Goods Shop	Green Intelligent Power Saver	Especially good, energy saving and power saving, especially good quality, good	Jingdong prefecture level city in Shandong
Dragon Quality Goods Shop	Green Intelligent Power Saver	Logistics is strong, the quality is very good, paste really save electricity, as described, will buy back!	Jingdong prefecture level city in Shandong
Dragon Quality Goods Shop	Green Intelligent Power Saver	Great stuff at a great price, worth the money!	Jingdong prefecture level city in Shandong
Dragon Quality Goods Shop	Green Intelligent Power Saver	Received the power saver, very good quality, very power saving!	Jingdong prefecture level city in Shandong
Dragon Quality Goods Shop	Green Intelligent Power Saver	There are no fruits, only samples...	Jingdong prefecture level city in Shandong
Dragon Quality Goods Shop	Green Intelligent Power Saver	Save a few dollars a month.	Jingdong prefecture level city in Shandong
Dragon Quality Goods Shop	Green Intelligent Power Saver	The quality is particularly good, it works well, and the seller has a good service attitude	Jingdong prefecture level city in Shandong
Dragon Quality Goods Shop	Green Intelligent Power Saver	I've compared them a couple of times and I don't feel any difference	Jingdong prefecture level city in Shandong
Dragon Quality Goods Shop	Green Intelligent Power Saver	I don't see the savings. It's a sham.	Jingdong prefecture level city in Shandong
Dragon Quality Goods Shop	Green Intelligent Power Saver	The power saving effect is very good, and the logistics is very fast. The seller's service attitude is very good	Jingdong prefecture level city in Shandong
Dragon Quality Goods Shop	Green Intelligent Power Saver	Received, well packaged, haven't tried it yet!	Jingdong prefecture level city in Shandong
Dragon Quality Goods Shop	Green Intelligent Power Saver	I don't know how it works, I'll comment after I use it.	Jingdong prefecture level city in Shandong
Dragon Quality Goods Shop	Green Intelligent Power Saver	The product doesn't work well enough to be of any use	Jingdong prefecture level city in Shandong
Dragon Quality Goods Shop	Green Intelligent Power Saver	On Trial 。。。。。。。	Jingdong prefecture level city in Shandong
Dragon Quality Goods Shop	Green Intelligent Power Saver	Being used with unknown results	Jingdong prefecture level city in Shandong
Dragon Quality Goods Shop	Green Intelligent Power Saver	Smart power saving and great quality	Jingdong prefecture level city in Shandong
Dragon Quality Goods Shop	Green Intelligent Power Saver	The saver was received, it works very well and the price is affordable!	Jingdong prefecture level city in Shandong
Dragon Quality Goods Shop	Green Intelligent Power Saver	How can I feel the power saving and how long does it last	Jingdong prefecture level city in Shandong
Dragon Quality Goods Shop	Green Intelligent Power Saver	And I know if it saves electricity or not.	Jingdong prefecture level city in Shandong
Dragon Quality Goods Shop	Green Intelligent Power Saver	It won't save any electricity. No.	Jingdong prefecture level city in Shandong
Dragon Quality Goods Shop	Green Intelligent Power Saver	Invalid. Fake.	Jingdong prefecture level city in Shandong
Dragon Quality Goods Shop	Green Intelligent Power Saver	It's good!	Jingdong prefecture level city in Shandong
Dragon Quality Goods Shop	Green Intelligent Power Saver	have no effect	Jingdong prefecture level city in Shandong
Dragon Quality Goods Shop	Green Intelligent Power Saver	Not used.	Jingdong prefecture level city in Shandong
Dragon Quality Goods Shop	Green Intelligent Power Saver	There is no load range, packaging thrift, ten days also did not develop invoices, received the goods with a receipt, hand-written receipt also has no financial stamp, communication for nearly a week a moment to say that the electronic invoice, a moment to say that the paper invoice, said it is mailed to me, ten days also did not see anything, the invoice invoice can not be found to say that did not stay on file, single single number can not be found, said that the warehouse more than one to a one to find out, in addition to the perfunctory or perfunctory. Products are no certificate of conformity, proper three	Jingdong prefecture level city in Shandong
Dragon Quality Goods Shop	Green Intelligent Power Saver	Satisfaction.	Jingdong prefecture level city in Shandong

Dragon Quality Goods Shop	Green Intelligent Power Saver	There is not a bit of power saving effect, and ringing up an increase in the electricity bill, because you have to add the cost of this machine's own electricity consumption and purchase, so I think it is better to use this machine instead of increasing the expenditure, uneconomical, rather than not use it.	Jingdong prefecture level city in Shandong
Dragon Quality Goods Shop	Green Intelligent Power Saver	I've been using it for two days now and I don't feel any effect.	Jingdong prefecture level city in Shandong
Dragon Quality Goods Shop	Green Intelligent Power Saver	What it looked like was nothing like what was sent.	Jingdong prefecture level city in Shandong
Dragon Quality Goods Shop	Green Intelligent Power Saver	It didn't work. It didn't work for a month or so.	Jingdong prefecture level city in Shandong
Dragon Quality Goods Shop	Green Intelligent Power Saver	Garbage products, physical and description is not the same, no effect at all, pit # no discussion, to buy the fine, garbage, garbage, garbage	Jingdong prefecture level city in Shandong
Dragon Quality Goods Shop	Green Intelligent Power Saver	general	Jingdong prefecture level city in Shandong
Dragon Quality Goods Shop	Green Intelligent Power Saver	IQ tax. Instead of saving electricity, it increases the power consumption of an appliance.	Jingdong prefecture level city in Shandong
Dragon Quality Goods Shop	Green Intelligent Power Saver	There's no trash at all.	Jingdong prefecture level city in Shandong
Dragon Quality Goods Shop	Green Intelligent Power Saver	It doesn't do anything.	Jingdong prefecture level city in Shandong
Dragon Quality Goods Shop	Green Intelligent Power Saver	(of an unmarried couple) be close	Jingdong prefecture level city in Shandong
Dragon Quality Goods Shop	Green Intelligent Power Saver	(of an unmarried couple) be close	Jingdong prefecture level city in Shandong
Dragon Quality Goods Shop	Green Intelligent Power Saver	no electricity saving	Jingdong prefecture level city in Shandong
Dragon Quality Goods Shop	Green Intelligent Power Saver	differ from	Jingdong prefecture level city in Shandong
Dragon Quality Goods Shop	Green Intelligent Power Saver	Saver has received, but real can save electricity, the quality is very good, I times I buy a few more, too good.	Jingdong prefecture level city in Shandong
Dragon Quality Goods Shop	Green Intelligent Power Saver	Great quality as always, a very favorite product, great! o(*￣︶￣*)o	Jingdong prefecture level city in Shandong
Dragon Quality Goods Shop	Green Intelligent Power Saver	If we can reduce the electricity bill, for individuals can save money, for the country can protect the environment, now the weather is getting hotter and hotter, to protect the environment to reduce emissions is very important. Already use the product, whether to reduce the electricity bill depends on next month!	Jingdong prefecture level city in Shandong
Dragon Quality Goods Shop	Green Intelligent Power Saver	Purchased a power saver received on the put into use, rent a room with many electrical appliances, power consumption is very large, we have a piece of electricity or 1.2 dollars a time, with this power saver is much better, every day can save a few cents, very convenient and practical!	Jingdong prefecture level city in Shandong
Dragon Quality Goods Shop	Green Intelligent Power Saver	The product is better to use before not to use when a month 1500 yuan electricity bill, now using the 5 second month electricity bill directly down 470 dollars or very good. A save money to buy several power saver money ZanZanZanZan👍	Jingdong prefecture level city in Shandong
Dragon Quality Goods Shop	Green Intelligent Power Saver	The moment I received the goods really satisfied with this product, not only high-grade packaging, the body are base alloy, up to the price, I was also introduced by a friend, he used last month did save a lot of electricity, but also recommended to me!	Jingdong prefecture level city in Shandong
Dragon Quality Goods Shop	Green Intelligent Power Saver	I got it. Good review. I'm very satisfied. Reasonable price.	Jingdong prefecture level city in Shandong
Dragon Quality Goods Shop	Green Intelligent Power Saver	Intelligent power saver received and used, looking at the quality is good, the quality of the plug sent is also very good. Expect the effect of the economizer is obvious, first five-star praise, with a period of time to follow up!	Jingdong prefecture level city in Shandong
Dragon Quality Goods Shop	Green Intelligent Power Saver	The saver has been received. It was plugged into an outlet and used immediately after receipt. Whether it really saves electricity is not clear yet, I believe that this brand of energy saver really saves	Jingdong prefecture level city in Shandong
Dragon Quality Goods Shop	Green Intelligent Power Saver	Okay, okay, okay. Thank you so much. Fast is good. Fast. Item was as described, mainly because the event was cheap and you can save a lot of money.	Jingdong prefecture level city in Shandong
Dragon Quality Goods Shop	Green Intelligent Power Saver	Received it a couple days ago! Plugged it in the same day! Let's see what the electric bill is in a month! Hopefully it works well oh!	Jingdong prefecture level city in Shandong
Dragon Quality Goods Shop	Green Intelligent Power Saver	Looking at the reviews good, energy-saving small products, compact, the results after 2 months to see, because the electricity bill season payment	Jingdong prefecture level city in Shandong
Dragon Quality Goods Shop	Green Intelligent Power Saver	I just received it and will try it out to see if it works well. I'll comment later.	Jingdong prefecture level city in Shandong
Dragon Quality Goods Shop	Green Intelligent Power Saver	It looks a little small and not very aesthetically pleasing, I don't know how effective it will be until I use it	Jingdong prefecture level city in Shandong
Dragon Quality Goods Shop	Green Intelligent Power Saver	Not only to the point of being useless yet	Jingdong prefecture level city in Shandong
Dragon Quality Goods Shop	Green Intelligent Power Saver	Used it for a month, nothing works, the meter you turn very fast. This is not a scam, but also sold on Jingdong. To cheat a bunch of people?	Jingdong prefecture level city in Shandong
Dragon Quality Goods Shop	Green Intelligent Power Saver	Very good to use, every day can save a little electricity, really good under the sample down a month to save a few dozen dollars in electricity, good before I did not know that there is this 👆 haha, the price is not expensive praise.	Jingdong prefecture level city in Shandong
Dragon Quality Goods Shop	Green Intelligent Power Saver	Tried it for ten days and the change is almost nothing, can't see the power saving yet, use it some more days and see how it goes.	Jingdong prefecture level city in Shandong
Dragon Quality Goods Shop	Green Intelligent Power Saver	Received very quickly, the quality is very good, really save electricity, business service attitude is also very good, I hope you do not miss this opportunity, I continue to buy a few in	Jingdong prefecture level city in Shandong
Dragon Quality Goods Shop	Green Intelligent Power Saver	Quality is very good Oh, the delivery is also very fast, a very favorite store, I want to crazy recommend to friends!	Jingdong prefecture level city in Shandong
Dragon Quality Goods Shop	Green Intelligent Power Saver	The goods have just received, fine workmanship, and the same as described by the merchant, to use a period of time to see if it can achieve the purpose of power saving.	Jingdong prefecture level city in Shandong
Dragon Quality Goods Shop	Green Intelligent Power Saver	Received the goods first use a period of time to see if there is no effect, the business commitment to ninety days invalid refund, first praise, good service!	Jingdong prefecture level city in Shandong
Dragon Quality Goods Shop	Green Intelligent Power Saver	The product is used for a month without energy-saving and power-saving effect does not have any effect	Jingdong prefecture level city in Shandong
Dragon Quality Goods Shop	Green Intelligent Power Saver	The baby is really great, I like it very much, my friends also say good to come, recommended to buy!	Jingdong prefecture level city in Shandong
Dragon Quality Goods Shop	Green Intelligent Power Saver	Logistics is fast, courier boy service is good, after using to see how the fruit, and then follow up.	Jingdong prefecture level city in Shandong
Dragon Quality Goods Shop	Green Intelligent Power Saver	Baby has been received to try, I believe that as the business advertised energy saving. The courier is very responsible.	Jingdong prefecture level city in Shandong
Dragon Quality Goods Shop	Green Intelligent Power Saver	Save Electric Energy King received, good quality, beautiful appearance, very satisfied!	Jingdong prefecture level city in Shandong
Dragon Quality Goods Shop	Green Intelligent Power Saver	Three no products no manufacturers no phone pure fraud induced consumption cheat	Jingdong prefecture level city in Shandong
Dragon Quality Goods Shop	Green Intelligent Power Saver	I'm using it now, I don't know if it's as good as it says, I'll follow up after a month	Jingdong prefecture level city in Shandong
Dragon Quality Goods Shop	Green Intelligent Power Saver	Good quality logistics and fast. Fine workmanship. The description is consistent cost-effective	Jingdong prefecture level city in Shandong
Dragon Quality Goods Shop	Green Intelligent Power Saver	Received, whether it is good or not, to wait a few days and then evaluate	Jingdong prefecture level city in Shandong
Dragon Quality Goods Shop	Green Intelligent Power Saver	I bought it and started using it, I won't know if it works well until I pay my electric bill	Jingdong prefecture level city in Shandong
Dragon Quality Goods Shop	Green Intelligent Power Saver	Especially good, energy saving and power saving, especially good quality, good	Jingdong prefecture level city in Shandong
Dragon Quality Goods Shop	Green Intelligent Power Saver	Logistics is strong, the quality is very good, paste really save electricity, as described, will buy back!	Jingdong prefecture level city in Shandong
Dragon Quality Goods Shop	Green Intelligent Power Saver	Great stuff at a great price, worth the money!	Jingdong prefecture level city in Shandong

Dragon Quality Goods Shop	Green Intelligent Power Saver	Received the power saver, very good quality, very power saving!	Jingdong prefecture level city in Shandong
Dragon Quality Goods Shop	Green Intelligent Power Saver	There are no fruits, only samples...	Jingdong prefecture level city in Shandong
Dragon Quality Goods Shop	Green Intelligent Power Saver	Save a few dollars a month.	Jingdong prefecture level city in Shandong
Dragon Quality Goods Shop	Green Intelligent Power Saver	The quality is particularly good, it works well, and the seller has a good service attitude	Jingdong prefecture level city in Shandong
Dragon Quality Goods Shop	Green Intelligent Power Saver	I've compared them a couple of times and I don't feel any difference	Jingdong prefecture level city in Shandong
Dragon Quality Goods Shop	Green Intelligent Power Saver	I don't see the savings. It's a sham.	Jingdong prefecture level city in Shandong
Dragon Quality Goods Shop	Green Intelligent Power Saver	The power saving effect is very good, and the logistics is very fast. The seller's service attitude is very good	Jingdong prefecture level city in Shandong
Dragon Quality Goods Shop	Green Intelligent Power Saver	Received, well packaged, haven't tried it yet!	Jingdong prefecture level city in Shandong
Dragon Quality Goods Shop	Green Intelligent Power Saver	I don't know how it works, I'll comment after I use it.	Jingdong prefecture level city in Shandong
Dragon Quality Goods Shop	Green Intelligent Power Saver	The product doesn't work well enough to be of any use	Jingdong prefecture level city in Shandong
Dragon Quality Goods Shop	Green Intelligent Power Saver	On Trial 。。。。。。。	Jingdong prefecture level city in Shandong
Dragon Quality Goods Shop	Green Intelligent Power Saver	Being used with unknown results	Jingdong prefecture level city in Shandong
Dragon Quality Goods Shop	Green Intelligent Power Saver	Smart power saving and great quality	Jingdong prefecture level city in Shandong
Dragon Quality Goods Shop	Green Intelligent Power Saver	The saver was received, it works very well and the price is affordable!	Jingdong prefecture level city in Shandong
Dragon Quality Goods Shop	Green Intelligent Power Saver	How can I feel the power saving and how long does it last	Jingdong prefecture level city in Shandong
Dragon Quality Goods Shop	Green Intelligent Power Saver	And I know if it saves electricity or not.	Jingdong prefecture level city in Shandong
Dragon Quality Goods Shop	Green Intelligent Power Saver	It won't save any electricity. No.	Jingdong prefecture level city in Shandong
Dragon Quality Goods Shop	Green Intelligent Power Saver	Invalid. Fake.	Jingdong prefecture level city in Shandong
Dragon Quality Goods Shop	Green Intelligent Power Saver	It's good!	Jingdong prefecture level city in Shandong
Dragon Quality Goods Shop	Green Intelligent Power Saver	have no effect	Jingdong prefecture level city in Shandong
Dragon Quality Goods Shop	Green Intelligent Power Saver	Not used.	Jingdong prefecture level city in Shandong
Dragon Quality Goods Shop	Green Intelligent Power Saver	There is no load range, packaging thrift, ten days also did not develop invoices, received the goods with a receipt, hand-written receipt also has no financial stamp, communication for nearly a week a moment to say that the electronic invoice, a moment to say that the paper invoice, said it is mailed to me, ten days also did not see anything, the invoice invoice can not be found to say that did not stay on file, single single number can not be found, said that the warehouse more than one to a one to find out, in addition to the perfunctory or perfunctory. Products are no certificate of conformity, proper three	Jingdong prefecture level city in Shandong
Dragon Quality Goods Shop	Green Intelligent Power Saver	Satisfaction.	Jingdong prefecture level city in Shandong
Dragon Quality Goods Shop	Green Intelligent Power Saver	There is not a bit of power saving effect, and ringing up an increase in the electricity bill, because you have to add the cost of this machine's own electricity consumption and purchase, so I think it is better to use this machine instead of increasing the expenditure, uneconomical, rather than not use it.	Jingdong prefecture level city in Shandong
Dragon Quality Goods Shop	Green Intelligent Power Saver	I've been using it for two days now and I don't feel any effect.	Jingdong prefecture level city in Shandong
Dragon Quality Goods Shop	Green Intelligent Power Saver	What it looked like was nothing like what was sent.	Jingdong prefecture level city in Shandong
Dragon Quality Goods Shop	Green Intelligent Power Saver	It didn't work. It didn't work for a month or so.	Jingdong prefecture level city in Shandong
Dragon Quality Goods Shop	Green Intelligent Power Saver	Garbage products, physical and description is not the same, no effect at all, pit # no discussion, to buy the fine, garbage, garbage, garbage	Jingdong prefecture level city in Shandong
Dragon Quality Goods Shop	Green Intelligent Power Saver	general	Jingdong prefecture level city in Shandong
Dragon Quality Goods Shop	Green Intelligent Power Saver	IQ tax. Instead of saving electricity, it increases the power consumption of an appliance.	Jingdong prefecture level city in Shandong
Dragon Quality Goods Shop	Green Intelligent Power Saver	There's no trash at all.	Jingdong prefecture level city in Shandong
Dragon Quality Goods Shop	Green Intelligent Power Saver	It doesn't do anything.	Jingdong prefecture level city in Shandong
Dragon Quality Goods Shop	Green Intelligent Power Saver	(of an unmarried couple) be close	Jingdong prefecture level city in Shandong
Dragon Quality Goods Shop	Green Intelligent Power Saver	(of an unmarried couple) be close	Jingdong prefecture level city in Shandong
Dragon Quality Goods Shop	Green Intelligent Power Saver	no electricity saving	Jingdong prefecture level city in Shandong
Dragon Quality Goods Shop	Green Intelligent Power Saver	differ from	Jingdong prefecture level city in Shandong
Dragon Quality Goods Shop	Green Intelligent Power Saver	Saver has received, but real can save electricity, the quality is very good, I times I buy a few more, too good.	Jingdong prefecture level city in Shandong
Dragon Quality Goods Shop	Green Intelligent Power Saver	Great quality as always, a very favorite product, great! o(*￣︶￣*)o	Jingdong prefecture level city in Shandong
Dragon Quality Goods Shop	Green Intelligent Power Saver	If we can reduce the electricity bill, for individuals can save money, for the country can protect the environment, now the weather is getting hotter and hotter, to protect the environment to reduce emissions is very important. Already use the product, whether to reduce the electricity bill depends on next month!	Jingdong prefecture level city in Shandong
Dragon Quality Goods Shop	Green Intelligent Power Saver	Purchased a power saver received on the put into use, rent a room with many electrical appliances, power consumption is very large, we have a piece of electricity or 1.2 dollars a time, with this power saver is much better, every day can save a few cents, very convenient and practical!	Jingdong prefecture level city in Shandong
Dragon Quality Goods Shop	Green Intelligent Power Saver	The product is better to use before not to use when a month 1500 yuan electricity bill, now using the 5 second month electricity bill directly down 470 dollars or very good. A save money to buy several power saver money ZanZanZanZan👍	Jingdong prefecture level city in Shandong
Dragon Quality Goods Shop	Green Intelligent Power Saver	The moment I received the goods really satisfied with this product, not only high-grade packaging, the body are base alloy, up to the price, I was also introduced by a friend, he used last month did save a lot of electricity, but also recommended to me!	Jingdong prefecture level city in Shandong
Dragon Quality Goods Shop	Green Intelligent Power Saver	I got it. Good review. I'm very satisfied. Reasonable price.	Jingdong prefecture level city in Shandong
Dragon Quality Goods Shop	Green Intelligent Power Saver	Intelligent power saver received and used, looking at the quality is good, the quality of the plug sent is also very good. Expect the effect of the economizer is obvious, first five-star praise, with a period of time to follow up!	Jingdong prefecture level city in Shandong
Dragon Quality Goods Shop	Green Intelligent Power Saver	The saver has been received. It was plugged into an outlet and used immediately after receipt. Whether it really saves electricity is not clear yet, I believe that this brand of energy saver really saves	Jingdong prefecture level city in Shandong
Dragon Quality Goods Shop	Green Intelligent Power Saver	Okay, okay, okay. Thank you so much. Fast is good. Fast. Item was as described, mainly because the event was cheap and you can save a lot of money.	Jingdong prefecture level city in Shandong

Dragon Quality Goods Shop	Green Intelligent Power Saver	Received it a couple days ago! Plugged it in the same day! Let's see what the electric bill is in a month! Hopefully it works well oh!	Jingdong prefecture level city in Shandong
Dragon Quality Goods Shop	Green Intelligent Power Saver	Looking at the reviews good, energy-saving small products, compact, the results after 2 months to see, because the electricity bill season payment	Jingdong prefecture level city in Shandong
Dragon Quality Goods Shop	Green Intelligent Power Saver	I just received it and will try it out to see if it works well. I'll comment later.	Jingdong prefecture level city in Shandong
Dragon Quality Goods Shop	Green Intelligent Power Saver	It looks a little small and not very aesthetically pleasing, I don't know how effective it will be until I use it	Jingdong prefecture level city in Shandong
Dragon Quality Goods Shop	Green Intelligent Power Saver	Not only to the point of being useless yet	Jingdong prefecture level city in Shandong
Dragon Quality Goods Shop	Green Intelligent Power Saver	Used it for a month, nothing works, the meter you turn very fast. This is not a scam, but also sold on Jingdong. To cheat a bunch of people?	Jingdong prefecture level city in Shandong
Dragon Quality Goods Shop	Green Intelligent Power Saver	Very good to use, every day can save a little electricity, really good under the sample down a month to save a few dozen dollars in electricity, good before I did not know that there is this haha, the price is not expensive praise.	Jingdong prefecture level city in Shandong
Dragon Quality Goods Shop	Green Intelligent Power Saver	Tried it for ten days and the change is almost nothing, can't see the power saving yet, use it some more days and see how it goes.	Jingdong prefecture level city in Shandong
Dragon Quality Goods Shop	Green Intelligent Power Saver	Received very quickly, the quality is very good, really save electricity, business service attitude is also very good, I hope you do not miss this opportunity, I continue to buy a few in	Jingdong prefecture level city in Shandong
Dragon Quality Goods Shop	Green Intelligent Power Saver	Quality is very good Oh, the delivery is also very fast, a very favorite store, I want to crazy recommend to friends!	Jingdong prefecture level city in Shandong
Dragon Quality Goods Shop	Green Intelligent Power Saver	The goods have just received, fine workmanship, and the same as described by the merchant, to use a period of time to see if it can achieve the purpose of power saving.	Jingdong prefecture level city in Shandong
Dragon Quality Goods Shop	Green Intelligent Power Saver	Received the goods first use a period of time to see if there is no effect, the business commitment to ninety days invalid refund, first praise, good service!	Jingdong prefecture level city in Shandong
Dragon Quality Goods Shop	Green Intelligent Power Saver	The product is used for a month without energy-saving and power-saving effect does not have any effect	Jingdong prefecture level city in Shandong
Dragon Quality Goods Shop	Green Intelligent Power Saver	The baby is really great, I like it very much, my friends also say good to come, recommended to buy!	Jingdong prefecture level city in Shandong
Dragon Quality Goods Shop	Green Intelligent Power Saver	Logistics is fast, courier boy service is good, after using to see how the fruit, and then follow up.	Jingdong prefecture level city in Shandong
Dragon Quality Goods Shop	Green Intelligent Power Saver	Baby has been received to try, I believe that as the business advertised energy saving. The courier is very responsible.	Jingdong prefecture level city in Shandong
Dragon Quality Goods Shop	Green Intelligent Power Saver	Save Electric Energy King received, good quality, beautiful appearance, very satisfied!	Jingdong prefecture level city in Shandong
Dragon Quality Goods Shop	Green Intelligent Power Saver	Three no products no manufacturers no phone pure fraud induced consumption cheat	Jingdong prefecture level city in Shandong
Dragon Quality Goods Shop	Green Intelligent Power Saver	I'm using it now, I don't know if it's as good as it says, I'll follow up after a month	Jingdong prefecture level city in Shandong
Dragon Quality Goods Shop	Green Intelligent Power Saver	Good quality logistics and fast. Fine workmanship. The description is consistent cost-effective	Jingdong prefecture level city in Shandong
Dragon Quality Goods Shop	Green Intelligent Power Saver	Received, whether it is good or not, to wait a few days and then evaluate	Jingdong prefecture level city in Shandong
Dragon Quality Goods Shop	Green Intelligent Power Saver	I bought it and started using it, I won't know if it works well until I pay my electric bill	Jingdong prefecture level city in Shandong
Dragon Quality Goods Shop	Green Intelligent Power Saver	Especially good, energy saving and power saving, especially good quality, good	Jingdong prefecture level city in Shandong
Dragon Quality Goods Shop	Green Intelligent Power Saver	Logistics is strong, the quality is very good, paste really save electricity, as described, will buy back!	Jingdong prefecture level city in Shandong
Dragon Quality Goods Shop	Green Intelligent Power Saver	Great stuff at a great price, worth the money!	Jingdong prefecture level city in Shandong
Dragon Quality Goods Shop	Green Intelligent Power Saver	Received the power saver, very good quality, very power saving!	Jingdong prefecture level city in Shandong
Dragon Quality Goods Shop	Green Intelligent Power Saver	There are no fruits, only samples...	Jingdong prefecture level city in Shandong
Dragon Quality Goods Shop	Green Intelligent Power Saver	Save a few dollars a month.	Jingdong prefecture level city in Shandong
Dragon Quality Goods Shop	Green Intelligent Power Saver	The quality is particularly good, it works well, and the seller has a good service attitude	Jingdong prefecture level city in Shandong
Dragon Quality Goods Shop	Green Intelligent Power Saver	I've compared them a couple of times and I don't feel any difference	Jingdong prefecture level city in Shandong
Dragon Quality Goods Shop	Green Intelligent Power Saver	I don't see the savings. It's a sham.	Jingdong prefecture level city in Shandong
Dragon Quality Goods Shop	Green Intelligent Power Saver	The power saving effect is very good, and the logistics is very fast. The seller's service attitude is very good	Jingdong prefecture level city in Shandong
Dragon Quality Goods Shop	Green Intelligent Power Saver	Received, well packaged, haven't tried it yet!	Jingdong prefecture level city in Shandong
Dragon Quality Goods Shop	Green Intelligent Power Saver	I don't know how it works, I'll comment after I use it.	Jingdong prefecture level city in Shandong
Dragon Quality Goods Shop	Green Intelligent Power Saver	The product doesn't work well enough to be of any use	Jingdong prefecture level city in Shandong
Dragon Quality Goods Shop	Green Intelligent Power Saver	On Trial 。。。。。。。	Jingdong prefecture level city in Shandong
Dragon Quality Goods Shop	Green Intelligent Power Saver	Being used with unknown results	Jingdong prefecture level city in Shandong
Dragon Quality Goods Shop	Green Intelligent Power Saver	Smart power saving and great quality	Jingdong prefecture level city in Shandong
Dragon Quality Goods Shop	Green Intelligent Power Saver	The saver was received, it works very well and the price is affordable!	Jingdong prefecture level city in Shandong
Dragon Quality Goods Shop	Green Intelligent Power Saver	How can I feel the power saving and how long does it last	Jingdong prefecture level city in Shandong
Dragon Quality Goods Shop	Green Intelligent Power Saver	And I know if it saves electricity or not.	Jingdong prefecture level city in Shandong
Dragon Quality Goods Shop	Green Intelligent Power Saver	It won't save any electricity. No.	Jingdong prefecture level city in Shandong
Dragon Quality Goods Shop	Green Intelligent Power Saver	Invalid. Fake.	Jingdong prefecture level city in Shandong
Dragon Quality Goods Shop	Green Intelligent Power Saver	It's good!	Jingdong prefecture level city in Shandong
Dragon Quality Goods Shop	Green Intelligent Power Saver	have no effect	Jingdong prefecture level city in Shandong
Dragon Quality Goods Shop	Green Intelligent Power Saver	Not used.	Jingdong prefecture level city in Shandong
Dragon Quality Goods Shop	Green Intelligent Power Saver	There is no load range, packaging thrift, ten days also did not develop invoices, received the goods with a receipt, hand-written receipt also has no financial stamp, communication for nearly a week a moment to say that the electronic invoice, a moment to say that the paper invoice, said it is mailed to me, ten days also did not see anything, the invoice invoice can not be found to say that did not stay on file, single single number can not be found, said that the warehouse more than one to a one to find out, in addition to the perfunctory or perfunctory. Products are no certificate of conformity, proper three	Jingdong prefecture level city in Shandong
Dragon Quality Goods Shop	Green Intelligent Power Saver	Satisfaction.	Jingdong prefecture level city in Shandong

Dragon Quality Goods Shop	Green Intelligent Power Saver	There is not a bit of power saving effect, and ringing up an increase in the electricity bill, because you have to add the cost of this machine's own electricity consumption and purchase, so I think it is better to use this machine instead of increasing the expenditure, uneconomical, rather than not use it.	Jingdong prefecture level city in Shandong
Dragon Quality Goods Shop	Green Intelligent Power Saver	I've been using it for two days now and I don't feel any effect.	Jingdong prefecture level city in Shandong
Dragon Quality Goods Shop	Green Intelligent Power Saver	What it looked like was nothing like what was sent.	Jingdong prefecture level city in Shandong
Dragon Quality Goods Shop	Green Intelligent Power Saver	It didn't work. It didn't work for a month or so.	Jingdong prefecture level city in Shandong
Dragon Quality Goods Shop	Green Intelligent Power Saver	Garbage products, physical and description is not the same, no effect at all, pit # no discussion, to buy the fine, garbage, garbage, garbage	Jingdong prefecture level city in Shandong
Dragon Quality Goods Shop	Green Intelligent Power Saver	general	Jingdong prefecture level city in Shandong
Dragon Quality Goods Shop	Green Intelligent Power Saver	IQ tax. Instead of saving electricity, it increases the power consumption of an appliance.	Jingdong prefecture level city in Shandong
Dragon Quality Goods Shop	Green Intelligent Power Saver	There's no trash at all.	Jingdong prefecture level city in Shandong
Dragon Quality Goods Shop	Green Intelligent Power Saver	It doesn't do anything.	Jingdong prefecture level city in Shandong
Dragon Quality Goods Shop	Green Intelligent Power Saver	(of an unmarried couple) be close	Jingdong prefecture level city in Shandong
Dragon Quality Goods Shop	Green Intelligent Power Saver	(of an unmarried couple) be close	Jingdong prefecture level city in Shandong
Dragon Quality Goods Shop	Green Intelligent Power Saver	no electricity saving	Jingdong prefecture level city in Shandong
Dragon Quality Goods Shop	Green Intelligent Power Saver	differ from	Jingdong prefecture level city in Shandong
Dragon Quality Goods Shop	Green Intelligent Power Saver	Saver has received, but real can save electricity, the quality is very good, I times I buy a few more, too good.	Jingdong prefecture level city in Shandong
Dragon Quality Goods Shop	Green Intelligent Power Saver	Great quality as always, a very favorite product, great! o(*￣︶￣*)o	Jingdong prefecture level city in Shandong
Dragon Quality Goods Shop	Green Intelligent Power Saver	If we can reduce the electricity bill, for individuals can save money, for the country can protect the environment, now the weather is getting hotter and hotter, to protect the environment to reduce emissions is very important. Already use the product, whether to reduce the electricity bill depends on next month!	Jingdong prefecture level city in Shandong
Dragon Quality Goods Shop	Green Intelligent Power Saver	Purchased a power saver received on the put into use, rent a room with many electrical appliances, power consumption is very large, we have a piece of electricity or 1.2 dollars a time, with this power saver is much better, every day can save a few cents, very convenient and practical!	Jingdong prefecture level city in Shandong
Dragon Quality Goods Shop	Green Intelligent Power Saver	The product is better to use before not to use when a month 1500 yuan electricity bill, now using the 5 second month electricity bill directly down 470 dollars or very good. A save money to buy several power saver money ZanZanZanZan👍	Jingdong prefecture level city in Shandong
Dragon Quality Goods Shop	Green Intelligent Power Saver	The moment I received the goods really satisfied with this product, not only high-grade packaging, the body are base alloy, up to the price, I was also introduced by a friend, he used last month did save a lot of electricity, but also recommended to me!	Jingdong prefecture level city in Shandong
Dragon Quality Goods Shop	Green Intelligent Power Saver	I got it. Good review. I'm very satisfied. Reasonable price.	Jingdong prefecture level city in Shandong
Dragon Quality Goods Shop	Green Intelligent Power Saver	Intelligent power saver received and used, looking at the quality is good, the quality of the plug sent is also very good. Expect the effect of the economizer is obvious, first five-star praise, with a period of time to follow up!	Jingdong prefecture level city in Shandong
Dragon Quality Goods Shop	Green Intelligent Power Saver	The saver has been received. It was plugged into an outlet and used immediately after receipt. Whether it really saves electricity is not clear yet, I believe that this brand of energy saver really saves	Jingdong prefecture level city in Shandong
Dragon Quality Goods Shop	Green Intelligent Power Saver	Okay, okay, okay. Thank you so much. Fast is good. Fast. Item was as described, mainly because the event was cheap and you can save a lot of money.	Jingdong prefecture level city in Shandong
Dragon Quality Goods Shop	Green Intelligent Power Saver	Received it a couple days ago! Plugged it in the same day! Let's see what the electric bill is in a month! Hopefully it works well oh!	Jingdong prefecture level city in Shandong
Dragon Quality Goods Shop	Green Intelligent Power Saver	Looking at the reviews good, energy-saving small products, compact, the results after 2 months to see, because the electricity bill season payment	Jingdong prefecture level city in Shandong
Dragon Quality Goods Shop	Green Intelligent Power Saver	I just received it and will try it out to see if it works well. I'll comment later.	Jingdong prefecture level city in Shandong
Dragon Quality Goods Shop	Green Intelligent Power Saver	It looks a little small and not very aesthetically pleasing, I don't know how effective it will be until I use it	Jingdong prefecture level city in Shandong
Dragon Quality Goods Shop	Green Intelligent Power Saver	Not only to the point of being useless yet	Jingdong prefecture level city in Shandong
Dragon Quality Goods Shop	Green Intelligent Power Saver	Used it for a month, nothing works, the meter you turn very fast. This is not a scam, but also sold on Jingdong. To cheat a bunch of people?	Jingdong prefecture level city in Shandong
Dragon Quality Goods Shop	Green Intelligent Power Saver	Very good to use, every day can save a little electricity, really good under the sample down a month to save a few dozen dollars in electricity, good before I did not know that there is this 👆 haha, the price is not expensive praise.	Jingdong prefecture level city in Shandong
Dragon Quality Goods Shop	Green Intelligent Power Saver	Tried it for ten days and the change is almost nothing, can't see the power saving yet, use it some more days and see how it goes.	Jingdong prefecture level city in Shandong
Dragon Quality Goods Shop	Green Intelligent Power Saver	Received very quickly, the quality is very good, really save electricity, business service attitude is also very good, I hope you do not miss this opportunity, I continue to buy a few in	Jingdong prefecture level city in Shandong
Dragon Quality Goods Shop	Green Intelligent Power Saver	Quality is very good Oh, the delivery is also very fast, a very favorite store, I want to crazy recommend to friends!	Jingdong prefecture level city in Shandong
Dragon Quality Goods Shop	Green Intelligent Power Saver	The goods have just received, fine workmanship, and the same as described by the merchant, to use a period of time to see if it can achieve the purpose of power saving.	Jingdong prefecture level city in Shandong
Dragon Quality Goods Shop	Green Intelligent Power Saver	Received the goods first use a period of time to see if there is no effect, the business commitment to ninety days invalid refund, first praise, good service!	Jingdong prefecture level city in Shandong
Dragon Quality Goods Shop	Green Intelligent Power Saver	The product is used for a month without energy-saving and power-saving effect does not have any effect	Jingdong prefecture level city in Shandong
Dragon Quality Goods Shop	Green Intelligent Power Saver	The baby is really great, I like it very much, my friends also say good to come, recommended to buy!	Jingdong prefecture level city in Shandong
Dragon Quality Goods Shop	Green Intelligent Power Saver	Logistics is fast, courier boy service is good, after using to see how the fruit, and then follow up.	Jingdong prefecture level city in Shandong
Dragon Quality Goods Shop	Green Intelligent Power Saver	Baby has been received to try, I believe that as the business advertised energy saving. The courier is very responsible.	Jingdong prefecture level city in Shandong
Dragon Quality Goods Shop	Green Intelligent Power Saver	Save Electric Energy King received, good quality, beautiful appearance, very satisfied!	Jingdong prefecture level city in Shandong
Dragon Quality Goods Shop	Green Intelligent Power Saver	Three no products no manufacturers no phone pure fraud induced consumption cheat	Jingdong prefecture level city in Shandong
Dragon Quality Goods Shop	Green Intelligent Power Saver	I'm using it now, I don't know if it's as good as it says, I'll follow up after a month	Jingdong prefecture level city in Shandong
Dragon Quality Goods Shop	Green Intelligent Power Saver	Good quality logistics and fast. Fine workmanship. The description is consistent cost-effective	Jingdong prefecture level city in Shandong
Dragon Quality Goods Shop	Green Intelligent Power Saver	Received, whether it is good or not, to wait a few days and then evaluate	Jingdong prefecture level city in Shandong
Dragon Quality Goods Shop	Green Intelligent Power Saver	I bought it and started using it, I won't know if it works well until I pay my electric bill	Jingdong prefecture level city in Shandong
Dragon Quality Goods Shop	Green Intelligent Power Saver	Especially good, energy saving and power saving, especially good quality, good	Jingdong prefecture level city in Shandong
Dragon Quality Goods Shop	Green Intelligent Power Saver	Logistics is strong, the quality is very good, paste really save electricity, as described, will buy back!	Jingdong prefecture level city in Shandong
Dragon Quality Goods Shop	Green Intelligent Power Saver	Great stuff at a great price, worth the money!	Jingdong prefecture level city in Shandong

Dragon Quality Goods Shop	Green Intelligent Power Saver	Received the power saver, very good quality, very power saving!	Jingdong prefecture level city in Shandong
Dragon Quality Goods Shop	Green Intelligent Power Saver	There are no fruits, only samples...	Jingdong prefecture level city in Shandong
Dragon Quality Goods Shop	Green Intelligent Power Saver	Save a few dollars a month.	Jingdong prefecture level city in Shandong
Dragon Quality Goods Shop	Green Intelligent Power Saver	The quality is particularly good, it works well, and the seller has a good service attitude	Jingdong prefecture level city in Shandong
Dragon Quality Goods Shop	Green Intelligent Power Saver	I've compared them a couple of times and I don't feel any difference	Jingdong prefecture level city in Shandong
Dragon Quality Goods Shop	Green Intelligent Power Saver	I don't see the savings. It's a sham.	Jingdong prefecture level city in Shandong
Dragon Quality Goods Shop	Green Intelligent Power Saver	The power saving effect is very good, and the logistics is very fast. The seller's service attitude is very good	Jingdong prefecture level city in Shandong
Dragon Quality Goods Shop	Green Intelligent Power Saver	Received, well packaged, haven't tried it yet!	Jingdong prefecture level city in Shandong
Dragon Quality Goods Shop	Green Intelligent Power Saver	I don't know how it works, I'll comment after I use it.	Jingdong prefecture level city in Shandong
Dragon Quality Goods Shop	Green Intelligent Power Saver	The product doesn't work well enough to be of any use	Jingdong prefecture level city in Shandong
Dragon Quality Goods Shop	Green Intelligent Power Saver	On Trial 。。。。。。。	Jingdong prefecture level city in Shandong
Dragon Quality Goods Shop	Green Intelligent Power Saver	Being used with unknown results	Jingdong prefecture level city in Shandong
Dragon Quality Goods Shop	Green Intelligent Power Saver	Smart power saving and great quality	Jingdong prefecture level city in Shandong
Dragon Quality Goods Shop	Green Intelligent Power Saver	The saver was received, it works very well and the price is affordable!	Jingdong prefecture level city in Shandong
Dragon Quality Goods Shop	Green Intelligent Power Saver	How can I feel the power saving and how long does it last	Jingdong prefecture level city in Shandong
Dragon Quality Goods Shop	Green Intelligent Power Saver	And I know if it saves electricity or not.	Jingdong prefecture level city in Shandong
Dragon Quality Goods Shop	Green Intelligent Power Saver	It won't save any electricity. No.	Jingdong prefecture level city in Shandong
Dragon Quality Goods Shop	Green Intelligent Power Saver	Invalid. Fake.	Jingdong prefecture level city in Shandong
Dragon Quality Goods Shop	Green Intelligent Power Saver	It's good!	Jingdong prefecture level city in Shandong
Dragon Quality Goods Shop	Green Intelligent Power Saver	have no effect	Jingdong prefecture level city in Shandong
Dragon Quality Goods Shop	Green Intelligent Power Saver	Not used.	Jingdong prefecture level city in Shandong
Dragon Quality Goods Shop	Green Intelligent Power Saver	There is no load range, packaging thrift, ten days also did not develop invoices, received the goods with a receipt, hand-written receipt also has no financial stamp, communication for nearly a week a moment to say that the electronic invoice, a moment to say that the paper invoice, said it is mailed to me, ten days also did not see anything, the invoice invoice can not be found to say that did not stay on file, single single number can not be found, said that the warehouse more than one to a one to find out, in addition to the perfunctory or perfunctory. Products are no certificate of conformity, proper three	Jingdong prefecture level city in Shandong
Dragon Quality Goods Shop	Green Intelligent Power Saver	Satisfaction.	Jingdong prefecture level city in Shandong
Dragon Quality Goods Shop	Green Intelligent Power Saver	There is not a bit of power saving effect, and ringing up an increase in the electricity bill, because you have to add the cost of this machine's own electricity consumption and purchase, so I think it is better to use this machine instead of increasing the expenditure, uneconomical, rather than not use it.	Jingdong prefecture level city in Shandong
Dragon Quality Goods Shop	Green Intelligent Power Saver	I've been using it for two days now and I don't feel any effect.	Jingdong prefecture level city in Shandong
Dragon Quality Goods Shop	Green Intelligent Power Saver	What it looked like was nothing like what was sent.	Jingdong prefecture level city in Shandong
Dragon Quality Goods Shop	Green Intelligent Power Saver	It didn't work. It didn't work for a month or so.	Jingdong prefecture level city in Shandong
Dragon Quality Goods Shop	Green Intelligent Power Saver	Garbage products, physical and description is not the same, no effect at all, pit # no discussion, to buy the fine, garbage, garbage, garbage	Jingdong prefecture level city in Shandong
Dragon Quality Goods Shop	Green Intelligent Power Saver	general	Jingdong prefecture level city in Shandong
Dragon Quality Goods Shop	Green Intelligent Power Saver	IQ tax. Instead of saving electricity, it increases the power consumption of an appliance.	Jingdong prefecture level city in Shandong
Dragon Quality Goods Shop	Green Intelligent Power Saver	There's no trash at all.	Jingdong prefecture level city in Shandong
Dragon Quality Goods Shop	Green Intelligent Power Saver	It doesn't do anything.	Jingdong prefecture level city in Shandong
Dragon Quality Goods Shop	Green Intelligent Power Saver	(of an unmarried couple) be close	Jingdong prefecture level city in Shandong
Dragon Quality Goods Shop	Green Intelligent Power Saver	(of an unmarried couple) be close	Jingdong prefecture level city in Shandong
Dragon Quality Goods Shop	Green Intelligent Power Saver	no electricity saving	Jingdong prefecture level city in Shandong
Dragon Quality Goods Shop	Green Intelligent Power Saver	differ from	Jingdong prefecture level city in Shandong
Dragon Quality Goods Shop	Green Intelligent Power Saver	Saver has received, but real can save electricity, the quality is very good, I times I buy a few more, too good.	Jingdong prefecture level city in Shandong
Dragon Quality Goods Shop	Green Intelligent Power Saver	Great quality as always, a very favorite product, great! o(*￣︶￣*)o	Jingdong prefecture level city in Shandong
Dragon Quality Goods Shop	Green Intelligent Power Saver	If we can reduce the electricity bill, for individuals can save money, for the country can protect the environment, now the weather is getting hotter and hotter, to protect the environment to reduce emissions is very important. Already use the product, whether to reduce the electricity bill depends on next month!	Jingdong prefecture level city in Shandong
Dragon Quality Goods Shop	Green Intelligent Power Saver	Purchased a power saver received on the put into use, rent a room with many electrical appliances, power consumption is very large, we have a piece of electricity or 1.2 dollars a time, with this power saver is much better, every day can save a few cents, very convenient and practical!	Jingdong prefecture level city in Shandong
Dragon Quality Goods Shop	Green Intelligent Power Saver	The product is better to use before not to use when a month 1500 yuan electricity bill, now using the 5 second month electricity bill directly down 470 dollars or very good. A save money to buy several power saver money ZanZanZanZan👍	Jingdong prefecture level city in Shandong
Dragon Quality Goods Shop	Green Intelligent Power Saver	The moment I received the goods really satisfied with this product, not only high-grade packaging, the body are base alloy, up to the price, I was also introduced by a friend, he used last month did save a lot of electricity, but also recommended to me!	Jingdong prefecture level city in Shandong
Dragon Quality Goods Shop	Green Intelligent Power Saver	I got it. Good review. I'm very satisfied. Reasonable price.	Jingdong prefecture level city in Shandong
Dragon Quality Goods Shop	Green Intelligent Power Saver	Intelligent power saver received and used, looking at the quality is good, the quality of the plug sent is also very good. Expect the effect of the economizer is obvious, first five-star praise, with a period of time to follow up!	Jingdong prefecture level city in Shandong
Dragon Quality Goods Shop	Green Intelligent Power Saver	The saver has been received. It was plugged into an outlet and used immediately after receipt. Whether it really saves electricity is not clear yet, I believe that this brand of energy saver really saves	Jingdong prefecture level city in Shandong
Dragon Quality Goods Shop	Green Intelligent Power Saver	Okay, okay, okay. Thank you so much. Fast is good. Fast. Item was as described, mainly because the event was cheap and you can save a lot of money.	Jingdong prefecture level city in Shandong

Dragon Quality Goods Shop	Green Intelligent Power Saver	Received it a couple days ago! Plugged it in the same day! Let's see what the electric bill is in a month! Hopefully it works well oh!	Jingdong prefecture level city in Shandong
Dragon Quality Goods Shop	Green Intelligent Power Saver	Looking at the reviews good, energy-saving small products, compact, the results after 2 months to see, because the electricity bill season payment	Jingdong prefecture level city in Shandong
Dragon Quality Goods Shop	Green Intelligent Power Saver	I just received it and will try it out to see if it works well. I'll comment later.	Jingdong prefecture level city in Shandong
Dragon Quality Goods Shop	Green Intelligent Power Saver	It looks a little small and not very aesthetically pleasing, I don't know how effective it will be until I use it	Jingdong prefecture level city in Shandong
Dragon Quality Goods Shop	Green Intelligent Power Saver	Not only to the point of being useless yet	Jingdong prefecture level city in Shandong
Dragon Quality Goods Shop	Green Intelligent Power Saver	Used it for a month, nothing works, the meter you turn very fast. This is not a scam, but also sold on Jingdong. To cheat a bunch of people?	Jingdong prefecture level city in Shandong
Dragon Quality Goods Shop	Green Intelligent Power Saver	Very good to use, every day can save a little electricity, really good under the sample down a month to save a few dozen dollars in electricity, good before I did not know that there is this haha, the price is not expensive praise.	Jingdong prefecture level city in Shandong
Dragon Quality Goods Shop	Green Intelligent Power Saver	Tried it for ten days and the change is almost nothing, can't see the power saving yet, use it some more days and see how it goes.	Jingdong prefecture level city in Shandong
Dragon Quality Goods Shop	Green Intelligent Power Saver	Received very quickly, the quality is very good, really save electricity, business service attitude is also very good, I hope you do not miss this opportunity, I continue to buy a few in	Jingdong prefecture level city in Shandong
Dragon Quality Goods Shop	Green Intelligent Power Saver	Quality is very good Oh, the delivery is also very fast, a very favorite store, I want to crazy recommend to friends!	Jingdong prefecture level city in Shandong
Dragon Quality Goods Shop	Green Intelligent Power Saver	The goods have just received, fine workmanship, and the same as described by the merchant, to use a period of time to see if it can achieve the purpose of power saving.	Jingdong prefecture level city in Shandong
Dragon Quality Goods Shop	Green Intelligent Power Saver	Received the goods first use a period of time to see if there is no effect, the business commitment to ninety days invalid refund, first praise, good service!	Jingdong prefecture level city in Shandong
Dragon Quality Goods Shop	Green Intelligent Power Saver	The product is used for a month without energy-saving and power-saving effect does not have any effect	Jingdong prefecture level city in Shandong
Dragon Quality Goods Shop	Green Intelligent Power Saver	The baby is really great, I like it very much, my friends also say good to come, recommended to buy!	Jingdong prefecture level city in Shandong
Dragon Quality Goods Shop	Green Intelligent Power Saver	Logistics is fast, courier boy service is good, after using to see how the fruit, and then follow up.	Jingdong prefecture level city in Shandong
Dragon Quality Goods Shop	Green Intelligent Power Saver	Baby has been received to try, I believe that as the business advertised energy saving. The courier is very responsible.	Jingdong prefecture level city in Shandong
Dragon Quality Goods Shop	Green Intelligent Power Saver	Save Electric Energy King received, good quality, beautiful appearance, very satisfied!	Jingdong prefecture level city in Shandong
Dragon Quality Goods Shop	Green Intelligent Power Saver	Three no products no manufacturers no phone pure fraud induced consumption cheat	Jingdong prefecture level city in Shandong
Dragon Quality Goods Shop	Green Intelligent Power Saver	I'm using it now, I don't know if it's as good as it says, I'll follow up after a month	Jingdong prefecture level city in Shandong
Dragon Quality Goods Shop	Green Intelligent Power Saver	Good quality logistics and fast. Fine workmanship. The description is consistent cost-effective	Jingdong prefecture level city in Shandong
Dragon Quality Goods Shop	Green Intelligent Power Saver	Received, whether it is good or not, to wait a few days and then evaluate	Jingdong prefecture level city in Shandong
Dragon Quality Goods Shop	Green Intelligent Power Saver	I bought it and started using it, I won't know if it works well until I pay my electric bill	Jingdong prefecture level city in Shandong
Dragon Quality Goods Shop	Green Intelligent Power Saver	Especially good, energy saving and power saving, especially good quality, good	Jingdong prefecture level city in Shandong
Dragon Quality Goods Shop	Green Intelligent Power Saver	Logistics is strong, the quality is very good, paste really save electricity, as described, will buy back!	Jingdong prefecture level city in Shandong
Dragon Quality Goods Shop	Green Intelligent Power Saver	Great stuff at a great price, worth the money!	Jingdong prefecture level city in Shandong
Dragon Quality Goods Shop	Green Intelligent Power Saver	Received the power saver, very good quality, very power saving!	Jingdong prefecture level city in Shandong
Dragon Quality Goods Shop	Green Intelligent Power Saver	There are no fruits, only samples...	Jingdong prefecture level city in Shandong
Dragon Quality Goods Shop	Green Intelligent Power Saver	Save a few dollars a month.	Jingdong prefecture level city in Shandong
Dragon Quality Goods Shop	Green Intelligent Power Saver	The quality is particularly good, it works well, and the seller has a good service attitude	Jingdong prefecture level city in Shandong
Dragon Quality Goods Shop	Green Intelligent Power Saver	I've compared them a couple of times and I don't feel any difference	Jingdong prefecture level city in Shandong
Dragon Quality Goods Shop	Green Intelligent Power Saver	I don't see the savings. It's a sham.	Jingdong prefecture level city in Shandong
Dragon Quality Goods Shop	Green Intelligent Power Saver	The power saving effect is very good, and the logistics is very fast. The seller's service attitude is very good	Jingdong prefecture level city in Shandong
Dragon Quality Goods Shop	Green Intelligent Power Saver	Received, well packaged, haven't tried it yet!	Jingdong prefecture level city in Shandong
Dragon Quality Goods Shop	Green Intelligent Power Saver	I don't know how it works, I'll comment after I use it.	Jingdong prefecture level city in Shandong
Dragon Quality Goods Shop	Green Intelligent Power Saver	The product doesn't work well enough to be of any use	Jingdong prefecture level city in Shandong
Dragon Quality Goods Shop	Green Intelligent Power Saver	On Trial 。。。。。。。	Jingdong prefecture level city in Shandong
Dragon Quality Goods Shop	Green Intelligent Power Saver	Being used with unknown results	Jingdong prefecture level city in Shandong
Dragon Quality Goods Shop	Green Intelligent Power Saver	Smart power saving and great quality	Jingdong prefecture level city in Shandong
Dragon Quality Goods Shop	Green Intelligent Power Saver	The saver was received, it works very well and the price is affordable!	Jingdong prefecture level city in Shandong
Dragon Quality Goods Shop	Green Intelligent Power Saver	How can I feel the power saving and how long does it last	Jingdong prefecture level city in Shandong
Dragon Quality Goods Shop	Green Intelligent Power Saver	And I know if it saves electricity or not.	Jingdong prefecture level city in Shandong
Dragon Quality Goods Shop	Green Intelligent Power Saver	It won't save any electricity. No.	Jingdong prefecture level city in Shandong
Dragon Quality Goods Shop	Green Intelligent Power Saver	Invalid. Fake.	Jingdong prefecture level city in Shandong
Dragon Quality Goods Shop	Green Intelligent Power Saver	It's good!	Jingdong prefecture level city in Shandong
Dragon Quality Goods Shop	Green Intelligent Power Saver	have no effect	Jingdong prefecture level city in Shandong
Dragon Quality Goods Shop	Green Intelligent Power Saver	Not used.	Jingdong prefecture level city in Shandong
Dragon Quality Goods Shop	Green Intelligent Power Saver	There is no load range, packaging thrift, ten days also did not develop invoices, received the goods with a receipt, hand-written receipt also has no financial stamp, communication for nearly a week a moment to say that the electronic invoice, a moment to say that the paper invoice, said it is mailed to me, ten days also did not see anything, the invoice invoice can not be found to say that did not stay on file, single single number can not be found, said that the warehouse more than one to a one to find out, in addition to the perfunctory or perfunctory. Products are no certificate of conformity, proper three	Jingdong prefecture level city in Shandong
Dragon Quality Goods Shop	Green Intelligent Power Saver	Satisfaction.	Jingdong prefecture level city in Shandong

Dragon Quality Goods Shop	Green Intelligent Power Saver	There is not a bit of power saving effect, and ringing up an increase in the electricity bill, because you have to add the cost of this machine's own electricity consumption and purchase, so I think it is better to use this machine instead of increasing the expenditure, uneconomical, rather than not use it.	Jingdong prefecture level city in Shandong
Dragon Quality Goods Shop	Green Intelligent Power Saver	I've been using it for two days now and I don't feel any effect.	Jingdong prefecture level city in Shandong
Dragon Quality Goods Shop	Green Intelligent Power Saver	What it looked like was nothing like what was sent.	Jingdong prefecture level city in Shandong
Dragon Quality Goods Shop	Green Intelligent Power Saver	It didn't work. It didn't work for a month or so.	Jingdong prefecture level city in Shandong
Dragon Quality Goods Shop	Green Intelligent Power Saver	Garbage products, physical and description is not the same, no effect at all, pit # no discussion, to buy the fine, garbage, garbage, garbage	Jingdong prefecture level city in Shandong
Dragon Quality Goods Shop	Green Intelligent Power Saver	general	Jingdong prefecture level city in Shandong
Dragon Quality Goods Shop	Green Intelligent Power Saver	IQ tax. Instead of saving electricity, it increases the power consumption of an appliance.	Jingdong prefecture level city in Shandong
Dragon Quality Goods Shop	Green Intelligent Power Saver	There's no trash at all.	Jingdong prefecture level city in Shandong
Dragon Quality Goods Shop	Green Intelligent Power Saver	It doesn't do anything.	Jingdong prefecture level city in Shandong
Dragon Quality Goods Shop	Green Intelligent Power Saver	(of an unmarried couple) be close	Jingdong prefecture level city in Shandong
Dragon Quality Goods Shop	Green Intelligent Power Saver	(of an unmarried couple) be close	Jingdong prefecture level city in Shandong
Dragon Quality Goods Shop	Green Intelligent Power Saver	no electricity saving	Jingdong prefecture level city in Shandong
Dragon Quality Goods Shop	Green Intelligent Power Saver	differ from	Jingdong prefecture level city in Shandong
Dragon Quality Goods Shop	Green Intelligent Power Saver	Saver has received, but real can save electricity, the quality is very good, I times I buy a few more, too good.	Jingdong prefecture level city in Shandong
Dragon Quality Goods Shop	Green Intelligent Power Saver	Great quality as always, a very favorite product, great! o(*￣︶￣*)o	Jingdong prefecture level city in Shandong
Dragon Quality Goods Shop	Green Intelligent Power Saver	If we can reduce the electricity bill, for individuals can save money, for the country can protect the environment, now the weather is getting hotter and hotter, to protect the environment to reduce emissions is very important. Already use the product, whether to reduce the electricity bill depends on next month!	Jingdong prefecture level city in Shandong
Dragon Quality Goods Shop	Green Intelligent Power Saver	Purchased a power saver received on the put into use, rent a room with many electrical appliances, power consumption is very large, we have a piece of electricity or 1.2 dollars a time, with this power saver is much better, every day can save a few cents, very convenient and practical!	Jingdong prefecture level city in Shandong
Dragon Quality Goods Shop	Green Intelligent Power Saver	The product is better to use before not to use when a month 1500 yuan electricity bill, now using the 5 second month electricity bill directly down 470 dollars or very good. A save money to buy several power saver money ZanZanZanZan👍	Jingdong prefecture level city in Shandong
Dragon Quality Goods Shop	Green Intelligent Power Saver	The moment I received the goods really satisfied with this product, not only high-grade packaging, the body are base alloy, up to the price, I was also introduced by a friend, he used last month did save a lot of electricity, but also recommended to me!	Jingdong prefecture level city in Shandong
Dragon Quality Goods Shop	Green Intelligent Power Saver	I got it. Good review. I'm very satisfied. Reasonable price.	Jingdong prefecture level city in Shandong
Dragon Quality Goods Shop	Green Intelligent Power Saver	Intelligent power saver received and used, looking at the quality is good, the quality of the plug sent is also very good. Expect the effect of the economizer is obvious, first five-star praise, with a period of time to follow up!	Jingdong prefecture level city in Shandong
Dragon Quality Goods Shop	Green Intelligent Power Saver	The saver has been received. It was plugged into an outlet and used immediately after receipt. Whether it really saves electricity is not clear yet, I believe that this brand of energy saver really saves	Jingdong prefecture level city in Shandong
Dragon Quality Goods Shop	Green Intelligent Power Saver	Okay, okay, okay. Thank you so much. Fast is good. Fast. Item was as described, mainly because the event was cheap and you can save a lot of money.	Jingdong prefecture level city in Shandong
Dragon Quality Goods Shop	Green Intelligent Power Saver	Received it a couple days ago! Plugged it in the same day! Let's see what the electric bill is in a month! Hopefully it works well oh!	Jingdong prefecture level city in Shandong
Dragon Quality Goods Shop	Green Intelligent Power Saver	Looking at the reviews good, energy-saving small products, compact, the results after 2 months to see, because the electricity bill season payment	Jingdong prefecture level city in Shandong
Dragon Quality Goods Shop	Green Intelligent Power Saver	I just received it and will try it out to see if it works well. I'll comment later.	Jingdong prefecture level city in Shandong
Dragon Quality Goods Shop	Green Intelligent Power Saver	It looks a little small and not very aesthetically pleasing, I don't know how effective it will be until I use it	Jingdong prefecture level city in Shandong
Dragon Quality Goods Shop	Green Intelligent Power Saver	Not only to the point of being useless yet	Jingdong prefecture level city in Shandong
Dragon Quality Goods Shop	Green Intelligent Power Saver	Used it for a month, nothing works, the meter you turn very fast. This is not a scam, but also sold on Jingdong. To cheat a bunch of people?	Jingdong prefecture level city in Shandong
Dragon Quality Goods Shop	Green Intelligent Power Saver	Very good to use, every day can save a little electricity, really good under the sample down a month to save a few dozen dollars in electricity, good before I did not know that there is this 👆 haha, the price is not expensive praise.	Jingdong prefecture level city in Shandong
Dragon Quality Goods Shop	Green Intelligent Power Saver	Tried it for ten days and the change is almost nothing, can't see the power saving yet, use it some more days and see how it goes.	Jingdong prefecture level city in Shandong
Dragon Quality Goods Shop	Green Intelligent Power Saver	Received very quickly, the quality is very good, really save electricity, business service attitude is also very good, I hope you do not miss this opportunity, I continue to buy a few in	Jingdong prefecture level city in Shandong
Dragon Quality Goods Shop	Green Intelligent Power Saver	Quality is very good Oh, the delivery is also very fast, a very favorite store, I want to crazy recommend to friends!	Jingdong prefecture level city in Shandong
Dragon Quality Goods Shop	Green Intelligent Power Saver	The goods have just received, fine workmanship, and the same as described by the merchant, to use a period of time to see if it can achieve the purpose of power saving.	Jingdong prefecture level city in Shandong
Dragon Quality Goods Shop	Green Intelligent Power Saver	Received the goods first use a period of time to see if there is no effect, the business commitment to ninety days invalid refund, first praise, good service!	Jingdong prefecture level city in Shandong
Dragon Quality Goods Shop	Green Intelligent Power Saver	The product is used for a month without energy-saving and power-saving effect does not have any effect	Jingdong prefecture level city in Shandong
Dragon Quality Goods Shop	Green Intelligent Power Saver	The baby is really great, I like it very much, my friends also say good to come, recommended to buy!	Jingdong prefecture level city in Shandong
Dragon Quality Goods Shop	Green Intelligent Power Saver	Logistics is fast, courier boy service is good, after using to see how the fruit, and then follow up.	Jingdong prefecture level city in Shandong
Dragon Quality Goods Shop	Green Intelligent Power Saver	Baby has been received to try, I believe that as the business advertised energy saving. The courier is very responsible.	Jingdong prefecture level city in Shandong
Dragon Quality Goods Shop	Green Intelligent Power Saver	Save Electric Energy King received, good quality, beautiful appearance, very satisfied!	Jingdong prefecture level city in Shandong
Dragon Quality Goods Shop	Green Intelligent Power Saver	Three no products no manufacturers no phone pure fraud induced consumption cheat	Jingdong prefecture level city in Shandong
Dragon Quality Goods Shop	Green Intelligent Power Saver	I'm using it now, I don't know if it's as good as it says, I'll follow up after a month	Jingdong prefecture level city in Shandong
Dragon Quality Goods Shop	Green Intelligent Power Saver	Good quality logistics and fast. Fine workmanship. The description is consistent cost-effective	Jingdong prefecture level city in Shandong
Dragon Quality Goods Shop	Green Intelligent Power Saver	Received, whether it is good or not, to wait a few days and then evaluate	Jingdong prefecture level city in Shandong
Dragon Quality Goods Shop	Green Intelligent Power Saver	I bought it and started using it, I won't know if it works well until I pay my electric bill	Jingdong prefecture level city in Shandong
Dragon Quality Goods Shop	Green Intelligent Power Saver	Especially good, energy saving and power saving, especially good quality, good	Jingdong prefecture level city in Shandong
Dragon Quality Goods Shop	Green Intelligent Power Saver	Logistics is strong, the quality is very good, paste really save electricity, as described, will buy back!	Jingdong prefecture level city in Shandong
Dragon Quality Goods Shop	Green Intelligent Power Saver	Great stuff at a great price, worth the money!	Jingdong prefecture level city in Shandong

Dragon Quality Goods Shop	Green Intelligent Power Saver	Received the power saver, very good quality, very power saving!	Jingdong prefecture level city in Shandong
Dragon Quality Goods Shop	Green Intelligent Power Saver	There are no fruits, only samples...	Jingdong prefecture level city in Shandong
Dragon Quality Goods Shop	Green Intelligent Power Saver	Save a few dollars a month.	Jingdong prefecture level city in Shandong
Dragon Quality Goods Shop	Green Intelligent Power Saver	The quality is particularly good, it works well, and the seller has a good service attitude	Jingdong prefecture level city in Shandong
Dragon Quality Goods Shop	Green Intelligent Power Saver	I've compared them a couple of times and I don't feel any difference	Jingdong prefecture level city in Shandong
Dragon Quality Goods Shop	Green Intelligent Power Saver	I don't see the savings. It's a sham.	Jingdong prefecture level city in Shandong
Dragon Quality Goods Shop	Green Intelligent Power Saver	The power saving effect is very good, and the logistics is very fast. The seller's service attitude is very good	Jingdong prefecture level city in Shandong
Dragon Quality Goods Shop	Green Intelligent Power Saver	Received, well packaged, haven't tried it yet!	Jingdong prefecture level city in Shandong
Dragon Quality Goods Shop	Green Intelligent Power Saver	I don't know how it works, I'll comment after I use it.	Jingdong prefecture level city in Shandong
Dragon Quality Goods Shop	Green Intelligent Power Saver	The product doesn't work well enough to be of any use	Jingdong prefecture level city in Shandong
Dragon Quality Goods Shop	Green Intelligent Power Saver	On Trial 。。。。。。。	Jingdong prefecture level city in Shandong
Dragon Quality Goods Shop	Green Intelligent Power Saver	Being used with unknown results	Jingdong prefecture level city in Shandong
Dragon Quality Goods Shop	Green Intelligent Power Saver	Smart power saving and great quality	Jingdong prefecture level city in Shandong
Dragon Quality Goods Shop	Green Intelligent Power Saver	The saver was received, it works very well and the price is affordable!	Jingdong prefecture level city in Shandong
Dragon Quality Goods Shop	Green Intelligent Power Saver	How can I feel the power saving and how long does it last	Jingdong prefecture level city in Shandong
Dragon Quality Goods Shop	Green Intelligent Power Saver	And I know if it saves electricity or not.	Jingdong prefecture level city in Shandong
Dragon Quality Goods Shop	Green Intelligent Power Saver	It won't save any electricity. No.	Jingdong prefecture level city in Shandong
Dragon Quality Goods Shop	Green Intelligent Power Saver	Invalid. Fake.	Jingdong prefecture level city in Shandong
Dragon Quality Goods Shop	Green Intelligent Power Saver	It's good!	Jingdong prefecture level city in Shandong
Dragon Quality Goods Shop	Green Intelligent Power Saver	have no effect	Jingdong prefecture level city in Shandong
Dragon Quality Goods Shop	Green Intelligent Power Saver	Not used.	Jingdong prefecture level city in Shandong
Dragon Quality Goods Shop	Green Intelligent Power Saver	There is no load range, packaging thrift, ten days also did not develop invoices, received the goods with a receipt, hand-written receipt also has no financial stamp, communication for nearly a week a moment to say that the electronic invoice, a moment to say that the paper invoice, said it is mailed to me, ten days also did not see anything, the invoice invoice can not be found to say that did not stay on file, single single number can not be found, said that the warehouse more than one to a one to find out, in addition to the perfunctory or perfunctory. Products are no certificate of conformity, proper three	Jingdong prefecture level city in Shandong
Dragon Quality Goods Shop	Green Intelligent Power Saver	Satisfaction.	Jingdong prefecture level city in Shandong
Dragon Quality Goods Shop	Green Intelligent Power Saver	There is not a bit of power saving effect, and ringing up an increase in the electricity bill, because you have to add the cost of this machine's own electricity consumption and purchase, so I think it is better to use this machine instead of increasing the expenditure, uneconomical, rather than not use it.	Jingdong prefecture level city in Shandong
Dragon Quality Goods Shop	Green Intelligent Power Saver	I've been using it for two days now and I don't feel any effect.	Jingdong prefecture level city in Shandong
Dragon Quality Goods Shop	Green Intelligent Power Saver	What it looked like was nothing like what was sent.	Jingdong prefecture level city in Shandong
Dragon Quality Goods Shop	Green Intelligent Power Saver	It didn't work. It didn't work for a month or so.	Jingdong prefecture level city in Shandong
Dragon Quality Goods Shop	Green Intelligent Power Saver	Garbage products, physical and description is not the same, no effect at all, pit # no discussion, to buy the fine, garbage, garbage, garbage	Jingdong prefecture level city in Shandong
Dragon Quality Goods Shop	Green Intelligent Power Saver	general	Jingdong prefecture level city in Shandong
Dragon Quality Goods Shop	Green Intelligent Power Saver	IQ tax. Instead of saving electricity, it increases the power consumption of an appliance.	Jingdong prefecture level city in Shandong
Dragon Quality Goods Shop	Green Intelligent Power Saver	There's no trash at all.	Jingdong prefecture level city in Shandong
Dragon Quality Goods Shop	Green Intelligent Power Saver	It doesn't do anything.	Jingdong prefecture level city in Shandong
Dragon Quality Goods Shop	Green Intelligent Power Saver	(of an unmarried couple) be close	Jingdong prefecture level city in Shandong
Dragon Quality Goods Shop	Green Intelligent Power Saver	(of an unmarried couple) be close	Jingdong prefecture level city in Shandong
Dragon Quality Goods Shop	Green Intelligent Power Saver	no electricity saving	Jingdong prefecture level city in Shandong
Dragon Quality Goods Shop	Green Intelligent Power Saver	differ from	Jingdong prefecture level city in Shandong
Dragon Quality Goods Shop	Green Intelligent Power Saver	Saver has received, but real can save electricity, the quality is very good, I times I buy a few more, too good.	Jingdong prefecture level city in Shandong
Dragon Quality Goods Shop	Green Intelligent Power Saver	Great quality as always, a very favorite product, great! o(*￣︶￣*)o	Jingdong prefecture level city in Shandong
Dragon Quality Goods Shop	Green Intelligent Power Saver	If we can reduce the electricity bill, for individuals can save money, for the country can protect the environment, now the weather is getting hotter and hotter, to protect the environment to reduce emissions is very important. Already use the product, whether to reduce the electricity bill depends on next month!	Jingdong prefecture level city in Shandong
Dragon Quality Goods Shop	Green Intelligent Power Saver	Purchased a power saver received on the put into use, rent a room with many electrical appliances, power consumption is very large, we have a piece of electricity or 1.2 dollars a time, with this power saver is much better, every day can save a few cents, very convenient and practical!	Jingdong prefecture level city in Shandong
Dragon Quality Goods Shop	Green Intelligent Power Saver	The product is better to use before not to use when a month 1500 yuan electricity bill, now using the 5 second month electricity bill directly down 470 dollars or very good. A save money to buy several power saver money ZanZanZanZan👍	Jingdong prefecture level city in Shandong
Dragon Quality Goods Shop	Green Intelligent Power Saver	The moment I received the goods really satisfied with this product, not only high-grade packaging, the body are base alloy, up to the price, I was also introduced by a friend, he used last month did save a lot of electricity, but also recommended to me!	Jingdong prefecture level city in Shandong
Dragon Quality Goods Shop	Green Intelligent Power Saver	I got it. Good review. I'm very satisfied. Reasonable price.	Jingdong prefecture level city in Shandong
Dragon Quality Goods Shop	Green Intelligent Power Saver	Intelligent power saver received and used, looking at the quality is good, the quality of the plug sent is also very good. Expect the effect of the economizer is obvious, first five-star praise, with a period of time to follow up!	Jingdong prefecture level city in Shandong
Dragon Quality Goods Shop	Green Intelligent Power Saver	The saver has been received. It was plugged into an outlet and used immediately after receipt. Whether it really saves electricity is not clear yet, I believe that this brand of energy saver really saves	Jingdong prefecture level city in Shandong
Dragon Quality Goods Shop	Green Intelligent Power Saver	Okay, okay, okay. Thank you so much. Fast is good. Fast. Item was as described, mainly because the event was cheap and you can save a lot of money.	Jingdong prefecture level city in Shandong

Dragon Quality Goods Shop	Green Intelligent Power Saver	Received it a couple days ago! Plugged it in the same day! Let's see what the electric bill is in a month! Hopefully it works well oh!	Jingdong prefecture level city in Shandong
Dragon Quality Goods Shop	Green Intelligent Power Saver	Looking at the reviews good, energy-saving small products, compact, the results after 2 months to see, because the electricity bill season payment	Jingdong prefecture level city in Shandong
Dragon Quality Goods Shop	Green Intelligent Power Saver	I just received it and will try it out to see if it works well. I'll comment later.	Jingdong prefecture level city in Shandong
Dragon Quality Goods Shop	Green Intelligent Power Saver	It looks a little small and not very aesthetically pleasing, I don't know how effective it will be until I use it	Jingdong prefecture level city in Shandong
Dragon Quality Goods Shop	Green Intelligent Power Saver	Not only to the point of being useless yet	Jingdong prefecture level city in Shandong
Dragon Quality Goods Shop	Green Intelligent Power Saver	Used it for a month, nothing works, the meter you turn very fast. This is not a scam, but also sold on Jingdong. To cheat a bunch of people?	Jingdong prefecture level city in Shandong
Dragon Quality Goods Shop	Green Intelligent Power Saver	Very good to use, every day can save a little electricity, really good under the sample down a month to save a few dozen dollars in electricity, good before I did not know that there is this haha, the price is not expensive praise.	Jingdong prefecture level city in Shandong
Dragon Quality Goods Shop	Green Intelligent Power Saver	Tried it for ten days and the change is almost nothing, can't see the power saving yet, use it some more days and see how it goes.	Jingdong prefecture level city in Shandong
Dragon Quality Goods Shop	Green Intelligent Power Saver	Received very quickly, the quality is very good, really save electricity, business service attitude is also very good, I hope you do not miss this opportunity, I continue to buy a few in	Jingdong prefecture level city in Shandong
Dragon Quality Goods Shop	Green Intelligent Power Saver	Quality is very good Oh, the delivery is also very fast, a very favorite store, I want to crazy recommend to friends!	Jingdong prefecture level city in Shandong
Dragon Quality Goods Shop	Green Intelligent Power Saver	The goods have just received, fine workmanship, and the same as described by the merchant, to use a period of time to see if it can achieve the purpose of power saving.	Jingdong prefecture level city in Shandong
Dragon Quality Goods Shop	Green Intelligent Power Saver	Received the goods first use a period of time to see if there is no effect, the business commitment to ninety days invalid refund, first praise, good service!	Jingdong prefecture level city in Shandong
Dragon Quality Goods Shop	Green Intelligent Power Saver	The product is used for a month without energy-saving and power-saving effect does not have any effect	Jingdong prefecture level city in Shandong
Dragon Quality Goods Shop	Green Intelligent Power Saver	The baby is really great, I like it very much, my friends also say good to come, recommended to buy!	Jingdong prefecture level city in Shandong
Dragon Quality Goods Shop	Green Intelligent Power Saver	Logistics is fast, courier boy service is good, after using to see how the fruit, and then follow up.	Jingdong prefecture level city in Shandong
Dragon Quality Goods Shop	Green Intelligent Power Saver	Baby has been received to try, I believe that as the business advertised energy saving. The courier is very responsible.	Jingdong prefecture level city in Shandong
Dragon Quality Goods Shop	Green Intelligent Power Saver	Save Electric Energy King received, good quality, beautiful appearance, very satisfied!	Jingdong prefecture level city in Shandong
Dragon Quality Goods Shop	Green Intelligent Power Saver	Three no products no manufacturers no phone pure fraud induced consumption cheat	Jingdong prefecture level city in Shandong
Dragon Quality Goods Shop	Green Intelligent Power Saver	I'm using it now, I don't know if it's as good as it says, I'll follow up after a month	Jingdong prefecture level city in Shandong
Dragon Quality Goods Shop	Green Intelligent Power Saver	Good quality logistics and fast. Fine workmanship. The description is consistent cost-effective	Jingdong prefecture level city in Shandong
Dragon Quality Goods Shop	Green Intelligent Power Saver	Received, whether it is good or not, to wait a few days and then evaluate	Jingdong prefecture level city in Shandong
Dragon Quality Goods Shop	Green Intelligent Power Saver	I bought it and started using it, I won't know if it works well until I pay my electric bill	Jingdong prefecture level city in Shandong
Dragon Quality Goods Shop	Green Intelligent Power Saver	Especially good, energy saving and power saving, especially good quality, good	Jingdong prefecture level city in Shandong
Dragon Quality Goods Shop	Green Intelligent Power Saver	Logistics is strong, the quality is very good, paste really save electricity, as described, will buy back!	Jingdong prefecture level city in Shandong
Dragon Quality Goods Shop	Green Intelligent Power Saver	Great stuff at a great price, worth the money!	Jingdong prefecture level city in Shandong
Dragon Quality Goods Shop	Green Intelligent Power Saver	Received the power saver, very good quality, very power saving!	Jingdong prefecture level city in Shandong
Dragon Quality Goods Shop	Green Intelligent Power Saver	There are no fruits, only samples...	Jingdong prefecture level city in Shandong
Dragon Quality Goods Shop	Green Intelligent Power Saver	Save a few dollars a month.	Jingdong prefecture level city in Shandong
Dragon Quality Goods Shop	Green Intelligent Power Saver	The quality is particularly good, it works well, and the seller has a good service attitude	Jingdong prefecture level city in Shandong
Dragon Quality Goods Shop	Green Intelligent Power Saver	I've compared them a couple of times and I don't feel any difference	Jingdong prefecture level city in Shandong
Dragon Quality Goods Shop	Green Intelligent Power Saver	I don't see the savings. It's a sham.	Jingdong prefecture level city in Shandong
Dragon Quality Goods Shop	Green Intelligent Power Saver	The power saving effect is very good, and the logistics is very fast. The seller's service attitude is very good	Jingdong prefecture level city in Shandong
Dragon Quality Goods Shop	Green Intelligent Power Saver	Received, well packaged, haven't tried it yet!	Jingdong prefecture level city in Shandong
Dragon Quality Goods Shop	Green Intelligent Power Saver	I don't know how it works, I'll comment after I use it.	Jingdong prefecture level city in Shandong
Dragon Quality Goods Shop	Green Intelligent Power Saver	The product doesn't work well enough to be of any use	Jingdong prefecture level city in Shandong
Dragon Quality Goods Shop	Green Intelligent Power Saver	On Trial 。。。。。。。	Jingdong prefecture level city in Shandong
Dragon Quality Goods Shop	Green Intelligent Power Saver	Being used with unknown results	Jingdong prefecture level city in Shandong
Dragon Quality Goods Shop	Green Intelligent Power Saver	Smart power saving and great quality	Jingdong prefecture level city in Shandong
Dragon Quality Goods Shop	Green Intelligent Power Saver	The saver was received, it works very well and the price is affordable!	Jingdong prefecture level city in Shandong
Dragon Quality Goods Shop	Green Intelligent Power Saver	How can I feel the power saving and how long does it last	Jingdong prefecture level city in Shandong
Dragon Quality Goods Shop	Green Intelligent Power Saver	And I know if it saves electricity or not.	Jingdong prefecture level city in Shandong
Dragon Quality Goods Shop	Green Intelligent Power Saver	It won't save any electricity. No.	Jingdong prefecture level city in Shandong
Dragon Quality Goods Shop	Green Intelligent Power Saver	Invalid. Fake.	Jingdong prefecture level city in Shandong
Dragon Quality Goods Shop	Green Intelligent Power Saver	It's good!	Jingdong prefecture level city in Shandong
Dragon Quality Goods Shop	Green Intelligent Power Saver	have no effect	Jingdong prefecture level city in Shandong
Dragon Quality Goods Shop	Green Intelligent Power Saver	Not used.	Jingdong prefecture level city in Shandong
Dragon Quality Goods Shop	Green Intelligent Power Saver	There is no load range, packaging thrift, ten days also did not develop invoices, received the goods with a receipt, hand-written receipt also has no financial stamp, communication for nearly a week a moment to say that the electronic invoice, a moment to say that the paper invoice, said it is mailed to me, ten days also did not see anything, the invoice invoice can not be found to say that did not stay on file, single single number can not be found, said that the warehouse more than one to a one to find out, in addition to the perfunctory or perfunctory. Products are no certificate of conformity, proper three	Jingdong prefecture level city in Shandong
Dragon Quality Goods Shop	Green Intelligent Power Saver	Satisfaction.	Jingdong prefecture level city in Shandong

Dragon Quality Goods Shop	Green Intelligent Power Saver	There is not a bit of power saving effect, and ringing up an increase in the electricity bill, because you have to add the cost of this machine's own electricity consumption and purchase, so I think it is better to use this machine instead of increasing the expenditure, uneconomical, rather than not use it.	Jingdong prefecture level city in Shandong
Dragon Quality Goods Shop	Green Intelligent Power Saver	I've been using it for two days now and I don't feel any effect.	Jingdong prefecture level city in Shandong
Dragon Quality Goods Shop	Green Intelligent Power Saver	What it looked like was nothing like what was sent.	Jingdong prefecture level city in Shandong
Dragon Quality Goods Shop	Green Intelligent Power Saver	It didn't work. It didn't work for a month or so.	Jingdong prefecture level city in Shandong
Dragon Quality Goods Shop	Green Intelligent Power Saver	Garbage products, physical and description is not the same, no effect at all, pit # no discussion, to buy the fine, garbage, garbage, garbage	Jingdong prefecture level city in Shandong
Dragon Quality Goods Shop	Green Intelligent Power Saver	general	Jingdong prefecture level city in Shandong
Dragon Quality Goods Shop	Green Intelligent Power Saver	IQ tax. Instead of saving electricity, it increases the power consumption of an appliance.	Jingdong prefecture level city in Shandong
Dragon Quality Goods Shop	Green Intelligent Power Saver	There's no trash at all.	Jingdong prefecture level city in Shandong
Dragon Quality Goods Shop	Green Intelligent Power Saver	It doesn't do anything.	Jingdong prefecture level city in Shandong
Dragon Quality Goods Shop	Green Intelligent Power Saver	(of an unmarried couple) be close	Jingdong prefecture level city in Shandong
Dragon Quality Goods Shop	Green Intelligent Power Saver	(of an unmarried couple) be close	Jingdong prefecture level city in Shandong
Dragon Quality Goods Shop	Green Intelligent Power Saver	no electricity saving	Jingdong prefecture level city in Shandong
Dragon Quality Goods Shop	Green Intelligent Power Saver	differ from	Jingdong prefecture level city in Shandong
Dragon Quality Goods Shop	Green Intelligent Power Saver	Saver has received, but real can save electricity, the quality is very good, I times I buy a few more, too good.	Jingdong prefecture level city in Shandong
Dragon Quality Goods Shop	Green Intelligent Power Saver	Great quality as always, a very favorite product, great! o(*￣︶￣*)o	Jingdong prefecture level city in Shandong
Dragon Quality Goods Shop	Green Intelligent Power Saver	If we can reduce the electricity bill, for individuals can save money, for the country can protect the environment, now the weather is getting hotter and hotter, to protect the environment to reduce emissions is very important. Already use the product, whether to reduce the electricity bill depends on next month!	Jingdong prefecture level city in Shandong
Dragon Quality Goods Shop	Green Intelligent Power Saver	Purchased a power saver received on the put into use, rent a room with many electrical appliances, power consumption is very large, we have a piece of electricity or 1.2 dollars a time, with this power saver is much better, every day can save a few cents, very convenient and practical!	Jingdong prefecture level city in Shandong
Dragon Quality Goods Shop	Green Intelligent Power Saver	The product is better to use before not to use when a month 1500 yuan electricity bill, now using the 5 second month electricity bill directly down 470 dollars or very good. A save money to buy several power saver money ZanZanZanZan👍	Jingdong prefecture level city in Shandong
Dragon Quality Goods Shop	Green Intelligent Power Saver	The moment I received the goods really satisfied with this product, not only high-grade packaging, the body are base alloy, up to the price, I was also introduced by a friend, he used last month did save a lot of electricity, but also recommended to me!	Jingdong prefecture level city in Shandong
Dragon Quality Goods Shop	Green Intelligent Power Saver	I got it. Good review. I'm very satisfied. Reasonable price.	Jingdong prefecture level city in Shandong
Dragon Quality Goods Shop	Green Intelligent Power Saver	Intelligent power saver received and used, looking at the quality is good, the quality of the plug sent is also very good. Expect the effect of the economizer is obvious, first five-star praise, with a period of time to follow up!	Jingdong prefecture level city in Shandong
Dragon Quality Goods Shop	Green Intelligent Power Saver	The saver has been received. It was plugged into an outlet and used immediately after receipt. Whether it really saves electricity is not clear yet, I believe that this brand of energy saver really saves	Jingdong prefecture level city in Shandong
Dragon Quality Goods Shop	Green Intelligent Power Saver	Okay, okay, okay. Thank you so much. Fast is good. Fast. Item was as described, mainly because the event was cheap and you can save a lot of money.	Jingdong prefecture level city in Shandong
Dragon Quality Goods Shop	Green Intelligent Power Saver	Received it a couple days ago! Plugged it in the same day! Let's see what the electric bill is in a month! Hopefully it works well oh!	Jingdong prefecture level city in Shandong
Dragon Quality Goods Shop	Green Intelligent Power Saver	Looking at the reviews good, energy-saving small products, compact, the results after 2 months to see, because the electricity bill season payment	Jingdong prefecture level city in Shandong
Dragon Quality Goods Shop	Green Intelligent Power Saver	I just received it and will try it out to see if it works well. I'll comment later.	Jingdong prefecture level city in Shandong
Dragon Quality Goods Shop	Green Intelligent Power Saver	It looks a little small and not very aesthetically pleasing, I don't know how effective it will be until I use it	Jingdong prefecture level city in Shandong
Dragon Quality Goods Shop	Green Intelligent Power Saver	Not only to the point of being useless yet	Jingdong prefecture level city in Shandong
Dragon Quality Goods Shop	Green Intelligent Power Saver	Used it for a month, nothing works, the meter you turn very fast. This is not a scam, but also sold on Jingdong. To cheat a bunch of people?	Jingdong prefecture level city in Shandong
Dragon Quality Goods Shop	Green Intelligent Power Saver	Very good to use, every day can save a little electricity, really good under the sample down a month to save a few dozen dollars in electricity, good before I did not know that there is this 👆 haha, the price is not expensive praise.	Jingdong prefecture level city in Shandong
Dragon Quality Goods Shop	Green Intelligent Power Saver	Tried it for ten days and the change is almost nothing, can't see the power saving yet, use it some more days and see how it goes.	Jingdong prefecture level city in Shandong
Dragon Quality Goods Shop	Green Intelligent Power Saver	Received very quickly, the quality is very good, really save electricity, business service attitude is also very good, I hope you do not miss this opportunity, I continue to buy a few in	Jingdong prefecture level city in Shandong
Dragon Quality Goods Shop	Green Intelligent Power Saver	Quality is very good Oh, the delivery is also very fast, a very favorite store, I want to crazy recommend to friends!	Jingdong prefecture level city in Shandong
Dragon Quality Goods Shop	Green Intelligent Power Saver	The goods have just received, fine workmanship, and the same as described by the merchant, to use a period of time to see if it can achieve the purpose of power saving.	Jingdong prefecture level city in Shandong
Dragon Quality Goods Shop	Green Intelligent Power Saver	Received the goods first use a period of time to see if there is no effect, the business commitment to ninety days invalid refund, first praise, good service!	Jingdong prefecture level city in Shandong
Dragon Quality Goods Shop	Green Intelligent Power Saver	The product is used for a month without energy-saving and power-saving effect does not have any effect	Jingdong prefecture level city in Shandong
Dragon Quality Goods Shop	Green Intelligent Power Saver	The baby is really great, I like it very much, my friends also say good to come, recommended to buy!	Jingdong prefecture level city in Shandong
Dragon Quality Goods Shop	Green Intelligent Power Saver	Logistics is fast, courier boy service is good, after using to see how the fruit, and then follow up.	Jingdong prefecture level city in Shandong
Dragon Quality Goods Shop	Green Intelligent Power Saver	Baby has been received to try, I believe that as the business advertised energy saving. The courier is very responsible.	Jingdong prefecture level city in Shandong
Dragon Quality Goods Shop	Green Intelligent Power Saver	Save Electric Energy King received, good quality, beautiful appearance, very satisfied!	Jingdong prefecture level city in Shandong
Dragon Quality Goods Shop	Green Intelligent Power Saver	Three no products no manufacturers no phone pure fraud induced consumption cheat	Jingdong prefecture level city in Shandong
Dragon Quality Goods Shop	Green Intelligent Power Saver	I'm using it now, I don't know if it's as good as it says, I'll follow up after a month	Jingdong prefecture level city in Shandong
Dragon Quality Goods Shop	Green Intelligent Power Saver	Good quality logistics and fast. Fine workmanship. The description is consistent cost-effective	Jingdong prefecture level city in Shandong
Dragon Quality Goods Shop	Green Intelligent Power Saver	Received, whether it is good or not, to wait a few days and then evaluate	Jingdong prefecture level city in Shandong
Dragon Quality Goods Shop	Green Intelligent Power Saver	I bought it and started using it, I won't know if it works well until I pay my electric bill	Jingdong prefecture level city in Shandong
Dragon Quality Goods Shop	Green Intelligent Power Saver	Especially good, energy saving and power saving, especially good quality, good	Jingdong prefecture level city in Shandong
Dragon Quality Goods Shop	Green Intelligent Power Saver	Logistics is strong, the quality is very good, paste really save electricity, as described, will buy back!	Jingdong prefecture level city in Shandong
Dragon Quality Goods Shop	Green Intelligent Power Saver	Great stuff at a great price, worth the money!	Jingdong prefecture level city in Shandong

Dragon Quality Goods Shop	Green Intelligent Power Saver	Received the power saver, very good quality, very power saving!	Jingdong prefecture level city in Shandong
Dragon Quality Goods Shop	Green Intelligent Power Saver	There are no fruits, only samples...	Jingdong prefecture level city in Shandong
Dragon Quality Goods Shop	Green Intelligent Power Saver	Save a few dollars a month.	Jingdong prefecture level city in Shandong
Dragon Quality Goods Shop	Green Intelligent Power Saver	The quality is particularly good, it works well, and the seller has a good service attitude	Jingdong prefecture level city in Shandong
Dragon Quality Goods Shop	Green Intelligent Power Saver	I've compared them a couple of times and I don't feel any difference	Jingdong prefecture level city in Shandong
Dragon Quality Goods Shop	Green Intelligent Power Saver	I don't see the savings. It's a sham.	Jingdong prefecture level city in Shandong
Dragon Quality Goods Shop	Green Intelligent Power Saver	The power saving effect is very good, and the logistics is very fast. The seller's service attitude is very good	Jingdong prefecture level city in Shandong
Dragon Quality Goods Shop	Green Intelligent Power Saver	Received, well packaged, haven't tried it yet!	Jingdong prefecture level city in Shandong
Dragon Quality Goods Shop	Green Intelligent Power Saver	I don't know how it works, I'll comment after I use it.	Jingdong prefecture level city in Shandong
Dragon Quality Goods Shop	Green Intelligent Power Saver	The product doesn't work well enough to be of any use	Jingdong prefecture level city in Shandong
Dragon Quality Goods Shop	Green Intelligent Power Saver	On Trial 。。。。。。。	Jingdong prefecture level city in Shandong
Dragon Quality Goods Shop	Green Intelligent Power Saver	Being used with unknown results	Jingdong prefecture level city in Shandong
Dragon Quality Goods Shop	Green Intelligent Power Saver	Smart power saving and great quality	Jingdong prefecture level city in Shandong
Dragon Quality Goods Shop	Green Intelligent Power Saver	The saver was received, it works very well and the price is affordable!	Jingdong prefecture level city in Shandong
Dragon Quality Goods Shop	Green Intelligent Power Saver	How can I feel the power saving and how long does it last	Jingdong prefecture level city in Shandong
Dragon Quality Goods Shop	Green Intelligent Power Saver	And I know if it saves electricity or not.	Jingdong prefecture level city in Shandong
Dragon Quality Goods Shop	Green Intelligent Power Saver	It won't save any electricity. No.	Jingdong prefecture level city in Shandong
Dragon Quality Goods Shop	Green Intelligent Power Saver	Invalid. Fake.	Jingdong prefecture level city in Shandong
Dragon Quality Goods Shop	Green Intelligent Power Saver	It's good!	Jingdong prefecture level city in Shandong
Dragon Quality Goods Shop	Green Intelligent Power Saver	have no effect	Jingdong prefecture level city in Shandong
Dragon Quality Goods Shop	Green Intelligent Power Saver	Not used.	Jingdong prefecture level city in Shandong
Dragon Quality Goods Shop	Green Intelligent Power Saver	There is no load range, packaging thrift, ten days also did not develop invoices, received the goods with a receipt, hand-written receipt also has no financial stamp, communication for nearly a week a moment to say that the electronic invoice, a moment to say that the paper invoice, said it is mailed to me, ten days also did not see anything, the invoice invoice can not be found to say that did not stay on file, single single number can not be found, said that the warehouse more than one to a one to find out, in addition to the perfunctory or perfunctory. Products are no certificate of conformity, proper three	Jingdong prefecture level city in Shandong
Dragon Quality Goods Shop	Green Intelligent Power Saver	Satisfaction.	Jingdong prefecture level city in Shandong
Dragon Quality Goods Shop	Green Intelligent Power Saver	There is not a bit of power saving effect, and ringing up an increase in the electricity bill, because you have to add the cost of this machine's own electricity consumption and purchase, so I think it is better to use this machine instead of increasing the expenditure, uneconomical, rather than not use it.	Jingdong prefecture level city in Shandong
Dragon Quality Goods Shop	Green Intelligent Power Saver	I've been using it for two days now and I don't feel any effect.	Jingdong prefecture level city in Shandong
Dragon Quality Goods Shop	Green Intelligent Power Saver	What it looked like was nothing like what was sent.	Jingdong prefecture level city in Shandong
Dragon Quality Goods Shop	Green Intelligent Power Saver	It didn't work. It didn't work for a month or so.	Jingdong prefecture level city in Shandong
Dragon Quality Goods Shop	Green Intelligent Power Saver	Garbage products, physical and description is not the same, no effect at all, pit # no discussion, to buy the fine, garbage, garbage, garbage	Jingdong prefecture level city in Shandong
Dragon Quality Goods Shop	Green Intelligent Power Saver	general	Jingdong prefecture level city in Shandong
Dragon Quality Goods Shop	Green Intelligent Power Saver	IQ tax. Instead of saving electricity, it increases the power consumption of an appliance.	Jingdong prefecture level city in Shandong
Dragon Quality Goods Shop	Green Intelligent Power Saver	There's no trash at all.	Jingdong prefecture level city in Shandong
Dragon Quality Goods Shop	Green Intelligent Power Saver	It doesn't do anything.	Jingdong prefecture level city in Shandong
Dragon Quality Goods Shop	Green Intelligent Power Saver	(of an unmarried couple) be close	Jingdong prefecture level city in Shandong
Dragon Quality Goods Shop	Green Intelligent Power Saver	(of an unmarried couple) be close	Jingdong prefecture level city in Shandong
Dragon Quality Goods Shop	Green Intelligent Power Saver	no electricity saving	Jingdong prefecture level city in Shandong
Dragon Quality Goods Shop	Green Intelligent Power Saver	differ from	Jingdong prefecture level city in Shandong
Dragon Quality Goods Shop	Green Intelligent Power Saver	Saver has received, but real can save electricity, the quality is very good, I times I buy a few more, too good.	Jingdong prefecture level city in Shandong
Dragon Quality Goods Shop	Green Intelligent Power Saver	Great quality as always, a very favorite product, great! o(*￣︶￣*)o	Jingdong prefecture level city in Shandong
Dragon Quality Goods Shop	Green Intelligent Power Saver	If we can reduce the electricity bill, for individuals can save money, for the country can protect the environment, now the weather is getting hotter and hotter, to protect the environment to reduce emissions is very important. Already use the product, whether to reduce the electricity bill depends on next month!	Jingdong prefecture level city in Shandong
Dragon Quality Goods Shop	Green Intelligent Power Saver	Purchased a power saver received on the put into use, rent a room with many electrical appliances, power consumption is very large, we have a piece of electricity or 1.2 dollars a time, with this power saver is much better, every day can save a few cents, very convenient and practical!	Jingdong prefecture level city in Shandong
Dragon Quality Goods Shop	Green Intelligent Power Saver	The product is better to use before not to use when a month 1500 yuan electricity bill, now using the 5 second month electricity bill directly down 470 dollars or very good. A save money to buy several power saver money ZanZanZanZan👍	Jingdong prefecture level city in Shandong
Dragon Quality Goods Shop	Green Intelligent Power Saver	The moment I received the goods really satisfied with this product, not only high-grade packaging, the body are base alloy, up to the price, I was also introduced by a friend, he used last month did save a lot of electricity, but also recommended to me!	Jingdong prefecture level city in Shandong
Dragon Quality Goods Shop	Green Intelligent Power Saver	I got it. Good review. I'm very satisfied. Reasonable price.	Jingdong prefecture level city in Shandong
Dragon Quality Goods Shop	Green Intelligent Power Saver	Intelligent power saver received and used, looking at the quality is good, the quality of the plug sent is also very good. Expect the effect of the economizer is obvious, first five-star praise, with a period of time to follow up!	Jingdong prefecture level city in Shandong
Dragon Quality Goods Shop	Green Intelligent Power Saver	The saver has been received. It was plugged into an outlet and used immediately after receipt. Whether it really saves electricity is not clear yet, I believe that this brand of energy saver really saves	Jingdong prefecture level city in Shandong
Dragon Quality Goods Shop	Green Intelligent Power Saver	Okay, okay, okay. Thank you so much. Fast is good. Fast. Item was as described, mainly because the event was cheap and you can save a lot of money.	Jingdong prefecture level city in Shandong

Dragon Quality Goods Shop	Green Intelligent Power Saver	Received it a couple days ago! Plugged it in the same day! Let's see what the electric bill is in a month! Hopefully it works well oh!	Jingdong prefecture level city in Shandong
Dragon Quality Goods Shop	Green Intelligent Power Saver	Looking at the reviews good, energy-saving small products, compact, the results after 2 months to see, because the electricity bill season payment	Jingdong prefecture level city in Shandong
Dragon Quality Goods Shop	Green Intelligent Power Saver	I just received it and will try it out to see if it works well. I'll comment later.	Jingdong prefecture level city in Shandong
Dragon Quality Goods Shop	Green Intelligent Power Saver	It looks a little small and not very aesthetically pleasing, I don't know how effective it will be until I use it	Jingdong prefecture level city in Shandong
Dragon Quality Goods Shop	Green Intelligent Power Saver	Not only to the point of being useless yet	Jingdong prefecture level city in Shandong
Dragon Quality Goods Shop	Green Intelligent Power Saver	Used it for a month, nothing works, the meter you turn very fast. This is not a scam, but also sold on Jingdong. To cheat a bunch of people?	Jingdong prefecture level city in Shandong
Dragon Quality Goods Shop	Green Intelligent Power Saver	Very good to use, every day can save a little electricity, really good under the sample down a month to save a few dozen dollars in electricity, good before I did not know that there is this haha, the price is not expensive praise.	Jingdong prefecture level city in Shandong
Dragon Quality Goods Shop	Green Intelligent Power Saver	Tried it for ten days and the change is almost nothing, can't see the power saving yet, use it some more days and see how it goes.	Jingdong prefecture level city in Shandong
Dragon Quality Goods Shop	Green Intelligent Power Saver	Received very quickly, the quality is very good, really save electricity, business service attitude is also very good, I hope you do not miss this opportunity, I continue to buy a few in	Jingdong prefecture level city in Shandong
Dragon Quality Goods Shop	Green Intelligent Power Saver	Quality is very good Oh, the delivery is also very fast, a very favorite store, I want to crazy recommend to friends!	Jingdong prefecture level city in Shandong
Dragon Quality Goods Shop	Green Intelligent Power Saver	The goods have just received, fine workmanship, and the same as described by the merchant, to use a period of time to see if it can achieve the purpose of power saving.	Jingdong prefecture level city in Shandong
Dragon Quality Goods Shop	Green Intelligent Power Saver	Received the goods first use a period of time to see if there is no effect, the business commitment to ninety days invalid refund, first praise, good service!	Jingdong prefecture level city in Shandong
Dragon Quality Goods Shop	Green Intelligent Power Saver	The product is used for a month without energy-saving and power-saving effect does not have any effect	Jingdong prefecture level city in Shandong
Dragon Quality Goods Shop	Green Intelligent Power Saver	The baby is really great, I like it very much, my friends also say good to come, recommended to buy!	Jingdong prefecture level city in Shandong
Dragon Quality Goods Shop	Green Intelligent Power Saver	Logistics is fast, courier boy service is good, after using to see how the fruit, and then follow up.	Jingdong prefecture level city in Shandong
Dragon Quality Goods Shop	Green Intelligent Power Saver	Baby has been received to try, I believe that as the business advertised energy saving. The courier is very responsible.	Jingdong prefecture level city in Shandong
Dragon Quality Goods Shop	Green Intelligent Power Saver	Save Electric Energy King received, good quality, beautiful appearance, very satisfied!	Jingdong prefecture level city in Shandong
Dragon Quality Goods Shop	Green Intelligent Power Saver	Three no products no manufacturers no phone pure fraud induced consumption cheat	Jingdong prefecture level city in Shandong
Dragon Quality Goods Shop	Green Intelligent Power Saver	I'm using it now, I don't know if it's as good as it says, I'll follow up after a month	Jingdong prefecture level city in Shandong
Dragon Quality Goods Shop	Green Intelligent Power Saver	Good quality logistics and fast. Fine workmanship. The description is consistent cost-effective	Jingdong prefecture level city in Shandong
Dragon Quality Goods Shop	Green Intelligent Power Saver	Received, whether it is good or not, to wait a few days and then evaluate	Jingdong prefecture level city in Shandong
Dragon Quality Goods Shop	Green Intelligent Power Saver	I bought it and started using it, I won't know if it works well until I pay my electric bill	Jingdong prefecture level city in Shandong
Dragon Quality Goods Shop	Green Intelligent Power Saver	Especially good, energy saving and power saving, especially good quality, good	Jingdong prefecture level city in Shandong
Dragon Quality Goods Shop	Green Intelligent Power Saver	Logistics is strong, the quality is very good, paste really save electricity, as described, will buy back!	Jingdong prefecture level city in Shandong
Dragon Quality Goods Shop	Green Intelligent Power Saver	Great stuff at a great price, worth the money!	Jingdong prefecture level city in Shandong
Dragon Quality Goods Shop	Green Intelligent Power Saver	Received the power saver, very good quality, very power saving!	Jingdong prefecture level city in Shandong
Dragon Quality Goods Shop	Green Intelligent Power Saver	There are no fruits, only samples...	Jingdong prefecture level city in Shandong
Dragon Quality Goods Shop	Green Intelligent Power Saver	Save a few dollars a month.	Jingdong prefecture level city in Shandong
Dragon Quality Goods Shop	Green Intelligent Power Saver	The quality is particularly good, it works well, and the seller has a good service attitude	Jingdong prefecture level city in Shandong
Dragon Quality Goods Shop	Green Intelligent Power Saver	I've compared them a couple of times and I don't feel any difference	Jingdong prefecture level city in Shandong
Dragon Quality Goods Shop	Green Intelligent Power Saver	I don't see the savings. It's a sham.	Jingdong prefecture level city in Shandong
Dragon Quality Goods Shop	Green Intelligent Power Saver	The power saving effect is very good, and the logistics is very fast. The seller's service attitude is very good	Jingdong prefecture level city in Shandong
Dragon Quality Goods Shop	Green Intelligent Power Saver	Received, well packaged, haven't tried it yet!	Jingdong prefecture level city in Shandong
Dragon Quality Goods Shop	Green Intelligent Power Saver	I don't know how it works, I'll comment after I use it.	Jingdong prefecture level city in Shandong
Dragon Quality Goods Shop	Green Intelligent Power Saver	The product doesn't work well enough to be of any use	Jingdong prefecture level city in Shandong
Dragon Quality Goods Shop	Green Intelligent Power Saver	On Trial 。。。。。。。	Jingdong prefecture level city in Shandong
Dragon Quality Goods Shop	Green Intelligent Power Saver	Being used with unknown results	Jingdong prefecture level city in Shandong
Dragon Quality Goods Shop	Green Intelligent Power Saver	Smart power saving and great quality	Jingdong prefecture level city in Shandong
Dragon Quality Goods Shop	Green Intelligent Power Saver	The saver was received, it works very well and the price is affordable!	Jingdong prefecture level city in Shandong
Dragon Quality Goods Shop	Green Intelligent Power Saver	How can I feel the power saving and how long does it last	Jingdong prefecture level city in Shandong
Dragon Quality Goods Shop	Green Intelligent Power Saver	And I know if it saves electricity or not.	Jingdong prefecture level city in Shandong
Dragon Quality Goods Shop	Green Intelligent Power Saver	It won't save any electricity. No.	Jingdong prefecture level city in Shandong
Dragon Quality Goods Shop	Green Intelligent Power Saver	Invalid. Fake.	Jingdong prefecture level city in Shandong
Dragon Quality Goods Shop	Green Intelligent Power Saver	It's good!	Jingdong prefecture level city in Shandong
Dragon Quality Goods Shop	Green Intelligent Power Saver	have no effect	Jingdong prefecture level city in Shandong
Dragon Quality Goods Shop	Green Intelligent Power Saver	Not used.	Jingdong prefecture level city in Shandong
Dragon Quality Goods Shop	Green Intelligent Power Saver	There is no load range, packaging thrift, ten days also did not develop invoices, received the goods with a receipt, hand-written receipt also has no financial stamp, communication for nearly a week a moment to say that the electronic invoice, a moment to say that the paper invoice, said it is mailed to me, ten days also did not see anything, the invoice invoice can not be found to say that did not stay on file, single single number can not be found, said that the warehouse more than one to a one to find out, in addition to the perfunctory or perfunctory. Products are no certificate of conformity, proper three	Jingdong prefecture level city in Shandong
Dragon Quality Goods Shop	Green Intelligent Power Saver	Satisfaction.	Jingdong prefecture level city in Shandong

Dragon Quality Goods Shop	Green Intelligent Power Saver	There is not a bit of power saving effect, and ringing up an increase in the electricity bill, because you have to add the cost of this machine's own electricity consumption and purchase, so I think it is better to use this machine instead of increasing the expenditure, uneconomical, rather than not use it.	Jingdong prefecture level city in Shandong
Dragon Quality Goods Shop	Green Intelligent Power Saver	I've been using it for two days now and I don't feel any effect.	Jingdong prefecture level city in Shandong
Dragon Quality Goods Shop	Green Intelligent Power Saver	What it looked like was nothing like what was sent.	Jingdong prefecture level city in Shandong
Dragon Quality Goods Shop	Green Intelligent Power Saver	It didn't work. It didn't work for a month or so.	Jingdong prefecture level city in Shandong
Dragon Quality Goods Shop	Green Intelligent Power Saver	Garbage products, physical and description is not the same, no effect at all, pit # no discussion, to buy the fine, garbage, garbage, garbage	Jingdong prefecture level city in Shandong
Dragon Quality Goods Shop	Green Intelligent Power Saver	general	Jingdong prefecture level city in Shandong
Dragon Quality Goods Shop	Green Intelligent Power Saver	IQ tax. Instead of saving electricity, it increases the power consumption of an appliance.	Jingdong prefecture level city in Shandong
Dragon Quality Goods Shop	Green Intelligent Power Saver	There's no trash at all.	Jingdong prefecture level city in Shandong
Dragon Quality Goods Shop	Green Intelligent Power Saver	It doesn't do anything.	Jingdong prefecture level city in Shandong
Dragon Quality Goods Shop	Green Intelligent Power Saver	(of an unmarried couple) be close	Jingdong prefecture level city in Shandong
Dragon Quality Goods Shop	Green Intelligent Power Saver	(of an unmarried couple) be close	Jingdong prefecture level city in Shandong
Dragon Quality Goods Shop	Green Intelligent Power Saver	no electricity saving	Jingdong prefecture level city in Shandong
Dragon Quality Goods Shop	Green Intelligent Power Saver	differ from	Jingdong prefecture level city in Shandong
Dragon Quality Goods Shop	Green Intelligent Power Saver	Saver has received, but real can save electricity, the quality is very good, I times I buy a few more, too good.	Jingdong prefecture level city in Shandong
Dragon Quality Goods Shop	Green Intelligent Power Saver	Great quality as always, a very favorite product, great! o(*￣︶￣*)o	Jingdong prefecture level city in Shandong
Dragon Quality Goods Shop	Green Intelligent Power Saver	If we can reduce the electricity bill, for individuals can save money, for the country can protect the environment, now the weather is getting hotter and hotter, to protect the environment to reduce emissions is very important. Already use the product, whether to reduce the electricity bill depends on next month!	Jingdong prefecture level city in Shandong
Dragon Quality Goods Shop	Green Intelligent Power Saver	Purchased a power saver received on the put into use, rent a room with many electrical appliances, power consumption is very large, we have a piece of electricity or 1.2 dollars a time, with this power saver is much better, every day can save a few cents, very convenient and practical!	Jingdong prefecture level city in Shandong
Dragon Quality Goods Shop	Green Intelligent Power Saver	The product is better to use before not to use when a month 1500 yuan electricity bill, now using the 5 second month electricity bill directly down 470 dollars or very good. A save money to buy several power saver money ZanZanZanZan👍	Jingdong prefecture level city in Shandong
Dragon Quality Goods Shop	Green Intelligent Power Saver	The moment I received the goods really satisfied with this product, not only high-grade packaging, the body are base alloy, up to the price, I was also introduced by a friend, he used last month did save a lot of electricity, but also recommended to me!	Jingdong prefecture level city in Shandong
Dragon Quality Goods Shop	Green Intelligent Power Saver	I got it. Good review. I'm very satisfied. Reasonable price.	Jingdong prefecture level city in Shandong
Dragon Quality Goods Shop	Green Intelligent Power Saver	Intelligent power saver received and used, looking at the quality is good, the quality of the plug sent is also very good. Expect the effect of the economizer is obvious, first five-star praise, with a period of time to follow up!	Jingdong prefecture level city in Shandong
Dragon Quality Goods Shop	Green Intelligent Power Saver	The saver has been received. It was plugged into an outlet and used immediately after receipt. Whether it really saves electricity is not clear yet, I believe that this brand of energy saver really saves	Jingdong prefecture level city in Shandong
Dragon Quality Goods Shop	Green Intelligent Power Saver	Okay, okay, okay. Thank you so much. Fast is good. Fast. Item was as described, mainly because the event was cheap and you can save a lot of money.	Jingdong prefecture level city in Shandong
Dragon Quality Goods Shop	Green Intelligent Power Saver	Received it a couple days ago! Plugged it in the same day! Let's see what the electric bill is in a month! Hopefully it works well oh!	Jingdong prefecture level city in Shandong
Dragon Quality Goods Shop	Green Intelligent Power Saver	Looking at the reviews good, energy-saving small products, compact, the results after 2 months to see, because the electricity bill season payment	Jingdong prefecture level city in Shandong
Dragon Quality Goods Shop	Green Intelligent Power Saver	I just received it and will try it out to see if it works well. I'll comment later.	Jingdong prefecture level city in Shandong
Dragon Quality Goods Shop	Green Intelligent Power Saver	It looks a little small and not very aesthetically pleasing, I don't know how effective it will be until I use it	Jingdong prefecture level city in Shandong
Dragon Quality Goods Shop	Green Intelligent Power Saver	Not only to the point of being useless yet	Jingdong prefecture level city in Shandong
Dragon Quality Goods Shop	Green Intelligent Power Saver	Used it for a month, nothing works, the meter you turn very fast. This is not a scam, but also sold on Jingdong. To cheat a bunch of people?	Jingdong prefecture level city in Shandong
Dragon Quality Goods Shop	Green Intelligent Power Saver	Very good to use, every day can save a little electricity, really good under the sample down a month to save a few dozen dollars in electricity, good before I did not know that there is this 👆 haha, the price is not expensive praise.	Jingdong prefecture level city in Shandong
Dragon Quality Goods Shop	Green Intelligent Power Saver	Tried it for ten days and the change is almost nothing, can't see the power saving yet, use it some more days and see how it goes.	Jingdong prefecture level city in Shandong
Dragon Quality Goods Shop	Green Intelligent Power Saver	Received very quickly, the quality is very good, really save electricity, business service attitude is also very good, I hope you do not miss this opportunity, I continue to buy a few in	Jingdong prefecture level city in Shandong
Dragon Quality Goods Shop	Green Intelligent Power Saver	Quality is very good Oh, the delivery is also very fast, a very favorite store, I want to crazy recommend to friends!	Jingdong prefecture level city in Shandong
Dragon Quality Goods Shop	Green Intelligent Power Saver	The goods have just received, fine workmanship, and the same as described by the merchant, to use a period of time to see if it can achieve the purpose of power saving.	Jingdong prefecture level city in Shandong
Dragon Quality Goods Shop	Green Intelligent Power Saver	Received the goods first use a period of time to see if there is no effect, the business commitment to ninety days invalid refund, first praise, good service!	Jingdong prefecture level city in Shandong
Dragon Quality Goods Shop	Green Intelligent Power Saver	The product is used for a month without energy-saving and power-saving effect does not have any effect	Jingdong prefecture level city in Shandong
Dragon Quality Goods Shop	Green Intelligent Power Saver	The baby is really great, I like it very much, my friends also say good to come, recommended to buy!	Jingdong prefecture level city in Shandong
Dragon Quality Goods Shop	Green Intelligent Power Saver	Logistics is fast, courier boy service is good, after using to see how the fruit, and then follow up.	Jingdong prefecture level city in Shandong
Dragon Quality Goods Shop	Green Intelligent Power Saver	Baby has been received to try, I believe that as the business advertised energy saving. The courier is very responsible.	Jingdong prefecture level city in Shandong
Dragon Quality Goods Shop	Green Intelligent Power Saver	Save Electric Energy King received, good quality, beautiful appearance, very satisfied!	Jingdong prefecture level city in Shandong
Dragon Quality Goods Shop	Green Intelligent Power Saver	Three no products no manufacturers no phone pure fraud induced consumption cheat	Jingdong prefecture level city in Shandong
Dragon Quality Goods Shop	Green Intelligent Power Saver	I'm using it now, I don't know if it's as good as it says, I'll follow up after a month	Jingdong prefecture level city in Shandong
Dragon Quality Goods Shop	Green Intelligent Power Saver	Good quality logistics and fast. Fine workmanship. The description is consistent cost-effective	Jingdong prefecture level city in Shandong
Dragon Quality Goods Shop	Green Intelligent Power Saver	Received, whether it is good or not, to wait a few days and then evaluate	Jingdong prefecture level city in Shandong
Dragon Quality Goods Shop	Green Intelligent Power Saver	I bought it and started using it, I won't know if it works well until I pay my electric bill	Jingdong prefecture level city in Shandong
Dragon Quality Goods Shop	Green Intelligent Power Saver	Especially good, energy saving and power saving, especially good quality, good	Jingdong prefecture level city in Shandong
Dragon Quality Goods Shop	Green Intelligent Power Saver	Logistics is strong, the quality is very good, paste really save electricity, as described, will buy back!	Jingdong prefecture level city in Shandong
Dragon Quality Goods Shop	Green Intelligent Power Saver	Great stuff at a great price, worth the money!	Jingdong prefecture level city in Shandong

Dragon Quality Goods Shop	Green Intelligent Power Saver	Received the power saver, very good quality, very power saving!	Jingdong prefecture level city in Shandong
Dragon Quality Goods Shop	Green Intelligent Power Saver	There are no fruits, only samples...	Jingdong prefecture level city in Shandong
Dragon Quality Goods Shop	Green Intelligent Power Saver	Save a few dollars a month.	Jingdong prefecture level city in Shandong
Dragon Quality Goods Shop	Green Intelligent Power Saver	The quality is particularly good, it works well, and the seller has a good service attitude	Jingdong prefecture level city in Shandong
Dragon Quality Goods Shop	Green Intelligent Power Saver	I've compared them a couple of times and I don't feel any difference	Jingdong prefecture level city in Shandong
Dragon Quality Goods Shop	Green Intelligent Power Saver	I don't see the savings. It's a sham.	Jingdong prefecture level city in Shandong
Dragon Quality Goods Shop	Green Intelligent Power Saver	The power saving effect is very good, and the logistics is very fast. The seller's service attitude is very good	Jingdong prefecture level city in Shandong
Dragon Quality Goods Shop	Green Intelligent Power Saver	Received, well packaged, haven't tried it yet!	Jingdong prefecture level city in Shandong
Dragon Quality Goods Shop	Green Intelligent Power Saver	I don't know how it works, I'll comment after I use it.	Jingdong prefecture level city in Shandong
Dragon Quality Goods Shop	Green Intelligent Power Saver	The product doesn't work well enough to be of any use	Jingdong prefecture level city in Shandong
Dragon Quality Goods Shop	Green Intelligent Power Saver	On Trial 。。。。。。。	Jingdong prefecture level city in Shandong
Dragon Quality Goods Shop	Green Intelligent Power Saver	Being used with unknown results	Jingdong prefecture level city in Shandong
Dragon Quality Goods Shop	Green Intelligent Power Saver	Smart power saving and great quality	Jingdong prefecture level city in Shandong
Dragon Quality Goods Shop	Green Intelligent Power Saver	The saver was received, it works very well and the price is affordable!	Jingdong prefecture level city in Shandong
Dragon Quality Goods Shop	Green Intelligent Power Saver	How can I feel the power saving and how long does it last	Jingdong prefecture level city in Shandong
Dragon Quality Goods Shop	Green Intelligent Power Saver	And I know if it saves electricity or not.	Jingdong prefecture level city in Shandong
Dragon Quality Goods Shop	Green Intelligent Power Saver	It won't save any electricity. No.	Jingdong prefecture level city in Shandong
Dragon Quality Goods Shop	Green Intelligent Power Saver	Invalid. Fake.	Jingdong prefecture level city in Shandong
Dragon Quality Goods Shop	Green Intelligent Power Saver	It's good!	Jingdong prefecture level city in Shandong
Dragon Quality Goods Shop	Green Intelligent Power Saver	have no effect	Jingdong prefecture level city in Shandong
Dragon Quality Goods Shop	Green Intelligent Power Saver	Not used.	Jingdong prefecture level city in Shandong
Dragon Quality Goods Shop	Green Intelligent Power Saver	There is no load range, packaging thrift, ten days also did not develop invoices, received the goods with a receipt, hand-written receipt also has no financial stamp, communication for nearly a week a moment to say that the electronic invoice, a moment to say that the paper invoice, said it is mailed to me, ten days also did not see anything, the invoice invoice can not be found to say that did not stay on file, single single number can not be found, said that the warehouse more than one to a one to find out, in addition to the perfunctory or perfunctory. Products are no certificate of conformity, proper three	Jingdong prefecture level city in Shandong
Dragon Quality Goods Shop	Green Intelligent Power Saver	Satisfaction.	Jingdong prefecture level city in Shandong
Dragon Quality Goods Shop	Green Intelligent Power Saver	There is not a bit of power saving effect, and ringing up an increase in the electricity bill, because you have to add the cost of this machine's own electricity consumption and purchase, so I think it is better to use this machine instead of increasing the expenditure, uneconomical, rather than not use it.	Jingdong prefecture level city in Shandong
Dragon Quality Goods Shop	Green Intelligent Power Saver	I've been using it for two days now and I don't feel any effect.	Jingdong prefecture level city in Shandong
Dragon Quality Goods Shop	Green Intelligent Power Saver	What it looked like was nothing like what was sent.	Jingdong prefecture level city in Shandong
Dragon Quality Goods Shop	Green Intelligent Power Saver	It didn't work. It didn't work for a month or so.	Jingdong prefecture level city in Shandong
Dragon Quality Goods Shop	Green Intelligent Power Saver	Garbage products, physical and description is not the same, no effect at all, pit # no discussion, to buy the fine, garbage, garbage, garbage	Jingdong prefecture level city in Shandong
Dragon Quality Goods Shop	Green Intelligent Power Saver	general	Jingdong prefecture level city in Shandong
Dragon Quality Goods Shop	Green Intelligent Power Saver	IQ tax. Instead of saving electricity, it increases the power consumption of an appliance.	Jingdong prefecture level city in Shandong
Dragon Quality Goods Shop	Green Intelligent Power Saver	There's no trash at all.	Jingdong prefecture level city in Shandong
Dragon Quality Goods Shop	Green Intelligent Power Saver	It doesn't do anything.	Jingdong prefecture level city in Shandong
Dragon Quality Goods Shop	Green Intelligent Power Saver	(of an unmarried couple) be close	Jingdong prefecture level city in Shandong
Dragon Quality Goods Shop	Green Intelligent Power Saver	(of an unmarried couple) be close	Jingdong prefecture level city in Shandong
Dragon Quality Goods Shop	Green Intelligent Power Saver	no electricity saving	Jingdong prefecture level city in Shandong
Dragon Quality Goods Shop	Green Intelligent Power Saver	differ from	Jingdong prefecture level city in Shandong
Dragon Quality Goods Shop	Green Intelligent Power Saver	Saver has received, but real can save electricity, the quality is very good, I times I buy a few more, too good.	Jingdong prefecture level city in Shandong
Dragon Quality Goods Shop	Green Intelligent Power Saver	Great quality as always, a very favorite product, great! o(*￣︶￣*)o	Jingdong prefecture level city in Shandong
Dragon Quality Goods Shop	Green Intelligent Power Saver	If we can reduce the electricity bill, for individuals can save money, for the country can protect the environment, now the weather is getting hotter and hotter, to protect the environment to reduce emissions is very important. Already use the product, whether to reduce the electricity bill depends on next month!	Jingdong prefecture level city in Shandong
Dragon Quality Goods Shop	Green Intelligent Power Saver	Purchased a power saver received on the put into use, rent a room with many electrical appliances, power consumption is very large, we have a piece of electricity or 1.2 dollars a time, with this power saver is much better, every day can save a few cents, very convenient and practical!	Jingdong prefecture level city in Shandong
Dragon Quality Goods Shop	Green Intelligent Power Saver	The product is better to use before not to use when a month 1500 yuan electricity bill, now using the 5 second month electricity bill directly down 470 dollars or very good. A save money to buy several power saver money ZanZanZanZan👍	Jingdong prefecture level city in Shandong
Dragon Quality Goods Shop	Green Intelligent Power Saver	The moment I received the goods really satisfied with this product, not only high-grade packaging, the body are base alloy, up to the price, I was also introduced by a friend, he used last month did save a lot of electricity, but also recommended to me!	Jingdong prefecture level city in Shandong
Dragon Quality Goods Shop	Green Intelligent Power Saver	I got it. Good review. I'm very satisfied. Reasonable price.	Jingdong prefecture level city in Shandong
Dragon Quality Goods Shop	Green Intelligent Power Saver	Intelligent power saver received and used, looking at the quality is good, the quality of the plug sent is also very good. Expect the effect of the economizer is obvious, first five-star praise, with a period of time to follow up!	Jingdong prefecture level city in Shandong
Dragon Quality Goods Shop	Green Intelligent Power Saver	The saver has been received. It was plugged into an outlet and used immediately after receipt. Whether it really saves electricity is not clear yet, I believe that this brand of energy saver really saves	Jingdong prefecture level city in Shandong
Dragon Quality Goods Shop	Green Intelligent Power Saver	Okay, okay, okay. Thank you so much. Fast is good. Fast. Item was as described, mainly because the event was cheap and you can save a lot of money.	Jingdong prefecture level city in Shandong

Dragon Quality Goods Shop	Green Intelligent Power Saver	Received it a couple days ago! Plugged it in the same day! Let's see what the electric bill is in a month! Hopefully it works well oh!	Jingdong prefecture level city in Shandong
Dragon Quality Goods Shop	Green Intelligent Power Saver	Looking at the reviews good, energy-saving small products, compact, the results after 2 months to see, because the electricity bill season payment	Jingdong prefecture level city in Shandong
Dragon Quality Goods Shop	Green Intelligent Power Saver	I just received it and will try it out to see if it works well. I'll comment later.	Jingdong prefecture level city in Shandong
Dragon Quality Goods Shop	Green Intelligent Power Saver	It looks a little small and not very aesthetically pleasing, I don't know how effective it will be until I use it	Jingdong prefecture level city in Shandong
Dragon Quality Goods Shop	Green Intelligent Power Saver	Not only to the point of being useless yet	Jingdong prefecture level city in Shandong
Dragon Quality Goods Shop	Green Intelligent Power Saver	Used it for a month, nothing works, the meter you turn very fast. This is not a scam, but also sold on Jingdong. To cheat a bunch of people?	Jingdong prefecture level city in Shandong
Dragon Quality Goods Shop	Green Intelligent Power Saver	Very good to use, every day can save a little electricity, really good under the sample down a month to save a few dozen dollars in electricity, good before I did not know that there is this haha, the price is not expensive praise.	Jingdong prefecture level city in Shandong
Dragon Quality Goods Shop	Green Intelligent Power Saver	Tried it for ten days and the change is almost nothing, can't see the power saving yet, use it some more days and see how it goes.	Jingdong prefecture level city in Shandong
Dragon Quality Goods Shop	Green Intelligent Power Saver	Received very quickly, the quality is very good, really save electricity, business service attitude is also very good, I hope you do not miss this opportunity, I continue to buy a few in	Jingdong prefecture level city in Shandong
Dragon Quality Goods Shop	Green Intelligent Power Saver	Quality is very good Oh, the delivery is also very fast, a very favorite store, I want to crazy recommend to friends!	Jingdong prefecture level city in Shandong
Dragon Quality Goods Shop	Green Intelligent Power Saver	The goods have just received, fine workmanship, and the same as described by the merchant, to use a period of time to see if it can achieve the purpose of power saving.	Jingdong prefecture level city in Shandong
Dragon Quality Goods Shop	Green Intelligent Power Saver	Received the goods first use a period of time to see if there is no effect, the business commitment to ninety days invalid refund, first praise, good service!	Jingdong prefecture level city in Shandong
Dragon Quality Goods Shop	Green Intelligent Power Saver	The product is used for a month without energy-saving and power-saving effect does not have any effect	Jingdong prefecture level city in Shandong
Dragon Quality Goods Shop	Green Intelligent Power Saver	The baby is really great, I like it very much, my friends also say good to come, recommended to buy!	Jingdong prefecture level city in Shandong
Dragon Quality Goods Shop	Green Intelligent Power Saver	Logistics is fast, courier boy service is good, after using to see how the fruit, and then follow up.	Jingdong prefecture level city in Shandong
Dragon Quality Goods Shop	Green Intelligent Power Saver	Baby has been received to try, I believe that as the business advertised energy saving. The courier is very responsible.	Jingdong prefecture level city in Shandong
Dragon Quality Goods Shop	Green Intelligent Power Saver	Save Electric Energy King received, good quality, beautiful appearance, very satisfied!	Jingdong prefecture level city in Shandong
Dragon Quality Goods Shop	Green Intelligent Power Saver	Three no products no manufacturers no phone pure fraud induced consumption cheat	Jingdong prefecture level city in Shandong
Dragon Quality Goods Shop	Green Intelligent Power Saver	I'm using it now, I don't know if it's as good as it says, I'll follow up after a month	Jingdong prefecture level city in Shandong
Dragon Quality Goods Shop	Green Intelligent Power Saver	Good quality logistics and fast. Fine workmanship. The description is consistent cost-effective	Jingdong prefecture level city in Shandong
Dragon Quality Goods Shop	Green Intelligent Power Saver	Received, whether it is good or not, to wait a few days and then evaluate	Jingdong prefecture level city in Shandong
Dragon Quality Goods Shop	Green Intelligent Power Saver	I bought it and started using it, I won't know if it works well until I pay my electric bill	Jingdong prefecture level city in Shandong
Dragon Quality Goods Shop	Green Intelligent Power Saver	Especially good, energy saving and power saving, especially good quality, good	Jingdong prefecture level city in Shandong
Dragon Quality Goods Shop	Green Intelligent Power Saver	Logistics is strong, the quality is very good, paste really save electricity, as described, will buy back!	Jingdong prefecture level city in Shandong
Dragon Quality Goods Shop	Green Intelligent Power Saver	Great stuff at a great price, worth the money!	Jingdong prefecture level city in Shandong
Dragon Quality Goods Shop	Green Intelligent Power Saver	Received the power saver, very good quality, very power saving!	Jingdong prefecture level city in Shandong
Dragon Quality Goods Shop	Green Intelligent Power Saver	There are no fruits, only samples...	Jingdong prefecture level city in Shandong
Dragon Quality Goods Shop	Green Intelligent Power Saver	Save a few dollars a month.	Jingdong prefecture level city in Shandong
Dragon Quality Goods Shop	Green Intelligent Power Saver	The quality is particularly good, it works well, and the seller has a good service attitude	Jingdong prefecture level city in Shandong
Dragon Quality Goods Shop	Green Intelligent Power Saver	I've compared them a couple of times and I don't feel any difference	Jingdong prefecture level city in Shandong
Dragon Quality Goods Shop	Green Intelligent Power Saver	I don't see the savings. It's a sham.	Jingdong prefecture level city in Shandong
Dragon Quality Goods Shop	Green Intelligent Power Saver	The power saving effect is very good, and the logistics is very fast. The seller's service attitude is very good	Jingdong prefecture level city in Shandong
Dragon Quality Goods Shop	Green Intelligent Power Saver	Received, well packaged, haven't tried it yet!	Jingdong prefecture level city in Shandong
Dragon Quality Goods Shop	Green Intelligent Power Saver	I don't know how it works, I'll comment after I use it.	Jingdong prefecture level city in Shandong
Dragon Quality Goods Shop	Green Intelligent Power Saver	The product doesn't work well enough to be of any use	Jingdong prefecture level city in Shandong
Dragon Quality Goods Shop	Green Intelligent Power Saver	On Trial 。。。。。。。	Jingdong prefecture level city in Shandong
Dragon Quality Goods Shop	Green Intelligent Power Saver	Being used with unknown results	Jingdong prefecture level city in Shandong
Dragon Quality Goods Shop	Green Intelligent Power Saver	Smart power saving and great quality	Jingdong prefecture level city in Shandong
Dragon Quality Goods Shop	Green Intelligent Power Saver	The saver was received, it works very well and the price is affordable!	Jingdong prefecture level city in Shandong
Dragon Quality Goods Shop	Green Intelligent Power Saver	How can I feel the power saving and how long does it last	Jingdong prefecture level city in Shandong
Dragon Quality Goods Shop	Green Intelligent Power Saver	And I know if it saves electricity or not.	Jingdong prefecture level city in Shandong
Dragon Quality Goods Shop	Green Intelligent Power Saver	It won't save any electricity. No.	Jingdong prefecture level city in Shandong
Dragon Quality Goods Shop	Green Intelligent Power Saver	Invalid. Fake.	Jingdong prefecture level city in Shandong
Dragon Quality Goods Shop	Green Intelligent Power Saver	It's good!	Jingdong prefecture level city in Shandong
Dragon Quality Goods Shop	Green Intelligent Power Saver	have no effect	Jingdong prefecture level city in Shandong
Dragon Quality Goods Shop	Green Intelligent Power Saver	Not used.	Jingdong prefecture level city in Shandong
Dragon Quality Goods Shop	Green Intelligent Power Saver	There is no load range, packaging thrift, ten days also did not develop invoices, received the goods with a receipt, hand-written receipt also has no financial stamp, communication for nearly a week a moment to say that the electronic invoice, a moment to say that the paper invoice, said it is mailed to me, ten days also did not see anything, the invoice invoice can not be found to say that did not stay on file, single single number can not be found, said that the warehouse more than one to a one to find out, in addition to the perfunctory or perfunctory. Products are no certificate of conformity, proper three	Jingdong prefecture level city in Shandong
Dragon Quality Goods Shop	Green Intelligent Power Saver	Satisfaction.	Jingdong prefecture level city in Shandong

Dragon Quality Goods Shop	Green Intelligent Power Saver	There is not a bit of power saving effect, and ringing up an increase in the electricity bill, because you have to add the cost of this machine's own electricity consumption and purchase, so I think it is better to use this machine instead of increasing the expenditure, uneconomical, rather than not use it.	Jingdong prefecture level city in Shandong
Dragon Quality Goods Shop	Green Intelligent Power Saver	I've been using it for two days now and I don't feel any effect.	Jingdong prefecture level city in Shandong
Dragon Quality Goods Shop	Green Intelligent Power Saver	What it looked like was nothing like what was sent.	Jingdong prefecture level city in Shandong
Dragon Quality Goods Shop	Green Intelligent Power Saver	It didn't work. It didn't work for a month or so.	Jingdong prefecture level city in Shandong
Dragon Quality Goods Shop	Green Intelligent Power Saver	Garbage products, physical and description is not the same, no effect at all, pit # no discussion, to buy the fine, garbage, garbage, garbage	Jingdong prefecture level city in Shandong
Dragon Quality Goods Shop	Green Intelligent Power Saver	general	Jingdong prefecture level city in Shandong
Dragon Quality Goods Shop	Green Intelligent Power Saver	IQ tax. Instead of saving electricity, it increases the power consumption of an appliance.	Jingdong prefecture level city in Shandong
Dragon Quality Goods Shop	Green Intelligent Power Saver	There's no trash at all.	Jingdong prefecture level city in Shandong
Dragon Quality Goods Shop	Green Intelligent Power Saver	It doesn't do anything.	Jingdong prefecture level city in Shandong
Dragon Quality Goods Shop	Green Intelligent Power Saver	(of an unmarried couple) be close	Jingdong prefecture level city in Shandong
Dragon Quality Goods Shop	Green Intelligent Power Saver	(of an unmarried couple) be close	Jingdong prefecture level city in Shandong
Dragon Quality Goods Shop	Green Intelligent Power Saver	no electricity saving	Jingdong prefecture level city in Shandong
Dragon Quality Goods Shop	Green Intelligent Power Saver	differ from	Jingdong prefecture level city in Shandong
Dragon Quality Goods Shop	Green Intelligent Power Saver	Saver has received, but real can save electricity, the quality is very good, I times I buy a few more, too good.	Jingdong prefecture level city in Shandong
Dragon Quality Goods Shop	Green Intelligent Power Saver	Great quality as always, a very favorite product, great! o(*￣︶￣*)o	Jingdong prefecture level city in Shandong
Dragon Quality Goods Shop	Green Intelligent Power Saver	If we can reduce the electricity bill, for individuals can save money, for the country can protect the environment, now the weather is getting hotter and hotter, to protect the environment to reduce emissions is very important. Already use the product, whether to reduce the electricity bill depends on next month!	Jingdong prefecture level city in Shandong
Dragon Quality Goods Shop	Green Intelligent Power Saver	Purchased a power saver received on the put into use, rent a room with many electrical appliances, power consumption is very large, we have a piece of electricity or 1.2 dollars a time, with this power saver is much better, every day can save a few cents, very convenient and practical!	Jingdong prefecture level city in Shandong
Dragon Quality Goods Shop	Green Intelligent Power Saver	The product is better to use before not to use when a month 1500 yuan electricity bill, now using the 5 second month electricity bill directly down 470 dollars or very good. A save money to buy several power saver money ZanZanZanZan👍	Jingdong prefecture level city in Shandong
Dragon Quality Goods Shop	Green Intelligent Power Saver	The moment I received the goods really satisfied with this product, not only high-grade packaging, the body are base alloy, up to the price, I was also introduced by a friend, he used last month did save a lot of electricity, but also recommended to me!	Jingdong prefecture level city in Shandong
Dragon Quality Goods Shop	Green Intelligent Power Saver	I got it. Good review. I'm very satisfied. Reasonable price.	Jingdong prefecture level city in Shandong
Dragon Quality Goods Shop	Green Intelligent Power Saver	Intelligent power saver received and used, looking at the quality is good, the quality of the plug sent is also very good. Expect the effect of the economizer is obvious, first five-star praise, with a period of time to follow up!	Jingdong prefecture level city in Shandong
Dragon Quality Goods Shop	Green Intelligent Power Saver	The saver has been received. It was plugged into an outlet and used immediately after receipt. Whether it really saves electricity is not clear yet, I believe that this brand of energy saver really saves	Jingdong prefecture level city in Shandong
Dragon Quality Goods Shop	Green Intelligent Power Saver	Okay, okay, okay. Thank you so much. Fast is good. Fast. Item was as described, mainly because the event was cheap and you can save a lot of money.	Jingdong prefecture level city in Shandong
Dragon Quality Goods Shop	Green Intelligent Power Saver	Received it a couple days ago! Plugged it in the same day! Let's see what the electric bill is in a month! Hopefully it works well oh!	Jingdong prefecture level city in Shandong
Dragon Quality Goods Shop	Green Intelligent Power Saver	Looking at the reviews good, energy-saving small products, compact, the results after 2 months to see, because the electricity bill season payment	Jingdong prefecture level city in Shandong
Dragon Quality Goods Shop	Green Intelligent Power Saver	I just received it and will try it out to see if it works well. I'll comment later.	Jingdong prefecture level city in Shandong
Dragon Quality Goods Shop	Green Intelligent Power Saver	It looks a little small and not very aesthetically pleasing, I don't know how effective it will be until I use it	Jingdong prefecture level city in Shandong
Dragon Quality Goods Shop	Green Intelligent Power Saver	Not only to the point of being useless yet	Jingdong prefecture level city in Shandong
Dragon Quality Goods Shop	Green Intelligent Power Saver	Used it for a month, nothing works, the meter you turn very fast. This is not a scam, but also sold on Jingdong. To cheat a bunch of people?	Jingdong prefecture level city in Shandong
Dragon Quality Goods Shop	Green Intelligent Power Saver	Very good to use, every day can save a little electricity, really good under the sample down a month to save a few dozen dollars in electricity, good before I did not know that there is this 👆 haha, the price is not expensive praise.	Jingdong prefecture level city in Shandong
Dragon Quality Goods Shop	Green Intelligent Power Saver	Tried it for ten days and the change is almost nothing, can't see the power saving yet, use it some more days and see how it goes.	Jingdong prefecture level city in Shandong
Dragon Quality Goods Shop	Green Intelligent Power Saver	Received very quickly, the quality is very good, really save electricity, business service attitude is also very good, I hope you do not miss this opportunity, I continue to buy a few in	Jingdong prefecture level city in Shandong
Dragon Quality Goods Shop	Green Intelligent Power Saver	Quality is very good Oh, the delivery is also very fast, a very favorite store, I want to crazy recommend to friends!	Jingdong prefecture level city in Shandong
Dragon Quality Goods Shop	Green Intelligent Power Saver	The goods have just received, fine workmanship, and the same as described by the merchant, to use a period of time to see if it can achieve the purpose of power saving.	Jingdong prefecture level city in Shandong
Dragon Quality Goods Shop	Green Intelligent Power Saver	Received the goods first use a period of time to see if there is no effect, the business commitment to ninety days invalid refund, first praise, good service!	Jingdong prefecture level city in Shandong
Dragon Quality Goods Shop	Green Intelligent Power Saver	The product is used for a month without energy-saving and power-saving effect does not have any effect	Jingdong prefecture level city in Shandong
Dragon Quality Goods Shop	Green Intelligent Power Saver	The baby is really great, I like it very much, my friends also say good to come, recommended to buy!	Jingdong prefecture level city in Shandong
Dragon Quality Goods Shop	Green Intelligent Power Saver	Logistics is fast, courier boy service is good, after using to see how the fruit, and then follow up.	Jingdong prefecture level city in Shandong
Dragon Quality Goods Shop	Green Intelligent Power Saver	Baby has been received to try, I believe that as the business advertised energy saving. The courier is very responsible.	Jingdong prefecture level city in Shandong
Dragon Quality Goods Shop	Green Intelligent Power Saver	Save Electric Energy King received, good quality, beautiful appearance, very satisfied!	Jingdong prefecture level city in Shandong
Dragon Quality Goods Shop	Green Intelligent Power Saver	Three no products no manufacturers no phone pure fraud induced consumption cheat	Jingdong prefecture level city in Shandong
Dragon Quality Goods Shop	Green Intelligent Power Saver	I'm using it now, I don't know if it's as good as it says, I'll follow up after a month	Jingdong prefecture level city in Shandong
Dragon Quality Goods Shop	Green Intelligent Power Saver	Good quality logistics and fast. Fine workmanship. The description is consistent cost-effective	Jingdong prefecture level city in Shandong
Dragon Quality Goods Shop	Green Intelligent Power Saver	Received, whether it is good or not, to wait a few days and then evaluate	Jingdong prefecture level city in Shandong
Dragon Quality Goods Shop	Green Intelligent Power Saver	I bought it and started using it, I won't know if it works well until I pay my electric bill	Jingdong prefecture level city in Shandong
Dragon Quality Goods Shop	Green Intelligent Power Saver	Especially good, energy saving and power saving, especially good quality, good	Jingdong prefecture level city in Shandong
Dragon Quality Goods Shop	Green Intelligent Power Saver	Logistics is strong, the quality is very good, paste really save electricity, as described, will buy back!	Jingdong prefecture level city in Shandong
Dragon Quality Goods Shop	Green Intelligent Power Saver	Great stuff at a great price, worth the money!	Jingdong prefecture level city in Shandong

Dragon Quality Goods Shop	Green Intelligent Power Saver	Received the power saver, very good quality, very power saving!	Jingdong prefecture level city in Shandong
Dragon Quality Goods Shop	Green Intelligent Power Saver	There are no fruits, only samples...	Jingdong prefecture level city in Shandong
Dragon Quality Goods Shop	Green Intelligent Power Saver	Save a few dollars a month.	Jingdong prefecture level city in Shandong
Dragon Quality Goods Shop	Green Intelligent Power Saver	The quality is particularly good, it works well, and the seller has a good service attitude	Jingdong prefecture level city in Shandong
Dragon Quality Goods Shop	Green Intelligent Power Saver	I've compared them a couple of times and I don't feel any difference	Jingdong prefecture level city in Shandong
Dragon Quality Goods Shop	Green Intelligent Power Saver	I don't see the savings. It's a sham.	Jingdong prefecture level city in Shandong
Dragon Quality Goods Shop	Green Intelligent Power Saver	The power saving effect is very good, and the logistics is very fast. The seller's service attitude is very good	Jingdong prefecture level city in Shandong
Dragon Quality Goods Shop	Green Intelligent Power Saver	Received, well packaged, haven't tried it yet!	Jingdong prefecture level city in Shandong
Dragon Quality Goods Shop	Green Intelligent Power Saver	I don't know how it works, I'll comment after I use it.	Jingdong prefecture level city in Shandong
Dragon Quality Goods Shop	Green Intelligent Power Saver	The product doesn't work well enough to be of any use	Jingdong prefecture level city in Shandong
Dragon Quality Goods Shop	Green Intelligent Power Saver	On Trial 。。。。。。。	Jingdong prefecture level city in Shandong
Dragon Quality Goods Shop	Green Intelligent Power Saver	Being used with unknown results	Jingdong prefecture level city in Shandong
Dragon Quality Goods Shop	Green Intelligent Power Saver	Smart power saving and great quality	Jingdong prefecture level city in Shandong
Dragon Quality Goods Shop	Green Intelligent Power Saver	The saver was received, it works very well and the price is affordable!	Jingdong prefecture level city in Shandong
Dragon Quality Goods Shop	Green Intelligent Power Saver	How can I feel the power saving and how long does it last	Jingdong prefecture level city in Shandong
Dragon Quality Goods Shop	Green Intelligent Power Saver	And I know if it saves electricity or not.	Jingdong prefecture level city in Shandong
Dragon Quality Goods Shop	Green Intelligent Power Saver	It won't save any electricity. No.	Jingdong prefecture level city in Shandong
Dragon Quality Goods Shop	Green Intelligent Power Saver	Invalid. Fake.	Jingdong prefecture level city in Shandong
Dragon Quality Goods Shop	Green Intelligent Power Saver	It's good!	Jingdong prefecture level city in Shandong
Dragon Quality Goods Shop	Green Intelligent Power Saver	have no effect	Jingdong prefecture level city in Shandong
Dragon Quality Goods Shop	Green Intelligent Power Saver	Not used.	Jingdong prefecture level city in Shandong
Dragon Quality Goods Shop	Green Intelligent Power Saver	There is no load range, packaging thrift, ten days also did not develop invoices, received the goods with a receipt, hand-written receipt also has no financial stamp, communication for nearly a week a moment to say that the electronic invoice, a moment to say that the paper invoice, said it is mailed to me, ten days also did not see anything, the invoice invoice can not be found to say that did not stay on file, single single number can not be found, said that the warehouse more than one to a one to find out, in addition to the perfunctory or perfunctory. Products are no certificate of conformity, proper three	Jingdong prefecture level city in Shandong
Dragon Quality Goods Shop	Green Intelligent Power Saver	Satisfaction.	Jingdong prefecture level city in Shandong
Dragon Quality Goods Shop	Green Intelligent Power Saver	There is not a bit of power saving effect, and ringing up an increase in the electricity bill, because you have to add the cost of this machine's own electricity consumption and purchase, so I think it is better to use this machine instead of increasing the expenditure, uneconomical, rather than not use it.	Jingdong prefecture level city in Shandong
Dragon Quality Goods Shop	Green Intelligent Power Saver	I've been using it for two days now and I don't feel any effect.	Jingdong prefecture level city in Shandong
Dragon Quality Goods Shop	Green Intelligent Power Saver	What it looked like was nothing like what was sent.	Jingdong prefecture level city in Shandong
Dragon Quality Goods Shop	Green Intelligent Power Saver	It didn't work. It didn't work for a month or so.	Jingdong prefecture level city in Shandong
Dragon Quality Goods Shop	Green Intelligent Power Saver	Garbage products, physical and description is not the same, no effect at all, pit # no discussion, to buy the fine, garbage, garbage, garbage	Jingdong prefecture level city in Shandong
Dragon Quality Goods Shop	Green Intelligent Power Saver	general	Jingdong prefecture level city in Shandong
Dragon Quality Goods Shop	Green Intelligent Power Saver	IQ tax. Instead of saving electricity, it increases the power consumption of an appliance.	Jingdong prefecture level city in Shandong
Dragon Quality Goods Shop	Green Intelligent Power Saver	There's no trash at all.	Jingdong prefecture level city in Shandong
Dragon Quality Goods Shop	Green Intelligent Power Saver	It doesn't do anything.	Jingdong prefecture level city in Shandong
Dragon Quality Goods Shop	Green Intelligent Power Saver	(of an unmarried couple) be close	Jingdong prefecture level city in Shandong
Dragon Quality Goods Shop	Green Intelligent Power Saver	(of an unmarried couple) be close	Jingdong prefecture level city in Shandong
Dragon Quality Goods Shop	Green Intelligent Power Saver	no electricity saving	Jingdong prefecture level city in Shandong
Dragon Quality Goods Shop	Green Intelligent Power Saver	differ from	Jingdong prefecture level city in Shandong
Dragon Quality Goods Shop	Green Intelligent Power Saver	Saver has received, but real can save electricity, the quality is very good, I times I buy a few more, too good.	Jingdong prefecture level city in Shandong
Dragon Quality Goods Shop	Green Intelligent Power Saver	Great quality as always, a very favorite product, great! o(*￣︶￣*)o	Jingdong prefecture level city in Shandong
Dragon Quality Goods Shop	Green Intelligent Power Saver	If we can reduce the electricity bill, for individuals can save money, for the country can protect the environment, now the weather is getting hotter and hotter, to protect the environment to reduce emissions is very important. Already use the product, whether to reduce the electricity bill depends on next month!	Jingdong prefecture level city in Shandong
Dragon Quality Goods Shop	Green Intelligent Power Saver	Purchased a power saver received on the put into use, rent a room with many electrical appliances, power consumption is very large, we have a piece of electricity or 1.2 dollars a time, with this power saver is much better, every day can save a few cents, very convenient and practical!	Jingdong prefecture level city in Shandong
Dragon Quality Goods Shop	Green Intelligent Power Saver	The product is better to use before not to use when a month 1500 yuan electricity bill, now using the 5 second month electricity bill directly down 470 dollars or very good. A save money to buy several power saver money ZanZanZanZan👍	Jingdong prefecture level city in Shandong
Dragon Quality Goods Shop	Green Intelligent Power Saver	The moment I received the goods really satisfied with this product, not only high-grade packaging, the body are base alloy, up to the price, I was also introduced by a friend, he used last month did save a lot of electricity, but also recommended to me!	Jingdong prefecture level city in Shandong
Dragon Quality Goods Shop	Green Intelligent Power Saver	I got it. Good review. I'm very satisfied. Reasonable price.	Jingdong prefecture level city in Shandong
Dragon Quality Goods Shop	Green Intelligent Power Saver	Intelligent power saver received and used, looking at the quality is good, the quality of the plug sent is also very good. Expect the effect of the economizer is obvious, first five-star praise, with a period of time to follow up!	Jingdong prefecture level city in Shandong
Dragon Quality Goods Shop	Green Intelligent Power Saver	The saver has been received. It was plugged into an outlet and used immediately after receipt. Whether it really saves electricity is not clear yet, I believe that this brand of energy saver really saves	Jingdong prefecture level city in Shandong
Dragon Quality Goods Shop	Green Intelligent Power Saver	Okay, okay, okay. Thank you so much. Fast is good. Fast. Item was as described, mainly because the event was cheap and you can save a lot of money.	Jingdong prefecture level city in Shandong

Dragon Quality Goods Shop	Green Intelligent Power Saver	Received it a couple days ago! Plugged it in the same day! Let's see what the electric bill is in a month! Hopefully it works well oh!	Jingdong prefecture level city in Shandong
Dragon Quality Goods Shop	Green Intelligent Power Saver	Looking at the reviews good, energy-saving small products, compact, the results after 2 months to see, because the electricity bill season payment	Jingdong prefecture level city in Shandong
Dragon Quality Goods Shop	Green Intelligent Power Saver	I just received it and will try it out to see if it works well. I'll comment later.	Jingdong prefecture level city in Shandong
Dragon Quality Goods Shop	Green Intelligent Power Saver	It looks a little small and not very aesthetically pleasing, I don't know how effective it will be until I use it	Jingdong prefecture level city in Shandong
Dragon Quality Goods Shop	Green Intelligent Power Saver	Not only to the point of being useless yet	Jingdong prefecture level city in Shandong
Dragon Quality Goods Shop	Green Intelligent Power Saver	Used it for a month, nothing works, the meter you turn very fast. This is not a scam, but also sold on Jingdong. To cheat a bunch of people?	Jingdong prefecture level city in Shandong
Dragon Quality Goods Shop	Green Intelligent Power Saver	Very good to use, every day can save a little electricity, really good under the sample down a month to save a few dozen dollars in electricity, good before I did not know that there is this 👆 haha, the price is not expensive praise.	Jingdong prefecture level city in Shandong
Dragon Quality Goods Shop	Green Intelligent Power Saver	Tried it for ten days and the change is almost nothing, can't see the power saving yet, use it some more days and see how it goes.	Jingdong prefecture level city in Shandong
Dragon Quality Goods Shop	Green Intelligent Power Saver	Received very quickly, the quality is very good, really save electricity, business service attitude is also very good, I hope you do not miss this opportunity, I continue to buy a few in	Jingdong prefecture level city in Shandong
Dragon Quality Goods Shop	Green Intelligent Power Saver	Quality is very good Oh, the delivery is also very fast, a very favorite store, I want to crazy recommend to friends!	Jingdong prefecture level city in Shandong
Dragon Quality Goods Shop	Green Intelligent Power Saver	The goods have just received, fine workmanship, and the same as described by the merchant, to use a period of time to see if it can achieve the purpose of power saving.	Jingdong prefecture level city in Shandong
Dragon Quality Goods Shop	Green Intelligent Power Saver	Received the goods first use a period of time to see if there is no effect, the business commitment to ninety days invalid refund, first praise, good service!	Jingdong prefecture level city in Shandong
Dragon Quality Goods Shop	Green Intelligent Power Saver	The product is used for a month without energy-saving and power-saving effect does not have any effect	Jingdong prefecture level city in Shandong
Dragon Quality Goods Shop	Green Intelligent Power Saver	The baby is really great, I like it very much, my friends also say good to come, recommended to buy!	Jingdong prefecture level city in Shandong
Dragon Quality Goods Shop	Green Intelligent Power Saver	Logistics is fast, courier boy service is good, after using to see how the fruit, and then follow up.	Jingdong prefecture level city in Shandong
Dragon Quality Goods Shop	Green Intelligent Power Saver	Baby has been received to try, I believe that as the business advertised energy saving. The courier is very responsible.	Jingdong prefecture level city in Shandong
Dragon Quality Goods Shop	Green Intelligent Power Saver	Save Electric Energy King received, good quality, beautiful appearance, very satisfied!	Jingdong prefecture level city in Shandong
Dragon Quality Goods Shop	Green Intelligent Power Saver	Three no products no manufacturers no phone pure fraud induced consumption cheat	Jingdong prefecture level city in Shandong
Dragon Quality Goods Shop	Green Intelligent Power Saver	I'm using it now, I don't know if it's as good as it says, I'll follow up after a month	Jingdong prefecture level city in Shandong
Dragon Quality Goods Shop	Green Intelligent Power Saver	Good quality logistics and fast. Fine workmanship. The description is consistent cost-effective	Jingdong prefecture level city in Shandong
Dragon Quality Goods Shop	Green Intelligent Power Saver	Received, whether it is good or not, to wait a few days and then evaluate	Jingdong prefecture level city in Shandong
Dragon Quality Goods Shop	Green Intelligent Power Saver	I bought it and started using it, I won't know if it works well until I pay my electric bill	Jingdong prefecture level city in Shandong
Dragon Quality Goods Shop	Green Intelligent Power Saver	Especially good, energy saving and power saving, especially good quality, good	Jingdong prefecture level city in Shandong
Dragon Quality Goods Shop	Green Intelligent Power Saver	Logistics is strong, the quality is very good, paste really save electricity, as described, will buy back!	Jingdong prefecture level city in Shandong
Dragon Quality Goods Shop	Green Intelligent Power Saver	Great stuff at a great price, worth the money!	Jingdong prefecture level city in Shandong
Dragon Quality Goods Shop	Green Intelligent Power Saver	Received the power saver, very good quality, very power saving!	Jingdong prefecture level city in Shandong
Dragon Quality Goods Shop	Green Intelligent Power Saver	There are no fruits, only samples...	Jingdong prefecture level city in Shandong
Dragon Quality Goods Shop	Green Intelligent Power Saver	Save a few dollars a month.	Jingdong prefecture level city in Shandong
Dragon Quality Goods Shop	Green Intelligent Power Saver	The quality is particularly good, it works well, and the seller has a good service attitude	Jingdong prefecture level city in Shandong
Dragon Quality Goods Shop	Green Intelligent Power Saver	I've compared them a couple of times and I don't feel any difference	Jingdong prefecture level city in Shandong
Dragon Quality Goods Shop	Green Intelligent Power Saver	I don't see the savings. It's a sham.	Jingdong prefecture level city in Shandong
Dragon Quality Goods Shop	Green Intelligent Power Saver	The power saving effect is very good, and the logistics is very fast. The seller's service attitude is very good	Jingdong prefecture level city in Shandong
Dragon Quality Goods Shop	Green Intelligent Power Saver	Received, well packaged, haven't tried it yet!	Jingdong prefecture level city in Shandong
Dragon Quality Goods Shop	Green Intelligent Power Saver	I don't know how it works, I'll comment after I use it.	Jingdong prefecture level city in Shandong
Dragon Quality Goods Shop	Green Intelligent Power Saver	The product doesn't work well enough to be of any use	Jingdong prefecture level city in Shandong
Dragon Quality Goods Shop	Green Intelligent Power Saver	On Trial 。。。。。。。	Jingdong prefecture level city in Shandong
Dragon Quality Goods Shop	Green Intelligent Power Saver	Being used with unknown results	Jingdong prefecture level city in Shandong
Dragon Quality Goods Shop	Green Intelligent Power Saver	Smart power saving and great quality	Jingdong prefecture level city in Shandong
Dragon Quality Goods Shop	Green Intelligent Power Saver	The saver was received, it works very well and the price is affordable!	Jingdong prefecture level city in Shandong
Dragon Quality Goods Shop	Green Intelligent Power Saver	How can I feel the power saving and how long does it last	Jingdong prefecture level city in Shandong
Dragon Quality Goods Shop	Green Intelligent Power Saver	And I know if it saves electricity or not.	Jingdong prefecture level city in Shandong
Dragon Quality Goods Shop	Green Intelligent Power Saver	It won't save any electricity. No.	Jingdong prefecture level city in Shandong
Dragon Quality Goods Shop	Green Intelligent Power Saver	Invalid. Fake.	Jingdong prefecture level city in Shandong
Dragon Quality Goods Shop	Green Intelligent Power Saver	It's good!	Jingdong prefecture level city in Shandong
Dragon Quality Goods Shop	Green Intelligent Power Saver	have no effect	Jingdong prefecture level city in Shandong
Dragon Quality Goods Shop	Green Intelligent Power Saver	Not used.	Jingdong prefecture level city in Shandong
Dragon Quality Goods Shop	Green Intelligent Power Saver	There is no load range, packaging thrift, ten days also did not develop invoices, received the goods with a receipt, hand-written receipt also has no financial stamp, communication for nearly a week a moment to say that the electronic invoice, a moment to say that the paper invoice, said it is mailed to me, ten days also did not see anything, the invoice invoice can not be found to say that did not stay on file, single single number can not be found, said that the warehouse more than one to a one to find out, in addition to the perfunctory or perfunctory. Products are no certificate of conformity, proper three	Jingdong prefecture level city in Shandong
Dragon Quality Goods Shop	Green Intelligent Power Saver	Satisfaction.	Jingdong prefecture level city in Shandong

Dragon Quality Goods Shop	Green Intelligent Power Saver	There is not a bit of power saving effect, and ringing up an increase in the electricity bill, because you have to add the cost of this machine's own electricity consumption and purchase, so I think it is better to use this machine instead of increasing the expenditure, uneconomical, rather than not use it.	Jingdong prefecture level city in Shandong
Dragon Quality Goods Shop	Green Intelligent Power Saver	I've been using it for two days now and I don't feel any effect.	Jingdong prefecture level city in Shandong
Dragon Quality Goods Shop	Green Intelligent Power Saver	What it looked like was nothing like what was sent.	Jingdong prefecture level city in Shandong
Dragon Quality Goods Shop	Green Intelligent Power Saver	It didn't work. It didn't work for a month or so.	Jingdong prefecture level city in Shandong
Dragon Quality Goods Shop	Green Intelligent Power Saver	Garbage products, physical and description is not the same, no effect at all, pit # no discussion, to buy the fine, garbage, garbage, garbage	Jingdong prefecture level city in Shandong
Dragon Quality Goods Shop	Green Intelligent Power Saver	general	Jingdong prefecture level city in Shandong
Dragon Quality Goods Shop	Green Intelligent Power Saver	IQ tax. Instead of saving electricity, it increases the power consumption of an appliance.	Jingdong prefecture level city in Shandong
Dragon Quality Goods Shop	Green Intelligent Power Saver	There's no trash at all.	Jingdong prefecture level city in Shandong
Dragon Quality Goods Shop	Green Intelligent Power Saver	It doesn't do anything.	Jingdong prefecture level city in Shandong
Dragon Quality Goods Shop	Green Intelligent Power Saver	(of an unmarried couple) be close	Jingdong prefecture level city in Shandong
Dragon Quality Goods Shop	Green Intelligent Power Saver	(of an unmarried couple) be close	Jingdong prefecture level city in Shandong
Dragon Quality Goods Shop	Green Intelligent Power Saver	no electricity saving	Jingdong prefecture level city in Shandong
Dragon Quality Goods Shop	Green Intelligent Power Saver	differ from	Jingdong prefecture level city in Shandong
Dragon Quality Goods Shop	Green Intelligent Power Saver	Saver has received, but real can save electricity, the quality is very good, I times I buy a few more, too good.	Jingdong prefecture level city in Shandong
Dragon Quality Goods Shop	Green Intelligent Power Saver	Great quality as always, a very favorite product, great! o(*￣︶￣*)o	Jingdong prefecture level city in Shandong
Dragon Quality Goods Shop	Green Intelligent Power Saver	If we can reduce the electricity bill, for individuals can save money, for the country can protect the environment, now the weather is getting hotter and hotter, to protect the environment to reduce emissions is very important. Already use the product, whether to reduce the electricity bill depends on next month!	Jingdong prefecture level city in Shandong
Dragon Quality Goods Shop	Green Intelligent Power Saver	Purchased a power saver received on the put into use, rent a room with many electrical appliances, power consumption is very large, we have a piece of electricity or 1.2 dollars a time, with this power saver is much better, every day can save a few cents, very convenient and practical!	Jingdong prefecture level city in Shandong
Dragon Quality Goods Shop	Green Intelligent Power Saver	The product is better to use before not to use when a month 1500 yuan electricity bill, now using the 5 second month electricity bill directly down 470 dollars or very good. A save money to buy several power saver money ZanZanZanZan👍	Jingdong prefecture level city in Shandong
Dragon Quality Goods Shop	Green Intelligent Power Saver	The moment I received the goods really satisfied with this product, not only high-grade packaging, the body are base alloy, up to the price, I was also introduced by a friend, he used last month did save a lot of electricity, but also recommended to me!	Jingdong prefecture level city in Shandong
Dragon Quality Goods Shop	Green Intelligent Power Saver	I got it. Good review. I'm very satisfied. Reasonable price.	Jingdong prefecture level city in Shandong
Dragon Quality Goods Shop	Green Intelligent Power Saver	Intelligent power saver received and used, looking at the quality is good, the quality of the plug sent is also very good. Expect the effect of the economizer is obvious, first five-star praise, with a period of time to follow up!	Jingdong prefecture level city in Shandong
Dragon Quality Goods Shop	Green Intelligent Power Saver	The saver has been received. It was plugged into an outlet and used immediately after receipt. Whether it really saves electricity is not clear yet, I believe that this brand of energy saver really saves	Jingdong prefecture level city in Shandong
Dragon Quality Goods Shop	Green Intelligent Power Saver	Okay, okay, okay. Thank you so much. Fast is good. Fast. Item was as described, mainly because the event was cheap and you can save a lot of money.	Jingdong prefecture level city in Shandong
Dragon Quality Goods Shop	Green Intelligent Power Saver	Received it a couple days ago! Plugged it in the same day! Let's see what the electric bill is in a month! Hopefully it works well oh!	Jingdong prefecture level city in Shandong
Dragon Quality Goods Shop	Green Intelligent Power Saver	Looking at the reviews good, energy-saving small products, compact, the results after 2 months to see, because the electricity bill season payment	Jingdong prefecture level city in Shandong
Dragon Quality Goods Shop	Green Intelligent Power Saver	I just received it and will try it out to see if it works well. I'll comment later.	Jingdong prefecture level city in Shandong
Dragon Quality Goods Shop	Green Intelligent Power Saver	It looks a little small and not very aesthetically pleasing, I don't know how effective it will be until I use it	Jingdong prefecture level city in Shandong
Dragon Quality Goods Shop	Green Intelligent Power Saver	Not only to the point of being useless yet	Jingdong prefecture level city in Shandong
Dragon Quality Goods Shop	Green Intelligent Power Saver	Used it for a month, nothing works, the meter you turn very fast. This is not a scam, but also sold on Jingdong. To cheat a bunch of people?	Jingdong prefecture level city in Shandong
Dragon Quality Goods Shop	Green Intelligent Power Saver	Very good to use, every day can save a little electricity, really good under the sample down a month to save a few dozen dollars in electricity, good before I did not know that there is this 👆 haha, the price is not expensive praise.	Jingdong prefecture level city in Shandong
Dragon Quality Goods Shop	Green Intelligent Power Saver	Tried it for ten days and the change is almost nothing, can't see the power saving yet, use it some more days and see how it goes.	Jingdong prefecture level city in Shandong
Dragon Quality Goods Shop	Green Intelligent Power Saver	Received very quickly, the quality is very good, really save electricity, business service attitude is also very good, I hope you do not miss this opportunity, I continue to buy a few in	Jingdong prefecture level city in Shandong
Dragon Quality Goods Shop	Green Intelligent Power Saver	Quality is very good Oh, the delivery is also very fast, a very favorite store, I want to crazy recommend to friends!	Jingdong prefecture level city in Shandong
Dragon Quality Goods Shop	Green Intelligent Power Saver	The goods have just received, fine workmanship, and the same as described by the merchant, to use a period of time to see if it can achieve the purpose of power saving.	Jingdong prefecture level city in Shandong
Dragon Quality Goods Shop	Green Intelligent Power Saver	Received the goods first use a period of time to see if there is no effect, the business commitment to ninety days invalid refund, first praise, good service!	Jingdong prefecture level city in Shandong
Dragon Quality Goods Shop	Green Intelligent Power Saver	The product is used for a month without energy-saving and power-saving effect does not have any effect	Jingdong prefecture level city in Shandong
Dragon Quality Goods Shop	Green Intelligent Power Saver	The baby is really great, I like it very much, my friends also say good to come, recommended to buy!	Jingdong prefecture level city in Shandong
Dragon Quality Goods Shop	Green Intelligent Power Saver	Logistics is fast, courier boy service is good, after using to see how the fruit, and then follow up.	Jingdong prefecture level city in Shandong
Dragon Quality Goods Shop	Green Intelligent Power Saver	Baby has been received to try, I believe that as the business advertised energy saving. The courier is very responsible.	Jingdong prefecture level city in Shandong
Dragon Quality Goods Shop	Green Intelligent Power Saver	Save Electric Energy King received, good quality, beautiful appearance, very satisfied!	Jingdong prefecture level city in Shandong
Dragon Quality Goods Shop	Green Intelligent Power Saver	Three no products no manufacturers no phone pure fraud induced consumption cheat	Jingdong prefecture level city in Shandong
Dragon Quality Goods Shop	Green Intelligent Power Saver	I'm using it now, I don't know if it's as good as it says, I'll follow up after a month	Jingdong prefecture level city in Shandong
Dragon Quality Goods Shop	Green Intelligent Power Saver	Good quality logistics and fast. Fine workmanship. The description is consistent cost-effective	Jingdong prefecture level city in Shandong
Dragon Quality Goods Shop	Green Intelligent Power Saver	Received, whether it is good or not, to wait a few days and then evaluate	Jingdong prefecture level city in Shandong
Dragon Quality Goods Shop	Green Intelligent Power Saver	I bought it and started using it, I won't know if it works well until I pay my electric bill	Jingdong prefecture level city in Shandong
Dragon Quality Goods Shop	Green Intelligent Power Saver	Especially good, energy saving and power saving, especially good quality, good	Jingdong prefecture level city in Shandong
Dragon Quality Goods Shop	Green Intelligent Power Saver	Logistics is strong, the quality is very good, paste really save electricity, as described, will buy back!	Jingdong prefecture level city in Shandong
Dragon Quality Goods Shop	Green Intelligent Power Saver	Great stuff at a great price, worth the money!	Jingdong prefecture level city in Shandong

Dragon Quality Goods Shop	Green Intelligent Power Saver	Received the power saver, very good quality, very power saving!	Jingdong prefecture level city in Shandong
Dragon Quality Goods Shop	Green Intelligent Power Saver	There are no fruits, only samples...	Jingdong prefecture level city in Shandong
Dragon Quality Goods Shop	Green Intelligent Power Saver	Save a few dollars a month.	Jingdong prefecture level city in Shandong
Dragon Quality Goods Shop	Green Intelligent Power Saver	The quality is particularly good, it works well, and the seller has a good service attitude	Jingdong prefecture level city in Shandong
Dragon Quality Goods Shop	Green Intelligent Power Saver	I've compared them a couple of times and I don't feel any difference	Jingdong prefecture level city in Shandong
Dragon Quality Goods Shop	Green Intelligent Power Saver	I don't see the savings. It's a sham.	Jingdong prefecture level city in Shandong
Dragon Quality Goods Shop	Green Intelligent Power Saver	The power saving effect is very good, and the logistics is very fast. The seller's service attitude is very good	Jingdong prefecture level city in Shandong
Dragon Quality Goods Shop	Green Intelligent Power Saver	Received, well packaged, haven't tried it yet!	Jingdong prefecture level city in Shandong
Dragon Quality Goods Shop	Green Intelligent Power Saver	I don't know how it works, I'll comment after I use it.	Jingdong prefecture level city in Shandong
Dragon Quality Goods Shop	Green Intelligent Power Saver	The product doesn't work well enough to be of any use	Jingdong prefecture level city in Shandong
Dragon Quality Goods Shop	Green Intelligent Power Saver	On Trial 。。。。。。。	Jingdong prefecture level city in Shandong
Dragon Quality Goods Shop	Green Intelligent Power Saver	Being used with unknown results	Jingdong prefecture level city in Shandong
Dragon Quality Goods Shop	Green Intelligent Power Saver	Smart power saving and great quality	Jingdong prefecture level city in Shandong
Dragon Quality Goods Shop	Green Intelligent Power Saver	The saver was received, it works very well and the price is affordable!	Jingdong prefecture level city in Shandong
Dragon Quality Goods Shop	Green Intelligent Power Saver	How can I feel the power saving and how long does it last	Jingdong prefecture level city in Shandong
Dragon Quality Goods Shop	Green Intelligent Power Saver	And I know if it saves electricity or not.	Jingdong prefecture level city in Shandong
Dragon Quality Goods Shop	Green Intelligent Power Saver	It won't save any electricity. No.	Jingdong prefecture level city in Shandong
Dragon Quality Goods Shop	Green Intelligent Power Saver	Invalid. Fake.	Jingdong prefecture level city in Shandong
Dragon Quality Goods Shop	Green Intelligent Power Saver	It's good!	Jingdong prefecture level city in Shandong
Dragon Quality Goods Shop	Green Intelligent Power Saver	have no effect	Jingdong prefecture level city in Shandong
Dragon Quality Goods Shop	Green Intelligent Power Saver	Not used.	Jingdong prefecture level city in Shandong
Dragon Quality Goods Shop	Green Intelligent Power Saver	There is no load range, packaging thrift, ten days also did not develop invoices, received the goods with a receipt, hand-written receipt also has no financial stamp, communication for nearly a week a moment to say that the electronic invoice, a moment to say that the paper invoice, said it is mailed to me, ten days also did not see anything, the invoice invoice can not be found to say that did not stay on file, single single number can not be found, said that the warehouse more than one to a one to find out, in addition to the perfunctory or perfunctory. Products are no certificate of conformity, proper three	Jingdong prefecture level city in Shandong
Dragon Quality Goods Shop	Green Intelligent Power Saver	Satisfaction.	Jingdong prefecture level city in Shandong
Dragon Quality Goods Shop	Green Intelligent Power Saver	There is not a bit of power saving effect, and ringing up an increase in the electricity bill, because you have to add the cost of this machine's own electricity consumption and purchase, so I think it is better to use this machine instead of increasing the expenditure, uneconomical, rather than not use it.	Jingdong prefecture level city in Shandong
Dragon Quality Goods Shop	Green Intelligent Power Saver	I've been using it for two days now and I don't feel any effect.	Jingdong prefecture level city in Shandong
Dragon Quality Goods Shop	Green Intelligent Power Saver	What it looked like was nothing like what was sent.	Jingdong prefecture level city in Shandong
Dragon Quality Goods Shop	Green Intelligent Power Saver	It didn't work. It didn't work for a month or so.	Jingdong prefecture level city in Shandong
Dragon Quality Goods Shop	Green Intelligent Power Saver	Garbage products, physical and description is not the same, no effect at all, pit # no discussion, to buy the fine, garbage, garbage, garbage	Jingdong prefecture level city in Shandong
Dragon Quality Goods Shop	Green Intelligent Power Saver	general	Jingdong prefecture level city in Shandong
Dragon Quality Goods Shop	Green Intelligent Power Saver	IQ tax. Instead of saving electricity, it increases the power consumption of an appliance.	Jingdong prefecture level city in Shandong
Dragon Quality Goods Shop	Green Intelligent Power Saver	There's no trash at all.	Jingdong prefecture level city in Shandong
Dragon Quality Goods Shop	Green Intelligent Power Saver	It doesn't do anything.	Jingdong prefecture level city in Shandong
Dragon Quality Goods Shop	Green Intelligent Power Saver	(of an unmarried couple) be close	Jingdong prefecture level city in Shandong
Dragon Quality Goods Shop	Green Intelligent Power Saver	(of an unmarried couple) be close	Jingdong prefecture level city in Shandong
Dragon Quality Goods Shop	Green Intelligent Power Saver	no electricity saving	Jingdong prefecture level city in Shandong
Dragon Quality Goods Shop	Green Intelligent Power Saver	differ from	Jingdong prefecture level city in Shandong
Dragon Quality Goods Shop	Green Intelligent Power Saver	Saver has received, but real can save electricity, the quality is very good, I times I buy a few more, too good.	Jingdong prefecture level city in Shandong
Dragon Quality Goods Shop	Green Intelligent Power Saver	Great quality as always, a very favorite product, great! o(*￣︶￣*)o	Jingdong prefecture level city in Shandong
Dragon Quality Goods Shop	Green Intelligent Power Saver	If we can reduce the electricity bill, for individuals can save money, for the country can protect the environment, now the weather is getting hotter and hotter, to protect the environment to reduce emissions is very important. Already use the product, whether to reduce the electricity bill depends on next month!	Jingdong prefecture level city in Shandong
Dragon Quality Goods Shop	Green Intelligent Power Saver	Purchased a power saver received on the put into use, rent a room with many electrical appliances, power consumption is very large, we have a piece of electricity or 1.2 dollars a time, with this power saver is much better, every day can save a few cents, very convenient and practical!	Jingdong prefecture level city in Shandong
Dragon Quality Goods Shop	Green Intelligent Power Saver	The product is better to use before not to use when a month 1500 yuan electricity bill, now using the 5 second month electricity bill directly down 470 dollars or very good. A save money to buy several power saver money ZanZanZanZan👍	Jingdong prefecture level city in Shandong
Dragon Quality Goods Shop	Green Intelligent Power Saver	The moment I received the goods really satisfied with this product, not only high-grade packaging, the body are base alloy, up to the price, I was also introduced by a friend, he used last month did save a lot of electricity, but also recommended to me!	Jingdong prefecture level city in Shandong
Dragon Quality Goods Shop	Green Intelligent Power Saver	I got it. Good review. I'm very satisfied. Reasonable price.	Jingdong prefecture level city in Shandong
Dragon Quality Goods Shop	Green Intelligent Power Saver	Intelligent power saver received and used, looking at the quality is good, the quality of the plug sent is also very good. Expect the effect of the economizer is obvious, first five-star praise, with a period of time to follow up!	Jingdong prefecture level city in Shandong
Dragon Quality Goods Shop	Green Intelligent Power Saver	The saver has been received. It was plugged into an outlet and used immediately after receipt. Whether it really saves electricity is not clear yet, I believe that this brand of energy saver really saves	Jingdong prefecture level city in Shandong
Dragon Quality Goods Shop	Green Intelligent Power Saver	Okay, okay, okay. Thank you so much. Fast is good. Fast. Item was as described, mainly because the event was cheap and you can save a lot of money.	Jingdong prefecture level city in Shandong

Dragon Quality Goods Shop	Green Intelligent Power Saver	Received it a couple days ago! Plugged it in the same day! Let's see what the electric bill is in a month! Hopefully it works well oh!	Jingdong prefecture level city in Shandong
Dragon Quality Goods Shop	Green Intelligent Power Saver	Looking at the reviews good, energy-saving small products, compact, the results after 2 months to see, because the electricity bill season payment	Jingdong prefecture level city in Shandong
Dragon Quality Goods Shop	Green Intelligent Power Saver	I just received it and will try it out to see if it works well. I'll comment later.	Jingdong prefecture level city in Shandong
Dragon Quality Goods Shop	Green Intelligent Power Saver	It looks a little small and not very aesthetically pleasing, I don't know how effective it will be until I use it	Jingdong prefecture level city in Shandong
Dragon Quality Goods Shop	Green Intelligent Power Saver	Not only to the point of being useless yet	Jingdong prefecture level city in Shandong
Dragon Quality Goods Shop	Green Intelligent Power Saver	Used it for a month, nothing works, the meter you turn very fast. This is not a scam, but also sold on Jingdong. To cheat a bunch of people?	Jingdong prefecture level city in Shandong
Dragon Quality Goods Shop	Green Intelligent Power Saver	Very good to use, every day can save a little electricity, really good under the sample down a month to save a few dozen dollars in electricity, good before I did not know that there is this ☝ haha, the price is not expensive praise.	Jingdong prefecture level city in Shandong
Dragon Quality Goods Shop	Green Intelligent Power Saver	Tried it for ten days and the change is almost nothing, can't see the power saving yet, use it some more days and see how it goes.	Jingdong prefecture level city in Shandong
Dragon Quality Goods Shop	Green Intelligent Power Saver	Received very quickly, the quality is very good, really save electricity, business service attitude is also very good, I hope you do not miss this opportunity, I continue to buy a few in	Jingdong prefecture level city in Shandong
Dragon Quality Goods Shop	Green Intelligent Power Saver	Quality is very good Oh, the delivery is also very fast, a very favorite store, I want to crazy recommend to friends!	Jingdong prefecture level city in Shandong
Dragon Quality Goods Shop	Green Intelligent Power Saver	The goods have just received, fine workmanship, and the same as described by the merchant, to use a period of time to see if it can achieve the purpose of power saving.	Jingdong prefecture level city in Shandong
Dragon Quality Goods Shop	Green Intelligent Power Saver	Received the goods first use a period of time to see if there is no effect, the business commitment to ninety days invalid refund, first praise, good service!	Jingdong prefecture level city in Shandong
Dragon Quality Goods Shop	Green Intelligent Power Saver	The product is used for a month without energy-saving and power-saving effect does not have any effect	Jingdong prefecture level city in Shandong
Dragon Quality Goods Shop	Green Intelligent Power Saver	The baby is really great, I like it very much, my friends also say good to come, recommended to buy!	Jingdong prefecture level city in Shandong
Dragon Quality Goods Shop	Green Intelligent Power Saver	Logistics is fast, courier boy service is good, after using to see how the fruit, and then follow up.	Jingdong prefecture level city in Shandong
Dragon Quality Goods Shop	Green Intelligent Power Saver	Baby has been received to try, I believe that as the business advertised energy saving. The courier is very responsible.	Jingdong prefecture level city in Shandong
Dragon Quality Goods Shop	Green Intelligent Power Saver	Save Electric Energy King received, good quality, beautiful appearance, very satisfied!	Jingdong prefecture level city in Shandong
Dragon Quality Goods Shop	Green Intelligent Power Saver	Three no products no manufacturers no phone pure fraud induced consumption cheat	Jingdong prefecture level city in Shandong
Dragon Quality Goods Shop	Green Intelligent Power Saver	I'm using it now, I don't know if it's as good as it says, I'll follow up after a month	Jingdong prefecture level city in Shandong
Dragon Quality Goods Shop	Green Intelligent Power Saver	Good quality logistics and fast. Fine workmanship. The description is consistent cost-effective	Jingdong prefecture level city in Shandong
Dragon Quality Goods Shop	Green Intelligent Power Saver	Received, whether it is good or not, to wait a few days and then evaluate	Jingdong prefecture level city in Shandong
Dragon Quality Goods Shop	Green Intelligent Power Saver	I bought it and started using it, I won't know if it works well until I pay my electric bill	Jingdong prefecture level city in Shandong
Dragon Quality Goods Shop	Green Intelligent Power Saver	Especially good, energy saving and power saving, especially good quality, good	Jingdong prefecture level city in Shandong
Dragon Quality Goods Shop	Green Intelligent Power Saver	Logistics is strong, the quality is very good, paste really save electricity, as described, will buy back!	Jingdong prefecture level city in Shandong
Dragon Quality Goods Shop	Green Intelligent Power Saver	Great stuff at a great price, worth the money!	Jingdong prefecture level city in Shandong
Dragon Quality Goods Shop	Green Intelligent Power Saver	Received the power saver, very good quality, very power saving!	Jingdong prefecture level city in Shandong
Dragon Quality Goods Shop	Green Intelligent Power Saver	There are no fruits, only samples...	Jingdong prefecture level city in Shandong
Dragon Quality Goods Shop	Green Intelligent Power Saver	Save a few dollars a month.	Jingdong prefecture level city in Shandong
Dragon Quality Goods Shop	Green Intelligent Power Saver	The quality is particularly good, it works well, and the seller has a good service attitude	Jingdong prefecture level city in Shandong
Dragon Quality Goods Shop	Green Intelligent Power Saver	I've compared them a couple of times and I don't feel any difference	Jingdong prefecture level city in Shandong
Dragon Quality Goods Shop	Green Intelligent Power Saver	I don't see the savings. It's a sham.	Jingdong prefecture level city in Shandong
Dragon Quality Goods Shop	Green Intelligent Power Saver	The power saving effect is very good, and the logistics is very fast. The seller's service attitude is very good	Jingdong prefecture level city in Shandong
Dragon Quality Goods Shop	Green Intelligent Power Saver	Received, well packaged, haven't tried it yet!	Jingdong prefecture level city in Shandong
Dragon Quality Goods Shop	Green Intelligent Power Saver	I don't know how it works, I'll comment after I use it.	Jingdong prefecture level city in Shandong
Dragon Quality Goods Shop	Green Intelligent Power Saver	The product doesn't work well enough to be of any use	Jingdong prefecture level city in Shandong
Dragon Quality Goods Shop	Green Intelligent Power Saver	On Trial 。。。。。。。	Jingdong prefecture level city in Shandong
Dragon Quality Goods Shop	Green Intelligent Power Saver	Being used with unknown results	Jingdong prefecture level city in Shandong
Dragon Quality Goods Shop	Green Intelligent Power Saver	Smart power saving and great quality	Jingdong prefecture level city in Shandong
Dragon Quality Goods Shop	Green Intelligent Power Saver	The saver was received, it works very well and the price is affordable!	Jingdong prefecture level city in Shandong
Dragon Quality Goods Shop	Green Intelligent Power Saver	How can I feel the power saving and how long does it last	Jingdong prefecture level city in Shandong
Dragon Quality Goods Shop	Green Intelligent Power Saver	And I know if it saves electricity or not.	Jingdong prefecture level city in Shandong
Dragon Quality Goods Shop	Green Intelligent Power Saver	It won't save any electricity. No.	Jingdong prefecture level city in Shandong
Dragon Quality Goods Shop	Green Intelligent Power Saver	Invalid. Fake.	Jingdong prefecture level city in Shandong
Dragon Quality Goods Shop	Green Intelligent Power Saver	It's good!	Jingdong prefecture level city in Shandong
Dragon Quality Goods Shop	Green Intelligent Power Saver	have no effect	Jingdong prefecture level city in Shandong
Dragon Quality Goods Shop	Green Intelligent Power Saver	Not used.	Jingdong prefecture level city in Shandong
Dragon Quality Goods Shop	Green Intelligent Power Saver	There is no load range, packaging thrift, ten days also did not develop invoices, received the goods with a receipt, hand-written receipt also has no financial stamp, communication for nearly a week a moment to say that the electronic invoice, a moment to say that the paper invoice, said it is mailed to me, ten days also did not see anything, the invoice invoice can not be found to say that did not stay on file, single single number can not be found, said that the warehouse more than one to a one to find out, in addition to the perfunctory or perfunctory. Products are no certificate of conformity, proper three	Jingdong prefecture level city in Shandong
Dragon Quality Goods Shop	Green Intelligent Power Saver	Satisfaction.	Jingdong prefecture level city in Shandong

Dragon Quality Goods Shop	Green Intelligent Power Saver	There is not a bit of power saving effect, and ringing up an increase in the electricity bill, because you have to add the cost of this machine's own electricity consumption and purchase, so I think it is better to use this machine instead of increasing the expenditure, uneconomical, rather than not use it.	Jingdong prefecture level city in Shandong
Dragon Quality Goods Shop	Green Intelligent Power Saver	I've been using it for two days now and I don't feel any effect.	Jingdong prefecture level city in Shandong
Dragon Quality Goods Shop	Green Intelligent Power Saver	What it looked like was nothing like what was sent.	Jingdong prefecture level city in Shandong
Dragon Quality Goods Shop	Green Intelligent Power Saver	It didn't work. It didn't work for a month or so.	Jingdong prefecture level city in Shandong
Dragon Quality Goods Shop	Green Intelligent Power Saver	Garbage products, physical and description is not the same, no effect at all, pit # no discussion, to buy the fine, garbage, garbage, garbage	Jingdong prefecture level city in Shandong
Dragon Quality Goods Shop	Green Intelligent Power Saver	general	Jingdong prefecture level city in Shandong
Dragon Quality Goods Shop	Green Intelligent Power Saver	IQ tax. Instead of saving electricity, it increases the power consumption of an appliance.	Jingdong prefecture level city in Shandong
Dragon Quality Goods Shop	Green Intelligent Power Saver	There's no trash at all.	Jingdong prefecture level city in Shandong
Dragon Quality Goods Shop	Green Intelligent Power Saver	It doesn't do anything.	Jingdong prefecture level city in Shandong
Dragon Quality Goods Shop	Green Intelligent Power Saver	(of an unmarried couple) be close	Jingdong prefecture level city in Shandong
Dragon Quality Goods Shop	Green Intelligent Power Saver	(of an unmarried couple) be close	Jingdong prefecture level city in Shandong
Dragon Quality Goods Shop	Green Intelligent Power Saver	no electricity saving	Jingdong prefecture level city in Shandong
Dragon Quality Goods Shop	Green Intelligent Power Saver	differ from	Jingdong prefecture level city in Shandong
Dragon Quality Goods Shop	Green Intelligent Power Saver	Saver has received, but real can save electricity, the quality is very good, I times I buy a few more, too good.	Jingdong prefecture level city in Shandong
Dragon Quality Goods Shop	Green Intelligent Power Saver	Great quality as always, a very favorite product, great! o(*￣︶￣*)o	Jingdong prefecture level city in Shandong
Dragon Quality Goods Shop	Green Intelligent Power Saver	If we can reduce the electricity bill, for individuals can save money, for the country can protect the environment, now the weather is getting hotter and hotter, to protect the environment to reduce emissions is very important. Already use the product, whether to reduce the electricity bill depends on next month!	Jingdong prefecture level city in Shandong
Dragon Quality Goods Shop	Green Intelligent Power Saver	Purchased a power saver received on the put into use, rent a room with many electrical appliances, power consumption is very large, we have a piece of electricity or 1.2 dollars a time, with this power saver is much better, every day can save a few cents, very convenient and practical!	Jingdong prefecture level city in Shandong
Dragon Quality Goods Shop	Green Intelligent Power Saver	The product is better to use before not to use when a month 1500 yuan electricity bill, now using the 5 second month electricity bill directly down 470 dollars or very good. A save money to buy several power saver money ZanZanZanZan👍	Jingdong prefecture level city in Shandong
Dragon Quality Goods Shop	Green Intelligent Power Saver	The moment I received the goods really satisfied with this product, not only high-grade packaging, the body are base alloy, up to the price, I was also introduced by a friend, he used last month did save a lot of electricity, but also recommended to me!	Jingdong prefecture level city in Shandong
Dragon Quality Goods Shop	Green Intelligent Power Saver	I got it. Good review. I'm very satisfied. Reasonable price.	Jingdong prefecture level city in Shandong
Dragon Quality Goods Shop	Green Intelligent Power Saver	Intelligent power saver received and used, looking at the quality is good, the quality of the plug sent is also very good. Expect the effect of the economizer is obvious, first five-star praise, with a period of time to follow up!	Jingdong prefecture level city in Shandong
Dragon Quality Goods Shop	Green Intelligent Power Saver	The saver has been received. It was plugged into an outlet and used immediately after receipt. Whether it really saves electricity is not clear yet, I believe that this brand of energy saver really saves	Jingdong prefecture level city in Shandong
Dragon Quality Goods Shop	Green Intelligent Power Saver	Okay, okay, okay. Thank you so much. Fast is good. Fast. Item was as described, mainly because the event was cheap and you can save a lot of money.	Jingdong prefecture level city in Shandong
Dragon Quality Goods Shop	Green Intelligent Power Saver	Received it a couple days ago! Plugged it in the same day! Let's see what the electric bill is in a month! Hopefully it works well oh!	Jingdong prefecture level city in Shandong
Dragon Quality Goods Shop	Green Intelligent Power Saver	Looking at the reviews good, energy-saving small products, compact, the results after 2 months to see, because the electricity bill season payment	Jingdong prefecture level city in Shandong
Dragon Quality Goods Shop	Green Intelligent Power Saver	I just received it and will try it out to see if it works well. I'll comment later.	Jingdong prefecture level city in Shandong
Dragon Quality Goods Shop	Green Intelligent Power Saver	It looks a little small and not very aesthetically pleasing, I don't know how effective it will be until I use it	Jingdong prefecture level city in Shandong
Dragon Quality Goods Shop	Green Intelligent Power Saver	Not only to the point of being useless yet	Jingdong prefecture level city in Shandong
Dragon Quality Goods Shop	Green Intelligent Power Saver	Used it for a month, nothing works, the meter you turn very fast. This is not a scam, but also sold on Jingdong. To cheat a bunch of people?	Jingdong prefecture level city in Shandong
Dragon Quality Goods Shop	Green Intelligent Power Saver	Very good to use, every day can save a little electricity, really good under the sample down a month to save a few dozen dollars in electricity, good before I did not know that there is this 👆 haha, the price is not expensive praise.	Jingdong prefecture level city in Shandong
Dragon Quality Goods Shop	Green Intelligent Power Saver	Tried it for ten days and the change is almost nothing, can't see the power saving yet, use it some more days and see how it goes.	Jingdong prefecture level city in Shandong
Dragon Quality Goods Shop	Green Intelligent Power Saver	Received very quickly, the quality is very good, really save electricity, business service attitude is also very good, I hope you do not miss this opportunity, I continue to buy a few in	Jingdong prefecture level city in Shandong
Dragon Quality Goods Shop	Green Intelligent Power Saver	Quality is very good Oh, the delivery is also very fast, a very favorite store, I want to crazy recommend to friends!	Jingdong prefecture level city in Shandong
Dragon Quality Goods Shop	Green Intelligent Power Saver	The goods have just received, fine workmanship, and the same as described by the merchant, to use a period of time to see if it can achieve the purpose of power saving.	Jingdong prefecture level city in Shandong
Dragon Quality Goods Shop	Green Intelligent Power Saver	Received the goods first use a period of time to see if there is no effect, the business commitment to ninety days invalid refund, first praise, good service!	Jingdong prefecture level city in Shandong
Dragon Quality Goods Shop	Green Intelligent Power Saver	The product is used for a month without energy-saving and power-saving effect does not have any effect	Jingdong prefecture level city in Shandong
Dragon Quality Goods Shop	Green Intelligent Power Saver	The baby is really great, I like it very much, my friends also say good to come, recommended to buy!	Jingdong prefecture level city in Shandong
Dragon Quality Goods Shop	Green Intelligent Power Saver	Logistics is fast, courier boy service is good, after using to see how the fruit, and then follow up.	Jingdong prefecture level city in Shandong
Dragon Quality Goods Shop	Green Intelligent Power Saver	Baby has been received to try, I believe that as the business advertised energy saving. The courier is very responsible.	Jingdong prefecture level city in Shandong
Dragon Quality Goods Shop	Green Intelligent Power Saver	Save Electric Energy King received, good quality, beautiful appearance, very satisfied!	Jingdong prefecture level city in Shandong
Dragon Quality Goods Shop	Green Intelligent Power Saver	Three no products no manufacturers no phone pure fraud induced consumption cheat	Jingdong prefecture level city in Shandong
Dragon Quality Goods Shop	Green Intelligent Power Saver	I'm using it now, I don't know if it's as good as it says, I'll follow up after a month	Jingdong prefecture level city in Shandong
Dragon Quality Goods Shop	Green Intelligent Power Saver	Good quality logistics and fast. Fine workmanship. The description is consistent cost-effective	Jingdong prefecture level city in Shandong
Dragon Quality Goods Shop	Green Intelligent Power Saver	Received, whether it is good or not, to wait a few days and then evaluate	Jingdong prefecture level city in Shandong
Dragon Quality Goods Shop	Green Intelligent Power Saver	I bought it and started using it, I won't know if it works well until I pay my electric bill	Jingdong prefecture level city in Shandong
Dragon Quality Goods Shop	Green Intelligent Power Saver	Especially good, energy saving and power saving, especially good quality, good	Jingdong prefecture level city in Shandong
Dragon Quality Goods Shop	Green Intelligent Power Saver	Logistics is strong, the quality is very good, paste really save electricity, as described, will buy back!	Jingdong prefecture level city in Shandong
Dragon Quality Goods Shop	Green Intelligent Power Saver	Great stuff at a great price, worth the money!	Jingdong prefecture level city in Shandong

Dragon Quality Goods Shop	Green Intelligent Power Saver	Received the power saver, very good quality, very power saving!	Jingdong prefecture level city in Shandong
Dragon Quality Goods Shop	Green Intelligent Power Saver	There are no fruits, only samples...	Jingdong prefecture level city in Shandong
Dragon Quality Goods Shop	Green Intelligent Power Saver	Save a few dollars a month.	Jingdong prefecture level city in Shandong
Dragon Quality Goods Shop	Green Intelligent Power Saver	The quality is particularly good, it works well, and the seller has a good service attitude	Jingdong prefecture level city in Shandong
Dragon Quality Goods Shop	Green Intelligent Power Saver	I've compared them a couple of times and I don't feel any difference	Jingdong prefecture level city in Shandong
Dragon Quality Goods Shop	Green Intelligent Power Saver	I don't see the savings. It's a sham.	Jingdong prefecture level city in Shandong
Dragon Quality Goods Shop	Green Intelligent Power Saver	The power saving effect is very good, and the logistics is very fast. The seller's service attitude is very good	Jingdong prefecture level city in Shandong
Dragon Quality Goods Shop	Green Intelligent Power Saver	Received, well packaged, haven't tried it yet!	Jingdong prefecture level city in Shandong
Dragon Quality Goods Shop	Green Intelligent Power Saver	I don't know how it works, I'll comment after I use it.	Jingdong prefecture level city in Shandong
Dragon Quality Goods Shop	Green Intelligent Power Saver	The product doesn't work well enough to be of any use	Jingdong prefecture level city in Shandong
Dragon Quality Goods Shop	Green Intelligent Power Saver	On Trial 。。。。。。。	Jingdong prefecture level city in Shandong
Dragon Quality Goods Shop	Green Intelligent Power Saver	Being used with unknown results	Jingdong prefecture level city in Shandong
Dragon Quality Goods Shop	Green Intelligent Power Saver	Smart power saving and great quality	Jingdong prefecture level city in Shandong
Dragon Quality Goods Shop	Green Intelligent Power Saver	The saver was received, it works very well and the price is affordable!	Jingdong prefecture level city in Shandong
Dragon Quality Goods Shop	Green Intelligent Power Saver	How can I feel the power saving and how long does it last	Jingdong prefecture level city in Shandong
Dragon Quality Goods Shop	Green Intelligent Power Saver	And I know if it saves electricity or not.	Jingdong prefecture level city in Shandong
Dragon Quality Goods Shop	Green Intelligent Power Saver	It won't save any electricity. No.	Jingdong prefecture level city in Shandong
Dragon Quality Goods Shop	Green Intelligent Power Saver	Invalid. Fake.	Jingdong prefecture level city in Shandong
Dragon Quality Goods Shop	Green Intelligent Power Saver	It's good!	Jingdong prefecture level city in Shandong
Dragon Quality Goods Shop	Green Intelligent Power Saver	have no effect	Jingdong prefecture level city in Shandong
Dragon Quality Goods Shop	Green Intelligent Power Saver	Not used.	Jingdong prefecture level city in Shandong
Dragon Quality Goods Shop	Green Intelligent Power Saver	There is no load range, packaging thrift, ten days also did not develop invoices, received the goods with a receipt, hand-written receipt also has no financial stamp, communication for nearly a week a moment to say that the electronic invoice, a moment to say that the paper invoice, said it is mailed to me, ten days also did not see anything, the invoice invoice can not be found to say that did not stay on file, single single number can not be found, said that the warehouse more than one to a one to find out, in addition to the perfunctory or perfunctory. Products are no certificate of conformity, proper three	Jingdong prefecture level city in Shandong
Dragon Quality Goods Shop	Green Intelligent Power Saver	Satisfaction.	Jingdong prefecture level city in Shandong
Dragon Quality Goods Shop	Green Intelligent Power Saver	There is not a bit of power saving effect, and ringing up an increase in the electricity bill, because you have to add the cost of this machine's own electricity consumption and purchase, so I think it is better to use this machine instead of increasing the expenditure, uneconomical, rather than not use it.	Jingdong prefecture level city in Shandong
Dragon Quality Goods Shop	Green Intelligent Power Saver	I've been using it for two days now and I don't feel any effect.	Jingdong prefecture level city in Shandong
Dragon Quality Goods Shop	Green Intelligent Power Saver	What it looked like was nothing like what was sent.	Jingdong prefecture level city in Shandong
Dragon Quality Goods Shop	Green Intelligent Power Saver	It didn't work. It didn't work for a month or so.	Jingdong prefecture level city in Shandong
Dragon Quality Goods Shop	Green Intelligent Power Saver	Garbage products, physical and description is not the same, no effect at all, pit # no discussion, to buy the fine, garbage, garbage, garbage	Jingdong prefecture level city in Shandong
Dragon Quality Goods Shop	Green Intelligent Power Saver	general	Jingdong prefecture level city in Shandong
Dragon Quality Goods Shop	Green Intelligent Power Saver	IQ tax. Instead of saving electricity, it increases the power consumption of an appliance.	Jingdong prefecture level city in Shandong
Dragon Quality Goods Shop	Green Intelligent Power Saver	There's no trash at all.	Jingdong prefecture level city in Shandong
Dragon Quality Goods Shop	Green Intelligent Power Saver	It doesn't do anything.	Jingdong prefecture level city in Shandong
Dragon Quality Goods Shop	Green Intelligent Power Saver	(of an unmarried couple) be close	Jingdong prefecture level city in Shandong
Dragon Quality Goods Shop	Green Intelligent Power Saver	(of an unmarried couple) be close	Jingdong prefecture level city in Shandong
Dragon Quality Goods Shop	Green Intelligent Power Saver	no electricity saving	Jingdong prefecture level city in Shandong
Dragon Quality Goods Shop	Green Intelligent Power Saver	differ from	Jingdong prefecture level city in Shandong
Dragon Quality Goods Shop	Green Intelligent Power Saver	Saver has received, but real can save electricity, the quality is very good, I times I buy a few more, too good.	Jingdong prefecture level city in Shandong
Dragon Quality Goods Shop	Green Intelligent Power Saver	Great quality as always, a very favorite product, great! o(*￣︶￣*)o	Jingdong prefecture level city in Shandong
Dragon Quality Goods Shop	Green Intelligent Power Saver	If we can reduce the electricity bill, for individuals can save money, for the country can protect the environment, now the weather is getting hotter and hotter, to protect the environment to reduce emissions is very important. Already use the product, whether to reduce the electricity bill depends on next month!	Jingdong prefecture level city in Shandong
Dragon Quality Goods Shop	Green Intelligent Power Saver	Purchased a power saver received on the put into use, rent a room with many electrical appliances, power consumption is very large, we have a piece of electricity or 1.2 dollars a time, with this power saver is much better, every day can save a few cents, very convenient and practical!	Jingdong prefecture level city in Shandong
Dragon Quality Goods Shop	Green Intelligent Power Saver	The product is better to use before not to use when a month 1500 yuan electricity bill, now using the 5 second month electricity bill directly down 470 dollars or very good. A save money to buy several power saver money ZanZanZanZan👍	Jingdong prefecture level city in Shandong
Dragon Quality Goods Shop	Green Intelligent Power Saver	The moment I received the goods really satisfied with this product, not only high-grade packaging, the body are base alloy, up to the price, I was also introduced by a friend, he used last month did save a lot of electricity, but also recommended to me!	Jingdong prefecture level city in Shandong
Dragon Quality Goods Shop	Green Intelligent Power Saver	I got it. Good review. I'm very satisfied. Reasonable price.	Jingdong prefecture level city in Shandong
Dragon Quality Goods Shop	Green Intelligent Power Saver	Intelligent power saver received and used, looking at the quality is good, the quality of the plug sent is also very good. Expect the effect of the economizer is obvious, first five-star praise, with a period of time to follow up!	Jingdong prefecture level city in Shandong
Dragon Quality Goods Shop	Green Intelligent Power Saver	The saver has been received. It was plugged into an outlet and used immediately after receipt. Whether it really saves electricity is not clear yet, I believe that this brand of energy saver really saves	Jingdong prefecture level city in Shandong
Dragon Quality Goods Shop	Green Intelligent Power Saver	Okay, okay, okay. Thank you so much. Fast is good. Fast. Item was as described, mainly because the event was cheap and you can save a lot of money.	Jingdong prefecture level city in Shandong

Dragon Quality Goods Shop	Green Intelligent Power Saver	Received it a couple days ago! Plugged it in the same day! Let's see what the electric bill is in a month! Hopefully it works well oh!	Jingdong prefecture level city in Shandong
Dragon Quality Goods Shop	Green Intelligent Power Saver	Looking at the reviews good, energy-saving small products, compact, the results after 2 months to see, because the electricity bill season payment	Jingdong prefecture level city in Shandong
Dragon Quality Goods Shop	Green Intelligent Power Saver	I just received it and will try it out to see if it works well. I'll comment later.	Jingdong prefecture level city in Shandong
Dragon Quality Goods Shop	Green Intelligent Power Saver	It looks a little small and not very aesthetically pleasing, I don't know how effective it will be until I use it	Jingdong prefecture level city in Shandong
Dragon Quality Goods Shop	Green Intelligent Power Saver	Not only to the point of being useless yet	Jingdong prefecture level city in Shandong
Dragon Quality Goods Shop	Green Intelligent Power Saver	Used it for a month, nothing works, the meter you turn very fast. This is not a scam, but also sold on Jingdong. To cheat a bunch of people?	Jingdong prefecture level city in Shandong
Dragon Quality Goods Shop	Green Intelligent Power Saver	Very good to use, every day can save a little electricity, really good under the sample down a month to save a few dozen dollars in electricity, good before I did not know that there is this haha, the price is not expensive praise.	Jingdong prefecture level city in Shandong
Dragon Quality Goods Shop	Green Intelligent Power Saver	Tried it for ten days and the change is almost nothing, can't see the power saving yet, use it some more days and see how it goes.	Jingdong prefecture level city in Shandong
Dragon Quality Goods Shop	Green Intelligent Power Saver	Received very quickly, the quality is very good, really save electricity, business service attitude is also very good, I hope you do not miss this opportunity, I continue to buy a few in	Jingdong prefecture level city in Shandong
Dragon Quality Goods Shop	Green Intelligent Power Saver	Quality is very good Oh, the delivery is also very fast, a very favorite store, I want to crazy recommend to friends!	Jingdong prefecture level city in Shandong
Dragon Quality Goods Shop	Green Intelligent Power Saver	The goods have just received, fine workmanship, and the same as described by the merchant, to use a period of time to see if it can achieve the purpose of power saving.	Jingdong prefecture level city in Shandong
Dragon Quality Goods Shop	Green Intelligent Power Saver	Received the goods first use a period of time to see if there is no effect, the business commitment to ninety days invalid refund, first praise, good service!	Jingdong prefecture level city in Shandong
Dragon Quality Goods Shop	Green Intelligent Power Saver	The product is used for a month without energy-saving and power-saving effect does not have any effect	Jingdong prefecture level city in Shandong
Dragon Quality Goods Shop	Green Intelligent Power Saver	The baby is really great, I like it very much, my friends also say good to come, recommended to buy!	Jingdong prefecture level city in Shandong
Dragon Quality Goods Shop	Green Intelligent Power Saver	Logistics is fast, courier boy service is good, after using to see how the fruit, and then follow up.	Jingdong prefecture level city in Shandong
Dragon Quality Goods Shop	Green Intelligent Power Saver	Baby has been received to try, I believe that as the business advertised energy saving. The courier is very responsible.	Jingdong prefecture level city in Shandong
Dragon Quality Goods Shop	Green Intelligent Power Saver	Save Electric Energy King received, good quality, beautiful appearance, very satisfied!	Jingdong prefecture level city in Shandong
Dragon Quality Goods Shop	Green Intelligent Power Saver	Three no products no manufacturers no phone pure fraud induced consumption cheat	Jingdong prefecture level city in Shandong
Dragon Quality Goods Shop	Green Intelligent Power Saver	I'm using it now, I don't know if it's as good as it says, I'll follow up after a month	Jingdong prefecture level city in Shandong
Dragon Quality Goods Shop	Green Intelligent Power Saver	Good quality logistics and fast. Fine workmanship. The description is consistent cost-effective	Jingdong prefecture level city in Shandong
Dragon Quality Goods Shop	Green Intelligent Power Saver	Received, whether it is good or not, to wait a few days and then evaluate	Jingdong prefecture level city in Shandong
Dragon Quality Goods Shop	Green Intelligent Power Saver	I bought it and started using it, I won't know if it works well until I pay my electric bill	Jingdong prefecture level city in Shandong
Dragon Quality Goods Shop	Green Intelligent Power Saver	Especially good, energy saving and power saving, especially good quality, good	Jingdong prefecture level city in Shandong
Dragon Quality Goods Shop	Green Intelligent Power Saver	Logistics is strong, the quality is very good, paste really save electricity, as described, will buy back!	Jingdong prefecture level city in Shandong
Dragon Quality Goods Shop	Green Intelligent Power Saver	Great stuff at a great price, worth the money!	Jingdong prefecture level city in Shandong
Dragon Quality Goods Shop	Green Intelligent Power Saver	Received the power saver, very good quality, very power saving!	Jingdong prefecture level city in Shandong
Dragon Quality Goods Shop	Green Intelligent Power Saver	There are no fruits, only samples...	Jingdong prefecture level city in Shandong
Dragon Quality Goods Shop	Green Intelligent Power Saver	Save a few dollars a month.	Jingdong prefecture level city in Shandong
Dragon Quality Goods Shop	Green Intelligent Power Saver	The quality is particularly good, it works well, and the seller has a good service attitude	Jingdong prefecture level city in Shandong
Dragon Quality Goods Shop	Green Intelligent Power Saver	I've compared them a couple of times and I don't feel any difference	Jingdong prefecture level city in Shandong
Dragon Quality Goods Shop	Green Intelligent Power Saver	I don't see the savings. It's a sham.	Jingdong prefecture level city in Shandong
Dragon Quality Goods Shop	Green Intelligent Power Saver	The power saving effect is very good, and the logistics is very fast. The seller's service attitude is very good	Jingdong prefecture level city in Shandong
Dragon Quality Goods Shop	Green Intelligent Power Saver	Received, well packaged, haven't tried it yet!	Jingdong prefecture level city in Shandong
Dragon Quality Goods Shop	Green Intelligent Power Saver	I don't know how it works, I'll comment after I use it.	Jingdong prefecture level city in Shandong
Dragon Quality Goods Shop	Green Intelligent Power Saver	The product doesn't work well enough to be of any use	Jingdong prefecture level city in Shandong
Dragon Quality Goods Shop	Green Intelligent Power Saver	On Trial 。。。。。。。	Jingdong prefecture level city in Shandong
Dragon Quality Goods Shop	Green Intelligent Power Saver	Being used with unknown results	Jingdong prefecture level city in Shandong
Dragon Quality Goods Shop	Green Intelligent Power Saver	Smart power saving and great quality	Jingdong prefecture level city in Shandong
Dragon Quality Goods Shop	Green Intelligent Power Saver	The saver was received, it works very well and the price is affordable!	Jingdong prefecture level city in Shandong
Dragon Quality Goods Shop	Green Intelligent Power Saver	How can I feel the power saving and how long does it last	Jingdong prefecture level city in Shandong
Dragon Quality Goods Shop	Green Intelligent Power Saver	And I know if it saves electricity or not.	Jingdong prefecture level city in Shandong
Dragon Quality Goods Shop	Green Intelligent Power Saver	It won't save any electricity. No.	Jingdong prefecture level city in Shandong
Dragon Quality Goods Shop	Green Intelligent Power Saver	Invalid. Fake.	Jingdong prefecture level city in Shandong
Dragon Quality Goods Shop	Green Intelligent Power Saver	It's good!	Jingdong prefecture level city in Shandong
Dragon Quality Goods Shop	Green Intelligent Power Saver	have no effect	Jingdong prefecture level city in Shandong
Dragon Quality Goods Shop	Green Intelligent Power Saver	Not used.	Jingdong prefecture level city in Shandong
Dragon Quality Goods Shop	Green Intelligent Power Saver	There is no load range, packaging thrift, ten days also did not develop invoices, received the goods with a receipt, hand-written receipt also has no financial stamp, communication for nearly a week a moment to say that the electronic invoice, a moment to say that the paper invoice, said it is mailed to me, ten days also did not see anything, the invoice invoice can not be found to say that did not stay on file, single single number can not be found, said that the warehouse more than one to a one to find out, in addition to the perfunctory or perfunctory. Products are no certificate of conformity, proper three	Jingdong prefecture level city in Shandong
Dragon Quality Goods Shop	Green Intelligent Power Saver	Satisfaction.	Jingdong prefecture level city in Shandong

Dragon Quality Goods Shop	Green Intelligent Power Saver	There is not a bit of power saving effect, and ringing up an increase in the electricity bill, because you have to add the cost of this machine's own electricity consumption and purchase, so I think it is better to use this machine instead of increasing the expenditure, uneconomical, rather than not use it.	Jingdong prefecture level city in Shandong
Dragon Quality Goods Shop	Green Intelligent Power Saver	I've been using it for two days now and I don't feel any effect.	Jingdong prefecture level city in Shandong
Dragon Quality Goods Shop	Green Intelligent Power Saver	What it looked like was nothing like what was sent.	Jingdong prefecture level city in Shandong
Dragon Quality Goods Shop	Green Intelligent Power Saver	It didn't work. It didn't work for a month or so.	Jingdong prefecture level city in Shandong
Dragon Quality Goods Shop	Green Intelligent Power Saver	Garbage products, physical and description is not the same, no effect at all, pit # no discussion, to buy the fine, garbage, garbage, garbage	Jingdong prefecture level city in Shandong
Dragon Quality Goods Shop	Green Intelligent Power Saver	general	Jingdong prefecture level city in Shandong
Dragon Quality Goods Shop	Green Intelligent Power Saver	IQ tax. Instead of saving electricity, it increases the power consumption of an appliance.	Jingdong prefecture level city in Shandong
Dragon Quality Goods Shop	Green Intelligent Power Saver	There's no trash at all.	Jingdong prefecture level city in Shandong
Dragon Quality Goods Shop	Green Intelligent Power Saver	It doesn't do anything.	Jingdong prefecture level city in Shandong
Dragon Quality Goods Shop	Green Intelligent Power Saver	(of an unmarried couple) be close	Jingdong prefecture level city in Shandong
Dragon Quality Goods Shop	Green Intelligent Power Saver	(of an unmarried couple) be close	Jingdong prefecture level city in Shandong
Dragon Quality Goods Shop	Green Intelligent Power Saver	no electricity saving	Jingdong prefecture level city in Shandong
Dragon Quality Goods Shop	Green Intelligent Power Saver	differ from	Jingdong prefecture level city in Shandong
Dragon Quality Goods Shop	Green Intelligent Power Saver	Saver has received, but real can save electricity, the quality is very good, I times I buy a few more, too good.	Jingdong prefecture level city in Shandong
Dragon Quality Goods Shop	Green Intelligent Power Saver	Great quality as always, a very favorite product, great! o(*￣︶￣*)o	Jingdong prefecture level city in Shandong
Dragon Quality Goods Shop	Green Intelligent Power Saver	If we can reduce the electricity bill, for individuals can save money, for the country can protect the environment, now the weather is getting hotter and hotter, to protect the environment to reduce emissions is very important. Already use the product, whether to reduce the electricity bill depends on next month!	Jingdong prefecture level city in Shandong
Dragon Quality Goods Shop	Green Intelligent Power Saver	Purchased a power saver received on the put into use, rent a room with many electrical appliances, power consumption is very large, we have a piece of electricity or 1.2 dollars a time, with this power saver is much better, every day can save a few cents, very convenient and practical!	Jingdong prefecture level city in Shandong
Dragon Quality Goods Shop	Green Intelligent Power Saver	The product is better to use before not to use when a month 1500 yuan electricity bill, now using the 5 second month electricity bill directly down 470 dollars or very good. A save money to buy several power saver money ZanZanZanZan👍	Jingdong prefecture level city in Shandong
Dragon Quality Goods Shop	Green Intelligent Power Saver	The moment I received the goods really satisfied with this product, not only high-grade packaging, the body are base alloy, up to the price, I was also introduced by a friend, he used last month did save a lot of electricity, but also recommended to me!	Jingdong prefecture level city in Shandong
Dragon Quality Goods Shop	Green Intelligent Power Saver	I got it. Good review. I'm very satisfied. Reasonable price.	Jingdong prefecture level city in Shandong
Dragon Quality Goods Shop	Green Intelligent Power Saver	Intelligent power saver received and used, looking at the quality is good, the quality of the plug sent is also very good. Expect the effect of the economizer is obvious, first five-star praise, with a period of time to follow up!	Jingdong prefecture level city in Shandong
Dragon Quality Goods Shop	Green Intelligent Power Saver	The saver has been received. It was plugged into an outlet and used immediately after receipt. Whether it really saves electricity is not clear yet, I believe that this brand of energy saver really saves	Jingdong prefecture level city in Shandong
Dragon Quality Goods Shop	Green Intelligent Power Saver	Okay, okay, okay. Thank you so much. Fast is good. Fast. Item was as described, mainly because the event was cheap and you can save a lot of money.	Jingdong prefecture level city in Shandong
Dragon Quality Goods Shop	Green Intelligent Power Saver	Received it a couple days ago! Plugged it in the same day! Let's see what the electric bill is in a month! Hopefully it works well oh!	Jingdong prefecture level city in Shandong
Dragon Quality Goods Shop	Green Intelligent Power Saver	Looking at the reviews good, energy-saving small products, compact, the results after 2 months to see, because the electricity bill season payment	Jingdong prefecture level city in Shandong
Dragon Quality Goods Shop	Green Intelligent Power Saver	I just received it and will try it out to see if it works well. I'll comment later.	Jingdong prefecture level city in Shandong
Dragon Quality Goods Shop	Green Intelligent Power Saver	It looks a little small and not very aesthetically pleasing, I don't know how effective it will be until I use it	Jingdong prefecture level city in Shandong
Dragon Quality Goods Shop	Green Intelligent Power Saver	Not only to the point of being useless yet	Jingdong prefecture level city in Shandong
Dragon Quality Goods Shop	Green Intelligent Power Saver	Used it for a month, nothing works, the meter you turn very fast. This is not a scam, but also sold on Jingdong. To cheat a bunch of people?	Jingdong prefecture level city in Shandong
Dragon Quality Goods Shop	Green Intelligent Power Saver	Very good to use, every day can save a little electricity, really good under the sample down a month to save a few dozen dollars in electricity, good before I did not know that there is this 👆 haha, the price is not expensive praise.	Jingdong prefecture level city in Shandong
Dragon Quality Goods Shop	Green Intelligent Power Saver	Tried it for ten days and the change is almost nothing, can't see the power saving yet, use it some more days and see how it goes.	Jingdong prefecture level city in Shandong
Dragon Quality Goods Shop	Green Intelligent Power Saver	Received very quickly, the quality is very good, really save electricity, business service attitude is also very good, I hope you do not miss this opportunity, I continue to buy a few in	Jingdong prefecture level city in Shandong
Dragon Quality Goods Shop	Green Intelligent Power Saver	Quality is very good Oh, the delivery is also very fast, a very favorite store, I want to crazy recommend to friends!	Jingdong prefecture level city in Shandong
Dragon Quality Goods Shop	Green Intelligent Power Saver	The goods have just received, fine workmanship, and the same as described by the merchant, to use a period of time to see if it can achieve the purpose of power saving.	Jingdong prefecture level city in Shandong
Dragon Quality Goods Shop	Green Intelligent Power Saver	Received the goods first use a period of time to see if there is no effect, the business commitment to ninety days invalid refund, first praise, good service!	Jingdong prefecture level city in Shandong
Dragon Quality Goods Shop	Green Intelligent Power Saver	The product is used for a month without energy-saving and power-saving effect does not have any effect	Jingdong prefecture level city in Shandong
Dragon Quality Goods Shop	Green Intelligent Power Saver	The baby is really great, I like it very much, my friends also say good to come, recommended to buy!	Jingdong prefecture level city in Shandong
Dragon Quality Goods Shop	Green Intelligent Power Saver	Logistics is fast, courier boy service is good, after using to see how the fruit, and then follow up.	Jingdong prefecture level city in Shandong
Dragon Quality Goods Shop	Green Intelligent Power Saver	Baby has been received to try, I believe that as the business advertised energy saving. The courier is very responsible.	Jingdong prefecture level city in Shandong
Dragon Quality Goods Shop	Green Intelligent Power Saver	Save Electric Energy King received, good quality, beautiful appearance, very satisfied!	Jingdong prefecture level city in Shandong
Dragon Quality Goods Shop	Green Intelligent Power Saver	Three no products no manufacturers no phone pure fraud induced consumption cheat	Jingdong prefecture level city in Shandong
Dragon Quality Goods Shop	Green Intelligent Power Saver	I'm using it now, I don't know if it's as good as it says, I'll follow up after a month	Jingdong prefecture level city in Shandong
Dragon Quality Goods Shop	Green Intelligent Power Saver	Good quality logistics and fast. Fine workmanship. The description is consistent cost-effective	Jingdong prefecture level city in Shandong
Dragon Quality Goods Shop	Green Intelligent Power Saver	Received, whether it is good or not, to wait a few days and then evaluate	Jingdong prefecture level city in Shandong
Dragon Quality Goods Shop	Green Intelligent Power Saver	I bought it and started using it, I won't know if it works well until I pay my electric bill	Jingdong prefecture level city in Shandong
Dragon Quality Goods Shop	Green Intelligent Power Saver	Especially good, energy saving and power saving, especially good quality, good	Jingdong prefecture level city in Shandong
Dragon Quality Goods Shop	Green Intelligent Power Saver	Logistics is strong, the quality is very good, paste really save electricity, as described, will buy back!	Jingdong prefecture level city in Shandong
Dragon Quality Goods Shop	Green Intelligent Power Saver	Great stuff at a great price, worth the money!	Jingdong prefecture level city in Shandong

Dragon Quality Goods Shop	Green Intelligent Power Saver	Received the power saver, very good quality, very power saving!	Jingdong prefecture level city in Shandong
Dragon Quality Goods Shop	Green Intelligent Power Saver	There are no fruits, only samples...	Jingdong prefecture level city in Shandong
Dragon Quality Goods Shop	Green Intelligent Power Saver	Save a few dollars a month.	Jingdong prefecture level city in Shandong
Dragon Quality Goods Shop	Green Intelligent Power Saver	The quality is particularly good, it works well, and the seller has a good service attitude	Jingdong prefecture level city in Shandong
Dragon Quality Goods Shop	Green Intelligent Power Saver	I've compared them a couple of times and I don't feel any difference	Jingdong prefecture level city in Shandong
Dragon Quality Goods Shop	Green Intelligent Power Saver	I don't see the savings. It's a sham.	Jingdong prefecture level city in Shandong
Dragon Quality Goods Shop	Green Intelligent Power Saver	The power saving effect is very good, and the logistics is very fast. The seller's service attitude is very good	Jingdong prefecture level city in Shandong
Dragon Quality Goods Shop	Green Intelligent Power Saver	Received, well packaged, haven't tried it yet!	Jingdong prefecture level city in Shandong
Dragon Quality Goods Shop	Green Intelligent Power Saver	I don't know how it works, I'll comment after I use it.	Jingdong prefecture level city in Shandong
Dragon Quality Goods Shop	Green Intelligent Power Saver	The product doesn't work well enough to be of any use	Jingdong prefecture level city in Shandong
Dragon Quality Goods Shop	Green Intelligent Power Saver	On Trial 。。。。。。。	Jingdong prefecture level city in Shandong
Dragon Quality Goods Shop	Green Intelligent Power Saver	Being used with unknown results	Jingdong prefecture level city in Shandong
Dragon Quality Goods Shop	Green Intelligent Power Saver	Smart power saving and great quality	Jingdong prefecture level city in Shandong
Dragon Quality Goods Shop	Green Intelligent Power Saver	The saver was received, it works very well and the price is affordable!	Jingdong prefecture level city in Shandong
Dragon Quality Goods Shop	Green Intelligent Power Saver	How can I feel the power saving and how long does it last	Jingdong prefecture level city in Shandong
Dragon Quality Goods Shop	Green Intelligent Power Saver	And I know if it saves electricity or not.	Jingdong prefecture level city in Shandong
Dragon Quality Goods Shop	Green Intelligent Power Saver	It won't save any electricity. No.	Jingdong prefecture level city in Shandong
Dragon Quality Goods Shop	Green Intelligent Power Saver	Invalid. Fake.	Jingdong prefecture level city in Shandong
Dragon Quality Goods Shop	Green Intelligent Power Saver	It's good!	Jingdong prefecture level city in Shandong
Dragon Quality Goods Shop	Green Intelligent Power Saver	have no effect	Jingdong prefecture level city in Shandong
Dragon Quality Goods Shop	Green Intelligent Power Saver	Not used.	Jingdong prefecture level city in Shandong
Dragon Quality Goods Shop	Green Intelligent Power Saver	There is no load range, packaging thrift, ten days also did not develop invoices, received the goods with a receipt, hand-written receipt also has no financial stamp, communication for nearly a week a moment to say that the electronic invoice, a moment to say that the paper invoice, said it is mailed to me, ten days also did not see anything, the invoice invoice can not be found to say that did not stay on file, single single number can not be found, said that the warehouse more than one to a one to find out, in addition to the perfunctory or perfunctory. Products are no certificate of conformity, proper three	Jingdong prefecture level city in Shandong
Dragon Quality Goods Shop	Green Intelligent Power Saver	Satisfaction.	Jingdong prefecture level city in Shandong
Dragon Quality Goods Shop	Green Intelligent Power Saver	There is not a bit of power saving effect, and ringing up an increase in the electricity bill, because you have to add the cost of this machine's own electricity consumption and purchase, so I think it is better to use this machine instead of increasing the expenditure, uneconomical, rather than not use it.	Jingdong prefecture level city in Shandong
Dragon Quality Goods Shop	Green Intelligent Power Saver	I've been using it for two days now and I don't feel any effect.	Jingdong prefecture level city in Shandong
Dragon Quality Goods Shop	Green Intelligent Power Saver	What it looked like was nothing like what was sent.	Jingdong prefecture level city in Shandong
Dragon Quality Goods Shop	Green Intelligent Power Saver	It didn't work. It didn't work for a month or so.	Jingdong prefecture level city in Shandong
Dragon Quality Goods Shop	Green Intelligent Power Saver	Garbage products, physical and description is not the same, no effect at all, pit # no discussion, to buy the fine, garbage, garbage, garbage	Jingdong prefecture level city in Shandong
Dragon Quality Goods Shop	Green Intelligent Power Saver	general	Jingdong prefecture level city in Shandong
Dragon Quality Goods Shop	Green Intelligent Power Saver	IQ tax. Instead of saving electricity, it increases the power consumption of an appliance.	Jingdong prefecture level city in Shandong
Dragon Quality Goods Shop	Green Intelligent Power Saver	There's no trash at all.	Jingdong prefecture level city in Shandong
Dragon Quality Goods Shop	Green Intelligent Power Saver	It doesn't do anything.	Jingdong prefecture level city in Shandong
Dragon Quality Goods Shop	Green Intelligent Power Saver	(of an unmarried couple) be close	Jingdong prefecture level city in Shandong
Dragon Quality Goods Shop	Green Intelligent Power Saver	(of an unmarried couple) be close	Jingdong prefecture level city in Shandong
Dragon Quality Goods Shop	Green Intelligent Power Saver	no electricity saving	Jingdong prefecture level city in Shandong
Dragon Quality Goods Shop	Green Intelligent Power Saver	differ from	Jingdong prefecture level city in Shandong
Dragon Quality Goods Shop	Green Intelligent Power Saver	Saver has received, but real can save electricity, the quality is very good, I times I buy a few more, too good.	Jingdong prefecture level city in Shandong
Dragon Quality Goods Shop	Green Intelligent Power Saver	Great quality as always, a very favorite product, great! o(*￣︶￣*)o	Jingdong prefecture level city in Shandong
Dragon Quality Goods Shop	Green Intelligent Power Saver	If we can reduce the electricity bill, for individuals can save money, for the country can protect the environment, now the weather is getting hotter and hotter, to protect the environment to reduce emissions is very important. Already use the product, whether to reduce the electricity bill depends on next month!	Jingdong prefecture level city in Shandong
Dragon Quality Goods Shop	Green Intelligent Power Saver	Purchased a power saver received on the put into use, rent a room with many electrical appliances, power consumption is very large, we have a piece of electricity or 1.2 dollars a time, with this power saver is much better, every day can save a few cents, very convenient and practical!	Jingdong prefecture level city in Shandong
Dragon Quality Goods Shop	Green Intelligent Power Saver	The product is better to use before not to use when a month 1500 yuan electricity bill, now using the 5 second month electricity bill directly down 470 dollars or very good. A save money to buy several power saver money ZanZanZanZan👍	Jingdong prefecture level city in Shandong
Dragon Quality Goods Shop	Green Intelligent Power Saver	The moment I received the goods really satisfied with this product, not only high-grade packaging, the body are base alloy, up to the price, I was also introduced by a friend, he used last month did save a lot of electricity, but also recommended to me!	Jingdong prefecture level city in Shandong
Dragon Quality Goods Shop	Green Intelligent Power Saver	Okay, okay, okay. Thank you so much. Fast is good. Fast. Item was as described, mainly because the event was cheap and you can save a lot of money.	Jingdong prefecture level city in Shandong
Dragon Quality Goods Shop	Green Intelligent Power Saver	Intelligent power saver received and used, looking at the quality is good, the quality of the plug sent is also very good. Expect the effect of the economizer is obvious, first five-star praise, with a period of time to follow up!	Jingdong prefecture level city in Shandong
Dragon Quality Goods Shop	Green Intelligent Power Saver	The saver has been received. It was plugged into an outlet and used immediately after receipt. Whether it really saves electricity is not clear yet, I believe that this brand of energy saver really saves	Jingdong prefecture level city in Shandong
Dragon Quality Goods Shop	Green Intelligent Power Saver	I got it. Good review. I'm very satisfied. Reasonable price.	Jingdong prefecture level city in Shandong

Dragon Quality Goods Shop	Green Intelligent Power Saver	Received it a couple days ago! Plugged it in the same day! Let's see what the electric bill is in a month! Hopefully it works well oh!	Jingdong prefecture level city in Shandong
Dragon Quality Goods Shop	Green Intelligent Power Saver	Looking at the reviews good, energy-saving small products, compact, the results after 2 months to see, because the electricity bill season payment	Jingdong prefecture level city in Shandong
Dragon Quality Goods Shop	Green Intelligent Power Saver	I just received it and will try it out to see if it works well. I'll comment later.	Jingdong prefecture level city in Shandong
Dragon Quality Goods Shop	Green Intelligent Power Saver	It looks a little small and not very aesthetically pleasing, I don't know how effective it will be until I use it	Jingdong prefecture level city in Shandong
Dragon Quality Goods Shop	Green Intelligent Power Saver	Not only to the point of being useless yet	Jingdong prefecture level city in Shandong
Dragon Quality Goods Shop	Green Intelligent Power Saver	Used it for a month, nothing works, the meter you turn very fast. This is not a scam, but also sold on Jingdong. To cheat a bunch of people?	Jingdong prefecture level city in Shandong
Dragon Quality Goods Shop	Green Intelligent Power Saver	Very good to use, every day can save a little electricity, really good under the sample down a month to save a few dozen dollars in electricity, good before I did not know that there is this haha, the price is not expensive praise.	Jingdong prefecture level city in Shandong
Dragon Quality Goods Shop	Green Intelligent Power Saver	Tried it for ten days and the change is almost nothing, can't see the power saving yet, use it some more days and see how it goes.	Jingdong prefecture level city in Shandong
Dragon Quality Goods Shop	Green Intelligent Power Saver	Received very quickly, the quality is very good, really save electricity, business service attitude is also very good, I hope you do not miss this opportunity, I continue to buy a few in	Jingdong prefecture level city in Shandong
Dragon Quality Goods Shop	Green Intelligent Power Saver	Quality is very good Oh, the delivery is also very fast, a very favorite store, I want to crazy recommend to friends!	Jingdong prefecture level city in Shandong
Dragon Quality Goods Shop	Green Intelligent Power Saver	The goods have just received, fine workmanship, and the same as described by the merchant, to use a period of time to see if it can achieve the purpose of power saving.	Jingdong prefecture level city in Shandong
Dragon Quality Goods Shop	Green Intelligent Power Saver	Received the goods first use a period of time to see if there is no effect, the business commitment to ninety days invalid refund, first praise, good service!	Jingdong prefecture level city in Shandong
Dragon Quality Goods Shop	Green Intelligent Power Saver	The product is used for a month without energy-saving and power-saving effect does not have any effect	Jingdong prefecture level city in Shandong
Dragon Quality Goods Shop	Green Intelligent Power Saver	The baby is really great, I like it very much, my friends also say good to come, recommended to buy!	Jingdong prefecture level city in Shandong
Dragon Quality Goods Shop	Green Intelligent Power Saver	Logistics is fast, courier boy service is good, after using to see how the fruit, and then follow up.	Jingdong prefecture level city in Shandong
Dragon Quality Goods Shop	Green Intelligent Power Saver	Baby has been received to try, I believe that as the business advertised energy saving. The courier is very responsible.	Jingdong prefecture level city in Shandong
Dragon Quality Goods Shop	Green Intelligent Power Saver	Save Electric Energy King received, good quality, beautiful appearance, very satisfied!	Jingdong prefecture level city in Shandong
Dragon Quality Goods Shop	Green Intelligent Power Saver	Three no products no manufacturers no phone pure fraud induced consumption cheat	Jingdong prefecture level city in Shandong
Dragon Quality Goods Shop	Green Intelligent Power Saver	I'm using it now, I don't know if it's as good as it says, I'll follow up after a month	Jingdong prefecture level city in Shandong
Dragon Quality Goods Shop	Green Intelligent Power Saver	Good quality logistics and fast. Fine workmanship. The description is consistent cost-effective	Jingdong prefecture level city in Shandong
Dragon Quality Goods Shop	Green Intelligent Power Saver	Received, whether it is good or not, to wait a few days and then evaluate	Jingdong prefecture level city in Shandong
Dragon Quality Goods Shop	Green Intelligent Power Saver	I bought it and started using it, I won't know if it works well until I pay my electric bill	Jingdong prefecture level city in Shandong
Dragon Quality Goods Shop	Green Intelligent Power Saver	Especially good, energy saving and power saving, especially good quality, good	Jingdong prefecture level city in Shandong
Dragon Quality Goods Shop	Green Intelligent Power Saver	Logistics is strong, the quality is very good, paste really save electricity, as described, will buy back!	Jingdong prefecture level city in Shandong
Dragon Quality Goods Shop	Green Intelligent Power Saver	Great stuff at a great price, worth the money!	Jingdong prefecture level city in Shandong
Dragon Quality Goods Shop	Green Intelligent Power Saver	Received the power saver, very good quality, very power saving!	Jingdong prefecture level city in Shandong
Dragon Quality Goods Shop	Green Intelligent Power Saver	There are no fruits, only samples...	Jingdong prefecture level city in Shandong
Dragon Quality Goods Shop	Green Intelligent Power Saver	Save a few dollars a month.	Jingdong prefecture level city in Shandong
Dragon Quality Goods Shop	Green Intelligent Power Saver	The quality is particularly good, it works well, and the seller has a good service attitude	Jingdong prefecture level city in Shandong
Dragon Quality Goods Shop	Green Intelligent Power Saver	I've compared them a couple of times and I don't feel any difference	Jingdong prefecture level city in Shandong
Dragon Quality Goods Shop	Green Intelligent Power Saver	I don't see the savings. It's a sham.	Jingdong prefecture level city in Shandong
Dragon Quality Goods Shop	Green Intelligent Power Saver	The power saving effect is very good, and the logistics is very fast. The seller's service attitude is very good	Jingdong prefecture level city in Shandong
Dragon Quality Goods Shop	Green Intelligent Power Saver	Received, well packaged, haven't tried it yet!	Jingdong prefecture level city in Shandong
Dragon Quality Goods Shop	Green Intelligent Power Saver	I don't know how it works, I'll comment after I use it.	Jingdong prefecture level city in Shandong
Dragon Quality Goods Shop	Green Intelligent Power Saver	The product doesn't work well enough to be of any use	Jingdong prefecture level city in Shandong
Dragon Quality Goods Shop	Green Intelligent Power Saver	On Trial 。。。。。。。	Jingdong prefecture level city in Shandong
Dragon Quality Goods Shop	Green Intelligent Power Saver	Being used with unknown results	Jingdong prefecture level city in Shandong
Dragon Quality Goods Shop	Green Intelligent Power Saver	Smart power saving and great quality	Jingdong prefecture level city in Shandong
Dragon Quality Goods Shop	Green Intelligent Power Saver	The saver was received, it works very well and the price is affordable!	Jingdong prefecture level city in Shandong
Dragon Quality Goods Shop	Green Intelligent Power Saver	How can I feel the power saving and how long does it last	Jingdong prefecture level city in Shandong
Dragon Quality Goods Shop	Green Intelligent Power Saver	And I know if it saves electricity or not.	Jingdong prefecture level city in Shandong
Dragon Quality Goods Shop	Green Intelligent Power Saver	It won't save any electricity. No.	Jingdong prefecture level city in Shandong
Dragon Quality Goods Shop	Green Intelligent Power Saver	Invalid. Fake.	Jingdong prefecture level city in Shandong
Dragon Quality Goods Shop	Green Intelligent Power Saver	It's good!	Jingdong prefecture level city in Shandong
Dragon Quality Goods Shop	Green Intelligent Power Saver	have no effect	Jingdong prefecture level city in Shandong
Dragon Quality Goods Shop	Green Intelligent Power Saver	Not used.	Jingdong prefecture level city in Shandong
Dragon Quality Goods Shop	Green Intelligent Power Saver	There is no load range, packaging thrift, ten days also did not develop invoices, received the goods with a receipt, hand-written receipt also has no financial stamp, communication for nearly a week a moment to say that the electronic invoice, a moment to say that the paper invoice, said it is mailed to me, ten days also did not see anything, the invoice invoice can not be found to say that did not stay on file, single single number can not be found, said that the warehouse more than one to a one to find out, in addition to the perfunctory or perfunctory. Products are no certificate of conformity, proper three	Jingdong prefecture level city in Shandong
Dragon Quality Goods Shop	Green Intelligent Power Saver	Satisfaction.	Jingdong prefecture level city in Shandong

Dragon Quality Goods Shop	Green Intelligent Power Saver	There is not a bit of power saving effect, and ringing up an increase in the electricity bill, because you have to add the cost of this machine's own electricity consumption and purchase, so I think it is better to use this machine instead of increasing the expenditure, uneconomical, rather than not use it.	Jingdong prefecture level city in Shandong
Dragon Quality Goods Shop	Green Intelligent Power Saver	I've been using it for two days now and I don't feel any effect.	Jingdong prefecture level city in Shandong
Dragon Quality Goods Shop	Green Intelligent Power Saver	What it looked like was nothing like what was sent.	Jingdong prefecture level city in Shandong
Dragon Quality Goods Shop	Green Intelligent Power Saver	It didn't work. It didn't work for a month or so.	Jingdong prefecture level city in Shandong
Dragon Quality Goods Shop	Green Intelligent Power Saver	Garbage products, physical and description is not the same, no effect at all, pit # no discussion, to buy the fine, garbage, garbage, garbage	Jingdong prefecture level city in Shandong
Dragon Quality Goods Shop	Green Intelligent Power Saver	general	Jingdong prefecture level city in Shandong
Dragon Quality Goods Shop	Green Intelligent Power Saver	IQ tax. Instead of saving electricity, it increases the power consumption of an appliance.	Jingdong prefecture level city in Shandong
Dragon Quality Goods Shop	Green Intelligent Power Saver	There's no trash at all.	Jingdong prefecture level city in Shandong
Dragon Quality Goods Shop	Green Intelligent Power Saver	It doesn't do anything.	Jingdong prefecture level city in Shandong
Dragon Quality Goods Shop	Green Intelligent Power Saver	(of an unmarried couple) be close	Jingdong prefecture level city in Shandong
Dragon Quality Goods Shop	Green Intelligent Power Saver	(of an unmarried couple) be close	Jingdong prefecture level city in Shandong
Dragon Quality Goods Shop	Green Intelligent Power Saver	no electricity saving	Jingdong prefecture level city in Shandong
Dragon Quality Goods Shop	Green Intelligent Power Saver	differ from	Jingdong prefecture level city in Shandong
Dragon Quality Goods Shop	Green Intelligent Power Saver	Saver has received, but real can save electricity, the quality is very good, I times I buy a few more, too good.	Jingdong prefecture level city in Shandong
Dragon Quality Goods Shop	Green Intelligent Power Saver	Great quality as always, a very favorite product, great! o(*￣︶￣*)o	Jingdong prefecture level city in Shandong
Dragon Quality Goods Shop	Green Intelligent Power Saver	If we can reduce the electricity bill, for individuals can save money, for the country can protect the environment, now the weather is getting hotter and hotter, to protect the environment to reduce emissions is very important. Already use the product, whether to reduce the electricity bill depends on next month!	Jingdong prefecture level city in Shandong
Dragon Quality Goods Shop	Green Intelligent Power Saver	Purchased a power saver received on the put into use, rent a room with many electrical appliances, power consumption is very large, we have a piece of electricity or 1.2 dollars a time, with this power saver is much better, every day can save a few cents, very convenient and practical!	Jingdong prefecture level city in Shandong
Dragon Quality Goods Shop	Green Intelligent Power Saver	The product is better to use before not to use when a month 1500 yuan electricity bill, now using the 5 second month electricity bill directly down 470 dollars or very good. A save money to buy several power saver money ZanZanZanZan👍	Jingdong prefecture level city in Shandong
Dragon Quality Goods Shop	Green Intelligent Power Saver	The moment I received the goods really satisfied with this product, not only high-grade packaging, the body are base alloy, up to the price, I was also introduced by a friend, he used last month did save a lot of electricity, but also recommended to me!	Jingdong prefecture level city in Shandong
Dragon Quality Goods Shop	Green Intelligent Power Saver	I got it. Good review. I'm very satisfied. Reasonable price.	Jingdong prefecture level city in Shandong
Dragon Quality Goods Shop	Green Intelligent Power Saver	Intelligent power saver received and used, looking at the quality is good, the quality of the plug sent is also very good. Expect the effect of the economizer is obvious, first five-star praise, with a period of time to follow up!	Jingdong prefecture level city in Shandong
Dragon Quality Goods Shop	Green Intelligent Power Saver	The saver has been received. It was plugged into an outlet and used immediately after receipt. Whether it really saves electricity is not clear yet, I believe that this brand of energy saver really saves	Jingdong prefecture level city in Shandong
Dragon Quality Goods Shop	Green Intelligent Power Saver	Okay, okay, okay. Thank you so much. Fast is good. Fast. Item was as described, mainly because the event was cheap and you can save a lot of money.	Jingdong prefecture level city in Shandong
Dragon Quality Goods Shop	Green Intelligent Power Saver	Received it a couple days ago! Plugged it in the same day! Let's see what the electric bill is in a month! Hopefully it works well oh!	Jingdong prefecture level city in Shandong
Dragon Quality Goods Shop	Green Intelligent Power Saver	Looking at the reviews good, energy-saving small products, compact, the results after 2 months to see, because the electricity bill season payment	Jingdong prefecture level city in Shandong
Dragon Quality Goods Shop	Green Intelligent Power Saver	I just received it and will try it out to see if it works well. I'll comment later.	Jingdong prefecture level city in Shandong
Dragon Quality Goods Shop	Green Intelligent Power Saver	It looks a little small and not very aesthetically pleasing, I don't know how effective it will be until I use it	Jingdong prefecture level city in Shandong
Dragon Quality Goods Shop	Green Intelligent Power Saver	Not only to the point of being useless yet	Jingdong prefecture level city in Shandong
Dragon Quality Goods Shop	Green Intelligent Power Saver	Used it for a month, nothing works, the meter you turn very fast. This is not a scam, but also sold on Jingdong. To cheat a bunch of people?	Jingdong prefecture level city in Shandong
Dragon Quality Goods Shop	Green Intelligent Power Saver	Very good to use, every day can save a little electricity, really good under the sample down a month to save a few dozen dollars in electricity, good before I did not know that there is this 👆 haha, the price is not expensive praise.	Jingdong prefecture level city in Shandong
Dragon Quality Goods Shop	Green Intelligent Power Saver	Tried it for ten days and the change is almost nothing, can't see the power saving yet, use it some more days and see how it goes.	Jingdong prefecture level city in Shandong
Dragon Quality Goods Shop	Green Intelligent Power Saver	Received very quickly, the quality is very good, really save electricity, business service attitude is also very good, I hope you do not miss this opportunity, I continue to buy a few in	Jingdong prefecture level city in Shandong
Dragon Quality Goods Shop	Green Intelligent Power Saver	Quality is very good Oh, the delivery is also very fast, a very favorite store, I want to crazy recommend to friends!	Jingdong prefecture level city in Shandong
Dragon Quality Goods Shop	Green Intelligent Power Saver	The goods have just received, fine workmanship, and the same as described by the merchant, to use a period of time to see if it can achieve the purpose of power saving.	Jingdong prefecture level city in Shandong
Dragon Quality Goods Shop	Green Intelligent Power Saver	Received the goods first use a period of time to see if there is no effect, the business commitment to ninety days invalid refund, first praise, good service!	Jingdong prefecture level city in Shandong
Dragon Quality Goods Shop	Green Intelligent Power Saver	The product is used for a month without energy-saving and power-saving effect does not have any effect	Jingdong prefecture level city in Shandong
Dragon Quality Goods Shop	Green Intelligent Power Saver	The baby is really great, I like it very much, my friends also say good to come, recommended to buy!	Jingdong prefecture level city in Shandong
Dragon Quality Goods Shop	Green Intelligent Power Saver	Logistics is fast, courier boy service is good, after using to see how the fruit, and then follow up.	Jingdong prefecture level city in Shandong
Dragon Quality Goods Shop	Green Intelligent Power Saver	Baby has been received to try, I believe that as the business advertised energy saving. The courier is very responsible.	Jingdong prefecture level city in Shandong
Dragon Quality Goods Shop	Green Intelligent Power Saver	Save Electric Energy King received, good quality, beautiful appearance, very satisfied!	Jingdong prefecture level city in Shandong
Dragon Quality Goods Shop	Green Intelligent Power Saver	Three no products no manufacturers no phone pure fraud induced consumption cheat	Jingdong prefecture level city in Shandong
Dragon Quality Goods Shop	Green Intelligent Power Saver	I'm using it now, I don't know if it's as good as it says, I'll follow up after a month	Jingdong prefecture level city in Shandong
Dragon Quality Goods Shop	Green Intelligent Power Saver	Good quality logistics and fast. Fine workmanship. The description is consistent cost-effective	Jingdong prefecture level city in Shandong
Dragon Quality Goods Shop	Green Intelligent Power Saver	Received, whether it is good or not, to wait a few days and then evaluate	Jingdong prefecture level city in Shandong
Dragon Quality Goods Shop	Green Intelligent Power Saver	I bought it and started using it, I won't know if it works well until I pay my electric bill	Jingdong prefecture level city in Shandong
Dragon Quality Goods Shop	Green Intelligent Power Saver	Especially good, energy saving and power saving, especially good quality, good	Jingdong prefecture level city in Shandong
Dragon Quality Goods Shop	Green Intelligent Power Saver	Logistics is strong, the quality is very good, paste really save electricity, as described, will buy back!	Jingdong prefecture level city in Shandong
Dragon Quality Goods Shop	Green Intelligent Power Saver	Great stuff at a great price, worth the money!	Jingdong prefecture level city in Shandong

Dragon Quality Goods Shop	Green Intelligent Power Saver	Received the power saver, very good quality, very power saving!	Jingdong prefecture level city in Shandong
Dragon Quality Goods Shop	Green Intelligent Power Saver	There are no fruits, only samples...	Jingdong prefecture level city in Shandong
Dragon Quality Goods Shop	Green Intelligent Power Saver	Save a few dollars a month.	Jingdong prefecture level city in Shandong
Dragon Quality Goods Shop	Green Intelligent Power Saver	The quality is particularly good, it works well, and the seller has a good service attitude	Jingdong prefecture level city in Shandong
Dragon Quality Goods Shop	Green Intelligent Power Saver	I've compared them a couple of times and I don't feel any difference	Jingdong prefecture level city in Shandong
Dragon Quality Goods Shop	Green Intelligent Power Saver	I don't see the savings. It's a sham.	Jingdong prefecture level city in Shandong
Dragon Quality Goods Shop	Green Intelligent Power Saver	The power saving effect is very good, and the logistics is very fast. The seller's service attitude is very good	Jingdong prefecture level city in Shandong
Dragon Quality Goods Shop	Green Intelligent Power Saver	Received, well packaged, haven't tried it yet!	Jingdong prefecture level city in Shandong
Dragon Quality Goods Shop	Green Intelligent Power Saver	I don't know how it works, I'll comment after I use it.	Jingdong prefecture level city in Shandong
Dragon Quality Goods Shop	Green Intelligent Power Saver	The product doesn't work well enough to be of any use	Jingdong prefecture level city in Shandong
Dragon Quality Goods Shop	Green Intelligent Power Saver	On Trial 。。。。。。。	Jingdong prefecture level city in Shandong
Dragon Quality Goods Shop	Green Intelligent Power Saver	Being used with unknown results	Jingdong prefecture level city in Shandong
Dragon Quality Goods Shop	Green Intelligent Power Saver	Smart power saving and great quality	Jingdong prefecture level city in Shandong
Dragon Quality Goods Shop	Green Intelligent Power Saver	The saver was received, it works very well and the price is affordable!	Jingdong prefecture level city in Shandong
Dragon Quality Goods Shop	Green Intelligent Power Saver	How can I feel the power saving and how long does it last	Jingdong prefecture level city in Shandong
Dragon Quality Goods Shop	Green Intelligent Power Saver	And I know if it saves electricity or not.	Jingdong prefecture level city in Shandong
Dragon Quality Goods Shop	Green Intelligent Power Saver	It won't save any electricity. No.	Jingdong prefecture level city in Shandong
Dragon Quality Goods Shop	Green Intelligent Power Saver	Invalid. Fake.	Jingdong prefecture level city in Shandong
Dragon Quality Goods Shop	Green Intelligent Power Saver	It's good!	Jingdong prefecture level city in Shandong
Dragon Quality Goods Shop	Green Intelligent Power Saver	have no effect	Jingdong prefecture level city in Shandong
Dragon Quality Goods Shop	Green Intelligent Power Saver	Not used.	Jingdong prefecture level city in Shandong
Dragon Quality Goods Shop	Green Intelligent Power Saver	There is no load range, packaging thrift, ten days also did not develop invoices, received the goods with a receipt, hand-written receipt also has no financial stamp, communication for nearly a week a moment to say that the electronic invoice, a moment to say that the paper invoice, said it is mailed to me, ten days also did not see anything, the invoice invoice can not be found to say that did not stay on file, single single number can not be found, said that the warehouse more than one to a one to find out, in addition to the perfunctory or perfunctory. Products are no certificate of conformity, proper three	Jingdong prefecture level city in Shandong
Dragon Quality Goods Shop	Green Intelligent Power Saver	Satisfaction.	Jingdong prefecture level city in Shandong
Dragon Quality Goods Shop	Green Intelligent Power Saver	There is not a bit of power saving effect, and ringing up an increase in the electricity bill, because you have to add the cost of this machine's own electricity consumption and purchase, so I think it is better to use this machine instead of increasing the expenditure, uneconomical, rather than not use it.	Jingdong prefecture level city in Shandong
Dragon Quality Goods Shop	Green Intelligent Power Saver	I've been using it for two days now and I don't feel any effect.	Jingdong prefecture level city in Shandong
Dragon Quality Goods Shop	Green Intelligent Power Saver	What it looked like was nothing like what was sent.	Jingdong prefecture level city in Shandong
Dragon Quality Goods Shop	Green Intelligent Power Saver	It didn't work. It didn't work for a month or so.	Jingdong prefecture level city in Shandong
Dragon Quality Goods Shop	Green Intelligent Power Saver	Garbage products, physical and description is not the same, no effect at all, pit # no discussion, to buy the fine, garbage, garbage, garbage	Jingdong prefecture level city in Shandong
Dragon Quality Goods Shop	Green Intelligent Power Saver	general	Jingdong prefecture level city in Shandong
Dragon Quality Goods Shop	Green Intelligent Power Saver	IQ tax. Instead of saving electricity, it increases the power consumption of an appliance.	Jingdong prefecture level city in Shandong
Dragon Quality Goods Shop	Green Intelligent Power Saver	There's no trash at all.	Jingdong prefecture level city in Shandong
Dragon Quality Goods Shop	Green Intelligent Power Saver	It doesn't do anything.	Jingdong prefecture level city in Shandong
Dragon Quality Goods Shop	Green Intelligent Power Saver	(of an unmarried couple) be close	Jingdong prefecture level city in Shandong
Dragon Quality Goods Shop	Green Intelligent Power Saver	(of an unmarried couple) be close	Jingdong prefecture level city in Shandong
Dragon Quality Goods Shop	Green Intelligent Power Saver	no electricity saving	Jingdong prefecture level city in Shandong
Dragon Quality Goods Shop	Green Intelligent Power Saver	differ from	Jingdong prefecture level city in Shandong
Dragon Quality Goods Shop	Green Intelligent Power Saver	After the return of the power saver, then I used 48 hours, and then looked at the meter went two degrees of electricity and then still open the air conditioning to open the air conditioning for the night, and then there is cooking, I did not expect so much power saving, and then I bought two more directly thanks	Jingdong prefecture level city in Shandong
Dragon Quality Goods Shop	Green Intelligent Power Saver	Power Saver is just fine, save money and power by buying Power Saver King everyone 👍👍👍👍	Jingdong prefecture level city in Shandong
Dragon Quality Goods Shop	White Intelligent Power Saver	not have	Jingdong prefecture level city in Shandong
Dragon Quality Goods Shop	In-situ compensated economizer	not have	Jingdong prefecture level city in Shandong
Dragon Quality Goods Shop	King of Power Saving	Indeed very effective, because now are cell phone **** payment,, summer air conditioning with a month of 300, we use this energy-saving products is about 28 days less than 200 yuan of electricity, it is indeed save a lot, this time and to the old man bought two. Also recommended their relatives do business with a	Jingdong prefecture level city in Shandong
Dragon Quality Goods Shop	King of Power Saving	The power saving effect is very good: my supermarket monthly electricity bill is more than 7000 yuan. On May 1st someone recommended buying a couple of power savers, and I bought 5 of them first. The electricity bill was about 2000 less in June. This time, I bought 5 more. The seller said 800 degrees of electricity with one. I feel it's basically enough. A total of 10 units	Jingdong prefecture level city in Shandong
Dragon Quality Goods Shop	King of Power Saving	The effect is too good, the family has now several units, back to buy a number of times, the power saver received, power-saving effect is very good, fine workmanship, long warranty, but also purify the air, before buying a used for a period of time, with the okay, and then buy a, only now to chase the level,	Jingdong prefecture level city in Shandong
Dragon Quality Goods Shop	King of Power Saving	Save more than 500 this month, open the clothing store power consumption is still quite large, this power saver is new, intelligent upgrade, very good, than I bought before the high-grade much, and the quality is also good, the business also guaranteed me a warranty of five years, you can rest assured that use!	Jingdong prefecture level city in Shandong
Dragon Quality Goods Shop	King of Power Saving	Simple and convenient to use, good power saving effect, can save a lot of electricity	Jingdong prefecture level city in Shandong

Dragon Quality Goods Shop	King of Power Saving	There are a lot of big appliances at home, the air conditioner is very expensive in summer, I bought this in front of me, the effect on the air conditioner is very good, let me explain, I bought three in one breath, I use more electricity, a month down about 200 dollars less, very satisfied	Jingdong prefecture level city in Shandong
Dragon Quality Goods Shop	King of Power Saving	There are a lot of appliances in the house, the summer air conditioning is very costly, I bought this in front of the air conditioning effect is very good, I explain that I bought three in one breath, I use more electricity a month down to 200 or so, very satisfied.	Jingdong prefecture level city in Shandong
Dragon Quality Goods Shop	King of Power Saving	Today's summer open air conditioning is not afraid of, saving electricity in the immediate future, I now open the air conditioning is very costly, almost 700 yuan a month, I was introduced by a friend to buy, although it is only used for more than 20 days, really can be a lot of money less, quite good Oh!	Jingdong prefecture level city in Shandong
Dragon Quality Goods Shop	King of Power Saving	I bought this and now it works pretty well with the air conditioning on, when I didn't use it it was $600 a month, now it's almost $500,00, that's over $100 less all of a sudden.	Jingdong prefecture level city in Shandong
Dragon Quality Goods Shop	King of Power Saving	Can Oh, the weather is hot, open the air conditioning is really good, this product is good, has been used for a really long time, more ok, power saving effect is not bad.	Jingdong prefecture level city in Shandong
Dragon Quality Goods Shop	King of Power Saving	I paid my electric bill today, it's a few dozen dollars less, anyway, it's not costly, it's already very good, the boss said that it would be better to buy one more, I've already placed an order	Jingdong prefecture level city in Shandong
Dragon Quality Goods Shop	King of Power Saving	I'm on a smart meter, I save about 30% a month, personally I think it's pretty good, it works well	Jingdong prefecture level city in Shandong
Dragon Quality Goods Shop	King of Power Saving	My dad asked me to buy one, but after I paid the bill, I asked and he said it was a lot less this month, so he asked me to buy another one.	Jingdong prefecture level city in Shandong
Dragon Quality Goods Shop	King of Power Saving	Very satisfied with the purchase thanks to the store for the prosperous business.	Jingdong prefecture level city in Shandong
Dragon Quality Goods Shop	King of Power Saving	Good use, good quality, very satisfied with the purchase	Jingdong prefecture level city in Shandong
Dragon Quality Goods Shop	King of Power Saving	The goods have been received, the quality is very good, is the ideal effect, will continue to pay attention to the	Jingdong prefecture level city in Shandong
Dragon Quality Goods Shop	King of Power Saving	This brand has been used for many years, and this time I saw this upgraded version of the product and got it again. The purpose of this product is to reduce the loss of electricity for a long time, so as to achieve the purpose of saving electricity.	Jingdong prefecture level city in Shandong
Dragon Quality Goods Shop	King of Power Saving	I've been using it for almost a month, and I've reduced my electricity bill by over 100 bucks! I'm planning to get two more for my family.	Jingdong prefecture level city in Shandong
Dragon Quality Goods Shop	King of Power Saving	The effect is too good, the family has now several units, back to buy a number of times, the power saver received, power-saving effect is very good,, fine workmanship, long warranty, but also purify the air, before buying a used for a period of time, with the okay, and then bought a, and only now come to the	Jingdong prefecture level city in Shandong
Dragon Quality Goods Shop	King of Power Saving	Finally received the goods, very, very much like,, has been a lot of people praise, really too happy, has been his family's loyal fans, will continue to buy buy buy buy!	Jingdong prefecture level city in Shandong
Dragon Quality Goods Shop	King of Power Saving	📩 I've been wanting it for a long time, I decided to go for it, I feel great after using it, it's genuine and the delivery is great, it lives up to its name👍	Jingdong prefecture level city in Shandong
Dragon Quality Goods Shop	King of Power Saving	The quality is sure as other friends who bought it said, and it is also beautiful, thanks to the owner for the recommendation, thanks!	Jingdong prefecture level city in Shandong
Dragon Quality Goods Shop	King of Power Saving	Baby received the quality is really good face value is also very high especially practical especially especially special favorite	Jingdong prefecture level city in Shandong
Dragon Quality Goods Shop	King of Power Saving	It is genuine, trust the merchant, the quality is very good, the price is moderate, the use of the effect is very good!	Jingdong prefecture level city in Shandong
Dragon Quality Goods Shop	King of Power Saving	Energy saver received really good quality affordable and good energy saving and environmental protection is the best to use.	Jingdong prefecture level city in Shandong
Dragon Quality Goods Shop	King of Power Saving	Reasonable price logistics is very fast, the feeling is not bad more satisfied with a shopping experience	Jingdong prefecture level city in Shandong
Dragon Quality Goods Shop	King of Power Saving	The stuff is even more perfect than I imagined, very powerful and worth getting!	Jingdong prefecture level city in Shandong
Dragon Quality Goods Shop	King of Power Saving	Goods have been received, good quality, affordable price, good customer service, worth recommending!	Jingdong prefecture level city in Shandong
Dragon Quality Goods Shop	King of Power Saving	Fast shipping, works great, have already recommended it to friends.	Jingdong prefecture level city in Shandong
Dragon Quality Goods Shop	King of Power Saving	Good quality, fast logistics, reasonable price, worth 5 stars.	Jingdong prefecture level city in Shandong
Dragon Quality Goods Shop	King of Power Saving	Good quality, fast logistics, good value, well packaged, practical	Jingdong prefecture level city in Shandong
Dragon Quality Goods Shop	King of Power Saving	Fast shipping, affordable price, great quality, very satisfied!	Jingdong prefecture level city in Shandong
Dragon Quality Goods Shop	King of Power Saving	Regret knowing too late, it's really good stuff. Works well.	Jingdong prefecture level city in Shandong
Dragon Quality Goods Shop	King of Power Saving	Not bad, very good product, quite save electricity	Jingdong prefecture level city in Shandong
Dragon Quality Goods Shop	King of Power Saving	Good quality, fast logistics, overall very satisfied!	Jingdong prefecture level city in Shandong
Dragon Quality Goods Shop	King of Power Saving	Tightly packaged with good results	Jingdong prefecture level city in Shandong
Dragon Quality Goods Shop	King of Power Saving	very happy	Jingdong prefecture level city in Shandong
Dragon Quality Goods Shop	King of Power Saving	favorable criticism	Jingdong prefecture level city in Shandong
Dragon Quality Goods Shop	King of Power Saving	Now they all sign for the goods without receiving them, the Jingdong platform and merchants are very powerful, and the goods are not received before signing for the goods.	Jingdong prefecture level city in Shandong
Dragon Quality Goods Shop	King of Power Saving	low-quality commodities	Jingdong prefecture level city in Shandong
Dragon Quality Goods Shop	King of Power Saving	I bought this and now it works pretty well with the air conditioning on, when I didn't use it it was $600 a month, now it's almost $500,00, that's over $100 less all of a sudden.	Jingdong prefecture level city in Shandong
Dragon Quality Goods Shop	King of Power Saving	Can Oh, the weather is hot, open the air conditioning is really good, this product is good, has been used for a really long time, more ok, power saving effect is not bad.	Jingdong prefecture level city in Shandong
Dragon Quality Goods Shop	King of Power Saving	I paid my electric bill today, it's a few dozen dollars less, anyway, it's not costly, it's already very good, the boss said that it would be better to buy one more, I've already placed an order	Jingdong prefecture level city in Shandong
Dragon Quality Goods Shop	King of Power Saving	I'm on a smart meter, I save about 30% a month, personally I think it's pretty good, it works well	Jingdong prefecture level city in Shandong
Dragon Quality Goods Shop	King of Power Saving	My dad asked me to buy one, but after I paid the bill, I asked and he said it was a lot less this month, so he asked me to buy another one.	Jingdong prefecture level city in Shandong
Dragon Quality Goods Shop	King of Power Saving	Very satisfied with the purchase thanks to the store for the prosperous business.	Jingdong prefecture level city in Shandong
Dragon Quality Goods Shop	King of Power Saving	Good use, good quality, very satisfied with the purchase	Jingdong prefecture level city in Shandong
Dragon Quality Goods Shop	King of Power Saving	The goods have been received, the quality is very good, is the ideal effect, will continue to pay attention to the	Jingdong prefecture level city in Shandong
Dragon Quality Goods Shop	King of Power Saving	This brand has been used for many years, and this time I saw this upgraded version of the product and got it again. The purpose of this product is to reduce the loss of electricity for a long time, so as to achieve the purpose of saving electricity.	Jingdong prefecture level city in Shandong
Dragon Quality Goods Shop	King of Power Saving	I've been using it for almost a month, and I've reduced my electricity bill by over 100 bucks! I'm planning to get two more for my family.	Jingdong prefecture level city in Shandong
Dragon Quality Goods Shop	King of Power Saving	The effect is too good, the family has now several units, back to buy a number of times, the power saver received, power-saving effect is very good,, fine workmanship, long warranty, but also purify the air, before buying a used for a period of time, with the okay, and then bought a, and only now come to the	Jingdong prefecture level city in Shandong
Dragon Quality Goods Shop	King of Power Saving	Finally received the goods, very, very much like,, has been a lot of people praise, really too happy, has been his family's loyal fans, will continue to buy buy buy buy!	Jingdong prefecture level city in Shandong
Dragon Quality Goods Shop	King of Power Saving	📩 I've been wanting it for a long time, I decided to go for it, I feel great after using it, it's genuine and the delivery is great, it lives up to its name👍	Jingdong prefecture level city in Shandong

Dragon Quality Goods Shop	King of Power Saving	The quality is sure as other friends who bought it said, and it is also beautiful, thanks to the owner for the recommendation, thanks!	Jingdong prefecture level city in Shandong
Dragon Quality Goods Shop	King of Power Saving	Baby received the quality is really good face value is also very high especially practical especially especially special favorite	Jingdong prefecture level city in Shandong
Dragon Quality Goods Shop	King of Power Saving	It is genuine, trust the merchant, the quality is very good, the price is moderate, the use of the effect is very good!	Jingdong prefecture level city in Shandong
Dragon Quality Goods Shop	King of Power Saving	Energy saver received really good quality affordable and good energy saving and environmental protection is the best to use.	Jingdong prefecture level city in Shandong
Dragon Quality Goods Shop	King of Power Saving	Reasonable price logistics is very fast, the feeling is not bad more satisfied with a shopping experience	Jingdong prefecture level city in Shandong
Dragon Quality Goods Shop	King of Power Saving	The stuff is even more perfect than I imagined, very powerful and worth getting!	Jingdong prefecture level city in Shandong
Dragon Quality Goods Shop	King of Power Saving	Goods have been received, good quality, affordable price, good customer service, worth recommending!	Jingdong prefecture level city in Shandong
Dragon Quality Goods Shop	King of Power Saving	Fast shipping, works great, have already recommended it to friends.	Jingdong prefecture level city in Shandong
Dragon Quality Goods Shop	King of Power Saving	Good quality, fast logistics, reasonable price, worth 5 stars.	Jingdong prefecture level city in Shandong
Dragon Quality Goods Shop	King of Power Saving	Good quality, fast logistics, good value, well packaged, practical	Jingdong prefecture level city in Shandong
Dragon Quality Goods Shop	King of Power Saving	Fast shipping, affordable price, great quality, very satisfied!	Jingdong prefecture level city in Shandong
Dragon Quality Goods Shop	King of Power Saving	Regret knowing too late, it's really good stuff. Works well.	Jingdong prefecture level city in Shandong
Dragon Quality Goods Shop	King of Power Saving	Not bad, very good product, quite save electricity	Jingdong prefecture level city in Shandong
Dragon Quality Goods Shop	King of Power Saving	Good quality, fast logistics, overall very satisfied!	Jingdong prefecture level city in Shandong
Dragon Quality Goods Shop	King of Power Saving	Tightly packaged with good results	Jingdong prefecture level city in Shandong
Dragon Quality Goods Shop	King of Power Saving	very happy	Jingdong prefecture level city in Shandong
Dragon Quality Goods Shop	King of Power Saving	favorable criticism	Jingdong prefecture level city in Shandong
Dragon Quality Goods Shop	King of Power Saving	Now they all sign for the goods without receiving them, the Jingdong platform and merchants are very powerful, and the goods are not received before signing for the goods.	Jingdong prefecture level city in Shandong
Dragon Quality Goods Shop	King of Power Saving	low-quality commodities	Jingdong prefecture level city in Shandong
Dragon Quality Goods Shop	King of Power Saving	I bought this and now it works pretty well with the air conditioning on, when I didn't use it it was $600 a month, now it's almost $500.00, that's over $100 less all of a sudden.	Jingdong prefecture level city in Shandong
Dragon Quality Goods Shop	King of Power Saving	Can Oh, the weather is hot, open the air conditioning is really good, this product is good, has been used for a really long time, more ok, power saving effect is not bad.	Jingdong prefecture level city in Shandong
Dragon Quality Goods Shop	King of Power Saving	I paid my electric bill today, it's a few dozen dollars less, anyway, it's not costly, it's already very good, the boss said that it would be better to buy one more, I've already placed an order	Jingdong prefecture level city in Shandong
Dragon Quality Goods Shop	King of Power Saving	I'm on a smart meter, I save about 30% a month, personally I think it's pretty good, it works well	Jingdong prefecture level city in Shandong
Dragon Quality Goods Shop	King of Power Saving	My dad asked me to buy one, but after I paid the bill, I asked and he said it was a lot less this month, so he asked me to buy another one.	Jingdong prefecture level city in Shandong
Dragon Quality Goods Shop	King of Power Saving	Very satisfied with the purchase thanks to the store for the prosperous business.	Jingdong prefecture level city in Shandong
Dragon Quality Goods Shop	King of Power Saving	Good use, good quality, very satisfied with the purchase	Jingdong prefecture level city in Shandong
Dragon Quality Goods Shop	King of Power Saving	The goods have been received, the quality is very good, is the ideal effect, will continue to pay attention to the	Jingdong prefecture level city in Shandong
Dragon Quality Goods Shop	King of Power Saving	This brand has been used for many years, and this time I saw this upgraded version of the product and got it again. The purpose of this product is to reduce the loss of electricity for a long time, so as to achieve the purpose of saving electricity.	Jingdong prefecture level city in Shandong
Dragon Quality Goods Shop	King of Power Saving	I've been using it for almost a month, and I've reduced my electricity bill by over 100 bucks! I'm planning to get two more for my family.	Jingdong prefecture level city in Shandong
Dragon Quality Goods Shop	King of Power Saving	The effect is too good, the family has now several units, back to buy a number of times, the power saver received, power-saving effect is very good,, fine workmanship, long warranty, but also purify the air, before buying a used for a period of time, with the okay, and then bought a, and only now come to the	Jingdong prefecture level city in Shandong
Dragon Quality Goods Shop	King of Power Saving	Finally received the goods, very, very much like,, has been a lot of people praise, really too happy, has been his family's loyal fans, will continue to buy buy buy buy!	Jingdong prefecture level city in Shandong
Dragon Quality Goods Shop	King of Power Saving	📩 I've been wanting it for a long time, I decided to go for it, I feel great after using it, it's genuine and the delivery is great, it lives up to its name👍	Jingdong prefecture level city in Shandong
Dragon Quality Goods Shop	King of Power Saving	The quality is sure as other friends who bought it said, and it is also beautiful, thanks to the owner for the recommendation, thanks!	Jingdong prefecture level city in Shandong
Dragon Quality Goods Shop	King of Power Saving	Baby received the quality is really good face value is also very high especially practical especially especially special favorite	Jingdong prefecture level city in Shandong
Dragon Quality Goods Shop	King of Power Saving	It is genuine, trust the merchant, the quality is very good, the price is moderate, the use of the effect is very good!	Jingdong prefecture level city in Shandong
Dragon Quality Goods Shop	King of Power Saving	Energy saver received really good quality affordable and good energy saving and environmental protection is the best to use.	Jingdong prefecture level city in Shandong
Dragon Quality Goods Shop	King of Power Saving	Reasonable price logistics is very fast, the feeling is not bad more satisfied with a shopping experience	Jingdong prefecture level city in Shandong
Dragon Quality Goods Shop	King of Power Saving	The stuff is even more perfect than I imagined, very powerful and worth getting!	Jingdong prefecture level city in Shandong
Dragon Quality Goods Shop	King of Power Saving	Goods have been received, good quality, affordable price, good customer service, worth recommending!	Jingdong prefecture level city in Shandong
Dragon Quality Goods Shop	King of Power Saving	Fast shipping, works great, have already recommended it to friends.	Jingdong prefecture level city in Shandong
Dragon Quality Goods Shop	King of Power Saving	Good quality, fast logistics, reasonable price, worth 5 stars.	Jingdong prefecture level city in Shandong
Dragon Quality Goods Shop	King of Power Saving	Good quality, fast logistics, good value, well packaged, practical	Jingdong prefecture level city in Shandong
Dragon Quality Goods Shop	King of Power Saving	Fast shipping, affordable price, great quality, very satisfied!	Jingdong prefecture level city in Shandong
Dragon Quality Goods Shop	King of Power Saving	Regret knowing too late, it's really good stuff. Works well.	Jingdong prefecture level city in Shandong
Dragon Quality Goods Shop	King of Power Saving	Not bad, very good product, quite save electricity	Jingdong prefecture level city in Shandong
Dragon Quality Goods Shop	King of Power Saving	Good quality, fast logistics, overall very satisfied!	Jingdong prefecture level city in Shandong
Dragon Quality Goods Shop	King of Power Saving	Tightly packaged with good results	Jingdong prefecture level city in Shandong
Dragon Quality Goods Shop	King of Power Saving	very happy	Jingdong prefecture level city in Shandong
Dragon Quality Goods Shop	King of Power Saving	favorable criticism	Jingdong prefecture level city in Shandong
Dragon Quality Goods Shop	King of Power Saving	Now they all sign for the goods without receiving them, the Jingdong platform and merchants are very powerful, and the goods are not received before signing for the goods.	Jingdong prefecture level city in Shandong

Dragon Quality Goods Shop	King of Power Saving	low-quality commodities	Jingdong prefecture level city in Shandong
Dragon Quality Goods Shop	King of Power Saving	I bought this and now it works pretty well with the air conditioning on, when I didn't use it it was $600 a month, now it's almost $500,00, that's over $100 less all of a sudden.	Jingdong prefecture level city in Shandong
Dragon Quality Goods Shop	King of Power Saving	Can Oh, the weather is hot, open the air conditioning is really good, this product is good, has been used for a really long time, more ok, power saving effect is not bad.	Jingdong prefecture level city in Shandong
Dragon Quality Goods Shop	King of Power Saving	I paid my electric bill today, it's a few dozen dollars less, anyway, it's not costly, it's already very good, the boss said that it would be better to buy one more, I've already placed an order	Jingdong prefecture level city in Shandong
Dragon Quality Goods Shop	King of Power Saving	I'm on a smart meter, I save about 30% a month, personally I think it's pretty good, it works well	Jingdong prefecture level city in Shandong
Dragon Quality Goods Shop	King of Power Saving	My dad asked me to buy one, but after I paid the bill, I asked and he said it was a lot less this month, so he asked me to buy another one.	Jingdong prefecture level city in Shandong
Dragon Quality Goods Shop	King of Power Saving	Very satisfied with the purchase thanks to the store for the prosperous business.	Jingdong prefecture level city in Shandong
Dragon Quality Goods Shop	King of Power Saving	Good use, good quality, very satisfied with the purchase	Jingdong prefecture level city in Shandong
Dragon Quality Goods Shop	King of Power Saving	The goods have been received, the quality is very good, is the ideal effect, will continue to pay attention to the	Jingdong prefecture level city in Shandong
Dragon Quality Goods Shop	King of Power Saving	This brand has been used for many years, and this time I saw this upgraded version of the product and got it again. The purpose of this product is to reduce the loss of electricity for a long time, so as to achieve the purpose of saving electricity.	Jingdong prefecture level city in Shandong
Dragon Quality Goods Shop	King of Power Saving	I've been using it for almost a month, and I've reduced my electricity bill by over 100 bucks! I'm planning to get two more for my family.	Jingdong prefecture level city in Shandong
Dragon Quality Goods Shop	King of Power Saving	The effect is too good, the family has now several units, back to buy a number of times, the power saver received, power-saving effect is very good,, fine workmanship, long warranty, but also purify the air, before buying a used for a period of time, with the okay, and then bought a, and only now come to the	Jingdong prefecture level city in Shandong
Dragon Quality Goods Shop	King of Power Saving	Finally received the goods, very, very much like,, has been a lot of people praise, really too happy, has been his family's loyal fans, will continue to buy buy buy buy!	Jingdong prefecture level city in Shandong
Dragon Quality Goods Shop	King of Power Saving	📩 I've been wanting it for a long time, I decided to go for it, I feel great after using it, it's genuine and the delivery is great, it lives up to its name👍	Jingdong prefecture level city in Shandong
Dragon Quality Goods Shop	King of Power Saving	The quality is sure as other friends who bought it said, and it is also beautiful, thanks to the owner for the recommendation, thanks!	Jingdong prefecture level city in Shandong
Dragon Quality Goods Shop	King of Power Saving	Baby received the quality is really good face value is also very high especially practical especially especially special favorite	Jingdong prefecture level city in Shandong
Dragon Quality Goods Shop	King of Power Saving	It is genuine, trust the merchant, the quality is very good, the price is moderate, the use of the effect is very good!	Jingdong prefecture level city in Shandong
Dragon Quality Goods Shop	King of Power Saving	Energy saver received really good quality affordable and good energy saving and environmental protection is the best to use.	Jingdong prefecture level city in Shandong
Dragon Quality Goods Shop	King of Power Saving	Reasonable price logistics is very fast, the feeling is not bad more satisfied with a shopping experience	Jingdong prefecture level city in Shandong
Dragon Quality Goods Shop	King of Power Saving	The stuff is even more perfect than I imagined, very powerful and worth getting!	Jingdong prefecture level city in Shandong
Dragon Quality Goods Shop	King of Power Saving	Goods have been received, good quality, affordable price, good customer service, worth recommending!	Jingdong prefecture level city in Shandong
Dragon Quality Goods Shop	King of Power Saving	Fast shipping, works great, have already recommended it to friends.	Jingdong prefecture level city in Shandong
Dragon Quality Goods Shop	King of Power Saving	Good quality, fast logistics, reasonable price, worth 5 stars.	Jingdong prefecture level city in Shandong
Dragon Quality Goods Shop	King of Power Saving	Good quality, fast logistics, good value, well packaged, practical	Jingdong prefecture level city in Shandong
Dragon Quality Goods Shop	King of Power Saving	Fast shipping, affordable price, great quality, very satisfied!	Jingdong prefecture level city in Shandong
Dragon Quality Goods Shop	King of Power Saving	Regret knowing too late, it's really good stuff. Works well.	Jingdong prefecture level city in Shandong
Dragon Quality Goods Shop	King of Power Saving	Not bad, very good product, quite save electricity	Jingdong prefecture level city in Shandong
Dragon Quality Goods Shop	King of Power Saving	Good quality, fast logistics, overall very satisfied!	Jingdong prefecture level city in Shandong
Dragon Quality Goods Shop	King of Power Saving	Tightly packaged with good results	Jingdong prefecture level city in Shandong
Dragon Quality Goods Shop	King of Power Saving	very happy	Jingdong prefecture level city in Shandong
Dragon Quality Goods Shop	King of Power Saving	favorable criticism	Jingdong prefecture level city in Shandong
Dragon Quality Goods Shop	King of Power Saving	Now they all sign for the goods without receiving them, the Jingdong platform and merchants are very powerful, and the goods are not received before signing for the goods.	Jingdong prefecture level city in Shandong
Dragon Quality Goods Shop	King of Power Saving	low-quality commodities	Jingdong prefecture level city in Shandong
Dragon Quality Goods Shop	King of Power Saving	I bought this and now it works pretty well with the air conditioning on, when I didn't use it it was $600 a month, now it's almost $500,00, that's over $100 less all of a sudden.	Jingdong prefecture level city in Shandong
Dragon Quality Goods Shop	King of Power Saving	Can Oh, the weather is hot, open the air conditioning is really good, this product is good, has been used for a really long time, more ok, power saving effect is not bad.	Jingdong prefecture level city in Shandong
Dragon Quality Goods Shop	King of Power Saving	I paid my electric bill today, it's a few dozen dollars less, anyway, it's not costly, it's already very good, the boss said that it would be better to buy one more, I've already placed an order	Jingdong prefecture level city in Shandong
Dragon Quality Goods Shop	King of Power Saving	I'm on a smart meter, I save about 30% a month, personally I think it's pretty good, it works well	Jingdong prefecture level city in Shandong
Dragon Quality Goods Shop	King of Power Saving	My dad asked me to buy one, but after I paid the bill, I asked and he said it was a lot less this month, so he asked me to buy another one.	Jingdong prefecture level city in Shandong
Dragon Quality Goods Shop	King of Power Saving	Very satisfied with the purchase thanks to the store for the prosperous business.	Jingdong prefecture level city in Shandong
Dragon Quality Goods Shop	King of Power Saving	Good use, good quality, very satisfied with the purchase	Jingdong prefecture level city in Shandong
Dragon Quality Goods Shop	King of Power Saving	The goods have been received, the quality is very good, is the ideal effect, will continue to pay attention to the	Jingdong prefecture level city in Shandong
Dragon Quality Goods Shop	King of Power Saving	This brand has been used for many years, and this time I saw this upgraded version of the product and got it again. The purpose of this product is to reduce the loss of electricity for a long time, so as to achieve the purpose of saving electricity.	Jingdong prefecture level city in Shandong
Dragon Quality Goods Shop	King of Power Saving	I've been using it for almost a month, and I've reduced my electricity bill by over 100 bucks! I'm planning to get two more for my family.	Jingdong prefecture level city in Shandong
Dragon Quality Goods Shop	King of Power Saving	The effect is too good, the family has now several units, back to buy a number of times, the power saver received, power-saving effect is very good,, fine workmanship, long warranty, but also purify the air, before buying a used for a period of time, with the okay, and then bought a, and only now come to the	Jingdong prefecture level city in Shandong
Dragon Quality Goods Shop	King of Power Saving	Finally received the goods, very, very much like,, has been a lot of people praise, really too happy, has been his family's loyal fans, will continue to buy buy buy buy!	Jingdong prefecture level city in Shandong
Dragon Quality Goods Shop	King of Power Saving	📩 I've been wanting it for a long time, I decided to go for it, I feel great after using it, it's genuine and the delivery is great, it lives up to its name👍	Jingdong prefecture level city in Shandong
Dragon Quality Goods Shop	King of Power Saving	The quality is sure as other friends who bought it said, and it is also beautiful, thanks to the owner for the recommendation, thanks!	Jingdong prefecture level city in Shandong
Dragon Quality Goods Shop	King of Power Saving	Baby received the quality is really good face value is also very high especially practical especially especially special favorite	Jingdong prefecture level city in Shandong
Dragon Quality Goods Shop	King of Power Saving	It is genuine, trust the merchant, the quality is very good, the price is moderate, the use of the effect is very good!	Jingdong prefecture level city in Shandong

Dragon Quality Goods Shop	King of Power Saving	Energy saver received really good quality affordable and good energy saving and environmental protection is the best to use.	Jingdong prefecture level city in Shandong
Dragon Quality Goods Shop	King of Power Saving	The stuff is even more perfect than I imagined, very powerful and worth getting!	Jingdong prefecture level city in Shandong
Dragon Quality Goods Shop	King of Power Saving	Reasonable price logistics is very fast, the feeling is not bad more satisfied with a shopping experience	Jingdong prefecture level city in Shandong
Dragon Quality Goods Shop	King of Power Saving	Goods have been received, good quality, affordable price, good customer service, worth recommending!	Jingdong prefecture level city in Shandong
Dragon Quality Goods Shop	King of Power Saving	Fast shipping, works great, have already recommended it to friends.	Jingdong prefecture level city in Shandong
Dragon Quality Goods Shop	King of Power Saving	Good quality, fast logistics, reasonable price, worth 5 stars.	Jingdong prefecture level city in Shandong
Dragon Quality Goods Shop	King of Power Saving	Good quality, fast logistics, good value, well packaged, practical	Jingdong prefecture level city in Shandong
Dragon Quality Goods Shop	King of Power Saving	Fast shipping, affordable price, great quality, very satisfied!	Jingdong prefecture level city in Shandong
Dragon Quality Goods Shop	King of Power Saving	Regret knowing too late, it's really good stuff. Works well.	Jingdong prefecture level city in Shandong
Dragon Quality Goods Shop	King of Power Saving	Not bad, very good product, quite save electricity	Jingdong prefecture level city in Shandong
Dragon Quality Goods Shop	King of Power Saving	Good quality, fast logistics, overall very satisfied!	Jingdong prefecture level city in Shandong
Dragon Quality Goods Shop	King of Power Saving	Tightly packaged with good results	Jingdong prefecture level city in Shandong
Dragon Quality Goods Shop	King of Power Saving	very happy	Jingdong prefecture level city in Shandong
Dragon Quality Goods Shop	King of Power Saving	favorable criticism	Jingdong prefecture level city in Shandong
Dragon Quality Goods Shop	King of Power Saving	Now they all sign for the goods without receiving them, the Jingdong platform and merchants are very powerful, and the goods are not received before signing for the goods.	Jingdong prefecture level city in Shandong
Dragon Quality Goods Shop	King of Power Saving	low-quality commodities	Jingdong prefecture level city in Shandong
Dragon Quality Goods Shop	King of Power Saving	I bought this and now it works pretty well with the air conditioning on, when I didn't use it it was $600 a month, now it's almost $500.00, that's over $100 less all of a sudden.	Jingdong prefecture level city in Shandong
Dragon Quality Goods Shop	King of Power Saving	Can Oh, the weather is hot, open the air conditioning is really good, this product is good, has been used for a really long time, more ok, power saving effect is not bad.	Jingdong prefecture level city in Shandong
Dragon Quality Goods Shop	King of Power Saving	I paid my electric bill today, it's a few dozen dollars less, anyway, it's not costly, it's already very good, the boss said that it would be better to buy one more, I've already placed an order	Jingdong prefecture level city in Shandong
Dragon Quality Goods Shop	King of Power Saving	I'm on a smart meter, I save about 30% a month, personally I think it's pretty good, it works well	Jingdong prefecture level city in Shandong
Dragon Quality Goods Shop	King of Power Saving	My dad asked me to buy one, but after I paid the bill, I asked and he said it was a lot less this month, so he asked me to buy another one.	Jingdong prefecture level city in Shandong
Dragon Quality Goods Shop	King of Power Saving	Very satisfied with the purchase thanks to the store for the prosperous business.	Jingdong prefecture level city in Shandong
Dragon Quality Goods Shop	King of Power Saving	Good use, good quality, very satisfied with the purchase	Jingdong prefecture level city in Shandong
Dragon Quality Goods Shop	King of Power Saving	The goods have been received, the quality is very good, is the ideal effect, will continue to pay attention to the	Jingdong prefecture level city in Shandong
Dragon Quality Goods Shop	King of Power Saving	This brand has been used for many years, and this time I saw this upgraded version of the product and got it again. The purpose of this product is to reduce the loss of electricity for a long time, so as to achieve the purpose of saving electricity.	Jingdong prefecture level city in Shandong
Dragon Quality Goods Shop	King of Power Saving	I've been using it for almost a month, and I've reduced my electricity bill by over 100 bucks! I'm planning to get two more for my family.	Jingdong prefecture level city in Shandong
Dragon Quality Goods Shop	King of Power Saving	The effect is too good, the family has now several units, back to buy a number of times, the power saver received, power-saving effect is very good,, fine workmanship, long warranty, but also purify the air, before buying a used for a period of time, with the okay, and then bought a, and only now come to the	Jingdong prefecture level city in Shandong
Dragon Quality Goods Shop	King of Power Saving	Finally received the goods, very, very much like,, has been a lot of people praise, really too happy, has been his family's loyal fans, will continue to buy buy buy buy!	Jingdong prefecture level city in Shandong
Dragon Quality Goods Shop	King of Power Saving	📩 I've been wanting it for a long time, I decided to go for it, I feel great after using it, it's genuine and the delivery is great, it lives up to its name👍	Jingdong prefecture level city in Shandong
Dragon Quality Goods Shop	King of Power Saving	The quality is sure as other friends who bought it said, and it is also beautiful, thanks to the owner for the recommendation, thanks!	Jingdong prefecture level city in Shandong
Dragon Quality Goods Shop	King of Power Saving	Baby received the quality is really good face value is also very high especially practical especially especially special favorite	Jingdong prefecture level city in Shandong
Dragon Quality Goods Shop	King of Power Saving	It is genuine, trust the merchant, the quality is very good, the price is moderate, the use of the effect is very good!	Jingdong prefecture level city in Shandong
Dragon Quality Goods Shop	King of Power Saving	Energy saver received really good quality affordable and good energy saving and environmental protection is the best to use.	Jingdong prefecture level city in Shandong
Dragon Quality Goods Shop	King of Power Saving	Reasonable price logistics is very fast, the feeling is not bad more satisfied with a shopping experience	Jingdong prefecture level city in Shandong
Dragon Quality Goods Shop	King of Power Saving	The stuff is even more perfect than I imagined, very powerful and worth getting!	Jingdong prefecture level city in Shandong
Dragon Quality Goods Shop	King of Power Saving	Goods have been received, good quality, affordable price, good customer service, worth recommending!	Jingdong prefecture level city in Shandong
Dragon Quality Goods Shop	King of Power Saving	Fast shipping, works great, have already recommended it to friends.	Jingdong prefecture level city in Shandong
Dragon Quality Goods Shop	King of Power Saving	Good quality, fast logistics, reasonable price, worth 5 stars.	Jingdong prefecture level city in Shandong
Dragon Quality Goods Shop	King of Power Saving	Good quality, fast logistics, good value, well packaged, practical	Jingdong prefecture level city in Shandong
Dragon Quality Goods Shop	King of Power Saving	Fast shipping, affordable price, great quality, very satisfied!	Jingdong prefecture level city in Shandong
Dragon Quality Goods Shop	King of Power Saving	Regret knowing too late, it's really good stuff. Works well.	Jingdong prefecture level city in Shandong
Dragon Quality Goods Shop	King of Power Saving	Not bad, very good product, quite save electricity	Jingdong prefecture level city in Shandong
Dragon Quality Goods Shop	King of Power Saving	Good quality, fast logistics, overall very satisfied!	Jingdong prefecture level city in Shandong
Dragon Quality Goods Shop	King of Power Saving	Tightly packaged with good results	Jingdong prefecture level city in Shandong
Dragon Quality Goods Shop	King of Power Saving	very happy	Jingdong prefecture level city in Shandong
Dragon Quality Goods Shop	King of Power Saving	favorable criticism	Jingdong prefecture level city in Shandong
Dragon Quality Goods Shop	King of Power Saving	Now they all sign for the goods without receiving them, the Jingdong platform and merchants are very powerful, and the goods are not received before signing for the goods.	Jingdong prefecture level city in Shandong
Dragon Quality Goods Shop	King of Power Saving	low-quality commodities	Jingdong prefecture level city in Shandong
Dragon Quality Goods Shop	King of Power Saving	I bought this and now it works pretty well with the air conditioning on, when I didn't use it it was $600 a month, now it's almost $500.00, that's over $100 less all of a sudden.	Jingdong prefecture level city in Shandong
Dragon Quality Goods Shop	King of Power Saving	Can Oh, the weather is hot, open the air conditioning is really good, this product is good, has been used for a really long time, more ok, power saving effect is not bad.	Jingdong prefecture level city in Shandong

Dragon Quality Goods Shop	King of Power Saving	I paid my electric bill today, it's a few dozen dollars less, anyway, it's not costly, it's already very good, the boss said that it would be better to buy one more, I've already placed an order	Jingdong prefecture level city in Shandong
Dragon Quality Goods Shop	King of Power Saving	I'm on a smart meter, I save about 30% a month, personally I think it's pretty good, it works well	Jingdong prefecture level city in Shandong
Dragon Quality Goods Shop	King of Power Saving	My dad asked me to buy one, but after I paid the bill, I asked and he said it was a lot less this month, so he asked me to buy another one.	Jingdong prefecture level city in Shandong
Dragon Quality Goods Shop	King of Power Saving	Very satisfied with the purchase thanks to the store for the prosperous business.	Jingdong prefecture level city in Shandong
Dragon Quality Goods Shop	King of Power Saving	Good use, good quality, very satisfied with the purchase	Jingdong prefecture level city in Shandong
Dragon Quality Goods Shop	King of Power Saving	The goods have been received, the quality is very good, is the ideal effect, will continue to pay attention to the	Jingdong prefecture level city in Shandong
Dragon Quality Goods Shop	King of Power Saving	This brand has been used for many years, and this time I saw this upgraded version of the product and got it again. The purpose of this product is to reduce the loss of electricity for a long time, so as to achieve the purpose of saving electricity.	Jingdong prefecture level city in Shandong
Dragon Quality Goods Shop	King of Power Saving	I've been using it for almost a month, and I've reduced my electricity bill by over 100 bucks! I'm planning to get two more for my family.	Jingdong prefecture level city in Shandong
Dragon Quality Goods Shop	King of Power Saving	The effect is too good, the family has now several units, back to buy a number of times, the power saver received, power-saving effect is very good,, fine workmanship, long warranty, but also purify the air, before buying a used for a period of time, with the okay, and then bought a, and only now come to the	Jingdong prefecture level city in Shandong
Dragon Quality Goods Shop	King of Power Saving	Finally received the goods, very, very much like,, has been a lot of people praise, really too happy, has been his family's loyal fans, will continue to buy buy buy buy!	Jingdong prefecture level city in Shandong
Dragon Quality Goods Shop	King of Power Saving	I've been wanting it for a long time, I decided to go for it, I feel great after using it, it's genuine and the delivery is great, it lives up to its name	Jingdong prefecture level city in Shandong
Dragon Quality Goods Shop	King of Power Saving	The quality is sure as other friends who bought it said, and it is also beautiful, thanks to the owner for the recommendation, thanks!	Jingdong prefecture level city in Shandong
Dragon Quality Goods Shop	King of Power Saving	Baby received the quality is really good face value is also very high especially practical especially especially special favorite	Jingdong prefecture level city in Shandong
Dragon Quality Goods Shop	King of Power Saving	It is genuine, trust the merchant, the quality is very good, the price is moderate, the use of the effect is very good!	Jingdong prefecture level city in Shandong
Dragon Quality Goods Shop	King of Power Saving	Energy saver received really good quality affordable and good energy saving and environmental protection is the best to use.	Jingdong prefecture level city in Shandong
Dragon Quality Goods Shop	King of Power Saving	Reasonable price logistics is very fast, the feeling is not bad more satisfied with a shopping experience	Jingdong prefecture level city in Shandong
Dragon Quality Goods Shop	King of Power Saving	The stuff is even more perfect than I imagined, very powerful and worth getting!	Jingdong prefecture level city in Shandong
Dragon Quality Goods Shop	King of Power Saving	Goods have been received, good quality, affordable price, good customer service, worth recommending!	Jingdong prefecture level city in Shandong
Dragon Quality Goods Shop	King of Power Saving	Fast shipping, works great, have already recommended it to friends.	Jingdong prefecture level city in Shandong
Dragon Quality Goods Shop	King of Power Saving	Good quality, fast logistics, reasonable price, worth 5 stars.	Jingdong prefecture level city in Shandong
Dragon Quality Goods Shop	King of Power Saving	Good quality, fast logistics, good value, well packaged, practical	Jingdong prefecture level city in Shandong
Dragon Quality Goods Shop	King of Power Saving	Fast shipping, affordable price, great quality, very satisfied!	Jingdong prefecture level city in Shandong
Dragon Quality Goods Shop	King of Power Saving	Regret knowing too late, it's really good stuff. Works well.	Jingdong prefecture level city in Shandong
Dragon Quality Goods Shop	King of Power Saving	Not bad, very good product, quite save electricity	Jingdong prefecture level city in Shandong
Dragon Quality Goods Shop	King of Power Saving	Good quality, fast logistics, overall very satisfied!	Jingdong prefecture level city in Shandong
Dragon Quality Goods Shop	King of Power Saving	Tightly packaged with good results	Jingdong prefecture level city in Shandong
Dragon Quality Goods Shop	King of Power Saving	very happy	Jingdong prefecture level city in Shandong
Dragon Quality Goods Shop	King of Power Saving	favorable criticism	Jingdong prefecture level city in Shandong
Dragon Quality Goods Shop	King of Power Saving	Now they all sign for the goods without receiving them, the Jingdong platform and merchants are very powerful, and the goods are not received before signing for the goods.	Jingdong prefecture level city in Shandong
Dragon Quality Goods Shop	King of Power Saving	low-quality commodities	Jingdong prefecture level city in Shandong
Dragon Quality Goods Shop	King of Power Saving	I bought this and now it works pretty well with the air conditioning on, when I didn't use it it was $600 a month, now it's almost $500,00, that's over $100 less all of a sudden.	Jingdong prefecture level city in Shandong
Dragon Quality Goods Shop	King of Power Saving	Can Oh, the weather is hot, open the air conditioning is really good, this product is good, has been used for a really long time, more ok, power saving effect is not bad.	Jingdong prefecture level city in Shandong
Dragon Quality Goods Shop	King of Power Saving	I paid my electric bill today, it's a few dozen dollars less, anyway, it's not costly, it's already very good, the boss said that it would be better to buy one more, I've already placed an order	Jingdong prefecture level city in Shandong
Dragon Quality Goods Shop	King of Power Saving	I'm on a smart meter, I save about 30% a month, personally I think it's pretty good, it works well	Jingdong prefecture level city in Shandong
Dragon Quality Goods Shop	King of Power Saving	My dad asked me to buy one, but after I paid the bill, I asked and he said it was a lot less this month, so he asked me to buy another one.	Jingdong prefecture level city in Shandong
Dragon Quality Goods Shop	King of Power Saving	Very satisfied with the purchase thanks to the store for the prosperous business.	Jingdong prefecture level city in Shandong
Dragon Quality Goods Shop	King of Power Saving	Good use, good quality, very satisfied with the purchase	Jingdong prefecture level city in Shandong
Dragon Quality Goods Shop	King of Power Saving	The goods have been received, the quality is very good, is the ideal effect, will continue to pay attention to the	Jingdong prefecture level city in Shandong
Dragon Quality Goods Shop	King of Power Saving	This brand has been used for many years, and this time I saw this upgraded version of the product and got it again. The purpose of this product is to reduce the loss of electricity for a long time, so as to achieve the purpose of saving electricity.	Jingdong prefecture level city in Shandong
Dragon Quality Goods Shop	King of Power Saving	I've been using it for almost a month, and I've reduced my electricity bill by over 100 bucks! I'm planning to get two more for my family.	Jingdong prefecture level city in Shandong
Dragon Quality Goods Shop	King of Power Saving	The effect is too good, the family has now several units, back to buy a number of times, the power saver received, power-saving effect is very good,, fine workmanship, long warranty, but also purify the air, before buying a used for a period of time, with the okay, and then bought a, and only now come to the	Jingdong prefecture level city in Shandong
Dragon Quality Goods Shop	King of Power Saving	Finally received the goods, very, very much like,, has been a lot of people praise, really too happy, has been his family's loyal fans, will continue to buy buy buy buy!	Jingdong prefecture level city in Shandong
Dragon Quality Goods Shop	King of Power Saving	I've been wanting it for a long time, I decided to go for it, I feel great after using it, it's genuine and the delivery is great, it lives up to its name	Jingdong prefecture level city in Shandong
Dragon Quality Goods Shop	King of Power Saving	The quality is sure as other friends who bought it said, and it is also beautiful, thanks to the owner for the recommendation, thanks!	Jingdong prefecture level city in Shandong
Dragon Quality Goods Shop	King of Power Saving	Baby received the quality is really good face value is also very high especially practical especially especially special favorite	Jingdong prefecture level city in Shandong
Dragon Quality Goods Shop	King of Power Saving	It is genuine, trust the merchant, the quality is very good, the price is moderate, the use of the effect is very good!	Jingdong prefecture level city in Shandong
Dragon Quality Goods Shop	King of Power Saving	Energy saver received really good quality affordable and good energy saving and environmental protection is the best to use.	Jingdong prefecture level city in Shandong
Dragon Quality Goods Shop	King of Power Saving	Reasonable price logistics is very fast, the feeling is not bad more satisfied with a shopping experience	Jingdong prefecture level city in Shandong
Dragon Quality Goods Shop	King of Power Saving	The stuff is even more perfect than I imagined, very powerful and worth getting!	Jingdong prefecture level city in Shandong

Dragon Quality Goods Shop	King of Power Saving	Goods have been received, good quality, affordable price, good customer service, worth recommending!	Jingdong prefecture level city in Shandong
Dragon Quality Goods Shop	King of Power Saving	Fast shipping, works great, have already recommended it to friends.	Jingdong prefecture level city in Shandong
Dragon Quality Goods Shop	King of Power Saving	Good quality, fast logistics, reasonable price, worth 5 stars.	Jingdong prefecture level city in Shandong
Dragon Quality Goods Shop	King of Power Saving	Good quality, fast logistics, good value, well packaged, practical	Jingdong prefecture level city in Shandong
Dragon Quality Goods Shop	King of Power Saving	Fast shipping, affordable price, great quality, very satisfied!	Jingdong prefecture level city in Shandong
Dragon Quality Goods Shop	King of Power Saving	Regret knowing too late, it's really good stuff. Works well.	Jingdong prefecture level city in Shandong
Dragon Quality Goods Shop	King of Power Saving	Not bad, very good product, quite save electricity	Jingdong prefecture level city in Shandong
Dragon Quality Goods Shop	King of Power Saving	Good quality, fast logistics, overall very satisfied!	Jingdong prefecture level city in Shandong
Dragon Quality Goods Shop	King of Power Saving	Tightly packaged with good results	Jingdong prefecture level city in Shandong
Dragon Quality Goods Shop	King of Power Saving	very happy	Jingdong prefecture level city in Shandong
Dragon Quality Goods Shop	King of Power Saving	favorable criticism	Jingdong prefecture level city in Shandong
Dragon Quality Goods Shop	King of Power Saving	Now they all sign for the goods without receiving them, the Jingdong platform and merchants are very powerful, and the goods are not received before signing for the goods.	Jingdong prefecture level city in Shandong
Dragon Quality Goods Shop	King of Power Saving	low-quality commodities	Jingdong prefecture level city in Shandong
Dragon Quality Goods Shop	King of Power Saving	I bought this and now it works pretty well with the air conditioning on, when I didn't use it it was $600 a month, now it's almost $500,00, that's over $100 less all of a sudden.	Jingdong prefecture level city in Shandong
Dragon Quality Goods Shop	King of Power Saving	Can Oh, the weather is hot, open the air conditioning is really good, this product is good, has been used for a really long time, more ok, power saving effect is not bad.	Jingdong prefecture level city in Shandong
Dragon Quality Goods Shop	King of Power Saving	I'm on a smart meter, I save about 30% a month, personally I think it's pretty good, it works well	Jingdong prefecture level city in Shandong
Dragon Quality Goods Shop	King of Power Saving	I paid my electric bill today, it's a few dozen dollars less, anyway, it's not costly, it's already very good, the boss said that it would be better to buy one more, I've already placed an order	Jingdong prefecture level city in Shandong
Dragon Quality Goods Shop	King of Power Saving	My dad asked me to buy one, but after I paid the bill, I asked and he said it was a lot less this month, so he asked me to buy another one.	Jingdong prefecture level city in Shandong
Dragon Quality Goods Shop	King of Power Saving	Very satisfied with the purchase thanks to the store for the prosperous business.	Jingdong prefecture level city in Shandong
Dragon Quality Goods Shop	King of Power Saving	Good use, good quality, very satisfied with the purchase	Jingdong prefecture level city in Shandong
Dragon Quality Goods Shop	King of Power Saving	The goods have been received, the quality is very good, is the ideal effect, will continue to pay attention to the	Jingdong prefecture level city in Shandong
Dragon Quality Goods Shop	King of Power Saving	This brand has been used for many years, and this time I saw this upgraded version of the product and got it again. The purpose of this product is to reduce the loss of electricity for a long time, so as to achieve the purpose of saving electricity.	Jingdong prefecture level city in Shandong
Dragon Quality Goods Shop	King of Power Saving	Finally received the goods, very, very much like,, has been a lot of people praise, really too happy, has been his family's loyal fans, will continue to buy buy buy buy!	Jingdong prefecture level city in Shandong
Dragon Quality Goods Shop	King of Power Saving	📩 I've been wanting it for a long time, I decided to go for it, I feel great after using it, it's genuine and the delivery is great, it lives up to its name👍	Jingdong prefecture level city in Shandong
Dragon Quality Goods Shop	King of Power Saving	I've been using it for almost a month, and I've reduced my electricity bill by over 100 bucks! I'm planning to get two more for my family.	Jingdong prefecture level city in Shandong
Dragon Quality Goods Shop	King of Power Saving	The effect is too good, the family has now several units, back to buy a number of times, the power saver received, power-saving effect is very good,, fine workmanship, long warranty, but also purify the air, before buying a used for a period of time, with the okay, and then bought a, and only now come to the	Jingdong prefecture level city in Shandong
Dragon Quality Goods Shop	King of Power Saving	The quality is sure as other friends who bought it said, and it is also beautiful, thanks to the owner for the recommendation, thanks!	Jingdong prefecture level city in Shandong
Dragon Quality Goods Shop	King of Power Saving	Reasonable price logistics is very fast, the feeling is not bad more satisfied with a shopping experience	Jingdong prefecture level city in Shandong
Dragon Quality Goods Shop	King of Power Saving	Baby received the quality is really good face value is also very high especially practical especially especially special favorite	Jingdong prefecture level city in Shandong
Dragon Quality Goods Shop	King of Power Saving	It is genuine, trust the merchant, the quality is very good, the price is moderate, the use of the effect is very good!	Jingdong prefecture level city in Shandong
Dragon Quality Goods Shop	King of Power Saving	Energy saver received really good quality affordable and good energy saving and environmental protection is the best to use.	Jingdong prefecture level city in Shandong
Dragon Quality Goods Shop	King of Power Saving	The stuff is even more perfect than I imagined, very powerful and worth getting!	Jingdong prefecture level city in Shandong
Dragon Quality Goods Shop	King of Power Saving	Goods have been received, good quality, affordable price, good customer service, worth recommending!	Jingdong prefecture level city in Shandong
Dragon Quality Goods Shop	King of Power Saving	Good quality, fast logistics, good value, well packaged, practical	Jingdong prefecture level city in Shandong
Dragon Quality Goods Shop	King of Power Saving	Fast shipping, affordable price, great quality, very satisfied!	Jingdong prefecture level city in Shandong
Dragon Quality Goods Shop	King of Power Saving	Good quality, fast logistics, reasonable price, worth 5 stars.	Jingdong prefecture level city in Shandong
Dragon Quality Goods Shop	King of Power Saving	Regret knowing too late, it's really good stuff. Works well.	Jingdong prefecture level city in Shandong
Dragon Quality Goods Shop	King of Power Saving	Not bad, very good product, quite save electricity	Jingdong prefecture level city in Shandong
Dragon Quality Goods Shop	King of Power Saving	Fast shipping, works great, have already recommended it to friends.	Jingdong prefecture level city in Shandong
Dragon Quality Goods Shop	King of Power Saving	Good quality, fast logistics, overall very satisfied!	Jingdong prefecture level city in Shandong
Dragon Quality Goods Shop	King of Power Saving	Tightly packaged with good results	Jingdong prefecture level city in Shandong
Dragon Quality Goods Shop	King of Power Saving	very happy	Jingdong prefecture level city in Shandong
Dragon Quality Goods Shop	King of Power Saving	Now they all sign for the goods without receiving them, the Jingdong platform and merchants are very powerful, and the goods are not received before signing for the goods.	Jingdong prefecture level city in Shandong
Dragon Quality Goods Shop	King of Power Saving	favorable criticism	Jingdong prefecture level city in Shandong
Dragon Quality Goods Shop	King of Power Saving	low-quality commodities	Jingdong prefecture level city in Shandong
Dragon Quality Goods Shop	King of Power Saving	I bought this and now it works pretty well with the air conditioning on, when I didn't use it it was $600 a month, now it's almost $500,00, that's over $100 less all of a sudden.	Jingdong prefecture level city in Shandong
Dragon Quality Goods Shop	King of Power Saving	Can Oh, the weather is hot, open the air conditioning is really good, this product is good, has been used for a really long time, more ok, power saving effect is not bad.	Jingdong prefecture level city in Shandong
Dragon Quality Goods Shop	King of Power Saving	I paid my electric bill today, it's a few dozen dollars less, anyway, it's not costly, it's already very good, the boss said that it would be better to buy one more, I've already placed an order	Jingdong prefecture level city in Shandong
Dragon Quality Goods Shop	King of Power Saving	I'm on a smart meter, I save about 30% a month, personally I think it's pretty good, it works well	Jingdong prefecture level city in Shandong
Dragon Quality Goods Shop	King of Power Saving	My dad asked me to buy one, but after I paid the bill, I asked and he said it was a lot less this month, so he asked me to buy another one.	Jingdong prefecture level city in Shandong

Dragon Quality Goods Shop	King of Power Saving	Very satisfied with the purchase thanks to the store for the prosperous business.	Jingdong prefecture level city in Shandong
Dragon Quality Goods Shop	King of Power Saving	Good use, good quality, very satisfied with the purchase	Jingdong prefecture level city in Shandong
Dragon Quality Goods Shop	King of Power Saving	The goods have been received, the quality is very good, is the ideal effect, will continue to pay attention to the	Jingdong prefecture level city in Shandong
Dragon Quality Goods Shop	King of Power Saving	This brand has been used for many years, and this time I saw this upgraded version of the product and got it again. The purpose of this product is to reduce the loss of electricity for a long time, so as to achieve the purpose of saving electricity.	Jingdong prefecture level city in Shandong
Dragon Quality Goods Shop	King of Power Saving	I've been using it for almost a month, and I've reduced my electricity bill by over 100 bucks! I'm planning to get two more for my family.	Jingdong prefecture level city in Shandong
Dragon Quality Goods Shop	King of Power Saving	The effect is too good, the family has now several units, back to buy a number of times, the power saver received, power-saving effect is very good,, fine workmanship, long warranty, but also purify the air, before buying a used for a period of time, with the okay, and then bought a, and only now come to the	Jingdong prefecture level city in Shandong
Dragon Quality Goods Shop	King of Power Saving	Finally received the goods, very, very much like,, has been a lot of people praise, really too happy, has been his family's loyal fans, will continue to buy buy buy buy!	Jingdong prefecture level city in Shandong
Dragon Quality Goods Shop	King of Power Saving	📩 I've been wanting it for a long time, I decided to go for it, I feel great after using it, it's genuine and the delivery is great, it lives up to its name👍	Jingdong prefecture level city in Shandong
Dragon Quality Goods Shop	King of Power Saving	The quality is sure as other friends who bought it said, and it is also beautiful, thanks to the owner for the recommendation, thanks!	Jingdong prefecture level city in Shandong
Dragon Quality Goods Shop	King of Power Saving	Baby received the quality is really good face value is also very high especially practical especially especially special favorite	Jingdong prefecture level city in Shandong
Dragon Quality Goods Shop	King of Power Saving	It is genuine, trust the merchant, the quality is very good, the price is moderate, the use of the effect is very good!	Jingdong prefecture level city in Shandong
Dragon Quality Goods Shop	King of Power Saving	Energy saver received really good quality affordable and good energy saving and environmental protection is the best to use.	Jingdong prefecture level city in Shandong
Dragon Quality Goods Shop	King of Power Saving	Reasonable price logistics is very fast, the feeling is not bad more satisfied with a shopping experience	Jingdong prefecture level city in Shandong
Dragon Quality Goods Shop	King of Power Saving	The stuff is even more perfect than I imagined, very powerful and worth getting!	Jingdong prefecture level city in Shandong
Dragon Quality Goods Shop	King of Power Saving	Goods have been received, good quality, affordable price, good customer service, worth recommending!	Jingdong prefecture level city in Shandong
Dragon Quality Goods Shop	King of Power Saving	Fast shipping, works great, have already recommended it to friends.	Jingdong prefecture level city in Shandong
Dragon Quality Goods Shop	King of Power Saving	Good quality, fast logistics, reasonable price, worth 5 stars.	Jingdong prefecture level city in Shandong
Dragon Quality Goods Shop	King of Power Saving	Good quality, fast logistics, good value, well packaged, practical	Jingdong prefecture level city in Shandong
Dragon Quality Goods Shop	King of Power Saving	Fast shipping, affordable price, great quality, very satisfied!	Jingdong prefecture level city in Shandong
Dragon Quality Goods Shop	King of Power Saving	Regret knowing too late, it's really good stuff. Works well.	Jingdong prefecture level city in Shandong
Dragon Quality Goods Shop	King of Power Saving	Not bad, very good product, quite save electricity	Jingdong prefecture level city in Shandong
Dragon Quality Goods Shop	King of Power Saving	Good quality, fast logistics, overall very satisfied!	Jingdong prefecture level city in Shandong
Dragon Quality Goods Shop	King of Power Saving	Tightly packaged with good results	Jingdong prefecture level city in Shandong
Dragon Quality Goods Shop	King of Power Saving	very happy	Jingdong prefecture level city in Shandong
Dragon Quality Goods Shop	King of Power Saving	favorable criticism	Jingdong prefecture level city in Shandong
Dragon Quality Goods Shop	King of Power Saving	Now they all sign for the goods without receiving them, the Jingdong platform and merchants are very powerful, and the goods are not received before signing for the goods.	Jingdong prefecture level city in Shandong
Dragon Quality Goods Shop	King of Power Saving	low-quality commodities	Jingdong prefecture level city in Shandong
Dragon Quality Goods Shop	King of Power Saving	I bought this and now it works pretty well with the air conditioning on, when I didn't use it it was $600 a month, now it's almost $500.00, that's over $100 less all of a sudden.	Jingdong prefecture level city in Shandong
Dragon Quality Goods Shop	King of Power Saving	Can Oh, the weather is hot, open the air conditioning is really good, this product is good, has been used for a really long time, more ok, power saving effect is not bad.	Jingdong prefecture level city in Shandong
Dragon Quality Goods Shop	King of Power Saving	I paid my electric bill today, it's a few dozen dollars less, anyway, it's not costly, it's already very good, the boss said that it would be better to buy one more, I've already placed an order	Jingdong prefecture level city in Shandong
Dragon Quality Goods Shop	King of Power Saving	I'm on a smart meter, I save about 30% a month, personally I think it's pretty good, it works well	Jingdong prefecture level city in Shandong
Dragon Quality Goods Shop	King of Power Saving	My dad asked me to buy one, but after I paid the bill, I asked and he said it was a lot less this month, so he asked me to buy another one.	Jingdong prefecture level city in Shandong
Dragon Quality Goods Shop	King of Power Saving	Very satisfied with the purchase thanks to the store for the prosperous business.	Jingdong prefecture level city in Shandong
Dragon Quality Goods Shop	King of Power Saving	Good use, good quality, very satisfied with the purchase	Jingdong prefecture level city in Shandong
Dragon Quality Goods Shop	King of Power Saving	The goods have been received, the quality is very good, is the ideal effect, will continue to pay attention to the	Jingdong prefecture level city in Shandong
Dragon Quality Goods Shop	King of Power Saving	This brand has been used for many years, and this time I saw this upgraded version of the product and got it again. The purpose of this product is to reduce the loss of electricity for a long time, so as to achieve the purpose of saving electricity.	Jingdong prefecture level city in Shandong
Dragon Quality Goods Shop	King of Power Saving	I've been using it for almost a month, and I've reduced my electricity bill by over 100 bucks! I'm planning to get two more for my family.	Jingdong prefecture level city in Shandong
Dragon Quality Goods Shop	King of Power Saving	The effect is too good, the family has now several units, back to buy a number of times, the power saver received, power-saving effect is very good,, fine workmanship, long warranty, but also purify the air, before buying a used for a period of time, with the okay, and then bought a, and only now come to the	Jingdong prefecture level city in Shandong
Dragon Quality Goods Shop	King of Power Saving	Finally received the goods, very, very much like,, has been a lot of people praise, really too happy, has been his family's loyal fans, will continue to buy buy buy buy!	Jingdong prefecture level city in Shandong
Dragon Quality Goods Shop	King of Power Saving	📩 I've been wanting it for a long time, I decided to go for it, I feel great after using it, it's genuine and the delivery is great, it lives up to its name👍	Jingdong prefecture level city in Shandong
Dragon Quality Goods Shop	King of Power Saving	The quality is sure as other friends who bought it said, and it is also beautiful, thanks to the owner for the recommendation, thanks!	Jingdong prefecture level city in Shandong
Dragon Quality Goods Shop	King of Power Saving	Baby received the quality is really good face value is also very high especially practical especially especially special favorite	Jingdong prefecture level city in Shandong
Dragon Quality Goods Shop	King of Power Saving	It is genuine, trust the merchant, the quality is very good, the price is moderate, the use of the effect is very good!	Jingdong prefecture level city in Shandong
Dragon Quality Goods Shop	King of Power Saving	Energy saver received really good quality affordable and good energy saving and environmental protection is the best to use.	Jingdong prefecture level city in Shandong
Dragon Quality Goods Shop	King of Power Saving	Reasonable price logistics is very fast, the feeling is not bad more satisfied with a shopping experience	Jingdong prefecture level city in Shandong
Dragon Quality Goods Shop	King of Power Saving	The stuff is even more perfect than I imagined, very powerful and worth getting!	Jingdong prefecture level city in Shandong
Dragon Quality Goods Shop	King of Power Saving	Goods have been received, good quality, affordable price, good customer service, worth recommending!	Jingdong prefecture level city in Shandong
Dragon Quality Goods Shop	King of Power Saving	Fast shipping, works great, have already recommended it to friends.	Jingdong prefecture level city in Shandong
Dragon Quality Goods Shop	King of Power Saving	Good quality, fast logistics, reasonable price, worth 5 stars.	Jingdong prefecture level city in Shandong

Dragon Quality Goods Shop	King of Power Saving	Good quality, fast logistics, good value, well packaged, practical	Jingdong prefecture level city in Shandong
Dragon Quality Goods Shop	King of Power Saving	Fast shipping, affordable price, great quality, very satisfied!	Jingdong prefecture level city in Shandong
Dragon Quality Goods Shop	King of Power Saving	Regret knowing too late, it's really good stuff. Works well.	Jingdong prefecture level city in Shandong
Dragon Quality Goods Shop	King of Power Saving	Not bad, very good product, quite save electricity	Jingdong prefecture level city in Shandong
Dragon Quality Goods Shop	King of Power Saving	Good quality, fast logistics, overall very satisfied!	Jingdong prefecture level city in Shandong
Dragon Quality Goods Shop	King of Power Saving	Tightly packaged with good results	Jingdong prefecture level city in Shandong
Dragon Quality Goods Shop	King of Power Saving	very happy	Jingdong prefecture level city in Shandong
Dragon Quality Goods Shop	King of Power Saving	favorable criticism	Jingdong prefecture level city in Shandong
Dragon Quality Goods Shop	King of Power Saving	Now they all sign for the goods without receiving them, the Jingdong platform and merchants are very powerful, and the goods are not received before signing for the goods.	Jingdong prefecture level city in Shandong
Dragon Quality Goods Shop	King of Power Saving	low-quality commodities	Jingdong prefecture level city in Shandong
Dragon Quality Goods Shop	King of Power Saving	I bought this and now it works pretty well with the air conditioning on, when I didn't use it it was $600 a month, now it's almost $500.00, that's over $100 less all of a sudden.	Jingdong prefecture level city in Shandong
Dragon Quality Goods Shop	King of Power Saving	Can Oh, the weather is hot, open the air conditioning is really good, this product is good, has been used for a really long time, more ok, power saving effect is not bad.	Jingdong prefecture level city in Shandong
Dragon Quality Goods Shop	King of Power Saving	I paid my electric bill today, it's a few dozen dollars less, anyway, it's not costly, it's already very good, the boss said that it would be better to buy one more, I've already placed an order	Jingdong prefecture level city in Shandong
Dragon Quality Goods Shop	King of Power Saving	I'm on a smart meter, I save about 30% a month, personally I think it's pretty good, it works well	Jingdong prefecture level city in Shandong
Dragon Quality Goods Shop	King of Power Saving	My dad asked me to buy one, but after I paid the bill, I asked and he said it was a lot less this month, so he asked me to buy another one.	Jingdong prefecture level city in Shandong
Dragon Quality Goods Shop	King of Power Saving	Very satisfied with the purchase thanks to the store for the prosperous business.	Jingdong prefecture level city in Shandong
Dragon Quality Goods Shop	King of Power Saving	Good use, good quality, very satisfied with the purchase	Jingdong prefecture level city in Shandong
Dragon Quality Goods Shop	King of Power Saving	The goods have been received, the quality is very good, is the ideal effect, will continue to pay attention to the	Jingdong prefecture level city in Shandong
Dragon Quality Goods Shop	King of Power Saving	This brand has been used for many years, and this time I saw this upgraded version of the product and got it again. The purpose of this product is to reduce the loss of electricity for a long time, so as to achieve the purpose of saving electricity.	Jingdong prefecture level city in Shandong
Dragon Quality Goods Shop	King of Power Saving	I've been using it for almost a month, and I've reduced my electricity bill by over 100 bucks! I'm planning to get two more for my family.	Jingdong prefecture level city in Shandong
Dragon Quality Goods Shop	King of Power Saving	The effect is too good, the family has now several units, back to buy a number of times, the power saver received, power-saving effect is very good,, fine workmanship, long warranty, but also purify the air, before buying a used for a period of time, with the okay, and then bought a, and only now come to the	Jingdong prefecture level city in Shandong
Dragon Quality Goods Shop	King of Power Saving	Finally received the goods, very, very much like,, has been a lot of people praise, really too happy, has been his family's loyal fans, will continue to buy buy buy buy!	Jingdong prefecture level city in Shandong
Dragon Quality Goods Shop	King of Power Saving	💌 I've been wanting it for a long time, I decided to go for it, I feel great after using it, it's genuine and the delivery is great, it lives up to its name👍	Jingdong prefecture level city in Shandong
Dragon Quality Goods Shop	King of Power Saving	The quality is sure as other friends who bought it said, and it is also beautiful, thanks to the owner for the recommendation, thanks!	Jingdong prefecture level city in Shandong
Dragon Quality Goods Shop	King of Power Saving	Baby received the quality is really good face value is also very high especially practical especially especially special favorite	Jingdong prefecture level city in Shandong
Dragon Quality Goods Shop	King of Power Saving	It is genuine, trust the merchant, the quality is very good, the price is moderate, the use of the effect is very good!	Jingdong prefecture level city in Shandong
Dragon Quality Goods Shop	King of Power Saving	Energy saver received really good quality affordable and good energy saving and environmental protection is the best to use.	Jingdong prefecture level city in Shandong
Dragon Quality Goods Shop	King of Power Saving	Reasonable price logistics is very fast, the feeling is not bad more satisfied with a shopping experience	Jingdong prefecture level city in Shandong
Dragon Quality Goods Shop	King of Power Saving	The stuff is even more perfect than I imagined, very powerful and worth getting!	Jingdong prefecture level city in Shandong
Dragon Quality Goods Shop	King of Power Saving	Goods have been received, good quality, affordable price, good customer service, worth recommending!	Jingdong prefecture level city in Shandong
Dragon Quality Goods Shop	King of Power Saving	Fast shipping, works great, have already recommended it to friends.	Jingdong prefecture level city in Shandong
Dragon Quality Goods Shop	King of Power Saving	Good quality, fast logistics, reasonable price, worth 5 stars.	Jingdong prefecture level city in Shandong
Dragon Quality Goods Shop	King of Power Saving	Good quality, fast logistics, good value, well packaged, practical	Jingdong prefecture level city in Shandong
Dragon Quality Goods Shop	King of Power Saving	Fast shipping, affordable price, great quality, very satisfied!	Jingdong prefecture level city in Shandong
Dragon Quality Goods Shop	King of Power Saving	Regret knowing too late, it's really good stuff. Works well.	Jingdong prefecture level city in Shandong
Dragon Quality Goods Shop	King of Power Saving	Not bad, very good product, quite save electricity	Jingdong prefecture level city in Shandong
Dragon Quality Goods Shop	King of Power Saving	Good quality, fast logistics, overall very satisfied!	Jingdong prefecture level city in Shandong
Dragon Quality Goods Shop	King of Power Saving	Tightly packaged with good results	Jingdong prefecture level city in Shandong
Dragon Quality Goods Shop	King of Power Saving	very happy	Jingdong prefecture level city in Shandong
Dragon Quality Goods Shop	King of Power Saving	favorable criticism	Jingdong prefecture level city in Shandong
Dragon Quality Goods Shop	King of Power Saving	Now they all sign for the goods without receiving them, the Jingdong platform and merchants are very powerful, and the goods are not received before signing for the goods.	Jingdong prefecture level city in Shandong
Dragon Quality Goods Shop	King of Power Saving	low-quality commodities	Jingdong prefecture level city in Shandong
Dragon Quality Goods Shop	King of Power Saving	I bought this and now it works pretty well with the air conditioning on, when I didn't use it it was $600 a month, now it's almost $500.00, that's over $100 less all of a sudden.	Jingdong prefecture level city in Shandong
Dragon Quality Goods Shop	King of Power Saving	Can Oh, the weather is hot, open the air conditioning is really good, this product is good, has been used for a really long time, more ok, power saving effect is not bad.	Jingdong prefecture level city in Shandong
Dragon Quality Goods Shop	King of Power Saving	I paid my electric bill today, it's a few dozen dollars less, anyway, it's not costly, it's already very good, the boss said that it would be better to buy one more, I've already placed an order	Jingdong prefecture level city in Shandong
Dragon Quality Goods Shop	King of Power Saving	I'm on a smart meter, I save about 30% a month, personally I think it's pretty good, it works well	Jingdong prefecture level city in Shandong
Dragon Quality Goods Shop	King of Power Saving	My dad asked me to buy one, but after I paid the bill, I asked and he said it was a lot less this month, so he asked me to buy another one.	Jingdong prefecture level city in Shandong
Dragon Quality Goods Shop	King of Power Saving	Very satisfied with the purchase thanks to the store for the prosperous business.	Jingdong prefecture level city in Shandong
Dragon Quality Goods Shop	King of Power Saving	Good use, good quality, very satisfied with the purchase	Jingdong prefecture level city in Shandong
Dragon Quality Goods Shop	King of Power Saving	The goods have been received, the quality is very good, is the ideal effect, will continue to pay attention to the	Jingdong prefecture level city in Shandong

Dragon Quality Goods Shop	King of Power Saving	This brand has been used for many years, and this time I saw this upgraded version of the product and got it again. The purpose of this product is to reduce the loss of electricity for a long time, so as to achieve the purpose of saving electricity.	Jingdong prefecture level city in Shandong
Dragon Quality Goods Shop	King of Power Saving	I've been using it for almost a month, and I've reduced my electricity bill by over 100 bucks! I'm planning to get two more for my family.	Jingdong prefecture level city in Shandong
Dragon Quality Goods Shop	King of Power Saving	The effect is too good, the family has now several units, back to buy a number of times, the power saver received, power-saving effect is very good,, fine workmanship, long warranty, but also purify the air, before buying a used for a period of time, with the okay, and then bought a, and only now come to the	Jingdong prefecture level city in Shandong
Dragon Quality Goods Shop	King of Power Saving	Finally received the goods, very, very much like,, has been a lot of people praise, really too happy, has been his family's loyal fans, will continue to buy buy buy buy!	Jingdong prefecture level city in Shandong
Dragon Quality Goods Shop	King of Power Saving	📩 I've been wanting it for a long time, I decided to go for it, I feel great after using it, it's genuine and the delivery is great, it lives up to its name👍	Jingdong prefecture level city in Shandong
Dragon Quality Goods Shop	King of Power Saving	The quality is sure as other friends who bought it said, and it is also beautiful, thanks to the owner for the recommendation, thanks!	Jingdong prefecture level city in Shandong
Dragon Quality Goods Shop	King of Power Saving	Baby received the quality is really good face value is also very high especially practical especially especially special favorite	Jingdong prefecture level city in Shandong
Dragon Quality Goods Shop	King of Power Saving	It is genuine, trust the merchant, the quality is very good, the price is moderate, the use of the effect is very good!	Jingdong prefecture level city in Shandong
Dragon Quality Goods Shop	King of Power Saving	Energy saver received really good quality affordable and good energy saving and environmental protection is the best to use.	Jingdong prefecture level city in Shandong
Dragon Quality Goods Shop	King of Power Saving	Reasonable price logistics is very fast, the feeling is not bad more satisfied with a shopping experience	Jingdong prefecture level city in Shandong
Dragon Quality Goods Shop	King of Power Saving	The stuff is even more perfect than I imagined, very powerful and worth getting!	Jingdong prefecture level city in Shandong
Dragon Quality Goods Shop	King of Power Saving	Goods have been received, good quality, affordable price, good customer service, worth recommending!	Jingdong prefecture level city in Shandong
Dragon Quality Goods Shop	King of Power Saving	Fast shipping, works great, have already recommended it to friends.	Jingdong prefecture level city in Shandong
Dragon Quality Goods Shop	King of Power Saving	Good quality, fast logistics, reasonable price, worth 5 stars.	Jingdong prefecture level city in Shandong
Dragon Quality Goods Shop	King of Power Saving	Good quality, fast logistics, good value, well packaged, practical	Jingdong prefecture level city in Shandong
Dragon Quality Goods Shop	King of Power Saving	Fast shipping, affordable price, great quality, very satisfied!	Jingdong prefecture level city in Shandong
Dragon Quality Goods Shop	King of Power Saving	Regret knowing too late, it's really good stuff. Works well.	Jingdong prefecture level city in Shandong
Dragon Quality Goods Shop	King of Power Saving	Not bad, very good product, quite save electricity	Jingdong prefecture level city in Shandong
Dragon Quality Goods Shop	King of Power Saving	Good quality, fast logistics, overall very satisfied!	Jingdong prefecture level city in Shandong
Dragon Quality Goods Shop	King of Power Saving	Tightly packaged with good results	Jingdong prefecture level city in Shandong
Dragon Quality Goods Shop	King of Power Saving	very happy	Jingdong prefecture level city in Shandong
Dragon Quality Goods Shop	King of Power Saving	favorable criticism	Jingdong prefecture level city in Shandong
Dragon Quality Goods Shop	King of Power Saving	Now they all sign for the goods without receiving them, the Jingdong platform and merchants are very powerful, and the goods are not received before signing for the goods.	Jingdong prefecture level city in Shandong
Dragon Quality Goods Shop	King of Power Saving	low-quality commodities	Jingdong prefecture level city in Shandong
Dragon Quality Goods Shop	King of Power Saving	I bought this and now it works pretty well with the air conditioning on, when I didn't use it it was $600 a month, now it's almost $500,00, that's over $100 less all of a sudden.	Jingdong prefecture level city in Shandong
Dragon Quality Goods Shop	King of Power Saving	Can Oh, the weather is hot, open the air conditioning is really good, this product is good, has been used for a really long time, more ok, power saving effect is not bad.	Jingdong prefecture level city in Shandong
Dragon Quality Goods Shop	King of Power Saving	I paid my electric bill today, it's a few dozen dollars less, anyway, it's not costly, it's already very good, the boss said that it would be better to buy one more, I've already placed an order	Jingdong prefecture level city in Shandong
Dragon Quality Goods Shop	King of Power Saving	I'm on a smart meter, I save about 30% a month, personally I think it's pretty good, it works well	Jingdong prefecture level city in Shandong
Dragon Quality Goods Shop	King of Power Saving	My dad asked me to buy one, but after I paid the bill, I asked and he said it was a lot less this month, so he asked me to buy another one.	Jingdong prefecture level city in Shandong
Dragon Quality Goods Shop	King of Power Saving	Very satisfied with the purchase thanks to the store for the prosperous business.	Jingdong prefecture level city in Shandong
Dragon Quality Goods Shop	King of Power Saving	Good use, good quality, very satisfied with the purchase	Jingdong prefecture level city in Shandong
Dragon Quality Goods Shop	King of Power Saving	The goods have been received, the quality is very good, is the ideal effect, will continue to pay attention to the	Jingdong prefecture level city in Shandong
Dragon Quality Goods Shop	King of Power Saving	This brand has been used for many years, and this time I saw this upgraded version of the product and got it again. The purpose of this product is to reduce the loss of electricity for a long time, so as to achieve the purpose of saving electricity.	Jingdong prefecture level city in Shandong
Dragon Quality Goods Shop	King of Power Saving	I've been using it for almost a month, and I've reduced my electricity bill by over 100 bucks! I'm planning to get two more for my family.	Jingdong prefecture level city in Shandong
Dragon Quality Goods Shop	King of Power Saving	The effect is too good, the family has now several units, back to buy a number of times, the power saver received, power-saving effect is very good,, fine workmanship, long warranty, but also purify the air, before buying a used for a period of time, with the okay, and then bought a, and only now come to the	Jingdong prefecture level city in Shandong
Dragon Quality Goods Shop	King of Power Saving	Finally received the goods, very, very much like,, has been a lot of people praise, really too happy, has been his family's loyal fans, will continue to buy buy buy buy!	Jingdong prefecture level city in Shandong
Dragon Quality Goods Shop	King of Power Saving	📩 I've been wanting it for a long time, I decided to go for it, I feel great after using it, it's genuine and the delivery is great, it lives up to its name👍	Jingdong prefecture level city in Shandong
Dragon Quality Goods Shop	King of Power Saving	The quality is sure as other friends who bought it said, and it is also beautiful, thanks to the owner for the recommendation, thanks!	Jingdong prefecture level city in Shandong
Dragon Quality Goods Shop	King of Power Saving	Baby received the quality is really good face value is also very high especially practical especially especially special favorite	Jingdong prefecture level city in Shandong
Dragon Quality Goods Shop	King of Power Saving	It is genuine, trust the merchant, the quality is very good, the price is moderate, the use of the effect is very good!	Jingdong prefecture level city in Shandong
Dragon Quality Goods Shop	King of Power Saving	Energy saver received really good quality affordable and good energy saving and environmental protection is the best to use.	Jingdong prefecture level city in Shandong
Dragon Quality Goods Shop	King of Power Saving	Reasonable price logistics is very fast, the feeling is not bad more satisfied with a shopping experience	Jingdong prefecture level city in Shandong
Dragon Quality Goods Shop	King of Power Saving	The stuff is even more perfect than I imagined, very powerful and worth getting!	Jingdong prefecture level city in Shandong
Dragon Quality Goods Shop	King of Power Saving	Goods have been received, good quality, affordable price, good customer service, worth recommending!	Jingdong prefecture level city in Shandong
Dragon Quality Goods Shop	King of Power Saving	Fast shipping, works great, have already recommended it to friends.	Jingdong prefecture level city in Shandong
Dragon Quality Goods Shop	King of Power Saving	Good quality, fast logistics, reasonable price, worth 5 stars.	Jingdong prefecture level city in Shandong
Dragon Quality Goods Shop	King of Power Saving	Good quality, fast logistics, good value, well packaged, practical	Jingdong prefecture level city in Shandong
Dragon Quality Goods Shop	King of Power Saving	Fast shipping, affordable price, great quality, very satisfied!	Jingdong prefecture level city in Shandong
Dragon Quality Goods Shop	King of Power Saving	Regret knowing too late, it's really good stuff. Works well.	Jingdong prefecture level city in Shandong

Dragon Quality Goods Shop	King of Power Saving	Not bad, very good product, quite save electricity	Jingdong prefecture level city in Shandong
Dragon Quality Goods Shop	King of Power Saving	Good quality, fast logistics, overall very satisfied!	Jingdong prefecture level city in Shandong
Dragon Quality Goods Shop	King of Power Saving	Tightly packaged with good results	Jingdong prefecture level city in Shandong
Dragon Quality Goods Shop	King of Power Saving	very happy	Jingdong prefecture level city in Shandong
Dragon Quality Goods Shop	King of Power Saving	favorable criticism	Jingdong prefecture level city in Shandong
Dragon Quality Goods Shop	King of Power Saving	Now they all sign for the goods without receiving them, the Jingdong platform and merchants are very powerful, and the goods are not received before signing for the goods.	Jingdong prefecture level city in Shandong
Dragon Quality Goods Shop	King of Power Saving	low-quality commodities	Jingdong prefecture level city in Shandong
Dragon Quality Goods Shop	King of Power Saving	I bought this and now it works pretty well with the air conditioning on, when I didn't use it it was $600 a month, now it's almost $500.00, that's over $100 less all of a sudden.	Jingdong prefecture level city in Shandong
Dragon Quality Goods Shop	King of Power Saving	Can Oh, the weather is hot, open the air conditioning is really good, this product is good, has been used for a really long time, more ok, power saving effect is not bad.	Jingdong prefecture level city in Shandong
Dragon Quality Goods Shop	King of Power Saving	I paid my electric bill today, it's a few dozen dollars less, anyway, it's not costly, it's already very good, the boss said that it would be better to buy one more, I've already placed an order	Jingdong prefecture level city in Shandong
Dragon Quality Goods Shop	King of Power Saving	I'm on a smart meter, I save about 30% a month, personally I think it's pretty good, it works well	Jingdong prefecture level city in Shandong
Dragon Quality Goods Shop	King of Power Saving	My dad asked me to buy one, but after I paid the bill, I asked and he said it was a lot less this month, so he asked me to buy another one.	Jingdong prefecture level city in Shandong
Dragon Quality Goods Shop	King of Power Saving	Very satisfied with the purchase thanks to the store for the prosperous business.	Jingdong prefecture level city in Shandong
Dragon Quality Goods Shop	King of Power Saving	Good use, good quality, very satisfied with the purchase	Jingdong prefecture level city in Shandong
Dragon Quality Goods Shop	King of Power Saving	The goods have been received, the quality is very good, is the ideal effect, will continue to pay attention to the	Jingdong prefecture level city in Shandong
Dragon Quality Goods Shop	King of Power Saving	This brand has been used for many years, and this time I saw this upgraded version of the product and got it again. The purpose of this product is to reduce the loss of electricity for a long time, so as to achieve the purpose of saving electricity.	Jingdong prefecture level city in Shandong
Dragon Quality Goods Shop	King of Power Saving	I've been using it for almost a month, and I've reduced my electricity bill by over 100 bucks! I'm planning to get two more for my family.	Jingdong prefecture level city in Shandong
Dragon Quality Goods Shop	King of Power Saving	The effect is too good, the family has now several units, back to buy a number of times, the power saver received, power-saving effect is very good,, fine workmanship, long warranty, but also purify the air, before buying a used for a period of time, with the okay, and then bought a, and only now come to the	Jingdong prefecture level city in Shandong
Dragon Quality Goods Shop	King of Power Saving	Finally received the goods, very, very much like,, has been a lot of people praise, really too happy, has been his family's loyal fans, will continue to buy buy buy buy!	Jingdong prefecture level city in Shandong
Dragon Quality Goods Shop	King of Power Saving	📩 I've been wanting it for a long time, I decided to go for it, I feel great after using it, it's genuine and the delivery is great, it lives up to its name👍	Jingdong prefecture level city in Shandong
Dragon Quality Goods Shop	King of Power Saving	The quality is sure as other friends who bought it said, and it is also beautiful, thanks to the owner for the recommendation, thanks!	Jingdong prefecture level city in Shandong
Dragon Quality Goods Shop	King of Power Saving	Baby received the quality is really good face value is also very high especially practical especially especially special favorite	Jingdong prefecture level city in Shandong
Dragon Quality Goods Shop	King of Power Saving	It is genuine, trust the merchant, the quality is very good, the price is moderate, the use of the effect is very good!	Jingdong prefecture level city in Shandong
Dragon Quality Goods Shop	King of Power Saving	Energy saver received really good quality affordable and good energy saving and environmental protection is the best to use.	Jingdong prefecture level city in Shandong
Dragon Quality Goods Shop	King of Power Saving	Reasonable price logistics is very fast, the feeling is not bad more satisfied with a shopping experience	Jingdong prefecture level city in Shandong
Dragon Quality Goods Shop	King of Power Saving	The stuff is even more perfect than I imagined, very powerful and worth getting!	Jingdong prefecture level city in Shandong
Dragon Quality Goods Shop	King of Power Saving	Goods have been received, good quality, affordable price, good customer service, worth recommending!	Jingdong prefecture level city in Shandong
Dragon Quality Goods Shop	King of Power Saving	Fast shipping, works great, have already recommended it to friends.	Jingdong prefecture level city in Shandong
Dragon Quality Goods Shop	King of Power Saving	Good quality, fast logistics, reasonable price, worth 5 stars.	Jingdong prefecture level city in Shandong
Dragon Quality Goods Shop	King of Power Saving	Good quality, fast logistics, good value, well packaged, practical	Jingdong prefecture level city in Shandong
Dragon Quality Goods Shop	King of Power Saving	Fast shipping, affordable price, great quality, very satisfied!	Jingdong prefecture level city in Shandong
Dragon Quality Goods Shop	King of Power Saving	Regret knowing too late, it's really good stuff. Works well.	Jingdong prefecture level city in Shandong
Dragon Quality Goods Shop	King of Power Saving	Not bad, very good product, quite save electricity	Jingdong prefecture level city in Shandong
Dragon Quality Goods Shop	King of Power Saving	Good quality, fast logistics, overall very satisfied!	Jingdong prefecture level city in Shandong
Dragon Quality Goods Shop	King of Power Saving	Tightly packaged with good results	Jingdong prefecture level city in Shandong
Dragon Quality Goods Shop	King of Power Saving	very happy	Jingdong prefecture level city in Shandong
Dragon Quality Goods Shop	King of Power Saving	favorable criticism	Jingdong prefecture level city in Shandong
Dragon Quality Goods Shop	King of Power Saving	Now they all sign for the goods without receiving them, the Jingdong platform and merchants are very powerful, and the goods are not received before signing for the goods.	Jingdong prefecture level city in Shandong
Dragon Quality Goods Shop	King of Power Saving	low-quality commodities	Jingdong prefecture level city in Shandong
Dragon Quality Goods Shop	King of Power Saving	I bought this and now it works pretty well with the air conditioning on, when I didn't use it it was $600 a month, now it's almost $500.00, that's over $100 less all of a sudden.	Jingdong prefecture level city in Shandong
Dragon Quality Goods Shop	King of Power Saving	Can Oh, the weather is hot, open the air conditioning is really good, this product is good, has been used for a really long time, more ok, power saving effect is not bad.	Jingdong prefecture level city in Shandong
Dragon Quality Goods Shop	King of Power Saving	I paid my electric bill today, it's a few dozen dollars less, anyway, it's not costly, it's already very good, the boss said that it would be better to buy one more, I've already placed an order	Jingdong prefecture level city in Shandong
Dragon Quality Goods Shop	King of Power Saving	I'm on a smart meter, I save about 30% a month, personally I think it's pretty good, it works well	Jingdong prefecture level city in Shandong
Dragon Quality Goods Shop	King of Power Saving	My dad asked me to buy one, but after I paid the bill, I asked and he said it was a lot less this month, so he asked me to buy another one.	Jingdong prefecture level city in Shandong
Dragon Quality Goods Shop	King of Power Saving	Very satisfied with the purchase thanks to the store for the prosperous business.	Jingdong prefecture level city in Shandong
Dragon Quality Goods Shop	King of Power Saving	Good use, good quality, very satisfied with the purchase	Jingdong prefecture level city in Shandong
Dragon Quality Goods Shop	King of Power Saving	The goods have been received, the quality is very good, is the ideal effect, will continue to pay attention to the	Jingdong prefecture level city in Shandong
Dragon Quality Goods Shop	King of Power Saving	This brand has been used for many years, and this time I saw this upgraded version of the product and got it again. The purpose of this product is to reduce the loss of electricity for a long time, so as to achieve the purpose of saving electricity.	Jingdong prefecture level city in Shandong
Dragon Quality Goods Shop	King of Power Saving	I've been using it for almost a month, and I've reduced my electricity bill by over 100 bucks! I'm planning to get two more for my family.	Jingdong prefecture level city in Shandong

Dragon Quality Goods Shop	King of Power Saving	The effect is too good, the family has now several units, back to buy a number of times, the power saver received, power-saving effect is very good,, fine workmanship, long warranty, but also purify the air, before buying a used for a period of time, with the okay, and then bought a, and only now come to the	Jingdong prefecture level city in Shandong
Dragon Quality Goods Shop	King of Power Saving	Finally received the goods, very, very much like,, has been a lot of people praise, really too happy, has been his family's loyal fans, will continue to buy buy buy buy!	Jingdong prefecture level city in Shandong
Dragon Quality Goods Shop	King of Power Saving	📩 I've been wanting it for a long time, I decided to go for it, I feel great after using it, it's genuine and the delivery is great, it lives up to its name👍	Jingdong prefecture level city in Shandong
Dragon Quality Goods Shop	King of Power Saving	The quality is sure as other friends who bought it said, and it is also beautiful, thanks to the owner for the recommendation, thanks!	Jingdong prefecture level city in Shandong
Dragon Quality Goods Shop	King of Power Saving	Baby received the quality is really good face value is also very high especially practical especially especially special favorite	Jingdong prefecture level city in Shandong
Dragon Quality Goods Shop	King of Power Saving	It is genuine, trust the merchant, the quality is very good, the price is moderate, the use of the effect is very good!	Jingdong prefecture level city in Shandong
Dragon Quality Goods Shop	King of Power Saving	Energy saver received really good quality affordable and good energy saving and environmental protection is the best to use.	Jingdong prefecture level city in Shandong
Dragon Quality Goods Shop	King of Power Saving	Reasonable price logistics is very fast, the feeling is not bad more satisfied with a shopping experience	Jingdong prefecture level city in Shandong
Dragon Quality Goods Shop	King of Power Saving	The stuff is even more perfect than I imagined, very powerful and worth getting!	Jingdong prefecture level city in Shandong
Dragon Quality Goods Shop	King of Power Saving	Goods have been received, good quality, affordable price, good customer service, worth recommending!	Jingdong prefecture level city in Shandong
Dragon Quality Goods Shop	King of Power Saving	Fast shipping, works great, have already recommended it to friends.	Jingdong prefecture level city in Shandong
Dragon Quality Goods Shop	King of Power Saving	Good quality, fast logistics, reasonable price, worth 5 stars.	Jingdong prefecture level city in Shandong
Dragon Quality Goods Shop	King of Power Saving	Good quality, fast logistics, good value, well packaged, practical	Jingdong prefecture level city in Shandong
Dragon Quality Goods Shop	King of Power Saving	Fast shipping, affordable price, great quality, very satisfied!	Jingdong prefecture level city in Shandong
Dragon Quality Goods Shop	King of Power Saving	Regret knowing too late, it's really good stuff. Works well.	Jingdong prefecture level city in Shandong
Dragon Quality Goods Shop	King of Power Saving	Not bad, very good product, quite save electricity	Jingdong prefecture level city in Shandong
Dragon Quality Goods Shop	King of Power Saving	Good quality, fast logistics, overall very satisfied!	Jingdong prefecture level city in Shandong
Dragon Quality Goods Shop	King of Power Saving	Tightly packaged with good results	Jingdong prefecture level city in Shandong
Dragon Quality Goods Shop	King of Power Saving	very happy	Jingdong prefecture level city in Shandong
Dragon Quality Goods Shop	King of Power Saving	favorable criticism	Jingdong prefecture level city in Shandong
Dragon Quality Goods Shop	King of Power Saving	Now they all sign for the goods without receiving them, the Jingdong platform and merchants are very powerful, and the goods are not received before signing for the goods.	Jingdong prefecture level city in Shandong
Dragon Quality Goods Shop	King of Power Saving	low-quality commodities	Jingdong prefecture level city in Shandong
Dragon Quality Goods Shop	King of Power Saving	I bought this and now it works pretty well with the air conditioning on, when I didn't use it it was $600 a month, now it's almost $500.00, that's over $100 less all of a sudden.	Jingdong prefecture level city in Shandong
Dragon Quality Goods Shop	King of Power Saving	Can Oh, the weather is hot, open the air conditioning is really good, this product is good, has been used for a really long time, more ok, power saving effect is not bad.	Jingdong prefecture level city in Shandong
Dragon Quality Goods Shop	King of Power Saving	I paid my electric bill today, it's a few dozen dollars less, anyway, it's not costly, it's already very good, the boss said that it would be better to buy one more, I've already placed an order	Jingdong prefecture level city in Shandong
Dragon Quality Goods Shop	King of Power Saving	I'm on a smart meter, I save about 30% a month, personally I think it's pretty good, it works well	Jingdong prefecture level city in Shandong
Dragon Quality Goods Shop	King of Power Saving	My dad asked me to buy one, but after I paid the bill, I asked and he said it was a lot less this month, so he asked me to buy another one.	Jingdong prefecture level city in Shandong
Dragon Quality Goods Shop	King of Power Saving	Very satisfied with the purchase thanks to the store for the prosperous business.	Jingdong prefecture level city in Shandong
Dragon Quality Goods Shop	King of Power Saving	Good use, good quality, very satisfied with the purchase	Jingdong prefecture level city in Shandong
Dragon Quality Goods Shop	King of Power Saving	The goods have been received, the quality is very good, is the ideal effect, will continue to pay attention to the	Jingdong prefecture level city in Shandong
Dragon Quality Goods Shop	King of Power Saving	This brand has been used for many years, and this time I saw this upgraded version of the product and got it again. The purpose of this product is to reduce the loss of electricity for a long time, so as to achieve the purpose of saving electricity.	Jingdong prefecture level city in Shandong
Dragon Quality Goods Shop	King of Power Saving	I've been using it for almost a month, and I've reduced my electricity bill by over 100 bucks! I'm planning to get two more for my family.	Jingdong prefecture level city in Shandong
Dragon Quality Goods Shop	King of Power Saving	The effect is too good, the family has now several units, back to buy a number of times, the power saver received, power-saving effect is very good,, fine workmanship, long warranty, but also purify the air, before buying a used for a period of time, with the okay, and then bought a, and only now come to the	Jingdong prefecture level city in Shandong
Dragon Quality Goods Shop	King of Power Saving	Finally received the goods, very, very much like,, has been a lot of people praise, really too happy, has been his family's loyal fans, will continue to buy buy buy buy!	Jingdong prefecture level city in Shandong
Dragon Quality Goods Shop	King of Power Saving	📩 I've been wanting it for a long time, I decided to go for it, I feel great after using it, it's genuine and the delivery is great, it lives up to its name👍	Jingdong prefecture level city in Shandong
Dragon Quality Goods Shop	King of Power Saving	The quality is sure as other friends who bought it said, and it is also beautiful, thanks to the owner for the recommendation, thanks!	Jingdong prefecture level city in Shandong
Dragon Quality Goods Shop	King of Power Saving	Baby received the quality is really good face value is also very high especially practical especially especially special favorite	Jingdong prefecture level city in Shandong
Dragon Quality Goods Shop	King of Power Saving	It is genuine, trust the merchant, the quality is very good, the price is moderate, the use of the effect is very good!	Jingdong prefecture level city in Shandong
Dragon Quality Goods Shop	King of Power Saving	Energy saver received really good quality affordable and good energy saving and environmental protection is the best to use.	Jingdong prefecture level city in Shandong
Dragon Quality Goods Shop	King of Power Saving	Reasonable price logistics is very fast, the feeling is not bad more satisfied with a shopping experience	Jingdong prefecture level city in Shandong
Dragon Quality Goods Shop	King of Power Saving	The stuff is even more perfect than I imagined, very powerful and worth getting!	Jingdong prefecture level city in Shandong
Dragon Quality Goods Shop	King of Power Saving	Goods have been received, good quality, affordable price, good customer service, worth recommending!	Jingdong prefecture level city in Shandong
Dragon Quality Goods Shop	King of Power Saving	Fast shipping, works great, have already recommended it to friends.	Jingdong prefecture level city in Shandong
Dragon Quality Goods Shop	King of Power Saving	Good quality, fast logistics, reasonable price, worth 5 stars.	Jingdong prefecture level city in Shandong
Dragon Quality Goods Shop	King of Power Saving	Good quality, fast logistics, good value, well packaged, practical	Jingdong prefecture level city in Shandong
Dragon Quality Goods Shop	King of Power Saving	Fast shipping, affordable price, great quality, very satisfied!	Jingdong prefecture level city in Shandong
Dragon Quality Goods Shop	King of Power Saving	Regret knowing too late, it's really good stuff. Works well.	Jingdong prefecture level city in Shandong
Dragon Quality Goods Shop	King of Power Saving	Not bad, very good product, quite save electricity	Jingdong prefecture level city in Shandong
Dragon Quality Goods Shop	King of Power Saving	Good quality, fast logistics, overall very satisfied!	Jingdong prefecture level city in Shandong
Dragon Quality Goods Shop	King of Power Saving	Tightly packaged with good results	Jingdong prefecture level city in Shandong

Dragon Quality Goods Shop	King of Power Saving	very happy	Jingdong prefecture level city in Shandong
Dragon Quality Goods Shop	King of Power Saving	favorable criticism	Jingdong prefecture level city in Shandong
Dragon Quality Goods Shop	King of Power Saving	Now they all sign for the goods without receiving them, the Jingdong platform and merchants are very powerful, and the goods are not received before signing for the goods.	Jingdong prefecture level city in Shandong
Dragon Quality Goods Shop	King of Power Saving	low-quality commodities	Jingdong prefecture level city in Shandong
Dragon Quality Goods Shop	King of Power Saving	I bought this and now it works pretty well with the air conditioning on, when I didn't use it it was $600 a month, now it's almost $500,00, that's over $100 less all of a sudden.	Jingdong prefecture level city in Shandong
Dragon Quality Goods Shop	King of Power Saving	Can Oh, the weather is hot, open the air conditioning is really good, this product is good, has been used for a really long time, more ok, power saving effect is not bad.	Jingdong prefecture level city in Shandong
Dragon Quality Goods Shop	King of Power Saving	I paid my electric bill today, it's a few dozen dollars less, anyway, it's not costly, it's already very good, the boss said that it would be better to buy one more, I've already placed an order	Jingdong prefecture level city in Shandong
Dragon Quality Goods Shop	King of Power Saving	I'm on a smart meter, I save about 30% a month, personally I think it's pretty good, it works well	Jingdong prefecture level city in Shandong
Dragon Quality Goods Shop	King of Power Saving	My dad asked me to buy one, but after I paid the bill, I asked and he said it was a lot less this month, so he asked me to buy another one.	Jingdong prefecture level city in Shandong
Dragon Quality Goods Shop	King of Power Saving	Very satisfied with the purchase thanks to the store for the prosperous business.	Jingdong prefecture level city in Shandong
Dragon Quality Goods Shop	King of Power Saving	Good use, good quality, very satisfied with the purchase	Jingdong prefecture level city in Shandong
Dragon Quality Goods Shop	King of Power Saving	The goods have been received, the quality is very good, is the ideal effect, will continue to pay attention to the	Jingdong prefecture level city in Shandong
Dragon Quality Goods Shop	King of Power Saving	This brand has been used for many years, and this time I saw this upgraded version of the product and got it again. The purpose of this product is to reduce the loss of electricity for a long time, so as to achieve the purpose of saving electricity.	Jingdong prefecture level city in Shandong
Dragon Quality Goods Shop	King of Power Saving	I've been using it for almost a month, and I've reduced my electricity bill by over 100 bucks! I'm planning to get two more for my family.	Jingdong prefecture level city in Shandong
Dragon Quality Goods Shop	King of Power Saving	The effect is too good, the family has now several units, back to buy a number of times, the power saver received, power-saving effect is very good,, fine workmanship, long warranty, but also purify the air, before buying a used for a period of time, with the okay, and then bought a, and only now come to the	Jingdong prefecture level city in Shandong
Dragon Quality Goods Shop	King of Power Saving	Finally received the goods, very, very much like,, has been a lot of people praise, really too happy, has been his family's loyal fans, will continue to buy buy buy!	Jingdong prefecture level city in Shandong
Dragon Quality Goods Shop	King of Power Saving	📩 I've been wanting it for a long time, I decided to go for it, I feel great after using it, it's genuine and the delivery is great, it lives up to its name👍	Jingdong prefecture level city in Shandong
Dragon Quality Goods Shop	King of Power Saving	The quality is sure as other friends who bought it said, and it is also beautiful, thanks to the owner for the recommendation, thanks!	Jingdong prefecture level city in Shandong
Dragon Quality Goods Shop	King of Power Saving	Baby received the quality is really good face value is also very high especially practical especially especially special favorite	Jingdong prefecture level city in Shandong
Dragon Quality Goods Shop	King of Power Saving	It is genuine, trust the merchant, the quality is very good, the price is moderate, the use of the effect is very good!	Jingdong prefecture level city in Shandong
Dragon Quality Goods Shop	King of Power Saving	Energy saver received really good quality affordable and good energy saving and environmental protection is the best to use.	Jingdong prefecture level city in Shandong
Dragon Quality Goods Shop	King of Power Saving	Reasonable price logistics is very fast, the feeling is not bad more satisfied with a shopping experience	Jingdong prefecture level city in Shandong
Dragon Quality Goods Shop	King of Power Saving	The stuff is even more perfect than I imagined, very powerful and worth getting!	Jingdong prefecture level city in Shandong
Dragon Quality Goods Shop	King of Power Saving	Goods have been received, good quality, affordable price, good customer service, worth recommending!	Jingdong prefecture level city in Shandong
Dragon Quality Goods Shop	King of Power Saving	Fast shipping, works great, have already recommended it to friends.	Jingdong prefecture level city in Shandong
Dragon Quality Goods Shop	King of Power Saving	Good quality, fast logistics, reasonable price, worth 5 stars.	Jingdong prefecture level city in Shandong
Dragon Quality Goods Shop	King of Power Saving	Good quality, fast logistics, good value, well packaged, practical	Jingdong prefecture level city in Shandong
Dragon Quality Goods Shop	King of Power Saving	Fast shipping, affordable price, great quality, very satisfied!	Jingdong prefecture level city in Shandong
Dragon Quality Goods Shop	King of Power Saving	Regret knowing too late, it's really good stuff. Works well.	Jingdong prefecture level city in Shandong
Dragon Quality Goods Shop	King of Power Saving	Not bad, very good product, quite save electricity	Jingdong prefecture level city in Shandong
Dragon Quality Goods Shop	King of Power Saving	Good quality, fast logistics, overall very satisfied!	Jingdong prefecture level city in Shandong
Dragon Quality Goods Shop	King of Power Saving	Tightly packaged with good results	Jingdong prefecture level city in Shandong
Dragon Quality Goods Shop	King of Power Saving	very happy	Jingdong prefecture level city in Shandong
Dragon Quality Goods Shop	King of Power Saving	favorable criticism	Jingdong prefecture level city in Shandong
Dragon Quality Goods Shop	King of Power Saving	Now they all sign for the goods without receiving them, the Jingdong platform and merchants are very powerful, and the goods are not received before signing for the goods.	Jingdong prefecture level city in Shandong
Dragon Quality Goods Shop	King of Power Saving	low-quality commodities	Jingdong prefecture level city in Shandong
Dragon Quality Goods Shop	King of Power Saving	I bought this and now it works pretty well with the air conditioning on, when I didn't use it it was $600 a month, now it's almost $500,00, that's over $100 less all of a sudden.	Jingdong prefecture level city in Shandong
Dragon Quality Goods Shop	King of Power Saving	Can Oh, the weather is hot, open the air conditioning is really good, this product is good, has been used for a really long time, more ok, power saving effect is not bad.	Jingdong prefecture level city in Shandong
Dragon Quality Goods Shop	King of Power Saving	I paid my electric bill today, it's a few dozen dollars less, anyway, it's not costly, it's already very good, the boss said that it would be better to buy one more, I've already placed an order	Jingdong prefecture level city in Shandong
Dragon Quality Goods Shop	King of Power Saving	I'm on a smart meter, I save about 30% a month, personally I think it's pretty good, it works well	Jingdong prefecture level city in Shandong
Dragon Quality Goods Shop	King of Power Saving	My dad asked me to buy one, but after I paid the bill, I asked and he said it was a lot less this month, so he asked me to buy another one.	Jingdong prefecture level city in Shandong
Dragon Quality Goods Shop	King of Power Saving	Very satisfied with the purchase thanks to the store for the prosperous business.	Jingdong prefecture level city in Shandong
Dragon Quality Goods Shop	King of Power Saving	Good use, good quality, very satisfied with the purchase	Jingdong prefecture level city in Shandong
Dragon Quality Goods Shop	King of Power Saving	The goods have been received, the quality is very good, is the ideal effect, will continue to pay attention to the	Jingdong prefecture level city in Shandong
Dragon Quality Goods Shop	King of Power Saving	This brand has been used for many years, and this time I saw this upgraded version of the product and got it again. The purpose of this product is to reduce the loss of electricity for a long time, so as to achieve the purpose of saving electricity.	Jingdong prefecture level city in Shandong
Dragon Quality Goods Shop	King of Power Saving	I've been using it for almost a month, and I've reduced my electricity bill by over 100 bucks! I'm planning to get two more for my family.	Jingdong prefecture level city in Shandong
Dragon Quality Goods Shop	King of Power Saving	The effect is too good, the family has now several units, back to buy a number of times, the power saver received, power-saving effect is very good,, fine workmanship, long warranty, but also purify the air, before buying a used for a period of time, with the okay, and then bought a, and only now come to the	Jingdong prefecture level city in Shandong
Dragon Quality Goods Shop	King of Power Saving	Finally received the goods, very, very much like,, has been a lot of people praise, really too happy, has been his family's loyal fans, will continue to buy buy buy!	Jingdong prefecture level city in Shandong
Dragon Quality Goods Shop	King of Power Saving	📩 I've been wanting it for a long time, I decided to go for it, I feel great after using it, it's genuine and the delivery is great, it lives up to its name👍	Jingdong prefecture level city in Shandong

Dragon Quality Goods Shop	King of Power Saving	The quality is sure as other friends who bought it said, and it is also beautiful, thanks to the owner for the recommendation, thanks!	Jingdong prefecture level city in Shandong
Dragon Quality Goods Shop	King of Power Saving	Baby received the quality is really good face value is also very high especially practical especially especially special favorite	Jingdong prefecture level city in Shandong
Dragon Quality Goods Shop	King of Power Saving	It is genuine, trust the merchant, the quality is very good, the price is moderate, the use of the effect is very good!	Jingdong prefecture level city in Shandong
Dragon Quality Goods Shop	King of Power Saving	Energy saver received really good quality affordable and good energy saving and environmental protection is the best to use.	Jingdong prefecture level city in Shandong
Dragon Quality Goods Shop	King of Power Saving	Reasonable price logistics is very fast, the feeling is not bad more satisfied with a shopping experience	Jingdong prefecture level city in Shandong
Dragon Quality Goods Shop	King of Power Saving	The stuff is even more perfect than I imagined, very powerful and worth getting!	Jingdong prefecture level city in Shandong
Dragon Quality Goods Shop	King of Power Saving	Goods have been received, good quality, affordable price, good customer service, worth recommending!	Jingdong prefecture level city in Shandong
Dragon Quality Goods Shop	King of Power Saving	Fast shipping, works great, have already recommended it to friends.	Jingdong prefecture level city in Shandong
Dragon Quality Goods Shop	King of Power Saving	Good quality, fast logistics, reasonable price, worth 5 stars.	Jingdong prefecture level city in Shandong
Dragon Quality Goods Shop	King of Power Saving	Good quality, fast logistics, good value, well packaged, practical	Jingdong prefecture level city in Shandong
Dragon Quality Goods Shop	King of Power Saving	Fast shipping, affordable price, great quality, very satisfied!	Jingdong prefecture level city in Shandong
Dragon Quality Goods Shop	King of Power Saving	Regret knowing too late, it's really good stuff. Works well.	Jingdong prefecture level city in Shandong
Dragon Quality Goods Shop	King of Power Saving	Not bad, very good product, quite save electricity	Jingdong prefecture level city in Shandong
Dragon Quality Goods Shop	King of Power Saving	Good quality, fast logistics, overall very satisfied!	Jingdong prefecture level city in Shandong
Dragon Quality Goods Shop	King of Power Saving	Tightly packaged with good results	Jingdong prefecture level city in Shandong
Dragon Quality Goods Shop	King of Power Saving	very happy	Jingdong prefecture level city in Shandong
Dragon Quality Goods Shop	King of Power Saving	favorable criticism	Jingdong prefecture level city in Shandong
Dragon Quality Goods Shop	King of Power Saving	Now they all sign for the goods without receiving them, the Jingdong platform and merchants are very powerful, and the goods are not received before signing for the goods.	Jingdong prefecture level city in Shandong
Dragon Quality Goods Shop	King of Power Saving	low-quality commodities	Jingdong prefecture level city in Shandong
Dragon Quality Goods Shop	King of Power Saving	I bought this and now it works pretty well with the air conditioning on, when I didn't use it it was $600 a month, now it's almost $500,00, that's over $100 less all of a sudden.	Jingdong prefecture level city in Shandong
Dragon Quality Goods Shop	King of Power Saving	Can Oh, the weather is hot, open the air conditioning is really good, this product is good, has been used for a really long time, more ok, power saving effect is not bad.	Jingdong prefecture level city in Shandong
Dragon Quality Goods Shop	King of Power Saving	I paid my electric bill today, it's a few dozen dollars less, anyway, it's not costly, it's already very good, the boss said that it would be better to buy one more, I've already placed an order	Jingdong prefecture level city in Shandong
Dragon Quality Goods Shop	King of Power Saving	I'm on a smart meter, I save about 30% a month, personally I think it's pretty good, it works well	Jingdong prefecture level city in Shandong
Dragon Quality Goods Shop	King of Power Saving	My dad asked me to buy one, but after I paid the bill, I asked and he said it was a lot less this month, so he asked me to buy another one.	Jingdong prefecture level city in Shandong
Dragon Quality Goods Shop	King of Power Saving	Very satisfied with the purchase thanks to the store for the prosperous business.	Jingdong prefecture level city in Shandong
Dragon Quality Goods Shop	King of Power Saving	Good use, good quality, very satisfied with the purchase	Jingdong prefecture level city in Shandong
Dragon Quality Goods Shop	King of Power Saving	The goods have been received, the quality is very good, is the ideal effect, will continue to pay attention to the	Jingdong prefecture level city in Shandong
Dragon Quality Goods Shop	King of Power Saving	This brand has been used for many years, and this time I saw this upgraded version of the product and got it again. The purpose of this product is to reduce the loss of electricity for a long time, so as to achieve the purpose of saving electricity.	Jingdong prefecture level city in Shandong
Dragon Quality Goods Shop	King of Power Saving	I've been using it for almost a month, and I've reduced my electricity bill by over 100 bucks! I'm planning to get two more for my family.	Jingdong prefecture level city in Shandong
Dragon Quality Goods Shop	King of Power Saving	The effect is too good, the family has now several units, back to buy a number of times, the power saver received, power-saving effect is very good,, fine workmanship, long warranty, but also purify the air, before buying a used for a period of time, with the okay, and then bought a, and only now come to the	Jingdong prefecture level city in Shandong
Dragon Quality Goods Shop	King of Power Saving	Finally received the goods, very, very much like,, has been a lot of people praise, really too happy, has been his family's loyal fans, will continue to buy buy buy buy!	Jingdong prefecture level city in Shandong
Dragon Quality Goods Shop	King of Power Saving	📩 I've been wanting it for a long time, I decided to go for it, I feel great after using it, it's genuine and the delivery is great, it lives up to its name👍	Jingdong prefecture level city in Shandong
Dragon Quality Goods Shop	King of Power Saving	The quality is sure as other friends who bought it said, and it is also beautiful, thanks to the owner for the recommendation, thanks!	Jingdong prefecture level city in Shandong
Dragon Quality Goods Shop	King of Power Saving	Baby received the quality is really good face value is also very high especially practical especially especially special favorite	Jingdong prefecture level city in Shandong
Dragon Quality Goods Shop	King of Power Saving	It is genuine, trust the merchant, the quality is very good, the price is moderate, the use of the effect is very good!	Jingdong prefecture level city in Shandong
Dragon Quality Goods Shop	King of Power Saving	Energy saver received really good quality affordable and good energy saving and environmental protection is the best to use.	Jingdong prefecture level city in Shandong
Dragon Quality Goods Shop	King of Power Saving	Reasonable price logistics is very fast, the feeling is not bad more satisfied with a shopping experience	Jingdong prefecture level city in Shandong
Dragon Quality Goods Shop	King of Power Saving	The stuff is even more perfect than I imagined, very powerful and worth getting!	Jingdong prefecture level city in Shandong
Dragon Quality Goods Shop	King of Power Saving	Goods have been received, good quality, affordable price, good customer service, worth recommending!	Jingdong prefecture level city in Shandong
Dragon Quality Goods Shop	King of Power Saving	Fast shipping, works great, have already recommended it to friends.	Jingdong prefecture level city in Shandong
Dragon Quality Goods Shop	King of Power Saving	Good quality, fast logistics, reasonable price, worth 5 stars.	Jingdong prefecture level city in Shandong
Dragon Quality Goods Shop	King of Power Saving	Good quality, fast logistics, good value, well packaged, practical	Jingdong prefecture level city in Shandong
Dragon Quality Goods Shop	King of Power Saving	Fast shipping, affordable price, great quality, very satisfied!	Jingdong prefecture level city in Shandong
Dragon Quality Goods Shop	King of Power Saving	Regret knowing too late, it's really good stuff. Works well.	Jingdong prefecture level city in Shandong
Dragon Quality Goods Shop	King of Power Saving	Not bad, very good product, quite save electricity	Jingdong prefecture level city in Shandong
Dragon Quality Goods Shop	King of Power Saving	Good quality, fast logistics, overall very satisfied!	Jingdong prefecture level city in Shandong
Dragon Quality Goods Shop	King of Power Saving	Tightly packaged with good results	Jingdong prefecture level city in Shandong
Dragon Quality Goods Shop	King of Power Saving	very happy	Jingdong prefecture level city in Shandong
Dragon Quality Goods Shop	King of Power Saving	favorable criticism	Jingdong prefecture level city in Shandong
Dragon Quality Goods Shop	King of Power Saving	Now they all sign for the goods without receiving them, the Jingdong platform and merchants are very powerful, and the goods are not received before signing for the goods.	Jingdong prefecture level city in Shandong

Dragon Quality Goods Shop	King of Power Saving	low-quality commodities	Jingdong prefecture level city in Shandong
Dragon Quality Goods Shop	King of Power Saving	I bought this and now it works pretty well with the air conditioning on, when I didn't use it it was $600 a month, now it's almost $500.00, that's over $100 less all of a sudden.	Jingdong prefecture level city in Shandong
Dragon Quality Goods Shop	King of Power Saving	Can Oh, the weather is hot, open the air conditioning is really good, this product is good, has been used for a really long time, more ok, power saving effect is not bad.	Jingdong prefecture level city in Shandong
Dragon Quality Goods Shop	King of Power Saving	I paid my electric bill today, it's a few dozen dollars less, anyway, it's not costly, it's already very good, the boss said that it would be better to buy one more, I've already placed an order	Jingdong prefecture level city in Shandong
Dragon Quality Goods Shop	King of Power Saving	I'm on a smart meter, I save about 30% a month, personally I think it's pretty good, it works well	Jingdong prefecture level city in Shandong
Dragon Quality Goods Shop	King of Power Saving	My dad asked me to buy one, but after I paid the bill, I asked and he said it was a lot less this month, so he asked me to buy another one.	Jingdong prefecture level city in Shandong
Dragon Quality Goods Shop	King of Power Saving	Very satisfied with the purchase thanks to the store for the prosperous business.	Jingdong prefecture level city in Shandong
Dragon Quality Goods Shop	King of Power Saving	Good use, good quality, very satisfied with the purchase	Jingdong prefecture level city in Shandong
Dragon Quality Goods Shop	King of Power Saving	The goods have been received, the quality is very good, is the ideal effect, will continue to pay attention to the	Jingdong prefecture level city in Shandong
Dragon Quality Goods Shop	King of Power Saving	This brand has been used for many years, and this time I saw this upgraded version of the product and got it again. The purpose of this product is to reduce the loss of electricity for a long time, so as to achieve the purpose of saving electricity.	Jingdong prefecture level city in Shandong
Dragon Quality Goods Shop	King of Power Saving	I've been using it for almost a month, and I've reduced my electricity bill by over 100 bucks! I'm planning to get two more for my family.	Jingdong prefecture level city in Shandong
Dragon Quality Goods Shop	King of Power Saving	The effect is too good, the family has now several units, back to buy a number of times, the power saver received, power-saving effect is very good,, fine workmanship, long warranty, but also purify the air, before buying a used for a period of time, with the okay, and then bought a, and only now come to the	Jingdong prefecture level city in Shandong
Dragon Quality Goods Shop	King of Power Saving	Finally received the goods, very, very much like,, has been a lot of people praise, really too happy, has been his family's loyal fans, will continue to buy buy buy buy!	Jingdong prefecture level city in Shandong
Dragon Quality Goods Shop	King of Power Saving	📩 I've been wanting it for a long time, I decided to go for it, I feel great after using it, it's genuine and the delivery is great, it lives up to its name👍	Jingdong prefecture level city in Shandong
Dragon Quality Goods Shop	King of Power Saving	The quality is sure as other friends who bought it said, and it is also beautiful, thanks to the owner for the recommendation, thanks!	Jingdong prefecture level city in Shandong
Dragon Quality Goods Shop	King of Power Saving	Baby received the quality is really good face value is also very high especially practical especially especially special favorite	Jingdong prefecture level city in Shandong
Dragon Quality Goods Shop	King of Power Saving	It is genuine, trust the merchant, the quality is very good, the price is moderate, the use of the effect is very good!	Jingdong prefecture level city in Shandong
Dragon Quality Goods Shop	King of Power Saving	Energy saver received really good quality affordable and good energy saving and environmental protection is the best to use.	Jingdong prefecture level city in Shandong
Dragon Quality Goods Shop	King of Power Saving	Reasonable price logistics is very fast, the feeling is not bad more satisfied with a shopping experience	Jingdong prefecture level city in Shandong
Dragon Quality Goods Shop	King of Power Saving	The stuff is even more perfect than I imagined, very powerful and worth getting!	Jingdong prefecture level city in Shandong
Dragon Quality Goods Shop	King of Power Saving	Goods have been received, good quality, affordable price, good customer service, worth recommending!	Jingdong prefecture level city in Shandong
Dragon Quality Goods Shop	King of Power Saving	Fast shipping, works great, have already recommended it to friends.	Jingdong prefecture level city in Shandong
Dragon Quality Goods Shop	King of Power Saving	Good quality, fast logistics, reasonable price, worth 5 stars.	Jingdong prefecture level city in Shandong
Dragon Quality Goods Shop	King of Power Saving	Good quality, fast logistics, good value, well packaged, practical	Jingdong prefecture level city in Shandong
Dragon Quality Goods Shop	King of Power Saving	Fast shipping, affordable price, great quality, very satisfied!	Jingdong prefecture level city in Shandong
Dragon Quality Goods Shop	King of Power Saving	Regret knowing too late, it's really good stuff. Works well.	Jingdong prefecture level city in Shandong
Dragon Quality Goods Shop	King of Power Saving	Not bad, very good product, quite save electricity	Jingdong prefecture level city in Shandong
Dragon Quality Goods Shop	King of Power Saving	Good quality, fast logistics, overall very satisfied!	Jingdong prefecture level city in Shandong
Dragon Quality Goods Shop	King of Power Saving	Tightly packaged with good results	Jingdong prefecture level city in Shandong
Dragon Quality Goods Shop	King of Power Saving	very happy	Jingdong prefecture level city in Shandong
Dragon Quality Goods Shop	King of Power Saving	favorable criticism	Jingdong prefecture level city in Shandong
Dragon Quality Goods Shop	King of Power Saving	Now they all sign for the goods without receiving them, the Jingdong platform and merchants are very powerful, and the goods are not received before signing for the goods.	Jingdong prefecture level city in Shandong
Dragon Quality Goods Shop	King of Power Saving	low-quality commodities	Jingdong prefecture level city in Shandong
Dragon Quality Goods Shop	King of Power Saving	I bought this and now it works pretty well with the air conditioning on, when I didn't use it it was $600 a month, now it's almost $500.00, that's over $100 less all of a sudden.	Jingdong prefecture level city in Shandong
Dragon Quality Goods Shop	King of Power Saving	Can Oh, the weather is hot, open the air conditioning is really good, this product is good, has been used for a really long time, more ok, power saving effect is not bad.	Jingdong prefecture level city in Shandong
Dragon Quality Goods Shop	King of Power Saving	I paid my electric bill today, it's a few dozen dollars less, anyway, it's not costly, it's already very good, the boss said that it would be better to buy one more, I've already placed an order	Jingdong prefecture level city in Shandong
Dragon Quality Goods Shop	King of Power Saving	I'm on a smart meter, I save about 30% a month, personally I think it's pretty good, it works well	Jingdong prefecture level city in Shandong
Dragon Quality Goods Shop	King of Power Saving	My dad asked me to buy one, but after I paid the bill, I asked and he said it was a lot less this month, so he asked me to buy another one.	Jingdong prefecture level city in Shandong
Dragon Quality Goods Shop	King of Power Saving	Very satisfied with the purchase thanks to the store for the prosperous business.	Jingdong prefecture level city in Shandong
Dragon Quality Goods Shop	King of Power Saving	Good use, good quality, very satisfied with the purchase	Jingdong prefecture level city in Shandong
Dragon Quality Goods Shop	King of Power Saving	The goods have been received, the quality is very good, is the ideal effect, will continue to pay attention to the	Jingdong prefecture level city in Shandong
Dragon Quality Goods Shop	King of Power Saving	This brand has been used for many years, and this time I saw this upgraded version of the product and got it again. The purpose of this product is to reduce the loss of electricity for a long time, so as to achieve the purpose of saving electricity.	Jingdong prefecture level city in Shandong
Dragon Quality Goods Shop	King of Power Saving	I've been using it for almost a month, and I've reduced my electricity bill by over 100 bucks! I'm planning to get two more for my family.	Jingdong prefecture level city in Shandong
Dragon Quality Goods Shop	King of Power Saving	The effect is too good, the family has now several units, back to buy a number of times, the power saver received, power-saving effect is very good,, fine workmanship, long warranty, but also purify the air, before buying a used for a period of time, with the okay, and then bought a, and only now come to the	Jingdong prefecture level city in Shandong
Dragon Quality Goods Shop	King of Power Saving	Finally received the goods, very, very much like,, has been a lot of people praise, really too happy, has been his family's loyal fans, will continue to buy buy buy buy!	Jingdong prefecture level city in Shandong
Dragon Quality Goods Shop	King of Power Saving	📩 I've been wanting it for a long time, I decided to go for it, I feel great after using it, it's genuine and the delivery is great, it lives up to its name👍	Jingdong prefecture level city in Shandong
Dragon Quality Goods Shop	King of Power Saving	The quality is sure as other friends who bought it said, and it is also beautiful, thanks to the owner for the recommendation, thanks!	Jingdong prefecture level city in Shandong
Dragon Quality Goods Shop	King of Power Saving	Baby received the quality is really good face value is also very high especially practical especially especially special favorite	Jingdong prefecture level city in Shandong
Dragon Quality Goods Shop	King of Power Saving	It is genuine, trust the merchant, the quality is very good, the price is moderate, the use of the effect is very good!	Jingdong prefecture level city in Shandong

Dragon Quality Goods Shop	King of Power Saving	Energy saver received really good quality affordable and good energy saving and environmental protection is the best to use.	Jingdong prefecture level city in Shandong
Dragon Quality Goods Shop	King of Power Saving	Reasonable price logistics is very fast, the feeling is not bad more satisfied with a shopping experience	Jingdong prefecture level city in Shandong
Dragon Quality Goods Shop	King of Power Saving	The stuff is even more perfect than I imagined, very powerful and worth getting!	Jingdong prefecture level city in Shandong
Dragon Quality Goods Shop	King of Power Saving	Goods have been received, good quality, affordable price, good customer service, worth recommending!	Jingdong prefecture level city in Shandong
Dragon Quality Goods Shop	King of Power Saving	Fast shipping, works great, have already recommended it to friends.	Jingdong prefecture level city in Shandong
Dragon Quality Goods Shop	King of Power Saving	Good quality, fast logistics, reasonable price, worth 5 stars.	Jingdong prefecture level city in Shandong
Dragon Quality Goods Shop	King of Power Saving	Good quality, fast logistics, good value, well packaged, practical	Jingdong prefecture level city in Shandong
Dragon Quality Goods Shop	King of Power Saving	Fast shipping, affordable price, great quality, very satisfied!	Jingdong prefecture level city in Shandong
Dragon Quality Goods Shop	King of Power Saving	Regret knowing too late, it's really good stuff. Works well.	Jingdong prefecture level city in Shandong
Dragon Quality Goods Shop	King of Power Saving	Not bad, very good product, quite save electricity	Jingdong prefecture level city in Shandong
Dragon Quality Goods Shop	King of Power Saving	Good quality, fast logistics, overall very satisfied!	Jingdong prefecture level city in Shandong
Dragon Quality Goods Shop	King of Power Saving	Tightly packaged with good results	Jingdong prefecture level city in Shandong
Dragon Quality Goods Shop	King of Power Saving	very happy	Jingdong prefecture level city in Shandong
Dragon Quality Goods Shop	King of Power Saving	favorable criticism	Jingdong prefecture level city in Shandong
Dragon Quality Goods Shop	King of Power Saving	Now they all sign for the goods without receiving them, the Jingdong platform and merchants are very powerful, and the goods are not received before signing for the goods.	Jingdong prefecture level city in Shandong
Dragon Quality Goods Shop	King of Power Saving	low-quality commodities	Jingdong prefecture level city in Shandong
Dragon Quality Goods Shop	King of Power Saving	I bought this and now it works pretty well with the air conditioning on, when I didn't use it it was $600 a month, now it's almost $500,00, that's over $100 less all of a sudden.	Jingdong prefecture level city in Shandong
Dragon Quality Goods Shop	King of Power Saving	Can Oh, the weather is hot, open the air conditioning is really good, this product is good, has been used for a really long time, more ok, power saving effect is not bad.	Jingdong prefecture level city in Shandong
Dragon Quality Goods Shop	King of Power Saving	I paid my electric bill today, it's a few dozen dollars less, anyway, it's not costly, it's already very good, the boss said that it would be better to buy one more, I've already placed an order	Jingdong prefecture level city in Shandong
Dragon Quality Goods Shop	King of Power Saving	I'm on a smart meter, I save about 30% a month, personally I think it's pretty good, it works well	Jingdong prefecture level city in Shandong
Dragon Quality Goods Shop	King of Power Saving	My dad asked me to buy one, but after I paid the bill, I asked and he said it was a lot less this month, so he asked me to buy another one.	Jingdong prefecture level city in Shandong
Dragon Quality Goods Shop	King of Power Saving	Very satisfied with the purchase thanks to the store for the prosperous business.	Jingdong prefecture level city in Shandong
Dragon Quality Goods Shop	King of Power Saving	Good use, good quality, very satisfied with the purchase	Jingdong prefecture level city in Shandong
Dragon Quality Goods Shop	King of Power Saving	The goods have been received, the quality is very good, is the ideal effect, will continue to pay attention to the	Jingdong prefecture level city in Shandong
Dragon Quality Goods Shop	King of Power Saving	This brand has been used for many years, and this time I saw this upgraded version of the product and got it again. The purpose of this product is to reduce the loss of electricity for a long time, so as to achieve the purpose of saving electricity.	Jingdong prefecture level city in Shandong
Dragon Quality Goods Shop	King of Power Saving	I've been using it for almost a month, and I've reduced my electricity bill by over 100 bucks! I'm planning to get two more for my family.	Jingdong prefecture level city in Shandong
Dragon Quality Goods Shop	King of Power Saving	The effect is too good, the family has now several units, back to buy a number of times, the power saver received, power-saving effect is very good,, fine workmanship, long warranty, but also purify the air, before buying a used for a period of time, with the okay, and then bought a, and only now come to the	Jingdong prefecture level city in Shandong
Dragon Quality Goods Shop	King of Power Saving	Finally received the goods, very, very much like,, has been a lot of people praise, really too happy, has been his family's loyal fans, will continue to buy buy buy buy!	Jingdong prefecture level city in Shandong
Dragon Quality Goods Shop	King of Power Saving	📩 I've been wanting it for a long time, I decided to go for it, I feel great after using it, it's genuine and the delivery is great, it lives up to its name👍	Jingdong prefecture level city in Shandong
Dragon Quality Goods Shop	King of Power Saving	The quality is sure as other friends who bought it said, and it is also beautiful, thanks to the owner for the recommendation, thanks!	Jingdong prefecture level city in Shandong
Dragon Quality Goods Shop	King of Power Saving	Baby received the quality is really good face value is also very high especially practical especially especially special favorite	Jingdong prefecture level city in Shandong
Dragon Quality Goods Shop	King of Power Saving	It is genuine, trust the merchant, the quality is very good, the price is moderate, the use of the effect is very good!	Jingdong prefecture level city in Shandong
Dragon Quality Goods Shop	King of Power Saving	Energy saver received really good quality affordable and good energy saving and environmental protection is the best to use.	Jingdong prefecture level city in Shandong
Dragon Quality Goods Shop	King of Power Saving	Reasonable price logistics is very fast, the feeling is not bad more satisfied with a shopping experience	Jingdong prefecture level city in Shandong
Dragon Quality Goods Shop	King of Power Saving	The stuff is even more perfect than I imagined, very powerful and worth getting!	Jingdong prefecture level city in Shandong
Dragon Quality Goods Shop	King of Power Saving	Goods have been received, good quality, affordable price, good customer service, worth recommending!	Jingdong prefecture level city in Shandong
Dragon Quality Goods Shop	King of Power Saving	Fast shipping, works great, have already recommended it to friends.	Jingdong prefecture level city in Shandong
Dragon Quality Goods Shop	King of Power Saving	Good quality, fast logistics, reasonable price, worth 5 stars.	Jingdong prefecture level city in Shandong
Dragon Quality Goods Shop	King of Power Saving	Good quality, fast logistics, good value, well packaged, practical	Jingdong prefecture level city in Shandong
Dragon Quality Goods Shop	King of Power Saving	Fast shipping, affordable price, great quality, very satisfied!	Jingdong prefecture level city in Shandong
Dragon Quality Goods Shop	King of Power Saving	Regret knowing too late, it's really good stuff. Works well.	Jingdong prefecture level city in Shandong
Dragon Quality Goods Shop	King of Power Saving	Not bad, very good product, quite save electricity	Jingdong prefecture level city in Shandong
Dragon Quality Goods Shop	King of Power Saving	Good quality, fast logistics, overall very satisfied!	Jingdong prefecture level city in Shandong
Dragon Quality Goods Shop	King of Power Saving	Tightly packaged with good results	Jingdong prefecture level city in Shandong
Dragon Quality Goods Shop	King of Power Saving	very happy	Jingdong prefecture level city in Shandong
Dragon Quality Goods Shop	King of Power Saving	favorable criticism	Jingdong prefecture level city in Shandong
Dragon Quality Goods Shop	King of Power Saving	Now they all sign for the goods without receiving them, the Jingdong platform and merchants are very powerful, and the goods are not received before signing for the goods.	Jingdong prefecture level city in Shandong
Dragon Quality Goods Shop	King of Power Saving	low-quality commodities	Jingdong prefecture level city in Shandong
Dragon Quality Goods Shop	King of Power Saving	I bought this and now it works pretty well with the air conditioning on, when I didn't use it it was $600 a month, now it's almost $500,00, that's over $100 less all of a sudden.	Jingdong prefecture level city in Shandong
Dragon Quality Goods Shop	King of Power Saving	Can Oh, the weather is hot, open the air conditioning is really good, this product is good, has been used for a really long time, more ok, power saving effect is not bad.	Jingdong prefecture level city in Shandong

Dragon Quality Goods Shop	King of Power Saving	I paid my electric bill today, it's a few dozen dollars less, anyway, it's not costly, it's already very good, the boss said that it would be better to buy one more, I've already placed an order	Jingdong prefecture level city in Shandong
Dragon Quality Goods Shop	King of Power Saving	I'm on a smart meter, I save about 30% a month, personally I think it's pretty good, it works well	Jingdong prefecture level city in Shandong
Dragon Quality Goods Shop	King of Power Saving	My dad asked me to buy one, but after I paid the bill, I asked and he said it was a lot less this month, so he asked me to buy another one.	Jingdong prefecture level city in Shandong
Dragon Quality Goods Shop	King of Power Saving	Very satisfied with the purchase thanks to the store for the prosperous business.	Jingdong prefecture level city in Shandong
Dragon Quality Goods Shop	King of Power Saving	Good use, good quality, very satisfied with the purchase	Jingdong prefecture level city in Shandong
Dragon Quality Goods Shop	King of Power Saving	The goods have been received, the quality is very good, is the ideal effect, will continue to pay attention to the	Jingdong prefecture level city in Shandong
Dragon Quality Goods Shop	King of Power Saving	This brand has been used for many years, and this time I saw this upgraded version of the product and got it again. The purpose of this product is to reduce the loss of electricity for a long time, so as to achieve the purpose of saving electricity.	Jingdong prefecture level city in Shandong
Dragon Quality Goods Shop	King of Power Saving	I've been using it for almost a month, and I've reduced my electricity bill by over 100 bucks! I'm planning to get two more for my family.	Jingdong prefecture level city in Shandong
Dragon Quality Goods Shop	King of Power Saving	The effect is too good, the family has now several units, back to buy a number of times, the power saver received, power-saving effect is very good,, fine workmanship, long warranty, but also purify the air, before buying a used for a period of time, with the okay, and then bought a, and only now come to the	Jingdong prefecture level city in Shandong
Dragon Quality Goods Shop	King of Power Saving	Finally received the goods, very, very much like,, has been a lot of people praise, really too happy, has been his family's loyal fans, will continue to buy buy buy buy!	Jingdong prefecture level city in Shandong
Dragon Quality Goods Shop	King of Power Saving	📩 I've been wanting it for a long time, I decided to go for it, I feel great after using it, it's genuine and the delivery is great, it lives up to its name👍	Jingdong prefecture level city in Shandong
Dragon Quality Goods Shop	King of Power Saving	The quality is sure as other friends who bought it said, and it is also beautiful, thanks to the owner for the recommendation, thanks!	Jingdong prefecture level city in Shandong
Dragon Quality Goods Shop	King of Power Saving	Baby received the quality is really good face value is also very high especially practical especially especially special favorite	Jingdong prefecture level city in Shandong
Dragon Quality Goods Shop	King of Power Saving	It is genuine, trust the merchant, the quality is very good, the price is moderate, the use of the effect is very good!	Jingdong prefecture level city in Shandong
Dragon Quality Goods Shop	King of Power Saving	Energy saver received really good quality affordable and good energy saving and environmental protection is the best to use.	Jingdong prefecture level city in Shandong
Dragon Quality Goods Shop	King of Power Saving	Reasonable price logistics is very fast, the feeling is not bad more satisfied with a shopping experience	Jingdong prefecture level city in Shandong
Dragon Quality Goods Shop	King of Power Saving	The stuff is even more perfect than I imagined, very powerful and worth getting!	Jingdong prefecture level city in Shandong
Dragon Quality Goods Shop	King of Power Saving	Goods have been received, good quality, affordable price, good customer service, worth recommending!	Jingdong prefecture level city in Shandong
Dragon Quality Goods Shop	King of Power Saving	Fast shipping, works great, have already recommended it to friends.	Jingdong prefecture level city in Shandong
Dragon Quality Goods Shop	King of Power Saving	Good quality, fast logistics, reasonable price, worth 5 stars.	Jingdong prefecture level city in Shandong
Dragon Quality Goods Shop	King of Power Saving	Good quality, fast logistics, good value, well packaged, practical	Jingdong prefecture level city in Shandong
Dragon Quality Goods Shop	King of Power Saving	Fast shipping, affordable price, great quality, very satisfied!	Jingdong prefecture level city in Shandong
Dragon Quality Goods Shop	King of Power Saving	Regret knowing too late, it's really good stuff. Works well.	Jingdong prefecture level city in Shandong
Dragon Quality Goods Shop	King of Power Saving	Not bad, very good product, quite save electricity	Jingdong prefecture level city in Shandong
Dragon Quality Goods Shop	King of Power Saving	Good quality, fast logistics, overall very satisfied!	Jingdong prefecture level city in Shandong
Dragon Quality Goods Shop	King of Power Saving	Tightly packaged with good results	Jingdong prefecture level city in Shandong
Dragon Quality Goods Shop	King of Power Saving	very happy	Jingdong prefecture level city in Shandong
Dragon Quality Goods Shop	King of Power Saving	favorable criticism	Jingdong prefecture level city in Shandong
Dragon Quality Goods Shop	King of Power Saving	Now they all sign for the goods without receiving them, the Jingdong platform and merchants are very powerful, and the goods are not received before signing for the goods.	Jingdong prefecture level city in Shandong
Dragon Quality Goods Shop	King of Power Saving	low-quality commodities	Jingdong prefecture level city in Shandong
Dragon Quality Goods Shop	King of Power Saving	I bought this and now it works pretty well with the air conditioning on, when I didn't use it it was $600 a month, now it's almost $500,00, that's over $100 less all of a sudden.	Jingdong prefecture level city in Shandong
Dragon Quality Goods Shop	King of Power Saving	Can Oh, the weather is hot, open the air conditioning is really good, this product is good, has been used for a really long time, more ok, power saving effect is not bad.	Jingdong prefecture level city in Shandong
Dragon Quality Goods Shop	King of Power Saving	I paid my electric bill today, it's a few dozen dollars less, anyway, it's not costly, it's already very good, the boss said that it would be better to buy one more, I've already placed an order	Jingdong prefecture level city in Shandong
Dragon Quality Goods Shop	King of Power Saving	I'm on a smart meter, I save about 30% a month, personally I think it's pretty good, it works well	Jingdong prefecture level city in Shandong
Dragon Quality Goods Shop	King of Power Saving	My dad asked me to buy one, but after I paid the bill, I asked and he said it was a lot less this month, so he asked me to buy another one.	Jingdong prefecture level city in Shandong
Dragon Quality Goods Shop	King of Power Saving	Very satisfied with the purchase thanks to the store for the prosperous business.	Jingdong prefecture level city in Shandong
Dragon Quality Goods Shop	King of Power Saving	Good use, good quality, very satisfied with the purchase	Jingdong prefecture level city in Shandong
Dragon Quality Goods Shop	King of Power Saving	The goods have been received, the quality is very good, is the ideal effect, will continue to pay attention to the	Jingdong prefecture level city in Shandong
Dragon Quality Goods Shop	King of Power Saving	This brand has been used for many years, and this time I saw this upgraded version of the product and got it again. The purpose of this product is to reduce the loss of electricity for a long time, so as to achieve the purpose of saving electricity.	Jingdong prefecture level city in Shandong
Dragon Quality Goods Shop	King of Power Saving	I've been using it for almost a month, and I've reduced my electricity bill by over 100 bucks! I'm planning to get two more for my family.	Jingdong prefecture level city in Shandong
Dragon Quality Goods Shop	King of Power Saving	The effect is too good, the family has now several units, back to buy a number of times, the power saver received, power-saving effect is very good,, fine workmanship, long warranty, but also purify the air, before buying a used for a period of time, with the okay, and then bought a, and only now come to the	Jingdong prefecture level city in Shandong
Dragon Quality Goods Shop	King of Power Saving	Finally received the goods, very, very much like,, has been a lot of people praise, really too happy, has been his family's loyal fans, will continue to buy buy buy buy!	Jingdong prefecture level city in Shandong
Dragon Quality Goods Shop	King of Power Saving	📩 I've been wanting it for a long time, I decided to go for it, I feel great after using it, it's genuine and the delivery is great, it lives up to its name👍	Jingdong prefecture level city in Shandong
Dragon Quality Goods Shop	King of Power Saving	The quality is sure as other friends who bought it said, and it is also beautiful, thanks to the owner for the recommendation, thanks!	Jingdong prefecture level city in Shandong
Dragon Quality Goods Shop	King of Power Saving	Baby received the quality is really good face value is also very high especially practical especially especially special favorite	Jingdong prefecture level city in Shandong
Dragon Quality Goods Shop	King of Power Saving	It is genuine, trust the merchant, the quality is very good, the price is moderate, the use of the effect is very good!	Jingdong prefecture level city in Shandong
Dragon Quality Goods Shop	King of Power Saving	Energy saver received really good quality affordable and good energy saving and environmental protection is the best to use.	Jingdong prefecture level city in Shandong
Dragon Quality Goods Shop	King of Power Saving	Reasonable price logistics is very fast, the feeling is not bad more satisfied with a shopping experience	Jingdong prefecture level city in Shandong
Dragon Quality Goods Shop	King of Power Saving	The stuff is even more perfect than I imagined, very powerful and worth getting!	Jingdong prefecture level city in Shandong

Dragon Quality Goods Shop	King of Power Saving	Goods have been received, good quality, affordable price, good customer service, worth recommending!	Jingdong prefecture level city in Shandong
Dragon Quality Goods Shop	King of Power Saving	Fast shipping, works great, have already recommended it to friends.	Jingdong prefecture level city in Shandong
Dragon Quality Goods Shop	King of Power Saving	Good quality, fast logistics, reasonable price, worth 5 stars.	Jingdong prefecture level city in Shandong
Dragon Quality Goods Shop	King of Power Saving	Good quality, fast logistics, good value, well packaged, practical	Jingdong prefecture level city in Shandong
Dragon Quality Goods Shop	King of Power Saving	Fast shipping, affordable price, great quality, very satisfied!	Jingdong prefecture level city in Shandong
Dragon Quality Goods Shop	King of Power Saving	Regret knowing too late, it's really good stuff. Works well.	Jingdong prefecture level city in Shandong
Dragon Quality Goods Shop	King of Power Saving	Not bad, very good product, quite save electricity	Jingdong prefecture level city in Shandong
Dragon Quality Goods Shop	King of Power Saving	Good quality, fast logistics, overall very satisfied!	Jingdong prefecture level city in Shandong
Dragon Quality Goods Shop	King of Power Saving	Tightly packaged with good results	Jingdong prefecture level city in Shandong
Dragon Quality Goods Shop	King of Power Saving	very happy	Jingdong prefecture level city in Shandong
Dragon Quality Goods Shop	King of Power Saving	favorable criticism	Jingdong prefecture level city in Shandong
Dragon Quality Goods Shop	King of Power Saving	Now they all sign for the goods without receiving them, the Jingdong platform and merchants are very powerful, and the goods are not received before signing for the goods.	Jingdong prefecture level city in Shandong
Dragon Quality Goods Shop	King of Power Saving	low-quality commodities	Jingdong prefecture level city in Shandong
Dragon Quality Goods Shop	King of Power Saving	I bought this and now it works pretty well with the air conditioning on, when I didn't use it it was $600 a month, now it's almost $500.00, that's over $100 less all of a sudden.	Jingdong prefecture level city in Shandong
Dragon Quality Goods Shop	King of Power Saving	Can Oh, the weather is hot, open the air conditioning is really good, this product is good, has been used for a really long time, more ok, power saving effect is not bad.	Jingdong prefecture level city in Shandong
Dragon Quality Goods Shop	King of Power Saving	I paid my electric bill today, it's a few dozen dollars less, anyway, it's not costly, it's already very good, the boss said that it would be better to buy one more, I've already placed an order	Jingdong prefecture level city in Shandong
Dragon Quality Goods Shop	King of Power Saving	I'm on a smart meter, I save about 30% a month, personally I think it's pretty good, it works well	Jingdong prefecture level city in Shandong
Dragon Quality Goods Shop	King of Power Saving	My dad asked me to buy one, but after I paid the bill, I asked and he said it was a lot less this month, so he asked me to buy another one.	Jingdong prefecture level city in Shandong
Dragon Quality Goods Shop	King of Power Saving	Very satisfied with the purchase thanks to the store for the prosperous business.	Jingdong prefecture level city in Shandong
Dragon Quality Goods Shop	King of Power Saving	Good use, good quality, very satisfied with the purchase	Jingdong prefecture level city in Shandong
Dragon Quality Goods Shop	King of Power Saving	The goods have been received, the quality is very good, is the ideal effect, will continue to pay attention to the	Jingdong prefecture level city in Shandong
Dragon Quality Goods Shop	King of Power Saving	This brand has been used for many years, and this time I saw this upgraded version of the product and got it again. The purpose of this product is to reduce the loss of electricity for a long time, so as to achieve the purpose of saving electricity.	Jingdong prefecture level city in Shandong
Dragon Quality Goods Shop	King of Power Saving	I've been using it for almost a month, and I've reduced my electricity bill by over 100 bucks! I'm planning to get two more for my family.	Jingdong prefecture level city in Shandong
Dragon Quality Goods Shop	King of Power Saving	The effect is too good, the family has now several units, back to buy a number of times, the power saver received, power-saving effect is very good,, fine workmanship, long warranty, but also purify the air, before buying a used for a period of time, with the okay, and then bought a, and only now come to the	Jingdong prefecture level city in Shandong
Dragon Quality Goods Shop	King of Power Saving	Finally received the goods, very, very much like,, has been a lot of people praise, really too happy, has been his family's loyal fans, will continue to buy buy buy buy!	Jingdong prefecture level city in Shandong
Dragon Quality Goods Shop	King of Power Saving	I've been wanting it for a long time, I decided to go for it, I feel great after using it, it's genuine and the delivery is great, it lives up to its name	Jingdong prefecture level city in Shandong
Dragon Quality Goods Shop	King of Power Saving	The quality is sure as other friends who bought it said, and it is also beautiful, thanks to the owner for the recommendation, thanks!	Jingdong prefecture level city in Shandong
Dragon Quality Goods Shop	King of Power Saving	Baby received the quality is really good face value is also very high especially practical especially especially special favorite	Jingdong prefecture level city in Shandong
Dragon Quality Goods Shop	King of Power Saving	It is genuine, trust the merchant, the quality is very good, the price is moderate, the use of the effect is very good!	Jingdong prefecture level city in Shandong
Dragon Quality Goods Shop	King of Power Saving	Energy saver received really good quality affordable and good energy saving and environmental protection is the best to use.	Jingdong prefecture level city in Shandong
Dragon Quality Goods Shop	King of Power Saving	Reasonable price logistics is very fast, the feeling is not bad more satisfied with a shopping experience	Jingdong prefecture level city in Shandong
Dragon Quality Goods Shop	King of Power Saving	The stuff is even more perfect than I imagined, very powerful and worth getting!	Jingdong prefecture level city in Shandong
Dragon Quality Goods Shop	King of Power Saving	Goods have been received, good quality, affordable price, good customer service, worth recommending!	Jingdong prefecture level city in Shandong
Dragon Quality Goods Shop	King of Power Saving	Fast shipping, works great, have already recommended it to friends.	Jingdong prefecture level city in Shandong
Dragon Quality Goods Shop	King of Power Saving	Good quality, fast logistics, reasonable price, worth 5 stars.	Jingdong prefecture level city in Shandong
Dragon Quality Goods Shop	King of Power Saving	Good quality, fast logistics, good value, well packaged, practical	Jingdong prefecture level city in Shandong
Dragon Quality Goods Shop	King of Power Saving	Fast shipping, affordable price, great quality, very satisfied!	Jingdong prefecture level city in Shandong
Dragon Quality Goods Shop	King of Power Saving	Regret knowing too late, it's really good stuff. Works well.	Jingdong prefecture level city in Shandong
Dragon Quality Goods Shop	King of Power Saving	Not bad, very good product, quite save electricity	Jingdong prefecture level city in Shandong
Dragon Quality Goods Shop	King of Power Saving	Good quality, fast logistics, overall very satisfied!	Jingdong prefecture level city in Shandong
Dragon Quality Goods Shop	King of Power Saving	Tightly packaged with good results	Jingdong prefecture level city in Shandong
Dragon Quality Goods Shop	King of Power Saving	very happy	Jingdong prefecture level city in Shandong
Dragon Quality Goods Shop	King of Power Saving	favorable criticism	Jingdong prefecture level city in Shandong
Dragon Quality Goods Shop	King of Power Saving	Now they all sign for the goods without receiving them, the Jingdong platform and merchants are very powerful, and the goods are not received before signing for the goods.	Jingdong prefecture level city in Shandong
Dragon Quality Goods Shop	King of Power Saving	low-quality commodities	Jingdong prefecture level city in Shandong
Dragon Quality Goods Shop	King of Power Saving	I bought this and now it works pretty well with the air conditioning on, when I didn't use it it was $600 a month, now it's almost $500.00, that's over $100 less all of a sudden.	Jingdong prefecture level city in Shandong
Dragon Quality Goods Shop	King of Power Saving	Can Oh, the weather is hot, open the air conditioning is really good, this product is good, has been used for a really long time, more ok, power saving effect is not bad.	Jingdong prefecture level city in Shandong
Dragon Quality Goods Shop	King of Power Saving	I paid my electric bill today, it's a few dozen dollars less, anyway, it's not costly, it's already very good, the boss said that it would be better to buy one more, I've already placed an order	Jingdong prefecture level city in Shandong
Dragon Quality Goods Shop	King of Power Saving	I'm on a smart meter, I save about 30% a month, personally I think it's pretty good, it works well	Jingdong prefecture level city in Shandong
Dragon Quality Goods Shop	King of Power Saving	My dad asked me to buy one, but after I paid the bill, I asked and he said it was a lot less this month, so he asked me to buy another one.	Jingdong prefecture level city in Shandong

Dragon Quality Goods Shop	King of Power Saving	Very satisfied with the purchase thanks to the store for the prosperous business.	Jingdong prefecture level city in Shandong
Dragon Quality Goods Shop	King of Power Saving	Good use, good quality, very satisfied with the purchase	Jingdong prefecture level city in Shandong
Dragon Quality Goods Shop	King of Power Saving	The goods have been received, the quality is very good, is the ideal effect, will continue to pay attention to the	Jingdong prefecture level city in Shandong
Dragon Quality Goods Shop	King of Power Saving	This brand has been used for many years, and this time I saw this upgraded version of the product and got it again. The purpose of this product is to reduce the loss of electricity for a long time, so as to achieve the purpose of saving electricity.	Jingdong prefecture level city in Shandong
Dragon Quality Goods Shop	King of Power Saving	I've been using it for almost a month, and I've reduced my electricity bill by over 100 bucks! I'm planning to get two more for my family.	Jingdong prefecture level city in Shandong
Dragon Quality Goods Shop	King of Power Saving	The effect is too good, the family has now several units, back to buy a number of times, the power saver received, power-saving effect is very good,, fine workmanship, long warranty, but also purify the air, before buying a used for a period of time, with the okay, and then bought a, and only now come to the	Jingdong prefecture level city in Shandong
Dragon Quality Goods Shop	King of Power Saving	Finally received the goods, very, very much like,, has been a lot of people praise, really too happy, has been his family's loyal fans, will continue to buy buy buy buy!	Jingdong prefecture level city in Shandong
Dragon Quality Goods Shop	King of Power Saving	📩 I've been wanting it for a long time, I decided to go for it, I feel great after using it, it's genuine and the delivery is great, it lives up to its name👍	Jingdong prefecture level city in Shandong
Dragon Quality Goods Shop	King of Power Saving	The quality is sure as other friends who bought it said, and it is also beautiful, thanks to the owner for the recommendation, thanks!	Jingdong prefecture level city in Shandong
Dragon Quality Goods Shop	King of Power Saving	Baby received the quality is really good face value is also very high especially practical especially especially special favorite	Jingdong prefecture level city in Shandong
Dragon Quality Goods Shop	King of Power Saving	It is genuine, trust the merchant, the quality is very good, the price is moderate, the use of the effect is very good!	Jingdong prefecture level city in Shandong
Dragon Quality Goods Shop	King of Power Saving	Energy saver received really good quality affordable and good energy saving and environmental protection is the best to use.	Jingdong prefecture level city in Shandong
Dragon Quality Goods Shop	King of Power Saving	Reasonable price logistics is very fast, the feeling is not bad more satisfied with a shopping experience	Jingdong prefecture level city in Shandong
Dragon Quality Goods Shop	King of Power Saving	The stuff is even more perfect than I imagined, very powerful and worth getting!	Jingdong prefecture level city in Shandong
Dragon Quality Goods Shop	King of Power Saving	Goods have been received, good quality, affordable price, good customer service, worth recommending!	Jingdong prefecture level city in Shandong
Dragon Quality Goods Shop	King of Power Saving	Fast shipping, works great, have already recommended it to friends.	Jingdong prefecture level city in Shandong
Dragon Quality Goods Shop	King of Power Saving	Good quality, fast logistics, reasonable price, worth 5 stars.	Jingdong prefecture level city in Shandong
Dragon Quality Goods Shop	King of Power Saving	Good quality, fast logistics, good value, well packaged, practical	Jingdong prefecture level city in Shandong
Dragon Quality Goods Shop	King of Power Saving	Fast shipping, affordable price, great quality, very satisfied!	Jingdong prefecture level city in Shandong
Dragon Quality Goods Shop	King of Power Saving	Regret knowing too late, it's really good stuff. Works well.	Jingdong prefecture level city in Shandong
Dragon Quality Goods Shop	King of Power Saving	Not bad, very good product, quite save electricity	Jingdong prefecture level city in Shandong
Dragon Quality Goods Shop	King of Power Saving	Good quality, fast logistics, overall very satisfied!	Jingdong prefecture level city in Shandong
Dragon Quality Goods Shop	King of Power Saving	Tightly packaged with good results	Jingdong prefecture level city in Shandong
Dragon Quality Goods Shop	King of Power Saving	very happy	Jingdong prefecture level city in Shandong
Dragon Quality Goods Shop	King of Power Saving	favorable criticism	Jingdong prefecture level city in Shandong
Dragon Quality Goods Shop	King of Power Saving	Now they all sign for the goods without receiving them, the Jingdong platform and merchants are very powerful, and the goods are not received before signing for the goods.	Jingdong prefecture level city in Shandong
Dragon Quality Goods Shop	King of Power Saving	low-quality commodities	Jingdong prefecture level city in Shandong
Dragon Quality Goods Shop	King of Power Saving	I bought this and now it works pretty well with the air conditioning on, when I didn't use it it was $600 a month, now it's almost $500.00, that's over $100 less all of a sudden.	Jingdong prefecture level city in Shandong
Dragon Quality Goods Shop	King of Power Saving	Can Oh, the weather is hot, open the air conditioning is really good, this product is good, has been used for a really long time, more ok, power saving effect is not bad.	Jingdong prefecture level city in Shandong
Dragon Quality Goods Shop	King of Power Saving	I paid my electric bill today, it's a few dozen dollars less, anyway, it's not costly, it's already very good, the boss said that it would be better to buy one more, I've already placed an order	Jingdong prefecture level city in Shandong
Dragon Quality Goods Shop	King of Power Saving	I'm on a smart meter, I save about 30% a month, personally I think it's pretty good, it works well	Jingdong prefecture level city in Shandong
Dragon Quality Goods Shop	King of Power Saving	My dad asked me to buy one, but after I paid the bill, I asked and he said it was a lot less this month, so he asked me to buy another one.	Jingdong prefecture level city in Shandong
Dragon Quality Goods Shop	King of Power Saving	Very satisfied with the purchase thanks to the store for the prosperous business.	Jingdong prefecture level city in Shandong
Dragon Quality Goods Shop	King of Power Saving	Good use, good quality, very satisfied with the purchase	Jingdong prefecture level city in Shandong
Dragon Quality Goods Shop	King of Power Saving	The goods have been received, the quality is very good, is the ideal effect, will continue to pay attention to the	Jingdong prefecture level city in Shandong
Dragon Quality Goods Shop	King of Power Saving	This brand has been used for many years, and this time I saw this upgraded version of the product and got it again. The purpose of this product is to reduce the loss of electricity for a long time, so as to achieve the purpose of saving electricity.	Jingdong prefecture level city in Shandong
Dragon Quality Goods Shop	King of Power Saving	I've been using it for almost a month, and I've reduced my electricity bill by over 100 bucks! I'm planning to get two more for my family.	Jingdong prefecture level city in Shandong
Dragon Quality Goods Shop	King of Power Saving	The effect is too good, the family has now several units, back to buy a number of times, the power saver received, power-saving effect is very good,, fine workmanship, long warranty, but also purify the air, before buying a used for a period of time, with the okay, and then bought a, and only now come to the	Jingdong prefecture level city in Shandong
Dragon Quality Goods Shop	King of Power Saving	Finally received the goods, very, very much like,, has been a lot of people praise, really too happy, has been his family's loyal fans, will continue to buy buy buy buy!	Jingdong prefecture level city in Shandong
Dragon Quality Goods Shop	King of Power Saving	📩 I've been wanting it for a long time, I decided to go for it, I feel great after using it, it's genuine and the delivery is great, it lives up to its name👍	Jingdong prefecture level city in Shandong
Dragon Quality Goods Shop	King of Power Saving	The quality is sure as other friends who bought it said, and it is also beautiful, thanks to the owner for the recommendation, thanks!	Jingdong prefecture level city in Shandong
Dragon Quality Goods Shop	King of Power Saving	Baby received the quality is really good face value is also very high especially practical especially especially special favorite	Jingdong prefecture level city in Shandong
Dragon Quality Goods Shop	King of Power Saving	It is genuine, trust the merchant, the quality is very good, the price is moderate, the use of the effect is very good!	Jingdong prefecture level city in Shandong
Dragon Quality Goods Shop	King of Power Saving	Energy saver received really good quality affordable and good energy saving and environmental protection is the best to use.	Jingdong prefecture level city in Shandong
Dragon Quality Goods Shop	King of Power Saving	Reasonable price logistics is very fast, the feeling is not bad more satisfied with a shopping experience	Jingdong prefecture level city in Shandong
Dragon Quality Goods Shop	King of Power Saving	The stuff is even more perfect than I imagined, very powerful and worth getting!	Jingdong prefecture level city in Shandong
Dragon Quality Goods Shop	King of Power Saving	Goods have been received, good quality, affordable price, good customer service, worth recommending!	Jingdong prefecture level city in Shandong
Dragon Quality Goods Shop	King of Power Saving	Fast shipping, works great, have already recommended it to friends.	Jingdong prefecture level city in Shandong
Dragon Quality Goods Shop	King of Power Saving	Good quality, fast logistics, reasonable price, worth 5 stars.	Jingdong prefecture level city in Shandong

Dragon Quality Goods Shop	King of Power Saving	Good quality, fast logistics, good value, well packaged, practical	Jingdong prefecture level city in Shandong
Dragon Quality Goods Shop	King of Power Saving	Fast shipping, affordable price, great quality, very satisfied!	Jingdong prefecture level city in Shandong
Dragon Quality Goods Shop	King of Power Saving	Regret knowing too late, it's really good stuff. Works well.	Jingdong prefecture level city in Shandong
Dragon Quality Goods Shop	King of Power Saving	Not bad, very good product, quite save electricity	Jingdong prefecture level city in Shandong
Dragon Quality Goods Shop	King of Power Saving	Good quality, fast logistics, overall very satisfied!	Jingdong prefecture level city in Shandong
Dragon Quality Goods Shop	King of Power Saving	Tightly packaged with good results	Jingdong prefecture level city in Shandong
Dragon Quality Goods Shop	King of Power Saving	very happy	Jingdong prefecture level city in Shandong
Dragon Quality Goods Shop	King of Power Saving	favorable criticism	Jingdong prefecture level city in Shandong
Dragon Quality Goods Shop	King of Power Saving	Now they all sign for the goods without receiving them, the Jingdong platform and merchants are very powerful, and the goods are not received before signing for the goods.	Jingdong prefecture level city in Shandong
Dragon Quality Goods Shop	King of Power Saving	low-quality commodities	Jingdong prefecture level city in Shandong
Dragon Quality Goods Shop	King of Power Saving	I bought this and now it works pretty well with the air conditioning on, when I didn't use it it was $600 a month, now it's almost $500.00, that's over $100 less all of a sudden.	Jingdong prefecture level city in Shandong
Dragon Quality Goods Shop	King of Power Saving	Can Oh, the weather is hot, open the air conditioning is really good, this product is good, has been used for a really long time, more ok, power saving effect is not bad.	Jingdong prefecture level city in Shandong
Dragon Quality Goods Shop	King of Power Saving	I paid my electric bill today, it's a few dozen dollars less, anyway, it's not costly, it's already very good, the boss said that it would be better to buy one more, I've already placed an order	Jingdong prefecture level city in Shandong
Dragon Quality Goods Shop	King of Power Saving	I'm on a smart meter, I save about 30% a month, personally I think it's pretty good, it works well	Jingdong prefecture level city in Shandong
Dragon Quality Goods Shop	King of Power Saving	My dad asked me to buy one, but after I paid the bill, I asked and he said it was a lot less this month, so he asked me to buy another one.	Jingdong prefecture level city in Shandong
Dragon Quality Goods Shop	King of Power Saving	Very satisfied with the purchase thanks to the store for the prosperous business.	Jingdong prefecture level city in Shandong
Dragon Quality Goods Shop	King of Power Saving	Good use, good quality, very satisfied with the purchase	Jingdong prefecture level city in Shandong
Dragon Quality Goods Shop	King of Power Saving	The goods have been received, the quality is very good, is the ideal effect, will continue to pay attention to the	Jingdong prefecture level city in Shandong
Dragon Quality Goods Shop	King of Power Saving	This brand has been used for many years, and this time I saw this upgraded version of the product and got it again. The purpose of this product is to reduce the loss of electricity for a long time, so as to achieve the purpose of saving electricity.	Jingdong prefecture level city in Shandong
Dragon Quality Goods Shop	King of Power Saving	I've been using it for almost a month, and I've reduced my electricity bill by over 100 bucks! I'm planning to get two more for my family.	Jingdong prefecture level city in Shandong
Dragon Quality Goods Shop	King of Power Saving	The effect is too good, the family has now several units, back to buy a number of times, the power saver received, power-saving effect is very good,, fine workmanship, long warranty, but also purify the air, before buying a used for a period of time, with the okay, and then bought a, and only now come to the	Jingdong prefecture level city in Shandong
Dragon Quality Goods Shop	King of Power Saving	Finally received the goods, very, very much like,, has been a lot of people praise, really too happy, has been his family's loyal fans, will continue to buy buy buy buy!	Jingdong prefecture level city in Shandong
Dragon Quality Goods Shop	King of Power Saving	I've been wanting it for a long time, I decided to go for it, I feel great after using it, it's genuine and the delivery is great, it lives up to its name	Jingdong prefecture level city in Shandong
Dragon Quality Goods Shop	King of Power Saving	The quality is sure as other friends who bought it said, and it is also beautiful, thanks to the owner for the recommendation, thanks!	Jingdong prefecture level city in Shandong
Dragon Quality Goods Shop	King of Power Saving	Baby received the quality is really good face value is also very high especially practical especially especially special favorite	Jingdong prefecture level city in Shandong
Dragon Quality Goods Shop	King of Power Saving	It is genuine, trust the merchant, the quality is very good, the price is moderate, the use of the effect is very good!	Jingdong prefecture level city in Shandong
Dragon Quality Goods Shop	King of Power Saving	Energy saver received really good quality affordable and good energy saving and environmental protection is the best to use.	Jingdong prefecture level city in Shandong
Dragon Quality Goods Shop	King of Power Saving	Reasonable price logistics is very fast, the feeling is not bad more satisfied with a shopping experience	Jingdong prefecture level city in Shandong
Dragon Quality Goods Shop	King of Power Saving	The stuff is even more perfect than I imagined, very powerful and worth getting!	Jingdong prefecture level city in Shandong
Dragon Quality Goods Shop	King of Power Saving	Goods have been received, good quality, affordable price, good customer service, worth recommending!	Jingdong prefecture level city in Shandong
Dragon Quality Goods Shop	King of Power Saving	Fast shipping, works great, have already recommended it to friends.	Jingdong prefecture level city in Shandong
Dragon Quality Goods Shop	King of Power Saving	Good quality, fast logistics, reasonable price, worth 5 stars.	Jingdong prefecture level city in Shandong
Dragon Quality Goods Shop	King of Power Saving	Good quality, fast logistics, good value, well packaged, practical	Jingdong prefecture level city in Shandong
Dragon Quality Goods Shop	King of Power Saving	Fast shipping, affordable price, great quality, very satisfied!	Jingdong prefecture level city in Shandong
Dragon Quality Goods Shop	King of Power Saving	Regret knowing too late, it's really good stuff. Works well.	Jingdong prefecture level city in Shandong
Dragon Quality Goods Shop	King of Power Saving	Not bad, very good product, quite save electricity	Jingdong prefecture level city in Shandong
Dragon Quality Goods Shop	King of Power Saving	Good quality, fast logistics, overall very satisfied!	Jingdong prefecture level city in Shandong
Dragon Quality Goods Shop	King of Power Saving	Tightly packaged with good results	Jingdong prefecture level city in Shandong
Dragon Quality Goods Shop	King of Power Saving	very happy	Jingdong prefecture level city in Shandong
Dragon Quality Goods Shop	King of Power Saving	favorable criticism	Jingdong prefecture level city in Shandong
Dragon Quality Goods Shop	King of Power Saving	Now they all sign for the goods without receiving them, the Jingdong platform and merchants are very powerful, and the goods are not received before signing for the goods.	Jingdong prefecture level city in Shandong
Dragon Quality Goods Shop	King of Power Saving	low-quality commodities	Jingdong prefecture level city in Shandong
Dragon Quality Goods Shop	King of Power Saving	I bought this and now it works pretty well with the air conditioning on, when I didn't use it it was $600 a month, now it's almost $500.00, that's over $100 less all of a sudden.	Jingdong prefecture level city in Shandong
Dragon Quality Goods Shop	King of Power Saving	Can Oh, the weather is hot, open the air conditioning is really good, this product is good, has been used for a really long time, more ok, power saving effect is not bad.	Jingdong prefecture level city in Shandong
Dragon Quality Goods Shop	King of Power Saving	I paid my electric bill today, it's a few dozen dollars less, anyway, it's not costly, it's already very good, the boss said that it would be better to buy one more, I've already placed an order	Jingdong prefecture level city in Shandong
Dragon Quality Goods Shop	King of Power Saving	I'm on a smart meter, I save about 30% a month, personally I think it's pretty good, it works well	Jingdong prefecture level city in Shandong
Dragon Quality Goods Shop	King of Power Saving	My dad asked me to buy one, but after I paid the bill, I asked and he said it was a lot less this month, so he asked me to buy another one.	Jingdong prefecture level city in Shandong
Dragon Quality Goods Shop	King of Power Saving	Very satisfied with the purchase thanks to the store for the prosperous business.	Jingdong prefecture level city in Shandong
Dragon Quality Goods Shop	King of Power Saving	Good use, good quality, very satisfied with the purchase	Jingdong prefecture level city in Shandong
Dragon Quality Goods Shop	King of Power Saving	The goods have been received, the quality is very good, is the ideal effect, will continue to pay attention to the	Jingdong prefecture level city in Shandong

Dragon Quality Goods Shop	King of Power Saving	This brand has been used for many years, and this time I saw this upgraded version of the product and got it again. The purpose of this product is to reduce the loss of electricity for a long time, so as to achieve the purpose of saving electricity.	Jingdong prefecture level city in Shandong
Dragon Quality Goods Shop	King of Power Saving	I've been using it for almost a month, and I've reduced my electricity bill by over 100 bucks! I'm planning to get two more for my family.	Jingdong prefecture level city in Shandong
Dragon Quality Goods Shop	King of Power Saving	The effect is too good, the family has now several units, back to buy a number of times, the power saver received, power-saving effect is very good,, fine workmanship, long warranty, but also purify the air, before buying a used for a period of time, with the okay, and then bought a, and only now come to the	Jingdong prefecture level city in Shandong
Dragon Quality Goods Shop	King of Power Saving	Finally received the goods, very, very much like,, has been a lot of people praise, really too happy, has been his family's loyal fans, will continue to buy buy buy buy!	Jingdong prefecture level city in Shandong
Dragon Quality Goods Shop	King of Power Saving	📩 I've been wanting it for a long time, I decided to go for it, I feel great after using it, it's genuine and the delivery is great, it lives up to its name👍	Jingdong prefecture level city in Shandong
Dragon Quality Goods Shop	King of Power Saving	The quality is sure as other friends who bought it said, and it is also beautiful, thanks to the owner for the recommendation, thanks!	Jingdong prefecture level city in Shandong
Dragon Quality Goods Shop	King of Power Saving	Baby received the quality is really good face value is also very high especially practical especially especially special favorite	Jingdong prefecture level city in Shandong
Dragon Quality Goods Shop	King of Power Saving	It is genuine, trust the merchant, the quality is very good, the price is moderate, the use of the effect is very good!	Jingdong prefecture level city in Shandong
Dragon Quality Goods Shop	King of Power Saving	Energy saver received really good quality affordable and good energy saving and environmental protection is the best to use.	Jingdong prefecture level city in Shandong
Dragon Quality Goods Shop	King of Power Saving	Reasonable price logistics is very fast, the feeling is not bad more satisfied with a shopping experience	Jingdong prefecture level city in Shandong
Dragon Quality Goods Shop	King of Power Saving	The stuff is even more perfect than I imagined, very powerful and worth getting!	Jingdong prefecture level city in Shandong
Dragon Quality Goods Shop	King of Power Saving	Goods have been received, good quality, affordable price, good customer service, worth recommending!	Jingdong prefecture level city in Shandong
Dragon Quality Goods Shop	King of Power Saving	Fast shipping, works great, have already recommended it to friends.	Jingdong prefecture level city in Shandong
Dragon Quality Goods Shop	King of Power Saving	Good quality, fast logistics, reasonable price, worth 5 stars.	Jingdong prefecture level city in Shandong
Dragon Quality Goods Shop	King of Power Saving	Good quality, fast logistics, good value, well packaged, practical	Jingdong prefecture level city in Shandong
Dragon Quality Goods Shop	King of Power Saving	Fast shipping, affordable price, great quality, very satisfied!	Jingdong prefecture level city in Shandong
Dragon Quality Goods Shop	King of Power Saving	Regret knowing too late, it's really good stuff. Works well.	Jingdong prefecture level city in Shandong
Dragon Quality Goods Shop	King of Power Saving	Not bad, very good product, quite save electricity	Jingdong prefecture level city in Shandong
Dragon Quality Goods Shop	King of Power Saving	Good quality, fast logistics, overall very satisfied!	Jingdong prefecture level city in Shandong
Dragon Quality Goods Shop	King of Power Saving	Tightly packaged with good results	Jingdong prefecture level city in Shandong
Dragon Quality Goods Shop	King of Power Saving	very happy	Jingdong prefecture level city in Shandong
Dragon Quality Goods Shop	King of Power Saving	favorable criticism	Jingdong prefecture level city in Shandong
Dragon Quality Goods Shop	King of Power Saving	Now they all sign for the goods without receiving them, the Jingdong platform and merchants are very powerful, and the goods are not received before signing for the goods.	Jingdong prefecture level city in Shandong
Dragon Quality Goods Shop	King of Power Saving	low-quality commodities	Jingdong prefecture level city in Shandong
Dragon Quality Goods Shop	King of Power Saving	I bought this and now it works pretty well with the air conditioning on, when I didn't use it it was $600 a month, now it's almost $500,00, that's over $100 less all of a sudden.	Jingdong prefecture level city in Shandong
Dragon Quality Goods Shop	King of Power Saving	Can Oh, the weather is hot, open the air conditioning is really good, this product is good, has been used for a really long time, more ok, power saving effect is not bad.	Jingdong prefecture level city in Shandong
Dragon Quality Goods Shop	King of Power Saving	I paid my electric bill today, it's a few dozen dollars less, anyway, it's not costly, it's already very good, the boss said that it would be better to buy one more, I've already placed an order	Jingdong prefecture level city in Shandong
Dragon Quality Goods Shop	King of Power Saving	I'm on a smart meter, I save about 30% a month, personally I think it's pretty good, it works well	Jingdong prefecture level city in Shandong
Dragon Quality Goods Shop	King of Power Saving	My dad asked me to buy one, but after I paid the bill, I asked and he said it was a lot less this month, so he asked me to buy another one.	Jingdong prefecture level city in Shandong
Dragon Quality Goods Shop	King of Power Saving	Very satisfied with the purchase thanks to the store for the prosperous business.	Jingdong prefecture level city in Shandong
Dragon Quality Goods Shop	King of Power Saving	Good use, good quality, very satisfied with the purchase	Jingdong prefecture level city in Shandong
Dragon Quality Goods Shop	King of Power Saving	The goods have been received, the quality is very good, is the ideal effect, will continue to pay attention to the	Jingdong prefecture level city in Shandong
Dragon Quality Goods Shop	King of Power Saving	This brand has been used for many years, and this time I saw this upgraded version of the product and got it again. The purpose of this product is to reduce the loss of electricity for a long time, so as to achieve the purpose of saving electricity.	Jingdong prefecture level city in Shandong
Dragon Quality Goods Shop	King of Power Saving	I've been using it for almost a month, and I've reduced my electricity bill by over 100 bucks! I'm planning to get two more for my family.	Jingdong prefecture level city in Shandong
Dragon Quality Goods Shop	King of Power Saving	The effect is too good, the family has now several units, back to buy a number of times, the power saver received, power-saving effect is very good,, fine workmanship, long warranty, but also purify the air, before buying a used for a period of time, with the okay, and then bought a, and only now come to the	Jingdong prefecture level city in Shandong
Dragon Quality Goods Shop	King of Power Saving	Finally received the goods, very, very much like,, has been a lot of people praise, really too happy, has been his family's loyal fans, will continue to buy buy buy buy!	Jingdong prefecture level city in Shandong
Dragon Quality Goods Shop	King of Power Saving	📩 I've been wanting it for a long time, I decided to go for it, I feel great after using it, it's genuine and the delivery is great, it lives up to its name👍	Jingdong prefecture level city in Shandong
Dragon Quality Goods Shop	King of Power Saving	The quality is sure as other friends who bought it said, and it is also beautiful, thanks to the owner for the recommendation, thanks!	Jingdong prefecture level city in Shandong
Dragon Quality Goods Shop	King of Power Saving	Baby received the quality is really good face value is also very high especially practical especially especially special favorite	Jingdong prefecture level city in Shandong
Dragon Quality Goods Shop	King of Power Saving	It is genuine, trust the merchant, the quality is very good, the price is moderate, the use of the effect is very good!	Jingdong prefecture level city in Shandong
Dragon Quality Goods Shop	King of Power Saving	Energy saver received really good quality affordable and good energy saving and environmental protection is the best to use.	Jingdong prefecture level city in Shandong
Dragon Quality Goods Shop	King of Power Saving	Reasonable price logistics is very fast, the feeling is not bad more satisfied with a shopping experience	Jingdong prefecture level city in Shandong
Dragon Quality Goods Shop	King of Power Saving	The stuff is even more perfect than I imagined, very powerful and worth getting!	Jingdong prefecture level city in Shandong
Dragon Quality Goods Shop	King of Power Saving	Goods have been received, good quality, affordable price, good customer service, worth recommending!	Jingdong prefecture level city in Shandong
Dragon Quality Goods Shop	King of Power Saving	Fast shipping, works great, have already recommended it to friends.	Jingdong prefecture level city in Shandong
Dragon Quality Goods Shop	King of Power Saving	Good quality, fast logistics, reasonable price, worth 5 stars.	Jingdong prefecture level city in Shandong
Dragon Quality Goods Shop	King of Power Saving	Good quality, fast logistics, good value, well packaged, practical	Jingdong prefecture level city in Shandong
Dragon Quality Goods Shop	King of Power Saving	Fast shipping, affordable price, great quality, very satisfied!	Jingdong prefecture level city in Shandong
Dragon Quality Goods Shop	King of Power Saving	Regret knowing too late, it's really good stuff. Works well.	Jingdong prefecture level city in Shandong

Dragon Quality Goods Shop	King of Power Saving	Not bad, very good product, quite save electricity	Jingdong prefecture level city in Shandong
Dragon Quality Goods Shop	King of Power Saving	Good quality, fast logistics, overall very satisfied!	Jingdong prefecture level city in Shandong
Dragon Quality Goods Shop	King of Power Saving	Tightly packaged with good results	Jingdong prefecture level city in Shandong
Dragon Quality Goods Shop	King of Power Saving	very happy	Jingdong prefecture level city in Shandong
Dragon Quality Goods Shop	King of Power Saving	favorable criticism	Jingdong prefecture level city in Shandong
Dragon Quality Goods Shop	King of Power Saving	Now they all sign for the goods without receiving them, the Jingdong platform and merchants are very powerful, and the goods are not received before signing for the goods.	Jingdong prefecture level city in Shandong
Dragon Quality Goods Shop	King of Power Saving	low-quality commodities	Jingdong prefecture level city in Shandong
Dragon Quality Goods Shop	King of Power Saving	I bought this and now it works pretty well with the air conditioning on, when I didn't use it it was $600 a month, now it's almost $500,00, that's over $100 less all of a sudden.	Jingdong prefecture level city in Shandong
Dragon Quality Goods Shop	King of Power Saving	Can Oh, the weather is hot, open the air conditioning is really good, this product is good, has been used for a really long time, more ok, power saving effect is not bad.	Jingdong prefecture level city in Shandong
Dragon Quality Goods Shop	King of Power Saving	I paid my electric bill today, it's a few dozen dollars less, anyway, it's not costly, it's already very good, the boss said that it would be better to buy one more, I've already placed an order	Jingdong prefecture level city in Shandong
Dragon Quality Goods Shop	King of Power Saving	I'm on a smart meter, I save about 30% a month, personally I think it's pretty good, it works well	Jingdong prefecture level city in Shandong
Dragon Quality Goods Shop	King of Power Saving	My dad asked me to buy one, but after I paid the bill, I asked and he said it was a lot less this month, so he asked me to buy another one.	Jingdong prefecture level city in Shandong
Dragon Quality Goods Shop	King of Power Saving	Very satisfied with the purchase thanks to the store for the prosperous business.	Jingdong prefecture level city in Shandong
Dragon Quality Goods Shop	King of Power Saving	Good use, good quality, very satisfied with the purchase	Jingdong prefecture level city in Shandong
Dragon Quality Goods Shop	King of Power Saving	The goods have been received, the quality is very good, is the ideal effect, will continue to pay attention to the	Jingdong prefecture level city in Shandong
Dragon Quality Goods Shop	King of Power Saving	This brand has been used for many years, and this time I saw this upgraded version of the product and got it again. The purpose of this product is to reduce the loss of electricity for a long time, so as to achieve the purpose of saving electricity.	Jingdong prefecture level city in Shandong
Dragon Quality Goods Shop	King of Power Saving	I've been using it for almost a month, and I've reduced my electricity bill by over 100 bucks! I'm planning to get two more for my family.	Jingdong prefecture level city in Shandong
Dragon Quality Goods Shop	King of Power Saving	The effect is too good, the family has now several units, back to buy a number of times, the power saver received, power-saving effect is very good,, fine workmanship, long warranty, but also purify the air, before buying a used for a period of time, with the okay, and then bought a, and only now come to the	Jingdong prefecture level city in Shandong
Dragon Quality Goods Shop	King of Power Saving	Finally received the goods, very, very much like,, has been a lot of people praise, really too happy, has been his family's loyal fans, will continue to buy buy buy buy!	Jingdong prefecture level city in Shandong
Dragon Quality Goods Shop	King of Power Saving	📩 I've been wanting it for a long time, I decided to go for it, I feel great after using it, it's genuine and the delivery is great, it lives up to its name👍	Jingdong prefecture level city in Shandong
Dragon Quality Goods Shop	King of Power Saving	The quality is sure as other friends who bought it said, and it is also beautiful, thanks to the owner for the recommendation, thanks!	Jingdong prefecture level city in Shandong
Dragon Quality Goods Shop	King of Power Saving	Baby received the quality is really good face value is also very high especially practical especially especially special favorite	Jingdong prefecture level city in Shandong
Dragon Quality Goods Shop	King of Power Saving	It is genuine, trust the merchant, the quality is very good, the price is moderate, the use of the effect is very good!	Jingdong prefecture level city in Shandong
Dragon Quality Goods Shop	King of Power Saving	Energy saver received really good quality affordable and good energy saving and environmental protection is the best to use.	Jingdong prefecture level city in Shandong
Dragon Quality Goods Shop	King of Power Saving	Reasonable price logistics is very fast, the feeling is not bad more satisfied with a shopping experience	Jingdong prefecture level city in Shandong
Dragon Quality Goods Shop	King of Power Saving	The stuff is even more perfect than I imagined, very powerful and worth getting!	Jingdong prefecture level city in Shandong
Dragon Quality Goods Shop	King of Power Saving	Goods have been received, good quality, affordable price, good customer service, worth recommending!	Jingdong prefecture level city in Shandong
Dragon Quality Goods Shop	King of Power Saving	Fast shipping, works great, have already recommended it to friends.	Jingdong prefecture level city in Shandong
Dragon Quality Goods Shop	King of Power Saving	Good quality, fast logistics, reasonable price, worth 5 stars.	Jingdong prefecture level city in Shandong
Dragon Quality Goods Shop	King of Power Saving	Good quality, fast logistics, good value, well packaged, practical	Jingdong prefecture level city in Shandong
Dragon Quality Goods Shop	King of Power Saving	Fast shipping, affordable price, great quality, very satisfied!	Jingdong prefecture level city in Shandong
Dragon Quality Goods Shop	King of Power Saving	Regret knowing too late, it's really good stuff. Works well.	Jingdong prefecture level city in Shandong
Dragon Quality Goods Shop	King of Power Saving	Not bad, very good product, quite save electricity	Jingdong prefecture level city in Shandong
Dragon Quality Goods Shop	King of Power Saving	Good quality, fast logistics, overall very satisfied!	Jingdong prefecture level city in Shandong
Dragon Quality Goods Shop	King of Power Saving	Tightly packaged with good results	Jingdong prefecture level city in Shandong
Dragon Quality Goods Shop	King of Power Saving	very happy	Jingdong prefecture level city in Shandong
Dragon Quality Goods Shop	King of Power Saving	favorable criticism	Jingdong prefecture level city in Shandong
Dragon Quality Goods Shop	King of Power Saving	Now they all sign for the goods without receiving them, the Jingdong platform and merchants are very powerful, and the goods are not received before signing for the goods.	Jingdong prefecture level city in Shandong
Dragon Quality Goods Shop	King of Power Saving	low-quality commodities	Jingdong prefecture level city in Shandong
Dragon Quality Goods Shop	King of Power Saving	I bought this and now it works pretty well with the air conditioning on, when I didn't use it it was $600 a month, now it's almost $500,00, that's over $100 less all of a sudden.	Jingdong prefecture level city in Shandong
Dragon Quality Goods Shop	King of Power Saving	Can Oh, the weather is hot, open the air conditioning is really good, this product is good, has been used for a really long time, more ok, power saving effect is not bad.	Jingdong prefecture level city in Shandong
Dragon Quality Goods Shop	King of Power Saving	I paid my electric bill today, it's a few dozen dollars less, anyway, it's not costly, it's already very good, the boss said that it would be better to buy one more, I've already placed an order	Jingdong prefecture level city in Shandong
Dragon Quality Goods Shop	King of Power Saving	I'm on a smart meter, I save about 30% a month, personally I think it's pretty good, it works well	Jingdong prefecture level city in Shandong
Dragon Quality Goods Shop	King of Power Saving	My dad asked me to buy one, but after I paid the bill, I asked and he said it was a lot less this month, so he asked me to buy another one.	Jingdong prefecture level city in Shandong
Dragon Quality Goods Shop	King of Power Saving	Very satisfied with the purchase thanks to the store for the prosperous business.	Jingdong prefecture level city in Shandong
Dragon Quality Goods Shop	King of Power Saving	Good use, good quality, very satisfied with the purchase	Jingdong prefecture level city in Shandong
Dragon Quality Goods Shop	King of Power Saving	The goods have been received, the quality is very good, is the ideal effect, will continue to pay attention to the	Jingdong prefecture level city in Shandong
Dragon Quality Goods Shop	King of Power Saving	This brand has been used for many years, and this time I saw this upgraded version of the product and got it again. The purpose of this product is to reduce the loss of electricity for a long time, so as to achieve the purpose of saving electricity.	Jingdong prefecture level city in Shandong
Dragon Quality Goods Shop	King of Power Saving	I've been using it for almost a month, and I've reduced my electricity bill by over 100 bucks! I'm planning to get two more for my family.	Jingdong prefecture level city in Shandong

Dragon Quality Goods Shop	King of Power Saving	The effect is too good, the family has now several units, back to buy a number of times, the power saver received, power-saving effect is very good,, fine workmanship, long warranty, but also purify the air, before buying a used for a period of time, with the okay, and then bought a, and only now come to the	Jingdong prefecture level city in Shandong
Dragon Quality Goods Shop	King of Power Saving	Finally received the goods, very, very much like,, has been a lot of people praise, really too happy, has been his family's loyal fans, will continue to buy buy buy buy!	Jingdong prefecture level city in Shandong
Dragon Quality Goods Shop	King of Power Saving	💌 I've been wanting it for a long time, I decided to go for it, I feel great after using it, it's genuine and the delivery is great, it lives up to its name👍	Jingdong prefecture level city in Shandong
Dragon Quality Goods Shop	King of Power Saving	The quality is sure as other friends who bought it said, and it is also beautiful, thanks to the owner for the recommendation, thanks!	Jingdong prefecture level city in Shandong
Dragon Quality Goods Shop	King of Power Saving	Baby received the quality is really good face value is also very high especially practical especially especially special favorite	Jingdong prefecture level city in Shandong
Dragon Quality Goods Shop	King of Power Saving	It is genuine, trust the merchant, the quality is very good, the price is moderate, the use of the effect is very good!	Jingdong prefecture level city in Shandong
Dragon Quality Goods Shop	King of Power Saving	Energy saver received really good quality affordable and good energy saving and environmental protection is the best to use.	Jingdong prefecture level city in Shandong
Dragon Quality Goods Shop	King of Power Saving	Reasonable price logistics is very fast, the feeling is not bad more satisfied with a shopping experience	Jingdong prefecture level city in Shandong
Dragon Quality Goods Shop	King of Power Saving	The stuff is even more perfect than I imagined, very powerful and worth getting!	Jingdong prefecture level city in Shandong
Dragon Quality Goods Shop	King of Power Saving	Goods have been received, good quality, affordable price, good customer service, worth recommending!	Jingdong prefecture level city in Shandong
Dragon Quality Goods Shop	King of Power Saving	Fast shipping, works great, have already recommended it to friends.	Jingdong prefecture level city in Shandong
Dragon Quality Goods Shop	King of Power Saving	Good quality, fast logistics, reasonable price, worth 5 stars.	Jingdong prefecture level city in Shandong
Dragon Quality Goods Shop	King of Power Saving	Good quality, fast logistics, good value, well packaged, practical	Jingdong prefecture level city in Shandong
Dragon Quality Goods Shop	King of Power Saving	Fast shipping, affordable price, great quality, very satisfied!	Jingdong prefecture level city in Shandong
Dragon Quality Goods Shop	King of Power Saving	Regret knowing too late, it's really good stuff. Works well.	Jingdong prefecture level city in Shandong
Dragon Quality Goods Shop	King of Power Saving	Not bad, very good product, quite save electricity	Jingdong prefecture level city in Shandong
Dragon Quality Goods Shop	King of Power Saving	Good quality, fast logistics, overall very satisfied!	Jingdong prefecture level city in Shandong
Dragon Quality Goods Shop	King of Power Saving	Tightly packaged with good results	Jingdong prefecture level city in Shandong
Dragon Quality Goods Shop	King of Power Saving	very happy	Jingdong prefecture level city in Shandong
Dragon Quality Goods Shop	King of Power Saving	favorable criticism	Jingdong prefecture level city in Shandong
Dragon Quality Goods Shop	King of Power Saving	Now they all sign for the goods without receiving them, the Jingdong platform and merchants are very powerful, and the goods are not received before signing for the goods.	Jingdong prefecture level city in Shandong
Dragon Quality Goods Shop	King of Power Saving	low-quality commodities	Jingdong prefecture level city in Shandong
Dragon Quality Goods Shop	King of Power Saving	I bought this and now it works pretty well with the air conditioning on, when I didn't use it it was $600 a month, now it's almost $500.00, that's over $100 less all of a sudden.	Jingdong prefecture level city in Shandong
Dragon Quality Goods Shop	King of Power Saving	Can Oh, the weather is hot, open the air conditioning is really good, this product is good, has been used for a really long time, more ok, power saving effect is not bad.	Jingdong prefecture level city in Shandong
Dragon Quality Goods Shop	King of Power Saving	I paid my electric bill today, it's a few dozen dollars less, anyway, it's not costly, it's already very good, the boss said that it would be better to buy one more, I've already placed an order	Jingdong prefecture level city in Shandong
Dragon Quality Goods Shop	King of Power Saving	I'm on a smart meter, I save about 30% a month, personally I think it's pretty good, it works well	Jingdong prefecture level city in Shandong
Dragon Quality Goods Shop	King of Power Saving	My dad asked me to buy one, but after I paid the bill, I asked and he said it was a lot less this month, so he asked me to buy another one.	Jingdong prefecture level city in Shandong
Dragon Quality Goods Shop	King of Power Saving	Very satisfied with the purchase thanks to the store for the prosperous business.	Jingdong prefecture level city in Shandong
Dragon Quality Goods Shop	King of Power Saving	Good use, good quality, very satisfied with the purchase	Jingdong prefecture level city in Shandong
Dragon Quality Goods Shop	King of Power Saving	The goods have been received, the quality is very good, is the ideal effect, will continue to pay attention to the	Jingdong prefecture level city in Shandong
Dragon Quality Goods Shop	King of Power Saving	This brand has been used for many years, and this time I saw this upgraded version of the product and got it again. The purpose of this product is to reduce the loss of electricity for a long time, so as to achieve the purpose of saving electricity.	Jingdong prefecture level city in Shandong
Dragon Quality Goods Shop	King of Power Saving	I've been using it for almost a month, and I've reduced my electricity bill by over 100 bucks! I'm planning to get two more for my family.	Jingdong prefecture level city in Shandong
Dragon Quality Goods Shop	King of Power Saving	The effect is too good, the family has now several units, back to buy a number of times, the power saver received, power-saving effect is very good,, fine workmanship, long warranty, but also purify the air, before buying a used for a period of time, with the okay, and then bought a, and only now come to the	Jingdong prefecture level city in Shandong
Dragon Quality Goods Shop	King of Power Saving	Finally received the goods, very, very much like,, has been a lot of people praise, really too happy, has been his family's loyal fans, will continue to buy buy buy buy!	Jingdong prefecture level city in Shandong
Dragon Quality Goods Shop	King of Power Saving	💌 I've been wanting it for a long time, I decided to go for it, I feel great after using it, it's genuine and the delivery is great, it lives up to its name👍	Jingdong prefecture level city in Shandong
Dragon Quality Goods Shop	King of Power Saving	The quality is sure as other friends who bought it said, and it is also beautiful, thanks to the owner for the recommendation, thanks!	Jingdong prefecture level city in Shandong
Dragon Quality Goods Shop	King of Power Saving	Baby received the quality is really good face value is also very high especially practical especially especially special favorite	Jingdong prefecture level city in Shandong
Dragon Quality Goods Shop	King of Power Saving	It is genuine, trust the merchant, the quality is very good, the price is moderate, the use of the effect is very good!	Jingdong prefecture level city in Shandong
Dragon Quality Goods Shop	King of Power Saving	Energy saver received really good quality affordable and good energy saving and environmental protection is the best to use.	Jingdong prefecture level city in Shandong
Dragon Quality Goods Shop	King of Power Saving	Reasonable price logistics is very fast, the feeling is not bad more satisfied with a shopping experience	Jingdong prefecture level city in Shandong
Dragon Quality Goods Shop	King of Power Saving	The stuff is even more perfect than I imagined, very powerful and worth getting!	Jingdong prefecture level city in Shandong
Dragon Quality Goods Shop	King of Power Saving	Goods have been received, good quality, affordable price, good customer service, worth recommending!	Jingdong prefecture level city in Shandong
Dragon Quality Goods Shop	King of Power Saving	Fast shipping, works great, have already recommended it to friends.	Jingdong prefecture level city in Shandong
Dragon Quality Goods Shop	King of Power Saving	Good quality, fast logistics, reasonable price, worth 5 stars.	Jingdong prefecture level city in Shandong
Dragon Quality Goods Shop	King of Power Saving	Good quality, fast logistics, good value, well packaged, practical	Jingdong prefecture level city in Shandong
Dragon Quality Goods Shop	King of Power Saving	Fast shipping, affordable price, great quality, very satisfied!	Jingdong prefecture level city in Shandong
Dragon Quality Goods Shop	King of Power Saving	Regret knowing too late, it's really good stuff. Works well.	Jingdong prefecture level city in Shandong
Dragon Quality Goods Shop	King of Power Saving	Not bad, very good product, quite save electricity	Jingdong prefecture level city in Shandong
Dragon Quality Goods Shop	King of Power Saving	Good quality, fast logistics, overall very satisfied!	Jingdong prefecture level city in Shandong
Dragon Quality Goods Shop	King of Power Saving	Tightly packaged with good results	Jingdong prefecture level city in Shandong

Dragon Quality Goods Shop	King of Power Saving	very happy	Jingdong prefecture level city in Shandong
Dragon Quality Goods Shop	King of Power Saving	favorable criticism	Jingdong prefecture level city in Shandong
Dragon Quality Goods Shop	King of Power Saving	Now they all sign for the goods without receiving them, the Jingdong platform and merchants are very powerful, and the goods are not received before signing for the goods.	Jingdong prefecture level city in Shandong
Dragon Quality Goods Shop	King of Power Saving	low-quality commodities	Jingdong prefecture level city in Shandong
Dragon Quality Goods Shop	King of Power Saving	I bought this and now it works pretty well with the air conditioning on, when I didn't use it it was $600 a month, now it's almost $500,00, that's over $100 less all of a sudden.	Jingdong prefecture level city in Shandong
Dragon Quality Goods Shop	King of Power Saving	Can Oh, the weather is hot, open the air conditioning is really good, this product is good, has been used for a really long time, more ok, power saving effect is not bad.	Jingdong prefecture level city in Shandong
Dragon Quality Goods Shop	King of Power Saving	I paid my electric bill today, it's a few dozen dollars less, anyway, it's not costly, it's already very good, the boss said that it would be better to buy one more, I've already placed an order	Jingdong prefecture level city in Shandong
Dragon Quality Goods Shop	King of Power Saving	I'm on a smart meter, I save about 30% a month, personally I think it's pretty good, it works well	Jingdong prefecture level city in Shandong
Dragon Quality Goods Shop	King of Power Saving	My dad asked me to buy one, but after I paid the bill, I asked and he said it was a lot less this month, so he asked me to buy another one.	Jingdong prefecture level city in Shandong
Dragon Quality Goods Shop	King of Power Saving	Very satisfied with the purchase thanks to the store for the prosperous business.	Jingdong prefecture level city in Shandong
Dragon Quality Goods Shop	King of Power Saving	Good use, good quality, very satisfied with the purchase	Jingdong prefecture level city in Shandong
Dragon Quality Goods Shop	King of Power Saving	The goods have been received, the quality is very good, is the ideal effect, will continue to pay attention to the	Jingdong prefecture level city in Shandong
Dragon Quality Goods Shop	King of Power Saving	This brand has been used for many years, and this time I saw this upgraded version of the product and got it again. The purpose of this product is to reduce the loss of electricity for a long time, so as to achieve the purpose of saving electricity.	Jingdong prefecture level city in Shandong
Dragon Quality Goods Shop	King of Power Saving	I've been using it for almost a month, and I've reduced my electricity bill by over 100 bucks! I'm planning to get two more for my family.	Jingdong prefecture level city in Shandong
Dragon Quality Goods Shop	King of Power Saving	The effect is too good, the family has now several units, back to buy a number of times, the power saver received, power-saving effect is very good,, fine workmanship, long warranty, but also purify the air, before buying a used for a period of time, with the okay, and then bought a, and only now come to the	Jingdong prefecture level city in Shandong
Dragon Quality Goods Shop	King of Power Saving	Finally received the goods, very, very much like,, has been a lot of people praise, really too happy, has been his family's loyal fans, will continue to buy buy buy!	Jingdong prefecture level city in Shandong
Dragon Quality Goods Shop	King of Power Saving	📩 I've been wanting it for a long time, I decided to go for it, I feel great after using it, it's genuine and the delivery is great, it lives up to its name👍	Jingdong prefecture level city in Shandong
Dragon Quality Goods Shop	King of Power Saving	The quality is sure as other friends who bought it said, and it is also beautiful, thanks to the owner for the recommendation, thanks!	Jingdong prefecture level city in Shandong
Dragon Quality Goods Shop	King of Power Saving	Baby received the quality is really good face value is also very high especially practical especially especially special favorite	Jingdong prefecture level city in Shandong
Dragon Quality Goods Shop	King of Power Saving	It is genuine, trust the merchant, the quality is very good, the price is moderate, the use of the effect is very good!	Jingdong prefecture level city in Shandong
Dragon Quality Goods Shop	King of Power Saving	Energy saver received really good quality affordable and good energy saving and environmental protection is the best to use.	Jingdong prefecture level city in Shandong
Dragon Quality Goods Shop	King of Power Saving	Reasonable price logistics is very fast, the feeling is not bad more satisfied with a shopping experience	Jingdong prefecture level city in Shandong
Dragon Quality Goods Shop	King of Power Saving	The stuff is even more perfect than I imagined, very powerful and worth getting!	Jingdong prefecture level city in Shandong
Dragon Quality Goods Shop	King of Power Saving	Goods have been received, good quality, affordable price, good customer service, worth recommending!	Jingdong prefecture level city in Shandong
Dragon Quality Goods Shop	King of Power Saving	Fast shipping, works great, have already recommended it to friends.	Jingdong prefecture level city in Shandong
Dragon Quality Goods Shop	King of Power Saving	Good quality, fast logistics, reasonable price, worth 5 stars.	Jingdong prefecture level city in Shandong
Dragon Quality Goods Shop	King of Power Saving	Good quality, fast logistics, good value, well packaged, practical	Jingdong prefecture level city in Shandong
Dragon Quality Goods Shop	King of Power Saving	Fast shipping, affordable price, great quality, very satisfied!	Jingdong prefecture level city in Shandong
Dragon Quality Goods Shop	King of Power Saving	Regret knowing too late, it's really good stuff. Works well.	Jingdong prefecture level city in Shandong
Dragon Quality Goods Shop	King of Power Saving	Not bad, very good product, quite save electricity	Jingdong prefecture level city in Shandong
Dragon Quality Goods Shop	King of Power Saving	Good quality, fast logistics, overall very satisfied!	Jingdong prefecture level city in Shandong
Dragon Quality Goods Shop	King of Power Saving	Tightly packaged with good results	Jingdong prefecture level city in Shandong
Dragon Quality Goods Shop	King of Power Saving	very happy	Jingdong prefecture level city in Shandong
Dragon Quality Goods Shop	King of Power Saving	favorable criticism	Jingdong prefecture level city in Shandong
Dragon Quality Goods Shop	King of Power Saving	Now they all sign for the goods without receiving them, the Jingdong platform and merchants are very powerful, and the goods are not received before signing for the goods.	Jingdong prefecture level city in Shandong
Dragon Quality Goods Shop	King of Power Saving	low-quality commodities	Jingdong prefecture level city in Shandong
Dragon Quality Goods Shop	King of Power Saving	I bought this and now it works pretty well with the air conditioning on, when I didn't use it it was $600 a month, now it's almost $500,00, that's over $100 less all of a sudden.	Jingdong prefecture level city in Shandong
Dragon Quality Goods Shop	King of Power Saving	Can Oh, the weather is hot, open the air conditioning is really good, this product is good, has been used for a really long time, more ok, power saving effect is not bad.	Jingdong prefecture level city in Shandong
Dragon Quality Goods Shop	King of Power Saving	I paid my electric bill today, it's a few dozen dollars less, anyway, it's not costly, it's already very good, the boss said that it would be better to buy one more, I've already placed an order	Jingdong prefecture level city in Shandong
Dragon Quality Goods Shop	King of Power Saving	I'm on a smart meter, I save about 30% a month, personally I think it's pretty good, it works well	Jingdong prefecture level city in Shandong
Dragon Quality Goods Shop	King of Power Saving	My dad asked me to buy one, but after I paid the bill, I asked and he said it was a lot less this month, so he asked me to buy another one.	Jingdong prefecture level city in Shandong
Dragon Quality Goods Shop	King of Power Saving	Very satisfied with the purchase thanks to the store for the prosperous business.	Jingdong prefecture level city in Shandong
Dragon Quality Goods Shop	King of Power Saving	Good use, good quality, very satisfied with the purchase	Jingdong prefecture level city in Shandong
Dragon Quality Goods Shop	King of Power Saving	The goods have been received, the quality is very good, is the ideal effect, will continue to pay attention to the	Jingdong prefecture level city in Shandong
Dragon Quality Goods Shop	King of Power Saving	This brand has been used for many years, and this time I saw this upgraded version of the product and got it again. The purpose of this product is to reduce the loss of electricity for a long time, so as to achieve the purpose of saving electricity.	Jingdong prefecture level city in Shandong
Dragon Quality Goods Shop	King of Power Saving	I've been using it for almost a month, and I've reduced my electricity bill by over 100 bucks! I'm planning to get two more for my family.	Jingdong prefecture level city in Shandong
Dragon Quality Goods Shop	King of Power Saving	The effect is too good, the family has now several units, back to buy a number of times, the power saver received, power-saving effect is very good,, fine workmanship, long warranty, but also purify the air, before buying a used for a period of time, with the okay, and then bought a, and only now come to the	Jingdong prefecture level city in Shandong
Dragon Quality Goods Shop	King of Power Saving	Finally received the goods, very, very much like,, has been a lot of people praise, really too happy, has been his family's loyal fans, will continue to buy buy buy!	Jingdong prefecture level city in Shandong
Dragon Quality Goods Shop	King of Power Saving	📩 I've been wanting it for a long time, I decided to go for it, I feel great after using it, it's genuine and the delivery is great, it lives up to its name👍	Jingdong prefecture level city in Shandong

Dragon Quality Goods Shop	King of Power Saving	The quality is sure as other friends who bought it said, and it is also beautiful, thanks to the owner for the recommendation, thanks!	Jingdong prefecture level city in Shandong
Dragon Quality Goods Shop	King of Power Saving	Baby received the quality is really good face value is also very high especially practical especially especially special favorite	Jingdong prefecture level city in Shandong
Dragon Quality Goods Shop	King of Power Saving	It is genuine, trust the merchant, the quality is very good, the price is moderate, the use of the effect is very good!	Jingdong prefecture level city in Shandong
Dragon Quality Goods Shop	King of Power Saving	Energy saver received really good quality affordable and good energy saving and environmental protection is the best to use.	Jingdong prefecture level city in Shandong
Dragon Quality Goods Shop	King of Power Saving	Reasonable price logistics is very fast, the feeling is not bad more satisfied with a shopping experience	Jingdong prefecture level city in Shandong
Dragon Quality Goods Shop	King of Power Saving	The stuff is even more perfect than I imagined, very powerful and worth getting!	Jingdong prefecture level city in Shandong
Dragon Quality Goods Shop	King of Power Saving	Goods have been received, good quality, affordable price, good customer service, worth recommending!	Jingdong prefecture level city in Shandong
Dragon Quality Goods Shop	King of Power Saving	Fast shipping, works great, have already recommended it to friends.	Jingdong prefecture level city in Shandong
Dragon Quality Goods Shop	King of Power Saving	Good quality, fast logistics, reasonable price, worth 5 stars.	Jingdong prefecture level city in Shandong
Dragon Quality Goods Shop	King of Power Saving	Good quality, fast logistics, good value, well packaged, practical	Jingdong prefecture level city in Shandong
Dragon Quality Goods Shop	King of Power Saving	Fast shipping, affordable price, great quality, very satisfied!	Jingdong prefecture level city in Shandong
Dragon Quality Goods Shop	King of Power Saving	Regret knowing too late, it's really good stuff. Works well.	Jingdong prefecture level city in Shandong
Dragon Quality Goods Shop	King of Power Saving	Not bad, very good product, quite save electricity	Jingdong prefecture level city in Shandong
Dragon Quality Goods Shop	King of Power Saving	Good quality, fast logistics, overall very satisfied!	Jingdong prefecture level city in Shandong
Dragon Quality Goods Shop	King of Power Saving	Tightly packaged with good results	Jingdong prefecture level city in Shandong
Dragon Quality Goods Shop	King of Power Saving	very happy	Jingdong prefecture level city in Shandong
Dragon Quality Goods Shop	King of Power Saving	favorable criticism	Jingdong prefecture level city in Shandong
Dragon Quality Goods Shop	King of Power Saving	Now they all sign for the goods without receiving them, the Jingdong platform and merchants are very powerful, and the goods are not received before signing for the goods.	Jingdong prefecture level city in Shandong
Dragon Quality Goods Shop	King of Power Saving	low-quality commodities	Jingdong prefecture level city in Shandong
Dragon Quality Goods Shop	King of Power Saving	I bought this and now it works pretty well with the air conditioning on, when I didn't use it it was $600 a month, now it's almost $500.00, that's over $100 less all of a sudden.	Jingdong prefecture level city in Shandong
Dragon Quality Goods Shop	King of Power Saving	Can Oh, the weather is hot, open the air conditioning is really good, this product is good, has been used for a really long time, more ok, power saving effect is not bad.	Jingdong prefecture level city in Shandong
Dragon Quality Goods Shop	King of Power Saving	I paid my electric bill today, it's a few dozen dollars less, anyway, it's not costly, it's already very good, the boss said that it would be better to buy one more, I've already placed an order	Jingdong prefecture level city in Shandong
Dragon Quality Goods Shop	King of Power Saving	I'm on a smart meter, I save about 30% a month, personally I think it's pretty good, it works well	Jingdong prefecture level city in Shandong
Dragon Quality Goods Shop	King of Power Saving	My dad asked me to buy one, but after I paid the bill, I asked and he said it was a lot less this month, so he asked me to buy another one.	Jingdong prefecture level city in Shandong
Dragon Quality Goods Shop	King of Power Saving	Very satisfied with the purchase thanks to the store for the prosperous business.	Jingdong prefecture level city in Shandong
Dragon Quality Goods Shop	King of Power Saving	Good use, good quality, very satisfied with the purchase	Jingdong prefecture level city in Shandong
Dragon Quality Goods Shop	King of Power Saving	The goods have been received, the quality is very good, is the ideal effect, will continue to pay attention to the	Jingdong prefecture level city in Shandong
Dragon Quality Goods Shop	King of Power Saving	This brand has been used for many years, and this time I saw this upgraded version of the product and got it again. The purpose of this product is to reduce the loss of electricity for a long time, so as to achieve the purpose of saving electricity.	Jingdong prefecture level city in Shandong
Dragon Quality Goods Shop	King of Power Saving	I've been using it for almost a month, and I've reduced my electricity bill by over 100 bucks! I'm planning to get two more for my family.	Jingdong prefecture level city in Shandong
Dragon Quality Goods Shop	King of Power Saving	The effect is too good, the family has now several units, back to buy a number of times, the power saver received, power-saving effect is very good,, fine workmanship, long warranty, but also purify the air, before buying a used for a period of time, with the okay, and then bought a, and only now come to the	Jingdong prefecture level city in Shandong
Dragon Quality Goods Shop	King of Power Saving	Finally received the goods, very, very much like,, has been a lot of people praise, really too happy, has been his family's loyal fans, will continue to buy buy buy buy!	Jingdong prefecture level city in Shandong
Dragon Quality Goods Shop	King of Power Saving	📩 I've been wanting it for a long time, I decided to go for it, I feel great after using it, it's genuine and the delivery is great, it lives up to its name👍	Jingdong prefecture level city in Shandong
Dragon Quality Goods Shop	King of Power Saving	The quality is sure as other friends who bought it said, and it is also beautiful, thanks to the owner for the recommendation, thanks!	Jingdong prefecture level city in Shandong
Dragon Quality Goods Shop	King of Power Saving	Baby received the quality is really good face value is also very high especially practical especially especially special favorite	Jingdong prefecture level city in Shandong
Dragon Quality Goods Shop	King of Power Saving	It is genuine, trust the merchant, the quality is very good, the price is moderate, the use of the effect is very good!	Jingdong prefecture level city in Shandong
Dragon Quality Goods Shop	King of Power Saving	Energy saver received really good quality affordable and good energy saving and environmental protection is the best to use.	Jingdong prefecture level city in Shandong
Dragon Quality Goods Shop	King of Power Saving	Reasonable price logistics is very fast, the feeling is not bad more satisfied with a shopping experience	Jingdong prefecture level city in Shandong
Dragon Quality Goods Shop	King of Power Saving	The stuff is even more perfect than I imagined, very powerful and worth getting!	Jingdong prefecture level city in Shandong
Dragon Quality Goods Shop	King of Power Saving	Goods have been received, good quality, affordable price, good customer service, worth recommending!	Jingdong prefecture level city in Shandong
Dragon Quality Goods Shop	King of Power Saving	Fast shipping, works great, have already recommended it to friends.	Jingdong prefecture level city in Shandong
Dragon Quality Goods Shop	King of Power Saving	Good quality, fast logistics, reasonable price, worth 5 stars.	Jingdong prefecture level city in Shandong
Dragon Quality Goods Shop	King of Power Saving	Good quality, fast logistics, good value, well packaged, practical	Jingdong prefecture level city in Shandong
Dragon Quality Goods Shop	King of Power Saving	Fast shipping, affordable price, great quality, very satisfied!	Jingdong prefecture level city in Shandong
Dragon Quality Goods Shop	King of Power Saving	Regret knowing too late, it's really good stuff. Works well.	Jingdong prefecture level city in Shandong
Dragon Quality Goods Shop	King of Power Saving	Not bad, very good product, quite save electricity	Jingdong prefecture level city in Shandong
Dragon Quality Goods Shop	King of Power Saving	Good quality, fast logistics, overall very satisfied!	Jingdong prefecture level city in Shandong
Dragon Quality Goods Shop	King of Power Saving	Tightly packaged with good results	Jingdong prefecture level city in Shandong
Dragon Quality Goods Shop	King of Power Saving	very happy	Jingdong prefecture level city in Shandong
Dragon Quality Goods Shop	King of Power Saving	favorable criticism	Jingdong prefecture level city in Shandong
Dragon Quality Goods Shop	King of Power Saving	Now they all sign for the goods without receiving them, the Jingdong platform and merchants are very powerful, and the goods are not received before signing for the goods.	Jingdong prefecture level city in Shandong

Dragon Quality Goods Shop	King of Power Saving	low-quality commodities	Jingdong prefecture level city in Shandong
Dragon Quality Goods Shop	King of Power Saving	I bought this and now it works pretty well with the air conditioning on, when I didn't use it it was $600 a month, now it's almost $500.00, that's over $100 less all of a sudden.	Jingdong prefecture level city in Shandong
Dragon Quality Goods Shop	King of Power Saving	Can Oh, the weather is hot, open the air conditioning is really good, this product is good, has been used for a really long time, more ok, power saving effect is not bad.	Jingdong prefecture level city in Shandong
Dragon Quality Goods Shop	King of Power Saving	I paid my electric bill today, it's a few dozen dollars less, anyway, it's not costly, it's already very good, the boss said that it would be better to buy one more, I've already placed an order	Jingdong prefecture level city in Shandong
Dragon Quality Goods Shop	King of Power Saving	I'm on a smart meter, I save about 30% a month, personally I think it's pretty good, it works well	Jingdong prefecture level city in Shandong
Dragon Quality Goods Shop	King of Power Saving	My dad asked me to buy one, but after I paid the bill, I asked and he said it was a lot less this month, so he asked me to buy another one.	Jingdong prefecture level city in Shandong
Dragon Quality Goods Shop	King of Power Saving	Very satisfied with the purchase thanks to the store for the prosperous business.	Jingdong prefecture level city in Shandong
Dragon Quality Goods Shop	King of Power Saving	Good use, good quality, very satisfied with the purchase	Jingdong prefecture level city in Shandong
Dragon Quality Goods Shop	King of Power Saving	The goods have been received, the quality is very good, is the ideal effect, will continue to pay attention to the	Jingdong prefecture level city in Shandong
Dragon Quality Goods Shop	King of Power Saving	This brand has been used for many years, and this time I saw this upgraded version of the product and got it again. The purpose of this product is to reduce the loss of electricity for a long time, so as to achieve the purpose of saving electricity.	Jingdong prefecture level city in Shandong
Dragon Quality Goods Shop	King of Power Saving	I've been using it for almost a month, and I've reduced my electricity bill by over 100 bucks! I'm planning to get two more for my family.	Jingdong prefecture level city in Shandong
Dragon Quality Goods Shop	King of Power Saving	The effect is too good, the family has now several units, back to buy a number of times, the power saver received, power-saving effect is very good,, fine workmanship, long warranty, but also purify the air, before buying a used for a period of time, with the okay, and then bought a, and only now come to the	Jingdong prefecture level city in Shandong
Dragon Quality Goods Shop	King of Power Saving	Finally received the goods, very, very much like,, has been a lot of people praise, really too happy, has been his family's loyal fans, will continue to buy buy buy buy!	Jingdong prefecture level city in Shandong
Dragon Quality Goods Shop	King of Power Saving	📩 I've been wanting it for a long time, I decided to go for it, I feel great after using it, it's genuine and the delivery is great, it lives up to its name👍	Jingdong prefecture level city in Shandong
Dragon Quality Goods Shop	King of Power Saving	The quality is sure as other friends who bought it said, and it is also beautiful, thanks to the owner for the recommendation, thanks!	Jingdong prefecture level city in Shandong
Dragon Quality Goods Shop	King of Power Saving	Baby received the quality is really good face value is also very high especially practical especially especially special favorite	Jingdong prefecture level city in Shandong
Dragon Quality Goods Shop	King of Power Saving	It is genuine, trust the merchant, the quality is very good, the price is moderate, the use of the effect is very good!	Jingdong prefecture level city in Shandong
Dragon Quality Goods Shop	King of Power Saving	Energy saver received really good quality affordable and good energy saving and environmental protection is the best to use.	Jingdong prefecture level city in Shandong
Dragon Quality Goods Shop	King of Power Saving	Reasonable price logistics is very fast, the feeling is not bad more satisfied with a shopping experience	Jingdong prefecture level city in Shandong
Dragon Quality Goods Shop	King of Power Saving	The stuff is even more perfect than I imagined, very powerful and worth getting!	Jingdong prefecture level city in Shandong
Dragon Quality Goods Shop	King of Power Saving	Goods have been received, good quality, affordable price, good customer service, worth recommending!	Jingdong prefecture level city in Shandong
Dragon Quality Goods Shop	King of Power Saving	Fast shipping, works great, have already recommended it to friends.	Jingdong prefecture level city in Shandong
Dragon Quality Goods Shop	King of Power Saving	Good quality, fast logistics, reasonable price, worth 5 stars.	Jingdong prefecture level city in Shandong
Dragon Quality Goods Shop	King of Power Saving	Good quality, fast logistics, good value, well packaged, practical	Jingdong prefecture level city in Shandong
Dragon Quality Goods Shop	King of Power Saving	Fast shipping, affordable price, great quality, very satisfied!	Jingdong prefecture level city in Shandong
Dragon Quality Goods Shop	King of Power Saving	Regret knowing too late, it's really good stuff. Works well.	Jingdong prefecture level city in Shandong
Dragon Quality Goods Shop	King of Power Saving	Not bad, very good product, quite save electricity	Jingdong prefecture level city in Shandong
Dragon Quality Goods Shop	King of Power Saving	Good quality, fast logistics, overall very satisfied!	Jingdong prefecture level city in Shandong
Dragon Quality Goods Shop	King of Power Saving	Tightly packaged with good results	Jingdong prefecture level city in Shandong
Dragon Quality Goods Shop	King of Power Saving	very happy	Jingdong prefecture level city in Shandong
Dragon Quality Goods Shop	King of Power Saving	favorable criticism	Jingdong prefecture level city in Shandong
Dragon Quality Goods Shop	King of Power Saving	Now they all sign for the goods without receiving them, the Jingdong platform and merchants are very powerful, and the goods are not received before signing for the goods.	Jingdong prefecture level city in Shandong
Dragon Quality Goods Shop	King of Power Saving	low-quality commodities	Jingdong prefecture level city in Shandong
Dragon Quality Goods Shop	King of Power Saving	I bought this and now it works pretty well with the air conditioning on, when I didn't use it it was $600 a month, now it's almost $500.00, that's over $100 less all of a sudden.	Jingdong prefecture level city in Shandong
Dragon Quality Goods Shop	King of Power Saving	Can Oh, the weather is hot, open the air conditioning is really good, this product is good, has been used for a really long time, more ok, power saving effect is not bad.	Jingdong prefecture level city in Shandong
Dragon Quality Goods Shop	King of Power Saving	I paid my electric bill today, it's a few dozen dollars less, anyway, it's not costly, it's already very good, the boss said that it would be better to buy one more, I've already placed an order	Jingdong prefecture level city in Shandong
Dragon Quality Goods Shop	King of Power Saving	I'm on a smart meter, I save about 30% a month, personally I think it's pretty good, it works well	Jingdong prefecture level city in Shandong
Dragon Quality Goods Shop	King of Power Saving	My dad asked me to buy one, but after I paid the bill, I asked and he said it was a lot less this month, so he asked me to buy another one.	Jingdong prefecture level city in Shandong
Dragon Quality Goods Shop	King of Power Saving	Very satisfied with the purchase thanks to the store for the prosperous business.	Jingdong prefecture level city in Shandong
Dragon Quality Goods Shop	King of Power Saving	Good use, good quality, very satisfied with the purchase	Jingdong prefecture level city in Shandong
Dragon Quality Goods Shop	King of Power Saving	The goods have been received, the quality is very good, is the ideal effect, will continue to pay attention to the	Jingdong prefecture level city in Shandong
Dragon Quality Goods Shop	King of Power Saving	This brand has been used for many years, and this time I saw this upgraded version of the product and got it again. The purpose of this product is to reduce the loss of electricity for a long time, so as to achieve the purpose of saving electricity.	Jingdong prefecture level city in Shandong
Dragon Quality Goods Shop	King of Power Saving	I've been using it for almost a month, and I've reduced my electricity bill by over 100 bucks! I'm planning to get two more for my family.	Jingdong prefecture level city in Shandong
Dragon Quality Goods Shop	King of Power Saving	The effect is too good, the family has now several units, back to buy a number of times, the power saver received, power-saving effect is very good,, fine workmanship, long warranty, but also purify the air, before buying a used for a period of time, with the okay, and then bought a, and only now come to the	Jingdong prefecture level city in Shandong
Dragon Quality Goods Shop	King of Power Saving	Finally received the goods, very, very much like,, has been a lot of people praise, really too happy, has been his family's loyal fans, will continue to buy buy buy buy!	Jingdong prefecture level city in Shandong
Dragon Quality Goods Shop	King of Power Saving	📩 I've been wanting it for a long time, I decided to go for it, I feel great after using it, it's genuine and the delivery is great, it lives up to its name👍	Jingdong prefecture level city in Shandong
Dragon Quality Goods Shop	King of Power Saving	The quality is sure as other friends who bought it said, and it is also beautiful, thanks to the owner for the recommendation, thanks!	Jingdong prefecture level city in Shandong
Dragon Quality Goods Shop	King of Power Saving	Baby received the quality is really good face value is also very high especially practical especially especially special favorite	Jingdong prefecture level city in Shandong
Dragon Quality Goods Shop	King of Power Saving	It is genuine, trust the merchant, the quality is very good, the price is moderate, the use of the effect is very good!	Jingdong prefecture level city in Shandong

Dragon Quality Goods Shop	King of Power Saving	Energy saver received really good quality affordable and good energy saving and environmental protection is the best to use.	Jingdong prefecture level city in Shandong
Dragon Quality Goods Shop	King of Power Saving	Reasonable price logistics is very fast, the feeling is not bad more satisfied with a shopping experience	Jingdong prefecture level city in Shandong
Dragon Quality Goods Shop	King of Power Saving	The stuff is even more perfect than I imagined, very powerful and worth getting!	Jingdong prefecture level city in Shandong
Dragon Quality Goods Shop	King of Power Saving	Goods have been received, good quality, affordable price, good customer service, worth recommending!	Jingdong prefecture level city in Shandong
Dragon Quality Goods Shop	King of Power Saving	Fast shipping, works great, have already recommended it to friends.	Jingdong prefecture level city in Shandong
Dragon Quality Goods Shop	King of Power Saving	Good quality, fast logistics, reasonable price, worth 5 stars.	Jingdong prefecture level city in Shandong
Dragon Quality Goods Shop	King of Power Saving	Good quality, fast logistics, good value, well packaged, practical	Jingdong prefecture level city in Shandong
Dragon Quality Goods Shop	King of Power Saving	Fast shipping, affordable price, great quality, very satisfied!	Jingdong prefecture level city in Shandong
Dragon Quality Goods Shop	King of Power Saving	Regret knowing too late, it's really good stuff. Works well.	Jingdong prefecture level city in Shandong
Dragon Quality Goods Shop	King of Power Saving	Not bad, very good product, quite save electricity	Jingdong prefecture level city in Shandong
Dragon Quality Goods Shop	King of Power Saving	Good quality, fast logistics, overall very satisfied!	Jingdong prefecture level city in Shandong
Dragon Quality Goods Shop	King of Power Saving	Tightly packaged with good results	Jingdong prefecture level city in Shandong
Dragon Quality Goods Shop	King of Power Saving	very happy	Jingdong prefecture level city in Shandong
Dragon Quality Goods Shop	King of Power Saving	favorable criticism	Jingdong prefecture level city in Shandong
Dragon Quality Goods Shop	King of Power Saving	Now they all sign for the goods without receiving them, the Jingdong platform and merchants are very powerful, and the goods are not received before signing for the goods.	Jingdong prefecture level city in Shandong
Dragon Quality Goods Shop	King of Power Saving	low-quality commodities	Jingdong prefecture level city in Shandong
Dragon Quality Goods Shop	King of Power Saving	I bought this and now it works pretty well with the air conditioning on, when I didn't use it it was $600 a month, now it's almost $500.00, that's over $100 less all of a sudden.	Jingdong prefecture level city in Shandong
Dragon Quality Goods Shop	King of Power Saving	Can Oh, the weather is hot, open the air conditioning is really good, this product is good, has been used for a really long time, more ok, power saving effect is not bad.	Jingdong prefecture level city in Shandong
Dragon Quality Goods Shop	King of Power Saving	I paid my electric bill today, it's a few dozen dollars less, anyway, it's not costly, it's already very good, the boss said that it would be better to buy one more, I've already placed an order	Jingdong prefecture level city in Shandong
Dragon Quality Goods Shop	King of Power Saving	I'm on a smart meter, I save about 30% a month, personally I think it's pretty good, it works well	Jingdong prefecture level city in Shandong
Dragon Quality Goods Shop	King of Power Saving	My dad asked me to buy one, but after I paid the bill, I asked and he said it was a lot less this month, so he asked me to buy another one.	Jingdong prefecture level city in Shandong
Dragon Quality Goods Shop	King of Power Saving	Very satisfied with the purchase thanks to the store for the prosperous business.	Jingdong prefecture level city in Shandong
Dragon Quality Goods Shop	King of Power Saving	Good use, good quality, very satisfied with the purchase	Jingdong prefecture level city in Shandong
Dragon Quality Goods Shop	King of Power Saving	The goods have been received, the quality is very good, is the ideal effect, will continue to pay attention to the	Jingdong prefecture level city in Shandong
Dragon Quality Goods Shop	King of Power Saving	This brand has been used for many years, and this time I saw this upgraded version of the product and got it again. The purpose of this product is to reduce the loss of electricity for a long time, so as to achieve the purpose of saving electricity.	Jingdong prefecture level city in Shandong
Dragon Quality Goods Shop	King of Power Saving	I've been using it for almost a month, and I've reduced my electricity bill by over 100 bucks! I'm planning to get two more for my family.	Jingdong prefecture level city in Shandong
Dragon Quality Goods Shop	King of Power Saving	The effect is too good, the family has now several units, back to buy a number of times, the power saver received, power-saving effect is very good,, fine workmanship, long warranty, but also purify the air, before buying a used for a period of time, with the okay, and then bought a, and only now come to the	Jingdong prefecture level city in Shandong
Dragon Quality Goods Shop	King of Power Saving	Finally received the goods, very, very much like,, has been a lot of people praise, really too happy, has been his family's loyal fans, will continue to buy buy buy buy!	Jingdong prefecture level city in Shandong
Dragon Quality Goods Shop	King of Power Saving	📩 I've been wanting it for a long time, I decided to go for it, I feel great after using it, it's genuine and the delivery is great, it lives up to its name👍	Jingdong prefecture level city in Shandong
Dragon Quality Goods Shop	King of Power Saving	The quality is sure as other friends who bought it said, and it is also beautiful, thanks to the owner for the recommendation, thanks!	Jingdong prefecture level city in Shandong
Dragon Quality Goods Shop	King of Power Saving	Baby received the quality is really good face value is also very high especially practical especially especially special favorite	Jingdong prefecture level city in Shandong
Dragon Quality Goods Shop	King of Power Saving	It is genuine, trust the merchant, the quality is very good, the price is moderate, the use of the effect is very good!	Jingdong prefecture level city in Shandong
Dragon Quality Goods Shop	King of Power Saving	Energy saver received really good quality affordable and good energy saving and environmental protection is the best to use.	Jingdong prefecture level city in Shandong
Dragon Quality Goods Shop	King of Power Saving	Reasonable price logistics is very fast, the feeling is not bad more satisfied with a shopping experience	Jingdong prefecture level city in Shandong
Dragon Quality Goods Shop	King of Power Saving	The stuff is even more perfect than I imagined, very powerful and worth getting!	Jingdong prefecture level city in Shandong
Dragon Quality Goods Shop	King of Power Saving	Goods have been received, good quality, affordable price, good customer service, worth recommending!	Jingdong prefecture level city in Shandong
Dragon Quality Goods Shop	King of Power Saving	Fast shipping, works great, have already recommended it to friends.	Jingdong prefecture level city in Shandong
Dragon Quality Goods Shop	King of Power Saving	Good quality, fast logistics, reasonable price, worth 5 stars.	Jingdong prefecture level city in Shandong
Dragon Quality Goods Shop	King of Power Saving	Good quality, fast logistics, good value, well packaged, practical	Jingdong prefecture level city in Shandong
Dragon Quality Goods Shop	King of Power Saving	Fast shipping, affordable price, great quality, very satisfied!	Jingdong prefecture level city in Shandong
Dragon Quality Goods Shop	King of Power Saving	Regret knowing too late, it's really good stuff. Works well.	Jingdong prefecture level city in Shandong
Dragon Quality Goods Shop	King of Power Saving	Not bad, very good product, quite save electricity	Jingdong prefecture level city in Shandong
Dragon Quality Goods Shop	King of Power Saving	Good quality, fast logistics, overall very satisfied!	Jingdong prefecture level city in Shandong
Dragon Quality Goods Shop	King of Power Saving	Tightly packaged with good results	Jingdong prefecture level city in Shandong
Dragon Quality Goods Shop	King of Power Saving	very happy	Jingdong prefecture level city in Shandong
Dragon Quality Goods Shop	King of Power Saving	favorable criticism	Jingdong prefecture level city in Shandong
Dragon Quality Goods Shop	King of Power Saving	Now they all sign for the goods without receiving them, the Jingdong platform and merchants are very powerful, and the goods are not received before signing for the goods.	Jingdong prefecture level city in Shandong
Dragon Quality Goods Shop	King of Power Saving	low-quality commodities	Jingdong prefecture level city in Shandong
Dragon Quality Goods Shop	King of Power Saving	I bought this and now it works pretty well with the air conditioning on, when I didn't use it it was $600 a month, now it's almost $500.00, that's over $100 less all of a sudden.	Jingdong prefecture level city in Shandong
Dragon Quality Goods Shop	King of Power Saving	Can Oh, the weather is hot, open the air conditioning is really good, this product is good, has been used for a really long time, more ok, power saving effect is not bad.	Jingdong prefecture level city in Shandong

Dragon Quality Goods Shop	King of Power Saving	I paid my electric bill today, it's a few dozen dollars less, anyway, it's not costly, it's already very good, the boss said that it would be better to buy one more, I've already placed an order	Jingdong prefecture level city in Shandong
Dragon Quality Goods Shop	King of Power Saving	I'm on a smart meter, I save about 30% a month, personally I think it's pretty good, it works well	Jingdong prefecture level city in Shandong
Dragon Quality Goods Shop	King of Power Saving	My dad asked me to buy one, but after I paid the bill, I asked and he said it was a lot less this month, so he asked me to buy another one.	Jingdong prefecture level city in Shandong
Dragon Quality Goods Shop	King of Power Saving	Very satisfied with the purchase thanks to the store for the prosperous business.	Jingdong prefecture level city in Shandong
Dragon Quality Goods Shop	King of Power Saving	Good use, good quality, very satisfied with the purchase	Jingdong prefecture level city in Shandong
Dragon Quality Goods Shop	King of Power Saving	The goods have been received, the quality is very good, is the ideal effect, will continue to pay attention to the	Jingdong prefecture level city in Shandong
Dragon Quality Goods Shop	King of Power Saving	This brand has been used for many years, and this time I saw this upgraded version of the product and got it again. The purpose of this product is to reduce the loss of electricity for a long time, so as to achieve the purpose of saving electricity.	Jingdong prefecture level city in Shandong
Dragon Quality Goods Shop	King of Power Saving	I've been using it for almost a month, and I've reduced my electricity bill by over 100 bucks! I'm planning to get two more for my family.	Jingdong prefecture level city in Shandong
Dragon Quality Goods Shop	King of Power Saving	The effect is too good, the family has now several units, back to buy a number of times, the power saver received, power-saving effect is very good,, fine workmanship, long warranty, but also purify the air, before buying a used for a period of time, with the okay, and then bought a, and only now come to the	Jingdong prefecture level city in Shandong
Dragon Quality Goods Shop	King of Power Saving	Finally received the goods, very, very much like,, has been a lot of people praise, really too happy, has been his family's loyal fans, will continue to buy buy buy buy!	Jingdong prefecture level city in Shandong
Dragon Quality Goods Shop	King of Power Saving	💌 I've been wanting it for a long time, I decided to go for it, I feel great after using it, it's genuine and the delivery is great, it lives up to its name👍	Jingdong prefecture level city in Shandong
Dragon Quality Goods Shop	King of Power Saving	The quality is sure as other friends who bought it said, and it is also beautiful, thanks to the owner for the recommendation, thanks!	Jingdong prefecture level city in Shandong
Dragon Quality Goods Shop	King of Power Saving	Baby received the quality is really good face value is also very high especially practical especially especially special favorite	Jingdong prefecture level city in Shandong
Dragon Quality Goods Shop	King of Power Saving	It is genuine, trust the merchant, the quality is very good, the price is moderate, the use of the effect is very good!	Jingdong prefecture level city in Shandong
Dragon Quality Goods Shop	King of Power Saving	Energy saver received really good quality affordable and good energy saving and environmental protection is the best to use.	Jingdong prefecture level city in Shandong
Dragon Quality Goods Shop	King of Power Saving	Reasonable price logistics is very fast, the feeling is not bad more satisfied with a shopping experience	Jingdong prefecture level city in Shandong
Dragon Quality Goods Shop	King of Power Saving	The stuff is even more perfect than I imagined, very powerful and worth getting!	Jingdong prefecture level city in Shandong
Dragon Quality Goods Shop	King of Power Saving	Goods have been received, good quality, affordable price, good customer service, worth recommending!	Jingdong prefecture level city in Shandong
Dragon Quality Goods Shop	King of Power Saving	Fast shipping, works great, have already recommended it to friends.	Jingdong prefecture level city in Shandong
Dragon Quality Goods Shop	King of Power Saving	Good quality, fast logistics, reasonable price, worth 5 stars.	Jingdong prefecture level city in Shandong
Dragon Quality Goods Shop	King of Power Saving	Good quality, fast logistics, good value, well packaged, practical	Jingdong prefecture level city in Shandong
Dragon Quality Goods Shop	King of Power Saving	Fast shipping, affordable price, great quality, very satisfied!	Jingdong prefecture level city in Shandong
Dragon Quality Goods Shop	King of Power Saving	Regret knowing too late, it's really good stuff. Works well.	Jingdong prefecture level city in Shandong
Dragon Quality Goods Shop	King of Power Saving	Not bad, very good product, quite save electricity	Jingdong prefecture level city in Shandong
Dragon Quality Goods Shop	King of Power Saving	Good quality, fast logistics, overall very satisfied!	Jingdong prefecture level city in Shandong
Dragon Quality Goods Shop	King of Power Saving	Tightly packaged with good results	Jingdong prefecture level city in Shandong
Dragon Quality Goods Shop	King of Power Saving	very happy	Jingdong prefecture level city in Shandong
Dragon Quality Goods Shop	King of Power Saving	favorable criticism	Jingdong prefecture level city in Shandong
Dragon Quality Goods Shop	King of Power Saving	Now they all sign for the goods without receiving them, the Jingdong platform and merchants are very powerful, and the goods are not received before signing for the goods.	Jingdong prefecture level city in Shandong
Dragon Quality Goods Shop	King of Power Saving	low-quality commodities	Jingdong prefecture level city in Shandong
Dragon Quality Goods Shop	King of Power Saving	I bought this and now it works pretty well with the air conditioning on, when I didn't use it it was $600 a month, now it's almost $500,00, that's over $100 less all of a sudden.	Jingdong prefecture level city in Shandong
Dragon Quality Goods Shop	King of Power Saving	Can Oh, the weather is hot, open the air conditioning is really good, this product is good, has been used for a really long time, more ok, power saving effect is not bad.	Jingdong prefecture level city in Shandong
Dragon Quality Goods Shop	King of Power Saving	I paid my electric bill today, it's a few dozen dollars less, anyway, it's not costly, it's already very good, the boss said that it would be better to buy one more, I've already placed an order	Jingdong prefecture level city in Shandong
Dragon Quality Goods Shop	King of Power Saving	I'm on a smart meter, I save about 30% a month, personally I think it's pretty good, it works well	Jingdong prefecture level city in Shandong
Dragon Quality Goods Shop	King of Power Saving	My dad asked me to buy one, but after I paid the bill, I asked and he said it was a lot less this month, so he asked me to buy another one.	Jingdong prefecture level city in Shandong
Dragon Quality Goods Shop	King of Power Saving	Very satisfied with the purchase thanks to the store for the prosperous business.	Jingdong prefecture level city in Shandong
Dragon Quality Goods Shop	King of Power Saving	Good use, good quality, very satisfied with the purchase	Jingdong prefecture level city in Shandong
Dragon Quality Goods Shop	King of Power Saving	The goods have been received, the quality is very good, is the ideal effect, will continue to pay attention to the	Jingdong prefecture level city in Shandong
Dragon Quality Goods Shop	King of Power Saving	This brand has been used for many years, and this time I saw this upgraded version of the product and got it again. The purpose of this product is to reduce the loss of electricity for a long time, so as to achieve the purpose of saving electricity.	Jingdong prefecture level city in Shandong
Dragon Quality Goods Shop	King of Power Saving	I've been using it for almost a month, and I've reduced my electricity bill by over 100 bucks! I'm planning to get two more for my family.	Jingdong prefecture level city in Shandong
Dragon Quality Goods Shop	King of Power Saving	The effect is too good, the family has now several units, back to buy a number of times, the power saver received, power-saving effect is very good,, fine workmanship, long warranty, but also purify the air, before buying a used for a period of time, with the okay, and then bought a, and only now come to the	Jingdong prefecture level city in Shandong
Dragon Quality Goods Shop	King of Power Saving	Finally received the goods, very, very much like,, has been a lot of people praise, really too happy, has been his family's loyal fans, will continue to buy buy buy buy!	Jingdong prefecture level city in Shandong
Dragon Quality Goods Shop	King of Power Saving	💌 I've been wanting it for a long time, I decided to go for it, I feel great after using it, it's genuine and the delivery is great, it lives up to its name👍	Jingdong prefecture level city in Shandong
Dragon Quality Goods Shop	King of Power Saving	The quality is sure as other friends who bought it said, and it is also beautiful, thanks to the owner for the recommendation, thanks!	Jingdong prefecture level city in Shandong
Dragon Quality Goods Shop	King of Power Saving	Baby received the quality is really good face value is also very high especially practical especially especially special favorite	Jingdong prefecture level city in Shandong
Dragon Quality Goods Shop	King of Power Saving	It is genuine, trust the merchant, the quality is very good, the price is moderate, the use of the effect is very good!	Jingdong prefecture level city in Shandong
Dragon Quality Goods Shop	King of Power Saving	Energy saver received really good quality affordable and good energy saving and environmental protection is the best to use.	Jingdong prefecture level city in Shandong
Dragon Quality Goods Shop	King of Power Saving	Reasonable price logistics is very fast, the feeling is not bad more satisfied with a shopping experience	Jingdong prefecture level city in Shandong
Dragon Quality Goods Shop	King of Power Saving	The stuff is even more perfect than I imagined, very powerful and worth getting!	Jingdong prefecture level city in Shandong

Dragon Quality Goods Shop	King of Power Saving	Goods have been received, good quality, affordable price, good customer service, worth recommending!	Jingdong prefecture level city in Shandong
Dragon Quality Goods Shop	King of Power Saving	Fast shipping, works great, have already recommended it to friends.	Jingdong prefecture level city in Shandong
Dragon Quality Goods Shop	King of Power Saving	Good quality, fast logistics, reasonable price, worth 5 stars.	Jingdong prefecture level city in Shandong
Dragon Quality Goods Shop	King of Power Saving	Good quality, fast logistics, good value, well packaged, practical	Jingdong prefecture level city in Shandong
Dragon Quality Goods Shop	King of Power Saving	Fast shipping, affordable price, great quality, very satisfied!	Jingdong prefecture level city in Shandong
Dragon Quality Goods Shop	King of Power Saving	Regret knowing too late, it's really good stuff. Works well.	Jingdong prefecture level city in Shandong
Dragon Quality Goods Shop	King of Power Saving	Not bad, very good product, quite save electricity	Jingdong prefecture level city in Shandong
Dragon Quality Goods Shop	King of Power Saving	Good quality, fast logistics, overall very satisfied!	Jingdong prefecture level city in Shandong
Dragon Quality Goods Shop	King of Power Saving	Tightly packaged with good results	Jingdong prefecture level city in Shandong
Dragon Quality Goods Shop	King of Power Saving	very happy	Jingdong prefecture level city in Shandong
Dragon Quality Goods Shop	King of Power Saving	favorable criticism	Jingdong prefecture level city in Shandong
Dragon Quality Goods Shop	King of Power Saving	Now they all sign for the goods without receiving them, the Jingdong platform and merchants are very powerful, and the goods are not received before signing for the goods.	Jingdong prefecture level city in Shandong
Dragon Quality Goods Shop	King of Power Saving	low-quality commodities	Jingdong prefecture level city in Shandong
Dragon Quality Goods Shop	King of Power Saving	I bought this and now it works pretty well with the air conditioning on, when I didn't use it it was $600 a month, now it's almost $500.00, that's over $100 less all of a sudden.	Jingdong prefecture level city in Shandong
Dragon Quality Goods Shop	King of Power Saving	Can Oh, the weather is hot, open the air conditioning is really good, this product is good, has been used for a really long time, more ok, power saving effect is not bad.	Jingdong prefecture level city in Shandong
Dragon Quality Goods Shop	King of Power Saving	I paid my electric bill today, it's a few dozen dollars less, anyway, it's not costly, it's already very good, the boss said that it would be better to buy one more, I've already placed an order	Jingdong prefecture level city in Shandong
Dragon Quality Goods Shop	King of Power Saving	I'm on a smart meter, I save about 30% a month, personally I think it's pretty good, it works well	Jingdong prefecture level city in Shandong
Dragon Quality Goods Shop	King of Power Saving	My dad asked me to buy one, but after I paid the bill, I asked and he said it was a lot less this month, so he asked me to buy another one.	Jingdong prefecture level city in Shandong
Dragon Quality Goods Shop	King of Power Saving	Very satisfied with the purchase thanks to the store for the prosperous business.	Jingdong prefecture level city in Shandong
Dragon Quality Goods Shop	King of Power Saving	Good use, good quality, very satisfied with the purchase	Jingdong prefecture level city in Shandong
Dragon Quality Goods Shop	King of Power Saving	The goods have been received, the quality is very good, is the ideal effect, will continue to pay attention to the	Jingdong prefecture level city in Shandong
Dragon Quality Goods Shop	King of Power Saving	This brand has been used for many years, and this time I saw this upgraded version of the product and got it again. The purpose of this product is to reduce the loss of electricity for a long time, so as to achieve the purpose of saving electricity.	Jingdong prefecture level city in Shandong
Dragon Quality Goods Shop	King of Power Saving	I've been using it for almost a month, and I've reduced my electricity bill by over 100 bucks! I'm planning to get two more for my family.	Jingdong prefecture level city in Shandong
Dragon Quality Goods Shop	King of Power Saving	The effect is too good, the family has now several units, back to buy a number of times, the power saver received, power-saving effect is very good,, fine workmanship, long warranty, but also purify the air, before buying a used for a period of time, with the okay, and then bought a, and only now come to the	Jingdong prefecture level city in Shandong
Dragon Quality Goods Shop	King of Power Saving	Finally received the goods, very, very much like,, has been a lot of people praise, really too happy, has been his family's loyal fans, will continue to buy buy buy buy!	Jingdong prefecture level city in Shandong
Dragon Quality Goods Shop	King of Power Saving	📩 I've been wanting it for a long time, I decided to go for it, I feel great after using it, it's genuine and the delivery is great, it lives up to its name👍	Jingdong prefecture level city in Shandong
Dragon Quality Goods Shop	King of Power Saving	The quality is sure as other friends who bought it said, and it is also beautiful, thanks to the owner for the recommendation, thanks!	Jingdong prefecture level city in Shandong
Dragon Quality Goods Shop	King of Power Saving	Baby received the quality is really good face value is also very high especially practical especially especially special favorite	Jingdong prefecture level city in Shandong
Dragon Quality Goods Shop	King of Power Saving	It is genuine, trust the merchant, the quality is very good, the price is moderate, the use of the effect is very good!	Jingdong prefecture level city in Shandong
Dragon Quality Goods Shop	King of Power Saving	Energy saver received really good quality affordable and good energy saving and environmental protection is the best to use.	Jingdong prefecture level city in Shandong
Dragon Quality Goods Shop	King of Power Saving	Reasonable price logistics is very fast, the feeling is not bad more satisfied with a shopping experience	Jingdong prefecture level city in Shandong
Dragon Quality Goods Shop	King of Power Saving	The stuff is even more perfect than I imagined, very powerful and worth getting!	Jingdong prefecture level city in Shandong
Dragon Quality Goods Shop	King of Power Saving	Goods have been received, good quality, affordable price, good customer service, worth recommending!	Jingdong prefecture level city in Shandong
Dragon Quality Goods Shop	King of Power Saving	Fast shipping, works great, have already recommended it to friends.	Jingdong prefecture level city in Shandong
Dragon Quality Goods Shop	King of Power Saving	Good quality, fast logistics, reasonable price, worth 5 stars.	Jingdong prefecture level city in Shandong
Dragon Quality Goods Shop	King of Power Saving	Good quality, fast logistics, good value, well packaged, practical	Jingdong prefecture level city in Shandong
Dragon Quality Goods Shop	King of Power Saving	Fast shipping, affordable price, great quality, very satisfied!	Jingdong prefecture level city in Shandong
Dragon Quality Goods Shop	King of Power Saving	Regret knowing too late, it's really good stuff. Works well.	Jingdong prefecture level city in Shandong
Dragon Quality Goods Shop	King of Power Saving	Not bad, very good product, quite save electricity	Jingdong prefecture level city in Shandong
Dragon Quality Goods Shop	King of Power Saving	Good quality, fast logistics, overall very satisfied!	Jingdong prefecture level city in Shandong
Dragon Quality Goods Shop	King of Power Saving	Tightly packaged with good results	Jingdong prefecture level city in Shandong
Dragon Quality Goods Shop	King of Power Saving	very happy	Jingdong prefecture level city in Shandong
Dragon Quality Goods Shop	King of Power Saving	favorable criticism	Jingdong prefecture level city in Shandong
Dragon Quality Goods Shop	King of Power Saving	Now they all sign for the goods without receiving them, the Jingdong platform and merchants are very powerful, and the goods are not received before signing for the goods.	Jingdong prefecture level city in Shandong
Dragon Quality Goods Shop	King of Power Saving	low-quality commodities	Jingdong prefecture level city in Shandong
Dragon Quality Goods Shop	King of Power Saving	I bought this and now it works pretty well with the air conditioning on, when I didn't use it it was $600 a month, now it's almost $500.00, that's over $100 less all of a sudden.	Jingdong prefecture level city in Shandong
Dragon Quality Goods Shop	King of Power Saving	Can Oh, the weather is hot, open the air conditioning is really good, this product is good, has been used for a really long time, more ok, power saving effect is not bad.	Jingdong prefecture level city in Shandong
Dragon Quality Goods Shop	King of Power Saving	I paid my electric bill today, it's a few dozen dollars less, anyway, it's not costly, it's already very good, the boss said that it would be better to buy one more, I've already placed an order	Jingdong prefecture level city in Shandong
Dragon Quality Goods Shop	King of Power Saving	I'm on a smart meter, I save about 30% a month, personally I think it's pretty good, it works well	Jingdong prefecture level city in Shandong
Dragon Quality Goods Shop	King of Power Saving	My dad asked me to buy one, but after I paid the bill, I asked and he said it was a lot less this month, so he asked me to buy another one.	Jingdong prefecture level city in Shandong

Dragon Quality Goods Shop	King of Power Saving	Very satisfied with the purchase thanks to the store for the prosperous business.	Jingdong prefecture level city in Shandong
Dragon Quality Goods Shop	King of Power Saving	Good use, good quality, very satisfied with the purchase	Jingdong prefecture level city in Shandong
Dragon Quality Goods Shop	King of Power Saving	The goods have been received, the quality is very good, is the ideal effect, will continue to pay attention to the	Jingdong prefecture level city in Shandong
Dragon Quality Goods Shop	King of Power Saving	This brand has been used for many years, and this time I saw this upgraded version of the product and got it again. The purpose of this product is to reduce the loss of electricity for a long time, so as to achieve the purpose of saving electricity.	Jingdong prefecture level city in Shandong
Dragon Quality Goods Shop	King of Power Saving	I've been using it for almost a month, and I've reduced my electricity bill by over 100 bucks! I'm planning to get two more for my family.	Jingdong prefecture level city in Shandong
Dragon Quality Goods Shop	King of Power Saving	The effect is too good, the family has now several units, back to buy a number of times, the power saver received, power-saving effect is very good,, fine workmanship, long warranty, but also purify the air, before buying a used for a period of time, with the okay, and then bought a, and only now come to the	Jingdong prefecture level city in Shandong
Dragon Quality Goods Shop	King of Power Saving	Finally received the goods, very, very much like,, has been a lot of people praise, really too happy, has been his family's loyal fans, will continue to buy buy buy buy!	Jingdong prefecture level city in Shandong
Dragon Quality Goods Shop	King of Power Saving	📩 I've been wanting it for a long time, I decided to go for it, I feel great after using it, it's genuine and the delivery is great, it lives up to its name👍	Jingdong prefecture level city in Shandong
Dragon Quality Goods Shop	King of Power Saving	The quality is sure as other friends who bought it said, and it is also beautiful, thanks to the owner for the recommendation, thanks!	Jingdong prefecture level city in Shandong
Dragon Quality Goods Shop	King of Power Saving	Baby received the quality is really good face value is also very high especially practical especially especially special favorite	Jingdong prefecture level city in Shandong
Dragon Quality Goods Shop	King of Power Saving	It is genuine, trust the merchant, the quality is very good, the price is moderate, the use of the effect is very good!	Jingdong prefecture level city in Shandong
Dragon Quality Goods Shop	King of Power Saving	Energy saver received really good quality affordable and good energy saving and environmental protection is the best to use.	Jingdong prefecture level city in Shandong
Dragon Quality Goods Shop	King of Power Saving	Reasonable price logistics is very fast, the feeling is not bad more satisfied with a shopping experience	Jingdong prefecture level city in Shandong
Dragon Quality Goods Shop	King of Power Saving	The stuff is even more perfect than I imagined, very powerful and worth getting!	Jingdong prefecture level city in Shandong
Dragon Quality Goods Shop	King of Power Saving	Goods have been received, good quality, affordable price, good customer service, worth recommending!	Jingdong prefecture level city in Shandong
Dragon Quality Goods Shop	King of Power Saving	Fast shipping, works great, have already recommended it to friends.	Jingdong prefecture level city in Shandong
Dragon Quality Goods Shop	King of Power Saving	Good quality, fast logistics, reasonable price, worth 5 stars.	Jingdong prefecture level city in Shandong
Dragon Quality Goods Shop	King of Power Saving	Good quality, fast logistics, good value, well packaged, practical	Jingdong prefecture level city in Shandong
Dragon Quality Goods Shop	King of Power Saving	Fast shipping, affordable price, great quality, very satisfied!	Jingdong prefecture level city in Shandong
Dragon Quality Goods Shop	King of Power Saving	Regret knowing too late, it's really good stuff. Works well.	Jingdong prefecture level city in Shandong
Dragon Quality Goods Shop	King of Power Saving	Not bad, very good product, quite save electricity	Jingdong prefecture level city in Shandong
Dragon Quality Goods Shop	King of Power Saving	Good quality, fast logistics, overall very satisfied!	Jingdong prefecture level city in Shandong
Dragon Quality Goods Shop	King of Power Saving	Tightly packaged with good results	Jingdong prefecture level city in Shandong
Dragon Quality Goods Shop	King of Power Saving	very happy	Jingdong prefecture level city in Shandong
Dragon Quality Goods Shop	King of Power Saving	favorable criticism	Jingdong prefecture level city in Shandong
Dragon Quality Goods Shop	King of Power Saving	Now they all sign for the goods without receiving them, the Jingdong platform and merchants are very powerful, and the goods are not received before signing for the goods.	Jingdong prefecture level city in Shandong
Dragon Quality Goods Shop	King of Power Saving	low-quality commodities	Jingdong prefecture level city in Shandong
Dragon Quality Goods Shop	King of Power Saving	I bought this and now it works pretty well with the air conditioning on, when I didn't use it it was $600 a month, now it's almost $500.00, that's over $100 less all of a sudden.	Jingdong prefecture level city in Shandong
Dragon Quality Goods Shop	King of Power Saving	Can Oh, the weather is hot, open the air conditioning is really good, this product is good, has been used for a really long time, more ok, power saving effect is not bad.	Jingdong prefecture level city in Shandong
Dragon Quality Goods Shop	King of Power Saving	I paid my electric bill today, it's a few dozen dollars less, anyway, it's not costly, it's already very good, the boss said that it would be better to buy one more, I've already placed an order	Jingdong prefecture level city in Shandong
Dragon Quality Goods Shop	King of Power Saving	I'm on a smart meter, I save about 30% a month, personally I think it's pretty good, it works well	Jingdong prefecture level city in Shandong
Dragon Quality Goods Shop	King of Power Saving	My dad asked me to buy one, but after I paid the bill, I asked and he said it was a lot less this month, so he asked me to buy another one.	Jingdong prefecture level city in Shandong
Dragon Quality Goods Shop	King of Power Saving	Very satisfied with the purchase thanks to the store for the prosperous business.	Jingdong prefecture level city in Shandong
Dragon Quality Goods Shop	King of Power Saving	Good use, good quality, very satisfied with the purchase	Jingdong prefecture level city in Shandong
Dragon Quality Goods Shop	King of Power Saving	The goods have been received, the quality is very good, is the ideal effect, will continue to pay attention to the	Jingdong prefecture level city in Shandong
Dragon Quality Goods Shop	King of Power Saving	This brand has been used for many years, and this time I saw this upgraded version of the product and got it again. The purpose of this product is to reduce the loss of electricity for a long time, so as to achieve the purpose of saving electricity.	Jingdong prefecture level city in Shandong
Dragon Quality Goods Shop	King of Power Saving	I've been using it for almost a month, and I've reduced my electricity bill by over 100 bucks! I'm planning to get two more for my family.	Jingdong prefecture level city in Shandong
Dragon Quality Goods Shop	King of Power Saving	The effect is too good, the family has now several units, back to buy a number of times, the power saver received, power-saving effect is very good,, fine workmanship, long warranty, but also purify the air, before buying a used for a period of time, with the okay, and then bought a, and only now come to the	Jingdong prefecture level city in Shandong
Dragon Quality Goods Shop	King of Power Saving	Finally received the goods, very, very much like,, has been a lot of people praise, really too happy, has been his family's loyal fans, will continue to buy buy buy buy!	Jingdong prefecture level city in Shandong
Dragon Quality Goods Shop	King of Power Saving	📩 I've been wanting it for a long time, I decided to go for it, I feel great after using it, it's genuine and the delivery is great, it lives up to its name👍	Jingdong prefecture level city in Shandong
Dragon Quality Goods Shop	King of Power Saving	The quality is sure as other friends who bought it said, and it is also beautiful, thanks to the owner for the recommendation, thanks!	Jingdong prefecture level city in Shandong
Dragon Quality Goods Shop	King of Power Saving	Baby received the quality is really good face value is also very high especially practical especially especially special favorite	Jingdong prefecture level city in Shandong
Dragon Quality Goods Shop	King of Power Saving	It is genuine, trust the merchant, the quality is very good, the price is moderate, the use of the effect is very good!	Jingdong prefecture level city in Shandong
Dragon Quality Goods Shop	King of Power Saving	Energy saver received really good quality affordable and good energy saving and environmental protection is the best to use.	Jingdong prefecture level city in Shandong
Dragon Quality Goods Shop	King of Power Saving	Reasonable price logistics is very fast, the feeling is not bad more satisfied with a shopping experience	Jingdong prefecture level city in Shandong
Dragon Quality Goods Shop	King of Power Saving	The stuff is even more perfect than I imagined, very powerful and worth getting!	Jingdong prefecture level city in Shandong
Dragon Quality Goods Shop	King of Power Saving	Goods have been received, good quality, affordable price, good customer service, worth recommending!	Jingdong prefecture level city in Shandong
Dragon Quality Goods Shop	King of Power Saving	Fast shipping, works great, have already recommended it to friends.	Jingdong prefecture level city in Shandong
Dragon Quality Goods Shop	King of Power Saving	Good quality, fast logistics, reasonable price, worth 5 stars.	Jingdong prefecture level city in Shandong

Dragon Quality Goods Shop	King of Power Saving	Good quality, fast logistics, good value, well packaged, practical	Jingdong prefecture level city in Shandong
Dragon Quality Goods Shop	King of Power Saving	Fast shipping, affordable price, great quality, very satisfied!	Jingdong prefecture level city in Shandong
Dragon Quality Goods Shop	King of Power Saving	Regret knowing too late, it's really good stuff. Works well.	Jingdong prefecture level city in Shandong
Dragon Quality Goods Shop	King of Power Saving	Not bad, very good product, quite save electricity	Jingdong prefecture level city in Shandong
Dragon Quality Goods Shop	King of Power Saving	Good quality, fast logistics, overall very satisfied!	Jingdong prefecture level city in Shandong
Dragon Quality Goods Shop	King of Power Saving	Tightly packaged with good results	Jingdong prefecture level city in Shandong
Dragon Quality Goods Shop	King of Power Saving	very happy	Jingdong prefecture level city in Shandong
Dragon Quality Goods Shop	King of Power Saving	favorable criticism	Jingdong prefecture level city in Shandong
Dragon Quality Goods Shop	King of Power Saving	Now they all sign for the goods without receiving them, the Jingdong platform and merchants are very powerful, and the goods are not received before signing for the goods.	Jingdong prefecture level city in Shandong
Dragon Quality Goods Shop	King of Power Saving	low-quality commodities	Jingdong prefecture level city in Shandong
Dragon Quality Goods Shop	King of Power Saving	I bought this and now it works pretty well with the air conditioning on, when I didn't use it it was $600 a month, now it's almost $500.00, that's over $100 less all of a sudden.	Jingdong prefecture level city in Shandong
Dragon Quality Goods Shop	King of Power Saving	Can Oh, the weather is hot, open the air conditioning is really good, this product is good, has been used for a really long time, more ok, power saving effect is not bad.	Jingdong prefecture level city in Shandong
Dragon Quality Goods Shop	King of Power Saving	I paid my electric bill today, it's a few dozen dollars less, anyway, it's not costly, it's already very good, the boss said that it would be better to buy one more, I've already placed an order	Jingdong prefecture level city in Shandong
Dragon Quality Goods Shop	King of Power Saving	I'm on a smart meter, I save about 30% a month, personally I think it's pretty good, it works well	Jingdong prefecture level city in Shandong
Dragon Quality Goods Shop	King of Power Saving	My dad asked me to buy one, but after I paid the bill, I asked and he said it was a lot less this month, so he asked me to buy another one.	Jingdong prefecture level city in Shandong
Dragon Quality Goods Shop	King of Power Saving	Very satisfied with the purchase thanks to the store for the prosperous business.	Jingdong prefecture level city in Shandong
Dragon Quality Goods Shop	King of Power Saving	Good use, good quality, very satisfied with the purchase	Jingdong prefecture level city in Shandong
Dragon Quality Goods Shop	King of Power Saving	The goods have been received, the quality is very good, is the ideal effect, will continue to pay attention to the	Jingdong prefecture level city in Shandong
Dragon Quality Goods Shop	King of Power Saving	This brand has been used for many years, and this time I saw this upgraded version of the product and got it again. The purpose of this product is to reduce the loss of electricity for a long time, so as to achieve the purpose of saving electricity.	Jingdong prefecture level city in Shandong
Dragon Quality Goods Shop	King of Power Saving	I've been using it for almost a month, and I've reduced my electricity bill by over 100 bucks! I'm planning to get two more for my family.	Jingdong prefecture level city in Shandong
Dragon Quality Goods Shop	King of Power Saving	The effect is too good, the family has now several units, back to buy a number of times, the power saver received, power-saving effect is very good,, fine workmanship, long warranty, but also purify the air, before buying a used for a period of time, with the okay, and then bought a, and only now come to the	Jingdong prefecture level city in Shandong
Dragon Quality Goods Shop	King of Power Saving	Finally received the goods, very, very much like,, has been a lot of people praise, really too happy, has been his family's loyal fans, will continue to buy buy buy buy!	Jingdong prefecture level city in Shandong
Dragon Quality Goods Shop	King of Power Saving	I've been wanting it for a long time, I decided to go for it, I feel great after using it, it's genuine and the delivery is great, it lives up to its name	Jingdong prefecture level city in Shandong
Dragon Quality Goods Shop	King of Power Saving	The quality is sure as other friends who bought it said, and it is also beautiful, thanks to the owner for the recommendation, thanks!	Jingdong prefecture level city in Shandong
Dragon Quality Goods Shop	King of Power Saving	Baby received the quality is really good face value is also very high especially practical especially especially special favorite	Jingdong prefecture level city in Shandong
Dragon Quality Goods Shop	King of Power Saving	It is genuine, trust the merchant, the quality is very good, the price is moderate, the use of the effect is very good!	Jingdong prefecture level city in Shandong
Dragon Quality Goods Shop	King of Power Saving	Energy saver received really good quality affordable and good energy saving and environmental protection is the best to use.	Jingdong prefecture level city in Shandong
Dragon Quality Goods Shop	King of Power Saving	Reasonable price logistics is very fast, the feeling is not bad more satisfied with a shopping experience	Jingdong prefecture level city in Shandong
Dragon Quality Goods Shop	King of Power Saving	The stuff is even more perfect than I imagined, very powerful and worth getting!	Jingdong prefecture level city in Shandong
Dragon Quality Goods Shop	King of Power Saving	Goods have been received, good quality, affordable price, good customer service, worth recommending!	Jingdong prefecture level city in Shandong
Dragon Quality Goods Shop	King of Power Saving	Fast shipping, works great, have already recommended it to friends.	Jingdong prefecture level city in Shandong
Dragon Quality Goods Shop	King of Power Saving	Good quality, fast logistics, reasonable price, worth 5 stars.	Jingdong prefecture level city in Shandong
Dragon Quality Goods Shop	King of Power Saving	Good quality, fast logistics, good value, well packaged, practical	Jingdong prefecture level city in Shandong
Dragon Quality Goods Shop	King of Power Saving	Fast shipping, affordable price, great quality, very satisfied!	Jingdong prefecture level city in Shandong
Dragon Quality Goods Shop	King of Power Saving	Regret knowing too late, it's really good stuff. Works well.	Jingdong prefecture level city in Shandong
Dragon Quality Goods Shop	King of Power Saving	Not bad, very good product, quite save electricity	Jingdong prefecture level city in Shandong
Dragon Quality Goods Shop	King of Power Saving	Good quality, fast logistics, overall very satisfied!	Jingdong prefecture level city in Shandong
Dragon Quality Goods Shop	King of Power Saving	Tightly packaged with good results	Jingdong prefecture level city in Shandong
Dragon Quality Goods Shop	King of Power Saving	very happy	Jingdong prefecture level city in Shandong
Dragon Quality Goods Shop	King of Power Saving	favorable criticism	Jingdong prefecture level city in Shandong
Dragon Quality Goods Shop	King of Power Saving	Now they all sign for the goods without receiving them, the Jingdong platform and merchants are very powerful, and the goods are not received before signing for the goods.	Jingdong prefecture level city in Shandong
Dragon Quality Goods Shop	King of Power Saving	low-quality commodities	Jingdong prefecture level city in Shandong
Dragon Quality Goods Shop	King of Power Saving	I bought this and now it works pretty well with the air conditioning on, when I didn't use it it was $600 a month, now it's almost $500.00, that's over $100 less all of a sudden.	Jingdong prefecture level city in Shandong
Dragon Quality Goods Shop	King of Power Saving	Can Oh, the weather is hot, open the air conditioning is really good, this product is good, has been used for a really long time, more ok, power saving effect is not bad.	Jingdong prefecture level city in Shandong
Dragon Quality Goods Shop	King of Power Saving	I paid my electric bill today, it's a few dozen dollars less, anyway, it's not costly, it's already very good, the boss said that it would be better to buy one more, I've already placed an order	Jingdong prefecture level city in Shandong
Dragon Quality Goods Shop	King of Power Saving	I'm on a smart meter, I save about 30% a month, personally I think it's pretty good, it works well	Jingdong prefecture level city in Shandong
Dragon Quality Goods Shop	King of Power Saving	My dad asked me to buy one, but after I paid the bill, I asked and he said it was a lot less this month, so he asked me to buy another one.	Jingdong prefecture level city in Shandong
Dragon Quality Goods Shop	King of Power Saving	Very satisfied with the purchase thanks to the store for the prosperous business.	Jingdong prefecture level city in Shandong
Dragon Quality Goods Shop	King of Power Saving	Good use, good quality, very satisfied with the purchase	Jingdong prefecture level city in Shandong
Dragon Quality Goods Shop	King of Power Saving	The goods have been received, the quality is very good, is the ideal effect, will continue to pay attention to the	Jingdong prefecture level city in Shandong

Dragon Quality Goods Shop	King of Power Saving	This brand has been used for many years, and this time I saw this upgraded version of the product and got it again. The purpose of this product is to reduce the loss of electricity for a long time, so as to achieve the purpose of saving electricity.	Jingdong prefecture level city in Shandong
Dragon Quality Goods Shop	King of Power Saving	I've been using it for almost a month, and I've reduced my electricity bill by over 100 bucks! I'm planning to get two more for my family.	Jingdong prefecture level city in Shandong
Dragon Quality Goods Shop	King of Power Saving	The effect is too good, the family has now several units, back to buy a number of times, the power saver received, power-saving effect is very good,, fine workmanship, long warranty, but also purify the air, before buying a used for a period of time, with the okay, and then bought a, and only now come to the	Jingdong prefecture level city in Shandong
Dragon Quality Goods Shop	King of Power Saving	Finally received the goods, very, very much like,, has been a lot of people praise, really too happy, has been his family's loyal fans, will continue to buy buy buy buy!	Jingdong prefecture level city in Shandong
Dragon Quality Goods Shop	King of Power Saving	📩 I've been wanting it for a long time, I decided to go for it, I feel great after using it, it's genuine and the delivery is great, it lives up to its name👍	Jingdong prefecture level city in Shandong
Dragon Quality Goods Shop	King of Power Saving	The quality is sure as other friends who bought it said, and it is also beautiful, thanks to the owner for the recommendation, thanks!	Jingdong prefecture level city in Shandong
Dragon Quality Goods Shop	King of Power Saving	Baby received the quality is really good face value is also very high especially practical especially especially special favorite	Jingdong prefecture level city in Shandong
Dragon Quality Goods Shop	King of Power Saving	It is genuine, trust the merchant, the quality is very good, the price is moderate, the use of the effect is very good!	Jingdong prefecture level city in Shandong
Dragon Quality Goods Shop	King of Power Saving	Energy saver received really good quality affordable and good energy saving and environmental protection is the best to use.	Jingdong prefecture level city in Shandong
Dragon Quality Goods Shop	King of Power Saving	Reasonable price logistics is very fast, the feeling is not bad more satisfied with a shopping experience	Jingdong prefecture level city in Shandong
Dragon Quality Goods Shop	King of Power Saving	The stuff is even more perfect than I imagined, very powerful and worth getting!	Jingdong prefecture level city in Shandong
Dragon Quality Goods Shop	King of Power Saving	Goods have been received, good quality, affordable price, good customer service, worth recommending!	Jingdong prefecture level city in Shandong
Dragon Quality Goods Shop	King of Power Saving	Fast shipping, works great, have already recommended it to friends.	Jingdong prefecture level city in Shandong
Dragon Quality Goods Shop	King of Power Saving	Good quality, fast logistics, reasonable price, worth 5 stars.	Jingdong prefecture level city in Shandong
Dragon Quality Goods Shop	King of Power Saving	Good quality, fast logistics, good value, well packaged, practical	Jingdong prefecture level city in Shandong
Dragon Quality Goods Shop	King of Power Saving	Fast shipping, affordable price, great quality, very satisfied!	Jingdong prefecture level city in Shandong
Dragon Quality Goods Shop	King of Power Saving	Regret knowing too late, it's really good stuff. Works well.	Jingdong prefecture level city in Shandong
Dragon Quality Goods Shop	King of Power Saving	Not bad, very good product, quite save electricity	Jingdong prefecture level city in Shandong
Dragon Quality Goods Shop	King of Power Saving	Good quality, fast logistics, overall very satisfied!	Jingdong prefecture level city in Shandong
Dragon Quality Goods Shop	King of Power Saving	Tightly packaged with good results	Jingdong prefecture level city in Shandong
Dragon Quality Goods Shop	King of Power Saving	very happy	Jingdong prefecture level city in Shandong
Dragon Quality Goods Shop	King of Power Saving	favorable criticism	Jingdong prefecture level city in Shandong
Dragon Quality Goods Shop	King of Power Saving	Now they all sign for the goods without receiving them, the Jingdong platform and merchants are very powerful, and the goods are not received before signing for the goods.	Jingdong prefecture level city in Shandong
Dragon Quality Goods Shop	King of Power Saving	low-quality commodities	Jingdong prefecture level city in Shandong
Dragon Quality Goods Shop	King of Power Saving	I bought this and now it works pretty well with the air conditioning on, when I didn't use it it was $600 a month, now it's almost $500,00, that's over $100 less all of a sudden.	Jingdong prefecture level city in Shandong
Dragon Quality Goods Shop	King of Power Saving	Can Oh, the weather is hot, open the air conditioning is really good, this product is good, has been used for a really long time, more ok, power saving effect is not bad.	Jingdong prefecture level city in Shandong
Dragon Quality Goods Shop	King of Power Saving	I paid my electric bill today, it's a few dozen dollars less, anyway, it's not costly, it's already very good, the boss said that it would be better to buy one more, I've already placed an order	Jingdong prefecture level city in Shandong
Dragon Quality Goods Shop	King of Power Saving	I'm on a smart meter, I save about 30% a month, personally I think it's pretty good, it works well	Jingdong prefecture level city in Shandong
Dragon Quality Goods Shop	King of Power Saving	My dad asked me to buy one, but after I paid the bill, I asked and he said it was a lot less this month, so he asked me to buy another one.	Jingdong prefecture level city in Shandong
Dragon Quality Goods Shop	King of Power Saving	Very satisfied with the purchase thanks to the store for the prosperous business.	Jingdong prefecture level city in Shandong
Dragon Quality Goods Shop	King of Power Saving	Good use, good quality, very satisfied with the purchase	Jingdong prefecture level city in Shandong
Dragon Quality Goods Shop	King of Power Saving	The goods have been received, the quality is very good, is the ideal effect, will continue to pay attention to the	Jingdong prefecture level city in Shandong
Dragon Quality Goods Shop	King of Power Saving	This brand has been used for many years, and this time I saw this upgraded version of the product and got it again. The purpose of this product is to reduce the loss of electricity for a long time, so as to achieve the purpose of saving electricity.	Jingdong prefecture level city in Shandong
Dragon Quality Goods Shop	King of Power Saving	I've been using it for almost a month, and I've reduced my electricity bill by over 100 bucks! I'm planning to get two more for my family.	Jingdong prefecture level city in Shandong
Dragon Quality Goods Shop	King of Power Saving	The effect is too good, the family has now several units, back to buy a number of times, the power saver received, power-saving effect is very good,, fine workmanship, long warranty, but also purify the air, before buying a used for a period of time, with the okay, and then bought a, and only now come to the	Jingdong prefecture level city in Shandong
Dragon Quality Goods Shop	King of Power Saving	Finally received the goods, very, very much like,, has been a lot of people praise, really too happy, has been his family's loyal fans, will continue to buy buy buy buy!	Jingdong prefecture level city in Shandong
Dragon Quality Goods Shop	King of Power Saving	📩 I've been wanting it for a long time, I decided to go for it, I feel great after using it, it's genuine and the delivery is great, it lives up to its name👍	Jingdong prefecture level city in Shandong
Dragon Quality Goods Shop	King of Power Saving	The quality is sure as other friends who bought it said, and it is also beautiful, thanks to the owner for the recommendation, thanks!	Jingdong prefecture level city in Shandong
Dragon Quality Goods Shop	King of Power Saving	Baby received the quality is really good face value is also very high especially practical especially especially special favorite	Jingdong prefecture level city in Shandong
Dragon Quality Goods Shop	King of Power Saving	It is genuine, trust the merchant, the quality is very good, the price is moderate, the use of the effect is very good!	Jingdong prefecture level city in Shandong
Dragon Quality Goods Shop	King of Power Saving	Energy saver received really good quality affordable and good energy saving and environmental protection is the best to use.	Jingdong prefecture level city in Shandong
Dragon Quality Goods Shop	King of Power Saving	Reasonable price logistics is very fast, the feeling is not bad more satisfied with a shopping experience	Jingdong prefecture level city in Shandong
Dragon Quality Goods Shop	King of Power Saving	The stuff is even more perfect than I imagined, very powerful and worth getting!	Jingdong prefecture level city in Shandong
Dragon Quality Goods Shop	King of Power Saving	Goods have been received, good quality, affordable price, good customer service, worth recommending!	Jingdong prefecture level city in Shandong
Dragon Quality Goods Shop	King of Power Saving	Fast shipping, works great, have already recommended it to friends.	Jingdong prefecture level city in Shandong
Dragon Quality Goods Shop	King of Power Saving	Good quality, fast logistics, reasonable price, worth 5 stars.	Jingdong prefecture level city in Shandong
Dragon Quality Goods Shop	King of Power Saving	Good quality, fast logistics, good value, well packaged, practical	Jingdong prefecture level city in Shandong
Dragon Quality Goods Shop	King of Power Saving	Fast shipping, affordable price, great quality, very satisfied!	Jingdong prefecture level city in Shandong
Dragon Quality Goods Shop	King of Power Saving	Regret knowing too late, it's really good stuff. Works well.	Jingdong prefecture level city in Shandong

Dragon Quality Goods Shop	King of Power Saving	Not bad, very good product, quite save electricity	Jingdong prefecture level city in Shandong
Dragon Quality Goods Shop	King of Power Saving	Good quality, fast logistics, overall very satisfied!	Jingdong prefecture level city in Shandong
Dragon Quality Goods Shop	King of Power Saving	Tightly packaged with good results	Jingdong prefecture level city in Shandong
Dragon Quality Goods Shop	King of Power Saving	very happy	Jingdong prefecture level city in Shandong
Dragon Quality Goods Shop	King of Power Saving	favorable criticism	Jingdong prefecture level city in Shandong
Dragon Quality Goods Shop	King of Power Saving	Now they all sign for the goods without receiving them, the Jingdong platform and merchants are very powerful, and the goods are not received before signing for the goods.	Jingdong prefecture level city in Shandong
Dragon Quality Goods Shop	King of Power Saving	low-quality commodities	Jingdong prefecture level city in Shandong
Dragon Quality Goods Shop	King of Power Saving	I bought this and now it works pretty well with the air conditioning on, when I didn't use it it was $600 a month, now it's almost $500.00, that's over $100 less all of a sudden.	Jingdong prefecture level city in Shandong
Dragon Quality Goods Shop	King of Power Saving	Can Oh, the weather is hot, open the air conditioning is really good, this product is good, has been used for a really long time, more ok, power saving effect is not bad.	Jingdong prefecture level city in Shandong
Dragon Quality Goods Shop	King of Power Saving	I paid my electric bill today, it's a few dozen dollars less, anyway, it's not costly, it's already very good, the boss said that it would be better to buy one more, I've already placed an order	Jingdong prefecture level city in Shandong
Dragon Quality Goods Shop	King of Power Saving	I'm on a smart meter, I save about 30% a month, personally I think it's pretty good, it works well	Jingdong prefecture level city in Shandong
Dragon Quality Goods Shop	King of Power Saving	My dad asked me to buy one, but after I paid the bill, I asked and he said it was a lot less this month, so he asked me to buy another one.	Jingdong prefecture level city in Shandong
Dragon Quality Goods Shop	King of Power Saving	Very satisfied with the purchase thanks to the store for the prosperous business.	Jingdong prefecture level city in Shandong
Dragon Quality Goods Shop	King of Power Saving	Good use, good quality, very satisfied with the purchase	Jingdong prefecture level city in Shandong
Dragon Quality Goods Shop	King of Power Saving	The goods have been received, the quality is very good, is the ideal effect, will continue to pay attention to the	Jingdong prefecture level city in Shandong
Dragon Quality Goods Shop	King of Power Saving	This brand has been used for many years, and this time I saw this upgraded version of the product and got it again. The purpose of this product is to reduce the loss of electricity for a long time, so as to achieve the purpose of saving electricity.	Jingdong prefecture level city in Shandong
Dragon Quality Goods Shop	King of Power Saving	I've been using it for almost a month, and I've reduced my electricity bill by over 100 bucks! I'm planning to get two more for my family.	Jingdong prefecture level city in Shandong
Dragon Quality Goods Shop	King of Power Saving	The effect is too good, the family has now several units, back to buy a number of times, the power saver received, power-saving effect is very good,, fine workmanship, long warranty, but also purify the air, before buying a used for a period of time, with the okay, and then bought a, and only now come to the	Jingdong prefecture level city in Shandong
Dragon Quality Goods Shop	King of Power Saving	Finally received the goods, very, very much like,, has been a lot of people praise, really too happy, has been his family's loyal fans, will continue to buy buy buy buy!	Jingdong prefecture level city in Shandong
Dragon Quality Goods Shop	King of Power Saving	📩 I've been wanting it for a long time, I decided to go for it, I feel great after using it, it's genuine and the delivery is great, it lives up to its name👍	Jingdong prefecture level city in Shandong
Dragon Quality Goods Shop	King of Power Saving	The quality is sure as other friends who bought it said, and it is also beautiful, thanks to the owner for the recommendation, thanks!	Jingdong prefecture level city in Shandong
Dragon Quality Goods Shop	King of Power Saving	Baby received the quality is really good face value is also very high especially practical especially especially special favorite	Jingdong prefecture level city in Shandong
Dragon Quality Goods Shop	King of Power Saving	It is genuine, trust the merchant, the quality is very good, the price is moderate, the use of the effect is very good!	Jingdong prefecture level city in Shandong
Dragon Quality Goods Shop	King of Power Saving	Energy saver received really good quality affordable and good energy saving and environmental protection is the best to use.	Jingdong prefecture level city in Shandong
Dragon Quality Goods Shop	King of Power Saving	Reasonable price logistics is very fast, the feeling is not bad more satisfied with a shopping experience	Jingdong prefecture level city in Shandong
Dragon Quality Goods Shop	King of Power Saving	The stuff is even more perfect than I imagined, very powerful and worth getting!	Jingdong prefecture level city in Shandong
Dragon Quality Goods Shop	King of Power Saving	Goods have been received, good quality, affordable price, good customer service, worth recommending!	Jingdong prefecture level city in Shandong
Dragon Quality Goods Shop	King of Power Saving	Fast shipping, works great, have already recommended it to friends.	Jingdong prefecture level city in Shandong
Dragon Quality Goods Shop	King of Power Saving	Good quality, fast logistics, reasonable price, worth 5 stars.	Jingdong prefecture level city in Shandong
Dragon Quality Goods Shop	King of Power Saving	Good quality, fast logistics, good value, well packaged, practical	Jingdong prefecture level city in Shandong
Dragon Quality Goods Shop	King of Power Saving	Fast shipping, affordable price, great quality, very satisfied!	Jingdong prefecture level city in Shandong
Dragon Quality Goods Shop	King of Power Saving	Regret knowing too late, it's really good stuff. Works well.	Jingdong prefecture level city in Shandong
Dragon Quality Goods Shop	King of Power Saving	Not bad, very good product, quite save electricity	Jingdong prefecture level city in Shandong
Dragon Quality Goods Shop	King of Power Saving	Good quality, fast logistics, overall very satisfied!	Jingdong prefecture level city in Shandong
Dragon Quality Goods Shop	King of Power Saving	Tightly packaged with good results	Jingdong prefecture level city in Shandong
Dragon Quality Goods Shop	King of Power Saving	very happy	Jingdong prefecture level city in Shandong
Dragon Quality Goods Shop	King of Power Saving	favorable criticism	Jingdong prefecture level city in Shandong
Dragon Quality Goods Shop	King of Power Saving	Now they all sign for the goods without receiving them, the Jingdong platform and merchants are very powerful, and the goods are not received before signing for the goods.	Jingdong prefecture level city in Shandong
Dragon Quality Goods Shop	King of Power Saving	low-quality commodities	Jingdong prefecture level city in Shandong
Dragon Quality Goods Shop	King of Power Saving	I bought this and now it works pretty well with the air conditioning on, when I didn't use it it was $600 a month, now it's almost $500.00, that's over $100 less all of a sudden.	Jingdong prefecture level city in Shandong
Dragon Quality Goods Shop	King of Power Saving	Can Oh, the weather is hot, open the air conditioning is really good, this product is good, has been used for a really long time, more ok, power saving effect is not bad.	Jingdong prefecture level city in Shandong
Dragon Quality Goods Shop	King of Power Saving	I paid my electric bill today, it's a few dozen dollars less, anyway, it's not costly, it's already very good, the boss said that it would be better to buy one more, I've already placed an order	Jingdong prefecture level city in Shandong
Dragon Quality Goods Shop	King of Power Saving	I'm on a smart meter, I save about 30% a month, personally I think it's pretty good, it works well	Jingdong prefecture level city in Shandong
Dragon Quality Goods Shop	King of Power Saving	My dad asked me to buy one, but after I paid the bill, I asked and he said it was a lot less this month, so he asked me to buy another one.	Jingdong prefecture level city in Shandong
Dragon Quality Goods Shop	King of Power Saving	Very satisfied with the purchase thanks to the store for the prosperous business.	Jingdong prefecture level city in Shandong
Dragon Quality Goods Shop	King of Power Saving	Good use, good quality, very satisfied with the purchase	Jingdong prefecture level city in Shandong
Dragon Quality Goods Shop	King of Power Saving	The goods have been received, the quality is very good, is the ideal effect, will continue to pay attention to the	Jingdong prefecture level city in Shandong
Dragon Quality Goods Shop	King of Power Saving	This brand has been used for many years, and this time I saw this upgraded version of the product and got it again. The purpose of this product is to reduce the loss of electricity for a long time, so as to achieve the purpose of saving electricity.	Jingdong prefecture level city in Shandong
Dragon Quality Goods Shop	King of Power Saving	I've been using it for almost a month, and I've reduced my electricity bill by over 100 bucks! I'm planning to get two more for my family.	Jingdong prefecture level city in Shandong

Dragon Quality Goods Shop	King of Power Saving	The effect is too good, the family has now several units, back to buy a number of times, the power saver received, power-saving effect is very good,, fine workmanship, long warranty, but also purify the air, before buying a used for a period of time, with the okay, and then bought a, and only now come to the	Jingdong prefecture level city in Shandong
Dragon Quality Goods Shop	King of Power Saving	Finally received the goods, very, very much like,, has been a lot of people praise, really too happy, has been his family's loyal fans, will continue to buy buy buy buy!	Jingdong prefecture level city in Shandong
Dragon Quality Goods Shop	King of Power Saving	💌 I've been wanting it for a long time, I decided to go for it, I feel great after using it, it's genuine and the delivery is great, it lives up to its name👍	Jingdong prefecture level city in Shandong
Dragon Quality Goods Shop	King of Power Saving	The quality is sure as other friends who bought it said, and it is also beautiful, thanks to the owner for the recommendation, thanks!	Jingdong prefecture level city in Shandong
Dragon Quality Goods Shop	King of Power Saving	Baby received the quality is really good face value is also very high especially practical especially especially special favorite	Jingdong prefecture level city in Shandong
Dragon Quality Goods Shop	King of Power Saving	It is genuine, trust the merchant, the quality is very good, the price is moderate, the use of the effect is very good!	Jingdong prefecture level city in Shandong
Dragon Quality Goods Shop	King of Power Saving	Energy saver received really good quality affordable and good energy saving and environmental protection is the best to use.	Jingdong prefecture level city in Shandong
Dragon Quality Goods Shop	King of Power Saving	Reasonable price logistics is very fast, the feeling is not bad more satisfied with a shopping experience	Jingdong prefecture level city in Shandong
Dragon Quality Goods Shop	King of Power Saving	The stuff is even more perfect than I imagined, very powerful and worth getting!	Jingdong prefecture level city in Shandong
Dragon Quality Goods Shop	King of Power Saving	Goods have been received, good quality, affordable price, good customer service, worth recommending!	Jingdong prefecture level city in Shandong
Dragon Quality Goods Shop	King of Power Saving	Fast shipping, works great, have already recommended it to friends.	Jingdong prefecture level city in Shandong
Dragon Quality Goods Shop	King of Power Saving	Good quality, fast logistics, reasonable price, worth 5 stars.	Jingdong prefecture level city in Shandong
Dragon Quality Goods Shop	King of Power Saving	Good quality, fast logistics, good value, well packaged, practical	Jingdong prefecture level city in Shandong
Dragon Quality Goods Shop	King of Power Saving	Fast shipping, affordable price, great quality, very satisfied!	Jingdong prefecture level city in Shandong
Dragon Quality Goods Shop	King of Power Saving	Regret knowing too late, it's really good stuff. Works well.	Jingdong prefecture level city in Shandong
Dragon Quality Goods Shop	King of Power Saving	Not bad, very good product, quite save electricity	Jingdong prefecture level city in Shandong
Dragon Quality Goods Shop	King of Power Saving	Good quality, fast logistics, overall very satisfied!	Jingdong prefecture level city in Shandong
Dragon Quality Goods Shop	King of Power Saving	Tightly packaged with good results	Jingdong prefecture level city in Shandong
Dragon Quality Goods Shop	King of Power Saving	very happy	Jingdong prefecture level city in Shandong
Dragon Quality Goods Shop	King of Power Saving	favorable criticism	Jingdong prefecture level city in Shandong
Dragon Quality Goods Shop	King of Power Saving	Now they all sign for the goods without receiving them, the Jingdong platform and merchants are very powerful, and the goods are not received before signing for the goods.	Jingdong prefecture level city in Shandong
Dragon Quality Goods Shop	King of Power Saving	low-quality commodities	Jingdong prefecture level city in Shandong
Dragon Quality Goods Shop	King of Power Saving	I bought this and now it works pretty well with the air conditioning on, when I didn't use it it was $600 a month, now it's almost $500,00, that's over $100 less all of a sudden.	Jingdong prefecture level city in Shandong
Dragon Quality Goods Shop	King of Power Saving	Can Oh, the weather is hot, open the air conditioning is really good, this product is good, has been used for a really long time, more ok, power saving effect is not bad.	Jingdong prefecture level city in Shandong
Dragon Quality Goods Shop	King of Power Saving	I paid my electric bill today, it's a few dozen dollars less, anyway, it's not costly, it's already very good, the boss said that it would be better to buy one more, I've already placed an order	Jingdong prefecture level city in Shandong
Dragon Quality Goods Shop	King of Power Saving	I'm on a smart meter, I save about 30% a month, personally I think it's pretty good, it works well	Jingdong prefecture level city in Shandong
Dragon Quality Goods Shop	King of Power Saving	My dad asked me to buy one, but after I paid the bill, I asked and he said it was a lot less this month, so he asked me to buy another one.	Jingdong prefecture level city in Shandong
Dragon Quality Goods Shop	King of Power Saving	Very satisfied with the purchase thanks to the store for the prosperous business.	Jingdong prefecture level city in Shandong
Dragon Quality Goods Shop	King of Power Saving	Good use, good quality, very satisfied with the purchase	Jingdong prefecture level city in Shandong
Dragon Quality Goods Shop	King of Power Saving	The goods have been received, the quality is very good, is the ideal effect, will continue to pay attention to the	Jingdong prefecture level city in Shandong
Dragon Quality Goods Shop	King of Power Saving	This brand has been used for many years, and this time I saw this upgraded version of the product and got it again. The purpose of this product is to reduce the loss of electricity for a long time, so as to achieve the purpose of saving electricity.	Jingdong prefecture level city in Shandong
Dragon Quality Goods Shop	King of Power Saving	I've been using it for almost a month, and I've reduced my electricity bill by over 100 bucks! I'm planning to get two more for my family.	Jingdong prefecture level city in Shandong
Dragon Quality Goods Shop	King of Power Saving	The effect is too good, the family has now several units, back to buy a number of times, the power saver received, power-saving effect is very good,, fine workmanship, long warranty, but also purify the air, before buying a used for a period of time, with the okay, and then bought a, and only now come to the	Jingdong prefecture level city in Shandong
Dragon Quality Goods Shop	King of Power Saving	Finally received the goods, very, very much like,, has been a lot of people praise, really too happy, has been his family's loyal fans, will continue to buy buy buy buy!	Jingdong prefecture level city in Shandong
Dragon Quality Goods Shop	King of Power Saving	💌 I've been wanting it for a long time, I decided to go for it, I feel great after using it, it's genuine and the delivery is great, it lives up to its name👍	Jingdong prefecture level city in Shandong
Dragon Quality Goods Shop	King of Power Saving	The quality is sure as other friends who bought it said, and it is also beautiful, thanks to the owner for the recommendation, thanks!	Jingdong prefecture level city in Shandong
Dragon Quality Goods Shop	King of Power Saving	Baby received the quality is really good face value is also very high especially practical especially especially special favorite	Jingdong prefecture level city in Shandong
Dragon Quality Goods Shop	King of Power Saving	It is genuine, trust the merchant, the quality is very good, the price is moderate, the use of the effect is very good!	Jingdong prefecture level city in Shandong
Dragon Quality Goods Shop	King of Power Saving	Energy saver received really good quality affordable and good energy saving and environmental protection is the best to use.	Jingdong prefecture level city in Shandong
Dragon Quality Goods Shop	King of Power Saving	Reasonable price logistics is very fast, the feeling is not bad more satisfied with a shopping experience	Jingdong prefecture level city in Shandong
Dragon Quality Goods Shop	King of Power Saving	The stuff is even more perfect than I imagined, very powerful and worth getting!	Jingdong prefecture level city in Shandong
Dragon Quality Goods Shop	King of Power Saving	Goods have been received, good quality, affordable price, good customer service, worth recommending!	Jingdong prefecture level city in Shandong
Dragon Quality Goods Shop	King of Power Saving	Fast shipping, works great, have already recommended it to friends.	Jingdong prefecture level city in Shandong
Dragon Quality Goods Shop	King of Power Saving	Good quality, fast logistics, reasonable price, worth 5 stars.	Jingdong prefecture level city in Shandong
Dragon Quality Goods Shop	King of Power Saving	Good quality, fast logistics, good value, well packaged, practical	Jingdong prefecture level city in Shandong
Dragon Quality Goods Shop	King of Power Saving	Fast shipping, affordable price, great quality, very satisfied!	Jingdong prefecture level city in Shandong
Dragon Quality Goods Shop	King of Power Saving	Regret knowing too late, it's really good stuff. Works well.	Jingdong prefecture level city in Shandong
Dragon Quality Goods Shop	King of Power Saving	Not bad, very good product, quite save electricity	Jingdong prefecture level city in Shandong
Dragon Quality Goods Shop	King of Power Saving	Good quality, fast logistics, overall very satisfied!	Jingdong prefecture level city in Shandong
Dragon Quality Goods Shop	King of Power Saving	Tightly packaged with good results	Jingdong prefecture level city in Shandong

Dragon Quality Goods Shop	King of Power Saving	very happy	Jingdong prefecture level city in Shandong
Dragon Quality Goods Shop	King of Power Saving	favorable criticism	Jingdong prefecture level city in Shandong
Dragon Quality Goods Shop	King of Power Saving	Now they all sign for the goods without receiving them, the Jingdong platform and merchants are very powerful, and the goods are not received before signing for the goods.	Jingdong prefecture level city in Shandong
Dragon Quality Goods Shop	King of Power Saving	low-quality commodities	Jingdong prefecture level city in Shandong
Dragon Quality Goods Shop	King of Power Saving	I bought this and now it works pretty well with the air conditioning on, when I didn't use it it was $600 a month, now it's almost $500,00, that's over $100 less all of a sudden.	Jingdong prefecture level city in Shandong
Dragon Quality Goods Shop	King of Power Saving	Can Oh, the weather is hot, open the air conditioning is really good, this product is good, has been used for a really long time, more ok, power saving effect is not bad.	Jingdong prefecture level city in Shandong
Dragon Quality Goods Shop	King of Power Saving	I paid my electric bill today, it's a few dozen dollars less, anyway, it's not costly, it's already very good, the boss said that it would be better to buy one more, I've already placed an order	Jingdong prefecture level city in Shandong
Dragon Quality Goods Shop	King of Power Saving	I'm on a smart meter, I save about 30% a month, personally I think it's pretty good, it works well	Jingdong prefecture level city in Shandong
Dragon Quality Goods Shop	King of Power Saving	My dad asked me to buy one, but after I paid the bill, I asked and he said it was a lot less this month, so he asked me to buy another one.	Jingdong prefecture level city in Shandong
Dragon Quality Goods Shop	King of Power Saving	Very satisfied with the purchase thanks to the store for the prosperous business.	Jingdong prefecture level city in Shandong
Dragon Quality Goods Shop	King of Power Saving	Good use, good quality, very satisfied with the purchase	Jingdong prefecture level city in Shandong
Dragon Quality Goods Shop	King of Power Saving	The goods have been received, the quality is very good, is the ideal effect, will continue to pay attention to the	Jingdong prefecture level city in Shandong
Dragon Quality Goods Shop	King of Power Saving	This brand has been used for many years, and this time I saw this upgraded version of the product and got it again. The purpose of this product is to reduce the loss of electricity for a long time, so as to achieve the purpose of saving electricity.	Jingdong prefecture level city in Shandong
Dragon Quality Goods Shop	King of Power Saving	I've been using it for almost a month, and I've reduced my electricity bill by over 100 bucks! I'm planning to get two more for my family.	Jingdong prefecture level city in Shandong
Dragon Quality Goods Shop	King of Power Saving	The effect is too good, the family has now several units, back to buy a number of times, the power saver received, power-saving effect is very good,, fine workmanship, long warranty, but also purify the air, before buying a used for a period of time, with the okay, and then bought a, and only now come to the	Jingdong prefecture level city in Shandong
Dragon Quality Goods Shop	King of Power Saving	Finally received the goods, very, very much like,, has been a lot of people praise, really too happy, has been his family's loyal fans, will continue to buy buy buy buy!	Jingdong prefecture level city in Shandong
Dragon Quality Goods Shop	King of Power Saving	📩 I've been wanting it for a long time, I decided to go for it, I feel great after using it, it's genuine and the delivery is great, it lives up to its name👍	Jingdong prefecture level city in Shandong
Dragon Quality Goods Shop	King of Power Saving	The quality is sure as other friends who bought it said, and it is also beautiful, thanks to the owner for the recommendation, thanks!	Jingdong prefecture level city in Shandong
Dragon Quality Goods Shop	King of Power Saving	Baby received the quality is really good face value is also very high especially practical especially especially special favorite	Jingdong prefecture level city in Shandong
Dragon Quality Goods Shop	King of Power Saving	It is genuine, trust the merchant, the quality is very good, the price is moderate, the use of the effect is very good!	Jingdong prefecture level city in Shandong
Dragon Quality Goods Shop	King of Power Saving	Energy saver received really good quality affordable and good energy saving and environmental protection is the best to use.	Jingdong prefecture level city in Shandong
Dragon Quality Goods Shop	King of Power Saving	Reasonable price logistics is very fast, the feeling is not bad more satisfied with a shopping experience	Jingdong prefecture level city in Shandong
Dragon Quality Goods Shop	King of Power Saving	The stuff is even more perfect than I imagined, very powerful and worth getting!	Jingdong prefecture level city in Shandong
Dragon Quality Goods Shop	King of Power Saving	Goods have been received, good quality, affordable price, good customer service, worth recommending!	Jingdong prefecture level city in Shandong
Dragon Quality Goods Shop	King of Power Saving	Fast shipping, works great, have already recommended it to friends.	Jingdong prefecture level city in Shandong
Dragon Quality Goods Shop	King of Power Saving	Good quality, fast logistics, reasonable price, worth 5 stars.	Jingdong prefecture level city in Shandong
Dragon Quality Goods Shop	King of Power Saving	Good quality, fast logistics, good value, well packaged, practical	Jingdong prefecture level city in Shandong
Dragon Quality Goods Shop	King of Power Saving	Fast shipping, affordable price, great quality, very satisfied!	Jingdong prefecture level city in Shandong
Dragon Quality Goods Shop	King of Power Saving	Regret knowing too late, it's really good stuff. Works well.	Jingdong prefecture level city in Shandong
Dragon Quality Goods Shop	King of Power Saving	Not bad, very good product, quite save electricity	Jingdong prefecture level city in Shandong
Dragon Quality Goods Shop	King of Power Saving	Good quality, fast logistics, overall very satisfied!	Jingdong prefecture level city in Shandong
Dragon Quality Goods Shop	King of Power Saving	Tightly packaged with good results	Jingdong prefecture level city in Shandong
Dragon Quality Goods Shop	King of Power Saving	very happy	Jingdong prefecture level city in Shandong
Dragon Quality Goods Shop	King of Power Saving	favorable criticism	Jingdong prefecture level city in Shandong
Dragon Quality Goods Shop	King of Power Saving	Now they all sign for the goods without receiving them, the Jingdong platform and merchants are very powerful, and the goods are not received before signing for the goods.	Jingdong prefecture level city in Shandong
Dragon Quality Goods Shop	King of Power Saving	low-quality commodities	Jingdong prefecture level city in Shandong
Dragon Quality Goods Shop	King of Power Saving	I bought this and now it works pretty well with the air conditioning on, when I didn't use it it was $600 a month, now it's almost $500,00, that's over $100 less all of a sudden.	Jingdong prefecture level city in Shandong
Dragon Quality Goods Shop	King of Power Saving	Can Oh, the weather is hot, open the air conditioning is really good, this product is good, has been used for a really long time, more ok, power saving effect is not bad.	Jingdong prefecture level city in Shandong
Dragon Quality Goods Shop	King of Power Saving	I paid my electric bill today, it's a few dozen dollars less, anyway, it's not costly, it's already very good, the boss said that it would be better to buy one more, I've already placed an order	Jingdong prefecture level city in Shandong
Dragon Quality Goods Shop	King of Power Saving	I'm on a smart meter, I save about 30% a month, personally I think it's pretty good, it works well	Jingdong prefecture level city in Shandong
Dragon Quality Goods Shop	King of Power Saving	My dad asked me to buy one, but after I paid the bill, I asked and he said it was a lot less this month, so he asked me to buy another one.	Jingdong prefecture level city in Shandong
Dragon Quality Goods Shop	King of Power Saving	Very satisfied with the purchase thanks to the store for the prosperous business.	Jingdong prefecture level city in Shandong
Dragon Quality Goods Shop	King of Power Saving	Good use, good quality, very satisfied with the purchase	Jingdong prefecture level city in Shandong
Dragon Quality Goods Shop	King of Power Saving	The goods have been received, the quality is very good, is the ideal effect, will continue to pay attention to the	Jingdong prefecture level city in Shandong
Dragon Quality Goods Shop	King of Power Saving	This brand has been used for many years, and this time I saw this upgraded version of the product and got it again. The purpose of this product is to reduce the loss of electricity for a long time, so as to achieve the purpose of saving electricity.	Jingdong prefecture level city in Shandong
Dragon Quality Goods Shop	King of Power Saving	I've been using it for almost a month, and I've reduced my electricity bill by over 100 bucks! I'm planning to get two more for my family.	Jingdong prefecture level city in Shandong
Dragon Quality Goods Shop	King of Power Saving	The effect is too good, the family has now several units, back to buy a number of times, the power saver received, power-saving effect is very good,, fine workmanship, long warranty, but also purify the air, before buying a used for a period of time, with the okay, and then bought a, and only now come to the	Jingdong prefecture level city in Shandong
Dragon Quality Goods Shop	King of Power Saving	Finally received the goods, very, very much like,, has been a lot of people praise, really too happy, has been his family's loyal fans, will continue to buy buy buy buy!	Jingdong prefecture level city in Shandong
Dragon Quality Goods Shop	King of Power Saving	📩 I've been wanting it for a long time, I decided to go for it, I feel great after using it, it's genuine and the delivery is great, it lives up to its name👍	Jingdong prefecture level city in Shandong

Dragon Quality Goods Shop	King of Power Saving	The quality is sure as other friends who bought it said, and it is also beautiful, thanks to the owner for the recommendation, thanks!	Jingdong prefecture level city in Shandong
Dragon Quality Goods Shop	King of Power Saving	Baby received the quality is really good face value is also very high especially practical especially especially special favorite	Jingdong prefecture level city in Shandong
Dragon Quality Goods Shop	King of Power Saving	It is genuine, trust the merchant, the quality is very good, the price is moderate, the use of the effect is very good!	Jingdong prefecture level city in Shandong
Dragon Quality Goods Shop	King of Power Saving	Energy saver received really good quality affordable and good energy saving and environmental protection is the best to use.	Jingdong prefecture level city in Shandong
Dragon Quality Goods Shop	King of Power Saving	Reasonable price logistics is very fast, the feeling is not bad more satisfied with a shopping experience	Jingdong prefecture level city in Shandong
Dragon Quality Goods Shop	King of Power Saving	The stuff is even more perfect than I imagined, very powerful and worth getting!	Jingdong prefecture level city in Shandong
Dragon Quality Goods Shop	King of Power Saving	Goods have been received, good quality, affordable price, good customer service, worth recommending!	Jingdong prefecture level city in Shandong
Dragon Quality Goods Shop	King of Power Saving	Fast shipping, works great, have already recommended it to friends.	Jingdong prefecture level city in Shandong
Dragon Quality Goods Shop	King of Power Saving	Good quality, fast logistics, reasonable price, worth 5 stars.	Jingdong prefecture level city in Shandong
Dragon Quality Goods Shop	King of Power Saving	Good quality, fast logistics, good value, well packaged, practical	Jingdong prefecture level city in Shandong
Dragon Quality Goods Shop	King of Power Saving	Fast shipping, affordable price, great quality, very satisfied!	Jingdong prefecture level city in Shandong
Dragon Quality Goods Shop	King of Power Saving	Regret knowing too late, it's really good stuff. Works well.	Jingdong prefecture level city in Shandong
Dragon Quality Goods Shop	King of Power Saving	Not bad, very good product, quite save electricity	Jingdong prefecture level city in Shandong
Dragon Quality Goods Shop	King of Power Saving	Good quality, fast logistics, overall very satisfied!	Jingdong prefecture level city in Shandong
Dragon Quality Goods Shop	King of Power Saving	Tightly packaged with good results	Jingdong prefecture level city in Shandong
Dragon Quality Goods Shop	King of Power Saving	very happy	Jingdong prefecture level city in Shandong
Dragon Quality Goods Shop	King of Power Saving	favorable criticism	Jingdong prefecture level city in Shandong
Dragon Quality Goods Shop	King of Power Saving	Now they all sign for the goods without receiving them, the Jingdong platform and merchants are very powerful, and the goods are not received before signing for the goods.	Jingdong prefecture level city in Shandong
Dragon Quality Goods Shop	King of Power Saving	low-quality commodities	Jingdong prefecture level city in Shandong
Dragon Quality Goods Shop	King of Power Saving	I bought this and now it works pretty well with the air conditioning on, when I didn't use it it was $600 a month, now it's almost $500.00, that's over $100 less all of a sudden.	Jingdong prefecture level city in Shandong
Dragon Quality Goods Shop	King of Power Saving	Can Oh, the weather is hot, open the air conditioning is really good, this product is good, has been used for a really long time, more ok, power saving effect is not bad.	Jingdong prefecture level city in Shandong
Dragon Quality Goods Shop	King of Power Saving	I paid my electric bill today, it's a few dozen dollars less, anyway, it's not costly, it's already very good, the boss said that it would be better to buy one more, I've already placed an order	Jingdong prefecture level city in Shandong
Dragon Quality Goods Shop	King of Power Saving	I'm on a smart meter, I save about 30% a month, personally I think it's pretty good, it works well	Jingdong prefecture level city in Shandong
Dragon Quality Goods Shop	King of Power Saving	My dad asked me to buy one, but after I paid the bill, I asked and he said it was a lot less this month, so he asked me to buy another one.	Jingdong prefecture level city in Shandong
Dragon Quality Goods Shop	King of Power Saving	Very satisfied with the purchase thanks to the store for the prosperous business.	Jingdong prefecture level city in Shandong
Dragon Quality Goods Shop	King of Power Saving	Good use, good quality, very satisfied with the purchase	Jingdong prefecture level city in Shandong
Dragon Quality Goods Shop	King of Power Saving	The goods have been received, the quality is very good, is the ideal effect, will continue to pay attention to the	Jingdong prefecture level city in Shandong
Dragon Quality Goods Shop	King of Power Saving	This brand has been used for many years, and this time I saw this upgraded version of the product and got it again. The purpose of this product is to reduce the loss of electricity for a long time, so as to achieve the purpose of saving electricity.	Jingdong prefecture level city in Shandong
Dragon Quality Goods Shop	King of Power Saving	I've been using it for almost a month, and I've reduced my electricity bill by over 100 bucks! I'm planning to get two more for my family.	Jingdong prefecture level city in Shandong
Dragon Quality Goods Shop	King of Power Saving	The effect is too good, the family has now several units, back to buy a number of times, the power saver received, power-saving effect is very good,, fine workmanship, long warranty, but also purify the air, before buying a used for a period of time, with the okay, and then bought a, and only now come to the	Jingdong prefecture level city in Shandong
Dragon Quality Goods Shop	King of Power Saving	Finally received the goods, very, very much like,, has been a lot of people praise, really too happy, has been his family's loyal fans, will continue to buy buy buy buy!	Jingdong prefecture level city in Shandong
Dragon Quality Goods Shop	King of Power Saving	📩 I've been wanting it for a long time, I decided to go for it, I feel great after using it, it's genuine and the delivery is great, it lives up to its name👍	Jingdong prefecture level city in Shandong
Dragon Quality Goods Shop	King of Power Saving	The quality is sure as other friends who bought it said, and it is also beautiful, thanks to the owner for the recommendation, thanks!	Jingdong prefecture level city in Shandong
Dragon Quality Goods Shop	King of Power Saving	Baby received the quality is really good face value is also very high especially practical especially especially special favorite	Jingdong prefecture level city in Shandong
Dragon Quality Goods Shop	King of Power Saving	It is genuine, trust the merchant, the quality is very good, the price is moderate, the use of the effect is very good!	Jingdong prefecture level city in Shandong
Dragon Quality Goods Shop	King of Power Saving	Energy saver received really good quality affordable and good energy saving and environmental protection is the best to use.	Jingdong prefecture level city in Shandong
Dragon Quality Goods Shop	King of Power Saving	Reasonable price logistics is very fast, the feeling is not bad more satisfied with a shopping experience	Jingdong prefecture level city in Shandong
Dragon Quality Goods Shop	King of Power Saving	The stuff is even more perfect than I imagined, very powerful and worth getting!	Jingdong prefecture level city in Shandong
Dragon Quality Goods Shop	King of Power Saving	Goods have been received, good quality, affordable price, good customer service, worth recommending!	Jingdong prefecture level city in Shandong
Dragon Quality Goods Shop	King of Power Saving	Fast shipping, works great, have already recommended it to friends.	Jingdong prefecture level city in Shandong
Dragon Quality Goods Shop	King of Power Saving	Good quality, fast logistics, reasonable price, worth 5 stars.	Jingdong prefecture level city in Shandong
Dragon Quality Goods Shop	King of Power Saving	Good quality, fast logistics, good value, well packaged, practical	Jingdong prefecture level city in Shandong
Dragon Quality Goods Shop	King of Power Saving	Fast shipping, affordable price, great quality, very satisfied!	Jingdong prefecture level city in Shandong
Dragon Quality Goods Shop	King of Power Saving	Regret knowing too late, it's really good stuff. Works well.	Jingdong prefecture level city in Shandong
Dragon Quality Goods Shop	King of Power Saving	Not bad, very good product, quite save electricity	Jingdong prefecture level city in Shandong
Dragon Quality Goods Shop	King of Power Saving	Good quality, fast logistics, overall very satisfied!	Jingdong prefecture level city in Shandong
Dragon Quality Goods Shop	King of Power Saving	Tightly packaged with good results	Jingdong prefecture level city in Shandong
Dragon Quality Goods Shop	King of Power Saving	very happy	Jingdong prefecture level city in Shandong
Dragon Quality Goods Shop	King of Power Saving	favorable criticism	Jingdong prefecture level city in Shandong
Dragon Quality Goods Shop	King of Power Saving	Now they all sign for the goods without receiving them, the Jingdong platform and merchants are very powerful, and the goods are not received before signing for the goods.	Jingdong prefecture level city in Shandong

Dragon Quality Goods Shop	King of Power Saving	low-quality commodities	Jingdong prefecture level city in Shandong
Dragon Quality Goods Shop	King of Power Saving	I bought this and now it works pretty well with the air conditioning on, when I didn't use it it was $600 a month, now it's almost $500,00, that's over $100 less all of a sudden.	Jingdong prefecture level city in Shandong
Dragon Quality Goods Shop	King of Power Saving	Can Oh, the weather is hot, open the air conditioning is really good, this product is good, has been used for a really long time, more ok, power saving effect is not bad.	Jingdong prefecture level city in Shandong
Dragon Quality Goods Shop	King of Power Saving	I paid my electric bill today, it's a few dozen dollars less, anyway, it's not costly, it's already very good, the boss said that it would be better to buy one more, I've already placed an order	Jingdong prefecture level city in Shandong
Dragon Quality Goods Shop	King of Power Saving	I'm on a smart meter, I save about 30% a month, personally I think it's pretty good, it works well	Jingdong prefecture level city in Shandong
Dragon Quality Goods Shop	King of Power Saving	My dad asked me to buy one, but after I paid the bill, I asked and he said it was a lot less this month, so he asked me to buy another one.	Jingdong prefecture level city in Shandong
Dragon Quality Goods Shop	King of Power Saving	Very satisfied with the purchase thanks to the store for the prosperous business.	Jingdong prefecture level city in Shandong
Dragon Quality Goods Shop	King of Power Saving	Good use, good quality, very satisfied with the purchase	Jingdong prefecture level city in Shandong
Dragon Quality Goods Shop	King of Power Saving	The goods have been received, the quality is very good, is the ideal effect, will continue to pay attention to the	Jingdong prefecture level city in Shandong
Dragon Quality Goods Shop	King of Power Saving	This brand has been used for many years, and this time I saw this upgraded version of the product and got it again. The purpose of this product is to reduce the loss of electricity for a long time, so as to achieve the purpose of saving electricity.	Jingdong prefecture level city in Shandong
Dragon Quality Goods Shop	King of Power Saving	I've been using it for almost a month, and I've reduced my electricity bill by over 100 bucks! I'm planning to get two more for my family.	Jingdong prefecture level city in Shandong
Dragon Quality Goods Shop	King of Power Saving	The effect is too good, the family has now several units, back to buy a number of times, the power saver received, power-saving effect is very good,, fine workmanship, long warranty, but also purify the air, before buying a used for a period of time, with the okay, and then bought a, and only now come to the	Jingdong prefecture level city in Shandong
Dragon Quality Goods Shop	King of Power Saving	Finally received the goods, very, very much like,, has been a lot of people praise, really too happy, has been his family's loyal fans, will continue to buy buy buy buy!	Jingdong prefecture level city in Shandong
Dragon Quality Goods Shop	King of Power Saving	📩 I've been wanting it for a long time, I decided to go for it, I feel great after using it, it's genuine and the delivery is great, it lives up to its name👍	Jingdong prefecture level city in Shandong
Dragon Quality Goods Shop	King of Power Saving	The quality is sure as other friends who bought it said, and it is also beautiful, thanks to the owner for the recommendation, thanks!	Jingdong prefecture level city in Shandong
Dragon Quality Goods Shop	King of Power Saving	Baby received the quality is really good face value is also very high especially practical especially especially special favorite	Jingdong prefecture level city in Shandong
Dragon Quality Goods Shop	King of Power Saving	It is genuine, trust the merchant, the quality is very good, the price is moderate, the use of the effect is very good!	Jingdong prefecture level city in Shandong
Dragon Quality Goods Shop	King of Power Saving	Energy saver received really good quality affordable and good energy saving and environmental protection is the best to use.	Jingdong prefecture level city in Shandong
Dragon Quality Goods Shop	King of Power Saving	Reasonable price logistics is very fast, the feeling is not bad more satisfied with a shopping experience	Jingdong prefecture level city in Shandong
Dragon Quality Goods Shop	King of Power Saving	The stuff is even more perfect than I imagined, very powerful and worth getting!	Jingdong prefecture level city in Shandong
Dragon Quality Goods Shop	King of Power Saving	Goods have been received, good quality, affordable price, good customer service, worth recommending!	Jingdong prefecture level city in Shandong
Dragon Quality Goods Shop	King of Power Saving	Fast shipping, works great, have already recommended it to friends.	Jingdong prefecture level city in Shandong
Dragon Quality Goods Shop	King of Power Saving	Good quality, fast logistics, reasonable price, worth 5 stars.	Jingdong prefecture level city in Shandong
Dragon Quality Goods Shop	King of Power Saving	Good quality, fast logistics, good value, well packaged, practical	Jingdong prefecture level city in Shandong
Dragon Quality Goods Shop	King of Power Saving	Fast shipping, affordable price, great quality, very satisfied!	Jingdong prefecture level city in Shandong
Dragon Quality Goods Shop	King of Power Saving	Regret knowing too late, it's really good stuff. Works well.	Jingdong prefecture level city in Shandong
Dragon Quality Goods Shop	King of Power Saving	Not bad, very good product, quite save electricity	Jingdong prefecture level city in Shandong
Dragon Quality Goods Shop	King of Power Saving	Good quality, fast logistics, overall very satisfied!	Jingdong prefecture level city in Shandong
Dragon Quality Goods Shop	King of Power Saving	Tightly packaged with good results	Jingdong prefecture level city in Shandong
Dragon Quality Goods Shop	King of Power Saving	very happy	Jingdong prefecture level city in Shandong
Dragon Quality Goods Shop	King of Power Saving	favorable criticism	Jingdong prefecture level city in Shandong
Dragon Quality Goods Shop	King of Power Saving	Now they all sign for the goods without receiving them, the Jingdong platform and merchants are very powerful, and the goods are not received before signing for the goods.	Jingdong prefecture level city in Shandong
Dragon Quality Goods Shop	King of Power Saving	low-quality commodities	Jingdong prefecture level city in Shandong
Dragon Quality Goods Shop	King of Power Saving	I bought this and now it works pretty well with the air conditioning on, when I didn't use it it was $600 a month, now it's almost $500,00, that's over $100 less all of a sudden.	Jingdong prefecture level city in Shandong
Dragon Quality Goods Shop	King of Power Saving	Can Oh, the weather is hot, open the air conditioning is really good, this product is good, has been used for a really long time, more ok, power saving effect is not bad.	Jingdong prefecture level city in Shandong
Dragon Quality Goods Shop	King of Power Saving	I paid my electric bill today, it's a few dozen dollars less, anyway, it's not costly, it's already very good, the boss said that it would be better to buy one more, I've already placed an order	Jingdong prefecture level city in Shandong
Dragon Quality Goods Shop	King of Power Saving	I'm on a smart meter, I save about 30% a month, personally I think it's pretty good, it works well	Jingdong prefecture level city in Shandong
Dragon Quality Goods Shop	King of Power Saving	My dad asked me to buy one, but after I paid the bill, I asked and he said it was a lot less this month, so he asked me to buy another one.	Jingdong prefecture level city in Shandong
Dragon Quality Goods Shop	King of Power Saving	Very satisfied with the purchase thanks to the store for the prosperous business.	Jingdong prefecture level city in Shandong
Dragon Quality Goods Shop	King of Power Saving	Good use, good quality, very satisfied with the purchase	Jingdong prefecture level city in Shandong
Dragon Quality Goods Shop	King of Power Saving	The goods have been received, the quality is very good, is the ideal effect, will continue to pay attention to the	Jingdong prefecture level city in Shandong
Dragon Quality Goods Shop	King of Power Saving	This brand has been used for many years, and this time I saw this upgraded version of the product and got it again. The purpose of this product is to reduce the loss of electricity for a long time, so as to achieve the purpose of saving electricity.	Jingdong prefecture level city in Shandong
Dragon Quality Goods Shop	King of Power Saving	I've been using it for almost a month, and I've reduced my electricity bill by over 100 bucks! I'm planning to get two more for my family.	Jingdong prefecture level city in Shandong
Dragon Quality Goods Shop	King of Power Saving	The effect is too good, the family has now several units, back to buy a number of times, the power saver received, power-saving effect is very good,, fine workmanship, long warranty, but also purify the air, before buying a used for a period of time, with the okay, and then bought a, and only now come to the	Jingdong prefecture level city in Shandong
Dragon Quality Goods Shop	King of Power Saving	Finally received the goods, very, very much like,, has been a lot of people praise, really too happy, has been his family's loyal fans, will continue to buy buy buy buy!	Jingdong prefecture level city in Shandong
Dragon Quality Goods Shop	King of Power Saving	📩 I've been wanting it for a long time, I decided to go for it, I feel great after using it, it's genuine and the delivery is great, it lives up to its name👍	Jingdong prefecture level city in Shandong
Dragon Quality Goods Shop	King of Power Saving	The quality is sure as other friends who bought it said, and it is also beautiful, thanks to the owner for the recommendation, thanks!	Jingdong prefecture level city in Shandong
Dragon Quality Goods Shop	King of Power Saving	Baby received the quality is really good face value is also very high especially practical especially especially special favorite	Jingdong prefecture level city in Shandong
Dragon Quality Goods Shop	King of Power Saving	It is genuine, trust the merchant, the quality is very good, the price is moderate, the use of the effect is very good!	Jingdong prefecture level city in Shandong

Dragon Quality Goods Shop	King of Power Saving	Energy saver received really good quality affordable and good energy saving and environmental protection is the best to use.	Jingdong prefecture level city in Shandong
Dragon Quality Goods Shop	King of Power Saving	Reasonable price logistics is very fast, the feeling is not bad more satisfied with a shopping experience	Jingdong prefecture level city in Shandong
Dragon Quality Goods Shop	King of Power Saving	The stuff is even more perfect than I imagined, very powerful and worth getting!	Jingdong prefecture level city in Shandong
Dragon Quality Goods Shop	King of Power Saving	Goods have been received, good quality, affordable price, good customer service, worth recommending!	Jingdong prefecture level city in Shandong
Dragon Quality Goods Shop	King of Power Saving	Fast shipping, works great, have already recommended it to friends.	Jingdong prefecture level city in Shandong
Dragon Quality Goods Shop	King of Power Saving	Good quality, fast logistics, reasonable price, worth 5 stars.	Jingdong prefecture level city in Shandong
Dragon Quality Goods Shop	King of Power Saving	Good quality, fast logistics, good value, well packaged, practical	Jingdong prefecture level city in Shandong
Dragon Quality Goods Shop	King of Power Saving	Fast shipping, affordable price, great quality, very satisfied!	Jingdong prefecture level city in Shandong
Dragon Quality Goods Shop	King of Power Saving	Regret knowing too late, it's really good stuff. Works well.	Jingdong prefecture level city in Shandong
Dragon Quality Goods Shop	King of Power Saving	Not bad, very good product, quite save electricity	Jingdong prefecture level city in Shandong
Dragon Quality Goods Shop	King of Power Saving	Good quality, fast logistics, overall very satisfied!	Jingdong prefecture level city in Shandong
Dragon Quality Goods Shop	King of Power Saving	Tightly packaged with good results	Jingdong prefecture level city in Shandong
Dragon Quality Goods Shop	King of Power Saving	very happy	Jingdong prefecture level city in Shandong
Dragon Quality Goods Shop	King of Power Saving	favorable criticism	Jingdong prefecture level city in Shandong
Dragon Quality Goods Shop	King of Power Saving	Now they all sign for the goods without receiving them, the Jingdong platform and merchants are very powerful, and the goods are not received before signing for the goods.	Jingdong prefecture level city in Shandong
Dragon Quality Goods Shop	King of Power Saving	low-quality commodities	Jingdong prefecture level city in Shandong
Dragon Quality Goods Shop	King of Power Saving	I bought this and now it works pretty well with the air conditioning on, when I didn't use it it was $600 a month, now it's almost $500.00, that's over $100 less all of a sudden.	Jingdong prefecture level city in Shandong
Dragon Quality Goods Shop	King of Power Saving	Can Oh, the weather is hot, open the air conditioning is really good, this product is good, has been used for a really long time, more ok, power saving effect is not bad.	Jingdong prefecture level city in Shandong
Dragon Quality Goods Shop	King of Power Saving	I paid my electric bill today, it's a few dozen dollars less, anyway, it's not costly, it's already very good, the boss said that it would be better to buy one more, I've already placed an order	Jingdong prefecture level city in Shandong
Dragon Quality Goods Shop	King of Power Saving	I'm on a smart meter, I save about 30% a month, personally I think it's pretty good, it works well	Jingdong prefecture level city in Shandong
Dragon Quality Goods Shop	King of Power Saving	My dad asked me to buy one, but after I paid the bill, I asked and he said it was a lot less this month, so he asked me to buy another one.	Jingdong prefecture level city in Shandong
Dragon Quality Goods Shop	King of Power Saving	Very satisfied with the purchase thanks to the store for the prosperous business.	Jingdong prefecture level city in Shandong
Dragon Quality Goods Shop	King of Power Saving	Good use, good quality, very satisfied with the purchase	Jingdong prefecture level city in Shandong
Dragon Quality Goods Shop	King of Power Saving	The goods have been received, the quality is very good, is the ideal effect, will continue to pay attention to the	Jingdong prefecture level city in Shandong
Dragon Quality Goods Shop	King of Power Saving	This brand has been used for many years, and this time I saw this upgraded version of the product and got it again. The purpose of this product is to reduce the loss of electricity for a long time, so as to achieve the purpose of saving electricity.	Jingdong prefecture level city in Shandong
Dragon Quality Goods Shop	King of Power Saving	I've been using it for almost a month, and I've reduced my electricity bill by over 100 bucks! I'm planning to get two more for my family.	Jingdong prefecture level city in Shandong
Dragon Quality Goods Shop	King of Power Saving	The effect is too good, the family has now several units, back to buy a number of times, the power saver received, power-saving effect is very good,, fine workmanship, long warranty, but also purify the air, before buying a used for a period of time, with the okay, and then bought a, and only now come to the	Jingdong prefecture level city in Shandong
Dragon Quality Goods Shop	King of Power Saving	Finally received the goods, very, very much like,, has been a lot of people praise, really too happy, has been his family's loyal fans, will continue to buy buy buy buy!	Jingdong prefecture level city in Shandong
Dragon Quality Goods Shop	King of Power Saving	📩 I've been wanting it for a long time, I decided to go for it, I feel great after using it, it's genuine and the delivery is great, it lives up to its name👍	Jingdong prefecture level city in Shandong
Dragon Quality Goods Shop	King of Power Saving	The quality is sure as other friends who bought it said, and it is also beautiful, thanks to the owner for the recommendation, thanks!	Jingdong prefecture level city in Shandong
Dragon Quality Goods Shop	King of Power Saving	Baby received the quality is really good face value is also very high especially practical especially especially special favorite	Jingdong prefecture level city in Shandong
Dragon Quality Goods Shop	King of Power Saving	It is genuine, trust the merchant, the quality is very good, the price is moderate, the use of the effect is very good!	Jingdong prefecture level city in Shandong
Dragon Quality Goods Shop	King of Power Saving	Energy saver received really good quality affordable and good energy saving and environmental protection is the best to use.	Jingdong prefecture level city in Shandong
Dragon Quality Goods Shop	King of Power Saving	Reasonable price logistics is very fast, the feeling is not bad more satisfied with a shopping experience	Jingdong prefecture level city in Shandong
Dragon Quality Goods Shop	King of Power Saving	The stuff is even more perfect than I imagined, very powerful and worth getting!	Jingdong prefecture level city in Shandong
Dragon Quality Goods Shop	King of Power Saving	Goods have been received, good quality, affordable price, good customer service, worth recommending!	Jingdong prefecture level city in Shandong
Dragon Quality Goods Shop	King of Power Saving	Fast shipping, works great, have already recommended it to friends.	Jingdong prefecture level city in Shandong
Dragon Quality Goods Shop	King of Power Saving	Good quality, fast logistics, reasonable price, worth 5 stars.	Jingdong prefecture level city in Shandong
Dragon Quality Goods Shop	King of Power Saving	Good quality, fast logistics, good value, well packaged, practical	Jingdong prefecture level city in Shandong
Dragon Quality Goods Shop	King of Power Saving	Fast shipping, affordable price, great quality, very satisfied!	Jingdong prefecture level city in Shandong
Dragon Quality Goods Shop	King of Power Saving	Regret knowing too late, it's really good stuff. Works well.	Jingdong prefecture level city in Shandong
Dragon Quality Goods Shop	King of Power Saving	Not bad, very good product, quite save electricity	Jingdong prefecture level city in Shandong
Dragon Quality Goods Shop	King of Power Saving	Good quality, fast logistics, overall very satisfied!	Jingdong prefecture level city in Shandong
Dragon Quality Goods Shop	King of Power Saving	Tightly packaged with good results	Jingdong prefecture level city in Shandong
Dragon Quality Goods Shop	King of Power Saving	very happy	Jingdong prefecture level city in Shandong
Dragon Quality Goods Shop	King of Power Saving	favorable criticism	Jingdong prefecture level city in Shandong
Dragon Quality Goods Shop	King of Power Saving	Now they all sign for the goods without receiving them, the Jingdong platform and merchants are very powerful, and the goods are not received before signing for the goods.	Jingdong prefecture level city in Shandong
Dragon Quality Goods Shop	King of Power Saving	low-quality commodities	Jingdong prefecture level city in Shandong
Dragon Quality Goods Shop	King of Power Saving	I bought this and now it works pretty well with the air conditioning on, when I didn't use it it was $600 a month, now it's almost $500.00, that's over $100 less all of a sudden.	Jingdong prefecture level city in Shandong
Dragon Quality Goods Shop	King of Power Saving	Can Oh, the weather is hot, open the air conditioning is really good, this product is good, has been used for a really long time, more ok, power saving effect is not bad.	Jingdong prefecture level city in Shandong

Dragon Quality Goods Shop	King of Power Saving	I paid my electric bill today, it's a few dozen dollars less, anyway, it's not costly, it's already very good, the boss said that it would be better to buy one more, I've already placed an order	Jingdong prefecture level city in Shandong
Dragon Quality Goods Shop	King of Power Saving	I'm on a smart meter, I save about 30% a month, personally I think it's pretty good, it works well	Jingdong prefecture level city in Shandong
Dragon Quality Goods Shop	King of Power Saving	My dad asked me to buy one, but after I paid the bill, I asked and he said it was a lot less this month, so he asked me to buy another one.	Jingdong prefecture level city in Shandong
Dragon Quality Goods Shop	King of Power Saving	Very satisfied with the purchase thanks to the store for the prosperous business.	Jingdong prefecture level city in Shandong
Dragon Quality Goods Shop	King of Power Saving	Good use, good quality, very satisfied with the purchase	Jingdong prefecture level city in Shandong
Dragon Quality Goods Shop	King of Power Saving	The goods have been received, the quality is very good, is the ideal effect, will continue to pay attention to the	Jingdong prefecture level city in Shandong
Dragon Quality Goods Shop	King of Power Saving	This brand has been used for many years, and this time I saw this upgraded version of the product and got it again. The purpose of this product is to reduce the loss of electricity for a long time, so as to achieve the purpose of saving electricity.	Jingdong prefecture level city in Shandong
Dragon Quality Goods Shop	King of Power Saving	I've been using it for almost a month, and I've reduced my electricity bill by over 100 bucks! I'm planning to get two more for my family.	Jingdong prefecture level city in Shandong
Dragon Quality Goods Shop	King of Power Saving	The effect is too good, the family has now several units, back to buy a number of times, the power saver received, power-saving effect is very good,, fine workmanship, long warranty, but also purify the air, before buying a used for a period of time, with the okay, and then bought a, and only now come to the	Jingdong prefecture level city in Shandong
Dragon Quality Goods Shop	King of Power Saving	Finally received the goods, very, very much like,, has been a lot of people praise, really too happy, has been his family's loyal fans, will continue to buy buy buy buy!	Jingdong prefecture level city in Shandong
Dragon Quality Goods Shop	King of Power Saving	I've been wanting it for a long time, I decided to go for it, I feel great after using it, it's genuine and the delivery is great, it lives up to its name	Jingdong prefecture level city in Shandong
Dragon Quality Goods Shop	King of Power Saving	The quality is sure as other friends who bought it said, and it is also beautiful, thanks to the owner for the recommendation, thanks!	Jingdong prefecture level city in Shandong
Dragon Quality Goods Shop	King of Power Saving	Baby received the quality is really good face value is also very high especially practical especially especially special favorite	Jingdong prefecture level city in Shandong
Dragon Quality Goods Shop	King of Power Saving	It is genuine, trust the merchant, the quality is very good, the price is moderate, the use of the effect is very good!	Jingdong prefecture level city in Shandong
Dragon Quality Goods Shop	King of Power Saving	Energy saver received really good quality affordable and good energy saving and environmental protection is the best to use.	Jingdong prefecture level city in Shandong
Dragon Quality Goods Shop	King of Power Saving	Reasonable price logistics is very fast, the feeling is not bad more satisfied with a shopping experience	Jingdong prefecture level city in Shandong
Dragon Quality Goods Shop	King of Power Saving	The stuff is even more perfect than I imagined, very powerful and worth getting!	Jingdong prefecture level city in Shandong
Dragon Quality Goods Shop	King of Power Saving	Goods have been received, good quality, affordable price, good customer service, worth recommending!	Jingdong prefecture level city in Shandong
Dragon Quality Goods Shop	King of Power Saving	Fast shipping, works great, have already recommended it to friends.	Jingdong prefecture level city in Shandong
Dragon Quality Goods Shop	King of Power Saving	Good quality, fast logistics, reasonable price, worth 5 stars.	Jingdong prefecture level city in Shandong
Dragon Quality Goods Shop	King of Power Saving	Good quality, fast logistics, good value, well packaged, practical	Jingdong prefecture level city in Shandong
Dragon Quality Goods Shop	King of Power Saving	Fast shipping, affordable price, great quality, very satisfied!	Jingdong prefecture level city in Shandong
Dragon Quality Goods Shop	King of Power Saving	Regret knowing too late, it's really good stuff. Works well.	Jingdong prefecture level city in Shandong
Dragon Quality Goods Shop	King of Power Saving	Not bad, very good product, quite save electricity	Jingdong prefecture level city in Shandong
Dragon Quality Goods Shop	King of Power Saving	Good quality, fast logistics, overall very satisfied!	Jingdong prefecture level city in Shandong
Dragon Quality Goods Shop	King of Power Saving	Tightly packaged with good results	Jingdong prefecture level city in Shandong
Dragon Quality Goods Shop	King of Power Saving	very happy	Jingdong prefecture level city in Shandong
Dragon Quality Goods Shop	King of Power Saving	favorable criticism	Jingdong prefecture level city in Shandong
Dragon Quality Goods Shop	King of Power Saving	Now they all sign for the goods without receiving them, the Jingdong platform and merchants are very powerful, and the goods are not received before signing for the goods.	Jingdong prefecture level city in Shandong
Dragon Quality Goods Shop	King of Power Saving	low-quality commodities	Jingdong prefecture level city in Shandong
Dragon Quality Goods Shop	King of Power Saving	I bought this and now it works pretty well with the air conditioning on, when I didn't use it it was $600 a month, now it's almost $500,00, that's over $100 less all of a sudden.	Jingdong prefecture level city in Shandong
Dragon Quality Goods Shop	King of Power Saving	Can Oh, the weather is hot, open the air conditioning is really good, this product is good, has been used for a really long time, more ok, power saving effect is not bad.	Jingdong prefecture level city in Shandong
Dragon Quality Goods Shop	King of Power Saving	I paid my electric bill today, it's a few dozen dollars less, anyway, it's not costly, it's already very good, the boss said that it would be better to buy one more, I've already placed an order	Jingdong prefecture level city in Shandong
Dragon Quality Goods Shop	King of Power Saving	I'm on a smart meter, I save about 30% a month, personally I think it's pretty good, it works well	Jingdong prefecture level city in Shandong
Dragon Quality Goods Shop	King of Power Saving	My dad asked me to buy one, but after I paid the bill, I asked and he said it was a lot less this month, so he asked me to buy another one.	Jingdong prefecture level city in Shandong
Dragon Quality Goods Shop	King of Power Saving	Very satisfied with the purchase thanks to the store for the prosperous business.	Jingdong prefecture level city in Shandong
Dragon Quality Goods Shop	King of Power Saving	Good use, good quality, very satisfied with the purchase	Jingdong prefecture level city in Shandong
Dragon Quality Goods Shop	King of Power Saving	The goods have been received, the quality is very good, is the ideal effect, will continue to pay attention to the	Jingdong prefecture level city in Shandong
Dragon Quality Goods Shop	King of Power Saving	This brand has been used for many years, and this time I saw this upgraded version of the product and got it again. The purpose of this product is to reduce the loss of electricity for a long time, so as to achieve the purpose of saving electricity.	Jingdong prefecture level city in Shandong
Dragon Quality Goods Shop	King of Power Saving	I've been using it for almost a month, and I've reduced my electricity bill by over 100 bucks! I'm planning to get two more for my family.	Jingdong prefecture level city in Shandong
Dragon Quality Goods Shop	King of Power Saving	The effect is too good, the family has now several units, back to buy a number of times, the power saver received, power-saving effect is very good,, fine workmanship, long warranty, but also purify the air, before buying a used for a period of time, with the okay, and then bought a, and only now come to the	Jingdong prefecture level city in Shandong
Dragon Quality Goods Shop	King of Power Saving	Finally received the goods, very, very much like,, has been a lot of people praise, really too happy, has been his family's loyal fans, will continue to buy buy buy buy!	Jingdong prefecture level city in Shandong
Dragon Quality Goods Shop	King of Power Saving	I've been wanting it for a long time, I decided to go for it, I feel great after using it, it's genuine and the delivery is great, it lives up to its name	Jingdong prefecture level city in Shandong
Dragon Quality Goods Shop	King of Power Saving	The quality is sure as other friends who bought it said, and it is also beautiful, thanks to the owner for the recommendation, thanks!	Jingdong prefecture level city in Shandong
Dragon Quality Goods Shop	King of Power Saving	Baby received the quality is really good face value is also very high especially practical especially especially special favorite	Jingdong prefecture level city in Shandong
Dragon Quality Goods Shop	King of Power Saving	It is genuine, trust the merchant, the quality is very good, the price is moderate, the use of the effect is very good!	Jingdong prefecture level city in Shandong
Dragon Quality Goods Shop	King of Power Saving	Energy saver received really good quality affordable and good energy saving and environmental protection is the best to use.	Jingdong prefecture level city in Shandong
Dragon Quality Goods Shop	King of Power Saving	Reasonable price logistics is very fast, the feeling is not bad more satisfied with a shopping experience	Jingdong prefecture level city in Shandong
Dragon Quality Goods Shop	King of Power Saving	The stuff is even more perfect than I imagined, very powerful and worth getting!	Jingdong prefecture level city in Shandong

Dragon Quality Goods Shop	King of Power Saving	Goods have been received, good quality, affordable price, good customer service, worth recommending!	Jingdong prefecture level city in Shandong
Dragon Quality Goods Shop	King of Power Saving	Fast shipping, works great, have already recommended it to friends.	Jingdong prefecture level city in Shandong
Dragon Quality Goods Shop	King of Power Saving	Good quality, fast logistics, reasonable price, worth 5 stars.	Jingdong prefecture level city in Shandong
Dragon Quality Goods Shop	King of Power Saving	Good quality, fast logistics, good value, well packaged, practical	Jingdong prefecture level city in Shandong
Dragon Quality Goods Shop	King of Power Saving	Fast shipping, affordable price, great quality, very satisfied!	Jingdong prefecture level city in Shandong
Dragon Quality Goods Shop	King of Power Saving	Regret knowing too late, it's really good stuff. Works well.	Jingdong prefecture level city in Shandong
Dragon Quality Goods Shop	King of Power Saving	Not bad, very good product, quite save electricity	Jingdong prefecture level city in Shandong
Dragon Quality Goods Shop	King of Power Saving	Good quality, fast logistics, overall very satisfied!	Jingdong prefecture level city in Shandong
Dragon Quality Goods Shop	King of Power Saving	Tightly packaged with good results	Jingdong prefecture level city in Shandong
Dragon Quality Goods Shop	King of Power Saving	very happy	Jingdong prefecture level city in Shandong
Dragon Quality Goods Shop	King of Power Saving	favorable criticism	Jingdong prefecture level city in Shandong
Dragon Quality Goods Shop	King of Power Saving	Now they all sign for the goods without receiving them, the Jingdong platform and merchants are very powerful, and the goods are not received before signing for the goods.	Jingdong prefecture level city in Shandong
Dragon Quality Goods Shop	King of Power Saving	low-quality commodities	Jingdong prefecture level city in Shandong
Dragon Quality Goods Shop	King of Power Saving	I bought this and now it works pretty well with the air conditioning on, when I didn't use it it was $600 a month, now it's almost $500.00, that's over $100 less all of a sudden.	Jingdong prefecture level city in Shandong
Dragon Quality Goods Shop	King of Power Saving	Can Oh, the weather is hot, open the air conditioning is really good, this product is good, has been used for a really long time, more ok, power saving effect is not bad.	Jingdong prefecture level city in Shandong
Dragon Quality Goods Shop	King of Power Saving	I paid my electric bill today, it's a few dozen dollars less, anyway, it's not costly, it's already very good, the boss said that it would be better to buy one more, I've already placed an order	Jingdong prefecture level city in Shandong
Dragon Quality Goods Shop	King of Power Saving	I'm on a smart meter, I save about 30% a month, personally I think it's pretty good, it works well	Jingdong prefecture level city in Shandong
Dragon Quality Goods Shop	King of Power Saving	My dad asked me to buy one, but after I paid the bill, I asked and he said it was a lot less this month, so he asked me to buy another one.	Jingdong prefecture level city in Shandong
Dragon Quality Goods Shop	King of Power Saving	Very satisfied with the purchase thanks to the store for the prosperous business.	Jingdong prefecture level city in Shandong
Dragon Quality Goods Shop	King of Power Saving	Good use, good quality, very satisfied with the purchase	Jingdong prefecture level city in Shandong
Dragon Quality Goods Shop	King of Power Saving	The goods have been received, the quality is very good, is the ideal effect, will continue to pay attention to the	Jingdong prefecture level city in Shandong
Dragon Quality Goods Shop	King of Power Saving	This brand has been used for many years, and this time I saw this upgraded version of the product and got it again. The purpose of this product is to reduce the loss of electricity for a long time, so as to achieve the purpose of saving electricity.	Jingdong prefecture level city in Shandong
Dragon Quality Goods Shop	King of Power Saving	I've been using it for almost a month, and I've reduced my electricity bill by over 100 bucks! I'm planning to get two more for my family.	Jingdong prefecture level city in Shandong
Dragon Quality Goods Shop	King of Power Saving	The effect is too good, the family has now several units, back to buy a number of times, the power saver received, power-saving effect is very good,, fine workmanship, long warranty, but also purify the air, before buying a used for a period of time, with the okay, and then bought a, and only now come to the	Jingdong prefecture level city in Shandong
Dragon Quality Goods Shop	King of Power Saving	Finally received the goods, very, very much like,, has been a lot of people praise, really too happy, has been his family's loyal fans, will continue to buy buy buy buy!	Jingdong prefecture level city in Shandong
Dragon Quality Goods Shop	King of Power Saving	📩 I've been wanting it for a long time, I decided to go for it, I feel great after using it, it's genuine and the delivery is great, it lives up to its name👍	Jingdong prefecture level city in Shandong
Dragon Quality Goods Shop	King of Power Saving	The quality is sure as other friends who bought it said, and it is also beautiful, thanks to the owner for the recommendation, thanks!	Jingdong prefecture level city in Shandong
Dragon Quality Goods Shop	King of Power Saving	Baby received the quality is really good face value is also very high especially practical especially especially special favorite	Jingdong prefecture level city in Shandong
Dragon Quality Goods Shop	King of Power Saving	It is genuine, trust the merchant, the quality is very good, the price is moderate, the use of the effect is very good!	Jingdong prefecture level city in Shandong
Dragon Quality Goods Shop	King of Power Saving	Energy saver received really good quality affordable and good energy saving and environmental protection is the best to use.	Jingdong prefecture level city in Shandong
Dragon Quality Goods Shop	King of Power Saving	Reasonable price logistics is very fast, the feeling is not bad more satisfied with a shopping experience	Jingdong prefecture level city in Shandong
Dragon Quality Goods Shop	King of Power Saving	The stuff is even more perfect than I imagined, very powerful and worth getting!	Jingdong prefecture level city in Shandong
Dragon Quality Goods Shop	King of Power Saving	Goods have been received, good quality, affordable price, good customer service, worth recommending!	Jingdong prefecture level city in Shandong
Dragon Quality Goods Shop	King of Power Saving	Fast shipping, works great, have already recommended it to friends.	Jingdong prefecture level city in Shandong
Dragon Quality Goods Shop	King of Power Saving	Good quality, fast logistics, reasonable price, worth 5 stars.	Jingdong prefecture level city in Shandong
Dragon Quality Goods Shop	King of Power Saving	Good quality, fast logistics, good value, well packaged, practical	Jingdong prefecture level city in Shandong
Dragon Quality Goods Shop	King of Power Saving	Fast shipping, affordable price, great quality, very satisfied!	Jingdong prefecture level city in Shandong
Dragon Quality Goods Shop	King of Power Saving	Regret knowing too late, it's really good stuff. Works well.	Jingdong prefecture level city in Shandong
Dragon Quality Goods Shop	King of Power Saving	Not bad, very good product, quite save electricity	Jingdong prefecture level city in Shandong
Dragon Quality Goods Shop	King of Power Saving	Good quality, fast logistics, overall very satisfied!	Jingdong prefecture level city in Shandong
Dragon Quality Goods Shop	King of Power Saving	Tightly packaged with good results	Jingdong prefecture level city in Shandong
Dragon Quality Goods Shop	King of Power Saving	very happy	Jingdong prefecture level city in Shandong
Dragon Quality Goods Shop	King of Power Saving	favorable criticism	Jingdong prefecture level city in Shandong
Dragon Quality Goods Shop	King of Power Saving	Now they all sign for the goods without receiving them, the Jingdong platform and merchants are very powerful, and the goods are not received before signing for the goods.	Jingdong prefecture level city in Shandong
Dragon Quality Goods Shop	King of Power Saving	low-quality commodities	Jingdong prefecture level city in Shandong
Dragon Quality Goods Shop	King of Power Saving	I bought this and now it works pretty well with the air conditioning on, when I didn't use it it was $600 a month, now it's almost $500.00, that's over $100 less all of a sudden.	Jingdong prefecture level city in Shandong
Dragon Quality Goods Shop	King of Power Saving	Can Oh, the weather is hot, open the air conditioning is really good, this product is good, has been used for a really long time, more ok, power saving effect is not bad.	Jingdong prefecture level city in Shandong
Dragon Quality Goods Shop	King of Power Saving	I paid my electric bill today, it's a few dozen dollars less, anyway, it's not costly, it's already very good, the boss said that it would be better to buy one more, I've already placed an order	Jingdong prefecture level city in Shandong
Dragon Quality Goods Shop	King of Power Saving	I'm on a smart meter, I save about 30% a month, personally I think it's pretty good, it works well	Jingdong prefecture level city in Shandong
Dragon Quality Goods Shop	King of Power Saving	My dad asked me to buy one, but after I paid the bill, I asked and he said it was a lot less this month, so he asked me to buy another one.	Jingdong prefecture level city in Shandong

Dragon Quality Goods Shop	King of Power Saving	Very satisfied with the purchase thanks to the store for the prosperous business.	Jingdong prefecture level city in Shandong
Dragon Quality Goods Shop	King of Power Saving	Good use, good quality, very satisfied with the purchase	Jingdong prefecture level city in Shandong
Dragon Quality Goods Shop	King of Power Saving	The goods have been received, the quality is very good, is the ideal effect, will continue to pay attention to the	Jingdong prefecture level city in Shandong
Dragon Quality Goods Shop	King of Power Saving	This brand has been used for many years, and this time I saw this upgraded version of the product and got it again. The purpose of this product is to reduce the loss of electricity for a long time, so as to achieve the purpose of saving electricity.	Jingdong prefecture level city in Shandong
Dragon Quality Goods Shop	King of Power Saving	I've been using it for almost a month, and I've reduced my electricity bill by over 100 bucks! I'm planning to get two more for my family.	Jingdong prefecture level city in Shandong
Dragon Quality Goods Shop	King of Power Saving	The effect is too good, the family has now several units, back to buy a number of times, the power saver received, power-saving effect is very good,, fine workmanship, long warranty, but also purify the air, before buying a used for a period of time, with the okay, and then bought a, and only now come to the	Jingdong prefecture level city in Shandong
Dragon Quality Goods Shop	King of Power Saving	Finally received the goods, very, very much like,, has been a lot of people praise, really too happy, has been his family's loyal fans, will continue to buy buy buy buy!	Jingdong prefecture level city in Shandong
Dragon Quality Goods Shop	King of Power Saving	📩 I've been wanting it for a long time, I decided to go for it, I feel great after using it, it's genuine and the delivery is great, it lives up to its name👍	Jingdong prefecture level city in Shandong
Dragon Quality Goods Shop	King of Power Saving	The quality is sure as other friends who bought it said, and it is also beautiful, thanks to the owner for the recommendation, thanks!	Jingdong prefecture level city in Shandong
Dragon Quality Goods Shop	King of Power Saving	Baby received the quality is really good face value is also very high especially practical especially especially special favorite	Jingdong prefecture level city in Shandong
Dragon Quality Goods Shop	King of Power Saving	It is genuine, trust the merchant, the quality is very good, the price is moderate, the use of the effect is very good!	Jingdong prefecture level city in Shandong
Dragon Quality Goods Shop	King of Power Saving	Energy saver received really good quality affordable and good energy saving and environmental protection is the best to use.	Jingdong prefecture level city in Shandong
Dragon Quality Goods Shop	King of Power Saving	Reasonable price logistics is very fast, the feeling is not bad more satisfied with a shopping experience	Jingdong prefecture level city in Shandong
Dragon Quality Goods Shop	King of Power Saving	The stuff is even more perfect than I imagined, very powerful and worth getting!	Jingdong prefecture level city in Shandong
Dragon Quality Goods Shop	King of Power Saving	Goods have been received, good quality, affordable price, good customer service, worth recommending!	Jingdong prefecture level city in Shandong
Dragon Quality Goods Shop	King of Power Saving	Fast shipping, works great, have already recommended it to friends.	Jingdong prefecture level city in Shandong
Dragon Quality Goods Shop	King of Power Saving	Good quality, fast logistics, reasonable price, worth 5 stars.	Jingdong prefecture level city in Shandong
Dragon Quality Goods Shop	King of Power Saving	Good quality, fast logistics, good value, well packaged, practical	Jingdong prefecture level city in Shandong
Dragon Quality Goods Shop	King of Power Saving	Fast shipping, affordable price, great quality, very satisfied!	Jingdong prefecture level city in Shandong
Dragon Quality Goods Shop	King of Power Saving	Regret knowing too late, it's really good stuff. Works well.	Jingdong prefecture level city in Shandong
Dragon Quality Goods Shop	King of Power Saving	Not bad, very good product, quite save electricity	Jingdong prefecture level city in Shandong
Dragon Quality Goods Shop	King of Power Saving	Good quality, fast logistics, overall very satisfied!	Jingdong prefecture level city in Shandong
Dragon Quality Goods Shop	King of Power Saving	Tightly packaged with good results	Jingdong prefecture level city in Shandong
Dragon Quality Goods Shop	King of Power Saving	very happy	Jingdong prefecture level city in Shandong
Dragon Quality Goods Shop	King of Power Saving	favorable criticism	Jingdong prefecture level city in Shandong
Dragon Quality Goods Shop	King of Power Saving	Now they all sign for the goods without receiving them, the Jingdong platform and merchants are very powerful, and the goods are not received before signing for the goods.	Jingdong prefecture level city in Shandong
Dragon Quality Goods Shop	King of Power Saving	low-quality commodities	Jingdong prefecture level city in Shandong
Dragon Quality Goods Shop	King of Power Saving	I bought this and now it works pretty well with the air conditioning on, when I didn't use it it was $600 a month, now it's almost $500.00, that's over $100 less all of a sudden.	Jingdong prefecture level city in Shandong
Dragon Quality Goods Shop	King of Power Saving	Can Oh, the weather is hot, open the air conditioning is really good, this product is good, has been used for a really long time, more ok, power saving effect is not bad.	Jingdong prefecture level city in Shandong
Dragon Quality Goods Shop	King of Power Saving	I paid my electric bill today, it's a few dozen dollars less, anyway, it's not costly, it's already very good, the boss said that it would be better to buy one more, I've already placed an order	Jingdong prefecture level city in Shandong
Dragon Quality Goods Shop	King of Power Saving	I'm on a smart meter, I save about 30% a month, personally I think it's pretty good, it works well	Jingdong prefecture level city in Shandong
Dragon Quality Goods Shop	King of Power Saving	My dad asked me to buy one, but after I paid the bill, I asked and he said it was a lot less this month, so he asked me to buy another one.	Jingdong prefecture level city in Shandong
Dragon Quality Goods Shop	King of Power Saving	Very satisfied with the purchase thanks to the store for the prosperous business.	Jingdong prefecture level city in Shandong
Dragon Quality Goods Shop	King of Power Saving	Good use, good quality, very satisfied with the purchase	Jingdong prefecture level city in Shandong
Dragon Quality Goods Shop	King of Power Saving	The goods have been received, the quality is very good, is the ideal effect, will continue to pay attention to the	Jingdong prefecture level city in Shandong
Dragon Quality Goods Shop	King of Power Saving	This brand has been used for many years, and this time I saw this upgraded version of the product and got it again. The purpose of this product is to reduce the loss of electricity for a long time, so as to achieve the purpose of saving electricity.	Jingdong prefecture level city in Shandong
Dragon Quality Goods Shop	King of Power Saving	I've been using it for almost a month, and I've reduced my electricity bill by over 100 bucks! I'm planning to get two more for my family.	Jingdong prefecture level city in Shandong
Dragon Quality Goods Shop	King of Power Saving	The effect is too good, the family has now several units, back to buy a number of times, the power saver received, power-saving effect is very good,, fine workmanship, long warranty, but also purify the air, before buying a used for a period of time, with the okay, and then bought a, and only now come to the	Jingdong prefecture level city in Shandong
Dragon Quality Goods Shop	King of Power Saving	Finally received the goods, very, very much like,, has been a lot of people praise, really too happy, has been his family's loyal fans, will continue to buy buy buy buy!	Jingdong prefecture level city in Shandong
Dragon Quality Goods Shop	King of Power Saving	📩 I've been wanting it for a long time, I decided to go for it, I feel great after using it, it's genuine and the delivery is great, it lives up to its name👍	Jingdong prefecture level city in Shandong
Dragon Quality Goods Shop	King of Power Saving	The quality is sure as other friends who bought it said, and it is also beautiful, thanks to the owner for the recommendation, thanks!	Jingdong prefecture level city in Shandong
Dragon Quality Goods Shop	King of Power Saving	Baby received the quality is really good face value is also very high especially practical especially especially special favorite	Jingdong prefecture level city in Shandong
Dragon Quality Goods Shop	King of Power Saving	It is genuine, trust the merchant, the quality is very good, the price is moderate, the use of the effect is very good!	Jingdong prefecture level city in Shandong
Dragon Quality Goods Shop	King of Power Saving	Energy saver received really good quality affordable and good energy saving and environmental protection is the best to use.	Jingdong prefecture level city in Shandong
Dragon Quality Goods Shop	King of Power Saving	Reasonable price logistics is very fast, the feeling is not bad more satisfied with a shopping experience	Jingdong prefecture level city in Shandong
Dragon Quality Goods Shop	King of Power Saving	The stuff is even more perfect than I imagined, very powerful and worth getting!	Jingdong prefecture level city in Shandong
Dragon Quality Goods Shop	King of Power Saving	Goods have been received, good quality, affordable price, good customer service, worth recommending!	Jingdong prefecture level city in Shandong
Dragon Quality Goods Shop	King of Power Saving	Fast shipping, works great, have already recommended it to friends.	Jingdong prefecture level city in Shandong
Dragon Quality Goods Shop	King of Power Saving	Good quality, fast logistics, reasonable price, worth 5 stars.	Jingdong prefecture level city in Shandong

Dragon Quality Goods Shop	King of Power Saving	Good quality, fast logistics, good value, well packaged, practical	Jingdong prefecture level city in Shandong
Dragon Quality Goods Shop	King of Power Saving	Fast shipping, affordable price, great quality, very satisfied!	Jingdong prefecture level city in Shandong
Dragon Quality Goods Shop	King of Power Saving	Regret knowing too late, it's really good stuff. Works well.	Jingdong prefecture level city in Shandong
Dragon Quality Goods Shop	King of Power Saving	Not bad, very good product, quite save electricity	Jingdong prefecture level city in Shandong
Dragon Quality Goods Shop	King of Power Saving	Good quality, fast logistics, overall very satisfied!	Jingdong prefecture level city in Shandong
Dragon Quality Goods Shop	King of Power Saving	Tightly packaged with good results	Jingdong prefecture level city in Shandong
Dragon Quality Goods Shop	King of Power Saving	very happy	Jingdong prefecture level city in Shandong
Dragon Quality Goods Shop	King of Power Saving	favorable criticism	Jingdong prefecture level city in Shandong
Dragon Quality Goods Shop	King of Power Saving	Now they all sign for the goods without receiving them, the Jingdong platform and merchants are very powerful, and the goods are not received before signing for the goods.	Jingdong prefecture level city in Shandong
Dragon Quality Goods Shop	King of Power Saving	low-quality commodities	Jingdong prefecture level city in Shandong
Dragon Quality Goods Shop	King of Power Saving	I bought this and now it works pretty well with the air conditioning on, when I didn't use it it was $600 a month, now it's almost $500.00, that's over $100 less all of a sudden.	Jingdong prefecture level city in Shandong
Dragon Quality Goods Shop	King of Power Saving	Can Oh, the weather is hot, open the air conditioning is really good, this product is good, has been used for a really long time, more ok, power saving effect is not bad.	Jingdong prefecture level city in Shandong
Dragon Quality Goods Shop	King of Power Saving	I paid my electric bill today, it's a few dozen dollars less, anyway, it's not costly, it's already very good, the boss said that it would be better to buy one more, I've already placed an order	Jingdong prefecture level city in Shandong
Dragon Quality Goods Shop	King of Power Saving	I'm on a smart meter, I save about 30% a month, personally I think it's pretty good, it works well	Jingdong prefecture level city in Shandong
Dragon Quality Goods Shop	King of Power Saving	My dad asked me to buy one, but after I paid the bill, I asked and he said it was a lot less this month, so he asked me to buy another one.	Jingdong prefecture level city in Shandong
Dragon Quality Goods Shop	King of Power Saving	Very satisfied with the purchase thanks to the store for the prosperous business.	Jingdong prefecture level city in Shandong
Dragon Quality Goods Shop	King of Power Saving	Good use, good quality, very satisfied with the purchase	Jingdong prefecture level city in Shandong
Dragon Quality Goods Shop	King of Power Saving	The goods have been received, the quality is very good, is the ideal effect, will continue to pay attention to the	Jingdong prefecture level city in Shandong
Dragon Quality Goods Shop	King of Power Saving	This brand has been used for many years, and this time I saw this upgraded version of the product and got it again. The purpose of this product is to reduce the loss of electricity for a long time, so as to achieve the purpose of saving electricity.	Jingdong prefecture level city in Shandong
Dragon Quality Goods Shop	King of Power Saving	I've been using it for almost a month, and I've reduced my electricity bill by over 100 bucks! I'm planning to get two more for my family.	Jingdong prefecture level city in Shandong
Dragon Quality Goods Shop	King of Power Saving	The effect is too good, the family has now several units, back to buy a number of times, the power saver received, power-saving effect is very good,, fine workmanship, long warranty, but also purify the air, before buying a used for a period of time, with the okay, and then bought a, and only now come to the	Jingdong prefecture level city in Shandong
Dragon Quality Goods Shop	King of Power Saving	Finally received the goods, very, very much like,, has been a lot of people praise, really too happy, has been his family's loyal fans, will continue to buy buy buy buy!	Jingdong prefecture level city in Shandong
Dragon Quality Goods Shop	King of Power Saving	💌 I've been wanting it for a long time, I decided to go for it, I feel great after using it, it's genuine and the delivery is great, it lives up to its name👍	Jingdong prefecture level city in Shandong
Dragon Quality Goods Shop	King of Power Saving	The quality is sure as other friends who bought it said, and it is also beautiful, thanks to the owner for the recommendation, thanks!	Jingdong prefecture level city in Shandong
Dragon Quality Goods Shop	King of Power Saving	Baby received the quality is really good face value is also very high especially practical especially especially special favorite	Jingdong prefecture level city in Shandong
Dragon Quality Goods Shop	King of Power Saving	It is genuine, trust the merchant, the quality is very good, the price is moderate, the use of the effect is very good!	Jingdong prefecture level city in Shandong
Dragon Quality Goods Shop	King of Power Saving	Energy saver received really good quality affordable and good energy saving and environmental protection is the best to use.	Jingdong prefecture level city in Shandong
Dragon Quality Goods Shop	King of Power Saving	Reasonable price logistics is very fast, the feeling is not bad more satisfied with a shopping experience	Jingdong prefecture level city in Shandong
Dragon Quality Goods Shop	King of Power Saving	The stuff is even more perfect than I imagined, very powerful and worth getting!	Jingdong prefecture level city in Shandong
Dragon Quality Goods Shop	King of Power Saving	Goods have been received, good quality, affordable price, good customer service, worth recommending!	Jingdong prefecture level city in Shandong
Dragon Quality Goods Shop	King of Power Saving	Fast shipping, works great, have already recommended it to friends.	Jingdong prefecture level city in Shandong
Dragon Quality Goods Shop	King of Power Saving	Good quality, fast logistics, reasonable price, worth 5 stars.	Jingdong prefecture level city in Shandong
Dragon Quality Goods Shop	King of Power Saving	Good quality, fast logistics, good value, well packaged, practical	Jingdong prefecture level city in Shandong
Dragon Quality Goods Shop	King of Power Saving	Fast shipping, affordable price, great quality, very satisfied!	Jingdong prefecture level city in Shandong
Dragon Quality Goods Shop	King of Power Saving	Regret knowing too late, it's really good stuff. Works well.	Jingdong prefecture level city in Shandong
Dragon Quality Goods Shop	King of Power Saving	Not bad, very good product, quite save electricity	Jingdong prefecture level city in Shandong
Dragon Quality Goods Shop	King of Power Saving	Good quality, fast logistics, overall very satisfied!	Jingdong prefecture level city in Shandong
Dragon Quality Goods Shop	King of Power Saving	Tightly packaged with good results	Jingdong prefecture level city in Shandong
Dragon Quality Goods Shop	King of Power Saving	very happy	Jingdong prefecture level city in Shandong
Dragon Quality Goods Shop	King of Power Saving	favorable criticism	Jingdong prefecture level city in Shandong
Dragon Quality Goods Shop	King of Power Saving	Now they all sign for the goods without receiving them, the Jingdong platform and merchants are very powerful, and the goods are not received before signing for the goods.	Jingdong prefecture level city in Shandong
Dragon Quality Goods Shop	King of Power Saving	low-quality commodities	Jingdong prefecture level city in Shandong
Dragon Quality Goods Shop	King of Power Saving	I bought this and now it works pretty well with the air conditioning on, when I didn't use it it was $600 a month, now it's almost $500.00, that's over $100 less all of a sudden.	Jingdong prefecture level city in Shandong
Dragon Quality Goods Shop	King of Power Saving	Can Oh, the weather is hot, open the air conditioning is really good, this product is good, has been used for a really long time, more ok, power saving effect is not bad.	Jingdong prefecture level city in Shandong
Dragon Quality Goods Shop	King of Power Saving	I paid my electric bill today, it's a few dozen dollars less, anyway, it's not costly, it's already very good, the boss said that it would be better to buy one more, I've already placed an order	Jingdong prefecture level city in Shandong
Dragon Quality Goods Shop	King of Power Saving	I'm on a smart meter, I save about 30% a month, personally I think it's pretty good, it works well	Jingdong prefecture level city in Shandong
Dragon Quality Goods Shop	King of Power Saving	My dad asked me to buy one, but after I paid the bill, I asked and he said it was a lot less this month, so he asked me to buy another one.	Jingdong prefecture level city in Shandong
Dragon Quality Goods Shop	King of Power Saving	Very satisfied with the purchase thanks to the store for the prosperous business.	Jingdong prefecture level city in Shandong
Dragon Quality Goods Shop	King of Power Saving	Good use, good quality, very satisfied with the purchase	Jingdong prefecture level city in Shandong
Dragon Quality Goods Shop	King of Power Saving	The goods have been received, the quality is very good, is the ideal effect, will continue to pay attention to the	Jingdong prefecture level city in Shandong

Dragon Quality Goods Shop	King of Power Saving	This brand has been used for many years, and this time I saw this upgraded version of the product and got it again. The purpose of this product is to reduce the loss of electricity for a long time, so as to achieve the purpose of saving electricity.	Jingdong prefecture level city in Shandong
Dragon Quality Goods Shop	King of Power Saving	I've been using it for almost a month, and I've reduced my electricity bill by over 100 bucks! I'm planning to get two more for my family.	Jingdong prefecture level city in Shandong
Dragon Quality Goods Shop	King of Power Saving	The effect is too good, the family has now several units, back to buy a number of times, the power saver received, power-saving effect is very good,, fine workmanship, long warranty, but also purify the air, before buying a used for a period of time, with the okay, and then bought a, and only now come to the	Jingdong prefecture level city in Shandong
Dragon Quality Goods Shop	King of Power Saving	Finally received the goods, very, very much like,, has been a lot of people praise, really too happy, has been his family's loyal fans, will continue to buy buy buy buy!	Jingdong prefecture level city in Shandong
Dragon Quality Goods Shop	King of Power Saving	📩 I've been wanting it for a long time, I decided to go for it, I feel great after using it, it's genuine and the delivery is great, it lives up to its name👍	Jingdong prefecture level city in Shandong
Dragon Quality Goods Shop	King of Power Saving	The quality is sure as other friends who bought it said, and it is also beautiful, thanks to the owner for the recommendation, thanks!	Jingdong prefecture level city in Shandong
Dragon Quality Goods Shop	King of Power Saving	Baby received the quality is really good face value is also very high especially practical especially especially special favorite	Jingdong prefecture level city in Shandong
Dragon Quality Goods Shop	King of Power Saving	It is genuine, trust the merchant, the quality is very good, the price is moderate, the use of the effect is very good!	Jingdong prefecture level city in Shandong
Dragon Quality Goods Shop	King of Power Saving	Energy saver received really good quality affordable and good energy saving and environmental protection is the best to use.	Jingdong prefecture level city in Shandong
Dragon Quality Goods Shop	King of Power Saving	Reasonable price logistics is very fast, the feeling is not bad more satisfied with a shopping experience	Jingdong prefecture level city in Shandong
Dragon Quality Goods Shop	King of Power Saving	The stuff is even more perfect than I imagined, very powerful and worth getting!	Jingdong prefecture level city in Shandong
Dragon Quality Goods Shop	King of Power Saving	Goods have been received, good quality, affordable price, good customer service, worth recommending!	Jingdong prefecture level city in Shandong
Dragon Quality Goods Shop	King of Power Saving	Fast shipping, works great, have already recommended it to friends.	Jingdong prefecture level city in Shandong
Dragon Quality Goods Shop	King of Power Saving	Good quality, fast logistics, reasonable price, worth 5 stars.	Jingdong prefecture level city in Shandong
Dragon Quality Goods Shop	King of Power Saving	Good quality, fast logistics, good value, well packaged, practical	Jingdong prefecture level city in Shandong
Dragon Quality Goods Shop	King of Power Saving	Fast shipping, affordable price, great quality, very satisfied!	Jingdong prefecture level city in Shandong
Dragon Quality Goods Shop	King of Power Saving	Regret knowing too late, it's really good stuff. Works well.	Jingdong prefecture level city in Shandong
Dragon Quality Goods Shop	King of Power Saving	Not bad, very good product, quite save electricity	Jingdong prefecture level city in Shandong
Dragon Quality Goods Shop	King of Power Saving	Good quality, fast logistics, overall very satisfied!	Jingdong prefecture level city in Shandong
Dragon Quality Goods Shop	King of Power Saving	Tightly packaged with good results	Jingdong prefecture level city in Shandong
Dragon Quality Goods Shop	King of Power Saving	very happy	Jingdong prefecture level city in Shandong
Dragon Quality Goods Shop	King of Power Saving	favorable criticism	Jingdong prefecture level city in Shandong
Dragon Quality Goods Shop	King of Power Saving	Now they all sign for the goods without receiving them, the Jingdong platform and merchants are very powerful, and the goods are not received before signing for the goods.	Jingdong prefecture level city in Shandong
Dragon Quality Goods Shop	King of Power Saving	low-quality commodities	Jingdong prefecture level city in Shandong
Dragon Quality Goods Shop	King of Power Saving	I bought this and now it works pretty well with the air conditioning on, when I didn't use it it was $600 a month, now it's almost $500,00, that's over $100 less all of a sudden.	Jingdong prefecture level city in Shandong
Dragon Quality Goods Shop	King of Power Saving	Can Oh, the weather is hot, open the air conditioning is really good, this product is good, has been used for a really long time, more ok, power saving effect is not bad.	Jingdong prefecture level city in Shandong
Dragon Quality Goods Shop	King of Power Saving	I paid my electric bill today, it's a few dozen dollars less, anyway, it's not costly, it's already very good, the boss said that it would be better to buy one more, I've already placed an order	Jingdong prefecture level city in Shandong
Dragon Quality Goods Shop	King of Power Saving	I'm on a smart meter, I save about 30% a month, personally I think it's pretty good, it works well	Jingdong prefecture level city in Shandong
Dragon Quality Goods Shop	King of Power Saving	My dad asked me to buy one, but after I paid the bill, I asked and he said it was a lot less this month, so he asked me to buy another one.	Jingdong prefecture level city in Shandong
Dragon Quality Goods Shop	King of Power Saving	Very satisfied with the purchase thanks to the store for the prosperous business.	Jingdong prefecture level city in Shandong
Dragon Quality Goods Shop	King of Power Saving	Good use, good quality, very satisfied with the purchase	Jingdong prefecture level city in Shandong
Dragon Quality Goods Shop	King of Power Saving	The goods have been received, the quality is very good, is the ideal effect, will continue to pay attention to the	Jingdong prefecture level city in Shandong
Dragon Quality Goods Shop	King of Power Saving	This brand has been used for many years, and this time I saw this upgraded version of the product and got it again. The purpose of this product is to reduce the loss of electricity for a long time, so as to achieve the purpose of saving electricity.	Jingdong prefecture level city in Shandong
Dragon Quality Goods Shop	King of Power Saving	I've been using it for almost a month, and I've reduced my electricity bill by over 100 bucks! I'm planning to get two more for my family.	Jingdong prefecture level city in Shandong
Dragon Quality Goods Shop	King of Power Saving	The effect is too good, the family has now several units, back to buy a number of times, the power saver received, power-saving effect is very good,, fine workmanship, long warranty, but also purify the air, before buying a used for a period of time, with the okay, and then bought a, and only now come to the	Jingdong prefecture level city in Shandong
Dragon Quality Goods Shop	King of Power Saving	Finally received the goods, very, very much like,, has been a lot of people praise, really too happy, has been his family's loyal fans, will continue to buy buy buy buy!	Jingdong prefecture level city in Shandong
Dragon Quality Goods Shop	King of Power Saving	📩 I've been wanting it for a long time, I decided to go for it, I feel great after using it, it's genuine and the delivery is great, it lives up to its name👍	Jingdong prefecture level city in Shandong
Dragon Quality Goods Shop	King of Power Saving	The quality is sure as other friends who bought it said, and it is also beautiful, thanks to the owner for the recommendation, thanks!	Jingdong prefecture level city in Shandong
Dragon Quality Goods Shop	King of Power Saving	Baby received the quality is really good face value is also very high especially practical especially especially special favorite	Jingdong prefecture level city in Shandong
Dragon Quality Goods Shop	King of Power Saving	It is genuine, trust the merchant, the quality is very good, the price is moderate, the use of the effect is very good!	Jingdong prefecture level city in Shandong
Dragon Quality Goods Shop	King of Power Saving	Energy saver received really good quality affordable and good energy saving and environmental protection is the best to use.	Jingdong prefecture level city in Shandong
Dragon Quality Goods Shop	King of Power Saving	Reasonable price logistics is very fast, the feeling is not bad more satisfied with a shopping experience	Jingdong prefecture level city in Shandong
Dragon Quality Goods Shop	King of Power Saving	The stuff is even more perfect than I imagined, very powerful and worth getting!	Jingdong prefecture level city in Shandong
Dragon Quality Goods Shop	King of Power Saving	Goods have been received, good quality, affordable price, good customer service, worth recommending!	Jingdong prefecture level city in Shandong
Dragon Quality Goods Shop	King of Power Saving	Fast shipping, works great, have already recommended it to friends.	Jingdong prefecture level city in Shandong
Dragon Quality Goods Shop	King of Power Saving	Good quality, fast logistics, reasonable price, worth 5 stars.	Jingdong prefecture level city in Shandong
Dragon Quality Goods Shop	King of Power Saving	Good quality, fast logistics, good value, well packaged, practical	Jingdong prefecture level city in Shandong
Dragon Quality Goods Shop	King of Power Saving	Fast shipping, affordable price, great quality, very satisfied!	Jingdong prefecture level city in Shandong
Dragon Quality Goods Shop	King of Power Saving	Regret knowing too late, it's really good stuff. Works well.	Jingdong prefecture level city in Shandong

Dragon Quality Goods Shop	King of Power Saving	Not bad, very good product, quite save electricity	Jingdong prefecture level city in Shandong
Dragon Quality Goods Shop	King of Power Saving	Good quality, fast logistics, overall very satisfied!	Jingdong prefecture level city in Shandong
Dragon Quality Goods Shop	King of Power Saving	Tightly packaged with good results	Jingdong prefecture level city in Shandong
Dragon Quality Goods Shop	King of Power Saving	very happy	Jingdong prefecture level city in Shandong
Dragon Quality Goods Shop	King of Power Saving	favorable criticism	Jingdong prefecture level city in Shandong
Dragon Quality Goods Shop	King of Power Saving	Now they all sign for the goods without receiving them, the Jingdong platform and merchants are very powerful, and the goods are not received before signing for the goods.	Jingdong prefecture level city in Shandong
Dragon Quality Goods Shop	King of Power Saving	low-quality commodities	Jingdong prefecture level city in Shandong
Dragon Quality Goods Shop	King of Power Saving	I bought this and now it works pretty well with the air conditioning on, when I didn't use it it was $600 a month, now it's almost $500.00, that's over $100 less all of a sudden.	Jingdong prefecture level city in Shandong
Dragon Quality Goods Shop	King of Power Saving	Can Oh, the weather is hot, open the air conditioning is really good, this product is good, has been used for a really long time, more ok, power saving effect is not bad.	Jingdong prefecture level city in Shandong
Dragon Quality Goods Shop	King of Power Saving	I paid my electric bill today, it's a few dozen dollars less, anyway, it's not costly, it's already very good, the boss said that it would be better to buy one more, I've already placed an order	Jingdong prefecture level city in Shandong
Dragon Quality Goods Shop	King of Power Saving	I'm on a smart meter, I save about 30% a month, personally I think it's pretty good, it works well	Jingdong prefecture level city in Shandong
Dragon Quality Goods Shop	King of Power Saving	My dad asked me to buy one, but after I paid the bill, I asked and he said it was a lot less this month, so he asked me to buy another one.	Jingdong prefecture level city in Shandong
Dragon Quality Goods Shop	King of Power Saving	Very satisfied with the purchase thanks to the store for the prosperous business.	Jingdong prefecture level city in Shandong
Dragon Quality Goods Shop	King of Power Saving	Good use, good quality, very satisfied with the purchase	Jingdong prefecture level city in Shandong
Dragon Quality Goods Shop	King of Power Saving	The goods have been received, the quality is very good, is the ideal effect, will continue to pay attention to the	Jingdong prefecture level city in Shandong
Dragon Quality Goods Shop	King of Power Saving	This brand has been used for many years, and this time I saw this upgraded version of the product and got it again. The purpose of this product is to reduce the loss of electricity for a long time, so as to achieve the purpose of saving electricity.	Jingdong prefecture level city in Shandong
Dragon Quality Goods Shop	King of Power Saving	I've been using it for almost a month, and I've reduced my electricity bill by over 100 bucks! I'm planning to get two more for my family.	Jingdong prefecture level city in Shandong
Dragon Quality Goods Shop	King of Power Saving	The effect is too good, the family has now several units, back to buy a number of times, the power saver received, power-saving effect is very good,, fine workmanship, long warranty, but also purify the air, before buying a used for a period of time, with the okay, and then bought a, and only now come to the	Jingdong prefecture level city in Shandong
Dragon Quality Goods Shop	King of Power Saving	Finally received the goods, very, very much like,, has been a lot of people praise, really too happy, has been his family's loyal fans, will continue to buy buy buy buy!	Jingdong prefecture level city in Shandong
Dragon Quality Goods Shop	King of Power Saving	💌 I've been wanting it for a long time, I decided to go for it, I feel great after using it, it's genuine and the delivery is great, it lives up to its name👍	Jingdong prefecture level city in Shandong
Dragon Quality Goods Shop	King of Power Saving	The quality is sure as other friends who bought it said, and it is also beautiful, thanks to the owner for the recommendation, thanks!	Jingdong prefecture level city in Shandong
Dragon Quality Goods Shop	King of Power Saving	Baby received the quality is really good face value is also very high especially practical especially especially special favorite	Jingdong prefecture level city in Shandong
Dragon Quality Goods Shop	King of Power Saving	It is genuine, trust the merchant, the quality is very good, the price is moderate, the use of the effect is very good!	Jingdong prefecture level city in Shandong
Dragon Quality Goods Shop	King of Power Saving	Energy saver received really good quality affordable and good energy saving and environmental protection is the best to use.	Jingdong prefecture level city in Shandong
Dragon Quality Goods Shop	King of Power Saving	Reasonable price logistics is very fast, the feeling is not bad more satisfied with a shopping experience	Jingdong prefecture level city in Shandong
Dragon Quality Goods Shop	King of Power Saving	The stuff is even more perfect than I imagined, very powerful and worth getting!	Jingdong prefecture level city in Shandong
Dragon Quality Goods Shop	King of Power Saving	Goods have been received, good quality, affordable price, good customer service, worth recommending!	Jingdong prefecture level city in Shandong
Dragon Quality Goods Shop	King of Power Saving	Fast shipping, works great, have already recommended it to friends.	Jingdong prefecture level city in Shandong
Dragon Quality Goods Shop	King of Power Saving	Good quality, fast logistics, reasonable price, worth 5 stars.	Jingdong prefecture level city in Shandong
Dragon Quality Goods Shop	King of Power Saving	Good quality, fast logistics, good value, well packaged, practical	Jingdong prefecture level city in Shandong
Dragon Quality Goods Shop	King of Power Saving	Fast shipping, affordable price, great quality, very satisfied!	Jingdong prefecture level city in Shandong
Dragon Quality Goods Shop	King of Power Saving	Regret knowing too late, it's really good stuff. Works well.	Jingdong prefecture level city in Shandong
Dragon Quality Goods Shop	King of Power Saving	Not bad, very good product, quite save electricity	Jingdong prefecture level city in Shandong
Dragon Quality Goods Shop	King of Power Saving	Good quality, fast logistics, overall very satisfied!	Jingdong prefecture level city in Shandong
Dragon Quality Goods Shop	King of Power Saving	Tightly packaged with good results	Jingdong prefecture level city in Shandong
Dragon Quality Goods Shop	King of Power Saving	very happy	Jingdong prefecture level city in Shandong
Dragon Quality Goods Shop	King of Power Saving	favorable criticism	Jingdong prefecture level city in Shandong
Dragon Quality Goods Shop	King of Power Saving	Now they all sign for the goods without receiving them, the Jingdong platform and merchants are very powerful, and the goods are not received before signing for the goods.	Jingdong prefecture level city in Shandong
Dragon Quality Goods Shop	King of Power Saving	low-quality commodities	Jingdong prefecture level city in Shandong
Dragon Quality Goods Shop	King of Power Saving	I bought this and now it works pretty well with the air conditioning on, when I didn't use it it was $600 a month, now it's almost $500.00, that's over $100 less all of a sudden.	Jingdong prefecture level city in Shandong
Dragon Quality Goods Shop	King of Power Saving	Can Oh, the weather is hot, open the air conditioning is really good, this product is good, has been used for a really long time, more ok, power saving effect is not bad.	Jingdong prefecture level city in Shandong
Dragon Quality Goods Shop	King of Power Saving	I paid my electric bill today, it's a few dozen dollars less, anyway, it's not costly, it's already very good, the boss said that it would be better to buy one more, I've already placed an order	Jingdong prefecture level city in Shandong
Dragon Quality Goods Shop	King of Power Saving	I'm on a smart meter, I save about 30% a month, personally I think it's pretty good, it works well	Jingdong prefecture level city in Shandong
Dragon Quality Goods Shop	King of Power Saving	My dad asked me to buy one, but after I paid the bill, I asked and he said it was a lot less this month, so he asked me to buy another one.	Jingdong prefecture level city in Shandong
Dragon Quality Goods Shop	King of Power Saving	Very satisfied with the purchase thanks to the store for the prosperous business.	Jingdong prefecture level city in Shandong
Dragon Quality Goods Shop	King of Power Saving	Good use, good quality, very satisfied with the purchase	Jingdong prefecture level city in Shandong
Dragon Quality Goods Shop	King of Power Saving	The goods have been received, the quality is very good, is the ideal effect, will continue to pay attention to the	Jingdong prefecture level city in Shandong
Dragon Quality Goods Shop	King of Power Saving	This brand has been used for many years, and this time I saw this upgraded version of the product and got it again. The purpose of this product is to reduce the loss of electricity for a long time, so as to achieve the purpose of saving electricity.	Jingdong prefecture level city in Shandong
Dragon Quality Goods Shop	King of Power Saving	I've been using it for almost a month, and I've reduced my electricity bill by over 100 bucks! I'm planning to get two more for my family.	Jingdong prefecture level city in Shandong

Dragon Quality Goods Shop	King of Power Saving	The effect is too good, the family has now several units, back to buy a number of times, the power saver received, power-saving effect is very good,, fine workmanship, long warranty, but also purify the air, before buying a used for a period of time, with the okay, and then bought a, and only now come to the	Jingdong prefecture level city in Shandong
Dragon Quality Goods Shop	King of Power Saving	Finally received the goods, very, very much like,, has been a lot of people praise, really too happy, has been his family's loyal fans, will continue to buy buy buy buy!	Jingdong prefecture level city in Shandong
Dragon Quality Goods Shop	King of Power Saving	📩 I've been wanting it for a long time, I decided to go for it, I feel great after using it, it's genuine and the delivery is great, it lives up to its name👍	Jingdong prefecture level city in Shandong
Dragon Quality Goods Shop	King of Power Saving	The quality is sure as other friends who bought it said, and it is also beautiful, thanks to the owner for the recommendation, thanks!	Jingdong prefecture level city in Shandong
Dragon Quality Goods Shop	King of Power Saving	Baby received the quality is really good face value is also very high especially practical especially especially special favorite	Jingdong prefecture level city in Shandong
Dragon Quality Goods Shop	King of Power Saving	It is genuine, trust the merchant, the quality is very good, the price is moderate, the use of the effect is very good!	Jingdong prefecture level city in Shandong
Dragon Quality Goods Shop	King of Power Saving	Energy saver received really good quality affordable and good energy saving and environmental protection is the best to use.	Jingdong prefecture level city in Shandong
Dragon Quality Goods Shop	King of Power Saving	Reasonable price logistics is very fast, the feeling is not bad more satisfied with a shopping experience	Jingdong prefecture level city in Shandong
Dragon Quality Goods Shop	King of Power Saving	The stuff is even more perfect than I imagined, very powerful and worth getting!	Jingdong prefecture level city in Shandong
Dragon Quality Goods Shop	King of Power Saving	Goods have been received, good quality, affordable price, good customer service, worth recommending!	Jingdong prefecture level city in Shandong
Dragon Quality Goods Shop	King of Power Saving	Fast shipping, works great, have already recommended it to friends.	Jingdong prefecture level city in Shandong
Dragon Quality Goods Shop	King of Power Saving	Good quality, fast logistics, reasonable price, worth 5 stars.	Jingdong prefecture level city in Shandong
Dragon Quality Goods Shop	King of Power Saving	Good quality, fast logistics, good value, well packaged, practical	Jingdong prefecture level city in Shandong
Dragon Quality Goods Shop	King of Power Saving	Fast shipping, affordable price, great quality, very satisfied!	Jingdong prefecture level city in Shandong
Dragon Quality Goods Shop	King of Power Saving	Regret knowing too late, it's really good stuff. Works well.	Jingdong prefecture level city in Shandong
Dragon Quality Goods Shop	King of Power Saving	Not bad, very good product, quite save electricity	Jingdong prefecture level city in Shandong
Dragon Quality Goods Shop	King of Power Saving	Good quality, fast logistics, overall very satisfied!	Jingdong prefecture level city in Shandong
Dragon Quality Goods Shop	King of Power Saving	Tightly packaged with good results	Jingdong prefecture level city in Shandong
Dragon Quality Goods Shop	King of Power Saving	very happy	Jingdong prefecture level city in Shandong
Dragon Quality Goods Shop	King of Power Saving	favorable criticism	Jingdong prefecture level city in Shandong
Dragon Quality Goods Shop	King of Power Saving	Now they all sign for the goods without receiving them, the Jingdong platform and merchants are very powerful, and the goods are not received before signing for the goods.	Jingdong prefecture level city in Shandong
Dragon Quality Goods Shop	King of Power Saving	low-quality commodities	Jingdong prefecture level city in Shandong
Dragon Quality Goods Shop	King of Power Saving	I bought this and now it works pretty well with the air conditioning on, when I didn't use it it was $600 a month, now it's almost $500,00, that's over $100 less all of a sudden.	Jingdong prefecture level city in Shandong
Dragon Quality Goods Shop	King of Power Saving	Can Oh, the weather is hot, open the air conditioning is really good, this product is good, has been used for a really long time, more ok, power saving effect is not bad.	Jingdong prefecture level city in Shandong
Dragon Quality Goods Shop	King of Power Saving	I paid my electric bill today, it's a few dozen dollars less, anyway, it's not costly, it's already very good, the boss said that it would be better to buy one more, I've already placed an order	Jingdong prefecture level city in Shandong
Dragon Quality Goods Shop	King of Power Saving	I'm on a smart meter, I save about 30% a month, personally I think it's pretty good, it works well	Jingdong prefecture level city in Shandong
Dragon Quality Goods Shop	King of Power Saving	My dad asked me to buy one, but after I paid the bill, I asked and he said it was a lot less this month, so he asked me to buy another one.	Jingdong prefecture level city in Shandong
Dragon Quality Goods Shop	King of Power Saving	Very satisfied with the purchase thanks to the store for the prosperous business.	Jingdong prefecture level city in Shandong
Dragon Quality Goods Shop	King of Power Saving	Good use, good quality, very satisfied with the purchase	Jingdong prefecture level city in Shandong
Dragon Quality Goods Shop	King of Power Saving	The goods have been received, the quality is very good, is the ideal effect, will continue to pay attention to the	Jingdong prefecture level city in Shandong
Dragon Quality Goods Shop	King of Power Saving	This brand has been used for many years, and this time I saw this upgraded version of the product and got it again. The purpose of this product is to reduce the loss of electricity for a long time, so as to achieve the purpose of saving electricity.	Jingdong prefecture level city in Shandong
Dragon Quality Goods Shop	King of Power Saving	I've been using it for almost a month, and I've reduced my electricity bill by over 100 bucks! I'm planning to get two more for my family.	Jingdong prefecture level city in Shandong
Dragon Quality Goods Shop	King of Power Saving	The effect is too good, the family has now several units, back to buy a number of times, the power saver received, power-saving effect is very good,, fine workmanship, long warranty, but also purify the air, before buying a used for a period of time, with the okay, and then bought a, and only now come to the	Jingdong prefecture level city in Shandong
Dragon Quality Goods Shop	King of Power Saving	Finally received the goods, very, very much like,, has been a lot of people praise, really too happy, has been his family's loyal fans, will continue to buy buy buy buy!	Jingdong prefecture level city in Shandong
Dragon Quality Goods Shop	King of Power Saving	📩 I've been wanting it for a long time, I decided to go for it, I feel great after using it, it's genuine and the delivery is great, it lives up to its name👍	Jingdong prefecture level city in Shandong
Dragon Quality Goods Shop	King of Power Saving	The quality is sure as other friends who bought it said, and it is also beautiful, thanks to the owner for the recommendation, thanks!	Jingdong prefecture level city in Shandong
Dragon Quality Goods Shop	King of Power Saving	Baby received the quality is really good face value is also very high especially practical especially especially special favorite	Jingdong prefecture level city in Shandong
Dragon Quality Goods Shop	King of Power Saving	It is genuine, trust the merchant, the quality is very good, the price is moderate, the use of the effect is very good!	Jingdong prefecture level city in Shandong
Dragon Quality Goods Shop	King of Power Saving	Energy saver received really good quality affordable and good energy saving and environmental protection is the best to use.	Jingdong prefecture level city in Shandong
Dragon Quality Goods Shop	King of Power Saving	Reasonable price logistics is very fast, the feeling is not bad more satisfied with a shopping experience	Jingdong prefecture level city in Shandong
Dragon Quality Goods Shop	King of Power Saving	The stuff is even more perfect than I imagined, very powerful and worth getting!	Jingdong prefecture level city in Shandong
Dragon Quality Goods Shop	King of Power Saving	Goods have been received, good quality, affordable price, good customer service, worth recommending!	Jingdong prefecture level city in Shandong
Dragon Quality Goods Shop	King of Power Saving	Fast shipping, works great, have already recommended it to friends.	Jingdong prefecture level city in Shandong
Dragon Quality Goods Shop	King of Power Saving	Good quality, fast logistics, reasonable price, worth 5 stars.	Jingdong prefecture level city in Shandong
Dragon Quality Goods Shop	King of Power Saving	Good quality, fast logistics, good value, well packaged, practical	Jingdong prefecture level city in Shandong
Dragon Quality Goods Shop	King of Power Saving	Fast shipping, affordable price, great quality, very satisfied!	Jingdong prefecture level city in Shandong
Dragon Quality Goods Shop	King of Power Saving	Regret knowing too late, it's really good stuff. Works well.	Jingdong prefecture level city in Shandong
Dragon Quality Goods Shop	King of Power Saving	Not bad, very good product, quite save electricity	Jingdong prefecture level city in Shandong
Dragon Quality Goods Shop	King of Power Saving	Good quality, fast logistics, overall very satisfied!	Jingdong prefecture level city in Shandong
Dragon Quality Goods Shop	King of Power Saving	Tightly packaged with good results	Jingdong prefecture level city in Shandong

Dragon Quality Goods Shop	King of Power Saving	very happy	Jingdong prefecture level city in Shandong
Dragon Quality Goods Shop	King of Power Saving	favorable criticism	Jingdong prefecture level city in Shandong
Dragon Quality Goods Shop	King of Power Saving	Now they all sign for the goods without receiving them, the Jingdong platform and merchants are very powerful, and the goods are not received before signing for the goods.	Jingdong prefecture level city in Shandong
Dragon Quality Goods Shop	King of Power Saving	low-quality commodities	Jingdong prefecture level city in Shandong
Dragon Quality Goods Shop	King of Power Saving	I bought this and now it works pretty well with the air conditioning on, when I didn't use it it was $600 a month, now it's almost $500,00, that's over $100 less all of a sudden.	Jingdong prefecture level city in Shandong
Dragon Quality Goods Shop	King of Power Saving	Can Oh, the weather is hot, open the air conditioning is really good, this product is good, has been used for a really long time, more ok, power saving effect is not bad.	Jingdong prefecture level city in Shandong
Dragon Quality Goods Shop	King of Power Saving	I paid my electric bill today, it's a few dozen dollars less, anyway, it's not costly, it's already very good, the boss said that it would be better to buy one more, I've already placed an order	Jingdong prefecture level city in Shandong
Dragon Quality Goods Shop	King of Power Saving	I'm on a smart meter, I save about 30% a month, personally I think it's pretty good, it works well	Jingdong prefecture level city in Shandong
Dragon Quality Goods Shop	King of Power Saving	My dad asked me to buy one, but after I paid the bill, I asked and he said it was a lot less this month, so he asked me to buy another one.	Jingdong prefecture level city in Shandong
Dragon Quality Goods Shop	King of Power Saving	Very satisfied with the purchase thanks to the store for the prosperous business.	Jingdong prefecture level city in Shandong
Dragon Quality Goods Shop	King of Power Saving	Good use, good quality, very satisfied with the purchase	Jingdong prefecture level city in Shandong
Dragon Quality Goods Shop	King of Power Saving	The goods have been received, the quality is very good, is the ideal effect, will continue to pay attention to the	Jingdong prefecture level city in Shandong
Dragon Quality Goods Shop	King of Power Saving	This brand has been used for many years, and this time I saw this upgraded version of the product and got it again. The purpose of this product is to reduce the loss of electricity for a long time, so as to achieve the purpose of saving electricity.	Jingdong prefecture level city in Shandong
Dragon Quality Goods Shop	King of Power Saving	I've been using it for almost a month, and I've reduced my electricity bill by over 100 bucks! I'm planning to get two more for my family.	Jingdong prefecture level city in Shandong
Dragon Quality Goods Shop	King of Power Saving	The effect is too good, the family has now several units, back to buy a number of times, the power saver received, power-saving effect is very good,, fine workmanship, long warranty, but also purify the air, before buying a used for a period of time, with the okay, and then bought a, and only now come to the	Jingdong prefecture level city in Shandong
Dragon Quality Goods Shop	King of Power Saving	Finally received the goods, very, very much like,, has been a lot of people praise, really too happy, has been his family's loyal fans, will continue to buy buy buy!	Jingdong prefecture level city in Shandong
Dragon Quality Goods Shop	King of Power Saving	📩 I've been wanting it for a long time, I decided to go for it, I feel great after using it, it's genuine and the delivery is great, it lives up to its name👍	Jingdong prefecture level city in Shandong
Dragon Quality Goods Shop	King of Power Saving	The quality is sure as other friends who bought it said, and it is also beautiful, thanks to the owner for the recommendation, thanks!	Jingdong prefecture level city in Shandong
Dragon Quality Goods Shop	King of Power Saving	Baby received the quality is really good face value is also very high especially practical especially especially special favorite	Jingdong prefecture level city in Shandong
Dragon Quality Goods Shop	King of Power Saving	It is genuine, trust the merchant, the quality is very good, the price is moderate, the use of the effect is very good!	Jingdong prefecture level city in Shandong
Dragon Quality Goods Shop	King of Power Saving	Energy saver received really good quality affordable and good energy saving and environmental protection is the best to use.	Jingdong prefecture level city in Shandong
Dragon Quality Goods Shop	King of Power Saving	Reasonable price logistics is very fast, the feeling is not bad more satisfied with a shopping experience	Jingdong prefecture level city in Shandong
Dragon Quality Goods Shop	King of Power Saving	The stuff is even more perfect than I imagined, very powerful and worth getting!	Jingdong prefecture level city in Shandong
Dragon Quality Goods Shop	King of Power Saving	Goods have been received, good quality, affordable price, good customer service, worth recommending!	Jingdong prefecture level city in Shandong
Dragon Quality Goods Shop	King of Power Saving	Fast shipping, works great, have already recommended it to friends.	Jingdong prefecture level city in Shandong
Dragon Quality Goods Shop	King of Power Saving	Good quality, fast logistics, reasonable price, worth 5 stars.	Jingdong prefecture level city in Shandong
Dragon Quality Goods Shop	King of Power Saving	Good quality, fast logistics, good value, well packaged, practical	Jingdong prefecture level city in Shandong
Dragon Quality Goods Shop	King of Power Saving	Fast shipping, affordable price, great quality, very satisfied!	Jingdong prefecture level city in Shandong
Dragon Quality Goods Shop	King of Power Saving	Regret knowing too late, it's really good stuff. Works well.	Jingdong prefecture level city in Shandong
Dragon Quality Goods Shop	King of Power Saving	Not bad, very good product, quite save electricity	Jingdong prefecture level city in Shandong
Dragon Quality Goods Shop	King of Power Saving	Good quality, fast logistics, overall very satisfied!	Jingdong prefecture level city in Shandong
Dragon Quality Goods Shop	King of Power Saving	Tightly packaged with good results	Jingdong prefecture level city in Shandong
Dragon Quality Goods Shop	King of Power Saving	very happy	Jingdong prefecture level city in Shandong
Dragon Quality Goods Shop	King of Power Saving	favorable criticism	Jingdong prefecture level city in Shandong
Dragon Quality Goods Shop	King of Power Saving	Now they all sign for the goods without receiving them, the Jingdong platform and merchants are very powerful, and the goods are not received before signing for the goods.	Jingdong prefecture level city in Shandong
Dragon Quality Goods Shop	King of Power Saving	low-quality commodities	Jingdong prefecture level city in Shandong
Dragon Quality Goods Shop	King of Power Saving	I bought this and now it works pretty well with the air conditioning on, when I didn't use it it was $600 a month, now it's almost $500,00, that's over $100 less all of a sudden.	Jingdong prefecture level city in Shandong
Dragon Quality Goods Shop	King of Power Saving	Can Oh, the weather is hot, open the air conditioning is really good, this product is good, has been used for a really long time, more ok, power saving effect is not bad.	Jingdong prefecture level city in Shandong
Dragon Quality Goods Shop	King of Power Saving	I paid my electric bill today, it's a few dozen dollars less, anyway, it's not costly, it's already very good, the boss said that it would be better to buy one more, I've already placed an order	Jingdong prefecture level city in Shandong
Dragon Quality Goods Shop	King of Power Saving	I'm on a smart meter, I save about 30% a month, personally I think it's pretty good, it works well	Jingdong prefecture level city in Shandong
Dragon Quality Goods Shop	King of Power Saving	My dad asked me to buy one, but after I paid the bill, I asked and he said it was a lot less this month, so he asked me to buy another one.	Jingdong prefecture level city in Shandong
Dragon Quality Goods Shop	King of Power Saving	Very satisfied with the purchase thanks to the store for the prosperous business.	Jingdong prefecture level city in Shandong
Dragon Quality Goods Shop	King of Power Saving	Good use, good quality, very satisfied with the purchase	Jingdong prefecture level city in Shandong
Dragon Quality Goods Shop	King of Power Saving	The goods have been received, the quality is very good, is the ideal effect, will continue to pay attention to the	Jingdong prefecture level city in Shandong
Dragon Quality Goods Shop	King of Power Saving	This brand has been used for many years, and this time I saw this upgraded version of the product and got it again. The purpose of this product is to reduce the loss of electricity for a long time, so as to achieve the purpose of saving electricity.	Jingdong prefecture level city in Shandong
Dragon Quality Goods Shop	King of Power Saving	I've been using it for almost a month, and I've reduced my electricity bill by over 100 bucks! I'm planning to get two more for my family.	Jingdong prefecture level city in Shandong
Dragon Quality Goods Shop	King of Power Saving	The effect is too good, the family has now several units, back to buy a number of times, the power saver received, power-saving effect is very good,, fine workmanship, long warranty, but also purify the air, before buying a used for a period of time, with the okay, and then bought a, and only now come to the	Jingdong prefecture level city in Shandong
Dragon Quality Goods Shop	King of Power Saving	Finally received the goods, very, very much like,, has been a lot of people praise, really too happy, has been his family's loyal fans, will continue to buy buy buy!	Jingdong prefecture level city in Shandong
Dragon Quality Goods Shop	King of Power Saving	📩 I've been wanting it for a long time, I decided to go for it, I feel great after using it, it's genuine and the delivery is great, it lives up to its name👍	Jingdong prefecture level city in Shandong

Dragon Quality Goods Shop	King of Power Saving	The quality is sure as other friends who bought it said, and it is also beautiful, thanks to the owner for the recommendation, thanks!	Jingdong prefecture level city in Shandong
Dragon Quality Goods Shop	King of Power Saving	Baby received the quality is really good face value is also very high especially practical especially especially special favorite	Jingdong prefecture level city in Shandong
Dragon Quality Goods Shop	King of Power Saving	It is genuine, trust the merchant, the quality is very good, the price is moderate, the use of the effect is very good!	Jingdong prefecture level city in Shandong
Dragon Quality Goods Shop	King of Power Saving	Energy saver received really good quality affordable and good energy saving and environmental protection is the best to use.	Jingdong prefecture level city in Shandong
Dragon Quality Goods Shop	King of Power Saving	Reasonable price logistics is very fast, the feeling is not bad more satisfied with a shopping experience	Jingdong prefecture level city in Shandong
Dragon Quality Goods Shop	King of Power Saving	The stuff is even more perfect than I imagined, very powerful and worth getting!	Jingdong prefecture level city in Shandong
Dragon Quality Goods Shop	King of Power Saving	Goods have been received, good quality, affordable price, good customer service, worth recommending!	Jingdong prefecture level city in Shandong
Dragon Quality Goods Shop	King of Power Saving	Fast shipping, works great, have already recommended it to friends.	Jingdong prefecture level city in Shandong
Dragon Quality Goods Shop	King of Power Saving	Good quality, fast logistics, reasonable price, worth 5 stars.	Jingdong prefecture level city in Shandong
Dragon Quality Goods Shop	King of Power Saving	Good quality, fast logistics, good value, well packaged, practical	Jingdong prefecture level city in Shandong
Dragon Quality Goods Shop	King of Power Saving	Fast shipping, affordable price, great quality, very satisfied!	Jingdong prefecture level city in Shandong
Dragon Quality Goods Shop	King of Power Saving	Regret knowing too late, it's really good stuff. Works well.	Jingdong prefecture level city in Shandong
Dragon Quality Goods Shop	King of Power Saving	Not bad, very good product, quite save electricity	Jingdong prefecture level city in Shandong
Dragon Quality Goods Shop	King of Power Saving	Good quality, fast logistics, overall very satisfied!	Jingdong prefecture level city in Shandong
Dragon Quality Goods Shop	King of Power Saving	Tightly packaged with good results	Jingdong prefecture level city in Shandong
Dragon Quality Goods Shop	King of Power Saving	very happy	Jingdong prefecture level city in Shandong
Dragon Quality Goods Shop	King of Power Saving	favorable criticism	Jingdong prefecture level city in Shandong
Dragon Quality Goods Shop	King of Power Saving	Now they all sign for the goods without receiving them, the Jingdong platform and merchants are very powerful, and the goods are not received before signing for the goods.	Jingdong prefecture level city in Shandong
Dragon Quality Goods Shop	King of Power Saving	low-quality commodities	Jingdong prefecture level city in Shandong
Dragon Quality Goods Shop	King of Power Saving	I bought this and now it works pretty well with the air conditioning on, when I didn't use it it was $600 a month, now it's almost $500.00, that's over $100 less all of a sudden.	Jingdong prefecture level city in Shandong
Dragon Quality Goods Shop	King of Power Saving	Can Oh, the weather is hot, open the air conditioning is really good, this product is good, has been used for a really long time, more ok, power saving effect is not bad.	Jingdong prefecture level city in Shandong
Dragon Quality Goods Shop	King of Power Saving	I paid my electric bill today, it's a few dozen dollars less, anyway, it's not costly, it's already very good, the boss said that it would be better to buy one more, I've already placed an order	Jingdong prefecture level city in Shandong
Dragon Quality Goods Shop	King of Power Saving	I'm on a smart meter, I save about 30% a month, personally I think it's pretty good, it works well	Jingdong prefecture level city in Shandong
Dragon Quality Goods Shop	King of Power Saving	My dad asked me to buy one, but after I paid the bill, I asked and he said it was a lot less this month, so he asked me to buy another one.	Jingdong prefecture level city in Shandong
Dragon Quality Goods Shop	King of Power Saving	Very satisfied with the purchase thanks to the store for the prosperous business.	Jingdong prefecture level city in Shandong
Dragon Quality Goods Shop	King of Power Saving	Good use, good quality, very satisfied with the purchase	Jingdong prefecture level city in Shandong
Dragon Quality Goods Shop	King of Power Saving	The goods have been received, the quality is very good, is the ideal effect, will continue to pay attention to the	Jingdong prefecture level city in Shandong
Dragon Quality Goods Shop	King of Power Saving	This brand has been used for many years, and this time I saw this upgraded version of the product and got it again. The purpose of this product is to reduce the loss of electricity for a long time, so as to achieve the purpose of saving electricity.	Jingdong prefecture level city in Shandong
Dragon Quality Goods Shop	King of Power Saving	I've been using it for almost a month, and I've reduced my electricity bill by over 100 bucks! I'm planning to get two more for my family.	Jingdong prefecture level city in Shandong
Dragon Quality Goods Shop	King of Power Saving	The effect is too good, the family has now several units, back to buy a number of times, the power saver received, power-saving effect is very good,, fine workmanship, long warranty, but also purify the air, before buying a used for a period of time, with the okay, and then bought a, and only now come to the	Jingdong prefecture level city in Shandong
Dragon Quality Goods Shop	King of Power Saving	Finally received the goods, very, very much like,, has been a lot of people praise, really too happy, has been his family's loyal fans, will continue to buy buy buy buy!	Jingdong prefecture level city in Shandong
Dragon Quality Goods Shop	King of Power Saving	📩 I've been wanting it for a long time, I decided to go for it, I feel great after using it, it's genuine and the delivery is great, it lives up to its name👍	Jingdong prefecture level city in Shandong
Dragon Quality Goods Shop	King of Power Saving	The quality is sure as other friends who bought it said, and it is also beautiful, thanks to the owner for the recommendation, thanks!	Jingdong prefecture level city in Shandong
Dragon Quality Goods Shop	King of Power Saving	Baby received the quality is really good face value is also very high especially practical especially especially special favorite	Jingdong prefecture level city in Shandong
Dragon Quality Goods Shop	King of Power Saving	It is genuine, trust the merchant, the quality is very good, the price is moderate, the use of the effect is very good!	Jingdong prefecture level city in Shandong
Dragon Quality Goods Shop	King of Power Saving	Energy saver received really good quality affordable and good energy saving and environmental protection is the best to use.	Jingdong prefecture level city in Shandong
Dragon Quality Goods Shop	King of Power Saving	Reasonable price logistics is very fast, the feeling is not bad more satisfied with a shopping experience	Jingdong prefecture level city in Shandong
Dragon Quality Goods Shop	King of Power Saving	The stuff is even more perfect than I imagined, very powerful and worth getting!	Jingdong prefecture level city in Shandong
Dragon Quality Goods Shop	King of Power Saving	Goods have been received, good quality, affordable price, good customer service, worth recommending!	Jingdong prefecture level city in Shandong
Dragon Quality Goods Shop	King of Power Saving	Fast shipping, works great, have already recommended it to friends.	Jingdong prefecture level city in Shandong
Dragon Quality Goods Shop	King of Power Saving	Good quality, fast logistics, reasonable price, worth 5 stars.	Jingdong prefecture level city in Shandong
Dragon Quality Goods Shop	King of Power Saving	Good quality, fast logistics, good value, well packaged, practical	Jingdong prefecture level city in Shandong
Dragon Quality Goods Shop	King of Power Saving	Fast shipping, affordable price, great quality, very satisfied!	Jingdong prefecture level city in Shandong
Dragon Quality Goods Shop	King of Power Saving	Regret knowing too late, it's really good stuff. Works well.	Jingdong prefecture level city in Shandong
Dragon Quality Goods Shop	King of Power Saving	Not bad, very good product, quite save electricity	Jingdong prefecture level city in Shandong
Dragon Quality Goods Shop	King of Power Saving	Good quality, fast logistics, overall very satisfied!	Jingdong prefecture level city in Shandong
Dragon Quality Goods Shop	King of Power Saving	Tightly packaged with good results	Jingdong prefecture level city in Shandong
Dragon Quality Goods Shop	King of Power Saving	very happy	Jingdong prefecture level city in Shandong
Dragon Quality Goods Shop	King of Power Saving	favorable criticism	Jingdong prefecture level city in Shandong
Dragon Quality Goods Shop	King of Power Saving	Now they all sign for the goods without receiving them, the Jingdong platform and merchants are very powerful, and the goods are not received before signing for the goods.	Jingdong prefecture level city in Shandong

Dragon Quality Goods Shop	King of Power Saving	low-quality commodities	Jingdong prefecture level city in Shandong
Dragon Quality Goods Shop	King of Power Saving	I bought this and now it works pretty well with the air conditioning on, when I didn't use it it was $600 a month, now it's almost $500,00, that's over $100 less all of a sudden.	Jingdong prefecture level city in Shandong
Dragon Quality Goods Shop	King of Power Saving	Can Oh, the weather is hot, open the air conditioning is really good, this product is good, has been used for a really long time, more ok, power saving effect is not bad.	Jingdong prefecture level city in Shandong
Dragon Quality Goods Shop	King of Power Saving	I paid my electric bill today, it's a few dozen dollars less, anyway, it's not costly, it's already very good, the boss said that it would be better to buy one more, I've already placed an order	Jingdong prefecture level city in Shandong
Dragon Quality Goods Shop	King of Power Saving	I'm on a smart meter, I save about 30% a month, personally I think it's pretty good, it works well	Jingdong prefecture level city in Shandong
Dragon Quality Goods Shop	King of Power Saving	My dad asked me to buy one, but after I paid the bill, I asked and he said it was a lot less this month, so he asked me to buy another one.	Jingdong prefecture level city in Shandong
Dragon Quality Goods Shop	King of Power Saving	Very satisfied with the purchase thanks to the store for the prosperous business.	Jingdong prefecture level city in Shandong
Dragon Quality Goods Shop	King of Power Saving	Good use, good quality, very satisfied with the purchase	Jingdong prefecture level city in Shandong
Dragon Quality Goods Shop	King of Power Saving	The goods have been received, the quality is very good, is the ideal effect, will continue to pay attention to the	Jingdong prefecture level city in Shandong
Dragon Quality Goods Shop	King of Power Saving	This brand has been used for many years, and this time I saw this upgraded version of the product and got it again. The purpose of this product is to reduce the loss of electricity for a long time, so as to achieve the purpose of saving electricity.	Jingdong prefecture level city in Shandong
Dragon Quality Goods Shop	King of Power Saving	I've been using it for almost a month, and I've reduced my electricity bill by over 100 bucks! I'm planning to get two more for my family.	Jingdong prefecture level city in Shandong
Dragon Quality Goods Shop	King of Power Saving	The effect is too good, the family has now several units, back to buy a number of times, the power saver received, power-saving effect is very good,, fine workmanship, long warranty, but also purify the air, before buying a used for a period of time, with the okay, and then bought a, and only now come to the	Jingdong prefecture level city in Shandong
Dragon Quality Goods Shop	King of Power Saving	Finally received the goods, very, very much like,, has been a lot of people praise, really too happy, has been his family's loyal fans, will continue to buy buy buy buy!	Jingdong prefecture level city in Shandong
Dragon Quality Goods Shop	King of Power Saving	📩 I've been wanting it for a long time, I decided to go for it, I feel great after using it, it's genuine and the delivery is great, it lives up to its name👍	Jingdong prefecture level city in Shandong
Dragon Quality Goods Shop	King of Power Saving	The quality is sure as other friends who bought it said, and it is also beautiful, thanks to the owner for the recommendation, thanks!	Jingdong prefecture level city in Shandong
Dragon Quality Goods Shop	King of Power Saving	Baby received the quality is really good face value is also very high especially practical especially especially special favorite	Jingdong prefecture level city in Shandong
Dragon Quality Goods Shop	King of Power Saving	It is genuine, trust the merchant, the quality is very good, the price is moderate, the use of the effect is very good!	Jingdong prefecture level city in Shandong
Dragon Quality Goods Shop	King of Power Saving	Energy saver received really good quality affordable and good energy saving and environmental protection is the best to use.	Jingdong prefecture level city in Shandong
Dragon Quality Goods Shop	King of Power Saving	Reasonable price logistics is very fast, the feeling is not bad more satisfied with a shopping experience	Jingdong prefecture level city in Shandong
Dragon Quality Goods Shop	King of Power Saving	The stuff is even more perfect than I imagined, very powerful and worth getting!	Jingdong prefecture level city in Shandong
Dragon Quality Goods Shop	King of Power Saving	Goods have been received, good quality, affordable price, good customer service, worth recommending!	Jingdong prefecture level city in Shandong
Dragon Quality Goods Shop	King of Power Saving	Fast shipping, works great, have already recommended it to friends.	Jingdong prefecture level city in Shandong
Dragon Quality Goods Shop	King of Power Saving	Good quality, fast logistics, reasonable price, worth 5 stars.	Jingdong prefecture level city in Shandong
Dragon Quality Goods Shop	King of Power Saving	Good quality, fast logistics, good value, well packaged, practical	Jingdong prefecture level city in Shandong
Dragon Quality Goods Shop	King of Power Saving	Fast shipping, affordable price, great quality, very satisfied!	Jingdong prefecture level city in Shandong
Dragon Quality Goods Shop	King of Power Saving	Regret knowing too late, it's really good stuff. Works well.	Jingdong prefecture level city in Shandong
Dragon Quality Goods Shop	King of Power Saving	Not bad, very good product, quite save electricity	Jingdong prefecture level city in Shandong
Dragon Quality Goods Shop	King of Power Saving	Good quality, fast logistics, overall very satisfied!	Jingdong prefecture level city in Shandong
Dragon Quality Goods Shop	King of Power Saving	Tightly packaged with good results	Jingdong prefecture level city in Shandong
Dragon Quality Goods Shop	King of Power Saving	very happy	Jingdong prefecture level city in Shandong
Dragon Quality Goods Shop	King of Power Saving	favorable criticism	Jingdong prefecture level city in Shandong
Dragon Quality Goods Shop	King of Power Saving	Now they all sign for the goods without receiving them, the Jingdong platform and merchants are very powerful, and the goods are not received before signing for the goods.	Jingdong prefecture level city in Shandong
Dragon Quality Goods Shop	King of Power Saving	low-quality commodities	Jingdong prefecture level city in Shandong
Dragon Quality Goods Shop	King of Power Saving	I bought this and now it works pretty well with the air conditioning on, when I didn't use it it was $600 a month, now it's almost $500,00, that's over $100 less all of a sudden.	Jingdong prefecture level city in Shandong
Dragon Quality Goods Shop	King of Power Saving	Can Oh, the weather is hot, open the air conditioning is really good, this product is good, has been used for a really long time, more ok, power saving effect is not bad.	Jingdong prefecture level city in Shandong
Dragon Quality Goods Shop	King of Power Saving	I paid my electric bill today, it's a few dozen dollars less, anyway, it's not costly, it's already very good, the boss said that it would be better to buy one more, I've already placed an order	Jingdong prefecture level city in Shandong
Dragon Quality Goods Shop	King of Power Saving	I'm on a smart meter, I save about 30% a month, personally I think it's pretty good, it works well	Jingdong prefecture level city in Shandong
Dragon Quality Goods Shop	King of Power Saving	My dad asked me to buy one, but after I paid the bill, I asked and he said it was a lot less this month, so he asked me to buy another one.	Jingdong prefecture level city in Shandong
Dragon Quality Goods Shop	King of Power Saving	Very satisfied with the purchase thanks to the store for the prosperous business.	Jingdong prefecture level city in Shandong
Dragon Quality Goods Shop	King of Power Saving	Good use, good quality, very satisfied with the purchase	Jingdong prefecture level city in Shandong
Dragon Quality Goods Shop	King of Power Saving	The goods have been received, the quality is very good, is the ideal effect, will continue to pay attention to the	Jingdong prefecture level city in Shandong
Dragon Quality Goods Shop	King of Power Saving	This brand has been used for many years, and this time I saw this upgraded version of the product and got it again. The purpose of this product is to reduce the loss of electricity for a long time, so as to achieve the purpose of saving electricity.	Jingdong prefecture level city in Shandong
Dragon Quality Goods Shop	King of Power Saving	I've been using it for almost a month, and I've reduced my electricity bill by over 100 bucks! I'm planning to get two more for my family.	Jingdong prefecture level city in Shandong
Dragon Quality Goods Shop	King of Power Saving	The effect is too good, the family has now several units, back to buy a number of times, the power saver received, power-saving effect is very good,, fine workmanship, long warranty, but also purify the air, before buying a used for a period of time, with the okay, and then bought a, and only now come to the	Jingdong prefecture level city in Shandong
Dragon Quality Goods Shop	King of Power Saving	Finally received the goods, very, very much like,, has been a lot of people praise, really too happy, has been his family's loyal fans, will continue to buy buy buy buy!	Jingdong prefecture level city in Shandong
Dragon Quality Goods Shop	King of Power Saving	📩 I've been wanting it for a long time, I decided to go for it, I feel great after using it, it's genuine and the delivery is great, it lives up to its name👍	Jingdong prefecture level city in Shandong
Dragon Quality Goods Shop	King of Power Saving	The quality is sure as other friends who bought it said, and it is also beautiful, thanks to the owner for the recommendation, thanks!	Jingdong prefecture level city in Shandong
Dragon Quality Goods Shop	King of Power Saving	Baby received the quality is really good face value is also very high especially practical especially especially special favorite	Jingdong prefecture level city in Shandong
Dragon Quality Goods Shop	King of Power Saving	It is genuine, trust the merchant, the quality is very good, the price is moderate, the use of the effect is very good!	Jingdong prefecture level city in Shandong

Dragon Quality Goods Shop	King of Power Saving	Energy saver received really good quality affordable and good energy saving and environmental protection is the best to use.	Jingdong prefecture level city in Shandong
Dragon Quality Goods Shop	King of Power Saving	Reasonable price logistics is very fast, the feeling is not bad more satisfied with a shopping experience	Jingdong prefecture level city in Shandong
Dragon Quality Goods Shop	King of Power Saving	The stuff is even more perfect than I imagined, very powerful and worth getting!	Jingdong prefecture level city in Shandong
Dragon Quality Goods Shop	King of Power Saving	Goods have been received, good quality, affordable price, good customer service, worth recommending!	Jingdong prefecture level city in Shandong
Dragon Quality Goods Shop	King of Power Saving	Fast shipping, works great, have already recommended it to friends.	Jingdong prefecture level city in Shandong
Dragon Quality Goods Shop	King of Power Saving	Good quality, fast logistics, reasonable price, worth 5 stars.	Jingdong prefecture level city in Shandong
Dragon Quality Goods Shop	King of Power Saving	Good quality, fast logistics, good value, well packaged, practical	Jingdong prefecture level city in Shandong
Dragon Quality Goods Shop	King of Power Saving	Fast shipping, affordable price, great quality, very satisfied!	Jingdong prefecture level city in Shandong
Dragon Quality Goods Shop	King of Power Saving	Regret knowing too late, it's really good stuff. Works well.	Jingdong prefecture level city in Shandong
Dragon Quality Goods Shop	King of Power Saving	Not bad, very good product, quite save electricity	Jingdong prefecture level city in Shandong
Dragon Quality Goods Shop	King of Power Saving	Good quality, fast logistics, overall very satisfied!	Jingdong prefecture level city in Shandong
Dragon Quality Goods Shop	King of Power Saving	Tightly packaged with good results	Jingdong prefecture level city in Shandong
Dragon Quality Goods Shop	King of Power Saving	very happy	Jingdong prefecture level city in Shandong
Dragon Quality Goods Shop	King of Power Saving	favorable criticism	Jingdong prefecture level city in Shandong
Dragon Quality Goods Shop	King of Power Saving	Now they all sign for the goods without receiving them, the Jingdong platform and merchants are very powerful, and the goods are not received before signing for the goods.	Jingdong prefecture level city in Shandong
Dragon Quality Goods Shop	King of Power Saving	low-quality commodities	Jingdong prefecture level city in Shandong
Dragon Quality Goods Shop	King of Power Saving	I bought this and now it works pretty well with the air conditioning on, when I didn't use it it was $600 a month, now it's almost $500,00, that's over $100 less all of a sudden.	Jingdong prefecture level city in Shandong
Dragon Quality Goods Shop	King of Power Saving	Can Oh, the weather is hot, open the air conditioning is really good, this product is good, has been used for a really long time, more ok, power saving effect is not bad.	Jingdong prefecture level city in Shandong
Dragon Quality Goods Shop	King of Power Saving	I paid my electric bill today, it's a few dozen dollars less, anyway, it's not costly, it's already very good, the boss said that it would be better to buy one more, I've already placed an order	Jingdong prefecture level city in Shandong
Dragon Quality Goods Shop	King of Power Saving	I'm on a smart meter, I save about 30% a month, personally I think it's pretty good, it works well	Jingdong prefecture level city in Shandong
Dragon Quality Goods Shop	King of Power Saving	My dad asked me to buy one, but after I paid the bill, I asked and he said it was a lot less this month, so he asked me to buy another one.	Jingdong prefecture level city in Shandong
Dragon Quality Goods Shop	King of Power Saving	Very satisfied with the purchase thanks to the store for the prosperous business.	Jingdong prefecture level city in Shandong
Dragon Quality Goods Shop	King of Power Saving	Good use, good quality, very satisfied with the purchase	Jingdong prefecture level city in Shandong
Dragon Quality Goods Shop	King of Power Saving	The goods have been received, the quality is very good, is the ideal effect, will continue to pay attention to the	Jingdong prefecture level city in Shandong
Dragon Quality Goods Shop	King of Power Saving	This brand has been used for many years, and this time I saw this upgraded version of the product and got it again. The purpose of this product is to reduce the loss of electricity for a long time, so as to achieve the purpose of saving electricity.	Jingdong prefecture level city in Shandong
Dragon Quality Goods Shop	King of Power Saving	I've been using it for almost a month, and I've reduced my electricity bill by over 100 bucks! I'm planning to get two more for my family.	Jingdong prefecture level city in Shandong
Dragon Quality Goods Shop	King of Power Saving	The effect is too good, the family has now several units, back to buy a number of times, the power saver received, power-saving effect is very good,, fine workmanship, long warranty, but also purify the air, before buying a used for a period of time, with the okay, and then bought a, and only now come to the	Jingdong prefecture level city in Shandong
Dragon Quality Goods Shop	King of Power Saving	Finally received the goods, very, very much like,, has been a lot of people praise, really too happy, has been his family's loyal fans, will continue to buy buy buy buy!	Jingdong prefecture level city in Shandong
Dragon Quality Goods Shop	King of Power Saving	💌 I've been wanting it for a long time, I decided to go for it, I feel great after using it, it's genuine and the delivery is great, it lives up to its name👍	Jingdong prefecture level city in Shandong
Dragon Quality Goods Shop	King of Power Saving	The quality is sure as other friends who bought it said, and it is also beautiful, thanks to the owner for the recommendation, thanks!	Jingdong prefecture level city in Shandong
Dragon Quality Goods Shop	King of Power Saving	Baby received the quality is really good face value is also very high especially practical especially especially special favorite	Jingdong prefecture level city in Shandong
Dragon Quality Goods Shop	King of Power Saving	It is genuine, trust the merchant, the quality is very good, the price is moderate, the use of the effect is very good!	Jingdong prefecture level city in Shandong
Dragon Quality Goods Shop	King of Power Saving	Energy saver received really good quality affordable and good energy saving and environmental protection is the best to use.	Jingdong prefecture level city in Shandong
Dragon Quality Goods Shop	King of Power Saving	Reasonable price logistics is very fast, the feeling is not bad more satisfied with a shopping experience	Jingdong prefecture level city in Shandong
Dragon Quality Goods Shop	King of Power Saving	The stuff is even more perfect than I imagined, very powerful and worth getting!	Jingdong prefecture level city in Shandong
Dragon Quality Goods Shop	King of Power Saving	Goods have been received, good quality, affordable price, good customer service, worth recommending!	Jingdong prefecture level city in Shandong
Dragon Quality Goods Shop	King of Power Saving	Fast shipping, works great, have already recommended it to friends.	Jingdong prefecture level city in Shandong
Dragon Quality Goods Shop	King of Power Saving	Good quality, fast logistics, reasonable price, worth 5 stars.	Jingdong prefecture level city in Shandong
Dragon Quality Goods Shop	King of Power Saving	Good quality, fast logistics, good value, well packaged, practical	Jingdong prefecture level city in Shandong
Dragon Quality Goods Shop	King of Power Saving	Fast shipping, affordable price, great quality, very satisfied!	Jingdong prefecture level city in Shandong
Dragon Quality Goods Shop	King of Power Saving	Regret knowing too late, it's really good stuff. Works well.	Jingdong prefecture level city in Shandong
Dragon Quality Goods Shop	King of Power Saving	Not bad, very good product, quite save electricity	Jingdong prefecture level city in Shandong
Dragon Quality Goods Shop	King of Power Saving	Good quality, fast logistics, overall very satisfied!	Jingdong prefecture level city in Shandong
Dragon Quality Goods Shop	King of Power Saving	Tightly packaged with good results	Jingdong prefecture level city in Shandong
Dragon Quality Goods Shop	King of Power Saving	very happy	Jingdong prefecture level city in Shandong
Dragon Quality Goods Shop	King of Power Saving	favorable criticism	Jingdong prefecture level city in Shandong
Dragon Quality Goods Shop	King of Power Saving	Now they all sign for the goods without receiving them, the Jingdong platform and merchants are very powerful, and the goods are not received before signing for the goods.	Jingdong prefecture level city in Shandong
Dragon Quality Goods Shop	King of Power Saving	low-quality commodities	Jingdong prefecture level city in Shandong
Dragon Quality Goods Shop	King of Power Saving	I bought this and now it works pretty well with the air conditioning on, when I didn't use it it was $600 a month, now it's almost $500,00, that's over $100 less all of a sudden.	Jingdong prefecture level city in Shandong
Dragon Quality Goods Shop	King of Power Saving	Can Oh, the weather is hot, open the air conditioning is really good, this product is good, has been used for a really long time, more ok, power saving effect is not bad.	Jingdong prefecture level city in Shandong

Dragon Quality Goods Shop	King of Power Saving	I paid my electric bill today, it's a few dozen dollars less, anyway, it's not costly, it's already very good, the boss said that it would be better to buy one more, I've already placed an order	Jingdong prefecture level city in Shandong
Dragon Quality Goods Shop	King of Power Saving	I'm on a smart meter, I save about 30% a month, personally I think it's pretty good, it works well	Jingdong prefecture level city in Shandong
Dragon Quality Goods Shop	King of Power Saving	My dad asked me to buy one, but after I paid the bill, I asked and he said it was a lot less this month, so he asked me to buy another one.	Jingdong prefecture level city in Shandong
Dragon Quality Goods Shop	King of Power Saving	Very satisfied with the purchase thanks to the store for the prosperous business.	Jingdong prefecture level city in Shandong
Dragon Quality Goods Shop	King of Power Saving	Good use, good quality, very satisfied with the purchase	Jingdong prefecture level city in Shandong
Dragon Quality Goods Shop	King of Power Saving	The goods have been received, the quality is very good, is the ideal effect, will continue to pay attention to the	Jingdong prefecture level city in Shandong
Dragon Quality Goods Shop	King of Power Saving	This brand has been used for many years, and this time I saw this upgraded version of the product and got it again. The purpose of this product is to reduce the loss of electricity for a long time, so as to achieve the purpose of saving electricity.	Jingdong prefecture level city in Shandong
Dragon Quality Goods Shop	King of Power Saving	I've been using it for almost a month, and I've reduced my electricity bill by over 100 bucks! I'm planning to get two more for my family.	Jingdong prefecture level city in Shandong
Dragon Quality Goods Shop	King of Power Saving	The effect is too good, the family has now several units, back to buy a number of times, the power saver received, power-saving effect is very good,, fine workmanship, long warranty, but also purify the air, before buying a used for a period of time, with the okay, and then bought a, and only now come to the	Jingdong prefecture level city in Shandong
Dragon Quality Goods Shop	King of Power Saving	Finally received the goods, very, very much like,, has been a lot of people praise, really too happy, has been his family's loyal fans, will continue to buy buy buy buy!	Jingdong prefecture level city in Shandong
Dragon Quality Goods Shop	King of Power Saving	I've been wanting it for a long time, I decided to go for it, I feel great after using it, it's genuine and the delivery is great, it lives up to its name	Jingdong prefecture level city in Shandong
Dragon Quality Goods Shop	King of Power Saving	The quality is sure as other friends who bought it said, and it is also beautiful, thanks to the owner for the recommendation, thanks!	Jingdong prefecture level city in Shandong
Dragon Quality Goods Shop	King of Power Saving	Baby received the quality is really good face value is also very high especially practical especially especially special favorite	Jingdong prefecture level city in Shandong
Dragon Quality Goods Shop	King of Power Saving	It is genuine, trust the merchant, the quality is very good, the price is moderate, the use of the effect is very good!	Jingdong prefecture level city in Shandong
Dragon Quality Goods Shop	King of Power Saving	Energy saver received really good quality affordable and good energy saving and environmental protection is the best to use.	Jingdong prefecture level city in Shandong
Dragon Quality Goods Shop	King of Power Saving	Reasonable price logistics is very fast, the feeling is not bad more satisfied with a shopping experience	Jingdong prefecture level city in Shandong
Dragon Quality Goods Shop	King of Power Saving	The stuff is even more perfect than I imagined, very powerful and worth getting!	Jingdong prefecture level city in Shandong
Dragon Quality Goods Shop	King of Power Saving	Goods have been received, good quality, affordable price, good customer service, worth recommending!	Jingdong prefecture level city in Shandong
Dragon Quality Goods Shop	King of Power Saving	Fast shipping, works great, have already recommended it to friends.	Jingdong prefecture level city in Shandong
Dragon Quality Goods Shop	King of Power Saving	Good quality, fast logistics, reasonable price, worth 5 stars.	Jingdong prefecture level city in Shandong
Dragon Quality Goods Shop	King of Power Saving	Good quality, fast logistics, good value, well packaged, practical	Jingdong prefecture level city in Shandong
Dragon Quality Goods Shop	King of Power Saving	Fast shipping, affordable price, great quality, very satisfied!	Jingdong prefecture level city in Shandong
Dragon Quality Goods Shop	King of Power Saving	Regret knowing too late, it's really good stuff. Works well.	Jingdong prefecture level city in Shandong
Dragon Quality Goods Shop	King of Power Saving	Not bad, very good product, quite save electricity	Jingdong prefecture level city in Shandong
Dragon Quality Goods Shop	King of Power Saving	Good quality, fast logistics, overall very satisfied!	Jingdong prefecture level city in Shandong
Dragon Quality Goods Shop	King of Power Saving	Tightly packaged with good results	Jingdong prefecture level city in Shandong
Dragon Quality Goods Shop	King of Power Saving	very happy	Jingdong prefecture level city in Shandong
Dragon Quality Goods Shop	King of Power Saving	favorable criticism	Jingdong prefecture level city in Shandong
Dragon Quality Goods Shop	King of Power Saving	Now they all sign for the goods without receiving them, the Jingdong platform and merchants are very powerful, and the goods are not received before signing for the goods.	Jingdong prefecture level city in Shandong
Dragon Quality Goods Shop	King of Power Saving	low-quality commodities	Jingdong prefecture level city in Shandong
Dragon Quality Goods Shop	King of Power Saving	I bought this and now it works pretty well with the air conditioning on, when I didn't use it it was $600 a month, now it's almost $500,00, that's over $100 less all of a sudden.	Jingdong prefecture level city in Shandong
Dragon Quality Goods Shop	King of Power Saving	Can Oh, the weather is hot, open the air conditioning is really good, this product is good, has been used for a really long time, more ok, power saving effect is not bad.	Jingdong prefecture level city in Shandong
Dragon Quality Goods Shop	King of Power Saving	I paid my electric bill today, it's a few dozen dollars less, anyway, it's not costly, it's already very good, the boss said that it would be better to buy one more, I've already placed an order	Jingdong prefecture level city in Shandong
Dragon Quality Goods Shop	King of Power Saving	I'm on a smart meter, I save about 30% a month, personally I think it's pretty good, it works well	Jingdong prefecture level city in Shandong
Dragon Quality Goods Shop	King of Power Saving	My dad asked me to buy one, but after I paid the bill, I asked and he said it was a lot less this month, so he asked me to buy another one.	Jingdong prefecture level city in Shandong
Dragon Quality Goods Shop	King of Power Saving	Very satisfied with the purchase thanks to the store for the prosperous business.	Jingdong prefecture level city in Shandong
Dragon Quality Goods Shop	King of Power Saving	Good use, good quality, very satisfied with the purchase	Jingdong prefecture level city in Shandong
Dragon Quality Goods Shop	King of Power Saving	The goods have been received, the quality is very good, is the ideal effect, will continue to pay attention to the	Jingdong prefecture level city in Shandong
Dragon Quality Goods Shop	King of Power Saving	This brand has been used for many years, and this time I saw this upgraded version of the product and got it again. The purpose of this product is to reduce the loss of electricity for a long time, so as to achieve the purpose of saving electricity.	Jingdong prefecture level city in Shandong
Dragon Quality Goods Shop	King of Power Saving	I've been using it for almost a month, and I've reduced my electricity bill by over 100 bucks! I'm planning to get two more for my family.	Jingdong prefecture level city in Shandong
Dragon Quality Goods Shop	King of Power Saving	The effect is too good, the family has now several units, back to buy a number of times, the power saver received, power-saving effect is very good,, fine workmanship, long warranty, but also purify the air, before buying a used for a period of time, with the okay, and then bought a, and only now come to the	Jingdong prefecture level city in Shandong
Dragon Quality Goods Shop	King of Power Saving	Finally received the goods, very, very much like,, has been a lot of people praise, really too happy, has been his family's loyal fans, will continue to buy buy buy buy!	Jingdong prefecture level city in Shandong
Dragon Quality Goods Shop	King of Power Saving	I've been wanting it for a long time, I decided to go for it, I feel great after using it, it's genuine and the delivery is great, it lives up to its name	Jingdong prefecture level city in Shandong
Dragon Quality Goods Shop	King of Power Saving	The quality is sure as other friends who bought it said, and it is also beautiful, thanks to the owner for the recommendation, thanks!	Jingdong prefecture level city in Shandong
Dragon Quality Goods Shop	King of Power Saving	Baby received the quality is really good face value is also very high especially practical especially especially special favorite	Jingdong prefecture level city in Shandong
Dragon Quality Goods Shop	King of Power Saving	It is genuine, trust the merchant, the quality is very good, the price is moderate, the use of the effect is very good!	Jingdong prefecture level city in Shandong
Dragon Quality Goods Shop	King of Power Saving	Energy saver received really good quality affordable and good energy saving and environmental protection is the best to use.	Jingdong prefecture level city in Shandong
Dragon Quality Goods Shop	King of Power Saving	Reasonable price logistics is very fast, the feeling is not bad more satisfied with a shopping experience	Jingdong prefecture level city in Shandong
Dragon Quality Goods Shop	King of Power Saving	The stuff is even more perfect than I imagined, very powerful and worth getting!	Jingdong prefecture level city in Shandong

Dragon Quality Goods Shop	King of Power Saving	Goods have been received, good quality, affordable price, good customer service, worth recommending!	Jingdong prefecture level city in Shandong
Dragon Quality Goods Shop	King of Power Saving	Fast shipping, works great, have already recommended it to friends.	Jingdong prefecture level city in Shandong
Dragon Quality Goods Shop	King of Power Saving	Good quality, fast logistics, reasonable price, worth 5 stars.	Jingdong prefecture level city in Shandong
Dragon Quality Goods Shop	King of Power Saving	Good quality, fast logistics, good value, well packaged, practical	Jingdong prefecture level city in Shandong
Dragon Quality Goods Shop	King of Power Saving	Fast shipping, affordable price, great quality, very satisfied!	Jingdong prefecture level city in Shandong
Dragon Quality Goods Shop	King of Power Saving	Regret knowing too late, it's really good stuff. Works well.	Jingdong prefecture level city in Shandong
Dragon Quality Goods Shop	King of Power Saving	Not bad, very good product, quite save electricity	Jingdong prefecture level city in Shandong
Dragon Quality Goods Shop	King of Power Saving	Good quality, fast logistics, overall very satisfied!	Jingdong prefecture level city in Shandong
Dragon Quality Goods Shop	King of Power Saving	Tightly packaged with good results	Jingdong prefecture level city in Shandong
Dragon Quality Goods Shop	King of Power Saving	very happy	Jingdong prefecture level city in Shandong
Dragon Quality Goods Shop	King of Power Saving	favorable criticism	Jingdong prefecture level city in Shandong
Dragon Quality Goods Shop	King of Power Saving	Now they all sign for the goods without receiving them, the Jingdong platform and merchants are very powerful, and the goods are not received before signing for the goods.	Jingdong prefecture level city in Shandong
Dragon Quality Goods Shop	King of Power Saving	low-quality commodities	Jingdong prefecture level city in Shandong
Dragon Quality Goods Shop	King of Power Saving	I bought this and now it works pretty well with the air conditioning on, when I didn't use it it was $600 a month, now it's almost $500.00, that's over $100 less all of a sudden.	Jingdong prefecture level city in Shandong
Dragon Quality Goods Shop	King of Power Saving	Can Oh, the weather is hot, open the air conditioning is really good, this product is good, has been used for a really long time, more ok, power saving effect is not bad.	Jingdong prefecture level city in Shandong
Dragon Quality Goods Shop	King of Power Saving	I paid my electric bill today, it's a few dozen dollars less, anyway, it's not costly, it's already very good, the boss said that it would be better to buy one more, I've already placed an order	Jingdong prefecture level city in Shandong
Dragon Quality Goods Shop	King of Power Saving	I'm on a smart meter, I save about 30% a month, personally I think it's pretty good, it works well	Jingdong prefecture level city in Shandong
Dragon Quality Goods Shop	King of Power Saving	My dad asked me to buy one, but after I paid the bill, I asked and he said it was a lot less this month, so he asked me to buy another one.	Jingdong prefecture level city in Shandong
Dragon Quality Goods Shop	King of Power Saving	Very satisfied with the purchase thanks to the store for the prosperous business.	Jingdong prefecture level city in Shandong
Dragon Quality Goods Shop	King of Power Saving	Good use, good quality, very satisfied with the purchase	Jingdong prefecture level city in Shandong
Dragon Quality Goods Shop	King of Power Saving	The goods have been received, the quality is very good, is the ideal effect, will continue to pay attention to the	Jingdong prefecture level city in Shandong
Dragon Quality Goods Shop	King of Power Saving	This brand has been used for many years, and this time I saw this upgraded version of the product and got it again. The purpose of this product is to reduce the loss of electricity for a long time, so as to achieve the purpose of saving electricity.	Jingdong prefecture level city in Shandong
Dragon Quality Goods Shop	King of Power Saving	I've been using it for almost a month, and I've reduced my electricity bill by over 100 bucks! I'm planning to get two more for my family.	Jingdong prefecture level city in Shandong
Dragon Quality Goods Shop	King of Power Saving	The effect is too good, the family has now several units, back to buy a number of times, the power saver received, power-saving effect is very good,, fine workmanship, long warranty, but also purify the air, before buying a used for a period of time, with the okay, and then bought a, and only now come to the	Jingdong prefecture level city in Shandong
Dragon Quality Goods Shop	King of Power Saving	Finally received the goods, very, very much like,, has been a lot of people praise, really too happy, has been his family's loyal fans, will continue to buy buy buy buy!	Jingdong prefecture level city in Shandong
Dragon Quality Goods Shop	King of Power Saving	I've been wanting it for a long time, I decided to go for it, I feel great after using it, it's genuine and the delivery is great, it lives up to its name	Jingdong prefecture level city in Shandong
Dragon Quality Goods Shop	King of Power Saving	The quality is sure as other friends who bought it said, and it is also beautiful, thanks to the owner for the recommendation, thanks!	Jingdong prefecture level city in Shandong
Dragon Quality Goods Shop	King of Power Saving	Baby received the quality is really good face value is also very high especially practical especially especially special favorite	Jingdong prefecture level city in Shandong
Dragon Quality Goods Shop	King of Power Saving	It is genuine, trust the merchant, the quality is very good, the price is moderate, the use of the effect is very good!	Jingdong prefecture level city in Shandong
Dragon Quality Goods Shop	King of Power Saving	Energy saver received really good quality affordable and good energy saving and environmental protection is the best to use.	Jingdong prefecture level city in Shandong
Dragon Quality Goods Shop	King of Power Saving	Reasonable price logistics is very fast, the feeling is not bad more satisfied with a shopping experience	Jingdong prefecture level city in Shandong
Dragon Quality Goods Shop	King of Power Saving	The stuff is even more perfect than I imagined, very powerful and worth getting!	Jingdong prefecture level city in Shandong
Dragon Quality Goods Shop	King of Power Saving	Goods have been received, good quality, affordable price, good customer service, worth recommending!	Jingdong prefecture level city in Shandong
Dragon Quality Goods Shop	King of Power Saving	Fast shipping, works great, have already recommended it to friends.	Jingdong prefecture level city in Shandong
Dragon Quality Goods Shop	King of Power Saving	Good quality, fast logistics, reasonable price, worth 5 stars.	Jingdong prefecture level city in Shandong
Dragon Quality Goods Shop	King of Power Saving	Good quality, fast logistics, good value, well packaged, practical	Jingdong prefecture level city in Shandong
Dragon Quality Goods Shop	King of Power Saving	Fast shipping, affordable price, great quality, very satisfied!	Jingdong prefecture level city in Shandong
Dragon Quality Goods Shop	King of Power Saving	Regret knowing too late, it's really good stuff. Works well.	Jingdong prefecture level city in Shandong
Dragon Quality Goods Shop	King of Power Saving	Not bad, very good product, quite save electricity	Jingdong prefecture level city in Shandong
Dragon Quality Goods Shop	King of Power Saving	Good quality, fast logistics, overall very satisfied!	Jingdong prefecture level city in Shandong
Dragon Quality Goods Shop	King of Power Saving	Tightly packaged with good results	Jingdong prefecture level city in Shandong
Dragon Quality Goods Shop	King of Power Saving	very happy	Jingdong prefecture level city in Shandong
Dragon Quality Goods Shop	King of Power Saving	favorable criticism	Jingdong prefecture level city in Shandong
Dragon Quality Goods Shop	King of Power Saving	Now they all sign for the goods without receiving them, the Jingdong platform and merchants are very powerful, and the goods are not received before signing for the goods.	Jingdong prefecture level city in Shandong
Dragon Quality Goods Shop	King of Power Saving	low-quality commodities	Jingdong prefecture level city in Shandong
Dragon Quality Goods Shop	King of Power Saving	I bought this and now it works pretty well with the air conditioning on, when I didn't use it it was $600 a month, now it's almost $500.00, that's over $100 less all of a sudden.	Jingdong prefecture level city in Shandong
Dragon Quality Goods Shop	King of Power Saving	Can Oh, the weather is hot, open the air conditioning is really good, this product is good, has been used for a really long time, more ok, power saving effect is not bad.	Jingdong prefecture level city in Shandong
Dragon Quality Goods Shop	King of Power Saving	I paid my electric bill today, it's a few dozen dollars less, anyway, it's not costly, it's already very good, the boss said that it would be better to buy one more, I've already placed an order	Jingdong prefecture level city in Shandong
Dragon Quality Goods Shop	King of Power Saving	I'm on a smart meter, I save about 30% a month, personally I think it's pretty good, it works well	Jingdong prefecture level city in Shandong
Dragon Quality Goods Shop	King of Power Saving	My dad asked me to buy one, but after I paid the bill, I asked and he said it was a lot less this month, so he asked me to buy another one.	Jingdong prefecture level city in Shandong

Dragon Quality Goods Shop	King of Power Saving	Very satisfied with the purchase thanks to the store for the prosperous business.	Jingdong prefecture level city in Shandong
Dragon Quality Goods Shop	King of Power Saving	Good use, good quality, very satisfied with the purchase	Jingdong prefecture level city in Shandong
Dragon Quality Goods Shop	King of Power Saving	The goods have been received, the quality is very good, is the ideal effect, will continue to pay attention to the	Jingdong prefecture level city in Shandong
Dragon Quality Goods Shop	King of Power Saving	This brand has been used for many years, and this time I saw this upgraded version of the product and got it again. The purpose of this product is to reduce the loss of electricity for a long time, so as to achieve the purpose of saving electricity.	Jingdong prefecture level city in Shandong
Dragon Quality Goods Shop	King of Power Saving	I've been using it for almost a month, and I've reduced my electricity bill by over 100 bucks! I'm planning to get two more for my family.	Jingdong prefecture level city in Shandong
Dragon Quality Goods Shop	King of Power Saving	The effect is too good, the family has now several units, back to buy a number of times, the power saver received, power-saving effect is very good,, fine workmanship, long warranty, but also purify the air, before buying a used for a period of time, with the okay, and then bought a, and only now come to the	Jingdong prefecture level city in Shandong
Dragon Quality Goods Shop	King of Power Saving	Finally received the goods, very, very much like,, has been a lot of people praise, really too happy, has been his family's loyal fans, will continue to buy buy buy buy!	Jingdong prefecture level city in Shandong
Dragon Quality Goods Shop	King of Power Saving	📩 I've been wanting it for a long time, I decided to go for it, I feel great after using it, it's genuine and the delivery is great, it lives up to its name👍	Jingdong prefecture level city in Shandong
Dragon Quality Goods Shop	King of Power Saving	The quality is sure as other friends who bought it said, and it is also beautiful, thanks to the owner for the recommendation, thanks!	Jingdong prefecture level city in Shandong
Dragon Quality Goods Shop	King of Power Saving	Baby received the quality is really good face value is also very high especially practical especially especially special favorite	Jingdong prefecture level city in Shandong
Dragon Quality Goods Shop	King of Power Saving	It is genuine, trust the merchant, the quality is very good, the price is moderate, the use of the effect is very good!	Jingdong prefecture level city in Shandong
Dragon Quality Goods Shop	King of Power Saving	Energy saver received really good quality affordable and good energy saving and environmental protection is the best to use.	Jingdong prefecture level city in Shandong
Dragon Quality Goods Shop	King of Power Saving	Reasonable price logistics is very fast, the feeling is not bad more satisfied with a shopping experience	Jingdong prefecture level city in Shandong
Dragon Quality Goods Shop	King of Power Saving	The stuff is even more perfect than I imagined, very powerful and worth getting!	Jingdong prefecture level city in Shandong
Dragon Quality Goods Shop	King of Power Saving	Goods have been received, good quality, affordable price, good customer service, worth recommending!	Jingdong prefecture level city in Shandong
Dragon Quality Goods Shop	King of Power Saving	Fast shipping, works great, have already recommended it to friends.	Jingdong prefecture level city in Shandong
Dragon Quality Goods Shop	King of Power Saving	Good quality, fast logistics, reasonable price, worth 5 stars.	Jingdong prefecture level city in Shandong
Dragon Quality Goods Shop	King of Power Saving	Good quality, fast logistics, good value, well packaged, practical	Jingdong prefecture level city in Shandong
Dragon Quality Goods Shop	King of Power Saving	Fast shipping, affordable price, great quality, very satisfied!	Jingdong prefecture level city in Shandong
Dragon Quality Goods Shop	King of Power Saving	Regret knowing too late, it's really good stuff. Works well.	Jingdong prefecture level city in Shandong
Dragon Quality Goods Shop	King of Power Saving	Not bad, very good product, quite save electricity	Jingdong prefecture level city in Shandong
Dragon Quality Goods Shop	King of Power Saving	Good quality, fast logistics, overall very satisfied!	Jingdong prefecture level city in Shandong
Dragon Quality Goods Shop	King of Power Saving	Tightly packaged with good results	Jingdong prefecture level city in Shandong
Dragon Quality Goods Shop	King of Power Saving	very happy	Jingdong prefecture level city in Shandong
Dragon Quality Goods Shop	King of Power Saving	favorable criticism	Jingdong prefecture level city in Shandong
Dragon Quality Goods Shop	King of Power Saving	Now they all sign for the goods without receiving them, the Jingdong platform and merchants are very powerful, and the goods are not received before signing for the goods.	Jingdong prefecture level city in Shandong
Dragon Quality Goods Shop	King of Power Saving	low-quality commodities	Jingdong prefecture level city in Shandong
Dragon Quality Goods Shop	King of Power Saving	I bought this and now it works pretty well with the air conditioning on, when I didn't use it it was $600 a month, now it's almost $500.00, that's over $100 less all of a sudden.	Jingdong prefecture level city in Shandong
Dragon Quality Goods Shop	King of Power Saving	Can Oh, the weather is hot, open the air conditioning is really good, this product is good, has been used for a really long time, more ok, power saving effect is not bad.	Jingdong prefecture level city in Shandong
Dragon Quality Goods Shop	King of Power Saving	I paid my electric bill today, it's a few dozen dollars less, anyway, it's not costly, it's already very good, the boss said that it would be better to buy one more, I've already placed an order	Jingdong prefecture level city in Shandong
Dragon Quality Goods Shop	King of Power Saving	I'm on a smart meter, I save about 30% a month, personally I think it's pretty good, it works well	Jingdong prefecture level city in Shandong
Dragon Quality Goods Shop	King of Power Saving	My dad asked me to buy one, but after I paid the bill, I asked and he said it was a lot less this month, so he asked me to buy another one.	Jingdong prefecture level city in Shandong
Dragon Quality Goods Shop	King of Power Saving	Very satisfied with the purchase thanks to the store for the prosperous business.	Jingdong prefecture level city in Shandong
Dragon Quality Goods Shop	King of Power Saving	Good use, good quality, very satisfied with the purchase	Jingdong prefecture level city in Shandong
Dragon Quality Goods Shop	King of Power Saving	The goods have been received, the quality is very good, is the ideal effect, will continue to pay attention to the	Jingdong prefecture level city in Shandong
Dragon Quality Goods Shop	King of Power Saving	This brand has been used for many years, and this time I saw this upgraded version of the product and got it again. The purpose of this product is to reduce the loss of electricity for a long time, so as to achieve the purpose of saving electricity.	Jingdong prefecture level city in Shandong
Dragon Quality Goods Shop	King of Power Saving	I've been using it for almost a month, and I've reduced my electricity bill by over 100 bucks! I'm planning to get two more for my family.	Jingdong prefecture level city in Shandong
Dragon Quality Goods Shop	King of Power Saving	The effect is too good, the family has now several units, back to buy a number of times, the power saver received, power-saving effect is very good,, fine workmanship, long warranty, but also purify the air, before buying a used for a period of time, with the okay, and then bought a, and only now come to the	Jingdong prefecture level city in Shandong
Dragon Quality Goods Shop	King of Power Saving	Finally received the goods, very, very much like,, has been a lot of people praise, really too happy, has been his family's loyal fans, will continue to buy buy buy buy!	Jingdong prefecture level city in Shandong
Dragon Quality Goods Shop	King of Power Saving	📩 I've been wanting it for a long time, I decided to go for it, I feel great after using it, it's genuine and the delivery is great, it lives up to its name👍	Jingdong prefecture level city in Shandong
Dragon Quality Goods Shop	King of Power Saving	The quality is sure as other friends who bought it said, and it is also beautiful, thanks to the owner for the recommendation, thanks!	Jingdong prefecture level city in Shandong
Dragon Quality Goods Shop	King of Power Saving	Baby received the quality is really good face value is also very high especially practical especially especially special favorite	Jingdong prefecture level city in Shandong
Dragon Quality Goods Shop	King of Power Saving	It is genuine, trust the merchant, the quality is very good, the price is moderate, the use of the effect is very good!	Jingdong prefecture level city in Shandong
Dragon Quality Goods Shop	King of Power Saving	Energy saver received really good quality affordable and good energy saving and environmental protection is the best to use.	Jingdong prefecture level city in Shandong
Dragon Quality Goods Shop	King of Power Saving	Reasonable price logistics is very fast, the feeling is not bad more satisfied with a shopping experience	Jingdong prefecture level city in Shandong
Dragon Quality Goods Shop	King of Power Saving	The stuff is even more perfect than I imagined, very powerful and worth getting!	Jingdong prefecture level city in Shandong
Dragon Quality Goods Shop	King of Power Saving	Goods have been received, good quality, affordable price, good customer service, worth recommending!	Jingdong prefecture level city in Shandong
Dragon Quality Goods Shop	King of Power Saving	Fast shipping, works great, have already recommended it to friends.	Jingdong prefecture level city in Shandong
Dragon Quality Goods Shop	King of Power Saving	Good quality, fast logistics, reasonable price, worth 5 stars.	Jingdong prefecture level city in Shandong

Dragon Quality Goods Shop	King of Power Saving	Good quality, fast logistics, good value, well packaged, practical	Jingdong prefecture level city in Shandong
Dragon Quality Goods Shop	King of Power Saving	Fast shipping, affordable price, great quality, very satisfied!	Jingdong prefecture level city in Shandong
Dragon Quality Goods Shop	King of Power Saving	Regret knowing too late, it's really good stuff. Works well.	Jingdong prefecture level city in Shandong
Dragon Quality Goods Shop	King of Power Saving	Not bad, very good product, quite save electricity	Jingdong prefecture level city in Shandong
Dragon Quality Goods Shop	King of Power Saving	Good quality, fast logistics, overall very satisfied!	Jingdong prefecture level city in Shandong
Dragon Quality Goods Shop	King of Power Saving	Tightly packaged with good results	Jingdong prefecture level city in Shandong
Dragon Quality Goods Shop	King of Power Saving	very happy	Jingdong prefecture level city in Shandong
Dragon Quality Goods Shop	King of Power Saving	favorable criticism	Jingdong prefecture level city in Shandong
Dragon Quality Goods Shop	King of Power Saving	Now they all sign for the goods without receiving them, the Jingdong platform and merchants are very powerful, and the goods are not received before signing for the goods.	Jingdong prefecture level city in Shandong
Dragon Quality Goods Shop	King of Power Saving	low-quality commodities	Jingdong prefecture level city in Shandong
Dragon Quality Goods Shop	King of Power Saving	I bought this and now it works pretty well with the air conditioning on, when I didn't use it it was $600 a month, now it's almost $500.00, that's over $100 less all of a sudden.	Jingdong prefecture level city in Shandong
Dragon Quality Goods Shop	King of Power Saving	Can Oh, the weather is hot, open the air conditioning is really good, this product is good, has been used for a really long time, more ok, power saving effect is not bad.	Jingdong prefecture level city in Shandong
Dragon Quality Goods Shop	King of Power Saving	I paid my electric bill today, it's a few dozen dollars less, anyway, it's not costly, it's already very good, the boss said that it would be better to buy one more, I've already placed an order	Jingdong prefecture level city in Shandong
Dragon Quality Goods Shop	King of Power Saving	I'm on a smart meter, I save about 30% a month, personally I think it's pretty good, it works well	Jingdong prefecture level city in Shandong
Dragon Quality Goods Shop	King of Power Saving	My dad asked me to buy one, but after I paid the bill, I asked and he said it was a lot less this month, so he asked me to buy another one.	Jingdong prefecture level city in Shandong
Dragon Quality Goods Shop	King of Power Saving	Very satisfied with the purchase thanks to the store for the prosperous business.	Jingdong prefecture level city in Shandong
Dragon Quality Goods Shop	King of Power Saving	Good use, good quality, very satisfied with the purchase	Jingdong prefecture level city in Shandong
Dragon Quality Goods Shop	King of Power Saving	The goods have been received, the quality is very good, is the ideal effect, will continue to pay attention to the	Jingdong prefecture level city in Shandong
Dragon Quality Goods Shop	King of Power Saving	This brand has been used for many years, and this time I saw this upgraded version of the product and got it again. The purpose of this product is to reduce the loss of electricity for a long time, so as to achieve the purpose of saving electricity.	Jingdong prefecture level city in Shandong
Dragon Quality Goods Shop	King of Power Saving	I've been using it for almost a month, and I've reduced my electricity bill by over 100 bucks! I'm planning to get two more for my family.	Jingdong prefecture level city in Shandong
Dragon Quality Goods Shop	King of Power Saving	The effect is too good, the family has now several units, back to buy a number of times, the power saver received, power-saving effect is very good,, fine workmanship, long warranty, but also purify the air, before buying a used for a period of time, with the okay, and then bought a, and only now come to the	Jingdong prefecture level city in Shandong
Dragon Quality Goods Shop	King of Power Saving	Finally received the goods, very, very much like,, has been a lot of people praise, really too happy, has been his family's loyal fans, will continue to buy buy buy buy!	Jingdong prefecture level city in Shandong
Dragon Quality Goods Shop	King of Power Saving	I've been wanting it for a long time, I decided to go for it, I feel great after using it, it's genuine and the delivery is great, it lives up to its name	Jingdong prefecture level city in Shandong
Dragon Quality Goods Shop	King of Power Saving	The quality is sure as other friends who bought it said, and it is also beautiful, thanks to the owner for the recommendation, thanks!	Jingdong prefecture level city in Shandong
Dragon Quality Goods Shop	King of Power Saving	Baby received the quality is really good face value is also very high especially practical especially especially special favorite	Jingdong prefecture level city in Shandong
Dragon Quality Goods Shop	King of Power Saving	It is genuine, trust the merchant, the quality is very good, the price is moderate, the use of the effect is very good!	Jingdong prefecture level city in Shandong
Dragon Quality Goods Shop	King of Power Saving	Energy saver received really good quality affordable and good energy saving and environmental protection is the best to use.	Jingdong prefecture level city in Shandong
Dragon Quality Goods Shop	King of Power Saving	Reasonable price logistics is very fast, the feeling is not bad more satisfied with a shopping experience	Jingdong prefecture level city in Shandong
Dragon Quality Goods Shop	King of Power Saving	The stuff is even more perfect than I imagined, very powerful and worth getting!	Jingdong prefecture level city in Shandong
Dragon Quality Goods Shop	King of Power Saving	Goods have been received, good quality, affordable price, good customer service, worth recommending!	Jingdong prefecture level city in Shandong
Dragon Quality Goods Shop	King of Power Saving	Fast shipping, works great, have already recommended it to friends.	Jingdong prefecture level city in Shandong
Dragon Quality Goods Shop	King of Power Saving	Good quality, fast logistics, reasonable price, worth 5 stars.	Jingdong prefecture level city in Shandong
Dragon Quality Goods Shop	King of Power Saving	Good quality, fast logistics, good value, well packaged, practical	Jingdong prefecture level city in Shandong
Dragon Quality Goods Shop	King of Power Saving	Fast shipping, affordable price, great quality, very satisfied!	Jingdong prefecture level city in Shandong
Dragon Quality Goods Shop	King of Power Saving	Regret knowing too late, it's really good stuff. Works well.	Jingdong prefecture level city in Shandong
Dragon Quality Goods Shop	King of Power Saving	Not bad, very good product, quite save electricity	Jingdong prefecture level city in Shandong
Dragon Quality Goods Shop	King of Power Saving	Good quality, fast logistics, overall very satisfied!	Jingdong prefecture level city in Shandong
Dragon Quality Goods Shop	King of Power Saving	Tightly packaged with good results	Jingdong prefecture level city in Shandong
Dragon Quality Goods Shop	King of Power Saving	very happy	Jingdong prefecture level city in Shandong
Dragon Quality Goods Shop	King of Power Saving	favorable criticism	Jingdong prefecture level city in Shandong
Dragon Quality Goods Shop	King of Power Saving	Now they all sign for the goods without receiving them, the Jingdong platform and merchants are very powerful, and the goods are not received before signing for the goods.	Jingdong prefecture level city in Shandong
Dragon Quality Goods Shop	King of Power Saving	low-quality commodities	Jingdong prefecture level city in Shandong
Dragon Quality Goods Shop	King of Power Saving	I bought this and now it works pretty well with the air conditioning on, when I didn't use it it was $600 a month, now it's almost $500.00, that's over $100 less all of a sudden.	Jingdong prefecture level city in Shandong
Dragon Quality Goods Shop	King of Power Saving	Can Oh, the weather is hot, open the air conditioning is really good, this product is good, has been used for a really long time, more ok, power saving effect is not bad.	Jingdong prefecture level city in Shandong
Dragon Quality Goods Shop	King of Power Saving	I paid my electric bill today, it's a few dozen dollars less, anyway, it's not costly, it's already very good, the boss said that it would be better to buy one more, I've already placed an order	Jingdong prefecture level city in Shandong
Dragon Quality Goods Shop	King of Power Saving	I'm on a smart meter, I save about 30% a month, personally I think it's pretty good, it works well	Jingdong prefecture level city in Shandong
Dragon Quality Goods Shop	King of Power Saving	My dad asked me to buy one, but after I paid the bill, I asked and he said it was a lot less this month, so he asked me to buy another one.	Jingdong prefecture level city in Shandong
Dragon Quality Goods Shop	King of Power Saving	Very satisfied with the purchase thanks to the store for the prosperous business.	Jingdong prefecture level city in Shandong
Dragon Quality Goods Shop	King of Power Saving	Good use, good quality, very satisfied with the purchase	Jingdong prefecture level city in Shandong
Dragon Quality Goods Shop	King of Power Saving	The goods have been received, the quality is very good, is the ideal effect, will continue to pay attention to the	Jingdong prefecture level city in Shandong

Dragon Quality Goods Shop	King of Power Saving	This brand has been used for many years, and this time I saw this upgraded version of the product and got it again. The purpose of this product is to reduce the loss of electricity for a long time, so as to achieve the purpose of saving electricity.	Jingdong prefecture level city in Shandong
Dragon Quality Goods Shop	King of Power Saving	I've been using it for almost a month, and I've reduced my electricity bill by over 100 bucks! I'm planning to get two more for my family.	Jingdong prefecture level city in Shandong
Dragon Quality Goods Shop	King of Power Saving	The effect is too good, the family has now several units, back to buy a number of times, the power saver received, power-saving effect is very good,, fine workmanship, long warranty, but also purify the air, before buying a used for a period of time, with the okay, and then bought a, and only now come to the	Jingdong prefecture level city in Shandong
Dragon Quality Goods Shop	King of Power Saving	Finally received the goods, very, very much like,, has been a lot of people praise, really too happy, has been his family's loyal fans, will continue to buy buy buy buy!	Jingdong prefecture level city in Shandong
Dragon Quality Goods Shop	King of Power Saving	📩 I've been wanting it for a long time, I decided to go for it, I feel great after using it, it's genuine and the delivery is great, it lives up to its name👍	Jingdong prefecture level city in Shandong
Dragon Quality Goods Shop	King of Power Saving	The quality is sure as other friends who bought it said, and it is also beautiful, thanks to the owner for the recommendation, thanks!	Jingdong prefecture level city in Shandong
Dragon Quality Goods Shop	King of Power Saving	Baby received the quality is really good face value is also very high especially practical especially especially special favorite	Jingdong prefecture level city in Shandong
Dragon Quality Goods Shop	King of Power Saving	It is genuine, trust the merchant, the quality is very good, the price is moderate, the use of the effect is very good!	Jingdong prefecture level city in Shandong
Dragon Quality Goods Shop	King of Power Saving	Energy saver received really good quality affordable and good energy saving and environmental protection is the best to use.	Jingdong prefecture level city in Shandong
Dragon Quality Goods Shop	King of Power Saving	Reasonable price logistics is very fast, the feeling is not bad more satisfied with a shopping experience	Jingdong prefecture level city in Shandong
Dragon Quality Goods Shop	King of Power Saving	The stuff is even more perfect than I imagined, very powerful and worth getting!	Jingdong prefecture level city in Shandong
Dragon Quality Goods Shop	King of Power Saving	Goods have been received, good quality, affordable price, good customer service, worth recommending!	Jingdong prefecture level city in Shandong
Dragon Quality Goods Shop	King of Power Saving	Fast shipping, works great, have already recommended it to friends.	Jingdong prefecture level city in Shandong
Dragon Quality Goods Shop	King of Power Saving	Good quality, fast logistics, reasonable price, worth 5 stars.	Jingdong prefecture level city in Shandong
Dragon Quality Goods Shop	King of Power Saving	Good quality, fast logistics, good value, well packaged, practical	Jingdong prefecture level city in Shandong
Dragon Quality Goods Shop	King of Power Saving	Fast shipping, affordable price, great quality, very satisfied!	Jingdong prefecture level city in Shandong
Dragon Quality Goods Shop	King of Power Saving	Regret knowing too late, it's really good stuff. Works well.	Jingdong prefecture level city in Shandong
Dragon Quality Goods Shop	King of Power Saving	Not bad, very good product, quite save electricity	Jingdong prefecture level city in Shandong
Dragon Quality Goods Shop	King of Power Saving	Good quality, fast logistics, overall very satisfied!	Jingdong prefecture level city in Shandong
Dragon Quality Goods Shop	King of Power Saving	Tightly packaged with good results	Jingdong prefecture level city in Shandong
Dragon Quality Goods Shop	King of Power Saving	very happy	Jingdong prefecture level city in Shandong
Dragon Quality Goods Shop	King of Power Saving	favorable criticism	Jingdong prefecture level city in Shandong
Dragon Quality Goods Shop	King of Power Saving	Now they all sign for the goods without receiving them, the Jingdong platform and merchants are very powerful, and the goods are not received before signing for the goods.	Jingdong prefecture level city in Shandong
Dragon Quality Goods Shop	King of Power Saving	low-quality commodities	Jingdong prefecture level city in Shandong
Dragon Quality Goods Shop	King of Power Saving	I bought this and now it works pretty well with the air conditioning on, when I didn't use it it was $600 a month, now it's almost $500,00, that's over $100 less all of a sudden.	Jingdong prefecture level city in Shandong
Dragon Quality Goods Shop	King of Power Saving	Can Oh, the weather is hot, open the air conditioning is really good, this product is good, has been used for a really long time, more ok, power saving effect is not bad.	Jingdong prefecture level city in Shandong
Dragon Quality Goods Shop	King of Power Saving	I paid my electric bill today, it's a few dozen dollars less, anyway, it's not costly, it's already very good, the boss said that it would be better to buy one more, I've already placed an order	Jingdong prefecture level city in Shandong
Dragon Quality Goods Shop	King of Power Saving	I'm on a smart meter, I save about 30% a month, personally I think it's pretty good, it works well	Jingdong prefecture level city in Shandong
Dragon Quality Goods Shop	King of Power Saving	My dad asked me to buy one, but after I paid the bill, I asked and he said it was a lot less this month, so he asked me to buy another one.	Jingdong prefecture level city in Shandong
Dragon Quality Goods Shop	King of Power Saving	Very satisfied with the purchase thanks to the store for the prosperous business.	Jingdong prefecture level city in Shandong
Dragon Quality Goods Shop	King of Power Saving	Good use, good quality, very satisfied with the purchase	Jingdong prefecture level city in Shandong
Dragon Quality Goods Shop	King of Power Saving	The goods have been received, the quality is very good, is the ideal effect, will continue to pay attention to the	Jingdong prefecture level city in Shandong
Dragon Quality Goods Shop	King of Power Saving	This brand has been used for many years, and this time I saw this upgraded version of the product and got it again. The purpose of this product is to reduce the loss of electricity for a long time, so as to achieve the purpose of saving electricity.	Jingdong prefecture level city in Shandong
Dragon Quality Goods Shop	King of Power Saving	I've been using it for almost a month, and I've reduced my electricity bill by over 100 bucks! I'm planning to get two more for my family.	Jingdong prefecture level city in Shandong
Dragon Quality Goods Shop	King of Power Saving	The effect is too good, the family has now several units, back to buy a number of times, the power saver received, power-saving effect is very good,, fine workmanship, long warranty, but also purify the air, before buying a used for a period of time, with the okay, and then bought a, and only now come to the	Jingdong prefecture level city in Shandong
Dragon Quality Goods Shop	King of Power Saving	Finally received the goods, very, very much like,, has been a lot of people praise, really too happy, has been his family's loyal fans, will continue to buy buy buy buy!	Jingdong prefecture level city in Shandong
Dragon Quality Goods Shop	King of Power Saving	📩 I've been wanting it for a long time, I decided to go for it, I feel great after using it, it's genuine and the delivery is great, it lives up to its name👍	Jingdong prefecture level city in Shandong
Dragon Quality Goods Shop	King of Power Saving	The quality is sure as other friends who bought it said, and it is also beautiful, thanks to the owner for the recommendation, thanks!	Jingdong prefecture level city in Shandong
Dragon Quality Goods Shop	King of Power Saving	Baby received the quality is really good face value is also very high especially practical especially especially special favorite	Jingdong prefecture level city in Shandong
Dragon Quality Goods Shop	King of Power Saving	It is genuine, trust the merchant, the quality is very good, the price is moderate, the use of the effect is very good!	Jingdong prefecture level city in Shandong
Dragon Quality Goods Shop	King of Power Saving	Energy saver received really good quality affordable and good energy saving and environmental protection is the best to use.	Jingdong prefecture level city in Shandong
Dragon Quality Goods Shop	King of Power Saving	Reasonable price logistics is very fast, the feeling is not bad more satisfied with a shopping experience	Jingdong prefecture level city in Shandong
Dragon Quality Goods Shop	King of Power Saving	The stuff is even more perfect than I imagined, very powerful and worth getting!	Jingdong prefecture level city in Shandong
Dragon Quality Goods Shop	King of Power Saving	Goods have been received, good quality, affordable price, good customer service, worth recommending!	Jingdong prefecture level city in Shandong
Dragon Quality Goods Shop	King of Power Saving	Fast shipping, works great, have already recommended it to friends.	Jingdong prefecture level city in Shandong
Dragon Quality Goods Shop	King of Power Saving	Good quality, fast logistics, reasonable price, worth 5 stars.	Jingdong prefecture level city in Shandong
Dragon Quality Goods Shop	King of Power Saving	Good quality, fast logistics, good value, well packaged, practical	Jingdong prefecture level city in Shandong
Dragon Quality Goods Shop	King of Power Saving	Fast shipping, affordable price, great quality, very satisfied!	Jingdong prefecture level city in Shandong
Dragon Quality Goods Shop	King of Power Saving	Regret knowing too late, it's really good stuff. Works well.	Jingdong prefecture level city in Shandong

Dragon Quality Goods Shop	King of Power Saving	Not bad, very good product, quite save electricity	Jingdong prefecture level city in Shandong
Dragon Quality Goods Shop	King of Power Saving	Good quality, fast logistics, overall very satisfied!	Jingdong prefecture level city in Shandong
Dragon Quality Goods Shop	King of Power Saving	Tightly packaged with good results	Jingdong prefecture level city in Shandong
Dragon Quality Goods Shop	King of Power Saving	very happy	Jingdong prefecture level city in Shandong
Dragon Quality Goods Shop	King of Power Saving	favorable criticism	Jingdong prefecture level city in Shandong
Dragon Quality Goods Shop	King of Power Saving	Now they all sign for the goods without receiving them, the Jingdong platform and merchants are very powerful, and the goods are not received before signing for the goods.	Jingdong prefecture level city in Shandong
Dragon Quality Goods Shop	King of Power Saving	low-quality commodities	Jingdong prefecture level city in Shandong
Dragon Quality Goods Shop	King of Power Saving	I bought this and now it works pretty well with the air conditioning on, when I didn't use it it was $600 a month, now it's almost $500.00, that's over $100 less all of a sudden.	Jingdong prefecture level city in Shandong
Dragon Quality Goods Shop	King of Power Saving	Can Oh, the weather is hot, open the air conditioning is really good, this product is good, has been used for a really long time, more ok, power saving effect is not bad.	Jingdong prefecture level city in Shandong
Dragon Quality Goods Shop	King of Power Saving	I paid my electric bill today, it's a few dozen dollars less, anyway, it's not costly, it's already very good, the boss said that it would be better to buy one more, I've already placed an order	Jingdong prefecture level city in Shandong
Dragon Quality Goods Shop	King of Power Saving	I'm on a smart meter, I save about 30% a month, personally I think it's pretty good, it works well	Jingdong prefecture level city in Shandong
Dragon Quality Goods Shop	King of Power Saving	Good use, good quality, very satisfied with the purchase	Jingdong prefecture level city in Shandong
Dragon Quality Goods Shop	King of Power Saving	The goods have been received, the quality is very good, is the ideal effect, will continue to pay attention to the	Jingdong prefecture level city in Shandong
Dragon Quality Goods Shop	King of Power Saving	My dad asked me to buy one, but after I paid the bill, I asked and he said it was a lot less this month, so he asked me to buy another one.	Jingdong prefecture level city in Shandong
Dragon Quality Goods Shop	King of Power Saving	Very satisfied with the purchase thanks to the store for the prosperous business.	Jingdong prefecture level city in Shandong
Dragon Quality Goods Shop	King of Power Saving	This brand has been used for many years, and this time I saw this upgraded version of the product and got it again. The purpose of this product is to reduce the loss of electricity for a long time, so as to achieve the purpose of saving electricity.	Jingdong prefecture level city in Shandong
Dragon Quality Goods Shop	King of Power Saving	The effect is too good, the family has now several units, back to buy a number of times, the power saver received, power-saving effect is very good,, fine workmanship, long warranty, but also purify the air, before buying a used for a period of time, with the okay, and then bought a, and only now come to the	Jingdong prefecture level city in Shandong
Dragon Quality Goods Shop	King of Power Saving	I've been using it for almost a month, and I've reduced my electricity bill by over 100 bucks! I'm planning to get two more for my family.	Jingdong prefecture level city in Shandong
Dragon Quality Goods Shop	King of Power Saving	Finally received the goods, very, very much like,, has been a lot of people praise, really too happy, has been his family's loyal fans, will continue to buy buy buy buy!	Jingdong prefecture level city in Shandong
Dragon Quality Goods Shop	King of Power Saving	📩 I've been wanting it for a long time, I decided to go for it, I feel great after using it, it's genuine and the delivery is great, it lives up to its name👍	Jingdong prefecture level city in Shandong
Dragon Quality Goods Shop	King of Power Saving	The quality is sure as other friends who bought it said, and it is also beautiful, thanks to the owner for the recommendation, thanks!	Jingdong prefecture level city in Shandong
Dragon Quality Goods Shop	King of Power Saving	Baby received the quality is really good face value is also very high especially practical especially especially special favorite	Jingdong prefecture level city in Shandong
Dragon Quality Goods Shop	King of Power Saving	It is genuine, trust the merchant, the quality is very good, the price is moderate, the use of the effect is very good!	Jingdong prefecture level city in Shandong
Dragon Quality Goods Shop	King of Power Saving	Energy saver received really good quality affordable and good energy saving and environmental protection is the best to use.	Jingdong prefecture level city in Shandong
Dragon Quality Goods Shop	King of Power Saving	Reasonable price logistics is very fast, the feeling is not bad more satisfied with a shopping experience	Jingdong prefecture level city in Shandong
Dragon Quality Goods Shop	King of Power Saving	The stuff is even more perfect than I imagined, very powerful and worth getting!	Jingdong prefecture level city in Shandong
Dragon Quality Goods Shop	King of Power Saving	Goods have been received, good quality, affordable price, good customer service, worth recommending!	Jingdong prefecture level city in Shandong
Dragon Quality Goods Shop	King of Power Saving	Fast shipping, works great, have already recommended it to friends.	Jingdong prefecture level city in Shandong
Dragon Quality Goods Shop	King of Power Saving	Good quality, fast logistics, reasonable price, worth 5 stars.	Jingdong prefecture level city in Shandong
Dragon Quality Goods Shop	King of Power Saving	Good quality, fast logistics, good value, well packaged, practical	Jingdong prefecture level city in Shandong
Dragon Quality Goods Shop	King of Power Saving	Fast shipping, affordable price, great quality, very satisfied!	Jingdong prefecture level city in Shandong
Dragon Quality Goods Shop	King of Power Saving	Regret knowing too late, it's really good stuff. Works well.	Jingdong prefecture level city in Shandong
Dragon Quality Goods Shop	King of Power Saving	Not bad, very good product, quite save electricity	Jingdong prefecture level city in Shandong
Dragon Quality Goods Shop	King of Power Saving	Good quality, fast logistics, overall very satisfied!	Jingdong prefecture level city in Shandong
Dragon Quality Goods Shop	King of Power Saving	Tightly packaged with good results	Jingdong prefecture level city in Shandong
Dragon Quality Goods Shop	King of Power Saving	very happy	Jingdong prefecture level city in Shandong
Dragon Quality Goods Shop	King of Power Saving	favorable criticism	Jingdong prefecture level city in Shandong
Dragon Quality Goods Shop	King of Power Saving	Now they all sign for the goods without receiving them, the Jingdong platform and merchants are very powerful, and the goods are not received before signing for the goods.	Jingdong prefecture level city in Shandong
Dragon Quality Goods Shop	King of Power Saving	low-quality commodities	Jingdong prefecture level city in Shandong
Dragon Quality Goods Shop	King of Power Saving	I bought this and now it works pretty well with the air conditioning on, when I didn't use it it was $600 a month, now it's almost $500.00, that's over $100 less all of a sudden.	Jingdong prefecture level city in Shandong
Dragon Quality Goods Shop	King of Power Saving	Can Oh, the weather is hot, open the air conditioning is really good, this product is good, has been used for a really long time, more ok, power saving effect is not bad.	Jingdong prefecture level city in Shandong
Dragon Quality Goods Shop	King of Power Saving	I paid my electric bill today, it's a few dozen dollars less, anyway, it's not costly, it's already very good, the boss said that it would be better to buy one more, I've already placed an order	Jingdong prefecture level city in Shandong
Dragon Quality Goods Shop	King of Power Saving	I'm on a smart meter, I save about 30% a month, personally I think it's pretty good, it works well	Jingdong prefecture level city in Shandong
Dragon Quality Goods Shop	King of Power Saving	My dad asked me to buy one, but after I paid the bill, I asked and he said it was a lot less this month, so he asked me to buy another one.	Jingdong prefecture level city in Shandong
Dragon Quality Goods Shop	King of Power Saving	Very satisfied with the purchase thanks to the store for the prosperous business.	Jingdong prefecture level city in Shandong
Dragon Quality Goods Shop	King of Power Saving	Good use, good quality, very satisfied with the purchase	Jingdong prefecture level city in Shandong
Dragon Quality Goods Shop	King of Power Saving	The goods have been received, the quality is very good, is the ideal effect, will continue to pay attention to the	Jingdong prefecture level city in Shandong
Dragon Quality Goods Shop	King of Power Saving	This brand has been used for many years, and this time I saw this upgraded version of the product and got it again. The purpose of this product is to reduce the loss of electricity for a long time, so as to achieve the purpose of saving electricity.	Jingdong prefecture level city in Shandong
Dragon Quality Goods Shop	King of Power Saving	I've been using it for almost a month, and I've reduced my electricity bill by over 100 bucks! I'm planning to get two more for my family.	Jingdong prefecture level city in Shandong

Dragon Quality Goods Shop	King of Power Saving	The effect is too good, the family has now several units, back to buy a number of times, the power saver received, power-saving effect is very good,, fine workmanship, long warranty, but also purify the air, before buying a used for a period of time, with the okay, and then bought a, and only now come to the	Jingdong prefecture level city in Shandong
Dragon Quality Goods Shop	King of Power Saving	Finally received the goods, very, very much like,, has been a lot of people praise, really too happy, has been his family's loyal fans, will continue to buy buy buy buy!	Jingdong prefecture level city in Shandong
Dragon Quality Goods Shop	King of Power Saving	💌 I've been wanting it for a long time, I decided to go for it, I feel great after using it, it's genuine and the delivery is great, it lives up to its name👍	Jingdong prefecture level city in Shandong
Dragon Quality Goods Shop	King of Power Saving	The quality is sure as other friends who bought it said, and it is also beautiful, thanks to the owner for the recommendation, thanks!	Jingdong prefecture level city in Shandong
Dragon Quality Goods Shop	King of Power Saving	Baby received the quality is really good face value is also very high especially practical especially especially special favorite	Jingdong prefecture level city in Shandong
Dragon Quality Goods Shop	King of Power Saving	It is genuine, trust the merchant, the quality is very good, the price is moderate, the use of the effect is very good!	Jingdong prefecture level city in Shandong
Dragon Quality Goods Shop	King of Power Saving	Energy saver received really good quality affordable and good energy saving and environmental protection is the best to use.	Jingdong prefecture level city in Shandong
Dragon Quality Goods Shop	King of Power Saving	Reasonable price logistics is very fast, the feeling is not bad more satisfied with a shopping experience	Jingdong prefecture level city in Shandong
Dragon Quality Goods Shop	King of Power Saving	The stuff is even more perfect than I imagined, very powerful and worth getting!	Jingdong prefecture level city in Shandong
Dragon Quality Goods Shop	King of Power Saving	Goods have been received, good quality, affordable price, good customer service, worth recommending!	Jingdong prefecture level city in Shandong
Dragon Quality Goods Shop	King of Power Saving	Fast shipping, works great, have already recommended it to friends.	Jingdong prefecture level city in Shandong
Dragon Quality Goods Shop	King of Power Saving	Good quality, fast logistics, reasonable price, worth 5 stars.	Jingdong prefecture level city in Shandong
Dragon Quality Goods Shop	King of Power Saving	Good quality, fast logistics, good value, well packaged, practical	Jingdong prefecture level city in Shandong
Dragon Quality Goods Shop	King of Power Saving	Fast shipping, affordable price, great quality, very satisfied!	Jingdong prefecture level city in Shandong
Dragon Quality Goods Shop	King of Power Saving	Regret knowing too late, it's really good stuff. Works well.	Jingdong prefecture level city in Shandong
Dragon Quality Goods Shop	King of Power Saving	Not bad, very good product, quite save electricity	Jingdong prefecture level city in Shandong
Dragon Quality Goods Shop	King of Power Saving	Good quality, fast logistics, overall very satisfied!	Jingdong prefecture level city in Shandong
Dragon Quality Goods Shop	King of Power Saving	Tightly packaged with good results	Jingdong prefecture level city in Shandong
Dragon Quality Goods Shop	King of Power Saving	very happy	Jingdong prefecture level city in Shandong
Dragon Quality Goods Shop	King of Power Saving	favorable criticism	Jingdong prefecture level city in Shandong
Dragon Quality Goods Shop	King of Power Saving	Now they all sign for the goods without receiving them, the Jingdong platform and merchants are very powerful, and the goods are not received before signing for the goods.	Jingdong prefecture level city in Shandong
Dragon Quality Goods Shop	King of Power Saving	low-quality commodities	Jingdong prefecture level city in Shandong
Dragon Quality Goods Shop	King of Power Saving	I bought this and now it works pretty well with the air conditioning on, when I didn't use it it was $600 a month, now it's almost $500,00, that's over $100 less all of a sudden.	Jingdong prefecture level city in Shandong
Dragon Quality Goods Shop	King of Power Saving	Can Oh, the weather is hot, open the air conditioning is really good, this product is good, has been used for a really long time, more ok, power saving effect is not bad.	Jingdong prefecture level city in Shandong
Dragon Quality Goods Shop	King of Power Saving	I paid my electric bill today, it's a few dozen dollars less, anyway, it's not costly, it's already very good, the boss said that it would be better to buy one more, I've already placed an order	Jingdong prefecture level city in Shandong
Dragon Quality Goods Shop	King of Power Saving	I'm on a smart meter, I save about 30% a month, personally I think it's pretty good, it works well	Jingdong prefecture level city in Shandong
Dragon Quality Goods Shop	King of Power Saving	My dad asked me to buy one, but after I paid the bill, I asked and he said it was a lot less this month, so he asked me to buy another one.	Jingdong prefecture level city in Shandong
Dragon Quality Goods Shop	King of Power Saving	Very satisfied with the purchase thanks to the store for the prosperous business.	Jingdong prefecture level city in Shandong
Dragon Quality Goods Shop	King of Power Saving	Good use, good quality, very satisfied with the purchase	Jingdong prefecture level city in Shandong
Dragon Quality Goods Shop	King of Power Saving	The goods have been received, the quality is very good, is the ideal effect, will continue to pay attention to the	Jingdong prefecture level city in Shandong
Dragon Quality Goods Shop	King of Power Saving	This brand has been used for many years, and this time I saw this upgraded version of the product and got it again. The purpose of this product is to reduce the loss of electricity for a long time, so as to achieve the purpose of saving electricity.	Jingdong prefecture level city in Shandong
Dragon Quality Goods Shop	King of Power Saving	I've been using it for almost a month, and I've reduced my electricity bill by over 100 bucks! I'm planning to get two more for my family.	Jingdong prefecture level city in Shandong
Dragon Quality Goods Shop	King of Power Saving	The effect is too good, the family has now several units, back to buy a number of times, the power saver received, power-saving effect is very good,, fine workmanship, long warranty, but also purify the air, before buying a used for a period of time, with the okay, and then bought a, and only now come to the	Jingdong prefecture level city in Shandong
Dragon Quality Goods Shop	King of Power Saving	Finally received the goods, very, very much like,, has been a lot of people praise, really too happy, has been his family's loyal fans, will continue to buy buy buy buy!	Jingdong prefecture level city in Shandong
Dragon Quality Goods Shop	King of Power Saving	💌 I've been wanting it for a long time, I decided to go for it, I feel great after using it, it's genuine and the delivery is great, it lives up to its name👍	Jingdong prefecture level city in Shandong
Dragon Quality Goods Shop	King of Power Saving	The quality is sure as other friends who bought it said, and it is also beautiful, thanks to the owner for the recommendation, thanks!	Jingdong prefecture level city in Shandong
Dragon Quality Goods Shop	King of Power Saving	Baby received the quality is really good face value is also very high especially practical especially especially special favorite	Jingdong prefecture level city in Shandong
Dragon Quality Goods Shop	King of Power Saving	It is genuine, trust the merchant, the quality is very good, the price is moderate, the use of the effect is very good!	Jingdong prefecture level city in Shandong
Dragon Quality Goods Shop	King of Power Saving	Energy saver received really good quality affordable and good energy saving and environmental protection is the best to use.	Jingdong prefecture level city in Shandong
Dragon Quality Goods Shop	King of Power Saving	Reasonable price logistics is very fast, the feeling is not bad more satisfied with a shopping experience	Jingdong prefecture level city in Shandong
Dragon Quality Goods Shop	King of Power Saving	The stuff is even more perfect than I imagined, very powerful and worth getting!	Jingdong prefecture level city in Shandong
Dragon Quality Goods Shop	King of Power Saving	Goods have been received, good quality, affordable price, good customer service, worth recommending!	Jingdong prefecture level city in Shandong
Dragon Quality Goods Shop	King of Power Saving	Fast shipping, works great, have already recommended it to friends.	Jingdong prefecture level city in Shandong
Dragon Quality Goods Shop	King of Power Saving	Good quality, fast logistics, reasonable price, worth 5 stars.	Jingdong prefecture level city in Shandong
Dragon Quality Goods Shop	King of Power Saving	Good quality, fast logistics, good value, well packaged, practical	Jingdong prefecture level city in Shandong
Dragon Quality Goods Shop	King of Power Saving	Fast shipping, affordable price, great quality, very satisfied!	Jingdong prefecture level city in Shandong
Dragon Quality Goods Shop	King of Power Saving	Regret knowing too late, it's really good stuff. Works well.	Jingdong prefecture level city in Shandong
Dragon Quality Goods Shop	King of Power Saving	Not bad, very good product, quite save electricity	Jingdong prefecture level city in Shandong
Dragon Quality Goods Shop	King of Power Saving	Good quality, fast logistics, overall very satisfied!	Jingdong prefecture level city in Shandong
Dragon Quality Goods Shop	King of Power Saving	Tightly packaged with good results	Jingdong prefecture level city in Shandong

Dragon Quality Goods Shop	King of Power Saving	very happy	Jingdong prefecture level city in Shandong
Dragon Quality Goods Shop	King of Power Saving	favorable criticism	Jingdong prefecture level city in Shandong
Dragon Quality Goods Shop	King of Power Saving	Now they all sign for the goods without receiving them, the Jingdong platform and merchants are very powerful, and the goods are not received before signing for the goods.	Jingdong prefecture level city in Shandong
Dragon Quality Goods Shop	King of Power Saving	low-quality commodities	Jingdong prefecture level city in Shandong
Dragon Quality Goods Shop	King of Power Saving	I bought this and now it works pretty well with the air conditioning on, when I didn't use it it was $600 a month, now it's almost $500,00, that's over $100 less all of a sudden.	Jingdong prefecture level city in Shandong
Dragon Quality Goods Shop	King of Power Saving	Can Oh, the weather is hot, open the air conditioning is really good, this product is good, has been used for a really long time, more ok, power saving effect is not bad.	Jingdong prefecture level city in Shandong
Dragon Quality Goods Shop	King of Power Saving	I paid my electric bill today, it's a few dozen dollars less, anyway, it's not costly, it's already very good, the boss said that it would be better to buy one more, I've already placed an order	Jingdong prefecture level city in Shandong
Dragon Quality Goods Shop	King of Power Saving	I'm on a smart meter, I save about 30% a month, personally I think it's pretty good, it works well	Jingdong prefecture level city in Shandong
Dragon Quality Goods Shop	King of Power Saving	My dad asked me to buy one, but after I paid the bill, I asked and he said it was a lot less this month, so he asked me to buy another one.	Jingdong prefecture level city in Shandong
Dragon Quality Goods Shop	King of Power Saving	Very satisfied with the purchase thanks to the store for the prosperous business.	Jingdong prefecture level city in Shandong
Dragon Quality Goods Shop	King of Power Saving	Good use, good quality, very satisfied with the purchase	Jingdong prefecture level city in Shandong
Dragon Quality Goods Shop	King of Power Saving	The goods have been received, the quality is very good, is the ideal effect, will continue to pay attention to the	Jingdong prefecture level city in Shandong
Dragon Quality Goods Shop	King of Power Saving	This brand has been used for many years, and this time I saw this upgraded version of the product and got it again. The purpose of this product is to reduce the loss of electricity for a long time, so as to achieve the purpose of saving electricity.	Jingdong prefecture level city in Shandong
Dragon Quality Goods Shop	King of Power Saving	I've been using it for almost a month, and I've reduced my electricity bill by over 100 bucks! I'm planning to get two more for my family.	Jingdong prefecture level city in Shandong
Dragon Quality Goods Shop	King of Power Saving	The effect is too good, the family has now several units, back to buy a number of times, the power saver received, power-saving effect is very good,, fine workmanship, long warranty, but also purify the air, before buying a used for a period of time, with the okay, and then bought a, and only now come to the	Jingdong prefecture level city in Shandong
Dragon Quality Goods Shop	King of Power Saving	Finally received the goods, very, very much like,, has been a lot of people praise, really too happy, has been his family's loyal fans, will continue to buy buy buy buy!	Jingdong prefecture level city in Shandong
Dragon Quality Goods Shop	King of Power Saving	📩 I've been wanting it for a long time, I decided to go for it, I feel great after using it, it's genuine and the delivery is great, it lives up to its name👍	Jingdong prefecture level city in Shandong
Dragon Quality Goods Shop	King of Power Saving	The quality is sure as other friends who bought it said, and it is also beautiful, thanks to the owner for the recommendation, thanks!	Jingdong prefecture level city in Shandong
Dragon Quality Goods Shop	King of Power Saving	Baby received the quality is really good face value is also very high especially practical especially especially special favorite	Jingdong prefecture level city in Shandong
Dragon Quality Goods Shop	King of Power Saving	It is genuine, trust the merchant, the quality is very good, the price is moderate, the use of the effect is very good!	Jingdong prefecture level city in Shandong
Dragon Quality Goods Shop	King of Power Saving	Energy saver received really good quality affordable and good energy saving and environmental protection is the best to use.	Jingdong prefecture level city in Shandong
Dragon Quality Goods Shop	King of Power Saving	Reasonable price logistics is very fast, the feeling is not bad more satisfied with a shopping experience	Jingdong prefecture level city in Shandong
Dragon Quality Goods Shop	King of Power Saving	The stuff is even more perfect than I imagined, very powerful and worth getting!	Jingdong prefecture level city in Shandong
Dragon Quality Goods Shop	King of Power Saving	Goods have been received, good quality, affordable price, good customer service, worth recommending!	Jingdong prefecture level city in Shandong
Dragon Quality Goods Shop	King of Power Saving	Fast shipping, works great, have already recommended it to friends.	Jingdong prefecture level city in Shandong
Dragon Quality Goods Shop	King of Power Saving	Good quality, fast logistics, reasonable price, worth 5 stars.	Jingdong prefecture level city in Shandong
Dragon Quality Goods Shop	King of Power Saving	Good quality, fast logistics, good value, well packaged, practical	Jingdong prefecture level city in Shandong
Dragon Quality Goods Shop	King of Power Saving	Fast shipping, affordable price, great quality, very satisfied!	Jingdong prefecture level city in Shandong
Dragon Quality Goods Shop	King of Power Saving	Regret knowing too late, it's really good stuff. Works well.	Jingdong prefecture level city in Shandong
Dragon Quality Goods Shop	King of Power Saving	Not bad, very good product, quite save electricity	Jingdong prefecture level city in Shandong
Dragon Quality Goods Shop	King of Power Saving	Good quality, fast logistics, overall very satisfied!	Jingdong prefecture level city in Shandong
Dragon Quality Goods Shop	King of Power Saving	Tightly packaged with good results	Jingdong prefecture level city in Shandong
Dragon Quality Goods Shop	King of Power Saving	very happy	Jingdong prefecture level city in Shandong
Dragon Quality Goods Shop	King of Power Saving	favorable criticism	Jingdong prefecture level city in Shandong
Dragon Quality Goods Shop	King of Power Saving	Now they all sign for the goods without receiving them, the Jingdong platform and merchants are very powerful, and the goods are not received before signing for the goods.	Jingdong prefecture level city in Shandong
Dragon Quality Goods Shop	King of Power Saving	low-quality commodities	Jingdong prefecture level city in Shandong
Dragon Quality Goods Shop	King of Power Saving	I bought this and now it works pretty well with the air conditioning on, when I didn't use it it was $600 a month, now it's almost $500,00, that's over $100 less all of a sudden.	Jingdong prefecture level city in Shandong
Dragon Quality Goods Shop	King of Power Saving	Can Oh, the weather is hot, open the air conditioning is really good, this product is good, has been used for a really long time, more ok, power saving effect is not bad.	Jingdong prefecture level city in Shandong
Dragon Quality Goods Shop	King of Power Saving	I paid my electric bill today, it's a few dozen dollars less, anyway, it's not costly, it's already very good, the boss said that it would be better to buy one more, I've already placed an order	Jingdong prefecture level city in Shandong
Dragon Quality Goods Shop	King of Power Saving	I'm on a smart meter, I save about 30% a month, personally I think it's pretty good, it works well	Jingdong prefecture level city in Shandong
Dragon Quality Goods Shop	King of Power Saving	My dad asked me to buy one, but after I paid the bill, I asked and he said it was a lot less this month, so he asked me to buy another one.	Jingdong prefecture level city in Shandong
Dragon Quality Goods Shop	King of Power Saving	Very satisfied with the purchase thanks to the store for the prosperous business.	Jingdong prefecture level city in Shandong
Dragon Quality Goods Shop	King of Power Saving	Good use, good quality, very satisfied with the purchase	Jingdong prefecture level city in Shandong
Dragon Quality Goods Shop	King of Power Saving	The goods have been received, the quality is very good, is the ideal effect, will continue to pay attention to the	Jingdong prefecture level city in Shandong
Dragon Quality Goods Shop	King of Power Saving	This brand has been used for many years, and this time I saw this upgraded version of the product and got it again. The purpose of this product is to reduce the loss of electricity for a long time, so as to achieve the purpose of saving electricity.	Jingdong prefecture level city in Shandong
Dragon Quality Goods Shop	King of Power Saving	I've been using it for almost a month, and I've reduced my electricity bill by over 100 bucks! I'm planning to get two more for my family.	Jingdong prefecture level city in Shandong
Dragon Quality Goods Shop	King of Power Saving	The effect is too good, the family has now several units, back to buy a number of times, the power saver received, power-saving effect is very good,, fine workmanship, long warranty, but also purify the air, before buying a used for a period of time, with the okay, and then bought a, and only now come to the	Jingdong prefecture level city in Shandong
Dragon Quality Goods Shop	King of Power Saving	Finally received the goods, very, very much like,, has been a lot of people praise, really too happy, has been his family's loyal fans, will continue to buy buy buy buy!	Jingdong prefecture level city in Shandong
Dragon Quality Goods Shop	King of Power Saving	📩 I've been wanting it for a long time, I decided to go for it, I feel great after using it, it's genuine and the delivery is great, it lives up to its name👍	Jingdong prefecture level city in Shandong

Dragon Quality Goods Shop	King of Power Saving	The quality is sure as other friends who bought it said, and it is also beautiful, thanks to the owner for the recommendation, thanks!	Jingdong prefecture level city in Shandong
Dragon Quality Goods Shop	King of Power Saving	Baby received the quality is really good face value is also very high especially practical especially especially special favorite	Jingdong prefecture level city in Shandong
Dragon Quality Goods Shop	King of Power Saving	It is genuine, trust the merchant, the quality is very good, the price is moderate, the use of the effect is very good!	Jingdong prefecture level city in Shandong
Dragon Quality Goods Shop	King of Power Saving	Energy saver received really good quality affordable and good energy saving and environmental protection is the best to use.	Jingdong prefecture level city in Shandong
Dragon Quality Goods Shop	King of Power Saving	Reasonable price logistics is very fast, the feeling is not bad more satisfied with a shopping experience	Jingdong prefecture level city in Shandong
Dragon Quality Goods Shop	King of Power Saving	The stuff is even more perfect than I imagined, very powerful and worth getting!	Jingdong prefecture level city in Shandong
Dragon Quality Goods Shop	King of Power Saving	Goods have been received, good quality, affordable price, good customer service, worth recommending!	Jingdong prefecture level city in Shandong
Dragon Quality Goods Shop	King of Power Saving	Fast shipping, works great, have already recommended it to friends.	Jingdong prefecture level city in Shandong
Dragon Quality Goods Shop	King of Power Saving	Good quality, fast logistics, reasonable price, worth 5 stars.	Jingdong prefecture level city in Shandong
Dragon Quality Goods Shop	King of Power Saving	Good quality, fast logistics, good value, well packaged, practical	Jingdong prefecture level city in Shandong
Dragon Quality Goods Shop	King of Power Saving	Fast shipping, affordable price, great quality, very satisfied!	Jingdong prefecture level city in Shandong
Dragon Quality Goods Shop	King of Power Saving	Regret knowing too late, it's really good stuff. Works well.	Jingdong prefecture level city in Shandong
Dragon Quality Goods Shop	King of Power Saving	Not bad, very good product, quite save electricity	Jingdong prefecture level city in Shandong
Dragon Quality Goods Shop	King of Power Saving	Good quality, fast logistics, overall very satisfied!	Jingdong prefecture level city in Shandong
Dragon Quality Goods Shop	King of Power Saving	Tightly packaged with good results	Jingdong prefecture level city in Shandong
Dragon Quality Goods Shop	King of Power Saving	very happy	Jingdong prefecture level city in Shandong
Dragon Quality Goods Shop	King of Power Saving	favorable criticism	Jingdong prefecture level city in Shandong
Dragon Quality Goods Shop	King of Power Saving	Now they all sign for the goods without receiving them, the Jingdong platform and merchants are very powerful, and the goods are not received before signing for the goods.	Jingdong prefecture level city in Shandong
Dragon Quality Goods Shop	King of Power Saving	low-quality commodities	Jingdong prefecture level city in Shandong
Dragon Quality Goods Shop	King of Power Saving	I bought this and now it works pretty well with the air conditioning on, when I didn't use it it was $600 a month, now it's almost $500.00, that's over $100 less all of a sudden.	Jingdong prefecture level city in Shandong
Dragon Quality Goods Shop	King of Power Saving	Can Oh, the weather is hot, open the air conditioning is really good, this product is good, has been used for a really long time, more ok, power saving effect is not bad.	Jingdong prefecture level city in Shandong
Dragon Quality Goods Shop	King of Power Saving	I paid my electric bill today, it's a few dozen dollars less, anyway, it's not costly, it's already very good, the boss said that it would be better to buy one more, I've already placed an order	Jingdong prefecture level city in Shandong
Dragon Quality Goods Shop	King of Power Saving	I'm on a smart meter, I save about 30% a month, personally I think it's pretty good, it works well	Jingdong prefecture level city in Shandong
Dragon Quality Goods Shop	King of Power Saving	My dad asked me to buy one, but after I paid the bill, I asked and he said it was a lot less this month, so he asked me to buy another one.	Jingdong prefecture level city in Shandong
Dragon Quality Goods Shop	King of Power Saving	Very satisfied with the purchase thanks to the store for the prosperous business.	Jingdong prefecture level city in Shandong
Dragon Quality Goods Shop	King of Power Saving	Good use, good quality, very satisfied with the purchase	Jingdong prefecture level city in Shandong
Dragon Quality Goods Shop	King of Power Saving	The goods have been received, the quality is very good, is the ideal effect, will continue to pay attention to the	Jingdong prefecture level city in Shandong
Dragon Quality Goods Shop	King of Power Saving	This brand has been used for many years, and this time I saw this upgraded version of the product and got it again. The purpose of this product is to reduce the loss of electricity for a long time, so as to achieve the purpose of saving electricity.	Jingdong prefecture level city in Shandong
Dragon Quality Goods Shop	King of Power Saving	I've been using it for almost a month, and I've reduced my electricity bill by over 100 bucks! I'm planning to get two more for my family.	Jingdong prefecture level city in Shandong
Dragon Quality Goods Shop	King of Power Saving	The effect is too good, the family has now several units, back to buy a number of times, the power saver received, power-saving effect is very good,, fine workmanship, long warranty, but also purify the air, before buying a used for a period of time, with the okay, and then bought a, and only now come to the	Jingdong prefecture level city in Shandong
Dragon Quality Goods Shop	King of Power Saving	Finally received the goods, very, very much like,, has been a lot of people praise, really too happy, has been his family's loyal fans, will continue to buy buy buy buy!	Jingdong prefecture level city in Shandong
Dragon Quality Goods Shop	King of Power Saving	💌 I've been wanting it for a long time, I decided to go for it, I feel great after using it, it's genuine and the delivery is great, it lives up to its name👍	Jingdong prefecture level city in Shandong
Dragon Quality Goods Shop	King of Power Saving	The quality is sure as other friends who bought it said, and it is also beautiful, thanks to the owner for the recommendation, thanks!	Jingdong prefecture level city in Shandong
Dragon Quality Goods Shop	King of Power Saving	Baby received the quality is really good face value is also very high especially practical especially especially special favorite	Jingdong prefecture level city in Shandong
Dragon Quality Goods Shop	King of Power Saving	It is genuine, trust the merchant, the quality is very good, the price is moderate, the use of the effect is very good!	Jingdong prefecture level city in Shandong
Dragon Quality Goods Shop	King of Power Saving	Energy saver received really good quality affordable and good energy saving and environmental protection is the best to use.	Jingdong prefecture level city in Shandong
Dragon Quality Goods Shop	King of Power Saving	Reasonable price logistics is very fast, the feeling is not bad more satisfied with a shopping experience	Jingdong prefecture level city in Shandong
Dragon Quality Goods Shop	King of Power Saving	The stuff is even more perfect than I imagined, very powerful and worth getting!	Jingdong prefecture level city in Shandong
Dragon Quality Goods Shop	King of Power Saving	Goods have been received, good quality, affordable price, good customer service, worth recommending!	Jingdong prefecture level city in Shandong
Dragon Quality Goods Shop	King of Power Saving	Fast shipping, works great, have already recommended it to friends.	Jingdong prefecture level city in Shandong
Dragon Quality Goods Shop	King of Power Saving	Good quality, fast logistics, reasonable price, worth 5 stars.	Jingdong prefecture level city in Shandong
Dragon Quality Goods Shop	King of Power Saving	Good quality, fast logistics, good value, well packaged, practical	Jingdong prefecture level city in Shandong
Dragon Quality Goods Shop	King of Power Saving	Fast shipping, affordable price, great quality, very satisfied!	Jingdong prefecture level city in Shandong
Dragon Quality Goods Shop	King of Power Saving	Regret knowing too late, it's really good stuff. Works well.	Jingdong prefecture level city in Shandong
Dragon Quality Goods Shop	King of Power Saving	Not bad, very good product, quite save electricity	Jingdong prefecture level city in Shandong
Dragon Quality Goods Shop	King of Power Saving	Good quality, fast logistics, overall very satisfied!	Jingdong prefecture level city in Shandong
Dragon Quality Goods Shop	King of Power Saving	Tightly packaged with good results	Jingdong prefecture level city in Shandong
Dragon Quality Goods Shop	King of Power Saving	very happy	Jingdong prefecture level city in Shandong
Dragon Quality Goods Shop	King of Power Saving	favorable criticism	Jingdong prefecture level city in Shandong
Dragon Quality Goods Shop	King of Power Saving	Now they all sign for the goods without receiving them, the Jingdong platform and merchants are very powerful, and the goods are not received before signing for the goods.	Jingdong prefecture level city in Shandong

Dragon Quality Goods Shop	King of Power Saving	low-quality commodities	Jingdong prefecture level city in Shandong
Dragon Quality Goods Shop	King of Power Saving	I bought this and now it works pretty well with the air conditioning on, when I didn't use it it was $600 a month, now it's almost $500,00, that's over $100 less all of a sudden.	Jingdong prefecture level city in Shandong
Dragon Quality Goods Shop	King of Power Saving	Can Oh, the weather is hot, open the air conditioning is really good, this product is good, has been used for a really long time, more ok, power saving effect is not bad.	Jingdong prefecture level city in Shandong
Dragon Quality Goods Shop	King of Power Saving	I paid my electric bill today, it's a few dozen dollars less, anyway, it's not costly, it's already very good, the boss said that it would be better to buy one more, I've already placed an order	Jingdong prefecture level city in Shandong
Dragon Quality Goods Shop	King of Power Saving	I'm on a smart meter, I save about 30% a month, personally I think it's pretty good, it works well	Jingdong prefecture level city in Shandong
Dragon Quality Goods Shop	King of Power Saving	My dad asked me to buy one, but after I paid the bill, I asked and he said it was a lot less this month, so he asked me to buy another one.	Jingdong prefecture level city in Shandong
Dragon Quality Goods Shop	King of Power Saving	Very satisfied with the purchase thanks to the store for the prosperous business.	Jingdong prefecture level city in Shandong
Dragon Quality Goods Shop	King of Power Saving	Good use, good quality, very satisfied with the purchase	Jingdong prefecture level city in Shandong
Dragon Quality Goods Shop	King of Power Saving	The goods have been received, the quality is very good, is the ideal effect, will continue to pay attention to the	Jingdong prefecture level city in Shandong
Dragon Quality Goods Shop	King of Power Saving	This brand has been used for many years, and this time I saw this upgraded version of the product and got it again. The purpose of this product is to reduce the loss of electricity for a long time, so as to achieve the purpose of saving electricity.	Jingdong prefecture level city in Shandong
Dragon Quality Goods Shop	King of Power Saving	I've been using it for almost a month, and I've reduced my electricity bill by over 100 bucks! I'm planning to get two more for my family.	Jingdong prefecture level city in Shandong
Dragon Quality Goods Shop	King of Power Saving	The effect is too good, the family has now several units, back to buy a number of times, the power saver received, power-saving effect is very good,, fine workmanship, long warranty, but also purify the air, before buying a used for a period of time, with the okay, and then bought a, and only now come to the	Jingdong prefecture level city in Shandong
Dragon Quality Goods Shop	King of Power Saving	Finally received the goods, very, very much like,, has been a lot of people praise, really too happy, has been his family's loyal fans, will continue to buy buy buy buy!	Jingdong prefecture level city in Shandong
Dragon Quality Goods Shop	King of Power Saving	📩 I've been wanting it for a long time, I decided to go for it, I feel great after using it, it's genuine and the delivery is great, it lives up to its name👍	Jingdong prefecture level city in Shandong
Dragon Quality Goods Shop	King of Power Saving	The quality is sure as other friends who bought it said, and it is also beautiful, thanks to the owner for the recommendation, thanks!	Jingdong prefecture level city in Shandong
Dragon Quality Goods Shop	King of Power Saving	Baby received the quality is really good face value is also very high especially practical especially especially special favorite	Jingdong prefecture level city in Shandong
Dragon Quality Goods Shop	King of Power Saving	It is genuine, trust the merchant, the quality is very good, the price is moderate, the use of the effect is very good!	Jingdong prefecture level city in Shandong
Dragon Quality Goods Shop	King of Power Saving	Energy saver received really good quality affordable and good energy saving and environmental protection is the best to use.	Jingdong prefecture level city in Shandong
Dragon Quality Goods Shop	King of Power Saving	Reasonable price logistics is very fast, the feeling is not bad more satisfied with a shopping experience	Jingdong prefecture level city in Shandong
Dragon Quality Goods Shop	King of Power Saving	The stuff is even more perfect than I imagined, very powerful and worth getting!	Jingdong prefecture level city in Shandong
Dragon Quality Goods Shop	King of Power Saving	Goods have been received, good quality, affordable price, good customer service, worth recommending!	Jingdong prefecture level city in Shandong
Dragon Quality Goods Shop	King of Power Saving	Fast shipping, works great, have already recommended it to friends.	Jingdong prefecture level city in Shandong
Dragon Quality Goods Shop	King of Power Saving	Good quality, fast logistics, reasonable price, worth 5 stars.	Jingdong prefecture level city in Shandong
Dragon Quality Goods Shop	King of Power Saving	Good quality, fast logistics, good value, well packaged, practical	Jingdong prefecture level city in Shandong
Dragon Quality Goods Shop	King of Power Saving	Fast shipping, affordable price, great quality, very satisfied!	Jingdong prefecture level city in Shandong
Dragon Quality Goods Shop	King of Power Saving	Regret knowing too late, it's really good stuff. Works well.	Jingdong prefecture level city in Shandong
Dragon Quality Goods Shop	King of Power Saving	Not bad, very good product, quite save electricity	Jingdong prefecture level city in Shandong
Dragon Quality Goods Shop	King of Power Saving	Good quality, fast logistics, overall very satisfied!	Jingdong prefecture level city in Shandong
Dragon Quality Goods Shop	King of Power Saving	Tightly packaged with good results	Jingdong prefecture level city in Shandong
Dragon Quality Goods Shop	King of Power Saving	very happy	Jingdong prefecture level city in Shandong
Dragon Quality Goods Shop	King of Power Saving	favorable criticism	Jingdong prefecture level city in Shandong
Dragon Quality Goods Shop	King of Power Saving	Now they all sign for the goods without receiving them, the Jingdong platform and merchants are very powerful, and the goods are not received before signing for the goods.	Jingdong prefecture level city in Shandong
Dragon Quality Goods Shop	King of Power Saving	low-quality commodities	Jingdong prefecture level city in Shandong
Dragon Quality Goods Shop	King of Power Saving	I bought this and now it works pretty well with the air conditioning on, when I didn't use it it was $600 a month, now it's almost $500,00, that's over $100 less all of a sudden.	Jingdong prefecture level city in Shandong
Dragon Quality Goods Shop	King of Power Saving	Can Oh, the weather is hot, open the air conditioning is really good, this product is good, has been used for a really long time, more ok, power saving effect is not bad.	Jingdong prefecture level city in Shandong
Dragon Quality Goods Shop	King of Power Saving	I paid my electric bill today, it's a few dozen dollars less, anyway, it's not costly, it's already very good, the boss said that it would be better to buy one more, I've already placed an order	Jingdong prefecture level city in Shandong
Dragon Quality Goods Shop	King of Power Saving	I'm on a smart meter, I save about 30% a month, personally I think it's pretty good, it works well	Jingdong prefecture level city in Shandong
Dragon Quality Goods Shop	King of Power Saving	My dad asked me to buy one, but after I paid the bill, I asked and he said it was a lot less this month, so he asked me to buy another one.	Jingdong prefecture level city in Shandong
Dragon Quality Goods Shop	King of Power Saving	Very satisfied with the purchase thanks to the store for the prosperous business.	Jingdong prefecture level city in Shandong
Dragon Quality Goods Shop	King of Power Saving	Good use, good quality, very satisfied with the purchase	Jingdong prefecture level city in Shandong
Dragon Quality Goods Shop	King of Power Saving	The goods have been received, the quality is very good, is the ideal effect, will continue to pay attention to the	Jingdong prefecture level city in Shandong
Dragon Quality Goods Shop	King of Power Saving	This brand has been used for many years, and this time I saw this upgraded version of the product and got it again. The purpose of this product is to reduce the loss of electricity for a long time, so as to achieve the purpose of saving electricity.	Jingdong prefecture level city in Shandong
Dragon Quality Goods Shop	King of Power Saving	I've been using it for almost a month, and I've reduced my electricity bill by over 100 bucks! I'm planning to get two more for my family.	Jingdong prefecture level city in Shandong
Dragon Quality Goods Shop	King of Power Saving	The effect is too good, the family has now several units, back to buy a number of times, the power saver received, power-saving effect is very good,, fine workmanship, long warranty, but also purify the air, before buying a used for a period of time, with the okay, and then bought a, and only now come to the	Jingdong prefecture level city in Shandong
Dragon Quality Goods Shop	King of Power Saving	Finally received the goods, very, very much like,, has been a lot of people praise, really too happy, has been his family's loyal fans, will continue to buy buy buy buy!	Jingdong prefecture level city in Shandong
Dragon Quality Goods Shop	King of Power Saving	📩 I've been wanting it for a long time, I decided to go for it, I feel great after using it, it's genuine and the delivery is great, it lives up to its name👍	Jingdong prefecture level city in Shandong
Dragon Quality Goods Shop	King of Power Saving	The quality is sure as other friends who bought it said, and it is also beautiful, thanks to the owner for the recommendation, thanks!	Jingdong prefecture level city in Shandong
Dragon Quality Goods Shop	King of Power Saving	Baby received the quality is really good face value is also very high especially practical especially especially special favorite	Jingdong prefecture level city in Shandong
Dragon Quality Goods Shop	King of Power Saving	It is genuine, trust the merchant, the quality is very good, the price is moderate, the use of the effect is very good!	Jingdong prefecture level city in Shandong

Dragon Quality Goods Shop	King of Power Saving	Energy saver received really good quality affordable and good energy saving and environmental protection is the best to use.	Jingdong prefecture level city in Shandong
Dragon Quality Goods Shop	King of Power Saving	Reasonable price logistics is very fast, the feeling is not bad more satisfied with a shopping experience	Jingdong prefecture level city in Shandong
Dragon Quality Goods Shop	King of Power Saving	The stuff is even more perfect than I imagined, very powerful and worth getting!	Jingdong prefecture level city in Shandong
Dragon Quality Goods Shop	King of Power Saving	Goods have been received, good quality, affordable price, good customer service, worth recommending!	Jingdong prefecture level city in Shandong
Dragon Quality Goods Shop	King of Power Saving	Fast shipping, works great, have already recommended it to friends.	Jingdong prefecture level city in Shandong
Dragon Quality Goods Shop	King of Power Saving	Good quality, fast logistics, reasonable price, worth 5 stars.	Jingdong prefecture level city in Shandong
Dragon Quality Goods Shop	King of Power Saving	Good quality, fast logistics, good value, well packaged, practical	Jingdong prefecture level city in Shandong
Dragon Quality Goods Shop	King of Power Saving	Fast shipping, affordable price, great quality, very satisfied!	Jingdong prefecture level city in Shandong
Dragon Quality Goods Shop	King of Power Saving	Regret knowing too late, it's really good stuff. Works well.	Jingdong prefecture level city in Shandong
Dragon Quality Goods Shop	King of Power Saving	Not bad, very good product, quite save electricity	Jingdong prefecture level city in Shandong
Dragon Quality Goods Shop	King of Power Saving	Good quality, fast logistics, overall very satisfied!	Jingdong prefecture level city in Shandong
Dragon Quality Goods Shop	King of Power Saving	Tightly packaged with good results	Jingdong prefecture level city in Shandong
Dragon Quality Goods Shop	King of Power Saving	very happy	Jingdong prefecture level city in Shandong
Dragon Quality Goods Shop	King of Power Saving	favorable criticism	Jingdong prefecture level city in Shandong
Dragon Quality Goods Shop	King of Power Saving	Now they all sign for the goods without receiving them, the Jingdong platform and merchants are very powerful, and the goods are not received before signing for the goods.	Jingdong prefecture level city in Shandong
Dragon Quality Goods Shop	King of Power Saving	low-quality commodities	Jingdong prefecture level city in Shandong
Dragon Quality Goods Shop	King of Power Saving	I bought this and now it works pretty well with the air conditioning on, when I didn't use it it was $600 a month, now it's almost $500,00, that's over $100 less all of a sudden.	Jingdong prefecture level city in Shandong
Dragon Quality Goods Shop	King of Power Saving	Can Oh, the weather is hot, open the air conditioning is really good, this product is good, has been used for a really long time, more ok, power saving effect is not bad.	Jingdong prefecture level city in Shandong
Dragon Quality Goods Shop	King of Power Saving	I paid my electric bill today, it's a few dozen dollars less, anyway, it's not costly, it's already very good, the boss said that it would be better to buy one more, I've already placed an order	Jingdong prefecture level city in Shandong
Dragon Quality Goods Shop	King of Power Saving	I'm on a smart meter, I save about 30% a month, personally I think it's pretty good, it works well	Jingdong prefecture level city in Shandong
Dragon Quality Goods Shop	King of Power Saving	My dad asked me to buy one, but after I paid the bill, I asked and he said it was a lot less this month, so he asked me to buy another one.	Jingdong prefecture level city in Shandong
Dragon Quality Goods Shop	King of Power Saving	Very satisfied with the purchase thanks to the store for the prosperous business.	Jingdong prefecture level city in Shandong
Dragon Quality Goods Shop	King of Power Saving	Good use, good quality, very satisfied with the purchase	Jingdong prefecture level city in Shandong
Dragon Quality Goods Shop	King of Power Saving	The goods have been received, the quality is very good, is the ideal effect, will continue to pay attention to the	Jingdong prefecture level city in Shandong
Dragon Quality Goods Shop	King of Power Saving	This brand has been used for many years, and this time I saw this upgraded version of the product and got it again. The purpose of this product is to reduce the loss of electricity for a long time, so as to achieve the purpose of saving electricity.	Jingdong prefecture level city in Shandong
Dragon Quality Goods Shop	King of Power Saving	I've been using it for almost a month, and I've reduced my electricity bill by over 100 bucks! I'm planning to get two more for my family.	Jingdong prefecture level city in Shandong
Dragon Quality Goods Shop	King of Power Saving	The effect is too good, the family has now several units, back to buy a number of times, the power saver received, power-saving effect is very good,, fine workmanship, long warranty, but also purify the air, before buying a used for a period of time, with the okay, and then bought a, and only now come to the	Jingdong prefecture level city in Shandong
Dragon Quality Goods Shop	King of Power Saving	Finally received the goods, very, very much like,, has been a lot of people praise, really too happy, has been his family's loyal fans, will continue to buy buy buy buy!	Jingdong prefecture level city in Shandong
Dragon Quality Goods Shop	King of Power Saving	📩 I've been wanting it for a long time, I decided to go for it, I feel great after using it, it's genuine and the delivery is great, it lives up to its name👍	Jingdong prefecture level city in Shandong
Dragon Quality Goods Shop	King of Power Saving	The quality is sure as other friends who bought it said, and it is also beautiful, thanks to the owner for the recommendation, thanks!	Jingdong prefecture level city in Shandong
Dragon Quality Goods Shop	King of Power Saving	Baby received the quality is really good face value is also very high especially practical especially especially special favorite	Jingdong prefecture level city in Shandong
Dragon Quality Goods Shop	King of Power Saving	It is genuine, trust the merchant, the quality is very good, the price is moderate, the use of the effect is very good!	Jingdong prefecture level city in Shandong
Dragon Quality Goods Shop	King of Power Saving	Energy saver received really good quality affordable and good energy saving and environmental protection is the best to use.	Jingdong prefecture level city in Shandong
Dragon Quality Goods Shop	King of Power Saving	Reasonable price logistics is very fast, the feeling is not bad more satisfied with a shopping experience	Jingdong prefecture level city in Shandong
Dragon Quality Goods Shop	King of Power Saving	The stuff is even more perfect than I imagined, very powerful and worth getting!	Jingdong prefecture level city in Shandong
Dragon Quality Goods Shop	King of Power Saving	Goods have been received, good quality, affordable price, good customer service, worth recommending!	Jingdong prefecture level city in Shandong
Dragon Quality Goods Shop	King of Power Saving	Fast shipping, works great, have already recommended it to friends.	Jingdong prefecture level city in Shandong
Dragon Quality Goods Shop	King of Power Saving	Good quality, fast logistics, reasonable price, worth 5 stars.	Jingdong prefecture level city in Shandong
Dragon Quality Goods Shop	King of Power Saving	Good quality, fast logistics, good value, well packaged, practical	Jingdong prefecture level city in Shandong
Dragon Quality Goods Shop	King of Power Saving	Fast shipping, affordable price, great quality, very satisfied!	Jingdong prefecture level city in Shandong
Dragon Quality Goods Shop	King of Power Saving	Regret knowing too late, it's really good stuff. Works well.	Jingdong prefecture level city in Shandong
Dragon Quality Goods Shop	King of Power Saving	Not bad, very good product, quite save electricity	Jingdong prefecture level city in Shandong
Dragon Quality Goods Shop	King of Power Saving	Good quality, fast logistics, overall very satisfied!	Jingdong prefecture level city in Shandong
Dragon Quality Goods Shop	King of Power Saving	Tightly packaged with good results	Jingdong prefecture level city in Shandong
Dragon Quality Goods Shop	King of Power Saving	very happy	Jingdong prefecture level city in Shandong
Dragon Quality Goods Shop	King of Power Saving	favorable criticism	Jingdong prefecture level city in Shandong
Dragon Quality Goods Shop	King of Power Saving	Now they all sign for the goods without receiving them, the Jingdong platform and merchants are very powerful, and the goods are not received before signing for the goods.	Jingdong prefecture level city in Shandong
Dragon Quality Goods Shop	King of Power Saving	low-quality commodities	Jingdong prefecture level city in Shandong
Dragon Quality Goods Shop	King of Power Saving	I bought this and now it works pretty well with the air conditioning on, when I didn't use it it was $600 a month, now it's almost $500,00, that's over $100 less all of a sudden.	Jingdong prefecture level city in Shandong
Dragon Quality Goods Shop	King of Power Saving	Can Oh, the weather is hot, open the air conditioning is really good, this product is good, has been used for a really long time, more ok, power saving effect is not bad.	Jingdong prefecture level city in Shandong

Dragon Quality Goods Shop	King of Power Saving	I paid my electric bill today, it's a few dozen dollars less, anyway, it's not costly, it's already very good, the boss said that it would be better to buy one more, I've already placed an order	Jingdong prefecture level city in Shandong
Dragon Quality Goods Shop	King of Power Saving	I'm on a smart meter, I save about 30% a month, personally I think it's pretty good, it works well	Jingdong prefecture level city in Shandong
Dragon Quality Goods Shop	King of Power Saving	My dad asked me to buy one, but after I paid the bill, I asked and he said it was a lot less this month, so he asked me to buy another one.	Jingdong prefecture level city in Shandong
Dragon Quality Goods Shop	King of Power Saving	Very satisfied with the purchase thanks to the store for the prosperous business.	Jingdong prefecture level city in Shandong
Dragon Quality Goods Shop	King of Power Saving	Good use, good quality, very satisfied with the purchase	Jingdong prefecture level city in Shandong
Dragon Quality Goods Shop	King of Power Saving	The goods have been received, the quality is very good, is the ideal effect, will continue to pay attention to the	Jingdong prefecture level city in Shandong
Dragon Quality Goods Shop	King of Power Saving	This brand has been used for many years, and this time I saw this upgraded version of the product and got it again. The purpose of this product is to reduce the loss of electricity for a long time, so as to achieve the purpose of saving electricity.	Jingdong prefecture level city in Shandong
Dragon Quality Goods Shop	King of Power Saving	I've been using it for almost a month, and I've reduced my electricity bill by over 100 bucks! I'm planning to get two more for my family.	Jingdong prefecture level city in Shandong
Dragon Quality Goods Shop	King of Power Saving	The effect is too good, the family has now several units, back to buy a number of times, the power saver received, power-saving effect is very good,, fine workmanship, long warranty, but also purify the air, before buying a used for a period of time, with the okay, and then bought a, and only now come to the	Jingdong prefecture level city in Shandong
Dragon Quality Goods Shop	King of Power Saving	Finally received the goods, very, very much like,, has been a lot of people praise, really too happy, has been his family's loyal fans, will continue to buy buy buy buy!	Jingdong prefecture level city in Shandong
Dragon Quality Goods Shop	King of Power Saving	I've been wanting it for a long time, I decided to go for it, I feel great after using it, it's genuine and the delivery is great, it lives up to its name	Jingdong prefecture level city in Shandong
Dragon Quality Goods Shop	King of Power Saving	The quality is sure as other friends who bought it said, and it is also beautiful, thanks to the owner for the recommendation, thanks!	Jingdong prefecture level city in Shandong
Dragon Quality Goods Shop	King of Power Saving	Baby received the quality is really good face value is also very high especially practical especially especially special favorite	Jingdong prefecture level city in Shandong
Dragon Quality Goods Shop	King of Power Saving	It is genuine, trust the merchant, the quality is very good, the price is moderate, the use of the effect is very good!	Jingdong prefecture level city in Shandong
Dragon Quality Goods Shop	King of Power Saving	Energy saver received really good quality affordable and good energy saving and environmental protection is the best to use.	Jingdong prefecture level city in Shandong
Dragon Quality Goods Shop	King of Power Saving	Reasonable price logistics is very fast, the feeling is not bad more satisfied with a shopping experience	Jingdong prefecture level city in Shandong
Dragon Quality Goods Shop	King of Power Saving	The stuff is even more perfect than I imagined, very powerful and worth getting!	Jingdong prefecture level city in Shandong
Dragon Quality Goods Shop	King of Power Saving	Goods have been received, good quality, affordable price, good customer service, worth recommending!	Jingdong prefecture level city in Shandong
Dragon Quality Goods Shop	King of Power Saving	Fast shipping, works great, have already recommended it to friends.	Jingdong prefecture level city in Shandong
Dragon Quality Goods Shop	King of Power Saving	Good quality, fast logistics, reasonable price, worth 5 stars.	Jingdong prefecture level city in Shandong
Dragon Quality Goods Shop	King of Power Saving	Good quality, fast logistics, good value, well packaged, practical	Jingdong prefecture level city in Shandong
Dragon Quality Goods Shop	King of Power Saving	Fast shipping, affordable price, great quality, very satisfied!	Jingdong prefecture level city in Shandong
Dragon Quality Goods Shop	King of Power Saving	Regret knowing too late, it's really good stuff. Works well.	Jingdong prefecture level city in Shandong
Dragon Quality Goods Shop	King of Power Saving	Not bad, very good product, quite save electricity	Jingdong prefecture level city in Shandong
Dragon Quality Goods Shop	King of Power Saving	Good quality, fast logistics, overall very satisfied!	Jingdong prefecture level city in Shandong
Dragon Quality Goods Shop	King of Power Saving	Tightly packaged with good results	Jingdong prefecture level city in Shandong
Dragon Quality Goods Shop	King of Power Saving	very happy	Jingdong prefecture level city in Shandong
Dragon Quality Goods Shop	King of Power Saving	favorable criticism	Jingdong prefecture level city in Shandong
Dragon Quality Goods Shop	King of Power Saving	Now they all sign for the goods without receiving them, the Jingdong platform and merchants are very powerful, and the goods are not received before signing for the goods.	Jingdong prefecture level city in Shandong
Dragon Quality Goods Shop	King of Power Saving	low-quality commodities	Jingdong prefecture level city in Shandong
Dragon Quality Goods Shop	King of Power Saving	I bought this and now it works pretty well with the air conditioning on, when I didn't use it it was $600 a month, now it's almost $500,00, that's over $100 less all of a sudden.	Jingdong prefecture level city in Shandong
Dragon Quality Goods Shop	King of Power Saving	Can Oh, the weather is hot, open the air conditioning is really good, this product is good, has been used for a really long time, more ok, power saving effect is not bad.	Jingdong prefecture level city in Shandong
Dragon Quality Goods Shop	King of Power Saving	I paid my electric bill today, it's a few dozen dollars less, anyway, it's not costly, it's already very good, the boss said that it would be better to buy one more, I've already placed an order	Jingdong prefecture level city in Shandong
Dragon Quality Goods Shop	King of Power Saving	I'm on a smart meter, I save about 30% a month, personally I think it's pretty good, it works well	Jingdong prefecture level city in Shandong
Dragon Quality Goods Shop	King of Power Saving	My dad asked me to buy one, but after I paid the bill, I asked and he said it was a lot less this month, so he asked me to buy another one.	Jingdong prefecture level city in Shandong
Dragon Quality Goods Shop	King of Power Saving	Very satisfied with the purchase thanks to the store for the prosperous business.	Jingdong prefecture level city in Shandong
Dragon Quality Goods Shop	King of Power Saving	Good use, good quality, very satisfied with the purchase	Jingdong prefecture level city in Shandong
Dragon Quality Goods Shop	King of Power Saving	The goods have been received, the quality is very good, is the ideal effect, will continue to pay attention to the	Jingdong prefecture level city in Shandong
Dragon Quality Goods Shop	King of Power Saving	This brand has been used for many years, and this time I saw this upgraded version of the product and got it again. The purpose of this product is to reduce the loss of electricity for a long time, so as to achieve the purpose of saving electricity.	Jingdong prefecture level city in Shandong
Dragon Quality Goods Shop	King of Power Saving	I've been using it for almost a month, and I've reduced my electricity bill by over 100 bucks! I'm planning to get two more for my family.	Jingdong prefecture level city in Shandong
Dragon Quality Goods Shop	King of Power Saving	The effect is too good, the family has now several units, back to buy a number of times, the power saver received, power-saving effect is very good,, fine workmanship, long warranty, but also purify the air, before buying a used for a period of time, with the okay, and then bought a, and only now come to the	Jingdong prefecture level city in Shandong
Dragon Quality Goods Shop	King of Power Saving	Finally received the goods, very, very much like,, has been a lot of people praise, really too happy, has been his family's loyal fans, will continue to buy buy buy buy!	Jingdong prefecture level city in Shandong
Dragon Quality Goods Shop	King of Power Saving	I've been wanting it for a long time, I decided to go for it, I feel great after using it, it's genuine and the delivery is great, it lives up to its name	Jingdong prefecture level city in Shandong
Dragon Quality Goods Shop	King of Power Saving	The quality is sure as other friends who bought it said, and it is also beautiful, thanks to the owner for the recommendation, thanks!	Jingdong prefecture level city in Shandong
Dragon Quality Goods Shop	King of Power Saving	Baby received the quality is really good face value is also very high especially practical especially especially special favorite	Jingdong prefecture level city in Shandong
Dragon Quality Goods Shop	King of Power Saving	It is genuine, trust the merchant, the quality is very good, the price is moderate, the use of the effect is very good!	Jingdong prefecture level city in Shandong
Dragon Quality Goods Shop	King of Power Saving	Energy saver received really good quality affordable and good energy saving and environmental protection is the best to use.	Jingdong prefecture level city in Shandong
Dragon Quality Goods Shop	King of Power Saving	Reasonable price logistics is very fast, the feeling is not bad more satisfied with a shopping experience	Jingdong prefecture level city in Shandong
Dragon Quality Goods Shop	King of Power Saving	The stuff is even more perfect than I imagined, very powerful and worth getting!	Jingdong prefecture level city in Shandong

Dragon Quality Goods Shop	King of Power Saving	Goods have been received, good quality, affordable price, good customer service, worth recommending!	Jingdong prefecture level city in Shandong
Dragon Quality Goods Shop	King of Power Saving	Fast shipping, works great, have already recommended it to friends.	Jingdong prefecture level city in Shandong
Dragon Quality Goods Shop	King of Power Saving	Good quality, fast logistics, reasonable price, worth 5 stars.	Jingdong prefecture level city in Shandong
Dragon Quality Goods Shop	King of Power Saving	Good quality, fast logistics, good value, well packaged, practical	Jingdong prefecture level city in Shandong
Dragon Quality Goods Shop	King of Power Saving	Fast shipping, affordable price, great quality, very satisfied!	Jingdong prefecture level city in Shandong
Dragon Quality Goods Shop	King of Power Saving	Regret knowing too late, it's really good stuff. Works well.	Jingdong prefecture level city in Shandong
Dragon Quality Goods Shop	King of Power Saving	Not bad, very good product, quite save electricity	Jingdong prefecture level city in Shandong
Dragon Quality Goods Shop	King of Power Saving	Good quality, fast logistics, overall very satisfied!	Jingdong prefecture level city in Shandong
Dragon Quality Goods Shop	King of Power Saving	Tightly packaged with good results	Jingdong prefecture level city in Shandong
Dragon Quality Goods Shop	King of Power Saving	very happy	Jingdong prefecture level city in Shandong
Dragon Quality Goods Shop	King of Power Saving	favorable criticism	Jingdong prefecture level city in Shandong
Dragon Quality Goods Shop	King of Power Saving	Now they all sign for the goods without receiving them, the Jingdong platform and merchants are very powerful, and the goods are not received before signing for the goods.	Jingdong prefecture level city in Shandong
Dragon Quality Goods Shop	King of Power Saving	low-quality commodities	Jingdong prefecture level city in Shandong
Dragon Quality Goods Shop	King of Power Saving	I bought this and now it works pretty well with the air conditioning on, when I didn't use it it was $600 a month, now it's almost $500.00, that's over $100 less all of a sudden.	Jingdong prefecture level city in Shandong
Dragon Quality Goods Shop	King of Power Saving	Can Oh, the weather is hot, open the air conditioning is really good, this product is good, has been used for a really long time, more ok, power saving effect is not bad.	Jingdong prefecture level city in Shandong
Dragon Quality Goods Shop	King of Power Saving	I paid my electric bill today, it's a few dozen dollars less, anyway, it's not costly, it's already very good, the boss said that it would be better to buy one more, I've already placed an order	Jingdong prefecture level city in Shandong
Dragon Quality Goods Shop	King of Power Saving	I'm on a smart meter, I save about 30% a month, personally I think it's pretty good, it works well	Jingdong prefecture level city in Shandong
Dragon Quality Goods Shop	King of Power Saving	My dad asked me to buy one, but after I paid the bill, I asked and he said it was a lot less this month, so he asked me to buy another one.	Jingdong prefecture level city in Shandong
Dragon Quality Goods Shop	King of Power Saving	Very satisfied with the purchase thanks to the store for the prosperous business.	Jingdong prefecture level city in Shandong
Dragon Quality Goods Shop	King of Power Saving	Good use, good quality, very satisfied with the purchase	Jingdong prefecture level city in Shandong
Dragon Quality Goods Shop	King of Power Saving	The goods have been received, the quality is very good, is the ideal effect, will continue to pay attention to the	Jingdong prefecture level city in Shandong
Dragon Quality Goods Shop	King of Power Saving	This brand has been used for many years, and this time I saw this upgraded version of the product and got it again. The purpose of this product is to reduce the loss of electricity for a long time, so as to achieve the purpose of saving electricity.	Jingdong prefecture level city in Shandong
Dragon Quality Goods Shop	King of Power Saving	I've been using it for almost a month, and I've reduced my electricity bill by over 100 bucks! I'm planning to get two more for my family.	Jingdong prefecture level city in Shandong
Dragon Quality Goods Shop	King of Power Saving	The effect is too good, the family has now several units, back to buy a number of times, the power saver received, power-saving effect is very good,, fine workmanship, long warranty, but also purify the air, before buying a used for a period of time, with the okay, and then bought a, and only now come to the	Jingdong prefecture level city in Shandong
Dragon Quality Goods Shop	King of Power Saving	Finally received the goods, very, very much like,, has been a lot of people praise, really too happy, has been his family's loyal fans, will continue to buy buy buy buy!	Jingdong prefecture level city in Shandong
Dragon Quality Goods Shop	King of Power Saving	💌 I've been wanting it for a long time, I decided to go for it, I feel great after using it, it's genuine and the delivery is great, it lives up to its name👍	Jingdong prefecture level city in Shandong
Dragon Quality Goods Shop	King of Power Saving	The quality is sure as other friends who bought it said, and it is also beautiful, thanks to the owner for the recommendation, thanks!	Jingdong prefecture level city in Shandong
Dragon Quality Goods Shop	King of Power Saving	Baby received the quality is really good face value is also very high especially practical especially especially special favorite	Jingdong prefecture level city in Shandong
Dragon Quality Goods Shop	King of Power Saving	It is genuine, trust the merchant, the quality is very good, the price is moderate, the use of the effect is very good!	Jingdong prefecture level city in Shandong
Dragon Quality Goods Shop	King of Power Saving	Energy saver received really good quality affordable and good energy saving and environmental protection is the best to use.	Jingdong prefecture level city in Shandong
Dragon Quality Goods Shop	King of Power Saving	Reasonable price logistics is very fast, the feeling is not bad more satisfied with a shopping experience	Jingdong prefecture level city in Shandong
Dragon Quality Goods Shop	King of Power Saving	The stuff is even more perfect than I imagined, very powerful and worth getting!	Jingdong prefecture level city in Shandong
Dragon Quality Goods Shop	King of Power Saving	Goods have been received, good quality, affordable price, good customer service, worth recommending!	Jingdong prefecture level city in Shandong
Dragon Quality Goods Shop	King of Power Saving	Fast shipping, works great, have already recommended it to friends.	Jingdong prefecture level city in Shandong
Dragon Quality Goods Shop	King of Power Saving	Good quality, fast logistics, reasonable price, worth 5 stars.	Jingdong prefecture level city in Shandong
Dragon Quality Goods Shop	King of Power Saving	Good quality, fast logistics, good value, well packaged, practical	Jingdong prefecture level city in Shandong
Dragon Quality Goods Shop	King of Power Saving	Fast shipping, affordable price, great quality, very satisfied!	Jingdong prefecture level city in Shandong
Dragon Quality Goods Shop	King of Power Saving	Regret knowing too late, it's really good stuff. Works well.	Jingdong prefecture level city in Shandong
Dragon Quality Goods Shop	King of Power Saving	Not bad, very good product, quite save electricity	Jingdong prefecture level city in Shandong
Dragon Quality Goods Shop	King of Power Saving	Good quality, fast logistics, overall very satisfied!	Jingdong prefecture level city in Shandong
Dragon Quality Goods Shop	King of Power Saving	Tightly packaged with good results	Jingdong prefecture level city in Shandong
Dragon Quality Goods Shop	King of Power Saving	very happy	Jingdong prefecture level city in Shandong
Dragon Quality Goods Shop	King of Power Saving	favorable criticism	Jingdong prefecture level city in Shandong
Dragon Quality Goods Shop	King of Power Saving	Now they all sign for the goods without receiving them, the Jingdong platform and merchants are very powerful, and the goods are not received before signing for the goods.	Jingdong prefecture level city in Shandong
Dragon Quality Goods Shop	King of Power Saving	low-quality commodities	Jingdong prefecture level city in Shandong
Dragon Quality Goods Shop	King of Power Saving	I bought this and now it works pretty well with the air conditioning on, when I didn't use it it was $600 a month, now it's almost $500.00, that's over $100 less all of a sudden.	Jingdong prefecture level city in Shandong
Dragon Quality Goods Shop	King of Power Saving	Can Oh, the weather is hot, open the air conditioning is really good, this product is good, has been used for a really long time, more ok, power saving effect is not bad.	Jingdong prefecture level city in Shandong
Dragon Quality Goods Shop	King of Power Saving	I paid my electric bill today, it's a few dozen dollars less, anyway, it's not costly, it's already very good, the boss said that it would be better to buy one more, I've already placed an order	Jingdong prefecture level city in Shandong
Dragon Quality Goods Shop	King of Power Saving	I'm on a smart meter, I save about 30% a month, personally I think it's pretty good, it works well	Jingdong prefecture level city in Shandong
Dragon Quality Goods Shop	King of Power Saving	My dad asked me to buy one, but after I paid the bill, I asked and he said it was a lot less this month, so he asked me to buy another one.	Jingdong prefecture level city in Shandong

Dragon Quality Goods Shop	King of Power Saving	Very satisfied with the purchase thanks to the store for the prosperous business.	Jingdong prefecture level city in Shandong
Dragon Quality Goods Shop	King of Power Saving	Good use, good quality, very satisfied with the purchase	Jingdong prefecture level city in Shandong
Dragon Quality Goods Shop	King of Power Saving	The goods have been received, the quality is very good, is the ideal effect, will continue to pay attention to the	Jingdong prefecture level city in Shandong
Dragon Quality Goods Shop	King of Power Saving	This brand has been used for many years, and this time I saw this upgraded version of the product and got it again. The purpose of this product is to reduce the loss of electricity for a long time, so as to achieve the purpose of saving electricity.	Jingdong prefecture level city in Shandong
Dragon Quality Goods Shop	King of Power Saving	I've been using it for almost a month, and I've reduced my electricity bill by over 100 bucks! I'm planning to get two more for my family.	Jingdong prefecture level city in Shandong
Dragon Quality Goods Shop	King of Power Saving	The effect is too good, the family has now several units, back to buy a number of times, the power saver received, power-saving effect is very good,, fine workmanship, long warranty, but also purify the air, before buying a used for a period of time, with the okay, and then bought a, and only now come to the	Jingdong prefecture level city in Shandong
Dragon Quality Goods Shop	King of Power Saving	Finally received the goods, very, very much like,, has been a lot of people praise, really too happy, has been his family's loyal fans, will continue to buy buy buy buy!	Jingdong prefecture level city in Shandong
Dragon Quality Goods Shop	King of Power Saving	📩 I've been wanting it for a long time, I decided to go for it, I feel great after using it, it's genuine and the delivery is great, it lives up to its name👍	Jingdong prefecture level city in Shandong
Dragon Quality Goods Shop	King of Power Saving	The quality is sure as other friends who bought it said, and it is also beautiful, thanks to the owner for the recommendation, thanks!	Jingdong prefecture level city in Shandong
Dragon Quality Goods Shop	King of Power Saving	Baby received the quality is really good face value is also very high especially practical especially especially special favorite	Jingdong prefecture level city in Shandong
Dragon Quality Goods Shop	King of Power Saving	It is genuine, trust the merchant, the quality is very good, the price is moderate, the use of the effect is very good!	Jingdong prefecture level city in Shandong
Dragon Quality Goods Shop	King of Power Saving	Energy saver received really good quality affordable and good energy saving and environmental protection is the best to use.	Jingdong prefecture level city in Shandong
Dragon Quality Goods Shop	King of Power Saving	Reasonable price logistics is very fast, the feeling is not bad more satisfied with a shopping experience	Jingdong prefecture level city in Shandong
Dragon Quality Goods Shop	King of Power Saving	The stuff is even more perfect than I imagined, very powerful and worth getting!	Jingdong prefecture level city in Shandong
Dragon Quality Goods Shop	King of Power Saving	Goods have been received, good quality, affordable price, good customer service, worth recommending!	Jingdong prefecture level city in Shandong
Dragon Quality Goods Shop	King of Power Saving	Fast shipping, works great, have already recommended it to friends.	Jingdong prefecture level city in Shandong
Dragon Quality Goods Shop	King of Power Saving	Good quality, fast logistics, reasonable price, worth 5 stars.	Jingdong prefecture level city in Shandong
Dragon Quality Goods Shop	King of Power Saving	Good quality, fast logistics, good value, well packaged, practical	Jingdong prefecture level city in Shandong
Dragon Quality Goods Shop	King of Power Saving	Fast shipping, affordable price, great quality, very satisfied!	Jingdong prefecture level city in Shandong
Dragon Quality Goods Shop	King of Power Saving	Regret knowing too late, it's really good stuff. Works well.	Jingdong prefecture level city in Shandong
Dragon Quality Goods Shop	King of Power Saving	Not bad, very good product, quite save electricity	Jingdong prefecture level city in Shandong
Dragon Quality Goods Shop	King of Power Saving	Good quality, fast logistics, overall very satisfied!	Jingdong prefecture level city in Shandong
Dragon Quality Goods Shop	King of Power Saving	Tightly packaged with good results	Jingdong prefecture level city in Shandong
Dragon Quality Goods Shop	King of Power Saving	very happy	Jingdong prefecture level city in Shandong
Dragon Quality Goods Shop	King of Power Saving	favorable criticism	Jingdong prefecture level city in Shandong
Dragon Quality Goods Shop	King of Power Saving	Now they all sign for the goods without receiving them, the Jingdong platform and merchants are very powerful, and the goods are not received before signing for the goods.	Jingdong prefecture level city in Shandong
Dragon Quality Goods Shop	King of Power Saving	low-quality commodities	Jingdong prefecture level city in Shandong
Dragon Quality Goods Shop	King of Power Saving	I bought this and now it works pretty well with the air conditioning on, when I didn't use it it was $600 a month, now it's almost $500.00, that's over $100 less all of a sudden.	Jingdong prefecture level city in Shandong
Dragon Quality Goods Shop	King of Power Saving	Can Oh, the weather is hot, open the air conditioning is really good, this product is good, has been used for a really long time, more ok, power saving effect is not bad.	Jingdong prefecture level city in Shandong
Dragon Quality Goods Shop	King of Power Saving	I paid my electric bill today, it's a few dozen dollars less, anyway, it's not costly, it's already very good, the boss said that it would be better to buy one more, I've already placed an order	Jingdong prefecture level city in Shandong
Dragon Quality Goods Shop	King of Power Saving	I'm on a smart meter, I save about 30% a month, personally I think it's pretty good, it works well	Jingdong prefecture level city in Shandong
Dragon Quality Goods Shop	King of Power Saving	My dad asked me to buy one, but after I paid the bill, I asked and he said it was a lot less this month, so he asked me to buy another one.	Jingdong prefecture level city in Shandong
Dragon Quality Goods Shop	King of Power Saving	Very satisfied with the purchase thanks to the store for the prosperous business.	Jingdong prefecture level city in Shandong
Dragon Quality Goods Shop	King of Power Saving	Good use, good quality, very satisfied with the purchase	Jingdong prefecture level city in Shandong
Dragon Quality Goods Shop	King of Power Saving	The goods have been received, the quality is very good, is the ideal effect, will continue to pay attention to the	Jingdong prefecture level city in Shandong
Dragon Quality Goods Shop	King of Power Saving	This brand has been used for many years, and this time I saw this upgraded version of the product and got it again. The purpose of this product is to reduce the loss of electricity for a long time, so as to achieve the purpose of saving electricity.	Jingdong prefecture level city in Shandong
Dragon Quality Goods Shop	King of Power Saving	I've been using it for almost a month, and I've reduced my electricity bill by over 100 bucks! I'm planning to get two more for my family.	Jingdong prefecture level city in Shandong
Dragon Quality Goods Shop	King of Power Saving	The effect is too good, the family has now several units, back to buy a number of times, the power saver received, power-saving effect is very good,, fine workmanship, long warranty, but also purify the air, before buying a used for a period of time, with the okay, and then bought a, and only now come to the	Jingdong prefecture level city in Shandong
Dragon Quality Goods Shop	King of Power Saving	Finally received the goods, very, very much like,, has been a lot of people praise, really too happy, has been his family's loyal fans, will continue to buy buy buy buy!	Jingdong prefecture level city in Shandong
Dragon Quality Goods Shop	King of Power Saving	📩 I've been wanting it for a long time, I decided to go for it, I feel great after using it, it's genuine and the delivery is great, it lives up to its name👍	Jingdong prefecture level city in Shandong
Dragon Quality Goods Shop	King of Power Saving	The quality is sure as other friends who bought it said, and it is also beautiful, thanks to the owner for the recommendation, thanks!	Jingdong prefecture level city in Shandong
Dragon Quality Goods Shop	King of Power Saving	Baby received the quality is really good face value is also very high especially practical especially especially special favorite	Jingdong prefecture level city in Shandong
Dragon Quality Goods Shop	King of Power Saving	It is genuine, trust the merchant, the quality is very good, the price is moderate, the use of the effect is very good!	Jingdong prefecture level city in Shandong
Dragon Quality Goods Shop	King of Power Saving	Energy saver received really good quality affordable and good energy saving and environmental protection is the best to use.	Jingdong prefecture level city in Shandong
Dragon Quality Goods Shop	King of Power Saving	Reasonable price logistics is very fast, the feeling is not bad more satisfied with a shopping experience	Jingdong prefecture level city in Shandong
Dragon Quality Goods Shop	King of Power Saving	The stuff is even more perfect than I imagined, very powerful and worth getting!	Jingdong prefecture level city in Shandong
Dragon Quality Goods Shop	King of Power Saving	Goods have been received, good quality, affordable price, good customer service, worth recommending!	Jingdong prefecture level city in Shandong
Dragon Quality Goods Shop	King of Power Saving	Fast shipping, works great, have already recommended it to friends.	Jingdong prefecture level city in Shandong
Dragon Quality Goods Shop	King of Power Saving	Good quality, fast logistics, reasonable price, worth 5 stars.	Jingdong prefecture level city in Shandong

Dragon Quality Goods Shop	King of Power Saving	Good quality, fast logistics, good value, well packaged, practical	Jingdong prefecture level city in Shandong
Dragon Quality Goods Shop	King of Power Saving	Fast shipping, affordable price, great quality, very satisfied!	Jingdong prefecture level city in Shandong
Dragon Quality Goods Shop	King of Power Saving	Regret knowing too late, it's really good stuff. Works well.	Jingdong prefecture level city in Shandong
Dragon Quality Goods Shop	King of Power Saving	Not bad, very good product, quite save electricity	Jingdong prefecture level city in Shandong
Dragon Quality Goods Shop	King of Power Saving	Good quality, fast logistics, overall very satisfied!	Jingdong prefecture level city in Shandong
Dragon Quality Goods Shop	King of Power Saving	Tightly packaged with good results	Jingdong prefecture level city in Shandong
Dragon Quality Goods Shop	King of Power Saving	very happy	Jingdong prefecture level city in Shandong
Dragon Quality Goods Shop	King of Power Saving	favorable criticism	Jingdong prefecture level city in Shandong
Dragon Quality Goods Shop	King of Power Saving	Now they all sign for the goods without receiving them, the Jingdong platform and merchants are very powerful, and the goods are not received before signing for the goods.	Jingdong prefecture level city in Shandong
Dragon Quality Goods Shop	King of Power Saving	low-quality commodities	Jingdong prefecture level city in Shandong
Dragon Quality Goods Shop	King of Power Saving	I bought this and now it works pretty well with the air conditioning on, when I didn't use it it was $600 a month, now it's almost $500.00, that's over $100 less all of a sudden.	Jingdong prefecture level city in Shandong
Dragon Quality Goods Shop	King of Power Saving	Can Oh, the weather is hot, open the air conditioning is really good, this product is good, has been used for a really long time, more ok, power saving effect is not bad.	Jingdong prefecture level city in Shandong
Dragon Quality Goods Shop	King of Power Saving	I paid my electric bill today, it's a few dozen dollars less, anyway, it's not costly, it's already very good, the boss said that it would be better to buy one more, I've already placed an order	Jingdong prefecture level city in Shandong
Dragon Quality Goods Shop	King of Power Saving	I'm on a smart meter, I save about 30% a month, personally I think it's pretty good, it works well	Jingdong prefecture level city in Shandong
Dragon Quality Goods Shop	King of Power Saving	My dad asked me to buy one, but after I paid the bill, I asked and he said it was a lot less this month, so he asked me to buy another one.	Jingdong prefecture level city in Shandong
Dragon Quality Goods Shop	King of Power Saving	Very satisfied with the purchase thanks to the store for the prosperous business.	Jingdong prefecture level city in Shandong
Dragon Quality Goods Shop	King of Power Saving	Good use, good quality, very satisfied with the purchase	Jingdong prefecture level city in Shandong
Dragon Quality Goods Shop	King of Power Saving	The goods have been received, the quality is very good, is the ideal effect, will continue to pay attention to the	Jingdong prefecture level city in Shandong
Dragon Quality Goods Shop	King of Power Saving	This brand has been used for many years, and this time I saw this upgraded version of the product and got it again. The purpose of this product is to reduce the loss of electricity for a long time, so as to achieve the purpose of saving electricity.	Jingdong prefecture level city in Shandong
Dragon Quality Goods Shop	King of Power Saving	I've been using it for almost a month, and I've reduced my electricity bill by over 100 bucks! I'm planning to get two more for my family.	Jingdong prefecture level city in Shandong
Dragon Quality Goods Shop	King of Power Saving	The effect is too good, the family has now several units, back to buy a number of times, the power saver received, power-saving effect is very good,, fine workmanship, long warranty, but also purify the air, before buying a used for a period of time, with the okay, and then bought a, and only now come to the	Jingdong prefecture level city in Shandong
Dragon Quality Goods Shop	King of Power Saving	Finally received the goods, very, very much like,, has been a lot of people praise, really too happy, has been his family's loyal fans, will continue to buy buy buy buy!	Jingdong prefecture level city in Shandong
Dragon Quality Goods Shop	King of Power Saving	I've been wanting it for a long time, I decided to go for it, I feel great after using it, it's genuine and the delivery is great, it lives up to its name	Jingdong prefecture level city in Shandong
Dragon Quality Goods Shop	King of Power Saving	The quality is sure as other friends who bought it said, and it is also beautiful, thanks to the owner for the recommendation, thanks!	Jingdong prefecture level city in Shandong
Dragon Quality Goods Shop	King of Power Saving	Baby received the quality is really good face value is also very high especially practical especially especially special favorite	Jingdong prefecture level city in Shandong
Dragon Quality Goods Shop	King of Power Saving	It is genuine, trust the merchant, the quality is very good, the price is moderate, the use of the effect is very good!	Jingdong prefecture level city in Shandong
Dragon Quality Goods Shop	King of Power Saving	Energy saver received really good quality affordable and good energy saving and environmental protection is the best to use.	Jingdong prefecture level city in Shandong
Dragon Quality Goods Shop	King of Power Saving	Reasonable price logistics is very fast, the feeling is not bad more satisfied with a shopping experience	Jingdong prefecture level city in Shandong
Dragon Quality Goods Shop	King of Power Saving	The stuff is even more perfect than I imagined, very powerful and worth getting!	Jingdong prefecture level city in Shandong
Dragon Quality Goods Shop	King of Power Saving	Goods have been received, good quality, affordable price, good customer service, worth recommending!	Jingdong prefecture level city in Shandong
Dragon Quality Goods Shop	King of Power Saving	Fast shipping, works great, have already recommended it to friends.	Jingdong prefecture level city in Shandong
Dragon Quality Goods Shop	King of Power Saving	Good quality, fast logistics, reasonable price, worth 5 stars.	Jingdong prefecture level city in Shandong
Dragon Quality Goods Shop	King of Power Saving	Good quality, fast logistics, good value, well packaged, practical	Jingdong prefecture level city in Shandong
Dragon Quality Goods Shop	King of Power Saving	Fast shipping, affordable price, great quality, very satisfied!	Jingdong prefecture level city in Shandong
Dragon Quality Goods Shop	King of Power Saving	Regret knowing too late, it's really good stuff. Works well.	Jingdong prefecture level city in Shandong
Dragon Quality Goods Shop	King of Power Saving	Not bad, very good product, quite save electricity	Jingdong prefecture level city in Shandong
Dragon Quality Goods Shop	King of Power Saving	Good quality, fast logistics, overall very satisfied!	Jingdong prefecture level city in Shandong
Dragon Quality Goods Shop	King of Power Saving	Tightly packaged with good results	Jingdong prefecture level city in Shandong
Dragon Quality Goods Shop	King of Power Saving	very happy	Jingdong prefecture level city in Shandong
Dragon Quality Goods Shop	King of Power Saving	favorable criticism	Jingdong prefecture level city in Shandong
Dragon Quality Goods Shop	King of Power Saving	Now they all sign for the goods without receiving them, the Jingdong platform and merchants are very powerful, and the goods are not received before signing for the goods.	Jingdong prefecture level city in Shandong
Dragon Quality Goods Shop	King of Power Saving	low-quality commodities	Jingdong prefecture level city in Shandong
Dragon Quality Goods Shop	King of Power Saving	I bought this and now it works pretty well with the air conditioning on, when I didn't use it it was $600 a month, now it's almost $500.00, that's over $100 less all of a sudden.	Jingdong prefecture level city in Shandong
Dragon Quality Goods Shop	King of Power Saving	Can Oh, the weather is hot, open the air conditioning is really good, this product is good, has been used for a really long time, more ok, power saving effect is not bad.	Jingdong prefecture level city in Shandong
Dragon Quality Goods Shop	King of Power Saving	I paid my electric bill today, it's a few dozen dollars less, anyway, it's not costly, it's already very good, the boss said that it would be better to buy one more, I've already placed an order	Jingdong prefecture level city in Shandong
Dragon Quality Goods Shop	King of Power Saving	I'm on a smart meter, I save about 30% a month, personally I think it's pretty good, it works well	Jingdong prefecture level city in Shandong
Dragon Quality Goods Shop	King of Power Saving	My dad asked me to buy one, but after I paid the bill, I asked and he said it was a lot less this month, so he asked me to buy another one.	Jingdong prefecture level city in Shandong
Dragon Quality Goods Shop	King of Power Saving	Very satisfied with the purchase thanks to the store for the prosperous business.	Jingdong prefecture level city in Shandong
Dragon Quality Goods Shop	King of Power Saving	Good use, good quality, very satisfied with the purchase	Jingdong prefecture level city in Shandong
Dragon Quality Goods Shop	King of Power Saving	The goods have been received, the quality is very good, is the ideal effect, will continue to pay attention to the	Jingdong prefecture level city in Shandong

Dragon Quality Goods Shop	King of Power Saving	This brand has been used for many years, and this time I saw this upgraded version of the product and got it again. The purpose of this product is to reduce the loss of electricity for a long time, so as to achieve the purpose of saving electricity.	Jingdong prefecture level city in Shandong
Dragon Quality Goods Shop	King of Power Saving	I've been using it for almost a month, and I've reduced my electricity bill by over 100 bucks! I'm planning to get two more for my family.	Jingdong prefecture level city in Shandong
Dragon Quality Goods Shop	King of Power Saving	The effect is too good, the family has now several units, back to buy a number of times, the power saver received, power-saving effect is very good,, fine workmanship, long warranty, but also purify the air, before buying a used for a period of time, with the okay, and then bought a, and only now come to the	Jingdong prefecture level city in Shandong
Dragon Quality Goods Shop	King of Power Saving	Finally received the goods, very, very much like,, has been a lot of people praise, really too happy, has been his family's loyal fans, will continue to buy buy buy buy!	Jingdong prefecture level city in Shandong
Dragon Quality Goods Shop	King of Power Saving	I've been wanting it for a long time, I decided to go for it, I feel great after using it, it's genuine and the delivery is great, it lives up to its name	Jingdong prefecture level city in Shandong
Dragon Quality Goods Shop	King of Power Saving	The quality is sure as other friends who bought it said, and it is also beautiful, thanks to the owner for the recommendation, thanks!	Jingdong prefecture level city in Shandong
Dragon Quality Goods Shop	King of Power Saving	Baby received the quality is really good face value is also very high especially practical especially especially special favorite	Jingdong prefecture level city in Shandong
Dragon Quality Goods Shop	King of Power Saving	It is genuine, trust the merchant, the quality is very good, the price is moderate, the use of the effect is very good!	Jingdong prefecture level city in Shandong
Dragon Quality Goods Shop	King of Power Saving	Energy saver received really good quality affordable and good energy saving and environmental protection is the best to use.	Jingdong prefecture level city in Shandong
Dragon Quality Goods Shop	King of Power Saving	Reasonable price logistics is very fast, the feeling is not bad more satisfied with a shopping experience	Jingdong prefecture level city in Shandong
Dragon Quality Goods Shop	King of Power Saving	The stuff is even more perfect than I imagined, very powerful and worth getting!	Jingdong prefecture level city in Shandong
Dragon Quality Goods Shop	King of Power Saving	Goods have been received, good quality, affordable price, good customer service, worth recommending!	Jingdong prefecture level city in Shandong
Dragon Quality Goods Shop	King of Power Saving	Fast shipping, works great, have already recommended it to friends.	Jingdong prefecture level city in Shandong
Dragon Quality Goods Shop	King of Power Saving	Good quality, fast logistics, reasonable price, worth 5 stars.	Jingdong prefecture level city in Shandong
Dragon Quality Goods Shop	King of Power Saving	Good quality, fast logistics, good value, well packaged, practical	Jingdong prefecture level city in Shandong
Dragon Quality Goods Shop	King of Power Saving	Fast shipping, affordable price, great quality, very satisfied!	Jingdong prefecture level city in Shandong
Dragon Quality Goods Shop	King of Power Saving	Regret knowing too late, it's really good stuff. Works well.	Jingdong prefecture level city in Shandong
Dragon Quality Goods Shop	King of Power Saving	Not bad, very good product, quite save electricity	Jingdong prefecture level city in Shandong
Dragon Quality Goods Shop	King of Power Saving	Good quality, fast logistics, overall very satisfied!	Jingdong prefecture level city in Shandong
Dragon Quality Goods Shop	King of Power Saving	Tightly packaged with good results	Jingdong prefecture level city in Shandong
Dragon Quality Goods Shop	King of Power Saving	very happy	Jingdong prefecture level city in Shandong
Dragon Quality Goods Shop	King of Power Saving	favorable criticism	Jingdong prefecture level city in Shandong
Dragon Quality Goods Shop	King of Power Saving	Now they all sign for the goods without receiving them, the Jingdong platform and merchants are very powerful, and the goods are not received before signing for the goods.	Jingdong prefecture level city in Shandong
Dragon Quality Goods Shop	King of Power Saving	low-quality commodities	Jingdong prefecture level city in Shandong
Dragon Quality Goods Shop	King of Power Saving	I bought this and now it works pretty well with the air conditioning on, when I didn't use it it was $600 a month, now it's almost $500,00, that's over $100 less all of a sudden.	Jingdong prefecture level city in Shandong
Dragon Quality Goods Shop	King of Power Saving	Can Oh, the weather is hot, open the air conditioning is really good, this product is good, has been used for a really long time, more ok, power saving effect is not bad.	Jingdong prefecture level city in Shandong
Dragon Quality Goods Shop	King of Power Saving	I paid my electric bill today, it's a few dozen dollars less, anyway, it's not costly, it's already very good, the boss said that it would be better to buy one more, I've already placed an order	Jingdong prefecture level city in Shandong
Dragon Quality Goods Shop	King of Power Saving	I'm on a smart meter, I save about 30% a month, personally I think it's pretty good, it works well	Jingdong prefecture level city in Shandong
Dragon Quality Goods Shop	King of Power Saving	My dad asked me to buy one, but after I paid the bill, I asked and he said it was a lot less this month, so he asked me to buy another one.	Jingdong prefecture level city in Shandong
Dragon Quality Goods Shop	King of Power Saving	Very satisfied with the purchase thanks to the store for the prosperous business.	Jingdong prefecture level city in Shandong
Dragon Quality Goods Shop	King of Power Saving	Good use, good quality, very satisfied with the purchase	Jingdong prefecture level city in Shandong
Dragon Quality Goods Shop	King of Power Saving	The goods have been received, the quality is very good, is the ideal effect, will continue to pay attention to the	Jingdong prefecture level city in Shandong
Dragon Quality Goods Shop	King of Power Saving	This brand has been used for many years, and this time I saw this upgraded version of the product and got it again. The purpose of this product is to reduce the loss of electricity for a long time, so as to achieve the purpose of saving electricity.	Jingdong prefecture level city in Shandong
Dragon Quality Goods Shop	King of Power Saving	I've been using it for almost a month, and I've reduced my electricity bill by over 100 bucks! I'm planning to get two more for my family.	Jingdong prefecture level city in Shandong
Dragon Quality Goods Shop	King of Power Saving	The effect is too good, the family has now several units, back to buy a number of times, the power saver received, power-saving effect is very good,, fine workmanship, long warranty, but also purify the air, before buying a used for a period of time, with the okay, and then bought a, and only now come to the	Jingdong prefecture level city in Shandong
Dragon Quality Goods Shop	King of Power Saving	Finally received the goods, very, very much like,, has been a lot of people praise, really too happy, has been his family's loyal fans, will continue to buy buy buy buy!	Jingdong prefecture level city in Shandong
Dragon Quality Goods Shop	King of Power Saving	I've been wanting it for a long time, I decided to go for it, I feel great after using it, it's genuine and the delivery is great, it lives up to its name	Jingdong prefecture level city in Shandong
Dragon Quality Goods Shop	King of Power Saving	The quality is sure as other friends who bought it said, and it is also beautiful, thanks to the owner for the recommendation, thanks!	Jingdong prefecture level city in Shandong
Dragon Quality Goods Shop	King of Power Saving	Baby received the quality is really good face value is also very high especially practical especially especially special favorite	Jingdong prefecture level city in Shandong
Dragon Quality Goods Shop	King of Power Saving	It is genuine, trust the merchant, the quality is very good, the price is moderate, the use of the effect is very good!	Jingdong prefecture level city in Shandong
Dragon Quality Goods Shop	King of Power Saving	Energy saver received really good quality affordable and good energy saving and environmental protection is the best to use.	Jingdong prefecture level city in Shandong
Dragon Quality Goods Shop	King of Power Saving	Reasonable price logistics is very fast, the feeling is not bad more satisfied with a shopping experience	Jingdong prefecture level city in Shandong
Dragon Quality Goods Shop	King of Power Saving	The stuff is even more perfect than I imagined, very powerful and worth getting!	Jingdong prefecture level city in Shandong
Dragon Quality Goods Shop	King of Power Saving	Goods have been received, good quality, affordable price, good customer service, worth recommending!	Jingdong prefecture level city in Shandong
Dragon Quality Goods Shop	King of Power Saving	Fast shipping, works great, have already recommended it to friends.	Jingdong prefecture level city in Shandong
Dragon Quality Goods Shop	King of Power Saving	Good quality, fast logistics, reasonable price, worth 5 stars.	Jingdong prefecture level city in Shandong
Dragon Quality Goods Shop	King of Power Saving	Good quality, fast logistics, good value, well packaged, practical	Jingdong prefecture level city in Shandong
Dragon Quality Goods Shop	King of Power Saving	Fast shipping, affordable price, great quality, very satisfied!	Jingdong prefecture level city in Shandong
Dragon Quality Goods Shop	King of Power Saving	Regret knowing too late, it's really good stuff. Works well.	Jingdong prefecture level city in Shandong

Dragon Quality Goods Shop	King of Power Saving	Not bad, very good product, quite save electricity	Jingdong prefecture level city in Shandong
Dragon Quality Goods Shop	King of Power Saving	Good quality, fast logistics, overall very satisfied!	Jingdong prefecture level city in Shandong
Dragon Quality Goods Shop	King of Power Saving	Tightly packaged with good results	Jingdong prefecture level city in Shandong
Dragon Quality Goods Shop	King of Power Saving	very happy	Jingdong prefecture level city in Shandong
Dragon Quality Goods Shop	King of Power Saving	favorable criticism	Jingdong prefecture level city in Shandong
Dragon Quality Goods Shop	King of Power Saving	Now they all sign for the goods without receiving them, the Jingdong platform and merchants are very powerful, and the goods are not received before signing for the goods.	Jingdong prefecture level city in Shandong
Dragon Quality Goods Shop	King of Power Saving	low-quality commodities	Jingdong prefecture level city in Shandong
Dragon Quality Goods Shop	King of Power Saving	This high-power is not bad Oh, the first few months are 1000 degrees so, I casually brush in Taobao to this product, I bought back to try, I used a month after this month left 736 degrees, scared me, I also called to the grid to confirm, really is 736 degrees, the effect is very good, and today bought a go back to the old family to use	Jingdong prefecture level city in Shandong
Dragon Quality Goods Shop	King of Power Saving	Previously bought other brands of power savers, but found no effect at home, this time to see how the products on the Jingdong, inserted in the home socket, the LED display is blue, the night light is very nice to see, look at the instructions that have to be half a year in order to achieve the best results, try it, I believe that the quality of the Jingdong, it should not be wrong.	Jingdong prefecture level city in Shandong
Power Saving King Shop	Green Intelligent Power Saver	Received the goods, the first time to unpack, the quality is very good, with the seller's description, completely exceeded my expectations, the packaging is very careful, very strict, in general this is a very satisfied with the shopping	Jingdong prefecture level city in Shandong
Power Saving King Shop	Green Intelligent Power Saver	Huge power saver, cheap but effective. Not bad.	Jingdong prefecture level city in Shandong
Power Saving King Shop	Green Intelligent Power Saver	The power saving effect is very obvious, trying to pay 30% less electricity bill in a month than before, hahaha	Jingdong prefecture level city in Shandong
Power Saving King Shop	Green Intelligent Power Saver	Bringing power savings into millions of homes. Still a great gadget. Pretty good.	Jingdong prefecture level city in Shandong
Power Saving King Shop	Green Intelligent Power Saver	Nice, it's been here for two days now, used it overnight and it did save two kilowatts of electricity	Jingdong prefecture level city in Shandong
Power Saving King Shop	Green Intelligent Power Saver	Quite a good power saver. Worth having. Mainly really saves electricity	Jingdong prefecture level city in Shandong
Power Saving King Shop	Green Intelligent Power Saver	Packaging is very good, fine workmanship material is good, really very practical, logistics is also very fast	Jingdong prefecture level city in Shandong
Power Saving King Shop	Green Intelligent Power Saver	Overall it is still very good, the store has a good service attitude, and the quality is also very good, no odor	Jingdong prefecture level city in Shandong
Power Saving King Shop	Green Intelligent Power Saver	Power Saving King received a tightly packaged logistics, installed the effect is very good price is cheaper than the physical store!	Jingdong prefecture level city in Shandong
Power Saving King Shop	Green Intelligent Power Saver	Super Power Saving King is very good quality, new style, is genuine, the use of the effect is very good, the price is cheap, it is worth buying!	Jingdong prefecture level city in Shandong
Power Saving King Shop	Green Intelligent Power Saver	Packaging is very tight, easy to install, quite practical, worth buying!	Jingdong prefecture level city in Shandong
Power Saving King Shop	Green Intelligent Power Saver	Quality assurance, affordable price, very energy efficient, will repurchase if needed!	Jingdong prefecture level city in Shandong
Power Saving King Shop	Green Intelligent Power Saver	Quality assurance, very good, affordable, logistics is also very fast, I'm very satisfied with the	Jingdong prefecture level city in Shandong
Power Saving King Shop	Green Intelligent Power Saver	Energy saving. It's so good, already installed, you can save a lot of electricity every month	Jingdong prefecture level city in Shandong
Power Saving King Shop	Green Intelligent Power Saver	The material is thick and the workmanship is fine in place, the power saving effect is very good, value for money!	Jingdong prefecture level city in Shandong
Power Saving King Shop	Green Intelligent Power Saver	The effect is great, genuine, and match the description, the logistics is fast, the price is reasonable 😍	Jingdong prefecture level city in Shandong
Power Saving King Shop	Green Intelligent Power Saver	It's genuine, works well, easy to use, plugs in and works, really saves power	Jingdong prefecture level city in Shandong
Power Saving King Shop	Green Intelligent Power Saver	It's easy to use, effective, great service, and recommended!	Jingdong prefecture level city in Shandong
Power Saving King Shop	Green Intelligent Power Saver	🌈 Great quality, bought it to my liking 💝 Awesome! ✨ Buy with confidence if you need it!	Jingdong prefecture level city in Shandong
Power Saving King Shop	Green Intelligent Power Saver	Good quality, easy to install, very energy saving and power saving, good to use	Jingdong prefecture level city in Shandong
Power Saving King Shop	Green Intelligent Power Saver	The quality is very good, it does have the effect of saving electricity, the description matches, the price is affordable, it is worth recommending.	Jingdong prefecture level city in Shandong
Power Saving King Shop	Green Intelligent Power Saver	Received, the functionality is particularly good, it is genuine, the price is affordable	Jingdong prefecture level city in Shandong
Power Saving King Shop	Green Intelligent Power Saver	Good quality, very easy to use, energy efficient, home electricity bill is obviously much less	Jingdong prefecture level city in Shandong
Power Saving King Shop	Green Intelligent Power Saver	Easy to install, energy saving and power saving, very satisfied with a shopping	Jingdong prefecture level city in Shandong
Power Saving King Shop	Green Intelligent Power Saver	Received it and tried it out, it's great, works great and worth having! Five stars praise!	Jingdong prefecture level city in Shandong
Power Saving King Shop	Green Intelligent Power Saver	Power saving device, no problem at all, the quality of this product at this price, very affordable!	Jingdong prefecture level city in Shandong
Power Saving King Shop	Green Intelligent Power Saver	Received the goods, did not expect to be so fast, the quality is very good, especially power saving	Jingdong prefecture level city in Shandong
Power Saving King Shop	Green Intelligent Power Saver	The logistics is very fast, especially good, need to come back again	Jingdong prefecture level city in Shandong
Power Saving King Shop	Green Intelligent Power Saver	It's already working and it really saves power, so I'm very happy with it!	Jingdong prefecture level city in Shandong
Power Saving King Shop	Green Intelligent Power Saver	Great quality, works well, can recommend to friends to buy.	Jingdong prefecture level city in Shandong
Power Saving King Shop	Green Intelligent Power Saver	It's a good deal and worth the money.	Jingdong prefecture level city in Shandong
Power Saving King Shop	Green Intelligent Power Saver	I received it and I'm very happy with it, it's very good quality, guaranteed, and	Jingdong prefecture level city in Shandong
Power Saving King Shop	Green Intelligent Power Saver	Good quality, safe and secure purchase, ideal results and great price.	Jingdong prefecture level city in Shandong
Power Saving King Shop	Green Intelligent Power Saver	Simple operation plugged in the power supply can be, friends recommended looking forward to the effect of the	Jingdong prefecture level city in Shandong
Power Saving King Shop	Green Intelligent Power Saver	It's not bad, it saves electricity, and the material is sturdy and durable.	Jingdong prefecture level city in Shandong
Power Saving King Shop	Green Intelligent Power Saver	Plug and play, no management required, convenient and hassle-free!	Jingdong prefecture level city in Shandong
Power Saving King Shop	Green Intelligent Power Saver	The pictures are too lazy to go up, the power saving effect is still there!	Jingdong prefecture level city in Shandong
Power Saving King Shop	Green Intelligent Power Saver	Fast shipping. Affordable prices. Efficient	Jingdong prefecture level city in Shandong
Power Saving King Shop	Green Intelligent Power Saver	Good power saving, worth buying	Jingdong prefecture level city in Shandong
Power Saving King Shop	Green Intelligent Power Saver	Especially great, comparison shopping before buying, the store owner is very good, answering questions very patiently and detailed, there is a need to come back!	Jingdong prefecture level city in Shandong

Power Saving King Shop	Green Intelligent Power Saver	Very trustworthy brand, quality is guaranteed, the price is still affordable, quite satisfied!	Jingdong prefecture level city in Shandong
Power Saving King Shop	Green Intelligent Power Saver	Recommended by a friend. They say you can save money on air conditioning with this. Try it.	Jingdong prefecture level city in Shandong
Power Saving King Shop	Green Intelligent Power Saver	Works great, will be back next time, very happy with it and a great price!	Jingdong prefecture level city in Shandong
Power Saving King Shop	Green Intelligent Power Saver	The quality of the product is quite good, fast shipping, satisfied.	Jingdong prefecture level city in Shandong
Power Saving King Shop	Green Intelligent Power Saver	Good quality and fast logistics	Jingdong prefecture level city in Shandong
Power Saving King Shop	Green Intelligent Power Saver	It really saves power. I've tested it myself.	Jingdong prefecture level city in Shandong
Power Saving King Shop	Green Intelligent Power Saver	Really saves electricity!	Jingdong prefecture level city in Shandong
Power Saving King Shop	Green Intelligent Power Saver	pretty good	Jingdong prefecture level city in Shandong
Power Saving King Shop	Green Intelligent Power Saver	serviceable	Jingdong prefecture level city in Shandong
Power Saving King Shop	Green Intelligent Power Saver	Power Saving King received a tightly packaged logistics, installed the effect is very good price is cheaper than the physical store!	Jingdong prefecture level city in Shandong
Power Saving King Shop	Green Intelligent Power Saver	Super Power Saving King is very good quality, new style, is genuine, the use of the effect is very good, the price is cheap, it is worth buying!	Jingdong prefecture level city in Shandong
Power Saving King Shop	Green Intelligent Power Saver	Packaging is very tight, easy to install, quite practical, worth buying!	Jingdong prefecture level city in Shandong
Power Saving King Shop	Green Intelligent Power Saver	Quality assurance, affordable price, very energy efficient, will repurchase if needed!	Jingdong prefecture level city in Shandong
Power Saving King Shop	Green Intelligent Power Saver	Quality assurance, very good, affordable, logistics is also very fast, I'm very satisfied with the	Jingdong prefecture level city in Shandong
Power Saving King Shop	Green Intelligent Power Saver	Energy saving. It's so good, already installed, you can save a lot of electricity every month	Jingdong prefecture level city in Shandong
Power Saving King Shop	Green Intelligent Power Saver	The material is thick and the workmanship is fine in place, the power saving effect is very good, value for money!	Jingdong prefecture level city in Shandong
Power Saving King Shop	Green Intelligent Power Saver	The effect is great, genuine, and match the description, the logistics is fast, the price is reasonable 😍	Jingdong prefecture level city in Shandong
Power Saving King Shop	Green Intelligent Power Saver	It's genuine, works well, easy to use, plugs in and works, really saves power	Jingdong prefecture level city in Shandong
Power Saving King Shop	Green Intelligent Power Saver	It's easy to use, effective, great service, and recommended!	Jingdong prefecture level city in Shandong
Power Saving King Shop	Green Intelligent Power Saver	🌈 Great quality, bought it to my liking 💝 Awesome! ✨ Buy with confidence if you need it!	Jingdong prefecture level city in Shandong
Power Saving King Shop	Green Intelligent Power Saver	Good quality, easy to install, very energy saving and power saving, good to use	Jingdong prefecture level city in Shandong
Power Saving King Shop	Green Intelligent Power Saver	The quality is very good, it does have the effect of saving electricity, the description matches, the price is affordable, it is worth recommending.	Jingdong prefecture level city in Shandong
Power Saving King Shop	Green Intelligent Power Saver	Received, the functionality is particularly good, it is genuine, the price is affordable	Jingdong prefecture level city in Shandong
Power Saving King Shop	Green Intelligent Power Saver	Good quality, very easy to use, energy efficient, home electricity bill is obviously much less	Jingdong prefecture level city in Shandong
Power Saving King Shop	Green Intelligent Power Saver	Easy to install, energy saving and power saving, very satisfied with a shopping	Jingdong prefecture level city in Shandong
Power Saving King Shop	Green Intelligent Power Saver	Received it and tried it out, it's great, works great and worth having! Five stars praise!	Jingdong prefecture level city in Shandong
Power Saving King Shop	Green Intelligent Power Saver	Power saving device, no problem at all, the quality of this product at this price, very affordable!	Jingdong prefecture level city in Shandong
Power Saving King Shop	Green Intelligent Power Saver	Received the goods, did not expect to be so fast, the quality is very good, especially power saving	Jingdong prefecture level city in Shandong
Power Saving King Shop	Green Intelligent Power Saver	The logistics is very fast, especially good, need to come back again	Jingdong prefecture level city in Shandong
Power Saving King Shop	Green Intelligent Power Saver	It's already working and it really saves power, so I'm very happy with it!	Jingdong prefecture level city in Shandong
Power Saving King Shop	Green Intelligent Power Saver	Great quality, works well, can recommend to friends to buy.	Jingdong prefecture level city in Shandong
Power Saving King Shop	Green Intelligent Power Saver	It's a good deal and worth the money.	Jingdong prefecture level city in Shandong
Power Saving King Shop	Green Intelligent Power Saver	I received it and I'm very happy with it, it's very good quality, guaranteed, and	Jingdong prefecture level city in Shandong
Power Saving King Shop	Green Intelligent Power Saver	Good quality, safe and secure purchase, ideal results and great price.	Jingdong prefecture level city in Shandong
Power Saving King Shop	Green Intelligent Power Saver	Simple operation plugged in the power supply can be, friends recommended looking forward to the effect of the	Jingdong prefecture level city in Shandong
Power Saving King Shop	Green Intelligent Power Saver	It's not bad, it saves electricity, and the material is sturdy and durable.	Jingdong prefecture level city in Shandong
Power Saving King Shop	Green Intelligent Power Saver	Plug and play, no management required, convenient and hassle-free!	Jingdong prefecture level city in Shandong
Power Saving King Shop	Green Intelligent Power Saver	The pictures are too lazy to go up, the power saving effect is still there!	Jingdong prefecture level city in Shandong
Power Saving King Shop	Green Intelligent Power Saver	Fast shipping. Affordable prices. Efficient	Jingdong prefecture level city in Shandong
Power Saving King Shop	Green Intelligent Power Saver	Good power saving, worth buying	Jingdong prefecture level city in Shandong
Power Saving King Shop	Green Intelligent Power Saver	Especially great, comparison shopping before buying, the store owner is very good, answering questions very patiently and detailed, there is a need to come back!	Jingdong prefecture level city in Shandong
Power Saving King Shop	Green Intelligent Power Saver	Very trustworthy brand, quality is guaranteed, the price is still affordable, quite satisfied!	Jingdong prefecture level city in Shandong
Power Saving King Shop	Green Intelligent Power Saver	Recommended by a friend. They say you can save money on air conditioning with this. Try it.	Jingdong prefecture level city in Shandong
Power Saving King Shop	Green Intelligent Power Saver	Works great, will be back next time, very happy with it and a great price!	Jingdong prefecture level city in Shandong
Power Saving King Shop	Green Intelligent Power Saver	The quality of the product is quite good, fast shipping, satisfied.	Jingdong prefecture level city in Shandong
Power Saving King Shop	Green Intelligent Power Saver	Good quality and fast logistics	Jingdong prefecture level city in Shandong
Power Saving King Shop	Green Intelligent Power Saver	It really saves power. I've tested it myself.	Jingdong prefecture level city in Shandong
Power Saving King Shop	Green Intelligent Power Saver	Really saves electricity!	Jingdong prefecture level city in Shandong
Power Saving King Shop	Green Intelligent Power Saver	pretty good	Jingdong prefecture level city in Shandong
Power Saving King Shop	Green Intelligent Power Saver	serviceable	Jingdong prefecture level city in Shandong
Power Saving King Shop	Green Intelligent Power Saver	Power Saving King received a tightly packaged logistics, installed the effect is very good price is cheaper than the physical store!	Jingdong prefecture level city in Shandong

Power Saving King Shop	Green Intelligent Power Saver	Super Power Saving King is very good quality, new style, is genuine, the use of the effect is very good, the price is cheap, it is worth buying!	Jingdong prefecture level city in Shandong
Power Saving King Shop	Green Intelligent Power Saver	Packaging is very tight, easy to install, quite practical, worth buying!	Jingdong prefecture level city in Shandong
Power Saving King Shop	Green Intelligent Power Saver	Quality assurance, affordable price, very energy efficient, will repurchase if needed!	Jingdong prefecture level city in Shandong
Power Saving King Shop	Green Intelligent Power Saver	Quality assurance, very good, affordable, logistics is also very fast, I'm very satisfied with the	Jingdong prefecture level city in Shandong
Power Saving King Shop	Green Intelligent Power Saver	Energy saving. It's so good, already installed, you can save a lot of electricity every month	Jingdong prefecture level city in Shandong
Power Saving King Shop	Green Intelligent Power Saver	The material is thick and the workmanship is fine in place, the power saving effect is very good, value for money!	Jingdong prefecture level city in Shandong
Power Saving King Shop	Green Intelligent Power Saver	The effect is great, genuine, and match the description, the logistics is fast, the price is reasonable 😍	Jingdong prefecture level city in Shandong
Power Saving King Shop	Green Intelligent Power Saver	It's genuine, works well, easy to use, plugs in and works, really saves power	Jingdong prefecture level city in Shandong
Power Saving King Shop	Green Intelligent Power Saver	It's easy to use, effective, great service, and recommended!	Jingdong prefecture level city in Shandong
Power Saving King Shop	Green Intelligent Power Saver	🌈 Great quality, bought it to my liking 💝 Awesome! ✨ Buy with confidence if you need it!	Jingdong prefecture level city in Shandong
Power Saving King Shop	Green Intelligent Power Saver	Good quality, easy to install, very energy saving and power saving, good to use	Jingdong prefecture level city in Shandong
Power Saving King Shop	Green Intelligent Power Saver	Received, the functionality is particularly good, it is genuine, the price is affordable	Jingdong prefecture level city in Shandong
Power Saving King Shop	Green Intelligent Power Saver	The quality is very good, it does have the effect of saving electricity, the description matches, the price is affordable, it is worth recommending.	Jingdong prefecture level city in Shandong
Power Saving King Shop	Green Intelligent Power Saver	Good quality, very easy to use, energy efficient, home electricity bill is obviously much less	Jingdong prefecture level city in Shandong
Power Saving King Shop	Green Intelligent Power Saver	Received it and tried it out, it's great, works great and worth having! Five stars praise!	Jingdong prefecture level city in Shandong
Power Saving King Shop	Green Intelligent Power Saver	Easy to install, energy saving and power saving, very satisfied with a shopping	Jingdong prefecture level city in Shandong
Power Saving King Shop	Green Intelligent Power Saver	Power saving device, no problem at all, the quality of this product at this price, very affordable!	Jingdong prefecture level city in Shandong
Power Saving King Shop	Green Intelligent Power Saver	Received the goods, did not expect to be so fast, the quality is very good, especially power saving	Jingdong prefecture level city in Shandong
Power Saving King Shop	Green Intelligent Power Saver	It's already working and it really saves power, so I'm very happy with it!	Jingdong prefecture level city in Shandong
Power Saving King Shop	Green Intelligent Power Saver	The logistics is very fast, especially good, need to come back again	Jingdong prefecture level city in Shandong
Power Saving King Shop	Green Intelligent Power Saver	Great quality, works well, can recommend to friends to buy.	Jingdong prefecture level city in Shandong
Power Saving King Shop	Green Intelligent Power Saver	I received it and I'm very happy with it, it's very good quality, guaranteed, and	Jingdong prefecture level city in Shandong
Power Saving King Shop	Green Intelligent Power Saver	It's a good deal and worth the money.	Jingdong prefecture level city in Shandong
Power Saving King Shop	Green Intelligent Power Saver	Good quality, safe and secure purchase, ideal results and great price.	Jingdong prefecture level city in Shandong
Power Saving King Shop	Green Intelligent Power Saver	Simple operation plugged in the power supply can be, friends recommended looking forward to the effect of the	Jingdong prefecture level city in Shandong
Power Saving King Shop	Green Intelligent Power Saver	It's not bad, it saves electricity, and the material is sturdy and durable.	Jingdong prefecture level city in Shandong
Power Saving King Shop	Green Intelligent Power Saver	Fast shipping. Affordable prices. Efficient	Jingdong prefecture level city in Shandong
Power Saving King Shop	Green Intelligent Power Saver	The pictures are too lazy to go up, the power saving effect is still there!	Jingdong prefecture level city in Shandong
Power Saving King Shop	Green Intelligent Power Saver	Especially great, comparison shopping before buying, the store owner is very good, answering questions very patiently and detailed, there is a need to come back!	Jingdong prefecture level city in Shandong
Power Saving King Shop	Green Intelligent Power Saver	Good power saving, worth buying	Jingdong prefecture level city in Shandong
Power Saving King Shop	Green Intelligent Power Saver	Plug and play, no management required, convenient and hassle-free!	Jingdong prefecture level city in Shandong
Power Saving King Shop	Green Intelligent Power Saver	Very trustworthy brand, quality is guaranteed, the price is still affordable, quite satisfied!	Jingdong prefecture level city in Shandong
Power Saving King Shop	Green Intelligent Power Saver	Works great, will be back next time, very happy with it and a great price!	Jingdong prefecture level city in Shandong
Power Saving King Shop	Green Intelligent Power Saver	Good quality and fast logistics	Jingdong prefecture level city in Shandong
Power Saving King Shop	Green Intelligent Power Saver	Recommended by a friend. They say you can save money on air conditioning with this. Try it.	Jingdong prefecture level city in Shandong
Power Saving King Shop	Green Intelligent Power Saver	Really saves electricity!	Jingdong prefecture level city in Shandong
Power Saving King Shop	Green Intelligent Power Saver	The quality of the product is quite good, fast shipping, satisfied.	Jingdong prefecture level city in Shandong
Power Saving King Shop	Green Intelligent Power Saver	It really saves power. I've tested it myself.	Jingdong prefecture level city in Shandong
Power Saving King Shop	Green Intelligent Power Saver	pretty good	Jingdong prefecture level city in Shandong
Power Saving King Shop	Green Intelligent Power Saver	serviceable	Jingdong prefecture level city in Shandong
Power Saving King Shop	Green Intelligent Power Saver	Power Saving King received a tightly packaged logistics, installed the effect is very good price is cheaper than the physical store!	Jingdong prefecture level city in Shandong
Power Saving King Shop	Green Intelligent Power Saver	Super Power Saving King is very good quality, new style, is genuine, the use of the effect is very good, the price is cheap, it is worth buying!	Jingdong prefecture level city in Shandong
Power Saving King Shop	Green Intelligent Power Saver	Packaging is very tight, easy to install, quite practical, worth buying!	Jingdong prefecture level city in Shandong
Power Saving King Shop	Green Intelligent Power Saver	Quality assurance, affordable price, very energy efficient, will repurchase if needed!	Jingdong prefecture level city in Shandong
Power Saving King Shop	Green Intelligent Power Saver	Quality assurance, very good, affordable, logistics is also very fast, I'm very satisfied with the	Jingdong prefecture level city in Shandong
Power Saving King Shop	Green Intelligent Power Saver	Energy saving. It's so good, already installed, you can save a lot of electricity every month	Jingdong prefecture level city in Shandong
Power Saving King Shop	Green Intelligent Power Saver	The material is thick and the workmanship is fine in place, the power saving effect is very good, value for money!	Jingdong prefecture level city in Shandong
Power Saving King Shop	Green Intelligent Power Saver	The effect is great, genuine, and match the description, the logistics is fast, the price is reasonable 😍	Jingdong prefecture level city in Shandong
Power Saving King Shop	Green Intelligent Power Saver	It's genuine, works well, easy to use, plugs in and works, really saves power	Jingdong prefecture level city in Shandong
Power Saving King Shop	Green Intelligent Power Saver	It's easy to use, effective, great service, and recommended!	Jingdong prefecture level city in Shandong
Power Saving King Shop	Green Intelligent Power Saver	🌈 Great quality, bought it to my liking 💝 Awesome! ✨ Buy with confidence if you need it!	Jingdong prefecture level city in Shandong

Power Saving King Shop	Green Intelligent Power Saver	Received, the functionality is particularly good, it is genuine, the price is affordable	Jingdong prefecture level city in Shandong
Power Saving King Shop	Green Intelligent Power Saver	Good quality, easy to install, very energy saving and power saving, good to use	Jingdong prefecture level city in Shandong
Power Saving King Shop	Green Intelligent Power Saver	The quality is very good, it does have the effect of saving electricity, the description matches, the price is affordable, it is worth recommending.	Jingdong prefecture level city in Shandong
Power Saving King Shop	Green Intelligent Power Saver	Good quality, very easy to use, energy efficient, home electricity bill is obviously much less	Jingdong prefecture level city in Shandong
Power Saving King Shop	Green Intelligent Power Saver	Easy to install, energy saving and power saving, very satisfied with a shopping	Jingdong prefecture level city in Shandong
Power Saving King Shop	Green Intelligent Power Saver	Received it and tried it out, it's great, works great and worth having! Five stars praise!	Jingdong prefecture level city in Shandong
Power Saving King Shop	Green Intelligent Power Saver	Power saving device, no problem at all, the quality of this product at this price, very affordable!	Jingdong prefecture level city in Shandong
Power Saving King Shop	Green Intelligent Power Saver	The logistics is very fast, especially good, need to come back again	Jingdong prefecture level city in Shandong
Power Saving King Shop	Green Intelligent Power Saver	Received the goods, did not expect to be so fast, the quality is very good, especially power saving	Jingdong prefecture level city in Shandong
Power Saving King Shop	Green Intelligent Power Saver	It's already working and it really saves power, so I'm very happy with it!	Jingdong prefecture level city in Shandong
Power Saving King Shop	Green Intelligent Power Saver	Great quality, works well, can recommend to friends to buy.	Jingdong prefecture level city in Shandong
Power Saving King Shop	Green Intelligent Power Saver	It's a good deal and worth the money.	Jingdong prefecture level city in Shandong
Power Saving King Shop	Green Intelligent Power Saver	I received it and I'm very happy with it, it's very good quality, guaranteed, and	Jingdong prefecture level city in Shandong
Power Saving King Shop	Green Intelligent Power Saver	Good quality, safe and secure purchase, ideal results and great price.	Jingdong prefecture level city in Shandong
Power Saving King Shop	Green Intelligent Power Saver	Simple operation plugged in the power supply can be, friends recommended looking forward to the effect of the	Jingdong prefecture level city in Shandong
Power Saving King Shop	Green Intelligent Power Saver	It's not bad, it saves electricity, and the material is sturdy and durable.	Jingdong prefecture level city in Shandong
Power Saving King Shop	Green Intelligent Power Saver	Plug and play, no management required, convenient and hassle-free!	Jingdong prefecture level city in Shandong
Power Saving King Shop	Green Intelligent Power Saver	The pictures are too lazy to go up, the power saving effect is still there!	Jingdong prefecture level city in Shandong
Power Saving King Shop	Green Intelligent Power Saver	Fast shipping. Affordable prices. Efficient	Jingdong prefecture level city in Shandong
Power Saving King Shop	Green Intelligent Power Saver	Good power saving, worth buying	Jingdong prefecture level city in Shandong
Power Saving King Shop	Green Intelligent Power Saver	Especially great, comparison shopping before buying, the store owner is very good, answering questions very patiently and detailed, there is a need to come back!	Jingdong prefecture level city in Shandong
Power Saving King Shop	Green Intelligent Power Saver	Very trustworthy brand, quality is guaranteed, the price is still affordable, quite satisfied!	Jingdong prefecture level city in Shandong
Power Saving King Shop	Green Intelligent Power Saver	Recommended by a friend. They say you can save money on air conditioning with this. Try it.	Jingdong prefecture level city in Shandong
Power Saving King Shop	Green Intelligent Power Saver	Works great, will be back next time, very happy with it and a great price!	Jingdong prefecture level city in Shandong
Power Saving King Shop	Green Intelligent Power Saver	The quality of the product is quite good, fast shipping, satisfied.	Jingdong prefecture level city in Shandong
Power Saving King Shop	Green Intelligent Power Saver	Good quality and fast logistics	Jingdong prefecture level city in Shandong
Power Saving King Shop	Green Intelligent Power Saver	It really saves power. I've tested it myself.	Jingdong prefecture level city in Shandong
Power Saving King Shop	Green Intelligent Power Saver	pretty good	Jingdong prefecture level city in Shandong
Power Saving King Shop	Green Intelligent Power Saver	Really saves electricity!	Jingdong prefecture level city in Shandong
Power Saving King Shop	Green Intelligent Power Saver	serviceable	Jingdong prefecture level city in Shandong
Power Saving King Shop	Green Intelligent Power Saver	Power Saving King received a tightly packaged logistics, installed the effect is very good price is cheaper than the physical store!	Jingdong prefecture level city in Shandong
Power Saving King Shop	Green Intelligent Power Saver	Super Power Saving King is very good quality, new style, is genuine, the use of the effect is very good, the price is cheap, it is worth buying!	Jingdong prefecture level city in Shandong
Power Saving King Shop	Green Intelligent Power Saver	Packaging is very tight, easy to install, quite practical, worth buying!	Jingdong prefecture level city in Shandong
Power Saving King Shop	Green Intelligent Power Saver	Quality assurance, affordable price, very energy efficient, will repurchase if needed!	Jingdong prefecture level city in Shandong
Power Saving King Shop	Green Intelligent Power Saver	Quality assurance, very good, affordable, logistics is also very fast, I'm very satisfied with the	Jingdong prefecture level city in Shandong
Power Saving King Shop	Green Intelligent Power Saver	Energy saving. It's so good, already installed, you can save a lot of electricity every month	Jingdong prefecture level city in Shandong
Power Saving King Shop	Green Intelligent Power Saver	The material is thick and the workmanship is fine in place, the power saving effect is very good, value for money!	Jingdong prefecture level city in Shandong
Power Saving King Shop	Green Intelligent Power Saver	The effect is great, genuine, and match the description, the logistics is fast, the price is reasonable 😍	Jingdong prefecture level city in Shandong
Power Saving King Shop	Green Intelligent Power Saver	It's genuine, works well, easy to use, plugs in and works, really saves power	Jingdong prefecture level city in Shandong
Power Saving King Shop	Green Intelligent Power Saver	It's easy to use, effective, great service, and recommended!	Jingdong prefecture level city in Shandong
Power Saving King Shop	Green Intelligent Power Saver	🌈 Great quality, bought it to my liking 💝 Awesome! ✨ Buy with confidence if you need it!	Jingdong prefecture level city in Shandong
Power Saving King Shop	Green Intelligent Power Saver	Good quality, easy to install, very energy saving and power saving, good to use	Jingdong prefecture level city in Shandong
Power Saving King Shop	Green Intelligent Power Saver	The quality is very good, it does have the effect of saving electricity, the description matches, the price is affordable, it is worth recommending.	Jingdong prefecture level city in Shandong
Power Saving King Shop	Green Intelligent Power Saver	Received, the functionality is particularly good, it is genuine, the price is affordable	Jingdong prefecture level city in Shandong
Power Saving King Shop	Green Intelligent Power Saver	Good quality, very easy to use, energy efficient, home electricity bill is obviously much less	Jingdong prefecture level city in Shandong
Power Saving King Shop	Green Intelligent Power Saver	Easy to install, energy saving and power saving, very satisfied with a shopping	Jingdong prefecture level city in Shandong
Power Saving King Shop	Green Intelligent Power Saver	Received it and tried it out, it's great, works great and worth having! Five stars praise!	Jingdong prefecture level city in Shandong
Power Saving King Shop	Green Intelligent Power Saver	Power saving device, no problem at all, the quality of this product at this price, very affordable!	Jingdong prefecture level city in Shandong
Power Saving King Shop	Green Intelligent Power Saver	Received the goods, did not expect to be so fast, the quality is very good, especially power saving	Jingdong prefecture level city in Shandong
Power Saving King Shop	Green Intelligent Power Saver	The logistics is very fast, especially good, need to come back again	Jingdong prefecture level city in Shandong
Power Saving King Shop	Green Intelligent Power Saver	It's already working and it really saves power, so I'm very happy with it!	Jingdong prefecture level city in Shandong

Power Saving King Shop	Green Intelligent Power Saver	Great quality, works well, can recommend to friends to buy.	Jingdong prefecture level city in Shandong
Power Saving King Shop	Green Intelligent Power Saver	It's a good deal and worth the money.	Jingdong prefecture level city in Shandong
Power Saving King Shop	Green Intelligent Power Saver	I received it and I'm very happy with it, it's very good quality, guaranteed, and	Jingdong prefecture level city in Shandong
Power Saving King Shop	Green Intelligent Power Saver	Good quality, safe and secure purchase, ideal results and great price.	Jingdong prefecture level city in Shandong
Power Saving King Shop	Green Intelligent Power Saver	Simple operation plugged in the power supply can be, friends recommended looking forward to the effect of the	Jingdong prefecture level city in Shandong
Power Saving King Shop	Green Intelligent Power Saver	It's not bad, it saves electricity, and the material is sturdy and durable.	Jingdong prefecture level city in Shandong
Power Saving King Shop	Green Intelligent Power Saver	Plug and play, no management required, convenient and hassle-free!	Jingdong prefecture level city in Shandong
Power Saving King Shop	Green Intelligent Power Saver	The pictures are too lazy to go up, the power saving effect is still there!	Jingdong prefecture level city in Shandong
Power Saving King Shop	Green Intelligent Power Saver	Fast shipping. Affordable prices. Efficient	Jingdong prefecture level city in Shandong
Power Saving King Shop	Green Intelligent Power Saver	Good power saving, worth buying	Jingdong prefecture level city in Shandong
Power Saving King Shop	Green Intelligent Power Saver	Especially great, comparison shopping before buying, the store owner is very good, answering questions very patiently and detailed, there is a need to come back!	Jingdong prefecture level city in Shandong
Power Saving King Shop	Green Intelligent Power Saver	Very trustworthy brand, quality is guaranteed, the price is still affordable, quite satisfied!	Jingdong prefecture level city in Shandong
Power Saving King Shop	Green Intelligent Power Saver	Recommended by a friend. They say you can save money on air conditioning with this. Try it.	Jingdong prefecture level city in Shandong
Power Saving King Shop	Green Intelligent Power Saver	Works great, will be back next time, very happy with it and a great price!	Jingdong prefecture level city in Shandong
Power Saving King Shop	Green Intelligent Power Saver	The quality of the product is quite good, fast shipping, satisfied.	Jingdong prefecture level city in Shandong
Power Saving King Shop	Green Intelligent Power Saver	Good quality and fast logistics	Jingdong prefecture level city in Shandong
Power Saving King Shop	Green Intelligent Power Saver	It really saves power. I've tested it myself.	Jingdong prefecture level city in Shandong
Power Saving King Shop	Green Intelligent Power Saver	Really saves electricity!	Jingdong prefecture level city in Shandong
Power Saving King Shop	Green Intelligent Power Saver	pretty good	Jingdong prefecture level city in Shandong
Power Saving King Shop	Green Intelligent Power Saver	serviceable	Jingdong prefecture level city in Shandong
Power Saving King Shop	Green Intelligent Power Saver	Power Saving King received a tightly packaged logistics, installed the effect is very good price is cheaper than the physical store!	Jingdong prefecture level city in Shandong
Power Saving King Shop	Green Intelligent Power Saver	Super Power Saving King is very good quality, new style, is genuine, the use of the effect is very good, the price is cheap, it is worth buying!	Jingdong prefecture level city in Shandong
Power Saving King Shop	Green Intelligent Power Saver	Packaging is very tight, easy to install, quite practical, worth buying!	Jingdong prefecture level city in Shandong
Power Saving King Shop	Green Intelligent Power Saver	Quality assurance, very good, affordable, logistics is also very fast, I'm very satisfied with the	Jingdong prefecture level city in Shandong
Power Saving King Shop	Green Intelligent Power Saver	Quality assurance, affordable price, very energy efficient, will repurchase if needed!	Jingdong prefecture level city in Shandong
Power Saving King Shop	Green Intelligent Power Saver	Energy saving. It's so good, already installed, you can save a lot of electricity every month	Jingdong prefecture level city in Shandong
Power Saving King Shop	Green Intelligent Power Saver	The material is thick and the workmanship is fine in place, the power saving effect is very good, value for money!	Jingdong prefecture level city in Shandong
Power Saving King Shop	Green Intelligent Power Saver	The effect is great, genuine, and match the description, the logistics is fast, the price is reasonable 😍	Jingdong prefecture level city in Shandong
Power Saving King Shop	Green Intelligent Power Saver	It's genuine, works well, easy to use, plugs in and works, really saves power	Jingdong prefecture level city in Shandong
Power Saving King Shop	Green Intelligent Power Saver	It's easy to use, effective, great service, and recommended!	Jingdong prefecture level city in Shandong
Power Saving King Shop	Green Intelligent Power Saver	🌈 Great quality, bought it to my liking 💞 Awesome! ✨ Buy with confidence if you need it!	Jingdong prefecture level city in Shandong
Power Saving King Shop	Green Intelligent Power Saver	Good quality, easy to install, very energy saving and power saving, good to use	Jingdong prefecture level city in Shandong
Power Saving King Shop	Green Intelligent Power Saver	The quality is very good, it does have the effect of saving electricity, the description matches, the price is affordable, it is worth recommending.	Jingdong prefecture level city in Shandong
Power Saving King Shop	Green Intelligent Power Saver	Good quality, very easy to use, energy efficient, home electricity bill is obviously much less	Jingdong prefecture level city in Shandong
Power Saving King Shop	Green Intelligent Power Saver	Easy to install, energy saving and power saving, very satisfied with a shopping	Jingdong prefecture level city in Shandong
Power Saving King Shop	Green Intelligent Power Saver	Received it and tried it out, it's great, works great and worth having! Five stars praise!	Jingdong prefecture level city in Shandong
Power Saving King Shop	Green Intelligent Power Saver	Received, the functionality is particularly good, it is genuine, the price is affordable	Jingdong prefecture level city in Shandong
Power Saving King Shop	Green Intelligent Power Saver	Power saving device, no problem at all, the quality of this product at this price, very affordable!	Jingdong prefecture level city in Shandong
Power Saving King Shop	Green Intelligent Power Saver	Received the goods, did not expect to be so fast, the quality is very good, especially power saving	Jingdong prefecture level city in Shandong
Power Saving King Shop	Green Intelligent Power Saver	The logistics is very fast, especially good, need to come back again	Jingdong prefecture level city in Shandong
Power Saving King Shop	Green Intelligent Power Saver	It's already working and it really saves power, so I'm very happy with it!	Jingdong prefecture level city in Shandong
Power Saving King Shop	Green Intelligent Power Saver	Great quality, works well, can recommend to friends to buy.	Jingdong prefecture level city in Shandong
Power Saving King Shop	Green Intelligent Power Saver	It's a good deal and worth the money.	Jingdong prefecture level city in Shandong
Power Saving King Shop	Green Intelligent Power Saver	I received it and I'm very happy with it, it's very good quality, guaranteed, and	Jingdong prefecture level city in Shandong
Power Saving King Shop	Green Intelligent Power Saver	Good quality, safe and secure purchase, ideal results and great price.	Jingdong prefecture level city in Shandong
Power Saving King Shop	Green Intelligent Power Saver	Simple operation plugged in the power supply can be, friends recommended looking forward to the effect of the	Jingdong prefecture level city in Shandong
Power Saving King Shop	Green Intelligent Power Saver	It's not bad, it saves electricity, and the material is sturdy and durable.	Jingdong prefecture level city in Shandong
Power Saving King Shop	Green Intelligent Power Saver	Fast shipping. Affordable prices. Efficient	Jingdong prefecture level city in Shandong
Power Saving King Shop	Green Intelligent Power Saver	Plug and play, no management required, convenient and hassle-free!	Jingdong prefecture level city in Shandong
Power Saving King Shop	Green Intelligent Power Saver	The pictures are too lazy to go up, the power saving effect is still there!	Jingdong prefecture level city in Shandong
Power Saving King Shop	Green Intelligent Power Saver	Good power saving, worth buying	Jingdong prefecture level city in Shandong

Power Saving King Shop	Green Intelligent Power Saver	Very trustworthy brand, quality is guaranteed, the price is still affordable, quite satisfied!	Jingdong prefecture level city in Shandong
Power Saving King Shop	Green Intelligent Power Saver	Recommended by a friend. They say you can save money on air conditioning with this. Try it.	Jingdong prefecture level city in Shandong
Power Saving King Shop	Green Intelligent Power Saver	Especially great, comparison shopping before buying, the store owner is very good, answering questions very patiently and detailed, there is a need to come back!	Jingdong prefecture level city in Shandong
Power Saving King Shop	Green Intelligent Power Saver	Works great, will be back next time, very happy with it and a great price!	Jingdong prefecture level city in Shandong
Power Saving King Shop	Green Intelligent Power Saver	The quality of the product is quite good, fast shipping, satisfied.	Jingdong prefecture level city in Shandong
Power Saving King Shop	Green Intelligent Power Saver	Good quality and fast logistics	Jingdong prefecture level city in Shandong
Power Saving King Shop	Green Intelligent Power Saver	It really saves power. I've tested it myself.	Jingdong prefecture level city in Shandong
Power Saving King Shop	Green Intelligent Power Saver	Really saves electricity!	Jingdong prefecture level city in Shandong
Power Saving King Shop	Green Intelligent Power Saver	pretty good	Jingdong prefecture level city in Shandong
Power Saving King Shop	Green Intelligent Power Saver	serviceable	Jingdong prefecture level city in Shandong
Power Saving King Shop	Green Intelligent Power Saver	Power Saving King received a tightly packaged logistics, installed the effect is very good price is cheaper than the physical store!	Jingdong prefecture level city in Shandong
Power Saving King Shop	Green Intelligent Power Saver	Super Power Saving King is very good quality, new style, is genuine, the use of the effect is very good, the price is cheap, it is worth buying!	Jingdong prefecture level city in Shandong
Power Saving King Shop	Green Intelligent Power Saver	Packaging is very tight, easy to install, quite practical, worth buying!	Jingdong prefecture level city in Shandong
Power Saving King Shop	Green Intelligent Power Saver	Quality assurance, affordable price, very energy efficient, will repurchase if needed!	Jingdong prefecture level city in Shandong
Power Saving King Shop	Green Intelligent Power Saver	Quality assurance, very good, affordable, logistics is also very fast, I'm very satisfied with the	Jingdong prefecture level city in Shandong
Power Saving King Shop	Green Intelligent Power Saver	Energy saving. It's so good, already installed, you can save a lot of electricity every month	Jingdong prefecture level city in Shandong
Power Saving King Shop	Green Intelligent Power Saver	The material is thick and the workmanship is fine in place, the power saving effect is very good, value for money!	Jingdong prefecture level city in Shandong
Power Saving King Shop	Green Intelligent Power Saver	The effect is great, genuine, and match the description, the logistics is fast, the price is reasonable 😍	Jingdong prefecture level city in Shandong
Power Saving King Shop	Green Intelligent Power Saver	It's genuine, works well, easy to use, plugs in and works, really saves power	Jingdong prefecture level city in Shandong
Power Saving King Shop	Green Intelligent Power Saver	It's easy to use, effective, great service, and recommended!	Jingdong prefecture level city in Shandong
Power Saving King Shop	Green Intelligent Power Saver	🌈 Great quality, bought it to my liking 💝 Awesome! ✨ Buy with confidence if you need it!	Jingdong prefecture level city in Shandong
Power Saving King Shop	Green Intelligent Power Saver	Good quality, easy to install, very energy saving and power saving, good to use	Jingdong prefecture level city in Shandong
Power Saving King Shop	Green Intelligent Power Saver	The quality is very good, it does have the effect of saving electricity, the description matches, the price is affordable, it is worth recommending.	Jingdong prefecture level city in Shandong
Power Saving King Shop	Green Intelligent Power Saver	Received, the functionality is particularly good, it is genuine, the price is affordable	Jingdong prefecture level city in Shandong
Power Saving King Shop	Green Intelligent Power Saver	Good quality, very easy to use, energy efficient, home electricity bill is obviously much less	Jingdong prefecture level city in Shandong
Power Saving King Shop	Green Intelligent Power Saver	Easy to install, energy saving and power saving, very satisfied with a shopping	Jingdong prefecture level city in Shandong
Power Saving King Shop	Green Intelligent Power Saver	Received it and tried it out, it's great, works great and worth having! Five stars praise!	Jingdong prefecture level city in Shandong
Power Saving King Shop	Green Intelligent Power Saver	Power saving device, no problem at all, the quality of this product at this price, very affordable!	Jingdong prefecture level city in Shandong
Power Saving King Shop	Green Intelligent Power Saver	Received the goods, did not expect to be so fast, the quality is very good, especially power saving	Jingdong prefecture level city in Shandong
Power Saving King Shop	Green Intelligent Power Saver	The logistics is very fast, especially good, need to come back again	Jingdong prefecture level city in Shandong
Power Saving King Shop	Green Intelligent Power Saver	It's already working and it really saves power, so I'm very happy with it!	Jingdong prefecture level city in Shandong
Power Saving King Shop	Green Intelligent Power Saver	Great quality, works well, can recommend to friends to buy.	Jingdong prefecture level city in Shandong
Power Saving King Shop	Green Intelligent Power Saver	It's a good deal and worth the money.	Jingdong prefecture level city in Shandong
Power Saving King Shop	Green Intelligent Power Saver	I received it and I'm very happy with it, it's very good quality, guaranteed, and	Jingdong prefecture level city in Shandong
Power Saving King Shop	Green Intelligent Power Saver	Good quality, safe and secure purchase, ideal results and great price.	Jingdong prefecture level city in Shandong
Power Saving King Shop	Green Intelligent Power Saver	Simple operation plugged in the power supply can be, friends recommended looking forward to the effect of the	Jingdong prefecture level city in Shandong
Power Saving King Shop	Green Intelligent Power Saver	It's not bad, it saves electricity, and the material is sturdy and durable.	Jingdong prefecture level city in Shandong
Power Saving King Shop	Green Intelligent Power Saver	Plug and play, no management required, convenient and hassle-free!	Jingdong prefecture level city in Shandong
Power Saving King Shop	Green Intelligent Power Saver	The pictures are too lazy to go up, the power saving effect is still there!	Jingdong prefecture level city in Shandong
Power Saving King Shop	Green Intelligent Power Saver	Fast shipping. Affordable prices. Efficient	Jingdong prefecture level city in Shandong
Power Saving King Shop	Green Intelligent Power Saver	Good power saving, worth buying	Jingdong prefecture level city in Shandong
Power Saving King Shop	Green Intelligent Power Saver	Especially great, comparison shopping before buying, the store owner is very good, answering questions very patiently and detailed, there is a need to come back!	Jingdong prefecture level city in Shandong
Power Saving King Shop	Green Intelligent Power Saver	Very trustworthy brand, quality is guaranteed, the price is still affordable, quite satisfied!	Jingdong prefecture level city in Shandong
Power Saving King Shop	Green Intelligent Power Saver	Recommended by a friend. They say you can save money on air conditioning with this. Try it.	Jingdong prefecture level city in Shandong
Power Saving King Shop	Green Intelligent Power Saver	Works great, will be back next time, very happy with it and a great price!	Jingdong prefecture level city in Shandong
Power Saving King Shop	Green Intelligent Power Saver	The quality of the product is quite good, fast shipping, satisfied.	Jingdong prefecture level city in Shandong
Power Saving King Shop	Green Intelligent Power Saver	Good quality and fast logistics	Jingdong prefecture level city in Shandong
Power Saving King Shop	Green Intelligent Power Saver	It really saves power. I've tested it myself.	Jingdong prefecture level city in Shandong
Power Saving King Shop	Green Intelligent Power Saver	Really saves electricity!	Jingdong prefecture level city in Shandong
Power Saving King Shop	Green Intelligent Power Saver	pretty good	Jingdong prefecture level city in Shandong
Power Saving King Shop	Green Intelligent Power Saver	serviceable	Jingdong prefecture level city in Shandong

Power Saving King Shop	Green Intelligent Power Saver	Power Saving King received a tightly packaged logistics, installed the effect is very good price is cheaper than the physical store!	Jingdong prefecture level city in Shandong
Power Saving King Shop	Green Intelligent Power Saver	Super Power Saving King is very good quality, new style, is genuine, the use of the effect is very good, the price is cheap, it is worth buying!	Jingdong prefecture level city in Shandong
Power Saving King Shop	Green Intelligent Power Saver	Packaging is very tight, easy to install, quite practical, worth buying!	Jingdong prefecture level city in Shandong
Power Saving King Shop	Green Intelligent Power Saver	Quality assurance, affordable price, very energy efficient, will repurchase if needed!	Jingdong prefecture level city in Shandong
Power Saving King Shop	Green Intelligent Power Saver	Quality assurance, very good, affordable, logistics is also very fast, I'm very satisfied with the	Jingdong prefecture level city in Shandong
Power Saving King Shop	Green Intelligent Power Saver	Energy saving. It's so good, already installed, you can save a lot of electricity every month	Jingdong prefecture level city in Shandong
Power Saving King Shop	Green Intelligent Power Saver	The material is thick and the workmanship is fine in place, the power saving effect is very good, value for money!	Jingdong prefecture level city in Shandong
Power Saving King Shop	Green Intelligent Power Saver	The effect is great, genuine, and match the description, the logistics is fast, the price is reasonable 😍	Jingdong prefecture level city in Shandong
Power Saving King Shop	Green Intelligent Power Saver	It's genuine, works well, easy to use, plugs in and works, really saves power	Jingdong prefecture level city in Shandong
Power Saving King Shop	Green Intelligent Power Saver	It's easy to use, effective, great service, and recommended!	Jingdong prefecture level city in Shandong
Power Saving King Shop	Green Intelligent Power Saver	Good quality, easy to install, very energy saving and power saving, good to use	Jingdong prefecture level city in Shandong
Power Saving King Shop	Green Intelligent Power Saver	🌈 Great quality, bought it to my liking 💖 Awesome! ✨ Buy with confidence if you need it!	Jingdong prefecture level city in Shandong
Power Saving King Shop	Green Intelligent Power Saver	The quality is very good, it does have the effect of saving electricity, the description matches, the price is affordable, it is worth recommending.	Jingdong prefecture level city in Shandong
Power Saving King Shop	Green Intelligent Power Saver	Received, the functionality is particularly good, it is genuine, the price is affordable	Jingdong prefecture level city in Shandong
Power Saving King Shop	Green Intelligent Power Saver	Good quality, very easy to use, energy efficient, home electricity bill is obviously much less	Jingdong prefecture level city in Shandong
Power Saving King Shop	Green Intelligent Power Saver	Received it and tried it out, it's great, works great and worth having! Five stars praise!	Jingdong prefecture level city in Shandong
Power Saving King Shop	Green Intelligent Power Saver	Easy to install, energy saving and power saving, very satisfied with a shopping	Jingdong prefecture level city in Shandong
Power Saving King Shop	Green Intelligent Power Saver	Power saving device, no problem at all, the quality of this product at this price, very affordable!	Jingdong prefecture level city in Shandong
Power Saving King Shop	Green Intelligent Power Saver	Received the goods, did not expect to be so fast, the quality is very good, especially power saving	Jingdong prefecture level city in Shandong
Power Saving King Shop	Green Intelligent Power Saver	The logistics is very fast, especially good, need to come back again	Jingdong prefecture level city in Shandong
Power Saving King Shop	Green Intelligent Power Saver	It's already working and it really saves power, so I'm very happy with it!	Jingdong prefecture level city in Shandong
Power Saving King Shop	Green Intelligent Power Saver	Great quality, works well, can recommend to friends to buy.	Jingdong prefecture level city in Shandong
Power Saving King Shop	Green Intelligent Power Saver	It's a good deal and worth the money.	Jingdong prefecture level city in Shandong
Power Saving King Shop	Green Intelligent Power Saver	I received it and I'm very happy with it, it's very good quality, guaranteed, and	Jingdong prefecture level city in Shandong
Power Saving King Shop	Green Intelligent Power Saver	Good quality, safe and secure purchase, ideal results and great price.	Jingdong prefecture level city in Shandong
Power Saving King Shop	Green Intelligent Power Saver	It's not bad, it saves electricity, and the material is sturdy and durable.	Jingdong prefecture level city in Shandong
Power Saving King Shop	Green Intelligent Power Saver	Simple operation plugged in the power supply can be, friends recommended looking forward to the effect of the	Jingdong prefecture level city in Shandong
Power Saving King Shop	Green Intelligent Power Saver	Plug and play, no management required, convenient and hassle-free!	Jingdong prefecture level city in Shandong
Power Saving King Shop	Green Intelligent Power Saver	The pictures are too lazy to go up, the power saving effect is still there!	Jingdong prefecture level city in Shandong
Power Saving King Shop	Green Intelligent Power Saver	Fast shipping. Affordable prices. Efficient	Jingdong prefecture level city in Shandong
Power Saving King Shop	Green Intelligent Power Saver	Good power saving, worth buying	Jingdong prefecture level city in Shandong
Power Saving King Shop	Green Intelligent Power Saver	Especially great, comparison shopping before buying, the store owner is very good, answering questions very patiently and detailed, there is a need to come back!	Jingdong prefecture level city in Shandong
Power Saving King Shop	Green Intelligent Power Saver	Very trustworthy brand, quality is guaranteed, the price is still affordable, quite satisfied!	Jingdong prefecture level city in Shandong
Power Saving King Shop	Green Intelligent Power Saver	Recommended by a friend. They say you can save money on air conditioning with this. Try it.	Jingdong prefecture level city in Shandong
Power Saving King Shop	Green Intelligent Power Saver	Works great, will be back next time, very happy with it and a great price!	Jingdong prefecture level city in Shandong
Power Saving King Shop	Green Intelligent Power Saver	The quality of the product is quite good, fast shipping, satisfied.	Jingdong prefecture level city in Shandong
Power Saving King Shop	Green Intelligent Power Saver	Good quality and fast logistics	Jingdong prefecture level city in Shandong
Power Saving King Shop	Green Intelligent Power Saver	It really saves power. I've tested it myself.	Jingdong prefecture level city in Shandong
Power Saving King Shop	Green Intelligent Power Saver	Really saves electricity!	Jingdong prefecture level city in Shandong
Power Saving King Shop	Green Intelligent Power Saver	pretty good	Jingdong prefecture level city in Shandong
Power Saving King Shop	Green Intelligent Power Saver	serviceable	Jingdong prefecture level city in Shandong
Power Saving King Shop	Green Intelligent Power Saver	Power Saving King received a tightly packaged logistics, installed the effect is very good price is cheaper than the physical store!	Jingdong prefecture level city in Shandong
Power Saving King Shop	Green Intelligent Power Saver	Super Power Saving King is very good quality, new style, is genuine, the use of the effect is very good, the price is cheap, it is worth buying!	Jingdong prefecture level city in Shandong
Power Saving King Shop	Green Intelligent Power Saver	Packaging is very tight, easy to install, quite practical, worth buying!	Jingdong prefecture level city in Shandong
Power Saving King Shop	Green Intelligent Power Saver	Quality assurance, affordable price, very energy efficient, will repurchase if needed!	Jingdong prefecture level city in Shandong
Power Saving King Shop	Green Intelligent Power Saver	Quality assurance, very good, affordable, logistics is also very fast, I'm very satisfied with the	Jingdong prefecture level city in Shandong
Power Saving King Shop	Green Intelligent Power Saver	Energy saving. It's so good, already installed, you can save a lot of electricity every month	Jingdong prefecture level city in Shandong
Power Saving King Shop	Green Intelligent Power Saver	The material is thick and the workmanship is fine in place, the power saving effect is very good, value for money!	Jingdong prefecture level city in Shandong
Power Saving King Shop	Green Intelligent Power Saver	The effect is great, genuine, and match the description, the logistics is fast, the price is reasonable 😍	Jingdong prefecture level city in Shandong
Power Saving King Shop	Green Intelligent Power Saver	It's genuine, works well, easy to use, plugs in and works, really saves power	Jingdong prefecture level city in Shandong
Power Saving King Shop	Green Intelligent Power Saver	It's easy to use, effective, great service, and recommended!	Jingdong prefecture level city in Shandong

Power Saving King Shop	Green Intelligent Power Saver	🌈 Great quality, bought it to my liking 💛 Awesome! ✨ Buy with confidence if you need it!	Jingdong prefecture level city in Shandong
Power Saving King Shop	Green Intelligent Power Saver	Good quality, easy to install, very energy saving and power saving, good to use	Jingdong prefecture level city in Shandong
Power Saving King Shop	Green Intelligent Power Saver	The quality is very good, it does have the effect of saving electricity, the description matches, the price is affordable, it is worth recommending.	Jingdong prefecture level city in Shandong
Power Saving King Shop	Green Intelligent Power Saver	Received, the functionality is particularly good, it is genuine, the price is affordable	Jingdong prefecture level city in Shandong
Power Saving King Shop	Green Intelligent Power Saver	Good quality, very easy to use, energy efficient, home electricity bill is obviously much less	Jingdong prefecture level city in Shandong
Power Saving King Shop	Green Intelligent Power Saver	Easy to install, energy saving and power saving, very satisfied with a shopping	Jingdong prefecture level city in Shandong
Power Saving King Shop	Green Intelligent Power Saver	Received it and tried it out, it's great, works great and worth having! Five stars praise!	Jingdong prefecture level city in Shandong
Power Saving King Shop	Green Intelligent Power Saver	Power saving device, no problem at all, the quality of this product at this price, very affordable!	Jingdong prefecture level city in Shandong
Power Saving King Shop	Green Intelligent Power Saver	Received the goods, did not expect to be so fast, the quality is very good, especially power saving	Jingdong prefecture level city in Shandong
Power Saving King Shop	Green Intelligent Power Saver	The logistics is very fast, especially good, need to come back again	Jingdong prefecture level city in Shandong
Power Saving King Shop	Green Intelligent Power Saver	It's already working and it really saves power, so I'm very happy with it!	Jingdong prefecture level city in Shandong
Power Saving King Shop	Green Intelligent Power Saver	Great quality, works well, can recommend to friends to buy.	Jingdong prefecture level city in Shandong
Power Saving King Shop	Green Intelligent Power Saver	It's a good deal and worth the money.	Jingdong prefecture level city in Shandong
Power Saving King Shop	Green Intelligent Power Saver	I received it and I'm very happy with it, it's very good quality, guaranteed, and	Jingdong prefecture level city in Shandong
Power Saving King Shop	Green Intelligent Power Saver	Good quality, safe and secure purchase, ideal results and great price.	Jingdong prefecture level city in Shandong
Power Saving King Shop	Green Intelligent Power Saver	Simple operation plugged in the power supply can be, friends recommended looking forward to the effect of the	Jingdong prefecture level city in Shandong
Power Saving King Shop	Green Intelligent Power Saver	It's not bad, it saves electricity, and the material is sturdy and durable.	Jingdong prefecture level city in Shandong
Power Saving King Shop	Green Intelligent Power Saver	Plug and play, no management required, convenient and hassle-free!	Jingdong prefecture level city in Shandong
Power Saving King Shop	Green Intelligent Power Saver	The pictures are too lazy to go up, the power saving effect is still there!	Jingdong prefecture level city in Shandong
Power Saving King Shop	Green Intelligent Power Saver	Fast shipping. Affordable prices. Efficient	Jingdong prefecture level city in Shandong
Power Saving King Shop	Green Intelligent Power Saver	Good power saving, worth buying	Jingdong prefecture level city in Shandong
Power Saving King Shop	Green Intelligent Power Saver	Especially great, comparison shopping before buying, the store owner is very good, answering questions very patiently and detailed, there is a need to come back!	Jingdong prefecture level city in Shandong
Power Saving King Shop	Green Intelligent Power Saver	Very trustworthy brand, quality is guaranteed, the price is still affordable, quite satisfied!	Jingdong prefecture level city in Shandong
Power Saving King Shop	Green Intelligent Power Saver	Recommended by a friend. They say you can save money on air conditioning with this. Try it.	Jingdong prefecture level city in Shandong
Power Saving King Shop	Green Intelligent Power Saver	Works great, will be back next time, very happy with it and a great price!	Jingdong prefecture level city in Shandong
Power Saving King Shop	Green Intelligent Power Saver	The quality of the product is quite good, fast shipping, satisfied.	Jingdong prefecture level city in Shandong
Power Saving King Shop	Green Intelligent Power Saver	Good quality and fast logistics	Jingdong prefecture level city in Shandong
Power Saving King Shop	Green Intelligent Power Saver	It really saves power. I've tested it myself.	Jingdong prefecture level city in Shandong
Power Saving King Shop	Green Intelligent Power Saver	Really saves electricity!	Jingdong prefecture level city in Shandong
Power Saving King Shop	Green Intelligent Power Saver	pretty good	Jingdong prefecture level city in Shandong
Power Saving King Shop	Green Intelligent Power Saver	serviceable	Jingdong prefecture level city in Shandong
Power Saving King Shop	Green Intelligent Power Saver	Power Saving King received a tightly packaged logistics, installed the effect is very good price is cheaper than the physical store!	Jingdong prefecture level city in Shandong
Power Saving King Shop	Green Intelligent Power Saver	Super Power Saving King is very good quality, new style, is genuine, the use of the effect is very good, the price is cheap, it is worth buying!	Jingdong prefecture level city in Shandong
Power Saving King Shop	Green Intelligent Power Saver	Packaging is very tight, easy to install, quite practical, worth buying!	Jingdong prefecture level city in Shandong
Power Saving King Shop	Green Intelligent Power Saver	Quality assurance, affordable price, very energy efficient, will repurchase if needed!	Jingdong prefecture level city in Shandong
Power Saving King Shop	Green Intelligent Power Saver	Quality assurance, very good, affordable, logistics is also very fast, I'm very satisfied with the	Jingdong prefecture level city in Shandong
Power Saving King Shop	Green Intelligent Power Saver	Energy saving. It's so good, already installed, you can save a lot of electricity every month	Jingdong prefecture level city in Shandong
Power Saving King Shop	Green Intelligent Power Saver	The material is thick and the workmanship is fine in place, the power saving effect is very good, value for money!	Jingdong prefecture level city in Shandong
Power Saving King Shop	Green Intelligent Power Saver	The effect is great, genuine, and match the description, the logistics is fast, the price is reasonable 😍	Jingdong prefecture level city in Shandong
Power Saving King Shop	Green Intelligent Power Saver	It's genuine, works well, easy to use, plugs in and works, really saves power	Jingdong prefecture level city in Shandong
Power Saving King Shop	Green Intelligent Power Saver	It's easy to use, effective, great service, and recommended!	Jingdong prefecture level city in Shandong
Power Saving King Shop	Green Intelligent Power Saver	🌈 Great quality, bought it to my liking 💛 Awesome! ✨ Buy with confidence if you need it!	Jingdong prefecture level city in Shandong
Power Saving King Shop	Green Intelligent Power Saver	Good quality, easy to install, very energy saving and power saving, good to use	Jingdong prefecture level city in Shandong
Power Saving King Shop	Green Intelligent Power Saver	The quality is very good, it does have the effect of saving electricity, the description matches, the price is affordable, it is worth recommending.	Jingdong prefecture level city in Shandong
Power Saving King Shop	Green Intelligent Power Saver	Received, the functionality is particularly good, it is genuine, the price is affordable	Jingdong prefecture level city in Shandong
Power Saving King Shop	Green Intelligent Power Saver	Good quality, very easy to use, energy efficient, home electricity bill is obviously much less	Jingdong prefecture level city in Shandong
Power Saving King Shop	Green Intelligent Power Saver	Easy to install, energy saving and power saving, very satisfied with a shopping	Jingdong prefecture level city in Shandong
Power Saving King Shop	Green Intelligent Power Saver	Received it and tried it out, it's great, works great and worth having! Five stars praise!	Jingdong prefecture level city in Shandong
Power Saving King Shop	Green Intelligent Power Saver	Power saving device, no problem at all, the quality of this product at this price, very affordable!	Jingdong prefecture level city in Shandong
Power Saving King Shop	Green Intelligent Power Saver	Received the goods, did not expect to be so fast, the quality is very good, especially power saving	Jingdong prefecture level city in Shandong
Power Saving King Shop	Green Intelligent Power Saver	The logistics is very fast, especially good, need to come back again	Jingdong prefecture level city in Shandong

Power Saving King Shop	Green Intelligent Power Saver	It's already working and it really saves power, so I'm very happy with it!	Jingdong prefecture level city in Shandong
Power Saving King Shop	Green Intelligent Power Saver	Great quality, works well, can recommend to friends to buy.	Jingdong prefecture level city in Shandong
Power Saving King Shop	Green Intelligent Power Saver	It's a good deal and worth the money.	Jingdong prefecture level city in Shandong
Power Saving King Shop	Green Intelligent Power Saver	I received it and I'm very happy with it, it's very good quality, guaranteed, and	Jingdong prefecture level city in Shandong
Power Saving King Shop	Green Intelligent Power Saver	Good quality, safe and secure purchase, ideal results and great price.	Jingdong prefecture level city in Shandong
Power Saving King Shop	Green Intelligent Power Saver	Simple operation plugged in the power supply can be, friends recommended looking forward to the effect of the	Jingdong prefecture level city in Shandong
Power Saving King Shop	Green Intelligent Power Saver	It's not bad, it saves electricity, and the material is sturdy and durable.	Jingdong prefecture level city in Shandong
Power Saving King Shop	Green Intelligent Power Saver	Plug and play, no management required, convenient and hassle-free!	Jingdong prefecture level city in Shandong
Power Saving King Shop	Green Intelligent Power Saver	The pictures are too lazy to go up, the power saving effect is still there!	Jingdong prefecture level city in Shandong
Power Saving King Shop	Green Intelligent Power Saver	Fast shipping. Affordable prices. Efficient	Jingdong prefecture level city in Shandong
Power Saving King Shop	Green Intelligent Power Saver	Good power saving, worth buying	Jingdong prefecture level city in Shandong
Power Saving King Shop	Green Intelligent Power Saver	Especially great, comparison shopping before buying, the store owner is very good, answering questions very patiently and detailed, there is a need to come back!	Jingdong prefecture level city in Shandong
Power Saving King Shop	Green Intelligent Power Saver	Very trustworthy brand, quality is guaranteed, the price is still affordable, quite satisfied!	Jingdong prefecture level city in Shandong
Power Saving King Shop	Green Intelligent Power Saver	Recommended by a friend. They say you can save money on air conditioning with this. Try it.	Jingdong prefecture level city in Shandong
Power Saving King Shop	Green Intelligent Power Saver	Works great, will be back next time, very happy with it and a great price!	Jingdong prefecture level city in Shandong
Power Saving King Shop	Green Intelligent Power Saver	The quality of the product is quite good, fast shipping, satisfied.	Jingdong prefecture level city in Shandong
Power Saving King Shop	Green Intelligent Power Saver	Good quality and fast logistics	Jingdong prefecture level city in Shandong
Power Saving King Shop	Green Intelligent Power Saver	It really saves power. I've tested it myself.	Jingdong prefecture level city in Shandong
Power Saving King Shop	Green Intelligent Power Saver	Really saves electricity!	Jingdong prefecture level city in Shandong
Power Saving King Shop	Green Intelligent Power Saver	pretty good	Jingdong prefecture level city in Shandong
Power Saving King Shop	Green Intelligent Power Saver	serviceable	Jingdong prefecture level city in Shandong
Power Saving King Shop	Green Intelligent Power Saver	Power Saving King received a tightly packaged logistics, installed the effect is very good price is cheaper than the physical store!	Jingdong prefecture level city in Shandong
Power Saving King Shop	Green Intelligent Power Saver	Super Power Saving King is very good quality, new style, is genuine, the use of the effect is very good, the price is cheap, it is worth buying!	Jingdong prefecture level city in Shandong
Power Saving King Shop	Green Intelligent Power Saver	Packaging is very tight, easy to install, quite practical, worth buying!	Jingdong prefecture level city in Shandong
Power Saving King Shop	Green Intelligent Power Saver	Quality assurance, affordable price, very energy efficient, will repurchase if needed!	Jingdong prefecture level city in Shandong
Power Saving King Shop	Green Intelligent Power Saver	Quality assurance, very good, affordable, logistics is also very fast, I'm very satisfied with the	Jingdong prefecture level city in Shandong
Power Saving King Shop	Green Intelligent Power Saver	Energy saving. It's so good, already installed, you can save a lot of electricity every month	Jingdong prefecture level city in Shandong
Power Saving King Shop	Green Intelligent Power Saver	The material is thick and the workmanship is fine in place, the power saving effect is very good, value for money!	Jingdong prefecture level city in Shandong
Power Saving King Shop	Green Intelligent Power Saver	The effect is great, genuine, and match the description, the logistics is fast, the price is reasonable 😍	Jingdong prefecture level city in Shandong
Power Saving King Shop	Green Intelligent Power Saver	It's easy to use, effective, great service, and recommended!	Jingdong prefecture level city in Shandong
Power Saving King Shop	Green Intelligent Power Saver	It's genuine, works well, easy to use, plugs in and works, really saves power	Jingdong prefecture level city in Shandong
Power Saving King Shop	Green Intelligent Power Saver	🌈 Great quality, bought it to my liking 💝 Awesome! ✨ Buy with confidence if you need it!	Jingdong prefecture level city in Shandong
Power Saving King Shop	Green Intelligent Power Saver	Good quality, easy to install, very energy saving and power saving, good to use	Jingdong prefecture level city in Shandong
Power Saving King Shop	Green Intelligent Power Saver	The quality is very good, it does have the effect of saving electricity, the description matches, the price is affordable, it is worth recommending.	Jingdong prefecture level city in Shandong
Power Saving King Shop	Green Intelligent Power Saver	Received, the functionality is particularly good, it is genuine, the price is affordable	Jingdong prefecture level city in Shandong
Power Saving King Shop	Green Intelligent Power Saver	Good quality, very easy to use, energy efficient, home electricity bill is obviously much less	Jingdong prefecture level city in Shandong
Power Saving King Shop	Green Intelligent Power Saver	Easy to install, energy saving and power saving, very satisfied with a shopping	Jingdong prefecture level city in Shandong
Power Saving King Shop	Green Intelligent Power Saver	Received it and tried it out, it's great, works great and worth having! Five stars praise!	Jingdong prefecture level city in Shandong
Power Saving King Shop	Green Intelligent Power Saver	Power saving device, no problem at all, the quality of this product at this price, very affordable!	Jingdong prefecture level city in Shandong
Power Saving King Shop	Green Intelligent Power Saver	Received the goods, did not expect to be so fast, the quality is very good, especially power saving	Jingdong prefecture level city in Shandong
Power Saving King Shop	Green Intelligent Power Saver	The logistics is very fast, especially good, need to come back again	Jingdong prefecture level city in Shandong
Power Saving King Shop	Green Intelligent Power Saver	It's already working and it really saves power, so I'm very happy with it!	Jingdong prefecture level city in Shandong
Power Saving King Shop	Green Intelligent Power Saver	Great quality, works well, can recommend to friends to buy.	Jingdong prefecture level city in Shandong
Power Saving King Shop	Green Intelligent Power Saver	It's a good deal and worth the money.	Jingdong prefecture level city in Shandong
Power Saving King Shop	Green Intelligent Power Saver	I received it and I'm very happy with it, it's very good quality, guaranteed, and	Jingdong prefecture level city in Shandong
Power Saving King Shop	Green Intelligent Power Saver	Good quality, safe and secure purchase, ideal results and great price.	Jingdong prefecture level city in Shandong
Power Saving King Shop	Green Intelligent Power Saver	Simple operation plugged in the power supply can be, friends recommended looking forward to the effect of the	Jingdong prefecture level city in Shandong
Power Saving King Shop	Green Intelligent Power Saver	It's not bad, it saves electricity, and the material is sturdy and durable.	Jingdong prefecture level city in Shandong
Power Saving King Shop	Green Intelligent Power Saver	Plug and play, no management required, convenient and hassle-free!	Jingdong prefecture level city in Shandong
Power Saving King Shop	Green Intelligent Power Saver	The pictures are too lazy to go up, the power saving effect is still there!	Jingdong prefecture level city in Shandong
Power Saving King Shop	Green Intelligent Power Saver	Fast shipping. Affordable prices. Efficient	Jingdong prefecture level city in Shandong

Power Saving King Shop	Green Intelligent Power Saver	Good power saving, worth buying	Jingdong prefecture level city in Shandong
Power Saving King Shop	Green Intelligent Power Saver	Especially great, comparison shopping before buying, the store owner is very good, answering questions very patiently and detailed, there is a need to come back!	Jingdong prefecture level city in Shandong
Power Saving King Shop	Green Intelligent Power Saver	Very trustworthy brand, quality is guaranteed, the price is still affordable, quite satisfied!	Jingdong prefecture level city in Shandong
Power Saving King Shop	Green Intelligent Power Saver	Recommended by a friend. They say you can save money on air conditioning with this. Try it.	Jingdong prefecture level city in Shandong
Power Saving King Shop	Green Intelligent Power Saver	Works great, will be back next time, very happy with it and a great price!	Jingdong prefecture level city in Shandong
Power Saving King Shop	Green Intelligent Power Saver	The quality of the product is quite good, fast shipping, satisfied.	Jingdong prefecture level city in Shandong
Power Saving King Shop	Green Intelligent Power Saver	Good quality and fast logistics	Jingdong prefecture level city in Shandong
Power Saving King Shop	Green Intelligent Power Saver	It really saves power. I've tested it myself.	Jingdong prefecture level city in Shandong
Power Saving King Shop	Green Intelligent Power Saver	Really saves electricity!	Jingdong prefecture level city in Shandong
Power Saving King Shop	Green Intelligent Power Saver	pretty good	Jingdong prefecture level city in Shandong
Power Saving King Shop	Green Intelligent Power Saver	serviceable	Jingdong prefecture level city in Shandong
Power Saving King Shop	Green Intelligent Power Saver	Power Saving King received a tightly packaged logistics, installed the effect is very good price is cheaper than the physical store!	Jingdong prefecture level city in Shandong
Power Saving King Shop	Green Intelligent Power Saver	Super Power Saving King is very good quality, new style, is genuine, the use of the effect is very good, the price is cheap, it is worth buying!	Jingdong prefecture level city in Shandong
Power Saving King Shop	Green Intelligent Power Saver	Packaging is very tight, easy to install, quite practical, worth buying!	Jingdong prefecture level city in Shandong
Power Saving King Shop	Green Intelligent Power Saver	Quality assurance, affordable price, very energy efficient, will repurchase if needed!	Jingdong prefecture level city in Shandong
Power Saving King Shop	Green Intelligent Power Saver	Quality assurance, very good, affordable, logistics is also very fast, I'm very satisfied with the	Jingdong prefecture level city in Shandong
Power Saving King Shop	Green Intelligent Power Saver	Energy saving. It's so good, already installed, you can save a lot of electricity every month	Jingdong prefecture level city in Shandong
Power Saving King Shop	Green Intelligent Power Saver	The material is thick and the workmanship is fine in place, the power saving effect is very good, value for money!	Jingdong prefecture level city in Shandong
Power Saving King Shop	Green Intelligent Power Saver	The effect is great, genuine, and match the description, the logistics is fast, the price is reasonable 😍	Jingdong prefecture level city in Shandong
Power Saving King Shop	Green Intelligent Power Saver	It's genuine, works well, easy to use, plugs in and works, really saves power	Jingdong prefecture level city in Shandong
Power Saving King Shop	Green Intelligent Power Saver	It's easy to use, effective, great service, and recommended!	Jingdong prefecture level city in Shandong
Power Saving King Shop	Green Intelligent Power Saver	🌈 Great quality, bought it to my liking 💝 Awesome! ✨ Buy with confidence if you need it!	Jingdong prefecture level city in Shandong
Power Saving King Shop	Green Intelligent Power Saver	Good quality, easy to install, very energy saving and power saving, good to use	Jingdong prefecture level city in Shandong
Power Saving King Shop	Green Intelligent Power Saver	The quality is very good, it does have the effect of saving electricity, the description matches, the price is affordable, it is worth recommending.	Jingdong prefecture level city in Shandong
Power Saving King Shop	Green Intelligent Power Saver	Received, the functionality is particularly good, it is genuine, the price is affordable	Jingdong prefecture level city in Shandong
Power Saving King Shop	Green Intelligent Power Saver	Good quality, very easy to use, energy efficient, home electricity bill is obviously much less	Jingdong prefecture level city in Shandong
Power Saving King Shop	Green Intelligent Power Saver	Easy to install, energy saving and power saving, very satisfied with a shopping	Jingdong prefecture level city in Shandong
Power Saving King Shop	Green Intelligent Power Saver	Received it and tried it out, it's great, works great and worth having! Five stars praise!	Jingdong prefecture level city in Shandong
Power Saving King Shop	Green Intelligent Power Saver	Power saving device, no problem at all, the quality of this product at this price, very affordable!	Jingdong prefecture level city in Shandong
Power Saving King Shop	Green Intelligent Power Saver	Received the goods, did not expect to be so fast, the quality is very good, especially power saving	Jingdong prefecture level city in Shandong
Power Saving King Shop	Green Intelligent Power Saver	The logistics is very fast, especially good, need to come back again	Jingdong prefecture level city in Shandong
Power Saving King Shop	Green Intelligent Power Saver	It's already working and it really saves power, so I'm very happy with it!	Jingdong prefecture level city in Shandong
Power Saving King Shop	Green Intelligent Power Saver	Great quality, works well, can recommend to friends to buy.	Jingdong prefecture level city in Shandong
Power Saving King Shop	Green Intelligent Power Saver	It's a good deal and worth the money.	Jingdong prefecture level city in Shandong
Power Saving King Shop	Green Intelligent Power Saver	I received it and I'm very happy with it, it's very good quality, guaranteed, and	Jingdong prefecture level city in Shandong
Power Saving King Shop	Green Intelligent Power Saver	Good quality, safe and secure purchase, ideal results and great price.	Jingdong prefecture level city in Shandong
Power Saving King Shop	Green Intelligent Power Saver	Simple operation plugged in the power supply can be, friends recommended looking forward to the effect of the	Jingdong prefecture level city in Shandong
Power Saving King Shop	Green Intelligent Power Saver	It's not bad, it saves electricity, and the material is sturdy and durable.	Jingdong prefecture level city in Shandong
Power Saving King Shop	Green Intelligent Power Saver	Plug and play, no management required, convenient and hassle-free!	Jingdong prefecture level city in Shandong
Power Saving King Shop	Green Intelligent Power Saver	The pictures are too lazy to go up, the power saving effect is still there!	Jingdong prefecture level city in Shandong
Power Saving King Shop	Green Intelligent Power Saver	Fast shipping. Affordable prices. Efficient	Jingdong prefecture level city in Shandong
Power Saving King Shop	Green Intelligent Power Saver	Good power saving, worth buying	Jingdong prefecture level city in Shandong
Power Saving King Shop	Green Intelligent Power Saver	Especially great, comparison shopping before buying, the store owner is very good, answering questions very patiently and detailed, there is a need to come back!	Jingdong prefecture level city in Shandong
Power Saving King Shop	Green Intelligent Power Saver	Very trustworthy brand, quality is guaranteed, the price is still affordable, quite satisfied!	Jingdong prefecture level city in Shandong
Power Saving King Shop	Green Intelligent Power Saver	Recommended by a friend. They say you can save money on air conditioning with this. Try it.	Jingdong prefecture level city in Shandong
Power Saving King Shop	Green Intelligent Power Saver	Works great, will be back next time, very happy with it and a great price!	Jingdong prefecture level city in Shandong
Power Saving King Shop	Green Intelligent Power Saver	The quality of the product is quite good, fast shipping, satisfied.	Jingdong prefecture level city in Shandong
Power Saving King Shop	Green Intelligent Power Saver	Good quality and fast logistics	Jingdong prefecture level city in Shandong
Power Saving King Shop	Green Intelligent Power Saver	It really saves power. I've tested it myself.	Jingdong prefecture level city in Shandong
Power Saving King Shop	Green Intelligent Power Saver	pretty good	Jingdong prefecture level city in Shandong
Power Saving King Shop	Green Intelligent Power Saver	Really saves electricity!	Jingdong prefecture level city in Shandong

Power Saving King Shop	Green Intelligent Power Saver	serviceable	Jingdong prefecture level city in Shandong
Power Saving King Shop	Green Intelligent Power Saver	Power Saving King received a tightly packaged logistics, installed the effect is very good price is cheaper than the physical store!	Jingdong prefecture level city in Shandong
Power Saving King Shop	Green Intelligent Power Saver	Super Power Saving King is very good quality, new style, is genuine, the use of the effect is very good, the price is cheap, it is worth buying!	Jingdong prefecture level city in Shandong
Power Saving King Shop	Green Intelligent Power Saver	Packaging is very tight, easy to install, quite practical, worth buying!	Jingdong prefecture level city in Shandong
Power Saving King Shop	Green Intelligent Power Saver	Quality assurance, affordable price, very energy efficient, will repurchase if needed!	Jingdong prefecture level city in Shandong
Power Saving King Shop	Green Intelligent Power Saver	Quality assurance, very good, affordable, logistics is also very fast, I'm very satisfied with the	Jingdong prefecture level city in Shandong
Power Saving King Shop	Green Intelligent Power Saver	Energy saving. It's so good, already installed, you can save a lot of electricity every month	Jingdong prefecture level city in Shandong
Power Saving King Shop	Green Intelligent Power Saver	The material is thick and the workmanship is fine in place, the power saving effect is very good, value for money!	Jingdong prefecture level city in Shandong
Power Saving King Shop	Green Intelligent Power Saver	The effect is great, genuine, and match the description, the logistics is fast, the price is reasonable 😍	Jingdong prefecture level city in Shandong
Power Saving King Shop	Green Intelligent Power Saver	It's genuine, works well, easy to use, plugs in and works, really saves power	Jingdong prefecture level city in Shandong
Power Saving King Shop	Green Intelligent Power Saver	It's easy to use, effective, great service, and recommended!	Jingdong prefecture level city in Shandong
Power Saving King Shop	Green Intelligent Power Saver	🌈 Great quality, bought it to my liking 💗 Awesome! ✨ Buy with confidence if you need it!	Jingdong prefecture level city in Shandong
Power Saving King Shop	Green Intelligent Power Saver	Good quality, easy to install, very energy saving and power saving, good to use	Jingdong prefecture level city in Shandong
Power Saving King Shop	Green Intelligent Power Saver	The quality is very good, it does have the effect of saving electricity, the description matches, the price is affordable, it is worth recommending.	Jingdong prefecture level city in Shandong
Power Saving King Shop	Green Intelligent Power Saver	Received, the functionality is particularly good, it is genuine, the price is affordable	Jingdong prefecture level city in Shandong
Power Saving King Shop	Green Intelligent Power Saver	Good quality, very easy to use, energy efficient, home electricity bill is obviously much less	Jingdong prefecture level city in Shandong
Power Saving King Shop	Green Intelligent Power Saver	Easy to install, energy saving and power saving, very satisfied with a shopping	Jingdong prefecture level city in Shandong
Power Saving King Shop	Green Intelligent Power Saver	Received it and tried it out, it's great, works great and worth having! Five stars praise!	Jingdong prefecture level city in Shandong
Power Saving King Shop	Green Intelligent Power Saver	Power saving device, no problem at all, the quality of this product at this price, very affordable!	Jingdong prefecture level city in Shandong
Power Saving King Shop	Green Intelligent Power Saver	Received the goods, did not expect to be so fast, the quality is very good, especially power saving	Jingdong prefecture level city in Shandong
Power Saving King Shop	Green Intelligent Power Saver	The logistics is very fast, especially good, need to come back again	Jingdong prefecture level city in Shandong
Power Saving King Shop	Green Intelligent Power Saver	It's already working and it really saves power, so I'm very happy with it!	Jingdong prefecture level city in Shandong
Power Saving King Shop	Green Intelligent Power Saver	Great quality, works well, can recommend to friends to buy.	Jingdong prefecture level city in Shandong
Power Saving King Shop	Green Intelligent Power Saver	It's a good deal and worth the money.	Jingdong prefecture level city in Shandong
Power Saving King Shop	Green Intelligent Power Saver	I received it and I'm very happy with it, it's very good quality, guaranteed, and	Jingdong prefecture level city in Shandong
Power Saving King Shop	Green Intelligent Power Saver	Good quality, safe and secure purchase, ideal results and great price.	Jingdong prefecture level city in Shandong
Power Saving King Shop	Green Intelligent Power Saver	Simple operation plugged in the power supply can be, friends recommended looking forward to the effect of the	Jingdong prefecture level city in Shandong
Power Saving King Shop	Green Intelligent Power Saver	It's not bad, it saves electricity, and the material is sturdy and durable.	Jingdong prefecture level city in Shandong
Power Saving King Shop	Green Intelligent Power Saver	Plug and play, no management required, convenient and hassle-free!	Jingdong prefecture level city in Shandong
Power Saving King Shop	Green Intelligent Power Saver	The pictures are too lazy to go up, the power saving effect is still there!	Jingdong prefecture level city in Shandong
Power Saving King Shop	Green Intelligent Power Saver	Fast shipping. Affordable prices. Efficient	Jingdong prefecture level city in Shandong
Power Saving King Shop	Green Intelligent Power Saver	Good power saving, worth buying	Jingdong prefecture level city in Shandong
Power Saving King Shop	Green Intelligent Power Saver	Especially great, comparison shopping before buying, the store owner is very good, answering questions very patiently and detailed, there is a need to come back!	Jingdong prefecture level city in Shandong
Power Saving King Shop	Green Intelligent Power Saver	Very trustworthy brand, quality is guaranteed, the price is still affordable, quite satisfied!	Jingdong prefecture level city in Shandong
Power Saving King Shop	Green Intelligent Power Saver	Recommended by a friend. They say you can save money on air conditioning with this. Try it.	Jingdong prefecture level city in Shandong
Power Saving King Shop	Green Intelligent Power Saver	Works great, will be back next time, very happy with it and a great price!	Jingdong prefecture level city in Shandong
Power Saving King Shop	Green Intelligent Power Saver	The quality of the product is quite good, fast shipping, satisfied.	Jingdong prefecture level city in Shandong
Power Saving King Shop	Green Intelligent Power Saver	Good quality and fast logistics	Jingdong prefecture level city in Shandong
Power Saving King Shop	Green Intelligent Power Saver	It really saves power. I've tested it myself.	Jingdong prefecture level city in Shandong
Power Saving King Shop	Green Intelligent Power Saver	Really saves electricity!	Jingdong prefecture level city in Shandong
Power Saving King Shop	Green Intelligent Power Saver	pretty good	Jingdong prefecture level city in Shandong
Power Saving King Shop	Green Intelligent Power Saver	serviceable	Jingdong prefecture level city in Shandong
Power Saving King Shop	Green Intelligent Power Saver	Power Saving King received a tightly packaged logistics, installed the effect is very good price is cheaper than the physical store!	Jingdong prefecture level city in Shandong
Power Saving King Shop	Green Intelligent Power Saver	Super Power Saving King is very good quality, new style, is genuine, the use of the effect is very good, the price is cheap, it is worth buying!	Jingdong prefecture level city in Shandong
Power Saving King Shop	Green Intelligent Power Saver	Packaging is very tight, easy to install, quite practical, worth buying!	Jingdong prefecture level city in Shandong
Power Saving King Shop	Green Intelligent Power Saver	Quality assurance, affordable price, very energy efficient, will repurchase if needed!	Jingdong prefecture level city in Shandong
Power Saving King Shop	Green Intelligent Power Saver	Quality assurance, very good, affordable, logistics is also very fast, I'm very satisfied with the	Jingdong prefecture level city in Shandong
Power Saving King Shop	Green Intelligent Power Saver	Energy saving. It's so good, already installed, you can save a lot of electricity every month	Jingdong prefecture level city in Shandong
Power Saving King Shop	Green Intelligent Power Saver	The material is thick and the workmanship is fine in place, the power saving effect is very good, value for money!	Jingdong prefecture level city in Shandong
Power Saving King Shop	Green Intelligent Power Saver	The effect is great, genuine, and match the description, the logistics is fast, the price is reasonable 😍	Jingdong prefecture level city in Shandong
Power Saving King Shop	Green Intelligent Power Saver	It's genuine, works well, easy to use, plugs in and works, really saves power	Jingdong prefecture level city in Shandong

Power Saving King Shop	Green Intelligent Power Saver	It's easy to use, effective, great service, and recommended!	Jingdong prefecture level city in Shandong
Power Saving King Shop	Green Intelligent Power Saver	🌈 Great quality, bought it to my liking 💞 Awesome! ✨ Buy with confidence if you need it!	Jingdong prefecture level city in Shandong
Power Saving King Shop	Green Intelligent Power Saver	Good quality, easy to install, very energy saving and power saving, good to use	Jingdong prefecture level city in Shandong
Power Saving King Shop	Green Intelligent Power Saver	The quality is very good, it does have the effect of saving electricity, the description matches, the price is affordable, it is worth recommending.	Jingdong prefecture level city in Shandong
Power Saving King Shop	Green Intelligent Power Saver	Received, the functionality is particularly good, it is genuine, the price is affordable	Jingdong prefecture level city in Shandong
Power Saving King Shop	Green Intelligent Power Saver	Good quality, very easy to use, energy efficient, home electricity bill is obviously much less	Jingdong prefecture level city in Shandong
Power Saving King Shop	Green Intelligent Power Saver	Easy to install, energy saving and power saving, very satisfied with a shopping	Jingdong prefecture level city in Shandong
Power Saving King Shop	Green Intelligent Power Saver	Received it and tried it out, it's great, works great and worth having! Five stars praise!	Jingdong prefecture level city in Shandong
Power Saving King Shop	Green Intelligent Power Saver	Power saving device, no problem at all, the quality of this product at this price, very affordable!	Jingdong prefecture level city in Shandong
Power Saving King Shop	Green Intelligent Power Saver	Received the goods, did not expect to be so fast, the quality is very good, especially power saving	Jingdong prefecture level city in Shandong
Power Saving King Shop	Green Intelligent Power Saver	The logistics is very fast, especially good, need to come back again	Jingdong prefecture level city in Shandong
Power Saving King Shop	Green Intelligent Power Saver	It's already working and it really saves power, so I'm very happy with it!	Jingdong prefecture level city in Shandong
Power Saving King Shop	Green Intelligent Power Saver	Great quality, works well, can recommend to friends to buy.	Jingdong prefecture level city in Shandong
Power Saving King Shop	Green Intelligent Power Saver	It's a good deal and worth the money.	Jingdong prefecture level city in Shandong
Power Saving King Shop	Green Intelligent Power Saver	I received it and I'm very happy with it, it's very good quality, guaranteed, and	Jingdong prefecture level city in Shandong
Power Saving King Shop	Green Intelligent Power Saver	Good quality, safe and secure purchase, ideal results and great price.	Jingdong prefecture level city in Shandong
Power Saving King Shop	Green Intelligent Power Saver	Simple operation plugged in the power supply can be, friends recommended looking forward to the effect of the	Jingdong prefecture level city in Shandong
Power Saving King Shop	Green Intelligent Power Saver	It's not bad, it saves electricity, and the material is sturdy and durable.	Jingdong prefecture level city in Shandong
Power Saving King Shop	Green Intelligent Power Saver	Plug and play, no management required, convenient and hassle-free!	Jingdong prefecture level city in Shandong
Power Saving King Shop	Green Intelligent Power Saver	The pictures are too lazy to go up, the power saving effect is still there!	Jingdong prefecture level city in Shandong
Power Saving King Shop	Green Intelligent Power Saver	Fast shipping. Affordable prices. Efficient	Jingdong prefecture level city in Shandong
Power Saving King Shop	Green Intelligent Power Saver	Good power saving, worth buying	Jingdong prefecture level city in Shandong
Power Saving King Shop	Green Intelligent Power Saver	Especially great, comparison shopping before buying, the store owner is very good, answering questions very patiently and detailed, there is a need to come back!	Jingdong prefecture level city in Shandong
Power Saving King Shop	Green Intelligent Power Saver	Very trustworthy brand, quality is guaranteed, the price is still affordable, quite satisfied!	Jingdong prefecture level city in Shandong
Power Saving King Shop	Green Intelligent Power Saver	Recommended by a friend. They say you can save money on air conditioning with this. Try it.	Jingdong prefecture level city in Shandong
Power Saving King Shop	Green Intelligent Power Saver	Works great, will be back next time, very happy with it and a great price!	Jingdong prefecture level city in Shandong
Power Saving King Shop	Green Intelligent Power Saver	The quality of the product is quite good, fast shipping, satisfied.	Jingdong prefecture level city in Shandong
Power Saving King Shop	Green Intelligent Power Saver	Good quality and fast logistics	Jingdong prefecture level city in Shandong
Power Saving King Shop	Green Intelligent Power Saver	It really saves power. I've tested it myself.	Jingdong prefecture level city in Shandong
Power Saving King Shop	Green Intelligent Power Saver	Really saves electricity!	Jingdong prefecture level city in Shandong
Power Saving King Shop	Green Intelligent Power Saver	pretty good	Jingdong prefecture level city in Shandong
Power Saving King Shop	Green Intelligent Power Saver	serviceable	Jingdong prefecture level city in Shandong
Power Saving King Shop	Green Intelligent Power Saver	Power Saving King received a tightly packaged logistics, installed the effect is very good price is cheaper than the physical store!	Jingdong prefecture level city in Shandong
Power Saving King Shop	Green Intelligent Power Saver	Super Power Saving King is very good quality, new style, is genuine, the use of the effect is very good, the price is cheap, it is worth buying!	Jingdong prefecture level city in Shandong
Power Saving King Shop	Green Intelligent Power Saver	Packaging is very tight, easy to install, quite practical, worth buying!	Jingdong prefecture level city in Shandong
Power Saving King Shop	Green Intelligent Power Saver	Quality assurance, affordable price, very energy efficient, will repurchase if needed!	Jingdong prefecture level city in Shandong
Power Saving King Shop	Green Intelligent Power Saver	Quality assurance, very good, affordable, logistics is also very fast, I'm very satisfied with the	Jingdong prefecture level city in Shandong
Power Saving King Shop	Green Intelligent Power Saver	Energy saving. It's so good, already installed, you can save a lot of electricity every month	Jingdong prefecture level city in Shandong
Power Saving King Shop	Green Intelligent Power Saver	The material is thick and the workmanship is fine in place, the power saving effect is very good, value for money!	Jingdong prefecture level city in Shandong
Power Saving King Shop	Green Intelligent Power Saver	The effect is great, genuine, and match the description, the logistics is fast, the price is reasonable 😍	Jingdong prefecture level city in Shandong
Power Saving King Shop	Green Intelligent Power Saver	It's genuine, works well, easy to use, plugs in and works, really saves power	Jingdong prefecture level city in Shandong
Power Saving King Shop	Green Intelligent Power Saver	It's easy to use, effective, great service, and recommended!	Jingdong prefecture level city in Shandong
Power Saving King Shop	Green Intelligent Power Saver	🌈 Great quality, bought it to my liking 💞 Awesome! ✨ Buy with confidence if you need it!	Jingdong prefecture level city in Shandong
Power Saving King Shop	Green Intelligent Power Saver	Good quality, easy to install, very energy saving and power saving, good to use	Jingdong prefecture level city in Shandong
Power Saving King Shop	Green Intelligent Power Saver	The quality is very good, it does have the effect of saving electricity, the description matches, the price is affordable, it is worth recommending.	Jingdong prefecture level city in Shandong
Power Saving King Shop	Green Intelligent Power Saver	Received, the functionality is particularly good, it is genuine, the price is affordable	Jingdong prefecture level city in Shandong
Power Saving King Shop	Green Intelligent Power Saver	Good quality, very easy to use, energy efficient, home electricity bill is obviously much less	Jingdong prefecture level city in Shandong
Power Saving King Shop	Green Intelligent Power Saver	Easy to install, energy saving and power saving, very satisfied with a shopping	Jingdong prefecture level city in Shandong
Power Saving King Shop	Green Intelligent Power Saver	Received it and tried it out, it's great, works great and worth having! Five stars praise!	Jingdong prefecture level city in Shandong
Power Saving King Shop	Green Intelligent Power Saver	Power saving device, no problem at all, the quality of this product at this price, very affordable!	Jingdong prefecture level city in Shandong
Power Saving King Shop	Green Intelligent Power Saver	Received the goods, did not expect to be so fast, the quality is very good, especially power saving	Jingdong prefecture level city in Shandong

Power Saving King Shop	Green Intelligent Power Saver	The logistics is very fast, especially good, need to come back again	Jingdong prefecture level city in Shandong
Power Saving King Shop	Green Intelligent Power Saver	It's already working and it really saves power, so I'm very happy with it!	Jingdong prefecture level city in Shandong
Power Saving King Shop	Green Intelligent Power Saver	Great quality, works well, can recommend to friends to buy.	Jingdong prefecture level city in Shandong
Power Saving King Shop	Green Intelligent Power Saver	It's a good deal and worth the money.	Jingdong prefecture level city in Shandong
Power Saving King Shop	Green Intelligent Power Saver	I received it and I'm very happy with it, it's very good quality, guaranteed, and	Jingdong prefecture level city in Shandong
Power Saving King Shop	Green Intelligent Power Saver	Good quality, safe and secure purchase, ideal results and great price.	Jingdong prefecture level city in Shandong
Power Saving King Shop	Green Intelligent Power Saver	Simple operation plugged in the power supply can be, friends recommended looking forward to the effect of the	Jingdong prefecture level city in Shandong
Power Saving King Shop	Green Intelligent Power Saver	It's not bad, it saves electricity, and the material is sturdy and durable.	Jingdong prefecture level city in Shandong
Power Saving King Shop	Green Intelligent Power Saver	Plug and play, no management required, convenient and hassle-free!	Jingdong prefecture level city in Shandong
Power Saving King Shop	Green Intelligent Power Saver	The pictures are too lazy to go up, the power saving effect is still there!	Jingdong prefecture level city in Shandong
Power Saving King Shop	Green Intelligent Power Saver	Fast shipping. Affordable prices. Efficient	Jingdong prefecture level city in Shandong
Power Saving King Shop	Green Intelligent Power Saver	Good power saving, worth buying	Jingdong prefecture level city in Shandong
Power Saving King Shop	Green Intelligent Power Saver	Especially great, comparison shopping before buying, the store owner is very good, answering questions very patiently and detailed, there is a need to come back!	Jingdong prefecture level city in Shandong
Power Saving King Shop	Green Intelligent Power Saver	Very trustworthy brand, quality is guaranteed, the price is still affordable, quite satisfied!	Jingdong prefecture level city in Shandong
Power Saving King Shop	Green Intelligent Power Saver	Recommended by a friend. They say you can save money on air conditioning with this. Try it.	Jingdong prefecture level city in Shandong
Power Saving King Shop	Green Intelligent Power Saver	Works great, will be back next time, very happy with it and a great price!	Jingdong prefecture level city in Shandong
Power Saving King Shop	Green Intelligent Power Saver	The quality of the product is quite good, fast shipping, satisfied.	Jingdong prefecture level city in Shandong
Power Saving King Shop	Green Intelligent Power Saver	Good quality and fast logistics	Jingdong prefecture level city in Shandong
Power Saving King Shop	Green Intelligent Power Saver	It really saves power. I've tested it myself.	Jingdong prefecture level city in Shandong
Power Saving King Shop	Green Intelligent Power Saver	Really saves electricity!	Jingdong prefecture level city in Shandong
Power Saving King Shop	Green Intelligent Power Saver	pretty good	Jingdong prefecture level city in Shandong
Power Saving King Shop	Green Intelligent Power Saver	serviceable	Jingdong prefecture level city in Shandong
Power Saving King Shop	Green Intelligent Power Saver	Power Saving King received a tightly packaged logistics, installed the effect is very good price is cheaper than the physical store!	Jingdong prefecture level city in Shandong
Power Saving King Shop	Green Intelligent Power Saver	Super Power Saving King is very good quality, new style, is genuine, the use of the effect is very good, the price is cheap, it is worth buying!	Jingdong prefecture level city in Shandong
Power Saving King Shop	Green Intelligent Power Saver	Packaging is very tight, easy to install, quite practical, worth buying!	Jingdong prefecture level city in Shandong
Power Saving King Shop	Green Intelligent Power Saver	Quality assurance, affordable price, very energy efficient, will repurchase if needed!	Jingdong prefecture level city in Shandong
Power Saving King Shop	Green Intelligent Power Saver	Quality assurance, very good, affordable, logistics is also very fast, I'm very satisfied with the	Jingdong prefecture level city in Shandong
Power Saving King Shop	Green Intelligent Power Saver	Energy saving. It's so good, already installed, you can save a lot of electricity every month	Jingdong prefecture level city in Shandong
Power Saving King Shop	Green Intelligent Power Saver	The material is thick and the workmanship is fine in place, the power saving effect is very good, value for money!	Jingdong prefecture level city in Shandong
Power Saving King Shop	Green Intelligent Power Saver	The effect is great, genuine, and match the description, the logistics is fast, the price is reasonable 😍	Jingdong prefecture level city in Shandong
Power Saving King Shop	Green Intelligent Power Saver	It's genuine, works well, easy to use, plugs in and works, really saves power	Jingdong prefecture level city in Shandong
Power Saving King Shop	Green Intelligent Power Saver	It's easy to use, effective, great service, and recommended!	Jingdong prefecture level city in Shandong
Power Saving King Shop	Green Intelligent Power Saver	🌈 Great quality, bought it to my liking 💝 Awesome! ✨ Buy with confidence if you need it!	Jingdong prefecture level city in Shandong
Power Saving King Shop	Green Intelligent Power Saver	Good quality, easy to install, very energy saving and power saving, good to use	Jingdong prefecture level city in Shandong
Power Saving King Shop	Green Intelligent Power Saver	The quality is very good, it does have the effect of saving electricity, the description matches, the price is affordable, it is worth recommending.	Jingdong prefecture level city in Shandong
Power Saving King Shop	Green Intelligent Power Saver	Received, the functionality is particularly good, it is genuine, the price is affordable	Jingdong prefecture level city in Shandong
Power Saving King Shop	Green Intelligent Power Saver	Good quality, very easy to use, energy efficient, home electricity bill is obviously much less	Jingdong prefecture level city in Shandong
Power Saving King Shop	Green Intelligent Power Saver	Easy to install, energy saving and power saving, very satisfied with a shopping	Jingdong prefecture level city in Shandong
Power Saving King Shop	Green Intelligent Power Saver	Received it and tried it out, it's great, works great and worth having! Five stars praise!	Jingdong prefecture level city in Shandong
Power Saving King Shop	Green Intelligent Power Saver	Power saving device, no problem at all, the quality of this product at this price, very affordable!	Jingdong prefecture level city in Shandong
Power Saving King Shop	Green Intelligent Power Saver	Received the goods, did not expect to be so fast, the quality is very good, especially power saving	Jingdong prefecture level city in Shandong
Power Saving King Shop	Green Intelligent Power Saver	The logistics is very fast, especially good, need to come back again	Jingdong prefecture level city in Shandong
Power Saving King Shop	Green Intelligent Power Saver	It's already working and it really saves power, so I'm very happy with it!	Jingdong prefecture level city in Shandong
Power Saving King Shop	Green Intelligent Power Saver	Great quality, works well, can recommend to friends to buy.	Jingdong prefecture level city in Shandong
Power Saving King Shop	Green Intelligent Power Saver	It's a good deal and worth the money.	Jingdong prefecture level city in Shandong
Power Saving King Shop	Green Intelligent Power Saver	I received it and I'm very happy with it, it's very good quality, guaranteed, and	Jingdong prefecture level city in Shandong
Power Saving King Shop	Green Intelligent Power Saver	Good quality, safe and secure purchase, ideal results and great price.	Jingdong prefecture level city in Shandong
Power Saving King Shop	Green Intelligent Power Saver	Simple operation plugged in the power supply can be, friends recommended looking forward to the effect of the	Jingdong prefecture level city in Shandong
Power Saving King Shop	Green Intelligent Power Saver	It's not bad, it saves electricity, and the material is sturdy and durable.	Jingdong prefecture level city in Shandong
Power Saving King Shop	Green Intelligent Power Saver	Plug and play, no management required, convenient and hassle-free!	Jingdong prefecture level city in Shandong
Power Saving King Shop	Green Intelligent Power Saver	The pictures are too lazy to go up, the power saving effect is still there!	Jingdong prefecture level city in Shandong

Power Saving King Shop	Green Intelligent Power Saver	Fast shipping. Affordable prices. Efficient	Jingdong prefecture level city in Shandong
Power Saving King Shop	Green Intelligent Power Saver	Good power saving, worth buying	Jingdong prefecture level city in Shandong
Power Saving King Shop	Green Intelligent Power Saver	Especially great, comparison shopping before buying, the store owner is very good, answering questions very patiently and detailed, there is a need to come back!	Jingdong prefecture level city in Shandong
Power Saving King Shop	Green Intelligent Power Saver	Very trustworthy brand, quality is guaranteed, the price is still affordable, quite satisfied!	Jingdong prefecture level city in Shandong
Power Saving King Shop	Green Intelligent Power Saver	Recommended by a friend. They say you can save money on air conditioning with this. Try it.	Jingdong prefecture level city in Shandong
Power Saving King Shop	Green Intelligent Power Saver	Works great, will be back next time, very happy with it and a great price!	Jingdong prefecture level city in Shandong
Power Saving King Shop	Green Intelligent Power Saver	The quality of the product is quite good, fast shipping, satisfied.	Jingdong prefecture level city in Shandong
Power Saving King Shop	Green Intelligent Power Saver	Good quality and fast logistics	Jingdong prefecture level city in Shandong
Power Saving King Shop	Green Intelligent Power Saver	It really saves power. I've tested it myself.	Jingdong prefecture level city in Shandong
Power Saving King Shop	Green Intelligent Power Saver	Really saves electricity!	Jingdong prefecture level city in Shandong
Power Saving King Shop	Green Intelligent Power Saver	pretty good	Jingdong prefecture level city in Shandong
Power Saving King Shop	Green Intelligent Power Saver	serviceable	Jingdong prefecture level city in Shandong
Power Saving King Shop	Green Intelligent Power Saver	Power Saving King received a tightly packaged logistics, installed the effect is very good price is cheaper than the physical store!	Jingdong prefecture level city in Shandong
Power Saving King Shop	Green Intelligent Power Saver	Super Power Saving King is very good quality, new style, is genuine, the use of the effect is very good, the price is cheap, it is worth buying!	Jingdong prefecture level city in Shandong
Power Saving King Shop	Green Intelligent Power Saver	Packaging is very tight, easy to install, quite practical, worth buying!	Jingdong prefecture level city in Shandong
Power Saving King Shop	Green Intelligent Power Saver	Quality assurance, affordable price, very energy efficient, will repurchase if needed!	Jingdong prefecture level city in Shandong
Power Saving King Shop	Green Intelligent Power Saver	Quality assurance, very good, affordable, logistics is also very fast, I'm very satisfied with the	Jingdong prefecture level city in Shandong
Power Saving King Shop	Green Intelligent Power Saver	Energy saving. It's so good, already installed, you can save a lot of electricity every month	Jingdong prefecture level city in Shandong
Power Saving King Shop	Green Intelligent Power Saver	The material is thick and the workmanship is fine in place, the power saving effect is very good, value for money!	Jingdong prefecture level city in Shandong
Power Saving King Shop	Green Intelligent Power Saver	The effect is great, genuine, and match the description, the logistics is fast, the price is reasonable 😍	Jingdong prefecture level city in Shandong
Power Saving King Shop	Green Intelligent Power Saver	It's genuine, works well, easy to use, plugs in and works, really saves power	Jingdong prefecture level city in Shandong
Power Saving King Shop	Green Intelligent Power Saver	It's easy to use, effective, great service, and recommended!	Jingdong prefecture level city in Shandong
Power Saving King Shop	Green Intelligent Power Saver	🌈 Great quality, bought it to my liking 💖 Awesome! ✨ Buy with confidence if you need it!	Jingdong prefecture level city in Shandong
Power Saving King Shop	Green Intelligent Power Saver	Good quality, easy to install, very energy saving and power saving, good to use	Jingdong prefecture level city in Shandong
Power Saving King Shop	Green Intelligent Power Saver	The quality is very good, it does have the effect of saving electricity, the description matches, the price is affordable, it is worth recommending.	Jingdong prefecture level city in Shandong
Power Saving King Shop	Green Intelligent Power Saver	Received, the functionality is particularly good, it is genuine, the price is affordable	Jingdong prefecture level city in Shandong
Power Saving King Shop	Green Intelligent Power Saver	Good quality, very easy to use, energy efficient, home electricity bill is obviously much less	Jingdong prefecture level city in Shandong
Power Saving King Shop	Green Intelligent Power Saver	Easy to install, energy saving and power saving, very satisfied with a shopping	Jingdong prefecture level city in Shandong
Power Saving King Shop	Green Intelligent Power Saver	Received it and tried it out, it's great, works great and worth having! Five stars praise!	Jingdong prefecture level city in Shandong
Power Saving King Shop	Green Intelligent Power Saver	Power saving device, no problem at all, the quality of this product at this price, very affordable!	Jingdong prefecture level city in Shandong
Power Saving King Shop	Green Intelligent Power Saver	Received the goods, did not expect to be so fast, the quality is very good, especially power saving	Jingdong prefecture level city in Shandong
Power Saving King Shop	Green Intelligent Power Saver	The logistics is very fast, especially good, need to come back again	Jingdong prefecture level city in Shandong
Power Saving King Shop	Green Intelligent Power Saver	It's already working and it really saves power, so I'm very happy with it!	Jingdong prefecture level city in Shandong
Power Saving King Shop	Green Intelligent Power Saver	Great quality, works well, can recommend to friends to buy.	Jingdong prefecture level city in Shandong
Power Saving King Shop	Green Intelligent Power Saver	It's a good deal and worth the money.	Jingdong prefecture level city in Shandong
Power Saving King Shop	Green Intelligent Power Saver	I received it and I'm very happy with it, it's very good quality, guaranteed, and	Jingdong prefecture level city in Shandong
Power Saving King Shop	Green Intelligent Power Saver	Good quality, safe and secure purchase, ideal results and great price.	Jingdong prefecture level city in Shandong
Power Saving King Shop	Green Intelligent Power Saver	Simple operation plugged in the power supply can be, friends recommended looking forward to the effect of the	Jingdong prefecture level city in Shandong
Power Saving King Shop	Green Intelligent Power Saver	It's not bad, it saves electricity, and the material is sturdy and durable.	Jingdong prefecture level city in Shandong
Power Saving King Shop	Green Intelligent Power Saver	Plug and play, no management required, convenient and hassle-free!	Jingdong prefecture level city in Shandong
Power Saving King Shop	Green Intelligent Power Saver	The pictures are too lazy to go up, the power saving effect is still there!	Jingdong prefecture level city in Shandong
Power Saving King Shop	Green Intelligent Power Saver	Fast shipping. Affordable prices. Efficient	Jingdong prefecture level city in Shandong
Power Saving King Shop	Green Intelligent Power Saver	Good power saving, worth buying	Jingdong prefecture level city in Shandong
Power Saving King Shop	Green Intelligent Power Saver	Especially great, comparison shopping before buying, the store owner is very good, answering questions very patiently and detailed, there is a need to come back!	Jingdong prefecture level city in Shandong
Power Saving King Shop	Green Intelligent Power Saver	Very trustworthy brand, quality is guaranteed, the price is still affordable, quite satisfied!	Jingdong prefecture level city in Shandong
Power Saving King Shop	Green Intelligent Power Saver	Recommended by a friend. They say you can save money on air conditioning with this. Try it.	Jingdong prefecture level city in Shandong
Power Saving King Shop	Green Intelligent Power Saver	Works great, will be back next time, very happy with it and a great price!	Jingdong prefecture level city in Shandong
Power Saving King Shop	Green Intelligent Power Saver	The quality of the product is quite good, fast shipping, satisfied.	Jingdong prefecture level city in Shandong
Power Saving King Shop	Green Intelligent Power Saver	Good quality and fast logistics	Jingdong prefecture level city in Shandong
Power Saving King Shop	Green Intelligent Power Saver	It really saves power. I've tested it myself.	Jingdong prefecture level city in Shandong
Power Saving King Shop	Green Intelligent Power Saver	Really saves electricity!	Jingdong prefecture level city in Shandong

Power Saving King Shop	Green Intelligent Power Saver	pretty good	Jingdong prefecture level city in Shandong
Power Saving King Shop	Green Intelligent Power Saver	serviceable	Jingdong prefecture level city in Shandong
Power Saving King Shop	Green Intelligent Power Saver	Power Saving King received a tightly packaged logistics, installed the effect is very good price is cheaper than the physical store!	Jingdong prefecture level city in Shandong
Power Saving King Shop	Green Intelligent Power Saver	Super Power Saving King is very good quality, new style, is genuine, the use of the effect is very good, the price is cheap, it is worth buying!	Jingdong prefecture level city in Shandong
Power Saving King Shop	Green Intelligent Power Saver	Packaging is very tight, easy to install, quite practical, worth buying!	Jingdong prefecture level city in Shandong
Power Saving King Shop	Green Intelligent Power Saver	Quality assurance, affordable price, very energy efficient, will repurchase if needed!	Jingdong prefecture level city in Shandong
Power Saving King Shop	Green Intelligent Power Saver	Quality assurance, very good, affordable, logistics is also very fast, I'm very satisfied with the	Jingdong prefecture level city in Shandong
Power Saving King Shop	Green Intelligent Power Saver	Energy saving. It's so good, already installed, you can save a lot of electricity every month	Jingdong prefecture level city in Shandong
Power Saving King Shop	Green Intelligent Power Saver	The material is thick and the workmanship is fine in place, the power saving effect is very good, value for money!	Jingdong prefecture level city in Shandong
Power Saving King Shop	Green Intelligent Power Saver	The effect is great, genuine, and match the description, the logistics is fast, the price is reasonable 😍	Jingdong prefecture level city in Shandong
Power Saving King Shop	Green Intelligent Power Saver	It's genuine, works well, easy to use, plugs in and works, really saves power	Jingdong prefecture level city in Shandong
Power Saving King Shop	Green Intelligent Power Saver	It's easy to use, effective, great service, and recommended!	Jingdong prefecture level city in Shandong
Power Saving King Shop	Green Intelligent Power Saver	🌈 Great quality, bought it to my liking 💕 Awesome! ✨ Buy with confidence if you need it!	Jingdong prefecture level city in Shandong
Power Saving King Shop	Green Intelligent Power Saver	Good quality, easy to install, very energy saving and power saving, good to use	Jingdong prefecture level city in Shandong
Power Saving King Shop	Green Intelligent Power Saver	The quality is very good, it does have the effect of saving electricity, the description matches, the price is affordable, it is worth recommending.	Jingdong prefecture level city in Shandong
Power Saving King Shop	Green Intelligent Power Saver	Received, the functionality is particularly good, it is genuine, the price is affordable	Jingdong prefecture level city in Shandong
Power Saving King Shop	Green Intelligent Power Saver	Good quality, very easy to use, energy efficient, home electricity bill is obviously much less	Jingdong prefecture level city in Shandong
Power Saving King Shop	Green Intelligent Power Saver	Easy to install, energy saving and power saving, very satisfied with a shopping	Jingdong prefecture level city in Shandong
Power Saving King Shop	Green Intelligent Power Saver	Received it and tried it out, it's great, works great and worth having! Five stars praise!	Jingdong prefecture level city in Shandong
Power Saving King Shop	Green Intelligent Power Saver	Power saving device, no problem at all, the quality of this product at this price, very affordable!	Jingdong prefecture level city in Shandong
Power Saving King Shop	Green Intelligent Power Saver	Received the goods, did not expect to be so fast, the quality is very good, especially power saving	Jingdong prefecture level city in Shandong
Power Saving King Shop	Green Intelligent Power Saver	The logistics is very fast, especially good, need to come back again	Jingdong prefecture level city in Shandong
Power Saving King Shop	Green Intelligent Power Saver	It's already working and it really saves power, so I'm very happy with it!	Jingdong prefecture level city in Shandong
Power Saving King Shop	Green Intelligent Power Saver	Great quality, works well, can recommend to friends to buy.	Jingdong prefecture level city in Shandong
Power Saving King Shop	Green Intelligent Power Saver	It's a good deal and worth the money.	Jingdong prefecture level city in Shandong
Power Saving King Shop	Green Intelligent Power Saver	I received it and I'm very happy with it, it's very good quality, guaranteed, and	Jingdong prefecture level city in Shandong
Power Saving King Shop	Green Intelligent Power Saver	Good quality, safe and secure purchase, ideal results and great price.	Jingdong prefecture level city in Shandong
Power Saving King Shop	Green Intelligent Power Saver	Simple operation plugged in the power supply can be, friends recommended looking forward to the effect of the	Jingdong prefecture level city in Shandong
Power Saving King Shop	Green Intelligent Power Saver	It's not bad, it saves electricity, and the material is sturdy and durable.	Jingdong prefecture level city in Shandong
Power Saving King Shop	Green Intelligent Power Saver	Plug and play, no management required, convenient and hassle-free!	Jingdong prefecture level city in Shandong
Power Saving King Shop	Green Intelligent Power Saver	The pictures are too lazy to go up, the power saving effect is still there!	Jingdong prefecture level city in Shandong
Power Saving King Shop	Green Intelligent Power Saver	Fast shipping. Affordable prices. Efficient	Jingdong prefecture level city in Shandong
Power Saving King Shop	Green Intelligent Power Saver	Good power saving, worth buying	Jingdong prefecture level city in Shandong
Power Saving King Shop	Green Intelligent Power Saver	Especially great, comparison shopping before buying, the store owner is very good, answering questions very patiently and detailed, there is a need to come back!	Jingdong prefecture level city in Shandong
Power Saving King Shop	Green Intelligent Power Saver	Very trustworthy brand, quality is guaranteed, the price is still affordable, quite satisfied!	Jingdong prefecture level city in Shandong
Power Saving King Shop	Green Intelligent Power Saver	Recommended by a friend. They say you can save money on air conditioning with this. Try it.	Jingdong prefecture level city in Shandong
Power Saving King Shop	Green Intelligent Power Saver	Works great, will be back next time, very happy with it and a great price!	Jingdong prefecture level city in Shandong
Power Saving King Shop	Green Intelligent Power Saver	The quality of the product is quite good, fast shipping, satisfied.	Jingdong prefecture level city in Shandong
Power Saving King Shop	Green Intelligent Power Saver	Good quality and fast logistics	Jingdong prefecture level city in Shandong
Power Saving King Shop	Green Intelligent Power Saver	It really saves power. I've tested it myself.	Jingdong prefecture level city in Shandong
Power Saving King Shop	Green Intelligent Power Saver	Really saves electricity!	Jingdong prefecture level city in Shandong
Power Saving King Shop	Green Intelligent Power Saver	pretty good	Jingdong prefecture level city in Shandong
Power Saving King Shop	Green Intelligent Power Saver	serviceable	Jingdong prefecture level city in Shandong
Power Saving King Shop	Green Intelligent Power Saver	Power Saving King received a tightly packaged logistics, installed the effect is very good price is cheaper than the physical store!	Jingdong prefecture level city in Shandong
Power Saving King Shop	Green Intelligent Power Saver	Super Power Saving King is very good quality, new style, is genuine, the use of the effect is very good, the price is cheap, it is worth buying!	Jingdong prefecture level city in Shandong
Power Saving King Shop	Green Intelligent Power Saver	Packaging is very tight, easy to install, quite practical, worth buying!	Jingdong prefecture level city in Shandong
Power Saving King Shop	Green Intelligent Power Saver	Quality assurance, affordable price, very energy efficient, will repurchase if needed!	Jingdong prefecture level city in Shandong
Power Saving King Shop	Green Intelligent Power Saver	Quality assurance, very good, affordable, logistics is also very fast, I'm very satisfied with the	Jingdong prefecture level city in Shandong
Power Saving King Shop	Green Intelligent Power Saver	Energy saving. It's so good, already installed, you can save a lot of electricity every month	Jingdong prefecture level city in Shandong
Power Saving King Shop	Green Intelligent Power Saver	The material is thick and the workmanship is fine in place, the power saving effect is very good, value for money!	Jingdong prefecture level city in Shandong
Power Saving King Shop	Green Intelligent Power Saver	The effect is great, genuine, and match the description, the logistics is fast, the price is reasonable 😍	Jingdong prefecture level city in Shandong

Power Saving King Shop	Green Intelligent Power Saver	It's genuine, works well, easy to use, plugs in and works, really saves power	Jingdong prefecture level city in Shandong
Power Saving King Shop	Green Intelligent Power Saver	It's easy to use, effective, great service, and recommended!	Jingdong prefecture level city in Shandong
Power Saving King Shop	Green Intelligent Power Saver	🌈 Great quality, bought it to my liking 💛 Awesome! ✨ Buy with confidence if you need it!	Jingdong prefecture level city in Shandong
Power Saving King Shop	Green Intelligent Power Saver	Good quality, easy to install, very energy saving and power saving, good to use	Jingdong prefecture level city in Shandong
Power Saving King Shop	Green Intelligent Power Saver	The quality is very good, it does have the effect of saving electricity, the description matches, the price is affordable, it is worth recommending.	Jingdong prefecture level city in Shandong
Power Saving King Shop	Green Intelligent Power Saver	Received, the functionality is particularly good, it is genuine, the price is affordable	Jingdong prefecture level city in Shandong
Power Saving King Shop	Green Intelligent Power Saver	Good quality, very easy to use, energy efficient, home electricity bill is obviously much less	Jingdong prefecture level city in Shandong
Power Saving King Shop	Green Intelligent Power Saver	Easy to install, energy saving and power saving, very satisfied with a shopping	Jingdong prefecture level city in Shandong
Power Saving King Shop	Green Intelligent Power Saver	Received it and tried it out, it's great, works great and worth having! Five stars praise!	Jingdong prefecture level city in Shandong
Power Saving King Shop	Green Intelligent Power Saver	Power saving device, no problem at all, the quality of this product at this price, very affordable!	Jingdong prefecture level city in Shandong
Power Saving King Shop	Green Intelligent Power Saver	Received the goods, did not expect to be so fast, the quality is very good, especially power saving	Jingdong prefecture level city in Shandong
Power Saving King Shop	Green Intelligent Power Saver	The logistics is very fast, especially good, need to come back again	Jingdong prefecture level city in Shandong
Power Saving King Shop	Green Intelligent Power Saver	It's already working and it really saves power, so I'm very happy with it!	Jingdong prefecture level city in Shandong
Power Saving King Shop	Green Intelligent Power Saver	Great quality, works well, can recommend to friends to buy.	Jingdong prefecture level city in Shandong
Power Saving King Shop	Green Intelligent Power Saver	It's a good deal and worth the money.	Jingdong prefecture level city in Shandong
Power Saving King Shop	Green Intelligent Power Saver	I received it and I'm very happy with it, it's very good quality, guaranteed, and	Jingdong prefecture level city in Shandong
Power Saving King Shop	Green Intelligent Power Saver	Good quality, safe and secure purchase, ideal results and great price.	Jingdong prefecture level city in Shandong
Power Saving King Shop	Green Intelligent Power Saver	Simple operation plugged in the power supply can be, friends recommended looking forward to the effect of the	Jingdong prefecture level city in Shandong
Power Saving King Shop	Green Intelligent Power Saver	It's not bad, it saves electricity, and the material is sturdy and durable.	Jingdong prefecture level city in Shandong
Power Saving King Shop	Green Intelligent Power Saver	Plug and play, no management required, convenient and hassle-free!	Jingdong prefecture level city in Shandong
Power Saving King Shop	Green Intelligent Power Saver	The pictures are too lazy to go up, the power saving effect is still there!	Jingdong prefecture level city in Shandong
Power Saving King Shop	Green Intelligent Power Saver	Fast shipping. Affordable prices. Efficient	Jingdong prefecture level city in Shandong
Power Saving King Shop	Green Intelligent Power Saver	Good power saving, worth buying	Jingdong prefecture level city in Shandong
Power Saving King Shop	Green Intelligent Power Saver	Especially great, comparison shopping before buying, the store owner is very good, answering questions very patiently and detailed, there is a need to come back!	Jingdong prefecture level city in Shandong
Power Saving King Shop	Green Intelligent Power Saver	Very trustworthy brand, quality is guaranteed, the price is still affordable, quite satisfied!	Jingdong prefecture level city in Shandong
Power Saving King Shop	Green Intelligent Power Saver	Recommended by a friend. They say you can save money on air conditioning with this. Try it.	Jingdong prefecture level city in Shandong
Power Saving King Shop	Green Intelligent Power Saver	Works great, will be back next time, very happy with it and a great price!	Jingdong prefecture level city in Shandong
Power Saving King Shop	Green Intelligent Power Saver	The quality of the product is quite good, fast shipping, satisfied.	Jingdong prefecture level city in Shandong
Power Saving King Shop	Green Intelligent Power Saver	Good quality and fast logistics	Jingdong prefecture level city in Shandong
Power Saving King Shop	Green Intelligent Power Saver	It really saves power. I've tested it myself.	Jingdong prefecture level city in Shandong
Power Saving King Shop	Green Intelligent Power Saver	Really saves electricity!	Jingdong prefecture level city in Shandong
Power Saving King Shop	Green Intelligent Power Saver	pretty good	Jingdong prefecture level city in Shandong
Power Saving King Shop	Green Intelligent Power Saver	serviceable	Jingdong prefecture level city in Shandong
Power Saving King Shop	Green Intelligent Power Saver	Power Saving King received a tightly packaged logistics, installed the effect is very good price is cheaper than the physical store!	Jingdong prefecture level city in Shandong
Power Saving King Shop	Green Intelligent Power Saver	Super Power Saving King is very good quality, new style, is genuine, the use of the effect is very good, the price is cheap, it is worth buying!	Jingdong prefecture level city in Shandong
Power Saving King Shop	Green Intelligent Power Saver	Packaging is very tight, easy to install, quite practical, worth buying!	Jingdong prefecture level city in Shandong
Power Saving King Shop	Green Intelligent Power Saver	Quality assurance, affordable price, very energy efficient, will repurchase if needed!	Jingdong prefecture level city in Shandong
Power Saving King Shop	Green Intelligent Power Saver	Quality assurance, very good, affordable, logistics is also very fast, I'm very satisfied with the	Jingdong prefecture level city in Shandong
Power Saving King Shop	Green Intelligent Power Saver	Energy saving. It's so good, already installed, you can save a lot of electricity every month	Jingdong prefecture level city in Shandong
Power Saving King Shop	Green Intelligent Power Saver	The material is thick and the workmanship is fine in place, the power saving effect is very good, value for money!	Jingdong prefecture level city in Shandong
Power Saving King Shop	Green Intelligent Power Saver	The effect is great, genuine, and match the description, the logistics is fast, the price is reasonable 😍	Jingdong prefecture level city in Shandong
Power Saving King Shop	Green Intelligent Power Saver	It's genuine, works well, easy to use, plugs in and works, really saves power	Jingdong prefecture level city in Shandong
Power Saving King Shop	Green Intelligent Power Saver	It's easy to use, effective, great service, and recommended!	Jingdong prefecture level city in Shandong
Power Saving King Shop	Green Intelligent Power Saver	🌈 Great quality, bought it to my liking 💛 Awesome! ✨ Buy with confidence if you need it!	Jingdong prefecture level city in Shandong
Power Saving King Shop	Green Intelligent Power Saver	Good quality, easy to install, very energy saving and power saving, good to use	Jingdong prefecture level city in Shandong
Power Saving King Shop	Green Intelligent Power Saver	The quality is very good, it does have the effect of saving electricity, the description matches, the price is affordable, it is worth recommending.	Jingdong prefecture level city in Shandong
Power Saving King Shop	Green Intelligent Power Saver	Received, the functionality is particularly good, it is genuine, the price is affordable	Jingdong prefecture level city in Shandong
Power Saving King Shop	Green Intelligent Power Saver	Good quality, very easy to use, energy efficient, home electricity bill is obviously much less	Jingdong prefecture level city in Shandong
Power Saving King Shop	Green Intelligent Power Saver	Easy to install, energy saving and power saving, very satisfied with a shopping	Jingdong prefecture level city in Shandong
Power Saving King Shop	Green Intelligent Power Saver	Received it and tried it out, it's great, works great and worth having! Five stars praise!	Jingdong prefecture level city in Shandong
Power Saving King Shop	Green Intelligent Power Saver	Power saving device, no problem at all, the quality of this product at this price, very affordable!	Jingdong prefecture level city in Shandong

Power Saving King Shop	Green Intelligent Power Saver	Received the goods, did not expect to be so fast, the quality is very good, especially power saving	Jingdong prefecture level city in Shandong
Power Saving King Shop	Green Intelligent Power Saver	The logistics is very fast, especially good, need to come back again	Jingdong prefecture level city in Shandong
Power Saving King Shop	Green Intelligent Power Saver	It's already working and it really saves power, so I'm very happy with it!	Jingdong prefecture level city in Shandong
Power Saving King Shop	Green Intelligent Power Saver	Great quality, works well, can recommend to friends to buy.	Jingdong prefecture level city in Shandong
Power Saving King Shop	Green Intelligent Power Saver	It's a good deal and worth the money.	Jingdong prefecture level city in Shandong
Power Saving King Shop	Green Intelligent Power Saver	I received it and I'm very happy with it, it's very good quality, guaranteed, and	Jingdong prefecture level city in Shandong
Power Saving King Shop	Green Intelligent Power Saver	Good quality, safe and secure purchase, ideal results and great price.	Jingdong prefecture level city in Shandong
Power Saving King Shop	Green Intelligent Power Saver	Simple operation plugged in the power supply can be, friends recommended looking forward to the effect of the	Jingdong prefecture level city in Shandong
Power Saving King Shop	Green Intelligent Power Saver	It's not bad, it saves electricity, and the material is sturdy and durable.	Jingdong prefecture level city in Shandong
Power Saving King Shop	Green Intelligent Power Saver	Plug and play, no management required, convenient and hassle-free!	Jingdong prefecture level city in Shandong
Power Saving King Shop	Green Intelligent Power Saver	The pictures are too lazy to go up, the power saving effect is still there!	Jingdong prefecture level city in Shandong
Power Saving King Shop	Green Intelligent Power Saver	Fast shipping. Affordable prices. Efficient	Jingdong prefecture level city in Shandong
Power Saving King Shop	Green Intelligent Power Saver	Good power saving, worth buying	Jingdong prefecture level city in Shandong
Power Saving King Shop	Green Intelligent Power Saver	Especially great, comparison shopping before buying, the store owner is very good, answering questions very patiently and detailed, there is a need to come back!	Jingdong prefecture level city in Shandong
Power Saving King Shop	Green Intelligent Power Saver	Very trustworthy brand, quality is guaranteed, the price is still affordable, quite satisfied!	Jingdong prefecture level city in Shandong
Power Saving King Shop	Green Intelligent Power Saver	Recommended by a friend. They say you can save money on air conditioning with this. Try it.	Jingdong prefecture level city in Shandong
Power Saving King Shop	Green Intelligent Power Saver	Works great, will be back next time, very happy with it and a great price!	Jingdong prefecture level city in Shandong
Power Saving King Shop	Green Intelligent Power Saver	The quality of the product is quite good, fast shipping, satisfied.	Jingdong prefecture level city in Shandong
Power Saving King Shop	Green Intelligent Power Saver	Good quality and fast logistics	Jingdong prefecture level city in Shandong
Power Saving King Shop	Green Intelligent Power Saver	It really saves power. I've tested it myself.	Jingdong prefecture level city in Shandong
Power Saving King Shop	Green Intelligent Power Saver	Really saves electricity!	Jingdong prefecture level city in Shandong
Power Saving King Shop	Green Intelligent Power Saver	pretty good	Jingdong prefecture level city in Shandong
Power Saving King Shop	Green Intelligent Power Saver	serviceable	Jingdong prefecture level city in Shandong
Power Saving King Shop	Green Intelligent Power Saver	Power Saving King received a tightly packaged logistics, installed the effect is very good price is cheaper than the physical store!	Jingdong prefecture level city in Shandong
Power Saving King Shop	Green Intelligent Power Saver	Super Power Saving King is very good quality, new style, is genuine, the use of the effect is very good, the price is cheap, it is worth buying!	Jingdong prefecture level city in Shandong
Power Saving King Shop	Green Intelligent Power Saver	Packaging is very tight, easy to install, quite practical, worth buying!	Jingdong prefecture level city in Shandong
Power Saving King Shop	Green Intelligent Power Saver	Quality assurance, affordable price, very energy efficient, will repurchase if needed!	Jingdong prefecture level city in Shandong
Power Saving King Shop	Green Intelligent Power Saver	Quality assurance, very good, affordable, logistics is also very fast, I'm very satisfied with the	Jingdong prefecture level city in Shandong
Power Saving King Shop	Green Intelligent Power Saver	Energy saving. It's so good, already installed, you can save a lot of electricity every month	Jingdong prefecture level city in Shandong
Power Saving King Shop	Green Intelligent Power Saver	The material is thick and the workmanship is fine in place, the power saving effect is very good, value for money!	Jingdong prefecture level city in Shandong
Power Saving King Shop	Green Intelligent Power Saver	The effect is great, genuine, and match the description, the logistics is fast, the price is reasonable 😍	Jingdong prefecture level city in Shandong
Power Saving King Shop	Green Intelligent Power Saver	It's genuine, works well, easy to use, plugs in and works, really saves power	Jingdong prefecture level city in Shandong
Power Saving King Shop	Green Intelligent Power Saver	It's easy to use, effective, great service, and recommended!	Jingdong prefecture level city in Shandong
Power Saving King Shop	Green Intelligent Power Saver	🌈 Great quality, bought it to my liking 💝 Awesome! ✨ Buy with confidence if you need it!	Jingdong prefecture level city in Shandong
Power Saving King Shop	Green Intelligent Power Saver	Good quality, easy to install, very energy saving and power saving, good to use	Jingdong prefecture level city in Shandong
Power Saving King Shop	Green Intelligent Power Saver	The quality is very good, it does have the effect of saving electricity, the description matches, the price is affordable, it is worth recommending.	Jingdong prefecture level city in Shandong
Power Saving King Shop	Green Intelligent Power Saver	Received, the functionality is particularly good, it is genuine, the price is affordable	Jingdong prefecture level city in Shandong
Power Saving King Shop	Green Intelligent Power Saver	Good quality, very easy to use, energy efficient, home electricity bill is obviously much less	Jingdong prefecture level city in Shandong
Power Saving King Shop	Green Intelligent Power Saver	Easy to install, energy saving and power saving, very satisfied with a shopping	Jingdong prefecture level city in Shandong
Power Saving King Shop	Green Intelligent Power Saver	Received it and tried it out, it's great, works great and worth having! Five stars praise!	Jingdong prefecture level city in Shandong
Power Saving King Shop	Green Intelligent Power Saver	Power saving device, no problem at all, the quality of this product at this price, very affordable!	Jingdong prefecture level city in Shandong
Power Saving King Shop	Green Intelligent Power Saver	Received the goods, did not expect to be so fast, the quality is very good, especially power saving	Jingdong prefecture level city in Shandong
Power Saving King Shop	Green Intelligent Power Saver	The logistics is very fast, especially good, need to come back again	Jingdong prefecture level city in Shandong
Power Saving King Shop	Green Intelligent Power Saver	It's already working and it really saves power, so I'm very happy with it!	Jingdong prefecture level city in Shandong
Power Saving King Shop	Green Intelligent Power Saver	Great quality, works well, can recommend to friends to buy.	Jingdong prefecture level city in Shandong
Power Saving King Shop	Green Intelligent Power Saver	It's a good deal and worth the money.	Jingdong prefecture level city in Shandong
Power Saving King Shop	Green Intelligent Power Saver	I received it and I'm very happy with it, it's very good quality, guaranteed, and	Jingdong prefecture level city in Shandong
Power Saving King Shop	Green Intelligent Power Saver	Good quality, safe and secure purchase, ideal results and great price.	Jingdong prefecture level city in Shandong
Power Saving King Shop	Green Intelligent Power Saver	Simple operation plugged in the power supply can be, friends recommended looking forward to the effect of the	Jingdong prefecture level city in Shandong
Power Saving King Shop	Green Intelligent Power Saver	It's not bad, it saves electricity, and the material is sturdy and durable.	Jingdong prefecture level city in Shandong
Power Saving King Shop	Green Intelligent Power Saver	Plug and play, no management required, convenient and hassle-free!	Jingdong prefecture level city in Shandong

Power Saving King Shop	Green Intelligent Power Saver	The pictures are too lazy to go up, the power saving effect is still there!	Jingdong prefecture level city in Shandong
Power Saving King Shop	Green Intelligent Power Saver	Fast shipping. Affordable prices. Efficient	Jingdong prefecture level city in Shandong
Power Saving King Shop	Green Intelligent Power Saver	Good power saving, worth buying	Jingdong prefecture level city in Shandong
Power Saving King Shop	Green Intelligent Power Saver	Especially great, comparison shopping before buying, the store owner is very good, answering questions very patiently and detailed, there is a need to come back!	Jingdong prefecture level city in Shandong
Power Saving King Shop	Green Intelligent Power Saver	Very trustworthy brand, quality is guaranteed, the price is still affordable, quite satisfied!	Jingdong prefecture level city in Shandong
Power Saving King Shop	Green Intelligent Power Saver	Recommended by a friend. They say you can save money on air conditioning with this. Try it.	Jingdong prefecture level city in Shandong
Power Saving King Shop	Green Intelligent Power Saver	Works great, will be back next time, very happy with it and a great price!	Jingdong prefecture level city in Shandong
Power Saving King Shop	Green Intelligent Power Saver	The quality of the product is quite good, fast shipping, satisfied.	Jingdong prefecture level city in Shandong
Power Saving King Shop	Green Intelligent Power Saver	Good quality and fast logistics	Jingdong prefecture level city in Shandong
Power Saving King Shop	Green Intelligent Power Saver	It really saves power. I've tested it myself.	Jingdong prefecture level city in Shandong
Power Saving King Shop	Green Intelligent Power Saver	Really saves electricity!	Jingdong prefecture level city in Shandong
Power Saving King Shop	Green Intelligent Power Saver	pretty good	Jingdong prefecture level city in Shandong
Power Saving King Shop	Green Intelligent Power Saver	serviceable	Jingdong prefecture level city in Shandong
Power Saving King Shop	Green Intelligent Power Saver	Power Saving King received a tightly packaged logistics, installed the effect is very good price is cheaper than the physical store!	Jingdong prefecture level city in Shandong
Power Saving King Shop	Green Intelligent Power Saver	Super Power Saving King is very good quality, new style, is genuine, the use of the effect is very good, the price is cheap, it is worth buying!	Jingdong prefecture level city in Shandong
Power Saving King Shop	Green Intelligent Power Saver	Packaging is very tight, easy to install, quite practical, worth buying!	Jingdong prefecture level city in Shandong
Power Saving King Shop	Green Intelligent Power Saver	Quality assurance, affordable price, very energy efficient, will repurchase if needed!	Jingdong prefecture level city in Shandong
Power Saving King Shop	Green Intelligent Power Saver	Quality assurance, very good, affordable, logistics is also very fast, I'm very satisfied with the	Jingdong prefecture level city in Shandong
Power Saving King Shop	Green Intelligent Power Saver	Energy saving. It's so good, already installed, you can save a lot of electricity every month	Jingdong prefecture level city in Shandong
Power Saving King Shop	Green Intelligent Power Saver	The material is thick and the workmanship is fine in place, the power saving effect is very good, value for money!	Jingdong prefecture level city in Shandong
Power Saving King Shop	Green Intelligent Power Saver	The effect is great, genuine, and match the description, the logistics is fast, the price is reasonable 😍	Jingdong prefecture level city in Shandong
Power Saving King Shop	Green Intelligent Power Saver	It's genuine, works well, easy to use, plugs in and works, really saves power	Jingdong prefecture level city in Shandong
Power Saving King Shop	Green Intelligent Power Saver	It's easy to use, effective, great service, and recommended!	Jingdong prefecture level city in Shandong
Power Saving King Shop	Green Intelligent Power Saver	🌈 Great quality, bought it to my liking 💝 Awesome! ✨ Buy with confidence if you need it!	Jingdong prefecture level city in Shandong
Power Saving King Shop	Green Intelligent Power Saver	Good quality, easy to install, very energy saving and power saving, good to use	Jingdong prefecture level city in Shandong
Power Saving King Shop	Green Intelligent Power Saver	The quality is very good, it does have the effect of saving electricity, the description matches, the price is affordable, it is worth recommending.	Jingdong prefecture level city in Shandong
Power Saving King Shop	Green Intelligent Power Saver	Received, the functionality is particularly good, it is genuine, the price is affordable	Jingdong prefecture level city in Shandong
Power Saving King Shop	Green Intelligent Power Saver	Good quality, very easy to use, energy efficient, home electricity bill is obviously much less	Jingdong prefecture level city in Shandong
Power Saving King Shop	Green Intelligent Power Saver	Easy to install, energy saving and power saving, very satisfied with a shopping	Jingdong prefecture level city in Shandong
Power Saving King Shop	Green Intelligent Power Saver	Received it and tried it out, it's great, works great and worth having! Five stars praise!	Jingdong prefecture level city in Shandong
Power Saving King Shop	Green Intelligent Power Saver	Power saving device, no problem at all, the quality of this product at this price, very affordable!	Jingdong prefecture level city in Shandong
Power Saving King Shop	Green Intelligent Power Saver	Received the goods, did not expect to be so fast, the quality is very good, especially power saving	Jingdong prefecture level city in Shandong
Power Saving King Shop	Green Intelligent Power Saver	The logistics is very fast, especially good, need to come back again	Jingdong prefecture level city in Shandong
Power Saving King Shop	Green Intelligent Power Saver	It's already working and it really saves power, so I'm very happy with it!	Jingdong prefecture level city in Shandong
Power Saving King Shop	Green Intelligent Power Saver	Great quality, works well, can recommend to friends to buy.	Jingdong prefecture level city in Shandong
Power Saving King Shop	Green Intelligent Power Saver	It's a good deal and worth the money.	Jingdong prefecture level city in Shandong
Power Saving King Shop	Green Intelligent Power Saver	I received it and I'm very happy with it, it's very good quality, guaranteed, and	Jingdong prefecture level city in Shandong
Power Saving King Shop	Green Intelligent Power Saver	Good quality, safe and secure purchase, ideal results and great price.	Jingdong prefecture level city in Shandong
Power Saving King Shop	Green Intelligent Power Saver	Simple operation plugged in the power supply can be, friends recommended looking forward to the effect of the	Jingdong prefecture level city in Shandong
Power Saving King Shop	Green Intelligent Power Saver	It's not bad, it saves electricity, and the material is sturdy and durable.	Jingdong prefecture level city in Shandong
Power Saving King Shop	Green Intelligent Power Saver	Plug and play, no management required, convenient and hassle-free!	Jingdong prefecture level city in Shandong
Power Saving King Shop	Green Intelligent Power Saver	The pictures are too lazy to go up, the power saving effect is still there!	Jingdong prefecture level city in Shandong
Power Saving King Shop	Green Intelligent Power Saver	Fast shipping. Affordable prices. Efficient	Jingdong prefecture level city in Shandong
Power Saving King Shop	Green Intelligent Power Saver	Good power saving, worth buying	Jingdong prefecture level city in Shandong
Power Saving King Shop	Green Intelligent Power Saver	Especially great, comparison shopping before buying, the store owner is very good, answering questions very patiently and detailed, there is a need to come back!	Jingdong prefecture level city in Shandong
Power Saving King Shop	Green Intelligent Power Saver	Very trustworthy brand, quality is guaranteed, the price is still affordable, quite satisfied!	Jingdong prefecture level city in Shandong
Power Saving King Shop	Green Intelligent Power Saver	Recommended by a friend. They say you can save money on air conditioning with this. Try it.	Jingdong prefecture level city in Shandong
Power Saving King Shop	Green Intelligent Power Saver	Works great, will be back next time, very happy with it and a great price!	Jingdong prefecture level city in Shandong
Power Saving King Shop	Green Intelligent Power Saver	The quality of the product is quite good, fast shipping, satisfied.	Jingdong prefecture level city in Shandong
Power Saving King Shop	Green Intelligent Power Saver	Good quality and fast logistics	Jingdong prefecture level city in Shandong
Power Saving King Shop	Green Intelligent Power Saver	It really saves power. I've tested it myself.	Jingdong prefecture level city in Shandong

Power Saving King Shop	Green Intelligent Power Saver	Really saves electricity!	Jingdong prefecture level city in Shandong
Power Saving King Shop	Green Intelligent Power Saver	pretty good	Jingdong prefecture level city in Shandong
Power Saving King Shop	Green Intelligent Power Saver	serviceable	Jingdong prefecture level city in Shandong
Power Saving King Shop	Green Intelligent Power Saver	Power Saving King received a tightly packaged logistics, installed the effect is very good price is cheaper than the physical store!	Jingdong prefecture level city in Shandong
Power Saving King Shop	Green Intelligent Power Saver	Super Power Saving King is very good quality, new style, is genuine, the use of the effect is very good, the price is cheap, it is worth buying!	Jingdong prefecture level city in Shandong
Power Saving King Shop	Green Intelligent Power Saver	Packaging is very tight, easy to install, quite practical, worth buying!	Jingdong prefecture level city in Shandong
Power Saving King Shop	Green Intelligent Power Saver	Quality assurance, affordable price, very energy efficient, will repurchase if needed!	Jingdong prefecture level city in Shandong
Power Saving King Shop	Green Intelligent Power Saver	Quality assurance, very good, affordable, logistics is also very fast, I'm very satisfied with the	Jingdong prefecture level city in Shandong
Power Saving King Shop	Green Intelligent Power Saver	Energy saving. It's so good, already installed, you can save a lot of electricity every month	Jingdong prefecture level city in Shandong
Power Saving King Shop	Green Intelligent Power Saver	The material is thick and the workmanship is fine in place, the power saving effect is very good, value for money!	Jingdong prefecture level city in Shandong
Power Saving King Shop	Green Intelligent Power Saver	The effect is great, genuine, and match the description, the logistics is fast, the price is reasonable 😍	Jingdong prefecture level city in Shandong
Power Saving King Shop	Green Intelligent Power Saver	It's genuine, works well, easy to use, plugs in and works, really saves power	Jingdong prefecture level city in Shandong
Power Saving King Shop	Green Intelligent Power Saver	It's easy to use, effective, great service, and recommended!	Jingdong prefecture level city in Shandong
Power Saving King Shop	Green Intelligent Power Saver	🌈 Great quality, bought it to my liking 💝 Awesome! ✨ Buy with confidence if you need it!	Jingdong prefecture level city in Shandong
Power Saving King Shop	Green Intelligent Power Saver	Good quality, easy to install, very energy saving and power saving, good to use	Jingdong prefecture level city in Shandong
Power Saving King Shop	Green Intelligent Power Saver	The quality is very good, it does have the effect of saving electricity, the description matches, the price is affordable, it is worth recommending.	Jingdong prefecture level city in Shandong
Power Saving King Shop	Green Intelligent Power Saver	Received, the functionality is particularly good, it is genuine, the price is affordable	Jingdong prefecture level city in Shandong
Power Saving King Shop	Green Intelligent Power Saver	Good quality, very easy to use, energy efficient, home electricity bill is obviously much less	Jingdong prefecture level city in Shandong
Power Saving King Shop	Green Intelligent Power Saver	Easy to install, energy saving and power saving, very satisfied with a shopping	Jingdong prefecture level city in Shandong
Power Saving King Shop	Green Intelligent Power Saver	Received it and tried it out, it's great, works great and worth having! Five stars praise!	Jingdong prefecture level city in Shandong
Power Saving King Shop	Green Intelligent Power Saver	Power saving device, no problem at all, the quality of this product at this price, very affordable!	Jingdong prefecture level city in Shandong
Power Saving King Shop	Green Intelligent Power Saver	Received the goods, did not expect to be so fast, the quality is very good, especially power saving	Jingdong prefecture level city in Shandong
Power Saving King Shop	Green Intelligent Power Saver	The logistics is very fast, especially good, need to come back again	Jingdong prefecture level city in Shandong
Power Saving King Shop	Green Intelligent Power Saver	It's already working and it really saves power, so I'm very happy with it!	Jingdong prefecture level city in Shandong
Power Saving King Shop	Green Intelligent Power Saver	Great quality, works well, can recommend to friends to buy.	Jingdong prefecture level city in Shandong
Power Saving King Shop	Green Intelligent Power Saver	It's a good deal and worth the money.	Jingdong prefecture level city in Shandong
Power Saving King Shop	Green Intelligent Power Saver	I received it and I'm very happy with it, it's very good quality, guaranteed, and	Jingdong prefecture level city in Shandong
Power Saving King Shop	Green Intelligent Power Saver	Good quality, safe and secure purchase, ideal results and great price.	Jingdong prefecture level city in Shandong
Power Saving King Shop	Green Intelligent Power Saver	Simple operation plugged in the power supply can be, friends recommended looking forward to the effect of the	Jingdong prefecture level city in Shandong
Power Saving King Shop	Green Intelligent Power Saver	It's not bad, it saves electricity, and the material is sturdy and durable.	Jingdong prefecture level city in Shandong
Power Saving King Shop	Green Intelligent Power Saver	Plug and play, no management required, convenient and hassle-free!	Jingdong prefecture level city in Shandong
Power Saving King Shop	Green Intelligent Power Saver	The pictures are too lazy to go up, the power saving effect is still there!	Jingdong prefecture level city in Shandong
Power Saving King Shop	Green Intelligent Power Saver	Fast shipping. Affordable prices. Efficient	Jingdong prefecture level city in Shandong
Power Saving King Shop	Green Intelligent Power Saver	Good power saving, worth buying	Jingdong prefecture level city in Shandong
Power Saving King Shop	Green Intelligent Power Saver	Especially great, comparison shopping before buying, the store owner is very good, answering questions very patiently and detailed, there is a need to come back!	Jingdong prefecture level city in Shandong
Power Saving King Shop	Green Intelligent Power Saver	Very trustworthy brand, quality is guaranteed, the price is still affordable, quite satisfied!	Jingdong prefecture level city in Shandong
Power Saving King Shop	Green Intelligent Power Saver	Recommended by a friend. They say you can save money on air conditioning with this. Try it.	Jingdong prefecture level city in Shandong
Power Saving King Shop	Green Intelligent Power Saver	Works great, will be back next time, very happy with it and a great price!	Jingdong prefecture level city in Shandong
Power Saving King Shop	Green Intelligent Power Saver	The quality of the product is quite good, fast shipping, satisfied.	Jingdong prefecture level city in Shandong
Power Saving King Shop	Green Intelligent Power Saver	Good quality and fast logistics	Jingdong prefecture level city in Shandong
Power Saving King Shop	Green Intelligent Power Saver	It really saves power. I've tested it myself.	Jingdong prefecture level city in Shandong
Power Saving King Shop	Green Intelligent Power Saver	Really saves electricity!	Jingdong prefecture level city in Shandong
Power Saving King Shop	Green Intelligent Power Saver	pretty good	Jingdong prefecture level city in Shandong
Power Saving King Shop	Green Intelligent Power Saver	serviceable	Jingdong prefecture level city in Shandong
Power Saving King Shop	Green Intelligent Power Saver	Power Saving King received a tightly packaged logistics, installed the effect is very good price is cheaper than the physical store!	Jingdong prefecture level city in Shandong
Power Saving King Shop	Green Intelligent Power Saver	Super Power Saving King is very good quality, new style, is genuine, the use of the effect is very good, the price is cheap, it is worth buying!	Jingdong prefecture level city in Shandong
Power Saving King Shop	Green Intelligent Power Saver	Packaging is very tight, easy to install, quite practical, worth buying!	Jingdong prefecture level city in Shandong
Power Saving King Shop	Green Intelligent Power Saver	Quality assurance, affordable price, very energy efficient, will repurchase if needed!	Jingdong prefecture level city in Shandong
Power Saving King Shop	Green Intelligent Power Saver	Quality assurance, very good, affordable, logistics is also very fast, I'm very satisfied with the	Jingdong prefecture level city in Shandong
Power Saving King Shop	Green Intelligent Power Saver	Energy saving. It's so good, already installed, you can save a lot of electricity every month	Jingdong prefecture level city in Shandong
Power Saving King Shop	Green Intelligent Power Saver	The material is thick and the workmanship is fine in place, the power saving effect is very good, value for money!	Jingdong prefecture level city in Shandong

Power Saving King Shop	Green Intelligent Power Saver	The effect is great, genuine, and match the description, the logistics is fast, the price is reasonable 😍	Jingdong prefecture level city in Shandong
Power Saving King Shop	Green Intelligent Power Saver	It's genuine, works well, easy to use, plugs in and works, really saves power	Jingdong prefecture level city in Shandong
Power Saving King Shop	Green Intelligent Power Saver	It's easy to use, effective, great service, and recommended!	Jingdong prefecture level city in Shandong
Power Saving King Shop	Green Intelligent Power Saver	🌈 Great quality, bought it to my liking 💗 Awesome! ✨ Buy with confidence if you need it!	Jingdong prefecture level city in Shandong
Power Saving King Shop	Green Intelligent Power Saver	Good quality, easy to install, very energy saving and power saving, good to use	Jingdong prefecture level city in Shandong
Power Saving King Shop	Green Intelligent Power Saver	The quality is very good, it does have the effect of saving electricity, the description matches, the price is affordable, it is worth recommending.	Jingdong prefecture level city in Shandong
Power Saving King Shop	Green Intelligent Power Saver	Received, the functionality is particularly good, it is genuine, the price is affordable	Jingdong prefecture level city in Shandong
Power Saving King Shop	Green Intelligent Power Saver	Good quality, very easy to use, energy efficient, home electricity bill is obviously much less	Jingdong prefecture level city in Shandong
Power Saving King Shop	Green Intelligent Power Saver	Easy to install, energy saving and power saving, very satisfied with a shopping	Jingdong prefecture level city in Shandong
Power Saving King Shop	Green Intelligent Power Saver	Received it and tried it out, it's great, works great and worth having! Five stars praise!	Jingdong prefecture level city in Shandong
Power Saving King Shop	Green Intelligent Power Saver	Power saving device, no problem at all, the quality of this product at this price, very affordable!	Jingdong prefecture level city in Shandong
Power Saving King Shop	Green Intelligent Power Saver	Received the goods, did not expect to be so fast, the quality is very good, especially power saving	Jingdong prefecture level city in Shandong
Power Saving King Shop	Green Intelligent Power Saver	The logistics is very fast, especially good, need to come back again	Jingdong prefecture level city in Shandong
Power Saving King Shop	Green Intelligent Power Saver	It's already working and it really saves power, so I'm very happy with it!	Jingdong prefecture level city in Shandong
Power Saving King Shop	Green Intelligent Power Saver	Great quality, works well, can recommend to friends to buy.	Jingdong prefecture level city in Shandong
Power Saving King Shop	Green Intelligent Power Saver	It's a good deal and worth the money.	Jingdong prefecture level city in Shandong
Power Saving King Shop	Green Intelligent Power Saver	I received it and I'm very happy with it, it's very good quality, guaranteed, and	Jingdong prefecture level city in Shandong
Power Saving King Shop	Green Intelligent Power Saver	Good quality, safe and secure purchase, ideal results and great price.	Jingdong prefecture level city in Shandong
Power Saving King Shop	Green Intelligent Power Saver	Simple operation plugged in the power supply can be, friends recommended looking forward to the effect of the	Jingdong prefecture level city in Shandong
Power Saving King Shop	Green Intelligent Power Saver	It's not bad, it saves electricity, and the material is sturdy and durable.	Jingdong prefecture level city in Shandong
Power Saving King Shop	Green Intelligent Power Saver	Plug and play, no management required, convenient and hassle-free!	Jingdong prefecture level city in Shandong
Power Saving King Shop	Green Intelligent Power Saver	The pictures are too lazy to go up, the power saving effect is still there!	Jingdong prefecture level city in Shandong
Power Saving King Shop	Green Intelligent Power Saver	Fast shipping. Affordable prices. Efficient	Jingdong prefecture level city in Shandong
Power Saving King Shop	Green Intelligent Power Saver	Good power saving, worth buying	Jingdong prefecture level city in Shandong
Power Saving King Shop	Green Intelligent Power Saver	Especially great, comparison shopping before buying, the store owner is very good, answering questions very patiently and detailed, there is a need to come back!	Jingdong prefecture level city in Shandong
Power Saving King Shop	Green Intelligent Power Saver	Very trustworthy brand, quality is guaranteed, the price is still affordable, quite satisfied!	Jingdong prefecture level city in Shandong
Power Saving King Shop	Green Intelligent Power Saver	Recommended by a friend. They say you can save money on air conditioning with this. Try it.	Jingdong prefecture level city in Shandong
Power Saving King Shop	Green Intelligent Power Saver	Works great, will be back next time, very happy with it and a great price!	Jingdong prefecture level city in Shandong
Power Saving King Shop	Green Intelligent Power Saver	The quality of the product is quite good, fast shipping, satisfied.	Jingdong prefecture level city in Shandong
Power Saving King Shop	Green Intelligent Power Saver	Good quality and fast logistics	Jingdong prefecture level city in Shandong
Power Saving King Shop	Green Intelligent Power Saver	It really saves power. I've tested it myself.	Jingdong prefecture level city in Shandong
Power Saving King Shop	Green Intelligent Power Saver	Really saves electricity!	Jingdong prefecture level city in Shandong
Power Saving King Shop	Green Intelligent Power Saver	pretty good	Jingdong prefecture level city in Shandong
Power Saving King Shop	Green Intelligent Power Saver	serviceable	Jingdong prefecture level city in Shandong
Power Saving King Shop	Green Intelligent Power Saver	Power Saving King received a tightly packaged logistics, installed the effect is very good price is cheaper than the physical store!	Jingdong prefecture level city in Shandong
Power Saving King Shop	Green Intelligent Power Saver	Super Power Saving King is very good quality, new style, is genuine, the use of the effect is very good, the price is cheap, it is worth buying!	Jingdong prefecture level city in Shandong
Power Saving King Shop	Green Intelligent Power Saver	Packaging is very tight, easy to install, quite practical, worth buying!	Jingdong prefecture level city in Shandong
Power Saving King Shop	Green Intelligent Power Saver	Quality assurance, affordable price, very energy efficient, will repurchase if needed!	Jingdong prefecture level city in Shandong
Power Saving King Shop	Green Intelligent Power Saver	Quality assurance, very good, affordable, logistics is also very fast, I'm very satisfied with the	Jingdong prefecture level city in Shandong
Power Saving King Shop	Green Intelligent Power Saver	Energy saving. It's so good, already installed, you can save a lot of electricity every month	Jingdong prefecture level city in Shandong
Power Saving King Shop	Green Intelligent Power Saver	The material is thick and the workmanship is fine in place, the power saving effect is very good, value for money!	Jingdong prefecture level city in Shandong
Power Saving King Shop	Green Intelligent Power Saver	The effect is great, genuine, and match the description, the logistics is fast, the price is reasonable 😍	Jingdong prefecture level city in Shandong
Power Saving King Shop	Green Intelligent Power Saver	It's genuine, works well, easy to use, plugs in and works, really saves power	Jingdong prefecture level city in Shandong
Power Saving King Shop	Green Intelligent Power Saver	It's easy to use, effective, great service, and recommended!	Jingdong prefecture level city in Shandong
Power Saving King Shop	Green Intelligent Power Saver	🌈 Great quality, bought it to my liking 💗 Awesome! ✨ Buy with confidence if you need it!	Jingdong prefecture level city in Shandong
Power Saving King Shop	Green Intelligent Power Saver	Good quality, easy to install, very energy saving and power saving, good to use	Jingdong prefecture level city in Shandong
Power Saving King Shop	Green Intelligent Power Saver	The quality is very good, it does have the effect of saving electricity, the description matches, the price is affordable, it is worth recommending.	Jingdong prefecture level city in Shandong
Power Saving King Shop	Green Intelligent Power Saver	Received, the functionality is particularly good, it is genuine, the price is affordable	Jingdong prefecture level city in Shandong
Power Saving King Shop	Green Intelligent Power Saver	Good quality, very easy to use, energy efficient, home electricity bill is obviously much less	Jingdong prefecture level city in Shandong
Power Saving King Shop	Green Intelligent Power Saver	Easy to install, energy saving and power saving, very satisfied with a shopping	Jingdong prefecture level city in Shandong
Power Saving King Shop	Green Intelligent Power Saver	Received it and tried it out, it's great, works great and worth having! Five stars praise!	Jingdong prefecture level city in Shandong

Power Saving King Shop	Green Intelligent Power Saver	Power saving device, no problem at all, the quality of this product at this price, very affordable!	Jingdong prefecture level city in Shandong
Power Saving King Shop	Green Intelligent Power Saver	Received the goods, did not expect to be so fast, the quality is very good, especially power saving	Jingdong prefecture level city in Shandong
Power Saving King Shop	Green Intelligent Power Saver	The logistics is very fast, especially good, need to come back again	Jingdong prefecture level city in Shandong
Power Saving King Shop	Green Intelligent Power Saver	It's already working and it really saves power, so I'm very happy with it!	Jingdong prefecture level city in Shandong
Power Saving King Shop	Green Intelligent Power Saver	Great quality, works well, can recommend to friends to buy.	Jingdong prefecture level city in Shandong
Power Saving King Shop	Green Intelligent Power Saver	It's a good deal and worth the money.	Jingdong prefecture level city in Shandong
Power Saving King Shop	Green Intelligent Power Saver	I received it and I'm very happy with it, it's very good quality, guaranteed, and	Jingdong prefecture level city in Shandong
Power Saving King Shop	Green Intelligent Power Saver	Good quality, safe and secure purchase, ideal results and great price.	Jingdong prefecture level city in Shandong
Power Saving King Shop	Green Intelligent Power Saver	Simple operation plugged in the power supply can be, friends recommended looking forward to the effect of the	Jingdong prefecture level city in Shandong
Power Saving King Shop	Green Intelligent Power Saver	It's not bad, it saves electricity, and the material is sturdy and durable.	Jingdong prefecture level city in Shandong
Power Saving King Shop	Green Intelligent Power Saver	Plug and play, no management required, convenient and hassle-free!	Jingdong prefecture level city in Shandong
Power Saving King Shop	Green Intelligent Power Saver	The pictures are too lazy to go up, the power saving effect is still there!	Jingdong prefecture level city in Shandong
Power Saving King Shop	Green Intelligent Power Saver	Fast shipping. Affordable prices. Efficient	Jingdong prefecture level city in Shandong
Power Saving King Shop	Green Intelligent Power Saver	Good power saving, worth buying	Jingdong prefecture level city in Shandong
Power Saving King Shop	Green Intelligent Power Saver	Especially great, comparison shopping before buying, the store owner is very good, answering questions very patiently and detailed, there is a need to come back!	Jingdong prefecture level city in Shandong
Power Saving King Shop	Green Intelligent Power Saver	Very trustworthy brand, quality is guaranteed, the price is still affordable, quite satisfied!	Jingdong prefecture level city in Shandong
Power Saving King Shop	Green Intelligent Power Saver	Recommended by a friend. They say you can save money on air conditioning with this. Try it.	Jingdong prefecture level city in Shandong
Power Saving King Shop	Green Intelligent Power Saver	Works great, will be back next time, very happy with it and a great price!	Jingdong prefecture level city in Shandong
Power Saving King Shop	Green Intelligent Power Saver	The quality of the product is quite good, fast shipping, satisfied.	Jingdong prefecture level city in Shandong
Power Saving King Shop	Green Intelligent Power Saver	Good quality and fast logistics	Jingdong prefecture level city in Shandong
Power Saving King Shop	Green Intelligent Power Saver	It really saves power. I've tested it myself.	Jingdong prefecture level city in Shandong
Power Saving King Shop	Green Intelligent Power Saver	Really saves electricity!	Jingdong prefecture level city in Shandong
Power Saving King Shop	Green Intelligent Power Saver	pretty good	Jingdong prefecture level city in Shandong
Power Saving King Shop	Green Intelligent Power Saver	serviceable	Jingdong prefecture level city in Shandong
Power Saving King Shop	Green Intelligent Power Saver	Power Saving King received a tightly packaged logistics, installed the effect is very good price is cheaper than the physical store!	Jingdong prefecture level city in Shandong
Power Saving King Shop	Green Intelligent Power Saver	Super Power Saving King is very good quality, new style, is genuine, the use of the effect is very good, the price is cheap, it is worth buying!	Jingdong prefecture level city in Shandong
Power Saving King Shop	Green Intelligent Power Saver	Packaging is very tight, easy to install, quite practical, worth buying!	Jingdong prefecture level city in Shandong
Power Saving King Shop	Green Intelligent Power Saver	Quality assurance, affordable price, very energy efficient, will repurchase if needed!	Jingdong prefecture level city in Shandong
Power Saving King Shop	Green Intelligent Power Saver	Quality assurance, very good, affordable, logistics is also very fast, I'm very satisfied with the	Jingdong prefecture level city in Shandong
Power Saving King Shop	Green Intelligent Power Saver	Energy saving. It's so good, already installed, you can save a lot of electricity every month	Jingdong prefecture level city in Shandong
Power Saving King Shop	Green Intelligent Power Saver	The material is thick and the workmanship is fine in place, the power saving effect is very good, value for money!	Jingdong prefecture level city in Shandong
Power Saving King Shop	Green Intelligent Power Saver	The effect is great, genuine, and match the description, the logistics is fast, the price is reasonable 😍	Jingdong prefecture level city in Shandong
Power Saving King Shop	Green Intelligent Power Saver	It's genuine, works well, easy to use, plugs in and works, really saves power	Jingdong prefecture level city in Shandong
Power Saving King Shop	Green Intelligent Power Saver	It's easy to use, effective, great service, and recommended!	Jingdong prefecture level city in Shandong
Power Saving King Shop	Green Intelligent Power Saver	🌈 Great quality, bought it to my liking 💝 Awesome! ✨ Buy with confidence if you need it!	Jingdong prefecture level city in Shandong
Power Saving King Shop	Green Intelligent Power Saver	Good quality, easy to install, very energy saving and power saving, good to use	Jingdong prefecture level city in Shandong
Power Saving King Shop	Green Intelligent Power Saver	The quality is very good, it does have the effect of saving electricity, the description matches, the price is affordable, it is worth recommending.	Jingdong prefecture level city in Shandong
Power Saving King Shop	Green Intelligent Power Saver	Received, the functionality is particularly good, it is genuine, the price is affordable	Jingdong prefecture level city in Shandong
Power Saving King Shop	Green Intelligent Power Saver	Good quality, very easy to use, energy efficient, home electricity bill is obviously much less	Jingdong prefecture level city in Shandong
Power Saving King Shop	Green Intelligent Power Saver	Easy to install, energy saving and power saving, very satisfied with a shopping	Jingdong prefecture level city in Shandong
Power Saving King Shop	Green Intelligent Power Saver	Received it and tried it out, it's great, works great and worth having! Five stars praise!	Jingdong prefecture level city in Shandong
Power Saving King Shop	Green Intelligent Power Saver	Power saving device, no problem at all, the quality of this product at this price, very affordable!	Jingdong prefecture level city in Shandong
Power Saving King Shop	Green Intelligent Power Saver	Received the goods, did not expect to be so fast, the quality is very good, especially power saving	Jingdong prefecture level city in Shandong
Power Saving King Shop	Green Intelligent Power Saver	The logistics is very fast, especially good, need to come back again	Jingdong prefecture level city in Shandong
Power Saving King Shop	Green Intelligent Power Saver	It's already working and it really saves power, so I'm very happy with it!	Jingdong prefecture level city in Shandong
Power Saving King Shop	Green Intelligent Power Saver	Great quality, works well, can recommend to friends to buy.	Jingdong prefecture level city in Shandong
Power Saving King Shop	Green Intelligent Power Saver	It's a good deal and worth the money.	Jingdong prefecture level city in Shandong
Power Saving King Shop	Green Intelligent Power Saver	I received it and I'm very happy with it, it's very good quality, guaranteed, and	Jingdong prefecture level city in Shandong
Power Saving King Shop	Green Intelligent Power Saver	Good quality, safe and secure purchase, ideal results and great price.	Jingdong prefecture level city in Shandong
Power Saving King Shop	Green Intelligent Power Saver	Simple operation plugged in the power supply can be, friends recommended looking forward to the effect of the	Jingdong prefecture level city in Shandong
Power Saving King Shop	Green Intelligent Power Saver	It's not bad, it saves electricity, and the material is sturdy and durable.	Jingdong prefecture level city in Shandong

Power Saving King Shop	Green Intelligent Power Saver	Plug and play, no management required, convenient and hassle-free!	Jingdong prefecture level city in Shandong
Power Saving King Shop	Green Intelligent Power Saver	The pictures are too lazy to go up, the power saving effect is still there!	Jingdong prefecture level city in Shandong
Power Saving King Shop	Green Intelligent Power Saver	Fast shipping. Affordable prices. Efficient	Jingdong prefecture level city in Shandong
Power Saving King Shop	Green Intelligent Power Saver	Good power saving, worth buying	Jingdong prefecture level city in Shandong
Power Saving King Shop	Green Intelligent Power Saver	Especially great, comparison shopping before buying, the store owner is very good, answering questions very patiently and detailed, there is a need to come back!	Jingdong prefecture level city in Shandong
Power Saving King Shop	Green Intelligent Power Saver	Very trustworthy brand, quality is guaranteed, the price is still affordable, quite satisfied!	Jingdong prefecture level city in Shandong
Power Saving King Shop	Green Intelligent Power Saver	Recommended by a friend. They say you can save money on air conditioning with this. Try it.	Jingdong prefecture level city in Shandong
Power Saving King Shop	Green Intelligent Power Saver	Works great, will be back next time, very happy with it and a great price!	Jingdong prefecture level city in Shandong
Power Saving King Shop	Green Intelligent Power Saver	The quality of the product is quite good, fast shipping, satisfied.	Jingdong prefecture level city in Shandong
Power Saving King Shop	Green Intelligent Power Saver	Good quality and fast logistics	Jingdong prefecture level city in Shandong
Power Saving King Shop	Green Intelligent Power Saver	It really saves power. I've tested it myself.	Jingdong prefecture level city in Shandong
Power Saving King Shop	Green Intelligent Power Saver	Really saves electricity!	Jingdong prefecture level city in Shandong
Power Saving King Shop	Green Intelligent Power Saver	pretty good	Jingdong prefecture level city in Shandong
Power Saving King Shop	Green Intelligent Power Saver	serviceable	Jingdong prefecture level city in Shandong
Power Saving King Shop	Green Intelligent Power Saver	Power Saving King received a tightly packaged logistics, installed the effect is very good price is cheaper than the physical store!	Jingdong prefecture level city in Shandong
Power Saving King Shop	Green Intelligent Power Saver	Super Power Saving King is very good quality, new style, is genuine, the use of the effect is very good, the price is cheap, it is worth buying!	Jingdong prefecture level city in Shandong
Power Saving King Shop	Green Intelligent Power Saver	Packaging is very tight, easy to install, quite practical, worth buying!	Jingdong prefecture level city in Shandong
Power Saving King Shop	Green Intelligent Power Saver	Quality assurance, affordable price, very energy efficient, will repurchase if needed!	Jingdong prefecture level city in Shandong
Power Saving King Shop	Green Intelligent Power Saver	Quality assurance, very good, affordable, logistics is also very fast, I'm very satisfied with the	Jingdong prefecture level city in Shandong
Power Saving King Shop	Green Intelligent Power Saver	Energy saving. It's so good, already installed, you can save a lot of electricity every month	Jingdong prefecture level city in Shandong
Power Saving King Shop	Green Intelligent Power Saver	The material is thick and the workmanship is fine in place, the power saving effect is very good, value for money!	Jingdong prefecture level city in Shandong
Power Saving King Shop	Green Intelligent Power Saver	The effect is great, genuine, and match the description, the logistics is fast, the price is reasonable 😍	Jingdong prefecture level city in Shandong
Power Saving King Shop	Green Intelligent Power Saver	It's genuine, works well, easy to use, plugs in and works, really saves power	Jingdong prefecture level city in Shandong
Power Saving King Shop	Green Intelligent Power Saver	It's easy to use, effective, great service, and recommended!	Jingdong prefecture level city in Shandong
Power Saving King Shop	Green Intelligent Power Saver	🌈 Great quality, bought it to my liking 💝 Awesome! ✨ Buy with confidence if you need it!	Jingdong prefecture level city in Shandong
Power Saving King Shop	Green Intelligent Power Saver	Good quality, easy to install, very energy saving and power saving, good to use	Jingdong prefecture level city in Shandong
Power Saving King Shop	Green Intelligent Power Saver	The quality is very good, it does have the effect of saving electricity, the description matches, the price is affordable, it is worth recommending.	Jingdong prefecture level city in Shandong
Power Saving King Shop	Green Intelligent Power Saver	Received, the functionality is particularly good, it is genuine, the price is affordable	Jingdong prefecture level city in Shandong
Power Saving King Shop	Green Intelligent Power Saver	Good quality, very easy to use, energy efficient, home electricity bill is obviously much less	Jingdong prefecture level city in Shandong
Power Saving King Shop	Green Intelligent Power Saver	Easy to install, energy saving and power saving, very satisfied with a shopping	Jingdong prefecture level city in Shandong
Power Saving King Shop	Green Intelligent Power Saver	Received it and tried it out, it's great, works great and worth having! Five stars praise!	Jingdong prefecture level city in Shandong
Power Saving King Shop	Green Intelligent Power Saver	Power saving device, no problem at all, the quality of this product at this price, very affordable!	Jingdong prefecture level city in Shandong
Power Saving King Shop	Green Intelligent Power Saver	Received the goods, did not expect to be so fast, the quality is very good, especially power saving	Jingdong prefecture level city in Shandong
Power Saving King Shop	Green Intelligent Power Saver	The logistics is very fast, especially good, need to come back again	Jingdong prefecture level city in Shandong
Power Saving King Shop	Green Intelligent Power Saver	It's already working and it really saves power, so I'm very happy with it!	Jingdong prefecture level city in Shandong
Power Saving King Shop	Green Intelligent Power Saver	Great quality, works well, can recommend to friends to buy.	Jingdong prefecture level city in Shandong
Power Saving King Shop	Green Intelligent Power Saver	It's a good deal and worth the money.	Jingdong prefecture level city in Shandong
Power Saving King Shop	Green Intelligent Power Saver	I received it and I'm very happy with it, it's very good quality, guaranteed, and	Jingdong prefecture level city in Shandong
Power Saving King Shop	Green Intelligent Power Saver	Good quality, safe and secure purchase, ideal results and great price.	Jingdong prefecture level city in Shandong
Power Saving King Shop	Green Intelligent Power Saver	Simple operation plugged in the power supply can be, friends recommended looking forward to the effect of the	Jingdong prefecture level city in Shandong
Power Saving King Shop	Green Intelligent Power Saver	It's not bad, it saves electricity, and the material is sturdy and durable.	Jingdong prefecture level city in Shandong
Power Saving King Shop	Green Intelligent Power Saver	Plug and play, no management required, convenient and hassle-free!	Jingdong prefecture level city in Shandong
Power Saving King Shop	Green Intelligent Power Saver	The pictures are too lazy to go up, the power saving effect is still there!	Jingdong prefecture level city in Shandong
Power Saving King Shop	Green Intelligent Power Saver	Fast shipping. Affordable prices. Efficient	Jingdong prefecture level city in Shandong
Power Saving King Shop	Green Intelligent Power Saver	Good power saving, worth buying	Jingdong prefecture level city in Shandong
Power Saving King Shop	Green Intelligent Power Saver	Especially great, comparison shopping before buying, the store owner is very good, answering questions very patiently and detailed, there is a need to come back!	Jingdong prefecture level city in Shandong
Power Saving King Shop	Green Intelligent Power Saver	Very trustworthy brand, quality is guaranteed, the price is still affordable, quite satisfied!	Jingdong prefecture level city in Shandong
Power Saving King Shop	Green Intelligent Power Saver	Recommended by a friend. They say you can save money on air conditioning with this. Try it.	Jingdong prefecture level city in Shandong
Power Saving King Shop	Green Intelligent Power Saver	Works great, will be back next time, very happy with it and a great price!	Jingdong prefecture level city in Shandong
Power Saving King Shop	Green Intelligent Power Saver	The quality of the product is quite good, fast shipping, satisfied.	Jingdong prefecture level city in Shandong
Power Saving King Shop	Green Intelligent Power Saver	Good quality and fast logistics	Jingdong prefecture level city in Shandong

Power Saving King Shop	Green Intelligent Power Saver	It really saves power. I’ve tested it myself.	Jingdong prefecture level city in Shandong
Power Saving King Shop	Green Intelligent Power Saver	Really saves electricity!	Jingdong prefecture level city in Shandong
Power Saving King Shop	Green Intelligent Power Saver	pretty good	Jingdong prefecture level city in Shandong
Power Saving King Shop	Green Intelligent Power Saver	serviceable	Jingdong prefecture level city in Shandong
Power Saving King Shop	Green Intelligent Power Saver	Power Saving King received a tightly packaged logistics, installed the effect is very good price is cheaper than the physical store!	Jingdong prefecture level city in Shandong
Power Saving King Shop	Green Intelligent Power Saver	Super Power Saving King is very good quality, new style, is genuine, the use of the effect is very good, the price is cheap, it is worth buying!	Jingdong prefecture level city in Shandong
Power Saving King Shop	Green Intelligent Power Saver	Packaging is very tight, easy to install, quite practical, worth buying!	Jingdong prefecture level city in Shandong
Power Saving King Shop	Green Intelligent Power Saver	Quality assurance, affordable price, very energy efficient, will repurchase if needed!	Jingdong prefecture level city in Shandong
Power Saving King Shop	Green Intelligent Power Saver	Quality assurance, very good, affordable, logistics is also very fast, I’m very satisfied with the	Jingdong prefecture level city in Shandong
Power Saving King Shop	Green Intelligent Power Saver	Energy saving. It’s so good, already installed, you can save a lot of electricity every month	Jingdong prefecture level city in Shandong
Power Saving King Shop	Green Intelligent Power Saver	The material is thick and the workmanship is fine in place, the power saving effect is very good, value for money!	Jingdong prefecture level city in Shandong
Power Saving King Shop	Green Intelligent Power Saver	The effect is great, genuine, and match the description, the logistics is fast, the price is reasonable 😍	Jingdong prefecture level city in Shandong
Power Saving King Shop	Green Intelligent Power Saver	It’s genuine, works well, easy to use, plugs in and works, really saves power	Jingdong prefecture level city in Shandong
Power Saving King Shop	Green Intelligent Power Saver	It’s easy to use, effective, great service, and recommended!	Jingdong prefecture level city in Shandong
Power Saving King Shop	Green Intelligent Power Saver	🌈 Great quality, bought it to my liking 💝 Awesome! ✨ Buy with confidence if you need it!	Jingdong prefecture level city in Shandong
Power Saving King Shop	Green Intelligent Power Saver	Good quality, easy to install, very energy saving and power saving, good to use	Jingdong prefecture level city in Shandong
Power Saving King Shop	Green Intelligent Power Saver	The quality is very good, it does have the effect of saving electricity, the description matches, the price is affordable, it is worth recommending.	Jingdong prefecture level city in Shandong
Power Saving King Shop	Green Intelligent Power Saver	Received, the functionality is particularly good, it is genuine, the price is affordable	Jingdong prefecture level city in Shandong
Power Saving King Shop	Green Intelligent Power Saver	Good quality, very easy to use, energy efficient, home electricity bill is obviously much less	Jingdong prefecture level city in Shandong
Power Saving King Shop	Green Intelligent Power Saver	Easy to install, energy saving and power saving, very satisfied with a shopping	Jingdong prefecture level city in Shandong
Power Saving King Shop	Green Intelligent Power Saver	Received it and tried it out, it’s great, works great and worth having! Five stars praise!	Jingdong prefecture level city in Shandong
Power Saving King Shop	Green Intelligent Power Saver	Power saving device, no problem at all, the quality of this product at this price, very affordable!	Jingdong prefecture level city in Shandong
Power Saving King Shop	Green Intelligent Power Saver	Received the goods, did not expect to be so fast, the quality is very good, especially power saving	Jingdong prefecture level city in Shandong
Power Saving King Shop	Green Intelligent Power Saver	The logistics is very fast, especially good, need to come back again	Jingdong prefecture level city in Shandong
Power Saving King Shop	Green Intelligent Power Saver	It’s already working and it really saves power, so I’m very happy with it!	Jingdong prefecture level city in Shandong
Power Saving King Shop	Green Intelligent Power Saver	Great quality, works well, can recommend to friends to buy.	Jingdong prefecture level city in Shandong
Power Saving King Shop	Green Intelligent Power Saver	It’s a good deal and worth the money.	Jingdong prefecture level city in Shandong
Power Saving King Shop	Green Intelligent Power Saver	I received it and I’m very happy with it, it’s very good quality, guaranteed, and	Jingdong prefecture level city in Shandong
Power Saving King Shop	Green Intelligent Power Saver	Good quality, safe and secure purchase, ideal results and great price.	Jingdong prefecture level city in Shandong
Power Saving King Shop	Green Intelligent Power Saver	Simple operation plugged in the power supply can be, friends recommended looking forward to the effect of the	Jingdong prefecture level city in Shandong
Power Saving King Shop	Green Intelligent Power Saver	It’s not bad, it saves electricity, and the material is sturdy and durable.	Jingdong prefecture level city in Shandong
Power Saving King Shop	Green Intelligent Power Saver	Plug and play, no management required, convenient and hassle-free!	Jingdong prefecture level city in Shandong
Power Saving King Shop	Green Intelligent Power Saver	The pictures are too lazy to go up, the power saving effect is still there!	Jingdong prefecture level city in Shandong
Power Saving King Shop	Green Intelligent Power Saver	Fast shipping. Affordable prices. Efficient	Jingdong prefecture level city in Shandong
Power Saving King Shop	Green Intelligent Power Saver	Good power saving, worth buying	Jingdong prefecture level city in Shandong
Power Saving King Shop	Green Intelligent Power Saver	Especially great, comparison shopping before buying, the store owner is very good, answering questions very patiently and detailed, there is a need to come back!	Jingdong prefecture level city in Shandong
Power Saving King Shop	Green Intelligent Power Saver	Very trustworthy brand, quality is guaranteed, the price is still affordable, quite satisfied!	Jingdong prefecture level city in Shandong
Power Saving King Shop	Green Intelligent Power Saver	Recommended by a friend. They say you can save money on air conditioning with this. Try it.	Jingdong prefecture level city in Shandong
Power Saving King Shop	Green Intelligent Power Saver	Works great, will be back next time, very happy with it and a great price!	Jingdong prefecture level city in Shandong
Power Saving King Shop	Green Intelligent Power Saver	The quality of the product is quite good, fast shipping, satisfied.	Jingdong prefecture level city in Shandong
Power Saving King Shop	Green Intelligent Power Saver	Good quality and fast logistics	Jingdong prefecture level city in Shandong
Power Saving King Shop	Green Intelligent Power Saver	It really saves power. I’ve tested it myself.	Jingdong prefecture level city in Shandong
Power Saving King Shop	Green Intelligent Power Saver	Really saves electricity!	Jingdong prefecture level city in Shandong
Power Saving King Shop	Green Intelligent Power Saver	pretty good	Jingdong prefecture level city in Shandong
Power Saving King Shop	Green Intelligent Power Saver	serviceable	Jingdong prefecture level city in Shandong
Power Saving King Shop	Green Intelligent Power Saver	Power Saving King received a tightly packaged logistics, installed the effect is very good price is cheaper than the physical store!	Jingdong prefecture level city in Shandong
Power Saving King Shop	Green Intelligent Power Saver	Super Power Saving King is very good quality, new style, is genuine, the use of the effect is very good, the price is cheap, it is worth buying!	Jingdong prefecture level city in Shandong
Power Saving King Shop	Green Intelligent Power Saver	Packaging is very tight, easy to install, quite practical, worth buying!	Jingdong prefecture level city in Shandong
Power Saving King Shop	Green Intelligent Power Saver	Quality assurance, affordable price, very energy efficient, will repurchase if needed!	Jingdong prefecture level city in Shandong
Power Saving King Shop	Green Intelligent Power Saver	Quality assurance, very good, affordable, logistics is also very fast, I’m very satisfied with the	Jingdong prefecture level city in Shandong
Power Saving King Shop	Green Intelligent Power Saver	Energy saving. It’s so good, already installed, you can save a lot of electricity every month	Jingdong prefecture level city in Shandong

Power Saving King Shop	Green Intelligent Power Saver	The material is thick and the workmanship is fine in place, the power saving effect is very good, value for money!	Jingdong prefecture level city in Shandong
Power Saving King Shop	Green Intelligent Power Saver	The effect is great, genuine, and match the description, the logistics is fast, the price is reasonable 😍	Jingdong prefecture level city in Shandong
Power Saving King Shop	Green Intelligent Power Saver	It's genuine, works well, easy to use, plugs in and works, really saves power	Jingdong prefecture level city in Shandong
Power Saving King Shop	Green Intelligent Power Saver	It's easy to use, effective, great service, and recommended!	Jingdong prefecture level city in Shandong
Power Saving King Shop	Green Intelligent Power Saver	🌈 Great quality, bought it to my liking 💛 Awesome! ✨ Buy with confidence if you need it!	Jingdong prefecture level city in Shandong
Power Saving King Shop	Green Intelligent Power Saver	Good quality, easy to install, very energy saving and power saving, good to use	Jingdong prefecture level city in Shandong
Power Saving King Shop	Green Intelligent Power Saver	The quality is very good, it does have the effect of saving electricity, the description matches, the price is affordable, it is worth recommending.	Jingdong prefecture level city in Shandong
Power Saving King Shop	Green Intelligent Power Saver	Received, the functionality is particularly good, it is genuine, the price is affordable	Jingdong prefecture level city in Shandong
Power Saving King Shop	Green Intelligent Power Saver	Good quality, very easy to use, energy efficient, home electricity bill is obviously much less	Jingdong prefecture level city in Shandong
Power Saving King Shop	Green Intelligent Power Saver	Easy to install, energy saving and power saving, very satisfied with a shopping	Jingdong prefecture level city in Shandong
Power Saving King Shop	Green Intelligent Power Saver	Received it and tried it out, it's great, works great and worth having! Five stars praise!	Jingdong prefecture level city in Shandong
Power Saving King Shop	Green Intelligent Power Saver	Power saving device, no problem at all, the quality of this product at this price, very affordable!	Jingdong prefecture level city in Shandong
Power Saving King Shop	Green Intelligent Power Saver	Received the goods, did not expect to be so fast, the quality is very good, especially power saving	Jingdong prefecture level city in Shandong
Power Saving King Shop	Green Intelligent Power Saver	The logistics is very fast, especially good, need to come back again	Jingdong prefecture level city in Shandong
Power Saving King Shop	Green Intelligent Power Saver	It's already working and it really saves power, so I'm very happy with it!	Jingdong prefecture level city in Shandong
Power Saving King Shop	Green Intelligent Power Saver	Great quality, works well, can recommend to friends to buy.	Jingdong prefecture level city in Shandong
Power Saving King Shop	Green Intelligent Power Saver	It's a good deal and worth the money.	Jingdong prefecture level city in Shandong
Power Saving King Shop	Green Intelligent Power Saver	I received it and I'm very happy with it, it's very good quality, guaranteed, and	Jingdong prefecture level city in Shandong
Power Saving King Shop	Green Intelligent Power Saver	Good quality, safe and secure purchase, ideal results and great price.	Jingdong prefecture level city in Shandong
Power Saving King Shop	Green Intelligent Power Saver	Simple operation plugged in the power supply can be, friends recommended looking forward to the effect of the	Jingdong prefecture level city in Shandong
Power Saving King Shop	Green Intelligent Power Saver	It's not bad, it saves electricity, and the material is sturdy and durable.	Jingdong prefecture level city in Shandong
Power Saving King Shop	Green Intelligent Power Saver	Plug and play, no management required, convenient and hassle-free!	Jingdong prefecture level city in Shandong
Power Saving King Shop	Green Intelligent Power Saver	The pictures are too lazy to go up, the power saving effect is still there!	Jingdong prefecture level city in Shandong
Power Saving King Shop	Green Intelligent Power Saver	Fast shipping. Affordable prices. Efficient	Jingdong prefecture level city in Shandong
Power Saving King Shop	Green Intelligent Power Saver	Good power saving, worth buying	Jingdong prefecture level city in Shandong
Power Saving King Shop	Green Intelligent Power Saver	Especially great, comparison shopping before buying, the store owner is very good, answering questions very patiently and detailed, there is a need to come back!	Jingdong prefecture level city in Shandong
Power Saving King Shop	Green Intelligent Power Saver	Very trustworthy brand, quality is guaranteed, the price is still affordable, quite satisfied!	Jingdong prefecture level city in Shandong
Power Saving King Shop	Green Intelligent Power Saver	Recommended by a friend. They say you can save money on air conditioning with this. Try it.	Jingdong prefecture level city in Shandong
Power Saving King Shop	Green Intelligent Power Saver	Works great, will be back next time, very happy with it and a great price!	Jingdong prefecture level city in Shandong
Power Saving King Shop	Green Intelligent Power Saver	The quality of the product is quite good, fast shipping, satisfied.	Jingdong prefecture level city in Shandong
Power Saving King Shop	Green Intelligent Power Saver	Good quality and fast logistics	Jingdong prefecture level city in Shandong
Power Saving King Shop	Green Intelligent Power Saver	It really saves power. I've tested it myself.	Jingdong prefecture level city in Shandong
Power Saving King Shop	Green Intelligent Power Saver	Really saves electricity!	Jingdong prefecture level city in Shandong
Power Saving King Shop	Green Intelligent Power Saver	pretty good	Jingdong prefecture level city in Shandong
Power Saving King Shop	Green Intelligent Power Saver	serviceable	Jingdong prefecture level city in Shandong
Power Saving King Shop	Green Intelligent Power Saver	Power Saving King received a tightly packaged logistics, installed the effect is very good price is cheaper than the physical store!	Jingdong prefecture level city in Shandong
Power Saving King Shop	Green Intelligent Power Saver	Super Power Saving King is very good quality, new style, is genuine, the use of the effect is very good, the price is cheap, it is worth buying!	Jingdong prefecture level city in Shandong
Power Saving King Shop	Green Intelligent Power Saver	Packaging is very tight, easy to install, quite practical, worth buying!	Jingdong prefecture level city in Shandong
Power Saving King Shop	Green Intelligent Power Saver	Quality assurance, affordable price, very energy efficient, will repurchase if needed!	Jingdong prefecture level city in Shandong
Power Saving King Shop	Green Intelligent Power Saver	Quality assurance, very good, affordable, logistics is also very fast, I'm very satisfied with the	Jingdong prefecture level city in Shandong
Power Saving King Shop	Green Intelligent Power Saver	Energy saving. It's so good, already installed, you can save a lot of electricity every month	Jingdong prefecture level city in Shandong
Power Saving King Shop	Green Intelligent Power Saver	The material is thick and the workmanship is fine in place, the power saving effect is very good, value for money!	Jingdong prefecture level city in Shandong
Power Saving King Shop	Green Intelligent Power Saver	The effect is great, genuine, and match the description, the logistics is fast, the price is reasonable 😍	Jingdong prefecture level city in Shandong
Power Saving King Shop	Green Intelligent Power Saver	It's genuine, works well, easy to use, plugs in and works, really saves power	Jingdong prefecture level city in Shandong
Power Saving King Shop	Green Intelligent Power Saver	It's easy to use, effective, great service, and recommended!	Jingdong prefecture level city in Shandong
Power Saving King Shop	Green Intelligent Power Saver	🌈 Great quality, bought it to my liking 💛 Awesome! ✨ Buy with confidence if you need it!	Jingdong prefecture level city in Shandong
Power Saving King Shop	Green Intelligent Power Saver	Good quality, easy to install, very energy saving and power saving, good to use	Jingdong prefecture level city in Shandong
Power Saving King Shop	Green Intelligent Power Saver	The quality is very good, it does have the effect of saving electricity, the description matches, the price is affordable, it is worth recommending.	Jingdong prefecture level city in Shandong
Power Saving King Shop	Green Intelligent Power Saver	Received, the functionality is particularly good, it is genuine, the price is affordable	Jingdong prefecture level city in Shandong
Power Saving King Shop	Green Intelligent Power Saver	Good quality, very easy to use, energy efficient, home electricity bill is obviously much less	Jingdong prefecture level city in Shandong
Power Saving King Shop	Green Intelligent Power Saver	Easy to install, energy saving and power saving, very satisfied with a shopping	Jingdong prefecture level city in Shandong

Power Saving King Shop	Green Intelligent Power Saver	Received it and tried it out, it's great, works great and worth having! Five stars praise!	Jingdong prefecture level city in Shandong
Power Saving King Shop	Green Intelligent Power Saver	Power saving device, no problem at all, the quality of this product at this price, very affordable!	Jingdong prefecture level city in Shandong
Power Saving King Shop	Green Intelligent Power Saver	Received the goods, did not expect to be so fast, the quality is very good, especially power saving	Jingdong prefecture level city in Shandong
Power Saving King Shop	Green Intelligent Power Saver	The logistics is very fast, especially good, need to come back again	Jingdong prefecture level city in Shandong
Power Saving King Shop	Green Intelligent Power Saver	It's already working and it really saves power, so I'm very happy with it!	Jingdong prefecture level city in Shandong
Power Saving King Shop	Green Intelligent Power Saver	Great quality, works well, can recommend to friends to buy.	Jingdong prefecture level city in Shandong
Power Saving King Shop	Green Intelligent Power Saver	It's a good deal and worth the money.	Jingdong prefecture level city in Shandong
Power Saving King Shop	Green Intelligent Power Saver	I received it and I'm very happy with it, it's very good quality, guaranteed, and	Jingdong prefecture level city in Shandong
Power Saving King Shop	Green Intelligent Power Saver	Good quality, safe and secure purchase, ideal results and great price.	Jingdong prefecture level city in Shandong
Power Saving King Shop	Green Intelligent Power Saver	Simple operation plugged in the power supply can be, friends recommended looking forward to the effect of the	Jingdong prefecture level city in Shandong
Power Saving King Shop	Green Intelligent Power Saver	It's not bad, it saves electricity, and the material is sturdy and durable.	Jingdong prefecture level city in Shandong
Power Saving King Shop	Green Intelligent Power Saver	Plug and play, no management required, convenient and hassle-free!	Jingdong prefecture level city in Shandong
Power Saving King Shop	Green Intelligent Power Saver	The pictures are too lazy to go up, the power saving effect is still there!	Jingdong prefecture level city in Shandong
Power Saving King Shop	Green Intelligent Power Saver	Fast shipping. Affordable prices. Efficient	Jingdong prefecture level city in Shandong
Power Saving King Shop	Green Intelligent Power Saver	Good power saving, worth buying	Jingdong prefecture level city in Shandong
Power Saving King Shop	Green Intelligent Power Saver	Especially great, comparison shopping before buying, the store owner is very good, answering questions very patiently and detailed, there is a need to come back!	Jingdong prefecture level city in Shandong
Power Saving King Shop	Green Intelligent Power Saver	Very trustworthy brand, quality is guaranteed, the price is still affordable, quite satisfied!	Jingdong prefecture level city in Shandong
Power Saving King Shop	Green Intelligent Power Saver	Recommended by a friend. They say you can save money on air conditioning with this. Try it.	Jingdong prefecture level city in Shandong
Power Saving King Shop	Green Intelligent Power Saver	Works great, will be back next time, very happy with it and a great price!	Jingdong prefecture level city in Shandong
Power Saving King Shop	Green Intelligent Power Saver	The quality of the product is quite good, fast shipping, satisfied.	Jingdong prefecture level city in Shandong
Power Saving King Shop	Green Intelligent Power Saver	Good quality and fast logistics	Jingdong prefecture level city in Shandong
Power Saving King Shop	Green Intelligent Power Saver	It really saves power. I've tested it myself.	Jingdong prefecture level city in Shandong
Power Saving King Shop	Green Intelligent Power Saver	Really saves electricity!	Jingdong prefecture level city in Shandong
Power Saving King Shop	Green Intelligent Power Saver	pretty good	Jingdong prefecture level city in Shandong
Power Saving King Shop	Green Intelligent Power Saver	serviceable	Jingdong prefecture level city in Shandong
Power Saving King Shop	Green Intelligent Power Saver	Power Saving King received a tightly packaged logistics, installed the effect is very good price is cheaper than the physical store!	Jingdong prefecture level city in Shandong
Power Saving King Shop	Green Intelligent Power Saver	Super Power Saving King is very good quality, new style, is genuine, the use of the effect is very good, the price is cheap, it is worth buying!	Jingdong prefecture level city in Shandong
Power Saving King Shop	Green Intelligent Power Saver	Packaging is very tight, easy to install, quite practical, worth buying!	Jingdong prefecture level city in Shandong
Power Saving King Shop	Green Intelligent Power Saver	Quality assurance, affordable price, very energy efficient, will repurchase if needed!	Jingdong prefecture level city in Shandong
Power Saving King Shop	Green Intelligent Power Saver	Quality assurance, very good, affordable, logistics is also very fast, I'm very satisfied with the	Jingdong prefecture level city in Shandong
Power Saving King Shop	Green Intelligent Power Saver	Energy saving. It's so good, already installed, you can save a lot of electricity every month	Jingdong prefecture level city in Shandong
Power Saving King Shop	Green Intelligent Power Saver	The material is thick and the workmanship is fine in place, the power saving effect is very good, value for money!	Jingdong prefecture level city in Shandong
Power Saving King Shop	Green Intelligent Power Saver	The effect is great, genuine, and match the description, the logistics is fast, the price is reasonable 😍	Jingdong prefecture level city in Shandong
Power Saving King Shop	Green Intelligent Power Saver	It's genuine, works well, easy to use, plugs in and works, really saves power	Jingdong prefecture level city in Shandong
Power Saving King Shop	Green Intelligent Power Saver	It's easy to use, effective, great service, and recommended!	Jingdong prefecture level city in Shandong
Power Saving King Shop	Green Intelligent Power Saver	🌈 Great quality, bought it to my liking 💞 Awesome! ✨ Buy with confidence if you need it!	Jingdong prefecture level city in Shandong
Power Saving King Shop	Green Intelligent Power Saver	Good quality, easy to install, very energy saving and power saving, good to use	Jingdong prefecture level city in Shandong
Power Saving King Shop	Green Intelligent Power Saver	The quality is very good, it does have the effect of saving electricity, the description matches, the price is affordable, it is worth recommending.	Jingdong prefecture level city in Shandong
Power Saving King Shop	Green Intelligent Power Saver	Received, the functionality is particularly good, it is genuine, the price is affordable	Jingdong prefecture level city in Shandong
Power Saving King Shop	Green Intelligent Power Saver	Good quality, very easy to use, energy efficient, home electricity bill is obviously much less	Jingdong prefecture level city in Shandong
Power Saving King Shop	Green Intelligent Power Saver	Easy to install, energy saving and power saving, very satisfied with a shopping	Jingdong prefecture level city in Shandong
Power Saving King Shop	Green Intelligent Power Saver	Received it and tried it out, it's great, works great and worth having! Five stars praise!	Jingdong prefecture level city in Shandong
Power Saving King Shop	Green Intelligent Power Saver	Power saving device, no problem at all, the quality of this product at this price, very affordable!	Jingdong prefecture level city in Shandong
Power Saving King Shop	Green Intelligent Power Saver	Received the goods, did not expect to be so fast, the quality is very good, especially power saving	Jingdong prefecture level city in Shandong
Power Saving King Shop	Green Intelligent Power Saver	The logistics is very fast, especially good, need to come back again	Jingdong prefecture level city in Shandong
Power Saving King Shop	Green Intelligent Power Saver	It's already working and it really saves power, so I'm very happy with it!	Jingdong prefecture level city in Shandong
Power Saving King Shop	Green Intelligent Power Saver	Great quality, works well, can recommend to friends to buy.	Jingdong prefecture level city in Shandong
Power Saving King Shop	Green Intelligent Power Saver	It's a good deal and worth the money.	Jingdong prefecture level city in Shandong
Power Saving King Shop	Green Intelligent Power Saver	I received it and I'm very happy with it, it's very good quality, guaranteed, and	Jingdong prefecture level city in Shandong
Power Saving King Shop	Green Intelligent Power Saver	Good quality, safe and secure purchase, ideal results and great price.	Jingdong prefecture level city in Shandong
Power Saving King Shop	Green Intelligent Power Saver	Simple operation plugged in the power supply can be, friends recommended looking forward to the effect of the	Jingdong prefecture level city in Shandong

Power Saving King Shop	Green Intelligent Power Saver	It's not bad, it saves electricity, and the material is sturdy and durable.	Jingdong prefecture level city in Shandong
Power Saving King Shop	Green Intelligent Power Saver	Plug and play, no management required, convenient and hassle-free!	Jingdong prefecture level city in Shandong
Power Saving King Shop	Green Intelligent Power Saver	The pictures are too lazy to go up, the power saving effect is still there!	Jingdong prefecture level city in Shandong
Power Saving King Shop	Green Intelligent Power Saver	Fast shipping. Affordable prices. Efficient	Jingdong prefecture level city in Shandong
Power Saving King Shop	Green Intelligent Power Saver	Good power saving, worth buying	Jingdong prefecture level city in Shandong
Power Saving King Shop	Green Intelligent Power Saver	Especially great, comparison shopping before buying, the store owner is very good, answering questions very patiently and detailed, there is a need to come back!	Jingdong prefecture level city in Shandong
Power Saving King Shop	Green Intelligent Power Saver	Very trustworthy brand, quality is guaranteed, the price is still affordable, quite satisfied!	Jingdong prefecture level city in Shandong
Power Saving King Shop	Green Intelligent Power Saver	Recommended by a friend. They say you can save money on air conditioning with this. Try it.	Jingdong prefecture level city in Shandong
Power Saving King Shop	Green Intelligent Power Saver	Works great, will be back next time, very happy with it and a great price!	Jingdong prefecture level city in Shandong
Power Saving King Shop	Green Intelligent Power Saver	The quality of the product is quite good, fast shipping, satisfied.	Jingdong prefecture level city in Shandong
Power Saving King Shop	Green Intelligent Power Saver	Good quality and fast logistics	Jingdong prefecture level city in Shandong
Power Saving King Shop	Green Intelligent Power Saver	It really saves power. I've tested it myself.	Jingdong prefecture level city in Shandong
Power Saving King Shop	Green Intelligent Power Saver	Really saves electricity!	Jingdong prefecture level city in Shandong
Power Saving King Shop	Green Intelligent Power Saver	pretty good	Jingdong prefecture level city in Shandong
Power Saving King Shop	Green Intelligent Power Saver	serviceable	Jingdong prefecture level city in Shandong
Power Saving King Shop	Green Intelligent Power Saver	Power Saving King received a tightly packaged logistics, installed the effect is very good price is cheaper than the physical store!	Jingdong prefecture level city in Shandong
Power Saving King Shop	Green Intelligent Power Saver	Super Power Saving King is very good quality, new style, is genuine, the use of the effect is very good, the price is cheap, it is worth buying!	Jingdong prefecture level city in Shandong
Power Saving King Shop	Green Intelligent Power Saver	Packaging is very tight, easy to install, quite practical, worth buying!	Jingdong prefecture level city in Shandong
Power Saving King Shop	Green Intelligent Power Saver	Quality assurance, affordable price, very energy efficient, will repurchase if needed!	Jingdong prefecture level city in Shandong
Power Saving King Shop	Green Intelligent Power Saver	Quality assurance, very good, affordable, logistics is also very fast, I'm very satisfied with the	Jingdong prefecture level city in Shandong
Power Saving King Shop	Green Intelligent Power Saver	Energy saving. It's so good, already installed, you can save a lot of electricity every month	Jingdong prefecture level city in Shandong
Power Saving King Shop	Green Intelligent Power Saver	The material is thick and the workmanship is fine in place, the power saving effect is very good, value for money!	Jingdong prefecture level city in Shandong
Power Saving King Shop	Green Intelligent Power Saver	The effect is great, genuine, and match the description, the logistics is fast, the price is reasonable 😍	Jingdong prefecture level city in Shandong
Power Saving King Shop	Green Intelligent Power Saver	It's genuine, works well, easy to use, plugs in and works, really saves power	Jingdong prefecture level city in Shandong
Power Saving King Shop	Green Intelligent Power Saver	It's easy to use, effective, great service, and recommended!	Jingdong prefecture level city in Shandong
Power Saving King Shop	Green Intelligent Power Saver	🌈 Great quality, bought it to my liking 💝 Awesome! ✨ Buy with confidence if you need it!	Jingdong prefecture level city in Shandong
Power Saving King Shop	Green Intelligent Power Saver	Good quality, easy to install, very energy saving and power saving, good to use	Jingdong prefecture level city in Shandong
Power Saving King Shop	Green Intelligent Power Saver	The quality is very good, it does have the effect of saving electricity, the description matches, the price is affordable, it is worth recommending.	Jingdong prefecture level city in Shandong
Power Saving King Shop	Green Intelligent Power Saver	Received, the functionality is particularly good, it is genuine, the price is affordable	Jingdong prefecture level city in Shandong
Power Saving King Shop	Green Intelligent Power Saver	Good quality, very easy to use, energy efficient, home electricity bill is obviously much less	Jingdong prefecture level city in Shandong
Power Saving King Shop	Green Intelligent Power Saver	Easy to install, energy saving and power saving, very satisfied with a shopping	Jingdong prefecture level city in Shandong
Power Saving King Shop	Green Intelligent Power Saver	Received it and tried it out, it's great, works great and worth having! Five stars praise!	Jingdong prefecture level city in Shandong
Power Saving King Shop	Green Intelligent Power Saver	Power saving device, no problem at all, the quality of this product at this price, very affordable!	Jingdong prefecture level city in Shandong
Power Saving King Shop	Green Intelligent Power Saver	Received the goods, did not expect to be so fast, the quality is very good, especially power saving	Jingdong prefecture level city in Shandong
Power Saving King Shop	Green Intelligent Power Saver	The logistics is very fast, especially good, need to come back again	Jingdong prefecture level city in Shandong
Power Saving King Shop	Green Intelligent Power Saver	It's already working and it really saves power, so I'm very happy with it!	Jingdong prefecture level city in Shandong
Power Saving King Shop	Green Intelligent Power Saver	Great quality, works well, can recommend to friends to buy.	Jingdong prefecture level city in Shandong
Power Saving King Shop	Green Intelligent Power Saver	It's a good deal and worth the money.	Jingdong prefecture level city in Shandong
Power Saving King Shop	Green Intelligent Power Saver	I received it and I'm very happy with it, it's very good quality, guaranteed, and	Jingdong prefecture level city in Shandong
Power Saving King Shop	Green Intelligent Power Saver	Good quality, safe and secure purchase, ideal results and great price.	Jingdong prefecture level city in Shandong
Power Saving King Shop	Green Intelligent Power Saver	Simple operation plugged in the power supply can be, friends recommended looking forward to the effect of the	Jingdong prefecture level city in Shandong
Power Saving King Shop	Green Intelligent Power Saver	It's not bad, it saves electricity, and the material is sturdy and durable.	Jingdong prefecture level city in Shandong
Power Saving King Shop	Green Intelligent Power Saver	Plug and play, no management required, convenient and hassle-free!	Jingdong prefecture level city in Shandong
Power Saving King Shop	Green Intelligent Power Saver	The pictures are too lazy to go up, the power saving effect is still there!	Jingdong prefecture level city in Shandong
Power Saving King Shop	Green Intelligent Power Saver	Fast shipping. Affordable prices. Efficient	Jingdong prefecture level city in Shandong
Power Saving King Shop	Green Intelligent Power Saver	Good power saving, worth buying	Jingdong prefecture level city in Shandong
Power Saving King Shop	Green Intelligent Power Saver	Especially great, comparison shopping before buying, the store owner is very good, answering questions very patiently and detailed, there is a need to come back!	Jingdong prefecture level city in Shandong
Power Saving King Shop	Green Intelligent Power Saver	Very trustworthy brand, quality is guaranteed, the price is still affordable, quite satisfied!	Jingdong prefecture level city in Shandong
Power Saving King Shop	Green Intelligent Power Saver	Recommended by a friend. They say you can save money on air conditioning with this. Try it.	Jingdong prefecture level city in Shandong
Power Saving King Shop	Green Intelligent Power Saver	Works great, will be back next time, very happy with it and a great price!	Jingdong prefecture level city in Shandong
Power Saving King Shop	Green Intelligent Power Saver	The quality of the product is quite good, fast shipping, satisfied.	Jingdong prefecture level city in Shandong

Power Saving King Shop	Green Intelligent Power Saver	Good quality and fast logistics	Jingdong prefecture level city in Shandong
Power Saving King Shop	Green Intelligent Power Saver	It really saves power. I’ve tested it myself.	Jingdong prefecture level city in Shandong
Power Saving King Shop	Green Intelligent Power Saver	Really saves electricity!	Jingdong prefecture level city in Shandong
Power Saving King Shop	Green Intelligent Power Saver	pretty good	Jingdong prefecture level city in Shandong
Power Saving King Shop	Green Intelligent Power Saver	serviceable	Jingdong prefecture level city in Shandong
Power Saving King Shop	Green Intelligent Power Saver	Power Saving King received a tightly packaged logistics, installed the effect is very good price is cheaper than the physical store!	Jingdong prefecture level city in Shandong
Power Saving King Shop	Green Intelligent Power Saver	Super Power Saving King is very good quality, new style, is genuine, the use of the effect is very good, the price is cheap, it is worth buying!	Jingdong prefecture level city in Shandong
Power Saving King Shop	Green Intelligent Power Saver	Packaging is very tight, easy to install, quite practical, worth buying!	Jingdong prefecture level city in Shandong
Power Saving King Shop	Green Intelligent Power Saver	Quality assurance, affordable price, very energy efficient, will repurchase if needed!	Jingdong prefecture level city in Shandong
Power Saving King Shop	Green Intelligent Power Saver	Quality assurance, very good, affordable, logistics is also very fast, I’m very satisfied with the	Jingdong prefecture level city in Shandong
Power Saving King Shop	Green Intelligent Power Saver	Energy saving. It’s so good, already installed, you can save a lot of electricity every month	Jingdong prefecture level city in Shandong
Power Saving King Shop	Green Intelligent Power Saver	The material is thick and the workmanship is fine in place, the power saving effect is very good, value for money!	Jingdong prefecture level city in Shandong
Power Saving King Shop	Green Intelligent Power Saver	The effect is great, genuine, and match the description, the logistics is fast, the price is reasonable 😍	Jingdong prefecture level city in Shandong
Power Saving King Shop	Green Intelligent Power Saver	It’s genuine, works well, easy to use, plugs in and works, really saves power	Jingdong prefecture level city in Shandong
Power Saving King Shop	Green Intelligent Power Saver	It’s easy to use, effective, great service, and recommended!	Jingdong prefecture level city in Shandong
Power Saving King Shop	Green Intelligent Power Saver	🌈 Great quality, bought it to my liking 💕 Awesome! ✨ Buy with confidence if you need it!	Jingdong prefecture level city in Shandong
Power Saving King Shop	Green Intelligent Power Saver	Good quality, easy to install, very energy saving and power saving, good to use	Jingdong prefecture level city in Shandong
Power Saving King Shop	Green Intelligent Power Saver	The quality is very good, it does have the effect of saving electricity, the description matches, the price is affordable, it is worth recommending.	Jingdong prefecture level city in Shandong
Power Saving King Shop	Green Intelligent Power Saver	Received, the functionality is particularly good, it is genuine, the price is affordable	Jingdong prefecture level city in Shandong
Power Saving King Shop	Green Intelligent Power Saver	Good quality, very easy to use, energy efficient, home electricity bill is obviously much less	Jingdong prefecture level city in Shandong
Power Saving King Shop	Green Intelligent Power Saver	Easy to install, energy saving and power saving, very satisfied with a shopping	Jingdong prefecture level city in Shandong
Power Saving King Shop	Green Intelligent Power Saver	Received it and tried it out, it’s great, works great and worth having! Five stars praise!	Jingdong prefecture level city in Shandong
Power Saving King Shop	Green Intelligent Power Saver	Power saving device, no problem at all, the quality of this product at this price, very affordable!	Jingdong prefecture level city in Shandong
Power Saving King Shop	Green Intelligent Power Saver	Received the goods, did not expect to be so fast, the quality is very good, especially power saving	Jingdong prefecture level city in Shandong
Power Saving King Shop	Green Intelligent Power Saver	The logistics is very fast, especially good, need to come back again	Jingdong prefecture level city in Shandong
Power Saving King Shop	Green Intelligent Power Saver	It’s already working and it really saves power, so I’m very happy with it!	Jingdong prefecture level city in Shandong
Power Saving King Shop	Green Intelligent Power Saver	Great quality, works well, can recommend to friends to buy.	Jingdong prefecture level city in Shandong
Power Saving King Shop	Green Intelligent Power Saver	It’s a good deal and worth the money.	Jingdong prefecture level city in Shandong
Power Saving King Shop	Green Intelligent Power Saver	I received it and I’m very happy with it, it’s very good quality, guaranteed, and	Jingdong prefecture level city in Shandong
Power Saving King Shop	Green Intelligent Power Saver	Good quality, safe and secure purchase, ideal results and great price.	Jingdong prefecture level city in Shandong
Power Saving King Shop	Green Intelligent Power Saver	Simple operation plugged in the power supply can be, friends recommended looking forward to the effect of the	Jingdong prefecture level city in Shandong
Power Saving King Shop	Green Intelligent Power Saver	It’s not bad, it saves electricity, and the material is sturdy and durable.	Jingdong prefecture level city in Shandong
Power Saving King Shop	Green Intelligent Power Saver	Plug and play, no management required, convenient and hassle-free!	Jingdong prefecture level city in Shandong
Power Saving King Shop	Green Intelligent Power Saver	The pictures are too lazy to go up, the power saving effect is still there!	Jingdong prefecture level city in Shandong
Power Saving King Shop	Green Intelligent Power Saver	Fast shipping. Affordable prices. Efficient	Jingdong prefecture level city in Shandong
Power Saving King Shop	Green Intelligent Power Saver	Good power saving, worth buying	Jingdong prefecture level city in Shandong
Power Saving King Shop	Green Intelligent Power Saver	Especially great, comparison shopping before buying, the store owner is very good, answering questions very patiently and detailed, there is a need to come back!	Jingdong prefecture level city in Shandong
Power Saving King Shop	Green Intelligent Power Saver	Very trustworthy brand, quality is guaranteed, the price is still affordable, quite satisfied!	Jingdong prefecture level city in Shandong
Power Saving King Shop	Green Intelligent Power Saver	Recommended by a friend. They say you can save money on air conditioning with this. Try it.	Jingdong prefecture level city in Shandong
Power Saving King Shop	Green Intelligent Power Saver	Works great, will be back next time, very happy with it and a great price!	Jingdong prefecture level city in Shandong
Power Saving King Shop	Green Intelligent Power Saver	The quality of the product is quite good, fast shipping, satisfied.	Jingdong prefecture level city in Shandong
Power Saving King Shop	Green Intelligent Power Saver	Good quality and fast logistics	Jingdong prefecture level city in Shandong
Power Saving King Shop	Green Intelligent Power Saver	It really saves power. I’ve tested it myself.	Jingdong prefecture level city in Shandong
Power Saving King Shop	Green Intelligent Power Saver	Really saves electricity!	Jingdong prefecture level city in Shandong
Power Saving King Shop	Green Intelligent Power Saver	pretty good	Jingdong prefecture level city in Shandong
Power Saving King Shop	Green Intelligent Power Saver	serviceable	Jingdong prefecture level city in Shandong
Power Saving King Shop	Green Intelligent Power Saver	Power Saving King received a tightly packaged logistics, installed the effect is very good price is cheaper than the physical store!	Jingdong prefecture level city in Shandong
Power Saving King Shop	Green Intelligent Power Saver	Super Power Saving King is very good quality, new style, is genuine, the use of the effect is very good, the price is cheap, it is worth buying!	Jingdong prefecture level city in Shandong
Power Saving King Shop	Green Intelligent Power Saver	Packaging is very tight, easy to install, quite practical, worth buying!	Jingdong prefecture level city in Shandong
Power Saving King Shop	Green Intelligent Power Saver	Quality assurance, affordable price, very energy efficient, will repurchase if needed!	Jingdong prefecture level city in Shandong
Power Saving King Shop	Green Intelligent Power Saver	Quality assurance, very good, affordable, logistics is also very fast, I’m very satisfied with the	Jingdong prefecture level city in Shandong

Power Saving King Shop	Green Intelligent Power Saver	Energy saving. It's so good, already installed, you can save a lot of electricity every month	Jingdong prefecture level city in Shandong
Power Saving King Shop	Green Intelligent Power Saver	The material is thick and the workmanship is fine in place, the power saving effect is very good, value for money!	Jingdong prefecture level city in Shandong
Power Saving King Shop	Green Intelligent Power Saver	The effect is great, genuine, and match the description, the logistics is fast, the price is reasonable 😍	Jingdong prefecture level city in Shandong
Power Saving King Shop	Green Intelligent Power Saver	It's genuine, works well, easy to use, plugs in and works, really saves power	Jingdong prefecture level city in Shandong
Power Saving King Shop	Green Intelligent Power Saver	It's easy to use, effective, great service, and recommended!	Jingdong prefecture level city in Shandong
Power Saving King Shop	Green Intelligent Power Saver	🌈 Great quality, bought it to my liking 💝 Awesome! ✨ Buy with confidence if you need it!	Jingdong prefecture level city in Shandong
Power Saving King Shop	Green Intelligent Power Saver	Good quality, easy to install, very energy saving and power saving, good to use	Jingdong prefecture level city in Shandong
Power Saving King Shop	Green Intelligent Power Saver	The quality is very good, it does have the effect of saving electricity, the description matches, the price is affordable, it is worth recommending.	Jingdong prefecture level city in Shandong
Power Saving King Shop	Green Intelligent Power Saver	Received, the functionality is particularly good, it is genuine, the price is affordable	Jingdong prefecture level city in Shandong
Power Saving King Shop	Green Intelligent Power Saver	Good quality, very easy to use, energy efficient, home electricity bill is obviously much less	Jingdong prefecture level city in Shandong
Power Saving King Shop	Green Intelligent Power Saver	Easy to install, energy saving and power saving, very satisfied with a shopping	Jingdong prefecture level city in Shandong
Power Saving King Shop	Green Intelligent Power Saver	Received it and tried it out, it's great, works great and worth having! Five stars praise!	Jingdong prefecture level city in Shandong
Power Saving King Shop	Green Intelligent Power Saver	Power saving device, no problem at all, the quality of this product at this price, very affordable!	Jingdong prefecture level city in Shandong
Power Saving King Shop	Green Intelligent Power Saver	Received the goods, did not expect to be so fast, the quality is very good, especially power saving	Jingdong prefecture level city in Shandong
Power Saving King Shop	Green Intelligent Power Saver	The logistics is very fast, especially good, need to come back again	Jingdong prefecture level city in Shandong
Power Saving King Shop	Green Intelligent Power Saver	It's already working and it really saves power, so I'm very happy with it!	Jingdong prefecture level city in Shandong
Power Saving King Shop	Green Intelligent Power Saver	Great quality, works well, can recommend to friends to buy.	Jingdong prefecture level city in Shandong
Power Saving King Shop	Green Intelligent Power Saver	It's a good deal and worth the money.	Jingdong prefecture level city in Shandong
Power Saving King Shop	Green Intelligent Power Saver	I received it and I'm very happy with it, it's very good quality, guaranteed, and	Jingdong prefecture level city in Shandong
Power Saving King Shop	Green Intelligent Power Saver	Good quality, safe and secure purchase, ideal results and great price.	Jingdong prefecture level city in Shandong
Power Saving King Shop	Green Intelligent Power Saver	Simple operation plugged in the power supply can be, friends recommended looking forward to the effect of the	Jingdong prefecture level city in Shandong
Power Saving King Shop	Green Intelligent Power Saver	It's not bad, it saves electricity, and the material is sturdy and durable.	Jingdong prefecture level city in Shandong
Power Saving King Shop	Green Intelligent Power Saver	Plug and play, no management required, convenient and hassle-free!	Jingdong prefecture level city in Shandong
Power Saving King Shop	Green Intelligent Power Saver	The pictures are too lazy to go up, the power saving effect is still there!	Jingdong prefecture level city in Shandong
Power Saving King Shop	Green Intelligent Power Saver	Fast shipping. Affordable prices. Efficient	Jingdong prefecture level city in Shandong
Power Saving King Shop	Green Intelligent Power Saver	Good power saving, worth buying	Jingdong prefecture level city in Shandong
Power Saving King Shop	Green Intelligent Power Saver	Especially great, comparison shopping before buying, the store owner is very good, answering questions very patiently and detailed, there is a need to come back!	Jingdong prefecture level city in Shandong
Power Saving King Shop	Green Intelligent Power Saver	Very trustworthy brand, quality is guaranteed, the price is still affordable, quite satisfied!	Jingdong prefecture level city in Shandong
Power Saving King Shop	Green Intelligent Power Saver	Recommended by a friend. They say you can save money on air conditioning with this. Try it.	Jingdong prefecture level city in Shandong
Power Saving King Shop	Green Intelligent Power Saver	Works great, will be back next time, very happy with it and a great price!	Jingdong prefecture level city in Shandong
Power Saving King Shop	Green Intelligent Power Saver	The quality of the product is quite good, fast shipping, satisfied.	Jingdong prefecture level city in Shandong
Power Saving King Shop	Green Intelligent Power Saver	Good quality and fast logistics	Jingdong prefecture level city in Shandong
Power Saving King Shop	Green Intelligent Power Saver	It really saves power. I've tested it myself.	Jingdong prefecture level city in Shandong
Power Saving King Shop	Green Intelligent Power Saver	Really saves electricity!	Jingdong prefecture level city in Shandong
Power Saving King Shop	Green Intelligent Power Saver	pretty good	Jingdong prefecture level city in Shandong
Power Saving King Shop	Green Intelligent Power Saver	serviceable	Jingdong prefecture level city in Shandong
Power Saving King Shop	Green Intelligent Power Saver	Power Saving King received a tightly packaged logistics, installed the effect is very good price is cheaper than the physical store!	Jingdong prefecture level city in Shandong
Power Saving King Shop	Green Intelligent Power Saver	Super Power Saving King is very good quality, new style, is genuine, the use of the effect is very good, the price is cheap, it is worth buying!	Jingdong prefecture level city in Shandong
Power Saving King Shop	Green Intelligent Power Saver	Packaging is very tight, easy to install, quite practical, worth buying!	Jingdong prefecture level city in Shandong
Power Saving King Shop	Green Intelligent Power Saver	Quality assurance, affordable price, very energy efficient, will repurchase if needed!	Jingdong prefecture level city in Shandong
Power Saving King Shop	Green Intelligent Power Saver	Quality assurance, very good, affordable, logistics is also very fast, I'm very satisfied with the	Jingdong prefecture level city in Shandong
Power Saving King Shop	Green Intelligent Power Saver	Energy saving. It's so good, already installed, you can save a lot of electricity every month	Jingdong prefecture level city in Shandong
Power Saving King Shop	Green Intelligent Power Saver	The material is thick and the workmanship is fine in place, the power saving effect is very good, value for money!	Jingdong prefecture level city in Shandong
Power Saving King Shop	Green Intelligent Power Saver	The effect is great, genuine, and match the description, the logistics is fast, the price is reasonable 😍	Jingdong prefecture level city in Shandong
Power Saving King Shop	Green Intelligent Power Saver	It's genuine, works well, easy to use, plugs in and works, really saves power	Jingdong prefecture level city in Shandong
Power Saving King Shop	Green Intelligent Power Saver	It's easy to use, effective, great service, and recommended!	Jingdong prefecture level city in Shandong
Power Saving King Shop	Green Intelligent Power Saver	🌈 Great quality, bought it to my liking 💝 Awesome! ✨ Buy with confidence if you need it!	Jingdong prefecture level city in Shandong
Power Saving King Shop	Green Intelligent Power Saver	Good quality, easy to install, very energy saving and power saving, good to use	Jingdong prefecture level city in Shandong
Power Saving King Shop	Green Intelligent Power Saver	The quality is very good, it does have the effect of saving electricity, the description matches, the price is affordable, it is worth recommending.	Jingdong prefecture level city in Shandong
Power Saving King Shop	Green Intelligent Power Saver	Received, the functionality is particularly good, it is genuine, the price is affordable	Jingdong prefecture level city in Shandong
Power Saving King Shop	Green Intelligent Power Saver	Good quality, very easy to use, energy efficient, home electricity bill is obviously much less	Jingdong prefecture level city in Shandong

Power Saving King Shop	Green Intelligent Power Saver	Easy to install, energy saving and power saving, very satisfied with a shopping	Jingdong prefecture level city in Shandong
Power Saving King Shop	Green Intelligent Power Saver	Received it and tried it out, it's great, works great and worth having! Five stars praise!	Jingdong prefecture level city in Shandong
Power Saving King Shop	Green Intelligent Power Saver	Power saving device, no problem at all, the quality of this product at this price, very affordable!	Jingdong prefecture level city in Shandong
Power Saving King Shop	Green Intelligent Power Saver	Received the goods, did not expect to be so fast, the quality is very good, especially power saving	Jingdong prefecture level city in Shandong
Power Saving King Shop	Green Intelligent Power Saver	The logistics is very fast, especially good, need to come back again	Jingdong prefecture level city in Shandong
Power Saving King Shop	Green Intelligent Power Saver	It's already working and it really saves power, so I'm very happy with it!	Jingdong prefecture level city in Shandong
Power Saving King Shop	Green Intelligent Power Saver	Great quality, works well, can recommend to friends to buy.	Jingdong prefecture level city in Shandong
Power Saving King Shop	Green Intelligent Power Saver	It's a good deal and worth the money.	Jingdong prefecture level city in Shandong
Power Saving King Shop	Green Intelligent Power Saver	I received it and I'm very happy with it, it's very good quality, guaranteed, and	Jingdong prefecture level city in Shandong
Power Saving King Shop	Green Intelligent Power Saver	Good quality, safe and secure purchase, ideal results and great price.	Jingdong prefecture level city in Shandong
Power Saving King Shop	Green Intelligent Power Saver	Simple operation plugged in the power supply can be, friends recommended looking forward to the effect of the	Jingdong prefecture level city in Shandong
Power Saving King Shop	Green Intelligent Power Saver	It's not bad, it saves electricity, and the material is sturdy and durable.	Jingdong prefecture level city in Shandong
Power Saving King Shop	Green Intelligent Power Saver	Plug and play, no management required, convenient and hassle-free!	Jingdong prefecture level city in Shandong
Power Saving King Shop	Green Intelligent Power Saver	The pictures are too lazy to go up, the power saving effect is still there!	Jingdong prefecture level city in Shandong
Power Saving King Shop	Green Intelligent Power Saver	Fast shipping. Affordable prices. Efficient	Jingdong prefecture level city in Shandong
Power Saving King Shop	Green Intelligent Power Saver	Good power saving, worth buying	Jingdong prefecture level city in Shandong
Power Saving King Shop	Green Intelligent Power Saver	Especially great, comparison shopping before buying, the store owner is very good, answering questions very patiently and detailed, there is a need to come back!	Jingdong prefecture level city in Shandong
Power Saving King Shop	Green Intelligent Power Saver	Very trustworthy brand, quality is guaranteed, the price is still affordable, quite satisfied!	Jingdong prefecture level city in Shandong
Power Saving King Shop	Green Intelligent Power Saver	Recommended by a friend. They say you can save money on air conditioning with this. Try it.	Jingdong prefecture level city in Shandong
Power Saving King Shop	Green Intelligent Power Saver	Works great, will be back next time, very happy with it and a great price!	Jingdong prefecture level city in Shandong
Power Saving King Shop	Green Intelligent Power Saver	The quality of the product is quite good, fast shipping, satisfied.	Jingdong prefecture level city in Shandong
Power Saving King Shop	Green Intelligent Power Saver	Good quality and fast logistics	Jingdong prefecture level city in Shandong
Power Saving King Shop	Green Intelligent Power Saver	It really saves power. I've tested it myself.	Jingdong prefecture level city in Shandong
Power Saving King Shop	Green Intelligent Power Saver	Really saves electricity!	Jingdong prefecture level city in Shandong
Power Saving King Shop	Green Intelligent Power Saver	pretty good	Jingdong prefecture level city in Shandong
Power Saving King Shop	Green Intelligent Power Saver	serviceable	Jingdong prefecture level city in Shandong
Power Saving King Shop	Green Intelligent Power Saver	Power Saving King received a tightly packaged logistics, installed the effect is very good price is cheaper than the physical store!	Jingdong prefecture level city in Shandong
Power Saving King Shop	Green Intelligent Power Saver	Super Power Saving King is very good quality, new style, is genuine, the use of the effect is very good, the price is cheap, it is worth buying!	Jingdong prefecture level city in Shandong
Power Saving King Shop	Green Intelligent Power Saver	Packaging is very tight, easy to install, quite practical, worth buying!	Jingdong prefecture level city in Shandong
Power Saving King Shop	Green Intelligent Power Saver	Quality assurance, affordable price, very energy efficient, will repurchase if needed!	Jingdong prefecture level city in Shandong
Power Saving King Shop	Green Intelligent Power Saver	Quality assurance, very good, affordable, logistics is also very fast, I'm very satisfied with the	Jingdong prefecture level city in Shandong
Power Saving King Shop	Green Intelligent Power Saver	Energy saving. It's so good, already installed, you can save a lot of electricity every month	Jingdong prefecture level city in Shandong
Power Saving King Shop	Green Intelligent Power Saver	The material is thick and the workmanship is fine in place, the power saving effect is very good, value for money!	Jingdong prefecture level city in Shandong
Power Saving King Shop	Green Intelligent Power Saver	The effect is great, genuine, and match the description, the logistics is fast, the price is reasonable 😍	Jingdong prefecture level city in Shandong
Power Saving King Shop	Green Intelligent Power Saver	It's genuine, works well, easy to use, plugs in and works, really saves power	Jingdong prefecture level city in Shandong
Power Saving King Shop	Green Intelligent Power Saver	It's easy to use, effective, great service, and recommended!	Jingdong prefecture level city in Shandong
Power Saving King Shop	Green Intelligent Power Saver	🌈 Great quality, bought it to my liking 💛 Awesome! ✨ Buy with confidence if you need it!	Jingdong prefecture level city in Shandong
Power Saving King Shop	Green Intelligent Power Saver	Good quality, easy to install, very energy saving and power saving, good to use	Jingdong prefecture level city in Shandong
Power Saving King Shop	Green Intelligent Power Saver	The quality is very good, it does have the effect of saving electricity, the description matches, the price is affordable, it is worth recommending.	Jingdong prefecture level city in Shandong
Power Saving King Shop	Green Intelligent Power Saver	Received, the functionality is particularly good, it is genuine, the price is affordable	Jingdong prefecture level city in Shandong
Power Saving King Shop	Green Intelligent Power Saver	Good quality, very easy to use, energy efficient, home electricity bill is obviously much less	Jingdong prefecture level city in Shandong
Power Saving King Shop	Green Intelligent Power Saver	Easy to install, energy saving and power saving, very satisfied with a shopping	Jingdong prefecture level city in Shandong
Power Saving King Shop	Green Intelligent Power Saver	Received it and tried it out, it's great, works great and worth having! Five stars praise!	Jingdong prefecture level city in Shandong
Power Saving King Shop	Green Intelligent Power Saver	Power saving device, no problem at all, the quality of this product at this price, very affordable!	Jingdong prefecture level city in Shandong
Power Saving King Shop	Green Intelligent Power Saver	Received the goods, did not expect to be so fast, the quality is very good, especially power saving	Jingdong prefecture level city in Shandong
Power Saving King Shop	Green Intelligent Power Saver	The logistics is very fast, especially good, need to come back again	Jingdong prefecture level city in Shandong
Power Saving King Shop	Green Intelligent Power Saver	It's already working and it really saves power, so I'm very happy with it!	Jingdong prefecture level city in Shandong
Power Saving King Shop	Green Intelligent Power Saver	Great quality, works well, can recommend to friends to buy.	Jingdong prefecture level city in Shandong
Power Saving King Shop	Green Intelligent Power Saver	It's a good deal and worth the money.	Jingdong prefecture level city in Shandong
Power Saving King Shop	Green Intelligent Power Saver	I received it and I'm very happy with it, it's very good quality, guaranteed, and	Jingdong prefecture level city in Shandong
Power Saving King Shop	Green Intelligent Power Saver	Good quality, safe and secure purchase, ideal results and great price.	Jingdong prefecture level city in Shandong

Power Saving King Shop	Green Intelligent Power Saver	Simple operation plugged in the power supply can be, friends recommended looking forward to the effect of the	Jingdong prefecture level city in Shandong
Power Saving King Shop	Green Intelligent Power Saver	It's not bad, it saves electricity, and the material is sturdy and durable.	Jingdong prefecture level city in Shandong
Power Saving King Shop	Green Intelligent Power Saver	Plug and play, no management required, convenient and hassle-free!	Jingdong prefecture level city in Shandong
Power Saving King Shop	Green Intelligent Power Saver	The pictures are too lazy to go up, the power saving effect is still there!	Jingdong prefecture level city in Shandong
Power Saving King Shop	Green Intelligent Power Saver	Fast shipping. Affordable prices. Efficient	Jingdong prefecture level city in Shandong
Power Saving King Shop	Green Intelligent Power Saver	Good power saving, worth buying	Jingdong prefecture level city in Shandong
Power Saving King Shop	Green Intelligent Power Saver	Especially great, comparison shopping before buying, the store owner is very good, answering questions very patiently and detailed, there is a need to come back!	Jingdong prefecture level city in Shandong
Power Saving King Shop	Green Intelligent Power Saver	Very trustworthy brand, quality is guaranteed, the price is still affordable, quite satisfied!	Jingdong prefecture level city in Shandong
Power Saving King Shop	Green Intelligent Power Saver	Recommended by a friend. They say you can save money on air conditioning with this. Try it.	Jingdong prefecture level city in Shandong
Power Saving King Shop	Green Intelligent Power Saver	Works great, will be back next time, very happy with it and a great price!	Jingdong prefecture level city in Shandong
Power Saving King Shop	Green Intelligent Power Saver	The quality of the product is quite good, fast shipping, satisfied.	Jingdong prefecture level city in Shandong
Power Saving King Shop	Green Intelligent Power Saver	Good quality and fast logistics	Jingdong prefecture level city in Shandong
Power Saving King Shop	Green Intelligent Power Saver	It really saves power. I've tested it myself.	Jingdong prefecture level city in Shandong
Power Saving King Shop	Green Intelligent Power Saver	Really saves electricity!	Jingdong prefecture level city in Shandong
Power Saving King Shop	Green Intelligent Power Saver	pretty good	Jingdong prefecture level city in Shandong
Power Saving King Shop	Green Intelligent Power Saver	serviceable	Jingdong prefecture level city in Shandong
Power Saving King Shop	Green Intelligent Power Saver	Power Saving King received a tightly packaged logistics, installed the effect is very good price is cheaper than the physical store!	Jingdong prefecture level city in Shandong
Power Saving King Shop	Green Intelligent Power Saver	Super Power Saving King is very good quality, new style, is genuine, the use of the effect is very good, the price is cheap, it is worth buying!	Jingdong prefecture level city in Shandong
Power Saving King Shop	Green Intelligent Power Saver	Packaging is very tight, easy to install, quite practical, worth buying!	Jingdong prefecture level city in Shandong
Power Saving King Shop	Green Intelligent Power Saver	Quality assurance, affordable price, very energy efficient, will repurchase if needed!	Jingdong prefecture level city in Shandong
Power Saving King Shop	Green Intelligent Power Saver	Quality assurance, very good, affordable, logistics is also very fast, I'm very satisfied with the	Jingdong prefecture level city in Shandong
Power Saving King Shop	Green Intelligent Power Saver	Energy saving. It's so good, already installed, you can save a lot of electricity every month	Jingdong prefecture level city in Shandong
Power Saving King Shop	Green Intelligent Power Saver	The material is thick and the workmanship is fine in place, the power saving effect is very good, value for money!	Jingdong prefecture level city in Shandong
Power Saving King Shop	Green Intelligent Power Saver	The effect is great, genuine, and match the description, the logistics is fast, the price is reasonable 😍	Jingdong prefecture level city in Shandong
Power Saving King Shop	Green Intelligent Power Saver	It's genuine, works well, easy to use, plugs in and works, really saves power	Jingdong prefecture level city in Shandong
Power Saving King Shop	Green Intelligent Power Saver	It's easy to use, effective, great service, and recommended!	Jingdong prefecture level city in Shandong
Power Saving King Shop	Green Intelligent Power Saver	🌈 Great quality, bought it to my liking 💝 Awesome! ✨ Buy with confidence if you need it!	Jingdong prefecture level city in Shandong
Power Saving King Shop	Green Intelligent Power Saver	Good quality, easy to install, very energy saving and power saving, good to use	Jingdong prefecture level city in Shandong
Power Saving King Shop	Green Intelligent Power Saver	The quality is very good, it does have the effect of saving electricity, the description matches, the price is affordable, it is worth recommending.	Jingdong prefecture level city in Shandong
Power Saving King Shop	Green Intelligent Power Saver	Received, the functionality is particularly good, it is genuine, the price is affordable	Jingdong prefecture level city in Shandong
Power Saving King Shop	Green Intelligent Power Saver	Good quality, very easy to use, energy efficient, home electricity bill is obviously much less	Jingdong prefecture level city in Shandong
Power Saving King Shop	Green Intelligent Power Saver	Easy to install, energy saving and power saving, very satisfied with a shopping	Jingdong prefecture level city in Shandong
Power Saving King Shop	Green Intelligent Power Saver	Received it and tried it out, it's great, works great and worth having! Five stars praise!	Jingdong prefecture level city in Shandong
Power Saving King Shop	Green Intelligent Power Saver	Power saving device, no problem at all, the quality of this product at this price, very affordable!	Jingdong prefecture level city in Shandong
Power Saving King Shop	Green Intelligent Power Saver	Received the goods, did not expect to be so fast, the quality is very good, especially power saving	Jingdong prefecture level city in Shandong
Power Saving King Shop	Green Intelligent Power Saver	The logistics is very fast, especially good, need to come back again	Jingdong prefecture level city in Shandong
Power Saving King Shop	Green Intelligent Power Saver	It's already working and it really saves power, so I'm very happy with it!	Jingdong prefecture level city in Shandong
Power Saving King Shop	Green Intelligent Power Saver	Great quality, works well, can recommend to friends to buy.	Jingdong prefecture level city in Shandong
Power Saving King Shop	Green Intelligent Power Saver	It's a good deal and worth the money.	Jingdong prefecture level city in Shandong
Power Saving King Shop	Green Intelligent Power Saver	I received it and I'm very happy with it, it's very good quality, guaranteed, and	Jingdong prefecture level city in Shandong
Power Saving King Shop	Green Intelligent Power Saver	Good quality, safe and secure purchase, ideal results and great price.	Jingdong prefecture level city in Shandong
Power Saving King Shop	Green Intelligent Power Saver	Simple operation plugged in the power supply can be, friends recommended looking forward to the effect of the	Jingdong prefecture level city in Shandong
Power Saving King Shop	Green Intelligent Power Saver	It's not bad, it saves electricity, and the material is sturdy and durable.	Jingdong prefecture level city in Shandong
Power Saving King Shop	Green Intelligent Power Saver	Plug and play, no management required, convenient and hassle-free!	Jingdong prefecture level city in Shandong
Power Saving King Shop	Green Intelligent Power Saver	The pictures are too lazy to go up, the power saving effect is still there!	Jingdong prefecture level city in Shandong
Power Saving King Shop	Green Intelligent Power Saver	Fast shipping. Affordable prices. Efficient	Jingdong prefecture level city in Shandong
Power Saving King Shop	Green Intelligent Power Saver	Good power saving, worth buying	Jingdong prefecture level city in Shandong
Power Saving King Shop	Green Intelligent Power Saver	Especially great, comparison shopping before buying, the store owner is very good, answering questions very patiently and detailed, there is a need to come back!	Jingdong prefecture level city in Shandong
Power Saving King Shop	Green Intelligent Power Saver	Very trustworthy brand, quality is guaranteed, the price is still affordable, quite satisfied!	Jingdong prefecture level city in Shandong
Power Saving King Shop	Green Intelligent Power Saver	Recommended by a friend. They say you can save money on air conditioning with this. Try it.	Jingdong prefecture level city in Shandong
Power Saving King Shop	Green Intelligent Power Saver	Works great, will be back next time, very happy with it and a great price!	Jingdong prefecture level city in Shandong

Power Saving King Shop	Green Intelligent Power Saver	The quality of the product is quite good, fast shipping, satisfied.	Jingdong prefecture level city in Shandong
Power Saving King Shop	Green Intelligent Power Saver	Good quality and fast logistics	Jingdong prefecture level city in Shandong
Power Saving King Shop	Green Intelligent Power Saver	It really saves power. I've tested it myself.	Jingdong prefecture level city in Shandong
Power Saving King Shop	Green Intelligent Power Saver	Really saves electricity!	Jingdong prefecture level city in Shandong
Power Saving King Shop	Green Intelligent Power Saver	pretty good	Jingdong prefecture level city in Shandong
Power Saving King Shop	Green Intelligent Power Saver	serviceable	Jingdong prefecture level city in Shandong
Power Saving King Shop	Green Intelligent Power Saver	Power Saving King received a tightly packaged logistics, installed the effect is very good price is cheaper than the physical store!	Jingdong prefecture level city in Shandong
Power Saving King Shop	Green Intelligent Power Saver	Super Power Saving King is very good quality, new style, is genuine, the use of the effect is very good, the price is cheap, it is worth buying!	Jingdong prefecture level city in Shandong
Power Saving King Shop	Green Intelligent Power Saver	Packaging is very tight, easy to install, quite practical, worth buying!	Jingdong prefecture level city in Shandong
Power Saving King Shop	Green Intelligent Power Saver	Quality assurance, affordable price, very energy efficient, will repurchase if needed!	Jingdong prefecture level city in Shandong
Power Saving King Shop	Green Intelligent Power Saver	Quality assurance, very good, affordable, logistics is also very fast, I'm very satisfied with the	Jingdong prefecture level city in Shandong
Power Saving King Shop	Green Intelligent Power Saver	Energy saving. It's so good, already installed, you can save a lot of electricity every month	Jingdong prefecture level city in Shandong
Power Saving King Shop	Green Intelligent Power Saver	The material is thick and the workmanship is fine in place, the power saving effect is very good, value for money!	Jingdong prefecture level city in Shandong
Power Saving King Shop	Green Intelligent Power Saver	The effect is great, genuine, and match the description, the logistics is fast, the price is reasonable 😍	Jingdong prefecture level city in Shandong
Power Saving King Shop	Green Intelligent Power Saver	It's genuine, works well, easy to use, plugs in and works, really saves power	Jingdong prefecture level city in Shandong
Power Saving King Shop	Green Intelligent Power Saver	It's easy to use, effective, great service, and recommended!	Jingdong prefecture level city in Shandong
Power Saving King Shop	Green Intelligent Power Saver	🌈 Great quality, bought it to my liking 💝 Awesome! ✨ Buy with confidence if you need it!	Jingdong prefecture level city in Shandong
Power Saving King Shop	Green Intelligent Power Saver	Good quality, easy to install, very energy saving and power saving, good to use	Jingdong prefecture level city in Shandong
Power Saving King Shop	Green Intelligent Power Saver	The quality is very good, it does have the effect of saving electricity, the description matches, the price is affordable, it is worth recommending.	Jingdong prefecture level city in Shandong
Power Saving King Shop	Green Intelligent Power Saver	Received, the functionality is particularly good, it is genuine, the price is affordable	Jingdong prefecture level city in Shandong
Power Saving King Shop	Green Intelligent Power Saver	Good quality, very easy to use, energy efficient, home electricity bill is obviously much less	Jingdong prefecture level city in Shandong
Power Saving King Shop	Green Intelligent Power Saver	Easy to install, energy saving and power saving, very satisfied with a shopping	Jingdong prefecture level city in Shandong
Power Saving King Shop	Green Intelligent Power Saver	Received it and tried it out, it's great, works great and worth having! Five stars praise!	Jingdong prefecture level city in Shandong
Power Saving King Shop	Green Intelligent Power Saver	Power saving device, no problem at all, the quality of this product at this price, very affordable!	Jingdong prefecture level city in Shandong
Power Saving King Shop	Green Intelligent Power Saver	Received the goods, did not expect to be so fast, the quality is very good, especially power saving	Jingdong prefecture level city in Shandong
Power Saving King Shop	Green Intelligent Power Saver	The logistics is very fast, especially good, need to come back again	Jingdong prefecture level city in Shandong
Power Saving King Shop	Green Intelligent Power Saver	It's already working and it really saves power, so I'm very happy with it!	Jingdong prefecture level city in Shandong
Power Saving King Shop	Green Intelligent Power Saver	Great quality, works well, can recommend to friends to buy.	Jingdong prefecture level city in Shandong
Power Saving King Shop	Green Intelligent Power Saver	It's a good deal and worth the money.	Jingdong prefecture level city in Shandong
Power Saving King Shop	Green Intelligent Power Saver	I received it and I'm very happy with it, it's very good quality, guaranteed, and	Jingdong prefecture level city in Shandong
Power Saving King Shop	Green Intelligent Power Saver	Good quality, safe and secure purchase, ideal results and great price.	Jingdong prefecture level city in Shandong
Power Saving King Shop	Green Intelligent Power Saver	Simple operation plugged in the power supply can be, friends recommended looking forward to the effect of the	Jingdong prefecture level city in Shandong
Power Saving King Shop	Green Intelligent Power Saver	It's not bad, it saves electricity, and the material is sturdy and durable.	Jingdong prefecture level city in Shandong
Power Saving King Shop	Green Intelligent Power Saver	Plug and play, no management required, convenient and hassle-free!	Jingdong prefecture level city in Shandong
Power Saving King Shop	Green Intelligent Power Saver	The pictures are too lazy to go up, the power saving effect is still there!	Jingdong prefecture level city in Shandong
Power Saving King Shop	Green Intelligent Power Saver	Fast shipping. Affordable prices. Efficient	Jingdong prefecture level city in Shandong
Power Saving King Shop	Green Intelligent Power Saver	Good power saving, worth buying	Jingdong prefecture level city in Shandong
Power Saving King Shop	Green Intelligent Power Saver	Especially great, comparison shopping before buying, the store owner is very good, answering questions very patiently and detailed, there is a need to come back!	Jingdong prefecture level city in Shandong
Power Saving King Shop	Green Intelligent Power Saver	Very trustworthy brand, quality is guaranteed, the price is still affordable, quite satisfied!	Jingdong prefecture level city in Shandong
Power Saving King Shop	Green Intelligent Power Saver	Recommended by a friend. They say you can save money on air conditioning with this. Try it.	Jingdong prefecture level city in Shandong
Power Saving King Shop	Green Intelligent Power Saver	Works great, will be back next time, very happy with it and a great price!	Jingdong prefecture level city in Shandong
Power Saving King Shop	Green Intelligent Power Saver	The quality of the product is quite good, fast shipping, satisfied.	Jingdong prefecture level city in Shandong
Power Saving King Shop	Green Intelligent Power Saver	Good quality and fast logistics	Jingdong prefecture level city in Shandong
Power Saving King Shop	Green Intelligent Power Saver	It really saves power. I've tested it myself.	Jingdong prefecture level city in Shandong
Power Saving King Shop	Green Intelligent Power Saver	Really saves electricity!	Jingdong prefecture level city in Shandong
Power Saving King Shop	Green Intelligent Power Saver	pretty good	Jingdong prefecture level city in Shandong
Power Saving King Shop	Green Intelligent Power Saver	serviceable	Jingdong prefecture level city in Shandong
Power Saving King Shop	Green Intelligent Power Saver	Power Saving King received a tightly packaged logistics, installed the effect is very good price is cheaper than the physical store!	Jingdong prefecture level city in Shandong
Power Saving King Shop	Green Intelligent Power Saver	Super Power Saving King is very good quality, new style, is genuine, the use of the effect is very good, the price is cheap, it is worth buying!	Jingdong prefecture level city in Shandong
Power Saving King Shop	Green Intelligent Power Saver	Packaging is very tight, easy to install, quite practical, worth buying!	Jingdong prefecture level city in Shandong
Power Saving King Shop	Green Intelligent Power Saver	Quality assurance, affordable price, very energy efficient, will repurchase if needed!	Jingdong prefecture level city in Shandong

Power Saving King Shop	Green Intelligent Power Saver	Quality assurance, very good, affordable, logistics is also very fast, I'm very satisfied with the	Jingdong prefecture level city in Shandong
Power Saving King Shop	Green Intelligent Power Saver	Energy saving. It's so good, already installed, you can save a lot of electricity every month	Jingdong prefecture level city in Shandong
Power Saving King Shop	Green Intelligent Power Saver	The material is thick and the workmanship is fine in place, the power saving effect is very good, value for money!	Jingdong prefecture level city in Shandong
Power Saving King Shop	Green Intelligent Power Saver	The effect is great, genuine, and match the description, the logistics is fast, the price is reasonable 😍	Jingdong prefecture level city in Shandong
Power Saving King Shop	Green Intelligent Power Saver	It's genuine, works well, easy to use, plugs in and works, really saves power	Jingdong prefecture level city in Shandong
Power Saving King Shop	Green Intelligent Power Saver	It's easy to use, effective, great service, and recommended!	Jingdong prefecture level city in Shandong
Power Saving King Shop	Green Intelligent Power Saver	🌈 Great quality, bought it to my liking 💜 Awesome! ✨ Buy with confidence if you need it!	Jingdong prefecture level city in Shandong
Power Saving King Shop	Green Intelligent Power Saver	Good quality, easy to install, very energy saving and power saving, good to use	Jingdong prefecture level city in Shandong
Power Saving King Shop	Green Intelligent Power Saver	The quality is very good, it does have the effect of saving electricity, the description matches, the price is affordable, it is worth recommending.	Jingdong prefecture level city in Shandong
Power Saving King Shop	Green Intelligent Power Saver	Received, the functionality is particularly good, it is genuine, the price is affordable	Jingdong prefecture level city in Shandong
Power Saving King Shop	Green Intelligent Power Saver	Good quality, very easy to use, energy efficient, home electricity bill is obviously much less	Jingdong prefecture level city in Shandong
Power Saving King Shop	Green Intelligent Power Saver	Easy to install, energy saving and power saving, very satisfied with a shopping	Jingdong prefecture level city in Shandong
Power Saving King Shop	Green Intelligent Power Saver	Received it and tried it out, it's great, works great and worth having! Five stars praise!	Jingdong prefecture level city in Shandong
Power Saving King Shop	Green Intelligent Power Saver	Power saving device, no problem at all, the quality of this product at this price, very affordable!	Jingdong prefecture level city in Shandong
Power Saving King Shop	Green Intelligent Power Saver	Received the goods, did not expect to be so fast, the quality is very good, especially power saving	Jingdong prefecture level city in Shandong
Power Saving King Shop	Green Intelligent Power Saver	The logistics is very fast, especially good, need to come back again	Jingdong prefecture level city in Shandong
Power Saving King Shop	Green Intelligent Power Saver	It's already working and it really saves power, so I'm very happy with it!	Jingdong prefecture level city in Shandong
Power Saving King Shop	Green Intelligent Power Saver	Great quality, works well, can recommend to friends to buy.	Jingdong prefecture level city in Shandong
Power Saving King Shop	Green Intelligent Power Saver	It's a good deal and worth the money.	Jingdong prefecture level city in Shandong
Power Saving King Shop	Green Intelligent Power Saver	I received it and I'm very happy with it, it's very good quality, guaranteed, and	Jingdong prefecture level city in Shandong
Power Saving King Shop	Green Intelligent Power Saver	Good quality, safe and secure purchase, ideal results and great price.	Jingdong prefecture level city in Shandong
Power Saving King Shop	Green Intelligent Power Saver	Simple operation plugged in the power supply can be, friends recommended looking forward to the effect of the	Jingdong prefecture level city in Shandong
Power Saving King Shop	Green Intelligent Power Saver	It's not bad, it saves electricity, and the material is sturdy and durable.	Jingdong prefecture level city in Shandong
Power Saving King Shop	Green Intelligent Power Saver	Plug and play, no management required, convenient and hassle-free!	Jingdong prefecture level city in Shandong
Power Saving King Shop	Green Intelligent Power Saver	The pictures are too lazy to go up, the power saving effect is still there!	Jingdong prefecture level city in Shandong
Power Saving King Shop	Green Intelligent Power Saver	Fast shipping. Affordable prices. Efficient	Jingdong prefecture level city in Shandong
Power Saving King Shop	Green Intelligent Power Saver	Good power saving, worth buying	Jingdong prefecture level city in Shandong
Power Saving King Shop	Green Intelligent Power Saver	Especially great, comparison shopping before buying, the store owner is very good, answering questions very patiently and detailed, there is a need to come back!	Jingdong prefecture level city in Shandong
Power Saving King Shop	Green Intelligent Power Saver	Very trustworthy brand, quality is guaranteed, the price is still affordable, quite satisfied!	Jingdong prefecture level city in Shandong
Power Saving King Shop	Green Intelligent Power Saver	Recommended by a friend. They say you can save money on air conditioning with this. Try it.	Jingdong prefecture level city in Shandong
Power Saving King Shop	Green Intelligent Power Saver	Works great, will be back next time, very happy with it and a great price!	Jingdong prefecture level city in Shandong
Power Saving King Shop	Green Intelligent Power Saver	The quality of the product is quite good, fast shipping, satisfied.	Jingdong prefecture level city in Shandong
Power Saving King Shop	Green Intelligent Power Saver	Good quality and fast logistics	Jingdong prefecture level city in Shandong
Power Saving King Shop	Green Intelligent Power Saver	It really saves power. I've tested it myself.	Jingdong prefecture level city in Shandong
Power Saving King Shop	Green Intelligent Power Saver	Really saves electricity!	Jingdong prefecture level city in Shandong
Power Saving King Shop	Green Intelligent Power Saver	pretty good	Jingdong prefecture level city in Shandong
Power Saving King Shop	Green Intelligent Power Saver	serviceable	Jingdong prefecture level city in Shandong
Power Saving King Shop	Green Intelligent Power Saver	Power Saving King received a tightly packaged logistics, installed the effect is very good price is cheaper than the physical store!	Jingdong prefecture level city in Shandong
Power Saving King Shop	Green Intelligent Power Saver	Super Power Saving King is very good quality, new style, is genuine, the use of the effect is very good, the price is cheap, it is worth buying!	Jingdong prefecture level city in Shandong
Power Saving King Shop	Green Intelligent Power Saver	Packaging is very tight, easy to install, quite practical, worth buying!	Jingdong prefecture level city in Shandong
Power Saving King Shop	Green Intelligent Power Saver	Quality assurance, affordable price, very energy efficient, will repurchase if needed!	Jingdong prefecture level city in Shandong
Power Saving King Shop	Green Intelligent Power Saver	Quality assurance, very good, affordable, logistics is also very fast, I'm very satisfied with the	Jingdong prefecture level city in Shandong
Power Saving King Shop	Green Intelligent Power Saver	Energy saving. It's so good, already installed, you can save a lot of electricity every month	Jingdong prefecture level city in Shandong
Power Saving King Shop	Green Intelligent Power Saver	The material is thick and the workmanship is fine in place, the power saving effect is very good, value for money!	Jingdong prefecture level city in Shandong
Power Saving King Shop	Green Intelligent Power Saver	The effect is great, genuine, and match the description, the logistics is fast, the price is reasonable 😍	Jingdong prefecture level city in Shandong
Power Saving King Shop	Green Intelligent Power Saver	It's genuine, works well, easy to use, plugs in and works, really saves power	Jingdong prefecture level city in Shandong
Power Saving King Shop	Green Intelligent Power Saver	It's easy to use, effective, great service, and recommended!	Jingdong prefecture level city in Shandong
Power Saving King Shop	Green Intelligent Power Saver	🌈 Great quality, bought it to my liking 💜 Awesome! ✨ Buy with confidence if you need it!	Jingdong prefecture level city in Shandong
Power Saving King Shop	Green Intelligent Power Saver	Good quality, easy to install, very energy saving and power saving, good to use	Jingdong prefecture level city in Shandong
Power Saving King Shop	Green Intelligent Power Saver	The quality is very good, it does have the effect of saving electricity, the description matches, the price is affordable, it is worth recommending.	Jingdong prefecture level city in Shandong
Power Saving King Shop	Green Intelligent Power Saver	Received, the functionality is particularly good, it is genuine, the price is affordable	Jingdong prefecture level city in Shandong

Power Saving King Shop	Green Intelligent Power Saver	Good quality, very easy to use, energy efficient, home electricity bill is obviously much less	Jingdong prefecture level city in Shandong
Power Saving King Shop	Green Intelligent Power Saver	Easy to install, energy saving and power saving, very satisfied with a shopping	Jingdong prefecture level city in Shandong
Power Saving King Shop	Green Intelligent Power Saver	Received it and tried it out, it's great, works great and worth having! Five stars praise!	Jingdong prefecture level city in Shandong
Power Saving King Shop	Green Intelligent Power Saver	Power saving device, no problem at all, the quality of this product at this price, very affordable!	Jingdong prefecture level city in Shandong
Power Saving King Shop	Green Intelligent Power Saver	Received the goods, did not expect to be so fast, the quality is very good, especially power saving	Jingdong prefecture level city in Shandong
Power Saving King Shop	Green Intelligent Power Saver	The logistics is very fast, especially good, need to come back again	Jingdong prefecture level city in Shandong
Power Saving King Shop	Green Intelligent Power Saver	It's already working and it really saves power, so I'm very happy with it!	Jingdong prefecture level city in Shandong
Power Saving King Shop	Green Intelligent Power Saver	Great quality, works well, can recommend to friends to buy.	Jingdong prefecture level city in Shandong
Power Saving King Shop	Green Intelligent Power Saver	It's a good deal and worth the money.	Jingdong prefecture level city in Shandong
Power Saving King Shop	Green Intelligent Power Saver	I received it and I'm very happy with it, it's very good quality, guaranteed, and	Jingdong prefecture level city in Shandong
Power Saving King Shop	Green Intelligent Power Saver	Good quality, safe and secure purchase, ideal results and great price.	Jingdong prefecture level city in Shandong
Power Saving King Shop	Green Intelligent Power Saver	Simple operation plugged in the power supply can be, friends recommended looking forward to the effect of the	Jingdong prefecture level city in Shandong
Power Saving King Shop	Green Intelligent Power Saver	It's not bad, it saves electricity, and the material is sturdy and durable.	Jingdong prefecture level city in Shandong
Power Saving King Shop	Green Intelligent Power Saver	Plug and play, no management required, convenient and hassle-free!	Jingdong prefecture level city in Shandong
Power Saving King Shop	Green Intelligent Power Saver	The pictures are too lazy to go up, the power saving effect is still there!	Jingdong prefecture level city in Shandong
Power Saving King Shop	Green Intelligent Power Saver	Fast shipping. Affordable prices. Efficient	Jingdong prefecture level city in Shandong
Power Saving King Shop	Green Intelligent Power Saver	Good power saving, worth buying	Jingdong prefecture level city in Shandong
Power Saving King Shop	Green Intelligent Power Saver	Especially great, comparison shopping before buying, the store owner is very good, answering questions very patiently and detailed, there is a need to come back!	Jingdong prefecture level city in Shandong
Power Saving King Shop	Green Intelligent Power Saver	Very trustworthy brand, quality is guaranteed, the price is still affordable, quite satisfied!	Jingdong prefecture level city in Shandong
Power Saving King Shop	Green Intelligent Power Saver	Recommended by a friend. They say you can save money on air conditioning with this. Try it.	Jingdong prefecture level city in Shandong
Power Saving King Shop	Green Intelligent Power Saver	Works great, will be back next time, very happy with it and a great price!	Jingdong prefecture level city in Shandong
Power Saving King Shop	Green Intelligent Power Saver	The quality of the product is quite good, fast shipping, satisfied.	Jingdong prefecture level city in Shandong
Power Saving King Shop	Green Intelligent Power Saver	Good quality and fast logistics	Jingdong prefecture level city in Shandong
Power Saving King Shop	Green Intelligent Power Saver	It really saves power. I've tested it myself.	Jingdong prefecture level city in Shandong
Power Saving King Shop	Green Intelligent Power Saver	Really saves electricity!	Jingdong prefecture level city in Shandong
Power Saving King Shop	Green Intelligent Power Saver	pretty good	Jingdong prefecture level city in Shandong
Power Saving King Shop	Green Intelligent Power Saver	serviceable	Jingdong prefecture level city in Shandong
Power Saving King Shop	Green Intelligent Power Saver	Power Saving King received a tightly packaged logistics, installed the effect is very good price is cheaper than the physical store!	Jingdong prefecture level city in Shandong
Power Saving King Shop	Green Intelligent Power Saver	Super Power Saving King is very good quality, new style, is genuine, the use of the effect is very good, the price is cheap, it is worth buying!	Jingdong prefecture level city in Shandong
Power Saving King Shop	Green Intelligent Power Saver	Packaging is very tight, easy to install, quite practical, worth buying!	Jingdong prefecture level city in Shandong
Power Saving King Shop	Green Intelligent Power Saver	Quality assurance, affordable price, very energy efficient, will repurchase if needed!	Jingdong prefecture level city in Shandong
Power Saving King Shop	Green Intelligent Power Saver	Quality assurance, very good, affordable, logistics is also very fast, I'm very satisfied with the	Jingdong prefecture level city in Shandong
Power Saving King Shop	Green Intelligent Power Saver	Energy saving. It's so good, already installed, you can save a lot of electricity every month	Jingdong prefecture level city in Shandong
Power Saving King Shop	Green Intelligent Power Saver	The material is thick and the workmanship is fine in place, the power saving effect is very good, value for money!	Jingdong prefecture level city in Shandong
Power Saving King Shop	Green Intelligent Power Saver	The effect is great, genuine, and match the description, the logistics is fast, the price is reasonable 😍	Jingdong prefecture level city in Shandong
Power Saving King Shop	Green Intelligent Power Saver	It's genuine, works well, easy to use, plugs in and works, really saves power	Jingdong prefecture level city in Shandong
Power Saving King Shop	Green Intelligent Power Saver	It's easy to use, effective, great service, and recommended!	Jingdong prefecture level city in Shandong
Power Saving King Shop	Green Intelligent Power Saver	🌈 Great quality, bought it to my liking 💝 Awesome! ✨ Buy with confidence if you need it!	Jingdong prefecture level city in Shandong
Power Saving King Shop	Green Intelligent Power Saver	Good quality, easy to install, very energy saving and power saving, good to use	Jingdong prefecture level city in Shandong
Power Saving King Shop	Green Intelligent Power Saver	The quality is very good, it does have the effect of saving electricity, the description matches, the price is affordable, it is worth recommending.	Jingdong prefecture level city in Shandong
Power Saving King Shop	Green Intelligent Power Saver	Received, the functionality is particularly good, it is genuine, the price is affordable	Jingdong prefecture level city in Shandong
Power Saving King Shop	Green Intelligent Power Saver	Good quality, very easy to use, energy efficient, home electricity bill is obviously much less	Jingdong prefecture level city in Shandong
Power Saving King Shop	Green Intelligent Power Saver	Easy to install, energy saving and power saving, very satisfied with a shopping	Jingdong prefecture level city in Shandong
Power Saving King Shop	Green Intelligent Power Saver	Received it and tried it out, it's great, works great and worth having! Five stars praise!	Jingdong prefecture level city in Shandong
Power Saving King Shop	Green Intelligent Power Saver	Power saving device, no problem at all, the quality of this product at this price, very affordable!	Jingdong prefecture level city in Shandong
Power Saving King Shop	Green Intelligent Power Saver	Received the goods, did not expect to be so fast, the quality is very good, especially power saving	Jingdong prefecture level city in Shandong
Power Saving King Shop	Green Intelligent Power Saver	The logistics is very fast, especially good, need to come back again	Jingdong prefecture level city in Shandong
Power Saving King Shop	Green Intelligent Power Saver	It's already working and it really saves power, so I'm very happy with it!	Jingdong prefecture level city in Shandong
Power Saving King Shop	Green Intelligent Power Saver	Great quality, works well, can recommend to friends to buy.	Jingdong prefecture level city in Shandong
Power Saving King Shop	Green Intelligent Power Saver	It's a good deal and worth the money.	Jingdong prefecture level city in Shandong
Power Saving King Shop	Green Intelligent Power Saver	I received it and I'm very happy with it, it's very good quality, guaranteed, and	Jingdong prefecture level city in Shandong

Power Saving King Shop	Green Intelligent Power Saver	Good quality, safe and secure purchase, ideal results and great price.	Jingdong prefecture level city in Shandong
Power Saving King Shop	Green Intelligent Power Saver	Simple operation plugged in the power supply can be, friends recommended looking forward to the effect of the	Jingdong prefecture level city in Shandong
Power Saving King Shop	Green Intelligent Power Saver	It's not bad, it saves electricity, and the material is sturdy and durable.	Jingdong prefecture level city in Shandong
Power Saving King Shop	Green Intelligent Power Saver	Plug and play, no management required, convenient and hassle-free!	Jingdong prefecture level city in Shandong
Power Saving King Shop	Green Intelligent Power Saver	The pictures are too lazy to go up, the power saving effect is still there!	Jingdong prefecture level city in Shandong
Power Saving King Shop	Green Intelligent Power Saver	Fast shipping. Affordable prices. Efficient	Jingdong prefecture level city in Shandong
Power Saving King Shop	Green Intelligent Power Saver	Good power saving, worth buying	Jingdong prefecture level city in Shandong
Power Saving King Shop	Green Intelligent Power Saver	Especially great, comparison shopping before buying, the store owner is very good, answering questions very patiently and detailed, there is a need to come back!	Jingdong prefecture level city in Shandong
Power Saving King Shop	Green Intelligent Power Saver	Very trustworthy brand, quality is guaranteed, the price is still affordable, quite satisfied!	Jingdong prefecture level city in Shandong
Power Saving King Shop	Green Intelligent Power Saver	Recommended by a friend. They say you can save money on air conditioning with this. Try it.	Jingdong prefecture level city in Shandong
Power Saving King Shop	Green Intelligent Power Saver	Works great, will be back next time, very happy with it and a great price!	Jingdong prefecture level city in Shandong
Power Saving King Shop	Green Intelligent Power Saver	The quality of the product is quite good, fast shipping, satisfied.	Jingdong prefecture level city in Shandong
Power Saving King Shop	Green Intelligent Power Saver	Good quality and fast logistics	Jingdong prefecture level city in Shandong
Power Saving King Shop	Green Intelligent Power Saver	It really saves power. I've tested it myself.	Jingdong prefecture level city in Shandong
Power Saving King Shop	Green Intelligent Power Saver	Really saves electricity!	Jingdong prefecture level city in Shandong
Power Saving King Shop	Green Intelligent Power Saver	pretty good	Jingdong prefecture level city in Shandong
Power Saving King Shop	Green Intelligent Power Saver	serviceable	Jingdong prefecture level city in Shandong
Power Saving King Shop	Green Intelligent Power Saver	Power Saving King received a tightly packaged logistics, installed the effect is very good price is cheaper than the physical store!	Jingdong prefecture level city in Shandong
Power Saving King Shop	Green Intelligent Power Saver	Super Power Saving King is very good quality, new style, is genuine, the use of the effect is very good, the price is cheap, it is worth buying!	Jingdong prefecture level city in Shandong
Power Saving King Shop	Green Intelligent Power Saver	Packaging is very tight, easy to install, quite practical, worth buying!	Jingdong prefecture level city in Shandong
Power Saving King Shop	Green Intelligent Power Saver	Quality assurance, affordable price, very energy efficient, will repurchase if needed!	Jingdong prefecture level city in Shandong
Power Saving King Shop	Green Intelligent Power Saver	Quality assurance, very good, affordable, logistics is also very fast, I'm very satisfied with the	Jingdong prefecture level city in Shandong
Power Saving King Shop	Green Intelligent Power Saver	Energy saving. It's so good, already installed, you can save a lot of electricity every month	Jingdong prefecture level city in Shandong
Power Saving King Shop	Green Intelligent Power Saver	The material is thick and the workmanship is fine in place, the power saving effect is very good, value for money!	Jingdong prefecture level city in Shandong
Power Saving King Shop	Green Intelligent Power Saver	The effect is great, genuine, and match the description, the logistics is fast, the price is reasonable 😍	Jingdong prefecture level city in Shandong
Power Saving King Shop	Green Intelligent Power Saver	It's genuine, works well, easy to use, plugs in and works, really saves power	Jingdong prefecture level city in Shandong
Power Saving King Shop	Green Intelligent Power Saver	It's easy to use, effective, great service, and recommended!	Jingdong prefecture level city in Shandong
Power Saving King Shop	Green Intelligent Power Saver	🌈 Great quality, bought it to my liking 💝 Awesome! ✨ Buy with confidence if you need it!	Jingdong prefecture level city in Shandong
Power Saving King Shop	Green Intelligent Power Saver	Good quality, easy to install, very energy saving and power saving, good to use	Jingdong prefecture level city in Shandong
Power Saving King Shop	Green Intelligent Power Saver	The quality is very good, it does have the effect of saving electricity, the description matches, the price is affordable, it is worth recommending.	Jingdong prefecture level city in Shandong
Power Saving King Shop	Green Intelligent Power Saver	Received, the functionality is particularly good, it is genuine, the price is affordable	Jingdong prefecture level city in Shandong
Power Saving King Shop	Green Intelligent Power Saver	Good quality, very easy to use, energy efficient, home electricity bill is obviously much less	Jingdong prefecture level city in Shandong
Power Saving King Shop	Green Intelligent Power Saver	Easy to install, energy saving and power saving, very satisfied with a shopping	Jingdong prefecture level city in Shandong
Power Saving King Shop	Green Intelligent Power Saver	Received it and tried it out, it's great, works great and worth having! Five stars praise!	Jingdong prefecture level city in Shandong
Power Saving King Shop	Green Intelligent Power Saver	Power saving device, no problem at all, the quality of this product at this price, very affordable!	Jingdong prefecture level city in Shandong
Power Saving King Shop	Green Intelligent Power Saver	Received the goods, did not expect to be so fast, the quality is very good, especially power saving	Jingdong prefecture level city in Shandong
Power Saving King Shop	Green Intelligent Power Saver	The logistics is very fast, especially good, need to come back again	Jingdong prefecture level city in Shandong
Power Saving King Shop	Green Intelligent Power Saver	It's already working and it really saves power, so I'm very happy with it!	Jingdong prefecture level city in Shandong
Power Saving King Shop	Green Intelligent Power Saver	Great quality, works well, can recommend to friends to buy.	Jingdong prefecture level city in Shandong
Power Saving King Shop	Green Intelligent Power Saver	It's a good deal and worth the money.	Jingdong prefecture level city in Shandong
Power Saving King Shop	Green Intelligent Power Saver	I received it and I'm very happy with it, it's very good quality, guaranteed, and	Jingdong prefecture level city in Shandong
Power Saving King Shop	Green Intelligent Power Saver	Good quality, safe and secure purchase, ideal results and great price.	Jingdong prefecture level city in Shandong
Power Saving King Shop	Green Intelligent Power Saver	Simple operation plugged in the power supply can be, friends recommended looking forward to the effect of the	Jingdong prefecture level city in Shandong
Power Saving King Shop	Green Intelligent Power Saver	It's not bad, it saves electricity, and the material is sturdy and durable.	Jingdong prefecture level city in Shandong
Power Saving King Shop	Green Intelligent Power Saver	Plug and play, no management required, convenient and hassle-free!	Jingdong prefecture level city in Shandong
Power Saving King Shop	Green Intelligent Power Saver	The pictures are too lazy to go up, the power saving effect is still there!	Jingdong prefecture level city in Shandong
Power Saving King Shop	Green Intelligent Power Saver	Fast shipping. Affordable prices. Efficient	Jingdong prefecture level city in Shandong
Power Saving King Shop	Green Intelligent Power Saver	Good power saving, worth buying	Jingdong prefecture level city in Shandong
Power Saving King Shop	Green Intelligent Power Saver	Especially great, comparison shopping before buying, the store owner is very good, answering questions very patiently and detailed, there is a need to come back!	Jingdong prefecture level city in Shandong
Power Saving King Shop	Green Intelligent Power Saver	Very trustworthy brand, quality is guaranteed, the price is still affordable, quite satisfied!	Jingdong prefecture level city in Shandong
Power Saving King Shop	Green Intelligent Power Saver	Recommended by a friend. They say you can save money on air conditioning with this. Try it.	Jingdong prefecture level city in Shandong

Power Saving King Shop	Green Intelligent Power Saver	Works great, will be back next time, very happy with it and a great price!	Jingdong prefecture level city in Shandong
Power Saving King Shop	Green Intelligent Power Saver	The quality of the product is quite good, fast shipping, satisfied.	Jingdong prefecture level city in Shandong
Power Saving King Shop	Green Intelligent Power Saver	Good quality and fast logistics	Jingdong prefecture level city in Shandong
Power Saving King Shop	Green Intelligent Power Saver	It really saves power. I've tested it myself.	Jingdong prefecture level city in Shandong
Power Saving King Shop	Green Intelligent Power Saver	Really saves electricity!	Jingdong prefecture level city in Shandong
Power Saving King Shop	Green Intelligent Power Saver	pretty good	Jingdong prefecture level city in Shandong
Power Saving King Shop	Green Intelligent Power Saver	serviceable	Jingdong prefecture level city in Shandong
Power Saving King Shop	Green Intelligent Power Saver	Power Saving King received a tightly packaged logistics, installed the effect is very good price is cheaper than the physical store!	Jingdong prefecture level city in Shandong
Power Saving King Shop	Green Intelligent Power Saver	Super Power Saving King is very good quality, new style, is genuine, the use of the effect is very good, the price is cheap, it is worth buying!	Jingdong prefecture level city in Shandong
Power Saving King Shop	Green Intelligent Power Saver	Packaging is very tight, easy to install, quite practical, worth buying!	Jingdong prefecture level city in Shandong
Power Saving King Shop	Green Intelligent Power Saver	Quality assurance, affordable price, very energy efficient, will repurchase if needed!	Jingdong prefecture level city in Shandong
Power Saving King Shop	Green Intelligent Power Saver	Quality assurance, very good, affordable, logistics is also very fast, I'm very satisfied with the	Jingdong prefecture level city in Shandong
Power Saving King Shop	Green Intelligent Power Saver	Energy saving. It's so good, already installed, you can save a lot of electricity every month	Jingdong prefecture level city in Shandong
Power Saving King Shop	Green Intelligent Power Saver	The material is thick and the workmanship is fine in place, the power saving effect is very good, value for money!	Jingdong prefecture level city in Shandong
Power Saving King Shop	Green Intelligent Power Saver	The effect is great, genuine, and match the description, the logistics is fast, the price is reasonable 😍	Jingdong prefecture level city in Shandong
Power Saving King Shop	Green Intelligent Power Saver	It's genuine, works well, easy to use, plugs in and works, really saves power	Jingdong prefecture level city in Shandong
Power Saving King Shop	Green Intelligent Power Saver	It's easy to use, effective, great service, and recommended!	Jingdong prefecture level city in Shandong
Power Saving King Shop	Green Intelligent Power Saver	🌈 Great quality, bought it to my liking 💖 Awesome! ✨ Buy with confidence if you need it!	Jingdong prefecture level city in Shandong
Power Saving King Shop	Green Intelligent Power Saver	Good quality, easy to install, very energy saving and power saving, good to use	Jingdong prefecture level city in Shandong
Power Saving King Shop	Green Intelligent Power Saver	The quality is very good, it does have the effect of saving electricity, the description matches, the price is affordable, it is worth recommending.	Jingdong prefecture level city in Shandong
Power Saving King Shop	Green Intelligent Power Saver	Received, the functionality is particularly good, it is genuine, the price is affordable	Jingdong prefecture level city in Shandong
Power Saving King Shop	Green Intelligent Power Saver	Good quality, very easy to use, energy efficient, home electricity bill is obviously much less	Jingdong prefecture level city in Shandong
Power Saving King Shop	Green Intelligent Power Saver	Easy to install, energy saving and power saving, very satisfied with a shopping	Jingdong prefecture level city in Shandong
Power Saving King Shop	Green Intelligent Power Saver	Received it and tried it out, it's great, works great and worth having! Five stars praise!	Jingdong prefecture level city in Shandong
Power Saving King Shop	Green Intelligent Power Saver	Power saving device, no problem at all, the quality of this product at this price, very affordable!	Jingdong prefecture level city in Shandong
Power Saving King Shop	Green Intelligent Power Saver	Received the goods, did not expect to be so fast, the quality is very good, especially power saving	Jingdong prefecture level city in Shandong
Power Saving King Shop	Green Intelligent Power Saver	The logistics is very fast, especially good, need to come back again	Jingdong prefecture level city in Shandong
Power Saving King Shop	Green Intelligent Power Saver	It's already working and it really saves power, so I'm very happy with it!	Jingdong prefecture level city in Shandong
Power Saving King Shop	Green Intelligent Power Saver	Great quality, works well, can recommend to friends to buy.	Jingdong prefecture level city in Shandong
Power Saving King Shop	Green Intelligent Power Saver	It's a good deal and worth the money.	Jingdong prefecture level city in Shandong
Power Saving King Shop	Green Intelligent Power Saver	I received it and I'm very happy with it, it's very good quality, guaranteed, and	Jingdong prefecture level city in Shandong
Power Saving King Shop	Green Intelligent Power Saver	Good quality, safe and secure purchase, ideal results and great price.	Jingdong prefecture level city in Shandong
Power Saving King Shop	Green Intelligent Power Saver	Simple operation plugged in the power supply can be, friends recommended looking forward to the effect of the	Jingdong prefecture level city in Shandong
Power Saving King Shop	Green Intelligent Power Saver	It's not bad, it saves electricity, and the material is sturdy and durable.	Jingdong prefecture level city in Shandong
Power Saving King Shop	Green Intelligent Power Saver	Plug and play, no management required, convenient and hassle-free!	Jingdong prefecture level city in Shandong
Power Saving King Shop	Green Intelligent Power Saver	The pictures are too lazy to go up, the power saving effect is still there!	Jingdong prefecture level city in Shandong
Power Saving King Shop	Green Intelligent Power Saver	Fast shipping. Affordable prices. Efficient	Jingdong prefecture level city in Shandong
Power Saving King Shop	Green Intelligent Power Saver	Good power saving, worth buying	Jingdong prefecture level city in Shandong
Power Saving King Shop	Green Intelligent Power Saver	Especially great, comparison shopping before buying, the store owner is very good, answering questions very patiently and detailed, there is a need to come back!	Jingdong prefecture level city in Shandong
Power Saving King Shop	Green Intelligent Power Saver	Very trustworthy brand, quality is guaranteed, the price is still affordable, quite satisfied!	Jingdong prefecture level city in Shandong
Power Saving King Shop	Green Intelligent Power Saver	Recommended by a friend. They say you can save money on air conditioning with this. Try it.	Jingdong prefecture level city in Shandong
Power Saving King Shop	Green Intelligent Power Saver	Works great, will be back next time, very happy with it and a great price!	Jingdong prefecture level city in Shandong
Power Saving King Shop	Green Intelligent Power Saver	The quality of the product is quite good, fast shipping, satisfied.	Jingdong prefecture level city in Shandong
Power Saving King Shop	Green Intelligent Power Saver	Good quality and fast logistics	Jingdong prefecture level city in Shandong
Power Saving King Shop	Green Intelligent Power Saver	It really saves power. I've tested it myself.	Jingdong prefecture level city in Shandong
Power Saving King Shop	Green Intelligent Power Saver	Really saves electricity!	Jingdong prefecture level city in Shandong
Power Saving King Shop	Green Intelligent Power Saver	pretty good	Jingdong prefecture level city in Shandong
Power Saving King Shop	Green Intelligent Power Saver	serviceable	Jingdong prefecture level city in Shandong
Power Saving King Shop	Green Intelligent Power Saver	Power Saving King received a tightly packaged logistics, installed the effect is very good price is cheaper than the physical store!	Jingdong prefecture level city in Shandong
Power Saving King Shop	Green Intelligent Power Saver	Super Power Saving King is very good quality, new style, is genuine, the use of the effect is very good, the price is cheap, it is worth buying!	Jingdong prefecture level city in Shandong
Power Saving King Shop	Green Intelligent Power Saver	Packaging is very tight, easy to install, quite practical, worth buying!	Jingdong prefecture level city in Shandong

Power Saving King Shop	Green Intelligent Power Saver	Quality assurance, affordable price, very energy efficient, will repurchase if needed!	Jingdong prefecture level city in Shandong
Power Saving King Shop	Green Intelligent Power Saver	Quality assurance, very good, affordable, logistics is also very fast, I'm very satisfied with the	Jingdong prefecture level city in Shandong
Power Saving King Shop	Green Intelligent Power Saver	Energy saving. It's so good, already installed, you can save a lot of electricity every month	Jingdong prefecture level city in Shandong
Power Saving King Shop	Green Intelligent Power Saver	The material is thick and the workmanship is fine in place, the power saving effect is very good, value for money!	Jingdong prefecture level city in Shandong
Power Saving King Shop	Green Intelligent Power Saver	The effect is great, genuine, and match the description, the logistics is fast, the price is reasonable 😍	Jingdong prefecture level city in Shandong
Power Saving King Shop	Green Intelligent Power Saver	It's genuine, works well, easy to use, plugs in and works, really saves power	Jingdong prefecture level city in Shandong
Power Saving King Shop	Green Intelligent Power Saver	It's easy to use, effective, great service, and recommended!	Jingdong prefecture level city in Shandong
Power Saving King Shop	Green Intelligent Power Saver	🌈 Great quality, bought it to my liking 💗 Awesome! 🌟 Buy with confidence if you need it!	Jingdong prefecture level city in Shandong
Power Saving King Shop	Green Intelligent Power Saver	Good quality, easy to install, very energy saving and power saving, good to use	Jingdong prefecture level city in Shandong
Power Saving King Shop	Green Intelligent Power Saver	The quality is very good, it does have the effect of saving electricity, the description matches, the price is affordable, it is worth recommending.	Jingdong prefecture level city in Shandong
Power Saving King Shop	Green Intelligent Power Saver	Received, the functionality is particularly good, it is genuine, the price is affordable	Jingdong prefecture level city in Shandong
Power Saving King Shop	Green Intelligent Power Saver	Good quality, very easy to use, energy efficient, home electricity bill is obviously much less	Jingdong prefecture level city in Shandong
Power Saving King Shop	Green Intelligent Power Saver	Easy to install, energy saving and power saving, very satisfied with a shopping	Jingdong prefecture level city in Shandong
Power Saving King Shop	Green Intelligent Power Saver	Received it and tried it out, it's great, works great and worth having! Five stars praise!	Jingdong prefecture level city in Shandong
Power Saving King Shop	Green Intelligent Power Saver	Power saving device, no problem at all, the quality of this product at this price, very affordable!	Jingdong prefecture level city in Shandong
Power Saving King Shop	Green Intelligent Power Saver	Received the goods, did not expect to be so fast, the quality is very good, especially power saving	Jingdong prefecture level city in Shandong
Power Saving King Shop	Green Intelligent Power Saver	The logistics is very fast, especially good, need to come back again	Jingdong prefecture level city in Shandong
Power Saving King Shop	Green Intelligent Power Saver	It's already working and it really saves power, so I'm very happy with it!	Jingdong prefecture level city in Shandong
Power Saving King Shop	Green Intelligent Power Saver	Great quality, works well, can recommend to friends to buy.	Jingdong prefecture level city in Shandong
Power Saving King Shop	Green Intelligent Power Saver	It's a good deal and worth the money.	Jingdong prefecture level city in Shandong
Power Saving King Shop	Green Intelligent Power Saver	I received it and I'm very happy with it, it's very good quality, guaranteed, and	Jingdong prefecture level city in Shandong
Power Saving King Shop	Green Intelligent Power Saver	Good quality, safe and secure purchase, ideal results and great price.	Jingdong prefecture level city in Shandong
Power Saving King Shop	Green Intelligent Power Saver	Simple operation plugged in the power supply can be, friends recommended looking forward to the effect of the	Jingdong prefecture level city in Shandong
Power Saving King Shop	Green Intelligent Power Saver	It's not bad, it saves electricity, and the material is sturdy and durable.	Jingdong prefecture level city in Shandong
Power Saving King Shop	Green Intelligent Power Saver	Plug and play, no management required, convenient and hassle-free!	Jingdong prefecture level city in Shandong
Power Saving King Shop	Green Intelligent Power Saver	The pictures are too lazy to go up, the power saving effect is still there!	Jingdong prefecture level city in Shandong
Power Saving King Shop	Green Intelligent Power Saver	Fast shipping. Affordable prices. Efficient	Jingdong prefecture level city in Shandong
Power Saving King Shop	Green Intelligent Power Saver	Good power saving, worth buying	Jingdong prefecture level city in Shandong
Power Saving King Shop	Green Intelligent Power Saver	Especially great, comparison shopping before buying, the store owner is very good, answering questions very patiently and detailed, there is a need to come back!	Jingdong prefecture level city in Shandong
Power Saving King Shop	Green Intelligent Power Saver	Very trustworthy brand, quality is guaranteed, the price is still affordable, quite satisfied!	Jingdong prefecture level city in Shandong
Power Saving King Shop	Green Intelligent Power Saver	Recommended by a friend. They say you can save money on air conditioning with this. Try it.	Jingdong prefecture level city in Shandong
Power Saving King Shop	Green Intelligent Power Saver	Works great, will be back next time, very happy with it and a great price!	Jingdong prefecture level city in Shandong
Power Saving King Shop	Green Intelligent Power Saver	The quality of the product is quite good, fast shipping, satisfied.	Jingdong prefecture level city in Shandong
Power Saving King Shop	Green Intelligent Power Saver	Good quality and fast logistics	Jingdong prefecture level city in Shandong
Power Saving King Shop	Green Intelligent Power Saver	It really saves power. I've tested it myself.	Jingdong prefecture level city in Shandong
Power Saving King Shop	Green Intelligent Power Saver	Really saves electricity!	Jingdong prefecture level city in Shandong
Power Saving King Shop	Green Intelligent Power Saver	pretty good	Jingdong prefecture level city in Shandong
Power Saving King Shop	Green Intelligent Power Saver	serviceable	Jingdong prefecture level city in Shandong
Power Saving King Shop	Green Intelligent Power Saver	Power Saving King received a tightly packaged logistics, installed the effect is very good price is cheaper than the physical store!	Jingdong prefecture level city in Shandong
Power Saving King Shop	Green Intelligent Power Saver	Super Power Saving King is very good quality, new style, is genuine, the use of the effect is very good, the price is cheap, it is worth buying!	Jingdong prefecture level city in Shandong
Power Saving King Shop	Green Intelligent Power Saver	Packaging is very tight, easy to install, quite practical, worth buying!	Jingdong prefecture level city in Shandong
Power Saving King Shop	Green Intelligent Power Saver	Quality assurance, affordable price, very energy efficient, will repurchase if needed!	Jingdong prefecture level city in Shandong
Power Saving King Shop	Green Intelligent Power Saver	Quality assurance, very good, affordable, logistics is also very fast, I'm very satisfied with the	Jingdong prefecture level city in Shandong
Power Saving King Shop	Green Intelligent Power Saver	Energy saving. It's so good, already installed, you can save a lot of electricity every month	Jingdong prefecture level city in Shandong
Power Saving King Shop	Green Intelligent Power Saver	The material is thick and the workmanship is fine in place, the power saving effect is very good, value for money!	Jingdong prefecture level city in Shandong
Power Saving King Shop	Green Intelligent Power Saver	The effect is great, genuine, and match the description, the logistics is fast, the price is reasonable 😍	Jingdong prefecture level city in Shandong
Power Saving King Shop	Green Intelligent Power Saver	It's genuine, works well, easy to use, plugs in and works, really saves power	Jingdong prefecture level city in Shandong
Power Saving King Shop	Green Intelligent Power Saver	It's easy to use, effective, great service, and recommended!	Jingdong prefecture level city in Shandong
Power Saving King Shop	Green Intelligent Power Saver	🌈 Great quality, bought it to my liking 💗 Awesome! 🌟 Buy with confidence if you need it!	Jingdong prefecture level city in Shandong
Power Saving King Shop	Green Intelligent Power Saver	Good quality, easy to install, very energy saving and power saving, good to use	Jingdong prefecture level city in Shandong
Power Saving King Shop	Green Intelligent Power Saver	The quality is very good, it does have the effect of saving electricity, the description matches, the price is affordable, it is worth recommending.	Jingdong prefecture level city in Shandong

Power Saving King Shop	Green Intelligent Power Saver	Received, the functionality is particularly good, it is genuine, the price is affordable	Jingdong prefecture level city in Shandong
Power Saving King Shop	Green Intelligent Power Saver	Good quality, very easy to use, energy efficient, home electricity bill is obviously much less	Jingdong prefecture level city in Shandong
Power Saving King Shop	Green Intelligent Power Saver	Easy to install, energy saving and power saving, very satisfied with a shopping	Jingdong prefecture level city in Shandong
Power Saving King Shop	Green Intelligent Power Saver	Received it and tried it out, it's great, works great and worth having! Five stars praise!	Jingdong prefecture level city in Shandong
Power Saving King Shop	Green Intelligent Power Saver	Power saving device, no problem at all, the quality of this product at this price, very affordable!	Jingdong prefecture level city in Shandong
Power Saving King Shop	Green Intelligent Power Saver	Received the goods, did not expect to be so fast, the quality is very good, especially power saving	Jingdong prefecture level city in Shandong
Power Saving King Shop	Green Intelligent Power Saver	The logistics is very fast, especially good, need to come back again	Jingdong prefecture level city in Shandong
Power Saving King Shop	Green Intelligent Power Saver	It's already working and it really saves power, so I'm very happy with it!	Jingdong prefecture level city in Shandong
Power Saving King Shop	Green Intelligent Power Saver	Great quality, works well, can recommend to friends to buy.	Jingdong prefecture level city in Shandong
Power Saving King Shop	Green Intelligent Power Saver	It's a good deal and worth the money.	Jingdong prefecture level city in Shandong
Power Saving King Shop	Green Intelligent Power Saver	I received it and I'm very happy with it, it's very good quality, guaranteed, and	Jingdong prefecture level city in Shandong
Power Saving King Shop	Green Intelligent Power Saver	Good quality, safe and secure purchase, ideal results and great price.	Jingdong prefecture level city in Shandong
Power Saving King Shop	Green Intelligent Power Saver	Simple operation plugged in the power supply can be, friends recommended looking forward to the effect of the	Jingdong prefecture level city in Shandong
Power Saving King Shop	Green Intelligent Power Saver	It's not bad, it saves electricity, and the material is sturdy and durable.	Jingdong prefecture level city in Shandong
Power Saving King Shop	Green Intelligent Power Saver	Plug and play, no management required, convenient and hassle-free!	Jingdong prefecture level city in Shandong
Power Saving King Shop	Green Intelligent Power Saver	The pictures are too lazy to go up, the power saving effect is still there!	Jingdong prefecture level city in Shandong
Power Saving King Shop	Green Intelligent Power Saver	Fast shipping. Affordable prices. Efficient	Jingdong prefecture level city in Shandong
Power Saving King Shop	Green Intelligent Power Saver	Good power saving, worth buying	Jingdong prefecture level city in Shandong
Power Saving King Shop	Green Intelligent Power Saver	Especially great, comparison shopping before buying, the store owner is very good, answering questions very patiently and detailed, there is a need to come back!	Jingdong prefecture level city in Shandong
Power Saving King Shop	Green Intelligent Power Saver	Very trustworthy brand, quality is guaranteed, the price is still affordable, quite satisfied!	Jingdong prefecture level city in Shandong
Power Saving King Shop	Green Intelligent Power Saver	Recommended by a friend. They say you can save money on air conditioning with this. Try it.	Jingdong prefecture level city in Shandong
Power Saving King Shop	Green Intelligent Power Saver	Works great, will be back next time, very happy with it and a great price!	Jingdong prefecture level city in Shandong
Power Saving King Shop	Green Intelligent Power Saver	The quality of the product is quite good, fast shipping, satisfied.	Jingdong prefecture level city in Shandong
Power Saving King Shop	Green Intelligent Power Saver	Good quality and fast logistics	Jingdong prefecture level city in Shandong
Power Saving King Shop	Green Intelligent Power Saver	It really saves power. I've tested it myself.	Jingdong prefecture level city in Shandong
Power Saving King Shop	Green Intelligent Power Saver	Really saves electricity!	Jingdong prefecture level city in Shandong
Power Saving King Shop	Green Intelligent Power Saver	pretty good	Jingdong prefecture level city in Shandong
Power Saving King Shop	Green Intelligent Power Saver	serviceable	Jingdong prefecture level city in Shandong
Power Saving King Shop	Green Intelligent Power Saver	Power Saving King received a tightly packaged logistics, installed the effect is very good price is cheaper than the physical store!	Jingdong prefecture level city in Shandong
Power Saving King Shop	Green Intelligent Power Saver	Super Power Saving King is very good quality, new style, is genuine, the use of the effect is very good, the price is cheap, it is worth buying!	Jingdong prefecture level city in Shandong
Power Saving King Shop	Green Intelligent Power Saver	Packaging is very tight, easy to install, quite practical, worth buying!	Jingdong prefecture level city in Shandong
Power Saving King Shop	Green Intelligent Power Saver	Quality assurance, affordable price, very energy efficient, will repurchase if needed!	Jingdong prefecture level city in Shandong
Power Saving King Shop	Green Intelligent Power Saver	Quality assurance, very good, affordable, logistics is also very fast, I'm very satisfied with the	Jingdong prefecture level city in Shandong
Power Saving King Shop	Green Intelligent Power Saver	Energy saving. It's so good, already installed, you can save a lot of electricity every month	Jingdong prefecture level city in Shandong
Power Saving King Shop	Green Intelligent Power Saver	The material is thick and the workmanship is fine in place, the power saving effect is very good, value for money!	Jingdong prefecture level city in Shandong
Power Saving King Shop	Green Intelligent Power Saver	The effect is great, genuine, and match the description, the logistics is fast, the price is reasonable 😍	Jingdong prefecture level city in Shandong
Power Saving King Shop	Green Intelligent Power Saver	It's genuine, works well, easy to use, plugs in and works, really saves power	Jingdong prefecture level city in Shandong
Power Saving King Shop	Green Intelligent Power Saver	It's easy to use, effective, great service, and recommended!	Jingdong prefecture level city in Shandong
Power Saving King Shop	Green Intelligent Power Saver	🌈 Great quality, bought it to my liking 💕 Awesome! ✨ Buy with confidence if you need it!	Jingdong prefecture level city in Shandong
Power Saving King Shop	Green Intelligent Power Saver	Good quality, easy to install, very energy saving and power saving, good to use	Jingdong prefecture level city in Shandong
Power Saving King Shop	Green Intelligent Power Saver	The quality is very good, it does have the effect of saving electricity, the description matches, the price is affordable, it is worth recommending.	Jingdong prefecture level city in Shandong
Power Saving King Shop	Green Intelligent Power Saver	Received, the functionality is particularly good, it is genuine, the price is affordable	Jingdong prefecture level city in Shandong
Power Saving King Shop	Green Intelligent Power Saver	Good quality, very easy to use, energy efficient, home electricity bill is obviously much less	Jingdong prefecture level city in Shandong
Power Saving King Shop	Green Intelligent Power Saver	Easy to install, energy saving and power saving, very satisfied with a shopping	Jingdong prefecture level city in Shandong
Power Saving King Shop	Green Intelligent Power Saver	Received it and tried it out, it's great, works great and worth having! Five stars praise!	Jingdong prefecture level city in Shandong
Power Saving King Shop	Green Intelligent Power Saver	Power saving device, no problem at all, the quality of this product at this price, very affordable!	Jingdong prefecture level city in Shandong
Power Saving King Shop	Green Intelligent Power Saver	Received the goods, did not expect to be so fast, the quality is very good, especially power saving	Jingdong prefecture level city in Shandong
Power Saving King Shop	Green Intelligent Power Saver	The logistics is very fast, especially good, need to come back again	Jingdong prefecture level city in Shandong
Power Saving King Shop	Green Intelligent Power Saver	It's already working and it really saves power, so I'm very happy with it!	Jingdong prefecture level city in Shandong
Power Saving King Shop	Green Intelligent Power Saver	Great quality, works well, can recommend to friends to buy.	Jingdong prefecture level city in Shandong
Power Saving King Shop	Green Intelligent Power Saver	It's a good deal and worth the money.	Jingdong prefecture level city in Shandong

Power Saving King Shop	Green Intelligent Power Saver	I received it and I'm very happy with it, it's very good quality, guaranteed, and	Jingdong prefecture level city in Shandong
Power Saving King Shop	Green Intelligent Power Saver	Good quality, safe and secure purchase, ideal results and great price.	Jingdong prefecture level city in Shandong
Power Saving King Shop	Green Intelligent Power Saver	Simple operation plugged in the power supply can be, friends recommended looking forward to the effect of the	Jingdong prefecture level city in Shandong
Power Saving King Shop	Green Intelligent Power Saver	It's not bad, it saves electricity, and the material is sturdy and durable.	Jingdong prefecture level city in Shandong
Power Saving King Shop	Green Intelligent Power Saver	Plug and play, no management required, convenient and hassle-free!	Jingdong prefecture level city in Shandong
Power Saving King Shop	Green Intelligent Power Saver	The pictures are too lazy to go up, the power saving effect is still there!	Jingdong prefecture level city in Shandong
Power Saving King Shop	Green Intelligent Power Saver	Fast shipping. Affordable prices. Efficient	Jingdong prefecture level city in Shandong
Power Saving King Shop	Green Intelligent Power Saver	Good power saving, worth buying	Jingdong prefecture level city in Shandong
Power Saving King Shop	Green Intelligent Power Saver	Especially great, comparison shopping before buying, the store owner is very good, answering questions very patiently and detailed, there is a need to come back!	Jingdong prefecture level city in Shandong
Power Saving King Shop	Green Intelligent Power Saver	Very trustworthy brand, quality is guaranteed, the price is still affordable, quite satisfied!	Jingdong prefecture level city in Shandong
Power Saving King Shop	Green Intelligent Power Saver	Recommended by a friend. They say you can save money on air conditioning with this. Try it.	Jingdong prefecture level city in Shandong
Power Saving King Shop	Green Intelligent Power Saver	Works great, will be back next time, very happy with it and a great price!	Jingdong prefecture level city in Shandong
Power Saving King Shop	Green Intelligent Power Saver	The quality of the product is quite good, fast shipping, satisfied.	Jingdong prefecture level city in Shandong
Power Saving King Shop	Green Intelligent Power Saver	Good quality and fast logistics	Jingdong prefecture level city in Shandong
Power Saving King Shop	Green Intelligent Power Saver	It really saves power. I've tested it myself.	Jingdong prefecture level city in Shandong
Power Saving King Shop	Green Intelligent Power Saver	Really saves electricity!	Jingdong prefecture level city in Shandong
Power Saving King Shop	Green Intelligent Power Saver	pretty good	Jingdong prefecture level city in Shandong
Power Saving King Shop	Green Intelligent Power Saver	serviceable	Jingdong prefecture level city in Shandong
Power Saving King Shop	Green Intelligent Power Saver	Power Saving King received a tightly packaged logistics, installed the effect is very good price is cheaper than the physical store!	Jingdong prefecture level city in Shandong
Power Saving King Shop	Green Intelligent Power Saver	Super Power Saving King is very good quality, new style, is genuine, the use of the effect is very good, the price is cheap, it is worth buying!	Jingdong prefecture level city in Shandong
Power Saving King Shop	Green Intelligent Power Saver	Packaging is very tight, easy to install, quite practical, worth buying!	Jingdong prefecture level city in Shandong
Power Saving King Shop	Green Intelligent Power Saver	Quality assurance, affordable price, very energy efficient, will repurchase if needed!	Jingdong prefecture level city in Shandong
Power Saving King Shop	Green Intelligent Power Saver	Quality assurance, very good, affordable, logistics is also very fast, I'm very satisfied with the	Jingdong prefecture level city in Shandong
Power Saving King Shop	Green Intelligent Power Saver	Energy saving. It's so good, already installed, you can save a lot of electricity every month	Jingdong prefecture level city in Shandong
Power Saving King Shop	Green Intelligent Power Saver	The material is thick and the workmanship is fine in place, the power saving effect is very good, value for money!	Jingdong prefecture level city in Shandong
Power Saving King Shop	Green Intelligent Power Saver	The effect is great, genuine, and match the description, the logistics is fast, the price is reasonable 😍	Jingdong prefecture level city in Shandong
Power Saving King Shop	Green Intelligent Power Saver	It's genuine, works well, easy to use, plugs in and works, really saves power	Jingdong prefecture level city in Shandong
Power Saving King Shop	Green Intelligent Power Saver	It's easy to use, effective, great service, and recommended!	Jingdong prefecture level city in Shandong
Power Saving King Shop	Green Intelligent Power Saver	🌈 Great quality, bought it to my liking 💗 Awesome! ✨ Buy with confidence if you need it!	Jingdong prefecture level city in Shandong
Power Saving King Shop	Green Intelligent Power Saver	Good quality, easy to install, very energy saving and power saving, good to use	Jingdong prefecture level city in Shandong
Power Saving King Shop	Green Intelligent Power Saver	The quality is very good, it does have the effect of saving electricity, the description matches, the price is affordable, it is worth recommending.	Jingdong prefecture level city in Shandong
Power Saving King Shop	Green Intelligent Power Saver	Received, the functionality is particularly good, it is genuine, the price is affordable	Jingdong prefecture level city in Shandong
Power Saving King Shop	Green Intelligent Power Saver	Good quality, very easy to use, energy efficient, home electricity bill is obviously much less	Jingdong prefecture level city in Shandong
Power Saving King Shop	Green Intelligent Power Saver	Easy to install, energy saving and power saving, very satisfied with a shopping	Jingdong prefecture level city in Shandong
Power Saving King Shop	Green Intelligent Power Saver	Received it and tried it out, it's great, works great and worth having! Five stars praise!	Jingdong prefecture level city in Shandong
Power Saving King Shop	Green Intelligent Power Saver	Power saving device, no problem at all, the quality of this product at this price, very affordable!	Jingdong prefecture level city in Shandong
Power Saving King Shop	Green Intelligent Power Saver	Received the goods, did not expect to be so fast, the quality is very good, especially power saving	Jingdong prefecture level city in Shandong
Power Saving King Shop	Green Intelligent Power Saver	The logistics is very fast, especially good, need to come back again	Jingdong prefecture level city in Shandong
Power Saving King Shop	Green Intelligent Power Saver	It's already working and it really saves power, so I'm very happy with it!	Jingdong prefecture level city in Shandong
Power Saving King Shop	Green Intelligent Power Saver	Great quality, works well, can recommend to friends to buy.	Jingdong prefecture level city in Shandong
Power Saving King Shop	Green Intelligent Power Saver	It's a good deal and worth the money.	Jingdong prefecture level city in Shandong
Power Saving King Shop	Green Intelligent Power Saver	I received it and I'm very happy with it, it's very good quality, guaranteed, and	Jingdong prefecture level city in Shandong
Power Saving King Shop	Green Intelligent Power Saver	Good quality, safe and secure purchase, ideal results and great price.	Jingdong prefecture level city in Shandong
Power Saving King Shop	Green Intelligent Power Saver	Simple operation plugged in the power supply can be, friends recommended looking forward to the effect of the	Jingdong prefecture level city in Shandong
Power Saving King Shop	Green Intelligent Power Saver	It's not bad, it saves electricity, and the material is sturdy and durable.	Jingdong prefecture level city in Shandong
Power Saving King Shop	Green Intelligent Power Saver	Plug and play, no management required, convenient and hassle-free!	Jingdong prefecture level city in Shandong
Power Saving King Shop	Green Intelligent Power Saver	The pictures are too lazy to go up, the power saving effect is still there!	Jingdong prefecture level city in Shandong
Power Saving King Shop	Green Intelligent Power Saver	Fast shipping. Affordable prices. Efficient	Jingdong prefecture level city in Shandong
Power Saving King Shop	Green Intelligent Power Saver	Good power saving, worth buying	Jingdong prefecture level city in Shandong
Power Saving King Shop	Green Intelligent Power Saver	Especially great, comparison shopping before buying, the store owner is very good, answering questions very patiently and detailed, there is a need to come back!	Jingdong prefecture level city in Shandong
Power Saving King Shop	Green Intelligent Power Saver	Very trustworthy brand, quality is guaranteed, the price is still affordable, quite satisfied!	Jingdong prefecture level city in Shandong

Power Saving King Shop	Green Intelligent Power Saver	Recommended by a friend. They say you can save money on air conditioning with this. Try it.	Jingdong prefecture level city in Shandong
Power Saving King Shop	Green Intelligent Power Saver	Works great, will be back next time, very happy with it and a great price!	Jingdong prefecture level city in Shandong
Power Saving King Shop	Green Intelligent Power Saver	The quality of the product is quite good, fast shipping, satisfied.	Jingdong prefecture level city in Shandong
Power Saving King Shop	Green Intelligent Power Saver	Good quality and fast logistics	Jingdong prefecture level city in Shandong
Power Saving King Shop	Green Intelligent Power Saver	It really saves power. I’ve tested it myself.	Jingdong prefecture level city in Shandong
Power Saving King Shop	Green Intelligent Power Saver	Really saves electricity!	Jingdong prefecture level city in Shandong
Power Saving King Shop	Green Intelligent Power Saver	pretty good	Jingdong prefecture level city in Shandong
Power Saving King Shop	Green Intelligent Power Saver	serviceable	Jingdong prefecture level city in Shandong
Power Saving King Shop	Green Intelligent Power Saver	Power Saving King received a tightly packaged logistics, installed the effect is very good price is cheaper than the physical store!	Jingdong prefecture level city in Shandong
Power Saving King Shop	Green Intelligent Power Saver	Super Power Saving King is very good quality, new style, is genuine, the use of the effect is very good, the price is cheap, it is worth buying!	Jingdong prefecture level city in Shandong
Power Saving King Shop	Green Intelligent Power Saver	Packaging is very tight, easy to install, quite practical, worth buying!	Jingdong prefecture level city in Shandong
Power Saving King Shop	Green Intelligent Power Saver	Quality assurance, affordable price, very energy efficient, will repurchase if needed!	Jingdong prefecture level city in Shandong
Power Saving King Shop	Green Intelligent Power Saver	Quality assurance, very good, affordable, logistics is also very fast, I’m very satisfied with the	Jingdong prefecture level city in Shandong
Power Saving King Shop	Green Intelligent Power Saver	Energy saving. It’s so good, already installed, you can save a lot of electricity every month	Jingdong prefecture level city in Shandong
Power Saving King Shop	Green Intelligent Power Saver	The material is thick and the workmanship is fine in place, the power saving effect is very good, value for money!	Jingdong prefecture level city in Shandong
Power Saving King Shop	Green Intelligent Power Saver	The effect is great, genuine, and match the description, the logistics is fast, the price is reasonable 😍	Jingdong prefecture level city in Shandong
Power Saving King Shop	Green Intelligent Power Saver	It’s genuine, works well, easy to use, plugs in and works, really saves power	Jingdong prefecture level city in Shandong
Power Saving King Shop	Green Intelligent Power Saver	It’s easy to use, effective, great service, and recommended!	Jingdong prefecture level city in Shandong
Power Saving King Shop	Green Intelligent Power Saver	🌈 Great quality, bought it to my liking 💝 Awesome! ✨ Buy with confidence if you need it!	Jingdong prefecture level city in Shandong
Power Saving King Shop	Green Intelligent Power Saver	Good quality, easy to install, very energy saving and power saving, good to use	Jingdong prefecture level city in Shandong
Power Saving King Shop	Green Intelligent Power Saver	The quality is very good, it does have the effect of saving electricity, the description matches, the price is affordable, it is worth recommending.	Jingdong prefecture level city in Shandong
Power Saving King Shop	Green Intelligent Power Saver	Received, the functionality is particularly good, it is genuine, the price is affordable	Jingdong prefecture level city in Shandong
Power Saving King Shop	Green Intelligent Power Saver	Good quality, very easy to use, energy efficient, home electricity bill is obviously much less	Jingdong prefecture level city in Shandong
Power Saving King Shop	Green Intelligent Power Saver	Easy to install, energy saving and power saving, very satisfied with a shopping	Jingdong prefecture level city in Shandong
Power Saving King Shop	Green Intelligent Power Saver	Received it and tried it out, it’s great, works great and worth having! Five stars praise!	Jingdong prefecture level city in Shandong
Power Saving King Shop	Green Intelligent Power Saver	Power saving device, no problem at all, the quality of this product at this price, very affordable!	Jingdong prefecture level city in Shandong
Power Saving King Shop	Green Intelligent Power Saver	Received the goods, did not expect to be so fast, the quality is very good, especially power saving	Jingdong prefecture level city in Shandong
Power Saving King Shop	Green Intelligent Power Saver	The logistics is very fast, especially good, need to come back again	Jingdong prefecture level city in Shandong
Power Saving King Shop	Green Intelligent Power Saver	It’s already working and it really saves power, so I’m very happy with it!	Jingdong prefecture level city in Shandong
Power Saving King Shop	Green Intelligent Power Saver	Great quality, works well, can recommend to friends to buy.	Jingdong prefecture level city in Shandong
Power Saving King Shop	Green Intelligent Power Saver	It’s a good deal and worth the money.	Jingdong prefecture level city in Shandong
Power Saving King Shop	Green Intelligent Power Saver	I received it and I’m very happy with it, it’s very good quality, guaranteed, and	Jingdong prefecture level city in Shandong
Power Saving King Shop	Green Intelligent Power Saver	Good quality, safe and secure purchase, ideal results and great price.	Jingdong prefecture level city in Shandong
Power Saving King Shop	Green Intelligent Power Saver	Simple operation plugged in the power supply can be, friends recommended looking forward to the effect of the	Jingdong prefecture level city in Shandong
Power Saving King Shop	Green Intelligent Power Saver	It’s not bad, it saves electricity, and the material is sturdy and durable.	Jingdong prefecture level city in Shandong
Power Saving King Shop	Green Intelligent Power Saver	Plug and play, no management required, convenient and hassle-free!	Jingdong prefecture level city in Shandong
Power Saving King Shop	Green Intelligent Power Saver	The pictures are too lazy to go up, the power saving effect is still there!	Jingdong prefecture level city in Shandong
Power Saving King Shop	Green Intelligent Power Saver	Fast shipping. Affordable prices. Efficient	Jingdong prefecture level city in Shandong
Power Saving King Shop	Green Intelligent Power Saver	Good power saving, worth buying	Jingdong prefecture level city in Shandong
Power Saving King Shop	Green Intelligent Power Saver	Especially great, comparison shopping before buying, the store owner is very good, answering questions very patiently and detailed, there is a need to come back!	Jingdong prefecture level city in Shandong
Power Saving King Shop	Green Intelligent Power Saver	Very trustworthy brand, quality is guaranteed, the price is still affordable, quite satisfied!	Jingdong prefecture level city in Shandong
Power Saving King Shop	Green Intelligent Power Saver	Recommended by a friend. They say you can save money on air conditioning with this. Try it.	Jingdong prefecture level city in Shandong
Power Saving King Shop	Green Intelligent Power Saver	Works great, will be back next time, very happy with it and a great price!	Jingdong prefecture level city in Shandong
Power Saving King Shop	Green Intelligent Power Saver	The quality of the product is quite good, fast shipping, satisfied.	Jingdong prefecture level city in Shandong
Power Saving King Shop	Green Intelligent Power Saver	Good quality and fast logistics	Jingdong prefecture level city in Shandong
Power Saving King Shop	Green Intelligent Power Saver	It really saves power. I’ve tested it myself.	Jingdong prefecture level city in Shandong
Power Saving King Shop	Green Intelligent Power Saver	Really saves electricity!	Jingdong prefecture level city in Shandong
Power Saving King Shop	Green Intelligent Power Saver	pretty good	Jingdong prefecture level city in Shandong
Power Saving King Shop	Green Intelligent Power Saver	serviceable	Jingdong prefecture level city in Shandong
Power Saving King Shop	Green Intelligent Power Saver	Power Saving King received a tightly packaged logistics, installed the effect is very good price is cheaper than the physical store!	Jingdong prefecture level city in Shandong
Power Saving King Shop	Green Intelligent Power Saver	Super Power Saving King is very good quality, new style, is genuine, the use of the effect is very good, the price is cheap, it is worth buying!	Jingdong prefecture level city in Shandong

Power Saving King Shop	Green Intelligent Power Saver	Packaging is very tight, easy to install, quite practical, worth buying!	Jingdong prefecture level city in Shandong
Power Saving King Shop	Green Intelligent Power Saver	Quality assurance, affordable price, very energy efficient, will repurchase if needed!	Jingdong prefecture level city in Shandong
Power Saving King Shop	Green Intelligent Power Saver	Quality assurance, very good, affordable, logistics is also very fast, I’m very satisfied with the	Jingdong prefecture level city in Shandong
Power Saving King Shop	Green Intelligent Power Saver	Energy saving. It’s so good, already installed, you can save a lot of electricity every month	Jingdong prefecture level city in Shandong
Power Saving King Shop	Green Intelligent Power Saver	The material is thick and the workmanship is fine in place, the power saving effect is very good, value for money!	Jingdong prefecture level city in Shandong
Power Saving King Shop	Green Intelligent Power Saver	The effect is great, genuine, and match the description, the logistics is fast, the price is reasonable 😍	Jingdong prefecture level city in Shandong
Power Saving King Shop	Green Intelligent Power Saver	It’s genuine, works well, easy to use, plugs in and works, really saves power	Jingdong prefecture level city in Shandong
Power Saving King Shop	Green Intelligent Power Saver	It’s easy to use, effective, great service, and recommended!	Jingdong prefecture level city in Shandong
Power Saving King Shop	Green Intelligent Power Saver	🌈 Great quality, bought it to my liking 💗 Awesome! ✨ Buy with confidence if you need it!	Jingdong prefecture level city in Shandong
Power Saving King Shop	Green Intelligent Power Saver	Good quality, easy to install, very energy saving and power saving, good to use	Jingdong prefecture level city in Shandong
Power Saving King Shop	Green Intelligent Power Saver	The quality is very good, it does have the effect of saving electricity, the description matches, the price is affordable, it is worth recommending.	Jingdong prefecture level city in Shandong
Power Saving King Shop	Green Intelligent Power Saver	Received, the functionality is particularly good, it is genuine, the price is affordable	Jingdong prefecture level city in Shandong
Power Saving King Shop	Green Intelligent Power Saver	Good quality, very easy to use, energy efficient, home electricity bill is obviously much less	Jingdong prefecture level city in Shandong
Power Saving King Shop	Green Intelligent Power Saver	Easy to install, energy saving and power saving, very satisfied with a shopping	Jingdong prefecture level city in Shandong
Power Saving King Shop	Green Intelligent Power Saver	Received it and tried it out, it’s great, works great and worth having! Five stars praise!	Jingdong prefecture level city in Shandong
Power Saving King Shop	Green Intelligent Power Saver	Power saving device, no problem at all, the quality of this product at this price, very affordable!	Jingdong prefecture level city in Shandong
Power Saving King Shop	Green Intelligent Power Saver	Received the goods, did not expect to be so fast, the quality is very good, especially power saving	Jingdong prefecture level city in Shandong
Power Saving King Shop	Green Intelligent Power Saver	The logistics is very fast, especially good, need to come back again	Jingdong prefecture level city in Shandong
Power Saving King Shop	Green Intelligent Power Saver	It’s already working and it really saves power, so I’m very happy with it!	Jingdong prefecture level city in Shandong
Power Saving King Shop	Green Intelligent Power Saver	Great quality, works well, can recommend to friends to buy.	Jingdong prefecture level city in Shandong
Power Saving King Shop	Green Intelligent Power Saver	It’s a good deal and worth the money.	Jingdong prefecture level city in Shandong
Power Saving King Shop	Green Intelligent Power Saver	I received it and I’m very happy with it, it’s very good quality, guaranteed, and	Jingdong prefecture level city in Shandong
Power Saving King Shop	Green Intelligent Power Saver	Good quality, safe and secure purchase, ideal results and great price.	Jingdong prefecture level city in Shandong
Power Saving King Shop	Green Intelligent Power Saver	Simple operation plugged in the power supply can be, friends recommended looking forward to the effect of the	Jingdong prefecture level city in Shandong
Power Saving King Shop	Green Intelligent Power Saver	It’s not bad, it saves electricity, and the material is sturdy and durable.	Jingdong prefecture level city in Shandong
Power Saving King Shop	Green Intelligent Power Saver	Plug and play, no management required, convenient and hassle-free!	Jingdong prefecture level city in Shandong
Power Saving King Shop	Green Intelligent Power Saver	The pictures are too lazy to go up, the power saving effect is still there!	Jingdong prefecture level city in Shandong
Power Saving King Shop	Green Intelligent Power Saver	Fast shipping. Affordable prices. Efficient	Jingdong prefecture level city in Shandong
Power Saving King Shop	Green Intelligent Power Saver	Good power saving, worth buying	Jingdong prefecture level city in Shandong
Power Saving King Shop	Green Intelligent Power Saver	Especially great, comparison shopping before buying, the store owner is very good, answering questions very patiently and detailed, there is a need to come back!	Jingdong prefecture level city in Shandong
Power Saving King Shop	Green Intelligent Power Saver	Very trustworthy brand, quality is guaranteed, the price is still affordable, quite satisfied!	Jingdong prefecture level city in Shandong
Power Saving King Shop	Green Intelligent Power Saver	Recommended by a friend. They say you can save money on air conditioning with this. Try it.	Jingdong prefecture level city in Shandong
Power Saving King Shop	Green Intelligent Power Saver	Works great, will be back next time, very happy with it and a great price!	Jingdong prefecture level city in Shandong
Power Saving King Shop	Green Intelligent Power Saver	The quality of the product is quite good, fast shipping, satisfied.	Jingdong prefecture level city in Shandong
Power Saving King Shop	Green Intelligent Power Saver	Good quality and fast logistics	Jingdong prefecture level city in Shandong
Power Saving King Shop	Green Intelligent Power Saver	It really saves power. I’ve tested it myself.	Jingdong prefecture level city in Shandong
Power Saving King Shop	Green Intelligent Power Saver	Really saves electricity!	Jingdong prefecture level city in Shandong
Power Saving King Shop	Green Intelligent Power Saver	pretty good	Jingdong prefecture level city in Shandong
Power Saving King Shop	Green Intelligent Power Saver	serviceable	Jingdong prefecture level city in Shandong
Power Saving King Shop	Green Intelligent Power Saver	Power Saving King received a tightly packaged logistics, installed the effect is very good price is cheaper than the physical store!	Jingdong prefecture level city in Shandong
Power Saving King Shop	Green Intelligent Power Saver	Super Power Saving King is very good quality, new style, is genuine, the use of the effect is very good, the price is cheap, it is worth buying!	Jingdong prefecture level city in Shandong
Power Saving King Shop	Green Intelligent Power Saver	Packaging is very tight, easy to install, quite practical, worth buying!	Jingdong prefecture level city in Shandong
Power Saving King Shop	Green Intelligent Power Saver	Quality assurance, affordable price, very energy efficient, will repurchase if needed!	Jingdong prefecture level city in Shandong
Power Saving King Shop	Green Intelligent Power Saver	Quality assurance, very good, affordable, logistics is also very fast, I’m very satisfied with the	Jingdong prefecture level city in Shandong
Power Saving King Shop	Green Intelligent Power Saver	Energy saving. It’s so good, already installed, you can save a lot of electricity every month	Jingdong prefecture level city in Shandong
Power Saving King Shop	Green Intelligent Power Saver	The material is thick and the workmanship is fine in place, the power saving effect is very good, value for money!	Jingdong prefecture level city in Shandong
Power Saving King Shop	Green Intelligent Power Saver	The effect is great, genuine, and match the description, the logistics is fast, the price is reasonable 😍	Jingdong prefecture level city in Shandong
Power Saving King Shop	Green Intelligent Power Saver	It’s genuine, works well, easy to use, plugs in and works, really saves power	Jingdong prefecture level city in Shandong
Power Saving King Shop	Green Intelligent Power Saver	It’s easy to use, effective, great service, and recommended!	Jingdong prefecture level city in Shandong
Power Saving King Shop	Green Intelligent Power Saver	🌈 Great quality, bought it to my liking 💗 Awesome! ✨ Buy with confidence if you need it!	Jingdong prefecture level city in Shandong
Power Saving King Shop	Green Intelligent Power Saver	Good quality, easy to install, very energy saving and power saving, good to use	Jingdong prefecture level city in Shandong

Power Saving King Shop	Green Intelligent Power Saver	The quality is very good, it does have the effect of saving electricity, the description matches, the price is affordable, it is worth recommending.	Jingdong prefecture level city in Shandong
Power Saving King Shop	Green Intelligent Power Saver	Received, the functionality is particularly good, it is genuine, the price is affordable	Jingdong prefecture level city in Shandong
Power Saving King Shop	Green Intelligent Power Saver	Good quality, very easy to use, energy efficient, home electricity bill is obviously much less	Jingdong prefecture level city in Shandong
Power Saving King Shop	Green Intelligent Power Saver	Easy to install, energy saving and power saving, very satisfied with a shopping	Jingdong prefecture level city in Shandong
Power Saving King Shop	Green Intelligent Power Saver	Received it and tried it out, it's great, works great and worth having! Five stars praise!	Jingdong prefecture level city in Shandong
Power Saving King Shop	Green Intelligent Power Saver	Power saving device, no problem at all, the quality of this product at this price, very affordable!	Jingdong prefecture level city in Shandong
Power Saving King Shop	Green Intelligent Power Saver	Received the goods, did not expect to be so fast, the quality is very good, especially power saving	Jingdong prefecture level city in Shandong
Power Saving King Shop	Green Intelligent Power Saver	The logistics is very fast, especially good, need to come back again	Jingdong prefecture level city in Shandong
Power Saving King Shop	Green Intelligent Power Saver	It's already working and it really saves power, so I'm very happy with it!	Jingdong prefecture level city in Shandong
Power Saving King Shop	Green Intelligent Power Saver	Great quality, works well, can recommend to friends to buy.	Jingdong prefecture level city in Shandong
Power Saving King Shop	Green Intelligent Power Saver	It's a good deal and worth the money.	Jingdong prefecture level city in Shandong
Power Saving King Shop	Green Intelligent Power Saver	I received it and I'm very happy with it, it's very good quality, guaranteed, and	Jingdong prefecture level city in Shandong
Power Saving King Shop	Green Intelligent Power Saver	Good quality, safe and secure purchase, ideal results and great price.	Jingdong prefecture level city in Shandong
Power Saving King Shop	Green Intelligent Power Saver	Simple operation plugged in the power supply can be, friends recommended looking forward to the effect of the	Jingdong prefecture level city in Shandong
Power Saving King Shop	Green Intelligent Power Saver	It's not bad, it saves electricity, and the material is sturdy and durable.	Jingdong prefecture level city in Shandong
Power Saving King Shop	Green Intelligent Power Saver	Plug and play, no management required, convenient and hassle-free!	Jingdong prefecture level city in Shandong
Power Saving King Shop	Green Intelligent Power Saver	The pictures are too lazy to go up, the power saving effect is still there!	Jingdong prefecture level city in Shandong
Power Saving King Shop	Green Intelligent Power Saver	Fast shipping. Affordable prices. Efficient	Jingdong prefecture level city in Shandong
Power Saving King Shop	Green Intelligent Power Saver	Good power saving, worth buying	Jingdong prefecture level city in Shandong
Power Saving King Shop	Green Intelligent Power Saver	Especially great, comparison shopping before buying, the store owner is very good, answering questions very patiently and detailed, there is a need to come back!	Jingdong prefecture level city in Shandong
Power Saving King Shop	Green Intelligent Power Saver	Very trustworthy brand, quality is guaranteed, the price is still affordable, quite satisfied!	Jingdong prefecture level city in Shandong
Power Saving King Shop	Green Intelligent Power Saver	Recommended by a friend. They say you can save money on air conditioning with this. Try it.	Jingdong prefecture level city in Shandong
Power Saving King Shop	Green Intelligent Power Saver	Works great, will be back next time, very happy with it and a great price!	Jingdong prefecture level city in Shandong
Power Saving King Shop	Green Intelligent Power Saver	The quality of the product is quite good, fast shipping, satisfied.	Jingdong prefecture level city in Shandong
Power Saving King Shop	Green Intelligent Power Saver	Good quality and fast logistics	Jingdong prefecture level city in Shandong
Power Saving King Shop	Green Intelligent Power Saver	It really saves power. I've tested it myself.	Jingdong prefecture level city in Shandong
Power Saving King Shop	Green Intelligent Power Saver	Really saves electricity!	Jingdong prefecture level city in Shandong
Power Saving King Shop	Green Intelligent Power Saver	pretty good	Jingdong prefecture level city in Shandong
Power Saving King Shop	Green Intelligent Power Saver	serviceable	Jingdong prefecture level city in Shandong
Power Saving King Shop	Green Intelligent Power Saver	Power Saving King received a tightly packaged logistics, installed the effect is very good price is cheaper than the physical store!	Jingdong prefecture level city in Shandong
Power Saving King Shop	Green Intelligent Power Saver	Super Power Saving King is very good quality, new style, is genuine, the use of the effect is very good, the price is cheap, it is worth buying!	Jingdong prefecture level city in Shandong
Power Saving King Shop	Green Intelligent Power Saver	Packaging is very tight, easy to install, quite practical, worth buying!	Jingdong prefecture level city in Shandong
Power Saving King Shop	Green Intelligent Power Saver	Quality assurance, affordable price, very energy efficient, will repurchase if needed!	Jingdong prefecture level city in Shandong
Power Saving King Shop	Green Intelligent Power Saver	Quality assurance, very good, affordable, logistics is also very fast, I'm very satisfied with the	Jingdong prefecture level city in Shandong
Power Saving King Shop	Green Intelligent Power Saver	Energy saving. It's so good, already installed, you can save a lot of electricity every month	Jingdong prefecture level city in Shandong
Power Saving King Shop	Green Intelligent Power Saver	The material is thick and the workmanship is fine in place, the power saving effect is very good, value for money!	Jingdong prefecture level city in Shandong
Power Saving King Shop	Green Intelligent Power Saver	The effect is great, genuine, and match the description, the logistics is fast, the price is reasonable 😍	Jingdong prefecture level city in Shandong
Power Saving King Shop	Green Intelligent Power Saver	It's genuine, works well, easy to use, plugs in and works, really saves power	Jingdong prefecture level city in Shandong
Power Saving King Shop	Green Intelligent Power Saver	It's easy to use, effective, great service, and recommended!	Jingdong prefecture level city in Shandong
Power Saving King Shop	Green Intelligent Power Saver	🌈 Great quality, bought it to my liking 💕 Awesome! ✨ Buy with confidence if you need it!	Jingdong prefecture level city in Shandong
Power Saving King Shop	Green Intelligent Power Saver	Good quality, easy to install, very energy saving and power saving, good to use	Jingdong prefecture level city in Shandong
Power Saving King Shop	Green Intelligent Power Saver	The quality is very good, it does have the effect of saving electricity, the description matches, the price is affordable, it is worth recommending.	Jingdong prefecture level city in Shandong
Power Saving King Shop	Green Intelligent Power Saver	Received, the functionality is particularly good, it is genuine, the price is affordable	Jingdong prefecture level city in Shandong
Power Saving King Shop	Green Intelligent Power Saver	Good quality, very easy to use, energy efficient, home electricity bill is obviously much less	Jingdong prefecture level city in Shandong
Power Saving King Shop	Green Intelligent Power Saver	Easy to install, energy saving and power saving, very satisfied with a shopping	Jingdong prefecture level city in Shandong
Power Saving King Shop	Green Intelligent Power Saver	Received it and tried it out, it's great, works great and worth having! Five stars praise!	Jingdong prefecture level city in Shandong
Power Saving King Shop	Green Intelligent Power Saver	Power saving device, no problem at all, the quality of this product at this price, very affordable!	Jingdong prefecture level city in Shandong
Power Saving King Shop	Green Intelligent Power Saver	Received the goods, did not expect to be so fast, the quality is very good, especially power saving	Jingdong prefecture level city in Shandong
Power Saving King Shop	Green Intelligent Power Saver	The logistics is very fast, especially good, need to come back again	Jingdong prefecture level city in Shandong
Power Saving King Shop	Green Intelligent Power Saver	It's already working and it really saves power, so I'm very happy with it!	Jingdong prefecture level city in Shandong
Power Saving King Shop	Green Intelligent Power Saver	Great quality, works well, can recommend to friends to buy.	Jingdong prefecture level city in Shandong

Power Saving King Shop	Green Intelligent Power Saver	It's a good deal and worth the money.	Jingdong prefecture level city in Shandong
Power Saving King Shop	Green Intelligent Power Saver	I received it and I'm very happy with it, it's very good quality, guaranteed, and	Jingdong prefecture level city in Shandong
Power Saving King Shop	Green Intelligent Power Saver	Good quality, safe and secure purchase, ideal results and great price.	Jingdong prefecture level city in Shandong
Power Saving King Shop	Green Intelligent Power Saver	Simple operation plugged in the power supply can be, friends recommended looking forward to the effect of the	Jingdong prefecture level city in Shandong
Power Saving King Shop	Green Intelligent Power Saver	It's not bad, it saves electricity, and the material is sturdy and durable.	Jingdong prefecture level city in Shandong
Power Saving King Shop	Green Intelligent Power Saver	Plug and play, no management required, convenient and hassle-free!	Jingdong prefecture level city in Shandong
Power Saving King Shop	Green Intelligent Power Saver	The pictures are too lazy to go up, the power saving effect is still there!	Jingdong prefecture level city in Shandong
Power Saving King Shop	Green Intelligent Power Saver	Fast shipping. Affordable prices. Efficient	Jingdong prefecture level city in Shandong
Power Saving King Shop	Green Intelligent Power Saver	Good power saving, worth buying	Jingdong prefecture level city in Shandong
Power Saving King Shop	Green Intelligent Power Saver	Especially great, comparison shopping before buying, the store owner is very good, answering questions very patiently and detailed, there is a need to come back!	Jingdong prefecture level city in Shandong
Power Saving King Shop	Green Intelligent Power Saver	Very trustworthy brand, quality is guaranteed, the price is still affordable, quite satisfied!	Jingdong prefecture level city in Shandong
Power Saving King Shop	Green Intelligent Power Saver	Recommended by a friend. They say you can save money on air conditioning with this. Try it.	Jingdong prefecture level city in Shandong
Power Saving King Shop	Green Intelligent Power Saver	Works great, will be back next time, very happy with it and a great price!	Jingdong prefecture level city in Shandong
Power Saving King Shop	Green Intelligent Power Saver	The quality of the product is quite good, fast shipping, satisfied.	Jingdong prefecture level city in Shandong
Power Saving King Shop	Green Intelligent Power Saver	Good quality and fast logistics	Jingdong prefecture level city in Shandong
Power Saving King Shop	Green Intelligent Power Saver	It really saves power. I've tested it myself.	Jingdong prefecture level city in Shandong
Power Saving King Shop	Green Intelligent Power Saver	Really saves electricity!	Jingdong prefecture level city in Shandong
Power Saving King Shop	Green Intelligent Power Saver	pretty good	Jingdong prefecture level city in Shandong
Power Saving King Shop	Green Intelligent Power Saver	serviceable	Jingdong prefecture level city in Shandong
Power Saving King Shop	Green Intelligent Power Saver	Power Saving King received a tightly packaged logistics, installed the effect is very good price is cheaper than the physical store!	Jingdong prefecture level city in Shandong
Power Saving King Shop	Green Intelligent Power Saver	Super Power Saving King is very good quality, new style, is genuine, the use of the effect is very good, the price is cheap, it is worth buying!	Jingdong prefecture level city in Shandong
Power Saving King Shop	Green Intelligent Power Saver	Packaging is very tight, easy to install, quite practical, worth buying!	Jingdong prefecture level city in Shandong
Power Saving King Shop	Green Intelligent Power Saver	Quality assurance, affordable price, very energy efficient, will repurchase if needed!	Jingdong prefecture level city in Shandong
Power Saving King Shop	Green Intelligent Power Saver	Quality assurance, very good, affordable, logistics is also very fast, I'm very satisfied with the	Jingdong prefecture level city in Shandong
Power Saving King Shop	Green Intelligent Power Saver	Energy saving. It's so good, already installed, you can save a lot of electricity every month	Jingdong prefecture level city in Shandong
Power Saving King Shop	Green Intelligent Power Saver	The material is thick and the workmanship is fine in place, the power saving effect is very good, value for money!	Jingdong prefecture level city in Shandong
Power Saving King Shop	Green Intelligent Power Saver	The effect is great, genuine, and match the description, the logistics is fast, the price is reasonable 😍	Jingdong prefecture level city in Shandong
Power Saving King Shop	Green Intelligent Power Saver	It's genuine, works well, easy to use, plugs in and works, really saves power	Jingdong prefecture level city in Shandong
Power Saving King Shop	Green Intelligent Power Saver	It's easy to use, effective, great service, and recommended!	Jingdong prefecture level city in Shandong
Power Saving King Shop	Green Intelligent Power Saver	🌈 Great quality, bought it to my liking 💝 Awesome! ✨ Buy with confidence if you need it!	Jingdong prefecture level city in Shandong
Power Saving King Shop	Green Intelligent Power Saver	Good quality, easy to install, very energy saving and power saving, good to use	Jingdong prefecture level city in Shandong
Power Saving King Shop	Green Intelligent Power Saver	The quality is very good, it does have the effect of saving electricity, the description matches, the price is affordable, it is worth recommending.	Jingdong prefecture level city in Shandong
Power Saving King Shop	Green Intelligent Power Saver	Received, the functionality is particularly good, it is genuine, the price is affordable	Jingdong prefecture level city in Shandong
Power Saving King Shop	Green Intelligent Power Saver	Good quality, very easy to use, energy efficient, home electricity bill is obviously much less	Jingdong prefecture level city in Shandong
Power Saving King Shop	Green Intelligent Power Saver	Easy to install, energy saving and power saving, very satisfied with a shopping	Jingdong prefecture level city in Shandong
Power Saving King Shop	Green Intelligent Power Saver	Received it and tried it out, it's great, works great and worth having! Five stars praise!	Jingdong prefecture level city in Shandong
Power Saving King Shop	Green Intelligent Power Saver	Power saving device, no problem at all, the quality of this product at this price, very affordable!	Jingdong prefecture level city in Shandong
Power Saving King Shop	Green Intelligent Power Saver	Received the goods, did not expect to be so fast, the quality is very good, especially power saving	Jingdong prefecture level city in Shandong
Power Saving King Shop	Green Intelligent Power Saver	The logistics is very fast, especially good, need to come back again	Jingdong prefecture level city in Shandong
Power Saving King Shop	Green Intelligent Power Saver	It's already working and it really saves power, so I'm very happy with it!	Jingdong prefecture level city in Shandong
Power Saving King Shop	Green Intelligent Power Saver	Great quality, works well, can recommend to friends to buy.	Jingdong prefecture level city in Shandong
Power Saving King Shop	Green Intelligent Power Saver	It's a good deal and worth the money.	Jingdong prefecture level city in Shandong
Power Saving King Shop	Green Intelligent Power Saver	I received it and I'm very happy with it, it's very good quality, guaranteed, and	Jingdong prefecture level city in Shandong
Power Saving King Shop	Green Intelligent Power Saver	Good quality, safe and secure purchase, ideal results and great price.	Jingdong prefecture level city in Shandong
Power Saving King Shop	Green Intelligent Power Saver	Simple operation plugged in the power supply can be, friends recommended looking forward to the effect of the	Jingdong prefecture level city in Shandong
Power Saving King Shop	Green Intelligent Power Saver	It's not bad, it saves electricity, and the material is sturdy and durable.	Jingdong prefecture level city in Shandong
Power Saving King Shop	Green Intelligent Power Saver	Plug and play, no management required, convenient and hassle-free!	Jingdong prefecture level city in Shandong
Power Saving King Shop	Green Intelligent Power Saver	The pictures are too lazy to go up, the power saving effect is still there!	Jingdong prefecture level city in Shandong
Power Saving King Shop	Green Intelligent Power Saver	Fast shipping. Affordable prices. Efficient	Jingdong prefecture level city in Shandong
Power Saving King Shop	Green Intelligent Power Saver	Good power saving, worth buying	Jingdong prefecture level city in Shandong
Power Saving King Shop	Green Intelligent Power Saver	Especially great, comparison shopping before buying, the store owner is very good, answering questions very patiently and detailed, there is a need to come back!	Jingdong prefecture level city in Shandong

Power Saving King Shop	Green Intelligent Power Saver	Very trustworthy brand, quality is guaranteed, the price is still affordable, quite satisfied!	Jingdong prefecture level city in Shandong
Power Saving King Shop	Green Intelligent Power Saver	Recommended by a friend. They say you can save money on air conditioning with this. Try it.	Jingdong prefecture level city in Shandong
Power Saving King Shop	Green Intelligent Power Saver	Works great, will be back next time, very happy with it and a great price!	Jingdong prefecture level city in Shandong
Power Saving King Shop	Green Intelligent Power Saver	The quality of the product is quite good, fast shipping, satisfied.	Jingdong prefecture level city in Shandong
Power Saving King Shop	Green Intelligent Power Saver	Good quality and fast logistics	Jingdong prefecture level city in Shandong
Power Saving King Shop	Green Intelligent Power Saver	It really saves power. I've tested it myself.	Jingdong prefecture level city in Shandong
Power Saving King Shop	Green Intelligent Power Saver	Really saves electricity!	Jingdong prefecture level city in Shandong
Power Saving King Shop	Green Intelligent Power Saver	pretty good	Jingdong prefecture level city in Shandong
Power Saving King Shop	Green Intelligent Power Saver	serviceable	Jingdong prefecture level city in Shandong
Power Saving King Shop	Green Intelligent Power Saver	Power Saving King received a tightly packaged logistics, installed the effect is very good price is cheaper than the physical store!	Jingdong prefecture level city in Shandong
Power Saving King Shop	Green Intelligent Power Saver	Super Power Saving King is very good quality, new style, is genuine, the use of the effect is very good, the price is cheap, it is worth buying!	Jingdong prefecture level city in Shandong
Power Saving King Shop	Green Intelligent Power Saver	Packaging is very tight, easy to install, quite practical, worth buying!	Jingdong prefecture level city in Shandong
Power Saving King Shop	Green Intelligent Power Saver	Quality assurance, affordable price, very energy efficient, will repurchase if needed!	Jingdong prefecture level city in Shandong
Power Saving King Shop	Green Intelligent Power Saver	Quality assurance, very good, affordable, logistics is also very fast, I'm very satisfied with the	Jingdong prefecture level city in Shandong
Power Saving King Shop	Green Intelligent Power Saver	Energy saving. It's so good, already installed, you can save a lot of electricity every month	Jingdong prefecture level city in Shandong
Power Saving King Shop	Green Intelligent Power Saver	The material is thick and the workmanship is fine in place, the power saving effect is very good, value for money!	Jingdong prefecture level city in Shandong
Power Saving King Shop	Green Intelligent Power Saver	The effect is great, genuine, and match the description, the logistics is fast, the price is reasonable 😍	Jingdong prefecture level city in Shandong
Power Saving King Shop	Green Intelligent Power Saver	It's genuine, works well, easy to use, plugs in and works, really saves power	Jingdong prefecture level city in Shandong
Power Saving King Shop	Green Intelligent Power Saver	It's easy to use, effective, great service, and recommended!	Jingdong prefecture level city in Shandong
Power Saving King Shop	Green Intelligent Power Saver	🌈 Great quality, bought it to my liking 💝 Awesome! ✨ Buy with confidence if you need it!	Jingdong prefecture level city in Shandong
Power Saving King Shop	Green Intelligent Power Saver	Good quality, easy to install, very energy saving and power saving, good to use	Jingdong prefecture level city in Shandong
Power Saving King Shop	Green Intelligent Power Saver	The quality is very good, it does have the effect of saving electricity, the description matches, the price is affordable, it is worth recommending.	Jingdong prefecture level city in Shandong
Power Saving King Shop	Green Intelligent Power Saver	Received, the functionality is particularly good, it is genuine, the price is affordable	Jingdong prefecture level city in Shandong
Power Saving King Shop	Green Intelligent Power Saver	Good quality, very easy to use, energy efficient, home electricity bill is obviously much less	Jingdong prefecture level city in Shandong
Power Saving King Shop	Green Intelligent Power Saver	Easy to install, energy saving and power saving, very satisfied with a shopping	Jingdong prefecture level city in Shandong
Power Saving King Shop	Green Intelligent Power Saver	Received it and tried it out, it's great, works great and worth having! Five stars praise!	Jingdong prefecture level city in Shandong
Power Saving King Shop	Green Intelligent Power Saver	Power saving device, no problem at all, the quality of this product at this price, very affordable!	Jingdong prefecture level city in Shandong
Power Saving King Shop	Green Intelligent Power Saver	Received the goods, did not expect to be so fast, the quality is very good, especially power saving	Jingdong prefecture level city in Shandong
Power Saving King Shop	Green Intelligent Power Saver	The logistics is very fast, especially good, need to come back again	Jingdong prefecture level city in Shandong
Power Saving King Shop	Green Intelligent Power Saver	It's already working and it really saves power, so I'm very happy with it!	Jingdong prefecture level city in Shandong
Power Saving King Shop	Green Intelligent Power Saver	Great quality, works well, can recommend to friends to buy.	Jingdong prefecture level city in Shandong
Power Saving King Shop	Green Intelligent Power Saver	It's a good deal and worth the money.	Jingdong prefecture level city in Shandong
Power Saving King Shop	Green Intelligent Power Saver	I received it and I'm very happy with it, it's very good quality, guaranteed, and	Jingdong prefecture level city in Shandong
Power Saving King Shop	Green Intelligent Power Saver	Good quality, safe and secure purchase, ideal results and great price.	Jingdong prefecture level city in Shandong
Power Saving King Shop	Green Intelligent Power Saver	Simple operation plugged in the power supply can be, friends recommended looking forward to the effect of the	Jingdong prefecture level city in Shandong
Power Saving King Shop	Green Intelligent Power Saver	It's not bad, it saves electricity, and the material is sturdy and durable.	Jingdong prefecture level city in Shandong
Power Saving King Shop	Green Intelligent Power Saver	Plug and play, no management required, convenient and hassle-free!	Jingdong prefecture level city in Shandong
Power Saving King Shop	Green Intelligent Power Saver	The pictures are too lazy to go up, the power saving effect is still there!	Jingdong prefecture level city in Shandong
Power Saving King Shop	Green Intelligent Power Saver	Fast shipping. Affordable prices. Efficient	Jingdong prefecture level city in Shandong
Power Saving King Shop	Green Intelligent Power Saver	Good power saving, worth buying	Jingdong prefecture level city in Shandong
Power Saving King Shop	Green Intelligent Power Saver	Especially great, comparison shopping before buying, the store owner is very good, answering questions very patiently and detailed, there is a need to come back!	Jingdong prefecture level city in Shandong
Power Saving King Shop	Green Intelligent Power Saver	Very trustworthy brand, quality is guaranteed, the price is still affordable, quite satisfied!	Jingdong prefecture level city in Shandong
Power Saving King Shop	Green Intelligent Power Saver	Recommended by a friend. They say you can save money on air conditioning with this. Try it.	Jingdong prefecture level city in Shandong
Power Saving King Shop	Green Intelligent Power Saver	Works great, will be back next time, very happy with it and a great price!	Jingdong prefecture level city in Shandong
Power Saving King Shop	Green Intelligent Power Saver	The quality of the product is quite good, fast shipping, satisfied.	Jingdong prefecture level city in Shandong
Power Saving King Shop	Green Intelligent Power Saver	Good quality and fast logistics	Jingdong prefecture level city in Shandong
Power Saving King Shop	Green Intelligent Power Saver	It really saves power. I've tested it myself.	Jingdong prefecture level city in Shandong
Power Saving King Shop	Green Intelligent Power Saver	Really saves electricity!	Jingdong prefecture level city in Shandong
Power Saving King Shop	Green Intelligent Power Saver	pretty good	Jingdong prefecture level city in Shandong
Power Saving King Shop	Green Intelligent Power Saver	serviceable	Jingdong prefecture level city in Shandong
Power Saving King Shop	Green Intelligent Power Saver	Power Saving King received a tightly packaged logistics, installed the effect is very good price is cheaper than the physical store!	Jingdong prefecture level city in Shandong

Power Saving King Shop	Green Intelligent Power Saver	Super Power Saving King is very good quality, new style, is genuine, the use of the effect is very good, the price is cheap, it is worth buying!	Jingdong prefecture level city in Shandong
Power Saving King Shop	Green Intelligent Power Saver	Packaging is very tight, easy to install, quite practical, worth buying!	Jingdong prefecture level city in Shandong
Power Saving King Shop	Green Intelligent Power Saver	Quality assurance, affordable price, very energy efficient, will repurchase if needed!	Jingdong prefecture level city in Shandong
Power Saving King Shop	Green Intelligent Power Saver	Quality assurance, very good, affordable, logistics is also very fast, I'm very satisfied with the	Jingdong prefecture level city in Shandong
Power Saving King Shop	Green Intelligent Power Saver	Energy saving. It's so good, already installed, you can save a lot of electricity every month	Jingdong prefecture level city in Shandong
Power Saving King Shop	Green Intelligent Power Saver	The material is thick and the workmanship is fine in place, the power saving effect is very good, value for money!	Jingdong prefecture level city in Shandong
Power Saving King Shop	Green Intelligent Power Saver	The effect is great, genuine, and match the description, the logistics is fast, the price is reasonable 😍	Jingdong prefecture level city in Shandong
Power Saving King Shop	Green Intelligent Power Saver	It's genuine, works well, easy to use, plugs in and works, really saves power	Jingdong prefecture level city in Shandong
Power Saving King Shop	Green Intelligent Power Saver	It's easy to use, effective, great service, and recommended!	Jingdong prefecture level city in Shandong
Power Saving King Shop	Green Intelligent Power Saver	🌈 Great quality, bought it to my liking 💗 Awesome! ✨ Buy with confidence if you need it!	Jingdong prefecture level city in Shandong
Power Saving King Shop	Green Intelligent Power Saver	Good quality, easy to install, very energy saving and power saving, good to use	Jingdong prefecture level city in Shandong
Power Saving King Shop	Green Intelligent Power Saver	The quality is very good, it does have the effect of saving electricity, the description matches, the price is affordable, it is worth recommending.	Jingdong prefecture level city in Shandong
Power Saving King Shop	Green Intelligent Power Saver	Received, the functionality is particularly good, it is genuine, the price is affordable	Jingdong prefecture level city in Shandong
Power Saving King Shop	Green Intelligent Power Saver	Good quality, very easy to use, energy efficient, home electricity bill is obviously much less	Jingdong prefecture level city in Shandong
Power Saving King Shop	Green Intelligent Power Saver	Easy to install, energy saving and power saving, very satisfied with a shopping	Jingdong prefecture level city in Shandong
Power Saving King Shop	Green Intelligent Power Saver	Received it and tried it out, it's great, works great and worth having! Five stars praise!	Jingdong prefecture level city in Shandong
Power Saving King Shop	Green Intelligent Power Saver	Power saving device, no problem at all, the quality of this product at this price, very affordable!	Jingdong prefecture level city in Shandong
Power Saving King Shop	Green Intelligent Power Saver	Received the goods, did not expect to be so fast, the quality is very good, especially power saving	Jingdong prefecture level city in Shandong
Power Saving King Shop	Green Intelligent Power Saver	The logistics is very fast, especially good, need to come back again	Jingdong prefecture level city in Shandong
Power Saving King Shop	Green Intelligent Power Saver	It's already working and it really saves power, so I'm very happy with it!	Jingdong prefecture level city in Shandong
Power Saving King Shop	Green Intelligent Power Saver	Great quality, works well, can recommend to friends to buy.	Jingdong prefecture level city in Shandong
Power Saving King Shop	Green Intelligent Power Saver	It's a good deal and worth the money.	Jingdong prefecture level city in Shandong
Power Saving King Shop	Green Intelligent Power Saver	I received it and I'm very happy with it, it's very good quality, guaranteed, and	Jingdong prefecture level city in Shandong
Power Saving King Shop	Green Intelligent Power Saver	Good quality, safe and secure purchase, ideal results and great price.	Jingdong prefecture level city in Shandong
Power Saving King Shop	Green Intelligent Power Saver	Simple operation plugged in the power supply can be, friends recommended looking forward to the effect of the	Jingdong prefecture level city in Shandong
Power Saving King Shop	Green Intelligent Power Saver	It's not bad, it saves electricity, and the material is sturdy and durable.	Jingdong prefecture level city in Shandong
Power Saving King Shop	Green Intelligent Power Saver	Plug and play, no management required, convenient and hassle-free!	Jingdong prefecture level city in Shandong
Power Saving King Shop	Green Intelligent Power Saver	The pictures are too lazy to go up, the power saving effect is still there!	Jingdong prefecture level city in Shandong
Power Saving King Shop	Green Intelligent Power Saver	Fast shipping. Affordable prices. Efficient	Jingdong prefecture level city in Shandong
Power Saving King Shop	Green Intelligent Power Saver	Good power saving, worth buying	Jingdong prefecture level city in Shandong
Power Saving King Shop	Green Intelligent Power Saver	Especially great, comparison shopping before buying, the store owner is very good, answering questions very patiently and detailed, there is a need to come back!	Jingdong prefecture level city in Shandong
Power Saving King Shop	Green Intelligent Power Saver	Very trustworthy brand, quality is guaranteed, the price is still affordable, quite satisfied!	Jingdong prefecture level city in Shandong
Power Saving King Shop	Green Intelligent Power Saver	Recommended by a friend. They say you can save money on air conditioning with this. Try it.	Jingdong prefecture level city in Shandong
Power Saving King Shop	Green Intelligent Power Saver	Works great, will be back next time, very happy with it and a great price!	Jingdong prefecture level city in Shandong
Power Saving King Shop	Green Intelligent Power Saver	The quality of the product is quite good, fast shipping, satisfied.	Jingdong prefecture level city in Shandong
Power Saving King Shop	Green Intelligent Power Saver	Good quality and fast logistics	Jingdong prefecture level city in Shandong
Power Saving King Shop	Green Intelligent Power Saver	It really saves power. I've tested it myself.	Jingdong prefecture level city in Shandong
Power Saving King Shop	Green Intelligent Power Saver	Really saves electricity!	Jingdong prefecture level city in Shandong
Power Saving King Shop	Green Intelligent Power Saver	pretty good	Jingdong prefecture level city in Shandong
Power Saving King Shop	Green Intelligent Power Saver	serviceable	Jingdong prefecture level city in Shandong
Power Saving King Shop	Green Intelligent Power Saver	Power Saving King received a tightly packaged logistics, installed the effect is very good price is cheaper than the physical store!	Jingdong prefecture level city in Shandong
Power Saving King Shop	Green Intelligent Power Saver	Super Power Saving King is very good quality, new style, is genuine, the use of the effect is very good, the price is cheap, it is worth buying!	Jingdong prefecture level city in Shandong
Power Saving King Shop	Green Intelligent Power Saver	Packaging is very tight, easy to install, quite practical, worth buying!	Jingdong prefecture level city in Shandong
Power Saving King Shop	Green Intelligent Power Saver	Quality assurance, affordable price, very energy efficient, will repurchase if needed!	Jingdong prefecture level city in Shandong
Power Saving King Shop	Green Intelligent Power Saver	Quality assurance, very good, affordable, logistics is also very fast, I'm very satisfied with the	Jingdong prefecture level city in Shandong
Power Saving King Shop	Green Intelligent Power Saver	Energy saving. It's so good, already installed, you can save a lot of electricity every month	Jingdong prefecture level city in Shandong
Power Saving King Shop	Green Intelligent Power Saver	The material is thick and the workmanship is fine in place, the power saving effect is very good, value for money!	Jingdong prefecture level city in Shandong
Power Saving King Shop	Green Intelligent Power Saver	The effect is great, genuine, and match the description, the logistics is fast, the price is reasonable 😍	Jingdong prefecture level city in Shandong
Power Saving King Shop	Green Intelligent Power Saver	It's genuine, works well, easy to use, plugs in and works, really saves power	Jingdong prefecture level city in Shandong
Power Saving King Shop	Green Intelligent Power Saver	It's easy to use, effective, great service, and recommended!	Jingdong prefecture level city in Shandong
Power Saving King Shop	Green Intelligent Power Saver	🌈 Great quality, bought it to my liking 💗 Awesome! ✨ Buy with confidence if you need it!	Jingdong prefecture level city in Shandong

Power Saving King Shop	Green Intelligent Power Saver	Good quality, easy to install, very energy saving and power saving, good to use	Jingdong prefecture level city in Shandong
Power Saving King Shop	Green Intelligent Power Saver	The quality is very good, it does have the effect of saving electricity, the description matches, the price is affordable, it is worth recommending.	Jingdong prefecture level city in Shandong
Power Saving King Shop	Green Intelligent Power Saver	Received, the functionality is particularly good, it is genuine, the price is affordable	Jingdong prefecture level city in Shandong
Power Saving King Shop	Green Intelligent Power Saver	Good quality, very easy to use, energy efficient, home electricity bill is obviously much less	Jingdong prefecture level city in Shandong
Power Saving King Shop	Green Intelligent Power Saver	Easy to install, energy saving and power saving, very satisfied with a shopping	Jingdong prefecture level city in Shandong
Power Saving King Shop	Green Intelligent Power Saver	Received it and tried it out, it's great, works great and worth having! Five stars praise!	Jingdong prefecture level city in Shandong
Power Saving King Shop	Green Intelligent Power Saver	Power saving device, no problem at all, the quality of this product at this price, very affordable!	Jingdong prefecture level city in Shandong
Power Saving King Shop	Green Intelligent Power Saver	Received the goods, did not expect to be so fast, the quality is very good, especially power saving	Jingdong prefecture level city in Shandong
Power Saving King Shop	Green Intelligent Power Saver	The logistics is very fast, especially good, need to come back again	Jingdong prefecture level city in Shandong
Power Saving King Shop	Green Intelligent Power Saver	It's already working and it really saves power, so I'm very happy with it!	Jingdong prefecture level city in Shandong
Power Saving King Shop	Green Intelligent Power Saver	Great quality, works well, can recommend to friends to buy.	Jingdong prefecture level city in Shandong
Power Saving King Shop	Green Intelligent Power Saver	It's a good deal and worth the money.	Jingdong prefecture level city in Shandong
Power Saving King Shop	Green Intelligent Power Saver	I received it and I'm very happy with it, it's very good quality, guaranteed, and	Jingdong prefecture level city in Shandong
Power Saving King Shop	Green Intelligent Power Saver	Good quality, safe and secure purchase, ideal results and great price.	Jingdong prefecture level city in Shandong
Power Saving King Shop	Green Intelligent Power Saver	Simple operation plugged in the power supply can be, friends recommended looking forward to the effect of the	Jingdong prefecture level city in Shandong
Power Saving King Shop	Green Intelligent Power Saver	It's not bad, it saves electricity, and the material is sturdy and durable.	Jingdong prefecture level city in Shandong
Power Saving King Shop	Green Intelligent Power Saver	Plug and play, no management required, convenient and hassle-free!	Jingdong prefecture level city in Shandong
Power Saving King Shop	Green Intelligent Power Saver	The pictures are too lazy to go up, the power saving effect is still there!	Jingdong prefecture level city in Shandong
Power Saving King Shop	Green Intelligent Power Saver	Fast shipping. Affordable prices. Efficient	Jingdong prefecture level city in Shandong
Power Saving King Shop	Green Intelligent Power Saver	Good power saving, worth buying	Jingdong prefecture level city in Shandong
Power Saving King Shop	Green Intelligent Power Saver	Especially great, comparison shopping before buying, the store owner is very good, answering questions very patiently and detailed, there is a need to come back!	Jingdong prefecture level city in Shandong
Power Saving King Shop	Green Intelligent Power Saver	Very trustworthy brand, quality is guaranteed, the price is still affordable, quite satisfied!	Jingdong prefecture level city in Shandong
Power Saving King Shop	Green Intelligent Power Saver	Recommended by a friend. They say you can save money on air conditioning with this. Try it.	Jingdong prefecture level city in Shandong
Power Saving King Shop	Green Intelligent Power Saver	Works great, will be back next time, very happy with it and a great price!	Jingdong prefecture level city in Shandong
Power Saving King Shop	Green Intelligent Power Saver	The quality of the product is quite good, fast shipping, satisfied.	Jingdong prefecture level city in Shandong
Power Saving King Shop	Green Intelligent Power Saver	Good quality and fast logistics	Jingdong prefecture level city in Shandong
Power Saving King Shop	Green Intelligent Power Saver	It really saves power. I've tested it myself.	Jingdong prefecture level city in Shandong
Power Saving King Shop	Green Intelligent Power Saver	Really saves electricity!	Jingdong prefecture level city in Shandong
Power Saving King Shop	Green Intelligent Power Saver	pretty good	Jingdong prefecture level city in Shandong
Power Saving King Shop	Green Intelligent Power Saver	serviceable	Jingdong prefecture level city in Shandong
Power Saving King Shop	Green Intelligent Power Saver	Power Saving King received a tightly packaged logistics, installed the effect is very good price is cheaper than the physical store!	Jingdong prefecture level city in Shandong
Power Saving King Shop	Green Intelligent Power Saver	Super Power Saving King is very good quality, new style, is genuine, the use of the effect is very good, the price is cheap, it is worth buying!	Jingdong prefecture level city in Shandong
Power Saving King Shop	Green Intelligent Power Saver	Packaging is very tight, easy to install, quite practical, worth buying!	Jingdong prefecture level city in Shandong
Power Saving King Shop	Green Intelligent Power Saver	Quality assurance, affordable price, very energy efficient, will repurchase if needed!	Jingdong prefecture level city in Shandong
Power Saving King Shop	Green Intelligent Power Saver	Quality assurance, very good, affordable, logistics is also very fast, I'm very satisfied with the	Jingdong prefecture level city in Shandong
Power Saving King Shop	Green Intelligent Power Saver	Energy saving. It's so good, already installed, you can save a lot of electricity every month	Jingdong prefecture level city in Shandong
Power Saving King Shop	Green Intelligent Power Saver	The material is thick and the workmanship is fine in place, the power saving effect is very good, value for money!	Jingdong prefecture level city in Shandong
Power Saving King Shop	Green Intelligent Power Saver	The effect is great, genuine, and match the description, the logistics is fast, the price is reasonable 😍	Jingdong prefecture level city in Shandong
Power Saving King Shop	Green Intelligent Power Saver	It's genuine, works well, easy to use, plugs in and works, really saves power	Jingdong prefecture level city in Shandong
Power Saving King Shop	Green Intelligent Power Saver	It's easy to use, effective, great service, and recommended!	Jingdong prefecture level city in Shandong
Power Saving King Shop	Green Intelligent Power Saver	🌈 Great quality, bought it to my liking 💝 Awesome! ✨ Buy with confidence if you need it!	Jingdong prefecture level city in Shandong
Power Saving King Shop	Green Intelligent Power Saver	Good quality, easy to install, very energy saving and power saving, good to use	Jingdong prefecture level city in Shandong
Power Saving King Shop	Green Intelligent Power Saver	The quality is very good, it does have the effect of saving electricity, the description matches, the price is affordable, it is worth recommending.	Jingdong prefecture level city in Shandong
Power Saving King Shop	Green Intelligent Power Saver	Received, the functionality is particularly good, it is genuine, the price is affordable	Jingdong prefecture level city in Shandong
Power Saving King Shop	Green Intelligent Power Saver	Good quality, very easy to use, energy efficient, home electricity bill is obviously much less	Jingdong prefecture level city in Shandong
Power Saving King Shop	Green Intelligent Power Saver	Easy to install, energy saving and power saving, very satisfied with a shopping	Jingdong prefecture level city in Shandong
Power Saving King Shop	Green Intelligent Power Saver	Received it and tried it out, it's great, works great and worth having! Five stars praise!	Jingdong prefecture level city in Shandong
Power Saving King Shop	Green Intelligent Power Saver	Power saving device, no problem at all, the quality of this product at this price, very affordable!	Jingdong prefecture level city in Shandong
Power Saving King Shop	Green Intelligent Power Saver	Received the goods, did not expect to be so fast, the quality is very good, especially power saving	Jingdong prefecture level city in Shandong
Power Saving King Shop	Green Intelligent Power Saver	The logistics is very fast, especially good, need to come back again	Jingdong prefecture level city in Shandong
Power Saving King Shop	Green Intelligent Power Saver	It's already working and it really saves power, so I'm very happy with it!	Jingdong prefecture level city in Shandong

Power Saving King Shop	Green Intelligent Power Saver	Great quality, works well, can recommend to friends to buy.	Jingdong prefecture level city in Shandong
Power Saving King Shop	Green Intelligent Power Saver	It’s a good deal and worth the money.	Jingdong prefecture level city in Shandong
Power Saving King Shop	Green Intelligent Power Saver	I received it and I’m very happy with it, it’s very good quality, guaranteed, and	Jingdong prefecture level city in Shandong
Power Saving King Shop	Green Intelligent Power Saver	Good quality, safe and secure purchase, ideal results and great price.	Jingdong prefecture level city in Shandong
Power Saving King Shop	Green Intelligent Power Saver	Simple operation plugged in the power supply can be, friends recommended looking forward to the effect of the	Jingdong prefecture level city in Shandong
Power Saving King Shop	Green Intelligent Power Saver	It’s not bad, it saves electricity, and the material is sturdy and durable.	Jingdong prefecture level city in Shandong
Power Saving King Shop	Green Intelligent Power Saver	Plug and play, no management required, convenient and hassle-free!	Jingdong prefecture level city in Shandong
Power Saving King Shop	Green Intelligent Power Saver	The pictures are too lazy to go up, the power saving effect is still there!	Jingdong prefecture level city in Shandong
Power Saving King Shop	Green Intelligent Power Saver	Fast shipping. Affordable prices. Efficient	Jingdong prefecture level city in Shandong
Power Saving King Shop	Green Intelligent Power Saver	Good power saving, worth buying	Jingdong prefecture level city in Shandong
Power Saving King Shop	Green Intelligent Power Saver	Especially great, comparison shopping before buying, the store owner is very good, answering questions very patiently and detailed, there is a need to come back!	Jingdong prefecture level city in Shandong
Power Saving King Shop	Green Intelligent Power Saver	Very trustworthy brand, quality is guaranteed, the price is still affordable, quite satisfied!	Jingdong prefecture level city in Shandong
Power Saving King Shop	Green Intelligent Power Saver	Recommended by a friend. They say you can save money on air conditioning with this. Try it.	Jingdong prefecture level city in Shandong
Power Saving King Shop	Green Intelligent Power Saver	Works great, will be back next time, very happy with it and a great price!	Jingdong prefecture level city in Shandong
Power Saving King Shop	Green Intelligent Power Saver	The quality of the product is quite good, fast shipping, satisfied.	Jingdong prefecture level city in Shandong
Power Saving King Shop	Green Intelligent Power Saver	Good quality and fast logistics	Jingdong prefecture level city in Shandong
Power Saving King Shop	Green Intelligent Power Saver	It really saves power. I’ve tested it myself.	Jingdong prefecture level city in Shandong
Power Saving King Shop	Green Intelligent Power Saver	Really saves electricity!	Jingdong prefecture level city in Shandong
Power Saving King Shop	Green Intelligent Power Saver	pretty good	Jingdong prefecture level city in Shandong
Power Saving King Shop	Green Intelligent Power Saver	serviceable	Jingdong prefecture level city in Shandong
Power Saving King Shop	Green Intelligent Power Saver	Power Saving King received a tightly packaged logistics, installed the effect is very good price is cheaper than the physical store!	Jingdong prefecture level city in Shandong
Power Saving King Shop	Green Intelligent Power Saver	Super Power Saving King is very good quality, new style, is genuine, the use of the effect is very good, the price is cheap, it is worth buying!	Jingdong prefecture level city in Shandong
Power Saving King Shop	Green Intelligent Power Saver	Packaging is very tight, easy to install, quite practical, worth buying!	Jingdong prefecture level city in Shandong
Power Saving King Shop	Green Intelligent Power Saver	Quality assurance, affordable price, very energy efficient, will repurchase if needed!	Jingdong prefecture level city in Shandong
Power Saving King Shop	Green Intelligent Power Saver	Quality assurance, very good, affordable, logistics is also very fast, I’m very satisfied with the	Jingdong prefecture level city in Shandong
Power Saving King Shop	Green Intelligent Power Saver	Energy saving. It’s so good, already installed, you can save a lot of electricity every month	Jingdong prefecture level city in Shandong
Power Saving King Shop	Green Intelligent Power Saver	The material is thick and the workmanship is fine in place, the power saving effect is very good, value for money!	Jingdong prefecture level city in Shandong
Power Saving King Shop	Green Intelligent Power Saver	The effect is great, genuine, and match the description, the logistics is fast, the price is reasonable 😍	Jingdong prefecture level city in Shandong
Power Saving King Shop	Green Intelligent Power Saver	It’s genuine, works well, easy to use, plugs in and works, really saves power	Jingdong prefecture level city in Shandong
Power Saving King Shop	Green Intelligent Power Saver	It’s easy to use, effective, great service, and recommended!	Jingdong prefecture level city in Shandong
Power Saving King Shop	Green Intelligent Power Saver	🌈 Great quality, bought it to my liking 💝 Awesome! ✨ Buy with confidence if you need it!	Jingdong prefecture level city in Shandong
Power Saving King Shop	Green Intelligent Power Saver	Good quality, easy to install, very energy saving and power saving, good to use	Jingdong prefecture level city in Shandong
Power Saving King Shop	Green Intelligent Power Saver	The quality is very good, it does have the effect of saving electricity, the description matches, the price is affordable, it is worth recommending.	Jingdong prefecture level city in Shandong
Power Saving King Shop	Green Intelligent Power Saver	Received, the functionality is particularly good, it is genuine, the price is affordable	Jingdong prefecture level city in Shandong
Power Saving King Shop	Green Intelligent Power Saver	Good quality, very easy to use, energy efficient, home electricity bill is obviously much less	Jingdong prefecture level city in Shandong
Power Saving King Shop	Green Intelligent Power Saver	Easy to install, energy saving and power saving, very satisfied with a shopping	Jingdong prefecture level city in Shandong
Power Saving King Shop	Green Intelligent Power Saver	Received it and tried it out, it’s great, works great and worth having! Five stars praise!	Jingdong prefecture level city in Shandong
Power Saving King Shop	Green Intelligent Power Saver	Power saving device, no problem at all, the quality of this product at this price, very affordable!	Jingdong prefecture level city in Shandong
Power Saving King Shop	Green Intelligent Power Saver	Received the goods, did not expect to be so fast, the quality is very good, especially power saving	Jingdong prefecture level city in Shandong
Power Saving King Shop	Green Intelligent Power Saver	The logistics is very fast, especially good, need to come back again	Jingdong prefecture level city in Shandong
Power Saving King Shop	Green Intelligent Power Saver	It’s already working and it really saves power, so I’m very happy with it!	Jingdong prefecture level city in Shandong
Power Saving King Shop	Green Intelligent Power Saver	Great quality, works well, can recommend to friends to buy.	Jingdong prefecture level city in Shandong
Power Saving King Shop	Green Intelligent Power Saver	It’s a good deal and worth the money.	Jingdong prefecture level city in Shandong
Power Saving King Shop	Green Intelligent Power Saver	I received it and I’m very happy with it, it’s very good quality, guaranteed, and	Jingdong prefecture level city in Shandong
Power Saving King Shop	Green Intelligent Power Saver	Good quality, safe and secure purchase, ideal results and great price.	Jingdong prefecture level city in Shandong
Power Saving King Shop	Green Intelligent Power Saver	Simple operation plugged in the power supply can be, friends recommended looking forward to the effect of the	Jingdong prefecture level city in Shandong
Power Saving King Shop	Green Intelligent Power Saver	It’s not bad, it saves electricity, and the material is sturdy and durable.	Jingdong prefecture level city in Shandong
Power Saving King Shop	Green Intelligent Power Saver	Plug and play, no management required, convenient and hassle-free!	Jingdong prefecture level city in Shandong
Power Saving King Shop	Green Intelligent Power Saver	The pictures are too lazy to go up, the power saving effect is still there!	Jingdong prefecture level city in Shandong
Power Saving King Shop	Green Intelligent Power Saver	Fast shipping. Affordable prices. Efficient	Jingdong prefecture level city in Shandong
Power Saving King Shop	Green Intelligent Power Saver	Good power saving, worth buying	Jingdong prefecture level city in Shandong

Power Saving King Shop	Green Intelligent Power Saver	Especially great, comparison shopping before buying, the store owner is very good, answering questions very patiently and detailed, there is a need to come back!	Jingdong prefecture level city in Shandong
Power Saving King Shop	Green Intelligent Power Saver	Very trustworthy brand, quality is guaranteed, the price is still affordable, quite satisfied!	Jingdong prefecture level city in Shandong
Power Saving King Shop	Green Intelligent Power Saver	Recommended by a friend. They say you can save money on air conditioning with this. Try it.	Jingdong prefecture level city in Shandong
Power Saving King Shop	Green Intelligent Power Saver	Works great, will be back next time, very happy with it and a great price!	Jingdong prefecture level city in Shandong
Power Saving King Shop	Green Intelligent Power Saver	The quality of the product is quite good, fast shipping, satisfied.	Jingdong prefecture level city in Shandong
Power Saving King Shop	Green Intelligent Power Saver	Good quality and fast logistics	Jingdong prefecture level city in Shandong
Power Saving King Shop	Green Intelligent Power Saver	It really saves power. I've tested it myself.	Jingdong prefecture level city in Shandong
Power Saving King Shop	Green Intelligent Power Saver	Really saves electricity!	Jingdong prefecture level city in Shandong
Power Saving King Shop	Green Intelligent Power Saver	pretty good	Jingdong prefecture level city in Shandong
Power Saving King Shop	Green Intelligent Power Saver	serviceable	Jingdong prefecture level city in Shandong
Power Saving King Shop	Green Intelligent Power Saver	Power Saving King received a tightly packaged logistics, installed the effect is very good price is cheaper than the physical store!	Jingdong prefecture level city in Shandong
Power Saving King Shop	Green Intelligent Power Saver	Super Power Saving King is very good quality, new style, is genuine, the use of the effect is very good, the price is cheap, it is worth buying!	Jingdong prefecture level city in Shandong
Power Saving King Shop	Green Intelligent Power Saver	Packaging is very tight, easy to install, quite practical, worth buying!	Jingdong prefecture level city in Shandong
Power Saving King Shop	Green Intelligent Power Saver	Quality assurance, affordable price, very energy efficient, will repurchase if needed!	Jingdong prefecture level city in Shandong
Power Saving King Shop	Green Intelligent Power Saver	Quality assurance, very good, affordable, logistics is also very fast, I'm very satisfied with the	Jingdong prefecture level city in Shandong
Power Saving King Shop	Green Intelligent Power Saver	Energy saving. It's so good, already installed, you can save a lot of electricity every month	Jingdong prefecture level city in Shandong
Power Saving King Shop	Green Intelligent Power Saver	The material is thick and the workmanship is fine in place, the power saving effect is very good, value for money!	Jingdong prefecture level city in Shandong
Power Saving King Shop	Green Intelligent Power Saver	The effect is great, genuine, and match the description, the logistics is fast, the price is reasonable 😍	Jingdong prefecture level city in Shandong
Power Saving King Shop	Green Intelligent Power Saver	It's genuine, works well, easy to use, plugs in and works, really saves power	Jingdong prefecture level city in Shandong
Power Saving King Shop	Green Intelligent Power Saver	It's easy to use, effective, great service, and recommended!	Jingdong prefecture level city in Shandong
Power Saving King Shop	Green Intelligent Power Saver	🌈 Great quality, bought it to my liking 💕 Awesome! ✨ Buy with confidence if you need it!	Jingdong prefecture level city in Shandong
Power Saving King Shop	Green Intelligent Power Saver	Good quality, easy to install, very energy saving and power saving, good to use	Jingdong prefecture level city in Shandong
Power Saving King Shop	Green Intelligent Power Saver	The quality is very good, it does have the effect of saving electricity, the description matches, the price is affordable, it is worth recommending.	Jingdong prefecture level city in Shandong
Power Saving King Shop	Green Intelligent Power Saver	Received, the functionality is particularly good, it is genuine, the price is affordable	Jingdong prefecture level city in Shandong
Power Saving King Shop	Green Intelligent Power Saver	Good quality, very easy to use, energy efficient, home electricity bill is obviously much less	Jingdong prefecture level city in Shandong
Power Saving King Shop	Green Intelligent Power Saver	Easy to install, energy saving and power saving, very satisfied with a shopping	Jingdong prefecture level city in Shandong
Power Saving King Shop	Green Intelligent Power Saver	Received it and tried it out, it's great, works great and worth having! Five stars praise!	Jingdong prefecture level city in Shandong
Power Saving King Shop	Green Intelligent Power Saver	Power saving device, no problem at all, the quality of this product at this price, very affordable!	Jingdong prefecture level city in Shandong
Power Saving King Shop	Green Intelligent Power Saver	Received the goods, did not expect to be so fast, the quality is very good, especially power saving	Jingdong prefecture level city in Shandong
Power Saving King Shop	Green Intelligent Power Saver	The logistics is very fast, especially good, need to come back again	Jingdong prefecture level city in Shandong
Power Saving King Shop	Green Intelligent Power Saver	It's already working and it really saves power, so I'm very happy with it!	Jingdong prefecture level city in Shandong
Power Saving King Shop	Green Intelligent Power Saver	Great quality, works well, can recommend to friends to buy.	Jingdong prefecture level city in Shandong
Power Saving King Shop	Green Intelligent Power Saver	It's a good deal and worth the money.	Jingdong prefecture level city in Shandong
Power Saving King Shop	Green Intelligent Power Saver	I received it and I'm very happy with it, it's very good quality, guaranteed, and	Jingdong prefecture level city in Shandong
Power Saving King Shop	Green Intelligent Power Saver	Good quality, safe and secure purchase, ideal results and great price.	Jingdong prefecture level city in Shandong
Power Saving King Shop	Green Intelligent Power Saver	Simple operation plugged in the power supply can be, friends recommended looking forward to the effect of the	Jingdong prefecture level city in Shandong
Power Saving King Shop	Green Intelligent Power Saver	It's not bad, it saves electricity, and the material is sturdy and durable.	Jingdong prefecture level city in Shandong
Power Saving King Shop	Green Intelligent Power Saver	Plug and play, no management required, convenient and hassle-free!	Jingdong prefecture level city in Shandong
Power Saving King Shop	Green Intelligent Power Saver	The pictures are too lazy to go up, the power saving effect is still there!	Jingdong prefecture level city in Shandong
Power Saving King Shop	Green Intelligent Power Saver	Fast shipping. Affordable prices. Efficient	Jingdong prefecture level city in Shandong
Power Saving King Shop	Green Intelligent Power Saver	Good power saving, worth buying	Jingdong prefecture level city in Shandong
Power Saving King Shop	Green Intelligent Power Saver	Especially great, comparison shopping before buying, the store owner is very good, answering questions very patiently and detailed, there is a need to come back!	Jingdong prefecture level city in Shandong
Power Saving King Shop	Green Intelligent Power Saver	Very trustworthy brand, quality is guaranteed, the price is still affordable, quite satisfied!	Jingdong prefecture level city in Shandong
Power Saving King Shop	Green Intelligent Power Saver	Recommended by a friend. They say you can save money on air conditioning with this. Try it.	Jingdong prefecture level city in Shandong
Power Saving King Shop	Green Intelligent Power Saver	Works great, will be back next time, very happy with it and a great price!	Jingdong prefecture level city in Shandong
Power Saving King Shop	Green Intelligent Power Saver	The quality of the product is quite good, fast shipping, satisfied.	Jingdong prefecture level city in Shandong
Power Saving King Shop	Green Intelligent Power Saver	Good quality and fast logistics	Jingdong prefecture level city in Shandong
Power Saving King Shop	Green Intelligent Power Saver	It really saves power. I've tested it myself.	Jingdong prefecture level city in Shandong
Power Saving King Shop	Green Intelligent Power Saver	Really saves electricity!	Jingdong prefecture level city in Shandong
Power Saving King Shop	Green Intelligent Power Saver	pretty good	Jingdong prefecture level city in Shandong
Power Saving King Shop	Green Intelligent Power Saver	serviceable	Jingdong prefecture level city in Shandong

Power Saving King Shop	Green Intelligent Power Saver	Power Saving King received a tightly packaged logistics, installed the effect is very good price is cheaper than the physical store!	Jingdong prefecture level city in Shandong
Power Saving King Shop	Green Intelligent Power Saver	Super Power Saving King is very good quality, new style, is genuine, the use of the effect is very good, the price is cheap, it is worth buying!	Jingdong prefecture level city in Shandong
Power Saving King Shop	Green Intelligent Power Saver	Packaging is very tight, easy to install, quite practical, worth buying!	Jingdong prefecture level city in Shandong
Power Saving King Shop	Green Intelligent Power Saver	Quality assurance, affordable price, very energy efficient, will repurchase if needed!	Jingdong prefecture level city in Shandong
Power Saving King Shop	Green Intelligent Power Saver	Quality assurance, very good, affordable, logistics is also very fast, I'm very satisfied with the	Jingdong prefecture level city in Shandong
Power Saving King Shop	Green Intelligent Power Saver	Energy saving. It's so good, already installed, you can save a lot of electricity every month	Jingdong prefecture level city in Shandong
Power Saving King Shop	Green Intelligent Power Saver	The material is thick and the workmanship is fine in place, the power saving effect is very good, value for money!	Jingdong prefecture level city in Shandong
Power Saving King Shop	Green Intelligent Power Saver	The effect is great, genuine, and match the description, the logistics is fast, the price is reasonable 😍	Jingdong prefecture level city in Shandong
Power Saving King Shop	Green Intelligent Power Saver	It's genuine, works well, easy to use, plugs in and works, really saves power	Jingdong prefecture level city in Shandong
Power Saving King Shop	Green Intelligent Power Saver	It's easy to use, effective, great service, and recommended!	Jingdong prefecture level city in Shandong
Power Saving King Shop	Green Intelligent Power Saver	🌈 Great quality, bought it to my liking 💝 Awesome! ✨ Buy with confidence if you need it!	Jingdong prefecture level city in Shandong
Power Saving King Shop	Green Intelligent Power Saver	Good quality, easy to install, very energy saving and power saving, good to use	Jingdong prefecture level city in Shandong
Power Saving King Shop	Green Intelligent Power Saver	The quality is very good, it does have the effect of saving electricity, the description matches, the price is affordable, it is worth recommending.	Jingdong prefecture level city in Shandong
Power Saving King Shop	Green Intelligent Power Saver	Received, the functionality is particularly good, it is genuine, the price is affordable	Jingdong prefecture level city in Shandong
Power Saving King Shop	Green Intelligent Power Saver	Good quality, very easy to use, energy efficient, home electricity bill is obviously much less	Jingdong prefecture level city in Shandong
Power Saving King Shop	Green Intelligent Power Saver	Easy to install, energy saving and power saving, very satisfied with a shopping	Jingdong prefecture level city in Shandong
Power Saving King Shop	Green Intelligent Power Saver	Received it and tried it out, it's great, works great and worth having! Five stars praise!	Jingdong prefecture level city in Shandong
Power Saving King Shop	Green Intelligent Power Saver	Power saving device, no problem at all, the quality of this product at this price, very affordable!	Jingdong prefecture level city in Shandong
Power Saving King Shop	Green Intelligent Power Saver	Received the goods, did not expect to be so fast, the quality is very good, especially power saving	Jingdong prefecture level city in Shandong
Power Saving King Shop	Green Intelligent Power Saver	The logistics is very fast, especially good, need to come back again	Jingdong prefecture level city in Shandong
Power Saving King Shop	Green Intelligent Power Saver	It's already working and it really saves power, so I'm very happy with it!	Jingdong prefecture level city in Shandong
Power Saving King Shop	Green Intelligent Power Saver	Great quality, works well, can recommend to friends to buy.	Jingdong prefecture level city in Shandong
Power Saving King Shop	Green Intelligent Power Saver	It's a good deal and worth the money.	Jingdong prefecture level city in Shandong
Power Saving King Shop	Green Intelligent Power Saver	I received it and I'm very happy with it, it's very good quality, guaranteed, and	Jingdong prefecture level city in Shandong
Power Saving King Shop	Green Intelligent Power Saver	Good quality, safe and secure purchase, ideal results and great price.	Jingdong prefecture level city in Shandong
Power Saving King Shop	Green Intelligent Power Saver	Simple operation plugged in the power supply can be, friends recommended looking forward to the effect of the	Jingdong prefecture level city in Shandong
Power Saving King Shop	Green Intelligent Power Saver	It's not bad, it saves electricity, and the material is sturdy and durable.	Jingdong prefecture level city in Shandong
Power Saving King Shop	Green Intelligent Power Saver	Plug and play, no management required, convenient and hassle-free!	Jingdong prefecture level city in Shandong
Power Saving King Shop	Green Intelligent Power Saver	The pictures are too lazy to go up, the power saving effect is still there!	Jingdong prefecture level city in Shandong
Power Saving King Shop	Green Intelligent Power Saver	Fast shipping. Affordable prices. Efficient	Jingdong prefecture level city in Shandong
Power Saving King Shop	Green Intelligent Power Saver	Good power saving, worth buying	Jingdong prefecture level city in Shandong
Power Saving King Shop	Green Intelligent Power Saver	Especially great, comparison shopping before buying, the store owner is very good, answering questions very patiently and detailed, there is a need to come back!	Jingdong prefecture level city in Shandong
Power Saving King Shop	Green Intelligent Power Saver	Very trustworthy brand, quality is guaranteed, the price is still affordable, quite satisfied!	Jingdong prefecture level city in Shandong
Power Saving King Shop	Green Intelligent Power Saver	Recommended by a friend. They say you can save money on air conditioning with this. Try it.	Jingdong prefecture level city in Shandong
Power Saving King Shop	Green Intelligent Power Saver	Works great, will be back next time, very happy with it and a great price!	Jingdong prefecture level city in Shandong
Power Saving King Shop	Green Intelligent Power Saver	The quality of the product is quite good, fast shipping, satisfied.	Jingdong prefecture level city in Shandong
Power Saving King Shop	Green Intelligent Power Saver	Good quality and fast logistics	Jingdong prefecture level city in Shandong
Power Saving King Shop	Green Intelligent Power Saver	It really saves power. I've tested it myself.	Jingdong prefecture level city in Shandong
Power Saving King Shop	Green Intelligent Power Saver	Really saves electricity!	Jingdong prefecture level city in Shandong
Power Saving King Shop	Green Intelligent Power Saver	pretty good	Jingdong prefecture level city in Shandong
Power Saving King Shop	Green Intelligent Power Saver	serviceable	Jingdong prefecture level city in Shandong
Power Saving King Shop	Green Intelligent Power Saver	Power Saving King received a tightly packaged logistics, installed the effect is very good price is cheaper than the physical store!	Jingdong prefecture level city in Shandong
Power Saving King Shop	Green Intelligent Power Saver	Super Power Saving King is very good quality, new style, is genuine, the use of the effect is very good, the price is cheap, it is worth buying!	Jingdong prefecture level city in Shandong
Power Saving King Shop	Green Intelligent Power Saver	Packaging is very tight, easy to install, quite practical, worth buying!	Jingdong prefecture level city in Shandong
Power Saving King Shop	Green Intelligent Power Saver	Quality assurance, affordable price, very energy efficient, will repurchase if needed!	Jingdong prefecture level city in Shandong
Power Saving King Shop	Green Intelligent Power Saver	Quality assurance, very good, affordable, logistics is also very fast, I'm very satisfied with the	Jingdong prefecture level city in Shandong
Power Saving King Shop	Green Intelligent Power Saver	Energy saving. It's so good, already installed, you can save a lot of electricity every month	Jingdong prefecture level city in Shandong
Power Saving King Shop	Green Intelligent Power Saver	The material is thick and the workmanship is fine in place, the power saving effect is very good, value for money!	Jingdong prefecture level city in Shandong
Power Saving King Shop	Green Intelligent Power Saver	The effect is great, genuine, and match the description, the logistics is fast, the price is reasonable 😍	Jingdong prefecture level city in Shandong
Power Saving King Shop	Green Intelligent Power Saver	It's genuine, works well, easy to use, plugs in and works, really saves power	Jingdong prefecture level city in Shandong
Power Saving King Shop	Green Intelligent Power Saver	It's easy to use, effective, great service, and recommended!	Jingdong prefecture level city in Shandong

Power Saving King Shop	Green Intelligent Power Saver	🌈 Great quality, bought it to my liking 💛 Awesome! ✨ Buy with confidence if you need it!	Jingdong prefecture level city in Shandong
Power Saving King Shop	Green Intelligent Power Saver	Good quality, easy to install, very energy saving and power saving, good to use	Jingdong prefecture level city in Shandong
Power Saving King Shop	Green Intelligent Power Saver	The quality is very good, it does have the effect of saving electricity, the description matches, the price is affordable, it is worth recommending.	Jingdong prefecture level city in Shandong
Power Saving King Shop	Green Intelligent Power Saver	Received, the functionality is particularly good, it is genuine, the price is affordable	Jingdong prefecture level city in Shandong
Power Saving King Shop	Green Intelligent Power Saver	Good quality, very easy to use, energy efficient, home electricity bill is obviously much less	Jingdong prefecture level city in Shandong
Power Saving King Shop	Green Intelligent Power Saver	Easy to install, energy saving and power saving, very satisfied with a shopping	Jingdong prefecture level city in Shandong
Power Saving King Shop	Green Intelligent Power Saver	Received it and tried it out, it's great, works great and worth having! Five stars praise!	Jingdong prefecture level city in Shandong
Power Saving King Shop	Green Intelligent Power Saver	Power saving device, no problem at all, the quality of this product at this price, very affordable!	Jingdong prefecture level city in Shandong
Power Saving King Shop	Green Intelligent Power Saver	Received the goods, did not expect to be so fast, the quality is very good, especially power saving	Jingdong prefecture level city in Shandong
Power Saving King Shop	Green Intelligent Power Saver	The logistics is very fast, especially good, need to come back again	Jingdong prefecture level city in Shandong
Power Saving King Shop	Green Intelligent Power Saver	It's already working and it really saves power, so I'm very happy with it!	Jingdong prefecture level city in Shandong
Power Saving King Shop	Green Intelligent Power Saver	Great quality, works well, can recommend to friends to buy.	Jingdong prefecture level city in Shandong
Power Saving King Shop	Green Intelligent Power Saver	It's a good deal and worth the money.	Jingdong prefecture level city in Shandong
Power Saving King Shop	Green Intelligent Power Saver	I received it and I'm very happy with it, it's very good quality, guaranteed, and	Jingdong prefecture level city in Shandong
Power Saving King Shop	Green Intelligent Power Saver	Good quality, safe and secure purchase, ideal results and great price.	Jingdong prefecture level city in Shandong
Power Saving King Shop	Green Intelligent Power Saver	Simple operation plugged in the power supply can be, friends recommended looking forward to the effect of the	Jingdong prefecture level city in Shandong
Power Saving King Shop	Green Intelligent Power Saver	It's not bad, it saves electricity, and the material is sturdy and durable.	Jingdong prefecture level city in Shandong
Power Saving King Shop	Green Intelligent Power Saver	Plug and play, no management required, convenient and hassle-free!	Jingdong prefecture level city in Shandong
Power Saving King Shop	Green Intelligent Power Saver	The pictures are too lazy to go up, the power saving effect is still there!	Jingdong prefecture level city in Shandong
Power Saving King Shop	Green Intelligent Power Saver	Fast shipping. Affordable prices. Efficient	Jingdong prefecture level city in Shandong
Power Saving King Shop	Green Intelligent Power Saver	Good power saving, worth buying	Jingdong prefecture level city in Shandong
Power Saving King Shop	Green Intelligent Power Saver	Especially great, comparison shopping before buying, the store owner is very good, answering questions very patiently and detailed, there is a need to come back!	Jingdong prefecture level city in Shandong
Power Saving King Shop	Green Intelligent Power Saver	Very trustworthy brand, quality is guaranteed, the price is still affordable, quite satisfied!	Jingdong prefecture level city in Shandong
Power Saving King Shop	Green Intelligent Power Saver	Recommended by a friend. They say you can save money on air conditioning with this. Try it.	Jingdong prefecture level city in Shandong
Power Saving King Shop	Green Intelligent Power Saver	Works great, will be back next time, very happy with it and a great price!	Jingdong prefecture level city in Shandong
Power Saving King Shop	Green Intelligent Power Saver	The quality of the product is quite good, fast shipping, satisfied.	Jingdong prefecture level city in Shandong
Power Saving King Shop	Green Intelligent Power Saver	Good quality and fast logistics	Jingdong prefecture level city in Shandong
Power Saving King Shop	Green Intelligent Power Saver	It really saves power. I've tested it myself.	Jingdong prefecture level city in Shandong
Power Saving King Shop	Green Intelligent Power Saver	Really saves electricity!	Jingdong prefecture level city in Shandong
Power Saving King Shop	Green Intelligent Power Saver	pretty good	Jingdong prefecture level city in Shandong
Power Saving King Shop	Green Intelligent Power Saver	serviceable	Jingdong prefecture level city in Shandong
Power Saving King Shop	Green Intelligent Power Saver	Power Saving King received a tightly packaged logistics, installed the effect is very good price is cheaper than the physical store!	Jingdong prefecture level city in Shandong
Power Saving King Shop	Green Intelligent Power Saver	Super Power Saving King is very good quality, new style, is genuine, the use of the effect is very good, the price is cheap, it is worth buying!	Jingdong prefecture level city in Shandong
Power Saving King Shop	Green Intelligent Power Saver	Packaging is very tight, easy to install, quite practical, worth buying!	Jingdong prefecture level city in Shandong
Power Saving King Shop	Green Intelligent Power Saver	Quality assurance, affordable price, very energy efficient, will repurchase if needed!	Jingdong prefecture level city in Shandong
Power Saving King Shop	Green Intelligent Power Saver	Quality assurance, very good, affordable, logistics is also very fast, I'm very satisfied with the	Jingdong prefecture level city in Shandong
Power Saving King Shop	Green Intelligent Power Saver	Energy saving. It's so good, already installed, you can save a lot of electricity every month	Jingdong prefecture level city in Shandong
Power Saving King Shop	Green Intelligent Power Saver	The material is thick and the workmanship is fine in place, the power saving effect is very good, value for money!	Jingdong prefecture level city in Shandong
Power Saving King Shop	Green Intelligent Power Saver	The effect is great, genuine, and match the description, the logistics is fast, the price is reasonable 😍	Jingdong prefecture level city in Shandong
Power Saving King Shop	Green Intelligent Power Saver	It's genuine, works well, easy to use, plugs in and works, really saves power	Jingdong prefecture level city in Shandong
Power Saving King Shop	Green Intelligent Power Saver	It's easy to use, effective, great service, and recommended!	Jingdong prefecture level city in Shandong
Power Saving King Shop	Green Intelligent Power Saver	🌈 Great quality, bought it to my liking 💛 Awesome! ✨ Buy with confidence if you need it!	Jingdong prefecture level city in Shandong
Power Saving King Shop	Green Intelligent Power Saver	Good quality, easy to install, very energy saving and power saving, good to use	Jingdong prefecture level city in Shandong
Power Saving King Shop	Green Intelligent Power Saver	The quality is very good, it does have the effect of saving electricity, the description matches, the price is affordable, it is worth recommending.	Jingdong prefecture level city in Shandong
Power Saving King Shop	Green Intelligent Power Saver	Received, the functionality is particularly good, it is genuine, the price is affordable	Jingdong prefecture level city in Shandong
Power Saving King Shop	Green Intelligent Power Saver	Good quality, very easy to use, energy efficient, home electricity bill is obviously much less	Jingdong prefecture level city in Shandong
Power Saving King Shop	Green Intelligent Power Saver	Easy to install, energy saving and power saving, very satisfied with a shopping	Jingdong prefecture level city in Shandong
Power Saving King Shop	Green Intelligent Power Saver	Received it and tried it out, it's great, works great and worth having! Five stars praise!	Jingdong prefecture level city in Shandong
Power Saving King Shop	Green Intelligent Power Saver	Power saving device, no problem at all, the quality of this product at this price, very affordable!	Jingdong prefecture level city in Shandong
Power Saving King Shop	Green Intelligent Power Saver	Received the goods, did not expect to be so fast, the quality is very good, especially power saving	Jingdong prefecture level city in Shandong
Power Saving King Shop	Green Intelligent Power Saver	The logistics is very fast, especially good, need to come back again	Jingdong prefecture level city in Shandong

Power Saving King Shop	Green Intelligent Power Saver	It's already working and it really saves power, so I'm very happy with it!	Jingdong prefecture level city in Shandong
Power Saving King Shop	Green Intelligent Power Saver	Great quality, works well, can recommend to friends to buy.	Jingdong prefecture level city in Shandong
Power Saving King Shop	Green Intelligent Power Saver	It's a good deal and worth the money.	Jingdong prefecture level city in Shandong
Power Saving King Shop	Green Intelligent Power Saver	I received it and I'm very happy with it, it's very good quality, guaranteed, and	Jingdong prefecture level city in Shandong
Power Saving King Shop	Green Intelligent Power Saver	Good quality, safe and secure purchase, ideal results and great price.	Jingdong prefecture level city in Shandong
Power Saving King Shop	Green Intelligent Power Saver	Simple operation plugged in the power supply can be, friends recommended looking forward to the effect of the	Jingdong prefecture level city in Shandong
Power Saving King Shop	Green Intelligent Power Saver	It's not bad, it saves electricity, and the material is sturdy and durable.	Jingdong prefecture level city in Shandong
Power Saving King Shop	Green Intelligent Power Saver	Plug and play, no management required, convenient and hassle-free!	Jingdong prefecture level city in Shandong
Power Saving King Shop	Green Intelligent Power Saver	The pictures are too lazy to go up, the power saving effect is still there!	Jingdong prefecture level city in Shandong
Power Saving King Shop	Green Intelligent Power Saver	Fast shipping. Affordable prices. Efficient	Jingdong prefecture level city in Shandong
Power Saving King Shop	Green Intelligent Power Saver	Good power saving, worth buying	Jingdong prefecture level city in Shandong
Power Saving King Shop	Green Intelligent Power Saver	Especially great, comparison shopping before buying, the store owner is very good, answering questions very patiently and detailed, there is a need to come back!	Jingdong prefecture level city in Shandong
Power Saving King Shop	Green Intelligent Power Saver	Very trustworthy brand, quality is guaranteed, the price is still affordable, quite satisfied!	Jingdong prefecture level city in Shandong
Power Saving King Shop	Green Intelligent Power Saver	Recommended by a friend. They say you can save money on air conditioning with this. Try it.	Jingdong prefecture level city in Shandong
Power Saving King Shop	Green Intelligent Power Saver	Works great, will be back next time, very happy with it and a great price!	Jingdong prefecture level city in Shandong
Power Saving King Shop	Green Intelligent Power Saver	The quality of the product is quite good, fast shipping, satisfied.	Jingdong prefecture level city in Shandong
Power Saving King Shop	Green Intelligent Power Saver	Good quality and fast logistics	Jingdong prefecture level city in Shandong
Power Saving King Shop	Green Intelligent Power Saver	It really saves power. I've tested it myself.	Jingdong prefecture level city in Shandong
Power Saving King Shop	Green Intelligent Power Saver	Really saves electricity!	Jingdong prefecture level city in Shandong
Power Saving King Shop	Green Intelligent Power Saver	pretty good	Jingdong prefecture level city in Shandong
Power Saving King Shop	Green Intelligent Power Saver	serviceable	Jingdong prefecture level city in Shandong
Power Saving King Shop	Green Intelligent Power Saver	Power Saving King received a tightly packaged logistics, installed the effect is very good price is cheaper than the physical store!	Jingdong prefecture level city in Shandong
Power Saving King Shop	Green Intelligent Power Saver	Super Power Saving King is very good quality, new style, is genuine, the use of the effect is very good, the price is cheap, it is worth buying!	Jingdong prefecture level city in Shandong
Power Saving King Shop	Green Intelligent Power Saver	Packaging is very tight, easy to install, quite practical, worth buying!	Jingdong prefecture level city in Shandong
Power Saving King Shop	Green Intelligent Power Saver	Quality assurance, affordable price, very energy efficient, will repurchase if needed!	Jingdong prefecture level city in Shandong
Power Saving King Shop	Green Intelligent Power Saver	Quality assurance, very good, affordable, logistics is also very fast, I'm very satisfied with the	Jingdong prefecture level city in Shandong
Power Saving King Shop	Green Intelligent Power Saver	Energy saving. It's so good, already installed, you can save a lot of electricity every month	Jingdong prefecture level city in Shandong
Power Saving King Shop	Green Intelligent Power Saver	The material is thick and the workmanship is fine in place, the power saving effect is very good, value for money!	Jingdong prefecture level city in Shandong
Power Saving King Shop	Green Intelligent Power Saver	The effect is great, genuine, and match the description, the logistics is fast, the price is reasonable 😍	Jingdong prefecture level city in Shandong
Power Saving King Shop	Green Intelligent Power Saver	It's genuine, works well, easy to use, plugs in and works, really saves power	Jingdong prefecture level city in Shandong
Power Saving King Shop	Green Intelligent Power Saver	It's easy to use, effective, great service, and recommended!	Jingdong prefecture level city in Shandong
Power Saving King Shop	Green Intelligent Power Saver	🌈 Great quality, bought it to my liking 💗 Awesome! ✨ Buy with confidence if you need it!	Jingdong prefecture level city in Shandong
Power Saving King Shop	Green Intelligent Power Saver	Good quality, easy to install, very energy saving and power saving, good to use	Jingdong prefecture level city in Shandong
Power Saving King Shop	Green Intelligent Power Saver	The quality is very good, it does have the effect of saving electricity, the description matches, the price is affordable, it is worth recommending.	Jingdong prefecture level city in Shandong
Power Saving King Shop	Green Intelligent Power Saver	Received, the functionality is particularly good, it is genuine, the price is affordable	Jingdong prefecture level city in Shandong
Power Saving King Shop	Green Intelligent Power Saver	Good quality, very easy to use, energy efficient, home electricity bill is obviously much less	Jingdong prefecture level city in Shandong
Power Saving King Shop	Green Intelligent Power Saver	Easy to install, energy saving and power saving, very satisfied with a shopping	Jingdong prefecture level city in Shandong
Power Saving King Shop	Green Intelligent Power Saver	Received it and tried it out, it's great, works great and worth having! Five stars praise!	Jingdong prefecture level city in Shandong
Power Saving King Shop	Green Intelligent Power Saver	Power saving device, no problem at all, the quality of this product at this price, very affordable!	Jingdong prefecture level city in Shandong
Power Saving King Shop	Green Intelligent Power Saver	Received the goods, did not expect to be so fast, the quality is very good, especially power saving	Jingdong prefecture level city in Shandong
Power Saving King Shop	Green Intelligent Power Saver	The logistics is very fast, especially good, need to come back again	Jingdong prefecture level city in Shandong
Power Saving King Shop	Green Intelligent Power Saver	It's already working and it really saves power, so I'm very happy with it!	Jingdong prefecture level city in Shandong
Power Saving King Shop	Green Intelligent Power Saver	Great quality, works well, can recommend to friends to buy.	Jingdong prefecture level city in Shandong
Power Saving King Shop	Green Intelligent Power Saver	It's a good deal and worth the money.	Jingdong prefecture level city in Shandong
Power Saving King Shop	Green Intelligent Power Saver	I received it and I'm very happy with it, it's very good quality, guaranteed, and	Jingdong prefecture level city in Shandong
Power Saving King Shop	Green Intelligent Power Saver	Good quality, safe and secure purchase, ideal results and great price.	Jingdong prefecture level city in Shandong
Power Saving King Shop	Green Intelligent Power Saver	Simple operation plugged in the power supply can be, friends recommended looking forward to the effect of the	Jingdong prefecture level city in Shandong
Power Saving King Shop	Green Intelligent Power Saver	It's not bad, it saves electricity, and the material is sturdy and durable.	Jingdong prefecture level city in Shandong
Power Saving King Shop	Green Intelligent Power Saver	Plug and play, no management required, convenient and hassle-free!	Jingdong prefecture level city in Shandong
Power Saving King Shop	Green Intelligent Power Saver	The pictures are too lazy to go up, the power saving effect is still there!	Jingdong prefecture level city in Shandong
Power Saving King Shop	Green Intelligent Power Saver	Fast shipping. Affordable prices. Efficient	Jingdong prefecture level city in Shandong

Power Saving King Shop	Green Intelligent Power Saver	Good power saving, worth buying	Jingdong prefecture level city in Shandong
Power Saving King Shop	Green Intelligent Power Saver	Especially great, comparison shopping before buying, the store owner is very good, answering questions very patiently and detailed, there is a need to come back!	Jingdong prefecture level city in Shandong
Power Saving King Shop	Green Intelligent Power Saver	Very trustworthy brand, quality is guaranteed, the price is still affordable, quite satisfied!	Jingdong prefecture level city in Shandong
Power Saving King Shop	Green Intelligent Power Saver	Recommended by a friend. They say you can save money on air conditioning with this. Try it.	Jingdong prefecture level city in Shandong
Power Saving King Shop	Green Intelligent Power Saver	Works great, will be back next time, very happy with it and a great price!	Jingdong prefecture level city in Shandong
Power Saving King Shop	Green Intelligent Power Saver	The quality of the product is quite good, fast shipping, satisfied.	Jingdong prefecture level city in Shandong
Power Saving King Shop	Green Intelligent Power Saver	Good quality and fast logistics	Jingdong prefecture level city in Shandong
Power Saving King Shop	Green Intelligent Power Saver	It really saves power. I've tested it myself.	Jingdong prefecture level city in Shandong
Power Saving King Shop	Green Intelligent Power Saver	Really saves electricity!	Jingdong prefecture level city in Shandong
Power Saving King Shop	Green Intelligent Power Saver	pretty good	Jingdong prefecture level city in Shandong
Power Saving King Shop	Green Intelligent Power Saver	serviceable	Jingdong prefecture level city in Shandong
Power Saving King Shop	Green Intelligent Power Saver	Power Saving King received a tightly packaged logistics, installed the effect is very good price is cheaper than the physical store!	Jingdong prefecture level city in Shandong
Power Saving King Shop	Green Intelligent Power Saver	Super Power Saving King is very good quality, new style, is genuine, the use of the effect is very good, the price is cheap, it is worth buying!	Jingdong prefecture level city in Shandong
Power Saving King Shop	Green Intelligent Power Saver	Packaging is very tight, easy to install, quite practical, worth buying!	Jingdong prefecture level city in Shandong
Power Saving King Shop	Green Intelligent Power Saver	Quality assurance, affordable price, very energy efficient, will repurchase if needed!	Jingdong prefecture level city in Shandong
Power Saving King Shop	Green Intelligent Power Saver	Quality assurance, very good, affordable, logistics is also very fast, I'm very satisfied with the	Jingdong prefecture level city in Shandong
Power Saving King Shop	Green Intelligent Power Saver	Energy saving. It's so good, already installed, you can save a lot of electricity every month	Jingdong prefecture level city in Shandong
Power Saving King Shop	Green Intelligent Power Saver	The material is thick and the workmanship is fine in place, the power saving effect is very good, value for money!	Jingdong prefecture level city in Shandong
Power Saving King Shop	Green Intelligent Power Saver	The effect is great, genuine, and match the description, the logistics is fast, the price is reasonable 😍	Jingdong prefecture level city in Shandong
Power Saving King Shop	Green Intelligent Power Saver	It's genuine, works well, easy to use, plugs in and works, really saves power	Jingdong prefecture level city in Shandong
Power Saving King Shop	Green Intelligent Power Saver	It's easy to use, effective, great service, and recommended!	Jingdong prefecture level city in Shandong
Power Saving King Shop	Green Intelligent Power Saver	🌈 Great quality, bought it to my liking 💝 Awesome! ✨ Buy with confidence if you need it!	Jingdong prefecture level city in Shandong
Power Saving King Shop	Green Intelligent Power Saver	Good quality, easy to install, very energy saving and power saving, good to use	Jingdong prefecture level city in Shandong
Power Saving King Shop	Green Intelligent Power Saver	The quality is very good, it does have the effect of saving electricity, the description matches, the price is affordable, it is worth recommending.	Jingdong prefecture level city in Shandong
Power Saving King Shop	Green Intelligent Power Saver	Received, the functionality is particularly good, it is genuine, the price is affordable	Jingdong prefecture level city in Shandong
Power Saving King Shop	Green Intelligent Power Saver	Good quality, very easy to use, energy efficient, home electricity bill is obviously much less	Jingdong prefecture level city in Shandong
Power Saving King Shop	Green Intelligent Power Saver	Easy to install, energy saving and power saving, very satisfied with a shopping	Jingdong prefecture level city in Shandong
Power Saving King Shop	Green Intelligent Power Saver	Received it and tried it out, it's great, works great and worth having! Five stars praise!	Jingdong prefecture level city in Shandong
Power Saving King Shop	Green Intelligent Power Saver	Power saving device, no problem at all, the quality of this product at this price, very affordable!	Jingdong prefecture level city in Shandong
Power Saving King Shop	Green Intelligent Power Saver	Received the goods, did not expect to be so fast, the quality is very good, especially power saving	Jingdong prefecture level city in Shandong
Power Saving King Shop	Green Intelligent Power Saver	The logistics is very fast, especially good, need to come back again	Jingdong prefecture level city in Shandong
Power Saving King Shop	Green Intelligent Power Saver	It's already working and it really saves power, so I'm very happy with it!	Jingdong prefecture level city in Shandong
Power Saving King Shop	Green Intelligent Power Saver	Great quality, works well, can recommend to friends to buy.	Jingdong prefecture level city in Shandong
Power Saving King Shop	Green Intelligent Power Saver	It's a good deal and worth the money.	Jingdong prefecture level city in Shandong
Power Saving King Shop	Green Intelligent Power Saver	I received it and I'm very happy with it, it's very good quality, guaranteed, and	Jingdong prefecture level city in Shandong
Power Saving King Shop	Green Intelligent Power Saver	Good quality, safe and secure purchase, ideal results and great price.	Jingdong prefecture level city in Shandong
Power Saving King Shop	Green Intelligent Power Saver	Simple operation plugged in the power supply can be, friends recommended looking forward to the effect of the	Jingdong prefecture level city in Shandong
Power Saving King Shop	Green Intelligent Power Saver	It's not bad, it saves electricity, and the material is sturdy and durable.	Jingdong prefecture level city in Shandong
Power Saving King Shop	Green Intelligent Power Saver	Plug and play, no management required, convenient and hassle-free!	Jingdong prefecture level city in Shandong
Power Saving King Shop	Green Intelligent Power Saver	The pictures are too lazy to go up, the power saving effect is still there!	Jingdong prefecture level city in Shandong
Power Saving King Shop	Green Intelligent Power Saver	Fast shipping. Affordable prices. Efficient	Jingdong prefecture level city in Shandong
Power Saving King Shop	Green Intelligent Power Saver	Good power saving, worth buying	Jingdong prefecture level city in Shandong
Power Saving King Shop	Green Intelligent Power Saver	Especially great, comparison shopping before buying, the store owner is very good, answering questions very patiently and detailed, there is a need to come back!	Jingdong prefecture level city in Shandong
Power Saving King Shop	Green Intelligent Power Saver	Very trustworthy brand, quality is guaranteed, the price is still affordable, quite satisfied!	Jingdong prefecture level city in Shandong
Power Saving King Shop	Green Intelligent Power Saver	Recommended by a friend. They say you can save money on air conditioning with this. Try it.	Jingdong prefecture level city in Shandong
Power Saving King Shop	Green Intelligent Power Saver	Works great, will be back next time, very happy with it and a great price!	Jingdong prefecture level city in Shandong
Power Saving King Shop	Green Intelligent Power Saver	The quality of the product is quite good, fast shipping, satisfied.	Jingdong prefecture level city in Shandong
Power Saving King Shop	Green Intelligent Power Saver	Good quality and fast logistics	Jingdong prefecture level city in Shandong
Power Saving King Shop	Green Intelligent Power Saver	It really saves power. I've tested it myself.	Jingdong prefecture level city in Shandong
Power Saving King Shop	Green Intelligent Power Saver	Really saves electricity!	Jingdong prefecture level city in Shandong
Power Saving King Shop	Green Intelligent Power Saver	pretty good	Jingdong prefecture level city in Shandong

Power Saving King Shop	Green Intelligent Power Saver	serviceable	Jingdong prefecture level city in Shandong
Power Saving King Shop	Green Intelligent Power Saver	Power Saving King received a tightly packaged logistics, installed the effect is very good price is cheaper than the physical store!	Jingdong prefecture level city in Shandong
Power Saving King Shop	Green Intelligent Power Saver	Super Power Saving King is very good quality, new style, is genuine, the use of the effect is very good, the price is cheap, it is worth buying!	Jingdong prefecture level city in Shandong
Power Saving King Shop	Green Intelligent Power Saver	Packaging is very tight, easy to install, quite practical, worth buying!	Jingdong prefecture level city in Shandong
Power Saving King Shop	Green Intelligent Power Saver	Quality assurance, affordable price, very energy efficient, will repurchase if needed!	Jingdong prefecture level city in Shandong
Power Saving King Shop	Green Intelligent Power Saver	Quality assurance, very good, affordable, logistics is also very fast, I'm very satisfied with the	Jingdong prefecture level city in Shandong
Power Saving King Shop	Green Intelligent Power Saver	Energy saving. It's so good, already installed, you can save a lot of electricity every month	Jingdong prefecture level city in Shandong
Power Saving King Shop	Green Intelligent Power Saver	The material is thick and the workmanship is fine in place, the power saving effect is very good, value for money!	Jingdong prefecture level city in Shandong
Power Saving King Shop	Green Intelligent Power Saver	The effect is great, genuine, and match the description, the logistics is fast, the price is reasonable 😍	Jingdong prefecture level city in Shandong
Power Saving King Shop	Green Intelligent Power Saver	It's genuine, works well, easy to use, plugs in and works, really saves power	Jingdong prefecture level city in Shandong
Power Saving King Shop	Green Intelligent Power Saver	It's easy to use, effective, great service, and recommended!	Jingdong prefecture level city in Shandong
Power Saving King Shop	Green Intelligent Power Saver	🌈 Great quality, bought it to my liking 💕 Awesome! ✨ Buy with confidence if you need it!	Jingdong prefecture level city in Shandong
Power Saving King Shop	Green Intelligent Power Saver	Good quality, easy to install, very energy saving and power saving, good to use	Jingdong prefecture level city in Shandong
Power Saving King Shop	Green Intelligent Power Saver	The quality is very good, it does have the effect of saving electricity, the description matches, the price is affordable, it is worth recommending.	Jingdong prefecture level city in Shandong
Power Saving King Shop	Green Intelligent Power Saver	Received, the functionality is particularly good, it is genuine, the price is affordable	Jingdong prefecture level city in Shandong
Power Saving King Shop	Green Intelligent Power Saver	Good quality, very easy to use, energy efficient, home electricity bill is obviously much less	Jingdong prefecture level city in Shandong
Power Saving King Shop	Green Intelligent Power Saver	Easy to install, energy saving and power saving, very satisfied with a shopping	Jingdong prefecture level city in Shandong
Power Saving King Shop	Green Intelligent Power Saver	Received it and tried it out, it's great, works great and worth having! Five stars praise!	Jingdong prefecture level city in Shandong
Power Saving King Shop	Green Intelligent Power Saver	Power saving device, no problem at all, the quality of this product at this price, very affordable!	Jingdong prefecture level city in Shandong
Power Saving King Shop	Green Intelligent Power Saver	Received the goods, did not expect to be so fast, the quality is very good, especially power saving	Jingdong prefecture level city in Shandong
Power Saving King Shop	Green Intelligent Power Saver	The logistics is very fast, especially good, need to come back again	Jingdong prefecture level city in Shandong
Power Saving King Shop	Green Intelligent Power Saver	It's already working and it really saves power, so I'm very happy with it!	Jingdong prefecture level city in Shandong
Power Saving King Shop	Green Intelligent Power Saver	Great quality, works well, can recommend to friends to buy.	Jingdong prefecture level city in Shandong
Power Saving King Shop	Green Intelligent Power Saver	It's a good deal and worth the money.	Jingdong prefecture level city in Shandong
Power Saving King Shop	Green Intelligent Power Saver	I received it and I'm very happy with it, it's very good quality, guaranteed, and	Jingdong prefecture level city in Shandong
Power Saving King Shop	Green Intelligent Power Saver	Good quality, safe and secure purchase, ideal results and great price.	Jingdong prefecture level city in Shandong
Power Saving King Shop	Green Intelligent Power Saver	Simple operation plugged in the power supply can be, friends recommended looking forward to the effect of the	Jingdong prefecture level city in Shandong
Power Saving King Shop	Green Intelligent Power Saver	It's not bad, it saves electricity, and the material is sturdy and durable.	Jingdong prefecture level city in Shandong
Power Saving King Shop	Green Intelligent Power Saver	Plug and play, no management required, convenient and hassle-free!	Jingdong prefecture level city in Shandong
Power Saving King Shop	Green Intelligent Power Saver	The pictures are too lazy to go up, the power saving effect is still there!	Jingdong prefecture level city in Shandong
Power Saving King Shop	Green Intelligent Power Saver	Fast shipping. Affordable prices. Efficient	Jingdong prefecture level city in Shandong
Power Saving King Shop	Green Intelligent Power Saver	Good power saving, worth buying	Jingdong prefecture level city in Shandong
Power Saving King Shop	Green Intelligent Power Saver	Especially great, comparison shopping before buying, the store owner is very good, answering questions very patiently and detailed, there is a need to come back!	Jingdong prefecture level city in Shandong
Power Saving King Shop	Green Intelligent Power Saver	Very trustworthy brand, quality is guaranteed, the price is still affordable, quite satisfied!	Jingdong prefecture level city in Shandong
Power Saving King Shop	Green Intelligent Power Saver	Recommended by a friend. They say you can save money on air conditioning with this. Try it.	Jingdong prefecture level city in Shandong
Power Saving King Shop	Green Intelligent Power Saver	Works great, will be back next time, very happy with it and a great price!	Jingdong prefecture level city in Shandong
Power Saving King Shop	Green Intelligent Power Saver	The quality of the product is quite good, fast shipping, satisfied.	Jingdong prefecture level city in Shandong
Power Saving King Shop	Green Intelligent Power Saver	Good quality and fast logistics	Jingdong prefecture level city in Shandong
Power Saving King Shop	Green Intelligent Power Saver	It really saves power. I've tested it myself.	Jingdong prefecture level city in Shandong
Power Saving King Shop	Green Intelligent Power Saver	Really saves electricity!	Jingdong prefecture level city in Shandong
Power Saving King Shop	Green Intelligent Power Saver	pretty good	Jingdong prefecture level city in Shandong
Power Saving King Shop	Green Intelligent Power Saver	serviceable	Jingdong prefecture level city in Shandong
Power Saving King Shop	Green Intelligent Power Saver	Power Saving King received a tightly packaged logistics, installed the effect is very good price is cheaper than the physical store!	Jingdong prefecture level city in Shandong
Power Saving King Shop	Green Intelligent Power Saver	Super Power Saving King is very good quality, new style, is genuine, the use of the effect is very good, the price is cheap, it is worth buying!	Jingdong prefecture level city in Shandong
Power Saving King Shop	Green Intelligent Power Saver	Packaging is very tight, easy to install, quite practical, worth buying!	Jingdong prefecture level city in Shandong
Power Saving King Shop	Green Intelligent Power Saver	Quality assurance, affordable price, very energy efficient, will repurchase if needed!	Jingdong prefecture level city in Shandong
Power Saving King Shop	Green Intelligent Power Saver	Quality assurance, very good, affordable, logistics is also very fast, I'm very satisfied with the	Jingdong prefecture level city in Shandong
Power Saving King Shop	Green Intelligent Power Saver	Energy saving. It's so good, already installed, you can save a lot of electricity every month	Jingdong prefecture level city in Shandong
Power Saving King Shop	Green Intelligent Power Saver	The material is thick and the workmanship is fine in place, the power saving effect is very good, value for money!	Jingdong prefecture level city in Shandong
Power Saving King Shop	Green Intelligent Power Saver	The effect is great, genuine, and match the description, the logistics is fast, the price is reasonable 😍	Jingdong prefecture level city in Shandong
Power Saving King Shop	Green Intelligent Power Saver	It's genuine, works well, easy to use, plugs in and works, really saves power	Jingdong prefecture level city in Shandong

Power Saving King Shop	Green Intelligent Power Saver	It's easy to use, effective, great service, and recommended!	Jingdong prefecture level city in Shandong
Power Saving King Shop	Green Intelligent Power Saver	🌈 Great quality, bought it to my liking 💝 Awesome! ✨ Buy with confidence if you need it!	Jingdong prefecture level city in Shandong
Power Saving King Shop	Green Intelligent Power Saver	Good quality, easy to install, very energy saving and power saving, good to use	Jingdong prefecture level city in Shandong
Power Saving King Shop	Green Intelligent Power Saver	The quality is very good, it does have the effect of saving electricity, the description matches, the price is affordable, it is worth recommending.	Jingdong prefecture level city in Shandong
Power Saving King Shop	Green Intelligent Power Saver	Received, the functionality is particularly good, it is genuine, the price is affordable	Jingdong prefecture level city in Shandong
Power Saving King Shop	Green Intelligent Power Saver	Good quality, very easy to use, energy efficient, home electricity bill is obviously much less	Jingdong prefecture level city in Shandong
Power Saving King Shop	Green Intelligent Power Saver	Easy to install, energy saving and power saving, very satisfied with a shopping	Jingdong prefecture level city in Shandong
Power Saving King Shop	Green Intelligent Power Saver	Received it and tried it out, it's great, works great and worth having! Five stars praise!	Jingdong prefecture level city in Shandong
Power Saving King Shop	Green Intelligent Power Saver	Power saving device, no problem at all, the quality of this product at this price, very affordable!	Jingdong prefecture level city in Shandong
Power Saving King Shop	Green Intelligent Power Saver	Received the goods, did not expect to be so fast, the quality is very good, especially power saving	Jingdong prefecture level city in Shandong
Power Saving King Shop	Green Intelligent Power Saver	The logistics is very fast, especially good, need to come back again	Jingdong prefecture level city in Shandong
Power Saving King Shop	Green Intelligent Power Saver	It's already working and it really saves power, so I'm very happy with it!	Jingdong prefecture level city in Shandong
Power Saving King Shop	Green Intelligent Power Saver	Great quality, works well, can recommend to friends to buy.	Jingdong prefecture level city in Shandong
Power Saving King Shop	Green Intelligent Power Saver	It's a good deal and worth the money.	Jingdong prefecture level city in Shandong
Power Saving King Shop	Green Intelligent Power Saver	I received it and I'm very happy with it, it's very good quality, guaranteed, and	Jingdong prefecture level city in Shandong
Power Saving King Shop	Green Intelligent Power Saver	Good quality, safe and secure purchase, ideal results and great price.	Jingdong prefecture level city in Shandong
Power Saving King Shop	Green Intelligent Power Saver	Simple operation plugged in the power supply can be, friends recommended looking forward to the effect of the	Jingdong prefecture level city in Shandong
Power Saving King Shop	Green Intelligent Power Saver	It's not bad, it saves electricity, and the material is sturdy and durable.	Jingdong prefecture level city in Shandong
Power Saving King Shop	Green Intelligent Power Saver	Plug and play, no management required, convenient and hassle-free!	Jingdong prefecture level city in Shandong
Power Saving King Shop	Green Intelligent Power Saver	The pictures are too lazy to go up, the power saving effect is still there!	Jingdong prefecture level city in Shandong
Power Saving King Shop	Green Intelligent Power Saver	Fast shipping. Affordable prices. Efficient	Jingdong prefecture level city in Shandong
Power Saving King Shop	Green Intelligent Power Saver	Good power saving, worth buying	Jingdong prefecture level city in Shandong
Power Saving King Shop	Green Intelligent Power Saver	Especially great, comparison shopping before buying, the store owner is very good, answering questions very patiently and detailed, there is a need to come back!	Jingdong prefecture level city in Shandong
Power Saving King Shop	Green Intelligent Power Saver	Very trustworthy brand, quality is guaranteed, the price is still affordable, quite satisfied!	Jingdong prefecture level city in Shandong
Power Saving King Shop	Green Intelligent Power Saver	Recommended by a friend. They say you can save money on air conditioning with this. Try it.	Jingdong prefecture level city in Shandong
Power Saving King Shop	Green Intelligent Power Saver	Works great, will be back next time, very happy with it and a great price!	Jingdong prefecture level city in Shandong
Power Saving King Shop	Green Intelligent Power Saver	The quality of the product is quite good, fast shipping, satisfied.	Jingdong prefecture level city in Shandong
Power Saving King Shop	Green Intelligent Power Saver	Good quality and fast logistics	Jingdong prefecture level city in Shandong
Power Saving King Shop	Green Intelligent Power Saver	It really saves power. I've tested it myself.	Jingdong prefecture level city in Shandong
Power Saving King Shop	Green Intelligent Power Saver	Really saves electricity!	Jingdong prefecture level city in Shandong
Power Saving King Shop	Green Intelligent Power Saver	pretty good	Jingdong prefecture level city in Shandong
Power Saving King Shop	Green Intelligent Power Saver	serviceable	Jingdong prefecture level city in Shandong
Power Saving King Shop	Green Intelligent Power Saver	Power Saving King received a tightly packaged logistics, installed the effect is very good price is cheaper than the physical store!	Jingdong prefecture level city in Shandong
Power Saving King Shop	Green Intelligent Power Saver	Super Power Saving King is very good quality, new style, is genuine, the use of the effect is very good, the price is cheap, it is worth buying!	Jingdong prefecture level city in Shandong
Power Saving King Shop	Green Intelligent Power Saver	Packaging is very tight, easy to install, quite practical, worth buying!	Jingdong prefecture level city in Shandong
Power Saving King Shop	Green Intelligent Power Saver	Quality assurance, affordable price, very energy efficient, will repurchase if needed!	Jingdong prefecture level city in Shandong
Power Saving King Shop	Green Intelligent Power Saver	Quality assurance, very good, affordable, logistics is also very fast, I'm very satisfied with the	Jingdong prefecture level city in Shandong
Power Saving King Shop	Green Intelligent Power Saver	Energy saving. It's so good, already installed, you can save a lot of electricity every month	Jingdong prefecture level city in Shandong
Power Saving King Shop	Green Intelligent Power Saver	The material is thick and the workmanship is fine in place, the power saving effect is very good, value for money!	Jingdong prefecture level city in Shandong
Power Saving King Shop	Green Intelligent Power Saver	The effect is great, genuine, and match the description, the logistics is fast, the price is reasonable 😍	Jingdong prefecture level city in Shandong
Power Saving King Shop	Green Intelligent Power Saver	It's genuine, works well, easy to use, plugs in and works, really saves power	Jingdong prefecture level city in Shandong
Power Saving King Shop	Green Intelligent Power Saver	It's easy to use, effective, great service, and recommended!	Jingdong prefecture level city in Shandong
Power Saving King Shop	Green Intelligent Power Saver	🌈 Great quality, bought it to my liking 💝 Awesome! ✨ Buy with confidence if you need it!	Jingdong prefecture level city in Shandong
Power Saving King Shop	Green Intelligent Power Saver	Good quality, easy to install, very energy saving and power saving, good to use	Jingdong prefecture level city in Shandong
Power Saving King Shop	Green Intelligent Power Saver	The quality is very good, it does have the effect of saving electricity, the description matches, the price is affordable, it is worth recommending.	Jingdong prefecture level city in Shandong
Power Saving King Shop	Green Intelligent Power Saver	Received, the functionality is particularly good, it is genuine, the price is affordable	Jingdong prefecture level city in Shandong
Power Saving King Shop	Green Intelligent Power Saver	Good quality, very easy to use, energy efficient, home electricity bill is obviously much less	Jingdong prefecture level city in Shandong
Power Saving King Shop	Green Intelligent Power Saver	Easy to install, energy saving and power saving, very satisfied with a shopping	Jingdong prefecture level city in Shandong
Power Saving King Shop	Green Intelligent Power Saver	Received it and tried it out, it's great, works great and worth having! Five stars praise!	Jingdong prefecture level city in Shandong
Power Saving King Shop	Green Intelligent Power Saver	Power saving device, no problem at all, the quality of this product at this price, very affordable!	Jingdong prefecture level city in Shandong
Power Saving King Shop	Green Intelligent Power Saver	Received the goods, did not expect to be so fast, the quality is very good, especially power saving	Jingdong prefecture level city in Shandong

Power Saving King Shop	Green Intelligent Power Saver	The logistics is very fast, especially good, need to come back again	Jingdong prefecture level city in Shandong
Power Saving King Shop	Green Intelligent Power Saver	It's already working and it really saves power, so I'm very happy with it!	Jingdong prefecture level city in Shandong
Power Saving King Shop	Green Intelligent Power Saver	Great quality, works well, can recommend to friends to buy.	Jingdong prefecture level city in Shandong
Power Saving King Shop	Green Intelligent Power Saver	It's a good deal and worth the money.	Jingdong prefecture level city in Shandong
Power Saving King Shop	Green Intelligent Power Saver	I received it and I'm very happy with it, it's very good quality, guaranteed, and	Jingdong prefecture level city in Shandong
Power Saving King Shop	Green Intelligent Power Saver	Good quality, safe and secure purchase, ideal results and great price.	Jingdong prefecture level city in Shandong
Power Saving King Shop	Green Intelligent Power Saver	Simple operation plugged in the power supply can be, friends recommended looking forward to the effect of the	Jingdong prefecture level city in Shandong
Power Saving King Shop	Green Intelligent Power Saver	It's not bad, it saves electricity, and the material is sturdy and durable.	Jingdong prefecture level city in Shandong
Power Saving King Shop	Green Intelligent Power Saver	Plug and play, no management required, convenient and hassle-free!	Jingdong prefecture level city in Shandong
Power Saving King Shop	Green Intelligent Power Saver	The pictures are too lazy to go up, the power saving effect is still there!	Jingdong prefecture level city in Shandong
Power Saving King Shop	Green Intelligent Power Saver	Fast shipping. Affordable prices. Efficient	Jingdong prefecture level city in Shandong
Power Saving King Shop	Green Intelligent Power Saver	Good power saving, worth buying	Jingdong prefecture level city in Shandong
Power Saving King Shop	Green Intelligent Power Saver	Especially great, comparison shopping before buying, the store owner is very good, answering questions very patiently and detailed, there is a need to come back!	Jingdong prefecture level city in Shandong
Power Saving King Shop	Green Intelligent Power Saver	Very trustworthy brand, quality is guaranteed, the price is still affordable, quite satisfied!	Jingdong prefecture level city in Shandong
Power Saving King Shop	Green Intelligent Power Saver	Recommended by a friend. They say you can save money on air conditioning with this. Try it.	Jingdong prefecture level city in Shandong
Power Saving King Shop	Green Intelligent Power Saver	Works great, will be back next time, very happy with it and a great price!	Jingdong prefecture level city in Shandong
Power Saving King Shop	Green Intelligent Power Saver	The quality of the product is quite good, fast shipping, satisfied.	Jingdong prefecture level city in Shandong
Power Saving King Shop	Green Intelligent Power Saver	Good quality and fast logistics	Jingdong prefecture level city in Shandong
Power Saving King Shop	Green Intelligent Power Saver	It really saves power. I've tested it myself.	Jingdong prefecture level city in Shandong
Power Saving King Shop	Green Intelligent Power Saver	Really saves electricity!	Jingdong prefecture level city in Shandong
Power Saving King Shop	Green Intelligent Power Saver	pretty good	Jingdong prefecture level city in Shandong
Power Saving King Shop	Green Intelligent Power Saver	serviceable	Jingdong prefecture level city in Shandong
Power Saving King Shop	Green Intelligent Power Saver	Power Saving King received a tightly packaged logistics, installed the effect is very good price is cheaper than the physical store!	Jingdong prefecture level city in Shandong
Power Saving King Shop	Green Intelligent Power Saver	Super Power Saving King is very good quality, new style, is genuine, the use of the effect is very good, the price is cheap, it is worth buying!	Jingdong prefecture level city in Shandong
Power Saving King Shop	Green Intelligent Power Saver	Packaging is very tight, easy to install, quite practical, worth buying!	Jingdong prefecture level city in Shandong
Power Saving King Shop	Green Intelligent Power Saver	Quality assurance, affordable price, very energy efficient, will repurchase if needed!	Jingdong prefecture level city in Shandong
Power Saving King Shop	Green Intelligent Power Saver	Quality assurance, very good, affordable, logistics is also very fast, I'm very satisfied with the	Jingdong prefecture level city in Shandong
Power Saving King Shop	Green Intelligent Power Saver	Energy saving. It's so good, already installed, you can save a lot of electricity every month	Jingdong prefecture level city in Shandong
Power Saving King Shop	Green Intelligent Power Saver	The material is thick and the workmanship is fine in place, the power saving effect is very good, value for money!	Jingdong prefecture level city in Shandong
Power Saving King Shop	Green Intelligent Power Saver	The effect is great, genuine, and match the description, the logistics is fast, the price is reasonable 😍	Jingdong prefecture level city in Shandong
Power Saving King Shop	Green Intelligent Power Saver	It's genuine, works well, easy to use, plugs in and works, really saves power	Jingdong prefecture level city in Shandong
Power Saving King Shop	Green Intelligent Power Saver	It's easy to use, effective, great service, and recommended!	Jingdong prefecture level city in Shandong
Power Saving King Shop	Green Intelligent Power Saver	🌈 Great quality, bought it to my liking 💝 Awesome! ✨ Buy with confidence if you need it!	Jingdong prefecture level city in Shandong
Power Saving King Shop	Green Intelligent Power Saver	Good quality, easy to install, very energy saving and power saving, good to use	Jingdong prefecture level city in Shandong
Power Saving King Shop	Green Intelligent Power Saver	The quality is very good, it does have the effect of saving electricity, the description matches, the price is affordable, it is worth recommending.	Jingdong prefecture level city in Shandong
Power Saving King Shop	Green Intelligent Power Saver	Received, the functionality is particularly good, it is genuine, the price is affordable	Jingdong prefecture level city in Shandong
Power Saving King Shop	Green Intelligent Power Saver	Good quality, very easy to use, energy efficient, home electricity bill is obviously much less	Jingdong prefecture level city in Shandong
Power Saving King Shop	Green Intelligent Power Saver	Easy to install, energy saving and power saving, very satisfied with a shopping	Jingdong prefecture level city in Shandong
Power Saving King Shop	Green Intelligent Power Saver	Received it and tried it out, it's great, works great and worth having! Five stars praise!	Jingdong prefecture level city in Shandong
Power Saving King Shop	Green Intelligent Power Saver	Power saving device, no problem at all, the quality of this product at this price, very affordable!	Jingdong prefecture level city in Shandong
Power Saving King Shop	Green Intelligent Power Saver	Received the goods, did not expect to be so fast, the quality is very good, especially power saving	Jingdong prefecture level city in Shandong
Power Saving King Shop	Green Intelligent Power Saver	The logistics is very fast, especially good, need to come back again	Jingdong prefecture level city in Shandong
Power Saving King Shop	Green Intelligent Power Saver	It's already working and it really saves power, so I'm very happy with it!	Jingdong prefecture level city in Shandong
Power Saving King Shop	Green Intelligent Power Saver	Great quality, works well, can recommend to friends to buy.	Jingdong prefecture level city in Shandong
Power Saving King Shop	Green Intelligent Power Saver	It's a good deal and worth the money.	Jingdong prefecture level city in Shandong
Power Saving King Shop	Green Intelligent Power Saver	I received it and I'm very happy with it, it's very good quality, guaranteed, and	Jingdong prefecture level city in Shandong
Power Saving King Shop	Green Intelligent Power Saver	Good quality, safe and secure purchase, ideal results and great price.	Jingdong prefecture level city in Shandong
Power Saving King Shop	Green Intelligent Power Saver	Simple operation plugged in the power supply can be, friends recommended looking forward to the effect of the	Jingdong prefecture level city in Shandong
Power Saving King Shop	Green Intelligent Power Saver	It's not bad, it saves electricity, and the material is sturdy and durable.	Jingdong prefecture level city in Shandong
Power Saving King Shop	Green Intelligent Power Saver	Plug and play, no management required, convenient and hassle-free!	Jingdong prefecture level city in Shandong
Power Saving King Shop	Green Intelligent Power Saver	The pictures are too lazy to go up, the power saving effect is still there!	Jingdong prefecture level city in Shandong

Power Saving King Shop	Green Intelligent Power Saver	Fast shipping. Affordable prices. Efficient	Jingdong prefecture level city in Shandong
Power Saving King Shop	Green Intelligent Power Saver	Good power saving, worth buying	Jingdong prefecture level city in Shandong
Power Saving King Shop	Green Intelligent Power Saver	Especially great, comparison shopping before buying, the store owner is very good, answering questions very patiently and detailed, there is a need to come back!	Jingdong prefecture level city in Shandong
Power Saving King Shop	Green Intelligent Power Saver	Very trustworthy brand, quality is guaranteed, the price is still affordable, quite satisfied!	Jingdong prefecture level city in Shandong
Power Saving King Shop	Green Intelligent Power Saver	Recommended by a friend. They say you can save money on air conditioning with this. Try it.	Jingdong prefecture level city in Shandong
Power Saving King Shop	Green Intelligent Power Saver	Works great, will be back next time, very happy with it and a great price!	Jingdong prefecture level city in Shandong
Power Saving King Shop	Green Intelligent Power Saver	The quality of the product is quite good, fast shipping, satisfied.	Jingdong prefecture level city in Shandong
Power Saving King Shop	Green Intelligent Power Saver	Good quality and fast logistics	Jingdong prefecture level city in Shandong
Power Saving King Shop	Green Intelligent Power Saver	It really saves power. I've tested it myself.	Jingdong prefecture level city in Shandong
Power Saving King Shop	Green Intelligent Power Saver	Really saves electricity!	Jingdong prefecture level city in Shandong
Power Saving King Shop	Green Intelligent Power Saver	pretty good	Jingdong prefecture level city in Shandong
Power Saving King Shop	Green Intelligent Power Saver	serviceable	Jingdong prefecture level city in Shandong
Power Saving King Shop	Green Intelligent Power Saver	Power Saving King received a tightly packaged logistics, installed the effect is very good price is cheaper than the physical store!	Jingdong prefecture level city in Shandong
Power Saving King Shop	Green Intelligent Power Saver	Super Power Saving King is very good quality, new style, is genuine, the use of the effect is very good, the price is cheap, it is worth buying!	Jingdong prefecture level city in Shandong
Power Saving King Shop	Green Intelligent Power Saver	Packaging is very tight, easy to install, quite practical, worth buying!	Jingdong prefecture level city in Shandong
Power Saving King Shop	Green Intelligent Power Saver	Quality assurance, affordable price, very energy efficient, will repurchase if needed!	Jingdong prefecture level city in Shandong
Power Saving King Shop	Green Intelligent Power Saver	Quality assurance, very good, affordable, logistics is also very fast, I'm very satisfied with the	Jingdong prefecture level city in Shandong
Power Saving King Shop	Green Intelligent Power Saver	Energy saving. It's so good, already installed, you can save a lot of electricity every month	Jingdong prefecture level city in Shandong
Power Saving King Shop	Green Intelligent Power Saver	The material is thick and the workmanship is fine in place, the power saving effect is very good, value for money!	Jingdong prefecture level city in Shandong
Power Saving King Shop	Green Intelligent Power Saver	The effect is great, genuine, and match the description, the logistics is fast, the price is reasonable 😍	Jingdong prefecture level city in Shandong
Power Saving King Shop	Green Intelligent Power Saver	It's genuine, works well, easy to use, plugs in and works, really saves power	Jingdong prefecture level city in Shandong
Power Saving King Shop	Green Intelligent Power Saver	It's easy to use, effective, great service, and recommended!	Jingdong prefecture level city in Shandong
Power Saving King Shop	Green Intelligent Power Saver	🌈 Great quality, bought it to my liking 💗 Awesome! ✨ Buy with confidence if you need it!	Jingdong prefecture level city in Shandong
Power Saving King Shop	Green Intelligent Power Saver	Good quality, easy to install, very energy saving and power saving, good to use	Jingdong prefecture level city in Shandong
Power Saving King Shop	Green Intelligent Power Saver	The quality is very good, it does have the effect of saving electricity, the description matches, the price is affordable, it is worth recommending.	Jingdong prefecture level city in Shandong
Power Saving King Shop	Green Intelligent Power Saver	Received, the functionality is particularly good, it is genuine, the price is affordable	Jingdong prefecture level city in Shandong
Power Saving King Shop	Green Intelligent Power Saver	Good quality, very easy to use, energy efficient, home electricity bill is obviously much less	Jingdong prefecture level city in Shandong
Power Saving King Shop	Green Intelligent Power Saver	Easy to install, energy saving and power saving, very satisfied with a shopping	Jingdong prefecture level city in Shandong
Power Saving King Shop	Green Intelligent Power Saver	Received it and tried it out, it's great, works great and worth having! Five stars praise!	Jingdong prefecture level city in Shandong
Power Saving King Shop	Green Intelligent Power Saver	Power saving device, no problem at all, the quality of this product at this price, very affordable!	Jingdong prefecture level city in Shandong
Power Saving King Shop	Green Intelligent Power Saver	Received the goods, did not expect to be so fast, the quality is very good, especially power saving	Jingdong prefecture level city in Shandong
Power Saving King Shop	Green Intelligent Power Saver	The logistics is very fast, especially good, need to come back again	Jingdong prefecture level city in Shandong
Power Saving King Shop	Green Intelligent Power Saver	It's already working and it really saves power, so I'm very happy with it!	Jingdong prefecture level city in Shandong
Power Saving King Shop	Green Intelligent Power Saver	Great quality, works well, can recommend to friends to buy.	Jingdong prefecture level city in Shandong
Power Saving King Shop	Green Intelligent Power Saver	It's a good deal and worth the money.	Jingdong prefecture level city in Shandong
Power Saving King Shop	Green Intelligent Power Saver	I received it and I'm very happy with it, it's very good quality, guaranteed, and	Jingdong prefecture level city in Shandong
Power Saving King Shop	Green Intelligent Power Saver	Good quality, safe and secure purchase, ideal results and great price.	Jingdong prefecture level city in Shandong
Power Saving King Shop	Green Intelligent Power Saver	Simple operation plugged in the power supply can be, friends recommended looking forward to the effect of the	Jingdong prefecture level city in Shandong
Power Saving King Shop	Green Intelligent Power Saver	It's not bad, it saves electricity, and the material is sturdy and durable.	Jingdong prefecture level city in Shandong
Power Saving King Shop	Green Intelligent Power Saver	Plug and play, no management required, convenient and hassle-free!	Jingdong prefecture level city in Shandong
Power Saving King Shop	Green Intelligent Power Saver	The pictures are too lazy to go up, the power saving effect is still there!	Jingdong prefecture level city in Shandong
Power Saving King Shop	Green Intelligent Power Saver	Fast shipping. Affordable prices. Efficient	Jingdong prefecture level city in Shandong
Power Saving King Shop	Green Intelligent Power Saver	Good power saving, worth buying	Jingdong prefecture level city in Shandong
Power Saving King Shop	Green Intelligent Power Saver	Especially great, comparison shopping before buying, the store owner is very good, answering questions very patiently and detailed, there is a need to come back!	Jingdong prefecture level city in Shandong
Power Saving King Shop	Green Intelligent Power Saver	Very trustworthy brand, quality is guaranteed, the price is still affordable, quite satisfied!	Jingdong prefecture level city in Shandong
Power Saving King Shop	Green Intelligent Power Saver	Recommended by a friend. They say you can save money on air conditioning with this. Try it.	Jingdong prefecture level city in Shandong
Power Saving King Shop	Green Intelligent Power Saver	Works great, will be back next time, very happy with it and a great price!	Jingdong prefecture level city in Shandong
Power Saving King Shop	Green Intelligent Power Saver	The quality of the product is quite good, fast shipping, satisfied.	Jingdong prefecture level city in Shandong
Power Saving King Shop	Green Intelligent Power Saver	Good quality and fast logistics	Jingdong prefecture level city in Shandong
Power Saving King Shop	Green Intelligent Power Saver	It really saves power. I've tested it myself.	Jingdong prefecture level city in Shandong
Power Saving King Shop	Green Intelligent Power Saver	Really saves electricity!	Jingdong prefecture level city in Shandong

Power Saving King Shop	Green Intelligent Power Saver	pretty good	Jingdong prefecture level city in Shandong
Power Saving King Shop	Green Intelligent Power Saver	serviceable	Jingdong prefecture level city in Shandong
Power Saving King Shop	Green Intelligent Power Saver	Power Saving King received a tightly packaged logistics, installed the effect is very good price is cheaper than the physical store!	Jingdong prefecture level city in Shandong
Power Saving King Shop	Green Intelligent Power Saver	Super Power Saving King is very good quality, new style, is genuine, the use of the effect is very good, the price is cheap, it is worth buying!	Jingdong prefecture level city in Shandong
Power Saving King Shop	Green Intelligent Power Saver	Packaging is very tight, easy to install, quite practical, worth buying!	Jingdong prefecture level city in Shandong
Power Saving King Shop	Green Intelligent Power Saver	Quality assurance, affordable price, very energy efficient, will repurchase if needed!	Jingdong prefecture level city in Shandong
Power Saving King Shop	Green Intelligent Power Saver	Quality assurance, very good, affordable, logistics is also very fast, I'm very satisfied with the	Jingdong prefecture level city in Shandong
Power Saving King Shop	Green Intelligent Power Saver	Energy saving. It's so good, already installed, you can save a lot of electricity every month	Jingdong prefecture level city in Shandong
Power Saving King Shop	Green Intelligent Power Saver	The material is thick and the workmanship is fine in place, the power saving effect is very good, value for money!	Jingdong prefecture level city in Shandong
Power Saving King Shop	Green Intelligent Power Saver	The effect is great, genuine, and match the description, the logistics is fast, the price is reasonable 😍	Jingdong prefecture level city in Shandong
Power Saving King Shop	Green Intelligent Power Saver	It's genuine, works well, easy to use, plugs in and works, really saves power	Jingdong prefecture level city in Shandong
Power Saving King Shop	Green Intelligent Power Saver	It's easy to use, effective, great service, and recommended!	Jingdong prefecture level city in Shandong
Power Saving King Shop	Green Intelligent Power Saver	🌈 Great quality, bought it to my liking 💝 Awesome! ✨ Buy with confidence if you need it!	Jingdong prefecture level city in Shandong
Power Saving King Shop	Green Intelligent Power Saver	Good quality, easy to install, very energy saving and power saving, good to use	Jingdong prefecture level city in Shandong
Power Saving King Shop	Green Intelligent Power Saver	The quality is very good, it does have the effect of saving electricity, the description matches, the price is affordable, it is worth recommending.	Jingdong prefecture level city in Shandong
Power Saving King Shop	Green Intelligent Power Saver	Received, the functionality is particularly good, it is genuine, the price is affordable	Jingdong prefecture level city in Shandong
Power Saving King Shop	Green Intelligent Power Saver	Good quality, very easy to use, energy efficient, home electricity bill is obviously much less	Jingdong prefecture level city in Shandong
Power Saving King Shop	Green Intelligent Power Saver	Easy to install, energy saving and power saving, very satisfied with a shopping	Jingdong prefecture level city in Shandong
Power Saving King Shop	Green Intelligent Power Saver	Received it and tried it out, it's great, works great and worth having! Five stars praise!	Jingdong prefecture level city in Shandong
Power Saving King Shop	Green Intelligent Power Saver	Power saving device, no problem at all, the quality of this product at this price, very affordable!	Jingdong prefecture level city in Shandong
Power Saving King Shop	Green Intelligent Power Saver	Received the goods, did not expect to be so fast, the quality is very good, especially power saving	Jingdong prefecture level city in Shandong
Power Saving King Shop	Green Intelligent Power Saver	The logistics is very fast, especially good, need to come back again	Jingdong prefecture level city in Shandong
Power Saving King Shop	Green Intelligent Power Saver	It's already working and it really saves power, so I'm very happy with it!	Jingdong prefecture level city in Shandong
Power Saving King Shop	Green Intelligent Power Saver	Great quality, works well, can recommend to friends to buy.	Jingdong prefecture level city in Shandong
Power Saving King Shop	Green Intelligent Power Saver	It's a good deal and worth the money.	Jingdong prefecture level city in Shandong
Power Saving King Shop	Green Intelligent Power Saver	I received it and I'm very happy with it, it's very good quality, guaranteed, and	Jingdong prefecture level city in Shandong
Power Saving King Shop	Green Intelligent Power Saver	Good quality, safe and secure purchase, ideal results and great price.	Jingdong prefecture level city in Shandong
Power Saving King Shop	Green Intelligent Power Saver	Simple operation plugged in the power supply can be, friends recommended looking forward to the effect of the	Jingdong prefecture level city in Shandong
Power Saving King Shop	Green Intelligent Power Saver	It's not bad, it saves electricity, and the material is sturdy and durable.	Jingdong prefecture level city in Shandong
Power Saving King Shop	Green Intelligent Power Saver	Plug and play, no management required, convenient and hassle-free!	Jingdong prefecture level city in Shandong
Power Saving King Shop	Green Intelligent Power Saver	The pictures are too lazy to go up, the power saving effect is still there!	Jingdong prefecture level city in Shandong
Power Saving King Shop	Green Intelligent Power Saver	Fast shipping. Affordable prices. Efficient	Jingdong prefecture level city in Shandong
Power Saving King Shop	Green Intelligent Power Saver	Good power saving, worth buying	Jingdong prefecture level city in Shandong
Power Saving King Shop	Green Intelligent Power Saver	Especially great, comparison shopping before buying, the store owner is very good, answering questions very patiently and detailed, there is a need to come back!	Jingdong prefecture level city in Shandong
Power Saving King Shop	Green Intelligent Power Saver	Very trustworthy brand, quality is guaranteed, the price is still affordable, quite satisfied!	Jingdong prefecture level city in Shandong
Power Saving King Shop	Green Intelligent Power Saver	Recommended by a friend. They say you can save money on air conditioning with this. Try it.	Jingdong prefecture level city in Shandong
Power Saving King Shop	Green Intelligent Power Saver	Works great, will be back next time, very happy with it and a great price!	Jingdong prefecture level city in Shandong
Power Saving King Shop	Green Intelligent Power Saver	The quality of the product is quite good, fast shipping, satisfied.	Jingdong prefecture level city in Shandong
Power Saving King Shop	Green Intelligent Power Saver	Good quality and fast logistics	Jingdong prefecture level city in Shandong
Power Saving King Shop	Green Intelligent Power Saver	It really saves power. I've tested it myself.	Jingdong prefecture level city in Shandong
Power Saving King Shop	Green Intelligent Power Saver	Really saves electricity!	Jingdong prefecture level city in Shandong
Power Saving King Shop	Green Intelligent Power Saver	pretty good	Jingdong prefecture level city in Shandong
Power Saving King Shop	Green Intelligent Power Saver	serviceable	Jingdong prefecture level city in Shandong
Power Saving King Shop	Green Intelligent Power Saver	Power Saving King received a tightly packaged logistics, installed the effect is very good price is cheaper than the physical store!	Jingdong prefecture level city in Shandong
Power Saving King Shop	Green Intelligent Power Saver	Super Power Saving King is very good quality, new style, is genuine, the use of the effect is very good, the price is cheap, it is worth buying!	Jingdong prefecture level city in Shandong
Power Saving King Shop	Green Intelligent Power Saver	Packaging is very tight, easy to install, quite practical, worth buying!	Jingdong prefecture level city in Shandong
Power Saving King Shop	Green Intelligent Power Saver	Quality assurance, affordable price, very energy efficient, will repurchase if needed!	Jingdong prefecture level city in Shandong
Power Saving King Shop	Green Intelligent Power Saver	Quality assurance, very good, affordable, logistics is also very fast, I'm very satisfied with the	Jingdong prefecture level city in Shandong
Power Saving King Shop	Green Intelligent Power Saver	Energy saving. It's so good, already installed, you can save a lot of electricity every month	Jingdong prefecture level city in Shandong
Power Saving King Shop	Green Intelligent Power Saver	The material is thick and the workmanship is fine in place, the power saving effect is very good, value for money!	Jingdong prefecture level city in Shandong
Power Saving King Shop	Green Intelligent Power Saver	The effect is great, genuine, and match the description, the logistics is fast, the price is reasonable 😍	Jingdong prefecture level city in Shandong

Power Saving King Shop	Green Intelligent Power Saver	It’s genuine, works well, easy to use, plugs in and works, really saves power	Jingdong prefecture level city in Shandong
Power Saving King Shop	Green Intelligent Power Saver	It’s easy to use, effective, great service, and recommended!	Jingdong prefecture level city in Shandong
Power Saving King Shop	Green Intelligent Power Saver	🌈 Great quality, bought it to my liking 💞 Awesome! ✨ Buy with confidence if you need it!	Jingdong prefecture level city in Shandong
Power Saving King Shop	Green Intelligent Power Saver	Good quality, easy to install, very energy saving and power saving, good to use	Jingdong prefecture level city in Shandong
Power Saving King Shop	Green Intelligent Power Saver	The quality is very good, it does have the effect of saving electricity, the description matches, the price is affordable, it is worth recommending.	Jingdong prefecture level city in Shandong
Power Saving King Shop	Green Intelligent Power Saver	Received, the functionality is particularly good, it is genuine, the price is affordable	Jingdong prefecture level city in Shandong
Power Saving King Shop	Green Intelligent Power Saver	Good quality, very easy to use, energy efficient, home electricity bill is obviously much less	Jingdong prefecture level city in Shandong
Power Saving King Shop	Green Intelligent Power Saver	Easy to install, energy saving and power saving, very satisfied with a shopping	Jingdong prefecture level city in Shandong
Power Saving King Shop	Green Intelligent Power Saver	Received it and tried it out, it’s great, works great and worth having! Five stars praise!	Jingdong prefecture level city in Shandong
Power Saving King Shop	Green Intelligent Power Saver	Power saving device, no problem at all, the quality of this product at this price, very affordable!	Jingdong prefecture level city in Shandong
Power Saving King Shop	Green Intelligent Power Saver	Received the goods, did not expect to be so fast, the quality is very good, especially power saving	Jingdong prefecture level city in Shandong
Power Saving King Shop	Green Intelligent Power Saver	The logistics is very fast, especially good, need to come back again	Jingdong prefecture level city in Shandong
Power Saving King Shop	Green Intelligent Power Saver	It’s already working and it really saves power, so I’m very happy with it!	Jingdong prefecture level city in Shandong
Power Saving King Shop	Green Intelligent Power Saver	Great quality, works well, can recommend to friends to buy.	Jingdong prefecture level city in Shandong
Power Saving King Shop	Green Intelligent Power Saver	It’s a good deal and worth the money.	Jingdong prefecture level city in Shandong
Power Saving King Shop	Green Intelligent Power Saver	I received it and I’m very happy with it, it’s very good quality, guaranteed, and	Jingdong prefecture level city in Shandong
Power Saving King Shop	Green Intelligent Power Saver	Good quality, safe and secure purchase, ideal results and great price.	Jingdong prefecture level city in Shandong
Power Saving King Shop	Green Intelligent Power Saver	Simple operation plugged in the power supply can be, friends recommended looking forward to the effect of the	Jingdong prefecture level city in Shandong
Power Saving King Shop	Green Intelligent Power Saver	It’s not bad, it saves electricity, and the material is sturdy and durable.	Jingdong prefecture level city in Shandong
Power Saving King Shop	Green Intelligent Power Saver	Plug and play, no management required, convenient and hassle-free!	Jingdong prefecture level city in Shandong
Power Saving King Shop	Green Intelligent Power Saver	The pictures are too lazy to go up, the power saving effect is still there!	Jingdong prefecture level city in Shandong
Power Saving King Shop	Green Intelligent Power Saver	Fast shipping. Affordable prices. Efficient	Jingdong prefecture level city in Shandong
Power Saving King Shop	Green Intelligent Power Saver	Good power saving, worth buying	Jingdong prefecture level city in Shandong
Power Saving King Shop	Green Intelligent Power Saver	Especially great, comparison shopping before buying, the store owner is very good, answering questions very patiently and detailed, there is a need to come back!	Jingdong prefecture level city in Shandong
Power Saving King Shop	Green Intelligent Power Saver	Very trustworthy brand, quality is guaranteed, the price is still affordable, quite satisfied!	Jingdong prefecture level city in Shandong
Power Saving King Shop	Green Intelligent Power Saver	Recommended by a friend. They say you can save money on air conditioning with this. Try it.	Jingdong prefecture level city in Shandong
Power Saving King Shop	Green Intelligent Power Saver	Works great, will be back next time, very happy with it and a great price!	Jingdong prefecture level city in Shandong
Power Saving King Shop	Green Intelligent Power Saver	The quality of the product is quite good, fast shipping, satisfied.	Jingdong prefecture level city in Shandong
Power Saving King Shop	Green Intelligent Power Saver	Good quality and fast logistics	Jingdong prefecture level city in Shandong
Power Saving King Shop	Green Intelligent Power Saver	It really saves power. I've tested it myself.	Jingdong prefecture level city in Shandong
Power Saving King Shop	Green Intelligent Power Saver	Really saves electricity!	Jingdong prefecture level city in Shandong
Power Saving King Shop	Green Intelligent Power Saver	pretty good	Jingdong prefecture level city in Shandong
Power Saving King Shop	Green Intelligent Power Saver	serviceable	Jingdong prefecture level city in Shandong
Power Saving King Shop	Green Intelligent Power Saver	Power Saving King received a tightly packaged logistics, installed the effect is very good price is cheaper than the physical store!	Jingdong prefecture level city in Shandong
Power Saving King Shop	Green Intelligent Power Saver	Super Power Saving King is very good quality, new style, is genuine, the use of the effect is very good, the price is cheap, it is worth buying!	Jingdong prefecture level city in Shandong
Power Saving King Shop	Green Intelligent Power Saver	Packaging is very tight, easy to install, quite practical, worth buying!	Jingdong prefecture level city in Shandong
Power Saving King Shop	Green Intelligent Power Saver	Quality assurance, affordable price, very energy efficient, will repurchase if needed!	Jingdong prefecture level city in Shandong
Power Saving King Shop	Green Intelligent Power Saver	Quality assurance, very good, affordable, logistics is also very fast, I’m very satisfied with the	Jingdong prefecture level city in Shandong
Power Saving King Shop	Green Intelligent Power Saver	Energy saving. It’s so good, already installed, you can save a lot of electricity every month	Jingdong prefecture level city in Shandong
Power Saving King Shop	Green Intelligent Power Saver	The material is thick and the workmanship is fine in place, the power saving effect is very good, value for money!	Jingdong prefecture level city in Shandong
Power Saving King Shop	Green Intelligent Power Saver	The effect is great, genuine, and match the description, the logistics is fast, the price is reasonable 😍	Jingdong prefecture level city in Shandong
Power Saving King Shop	Green Intelligent Power Saver	It’s genuine, works well, easy to use, plugs in and works, really saves power	Jingdong prefecture level city in Shandong
Power Saving King Shop	Green Intelligent Power Saver	It’s easy to use, effective, great service, and recommended!	Jingdong prefecture level city in Shandong
Power Saving King Shop	Green Intelligent Power Saver	🌈 Great quality, bought it to my liking 💞 Awesome! ✨ Buy with confidence if you need it!	Jingdong prefecture level city in Shandong
Power Saving King Shop	Green Intelligent Power Saver	Good quality, easy to install, very energy saving and power saving, good to use	Jingdong prefecture level city in Shandong
Power Saving King Shop	Green Intelligent Power Saver	The quality is very good, it does have the effect of saving electricity, the description matches, the price is affordable, it is worth recommending.	Jingdong prefecture level city in Shandong
Power Saving King Shop	Green Intelligent Power Saver	Received, the functionality is particularly good, it is genuine, the price is affordable	Jingdong prefecture level city in Shandong
Power Saving King Shop	Green Intelligent Power Saver	Good quality, very easy to use, energy efficient, home electricity bill is obviously much less	Jingdong prefecture level city in Shandong
Power Saving King Shop	Green Intelligent Power Saver	Easy to install, energy saving and power saving, very satisfied with a shopping	Jingdong prefecture level city in Shandong
Power Saving King Shop	Green Intelligent Power Saver	Received it and tried it out, it’s great, works great and worth having! Five stars praise!	Jingdong prefecture level city in Shandong
Power Saving King Shop	Green Intelligent Power Saver	Power saving device, no problem at all, the quality of this product at this price, very affordable!	Jingdong prefecture level city in Shandong

Power Saving King Shop	Green Intelligent Power Saver	Received the goods, did not expect to be so fast, the quality is very good, especially power saving	Jingdong prefecture level city in Shandong
Power Saving King Shop	Green Intelligent Power Saver	The logistics is very fast, especially good, need to come back again	Jingdong prefecture level city in Shandong
Power Saving King Shop	Green Intelligent Power Saver	It's already working and it really saves power, so I'm very happy with it!	Jingdong prefecture level city in Shandong
Power Saving King Shop	Green Intelligent Power Saver	Great quality, works well, can recommend to friends to buy.	Jingdong prefecture level city in Shandong
Power Saving King Shop	Green Intelligent Power Saver	It's a good deal and worth the money.	Jingdong prefecture level city in Shandong
Power Saving King Shop	Green Intelligent Power Saver	I received it and I'm very happy with it, it's very good quality, guaranteed, and	Jingdong prefecture level city in Shandong
Power Saving King Shop	Green Intelligent Power Saver	Good quality, safe and secure purchase, ideal results and great price.	Jingdong prefecture level city in Shandong
Power Saving King Shop	Green Intelligent Power Saver	Simple operation plugged in the power supply can be, friends recommended looking forward to the effect of the	Jingdong prefecture level city in Shandong
Power Saving King Shop	Green Intelligent Power Saver	It's not bad, it saves electricity, and the material is sturdy and durable.	Jingdong prefecture level city in Shandong
Power Saving King Shop	Green Intelligent Power Saver	Plug and play, no management required, convenient and hassle-free!	Jingdong prefecture level city in Shandong
Power Saving King Shop	Green Intelligent Power Saver	The pictures are too lazy to go up, the power saving effect is still there!	Jingdong prefecture level city in Shandong
Power Saving King Shop	Green Intelligent Power Saver	Fast shipping. Affordable prices. Efficient	Jingdong prefecture level city in Shandong
Power Saving King Shop	Green Intelligent Power Saver	Good power saving, worth buying	Jingdong prefecture level city in Shandong
Power Saving King Shop	Green Intelligent Power Saver	Especially great, comparison shopping before buying, the store owner is very good, answering questions very patiently and detailed, there is a need to come back!	Jingdong prefecture level city in Shandong
Power Saving King Shop	Green Intelligent Power Saver	Very trustworthy brand, quality is guaranteed, the price is still affordable, quite satisfied!	Jingdong prefecture level city in Shandong
Power Saving King Shop	Green Intelligent Power Saver	Recommended by a friend. They say you can save money on air conditioning with this. Try it.	Jingdong prefecture level city in Shandong
Power Saving King Shop	Green Intelligent Power Saver	Works great, will be back next time, very happy with it and a great price!	Jingdong prefecture level city in Shandong
Power Saving King Shop	Green Intelligent Power Saver	The quality of the product is quite good, fast shipping, satisfied.	Jingdong prefecture level city in Shandong
Power Saving King Shop	Green Intelligent Power Saver	Good quality and fast logistics	Jingdong prefecture level city in Shandong
Power Saving King Shop	Green Intelligent Power Saver	It really saves power. I've tested it myself.	Jingdong prefecture level city in Shandong
Power Saving King Shop	Green Intelligent Power Saver	Really saves electricity!	Jingdong prefecture level city in Shandong
Power Saving King Shop	Green Intelligent Power Saver	pretty good	Jingdong prefecture level city in Shandong
Power Saving King Shop	Green Intelligent Power Saver	serviceable	Jingdong prefecture level city in Shandong
Power Saving King Shop	Green Intelligent Power Saver	Power Saving King received a tightly packaged logistics, installed the effect is very good price is cheaper than the physical store!	Jingdong prefecture level city in Shandong
Power Saving King Shop	Green Intelligent Power Saver	Super Power Saving King is very good quality, new style, is genuine, the use of the effect is very good, the price is cheap, it is worth buying!	Jingdong prefecture level city in Shandong
Power Saving King Shop	Green Intelligent Power Saver	Packaging is very tight, easy to install, quite practical, worth buying!	Jingdong prefecture level city in Shandong
Power Saving King Shop	Green Intelligent Power Saver	Quality assurance, affordable price, very energy efficient, will repurchase if needed!	Jingdong prefecture level city in Shandong
Power Saving King Shop	Green Intelligent Power Saver	Quality assurance, very good, affordable, logistics is also very fast, I'm very satisfied with the	Jingdong prefecture level city in Shandong
Power Saving King Shop	Green Intelligent Power Saver	Energy saving. It's so good, already installed, you can save a lot of electricity every month	Jingdong prefecture level city in Shandong
Power Saving King Shop	Green Intelligent Power Saver	The material is thick and the workmanship is fine in place, the power saving effect is very good, value for money!	Jingdong prefecture level city in Shandong
Power Saving King Shop	Green Intelligent Power Saver	The effect is great, genuine, and match the description, the logistics is fast, the price is reasonable 😍	Jingdong prefecture level city in Shandong
Power Saving King Shop	Green Intelligent Power Saver	It's genuine, works well, easy to use, plugs in and works, really saves power	Jingdong prefecture level city in Shandong
Power Saving King Shop	Green Intelligent Power Saver	It's easy to use, effective, great service, and recommended!	Jingdong prefecture level city in Shandong
Power Saving King Shop	Green Intelligent Power Saver	🌈 Great quality, bought it to my liking 💖 Awesome! ✨ Buy with confidence if you need it!	Jingdong prefecture level city in Shandong
Power Saving King Shop	Green Intelligent Power Saver	Good quality, easy to install, very energy saving and power saving, good to use	Jingdong prefecture level city in Shandong
Power Saving King Shop	Green Intelligent Power Saver	The quality is very good, it does have the effect of saving electricity, the description matches, the price is affordable, it is worth recommending.	Jingdong prefecture level city in Shandong
Power Saving King Shop	Green Intelligent Power Saver	Received, the functionality is particularly good, it is genuine, the price is affordable	Jingdong prefecture level city in Shandong
Power Saving King Shop	Green Intelligent Power Saver	Good quality, very easy to use, energy efficient, home electricity bill is obviously much less	Jingdong prefecture level city in Shandong
Power Saving King Shop	Green Intelligent Power Saver	Easy to install, energy saving and power saving, very satisfied with a shopping	Jingdong prefecture level city in Shandong
Power Saving King Shop	Green Intelligent Power Saver	Received it and tried it out, it's great, works great and worth having! Five stars praise!	Jingdong prefecture level city in Shandong
Power Saving King Shop	Green Intelligent Power Saver	Power saving device, no problem at all, the quality of this product at this price, very affordable!	Jingdong prefecture level city in Shandong
Power Saving King Shop	Green Intelligent Power Saver	Received the goods, did not expect to be so fast, the quality is very good, especially power saving	Jingdong prefecture level city in Shandong
Power Saving King Shop	Green Intelligent Power Saver	The logistics is very fast, especially good, need to come back again	Jingdong prefecture level city in Shandong
Power Saving King Shop	Green Intelligent Power Saver	It's already working and it really saves power, so I'm very happy with it!	Jingdong prefecture level city in Shandong
Power Saving King Shop	Green Intelligent Power Saver	Great quality, works well, can recommend to friends to buy.	Jingdong prefecture level city in Shandong
Power Saving King Shop	Green Intelligent Power Saver	It's a good deal and worth the money.	Jingdong prefecture level city in Shandong
Power Saving King Shop	Green Intelligent Power Saver	I received it and I'm very happy with it, it's very good quality, guaranteed, and	Jingdong prefecture level city in Shandong
Power Saving King Shop	Green Intelligent Power Saver	Good quality, safe and secure purchase, ideal results and great price.	Jingdong prefecture level city in Shandong
Power Saving King Shop	Green Intelligent Power Saver	Simple operation plugged in the power supply can be, friends recommended looking forward to the effect of the	Jingdong prefecture level city in Shandong
Power Saving King Shop	Green Intelligent Power Saver	It's not bad, it saves electricity, and the material is sturdy and durable.	Jingdong prefecture level city in Shandong
Power Saving King Shop	Green Intelligent Power Saver	Plug and play, no management required, convenient and hassle-free!	Jingdong prefecture level city in Shandong

Power Saving King Shop	Green Intelligent Power Saver	The pictures are too lazy to go up, the power saving effect is still there!	Jingdong prefecture level city in Shandong
Power Saving King Shop	Green Intelligent Power Saver	Fast shipping. Affordable prices. Efficient	Jingdong prefecture level city in Shandong
Power Saving King Shop	Green Intelligent Power Saver	Good power saving, worth buying	Jingdong prefecture level city in Shandong
Power Saving King Shop	Green Intelligent Power Saver	Especially great, comparison shopping before buying, the store owner is very good, answering questions very patiently and detailed, there is a need to come back!	Jingdong prefecture level city in Shandong
Power Saving King Shop	Green Intelligent Power Saver	Very trustworthy brand, quality is guaranteed, the price is still affordable, quite satisfied!	Jingdong prefecture level city in Shandong
Power Saving King Shop	Green Intelligent Power Saver	Recommended by a friend. They say you can save money on air conditioning with this. Try it.	Jingdong prefecture level city in Shandong
Power Saving King Shop	Green Intelligent Power Saver	Works great, will be back next time, very happy with it and a great price!	Jingdong prefecture level city in Shandong
Power Saving King Shop	Green Intelligent Power Saver	The quality of the product is quite good, fast shipping, satisfied.	Jingdong prefecture level city in Shandong
Power Saving King Shop	Green Intelligent Power Saver	Good quality and fast logistics	Jingdong prefecture level city in Shandong
Power Saving King Shop	Green Intelligent Power Saver	It really saves power. I've tested it myself.	Jingdong prefecture level city in Shandong
Power Saving King Shop	Green Intelligent Power Saver	Really saves electricity!	Jingdong prefecture level city in Shandong
Power Saving King Shop	Green Intelligent Power Saver	pretty good	Jingdong prefecture level city in Shandong
Power Saving King Shop	Green Intelligent Power Saver	serviceable	Jingdong prefecture level city in Shandong
Power Saving King Shop	Green Intelligent Power Saver	Power Saving King received a tightly packaged logistics, installed the effect is very good price is cheaper than the physical store!	Jingdong prefecture level city in Shandong
Power Saving King Shop	Green Intelligent Power Saver	Super Power Saving King is very good quality, new style, is genuine, the use of the effect is very good, the price is cheap, it is worth buying!	Jingdong prefecture level city in Shandong
Power Saving King Shop	Green Intelligent Power Saver	Packaging is very tight, easy to install, quite practical, worth buying!	Jingdong prefecture level city in Shandong
Power Saving King Shop	Green Intelligent Power Saver	Quality assurance, affordable price, very energy efficient, will repurchase if needed!	Jingdong prefecture level city in Shandong
Power Saving King Shop	Green Intelligent Power Saver	Quality assurance, very good, affordable, logistics is also very fast, I'm very satisfied with the	Jingdong prefecture level city in Shandong
Power Saving King Shop	Green Intelligent Power Saver	Energy saving. It's so good, already installed, you can save a lot of electricity every month	Jingdong prefecture level city in Shandong
Power Saving King Shop	Green Intelligent Power Saver	The material is thick and the workmanship is fine in place, the power saving effect is very good, value for money!	Jingdong prefecture level city in Shandong
Power Saving King Shop	Green Intelligent Power Saver	The effect is great, genuine, and match the description, the logistics is fast, the price is reasonable 😍	Jingdong prefecture level city in Shandong
Power Saving King Shop	Green Intelligent Power Saver	It's genuine, works well, easy to use, plugs in and works, really saves power	Jingdong prefecture level city in Shandong
Power Saving King Shop	Green Intelligent Power Saver	It's easy to use, effective, great service, and recommended!	Jingdong prefecture level city in Shandong
Power Saving King Shop	Green Intelligent Power Saver	🌈 Great quality, bought it to my liking 💗 Awesome! ✨ Buy with confidence if you need it!	Jingdong prefecture level city in Shandong
Power Saving King Shop	Green Intelligent Power Saver	Good quality, easy to install, very energy saving and power saving, good to use	Jingdong prefecture level city in Shandong
Power Saving King Shop	Green Intelligent Power Saver	The quality is very good, it does have the effect of saving electricity, the description matches, the price is affordable, it is worth recommending.	Jingdong prefecture level city in Shandong
Power Saving King Shop	Green Intelligent Power Saver	Received, the functionality is particularly good, it is genuine, the price is affordable	Jingdong prefecture level city in Shandong
Power Saving King Shop	Green Intelligent Power Saver	Good quality, very easy to use, energy efficient, home electricity bill is obviously much less	Jingdong prefecture level city in Shandong
Power Saving King Shop	Green Intelligent Power Saver	Easy to install, energy saving and power saving, very satisfied with a shopping	Jingdong prefecture level city in Shandong
Power Saving King Shop	Green Intelligent Power Saver	Received it and tried it out, it's great, works great and worth having! Five stars praise!	Jingdong prefecture level city in Shandong
Power Saving King Shop	Green Intelligent Power Saver	Power saving device, no problem at all, the quality of this product at this price, very affordable!	Jingdong prefecture level city in Shandong
Power Saving King Shop	Green Intelligent Power Saver	Received the goods, did not expect to be so fast, the quality is very good, especially power saving	Jingdong prefecture level city in Shandong
Power Saving King Shop	Green Intelligent Power Saver	The logistics is very fast, especially good, need to come back again	Jingdong prefecture level city in Shandong
Power Saving King Shop	Green Intelligent Power Saver	It's already working and it really saves power, so I'm very happy with it!	Jingdong prefecture level city in Shandong
Power Saving King Shop	Green Intelligent Power Saver	Great quality, works well, can recommend to friends to buy.	Jingdong prefecture level city in Shandong
Power Saving King Shop	Green Intelligent Power Saver	It's a good deal and worth the money.	Jingdong prefecture level city in Shandong
Power Saving King Shop	Green Intelligent Power Saver	I received it and I'm very happy with it, it's very good quality, guaranteed, and	Jingdong prefecture level city in Shandong
Power Saving King Shop	Green Intelligent Power Saver	Good quality, safe and secure purchase, ideal results and great price.	Jingdong prefecture level city in Shandong
Power Saving King Shop	Green Intelligent Power Saver	Simple operation plugged in the power supply can be, friends recommended looking forward to the effect of the	Jingdong prefecture level city in Shandong
Power Saving King Shop	Green Intelligent Power Saver	It's not bad, it saves electricity, and the material is sturdy and durable.	Jingdong prefecture level city in Shandong
Power Saving King Shop	Green Intelligent Power Saver	Plug and play, no management required, convenient and hassle-free!	Jingdong prefecture level city in Shandong
Power Saving King Shop	Green Intelligent Power Saver	The pictures are too lazy to go up, the power saving effect is still there!	Jingdong prefecture level city in Shandong
Power Saving King Shop	Green Intelligent Power Saver	Fast shipping. Affordable prices. Efficient	Jingdong prefecture level city in Shandong
Power Saving King Shop	Green Intelligent Power Saver	Good power saving, worth buying	Jingdong prefecture level city in Shandong
Power Saving King Shop	Green Intelligent Power Saver	Especially great, comparison shopping before buying, the store owner is very good, answering questions very patiently and detailed, there is a need to come back!	Jingdong prefecture level city in Shandong
Power Saving King Shop	Green Intelligent Power Saver	Very trustworthy brand, quality is guaranteed, the price is still affordable, quite satisfied!	Jingdong prefecture level city in Shandong
Power Saving King Shop	Green Intelligent Power Saver	Recommended by a friend. They say you can save money on air conditioning with this. Try it.	Jingdong prefecture level city in Shandong
Power Saving King Shop	Green Intelligent Power Saver	Works great, will be back next time, very happy with it and a great price!	Jingdong prefecture level city in Shandong
Power Saving King Shop	Green Intelligent Power Saver	The quality of the product is quite good, fast shipping, satisfied.	Jingdong prefecture level city in Shandong
Power Saving King Shop	Green Intelligent Power Saver	Good quality and fast logistics	Jingdong prefecture level city in Shandong
Power Saving King Shop	Green Intelligent Power Saver	It really saves power. I've tested it myself.	Jingdong prefecture level city in Shandong

Power Saving King Shop	Green Intelligent Power Saver	Really saves electricity!	Jingdong prefecture level city in Shandong
Power Saving King Shop	Green Intelligent Power Saver	pretty good	Jingdong prefecture level city in Shandong
Power Saving King Shop	Green Intelligent Power Saver	serviceable	Jingdong prefecture level city in Shandong
Power Saving King Shop	Green Intelligent Power Saver	Power Saving King received a tightly packaged logistics, installed the effect is very good price is cheaper than the physical store!	Jingdong prefecture level city in Shandong
Power Saving King Shop	Green Intelligent Power Saver	Super Power Saving King is very good quality, new style, is genuine, the use of the effect is very good, the price is cheap, it is worth buying!	Jingdong prefecture level city in Shandong
Power Saving King Shop	Green Intelligent Power Saver	Packaging is very tight, easy to install, quite practical, worth buying!	Jingdong prefecture level city in Shandong
Power Saving King Shop	Green Intelligent Power Saver	Quality assurance, affordable price, very energy efficient, will repurchase if needed!	Jingdong prefecture level city in Shandong
Power Saving King Shop	Green Intelligent Power Saver	Quality assurance, very good, affordable, logistics is also very fast, I'm very satisfied with the	Jingdong prefecture level city in Shandong
Power Saving King Shop	Green Intelligent Power Saver	Energy saving. It's so good, already installed, you can save a lot of electricity every month	Jingdong prefecture level city in Shandong
Power Saving King Shop	Green Intelligent Power Saver	The material is thick and the workmanship is fine in place, the power saving effect is very good, value for money!	Jingdong prefecture level city in Shandong
Power Saving King Shop	Green Intelligent Power Saver	The effect is great, genuine, and match the description, the logistics is fast, the price is reasonable 😍	Jingdong prefecture level city in Shandong
Power Saving King Shop	Green Intelligent Power Saver	It's genuine, works well, easy to use, plugs in and works, really saves power	Jingdong prefecture level city in Shandong
Power Saving King Shop	Green Intelligent Power Saver	It's easy to use, effective, great service, and recommended!	Jingdong prefecture level city in Shandong
Power Saving King Shop	Green Intelligent Power Saver	🌈 Great quality, bought it to my liking 💝 Awesome! ✨ Buy with confidence if you need it!	Jingdong prefecture level city in Shandong
Power Saving King Shop	Green Intelligent Power Saver	Good quality, easy to install, very energy saving and power saving, good to use	Jingdong prefecture level city in Shandong
Power Saving King Shop	Green Intelligent Power Saver	The quality is very good, it does have the effect of saving electricity, the description matches, the price is affordable, it is worth recommending.	Jingdong prefecture level city in Shandong
Power Saving King Shop	Green Intelligent Power Saver	Received, the functionality is particularly good, it is genuine, the price is affordable	Jingdong prefecture level city in Shandong
Power Saving King Shop	Green Intelligent Power Saver	Good quality, very easy to use, energy efficient, home electricity bill is obviously much less	Jingdong prefecture level city in Shandong
Power Saving King Shop	Green Intelligent Power Saver	Easy to install, energy saving and power saving, very satisfied with a shopping	Jingdong prefecture level city in Shandong
Power Saving King Shop	Green Intelligent Power Saver	Received it and tried it out, it's great, works great and worth having! Five stars praise!	Jingdong prefecture level city in Shandong
Power Saving King Shop	Green Intelligent Power Saver	Power saving device, no problem at all, the quality of this product at this price, very affordable!	Jingdong prefecture level city in Shandong
Power Saving King Shop	Green Intelligent Power Saver	Received the goods, did not expect to be so fast, the quality is very good, especially power saving	Jingdong prefecture level city in Shandong
Power Saving King Shop	Green Intelligent Power Saver	The logistics is very fast, especially good, need to come back again	Jingdong prefecture level city in Shandong
Power Saving King Shop	Green Intelligent Power Saver	It's already working and it really saves power, so I'm very happy with it!	Jingdong prefecture level city in Shandong
Power Saving King Shop	Green Intelligent Power Saver	Great quality, works well, can recommend to friends to buy.	Jingdong prefecture level city in Shandong
Power Saving King Shop	Green Intelligent Power Saver	It's a good deal and worth the money.	Jingdong prefecture level city in Shandong
Power Saving King Shop	Green Intelligent Power Saver	I received it and I'm very happy with it, it's very good quality, guaranteed, and	Jingdong prefecture level city in Shandong
Power Saving King Shop	Green Intelligent Power Saver	Good quality, safe and secure purchase, ideal results and great price.	Jingdong prefecture level city in Shandong
Power Saving King Shop	Green Intelligent Power Saver	Simple operation plugged in the power supply can be, friends recommended looking forward to the effect of the	Jingdong prefecture level city in Shandong
Power Saving King Shop	Green Intelligent Power Saver	It's not bad, it saves electricity, and the material is sturdy and durable.	Jingdong prefecture level city in Shandong
Power Saving King Shop	Green Intelligent Power Saver	Plug and play, no management required, convenient and hassle-free!	Jingdong prefecture level city in Shandong
Power Saving King Shop	Green Intelligent Power Saver	The pictures are too lazy to go up, the power saving effect is still there!	Jingdong prefecture level city in Shandong
Power Saving King Shop	Green Intelligent Power Saver	Fast shipping. Affordable prices. Efficient	Jingdong prefecture level city in Shandong
Power Saving King Shop	Green Intelligent Power Saver	Good power saving, worth buying	Jingdong prefecture level city in Shandong
Power Saving King Shop	Green Intelligent Power Saver	Especially great, comparison shopping before buying, the store owner is very good, answering questions very patiently and detailed, there is a need to come back!	Jingdong prefecture level city in Shandong
Power Saving King Shop	Green Intelligent Power Saver	Very trustworthy brand, quality is guaranteed, the price is still affordable, quite satisfied!	Jingdong prefecture level city in Shandong
Power Saving King Shop	Green Intelligent Power Saver	Recommended by a friend. They say you can save money on air conditioning with this. Try it.	Jingdong prefecture level city in Shandong
Power Saving King Shop	Green Intelligent Power Saver	Works great, will be back next time, very happy with it and a great price!	Jingdong prefecture level city in Shandong
Power Saving King Shop	Green Intelligent Power Saver	The quality of the product is quite good, fast shipping, satisfied.	Jingdong prefecture level city in Shandong
Power Saving King Shop	Green Intelligent Power Saver	Good quality and fast logistics	Jingdong prefecture level city in Shandong
Power Saving King Shop	Green Intelligent Power Saver	It really saves power. I've tested it myself.	Jingdong prefecture level city in Shandong
Power Saving King Shop	Green Intelligent Power Saver	Really saves electricity!	Jingdong prefecture level city in Shandong
Power Saving King Shop	Green Intelligent Power Saver	pretty good	Jingdong prefecture level city in Shandong
Power Saving King Shop	Green Intelligent Power Saver	serviceable	Jingdong prefecture level city in Shandong
Power Saving King Shop	Green Intelligent Power Saver	Power Saving King received a tightly packaged logistics, installed the effect is very good price is cheaper than the physical store!	Jingdong prefecture level city in Shandong
Power Saving King Shop	Green Intelligent Power Saver	Super Power Saving King is very good quality, new style, is genuine, the use of the effect is very good, the price is cheap, it is worth buying!	Jingdong prefecture level city in Shandong
Power Saving King Shop	Green Intelligent Power Saver	Packaging is very tight, easy to install, quite practical, worth buying!	Jingdong prefecture level city in Shandong
Power Saving King Shop	Green Intelligent Power Saver	Quality assurance, affordable price, very energy efficient, will repurchase if needed!	Jingdong prefecture level city in Shandong
Power Saving King Shop	Green Intelligent Power Saver	Quality assurance, very good, affordable, logistics is also very fast, I'm very satisfied with the	Jingdong prefecture level city in Shandong
Power Saving King Shop	Green Intelligent Power Saver	Energy saving. It's so good, already installed, you can save a lot of electricity every month	Jingdong prefecture level city in Shandong
Power Saving King Shop	Green Intelligent Power Saver	The material is thick and the workmanship is fine in place, the power saving effect is very good, value for money!	Jingdong prefecture level city in Shandong

Power Saving King Shop	Green Intelligent Power Saver	The effect is great, genuine, and match the description, the logistics is fast, the price is reasonable 😍	Jingdong prefecture level city in Shandong
Power Saving King Shop	Green Intelligent Power Saver	It's genuine, works well, easy to use, plugs in and works, really saves power	Jingdong prefecture level city in Shandong
Power Saving King Shop	Green Intelligent Power Saver	It's easy to use, effective, great service, and recommended!	Jingdong prefecture level city in Shandong
Power Saving King Shop	Green Intelligent Power Saver	🌈 Great quality, bought it to my liking 💝 Awesome! ✨ Buy with confidence if you need it!	Jingdong prefecture level city in Shandong
Power Saving King Shop	Green Intelligent Power Saver	Good quality, easy to install, very energy saving and power saving, good to use	Jingdong prefecture level city in Shandong
Power Saving King Shop	Green Intelligent Power Saver	The quality is very good, it does have the effect of saving electricity, the description matches, the price is affordable, it is worth recommending.	Jingdong prefecture level city in Shandong
Power Saving King Shop	Green Intelligent Power Saver	Received, the functionality is particularly good, it is genuine, the price is affordable	Jingdong prefecture level city in Shandong
Power Saving King Shop	Green Intelligent Power Saver	Good quality, very easy to use, energy efficient, home electricity bill is obviously much less	Jingdong prefecture level city in Shandong
Power Saving King Shop	Green Intelligent Power Saver	Easy to install, energy saving and power saving, very satisfied with a shopping	Jingdong prefecture level city in Shandong
Power Saving King Shop	Green Intelligent Power Saver	Received it and tried it out, it's great, works great and worth having! Five stars praise!	Jingdong prefecture level city in Shandong
Power Saving King Shop	Green Intelligent Power Saver	Power saving device, no problem at all, the quality of this product at this price, very affordable!	Jingdong prefecture level city in Shandong
Power Saving King Shop	Green Intelligent Power Saver	Received the goods, did not expect to be so fast, the quality is very good, especially power saving	Jingdong prefecture level city in Shandong
Power Saving King Shop	Green Intelligent Power Saver	The logistics is very fast, especially good, need to come back again	Jingdong prefecture level city in Shandong
Power Saving King Shop	Green Intelligent Power Saver	It's already working and it really saves power, so I'm very happy with it!	Jingdong prefecture level city in Shandong
Power Saving King Shop	Green Intelligent Power Saver	Great quality, works well, can recommend to friends to buy.	Jingdong prefecture level city in Shandong
Power Saving King Shop	Green Intelligent Power Saver	It's a good deal and worth the money.	Jingdong prefecture level city in Shandong
Power Saving King Shop	Green Intelligent Power Saver	I received it and I'm very happy with it, it's very good quality, guaranteed, and	Jingdong prefecture level city in Shandong
Power Saving King Shop	Green Intelligent Power Saver	Good quality, safe and secure purchase, ideal results and great price.	Jingdong prefecture level city in Shandong
Power Saving King Shop	Green Intelligent Power Saver	Simple operation plugged in the power supply can be, friends recommended looking forward to the effect of the	Jingdong prefecture level city in Shandong
Power Saving King Shop	Green Intelligent Power Saver	It's not bad, it saves electricity, and the material is sturdy and durable.	Jingdong prefecture level city in Shandong
Power Saving King Shop	Green Intelligent Power Saver	Plug and play, no management required, convenient and hassle-free!	Jingdong prefecture level city in Shandong
Power Saving King Shop	Green Intelligent Power Saver	The pictures are too lazy to go up, the power saving effect is still there!	Jingdong prefecture level city in Shandong
Power Saving King Shop	Green Intelligent Power Saver	Fast shipping. Affordable prices. Efficient	Jingdong prefecture level city in Shandong
Power Saving King Shop	Green Intelligent Power Saver	Good power saving, worth buying	Jingdong prefecture level city in Shandong
Power Saving King Shop	Green Intelligent Power Saver	Especially great, comparison shopping before buying, the store owner is very good, answering questions very patiently and detailed, there is a need to come back!	Jingdong prefecture level city in Shandong
Power Saving King Shop	Green Intelligent Power Saver	Very trustworthy brand, quality is guaranteed, the price is still affordable, quite satisfied!	Jingdong prefecture level city in Shandong
Power Saving King Shop	Green Intelligent Power Saver	Recommended by a friend. They say you can save money on air conditioning with this. Try it.	Jingdong prefecture level city in Shandong
Power Saving King Shop	Green Intelligent Power Saver	Works great, will be back next time, very happy with it and a great price!	Jingdong prefecture level city in Shandong
Power Saving King Shop	Green Intelligent Power Saver	The quality of the product is quite good, fast shipping, satisfied.	Jingdong prefecture level city in Shandong
Power Saving King Shop	Green Intelligent Power Saver	Good quality and fast logistics	Jingdong prefecture level city in Shandong
Power Saving King Shop	Green Intelligent Power Saver	It really saves power. I've tested it myself.	Jingdong prefecture level city in Shandong
Power Saving King Shop	Green Intelligent Power Saver	Really saves electricity!	Jingdong prefecture level city in Shandong
Power Saving King Shop	Green Intelligent Power Saver	pretty good	Jingdong prefecture level city in Shandong
Power Saving King Shop	Green Intelligent Power Saver	serviceable	Jingdong prefecture level city in Shandong
Power Saving King Shop	Green Intelligent Power Saver	Power Saving King received a tightly packaged logistics, installed the effect is very good price is cheaper than the physical store!	Jingdong prefecture level city in Shandong
Power Saving King Shop	Green Intelligent Power Saver	Super Power Saving King is very good quality, new style, is genuine, the use of the effect is very good, the price is cheap, it is worth buying!	Jingdong prefecture level city in Shandong
Power Saving King Shop	Green Intelligent Power Saver	Packaging is very tight, easy to install, quite practical, worth buying!	Jingdong prefecture level city in Shandong
Power Saving King Shop	Green Intelligent Power Saver	Quality assurance, affordable price, very energy efficient, will repurchase if needed!	Jingdong prefecture level city in Shandong
Power Saving King Shop	Green Intelligent Power Saver	Quality assurance, very good, affordable, logistics is also very fast, I'm very satisfied with the	Jingdong prefecture level city in Shandong
Power Saving King Shop	Green Intelligent Power Saver	Energy saving. It's so good, already installed, you can save a lot of electricity every month	Jingdong prefecture level city in Shandong
Power Saving King Shop	Green Intelligent Power Saver	The material is thick and the workmanship is fine in place, the power saving effect is very good, value for money!	Jingdong prefecture level city in Shandong
Power Saving King Shop	Green Intelligent Power Saver	The effect is great, genuine, and match the description, the logistics is fast, the price is reasonable 😍	Jingdong prefecture level city in Shandong
Power Saving King Shop	Green Intelligent Power Saver	It's genuine, works well, easy to use, plugs in and works, really saves power	Jingdong prefecture level city in Shandong
Power Saving King Shop	Green Intelligent Power Saver	It's easy to use, effective, great service, and recommended!	Jingdong prefecture level city in Shandong
Power Saving King Shop	Green Intelligent Power Saver	🌈 Great quality, bought it to my liking 💝 Awesome! ✨ Buy with confidence if you need it!	Jingdong prefecture level city in Shandong
Power Saving King Shop	Green Intelligent Power Saver	Good quality, easy to install, very energy saving and power saving, good to use	Jingdong prefecture level city in Shandong
Power Saving King Shop	Green Intelligent Power Saver	The quality is very good, it does have the effect of saving electricity, the description matches, the price is affordable, it is worth recommending.	Jingdong prefecture level city in Shandong
Power Saving King Shop	Green Intelligent Power Saver	Received, the functionality is particularly good, it is genuine, the price is affordable	Jingdong prefecture level city in Shandong
Power Saving King Shop	Green Intelligent Power Saver	Good quality, very easy to use, energy efficient, home electricity bill is obviously much less	Jingdong prefecture level city in Shandong
Power Saving King Shop	Green Intelligent Power Saver	Easy to install, energy saving and power saving, very satisfied with a shopping	Jingdong prefecture level city in Shandong
Power Saving King Shop	Green Intelligent Power Saver	Received it and tried it out, it's great, works great and worth having! Five stars praise!	Jingdong prefecture level city in Shandong

Power Saving King Shop	Green Intelligent Power Saver	Power saving device, no problem at all, the quality of this product at this price, very affordable!	Jingdong prefecture level city in Shandong
Power Saving King Shop	Green Intelligent Power Saver	Received the goods, did not expect to be so fast, the quality is very good, especially power saving	Jingdong prefecture level city in Shandong
Power Saving King Shop	Green Intelligent Power Saver	The logistics is very fast, especially good, need to come back again	Jingdong prefecture level city in Shandong
Power Saving King Shop	Green Intelligent Power Saver	It's already working and it really saves power, so I'm very happy with it!	Jingdong prefecture level city in Shandong
Power Saving King Shop	Green Intelligent Power Saver	Great quality, works well, can recommend to friends to buy.	Jingdong prefecture level city in Shandong
Power Saving King Shop	Green Intelligent Power Saver	It's a good deal and worth the money.	Jingdong prefecture level city in Shandong
Power Saving King Shop	Green Intelligent Power Saver	I received it and I'm very happy with it, it's very good quality, guaranteed, and	Jingdong prefecture level city in Shandong
Power Saving King Shop	Green Intelligent Power Saver	Good quality, safe and secure purchase, ideal results and great price.	Jingdong prefecture level city in Shandong
Power Saving King Shop	Green Intelligent Power Saver	Simple operation plugged in the power supply can be, friends recommended looking forward to the effect of the	Jingdong prefecture level city in Shandong
Power Saving King Shop	Green Intelligent Power Saver	It's not bad, it saves electricity, and the material is sturdy and durable.	Jingdong prefecture level city in Shandong
Power Saving King Shop	Green Intelligent Power Saver	Plug and play, no management required, convenient and hassle-free!	Jingdong prefecture level city in Shandong
Power Saving King Shop	Green Intelligent Power Saver	The pictures are too lazy to go up, the power saving effect is still there!	Jingdong prefecture level city in Shandong
Power Saving King Shop	Green Intelligent Power Saver	Fast shipping. Affordable prices. Efficient	Jingdong prefecture level city in Shandong
Power Saving King Shop	Green Intelligent Power Saver	Good power saving, worth buying	Jingdong prefecture level city in Shandong
Power Saving King Shop	Green Intelligent Power Saver	Especially great, comparison shopping before buying, the store owner is very good, answering questions very patiently and detailed, there is a need to come back!	Jingdong prefecture level city in Shandong
Power Saving King Shop	Green Intelligent Power Saver	Very trustworthy brand, quality is guaranteed, the price is still affordable, quite satisfied!	Jingdong prefecture level city in Shandong
Power Saving King Shop	Green Intelligent Power Saver	Recommended by a friend. They say you can save money on air conditioning with this. Try it.	Jingdong prefecture level city in Shandong
Power Saving King Shop	Green Intelligent Power Saver	Works great, will be back next time, very happy with it and a great price!	Jingdong prefecture level city in Shandong
Power Saving King Shop	Green Intelligent Power Saver	The quality of the product is quite good, fast shipping, satisfied.	Jingdong prefecture level city in Shandong
Power Saving King Shop	Green Intelligent Power Saver	Good quality and fast logistics	Jingdong prefecture level city in Shandong
Power Saving King Shop	Green Intelligent Power Saver	It really saves power. I've tested it myself.	Jingdong prefecture level city in Shandong
Power Saving King Shop	Green Intelligent Power Saver	Really saves electricity!	Jingdong prefecture level city in Shandong
Power Saving King Shop	Green Intelligent Power Saver	pretty good	Jingdong prefecture level city in Shandong
Power Saving King Shop	Green Intelligent Power Saver	serviceable	Jingdong prefecture level city in Shandong
Power Saving King Shop	Green Intelligent Power Saver	Power Saving King received a tightly packaged logistics, installed the effect is very good price is cheaper than the physical store!	Jingdong prefecture level city in Shandong
Power Saving King Shop	Green Intelligent Power Saver	Super Power Saving King is very good quality, new style, is genuine, the use of the effect is very good, the price is cheap, it is worth buying!	Jingdong prefecture level city in Shandong
Power Saving King Shop	Green Intelligent Power Saver	Packaging is very tight, easy to install, quite practical, worth buying!	Jingdong prefecture level city in Shandong
Power Saving King Shop	Green Intelligent Power Saver	Quality assurance, affordable price, very energy efficient, will repurchase if needed!	Jingdong prefecture level city in Shandong
Power Saving King Shop	Green Intelligent Power Saver	Quality assurance, very good, affordable, logistics is also very fast, I'm very satisfied with the	Jingdong prefecture level city in Shandong
Power Saving King Shop	Green Intelligent Power Saver	Energy saving. It's so good, already installed, you can save a lot of electricity every month	Jingdong prefecture level city in Shandong
Power Saving King Shop	Green Intelligent Power Saver	The material is thick and the workmanship is fine in place, the power saving effect is very good, value for money!	Jingdong prefecture level city in Shandong
Power Saving King Shop	Green Intelligent Power Saver	The effect is great, genuine, and match the description, the logistics is fast, the price is reasonable 😍	Jingdong prefecture level city in Shandong
Power Saving King Shop	Green Intelligent Power Saver	It's genuine, works well, easy to use, plugs in and works, really saves power	Jingdong prefecture level city in Shandong
Power Saving King Shop	Green Intelligent Power Saver	It's easy to use, effective, great service, and recommended!	Jingdong prefecture level city in Shandong
Power Saving King Shop	Green Intelligent Power Saver	🌈 Great quality, bought it to my liking 💝 Awesome! ✨ Buy with confidence if you need it!	Jingdong prefecture level city in Shandong
Power Saving King Shop	Green Intelligent Power Saver	Good quality, easy to install, very energy saving and power saving, good to use	Jingdong prefecture level city in Shandong
Power Saving King Shop	Green Intelligent Power Saver	The quality is very good, it does have the effect of saving electricity, the description matches, the price is affordable, it is worth recommending.	Jingdong prefecture level city in Shandong
Power Saving King Shop	Green Intelligent Power Saver	Received, the functionality is particularly good, it is genuine, the price is affordable	Jingdong prefecture level city in Shandong
Power Saving King Shop	Green Intelligent Power Saver	Good quality, very easy to use, energy efficient, home electricity bill is obviously much less	Jingdong prefecture level city in Shandong
Power Saving King Shop	Green Intelligent Power Saver	Easy to install, energy saving and power saving, very satisfied with a shopping	Jingdong prefecture level city in Shandong
Power Saving King Shop	Green Intelligent Power Saver	Received it and tried it out, it's great, works great and worth having! Five stars praise!	Jingdong prefecture level city in Shandong
Power Saving King Shop	Green Intelligent Power Saver	Power saving device, no problem at all, the quality of this product at this price, very affordable!	Jingdong prefecture level city in Shandong
Power Saving King Shop	Green Intelligent Power Saver	Received the goods, did not expect to be so fast, the quality is very good, especially power saving	Jingdong prefecture level city in Shandong
Power Saving King Shop	Green Intelligent Power Saver	The logistics is very fast, especially good, need to come back again	Jingdong prefecture level city in Shandong
Power Saving King Shop	Green Intelligent Power Saver	It's already working and it really saves power, so I'm very happy with it!	Jingdong prefecture level city in Shandong
Power Saving King Shop	Green Intelligent Power Saver	Great quality, works well, can recommend to friends to buy.	Jingdong prefecture level city in Shandong
Power Saving King Shop	Green Intelligent Power Saver	It's a good deal and worth the money.	Jingdong prefecture level city in Shandong
Power Saving King Shop	Green Intelligent Power Saver	I received it and I'm very happy with it, it's very good quality, guaranteed, and	Jingdong prefecture level city in Shandong
Power Saving King Shop	Green Intelligent Power Saver	Good quality, safe and secure purchase, ideal results and great price.	Jingdong prefecture level city in Shandong
Power Saving King Shop	Green Intelligent Power Saver	Simple operation plugged in the power supply can be, friends recommended looking forward to the effect of the	Jingdong prefecture level city in Shandong
Power Saving King Shop	Green Intelligent Power Saver	It's not bad, it saves electricity, and the material is sturdy and durable.	Jingdong prefecture level city in Shandong

Power Saving King Shop	Green Intelligent Power Saver	Plug and play, no management required, convenient and hassle-free!	Jingdong prefecture level city in Shandong
Power Saving King Shop	Green Intelligent Power Saver	The pictures are too lazy to go up, the power saving effect is still there!	Jingdong prefecture level city in Shandong
Power Saving King Shop	Green Intelligent Power Saver	Fast shipping. Affordable prices. Efficient	Jingdong prefecture level city in Shandong
Power Saving King Shop	Green Intelligent Power Saver	Good power saving, worth buying	Jingdong prefecture level city in Shandong
Power Saving King Shop	Green Intelligent Power Saver	Especially great, comparison shopping before buying, the store owner is very good, answering questions very patiently and detailed, there is a need to come back!	Jingdong prefecture level city in Shandong
Power Saving King Shop	Green Intelligent Power Saver	Very trustworthy brand, quality is guaranteed, the price is still affordable, quite satisfied!	Jingdong prefecture level city in Shandong
Power Saving King Shop	Green Intelligent Power Saver	Recommended by a friend. They say you can save money on air conditioning with this. Try it.	Jingdong prefecture level city in Shandong
Power Saving King Shop	Green Intelligent Power Saver	Works great, will be back next time, very happy with it and a great price!	Jingdong prefecture level city in Shandong
Power Saving King Shop	Green Intelligent Power Saver	The quality of the product is quite good, fast shipping, satisfied.	Jingdong prefecture level city in Shandong
Power Saving King Shop	Green Intelligent Power Saver	Good quality and fast logistics	Jingdong prefecture level city in Shandong
Power Saving King Shop	Green Intelligent Power Saver	It really saves power. I've tested it myself.	Jingdong prefecture level city in Shandong
Power Saving King Shop	Green Intelligent Power Saver	Really saves electricity!	Jingdong prefecture level city in Shandong
Power Saving King Shop	Green Intelligent Power Saver	pretty good	Jingdong prefecture level city in Shandong
Power Saving King Shop	Green Intelligent Power Saver	serviceable	Jingdong prefecture level city in Shandong
Power Saving King Shop	Green Intelligent Power Saver	Power Saving King received a tightly packaged logistics, installed the effect is very good price is cheaper than the physical store!	Jingdong prefecture level city in Shandong
Power Saving King Shop	Green Intelligent Power Saver	Super Power Saving King is very good quality, new style, is genuine, the use of the effect is very good, the price is cheap, it is worth buying!	Jingdong prefecture level city in Shandong
Power Saving King Shop	Green Intelligent Power Saver	Packaging is very tight, easy to install, quite practical, worth buying!	Jingdong prefecture level city in Shandong
Power Saving King Shop	Green Intelligent Power Saver	Quality assurance, affordable price, very energy efficient, will repurchase if needed!	Jingdong prefecture level city in Shandong
Power Saving King Shop	Green Intelligent Power Saver	Quality assurance, very good, affordable, logistics is also very fast, I'm very satisfied with the	Jingdong prefecture level city in Shandong
Power Saving King Shop	Green Intelligent Power Saver	Energy saving. It's so good, already installed, you can save a lot of electricity every month	Jingdong prefecture level city in Shandong
Power Saving King Shop	Green Intelligent Power Saver	The material is thick and the workmanship is fine in place, the power saving effect is very good, value for money!	Jingdong prefecture level city in Shandong
Power Saving King Shop	Green Intelligent Power Saver	The effect is great, genuine, and match the description, the logistics is fast, the price is reasonable 😍	Jingdong prefecture level city in Shandong
Power Saving King Shop	Green Intelligent Power Saver	It's genuine, works well, easy to use, plugs in and works, really saves power	Jingdong prefecture level city in Shandong
Power Saving King Shop	Green Intelligent Power Saver	It's easy to use, effective, great service, and recommended!	Jingdong prefecture level city in Shandong
Power Saving King Shop	Green Intelligent Power Saver	🌈 Great quality, bought it to my liking 💝 Awesome! 🌟 Buy with confidence if you need it!	Jingdong prefecture level city in Shandong
Power Saving King Shop	Green Intelligent Power Saver	Good quality, easy to install, very energy saving and power saving, good to use	Jingdong prefecture level city in Shandong
Power Saving King Shop	Green Intelligent Power Saver	The quality is very good, it does have the effect of saving electricity, the description matches, the price is affordable, it is worth recommending.	Jingdong prefecture level city in Shandong
Power Saving King Shop	Green Intelligent Power Saver	Received, the functionality is particularly good, it is genuine, the price is affordable	Jingdong prefecture level city in Shandong
Power Saving King Shop	Green Intelligent Power Saver	Good quality, very easy to use, energy efficient, home electricity bill is obviously much less	Jingdong prefecture level city in Shandong
Power Saving King Shop	Green Intelligent Power Saver	Easy to install, energy saving and power saving, very satisfied with a shopping	Jingdong prefecture level city in Shandong
Power Saving King Shop	Green Intelligent Power Saver	Received it and tried it out, it's great, works great and worth having! Five stars praise!	Jingdong prefecture level city in Shandong
Power Saving King Shop	Green Intelligent Power Saver	Power saving device, no problem at all, the quality of this product at this price, very affordable!	Jingdong prefecture level city in Shandong
Power Saving King Shop	Green Intelligent Power Saver	Received the goods, did not expect to be so fast, the quality is very good, especially power saving	Jingdong prefecture level city in Shandong
Power Saving King Shop	Green Intelligent Power Saver	The logistics is very fast, especially good, need to come back again	Jingdong prefecture level city in Shandong
Power Saving King Shop	Green Intelligent Power Saver	It's already working and it really saves power, so I'm very happy with it!	Jingdong prefecture level city in Shandong
Power Saving King Shop	Green Intelligent Power Saver	Great quality, works well, can recommend to friends to buy.	Jingdong prefecture level city in Shandong
Power Saving King Shop	Green Intelligent Power Saver	It's a good deal and worth the money.	Jingdong prefecture level city in Shandong
Power Saving King Shop	Green Intelligent Power Saver	I received it and I'm very happy with it, it's very good quality, guaranteed, and	Jingdong prefecture level city in Shandong
Power Saving King Shop	Green Intelligent Power Saver	Good quality, safe and secure purchase, ideal results and great price.	Jingdong prefecture level city in Shandong
Power Saving King Shop	Green Intelligent Power Saver	Simple operation plugged in the power supply can be, friends recommended looking forward to the effect of the	Jingdong prefecture level city in Shandong
Power Saving King Shop	Green Intelligent Power Saver	It's not bad, it saves electricity, and the material is sturdy and durable.	Jingdong prefecture level city in Shandong
Power Saving King Shop	Green Intelligent Power Saver	Plug and play, no management required, convenient and hassle-free!	Jingdong prefecture level city in Shandong
Power Saving King Shop	Green Intelligent Power Saver	The pictures are too lazy to go up, the power saving effect is still there!	Jingdong prefecture level city in Shandong
Power Saving King Shop	Green Intelligent Power Saver	Fast shipping. Affordable prices. Efficient	Jingdong prefecture level city in Shandong
Power Saving King Shop	Green Intelligent Power Saver	Good power saving, worth buying	Jingdong prefecture level city in Shandong
Power Saving King Shop	Green Intelligent Power Saver	Especially great, comparison shopping before buying, the store owner is very good, answering questions very patiently and detailed, there is a need to come back!	Jingdong prefecture level city in Shandong
Power Saving King Shop	Green Intelligent Power Saver	Very trustworthy brand, quality is guaranteed, the price is still affordable, quite satisfied!	Jingdong prefecture level city in Shandong
Power Saving King Shop	Green Intelligent Power Saver	Recommended by a friend. They say you can save money on air conditioning with this. Try it.	Jingdong prefecture level city in Shandong
Power Saving King Shop	Green Intelligent Power Saver	Works great, will be back next time, very happy with it and a great price!	Jingdong prefecture level city in Shandong
Power Saving King Shop	Green Intelligent Power Saver	The quality of the product is quite good, fast shipping, satisfied.	Jingdong prefecture level city in Shandong
Power Saving King Shop	Green Intelligent Power Saver	Good quality and fast logistics	Jingdong prefecture level city in Shandong

Power Saving King Shop	Green Intelligent Power Saver	It really saves power. I've tested it myself.	Jingdong prefecture level city in Shandong
Power Saving King Shop	Green Intelligent Power Saver	Really saves electricity!	Jingdong prefecture level city in Shandong
Power Saving King Shop	Green Intelligent Power Saver	pretty good	Jingdong prefecture level city in Shandong
Power Saving King Shop	Green Intelligent Power Saver	serviceable	Jingdong prefecture level city in Shandong
Power Saving King Shop	Green Intelligent Power Saver	Power Saving King received a tightly packaged logistics, installed the effect is very good price is cheaper than the physical store!	Jingdong prefecture level city in Shandong
Power Saving King Shop	Green Intelligent Power Saver	Super Power Saving King is very good quality, new style, is genuine, the use of the effect is very good, the price is cheap, it is worth buying!	Jingdong prefecture level city in Shandong
Power Saving King Shop	Green Intelligent Power Saver	Packaging is very tight, easy to install, quite practical, worth buying!	Jingdong prefecture level city in Shandong
Power Saving King Shop	Green Intelligent Power Saver	Quality assurance, affordable price, very energy efficient, will repurchase if needed!	Jingdong prefecture level city in Shandong
Power Saving King Shop	Green Intelligent Power Saver	Quality assurance, very good, affordable, logistics is also very fast, I'm very satisfied with the	Jingdong prefecture level city in Shandong
Power Saving King Shop	Green Intelligent Power Saver	Energy saving. It's so good, already installed, you can save a lot of electricity every month	Jingdong prefecture level city in Shandong
Power Saving King Shop	Green Intelligent Power Saver	The material is thick and the workmanship is fine in place, the power saving effect is very good, value for money!	Jingdong prefecture level city in Shandong
Power Saving King Shop	Green Intelligent Power Saver	The effect is great, genuine, and match the description, the logistics is fast, the price is reasonable 😍	Jingdong prefecture level city in Shandong
Power Saving King Shop	Green Intelligent Power Saver	It's genuine, works well, easy to use, plugs in and works, really saves power	Jingdong prefecture level city in Shandong
Power Saving King Shop	Green Intelligent Power Saver	It's easy to use, effective, great service, and recommended!	Jingdong prefecture level city in Shandong
Power Saving King Shop	Green Intelligent Power Saver	🌈 Great quality, bought it to my liking 💝 Awesome! ✨ Buy with confidence if you need it!	Jingdong prefecture level city in Shandong
Power Saving King Shop	Green Intelligent Power Saver	Good quality, easy to install, very energy saving and power saving, good to use	Jingdong prefecture level city in Shandong
Power Saving King Shop	Green Intelligent Power Saver	The quality is very good, it does have the effect of saving electricity, the description matches, the price is affordable, it is worth recommending.	Jingdong prefecture level city in Shandong
Power Saving King Shop	Green Intelligent Power Saver	Received, the functionality is particularly good, it is genuine, the price is affordable	Jingdong prefecture level city in Shandong
Power Saving King Shop	Green Intelligent Power Saver	Good quality, very easy to use, energy efficient, home electricity bill is obviously much less	Jingdong prefecture level city in Shandong
Power Saving King Shop	Green Intelligent Power Saver	Easy to install, energy saving and power saving, very satisfied with a shopping	Jingdong prefecture level city in Shandong
Power Saving King Shop	Green Intelligent Power Saver	Received it and tried it out, it's great, works great and worth having! Five stars praise!	Jingdong prefecture level city in Shandong
Power Saving King Shop	Green Intelligent Power Saver	Power saving device, no problem at all, the quality of this product at this price, very affordable!	Jingdong prefecture level city in Shandong
Power Saving King Shop	Green Intelligent Power Saver	Received the goods, did not expect to be so fast, the quality is very good, especially power saving	Jingdong prefecture level city in Shandong
Power Saving King Shop	Green Intelligent Power Saver	The logistics is very fast, especially good, need to come back again	Jingdong prefecture level city in Shandong
Power Saving King Shop	Green Intelligent Power Saver	It's already working and it really saves power, so I'm very happy with it!	Jingdong prefecture level city in Shandong
Power Saving King Shop	Green Intelligent Power Saver	Great quality, works well, can recommend to friends to buy.	Jingdong prefecture level city in Shandong
Power Saving King Shop	Green Intelligent Power Saver	It's a good deal and worth the money.	Jingdong prefecture level city in Shandong
Power Saving King Shop	Green Intelligent Power Saver	I received it and I'm very happy with it, it's very good quality, guaranteed, and	Jingdong prefecture level city in Shandong
Power Saving King Shop	Green Intelligent Power Saver	Good quality, safe and secure purchase, ideal results and great price.	Jingdong prefecture level city in Shandong
Power Saving King Shop	Green Intelligent Power Saver	Simple operation plugged in the power supply can be, friends recommended looking forward to the effect of the	Jingdong prefecture level city in Shandong
Power Saving King Shop	Green Intelligent Power Saver	It's not bad, it saves electricity, and the material is sturdy and durable.	Jingdong prefecture level city in Shandong
Power Saving King Shop	Green Intelligent Power Saver	Plug and play, no management required, convenient and hassle-free!	Jingdong prefecture level city in Shandong
Power Saving King Shop	Green Intelligent Power Saver	The pictures are too lazy to go up, the power saving effect is still there!	Jingdong prefecture level city in Shandong
Power Saving King Shop	Green Intelligent Power Saver	Fast shipping. Affordable prices. Efficient	Jingdong prefecture level city in Shandong
Power Saving King Shop	Green Intelligent Power Saver	Good power saving, worth buying	Jingdong prefecture level city in Shandong
Power Saving King Shop	Green Intelligent Power Saver	Especially great, comparison shopping before buying, the store owner is very good, answering questions very patiently and detailed, there is a need to come back!	Jingdong prefecture level city in Shandong
Power Saving King Shop	Green Intelligent Power Saver	Very trustworthy brand, quality is guaranteed, the price is still affordable, quite satisfied!	Jingdong prefecture level city in Shandong
Power Saving King Shop	Green Intelligent Power Saver	Recommended by a friend. They say you can save money on air conditioning with this. Try it.	Jingdong prefecture level city in Shandong
Power Saving King Shop	Green Intelligent Power Saver	Works great, will be back next time, very happy with it and a great price!	Jingdong prefecture level city in Shandong
Power Saving King Shop	Green Intelligent Power Saver	The quality of the product is quite good, fast shipping, satisfied.	Jingdong prefecture level city in Shandong
Power Saving King Shop	Green Intelligent Power Saver	Good quality and fast logistics	Jingdong prefecture level city in Shandong
Power Saving King Shop	Green Intelligent Power Saver	It really saves power. I've tested it myself.	Jingdong prefecture level city in Shandong
Power Saving King Shop	Green Intelligent Power Saver	Really saves electricity!	Jingdong prefecture level city in Shandong
Power Saving King Shop	Green Intelligent Power Saver	pretty good	Jingdong prefecture level city in Shandong
Power Saving King Shop	Green Intelligent Power Saver	serviceable	Jingdong prefecture level city in Shandong
Power Saving King Shop	Green Intelligent Power Saver	Power Saving King received a tightly packaged logistics, installed the effect is very good price is cheaper than the physical store!	Jingdong prefecture level city in Shandong
Power Saving King Shop	Green Intelligent Power Saver	Super Power Saving King is very good quality, new style, is genuine, the use of the effect is very good, the price is cheap, it is worth buying!	Jingdong prefecture level city in Shandong
Power Saving King Shop	Green Intelligent Power Saver	Packaging is very tight, easy to install, quite practical, worth buying!	Jingdong prefecture level city in Shandong
Power Saving King Shop	Green Intelligent Power Saver	Quality assurance, affordable price, very energy efficient, will repurchase if needed!	Jingdong prefecture level city in Shandong
Power Saving King Shop	Green Intelligent Power Saver	Quality assurance, very good, affordable, logistics is also very fast, I'm very satisfied with the	Jingdong prefecture level city in Shandong
Power Saving King Shop	Green Intelligent Power Saver	Energy saving. It's so good, already installed, you can save a lot of electricity every month	Jingdong prefecture level city in Shandong

Power Saving King Shop	Green Intelligent Power Saver	The material is thick and the workmanship is fine in place, the power saving effect is very good, value for money!	Jingdong prefecture level city in Shandong
Power Saving King Shop	Green Intelligent Power Saver	The effect is great, genuine, and match the description, the logistics is fast, the price is reasonable 😍	Jingdong prefecture level city in Shandong
Power Saving King Shop	Green Intelligent Power Saver	It's genuine, works well, easy to use, plugs in and works, really saves power	Jingdong prefecture level city in Shandong
Power Saving King Shop	Green Intelligent Power Saver	It's easy to use, effective, great service, and recommended!	Jingdong prefecture level city in Shandong
Power Saving King Shop	Green Intelligent Power Saver	🌈 Great quality, bought it to my liking 💗 Awesome! ✨ Buy with confidence if you need it!	Jingdong prefecture level city in Shandong
Power Saving King Shop	Green Intelligent Power Saver	Good quality, easy to install, very energy saving and power saving, good to use	Jingdong prefecture level city in Shandong
Power Saving King Shop	Green Intelligent Power Saver	The quality is very good, it does have the effect of saving electricity, the description matches, the price is affordable, it is worth recommending.	Jingdong prefecture level city in Shandong
Power Saving King Shop	Green Intelligent Power Saver	Received, the functionality is particularly good, it is genuine, the price is affordable	Jingdong prefecture level city in Shandong
Power Saving King Shop	Green Intelligent Power Saver	Good quality, very easy to use, energy efficient, home electricity bill is obviously much less	Jingdong prefecture level city in Shandong
Power Saving King Shop	Green Intelligent Power Saver	Easy to install, energy saving and power saving, very satisfied with a shopping	Jingdong prefecture level city in Shandong
Power Saving King Shop	Green Intelligent Power Saver	Received it and tried it out, it's great, works great and worth having! Five stars praise!	Jingdong prefecture level city in Shandong
Power Saving King Shop	Green Intelligent Power Saver	Power saving device, no problem at all, the quality of this product at this price, very affordable!	Jingdong prefecture level city in Shandong
Power Saving King Shop	Green Intelligent Power Saver	Received the goods, did not expect to be so fast, the quality is very good, especially power saving	Jingdong prefecture level city in Shandong
Power Saving King Shop	Green Intelligent Power Saver	The logistics is very fast, especially good, need to come back again	Jingdong prefecture level city in Shandong
Power Saving King Shop	Green Intelligent Power Saver	It's already working and it really saves power, so I'm very happy with it!	Jingdong prefecture level city in Shandong
Power Saving King Shop	Green Intelligent Power Saver	Great quality, works well, can recommend to friends to buy.	Jingdong prefecture level city in Shandong
Power Saving King Shop	Green Intelligent Power Saver	It's a good deal and worth the money.	Jingdong prefecture level city in Shandong
Power Saving King Shop	Green Intelligent Power Saver	I received it and I'm very happy with it, it's very good quality, guaranteed, and	Jingdong prefecture level city in Shandong
Power Saving King Shop	Green Intelligent Power Saver	Good quality, safe and secure purchase, ideal results and great price.	Jingdong prefecture level city in Shandong
Power Saving King Shop	Green Intelligent Power Saver	Simple operation plugged in the power supply can be, friends recommended looking forward to the effect of the	Jingdong prefecture level city in Shandong
Power Saving King Shop	Green Intelligent Power Saver	It's not bad, it saves electricity, and the material is sturdy and durable.	Jingdong prefecture level city in Shandong
Power Saving King Shop	Green Intelligent Power Saver	Plug and play, no management required, convenient and hassle-free!	Jingdong prefecture level city in Shandong
Power Saving King Shop	Green Intelligent Power Saver	The pictures are too lazy to go up, the power saving effect is still there!	Jingdong prefecture level city in Shandong
Power Saving King Shop	Green Intelligent Power Saver	Fast shipping. Affordable prices. Efficient	Jingdong prefecture level city in Shandong
Power Saving King Shop	Green Intelligent Power Saver	Good power saving, worth buying	Jingdong prefecture level city in Shandong
Power Saving King Shop	Green Intelligent Power Saver	Especially great, comparison shopping before buying, the store owner is very good, answering questions very patiently and detailed, there is a need to come back!	Jingdong prefecture level city in Shandong
Power Saving King Shop	Green Intelligent Power Saver	Very trustworthy brand, quality is guaranteed, the price is still affordable, quite satisfied!	Jingdong prefecture level city in Shandong
Power Saving King Shop	Green Intelligent Power Saver	Recommended by a friend. They say you can save money on air conditioning with this. Try it.	Jingdong prefecture level city in Shandong
Power Saving King Shop	Green Intelligent Power Saver	Works great, will be back next time, very happy with it and a great price!	Jingdong prefecture level city in Shandong
Power Saving King Shop	Green Intelligent Power Saver	The quality of the product is quite good, fast shipping, satisfied.	Jingdong prefecture level city in Shandong
Power Saving King Shop	Green Intelligent Power Saver	Good quality and fast logistics	Jingdong prefecture level city in Shandong
Power Saving King Shop	Green Intelligent Power Saver	It really saves power. I've tested it myself.	Jingdong prefecture level city in Shandong
Power Saving King Shop	Green Intelligent Power Saver	Really saves electricity!	Jingdong prefecture level city in Shandong
Power Saving King Shop	Green Intelligent Power Saver	pretty good	Jingdong prefecture level city in Shandong
Power Saving King Shop	Green Intelligent Power Saver	serviceable	Jingdong prefecture level city in Shandong
Power Saving King Shop	Green Intelligent Power Saver	Power Saving King received a tightly packaged logistics, installed the effect is very good price is cheaper than the physical store!	Jingdong prefecture level city in Shandong
Power Saving King Shop	Green Intelligent Power Saver	Super Power Saving King is very good quality, new style, is genuine, the use of the effect is very good, the price is cheap, it is worth buying!	Jingdong prefecture level city in Shandong
Power Saving King Shop	Green Intelligent Power Saver	Packaging is very tight, easy to install, quite practical, worth buying!	Jingdong prefecture level city in Shandong
Power Saving King Shop	Green Intelligent Power Saver	Quality assurance, affordable price, very energy efficient, will repurchase if needed!	Jingdong prefecture level city in Shandong
Power Saving King Shop	Green Intelligent Power Saver	Quality assurance, very good, affordable, logistics is also very fast, I'm very satisfied with the	Jingdong prefecture level city in Shandong
Power Saving King Shop	Green Intelligent Power Saver	Energy saving. It's so good, already installed, you can save a lot of electricity every month	Jingdong prefecture level city in Shandong
Power Saving King Shop	Green Intelligent Power Saver	The material is thick and the workmanship is fine in place, the power saving effect is very good, value for money!	Jingdong prefecture level city in Shandong
Power Saving King Shop	Green Intelligent Power Saver	The effect is great, genuine, and match the description, the logistics is fast, the price is reasonable 😍	Jingdong prefecture level city in Shandong
Power Saving King Shop	Green Intelligent Power Saver	It's genuine, works well, easy to use, plugs in and works, really saves power	Jingdong prefecture level city in Shandong
Power Saving King Shop	Green Intelligent Power Saver	It's easy to use, effective, great service, and recommended!	Jingdong prefecture level city in Shandong
Power Saving King Shop	Green Intelligent Power Saver	🌈 Great quality, bought it to my liking 💗 Awesome! ✨ Buy with confidence if you need it!	Jingdong prefecture level city in Shandong
Power Saving King Shop	Green Intelligent Power Saver	Good quality, easy to install, very energy saving and power saving, good to use	Jingdong prefecture level city in Shandong
Power Saving King Shop	Green Intelligent Power Saver	The quality is very good, it does have the effect of saving electricity, the description matches, the price is affordable, it is worth recommending.	Jingdong prefecture level city in Shandong
Power Saving King Shop	Green Intelligent Power Saver	Received, the functionality is particularly good, it is genuine, the price is affordable	Jingdong prefecture level city in Shandong
Power Saving King Shop	Green Intelligent Power Saver	Good quality, very easy to use, energy efficient, home electricity bill is obviously much less	Jingdong prefecture level city in Shandong
Power Saving King Shop	Green Intelligent Power Saver	Easy to install, energy saving and power saving, very satisfied with a shopping	Jingdong prefecture level city in Shandong

Power Saving King Shop	Green Intelligent Power Saver	Received it and tried it out, it's great, works great and worth having! Five stars praise!	Jingdong prefecture level city in Shandong
Power Saving King Shop	Green Intelligent Power Saver	Power saving device, no problem at all, the quality of this product at this price, very affordable!	Jingdong prefecture level city in Shandong
Power Saving King Shop	Green Intelligent Power Saver	Received the goods, did not expect to be so fast, the quality is very good, especially power saving	Jingdong prefecture level city in Shandong
Power Saving King Shop	Green Intelligent Power Saver	The logistics is very fast, especially good, need to come back again	Jingdong prefecture level city in Shandong
Power Saving King Shop	Green Intelligent Power Saver	It's already working and it really saves power, so I'm very happy with it!	Jingdong prefecture level city in Shandong
Power Saving King Shop	Green Intelligent Power Saver	Great quality, works well, can recommend to friends to buy.	Jingdong prefecture level city in Shandong
Power Saving King Shop	Green Intelligent Power Saver	It's a good deal and worth the money.	Jingdong prefecture level city in Shandong
Power Saving King Shop	Green Intelligent Power Saver	I received it and I'm very happy with it, it's very good quality, guaranteed, and	Jingdong prefecture level city in Shandong
Power Saving King Shop	Green Intelligent Power Saver	Good quality, safe and secure purchase, ideal results and great price.	Jingdong prefecture level city in Shandong
Power Saving King Shop	Green Intelligent Power Saver	Simple operation plugged in the power supply can be, friends recommended looking forward to the effect of the	Jingdong prefecture level city in Shandong
Power Saving King Shop	Green Intelligent Power Saver	It's not bad, it saves electricity, and the material is sturdy and durable.	Jingdong prefecture level city in Shandong
Power Saving King Shop	Green Intelligent Power Saver	Plug and play, no management required, convenient and hassle-free!	Jingdong prefecture level city in Shandong
Power Saving King Shop	Green Intelligent Power Saver	The pictures are too lazy to go up, the power saving effect is still there!	Jingdong prefecture level city in Shandong
Power Saving King Shop	Green Intelligent Power Saver	Fast shipping. Affordable prices. Efficient	Jingdong prefecture level city in Shandong
Power Saving King Shop	Green Intelligent Power Saver	Good power saving, worth buying	Jingdong prefecture level city in Shandong
Power Saving King Shop	Green Intelligent Power Saver	Especially great, comparison shopping before buying, the store owner is very good, answering questions very patiently and detailed, there is a need to come back!	Jingdong prefecture level city in Shandong
Power Saving King Shop	Green Intelligent Power Saver	Very trustworthy brand, quality is guaranteed, the price is still affordable, quite satisfied!	Jingdong prefecture level city in Shandong
Power Saving King Shop	Green Intelligent Power Saver	Recommended by a friend. They say you can save money on air conditioning with this. Try it.	Jingdong prefecture level city in Shandong
Power Saving King Shop	Green Intelligent Power Saver	Works great, will be back next time, very happy with it and a great price!	Jingdong prefecture level city in Shandong
Power Saving King Shop	Green Intelligent Power Saver	The quality of the product is quite good, fast shipping, satisfied.	Jingdong prefecture level city in Shandong
Power Saving King Shop	Green Intelligent Power Saver	Good quality and fast logistics	Jingdong prefecture level city in Shandong
Power Saving King Shop	Green Intelligent Power Saver	It really saves power. I've tested it myself.	Jingdong prefecture level city in Shandong
Power Saving King Shop	Green Intelligent Power Saver	Really saves electricity!	Jingdong prefecture level city in Shandong
Power Saving King Shop	Green Intelligent Power Saver	pretty good	Jingdong prefecture level city in Shandong
Power Saving King Shop	Green Intelligent Power Saver	serviceable	Jingdong prefecture level city in Shandong
Power Saving King Shop	Green Intelligent Power Saver	Power Saving King received a tightly packaged logistics, installed the effect is very good price is cheaper than the physical store!	Jingdong prefecture level city in Shandong
Power Saving King Shop	Green Intelligent Power Saver	Super Power Saving King is very good quality, new style, is genuine, the use of the effect is very good, the price is cheap, it is worth buying!	Jingdong prefecture level city in Shandong
Power Saving King Shop	Green Intelligent Power Saver	Packaging is very tight, easy to install, quite practical, worth buying!	Jingdong prefecture level city in Shandong
Power Saving King Shop	Green Intelligent Power Saver	Quality assurance, affordable price, very energy efficient, will repurchase if needed!	Jingdong prefecture level city in Shandong
Power Saving King Shop	Green Intelligent Power Saver	Quality assurance, very good, affordable, logistics is also very fast, I'm very satisfied with the	Jingdong prefecture level city in Shandong
Power Saving King Shop	Green Intelligent Power Saver	Energy saving. It's so good, already installed, you can save a lot of electricity every month	Jingdong prefecture level city in Shandong
Power Saving King Shop	Green Intelligent Power Saver	The material is thick and the workmanship is fine in place, the power saving effect is very good, value for money!	Jingdong prefecture level city in Shandong
Power Saving King Shop	Green Intelligent Power Saver	The effect is great, genuine, and match the description, the logistics is fast, the price is reasonable 😍	Jingdong prefecture level city in Shandong
Power Saving King Shop	Green Intelligent Power Saver	It's genuine, works well, easy to use, plugs in and works, really saves power	Jingdong prefecture level city in Shandong
Power Saving King Shop	Green Intelligent Power Saver	It's easy to use, effective, great service, and recommended!	Jingdong prefecture level city in Shandong
Power Saving King Shop	Green Intelligent Power Saver	🌈 Great quality, bought it to my liking 💖 Awesome! ✨ Buy with confidence if you need it!	Jingdong prefecture level city in Shandong
Power Saving King Shop	Green Intelligent Power Saver	Good quality, easy to install, very energy saving and power saving, good to use	Jingdong prefecture level city in Shandong
Power Saving King Shop	Green Intelligent Power Saver	The quality is very good, it does have the effect of saving electricity, the description matches, the price is affordable, it is worth recommending.	Jingdong prefecture level city in Shandong
Power Saving King Shop	Green Intelligent Power Saver	Received, the functionality is particularly good, it is genuine, the price is affordable	Jingdong prefecture level city in Shandong
Power Saving King Shop	Green Intelligent Power Saver	Good quality, very easy to use, energy efficient, home electricity bill is obviously much less	Jingdong prefecture level city in Shandong
Power Saving King Shop	Green Intelligent Power Saver	Easy to install, energy saving and power saving, very satisfied with a shopping	Jingdong prefecture level city in Shandong
Power Saving King Shop	Green Intelligent Power Saver	Received it and tried it out, it's great, works great and worth having! Five stars praise!	Jingdong prefecture level city in Shandong
Power Saving King Shop	Green Intelligent Power Saver	Power saving device, no problem at all, the quality of this product at this price, very affordable!	Jingdong prefecture level city in Shandong
Power Saving King Shop	Green Intelligent Power Saver	Received the goods, did not expect to be so fast, the quality is very good, especially power saving	Jingdong prefecture level city in Shandong
Power Saving King Shop	Green Intelligent Power Saver	The logistics is very fast, especially good, need to come back again	Jingdong prefecture level city in Shandong
Power Saving King Shop	Green Intelligent Power Saver	It's already working and it really saves power, so I'm very happy with it!	Jingdong prefecture level city in Shandong
Power Saving King Shop	Green Intelligent Power Saver	Great quality, works well, can recommend to friends to buy.	Jingdong prefecture level city in Shandong
Power Saving King Shop	Green Intelligent Power Saver	It's a good deal and worth the money.	Jingdong prefecture level city in Shandong
Power Saving King Shop	Green Intelligent Power Saver	I received it and I'm very happy with it, it's very good quality, guaranteed, and	Jingdong prefecture level city in Shandong
Power Saving King Shop	Green Intelligent Power Saver	Good quality, safe and secure purchase, ideal results and great price.	Jingdong prefecture level city in Shandong
Power Saving King Shop	Green Intelligent Power Saver	Simple operation plugged in the power supply can be, friends recommended looking forward to the effect of the	Jingdong prefecture level city in Shandong

Power Saving King Shop	Green Intelligent Power Saver	It's not bad, it saves electricity, and the material is sturdy and durable.	Jingdong prefecture level city in Shandong
Power Saving King Shop	Green Intelligent Power Saver	Plug and play, no management required, convenient and hassle-free!	Jingdong prefecture level city in Shandong
Power Saving King Shop	Green Intelligent Power Saver	The pictures are too lazy to go up, the power saving effect is still there!	Jingdong prefecture level city in Shandong
Power Saving King Shop	Green Intelligent Power Saver	Fast shipping. Affordable prices. Efficient	Jingdong prefecture level city in Shandong
Power Saving King Shop	Green Intelligent Power Saver	Good power saving, worth buying	Jingdong prefecture level city in Shandong
Power Saving King Shop	Green Intelligent Power Saver	Especially great, comparison shopping before buying, the store owner is very good, answering questions very patiently and detailed, there is a need to come back!	Jingdong prefecture level city in Shandong
Power Saving King Shop	Green Intelligent Power Saver	Very trustworthy brand, quality is guaranteed, the price is still affordable, quite satisfied!	Jingdong prefecture level city in Shandong
Power Saving King Shop	Green Intelligent Power Saver	Recommended by a friend. They say you can save money on air conditioning with this. Try it.	Jingdong prefecture level city in Shandong
Power Saving King Shop	Green Intelligent Power Saver	Works great, will be back next time, very happy with it and a great price!	Jingdong prefecture level city in Shandong
Power Saving King Shop	Green Intelligent Power Saver	The quality of the product is quite good, fast shipping, satisfied.	Jingdong prefecture level city in Shandong
Power Saving King Shop	Green Intelligent Power Saver	Good quality and fast logistics	Jingdong prefecture level city in Shandong
Power Saving King Shop	Green Intelligent Power Saver	It really saves power. I've tested it myself.	Jingdong prefecture level city in Shandong
Power Saving King Shop	Green Intelligent Power Saver	Really saves electricity!	Jingdong prefecture level city in Shandong
Power Saving King Shop	Green Intelligent Power Saver	pretty good	Jingdong prefecture level city in Shandong
Power Saving King Shop	Green Intelligent Power Saver	serviceable	Jingdong prefecture level city in Shandong
Power Saving King Shop	Green Intelligent Power Saver	Power Saving King received a tightly packaged logistics, installed the effect is very good price is cheaper than the physical store!	Jingdong prefecture level city in Shandong
Power Saving King Shop	Green Intelligent Power Saver	Super Power Saving King is very good quality, new style, is genuine, the use of the effect is very good, the price is cheap, it is worth buying!	Jingdong prefecture level city in Shandong
Power Saving King Shop	Green Intelligent Power Saver	Packaging is very tight, easy to install, quite practical, worth buying!	Jingdong prefecture level city in Shandong
Power Saving King Shop	Green Intelligent Power Saver	Quality assurance, affordable price, very energy efficient, will repurchase if needed!	Jingdong prefecture level city in Shandong
Power Saving King Shop	Green Intelligent Power Saver	Quality assurance, very good, affordable, logistics is also very fast, I'm very satisfied with the	Jingdong prefecture level city in Shandong
Power Saving King Shop	Green Intelligent Power Saver	Energy saving. It's so good, already installed, you can save a lot of electricity every month	Jingdong prefecture level city in Shandong
Power Saving King Shop	Green Intelligent Power Saver	The material is thick and the workmanship is fine in place, the power saving effect is very good, value for money!	Jingdong prefecture level city in Shandong
Power Saving King Shop	Green Intelligent Power Saver	The effect is great, genuine, and match the description, the logistics is fast, the price is reasonable 😍	Jingdong prefecture level city in Shandong
Power Saving King Shop	Green Intelligent Power Saver	It's genuine, works well, easy to use, plugs in and works, really saves power	Jingdong prefecture level city in Shandong
Power Saving King Shop	Green Intelligent Power Saver	It's easy to use, effective, great service, and recommended!	Jingdong prefecture level city in Shandong
Power Saving King Shop	Green Intelligent Power Saver	🌈 Great quality, bought it to my liking 💞 Awesome! ✨ Buy with confidence if you need it!	Jingdong prefecture level city in Shandong
Power Saving King Shop	Green Intelligent Power Saver	Good quality, easy to install, very energy saving and power saving, good to use	Jingdong prefecture level city in Shandong
Power Saving King Shop	Green Intelligent Power Saver	The quality is very good, it does have the effect of saving electricity, the description matches, the price is affordable, it is worth recommending.	Jingdong prefecture level city in Shandong
Power Saving King Shop	Green Intelligent Power Saver	Received, the functionality is particularly good, it is genuine, the price is affordable	Jingdong prefecture level city in Shandong
Power Saving King Shop	Green Intelligent Power Saver	Good quality, very easy to use, energy efficient, home electricity bill is obviously much less	Jingdong prefecture level city in Shandong
Power Saving King Shop	Green Intelligent Power Saver	Easy to install, energy saving and power saving, very satisfied with a shopping	Jingdong prefecture level city in Shandong
Power Saving King Shop	Green Intelligent Power Saver	Received it and tried it out, it's great, works great and worth having! Five stars praise!	Jingdong prefecture level city in Shandong
Power Saving King Shop	Green Intelligent Power Saver	Power saving device, no problem at all, the quality of this product at this price, very affordable!	Jingdong prefecture level city in Shandong
Power Saving King Shop	Green Intelligent Power Saver	Received the goods, did not expect to be so fast, the quality is very good, especially power saving	Jingdong prefecture level city in Shandong
Power Saving King Shop	Green Intelligent Power Saver	The logistics is very fast, especially good, need to come back again	Jingdong prefecture level city in Shandong
Power Saving King Shop	Green Intelligent Power Saver	It's already working and it really saves power, so I'm very happy with it!	Jingdong prefecture level city in Shandong
Power Saving King Shop	Green Intelligent Power Saver	Great quality, works well, can recommend to friends to buy.	Jingdong prefecture level city in Shandong
Power Saving King Shop	Green Intelligent Power Saver	It's a good deal and worth the money.	Jingdong prefecture level city in Shandong
Power Saving King Shop	Green Intelligent Power Saver	I received it and I'm very happy with it, it's very good quality, guaranteed, and	Jingdong prefecture level city in Shandong
Power Saving King Shop	Green Intelligent Power Saver	Good quality, safe and secure purchase, ideal results and great price.	Jingdong prefecture level city in Shandong
Power Saving King Shop	Green Intelligent Power Saver	Simple operation plugged in the power supply can be, friends recommended looking forward to the effect of the	Jingdong prefecture level city in Shandong
Power Saving King Shop	Green Intelligent Power Saver	It's not bad, it saves electricity, and the material is sturdy and durable.	Jingdong prefecture level city in Shandong
Power Saving King Shop	Green Intelligent Power Saver	Plug and play, no management required, convenient and hassle-free!	Jingdong prefecture level city in Shandong
Power Saving King Shop	Green Intelligent Power Saver	The pictures are too lazy to go up, the power saving effect is still there!	Jingdong prefecture level city in Shandong
Power Saving King Shop	Green Intelligent Power Saver	Fast shipping. Affordable prices. Efficient	Jingdong prefecture level city in Shandong
Power Saving King Shop	Green Intelligent Power Saver	Good power saving, worth buying	Jingdong prefecture level city in Shandong
Power Saving King Shop	Green Intelligent Power Saver	Especially great, comparison shopping before buying, the store owner is very good, answering questions very patiently and detailed, there is a need to come back!	Jingdong prefecture level city in Shandong
Power Saving King Shop	Green Intelligent Power Saver	Very trustworthy brand, quality is guaranteed, the price is still affordable, quite satisfied!	Jingdong prefecture level city in Shandong
Power Saving King Shop	Green Intelligent Power Saver	Recommended by a friend. They say you can save money on air conditioning with this. Try it.	Jingdong prefecture level city in Shandong
Power Saving King Shop	Green Intelligent Power Saver	Works great, will be back next time, very happy with it and a great price!	Jingdong prefecture level city in Shandong
Power Saving King Shop	Green Intelligent Power Saver	The quality of the product is quite good, fast shipping, satisfied.	Jingdong prefecture level city in Shandong

Power Saving King Shop	Green Intelligent Power Saver	Good quality and fast logistics	Jingdong prefecture level city in Shandong
Power Saving King Shop	Green Intelligent Power Saver	It really saves power. I’ve tested it myself.	Jingdong prefecture level city in Shandong
Power Saving King Shop	Green Intelligent Power Saver	Really saves electricity!	Jingdong prefecture level city in Shandong
Power Saving King Shop	Green Intelligent Power Saver	pretty good	Jingdong prefecture level city in Shandong
Power Saving King Shop	Green Intelligent Power Saver	serviceable	Jingdong prefecture level city in Shandong
Power Saving King Shop	Green Intelligent Power Saver	Power Saving King received a tightly packaged logistics, installed the effect is very good price is cheaper than the physical store!	Jingdong prefecture level city in Shandong
Power Saving King Shop	Green Intelligent Power Saver	Super Power Saving King is very good quality, new style, is genuine, the use of the effect is very good, the price is cheap, it is worth buying!	Jingdong prefecture level city in Shandong
Power Saving King Shop	Green Intelligent Power Saver	Packaging is very tight, easy to install, quite practical, worth buying!	Jingdong prefecture level city in Shandong
Power Saving King Shop	Green Intelligent Power Saver	Quality assurance, affordable price, very energy efficient, will repurchase if needed!	Jingdong prefecture level city in Shandong
Power Saving King Shop	Green Intelligent Power Saver	Quality assurance, very good, affordable, logistics is also very fast, I’m very satisfied with the	Jingdong prefecture level city in Shandong
Power Saving King Shop	Green Intelligent Power Saver	Energy saving. It’s so good, already installed, you can save a lot of electricity every month	Jingdong prefecture level city in Shandong
Power Saving King Shop	Green Intelligent Power Saver	The material is thick and the workmanship is fine in place, the power saving effect is very good, value for money!	Jingdong prefecture level city in Shandong
Power Saving King Shop	Green Intelligent Power Saver	The effect is great, genuine, and match the description, the logistics is fast, the price is reasonable 😍	Jingdong prefecture level city in Shandong
Power Saving King Shop	Green Intelligent Power Saver	It’s genuine, works well, easy to use, plugs in and works, really saves power	Jingdong prefecture level city in Shandong
Power Saving King Shop	Green Intelligent Power Saver	It’s easy to use, effective, great service, and recommended!	Jingdong prefecture level city in Shandong
Power Saving King Shop	Green Intelligent Power Saver	🌈 Great quality, bought it to my liking 💗 Awesome! ✨ Buy with confidence if you need it!	Jingdong prefecture level city in Shandong
Power Saving King Shop	Green Intelligent Power Saver	Good quality, easy to install, very energy saving and power saving, good to use	Jingdong prefecture level city in Shandong
Power Saving King Shop	Green Intelligent Power Saver	The quality is very good, it does have the effect of saving electricity, the description matches, the price is affordable, it is worth recommending.	Jingdong prefecture level city in Shandong
Power Saving King Shop	Green Intelligent Power Saver	Received, the functionality is particularly good, it is genuine, the price is affordable	Jingdong prefecture level city in Shandong
Power Saving King Shop	Green Intelligent Power Saver	Good quality, very easy to use, energy efficient, home electricity bill is obviously much less	Jingdong prefecture level city in Shandong
Power Saving King Shop	Green Intelligent Power Saver	Easy to install, energy saving and power saving, very satisfied with a shopping	Jingdong prefecture level city in Shandong
Power Saving King Shop	Green Intelligent Power Saver	Received it and tried it out, it’s great, works great and worth having! Five stars praise!	Jingdong prefecture level city in Shandong
Power Saving King Shop	Green Intelligent Power Saver	Power saving device, no problem at all, the quality of this product at this price, very affordable!	Jingdong prefecture level city in Shandong
Power Saving King Shop	Green Intelligent Power Saver	Received the goods, did not expect to be so fast, the quality is very good, especially power saving	Jingdong prefecture level city in Shandong
Power Saving King Shop	Green Intelligent Power Saver	The logistics is very fast, especially good, need to come back again	Jingdong prefecture level city in Shandong
Power Saving King Shop	Green Intelligent Power Saver	It’s already working and it really saves power, so I’m very happy with it!	Jingdong prefecture level city in Shandong
Power Saving King Shop	Green Intelligent Power Saver	Great quality, works well, can recommend to friends to buy.	Jingdong prefecture level city in Shandong
Power Saving King Shop	Green Intelligent Power Saver	It’s a good deal and worth the money.	Jingdong prefecture level city in Shandong
Power Saving King Shop	Green Intelligent Power Saver	I received it and I’m very happy with it, it’s very good quality, guaranteed, and	Jingdong prefecture level city in Shandong
Power Saving King Shop	Green Intelligent Power Saver	Good quality, safe and secure purchase, ideal results and great price.	Jingdong prefecture level city in Shandong
Power Saving King Shop	Green Intelligent Power Saver	Simple operation plugged in the power supply can be, friends recommended looking forward to the effect of the	Jingdong prefecture level city in Shandong
Power Saving King Shop	Green Intelligent Power Saver	It’s not bad, it saves electricity, and the material is sturdy and durable.	Jingdong prefecture level city in Shandong
Power Saving King Shop	Green Intelligent Power Saver	Plug and play, no management required, convenient and hassle-free!	Jingdong prefecture level city in Shandong
Power Saving King Shop	Green Intelligent Power Saver	The pictures are too lazy to go up, the power saving effect is still there!	Jingdong prefecture level city in Shandong
Power Saving King Shop	Green Intelligent Power Saver	Fast shipping. Affordable prices. Efficient	Jingdong prefecture level city in Shandong
Power Saving King Shop	Green Intelligent Power Saver	Good power saving, worth buying	Jingdong prefecture level city in Shandong
Power Saving King Shop	Green Intelligent Power Saver	Especially great, comparison shopping before buying, the store owner is very good, answering questions very patiently and detailed, there is a need to come back!	Jingdong prefecture level city in Shandong
Power Saving King Shop	Green Intelligent Power Saver	Very trustworthy brand, quality is guaranteed, the price is still affordable, quite satisfied!	Jingdong prefecture level city in Shandong
Power Saving King Shop	Green Intelligent Power Saver	Recommended by a friend. They say you can save money on air conditioning with this. Try it.	Jingdong prefecture level city in Shandong
Power Saving King Shop	Green Intelligent Power Saver	Works great, will be back next time, very happy with it and a great price!	Jingdong prefecture level city in Shandong
Power Saving King Shop	Green Intelligent Power Saver	The quality of the product is quite good, fast shipping, satisfied.	Jingdong prefecture level city in Shandong
Power Saving King Shop	Green Intelligent Power Saver	Good quality and fast logistics	Jingdong prefecture level city in Shandong
Power Saving King Shop	Green Intelligent Power Saver	It really saves power. I’ve tested it myself.	Jingdong prefecture level city in Shandong
Power Saving King Shop	Green Intelligent Power Saver	Really saves electricity!	Jingdong prefecture level city in Shandong
Power Saving King Shop	Green Intelligent Power Saver	pretty good	Jingdong prefecture level city in Shandong
Power Saving King Shop	Green Intelligent Power Saver	serviceable	Jingdong prefecture level city in Shandong
Power Saving King Shop	Green Intelligent Power Saver	Power Saving King received a tightly packaged logistics, installed the effect is very good price is cheaper than the physical store!	Jingdong prefecture level city in Shandong
Power Saving King Shop	Green Intelligent Power Saver	Super Power Saving King is very good quality, new style, is genuine, the use of the effect is very good, the price is cheap, it is worth buying!	Jingdong prefecture level city in Shandong
Power Saving King Shop	Green Intelligent Power Saver	Packaging is very tight, easy to install, quite practical, worth buying!	Jingdong prefecture level city in Shandong
Power Saving King Shop	Green Intelligent Power Saver	Quality assurance, affordable price, very energy efficient, will repurchase if needed!	Jingdong prefecture level city in Shandong
Power Saving King Shop	Green Intelligent Power Saver	Quality assurance, very good, affordable, logistics is also very fast, I’m very satisfied with the	Jingdong prefecture level city in Shandong

Power Saving King Shop	Green Intelligent Power Saver	Energy saving. It's so good, already installed, you can save a lot of electricity every month	Jingdong prefecture level city in Shandong
Power Saving King Shop	Green Intelligent Power Saver	The material is thick and the workmanship is fine in place, the power saving effect is very good, value for money!	Jingdong prefecture level city in Shandong
Power Saving King Shop	Green Intelligent Power Saver	The effect is great, genuine, and match the description, the logistics is fast, the price is reasonable 😍	Jingdong prefecture level city in Shandong
Power Saving King Shop	Green Intelligent Power Saver	It's genuine, works well, easy to use, plugs in and works, really saves power	Jingdong prefecture level city in Shandong
Power Saving King Shop	Green Intelligent Power Saver	It's easy to use, effective, great service, and recommended!	Jingdong prefecture level city in Shandong
Power Saving King Shop	Green Intelligent Power Saver	🌈 Great quality, bought it to my liking 💛 Awesome! ✨ Buy with confidence if you need it!	Jingdong prefecture level city in Shandong
Power Saving King Shop	Green Intelligent Power Saver	Good quality, easy to install, very energy saving and power saving, good to use	Jingdong prefecture level city in Shandong
Power Saving King Shop	Green Intelligent Power Saver	The quality is very good, it does have the effect of saving electricity, the description matches, the price is affordable, it is worth recommending.	Jingdong prefecture level city in Shandong
Power Saving King Shop	Green Intelligent Power Saver	Received, the functionality is particularly good, it is genuine, the price is affordable	Jingdong prefecture level city in Shandong
Power Saving King Shop	Green Intelligent Power Saver	Good quality, very easy to use, energy efficient, home electricity bill is obviously much less	Jingdong prefecture level city in Shandong
Power Saving King Shop	Green Intelligent Power Saver	Easy to install, energy saving and power saving, very satisfied with a shopping	Jingdong prefecture level city in Shandong
Power Saving King Shop	Green Intelligent Power Saver	Received it and tried it out, it's great, works great and worth having! Five stars praise!	Jingdong prefecture level city in Shandong
Power Saving King Shop	Green Intelligent Power Saver	Power saving device, no problem at all, the quality of this product at this price, very affordable!	Jingdong prefecture level city in Shandong
Power Saving King Shop	Green Intelligent Power Saver	Received the goods, did not expect to be so fast, the quality is very good, especially power saving	Jingdong prefecture level city in Shandong
Power Saving King Shop	Green Intelligent Power Saver	The logistics is very fast, especially good, need to come back again	Jingdong prefecture level city in Shandong
Power Saving King Shop	Green Intelligent Power Saver	It's already working and it really saves power, so I'm very happy with it!	Jingdong prefecture level city in Shandong
Power Saving King Shop	Green Intelligent Power Saver	Great quality, works well, can recommend to friends to buy.	Jingdong prefecture level city in Shandong
Power Saving King Shop	Green Intelligent Power Saver	It's a good deal and worth the money.	Jingdong prefecture level city in Shandong
Power Saving King Shop	Green Intelligent Power Saver	I received it and I'm very happy with it, it's very good quality, guaranteed, and	Jingdong prefecture level city in Shandong
Power Saving King Shop	Green Intelligent Power Saver	Good quality, safe and secure purchase, ideal results and great price.	Jingdong prefecture level city in Shandong
Power Saving King Shop	Green Intelligent Power Saver	Simple operation plugged in the power supply can be, friends recommended looking forward to the effect of the	Jingdong prefecture level city in Shandong
Power Saving King Shop	Green Intelligent Power Saver	It's not bad, it saves electricity, and the material is sturdy and durable.	Jingdong prefecture level city in Shandong
Power Saving King Shop	Green Intelligent Power Saver	Plug and play, no management required, convenient and hassle-free!	Jingdong prefecture level city in Shandong
Power Saving King Shop	Green Intelligent Power Saver	The pictures are too lazy to go up, the power saving effect is still there!	Jingdong prefecture level city in Shandong
Power Saving King Shop	Green Intelligent Power Saver	Fast shipping. Affordable prices. Efficient	Jingdong prefecture level city in Shandong
Power Saving King Shop	Green Intelligent Power Saver	Good power saving, worth buying	Jingdong prefecture level city in Shandong
Power Saving King Shop	Green Intelligent Power Saver	Especially great, comparison shopping before buying, the store owner is very good, answering questions very patiently and detailed, there is a need to come back!	Jingdong prefecture level city in Shandong
Power Saving King Shop	Green Intelligent Power Saver	Very trustworthy brand, quality is guaranteed, the price is still affordable, quite satisfied!	Jingdong prefecture level city in Shandong
Power Saving King Shop	Green Intelligent Power Saver	Recommended by a friend. They say you can save money on air conditioning with this. Try it.	Jingdong prefecture level city in Shandong
Power Saving King Shop	Green Intelligent Power Saver	Works great, will be back next time, very happy with it and a great price!	Jingdong prefecture level city in Shandong
Power Saving King Shop	Green Intelligent Power Saver	The quality of the product is quite good, fast shipping, satisfied.	Jingdong prefecture level city in Shandong
Power Saving King Shop	Green Intelligent Power Saver	Good quality and fast logistics	Jingdong prefecture level city in Shandong
Power Saving King Shop	Green Intelligent Power Saver	It really saves power. I've tested it myself.	Jingdong prefecture level city in Shandong
Power Saving King Shop	Green Intelligent Power Saver	Really saves electricity!	Jingdong prefecture level city in Shandong
Power Saving King Shop	Green Intelligent Power Saver	pretty good	Jingdong prefecture level city in Shandong
Power Saving King Shop	Green Intelligent Power Saver	serviceable	Jingdong prefecture level city in Shandong
Power Saving King Shop	Green Intelligent Power Saver	Power Saving King received a tightly packaged logistics, installed the effect is very good price is cheaper than the physical store!	Jingdong prefecture level city in Shandong
Power Saving King Shop	Green Intelligent Power Saver	Super Power Saving King is very good quality, new style, is genuine, the use of the effect is very good, the price is cheap, it is worth buying!	Jingdong prefecture level city in Shandong
Power Saving King Shop	Green Intelligent Power Saver	Packaging is very tight, easy to install, quite practical, worth buying!	Jingdong prefecture level city in Shandong
Power Saving King Shop	Green Intelligent Power Saver	Quality assurance, affordable price, very energy efficient, will repurchase if needed!	Jingdong prefecture level city in Shandong
Power Saving King Shop	Green Intelligent Power Saver	Quality assurance, very good, affordable, logistics is also very fast, I'm very satisfied with the	Jingdong prefecture level city in Shandong
Power Saving King Shop	Green Intelligent Power Saver	Energy saving. It's so good, already installed, you can save a lot of electricity every month	Jingdong prefecture level city in Shandong
Power Saving King Shop	Green Intelligent Power Saver	The material is thick and the workmanship is fine in place, the power saving effect is very good, value for money!	Jingdong prefecture level city in Shandong
Power Saving King Shop	Green Intelligent Power Saver	The effect is great, genuine, and match the description, the logistics is fast, the price is reasonable 😍	Jingdong prefecture level city in Shandong
Power Saving King Shop	Green Intelligent Power Saver	It's genuine, works well, easy to use, plugs in and works, really saves power	Jingdong prefecture level city in Shandong
Power Saving King Shop	Green Intelligent Power Saver	It's easy to use, effective, great service, and recommended!	Jingdong prefecture level city in Shandong
Power Saving King Shop	Green Intelligent Power Saver	🌈 Great quality, bought it to my liking 💛 Awesome! ✨ Buy with confidence if you need it!	Jingdong prefecture level city in Shandong
Power Saving King Shop	Green Intelligent Power Saver	Good quality, easy to install, very energy saving and power saving, good to use	Jingdong prefecture level city in Shandong
Power Saving King Shop	Green Intelligent Power Saver	The quality is very good, it does have the effect of saving electricity, the description matches, the price is affordable, it is worth recommending.	Jingdong prefecture level city in Shandong
Power Saving King Shop	Green Intelligent Power Saver	Received, the functionality is particularly good, it is genuine, the price is affordable	Jingdong prefecture level city in Shandong
Power Saving King Shop	Green Intelligent Power Saver	Good quality, very easy to use, energy efficient, home electricity bill is obviously much less	Jingdong prefecture level city in Shandong

Power Saving King Shop	Green Intelligent Power Saver	Easy to install, energy saving and power saving, very satisfied with a shopping	Jingdong prefecture level city in Shandong
Power Saving King Shop	Green Intelligent Power Saver	Received it and tried it out, it's great, works great and worth having! Five stars praise!	Jingdong prefecture level city in Shandong
Power Saving King Shop	Green Intelligent Power Saver	Power saving device, no problem at all, the quality of this product at this price, very affordable!	Jingdong prefecture level city in Shandong
Power Saving King Shop	Green Intelligent Power Saver	Received the goods, did not expect to be so fast, the quality is very good, especially power saving	Jingdong prefecture level city in Shandong
Power Saving King Shop	Green Intelligent Power Saver	The logistics is very fast, especially good, need to come back again	Jingdong prefecture level city in Shandong
Power Saving King Shop	Green Intelligent Power Saver	It's already working and it really saves power, so I'm very happy with it!	Jingdong prefecture level city in Shandong
Power Saving King Shop	Green Intelligent Power Saver	Great quality, works well, can recommend to friends to buy.	Jingdong prefecture level city in Shandong
Power Saving King Shop	Green Intelligent Power Saver	It's a good deal and worth the money.	Jingdong prefecture level city in Shandong
Power Saving King Shop	Green Intelligent Power Saver	I received it and I'm very happy with it, it's very good quality, guaranteed, and	Jingdong prefecture level city in Shandong
Power Saving King Shop	Green Intelligent Power Saver	Good quality, safe and secure purchase, ideal results and great price.	Jingdong prefecture level city in Shandong
Power Saving King Shop	Green Intelligent Power Saver	Simple operation plugged in the power supply can be, friends recommended looking forward to the effect of the	Jingdong prefecture level city in Shandong
Power Saving King Shop	Green Intelligent Power Saver	It's not bad, it saves electricity, and the material is sturdy and durable.	Jingdong prefecture level city in Shandong
Power Saving King Shop	Green Intelligent Power Saver	Plug and play, no management required, convenient and hassle-free!	Jingdong prefecture level city in Shandong
Power Saving King Shop	Green Intelligent Power Saver	The pictures are too lazy to go up, the power saving effect is still there!	Jingdong prefecture level city in Shandong
Power Saving King Shop	Green Intelligent Power Saver	Fast shipping. Affordable prices. Efficient	Jingdong prefecture level city in Shandong
Power Saving King Shop	Green Intelligent Power Saver	Good power saving, worth buying	Jingdong prefecture level city in Shandong
Power Saving King Shop	Green Intelligent Power Saver	Especially great, comparison shopping before buying, the store owner is very good, answering questions very patiently and detailed, there is a need to come back!	Jingdong prefecture level city in Shandong
Power Saving King Shop	Green Intelligent Power Saver	Very trustworthy brand, quality is guaranteed, the price is still affordable, quite satisfied!	Jingdong prefecture level city in Shandong
Power Saving King Shop	Green Intelligent Power Saver	Recommended by a friend. They say you can save money on air conditioning with this. Try it.	Jingdong prefecture level city in Shandong
Power Saving King Shop	Green Intelligent Power Saver	Works great, will be back next time, very happy with it and a great price!	Jingdong prefecture level city in Shandong
Power Saving King Shop	Green Intelligent Power Saver	The quality of the product is quite good, fast shipping, satisfied.	Jingdong prefecture level city in Shandong
Power Saving King Shop	Green Intelligent Power Saver	Good quality and fast logistics	Jingdong prefecture level city in Shandong
Power Saving King Shop	Green Intelligent Power Saver	It really saves power. I've tested it myself.	Jingdong prefecture level city in Shandong
Power Saving King Shop	Green Intelligent Power Saver	Really saves electricity!	Jingdong prefecture level city in Shandong
Power Saving King Shop	Green Intelligent Power Saver	pretty good	Jingdong prefecture level city in Shandong
Power Saving King Shop	Green Intelligent Power Saver	serviceable	Jingdong prefecture level city in Shandong
Power Saving King Shop	Green Intelligent Power Saver	Power Saving King received a tightly packaged logistics, installed the effect is very good price is cheaper than the physical store!	Jingdong prefecture level city in Shandong
Power Saving King Shop	Green Intelligent Power Saver	Super Power Saving King is very good quality, new style, is genuine, the use of the effect is very good, the price is cheap, it is worth buying!	Jingdong prefecture level city in Shandong
Power Saving King Shop	Green Intelligent Power Saver	Packaging is very tight, easy to install, quite practical, worth buying!	Jingdong prefecture level city in Shandong
Power Saving King Shop	Green Intelligent Power Saver	Quality assurance, affordable price, very energy efficient, will repurchase if needed!	Jingdong prefecture level city in Shandong
Power Saving King Shop	Green Intelligent Power Saver	Quality assurance, very good, affordable, logistics is also very fast, I'm very satisfied with the	Jingdong prefecture level city in Shandong
Power Saving King Shop	Green Intelligent Power Saver	Energy saving. It's so good, already installed, you can save a lot of electricity every month	Jingdong prefecture level city in Shandong
Power Saving King Shop	Green Intelligent Power Saver	The material is thick and the workmanship is fine in place, the power saving effect is very good, value for money!	Jingdong prefecture level city in Shandong
Power Saving King Shop	Green Intelligent Power Saver	The effect is great, genuine, and match the description, the logistics is fast, the price is reasonable 😍	Jingdong prefecture level city in Shandong
Power Saving King Shop	Green Intelligent Power Saver	It's genuine, works well, easy to use, plugs in and works, really saves power	Jingdong prefecture level city in Shandong
Power Saving King Shop	Green Intelligent Power Saver	It's easy to use, effective, great service, and recommended!	Jingdong prefecture level city in Shandong
Power Saving King Shop	Green Intelligent Power Saver	🌈 Great quality, bought it to my liking 💝 Awesome! ✨ Buy with confidence if you need it!	Jingdong prefecture level city in Shandong
Power Saving King Shop	Green Intelligent Power Saver	Good quality, easy to install, very energy saving and power saving, good to use	Jingdong prefecture level city in Shandong
Power Saving King Shop	Green Intelligent Power Saver	The quality is very good, it does have the effect of saving electricity, the description matches, the price is affordable, it is worth recommending.	Jingdong prefecture level city in Shandong
Power Saving King Shop	Green Intelligent Power Saver	Received, the functionality is particularly good, it is genuine, the price is affordable	Jingdong prefecture level city in Shandong
Power Saving King Shop	Green Intelligent Power Saver	Good quality, very easy to use, energy efficient, home electricity bill is obviously much less	Jingdong prefecture level city in Shandong
Power Saving King Shop	Green Intelligent Power Saver	Easy to install, energy saving and power saving, very satisfied with a shopping	Jingdong prefecture level city in Shandong
Power Saving King Shop	Green Intelligent Power Saver	Received it and tried it out, it's great, works great and worth having! Five stars praise!	Jingdong prefecture level city in Shandong
Power Saving King Shop	Green Intelligent Power Saver	Power saving device, no problem at all, the quality of this product at this price, very affordable!	Jingdong prefecture level city in Shandong
Power Saving King Shop	Green Intelligent Power Saver	Received the goods, did not expect to be so fast, the quality is very good, especially power saving	Jingdong prefecture level city in Shandong
Power Saving King Shop	Green Intelligent Power Saver	The logistics is very fast, especially good, need to come back again	Jingdong prefecture level city in Shandong
Power Saving King Shop	Green Intelligent Power Saver	It's already working and it really saves power, so I'm very happy with it!	Jingdong prefecture level city in Shandong
Power Saving King Shop	Green Intelligent Power Saver	Great quality, works well, can recommend to friends to buy.	Jingdong prefecture level city in Shandong
Power Saving King Shop	Green Intelligent Power Saver	It's a good deal and worth the money.	Jingdong prefecture level city in Shandong
Power Saving King Shop	Green Intelligent Power Saver	I received it and I'm very happy with it, it's very good quality, guaranteed, and	Jingdong prefecture level city in Shandong
Power Saving King Shop	Green Intelligent Power Saver	Good quality, safe and secure purchase, ideal results and great price.	Jingdong prefecture level city in Shandong

Power Saving King Shop	Green Intelligent Power Saver	Simple operation plugged in the power supply can be, friends recommended looking forward to the effect of the	Jingdong prefecture level city in Shandong
Power Saving King Shop	Green Intelligent Power Saver	It’s not bad, it saves electricity, and the material is sturdy and durable.	Jingdong prefecture level city in Shandong
Power Saving King Shop	Green Intelligent Power Saver	Plug and play, no management required, convenient and hassle-free!	Jingdong prefecture level city in Shandong
Power Saving King Shop	Green Intelligent Power Saver	The pictures are too lazy to go up, the power saving effect is still there!	Jingdong prefecture level city in Shandong
Power Saving King Shop	Green Intelligent Power Saver	Fast shipping. Affordable prices. Efficient	Jingdong prefecture level city in Shandong
Power Saving King Shop	Green Intelligent Power Saver	Good power saving, worth buying	Jingdong prefecture level city in Shandong
Power Saving King Shop	Green Intelligent Power Saver	Especially great, comparison shopping before buying, the store owner is very good, answering questions very patiently and detailed, there is a need to come back!	Jingdong prefecture level city in Shandong
Power Saving King Shop	Green Intelligent Power Saver	Very trustworthy brand, quality is guaranteed, the price is still affordable, quite satisfied!	Jingdong prefecture level city in Shandong
Power Saving King Shop	Green Intelligent Power Saver	Recommended by a friend. They say you can save money on air conditioning with this. Try it.	Jingdong prefecture level city in Shandong
Power Saving King Shop	Green Intelligent Power Saver	Works great, will be back next time, very happy with it and a great price!	Jingdong prefecture level city in Shandong
Power Saving King Shop	Green Intelligent Power Saver	The quality of the product is quite good, fast shipping, satisfied.	Jingdong prefecture level city in Shandong
Power Saving King Shop	Green Intelligent Power Saver	Good quality and fast logistics	Jingdong prefecture level city in Shandong
Power Saving King Shop	Green Intelligent Power Saver	It really saves power. I’ve tested it myself.	Jingdong prefecture level city in Shandong
Power Saving King Shop	Green Intelligent Power Saver	Really saves electricity!	Jingdong prefecture level city in Shandong
Power Saving King Shop	Green Intelligent Power Saver	pretty good	Jingdong prefecture level city in Shandong
Power Saving King Shop	Green Intelligent Power Saver	serviceable	Jingdong prefecture level city in Shandong
Power Saving King Shop	Green Intelligent Power Saver	Power Saving King received a tightly packaged logistics, installed the effect is very good price is cheaper than the physical store!	Jingdong prefecture level city in Shandong
Power Saving King Shop	Green Intelligent Power Saver	Super Power Saving King is very good quality, new style, is genuine, the use of the effect is very good, the price is cheap, it is worth buying!	Jingdong prefecture level city in Shandong
Power Saving King Shop	Green Intelligent Power Saver	Packaging is very tight, easy to install, quite practical, worth buying!	Jingdong prefecture level city in Shandong
Power Saving King Shop	Green Intelligent Power Saver	Quality assurance, affordable price, very energy efficient, will repurchase if needed!	Jingdong prefecture level city in Shandong
Power Saving King Shop	Green Intelligent Power Saver	Quality assurance, very good, affordable, logistics is also very fast, I’m very satisfied with the	Jingdong prefecture level city in Shandong
Power Saving King Shop	Green Intelligent Power Saver	Energy saving. It’s so good, already installed, you can save a lot of electricity every month	Jingdong prefecture level city in Shandong
Power Saving King Shop	Green Intelligent Power Saver	The material is thick and the workmanship is fine in place, the power saving effect is very good, value for money!	Jingdong prefecture level city in Shandong
Power Saving King Shop	Green Intelligent Power Saver	The effect is great, genuine, and match the description, the logistics is fast, the price is reasonable 😍	Jingdong prefecture level city in Shandong
Power Saving King Shop	Green Intelligent Power Saver	It’s genuine, works well, easy to use, plugs in and works, really saves power	Jingdong prefecture level city in Shandong
Power Saving King Shop	Green Intelligent Power Saver	It’s easy to use, effective, great service, and recommended!	Jingdong prefecture level city in Shandong
Power Saving King Shop	Green Intelligent Power Saver	🌈 Great quality, bought it to my liking 🤍 Awesome! ✨ Buy with confidence if you need it!	Jingdong prefecture level city in Shandong
Power Saving King Shop	Green Intelligent Power Saver	Good quality, easy to install, very energy saving and power saving, good to use	Jingdong prefecture level city in Shandong
Power Saving King Shop	Green Intelligent Power Saver	The quality is very good, it does have the effect of saving electricity, the description matches, the price is affordable, it is worth recommending.	Jingdong prefecture level city in Shandong
Power Saving King Shop	Green Intelligent Power Saver	Received, the functionality is particularly good, it is genuine, the price is affordable	Jingdong prefecture level city in Shandong
Power Saving King Shop	Green Intelligent Power Saver	Good quality, very easy to use, energy efficient, home electricity bill is obviously much less	Jingdong prefecture level city in Shandong
Power Saving King Shop	Green Intelligent Power Saver	Easy to install, energy saving and power saving, very satisfied with a shopping	Jingdong prefecture level city in Shandong
Power Saving King Shop	Green Intelligent Power Saver	Received it and tried it out, it’s great, works great and worth having! Five stars praise!	Jingdong prefecture level city in Shandong
Power Saving King Shop	Green Intelligent Power Saver	Power saving device, no problem at all, the quality of this product at this price, very affordable!	Jingdong prefecture level city in Shandong
Power Saving King Shop	Green Intelligent Power Saver	Received the goods, did not expect to be so fast, the quality is very good, especially power saving	Jingdong prefecture level city in Shandong
Power Saving King Shop	Green Intelligent Power Saver	The logistics is very fast, especially good, need to come back again	Jingdong prefecture level city in Shandong
Power Saving King Shop	Green Intelligent Power Saver	It’s already working and it really saves power, so I’m very happy with it!	Jingdong prefecture level city in Shandong
Power Saving King Shop	Green Intelligent Power Saver	Great quality, works well, can recommend to friends to buy.	Jingdong prefecture level city in Shandong
Power Saving King Shop	Green Intelligent Power Saver	It’s a good deal and worth the money.	Jingdong prefecture level city in Shandong
Power Saving King Shop	Green Intelligent Power Saver	I received it and I’m very happy with it, it’s very good quality, guaranteed, and	Jingdong prefecture level city in Shandong
Power Saving King Shop	Green Intelligent Power Saver	Good quality, safe and secure purchase, ideal results and great price.	Jingdong prefecture level city in Shandong
Power Saving King Shop	Green Intelligent Power Saver	Simple operation plugged in the power supply can be, friends recommended looking forward to the effect of the	Jingdong prefecture level city in Shandong
Power Saving King Shop	Green Intelligent Power Saver	It’s not bad, it saves electricity, and the material is sturdy and durable.	Jingdong prefecture level city in Shandong
Power Saving King Shop	Green Intelligent Power Saver	Plug and play, no management required, convenient and hassle-free!	Jingdong prefecture level city in Shandong
Power Saving King Shop	Green Intelligent Power Saver	The pictures are too lazy to go up, the power saving effect is still there!	Jingdong prefecture level city in Shandong
Power Saving King Shop	Green Intelligent Power Saver	Fast shipping. Affordable prices. Efficient	Jingdong prefecture level city in Shandong
Power Saving King Shop	Green Intelligent Power Saver	Good power saving, worth buying	Jingdong prefecture level city in Shandong
Power Saving King Shop	Green Intelligent Power Saver	Especially great, comparison shopping before buying, the store owner is very good, answering questions very patiently and detailed, there is a need to come back!	Jingdong prefecture level city in Shandong
Power Saving King Shop	Green Intelligent Power Saver	Very trustworthy brand, quality is guaranteed, the price is still affordable, quite satisfied!	Jingdong prefecture level city in Shandong
Power Saving King Shop	Green Intelligent Power Saver	Recommended by a friend. They say you can save money on air conditioning with this. Try it.	Jingdong prefecture level city in Shandong
Power Saving King Shop	Green Intelligent Power Saver	Works great, will be back next time, very happy with it and a great price!	Jingdong prefecture level city in Shandong

Power Saving King Shop	Green Intelligent Power Saver	The quality of the product is quite good, fast shipping, satisfied.	Jingdong prefecture level city in Shandong
Power Saving King Shop	Green Intelligent Power Saver	Good quality and fast logistics	Jingdong prefecture level city in Shandong
Power Saving King Shop	Green Intelligent Power Saver	It really saves power. I've tested it myself.	Jingdong prefecture level city in Shandong
Power Saving King Shop	Green Intelligent Power Saver	Really saves electricity!	Jingdong prefecture level city in Shandong
Power Saving King Shop	Green Intelligent Power Saver	pretty good	Jingdong prefecture level city in Shandong
Power Saving King Shop	Green Intelligent Power Saver	serviceable	Jingdong prefecture level city in Shandong
Power Saving King Shop	Green Intelligent Power Saver	Power Saving King received a tightly packaged logistics, installed the effect is very good price is cheaper than the physical store!	Jingdong prefecture level city in Shandong
Power Saving King Shop	Green Intelligent Power Saver	Super Power Saving King is very good quality, new style, is genuine, the use of the effect is very good, the price is cheap, it is worth buying!	Jingdong prefecture level city in Shandong
Power Saving King Shop	Green Intelligent Power Saver	Packaging is very tight, easy to install, quite practical, worth buying!	Jingdong prefecture level city in Shandong
Power Saving King Shop	Green Intelligent Power Saver	Quality assurance, affordable price, very energy efficient, will repurchase if needed!	Jingdong prefecture level city in Shandong
Power Saving King Shop	Green Intelligent Power Saver	Quality assurance, very good, affordable, logistics is also very fast, I'm very satisfied with the	Jingdong prefecture level city in Shandong
Power Saving King Shop	Green Intelligent Power Saver	Energy saving. It's so good, already installed, you can save a lot of electricity every month	Jingdong prefecture level city in Shandong
Power Saving King Shop	Green Intelligent Power Saver	The material is thick and the workmanship is fine in place, the power saving effect is very good, value for money!	Jingdong prefecture level city in Shandong
Power Saving King Shop	Green Intelligent Power Saver	The effect is great, genuine, and match the description, the logistics is fast, the price is reasonable 😍	Jingdong prefecture level city in Shandong
Power Saving King Shop	Green Intelligent Power Saver	It's genuine, works well, easy to use, plugs in and works, really saves power	Jingdong prefecture level city in Shandong
Power Saving King Shop	Green Intelligent Power Saver	It's easy to use, effective, great service, and recommended!	Jingdong prefecture level city in Shandong
Power Saving King Shop	Green Intelligent Power Saver	🌈 Great quality, bought it to my liking 💞 Awesome! ✨ Buy with confidence if you need it!	Jingdong prefecture level city in Shandong
Power Saving King Shop	Green Intelligent Power Saver	Good quality, easy to install, very energy saving and power saving, good to use	Jingdong prefecture level city in Shandong
Power Saving King Shop	Green Intelligent Power Saver	The quality is very good, it does have the effect of saving electricity, the description matches, the price is affordable, it is worth recommending.	Jingdong prefecture level city in Shandong
Power Saving King Shop	Green Intelligent Power Saver	Received, the functionality is particularly good, it is genuine, the price is affordable	Jingdong prefecture level city in Shandong
Power Saving King Shop	Green Intelligent Power Saver	Good quality, very easy to use, energy efficient, home electricity bill is obviously much less	Jingdong prefecture level city in Shandong
Power Saving King Shop	Green Intelligent Power Saver	Easy to install, energy saving and power saving, very satisfied with a shopping	Jingdong prefecture level city in Shandong
Power Saving King Shop	Green Intelligent Power Saver	Received it and tried it out, it's great, works great and worth having! Five stars praise!	Jingdong prefecture level city in Shandong
Power Saving King Shop	Green Intelligent Power Saver	Power saving device, no problem at all, the quality of this product at this price, very affordable!	Jingdong prefecture level city in Shandong
Power Saving King Shop	Green Intelligent Power Saver	Received the goods, did not expect to be so fast, the quality is very good, especially power saving	Jingdong prefecture level city in Shandong
Power Saving King Shop	Green Intelligent Power Saver	The logistics is very fast, especially good, need to come back again	Jingdong prefecture level city in Shandong
Power Saving King Shop	Green Intelligent Power Saver	It's already working and it really saves power, so I'm very happy with it!	Jingdong prefecture level city in Shandong
Power Saving King Shop	Green Intelligent Power Saver	Great quality, works well, can recommend to friends to buy.	Jingdong prefecture level city in Shandong
Power Saving King Shop	Green Intelligent Power Saver	It's a good deal and worth the money.	Jingdong prefecture level city in Shandong
Power Saving King Shop	Green Intelligent Power Saver	I received it and I'm very happy with it, it's very good quality, guaranteed, and	Jingdong prefecture level city in Shandong
Power Saving King Shop	Green Intelligent Power Saver	Good quality, safe and secure purchase, ideal results and great price.	Jingdong prefecture level city in Shandong
Power Saving King Shop	Green Intelligent Power Saver	Simple operation plugged in the power supply can be, friends recommended looking forward to the effect of the	Jingdong prefecture level city in Shandong
Power Saving King Shop	Green Intelligent Power Saver	It's not bad, it saves electricity, and the material is sturdy and durable.	Jingdong prefecture level city in Shandong
Power Saving King Shop	Green Intelligent Power Saver	Plug and play, no management required, convenient and hassle-free!	Jingdong prefecture level city in Shandong
Power Saving King Shop	Green Intelligent Power Saver	The pictures are too lazy to go up, the power saving effect is still there!	Jingdong prefecture level city in Shandong
Power Saving King Shop	Green Intelligent Power Saver	Fast shipping. Affordable prices. Efficient	Jingdong prefecture level city in Shandong
Power Saving King Shop	Green Intelligent Power Saver	Good power saving, worth buying	Jingdong prefecture level city in Shandong
Power Saving King Shop	Green Intelligent Power Saver	Especially great, comparison shopping before buying, the store owner is very good, answering questions very patiently and detailed, there is a need to come back!	Jingdong prefecture level city in Shandong
Power Saving King Shop	Green Intelligent Power Saver	Very trustworthy brand, quality is guaranteed, the price is still affordable, quite satisfied!	Jingdong prefecture level city in Shandong
Power Saving King Shop	Green Intelligent Power Saver	Recommended by a friend. They say you can save money on air conditioning with this. Try it.	Jingdong prefecture level city in Shandong
Power Saving King Shop	Green Intelligent Power Saver	Works great, will be back next time, very happy with it and a great price!	Jingdong prefecture level city in Shandong
Power Saving King Shop	Green Intelligent Power Saver	The quality of the product is quite good, fast shipping, satisfied.	Jingdong prefecture level city in Shandong
Power Saving King Shop	Green Intelligent Power Saver	Good quality and fast logistics	Jingdong prefecture level city in Shandong
Power Saving King Shop	Green Intelligent Power Saver	It really saves power. I've tested it myself.	Jingdong prefecture level city in Shandong
Power Saving King Shop	Green Intelligent Power Saver	Really saves electricity!	Jingdong prefecture level city in Shandong
Power Saving King Shop	Green Intelligent Power Saver	pretty good	Jingdong prefecture level city in Shandong
Power Saving King Shop	Green Intelligent Power Saver	serviceable	Jingdong prefecture level city in Shandong
Power Saving King Shop	Green Intelligent Power Saver	Power Saving King received a tightly packaged logistics, installed the effect is very good price is cheaper than the physical store!	Jingdong prefecture level city in Shandong
Power Saving King Shop	Green Intelligent Power Saver	Super Power Saving King is very good quality, new style, is genuine, the use of the effect is very good, the price is cheap, it is worth buying!	Jingdong prefecture level city in Shandong
Power Saving King Shop	Green Intelligent Power Saver	Packaging is very tight, easy to install, quite practical, worth buying!	Jingdong prefecture level city in Shandong
Power Saving King Shop	Green Intelligent Power Saver	Quality assurance, affordable price, very energy efficient, will repurchase if needed!	Jingdong prefecture level city in Shandong

Power Saving King Shop	Green Intelligent Power Saver	Quality assurance, very good, affordable, logistics is also very fast, I'm very satisfied with the	Jingdong prefecture level city in Shandong
Power Saving King Shop	Green Intelligent Power Saver	Energy saving. It's so good, already installed, you can save a lot of electricity every month	Jingdong prefecture level city in Shandong
Power Saving King Shop	Green Intelligent Power Saver	The material is thick and the workmanship is fine in place, the power saving effect is very good, value for money!	Jingdong prefecture level city in Shandong
Power Saving King Shop	Green Intelligent Power Saver	The effect is great, genuine, and match the description, the logistics is fast, the price is reasonable 😍	Jingdong prefecture level city in Shandong
Power Saving King Shop	Green Intelligent Power Saver	It's genuine, works well, easy to use, plugs in and works, really saves power	Jingdong prefecture level city in Shandong
Power Saving King Shop	Green Intelligent Power Saver	It's easy to use, effective, great service, and recommended!	Jingdong prefecture level city in Shandong
Power Saving King Shop	Green Intelligent Power Saver	🌈 Great quality, bought it to my liking 💝 Awesome! ✨ Buy with confidence if you need it!	Jingdong prefecture level city in Shandong
Power Saving King Shop	Green Intelligent Power Saver	Good quality, easy to install, very energy saving and power saving, good to use	Jingdong prefecture level city in Shandong
Power Saving King Shop	Green Intelligent Power Saver	The quality is very good, it does have the effect of saving electricity, the description matches, the price is affordable, it is worth recommending.	Jingdong prefecture level city in Shandong
Power Saving King Shop	Green Intelligent Power Saver	Received, the functionality is particularly good, it is genuine, the price is affordable	Jingdong prefecture level city in Shandong
Power Saving King Shop	Green Intelligent Power Saver	Good quality, very easy to use, energy efficient, home electricity bill is obviously much less	Jingdong prefecture level city in Shandong
Power Saving King Shop	Green Intelligent Power Saver	Easy to install, energy saving and power saving, very satisfied with a shopping	Jingdong prefecture level city in Shandong
Power Saving King Shop	Green Intelligent Power Saver	Received it and tried it out, it's great, works great and worth having! Five stars praise!	Jingdong prefecture level city in Shandong
Power Saving King Shop	Green Intelligent Power Saver	Power saving device, no problem at all, the quality of this product at this price, very affordable!	Jingdong prefecture level city in Shandong
Power Saving King Shop	Green Intelligent Power Saver	Received the goods, did not expect to be so fast, the quality is very good, especially power saving	Jingdong prefecture level city in Shandong
Power Saving King Shop	Green Intelligent Power Saver	The logistics is very fast, especially good, need to come back again	Jingdong prefecture level city in Shandong
Power Saving King Shop	Green Intelligent Power Saver	It's already working and it really saves power, so I'm very happy with it!	Jingdong prefecture level city in Shandong
Power Saving King Shop	Green Intelligent Power Saver	Great quality, works well, can recommend to friends to buy.	Jingdong prefecture level city in Shandong
Power Saving King Shop	Green Intelligent Power Saver	It's a good deal and worth the money.	Jingdong prefecture level city in Shandong
Power Saving King Shop	Green Intelligent Power Saver	I received it and I'm very happy with it, it's very good quality, guaranteed, and	Jingdong prefecture level city in Shandong
Power Saving King Shop	Green Intelligent Power Saver	Good quality, safe and secure purchase, ideal results and great price.	Jingdong prefecture level city in Shandong
Power Saving King Shop	Green Intelligent Power Saver	Simple operation plugged in the power supply can be, friends recommended looking forward to the effect of the	Jingdong prefecture level city in Shandong
Power Saving King Shop	Green Intelligent Power Saver	It's not bad, it saves electricity, and the material is sturdy and durable.	Jingdong prefecture level city in Shandong
Power Saving King Shop	Green Intelligent Power Saver	Plug and play, no management required, convenient and hassle-free!	Jingdong prefecture level city in Shandong
Power Saving King Shop	Green Intelligent Power Saver	The pictures are too lazy to go up, the power saving effect is still there!	Jingdong prefecture level city in Shandong
Power Saving King Shop	Green Intelligent Power Saver	Fast shipping. Affordable prices. Efficient	Jingdong prefecture level city in Shandong
Power Saving King Shop	Green Intelligent Power Saver	Good power saving, worth buying	Jingdong prefecture level city in Shandong
Power Saving King Shop	Green Intelligent Power Saver	Especially great, comparison shopping before buying, the store owner is very good, answering questions very patiently and detailed, there is a need to come back!	Jingdong prefecture level city in Shandong
Power Saving King Shop	Green Intelligent Power Saver	Very trustworthy brand, quality is guaranteed, the price is still affordable, quite satisfied!	Jingdong prefecture level city in Shandong
Power Saving King Shop	Green Intelligent Power Saver	Recommended by a friend. They say you can save money on air conditioning with this. Try it.	Jingdong prefecture level city in Shandong
Power Saving King Shop	Green Intelligent Power Saver	Works great, will be back next time, very happy with it and a great price!	Jingdong prefecture level city in Shandong
Power Saving King Shop	Green Intelligent Power Saver	The quality of the product is quite good, fast shipping, satisfied.	Jingdong prefecture level city in Shandong
Power Saving King Shop	Green Intelligent Power Saver	Good quality and fast logistics	Jingdong prefecture level city in Shandong
Power Saving King Shop	Green Intelligent Power Saver	It really saves power. I've tested it myself.	Jingdong prefecture level city in Shandong
Power Saving King Shop	Green Intelligent Power Saver	Really saves electricity!	Jingdong prefecture level city in Shandong
Power Saving King Shop	Green Intelligent Power Saver	pretty good	Jingdong prefecture level city in Shandong
Power Saving King Shop	Green Intelligent Power Saver	serviceable	Jingdong prefecture level city in Shandong
Power Saving King Shop	Green Intelligent Power Saver	Power Saving King received a tightly packaged logistics, installed the effect is very good price is cheaper than the physical store!	Jingdong prefecture level city in Shandong
Power Saving King Shop	Green Intelligent Power Saver	Super Power Saving King is very good quality, new style, is genuine, the use of the effect is very good, the price is cheap, it is worth buying!	Jingdong prefecture level city in Shandong
Power Saving King Shop	Green Intelligent Power Saver	Packaging is very tight, easy to install, quite practical, worth buying!	Jingdong prefecture level city in Shandong
Power Saving King Shop	Green Intelligent Power Saver	Quality assurance, affordable price, very energy efficient, will repurchase if needed!	Jingdong prefecture level city in Shandong
Power Saving King Shop	Green Intelligent Power Saver	Quality assurance, very good, affordable, logistics is also very fast, I'm very satisfied with the	Jingdong prefecture level city in Shandong
Power Saving King Shop	Green Intelligent Power Saver	Energy saving. It's so good, already installed, you can save a lot of electricity every month	Jingdong prefecture level city in Shandong
Power Saving King Shop	Green Intelligent Power Saver	The material is thick and the workmanship is fine in place, the power saving effect is very good, value for money!	Jingdong prefecture level city in Shandong
Power Saving King Shop	Green Intelligent Power Saver	The effect is great, genuine, and match the description, the logistics is fast, the price is reasonable 😍	Jingdong prefecture level city in Shandong
Power Saving King Shop	Green Intelligent Power Saver	It's genuine, works well, easy to use, plugs in and works, really saves power	Jingdong prefecture level city in Shandong
Power Saving King Shop	Green Intelligent Power Saver	It's easy to use, effective, great service, and recommended!	Jingdong prefecture level city in Shandong
Power Saving King Shop	Green Intelligent Power Saver	🌈 Great quality, bought it to my liking 💝 Awesome! ✨ Buy with confidence if you need it!	Jingdong prefecture level city in Shandong
Power Saving King Shop	Green Intelligent Power Saver	Good quality, easy to install, very energy saving and power saving, good to use	Jingdong prefecture level city in Shandong
Power Saving King Shop	Green Intelligent Power Saver	The quality is very good, it does have the effect of saving electricity, the description matches, the price is affordable, it is worth recommending.	Jingdong prefecture level city in Shandong
Power Saving King Shop	Green Intelligent Power Saver	Received, the functionality is particularly good, it is genuine, the price is affordable	Jingdong prefecture level city in Shandong

Power Saving King Shop	Green Intelligent Power Saver	Good quality, very easy to use, energy efficient, home electricity bill is obviously much less	Jingdong prefecture level city in Shandong
Power Saving King Shop	Green Intelligent Power Saver	Easy to install, energy saving and power saving, very satisfied with a shopping	Jingdong prefecture level city in Shandong
Power Saving King Shop	Green Intelligent Power Saver	Received it and tried it out, it’s great, works great and worth having! Five stars praise!	Jingdong prefecture level city in Shandong
Power Saving King Shop	Green Intelligent Power Saver	Power saving device, no problem at all, the quality of this product at this price, very affordable!	Jingdong prefecture level city in Shandong
Power Saving King Shop	Green Intelligent Power Saver	Received the goods, did not expect to be so fast, the quality is very good, especially power saving	Jingdong prefecture level city in Shandong
Power Saving King Shop	Green Intelligent Power Saver	The logistics is very fast, especially good, need to come back again	Jingdong prefecture level city in Shandong
Power Saving King Shop	Green Intelligent Power Saver	It’s already working and it really saves power, so I’m very happy with it!	Jingdong prefecture level city in Shandong
Power Saving King Shop	Green Intelligent Power Saver	Great quality, works well, can recommend to friends to buy.	Jingdong prefecture level city in Shandong
Power Saving King Shop	Green Intelligent Power Saver	It’s a good deal and worth the money.	Jingdong prefecture level city in Shandong
Power Saving King Shop	Green Intelligent Power Saver	I received it and I’m very happy with it, it’s very good quality, guaranteed, and	Jingdong prefecture level city in Shandong
Power Saving King Shop	Green Intelligent Power Saver	Good quality, safe and secure purchase, ideal results and great price.	Jingdong prefecture level city in Shandong
Power Saving King Shop	Green Intelligent Power Saver	Simple operation plugged in the power supply can be, friends recommended looking forward to the effect of the	Jingdong prefecture level city in Shandong
Power Saving King Shop	Green Intelligent Power Saver	It’s not bad, it saves electricity, and the material is sturdy and durable.	Jingdong prefecture level city in Shandong
Power Saving King Shop	Green Intelligent Power Saver	Plug and play, no management required, convenient and hassle-free!	Jingdong prefecture level city in Shandong
Power Saving King Shop	Green Intelligent Power Saver	The pictures are too lazy to go up, the power saving effect is still there!	Jingdong prefecture level city in Shandong
Power Saving King Shop	Green Intelligent Power Saver	Fast shipping. Affordable prices. Efficient	Jingdong prefecture level city in Shandong
Power Saving King Shop	Green Intelligent Power Saver	Good power saving, worth buying	Jingdong prefecture level city in Shandong
Power Saving King Shop	Green Intelligent Power Saver	Especially great, comparison shopping before buying, the store owner is very good, answering questions very patiently and detailed, there is a need to come back!	Jingdong prefecture level city in Shandong
Power Saving King Shop	Green Intelligent Power Saver	Very trustworthy brand, quality is guaranteed, the price is still affordable, quite satisfied!	Jingdong prefecture level city in Shandong
Power Saving King Shop	Green Intelligent Power Saver	Recommended by a friend. They say you can save money on air conditioning with this. Try it.	Jingdong prefecture level city in Shandong
Power Saving King Shop	Green Intelligent Power Saver	Works great, will be back next time, very happy with it and a great price!	Jingdong prefecture level city in Shandong
Power Saving King Shop	Green Intelligent Power Saver	The quality of the product is quite good, fast shipping, satisfied.	Jingdong prefecture level city in Shandong
Power Saving King Shop	Green Intelligent Power Saver	Good quality and fast logistics	Jingdong prefecture level city in Shandong
Power Saving King Shop	Green Intelligent Power Saver	It really saves power. I’ve tested it myself.	Jingdong prefecture level city in Shandong
Power Saving King Shop	Green Intelligent Power Saver	Really saves electricity!	Jingdong prefecture level city in Shandong
Power Saving King Shop	Green Intelligent Power Saver	pretty good	Jingdong prefecture level city in Shandong
Power Saving King Shop	Green Intelligent Power Saver	serviceable	Jingdong prefecture level city in Shandong
Power Saving King Shop	Green Intelligent Power Saver	Power Saving King received a tightly packaged logistics, installed the effect is very good price is cheaper than the physical store!	Jingdong prefecture level city in Shandong
Power Saving King Shop	Green Intelligent Power Saver	Super Power Saving King is very good quality, new style, is genuine, the use of the effect is very good, the price is cheap, it is worth buying!	Jingdong prefecture level city in Shandong
Power Saving King Shop	Green Intelligent Power Saver	Packaging is very tight, easy to install, quite practical, worth buying!	Jingdong prefecture level city in Shandong
Power Saving King Shop	Green Intelligent Power Saver	Quality assurance, affordable price, very energy efficient, will repurchase if needed!	Jingdong prefecture level city in Shandong
Power Saving King Shop	Green Intelligent Power Saver	Quality assurance, very good, affordable, logistics is also very fast, I’m very satisfied with the	Jingdong prefecture level city in Shandong
Power Saving King Shop	Green Intelligent Power Saver	Energy saving. It’s so good, already installed, you can save a lot of electricity every month	Jingdong prefecture level city in Shandong
Power Saving King Shop	Green Intelligent Power Saver	The material is thick and the workmanship is fine in place, the power saving effect is very good, value for money!	Jingdong prefecture level city in Shandong
Power Saving King Shop	Green Intelligent Power Saver	The effect is great, genuine, and match the description, the logistics is fast, the price is reasonable 😍	Jingdong prefecture level city in Shandong
Power Saving King Shop	Green Intelligent Power Saver	It’s genuine, works well, easy to use, plugs in and works, really saves power	Jingdong prefecture level city in Shandong
Power Saving King Shop	Green Intelligent Power Saver	It’s easy to use, effective, great service, and recommended!	Jingdong prefecture level city in Shandong
Power Saving King Shop	Green Intelligent Power Saver	🌈 Great quality, bought it to my liking 💕 Awesome! ✨ Buy with confidence if you need it!	Jingdong prefecture level city in Shandong
Power Saving King Shop	Green Intelligent Power Saver	Good quality, easy to install, very energy saving and power saving, good to use	Jingdong prefecture level city in Shandong
Power Saving King Shop	Green Intelligent Power Saver	The quality is very good, it does have the effect of saving electricity, the description matches, the price is affordable, it is worth recommending.	Jingdong prefecture level city in Shandong
Power Saving King Shop	Green Intelligent Power Saver	Received, the functionality is particularly good, it is genuine, the price is affordable	Jingdong prefecture level city in Shandong
Power Saving King Shop	Green Intelligent Power Saver	Good quality, very easy to use, energy efficient, home electricity bill is obviously much less	Jingdong prefecture level city in Shandong
Power Saving King Shop	Green Intelligent Power Saver	Easy to install, energy saving and power saving, very satisfied with a shopping	Jingdong prefecture level city in Shandong
Power Saving King Shop	Green Intelligent Power Saver	Received it and tried it out, it’s great, works great and worth having! Five stars praise!	Jingdong prefecture level city in Shandong
Power Saving King Shop	Green Intelligent Power Saver	Power saving device, no problem at all, the quality of this product at this price, very affordable!	Jingdong prefecture level city in Shandong
Power Saving King Shop	Green Intelligent Power Saver	Received the goods, did not expect to be so fast, the quality is very good, especially power saving	Jingdong prefecture level city in Shandong
Power Saving King Shop	Green Intelligent Power Saver	The logistics is very fast, especially good, need to come back again	Jingdong prefecture level city in Shandong
Power Saving King Shop	Green Intelligent Power Saver	It’s already working and it really saves power, so I’m very happy with it!	Jingdong prefecture level city in Shandong
Power Saving King Shop	Green Intelligent Power Saver	Great quality, works well, can recommend to friends to buy.	Jingdong prefecture level city in Shandong
Power Saving King Shop	Green Intelligent Power Saver	It’s a good deal and worth the money.	Jingdong prefecture level city in Shandong
Power Saving King Shop	Green Intelligent Power Saver	I received it and I’m very happy with it, it’s very good quality, guaranteed, and	Jingdong prefecture level city in Shandong

Power Saving King Shop	Green Intelligent Power Saver	Good quality, safe and secure purchase, ideal results and great price.	Jingdong prefecture level city in Shandong
Power Saving King Shop	Green Intelligent Power Saver	Simple operation plugged in the power supply can be, friends recommended looking forward to the effect of the	Jingdong prefecture level city in Shandong
Power Saving King Shop	Green Intelligent Power Saver	It's not bad, it saves electricity, and the material is sturdy and durable.	Jingdong prefecture level city in Shandong
Power Saving King Shop	Green Intelligent Power Saver	Plug and play, no management required, convenient and hassle-free!	Jingdong prefecture level city in Shandong
Power Saving King Shop	Green Intelligent Power Saver	The pictures are too lazy to go up, the power saving effect is still there!	Jingdong prefecture level city in Shandong
Power Saving King Shop	Green Intelligent Power Saver	Fast shipping. Affordable prices. Efficient	Jingdong prefecture level city in Shandong
Power Saving King Shop	Green Intelligent Power Saver	Good power saving, worth buying	Jingdong prefecture level city in Shandong
Power Saving King Shop	Green Intelligent Power Saver	Especially great, comparison shopping before buying, the store owner is very good, answering questions very patiently and detailed, there is a need to come back!	Jingdong prefecture level city in Shandong
Power Saving King Shop	Green Intelligent Power Saver	Very trustworthy brand, quality is guaranteed, the price is still affordable, quite satisfied!	Jingdong prefecture level city in Shandong
Power Saving King Shop	Green Intelligent Power Saver	Recommended by a friend. They say you can save money on air conditioning with this. Try it.	Jingdong prefecture level city in Shandong
Power Saving King Shop	Green Intelligent Power Saver	Works great, will be back next time, very happy with it and a great price!	Jingdong prefecture level city in Shandong
Power Saving King Shop	Green Intelligent Power Saver	The quality of the product is quite good, fast shipping, satisfied.	Jingdong prefecture level city in Shandong
Power Saving King Shop	Green Intelligent Power Saver	Good quality and fast logistics	Jingdong prefecture level city in Shandong
Power Saving King Shop	Green Intelligent Power Saver	It really saves power. I've tested it myself.	Jingdong prefecture level city in Shandong
Power Saving King Shop	Green Intelligent Power Saver	Really saves electricity!	Jingdong prefecture level city in Shandong
Power Saving King Shop	Green Intelligent Power Saver	pretty good	Jingdong prefecture level city in Shandong
Power Saving King Shop	Green Intelligent Power Saver	serviceable	Jingdong prefecture level city in Shandong
Power Saving King Shop	Green Intelligent Power Saver	Power Saving King received a tightly packaged logistics, installed the effect is very good price is cheaper than the physical store!	Jingdong prefecture level city in Shandong
Power Saving King Shop	Green Intelligent Power Saver	Super Power Saving King is very good quality, new style, is genuine, the use of the effect is very good, the price is cheap, it is worth buying!	Jingdong prefecture level city in Shandong
Power Saving King Shop	Green Intelligent Power Saver	Packaging is very tight, easy to install, quite practical, worth buying!	Jingdong prefecture level city in Shandong
Power Saving King Shop	Green Intelligent Power Saver	Quality assurance, affordable price, very energy efficient, will repurchase if needed!	Jingdong prefecture level city in Shandong
Power Saving King Shop	Green Intelligent Power Saver	Quality assurance, very good, affordable, logistics is also very fast, I'm very satisfied with the	Jingdong prefecture level city in Shandong
Power Saving King Shop	Green Intelligent Power Saver	Energy saving. It's so good, already installed, you can save a lot of electricity every month	Jingdong prefecture level city in Shandong
Power Saving King Shop	Green Intelligent Power Saver	The material is thick and the workmanship is fine in place, the power saving effect is very good, value for money!	Jingdong prefecture level city in Shandong
Power Saving King Shop	Green Intelligent Power Saver	The effect is great, genuine, and match the description, the logistics is fast, the price is reasonable 😍	Jingdong prefecture level city in Shandong
Power Saving King Shop	Green Intelligent Power Saver	It's genuine, works well, easy to use, plugs in and works, really saves power	Jingdong prefecture level city in Shandong
Power Saving King Shop	Green Intelligent Power Saver	It's easy to use, effective, great service, and recommended!	Jingdong prefecture level city in Shandong
Power Saving King Shop	Green Intelligent Power Saver	🌈 Great quality, bought it to my liking 💝 Awesome! ✨ Buy with confidence if you need it!	Jingdong prefecture level city in Shandong
Power Saving King Shop	Green Intelligent Power Saver	Good quality, easy to install, very energy saving and power saving, good to use	Jingdong prefecture level city in Shandong
Power Saving King Shop	Green Intelligent Power Saver	The quality is very good, it does have the effect of saving electricity, the description matches, the price is affordable, it is worth recommending.	Jingdong prefecture level city in Shandong
Power Saving King Shop	Green Intelligent Power Saver	Received, the functionality is particularly good, it is genuine, the price is affordable	Jingdong prefecture level city in Shandong
Power Saving King Shop	Green Intelligent Power Saver	Good quality, very easy to use, energy efficient, home electricity bill is obviously much less	Jingdong prefecture level city in Shandong
Power Saving King Shop	Green Intelligent Power Saver	Easy to install, energy saving and power saving, very satisfied with a shopping	Jingdong prefecture level city in Shandong
Power Saving King Shop	Green Intelligent Power Saver	Received it and tried it out, it's great, works great and worth having! Five stars praise!	Jingdong prefecture level city in Shandong
Power Saving King Shop	Green Intelligent Power Saver	Power saving device, no problem at all, the quality of this product at this price, very affordable!	Jingdong prefecture level city in Shandong
Power Saving King Shop	Green Intelligent Power Saver	Received the goods, did not expect to be so fast, the quality is very good, especially power saving	Jingdong prefecture level city in Shandong
Power Saving King Shop	Green Intelligent Power Saver	The logistics is very fast, especially good, need to come back again	Jingdong prefecture level city in Shandong
Power Saving King Shop	Green Intelligent Power Saver	It's already working and it really saves power, so I'm very happy with it!	Jingdong prefecture level city in Shandong
Power Saving King Shop	Green Intelligent Power Saver	Great quality, works well, can recommend to friends to buy.	Jingdong prefecture level city in Shandong
Power Saving King Shop	Green Intelligent Power Saver	It's a good deal and worth the money.	Jingdong prefecture level city in Shandong
Power Saving King Shop	Green Intelligent Power Saver	I received it and I'm very happy with it, it's very good quality, guaranteed, and	Jingdong prefecture level city in Shandong
Power Saving King Shop	Green Intelligent Power Saver	Good quality, safe and secure purchase, ideal results and great price.	Jingdong prefecture level city in Shandong
Power Saving King Shop	Green Intelligent Power Saver	Simple operation plugged in the power supply can be, friends recommended looking forward to the effect of the	Jingdong prefecture level city in Shandong
Power Saving King Shop	Green Intelligent Power Saver	It's not bad, it saves electricity, and the material is sturdy and durable.	Jingdong prefecture level city in Shandong
Power Saving King Shop	Green Intelligent Power Saver	Plug and play, no management required, convenient and hassle-free!	Jingdong prefecture level city in Shandong
Power Saving King Shop	Green Intelligent Power Saver	The pictures are too lazy to go up, the power saving effect is still there!	Jingdong prefecture level city in Shandong
Power Saving King Shop	Green Intelligent Power Saver	Fast shipping. Affordable prices. Efficient	Jingdong prefecture level city in Shandong
Power Saving King Shop	Green Intelligent Power Saver	Good power saving, worth buying	Jingdong prefecture level city in Shandong
Power Saving King Shop	Green Intelligent Power Saver	Especially great, comparison shopping before buying, the store owner is very good, answering questions very patiently and detailed, there is a need to come back!	Jingdong prefecture level city in Shandong
Power Saving King Shop	Green Intelligent Power Saver	Very trustworthy brand, quality is guaranteed, the price is still affordable, quite satisfied!	Jingdong prefecture level city in Shandong
Power Saving King Shop	Green Intelligent Power Saver	Recommended by a friend. They say you can save money on air conditioning with this. Try it.	Jingdong prefecture level city in Shandong

Power Saving King Shop	Green Intelligent Power Saver	Works great, will be back next time, very happy with it and a great price!	Jingdong prefecture level city in Shandong
Power Saving King Shop	Green Intelligent Power Saver	The quality of the product is quite good, fast shipping, satisfied.	Jingdong prefecture level city in Shandong
Power Saving King Shop	Green Intelligent Power Saver	Good quality and fast logistics	Jingdong prefecture level city in Shandong
Power Saving King Shop	Green Intelligent Power Saver	It really saves power. I've tested it myself.	Jingdong prefecture level city in Shandong
Power Saving King Shop	Green Intelligent Power Saver	Really saves electricity!	Jingdong prefecture level city in Shandong
Power Saving King Shop	Green Intelligent Power Saver	pretty good	Jingdong prefecture level city in Shandong
Power Saving King Shop	Green Intelligent Power Saver	serviceable	Jingdong prefecture level city in Shandong
Power Saving King Shop	Green Intelligent Power Saver	Power Saving King received a tightly packaged logistics, installed the effect is very good price is cheaper than the physical store!	Jingdong prefecture level city in Shandong
Power Saving King Shop	Green Intelligent Power Saver	Super Power Saving King is very good quality, new style, is genuine, the use of the effect is very good, the price is cheap, it is worth buying!	Jingdong prefecture level city in Shandong
Power Saving King Shop	Green Intelligent Power Saver	Packaging is very tight, easy to install, quite practical, worth buying!	Jingdong prefecture level city in Shandong
Power Saving King Shop	Green Intelligent Power Saver	Quality assurance, affordable price, very energy efficient, will repurchase if needed!	Jingdong prefecture level city in Shandong
Power Saving King Shop	Green Intelligent Power Saver	Quality assurance, very good, affordable, logistics is also very fast, I'm very satisfied with the	Jingdong prefecture level city in Shandong
Power Saving King Shop	Green Intelligent Power Saver	Energy saving. It's so good, already installed, you can save a lot of electricity every month	Jingdong prefecture level city in Shandong
Power Saving King Shop	Green Intelligent Power Saver	The material is thick and the workmanship is fine in place, the power saving effect is very good, value for money!	Jingdong prefecture level city in Shandong
Power Saving King Shop	Green Intelligent Power Saver	The effect is great, genuine, and match the description, the logistics is fast, the price is reasonable 😍	Jingdong prefecture level city in Shandong
Power Saving King Shop	Green Intelligent Power Saver	It's genuine, works well, easy to use, plugs in and works, really saves power	Jingdong prefecture level city in Shandong
Power Saving King Shop	Green Intelligent Power Saver	It's easy to use, effective, great service, and recommended!	Jingdong prefecture level city in Shandong
Power Saving King Shop	Green Intelligent Power Saver	🌈 Great quality, bought it to my liking 💕 Awesome! ✨ Buy with confidence if you need it!	Jingdong prefecture level city in Shandong
Power Saving King Shop	Green Intelligent Power Saver	Good quality, easy to install, very energy saving and power saving, good to use	Jingdong prefecture level city in Shandong
Power Saving King Shop	Green Intelligent Power Saver	The quality is very good, it does have the effect of saving electricity, the description matches, the price is affordable, it is worth recommending.	Jingdong prefecture level city in Shandong
Power Saving King Shop	Green Intelligent Power Saver	Received, the functionality is particularly good, it is genuine, the price is affordable	Jingdong prefecture level city in Shandong
Power Saving King Shop	Green Intelligent Power Saver	Good quality, very easy to use, energy efficient, home electricity bill is obviously much less	Jingdong prefecture level city in Shandong
Power Saving King Shop	Green Intelligent Power Saver	Easy to install, energy saving and power saving, very satisfied with a shopping	Jingdong prefecture level city in Shandong
Power Saving King Shop	Green Intelligent Power Saver	Received it and tried it out, it's great, works great and worth having! Five stars praise!	Jingdong prefecture level city in Shandong
Power Saving King Shop	Green Intelligent Power Saver	Power saving device, no problem at all, the quality of this product at this price, very affordable!	Jingdong prefecture level city in Shandong
Power Saving King Shop	Green Intelligent Power Saver	Received the goods, did not expect to be so fast, the quality is very good, especially power saving	Jingdong prefecture level city in Shandong
Power Saving King Shop	Green Intelligent Power Saver	The logistics is very fast, especially good, need to come back again	Jingdong prefecture level city in Shandong
Power Saving King Shop	Green Intelligent Power Saver	It's already working and it really saves power, so I'm very happy with it!	Jingdong prefecture level city in Shandong
Power Saving King Shop	Green Intelligent Power Saver	Great quality, works well, can recommend to friends to buy.	Jingdong prefecture level city in Shandong
Power Saving King Shop	Green Intelligent Power Saver	It's a good deal and worth the money.	Jingdong prefecture level city in Shandong
Power Saving King Shop	Green Intelligent Power Saver	I received it and I'm very happy with it, it's very good quality, guaranteed, and	Jingdong prefecture level city in Shandong
Power Saving King Shop	Green Intelligent Power Saver	Good quality, safe and secure purchase, ideal results and great price.	Jingdong prefecture level city in Shandong
Power Saving King Shop	Green Intelligent Power Saver	Simple operation plugged in the power supply can be, friends recommended looking forward to the effect of the	Jingdong prefecture level city in Shandong
Power Saving King Shop	Green Intelligent Power Saver	It's not bad, it saves electricity, and the material is sturdy and durable.	Jingdong prefecture level city in Shandong
Power Saving King Shop	Green Intelligent Power Saver	Plug and play, no management required, convenient and hassle-free!	Jingdong prefecture level city in Shandong
Power Saving King Shop	Green Intelligent Power Saver	The pictures are too lazy to go up, the power saving effect is still there!	Jingdong prefecture level city in Shandong
Power Saving King Shop	Green Intelligent Power Saver	Fast shipping. Affordable prices. Efficient	Jingdong prefecture level city in Shandong
Power Saving King Shop	Green Intelligent Power Saver	Good power saving, worth buying	Jingdong prefecture level city in Shandong
Power Saving King Shop	Green Intelligent Power Saver	Especially great, comparison shopping before buying, the store owner is very good, answering questions very patiently and detailed, there is a need to come back!	Jingdong prefecture level city in Shandong
Power Saving King Shop	Green Intelligent Power Saver	Very trustworthy brand, quality is guaranteed, the price is still affordable, quite satisfied!	Jingdong prefecture level city in Shandong
Power Saving King Shop	Green Intelligent Power Saver	Recommended by a friend. They say you can save money on air conditioning with this. Try it.	Jingdong prefecture level city in Shandong
Power Saving King Shop	Green Intelligent Power Saver	Works great, will be back next time, very happy with it and a great price!	Jingdong prefecture level city in Shandong
Power Saving King Shop	Green Intelligent Power Saver	The quality of the product is quite good, fast shipping, satisfied.	Jingdong prefecture level city in Shandong
Power Saving King Shop	Green Intelligent Power Saver	Good quality and fast logistics	Jingdong prefecture level city in Shandong
Power Saving King Shop	Green Intelligent Power Saver	It really saves power. I've tested it myself.	Jingdong prefecture level city in Shandong
Power Saving King Shop	Green Intelligent Power Saver	Really saves electricity!	Jingdong prefecture level city in Shandong
Power Saving King Shop	Green Intelligent Power Saver	pretty good	Jingdong prefecture level city in Shandong
Power Saving King Shop	Green Intelligent Power Saver	serviceable	Jingdong prefecture level city in Shandong
Power Saving King Shop	Green Intelligent Power Saver	Power Saving King received a tightly packaged logistics, installed the effect is very good price is cheaper than the physical store!	Jingdong prefecture level city in Shandong
Power Saving King Shop	Green Intelligent Power Saver	Super Power Saving King is very good quality, new style, is genuine, the use of the effect is very good, the price is cheap, it is worth buying!	Jingdong prefecture level city in Shandong
Power Saving King Shop	Green Intelligent Power Saver	Packaging is very tight, easy to install, quite practical, worth buying!	Jingdong prefecture level city in Shandong

Power Saving King Shop	Green Intelligent Power Saver	Quality assurance, affordable price, very energy efficient, will repurchase if needed!	Jingdong prefecture level city in Shandong
Power Saving King Shop	Green Intelligent Power Saver	Quality assurance, very good, affordable, logistics is also very fast, I'm very satisfied with the	Jingdong prefecture level city in Shandong
Power Saving King Shop	Green Intelligent Power Saver	Energy saving. It's so good, already installed, you can save a lot of electricity every month	Jingdong prefecture level city in Shandong
Power Saving King Shop	Green Intelligent Power Saver	The material is thick and the workmanship is fine in place, the power saving effect is very good, value for money!	Jingdong prefecture level city in Shandong
Power Saving King Shop	Green Intelligent Power Saver	The effect is great, genuine, and match the description, the logistics is fast, the price is reasonable 😍	Jingdong prefecture level city in Shandong
Power Saving King Shop	Green Intelligent Power Saver	It's genuine, works well, easy to use, plugs in and works, really saves power	Jingdong prefecture level city in Shandong
Power Saving King Shop	Green Intelligent Power Saver	It's easy to use, effective, great service, and recommended!	Jingdong prefecture level city in Shandong
Power Saving King Shop	Green Intelligent Power Saver	🌈 Great quality, bought it to my liking 💝 Awesome! ✨ Buy with confidence if you need it!	Jingdong prefecture level city in Shandong
Power Saving King Shop	Green Intelligent Power Saver	Good quality, easy to install, very energy saving and power saving, good to use	Jingdong prefecture level city in Shandong
Power Saving King Shop	Green Intelligent Power Saver	The quality is very good, it does have the effect of saving electricity, the description matches, the price is affordable, it is worth recommending.	Jingdong prefecture level city in Shandong
Power Saving King Shop	Green Intelligent Power Saver	Received, the functionality is particularly good, it is genuine, the price is affordable	Jingdong prefecture level city in Shandong
Power Saving King Shop	Green Intelligent Power Saver	Good quality, very easy to use, energy efficient, home electricity bill is obviously much less	Jingdong prefecture level city in Shandong
Power Saving King Shop	Green Intelligent Power Saver	Easy to install, energy saving and power saving, very satisfied with a shopping	Jingdong prefecture level city in Shandong
Power Saving King Shop	Green Intelligent Power Saver	Received it and tried it out, it's great, works great and worth having! Five stars praise!	Jingdong prefecture level city in Shandong
Power Saving King Shop	Green Intelligent Power Saver	Power saving device, no problem at all, the quality of this product at this price, very affordable!	Jingdong prefecture level city in Shandong
Power Saving King Shop	Green Intelligent Power Saver	Received the goods, did not expect to be so fast, the quality is very good, especially power saving	Jingdong prefecture level city in Shandong
Power Saving King Shop	Green Intelligent Power Saver	The logistics is very fast, especially good, need to come back again	Jingdong prefecture level city in Shandong
Power Saving King Shop	Green Intelligent Power Saver	It's already working and it really saves power, so I'm very happy with it!	Jingdong prefecture level city in Shandong
Power Saving King Shop	Green Intelligent Power Saver	Great quality, works well, can recommend to friends to buy.	Jingdong prefecture level city in Shandong
Power Saving King Shop	Green Intelligent Power Saver	It's a good deal and worth the money.	Jingdong prefecture level city in Shandong
Power Saving King Shop	Green Intelligent Power Saver	I received it and I'm very happy with it, it's very good quality, guaranteed, and	Jingdong prefecture level city in Shandong
Power Saving King Shop	Green Intelligent Power Saver	Good quality, safe and secure purchase, ideal results and great price.	Jingdong prefecture level city in Shandong
Power Saving King Shop	Green Intelligent Power Saver	Simple operation plugged in the power supply can be, friends recommended looking forward to the effect of the	Jingdong prefecture level city in Shandong
Power Saving King Shop	Green Intelligent Power Saver	It's not bad, it saves electricity, and the material is sturdy and durable.	Jingdong prefecture level city in Shandong
Power Saving King Shop	Green Intelligent Power Saver	Plug and play, no management required, convenient and hassle-free!	Jingdong prefecture level city in Shandong
Power Saving King Shop	Green Intelligent Power Saver	The pictures are too lazy to go up, the power saving effect is still there!	Jingdong prefecture level city in Shandong
Power Saving King Shop	Green Intelligent Power Saver	Fast shipping. Affordable prices. Efficient	Jingdong prefecture level city in Shandong
Power Saving King Shop	Green Intelligent Power Saver	Good power saving, worth buying	Jingdong prefecture level city in Shandong
Power Saving King Shop	Green Intelligent Power Saver	Especially great, comparison shopping before buying, the store owner is very good, answering questions very patiently and detailed, there is a need to come back!	Jingdong prefecture level city in Shandong
Power Saving King Shop	Green Intelligent Power Saver	Very trustworthy brand, quality is guaranteed, the price is still affordable, quite satisfied!	Jingdong prefecture level city in Shandong
Power Saving King Shop	Green Intelligent Power Saver	Recommended by a friend. They say you can save money on air conditioning with this. Try it.	Jingdong prefecture level city in Shandong
Power Saving King Shop	Green Intelligent Power Saver	Works great, will be back next time, very happy with it and a great price!	Jingdong prefecture level city in Shandong
Power Saving King Shop	Green Intelligent Power Saver	The quality of the product is quite good, fast shipping, satisfied.	Jingdong prefecture level city in Shandong
Power Saving King Shop	Green Intelligent Power Saver	Good quality and fast logistics	Jingdong prefecture level city in Shandong
Power Saving King Shop	Green Intelligent Power Saver	It really saves power. I've tested it myself.	Jingdong prefecture level city in Shandong
Power Saving King Shop	Green Intelligent Power Saver	Really saves electricity!	Jingdong prefecture level city in Shandong
Power Saving King Shop	Green Intelligent Power Saver	pretty good	Jingdong prefecture level city in Shandong
Power Saving King Shop	Green Intelligent Power Saver	serviceable	Jingdong prefecture level city in Shandong
Power Saving King Shop	Green Intelligent Power Saver	Power Saving King received a tightly packaged logistics, installed the effect is very good price is cheaper than the physical store!	Jingdong prefecture level city in Shandong
Power Saving King Shop	Green Intelligent Power Saver	Super Power Saving King is very good quality, new style, is genuine, the use of the effect is very good, the price is cheap, it is worth buying!	Jingdong prefecture level city in Shandong
Power Saving King Shop	Green Intelligent Power Saver	Packaging is very tight, easy to install, quite practical, worth buying!	Jingdong prefecture level city in Shandong
Power Saving King Shop	Green Intelligent Power Saver	Quality assurance, affordable price, very energy efficient, will repurchase if needed!	Jingdong prefecture level city in Shandong
Power Saving King Shop	Green Intelligent Power Saver	Quality assurance, very good, affordable, logistics is also very fast, I'm very satisfied with the	Jingdong prefecture level city in Shandong
Power Saving King Shop	Green Intelligent Power Saver	Energy saving. It's so good, already installed, you can save a lot of electricity every month	Jingdong prefecture level city in Shandong
Power Saving King Shop	Green Intelligent Power Saver	The material is thick and the workmanship is fine in place, the power saving effect is very good, value for money!	Jingdong prefecture level city in Shandong
Power Saving King Shop	Green Intelligent Power Saver	The effect is great, genuine, and match the description, the logistics is fast, the price is reasonable 😍	Jingdong prefecture level city in Shandong
Power Saving King Shop	Green Intelligent Power Saver	It's genuine, works well, easy to use, plugs in and works, really saves power	Jingdong prefecture level city in Shandong
Power Saving King Shop	Green Intelligent Power Saver	It's easy to use, effective, great service, and recommended!	Jingdong prefecture level city in Shandong
Power Saving King Shop	Green Intelligent Power Saver	🌈 Great quality, bought it to my liking 💝 Awesome! ✨ Buy with confidence if you need it!	Jingdong prefecture level city in Shandong
Power Saving King Shop	Green Intelligent Power Saver	Good quality, easy to install, very energy saving and power saving, good to use	Jingdong prefecture level city in Shandong
Power Saving King Shop	Green Intelligent Power Saver	The quality is very good, it does have the effect of saving electricity, the description matches, the price is affordable, it is worth recommending.	Jingdong prefecture level city in Shandong

Power Saving King Shop	Green Intelligent Power Saver	Received, the functionality is particularly good, it is genuine, the price is affordable	Jingdong prefecture level city in Shandong
Power Saving King Shop	Green Intelligent Power Saver	Good quality, very easy to use, energy efficient, home electricity bill is obviously much less	Jingdong prefecture level city in Shandong
Power Saving King Shop	Green Intelligent Power Saver	Easy to install, energy saving and power saving, very satisfied with a shopping	Jingdong prefecture level city in Shandong
Power Saving King Shop	Green Intelligent Power Saver	Received it and tried it out, it's great, works great and worth having! Five stars praise!	Jingdong prefecture level city in Shandong
Power Saving King Shop	Green Intelligent Power Saver	Power saving device, no problem at all, the quality of this product at this price, very affordable!	Jingdong prefecture level city in Shandong
Power Saving King Shop	Green Intelligent Power Saver	Received the goods, did not expect to be so fast, the quality is very good, especially power saving	Jingdong prefecture level city in Shandong
Power Saving King Shop	Green Intelligent Power Saver	The logistics is very fast, especially good, need to come back again	Jingdong prefecture level city in Shandong
Power Saving King Shop	Green Intelligent Power Saver	It's already working and it really saves power, so I'm very happy with it!	Jingdong prefecture level city in Shandong
Power Saving King Shop	Green Intelligent Power Saver	Great quality, works well, can recommend to friends to buy.	Jingdong prefecture level city in Shandong
Power Saving King Shop	Green Intelligent Power Saver	It's a good deal and worth the money.	Jingdong prefecture level city in Shandong
Power Saving King Shop	Green Intelligent Power Saver	I received it and I'm very happy with it, it's very good quality, guaranteed, and	Jingdong prefecture level city in Shandong
Power Saving King Shop	Green Intelligent Power Saver	Good quality, safe and secure purchase, ideal results and great price.	Jingdong prefecture level city in Shandong
Power Saving King Shop	Green Intelligent Power Saver	Simple operation plugged in the power supply can be, friends recommended looking forward to the effect of the	Jingdong prefecture level city in Shandong
Power Saving King Shop	Green Intelligent Power Saver	It's not bad, it saves electricity, and the material is sturdy and durable.	Jingdong prefecture level city in Shandong
Power Saving King Shop	Green Intelligent Power Saver	Plug and play, no management required, convenient and hassle-free!	Jingdong prefecture level city in Shandong
Power Saving King Shop	Green Intelligent Power Saver	The pictures are too lazy to go up, the power saving effect is still there!	Jingdong prefecture level city in Shandong
Power Saving King Shop	Green Intelligent Power Saver	Fast shipping. Affordable prices. Efficient	Jingdong prefecture level city in Shandong
Power Saving King Shop	Green Intelligent Power Saver	Good power saving, worth buying	Jingdong prefecture level city in Shandong
Power Saving King Shop	Green Intelligent Power Saver	Especially great, comparison shopping before buying, the store owner is very good, answering questions very patiently and detailed, there is a need to come back!	Jingdong prefecture level city in Shandong
Power Saving King Shop	Green Intelligent Power Saver	Very trustworthy brand, quality is guaranteed, the price is still affordable, quite satisfied!	Jingdong prefecture level city in Shandong
Power Saving King Shop	Green Intelligent Power Saver	Recommended by a friend. They say you can save money on air conditioning with this. Try it.	Jingdong prefecture level city in Shandong
Power Saving King Shop	Green Intelligent Power Saver	Works great, will be back next time, very happy with it and a great price!	Jingdong prefecture level city in Shandong
Power Saving King Shop	Green Intelligent Power Saver	The quality of the product is quite good, fast shipping, satisfied.	Jingdong prefecture level city in Shandong
Power Saving King Shop	Green Intelligent Power Saver	Good quality and fast logistics	Jingdong prefecture level city in Shandong
Power Saving King Shop	Green Intelligent Power Saver	It really saves power. I've tested it myself.	Jingdong prefecture level city in Shandong
Power Saving King Shop	Green Intelligent Power Saver	Really saves electricity!	Jingdong prefecture level city in Shandong
Power Saving King Shop	Green Intelligent Power Saver	pretty good	Jingdong prefecture level city in Shandong
Power Saving King Shop	Green Intelligent Power Saver	serviceable	Jingdong prefecture level city in Shandong
Power Saving King Shop	Green Intelligent Power Saver	Power Saving King received a tightly packaged logistics, installed the effect is very good price is cheaper than the physical store!	Jingdong prefecture level city in Shandong
Power Saving King Shop	Green Intelligent Power Saver	Super Power Saving King is very good quality, new style, is genuine, the use of the effect is very good, the price is cheap, it is worth buying!	Jingdong prefecture level city in Shandong
Power Saving King Shop	Green Intelligent Power Saver	Packaging is very tight, easy to install, quite practical, worth buying!	Jingdong prefecture level city in Shandong
Power Saving King Shop	Green Intelligent Power Saver	Quality assurance, affordable price, very energy efficient, will repurchase if needed!	Jingdong prefecture level city in Shandong
Power Saving King Shop	Green Intelligent Power Saver	Quality assurance, very good, affordable, logistics is also very fast, I'm very satisfied with the	Jingdong prefecture level city in Shandong
Power Saving King Shop	Green Intelligent Power Saver	Energy saving. It's so good, already installed, you can save a lot of electricity every month	Jingdong prefecture level city in Shandong
Power Saving King Shop	Green Intelligent Power Saver	The material is thick and the workmanship is fine in place, the power saving effect is very good, value for money!	Jingdong prefecture level city in Shandong
Power Saving King Shop	Green Intelligent Power Saver	The effect is great, genuine, and match the description, the logistics is fast, the price is reasonable 😍	Jingdong prefecture level city in Shandong
Power Saving King Shop	Green Intelligent Power Saver	It's genuine, works well, easy to use, plugs in and works, really saves power	Jingdong prefecture level city in Shandong
Power Saving King Shop	Green Intelligent Power Saver	It's easy to use, effective, great service, and recommended!	Jingdong prefecture level city in Shandong
Power Saving King Shop	Green Intelligent Power Saver	🌈 Great quality, bought it to my liking 💖 Awesome! ✨ Buy with confidence if you need it!	Jingdong prefecture level city in Shandong
Power Saving King Shop	Green Intelligent Power Saver	Good quality, easy to install, very energy saving and power saving, good to use	Jingdong prefecture level city in Shandong
Power Saving King Shop	Green Intelligent Power Saver	The quality is very good, it does have the effect of saving electricity, the description matches, the price is affordable, it is worth recommending.	Jingdong prefecture level city in Shandong
Power Saving King Shop	Green Intelligent Power Saver	Received, the functionality is particularly good, it is genuine, the price is affordable	Jingdong prefecture level city in Shandong
Power Saving King Shop	Green Intelligent Power Saver	Good quality, very easy to use, energy efficient, home electricity bill is obviously much less	Jingdong prefecture level city in Shandong
Power Saving King Shop	Green Intelligent Power Saver	Easy to install, energy saving and power saving, very satisfied with a shopping	Jingdong prefecture level city in Shandong
Power Saving King Shop	Green Intelligent Power Saver	Received it and tried it out, it's great, works great and worth having! Five stars praise!	Jingdong prefecture level city in Shandong
Power Saving King Shop	Green Intelligent Power Saver	Power saving device, no problem at all, the quality of this product at this price, very affordable!	Jingdong prefecture level city in Shandong
Power Saving King Shop	Green Intelligent Power Saver	Received the goods, did not expect to be so fast, the quality is very good, especially power saving	Jingdong prefecture level city in Shandong
Power Saving King Shop	Green Intelligent Power Saver	The logistics is very fast, especially good, need to come back again	Jingdong prefecture level city in Shandong
Power Saving King Shop	Green Intelligent Power Saver	It's already working and it really saves power, so I'm very happy with it!	Jingdong prefecture level city in Shandong
Power Saving King Shop	Green Intelligent Power Saver	Great quality, works well, can recommend to friends to buy.	Jingdong prefecture level city in Shandong
Power Saving King Shop	Green Intelligent Power Saver	It's a good deal and worth the money.	Jingdong prefecture level city in Shandong

Power Saving King Shop	Green Intelligent Power Saver	I received it and I’m very happy with it, it’s very good quality, guaranteed, and	Jingdong prefecture level city in Shandong
Power Saving King Shop	Green Intelligent Power Saver	Good quality, safe and secure purchase, ideal results and great price.	Jingdong prefecture level city in Shandong
Power Saving King Shop	Green Intelligent Power Saver	Simple operation plugged in the power supply can be, friends recommended looking forward to the effect of the	Jingdong prefecture level city in Shandong
Power Saving King Shop	Green Intelligent Power Saver	It’s not bad, it saves electricity, and the material is sturdy and durable.	Jingdong prefecture level city in Shandong
Power Saving King Shop	Green Intelligent Power Saver	Plug and play, no management required, convenient and hassle-free!	Jingdong prefecture level city in Shandong
Power Saving King Shop	Green Intelligent Power Saver	The pictures are too lazy to go up, the power saving effect is still there!	Jingdong prefecture level city in Shandong
Power Saving King Shop	Green Intelligent Power Saver	Fast shipping. Affordable prices. Efficient	Jingdong prefecture level city in Shandong
Power Saving King Shop	Green Intelligent Power Saver	Good power saving, worth buying	Jingdong prefecture level city in Shandong
Power Saving King Shop	Green Intelligent Power Saver	Especially great, comparison shopping before buying, the store owner is very good, answering questions very patiently and detailed, there is a need to come back!	Jingdong prefecture level city in Shandong
Power Saving King Shop	Green Intelligent Power Saver	Very trustworthy brand, quality is guaranteed, the price is still affordable, quite satisfied!	Jingdong prefecture level city in Shandong
Power Saving King Shop	Green Intelligent Power Saver	Recommended by a friend. They say you can save money on air conditioning with this. Try it.	Jingdong prefecture level city in Shandong
Power Saving King Shop	Green Intelligent Power Saver	Works great, will be back next time, very happy with it and a great price!	Jingdong prefecture level city in Shandong
Power Saving King Shop	Green Intelligent Power Saver	The quality of the product is quite good, fast shipping, satisfied.	Jingdong prefecture level city in Shandong
Power Saving King Shop	Green Intelligent Power Saver	Good quality and fast logistics	Jingdong prefecture level city in Shandong
Power Saving King Shop	Green Intelligent Power Saver	It really saves power. I’ve tested it myself.	Jingdong prefecture level city in Shandong
Power Saving King Shop	Green Intelligent Power Saver	Really saves electricity!	Jingdong prefecture level city in Shandong
Power Saving King Shop	Green Intelligent Power Saver	pretty good	Jingdong prefecture level city in Shandong
Power Saving King Shop	Green Intelligent Power Saver	serviceable	Jingdong prefecture level city in Shandong
Power Saving King Shop	Green Intelligent Power Saver	Power Saving King received a tightly packaged logistics, installed the effect is very good price is cheaper than the physical store!	Jingdong prefecture level city in Shandong
Power Saving King Shop	Green Intelligent Power Saver	Super Power Saving King is very good quality, new style, is genuine, the use of the effect is very good, the price is cheap, it is worth buying!	Jingdong prefecture level city in Shandong
Power Saving King Shop	Green Intelligent Power Saver	Packaging is very tight, easy to install, quite practical, worth buying!	Jingdong prefecture level city in Shandong
Power Saving King Shop	Green Intelligent Power Saver	Quality assurance, affordable price, very energy efficient, will repurchase if needed!	Jingdong prefecture level city in Shandong
Power Saving King Shop	Green Intelligent Power Saver	Quality assurance, very good, affordable, logistics is also very fast, I’m very satisfied with the	Jingdong prefecture level city in Shandong
Power Saving King Shop	Green Intelligent Power Saver	Energy saving. It’s so good, already installed, you can save a lot of electricity every month	Jingdong prefecture level city in Shandong
Power Saving King Shop	Green Intelligent Power Saver	The material is thick and the workmanship is fine in place, the power saving effect is very good, value for money!	Jingdong prefecture level city in Shandong
Power Saving King Shop	Green Intelligent Power Saver	The effect is great, genuine, and match the description, the logistics is fast, the price is reasonable 😍	Jingdong prefecture level city in Shandong
Power Saving King Shop	Green Intelligent Power Saver	It’s genuine, works well, easy to use, plugs in and works, really saves power	Jingdong prefecture level city in Shandong
Power Saving King Shop	Green Intelligent Power Saver	It’s easy to use, effective, great service, and recommended!	Jingdong prefecture level city in Shandong
Power Saving King Shop	Green Intelligent Power Saver	🌈 Great quality, bought it to my liking 💞 Awesome! ✨ Buy with confidence if you need it!	Jingdong prefecture level city in Shandong
Power Saving King Shop	Green Intelligent Power Saver	Good quality, easy to install, very energy saving and power saving, good to use	Jingdong prefecture level city in Shandong
Power Saving King Shop	Green Intelligent Power Saver	The quality is very good, it does have the effect of saving electricity, the description matches, the price is affordable, it is worth recommending.	Jingdong prefecture level city in Shandong
Power Saving King Shop	Green Intelligent Power Saver	Received, the functionality is particularly good, it is genuine, the price is affordable	Jingdong prefecture level city in Shandong
Power Saving King Shop	Green Intelligent Power Saver	Good quality, very easy to use, energy efficient, home electricity bill is obviously much less	Jingdong prefecture level city in Shandong
Power Saving King Shop	Green Intelligent Power Saver	Easy to install, energy saving and power saving, very satisfied with a shopping	Jingdong prefecture level city in Shandong
Power Saving King Shop	Green Intelligent Power Saver	Received it and tried it out, it’s great, works great and worth having! Five stars praise!	Jingdong prefecture level city in Shandong
Power Saving King Shop	Green Intelligent Power Saver	Power saving device, no problem at all, the quality of this product at this price, very affordable!	Jingdong prefecture level city in Shandong
Power Saving King Shop	Green Intelligent Power Saver	Received the goods, did not expect to be so fast, the quality is very good, especially power saving	Jingdong prefecture level city in Shandong
Power Saving King Shop	Green Intelligent Power Saver	The logistics is very fast, especially good, need to come back again	Jingdong prefecture level city in Shandong
Power Saving King Shop	Green Intelligent Power Saver	It’s already working and it really saves power, so I’m very happy with it!	Jingdong prefecture level city in Shandong
Power Saving King Shop	Green Intelligent Power Saver	Great quality, works well, can recommend to friends to buy.	Jingdong prefecture level city in Shandong
Power Saving King Shop	Green Intelligent Power Saver	It’s a good deal and worth the money.	Jingdong prefecture level city in Shandong
Power Saving King Shop	Green Intelligent Power Saver	I received it and I’m very happy with it, it’s very good quality, guaranteed, and	Jingdong prefecture level city in Shandong
Power Saving King Shop	Green Intelligent Power Saver	Good quality, safe and secure purchase, ideal results and great price.	Jingdong prefecture level city in Shandong
Power Saving King Shop	Green Intelligent Power Saver	Simple operation plugged in the power supply can be, friends recommended looking forward to the effect of the	Jingdong prefecture level city in Shandong
Power Saving King Shop	Green Intelligent Power Saver	It’s not bad, it saves electricity, and the material is sturdy and durable.	Jingdong prefecture level city in Shandong
Power Saving King Shop	Green Intelligent Power Saver	Plug and play, no management required, convenient and hassle-free!	Jingdong prefecture level city in Shandong
Power Saving King Shop	Green Intelligent Power Saver	The pictures are too lazy to go up, the power saving effect is still there!	Jingdong prefecture level city in Shandong
Power Saving King Shop	Green Intelligent Power Saver	Fast shipping. Affordable prices. Efficient	Jingdong prefecture level city in Shandong
Power Saving King Shop	Green Intelligent Power Saver	Good power saving, worth buying	Jingdong prefecture level city in Shandong
Power Saving King Shop	Green Intelligent Power Saver	Especially great, comparison shopping before buying, the store owner is very good, answering questions very patiently and detailed, there is a need to come back!	Jingdong prefecture level city in Shandong
Power Saving King Shop	Green Intelligent Power Saver	Very trustworthy brand, quality is guaranteed, the price is still affordable, quite satisfied!	Jingdong prefecture level city in Shandong

Power Saving King Shop	Green Intelligent Power Saver	Recommended by a friend. They say you can save money on air conditioning with this. Try it.	Jingdong prefecture level city in Shandong
Power Saving King Shop	Green Intelligent Power Saver	Works great, will be back next time, very happy with it and a great price!	Jingdong prefecture level city in Shandong
Power Saving King Shop	Green Intelligent Power Saver	The quality of the product is quite good, fast shipping, satisfied.	Jingdong prefecture level city in Shandong
Power Saving King Shop	Green Intelligent Power Saver	Good quality and fast logistics	Jingdong prefecture level city in Shandong
Power Saving King Shop	Green Intelligent Power Saver	It really saves power. I've tested it myself.	Jingdong prefecture level city in Shandong
Power Saving King Shop	Green Intelligent Power Saver	Really saves electricity!	Jingdong prefecture level city in Shandong
Power Saving King Shop	Green Intelligent Power Saver	pretty good	Jingdong prefecture level city in Shandong
Power Saving King Shop	Green Intelligent Power Saver	serviceable	Jingdong prefecture level city in Shandong
Power Saving King Shop	Green Intelligent Power Saver	Power Saving King received a tightly packaged logistics, installed the effect is very good price is cheaper than the physical store!	Jingdong prefecture level city in Shandong
Power Saving King Shop	Green Intelligent Power Saver	Super Power Saving King is very good quality, new style, is genuine, the use of the effect is very good, the price is cheap, it is worth buying!	Jingdong prefecture level city in Shandong
Power Saving King Shop	Green Intelligent Power Saver	Packaging is very tight, easy to install, quite practical, worth buying!	Jingdong prefecture level city in Shandong
Power Saving King Shop	Green Intelligent Power Saver	Quality assurance, affordable price, very energy efficient, will repurchase if needed!	Jingdong prefecture level city in Shandong
Power Saving King Shop	Green Intelligent Power Saver	Quality assurance, very good, affordable, logistics is also very fast, I'm very satisfied with the	Jingdong prefecture level city in Shandong
Power Saving King Shop	Green Intelligent Power Saver	Energy saving. It's so good, already installed, you can save a lot of electricity every month	Jingdong prefecture level city in Shandong
Power Saving King Shop	Green Intelligent Power Saver	The material is thick and the workmanship is fine in place, the power saving effect is very good, value for money!	Jingdong prefecture level city in Shandong
Power Saving King Shop	Green Intelligent Power Saver	The effect is great, genuine, and match the description, the logistics is fast, the price is reasonable 😍	Jingdong prefecture level city in Shandong
Power Saving King Shop	Green Intelligent Power Saver	It's genuine, works well, easy to use, plugs in and works, really saves power	Jingdong prefecture level city in Shandong
Power Saving King Shop	Green Intelligent Power Saver	It's easy to use, effective, great service, and recommended!	Jingdong prefecture level city in Shandong
Power Saving King Shop	Green Intelligent Power Saver	🌈 Great quality, bought it to my liking 💝 Awesome! ✨ Buy with confidence if you need it!	Jingdong prefecture level city in Shandong
Power Saving King Shop	Green Intelligent Power Saver	Good quality, easy to install, very energy saving and power saving, good to use	Jingdong prefecture level city in Shandong
Power Saving King Shop	Green Intelligent Power Saver	The quality is very good, it does have the effect of saving electricity, the description matches, the price is affordable, it is worth recommending.	Jingdong prefecture level city in Shandong
Power Saving King Shop	Green Intelligent Power Saver	Received, the functionality is particularly good, it is genuine, the price is affordable	Jingdong prefecture level city in Shandong
Power Saving King Shop	Green Intelligent Power Saver	Good quality, very easy to use, energy efficient, home electricity bill is obviously much less	Jingdong prefecture level city in Shandong
Power Saving King Shop	Green Intelligent Power Saver	Easy to install, energy saving and power saving, very satisfied with a shopping	Jingdong prefecture level city in Shandong
Power Saving King Shop	Green Intelligent Power Saver	Received it and tried it out, it's great, works great and worth having! Five stars praise!	Jingdong prefecture level city in Shandong
Power Saving King Shop	Green Intelligent Power Saver	Power saving device, no problem at all, the quality of this product at this price, very affordable!	Jingdong prefecture level city in Shandong
Power Saving King Shop	Green Intelligent Power Saver	Received the goods, did not expect to be so fast, the quality is very good, especially power saving	Jingdong prefecture level city in Shandong
Power Saving King Shop	Green Intelligent Power Saver	The logistics is very fast, especially good, need to come back again	Jingdong prefecture level city in Shandong
Power Saving King Shop	Green Intelligent Power Saver	It's already working and it really saves power, so I'm very happy with it!	Jingdong prefecture level city in Shandong
Power Saving King Shop	Green Intelligent Power Saver	Great quality, works well, can recommend to friends to buy.	Jingdong prefecture level city in Shandong
Power Saving King Shop	Green Intelligent Power Saver	It's a good deal and worth the money.	Jingdong prefecture level city in Shandong
Power Saving King Shop	Green Intelligent Power Saver	I received it and I'm very happy with it, it's very good quality, guaranteed, and	Jingdong prefecture level city in Shandong
Power Saving King Shop	Green Intelligent Power Saver	Good quality, safe and secure purchase, ideal results and great price.	Jingdong prefecture level city in Shandong
Power Saving King Shop	Green Intelligent Power Saver	Simple operation plugged in the power supply can be, friends recommended looking forward to the effect of the	Jingdong prefecture level city in Shandong
Power Saving King Shop	Green Intelligent Power Saver	It's not bad, it saves electricity, and the material is sturdy and durable.	Jingdong prefecture level city in Shandong
Power Saving King Shop	Green Intelligent Power Saver	Plug and play, no management required, convenient and hassle-free!	Jingdong prefecture level city in Shandong
Power Saving King Shop	Green Intelligent Power Saver	The pictures are too lazy to go up, the power saving effect is still there!	Jingdong prefecture level city in Shandong
Power Saving King Shop	Green Intelligent Power Saver	Fast shipping. Affordable prices. Efficient	Jingdong prefecture level city in Shandong
Power Saving King Shop	Green Intelligent Power Saver	Good power saving, worth buying	Jingdong prefecture level city in Shandong
Power Saving King Shop	Green Intelligent Power Saver	Especially great, comparison shopping before buying, the store owner is very good, answering questions very patiently and detailed, there is a need to come back!	Jingdong prefecture level city in Shandong
Power Saving King Shop	Green Intelligent Power Saver	Very trustworthy brand, quality is guaranteed, the price is still affordable, quite satisfied!	Jingdong prefecture level city in Shandong
Power Saving King Shop	Green Intelligent Power Saver	Recommended by a friend. They say you can save money on air conditioning with this. Try it.	Jingdong prefecture level city in Shandong
Power Saving King Shop	Green Intelligent Power Saver	Works great, will be back next time, very happy with it and a great price!	Jingdong prefecture level city in Shandong
Power Saving King Shop	Green Intelligent Power Saver	The quality of the product is quite good, fast shipping, satisfied.	Jingdong prefecture level city in Shandong
Power Saving King Shop	Green Intelligent Power Saver	Good quality and fast logistics	Jingdong prefecture level city in Shandong
Power Saving King Shop	Green Intelligent Power Saver	It really saves power. I've tested it myself.	Jingdong prefecture level city in Shandong
Power Saving King Shop	Green Intelligent Power Saver	Really saves electricity!	Jingdong prefecture level city in Shandong
Power Saving King Shop	Green Intelligent Power Saver	pretty good	Jingdong prefecture level city in Shandong
Power Saving King Shop	Green Intelligent Power Saver	serviceable	Jingdong prefecture level city in Shandong
Power Saving King Shop	Green Intelligent Power Saver	Quality is very good, and the seller described exactly the same, very satisfied, really like, completely exceeded expectations, the delivery speed is very fast, the packaging is very careful and strict, the logistics company has a very good service attitude, the delivery speed is very fast, very satisfied with	Jingdong prefecture level city in Shandong
Power Saving King Shop	Green Intelligent Power Saver	Energy saver has been used, rental house inside the use of the meter is always running fast, with this can save electricity, easy to use, plug it can be, very satisfied!	Jingdong prefecture level city in Shandong

Power Saving King Shop	King of Power Saving	Power saving is still very powerful, purposely use at night, turn off all electrical appliances, leave an air conditioner, compared to two nights, or power saving, usually a night landlord's five energy consumption of air conditioning, power consumption of 5 degrees a night, with this save two degrees of electricity, a month down properly more than a hundred points fee saved.	Jingdong prefecture level city in Shandong
Power Saving King Shop	King of Power Saving	System recommended, bought with skepticism, received the goods feel measured for a day as if the meter is slowed down. That is not afraid of the landlord 1.5 of the electricity bill.	Jingdong prefecture level city in Shandong
Power Saving King Shop	King of Power Saving	It's a real saver. It's not bad. It's a meter with a meter on it, a real saver.	Jingdong prefecture level city in Shandong
Power Saving King Shop	King of Power Saving	Nice, the electric bill came out. Really saves electricity.	Jingdong prefecture level city in Shandong
Power Saving King Shop	King of Power Saving	Received it, using it today, if there is a chaser, that means this is not great, if not, that means it is satisfactory. Today is the 8.21 review.	Jingdong prefecture level city in Shandong
Power Saving King Shop	King of Power Saving	You won't be afraid to pay your electric bill with this power saver. It's a great deal.	Jingdong prefecture level city in Shandong
Power Saving King Shop	King of Power Saving	Go buy one with skepticism, it's not expensive. It really does save a few kilowatts a night	Jingdong prefecture level city in Shandong
Power Saving King Shop	King of Power Saving	The power saving is good. Worth a good review.	Jingdong prefecture level city in Shandong
Power Saving King Shop	King of Power Saving	Received intact, open a super good look beautiful and generous, ~ bit of high quality is recommended to buy, the need for friends rest assured that the next order	Jingdong prefecture level city in Shandong
Power Saving King Shop	King of Power Saving	Very good to use, friends introduced, save electricity and money, long-lasting products cost-effective, large factory production, good quality, fine workmanship cost-effective!	Jingdong prefecture level city in Shandong
Power Saving King Shop	King of Power Saving	The power saving effect is very obvious, trying to pay 30% less electricity bill in a month than before, hahaha	Jingdong prefecture level city in Shandong
Power Saving King Shop	King of Power Saving	It's such a power saver to use this, it saves money on kids snacks for a month. Kudos!	Jingdong prefecture level city in Shandong
Power Saving King Shop	King of Power Saving	Plug and play, stable voltage, the screen has a display when working, this winter home appliances, need more electricity, just buy to use, save electricity and save money, with instructions, not too big and does not take up space, the quality is very good, and the seller describes exactly the same!	Jingdong prefecture level city in Shandong
Power Saving King Shop	King of Power Saving	A must have for opening a store. Save over a thousand dollars a month on your electric bill	Jingdong prefecture level city in Shandong
Power Saving King Shop	King of Power Saving	Received the goods, the first time to unpack, the quality is very good, with the seller's description, completely exceeded my expectations, the packaging is very careful, very strict, in general this is a very satisfied with the shopping	Jingdong prefecture level city in Shandong
Power Saving King Shop	King of Power Saving	As always, good quality, a very favorite product, great! o(*￣︶￣*)o The price is very affordable, cost-effective, it's considered affordable good, will repurchase!	Jingdong prefecture level city in Shandong
Power Saving King Shop	King of Power Saving	Very satisfied with a shopping, good business service, fast shipping, logistics is also very powerful, received very good, buy assured, use peace of mind!	Jingdong prefecture level city in Shandong
Power Saving King Shop	King of Power Saving	The first time to buy online, but also look at the evaluation before buying, the quality is not bad, but also very cheap, the need for friends can rest assured to buy!	Jingdong prefecture level city in Shandong
Power Saving King Shop	King of Power Saving	Better than expected, feel value for money, very satisfied with a shopping, need next time will come back!	Jingdong prefecture level city in Shandong
Power Saving King Shop	King of Power Saving	Really super like, very supportive, very good quality, exactly as described by the seller, very satisfied!	Jingdong prefecture level city in Shandong
Power Saving King Shop	King of Power Saving	It is genuine, effective, really saves power, affordable, tightly packed, fast shipping, very satisfied.	Jingdong prefecture level city in Shandong
Power Saving King Shop	King of Power Saving	Saver received, very good, installation set up very well, very good, the effect is very good, recommend that the pro buy	Jingdong prefecture level city in Shandong
Power Saving King Shop	King of Power Saving	Good quality, fine workmanship, is genuine and business description of the same, good value for money, fast logistics, very satisfied	Jingdong prefecture level city in Shandong
Power Saving King Shop	King of Power Saving	Electromagnetic good, high power convenient, enough force, the price is right it, quality can be 😊 😊 😊 😊 😊	Jingdong prefecture level city in Shandong
Power Saving King Shop	King of Power Saving	First-class quality, packaging is also good, logistics is very fast, and the price is reasonable, very affordable, I like it!	Jingdong prefecture level city in Shandong
Power Saving King Shop	King of Power Saving	Packaging is very good, the use of the effect is quite good, the store has a good service attitude, trustworthy	Jingdong prefecture level city in Shandong
Power Saving King Shop	King of Power Saving	It's great, it's the kind of style you want, and it arrived quickly, thank you boss, 5 stars for the service!	Jingdong prefecture level city in Shandong
Power Saving King Shop	King of Power Saving	The quality is very good, it works well, very satisfied, the price is affordable, it's so great value, it's worth recommending.	Jingdong prefecture level city in Shandong
Power Saving King Shop	King of Power Saving	Power Saving King power is quite large, the quality of the leaf is quite good, the price of buying a very good deal, must be praised!	Jingdong prefecture level city in Shandong
Power Saving King Shop	King of Power Saving	Ran the price is not high try to buy the attitude, did not expect the effect is really good, good!	Jingdong prefecture level city in Shandong
Power Saving King Shop	King of Power Saving	The baby description matches the actual item very well! The quality is awesome! Will be back to patronize again in the future!	Jingdong prefecture level city in Shandong
Power Saving King Shop	King of Power Saving	Good product quality is the king of power-saving products trustworthy as always good to recommend!	Jingdong prefecture level city in Shandong
Power Saving King Shop	King of Power Saving	Shipping was pretty fast and affordable, not bad 👍	Jingdong prefecture level city in Shandong
Power Saving King Shop	King of Power Saving	Works great, very affordable, shipped pretty fast, comfortable to use	Jingdong prefecture level city in Shandong
Power Saving King Shop	King of Power Saving	Digital voltage display, voltage at a glance, plug and play, safe and simple!	Jingdong prefecture level city in Shandong
Power Saving King Shop	King of Power Saving	Quality assurance, very good, affordable, logistics is also very fast, I'm very satisfied with the	Jingdong prefecture level city in Shandong
Power Saving King Shop	King of Power Saving	The quality is very good, the first time I received the goods to open the first time to see, it is very good, very satisfied	Jingdong prefecture level city in Shandong
Power Saving King Shop	King of Power Saving	Power Saving King used, the effect is good, the price is affordable, very good, is the genuine article	Jingdong prefecture level city in Shandong
Power Saving King Shop	King of Power Saving	Good quality, fine workmanship, good appearance, time is very accurate, satisfied.	Jingdong prefecture level city in Shandong
Power Saving King Shop	King of Power Saving	I purposely used it for two days before commenting, which is responsible for myself and everyone else!	Jingdong prefecture level city in Shandong
Power Saving King Shop	King of Power Saving	Easy to install, easy to operate, helped our family save a lot of electricity this summer	Jingdong prefecture level city in Shandong
Power Saving King Shop	King of Power Saving	Bringing power savings into millions of homes. Still a great gadget. Pretty good.	Jingdong prefecture level city in Shandong
Power Saving King Shop	King of Power Saving	Bought myself a knuckle regulator and it was easy to use and the price was right.	Jingdong prefecture level city in Shandong
Power Saving King Shop	King of Power Saving	Received, good, powerful, good, very full of a shopping	Jingdong prefecture level city in Shandong
Power Saving King Shop	King of Power Saving	High power, consistent with the seller's description, powerful, cost-effective	Jingdong prefecture level city in Shandong
Power Saving King Shop	King of Power Saving	Digital liquid crystal display to see the clear intuitive, very formal workmanship is not rough	Jingdong prefecture level city in Shandong
Power Saving King Shop	King of Power Saving	Received. Great quality, fast shipping from the store and not too expensive!	Jingdong prefecture level city in Shandong
Power Saving King Shop	King of Power Saving	It works very well and also saves power at an affordable price a great deal.	Jingdong prefecture level city in Shandong
Power Saving King Shop	King of Power Saving	Good quality, good can recommend to friends to buy.	Jingdong prefecture level city in Shandong

Power Saving King Shop	King of Power Saving	Will repurchase, quality is guaranteed, safe and secure purchase.	Jingdong prefecture level city in Shandong
Power Saving King Shop	King of Power Saving	The quality is very good is genuine, cost-effective recommended	Jingdong prefecture level city in Shandong
Power Saving King Shop	King of Power Saving	Quality Assurance. Favorable price. Very practical and durable	Jingdong prefecture level city in Shandong
Power Saving King Shop	King of Power Saving	The logistics is very fast, the thing is also good to use, energy saving power rely on it!	Jingdong prefecture level city in Shandong
Power Saving King Shop	King of Power Saving	Power Saver is good quality and works very well, worth buying!	Jingdong prefecture level city in Shandong
Power Saving King Shop	King of Power Saving	Buy it for your rental apartment and never worry about your landlord charging you too much.	Jingdong prefecture level city in Shandong
Power Saving King Shop	King of Power Saving	Good value for money. Conscientious businessman. Recommended to everyone	Jingdong prefecture level city in Shandong
Power Saving King Shop	King of Power Saving	Nice and affordable, will repurchase next time	Jingdong prefecture level city in Shandong
Power Saving King Shop	King of Power Saving	Good quality, high power easy to use	Jingdong prefecture level city in Shandong
Power Saving King Shop	King of Power Saving	Power saving little helper. Deserves a favorable review.	Jingdong prefecture level city in Shandong
Power Saving King Shop	King of Power Saving	It's very energy efficient. I've been using it for a few days now.	Jingdong prefecture level city in Shandong
Power Saving King Shop	King of Power Saving	Power saving is proper! It's good.	Jingdong prefecture level city in Shandong
Power Saving King Shop	King of Power Saving	Power saving. Works well. Not bad.	Jingdong prefecture level city in Shandong
Power Saving King Shop	King of Power Saving	Great value for money, great value for money.	Jingdong prefecture level city in Shandong
Power Saving King Shop	King of Power Saving	Works well and as described by the seller	Jingdong prefecture level city in Shandong
Power Saving King Shop	King of Power Saving	Satisfied with power saving	Jingdong prefecture level city in Shandong
Power Saving King Shop	King of Power Saving	Received intact, open a super good look beautiful and generous, ~ bit of high quality is recommended to buy, the need for friends rest assured that the next order	Jingdong prefecture level city in Shandong
Power Saving King Shop	King of Power Saving	Very good to use, friends introduced, save electricity and money, long-lasting products cost-effective, large factory production, good quality, fine workmanship cost-effective!	Jingdong prefecture level city in Shandong
Power Saving King Shop	King of Power Saving	The power saving effect is very obvious, trying to pay 30% less electricity bill in a month than before, hahaha	Jingdong prefecture level city in Shandong
Power Saving King Shop	King of Power Saving	It's such a power saver to use this, it saves money on kids snacks for a month. Kudos!	Jingdong prefecture level city in Shandong
Power Saving King Shop	King of Power Saving	Plug and play, stable voltage, the screen has a display when working, this winter home appliances, need more electricity, just buy to use, save electricity and save money, with instructions, not too big and does not take up space, the quality is very good, and the seller describes exactly the same!	Jingdong prefecture level city in Shandong
Power Saving King Shop	King of Power Saving	A must have for opening a store. Save over a thousand dollars a month on your electric bill	Jingdong prefecture level city in Shandong
Power Saving King Shop	King of Power Saving	Received the goods, the first time to unpack, the quality is very good, with the seller's description, completely exceeded my expectations, the packaging is very careful, very strict, in general this is a very satisfied with the shopping	Jingdong prefecture level city in Shandong
Power Saving King Shop	King of Power Saving	As always, good quality, a very favorite product, great! o(*￣︶￣*)o The price is very affordable, cost-effective, it's considered affordable good, will repurchase!	Jingdong prefecture level city in Shandong
Power Saving King Shop	King of Power Saving	Very satisfied with a shopping, good business service, fast shipping, logistics is also very powerful, received very good, buy assured, use peace of mind!	Jingdong prefecture level city in Shandong
Power Saving King Shop	King of Power Saving	The first time to buy online, but also look at the evaluation before buying, the quality is not bad, but also very cheap, the need for friends can rest assured to buy!	Jingdong prefecture level city in Shandong
Power Saving King Shop	King of Power Saving	Better than expected, feel value for money, very satisfied with a shopping, need next time will come back!	Jingdong prefecture level city in Shandong
Power Saving King Shop	King of Power Saving	Really super like, very supportive, very good quality, exactly as described by the seller, very satisfied!	Jingdong prefecture level city in Shandong
Power Saving King Shop	King of Power Saving	It is genuine, effective, really saves power, affordable, tightly packed, fast shipping, very satisfied.	Jingdong prefecture level city in Shandong
Power Saving King Shop	King of Power Saving	Good quality, fine workmanship, is genuine and business description of the same, good value for money, fast logistics, very satisfied	Jingdong prefecture level city in Shandong
Power Saving King Shop	King of Power Saving	Saver received, very good, installation set up very well, very good, the effect is very good, recommend that the pro buy	Jingdong prefecture level city in Shandong
Power Saving King Shop	King of Power Saving	Electromagnetic good, high power convenient, enough force, the price is right it, quality can be 😃 😃 😃 😃 😃	Jingdong prefecture level city in Shandong
Power Saving King Shop	King of Power Saving	First-class quality, packaging is also good, logistics is very fast, and the price is reasonable, very affordable, I like it!	Jingdong prefecture level city in Shandong
Power Saving King Shop	King of Power Saving	Packaging is very good, the use of the effect is quite good, the store has a good service attitude, trustworthy	Jingdong prefecture level city in Shandong
Power Saving King Shop	King of Power Saving	It's great, it's the kind of style you want, and it arrived quickly, thank you boss, 5 stars for the service!	Jingdong prefecture level city in Shandong
Power Saving King Shop	King of Power Saving	The quality is very good, it works well, very satisfied, the price is affordable, it's so great value, it's worth recommending.	Jingdong prefecture level city in Shandong
Power Saving King Shop	King of Power Saving	The baby description matches the actual item very well! The quality is awesome! Will be back to patronize again in the future!	Jingdong prefecture level city in Shandong
Power Saving King Shop	King of Power Saving	Ran the price is not high try to buy the attitude, did not expect the effect is really good, good!	Jingdong prefecture level city in Shandong
Power Saving King Shop	King of Power Saving	Works great, very affordable, shipped pretty fast, comfortable to use	Jingdong prefecture level city in Shandong
Power Saving King Shop	King of Power Saving	Good product quality is the king of power-saving products trustworthy as always good to recommend!	Jingdong prefecture level city in Shandong
Power Saving King Shop	King of Power Saving	Power Saving King power is quite large, the quality of the leaf is quite good, the price of buying a very good deal, must be praised!	Jingdong prefecture level city in Shandong
Power Saving King Shop	King of Power Saving	Shipping was pretty fast and affordable, not bad 👍	Jingdong prefecture level city in Shandong
Power Saving King Shop	King of Power Saving	Digital voltage display, voltage at a glance, plug and play, safe and simple!	Jingdong prefecture level city in Shandong
Power Saving King Shop	King of Power Saving	Quality assurance, very good, affordable, logistics is also very fast, I'm very satisfied with the	Jingdong prefecture level city in Shandong
Power Saving King Shop	King of Power Saving	The quality is very good, the first time I received the goods to open the first time to see, it is very good, very satisfied	Jingdong prefecture level city in Shandong
Power Saving King Shop	King of Power Saving	Power Saving King used, the effect is good, the price is affordable, very good, is the genuine article	Jingdong prefecture level city in Shandong
Power Saving King Shop	King of Power Saving	Good quality, fine workmanship, good appearance, time is very accurate, satisfied.	Jingdong prefecture level city in Shandong
Power Saving King Shop	King of Power Saving	I purposely used it for two days before commenting, which is responsible for myself and everyone else!	Jingdong prefecture level city in Shandong
Power Saving King Shop	King of Power Saving	Bringing power savings into millions of homes. Still a great gadget. Pretty good.	Jingdong prefecture level city in Shandong
Power Saving King Shop	King of Power Saving	Easy to install, easy to operate, helped our family save a lot of electricity this summer	Jingdong prefecture level city in Shandong

Power Saving King Shop	King of Power Saving	Digital liquid crystal display to see the clear intuitive, very formal workmanship is not rough	Jingdong prefecture level city in Shandong
Power Saving King Shop	King of Power Saving	Received. Great quality, fast shipping from the store and not too expensive!	Jingdong prefecture level city in Shandong
Power Saving King Shop	King of Power Saving	Bought myself a knuckle regulator and it was easy to use and the price was right.	Jingdong prefecture level city in Shandong
Power Saving King Shop	King of Power Saving	Received, good, powerful, good, very full of a shopping	Jingdong prefecture level city in Shandong
Power Saving King Shop	King of Power Saving	High power, consistent with the seller's description, powerful, cost-effective	Jingdong prefecture level city in Shandong
Power Saving King Shop	King of Power Saving	It works very well and also saves power at an affordable price a great deal.	Jingdong prefecture level city in Shandong
Power Saving King Shop	King of Power Saving	Good quality, good can recommend to friends to buy.	Jingdong prefecture level city in Shandong
Power Saving King Shop	King of Power Saving	The logistics is very fast, the thing is also good to use, energy saving power rely on it!	Jingdong prefecture level city in Shandong
Power Saving King Shop	King of Power Saving	Will repurchase, quality is guaranteed, safe and secure purchase.	Jingdong prefecture level city in Shandong
Power Saving King Shop	King of Power Saving	Power Saver is good quality and works very well, worth buying!	Jingdong prefecture level city in Shandong
Power Saving King Shop	King of Power Saving	The quality is very good is genuine, cost-effective recommended	Jingdong prefecture level city in Shandong
Power Saving King Shop	King of Power Saving	Good value for money. Conscientious businessman. Recommended to everyone	Jingdong prefecture level city in Shandong
Power Saving King Shop	King of Power Saving	Buy it for your rental apartment and never worry about your landlord charging you too much.	Jingdong prefecture level city in Shandong
Power Saving King Shop	King of Power Saving	Nice and affordable, will repurchase next time	Jingdong prefecture level city in Shandong
Power Saving King Shop	King of Power Saving	It's very energy efficient. I've been using it for a few days now.	Jingdong prefecture level city in Shandong
Power Saving King Shop	King of Power Saving	Quality Assurance. Favorable price. Very practical and durable	Jingdong prefecture level city in Shandong
Power Saving King Shop	King of Power Saving	Good quality, high power easy to use	Jingdong prefecture level city in Shandong
Power Saving King Shop	King of Power Saving	Power saving little helper. Deserves a favorable review.	Jingdong prefecture level city in Shandong
Power Saving King Shop	King of Power Saving	Great value for money, great value for money.	Jingdong prefecture level city in Shandong
Power Saving King Shop	King of Power Saving	Works well and as described by the seller	Jingdong prefecture level city in Shandong
Power Saving King Shop	King of Power Saving	Power saving. Works well. Not bad.	Jingdong prefecture level city in Shandong
Power Saving King Shop	King of Power Saving	Power saving is proper! It's good.	Jingdong prefecture level city in Shandong
Power Saving King Shop	King of Power Saving	Satisfied with power saving	Jingdong prefecture level city in Shandong
Power Saving King Shop	King of Power Saving	Received intact, open a super good look beautiful and generous, ~ bit of high quality is recommended to buy, the need for friends rest assured that the next order	Jingdong prefecture level city in Shandong
Power Saving King Shop	King of Power Saving	Very good to use, friends introduced, save electricity and money, long-lasting products cost-effective, large factory production, good quality, fine workmanship cost-effective!	Jingdong prefecture level city in Shandong
Power Saving King Shop	King of Power Saving	The power saving effect is very obvious, trying to pay 30% less electricity bill in a month than before, hahaha	Jingdong prefecture level city in Shandong
Power Saving King Shop	King of Power Saving	It's such a power saver to use this, it saves money on kids snacks for a month. Kudos!	Jingdong prefecture level city in Shandong
Power Saving King Shop	King of Power Saving	Plug and play, stable voltage, the screen has a display when working, this winter home appliances, need more electricity, just buy to use, save electricity and save money, with instructions, not too big and does not take up space, the quality is very good, and the seller describes exactly the same!	Jingdong prefecture level city in Shandong
Power Saving King Shop	King of Power Saving	Received the goods, the first time to unpack, the quality is very good, with the seller's description, completely exceeded my expectations, the packaging is very careful, very strict, in general this is a very satisfied with the shopping	Jingdong prefecture level city in Shandong
Power Saving King Shop	King of Power Saving	A must have for opening a store. Save over a thousand dollars a month on your electric bill	Jingdong prefecture level city in Shandong
Power Saving King Shop	King of Power Saving	As always, good quality, a very favorite product, great! o(*￣︶￣*)o The price is very affordable, cost-effective, it's considered affordable good, will repurchase!	Jingdong prefecture level city in Shandong
Power Saving King Shop	King of Power Saving	Very satisfied with a shopping, good business service, fast shipping, logistics is also very powerful, received very good, buy assured, use peace of mind!	Jingdong prefecture level city in Shandong
Power Saving King Shop	King of Power Saving	The first time to buy online, but also look at the evaluation before buying, the quality is not bad, but also very cheap, the need for friends can rest assured to buy!	Jingdong prefecture level city in Shandong
Power Saving King Shop	King of Power Saving	Better than expected, feel value for money, very satisfied with a shopping, need next time will come back!	Jingdong prefecture level city in Shandong
Power Saving King Shop	King of Power Saving	Really super like, very supportive, very good quality, exactly as described by the seller, very satisfied!	Jingdong prefecture level city in Shandong
Power Saving King Shop	King of Power Saving	It is genuine, effective, really saves power, affordable, tightly packed, fast shipping, very satisfied.	Jingdong prefecture level city in Shandong
Power Saving King Shop	King of Power Saving	Good quality, fine workmanship, is genuine and business description of the same, good value for money, fast logistics, very satisfied	Jingdong prefecture level city in Shandong
Power Saving King Shop	King of Power Saving	Saver received, very good, installation set up very well, very good, the effect is very good, recommend that the pro buy	Jingdong prefecture level city in Shandong
Power Saving King Shop	King of Power Saving	Electromagnetic good, high power convenient, enough force, the price is right it, quality can be 😃 😃 😃 😃 😃	Jingdong prefecture level city in Shandong
Power Saving King Shop	King of Power Saving	First-class quality, packaging is also good, logistics is very fast, and the price is reasonable, very affordable, I like it!	Jingdong prefecture level city in Shandong
Power Saving King Shop	King of Power Saving	It's great, it's the kind of style you want, and it arrived quickly, thank you boss, 5 stars for the service!	Jingdong prefecture level city in Shandong
Power Saving King Shop	King of Power Saving	Packaging is very good, the use of the effect is quite good, the store has a good service attitude, trustworthy	Jingdong prefecture level city in Shandong
Power Saving King Shop	King of Power Saving	The quality is very good, it works well, very satisfied, the price is affordable, it's so great value, it's worth recommending.	Jingdong prefecture level city in Shandong
Power Saving King Shop	King of Power Saving	The baby description matches the actual item very well! The quality is awesome! Will be back to patronize again in the future!	Jingdong prefecture level city in Shandong
Power Saving King Shop	King of Power Saving	Ran the price is not high try to buy the attitude, did not expect the effect is really good, good!	Jingdong prefecture level city in Shandong
Power Saving King Shop	King of Power Saving	Good product quality is the king of power-saving products trustworthy as always good to recommend!	Jingdong prefecture level city in Shandong
Power Saving King Shop	King of Power Saving	Power Saving King power is quite large, the quality of the leaf is quite good, the price of buying a very good deal, must be praised!	Jingdong prefecture level city in Shandong
Power Saving King Shop	King of Power Saving	Works great, very affordable, shipped pretty fast, comfortable to use	Jingdong prefecture level city in Shandong
Power Saving King Shop	King of Power Saving	Shipping was pretty fast and affordable, not bad 👍	Jingdong prefecture level city in Shandong
Power Saving King Shop	King of Power Saving	Digital voltage display, voltage at a glance, plug and play, safe and simple!	Jingdong prefecture level city in Shandong

Power Saving King Shop	King of Power Saving	Quality assurance, very good, affordable, logistics is also very fast, I'm very satisfied with the	Jingdong prefecture level city in Shandong
Power Saving King Shop	King of Power Saving	The quality is very good, the first time I received the goods to open the first time to see, it is very good, very satisfied	Jingdong prefecture level city in Shandong
Power Saving King Shop	King of Power Saving	Power Saving King used, the effect is good, the price is affordable, very good, is the genuine article	Jingdong prefecture level city in Shandong
Power Saving King Shop	King of Power Saving	Good quality, fine workmanship, good appearance, time is very accurate, satisfied.	Jingdong prefecture level city in Shandong
Power Saving King Shop	King of Power Saving	I purposely used it for two days before commenting, which is responsible for myself and everyone else!	Jingdong prefecture level city in Shandong
Power Saving King Shop	King of Power Saving	Easy to install, easy to operate, helped our family save a lot of electricity this summer	Jingdong prefecture level city in Shandong
Power Saving King Shop	King of Power Saving	Bringing power savings into millions of homes. Still a great gadget. Pretty good.	Jingdong prefecture level city in Shandong
Power Saving King Shop	King of Power Saving	Digital liquid crystal display to see the clear intuitive, very formal workmanship is not rough	Jingdong prefecture level city in Shandong
Power Saving King Shop	King of Power Saving	Received. Great quality, fast shipping from the store and not too expensive!	Jingdong prefecture level city in Shandong
Power Saving King Shop	King of Power Saving	Bought myself a knuckle regulator and it was easy to use and the price was right.	Jingdong prefecture level city in Shandong
Power Saving King Shop	King of Power Saving	Received, good, powerful, good, very full of a shopping	Jingdong prefecture level city in Shandong
Power Saving King Shop	King of Power Saving	High power, consistent with the seller's description, powerful, cost-effective	Jingdong prefecture level city in Shandong
Power Saving King Shop	King of Power Saving	It works very well and also saves power at an affordable price a great deal.	Jingdong prefecture level city in Shandong
Power Saving King Shop	King of Power Saving	Good quality, good can recommend to friends to buy.	Jingdong prefecture level city in Shandong
Power Saving King Shop	King of Power Saving	Will repurchase, quality is guaranteed, safe and secure purchase.	Jingdong prefecture level city in Shandong
Power Saving King Shop	King of Power Saving	The logistics is very fast, the thing is also good to use, energy saving power rely on it!	Jingdong prefecture level city in Shandong
Power Saving King Shop	King of Power Saving	Power Saver is good quality and works very well, worth buying!	Jingdong prefecture level city in Shandong
Power Saving King Shop	King of Power Saving	The quality is very good is genuine, cost-effective recommended	Jingdong prefecture level city in Shandong
Power Saving King Shop	King of Power Saving	Quality Assurance. Favorable price. Very practical and durable	Jingdong prefecture level city in Shandong
Power Saving King Shop	King of Power Saving	Good value for money. Conscientious businessman. Recommended to everyone	Jingdong prefecture level city in Shandong
Power Saving King Shop	King of Power Saving	Buy it for your rental apartment and never worry about your landlord charging you too much.	Jingdong prefecture level city in Shandong
Power Saving King Shop	King of Power Saving	Nice and affordable, will repurchase next time	Jingdong prefecture level city in Shandong
Power Saving King Shop	King of Power Saving	It's very energy efficient. I've been using it for a few days now.	Jingdong prefecture level city in Shandong
Power Saving King Shop	King of Power Saving	Good quality, high power easy to use	Jingdong prefecture level city in Shandong
Power Saving King Shop	King of Power Saving	Power saving little helper. Deserves a favorable review.	Jingdong prefecture level city in Shandong
Power Saving King Shop	King of Power Saving	Great value for money, great value for money.	Jingdong prefecture level city in Shandong
Power Saving King Shop	King of Power Saving	Works well and as described by the seller	Jingdong prefecture level city in Shandong
Power Saving King Shop	King of Power Saving	Power saving is proper! It's good.	Jingdong prefecture level city in Shandong
Power Saving King Shop	King of Power Saving	Power saving. Works well. Not bad.	Jingdong prefecture level city in Shandong
Power Saving King Shop	King of Power Saving	Satisfied with power saving	Jingdong prefecture level city in Shandong
Power Saving King Shop	King of Power Saving	Received intact, open a super good look beautiful and generous, ~ bit of high quality is recommended to buy, the need for friends rest assured that the next order	Jingdong prefecture level city in Shandong
Power Saving King Shop	King of Power Saving	Very good to use, friends introduced, save electricity and money, long-lasting products cost-effective, large factory production, good quality, fine workmanship cost-effective!	Jingdong prefecture level city in Shandong
Power Saving King Shop	King of Power Saving	The power saving effect is very obvious, trying to pay 30% less electricity bill in a month than before, hahaha	Jingdong prefecture level city in Shandong
Power Saving King Shop	King of Power Saving	Plug and play, stable voltage, the screen has a display when working, this winter home appliances, need more electricity, just buy to use, save electricity and save money, with instructions, not too big and does not take up space, the quality is very good, and the seller describes exactly the same!	Jingdong prefecture level city in Shandong
Power Saving King Shop	King of Power Saving	It's such a power saver to use this, it saves money on kids snacks for a month. Kudos!	Jingdong prefecture level city in Shandong
Power Saving King Shop	King of Power Saving	Received the goods, the first time to unpack, the quality is very good, with the seller's description, completely exceeded my expectations, the packaging is very careful, very strict, in general this is a very satisfied with the shopping	Jingdong prefecture level city in Shandong
Power Saving King Shop	King of Power Saving	A must have for opening a store. Save over a thousand dollars a month on your electric bill	Jingdong prefecture level city in Shandong
Power Saving King Shop	King of Power Saving	As always, good quality, a very favorite product, great! o(*￣︶￣*)o The price is very affordable, cost-effective, it's considered affordable good, will repurchase!	Jingdong prefecture level city in Shandong
Power Saving King Shop	King of Power Saving	Very satisfied with a shopping, good business service, fast shipping, logistics is also very powerful, received very good, buy assured, use peace of mind!	Jingdong prefecture level city in Shandong
Power Saving King Shop	King of Power Saving	The first time to buy online, but also look at the evaluation before buying, the quality is not bad, but also very cheap, the need for friends can rest assured to buy!	Jingdong prefecture level city in Shandong
Power Saving King Shop	King of Power Saving	Better than expected, feel value for money, very satisfied with a shopping, need next time will come back!	Jingdong prefecture level city in Shandong
Power Saving King Shop	King of Power Saving	Really super like, very supportive, very good quality, exactly as described by the seller, very satisfied!	Jingdong prefecture level city in Shandong
Power Saving King Shop	King of Power Saving	It is genuine, effective, really saves power, affordable, tightly packed, fast shipping, very satisfied.	Jingdong prefecture level city in Shandong
Power Saving King Shop	King of Power Saving	Good quality, fine workmanship, is genuine and business description of the same, good value for money, fast logistics, very satisfied	Jingdong prefecture level city in Shandong
Power Saving King Shop	King of Power Saving	Saver received, very good, installation set up very well, very good, the effect is very good, recommend that the pro buy	Jingdong prefecture level city in Shandong
Power Saving King Shop	King of Power Saving	First-class quality, packaging is also good, logistics is very fast, and the price is reasonable, very affordable, I like it!	Jingdong prefecture level city in Shandong
Power Saving King Shop	King of Power Saving	Electromagnetic good, high power convenient, enough force, the price is right it, quality can be ☺ ☺ ☺ ☺ ☺	Jingdong prefecture level city in Shandong
Power Saving King Shop	King of Power Saving	The quality is very good, it works well, very satisfied, the price is affordable, it's so great value, it's worth recommending.	Jingdong prefecture level city in Shandong
Power Saving King Shop	King of Power Saving	It's great, it's the kind of style you want, and it arrived quickly, thank you boss, 5 stars for the service!	Jingdong prefecture level city in Shandong
Power Saving King Shop	King of Power Saving	Packaging is very good, the use of the effect is quite good, the store has a good service attitude, trustworthy	Jingdong prefecture level city in Shandong

Power Saving King Shop	King of Power Saving	The baby description matches the actual item very well! The quality is awesome! Will be back to patronize again in the future!	Jingdong prefecture level city in Shandong
Power Saving King Shop	King of Power Saving	Ran the price is not high try to buy the attitude, did not expect the effect is really good, good!	Jingdong prefecture level city in Shandong
Power Saving King Shop	King of Power Saving	Good product quality is the king of power-saving products trustworthy as always good to recommend!	Jingdong prefecture level city in Shandong
Power Saving King Shop	King of Power Saving	Power Saving King power is quite large, the quality of the leaf is quite good, the price of buying a very good deal, must be praised!	Jingdong prefecture level city in Shandong
Power Saving King Shop	King of Power Saving	Works great, very affordable, shipped pretty fast, comfortable to use	Jingdong prefecture level city in Shandong
Power Saving King Shop	King of Power Saving	Shipping was pretty fast and affordable, not bad 👍	Jingdong prefecture level city in Shandong
Power Saving King Shop	King of Power Saving	The quality is very good, the first time I received the goods to open the first time to see, it is very good, very satisfied	Jingdong prefecture level city in Shandong
Power Saving King Shop	King of Power Saving	Quality assurance, very good, affordable, logistics is also very fast, I'm very satisfied with the	Jingdong prefecture level city in Shandong
Power Saving King Shop	King of Power Saving	Digital voltage display, voltage at a glance, plug and play, safe and simple!	Jingdong prefecture level city in Shandong
Power Saving King Shop	King of Power Saving	Power Saving King used, the effect is good, the price is affordable, very good, is the genuine article	Jingdong prefecture level city in Shandong
Power Saving King Shop	King of Power Saving	Good quality, fine workmanship, good appearance, time is very accurate, satisfied.	Jingdong prefecture level city in Shandong
Power Saving King Shop	King of Power Saving	Bringing power savings into millions of homes. Still a great gadget. Pretty good.	Jingdong prefecture level city in Shandong
Power Saving King Shop	King of Power Saving	Digital liquid crystal display to see the clear intuitive, very formal workmanship is not rough	Jingdong prefecture level city in Shandong
Power Saving King Shop	King of Power Saving	Received. Great quality, fast shipping from the store and not too expensive!	Jingdong prefecture level city in Shandong
Power Saving King Shop	King of Power Saving	Bought myself a knuckle regulator and it was easy to use and the price was right.	Jingdong prefecture level city in Shandong
Power Saving King Shop	King of Power Saving	I purposely used it for two days before commenting, which is responsible for myself and everyone else!	Jingdong prefecture level city in Shandong
Power Saving King Shop	King of Power Saving	Easy to install, easy to operate, helped our family save a lot of electricity this summer	Jingdong prefecture level city in Shandong
Power Saving King Shop	King of Power Saving	Received, good, powerful, good, very full of a shopping	Jingdong prefecture level city in Shandong
Power Saving King Shop	King of Power Saving	High power, consistent with the seller's description, powerful, cost-effective	Jingdong prefecture level city in Shandong
Power Saving King Shop	King of Power Saving	It works very well and also saves power at an affordable price a great deal.	Jingdong prefecture level city in Shandong
Power Saving King Shop	King of Power Saving	Will repurchase, quality is guaranteed, safe and secure purchase.	Jingdong prefecture level city in Shandong
Power Saving King Shop	King of Power Saving	Power Saver is good quality and works very well, worth buying!	Jingdong prefecture level city in Shandong
Power Saving King Shop	King of Power Saving	Good quality, good can recommend to friends to buy.	Jingdong prefecture level city in Shandong
Power Saving King Shop	King of Power Saving	The logistics is very fast, the thing is also good to use, energy saving power rely on it!	Jingdong prefecture level city in Shandong
Power Saving King Shop	King of Power Saving	Good value for money. Conscientious businessman. Recommended to everyone	Jingdong prefecture level city in Shandong
Power Saving King Shop	King of Power Saving	Quality Assurance. Favorable price. Very practical and durable	Jingdong prefecture level city in Shandong
Power Saving King Shop	King of Power Saving	The quality is very good is genuine, cost-effective recommended	Jingdong prefecture level city in Shandong
Power Saving King Shop	King of Power Saving	Nice and affordable, will repurchase next time	Jingdong prefecture level city in Shandong
Power Saving King Shop	King of Power Saving	Buy it for your rental apartment and never worry about your landlord charging you too much.	Jingdong prefecture level city in Shandong
Power Saving King Shop	King of Power Saving	Power saving little helper. Deserves a favorable review.	Jingdong prefecture level city in Shandong
Power Saving King Shop	King of Power Saving	Great value for money, great value for money.	Jingdong prefecture level city in Shandong
Power Saving King Shop	King of Power Saving	Works well and as described by the seller	Jingdong prefecture level city in Shandong
Power Saving King Shop	King of Power Saving	Power saving is proper! It's good.	Jingdong prefecture level city in Shandong
Power Saving King Shop	King of Power Saving	Good quality, high power easy to use	Jingdong prefecture level city in Shandong
Power Saving King Shop	King of Power Saving	It's very energy efficient. I've been using it for a few days now.	Jingdong prefecture level city in Shandong
Power Saving King Shop	King of Power Saving	Power saving. Works well. Not bad.	Jingdong prefecture level city in Shandong
Power Saving King Shop	King of Power Saving	Satisfied with power saving	Jingdong prefecture level city in Shandong
Power Saving King Shop	King of Power Saving	Received intact, open a super good look beautiful and generous, ~ bit of high quality is recommended to buy, the need for friends rest assured that the next order	Jingdong prefecture level city in Shandong
Power Saving King Shop	King of Power Saving	Very good to use, friends introduced, save electricity and money, long-lasting products cost-effective, large factory production, good quality, fine workmanship cost-effective!	Jingdong prefecture level city in Shandong
Power Saving King Shop	King of Power Saving	The power saving effect is very obvious, trying to pay 30% less electricity bill in a month than before, hahaha	Jingdong prefecture level city in Shandong
Power Saving King Shop	King of Power Saving	Plug and play, stable voltage, the screen has a display when working, this winter home appliances, need more electricity, just buy to use, save electricity and save money, with instructions, not too big and does not take up space, the quality is very good, and the seller describes exactly the same!	Jingdong prefecture level city in Shandong
Power Saving King Shop	King of Power Saving	It's such a power saver to use this, it saves money on kids snacks for a month. Kudos!	Jingdong prefecture level city in Shandong
Power Saving King Shop	King of Power Saving	A must have for opening a store. Save over a thousand dollars a month on your electric bill	Jingdong prefecture level city in Shandong
Power Saving King Shop	King of Power Saving	Received the goods, the first time to unpack, the quality is very good, with the seller's description, completely exceeded my expectations, the packaging is very careful, very strict, in general this is a very satisfied with the shopping	Jingdong prefecture level city in Shandong
Power Saving King Shop	King of Power Saving	As always, good quality, a very favorite product, great! o(*￣︶￣*)o The price is very affordable, cost-effective, it's considered affordable good, will repurchase!	Jingdong prefecture level city in Shandong
Power Saving King Shop	King of Power Saving	Very satisfied with a shopping, good business service, fast shipping, logistics is also very powerful, received very good, buy assured, use peace of mind!	Jingdong prefecture level city in Shandong
Power Saving King Shop	King of Power Saving	The first time to buy online, but also look at the evaluation before buying, the quality is not bad, but also very cheap, the need for friends can rest assured to buy!	Jingdong prefecture level city in Shandong
Power Saving King Shop	King of Power Saving	Better than expected, feel value for money, very satisfied with a shopping, need next time will come back!	Jingdong prefecture level city in Shandong
Power Saving King Shop	King of Power Saving	Really super like, very supportive, very good quality, exactly as described by the seller, very satisfied!	Jingdong prefecture level city in Shandong
Power Saving King Shop	King of Power Saving	It is genuine, effective, really saves power, affordable, tightly packed, fast shipping, very satisfied.	Jingdong prefecture level city in Shandong

Power Saving King Shop	King of Power Saving	Good quality, fine workmanship, is genuine and business description of the same, good value for money, fast logistics, very satisfied	Jingdong prefecture level city in Shandong
Power Saving King Shop	King of Power Saving	Saver received, very good, installation set up very well, very good, the effect is very good, recommend that the pro buy	Jingdong prefecture level city in Shandong
Power Saving King Shop	King of Power Saving	Electromagnetic good, high power convenient, enough force, the price is right it, quality can be 😀 😀 😀 😀 😀	Jingdong prefecture level city in Shandong
Power Saving King Shop	King of Power Saving	First-class quality, packaging is also good, logistics is very fast, and the price is reasonable, very affordable, I like it!	Jingdong prefecture level city in Shandong
Power Saving King Shop	King of Power Saving	It's great, it's the kind of style you want, and it arrived quickly, thank you boss, 5 stars for the service!	Jingdong prefecture level city in Shandong
Power Saving King Shop	King of Power Saving	Packaging is very good, the use of the effect is quite good, the store has a good service attitude, trustworthy	Jingdong prefecture level city in Shandong
Power Saving King Shop	King of Power Saving	The quality is very good, it works well, very satisfied, the price is affordable, it's so great value, it's worth recommending.	Jingdong prefecture level city in Shandong
Power Saving King Shop	King of Power Saving	The baby description matches the actual item very well! The quality is awesome! Will be back to patronize again in the future!	Jingdong prefecture level city in Shandong
Power Saving King Shop	King of Power Saving	Ran the price is not high try to buy the attitude, did not expect the effect is really good, good!	Jingdong prefecture level city in Shandong
Power Saving King Shop	King of Power Saving	Power Saving King power is quite large, the quality of the leaf is quite good, the price of buying a very good deal, must be praised!	Jingdong prefecture level city in Shandong
Power Saving King Shop	King of Power Saving	Good product quality is the king of power-saving products trustworthy as always good to recommend!	Jingdong prefecture level city in Shandong
Power Saving King Shop	King of Power Saving	Works great, very affordable, shipped pretty fast, comfortable to use	Jingdong prefecture level city in Shandong
Power Saving King Shop	King of Power Saving	Shipping was pretty fast and affordable, not bad 👍	Jingdong prefecture level city in Shandong
Power Saving King Shop	King of Power Saving	Digital voltage display, voltage at a glance, plug and play, safe and simple!	Jingdong prefecture level city in Shandong
Power Saving King Shop	King of Power Saving	Power Saving King used, the effect is good, the price is affordable, very good, is the genuine article	Jingdong prefecture level city in Shandong
Power Saving King Shop	King of Power Saving	Quality assurance, very good, affordable, logistics is also very fast, I'm very satisfied with the	Jingdong prefecture level city in Shandong
Power Saving King Shop	King of Power Saving	The quality is very good, the first time I received the goods to open the first time to see, it is very good, very satisfied	Jingdong prefecture level city in Shandong
Power Saving King Shop	King of Power Saving	Good quality, fine workmanship, good appearance, time is very accurate, satisfied.	Jingdong prefecture level city in Shandong
Power Saving King Shop	King of Power Saving	I purposely used it for two days before commenting, which is responsible for myself and everyone else!	Jingdong prefecture level city in Shandong
Power Saving King Shop	King of Power Saving	Digital liquid crystal display to see the clear intuitive, very formal workmanship is not rough	Jingdong prefecture level city in Shandong
Power Saving King Shop	King of Power Saving	Bringing power savings into millions of homes. Still a great gadget. Pretty good.	Jingdong prefecture level city in Shandong
Power Saving King Shop	King of Power Saving	Easy to install, easy to operate, helped our family save a lot of electricity this summer	Jingdong prefecture level city in Shandong
Power Saving King Shop	King of Power Saving	Received. Great quality, fast shipping from the store and not too expensive!	Jingdong prefecture level city in Shandong
Power Saving King Shop	King of Power Saving	Received, good, powerful, good, very full of a shopping	Jingdong prefecture level city in Shandong
Power Saving King Shop	King of Power Saving	High power, consistent with the seller's description, powerful, cost-effective	Jingdong prefecture level city in Shandong
Power Saving King Shop	King of Power Saving	Bought myself a knuckle regulator and it was easy to use and the price was right.	Jingdong prefecture level city in Shandong
Power Saving King Shop	King of Power Saving	It works very well and also saves power at an affordable price a great deal.	Jingdong prefecture level city in Shandong
Power Saving King Shop	King of Power Saving	Good quality, good can recommend to friends to buy.	Jingdong prefecture level city in Shandong
Power Saving King Shop	King of Power Saving	Will repurchase, quality is guaranteed, safe and secure purchase.	Jingdong prefecture level city in Shandong
Power Saving King Shop	King of Power Saving	The logistics is very fast, the thing is also good to use, energy saving power rely on it!	Jingdong prefecture level city in Shandong
Power Saving King Shop	King of Power Saving	Power Saver is good quality and works very well, worth buying!	Jingdong prefecture level city in Shandong
Power Saving King Shop	King of Power Saving	The quality is very good is genuine, cost-effective recommended	Jingdong prefecture level city in Shandong
Power Saving King Shop	King of Power Saving	Nice and affordable, will repurchase next time	Jingdong prefecture level city in Shandong
Power Saving King Shop	King of Power Saving	It's very energy efficient. I've been using it for a few days now.	Jingdong prefecture level city in Shandong
Power Saving King Shop	King of Power Saving	Buy it for your rental apartment and never worry about your landlord charging you too much.	Jingdong prefecture level city in Shandong
Power Saving King Shop	King of Power Saving	Quality Assurance. Favorable price. Very practical and durable	Jingdong prefecture level city in Shandong
Power Saving King Shop	King of Power Saving	Good value for money. Conscientious businessman. Recommended to everyone	Jingdong prefecture level city in Shandong
Power Saving King Shop	King of Power Saving	Good quality, high power easy to use	Jingdong prefecture level city in Shandong
Power Saving King Shop	King of Power Saving	Power saving little helper. Deserves a favorable review.	Jingdong prefecture level city in Shandong
Power Saving King Shop	King of Power Saving	Great value for money, great value for money.	Jingdong prefecture level city in Shandong
Power Saving King Shop	King of Power Saving	Power saving is proper! It's good.	Jingdong prefecture level city in Shandong
Power Saving King Shop	King of Power Saving	Works well and as described by the seller	Jingdong prefecture level city in Shandong
Power Saving King Shop	King of Power Saving	Power saving. Works well. Not bad.	Jingdong prefecture level city in Shandong
Power Saving King Shop	King of Power Saving	Satisfied with power saving	Jingdong prefecture level city in Shandong
Power Saving King Shop	King of Power Saving	Received intact, open a super good look beautiful and generous, ~ bit of high quality is recommended to buy, the need for friends rest assured that the next order	Jingdong prefecture level city in Shandong
Power Saving King Shop	King of Power Saving	Very good to use, friends introduced, save electricity and money, long-lasting products cost-effective, large factory production, good quality, fine workmanship cost-effective!	Jingdong prefecture level city in Shandong
Power Saving King Shop	King of Power Saving	The power saving effect is very obvious, trying to pay 30% less electricity bill in a month than before, hahaha	Jingdong prefecture level city in Shandong
Power Saving King Shop	King of Power Saving	It's such a power saver to use this, it saves money on kids snacks for a month. Kudos!	Jingdong prefecture level city in Shandong
Power Saving King Shop	King of Power Saving	Plug and play, stable voltage, the screen has a display when working, this winter home appliances, need more electricity, just buy to use, save electricity and save money, with instructions, not too big and does not take up space, the quality is very good, and the seller describes exactly the same!	Jingdong prefecture level city in Shandong
Power Saving King Shop	King of Power Saving	A must have for opening a store. Save over a thousand dollars a month on your electric bill	Jingdong prefecture level city in Shandong

Power Saving King Shop	King of Power Saving	Received the goods, the first time to unpack, the quality is very good, with the seller's description, completely exceeded my expectations, the packaging is very careful, very strict, in general this is a very satisfied with the shopping	Jingdong prefecture level city in Shandong
Power Saving King Shop	King of Power Saving	As always, good quality, a very favorite product, great! o(*￣︶￣*)o The price is very affordable, cost-effective, it's considered affordable good, will repurchase!	Jingdong prefecture level city in Shandong
Power Saving King Shop	King of Power Saving	Very satisfied with a shopping, good business service, fast shipping, logistics is also very powerful, received very good, buy assured, use peace of mind!	Jingdong prefecture level city in Shandong
Power Saving King Shop	King of Power Saving	The first time to buy online, but also look at the evaluation before buying, the quality is not bad, but also very cheap, the need for friends can rest assured to buy!	Jingdong prefecture level city in Shandong
Power Saving King Shop	King of Power Saving	Better than expected, feel value for money, very satisfied with a shopping, need next time will come back!	Jingdong prefecture level city in Shandong
Power Saving King Shop	King of Power Saving	Really super like, very supportive, very good quality, exactly as described by the seller, very satisfied!	Jingdong prefecture level city in Shandong
Power Saving King Shop	King of Power Saving	It is genuine, effective, really saves power, affordable, tightly packed, fast shipping, very satisfied.	Jingdong prefecture level city in Shandong
Power Saving King Shop	King of Power Saving	Good quality, fine workmanship, is genuine and business description of the same, good value for money, fast logistics, very satisfied	Jingdong prefecture level city in Shandong
Power Saving King Shop	King of Power Saving	Saver received, very good, installation set up very well, very good, the effect is very good, recommend that the pro buy	Jingdong prefecture level city in Shandong
Power Saving King Shop	King of Power Saving	Electromagnetic good, high power convenient, enough force, the price is right it, quality can be 😃 😃 😃 😃 😃	Jingdong prefecture level city in Shandong
Power Saving King Shop	King of Power Saving	First-class quality, packaging is also good, logistics is very fast, and the price is reasonable, very affordable, I like it!	Jingdong prefecture level city in Shandong
Power Saving King Shop	King of Power Saving	It's great, it's the kind of style you want, and it arrived quickly, thank you boss, 5 stars for the service!	Jingdong prefecture level city in Shandong
Power Saving King Shop	King of Power Saving	Packaging is very good, the use of the effect is quite good, the store has a good service attitude, trustworthy	Jingdong prefecture level city in Shandong
Power Saving King Shop	King of Power Saving	The quality is very good, it works well, very satisfied, the price is affordable, it's so great value, it's worth recommending.	Jingdong prefecture level city in Shandong
Power Saving King Shop	King of Power Saving	The baby description matches the actual item very well! The quality is awesome! Will be back to patronize again in the future!	Jingdong prefecture level city in Shandong
Power Saving King Shop	King of Power Saving	Ran the price is not high try to buy the attitude, did not expect the effect is really good, good!	Jingdong prefecture level city in Shandong
Power Saving King Shop	King of Power Saving	Power Saving King power is quite large, the quality of the leaf is quite good, the price of buying a very good deal, must be praised!	Jingdong prefecture level city in Shandong
Power Saving King Shop	King of Power Saving	Good product quality is the king of power-saving products trustworthy as always good to recommend!	Jingdong prefecture level city in Shandong
Power Saving King Shop	King of Power Saving	Works great, very affordable, shipped pretty fast, comfortable to use	Jingdong prefecture level city in Shandong
Power Saving King Shop	King of Power Saving	Shipping was pretty fast and affordable, not bad 👍	Jingdong prefecture level city in Shandong
Power Saving King Shop	King of Power Saving	Digital voltage display, voltage at a glance, plug and play, safe and simple!	Jingdong prefecture level city in Shandong
Power Saving King Shop	King of Power Saving	Quality assurance, very good, affordable, logistics is also very fast, I'm very satisfied with the	Jingdong prefecture level city in Shandong
Power Saving King Shop	King of Power Saving	The quality is very good, the first time I received the goods to open the first time to see, it is very good, very satisfied	Jingdong prefecture level city in Shandong
Power Saving King Shop	King of Power Saving	Power Saving King used, the effect is good, the price is affordable, very good, is the genuine article	Jingdong prefecture level city in Shandong
Power Saving King Shop	King of Power Saving	Good quality, fine workmanship, good appearance, time is very accurate, satisfied.	Jingdong prefecture level city in Shandong
Power Saving King Shop	King of Power Saving	I purposely used it for two days before commenting, which is responsible for myself and everyone else!	Jingdong prefecture level city in Shandong
Power Saving King Shop	King of Power Saving	Easy to install, easy to operate, helped our family save a lot of electricity this summer	Jingdong prefecture level city in Shandong
Power Saving King Shop	King of Power Saving	Bringing power savings into millions of homes. Still a great gadget. Pretty good.	Jingdong prefecture level city in Shandong
Power Saving King Shop	King of Power Saving	Digital liquid crystal display to see the clear intuitive, very formal workmanship is not rough	Jingdong prefecture level city in Shandong
Power Saving King Shop	King of Power Saving	Received. Great quality, fast shipping from the store and not too expensive!	Jingdong prefecture level city in Shandong
Power Saving King Shop	King of Power Saving	Bought myself a knuckle regulator and it was easy to use and the price was right.	Jingdong prefecture level city in Shandong
Power Saving King Shop	King of Power Saving	Received, good, powerful, good, very full of a shopping	Jingdong prefecture level city in Shandong
Power Saving King Shop	King of Power Saving	High power, consistent with the seller's description, powerful, cost-effective	Jingdong prefecture level city in Shandong
Power Saving King Shop	King of Power Saving	It works very well and also saves power at an affordable price a great deal.	Jingdong prefecture level city in Shandong
Power Saving King Shop	King of Power Saving	Good quality, good can recommend to friends to buy.	Jingdong prefecture level city in Shandong
Power Saving King Shop	King of Power Saving	Will repurchase, quality is guaranteed, safe and secure purchase.	Jingdong prefecture level city in Shandong
Power Saving King Shop	King of Power Saving	The logistics is very fast, the thing is also good to use, energy saving power rely on it!	Jingdong prefecture level city in Shandong
Power Saving King Shop	King of Power Saving	Power Saver is good quality and works very well, worth buying!	Jingdong prefecture level city in Shandong
Power Saving King Shop	King of Power Saving	The quality is very good is genuine, cost-effective recommended	Jingdong prefecture level city in Shandong
Power Saving King Shop	King of Power Saving	Quality Assurance. Favorable price. Very practical and durable	Jingdong prefecture level city in Shandong
Power Saving King Shop	King of Power Saving	Good value for money. Conscientious businessman. Recommended to everyone	Jingdong prefecture level city in Shandong
Power Saving King Shop	King of Power Saving	Buy it for your rental apartment and never worry about your landlord charging you too much.	Jingdong prefecture level city in Shandong
Power Saving King Shop	King of Power Saving	Nice and affordable, will repurchase next time	Jingdong prefecture level city in Shandong
Power Saving King Shop	King of Power Saving	It's very energy efficient. I've been using it for a few days now.	Jingdong prefecture level city in Shandong
Power Saving King Shop	King of Power Saving	Good quality, high power easy to use	Jingdong prefecture level city in Shandong
Power Saving King Shop	King of Power Saving	Power saving little helper. Deserves a favorable review.	Jingdong prefecture level city in Shandong
Power Saving King Shop	King of Power Saving	Great value for money, great value for money.	Jingdong prefecture level city in Shandong
Power Saving King Shop	King of Power Saving	Works well and as described by the seller	Jingdong prefecture level city in Shandong
Power Saving King Shop	King of Power Saving	Power saving is proper! It's good.	Jingdong prefecture level city in Shandong
Power Saving King Shop	King of Power Saving	Power saving. Works well. Not bad.	Jingdong prefecture level city in Shandong
Power Saving King Shop	King of Power Saving	Satisfied with power saving	Jingdong prefecture level city in Shandong

Power Saving King Shop	King of Power Saving	Received intact, open a super good look beautiful and generous, ~ bit of high quality is recommended to buy, the need for friends rest assured that the next order	Jingdong prefecture level city in Shandong
Power Saving King Shop	King of Power Saving	Very good to use, friends introduced, save electricity and money, long-lasting products cost-effective, large factory production, good quality, fine workmanship cost-effective!	Jingdong prefecture level city in Shandong
Power Saving King Shop	King of Power Saving	The power saving effect is very obvious, trying to pay 30% less electricity bill in a month than before, hahaha	Jingdong prefecture level city in Shandong
Power Saving King Shop	King of Power Saving	It's such a power saver to use this, it saves money on kids snacks for a month. Kudos!	Jingdong prefecture level city in Shandong
Power Saving King Shop	King of Power Saving	Plug and play, stable voltage, the screen has a display when working, this winter home appliances, need more electricity, just buy to use, save electricity and save money, with instructions, not too big and does not take up space, the quality is very good, and the seller describes exactly the same!	Jingdong prefecture level city in Shandong
Power Saving King Shop	King of Power Saving	A must have for opening a store. Save over a thousand dollars a month on your electric bill	Jingdong prefecture level city in Shandong
Power Saving King Shop	King of Power Saving	Received the goods, the first time to unpack, the quality is very good, with the seller's description, completely exceeded my expectations, the packaging is very careful, very strict, in general this is a very satisfied with the shopping	Jingdong prefecture level city in Shandong
Power Saving King Shop	King of Power Saving	As always, good quality, a very favorite product, great! o(*￣︶￣*)o The price is very affordable, cost-effective, it's considered affordable good, will repurchase!	Jingdong prefecture level city in Shandong
Power Saving King Shop	King of Power Saving	Very satisfied with a shopping, good business service, fast shipping, logistics is also very powerful, received very good, buy assured, use peace of mind!	Jingdong prefecture level city in Shandong
Power Saving King Shop	King of Power Saving	The first time to buy online, but also look at the evaluation before buying, the quality is not bad, but also very cheap, the need for friends can rest assured to buy!	Jingdong prefecture level city in Shandong
Power Saving King Shop	King of Power Saving	Better than expected, feel value for money, very satisfied with a shopping, need next time will come back!	Jingdong prefecture level city in Shandong
Power Saving King Shop	King of Power Saving	Really super like, very supportive, very good quality, exactly as described by the seller, very satisfied!	Jingdong prefecture level city in Shandong
Power Saving King Shop	King of Power Saving	It is genuine, effective, really saves power, affordable, tightly packed, fast shipping, very satisfied.	Jingdong prefecture level city in Shandong
Power Saving King Shop	King of Power Saving	Good quality, fine workmanship, is genuine and business description of the same, good value for money, fast logistics, very satisfied	Jingdong prefecture level city in Shandong
Power Saving King Shop	King of Power Saving	Saver received, very good, installation set up very well, very good, the effect is very good, recommend that the pro buy	Jingdong prefecture level city in Shandong
Power Saving King Shop	King of Power Saving	Electromagnetic good, high power convenient, enough force, the price is right it, quality can be 😃 😃 😃 😃 😃	Jingdong prefecture level city in Shandong
Power Saving King Shop	King of Power Saving	First-class quality, packaging is also good, logistics is very fast, and the price is reasonable, very affordable, I like it!	Jingdong prefecture level city in Shandong
Power Saving King Shop	King of Power Saving	It's great, it's the kind of style you want, and it arrived quickly, thank you boss, 5 stars for the service!	Jingdong prefecture level city in Shandong
Power Saving King Shop	King of Power Saving	Packaging is very good, the use of the effect is quite good, the store has a good service attitude, trustworthy	Jingdong prefecture level city in Shandong
Power Saving King Shop	King of Power Saving	The quality is very good, it works well, very satisfied, the price is affordable, it's so great value, it's worth recommending.	Jingdong prefecture level city in Shandong
Power Saving King Shop	King of Power Saving	The baby description matches the actual item very well! The quality is awesome! Will be back to patronize again in the future!	Jingdong prefecture level city in Shandong
Power Saving King Shop	King of Power Saving	Ran the price is not high try to buy the attitude, did not expect the effect is really good, good!	Jingdong prefecture level city in Shandong
Power Saving King Shop	King of Power Saving	Power Saving King power is quite large, the quality of the leaf is quite good, the price of buying a very good deal, must be praised!	Jingdong prefecture level city in Shandong
Power Saving King Shop	King of Power Saving	Good product quality is the king of power-saving products trustworthy as always good to recommend!	Jingdong prefecture level city in Shandong
Power Saving King Shop	King of Power Saving	Works great, very affordable, shipped pretty fast, comfortable to use	Jingdong prefecture level city in Shandong
Power Saving King Shop	King of Power Saving	Shipping was pretty fast and affordable, not bad 👍	Jingdong prefecture level city in Shandong
Power Saving King Shop	King of Power Saving	Digital voltage display, voltage at a glance, plug and play, safe and simple!	Jingdong prefecture level city in Shandong
Power Saving King Shop	King of Power Saving	Quality assurance, very good, affordable, logistics is also very fast, I'm very satisfied with the	Jingdong prefecture level city in Shandong
Power Saving King Shop	King of Power Saving	The quality is very good, the first time I received the goods to open the first time to see, it is very good, very satisfied	Jingdong prefecture level city in Shandong
Power Saving King Shop	King of Power Saving	Power Saving King used, the effect is good, the price is affordable, very good, is the genuine article	Jingdong prefecture level city in Shandong
Power Saving King Shop	King of Power Saving	Good quality, fine workmanship, good appearance, time is very accurate, satisfied.	Jingdong prefecture level city in Shandong
Power Saving King Shop	King of Power Saving	I purposely used it for two days before commenting, which is responsible for myself and everyone else!	Jingdong prefecture level city in Shandong
Power Saving King Shop	King of Power Saving	Easy to install, easy to operate, helped our family save a lot of electricity this summer	Jingdong prefecture level city in Shandong
Power Saving King Shop	King of Power Saving	Bringing power savings into millions of homes. Still a great gadget. Pretty good.	Jingdong prefecture level city in Shandong
Power Saving King Shop	King of Power Saving	Digital liquid crystal display to see the clear intuitive, very formal workmanship is not rough	Jingdong prefecture level city in Shandong
Power Saving King Shop	King of Power Saving	Received. Great quality, fast shipping from the store and not too expensive!	Jingdong prefecture level city in Shandong
Power Saving King Shop	King of Power Saving	Bought myself a knuckle regulator and it was easy to use and the price was right.	Jingdong prefecture level city in Shandong
Power Saving King Shop	King of Power Saving	Received, good, powerful, good, very full of a shopping	Jingdong prefecture level city in Shandong
Power Saving King Shop	King of Power Saving	High power, consistent with the seller's description, powerful, cost-effective	Jingdong prefecture level city in Shandong
Power Saving King Shop	King of Power Saving	It works very well and also saves power at an affordable price a great deal.	Jingdong prefecture level city in Shandong
Power Saving King Shop	King of Power Saving	Good quality, good can recommend to friends to buy.	Jingdong prefecture level city in Shandong
Power Saving King Shop	King of Power Saving	Will repurchase, quality is guaranteed, safe and secure purchase.	Jingdong prefecture level city in Shandong
Power Saving King Shop	King of Power Saving	The logistics is very fast, the thing is also good to use, energy saving power rely on it!	Jingdong prefecture level city in Shandong
Power Saving King Shop	King of Power Saving	Power Saver is good quality and works very well, worth buying!	Jingdong prefecture level city in Shandong
Power Saving King Shop	King of Power Saving	The quality is very good is genuine, cost-effective recommended	Jingdong prefecture level city in Shandong
Power Saving King Shop	King of Power Saving	Quality Assurance. Favorable price. Very practical and durable	Jingdong prefecture level city in Shandong
Power Saving King Shop	King of Power Saving	Good value for money. Conscientious businessman. Recommended to everyone	Jingdong prefecture level city in Shandong
Power Saving King Shop	King of Power Saving	Buy it for your rental apartment and never worry about your landlord charging you too much.	Jingdong prefecture level city in Shandong
Power Saving King Shop	King of Power Saving	Nice and affordable, will repurchase next time	Jingdong prefecture level city in Shandong
Power Saving King Shop	King of Power Saving	It's very energy efficient. I've been using it for a few days now.	Jingdong prefecture level city in Shandong

Power Saving King Shop	King of Power Saving	Good quality, high power easy to use	Jingdong prefecture level city in Shandong
Power Saving King Shop	King of Power Saving	Power saving little helper. Deserves a favorable review.	Jingdong prefecture level city in Shandong
Power Saving King Shop	King of Power Saving	Great value for money, great value for money.	Jingdong prefecture level city in Shandong
Power Saving King Shop	King of Power Saving	Works well and as described by the seller	Jingdong prefecture level city in Shandong
Power Saving King Shop	King of Power Saving	Power saving is proper! It's good.	Jingdong prefecture level city in Shandong
Power Saving King Shop	King of Power Saving	Power saving. Works well. Not bad.	Jingdong prefecture level city in Shandong
Power Saving King Shop	King of Power Saving	Satisfied with power saving	Jingdong prefecture level city in Shandong
Power Saving King Shop	King of Power Saving	Received intact, open a super good look beautiful and generous, ~ bit of high quality is recommended to buy, the need for friends rest assured that the next order	Jingdong prefecture level city in Shandong
Power Saving King Shop	King of Power Saving	Very good to use, friends introduced, save electricity and money, long-lasting products cost-effective, large factory production, good quality, fine workmanship cost-effective!	Jingdong prefecture level city in Shandong
Power Saving King Shop	King of Power Saving	The power saving effect is very obvious, trying to pay 30% less electricity bill in a month than before, hahaha	Jingdong prefecture level city in Shandong
Power Saving King Shop	King of Power Saving	It's such a power saver to use this, it saves money on kids snacks for a month. Kudos!	Jingdong prefecture level city in Shandong
Power Saving King Shop	King of Power Saving	Plug and play, stable voltage, the screen has a display when working, this winter home appliances, need more electricity, just buy to use, save electricity and save money, with instructions, not too big and does not take up space, the quality is very good, and the seller describes exactly the same!	Jingdong prefecture level city in Shandong
Power Saving King Shop	King of Power Saving	A must have for opening a store. Save over a thousand dollars a month on your electric bill	Jingdong prefecture level city in Shandong
Power Saving King Shop	King of Power Saving	Received the goods, the first time to unpack, the quality is very good, with the seller's description, completely exceeded my expectations, the packaging is very careful, very strict, in general this is a very satisfied with the shopping	Jingdong prefecture level city in Shandong
Power Saving King Shop	King of Power Saving	As always, good quality, a very favorite product, great! o(*￣︶￣*)o The price is very affordable, cost-effective, it's considered affordable good, will repurchase!	Jingdong prefecture level city in Shandong
Power Saving King Shop	King of Power Saving	Very satisfied with a shopping, good business service, fast shipping, logistics is also very powerful, received very good, buy assured, use peace of mind!	Jingdong prefecture level city in Shandong
Power Saving King Shop	King of Power Saving	The first time to buy online, but also look at the evaluation before buying, the quality is not bad, but also very cheap, the need for friends can rest assured to buy!	Jingdong prefecture level city in Shandong
Power Saving King Shop	King of Power Saving	Better than expected, feel value for money, very satisfied with a shopping, need next time will come back!	Jingdong prefecture level city in Shandong
Power Saving King Shop	King of Power Saving	Really super like, very supportive, very good quality, exactly as described by the seller, very satisfied!	Jingdong prefecture level city in Shandong
Power Saving King Shop	King of Power Saving	It is genuine, effective, really saves power, affordable, tightly packed, fast shipping, very satisfied.	Jingdong prefecture level city in Shandong
Power Saving King Shop	King of Power Saving	Good quality, fine workmanship, is genuine and business description of the same, good value for money, fast logistics, very satisfied	Jingdong prefecture level city in Shandong
Power Saving King Shop	King of Power Saving	Saver received, very good, installation set up very well, very good, the effect is very good, recommend that the pro buy	Jingdong prefecture level city in Shandong
Power Saving King Shop	King of Power Saving	Electromagnetic good, high power convenient, enough force, the price is right it, quality can be 😃 😃 😃 😃 😃	Jingdong prefecture level city in Shandong
Power Saving King Shop	King of Power Saving	First-class quality, packaging is also good, logistics is very fast, and the price is reasonable, very affordable, I like it!	Jingdong prefecture level city in Shandong
Power Saving King Shop	King of Power Saving	It's great, it's the kind of style you want, and it arrived quickly, thank you boss, 5 stars for the service!	Jingdong prefecture level city in Shandong
Power Saving King Shop	King of Power Saving	Packaging is very good, the use of the effect is quite good, the store has a good service attitude, trustworthy	Jingdong prefecture level city in Shandong
Power Saving King Shop	King of Power Saving	The quality is very good, it works well, very satisfied, the price is affordable, it's so great value, it's worth recommending.	Jingdong prefecture level city in Shandong
Power Saving King Shop	King of Power Saving	The baby description matches the actual item very well! The quality is awesome! Will be back to patronize again in the future!	Jingdong prefecture level city in Shandong
Power Saving King Shop	King of Power Saving	Ran the price is not high try to buy the attitude, did not expect the effect is really good, good!	Jingdong prefecture level city in Shandong
Power Saving King Shop	King of Power Saving	Power Saving King power is quite large, the quality of the leaf is quite good, the price of buying a very good deal, must be praised!	Jingdong prefecture level city in Shandong
Power Saving King Shop	King of Power Saving	Good product quality is the king of power-saving products trustworthy as always good to recommend!	Jingdong prefecture level city in Shandong
Power Saving King Shop	King of Power Saving	Works great, very affordable, shipped pretty fast, comfortable to use	Jingdong prefecture level city in Shandong
Power Saving King Shop	King of Power Saving	Shipping was pretty fast and affordable, not bad 👍	Jingdong prefecture level city in Shandong
Power Saving King Shop	King of Power Saving	Digital voltage display, voltage at a glance, plug and play, safe and simple!	Jingdong prefecture level city in Shandong
Power Saving King Shop	King of Power Saving	Quality assurance, very good, affordable, logistics is also very fast, I'm very satisfied with the	Jingdong prefecture level city in Shandong
Power Saving King Shop	King of Power Saving	The quality is very good, the first time I received the goods to open the first time to see, it is very good, very satisfied	Jingdong prefecture level city in Shandong
Power Saving King Shop	King of Power Saving	Power Saving King used, the effect is good, the price is affordable, very good, is the genuine article	Jingdong prefecture level city in Shandong
Power Saving King Shop	King of Power Saving	Good quality, fine workmanship, good appearance, time is very accurate, satisfied.	Jingdong prefecture level city in Shandong
Power Saving King Shop	King of Power Saving	I purposely used it for two days before commenting, which is responsible for myself and everyone else!	Jingdong prefecture level city in Shandong
Power Saving King Shop	King of Power Saving	Easy to install, easy to operate, helped our family save a lot of electricity this summer	Jingdong prefecture level city in Shandong
Power Saving King Shop	King of Power Saving	Bringing power savings into millions of homes. Still a great gadget. Pretty good.	Jingdong prefecture level city in Shandong
Power Saving King Shop	King of Power Saving	Digital liquid crystal display to see the clear intuitive, very formal workmanship is not rough	Jingdong prefecture level city in Shandong
Power Saving King Shop	King of Power Saving	Received. Great quality, fast shipping from the store and not too expensive!	Jingdong prefecture level city in Shandong
Power Saving King Shop	King of Power Saving	Bought myself a knuckle regulator and it was easy to use and the price was right.	Jingdong prefecture level city in Shandong
Power Saving King Shop	King of Power Saving	Received, good, powerful, good, very full of a shopping	Jingdong prefecture level city in Shandong
Power Saving King Shop	King of Power Saving	High power, consistent with the seller's description, powerful, cost-effective	Jingdong prefecture level city in Shandong
Power Saving King Shop	King of Power Saving	It works very well and also saves power at an affordable price a great deal.	Jingdong prefecture level city in Shandong
Power Saving King Shop	King of Power Saving	Good quality, good can recommend to friends to buy.	Jingdong prefecture level city in Shandong
Power Saving King Shop	King of Power Saving	Will repurchase, quality is guaranteed, safe and secure purchase.	Jingdong prefecture level city in Shandong
Power Saving King Shop	King of Power Saving	The logistics is very fast, the thing is also good to use, energy saving power rely on it!	Jingdong prefecture level city in Shandong

Power Saving King Shop	King of Power Saving	Power Saver is good quality and works very well, worth buying!	Jingdong prefecture level city in Shandong
Power Saving King Shop	King of Power Saving	The quality is very good is genuine, cost-effective recommended	Jingdong prefecture level city in Shandong
Power Saving King Shop	King of Power Saving	Quality Assurance. Favorable price. Very practical and durable	Jingdong prefecture level city in Shandong
Power Saving King Shop	King of Power Saving	Good value for money. Conscientious businessman. Recommended to everyone	Jingdong prefecture level city in Shandong
Power Saving King Shop	King of Power Saving	Buy it for your rental apartment and never worry about your landlord charging you too much.	Jingdong prefecture level city in Shandong
Power Saving King Shop	King of Power Saving	Nice and affordable, will repurchase next time	Jingdong prefecture level city in Shandong
Power Saving King Shop	King of Power Saving	It's very energy efficient. I've been using it for a few days now.	Jingdong prefecture level city in Shandong
Power Saving King Shop	King of Power Saving	Good quality, high power easy to use	Jingdong prefecture level city in Shandong
Power Saving King Shop	King of Power Saving	Power saving little helper. Deserves a favorable review.	Jingdong prefecture level city in Shandong
Power Saving King Shop	King of Power Saving	Great value for money, great value for money.	Jingdong prefecture level city in Shandong
Power Saving King Shop	King of Power Saving	Works well and as described by the seller	Jingdong prefecture level city in Shandong
Power Saving King Shop	King of Power Saving	Power saving is proper! It's good.	Jingdong prefecture level city in Shandong
Power Saving King Shop	King of Power Saving	Power saving. Works well. Not bad.	Jingdong prefecture level city in Shandong
Power Saving King Shop	King of Power Saving	Satisfied with power saving	Jingdong prefecture level city in Shandong
Power Saving King Shop	King of Power Saving	Received intact, open a super good look beautiful and generous, ~ bit of high quality is recommended to buy, the need for friends rest assured that the next order	Jingdong prefecture level city in Shandong
Power Saving King Shop	King of Power Saving	Very good to use, friends introduced, save electricity and money, long-lasting products cost-effective, large factory production, good quality, fine workmanship cost-effective!	Jingdong prefecture level city in Shandong
Power Saving King Shop	King of Power Saving	The power saving effect is very obvious, trying to pay 30% less electricity bill in a month than before, hahaha	Jingdong prefecture level city in Shandong
Power Saving King Shop	King of Power Saving	It's such a power saver to use this, it saves money on kids snacks for a month. Kudos!	Jingdong prefecture level city in Shandong
Power Saving King Shop	King of Power Saving	Plug and play, stable voltage, the screen has a display when working, this winter home appliances, need more electricity, just buy to use, save electricity and save money, with instructions, not too big and does not take up space, the quality is very good, and the seller describes exactly the same!	Jingdong prefecture level city in Shandong
Power Saving King Shop	King of Power Saving	A must have for opening a store. Save over a thousand dollars a month on your electric bill	Jingdong prefecture level city in Shandong
Power Saving King Shop	King of Power Saving	Received the goods, the first time to unpack, the quality is very good, with the seller's description, completely exceeded my expectations, the packaging is very careful, very strict, in general this is a very satisfied with the shopping	Jingdong prefecture level city in Shandong
Power Saving King Shop	King of Power Saving	As always, good quality, a very favorite product, great! o(*￣︶￣*)o The price is very affordable, cost-effective, it's considered affordable good, will repurchase!	Jingdong prefecture level city in Shandong
Power Saving King Shop	King of Power Saving	Very satisfied with a shopping, good business service, fast shipping, logistics is also very powerful, received very good, buy assured, use peace of mind!	Jingdong prefecture level city in Shandong
Power Saving King Shop	King of Power Saving	The first time to buy online, but also look at the evaluation before buying, the quality is not bad, but also very cheap, the need for friends can rest assured to buy!	Jingdong prefecture level city in Shandong
Power Saving King Shop	King of Power Saving	Better than expected, feel value for money, very satisfied with a shopping, need next time will come back!	Jingdong prefecture level city in Shandong
Power Saving King Shop	King of Power Saving	Really super like, very supportive, very good quality, exactly as described by the seller, very satisfied!	Jingdong prefecture level city in Shandong
Power Saving King Shop	King of Power Saving	It is genuine, effective, really saves power, affordable, tightly packed, fast shipping, very satisfied.	Jingdong prefecture level city in Shandong
Power Saving King Shop	King of Power Saving	Good quality, fine workmanship, is genuine and business description of the same, good value for money, fast logistics, very satisfied	Jingdong prefecture level city in Shandong
Power Saving King Shop	King of Power Saving	Saver received, very good, installation set up very well, very good, the effect is very good, recommend that the pro buy	Jingdong prefecture level city in Shandong
Power Saving King Shop	King of Power Saving	Electromagnetic good, high power convenient, enough force, the price is right it, quality can be 😀 😀 😀 😀 😀	Jingdong prefecture level city in Shandong
Power Saving King Shop	King of Power Saving	First-class quality, packaging is also good, logistics is very fast, and the price is reasonable, very affordable, I like it!	Jingdong prefecture level city in Shandong
Power Saving King Shop	King of Power Saving	It's great, it's the kind of style you want, and it arrived quickly, thank you boss, 5 stars for the service!	Jingdong prefecture level city in Shandong
Power Saving King Shop	King of Power Saving	Packaging is very good, the use of the effect is quite good, the store has a good service attitude, trustworthy	Jingdong prefecture level city in Shandong
Power Saving King Shop	King of Power Saving	The quality is very good, it works well, very satisfied, the price is affordable, it's so great value, it's worth recommending.	Jingdong prefecture level city in Shandong
Power Saving King Shop	King of Power Saving	The baby description matches the actual item very well! The quality is awesome! Will be back to patronize again in the future!	Jingdong prefecture level city in Shandong
Power Saving King Shop	King of Power Saving	Ran the price is not high try to buy the attitude, did not expect the effect is really good, good!	Jingdong prefecture level city in Shandong
Power Saving King Shop	King of Power Saving	Power Saving King power is quite large, the quality of the leaf is quite good, the price of buying a very good deal, must be praised!	Jingdong prefecture level city in Shandong
Power Saving King Shop	King of Power Saving	Good product quality is the king of power-saving products trustworthy as always good to recommend!	Jingdong prefecture level city in Shandong
Power Saving King Shop	King of Power Saving	Works great, very affordable, shipped pretty fast, comfortable to use	Jingdong prefecture level city in Shandong
Power Saving King Shop	King of Power Saving	Shipping was pretty fast and affordable, not bad 👍	Jingdong prefecture level city in Shandong
Power Saving King Shop	King of Power Saving	Digital voltage display, voltage at a glance, plug and play, safe and simple!	Jingdong prefecture level city in Shandong
Power Saving King Shop	King of Power Saving	Quality assurance, very good, affordable, logistics is also very fast, I'm very satisfied with the	Jingdong prefecture level city in Shandong
Power Saving King Shop	King of Power Saving	The quality is very good, the first time I received the goods to open the first time to see, it is very good, very satisfied	Jingdong prefecture level city in Shandong
Power Saving King Shop	King of Power Saving	Power Saving King used, the effect is good, the price is affordable, very good, is the genuine article	Jingdong prefecture level city in Shandong
Power Saving King Shop	King of Power Saving	Good quality, fine workmanship, good appearance, time is very accurate, satisfied.	Jingdong prefecture level city in Shandong
Power Saving King Shop	King of Power Saving	I purposely used it for two days before commenting, which is responsible for myself and everyone else!	Jingdong prefecture level city in Shandong
Power Saving King Shop	King of Power Saving	Easy to install, easy to operate, helped our family save a lot of electricity this summer	Jingdong prefecture level city in Shandong
Power Saving King Shop	King of Power Saving	Bringing power savings into millions of homes. Still a great gadget. Pretty good.	Jingdong prefecture level city in Shandong
Power Saving King Shop	King of Power Saving	Digital liquid crystal display to see the clear intuitive, very formal workmanship is not rough	Jingdong prefecture level city in Shandong
Power Saving King Shop	King of Power Saving	Received. Great quality, fast shipping from the store and not too expensive!	Jingdong prefecture level city in Shandong

Power Saving King Shop	King of Power Saving	Bought myself a knuckle regulator and it was easy to use and the price was right.	Jingdong prefecture level city in Shandong
Power Saving King Shop	King of Power Saving	Received, good, powerful, good, very full of a shopping	Jingdong prefecture level city in Shandong
Power Saving King Shop	King of Power Saving	High power, consistent with the seller's description, powerful, cost-effective	Jingdong prefecture level city in Shandong
Power Saving King Shop	King of Power Saving	It works very well and also saves power at an affordable price a great deal.	Jingdong prefecture level city in Shandong
Power Saving King Shop	King of Power Saving	Good quality, good can recommend to friends to buy.	Jingdong prefecture level city in Shandong
Power Saving King Shop	King of Power Saving	Will repurchase, quality is guaranteed, safe and secure purchase.	Jingdong prefecture level city in Shandong
Power Saving King Shop	King of Power Saving	The logistics is very fast, the thing is also good to use, energy saving power rely on it!	Jingdong prefecture level city in Shandong
Power Saving King Shop	King of Power Saving	Power Saver is good quality and works very well, worth buying!	Jingdong prefecture level city in Shandong
Power Saving King Shop	King of Power Saving	The quality is very good is genuine, cost-effective recommended	Jingdong prefecture level city in Shandong
Power Saving King Shop	King of Power Saving	Quality Assurance. Favorable price. Very practical and durable	Jingdong prefecture level city in Shandong
Power Saving King Shop	King of Power Saving	Good value for money. Conscientious businessman. Recommended to everyone	Jingdong prefecture level city in Shandong
Power Saving King Shop	King of Power Saving	Buy it for your rental apartment and never worry about your landlord charging you too much.	Jingdong prefecture level city in Shandong
Power Saving King Shop	King of Power Saving	Nice and affordable, will repurchase next time	Jingdong prefecture level city in Shandong
Power Saving King Shop	King of Power Saving	It's very energy efficient. I've been using it for a few days now.	Jingdong prefecture level city in Shandong
Power Saving King Shop	King of Power Saving	Good quality, high power easy to use	Jingdong prefecture level city in Shandong
Power Saving King Shop	King of Power Saving	Power saving little helper. Deserves a favorable review.	Jingdong prefecture level city in Shandong
Power Saving King Shop	King of Power Saving	Great value for money, great value for money.	Jingdong prefecture level city in Shandong
Power Saving King Shop	King of Power Saving	Works well and as described by the seller	Jingdong prefecture level city in Shandong
Power Saving King Shop	King of Power Saving	Power saving is proper! It's good.	Jingdong prefecture level city in Shandong
Power Saving King Shop	King of Power Saving	Power saving. Works well. Not bad.	Jingdong prefecture level city in Shandong
Power Saving King Shop	King of Power Saving	Satisfied with power saving	Jingdong prefecture level city in Shandong
Power Saving King Shop	King of Power Saving	Received intact, open a super good look beautiful and generous, ~ bit of high quality is recommended to buy, the need for friends rest assured that the next order	Jingdong prefecture level city in Shandong
Power Saving King Shop	King of Power Saving	Very good to use, friends introduced, save electricity and money, long-lasting products cost-effective, large factory production, good quality, fine workmanship cost-effective!	Jingdong prefecture level city in Shandong
Power Saving King Shop	King of Power Saving	The power saving effect is very obvious, trying to pay 30% less electricity bill in a month than before, hahaha	Jingdong prefecture level city in Shandong
Power Saving King Shop	King of Power Saving	It's such a power saver to use this, it saves money on kids snacks for a month. Kudos!	Jingdong prefecture level city in Shandong
Power Saving King Shop	King of Power Saving	Plug and play, stable voltage, the screen has a display when working, this winter home appliances, need more electricity, just buy to use, save electricity and save money, with instructions, not too big and does not take up space, the quality is very good, and the seller describes exactly the same!	Jingdong prefecture level city in Shandong
Power Saving King Shop	King of Power Saving	A must have for opening a store. Save over a thousand dollars a month on your electric bill	Jingdong prefecture level city in Shandong
Power Saving King Shop	King of Power Saving	Received the goods, the first time to unpack, the quality is very good, with the seller's description, completely exceeded my expectations, the packaging is very careful, very strict, in general this is a very satisfied with the shopping	Jingdong prefecture level city in Shandong
Power Saving King Shop	King of Power Saving	As always, good quality, a very favorite product, great! o(*￣︶￣*)o The price is very affordable, cost-effective, it's considered affordable good, will repurchase!	Jingdong prefecture level city in Shandong
Power Saving King Shop	King of Power Saving	Very satisfied with a shopping, good business service, fast shipping, logistics is also very powerful, received very good, buy assured, use peace of mind!	Jingdong prefecture level city in Shandong
Power Saving King Shop	King of Power Saving	The first time to buy online, but also look at the evaluation before buying, the quality is not bad, but also very cheap, the need for friends can rest assured to buy!	Jingdong prefecture level city in Shandong
Power Saving King Shop	King of Power Saving	Better than expected, feel value for money, very satisfied with a shopping, need next time will come back!	Jingdong prefecture level city in Shandong
Power Saving King Shop	King of Power Saving	Really super like, very supportive, very good quality, exactly as described by the seller, very satisfied!	Jingdong prefecture level city in Shandong
Power Saving King Shop	King of Power Saving	It is genuine, effective, really saves power, affordable, tightly packed, fast shipping, very satisfied.	Jingdong prefecture level city in Shandong
Power Saving King Shop	King of Power Saving	Good quality, fine workmanship, is genuine and business description of the same, good value for money, fast logistics, very satisfied	Jingdong prefecture level city in Shandong
Power Saving King Shop	King of Power Saving	Saver received, very good, installation set up very well, very good, the effect is very good, recommend that the pro buy	Jingdong prefecture level city in Shandong
Power Saving King Shop	King of Power Saving	Electromagnetic good, high power convenient, enough force, the price is right it, quality can be 😊 😊 😊 😊 😊	Jingdong prefecture level city in Shandong
Power Saving King Shop	King of Power Saving	First-class quality, packaging is also good, logistics is very fast, and the price is reasonable, very affordable, I like it!	Jingdong prefecture level city in Shandong
Power Saving King Shop	King of Power Saving	It's great, it's the kind of style you want, and it arrived quickly, thank you boss, 5 stars for the service!	Jingdong prefecture level city in Shandong
Power Saving King Shop	King of Power Saving	Packaging is very good, the use of the effect is quite good, the store has a good service attitude, trustworthy	Jingdong prefecture level city in Shandong
Power Saving King Shop	King of Power Saving	The quality is very good, it works well, very satisfied, the price is affordable, it's so great value, it's worth recommending.	Jingdong prefecture level city in Shandong
Power Saving King Shop	King of Power Saving	The baby description matches the actual item very well! The quality is awesome! Will be back to patronize again in the future!	Jingdong prefecture level city in Shandong
Power Saving King Shop	King of Power Saving	Ran the price is not high try to buy the attitude, did not expect the effect is really good, good!	Jingdong prefecture level city in Shandong
Power Saving King Shop	King of Power Saving	Power Saving King power is quite large, the quality of the leaf is quite good, the price of buying a very good deal, must be praised!	Jingdong prefecture level city in Shandong
Power Saving King Shop	King of Power Saving	Good product quality is the king of power-saving products trustworthy as always good to recommend!	Jingdong prefecture level city in Shandong
Power Saving King Shop	King of Power Saving	Works great, very affordable, shipped pretty fast, comfortable to use	Jingdong prefecture level city in Shandong
Power Saving King Shop	King of Power Saving	Shipping was pretty fast and affordable, not bad 👍	Jingdong prefecture level city in Shandong
Power Saving King Shop	King of Power Saving	Digital voltage display, voltage at a glance, plug and play, safe and simple!	Jingdong prefecture level city in Shandong
Power Saving King Shop	King of Power Saving	Quality assurance, very good, affordable, logistics is also very fast, I'm very satisfied with the	Jingdong prefecture level city in Shandong
Power Saving King Shop	King of Power Saving	The quality is very good, the first time I received the goods to open the first time to see, it is very good, very satisfied	Jingdong prefecture level city in Shandong

Power Saving King Shop	King of Power Saving	Power Saving King used, the effect is good, the price is affordable, very good, is the genuine article	Jingdong prefecture level city in Shandong
Power Saving King Shop	King of Power Saving	Good quality, fine workmanship, good appearance, time is very accurate, satisfied.	Jingdong prefecture level city in Shandong
Power Saving King Shop	King of Power Saving	I purposely used it for two days before commenting, which is responsible for myself and everyone else!	Jingdong prefecture level city in Shandong
Power Saving King Shop	King of Power Saving	Easy to install, easy to operate, helped our family save a lot of electricity this summer	Jingdong prefecture level city in Shandong
Power Saving King Shop	King of Power Saving	Bringing power savings into millions of homes. Still a great gadget. Pretty good.	Jingdong prefecture level city in Shandong
Power Saving King Shop	King of Power Saving	Digital liquid crystal display to see the clear intuitive, very formal workmanship is not rough	Jingdong prefecture level city in Shandong
Power Saving King Shop	King of Power Saving	Received. Great quality, fast shipping from the store and not too expensive!	Jingdong prefecture level city in Shandong
Power Saving King Shop	King of Power Saving	Bought myself a knuckle regulator and it was easy to use and the price was right.	Jingdong prefecture level city in Shandong
Power Saving King Shop	King of Power Saving	Received, good, powerful, good, very full of a shopping	Jingdong prefecture level city in Shandong
Power Saving King Shop	King of Power Saving	High power, consistent with the seller's description, powerful, cost-effective	Jingdong prefecture level city in Shandong
Power Saving King Shop	King of Power Saving	It works very well and also saves power at an affordable price a great deal.	Jingdong prefecture level city in Shandong
Power Saving King Shop	King of Power Saving	Good quality, good can recommend to friends to buy.	Jingdong prefecture level city in Shandong
Power Saving King Shop	King of Power Saving	Will repurchase, quality is guaranteed, safe and secure purchase.	Jingdong prefecture level city in Shandong
Power Saving King Shop	King of Power Saving	The logistics is very fast, the thing is also good to use, energy saving power rely on it!	Jingdong prefecture level city in Shandong
Power Saving King Shop	King of Power Saving	Power Saver is good quality and works very well, worth buying!	Jingdong prefecture level city in Shandong
Power Saving King Shop	King of Power Saving	The quality is very good is genuine, cost-effective recommended	Jingdong prefecture level city in Shandong
Power Saving King Shop	King of Power Saving	Quality Assurance. Favorable price. Very practical and durable	Jingdong prefecture level city in Shandong
Power Saving King Shop	King of Power Saving	Good value for money. Conscientious businessman. Recommended to everyone	Jingdong prefecture level city in Shandong
Power Saving King Shop	King of Power Saving	Buy it for your rental apartment and never worry about your landlord charging you too much.	Jingdong prefecture level city in Shandong
Power Saving King Shop	King of Power Saving	Nice and affordable, will repurchase next time	Jingdong prefecture level city in Shandong
Power Saving King Shop	King of Power Saving	It's very energy efficient. I've been using it for a few days now.	Jingdong prefecture level city in Shandong
Power Saving King Shop	King of Power Saving	Good quality, high power easy to use	Jingdong prefecture level city in Shandong
Power Saving King Shop	King of Power Saving	Power saving little helper. Deserves a favorable review.	Jingdong prefecture level city in Shandong
Power Saving King Shop	King of Power Saving	Great value for money, great value for money.	Jingdong prefecture level city in Shandong
Power Saving King Shop	King of Power Saving	Works well and as described by the seller	Jingdong prefecture level city in Shandong
Power Saving King Shop	King of Power Saving	Power saving is proper! It's good.	Jingdong prefecture level city in Shandong
Power Saving King Shop	King of Power Saving	Power saving. Works well. Not bad.	Jingdong prefecture level city in Shandong
Power Saving King Shop	King of Power Saving	Satisfied with power saving	Jingdong prefecture level city in Shandong
Power Saving King Shop	King of Power Saving	Received intact, open a super good look beautiful and generous, ~ bit of high quality is recommended to buy, the need for friends rest assured that the next order	Jingdong prefecture level city in Shandong
Power Saving King Shop	King of Power Saving	Very good to use, friends introduced, save electricity and money, long-lasting products cost-effective, large factory production, good quality, fine workmanship cost-effective!	Jingdong prefecture level city in Shandong
Power Saving King Shop	King of Power Saving	The power saving effect is very obvious, trying to pay 30% less electricity bill in a month than before, hahaha	Jingdong prefecture level city in Shandong
Power Saving King Shop	King of Power Saving	It's such a power saver to use this, it saves money on kids snacks for a month. Kudos!	Jingdong prefecture level city in Shandong
Power Saving King Shop	King of Power Saving	Plug and play, stable voltage, the screen has a display when working, this winter home appliances, need more electricity, just buy to use, save electricity and save money, with instructions, not too big and does not take up space, the quality is very good, and the seller describes exactly the same!	Jingdong prefecture level city in Shandong
Power Saving King Shop	King of Power Saving	A must have for opening a store. Save over a thousand dollars a month on your electric bill	Jingdong prefecture level city in Shandong
Power Saving King Shop	King of Power Saving	Received the goods, the first time to unpack, the quality is very good, with the seller's description, completely exceeded my expectations, the packaging is very careful, very strict, in general this is a very satisfied with the shopping	Jingdong prefecture level city in Shandong
Power Saving King Shop	King of Power Saving	As always, good quality, a very favorite product, great! o(*￣︶￣*)o The price is very affordable, cost-effective, it's considered affordable good, will repurchase!	Jingdong prefecture level city in Shandong
Power Saving King Shop	King of Power Saving	Very satisfied with a shopping, good business service, fast shipping, logistics is also very powerful, received very good, buy assured, use peace of mind!	Jingdong prefecture level city in Shandong
Power Saving King Shop	King of Power Saving	The first time to buy online, but also look at the evaluation before buying, the quality is not bad, but also very cheap, the need for friends can rest assured to buy!	Jingdong prefecture level city in Shandong
Power Saving King Shop	King of Power Saving	Better than expected, feel value for money, very satisfied with a shopping, need next time will come back!	Jingdong prefecture level city in Shandong
Power Saving King Shop	King of Power Saving	Really super like, very supportive, very good quality, exactly as described by the seller, very satisfied!	Jingdong prefecture level city in Shandong
Power Saving King Shop	King of Power Saving	It is genuine, effective, really saves power, affordable, tightly packed, fast shipping, very satisfied.	Jingdong prefecture level city in Shandong
Power Saving King Shop	King of Power Saving	Good quality, fine workmanship, is genuine and business description of the same, good value for money, fast logistics, very satisfied	Jingdong prefecture level city in Shandong
Power Saving King Shop	King of Power Saving	Saver received, very good, installation set up very well, very good, the effect is very good, recommend that the pro buy	Jingdong prefecture level city in Shandong
Power Saving King Shop	King of Power Saving	Electromagnetic good, high power convenient, enough force, the price is right it, quality can be 😀 😀 😀 😀 😀	Jingdong prefecture level city in Shandong
Power Saving King Shop	King of Power Saving	First-class quality, packaging is also good, logistics is very fast, and the price is reasonable, very affordable, I like it!	Jingdong prefecture level city in Shandong
Power Saving King Shop	King of Power Saving	It's great, it's the kind of style you want, and it arrived quickly, thank you boss, 5 stars for the service!	Jingdong prefecture level city in Shandong
Power Saving King Shop	King of Power Saving	Packaging is very good, the use of the effect is quite good, the store has a good service attitude, trustworthy	Jingdong prefecture level city in Shandong
Power Saving King Shop	King of Power Saving	The quality is very good, it works well, very satisfied, the price is affordable, it's so great value, it's worth recommending.	Jingdong prefecture level city in Shandong
Power Saving King Shop	King of Power Saving	The baby description matches the actual item very well! The quality is awesome! Will be back to patronize again in the future!	Jingdong prefecture level city in Shandong
Power Saving King Shop	King of Power Saving	Ran the price is not high try to buy the attitude, did not expect the effect is really good, good!	Jingdong prefecture level city in Shandong

Power Saving King Shop	King of Power Saving	Power Saving King power is quite large, the quality of the leaf is quite good, the price of buying a very good deal, must be praised!	Jingdong prefecture level city in Shandong
Power Saving King Shop	King of Power Saving	Good product quality is the king of power-saving products trustworthy as always good to recommend!	Jingdong prefecture level city in Shandong
Power Saving King Shop	King of Power Saving	Works great, very affordable, shipped pretty fast, comfortable to use	Jingdong prefecture level city in Shandong
Power Saving King Shop	King of Power Saving	Shipping was pretty fast and affordable, not bad 👍	Jingdong prefecture level city in Shandong
Power Saving King Shop	King of Power Saving	Digital voltage display, voltage at a glance, plug and play, safe and simple!	Jingdong prefecture level city in Shandong
Power Saving King Shop	King of Power Saving	Quality assurance, very good, affordable, logistics is also very fast, I'm very satisfied with the	Jingdong prefecture level city in Shandong
Power Saving King Shop	King of Power Saving	The quality is very good, the first time I received the goods to open the first time to see, it is very good, very satisfied	Jingdong prefecture level city in Shandong
Power Saving King Shop	King of Power Saving	Power Saving King used, the effect is good, the price is affordable, very good, is the genuine article	Jingdong prefecture level city in Shandong
Power Saving King Shop	King of Power Saving	Good quality, fine workmanship, good appearance, time is very accurate, satisfied.	Jingdong prefecture level city in Shandong
Power Saving King Shop	King of Power Saving	I purposely used it for two days before commenting, which is responsible for myself and everyone else!	Jingdong prefecture level city in Shandong
Power Saving King Shop	King of Power Saving	Easy to install, easy to operate, helped our family save a lot of electricity this summer	Jingdong prefecture level city in Shandong
Power Saving King Shop	King of Power Saving	Bringing power savings into millions of homes. Still a great gadget. Pretty good.	Jingdong prefecture level city in Shandong
Power Saving King Shop	King of Power Saving	Digital liquid crystal display to see the clear intuitive, very formal workmanship is not rough	Jingdong prefecture level city in Shandong
Power Saving King Shop	King of Power Saving	Received. Great quality, fast shipping from the store and not too expensive!	Jingdong prefecture level city in Shandong
Power Saving King Shop	King of Power Saving	Bought myself a knuckle regulator and it was easy to use and the price was right.	Jingdong prefecture level city in Shandong
Power Saving King Shop	King of Power Saving	Received, good, powerful, good, very full of a shopping	Jingdong prefecture level city in Shandong
Power Saving King Shop	King of Power Saving	High power, consistent with the seller's description, powerful, cost-effective	Jingdong prefecture level city in Shandong
Power Saving King Shop	King of Power Saving	It works very well and also saves power at an affordable price a great deal.	Jingdong prefecture level city in Shandong
Power Saving King Shop	King of Power Saving	Good quality, good can recommend to friends to buy.	Jingdong prefecture level city in Shandong
Power Saving King Shop	King of Power Saving	Will repurchase, quality is guaranteed, safe and secure purchase.	Jingdong prefecture level city in Shandong
Power Saving King Shop	King of Power Saving	The logistics is very fast, the thing is also good to use, energy saving power rely on it!	Jingdong prefecture level city in Shandong
Power Saving King Shop	King of Power Saving	Power Saver is good quality and works very well, worth buying!	Jingdong prefecture level city in Shandong
Power Saving King Shop	King of Power Saving	The quality is very good is genuine, cost-effective recommended	Jingdong prefecture level city in Shandong
Power Saving King Shop	King of Power Saving	Quality Assurance. Favorable price. Very practical and durable	Jingdong prefecture level city in Shandong
Power Saving King Shop	King of Power Saving	Good value for money. Conscientious businessman. Recommended to everyone	Jingdong prefecture level city in Shandong
Power Saving King Shop	King of Power Saving	Buy it for your rental apartment and never worry about your landlord charging you too much.	Jingdong prefecture level city in Shandong
Power Saving King Shop	King of Power Saving	Nice and affordable, will repurchase next time	Jingdong prefecture level city in Shandong
Power Saving King Shop	King of Power Saving	It's very energy efficient. I've been using it for a few days now.	Jingdong prefecture level city in Shandong
Power Saving King Shop	King of Power Saving	Good quality, high power easy to use	Jingdong prefecture level city in Shandong
Power Saving King Shop	King of Power Saving	Power saving little helper. Deserves a favorable review.	Jingdong prefecture level city in Shandong
Power Saving King Shop	King of Power Saving	Great value for money, great value for money.	Jingdong prefecture level city in Shandong
Power Saving King Shop	King of Power Saving	Works well and as described by the seller	Jingdong prefecture level city in Shandong
Power Saving King Shop	King of Power Saving	Power saving is proper! It's good.	Jingdong prefecture level city in Shandong
Power Saving King Shop	King of Power Saving	Power saving. Works well. Not bad.	Jingdong prefecture level city in Shandong
Power Saving King Shop	King of Power Saving	Satisfied with power saving	Jingdong prefecture level city in Shandong
Power Saving King Shop	King of Power Saving	Received intact, open a super good look beautiful and generous, ~ bit of high quality is recommended to buy, the need for friends rest assured that the next order	Jingdong prefecture level city in Shandong
Power Saving King Shop	King of Power Saving	Very good to use, friends introduced, save electricity and money, long-lasting products cost-effective, large factory production, good quality, fine workmanship cost-effective!	Jingdong prefecture level city in Shandong
Power Saving King Shop	King of Power Saving	The power saving effect is very obvious, trying to pay 30% less electricity bill in a month than before, hahaha	Jingdong prefecture level city in Shandong
Power Saving King Shop	King of Power Saving	It's such a power saver to use this, it saves money on kids snacks for a month. Kudos!	Jingdong prefecture level city in Shandong
Power Saving King Shop	King of Power Saving	Plug and play, stable voltage, the screen has a display when working, this winter home appliances, need more electricity, just buy to use, save electricity and save money, with instructions, not too big and does not take up space, the quality is very good, and the seller describes exactly the same!	Jingdong prefecture level city in Shandong
Power Saving King Shop	King of Power Saving	A must have for opening a store. Save over a thousand dollars a month on your electric bill	Jingdong prefecture level city in Shandong
Power Saving King Shop	King of Power Saving	Received the goods, the first time to unpack, the quality is very good, with the seller's description, completely exceeded my expectations, the packaging is very careful, very strict, in general this is a very satisfied with the shopping	Jingdong prefecture level city in Shandong
Power Saving King Shop	King of Power Saving	As always, good quality, a very favorite product, great! o(*￣︶￣*)o The price is very affordable, cost-effective, it's considered affordable good, will repurchase!	Jingdong prefecture level city in Shandong
Power Saving King Shop	King of Power Saving	Very satisfied with a shopping, good business service, fast shipping, logistics is also very powerful, received very good, buy assured, use peace of mind!	Jingdong prefecture level city in Shandong
Power Saving King Shop	King of Power Saving	The first time to buy online, but also look at the evaluation before buying, the quality is not bad, but also very cheap, the need for friends can rest assured to buy!	Jingdong prefecture level city in Shandong
Power Saving King Shop	King of Power Saving	Better than expected, feel value for money, very satisfied with a shopping, need next time will come back!	Jingdong prefecture level city in Shandong
Power Saving King Shop	King of Power Saving	Really super like, very supportive, very good quality, exactly as described by the seller, very satisfied!	Jingdong prefecture level city in Shandong
Power Saving King Shop	King of Power Saving	It is genuine, effective, really saves power, affordable, tightly packed, fast shipping, very satisfied.	Jingdong prefecture level city in Shandong
Power Saving King Shop	King of Power Saving	Good quality, fine workmanship, is genuine and business description of the same, good value for money, fast logistics, very satisfied	Jingdong prefecture level city in Shandong
Power Saving King Shop	King of Power Saving	Saver received, very good, installation set up very well, very good, the effect is very good, recommend that the pro buy	Jingdong prefecture level city in Shandong

Power Saving King Shop	King of Power Saving	Electromagnetic good, high power convenient, enough force, the price is right it, quality can be 😀 😀 😀 😀 😀	Jingdong prefecture level city in Shandong
Power Saving King Shop	King of Power Saving	First-class quality, packaging is also good, logistics is very fast, and the price is reasonable, very affordable, I like it!	Jingdong prefecture level city in Shandong
Power Saving King Shop	King of Power Saving	It's great, it's the kind of style you want, and it arrived quickly, thank you boss, 5 stars for the service!	Jingdong prefecture level city in Shandong
Power Saving King Shop	King of Power Saving	Packaging is very good, the use of the effect is quite good, the store has a good service attitude, trustworthy	Jingdong prefecture level city in Shandong
Power Saving King Shop	King of Power Saving	The quality is very good, it works well, very satisfied, the price is affordable, it's so great value, it's worth recommending.	Jingdong prefecture level city in Shandong
Power Saving King Shop	King of Power Saving	The baby description matches the actual item very well! The quality is awesome! Will be back to patronize again in the future!	Jingdong prefecture level city in Shandong
Power Saving King Shop	King of Power Saving	Ran the price is not high try to buy the attitude, did not expect the effect is really good, good!	Jingdong prefecture level city in Shandong
Power Saving King Shop	King of Power Saving	Power Saving King power is quite large, the quality of the leaf is quite good, the price of buying a very good deal, must be praised!	Jingdong prefecture level city in Shandong
Power Saving King Shop	King of Power Saving	Good product quality is the king of power-saving products trustworthy as always good to recommend!	Jingdong prefecture level city in Shandong
Power Saving King Shop	King of Power Saving	Works great, very affordable, shipped pretty fast, comfortable to use	Jingdong prefecture level city in Shandong
Power Saving King Shop	King of Power Saving	Shipping was pretty fast and affordable, not bad 👍	Jingdong prefecture level city in Shandong
Power Saving King Shop	King of Power Saving	Digital voltage display, voltage at a glance, plug and play, safe and simple!	Jingdong prefecture level city in Shandong
Power Saving King Shop	King of Power Saving	Quality assurance, very good, affordable, logistics is also very fast, I'm very satisfied with the	Jingdong prefecture level city in Shandong
Power Saving King Shop	King of Power Saving	The quality is very good, the first time I received the goods to open the first time to see, it is very good, very satisfied	Jingdong prefecture level city in Shandong
Power Saving King Shop	King of Power Saving	Power Saving King used, the effect is good, the price is affordable, very good, is the genuine article	Jingdong prefecture level city in Shandong
Power Saving King Shop	King of Power Saving	Good quality, fine workmanship, good appearance, time is very accurate, satisfied.	Jingdong prefecture level city in Shandong
Power Saving King Shop	King of Power Saving	I purposely used it for two days before commenting, which is responsible for myself and everyone else!	Jingdong prefecture level city in Shandong
Power Saving King Shop	King of Power Saving	Easy to install, easy to operate, helped our family save a lot of electricity this summer	Jingdong prefecture level city in Shandong
Power Saving King Shop	King of Power Saving	Bringing power savings into millions of homes. Still a great gadget. Pretty good.	Jingdong prefecture level city in Shandong
Power Saving King Shop	King of Power Saving	Digital liquid crystal display to see the clear intuitive, very formal workmanship is not rough	Jingdong prefecture level city in Shandong
Power Saving King Shop	King of Power Saving	Received. Great quality, fast shipping from the store and not too expensive!	Jingdong prefecture level city in Shandong
Power Saving King Shop	King of Power Saving	Bought myself a knuckle regulator and it was easy to use and the price was right.	Jingdong prefecture level city in Shandong
Power Saving King Shop	King of Power Saving	Received, good, powerful, good, very full of a shopping	Jingdong prefecture level city in Shandong
Power Saving King Shop	King of Power Saving	High power, consistent with the seller's description, powerful, cost-effective	Jingdong prefecture level city in Shandong
Power Saving King Shop	King of Power Saving	It works very well and also saves power at an affordable price a great deal.	Jingdong prefecture level city in Shandong
Power Saving King Shop	King of Power Saving	Good quality, good can recommend to friends to buy.	Jingdong prefecture level city in Shandong
Power Saving King Shop	King of Power Saving	Will repurchase, quality is guaranteed, safe and secure purchase.	Jingdong prefecture level city in Shandong
Power Saving King Shop	King of Power Saving	The logistics is very fast, the thing is also good to use, energy saving power rely on it!	Jingdong prefecture level city in Shandong
Power Saving King Shop	King of Power Saving	Power Saver is good quality and works very well, worth buying!	Jingdong prefecture level city in Shandong
Power Saving King Shop	King of Power Saving	The quality is very good is genuine, cost-effective recommended	Jingdong prefecture level city in Shandong
Power Saving King Shop	King of Power Saving	Quality Assurance. Favorable price. Very practical and durable	Jingdong prefecture level city in Shandong
Power Saving King Shop	King of Power Saving	Good value for money. Conscientious businessman. Recommended to everyone	Jingdong prefecture level city in Shandong
Power Saving King Shop	King of Power Saving	Buy it for your rental apartment and never worry about your landlord charging you too much.	Jingdong prefecture level city in Shandong
Power Saving King Shop	King of Power Saving	Nice and affordable, will repurchase next time	Jingdong prefecture level city in Shandong
Power Saving King Shop	King of Power Saving	It's very energy efficient. I've been using it for a few days now.	Jingdong prefecture level city in Shandong
Power Saving King Shop	King of Power Saving	Good quality, high power easy to use	Jingdong prefecture level city in Shandong
Power Saving King Shop	King of Power Saving	Power saving little helper. Deserves a favorable review.	Jingdong prefecture level city in Shandong
Power Saving King Shop	King of Power Saving	Great value for money, great value for money.	Jingdong prefecture level city in Shandong
Power Saving King Shop	King of Power Saving	Works well and as described by the seller	Jingdong prefecture level city in Shandong
Power Saving King Shop	King of Power Saving	Power saving is proper! It's good.	Jingdong prefecture level city in Shandong
Power Saving King Shop	King of Power Saving	Power saving. Works well. Not bad.	Jingdong prefecture level city in Shandong
Power Saving King Shop	King of Power Saving	Satisfied with power saving	Jingdong prefecture level city in Shandong
Power Saving King Shop	King of Power Saving	Received intact, open a super good look beautiful and generous, ~ bit of high quality is recommended to buy, the need for friends rest assured that the next order	Jingdong prefecture level city in Shandong
Power Saving King Shop	King of Power Saving	Very good to use, friends introduced, save electricity and money, long-lasting products cost-effective, large factory production, good quality, fine workmanship cost-effective!	Jingdong prefecture level city in Shandong
Power Saving King Shop	King of Power Saving	The power saving effect is very obvious, trying to pay 30% less electricity bill in a month than before, hahaha	Jingdong prefecture level city in Shandong
Power Saving King Shop	King of Power Saving	It's such a power saver to use this, it saves money on kids snacks for a month. Kudos!	Jingdong prefecture level city in Shandong
Power Saving King Shop	King of Power Saving	Plug and play, stable voltage, the screen has a display when working, this winter home appliances, need more electricity, just buy to use, save electricity and save money, with instructions, not too big and does not take up space, the quality is very good, and the seller describes exactly the same!	Jingdong prefecture level city in Shandong
Power Saving King Shop	King of Power Saving	A must have for opening a store. Save over a thousand dollars a month on your electric bill	Jingdong prefecture level city in Shandong
Power Saving King Shop	King of Power Saving	Received the goods, the first time to unpack, the quality is very good, with the seller's description, completely exceeded my expectations, the packaging is very careful, very strict, in general this is a very satisfied with the shopping	Jingdong prefecture level city in Shandong
Power Saving King Shop	King of Power Saving	As always, good quality, a very favorite product, great! o(*￣︶￣*)o The price is very affordable, cost-effective, it's considered affordable good, will repurchase!	Jingdong prefecture level city in Shandong

Power Saving King Shop	King of Power Saving	Very satisfied with a shopping, good business service, fast shipping, logistics is also very powerful, received very good, buy assured, use peace of mind!	Jingdong prefecture level city in Shandong
Power Saving King Shop	King of Power Saving	The first time to buy online, but also look at the evaluation before buying, the quality is not bad, but also very cheap, the need for friends can rest assured to buy!	Jingdong prefecture level city in Shandong
Power Saving King Shop	King of Power Saving	Better than expected, feel value for money, very satisfied with a shopping, need next time will come back!	Jingdong prefecture level city in Shandong
Power Saving King Shop	King of Power Saving	Really super like, very supportive, very good quality, exactly as described by the seller, very satisfied!	Jingdong prefecture level city in Shandong
Power Saving King Shop	King of Power Saving	It is genuine, effective, really saves power, affordable, tightly packed, fast shipping, very satisfied.	Jingdong prefecture level city in Shandong
Power Saving King Shop	King of Power Saving	Good quality, fine workmanship, is genuine and business description of the same, good value for money, fast logistics, very satisfied	Jingdong prefecture level city in Shandong
Power Saving King Shop	King of Power Saving	Saver received, very good, installation set up very well, very good, the effect is very good, recommend that the pro buy	Jingdong prefecture level city in Shandong
Power Saving King Shop	King of Power Saving	Electromagnetic good, high power convenient, enough force, the price is right it, quality can be 😃 😃 😃 😃 😃	Jingdong prefecture level city in Shandong
Power Saving King Shop	King of Power Saving	First-class quality, packaging is also good, logistics is very fast, and the price is reasonable, very affordable, I like it!	Jingdong prefecture level city in Shandong
Power Saving King Shop	King of Power Saving	It's great, it's the kind of style you want, and it arrived quickly, thank you boss, 5 stars for the service!	Jingdong prefecture level city in Shandong
Power Saving King Shop	King of Power Saving	Packaging is very good, the use of the effect is quite good, the store has a good service attitude, trustworthy	Jingdong prefecture level city in Shandong
Power Saving King Shop	King of Power Saving	The quality is very good, it works well, very satisfied, the price is affordable, it's so great value, it's worth recommending.	Jingdong prefecture level city in Shandong
Power Saving King Shop	King of Power Saving	The baby description matches the actual item very well! The quality is awesome! Will be back to patronize again in the future!	Jingdong prefecture level city in Shandong
Power Saving King Shop	King of Power Saving	Ran the price is not high try to buy the attitude, did not expect the effect is really good, good!	Jingdong prefecture level city in Shandong
Power Saving King Shop	King of Power Saving	Power Saving King power is quite large, the quality of the leaf is quite good, the price of buying a very good deal, must be praised!	Jingdong prefecture level city in Shandong
Power Saving King Shop	King of Power Saving	Good product quality is the king of power-saving products trustworthy as always good to recommend!	Jingdong prefecture level city in Shandong
Power Saving King Shop	King of Power Saving	Works great, very affordable, shipped pretty fast, comfortable to use	Jingdong prefecture level city in Shandong
Power Saving King Shop	King of Power Saving	Shipping was pretty fast and affordable, not bad 👍	Jingdong prefecture level city in Shandong
Power Saving King Shop	King of Power Saving	Digital voltage display, voltage at a glance, plug and play, safe and simple!	Jingdong prefecture level city in Shandong
Power Saving King Shop	King of Power Saving	Quality assurance, very good, affordable, logistics is also very fast, I'm very satisfied with the	Jingdong prefecture level city in Shandong
Power Saving King Shop	King of Power Saving	The quality is very good, the first time I received the goods to open the first time to see, it is very good, very satisfied	Jingdong prefecture level city in Shandong
Power Saving King Shop	King of Power Saving	Power Saving King used, the effect is good, the price is affordable, very good, is the genuine article	Jingdong prefecture level city in Shandong
Power Saving King Shop	King of Power Saving	Good quality, fine workmanship, good appearance, time is very accurate, satisfied.	Jingdong prefecture level city in Shandong
Power Saving King Shop	King of Power Saving	I purposely used it for two days before commenting, which is responsible for myself and everyone else!	Jingdong prefecture level city in Shandong
Power Saving King Shop	King of Power Saving	Easy to install, easy to operate, helped our family save a lot of electricity this summer	Jingdong prefecture level city in Shandong
Power Saving King Shop	King of Power Saving	Bringing power savings into millions of homes. Still a great gadget. Pretty good.	Jingdong prefecture level city in Shandong
Power Saving King Shop	King of Power Saving	Digital liquid crystal display to see the clear intuitive, very formal workmanship is not rough	Jingdong prefecture level city in Shandong
Power Saving King Shop	King of Power Saving	Received. Great quality, fast shipping from the store and not too expensive!	Jingdong prefecture level city in Shandong
Power Saving King Shop	King of Power Saving	Bought myself a knuckle regulator and it was easy to use and the price was right.	Jingdong prefecture level city in Shandong
Power Saving King Shop	King of Power Saving	Received, good, powerful, good, very full of a shopping	Jingdong prefecture level city in Shandong
Power Saving King Shop	King of Power Saving	High power, consistent with the seller's description, powerful, cost-effective	Jingdong prefecture level city in Shandong
Power Saving King Shop	King of Power Saving	It works very well and also saves power at an affordable price a great deal.	Jingdong prefecture level city in Shandong
Power Saving King Shop	King of Power Saving	Good quality, good can recommend to friends to buy.	Jingdong prefecture level city in Shandong
Power Saving King Shop	King of Power Saving	Will repurchase, quality is guaranteed, safe and secure purchase.	Jingdong prefecture level city in Shandong
Power Saving King Shop	King of Power Saving	The logistics is very fast, the thing is also good to use, energy saving power rely on it!	Jingdong prefecture level city in Shandong
Power Saving King Shop	King of Power Saving	Power Saver is good quality and works very well, worth buying!	Jingdong prefecture level city in Shandong
Power Saving King Shop	King of Power Saving	The quality is very good is genuine, cost-effective recommended	Jingdong prefecture level city in Shandong
Power Saving King Shop	King of Power Saving	Quality Assurance. Favorable price. Very practical and durable	Jingdong prefecture level city in Shandong
Power Saving King Shop	King of Power Saving	Good value for money. Conscientious businessman. Recommended to everyone	Jingdong prefecture level city in Shandong
Power Saving King Shop	King of Power Saving	Buy it for your rental apartment and never worry about your landlord charging you too much.	Jingdong prefecture level city in Shandong
Power Saving King Shop	King of Power Saving	Nice and affordable, will repurchase next time	Jingdong prefecture level city in Shandong
Power Saving King Shop	King of Power Saving	It's very energy efficient. I've been using it for a few days now.	Jingdong prefecture level city in Shandong
Power Saving King Shop	King of Power Saving	Good quality, high power easy to use	Jingdong prefecture level city in Shandong
Power Saving King Shop	King of Power Saving	Power saving little helper. Deserves a favorable review.	Jingdong prefecture level city in Shandong
Power Saving King Shop	King of Power Saving	Great value for money, great value for money.	Jingdong prefecture level city in Shandong
Power Saving King Shop	King of Power Saving	Works well and as described by the seller	Jingdong prefecture level city in Shandong
Power Saving King Shop	King of Power Saving	Power saving is proper! It's good.	Jingdong prefecture level city in Shandong
Power Saving King Shop	King of Power Saving	Power saving. Works well. Not bad.	Jingdong prefecture level city in Shandong
Power Saving King Shop	King of Power Saving	Satisfied with power saving	Jingdong prefecture level city in Shandong
Power Saving King Shop	King of Power Saving	Received intact, open a super good look beautiful and generous, ~ bit of high quality is recommended to buy, the need for friends rest assured that the next order	Jingdong prefecture level city in Shandong
Power Saving King Shop	King of Power Saving	Very good to use, friends introduced, save electricity and money, long-lasting products cost-effective, large factory production, good quality, fine workmanship cost-effective!	Jingdong prefecture level city in Shandong

Power Saving King Shop	King of Power Saving	The power saving effect is very obvious, trying to pay 30% less electricity bill in a month than before, hahaha	Jingdong prefecture level city in Shandong
Power Saving King Shop	King of Power Saving	It's such a power saver to use this, it saves money on kids snacks for a month. Kudos!	Jingdong prefecture level city in Shandong
Power Saving King Shop	King of Power Saving	Plug and play, stable voltage, the screen has a display when working, this winter home appliances, need more electricity, just buy to use, save electricity and save money, with instructions, not too big and does not take up space, the quality is very good, and the seller describes exactly the same!	Jingdong prefecture level city in Shandong
Power Saving King Shop	King of Power Saving	A must have for opening a store. Save over a thousand dollars a month on your electric bill	Jingdong prefecture level city in Shandong
Power Saving King Shop	King of Power Saving	Received the goods, the first time to unpack, the quality is very good, with the seller's description, completely exceeded my expectations, the packaging is very careful, very strict, in general this is a very satisfied with the shopping	Jingdong prefecture level city in Shandong
Power Saving King Shop	King of Power Saving	As always, good quality, a very favorite product, great! o(*￣︶￣*)o The price is very affordable, cost-effective, it's considered affordable good, will repurchase!	Jingdong prefecture level city in Shandong
Power Saving King Shop	King of Power Saving	Very satisfied with a shopping, good business service, fast shipping, logistics is also very powerful, received very good, buy assured, use peace of mind!	Jingdong prefecture level city in Shandong
Power Saving King Shop	King of Power Saving	The first time to buy online, but also look at the evaluation before buying, the quality is not bad, but also very cheap, the need for friends can rest assured to buy!	Jingdong prefecture level city in Shandong
Power Saving King Shop	King of Power Saving	Better than expected, feel value for money, very satisfied with a shopping, need next time will come back!	Jingdong prefecture level city in Shandong
Power Saving King Shop	King of Power Saving	Really super like, very supportive, very good quality, exactly as described by the seller, very satisfied!	Jingdong prefecture level city in Shandong
Power Saving King Shop	King of Power Saving	It is genuine, effective, really saves power, affordable, tightly packed, fast shipping, very satisfied.	Jingdong prefecture level city in Shandong
Power Saving King Shop	King of Power Saving	Good quality, fine workmanship, is genuine and business description of the same, good value for money, fast logistics, very satisfied	Jingdong prefecture level city in Shandong
Power Saving King Shop	King of Power Saving	Saver received, very good, installation set up very well, very good, the effect is very good, recommend that the pro buy	Jingdong prefecture level city in Shandong
Power Saving King Shop	King of Power Saving	Electromagnetic good, high power convenient, enough force, the price is right it, quality can be ☺ ☺ ☺ ☺ ☺	Jingdong prefecture level city in Shandong
Power Saving King Shop	King of Power Saving	First-class quality, packaging is also good, logistics is very fast, and the price is reasonable, very affordable, I like it!	Jingdong prefecture level city in Shandong
Power Saving King Shop	King of Power Saving	It's great, it's the kind of style you want, and it arrived quickly, thank you boss, 5 stars for the service!	Jingdong prefecture level city in Shandong
Power Saving King Shop	King of Power Saving	Packaging is very good, the use of the effect is quite good, the store has a good service attitude, trustworthy	Jingdong prefecture level city in Shandong
Power Saving King Shop	King of Power Saving	The quality is very good, it works well, very satisfied, the price is affordable, it's so great value, it's worth recommending.	Jingdong prefecture level city in Shandong
Power Saving King Shop	King of Power Saving	The baby description matches the actual item very well! The quality is awesome! Will be back to patronize again in the future!	Jingdong prefecture level city in Shandong
Power Saving King Shop	King of Power Saving	Ran the price is not high try to buy the attitude, did not expect the effect is really good, good!	Jingdong prefecture level city in Shandong
Power Saving King Shop	King of Power Saving	Power Saving King power is quite large, the quality of the leaf is quite good, the price of buying a very good deal, must be praised!	Jingdong prefecture level city in Shandong
Power Saving King Shop	King of Power Saving	Good product quality is the king of power-saving products trustworthy as always good to recommend!	Jingdong prefecture level city in Shandong
Power Saving King Shop	King of Power Saving	Works great, very affordable, shipped pretty fast, comfortable to use	Jingdong prefecture level city in Shandong
Power Saving King Shop	King of Power Saving	Shipping was pretty fast and affordable, not bad 👍	Jingdong prefecture level city in Shandong
Power Saving King Shop	King of Power Saving	Digital voltage display, voltage at a glance, plug and play, safe and simple!	Jingdong prefecture level city in Shandong
Power Saving King Shop	King of Power Saving	Quality assurance, very good, affordable, logistics is also very fast, I'm very satisfied with the	Jingdong prefecture level city in Shandong
Power Saving King Shop	King of Power Saving	The quality is very good, the first time I received the goods to open the first time to see, it is very good, very satisfied	Jingdong prefecture level city in Shandong
Power Saving King Shop	King of Power Saving	Power Saving King used, the effect is good, the price is affordable, very good, is the genuine article	Jingdong prefecture level city in Shandong
Power Saving King Shop	King of Power Saving	Good quality, fine workmanship, good appearance, time is very accurate, satisfied.	Jingdong prefecture level city in Shandong
Power Saving King Shop	King of Power Saving	I purposely used it for two days before commenting, which is responsible for myself and everyone else!	Jingdong prefecture level city in Shandong
Power Saving King Shop	King of Power Saving	Easy to install, easy to operate, helped our family save a lot of electricity this summer	Jingdong prefecture level city in Shandong
Power Saving King Shop	King of Power Saving	Bringing power savings into millions of homes. Still a great gadget. Pretty good.	Jingdong prefecture level city in Shandong
Power Saving King Shop	King of Power Saving	Digital liquid crystal display to see the clear intuitive, very formal workmanship is not rough	Jingdong prefecture level city in Shandong
Power Saving King Shop	King of Power Saving	Received. Great quality, fast shipping from the store and not too expensive!	Jingdong prefecture level city in Shandong
Power Saving King Shop	King of Power Saving	Bought myself a knuckle regulator and it was easy to use and the price was right.	Jingdong prefecture level city in Shandong
Power Saving King Shop	King of Power Saving	Received, good, powerful, good, very full of a shopping	Jingdong prefecture level city in Shandong
Power Saving King Shop	King of Power Saving	High power, consistent with the seller's description, powerful, cost-effective	Jingdong prefecture level city in Shandong
Power Saving King Shop	King of Power Saving	It works very well and also saves power at an affordable price a great deal.	Jingdong prefecture level city in Shandong
Power Saving King Shop	King of Power Saving	Good quality, good can recommend to friends to buy.	Jingdong prefecture level city in Shandong
Power Saving King Shop	King of Power Saving	Will repurchase, quality is guaranteed, safe and secure purchase.	Jingdong prefecture level city in Shandong
Power Saving King Shop	King of Power Saving	The logistics is very fast, the thing is also good to use, energy saving power rely on it!	Jingdong prefecture level city in Shandong
Power Saving King Shop	King of Power Saving	Power Saver is good quality and works very well, worth buying!	Jingdong prefecture level city in Shandong
Power Saving King Shop	King of Power Saving	The quality is very good is genuine, cost-effective recommended	Jingdong prefecture level city in Shandong
Power Saving King Shop	King of Power Saving	Quality Assurance. Favorable price. Very practical and durable	Jingdong prefecture level city in Shandong
Power Saving King Shop	King of Power Saving	Good value for money. Conscientious businessman. Recommended to everyone	Jingdong prefecture level city in Shandong
Power Saving King Shop	King of Power Saving	Buy it for your rental apartment and never worry about your landlord charging you too much.	Jingdong prefecture level city in Shandong
Power Saving King Shop	King of Power Saving	Nice and affordable, will repurchase next time	Jingdong prefecture level city in Shandong
Power Saving King Shop	King of Power Saving	It's very energy efficient. I've been using it for a few days now.	Jingdong prefecture level city in Shandong
Power Saving King Shop	King of Power Saving	Good quality, high power easy to use	Jingdong prefecture level city in Shandong
Power Saving King Shop	King of Power Saving	Power saving little helper. Deserves a favorable review.	Jingdong prefecture level city in Shandong

Power Saving King Shop	King of Power Saving	Great value for money, great value for money.	Jingdong prefecture level city in Shandong
Power Saving King Shop	King of Power Saving	Works well and as described by the seller	Jingdong prefecture level city in Shandong
Power Saving King Shop	King of Power Saving	Power saving is proper! It's good.	Jingdong prefecture level city in Shandong
Power Saving King Shop	King of Power Saving	Power saving. Works well. Not bad.	Jingdong prefecture level city in Shandong
Power Saving King Shop	King of Power Saving	Satisfied with power saving	Jingdong prefecture level city in Shandong
Power Saving King Shop	King of Power Saving	Received intact, open a super good look beautiful and generous, ~ bit of high quality is recommended to buy, the need for friends rest assured that the next order	Jingdong prefecture level city in Shandong
Power Saving King Shop	King of Power Saving	Very good to use, friends introduced, save electricity and money, long-lasting products cost-effective, large factory production, good quality, fine workmanship cost-effective!	Jingdong prefecture level city in Shandong
Power Saving King Shop	King of Power Saving	The power saving effect is very obvious, trying to pay 30% less electricity bill in a month than before, hahaha	Jingdong prefecture level city in Shandong
Power Saving King Shop	King of Power Saving	It's such a power saver to use this, it saves money on kids snacks for a month. Kudos!	Jingdong prefecture level city in Shandong
Power Saving King Shop	King of Power Saving	Plug and play, stable voltage, the screen has a display when working, this winter home appliances, need more electricity, just buy to use, save electricity and save money, with instructions, not too big and does not take up space, the quality is very good, and the seller describes exactly the same!	Jingdong prefecture level city in Shandong
Power Saving King Shop	King of Power Saving	A must have for opening a store. Save over a thousand dollars a month on your electric bill	Jingdong prefecture level city in Shandong
Power Saving King Shop	King of Power Saving	Received the goods, the first time to unpack, the quality is very good, with the seller's description, completely exceeded my expectations, the packaging is very careful, very strict, in general this is a very satisfied with the shopping	Jingdong prefecture level city in Shandong
Power Saving King Shop	King of Power Saving	As always, good quality, a very favorite product, great! o(*￣︶￣*)o The price is very affordable, cost-effective, it's considered affordable good, will repurchase!	Jingdong prefecture level city in Shandong
Power Saving King Shop	King of Power Saving	Very satisfied with a shopping, good business service, fast shipping, logistics is also very powerful, received very good, buy assured, use peace of mind!	Jingdong prefecture level city in Shandong
Power Saving King Shop	King of Power Saving	The first time to buy online, but also look at the evaluation before buying, the quality is not bad, but also very cheap, the need for friends can rest assured to buy!	Jingdong prefecture level city in Shandong
Power Saving King Shop	King of Power Saving	Better than expected, feel value for money, very satisfied with a shopping, need next time will come back!	Jingdong prefecture level city in Shandong
Power Saving King Shop	King of Power Saving	Really super like, very supportive, very good quality, exactly as described by the seller, very satisfied!	Jingdong prefecture level city in Shandong
Power Saving King Shop	King of Power Saving	It is genuine, effective, really saves power, affordable, tightly packed, fast shipping, very satisfied.	Jingdong prefecture level city in Shandong
Power Saving King Shop	King of Power Saving	Good quality, fine workmanship, is genuine and business description of the same, good value for money, fast logistics, very satisfied	Jingdong prefecture level city in Shandong
Power Saving King Shop	King of Power Saving	Saver received, very good, installation set up very well, very good, the effect is very good, recommend that the pro buy	Jingdong prefecture level city in Shandong
Power Saving King Shop	King of Power Saving	Electromagnetic good, high power convenient, enough force, the price is right it, quality can be 😀 😀 😀 😀 😀	Jingdong prefecture level city in Shandong
Power Saving King Shop	King of Power Saving	First-class quality, packaging is also good, logistics is very fast, and the price is reasonable, very affordable, I like it!	Jingdong prefecture level city in Shandong
Power Saving King Shop	King of Power Saving	It's great, it's the kind of style you want, and it arrived quickly, thank you boss, 5 stars for the service!	Jingdong prefecture level city in Shandong
Power Saving King Shop	King of Power Saving	Packaging is very good, the use of the effect is quite good, the store has a good service attitude, trustworthy	Jingdong prefecture level city in Shandong
Power Saving King Shop	King of Power Saving	The quality is very good, it works well, very satisfied, the price is affordable, it's so great value, it's worth recommending.	Jingdong prefecture level city in Shandong
Power Saving King Shop	King of Power Saving	The baby description matches the actual item very well! The quality is awesome! Will be back to patronize again in the future!	Jingdong prefecture level city in Shandong
Power Saving King Shop	King of Power Saving	Ran the price is not high try to buy the attitude, did not expect the effect is really good, good!	Jingdong prefecture level city in Shandong
Power Saving King Shop	King of Power Saving	Power Saving King power is quite large, the quality of the leaf is quite good, the price of buying a very good deal, must be praised!	Jingdong prefecture level city in Shandong
Power Saving King Shop	King of Power Saving	Good product quality is the king of power-saving products trustworthy as always good to recommend!	Jingdong prefecture level city in Shandong
Power Saving King Shop	King of Power Saving	Works great, very affordable, shipped pretty fast, comfortable to use	Jingdong prefecture level city in Shandong
Power Saving King Shop	King of Power Saving	Shipping was pretty fast and affordable, not bad 👍	Jingdong prefecture level city in Shandong
Power Saving King Shop	King of Power Saving	Digital voltage display, voltage at a glance, plug and play, safe and simple!	Jingdong prefecture level city in Shandong
Power Saving King Shop	King of Power Saving	Quality assurance, very good, affordable, logistics is also very fast, I'm very satisfied with the	Jingdong prefecture level city in Shandong
Power Saving King Shop	King of Power Saving	The quality is very good, the first time I received the goods to open the first time to see, it is very good, very satisfied	Jingdong prefecture level city in Shandong
Power Saving King Shop	King of Power Saving	Power Saving King used, the effect is good, the price is affordable, very good, is the genuine article	Jingdong prefecture level city in Shandong
Power Saving King Shop	King of Power Saving	Good quality, fine workmanship, good appearance, time is very accurate, satisfied.	Jingdong prefecture level city in Shandong
Power Saving King Shop	King of Power Saving	I purposely used it for two days before commenting, which is responsible for myself and everyone else!	Jingdong prefecture level city in Shandong
Power Saving King Shop	King of Power Saving	Easy to install, easy to operate, helped our family save a lot of electricity this summer	Jingdong prefecture level city in Shandong
Power Saving King Shop	King of Power Saving	Bringing power savings into millions of homes. Still a great gadget. Pretty good.	Jingdong prefecture level city in Shandong
Power Saving King Shop	King of Power Saving	Digital liquid crystal display to see the clear intuitive, very formal workmanship is not rough	Jingdong prefecture level city in Shandong
Power Saving King Shop	King of Power Saving	Received. Great quality, fast shipping from the store and not too expensive!	Jingdong prefecture level city in Shandong
Power Saving King Shop	King of Power Saving	Bought myself a knuckle regulator and it was easy to use and the price was right.	Jingdong prefecture level city in Shandong
Power Saving King Shop	King of Power Saving	Received, good, powerful, good, very full of a shopping	Jingdong prefecture level city in Shandong
Power Saving King Shop	King of Power Saving	High power, consistent with the seller's description, powerful, cost-effective	Jingdong prefecture level city in Shandong
Power Saving King Shop	King of Power Saving	It works very well and also saves power at an affordable price a great deal.	Jingdong prefecture level city in Shandong
Power Saving King Shop	King of Power Saving	Good quality, good can recommend to friends to buy.	Jingdong prefecture level city in Shandong
Power Saving King Shop	King of Power Saving	Will repurchase, quality is guaranteed, safe and secure purchase.	Jingdong prefecture level city in Shandong
Power Saving King Shop	King of Power Saving	The logistics is very fast, the thing is also good to use, energy saving power rely on it!	Jingdong prefecture level city in Shandong
Power Saving King Shop	King of Power Saving	Power Saver is good quality and works very well, worth buying!	Jingdong prefecture level city in Shandong
Power Saving King Shop	King of Power Saving	The quality is very good is genuine, cost-effective recommended	Jingdong prefecture level city in Shandong

Power Saving King Shop	King of Power Saving	Quality Assurance. Favorable price. Very practical and durable	Jingdong prefecture level city in Shandong
Power Saving King Shop	King of Power Saving	Good value for money. Conscientious businessman. Recommended to everyone	Jingdong prefecture level city in Shandong
Power Saving King Shop	King of Power Saving	Buy it for your rental apartment and never worry about your landlord charging you too much.	Jingdong prefecture level city in Shandong
Power Saving King Shop	King of Power Saving	Nice and affordable, will repurchase next time	Jingdong prefecture level city in Shandong
Power Saving King Shop	King of Power Saving	It's very energy efficient. I've been using it for a few days now.	Jingdong prefecture level city in Shandong
Power Saving King Shop	King of Power Saving	Good quality, high power easy to use	Jingdong prefecture level city in Shandong
Power Saving King Shop	King of Power Saving	Great value for money, great value for money.	Jingdong prefecture level city in Shandong
Power Saving King Shop	King of Power Saving	Power saving little helper. Deserves a favorable review.	Jingdong prefecture level city in Shandong
Power Saving King Shop	King of Power Saving	Works well and as described by the seller	Jingdong prefecture level city in Shandong
Power Saving King Shop	King of Power Saving	Power saving is proper! It's good.	Jingdong prefecture level city in Shandong
Power Saving King Shop	King of Power Saving	Power saving. Works well. Not bad.	Jingdong prefecture level city in Shandong
Power Saving King Shop	King of Power Saving	Satisfied with power saving	Jingdong prefecture level city in Shandong
Power Saving King Shop	King of Power Saving	Received intact, open a super good look beautiful and generous, ~ bit of high quality is recommended to buy, the need for friends rest assured that the next order	Jingdong prefecture level city in Shandong
Power Saving King Shop	King of Power Saving	Very good to use, friends introduced, save electricity and money, long-lasting products cost-effective, large factory production, good quality, fine workmanship cost-effective!	Jingdong prefecture level city in Shandong
Power Saving King Shop	King of Power Saving	The power saving effect is very obvious, trying to pay 30% less electricity bill in a month than before, hahaha	Jingdong prefecture level city in Shandong
Power Saving King Shop	King of Power Saving	It's such a power saver to use this, it saves money on kids snacks for a month. Kudos!	Jingdong prefecture level city in Shandong
Power Saving King Shop	King of Power Saving	Plug and play, stable voltage, the screen has a display when working, this winter home appliances, need more electricity, just buy to use, save electricity and save money, with instructions, not too big and does not take up space, the quality is very good, and the seller describes exactly the same!	Jingdong prefecture level city in Shandong
Power Saving King Shop	King of Power Saving	A must have for opening a store. Save over a thousand dollars a month on your electric bill	Jingdong prefecture level city in Shandong
Power Saving King Shop	King of Power Saving	Received the goods, the first time to unpack, the quality is very good, with the seller's description, completely exceeded my expectations, the packaging is very careful, very strict, in general this is a very satisfied with the shopping	Jingdong prefecture level city in Shandong
Power Saving King Shop	King of Power Saving	As always, good quality, a very favorite product, great! o(*￣︶￣*)o The price is very affordable, cost-effective, it's considered affordable good, will repurchase!	Jingdong prefecture level city in Shandong
Power Saving King Shop	King of Power Saving	Very satisfied with a shopping, good business service, fast shipping, logistics is also very powerful, received very good, buy assured, use peace of mind!	Jingdong prefecture level city in Shandong
Power Saving King Shop	King of Power Saving	The first time to buy online, but also look at the evaluation before buying, the quality is not bad, but also very cheap, the need for friends can rest assured to buy!	Jingdong prefecture level city in Shandong
Power Saving King Shop	King of Power Saving	Better than expected, feel value for money, very satisfied with a shopping, need next time will come back!	Jingdong prefecture level city in Shandong
Power Saving King Shop	King of Power Saving	Really super like, very supportive, very good quality, exactly as described by the seller, very satisfied!	Jingdong prefecture level city in Shandong
Power Saving King Shop	King of Power Saving	It is genuine, effective, really saves power, affordable, tightly packed, fast shipping, very satisfied.	Jingdong prefecture level city in Shandong
Power Saving King Shop	King of Power Saving	Good quality, fine workmanship, is genuine and business description of the same, good value for money, fast logistics, very satisfied	Jingdong prefecture level city in Shandong
Power Saving King Shop	King of Power Saving	Saver received, very good, installation set up very well, very good, the effect is very good, recommend that the pro buy	Jingdong prefecture level city in Shandong
Power Saving King Shop	King of Power Saving	Electromagnetic good, high power convenient, enough force, the price is right it, quality can be ☺ ☺ ☺ ☺ ☺	Jingdong prefecture level city in Shandong
Power Saving King Shop	King of Power Saving	First-class quality, packaging is also good, logistics is very fast, and the price is reasonable, very affordable, I like it!	Jingdong prefecture level city in Shandong
Power Saving King Shop	King of Power Saving	It's great, it's the kind of style you want, and it arrived quickly, thank you boss, 5 stars for the service!	Jingdong prefecture level city in Shandong
Power Saving King Shop	King of Power Saving	Packaging is very good, the use of the effect is quite good, the store has a good service attitude, trustworthy	Jingdong prefecture level city in Shandong
Power Saving King Shop	King of Power Saving	The quality is very good, it works well, very satisfied, the price is affordable, it's so great value, it's worth recommending.	Jingdong prefecture level city in Shandong
Power Saving King Shop	King of Power Saving	The baby description matches the actual item very well! The quality is awesome! Will be back to patronize again in the future!	Jingdong prefecture level city in Shandong
Power Saving King Shop	King of Power Saving	Ran the price is not high try to buy the attitude, did not expect the effect is really good, good!	Jingdong prefecture level city in Shandong
Power Saving King Shop	King of Power Saving	Power Saving King power is quite large, the quality of the leaf is quite good, the price of buying a very good deal, must be praised!	Jingdong prefecture level city in Shandong
Power Saving King Shop	King of Power Saving	Good product quality is the king of power-saving products trustworthy as always good to recommend!	Jingdong prefecture level city in Shandong
Power Saving King Shop	King of Power Saving	Works great, very affordable, shipped pretty fast, comfortable to use	Jingdong prefecture level city in Shandong
Power Saving King Shop	King of Power Saving	Shipping was pretty fast and affordable, not bad 👍	Jingdong prefecture level city in Shandong
Power Saving King Shop	King of Power Saving	Digital voltage display, voltage at a glance, plug and play, safe and simple!	Jingdong prefecture level city in Shandong
Power Saving King Shop	King of Power Saving	Quality assurance, very good, affordable, logistics is also very fast, I'm very satisfied with the	Jingdong prefecture level city in Shandong
Power Saving King Shop	King of Power Saving	The quality is very good, the first time I received the goods to open the first time to see, it is very good, very satisfied	Jingdong prefecture level city in Shandong
Power Saving King Shop	King of Power Saving	Power Saving King used, the effect is good, the price is affordable, very good, is the genuine article	Jingdong prefecture level city in Shandong
Power Saving King Shop	King of Power Saving	Good quality, fine workmanship, good appearance, time is very accurate, satisfied.	Jingdong prefecture level city in Shandong
Power Saving King Shop	King of Power Saving	I purposely used it for two days before commenting, which is responsible for myself and everyone else!	Jingdong prefecture level city in Shandong
Power Saving King Shop	King of Power Saving	Easy to install, easy to operate, helped our family save a lot of electricity this summer	Jingdong prefecture level city in Shandong
Power Saving King Shop	King of Power Saving	Bringing power savings into millions of homes. Still a great gadget. Pretty good.	Jingdong prefecture level city in Shandong
Power Saving King Shop	King of Power Saving	Digital liquid crystal display to see the clear intuitive, very formal workmanship is not rough	Jingdong prefecture level city in Shandong
Power Saving King Shop	King of Power Saving	Received. Great quality, fast shipping from the store and not too expensive!	Jingdong prefecture level city in Shandong
Power Saving King Shop	King of Power Saving	Bought myself a knuckle regulator and it was easy to use and the price was right.	Jingdong prefecture level city in Shandong
Power Saving King Shop	King of Power Saving	Received, good, powerful, good, very full of a shopping	Jingdong prefecture level city in Shandong

Power Saving King Shop	King of Power Saving	High power, consistent with the seller's description, powerful, cost-effective	Jingdong prefecture level city in Shandong
Power Saving King Shop	King of Power Saving	It works very well and also saves power at an affordable price a great deal.	Jingdong prefecture level city in Shandong
Power Saving King Shop	King of Power Saving	Good quality, good can recommend to friends to buy.	Jingdong prefecture level city in Shandong
Power Saving King Shop	King of Power Saving	Will repurchase, quality is guaranteed, safe and secure purchase.	Jingdong prefecture level city in Shandong
Power Saving King Shop	King of Power Saving	The logistics is very fast, the thing is also good to use, energy saving power rely on it!	Jingdong prefecture level city in Shandong
Power Saving King Shop	King of Power Saving	Power Saver is good quality and works very well, worth buying!	Jingdong prefecture level city in Shandong
Power Saving King Shop	King of Power Saving	The quality is very good is genuine, cost-effective recommended	Jingdong prefecture level city in Shandong
Power Saving King Shop	King of Power Saving	Quality Assurance. Favorable price. Very practical and durable	Jingdong prefecture level city in Shandong
Power Saving King Shop	King of Power Saving	Good value for money. Conscientious businessman. Recommended to everyone	Jingdong prefecture level city in Shandong
Power Saving King Shop	King of Power Saving	Buy it for your rental apartment and never worry about your landlord charging you too much.	Jingdong prefecture level city in Shandong
Power Saving King Shop	King of Power Saving	Nice and affordable, will repurchase next time	Jingdong prefecture level city in Shandong
Power Saving King Shop	King of Power Saving	It's very energy efficient. I've been using it for a few days now.	Jingdong prefecture level city in Shandong
Power Saving King Shop	King of Power Saving	Good quality, high power easy to use	Jingdong prefecture level city in Shandong
Power Saving King Shop	King of Power Saving	Power saving little helper. Deserves a favorable review.	Jingdong prefecture level city in Shandong
Power Saving King Shop	King of Power Saving	Great value for money, great value for money.	Jingdong prefecture level city in Shandong
Power Saving King Shop	King of Power Saving	Works well and as described by the seller	Jingdong prefecture level city in Shandong
Power Saving King Shop	King of Power Saving	Power saving is proper! It's good.	Jingdong prefecture level city in Shandong
Power Saving King Shop	King of Power Saving	Power saving. Works well. Not bad.	Jingdong prefecture level city in Shandong
Power Saving King Shop	King of Power Saving	Satisfied with power saving	Jingdong prefecture level city in Shandong
Power Saving King Shop	King of Power Saving	Received intact, open a super good look beautiful and generous, ~ bit of high quality is recommended to buy, the need for friends rest assured that the next order	Jingdong prefecture level city in Shandong
Power Saving King Shop	King of Power Saving	Very good to use, friends introduced, save electricity and money, long-lasting products cost-effective, large factory production, good quality, fine workmanship cost-effective!	Jingdong prefecture level city in Shandong
Power Saving King Shop	King of Power Saving	The power saving effect is very obvious, trying to pay 30% less electricity bill in a month than before, hahaha	Jingdong prefecture level city in Shandong
Power Saving King Shop	King of Power Saving	It's such a power saver to use this, it saves money on kids snacks for a month. Kudos!	Jingdong prefecture level city in Shandong
Power Saving King Shop	King of Power Saving	Plug and play, stable voltage, the screen has a display when working, this winter home appliances, need more electricity, just buy to use, save electricity and save money, with instructions, not too big and does not take up space, the quality is very good, and the seller describes exactly the same!	Jingdong prefecture level city in Shandong
Power Saving King Shop	King of Power Saving	A must have for opening a store. Save over a thousand dollars a month on your electric bill	Jingdong prefecture level city in Shandong
Power Saving King Shop	King of Power Saving	Received the goods, the first time to unpack, the quality is very good, with the seller's description, completely exceeded my expectations, the packaging is very careful, very strict, in general this is a very satisfied with the shopping	Jingdong prefecture level city in Shandong
Power Saving King Shop	King of Power Saving	As always, good quality, a very favorite product, great! o(*￣︶￣*)o The price is very affordable, cost-effective, it's considered affordable good, will repurchase!	Jingdong prefecture level city in Shandong
Power Saving King Shop	King of Power Saving	Very satisfied with a shopping, good business service, fast shipping, logistics is also very powerful, received very good, buy assured, use peace of mind!	Jingdong prefecture level city in Shandong
Power Saving King Shop	King of Power Saving	The first time to buy online, but also look at the evaluation before buying, the quality is not bad, but also very cheap, the need for friends can rest assured to buy!	Jingdong prefecture level city in Shandong
Power Saving King Shop	King of Power Saving	Better than expected, feel value for money, very satisfied with a shopping, need next time will come back!	Jingdong prefecture level city in Shandong
Power Saving King Shop	King of Power Saving	Really super like, very supportive, very good quality, exactly as described by the seller, very satisfied!	Jingdong prefecture level city in Shandong
Power Saving King Shop	King of Power Saving	It is genuine, effective, really saves power, affordable, tightly packed, fast shipping, very satisfied.	Jingdong prefecture level city in Shandong
Power Saving King Shop	King of Power Saving	Good quality, fine workmanship, is genuine and business description of the same, good value for money, fast logistics, very satisfied	Jingdong prefecture level city in Shandong
Power Saving King Shop	King of Power Saving	Saver received, very good, installation set up very well, very good, the effect is very good, recommend that the pro buy	Jingdong prefecture level city in Shandong
Power Saving King Shop	King of Power Saving	Electromagnetic good, high power convenient, enough force, the price is right it, quality can be 😃 😃 😃 😃 😃	Jingdong prefecture level city in Shandong
Power Saving King Shop	King of Power Saving	First-class quality, packaging is also good, logistics is very fast, and the price is reasonable, very affordable, I like it!	Jingdong prefecture level city in Shandong
Power Saving King Shop	King of Power Saving	It's great, it's the kind of style you want, and it arrived quickly, thank you boss, 5 stars for the service!	Jingdong prefecture level city in Shandong
Power Saving King Shop	King of Power Saving	Packaging is very good, the use of the effect is quite good, the store has a good service attitude, trustworthy	Jingdong prefecture level city in Shandong
Power Saving King Shop	King of Power Saving	The quality is very good, it works well, very satisfied, the price is affordable, it's so great value, it's worth recommending.	Jingdong prefecture level city in Shandong
Power Saving King Shop	King of Power Saving	The baby description matches the actual item very well! The quality is awesome! Will be back to patronize again in the future!	Jingdong prefecture level city in Shandong
Power Saving King Shop	King of Power Saving	Ran the price is not high try to buy the attitude, did not expect the effect is really good, good!	Jingdong prefecture level city in Shandong
Power Saving King Shop	King of Power Saving	Power Saving King power is quite large, the quality of the leaf is quite good, the price of buying a very good deal, must be praised!	Jingdong prefecture level city in Shandong
Power Saving King Shop	King of Power Saving	Good product quality is the king of power-saving products trustworthy as always good to recommend!	Jingdong prefecture level city in Shandong
Power Saving King Shop	King of Power Saving	Works great, very affordable, shipped pretty fast, comfortable to use	Jingdong prefecture level city in Shandong
Power Saving King Shop	King of Power Saving	Shipping was pretty fast and affordable, not bad 👍	Jingdong prefecture level city in Shandong
Power Saving King Shop	King of Power Saving	Digital voltage display, voltage at a glance, plug and play, safe and simple!	Jingdong prefecture level city in Shandong
Power Saving King Shop	King of Power Saving	Quality assurance, very good, affordable, logistics is also very fast, I'm very satisfied with the	Jingdong prefecture level city in Shandong
Power Saving King Shop	King of Power Saving	The quality is very good, the first time I received the goods to open the first time to see, it is very good, very satisfied	Jingdong prefecture level city in Shandong
Power Saving King Shop	King of Power Saving	Power Saving King used, the effect is good, the price is affordable, very good, is the genuine article	Jingdong prefecture level city in Shandong
Power Saving King Shop	King of Power Saving	Good quality, fine workmanship, good appearance, time is very accurate, satisfied.	Jingdong prefecture level city in Shandong

Power Saving King Shop	King of Power Saving	I purposely used it for two days before commenting, which is responsible for myself and everyone else!	Jingdong prefecture level city in Shandong
Power Saving King Shop	King of Power Saving	Easy to install, easy to operate, helped our family save a lot of electricity this summer	Jingdong prefecture level city in Shandong
Power Saving King Shop	King of Power Saving	Bringing power savings into millions of homes. Still a great gadget. Pretty good.	Jingdong prefecture level city in Shandong
Power Saving King Shop	King of Power Saving	Digital liquid crystal display to see the clear intuitive, very formal workmanship is not rough	Jingdong prefecture level city in Shandong
Power Saving King Shop	King of Power Saving	Received. Great quality, fast shipping from the store and not too expensive!	Jingdong prefecture level city in Shandong
Power Saving King Shop	King of Power Saving	Bought myself a knuckle regulator and it was easy to use and the price was right.	Jingdong prefecture level city in Shandong
Power Saving King Shop	King of Power Saving	Received, good, powerful, good, very full of a shopping	Jingdong prefecture level city in Shandong
Power Saving King Shop	King of Power Saving	High power, consistent with the seller's description, powerful, cost-effective	Jingdong prefecture level city in Shandong
Power Saving King Shop	King of Power Saving	It works very well and also saves power at an affordable price a great deal.	Jingdong prefecture level city in Shandong
Power Saving King Shop	King of Power Saving	Good quality, good can recommend to friends to buy.	Jingdong prefecture level city in Shandong
Power Saving King Shop	King of Power Saving	Will repurchase, quality is guaranteed, safe and secure purchase.	Jingdong prefecture level city in Shandong
Power Saving King Shop	King of Power Saving	The logistics is very fast, the thing is also good to use, energy saving power rely on it!	Jingdong prefecture level city in Shandong
Power Saving King Shop	King of Power Saving	Power Saver is good quality and works very well, worth buying!	Jingdong prefecture level city in Shandong
Power Saving King Shop	King of Power Saving	The quality is very good is genuine, cost-effective recommended	Jingdong prefecture level city in Shandong
Power Saving King Shop	King of Power Saving	Quality Assurance. Favorable price. Very practical and durable	Jingdong prefecture level city in Shandong
Power Saving King Shop	King of Power Saving	Good value for money. Conscientious businessman. Recommended to everyone	Jingdong prefecture level city in Shandong
Power Saving King Shop	King of Power Saving	Buy it for your rental apartment and never worry about your landlord charging you too much.	Jingdong prefecture level city in Shandong
Power Saving King Shop	King of Power Saving	Nice and affordable, will repurchase next time	Jingdong prefecture level city in Shandong
Power Saving King Shop	King of Power Saving	It's very energy efficient. I've been using it for a few days now.	Jingdong prefecture level city in Shandong
Power Saving King Shop	King of Power Saving	Good quality, high power easy to use	Jingdong prefecture level city in Shandong
Power Saving King Shop	King of Power Saving	Power saving little helper. Deserves a favorable review.	Jingdong prefecture level city in Shandong
Power Saving King Shop	King of Power Saving	Great value for money, great value for money.	Jingdong prefecture level city in Shandong
Power Saving King Shop	King of Power Saving	Works well and as described by the seller	Jingdong prefecture level city in Shandong
Power Saving King Shop	King of Power Saving	Power saving is proper! It's good.	Jingdong prefecture level city in Shandong
Power Saving King Shop	King of Power Saving	Power saving. Works well. Not bad.	Jingdong prefecture level city in Shandong
Power Saving King Shop	King of Power Saving	Satisfied with power saving	Jingdong prefecture level city in Shandong
Power Saving King Shop	King of Power Saving	Received intact, open a super good look beautiful and generous, ~ bit of high quality is recommended to buy, the need for friends rest assured that the next order	Jingdong prefecture level city in Shandong
Power Saving King Shop	King of Power Saving	Very good to use, friends introduced, save electricity and money, long-lasting products cost-effective, large factory production, good quality, fine workmanship cost-effective!	Jingdong prefecture level city in Shandong
Power Saving King Shop	King of Power Saving	The power saving effect is very obvious, trying to pay 30% less electricity bill in a month than before, hahaha	Jingdong prefecture level city in Shandong
Power Saving King Shop	King of Power Saving	It's such a power saver to use this, it saves money on kids snacks for a month. Kudos!	Jingdong prefecture level city in Shandong
Power Saving King Shop	King of Power Saving	Plug and play, stable voltage, the screen has a display when working, this winter home appliances, need more electricity, just buy to use, save electricity and save money, with instructions, not too big and does not take up space, the quality is very good, and the seller describes exactly the same!	Jingdong prefecture level city in Shandong
Power Saving King Shop	King of Power Saving	A must have for opening a store. Save over a thousand dollars a month on your electric bill	Jingdong prefecture level city in Shandong
Power Saving King Shop	King of Power Saving	Received the goods, the first time to unpack, the quality is very good, with the seller's description, completely exceeded my expectations, the packaging is very careful, very strict, in general this is a very satisfied with the shopping	Jingdong prefecture level city in Shandong
Power Saving King Shop	King of Power Saving	As always, good quality, a very favorite product, great! o(*￣︶￣*)o The price is very affordable, cost-effective, it's considered affordable good, will repurchase!	Jingdong prefecture level city in Shandong
Power Saving King Shop	King of Power Saving	Very satisfied with a shopping, good business service, fast shipping, logistics is also very powerful, received very good, buy assured, use peace of mind!	Jingdong prefecture level city in Shandong
Power Saving King Shop	King of Power Saving	The first time to buy online, but also look at the evaluation before buying, the quality is not bad, but also very cheap, the need for friends can rest assured to buy!	Jingdong prefecture level city in Shandong
Power Saving King Shop	King of Power Saving	Better than expected, feel value for money, very satisfied with a shopping, need next time will come back!	Jingdong prefecture level city in Shandong
Power Saving King Shop	King of Power Saving	Really super like, very supportive, very good quality, exactly as described by the seller, very satisfied!	Jingdong prefecture level city in Shandong
Power Saving King Shop	King of Power Saving	It is genuine, effective, really saves power, affordable, tightly packed, fast shipping, very satisfied.	Jingdong prefecture level city in Shandong
Power Saving King Shop	King of Power Saving	Good quality, fine workmanship, is genuine and business description of the same, good value for money, fast logistics, very satisfied	Jingdong prefecture level city in Shandong
Power Saving King Shop	King of Power Saving	Saver received, very good, installation set up very well, very good, the effect is very good, recommend that the pro buy	Jingdong prefecture level city in Shandong
Power Saving King Shop	King of Power Saving	Electromagnetic good, high power convenient, enough force, the price is right it, quality can be 😀 😀 😀 😀 😀	Jingdong prefecture level city in Shandong
Power Saving King Shop	King of Power Saving	First-class quality, packaging is also good, logistics is very fast, and the price is reasonable, very affordable, I like it!	Jingdong prefecture level city in Shandong
Power Saving King Shop	King of Power Saving	It's great, it's the kind of style you want, and it arrived quickly, thank you boss, 5 stars for the service!	Jingdong prefecture level city in Shandong
Power Saving King Shop	King of Power Saving	Packaging is very good, the use of the effect is quite good, the store has a good service attitude, trustworthy	Jingdong prefecture level city in Shandong
Power Saving King Shop	King of Power Saving	The quality is very good, it works well, very satisfied, the price is affordable, it's so great value, it's worth recommending.	Jingdong prefecture level city in Shandong
Power Saving King Shop	King of Power Saving	The baby description matches the actual item very well! The quality is awesome! Will be back to patronize again in the future!	Jingdong prefecture level city in Shandong
Power Saving King Shop	King of Power Saving	Ran the price is not high try to buy the attitude, did not expect the effect is really good, good!	Jingdong prefecture level city in Shandong
Power Saving King Shop	King of Power Saving	Power Saving King power is quite large, the quality of the leaf is quite good, the price of buying a very good deal, must be praised!	Jingdong prefecture level city in Shandong
Power Saving King Shop	King of Power Saving	Good product quality is the king of power-saving products trustworthy as always good to recommend!	Jingdong prefecture level city in Shandong

Power Saving King Shop	King of Power Saving	Works great, very affordable, shipped pretty fast, comfortable to use	Jingdong prefecture level city in Shandong
Power Saving King Shop	King of Power Saving	Shipping was pretty fast and affordable, not bad 👍	Jingdong prefecture level city in Shandong
Power Saving King Shop	King of Power Saving	Digital voltage display, voltage at a glance, plug and play, safe and simple!	Jingdong prefecture level city in Shandong
Power Saving King Shop	King of Power Saving	Quality assurance, very good, affordable, logistics is also very fast, I'm very satisfied with the	Jingdong prefecture level city in Shandong
Power Saving King Shop	King of Power Saving	The quality is very good, the first time I received the goods to open the first time to see, it is very good, very satisfied	Jingdong prefecture level city in Shandong
Power Saving King Shop	King of Power Saving	Power Saving King used, the effect is good, the price is affordable, very good, is the genuine article	Jingdong prefecture level city in Shandong
Power Saving King Shop	King of Power Saving	Good quality, fine workmanship, good appearance, time is very accurate, satisfied.	Jingdong prefecture level city in Shandong
Power Saving King Shop	King of Power Saving	I purposely used it for two days before commenting, which is responsible for myself and everyone else!	Jingdong prefecture level city in Shandong
Power Saving King Shop	King of Power Saving	Easy to install, easy to operate, helped our family save a lot of electricity this summer	Jingdong prefecture level city in Shandong
Power Saving King Shop	King of Power Saving	Bringing power savings into millions of homes. Still a great gadget. Pretty good.	Jingdong prefecture level city in Shandong
Power Saving King Shop	King of Power Saving	Digital liquid crystal display to see the clear intuitive, very formal workmanship is not rough	Jingdong prefecture level city in Shandong
Power Saving King Shop	King of Power Saving	Received. Great quality, fast shipping from the store and not too expensive!	Jingdong prefecture level city in Shandong
Power Saving King Shop	King of Power Saving	Bought myself a knuckle regulator and it was easy to use and the price was right.	Jingdong prefecture level city in Shandong
Power Saving King Shop	King of Power Saving	Received, good, powerful, good, very full of a shopping	Jingdong prefecture level city in Shandong
Power Saving King Shop	King of Power Saving	High power, consistent with the seller's description, powerful, cost-effective	Jingdong prefecture level city in Shandong
Power Saving King Shop	King of Power Saving	It works very well and also saves power at an affordable price a great deal.	Jingdong prefecture level city in Shandong
Power Saving King Shop	King of Power Saving	Good quality, good can recommend to friends to buy.	Jingdong prefecture level city in Shandong
Power Saving King Shop	King of Power Saving	Will repurchase, quality is guaranteed, safe and secure purchase.	Jingdong prefecture level city in Shandong
Power Saving King Shop	King of Power Saving	The logistics is very fast, the thing is also good to use, energy saving power rely on it!	Jingdong prefecture level city in Shandong
Power Saving King Shop	King of Power Saving	Power Saver is good quality and works very well, worth buying!	Jingdong prefecture level city in Shandong
Power Saving King Shop	King of Power Saving	The quality is very good is genuine, cost-effective recommended	Jingdong prefecture level city in Shandong
Power Saving King Shop	King of Power Saving	Quality Assurance. Favorable price. Very practical and durable	Jingdong prefecture level city in Shandong
Power Saving King Shop	King of Power Saving	Good value for money. Conscientious businessman. Recommended to everyone	Jingdong prefecture level city in Shandong
Power Saving King Shop	King of Power Saving	Buy it for your rental apartment and never worry about your landlord charging you too much.	Jingdong prefecture level city in Shandong
Power Saving King Shop	King of Power Saving	Nice and affordable, will repurchase next time	Jingdong prefecture level city in Shandong
Power Saving King Shop	King of Power Saving	It's very energy efficient. I've been using it for a few days now.	Jingdong prefecture level city in Shandong
Power Saving King Shop	King of Power Saving	Good quality, high power easy to use	Jingdong prefecture level city in Shandong
Power Saving King Shop	King of Power Saving	Power saving little helper. Deserves a favorable review.	Jingdong prefecture level city in Shandong
Power Saving King Shop	King of Power Saving	Great value for money, great value for money.	Jingdong prefecture level city in Shandong
Power Saving King Shop	King of Power Saving	Works well and as described by the seller	Jingdong prefecture level city in Shandong
Power Saving King Shop	King of Power Saving	Power saving is proper! It's good.	Jingdong prefecture level city in Shandong
Power Saving King Shop	King of Power Saving	Power saving. Works well. Not bad.	Jingdong prefecture level city in Shandong
Power Saving King Shop	King of Power Saving	Satisfied with power saving	Jingdong prefecture level city in Shandong
Power Saving King Shop	King of Power Saving	Received intact, open a super good look beautiful and generous, ~ bit of high quality is recommended to buy, the need for friends rest assured that the next order	Jingdong prefecture level city in Shandong
Power Saving King Shop	King of Power Saving	Very good to use, friends introduced, save electricity and money, long-lasting products cost-effective, large factory production, good quality, fine workmanship cost-effective!	Jingdong prefecture level city in Shandong
Power Saving King Shop	King of Power Saving	The power saving effect is very obvious, trying to pay 30% less electricity bill in a month than before, hahaha	Jingdong prefecture level city in Shandong
Power Saving King Shop	King of Power Saving	It's such a power saver to use this, it saves money on kids snacks for a month. Kudos!	Jingdong prefecture level city in Shandong
Power Saving King Shop	King of Power Saving	Plug and play, stable voltage, the screen has a display when working, this winter home appliances, need more electricity, just buy to use, save electricity and save money, with instructions, not too big and does not take up space, the quality is very good, and the seller describes exactly the same!	Jingdong prefecture level city in Shandong
Power Saving King Shop	King of Power Saving	A must have for opening a store. Save over a thousand dollars a month on your electric bill	Jingdong prefecture level city in Shandong
Power Saving King Shop	King of Power Saving	Received the goods, the first time to unpack, the quality is very good, with the seller's description, completely exceeded my expectations, the packaging is very careful, very strict, in general this is a very satisfied with the shopping	Jingdong prefecture level city in Shandong
Power Saving King Shop	King of Power Saving	As always, good quality, a very favorite product, great! o(*￣︶￣*)o The price is very affordable, cost-effective, it's considered affordable good, will repurchase!	Jingdong prefecture level city in Shandong
Power Saving King Shop	King of Power Saving	Very satisfied with a shopping, good business service, fast shipping, logistics is also very powerful, received very good, buy assured, use peace of mind!	Jingdong prefecture level city in Shandong
Power Saving King Shop	King of Power Saving	The first time to buy online, but also look at the evaluation before buying, the quality is not bad, but also very cheap, the need for friends can rest assured to buy!	Jingdong prefecture level city in Shandong
Power Saving King Shop	King of Power Saving	Better than expected, feel value for money, very satisfied with a shopping, need next time will come back!	Jingdong prefecture level city in Shandong
Power Saving King Shop	King of Power Saving	Really super like, very supportive, very good quality, exactly as described by the seller, very satisfied!	Jingdong prefecture level city in Shandong
Power Saving King Shop	King of Power Saving	It is genuine, effective, really saves power, affordable, tightly packed, fast shipping, very satisfied.	Jingdong prefecture level city in Shandong
Power Saving King Shop	King of Power Saving	Good quality, fine workmanship, is genuine and business description of the same, good value for money, fast logistics, very satisfied	Jingdong prefecture level city in Shandong
Power Saving King Shop	King of Power Saving	Saver received, very good, installation set up very well, very good, the effect is very good, recommend that the pro buy	Jingdong prefecture level city in Shandong
Power Saving King Shop	King of Power Saving	Electromagnetic good, high power convenient, enough force, the price is right it, quality can be 😃 😃 😃 😃 😃	Jingdong prefecture level city in Shandong
Power Saving King Shop	King of Power Saving	First-class quality, packaging is also good, logistics is very fast, and the price is reasonable, very affordable, I like it!	Jingdong prefecture level city in Shandong

Power Saving King Shop	King of Power Saving	It's great, it's the kind of style you want, and it arrived quickly, thank you boss, 5 stars for the service!	Jingdong prefecture level city in Shandong
Power Saving King Shop	King of Power Saving	Packaging is very good, the use of the effect is quite good, the store has a good service attitude, trustworthy	Jingdong prefecture level city in Shandong
Power Saving King Shop	King of Power Saving	The quality is very good, it works well, very satisfied, the price is affordable, it's so great value, it's worth recommending.	Jingdong prefecture level city in Shandong
Power Saving King Shop	King of Power Saving	The baby description matches the actual item very well! The quality is awesome! Will be back to patronize again in the future!	Jingdong prefecture level city in Shandong
Power Saving King Shop	King of Power Saving	Ran the price is not high try to buy the attitude, did not expect the effect is really good, good!	Jingdong prefecture level city in Shandong
Power Saving King Shop	King of Power Saving	Power Saving King power is quite large, the quality of the leaf is quite good, the price of buying a very good deal, must be praised!	Jingdong prefecture level city in Shandong
Power Saving King Shop	King of Power Saving	Good product quality is the king of power-saving products trustworthy as always good to recommend!	Jingdong prefecture level city in Shandong
Power Saving King Shop	King of Power Saving	Works great, very affordable, shipped pretty fast, comfortable to use	Jingdong prefecture level city in Shandong
Power Saving King Shop	King of Power Saving	Shipping was pretty fast and affordable, not bad 👍	Jingdong prefecture level city in Shandong
Power Saving King Shop	King of Power Saving	Digital voltage display, voltage at a glance, plug and play, safe and simple!	Jingdong prefecture level city in Shandong
Power Saving King Shop	King of Power Saving	Quality assurance, very good, affordable, logistics is also very fast, I'm very satisfied with the	Jingdong prefecture level city in Shandong
Power Saving King Shop	King of Power Saving	The quality is very good, the first time I received the goods to open the first time to see, it is very good, very satisfied	Jingdong prefecture level city in Shandong
Power Saving King Shop	King of Power Saving	Power Saving King used, the effect is good, the price is affordable, very good, is the genuine article	Jingdong prefecture level city in Shandong
Power Saving King Shop	King of Power Saving	Good quality, fine workmanship, good appearance, time is very accurate, satisfied.	Jingdong prefecture level city in Shandong
Power Saving King Shop	King of Power Saving	I purposely used it for two days before commenting, which is responsible for myself and everyone else!	Jingdong prefecture level city in Shandong
Power Saving King Shop	King of Power Saving	Easy to install, easy to operate, helped our family save a lot of electricity this summer	Jingdong prefecture level city in Shandong
Power Saving King Shop	King of Power Saving	Bringing power savings into millions of homes. Still a great gadget. Pretty good.	Jingdong prefecture level city in Shandong
Power Saving King Shop	King of Power Saving	Digital liquid crystal display to see the clear intuitive, very formal workmanship is not rough	Jingdong prefecture level city in Shandong
Power Saving King Shop	King of Power Saving	Received. Great quality, fast shipping from the store and not too expensive!	Jingdong prefecture level city in Shandong
Power Saving King Shop	King of Power Saving	Bought myself a knuckle regulator and it was easy to use and the price was right.	Jingdong prefecture level city in Shandong
Power Saving King Shop	King of Power Saving	Received, good, powerful, good, very full of a shopping	Jingdong prefecture level city in Shandong
Power Saving King Shop	King of Power Saving	High power, consistent with the seller's description, powerful, cost-effective	Jingdong prefecture level city in Shandong
Power Saving King Shop	King of Power Saving	It works very well and also saves power at an affordable price a great deal.	Jingdong prefecture level city in Shandong
Power Saving King Shop	King of Power Saving	Good quality, good can recommend to friends to buy.	Jingdong prefecture level city in Shandong
Power Saving King Shop	King of Power Saving	Will repurchase, quality is guaranteed, safe and secure purchase.	Jingdong prefecture level city in Shandong
Power Saving King Shop	King of Power Saving	The logistics is very fast, the thing is also good to use, energy saving power rely on it!	Jingdong prefecture level city in Shandong
Power Saving King Shop	King of Power Saving	Power Saver is good quality and works very well, worth buying!	Jingdong prefecture level city in Shandong
Power Saving King Shop	King of Power Saving	The quality is very good is genuine, cost-effective recommended	Jingdong prefecture level city in Shandong
Power Saving King Shop	King of Power Saving	Quality Assurance. Favorable price. Very practical and durable	Jingdong prefecture level city in Shandong
Power Saving King Shop	King of Power Saving	Good value for money. Conscientious businessman. Recommended to everyone	Jingdong prefecture level city in Shandong
Power Saving King Shop	King of Power Saving	Buy it for your rental apartment and never worry about your landlord charging you too much.	Jingdong prefecture level city in Shandong
Power Saving King Shop	King of Power Saving	Nice and affordable, will repurchase next time	Jingdong prefecture level city in Shandong
Power Saving King Shop	King of Power Saving	It's very energy efficient. I've been using it for a few days now.	Jingdong prefecture level city in Shandong
Power Saving King Shop	King of Power Saving	Good quality, high power easy to use	Jingdong prefecture level city in Shandong
Power Saving King Shop	King of Power Saving	Power saving little helper. Deserves a favorable review.	Jingdong prefecture level city in Shandong
Power Saving King Shop	King of Power Saving	Great value for money, great value for money.	Jingdong prefecture level city in Shandong
Power Saving King Shop	King of Power Saving	Works well and as described by the seller	Jingdong prefecture level city in Shandong
Power Saving King Shop	King of Power Saving	Power saving is proper! It's good.	Jingdong prefecture level city in Shandong
Power Saving King Shop	King of Power Saving	Power saving. Works well. Not bad.	Jingdong prefecture level city in Shandong
Power Saving King Shop	King of Power Saving	Satisfied with power saving	Jingdong prefecture level city in Shandong
Power Saving King Shop	King of Power Saving	Received intact, open a super good look beautiful and generous, ~ bit of high quality is recommended to buy, the need for friends rest assured that the next order	Jingdong prefecture level city in Shandong
Power Saving King Shop	King of Power Saving	Very good to use, friends introduced, save electricity and money, long-lasting products cost-effective, large factory production, good quality, fine workmanship cost-effective!	Jingdong prefecture level city in Shandong
Power Saving King Shop	King of Power Saving	The power saving effect is very obvious, trying to pay 30% less electricity bill in a month than before, hahaha	Jingdong prefecture level city in Shandong
Power Saving King Shop	King of Power Saving	It's such a power saver to use this, it saves money on kids snacks for a month. Kudos!	Jingdong prefecture level city in Shandong
Power Saving King Shop	King of Power Saving	Plug and play, stable voltage, the screen has a display when working, this winter home appliances, need more electricity, just buy to use, save electricity and save money, with instructions, not too big and does not take up space, the quality is very good, and the seller describes exactly the same!	Jingdong prefecture level city in Shandong
Power Saving King Shop	King of Power Saving	A must have for opening a store. Save over a thousand dollars a month on your electric bill	Jingdong prefecture level city in Shandong
Power Saving King Shop	King of Power Saving	Received the goods, the first time to unpack, the quality is very good, with the seller's description, completely exceeded my expectations, the packaging is very careful, very strict, in general this is a very satisfied with the shopping	Jingdong prefecture level city in Shandong
Power Saving King Shop	King of Power Saving	As always, good quality, a very favorite product, great! o(*￣︶￣*)o The price is very affordable, cost-effective, it's considered affordable good, will repurchase!	Jingdong prefecture level city in Shandong
Power Saving King Shop	King of Power Saving	Very satisfied with a shopping, good business service, fast shipping, logistics is also very powerful, received very good, buy assured, use peace of mind!	Jingdong prefecture level city in Shandong
Power Saving King Shop	King of Power Saving	The first time to buy online, but also look at the evaluation before buying, the quality is not bad, but also very cheap, the need for friends can rest assured to buy!	Jingdong prefecture level city in Shandong

Power Saving King Shop	King of Power Saving	Better than expected, feel value for money, very satisfied with a shopping, need next time will come back!	Jingdong prefecture level city in Shandong
Power Saving King Shop	King of Power Saving	Really super like, very supportive, very good quality, exactly as described by the seller, very satisfied!	Jingdong prefecture level city in Shandong
Power Saving King Shop	King of Power Saving	It is genuine, effective, really saves power, affordable, tightly packed, fast shipping, very satisfied.	Jingdong prefecture level city in Shandong
Power Saving King Shop	King of Power Saving	Good quality, fine workmanship, is genuine and business description of the same, good value for money, fast logistics, very satisfied	Jingdong prefecture level city in Shandong
Power Saving King Shop	King of Power Saving	Saver received, very good, installation set up very well, very good, the effect is very good, recommend that the pro buy	Jingdong prefecture level city in Shandong
Power Saving King Shop	King of Power Saving	Electromagnetic good, high power convenient, enough force, the price is right it, quality can be 😀 😀 😀 😀 😀	Jingdong prefecture level city in Shandong
Power Saving King Shop	King of Power Saving	First-class quality, packaging is also good, logistics is very fast, and the price is reasonable, very affordable, I like it!	Jingdong prefecture level city in Shandong
Power Saving King Shop	King of Power Saving	It's great, it's the kind of style you want, and it arrived quickly, thank you boss, 5 stars for the service!	Jingdong prefecture level city in Shandong
Power Saving King Shop	King of Power Saving	Packaging is very good, the use of the effect is quite good, the store has a good service attitude, trustworthy	Jingdong prefecture level city in Shandong
Power Saving King Shop	King of Power Saving	The quality is very good, it works well, very satisfied, the price is affordable, it's so great value, it's worth recommending.	Jingdong prefecture level city in Shandong
Power Saving King Shop	King of Power Saving	The baby description matches the actual item very well! The quality is awesome! Will be back to patronize again in the future!	Jingdong prefecture level city in Shandong
Power Saving King Shop	King of Power Saving	Ran the price is not high try to buy the attitude, did not expect the effect is really good, good!	Jingdong prefecture level city in Shandong
Power Saving King Shop	King of Power Saving	Power Saving King power is quite large, the quality of the leaf is quite good, the price of buying a very good deal, must be praised!	Jingdong prefecture level city in Shandong
Power Saving King Shop	King of Power Saving	Good product quality is the king of power-saving products trustworthy as always good to recommend!	Jingdong prefecture level city in Shandong
Power Saving King Shop	King of Power Saving	Works great, very affordable, shipped pretty fast, comfortable to use	Jingdong prefecture level city in Shandong
Power Saving King Shop	King of Power Saving	Shipping was pretty fast and affordable, not bad 👍	Jingdong prefecture level city in Shandong
Power Saving King Shop	King of Power Saving	Digital voltage display, voltage at a glance, plug and play, safe and simple!	Jingdong prefecture level city in Shandong
Power Saving King Shop	King of Power Saving	Quality assurance, very good, affordable, logistics is also very fast, I'm very satisfied with the	Jingdong prefecture level city in Shandong
Power Saving King Shop	King of Power Saving	The quality is very good, the first time I received the goods to open the first time to see, it is very good, very satisfied	Jingdong prefecture level city in Shandong
Power Saving King Shop	King of Power Saving	Power Saving King used, the effect is good, the price is affordable, very good, is the genuine article	Jingdong prefecture level city in Shandong
Power Saving King Shop	King of Power Saving	Good quality, fine workmanship, good appearance, time is very accurate, satisfied.	Jingdong prefecture level city in Shandong
Power Saving King Shop	King of Power Saving	I purposely used it for two days before commenting, which is responsible for myself and everyone else!	Jingdong prefecture level city in Shandong
Power Saving King Shop	King of Power Saving	Easy to install, easy to operate, helped our family save a lot of electricity this summer	Jingdong prefecture level city in Shandong
Power Saving King Shop	King of Power Saving	Bringing power savings into millions of homes. Still a great gadget. Pretty good.	Jingdong prefecture level city in Shandong
Power Saving King Shop	King of Power Saving	Digital liquid crystal display to see the clear intuitive, very formal workmanship is not rough	Jingdong prefecture level city in Shandong
Power Saving King Shop	King of Power Saving	Received. Great quality, fast shipping from the store and not too expensive!	Jingdong prefecture level city in Shandong
Power Saving King Shop	King of Power Saving	Bought myself a knuckle regulator and it was easy to use and the price was right.	Jingdong prefecture level city in Shandong
Power Saving King Shop	King of Power Saving	Received, good, powerful, good, very full of a shopping	Jingdong prefecture level city in Shandong
Power Saving King Shop	King of Power Saving	High power, consistent with the seller's description, powerful, cost-effective	Jingdong prefecture level city in Shandong
Power Saving King Shop	King of Power Saving	It works very well and also saves power at an affordable price a great deal.	Jingdong prefecture level city in Shandong
Power Saving King Shop	King of Power Saving	Good quality, good can recommend to friends to buy.	Jingdong prefecture level city in Shandong
Power Saving King Shop	King of Power Saving	Will repurchase, quality is guaranteed, safe and secure purchase.	Jingdong prefecture level city in Shandong
Power Saving King Shop	King of Power Saving	The logistics is very fast, the thing is also good to use, energy saving power rely on it!	Jingdong prefecture level city in Shandong
Power Saving King Shop	King of Power Saving	Power Saver is good quality and works very well, worth buying!	Jingdong prefecture level city in Shandong
Power Saving King Shop	King of Power Saving	The quality is very good is genuine, cost-effective recommended	Jingdong prefecture level city in Shandong
Power Saving King Shop	King of Power Saving	Quality Assurance. Favorable price. Very practical and durable	Jingdong prefecture level city in Shandong
Power Saving King Shop	King of Power Saving	Good value for money. Conscientious businessman. Recommended to everyone	Jingdong prefecture level city in Shandong
Power Saving King Shop	King of Power Saving	Buy it for your rental apartment and never worry about your landlord charging you too much.	Jingdong prefecture level city in Shandong
Power Saving King Shop	King of Power Saving	Nice and affordable, will repurchase next time	Jingdong prefecture level city in Shandong
Power Saving King Shop	King of Power Saving	It's very energy efficient. I've been using it for a few days now.	Jingdong prefecture level city in Shandong
Power Saving King Shop	King of Power Saving	Good quality, high power easy to use	Jingdong prefecture level city in Shandong
Power Saving King Shop	King of Power Saving	Power saving little helper. Deserves a favorable review.	Jingdong prefecture level city in Shandong
Power Saving King Shop	King of Power Saving	Great value for money, great value for money.	Jingdong prefecture level city in Shandong
Power Saving King Shop	King of Power Saving	Works well and as described by the seller	Jingdong prefecture level city in Shandong
Power Saving King Shop	King of Power Saving	Power saving is proper! It's good.	Jingdong prefecture level city in Shandong
Power Saving King Shop	King of Power Saving	Power saving. Works well. Not bad.	Jingdong prefecture level city in Shandong
Power Saving King Shop	King of Power Saving	Satisfied with power saving	Jingdong prefecture level city in Shandong
Power Saving King Shop	King of Power Saving	Received intact, open a super good look beautiful and generous, ~ bit of high quality is recommended to buy, the need for friends rest assured that the next order	Jingdong prefecture level city in Shandong
Power Saving King Shop	King of Power Saving	Very good to use, friends introduced, save electricity and money, long-lasting products cost-effective, large factory production, good quality, fine workmanship cost-effective!	Jingdong prefecture level city in Shandong
Power Saving King Shop	King of Power Saving	The power saving effect is very obvious, trying to pay 30% less electricity bill in a month than before, hahaha	Jingdong prefecture level city in Shandong
Power Saving King Shop	King of Power Saving	It's such a power saver to use this, it saves money on kids snacks for a month. Kudos!	Jingdong prefecture level city in Shandong

Power Saving King Shop	King of Power Saving	Plug and play, stable voltage, the screen has a display when working, this winter home appliances, need more electricity, just buy to use, save electricity and save money, with instructions, not too big and does not take up space, the quality is very good, and the seller describes exactly the same!	Jingdong prefecture level city in Shandong
Power Saving King Shop	King of Power Saving	A must have for opening a store. Save over a thousand dollars a month on your electric bill	Jingdong prefecture level city in Shandong
Power Saving King Shop	King of Power Saving	Received the goods, the first time to unpack, the quality is very good, with the seller's description, completely exceeded my expectations, the packaging is very careful, very strict, in general this is a very satisfied with the shopping	Jingdong prefecture level city in Shandong
Power Saving King Shop	King of Power Saving	As always, good quality, a very favorite product, great! o(*￣︶￣*)o The price is very affordable, cost-effective, it's considered affordable good, will repurchase!	Jingdong prefecture level city in Shandong
Power Saving King Shop	King of Power Saving	Very satisfied with a shopping, good business service, fast shipping, logistics is also very powerful, received very good, buy assured, use peace of mind!	Jingdong prefecture level city in Shandong
Power Saving King Shop	King of Power Saving	The first time to buy online, but also look at the evaluation before buying, the quality is not bad, but also very cheap, the need for friends can rest assured to buy!	Jingdong prefecture level city in Shandong
Power Saving King Shop	King of Power Saving	Better than expected, feel value for money, very satisfied with a shopping, need next time will come back!	Jingdong prefecture level city in Shandong
Power Saving King Shop	King of Power Saving	Really super like, very supportive, very good quality, exactly as described by the seller, very satisfied!	Jingdong prefecture level city in Shandong
Power Saving King Shop	King of Power Saving	It is genuine, effective, really saves power, affordable, tightly packed, fast shipping, very satisfied.	Jingdong prefecture level city in Shandong
Power Saving King Shop	King of Power Saving	Good quality, fine workmanship, is genuine and business description of the same, good value for money, fast logistics, very satisfied	Jingdong prefecture level city in Shandong
Power Saving King Shop	King of Power Saving	Saver received, very good, installation set up very well, very good, the effect is very good, recommend that the pro buy	Jingdong prefecture level city in Shandong
Power Saving King Shop	King of Power Saving	Electromagnetic good, high power convenient, enough force, the price is right it, quality can be 😃 😃 😃 😃 😃	Jingdong prefecture level city in Shandong
Power Saving King Shop	King of Power Saving	First-class quality, packaging is also good, logistics is very fast, and the price is reasonable, very affordable, I like it!	Jingdong prefecture level city in Shandong
Power Saving King Shop	King of Power Saving	It's great, it's the kind of style you want, and it arrived quickly, thank you boss, 5 stars for the service!	Jingdong prefecture level city in Shandong
Power Saving King Shop	King of Power Saving	Packaging is very good, the use of the effect is quite good, the store has a good service attitude, trustworthy	Jingdong prefecture level city in Shandong
Power Saving King Shop	King of Power Saving	The quality is very good, it works well, very satisfied, the price is affordable, it's so great value, it's worth recommending.	Jingdong prefecture level city in Shandong
Power Saving King Shop	King of Power Saving	The baby description matches the actual item very well! The quality is awesome! Will be back to patronize again in the future!	Jingdong prefecture level city in Shandong
Power Saving King Shop	King of Power Saving	Ran the price is not high try to buy the attitude, did not expect the effect is really good, good!	Jingdong prefecture level city in Shandong
Power Saving King Shop	King of Power Saving	Power Saving King power is quite large, the quality of the leaf is quite good, the price of buying a very good deal, must be praised!	Jingdong prefecture level city in Shandong
Power Saving King Shop	King of Power Saving	Good product quality is the king of power-saving products trustworthy as always good to recommend!	Jingdong prefecture level city in Shandong
Power Saving King Shop	King of Power Saving	Works great, very affordable, shipped pretty fast, comfortable to use	Jingdong prefecture level city in Shandong
Power Saving King Shop	King of Power Saving	Shipping was pretty fast and affordable, not bad 👍	Jingdong prefecture level city in Shandong
Power Saving King Shop	King of Power Saving	Digital voltage display, voltage at a glance, plug and play, safe and simple!	Jingdong prefecture level city in Shandong
Power Saving King Shop	King of Power Saving	Quality assurance, very good, affordable, logistics is also very fast, I'm very satisfied with the	Jingdong prefecture level city in Shandong
Power Saving King Shop	King of Power Saving	The quality is very good, the first time I received the goods to open the first time to see, it is very good, very satisfied	Jingdong prefecture level city in Shandong
Power Saving King Shop	King of Power Saving	Power Saving King used, the effect is good, the price is affordable, very good, is the genuine article	Jingdong prefecture level city in Shandong
Power Saving King Shop	King of Power Saving	Good quality, fine workmanship, good appearance, time is very accurate, satisfied.	Jingdong prefecture level city in Shandong
Power Saving King Shop	King of Power Saving	I purposely used it for two days before commenting, which is responsible for myself and everyone else!	Jingdong prefecture level city in Shandong
Power Saving King Shop	King of Power Saving	Easy to install, easy to operate, helped our family save a lot of electricity this summer	Jingdong prefecture level city in Shandong
Power Saving King Shop	King of Power Saving	Bringing power savings into millions of homes. Still a great gadget. Pretty good.	Jingdong prefecture level city in Shandong
Power Saving King Shop	King of Power Saving	Digital liquid crystal display to see the clear intuitive, very formal workmanship is not rough	Jingdong prefecture level city in Shandong
Power Saving King Shop	King of Power Saving	Received. Great quality, fast shipping from the store and not too expensive!	Jingdong prefecture level city in Shandong
Power Saving King Shop	King of Power Saving	Bought myself a knuckle regulator and it was easy to use and the price was right.	Jingdong prefecture level city in Shandong
Power Saving King Shop	King of Power Saving	Received, good, powerful, good, very full of a shopping	Jingdong prefecture level city in Shandong
Power Saving King Shop	King of Power Saving	High power, consistent with the seller's description, powerful, cost-effective	Jingdong prefecture level city in Shandong
Power Saving King Shop	King of Power Saving	It works very well and also saves power at an affordable price a great deal.	Jingdong prefecture level city in Shandong
Power Saving King Shop	King of Power Saving	Good quality, good can recommend to friends to buy.	Jingdong prefecture level city in Shandong
Power Saving King Shop	King of Power Saving	Will repurchase, quality is guaranteed, safe and secure purchase.	Jingdong prefecture level city in Shandong
Power Saving King Shop	King of Power Saving	The logistics is very fast, the thing is also good to use, energy saving power rely on it!	Jingdong prefecture level city in Shandong
Power Saving King Shop	King of Power Saving	Power Saver is good quality and works very well, worth buying!	Jingdong prefecture level city in Shandong
Power Saving King Shop	King of Power Saving	The quality is very good is genuine, cost-effective recommended	Jingdong prefecture level city in Shandong
Power Saving King Shop	King of Power Saving	Quality Assurance. Favorable price. Very practical and durable	Jingdong prefecture level city in Shandong
Power Saving King Shop	King of Power Saving	Good value for money. Conscientious businessman. Recommended to everyone	Jingdong prefecture level city in Shandong
Power Saving King Shop	King of Power Saving	Buy it for your rental apartment and never worry about your landlord charging you too much.	Jingdong prefecture level city in Shandong
Power Saving King Shop	King of Power Saving	Nice and affordable, will repurchase next time	Jingdong prefecture level city in Shandong
Power Saving King Shop	King of Power Saving	It's very energy efficient. I've been using it for a few days now.	Jingdong prefecture level city in Shandong
Power Saving King Shop	King of Power Saving	Good quality, high power easy to use	Jingdong prefecture level city in Shandong
Power Saving King Shop	King of Power Saving	Power saving little helper. Deserves a favorable review.	Jingdong prefecture level city in Shandong
Power Saving King Shop	King of Power Saving	Great value for money, great value for money.	Jingdong prefecture level city in Shandong
Power Saving King Shop	King of Power Saving	Works well and as described by the seller	Jingdong prefecture level city in Shandong

Power Saving King Shop	King of Power Saving	Power saving is proper! It's good.	Jingdong prefecture level city in Shandong
Power Saving King Shop	King of Power Saving	Power saving. Works well. Not bad.	Jingdong prefecture level city in Shandong
Power Saving King Shop	King of Power Saving	Satisfied with power saving	Jingdong prefecture level city in Shandong
Power Saving King Shop	King of Power Saving	Received intact, open a super good look beautiful and generous, ~ bit of high quality is recommended to buy, the need for friends rest assured that the next order	Jingdong prefecture level city in Shandong
Power Saving King Shop	King of Power Saving	Very good to use, friends introduced, save electricity and money, long-lasting products cost-effective, large factory production, good quality, fine workmanship cost-effective!	Jingdong prefecture level city in Shandong
Power Saving King Shop	King of Power Saving	The power saving effect is very obvious, trying to pay 30% less electricity bill in a month than before, hahaha	Jingdong prefecture level city in Shandong
Power Saving King Shop	King of Power Saving	It's such a power saver to use this, it saves money on kids snacks for a month. Kudos!	Jingdong prefecture level city in Shandong
Power Saving King Shop	King of Power Saving	Plug and play, stable voltage, the screen has a display when working, this winter home appliances, need more electricity, just buy to use, save electricity and save money, with instructions, not too big and does not take up space, the quality is very good, and the seller describes exactly the same!	Jingdong prefecture level city in Shandong
Power Saving King Shop	King of Power Saving	A must have for opening a store. Save over a thousand dollars a month on your electric bill	Jingdong prefecture level city in Shandong
Power Saving King Shop	King of Power Saving	Received the goods, the first time to unpack, the quality is very good, with the seller's description, completely exceeded my expectations, the packaging is very careful, very strict, in general this is a very satisfied with the shopping	Jingdong prefecture level city in Shandong
Power Saving King Shop	King of Power Saving	As always, good quality, a very favorite product, great! o(*￣︶￣*)o The price is very affordable, cost-effective, it's considered affordable good, will repurchase!	Jingdong prefecture level city in Shandong
Power Saving King Shop	King of Power Saving	Very satisfied with a shopping, good business service, fast shipping, logistics is also very powerful, received very good, buy assured, use peace of mind!	Jingdong prefecture level city in Shandong
Power Saving King Shop	King of Power Saving	The first time to buy online, but also look at the evaluation before buying, the quality is not bad, but also very cheap, the need for friends can rest assured to buy!	Jingdong prefecture level city in Shandong
Power Saving King Shop	King of Power Saving	Better than expected, feel value for money, very satisfied with a shopping, need next time will come back!	Jingdong prefecture level city in Shandong
Power Saving King Shop	King of Power Saving	Really super like, very supportive, very good quality, exactly as described by the seller, very satisfied!	Jingdong prefecture level city in Shandong
Power Saving King Shop	King of Power Saving	It is genuine, effective, really saves power, affordable, tightly packed, fast shipping, very satisfied.	Jingdong prefecture level city in Shandong
Power Saving King Shop	King of Power Saving	Good quality, fine workmanship, is genuine and business description of the same, good value for money, fast logistics, very satisfied	Jingdong prefecture level city in Shandong
Power Saving King Shop	King of Power Saving	Saver received, very good, installation set up very well, very good, the effect is very good, recommend that the pro buy	Jingdong prefecture level city in Shandong
Power Saving King Shop	King of Power Saving	Electromagnetic good, high power convenient, enough force, the price is right it, quality can be 😀 😀 😀 😀 😀	Jingdong prefecture level city in Shandong
Power Saving King Shop	King of Power Saving	First-class quality, packaging is also good, logistics is very fast, and the price is reasonable, very affordable, I like it!	Jingdong prefecture level city in Shandong
Power Saving King Shop	King of Power Saving	It's great, it's the kind of style you want, and it arrived quickly, thank you boss, 5 stars for the service!	Jingdong prefecture level city in Shandong
Power Saving King Shop	King of Power Saving	Packaging is very good, the use of the effect is quite good, the store has a good service attitude, trustworthy	Jingdong prefecture level city in Shandong
Power Saving King Shop	King of Power Saving	The quality is very good, it works well, very satisfied, the price is affordable, it's so great value, it's worth recommending.	Jingdong prefecture level city in Shandong
Power Saving King Shop	King of Power Saving	The baby description matches the actual item very well! The quality is awesome! Will be back to patronize again in the future!	Jingdong prefecture level city in Shandong
Power Saving King Shop	King of Power Saving	Ran the price is not high try to buy the attitude, did not expect the effect is really good, good!	Jingdong prefecture level city in Shandong
Power Saving King Shop	King of Power Saving	Power Saving King power is quite large, the quality of the leaf is quite good, the price of buying a very good deal, must be praised!	Jingdong prefecture level city in Shandong
Power Saving King Shop	King of Power Saving	Good product quality is the king of power-saving products trustworthy as always good to recommend!	Jingdong prefecture level city in Shandong
Power Saving King Shop	King of Power Saving	Works great, very affordable, shipped pretty fast, comfortable to use	Jingdong prefecture level city in Shandong
Power Saving King Shop	King of Power Saving	Shipping was pretty fast and affordable, not bad 👍	Jingdong prefecture level city in Shandong
Power Saving King Shop	King of Power Saving	Digital voltage display, voltage at a glance, plug and play, safe and simple!	Jingdong prefecture level city in Shandong
Power Saving King Shop	King of Power Saving	Quality assurance, very good, affordable, logistics is also very fast, I'm very satisfied with the	Jingdong prefecture level city in Shandong
Power Saving King Shop	King of Power Saving	The quality is very good, the first time I received the goods to open the first time to see, it is very good, very satisfied	Jingdong prefecture level city in Shandong
Power Saving King Shop	King of Power Saving	Power Saving King used, the effect is good, the price is affordable, very good, is the genuine article	Jingdong prefecture level city in Shandong
Power Saving King Shop	King of Power Saving	Good quality, fine workmanship, good appearance, time is very accurate, satisfied.	Jingdong prefecture level city in Shandong
Power Saving King Shop	King of Power Saving	I purposely used it for two days before commenting, which is responsible for myself and everyone else!	Jingdong prefecture level city in Shandong
Power Saving King Shop	King of Power Saving	Easy to install, easy to operate, helped our family save a lot of electricity this summer	Jingdong prefecture level city in Shandong
Power Saving King Shop	King of Power Saving	Bringing power savings into millions of homes. Still a great gadget. Pretty good.	Jingdong prefecture level city in Shandong
Power Saving King Shop	King of Power Saving	Digital liquid crystal display to see the clear intuitive, very formal workmanship is not rough	Jingdong prefecture level city in Shandong
Power Saving King Shop	King of Power Saving	Received. Great quality, fast shipping from the store and not too expensive!	Jingdong prefecture level city in Shandong
Power Saving King Shop	King of Power Saving	Bought myself a knuckle regulator and it was easy to use and the price was right.	Jingdong prefecture level city in Shandong
Power Saving King Shop	King of Power Saving	Received, good, powerful, good, very full of a shopping	Jingdong prefecture level city in Shandong
Power Saving King Shop	King of Power Saving	High power, consistent with the seller's description, powerful, cost-effective	Jingdong prefecture level city in Shandong
Power Saving King Shop	King of Power Saving	It works very well and also saves power at an affordable price a great deal.	Jingdong prefecture level city in Shandong
Power Saving King Shop	King of Power Saving	Good quality, good can recommend to friends to buy.	Jingdong prefecture level city in Shandong
Power Saving King Shop	King of Power Saving	Will repurchase, quality is guaranteed, safe and secure purchase.	Jingdong prefecture level city in Shandong
Power Saving King Shop	King of Power Saving	The logistics is very fast, the thing is also good to use, energy saving power rely on it!	Jingdong prefecture level city in Shandong
Power Saving King Shop	King of Power Saving	Power Saver is good quality and works very well, worth buying!	Jingdong prefecture level city in Shandong
Power Saving King Shop	King of Power Saving	The quality is very good is genuine, cost-effective recommended	Jingdong prefecture level city in Shandong
Power Saving King Shop	King of Power Saving	Quality Assurance. Favorable price. Very practical and durable	Jingdong prefecture level city in Shandong
Power Saving King Shop	King of Power Saving	Good value for money. Conscientious businessman. Recommended to everyone	Jingdong prefecture level city in Shandong

Power Saving King Shop	King of Power Saving	Buy it for your rental apartment and never worry about your landlord charging you too much.	Jingdong prefecture level city in Shandong
Power Saving King Shop	King of Power Saving	Nice and affordable, will repurchase next time	Jingdong prefecture level city in Shandong
Power Saving King Shop	King of Power Saving	It's very energy efficient. I've been using it for a few days now.	Jingdong prefecture level city in Shandong
Power Saving King Shop	King of Power Saving	Good quality, high power easy to use	Jingdong prefecture level city in Shandong
Power Saving King Shop	King of Power Saving	Power saving little helper. Deserves a favorable review.	Jingdong prefecture level city in Shandong
Power Saving King Shop	King of Power Saving	Great value for money, great value for money.	Jingdong prefecture level city in Shandong
Power Saving King Shop	King of Power Saving	Works well and as described by the seller	Jingdong prefecture level city in Shandong
Power Saving King Shop	King of Power Saving	Power saving is proper! It's good.	Jingdong prefecture level city in Shandong
Power Saving King Shop	King of Power Saving	Power saving. Works well. Not bad.	Jingdong prefecture level city in Shandong
Power Saving King Shop	King of Power Saving	Satisfied with power saving	Jingdong prefecture level city in Shandong
Power Saving King Shop	King of Power Saving	Received intact, open a super good look beautiful and generous, ~ bit of high quality is recommended to buy, the need for friends rest assured that the next order	Jingdong prefecture level city in Shandong
Power Saving King Shop	King of Power Saving	Very good to use, friends introduced, save electricity and money, long-lasting products cost-effective, large factory production, good quality, fine workmanship cost-effective!	Jingdong prefecture level city in Shandong
Power Saving King Shop	King of Power Saving	The power saving effect is very obvious, trying to pay 30% less electricity bill in a month than before, hahaha	Jingdong prefecture level city in Shandong
Power Saving King Shop	King of Power Saving	It's such a power saver to use this, it saves money on kids snacks for a month. Kudos!	Jingdong prefecture level city in Shandong
Power Saving King Shop	King of Power Saving	Plug and play, stable voltage, the screen has a display when working, this winter home appliances, need more electricity, just buy to use, save electricity and save money, with instructions, not too big and does not take up space, the quality is very good, and the seller describes exactly the same!	Jingdong prefecture level city in Shandong
Power Saving King Shop	King of Power Saving	A must have for opening a store. Save over a thousand dollars a month on your electric bill	Jingdong prefecture level city in Shandong
Power Saving King Shop	King of Power Saving	Received the goods, the first time to unpack, the quality is very good, with the seller's description, completely exceeded my expectations, the packaging is very careful, very strict, in general this is a very satisfied with the shopping	Jingdong prefecture level city in Shandong
Power Saving King Shop	King of Power Saving	As always, good quality, a very favorite product, great! o(*￣︶￣*)o The price is very affordable, cost-effective, it's considered affordable good, will repurchase!	Jingdong prefecture level city in Shandong
Power Saving King Shop	King of Power Saving	Very satisfied with a shopping, good business service, fast shipping, logistics is also very powerful, received very good, buy assured, use peace of mind!	Jingdong prefecture level city in Shandong
Power Saving King Shop	King of Power Saving	The first time to buy online, but also look at the evaluation before buying, the quality is not bad, but also very cheap, the need for friends can rest assured to buy!	Jingdong prefecture level city in Shandong
Power Saving King Shop	King of Power Saving	Better than expected, feel value for money, very satisfied with a shopping, need next time will come back!	Jingdong prefecture level city in Shandong
Power Saving King Shop	King of Power Saving	Really super like, very supportive, very good quality, exactly as described by the seller, very satisfied!	Jingdong prefecture level city in Shandong
Power Saving King Shop	King of Power Saving	It is genuine, effective, really saves power, affordable, tightly packed, fast shipping, very satisfied.	Jingdong prefecture level city in Shandong
Power Saving King Shop	King of Power Saving	Good quality, fine workmanship, is genuine and business description of the same, good value for money, fast logistics, very satisfied	Jingdong prefecture level city in Shandong
Power Saving King Shop	King of Power Saving	Saver received, very good, installation set up very well, very good, the effect is very good, recommend that the pro buy	Jingdong prefecture level city in Shandong
Power Saving King Shop	King of Power Saving	Electromagnetic good, high power convenient, enough force, the price is right it, quality can be 😀 😀 😀 😀 😀	Jingdong prefecture level city in Shandong
Power Saving King Shop	King of Power Saving	First-class quality, packaging is also good, logistics is very fast, and the price is reasonable, very affordable, I like it!	Jingdong prefecture level city in Shandong
Power Saving King Shop	King of Power Saving	It's great, it's the kind of style you want, and it arrived quickly, thank you boss, 5 stars for the service!	Jingdong prefecture level city in Shandong
Power Saving King Shop	King of Power Saving	Packaging is very good, the use of the effect is quite good, the store has a good service attitude, trustworthy	Jingdong prefecture level city in Shandong
Power Saving King Shop	King of Power Saving	The quality is very good, it works well, very satisfied, the price is affordable, it's so great value, it's worth recommending.	Jingdong prefecture level city in Shandong
Power Saving King Shop	King of Power Saving	The baby description matches the actual item very well! The quality is awesome! Will be back to patronize again in the future!	Jingdong prefecture level city in Shandong
Power Saving King Shop	King of Power Saving	Ran the price is not high try to buy the attitude, did not expect the effect is really good, good!	Jingdong prefecture level city in Shandong
Power Saving King Shop	King of Power Saving	Power Saving King power is quite large, the quality of the leaf is quite good, the price of buying a very good deal, must be praised!	Jingdong prefecture level city in Shandong
Power Saving King Shop	King of Power Saving	Good product quality is the king of power-saving products trustworthy as always good to recommend!	Jingdong prefecture level city in Shandong
Power Saving King Shop	King of Power Saving	Works great, very affordable, shipped pretty fast, comfortable to use	Jingdong prefecture level city in Shandong
Power Saving King Shop	King of Power Saving	Shipping was pretty fast and affordable, not bad 👍	Jingdong prefecture level city in Shandong
Power Saving King Shop	King of Power Saving	Digital voltage display, voltage at a glance, plug and play, safe and simple!	Jingdong prefecture level city in Shandong
Power Saving King Shop	King of Power Saving	Quality assurance, very good, affordable, logistics is also very fast, I'm very satisfied with the	Jingdong prefecture level city in Shandong
Power Saving King Shop	King of Power Saving	The quality is very good, the first time I received the goods to open the first time to see, it is very good, very satisfied	Jingdong prefecture level city in Shandong
Power Saving King Shop	King of Power Saving	Power Saving King used, the effect is good, the price is affordable, very good, is the genuine article	Jingdong prefecture level city in Shandong
Power Saving King Shop	King of Power Saving	Good quality, fine workmanship, good appearance, time is very accurate, satisfied.	Jingdong prefecture level city in Shandong
Power Saving King Shop	King of Power Saving	I purposely used it for two days before commenting, which is responsible for myself and everyone else!	Jingdong prefecture level city in Shandong
Power Saving King Shop	King of Power Saving	Easy to install, easy to operate, helped our family save a lot of electricity this summer	Jingdong prefecture level city in Shandong
Power Saving King Shop	King of Power Saving	Bringing power savings into millions of homes. Still a great gadget. Pretty good.	Jingdong prefecture level city in Shandong
Power Saving King Shop	King of Power Saving	Digital liquid crystal display to see the clear intuitive, very formal workmanship is not rough	Jingdong prefecture level city in Shandong
Power Saving King Shop	King of Power Saving	Received. Great quality, fast shipping from the store and not too expensive!	Jingdong prefecture level city in Shandong
Power Saving King Shop	King of Power Saving	Bought myself a knuckle regulator and it was easy to use and the price was right.	Jingdong prefecture level city in Shandong
Power Saving King Shop	King of Power Saving	Received, good, powerful, good, very full of a shopping	Jingdong prefecture level city in Shandong
Power Saving King Shop	King of Power Saving	High power, consistent with the seller's description, powerful, cost-effective	Jingdong prefecture level city in Shandong
Power Saving King Shop	King of Power Saving	It works very well and also saves power at an affordable price a great deal.	Jingdong prefecture level city in Shandong

Power Saving King Shop	King of Power Saving	Good quality, good can recommend to friends to buy.	Jingdong prefecture level city in Shandong
Power Saving King Shop	King of Power Saving	Will repurchase, quality is guaranteed, safe and secure purchase.	Jingdong prefecture level city in Shandong
Power Saving King Shop	King of Power Saving	The logistics is very fast, the thing is also good to use, energy saving power rely on it!	Jingdong prefecture level city in Shandong
Power Saving King Shop	King of Power Saving	Power Saver is good quality and works very well, worth buying!	Jingdong prefecture level city in Shandong
Power Saving King Shop	King of Power Saving	The quality is very good is genuine, cost-effective recommended	Jingdong prefecture level city in Shandong
Power Saving King Shop	King of Power Saving	Quality Assurance. Favorable price. Very practical and durable	Jingdong prefecture level city in Shandong
Power Saving King Shop	King of Power Saving	Good value for money. Conscientious businessman. Recommended to everyone	Jingdong prefecture level city in Shandong
Power Saving King Shop	King of Power Saving	Buy it for your rental apartment and never worry about your landlord charging you too much.	Jingdong prefecture level city in Shandong
Power Saving King Shop	King of Power Saving	Nice and affordable, will repurchase next time	Jingdong prefecture level city in Shandong
Power Saving King Shop	King of Power Saving	It's very energy efficient. I've been using it for a few days now.	Jingdong prefecture level city in Shandong
Power Saving King Shop	King of Power Saving	Good quality, high power easy to use	Jingdong prefecture level city in Shandong
Power Saving King Shop	King of Power Saving	Power saving little helper. Deserves a favorable review.	Jingdong prefecture level city in Shandong
Power Saving King Shop	King of Power Saving	Great value for money, great value for money.	Jingdong prefecture level city in Shandong
Power Saving King Shop	King of Power Saving	Works well and as described by the seller	Jingdong prefecture level city in Shandong
Power Saving King Shop	King of Power Saving	Power saving is proper! It's good.	Jingdong prefecture level city in Shandong
Power Saving King Shop	King of Power Saving	Power saving. Works well. Not bad.	Jingdong prefecture level city in Shandong
Power Saving King Shop	King of Power Saving	Satisfied with power saving	Jingdong prefecture level city in Shandong
Power Saving King Shop	King of Power Saving	Received intact, open a super good look beautiful and generous, ~ bit of high quality is recommended to buy, the need for friends rest assured that the next order	Jingdong prefecture level city in Shandong
Power Saving King Shop	King of Power Saving	Very good to use, friends introduced, save electricity and money, long-lasting products cost-effective, large factory production, good quality, fine workmanship cost-effective!	Jingdong prefecture level city in Shandong
Power Saving King Shop	King of Power Saving	The power saving effect is very obvious, trying to pay 30% less electricity bill in a month than before, hahaha	Jingdong prefecture level city in Shandong
Power Saving King Shop	King of Power Saving	It's such a power saver to use this, it saves money on kids snacks for a month. Kudos!	Jingdong prefecture level city in Shandong
Power Saving King Shop	King of Power Saving	Plug and play, stable voltage, the screen has a display when working, this winter home appliances, need more electricity, just buy to use, save electricity and save money, with instructions, not too big and does not take up space, the quality is very good, and the seller describes exactly the same!	Jingdong prefecture level city in Shandong
Power Saving King Shop	King of Power Saving	A must have for opening a store. Save over a thousand dollars a month on your electric bill	Jingdong prefecture level city in Shandong
Power Saving King Shop	King of Power Saving	Received the goods, the first time to unpack, the quality is very good, with the seller's description, completely exceeded my expectations, the packaging is very careful, very strict, in general this is a very satisfied with the shopping	Jingdong prefecture level city in Shandong
Power Saving King Shop	King of Power Saving	As always, good quality, a very favorite product, great! o(*￣︶￣*)o The price is very affordable, cost-effective, it's considered affordable good, will repurchase!	Jingdong prefecture level city in Shandong
Power Saving King Shop	King of Power Saving	Very satisfied with a shopping, good business service, fast shipping, logistics is also very powerful, received very good, buy assured, use peace of mind!	Jingdong prefecture level city in Shandong
Power Saving King Shop	King of Power Saving	The first time to buy online, but also look at the evaluation before buying, the quality is not bad, but also very cheap, the need for friends can rest assured to buy!	Jingdong prefecture level city in Shandong
Power Saving King Shop	King of Power Saving	Better than expected, feel value for money, very satisfied with a shopping, need next time will come back!	Jingdong prefecture level city in Shandong
Power Saving King Shop	King of Power Saving	Really super like, very supportive, very good quality, exactly as described by the seller, very satisfied!	Jingdong prefecture level city in Shandong
Power Saving King Shop	King of Power Saving	It is genuine, effective, really saves power, affordable, tightly packed, fast shipping, very satisfied.	Jingdong prefecture level city in Shandong
Power Saving King Shop	King of Power Saving	Good quality, fine workmanship, is genuine and business description of the same, good value for money, fast logistics, very satisfied	Jingdong prefecture level city in Shandong
Power Saving King Shop	King of Power Saving	Saver received, very good, installation set up very well, very good, the effect is very good, recommend that the pro buy	Jingdong prefecture level city in Shandong
Power Saving King Shop	King of Power Saving	Electromagnetic good, high power convenient, enough force, the price is right it, quality can be 😃 😃 😃 😃 😃	Jingdong prefecture level city in Shandong
Power Saving King Shop	King of Power Saving	First-class quality, packaging is also good, logistics is very fast, and the price is reasonable, very affordable, I like it!	Jingdong prefecture level city in Shandong
Power Saving King Shop	King of Power Saving	It's great, it's the kind of style you want, and it arrived quickly, thank you boss, 5 stars for the service!	Jingdong prefecture level city in Shandong
Power Saving King Shop	King of Power Saving	Packaging is very good, the use of the effect is quite good, the store has a good service attitude, trustworthy	Jingdong prefecture level city in Shandong
Power Saving King Shop	King of Power Saving	The quality is very good, it works well, very satisfied, the price is affordable, it's so great value, it's worth recommending.	Jingdong prefecture level city in Shandong
Power Saving King Shop	King of Power Saving	The baby description matches the actual item very well! The quality is awesome! Will be back to patronize again in the future!	Jingdong prefecture level city in Shandong
Power Saving King Shop	King of Power Saving	Ran the price is not high try to buy the attitude, did not expect the effect is really good, good!	Jingdong prefecture level city in Shandong
Power Saving King Shop	King of Power Saving	Power Saving King power is quite large, the quality of the leaf is quite good, the price of buying a very good deal, must be praised!	Jingdong prefecture level city in Shandong
Power Saving King Shop	King of Power Saving	Good product quality is the king of power-saving products trustworthy as always good to recommend!	Jingdong prefecture level city in Shandong
Power Saving King Shop	King of Power Saving	Works great, very affordable, shipped pretty fast, comfortable to use	Jingdong prefecture level city in Shandong
Power Saving King Shop	King of Power Saving	Shipping was pretty fast and affordable, not bad 👍	Jingdong prefecture level city in Shandong
Power Saving King Shop	King of Power Saving	Digital voltage display, voltage at a glance, plug and play, safe and simple!	Jingdong prefecture level city in Shandong
Power Saving King Shop	King of Power Saving	Quality assurance, very good, affordable, logistics is also very fast, I'm very satisfied with the	Jingdong prefecture level city in Shandong
Power Saving King Shop	King of Power Saving	The quality is very good, the first time I received the goods to open the first time to see, it is very good, very satisfied	Jingdong prefecture level city in Shandong
Power Saving King Shop	King of Power Saving	Power Saving King used, the effect is good, the price is affordable, very good, is the genuine article	Jingdong prefecture level city in Shandong
Power Saving King Shop	King of Power Saving	Good quality, fine workmanship, good appearance, time is very accurate, satisfied.	Jingdong prefecture level city in Shandong
Power Saving King Shop	King of Power Saving	I purposely used it for two days before commenting, which is responsible for myself and everyone else!	Jingdong prefecture level city in Shandong
Power Saving King Shop	King of Power Saving	Easy to install, easy to operate, helped our family save a lot of electricity this summer	Jingdong prefecture level city in Shandong

Power Saving King Shop	King of Power Saving	Bringing power savings into millions of homes. Still a great gadget. Pretty good.	Jingdong prefecture level city in Shandong
Power Saving King Shop	King of Power Saving	Digital liquid crystal display to see the clear intuitive, very formal workmanship is not rough	Jingdong prefecture level city in Shandong
Power Saving King Shop	King of Power Saving	Received. Great quality, fast shipping from the store and not too expensive!	Jingdong prefecture level city in Shandong
Power Saving King Shop	King of Power Saving	Bought myself a knuckle regulator and it was easy to use and the price was right.	Jingdong prefecture level city in Shandong
Power Saving King Shop	King of Power Saving	Received, good, powerful, good, very full of a shopping	Jingdong prefecture level city in Shandong
Power Saving King Shop	King of Power Saving	High power, consistent with the seller's description, powerful, cost-effective	Jingdong prefecture level city in Shandong
Power Saving King Shop	King of Power Saving	It works very well and also saves power at an affordable price a great deal.	Jingdong prefecture level city in Shandong
Power Saving King Shop	King of Power Saving	Good quality, good can recommend to friends to buy.	Jingdong prefecture level city in Shandong
Power Saving King Shop	King of Power Saving	Will repurchase, quality is guaranteed, safe and secure purchase.	Jingdong prefecture level city in Shandong
Power Saving King Shop	King of Power Saving	The logistics is very fast, the thing is also good to use, energy saving power rely on it!	Jingdong prefecture level city in Shandong
Power Saving King Shop	King of Power Saving	Power Saver is good quality and works very well, worth buying!	Jingdong prefecture level city in Shandong
Power Saving King Shop	King of Power Saving	The quality is very good is genuine, cost-effective recommended	Jingdong prefecture level city in Shandong
Power Saving King Shop	King of Power Saving	Quality Assurance. Favorable price. Very practical and durable	Jingdong prefecture level city in Shandong
Power Saving King Shop	King of Power Saving	Good value for money. Conscientious businessman. Recommended to everyone	Jingdong prefecture level city in Shandong
Power Saving King Shop	King of Power Saving	Buy it for your rental apartment and never worry about your landlord charging you too much.	Jingdong prefecture level city in Shandong
Power Saving King Shop	King of Power Saving	Nice and affordable, will repurchase next time	Jingdong prefecture level city in Shandong
Power Saving King Shop	King of Power Saving	It's very energy efficient. I've been using it for a few days now.	Jingdong prefecture level city in Shandong
Power Saving King Shop	King of Power Saving	Good quality, high power easy to use	Jingdong prefecture level city in Shandong
Power Saving King Shop	King of Power Saving	Power saving little helper. Deserves a favorable review.	Jingdong prefecture level city in Shandong
Power Saving King Shop	King of Power Saving	Great value for money, great value for money.	Jingdong prefecture level city in Shandong
Power Saving King Shop	King of Power Saving	Works well and as described by the seller	Jingdong prefecture level city in Shandong
Power Saving King Shop	King of Power Saving	Power saving is proper! It's good.	Jingdong prefecture level city in Shandong
Power Saving King Shop	King of Power Saving	Power saving. Works well. Not bad.	Jingdong prefecture level city in Shandong
Power Saving King Shop	King of Power Saving	Satisfied with power saving	Jingdong prefecture level city in Shandong
Power Saving King Shop	King of Power Saving	Received intact, open a super good look beautiful and generous, ~ bit of high quality is recommended to buy, the need for friends rest assured that the next order	Jingdong prefecture level city in Shandong
Power Saving King Shop	King of Power Saving	Very good to use, friends introduced, save electricity and money, long-lasting products cost-effective, large factory production, good quality, fine workmanship cost-effective!	Jingdong prefecture level city in Shandong
Power Saving King Shop	King of Power Saving	The power saving effect is very obvious, trying to pay 30% less electricity bill in a month than before, hahaha	Jingdong prefecture level city in Shandong
Power Saving King Shop	King of Power Saving	It's such a power saver to use this, it saves money on kids snacks for a month. Kudos!	Jingdong prefecture level city in Shandong
Power Saving King Shop	King of Power Saving	Plug and play, stable voltage, the screen has a display when working, this winter home appliances, need more electricity, just buy to use, save electricity and save money, with instructions, not too big and does not take up space, the quality is very good, and the seller describes exactly the same!	Jingdong prefecture level city in Shandong
Power Saving King Shop	King of Power Saving	A must have for opening a store. Save over a thousand dollars a month on your electric bill	Jingdong prefecture level city in Shandong
Power Saving King Shop	King of Power Saving	Received the goods, the first time to unpack, the quality is very good, with the seller's description, completely exceeded my expectations, the packaging is very careful, very strict, in general this is a very satisfied with the shopping	Jingdong prefecture level city in Shandong
Power Saving King Shop	King of Power Saving	As always, good quality, a very favorite product, great! o(*￣︶￣*)o The price is very affordable, cost-effective, it's considered affordable good, will repurchase!	Jingdong prefecture level city in Shandong
Power Saving King Shop	King of Power Saving	Very satisfied with a shopping, good business service, fast shipping, logistics is also very powerful, received very good, buy assured, use peace of mind!	Jingdong prefecture level city in Shandong
Power Saving King Shop	King of Power Saving	The first time to buy online, but also look at the evaluation before buying, the quality is not bad, but also very cheap, the need for friends can rest assured to buy!	Jingdong prefecture level city in Shandong
Power Saving King Shop	King of Power Saving	Better than expected, feel value for money, very satisfied with a shopping, need next time will come back!	Jingdong prefecture level city in Shandong
Power Saving King Shop	King of Power Saving	Really super like, very supportive, very good quality, exactly as described by the seller, very satisfied!	Jingdong prefecture level city in Shandong
Power Saving King Shop	King of Power Saving	It is genuine, effective, really saves power, affordable, tightly packed, fast shipping, very satisfied.	Jingdong prefecture level city in Shandong
Power Saving King Shop	King of Power Saving	Good quality, fine workmanship, is genuine and business description of the same, good value for money, fast logistics, very satisfied	Jingdong prefecture level city in Shandong
Power Saving King Shop	King of Power Saving	Saver received, very good, installation set up very well, very good, the effect is very good, recommend that the pro buy	Jingdong prefecture level city in Shandong
Power Saving King Shop	King of Power Saving	Electromagnetic good, high power convenient, enough force, the price is right it, quality can be 😃 😃 😃 😃 😃	Jingdong prefecture level city in Shandong
Power Saving King Shop	King of Power Saving	First-class quality, packaging is also good, logistics is very fast, and the price is reasonable, very affordable, I like it!	Jingdong prefecture level city in Shandong
Power Saving King Shop	King of Power Saving	It's great, it's the kind of style you want, and it arrived quickly, thank you boss, 5 stars for the service!	Jingdong prefecture level city in Shandong
Power Saving King Shop	King of Power Saving	Packaging is very good, the use of the effect is quite good, the store has a good service attitude, trustworthy	Jingdong prefecture level city in Shandong
Power Saving King Shop	King of Power Saving	The quality is very good, it works well, very satisfied, the price is affordable, it's so great value, it's worth recommending.	Jingdong prefecture level city in Shandong
Power Saving King Shop	King of Power Saving	The baby description matches the actual item very well! The quality is awesome! Will be back to patronize again in the future!	Jingdong prefecture level city in Shandong
Power Saving King Shop	King of Power Saving	Ran the price is not high try to buy the attitude, did not expect the effect is really good, good!	Jingdong prefecture level city in Shandong
Power Saving King Shop	King of Power Saving	Power Saving King power is quite large, the quality of the leaf is quite good, the price of buying a very good deal, must be praised!	Jingdong prefecture level city in Shandong
Power Saving King Shop	King of Power Saving	Good product quality is the king of power-saving products trustworthy as always good to recommend!	Jingdong prefecture level city in Shandong
Power Saving King Shop	King of Power Saving	Works great, very affordable, shipped pretty fast, comfortable to use	Jingdong prefecture level city in Shandong
Power Saving King Shop	King of Power Saving	Shipping was pretty fast and affordable, not bad 👍	Jingdong prefecture level city in Shandong

Power Saving King Shop	King of Power Saving	Digital voltage display, voltage at a glance, plug and play, safe and simple!	Jingdong prefecture level city in Shandong
Power Saving King Shop	King of Power Saving	Quality assurance, very good, affordable, logistics is also very fast, I'm very satisfied with the	Jingdong prefecture level city in Shandong
Power Saving King Shop	King of Power Saving	The quality is very good, the first time I received the goods to open the first time to see, it is very good, very satisfied	Jingdong prefecture level city in Shandong
Power Saving King Shop	King of Power Saving	Power Saving King used, the effect is good, the price is affordable, very good, is the genuine article	Jingdong prefecture level city in Shandong
Power Saving King Shop	King of Power Saving	Good quality, fine workmanship, good appearance, time is very accurate, satisfied.	Jingdong prefecture level city in Shandong
Power Saving King Shop	King of Power Saving	I purposely used it for two days before commenting, which is responsible for myself and everyone else!	Jingdong prefecture level city in Shandong
Power Saving King Shop	King of Power Saving	Easy to install, easy to operate, helped our family save a lot of electricity this summer	Jingdong prefecture level city in Shandong
Power Saving King Shop	King of Power Saving	Bringing power savings into millions of homes. Still a great gadget. Pretty good.	Jingdong prefecture level city in Shandong
Power Saving King Shop	King of Power Saving	Digital liquid crystal display to see the clear intuitive, very formal workmanship is not rough	Jingdong prefecture level city in Shandong
Power Saving King Shop	King of Power Saving	Received. Great quality, fast shipping from the store and not too expensive!	Jingdong prefecture level city in Shandong
Power Saving King Shop	King of Power Saving	Bought myself a knuckle regulator and it was easy to use and the price was right.	Jingdong prefecture level city in Shandong
Power Saving King Shop	King of Power Saving	Received, good, powerful, good, very full of a shopping	Jingdong prefecture level city in Shandong
Power Saving King Shop	King of Power Saving	High power, consistent with the seller's description, powerful, cost-effective	Jingdong prefecture level city in Shandong
Power Saving King Shop	King of Power Saving	It works very well and also saves power at an affordable price a great deal.	Jingdong prefecture level city in Shandong
Power Saving King Shop	King of Power Saving	Good quality, good can recommend to friends to buy.	Jingdong prefecture level city in Shandong
Power Saving King Shop	King of Power Saving	Will repurchase, quality is guaranteed, safe and secure purchase.	Jingdong prefecture level city in Shandong
Power Saving King Shop	King of Power Saving	The logistics is very fast, the thing is also good to use, energy saving power rely on it!	Jingdong prefecture level city in Shandong
Power Saving King Shop	King of Power Saving	Power Saver is good quality and works very well, worth buying!	Jingdong prefecture level city in Shandong
Power Saving King Shop	King of Power Saving	The quality is very good is genuine, cost-effective recommended	Jingdong prefecture level city in Shandong
Power Saving King Shop	King of Power Saving	Quality Assurance. Favorable price. Very practical and durable	Jingdong prefecture level city in Shandong
Power Saving King Shop	King of Power Saving	Good value for money. Conscientious businessman. Recommended to everyone	Jingdong prefecture level city in Shandong
Power Saving King Shop	King of Power Saving	Buy it for your rental apartment and never worry about your landlord charging you too much.	Jingdong prefecture level city in Shandong
Power Saving King Shop	King of Power Saving	Nice and affordable, will repurchase next time	Jingdong prefecture level city in Shandong
Power Saving King Shop	King of Power Saving	It's very energy efficient. I've been using it for a few days now.	Jingdong prefecture level city in Shandong
Power Saving King Shop	King of Power Saving	Good quality, high power easy to use	Jingdong prefecture level city in Shandong
Power Saving King Shop	King of Power Saving	Power saving little helper. Deserves a favorable review.	Jingdong prefecture level city in Shandong
Power Saving King Shop	King of Power Saving	Works well and as described by the seller	Jingdong prefecture level city in Shandong
Power Saving King Shop	King of Power Saving	Great value for money, great value for money.	Jingdong prefecture level city in Shandong
Power Saving King Shop	King of Power Saving	Power saving is proper! It's good.	Jingdong prefecture level city in Shandong
Power Saving King Shop	King of Power Saving	Power saving. Works well. Not bad.	Jingdong prefecture level city in Shandong
Power Saving King Shop	King of Power Saving	Satisfied with power saving	Jingdong prefecture level city in Shandong
Power Saving King Shop	King of Power Saving	Received intact, open a super good look beautiful and generous, ~ bit of high quality is recommended to buy, the need for friends rest assured that the next order	Jingdong prefecture level city in Shandong
Power Saving King Shop	King of Power Saving	Very good to use, friends introduced, save electricity and money, long-lasting products cost-effective, large factory production, good quality, fine workmanship cost-effective!	Jingdong prefecture level city in Shandong
Power Saving King Shop	King of Power Saving	The power saving effect is very obvious, trying to pay 30% less electricity bill in a month than before, hahaha	Jingdong prefecture level city in Shandong
Power Saving King Shop	King of Power Saving	It's such a power saver to use this, it saves money on kids snacks for a month. Kudos!	Jingdong prefecture level city in Shandong
Power Saving King Shop	King of Power Saving	Plug and play, stable voltage, the screen has a display when working, this winter home appliances, need more electricity, just buy to use, save electricity and save money, with instructions, not too big and does not take up space, the quality is very good, and the seller describes exactly the same!	Jingdong prefecture level city in Shandong
Power Saving King Shop	King of Power Saving	Received the goods, the first time to unpack, the quality is very good, with the seller's description, completely exceeded my expectations, the packaging is very careful, very strict, in general this is a very satisfied with the shopping	Jingdong prefecture level city in Shandong
Power Saving King Shop	King of Power Saving	A must have for opening a store. Save over a thousand dollars a month on your electric bill	Jingdong prefecture level city in Shandong
Power Saving King Shop	King of Power Saving	As always, good quality, a very favorite product, great! o(*￣︶￣*)o The price is very affordable, cost-effective, it's considered affordable good, will repurchase!	Jingdong prefecture level city in Shandong
Power Saving King Shop	King of Power Saving	The first time to buy online, but also look at the evaluation before buying, the quality is not bad, but also very cheap, the need for friends can rest assured to buy!	Jingdong prefecture level city in Shandong
Power Saving King Shop	King of Power Saving	Very satisfied with a shopping, good business service, fast shipping, logistics is also very powerful, received very good, buy assured, use peace of mind!	Jingdong prefecture level city in Shandong
Power Saving King Shop	King of Power Saving	Better than expected, feel value for money, very satisfied with a shopping, need next time will come back!	Jingdong prefecture level city in Shandong
Power Saving King Shop	King of Power Saving	It is genuine, effective, really saves power, affordable, tightly packed, fast shipping, very satisfied.	Jingdong prefecture level city in Shandong
Power Saving King Shop	King of Power Saving	Really super like, very supportive, very good quality, exactly as described by the seller, very satisfied!	Jingdong prefecture level city in Shandong
Power Saving King Shop	King of Power Saving	Good quality, fine workmanship, is genuine and business description of the same, good value for money, fast logistics, very satisfied	Jingdong prefecture level city in Shandong
Power Saving King Shop	King of Power Saving	Saver received, very good, installation set up very well, very good, the effect is very good, recommend that the pro buy	Jingdong prefecture level city in Shandong
Power Saving King Shop	King of Power Saving	Electromagnetic good, high power convenient, enough force, the price is right it, quality can be 😃 😃 😃 😃 😃	Jingdong prefecture level city in Shandong
Power Saving King Shop	King of Power Saving	First-class quality, packaging is also good, logistics is very fast, and the price is reasonable, very affordable, I like it!	Jingdong prefecture level city in Shandong
Power Saving King Shop	King of Power Saving	It's great, it's the kind of style you want, and it arrived quickly, thank you boss, 5 stars for the service!	Jingdong prefecture level city in Shandong
Power Saving King Shop	King of Power Saving	Packaging is very good, the use of the effect is quite good, the store has a good service attitude, trustworthy	Jingdong prefecture level city in Shandong

Power Saving King Shop	King of Power Saving	The quality is very good, it works well, very satisfied, the price is affordable, it's so great value, it's worth recommending.	Jingdong prefecture level city in Shandong
Power Saving King Shop	King of Power Saving	The baby description matches the actual item very well! The quality is awesome! Will be back to patronize again in the future!	Jingdong prefecture level city in Shandong
Power Saving King Shop	King of Power Saving	Ran the price is not high try to buy the attitude, did not expect the effect is really good, good!	Jingdong prefecture level city in Shandong
Power Saving King Shop	King of Power Saving	Power Saving King power is quite large, the quality of the leaf is quite good, the price of buying a very good deal, must be praised!	Jingdong prefecture level city in Shandong
Power Saving King Shop	King of Power Saving	Good product quality is the king of power-saving products trustworthy as always good to recommend!	Jingdong prefecture level city in Shandong
Power Saving King Shop	King of Power Saving	Works great, very affordable, shipped pretty fast, comfortable to use	Jingdong prefecture level city in Shandong
Power Saving King Shop	King of Power Saving	Shipping was pretty fast and affordable, not bad 👍	Jingdong prefecture level city in Shandong
Power Saving King Shop	King of Power Saving	Digital voltage display, voltage at a glance, plug and play, safe and simple!	Jingdong prefecture level city in Shandong
Power Saving King Shop	King of Power Saving	Quality assurance, very good, affordable, logistics is also very fast, I'm very satisfied with the	Jingdong prefecture level city in Shandong
Power Saving King Shop	King of Power Saving	The quality is very good, the first time I received the goods to open the first time to see, it is very good, very satisfied	Jingdong prefecture level city in Shandong
Power Saving King Shop	King of Power Saving	Power Saving King used, the effect is good, the price is affordable, very good, is the genuine article	Jingdong prefecture level city in Shandong
Power Saving King Shop	King of Power Saving	Good quality, fine workmanship, good appearance, time is very accurate, satisfied.	Jingdong prefecture level city in Shandong
Power Saving King Shop	King of Power Saving	I purposely used it for two days before commenting, which is responsible for myself and everyone else!	Jingdong prefecture level city in Shandong
Power Saving King Shop	King of Power Saving	Easy to install, easy to operate, helped our family save a lot of electricity this summer	Jingdong prefecture level city in Shandong
Power Saving King Shop	King of Power Saving	Bringing power savings into millions of homes. Still a great gadget. Pretty good.	Jingdong prefecture level city in Shandong
Power Saving King Shop	King of Power Saving	Digital liquid crystal display to see the clear intuitive, very formal workmanship is not rough	Jingdong prefecture level city in Shandong
Power Saving King Shop	King of Power Saving	Received. Great quality, fast shipping from the store and not too expensive!	Jingdong prefecture level city in Shandong
Power Saving King Shop	King of Power Saving	Bought myself a knuckle regulator and it was easy to use and the price was right.	Jingdong prefecture level city in Shandong
Power Saving King Shop	King of Power Saving	Received, good, powerful, good, very full of a shopping	Jingdong prefecture level city in Shandong
Power Saving King Shop	King of Power Saving	High power, consistent with the seller's description, powerful, cost-effective	Jingdong prefecture level city in Shandong
Power Saving King Shop	King of Power Saving	It works very well and also saves power at an affordable price a great deal.	Jingdong prefecture level city in Shandong
Power Saving King Shop	King of Power Saving	Good quality, good can recommend to friends to buy.	Jingdong prefecture level city in Shandong
Power Saving King Shop	King of Power Saving	Will repurchase, quality is guaranteed, safe and secure purchase.	Jingdong prefecture level city in Shandong
Power Saving King Shop	King of Power Saving	The logistics is very fast, the thing is also good to use, energy saving power rely on it!	Jingdong prefecture level city in Shandong
Power Saving King Shop	King of Power Saving	Power Saver is good quality and works very well, worth buying!	Jingdong prefecture level city in Shandong
Power Saving King Shop	King of Power Saving	The quality is very good is genuine, cost-effective recommended	Jingdong prefecture level city in Shandong
Power Saving King Shop	King of Power Saving	Quality Assurance. Favorable price. Very practical and durable	Jingdong prefecture level city in Shandong
Power Saving King Shop	King of Power Saving	Good value for money. Conscientious businessman. Recommended to everyone	Jingdong prefecture level city in Shandong
Power Saving King Shop	King of Power Saving	Buy it for your rental apartment and never worry about your landlord charging you too much.	Jingdong prefecture level city in Shandong
Power Saving King Shop	King of Power Saving	Nice and affordable, will repurchase next time	Jingdong prefecture level city in Shandong
Power Saving King Shop	King of Power Saving	It's very energy efficient. I've been using it for a few days now.	Jingdong prefecture level city in Shandong
Power Saving King Shop	King of Power Saving	Good quality, high power easy to use	Jingdong prefecture level city in Shandong
Power Saving King Shop	King of Power Saving	Power saving little helper. Deserves a favorable review.	Jingdong prefecture level city in Shandong
Power Saving King Shop	King of Power Saving	Great value for money, great value for money.	Jingdong prefecture level city in Shandong
Power Saving King Shop	King of Power Saving	Works well and as described by the seller	Jingdong prefecture level city in Shandong
Power Saving King Shop	King of Power Saving	Power saving is proper! It's good.	Jingdong prefecture level city in Shandong
Power Saving King Shop	King of Power Saving	Power saving. Works well. Not bad.	Jingdong prefecture level city in Shandong
Power Saving King Shop	King of Power Saving	Satisfied with power saving	Jingdong prefecture level city in Shandong
Power Saving King Shop	King of Power Saving	Received intact, open a super good look beautiful and generous, ~ bit of high quality is recommended to buy, the need for friends rest assured that the next order	Jingdong prefecture level city in Shandong
Power Saving King Shop	King of Power Saving	Very good to use, friends introduced, save electricity and money, long-lasting products cost-effective, large factory production, good quality, fine workmanship cost-effective!	Jingdong prefecture level city in Shandong
Power Saving King Shop	King of Power Saving	The power saving effect is very obvious, trying to pay 30% less electricity bill in a month than before, hahaha	Jingdong prefecture level city in Shandong
Power Saving King Shop	King of Power Saving	It's such a power saver to use this, it saves money on kids snacks for a month. Kudos!	Jingdong prefecture level city in Shandong
Power Saving King Shop	King of Power Saving	Plug and play, stable voltage, the screen has a display when working, this winter home appliances, need more electricity, just buy to use, save electricity and save money, with instructions, not too big and does not take up space, the quality is very good, and the seller describes exactly the same!	Jingdong prefecture level city in Shandong
Power Saving King Shop	King of Power Saving	A must have for opening a store. Save over a thousand dollars a month on your electric bill	Jingdong prefecture level city in Shandong
Power Saving King Shop	King of Power Saving	Received the goods, the first time to unpack, the quality is very good, with the seller's description, completely exceeded my expectations, the packaging is very careful, very strict, in general this is a very satisfied with the shopping	Jingdong prefecture level city in Shandong
Power Saving King Shop	King of Power Saving	As always, good quality, a very favorite product, great! o(*￣︶￣*)o The price is very affordable, cost-effective, it's considered affordable good, will repurchase!	Jingdong prefecture level city in Shandong
Power Saving King Shop	King of Power Saving	Very satisfied with a shopping, good business service, fast shipping, logistics is also very powerful, received very good, buy assured, use peace of mind!	Jingdong prefecture level city in Shandong
Power Saving King Shop	King of Power Saving	The first time to buy online, but also look at the evaluation before buying, the quality is not bad, but also very cheap, the need for friends can rest assured to buy!	Jingdong prefecture level city in Shandong
Power Saving King Shop	King of Power Saving	Better than expected, feel value for money, very satisfied with a shopping, need next time will come back!	Jingdong prefecture level city in Shandong
Power Saving King Shop	King of Power Saving	Really super like, very supportive, very good quality, exactly as described by the seller, very satisfied!	Jingdong prefecture level city in Shandong

Power Saving King Shop	King of Power Saving	It is genuine, effective, really saves power, affordable, tightly packed, fast shipping, very satisfied.	Jingdong prefecture level city in Shandong
Power Saving King Shop	King of Power Saving	Good quality, fine workmanship, is genuine and business description of the same, good value for money, fast logistics, very satisfied	Jingdong prefecture level city in Shandong
Power Saving King Shop	King of Power Saving	Saver received, very good, installation set up very well, very good, the effect is very good, recommend that the pro buy	Jingdong prefecture level city in Shandong
Power Saving King Shop	King of Power Saving	Electromagnetic good, high power convenient, enough force, the price is right it, quality can be 😀 😀 😀 😀 😀	Jingdong prefecture level city in Shandong
Power Saving King Shop	King of Power Saving	First-class quality, packaging is also good, logistics is very fast, and the price is reasonable, very affordable, I like it!	Jingdong prefecture level city in Shandong
Power Saving King Shop	King of Power Saving	It's great, it's the kind of style you want, and it arrived quickly, thank you boss, 5 stars for the service!	Jingdong prefecture level city in Shandong
Power Saving King Shop	King of Power Saving	Packaging is very good, the use of the effect is quite good, the store has a good service attitude, trustworthy	Jingdong prefecture level city in Shandong
Power Saving King Shop	King of Power Saving	The quality is very good, it works well, very satisfied, the price is affordable, it's so great value, it's worth recommending.	Jingdong prefecture level city in Shandong
Power Saving King Shop	King of Power Saving	The baby description matches the actual item very well! The quality is awesome! Will be back to patronize again in the future!	Jingdong prefecture level city in Shandong
Power Saving King Shop	King of Power Saving	Ran the price is not high try to buy the attitude, did not expect the effect is really good, good!	Jingdong prefecture level city in Shandong
Power Saving King Shop	King of Power Saving	Power Saving King power is quite large, the quality of the leaf is quite good, the price of buying a very good deal, must be praised!	Jingdong prefecture level city in Shandong
Power Saving King Shop	King of Power Saving	Good product quality is the king of power-saving products trustworthy as always good to recommend!	Jingdong prefecture level city in Shandong
Power Saving King Shop	King of Power Saving	Works great, very affordable, shipped pretty fast, comfortable to use	Jingdong prefecture level city in Shandong
Power Saving King Shop	King of Power Saving	Shipping was pretty fast and affordable, not bad 👍	Jingdong prefecture level city in Shandong
Power Saving King Shop	King of Power Saving	Digital voltage display, voltage at a glance, plug and play, safe and simple!	Jingdong prefecture level city in Shandong
Power Saving King Shop	King of Power Saving	Quality assurance, very good, affordable, logistics is also very fast, I'm very satisfied with the	Jingdong prefecture level city in Shandong
Power Saving King Shop	King of Power Saving	The quality is very good, the first time I received the goods to open the first time to see, it is very good, very satisfied	Jingdong prefecture level city in Shandong
Power Saving King Shop	King of Power Saving	Power Saving King used, the effect is good, the price is affordable, very good, is the genuine article	Jingdong prefecture level city in Shandong
Power Saving King Shop	King of Power Saving	Good quality, fine workmanship, good appearance, time is very accurate, satisfied.	Jingdong prefecture level city in Shandong
Power Saving King Shop	King of Power Saving	I purposely used it for two days before commenting, which is responsible for myself and everyone else!	Jingdong prefecture level city in Shandong
Power Saving King Shop	King of Power Saving	Easy to install, easy to operate, helped our family save a lot of electricity this summer	Jingdong prefecture level city in Shandong
Power Saving King Shop	King of Power Saving	Bringing power savings into millions of homes. Still a great gadget. Pretty good.	Jingdong prefecture level city in Shandong
Power Saving King Shop	King of Power Saving	Digital liquid crystal display to see the clear intuitive, very formal workmanship is not rough	Jingdong prefecture level city in Shandong
Power Saving King Shop	King of Power Saving	Received. Great quality, fast shipping from the store and not too expensive!	Jingdong prefecture level city in Shandong
Power Saving King Shop	King of Power Saving	Bought myself a knuckle regulator and it was easy to use and the price was right.	Jingdong prefecture level city in Shandong
Power Saving King Shop	King of Power Saving	Received, good, powerful, good, very full of a shopping	Jingdong prefecture level city in Shandong
Power Saving King Shop	King of Power Saving	High power, consistent with the seller's description, powerful, cost-effective	Jingdong prefecture level city in Shandong
Power Saving King Shop	King of Power Saving	It works very well and also saves power at an affordable price a great deal.	Jingdong prefecture level city in Shandong
Power Saving King Shop	King of Power Saving	Good quality, good can recommend to friends to buy.	Jingdong prefecture level city in Shandong
Power Saving King Shop	King of Power Saving	Will repurchase, quality is guaranteed, safe and secure purchase.	Jingdong prefecture level city in Shandong
Power Saving King Shop	King of Power Saving	The logistics is very fast, the thing is also good to use, energy saving power rely on it!	Jingdong prefecture level city in Shandong
Power Saving King Shop	King of Power Saving	Power Saver is good quality and works very well, worth buying!	Jingdong prefecture level city in Shandong
Power Saving King Shop	King of Power Saving	The quality is very good is genuine, cost-effective recommended	Jingdong prefecture level city in Shandong
Power Saving King Shop	King of Power Saving	Quality Assurance. Favorable price. Very practical and durable	Jingdong prefecture level city in Shandong
Power Saving King Shop	King of Power Saving	Good value for money. Conscientious businessman. Recommended to everyone	Jingdong prefecture level city in Shandong
Power Saving King Shop	King of Power Saving	Buy it for your rental apartment and never worry about your landlord charging you too much.	Jingdong prefecture level city in Shandong
Power Saving King Shop	King of Power Saving	Nice and affordable, will repurchase next time	Jingdong prefecture level city in Shandong
Power Saving King Shop	King of Power Saving	It's very energy efficient. I've been using it for a few days now.	Jingdong prefecture level city in Shandong
Power Saving King Shop	King of Power Saving	Good quality, high power easy to use	Jingdong prefecture level city in Shandong
Power Saving King Shop	King of Power Saving	Power saving little helper. Deserves a favorable review.	Jingdong prefecture level city in Shandong
Power Saving King Shop	King of Power Saving	Great value for money, great value for money.	Jingdong prefecture level city in Shandong
Power Saving King Shop	King of Power Saving	Works well and as described by the seller	Jingdong prefecture level city in Shandong
Power Saving King Shop	King of Power Saving	Power saving is proper! It's good.	Jingdong prefecture level city in Shandong
Power Saving King Shop	King of Power Saving	Power saving. Works well. Not bad.	Jingdong prefecture level city in Shandong
Power Saving King Shop	King of Power Saving	Satisfied with power saving	Jingdong prefecture level city in Shandong
Power Saving King Shop	King of Power Saving	Received intact, open a super good look beautiful and generous, ~ bit of high quality is recommended to buy, the need for friends rest assured that the next order	Jingdong prefecture level city in Shandong
Power Saving King Shop	King of Power Saving	Very good to use, friends introduced, save electricity and money, long-lasting products cost-effective, large factory production, good quality, fine workmanship cost-effective!	Jingdong prefecture level city in Shandong
Power Saving King Shop	King of Power Saving	The power saving effect is very obvious, trying to pay 30% less electricity bill in a month than before, hahaha	Jingdong prefecture level city in Shandong
Power Saving King Shop	King of Power Saving	It's such a power saver to use this, it saves money on kids snacks for a month. Kudos!	Jingdong prefecture level city in Shandong
Power Saving King Shop	King of Power Saving	Plug and play, stable voltage, the screen has a display when working, this winter home appliances, need more electricity, just buy to use, save electricity and save money, with instructions, not too big and does not take up space, the quality is very good, and the seller describes exactly the same!	Jingdong prefecture level city in Shandong
Power Saving King Shop	King of Power Saving	A must have for opening a store. Save over a thousand dollars a month on your electric bill	Jingdong prefecture level city in Shandong

Power Saving King Shop	King of Power Saving	Received the goods, the first time to unpack, the quality is very good, with the seller's description, completely exceeded my expectations, the packaging is very careful, very strict, in general this is a very satisfied with the shopping	Jingdong prefecture level city in Shandong
Power Saving King Shop	King of Power Saving	As always, good quality, a very favorite product, great! o(*￣︶￣*)o The price is very affordable, cost-effective, it's considered affordable good, will repurchase!	Jingdong prefecture level city in Shandong
Power Saving King Shop	King of Power Saving	Very satisfied with a shopping, good business service, fast shipping, logistics is also very powerful, received very good, buy assured, use peace of mind!	Jingdong prefecture level city in Shandong
Power Saving King Shop	King of Power Saving	The first time to buy online, but also look at the evaluation before buying, the quality is not bad, but also very cheap, the need for friends can rest assured to buy!	Jingdong prefecture level city in Shandong
Power Saving King Shop	King of Power Saving	Better than expected, feel value for money, very satisfied with a shopping, need next time will come back!	Jingdong prefecture level city in Shandong
Power Saving King Shop	King of Power Saving	Really super like, very supportive, very good quality, exactly as described by the seller, very satisfied!	Jingdong prefecture level city in Shandong
Power Saving King Shop	King of Power Saving	It is genuine, effective, really saves power, affordable, tightly packed, fast shipping, very satisfied.	Jingdong prefecture level city in Shandong
Power Saving King Shop	King of Power Saving	Good quality, fine workmanship, is genuine and business description of the same, good value for money, fast logistics, very satisfied	Jingdong prefecture level city in Shandong
Power Saving King Shop	King of Power Saving	Saver received, very good, installation set up very well, very good, the effect is very good, recommend that the pro buy	Jingdong prefecture level city in Shandong
Power Saving King Shop	King of Power Saving	Electromagnetic good, high power convenient, enough force, the price is right it, quality can be 😀 😀 😀 😀 😀	Jingdong prefecture level city in Shandong
Power Saving King Shop	King of Power Saving	First-class quality, packaging is also good, logistics is very fast, and the price is reasonable, very affordable, I like it!	Jingdong prefecture level city in Shandong
Power Saving King Shop	King of Power Saving	It's great, it's the kind of style you want, and it arrived quickly, thank you boss, 5 stars for the service!	Jingdong prefecture level city in Shandong
Power Saving King Shop	King of Power Saving	Packaging is very good, the use of the effect is quite good, the store has a good service attitude, trustworthy	Jingdong prefecture level city in Shandong
Power Saving King Shop	King of Power Saving	The quality is very good, it works well, very satisfied, the price is affordable, it's so great value, it's worth recommending.	Jingdong prefecture level city in Shandong
Power Saving King Shop	King of Power Saving	The baby description matches the actual item very well! The quality is awesome! Will be back to patronize again in the future!	Jingdong prefecture level city in Shandong
Power Saving King Shop	King of Power Saving	Ran the price is not high try to buy the attitude, did not expect the effect is really good, good!	Jingdong prefecture level city in Shandong
Power Saving King Shop	King of Power Saving	Power Saving King power is quite large, the quality of the leaf is quite good, the price of buying a very good deal, must be praised!	Jingdong prefecture level city in Shandong
Power Saving King Shop	King of Power Saving	Good product quality is the king of power-saving products trustworthy as always good to recommend!	Jingdong prefecture level city in Shandong
Power Saving King Shop	King of Power Saving	Works great, very affordable, shipped pretty fast, comfortable to use	Jingdong prefecture level city in Shandong
Power Saving King Shop	King of Power Saving	Shipping was pretty fast and affordable, not bad 👍	Jingdong prefecture level city in Shandong
Power Saving King Shop	King of Power Saving	Digital voltage display, voltage at a glance, plug and play, safe and simple!	Jingdong prefecture level city in Shandong
Power Saving King Shop	King of Power Saving	Quality assurance, very good, affordable, logistics is also very fast, I'm very satisfied with the	Jingdong prefecture level city in Shandong
Power Saving King Shop	King of Power Saving	The quality is very good, the first time I received the goods to open the first time to see, it is very good, very satisfied	Jingdong prefecture level city in Shandong
Power Saving King Shop	King of Power Saving	Power Saving King used, the effect is good, the price is affordable, very good, is the genuine article	Jingdong prefecture level city in Shandong
Power Saving King Shop	King of Power Saving	Good quality, fine workmanship, good appearance, time is very accurate, satisfied.	Jingdong prefecture level city in Shandong
Power Saving King Shop	King of Power Saving	I purposely used it for two days before commenting, which is responsible for myself and everyone else!	Jingdong prefecture level city in Shandong
Power Saving King Shop	King of Power Saving	Easy to install, easy to operate, helped our family save a lot of electricity this summer	Jingdong prefecture level city in Shandong
Power Saving King Shop	King of Power Saving	Bringing power savings into millions of homes. Still a great gadget. Pretty good.	Jingdong prefecture level city in Shandong
Power Saving King Shop	King of Power Saving	Digital liquid crystal display to see the clear intuitive, very formal workmanship is not rough	Jingdong prefecture level city in Shandong
Power Saving King Shop	King of Power Saving	Received. Great quality, fast shipping from the store and not too expensive!	Jingdong prefecture level city in Shandong
Power Saving King Shop	King of Power Saving	Bought myself a knuckle regulator and it was easy to use and the price was right.	Jingdong prefecture level city in Shandong
Power Saving King Shop	King of Power Saving	Received, good, powerful, good, very full of a shopping	Jingdong prefecture level city in Shandong
Power Saving King Shop	King of Power Saving	High power, consistent with the seller's description, powerful, cost-effective	Jingdong prefecture level city in Shandong
Power Saving King Shop	King of Power Saving	It works very well and also saves power at an affordable price a great deal.	Jingdong prefecture level city in Shandong
Power Saving King Shop	King of Power Saving	Good quality, good can recommend to friends to buy.	Jingdong prefecture level city in Shandong
Power Saving King Shop	King of Power Saving	Will repurchase, quality is guaranteed, safe and secure purchase.	Jingdong prefecture level city in Shandong
Power Saving King Shop	King of Power Saving	The logistics is very fast, the thing is also good to use, energy saving power rely on it!	Jingdong prefecture level city in Shandong
Power Saving King Shop	King of Power Saving	Power Saver is good quality and works very well, worth buying!	Jingdong prefecture level city in Shandong
Power Saving King Shop	King of Power Saving	The quality is very good is genuine, cost-effective recommended	Jingdong prefecture level city in Shandong
Power Saving King Shop	King of Power Saving	Quality Assurance. Favorable price. Very practical and durable	Jingdong prefecture level city in Shandong
Power Saving King Shop	King of Power Saving	Good value for money. Conscientious businessman. Recommended to everyone	Jingdong prefecture level city in Shandong
Power Saving King Shop	King of Power Saving	Buy it for your rental apartment and never worry about your landlord charging you too much.	Jingdong prefecture level city in Shandong
Power Saving King Shop	King of Power Saving	Nice and affordable, will repurchase next time	Jingdong prefecture level city in Shandong
Power Saving King Shop	King of Power Saving	It's very energy efficient. I've been using it for a few days now.	Jingdong prefecture level city in Shandong
Power Saving King Shop	King of Power Saving	Good quality, high power easy to use	Jingdong prefecture level city in Shandong
Power Saving King Shop	King of Power Saving	Power saving little helper. Deserves a favorable review.	Jingdong prefecture level city in Shandong
Power Saving King Shop	King of Power Saving	Great value for money, great value for money.	Jingdong prefecture level city in Shandong
Power Saving King Shop	King of Power Saving	Works well and as described by the seller	Jingdong prefecture level city in Shandong
Power Saving King Shop	King of Power Saving	Power saving is proper! It's good.	Jingdong prefecture level city in Shandong
Power Saving King Shop	King of Power Saving	Power saving. Works well. Not bad.	Jingdong prefecture level city in Shandong
Power Saving King Shop	King of Power Saving	Satisfied with power saving	Jingdong prefecture level city in Shandong

Power Saving King Shop	King of Power Saving	Received intact, open a super good look beautiful and generous, ~ bit of high quality is recommended to buy, the need for friends rest assured that the next order	Jingdong prefecture level city in Shandong
Power Saving King Shop	King of Power Saving	Very good to use, friends introduced, save electricity and money, long-lasting products cost-effective, large factory production, good quality, fine workmanship cost-effective!	Jingdong prefecture level city in Shandong
Power Saving King Shop	King of Power Saving	The power saving effect is very obvious, trying to pay 30% less electricity bill in a month than before, hahaha	Jingdong prefecture level city in Shandong
Power Saving King Shop	King of Power Saving	It's such a power saver to use this, it saves money on kids snacks for a month. Kudos!	Jingdong prefecture level city in Shandong
Power Saving King Shop	King of Power Saving	Plug and play, stable voltage, the screen has a display when working, this winter home appliances, need more electricity, just buy to use, save electricity and save money, with instructions, not too big and does not take up space, the quality is very good, and the seller describes exactly the same!	Jingdong prefecture level city in Shandong
Power Saving King Shop	King of Power Saving	A must have for opening a store. Save over a thousand dollars a month on your electric bill	Jingdong prefecture level city in Shandong
Power Saving King Shop	King of Power Saving	Received the goods, the first time to unpack, the quality is very good, with the seller's description, completely exceeded my expectations, the packaging is very careful, very strict, in general this is a very satisfied with the shopping	Jingdong prefecture level city in Shandong
Power Saving King Shop	King of Power Saving	As always, good quality, a very favorite product, great! o(* ︶ *)o The price is very affordable, cost-effective, it's considered affordable good, will repurchase!	Jingdong prefecture level city in Shandong
Power Saving King Shop	King of Power Saving	Very satisfied with a shopping, good business service, fast shipping, logistics is also very powerful, received very good, buy assured, use peace of mind!	Jingdong prefecture level city in Shandong
Power Saving King Shop	King of Power Saving	The first time to buy online, but also look at the evaluation before buying, the quality is not bad, but also very cheap, the need for friends can rest assured to buy!	Jingdong prefecture level city in Shandong
Power Saving King Shop	King of Power Saving	Better than expected, feel value for money, very satisfied with a shopping, need next time will come back!	Jingdong prefecture level city in Shandong
Power Saving King Shop	King of Power Saving	Really super like, very supportive, very good quality, exactly as described by the seller, very satisfied!	Jingdong prefecture level city in Shandong
Power Saving King Shop	King of Power Saving	It is genuine, effective, really saves power, affordable, tightly packed, fast shipping, very satisfied.	Jingdong prefecture level city in Shandong
Power Saving King Shop	King of Power Saving	Good quality, fine workmanship, is genuine and business description of the same, good value for money, fast logistics, very satisfied	Jingdong prefecture level city in Shandong
Power Saving King Shop	King of Power Saving	Saver received, very good, installation set up very well, very good, the effect is very good, recommend that the pro buy	Jingdong prefecture level city in Shandong
Power Saving King Shop	King of Power Saving	Electromagnetic good, high power convenient, enough force, the price is right it, quality can be 😀 😀 😀 😀 😀	Jingdong prefecture level city in Shandong
Power Saving King Shop	King of Power Saving	First-class quality, packaging is also good, logistics is very fast, and the price is reasonable, very affordable, I like it!	Jingdong prefecture level city in Shandong
Power Saving King Shop	King of Power Saving	It's great, it's the kind of style you want, and it arrived quickly, thank you boss, 5 stars for the service!	Jingdong prefecture level city in Shandong
Power Saving King Shop	King of Power Saving	Packaging is very good, the use of the effect is quite good, the store has a good service attitude, trustworthy	Jingdong prefecture level city in Shandong
Power Saving King Shop	King of Power Saving	The quality is very good, it works well, very satisfied, the price is affordable, it's so great value, it's worth recommending.	Jingdong prefecture level city in Shandong
Power Saving King Shop	King of Power Saving	The baby description matches the actual item very well! The quality is awesome! Will be back to patronize again in the future!	Jingdong prefecture level city in Shandong
Power Saving King Shop	King of Power Saving	Ran the price is not high try to buy the attitude, did not expect the effect is really good, good!	Jingdong prefecture level city in Shandong
Power Saving King Shop	King of Power Saving	Power Saving King power is quite large, the quality of the leaf is quite good, the price of buying a very good deal, must be praised!	Jingdong prefecture level city in Shandong
Power Saving King Shop	King of Power Saving	Good product quality is the king of power-saving products trustworthy as always good to recommend!	Jingdong prefecture level city in Shandong
Power Saving King Shop	King of Power Saving	Works great, very affordable, shipped pretty fast, comfortable to use	Jingdong prefecture level city in Shandong
Power Saving King Shop	King of Power Saving	Shipping was pretty fast and affordable, not bad 👍	Jingdong prefecture level city in Shandong
Power Saving King Shop	King of Power Saving	Digital voltage display, voltage at a glance, plug and play, safe and simple!	Jingdong prefecture level city in Shandong
Power Saving King Shop	King of Power Saving	Quality assurance, very good, affordable, logistics is also very fast, I'm very satisfied with the	Jingdong prefecture level city in Shandong
Power Saving King Shop	King of Power Saving	The quality is very good, the first time I received the goods to open the first time to see, it is very good, very satisfied	Jingdong prefecture level city in Shandong
Power Saving King Shop	King of Power Saving	Power Saving King used, the effect is good, the price is affordable, very good, is the genuine article	Jingdong prefecture level city in Shandong
Power Saving King Shop	King of Power Saving	Good quality, fine workmanship, good appearance, time is very accurate, satisfied.	Jingdong prefecture level city in Shandong
Power Saving King Shop	King of Power Saving	I purposely used it for two days before commenting, which is responsible for myself and everyone else!	Jingdong prefecture level city in Shandong
Power Saving King Shop	King of Power Saving	Easy to install, easy to operate, helped our family save a lot of electricity this summer	Jingdong prefecture level city in Shandong
Power Saving King Shop	King of Power Saving	Bringing power savings into millions of homes. Still a great gadget. Pretty good.	Jingdong prefecture level city in Shandong
Power Saving King Shop	King of Power Saving	Digital liquid crystal display to see the clear intuitive, very formal workmanship is not rough	Jingdong prefecture level city in Shandong
Power Saving King Shop	King of Power Saving	Received. Great quality, fast shipping from the store and not too expensive!	Jingdong prefecture level city in Shandong
Power Saving King Shop	King of Power Saving	Bought myself a knuckle regulator and it was easy to use and the price was right.	Jingdong prefecture level city in Shandong
Power Saving King Shop	King of Power Saving	Received, good, powerful, good, very full of a shopping	Jingdong prefecture level city in Shandong
Power Saving King Shop	King of Power Saving	High power, consistent with the seller's description, powerful, cost-effective	Jingdong prefecture level city in Shandong
Power Saving King Shop	King of Power Saving	It works very well and also saves power at an affordable price a great deal.	Jingdong prefecture level city in Shandong
Power Saving King Shop	King of Power Saving	Good quality, good can recommend to friends to buy.	Jingdong prefecture level city in Shandong
Power Saving King Shop	King of Power Saving	Will repurchase, quality is guaranteed, safe and secure purchase.	Jingdong prefecture level city in Shandong
Power Saving King Shop	King of Power Saving	The logistics is very fast, the thing is also good to use, energy saving power rely on it!	Jingdong prefecture level city in Shandong
Power Saving King Shop	King of Power Saving	Power Saver is good quality and works very well, worth buying!	Jingdong prefecture level city in Shandong
Power Saving King Shop	King of Power Saving	The quality is very good is genuine, cost-effective recommended	Jingdong prefecture level city in Shandong
Power Saving King Shop	King of Power Saving	Quality Assurance. Favorable price. Very practical and durable	Jingdong prefecture level city in Shandong
Power Saving King Shop	King of Power Saving	Good value for money. Conscientious businessman. Recommended to everyone	Jingdong prefecture level city in Shandong
Power Saving King Shop	King of Power Saving	Buy it for your rental apartment and never worry about your landlord charging you too much.	Jingdong prefecture level city in Shandong
Power Saving King Shop	King of Power Saving	Nice and affordable, will repurchase next time	Jingdong prefecture level city in Shandong
Power Saving King Shop	King of Power Saving	It's very energy efficient. I've been using it for a few days now.	Jingdong prefecture level city in Shandong

Power Saving King Shop	King of Power Saving	Good quality, high power easy to use	Jingdong prefecture level city in Shandong
Power Saving King Shop	King of Power Saving	Power saving little helper. Deserves a favorable review.	Jingdong prefecture level city in Shandong
Power Saving King Shop	King of Power Saving	Great value for money, great value for money.	Jingdong prefecture level city in Shandong
Power Saving King Shop	King of Power Saving	Works well and as described by the seller	Jingdong prefecture level city in Shandong
Power Saving King Shop	King of Power Saving	Power saving is proper! It’s good.	Jingdong prefecture level city in Shandong
Power Saving King Shop	King of Power Saving	Power saving. Works well. Not bad.	Jingdong prefecture level city in Shandong
Power Saving King Shop	King of Power Saving	Satisfied with power saving	Jingdong prefecture level city in Shandong
Power Saving King Shop	King of Power Saving	Received intact, open a super good look beautiful and generous, ~ bit of high quality is recommended to buy, the need for friends rest assured that the next order	Jingdong prefecture level city in Shandong
Power Saving King Shop	King of Power Saving	Very good to use, friends introduced, save electricity and money, long-lasting products cost-effective, large factory production, good quality, fine workmanship cost-effective!	Jingdong prefecture level city in Shandong
Power Saving King Shop	King of Power Saving	The power saving effect is very obvious, trying to pay 30% less electricity bill in a month than before, hahaha	Jingdong prefecture level city in Shandong
Power Saving King Shop	King of Power Saving	It’s such a power saver to use this, it saves money on kids snacks for a month. Kudos!	Jingdong prefecture level city in Shandong
Power Saving King Shop	King of Power Saving	Plug and play, stable voltage, the screen has a display when working, this winter home appliances, need more electricity, just buy to use, save electricity and save money, with instructions, not too big and does not take up space, the quality is very good, and the seller describes exactly the same!	Jingdong prefecture level city in Shandong
Power Saving King Shop	King of Power Saving	A must have for opening a store. Save over a thousand dollars a month on your electric bill	Jingdong prefecture level city in Shandong
Power Saving King Shop	King of Power Saving	Received the goods, the first time to unpack, the quality is very good, with the seller’s description, completely exceeded my expectations, the packaging is very careful, very strict, in general this is a very satisfied with the shopping	Jingdong prefecture level city in Shandong
Power Saving King Shop	King of Power Saving	As always, good quality, a very favorite product, great! o(*￣︶￣*)o The price is very affordable, cost-effective, it’s considered affordable good, will repurchase!	Jingdong prefecture level city in Shandong
Power Saving King Shop	King of Power Saving	Very satisfied with a shopping, good business service, fast shipping, logistics is also very powerful, received very good, buy assured, use peace of mind!	Jingdong prefecture level city in Shandong
Power Saving King Shop	King of Power Saving	The first time to buy online, but also look at the evaluation before buying, the quality is not bad, but also very cheap, the need for friends can rest assured to buy!	Jingdong prefecture level city in Shandong
Power Saving King Shop	King of Power Saving	Better than expected, feel value for money, very satisfied with a shopping, need next time will come back!	Jingdong prefecture level city in Shandong
Power Saving King Shop	King of Power Saving	Really super like, very supportive, very good quality, exactly as described by the seller, very satisfied!	Jingdong prefecture level city in Shandong
Power Saving King Shop	King of Power Saving	It is genuine, effective, really saves power, affordable, tightly packed, fast shipping, very satisfied.	Jingdong prefecture level city in Shandong
Power Saving King Shop	King of Power Saving	Good quality, fine workmanship, is genuine and business description of the same, good value for money, fast logistics, very satisfied	Jingdong prefecture level city in Shandong
Power Saving King Shop	King of Power Saving	Saver received, very good, installation set up very well, very good, the effect is very good, recommend that the pro buy	Jingdong prefecture level city in Shandong
Power Saving King Shop	King of Power Saving	Electromagnetic good, high power convenient, enough force, the price is right it, quality can be 😃 😃 😃 😃 😃	Jingdong prefecture level city in Shandong
Power Saving King Shop	King of Power Saving	First-class quality, packaging is also good, logistics is very fast, and the price is reasonable, very affordable, I like it!	Jingdong prefecture level city in Shandong
Power Saving King Shop	King of Power Saving	It’s great, it’s the kind of style you want, and it arrived quickly, thank you boss, 5 stars for the service!	Jingdong prefecture level city in Shandong
Power Saving King Shop	King of Power Saving	Packaging is very good, the use of the effect is quite good, the store has a good service attitude, trustworthy	Jingdong prefecture level city in Shandong
Power Saving King Shop	King of Power Saving	The quality is very good, it works well, very satisfied, the price is affordable, it’s so great value, it’s worth recommending.	Jingdong prefecture level city in Shandong
Power Saving King Shop	King of Power Saving	The baby description matches the actual item very well! The quality is awesome! Will be back to patronize again in the future!	Jingdong prefecture level city in Shandong
Power Saving King Shop	King of Power Saving	Ran the price is not high try to buy the attitude, did not expect the effect is really good, good!	Jingdong prefecture level city in Shandong
Power Saving King Shop	King of Power Saving	Power Saving King power is quite large, the quality of the leaf is quite good, the price of buying a very good deal, must be praised!	Jingdong prefecture level city in Shandong
Power Saving King Shop	King of Power Saving	Good product quality is the king of power-saving products trustworthy as always good to recommend!	Jingdong prefecture level city in Shandong
Power Saving King Shop	King of Power Saving	Works great, very affordable, shipped pretty fast, comfortable to use	Jingdong prefecture level city in Shandong
Power Saving King Shop	King of Power Saving	Shipping was pretty fast and affordable, not bad 👍	Jingdong prefecture level city in Shandong
Power Saving King Shop	King of Power Saving	Digital voltage display, voltage at a glance, plug and play, safe and simple!	Jingdong prefecture level city in Shandong
Power Saving King Shop	King of Power Saving	Quality assurance, very good, affordable, logistics is also very fast, I’m very satisfied with the	Jingdong prefecture level city in Shandong
Power Saving King Shop	King of Power Saving	The quality is very good, the first time I received the goods to open the first time to see, it is very good, very satisfied	Jingdong prefecture level city in Shandong
Power Saving King Shop	King of Power Saving	Power Saving King used, the effect is good, the price is affordable, very good, is the genuine article	Jingdong prefecture level city in Shandong
Power Saving King Shop	King of Power Saving	Good quality, fine workmanship, good appearance, time is very accurate, satisfied.	Jingdong prefecture level city in Shandong
Power Saving King Shop	King of Power Saving	I purposely used it for two days before commenting, which is responsible for myself and everyone else!	Jingdong prefecture level city in Shandong
Power Saving King Shop	King of Power Saving	Easy to install, easy to operate, helped our family save a lot of electricity this summer	Jingdong prefecture level city in Shandong
Power Saving King Shop	King of Power Saving	Bringing power savings into millions of homes. Still a great gadget. Pretty good.	Jingdong prefecture level city in Shandong
Power Saving King Shop	King of Power Saving	Digital liquid crystal display to see the clear intuitive, very formal workmanship is not rough	Jingdong prefecture level city in Shandong
Power Saving King Shop	King of Power Saving	Received. Great quality, fast shipping from the store and not too expensive!	Jingdong prefecture level city in Shandong
Power Saving King Shop	King of Power Saving	Bought myself a knuckle regulator and it was easy to use and the price was right.	Jingdong prefecture level city in Shandong
Power Saving King Shop	King of Power Saving	Received, good, powerful, good, very full of a shopping	Jingdong prefecture level city in Shandong
Power Saving King Shop	King of Power Saving	High power, consistent with the seller’s description, powerful, cost-effective	Jingdong prefecture level city in Shandong
Power Saving King Shop	King of Power Saving	It works very well and also saves power at an affordable price a great deal.	Jingdong prefecture level city in Shandong
Power Saving King Shop	King of Power Saving	Good quality, good can recommend to friends to buy.	Jingdong prefecture level city in Shandong
Power Saving King Shop	King of Power Saving	Will repurchase, quality is guaranteed, safe and secure purchase.	Jingdong prefecture level city in Shandong
Power Saving King Shop	King of Power Saving	The logistics is very fast, the thing is also good to use, energy saving power rely on it!	Jingdong prefecture level city in Shandong

Power Saving King Shop	King of Power Saving	Power Saver is good quality and works very well, worth buying!	Jingdong prefecture level city in Shandong
Power Saving King Shop	King of Power Saving	The quality is very good is genuine, cost-effective recommended	Jingdong prefecture level city in Shandong
Power Saving King Shop	King of Power Saving	Quality Assurance. Favorable price. Very practical and durable	Jingdong prefecture level city in Shandong
Power Saving King Shop	King of Power Saving	Good value for money. Conscientious businessman. Recommended to everyone	Jingdong prefecture level city in Shandong
Power Saving King Shop	King of Power Saving	Buy it for your rental apartment and never worry about your landlord charging you too much.	Jingdong prefecture level city in Shandong
Power Saving King Shop	King of Power Saving	Nice and affordable, will repurchase next time	Jingdong prefecture level city in Shandong
Power Saving King Shop	King of Power Saving	It's very energy efficient. I've been using it for a few days now.	Jingdong prefecture level city in Shandong
Power Saving King Shop	King of Power Saving	Good quality, high power easy to use	Jingdong prefecture level city in Shandong
Power Saving King Shop	King of Power Saving	Power saving little helper. Deserves a favorable review.	Jingdong prefecture level city in Shandong
Power Saving King Shop	King of Power Saving	Great value for money, great value for money.	Jingdong prefecture level city in Shandong
Power Saving King Shop	King of Power Saving	Works well and as described by the seller	Jingdong prefecture level city in Shandong
Power Saving King Shop	King of Power Saving	Power saving is proper! It's good.	Jingdong prefecture level city in Shandong
Power Saving King Shop	King of Power Saving	Power saving. Works well. Not bad.	Jingdong prefecture level city in Shandong
Power Saving King Shop	King of Power Saving	Satisfied with power saving	Jingdong prefecture level city in Shandong
Power Saving King Shop	King of Power Saving	Received intact, open a super good look beautiful and generous, ~ bit of high quality is recommended to buy, the need for friends rest assured that the next order	Jingdong prefecture level city in Shandong
Power Saving King Shop	King of Power Saving	Very good to use, friends introduced, save electricity and money, long-lasting products cost-effective, large factory production, good quality, fine workmanship cost-effective!	Jingdong prefecture level city in Shandong
Power Saving King Shop	King of Power Saving	The power saving effect is very obvious, trying to pay 30% less electricity bill in a month than before, hahaha	Jingdong prefecture level city in Shandong
Power Saving King Shop	King of Power Saving	It's such a power saver to use this, it saves money on kids snacks for a month. Kudos!	Jingdong prefecture level city in Shandong
Power Saving King Shop	King of Power Saving	Plug and play, stable voltage, the screen has a display when working, this winter home appliances, need more electricity, just buy to use, save electricity and save money, with instructions, not too big and does not take up space, the quality is very good, and the seller describes exactly the same!	Jingdong prefecture level city in Shandong
Power Saving King Shop	King of Power Saving	A must have for opening a store. Save over a thousand dollars a month on your electric bill	Jingdong prefecture level city in Shandong
Power Saving King Shop	King of Power Saving	Received the goods, the first time to unpack, the quality is very good, with the seller's description, completely exceeded my expectations, the packaging is very careful, very strict, in general this is a very satisfied with the shopping	Jingdong prefecture level city in Shandong
Power Saving King Shop	King of Power Saving	As always, good quality, a very favorite product, great! o(*￣︶￣*)o The price is very affordable, cost-effective, it's considered affordable good, will repurchase!	Jingdong prefecture level city in Shandong
Power Saving King Shop	King of Power Saving	Very satisfied with a shopping, good business service, fast shipping, logistics is also very powerful, received very good, buy assured, use peace of mind!	Jingdong prefecture level city in Shandong
Power Saving King Shop	King of Power Saving	The first time to buy online, but also look at the evaluation before buying, the quality is not bad, but also very cheap, the need for friends can rest assured to buy!	Jingdong prefecture level city in Shandong
Power Saving King Shop	King of Power Saving	Better than expected, feel value for money, very satisfied with a shopping, need next time will come back!	Jingdong prefecture level city in Shandong
Power Saving King Shop	King of Power Saving	Really super like, very supportive, very good quality, exactly as described by the seller, very satisfied!	Jingdong prefecture level city in Shandong
Power Saving King Shop	King of Power Saving	It is genuine, effective, really saves power, affordable, tightly packed, fast shipping, very satisfied.	Jingdong prefecture level city in Shandong
Power Saving King Shop	King of Power Saving	Good quality, fine workmanship, is genuine and business description of the same, good value for money, fast logistics, very satisfied	Jingdong prefecture level city in Shandong
Power Saving King Shop	King of Power Saving	Saver received, very good, installation set up very well, very good, the effect is very good, recommend that the pro buy	Jingdong prefecture level city in Shandong
Power Saving King Shop	King of Power Saving	Electromagnetic good, high power convenient, enough force, the price is right it, quality can be 😀 😀 😀 😀 😀	Jingdong prefecture level city in Shandong
Power Saving King Shop	King of Power Saving	First-class quality, packaging is also good, logistics is very fast, and the price is reasonable, very affordable, I like it!	Jingdong prefecture level city in Shandong
Power Saving King Shop	King of Power Saving	It's great, it's the kind of style you want, and it arrived quickly, thank you boss, 5 stars for the service!	Jingdong prefecture level city in Shandong
Power Saving King Shop	King of Power Saving	Packaging is very good, the use of the effect is quite good, the store has a good service attitude, trustworthy	Jingdong prefecture level city in Shandong
Power Saving King Shop	King of Power Saving	The quality is very good, it works well, very satisfied, the price is affordable, it's so great value, it's worth recommending.	Jingdong prefecture level city in Shandong
Power Saving King Shop	King of Power Saving	The baby description matches the actual item very well! The quality is awesome! Will be back to patronize again in the future!	Jingdong prefecture level city in Shandong
Power Saving King Shop	King of Power Saving	Ran the price is not high try to buy the attitude, did not expect the effect is really good, good!	Jingdong prefecture level city in Shandong
Power Saving King Shop	King of Power Saving	Power Saving King power is quite large, the quality of the leaf is quite good, the price of buying a very good deal, must be praised!	Jingdong prefecture level city in Shandong
Power Saving King Shop	King of Power Saving	Good product quality is the king of power-saving products trustworthy as always good to recommend!	Jingdong prefecture level city in Shandong
Power Saving King Shop	King of Power Saving	Works great, very affordable, shipped pretty fast, comfortable to use	Jingdong prefecture level city in Shandong
Power Saving King Shop	King of Power Saving	Shipping was pretty fast and affordable, not bad 👍	Jingdong prefecture level city in Shandong
Power Saving King Shop	King of Power Saving	Digital voltage display, voltage at a glance, plug and play, safe and simple!	Jingdong prefecture level city in Shandong
Power Saving King Shop	King of Power Saving	Quality assurance, very good, affordable, logistics is also very fast, I'm very satisfied with the	Jingdong prefecture level city in Shandong
Power Saving King Shop	King of Power Saving	The quality is very good, the first time I received the goods to open the first time to see, it is very good, very satisfied	Jingdong prefecture level city in Shandong
Power Saving King Shop	King of Power Saving	Power Saving King used, the effect is good, the price is affordable, very good, is the genuine article	Jingdong prefecture level city in Shandong
Power Saving King Shop	King of Power Saving	Good quality, fine workmanship, good appearance, time is very accurate, satisfied.	Jingdong prefecture level city in Shandong
Power Saving King Shop	King of Power Saving	I purposely used it for two days before commenting, which is responsible for myself and everyone else!	Jingdong prefecture level city in Shandong
Power Saving King Shop	King of Power Saving	Easy to install, easy to operate, helped our family save a lot of electricity this summer	Jingdong prefecture level city in Shandong
Power Saving King Shop	King of Power Saving	Bringing power savings into millions of homes. Still a great gadget. Pretty good.	Jingdong prefecture level city in Shandong
Power Saving King Shop	King of Power Saving	Digital liquid crystal display to see the clear intuitive, very formal workmanship is not rough	Jingdong prefecture level city in Shandong
Power Saving King Shop	King of Power Saving	Received. Great quality, fast shipping from the store and not too expensive!	Jingdong prefecture level city in Shandong

Power Saving King Shop	King of Power Saving	Bought myself a knuckle regulator and it was easy to use and the price was right.	Jingdong prefecture level city in Shandong
Power Saving King Shop	King of Power Saving	Received, good, powerful, good, very full of a shopping	Jingdong prefecture level city in Shandong
Power Saving King Shop	King of Power Saving	High power, consistent with the seller's description, powerful, cost-effective	Jingdong prefecture level city in Shandong
Power Saving King Shop	King of Power Saving	It works very well and also saves power at an affordable price a great deal.	Jingdong prefecture level city in Shandong
Power Saving King Shop	King of Power Saving	Good quality, good can recommend to friends to buy.	Jingdong prefecture level city in Shandong
Power Saving King Shop	King of Power Saving	Will repurchase, quality is guaranteed, safe and secure purchase.	Jingdong prefecture level city in Shandong
Power Saving King Shop	King of Power Saving	The logistics is very fast, the thing is also good to use, energy saving power rely on it!	Jingdong prefecture level city in Shandong
Power Saving King Shop	King of Power Saving	Power Saver is good quality and works very well, worth buying!	Jingdong prefecture level city in Shandong
Power Saving King Shop	King of Power Saving	The quality is very good is genuine, cost-effective recommended	Jingdong prefecture level city in Shandong
Power Saving King Shop	King of Power Saving	Quality Assurance. Favorable price. Very practical and durable	Jingdong prefecture level city in Shandong
Power Saving King Shop	King of Power Saving	Good value for money. Conscientious businessman. Recommended to everyone	Jingdong prefecture level city in Shandong
Power Saving King Shop	King of Power Saving	Buy it for your rental apartment and never worry about your landlord charging you too much.	Jingdong prefecture level city in Shandong
Power Saving King Shop	King of Power Saving	Nice and affordable, will repurchase next time	Jingdong prefecture level city in Shandong
Power Saving King Shop	King of Power Saving	It's very energy efficient. I've been using it for a few days now.	Jingdong prefecture level city in Shandong
Power Saving King Shop	King of Power Saving	Good quality, high power easy to use	Jingdong prefecture level city in Shandong
Power Saving King Shop	King of Power Saving	Power saving little helper. Deserves a favorable review.	Jingdong prefecture level city in Shandong
Power Saving King Shop	King of Power Saving	Great value for money, great value for money.	Jingdong prefecture level city in Shandong
Power Saving King Shop	King of Power Saving	Works well and as described by the seller	Jingdong prefecture level city in Shandong
Power Saving King Shop	King of Power Saving	Power saving is proper! It's good.	Jingdong prefecture level city in Shandong
Power Saving King Shop	King of Power Saving	Power saving. Works well. Not bad.	Jingdong prefecture level city in Shandong
Power Saving King Shop	King of Power Saving	Satisfied with power saving	Jingdong prefecture level city in Shandong
Power Saving King Shop	King of Power Saving	Received intact, open a super good look beautiful and generous, ~ bit of high quality is recommended to buy, the need for friends rest assured that the next order	Jingdong prefecture level city in Shandong
Power Saving King Shop	King of Power Saving	Very good to use, friends introduced, save electricity and money, long-lasting products cost-effective, large factory production, good quality, fine workmanship cost-effective!	Jingdong prefecture level city in Shandong
Power Saving King Shop	King of Power Saving	The power saving effect is very obvious, trying to pay 30% less electricity bill in a month than before, hahaha	Jingdong prefecture level city in Shandong
Power Saving King Shop	King of Power Saving	It's such a power saver to use this, it saves money on kids snacks for a month. Kudos!	Jingdong prefecture level city in Shandong
Power Saving King Shop	King of Power Saving	Plug and play, stable voltage, the screen has a display when working, this winter home appliances, need more electricity, just buy to use, save electricity and save money, with instructions, not too big and does not take up space, the quality is very good, and the seller describes exactly the same!	Jingdong prefecture level city in Shandong
Power Saving King Shop	King of Power Saving	A must have for opening a store. Save over a thousand dollars a month on your electric bill	Jingdong prefecture level city in Shandong
Power Saving King Shop	King of Power Saving	Received the goods, the first time to unpack, the quality is very good, with the seller's description, completely exceeded my expectations, the packaging is very careful, very strict, in general this is a very satisfied with the shopping	Jingdong prefecture level city in Shandong
Power Saving King Shop	King of Power Saving	As always, good quality, a very favorite product, great! o(*￣︶￣*)o The price is very affordable, cost-effective, it's considered affordable good, will repurchase!	Jingdong prefecture level city in Shandong
Power Saving King Shop	King of Power Saving	Very satisfied with a shopping, good business service, fast shipping, logistics is also very powerful, received very good, buy assured, use peace of mind!	Jingdong prefecture level city in Shandong
Power Saving King Shop	King of Power Saving	The first time to buy online, but also look at the evaluation before buying, the quality is not bad, but also very cheap, the need for friends can rest assured to buy!	Jingdong prefecture level city in Shandong
Power Saving King Shop	King of Power Saving	Better than expected, feel value for money, very satisfied with a shopping, need next time will come back!	Jingdong prefecture level city in Shandong
Power Saving King Shop	King of Power Saving	Really super like, very supportive, very good quality, exactly as described by the seller, very satisfied!	Jingdong prefecture level city in Shandong
Power Saving King Shop	King of Power Saving	It is genuine, effective, really saves power, affordable, tightly packed, fast shipping, very satisfied.	Jingdong prefecture level city in Shandong
Power Saving King Shop	King of Power Saving	Good quality, fine workmanship, is genuine and business description of the same, good value for money, fast logistics, very satisfied	Jingdong prefecture level city in Shandong
Power Saving King Shop	King of Power Saving	Saver received, very good, installation set up very well, very good, the effect is very good, recommend that the pro buy	Jingdong prefecture level city in Shandong
Power Saving King Shop	King of Power Saving	Electromagnetic good, high power convenient, enough force, the price is right it, quality can be 😊 😊 😊 😊 😊	Jingdong prefecture level city in Shandong
Power Saving King Shop	King of Power Saving	First-class quality, packaging is also good, logistics is very fast, and the price is reasonable, very affordable, I like it!	Jingdong prefecture level city in Shandong
Power Saving King Shop	King of Power Saving	It's great, it's the kind of style you want, and it arrived quickly, thank you boss, 5 stars for the service!	Jingdong prefecture level city in Shandong
Power Saving King Shop	King of Power Saving	Packaging is very good, the use of the effect is quite good, the store has a good service attitude, trustworthy	Jingdong prefecture level city in Shandong
Power Saving King Shop	King of Power Saving	The quality is very good, it works well, very satisfied, the price is affordable, it's so great value, it's worth recommending.	Jingdong prefecture level city in Shandong
Power Saving King Shop	King of Power Saving	The baby description matches the actual item very well! The quality is awesome! Will be back to patronize again in the future!	Jingdong prefecture level city in Shandong
Power Saving King Shop	King of Power Saving	Ran the price is not high try to buy the attitude, did not expect the effect is really good, good!	Jingdong prefecture level city in Shandong
Power Saving King Shop	King of Power Saving	Power Saving King power is quite large, the quality of the leaf is quite good, the price of buying a very good deal, must be praised!	Jingdong prefecture level city in Shandong
Power Saving King Shop	King of Power Saving	Good product quality is the king of power-saving products trustworthy as always good to recommend!	Jingdong prefecture level city in Shandong
Power Saving King Shop	King of Power Saving	Works great, very affordable, shipped pretty fast, comfortable to use	Jingdong prefecture level city in Shandong
Power Saving King Shop	King of Power Saving	Shipping was pretty fast and affordable, not bad 👍	Jingdong prefecture level city in Shandong
Power Saving King Shop	King of Power Saving	Digital voltage display, voltage at a glance, plug and play, safe and simple!	Jingdong prefecture level city in Shandong
Power Saving King Shop	King of Power Saving	Quality assurance, very good, affordable, logistics is also very fast, I'm very satisfied with the	Jingdong prefecture level city in Shandong
Power Saving King Shop	King of Power Saving	The quality is very good, the first time I received the goods to open the first time to see, it is very good, very satisfied	Jingdong prefecture level city in Shandong

Power Saving King Shop	King of Power Saving	Power Saving King used, the effect is good, the price is affordable, very good, is the genuine article	Jingdong prefecture level city in Shandong
Power Saving King Shop	King of Power Saving	Good quality, fine workmanship, good appearance, time is very accurate, satisfied.	Jingdong prefecture level city in Shandong
Power Saving King Shop	King of Power Saving	I purposely used it for two days before commenting, which is responsible for myself and everyone else!	Jingdong prefecture level city in Shandong
Power Saving King Shop	King of Power Saving	Easy to install, easy to operate, helped our family save a lot of electricity this summer	Jingdong prefecture level city in Shandong
Power Saving King Shop	King of Power Saving	Bringing power savings into millions of homes. Still a great gadget. Pretty good.	Jingdong prefecture level city in Shandong
Power Saving King Shop	King of Power Saving	Digital liquid crystal display to see the clear intuitive, very formal workmanship is not rough	Jingdong prefecture level city in Shandong
Power Saving King Shop	King of Power Saving	Received. Great quality, fast shipping from the store and not too expensive!	Jingdong prefecture level city in Shandong
Power Saving King Shop	King of Power Saving	Bought myself a knuckle regulator and it was easy to use and the price was right.	Jingdong prefecture level city in Shandong
Power Saving King Shop	King of Power Saving	Received, good, powerful, good, very full of a shopping	Jingdong prefecture level city in Shandong
Power Saving King Shop	King of Power Saving	High power, consistent with the seller's description, powerful, cost-effective	Jingdong prefecture level city in Shandong
Power Saving King Shop	King of Power Saving	It works very well and also saves power at an affordable price a great deal.	Jingdong prefecture level city in Shandong
Power Saving King Shop	King of Power Saving	Good quality, good can recommend to friends to buy.	Jingdong prefecture level city in Shandong
Power Saving King Shop	King of Power Saving	Will repurchase, quality is guaranteed, safe and secure purchase.	Jingdong prefecture level city in Shandong
Power Saving King Shop	King of Power Saving	The logistics is very fast, the thing is also good to use, energy saving power rely on it!	Jingdong prefecture level city in Shandong
Power Saving King Shop	King of Power Saving	Power Saver is good quality and works very well, worth buying!	Jingdong prefecture level city in Shandong
Power Saving King Shop	King of Power Saving	The quality is very good is genuine, cost-effective recommended	Jingdong prefecture level city in Shandong
Power Saving King Shop	King of Power Saving	Quality Assurance. Favorable price. Very practical and durable	Jingdong prefecture level city in Shandong
Power Saving King Shop	King of Power Saving	Good value for money. Conscientious businessman. Recommended to everyone	Jingdong prefecture level city in Shandong
Power Saving King Shop	King of Power Saving	Buy it for your rental apartment and never worry about your landlord charging you too much.	Jingdong prefecture level city in Shandong
Power Saving King Shop	King of Power Saving	Nice and affordable, will repurchase next time	Jingdong prefecture level city in Shandong
Power Saving King Shop	King of Power Saving	It's very energy efficient. I've been using it for a few days now.	Jingdong prefecture level city in Shandong
Power Saving King Shop	King of Power Saving	Good quality, high power easy to use	Jingdong prefecture level city in Shandong
Power Saving King Shop	King of Power Saving	Power saving little helper. Deserves a favorable review.	Jingdong prefecture level city in Shandong
Power Saving King Shop	King of Power Saving	Great value for money, great value for money.	Jingdong prefecture level city in Shandong
Power Saving King Shop	King of Power Saving	Works well and as described by the seller	Jingdong prefecture level city in Shandong
Power Saving King Shop	King of Power Saving	Power saving is proper! It's good.	Jingdong prefecture level city in Shandong
Power Saving King Shop	King of Power Saving	Power saving. Works well. Not bad.	Jingdong prefecture level city in Shandong
Power Saving King Shop	King of Power Saving	Satisfied with power saving	Jingdong prefecture level city in Shandong
Power Saving King Shop	King of Power Saving	Received intact, open a super good look beautiful and generous, ~ bit of high quality is recommended to buy, the need for friends rest assured that the next order	Jingdong prefecture level city in Shandong
Power Saving King Shop	King of Power Saving	Very good to use, friends introduced, save electricity and money, long-lasting products cost-effective, large factory production, good quality, fine workmanship cost-effective!	Jingdong prefecture level city in Shandong
Power Saving King Shop	King of Power Saving	The power saving effect is very obvious, trying to pay 30% less electricity bill in a month than before, hahaha	Jingdong prefecture level city in Shandong
Power Saving King Shop	King of Power Saving	It's such a power saver to use this, it saves money on kids snacks for a month. Kudos!	Jingdong prefecture level city in Shandong
Power Saving King Shop	King of Power Saving	Plug and play, stable voltage, the screen has a display when working, this winter home appliances, need more electricity, just buy to use, save electricity and save money, with instructions, not too big and does not take up space, the quality is very good, and the seller describes exactly the same!	Jingdong prefecture level city in Shandong
Power Saving King Shop	King of Power Saving	A must have for opening a store. Save over a thousand dollars a month on your electric bill	Jingdong prefecture level city in Shandong
Power Saving King Shop	King of Power Saving	Received the goods, the first time to unpack, the quality is very good, with the seller's description, completely exceeded my expectations, the packaging is very careful, very strict, in general this is a very satisfied with the shopping	Jingdong prefecture level city in Shandong
Power Saving King Shop	King of Power Saving	As always, good quality, a very favorite product, great! o(*￣︶￣*)o The price is very affordable, cost-effective, it's considered affordable good, will repurchase!	Jingdong prefecture level city in Shandong
Power Saving King Shop	King of Power Saving	Very satisfied with a shopping, good business service, fast shipping, logistics is also very powerful, received very good, buy assured, use peace of mind!	Jingdong prefecture level city in Shandong
Power Saving King Shop	King of Power Saving	The first time to buy online, but also look at the evaluation before buying, the quality is not bad, but also very cheap, the need for friends can rest assured to buy!	Jingdong prefecture level city in Shandong
Power Saving King Shop	King of Power Saving	Better than expected, feel value for money, very satisfied with a shopping, need next time will come back!	Jingdong prefecture level city in Shandong
Power Saving King Shop	King of Power Saving	Really super like, very supportive, very good quality, exactly as described by the seller, very satisfied!	Jingdong prefecture level city in Shandong
Power Saving King Shop	King of Power Saving	It is genuine, effective, really saves power, affordable, tightly packed, fast shipping, very satisfied.	Jingdong prefecture level city in Shandong
Power Saving King Shop	King of Power Saving	Good quality, fine workmanship, is genuine and business description of the same, good value for money, fast logistics, very satisfied	Jingdong prefecture level city in Shandong
Power Saving King Shop	King of Power Saving	Saver received, very good, installation set up very well, very good, the effect is very good, recommend that the pro buy	Jingdong prefecture level city in Shandong
Power Saving King Shop	King of Power Saving	Electromagnetic good, high power convenient, enough force, the price is right it, quality can be ☺ ☺ ☺ ☺ ☺	Jingdong prefecture level city in Shandong
Power Saving King Shop	King of Power Saving	First-class quality, packaging is also good, logistics is very fast, and the price is reasonable, very affordable, I like it!	Jingdong prefecture level city in Shandong
Power Saving King Shop	King of Power Saving	It's great, it's the kind of style you want, and it arrived quickly, thank you boss, 5 stars for the service!	Jingdong prefecture level city in Shandong
Power Saving King Shop	King of Power Saving	Packaging is very good, the use of the effect is quite good, the store has a good service attitude, trustworthy	Jingdong prefecture level city in Shandong
Power Saving King Shop	King of Power Saving	The quality is very good, it works well, very satisfied, the price is affordable, it's so great value, it's worth recommending.	Jingdong prefecture level city in Shandong
Power Saving King Shop	King of Power Saving	The baby description matches the actual item very well! The quality is awesome! Will be back to patronize again in the future!	Jingdong prefecture level city in Shandong
Power Saving King Shop	King of Power Saving	Ran the price is not high try to buy the attitude, did not expect the effect is really good, good!	Jingdong prefecture level city in Shandong

Power Saving King Shop	King of Power Saving	Power Saving King power is quite large, the quality of the leaf is quite good, the price of buying a very good deal, must be praised!	Jingdong prefecture level city in Shandong
Power Saving King Shop	King of Power Saving	Good product quality is the king of power-saving products trustworthy as always good to recommend!	Jingdong prefecture level city in Shandong
Power Saving King Shop	King of Power Saving	Works great, very affordable, shipped pretty fast, comfortable to use	Jingdong prefecture level city in Shandong
Power Saving King Shop	King of Power Saving	Shipping was pretty fast and affordable, not bad	Jingdong prefecture level city in Shandong
Power Saving King Shop	King of Power Saving	Digital voltage display, voltage at a glance, plug and play, safe and simple!	Jingdong prefecture level city in Shandong
Power Saving King Shop	King of Power Saving	Quality assurance, very good, affordable, logistics is also very fast, I'm very satisfied with the	Jingdong prefecture level city in Shandong
Power Saving King Shop	King of Power Saving	The quality is very good, the first time I received the goods to open the first time to see, it is very good, very satisfied	Jingdong prefecture level city in Shandong
Power Saving King Shop	King of Power Saving	Power Saving King used, the effect is good, the price is affordable, very good, is the genuine article	Jingdong prefecture level city in Shandong
Power Saving King Shop	King of Power Saving	Good quality, fine workmanship, good appearance, time is very accurate, satisfied.	Jingdong prefecture level city in Shandong
Power Saving King Shop	King of Power Saving	I purposely used it for two days before commenting, which is responsible for myself and everyone else!	Jingdong prefecture level city in Shandong
Power Saving King Shop	King of Power Saving	Easy to install, easy to operate, helped our family save a lot of electricity this summer	Jingdong prefecture level city in Shandong
Power Saving King Shop	King of Power Saving	Bringing power savings into millions of homes. Still a great gadget. Pretty good.	Jingdong prefecture level city in Shandong
Power Saving King Shop	King of Power Saving	Digital liquid crystal display to see the clear intuitive, very formal workmanship is not rough	Jingdong prefecture level city in Shandong
Power Saving King Shop	King of Power Saving	Received. Great quality, fast shipping from the store and not too expensive!	Jingdong prefecture level city in Shandong
Power Saving King Shop	King of Power Saving	Bought myself a knuckle regulator and it was easy to use and the price was right.	Jingdong prefecture level city in Shandong
Power Saving King Shop	King of Power Saving	Received, good, powerful, good, very full of a shopping	Jingdong prefecture level city in Shandong
Power Saving King Shop	King of Power Saving	High power, consistent with the seller's description, powerful, cost-effective	Jingdong prefecture level city in Shandong
Power Saving King Shop	King of Power Saving	It works very well and also saves power at an affordable price a great deal.	Jingdong prefecture level city in Shandong
Power Saving King Shop	King of Power Saving	Good quality, good can recommend to friends to buy.	Jingdong prefecture level city in Shandong
Power Saving King Shop	King of Power Saving	Will repurchase, quality is guaranteed, safe and secure purchase.	Jingdong prefecture level city in Shandong
Power Saving King Shop	King of Power Saving	The logistics is very fast, the thing is also good to use, energy saving power rely on it!	Jingdong prefecture level city in Shandong
Power Saving King Shop	King of Power Saving	Power Saver is good quality and works very well, worth buying!	Jingdong prefecture level city in Shandong
Power Saving King Shop	King of Power Saving	The quality is very good is genuine, cost-effective recommended	Jingdong prefecture level city in Shandong
Power Saving King Shop	King of Power Saving	Quality Assurance. Favorable price. Very practical and durable	Jingdong prefecture level city in Shandong
Power Saving King Shop	King of Power Saving	Good value for money. Conscientious businessman. Recommended to everyone	Jingdong prefecture level city in Shandong
Power Saving King Shop	King of Power Saving	Buy it for your rental apartment and never worry about your landlord charging you too much.	Jingdong prefecture level city in Shandong
Power Saving King Shop	King of Power Saving	Nice and affordable, will repurchase next time	Jingdong prefecture level city in Shandong
Power Saving King Shop	King of Power Saving	It's very energy efficient. I've been using it for a few days now.	Jingdong prefecture level city in Shandong
Power Saving King Shop	King of Power Saving	Good quality, high power easy to use	Jingdong prefecture level city in Shandong
Power Saving King Shop	King of Power Saving	Power saving little helper. Deserves a favorable review.	Jingdong prefecture level city in Shandong
Power Saving King Shop	King of Power Saving	Great value for money, great value for money.	Jingdong prefecture level city in Shandong
Power Saving King Shop	King of Power Saving	Works well and as described by the seller	Jingdong prefecture level city in Shandong
Power Saving King Shop	King of Power Saving	Power saving is proper! It's good.	Jingdong prefecture level city in Shandong
Power Saving King Shop	King of Power Saving	Power saving. Works well. Not bad.	Jingdong prefecture level city in Shandong
Power Saving King Shop	King of Power Saving	Satisfied with power saving	Jingdong prefecture level city in Shandong
Power Saving King Shop	King of Power Saving	Received intact, open a super good look beautiful and generous, ~ bit of high quality is recommended to buy, the need for friends rest assured that the next order	Jingdong prefecture level city in Shandong
Power Saving King Shop	King of Power Saving	Very good to use, friends introduced, save electricity and money, long-lasting products cost-effective, large factory production, good quality, fine workmanship cost-effective!	Jingdong prefecture level city in Shandong
Power Saving King Shop	King of Power Saving	The power saving effect is very obvious, trying to pay 30% less electricity bill in a month than before, hahaha	Jingdong prefecture level city in Shandong
Power Saving King Shop	King of Power Saving	It's such a power saver to use this, it saves money on kids snacks for a month. Kudos!	Jingdong prefecture level city in Shandong
Power Saving King Shop	King of Power Saving	Plug and play, stable voltage, the screen has a display when working, this winter home appliances, need more electricity, just buy to use, save electricity and save money, with instructions, not too big and does not take up space, the quality is very good, and the seller describes exactly the same!	Jingdong prefecture level city in Shandong
Power Saving King Shop	King of Power Saving	A must have for opening a store. Save over a thousand dollars a month on your electric bill	Jingdong prefecture level city in Shandong
Power Saving King Shop	King of Power Saving	Received the goods, the first time to unpack, the quality is very good, with the seller's description, completely exceeded my expectations, the packaging is very careful, very strict, in general this is a very satisfied with the shopping	Jingdong prefecture level city in Shandong
Power Saving King Shop	King of Power Saving	As always, good quality, a very favorite product, great! o(*￣︶￣*)o The price is very affordable, cost-effective, it's considered affordable good, will repurchase!	Jingdong prefecture level city in Shandong
Power Saving King Shop	King of Power Saving	Very satisfied with a shopping, good business service, fast shipping, logistics is also very powerful, received very good, buy assured, use peace of mind!	Jingdong prefecture level city in Shandong
Power Saving King Shop	King of Power Saving	The first time to buy online, but also look at the evaluation before buying, the quality is not bad, but also very cheap, the need for friends can rest assured to buy!	Jingdong prefecture level city in Shandong
Power Saving King Shop	King of Power Saving	Better than expected, feel value for money, very satisfied with a shopping, need next time will come back!	Jingdong prefecture level city in Shandong
Power Saving King Shop	King of Power Saving	Really super like, very supportive, very good quality, exactly as described by the seller, very satisfied!	Jingdong prefecture level city in Shandong
Power Saving King Shop	King of Power Saving	It is genuine, effective, really saves power, affordable, tightly packed, fast shipping, very satisfied.	Jingdong prefecture level city in Shandong
Power Saving King Shop	King of Power Saving	Good quality, fine workmanship, is genuine and business description of the same, good value for money, fast logistics, very satisfied	Jingdong prefecture level city in Shandong
Power Saving King Shop	King of Power Saving	Saver received, very good, installation set up very well, very good, the effect is very good, recommend that the pro buy	Jingdong prefecture level city in Shandong

Power Saving King Shop	King of Power Saving	Electromagnetic good, high power convenient, enough force, the price is right it, quality can be 😃 😃 😃 😃 😃	Jingdong prefecture level city in Shandong
Power Saving King Shop	King of Power Saving	First-class quality, packaging is also good, logistics is very fast, and the price is reasonable, very affordable, I like it!	Jingdong prefecture level city in Shandong
Power Saving King Shop	King of Power Saving	It's great, it's the kind of style you want, and it arrived quickly, thank you boss, 5 stars for the service!	Jingdong prefecture level city in Shandong
Power Saving King Shop	King of Power Saving	Packaging is very good, the use of the effect is quite good, the store has a good service attitude, trustworthy	Jingdong prefecture level city in Shandong
Power Saving King Shop	King of Power Saving	The quality is very good, it works well, very satisfied, the price is affordable, it's so great value, it's worth recommending.	Jingdong prefecture level city in Shandong
Power Saving King Shop	King of Power Saving	The baby description matches the actual item very well! The quality is awesome! Will be back to patronize again in the future!	Jingdong prefecture level city in Shandong
Power Saving King Shop	King of Power Saving	Ran the price is not high try to buy the attitude, did not expect the effect is really good, good!	Jingdong prefecture level city in Shandong
Power Saving King Shop	King of Power Saving	Power Saving King power is quite large, the quality of the leaf is quite good, the price of buying a very good deal, must be praised!	Jingdong prefecture level city in Shandong
Power Saving King Shop	King of Power Saving	Good product quality is the king of power-saving products trustworthy as always good to recommend!	Jingdong prefecture level city in Shandong
Power Saving King Shop	King of Power Saving	Works great, very affordable, shipped pretty fast, comfortable to use	Jingdong prefecture level city in Shandong
Power Saving King Shop	King of Power Saving	Shipping was pretty fast and affordable, not bad 👍	Jingdong prefecture level city in Shandong
Power Saving King Shop	King of Power Saving	Digital voltage display, voltage at a glance, plug and play, safe and simple!	Jingdong prefecture level city in Shandong
Power Saving King Shop	King of Power Saving	Quality assurance, very good, affordable, logistics is also very fast, I'm very satisfied with the	Jingdong prefecture level city in Shandong
Power Saving King Shop	King of Power Saving	The quality is very good, the first time I received the goods to open the first time to see, it is very good, very satisfied	Jingdong prefecture level city in Shandong
Power Saving King Shop	King of Power Saving	Power Saving King used, the effect is good, the price is affordable, very good, is the genuine article	Jingdong prefecture level city in Shandong
Power Saving King Shop	King of Power Saving	Good quality, fine workmanship, good appearance, time is very accurate, satisfied.	Jingdong prefecture level city in Shandong
Power Saving King Shop	King of Power Saving	I purposely used it for two days before commenting, which is responsible for myself and everyone else!	Jingdong prefecture level city in Shandong
Power Saving King Shop	King of Power Saving	Easy to install, easy to operate, helped our family save a lot of electricity this summer	Jingdong prefecture level city in Shandong
Power Saving King Shop	King of Power Saving	Bringing power savings into millions of homes. Still a great gadget. Pretty good.	Jingdong prefecture level city in Shandong
Power Saving King Shop	King of Power Saving	Digital liquid crystal display to see the clear intuitive, very formal workmanship is not rough	Jingdong prefecture level city in Shandong
Power Saving King Shop	King of Power Saving	Received. Great quality, fast shipping from the store and not too expensive!	Jingdong prefecture level city in Shandong
Power Saving King Shop	King of Power Saving	Bought myself a knuckle regulator and it was easy to use and the price was right.	Jingdong prefecture level city in Shandong
Power Saving King Shop	King of Power Saving	Received, good, powerful, good, very full of a shopping	Jingdong prefecture level city in Shandong
Power Saving King Shop	King of Power Saving	High power, consistent with the seller's description, powerful, cost-effective	Jingdong prefecture level city in Shandong
Power Saving King Shop	King of Power Saving	It works very well and also saves power at an affordable price a great deal.	Jingdong prefecture level city in Shandong
Power Saving King Shop	King of Power Saving	Good quality, good can recommend to friends to buy.	Jingdong prefecture level city in Shandong
Power Saving King Shop	King of Power Saving	Will repurchase, quality is guaranteed, safe and secure purchase.	Jingdong prefecture level city in Shandong
Power Saving King Shop	King of Power Saving	The logistics is very fast, the thing is also good to use, energy saving power rely on it!	Jingdong prefecture level city in Shandong
Power Saving King Shop	King of Power Saving	Power Saver is good quality and works very well, worth buying!	Jingdong prefecture level city in Shandong
Power Saving King Shop	King of Power Saving	The quality is very good is genuine, cost-effective recommended	Jingdong prefecture level city in Shandong
Power Saving King Shop	King of Power Saving	Quality Assurance. Favorable price. Very practical and durable	Jingdong prefecture level city in Shandong
Power Saving King Shop	King of Power Saving	Good value for money. Conscientious businessman. Recommended to everyone	Jingdong prefecture level city in Shandong
Power Saving King Shop	King of Power Saving	Buy it for your rental apartment and never worry about your landlord charging you too much.	Jingdong prefecture level city in Shandong
Power Saving King Shop	King of Power Saving	Nice and affordable, will repurchase next time	Jingdong prefecture level city in Shandong
Power Saving King Shop	King of Power Saving	It's very energy efficient. I've been using it for a few days now.	Jingdong prefecture level city in Shandong
Power Saving King Shop	King of Power Saving	Good quality, high power easy to use	Jingdong prefecture level city in Shandong
Power Saving King Shop	King of Power Saving	Power saving little helper. Deserves a favorable review.	Jingdong prefecture level city in Shandong
Power Saving King Shop	King of Power Saving	Great value for money, great value for money.	Jingdong prefecture level city in Shandong
Power Saving King Shop	King of Power Saving	Works well and as described by the seller	Jingdong prefecture level city in Shandong
Power Saving King Shop	King of Power Saving	Power saving is proper! It's good.	Jingdong prefecture level city in Shandong
Power Saving King Shop	King of Power Saving	Power saving. Works well. Not bad.	Jingdong prefecture level city in Shandong
Power Saving King Shop	King of Power Saving	Satisfied with power saving	Jingdong prefecture level city in Shandong
Power Saving King Shop	King of Power Saving	Received intact, open a super good look beautiful and generous, ~ bit of high quality is recommended to buy, the need for friends rest assured that the next order	Jingdong prefecture level city in Shandong
Power Saving King Shop	King of Power Saving	Very good to use, friends introduced, save electricity and money, long-lasting products cost-effective, large factory production, good quality, fine workmanship cost-effective!	Jingdong prefecture level city in Shandong
Power Saving King Shop	King of Power Saving	The power saving effect is very obvious, trying to pay 30% less electricity bill in a month than before, hahaha	Jingdong prefecture level city in Shandong
Power Saving King Shop	King of Power Saving	It's such a power saver to use this, it saves money on kids snacks for a month. Kudos!	Jingdong prefecture level city in Shandong
Power Saving King Shop	King of Power Saving	Plug and play, stable voltage, the screen has a display when working, this winter home appliances, need more electricity, just buy to use, save electricity and save money, with instructions, not too big and does not take up space, the quality is very good, and the seller describes exactly the same!	Jingdong prefecture level city in Shandong
Power Saving King Shop	King of Power Saving	A must have for opening a store. Save over a thousand dollars a month on your electric bill	Jingdong prefecture level city in Shandong
Power Saving King Shop	King of Power Saving	Received the goods, the first time to unpack, the quality is very good, with the seller's description, completely exceeded my expectations, the packaging is very careful, very strict, in general this is a very satisfied with the shopping	Jingdong prefecture level city in Shandong
Power Saving King Shop	King of Power Saving	As always, good quality, a very favorite product, great! o(*￣︶￣*)o The price is very affordable, cost-effective, it's considered affordable good, will repurchase!	Jingdong prefecture level city in Shandong

Power Saving King Shop	King of Power Saving	Very satisfied with a shopping, good business service, fast shipping, logistics is also very powerful, received very good, buy assured, use peace of mind!	Jingdong prefecture level city in Shandong
Power Saving King Shop	King of Power Saving	The first time to buy online, but also look at the evaluation before buying, the quality is not bad, but also very cheap, the need for friends can rest assured to buy!	Jingdong prefecture level city in Shandong
Power Saving King Shop	King of Power Saving	Better than expected, feel value for money, very satisfied with a shopping, need next time will come back!	Jingdong prefecture level city in Shandong
Power Saving King Shop	King of Power Saving	Really super like, very supportive, very good quality, exactly as described by the seller, very satisfied!	Jingdong prefecture level city in Shandong
Power Saving King Shop	King of Power Saving	It is genuine, effective, really saves power, affordable, tightly packed, fast shipping, very satisfied.	Jingdong prefecture level city in Shandong
Power Saving King Shop	King of Power Saving	Good quality, fine workmanship, is genuine and business description of the same, good value for money, fast logistics, very satisfied	Jingdong prefecture level city in Shandong
Power Saving King Shop	King of Power Saving	Saver received, very good, installation set up very well, very good, the effect is very good, recommend that the pro buy	Jingdong prefecture level city in Shandong
Power Saving King Shop	King of Power Saving	Electromagnetic good, high power convenient, enough force, the price is right it, quality can be 😀 😀 😀 😀 😀	Jingdong prefecture level city in Shandong
Power Saving King Shop	King of Power Saving	First-class quality, packaging is also good, logistics is very fast, and the price is reasonable, very affordable, I like it!	Jingdong prefecture level city in Shandong
Power Saving King Shop	King of Power Saving	It's great, it's the kind of style you want, and it arrived quickly, thank you boss, 5 stars for the service!	Jingdong prefecture level city in Shandong
Power Saving King Shop	King of Power Saving	Packaging is very good, the use of the effect is quite good, the store has a good service attitude, trustworthy	Jingdong prefecture level city in Shandong
Power Saving King Shop	King of Power Saving	The quality is very good, it works well, very satisfied, the price is affordable, it's so great value, it's worth recommending.	Jingdong prefecture level city in Shandong
Power Saving King Shop	King of Power Saving	The baby description matches the actual item very well! The quality is awesome! Will be back to patronize again in the future!	Jingdong prefecture level city in Shandong
Power Saving King Shop	King of Power Saving	Ran the price is not high try to buy the attitude, did not expect the effect is really good, good!	Jingdong prefecture level city in Shandong
Power Saving King Shop	King of Power Saving	Power Saving King power is quite large, the quality of the leaf is quite good, the price of buying a very good deal, must be praised!	Jingdong prefecture level city in Shandong
Power Saving King Shop	King of Power Saving	Good product quality is the king of power-saving products trustworthy as always good to recommend!	Jingdong prefecture level city in Shandong
Power Saving King Shop	King of Power Saving	Works great, very affordable, shipped pretty fast, comfortable to use	Jingdong prefecture level city in Shandong
Power Saving King Shop	King of Power Saving	Shipping was pretty fast and affordable, not bad 👍	Jingdong prefecture level city in Shandong
Power Saving King Shop	King of Power Saving	Digital voltage display, voltage at a glance, plug and play, safe and simple!	Jingdong prefecture level city in Shandong
Power Saving King Shop	King of Power Saving	Quality assurance, very good, affordable, logistics is also very fast, I'm very satisfied with the	Jingdong prefecture level city in Shandong
Power Saving King Shop	King of Power Saving	The quality is very good, the first time I received the goods to open the first time to see, it is very good, very satisfied	Jingdong prefecture level city in Shandong
Power Saving King Shop	King of Power Saving	Power Saving King used, the effect is good, the price is affordable, very good, is the genuine article	Jingdong prefecture level city in Shandong
Power Saving King Shop	King of Power Saving	Good quality, fine workmanship, good appearance, time is very accurate, satisfied.	Jingdong prefecture level city in Shandong
Power Saving King Shop	King of Power Saving	I purposely used it for two days before commenting, which is responsible for myself and everyone else!	Jingdong prefecture level city in Shandong
Power Saving King Shop	King of Power Saving	Easy to install, easy to operate, helped our family save a lot of electricity this summer	Jingdong prefecture level city in Shandong
Power Saving King Shop	King of Power Saving	Bringing power savings into millions of homes. Still a great gadget. Pretty good.	Jingdong prefecture level city in Shandong
Power Saving King Shop	King of Power Saving	Digital liquid crystal display to see the clear intuitive, very formal workmanship is not rough	Jingdong prefecture level city in Shandong
Power Saving King Shop	King of Power Saving	Received. Great quality, fast shipping from the store and not too expensive!	Jingdong prefecture level city in Shandong
Power Saving King Shop	King of Power Saving	Bought myself a knuckle regulator and it was easy to use and the price was right.	Jingdong prefecture level city in Shandong
Power Saving King Shop	King of Power Saving	Received, good, powerful, good, very full of a shopping	Jingdong prefecture level city in Shandong
Power Saving King Shop	King of Power Saving	High power, consistent with the seller's description, powerful, cost-effective	Jingdong prefecture level city in Shandong
Power Saving King Shop	King of Power Saving	It works very well and also saves power at an affordable price a great deal.	Jingdong prefecture level city in Shandong
Power Saving King Shop	King of Power Saving	Good quality, good can recommend to friends to buy.	Jingdong prefecture level city in Shandong
Power Saving King Shop	King of Power Saving	Will repurchase, quality is guaranteed, safe and secure purchase.	Jingdong prefecture level city in Shandong
Power Saving King Shop	King of Power Saving	The logistics is very fast, the thing is also good to use, energy saving power rely on it!	Jingdong prefecture level city in Shandong
Power Saving King Shop	King of Power Saving	Power Saver is good quality and works very well, worth buying!	Jingdong prefecture level city in Shandong
Power Saving King Shop	King of Power Saving	The quality is very good is genuine, cost-effective recommended	Jingdong prefecture level city in Shandong
Power Saving King Shop	King of Power Saving	Quality Assurance. Favorable price. Very practical and durable	Jingdong prefecture level city in Shandong
Power Saving King Shop	King of Power Saving	Good value for money. Conscientious businessman. Recommended to everyone	Jingdong prefecture level city in Shandong
Power Saving King Shop	King of Power Saving	Buy it for your rental apartment and never worry about your landlord charging you too much.	Jingdong prefecture level city in Shandong
Power Saving King Shop	King of Power Saving	Nice and affordable, will repurchase next time	Jingdong prefecture level city in Shandong
Power Saving King Shop	King of Power Saving	It's very energy efficient. I've been using it for a few days now.	Jingdong prefecture level city in Shandong
Power Saving King Shop	King of Power Saving	Good quality, high power easy to use	Jingdong prefecture level city in Shandong
Power Saving King Shop	King of Power Saving	Power saving little helper. Deserves a favorable review.	Jingdong prefecture level city in Shandong
Power Saving King Shop	King of Power Saving	Great value for money, great value for money.	Jingdong prefecture level city in Shandong
Power Saving King Shop	King of Power Saving	Works well and as described by the seller	Jingdong prefecture level city in Shandong
Power Saving King Shop	King of Power Saving	Power saving is proper! It's good.	Jingdong prefecture level city in Shandong
Power Saving King Shop	King of Power Saving	Power saving. Works well. Not bad.	Jingdong prefecture level city in Shandong
Power Saving King Shop	King of Power Saving	Satisfied with power saving	Jingdong prefecture level city in Shandong
Power Saving King Shop	King of Power Saving	Received intact, open a super good look beautiful and generous, ~ bit of high quality is recommended to buy, the need for friends rest assured that the next order	Jingdong prefecture level city in Shandong
Power Saving King Shop	King of Power Saving	Very good to use, friends introduced, save electricity and money, long-lasting products cost-effective, large factory production, good quality, fine workmanship cost-effective!	Jingdong prefecture level city in Shandong

Power Saving King Shop	King of Power Saving	The power saving effect is very obvious, trying to pay 30% less electricity bill in a month than before, hahaha	Jingdong prefecture level city in Shandong
Power Saving King Shop	King of Power Saving	It's such a power saver to use this, it saves money on kids snacks for a month. Kudos!	Jingdong prefecture level city in Shandong
Power Saving King Shop	King of Power Saving	Plug and play, stable voltage, the screen has a display when working, this winter home appliances, need more electricity, just buy to use, save electricity and save money, with instructions, not too big and does not take up space, the quality is very good, and the seller describes exactly the same!	Jingdong prefecture level city in Shandong
Power Saving King Shop	King of Power Saving	A must have for opening a store. Save over a thousand dollars a month on your electric bill	Jingdong prefecture level city in Shandong
Power Saving King Shop	King of Power Saving	Received the goods, the first time to unpack, the quality is very good, with the seller's description, completely exceeded my expectations, the packaging is very careful, very strict, in general this is a very satisfied with the shopping	Jingdong prefecture level city in Shandong
Power Saving King Shop	King of Power Saving	As always, good quality, a very favorite product, great! o(*￣︶￣*)o The price is very affordable, cost-effective, it's considered affordable good, will repurchase!	Jingdong prefecture level city in Shandong
Power Saving King Shop	King of Power Saving	Very satisfied with a shopping, good business service, fast shipping, logistics is also very powerful, received very good, buy assured, use peace of mind!	Jingdong prefecture level city in Shandong
Power Saving King Shop	King of Power Saving	The first time to buy online, but also look at the evaluation before buying, the quality is not bad, but also very cheap, the need for friends can rest assured to buy!	Jingdong prefecture level city in Shandong
Power Saving King Shop	King of Power Saving	Better than expected, feel value for money, very satisfied with a shopping, need next time will come back!	Jingdong prefecture level city in Shandong
Power Saving King Shop	King of Power Saving	Really super like, very supportive, very good quality, exactly as described by the seller, very satisfied!	Jingdong prefecture level city in Shandong
Power Saving King Shop	King of Power Saving	It is genuine, effective, really saves power, affordable, tightly packed, fast shipping, very satisfied.	Jingdong prefecture level city in Shandong
Power Saving King Shop	King of Power Saving	Good quality, fine workmanship, is genuine and business description of the same, good value for money, fast logistics, very satisfied	Jingdong prefecture level city in Shandong
Power Saving King Shop	King of Power Saving	Saver received, very good, installation set up very well, very good, the effect is very good, recommend that the pro buy	Jingdong prefecture level city in Shandong
Power Saving King Shop	King of Power Saving	Electromagnetic good, high power convenient, enough force, the price is right it, quality can be ☺ ☺ ☺ ☺ ☺	Jingdong prefecture level city in Shandong
Power Saving King Shop	King of Power Saving	First-class quality, packaging is also good, logistics is very fast, and the price is reasonable, very affordable, I like it!	Jingdong prefecture level city in Shandong
Power Saving King Shop	King of Power Saving	It's great, it's the kind of style you want, and it arrived quickly, thank you boss, 5 stars for the service!	Jingdong prefecture level city in Shandong
Power Saving King Shop	King of Power Saving	Packaging is very good, the use of the effect is quite good, the store has a good service attitude, trustworthy	Jingdong prefecture level city in Shandong
Power Saving King Shop	King of Power Saving	The quality is very good, it works well, very satisfied, the price is affordable, it's so great value, it's worth recommending.	Jingdong prefecture level city in Shandong
Power Saving King Shop	King of Power Saving	The baby description matches the actual item very well! The quality is awesome! Will be back to patronize again in the future!	Jingdong prefecture level city in Shandong
Power Saving King Shop	King of Power Saving	Ran the price is not high try to buy the attitude, did not expect the effect is really good, good!	Jingdong prefecture level city in Shandong
Power Saving King Shop	King of Power Saving	Power Saving King power is quite large, the quality of the leaf is quite good, the price of buying a very good deal, must be praised!	Jingdong prefecture level city in Shandong
Power Saving King Shop	King of Power Saving	Good product quality is the king of power-saving products trustworthy as always good to recommend!	Jingdong prefecture level city in Shandong
Power Saving King Shop	King of Power Saving	Works great, very affordable, shipped pretty fast, comfortable to use	Jingdong prefecture level city in Shandong
Power Saving King Shop	King of Power Saving	Shipping was pretty fast and affordable, not bad 👍	Jingdong prefecture level city in Shandong
Power Saving King Shop	King of Power Saving	Digital voltage display, voltage at a glance, plug and play, safe and simple!	Jingdong prefecture level city in Shandong
Power Saving King Shop	King of Power Saving	Quality assurance, very good, affordable, logistics is also very fast, I'm very satisfied with the	Jingdong prefecture level city in Shandong
Power Saving King Shop	King of Power Saving	The quality is very good, the first time I received the goods to open the first time to see, it is very good, very satisfied	Jingdong prefecture level city in Shandong
Power Saving King Shop	King of Power Saving	Power Saving King used, the effect is good, the price is affordable, very good, is the genuine article	Jingdong prefecture level city in Shandong
Power Saving King Shop	King of Power Saving	Good quality, fine workmanship, good appearance, time is very accurate, satisfied.	Jingdong prefecture level city in Shandong
Power Saving King Shop	King of Power Saving	I purposely used it for two days before commenting, which is responsible for myself and everyone else!	Jingdong prefecture level city in Shandong
Power Saving King Shop	King of Power Saving	Easy to install, easy to operate, helped our family save a lot of electricity this summer	Jingdong prefecture level city in Shandong
Power Saving King Shop	King of Power Saving	Bringing power savings into millions of homes. Still a great gadget. Pretty good.	Jingdong prefecture level city in Shandong
Power Saving King Shop	King of Power Saving	Digital liquid crystal display to see the clear intuitive, very formal workmanship is not rough	Jingdong prefecture level city in Shandong
Power Saving King Shop	King of Power Saving	Received. Great quality, fast shipping from the store and not too expensive!	Jingdong prefecture level city in Shandong
Power Saving King Shop	King of Power Saving	Bought myself a knuckle regulator and it was easy to use and the price was right.	Jingdong prefecture level city in Shandong
Power Saving King Shop	King of Power Saving	Received, good, powerful, good, very full of a shopping	Jingdong prefecture level city in Shandong
Power Saving King Shop	King of Power Saving	High power, consistent with the seller's description, powerful, cost-effective	Jingdong prefecture level city in Shandong
Power Saving King Shop	King of Power Saving	It works very well and also saves power at an affordable price a great deal.	Jingdong prefecture level city in Shandong
Power Saving King Shop	King of Power Saving	Good quality, good can recommend to friends to buy.	Jingdong prefecture level city in Shandong
Power Saving King Shop	King of Power Saving	Will repurchase, quality is guaranteed, safe and secure purchase.	Jingdong prefecture level city in Shandong
Power Saving King Shop	King of Power Saving	The logistics is very fast, the thing is also good to use, energy saving power rely on it!	Jingdong prefecture level city in Shandong
Power Saving King Shop	King of Power Saving	Power Saver is good quality and works very well, worth buying!	Jingdong prefecture level city in Shandong
Power Saving King Shop	King of Power Saving	The quality is very good is genuine, cost-effective recommended	Jingdong prefecture level city in Shandong
Power Saving King Shop	King of Power Saving	Quality Assurance. Favorable price. Very practical and durable	Jingdong prefecture level city in Shandong
Power Saving King Shop	King of Power Saving	Good value for money. Conscientious businessman. Recommended to everyone	Jingdong prefecture level city in Shandong
Power Saving King Shop	King of Power Saving	Buy it for your rental apartment and never worry about your landlord charging you too much.	Jingdong prefecture level city in Shandong
Power Saving King Shop	King of Power Saving	Nice and affordable, will repurchase next time	Jingdong prefecture level city in Shandong
Power Saving King Shop	King of Power Saving	It's very energy efficient. I've been using it for a few days now.	Jingdong prefecture level city in Shandong
Power Saving King Shop	King of Power Saving	Good quality, high power easy to use	Jingdong prefecture level city in Shandong
Power Saving King Shop	King of Power Saving	Power saving little helper. Deserves a favorable review.	Jingdong prefecture level city in Shandong

Power Saving King Shop	King of Power Saving	Great value for money, great value for money.	Jingdong prefecture level city in Shandong
Power Saving King Shop	King of Power Saving	Works well and as described by the seller	Jingdong prefecture level city in Shandong
Power Saving King Shop	King of Power Saving	Power saving is proper! It's good.	Jingdong prefecture level city in Shandong
Power Saving King Shop	King of Power Saving	Power saving. Works well. Not bad.	Jingdong prefecture level city in Shandong
Power Saving King Shop	King of Power Saving	Satisfied with power saving	Jingdong prefecture level city in Shandong
Power Saving King Shop	King of Power Saving	Received intact, open a super good look beautiful and generous, ~ bit of high quality is recommended to buy, the need for friends rest assured that the next order	Jingdong prefecture level city in Shandong
Power Saving King Shop	King of Power Saving	Very good to use, friends introduced, save electricity and money, long-lasting products cost-effective, large factory production, good quality, fine workmanship cost-effective!	Jingdong prefecture level city in Shandong
Power Saving King Shop	King of Power Saving	The power saving effect is very obvious, trying to pay 30% less electricity bill in a month than before, hahaha	Jingdong prefecture level city in Shandong
Power Saving King Shop	King of Power Saving	It's such a power saver to use this, it saves money on kids snacks for a month. Kudos!	Jingdong prefecture level city in Shandong
Power Saving King Shop	King of Power Saving	Plug and play, stable voltage, the screen has a display when working, this winter home appliances, need more electricity, just buy to use, save electricity and save money, with instructions, not too big and does not take up space, the quality is very good, and the seller describes exactly the same!	Jingdong prefecture level city in Shandong
Power Saving King Shop	King of Power Saving	A must have for opening a store. Save over a thousand dollars a month on your electric bill	Jingdong prefecture level city in Shandong
Power Saving King Shop	King of Power Saving	Received the goods, the first time to unpack, the quality is very good, with the seller's description, completely exceeded my expectations, the packaging is very careful, very strict, in general this is a very satisfied with the shopping	Jingdong prefecture level city in Shandong
Power Saving King Shop	King of Power Saving	As always, good quality, a very favorite product, great! o(*￣︶￣*)o The price is very affordable, cost-effective, it's considered affordable good, will repurchase!	Jingdong prefecture level city in Shandong
Power Saving King Shop	King of Power Saving	Very satisfied with a shopping, good business service, fast shipping, logistics is also very powerful, received very good, buy assured, use peace of mind!	Jingdong prefecture level city in Shandong
Power Saving King Shop	King of Power Saving	The first time to buy online, but also look at the evaluation before buying, the quality is not bad, but also very cheap, the need for friends can rest assured to buy!	Jingdong prefecture level city in Shandong
Power Saving King Shop	King of Power Saving	Better than expected, feel value for money, very satisfied with a shopping, need next time will come back!	Jingdong prefecture level city in Shandong
Power Saving King Shop	King of Power Saving	Really super like, very supportive, very good quality, exactly as described by the seller, very satisfied!	Jingdong prefecture level city in Shandong
Power Saving King Shop	King of Power Saving	It is genuine, effective, really saves power, affordable, tightly packed, fast shipping, very satisfied.	Jingdong prefecture level city in Shandong
Power Saving King Shop	King of Power Saving	Good quality, fine workmanship, is genuine and business description of the same, good value for money, fast logistics, very satisfied	Jingdong prefecture level city in Shandong
Power Saving King Shop	King of Power Saving	Saver received, very good, installation set up very well, very good, the effect is very good, recommend that the pro buy	Jingdong prefecture level city in Shandong
Power Saving King Shop	King of Power Saving	Electromagnetic good, high power convenient, enough force, the price is right it, quality can be 😃 😃 😃 😃 😃	Jingdong prefecture level city in Shandong
Power Saving King Shop	King of Power Saving	First-class quality, packaging is also good, logistics is very fast, and the price is reasonable, very affordable, I like it!	Jingdong prefecture level city in Shandong
Power Saving King Shop	King of Power Saving	It's great, it's the kind of style you want, and it arrived quickly, thank you boss, 5 stars for the service!	Jingdong prefecture level city in Shandong
Power Saving King Shop	King of Power Saving	Packaging is very good, the use of the effect is quite good, the store has a good service attitude, trustworthy	Jingdong prefecture level city in Shandong
Power Saving King Shop	King of Power Saving	The quality is very good, it works well, very satisfied, the price is affordable, it's so great value, it's worth recommending.	Jingdong prefecture level city in Shandong
Power Saving King Shop	King of Power Saving	The baby description matches the actual item very well! The quality is awesome! Will be back to patronize again in the future!	Jingdong prefecture level city in Shandong
Power Saving King Shop	King of Power Saving	Ran the price is not high try to buy the attitude, did not expect the effect is really good, good!	Jingdong prefecture level city in Shandong
Power Saving King Shop	King of Power Saving	Power Saving King power is quite large, the quality of the leaf is quite good, the price of buying a very good deal, must be praised!	Jingdong prefecture level city in Shandong
Power Saving King Shop	King of Power Saving	Good product quality is the king of power-saving products trustworthy as always good to recommend!	Jingdong prefecture level city in Shandong
Power Saving King Shop	King of Power Saving	Works great, very affordable, shipped pretty fast, comfortable to use	Jingdong prefecture level city in Shandong
Power Saving King Shop	King of Power Saving	Shipping was pretty fast and affordable, not bad 👍	Jingdong prefecture level city in Shandong
Power Saving King Shop	King of Power Saving	Digital voltage display, voltage at a glance, plug and play, safe and simple!	Jingdong prefecture level city in Shandong
Power Saving King Shop	King of Power Saving	Quality assurance, very good, affordable, logistics is also very fast, I'm very satisfied with the	Jingdong prefecture level city in Shandong
Power Saving King Shop	King of Power Saving	The quality is very good, the first time I received the goods to open the first time to see, it is very good, very satisfied	Jingdong prefecture level city in Shandong
Power Saving King Shop	King of Power Saving	Power Saving King used, the effect is good, the price is affordable, very good, is the genuine article	Jingdong prefecture level city in Shandong
Power Saving King Shop	King of Power Saving	Good quality, fine workmanship, good appearance, time is very accurate, satisfied.	Jingdong prefecture level city in Shandong
Power Saving King Shop	King of Power Saving	I purposely used it for two days before commenting, which is responsible for myself and everyone else!	Jingdong prefecture level city in Shandong
Power Saving King Shop	King of Power Saving	Easy to install, easy to operate, helped our family save a lot of electricity this summer	Jingdong prefecture level city in Shandong
Power Saving King Shop	King of Power Saving	Bringing power savings into millions of homes. Still a great gadget. Pretty good.	Jingdong prefecture level city in Shandong
Power Saving King Shop	King of Power Saving	Digital liquid crystal display to see the clear intuitive, very formal workmanship is not rough	Jingdong prefecture level city in Shandong
Power Saving King Shop	King of Power Saving	Received. Great quality, fast shipping from the store and not too expensive!	Jingdong prefecture level city in Shandong
Power Saving King Shop	King of Power Saving	Bought myself a knuckle regulator and it was easy to use and the price was right.	Jingdong prefecture level city in Shandong
Power Saving King Shop	King of Power Saving	Received, good, powerful, good, very full of a shopping	Jingdong prefecture level city in Shandong
Power Saving King Shop	King of Power Saving	High power, consistent with the seller's description, powerful, cost-effective	Jingdong prefecture level city in Shandong
Power Saving King Shop	King of Power Saving	It works very well and also saves power at an affordable price a great deal.	Jingdong prefecture level city in Shandong
Power Saving King Shop	King of Power Saving	Good quality, good can recommend to friends to buy.	Jingdong prefecture level city in Shandong
Power Saving King Shop	King of Power Saving	Will repurchase, quality is guaranteed, safe and secure purchase.	Jingdong prefecture level city in Shandong
Power Saving King Shop	King of Power Saving	The logistics is very fast, the thing is also good to use, energy saving power rely on it!	Jingdong prefecture level city in Shandong
Power Saving King Shop	King of Power Saving	Power Saver is good quality and works very well, worth buying!	Jingdong prefecture level city in Shandong
Power Saving King Shop	King of Power Saving	The quality is very good is genuine, cost-effective recommended	Jingdong prefecture level city in Shandong

Power Saving King Shop	King of Power Saving	Quality Assurance. Favorable price. Very practical and durable	Jingdong prefecture level city in Shandong
Power Saving King Shop	King of Power Saving	Good value for money. Conscientious businessman. Recommended to everyone	Jingdong prefecture level city in Shandong
Power Saving King Shop	King of Power Saving	Buy it for your rental apartment and never worry about your landlord charging you too much.	Jingdong prefecture level city in Shandong
Power Saving King Shop	King of Power Saving	Nice and affordable, will repurchase next time	Jingdong prefecture level city in Shandong
Power Saving King Shop	King of Power Saving	It's very energy efficient. I've been using it for a few days now.	Jingdong prefecture level city in Shandong
Power Saving King Shop	King of Power Saving	Good quality, high power easy to use	Jingdong prefecture level city in Shandong
Power Saving King Shop	King of Power Saving	Power saving little helper. Deserves a favorable review.	Jingdong prefecture level city in Shandong
Power Saving King Shop	King of Power Saving	Great value for money, great value for money.	Jingdong prefecture level city in Shandong
Power Saving King Shop	King of Power Saving	Works well and as described by the seller	Jingdong prefecture level city in Shandong
Power Saving King Shop	King of Power Saving	Power saving is proper! It's good.	Jingdong prefecture level city in Shandong
Power Saving King Shop	King of Power Saving	Power saving. Works well. Not bad.	Jingdong prefecture level city in Shandong
Power Saving King Shop	King of Power Saving	Satisfied with power saving	Jingdong prefecture level city in Shandong
Power Saving King Shop	King of Power Saving	Received intact, open a super good look beautiful and generous, ~ bit of high quality is recommended to buy, the need for friends rest assured that the next order	Jingdong prefecture level city in Shandong
Power Saving King Shop	King of Power Saving	Very good to use, friends introduced, save electricity and money, long-lasting products cost-effective, large factory production, good quality, fine workmanship cost-effective!	Jingdong prefecture level city in Shandong
Power Saving King Shop	King of Power Saving	The power saving effect is very obvious, trying to pay 30% less electricity bill in a month than before, hahaha	Jingdong prefecture level city in Shandong
Power Saving King Shop	King of Power Saving	It's such a power saver to use this, it saves money on kids snacks for a month. Kudos!	Jingdong prefecture level city in Shandong
Power Saving King Shop	King of Power Saving	Plug and play, stable voltage, the screen has a display when working, this winter home appliances, need more electricity, just buy to use, save electricity and save money, with instructions, not too big and does not take up space, the quality is very good, and the seller describes exactly the same!	Jingdong prefecture level city in Shandong
Power Saving King Shop	King of Power Saving	A must have for opening a store. Save over a thousand dollars a month on your electric bill	Jingdong prefecture level city in Shandong
Power Saving King Shop	King of Power Saving	Received the goods, the first time to unpack, the quality is very good, with the seller's description, completely exceeded my expectations, the packaging is very careful, very strict, in general this is a very satisfied with the shopping	Jingdong prefecture level city in Shandong
Power Saving King Shop	King of Power Saving	As always, good quality, a very favorite product, great! o(*￣︶￣*)o The price is very affordable, cost-effective, it's considered affordable good, will repurchase!	Jingdong prefecture level city in Shandong
Power Saving King Shop	King of Power Saving	Very satisfied with a shopping, good business service, fast shipping, logistics is also very powerful, received very good, buy assured, use peace of mind!	Jingdong prefecture level city in Shandong
Power Saving King Shop	King of Power Saving	The first time to buy online, but also look at the evaluation before buying, the quality is not bad, but also very cheap, the need for friends can rest assured to buy!	Jingdong prefecture level city in Shandong
Power Saving King Shop	King of Power Saving	Better than expected, feel value for money, very satisfied with a shopping, need next time will come back!	Jingdong prefecture level city in Shandong
Power Saving King Shop	King of Power Saving	Really super like, very supportive, very good quality, exactly as described by the seller, very satisfied!	Jingdong prefecture level city in Shandong
Power Saving King Shop	King of Power Saving	It is genuine, effective, really saves power, affordable, tightly packed, fast shipping, very satisfied.	Jingdong prefecture level city in Shandong
Power Saving King Shop	King of Power Saving	Good quality, fine workmanship, is genuine and business description of the same, good value for money, fast logistics, very satisfied	Jingdong prefecture level city in Shandong
Power Saving King Shop	King of Power Saving	Saver received, very good, installation set up very well, very good, the effect is very good, recommend that the pro buy	Jingdong prefecture level city in Shandong
Power Saving King Shop	King of Power Saving	Electromagnetic good, high power convenient, enough force, the price is right it, quality can be ☺ ☺ ☺ ☺ ☺	Jingdong prefecture level city in Shandong
Power Saving King Shop	King of Power Saving	First-class quality, packaging is also good, logistics is very fast, and the price is reasonable, very affordable, I like it!	Jingdong prefecture level city in Shandong
Power Saving King Shop	King of Power Saving	It's great, it's the kind of style you want, and it arrived quickly, thank you boss, 5 stars for the service!	Jingdong prefecture level city in Shandong
Power Saving King Shop	King of Power Saving	Packaging is very good, the use of the effect is quite good, the store has a good service attitude, trustworthy	Jingdong prefecture level city in Shandong
Power Saving King Shop	King of Power Saving	The quality is very good, it works well, very satisfied, the price is affordable, it's so great value, it's worth recommending.	Jingdong prefecture level city in Shandong
Power Saving King Shop	King of Power Saving	The baby description matches the actual item very well! The quality is awesome! Will be back to patronize again in the future!	Jingdong prefecture level city in Shandong
Power Saving King Shop	King of Power Saving	Ran the price is not high try to buy the attitude, did not expect the effect is really good, good!	Jingdong prefecture level city in Shandong
Power Saving King Shop	King of Power Saving	Power Saving King power is quite large, the quality of the leaf is quite good, the price of buying a very good deal, must be praised!	Jingdong prefecture level city in Shandong
Power Saving King Shop	King of Power Saving	Good product quality is the king of power-saving products trustworthy as always good to recommend!	Jingdong prefecture level city in Shandong
Power Saving King Shop	King of Power Saving	Works great, very affordable, shipped pretty fast, comfortable to use	Jingdong prefecture level city in Shandong
Power Saving King Shop	King of Power Saving	Shipping was pretty fast and affordable, not bad 👍	Jingdong prefecture level city in Shandong
Power Saving King Shop	King of Power Saving	Digital voltage display, voltage at a glance, plug and play, safe and simple!	Jingdong prefecture level city in Shandong
Power Saving King Shop	King of Power Saving	Quality assurance, very good, affordable, logistics is also very fast, I'm very satisfied with the	Jingdong prefecture level city in Shandong
Power Saving King Shop	King of Power Saving	The quality is very good, the first time I received the goods to open the first time to see, it is very good, very satisfied	Jingdong prefecture level city in Shandong
Power Saving King Shop	King of Power Saving	Power Saving King used, the effect is good, the price is affordable, very good, is the genuine article	Jingdong prefecture level city in Shandong
Power Saving King Shop	King of Power Saving	Good quality, fine workmanship, good appearance, time is very accurate, satisfied.	Jingdong prefecture level city in Shandong
Power Saving King Shop	King of Power Saving	I purposely used it for two days before commenting, which is responsible for myself and everyone else!	Jingdong prefecture level city in Shandong
Power Saving King Shop	King of Power Saving	Easy to install, easy to operate, helped our family save a lot of electricity this summer	Jingdong prefecture level city in Shandong
Power Saving King Shop	King of Power Saving	Bringing power savings into millions of homes. Still a great gadget. Pretty good.	Jingdong prefecture level city in Shandong
Power Saving King Shop	King of Power Saving	Digital liquid crystal display to see the clear intuitive, very formal workmanship is not rough	Jingdong prefecture level city in Shandong
Power Saving King Shop	King of Power Saving	Received. Great quality, fast shipping from the store and not too expensive!	Jingdong prefecture level city in Shandong
Power Saving King Shop	King of Power Saving	Bought myself a knuckle regulator and it was easy to use and the price was right.	Jingdong prefecture level city in Shandong
Power Saving King Shop	King of Power Saving	Received, good, powerful, good, very full of a shopping	Jingdong prefecture level city in Shandong

Power Saving King Shop	King of Power Saving	High power, consistent with the seller's description, powerful, cost-effective	Jingdong prefecture level city in Shandong
Power Saving King Shop	King of Power Saving	It works very well and also saves power at an affordable price a great deal.	Jingdong prefecture level city in Shandong
Power Saving King Shop	King of Power Saving	Good quality, good can recommend to friends to buy.	Jingdong prefecture level city in Shandong
Power Saving King Shop	King of Power Saving	Will repurchase, quality is guaranteed, safe and secure purchase.	Jingdong prefecture level city in Shandong
Power Saving King Shop	King of Power Saving	The logistics is very fast, the thing is also good to use, energy saving power rely on it!	Jingdong prefecture level city in Shandong
Power Saving King Shop	King of Power Saving	Power Saver is good quality and works very well, worth buying!	Jingdong prefecture level city in Shandong
Power Saving King Shop	King of Power Saving	The quality is very good is genuine, cost-effective recommended	Jingdong prefecture level city in Shandong
Power Saving King Shop	King of Power Saving	Quality Assurance. Favorable price. Very practical and durable	Jingdong prefecture level city in Shandong
Power Saving King Shop	King of Power Saving	Good value for money. Conscientious businessman. Recommended to everyone	Jingdong prefecture level city in Shandong
Power Saving King Shop	King of Power Saving	Buy it for your rental apartment and never worry about your landlord charging you too much.	Jingdong prefecture level city in Shandong
Power Saving King Shop	King of Power Saving	Nice and affordable, will repurchase next time	Jingdong prefecture level city in Shandong
Power Saving King Shop	King of Power Saving	It's very energy efficient. I've been using it for a few days now.	Jingdong prefecture level city in Shandong
Power Saving King Shop	King of Power Saving	Good quality, high power easy to use	Jingdong prefecture level city in Shandong
Power Saving King Shop	King of Power Saving	Power saving little helper. Deserves a favorable review.	Jingdong prefecture level city in Shandong
Power Saving King Shop	King of Power Saving	Great value for money, great value for money.	Jingdong prefecture level city in Shandong
Power Saving King Shop	King of Power Saving	Works well and as described by the seller	Jingdong prefecture level city in Shandong
Power Saving King Shop	King of Power Saving	Power saving is proper! It's good.	Jingdong prefecture level city in Shandong
Power Saving King Shop	King of Power Saving	Power saving. Works well. Not bad.	Jingdong prefecture level city in Shandong
Power Saving King Shop	King of Power Saving	Satisfied with power saving	Jingdong prefecture level city in Shandong
Power Saving King Shop	King of Power Saving	Received intact, open a super good look beautiful and generous, ~ bit of high quality is recommended to buy, the need for friends rest assured that the next order	Jingdong prefecture level city in Shandong
Power Saving King Shop	King of Power Saving	Very good to use, friends introduced, save electricity and money, long-lasting products cost-effective, large factory production, good quality, fine workmanship cost-effective!	Jingdong prefecture level city in Shandong
Power Saving King Shop	King of Power Saving	The power saving effect is very obvious, trying to pay 30% less electricity bill in a month than before, hahaha	Jingdong prefecture level city in Shandong
Power Saving King Shop	King of Power Saving	It's such a power saver to use this, it saves money on kids snacks for a month. Kudos!	Jingdong prefecture level city in Shandong
Power Saving King Shop	King of Power Saving	Plug and play, stable voltage, the screen has a display when working, this winter home appliances, need more electricity, just buy to use, save electricity and save money, with instructions, not too big and does not take up space, the quality is very good, and the seller describes exactly the same!	Jingdong prefecture level city in Shandong
Power Saving King Shop	King of Power Saving	A must have for opening a store. Save over a thousand dollars a month on your electric bill	Jingdong prefecture level city in Shandong
Power Saving King Shop	King of Power Saving	Received the goods, the first time to unpack, the quality is very good, with the seller's description, completely exceeded my expectations, the packaging is very careful, very strict, in general this is a very satisfied with the shopping	Jingdong prefecture level city in Shandong
Power Saving King Shop	King of Power Saving	As always, good quality, a very favorite product, great! o(*￣︶￣*)o The price is very affordable, cost-effective, it's considered affordable good, will repurchase!	Jingdong prefecture level city in Shandong
Power Saving King Shop	King of Power Saving	Very satisfied with a shopping, good business service, fast shipping, logistics is also very powerful, received very good, buy assured, use peace of mind!	Jingdong prefecture level city in Shandong
Power Saving King Shop	King of Power Saving	The first time to buy online, but also look at the evaluation before buying, the quality is not bad, but also very cheap, the need for friends can rest assured to buy!	Jingdong prefecture level city in Shandong
Power Saving King Shop	King of Power Saving	Better than expected, feel value for money, very satisfied with a shopping, need next time will come back!	Jingdong prefecture level city in Shandong
Power Saving King Shop	King of Power Saving	Really super like, very supportive, very good quality, exactly as described by the seller, very satisfied!	Jingdong prefecture level city in Shandong
Power Saving King Shop	King of Power Saving	It is genuine, effective, really saves power, affordable, tightly packed, fast shipping, very satisfied.	Jingdong prefecture level city in Shandong
Power Saving King Shop	King of Power Saving	Good quality, fine workmanship, is genuine and business description of the same, good value for money, fast logistics, very satisfied	Jingdong prefecture level city in Shandong
Power Saving King Shop	King of Power Saving	Saver received, very good, installation set up very well, very good, the effect is very good, recommend that the pro buy	Jingdong prefecture level city in Shandong
Power Saving King Shop	King of Power Saving	Electromagnetic good, high power convenient, enough force, the price is right it, quality can be 😃 😃 😃 😃 😃	Jingdong prefecture level city in Shandong
Power Saving King Shop	King of Power Saving	First-class quality, packaging is also good, logistics is very fast, and the price is reasonable, very affordable, I like it!	Jingdong prefecture level city in Shandong
Power Saving King Shop	King of Power Saving	It's great, it's the kind of style you want, and it arrived quickly, thank you boss, 5 stars for the service!	Jingdong prefecture level city in Shandong
Power Saving King Shop	King of Power Saving	Packaging is very good, the use of the effect is quite good, the store has a good service attitude, trustworthy	Jingdong prefecture level city in Shandong
Power Saving King Shop	King of Power Saving	The quality is very good, it works well, very satisfied, the price is affordable, it's so great value, it's worth recommending.	Jingdong prefecture level city in Shandong
Power Saving King Shop	King of Power Saving	The baby description matches the actual item very well! The quality is awesome! Will be back to patronize again in the future!	Jingdong prefecture level city in Shandong
Power Saving King Shop	King of Power Saving	Ran the price is not high try to buy the attitude, did not expect the effect is really good, good!	Jingdong prefecture level city in Shandong
Power Saving King Shop	King of Power Saving	Power Saving King power is quite large, the quality of the leaf is quite good, the price of buying a very good deal, must be praised!	Jingdong prefecture level city in Shandong
Power Saving King Shop	King of Power Saving	Good product quality is the king of power-saving products trustworthy as always good to recommend!	Jingdong prefecture level city in Shandong
Power Saving King Shop	King of Power Saving	Works great, very affordable, shipped pretty fast, comfortable to use	Jingdong prefecture level city in Shandong
Power Saving King Shop	King of Power Saving	Shipping was pretty fast and affordable, not bad 👍	Jingdong prefecture level city in Shandong
Power Saving King Shop	King of Power Saving	Digital voltage display, voltage at a glance, plug and play, safe and simple!	Jingdong prefecture level city in Shandong
Power Saving King Shop	King of Power Saving	Quality assurance, very good, affordable, logistics is also very fast, I'm very satisfied with the	Jingdong prefecture level city in Shandong
Power Saving King Shop	King of Power Saving	The quality is very good, the first time I received the goods to open the first time to see, it is very good, very satisfied	Jingdong prefecture level city in Shandong
Power Saving King Shop	King of Power Saving	Power Saving King used, the effect is good, the price is affordable, very good, is the genuine article	Jingdong prefecture level city in Shandong
Power Saving King Shop	King of Power Saving	Good quality, fine workmanship, good appearance, time is very accurate, satisfied.	Jingdong prefecture level city in Shandong

Power Saving King Shop	King of Power Saving	I purposely used it for two days before commenting, which is responsible for myself and everyone else!	Jingdong prefecture level city in Shandong
Power Saving King Shop	King of Power Saving	Easy to install, easy to operate, helped our family save a lot of electricity this summer	Jingdong prefecture level city in Shandong
Power Saving King Shop	King of Power Saving	Bringing power savings into millions of homes. Still a great gadget. Pretty good.	Jingdong prefecture level city in Shandong
Power Saving King Shop	King of Power Saving	Digital liquid crystal display to see the clear intuitive, very formal workmanship is not rough	Jingdong prefecture level city in Shandong
Power Saving King Shop	King of Power Saving	Received. Great quality, fast shipping from the store and not too expensive!	Jingdong prefecture level city in Shandong
Power Saving King Shop	King of Power Saving	Bought myself a knuckle regulator and it was easy to use and the price was right.	Jingdong prefecture level city in Shandong
Power Saving King Shop	King of Power Saving	Received, good, powerful, good, very full of a shopping	Jingdong prefecture level city in Shandong
Power Saving King Shop	King of Power Saving	High power, consistent with the seller's description, powerful, cost-effective	Jingdong prefecture level city in Shandong
Power Saving King Shop	King of Power Saving	It works very well and also saves power at an affordable price a great deal.	Jingdong prefecture level city in Shandong
Power Saving King Shop	King of Power Saving	Good quality, good can recommend to friends to buy.	Jingdong prefecture level city in Shandong
Power Saving King Shop	King of Power Saving	Will repurchase, quality is guaranteed, safe and secure purchase.	Jingdong prefecture level city in Shandong
Power Saving King Shop	King of Power Saving	The logistics is very fast, the thing is also good to use, energy saving power rely on it!	Jingdong prefecture level city in Shandong
Power Saving King Shop	King of Power Saving	Power Saver is good quality and works very well, worth buying!	Jingdong prefecture level city in Shandong
Power Saving King Shop	King of Power Saving	The quality is very good is genuine, cost-effective recommended	Jingdong prefecture level city in Shandong
Power Saving King Shop	King of Power Saving	Quality Assurance. Favorable price. Very practical and durable	Jingdong prefecture level city in Shandong
Power Saving King Shop	King of Power Saving	Good value for money. Conscientious businessman. Recommended to everyone	Jingdong prefecture level city in Shandong
Power Saving King Shop	King of Power Saving	Buy it for your rental apartment and never worry about your landlord charging you too much.	Jingdong prefecture level city in Shandong
Power Saving King Shop	King of Power Saving	Nice and affordable, will repurchase next time	Jingdong prefecture level city in Shandong
Power Saving King Shop	King of Power Saving	It's very energy efficient. I've been using it for a few days now.	Jingdong prefecture level city in Shandong
Power Saving King Shop	King of Power Saving	Good quality, high power easy to use	Jingdong prefecture level city in Shandong
Power Saving King Shop	King of Power Saving	Power saving little helper. Deserves a favorable review.	Jingdong prefecture level city in Shandong
Power Saving King Shop	King of Power Saving	Great value for money, great value for money.	Jingdong prefecture level city in Shandong
Power Saving King Shop	King of Power Saving	Works well and as described by the seller	Jingdong prefecture level city in Shandong
Power Saving King Shop	King of Power Saving	Power saving is proper! It's good.	Jingdong prefecture level city in Shandong
Power Saving King Shop	King of Power Saving	Power saving. Works well. Not bad.	Jingdong prefecture level city in Shandong
Power Saving King Shop	King of Power Saving	Satisfied with power saving	Jingdong prefecture level city in Shandong
Power Saving King Shop	King of Power Saving	Received intact, open a super good look beautiful and generous, ~ bit of high quality is recommended to buy, the need for friends rest assured that the next order	Jingdong prefecture level city in Shandong
Power Saving King Shop	King of Power Saving	Very good to use, friends introduced, save electricity and money, long-lasting products cost-effective, large factory production, good quality, fine workmanship cost-effective!	Jingdong prefecture level city in Shandong
Power Saving King Shop	King of Power Saving	The power saving effect is very obvious, trying to pay 30% less electricity bill in a month than before, hahaha	Jingdong prefecture level city in Shandong
Power Saving King Shop	King of Power Saving	It's such a power saver to use this, it saves money on kids snacks for a month. Kudos!	Jingdong prefecture level city in Shandong
Power Saving King Shop	King of Power Saving	Plug and play, stable voltage, the screen has a display when working, this winter home appliances, need more electricity, just buy to use, save electricity and save money, with instructions, not too big and does not take up space, the quality is very good, and the seller describes exactly the same!	Jingdong prefecture level city in Shandong
Power Saving King Shop	King of Power Saving	A must have for opening a store. Save over a thousand dollars a month on your electric bill	Jingdong prefecture level city in Shandong
Power Saving King Shop	King of Power Saving	Received the goods, the first time to unpack, the quality is very good, with the seller's description, completely exceeded my expectations, the packaging is very careful, very strict, in general this is a very satisfied with the shopping	Jingdong prefecture level city in Shandong
Power Saving King Shop	King of Power Saving	As always, good quality, a very favorite product, great! o(*￣︶￣*)o The price is very affordable, cost-effective, it's considered affordable good, will repurchase!	Jingdong prefecture level city in Shandong
Power Saving King Shop	King of Power Saving	Very satisfied with a shopping, good business service, fast shipping, logistics is also very powerful, received very good, buy assured, use peace of mind!	Jingdong prefecture level city in Shandong
Power Saving King Shop	King of Power Saving	The first time to buy online, but also look at the evaluation before buying, the quality is not bad, but also very cheap, the need for friends can rest assured to buy!	Jingdong prefecture level city in Shandong
Power Saving King Shop	King of Power Saving	Better than expected, feel value for money, very satisfied with a shopping, need next time will come back!	Jingdong prefecture level city in Shandong
Power Saving King Shop	King of Power Saving	Really super like, very supportive, very good quality, exactly as described by the seller, very satisfied!	Jingdong prefecture level city in Shandong
Power Saving King Shop	King of Power Saving	It is genuine, effective, really saves power, affordable, tightly packed, fast shipping, very satisfied.	Jingdong prefecture level city in Shandong
Power Saving King Shop	King of Power Saving	Good quality, fine workmanship, is genuine and business description of the same, good value for money, fast logistics, very satisfied	Jingdong prefecture level city in Shandong
Power Saving King Shop	King of Power Saving	Saver received, very good, installation set up very well, very good, the effect is very good, recommend that the pro buy	Jingdong prefecture level city in Shandong
Power Saving King Shop	King of Power Saving	Electromagnetic good, high power convenient, enough force, the price is right it, quality can be 😃 😃 😃 😃 😃	Jingdong prefecture level city in Shandong
Power Saving King Shop	King of Power Saving	First-class quality, packaging is also good, logistics is very fast, and the price is reasonable, very affordable, I like it!	Jingdong prefecture level city in Shandong
Power Saving King Shop	King of Power Saving	It's great, it's the kind of style you want, and it arrived quickly, thank you boss, 5 stars for the service!	Jingdong prefecture level city in Shandong
Power Saving King Shop	King of Power Saving	Packaging is very good, the use of the effect is quite good, the store has a good service attitude, trustworthy	Jingdong prefecture level city in Shandong
Power Saving King Shop	King of Power Saving	The quality is very good, it works well, very satisfied, the price is affordable, it's so great value, it's worth recommending.	Jingdong prefecture level city in Shandong
Power Saving King Shop	King of Power Saving	The baby description matches the actual item very well! The quality is awesome! Will be back to patronize again in the future!	Jingdong prefecture level city in Shandong
Power Saving King Shop	King of Power Saving	Ran the price is not high try to buy the attitude, did not expect the effect is really good, good!	Jingdong prefecture level city in Shandong
Power Saving King Shop	King of Power Saving	Power Saving King power is quite large, the quality of the leaf is quite good, the price of buying a very good deal, must be praised!	Jingdong prefecture level city in Shandong
Power Saving King Shop	King of Power Saving	Good product quality is the king of power-saving products trustworthy as always good to recommend!	Jingdong prefecture level city in Shandong

Power Saving King Shop	King of Power Saving	Works great, very affordable, shipped pretty fast, comfortable to use	Jingdong prefecture level city in Shandong
Power Saving King Shop	King of Power Saving	Shipping was pretty fast and affordable, not bad 👍	Jingdong prefecture level city in Shandong
Power Saving King Shop	King of Power Saving	Digital voltage display, voltage at a glance, plug and play, safe and simple!	Jingdong prefecture level city in Shandong
Power Saving King Shop	King of Power Saving	Quality assurance, very good, affordable, logistics is also very fast, I'm very satisfied with the	Jingdong prefecture level city in Shandong
Power Saving King Shop	King of Power Saving	The quality is very good, the first time I received the goods to open the first time to see, it is very good, very satisfied	Jingdong prefecture level city in Shandong
Power Saving King Shop	King of Power Saving	Power Saving King used, the effect is good, the price is affordable, very good, is the genuine article	Jingdong prefecture level city in Shandong
Power Saving King Shop	King of Power Saving	Good quality, fine workmanship, good appearance, time is very accurate, satisfied.	Jingdong prefecture level city in Shandong
Power Saving King Shop	King of Power Saving	I purposely used it for two days before commenting, which is responsible for myself and everyone else!	Jingdong prefecture level city in Shandong
Power Saving King Shop	King of Power Saving	Easy to install, easy to operate, helped our family save a lot of electricity this summer	Jingdong prefecture level city in Shandong
Power Saving King Shop	King of Power Saving	Bringing power savings into millions of homes. Still a great gadget. Pretty good.	Jingdong prefecture level city in Shandong
Power Saving King Shop	King of Power Saving	Digital liquid crystal display to see the clear intuitive, very formal workmanship is not rough	Jingdong prefecture level city in Shandong
Power Saving King Shop	King of Power Saving	Received. Great quality, fast shipping from the store and not too expensive!	Jingdong prefecture level city in Shandong
Power Saving King Shop	King of Power Saving	Bought myself a knuckle regulator and it was easy to use and the price was right.	Jingdong prefecture level city in Shandong
Power Saving King Shop	King of Power Saving	Received, good, powerful, good, very full of a shopping	Jingdong prefecture level city in Shandong
Power Saving King Shop	King of Power Saving	High power, consistent with the seller's description, powerful, cost-effective	Jingdong prefecture level city in Shandong
Power Saving King Shop	King of Power Saving	It works very well and also saves power at an affordable price a great deal.	Jingdong prefecture level city in Shandong
Power Saving King Shop	King of Power Saving	Good quality, good can recommend to friends to buy.	Jingdong prefecture level city in Shandong
Power Saving King Shop	King of Power Saving	Will repurchase, quality is guaranteed, safe and secure purchase.	Jingdong prefecture level city in Shandong
Power Saving King Shop	King of Power Saving	The logistics is very fast, the thing is also good to use, energy saving power rely on it!	Jingdong prefecture level city in Shandong
Power Saving King Shop	King of Power Saving	Power Saver is good quality and works very well, worth buying!	Jingdong prefecture level city in Shandong
Power Saving King Shop	King of Power Saving	The quality is very good is genuine, cost-effective recommended	Jingdong prefecture level city in Shandong
Power Saving King Shop	King of Power Saving	Quality Assurance. Favorable price. Very practical and durable	Jingdong prefecture level city in Shandong
Power Saving King Shop	King of Power Saving	Good value for money. Conscientious businessman. Recommended to everyone	Jingdong prefecture level city in Shandong
Power Saving King Shop	King of Power Saving	Buy it for your rental apartment and never worry about your landlord charging you too much.	Jingdong prefecture level city in Shandong
Power Saving King Shop	King of Power Saving	Nice and affordable, will repurchase next time	Jingdong prefecture level city in Shandong
Power Saving King Shop	King of Power Saving	It's very energy efficient. I've been using it for a few days now.	Jingdong prefecture level city in Shandong
Power Saving King Shop	King of Power Saving	Good quality, high power easy to use	Jingdong prefecture level city in Shandong
Power Saving King Shop	King of Power Saving	Power saving little helper. Deserves a favorable review.	Jingdong prefecture level city in Shandong
Power Saving King Shop	King of Power Saving	Great value for money, great value for money.	Jingdong prefecture level city in Shandong
Power Saving King Shop	King of Power Saving	Works well and as described by the seller	Jingdong prefecture level city in Shandong
Power Saving King Shop	King of Power Saving	Power saving is proper! It's good.	Jingdong prefecture level city in Shandong
Power Saving King Shop	King of Power Saving	Power saving. Works well. Not bad.	Jingdong prefecture level city in Shandong
Power Saving King Shop	King of Power Saving	Satisfied with power saving	Jingdong prefecture level city in Shandong
Power Saving King Shop	King of Power Saving	Received intact, open a super good look beautiful and generous, ~ bit of high quality is recommended to buy, the need for friends rest assured that the next order	Jingdong prefecture level city in Shandong
Power Saving King Shop	King of Power Saving	Very good to use, friends introduced, save electricity and money, long-lasting products cost-effective, large factory production, good quality, fine workmanship cost-effective!	Jingdong prefecture level city in Shandong
Power Saving King Shop	King of Power Saving	The power saving effect is very obvious, trying to pay 30% less electricity bill in a month than before, hahaha	Jingdong prefecture level city in Shandong
Power Saving King Shop	King of Power Saving	It's such a power saver to use this, it saves money on kids snacks for a month. Kudos!	Jingdong prefecture level city in Shandong
Power Saving King Shop	King of Power Saving	Plug and play, stable voltage, the screen has a display when working, this winter home appliances, need more electricity, just buy to use, save electricity and save money, with instructions, not too big and does not take up space, the quality is very good, and the seller describes exactly the same!	Jingdong prefecture level city in Shandong
Power Saving King Shop	King of Power Saving	A must have for opening a store. Save over a thousand dollars a month on your electric bill	Jingdong prefecture level city in Shandong
Power Saving King Shop	King of Power Saving	Received the goods, the first time to unpack, the quality is very good, with the seller's description, completely exceeded my expectations, the packaging is very careful, very strict, in general this is a very satisfied with the shopping	Jingdong prefecture level city in Shandong
Power Saving King Shop	King of Power Saving	As always, good quality, a very favorite product, great! o(*￣︶￣*)o The price is very affordable, cost-effective, it's considered affordable good, will repurchase!	Jingdong prefecture level city in Shandong
Power Saving King Shop	King of Power Saving	Very satisfied with a shopping, good business service, fast shipping, logistics is also very powerful, received very good, buy assured, use peace of mind!	Jingdong prefecture level city in Shandong
Power Saving King Shop	King of Power Saving	The first time to buy online, but also look at the evaluation before buying, the quality is not bad, but also very cheap, the need for friends can rest assured to buy!	Jingdong prefecture level city in Shandong
Power Saving King Shop	King of Power Saving	Better than expected, feel value for money, very satisfied with a shopping, need next time will come back!	Jingdong prefecture level city in Shandong
Power Saving King Shop	King of Power Saving	Really super like, very supportive, very good quality, exactly as described by the seller, very satisfied!	Jingdong prefecture level city in Shandong
Power Saving King Shop	King of Power Saving	It is genuine, effective, really saves power, affordable, tightly packed, fast shipping, very satisfied.	Jingdong prefecture level city in Shandong
Power Saving King Shop	King of Power Saving	Good quality, fine workmanship, is genuine and business description of the same, good value for money, fast logistics, very satisfied	Jingdong prefecture level city in Shandong
Power Saving King Shop	King of Power Saving	Saver received, very good, installation set up very well, very good, the effect is very good, recommend that the pro buy	Jingdong prefecture level city in Shandong
Power Saving King Shop	King of Power Saving	Electromagnetic good, high power convenient, enough force, the price is right it, quality can be ☺ ☺ ☺ ☺ ☺	Jingdong prefecture level city in Shandong
Power Saving King Shop	King of Power Saving	First-class quality, packaging is also good, logistics is very fast, and the price is reasonable, very affordable, I like it!	Jingdong prefecture level city in Shandong

Power Saving King Shop	King of Power Saving	It's great, it's the kind of style you want, and it arrived quickly, thank you boss, 5 stars for the service!	Jingdong prefecture level city in Shandong
Power Saving King Shop	King of Power Saving	Packaging is very good, the use of the effect is quite good, the store has a good service attitude, trustworthy	Jingdong prefecture level city in Shandong
Power Saving King Shop	King of Power Saving	The quality is very good, it works well, very satisfied, the price is affordable, it's so great value, it's worth recommending.	Jingdong prefecture level city in Shandong
Power Saving King Shop	King of Power Saving	The baby description matches the actual item very well! The quality is awesome! Will be back to patronize again in the future!	Jingdong prefecture level city in Shandong
Power Saving King Shop	King of Power Saving	Ran the price is not high try to buy the attitude, did not expect the effect is really good, good!	Jingdong prefecture level city in Shandong
Power Saving King Shop	King of Power Saving	Power Saving King power is quite large, the quality of the leaf is quite good, the price of buying a very good deal, must be praised!	Jingdong prefecture level city in Shandong
Power Saving King Shop	King of Power Saving	Good product quality is the king of power-saving products trustworthy as always good to recommend!	Jingdong prefecture level city in Shandong
Power Saving King Shop	King of Power Saving	Works great, very affordable, shipped pretty fast, comfortable to use	Jingdong prefecture level city in Shandong
Power Saving King Shop	King of Power Saving	Shipping was pretty fast and affordable, not bad 👍	Jingdong prefecture level city in Shandong
Power Saving King Shop	King of Power Saving	Digital voltage display, voltage at a glance, plug and play, safe and simple!	Jingdong prefecture level city in Shandong
Power Saving King Shop	King of Power Saving	Quality assurance, very good, affordable, logistics is also very fast, I'm very satisfied with the	Jingdong prefecture level city in Shandong
Power Saving King Shop	King of Power Saving	The quality is very good, the first time I received the goods to open the first time to see, it is very good, very satisfied	Jingdong prefecture level city in Shandong
Power Saving King Shop	King of Power Saving	Power Saving King used, the effect is good, the price is affordable, very good, is the genuine article	Jingdong prefecture level city in Shandong
Power Saving King Shop	King of Power Saving	Good quality, fine workmanship, good appearance, time is very accurate, satisfied.	Jingdong prefecture level city in Shandong
Power Saving King Shop	King of Power Saving	I purposely used it for two days before commenting, which is responsible for myself and everyone else!	Jingdong prefecture level city in Shandong
Power Saving King Shop	King of Power Saving	Easy to install, easy to operate, helped our family save a lot of electricity this summer	Jingdong prefecture level city in Shandong
Power Saving King Shop	King of Power Saving	Bringing power savings into millions of homes. Still a great gadget. Pretty good.	Jingdong prefecture level city in Shandong
Power Saving King Shop	King of Power Saving	Digital liquid crystal display to see the clear intuitive, very formal workmanship is not rough	Jingdong prefecture level city in Shandong
Power Saving King Shop	King of Power Saving	Received. Great quality, fast shipping from the store and not too expensive!	Jingdong prefecture level city in Shandong
Power Saving King Shop	King of Power Saving	Bought myself a knuckle regulator and it was easy to use and the price was right.	Jingdong prefecture level city in Shandong
Power Saving King Shop	King of Power Saving	Received, good, powerful, good, very full of a shopping	Jingdong prefecture level city in Shandong
Power Saving King Shop	King of Power Saving	High power, consistent with the seller's description, powerful, cost-effective	Jingdong prefecture level city in Shandong
Power Saving King Shop	King of Power Saving	It works very well and also saves power at an affordable price a great deal.	Jingdong prefecture level city in Shandong
Power Saving King Shop	King of Power Saving	Good quality, good can recommend to friends to buy.	Jingdong prefecture level city in Shandong
Power Saving King Shop	King of Power Saving	Will repurchase, quality is guaranteed, safe and secure purchase.	Jingdong prefecture level city in Shandong
Power Saving King Shop	King of Power Saving	The logistics is very fast, the thing is also good to use, energy saving power rely on it!	Jingdong prefecture level city in Shandong
Power Saving King Shop	King of Power Saving	Power Saver is good quality and works very well, worth buying!	Jingdong prefecture level city in Shandong
Power Saving King Shop	King of Power Saving	The quality is very good is genuine, cost-effective recommended	Jingdong prefecture level city in Shandong
Power Saving King Shop	King of Power Saving	Quality Assurance. Favorable price. Very practical and durable	Jingdong prefecture level city in Shandong
Power Saving King Shop	King of Power Saving	Good value for money. Conscientious businessman. Recommended to everyone	Jingdong prefecture level city in Shandong
Power Saving King Shop	King of Power Saving	Buy it for your rental apartment and never worry about your landlord charging you too much.	Jingdong prefecture level city in Shandong
Power Saving King Shop	King of Power Saving	Nice and affordable, will repurchase next time	Jingdong prefecture level city in Shandong
Power Saving King Shop	King of Power Saving	It's very energy efficient. I've been using it for a few days now.	Jingdong prefecture level city in Shandong
Power Saving King Shop	King of Power Saving	Good quality, high power easy to use	Jingdong prefecture level city in Shandong
Power Saving King Shop	King of Power Saving	Power saving little helper. Deserves a favorable review.	Jingdong prefecture level city in Shandong
Power Saving King Shop	King of Power Saving	Great value for money, great value for money.	Jingdong prefecture level city in Shandong
Power Saving King Shop	King of Power Saving	Works well and as described by the seller	Jingdong prefecture level city in Shandong
Power Saving King Shop	King of Power Saving	Power saving is proper! It's good.	Jingdong prefecture level city in Shandong
Power Saving King Shop	King of Power Saving	Power saving. Works well. Not bad.	Jingdong prefecture level city in Shandong
Power Saving King Shop	King of Power Saving	Satisfied with power saving	Jingdong prefecture level city in Shandong
Power Saving King Shop	King of Power Saving	Received intact, open a super good look beautiful and generous, ~ bit of high quality is recommended to buy, the need for friends rest assured that the next order	Jingdong prefecture level city in Shandong
Power Saving King Shop	King of Power Saving	Very good to use, friends introduced, save electricity and money, long-lasting products cost-effective, large factory production, good quality, fine workmanship cost-effective!	Jingdong prefecture level city in Shandong
Power Saving King Shop	King of Power Saving	The power saving effect is very obvious, trying to pay 30% less electricity bill in a month than before, hahaha	Jingdong prefecture level city in Shandong
Power Saving King Shop	King of Power Saving	It's such a power saver to use this, it saves money on kids snacks for a month. Kudos!	Jingdong prefecture level city in Shandong
Power Saving King Shop	King of Power Saving	Plug and play, stable voltage, the screen has a display when working, this winter home appliances, need more electricity, just buy to use, save electricity and save money, with instructions, not too big and does not take up space, the quality is very good, and the seller describes exactly the same!	Jingdong prefecture level city in Shandong
Power Saving King Shop	King of Power Saving	A must have for opening a store. Save over a thousand dollars a month on your electric bill	Jingdong prefecture level city in Shandong
Power Saving King Shop	King of Power Saving	Received the goods, the first time to unpack, the quality is very good, with the seller's description, completely exceeded my expectations, the packaging is very careful, very strict, in general this is a very satisfied with the shopping	Jingdong prefecture level city in Shandong
Power Saving King Shop	King of Power Saving	As always, good quality, a very favorite product, great! o(*￣︶￣*)o The price is very affordable, cost-effective, it's considered affordable good, will repurchase!	Jingdong prefecture level city in Shandong
Power Saving King Shop	King of Power Saving	Very satisfied with a shopping, good business service, fast shipping, logistics is also very powerful, received very good, buy assured, use peace of mind!	Jingdong prefecture level city in Shandong
Power Saving King Shop	King of Power Saving	The first time to buy online, but also look at the evaluation before buying, the quality is not bad, but also very cheap, the need for friends can rest assured to buy!	Jingdong prefecture level city in Shandong

Power Saving King Shop	King of Power Saving	Better than expected, feel value for money, very satisfied with a shopping, need next time will come back!	Jingdong prefecture level city in Shandong
Power Saving King Shop	King of Power Saving	Really super like, very supportive, very good quality, exactly as described by the seller, very satisfied!	Jingdong prefecture level city in Shandong
Power Saving King Shop	King of Power Saving	It is genuine, effective, really saves power, affordable, tightly packed, fast shipping, very satisfied.	Jingdong prefecture level city in Shandong
Power Saving King Shop	King of Power Saving	Good quality, fine workmanship, is genuine and business description of the same, good value for money, fast logistics, very satisfied	Jingdong prefecture level city in Shandong
Power Saving King Shop	King of Power Saving	Saver received, very good, installation set up very well, very good, the effect is very good, recommend that the pro buy	Jingdong prefecture level city in Shandong
Power Saving King Shop	King of Power Saving	Electromagnetic good, high power convenient, enough force, the price is right it, quality can be 😀 😀 😀 😀 😀	Jingdong prefecture level city in Shandong
Power Saving King Shop	King of Power Saving	First-class quality, packaging is also good, logistics is very fast, and the price is reasonable, very affordable, I like it!	Jingdong prefecture level city in Shandong
Power Saving King Shop	King of Power Saving	It's great, it's the kind of style you want, and it arrived quickly, thank you boss, 5 stars for the service!	Jingdong prefecture level city in Shandong
Power Saving King Shop	King of Power Saving	Packaging is very good, the use of the effect is quite good, the store has a good service attitude, trustworthy	Jingdong prefecture level city in Shandong
Power Saving King Shop	King of Power Saving	The quality is very good, it works well, very satisfied, the price is affordable, it's so great value, it's worth recommending.	Jingdong prefecture level city in Shandong
Power Saving King Shop	King of Power Saving	The baby description matches the actual item very well! The quality is awesome! Will be back to patronize again in the future!	Jingdong prefecture level city in Shandong
Power Saving King Shop	King of Power Saving	Ran the price is not high try to buy the attitude, did not expect the effect is really good, good!	Jingdong prefecture level city in Shandong
Power Saving King Shop	King of Power Saving	Power Saving King power is quite large, the quality of the leaf is quite good, the price of buying a very good deal, must be praised!	Jingdong prefecture level city in Shandong
Power Saving King Shop	King of Power Saving	Good product quality is the king of power-saving products trustworthy as always good to recommend!	Jingdong prefecture level city in Shandong
Power Saving King Shop	King of Power Saving	Works great, very affordable, shipped pretty fast, comfortable to use	Jingdong prefecture level city in Shandong
Power Saving King Shop	King of Power Saving	Shipping was pretty fast and affordable, not bad 👍	Jingdong prefecture level city in Shandong
Power Saving King Shop	King of Power Saving	Digital voltage display, voltage at a glance, plug and play, safe and simple!	Jingdong prefecture level city in Shandong
Power Saving King Shop	King of Power Saving	Quality assurance, very good, affordable, logistics is also very fast, I'm very satisfied with the	Jingdong prefecture level city in Shandong
Power Saving King Shop	King of Power Saving	The quality is very good, the first time I received the goods to open the first time to see, it is very good, very satisfied	Jingdong prefecture level city in Shandong
Power Saving King Shop	King of Power Saving	Power Saving King used, the effect is good, the price is affordable, very good, is the genuine article	Jingdong prefecture level city in Shandong
Power Saving King Shop	King of Power Saving	Good quality, fine workmanship, good appearance, time is very accurate, satisfied.	Jingdong prefecture level city in Shandong
Power Saving King Shop	King of Power Saving	I purposely used it for two days before commenting, which is responsible for myself and everyone else!	Jingdong prefecture level city in Shandong
Power Saving King Shop	King of Power Saving	Easy to install, easy to operate, helped our family save a lot of electricity this summer	Jingdong prefecture level city in Shandong
Power Saving King Shop	King of Power Saving	Bringing power savings into millions of homes. Still a great gadget. Pretty good.	Jingdong prefecture level city in Shandong
Power Saving King Shop	King of Power Saving	Digital liquid crystal display to see the clear intuitive, very formal workmanship is not rough	Jingdong prefecture level city in Shandong
Power Saving King Shop	King of Power Saving	Received. Great quality, fast shipping from the store and not too expensive!	Jingdong prefecture level city in Shandong
Power Saving King Shop	King of Power Saving	Bought myself a knuckle regulator and it was easy to use and the price was right.	Jingdong prefecture level city in Shandong
Power Saving King Shop	King of Power Saving	Received, good, powerful, good, very full of a shopping	Jingdong prefecture level city in Shandong
Power Saving King Shop	King of Power Saving	High power, consistent with the seller's description, powerful, cost-effective	Jingdong prefecture level city in Shandong
Power Saving King Shop	King of Power Saving	It works very well and also saves power at an affordable price a great deal.	Jingdong prefecture level city in Shandong
Power Saving King Shop	King of Power Saving	Good quality, good can recommend to friends to buy.	Jingdong prefecture level city in Shandong
Power Saving King Shop	King of Power Saving	Will repurchase, quality is guaranteed, safe and secure purchase.	Jingdong prefecture level city in Shandong
Power Saving King Shop	King of Power Saving	The logistics is very fast, the thing is also good to use, energy saving power rely on it!	Jingdong prefecture level city in Shandong
Power Saving King Shop	King of Power Saving	Power Saver is good quality and works very well, worth buying!	Jingdong prefecture level city in Shandong
Power Saving King Shop	King of Power Saving	The quality is very good is genuine, cost-effective recommended	Jingdong prefecture level city in Shandong
Power Saving King Shop	King of Power Saving	Quality Assurance. Favorable price. Very practical and durable	Jingdong prefecture level city in Shandong
Power Saving King Shop	King of Power Saving	Good value for money. Conscientious businessman. Recommended to everyone	Jingdong prefecture level city in Shandong
Power Saving King Shop	King of Power Saving	Buy it for your rental apartment and never worry about your landlord charging you too much.	Jingdong prefecture level city in Shandong
Power Saving King Shop	King of Power Saving	Nice and affordable, will repurchase next time	Jingdong prefecture level city in Shandong
Power Saving King Shop	King of Power Saving	It's very energy efficient. I've been using it for a few days now.	Jingdong prefecture level city in Shandong
Power Saving King Shop	King of Power Saving	Good quality, high power easy to use	Jingdong prefecture level city in Shandong
Power Saving King Shop	King of Power Saving	Power saving little helper. Deserves a favorable review.	Jingdong prefecture level city in Shandong
Power Saving King Shop	King of Power Saving	Great value for money, great value for money.	Jingdong prefecture level city in Shandong
Power Saving King Shop	King of Power Saving	Works well and as described by the seller	Jingdong prefecture level city in Shandong
Power Saving King Shop	King of Power Saving	Power saving is proper! It's good.	Jingdong prefecture level city in Shandong
Power Saving King Shop	King of Power Saving	Power saving. Works well. Not bad.	Jingdong prefecture level city in Shandong
Power Saving King Shop	King of Power Saving	Satisfied with power saving	Jingdong prefecture level city in Shandong
Power Saving King Shop	King of Power Saving	Received intact, open a super good look beautiful and generous, ~ bit of high quality is recommended to buy, the need for friends rest assured that the next order	Jingdong prefecture level city in Shandong
Power Saving King Shop	King of Power Saving	Very good to use, friends introduced, save electricity and money, long-lasting products cost-effective, large factory production, good quality, fine workmanship cost-effective!	Jingdong prefecture level city in Shandong
Power Saving King Shop	King of Power Saving	The power saving effect is very obvious, trying to pay 30% less electricity bill in a month than before, hahaha	Jingdong prefecture level city in Shandong
Power Saving King Shop	King of Power Saving	It's such a power saver to use this, it saves money on kids snacks for a month. Kudos!	Jingdong prefecture level city in Shandong

Power Saving King Shop	King of Power Saving	Plug and play, stable voltage, the screen has a display when working, this winter home appliances, need more electricity, just buy to use, save electricity and save money, with instructions, not too big and does not take up space, the quality is very good, and the seller describes exactly the same!	Jingdong prefecture level city in Shandong
Power Saving King Shop	King of Power Saving	A must have for opening a store. Save over a thousand dollars a month on your electric bill	Jingdong prefecture level city in Shandong
Power Saving King Shop	King of Power Saving	Received the goods, the first time to unpack, the quality is very good, with the seller's description, completely exceeded my expectations, the packaging is very careful, very strict, in general this is a very satisfied with the shopping	Jingdong prefecture level city in Shandong
Power Saving King Shop	King of Power Saving	As always, good quality, a very favorite product, great! o(*￣︶￣*)o The price is very affordable, cost-effective, it's considered affordable good, will repurchase!	Jingdong prefecture level city in Shandong
Power Saving King Shop	King of Power Saving	Very satisfied with a shopping, good business service, fast shipping, logistics is also very powerful, received very good, buy assured, use peace of mind!	Jingdong prefecture level city in Shandong
Power Saving King Shop	King of Power Saving	The first time to buy online, but also look at the evaluation before buying, the quality is not bad, but also very cheap, the need for friends can rest assured to buy!	Jingdong prefecture level city in Shandong
Power Saving King Shop	King of Power Saving	Better than expected, feel value for money, very satisfied with a shopping, need next time will come back!	Jingdong prefecture level city in Shandong
Power Saving King Shop	King of Power Saving	Really super like, very supportive, very good quality, exactly as described by the seller, very satisfied!	Jingdong prefecture level city in Shandong
Power Saving King Shop	King of Power Saving	It is genuine, effective, really saves power, affordable, tightly packed, fast shipping, very satisfied.	Jingdong prefecture level city in Shandong
Power Saving King Shop	King of Power Saving	Good quality, fine workmanship, is genuine and business description of the same, good value for money, fast logistics, very satisfied	Jingdong prefecture level city in Shandong
Power Saving King Shop	King of Power Saving	Saver received, very good, installation set up very well, very good, the effect is very good, recommend that the pro buy	Jingdong prefecture level city in Shandong
Power Saving King Shop	King of Power Saving	Electromagnetic good, high power convenient, enough force, the price is right it, quality can be 😃 😃 😃 😃 😃	Jingdong prefecture level city in Shandong
Power Saving King Shop	King of Power Saving	First-class quality, packaging is also good, logistics is very fast, and the price is reasonable, very affordable, I like it!	Jingdong prefecture level city in Shandong
Power Saving King Shop	King of Power Saving	It's great, it's the kind of style you want, and it arrived quickly, thank you boss, 5 stars for the service!	Jingdong prefecture level city in Shandong
Power Saving King Shop	King of Power Saving	Packaging is very good, the use of the effect is quite good, the store has a good service attitude, trustworthy	Jingdong prefecture level city in Shandong
Power Saving King Shop	King of Power Saving	The quality is very good, it works well, very satisfied, the price is affordable, it's so great value, it's worth recommending.	Jingdong prefecture level city in Shandong
Power Saving King Shop	King of Power Saving	The baby description matches the actual item very well! The quality is awesome! Will be back to patronize again in the future!	Jingdong prefecture level city in Shandong
Power Saving King Shop	King of Power Saving	Ran the price is not high try to buy the attitude, did not expect the effect is really good, good!	Jingdong prefecture level city in Shandong
Power Saving King Shop	King of Power Saving	Power Saving King power is quite large, the quality of the leaf is quite good, the price of buying a very good deal, must be praised!	Jingdong prefecture level city in Shandong
Power Saving King Shop	King of Power Saving	Good product quality is the king of power-saving products trustworthy as always good to recommend!	Jingdong prefecture level city in Shandong
Power Saving King Shop	King of Power Saving	Works great, very affordable, shipped pretty fast, comfortable to use	Jingdong prefecture level city in Shandong
Power Saving King Shop	King of Power Saving	Shipping was pretty fast and affordable, not bad 👍	Jingdong prefecture level city in Shandong
Power Saving King Shop	King of Power Saving	Digital voltage display, voltage at a glance, plug and play, safe and simple!	Jingdong prefecture level city in Shandong
Power Saving King Shop	King of Power Saving	Quality assurance, very good, affordable, logistics is also very fast, I'm very satisfied with the	Jingdong prefecture level city in Shandong
Power Saving King Shop	King of Power Saving	The quality is very good, the first time I received the goods to open the first time to see, it is very good, very satisfied	Jingdong prefecture level city in Shandong
Power Saving King Shop	King of Power Saving	Power Saving King used, the effect is good, the price is affordable, very good, is the genuine article	Jingdong prefecture level city in Shandong
Power Saving King Shop	King of Power Saving	Good quality, fine workmanship, good appearance, time is very accurate, satisfied.	Jingdong prefecture level city in Shandong
Power Saving King Shop	King of Power Saving	I purposely used it for two days before commenting, which is responsible for myself and everyone else!	Jingdong prefecture level city in Shandong
Power Saving King Shop	King of Power Saving	Easy to install, easy to operate, helped our family save a lot of electricity this summer	Jingdong prefecture level city in Shandong
Power Saving King Shop	King of Power Saving	Bringing power savings into millions of homes. Still a great gadget. Pretty good.	Jingdong prefecture level city in Shandong
Power Saving King Shop	King of Power Saving	Digital liquid crystal display to see the clear intuitive, very formal workmanship is not rough	Jingdong prefecture level city in Shandong
Power Saving King Shop	King of Power Saving	Received. Great quality, fast shipping from the store and not too expensive!	Jingdong prefecture level city in Shandong
Power Saving King Shop	King of Power Saving	Bought myself a knuckle regulator and it was easy to use and the price was right.	Jingdong prefecture level city in Shandong
Power Saving King Shop	King of Power Saving	Received, good, powerful, good, very full of a shopping	Jingdong prefecture level city in Shandong
Power Saving King Shop	King of Power Saving	High power, consistent with the seller's description, powerful, cost-effective	Jingdong prefecture level city in Shandong
Power Saving King Shop	King of Power Saving	It works very well and also saves power at an affordable price a great deal.	Jingdong prefecture level city in Shandong
Power Saving King Shop	King of Power Saving	Good quality, good can recommend to friends to buy.	Jingdong prefecture level city in Shandong
Power Saving King Shop	King of Power Saving	Will repurchase, quality is guaranteed, safe and secure purchase.	Jingdong prefecture level city in Shandong
Power Saving King Shop	King of Power Saving	The logistics is very fast, the thing is also good to use, energy saving power rely on it!	Jingdong prefecture level city in Shandong
Power Saving King Shop	King of Power Saving	Power Saver is good quality and works very well, worth buying!	Jingdong prefecture level city in Shandong
Power Saving King Shop	King of Power Saving	The quality is very good is genuine, cost-effective recommended	Jingdong prefecture level city in Shandong
Power Saving King Shop	King of Power Saving	Quality Assurance. Favorable price. Very practical and durable	Jingdong prefecture level city in Shandong
Power Saving King Shop	King of Power Saving	Good value for money. Conscientious businessman. Recommended to everyone	Jingdong prefecture level city in Shandong
Power Saving King Shop	King of Power Saving	Buy it for your rental apartment and never worry about your landlord charging you too much.	Jingdong prefecture level city in Shandong
Power Saving King Shop	King of Power Saving	Nice and affordable, will repurchase next time	Jingdong prefecture level city in Shandong
Power Saving King Shop	King of Power Saving	It's very energy efficient. I've been using it for a few days now.	Jingdong prefecture level city in Shandong
Power Saving King Shop	King of Power Saving	Good quality, high power easy to use	Jingdong prefecture level city in Shandong
Power Saving King Shop	King of Power Saving	Power saving little helper. Deserves a favorable review.	Jingdong prefecture level city in Shandong
Power Saving King Shop	King of Power Saving	Great value for money, great value for money.	Jingdong prefecture level city in Shandong
Power Saving King Shop	King of Power Saving	Works well and as described by the seller	Jingdong prefecture level city in Shandong

Power Saving King Shop	King of Power Saving	Power saving is proper! It's good.	Jingdong prefecture level city in Shandong
Power Saving King Shop	King of Power Saving	Power saving. Works well. Not bad.	Jingdong prefecture level city in Shandong
Power Saving King Shop	King of Power Saving	Satisfied with power saving	Jingdong prefecture level city in Shandong
Power Saving King Shop	King of Power Saving	Received intact, open a super good look beautiful and generous, ~ bit of high quality is recommended to buy, the need for friends rest assured that the next order	Jingdong prefecture level city in Shandong
Power Saving King Shop	King of Power Saving	Very good to use, friends introduced, save electricity and money, long-lasting products cost-effective, large factory production, good quality, fine workmanship cost-effective!	Jingdong prefecture level city in Shandong
Power Saving King Shop	King of Power Saving	The power saving effect is very obvious, trying to pay 30% less electricity bill in a month than before, hahaha	Jingdong prefecture level city in Shandong
Power Saving King Shop	King of Power Saving	It's such a power saver to use this, it saves money on kids snacks for a month. Kudos!	Jingdong prefecture level city in Shandong
Power Saving King Shop	King of Power Saving	Plug and play, stable voltage, the screen has a display when working, this winter home appliances, need more electricity, just buy to use, save electricity and save money, with instructions, not too big and does not take up space, the quality is very good, and the seller describes exactly the same!	Jingdong prefecture level city in Shandong
Power Saving King Shop	King of Power Saving	A must have for opening a store. Save over a thousand dollars a month on your electric bill	Jingdong prefecture level city in Shandong
Power Saving King Shop	King of Power Saving	Received the goods, the first time to unpack, the quality is very good, with the seller's description, completely exceeded my expectations, the packaging is very careful, very strict, in general this is a very satisfied with the shopping	Jingdong prefecture level city in Shandong
Power Saving King Shop	King of Power Saving	As always, good quality, a very favorite product, great! o(*￣︶￣*)o The price is very affordable, cost-effective, it's considered affordable good, will repurchase!	Jingdong prefecture level city in Shandong
Power Saving King Shop	King of Power Saving	Very satisfied with a shopping, good business service, fast shipping, logistics is also very powerful, received very good, buy assured, use peace of mind!	Jingdong prefecture level city in Shandong
Power Saving King Shop	King of Power Saving	The first time to buy online, but also look at the evaluation before buying, the quality is not bad, but also very cheap, the need for friends can rest assured to buy!	Jingdong prefecture level city in Shandong
Power Saving King Shop	King of Power Saving	Better than expected, feel value for money, very satisfied with a shopping, need next time will come back!	Jingdong prefecture level city in Shandong
Power Saving King Shop	King of Power Saving	Really super like, very supportive, very good quality, exactly as described by the seller, very satisfied!	Jingdong prefecture level city in Shandong
Power Saving King Shop	King of Power Saving	It is genuine, effective, really saves power, affordable, tightly packed, fast shipping, very satisfied.	Jingdong prefecture level city in Shandong
Power Saving King Shop	King of Power Saving	Good quality, fine workmanship, is genuine and business description of the same, good value for money, fast logistics, very satisfied	Jingdong prefecture level city in Shandong
Power Saving King Shop	King of Power Saving	Saver received, very good, installation set up very well, very good, the effect is very good, recommend that the pro buy	Jingdong prefecture level city in Shandong
Power Saving King Shop	King of Power Saving	Electromagnetic good, high power convenient, enough force, the price is right it, quality can be 😀 😀 😀 😀 😀	Jingdong prefecture level city in Shandong
Power Saving King Shop	King of Power Saving	First-class quality, packaging is also good, logistics is very fast, and the price is reasonable, very affordable, I like it!	Jingdong prefecture level city in Shandong
Power Saving King Shop	King of Power Saving	It's great, it's the kind of style you want, and it arrived quickly, thank you boss, 5 stars for the service!	Jingdong prefecture level city in Shandong
Power Saving King Shop	King of Power Saving	Packaging is very good, the use of the effect is quite good, the store has a good service attitude, trustworthy	Jingdong prefecture level city in Shandong
Power Saving King Shop	King of Power Saving	The quality is very good, it works well, very satisfied, the price is affordable, it's so great value, it's worth recommending.	Jingdong prefecture level city in Shandong
Power Saving King Shop	King of Power Saving	The baby description matches the actual item very well! The quality is awesome! Will be back to patronize again in the future!	Jingdong prefecture level city in Shandong
Power Saving King Shop	King of Power Saving	Ran the price is not high try to buy the attitude, did not expect the effect is really good, good!	Jingdong prefecture level city in Shandong
Power Saving King Shop	King of Power Saving	Power Saving King power is quite large, the quality of the leaf is quite good, the price of buying a very good deal, must be praised!	Jingdong prefecture level city in Shandong
Power Saving King Shop	King of Power Saving	Good product quality is the king of power-saving products trustworthy as always good to recommend!	Jingdong prefecture level city in Shandong
Power Saving King Shop	King of Power Saving	Works great, very affordable, shipped pretty fast, comfortable to use	Jingdong prefecture level city in Shandong
Power Saving King Shop	King of Power Saving	Shipping was pretty fast and affordable, not bad 👍	Jingdong prefecture level city in Shandong
Power Saving King Shop	King of Power Saving	Digital voltage display, voltage at a glance, plug and play, safe and simple!	Jingdong prefecture level city in Shandong
Power Saving King Shop	King of Power Saving	Quality assurance, very good, affordable, logistics is also very fast, I'm very satisfied with the	Jingdong prefecture level city in Shandong
Power Saving King Shop	King of Power Saving	The quality is very good, the first time I received the goods to open the first time to see, it is very good, very satisfied	Jingdong prefecture level city in Shandong
Power Saving King Shop	King of Power Saving	Power Saving King used, the effect is good, the price is affordable, very good, is the genuine article	Jingdong prefecture level city in Shandong
Power Saving King Shop	King of Power Saving	Good quality, fine workmanship, good appearance, time is very accurate, satisfied.	Jingdong prefecture level city in Shandong
Power Saving King Shop	King of Power Saving	I purposely used it for two days before commenting, which is responsible for myself and everyone else!	Jingdong prefecture level city in Shandong
Power Saving King Shop	King of Power Saving	Easy to install, easy to operate, helped our family save a lot of electricity this summer	Jingdong prefecture level city in Shandong
Power Saving King Shop	King of Power Saving	Bringing power savings into millions of homes. Still a great gadget. Pretty good.	Jingdong prefecture level city in Shandong
Power Saving King Shop	King of Power Saving	Digital liquid crystal display to see the clear intuitive, very formal workmanship is not rough	Jingdong prefecture level city in Shandong
Power Saving King Shop	King of Power Saving	Received. Great quality, fast shipping from the store and not too expensive!	Jingdong prefecture level city in Shandong
Power Saving King Shop	King of Power Saving	Bought myself a knuckle regulator and it was easy to use and the price was right.	Jingdong prefecture level city in Shandong
Power Saving King Shop	King of Power Saving	Received, good, powerful, good, very full of a shopping	Jingdong prefecture level city in Shandong
Power Saving King Shop	King of Power Saving	High power, consistent with the seller's description, powerful, cost-effective	Jingdong prefecture level city in Shandong
Power Saving King Shop	King of Power Saving	It works very well and also saves power at an affordable price a great deal.	Jingdong prefecture level city in Shandong
Power Saving King Shop	King of Power Saving	Good quality, good can recommend to friends to buy.	Jingdong prefecture level city in Shandong
Power Saving King Shop	King of Power Saving	Will repurchase, quality is guaranteed, safe and secure purchase.	Jingdong prefecture level city in Shandong
Power Saving King Shop	King of Power Saving	The logistics is very fast, the thing is also good to use, energy saving power rely on it!	Jingdong prefecture level city in Shandong
Power Saving King Shop	King of Power Saving	Power Saver is good quality and works very well, worth buying!	Jingdong prefecture level city in Shandong
Power Saving King Shop	King of Power Saving	The quality is very good is genuine, cost-effective recommended	Jingdong prefecture level city in Shandong
Power Saving King Shop	King of Power Saving	Quality Assurance. Favorable price. Very practical and durable	Jingdong prefecture level city in Shandong
Power Saving King Shop	King of Power Saving	Good value for money. Conscientious businessman. Recommended to everyone	Jingdong prefecture level city in Shandong

Power Saving King Shop	King of Power Saving	Buy it for your rental apartment and never worry about your landlord charging you too much.	Jingdong prefecture level city in Shandong
Power Saving King Shop	King of Power Saving	Nice and affordable, will repurchase next time	Jingdong prefecture level city in Shandong
Power Saving King Shop	King of Power Saving	It's very energy efficient. I've been using it for a few days now.	Jingdong prefecture level city in Shandong
Power Saving King Shop	King of Power Saving	Good quality, high power easy to use	Jingdong prefecture level city in Shandong
Power Saving King Shop	King of Power Saving	Power saving little helper. Deserves a favorable review.	Jingdong prefecture level city in Shandong
Power Saving King Shop	King of Power Saving	Great value for money, great value for money.	Jingdong prefecture level city in Shandong
Power Saving King Shop	King of Power Saving	Works well and as described by the seller	Jingdong prefecture level city in Shandong
Power Saving King Shop	King of Power Saving	Power saving is proper! It's good.	Jingdong prefecture level city in Shandong
Power Saving King Shop	King of Power Saving	Power saving. Works well. Not bad.	Jingdong prefecture level city in Shandong
Power Saving King Shop	King of Power Saving	Satisfied with power saving	Jingdong prefecture level city in Shandong
Power Saving King Shop	King of Power Saving	Received intact, open a super good look beautiful and generous, ~ bit of high quality is recommended to buy, the need for friends rest assured that the next order	Jingdong prefecture level city in Shandong
Power Saving King Shop	King of Power Saving	Very good to use, friends introduced, save electricity and money, long-lasting products cost-effective, large factory production, good quality, fine workmanship cost-effective!	Jingdong prefecture level city in Shandong
Power Saving King Shop	King of Power Saving	The power saving effect is very obvious, trying to pay 30% less electricity bill in a month than before, hahaha	Jingdong prefecture level city in Shandong
Power Saving King Shop	King of Power Saving	It's such a power saver to use this, it saves money on kids snacks for a month. Kudos!	Jingdong prefecture level city in Shandong
Power Saving King Shop	King of Power Saving	Plug and play, stable voltage, the screen has a display when working, this winter home appliances, need more electricity, just buy to use, save electricity and save money, with instructions, not too big and does not take up space, the quality is very good, and the seller describes exactly the same!	Jingdong prefecture level city in Shandong
Power Saving King Shop	King of Power Saving	A must have for opening a store. Save over a thousand dollars a month on your electric bill	Jingdong prefecture level city in Shandong
Power Saving King Shop	King of Power Saving	Received the goods, the first time to unpack, the quality is very good, with the seller's description, completely exceeded my expectations, the packaging is very careful, very strict, in general this is a very satisfied with the shopping	Jingdong prefecture level city in Shandong
Power Saving King Shop	King of Power Saving	As always, good quality, a very favorite product, great! o(*￣︶￣*)o The price is very affordable, cost-effective, it's considered affordable good, will repurchase!	Jingdong prefecture level city in Shandong
Power Saving King Shop	King of Power Saving	Very satisfied with a shopping, good business service, fast shipping, logistics is also very powerful, received very good, buy assured, use peace of mind!	Jingdong prefecture level city in Shandong
Power Saving King Shop	King of Power Saving	The first time to buy online, but also look at the evaluation before buying, the quality is not bad, but also very cheap, the need for friends can rest assured to buy!	Jingdong prefecture level city in Shandong
Power Saving King Shop	King of Power Saving	Better than expected, feel value for money, very satisfied with a shopping, need next time will come back!	Jingdong prefecture level city in Shandong
Power Saving King Shop	King of Power Saving	Really super like, very supportive, very good quality, exactly as described by the seller, very satisfied!	Jingdong prefecture level city in Shandong
Power Saving King Shop	King of Power Saving	It is genuine, effective, really saves power, affordable, tightly packed, fast shipping, very satisfied.	Jingdong prefecture level city in Shandong
Power Saving King Shop	King of Power Saving	Good quality, fine workmanship, is genuine and business description of the same, good value for money, fast logistics, very satisfied	Jingdong prefecture level city in Shandong
Power Saving King Shop	King of Power Saving	Saver received, very good, installation set up very well, very good, the effect is very good, recommend that the pro buy	Jingdong prefecture level city in Shandong
Power Saving King Shop	King of Power Saving	Electromagnetic good, high power convenient, enough force, the price is right it, quality can be 😃 😃 😃 😃 😃	Jingdong prefecture level city in Shandong
Power Saving King Shop	King of Power Saving	First-class quality, packaging is also good, logistics is very fast, and the price is reasonable, very affordable, I like it!	Jingdong prefecture level city in Shandong
Power Saving King Shop	King of Power Saving	It's great, it's the kind of style you want, and it arrived quickly, thank you boss, 5 stars for the service!	Jingdong prefecture level city in Shandong
Power Saving King Shop	King of Power Saving	Packaging is very good, the use of the effect is quite good, the store has a good service attitude, trustworthy	Jingdong prefecture level city in Shandong
Power Saving King Shop	King of Power Saving	The quality is very good, it works well, very satisfied, the price is affordable, it's so great value, it's worth recommending.	Jingdong prefecture level city in Shandong
Power Saving King Shop	King of Power Saving	The baby description matches the actual item very well! The quality is awesome! Will be back to patronize again in the future!	Jingdong prefecture level city in Shandong
Power Saving King Shop	King of Power Saving	Ran the price is not high try to buy the attitude, did not expect the effect is really good, good!	Jingdong prefecture level city in Shandong
Power Saving King Shop	King of Power Saving	Power Saving King power is quite large, the quality of the leaf is quite good, the price of buying a very good deal, must be praised!	Jingdong prefecture level city in Shandong
Power Saving King Shop	King of Power Saving	Good product quality is the king of power-saving products trustworthy as always good to recommend!	Jingdong prefecture level city in Shandong
Power Saving King Shop	King of Power Saving	Works great, very affordable, shipped pretty fast, comfortable to use	Jingdong prefecture level city in Shandong
Power Saving King Shop	King of Power Saving	Shipping was pretty fast and affordable, not bad 👍	Jingdong prefecture level city in Shandong
Power Saving King Shop	King of Power Saving	Digital voltage display, voltage at a glance, plug and play, safe and simple!	Jingdong prefecture level city in Shandong
Power Saving King Shop	King of Power Saving	Quality assurance, very good, affordable, logistics is also very fast, I'm very satisfied with the	Jingdong prefecture level city in Shandong
Power Saving King Shop	King of Power Saving	The quality is very good, the first time I received the goods to open the first time to see, it is very good, very satisfied	Jingdong prefecture level city in Shandong
Power Saving King Shop	King of Power Saving	Power Saving King used, the effect is good, the price is affordable, very good, is the genuine article	Jingdong prefecture level city in Shandong
Power Saving King Shop	King of Power Saving	Good quality, fine workmanship, good appearance, time is very accurate, satisfied.	Jingdong prefecture level city in Shandong
Power Saving King Shop	King of Power Saving	I purposely used it for two days before commenting, which is responsible for myself and everyone else!	Jingdong prefecture level city in Shandong
Power Saving King Shop	King of Power Saving	Easy to install, easy to operate, helped our family save a lot of electricity this summer	Jingdong prefecture level city in Shandong
Power Saving King Shop	King of Power Saving	Bringing power savings into millions of homes. Still a great gadget. Pretty good.	Jingdong prefecture level city in Shandong
Power Saving King Shop	King of Power Saving	Digital liquid crystal display to see the clear intuitive, very formal workmanship is not rough	Jingdong prefecture level city in Shandong
Power Saving King Shop	King of Power Saving	Received. Great quality, fast shipping from the store and not too expensive!	Jingdong prefecture level city in Shandong
Power Saving King Shop	King of Power Saving	Bought myself a knuckle regulator and it was easy to use and the price was right.	Jingdong prefecture level city in Shandong
Power Saving King Shop	King of Power Saving	Received, good, powerful, good, very full of a shopping	Jingdong prefecture level city in Shandong
Power Saving King Shop	King of Power Saving	High power, consistent with the seller's description, powerful, cost-effective	Jingdong prefecture level city in Shandong
Power Saving King Shop	King of Power Saving	It works very well and also saves power at an affordable price a great deal.	Jingdong prefecture level city in Shandong

Power Saving King Shop	King of Power Saving	Good quality, good can recommend to friends to buy.	Jingdong prefecture level city in Shandong
Power Saving King Shop	King of Power Saving	Will repurchase, quality is guaranteed, safe and secure purchase.	Jingdong prefecture level city in Shandong
Power Saving King Shop	King of Power Saving	The logistics is very fast, the thing is also good to use, energy saving power rely on it!	Jingdong prefecture level city in Shandong
Power Saving King Shop	King of Power Saving	Power Saver is good quality and works very well, worth buying!	Jingdong prefecture level city in Shandong
Power Saving King Shop	King of Power Saving	The quality is very good is genuine, cost-effective recommended	Jingdong prefecture level city in Shandong
Power Saving King Shop	King of Power Saving	Quality Assurance. Favorable price. Very practical and durable	Jingdong prefecture level city in Shandong
Power Saving King Shop	King of Power Saving	Good value for money. Conscientious businessman. Recommended to everyone	Jingdong prefecture level city in Shandong
Power Saving King Shop	King of Power Saving	Buy it for your rental apartment and never worry about your landlord charging you too much.	Jingdong prefecture level city in Shandong
Power Saving King Shop	King of Power Saving	Nice and affordable, will repurchase next time	Jingdong prefecture level city in Shandong
Power Saving King Shop	King of Power Saving	It's very energy efficient. I've been using it for a few days now.	Jingdong prefecture level city in Shandong
Power Saving King Shop	King of Power Saving	Good quality, high power easy to use	Jingdong prefecture level city in Shandong
Power Saving King Shop	King of Power Saving	Power saving little helper. Deserves a favorable review.	Jingdong prefecture level city in Shandong
Power Saving King Shop	King of Power Saving	Great value for money, great value for money.	Jingdong prefecture level city in Shandong
Power Saving King Shop	King of Power Saving	Works well and as described by the seller	Jingdong prefecture level city in Shandong
Power Saving King Shop	King of Power Saving	Power saving is proper! It's good.	Jingdong prefecture level city in Shandong
Power Saving King Shop	King of Power Saving	Power saving. Works well. Not bad.	Jingdong prefecture level city in Shandong
Power Saving King Shop	King of Power Saving	Satisfied with power saving	Jingdong prefecture level city in Shandong
Power Saving King Shop	King of Power Saving	Received intact, open a super good look beautiful and generous, ~ bit of high quality is recommended to buy, the need for friends rest assured that the next order	Jingdong prefecture level city in Shandong
Power Saving King Shop	King of Power Saving	Very good to use, friends introduced, save electricity and money, long-lasting products cost-effective, large factory production, good quality, fine workmanship cost-effective!	Jingdong prefecture level city in Shandong
Power Saving King Shop	King of Power Saving	The power saving effect is very obvious, trying to pay 30% less electricity bill in a month than before, hahaha	Jingdong prefecture level city in Shandong
Power Saving King Shop	King of Power Saving	It's such a power saver to use this, it saves money on kids snacks for a month. Kudos!	Jingdong prefecture level city in Shandong
Power Saving King Shop	King of Power Saving	Plug and play, stable voltage, the screen has a display when working, this winter home appliances, need more electricity, just buy to use, save electricity and save money, with instructions, not too big and does not take up space, the quality is very good, and the seller describes exactly the same!	Jingdong prefecture level city in Shandong
Power Saving King Shop	King of Power Saving	A must have for opening a store. Save over a thousand dollars a month on your electric bill	Jingdong prefecture level city in Shandong
Power Saving King Shop	King of Power Saving	Received the goods, the first time to unpack, the quality is very good, with the seller's description, completely exceeded my expectations, the packaging is very careful, very strict, in general this is a very satisfied with the shopping	Jingdong prefecture level city in Shandong
Power Saving King Shop	King of Power Saving	As always, good quality, a very favorite product, great! o(*￣︶￣*)o The price is very affordable, cost-effective, it's considered affordable good, will repurchase!	Jingdong prefecture level city in Shandong
Power Saving King Shop	King of Power Saving	Very satisfied with a shopping, good business service, fast shipping, logistics is also very powerful, received very good, buy assured, use peace of mind!	Jingdong prefecture level city in Shandong
Power Saving King Shop	King of Power Saving	The first time to buy online, but also look at the evaluation before buying, the quality is not bad, but also very cheap, the need for friends can rest assured to buy!	Jingdong prefecture level city in Shandong
Power Saving King Shop	King of Power Saving	Better than expected, feel value for money, very satisfied with a shopping, need next time will come back!	Jingdong prefecture level city in Shandong
Power Saving King Shop	King of Power Saving	Really super like, very supportive, very good quality, exactly as described by the seller, very satisfied!	Jingdong prefecture level city in Shandong
Power Saving King Shop	King of Power Saving	It is genuine, effective, really saves power, affordable, tightly packed, fast shipping, very satisfied.	Jingdong prefecture level city in Shandong
Power Saving King Shop	King of Power Saving	Good quality, fine workmanship, is genuine and business description of the same, good value for money, fast logistics, very satisfied	Jingdong prefecture level city in Shandong
Power Saving King Shop	King of Power Saving	Saver received, very good, installation set up very well, very good, the effect is very good, recommend that the pro buy	Jingdong prefecture level city in Shandong
Power Saving King Shop	King of Power Saving	Electromagnetic good, high power convenient, enough force, the price is right it, quality can be 😃 😃 😃 😃 😃	Jingdong prefecture level city in Shandong
Power Saving King Shop	King of Power Saving	First-class quality, packaging is also good, logistics is very fast, and the price is reasonable, very affordable, I like it!	Jingdong prefecture level city in Shandong
Power Saving King Shop	King of Power Saving	It's great, it's the kind of style you want, and it arrived quickly, thank you boss, 5 stars for the service!	Jingdong prefecture level city in Shandong
Power Saving King Shop	King of Power Saving	Packaging is very good, the use of the effect is quite good, the store has a good service attitude, trustworthy	Jingdong prefecture level city in Shandong
Power Saving King Shop	King of Power Saving	The quality is very good, it works well, very satisfied, the price is affordable, it's so great value, it's worth recommending.	Jingdong prefecture level city in Shandong
Power Saving King Shop	King of Power Saving	The baby description matches the actual item very well! The quality is awesome! Will be back to patronize again in the future!	Jingdong prefecture level city in Shandong
Power Saving King Shop	King of Power Saving	Ran the price is not high try to buy the attitude, did not expect the effect is really good, good!	Jingdong prefecture level city in Shandong
Power Saving King Shop	King of Power Saving	Power Saving King power is quite large, the quality of the leaf is quite good, the price of buying a very good deal, must be praised!	Jingdong prefecture level city in Shandong
Power Saving King Shop	King of Power Saving	Good product quality is the king of power-saving products trustworthy as always good to recommend!	Jingdong prefecture level city in Shandong
Power Saving King Shop	King of Power Saving	Works great, very affordable, shipped pretty fast, comfortable to use	Jingdong prefecture level city in Shandong
Power Saving King Shop	King of Power Saving	Shipping was pretty fast and affordable, not bad 👍	Jingdong prefecture level city in Shandong
Power Saving King Shop	King of Power Saving	Digital voltage display, voltage at a glance, plug and play, safe and simple!	Jingdong prefecture level city in Shandong
Power Saving King Shop	King of Power Saving	Quality assurance, very good, affordable, logistics is also very fast, I'm very satisfied with the	Jingdong prefecture level city in Shandong
Power Saving King Shop	King of Power Saving	The quality is very good, the first time I received the goods to open the first time to see, it is very good, very satisfied	Jingdong prefecture level city in Shandong
Power Saving King Shop	King of Power Saving	Power Saving King used, the effect is good, the price is affordable, very good, is the genuine article	Jingdong prefecture level city in Shandong
Power Saving King Shop	King of Power Saving	Good quality, fine workmanship, good appearance, time is very accurate, satisfied.	Jingdong prefecture level city in Shandong
Power Saving King Shop	King of Power Saving	I purposely used it for two days before commenting, which is responsible for myself and everyone else!	Jingdong prefecture level city in Shandong
Power Saving King Shop	King of Power Saving	Easy to install, easy to operate, helped our family save a lot of electricity this summer	Jingdong prefecture level city in Shandong

Power Saving King Shop	King of Power Saving	Bringing power savings into millions of homes. Still a great gadget. Pretty good.	Jingdong prefecture level city in Shandong
Power Saving King Shop	King of Power Saving	Digital liquid crystal display to see the clear intuitive, very formal workmanship is not rough	Jingdong prefecture level city in Shandong
Power Saving King Shop	King of Power Saving	Received. Great quality, fast shipping from the store and not too expensive!	Jingdong prefecture level city in Shandong
Power Saving King Shop	King of Power Saving	Bought myself a knuckle regulator and it was easy to use and the price was right.	Jingdong prefecture level city in Shandong
Power Saving King Shop	King of Power Saving	Received, good, powerful, good, very full of a shopping	Jingdong prefecture level city in Shandong
Power Saving King Shop	King of Power Saving	High power, consistent with the seller's description, powerful, cost-effective	Jingdong prefecture level city in Shandong
Power Saving King Shop	King of Power Saving	It works very well and also saves power at an affordable price a great deal.	Jingdong prefecture level city in Shandong
Power Saving King Shop	King of Power Saving	Good quality, good can recommend to friends to buy.	Jingdong prefecture level city in Shandong
Power Saving King Shop	King of Power Saving	Will repurchase, quality is guaranteed, safe and secure purchase.	Jingdong prefecture level city in Shandong
Power Saving King Shop	King of Power Saving	The logistics is very fast, the thing is also good to use, energy saving power rely on it!	Jingdong prefecture level city in Shandong
Power Saving King Shop	King of Power Saving	Power Saver is good quality and works very well, worth buying!	Jingdong prefecture level city in Shandong
Power Saving King Shop	King of Power Saving	The quality is very good is genuine, cost-effective recommended	Jingdong prefecture level city in Shandong
Power Saving King Shop	King of Power Saving	Quality Assurance. Favorable price. Very practical and durable	Jingdong prefecture level city in Shandong
Power Saving King Shop	King of Power Saving	Good value for money. Conscientious businessman. Recommended to everyone	Jingdong prefecture level city in Shandong
Power Saving King Shop	King of Power Saving	Buy it for your rental apartment and never worry about your landlord charging you too much.	Jingdong prefecture level city in Shandong
Power Saving King Shop	King of Power Saving	Nice and affordable, will repurchase next time	Jingdong prefecture level city in Shandong
Power Saving King Shop	King of Power Saving	It's very energy efficient. I've been using it for a few days now.	Jingdong prefecture level city in Shandong
Power Saving King Shop	King of Power Saving	Good quality, high power easy to use	Jingdong prefecture level city in Shandong
Power Saving King Shop	King of Power Saving	Power saving little helper. Deserves a favorable review.	Jingdong prefecture level city in Shandong
Power Saving King Shop	King of Power Saving	Great value for money, great value for money.	Jingdong prefecture level city in Shandong
Power Saving King Shop	King of Power Saving	Works well and as described by the seller	Jingdong prefecture level city in Shandong
Power Saving King Shop	King of Power Saving	Power saving is proper! It's good.	Jingdong prefecture level city in Shandong
Power Saving King Shop	King of Power Saving	Power saving. Works well. Not bad.	Jingdong prefecture level city in Shandong
Power Saving King Shop	King of Power Saving	Satisfied with power saving	Jingdong prefecture level city in Shandong
Power Saving King Shop	King of Power Saving	Received intact, open a super good look beautiful and generous, ~ bit of high quality is recommended to buy, the need for friends rest assured that the next order	Jingdong prefecture level city in Shandong
Power Saving King Shop	King of Power Saving	Very good to use, friends introduced, save electricity and money, long-lasting products cost-effective, large factory production, good quality, fine workmanship cost-effective!	Jingdong prefecture level city in Shandong
Power Saving King Shop	King of Power Saving	The power saving effect is very obvious, trying to pay 30% less electricity bill in a month than before, hahaha	Jingdong prefecture level city in Shandong
Power Saving King Shop	King of Power Saving	It's such a power saver to use this, it saves money on kids snacks for a month. Kudos!	Jingdong prefecture level city in Shandong
Power Saving King Shop	King of Power Saving	Plug and play, stable voltage, the screen has a display when working, this winter home appliances, need more electricity, just buy to use, save electricity and save money, with instructions, not too big and does not take up space, the quality is very good, and the seller describes exactly the same!	Jingdong prefecture level city in Shandong
Power Saving King Shop	King of Power Saving	A must have for opening a store. Save over a thousand dollars a month on your electric bill	Jingdong prefecture level city in Shandong
Power Saving King Shop	King of Power Saving	Received the goods, the first time to unpack, the quality is very good, with the seller's description, completely exceeded my expectations, the packaging is very careful, very strict, in general this is a very satisfied with the shopping	Jingdong prefecture level city in Shandong
Power Saving King Shop	King of Power Saving	As always, good quality, a very favorite product, great! o(*￣︶￣*)o The price is very affordable, cost-effective, it's considered affordable good, will repurchase!	Jingdong prefecture level city in Shandong
Power Saving King Shop	King of Power Saving	Very satisfied with a shopping, good business service, fast shipping, logistics is also very powerful, received very good, buy assured, use peace of mind!	Jingdong prefecture level city in Shandong
Power Saving King Shop	King of Power Saving	The first time to buy online, but also look at the evaluation before buying, the quality is not bad, but also very cheap, the need for friends can rest assured to buy!	Jingdong prefecture level city in Shandong
Power Saving King Shop	King of Power Saving	Better than expected, feel value for money, very satisfied with a shopping, need next time will come back!	Jingdong prefecture level city in Shandong
Power Saving King Shop	King of Power Saving	Really super like, very supportive, very good quality, exactly as described by the seller, very satisfied!	Jingdong prefecture level city in Shandong
Power Saving King Shop	King of Power Saving	It is genuine, effective, really saves power, affordable, tightly packed, fast shipping, very satisfied.	Jingdong prefecture level city in Shandong
Power Saving King Shop	King of Power Saving	Good quality, fine workmanship, is genuine and business description of the same, good value for money, fast logistics, very satisfied	Jingdong prefecture level city in Shandong
Power Saving King Shop	King of Power Saving	Saver received, very good, installation set up very well, very good, the effect is very good, recommend that the pro buy	Jingdong prefecture level city in Shandong
Power Saving King Shop	King of Power Saving	Electromagnetic good, high power convenient, enough force, the price is right it, quality can be 😃 😃 😃 😃 😃	Jingdong prefecture level city in Shandong
Power Saving King Shop	King of Power Saving	First-class quality, packaging is also good, logistics is very fast, and the price is reasonable, very affordable, I like it!	Jingdong prefecture level city in Shandong
Power Saving King Shop	King of Power Saving	It's great, it's the kind of style you want, and it arrived quickly, thank you boss, 5 stars for the service!	Jingdong prefecture level city in Shandong
Power Saving King Shop	King of Power Saving	Packaging is very good, the use of the effect is quite good, the store has a good service attitude, trustworthy	Jingdong prefecture level city in Shandong
Power Saving King Shop	King of Power Saving	The quality is very good, it works well, very satisfied, the price is affordable, it's so great value, it's worth recommending.	Jingdong prefecture level city in Shandong
Power Saving King Shop	King of Power Saving	The baby description matches the actual item very well! The quality is awesome! Will be back to patronize again in the future!	Jingdong prefecture level city in Shandong
Power Saving King Shop	King of Power Saving	Ran the price is not high try to buy the attitude, did not expect the effect is really good, good!	Jingdong prefecture level city in Shandong
Power Saving King Shop	King of Power Saving	Power Saving King power is quite large, the quality of the leaf is quite good, the price of buying a very good deal, must be praised!	Jingdong prefecture level city in Shandong
Power Saving King Shop	King of Power Saving	Good product quality is the king of power-saving products trustworthy as always good to recommend!	Jingdong prefecture level city in Shandong
Power Saving King Shop	King of Power Saving	Works great, very affordable, shipped pretty fast, comfortable to use	Jingdong prefecture level city in Shandong
Power Saving King Shop	King of Power Saving	Shipping was pretty fast and affordable, not bad 👍	Jingdong prefecture level city in Shandong

Power Saving King Shop	King of Power Saving	Digital voltage display, voltage at a glance, plug and play, safe and simple!	Jingdong prefecture level city in Shandong
Power Saving King Shop	King of Power Saving	Quality assurance, very good, affordable, logistics is also very fast, I'm very satisfied with the	Jingdong prefecture level city in Shandong
Power Saving King Shop	King of Power Saving	The quality is very good, the first time I received the goods to open the first time to see, it is very good, very satisfied	Jingdong prefecture level city in Shandong
Power Saving King Shop	King of Power Saving	Power Saving King used, the effect is good, the price is affordable, very good, is the genuine article	Jingdong prefecture level city in Shandong
Power Saving King Shop	King of Power Saving	Good quality, fine workmanship, good appearance, time is very accurate, satisfied.	Jingdong prefecture level city in Shandong
Power Saving King Shop	King of Power Saving	I purposely used it for two days before commenting, which is responsible for myself and everyone else!	Jingdong prefecture level city in Shandong
Power Saving King Shop	King of Power Saving	Easy to install, easy to operate, helped our family save a lot of electricity this summer	Jingdong prefecture level city in Shandong
Power Saving King Shop	King of Power Saving	Bringing power savings into millions of homes. Still a great gadget. Pretty good.	Jingdong prefecture level city in Shandong
Power Saving King Shop	King of Power Saving	Digital liquid crystal display to see the clear intuitive, very formal workmanship is not rough	Jingdong prefecture level city in Shandong
Power Saving King Shop	King of Power Saving	Received. Great quality, fast shipping from the store and not too expensive!	Jingdong prefecture level city in Shandong
Power Saving King Shop	King of Power Saving	Bought myself a knuckle regulator and it was easy to use and the price was right.	Jingdong prefecture level city in Shandong
Power Saving King Shop	King of Power Saving	Received, good, powerful, good, very full of a shopping	Jingdong prefecture level city in Shandong
Power Saving King Shop	King of Power Saving	High power, consistent with the seller's description, powerful, cost-effective	Jingdong prefecture level city in Shandong
Power Saving King Shop	King of Power Saving	It works very well and also saves power at an affordable price a great deal.	Jingdong prefecture level city in Shandong
Power Saving King Shop	King of Power Saving	Good quality, good can recommend to friends to buy.	Jingdong prefecture level city in Shandong
Power Saving King Shop	King of Power Saving	Will repurchase, quality is guaranteed, safe and secure purchase.	Jingdong prefecture level city in Shandong
Power Saving King Shop	King of Power Saving	The logistics is very fast, the thing is also good to use, energy saving power rely on it!	Jingdong prefecture level city in Shandong
Power Saving King Shop	King of Power Saving	Power Saver is good quality and works very well, worth buying!	Jingdong prefecture level city in Shandong
Power Saving King Shop	King of Power Saving	The quality is very good is genuine, cost-effective recommended	Jingdong prefecture level city in Shandong
Power Saving King Shop	King of Power Saving	Quality Assurance. Favorable price. Very practical and durable	Jingdong prefecture level city in Shandong
Power Saving King Shop	King of Power Saving	Good value for money. Conscientious businessman. Recommended to everyone	Jingdong prefecture level city in Shandong
Power Saving King Shop	King of Power Saving	Buy it for your rental apartment and never worry about your landlord charging you too much.	Jingdong prefecture level city in Shandong
Power Saving King Shop	King of Power Saving	Nice and affordable, will repurchase next time	Jingdong prefecture level city in Shandong
Power Saving King Shop	King of Power Saving	It's very energy efficient. I've been using it for a few days now.	Jingdong prefecture level city in Shandong
Power Saving King Shop	King of Power Saving	Good quality, high power easy to use	Jingdong prefecture level city in Shandong
Power Saving King Shop	King of Power Saving	Power saving little helper. Deserves a favorable review.	Jingdong prefecture level city in Shandong
Power Saving King Shop	King of Power Saving	Great value for money, great value for money.	Jingdong prefecture level city in Shandong
Power Saving King Shop	King of Power Saving	Works well and as described by the seller	Jingdong prefecture level city in Shandong
Power Saving King Shop	King of Power Saving	Power saving is proper! It's good.	Jingdong prefecture level city in Shandong
Power Saving King Shop	King of Power Saving	Power saving. Works well. Not bad.	Jingdong prefecture level city in Shandong
Power Saving King Shop	King of Power Saving	Satisfied with power saving	Jingdong prefecture level city in Shandong
Power Saving King Shop	King of Power Saving	Received intact, open a super good look beautiful and generous, ~ bit of high quality is recommended to buy, the need for friends rest assured that the next order	Jingdong prefecture level city in Shandong
Power Saving King Shop	King of Power Saving	Very good to use, friends introduced, save electricity and money, long-lasting products cost-effective, large factory production, good quality, fine workmanship cost-effective!	Jingdong prefecture level city in Shandong
Power Saving King Shop	King of Power Saving	The power saving effect is very obvious, trying to pay 30% less electricity bill in a month than before, hahaha	Jingdong prefecture level city in Shandong
Power Saving King Shop	King of Power Saving	It's such a power saver to use this, it saves money on kids snacks for a month. Kudos!	Jingdong prefecture level city in Shandong
Power Saving King Shop	King of Power Saving	Plug and play, stable voltage, the screen has a display when working, this winter home appliances, need more electricity, just buy to use, save electricity and save money, with instructions, not too big and does not take up space, the quality is very good, and the seller describes exactly the same!	Jingdong prefecture level city in Shandong
Power Saving King Shop	King of Power Saving	A must have for opening a store. Save over a thousand dollars a month on your electric bill	Jingdong prefecture level city in Shandong
Power Saving King Shop	King of Power Saving	Received the goods, the first time to unpack, the quality is very good, with the seller's description, completely exceeded my expectations, the packaging is very careful, very strict, in general this is a very satisfied with the shopping	Jingdong prefecture level city in Shandong
Power Saving King Shop	King of Power Saving	As always, good quality, a very favorite product, great! o(*￣︶￣*)o The price is very affordable, cost-effective, it's considered affordable good, will repurchase!	Jingdong prefecture level city in Shandong
Power Saving King Shop	King of Power Saving	Very satisfied with a shopping, good business service, fast shipping, logistics is also very powerful, received very good, buy assured, use peace of mind!	Jingdong prefecture level city in Shandong
Power Saving King Shop	King of Power Saving	The first time to buy online, but also look at the evaluation before buying, the quality is not bad, but also very cheap, the need for friends can rest assured to buy!	Jingdong prefecture level city in Shandong
Power Saving King Shop	King of Power Saving	Better than expected, feel value for money, very satisfied with a shopping, need next time will come back!	Jingdong prefecture level city in Shandong
Power Saving King Shop	King of Power Saving	Really super like, very supportive, very good quality, exactly as described by the seller, very satisfied!	Jingdong prefecture level city in Shandong
Power Saving King Shop	King of Power Saving	It is genuine, effective, really saves power, affordable, tightly packed, fast shipping, very satisfied.	Jingdong prefecture level city in Shandong
Power Saving King Shop	King of Power Saving	Good quality, fine workmanship, is genuine and business description of the same, good value for money, fast logistics, very satisfied	Jingdong prefecture level city in Shandong
Power Saving King Shop	King of Power Saving	Saver received, very good, installation set up very well, very good, the effect is very good, recommend that the pro buy	Jingdong prefecture level city in Shandong
Power Saving King Shop	King of Power Saving	Electromagnetic good, high power convenient, enough force, the price is right it, quality can be ☺ ☺ ☺ ☺ ☺	Jingdong prefecture level city in Shandong
Power Saving King Shop	King of Power Saving	First-class quality, packaging is also good, logistics is very fast, and the price is reasonable, very affordable, I like it!	Jingdong prefecture level city in Shandong
Power Saving King Shop	King of Power Saving	It's great, it's the kind of style you want, and it arrived quickly, thank you boss, 5 stars for the service!	Jingdong prefecture level city in Shandong
Power Saving King Shop	King of Power Saving	Packaging is very good, the use of the effect is quite good, the store has a good service attitude, trustworthy	Jingdong prefecture level city in Shandong

Power Saving King Shop	King of Power Saving	The quality is very good, it works well, very satisfied, the price is affordable, it's so great value, it's worth recommending.	Jingdong prefecture level city in Shandong
Power Saving King Shop	King of Power Saving	The baby description matches the actual item very well! The quality is awesome! Will be back to patronize again in the future!	Jingdong prefecture level city in Shandong
Power Saving King Shop	King of Power Saving	Ran the price is not high try to buy the attitude, did not expect the effect is really good, good!	Jingdong prefecture level city in Shandong
Power Saving King Shop	King of Power Saving	Power Saving King power is quite large, the quality of the leaf is quite good, the price of buying a very good deal, must be praised!	Jingdong prefecture level city in Shandong
Power Saving King Shop	King of Power Saving	Good product quality is the king of power-saving products trustworthy as always good to recommend!	Jingdong prefecture level city in Shandong
Power Saving King Shop	King of Power Saving	Works great, very affordable, shipped pretty fast, comfortable to use	Jingdong prefecture level city in Shandong
Power Saving King Shop	King of Power Saving	Shipping was pretty fast and affordable, not bad 👍	Jingdong prefecture level city in Shandong
Power Saving King Shop	King of Power Saving	Digital voltage display, voltage at a glance, plug and play, safe and simple!	Jingdong prefecture level city in Shandong
Power Saving King Shop	King of Power Saving	Quality assurance, very good, affordable, logistics is also very fast, I'm very satisfied with the	Jingdong prefecture level city in Shandong
Power Saving King Shop	King of Power Saving	The quality is very good, the first time I received the goods to open the first time to see, it is very good, very satisfied	Jingdong prefecture level city in Shandong
Power Saving King Shop	King of Power Saving	Power Saving King used, the effect is good, the price is affordable, very good, is the genuine article	Jingdong prefecture level city in Shandong
Power Saving King Shop	King of Power Saving	Good quality, fine workmanship, good appearance, time is very accurate, satisfied.	Jingdong prefecture level city in Shandong
Power Saving King Shop	King of Power Saving	I purposely used it for two days before commenting, which is responsible for myself and everyone else!	Jingdong prefecture level city in Shandong
Power Saving King Shop	King of Power Saving	Easy to install, easy to operate, helped our family save a lot of electricity this summer	Jingdong prefecture level city in Shandong
Power Saving King Shop	King of Power Saving	Bringing power savings into millions of homes. Still a great gadget. Pretty good.	Jingdong prefecture level city in Shandong
Power Saving King Shop	King of Power Saving	Digital liquid crystal display to see the clear intuitive, very formal workmanship is not rough	Jingdong prefecture level city in Shandong
Power Saving King Shop	King of Power Saving	Received. Great quality, fast shipping from the store and not too expensive!	Jingdong prefecture level city in Shandong
Power Saving King Shop	King of Power Saving	Bought myself a knuckle regulator and it was easy to use and the price was right.	Jingdong prefecture level city in Shandong
Power Saving King Shop	King of Power Saving	Received, good, powerful, good, very full of a shopping	Jingdong prefecture level city in Shandong
Power Saving King Shop	King of Power Saving	High power, consistent with the seller's description, powerful, cost-effective	Jingdong prefecture level city in Shandong
Power Saving King Shop	King of Power Saving	It works very well and also saves power at an affordable price a great deal.	Jingdong prefecture level city in Shandong
Power Saving King Shop	King of Power Saving	Good quality, good can recommend to friends to buy.	Jingdong prefecture level city in Shandong
Power Saving King Shop	King of Power Saving	Will repurchase, quality is guaranteed, safe and secure purchase.	Jingdong prefecture level city in Shandong
Power Saving King Shop	King of Power Saving	The logistics is very fast, the thing is also good to use, energy saving power rely on it!	Jingdong prefecture level city in Shandong
Power Saving King Shop	King of Power Saving	Power Saver is good quality and works very well, worth buying!	Jingdong prefecture level city in Shandong
Power Saving King Shop	King of Power Saving	The quality is very good is genuine, cost-effective recommended	Jingdong prefecture level city in Shandong
Power Saving King Shop	King of Power Saving	Quality Assurance. Favorable price. Very practical and durable	Jingdong prefecture level city in Shandong
Power Saving King Shop	King of Power Saving	Good value for money. Conscientious businessman. Recommended to everyone	Jingdong prefecture level city in Shandong
Power Saving King Shop	King of Power Saving	Buy it for your rental apartment and never worry about your landlord charging you too much.	Jingdong prefecture level city in Shandong
Power Saving King Shop	King of Power Saving	Nice and affordable, will repurchase next time	Jingdong prefecture level city in Shandong
Power Saving King Shop	King of Power Saving	It's very energy efficient. I've been using it for a few days now.	Jingdong prefecture level city in Shandong
Power Saving King Shop	King of Power Saving	Good quality, high power easy to use	Jingdong prefecture level city in Shandong
Power Saving King Shop	King of Power Saving	Power saving little helper. Deserves a favorable review.	Jingdong prefecture level city in Shandong
Power Saving King Shop	King of Power Saving	Great value for money, great value for money.	Jingdong prefecture level city in Shandong
Power Saving King Shop	King of Power Saving	Works well and as described by the seller	Jingdong prefecture level city in Shandong
Power Saving King Shop	King of Power Saving	Power saving is proper! It's good.	Jingdong prefecture level city in Shandong
Power Saving King Shop	King of Power Saving	Power saving. Works well. Not bad.	Jingdong prefecture level city in Shandong
Power Saving King Shop	King of Power Saving	Satisfied with power saving	Jingdong prefecture level city in Shandong
Power Saving King Shop	King of Power Saving	Received intact, open a super good look beautiful and generous, ~ bit of high quality is recommended to buy, the need for friends rest assured that the next order	Jingdong prefecture level city in Shandong
Power Saving King Shop	King of Power Saving	Very good to use, friends introduced, save electricity and money, long-lasting products cost-effective, large factory production, good quality, fine workmanship cost-effective!	Jingdong prefecture level city in Shandong
Power Saving King Shop	King of Power Saving	The power saving effect is very obvious, trying to pay 30% less electricity bill in a month than before, hahaha	Jingdong prefecture level city in Shandong
Power Saving King Shop	King of Power Saving	It's such a power saver to use this, it saves money on kids snacks for a month. Kudos!	Jingdong prefecture level city in Shandong
Power Saving King Shop	King of Power Saving	Plug and play, stable voltage, the screen has a display when working, this winter home appliances, need more electricity, just buy to use, save electricity and save money, with instructions, not too big and does not take up space, the quality is very good, and the seller describes exactly the same!	Jingdong prefecture level city in Shandong
Power Saving King Shop	King of Power Saving	A must have for opening a store. Save over a thousand dollars a month on your electric bill	Jingdong prefecture level city in Shandong
Power Saving King Shop	King of Power Saving	Received the goods, the first time to unpack, the quality is very good, with the seller's description, completely exceeded my expectations, the packaging is very careful, very strict, in general this is a very satisfied with the shopping	Jingdong prefecture level city in Shandong
Power Saving King Shop	King of Power Saving	As always, good quality, a very favorite product, great! o(* ￣︶￣ *)o The price is very affordable, cost-effective, it's considered affordable good, will repurchase!	Jingdong prefecture level city in Shandong
Power Saving King Shop	King of Power Saving	Very satisfied with a shopping, good business service, fast shipping, logistics is also very powerful, received very good, buy assured, use peace of mind!	Jingdong prefecture level city in Shandong
Power Saving King Shop	King of Power Saving	The first time to buy online, but also look at the evaluation before buying, the quality is not bad, but also very cheap, the need for friends can rest assured to buy!	Jingdong prefecture level city in Shandong
Power Saving King Shop	King of Power Saving	Better than expected, feel value for money, very satisfied with a shopping, need next time will come back!	Jingdong prefecture level city in Shandong
Power Saving King Shop	King of Power Saving	Really super like, very supportive, very good quality, exactly as described by the seller, very satisfied!	Jingdong prefecture level city in Shandong

Power Saving King Shop	King of Power Saving	It is genuine, effective, really saves power, affordable, tightly packed, fast shipping, very satisfied.	Jingdong prefecture level city in Shandong
Power Saving King Shop	King of Power Saving	Good quality, fine workmanship, is genuine and business description of the same, good value for money, fast logistics, very satisfied	Jingdong prefecture level city in Shandong
Power Saving King Shop	King of Power Saving	Saver received, very good, installation set up very well, very good, the effect is very good, recommend that the pro buy	Jingdong prefecture level city in Shandong
Power Saving King Shop	King of Power Saving	Electromagnetic good, high power convenient, enough force, the price is right it, quality can be 😀 😀 😀 😀 😀	Jingdong prefecture level city in Shandong
Power Saving King Shop	King of Power Saving	First-class quality, packaging is also good, logistics is very fast, and the price is reasonable, very affordable, I like it!	Jingdong prefecture level city in Shandong
Power Saving King Shop	King of Power Saving	It's great, it's the kind of style you want, and it arrived quickly, thank you boss, 5 stars for the service!	Jingdong prefecture level city in Shandong
Power Saving King Shop	King of Power Saving	Packaging is very good, the use of the effect is quite good, the store has a good service attitude, trustworthy	Jingdong prefecture level city in Shandong
Power Saving King Shop	King of Power Saving	The quality is very good, it works well, very satisfied, the price is affordable, it's so great value, it's worth recommending.	Jingdong prefecture level city in Shandong
Power Saving King Shop	King of Power Saving	The baby description matches the actual item very well! The quality is awesome! Will be back to patronize again in the future!	Jingdong prefecture level city in Shandong
Power Saving King Shop	King of Power Saving	Ran the price is not high try to buy the attitude, did not expect the effect is really good, good!	Jingdong prefecture level city in Shandong
Power Saving King Shop	King of Power Saving	Power Saving King power is quite large, the quality of the leaf is quite good, the price of buying a very good deal, must be praised!	Jingdong prefecture level city in Shandong
Power Saving King Shop	King of Power Saving	Good product quality is the king of power-saving products trustworthy as always good to recommend!	Jingdong prefecture level city in Shandong
Power Saving King Shop	King of Power Saving	Works great, very affordable, shipped pretty fast, comfortable to use	Jingdong prefecture level city in Shandong
Power Saving King Shop	King of Power Saving	Shipping was pretty fast and affordable, not bad 👍	Jingdong prefecture level city in Shandong
Power Saving King Shop	King of Power Saving	Digital voltage display, voltage at a glance, plug and play, safe and simple!	Jingdong prefecture level city in Shandong
Power Saving King Shop	King of Power Saving	Quality assurance, very good, affordable, logistics is also very fast, I'm very satisfied with the	Jingdong prefecture level city in Shandong
Power Saving King Shop	King of Power Saving	The quality is very good, the first time I received the goods to open the first time to see, it is very good, very satisfied	Jingdong prefecture level city in Shandong
Power Saving King Shop	King of Power Saving	Power Saving King used, the effect is good, the price is affordable, very good, is the genuine article	Jingdong prefecture level city in Shandong
Power Saving King Shop	King of Power Saving	Good quality, fine workmanship, good appearance, time is very accurate, satisfied.	Jingdong prefecture level city in Shandong
Power Saving King Shop	King of Power Saving	I purposely used it for two days before commenting, which is responsible for myself and everyone else!	Jingdong prefecture level city in Shandong
Power Saving King Shop	King of Power Saving	Easy to install, easy to operate, helped our family save a lot of electricity this summer	Jingdong prefecture level city in Shandong
Power Saving King Shop	King of Power Saving	Bringing power savings into millions of homes. Still a great gadget. Pretty good.	Jingdong prefecture level city in Shandong
Power Saving King Shop	King of Power Saving	Digital liquid crystal display to see the clear intuitive, very formal workmanship is not rough	Jingdong prefecture level city in Shandong
Power Saving King Shop	King of Power Saving	Received. Great quality, fast shipping from the store and not too expensive!	Jingdong prefecture level city in Shandong
Power Saving King Shop	King of Power Saving	Bought myself a knuckle regulator and it was easy to use and the price was right.	Jingdong prefecture level city in Shandong
Power Saving King Shop	King of Power Saving	Received, good, powerful, good, very full of a shopping	Jingdong prefecture level city in Shandong
Power Saving King Shop	King of Power Saving	High power, consistent with the seller's description, powerful, cost-effective	Jingdong prefecture level city in Shandong
Power Saving King Shop	King of Power Saving	It works very well and also saves power at an affordable price a great deal.	Jingdong prefecture level city in Shandong
Power Saving King Shop	King of Power Saving	Good quality, good can recommend to friends to buy.	Jingdong prefecture level city in Shandong
Power Saving King Shop	King of Power Saving	Will repurchase, quality is guaranteed, safe and secure purchase.	Jingdong prefecture level city in Shandong
Power Saving King Shop	King of Power Saving	The logistics is very fast, the thing is also good to use, energy saving power rely on it!	Jingdong prefecture level city in Shandong
Power Saving King Shop	King of Power Saving	Power Saver is good quality and works very well, worth buying!	Jingdong prefecture level city in Shandong
Power Saving King Shop	King of Power Saving	The quality is very good is genuine, cost-effective recommended	Jingdong prefecture level city in Shandong
Power Saving King Shop	King of Power Saving	Quality Assurance. Favorable price. Very practical and durable	Jingdong prefecture level city in Shandong
Power Saving King Shop	King of Power Saving	Good value for money. Conscientious businessman. Recommended to everyone	Jingdong prefecture level city in Shandong
Power Saving King Shop	King of Power Saving	Buy it for your rental apartment and never worry about your landlord charging you too much.	Jingdong prefecture level city in Shandong
Power Saving King Shop	King of Power Saving	Nice and affordable, will repurchase next time	Jingdong prefecture level city in Shandong
Power Saving King Shop	King of Power Saving	It's very energy efficient. I've been using it for a few days now.	Jingdong prefecture level city in Shandong
Power Saving King Shop	King of Power Saving	Good quality, high power easy to use	Jingdong prefecture level city in Shandong
Power Saving King Shop	King of Power Saving	Power saving little helper. Deserves a favorable review.	Jingdong prefecture level city in Shandong
Power Saving King Shop	King of Power Saving	Great value for money, great value for money.	Jingdong prefecture level city in Shandong
Power Saving King Shop	King of Power Saving	Works well and as described by the seller	Jingdong prefecture level city in Shandong
Power Saving King Shop	King of Power Saving	Power saving is proper! It's good.	Jingdong prefecture level city in Shandong
Power Saving King Shop	King of Power Saving	Power saving. Works well. Not bad.	Jingdong prefecture level city in Shandong
Power Saving King Shop	King of Power Saving	Satisfied with power saving	Jingdong prefecture level city in Shandong
Power Saving King Shop	King of Power Saving	Received intact, open a super good look beautiful and generous, ~ bit of high quality is recommended to buy, the need for friends rest assured that the next order	Jingdong prefecture level city in Shandong
Power Saving King Shop	King of Power Saving	Very good to use, friends introduced, save electricity and money, long-lasting products cost-effective, large factory production, good quality, fine workmanship cost-effective!	Jingdong prefecture level city in Shandong
Power Saving King Shop	King of Power Saving	The power saving effect is very obvious, trying to pay 30% less electricity bill in a month than before, hahaha	Jingdong prefecture level city in Shandong
Power Saving King Shop	King of Power Saving	It's such a power saver to use this, it saves money on kids snacks for a month. Kudos!	Jingdong prefecture level city in Shandong
Power Saving King Shop	King of Power Saving	Plug and play, stable voltage, the screen has a display when working, this winter home appliances, need more electricity, just buy to use, save electricity and save money, with instructions, not too big and does not take up space, the quality is very good, and the seller describes exactly the same!	Jingdong prefecture level city in Shandong
Power Saving King Shop	King of Power Saving	A must have for opening a store. Save over a thousand dollars a month on your electric bill	Jingdong prefecture level city in Shandong

Power Saving King Shop	King of Power Saving	Received the goods, the first time to unpack, the quality is very good, with the seller's description, completely exceeded my expectations, the packaging is very careful, very strict, in general this is a very satisfied with the shopping	Jingdong prefecture level city in Shandong
Power Saving King Shop	King of Power Saving	As always, good quality, a very favorite product, great! o(*￣︶￣*)o The price is very affordable, cost-effective, it's considered affordable good, will repurchase!	Jingdong prefecture level city in Shandong
Power Saving King Shop	King of Power Saving	Very satisfied with a shopping, good business service, fast shipping, logistics is also very powerful, received very good, buy assured, use peace of mind!	Jingdong prefecture level city in Shandong
Power Saving King Shop	King of Power Saving	The first time to buy online, but also look at the evaluation before buying, the quality is not bad, but also very cheap, the need for friends can rest assured to buy!	Jingdong prefecture level city in Shandong
Power Saving King Shop	King of Power Saving	Better than expected, feel value for money, very satisfied with a shopping, need next time will come back!	Jingdong prefecture level city in Shandong
Power Saving King Shop	King of Power Saving	Really super like, very supportive, very good quality, exactly as described by the seller, very satisfied!	Jingdong prefecture level city in Shandong
Power Saving King Shop	King of Power Saving	It is genuine, effective, really saves power, affordable, tightly packed, fast shipping, very satisfied.	Jingdong prefecture level city in Shandong
Power Saving King Shop	King of Power Saving	Good quality, fine workmanship, is genuine and business description of the same, good value for money, fast logistics, very satisfied	Jingdong prefecture level city in Shandong
Power Saving King Shop	King of Power Saving	Saver received, very good, installation set up very well, very good, the effect is very good, recommend that the pro buy	Jingdong prefecture level city in Shandong
Power Saving King Shop	King of Power Saving	Electromagnetic good, high power convenient, enough force, the price is right it, quality can be 😃 😃 😃 😃 😃	Jingdong prefecture level city in Shandong
Power Saving King Shop	King of Power Saving	First-class quality, packaging is also good, logistics is very fast, and the price is reasonable, very affordable, I like it!	Jingdong prefecture level city in Shandong
Power Saving King Shop	King of Power Saving	It's great, it's the kind of style you want, and it arrived quickly, thank you boss, 5 stars for the service!	Jingdong prefecture level city in Shandong
Power Saving King Shop	King of Power Saving	Packaging is very good, the use of the effect is quite good, the store has a good service attitude, trustworthy	Jingdong prefecture level city in Shandong
Power Saving King Shop	King of Power Saving	The quality is very good, it works well, very satisfied, the price is affordable, it's so great value, it's worth recommending.	Jingdong prefecture level city in Shandong
Power Saving King Shop	King of Power Saving	The baby description matches the actual item very well! The quality is awesome! Will be back to patronize again in the future!	Jingdong prefecture level city in Shandong
Power Saving King Shop	King of Power Saving	Ran the price is not high try to buy the attitude, did not expect the effect is really good, good!	Jingdong prefecture level city in Shandong
Power Saving King Shop	King of Power Saving	Power Saving King power is quite large, the quality of the leaf is quite good, the price of buying a very good deal, must be praised!	Jingdong prefecture level city in Shandong
Power Saving King Shop	King of Power Saving	Good product quality is the king of power-saving products trustworthy as always good to recommend!	Jingdong prefecture level city in Shandong
Power Saving King Shop	King of Power Saving	Works great, very affordable, shipped pretty fast, comfortable to use	Jingdong prefecture level city in Shandong
Power Saving King Shop	King of Power Saving	Shipping was pretty fast and affordable, not bad 👍	Jingdong prefecture level city in Shandong
Power Saving King Shop	King of Power Saving	Digital voltage display, voltage at a glance, plug and play, safe and simple!	Jingdong prefecture level city in Shandong
Power Saving King Shop	King of Power Saving	Quality assurance, very good, affordable, logistics is also very fast, I'm very satisfied with the	Jingdong prefecture level city in Shandong
Power Saving King Shop	King of Power Saving	The quality is very good, the first time I received the goods to open the first time to see, it is very good, very satisfied	Jingdong prefecture level city in Shandong
Power Saving King Shop	King of Power Saving	Power Saving King used, the effect is good, the price is affordable, very good, is the genuine article	Jingdong prefecture level city in Shandong
Power Saving King Shop	King of Power Saving	Good quality, fine workmanship, good appearance, time is very accurate, satisfied.	Jingdong prefecture level city in Shandong
Power Saving King Shop	King of Power Saving	I purposely used it for two days before commenting, which is responsible for myself and everyone else!	Jingdong prefecture level city in Shandong
Power Saving King Shop	King of Power Saving	Easy to install, easy to operate, helped our family save a lot of electricity this summer	Jingdong prefecture level city in Shandong
Power Saving King Shop	King of Power Saving	Bringing power savings into millions of homes. Still a great gadget. Pretty good.	Jingdong prefecture level city in Shandong
Power Saving King Shop	King of Power Saving	Digital liquid crystal display to see the clear intuitive, very formal workmanship is not rough	Jingdong prefecture level city in Shandong
Power Saving King Shop	King of Power Saving	Received. Great quality, fast shipping from the store and not too expensive!	Jingdong prefecture level city in Shandong
Power Saving King Shop	King of Power Saving	Bought myself a knuckle regulator and it was easy to use and the price was right.	Jingdong prefecture level city in Shandong
Power Saving King Shop	King of Power Saving	Received, good, powerful, good, very full of a shopping	Jingdong prefecture level city in Shandong
Power Saving King Shop	King of Power Saving	High power, consistent with the seller's description, powerful, cost-effective	Jingdong prefecture level city in Shandong
Power Saving King Shop	King of Power Saving	It works very well and also saves power at an affordable price a great deal.	Jingdong prefecture level city in Shandong
Power Saving King Shop	King of Power Saving	Good quality, good can recommend to friends to buy.	Jingdong prefecture level city in Shandong
Power Saving King Shop	King of Power Saving	Will repurchase, quality is guaranteed, safe and secure purchase.	Jingdong prefecture level city in Shandong
Power Saving King Shop	King of Power Saving	The logistics is very fast, the thing is also good to use, energy saving power rely on it!	Jingdong prefecture level city in Shandong
Power Saving King Shop	King of Power Saving	Power Saver is good quality and works very well, worth buying!	Jingdong prefecture level city in Shandong
Power Saving King Shop	King of Power Saving	The quality is very good is genuine, cost-effective recommended	Jingdong prefecture level city in Shandong
Power Saving King Shop	King of Power Saving	Quality Assurance. Favorable price. Very practical and durable	Jingdong prefecture level city in Shandong
Power Saving King Shop	King of Power Saving	Good value for money. Conscientious businessman. Recommended to everyone	Jingdong prefecture level city in Shandong
Power Saving King Shop	King of Power Saving	Buy it for your rental apartment and never worry about your landlord charging you too much.	Jingdong prefecture level city in Shandong
Power Saving King Shop	King of Power Saving	Nice and affordable, will repurchase next time	Jingdong prefecture level city in Shandong
Power Saving King Shop	King of Power Saving	It's very energy efficient. I've been using it for a few days now.	Jingdong prefecture level city in Shandong
Power Saving King Shop	King of Power Saving	Good quality, high power easy to use	Jingdong prefecture level city in Shandong
Power Saving King Shop	King of Power Saving	Power saving little helper. Deserves a favorable review.	Jingdong prefecture level city in Shandong
Power Saving King Shop	King of Power Saving	Great value for money, great value for money.	Jingdong prefecture level city in Shandong
Power Saving King Shop	King of Power Saving	Works well and as described by the seller	Jingdong prefecture level city in Shandong
Power Saving King Shop	King of Power Saving	Power saving is proper! It's good.	Jingdong prefecture level city in Shandong
Power Saving King Shop	King of Power Saving	Power saving. Works well. Not bad.	Jingdong prefecture level city in Shandong
Power Saving King Shop	King of Power Saving	Satisfied with power saving	Jingdong prefecture level city in Shandong

Power Saving King Shop	King of Power Saving	Received intact, open a super good look beautiful and generous, ~ bit of high quality is recommended to buy, the need for friends rest assured that the next order	Jingdong prefecture level city in Shandong
Power Saving King Shop	King of Power Saving	Very good to use, friends introduced, save electricity and money, long-lasting products cost-effective, large factory production, good quality, fine workmanship cost-effective!	Jingdong prefecture level city in Shandong
Power Saving King Shop	King of Power Saving	The power saving effect is very obvious, trying to pay 30% less electricity bill in a month than before, hahaha	Jingdong prefecture level city in Shandong
Power Saving King Shop	King of Power Saving	It's such a power saver to use this, it saves money on kids snacks for a month. Kudos!	Jingdong prefecture level city in Shandong
Power Saving King Shop	King of Power Saving	Plug and play, stable voltage, the screen has a display when working, this winter home appliances, need more electricity, just buy to use, save electricity and save money, with instructions, not too big and does not take up space, the quality is very good, and the seller describes exactly the same!	Jingdong prefecture level city in Shandong
Power Saving King Shop	King of Power Saving	A must have for opening a store. Save over a thousand dollars a month on your electric bill	Jingdong prefecture level city in Shandong
Power Saving King Shop	King of Power Saving	Received the goods, the first time to unpack, the quality is very good, with the seller's description, completely exceeded my expectations, the packaging is very careful, very strict, in general this is a very satisfied with the shopping	Jingdong prefecture level city in Shandong
Power Saving King Shop	King of Power Saving	As always, good quality, a very favorite product, great! o(*￣︶￣*)o The price is very affordable, cost-effective, it's considered affordable good, will repurchase!	Jingdong prefecture level city in Shandong
Power Saving King Shop	King of Power Saving	Very satisfied with a shopping, good business service, fast shipping, logistics is also very powerful, received very good, buy assured, use peace of mind!	Jingdong prefecture level city in Shandong
Power Saving King Shop	King of Power Saving	The first time to buy online, but also look at the evaluation before buying, the quality is not bad, but also very cheap, the need for friends can rest assured to buy!	Jingdong prefecture level city in Shandong
Power Saving King Shop	King of Power Saving	Better than expected, feel value for money, very satisfied with a shopping, need next time will come back!	Jingdong prefecture level city in Shandong
Power Saving King Shop	King of Power Saving	Really super like, very supportive, very good quality, exactly as described by the seller, very satisfied!	Jingdong prefecture level city in Shandong
Power Saving King Shop	King of Power Saving	It is genuine, effective, really saves power, affordable, tightly packed, fast shipping, very satisfied.	Jingdong prefecture level city in Shandong
Power Saving King Shop	King of Power Saving	Good quality, fine workmanship, is genuine and business description of the same, good value for money, fast logistics, very satisfied	Jingdong prefecture level city in Shandong
Power Saving King Shop	King of Power Saving	Saver received, very good, installation set up very well, very good, the effect is very good, recommend that the pro buy	Jingdong prefecture level city in Shandong
Power Saving King Shop	King of Power Saving	Electromagnetic good, high power convenient, enough force, the price is right it, quality can be 😀 😀 😀 😀 😀	Jingdong prefecture level city in Shandong
Power Saving King Shop	King of Power Saving	First-class quality, packaging is also good, logistics is very fast, and the price is reasonable, very affordable, I like it!	Jingdong prefecture level city in Shandong
Power Saving King Shop	King of Power Saving	It's great, it's the kind of style you want, and it arrived quickly, thank you boss, 5 stars for the service!	Jingdong prefecture level city in Shandong
Power Saving King Shop	King of Power Saving	Packaging is very good, the use of the effect is quite good, the store has a good service attitude, trustworthy	Jingdong prefecture level city in Shandong
Power Saving King Shop	King of Power Saving	The quality is very good, it works well, very satisfied, the price is affordable, it's so great value, it's worth recommending.	Jingdong prefecture level city in Shandong
Power Saving King Shop	King of Power Saving	The baby description matches the actual item very well! The quality is awesome! Will be back to patronize again in the future!	Jingdong prefecture level city in Shandong
Power Saving King Shop	King of Power Saving	Ran the price is not high try to buy the attitude, did not expect the effect is really good, good!	Jingdong prefecture level city in Shandong
Power Saving King Shop	King of Power Saving	Power Saving King power is quite large, the quality of the leaf is quite good, the price of buying a very good deal, must be praised!	Jingdong prefecture level city in Shandong
Power Saving King Shop	King of Power Saving	Good product quality is the king of power-saving products trustworthy as always good to recommend!	Jingdong prefecture level city in Shandong
Power Saving King Shop	King of Power Saving	Works great, very affordable, shipped pretty fast, comfortable to use	Jingdong prefecture level city in Shandong
Power Saving King Shop	King of Power Saving	Shipping was pretty fast and affordable, not bad 👍	Jingdong prefecture level city in Shandong
Power Saving King Shop	King of Power Saving	Digital voltage display, voltage at a glance, plug and play, safe and simple!	Jingdong prefecture level city in Shandong
Power Saving King Shop	King of Power Saving	Quality assurance, very good, affordable, logistics is also very fast, I'm very satisfied with the	Jingdong prefecture level city in Shandong
Power Saving King Shop	King of Power Saving	The quality is very good, the first time I received the goods to open the first time to see, it is very good, very satisfied	Jingdong prefecture level city in Shandong
Power Saving King Shop	King of Power Saving	Power Saving King used, the effect is good, the price is affordable, very good, is the genuine article	Jingdong prefecture level city in Shandong
Power Saving King Shop	King of Power Saving	Good quality, fine workmanship, good appearance, time is very accurate, satisfied.	Jingdong prefecture level city in Shandong
Power Saving King Shop	King of Power Saving	I purposely used it for two days before commenting, which is responsible for myself and everyone else!	Jingdong prefecture level city in Shandong
Power Saving King Shop	King of Power Saving	Easy to install, easy to operate, helped our family save a lot of electricity this summer	Jingdong prefecture level city in Shandong
Power Saving King Shop	King of Power Saving	Bringing power savings into millions of homes. Still a great gadget. Pretty good.	Jingdong prefecture level city in Shandong
Power Saving King Shop	King of Power Saving	Digital liquid crystal display to see the clear intuitive, very formal workmanship is not rough	Jingdong prefecture level city in Shandong
Power Saving King Shop	King of Power Saving	Received. Great quality, fast shipping from the store and not too expensive!	Jingdong prefecture level city in Shandong
Power Saving King Shop	King of Power Saving	Bought myself a knuckle regulator and it was easy to use and the price was right.	Jingdong prefecture level city in Shandong
Power Saving King Shop	King of Power Saving	Received, good, powerful, good, very full of a shopping	Jingdong prefecture level city in Shandong
Power Saving King Shop	King of Power Saving	High power, consistent with the seller's description, powerful, cost-effective	Jingdong prefecture level city in Shandong
Power Saving King Shop	King of Power Saving	It works very well and also saves power at an affordable price a great deal.	Jingdong prefecture level city in Shandong
Power Saving King Shop	King of Power Saving	Good quality, good can recommend to friends to buy.	Jingdong prefecture level city in Shandong
Power Saving King Shop	King of Power Saving	Will repurchase, quality is guaranteed, safe and secure purchase.	Jingdong prefecture level city in Shandong
Power Saving King Shop	King of Power Saving	The logistics is very fast, the thing is also good to use, energy saving power rely on it!	Jingdong prefecture level city in Shandong
Power Saving King Shop	King of Power Saving	Power Saver is good quality and works very well, worth buying!	Jingdong prefecture level city in Shandong
Power Saving King Shop	King of Power Saving	The quality is very good is genuine, cost-effective recommended	Jingdong prefecture level city in Shandong
Power Saving King Shop	King of Power Saving	Quality Assurance. Favorable price. Very practical and durable	Jingdong prefecture level city in Shandong
Power Saving King Shop	King of Power Saving	Good value for money. Conscientious businessman. Recommended to everyone	Jingdong prefecture level city in Shandong
Power Saving King Shop	King of Power Saving	Buy it for your rental apartment and never worry about your landlord charging you too much.	Jingdong prefecture level city in Shandong
Power Saving King Shop	King of Power Saving	Nice and affordable, will repurchase next time	Jingdong prefecture level city in Shandong
Power Saving King Shop	King of Power Saving	It's very energy efficient. I've been using it for a few days now.	Jingdong prefecture level city in Shandong

Power Saving King Shop	King of Power Saving	Good quality, high power easy to use	Jingdong prefecture level city in Shandong
Power Saving King Shop	King of Power Saving	Power saving little helper. Deserves a favorable review.	Jingdong prefecture level city in Shandong
Power Saving King Shop	King of Power Saving	Great value for money, great value for money.	Jingdong prefecture level city in Shandong
Power Saving King Shop	King of Power Saving	Works well and as described by the seller	Jingdong prefecture level city in Shandong
Power Saving King Shop	King of Power Saving	Power saving is proper! It's good.	Jingdong prefecture level city in Shandong
Power Saving King Shop	King of Power Saving	Power saving. Works well. Not bad.	Jingdong prefecture level city in Shandong
Power Saving King Shop	King of Power Saving	Satisfied with power saving	Jingdong prefecture level city in Shandong
Power Saving King Shop	King of Power Saving	Received intact, open a super good look beautiful and generous, ~ bit of high quality is recommended to buy, the need for friends rest assured that the next order	Jingdong prefecture level city in Shandong
Power Saving King Shop	King of Power Saving	Very good to use, friends introduced, save electricity and money, long-lasting products cost-effective, large factory production, good quality, fine workmanship cost-effective!	Jingdong prefecture level city in Shandong
Power Saving King Shop	King of Power Saving	The power saving effect is very obvious, trying to pay 30% less electricity bill in a month than before, hahaha	Jingdong prefecture level city in Shandong
Power Saving King Shop	King of Power Saving	It's such a power saver to use this, it saves money on kids snacks for a month. Kudos!	Jingdong prefecture level city in Shandong
Power Saving King Shop	King of Power Saving	Plug and play, stable voltage, the screen has a display when working, this winter home appliances, need more electricity, just buy to use, save electricity and save money, with instructions, not too big and does not take up space, the quality is very good, and the seller describes exactly the same!	Jingdong prefecture level city in Shandong
Power Saving King Shop	King of Power Saving	A must have for opening a store. Save over a thousand dollars a month on your electric bill	Jingdong prefecture level city in Shandong
Power Saving King Shop	King of Power Saving	Received the goods, the first time to unpack, the quality is very good, with the seller's description, completely exceeded my expectations, the packaging is very careful, very strict, in general this is a very satisfied with the shopping	Jingdong prefecture level city in Shandong
Power Saving King Shop	King of Power Saving	As always, good quality, a very favorite product, great! o(*￣︶￣*)o The price is very affordable, cost-effective, it's considered affordable good, will repurchase!	Jingdong prefecture level city in Shandong
Power Saving King Shop	King of Power Saving	Very satisfied with a shopping, good business service, fast shipping, logistics is also very powerful, received very good, buy assured, use peace of mind!	Jingdong prefecture level city in Shandong
Power Saving King Shop	King of Power Saving	The first time to buy online, but also look at the evaluation before buying, the quality is not bad, but also very cheap, the need for friends can rest assured to buy!	Jingdong prefecture level city in Shandong
Power Saving King Shop	King of Power Saving	Better than expected, feel value for money, very satisfied with a shopping, need next time will come back!	Jingdong prefecture level city in Shandong
Power Saving King Shop	King of Power Saving	Really super like, very supportive, very good quality, exactly as described by the seller, very satisfied!	Jingdong prefecture level city in Shandong
Power Saving King Shop	King of Power Saving	It is genuine, effective, really saves power, affordable, tightly packed, fast shipping, very satisfied.	Jingdong prefecture level city in Shandong
Power Saving King Shop	King of Power Saving	Good quality, fine workmanship, is genuine and business description of the same, good value for money, fast logistics, very satisfied	Jingdong prefecture level city in Shandong
Power Saving King Shop	King of Power Saving	Saver received, very good, installation set up very well, very good, the effect is very good, recommend that the pro buy	Jingdong prefecture level city in Shandong
Power Saving King Shop	King of Power Saving	Electromagnetic good, high power convenient, enough force, the price is right it, quality can be 😃 😃 😃 😃 😃	Jingdong prefecture level city in Shandong
Power Saving King Shop	King of Power Saving	First-class quality, packaging is also good, logistics is very fast, and the price is reasonable, very affordable, I like it!	Jingdong prefecture level city in Shandong
Power Saving King Shop	King of Power Saving	It's great, it's the kind of style you want, and it arrived quickly, thank you boss, 5 stars for the service!	Jingdong prefecture level city in Shandong
Power Saving King Shop	King of Power Saving	Packaging is very good, the use of the effect is quite good, the store has a good service attitude, trustworthy	Jingdong prefecture level city in Shandong
Power Saving King Shop	King of Power Saving	The quality is very good, it works well, very satisfied, the price is affordable, it's so great value, it's worth recommending.	Jingdong prefecture level city in Shandong
Power Saving King Shop	King of Power Saving	The baby description matches the actual item very well! The quality is awesome! Will be back to patronize again in the future!	Jingdong prefecture level city in Shandong
Power Saving King Shop	King of Power Saving	Ran the price is not high try to buy the attitude, did not expect the effect is really good, good!	Jingdong prefecture level city in Shandong
Power Saving King Shop	King of Power Saving	Power Saving King power is quite large, the quality of the leaf is quite good, the price of buying a very good deal, must be praised!	Jingdong prefecture level city in Shandong
Power Saving King Shop	King of Power Saving	Good product quality is the king of power-saving products trustworthy as always good to recommend!	Jingdong prefecture level city in Shandong
Power Saving King Shop	King of Power Saving	Works great, very affordable, shipped pretty fast, comfortable to use	Jingdong prefecture level city in Shandong
Power Saving King Shop	King of Power Saving	Shipping was pretty fast and affordable, not bad 👍	Jingdong prefecture level city in Shandong
Power Saving King Shop	King of Power Saving	Digital voltage display, voltage at a glance, plug and play, safe and simple!	Jingdong prefecture level city in Shandong
Power Saving King Shop	King of Power Saving	Quality assurance, very good, affordable, logistics is also very fast, I'm very satisfied with the	Jingdong prefecture level city in Shandong
Power Saving King Shop	King of Power Saving	The quality is very good, the first time I received the goods to open the first time to see, it is very good, very satisfied	Jingdong prefecture level city in Shandong
Power Saving King Shop	King of Power Saving	Power Saving King used, the effect is good, the price is affordable, very good, is the genuine article	Jingdong prefecture level city in Shandong
Power Saving King Shop	King of Power Saving	Good quality, fine workmanship, good appearance, time is very accurate, satisfied.	Jingdong prefecture level city in Shandong
Power Saving King Shop	King of Power Saving	I purposely used it for two days before commenting, which is responsible for myself and everyone else!	Jingdong prefecture level city in Shandong
Power Saving King Shop	King of Power Saving	Easy to install, easy to operate, helped our family save a lot of electricity this summer	Jingdong prefecture level city in Shandong
Power Saving King Shop	King of Power Saving	Bringing power savings into millions of homes. Still a great gadget. Pretty good.	Jingdong prefecture level city in Shandong
Power Saving King Shop	King of Power Saving	Digital liquid crystal display to see the clear intuitive, very formal workmanship is not rough	Jingdong prefecture level city in Shandong
Power Saving King Shop	King of Power Saving	Received. Great quality, fast shipping from the store and not too expensive!	Jingdong prefecture level city in Shandong
Power Saving King Shop	King of Power Saving	Bought myself a knuckle regulator and it was easy to use and the price was right.	Jingdong prefecture level city in Shandong
Power Saving King Shop	King of Power Saving	Received, good, powerful, good, very full of a shopping	Jingdong prefecture level city in Shandong
Power Saving King Shop	King of Power Saving	High power, consistent with the seller's description, powerful, cost-effective	Jingdong prefecture level city in Shandong
Power Saving King Shop	King of Power Saving	It works very well and also saves power at an affordable price a great deal.	Jingdong prefecture level city in Shandong
Power Saving King Shop	King of Power Saving	Good quality, good can recommend to friends to buy.	Jingdong prefecture level city in Shandong
Power Saving King Shop	King of Power Saving	Will repurchase, quality is guaranteed, safe and secure purchase.	Jingdong prefecture level city in Shandong
Power Saving King Shop	King of Power Saving	The logistics is very fast, the thing is also good to use, energy saving power rely on it!	Jingdong prefecture level city in Shandong

Power Saving King Shop	King of Power Saving	Power Saver is good quality and works very well, worth buying!	Jingdong prefecture level city in Shandong
Power Saving King Shop	King of Power Saving	The quality is very good is genuine, cost-effective recommended	Jingdong prefecture level city in Shandong
Power Saving King Shop	King of Power Saving	Quality Assurance. Favorable price. Very practical and durable	Jingdong prefecture level city in Shandong
Power Saving King Shop	King of Power Saving	Good value for money. Conscientious businessman. Recommended to everyone	Jingdong prefecture level city in Shandong
Power Saving King Shop	King of Power Saving	Buy it for your rental apartment and never worry about your landlord charging you too much.	Jingdong prefecture level city in Shandong
Power Saving King Shop	King of Power Saving	Nice and affordable, will repurchase next time	Jingdong prefecture level city in Shandong
Power Saving King Shop	King of Power Saving	It's very energy efficient. I've been using it for a few days now.	Jingdong prefecture level city in Shandong
Power Saving King Shop	King of Power Saving	Good quality, high power easy to use	Jingdong prefecture level city in Shandong
Power Saving King Shop	King of Power Saving	Power saving little helper. Deserves a favorable review.	Jingdong prefecture level city in Shandong
Power Saving King Shop	King of Power Saving	Great value for money, great value for money.	Jingdong prefecture level city in Shandong
Power Saving King Shop	King of Power Saving	Works well and as described by the seller	Jingdong prefecture level city in Shandong
Power Saving King Shop	King of Power Saving	Power saving is proper! It's good.	Jingdong prefecture level city in Shandong
Power Saving King Shop	King of Power Saving	Power saving. Works well. Not bad.	Jingdong prefecture level city in Shandong
Power Saving King Shop	King of Power Saving	Satisfied with power saving	Jingdong prefecture level city in Shandong
Power Saving King Shop	King of Power Saving	Received intact, open a super good look beautiful and generous, ~ bit of high quality is recommended to buy, the need for friends rest assured that the next order	Jingdong prefecture level city in Shandong
Power Saving King Shop	King of Power Saving	Very good to use, friends introduced, save electricity and money, long-lasting products cost-effective, large factory production, good quality, fine workmanship cost-effective!	Jingdong prefecture level city in Shandong
Power Saving King Shop	King of Power Saving	The power saving effect is very obvious, trying to pay 30% less electricity bill in a month than before, hahaha	Jingdong prefecture level city in Shandong
Power Saving King Shop	King of Power Saving	It's such a power saver to use this, it saves money on kids snacks for a month. Kudos!	Jingdong prefecture level city in Shandong
Power Saving King Shop	King of Power Saving	Plug and play, stable voltage, the screen has a display when working, this winter home appliances, need more electricity, just buy to use, save electricity and save money, with instructions, not too big and does not take up space, the quality is very good, and the seller describes exactly the same!	Jingdong prefecture level city in Shandong
Power Saving King Shop	King of Power Saving	A must have for opening a store. Save over a thousand dollars a month on your electric bill	Jingdong prefecture level city in Shandong
Power Saving King Shop	King of Power Saving	Received the goods, the first time to unpack, the quality is very good, with the seller's description, completely exceeded my expectations, the packaging is very careful, very strict, in general this is a very satisfied with the shopping	Jingdong prefecture level city in Shandong
Power Saving King Shop	King of Power Saving	As always, good quality, a very favorite product, great! o(*￣︶￣*)o The price is very affordable, cost-effective, it's considered affordable good, will repurchase!	Jingdong prefecture level city in Shandong
Power Saving King Shop	King of Power Saving	Very satisfied with a shopping, good business service, fast shipping, logistics is also very powerful, received very good, buy assured, use peace of mind!	Jingdong prefecture level city in Shandong
Power Saving King Shop	King of Power Saving	The first time to buy online, but also look at the evaluation before buying, the quality is not bad, but also very cheap, the need for friends can rest assured to buy!	Jingdong prefecture level city in Shandong
Power Saving King Shop	King of Power Saving	Better than expected, feel value for money, very satisfied with a shopping, need next time will come back!	Jingdong prefecture level city in Shandong
Power Saving King Shop	King of Power Saving	Really super like, very supportive, very good quality, exactly as described by the seller, very satisfied!	Jingdong prefecture level city in Shandong
Power Saving King Shop	King of Power Saving	It is genuine, effective, really saves power, affordable, tightly packed, fast shipping, very satisfied.	Jingdong prefecture level city in Shandong
Power Saving King Shop	King of Power Saving	Good quality, fine workmanship, is genuine and business description of the same, good value for money, fast logistics, very satisfied	Jingdong prefecture level city in Shandong
Power Saving King Shop	King of Power Saving	Saver received, very good, installation set up very well, very good, the effect is very good, recommend that the pro buy	Jingdong prefecture level city in Shandong
Power Saving King Shop	King of Power Saving	Electromagnetic good, high power convenient, enough force, the price is right it, quality can be 😀 😀 😀 😀 😀	Jingdong prefecture level city in Shandong
Power Saving King Shop	King of Power Saving	First-class quality, packaging is also good, logistics is very fast, and the price is reasonable, very affordable, I like it!	Jingdong prefecture level city in Shandong
Power Saving King Shop	King of Power Saving	It's great, it's the kind of style you want, and it arrived quickly, thank you boss, 5 stars for the service!	Jingdong prefecture level city in Shandong
Power Saving King Shop	King of Power Saving	Packaging is very good, the use of the effect is quite good, the store has a good service attitude, trustworthy	Jingdong prefecture level city in Shandong
Power Saving King Shop	King of Power Saving	The quality is very good, it works well, very satisfied, the price is affordable, it's so great value, it's worth recommending.	Jingdong prefecture level city in Shandong
Power Saving King Shop	King of Power Saving	The baby description matches the actual item very well! The quality is awesome! Will be back to patronize again in the future!	Jingdong prefecture level city in Shandong
Power Saving King Shop	King of Power Saving	Ran the price is not high try to buy the attitude, did not expect the effect is really good, good!	Jingdong prefecture level city in Shandong
Power Saving King Shop	King of Power Saving	Power Saving King power is quite large, the quality of the leaf is quite good, the price of buying a very good deal, must be praised!	Jingdong prefecture level city in Shandong
Power Saving King Shop	King of Power Saving	Good product quality is the king of power-saving products trustworthy as always good to recommend!	Jingdong prefecture level city in Shandong
Power Saving King Shop	King of Power Saving	Works great, very affordable, shipped pretty fast, comfortable to use	Jingdong prefecture level city in Shandong
Power Saving King Shop	King of Power Saving	Shipping was pretty fast and affordable, not bad 👍	Jingdong prefecture level city in Shandong
Power Saving King Shop	King of Power Saving	Digital voltage display, voltage at a glance, plug and play, safe and simple!	Jingdong prefecture level city in Shandong
Power Saving King Shop	King of Power Saving	Quality assurance, very good, affordable, logistics is also very fast, I'm very satisfied with the	Jingdong prefecture level city in Shandong
Power Saving King Shop	King of Power Saving	The quality is very good, the first time I received the goods to open the first time to see, it is very good, very satisfied	Jingdong prefecture level city in Shandong
Power Saving King Shop	King of Power Saving	Power Saving King used, the effect is good, the price is affordable, very good, is the genuine article	Jingdong prefecture level city in Shandong
Power Saving King Shop	King of Power Saving	Good quality, fine workmanship, good appearance, time is very accurate, satisfied.	Jingdong prefecture level city in Shandong
Power Saving King Shop	King of Power Saving	I purposely used it for two days before commenting, which is responsible for myself and everyone else!	Jingdong prefecture level city in Shandong
Power Saving King Shop	King of Power Saving	Easy to install, easy to operate, helped our family save a lot of electricity this summer	Jingdong prefecture level city in Shandong
Power Saving King Shop	King of Power Saving	Bringing power savings into millions of homes. Still a great gadget. Pretty good.	Jingdong prefecture level city in Shandong
Power Saving King Shop	King of Power Saving	Digital liquid crystal display to see the clear intuitive, very formal workmanship is not rough	Jingdong prefecture level city in Shandong
Power Saving King Shop	King of Power Saving	Received. Great quality, fast shipping from the store and not too expensive!	Jingdong prefecture level city in Shandong

Power Saving King Shop	King of Power Saving	Bought myself a knuckle regulator and it was easy to use and the price was right.	Jingdong prefecture level city in Shandong
Power Saving King Shop	King of Power Saving	Received, good, powerful, good, very full of a shopping	Jingdong prefecture level city in Shandong
Power Saving King Shop	King of Power Saving	High power, consistent with the seller's description, powerful, cost-effective	Jingdong prefecture level city in Shandong
Power Saving King Shop	King of Power Saving	It works very well and also saves power at an affordable price a great deal.	Jingdong prefecture level city in Shandong
Power Saving King Shop	King of Power Saving	Good quality, good can recommend to friends to buy.	Jingdong prefecture level city in Shandong
Power Saving King Shop	King of Power Saving	Will repurchase, quality is guaranteed, safe and secure purchase.	Jingdong prefecture level city in Shandong
Power Saving King Shop	King of Power Saving	The logistics is very fast, the thing is also good to use, energy saving power rely on it!	Jingdong prefecture level city in Shandong
Power Saving King Shop	King of Power Saving	Power Saver is good quality and works very well, worth buying!	Jingdong prefecture level city in Shandong
Power Saving King Shop	King of Power Saving	The quality is very good is genuine, cost-effective recommended	Jingdong prefecture level city in Shandong
Power Saving King Shop	King of Power Saving	Quality Assurance. Favorable price. Very practical and durable	Jingdong prefecture level city in Shandong
Power Saving King Shop	King of Power Saving	Good value for money. Conscientious businessman. Recommended to everyone	Jingdong prefecture level city in Shandong
Power Saving King Shop	King of Power Saving	Buy it for your rental apartment and never worry about your landlord charging you too much.	Jingdong prefecture level city in Shandong
Power Saving King Shop	King of Power Saving	Nice and affordable, will repurchase next time	Jingdong prefecture level city in Shandong
Power Saving King Shop	King of Power Saving	It's very energy efficient. I've been using it for a few days now.	Jingdong prefecture level city in Shandong
Power Saving King Shop	King of Power Saving	Good quality, high power easy to use	Jingdong prefecture level city in Shandong
Power Saving King Shop	King of Power Saving	Power saving little helper. Deserves a favorable review.	Jingdong prefecture level city in Shandong
Power Saving King Shop	King of Power Saving	Great value for money, great value for money.	Jingdong prefecture level city in Shandong
Power Saving King Shop	King of Power Saving	Works well and as described by the seller	Jingdong prefecture level city in Shandong
Power Saving King Shop	King of Power Saving	Power saving is proper! It's good.	Jingdong prefecture level city in Shandong
Power Saving King Shop	King of Power Saving	Power saving. Works well. Not bad.	Jingdong prefecture level city in Shandong
Power Saving King Shop	King of Power Saving	Satisfied with power saving	Jingdong prefecture level city in Shandong
Power Saving King Shop	King of Power Saving	Received intact, open a super good look beautiful and generous, ~ bit of high quality is recommended to buy, the need for friends rest assured that the next order	Jingdong prefecture level city in Shandong
Power Saving King Shop	King of Power Saving	Very good to use, friends introduced, save electricity and money, long-lasting products cost-effective, large factory production, good quality, fine workmanship cost-effective!	Jingdong prefecture level city in Shandong
Power Saving King Shop	King of Power Saving	The power saving effect is very obvious, trying to pay 30% less electricity bill in a month than before, hahaha	Jingdong prefecture level city in Shandong
Power Saving King Shop	King of Power Saving	It's such a power saver to use this, it saves money on kids snacks for a month. Kudos!	Jingdong prefecture level city in Shandong
Power Saving King Shop	King of Power Saving	Plug and play, stable voltage, the screen has a display when working, this winter home appliances, need more electricity, just buy to use, save electricity and save money, with instructions, not too big and does not take up space, the quality is very good, and the seller describes exactly the same!	Jingdong prefecture level city in Shandong
Power Saving King Shop	King of Power Saving	A must have for opening a store. Save over a thousand dollars a month on your electric bill	Jingdong prefecture level city in Shandong
Power Saving King Shop	King of Power Saving	Received the goods, the first time to unpack, the quality is very good, with the seller's description, completely exceeded my expectations, the packaging is very careful, very strict, in general this is a very satisfied with the shopping	Jingdong prefecture level city in Shandong
Power Saving King Shop	King of Power Saving	As always, good quality, a very favorite product, great! o(*￣︶￣*)o The price is very affordable, cost-effective, it's considered affordable good, will repurchase!	Jingdong prefecture level city in Shandong
Power Saving King Shop	King of Power Saving	Very satisfied with a shopping, good business service, fast shipping, logistics is also very powerful, received very good, buy assured, use peace of mind!	Jingdong prefecture level city in Shandong
Power Saving King Shop	King of Power Saving	The first time to buy online, but also look at the evaluation before buying, the quality is not bad, but also very cheap, the need for friends can rest assured to buy!	Jingdong prefecture level city in Shandong
Power Saving King Shop	King of Power Saving	Better than expected, feel value for money, very satisfied with a shopping, need next time will come back!	Jingdong prefecture level city in Shandong
Power Saving King Shop	King of Power Saving	Really super like, very supportive, very good quality, exactly as described by the seller, very satisfied!	Jingdong prefecture level city in Shandong
Power Saving King Shop	King of Power Saving	It is genuine, effective, really saves power, affordable, tightly packed, fast shipping, very satisfied.	Jingdong prefecture level city in Shandong
Power Saving King Shop	King of Power Saving	Good quality, fine workmanship, is genuine and business description of the same, good value for money, fast logistics, very satisfied	Jingdong prefecture level city in Shandong
Power Saving King Shop	King of Power Saving	Saver received, very good, installation set up very well, very good, the effect is very good, recommend that the pro buy	Jingdong prefecture level city in Shandong
Power Saving King Shop	King of Power Saving	Electromagnetic good, high power convenient, enough force, the price is right it, quality can be 😊 😊 😊 😊 😊	Jingdong prefecture level city in Shandong
Power Saving King Shop	King of Power Saving	First-class quality, packaging is also good, logistics is very fast, and the price is reasonable, very affordable, I like it!	Jingdong prefecture level city in Shandong
Power Saving King Shop	King of Power Saving	It's great, it's the kind of style you want, and it arrived quickly, thank you boss, 5 stars for the service!	Jingdong prefecture level city in Shandong
Power Saving King Shop	King of Power Saving	Packaging is very good, the use of the effect is quite good, the store has a good service attitude, trustworthy	Jingdong prefecture level city in Shandong
Power Saving King Shop	King of Power Saving	The quality is very good, it works well, very satisfied, the price is affordable, it's so great value, it's worth recommending.	Jingdong prefecture level city in Shandong
Power Saving King Shop	King of Power Saving	The baby description matches the actual item very well! The quality is awesome! Will be back to patronize again in the future!	Jingdong prefecture level city in Shandong
Power Saving King Shop	King of Power Saving	Ran the price is not high try to buy the attitude, did not expect the effect is really good, good!	Jingdong prefecture level city in Shandong
Power Saving King Shop	King of Power Saving	Power Saving King power is quite large, the quality of the leaf is quite good, the price of buying a very good deal, must be praised!	Jingdong prefecture level city in Shandong
Power Saving King Shop	King of Power Saving	Good product quality is the king of power-saving products trustworthy as always good to recommend!	Jingdong prefecture level city in Shandong
Power Saving King Shop	King of Power Saving	Works great, very affordable, shipped pretty fast, comfortable to use	Jingdong prefecture level city in Shandong
Power Saving King Shop	King of Power Saving	Shipping was pretty fast and affordable, not bad 👍	Jingdong prefecture level city in Shandong
Power Saving King Shop	King of Power Saving	Digital voltage display, voltage at a glance, plug and play, safe and simple!	Jingdong prefecture level city in Shandong
Power Saving King Shop	King of Power Saving	Quality assurance, very good, affordable, logistics is also very fast, I'm very satisfied with the	Jingdong prefecture level city in Shandong
Power Saving King Shop	King of Power Saving	The quality is very good, the first time I received the goods to open the first time to see, it is very good, very satisfied	Jingdong prefecture level city in Shandong

Power Saving King Shop	King of Power Saving	Power Saving King used, the effect is good, the price is affordable, very good, is the genuine article	Jingdong prefecture level city in Shandong
Power Saving King Shop	King of Power Saving	Good quality, fine workmanship, good appearance, time is very accurate, satisfied.	Jingdong prefecture level city in Shandong
Power Saving King Shop	King of Power Saving	I purposely used it for two days before commenting, which is responsible for myself and everyone else!	Jingdong prefecture level city in Shandong
Power Saving King Shop	King of Power Saving	Easy to install, easy to operate, helped our family save a lot of electricity this summer	Jingdong prefecture level city in Shandong
Power Saving King Shop	King of Power Saving	Bringing power savings into millions of homes. Still a great gadget. Pretty good.	Jingdong prefecture level city in Shandong
Power Saving King Shop	King of Power Saving	Digital liquid crystal display to see the clear intuitive, very formal workmanship is not rough	Jingdong prefecture level city in Shandong
Power Saving King Shop	King of Power Saving	Received. Great quality, fast shipping from the store and not too expensive!	Jingdong prefecture level city in Shandong
Power Saving King Shop	King of Power Saving	Bought myself a knuckle regulator and it was easy to use and the price was right.	Jingdong prefecture level city in Shandong
Power Saving King Shop	King of Power Saving	Received, good, powerful, good, very full of a shopping	Jingdong prefecture level city in Shandong
Power Saving King Shop	King of Power Saving	High power, consistent with the seller's description, powerful, cost-effective	Jingdong prefecture level city in Shandong
Power Saving King Shop	King of Power Saving	It works very well and also saves power at an affordable price a great deal.	Jingdong prefecture level city in Shandong
Power Saving King Shop	King of Power Saving	Good quality, good can recommend to friends to buy.	Jingdong prefecture level city in Shandong
Power Saving King Shop	King of Power Saving	Will repurchase, quality is guaranteed, safe and secure purchase.	Jingdong prefecture level city in Shandong
Power Saving King Shop	King of Power Saving	The logistics is very fast, the thing is also good to use, energy saving power rely on it!	Jingdong prefecture level city in Shandong
Power Saving King Shop	King of Power Saving	Power Saver is good quality and works very well, worth buying!	Jingdong prefecture level city in Shandong
Power Saving King Shop	King of Power Saving	The quality is very good is genuine, cost-effective recommended	Jingdong prefecture level city in Shandong
Power Saving King Shop	King of Power Saving	Quality Assurance. Favorable price. Very practical and durable	Jingdong prefecture level city in Shandong
Power Saving King Shop	King of Power Saving	Good value for money. Conscientious businessman. Recommended to everyone	Jingdong prefecture level city in Shandong
Power Saving King Shop	King of Power Saving	Buy it for your rental apartment and never worry about your landlord charging you too much.	Jingdong prefecture level city in Shandong
Power Saving King Shop	King of Power Saving	Nice and affordable, will repurchase next time	Jingdong prefecture level city in Shandong
Power Saving King Shop	King of Power Saving	It's very energy efficient. I've been using it for a few days now.	Jingdong prefecture level city in Shandong
Power Saving King Shop	King of Power Saving	Good quality, high power easy to use	Jingdong prefecture level city in Shandong
Power Saving King Shop	King of Power Saving	Power saving little helper. Deserves a favorable review.	Jingdong prefecture level city in Shandong
Power Saving King Shop	King of Power Saving	Great value for money, great value for money.	Jingdong prefecture level city in Shandong
Power Saving King Shop	King of Power Saving	Works well and as described by the seller	Jingdong prefecture level city in Shandong
Power Saving King Shop	King of Power Saving	Power saving is proper! It's good.	Jingdong prefecture level city in Shandong
Power Saving King Shop	King of Power Saving	Power saving. Works well. Not bad.	Jingdong prefecture level city in Shandong
Power Saving King Shop	King of Power Saving	Satisfied with power saving	Jingdong prefecture level city in Shandong
Power Saving King Shop	King of Power Saving	Received intact, open a super good look beautiful and generous, ~ bit of high quality is recommended to buy, the need for friends rest assured that the next order	Jingdong prefecture level city in Shandong
Power Saving King Shop	King of Power Saving	Very good to use, friends introduced, save electricity and money, long-lasting products cost-effective, large factory production, good quality, fine workmanship cost-effective!	Jingdong prefecture level city in Shandong
Power Saving King Shop	King of Power Saving	The power saving effect is very obvious, trying to pay 30% less electricity bill in a month than before, hahaha	Jingdong prefecture level city in Shandong
Power Saving King Shop	King of Power Saving	It's such a power saver to use this, it saves money on kids snacks for a month. Kudos!	Jingdong prefecture level city in Shandong
Power Saving King Shop	King of Power Saving	Plug and play, stable voltage, the screen has a display when working, this winter home appliances, need more electricity, just buy to use, save electricity and save money, with instructions, not too big and does not take up space, the quality is very good, and the seller describes exactly the same!	Jingdong prefecture level city in Shandong
Power Saving King Shop	King of Power Saving	A must have for opening a store. Save over a thousand dollars a month on your electric bill	Jingdong prefecture level city in Shandong
Power Saving King Shop	King of Power Saving	Received the goods, the first time to unpack, the quality is very good, with the seller's description, completely exceeded my expectations, the packaging is very careful, very strict, in general this is a very satisfied with the shopping	Jingdong prefecture level city in Shandong
Power Saving King Shop	King of Power Saving	As always, good quality, a very favorite product, great! o(*￣︶￣*)o The price is very affordable, cost-effective, it's considered affordable good, will repurchase!	Jingdong prefecture level city in Shandong
Power Saving King Shop	King of Power Saving	Very satisfied with a shopping, good business service, fast shipping, logistics is also very powerful, received very good, buy assured, use peace of mind!	Jingdong prefecture level city in Shandong
Power Saving King Shop	King of Power Saving	The first time to buy online, but also look at the evaluation before buying, the quality is not bad, but also very cheap, the need for friends can rest assured to buy!	Jingdong prefecture level city in Shandong
Power Saving King Shop	King of Power Saving	Better than expected, feel value for money, very satisfied with a shopping, need next time will come back!	Jingdong prefecture level city in Shandong
Power Saving King Shop	King of Power Saving	Really super like, very supportive, very good quality, exactly as described by the seller, very satisfied!	Jingdong prefecture level city in Shandong
Power Saving King Shop	King of Power Saving	It is genuine, effective, really saves power, affordable, tightly packed, fast shipping, very satisfied.	Jingdong prefecture level city in Shandong
Power Saving King Shop	King of Power Saving	Good quality, fine workmanship, is genuine and business description of the same, good value for money, fast logistics, very satisfied	Jingdong prefecture level city in Shandong
Power Saving King Shop	King of Power Saving	Saver received, very good, installation set up very well, very good, the effect is very good, recommend that the pro buy	Jingdong prefecture level city in Shandong
Power Saving King Shop	King of Power Saving	Electromagnetic good, high power convenient, enough force, the price is right it, quality can be ☺ ☺ ☺ ☺ ☺	Jingdong prefecture level city in Shandong
Power Saving King Shop	King of Power Saving	First-class quality, packaging is also good, logistics is very fast, and the price is reasonable, very affordable, I like it!	Jingdong prefecture level city in Shandong
Power Saving King Shop	King of Power Saving	It's great, it's the kind of style you want, and it arrived quickly, thank you boss, 5 stars for the service!	Jingdong prefecture level city in Shandong
Power Saving King Shop	King of Power Saving	Packaging is very good, the use of the effect is quite good, the store has a good service attitude, trustworthy	Jingdong prefecture level city in Shandong
Power Saving King Shop	King of Power Saving	The quality is very good, it works well, very satisfied, the price is affordable, it's so great value, it's worth recommending.	Jingdong prefecture level city in Shandong
Power Saving King Shop	King of Power Saving	The baby description matches the actual item very well! The quality is awesome! Will be back to patronize again in the future!	Jingdong prefecture level city in Shandong
Power Saving King Shop	King of Power Saving	Ran the price is not high try to buy the attitude, did not expect the effect is really good, good!	Jingdong prefecture level city in Shandong

Power Saving King Shop	King of Power Saving	Power Saving King power is quite large, the quality of the leaf is quite good, the price of buying a very good deal, must be praised!	Jingdong prefecture level city in Shandong
Power Saving King Shop	King of Power Saving	Good product quality is the king of power-saving products trustworthy as always good to recommend!	Jingdong prefecture level city in Shandong
Power Saving King Shop	King of Power Saving	Works great, very affordable, shipped pretty fast, comfortable to use	Jingdong prefecture level city in Shandong
Power Saving King Shop	King of Power Saving	Shipping was pretty fast and affordable, not bad	Jingdong prefecture level city in Shandong
Power Saving King Shop	King of Power Saving	Digital voltage display, voltage at a glance, plug and play, safe and simple!	Jingdong prefecture level city in Shandong
Power Saving King Shop	King of Power Saving	Quality assurance, very good, affordable, logistics is also very fast, I'm very satisfied with the	Jingdong prefecture level city in Shandong
Power Saving King Shop	King of Power Saving	The quality is very good, the first time I received the goods to open the first time to see, it is very good, very satisfied	Jingdong prefecture level city in Shandong
Power Saving King Shop	King of Power Saving	Power Saving King used, the effect is good, the price is affordable, very good, is the genuine article	Jingdong prefecture level city in Shandong
Power Saving King Shop	King of Power Saving	Good quality, fine workmanship, good appearance, time is very accurate, satisfied.	Jingdong prefecture level city in Shandong
Power Saving King Shop	King of Power Saving	I purposely used it for two days before commenting, which is responsible for myself and everyone else!	Jingdong prefecture level city in Shandong
Power Saving King Shop	King of Power Saving	Easy to install, easy to operate, helped our family save a lot of electricity this summer	Jingdong prefecture level city in Shandong
Power Saving King Shop	King of Power Saving	Bringing power savings into millions of homes. Still a great gadget. Pretty good.	Jingdong prefecture level city in Shandong
Power Saving King Shop	King of Power Saving	Digital liquid crystal display to see the clear intuitive, very formal workmanship is not rough	Jingdong prefecture level city in Shandong
Power Saving King Shop	King of Power Saving	Received. Great quality, fast shipping from the store and not too expensive!	Jingdong prefecture level city in Shandong
Power Saving King Shop	King of Power Saving	Bought myself a knuckle regulator and it was easy to use and the price was right.	Jingdong prefecture level city in Shandong
Power Saving King Shop	King of Power Saving	Received, good, powerful, good, very full of a shopping	Jingdong prefecture level city in Shandong
Power Saving King Shop	King of Power Saving	High power, consistent with the seller's description, powerful, cost-effective	Jingdong prefecture level city in Shandong
Power Saving King Shop	King of Power Saving	It works very well and also saves power at an affordable price a great deal.	Jingdong prefecture level city in Shandong
Power Saving King Shop	King of Power Saving	Good quality, good can recommend to friends to buy.	Jingdong prefecture level city in Shandong
Power Saving King Shop	King of Power Saving	Will repurchase, quality is guaranteed, safe and secure purchase.	Jingdong prefecture level city in Shandong
Power Saving King Shop	King of Power Saving	The logistics is very fast, the thing is also good to use, energy saving power rely on it!	Jingdong prefecture level city in Shandong
Power Saving King Shop	King of Power Saving	Power Saver is good quality and works very well, worth buying!	Jingdong prefecture level city in Shandong
Power Saving King Shop	King of Power Saving	The quality is very good is genuine, cost-effective recommended	Jingdong prefecture level city in Shandong
Power Saving King Shop	King of Power Saving	Quality Assurance. Favorable price. Very practical and durable	Jingdong prefecture level city in Shandong
Power Saving King Shop	King of Power Saving	Good value for money. Conscientious businessman. Recommended to everyone	Jingdong prefecture level city in Shandong
Power Saving King Shop	King of Power Saving	Buy it for your rental apartment and never worry about your landlord charging you too much.	Jingdong prefecture level city in Shandong
Power Saving King Shop	King of Power Saving	Nice and affordable, will repurchase next time	Jingdong prefecture level city in Shandong
Power Saving King Shop	King of Power Saving	It's very energy efficient. I've been using it for a few days now.	Jingdong prefecture level city in Shandong
Power Saving King Shop	King of Power Saving	Good quality, high power easy to use	Jingdong prefecture level city in Shandong
Power Saving King Shop	King of Power Saving	Power saving little helper. Deserves a favorable review.	Jingdong prefecture level city in Shandong
Power Saving King Shop	King of Power Saving	Great value for money, great value for money.	Jingdong prefecture level city in Shandong
Power Saving King Shop	King of Power Saving	Works well and as described by the seller	Jingdong prefecture level city in Shandong
Power Saving King Shop	King of Power Saving	Power saving is proper! It's good.	Jingdong prefecture level city in Shandong
Power Saving King Shop	King of Power Saving	Power saving. Works well. Not bad.	Jingdong prefecture level city in Shandong
Power Saving King Shop	King of Power Saving	Satisfied with power saving	Jingdong prefecture level city in Shandong
Power Saving King Shop	King of Power Saving	Received intact, open a super good look beautiful and generous, ~ bit of high quality is recommended to buy, the need for friends rest assured that the next order	Jingdong prefecture level city in Shandong
Power Saving King Shop	King of Power Saving	Very good to use, friends introduced, save electricity and money, long-lasting products cost-effective, large factory production, good quality, fine workmanship cost-effective!	Jingdong prefecture level city in Shandong
Power Saving King Shop	King of Power Saving	The power saving effect is very obvious, trying to pay 30% less electricity bill in a month than before, hahaha	Jingdong prefecture level city in Shandong
Power Saving King Shop	King of Power Saving	It's such a power saver to use this, it saves money on kids snacks for a month. Kudos!	Jingdong prefecture level city in Shandong
Power Saving King Shop	King of Power Saving	Plug and play, stable voltage, the screen has a display when working, this winter home appliances, need more electricity, just buy to use, save electricity and save money, with instructions, not too big and does not take up space, the quality is very good, and the seller describes exactly the same!	Jingdong prefecture level city in Shandong
Power Saving King Shop	King of Power Saving	A must have for opening a store. Save over a thousand dollars a month on your electric bill	Jingdong prefecture level city in Shandong
Power Saving King Shop	King of Power Saving	Received the goods, the first time to unpack, the quality is very good, with the seller's description, completely exceeded my expectations, the packaging is very careful, very strict, in general this is a very satisfied with the shopping	Jingdong prefecture level city in Shandong
Power Saving King Shop	King of Power Saving	As always, good quality, a very favorite product, great! o(*￣︶￣*)o The price is very affordable, cost-effective, it's considered affordable good, will repurchase!	Jingdong prefecture level city in Shandong
Power Saving King Shop	King of Power Saving	Very satisfied with a shopping, good business service, fast shipping, logistics is also very powerful, received very good, buy assured, use peace of mind!	Jingdong prefecture level city in Shandong
Power Saving King Shop	King of Power Saving	The first time to buy online, but also look at the evaluation before buying, the quality is not bad, but also very cheap, the need for friends can rest assured to buy!	Jingdong prefecture level city in Shandong
Power Saving King Shop	King of Power Saving	Better than expected, feel value for money, very satisfied with a shopping, need next time will come back!	Jingdong prefecture level city in Shandong
Power Saving King Shop	King of Power Saving	Really super like, very supportive, very good quality, exactly as described by the seller, very satisfied!	Jingdong prefecture level city in Shandong
Power Saving King Shop	King of Power Saving	It is genuine, effective, really saves power, affordable, tightly packed, fast shipping, very satisfied.	Jingdong prefecture level city in Shandong
Power Saving King Shop	King of Power Saving	Good quality, fine workmanship, is genuine and business description of the same, good value for money, fast logistics, very satisfied	Jingdong prefecture level city in Shandong
Power Saving King Shop	King of Power Saving	Saver received, very good, installation set up very well, very good, the effect is very good, recommend that the pro buy	Jingdong prefecture level city in Shandong

Power Saving King Shop	King of Power Saving	Electromagnetic good, high power convenient, enough force, the price is right it, quality can be 😀 😀 😀 😀 😀	Jingdong prefecture level city in Shandong
Power Saving King Shop	King of Power Saving	First-class quality, packaging is also good, logistics is very fast, and the price is reasonable, very affordable, I like it!	Jingdong prefecture level city in Shandong
Power Saving King Shop	King of Power Saving	It's great, it's the kind of style you want, and it arrived quickly, thank you boss, 5 stars for the service!	Jingdong prefecture level city in Shandong
Power Saving King Shop	King of Power Saving	Packaging is very good, the use of the effect is quite good, the store has a good service attitude, trustworthy	Jingdong prefecture level city in Shandong
Power Saving King Shop	King of Power Saving	The quality is very good, it works well, very satisfied, the price is affordable, it's so great value, it's worth recommending.	Jingdong prefecture level city in Shandong
Power Saving King Shop	King of Power Saving	The baby description matches the actual item very well! The quality is awesome! Will be back to patronize again in the future!	Jingdong prefecture level city in Shandong
Power Saving King Shop	King of Power Saving	Ran the price is not high try to buy the attitude, did not expect the effect is really good, good!	Jingdong prefecture level city in Shandong
Power Saving King Shop	King of Power Saving	Power Saving King power is quite large, the quality of the leaf is quite good, the price of buying a very good deal, must be praised!	Jingdong prefecture level city in Shandong
Power Saving King Shop	King of Power Saving	Good product quality is the king of power-saving products trustworthy as always good to recommend!	Jingdong prefecture level city in Shandong
Power Saving King Shop	King of Power Saving	Works great, very affordable, shipped pretty fast, comfortable to use	Jingdong prefecture level city in Shandong
Power Saving King Shop	King of Power Saving	Shipping was pretty fast and affordable, not bad 👍	Jingdong prefecture level city in Shandong
Power Saving King Shop	King of Power Saving	Digital voltage display, voltage at a glance, plug and play, safe and simple!	Jingdong prefecture level city in Shandong
Power Saving King Shop	King of Power Saving	Quality assurance, very good, affordable, logistics is also very fast, I'm very satisfied with the	Jingdong prefecture level city in Shandong
Power Saving King Shop	King of Power Saving	The quality is very good, the first time I received the goods to open the first time to see, it is very good, very satisfied	Jingdong prefecture level city in Shandong
Power Saving King Shop	King of Power Saving	Power Saving King used, the effect is good, the price is affordable, very good, is the genuine article	Jingdong prefecture level city in Shandong
Power Saving King Shop	King of Power Saving	Good quality, fine workmanship, good appearance, time is very accurate, satisfied.	Jingdong prefecture level city in Shandong
Power Saving King Shop	King of Power Saving	I purposely used it for two days before commenting, which is responsible for myself and everyone else!	Jingdong prefecture level city in Shandong
Power Saving King Shop	King of Power Saving	Easy to install, easy to operate, helped our family save a lot of electricity this summer	Jingdong prefecture level city in Shandong
Power Saving King Shop	King of Power Saving	Bringing power savings into millions of homes. Still a great gadget. Pretty good.	Jingdong prefecture level city in Shandong
Power Saving King Shop	King of Power Saving	Digital liquid crystal display to see the clear intuitive, very formal workmanship is not rough	Jingdong prefecture level city in Shandong
Power Saving King Shop	King of Power Saving	Received. Great quality, fast shipping from the store and not too expensive!	Jingdong prefecture level city in Shandong
Power Saving King Shop	King of Power Saving	Bought myself a knuckle regulator and it was easy to use and the price was right.	Jingdong prefecture level city in Shandong
Power Saving King Shop	King of Power Saving	Received, good, powerful, good, very full of a shopping	Jingdong prefecture level city in Shandong
Power Saving King Shop	King of Power Saving	High power, consistent with the seller's description, powerful, cost-effective	Jingdong prefecture level city in Shandong
Power Saving King Shop	King of Power Saving	It works very well and also saves power at an affordable price a great deal.	Jingdong prefecture level city in Shandong
Power Saving King Shop	King of Power Saving	Good quality, good can recommend to friends to buy.	Jingdong prefecture level city in Shandong
Power Saving King Shop	King of Power Saving	Will repurchase, quality is guaranteed, safe and secure purchase.	Jingdong prefecture level city in Shandong
Power Saving King Shop	King of Power Saving	The logistics is very fast, the thing is also good to use, energy saving power rely on it!	Jingdong prefecture level city in Shandong
Power Saving King Shop	King of Power Saving	Power Saver is good quality and works very well, worth buying!	Jingdong prefecture level city in Shandong
Power Saving King Shop	King of Power Saving	The quality is very good is genuine, cost-effective recommended	Jingdong prefecture level city in Shandong
Power Saving King Shop	King of Power Saving	Quality Assurance. Favorable price. Very practical and durable	Jingdong prefecture level city in Shandong
Power Saving King Shop	King of Power Saving	Good value for money. Conscientious businessman. Recommended to everyone	Jingdong prefecture level city in Shandong
Power Saving King Shop	King of Power Saving	Buy it for your rental apartment and never worry about your landlord charging you too much.	Jingdong prefecture level city in Shandong
Power Saving King Shop	King of Power Saving	Nice and affordable, will repurchase next time	Jingdong prefecture level city in Shandong
Power Saving King Shop	King of Power Saving	It's very energy efficient. I've been using it for a few days now.	Jingdong prefecture level city in Shandong
Power Saving King Shop	King of Power Saving	Good quality, high power easy to use	Jingdong prefecture level city in Shandong
Power Saving King Shop	King of Power Saving	Power saving little helper. Deserves a favorable review.	Jingdong prefecture level city in Shandong
Power Saving King Shop	King of Power Saving	Great value for money, great value for money.	Jingdong prefecture level city in Shandong
Power Saving King Shop	King of Power Saving	Works well and as described by the seller	Jingdong prefecture level city in Shandong
Power Saving King Shop	King of Power Saving	Power saving is proper! It's good.	Jingdong prefecture level city in Shandong
Power Saving King Shop	King of Power Saving	Power saving. Works well. Not bad.	Jingdong prefecture level city in Shandong
Power Saving King Shop	King of Power Saving	Satisfied with power saving	Jingdong prefecture level city in Shandong
Power Saving King Shop	King of Power Saving	Received intact, open a super good look beautiful and generous, ~ bit of high quality is recommended to buy, the need for friends rest assured that the next order	Jingdong prefecture level city in Shandong
Power Saving King Shop	King of Power Saving	Very good to use, friends introduced, save electricity and money, long-lasting products cost-effective, large factory production, good quality, fine workmanship cost-effective!	Jingdong prefecture level city in Shandong
Power Saving King Shop	King of Power Saving	The power saving effect is very obvious, trying to pay 30% less electricity bill in a month than before, hahaha	Jingdong prefecture level city in Shandong
Power Saving King Shop	King of Power Saving	It's such a power saver to use this, it saves money on kids snacks for a month. Kudos!	Jingdong prefecture level city in Shandong
Power Saving King Shop	King of Power Saving	Plug and play, stable voltage, the screen has a display when working, this winter home appliances, need more electricity, just buy to use, save electricity and save money, with instructions, not too big and does not take up space, the quality is very good, and the seller describes exactly the same!	Jingdong prefecture level city in Shandong
Power Saving King Shop	King of Power Saving	A must have for opening a store. Save over a thousand dollars a month on your electric bill	Jingdong prefecture level city in Shandong
Power Saving King Shop	King of Power Saving	Received the goods, the first time to unpack, the quality is very good, with the seller's description, completely exceeded my expectations, the packaging is very careful, very strict, in general this is a very satisfied with the shopping	Jingdong prefecture level city in Shandong
Power Saving King Shop	King of Power Saving	As always, good quality, a very favorite product, great! o(*￣︶￣*)o The price is very affordable, cost-effective, it's considered affordable good, will repurchase!	Jingdong prefecture level city in Shandong

Power Saving King Shop	King of Power Saving	Very satisfied with a shopping, good business service, fast shipping, logistics is also very powerful, received very good, buy assured, use peace of mind!	Jingdong prefecture level city in Shandong
Power Saving King Shop	King of Power Saving	The first time to buy online, but also look at the evaluation before buying, the quality is not bad, but also very cheap, the need for friends can rest assured to buy!	Jingdong prefecture level city in Shandong
Power Saving King Shop	King of Power Saving	Better than expected, feel value for money, very satisfied with a shopping, need next time will come back!	Jingdong prefecture level city in Shandong
Power Saving King Shop	King of Power Saving	Really super like, very supportive, very good quality, exactly as described by the seller, very satisfied!	Jingdong prefecture level city in Shandong
Power Saving King Shop	King of Power Saving	It is genuine, effective, really saves power, affordable, tightly packed, fast shipping, very satisfied.	Jingdong prefecture level city in Shandong
Power Saving King Shop	King of Power Saving	Good quality, fine workmanship, is genuine and business description of the same, good value for money, fast logistics, very satisfied	Jingdong prefecture level city in Shandong
Power Saving King Shop	King of Power Saving	Saver received, very good, installation set up very well, very good, the effect is very good, recommend that the pro buy	Jingdong prefecture level city in Shandong
Power Saving King Shop	King of Power Saving	Electromagnetic good, high power convenient, enough force, the price is right it, quality can be 😃 😃 😃 😃 😃	Jingdong prefecture level city in Shandong
Power Saving King Shop	King of Power Saving	First-class quality, packaging is also good, logistics is very fast, and the price is reasonable, very affordable, I like it!	Jingdong prefecture level city in Shandong
Power Saving King Shop	King of Power Saving	It's great, it's the kind of style you want, and it arrived quickly, thank you boss, 5 stars for the service!	Jingdong prefecture level city in Shandong
Power Saving King Shop	King of Power Saving	Packaging is very good, the use of the effect is quite good, the store has a good service attitude, trustworthy	Jingdong prefecture level city in Shandong
Power Saving King Shop	King of Power Saving	The quality is very good, it works well, very satisfied, the price is affordable, it's so great value, it's worth recommending.	Jingdong prefecture level city in Shandong
Power Saving King Shop	King of Power Saving	The baby description matches the actual item very well! The quality is awesome! Will be back to patronize again in the future!	Jingdong prefecture level city in Shandong
Power Saving King Shop	King of Power Saving	Ran the price is not high try to buy the attitude, did not expect the effect is really good, good!	Jingdong prefecture level city in Shandong
Power Saving King Shop	King of Power Saving	Power Saving King power is quite large, the quality of the leaf is quite good, the price of buying a very good deal, must be praised!	Jingdong prefecture level city in Shandong
Power Saving King Shop	King of Power Saving	Good product quality is the king of power-saving products trustworthy as always good to recommend!	Jingdong prefecture level city in Shandong
Power Saving King Shop	King of Power Saving	Works great, very affordable, shipped pretty fast, comfortable to use	Jingdong prefecture level city in Shandong
Power Saving King Shop	King of Power Saving	Shipping was pretty fast and affordable, not bad 👍	Jingdong prefecture level city in Shandong
Power Saving King Shop	King of Power Saving	Digital voltage display, voltage at a glance, plug and play, safe and simple!	Jingdong prefecture level city in Shandong
Power Saving King Shop	King of Power Saving	Quality assurance, very good, affordable, logistics is also very fast, I'm very satisfied with the	Jingdong prefecture level city in Shandong
Power Saving King Shop	King of Power Saving	The quality is very good, the first time I received the goods to open the first time to see, it is very good, very satisfied	Jingdong prefecture level city in Shandong
Power Saving King Shop	King of Power Saving	Power Saving King used, the effect is good, the price is affordable, very good, is the genuine article	Jingdong prefecture level city in Shandong
Power Saving King Shop	King of Power Saving	Good quality, fine workmanship, good appearance, time is very accurate, satisfied.	Jingdong prefecture level city in Shandong
Power Saving King Shop	King of Power Saving	I purposely used it for two days before commenting, which is responsible for myself and everyone else!	Jingdong prefecture level city in Shandong
Power Saving King Shop	King of Power Saving	Easy to install, easy to operate, helped our family save a lot of electricity this summer	Jingdong prefecture level city in Shandong
Power Saving King Shop	King of Power Saving	Bringing power savings into millions of homes. Still a great gadget. Pretty good.	Jingdong prefecture level city in Shandong
Power Saving King Shop	King of Power Saving	Digital liquid crystal display to see the clear intuitive, very formal workmanship is not rough	Jingdong prefecture level city in Shandong
Power Saving King Shop	King of Power Saving	Received. Great quality, fast shipping from the store and not too expensive!	Jingdong prefecture level city in Shandong
Power Saving King Shop	King of Power Saving	Bought myself a knuckle regulator and it was easy to use and the price was right.	Jingdong prefecture level city in Shandong
Power Saving King Shop	King of Power Saving	Received, good, powerful, good, very full of a shopping	Jingdong prefecture level city in Shandong
Power Saving King Shop	King of Power Saving	High power, consistent with the seller's description, powerful, cost-effective	Jingdong prefecture level city in Shandong
Power Saving King Shop	King of Power Saving	It works very well and also saves power at an affordable price a great deal.	Jingdong prefecture level city in Shandong
Power Saving King Shop	King of Power Saving	Good quality, good can recommend to friends to buy.	Jingdong prefecture level city in Shandong
Power Saving King Shop	King of Power Saving	Will repurchase, quality is guaranteed, safe and secure purchase.	Jingdong prefecture level city in Shandong
Power Saving King Shop	King of Power Saving	The logistics is very fast, the thing is also good to use, energy saving power rely on it!	Jingdong prefecture level city in Shandong
Power Saving King Shop	King of Power Saving	Power Saver is good quality and works very well, worth buying!	Jingdong prefecture level city in Shandong
Power Saving King Shop	King of Power Saving	The quality is very good is genuine, cost-effective recommended	Jingdong prefecture level city in Shandong
Power Saving King Shop	King of Power Saving	Quality Assurance. Favorable price. Very practical and durable	Jingdong prefecture level city in Shandong
Power Saving King Shop	King of Power Saving	Good value for money. Conscientious businessman. Recommended to everyone	Jingdong prefecture level city in Shandong
Power Saving King Shop	King of Power Saving	Buy it for your rental apartment and never worry about your landlord charging you too much.	Jingdong prefecture level city in Shandong
Power Saving King Shop	King of Power Saving	Nice and affordable, will repurchase next time	Jingdong prefecture level city in Shandong
Power Saving King Shop	King of Power Saving	It's very energy efficient. I've been using it for a few days now.	Jingdong prefecture level city in Shandong
Power Saving King Shop	King of Power Saving	Good quality, high power easy to use	Jingdong prefecture level city in Shandong
Power Saving King Shop	King of Power Saving	Power saving little helper. Deserves a favorable review.	Jingdong prefecture level city in Shandong
Power Saving King Shop	King of Power Saving	Great value for money, great value for money.	Jingdong prefecture level city in Shandong
Power Saving King Shop	King of Power Saving	Works well and as described by the seller	Jingdong prefecture level city in Shandong
Power Saving King Shop	King of Power Saving	Power saving is proper! It's good.	Jingdong prefecture level city in Shandong
Power Saving King Shop	King of Power Saving	Power saving. Works well. Not bad.	Jingdong prefecture level city in Shandong
Power Saving King Shop	King of Power Saving	Satisfied with power saving	Jingdong prefecture level city in Shandong
Power Saving King Shop	King of Power Saving	Received intact, open a super good look beautiful and generous, ~ bit of high quality is recommended to buy, the need for friends rest assured that the next order	Jingdong prefecture level city in Shandong
Power Saving King Shop	King of Power Saving	Very good to use, friends introduced, save electricity and money, long-lasting products cost-effective, large factory production, good quality, fine workmanship cost-effective!	Jingdong prefecture level city in Shandong

Power Saving King Shop	King of Power Saving	The power saving effect is very obvious, trying to pay 30% less electricity bill in a month than before, hahaha	Jingdong prefecture level city in Shandong
Power Saving King Shop	King of Power Saving	It's such a power saver to use this, it saves money on kids snacks for a month. Kudos!	Jingdong prefecture level city in Shandong
Power Saving King Shop	King of Power Saving	Plug and play, stable voltage, the screen has a display when working, this winter home appliances, need more electricity, just buy to use, save electricity and save money, with instructions, not too big and does not take up space, the quality is very good, and the seller describes exactly the same!	Jingdong prefecture level city in Shandong
Power Saving King Shop	King of Power Saving	A must have for opening a store. Save over a thousand dollars a month on your electric bill	Jingdong prefecture level city in Shandong
Power Saving King Shop	King of Power Saving	Received the goods, the first time to unpack, the quality is very good, with the seller's description, completely exceeded my expectations, the packaging is very careful, very strict, in general this is a very satisfied with the shopping	Jingdong prefecture level city in Shandong
Power Saving King Shop	King of Power Saving	As always, good quality, a very favorite product, great! o(*￣︶￣*)o The price is very affordable, cost-effective, it's considered affordable good, will repurchase!	Jingdong prefecture level city in Shandong
Power Saving King Shop	King of Power Saving	Very satisfied with a shopping, good business service, fast shipping, logistics is also very powerful, received very good, buy assured, use peace of mind!	Jingdong prefecture level city in Shandong
Power Saving King Shop	King of Power Saving	The first time to buy online, but also look at the evaluation before buying, the quality is not bad, but also very cheap, the need for friends can rest assured to buy!	Jingdong prefecture level city in Shandong
Power Saving King Shop	King of Power Saving	Better than expected, feel value for money, very satisfied with a shopping, need next time will come back!	Jingdong prefecture level city in Shandong
Power Saving King Shop	King of Power Saving	Really super like, very supportive, very good quality, exactly as described by the seller, very satisfied!	Jingdong prefecture level city in Shandong
Power Saving King Shop	King of Power Saving	It is genuine, effective, really saves power, affordable, tightly packed, fast shipping, very satisfied.	Jingdong prefecture level city in Shandong
Power Saving King Shop	King of Power Saving	Good quality, fine workmanship, is genuine and business description of the same, good value for money, fast logistics, very satisfied	Jingdong prefecture level city in Shandong
Power Saving King Shop	King of Power Saving	Saver received, very good, installation set up very well, very good, the effect is very good, recommend that the pro buy	Jingdong prefecture level city in Shandong
Power Saving King Shop	King of Power Saving	Electromagnetic good, high power convenient, enough force, the price is right it, quality can be ☺ ☺ ☺ ☺ ☺	Jingdong prefecture level city in Shandong
Power Saving King Shop	King of Power Saving	First-class quality, packaging is also good, logistics is very fast, and the price is reasonable, very affordable, I like it!	Jingdong prefecture level city in Shandong
Power Saving King Shop	King of Power Saving	It's great, it's the kind of style you want, and it arrived quickly, thank you boss, 5 stars for the service!	Jingdong prefecture level city in Shandong
Power Saving King Shop	King of Power Saving	Packaging is very good, the use of the effect is quite good, the store has a good service attitude, trustworthy	Jingdong prefecture level city in Shandong
Power Saving King Shop	King of Power Saving	The quality is very good, it works well, very satisfied, the price is affordable, it's so great value, it's worth recommending.	Jingdong prefecture level city in Shandong
Power Saving King Shop	King of Power Saving	The baby description matches the actual item very well! The quality is awesome! Will be back to patronize again in the future!	Jingdong prefecture level city in Shandong
Power Saving King Shop	King of Power Saving	Ran the price is not high try to buy the attitude, did not expect the effect is really good, good!	Jingdong prefecture level city in Shandong
Power Saving King Shop	King of Power Saving	Power Saving King power is quite large, the quality of the leaf is quite good, the price of buying a very good deal, must be praised!	Jingdong prefecture level city in Shandong
Power Saving King Shop	King of Power Saving	Good product quality is the king of power-saving products trustworthy as always good to recommend!	Jingdong prefecture level city in Shandong
Power Saving King Shop	King of Power Saving	Works great, very affordable, shipped pretty fast, comfortable to use	Jingdong prefecture level city in Shandong
Power Saving King Shop	King of Power Saving	Shipping was pretty fast and affordable, not bad 👍	Jingdong prefecture level city in Shandong
Power Saving King Shop	King of Power Saving	Digital voltage display, voltage at a glance, plug and play, safe and simple!	Jingdong prefecture level city in Shandong
Power Saving King Shop	King of Power Saving	Quality assurance, very good, affordable, logistics is also very fast, I'm very satisfied with the	Jingdong prefecture level city in Shandong
Power Saving King Shop	King of Power Saving	The quality is very good, the first time I received the goods to open the first time to see, it is very good, very satisfied	Jingdong prefecture level city in Shandong
Power Saving King Shop	King of Power Saving	Power Saving King used, the effect is good, the price is affordable, very good, is the genuine article	Jingdong prefecture level city in Shandong
Power Saving King Shop	King of Power Saving	Good quality, fine workmanship, good appearance, time is very accurate, satisfied.	Jingdong prefecture level city in Shandong
Power Saving King Shop	King of Power Saving	I purposely used it for two days before commenting, which is responsible for myself and everyone else!	Jingdong prefecture level city in Shandong
Power Saving King Shop	King of Power Saving	Easy to install, easy to operate, helped our family save a lot of electricity this summer	Jingdong prefecture level city in Shandong
Power Saving King Shop	King of Power Saving	Bringing power savings into millions of homes. Still a great gadget. Pretty good.	Jingdong prefecture level city in Shandong
Power Saving King Shop	King of Power Saving	Digital liquid crystal display to see the clear intuitive, very formal workmanship is not rough	Jingdong prefecture level city in Shandong
Power Saving King Shop	King of Power Saving	Received. Great quality, fast shipping from the store and not too expensive!	Jingdong prefecture level city in Shandong
Power Saving King Shop	King of Power Saving	Bought myself a knuckle regulator and it was easy to use and the price was right.	Jingdong prefecture level city in Shandong
Power Saving King Shop	King of Power Saving	Received, good, powerful, good, very full of a shopping	Jingdong prefecture level city in Shandong
Power Saving King Shop	King of Power Saving	High power, consistent with the seller's description, powerful, cost-effective	Jingdong prefecture level city in Shandong
Power Saving King Shop	King of Power Saving	It works very well and also saves power at an affordable price a great deal.	Jingdong prefecture level city in Shandong
Power Saving King Shop	King of Power Saving	Good quality, good can recommend to friends to buy.	Jingdong prefecture level city in Shandong
Power Saving King Shop	King of Power Saving	Will repurchase, quality is guaranteed, safe and secure purchase.	Jingdong prefecture level city in Shandong
Power Saving King Shop	King of Power Saving	The logistics is very fast, the thing is also good to use, energy saving power rely on it!	Jingdong prefecture level city in Shandong
Power Saving King Shop	King of Power Saving	Power Saver is good quality and works very well, worth buying!	Jingdong prefecture level city in Shandong
Power Saving King Shop	King of Power Saving	The quality is very good is genuine, cost-effective recommended	Jingdong prefecture level city in Shandong
Power Saving King Shop	King of Power Saving	Quality Assurance. Favorable price. Very practical and durable	Jingdong prefecture level city in Shandong
Power Saving King Shop	King of Power Saving	Good value for money. Conscientious businessman. Recommended to everyone	Jingdong prefecture level city in Shandong
Power Saving King Shop	King of Power Saving	Buy it for your rental apartment and never worry about your landlord charging you too much.	Jingdong prefecture level city in Shandong
Power Saving King Shop	King of Power Saving	Nice and affordable, will repurchase next time	Jingdong prefecture level city in Shandong
Power Saving King Shop	King of Power Saving	It's very energy efficient. I've been using it for a few days now.	Jingdong prefecture level city in Shandong
Power Saving King Shop	King of Power Saving	Good quality, high power easy to use	Jingdong prefecture level city in Shandong
Power Saving King Shop	King of Power Saving	Power saving little helper. Deserves a favorable review.	Jingdong prefecture level city in Shandong

Power Saving King Shop	King of Power Saving	Great value for money, great value for money.	Jingdong prefecture level city in Shandong
Power Saving King Shop	King of Power Saving	Works well and as described by the seller	Jingdong prefecture level city in Shandong
Power Saving King Shop	King of Power Saving	Power saving is proper! It's good.	Jingdong prefecture level city in Shandong
Power Saving King Shop	King of Power Saving	Power saving. Works well. Not bad.	Jingdong prefecture level city in Shandong
Power Saving King Shop	King of Power Saving	Satisfied with power saving	Jingdong prefecture level city in Shandong
Power Saving King Shop	King of Power Saving	Received intact, open a super good look beautiful and generous, ~ bit of high quality is recommended to buy, the need for friends rest assured that the next order	Jingdong prefecture level city in Shandong
Power Saving King Shop	King of Power Saving	Very good to use, friends introduced, save electricity and money, long-lasting products cost-effective, large factory production, good quality, fine workmanship cost-effective!	Jingdong prefecture level city in Shandong
Power Saving King Shop	King of Power Saving	The power saving effect is very obvious, trying to pay 30% less electricity bill in a month than before, hahaha	Jingdong prefecture level city in Shandong
Power Saving King Shop	King of Power Saving	It's such a power saver to use this, it saves money on kids snacks for a month. Kudos!	Jingdong prefecture level city in Shandong
Power Saving King Shop	King of Power Saving	Plug and play, stable voltage, the screen has a display when working, this winter home appliances, need more electricity, just buy to use, save electricity and save money, with instructions, not too big and does not take up space, the quality is very good, and the seller describes exactly the same!	Jingdong prefecture level city in Shandong
Power Saving King Shop	King of Power Saving	A must have for opening a store. Save over a thousand dollars a month on your electric bill	Jingdong prefecture level city in Shandong
Power Saving King Shop	King of Power Saving	Received the goods, the first time to unpack, the quality is very good, with the seller's description, completely exceeded my expectations, the packaging is very careful, very strict, in general this is a very satisfied with the shopping	Jingdong prefecture level city in Shandong
Power Saving King Shop	King of Power Saving	As always, good quality, a very favorite product, great! o(*￣︶￣*)o The price is very affordable, cost-effective, it's considered affordable good, will repurchase!	Jingdong prefecture level city in Shandong
Power Saving King Shop	King of Power Saving	Very satisfied with a shopping, good business service, fast shipping, logistics is also very powerful, received very good, buy assured, use peace of mind!	Jingdong prefecture level city in Shandong
Power Saving King Shop	King of Power Saving	The first time to buy online, but also look at the evaluation before buying, the quality is not bad, but also very cheap, the need for friends can rest assured to buy!	Jingdong prefecture level city in Shandong
Power Saving King Shop	King of Power Saving	Better than expected, feel value for money, very satisfied with a shopping, need next time will come back!	Jingdong prefecture level city in Shandong
Power Saving King Shop	King of Power Saving	Really super like, very supportive, very good quality, exactly as described by the seller, very satisfied!	Jingdong prefecture level city in Shandong
Power Saving King Shop	King of Power Saving	It is genuine, effective, really saves power, affordable, tightly packed, fast shipping, very satisfied.	Jingdong prefecture level city in Shandong
Power Saving King Shop	King of Power Saving	Good quality, fine workmanship, is genuine and business description of the same, good value for money, fast logistics, very satisfied	Jingdong prefecture level city in Shandong
Power Saving King Shop	King of Power Saving	Saver received, very good, installation set up very well, very good, the effect is very good, recommend that the pro buy	Jingdong prefecture level city in Shandong
Power Saving King Shop	King of Power Saving	Electromagnetic good, high power convenient, enough force, the price is right it, quality can be 😃 😃 😃 😃 😃	Jingdong prefecture level city in Shandong
Power Saving King Shop	King of Power Saving	First-class quality, packaging is also good, logistics is very fast, and the price is reasonable, very affordable, I like it!	Jingdong prefecture level city in Shandong
Power Saving King Shop	King of Power Saving	It's great, it's the kind of style you want, and it arrived quickly, thank you boss, 5 stars for the service!	Jingdong prefecture level city in Shandong
Power Saving King Shop	King of Power Saving	Packaging is very good, the use of the effect is quite good, the store has a good service attitude, trustworthy	Jingdong prefecture level city in Shandong
Power Saving King Shop	King of Power Saving	The quality is very good, it works well, very satisfied, the price is affordable, it's so great value, it's worth recommending.	Jingdong prefecture level city in Shandong
Power Saving King Shop	King of Power Saving	The baby description matches the actual item very well! The quality is awesome! Will be back to patronize again in the future!	Jingdong prefecture level city in Shandong
Power Saving King Shop	King of Power Saving	Ran the price is not high try to buy the attitude, did not expect the effect is really good, good!	Jingdong prefecture level city in Shandong
Power Saving King Shop	King of Power Saving	Power Saving King power is quite large, the quality of the leaf is quite good, the price of buying a very good deal, must be praised!	Jingdong prefecture level city in Shandong
Power Saving King Shop	King of Power Saving	Good product quality is the king of power-saving products trustworthy as always good to recommend!	Jingdong prefecture level city in Shandong
Power Saving King Shop	King of Power Saving	Works great, very affordable, shipped pretty fast, comfortable to use	Jingdong prefecture level city in Shandong
Power Saving King Shop	King of Power Saving	Shipping was pretty fast and affordable, not bad 👍	Jingdong prefecture level city in Shandong
Power Saving King Shop	King of Power Saving	Digital voltage display, voltage at a glance, plug and play, safe and simple!	Jingdong prefecture level city in Shandong
Power Saving King Shop	King of Power Saving	Quality assurance, very good, affordable, logistics is also very fast, I'm very satisfied with the	Jingdong prefecture level city in Shandong
Power Saving King Shop	King of Power Saving	The quality is very good, the first time I received the goods to open the first time to see, it is very good, very satisfied	Jingdong prefecture level city in Shandong
Power Saving King Shop	King of Power Saving	Power Saving King used, the effect is good, the price is affordable, very good, is the genuine article	Jingdong prefecture level city in Shandong
Power Saving King Shop	King of Power Saving	Good quality, fine workmanship, good appearance, time is very accurate, satisfied.	Jingdong prefecture level city in Shandong
Power Saving King Shop	King of Power Saving	I purposely used it for two days before commenting, which is responsible for myself and everyone else!	Jingdong prefecture level city in Shandong
Power Saving King Shop	King of Power Saving	Easy to install, easy to operate, helped our family save a lot of electricity this summer	Jingdong prefecture level city in Shandong
Power Saving King Shop	King of Power Saving	Bringing power savings into millions of homes. Still a great gadget. Pretty good.	Jingdong prefecture level city in Shandong
Power Saving King Shop	King of Power Saving	Digital liquid crystal display to see the clear intuitive, very formal workmanship is not rough	Jingdong prefecture level city in Shandong
Power Saving King Shop	King of Power Saving	Received. Great quality, fast shipping from the store and not too expensive!	Jingdong prefecture level city in Shandong
Power Saving King Shop	King of Power Saving	Bought myself a knuckle regulator and it was easy to use and the price was right.	Jingdong prefecture level city in Shandong
Power Saving King Shop	King of Power Saving	Received, good, powerful, good, very full of a shopping	Jingdong prefecture level city in Shandong
Power Saving King Shop	King of Power Saving	High power, consistent with the seller's description, powerful, cost-effective	Jingdong prefecture level city in Shandong
Power Saving King Shop	King of Power Saving	It works very well and also saves power at an affordable price a great deal.	Jingdong prefecture level city in Shandong
Power Saving King Shop	King of Power Saving	Good quality, good can recommend to friends to buy.	Jingdong prefecture level city in Shandong
Power Saving King Shop	King of Power Saving	Will repurchase, quality is guaranteed, safe and secure purchase.	Jingdong prefecture level city in Shandong
Power Saving King Shop	King of Power Saving	The logistics is very fast, the thing is also good to use, energy saving power rely on it!	Jingdong prefecture level city in Shandong
Power Saving King Shop	King of Power Saving	Power Saver is good quality and works very well, worth buying!	Jingdong prefecture level city in Shandong
Power Saving King Shop	King of Power Saving	The quality is very good is genuine, cost-effective recommended	Jingdong prefecture level city in Shandong

Power Saving King Shop	King of Power Saving	Quality Assurance. Favorable price. Very practical and durable	Jingdong prefecture level city in Shandong
Power Saving King Shop	King of Power Saving	Good value for money. Conscientious businessman. Recommended to everyone	Jingdong prefecture level city in Shandong
Power Saving King Shop	King of Power Saving	Buy it for your rental apartment and never worry about your landlord charging you too much.	Jingdong prefecture level city in Shandong
Power Saving King Shop	King of Power Saving	Nice and affordable, will repurchase next time	Jingdong prefecture level city in Shandong
Power Saving King Shop	King of Power Saving	It's very energy efficient. I've been using it for a few days now.	Jingdong prefecture level city in Shandong
Power Saving King Shop	King of Power Saving	Good quality, high power easy to use	Jingdong prefecture level city in Shandong
Power Saving King Shop	King of Power Saving	Power saving little helper. Deserves a favorable review.	Jingdong prefecture level city in Shandong
Power Saving King Shop	King of Power Saving	Great value for money, great value for money.	Jingdong prefecture level city in Shandong
Power Saving King Shop	King of Power Saving	Works well and as described by the seller	Jingdong prefecture level city in Shandong
Power Saving King Shop	King of Power Saving	Power saving is proper! It's good.	Jingdong prefecture level city in Shandong
Power Saving King Shop	King of Power Saving	Power saving. Works well. Not bad.	Jingdong prefecture level city in Shandong
Power Saving King Shop	King of Power Saving	Satisfied with power saving	Jingdong prefecture level city in Shandong
Power Saving King Shop	King of Power Saving	Received intact, open a super good look beautiful and generous, ~ bit of high quality is recommended to buy, the need for friends rest assured that the next order	Jingdong prefecture level city in Shandong
Power Saving King Shop	King of Power Saving	Very good to use, friends introduced, save electricity and money, long-lasting products cost-effective, large factory production, good quality, fine workmanship cost-effective!	Jingdong prefecture level city in Shandong
Power Saving King Shop	King of Power Saving	The power saving effect is very obvious, trying to pay 30% less electricity bill in a month than before, hahaha	Jingdong prefecture level city in Shandong
Power Saving King Shop	King of Power Saving	It's such a power saver to use this, it saves money on kids snacks for a month. Kudos!	Jingdong prefecture level city in Shandong
Power Saving King Shop	King of Power Saving	Plug and play, stable voltage, the screen has a display when working, this winter home appliances, need more electricity, just buy to use, save electricity and save money, with instructions, not too big and does not take up space, the quality is very good, and the seller describes exactly the same!	Jingdong prefecture level city in Shandong
Power Saving King Shop	King of Power Saving	A must have for opening a store. Save over a thousand dollars a month on your electric bill	Jingdong prefecture level city in Shandong
Power Saving King Shop	King of Power Saving	Received the goods, the first time to unpack, the quality is very good, with the seller's description, completely exceeded my expectations, the packaging is very careful, very strict, in general this is a very satisfied with the shopping	Jingdong prefecture level city in Shandong
Power Saving King Shop	King of Power Saving	As always, good quality, a very favorite product, great! o(*￣︶￣*)o The price is very affordable, cost-effective, it's considered affordable good, will repurchase!	Jingdong prefecture level city in Shandong
Power Saving King Shop	King of Power Saving	Very satisfied with a shopping, good business service, fast shipping, logistics is also very powerful, received very good, buy assured, use peace of mind!	Jingdong prefecture level city in Shandong
Power Saving King Shop	King of Power Saving	The first time to buy online, but also look at the evaluation before buying, the quality is not bad, but also very cheap, the need for friends can rest assured to buy!	Jingdong prefecture level city in Shandong
Power Saving King Shop	King of Power Saving	Better than expected, feel value for money, very satisfied with a shopping, need next time will come back!	Jingdong prefecture level city in Shandong
Power Saving King Shop	King of Power Saving	Really super like, very supportive, very good quality, exactly as described by the seller, very satisfied!	Jingdong prefecture level city in Shandong
Power Saving King Shop	King of Power Saving	It is genuine, effective, really saves power, affordable, tightly packed, fast shipping, very satisfied.	Jingdong prefecture level city in Shandong
Power Saving King Shop	King of Power Saving	Good quality, fine workmanship, is genuine and business description of the same, good value for money, fast logistics, very satisfied	Jingdong prefecture level city in Shandong
Power Saving King Shop	King of Power Saving	Saver received, very good, installation set up very well, very good, the effect is very good, recommend that the pro buy	Jingdong prefecture level city in Shandong
Power Saving King Shop	King of Power Saving	Electromagnetic good, high power convenient, enough force, the price is right it, quality can be ☺ ☺ ☺ ☺ ☺	Jingdong prefecture level city in Shandong
Power Saving King Shop	King of Power Saving	First-class quality, packaging is also good, logistics is very fast, and the price is reasonable, very affordable, I like it!	Jingdong prefecture level city in Shandong
Power Saving King Shop	King of Power Saving	It's great, it's the kind of style you want, and it arrived quickly, thank you boss, 5 stars for the service!	Jingdong prefecture level city in Shandong
Power Saving King Shop	King of Power Saving	Packaging is very good, the use of the effect is quite good, the store has a good service attitude, trustworthy	Jingdong prefecture level city in Shandong
Power Saving King Shop	King of Power Saving	The quality is very good, it works well, very satisfied, the price is affordable, it's so great value, it's worth recommending.	Jingdong prefecture level city in Shandong
Power Saving King Shop	King of Power Saving	The baby description matches the actual item very well! The quality is awesome! Will be back to patronize again in the future!	Jingdong prefecture level city in Shandong
Power Saving King Shop	King of Power Saving	Ran the price is not high try to buy the attitude, did not expect the effect is really good, good!	Jingdong prefecture level city in Shandong
Power Saving King Shop	King of Power Saving	Power Saving King power is quite large, the quality of the leaf is quite good, the price of buying a very good deal, must be praised!	Jingdong prefecture level city in Shandong
Power Saving King Shop	King of Power Saving	Good product quality is the king of power-saving products trustworthy as always good to recommend!	Jingdong prefecture level city in Shandong
Power Saving King Shop	King of Power Saving	Works great, very affordable, shipped pretty fast, comfortable to use	Jingdong prefecture level city in Shandong
Power Saving King Shop	King of Power Saving	Shipping was pretty fast and affordable, not bad 👍	Jingdong prefecture level city in Shandong
Power Saving King Shop	King of Power Saving	Digital voltage display, voltage at a glance, plug and play, safe and simple!	Jingdong prefecture level city in Shandong
Power Saving King Shop	King of Power Saving	Quality assurance, very good, affordable, logistics is also very fast, I'm very satisfied with the	Jingdong prefecture level city in Shandong
Power Saving King Shop	King of Power Saving	The quality is very good, the first time I received the goods to open the first time to see, it is very good, very satisfied	Jingdong prefecture level city in Shandong
Power Saving King Shop	King of Power Saving	Power Saving King used, the effect is good, the price is affordable, very good, is the genuine article	Jingdong prefecture level city in Shandong
Power Saving King Shop	King of Power Saving	Good quality, fine workmanship, good appearance, time is very accurate, satisfied.	Jingdong prefecture level city in Shandong
Power Saving King Shop	King of Power Saving	I purposely used it for two days before commenting, which is responsible for myself and everyone else!	Jingdong prefecture level city in Shandong
Power Saving King Shop	King of Power Saving	Easy to install, easy to operate, helped our family save a lot of electricity this summer	Jingdong prefecture level city in Shandong
Power Saving King Shop	King of Power Saving	Bringing power savings into millions of homes. Still a great gadget. Pretty good.	Jingdong prefecture level city in Shandong
Power Saving King Shop	King of Power Saving	Digital liquid crystal display to see the clear intuitive, very formal workmanship is not rough	Jingdong prefecture level city in Shandong
Power Saving King Shop	King of Power Saving	Received. Great quality, fast shipping from the store and not too expensive!	Jingdong prefecture level city in Shandong
Power Saving King Shop	King of Power Saving	Bought myself a knuckle regulator and it was easy to use and the price was right.	Jingdong prefecture level city in Shandong
Power Saving King Shop	King of Power Saving	Received, good, powerful, good, very full of a shopping	Jingdong prefecture level city in Shandong

Power Saving King Shop	King of Power Saving	High power, consistent with the seller's description, powerful, cost-effective	Jingdong prefecture level city in Shandong
Power Saving King Shop	King of Power Saving	It works very well and also saves power at an affordable price a great deal.	Jingdong prefecture level city in Shandong
Power Saving King Shop	King of Power Saving	Good quality, good can recommend to friends to buy.	Jingdong prefecture level city in Shandong
Power Saving King Shop	King of Power Saving	Will repurchase, quality is guaranteed, safe and secure purchase.	Jingdong prefecture level city in Shandong
Power Saving King Shop	King of Power Saving	The logistics is very fast, the thing is also good to use, energy saving power rely on it!	Jingdong prefecture level city in Shandong
Power Saving King Shop	King of Power Saving	Power Saver is good quality and works very well, worth buying!	Jingdong prefecture level city in Shandong
Power Saving King Shop	King of Power Saving	The quality is very good is genuine, cost-effective recommended	Jingdong prefecture level city in Shandong
Power Saving King Shop	King of Power Saving	Quality Assurance. Favorable price. Very practical and durable	Jingdong prefecture level city in Shandong
Power Saving King Shop	King of Power Saving	Good value for money. Conscientious businessman. Recommended to everyone	Jingdong prefecture level city in Shandong
Power Saving King Shop	King of Power Saving	Buy it for your rental apartment and never worry about your landlord charging you too much.	Jingdong prefecture level city in Shandong
Power Saving King Shop	King of Power Saving	Nice and affordable, will repurchase next time	Jingdong prefecture level city in Shandong
Power Saving King Shop	King of Power Saving	It's very energy efficient. I've been using it for a few days now.	Jingdong prefecture level city in Shandong
Power Saving King Shop	King of Power Saving	Good quality, high power easy to use	Jingdong prefecture level city in Shandong
Power Saving King Shop	King of Power Saving	Power saving little helper. Deserves a favorable review.	Jingdong prefecture level city in Shandong
Power Saving King Shop	King of Power Saving	Great value for money, great value for money.	Jingdong prefecture level city in Shandong
Power Saving King Shop	King of Power Saving	Works well and as described by the seller	Jingdong prefecture level city in Shandong
Power Saving King Shop	King of Power Saving	Power saving is proper! It's good.	Jingdong prefecture level city in Shandong
Power Saving King Shop	King of Power Saving	Power saving. Works well. Not bad.	Jingdong prefecture level city in Shandong
Power Saving King Shop	King of Power Saving	Satisfied with power saving	Jingdong prefecture level city in Shandong
Power Saving King Shop	King of Power Saving	Received intact, open a super good look beautiful and generous, ˜ bit of high quality is recommended to buy, the need for friends rest assured that the next order	Jingdong prefecture level city in Shandong
Power Saving King Shop	King of Power Saving	Very good to use, friends introduced, save electricity and money, long-lasting products cost-effective, large factory production, good quality, fine workmanship cost-effective!	Jingdong prefecture level city in Shandong
Power Saving King Shop	King of Power Saving	The power saving effect is very obvious, trying to pay 30% less electricity bill in a month than before, hahaha	Jingdong prefecture level city in Shandong
Power Saving King Shop	King of Power Saving	It's such a power saver to use this, it saves money on kids snacks for a month. Kudos!	Jingdong prefecture level city in Shandong
Power Saving King Shop	King of Power Saving	Plug and play, stable voltage, the screen has a display when working, this winter home appliances, need more electricity, just buy to use, save electricity and save money, with instructions, not too big and does not take up space, the quality is very good, and the seller describes exactly the same!	Jingdong prefecture level city in Shandong
Power Saving King Shop	King of Power Saving	A must have for opening a store. Save over a thousand dollars a month on your electric bill	Jingdong prefecture level city in Shandong
Power Saving King Shop	King of Power Saving	Received the goods, the first time to unpack, the quality is very good, with the seller's description, completely exceeded my expectations, the packaging is very careful, very strict, in general this is a very satisfied with the shopping	Jingdong prefecture level city in Shandong
Power Saving King Shop	King of Power Saving	As always, good quality, a very favorite product, great! o(* ︶ *)o The price is very affordable, cost-effective, it's considered affordable good, will repurchase!	Jingdong prefecture level city in Shandong
Power Saving King Shop	King of Power Saving	Very satisfied with a shopping, good business service, fast shipping, logistics is also very powerful, received very good, buy assured, use peace of mind!	Jingdong prefecture level city in Shandong
Power Saving King Shop	King of Power Saving	The first time to buy online, but also look at the evaluation before buying, the quality is not bad, but also very cheap, the need for friends can rest assured to buy!	Jingdong prefecture level city in Shandong
Power Saving King Shop	King of Power Saving	Better than expected, feel value for money, very satisfied with a shopping, need next time will come back!	Jingdong prefecture level city in Shandong
Power Saving King Shop	King of Power Saving	Really super like, very supportive, very good quality, exactly as described by the seller, very satisfied!	Jingdong prefecture level city in Shandong
Power Saving King Shop	King of Power Saving	It is genuine, effective, really saves power, affordable, tightly packed, fast shipping, very satisfied.	Jingdong prefecture level city in Shandong
Power Saving King Shop	King of Power Saving	Good quality, fine workmanship, is genuine and business description of the same, good value for money, fast logistics, very satisfied	Jingdong prefecture level city in Shandong
Power Saving King Shop	King of Power Saving	Saver received, very good, installation set up very well, very good, the effect is very good, recommend that the pro buy	Jingdong prefecture level city in Shandong
Power Saving King Shop	King of Power Saving	Electromagnetic good, high power convenient, enough force, the price is right it, quality can be 😃 😃 😃 😃 😃	Jingdong prefecture level city in Shandong
Power Saving King Shop	King of Power Saving	First-class quality, packaging is also good, logistics is very fast, and the price is reasonable, very affordable, I like it!	Jingdong prefecture level city in Shandong
Power Saving King Shop	King of Power Saving	It's great, it's the kind of style you want, and it arrived quickly, thank you boss, 5 stars for the service!	Jingdong prefecture level city in Shandong
Power Saving King Shop	King of Power Saving	Packaging is very good, the use of the effect is quite good, the store has a good service attitude, trustworthy	Jingdong prefecture level city in Shandong
Power Saving King Shop	King of Power Saving	The quality is very good, it works well, very satisfied, the price is affordable, it's so great value, it's worth recommending.	Jingdong prefecture level city in Shandong
Power Saving King Shop	King of Power Saving	The baby description matches the actual item very well! The quality is awesome! Will be back to patronize again in the future!	Jingdong prefecture level city in Shandong
Power Saving King Shop	King of Power Saving	Ran the price is not high try to buy the attitude, did not expect the effect is really good, good!	Jingdong prefecture level city in Shandong
Power Saving King Shop	King of Power Saving	Power Saving King power is quite large, the quality of the leaf is quite good, the price of buying a very good deal, must be praised!	Jingdong prefecture level city in Shandong
Power Saving King Shop	King of Power Saving	Good product quality is the king of power-saving products trustworthy as always good to recommend!	Jingdong prefecture level city in Shandong
Power Saving King Shop	King of Power Saving	Works great, very affordable, shipped pretty fast, comfortable to use	Jingdong prefecture level city in Shandong
Power Saving King Shop	King of Power Saving	Shipping was pretty fast and affordable, not bad 👍	Jingdong prefecture level city in Shandong
Power Saving King Shop	King of Power Saving	Digital voltage display, voltage at a glance, plug and play, safe and simple!	Jingdong prefecture level city in Shandong
Power Saving King Shop	King of Power Saving	Quality assurance, very good, affordable, logistics is also very fast, I'm very satisfied with the	Jingdong prefecture level city in Shandong
Power Saving King Shop	King of Power Saving	The quality is very good, the first time I received the goods to open the first time to see, it is very good, very satisfied	Jingdong prefecture level city in Shandong
Power Saving King Shop	King of Power Saving	Power Saving King used, the effect is good, the price is affordable, very good, is the genuine article	Jingdong prefecture level city in Shandong
Power Saving King Shop	King of Power Saving	Good quality, fine workmanship, good appearance, time is very accurate, satisfied.	Jingdong prefecture level city in Shandong

Power Saving King Shop	King of Power Saving	I purposely used it for two days before commenting, which is responsible for myself and everyone else!	Jingdong prefecture level city in Shandong
Power Saving King Shop	King of Power Saving	Easy to install, easy to operate, helped our family save a lot of electricity this summer	Jingdong prefecture level city in Shandong
Power Saving King Shop	King of Power Saving	Bringing power savings into millions of homes. Still a great gadget. Pretty good.	Jingdong prefecture level city in Shandong
Power Saving King Shop	King of Power Saving	Digital liquid crystal display to see the clear intuitive, very formal workmanship is not rough	Jingdong prefecture level city in Shandong
Power Saving King Shop	King of Power Saving	Received. Great quality, fast shipping from the store and not too expensive!	Jingdong prefecture level city in Shandong
Power Saving King Shop	King of Power Saving	Bought myself a knuckle regulator and it was easy to use and the price was right.	Jingdong prefecture level city in Shandong
Power Saving King Shop	King of Power Saving	Received, good, powerful, good, very full of a shopping	Jingdong prefecture level city in Shandong
Power Saving King Shop	King of Power Saving	High power, consistent with the seller's description, powerful, cost-effective	Jingdong prefecture level city in Shandong
Power Saving King Shop	King of Power Saving	It works very well and also saves power at an affordable price a great deal.	Jingdong prefecture level city in Shandong
Power Saving King Shop	King of Power Saving	Good quality, good can recommend to friends to buy.	Jingdong prefecture level city in Shandong
Power Saving King Shop	King of Power Saving	Will repurchase, quality is guaranteed, safe and secure purchase.	Jingdong prefecture level city in Shandong
Power Saving King Shop	King of Power Saving	The logistics is very fast, the thing is also good to use, energy saving power rely on it!	Jingdong prefecture level city in Shandong
Power Saving King Shop	King of Power Saving	Power Saver is good quality and works very well, worth buying!	Jingdong prefecture level city in Shandong
Power Saving King Shop	King of Power Saving	The quality is very good is genuine, cost-effective recommended	Jingdong prefecture level city in Shandong
Power Saving King Shop	King of Power Saving	Quality Assurance. Favorable price. Very practical and durable	Jingdong prefecture level city in Shandong
Power Saving King Shop	King of Power Saving	Good value for money. Conscientious businessman. Recommended to everyone	Jingdong prefecture level city in Shandong
Power Saving King Shop	King of Power Saving	Buy it for your rental apartment and never worry about your landlord charging you too much.	Jingdong prefecture level city in Shandong
Power Saving King Shop	King of Power Saving	Nice and affordable, will repurchase next time	Jingdong prefecture level city in Shandong
Power Saving King Shop	King of Power Saving	It's very energy efficient. I've been using it for a few days now.	Jingdong prefecture level city in Shandong
Power Saving King Shop	King of Power Saving	Good quality, high power easy to use	Jingdong prefecture level city in Shandong
Power Saving King Shop	King of Power Saving	Power saving little helper. Deserves a favorable review.	Jingdong prefecture level city in Shandong
Power Saving King Shop	King of Power Saving	Great value for money, great value for money.	Jingdong prefecture level city in Shandong
Power Saving King Shop	King of Power Saving	Works well and as described by the seller	Jingdong prefecture level city in Shandong
Power Saving King Shop	King of Power Saving	Power saving is proper! It's good.	Jingdong prefecture level city in Shandong
Power Saving King Shop	King of Power Saving	Power saving. Works well. Not bad.	Jingdong prefecture level city in Shandong
Power Saving King Shop	King of Power Saving	Satisfied with power saving	Jingdong prefecture level city in Shandong
Power Saving King Shop	King of Power Saving	Received intact, open a super good look beautiful and generous, ~ bit of high quality is recommended to buy, the need for friends rest assured that the next order	Jingdong prefecture level city in Shandong
Power Saving King Shop	King of Power Saving	Very good to use, friends introduced, save electricity and money, long-lasting products cost-effective, large factory production, good quality, fine workmanship cost-effective!	Jingdong prefecture level city in Shandong
Power Saving King Shop	King of Power Saving	The power saving effect is very obvious, trying to pay 30% less electricity bill in a month than before, hahaha	Jingdong prefecture level city in Shandong
Power Saving King Shop	King of Power Saving	It's such a power saver to use this, it saves money on kids snacks for a month. Kudos!	Jingdong prefecture level city in Shandong
Power Saving King Shop	King of Power Saving	Plug and play, stable voltage, the screen has a display when working, this winter home appliances, need more electricity, just buy to use, save electricity and save money, with instructions, not too big and does not take up space, the quality is very good, and the seller describes exactly the same!	Jingdong prefecture level city in Shandong
Power Saving King Shop	King of Power Saving	A must have for opening a store. Save over a thousand dollars a month on your electric bill	Jingdong prefecture level city in Shandong
Power Saving King Shop	King of Power Saving	Received the goods, the first time to unpack, the quality is very good, with the seller's description, completely exceeded my expectations, the packaging is very careful, very strict, in general this is a very satisfied with the shopping	Jingdong prefecture level city in Shandong
Power Saving King Shop	King of Power Saving	As always, good quality, a very favorite product, great! o(*￣︶￣*)o The price is very affordable, cost-effective, it's considered affordable good, will repurchase!	Jingdong prefecture level city in Shandong
Power Saving King Shop	King of Power Saving	Very satisfied with a shopping, good business service, fast shipping, logistics is also very powerful, received very good, buy assured, use peace of mind!	Jingdong prefecture level city in Shandong
Power Saving King Shop	King of Power Saving	The first time to buy online, but also look at the evaluation before buying, the quality is not bad, but also very cheap, the need for friends can rest assured to buy!	Jingdong prefecture level city in Shandong
Power Saving King Shop	King of Power Saving	Better than expected, feel value for money, very satisfied with a shopping, need next time will come back!	Jingdong prefecture level city in Shandong
Power Saving King Shop	King of Power Saving	Really super like, very supportive, very good quality, exactly as described by the seller, very satisfied!	Jingdong prefecture level city in Shandong
Power Saving King Shop	King of Power Saving	It is genuine, effective, really saves power, affordable, tightly packed, fast shipping, very satisfied.	Jingdong prefecture level city in Shandong
Power Saving King Shop	King of Power Saving	Good quality, fine workmanship, is genuine and business description of the same, good value for money, fast logistics, very satisfied	Jingdong prefecture level city in Shandong
Power Saving King Shop	King of Power Saving	Saver received, very good, installation set up very well, very good, the effect is very good, recommend that the pro buy	Jingdong prefecture level city in Shandong
Power Saving King Shop	King of Power Saving	Electromagnetic good, high power convenient, enough force, the price is right it, quality can be 😃 😃 😃 😃 😃	Jingdong prefecture level city in Shandong
Power Saving King Shop	King of Power Saving	First-class quality, packaging is also good, logistics is very fast, and the price is reasonable, very affordable, I like it!	Jingdong prefecture level city in Shandong
Power Saving King Shop	King of Power Saving	It's great, it's the kind of style you want, and it arrived quickly, thank you boss, 5 stars for the service!	Jingdong prefecture level city in Shandong
Power Saving King Shop	King of Power Saving	Packaging is very good, the use of the effect is quite good, the store has a good service attitude, trustworthy	Jingdong prefecture level city in Shandong
Power Saving King Shop	King of Power Saving	The quality is very good, it works well, very satisfied, the price is affordable, it's so great value, it's worth recommending.	Jingdong prefecture level city in Shandong
Power Saving King Shop	King of Power Saving	The baby description matches the actual item very well! The quality is awesome! Will be back to patronize again in the future!	Jingdong prefecture level city in Shandong
Power Saving King Shop	King of Power Saving	Ran the price is not high try to buy the attitude, did not expect the effect is really good, good!	Jingdong prefecture level city in Shandong
Power Saving King Shop	King of Power Saving	Power Saving King power is quite large, the quality of the leaf is quite good, the price of buying a very good deal, must be praised!	Jingdong prefecture level city in Shandong
Power Saving King Shop	King of Power Saving	Good product quality is the king of power-saving products trustworthy as always good to recommend!	Jingdong prefecture level city in Shandong

Power Saving King Shop	King of Power Saving	Works great, very affordable, shipped pretty fast, comfortable to use	Jingdong prefecture level city in Shandong
Power Saving King Shop	King of Power Saving	Shipping was pretty fast and affordable, not bad 👍	Jingdong prefecture level city in Shandong
Power Saving King Shop	King of Power Saving	Digital voltage display, voltage at a glance, plug and play, safe and simple!	Jingdong prefecture level city in Shandong
Power Saving King Shop	King of Power Saving	Quality assurance, very good, affordable, logistics is also very fast, I'm very satisfied with the	Jingdong prefecture level city in Shandong
Power Saving King Shop	King of Power Saving	The quality is very good, the first time I received the goods to open the first time to see, it is very good, very satisfied	Jingdong prefecture level city in Shandong
Power Saving King Shop	King of Power Saving	Power Saving King used, the effect is good, the price is affordable, very good, is the genuine article	Jingdong prefecture level city in Shandong
Power Saving King Shop	King of Power Saving	Good quality, fine workmanship, good appearance, time is very accurate, satisfied.	Jingdong prefecture level city in Shandong
Power Saving King Shop	King of Power Saving	I purposely used it for two days before commenting, which is responsible for myself and everyone else!	Jingdong prefecture level city in Shandong
Power Saving King Shop	King of Power Saving	Easy to install, easy to operate, helped our family save a lot of electricity this summer	Jingdong prefecture level city in Shandong
Power Saving King Shop	King of Power Saving	Bringing power savings into millions of homes. Still a great gadget. Pretty good.	Jingdong prefecture level city in Shandong
Power Saving King Shop	King of Power Saving	Digital liquid crystal display to see the clear intuitive, very formal workmanship is not rough	Jingdong prefecture level city in Shandong
Power Saving King Shop	King of Power Saving	Received. Great quality, fast shipping from the store and not too expensive!	Jingdong prefecture level city in Shandong
Power Saving King Shop	King of Power Saving	Bought myself a knuckle regulator and it was easy to use and the price was right.	Jingdong prefecture level city in Shandong
Power Saving King Shop	King of Power Saving	Received, good, powerful, good, very full of a shopping	Jingdong prefecture level city in Shandong
Power Saving King Shop	King of Power Saving	High power, consistent with the seller's description, powerful, cost-effective	Jingdong prefecture level city in Shandong
Power Saving King Shop	King of Power Saving	It works very well and also saves power at an affordable price a great deal.	Jingdong prefecture level city in Shandong
Power Saving King Shop	King of Power Saving	Good quality, good can recommend to friends to buy.	Jingdong prefecture level city in Shandong
Power Saving King Shop	King of Power Saving	Will repurchase, quality is guaranteed, safe and secure purchase.	Jingdong prefecture level city in Shandong
Power Saving King Shop	King of Power Saving	The logistics is very fast, the thing is also good to use, energy saving power rely on it!	Jingdong prefecture level city in Shandong
Power Saving King Shop	King of Power Saving	Power Saver is good quality and works very well, worth buying!	Jingdong prefecture level city in Shandong
Power Saving King Shop	King of Power Saving	The quality is very good is genuine, cost-effective recommended	Jingdong prefecture level city in Shandong
Power Saving King Shop	King of Power Saving	Quality Assurance. Favorable price. Very practical and durable	Jingdong prefecture level city in Shandong
Power Saving King Shop	King of Power Saving	Good value for money. Conscientious businessman. Recommended to everyone	Jingdong prefecture level city in Shandong
Power Saving King Shop	King of Power Saving	Buy it for your rental apartment and never worry about your landlord charging you too much.	Jingdong prefecture level city in Shandong
Power Saving King Shop	King of Power Saving	Nice and affordable, will repurchase next time	Jingdong prefecture level city in Shandong
Power Saving King Shop	King of Power Saving	It's very energy efficient. I've been using it for a few days now.	Jingdong prefecture level city in Shandong
Power Saving King Shop	King of Power Saving	Good quality, high power easy to use	Jingdong prefecture level city in Shandong
Power Saving King Shop	King of Power Saving	Power saving little helper. Deserves a favorable review.	Jingdong prefecture level city in Shandong
Power Saving King Shop	King of Power Saving	Great value for money, great value for money.	Jingdong prefecture level city in Shandong
Power Saving King Shop	King of Power Saving	Works well and as described by the seller	Jingdong prefecture level city in Shandong
Power Saving King Shop	King of Power Saving	Power saving is proper! It's good.	Jingdong prefecture level city in Shandong
Power Saving King Shop	King of Power Saving	Power saving. Works well. Not bad.	Jingdong prefecture level city in Shandong
Power Saving King Shop	King of Power Saving	Satisfied with power saving	Jingdong prefecture level city in Shandong
Power Saving King Shop	King of Power Saving	Received intact, open a super good look beautiful and generous, ~ bit of high quality is recommended to buy, the need for friends rest assured that the next order	Jingdong prefecture level city in Shandong
Power Saving King Shop	King of Power Saving	Very good to use, friends introduced, save electricity and money, long-lasting products cost-effective, large factory production, good quality, fine workmanship cost-effective!	Jingdong prefecture level city in Shandong
Power Saving King Shop	King of Power Saving	The power saving effect is very obvious, trying to pay 30% less electricity bill in a month than before, hahaha	Jingdong prefecture level city in Shandong
Power Saving King Shop	King of Power Saving	It's such a power saver to use this, it saves money on kids snacks for a month. Kudos!	Jingdong prefecture level city in Shandong
Power Saving King Shop	King of Power Saving	Plug and play, stable voltage, the screen has a display when working, this winter home appliances, need more electricity, just buy to use, save electricity and save money, with instructions, not too big and does not take up space, the quality is very good, and the seller describes exactly the same!	Jingdong prefecture level city in Shandong
Power Saving King Shop	King of Power Saving	A must have for opening a store. Save over a thousand dollars a month on your electric bill	Jingdong prefecture level city in Shandong
Power Saving King Shop	King of Power Saving	Received the goods, the first time to unpack, the quality is very good, with the seller's description, completely exceeded my expectations, the packaging is very careful, very strict, in general this is a very satisfied with the shopping	Jingdong prefecture level city in Shandong
Power Saving King Shop	King of Power Saving	As always, good quality, a very favorite product, great! o(*￣︶￣*)o The price is very affordable, cost-effective, it's considered affordable good, will repurchase!	Jingdong prefecture level city in Shandong
Power Saving King Shop	King of Power Saving	Very satisfied with a shopping, good business service, fast shipping, logistics is also very powerful, received very good, buy assured, use peace of mind!	Jingdong prefecture level city in Shandong
Power Saving King Shop	King of Power Saving	The first time to buy online, but also look at the evaluation before buying, the quality is not bad, but also very cheap, the need for friends can rest assured to buy!	Jingdong prefecture level city in Shandong
Power Saving King Shop	King of Power Saving	Better than expected, feel value for money, very satisfied with a shopping, need next time will come back!	Jingdong prefecture level city in Shandong
Power Saving King Shop	King of Power Saving	Really super like, very supportive, very good quality, exactly as described by the seller, very satisfied!	Jingdong prefecture level city in Shandong
Power Saving King Shop	King of Power Saving	It is genuine, effective, really saves power, affordable, tightly packed, fast shipping, very satisfied.	Jingdong prefecture level city in Shandong
Power Saving King Shop	King of Power Saving	Good quality, fine workmanship, is genuine and business description of the same, good value for money, fast logistics, very satisfied	Jingdong prefecture level city in Shandong
Power Saving King Shop	King of Power Saving	Saver received, very good, installation set up very well, very good, the effect is very good, recommend that the pro buy	Jingdong prefecture level city in Shandong
Power Saving King Shop	King of Power Saving	Electromagnetic good, high power convenient, enough force, the price is right it, quality can be 😃 😃 😃 😃 😃	Jingdong prefecture level city in Shandong
Power Saving King Shop	King of Power Saving	First-class quality, packaging is also good, logistics is very fast, and the price is reasonable, very affordable, I like it!	Jingdong prefecture level city in Shandong

Power Saving King Shop	King of Power Saving	It's great, it's the kind of style you want, and it arrived quickly, thank you boss, 5 stars for the service!	Jingdong prefecture level city in Shandong
Power Saving King Shop	King of Power Saving	Packaging is very good, the use of the effect is quite good, the store has a good service attitude, trustworthy	Jingdong prefecture level city in Shandong
Power Saving King Shop	King of Power Saving	The quality is very good, it works well, very satisfied, the price is affordable, it's so great value, it's worth recommending.	Jingdong prefecture level city in Shandong
Power Saving King Shop	King of Power Saving	The baby description matches the actual item very well! The quality is awesome! Will be back to patronize again in the future!	Jingdong prefecture level city in Shandong
Power Saving King Shop	King of Power Saving	Ran the price is not high try to buy the attitude, did not expect the effect is really good, good!	Jingdong prefecture level city in Shandong
Power Saving King Shop	King of Power Saving	Power Saving King power is quite large, the quality of the leaf is quite good, the price of buying a very good deal, must be praised!	Jingdong prefecture level city in Shandong
Power Saving King Shop	King of Power Saving	Good product quality is the king of power-saving products trustworthy as always good to recommend!	Jingdong prefecture level city in Shandong
Power Saving King Shop	King of Power Saving	Works great, very affordable, shipped pretty fast, comfortable to use	Jingdong prefecture level city in Shandong
Power Saving King Shop	King of Power Saving	Shipping was pretty fast and affordable, not bad 👍	Jingdong prefecture level city in Shandong
Power Saving King Shop	King of Power Saving	Digital voltage display, voltage at a glance, plug and play, safe and simple!	Jingdong prefecture level city in Shandong
Power Saving King Shop	King of Power Saving	Quality assurance, very good, affordable, logistics is also very fast, I'm very satisfied with the	Jingdong prefecture level city in Shandong
Power Saving King Shop	King of Power Saving	The quality is very good, the first time I received the goods to open the first time to see, it is very good, very satisfied	Jingdong prefecture level city in Shandong
Power Saving King Shop	King of Power Saving	Power Saving King used, the effect is good, the price is affordable, very good, is the genuine article	Jingdong prefecture level city in Shandong
Power Saving King Shop	King of Power Saving	Good quality, fine workmanship, good appearance, time is very accurate, satisfied.	Jingdong prefecture level city in Shandong
Power Saving King Shop	King of Power Saving	I purposely used it for two days before commenting, which is responsible for myself and everyone else!	Jingdong prefecture level city in Shandong
Power Saving King Shop	King of Power Saving	Easy to install, easy to operate, helped our family save a lot of electricity this summer	Jingdong prefecture level city in Shandong
Power Saving King Shop	King of Power Saving	Bringing power savings into millions of homes. Still a great gadget. Pretty good.	Jingdong prefecture level city in Shandong
Power Saving King Shop	King of Power Saving	Digital liquid crystal display to see the clear intuitive, very formal workmanship is not rough	Jingdong prefecture level city in Shandong
Power Saving King Shop	King of Power Saving	Received. Great quality, fast shipping from the store and not too expensive!	Jingdong prefecture level city in Shandong
Power Saving King Shop	King of Power Saving	Bought myself a knuckle regulator and it was easy to use and the price was right.	Jingdong prefecture level city in Shandong
Power Saving King Shop	King of Power Saving	Received, good, powerful, good, very full of a shopping	Jingdong prefecture level city in Shandong
Power Saving King Shop	King of Power Saving	High power, consistent with the seller's description, powerful, cost-effective	Jingdong prefecture level city in Shandong
Power Saving King Shop	King of Power Saving	It works very well and also saves power at an affordable price a great deal.	Jingdong prefecture level city in Shandong
Power Saving King Shop	King of Power Saving	Good quality, good can recommend to friends to buy.	Jingdong prefecture level city in Shandong
Power Saving King Shop	King of Power Saving	Will repurchase, quality is guaranteed, safe and secure purchase.	Jingdong prefecture level city in Shandong
Power Saving King Shop	King of Power Saving	The logistics is very fast, the thing is also good to use, energy saving power rely on it!	Jingdong prefecture level city in Shandong
Power Saving King Shop	King of Power Saving	Power Saver is good quality and works very well, worth buying!	Jingdong prefecture level city in Shandong
Power Saving King Shop	King of Power Saving	The quality is very good is genuine, cost-effective recommended	Jingdong prefecture level city in Shandong
Power Saving King Shop	King of Power Saving	Quality Assurance. Favorable price. Very practical and durable	Jingdong prefecture level city in Shandong
Power Saving King Shop	King of Power Saving	Good value for money. Conscientious businessman. Recommended to everyone	Jingdong prefecture level city in Shandong
Power Saving King Shop	King of Power Saving	Buy it for your rental apartment and never worry about your landlord charging you too much.	Jingdong prefecture level city in Shandong
Power Saving King Shop	King of Power Saving	Nice and affordable, will repurchase next time	Jingdong prefecture level city in Shandong
Power Saving King Shop	King of Power Saving	It's very energy efficient. I've been using it for a few days now.	Jingdong prefecture level city in Shandong
Power Saving King Shop	King of Power Saving	Good quality, high power easy to use	Jingdong prefecture level city in Shandong
Power Saving King Shop	King of Power Saving	Power saving little helper. Deserves a favorable review.	Jingdong prefecture level city in Shandong
Power Saving King Shop	King of Power Saving	Great value for money, great value for money.	Jingdong prefecture level city in Shandong
Power Saving King Shop	King of Power Saving	Works well and as described by the seller	Jingdong prefecture level city in Shandong
Power Saving King Shop	King of Power Saving	Power saving is proper! It's good.	Jingdong prefecture level city in Shandong
Power Saving King Shop	King of Power Saving	Power saving. Works well. Not bad.	Jingdong prefecture level city in Shandong
Power Saving King Shop	King of Power Saving	Satisfied with power saving	Jingdong prefecture level city in Shandong
Power Saving King Shop	King of Power Saving	Received intact, open a super good look beautiful and generous, ~ bit of high quality is recommended to buy, the need for friends rest assured that the next order	Jingdong prefecture level city in Shandong
Power Saving King Shop	King of Power Saving	Very good to use, friends introduced, save electricity and money, long-lasting products cost-effective, large factory production, good quality, fine workmanship cost-effective!	Jingdong prefecture level city in Shandong
Power Saving King Shop	King of Power Saving	The power saving effect is very obvious, trying to pay 30% less electricity bill in a month than before, hahaha	Jingdong prefecture level city in Shandong
Power Saving King Shop	King of Power Saving	It's such a power saver to use this, it saves money on kids snacks for a month. Kudos!	Jingdong prefecture level city in Shandong
Power Saving King Shop	King of Power Saving	Plug and play, stable voltage, the screen has a display when working, this winter home appliances, need more electricity, just buy to use, save electricity and save money, with instructions, not too big and does not take up space, the quality is very good, and the seller describes exactly the same!	Jingdong prefecture level city in Shandong
Power Saving King Shop	King of Power Saving	A must have for opening a store. Save over a thousand dollars a month on your electric bill	Jingdong prefecture level city in Shandong
Power Saving King Shop	King of Power Saving	Received the goods, the first time to unpack, the quality is very good, with the seller's description, completely exceeded my expectations, the packaging is very careful, very strict, in general this is a very satisfied with the shopping	Jingdong prefecture level city in Shandong
Power Saving King Shop	King of Power Saving	As always, good quality, a very favorite product, great! o(*￣︶￣*)o The price is very affordable, cost-effective, it's considered affordable good, will repurchase!	Jingdong prefecture level city in Shandong
Power Saving King Shop	King of Power Saving	Very satisfied with a shopping, good business service, fast shipping, logistics is also very powerful, received very good, buy assured, use peace of mind!	Jingdong prefecture level city in Shandong
Power Saving King Shop	King of Power Saving	The first time to buy online, but also look at the evaluation before buying, the quality is not bad, but also very cheap, the need for friends can rest assured to buy!	Jingdong prefecture level city in Shandong

Power Saving King Shop	King of Power Saving	Better than expected, feel value for money, very satisfied with a shopping, need next time will come back!	Jingdong prefecture level city in Shandong
Power Saving King Shop	King of Power Saving	Really super like, very supportive, very good quality, exactly as described by the seller, very satisfied!	Jingdong prefecture level city in Shandong
Power Saving King Shop	King of Power Saving	It is genuine, effective, really saves power, affordable, tightly packed, fast shipping, very satisfied.	Jingdong prefecture level city in Shandong
Power Saving King Shop	King of Power Saving	Good quality, fine workmanship, is genuine and business description of the same, good value for money, fast logistics, very satisfied	Jingdong prefecture level city in Shandong
Power Saving King Shop	King of Power Saving	Saver received, very good, installation set up very well, very good, the effect is very good, recommend that the pro buy	Jingdong prefecture level city in Shandong
Power Saving King Shop	King of Power Saving	Electromagnetic good, high power convenient, enough force, the price is right it, quality can be 😃 😃 😃 😃 😃	Jingdong prefecture level city in Shandong
Power Saving King Shop	King of Power Saving	First-class quality, packaging is also good, logistics is very fast, and the price is reasonable, very affordable, I like it!	Jingdong prefecture level city in Shandong
Power Saving King Shop	King of Power Saving	It's great, it's the kind of style you want, and it arrived quickly, thank you boss, 5 stars for the service!	Jingdong prefecture level city in Shandong
Power Saving King Shop	King of Power Saving	Packaging is very good, the use of the effect is quite good, the store has a good service attitude, trustworthy	Jingdong prefecture level city in Shandong
Power Saving King Shop	King of Power Saving	The quality is very good, it works well, very satisfied, the price is affordable, it's so great value, it's worth recommending.	Jingdong prefecture level city in Shandong
Power Saving King Shop	King of Power Saving	The baby description matches the actual item very well! The quality is awesome! Will be back to patronize again in the future!	Jingdong prefecture level city in Shandong
Power Saving King Shop	King of Power Saving	Ran the price is not high try to buy the attitude, did not expect the effect is really good, good!	Jingdong prefecture level city in Shandong
Power Saving King Shop	King of Power Saving	Power Saving King power is quite large, the quality of the leaf is quite good, the price of buying a very good deal, must be praised!	Jingdong prefecture level city in Shandong
Power Saving King Shop	King of Power Saving	Good product quality is the king of power-saving products trustworthy as always good to recommend!	Jingdong prefecture level city in Shandong
Power Saving King Shop	King of Power Saving	Works great, very affordable, shipped pretty fast, comfortable to use	Jingdong prefecture level city in Shandong
Power Saving King Shop	King of Power Saving	Shipping was pretty fast and affordable, not bad 👍	Jingdong prefecture level city in Shandong
Power Saving King Shop	King of Power Saving	Digital voltage display, voltage at a glance, plug and play, safe and simple!	Jingdong prefecture level city in Shandong
Power Saving King Shop	King of Power Saving	Quality assurance, very good, affordable, logistics is also very fast, I'm very satisfied with the	Jingdong prefecture level city in Shandong
Power Saving King Shop	King of Power Saving	The quality is very good, the first time I received the goods to open the first time to see, it is very good, very satisfied	Jingdong prefecture level city in Shandong
Power Saving King Shop	King of Power Saving	Power Saving King used, the effect is good, the price is affordable, very good, is the genuine article	Jingdong prefecture level city in Shandong
Power Saving King Shop	King of Power Saving	Good quality, fine workmanship, good appearance, time is very accurate, satisfied.	Jingdong prefecture level city in Shandong
Power Saving King Shop	King of Power Saving	I purposely used it for two days before commenting, which is responsible for myself and everyone else!	Jingdong prefecture level city in Shandong
Power Saving King Shop	King of Power Saving	Easy to install, easy to operate, helped our family save a lot of electricity this summer	Jingdong prefecture level city in Shandong
Power Saving King Shop	King of Power Saving	Bringing power savings into millions of homes. Still a great gadget. Pretty good.	Jingdong prefecture level city in Shandong
Power Saving King Shop	King of Power Saving	Digital liquid crystal display to see the clear intuitive, very formal workmanship is not rough	Jingdong prefecture level city in Shandong
Power Saving King Shop	King of Power Saving	Received. Great quality, fast shipping from the store and not too expensive!	Jingdong prefecture level city in Shandong
Power Saving King Shop	King of Power Saving	Bought myself a knuckle regulator and it was easy to use and the price was right.	Jingdong prefecture level city in Shandong
Power Saving King Shop	King of Power Saving	Received, good, powerful, good, very full of a shopping	Jingdong prefecture level city in Shandong
Power Saving King Shop	King of Power Saving	High power, consistent with the seller's description, powerful, cost-effective	Jingdong prefecture level city in Shandong
Power Saving King Shop	King of Power Saving	It works very well and also saves power at an affordable price a great deal.	Jingdong prefecture level city in Shandong
Power Saving King Shop	King of Power Saving	Good quality, good can recommend to friends to buy.	Jingdong prefecture level city in Shandong
Power Saving King Shop	King of Power Saving	Will repurchase, quality is guaranteed, safe and secure purchase.	Jingdong prefecture level city in Shandong
Power Saving King Shop	King of Power Saving	The logistics is very fast, the thing is also good to use, energy saving power rely on it!	Jingdong prefecture level city in Shandong
Power Saving King Shop	King of Power Saving	Power Saver is good quality and works very well, worth buying!	Jingdong prefecture level city in Shandong
Power Saving King Shop	King of Power Saving	The quality is very good is genuine, cost-effective recommended	Jingdong prefecture level city in Shandong
Power Saving King Shop	King of Power Saving	Quality Assurance. Favorable price. Very practical and durable	Jingdong prefecture level city in Shandong
Power Saving King Shop	King of Power Saving	Good value for money. Conscientious businessman. Recommended to everyone	Jingdong prefecture level city in Shandong
Power Saving King Shop	King of Power Saving	Buy it for your rental apartment and never worry about your landlord charging you too much.	Jingdong prefecture level city in Shandong
Power Saving King Shop	King of Power Saving	Nice and affordable, will repurchase next time	Jingdong prefecture level city in Shandong
Power Saving King Shop	King of Power Saving	It's very energy efficient. I've been using it for a few days now.	Jingdong prefecture level city in Shandong
Power Saving King Shop	King of Power Saving	Good quality, high power easy to use	Jingdong prefecture level city in Shandong
Power Saving King Shop	King of Power Saving	Power saving little helper. Deserves a favorable review.	Jingdong prefecture level city in Shandong
Power Saving King Shop	King of Power Saving	Great value for money, great value for money.	Jingdong prefecture level city in Shandong
Power Saving King Shop	King of Power Saving	Works well and as described by the seller	Jingdong prefecture level city in Shandong
Power Saving King Shop	King of Power Saving	Power saving is proper! It's good.	Jingdong prefecture level city in Shandong
Power Saving King Shop	King of Power Saving	Power saving. Works well. Not bad.	Jingdong prefecture level city in Shandong
Power Saving King Shop	King of Power Saving	Satisfied with power saving	Jingdong prefecture level city in Shandong
Power Saving King Shop	King of Power Saving	Received intact, open a super good look beautiful and generous, ~ bit of high quality is recommended to buy, the need for friends rest assured that the next order	Jingdong prefecture level city in Shandong
Power Saving King Shop	King of Power Saving	Very good to use, friends introduced, save electricity and money, long-lasting products cost-effective, large factory production, good quality, fine workmanship cost-effective!	Jingdong prefecture level city in Shandong
Power Saving King Shop	King of Power Saving	The power saving effect is very obvious, trying to pay 30% less electricity bill in a month than before, hahaha	Jingdong prefecture level city in Shandong
Power Saving King Shop	King of Power Saving	It's such a power saver to use this, it saves money on kids snacks for a month. Kudos!	Jingdong prefecture level city in Shandong

Power Saving King Shop	King of Power Saving	Plug and play, stable voltage, the screen has a display when working, this winter home appliances, need more electricity, just buy to use, save electricity and save money, with instructions, not too big and does not take up space, the quality is very good, and the seller describes exactly the same!	Jingdong prefecture level city in Shandong
Power Saving King Shop	King of Power Saving	A must have for opening a store. Save over a thousand dollars a month on your electric bill	Jingdong prefecture level city in Shandong
Power Saving King Shop	King of Power Saving	Received the goods, the first time to unpack, the quality is very good, with the seller's description, completely exceeded my expectations, the packaging is very careful, very strict, in general this is a very satisfied with the shopping	Jingdong prefecture level city in Shandong
Power Saving King Shop	King of Power Saving	As always, good quality, a very favorite product, great! o(*￣︶￣*)o The price is very affordable, cost-effective, it's considered affordable good, will repurchase!	Jingdong prefecture level city in Shandong
Power Saving King Shop	King of Power Saving	Very satisfied with a shopping, good business service, fast shipping, logistics is also very powerful, received very good, buy assured, use peace of mind!	Jingdong prefecture level city in Shandong
Power Saving King Shop	King of Power Saving	The first time to buy online, but also look at the evaluation before buying, the quality is not bad, but also very cheap, the need for friends can rest assured to buy!	Jingdong prefecture level city in Shandong
Power Saving King Shop	King of Power Saving	Better than expected, feel value for money, very satisfied with a shopping, need next time will come back!	Jingdong prefecture level city in Shandong
Power Saving King Shop	King of Power Saving	Really super like, very supportive, very good quality, exactly as described by the seller, very satisfied!	Jingdong prefecture level city in Shandong
Power Saving King Shop	King of Power Saving	It is genuine, effective, really saves power, affordable, tightly packed, fast shipping, very satisfied.	Jingdong prefecture level city in Shandong
Power Saving King Shop	King of Power Saving	Good quality, fine workmanship, is genuine and business description of the same, good value for money, fast logistics, very satisfied	Jingdong prefecture level city in Shandong
Power Saving King Shop	King of Power Saving	Saver received, very good, installation set up very well, very good, the effect is very good, recommend that the pro buy	Jingdong prefecture level city in Shandong
Power Saving King Shop	King of Power Saving	Electromagnetic good, high power convenient, enough force, the price is right it, quality can be 😊 😊 😊 😊 😊	Jingdong prefecture level city in Shandong
Power Saving King Shop	King of Power Saving	First-class quality, packaging is also good, logistics is very fast, and the price is reasonable, very affordable, I like it!	Jingdong prefecture level city in Shandong
Power Saving King Shop	King of Power Saving	It's great, it's the kind of style you want, and it arrived quickly, thank you boss, 5 stars for the service!	Jingdong prefecture level city in Shandong
Power Saving King Shop	King of Power Saving	Packaging is very good, the use of the effect is quite good, the store has a good service attitude, trustworthy	Jingdong prefecture level city in Shandong
Power Saving King Shop	King of Power Saving	The quality is very good, it works well, very satisfied, the price is affordable, it's so great value, it's worth recommending.	Jingdong prefecture level city in Shandong
Power Saving King Shop	King of Power Saving	The baby description matches the actual item very well! The quality is awesome! Will be back to patronize again in the future!	Jingdong prefecture level city in Shandong
Power Saving King Shop	King of Power Saving	Ran the price is not high try to buy the attitude, did not expect the effect is really good, good!	Jingdong prefecture level city in Shandong
Power Saving King Shop	King of Power Saving	Power Saving King power is quite large, the quality of the leaf is quite good, the price of buying a very good deal, must be praised!	Jingdong prefecture level city in Shandong
Power Saving King Shop	King of Power Saving	Good product quality is the king of power-saving products trustworthy as always good to recommend!	Jingdong prefecture level city in Shandong
Power Saving King Shop	King of Power Saving	Works great, very affordable, shipped pretty fast, comfortable to use	Jingdong prefecture level city in Shandong
Power Saving King Shop	King of Power Saving	Shipping was pretty fast and affordable, not bad 👍	Jingdong prefecture level city in Shandong
Power Saving King Shop	King of Power Saving	Digital voltage display, voltage at a glance, plug and play, safe and simple!	Jingdong prefecture level city in Shandong
Power Saving King Shop	King of Power Saving	Quality assurance, very good, affordable, logistics is also very fast, I'm very satisfied with the	Jingdong prefecture level city in Shandong
Power Saving King Shop	King of Power Saving	The quality is very good, the first time I received the goods to open the first time to see, it is very good, very satisfied	Jingdong prefecture level city in Shandong
Power Saving King Shop	King of Power Saving	Power Saving King used, the effect is good, the price is affordable, very good, is the genuine article	Jingdong prefecture level city in Shandong
Power Saving King Shop	King of Power Saving	Good quality, fine workmanship, good appearance, time is very accurate, satisfied.	Jingdong prefecture level city in Shandong
Power Saving King Shop	King of Power Saving	I purposely used it for two days before commenting, which is responsible for myself and everyone else!	Jingdong prefecture level city in Shandong
Power Saving King Shop	King of Power Saving	Easy to install, easy to operate, helped our family save a lot of electricity this summer	Jingdong prefecture level city in Shandong
Power Saving King Shop	King of Power Saving	Bringing power savings into millions of homes. Still a great gadget. Pretty good.	Jingdong prefecture level city in Shandong
Power Saving King Shop	King of Power Saving	Digital liquid crystal display to see the clear intuitive, very formal workmanship is not rough	Jingdong prefecture level city in Shandong
Power Saving King Shop	King of Power Saving	Received. Great quality, fast shipping from the store and not too expensive!	Jingdong prefecture level city in Shandong
Power Saving King Shop	King of Power Saving	Bought myself a knuckle regulator and it was easy to use and the price was right.	Jingdong prefecture level city in Shandong
Power Saving King Shop	King of Power Saving	Received, good, powerful, good, very full of a shopping	Jingdong prefecture level city in Shandong
Power Saving King Shop	King of Power Saving	High power, consistent with the seller's description, powerful, cost-effective	Jingdong prefecture level city in Shandong
Power Saving King Shop	King of Power Saving	It works very well and also saves power at an affordable price a great deal.	Jingdong prefecture level city in Shandong
Power Saving King Shop	King of Power Saving	Good quality, good can recommend to friends to buy.	Jingdong prefecture level city in Shandong
Power Saving King Shop	King of Power Saving	Will repurchase, quality is guaranteed, safe and secure purchase.	Jingdong prefecture level city in Shandong
Power Saving King Shop	King of Power Saving	The logistics is very fast, the thing is also good to use, energy saving power rely on it!	Jingdong prefecture level city in Shandong
Power Saving King Shop	King of Power Saving	Power Saver is good quality and works very well, worth buying!	Jingdong prefecture level city in Shandong
Power Saving King Shop	King of Power Saving	The quality is very good is genuine, cost-effective recommended	Jingdong prefecture level city in Shandong
Power Saving King Shop	King of Power Saving	Quality Assurance. Favorable price. Very practical and durable	Jingdong prefecture level city in Shandong
Power Saving King Shop	King of Power Saving	Good value for money. Conscientious businessman. Recommended to everyone	Jingdong prefecture level city in Shandong
Power Saving King Shop	King of Power Saving	Buy it for your rental apartment and never worry about your landlord charging you too much.	Jingdong prefecture level city in Shandong
Power Saving King Shop	King of Power Saving	Nice and affordable, will repurchase next time	Jingdong prefecture level city in Shandong
Power Saving King Shop	King of Power Saving	It's very energy efficient. I've been using it for a few days now.	Jingdong prefecture level city in Shandong
Power Saving King Shop	King of Power Saving	Good quality, high power easy to use	Jingdong prefecture level city in Shandong
Power Saving King Shop	King of Power Saving	Power saving little helper. Deserves a favorable review.	Jingdong prefecture level city in Shandong
Power Saving King Shop	King of Power Saving	Great value for money, great value for money.	Jingdong prefecture level city in Shandong
Power Saving King Shop	King of Power Saving	Works well and as described by the seller	Jingdong prefecture level city in Shandong

Power Saving King Shop	King of Power Saving	Power saving is proper! It's good.	Jingdong prefecture level city in Shandong
Power Saving King Shop	King of Power Saving	Power saving. Works well. Not bad.	Jingdong prefecture level city in Shandong
Power Saving King Shop	King of Power Saving	Satisfied with power saving	Jingdong prefecture level city in Shandong
Power Saving King Shop	King of Power Saving	Received intact, open a super good look beautiful and generous, ~ bit of high quality is recommended to buy, the need for friends rest assured that the next order	Jingdong prefecture level city in Shandong
Power Saving King Shop	King of Power Saving	Very good to use, friends introduced, save electricity and money, long-lasting products cost-effective, large factory production, good quality, fine workmanship cost-effective!	Jingdong prefecture level city in Shandong
Power Saving King Shop	King of Power Saving	The power saving effect is very obvious, trying to pay 30% less electricity bill in a month than before, hahaha	Jingdong prefecture level city in Shandong
Power Saving King Shop	King of Power Saving	It's such a power saver to use this, it saves money on kids snacks for a month. Kudos!	Jingdong prefecture level city in Shandong
Power Saving King Shop	King of Power Saving	Plug and play, stable voltage, the screen has a display when working, this winter home appliances, need more electricity, just buy to use, save electricity and save money, with instructions, not too big and does not take up space, the quality is very good, and the seller describes exactly the same!	Jingdong prefecture level city in Shandong
Power Saving King Shop	King of Power Saving	A must have for opening a store. Save over a thousand dollars a month on your electric bill	Jingdong prefecture level city in Shandong
Power Saving King Shop	King of Power Saving	Received the goods, the first time to unpack, the quality is very good, with the seller's description, completely exceeded my expectations, the packaging is very careful, very strict, in general this is a very satisfied with the shopping	Jingdong prefecture level city in Shandong
Power Saving King Shop	King of Power Saving	As always, good quality, a very favorite product, great! o(*￣︶￣*)o The price is very affordable, cost-effective, it's considered affordable good, will repurchase!	Jingdong prefecture level city in Shandong
Power Saving King Shop	King of Power Saving	Very satisfied with a shopping, good business service, fast shipping, logistics is also very powerful, received very good, buy assured, use peace of mind!	Jingdong prefecture level city in Shandong
Power Saving King Shop	King of Power Saving	The first time to buy online, but also look at the evaluation before buying, the quality is not bad, but also very cheap, the need for friends can rest assured to buy!	Jingdong prefecture level city in Shandong
Power Saving King Shop	King of Power Saving	Better than expected, feel value for money, very satisfied with a shopping, need next time will come back!	Jingdong prefecture level city in Shandong
Power Saving King Shop	King of Power Saving	Really super like, very supportive, very good quality, exactly as described by the seller, very satisfied!	Jingdong prefecture level city in Shandong
Power Saving King Shop	King of Power Saving	It is genuine, effective, really saves power, affordable, tightly packed, fast shipping, very satisfied.	Jingdong prefecture level city in Shandong
Power Saving King Shop	King of Power Saving	Good quality, fine workmanship, is genuine and business description of the same, good value for money, fast logistics, very satisfied	Jingdong prefecture level city in Shandong
Power Saving King Shop	King of Power Saving	Saver received, very good, installation set up very well, very good, the effect is very good, recommend that the pro buy	Jingdong prefecture level city in Shandong
Power Saving King Shop	King of Power Saving	Electromagnetic good, high power convenient, enough force, the price is right it, quality can be 😀 😀 😀 😀 😀	Jingdong prefecture level city in Shandong
Power Saving King Shop	King of Power Saving	First-class quality, packaging is also good, logistics is very fast, and the price is reasonable, very affordable, I like it!	Jingdong prefecture level city in Shandong
Power Saving King Shop	King of Power Saving	It's great, it's the kind of style you want, and it arrived quickly, thank you boss, 5 stars for the service!	Jingdong prefecture level city in Shandong
Power Saving King Shop	King of Power Saving	Packaging is very good, the use of the effect is quite good, the store has a good service attitude, trustworthy	Jingdong prefecture level city in Shandong
Power Saving King Shop	King of Power Saving	The quality is very good, it works well, very satisfied, the price is affordable, it's so great value, it's worth recommending.	Jingdong prefecture level city in Shandong
Power Saving King Shop	King of Power Saving	The baby description matches the actual item very well! The quality is awesome! Will be back to patronize again in the future!	Jingdong prefecture level city in Shandong
Power Saving King Shop	King of Power Saving	Ran the price is not high try to buy the attitude, did not expect the effect is really good, good!	Jingdong prefecture level city in Shandong
Power Saving King Shop	King of Power Saving	Power Saving King power is quite large, the quality of the leaf is quite good, the price of buying a very good deal, must be praised!	Jingdong prefecture level city in Shandong
Power Saving King Shop	King of Power Saving	Good product quality is the king of power-saving products trustworthy as always good to recommend!	Jingdong prefecture level city in Shandong
Power Saving King Shop	King of Power Saving	Works great, very affordable, shipped pretty fast, comfortable to use	Jingdong prefecture level city in Shandong
Power Saving King Shop	King of Power Saving	Shipping was pretty fast and affordable, not bad 👍	Jingdong prefecture level city in Shandong
Power Saving King Shop	King of Power Saving	Digital voltage display, voltage at a glance, plug and play, safe and simple!	Jingdong prefecture level city in Shandong
Power Saving King Shop	King of Power Saving	Quality assurance, very good, affordable, logistics is also very fast, I'm very satisfied with the	Jingdong prefecture level city in Shandong
Power Saving King Shop	King of Power Saving	The quality is very good, the first time I received the goods to open the first time to see, it is very good, very satisfied	Jingdong prefecture level city in Shandong
Power Saving King Shop	King of Power Saving	Power Saving King used, the effect is good, the price is affordable, very good, is the genuine article	Jingdong prefecture level city in Shandong
Power Saving King Shop	King of Power Saving	Good quality, fine workmanship, good appearance, time is very accurate, satisfied.	Jingdong prefecture level city in Shandong
Power Saving King Shop	King of Power Saving	I purposely used it for two days before commenting, which is responsible for myself and everyone else!	Jingdong prefecture level city in Shandong
Power Saving King Shop	King of Power Saving	Easy to install, easy to operate, helped our family save a lot of electricity this summer	Jingdong prefecture level city in Shandong
Power Saving King Shop	King of Power Saving	Bringing power savings into millions of homes. Still a great gadget. Pretty good.	Jingdong prefecture level city in Shandong
Power Saving King Shop	King of Power Saving	Digital liquid crystal display to see the clear intuitive, very formal workmanship is not rough	Jingdong prefecture level city in Shandong
Power Saving King Shop	King of Power Saving	Received. Great quality, fast shipping from the store and not too expensive!	Jingdong prefecture level city in Shandong
Power Saving King Shop	King of Power Saving	Bought myself a knuckle regulator and it was easy to use and the price was right.	Jingdong prefecture level city in Shandong
Power Saving King Shop	King of Power Saving	Received, good, powerful, good, very full of a shopping	Jingdong prefecture level city in Shandong
Power Saving King Shop	King of Power Saving	High power, consistent with the seller's description, powerful, cost-effective	Jingdong prefecture level city in Shandong
Power Saving King Shop	King of Power Saving	It works very well and also saves power at an affordable price a great deal.	Jingdong prefecture level city in Shandong
Power Saving King Shop	King of Power Saving	Good quality, good can recommend to friends to buy.	Jingdong prefecture level city in Shandong
Power Saving King Shop	King of Power Saving	Will repurchase, quality is guaranteed, safe and secure purchase.	Jingdong prefecture level city in Shandong
Power Saving King Shop	King of Power Saving	The logistics is very fast, the thing is also good to use, energy saving power rely on it!	Jingdong prefecture level city in Shandong
Power Saving King Shop	King of Power Saving	Power Saver is good quality and works very well, worth buying!	Jingdong prefecture level city in Shandong
Power Saving King Shop	King of Power Saving	The quality is very good is genuine, cost-effective recommended	Jingdong prefecture level city in Shandong
Power Saving King Shop	King of Power Saving	Quality Assurance. Favorable price. Very practical and durable	Jingdong prefecture level city in Shandong
Power Saving King Shop	King of Power Saving	Good value for money. Conscientious businessman. Recommended to everyone	Jingdong prefecture level city in Shandong

Power Saving King Shop	King of Power Saving	Buy it for your rental apartment and never worry about your landlord charging you too much.	Jingdong prefecture level city in Shandong
Power Saving King Shop	King of Power Saving	Nice and affordable, will repurchase next time	Jingdong prefecture level city in Shandong
Power Saving King Shop	King of Power Saving	It's very energy efficient. I've been using it for a few days now.	Jingdong prefecture level city in Shandong
Power Saving King Shop	King of Power Saving	Good quality, high power easy to use	Jingdong prefecture level city in Shandong
Power Saving King Shop	King of Power Saving	Power saving little helper. Deserves a favorable review.	Jingdong prefecture level city in Shandong
Power Saving King Shop	King of Power Saving	Great value for money, great value for money.	Jingdong prefecture level city in Shandong
Power Saving King Shop	King of Power Saving	Works well and as described by the seller	Jingdong prefecture level city in Shandong
Power Saving King Shop	King of Power Saving	Power saving is proper! It's good.	Jingdong prefecture level city in Shandong
Power Saving King Shop	King of Power Saving	Power saving. Works well. Not bad.	Jingdong prefecture level city in Shandong
Power Saving King Shop	King of Power Saving	Satisfied with power saving	Jingdong prefecture level city in Shandong
Power Saving King Shop	King of Power Saving	Received intact, open a super good look beautiful and generous, ~ bit of high quality is recommended to buy, the need for friends rest assured that the next order	Jingdong prefecture level city in Shandong
Power Saving King Shop	King of Power Saving	Very good to use, friends introduced, save electricity and money, long-lasting products cost-effective, large factory production, good quality, fine workmanship cost-effective!	Jingdong prefecture level city in Shandong
Power Saving King Shop	King of Power Saving	The power saving effect is very obvious, trying to pay 30% less electricity bill in a month than before, hahaha	Jingdong prefecture level city in Shandong
Power Saving King Shop	King of Power Saving	It's such a power saver to use this, it saves money on kids snacks for a month. Kudos!	Jingdong prefecture level city in Shandong
Power Saving King Shop	King of Power Saving	Plug and play, stable voltage, the screen has a display when working, this winter home appliances, need more electricity, just buy to use, save electricity and save money, with instructions, not too big and does not take up space, the quality is very good, and the seller describes exactly the same!	Jingdong prefecture level city in Shandong
Power Saving King Shop	King of Power Saving	A must have for opening a store. Save over a thousand dollars a month on your electric bill	Jingdong prefecture level city in Shandong
Power Saving King Shop	King of Power Saving	Received the goods, the first time to unpack, the quality is very good, with the seller's description, completely exceeded my expectations, the packaging is very careful, very strict, in general this is a very satisfied with the shopping	Jingdong prefecture level city in Shandong
Power Saving King Shop	King of Power Saving	As always, good quality, a very favorite product, great! o(*￣︶￣*)o The price is very affordable, cost-effective, it's considered affordable good, will repurchase!	Jingdong prefecture level city in Shandong
Power Saving King Shop	King of Power Saving	Very satisfied with a shopping, good business service, fast shipping, logistics is also very powerful, received very good, buy assured, use peace of mind!	Jingdong prefecture level city in Shandong
Power Saving King Shop	King of Power Saving	The first time to buy online, but also look at the evaluation before buying, the quality is not bad, but also very cheap, the need for friends can rest assured to buy!	Jingdong prefecture level city in Shandong
Power Saving King Shop	King of Power Saving	Better than expected, feel value for money, very satisfied with a shopping, need next time will come back!	Jingdong prefecture level city in Shandong
Power Saving King Shop	King of Power Saving	Really super like, very supportive, very good quality, exactly as described by the seller, very satisfied!	Jingdong prefecture level city in Shandong
Power Saving King Shop	King of Power Saving	It is genuine, effective, really saves power, affordable, tightly packed, fast shipping, very satisfied.	Jingdong prefecture level city in Shandong
Power Saving King Shop	King of Power Saving	Good quality, fine workmanship, is genuine and business description of the same, good value for money, fast logistics, very satisfied	Jingdong prefecture level city in Shandong
Power Saving King Shop	King of Power Saving	Saver received, very good, installation set up very well, very good, the effect is very good, recommend that the pro buy	Jingdong prefecture level city in Shandong
Power Saving King Shop	King of Power Saving	Electromagnetic good, high power convenient, enough force, the price is right it, quality can be 😃 😃 😃 😃 😃	Jingdong prefecture level city in Shandong
Power Saving King Shop	King of Power Saving	First-class quality, packaging is also good, logistics is very fast, and the price is reasonable, very affordable, I like it!	Jingdong prefecture level city in Shandong
Power Saving King Shop	King of Power Saving	It's great, it's the kind of style you want, and it arrived quickly, thank you boss, 5 stars for the service!	Jingdong prefecture level city in Shandong
Power Saving King Shop	King of Power Saving	Packaging is very good, the use of the effect is quite good, the store has a good service attitude, trustworthy	Jingdong prefecture level city in Shandong
Power Saving King Shop	King of Power Saving	The quality is very good, it works well, very satisfied, the price is affordable, it's so great value, it's worth recommending.	Jingdong prefecture level city in Shandong
Power Saving King Shop	King of Power Saving	The baby description matches the actual item very well! The quality is awesome! Will be back to patronize again in the future!	Jingdong prefecture level city in Shandong
Power Saving King Shop	King of Power Saving	Ran the price is not high try to buy the attitude, did not expect the effect is really good, good!	Jingdong prefecture level city in Shandong
Power Saving King Shop	King of Power Saving	Power Saving King power is quite large, the quality of the leaf is quite good, the price of buying a very good deal, must be praised!	Jingdong prefecture level city in Shandong
Power Saving King Shop	King of Power Saving	Good product quality is the king of power-saving products trustworthy as always good to recommend!	Jingdong prefecture level city in Shandong
Power Saving King Shop	King of Power Saving	Works great, very affordable, shipped pretty fast, comfortable to use	Jingdong prefecture level city in Shandong
Power Saving King Shop	King of Power Saving	Shipping was pretty fast and affordable, not bad 👍	Jingdong prefecture level city in Shandong
Power Saving King Shop	King of Power Saving	Digital voltage display, voltage at a glance, plug and play, safe and simple!	Jingdong prefecture level city in Shandong
Power Saving King Shop	King of Power Saving	Quality assurance, very good, affordable, logistics is also very fast, I'm very satisfied with the	Jingdong prefecture level city in Shandong
Power Saving King Shop	King of Power Saving	The quality is very good, the first time I received the goods to open the first time to see, it is very good, very satisfied	Jingdong prefecture level city in Shandong
Power Saving King Shop	King of Power Saving	Power Saving King used, the effect is good, the price is affordable, very good, is the genuine article	Jingdong prefecture level city in Shandong
Power Saving King Shop	King of Power Saving	Good quality, fine workmanship, good appearance, time is very accurate, satisfied.	Jingdong prefecture level city in Shandong
Power Saving King Shop	King of Power Saving	I purposely used it for two days before commenting, which is responsible for myself and everyone else!	Jingdong prefecture level city in Shandong
Power Saving King Shop	King of Power Saving	Easy to install, easy to operate, helped our family save a lot of electricity this summer	Jingdong prefecture level city in Shandong
Power Saving King Shop	King of Power Saving	Bringing power savings into millions of homes. Still a great gadget. Pretty good.	Jingdong prefecture level city in Shandong
Power Saving King Shop	King of Power Saving	Digital liquid crystal display to see the clear intuitive, very formal workmanship is not rough	Jingdong prefecture level city in Shandong
Power Saving King Shop	King of Power Saving	Received. Great quality, fast shipping from the store and not too expensive!	Jingdong prefecture level city in Shandong
Power Saving King Shop	King of Power Saving	Bought myself a knuckle regulator and it was easy to use and the price was right.	Jingdong prefecture level city in Shandong
Power Saving King Shop	King of Power Saving	Received, good, powerful, good, very full of a shopping	Jingdong prefecture level city in Shandong
Power Saving King Shop	King of Power Saving	High power, consistent with the seller's description, powerful, cost-effective	Jingdong prefecture level city in Shandong
Power Saving King Shop	King of Power Saving	It works very well and also saves power at an affordable price a great deal.	Jingdong prefecture level city in Shandong

Power Saving King Shop	King of Power Saving	Good quality, good can recommend to friends to buy.	Jingdong prefecture level city in Shandong
Power Saving King Shop	King of Power Saving	Will repurchase, quality is guaranteed, safe and secure purchase.	Jingdong prefecture level city in Shandong
Power Saving King Shop	King of Power Saving	The logistics is very fast, the thing is also good to use, energy saving power rely on it!	Jingdong prefecture level city in Shandong
Power Saving King Shop	King of Power Saving	Power Saver is good quality and works very well, worth buying!	Jingdong prefecture level city in Shandong
Power Saving King Shop	King of Power Saving	The quality is very good is genuine, cost-effective recommended	Jingdong prefecture level city in Shandong
Power Saving King Shop	King of Power Saving	Quality Assurance. Favorable price. Very practical and durable	Jingdong prefecture level city in Shandong
Power Saving King Shop	King of Power Saving	Good value for money. Conscientious businessman. Recommended to everyone	Jingdong prefecture level city in Shandong
Power Saving King Shop	King of Power Saving	Buy it for your rental apartment and never worry about your landlord charging you too much.	Jingdong prefecture level city in Shandong
Power Saving King Shop	King of Power Saving	Nice and affordable, will repurchase next time	Jingdong prefecture level city in Shandong
Power Saving King Shop	King of Power Saving	It's very energy efficient. I've been using it for a few days now.	Jingdong prefecture level city in Shandong
Power Saving King Shop	King of Power Saving	Good quality, high power easy to use	Jingdong prefecture level city in Shandong
Power Saving King Shop	King of Power Saving	Power saving little helper. Deserves a favorable review.	Jingdong prefecture level city in Shandong
Power Saving King Shop	King of Power Saving	Great value for money, great value for money.	Jingdong prefecture level city in Shandong
Power Saving King Shop	King of Power Saving	Works well and as described by the seller	Jingdong prefecture level city in Shandong
Power Saving King Shop	King of Power Saving	Power saving is proper! It's good.	Jingdong prefecture level city in Shandong
Power Saving King Shop	King of Power Saving	Power saving. Works well. Not bad.	Jingdong prefecture level city in Shandong
Power Saving King Shop	King of Power Saving	Satisfied with power saving	Jingdong prefecture level city in Shandong
Power Saving King Shop	King of Power Saving	Received intact, open a super good look beautiful and generous, ~ bit of high quality is recommended to buy, the need for friends rest assured that the next order	Jingdong prefecture level city in Shandong
Power Saving King Shop	King of Power Saving	Very good to use, friends introduced, save electricity and money, long-lasting products cost-effective, large factory production, good quality, fine workmanship cost-effective!	Jingdong prefecture level city in Shandong
Power Saving King Shop	King of Power Saving	The power saving effect is very obvious, trying to pay 30% less electricity bill in a month than before, hahaha	Jingdong prefecture level city in Shandong
Power Saving King Shop	King of Power Saving	It's such a power saver to use this, it saves money on kids snacks for a month. Kudos!	Jingdong prefecture level city in Shandong
Power Saving King Shop	King of Power Saving	Plug and play, stable voltage, the screen has a display when working, this winter home appliances, need more electricity, just buy to use, save electricity and save money, with instructions, not too big and does not take up space, the quality is very good, and the seller describes exactly the same!	Jingdong prefecture level city in Shandong
Power Saving King Shop	King of Power Saving	A must have for opening a store. Save over a thousand dollars a month on your electric bill	Jingdong prefecture level city in Shandong
Power Saving King Shop	King of Power Saving	Received the goods, the first time to unpack, the quality is very good, with the seller's description, completely exceeded my expectations, the packaging is very careful, very strict, in general this is a very satisfied with the shopping	Jingdong prefecture level city in Shandong
Power Saving King Shop	King of Power Saving	As always, good quality, a very favorite product, great! o(*￣︶￣*)o The price is very affordable, cost-effective, it's considered affordable good, will repurchase!	Jingdong prefecture level city in Shandong
Power Saving King Shop	King of Power Saving	Very satisfied with a shopping, good business service, fast shipping, logistics is also very powerful, received very good, buy assured, use peace of mind!	Jingdong prefecture level city in Shandong
Power Saving King Shop	King of Power Saving	The first time to buy online, but also look at the evaluation before buying, the quality is not bad, but also very cheap, the need for friends can rest assured to buy!	Jingdong prefecture level city in Shandong
Power Saving King Shop	King of Power Saving	Better than expected, feel value for money, very satisfied with a shopping, need next time will come back!	Jingdong prefecture level city in Shandong
Power Saving King Shop	King of Power Saving	Really super like, very supportive, very good quality, exactly as described by the seller, very satisfied!	Jingdong prefecture level city in Shandong
Power Saving King Shop	King of Power Saving	It is genuine, effective, really saves power, affordable, tightly packed, fast shipping, very satisfied.	Jingdong prefecture level city in Shandong
Power Saving King Shop	King of Power Saving	Good quality, fine workmanship, is genuine and business description of the same, good value for money, fast logistics, very satisfied	Jingdong prefecture level city in Shandong
Power Saving King Shop	King of Power Saving	Saver received, very good, installation set up very well, very good, the effect is very good, recommend that the pro buy	Jingdong prefecture level city in Shandong
Power Saving King Shop	King of Power Saving	Electromagnetic good, high power convenient, enough force, the price is right it, quality can be 😊 😊 😊 😊 😊	Jingdong prefecture level city in Shandong
Power Saving King Shop	King of Power Saving	First-class quality, packaging is also good, logistics is very fast, and the price is reasonable, very affordable, I like it!	Jingdong prefecture level city in Shandong
Power Saving King Shop	King of Power Saving	It's great, it's the kind of style you want, and it arrived quickly, thank you boss, 5 stars for the service!	Jingdong prefecture level city in Shandong
Power Saving King Shop	King of Power Saving	Packaging is very good, the use of the effect is quite good, the store has a good service attitude, trustworthy	Jingdong prefecture level city in Shandong
Power Saving King Shop	King of Power Saving	The quality is very good, it works well, very satisfied, the price is affordable, it's so great value, it's worth recommending.	Jingdong prefecture level city in Shandong
Power Saving King Shop	King of Power Saving	The baby description matches the actual item very well! The quality is awesome! Will be back to patronize again in the future!	Jingdong prefecture level city in Shandong
Power Saving King Shop	King of Power Saving	Ran the price is not high try to buy the attitude, did not expect the effect is really good, good!	Jingdong prefecture level city in Shandong
Power Saving King Shop	King of Power Saving	Power Saving King power is quite large, the quality of the leaf is quite good, the price of buying a very good deal, must be praised!	Jingdong prefecture level city in Shandong
Power Saving King Shop	King of Power Saving	Good product quality is the king of power-saving products trustworthy as always good to recommend!	Jingdong prefecture level city in Shandong
Power Saving King Shop	King of Power Saving	Works great, very affordable, shipped pretty fast, comfortable to use	Jingdong prefecture level city in Shandong
Power Saving King Shop	King of Power Saving	Shipping was pretty fast and affordable, not bad 👍	Jingdong prefecture level city in Shandong
Power Saving King Shop	King of Power Saving	Digital voltage display, voltage at a glance, plug and play, safe and simple!	Jingdong prefecture level city in Shandong
Power Saving King Shop	King of Power Saving	Quality assurance, very good, affordable, logistics is also very fast, I'm very satisfied with the	Jingdong prefecture level city in Shandong
Power Saving King Shop	King of Power Saving	The quality is very good, the first time I received the goods to open the first time to see, it is very good, very satisfied	Jingdong prefecture level city in Shandong
Power Saving King Shop	King of Power Saving	Power Saving King used, the effect is good, the price is affordable, very good, is the genuine article	Jingdong prefecture level city in Shandong
Power Saving King Shop	King of Power Saving	Good quality, fine workmanship, good appearance, time is very accurate, satisfied.	Jingdong prefecture level city in Shandong
Power Saving King Shop	King of Power Saving	I purposely used it for two days before commenting, which is responsible for myself and everyone else!	Jingdong prefecture level city in Shandong
Power Saving King Shop	King of Power Saving	Easy to install, easy to operate, helped our family save a lot of electricity this summer	Jingdong prefecture level city in Shandong

Power Saving King Shop	King of Power Saving	Bringing power savings into millions of homes. Still a great gadget. Pretty good.	Jingdong prefecture level city in Shandong
Power Saving King Shop	King of Power Saving	Digital liquid crystal display to see the clear intuitive, very formal workmanship is not rough	Jingdong prefecture level city in Shandong
Power Saving King Shop	King of Power Saving	Received. Great quality, fast shipping from the store and not too expensive!	Jingdong prefecture level city in Shandong
Power Saving King Shop	King of Power Saving	Bought myself a knuckle regulator and it was easy to use and the price was right.	Jingdong prefecture level city in Shandong
Power Saving King Shop	King of Power Saving	Received, good, powerful, good, very full of a shopping	Jingdong prefecture level city in Shandong
Power Saving King Shop	King of Power Saving	High power, consistent with the seller's description, powerful, cost-effective	Jingdong prefecture level city in Shandong
Power Saving King Shop	King of Power Saving	It works very well and also saves power at an affordable price a great deal.	Jingdong prefecture level city in Shandong
Power Saving King Shop	King of Power Saving	Good quality, good can recommend to friends to buy.	Jingdong prefecture level city in Shandong
Power Saving King Shop	King of Power Saving	Will repurchase, quality is guaranteed, safe and secure purchase.	Jingdong prefecture level city in Shandong
Power Saving King Shop	King of Power Saving	The logistics is very fast, the thing is also good to use, energy saving power rely on it!	Jingdong prefecture level city in Shandong
Power Saving King Shop	King of Power Saving	Power Saver is good quality and works very well, worth buying!	Jingdong prefecture level city in Shandong
Power Saving King Shop	King of Power Saving	The quality is very good is genuine, cost-effective recommended	Jingdong prefecture level city in Shandong
Power Saving King Shop	King of Power Saving	Quality Assurance. Favorable price. Very practical and durable	Jingdong prefecture level city in Shandong
Power Saving King Shop	King of Power Saving	Good value for money. Conscientious businessman. Recommended to everyone	Jingdong prefecture level city in Shandong
Power Saving King Shop	King of Power Saving	Buy it for your rental apartment and never worry about your landlord charging you too much.	Jingdong prefecture level city in Shandong
Power Saving King Shop	King of Power Saving	Nice and affordable, will repurchase next time	Jingdong prefecture level city in Shandong
Power Saving King Shop	King of Power Saving	It's very energy efficient. I've been using it for a few days now.	Jingdong prefecture level city in Shandong
Power Saving King Shop	King of Power Saving	Good quality, high power easy to use	Jingdong prefecture level city in Shandong
Power Saving King Shop	King of Power Saving	Power saving little helper. Deserves a favorable review.	Jingdong prefecture level city in Shandong
Power Saving King Shop	King of Power Saving	Great value for money, great value for money.	Jingdong prefecture level city in Shandong
Power Saving King Shop	King of Power Saving	Works well and as described by the seller	Jingdong prefecture level city in Shandong
Power Saving King Shop	King of Power Saving	Power saving is proper! It's good.	Jingdong prefecture level city in Shandong
Power Saving King Shop	King of Power Saving	Power saving. Works well. Not bad.	Jingdong prefecture level city in Shandong
Power Saving King Shop	King of Power Saving	Satisfied with power saving	Jingdong prefecture level city in Shandong
Power Saving King Shop	King of Power Saving	Received intact, open a super good look beautiful and generous, ~ bit of high quality is recommended to buy, the need for friends rest assured that the next order	Jingdong prefecture level city in Shandong
Power Saving King Shop	King of Power Saving	Very good to use, friends introduced, save electricity and money, long-lasting products cost-effective, large factory production, good quality, fine workmanship cost-effective!	Jingdong prefecture level city in Shandong
Power Saving King Shop	King of Power Saving	The power saving effect is very obvious, trying to pay 30% less electricity bill in a month than before, hahaha	Jingdong prefecture level city in Shandong
Power Saving King Shop	King of Power Saving	It's such a power saver to use this, it saves money on kids snacks for a month. Kudos!	Jingdong prefecture level city in Shandong
Power Saving King Shop	King of Power Saving	Plug and play, stable voltage, the screen has a display when working, this winter home appliances, need more electricity, just buy to use, save electricity and save money, with instructions, not too big and does not take up space, the quality is very good, and the seller describes exactly the same!	Jingdong prefecture level city in Shandong
Power Saving King Shop	King of Power Saving	A must have for opening a store. Save over a thousand dollars a month on your electric bill	Jingdong prefecture level city in Shandong
Power Saving King Shop	King of Power Saving	Received the goods, the first time to unpack, the quality is very good, with the seller's description, completely exceeded my expectations, the packaging is very careful, very strict, in general this is a very satisfied with the shopping	Jingdong prefecture level city in Shandong
Power Saving King Shop	King of Power Saving	As always, good quality, a very favorite product, great! o(*￣︶￣*)o The price is very affordable, cost-effective, it's considered affordable good, will repurchase!	Jingdong prefecture level city in Shandong
Power Saving King Shop	King of Power Saving	Very satisfied with a shopping, good business service, fast shipping, logistics is also very powerful, received very good, buy assured, use peace of mind!	Jingdong prefecture level city in Shandong
Power Saving King Shop	King of Power Saving	The first time to buy online, but also look at the evaluation before buying, the quality is not bad, but also very cheap, the need for friends can rest assured to buy!	Jingdong prefecture level city in Shandong
Power Saving King Shop	King of Power Saving	Better than expected, feel value for money, very satisfied with a shopping, need next time will come back!	Jingdong prefecture level city in Shandong
Power Saving King Shop	King of Power Saving	Really super like, very supportive, very good quality, exactly as described by the seller, very satisfied!	Jingdong prefecture level city in Shandong
Power Saving King Shop	King of Power Saving	It is genuine, effective, really saves power, affordable, tightly packed, fast shipping, very satisfied.	Jingdong prefecture level city in Shandong
Power Saving King Shop	King of Power Saving	Good quality, fine workmanship, is genuine and business description of the same, good value for money, fast logistics, very satisfied	Jingdong prefecture level city in Shandong
Power Saving King Shop	King of Power Saving	Saver received, very good, installation set up very well, very good, the effect is very good, recommend that the pro buy	Jingdong prefecture level city in Shandong
Power Saving King Shop	King of Power Saving	Electromagnetic good, high power convenient, enough force, the price is right it, quality can be 😃 😃 😃 😃 😃	Jingdong prefecture level city in Shandong
Power Saving King Shop	King of Power Saving	First-class quality, packaging is also good, logistics is very fast, and the price is reasonable, very affordable, I like it!	Jingdong prefecture level city in Shandong
Power Saving King Shop	King of Power Saving	It's great, it's the kind of style you want, and it arrived quickly, thank you boss, 5 stars for the service!	Jingdong prefecture level city in Shandong
Power Saving King Shop	King of Power Saving	Packaging is very good, the use of the effect is quite good, the store has a good service attitude, trustworthy	Jingdong prefecture level city in Shandong
Power Saving King Shop	King of Power Saving	The quality is very good, it works well, very satisfied, the price is affordable, it's so great value, it's worth recommending.	Jingdong prefecture level city in Shandong
Power Saving King Shop	King of Power Saving	The baby description matches the actual item very well! The quality is awesome! Will be back to patronize again in the future!	Jingdong prefecture level city in Shandong
Power Saving King Shop	King of Power Saving	Ran the price is not high try to buy the attitude, did not expect the effect is really good, good!	Jingdong prefecture level city in Shandong
Power Saving King Shop	King of Power Saving	Power Saving King power is quite large, the quality of the leaf is quite good, the price of buying a very good deal, must be praised!	Jingdong prefecture level city in Shandong
Power Saving King Shop	King of Power Saving	Good product quality is the king of power-saving products trustworthy as always good to recommend!	Jingdong prefecture level city in Shandong
Power Saving King Shop	King of Power Saving	Works great, very affordable, shipped pretty fast, comfortable to use	Jingdong prefecture level city in Shandong
Power Saving King Shop	King of Power Saving	Shipping was pretty fast and affordable, not bad 👍	Jingdong prefecture level city in Shandong

Power Saving King Shop	King of Power Saving	Digital voltage display, voltage at a glance, plug and play, safe and simple!	Jingdong prefecture level city in Shandong
Power Saving King Shop	King of Power Saving	Quality assurance, very good, affordable, logistics is also very fast, I'm very satisfied with the	Jingdong prefecture level city in Shandong
Power Saving King Shop	King of Power Saving	The quality is very good, the first time I received the goods to open the first time to see, it is very good, very satisfied	Jingdong prefecture level city in Shandong
Power Saving King Shop	King of Power Saving	Power Saving King used, the effect is good, the price is affordable, very good, is the genuine article	Jingdong prefecture level city in Shandong
Power Saving King Shop	King of Power Saving	Good quality, fine workmanship, good appearance, time is very accurate, satisfied.	Jingdong prefecture level city in Shandong
Power Saving King Shop	King of Power Saving	I purposely used it for two days before commenting, which is responsible for myself and everyone else!	Jingdong prefecture level city in Shandong
Power Saving King Shop	King of Power Saving	Easy to install, easy to operate, helped our family save a lot of electricity this summer	Jingdong prefecture level city in Shandong
Power Saving King Shop	King of Power Saving	Bringing power savings into millions of homes. Still a great gadget. Pretty good.	Jingdong prefecture level city in Shandong
Power Saving King Shop	King of Power Saving	Digital liquid crystal display to see the clear intuitive, very formal workmanship is not rough	Jingdong prefecture level city in Shandong
Power Saving King Shop	King of Power Saving	Received. Great quality, fast shipping from the store and not too expensive!	Jingdong prefecture level city in Shandong
Power Saving King Shop	King of Power Saving	Bought myself a knuckle regulator and it was easy to use and the price was right.	Jingdong prefecture level city in Shandong
Power Saving King Shop	King of Power Saving	Received, good, powerful, good, very full of a shopping	Jingdong prefecture level city in Shandong
Power Saving King Shop	King of Power Saving	High power, consistent with the seller's description, powerful, cost-effective	Jingdong prefecture level city in Shandong
Power Saving King Shop	King of Power Saving	It works very well and also saves power at an affordable price a great deal.	Jingdong prefecture level city in Shandong
Power Saving King Shop	King of Power Saving	Good quality, good can recommend to friends to buy.	Jingdong prefecture level city in Shandong
Power Saving King Shop	King of Power Saving	Will repurchase, quality is guaranteed, safe and secure purchase.	Jingdong prefecture level city in Shandong
Power Saving King Shop	King of Power Saving	The logistics is very fast, the thing is also good to use, energy saving power rely on it!	Jingdong prefecture level city in Shandong
Power Saving King Shop	King of Power Saving	Power Saver is good quality and works very well, worth buying!	Jingdong prefecture level city in Shandong
Power Saving King Shop	King of Power Saving	The quality is very good is genuine, cost-effective recommended	Jingdong prefecture level city in Shandong
Power Saving King Shop	King of Power Saving	Quality Assurance. Favorable price. Very practical and durable	Jingdong prefecture level city in Shandong
Power Saving King Shop	King of Power Saving	Good value for money. Conscientious businessman. Recommended to everyone	Jingdong prefecture level city in Shandong
Power Saving King Shop	King of Power Saving	Buy it for your rental apartment and never worry about your landlord charging you too much.	Jingdong prefecture level city in Shandong
Power Saving King Shop	King of Power Saving	Nice and affordable, will repurchase next time	Jingdong prefecture level city in Shandong
Power Saving King Shop	King of Power Saving	It's very energy efficient. I've been using it for a few days now.	Jingdong prefecture level city in Shandong
Power Saving King Shop	King of Power Saving	Good quality, high power easy to use	Jingdong prefecture level city in Shandong
Power Saving King Shop	King of Power Saving	Power saving little helper. Deserves a favorable review.	Jingdong prefecture level city in Shandong
Power Saving King Shop	King of Power Saving	Great value for money, great value for money.	Jingdong prefecture level city in Shandong
Power Saving King Shop	King of Power Saving	Works well and as described by the seller	Jingdong prefecture level city in Shandong
Power Saving King Shop	King of Power Saving	Power saving is proper! It's good.	Jingdong prefecture level city in Shandong
Power Saving King Shop	King of Power Saving	Power saving. Works well. Not bad.	Jingdong prefecture level city in Shandong
Power Saving King Shop	King of Power Saving	Satisfied with power saving	Jingdong prefecture level city in Shandong
Power Saving King Shop	King of Power Saving	Received intact, open a super good look beautiful and generous, ~ bit of high quality is recommended to buy, the need for friends rest assured that the next order	Jingdong prefecture level city in Shandong
Power Saving King Shop	King of Power Saving	Very good to use, friends introduced, save electricity and money, long-lasting products cost-effective, large factory production, good quality, fine workmanship cost-effective!	Jingdong prefecture level city in Shandong
Power Saving King Shop	King of Power Saving	The power saving effect is very obvious, trying to pay 30% less electricity bill in a month than before, hahaha	Jingdong prefecture level city in Shandong
Power Saving King Shop	King of Power Saving	It's such a power saver to use this, it saves money on kids snacks for a month. Kudos!	Jingdong prefecture level city in Shandong
Power Saving King Shop	King of Power Saving	Plug and play, stable voltage, the screen has a display when working, this winter home appliances, need more electricity, just buy to use, save electricity and save money, with instructions, not too big and does not take up space, the quality is very good, and the seller describes exactly the same!	Jingdong prefecture level city in Shandong
Power Saving King Shop	King of Power Saving	A must have for opening a store. Save over a thousand dollars a month on your electric bill	Jingdong prefecture level city in Shandong
Power Saving King Shop	King of Power Saving	Received the goods, the first time to unpack, the quality is very good, with the seller's description, completely exceeded my expectations, the packaging is very careful, very strict, in general this is a very satisfied with the shopping	Jingdong prefecture level city in Shandong
Power Saving King Shop	King of Power Saving	As always, good quality, a very favorite product, great! o(*￣︶￣*)o The price is very affordable, cost-effective, it's considered affordable good, will repurchase!	Jingdong prefecture level city in Shandong
Power Saving King Shop	King of Power Saving	Very satisfied with a shopping, good business service, fast shipping, logistics is also very powerful, received very good, buy assured, use peace of mind!	Jingdong prefecture level city in Shandong
Power Saving King Shop	King of Power Saving	The first time to buy online, but also look at the evaluation before buying, the quality is not bad, but also very cheap, the need for friends can rest assured to buy!	Jingdong prefecture level city in Shandong
Power Saving King Shop	King of Power Saving	Better than expected, feel value for money, very satisfied with a shopping, need next time will come back!	Jingdong prefecture level city in Shandong
Power Saving King Shop	King of Power Saving	Really super like, very supportive, very good quality, exactly as described by the seller, very satisfied!	Jingdong prefecture level city in Shandong
Power Saving King Shop	King of Power Saving	It is genuine, effective, really saves power, affordable, tightly packed, fast shipping, very satisfied.	Jingdong prefecture level city in Shandong
Power Saving King Shop	King of Power Saving	Good quality, fine workmanship, is genuine and business description of the same, good value for money, fast logistics, very satisfied	Jingdong prefecture level city in Shandong
Power Saving King Shop	King of Power Saving	Saver received, very good, installation set up very well, very good, the effect is very good, recommend that the pro buy	Jingdong prefecture level city in Shandong
Power Saving King Shop	King of Power Saving	Electromagnetic good, high power convenient, enough force, the price is right it, quality can be ☺ ☺ ☺ ☺ ☺	Jingdong prefecture level city in Shandong
Power Saving King Shop	King of Power Saving	First-class quality, packaging is also good, logistics is very fast, and the price is reasonable, very affordable, I like it!	Jingdong prefecture level city in Shandong
Power Saving King Shop	King of Power Saving	It's great, it's the kind of style you want, and it arrived quickly, thank you boss, 5 stars for the service!	Jingdong prefecture level city in Shandong
Power Saving King Shop	King of Power Saving	Packaging is very good, the use of the effect is quite good, the store has a good service attitude, trustworthy	Jingdong prefecture level city in Shandong

Power Saving King Shop	King of Power Saving	The quality is very good, it works well, very satisfied, the price is affordable, it's so great value, it's worth recommending.	Jingdong prefecture level city in Shandong
Power Saving King Shop	King of Power Saving	The baby description matches the actual item very well! The quality is awesome! Will be back to patronize again in the future!	Jingdong prefecture level city in Shandong
Power Saving King Shop	King of Power Saving	Ran the price is not high try to buy the attitude, did not expect the effect is really good, good!	Jingdong prefecture level city in Shandong
Power Saving King Shop	King of Power Saving	Power Saving King power is quite large, the quality of the leaf is quite good, the price of buying a very good deal, must be praised!	Jingdong prefecture level city in Shandong
Power Saving King Shop	King of Power Saving	Good product quality is the king of power-saving products trustworthy as always good to recommend!	Jingdong prefecture level city in Shandong
Power Saving King Shop	King of Power Saving	Works great, very affordable, shipped pretty fast, comfortable to use	Jingdong prefecture level city in Shandong
Power Saving King Shop	King of Power Saving	Shipping was pretty fast and affordable, not bad 👍	Jingdong prefecture level city in Shandong
Power Saving King Shop	King of Power Saving	Digital voltage display, voltage at a glance, plug and play, safe and simple!	Jingdong prefecture level city in Shandong
Power Saving King Shop	King of Power Saving	Quality assurance, very good, affordable, logistics is also very fast, I'm very satisfied with the	Jingdong prefecture level city in Shandong
Power Saving King Shop	King of Power Saving	The quality is very good, the first time I received the goods to open the first time to see, it is very good, very satisfied	Jingdong prefecture level city in Shandong
Power Saving King Shop	King of Power Saving	Power Saving King used, the effect is good, the price is affordable, very good, is the genuine article	Jingdong prefecture level city in Shandong
Power Saving King Shop	King of Power Saving	Good quality, fine workmanship, good appearance, time is very accurate, satisfied.	Jingdong prefecture level city in Shandong
Power Saving King Shop	King of Power Saving	I purposely used it for two days before commenting, which is responsible for myself and everyone else!	Jingdong prefecture level city in Shandong
Power Saving King Shop	King of Power Saving	Easy to install, easy to operate, helped our family save a lot of electricity this summer	Jingdong prefecture level city in Shandong
Power Saving King Shop	King of Power Saving	Bringing power savings into millions of homes. Still a great gadget. Pretty good.	Jingdong prefecture level city in Shandong
Power Saving King Shop	King of Power Saving	Digital liquid crystal display to see the clear intuitive, very formal workmanship is not rough	Jingdong prefecture level city in Shandong
Power Saving King Shop	King of Power Saving	Received. Great quality, fast shipping from the store and not too expensive!	Jingdong prefecture level city in Shandong
Power Saving King Shop	King of Power Saving	Bought myself a knuckle regulator and it was easy to use and the price was right.	Jingdong prefecture level city in Shandong
Power Saving King Shop	King of Power Saving	Received, good, powerful, good, very full of a shopping	Jingdong prefecture level city in Shandong
Power Saving King Shop	King of Power Saving	High power, consistent with the seller's description, powerful, cost-effective	Jingdong prefecture level city in Shandong
Power Saving King Shop	King of Power Saving	It works very well and also saves power at an affordable price a great deal.	Jingdong prefecture level city in Shandong
Power Saving King Shop	King of Power Saving	Good quality, good can recommend to friends to buy.	Jingdong prefecture level city in Shandong
Power Saving King Shop	King of Power Saving	Will repurchase, quality is guaranteed, safe and secure purchase.	Jingdong prefecture level city in Shandong
Power Saving King Shop	King of Power Saving	The logistics is very fast, the thing is also good to use, energy saving power rely on it!	Jingdong prefecture level city in Shandong
Power Saving King Shop	King of Power Saving	Power Saver is good quality and works very well, worth buying!	Jingdong prefecture level city in Shandong
Power Saving King Shop	King of Power Saving	The quality is very good is genuine, cost-effective recommended	Jingdong prefecture level city in Shandong
Power Saving King Shop	King of Power Saving	Quality Assurance. Favorable price. Very practical and durable	Jingdong prefecture level city in Shandong
Power Saving King Shop	King of Power Saving	Good value for money. Conscientious businessman. Recommended to everyone	Jingdong prefecture level city in Shandong
Power Saving King Shop	King of Power Saving	Buy it for your rental apartment and never worry about your landlord charging you too much.	Jingdong prefecture level city in Shandong
Power Saving King Shop	King of Power Saving	Nice and affordable, will repurchase next time	Jingdong prefecture level city in Shandong
Power Saving King Shop	King of Power Saving	It's very energy efficient. I've been using it for a few days now.	Jingdong prefecture level city in Shandong
Power Saving King Shop	King of Power Saving	Good quality, high power easy to use	Jingdong prefecture level city in Shandong
Power Saving King Shop	King of Power Saving	Power saving little helper. Deserves a favorable review.	Jingdong prefecture level city in Shandong
Power Saving King Shop	King of Power Saving	Great value for money, great value for money.	Jingdong prefecture level city in Shandong
Power Saving King Shop	King of Power Saving	Works well and as described by the seller	Jingdong prefecture level city in Shandong
Power Saving King Shop	King of Power Saving	Power saving is proper! It's good.	Jingdong prefecture level city in Shandong
Power Saving King Shop	King of Power Saving	Power saving. Works well. Not bad.	Jingdong prefecture level city in Shandong
Power Saving King Shop	King of Power Saving	Satisfied with power saving	Jingdong prefecture level city in Shandong
Power Saving King Shop	King of Power Saving	Received intact, open a super good look beautiful and generous, ~ bit of high quality is recommended to buy, the need for friends rest assured that the next order	Jingdong prefecture level city in Shandong
Power Saving King Shop	King of Power Saving	Very good to use, friends introduced, save electricity and money, long-lasting products cost-effective, large factory production, good quality, fine workmanship cost-effective!	Jingdong prefecture level city in Shandong
Power Saving King Shop	King of Power Saving	The power saving effect is very obvious, trying to pay 30% less electricity bill in a month than before, hahaha	Jingdong prefecture level city in Shandong
Power Saving King Shop	King of Power Saving	It's such a power saver to use this, it saves money on kids snacks for a month. Kudos!	Jingdong prefecture level city in Shandong
Power Saving King Shop	King of Power Saving	Plug and play, stable voltage, the screen has a display when working, this winter home appliances, need more electricity, just buy to use, save electricity and save money, with instructions, not too big and does not take up space, the quality is very good, and the seller describes exactly the same!	Jingdong prefecture level city in Shandong
Power Saving King Shop	King of Power Saving	A must have for opening a store. Save over a thousand dollars a month on your electric bill	Jingdong prefecture level city in Shandong
Power Saving King Shop	King of Power Saving	Received the goods, the first time to unpack, the quality is very good, with the seller's description, completely exceeded my expectations, the packaging is very careful, very strict, in general this is a very satisfied with the shopping	Jingdong prefecture level city in Shandong
Power Saving King Shop	King of Power Saving	As always, good quality, a very favorite product, great! o(*￣︶￣*)o The price is very affordable, cost-effective, it's considered affordable good, will repurchase!	Jingdong prefecture level city in Shandong
Power Saving King Shop	King of Power Saving	Very satisfied with a shopping, good business service, fast shipping, logistics is also very powerful, received very good, buy assured, use peace of mind!	Jingdong prefecture level city in Shandong
Power Saving King Shop	King of Power Saving	The first time to buy online, but also look at the evaluation before buying, the quality is not bad, but also very cheap, the need for friends can rest assured to buy!	Jingdong prefecture level city in Shandong
Power Saving King Shop	King of Power Saving	Better than expected, feel value for money, very satisfied with a shopping, need next time will come back!	Jingdong prefecture level city in Shandong
Power Saving King Shop	King of Power Saving	Really super like, very supportive, very good quality, exactly as described by the seller, very satisfied!	Jingdong prefecture level city in Shandong

Power Saving King Shop	King of Power Saving	It is genuine, effective, really saves power, affordable, tightly packed, fast shipping, very satisfied.	Jingdong prefecture level city in Shandong
Power Saving King Shop	King of Power Saving	Good quality, fine workmanship, is genuine and business description of the same, good value for money, fast logistics, very satisfied	Jingdong prefecture level city in Shandong
Power Saving King Shop	King of Power Saving	Saver received, very good, installation set up very well, very good, the effect is very good, recommend that the pro buy	Jingdong prefecture level city in Shandong
Power Saving King Shop	King of Power Saving	Electromagnetic good, high power convenient, enough force, the price is right it, quality can be 😃 😃 😃 😃 😃	Jingdong prefecture level city in Shandong
Power Saving King Shop	King of Power Saving	First-class quality, packaging is also good, logistics is very fast, and the price is reasonable, very affordable, I like it!	Jingdong prefecture level city in Shandong
Power Saving King Shop	King of Power Saving	It's great, it's the kind of style you want, and it arrived quickly, thank you boss, 5 stars for the service!	Jingdong prefecture level city in Shandong
Power Saving King Shop	King of Power Saving	Packaging is very good, the use of the effect is quite good, the store has a good service attitude, trustworthy	Jingdong prefecture level city in Shandong
Power Saving King Shop	King of Power Saving	The quality is very good, it works well, very satisfied, the price is affordable, it's so great value, it's worth recommending.	Jingdong prefecture level city in Shandong
Power Saving King Shop	King of Power Saving	The baby description matches the actual item very well! The quality is awesome! Will be back to patronize again in the future!	Jingdong prefecture level city in Shandong
Power Saving King Shop	King of Power Saving	Ran the price is not high try to buy the attitude, did not expect the effect is really good, good!	Jingdong prefecture level city in Shandong
Power Saving King Shop	King of Power Saving	Power Saving King power is quite large, the quality of the leaf is quite good, the price of buying a very good deal, must be praised!	Jingdong prefecture level city in Shandong
Power Saving King Shop	King of Power Saving	Good product quality is the king of power-saving products trustworthy as always good to recommend!	Jingdong prefecture level city in Shandong
Power Saving King Shop	King of Power Saving	Works great, very affordable, shipped pretty fast, comfortable to use	Jingdong prefecture level city in Shandong
Power Saving King Shop	King of Power Saving	Shipping was pretty fast and affordable, not bad 👍	Jingdong prefecture level city in Shandong
Power Saving King Shop	King of Power Saving	Digital voltage display, voltage at a glance, plug and play, safe and simple!	Jingdong prefecture level city in Shandong
Power Saving King Shop	King of Power Saving	Quality assurance, very good, affordable, logistics is also very fast, I'm very satisfied with the	Jingdong prefecture level city in Shandong
Power Saving King Shop	King of Power Saving	The quality is very good, the first time I received the goods to open the first time to see, it is very good, very satisfied	Jingdong prefecture level city in Shandong
Power Saving King Shop	King of Power Saving	Power Saving King used, the effect is good, the price is affordable, very good, is the genuine article	Jingdong prefecture level city in Shandong
Power Saving King Shop	King of Power Saving	Good quality, fine workmanship, good appearance, time is very accurate, satisfied.	Jingdong prefecture level city in Shandong
Power Saving King Shop	King of Power Saving	I purposely used it for two days before commenting, which is responsible for myself and everyone else!	Jingdong prefecture level city in Shandong
Power Saving King Shop	King of Power Saving	Easy to install, easy to operate, helped our family save a lot of electricity this summer	Jingdong prefecture level city in Shandong
Power Saving King Shop	King of Power Saving	Bringing power savings into millions of homes. Still a great gadget. Pretty good.	Jingdong prefecture level city in Shandong
Power Saving King Shop	King of Power Saving	Digital liquid crystal display to see the clear intuitive, very formal workmanship is not rough	Jingdong prefecture level city in Shandong
Power Saving King Shop	King of Power Saving	Received. Great quality, fast shipping from the store and not too expensive!	Jingdong prefecture level city in Shandong
Power Saving King Shop	King of Power Saving	Bought myself a knuckle regulator and it was easy to use and the price was right.	Jingdong prefecture level city in Shandong
Power Saving King Shop	King of Power Saving	Received, good, powerful, good, very full of a shopping	Jingdong prefecture level city in Shandong
Power Saving King Shop	King of Power Saving	High power, consistent with the seller's description, powerful, cost-effective	Jingdong prefecture level city in Shandong
Power Saving King Shop	King of Power Saving	It works very well and also saves power at an affordable price a great deal.	Jingdong prefecture level city in Shandong
Power Saving King Shop	King of Power Saving	Good quality, good can recommend to friends to buy.	Jingdong prefecture level city in Shandong
Power Saving King Shop	King of Power Saving	Will repurchase, quality is guaranteed, safe and secure purchase.	Jingdong prefecture level city in Shandong
Power Saving King Shop	King of Power Saving	The logistics is very fast, the thing is also good to use, energy saving power rely on it!	Jingdong prefecture level city in Shandong
Power Saving King Shop	King of Power Saving	Power Saver is good quality and works very well, worth buying!	Jingdong prefecture level city in Shandong
Power Saving King Shop	King of Power Saving	The quality is very good is genuine, cost-effective recommended	Jingdong prefecture level city in Shandong
Power Saving King Shop	King of Power Saving	Quality Assurance. Favorable price. Very practical and durable	Jingdong prefecture level city in Shandong
Power Saving King Shop	King of Power Saving	Good value for money. Conscientious businessman. Recommended to everyone	Jingdong prefecture level city in Shandong
Power Saving King Shop	King of Power Saving	Buy it for your rental apartment and never worry about your landlord charging you too much.	Jingdong prefecture level city in Shandong
Power Saving King Shop	King of Power Saving	Nice and affordable, will repurchase next time	Jingdong prefecture level city in Shandong
Power Saving King Shop	King of Power Saving	It's very energy efficient. I've been using it for a few days now.	Jingdong prefecture level city in Shandong
Power Saving King Shop	King of Power Saving	Good quality, high power easy to use	Jingdong prefecture level city in Shandong
Power Saving King Shop	King of Power Saving	Power saving little helper. Deserves a favorable review.	Jingdong prefecture level city in Shandong
Power Saving King Shop	King of Power Saving	Great value for money, great value for money.	Jingdong prefecture level city in Shandong
Power Saving King Shop	King of Power Saving	Works well and as described by the seller	Jingdong prefecture level city in Shandong
Power Saving King Shop	King of Power Saving	Power saving is proper! It's good.	Jingdong prefecture level city in Shandong
Power Saving King Shop	King of Power Saving	Power saving. Works well. Not bad.	Jingdong prefecture level city in Shandong
Power Saving King Shop	King of Power Saving	Satisfied with power saving	Jingdong prefecture level city in Shandong
Power Saving King Shop	King of Power Saving	Received intact, open a super good look beautiful and generous, ~ bit of high quality is recommended to buy, the need for friends rest assured that the next order	Jingdong prefecture level city in Shandong
Power Saving King Shop	King of Power Saving	Very good to use, friends introduced, save electricity and money, long-lasting products cost-effective, large factory production, good quality, fine workmanship cost-effective!	Jingdong prefecture level city in Shandong
Power Saving King Shop	King of Power Saving	The power saving effect is very obvious, trying to pay 30% less electricity bill in a month than before, hahaha	Jingdong prefecture level city in Shandong
Power Saving King Shop	King of Power Saving	It's such a power saver to use this, it saves money on kids snacks for a month. Kudos!	Jingdong prefecture level city in Shandong
Power Saving King Shop	King of Power Saving	Plug and play, stable voltage, the screen has a display when working, this winter home appliances, need more electricity, just buy to use, save electricity and save money, with instructions, not too big and does not take up space, the quality is very good, and the seller describes exactly the same!	Jingdong prefecture level city in Shandong
Power Saving King Shop	King of Power Saving	A must have for opening a store. Save over a thousand dollars a month on your electric bill	Jingdong prefecture level city in Shandong

Power Saving King Shop	King of Power Saving	Received the goods, the first time to unpack, the quality is very good, with the seller's description, completely exceeded my expectations, the packaging is very careful, very strict, in general this is a very satisfied with the shopping	Jingdong prefecture level city in Shandong
Power Saving King Shop	King of Power Saving	As always, good quality, a very favorite product, great! o(*￣︶￣*)o The price is very affordable, cost-effective, it's considered affordable good, will repurchase!	Jingdong prefecture level city in Shandong
Power Saving King Shop	King of Power Saving	Very satisfied with a shopping, good business service, fast shipping, logistics is also very powerful, received very good, buy assured, use peace of mind!	Jingdong prefecture level city in Shandong
Power Saving King Shop	King of Power Saving	The first time to buy online, but also look at the evaluation before buying, the quality is not bad, but also very cheap, the need for friends can rest assured to buy!	Jingdong prefecture level city in Shandong
Power Saving King Shop	King of Power Saving	Better than expected, feel value for money, very satisfied with a shopping, need next time will come back!	Jingdong prefecture level city in Shandong
Power Saving King Shop	King of Power Saving	Really super like, very supportive, very good quality, exactly as described by the seller, very satisfied!	Jingdong prefecture level city in Shandong
Power Saving King Shop	King of Power Saving	It is genuine, effective, really saves power, affordable, tightly packed, fast shipping, very satisfied.	Jingdong prefecture level city in Shandong
Power Saving King Shop	King of Power Saving	Good quality, fine workmanship, is genuine and business description of the same, good value for money, fast logistics, very satisfied	Jingdong prefecture level city in Shandong
Power Saving King Shop	King of Power Saving	Saver received, very good, installation set up very well, very good, the effect is very good, recommend that the pro buy	Jingdong prefecture level city in Shandong
Power Saving King Shop	King of Power Saving	Electromagnetic good, high power convenient, enough force, the price is right it, quality can be 😃 😃 😃 😃 😃	Jingdong prefecture level city in Shandong
Power Saving King Shop	King of Power Saving	First-class quality, packaging is also good, logistics is very fast, and the price is reasonable, very affordable, I like it!	Jingdong prefecture level city in Shandong
Power Saving King Shop	King of Power Saving	It's great, it's the kind of style you want, and it arrived quickly, thank you boss, 5 stars for the service!	Jingdong prefecture level city in Shandong
Power Saving King Shop	King of Power Saving	Packaging is very good, the use of the effect is quite good, the store has a good service attitude, trustworthy	Jingdong prefecture level city in Shandong
Power Saving King Shop	King of Power Saving	The quality is very good, it works well, very satisfied, the price is affordable, it's so great value, it's worth recommending.	Jingdong prefecture level city in Shandong
Power Saving King Shop	King of Power Saving	The baby description matches the actual item very well! The quality is awesome! Will be back to patronize again in the future!	Jingdong prefecture level city in Shandong
Power Saving King Shop	King of Power Saving	Ran the price is not high try to buy the attitude, did not expect the effect is really good, good!	Jingdong prefecture level city in Shandong
Power Saving King Shop	King of Power Saving	Power Saving King power is quite large, the quality of the leaf is quite good, the price of buying a very good deal, must be praised!	Jingdong prefecture level city in Shandong
Power Saving King Shop	King of Power Saving	Good product quality is the king of power-saving products trustworthy as always good to recommend!	Jingdong prefecture level city in Shandong
Power Saving King Shop	King of Power Saving	Works great, very affordable, shipped pretty fast, comfortable to use	Jingdong prefecture level city in Shandong
Power Saving King Shop	King of Power Saving	Shipping was pretty fast and affordable, not bad 👍	Jingdong prefecture level city in Shandong
Power Saving King Shop	King of Power Saving	Digital voltage display, voltage at a glance, plug and play, safe and simple!	Jingdong prefecture level city in Shandong
Power Saving King Shop	King of Power Saving	Quality assurance, very good, affordable, logistics is also very fast, I'm very satisfied with the	Jingdong prefecture level city in Shandong
Power Saving King Shop	King of Power Saving	The quality is very good, the first time I received the goods to open the first time to see, it is very good, very satisfied	Jingdong prefecture level city in Shandong
Power Saving King Shop	King of Power Saving	Power Saving King used, the effect is good, the price is affordable, very good, is the genuine article	Jingdong prefecture level city in Shandong
Power Saving King Shop	King of Power Saving	Good quality, fine workmanship, good appearance, time is very accurate, satisfied.	Jingdong prefecture level city in Shandong
Power Saving King Shop	King of Power Saving	I purposely used it for two days before commenting, which is responsible for myself and everyone else!	Jingdong prefecture level city in Shandong
Power Saving King Shop	King of Power Saving	Easy to install, easy to operate, helped our family save a lot of electricity this summer	Jingdong prefecture level city in Shandong
Power Saving King Shop	King of Power Saving	Bringing power savings into millions of homes. Still a great gadget. Pretty good.	Jingdong prefecture level city in Shandong
Power Saving King Shop	King of Power Saving	Digital liquid crystal display to see the clear intuitive, very formal workmanship is not rough	Jingdong prefecture level city in Shandong
Power Saving King Shop	King of Power Saving	Received. Great quality, fast shipping from the store and not too expensive!	Jingdong prefecture level city in Shandong
Power Saving King Shop	King of Power Saving	Bought myself a knuckle regulator and it was easy to use and the price was right.	Jingdong prefecture level city in Shandong
Power Saving King Shop	King of Power Saving	Received, good, powerful, good, very full of a shopping	Jingdong prefecture level city in Shandong
Power Saving King Shop	King of Power Saving	High power, consistent with the seller's description, powerful, cost-effective	Jingdong prefecture level city in Shandong
Power Saving King Shop	King of Power Saving	It works very well and also saves power at an affordable price a great deal.	Jingdong prefecture level city in Shandong
Power Saving King Shop	King of Power Saving	Good quality, good can recommend to friends to buy.	Jingdong prefecture level city in Shandong
Power Saving King Shop	King of Power Saving	Will repurchase, quality is guaranteed, safe and secure purchase.	Jingdong prefecture level city in Shandong
Power Saving King Shop	King of Power Saving	The logistics is very fast, the thing is also good to use, energy saving power rely on it!	Jingdong prefecture level city in Shandong
Power Saving King Shop	King of Power Saving	Power Saver is good quality and works very well, worth buying!	Jingdong prefecture level city in Shandong
Power Saving King Shop	King of Power Saving	The quality is very good is genuine, cost-effective recommended	Jingdong prefecture level city in Shandong
Power Saving King Shop	King of Power Saving	Quality Assurance. Favorable price. Very practical and durable	Jingdong prefecture level city in Shandong
Power Saving King Shop	King of Power Saving	Good value for money. Conscientious businessman. Recommended to everyone	Jingdong prefecture level city in Shandong
Power Saving King Shop	King of Power Saving	Buy it for your rental apartment and never worry about your landlord charging you too much.	Jingdong prefecture level city in Shandong
Power Saving King Shop	King of Power Saving	Nice and affordable, will repurchase next time	Jingdong prefecture level city in Shandong
Power Saving King Shop	King of Power Saving	It's very energy efficient. I've been using it for a few days now.	Jingdong prefecture level city in Shandong
Power Saving King Shop	King of Power Saving	Good quality, high power easy to use	Jingdong prefecture level city in Shandong
Power Saving King Shop	King of Power Saving	Power saving little helper. Deserves a favorable review.	Jingdong prefecture level city in Shandong
Power Saving King Shop	King of Power Saving	Great value for money, great value for money.	Jingdong prefecture level city in Shandong
Power Saving King Shop	King of Power Saving	Works well and as described by the seller	Jingdong prefecture level city in Shandong
Power Saving King Shop	King of Power Saving	Power saving is proper! It's good.	Jingdong prefecture level city in Shandong
Power Saving King Shop	King of Power Saving	Power saving. Works well. Not bad.	Jingdong prefecture level city in Shandong
Power Saving King Shop	King of Power Saving	Satisfied with power saving	Jingdong prefecture level city in Shandong

Power Saving King Shop	King of Power Saving	Received intact, open a super good look beautiful and generous, ~ bit of high quality is recommended to buy, the need for friends rest assured that the next order	Jingdong prefecture level city in Shandong
Power Saving King Shop	King of Power Saving	Very good to use, friends introduced, save electricity and money, long-lasting products cost-effective, large factory production, good quality, fine workmanship cost-effective!	Jingdong prefecture level city in Shandong
Power Saving King Shop	King of Power Saving	The power saving effect is very obvious, trying to pay 30% less electricity bill in a month than before, hahaha	Jingdong prefecture level city in Shandong
Power Saving King Shop	King of Power Saving	It's such a power saver to use this, it saves money on kids snacks for a month. Kudos!	Jingdong prefecture level city in Shandong
Power Saving King Shop	King of Power Saving	Plug and play, stable voltage, the screen has a display when working, this winter home appliances, need more electricity, just buy to use, save electricity and save money, with instructions, not too big and does not take up space, the quality is very good, and the seller describes exactly the same!	Jingdong prefecture level city in Shandong
Power Saving King Shop	King of Power Saving	A must have for opening a store. Save over a thousand dollars a month on your electric bill	Jingdong prefecture level city in Shandong
Power Saving King Shop	King of Power Saving	Received the goods, the first time to unpack, the quality is very good, with the seller's description, completely exceeded my expectations, the packaging is very careful, very strict, in general this is a very satisfied with the shopping	Jingdong prefecture level city in Shandong
Power Saving King Shop	King of Power Saving	As always, good quality, a very favorite product, great! o(*￣︶￣*)o The price is very affordable, cost-effective, it's considered affordable good, will repurchase!	Jingdong prefecture level city in Shandong
Power Saving King Shop	King of Power Saving	Very satisfied with a shopping, good business service, fast shipping, logistics is also very powerful, received very good, buy assured, use peace of mind!	Jingdong prefecture level city in Shandong
Power Saving King Shop	King of Power Saving	The first time to buy online, but also look at the evaluation before buying, the quality is not bad, but also very cheap, the need for friends can rest assured to buy!	Jingdong prefecture level city in Shandong
Power Saving King Shop	King of Power Saving	Better than expected, feel value for money, very satisfied with a shopping, need next time will come back!	Jingdong prefecture level city in Shandong
Power Saving King Shop	King of Power Saving	Really super like, very supportive, very good quality, exactly as described by the seller, very satisfied!	Jingdong prefecture level city in Shandong
Power Saving King Shop	King of Power Saving	It is genuine, effective, really saves power, affordable, tightly packed, fast shipping, very satisfied.	Jingdong prefecture level city in Shandong
Power Saving King Shop	King of Power Saving	Good quality, fine workmanship, is genuine and business description of the same, good value for money, fast logistics, very satisfied	Jingdong prefecture level city in Shandong
Power Saving King Shop	King of Power Saving	Saver received, very good, installation set up very well, very good, the effect is very good, recommend that the pro buy	Jingdong prefecture level city in Shandong
Power Saving King Shop	King of Power Saving	Electromagnetic good, high power convenient, enough force, the price is right it, quality can be 😀 😀 😀 😀 😀	Jingdong prefecture level city in Shandong
Power Saving King Shop	King of Power Saving	First-class quality, packaging is also good, logistics is very fast, and the price is reasonable, very affordable, I like it!	Jingdong prefecture level city in Shandong
Power Saving King Shop	King of Power Saving	It's great, it's the kind of style you want, and it arrived quickly, thank you boss, 5 stars for the service!	Jingdong prefecture level city in Shandong
Power Saving King Shop	King of Power Saving	Packaging is very good, the use of the effect is quite good, the store has a good service attitude, trustworthy	Jingdong prefecture level city in Shandong
Power Saving King Shop	King of Power Saving	The quality is very good, it works well, very satisfied, the price is affordable, it's so great value, it's worth recommending.	Jingdong prefecture level city in Shandong
Power Saving King Shop	King of Power Saving	The baby description matches the actual item very well! The quality is awesome! Will be back to patronize again in the future!	Jingdong prefecture level city in Shandong
Power Saving King Shop	King of Power Saving	Ran the price is not high try to buy the attitude, did not expect the effect is really good, good!	Jingdong prefecture level city in Shandong
Power Saving King Shop	King of Power Saving	Power Saving King power is quite large, the quality of the leaf is quite good, the price of buying a very good deal, must be praised!	Jingdong prefecture level city in Shandong
Power Saving King Shop	King of Power Saving	Good product quality is the king of power-saving products trustworthy as always good to recommend!	Jingdong prefecture level city in Shandong
Power Saving King Shop	King of Power Saving	Works great, very affordable, shipped pretty fast, comfortable to use	Jingdong prefecture level city in Shandong
Power Saving King Shop	King of Power Saving	Shipping was pretty fast and affordable, not bad 👍	Jingdong prefecture level city in Shandong
Power Saving King Shop	King of Power Saving	Digital voltage display, voltage at a glance, plug and play, safe and simple!	Jingdong prefecture level city in Shandong
Power Saving King Shop	King of Power Saving	Quality assurance, very good, affordable, logistics is also very fast, I'm very satisfied with the	Jingdong prefecture level city in Shandong
Power Saving King Shop	King of Power Saving	The quality is very good, the first time I received the goods to open the first time to see, it is very good, very satisfied	Jingdong prefecture level city in Shandong
Power Saving King Shop	King of Power Saving	Power Saving King used, the effect is good, the price is affordable, very good, is the genuine article	Jingdong prefecture level city in Shandong
Power Saving King Shop	King of Power Saving	Good quality, fine workmanship, good appearance, time is very accurate, satisfied.	Jingdong prefecture level city in Shandong
Power Saving King Shop	King of Power Saving	I purposely used it for two days before commenting, which is responsible for myself and everyone else!	Jingdong prefecture level city in Shandong
Power Saving King Shop	King of Power Saving	Easy to install, easy to operate, helped our family save a lot of electricity this summer	Jingdong prefecture level city in Shandong
Power Saving King Shop	King of Power Saving	Bringing power savings into millions of homes. Still a great gadget. Pretty good.	Jingdong prefecture level city in Shandong
Power Saving King Shop	King of Power Saving	Digital liquid crystal display to see the clear intuitive, very formal workmanship is not rough	Jingdong prefecture level city in Shandong
Power Saving King Shop	King of Power Saving	Received. Great quality, fast shipping from the store and not too expensive!	Jingdong prefecture level city in Shandong
Power Saving King Shop	King of Power Saving	Bought myself a knuckle regulator and it was easy to use and the price was right.	Jingdong prefecture level city in Shandong
Power Saving King Shop	King of Power Saving	Received, good, powerful, good, very full of a shopping	Jingdong prefecture level city in Shandong
Power Saving King Shop	King of Power Saving	High power, consistent with the seller's description, powerful, cost-effective	Jingdong prefecture level city in Shandong
Power Saving King Shop	King of Power Saving	It works very well and also saves power at an affordable price a great deal.	Jingdong prefecture level city in Shandong
Power Saving King Shop	King of Power Saving	Good quality, good can recommend to friends to buy.	Jingdong prefecture level city in Shandong
Power Saving King Shop	King of Power Saving	Will repurchase, quality is guaranteed, safe and secure purchase.	Jingdong prefecture level city in Shandong
Power Saving King Shop	King of Power Saving	The logistics is very fast, the thing is also good to use, energy saving power rely on it!	Jingdong prefecture level city in Shandong
Power Saving King Shop	King of Power Saving	Power Saver is good quality and works very well, worth buying!	Jingdong prefecture level city in Shandong
Power Saving King Shop	King of Power Saving	The quality is very good is genuine, cost-effective recommended	Jingdong prefecture level city in Shandong
Power Saving King Shop	King of Power Saving	Quality Assurance. Favorable price. Very practical and durable	Jingdong prefecture level city in Shandong
Power Saving King Shop	King of Power Saving	Good value for money. Conscientious businessman. Recommended to everyone	Jingdong prefecture level city in Shandong
Power Saving King Shop	King of Power Saving	Buy it for your rental apartment and never worry about your landlord charging you too much.	Jingdong prefecture level city in Shandong
Power Saving King Shop	King of Power Saving	Nice and affordable, will repurchase next time	Jingdong prefecture level city in Shandong
Power Saving King Shop	King of Power Saving	It's very energy efficient. I've been using it for a few days now.	Jingdong prefecture level city in Shandong

Power Saving King Shop	King of Power Saving	Good quality, high power easy to use	Jingdong prefecture level city in Shandong
Power Saving King Shop	King of Power Saving	Power saving little helper. Deserves a favorable review.	Jingdong prefecture level city in Shandong
Power Saving King Shop	King of Power Saving	Great value for money, great value for money.	Jingdong prefecture level city in Shandong
Power Saving King Shop	King of Power Saving	Works well and as described by the seller	Jingdong prefecture level city in Shandong
Power Saving King Shop	King of Power Saving	Power saving is proper! It's good.	Jingdong prefecture level city in Shandong
Power Saving King Shop	King of Power Saving	Power saving. Works well. Not bad.	Jingdong prefecture level city in Shandong
Power Saving King Shop	King of Power Saving	Satisfied with power saving	Jingdong prefecture level city in Shandong
Power Saving King Shop	King of Power Saving	Received intact, open a super good look beautiful and generous, ~ bit of high quality is recommended to buy, the need for friends rest assured that the next order	Jingdong prefecture level city in Shandong
Power Saving King Shop	King of Power Saving	Very good to use, friends introduced, save electricity and money, long-lasting products cost-effective, large factory production, good quality, fine workmanship cost-effective!	Jingdong prefecture level city in Shandong
Power Saving King Shop	King of Power Saving	The power saving effect is very obvious, trying to pay 30% less electricity bill in a month than before, hahaha	Jingdong prefecture level city in Shandong
Power Saving King Shop	King of Power Saving	It's such a power saver to use this, it saves money on kids snacks for a month. Kudos!	Jingdong prefecture level city in Shandong
Power Saving King Shop	King of Power Saving	Plug and play, stable voltage, the screen has a display when working, this winter home appliances, need more electricity, just buy to use, save electricity and save money, with instructions, not too big and does not take up space, the quality is very good, and the seller describes exactly the same!	Jingdong prefecture level city in Shandong
Power Saving King Shop	King of Power Saving	A must have for opening a store. Save over a thousand dollars a month on your electric bill	Jingdong prefecture level city in Shandong
Power Saving King Shop	King of Power Saving	Received the goods, the first time to unpack, the quality is very good, with the seller's description, completely exceeded my expectations, the packaging is very careful, very strict, in general this is a very satisfied with the shopping	Jingdong prefecture level city in Shandong
Power Saving King Shop	King of Power Saving	As always, good quality, a very favorite product, great! o(*￣︶￣*)o The price is very affordable, cost-effective, it's considered affordable good, will repurchase!	Jingdong prefecture level city in Shandong
Power Saving King Shop	King of Power Saving	Very satisfied with a shopping, good business service, fast shipping, logistics is also very powerful, received very good, buy assured, use peace of mind!	Jingdong prefecture level city in Shandong
Power Saving King Shop	King of Power Saving	The first time to buy online, but also look at the evaluation before buying, the quality is not bad, but also very cheap, the need for friends can rest assured to buy!	Jingdong prefecture level city in Shandong
Power Saving King Shop	King of Power Saving	Better than expected, feel value for money, very satisfied with a shopping, need next time will come back!	Jingdong prefecture level city in Shandong
Power Saving King Shop	King of Power Saving	Really super like, very supportive, very good quality, exactly as described by the seller, very satisfied!	Jingdong prefecture level city in Shandong
Power Saving King Shop	King of Power Saving	It is genuine, effective, really saves power, affordable, tightly packed, fast shipping, very satisfied.	Jingdong prefecture level city in Shandong
Power Saving King Shop	King of Power Saving	Good quality, fine workmanship, is genuine and business description of the same, good value for money, fast logistics, very satisfied	Jingdong prefecture level city in Shandong
Power Saving King Shop	King of Power Saving	Saver received, very good, installation set up very well, very good, the effect is very good, recommend that the pro buy	Jingdong prefecture level city in Shandong
Power Saving King Shop	King of Power Saving	Electromagnetic good, high power convenient, enough force, the price is right it, quality can be 😃 😃 😃 😃 😃	Jingdong prefecture level city in Shandong
Power Saving King Shop	King of Power Saving	First-class quality, packaging is also good, logistics is very fast, and the price is reasonable, very affordable, I like it!	Jingdong prefecture level city in Shandong
Power Saving King Shop	King of Power Saving	It's great, it's the kind of style you want, and it arrived quickly, thank you boss, 5 stars for the service!	Jingdong prefecture level city in Shandong
Power Saving King Shop	King of Power Saving	Packaging is very good, the use of the effect is quite good, the store has a good service attitude, trustworthy	Jingdong prefecture level city in Shandong
Power Saving King Shop	King of Power Saving	The quality is very good, it works well, very satisfied, the price is affordable, it's so great value, it's worth recommending.	Jingdong prefecture level city in Shandong
Power Saving King Shop	King of Power Saving	The baby description matches the actual item very well! The quality is awesome! Will be back to patronize again in the future!	Jingdong prefecture level city in Shandong
Power Saving King Shop	King of Power Saving	Ran the price is not high try to buy the attitude, did not expect the effect is really good, good!	Jingdong prefecture level city in Shandong
Power Saving King Shop	King of Power Saving	Power Saving King power is quite large, the quality of the leaf is quite good, the price of buying a very good deal, must be praised!	Jingdong prefecture level city in Shandong
Power Saving King Shop	King of Power Saving	Good product quality is the king of power-saving products trustworthy as always good to recommend!	Jingdong prefecture level city in Shandong
Power Saving King Shop	King of Power Saving	Works great, very affordable, shipped pretty fast, comfortable to use	Jingdong prefecture level city in Shandong
Power Saving King Shop	King of Power Saving	Shipping was pretty fast and affordable, not bad 👍	Jingdong prefecture level city in Shandong
Power Saving King Shop	King of Power Saving	Digital voltage display, voltage at a glance, plug and play, safe and simple!	Jingdong prefecture level city in Shandong
Power Saving King Shop	King of Power Saving	Quality assurance, very good, affordable, logistics is also very fast, I'm very satisfied with the	Jingdong prefecture level city in Shandong
Power Saving King Shop	King of Power Saving	The quality is very good, the first time I received the goods to open the first time to see, it is very good, very satisfied	Jingdong prefecture level city in Shandong
Power Saving King Shop	King of Power Saving	Power Saving King used, the effect is good, the price is affordable, very good, is the genuine article	Jingdong prefecture level city in Shandong
Power Saving King Shop	King of Power Saving	Good quality, fine workmanship, good appearance, time is very accurate, satisfied.	Jingdong prefecture level city in Shandong
Power Saving King Shop	King of Power Saving	I purposely used it for two days before commenting, which is responsible for myself and everyone else!	Jingdong prefecture level city in Shandong
Power Saving King Shop	King of Power Saving	Easy to install, easy to operate, helped our family save a lot of electricity this summer	Jingdong prefecture level city in Shandong
Power Saving King Shop	King of Power Saving	Bringing power savings into millions of homes. Still a great gadget. Pretty good.	Jingdong prefecture level city in Shandong
Power Saving King Shop	King of Power Saving	Digital liquid crystal display to see the clear intuitive, very formal workmanship is not rough	Jingdong prefecture level city in Shandong
Power Saving King Shop	King of Power Saving	Received. Great quality, fast shipping from the store and not too expensive!	Jingdong prefecture level city in Shandong
Power Saving King Shop	King of Power Saving	Bought myself a knuckle regulator and it was easy to use and the price was right.	Jingdong prefecture level city in Shandong
Power Saving King Shop	King of Power Saving	Received, good, powerful, good, very full of a shopping	Jingdong prefecture level city in Shandong
Power Saving King Shop	King of Power Saving	High power, consistent with the seller's description, powerful, cost-effective	Jingdong prefecture level city in Shandong
Power Saving King Shop	King of Power Saving	It works very well and also saves power at an affordable price a great deal.	Jingdong prefecture level city in Shandong
Power Saving King Shop	King of Power Saving	Good quality, good can recommend to friends to buy.	Jingdong prefecture level city in Shandong
Power Saving King Shop	King of Power Saving	Will repurchase, quality is guaranteed, safe and secure purchase.	Jingdong prefecture level city in Shandong
Power Saving King Shop	King of Power Saving	The logistics is very fast, the thing is also good to use, energy saving power rely on it!	Jingdong prefecture level city in Shandong

Power Saving King Shop	King of Power Saving	Power Saver is good quality and works very well, worth buying!	Jingdong prefecture level city in Shandong
Power Saving King Shop	King of Power Saving	The quality is very good is genuine, cost-effective recommended	Jingdong prefecture level city in Shandong
Power Saving King Shop	King of Power Saving	Quality Assurance. Favorable price. Very practical and durable	Jingdong prefecture level city in Shandong
Power Saving King Shop	King of Power Saving	Good value for money. Conscientious businessman. Recommended to everyone	Jingdong prefecture level city in Shandong
Power Saving King Shop	King of Power Saving	Buy it for your rental apartment and never worry about your landlord charging you too much.	Jingdong prefecture level city in Shandong
Power Saving King Shop	King of Power Saving	Nice and affordable, will repurchase next time	Jingdong prefecture level city in Shandong
Power Saving King Shop	King of Power Saving	It's very energy efficient. I've been using it for a few days now.	Jingdong prefecture level city in Shandong
Power Saving King Shop	King of Power Saving	Good quality, high power easy to use	Jingdong prefecture level city in Shandong
Power Saving King Shop	King of Power Saving	Power saving little helper. Deserves a favorable review.	Jingdong prefecture level city in Shandong
Power Saving King Shop	King of Power Saving	Great value for money, great value for money.	Jingdong prefecture level city in Shandong
Power Saving King Shop	King of Power Saving	Works well and as described by the seller	Jingdong prefecture level city in Shandong
Power Saving King Shop	King of Power Saving	Power saving is proper! It's good.	Jingdong prefecture level city in Shandong
Power Saving King Shop	King of Power Saving	Power saving. Works well. Not bad.	Jingdong prefecture level city in Shandong
Power Saving King Shop	King of Power Saving	Satisfied with power saving	Jingdong prefecture level city in Shandong
Power Saving King Shop	King of Power Saving	Received intact, open a super good look beautiful and generous, ~ bit of high quality is recommended to buy, the need for friends rest assured that the next order	Jingdong prefecture level city in Shandong
Power Saving King Shop	King of Power Saving	Very good to use, friends introduced, save electricity and money, long-lasting products cost-effective, large factory production, good quality, fine workmanship cost-effective!	Jingdong prefecture level city in Shandong
Power Saving King Shop	King of Power Saving	The power saving effect is very obvious, trying to pay 30% less electricity bill in a month than before, hahaha	Jingdong prefecture level city in Shandong
Power Saving King Shop	King of Power Saving	It's such a power saver to use this, it saves money on kids snacks for a month. Kudos!	Jingdong prefecture level city in Shandong
Power Saving King Shop	King of Power Saving	Plug and play, stable voltage, the screen has a display when working, this winter home appliances, need more electricity, just buy to use, save electricity and save money, with instructions, not too big and does not take up space, the quality is very good, and the seller describes exactly the same!	Jingdong prefecture level city in Shandong
Power Saving King Shop	King of Power Saving	A must have for opening a store. Save over a thousand dollars a month on your electric bill	Jingdong prefecture level city in Shandong
Power Saving King Shop	King of Power Saving	Received the goods, the first time to unpack, the quality is very good, with the seller's description, completely exceeded my expectations, the packaging is very careful, very strict, in general this is a very satisfied with the shopping	Jingdong prefecture level city in Shandong
Power Saving King Shop	King of Power Saving	As always, good quality, a very favorite product, great! o(*￣︶￣*)o The price is very affordable, cost-effective, it's considered affordable good, will repurchase!	Jingdong prefecture level city in Shandong
Power Saving King Shop	King of Power Saving	Very satisfied with a shopping, good business service, fast shipping, logistics is also very powerful, received very good, buy assured, use peace of mind!	Jingdong prefecture level city in Shandong
Power Saving King Shop	King of Power Saving	The first time to buy online, but also look at the evaluation before buying, the quality is not bad, but also very cheap, the need for friends can rest assured to buy!	Jingdong prefecture level city in Shandong
Power Saving King Shop	King of Power Saving	Better than expected, feel value for money, very satisfied with a shopping, need next time will come back!	Jingdong prefecture level city in Shandong
Power Saving King Shop	King of Power Saving	Really super like, very supportive, very good quality, exactly as described by the seller, very satisfied!	Jingdong prefecture level city in Shandong
Power Saving King Shop	King of Power Saving	It is genuine, effective, really saves power, affordable, tightly packed, fast shipping, very satisfied.	Jingdong prefecture level city in Shandong
Power Saving King Shop	King of Power Saving	Good quality, fine workmanship, is genuine and business description of the same, good value for money, fast logistics, very satisfied	Jingdong prefecture level city in Shandong
Power Saving King Shop	King of Power Saving	Saver received, very good, installation set up very well, very good, the effect is very good, recommend that the pro buy	Jingdong prefecture level city in Shandong
Power Saving King Shop	King of Power Saving	Electromagnetic good, high power convenient, enough force, the price is right it, quality can be 😊 😊 😊 😊 😊	Jingdong prefecture level city in Shandong
Power Saving King Shop	King of Power Saving	First-class quality, packaging is also good, logistics is very fast, and the price is reasonable, very affordable, I like it!	Jingdong prefecture level city in Shandong
Power Saving King Shop	King of Power Saving	It's great, it's the kind of style you want, and it arrived quickly, thank you boss, 5 stars for the service!	Jingdong prefecture level city in Shandong
Power Saving King Shop	King of Power Saving	Packaging is very good, the use of the effect is quite good, the store has a good service attitude, trustworthy	Jingdong prefecture level city in Shandong
Power Saving King Shop	King of Power Saving	The quality is very good, it works well, very satisfied, the price is affordable, it's so great value, it's worth recommending.	Jingdong prefecture level city in Shandong
Power Saving King Shop	King of Power Saving	The baby description matches the actual item very well! The quality is awesome! Will be back to patronize again in the future!	Jingdong prefecture level city in Shandong
Power Saving King Shop	King of Power Saving	Ran the price is not high try to buy the attitude, did not expect the effect is really good, good!	Jingdong prefecture level city in Shandong
Power Saving King Shop	King of Power Saving	Power Saving King power is quite large, the quality of the leaf is quite good, the price of buying a very good deal, must be praised!	Jingdong prefecture level city in Shandong
Power Saving King Shop	King of Power Saving	Good product quality is the king of power-saving products trustworthy as always good to recommend!	Jingdong prefecture level city in Shandong
Power Saving King Shop	King of Power Saving	Works great, very affordable, shipped pretty fast, comfortable to use	Jingdong prefecture level city in Shandong
Power Saving King Shop	King of Power Saving	Shipping was pretty fast and affordable, not bad 👍	Jingdong prefecture level city in Shandong
Power Saving King Shop	King of Power Saving	Digital voltage display, voltage at a glance, plug and play, safe and simple!	Jingdong prefecture level city in Shandong
Power Saving King Shop	King of Power Saving	Quality assurance, very good, affordable, logistics is also very fast, I'm very satisfied with the	Jingdong prefecture level city in Shandong
Power Saving King Shop	King of Power Saving	The quality is very good, the first time I received the goods to open the first time to see, it is very good, very satisfied	Jingdong prefecture level city in Shandong
Power Saving King Shop	King of Power Saving	Power Saving King used, the effect is good, the price is affordable, very good, is the genuine article	Jingdong prefecture level city in Shandong
Power Saving King Shop	King of Power Saving	Good quality, fine workmanship, good appearance, time is very accurate, satisfied.	Jingdong prefecture level city in Shandong
Power Saving King Shop	King of Power Saving	I purposely used it for two days before commenting, which is responsible for myself and everyone else!	Jingdong prefecture level city in Shandong
Power Saving King Shop	King of Power Saving	Easy to install, easy to operate, helped our family save a lot of electricity this summer	Jingdong prefecture level city in Shandong
Power Saving King Shop	King of Power Saving	Bringing power savings into millions of homes. Still a great gadget. Pretty good.	Jingdong prefecture level city in Shandong
Power Saving King Shop	King of Power Saving	Digital liquid crystal display to see the clear intuitive, very formal workmanship is not rough	Jingdong prefecture level city in Shandong
Power Saving King Shop	King of Power Saving	Received. Great quality, fast shipping from the store and not too expensive!	Jingdong prefecture level city in Shandong

Power Saving King Shop	King of Power Saving	Bought myself a knuckle regulator and it was easy to use and the price was right.	Jingdong prefecture level city in Shandong
Power Saving King Shop	King of Power Saving	Received, good, powerful, good, very full of a shopping	Jingdong prefecture level city in Shandong
Power Saving King Shop	King of Power Saving	High power, consistent with the seller's description, powerful, cost-effective	Jingdong prefecture level city in Shandong
Power Saving King Shop	King of Power Saving	It works very well and also saves power at an affordable price a great deal.	Jingdong prefecture level city in Shandong
Power Saving King Shop	King of Power Saving	Good quality, good can recommend to friends to buy.	Jingdong prefecture level city in Shandong
Power Saving King Shop	King of Power Saving	Will repurchase, quality is guaranteed, safe and secure purchase.	Jingdong prefecture level city in Shandong
Power Saving King Shop	King of Power Saving	The logistics is very fast, the thing is also good to use, energy saving power rely on it!	Jingdong prefecture level city in Shandong
Power Saving King Shop	King of Power Saving	Power Saver is good quality and works very well, worth buying!	Jingdong prefecture level city in Shandong
Power Saving King Shop	King of Power Saving	The quality is very good is genuine, cost-effective recommended	Jingdong prefecture level city in Shandong
Power Saving King Shop	King of Power Saving	Quality Assurance. Favorable price. Very practical and durable	Jingdong prefecture level city in Shandong
Power Saving King Shop	King of Power Saving	Good value for money. Conscientious businessman. Recommended to everyone	Jingdong prefecture level city in Shandong
Power Saving King Shop	King of Power Saving	Buy it for your rental apartment and never worry about your landlord charging you too much.	Jingdong prefecture level city in Shandong
Power Saving King Shop	King of Power Saving	Nice and affordable, will repurchase next time	Jingdong prefecture level city in Shandong
Power Saving King Shop	King of Power Saving	It's very energy efficient. I've been using it for a few days now.	Jingdong prefecture level city in Shandong
Power Saving King Shop	King of Power Saving	Good quality, high power easy to use	Jingdong prefecture level city in Shandong
Power Saving King Shop	King of Power Saving	Power saving little helper. Deserves a favorable review.	Jingdong prefecture level city in Shandong
Power Saving King Shop	King of Power Saving	Great value for money, great value for money.	Jingdong prefecture level city in Shandong
Power Saving King Shop	King of Power Saving	Works well and as described by the seller	Jingdong prefecture level city in Shandong
Power Saving King Shop	King of Power Saving	Power saving is proper! It's good.	Jingdong prefecture level city in Shandong
Power Saving King Shop	King of Power Saving	Power saving. Works well. Not bad.	Jingdong prefecture level city in Shandong
Power Saving King Shop	King of Power Saving	Satisfied with power saving	Jingdong prefecture level city in Shandong
Power Saving King Shop	King of Power Saving	Received intact, open a super good look beautiful and generous, ~ bit of high quality is recommended to buy, the need for friends rest assured that the next order	Jingdong prefecture level city in Shandong
Power Saving King Shop	King of Power Saving	Very good to use, friends introduced, save electricity and money, long-lasting products cost-effective, large factory production, good quality, fine workmanship cost-effective!	Jingdong prefecture level city in Shandong
Power Saving King Shop	King of Power Saving	The power saving effect is very obvious, trying to pay 30% less electricity bill in a month than before, hahaha	Jingdong prefecture level city in Shandong
Power Saving King Shop	King of Power Saving	It's such a power saver to use this, it saves money on kids snacks for a month. Kudos!	Jingdong prefecture level city in Shandong
Power Saving King Shop	King of Power Saving	Plug and play, stable voltage, the screen has a display when working, this winter home appliances, need more electricity, just buy to use, save electricity and save money, with instructions, not too big and does not take up space, the quality is very good, and the seller describes exactly the same!	Jingdong prefecture level city in Shandong
Power Saving King Shop	King of Power Saving	A must have for opening a store. Save over a thousand dollars a month on your electric bill	Jingdong prefecture level city in Shandong
Power Saving King Shop	King of Power Saving	Received the goods, the first time to unpack, the quality is very good, with the seller's description, completely exceeded my expectations, the packaging is very careful, very strict, in general this is a very satisfied with the shopping	Jingdong prefecture level city in Shandong
Power Saving King Shop	King of Power Saving	As always, good quality, a very favorite product, great! o(*￣︶￣*)o The price is very affordable, cost-effective, it's considered affordable good, will repurchase!	Jingdong prefecture level city in Shandong
Power Saving King Shop	King of Power Saving	Very satisfied with a shopping, good business service, fast shipping, logistics is also very powerful, received very good, buy assured, use peace of mind!	Jingdong prefecture level city in Shandong
Power Saving King Shop	King of Power Saving	The first time to buy online, but also look at the evaluation before buying, the quality is not bad, but also very cheap, the need for friends can rest assured to buy!	Jingdong prefecture level city in Shandong
Power Saving King Shop	King of Power Saving	Better than expected, feel value for money, very satisfied with a shopping, need next time will come back!	Jingdong prefecture level city in Shandong
Power Saving King Shop	King of Power Saving	Really super like, very supportive, very good quality, exactly as described by the seller, very satisfied!	Jingdong prefecture level city in Shandong
Power Saving King Shop	King of Power Saving	It is genuine, effective, really saves power, affordable, tightly packed, fast shipping, very satisfied.	Jingdong prefecture level city in Shandong
Power Saving King Shop	King of Power Saving	Good quality, fine workmanship, is genuine and business description of the same, good value for money, fast logistics, very satisfied	Jingdong prefecture level city in Shandong
Power Saving King Shop	King of Power Saving	Saver received, very good, installation set up very well, very good, the effect is very good, recommend that the pro buy	Jingdong prefecture level city in Shandong
Power Saving King Shop	King of Power Saving	Electromagnetic good, high power convenient, enough force, the price is right it, quality can be 😃 😃 😃 😃 😃	Jingdong prefecture level city in Shandong
Power Saving King Shop	King of Power Saving	First-class quality, packaging is also good, logistics is very fast, and the price is reasonable, very affordable, I like it!	Jingdong prefecture level city in Shandong
Power Saving King Shop	King of Power Saving	It's great, it's the kind of style you want, and it arrived quickly, thank you boss, 5 stars for the service!	Jingdong prefecture level city in Shandong
Power Saving King Shop	King of Power Saving	Packaging is very good, the use of the effect is quite good, the store has a good service attitude, trustworthy	Jingdong prefecture level city in Shandong
Power Saving King Shop	King of Power Saving	The quality is very good, it works well, very satisfied, the price is affordable, it's so great value, it's worth recommending.	Jingdong prefecture level city in Shandong
Power Saving King Shop	King of Power Saving	The baby description matches the actual item very well! The quality is awesome! Will be back to patronize again in the future!	Jingdong prefecture level city in Shandong
Power Saving King Shop	King of Power Saving	Ran the price is not high try to buy the attitude, did not expect the effect is really good, good!	Jingdong prefecture level city in Shandong
Power Saving King Shop	King of Power Saving	Power Saving King power is quite large, the quality of the leaf is quite good, the price of buying a very good deal, must be praised!	Jingdong prefecture level city in Shandong
Power Saving King Shop	King of Power Saving	Good product quality is the king of power-saving products trustworthy as always good to recommend!	Jingdong prefecture level city in Shandong
Power Saving King Shop	King of Power Saving	Works great, very affordable, shipped pretty fast, comfortable to use	Jingdong prefecture level city in Shandong
Power Saving King Shop	King of Power Saving	Shipping was pretty fast and affordable, not bad 👍	Jingdong prefecture level city in Shandong
Power Saving King Shop	King of Power Saving	Digital voltage display, voltage at a glance, plug and play, safe and simple!	Jingdong prefecture level city in Shandong
Power Saving King Shop	King of Power Saving	Quality assurance, very good, affordable, logistics is also very fast, I'm very satisfied with the	Jingdong prefecture level city in Shandong
Power Saving King Shop	King of Power Saving	The quality is very good, the first time I received the goods to open the first time to see, it is very good, very satisfied	Jingdong prefecture level city in Shandong

Power Saving King Shop	King of Power Saving	Power Saving King used, the effect is good, the price is affordable, very good, is the genuine article	Jingdong prefecture level city in Shandong
Power Saving King Shop	King of Power Saving	Good quality, fine workmanship, good appearance, time is very accurate, satisfied.	Jingdong prefecture level city in Shandong
Power Saving King Shop	King of Power Saving	I purposely used it for two days before commenting, which is responsible for myself and everyone else!	Jingdong prefecture level city in Shandong
Power Saving King Shop	King of Power Saving	Easy to install, easy to operate, helped our family save a lot of electricity this summer	Jingdong prefecture level city in Shandong
Power Saving King Shop	King of Power Saving	Bringing power savings into millions of homes. Still a great gadget. Pretty good.	Jingdong prefecture level city in Shandong
Power Saving King Shop	King of Power Saving	Digital liquid crystal display to see the clear intuitive, very formal workmanship is not rough	Jingdong prefecture level city in Shandong
Power Saving King Shop	King of Power Saving	Received. Great quality, fast shipping from the store and not too expensive!	Jingdong prefecture level city in Shandong
Power Saving King Shop	King of Power Saving	Bought myself a knuckle regulator and it was easy to use and the price was right.	Jingdong prefecture level city in Shandong
Power Saving King Shop	King of Power Saving	Received, good, powerful, good, very full of a shopping	Jingdong prefecture level city in Shandong
Power Saving King Shop	King of Power Saving	High power, consistent with the seller's description, powerful, cost-effective	Jingdong prefecture level city in Shandong
Power Saving King Shop	King of Power Saving	It works very well and also saves power at an affordable price a great deal.	Jingdong prefecture level city in Shandong
Power Saving King Shop	King of Power Saving	Good quality, good can recommend to friends to buy.	Jingdong prefecture level city in Shandong
Power Saving King Shop	King of Power Saving	Will repurchase, quality is guaranteed, safe and secure purchase.	Jingdong prefecture level city in Shandong
Power Saving King Shop	King of Power Saving	The logistics is very fast, the thing is also good to use, energy saving power rely on it!	Jingdong prefecture level city in Shandong
Power Saving King Shop	King of Power Saving	Power Saver is good quality and works very well, worth buying!	Jingdong prefecture level city in Shandong
Power Saving King Shop	King of Power Saving	The quality is very good is genuine, cost-effective recommended	Jingdong prefecture level city in Shandong
Power Saving King Shop	King of Power Saving	Quality Assurance. Favorable price. Very practical and durable	Jingdong prefecture level city in Shandong
Power Saving King Shop	King of Power Saving	Good value for money. Conscientious businessman. Recommended to everyone	Jingdong prefecture level city in Shandong
Power Saving King Shop	King of Power Saving	Buy it for your rental apartment and never worry about your landlord charging you too much.	Jingdong prefecture level city in Shandong
Power Saving King Shop	King of Power Saving	Nice and affordable, will repurchase next time	Jingdong prefecture level city in Shandong
Power Saving King Shop	King of Power Saving	It's very energy efficient. I've been using it for a few days now.	Jingdong prefecture level city in Shandong
Power Saving King Shop	King of Power Saving	Good quality, high power easy to use	Jingdong prefecture level city in Shandong
Power Saving King Shop	King of Power Saving	Power saving little helper. Deserves a favorable review.	Jingdong prefecture level city in Shandong
Power Saving King Shop	King of Power Saving	Great value for money, great value for money.	Jingdong prefecture level city in Shandong
Power Saving King Shop	King of Power Saving	Works well and as described by the seller	Jingdong prefecture level city in Shandong
Power Saving King Shop	King of Power Saving	Power saving is proper! It's good.	Jingdong prefecture level city in Shandong
Power Saving King Shop	King of Power Saving	Power saving. Works well. Not bad.	Jingdong prefecture level city in Shandong
Power Saving King Shop	King of Power Saving	Satisfied with power saving	Jingdong prefecture level city in Shandong
Power Saving King Shop	King of Power Saving	Received intact, open a super good look beautiful and generous, ~ bit of high quality is recommended to buy, the need for friends rest assured that the next order	Jingdong prefecture level city in Shandong
Power Saving King Shop	King of Power Saving	Very good to use, friends introduced, save electricity and money, long-lasting products cost-effective, large factory production, good quality, fine workmanship cost-effective!	Jingdong prefecture level city in Shandong
Power Saving King Shop	King of Power Saving	The power saving effect is very obvious, trying to pay 30% less electricity bill in a month than before, hahaha	Jingdong prefecture level city in Shandong
Power Saving King Shop	King of Power Saving	It's such a power saver to use this, it saves money on kids snacks for a month. Kudos!	Jingdong prefecture level city in Shandong
Power Saving King Shop	King of Power Saving	Plug and play, stable voltage, the screen has a display when working, this winter home appliances, need more electricity, just buy to use, save electricity and save money, with instructions, not too big and does not take up space, the quality is very good, and the seller describes exactly the same!	Jingdong prefecture level city in Shandong
Power Saving King Shop	King of Power Saving	A must have for opening a store. Save over a thousand dollars a month on your electric bill	Jingdong prefecture level city in Shandong
Power Saving King Shop	King of Power Saving	Received the goods, the first time to unpack, the quality is very good, with the seller's description, completely exceeded my expectations, the packaging is very careful, very strict, in general this is a very satisfied with the shopping	Jingdong prefecture level city in Shandong
Power Saving King Shop	King of Power Saving	As always, good quality, a very favorite product, great! o(*￣︶￣*)o The price is very affordable, cost-effective, it's considered affordable good, will repurchase!	Jingdong prefecture level city in Shandong
Power Saving King Shop	King of Power Saving	Very satisfied with a shopping, good business service, fast shipping, logistics is also very powerful, received very good, buy assured, use peace of mind!	Jingdong prefecture level city in Shandong
Power Saving King Shop	King of Power Saving	The first time to buy online, but also look at the evaluation before buying, the quality is not bad, but also very cheap, the need for friends can rest assured to buy!	Jingdong prefecture level city in Shandong
Power Saving King Shop	King of Power Saving	Better than expected, feel value for money, very satisfied with a shopping, need next time will come back!	Jingdong prefecture level city in Shandong
Power Saving King Shop	King of Power Saving	Really super like, very supportive, very good quality, exactly as described by the seller, very satisfied!	Jingdong prefecture level city in Shandong
Power Saving King Shop	King of Power Saving	It is genuine, effective, really saves power, affordable, tightly packed, fast shipping, very satisfied.	Jingdong prefecture level city in Shandong
Power Saving King Shop	King of Power Saving	Good quality, fine workmanship, is genuine and business description of the same, good value for money, fast logistics, very satisfied	Jingdong prefecture level city in Shandong
Power Saving King Shop	King of Power Saving	Saver received, very good, installation set up very well, very good, the effect is very good, recommend that the pro buy	Jingdong prefecture level city in Shandong
Power Saving King Shop	King of Power Saving	Electromagnetic good, high power convenient, enough force, the price is right it, quality can be 😃 😃 😃 😃 😃	Jingdong prefecture level city in Shandong
Power Saving King Shop	King of Power Saving	First-class quality, packaging is also good, logistics is very fast, and the price is reasonable, very affordable, I like it!	Jingdong prefecture level city in Shandong
Power Saving King Shop	King of Power Saving	It's great, it's the kind of style you want, and it arrived quickly, thank you boss, 5 stars for the service!	Jingdong prefecture level city in Shandong
Power Saving King Shop	King of Power Saving	Packaging is very good, the use of the effect is quite good, the store has a good service attitude, trustworthy	Jingdong prefecture level city in Shandong
Power Saving King Shop	King of Power Saving	The quality is very good, it works well, very satisfied, the price is affordable, it's so great value, it's worth recommending.	Jingdong prefecture level city in Shandong
Power Saving King Shop	King of Power Saving	The baby description matches the actual item very well! The quality is awesome! Will be back to patronize again in the future!	Jingdong prefecture level city in Shandong
Power Saving King Shop	King of Power Saving	Ran the price is not high try to buy the attitude, did not expect the effect is really good, good!	Jingdong prefecture level city in Shandong

Power Saving King Shop	King of Power Saving	Power Saving King power is quite large, the quality of the leaf is quite good, the price of buying a very good deal, must be praised!	Jingdong prefecture level city in Shandong
Power Saving King Shop	King of Power Saving	Good product quality is the king of power-saving products trustworthy as always good to recommend!	Jingdong prefecture level city in Shandong
Power Saving King Shop	King of Power Saving	Works great, very affordable, shipped pretty fast, comfortable to use	Jingdong prefecture level city in Shandong
Power Saving King Shop	King of Power Saving	Shipping was pretty fast and affordable, not bad 👍	Jingdong prefecture level city in Shandong
Power Saving King Shop	King of Power Saving	Digital voltage display, voltage at a glance, plug and play, safe and simple!	Jingdong prefecture level city in Shandong
Power Saving King Shop	King of Power Saving	Quality assurance, very good, affordable, logistics is also very fast, I'm very satisfied with the	Jingdong prefecture level city in Shandong
Power Saving King Shop	King of Power Saving	The quality is very good, the first time I received the goods to open the first time to see, it is very good, very satisfied	Jingdong prefecture level city in Shandong
Power Saving King Shop	King of Power Saving	Power Saving King used, the effect is good, the price is affordable, very good, is the genuine article	Jingdong prefecture level city in Shandong
Power Saving King Shop	King of Power Saving	Good quality, fine workmanship, good appearance, time is very accurate, satisfied.	Jingdong prefecture level city in Shandong
Power Saving King Shop	King of Power Saving	I purposely used it for two days before commenting, which is responsible for myself and everyone else!	Jingdong prefecture level city in Shandong
Power Saving King Shop	King of Power Saving	Easy to install, easy to operate, helped our family save a lot of electricity this summer	Jingdong prefecture level city in Shandong
Power Saving King Shop	King of Power Saving	Bringing power savings into millions of homes. Still a great gadget. Pretty good.	Jingdong prefecture level city in Shandong
Power Saving King Shop	King of Power Saving	Digital liquid crystal display to see the clear intuitive, very formal workmanship is not rough	Jingdong prefecture level city in Shandong
Power Saving King Shop	King of Power Saving	Received. Great quality, fast shipping from the store and not too expensive!	Jingdong prefecture level city in Shandong
Power Saving King Shop	King of Power Saving	Bought myself a knuckle regulator and it was easy to use and the price was right.	Jingdong prefecture level city in Shandong
Power Saving King Shop	King of Power Saving	Received, good, powerful, good, very full of a shopping	Jingdong prefecture level city in Shandong
Power Saving King Shop	King of Power Saving	High power, consistent with the seller's description, powerful, cost-effective	Jingdong prefecture level city in Shandong
Power Saving King Shop	King of Power Saving	It works very well and also saves power at an affordable price a great deal.	Jingdong prefecture level city in Shandong
Power Saving King Shop	King of Power Saving	Good quality, good can recommend to friends to buy.	Jingdong prefecture level city in Shandong
Power Saving King Shop	King of Power Saving	Will repurchase, quality is guaranteed, safe and secure purchase.	Jingdong prefecture level city in Shandong
Power Saving King Shop	King of Power Saving	The logistics is very fast, the thing is also good to use, energy saving power rely on it!	Jingdong prefecture level city in Shandong
Power Saving King Shop	King of Power Saving	Power Saver is good quality and works very well, worth buying!	Jingdong prefecture level city in Shandong
Power Saving King Shop	King of Power Saving	The quality is very good is genuine, cost-effective recommended	Jingdong prefecture level city in Shandong
Power Saving King Shop	King of Power Saving	Quality Assurance. Favorable price. Very practical and durable	Jingdong prefecture level city in Shandong
Power Saving King Shop	King of Power Saving	Good value for money. Conscientious businessman. Recommended to everyone	Jingdong prefecture level city in Shandong
Power Saving King Shop	King of Power Saving	Buy it for your rental apartment and never worry about your landlord charging you too much.	Jingdong prefecture level city in Shandong
Power Saving King Shop	King of Power Saving	Nice and affordable, will repurchase next time	Jingdong prefecture level city in Shandong
Power Saving King Shop	King of Power Saving	It's very energy efficient. I've been using it for a few days now.	Jingdong prefecture level city in Shandong
Power Saving King Shop	King of Power Saving	Good quality, high power easy to use	Jingdong prefecture level city in Shandong
Power Saving King Shop	King of Power Saving	Power saving little helper. Deserves a favorable review.	Jingdong prefecture level city in Shandong
Power Saving King Shop	King of Power Saving	Great value for money, great value for money.	Jingdong prefecture level city in Shandong
Power Saving King Shop	King of Power Saving	Works well and as described by the seller	Jingdong prefecture level city in Shandong
Power Saving King Shop	King of Power Saving	Power saving is proper! It's good.	Jingdong prefecture level city in Shandong
Power Saving King Shop	King of Power Saving	Power saving. Works well. Not bad.	Jingdong prefecture level city in Shandong
Power Saving King Shop	King of Power Saving	Satisfied with power saving	Jingdong prefecture level city in Shandong
Power Saving King Shop	King of Power Saving	Received intact, open a super good look beautiful and generous, ~ bit of high quality is recommended to buy, the need for friends rest assured that the next order	Jingdong prefecture level city in Shandong
Power Saving King Shop	King of Power Saving	Very good to use, friends introduced, save electricity and money, long-lasting products cost-effective, large factory production, good quality, fine workmanship cost-effective!	Jingdong prefecture level city in Shandong
Power Saving King Shop	King of Power Saving	The power saving effect is very obvious, trying to pay 30% less electricity bill in a month than before, hahaha	Jingdong prefecture level city in Shandong
Power Saving King Shop	King of Power Saving	It's such a power saver to use this, it saves money on kids snacks for a month. Kudos!	Jingdong prefecture level city in Shandong
Power Saving King Shop	King of Power Saving	Plug and play, stable voltage, the screen has a display when working, this winter home appliances, need more electricity, just buy to use, save electricity and save money, with instructions, not too big and does not take up space, the quality is very good, and the seller describes exactly the same!	Jingdong prefecture level city in Shandong
Power Saving King Shop	King of Power Saving	A must have for opening a store. Save over a thousand dollars a month on your electric bill	Jingdong prefecture level city in Shandong
Power Saving King Shop	King of Power Saving	Received the goods, the first time to unpack, the quality is very good, with the seller's description, completely exceeded my expectations, the packaging is very careful, very strict, in general this is a very satisfied with the shopping	Jingdong prefecture level city in Shandong
Power Saving King Shop	King of Power Saving	As always, good quality, a very favorite product, great! o(*￣︶￣*)o The price is very affordable, cost-effective, it's considered affordable good, will repurchase!	Jingdong prefecture level city in Shandong
Power Saving King Shop	King of Power Saving	Very satisfied with a shopping, good business service, fast shipping, logistics is also very powerful, received very good, buy assured, use peace of mind!	Jingdong prefecture level city in Shandong
Power Saving King Shop	King of Power Saving	The first time to buy online, but also look at the evaluation before buying, the quality is not bad, but also very cheap, the need for friends can rest assured to buy!	Jingdong prefecture level city in Shandong
Power Saving King Shop	King of Power Saving	Better than expected, feel value for money, very satisfied with a shopping, need next time will come back!	Jingdong prefecture level city in Shandong
Power Saving King Shop	King of Power Saving	Really super like, very supportive, very good quality, exactly as described by the seller, very satisfied!	Jingdong prefecture level city in Shandong
Power Saving King Shop	King of Power Saving	It is genuine, effective, really saves power, affordable, tightly packed, fast shipping, very satisfied.	Jingdong prefecture level city in Shandong
Power Saving King Shop	King of Power Saving	Good quality, fine workmanship, is genuine and business description of the same, good value for money, fast logistics, very satisfied	Jingdong prefecture level city in Shandong
Power Saving King Shop	King of Power Saving	Saver received, very good, installation set up very well, very good, the effect is very good, recommend that the pro buy	Jingdong prefecture level city in Shandong

Power Saving King Shop	King of Power Saving	Electromagnetic good, high power convenient, enough force, the price is right it, quality can be 😃 😃 😃 😃 😃	Jingdong prefecture level city in Shandong
Power Saving King Shop	King of Power Saving	First-class quality, packaging is also good, logistics is very fast, and the price is reasonable, very affordable, I like it!	Jingdong prefecture level city in Shandong
Power Saving King Shop	King of Power Saving	It's great, it's the kind of style you want, and it arrived quickly, thank you boss, 5 stars for the service!	Jingdong prefecture level city in Shandong
Power Saving King Shop	King of Power Saving	Packaging is very good, the use of the effect is quite good, the store has a good service attitude, trustworthy	Jingdong prefecture level city in Shandong
Power Saving King Shop	King of Power Saving	The quality is very good, it works well, very satisfied, the price is affordable, it's so great value, it's worth recommending.	Jingdong prefecture level city in Shandong
Power Saving King Shop	King of Power Saving	The baby description matches the actual item very well! The quality is awesome! Will be back to patronize again in the future!	Jingdong prefecture level city in Shandong
Power Saving King Shop	King of Power Saving	Ran the price is not high try to buy the attitude, did not expect the effect is really good, good!	Jingdong prefecture level city in Shandong
Power Saving King Shop	King of Power Saving	Power Saving King power is quite large, the quality of the leaf is quite good, the price of buying a very good deal, must be praised!	Jingdong prefecture level city in Shandong
Power Saving King Shop	King of Power Saving	Good product quality is the king of power-saving products trustworthy as always good to recommend!	Jingdong prefecture level city in Shandong
Power Saving King Shop	King of Power Saving	Works great, very affordable, shipped pretty fast, comfortable to use	Jingdong prefecture level city in Shandong
Power Saving King Shop	King of Power Saving	Shipping was pretty fast and affordable, not bad 👍	Jingdong prefecture level city in Shandong
Power Saving King Shop	King of Power Saving	Digital voltage display, voltage at a glance, plug and play, safe and simple!	Jingdong prefecture level city in Shandong
Power Saving King Shop	King of Power Saving	Quality assurance, very good, affordable, logistics is also very fast, I'm very satisfied with the	Jingdong prefecture level city in Shandong
Power Saving King Shop	King of Power Saving	The quality is very good, the first time I received the goods to open the first time to see, it is very good, very satisfied	Jingdong prefecture level city in Shandong
Power Saving King Shop	King of Power Saving	Power Saving King used, the effect is good, the price is affordable, very good, is the genuine article	Jingdong prefecture level city in Shandong
Power Saving King Shop	King of Power Saving	Good quality, fine workmanship, good appearance, time is very accurate, satisfied.	Jingdong prefecture level city in Shandong
Power Saving King Shop	King of Power Saving	I purposely used it for two days before commenting, which is responsible for myself and everyone else!	Jingdong prefecture level city in Shandong
Power Saving King Shop	King of Power Saving	Easy to install, easy to operate, helped our family save a lot of electricity this summer	Jingdong prefecture level city in Shandong
Power Saving King Shop	King of Power Saving	Bringing power savings into millions of homes. Still a great gadget. Pretty good.	Jingdong prefecture level city in Shandong
Power Saving King Shop	King of Power Saving	Digital liquid crystal display to see the clear intuitive, very formal workmanship is not rough	Jingdong prefecture level city in Shandong
Power Saving King Shop	King of Power Saving	Received. Great quality, fast shipping from the store and not too expensive!	Jingdong prefecture level city in Shandong
Power Saving King Shop	King of Power Saving	Bought myself a knuckle regulator and it was easy to use and the price was right.	Jingdong prefecture level city in Shandong
Power Saving King Shop	King of Power Saving	Received, good, powerful, good, very full of a shopping	Jingdong prefecture level city in Shandong
Power Saving King Shop	King of Power Saving	High power, consistent with the seller's description, powerful, cost-effective	Jingdong prefecture level city in Shandong
Power Saving King Shop	King of Power Saving	It works very well and also saves power at an affordable price a great deal.	Jingdong prefecture level city in Shandong
Power Saving King Shop	King of Power Saving	Good quality, good can recommend to friends to buy.	Jingdong prefecture level city in Shandong
Power Saving King Shop	King of Power Saving	Will repurchase, quality is guaranteed, safe and secure purchase.	Jingdong prefecture level city in Shandong
Power Saving King Shop	King of Power Saving	The logistics is very fast, the thing is also good to use, energy saving power rely on it!	Jingdong prefecture level city in Shandong
Power Saving King Shop	King of Power Saving	Power Saver is good quality and works very well, worth buying!	Jingdong prefecture level city in Shandong
Power Saving King Shop	King of Power Saving	The quality is very good is genuine, cost-effective recommended	Jingdong prefecture level city in Shandong
Power Saving King Shop	King of Power Saving	Quality Assurance. Favorable price. Very practical and durable	Jingdong prefecture level city in Shandong
Power Saving King Shop	King of Power Saving	Good value for money. Conscientious businessman. Recommended to everyone	Jingdong prefecture level city in Shandong
Power Saving King Shop	King of Power Saving	Buy it for your rental apartment and never worry about your landlord charging you too much.	Jingdong prefecture level city in Shandong
Power Saving King Shop	King of Power Saving	Nice and affordable, will repurchase next time	Jingdong prefecture level city in Shandong
Power Saving King Shop	King of Power Saving	It's very energy efficient. I've been using it for a few days now.	Jingdong prefecture level city in Shandong
Power Saving King Shop	King of Power Saving	Good quality, high power easy to use	Jingdong prefecture level city in Shandong
Power Saving King Shop	King of Power Saving	Power saving little helper. Deserves a favorable review.	Jingdong prefecture level city in Shandong
Power Saving King Shop	King of Power Saving	Great value for money, great value for money.	Jingdong prefecture level city in Shandong
Power Saving King Shop	King of Power Saving	Works well and as described by the seller	Jingdong prefecture level city in Shandong
Power Saving King Shop	King of Power Saving	Power saving is proper! It's good.	Jingdong prefecture level city in Shandong
Power Saving King Shop	King of Power Saving	Power saving. Works well. Not bad.	Jingdong prefecture level city in Shandong
Power Saving King Shop	King of Power Saving	Satisfied with power saving	Jingdong prefecture level city in Shandong
Power Saving King Shop	King of Power Saving	Received intact, open a super good look beautiful and generous, ~ bit of high quality is recommended to buy, the need for friends rest assured that the next order	Jingdong prefecture level city in Shandong
Power Saving King Shop	King of Power Saving	Very good to use, friends introduced, save electricity and money, long-lasting products cost-effective, large factory production, good quality, fine workmanship cost-effective!	Jingdong prefecture level city in Shandong
Power Saving King Shop	King of Power Saving	The power saving effect is very obvious, trying to pay 30% less electricity bill in a month than before, hahaha	Jingdong prefecture level city in Shandong
Power Saving King Shop	King of Power Saving	It's such a power saver to use this, it saves money on kids snacks for a month. Kudos!	Jingdong prefecture level city in Shandong
Power Saving King Shop	King of Power Saving	Plug and play, stable voltage, the screen has a display when working, this winter home appliances, need more electricity, just buy to use, save electricity and save money, with instructions, not too big and does not take up space, the quality is very good, and the seller describes exactly the same!	Jingdong prefecture level city in Shandong
Power Saving King Shop	King of Power Saving	A must have for opening a store. Save over a thousand dollars a month on your electric bill	Jingdong prefecture level city in Shandong
Power Saving King Shop	King of Power Saving	Received the goods, the first time to unpack, the quality is very good, with the seller's description, completely exceeded my expectations, the packaging is very careful, very strict, in general this is a very satisfied with the shopping	Jingdong prefecture level city in Shandong
Power Saving King Shop	King of Power Saving	As always, good quality, a very favorite product, great! o(*￣︶￣*)o The price is very affordable, cost-effective, it's considered affordable good, will repurchase!	Jingdong prefecture level city in Shandong

Power Saving King Shop	King of Power Saving	Very satisfied with a shopping, good business service, fast shipping, logistics is also very powerful, received very good, buy assured, use peace of mind!	Jingdong prefecture level city in Shandong
Power Saving King Shop	King of Power Saving	The first time to buy online, but also look at the evaluation before buying, the quality is not bad, but also very cheap, the need for friends can rest assured to buy!	Jingdong prefecture level city in Shandong
Power Saving King Shop	King of Power Saving	Better than expected, feel value for money, very satisfied with a shopping, need next time will come back!	Jingdong prefecture level city in Shandong
Power Saving King Shop	King of Power Saving	Really super like, very supportive, very good quality, exactly as described by the seller, very satisfied!	Jingdong prefecture level city in Shandong
Power Saving King Shop	King of Power Saving	It is genuine, effective, really saves power, affordable, tightly packed, fast shipping, very satisfied.	Jingdong prefecture level city in Shandong
Power Saving King Shop	King of Power Saving	Good quality, fine workmanship, is genuine and business description of the same, good value for money, fast logistics, very satisfied	Jingdong prefecture level city in Shandong
Power Saving King Shop	King of Power Saving	Saver received, very good, installation set up very well, very good, the effect is very good, recommend that the pro buy	Jingdong prefecture level city in Shandong
Power Saving King Shop	King of Power Saving	Electromagnetic good, high power convenient, enough force, the price is right it, quality can be 😀 😀 😀 😀 😀	Jingdong prefecture level city in Shandong
Power Saving King Shop	King of Power Saving	First-class quality, packaging is also good, logistics is very fast, and the price is reasonable, very affordable, I like it!	Jingdong prefecture level city in Shandong
Power Saving King Shop	King of Power Saving	It's great, it's the kind of style you want, and it arrived quickly, thank you boss, 5 stars for the service!	Jingdong prefecture level city in Shandong
Power Saving King Shop	King of Power Saving	Packaging is very good, the use of the effect is quite good, the store has a good service attitude, trustworthy	Jingdong prefecture level city in Shandong
Power Saving King Shop	King of Power Saving	The quality is very good, it works well, very satisfied, the price is affordable, it's so great value, it's worth recommending.	Jingdong prefecture level city in Shandong
Power Saving King Shop	King of Power Saving	The baby description matches the actual item very well! The quality is awesome! Will be back to patronize again in the future!	Jingdong prefecture level city in Shandong
Power Saving King Shop	King of Power Saving	Ran the price is not high try to buy the attitude, did not expect the effect is really good, good!	Jingdong prefecture level city in Shandong
Power Saving King Shop	King of Power Saving	Power Saving King power is quite large, the quality of the leaf is quite good, the price of buying a very good deal, must be praised!	Jingdong prefecture level city in Shandong
Power Saving King Shop	King of Power Saving	Good product quality is the king of power-saving products trustworthy as always good to recommend!	Jingdong prefecture level city in Shandong
Power Saving King Shop	King of Power Saving	Works great, very affordable, shipped pretty fast, comfortable to use	Jingdong prefecture level city in Shandong
Power Saving King Shop	King of Power Saving	Shipping was pretty fast and affordable, not bad 👍	Jingdong prefecture level city in Shandong
Power Saving King Shop	King of Power Saving	Digital voltage display, voltage at a glance, plug and play, safe and simple!	Jingdong prefecture level city in Shandong
Power Saving King Shop	King of Power Saving	Quality assurance, very good, affordable, logistics is also very fast, I'm very satisfied with the	Jingdong prefecture level city in Shandong
Power Saving King Shop	King of Power Saving	The quality is very good, the first time I received the goods to open the first time to see, it is very good, very satisfied	Jingdong prefecture level city in Shandong
Power Saving King Shop	King of Power Saving	Power Saving King used, the effect is good, the price is affordable, very good, is the genuine article	Jingdong prefecture level city in Shandong
Power Saving King Shop	King of Power Saving	Good quality, fine workmanship, good appearance, time is very accurate, satisfied.	Jingdong prefecture level city in Shandong
Power Saving King Shop	King of Power Saving	I purposely used it for two days before commenting, which is responsible for myself and everyone else!	Jingdong prefecture level city in Shandong
Power Saving King Shop	King of Power Saving	Easy to install, easy to operate, helped our family save a lot of electricity this summer	Jingdong prefecture level city in Shandong
Power Saving King Shop	King of Power Saving	Bringing power savings into millions of homes. Still a great gadget. Pretty good.	Jingdong prefecture level city in Shandong
Power Saving King Shop	King of Power Saving	Digital liquid crystal display to see the clear intuitive, very formal workmanship is not rough	Jingdong prefecture level city in Shandong
Power Saving King Shop	King of Power Saving	Received. Great quality, fast shipping from the store and not too expensive!	Jingdong prefecture level city in Shandong
Power Saving King Shop	King of Power Saving	Bought myself a knuckle regulator and it was easy to use and the price was right.	Jingdong prefecture level city in Shandong
Power Saving King Shop	King of Power Saving	Received, good, powerful, good, very full of a shopping	Jingdong prefecture level city in Shandong
Power Saving King Shop	King of Power Saving	High power, consistent with the seller's description, powerful, cost-effective	Jingdong prefecture level city in Shandong
Power Saving King Shop	King of Power Saving	It works very well and also saves power at an affordable price a great deal.	Jingdong prefecture level city in Shandong
Power Saving King Shop	King of Power Saving	Good quality, good can recommend to friends to buy.	Jingdong prefecture level city in Shandong
Power Saving King Shop	King of Power Saving	Will repurchase, quality is guaranteed, safe and secure purchase.	Jingdong prefecture level city in Shandong
Power Saving King Shop	King of Power Saving	The logistics is very fast, the thing is also good to use, energy saving power rely on it!	Jingdong prefecture level city in Shandong
Power Saving King Shop	King of Power Saving	Power Saver is good quality and works very well, worth buying!	Jingdong prefecture level city in Shandong
Power Saving King Shop	King of Power Saving	The quality is very good is genuine, cost-effective recommended	Jingdong prefecture level city in Shandong
Power Saving King Shop	King of Power Saving	Quality Assurance. Favorable price. Very practical and durable	Jingdong prefecture level city in Shandong
Power Saving King Shop	King of Power Saving	Good value for money. Conscientious businessman. Recommended to everyone	Jingdong prefecture level city in Shandong
Power Saving King Shop	King of Power Saving	Buy it for your rental apartment and never worry about your landlord charging you too much.	Jingdong prefecture level city in Shandong
Power Saving King Shop	King of Power Saving	Nice and affordable, will repurchase next time	Jingdong prefecture level city in Shandong
Power Saving King Shop	King of Power Saving	It's very energy efficient. I've been using it for a few days now.	Jingdong prefecture level city in Shandong
Power Saving King Shop	King of Power Saving	Good quality, high power easy to use	Jingdong prefecture level city in Shandong
Power Saving King Shop	King of Power Saving	Power saving little helper. Deserves a favorable review.	Jingdong prefecture level city in Shandong
Power Saving King Shop	King of Power Saving	Great value for money, great value for money.	Jingdong prefecture level city in Shandong
Power Saving King Shop	King of Power Saving	Works well and as described by the seller	Jingdong prefecture level city in Shandong
Power Saving King Shop	King of Power Saving	Power saving is proper! It's good.	Jingdong prefecture level city in Shandong
Power Saving King Shop	King of Power Saving	Power saving. Works well. Not bad.	Jingdong prefecture level city in Shandong
Power Saving King Shop	King of Power Saving	Satisfied with power saving	Jingdong prefecture level city in Shandong
Power Saving King Shop	King of Power Saving	Received intact, open a super good look beautiful and generous, ~ bit of high quality is recommended to buy, the need for friends rest assured that the next order	Jingdong prefecture level city in Shandong
Power Saving King Shop	King of Power Saving	Very good to use, friends introduced, save electricity and money, long-lasting products cost-effective, large factory production, good quality, fine workmanship cost-effective!	Jingdong prefecture level city in Shandong

Power Saving King Shop	King of Power Saving	The power saving effect is very obvious, trying to pay 30% less electricity bill in a month than before, hahaha	Jingdong prefecture level city in Shandong
Power Saving King Shop	King of Power Saving	It's such a power saver to use this, it saves money on kids snacks for a month. Kudos!	Jingdong prefecture level city in Shandong
Power Saving King Shop	King of Power Saving	Plug and play, stable voltage, the screen has a display when working, this winter home appliances, need more electricity, just buy to use, save electricity and save money, with instructions, not too big and does not take up space, the quality is very good, and the seller describes exactly the same!	Jingdong prefecture level city in Shandong
Power Saving King Shop	King of Power Saving	A must have for opening a store. Save over a thousand dollars a month on your electric bill	Jingdong prefecture level city in Shandong
Power Saving King Shop	King of Power Saving	Received the goods, the first time to unpack, the quality is very good, with the seller's description, completely exceeded my expectations, the packaging is very careful, very strict, in general this is a very satisfied with the shopping	Jingdong prefecture level city in Shandong
Power Saving King Shop	King of Power Saving	As always, good quality, a very favorite product, great! o(*￣︶￣*)o The price is very affordable, cost-effective, it's considered affordable good, will repurchase!	Jingdong prefecture level city in Shandong
Power Saving King Shop	King of Power Saving	Very satisfied with a shopping, good business service, fast shipping, logistics is also very powerful, received very good, buy assured, use peace of mind!	Jingdong prefecture level city in Shandong
Power Saving King Shop	King of Power Saving	The first time to buy online, but also look at the evaluation before buying, the quality is not bad, but also very cheap, the need for friends can rest assured to buy!	Jingdong prefecture level city in Shandong
Power Saving King Shop	King of Power Saving	Better than expected, feel value for money, very satisfied with a shopping, need next time will come back!	Jingdong prefecture level city in Shandong
Power Saving King Shop	King of Power Saving	Really super like, very supportive, very good quality, exactly as described by the seller, very satisfied!	Jingdong prefecture level city in Shandong
Power Saving King Shop	King of Power Saving	It is genuine, effective, really saves power, affordable, tightly packed, fast shipping, very satisfied.	Jingdong prefecture level city in Shandong
Power Saving King Shop	King of Power Saving	Good quality, fine workmanship, is genuine and business description of the same, good value for money, fast logistics, very satisfied	Jingdong prefecture level city in Shandong
Power Saving King Shop	King of Power Saving	Saver received, very good, installation set up very well, very good, the effect is very good, recommend that the pro buy	Jingdong prefecture level city in Shandong
Power Saving King Shop	King of Power Saving	Electromagnetic good, high power convenient, enough force, the price is right it, quality can be ☺ ☺ ☺ ☺ ☺	Jingdong prefecture level city in Shandong
Power Saving King Shop	King of Power Saving	First-class quality, packaging is also good, logistics is very fast, and the price is reasonable, very affordable, I like it!	Jingdong prefecture level city in Shandong
Power Saving King Shop	King of Power Saving	It's great, it's the kind of style you want, and it arrived quickly, thank you boss, 5 stars for the service!	Jingdong prefecture level city in Shandong
Power Saving King Shop	King of Power Saving	Packaging is very good, the use of the effect is quite good, the store has a good service attitude, trustworthy	Jingdong prefecture level city in Shandong
Power Saving King Shop	King of Power Saving	The quality is very good, it works well, very satisfied, the price is affordable, it's so great value, it's worth recommending.	Jingdong prefecture level city in Shandong
Power Saving King Shop	King of Power Saving	The baby description matches the actual item very well! The quality is awesome! Will be back to patronize again in the future!	Jingdong prefecture level city in Shandong
Power Saving King Shop	King of Power Saving	Ran the price is not high try to buy the attitude, did not expect the effect is really good, good!	Jingdong prefecture level city in Shandong
Power Saving King Shop	King of Power Saving	Power Saving King power is quite large, the quality of the leaf is quite good, the price of buying a very good deal, must be praised!	Jingdong prefecture level city in Shandong
Power Saving King Shop	King of Power Saving	Good product quality is the king of power-saving products trustworthy as always good to recommend!	Jingdong prefecture level city in Shandong
Power Saving King Shop	King of Power Saving	Works great, very affordable, shipped pretty fast, comfortable to use	Jingdong prefecture level city in Shandong
Power Saving King Shop	King of Power Saving	Shipping was pretty fast and affordable, not bad 👍	Jingdong prefecture level city in Shandong
Power Saving King Shop	King of Power Saving	Digital voltage display, voltage at a glance, plug and play, safe and simple!	Jingdong prefecture level city in Shandong
Power Saving King Shop	King of Power Saving	Quality assurance, very good, affordable, logistics is also very fast, I'm very satisfied with the	Jingdong prefecture level city in Shandong
Power Saving King Shop	King of Power Saving	The quality is very good, the first time I received the goods to open the first time to see, it is very good, very satisfied	Jingdong prefecture level city in Shandong
Power Saving King Shop	King of Power Saving	Power Saving King used, the effect is good, the price is affordable, very good, is the genuine article	Jingdong prefecture level city in Shandong
Power Saving King Shop	King of Power Saving	Good quality, fine workmanship, good appearance, time is very accurate, satisfied.	Jingdong prefecture level city in Shandong
Power Saving King Shop	King of Power Saving	I purposely used it for two days before commenting, which is responsible for myself and everyone else!	Jingdong prefecture level city in Shandong
Power Saving King Shop	King of Power Saving	Easy to install, easy to operate, helped our family save a lot of electricity this summer	Jingdong prefecture level city in Shandong
Power Saving King Shop	King of Power Saving	Bringing power savings into millions of homes. Still a great gadget. Pretty good.	Jingdong prefecture level city in Shandong
Power Saving King Shop	King of Power Saving	Digital liquid crystal display to see the clear intuitive, very formal workmanship is not rough	Jingdong prefecture level city in Shandong
Power Saving King Shop	King of Power Saving	Received. Great quality, fast shipping from the store and not too expensive!	Jingdong prefecture level city in Shandong
Power Saving King Shop	King of Power Saving	Bought myself a knuckle regulator and it was easy to use and the price was right.	Jingdong prefecture level city in Shandong
Power Saving King Shop	King of Power Saving	Received, good, powerful, good, very full of a shopping	Jingdong prefecture level city in Shandong
Power Saving King Shop	King of Power Saving	High power, consistent with the seller's description, powerful, cost-effective	Jingdong prefecture level city in Shandong
Power Saving King Shop	King of Power Saving	It works very well and also saves power at an affordable price a great deal.	Jingdong prefecture level city in Shandong
Power Saving King Shop	King of Power Saving	Good quality, good can recommend to friends to buy.	Jingdong prefecture level city in Shandong
Power Saving King Shop	King of Power Saving	Will repurchase, quality is guaranteed, safe and secure purchase.	Jingdong prefecture level city in Shandong
Power Saving King Shop	King of Power Saving	The logistics is very fast, the thing is also good to use, energy saving power rely on it!	Jingdong prefecture level city in Shandong
Power Saving King Shop	King of Power Saving	Power Saver is good quality and works very well, worth buying!	Jingdong prefecture level city in Shandong
Power Saving King Shop	King of Power Saving	The quality is very good is genuine, cost-effective recommended	Jingdong prefecture level city in Shandong
Power Saving King Shop	King of Power Saving	Quality Assurance. Favorable price. Very practical and durable	Jingdong prefecture level city in Shandong
Power Saving King Shop	King of Power Saving	Good value for money. Conscientious businessman. Recommended to everyone	Jingdong prefecture level city in Shandong
Power Saving King Shop	King of Power Saving	Buy it for your rental apartment and never worry about your landlord charging you too much.	Jingdong prefecture level city in Shandong
Power Saving King Shop	King of Power Saving	Nice and affordable, will repurchase next time	Jingdong prefecture level city in Shandong
Power Saving King Shop	King of Power Saving	It's very energy efficient. I've been using it for a few days now.	Jingdong prefecture level city in Shandong
Power Saving King Shop	King of Power Saving	Good quality, high power easy to use	Jingdong prefecture level city in Shandong
Power Saving King Shop	King of Power Saving	Power saving little helper. Deserves a favorable review.	Jingdong prefecture level city in Shandong

Power Saving King Shop	King of Power Saving	Great value for money, great value for money.	Jingdong prefecture level city in Shandong
Power Saving King Shop	King of Power Saving	Works well and as described by the seller	Jingdong prefecture level city in Shandong
Power Saving King Shop	King of Power Saving	Power saving is proper! It's good.	Jingdong prefecture level city in Shandong
Power Saving King Shop	King of Power Saving	Power saving. Works well. Not bad.	Jingdong prefecture level city in Shandong
Power Saving King Shop	King of Power Saving	Satisfied with power saving	Jingdong prefecture level city in Shandong
Power Saving King Shop	King of Power Saving	Received intact, open a super good look beautiful and generous, ~ bit of high quality is recommended to buy, the need for friends rest assured that the next order	Jingdong prefecture level city in Shandong
Power Saving King Shop	King of Power Saving	Very good to use, friends introduced, save electricity and money, long-lasting products cost-effective, large factory production, good quality, fine workmanship cost-effective!	Jingdong prefecture level city in Shandong
Power Saving King Shop	King of Power Saving	The power saving effect is very obvious, trying to pay 30% less electricity bill in a month than before, hahaha	Jingdong prefecture level city in Shandong
Power Saving King Shop	King of Power Saving	It's such a power saver to use this, it saves money on kids snacks for a month. Kudos!	Jingdong prefecture level city in Shandong
Power Saving King Shop	King of Power Saving	Plug and play, stable voltage, the screen has a display when working, this winter home appliances, need more electricity, just buy to use, save electricity and save money, with instructions, not too big and does not take up space, the quality is very good, and the seller describes exactly the same!	Jingdong prefecture level city in Shandong
Power Saving King Shop	King of Power Saving	A must have for opening a store. Save over a thousand dollars a month on your electric bill	Jingdong prefecture level city in Shandong
Power Saving King Shop	King of Power Saving	Received the goods, the first time to unpack, the quality is very good, with the seller's description, completely exceeded my expectations, the packaging is very careful, very strict, in general this is a very satisfied with the shopping	Jingdong prefecture level city in Shandong
Power Saving King Shop	King of Power Saving	As always, good quality, a very favorite product, great! o(*￣︶￣*)o The price is very affordable, cost-effective, it's considered affordable good, will repurchase!	Jingdong prefecture level city in Shandong
Power Saving King Shop	King of Power Saving	Very satisfied with a shopping, good business service, fast shipping, logistics is also very powerful, received very good, buy assured, use peace of mind!	Jingdong prefecture level city in Shandong
Power Saving King Shop	King of Power Saving	The first time to buy online, but also look at the evaluation before buying, the quality is not bad, but also very cheap, the need for friends can rest assured to buy!	Jingdong prefecture level city in Shandong
Power Saving King Shop	King of Power Saving	Better than expected, feel value for money, very satisfied with a shopping, need next time will come back!	Jingdong prefecture level city in Shandong
Power Saving King Shop	King of Power Saving	Really super like, very supportive, very good quality, exactly as described by the seller, very satisfied!	Jingdong prefecture level city in Shandong
Power Saving King Shop	King of Power Saving	It is genuine, effective, really saves power, affordable, tightly packed, fast shipping, very satisfied.	Jingdong prefecture level city in Shandong
Power Saving King Shop	King of Power Saving	Good quality, fine workmanship, is genuine and business description of the same, good value for money, fast logistics, very satisfied	Jingdong prefecture level city in Shandong
Power Saving King Shop	King of Power Saving	Saver received, very good, installation set up very well, very good, the effect is very good, recommend that the pro buy	Jingdong prefecture level city in Shandong
Power Saving King Shop	King of Power Saving	Electromagnetic good, high power convenient, enough force, the price is right it, quality can be ☺ ☺ ☺ ☺ ☺	Jingdong prefecture level city in Shandong
Power Saving King Shop	King of Power Saving	First-class quality, packaging is also good, logistics is very fast, and the price is reasonable, very affordable, I like it!	Jingdong prefecture level city in Shandong
Power Saving King Shop	King of Power Saving	It's great, it's the kind of style you want, and it arrived quickly, thank you boss, 5 stars for the service!	Jingdong prefecture level city in Shandong
Power Saving King Shop	King of Power Saving	Packaging is very good, the use of the effect is quite good, the store has a good service attitude, trustworthy	Jingdong prefecture level city in Shandong
Power Saving King Shop	King of Power Saving	The quality is very good, it works well, very satisfied, the price is affordable, it's so great value, it's worth recommending.	Jingdong prefecture level city in Shandong
Power Saving King Shop	King of Power Saving	The baby description matches the actual item very well! The quality is awesome! Will be back to patronize again in the future!	Jingdong prefecture level city in Shandong
Power Saving King Shop	King of Power Saving	Ran the price is not high try to buy the attitude, did not expect the effect is really good, good!	Jingdong prefecture level city in Shandong
Power Saving King Shop	King of Power Saving	Power Saving King power is quite large, the quality of the leaf is quite good, the price of buying a very good deal, must be praised!	Jingdong prefecture level city in Shandong
Power Saving King Shop	King of Power Saving	Good product quality is the king of power-saving products trustworthy as always good to recommend!	Jingdong prefecture level city in Shandong
Power Saving King Shop	King of Power Saving	Works great, very affordable, shipped pretty fast, comfortable to use	Jingdong prefecture level city in Shandong
Power Saving King Shop	King of Power Saving	Shipping was pretty fast and affordable, not bad 👍	Jingdong prefecture level city in Shandong
Power Saving King Shop	King of Power Saving	Digital voltage display, voltage at a glance, plug and play, safe and simple!	Jingdong prefecture level city in Shandong
Power Saving King Shop	King of Power Saving	Quality assurance, very good, affordable, logistics is also very fast, I'm very satisfied with the	Jingdong prefecture level city in Shandong
Power Saving King Shop	King of Power Saving	The quality is very good, the first time I received the goods to open the first time to see, it is very good, very satisfied	Jingdong prefecture level city in Shandong
Power Saving King Shop	King of Power Saving	Power Saving King used, the effect is good, the price is affordable, very good, is the genuine article	Jingdong prefecture level city in Shandong
Power Saving King Shop	King of Power Saving	Good quality, fine workmanship, good appearance, time is very accurate, satisfied.	Jingdong prefecture level city in Shandong
Power Saving King Shop	King of Power Saving	I purposely used it for two days before commenting, which is responsible for myself and everyone else!	Jingdong prefecture level city in Shandong
Power Saving King Shop	King of Power Saving	Easy to install, easy to operate, helped our family save a lot of electricity this summer	Jingdong prefecture level city in Shandong
Power Saving King Shop	King of Power Saving	Bringing power savings into millions of homes. Still a great gadget. Pretty good.	Jingdong prefecture level city in Shandong
Power Saving King Shop	King of Power Saving	Digital liquid crystal display to see the clear intuitive, very formal workmanship is not rough	Jingdong prefecture level city in Shandong
Power Saving King Shop	King of Power Saving	Received. Great quality, fast shipping from the store and not too expensive!	Jingdong prefecture level city in Shandong
Power Saving King Shop	King of Power Saving	Bought myself a knuckle regulator and it was easy to use and the price was right.	Jingdong prefecture level city in Shandong
Power Saving King Shop	King of Power Saving	Received, good, powerful, good, very full of a shopping	Jingdong prefecture level city in Shandong
Power Saving King Shop	King of Power Saving	High power, consistent with the seller's description, powerful, cost-effective	Jingdong prefecture level city in Shandong
Power Saving King Shop	King of Power Saving	It works very well and also saves power at an affordable price a great deal.	Jingdong prefecture level city in Shandong
Power Saving King Shop	King of Power Saving	Good quality, good can recommend to friends to buy.	Jingdong prefecture level city in Shandong
Power Saving King Shop	King of Power Saving	Will repurchase, quality is guaranteed, safe and secure purchase.	Jingdong prefecture level city in Shandong
Power Saving King Shop	King of Power Saving	The logistics is very fast, the thing is also good to use, energy saving power rely on it!	Jingdong prefecture level city in Shandong
Power Saving King Shop	King of Power Saving	Power Saver is good quality and works very well, worth buying!	Jingdong prefecture level city in Shandong
Power Saving King Shop	King of Power Saving	The quality is very good is genuine, cost-effective recommended	Jingdong prefecture level city in Shandong

Power Saving King Shop	King of Power Saving	Quality Assurance. Favorable price. Very practical and durable	Jingdong prefecture level city in Shandong
Power Saving King Shop	King of Power Saving	Good value for money. Conscientious businessman. Recommended to everyone	Jingdong prefecture level city in Shandong
Power Saving King Shop	King of Power Saving	Buy it for your rental apartment and never worry about your landlord charging you too much.	Jingdong prefecture level city in Shandong
Power Saving King Shop	King of Power Saving	Nice and affordable, will repurchase next time	Jingdong prefecture level city in Shandong
Power Saving King Shop	King of Power Saving	It's very energy efficient. I've been using it for a few days now.	Jingdong prefecture level city in Shandong
Power Saving King Shop	King of Power Saving	Good quality, high power easy to use	Jingdong prefecture level city in Shandong
Power Saving King Shop	King of Power Saving	Power saving little helper. Deserves a favorable review.	Jingdong prefecture level city in Shandong
Power Saving King Shop	King of Power Saving	Great value for money, great value for money.	Jingdong prefecture level city in Shandong
Power Saving King Shop	King of Power Saving	Works well and as described by the seller	Jingdong prefecture level city in Shandong
Power Saving King Shop	King of Power Saving	Power saving is proper! It's good.	Jingdong prefecture level city in Shandong
Power Saving King Shop	King of Power Saving	Power saving. Works well. Not bad.	Jingdong prefecture level city in Shandong
Power Saving King Shop	King of Power Saving	Satisfied with power saving	Jingdong prefecture level city in Shandong
Power Saving King Shop	King of Power Saving	Received intact, open a super good look beautiful and generous, ~ bit of high quality is recommended to buy, the need for friends rest assured that the next order	Jingdong prefecture level city in Shandong
Power Saving King Shop	King of Power Saving	Very good to use, friends introduced, save electricity and money, long-lasting products cost-effective, large factory production, good quality, fine workmanship cost-effective!	Jingdong prefecture level city in Shandong
Power Saving King Shop	King of Power Saving	The power saving effect is very obvious, trying to pay 30% less electricity bill in a month than before, hahaha	Jingdong prefecture level city in Shandong
Power Saving King Shop	King of Power Saving	It's such a power saver to use this, it saves money on kids snacks for a month. Kudos!	Jingdong prefecture level city in Shandong
Power Saving King Shop	King of Power Saving	Plug and play, stable voltage, the screen has a display when working, this winter home appliances, need more electricity, just buy to use, save electricity and save money, with instructions, not too big and does not take up space, the quality is very good, and the seller describes exactly the same!	Jingdong prefecture level city in Shandong
Power Saving King Shop	King of Power Saving	A must have for opening a store. Save over a thousand dollars a month on your electric bill	Jingdong prefecture level city in Shandong
Power Saving King Shop	King of Power Saving	Received the goods, the first time to unpack, the quality is very good, with the seller's description, completely exceeded my expectations, the packaging is very careful, very strict, in general this is a very satisfied with the shopping	Jingdong prefecture level city in Shandong
Power Saving King Shop	King of Power Saving	As always, good quality, a very favorite product, great! o(*￣︶￣*)o The price is very affordable, cost-effective, it's considered affordable good, will repurchase!	Jingdong prefecture level city in Shandong
Power Saving King Shop	King of Power Saving	Very satisfied with a shopping, good business service, fast shipping, logistics is also very powerful, received very good, buy assured, use peace of mind!	Jingdong prefecture level city in Shandong
Power Saving King Shop	King of Power Saving	The first time to buy online, but also look at the evaluation before buying, the quality is not bad, but also very cheap, the need for friends can rest assured to buy!	Jingdong prefecture level city in Shandong
Power Saving King Shop	King of Power Saving	Better than expected, feel value for money, very satisfied with a shopping, need next time will come back!	Jingdong prefecture level city in Shandong
Power Saving King Shop	King of Power Saving	Really super like, very supportive, very good quality, exactly as described by the seller, very satisfied!	Jingdong prefecture level city in Shandong
Power Saving King Shop	King of Power Saving	It is genuine, effective, really saves power, affordable, tightly packed, fast shipping, very satisfied.	Jingdong prefecture level city in Shandong
Power Saving King Shop	King of Power Saving	Good quality, fine workmanship, is genuine and business description of the same, good value for money, fast logistics, very satisfied	Jingdong prefecture level city in Shandong
Power Saving King Shop	King of Power Saving	Saver received, very good, installation set up very well, very good, the effect is very good, recommend that the pro buy	Jingdong prefecture level city in Shandong
Power Saving King Shop	King of Power Saving	Electromagnetic good, high power convenient, enough force, the price is right it, quality can be 😊 😊 😊 😊 😊	Jingdong prefecture level city in Shandong
Power Saving King Shop	King of Power Saving	First-class quality, packaging is also good, logistics is very fast, and the price is reasonable, very affordable, I like it!	Jingdong prefecture level city in Shandong
Power Saving King Shop	King of Power Saving	It's great, it's the kind of style you want, and it arrived quickly, thank you boss, 5 stars for the service!	Jingdong prefecture level city in Shandong
Power Saving King Shop	King of Power Saving	Packaging is very good, the use of the effect is quite good, the store has a good service attitude, trustworthy	Jingdong prefecture level city in Shandong
Power Saving King Shop	King of Power Saving	The quality is very good, it works well, very satisfied, the price is affordable, it's so great value, it's worth recommending.	Jingdong prefecture level city in Shandong
Power Saving King Shop	King of Power Saving	The baby description matches the actual item very well! The quality is awesome! Will be back to patronize again in the future!	Jingdong prefecture level city in Shandong
Power Saving King Shop	King of Power Saving	Ran the price is not high try to buy the attitude, did not expect the effect is really good, good!	Jingdong prefecture level city in Shandong
Power Saving King Shop	King of Power Saving	Power Saving King power is quite large, the quality of the leaf is quite good, the price of buying a very good deal, must be praised!	Jingdong prefecture level city in Shandong
Power Saving King Shop	King of Power Saving	Good product quality is the king of power-saving products trustworthy as always good to recommend!	Jingdong prefecture level city in Shandong
Power Saving King Shop	King of Power Saving	Works great, very affordable, shipped pretty fast, comfortable to use	Jingdong prefecture level city in Shandong
Power Saving King Shop	King of Power Saving	Shipping was pretty fast and affordable, not bad 👍	Jingdong prefecture level city in Shandong
Power Saving King Shop	King of Power Saving	Digital voltage display, voltage at a glance, plug and play, safe and simple!	Jingdong prefecture level city in Shandong
Power Saving King Shop	King of Power Saving	Quality assurance, very good, affordable, logistics is also very fast, I'm very satisfied with the	Jingdong prefecture level city in Shandong
Power Saving King Shop	King of Power Saving	The quality is very good, the first time I received the goods to open the first time to see, it is very good, very satisfied	Jingdong prefecture level city in Shandong
Power Saving King Shop	King of Power Saving	Power Saving King used, the effect is good, the price is affordable, very good, is the genuine article	Jingdong prefecture level city in Shandong
Power Saving King Shop	King of Power Saving	Good quality, fine workmanship, good appearance, time is very accurate, satisfied.	Jingdong prefecture level city in Shandong
Power Saving King Shop	King of Power Saving	I purposely used it for two days before commenting, which is responsible for myself and everyone else!	Jingdong prefecture level city in Shandong
Power Saving King Shop	King of Power Saving	Easy to install, easy to operate, helped our family save a lot of electricity this summer	Jingdong prefecture level city in Shandong
Power Saving King Shop	King of Power Saving	Bringing power savings into millions of homes. Still a great gadget. Pretty good.	Jingdong prefecture level city in Shandong
Power Saving King Shop	King of Power Saving	Digital liquid crystal display to see the clear intuitive, very formal workmanship is not rough	Jingdong prefecture level city in Shandong
Power Saving King Shop	King of Power Saving	Received. Great quality, fast shipping from the store and not too expensive!	Jingdong prefecture level city in Shandong
Power Saving King Shop	King of Power Saving	Bought myself a knuckle regulator and it was easy to use and the price was right.	Jingdong prefecture level city in Shandong
Power Saving King Shop	King of Power Saving	Received, good, powerful, good, very full of a shopping	Jingdong prefecture level city in Shandong

Power Saving King Shop	King of Power Saving	High power, consistent with the seller's description, powerful, cost-effective	Jingdong prefecture level city in Shandong
Power Saving King Shop	King of Power Saving	It works very well and also saves power at an affordable price a great deal.	Jingdong prefecture level city in Shandong
Power Saving King Shop	King of Power Saving	Good quality, good can recommend to friends to buy.	Jingdong prefecture level city in Shandong
Power Saving King Shop	King of Power Saving	Will repurchase, quality is guaranteed, safe and secure purchase.	Jingdong prefecture level city in Shandong
Power Saving King Shop	King of Power Saving	The logistics is very fast, the thing is also good to use, energy saving power rely on it!	Jingdong prefecture level city in Shandong
Power Saving King Shop	King of Power Saving	Power Saver is good quality and works very well, worth buying!	Jingdong prefecture level city in Shandong
Power Saving King Shop	King of Power Saving	The quality is very good is genuine, cost-effective recommended	Jingdong prefecture level city in Shandong
Power Saving King Shop	King of Power Saving	Quality Assurance. Favorable price. Very practical and durable	Jingdong prefecture level city in Shandong
Power Saving King Shop	King of Power Saving	Good value for money. Conscientious businessman. Recommended to everyone	Jingdong prefecture level city in Shandong
Power Saving King Shop	King of Power Saving	Buy it for your rental apartment and never worry about your landlord charging you too much.	Jingdong prefecture level city in Shandong
Power Saving King Shop	King of Power Saving	Nice and affordable, will repurchase next time	Jingdong prefecture level city in Shandong
Power Saving King Shop	King of Power Saving	It's very energy efficient. I've been using it for a few days now.	Jingdong prefecture level city in Shandong
Power Saving King Shop	King of Power Saving	Good quality, high power easy to use	Jingdong prefecture level city in Shandong
Power Saving King Shop	King of Power Saving	Power saving little helper. Deserves a favorable review.	Jingdong prefecture level city in Shandong
Power Saving King Shop	King of Power Saving	Great value for money, great value for money.	Jingdong prefecture level city in Shandong
Power Saving King Shop	King of Power Saving	Works well and as described by the seller	Jingdong prefecture level city in Shandong
Power Saving King Shop	King of Power Saving	Power saving is proper! It's good.	Jingdong prefecture level city in Shandong
Power Saving King Shop	King of Power Saving	Power saving. Works well. Not bad.	Jingdong prefecture level city in Shandong
Power Saving King Shop	King of Power Saving	Satisfied with power saving	Jingdong prefecture level city in Shandong
Power Saving King Shop	King of Power Saving	Received intact, open a super good look beautiful and generous, ~ bit of high quality is recommended to buy, the need for friends rest assured that the next order	Jingdong prefecture level city in Shandong
Power Saving King Shop	King of Power Saving	Very good to use, friends introduced, save electricity and money, long-lasting products cost-effective, large factory production, good quality, fine workmanship cost-effective!	Jingdong prefecture level city in Shandong
Power Saving King Shop	King of Power Saving	The power saving effect is very obvious, trying to pay 30% less electricity bill in a month than before, hahaha	Jingdong prefecture level city in Shandong
Power Saving King Shop	King of Power Saving	It's such a power saver to use this, it saves money on kids snacks for a month. Kudos!	Jingdong prefecture level city in Shandong
Power Saving King Shop	King of Power Saving	Plug and play, stable voltage, the screen has a display when working, this winter home appliances, need more electricity, just buy to use, save electricity and save money, with instructions, not too big and does not take up space, the quality is very good, and the seller describes exactly the same!	Jingdong prefecture level city in Shandong
Power Saving King Shop	King of Power Saving	A must have for opening a store. Save over a thousand dollars a month on your electric bill	Jingdong prefecture level city in Shandong
Power Saving King Shop	King of Power Saving	Received the goods, the first time to unpack, the quality is very good, with the seller's description, completely exceeded my expectations, the packaging is very careful, very strict, in general this is a very satisfied with the shopping	Jingdong prefecture level city in Shandong
Power Saving King Shop	King of Power Saving	As always, good quality, a very favorite product, great! o(*￣︶￣*)o The price is very affordable, cost-effective, it's considered affordable good, will repurchase!	Jingdong prefecture level city in Shandong
Power Saving King Shop	King of Power Saving	Very satisfied with a shopping, good business service, fast shipping, logistics is also very powerful, received very good, buy assured, use peace of mind!	Jingdong prefecture level city in Shandong
Power Saving King Shop	King of Power Saving	The first time to buy online, but also look at the evaluation before buying, the quality is not bad, but also very cheap, the need for friends can rest assured to buy!	Jingdong prefecture level city in Shandong
Power Saving King Shop	King of Power Saving	Better than expected, feel value for money, very satisfied with a shopping, need next time will come back!	Jingdong prefecture level city in Shandong
Power Saving King Shop	King of Power Saving	Really super like, very supportive, very good quality, exactly as described by the seller, very satisfied!	Jingdong prefecture level city in Shandong
Power Saving King Shop	King of Power Saving	It is genuine, effective, really saves power, affordable, tightly packed, fast shipping, very satisfied.	Jingdong prefecture level city in Shandong
Power Saving King Shop	King of Power Saving	Good quality, fine workmanship, is genuine and business description of the same, good value for money, fast logistics, very satisfied	Jingdong prefecture level city in Shandong
Power Saving King Shop	King of Power Saving	Saver received, very good, installation set up very well, very good, the effect is very good, recommend that the pro buy	Jingdong prefecture level city in Shandong
Power Saving King Shop	King of Power Saving	Electromagnetic good, high power convenient, enough force, the price is right it, quality can be 😃 😃 😃 😃 😃	Jingdong prefecture level city in Shandong
Power Saving King Shop	King of Power Saving	First-class quality, packaging is also good, logistics is very fast, and the price is reasonable, very affordable, I like it!	Jingdong prefecture level city in Shandong
Power Saving King Shop	King of Power Saving	It's great, it's the kind of style you want, and it arrived quickly, thank you boss, 5 stars for the service!	Jingdong prefecture level city in Shandong
Power Saving King Shop	King of Power Saving	Packaging is very good, the use of the effect is quite good, the store has a good service attitude, trustworthy	Jingdong prefecture level city in Shandong
Power Saving King Shop	King of Power Saving	The quality is very good, it works well, very satisfied, the price is affordable, it's so great value, it's worth recommending.	Jingdong prefecture level city in Shandong
Power Saving King Shop	King of Power Saving	The baby description matches the actual item very well! The quality is awesome! Will be back to patronize again in the future!	Jingdong prefecture level city in Shandong
Power Saving King Shop	King of Power Saving	Ran the price is not high try to buy the attitude, did not expect the effect is really good, good!	Jingdong prefecture level city in Shandong
Power Saving King Shop	King of Power Saving	Power Saving King power is quite large, the quality of the leaf is quite good, the price of buying a very good deal, must be praised!	Jingdong prefecture level city in Shandong
Power Saving King Shop	King of Power Saving	Good product quality is the king of power-saving products trustworthy as always good to recommend!	Jingdong prefecture level city in Shandong
Power Saving King Shop	King of Power Saving	Works great, very affordable, shipped pretty fast, comfortable to use	Jingdong prefecture level city in Shandong
Power Saving King Shop	King of Power Saving	Shipping was pretty fast and affordable, not bad 👍	Jingdong prefecture level city in Shandong
Power Saving King Shop	King of Power Saving	Digital voltage display, voltage at a glance, plug and play, safe and simple!	Jingdong prefecture level city in Shandong
Power Saving King Shop	King of Power Saving	Quality assurance, very good, affordable, logistics is also very fast, I'm very satisfied with the	Jingdong prefecture level city in Shandong
Power Saving King Shop	King of Power Saving	The quality is very good, the first time I received the goods to open the first time to see, it is very good, very satisfied	Jingdong prefecture level city in Shandong
Power Saving King Shop	King of Power Saving	Power Saving King used, the effect is good, the price is affordable, very good, is the genuine article	Jingdong prefecture level city in Shandong
Power Saving King Shop	King of Power Saving	Good quality, fine workmanship, good appearance, time is very accurate, satisfied.	Jingdong prefecture level city in Shandong

Power Saving King Shop	King of Power Saving	I purposely used it for two days before commenting, which is responsible for myself and everyone else!	Jingdong prefecture level city in Shandong
Power Saving King Shop	King of Power Saving	Easy to install, easy to operate, helped our family save a lot of electricity this summer	Jingdong prefecture level city in Shandong
Power Saving King Shop	King of Power Saving	Bringing power savings into millions of homes. Still a great gadget. Pretty good.	Jingdong prefecture level city in Shandong
Power Saving King Shop	King of Power Saving	Digital liquid crystal display to see the clear intuitive, very formal workmanship is not rough	Jingdong prefecture level city in Shandong
Power Saving King Shop	King of Power Saving	Received. Great quality, fast shipping from the store and not too expensive!	Jingdong prefecture level city in Shandong
Power Saving King Shop	King of Power Saving	Bought myself a knuckle regulator and it was easy to use and the price was right.	Jingdong prefecture level city in Shandong
Power Saving King Shop	King of Power Saving	Received, good, powerful, good, very full of a shopping	Jingdong prefecture level city in Shandong
Power Saving King Shop	King of Power Saving	High power, consistent with the seller's description, powerful, cost-effective	Jingdong prefecture level city in Shandong
Power Saving King Shop	King of Power Saving	It works very well and also saves power at an affordable price a great deal.	Jingdong prefecture level city in Shandong
Power Saving King Shop	King of Power Saving	Good quality, good can recommend to friends to buy.	Jingdong prefecture level city in Shandong
Power Saving King Shop	King of Power Saving	Will repurchase, quality is guaranteed, safe and secure purchase.	Jingdong prefecture level city in Shandong
Power Saving King Shop	King of Power Saving	The logistics is very fast, the thing is also good to use, energy saving power rely on it!	Jingdong prefecture level city in Shandong
Power Saving King Shop	King of Power Saving	Power Saver is good quality and works very well, worth buying!	Jingdong prefecture level city in Shandong
Power Saving King Shop	King of Power Saving	The quality is very good is genuine, cost-effective recommended	Jingdong prefecture level city in Shandong
Power Saving King Shop	King of Power Saving	Quality Assurance. Favorable price. Very practical and durable	Jingdong prefecture level city in Shandong
Power Saving King Shop	King of Power Saving	Good value for money. Conscientious businessman. Recommended to everyone	Jingdong prefecture level city in Shandong
Power Saving King Shop	King of Power Saving	Buy it for your rental apartment and never worry about your landlord charging you too much.	Jingdong prefecture level city in Shandong
Power Saving King Shop	King of Power Saving	Nice and affordable, will repurchase next time	Jingdong prefecture level city in Shandong
Power Saving King Shop	King of Power Saving	It's very energy efficient. I've been using it for a few days now.	Jingdong prefecture level city in Shandong
Power Saving King Shop	King of Power Saving	Good quality, high power easy to use	Jingdong prefecture level city in Shandong
Power Saving King Shop	King of Power Saving	Power saving little helper. Deserves a favorable review.	Jingdong prefecture level city in Shandong
Power Saving King Shop	King of Power Saving	Great value for money, great value for money.	Jingdong prefecture level city in Shandong
Power Saving King Shop	King of Power Saving	Works well and as described by the seller	Jingdong prefecture level city in Shandong
Power Saving King Shop	King of Power Saving	Power saving is proper! It's good.	Jingdong prefecture level city in Shandong
Power Saving King Shop	King of Power Saving	Power saving. Works well. Not bad.	Jingdong prefecture level city in Shandong
Power Saving King Shop	King of Power Saving	Satisfied with power saving	Jingdong prefecture level city in Shandong
Power Saving King Shop	King of Power Saving	Received intact, open a super good look beautiful and generous, ~ bit of high quality is recommended to buy, the need for friends rest assured that the next order	Jingdong prefecture level city in Shandong
Power Saving King Shop	King of Power Saving	Very good to use, friends introduced, save electricity and money, long-lasting products cost-effective, large factory production, good quality, fine workmanship cost-effective!	Jingdong prefecture level city in Shandong
Power Saving King Shop	King of Power Saving	The power saving effect is very obvious, trying to pay 30% less electricity bill in a month than before, hahaha	Jingdong prefecture level city in Shandong
Power Saving King Shop	King of Power Saving	It's such a power saver to use this, it saves money on kids snacks for a month. Kudos!	Jingdong prefecture level city in Shandong
Power Saving King Shop	King of Power Saving	Plug and play, stable voltage, the screen has a display when working, this winter home appliances, need more electricity, just buy to use, save electricity and save money, with instructions, not too big and does not take up space, the quality is very good, and the seller describes exactly the same!	Jingdong prefecture level city in Shandong
Power Saving King Shop	King of Power Saving	A must have for opening a store. Save over a thousand dollars a month on your electric bill	Jingdong prefecture level city in Shandong
Power Saving King Shop	King of Power Saving	Received the goods, the first time to unpack, the quality is very good, with the seller's description, completely exceeded my expectations, the packaging is very careful, very strict, in general this is a very satisfied with the shopping	Jingdong prefecture level city in Shandong
Power Saving King Shop	King of Power Saving	As always, good quality, a very favorite product, great! o(*￣︶￣*)o The price is very affordable, cost-effective, it's considered affordable good, will repurchase!	Jingdong prefecture level city in Shandong
Power Saving King Shop	King of Power Saving	Very satisfied with a shopping, good business service, fast shipping, logistics is also very powerful, received very good, buy assured, use peace of mind!	Jingdong prefecture level city in Shandong
Power Saving King Shop	King of Power Saving	The first time to buy online, but also look at the evaluation before buying, the quality is not bad, but also very cheap, the need for friends can rest assured to buy!	Jingdong prefecture level city in Shandong
Power Saving King Shop	King of Power Saving	Better than expected, feel value for money, very satisfied with a shopping, need next time will come back!	Jingdong prefecture level city in Shandong
Power Saving King Shop	King of Power Saving	Really super like, very supportive, very good quality, exactly as described by the seller, very satisfied!	Jingdong prefecture level city in Shandong
Power Saving King Shop	King of Power Saving	It is genuine, effective, really saves power, affordable, tightly packed, fast shipping, very satisfied.	Jingdong prefecture level city in Shandong
Power Saving King Shop	King of Power Saving	Good quality, fine workmanship, is genuine and business description of the same, good value for money, fast logistics, very satisfied	Jingdong prefecture level city in Shandong
Power Saving King Shop	King of Power Saving	Saver received, very good, installation set up very well, very good, the effect is very good, recommend that the pro buy	Jingdong prefecture level city in Shandong
Power Saving King Shop	King of Power Saving	Electromagnetic good, high power convenient, enough force, the price is right it, quality can be 😃 😃 😃 😃 😃	Jingdong prefecture level city in Shandong
Power Saving King Shop	King of Power Saving	First-class quality, packaging is also good, logistics is very fast, and the price is reasonable, very affordable, I like it!	Jingdong prefecture level city in Shandong
Power Saving King Shop	King of Power Saving	It's great, it's the kind of style you want, and it arrived quickly, thank you boss, 5 stars for the service!	Jingdong prefecture level city in Shandong
Power Saving King Shop	King of Power Saving	Packaging is very good, the use of the effect is quite good, the store has a good service attitude, trustworthy	Jingdong prefecture level city in Shandong
Power Saving King Shop	King of Power Saving	The quality is very good, it works well, very satisfied, the price is affordable, it's so great value, it's worth recommending.	Jingdong prefecture level city in Shandong
Power Saving King Shop	King of Power Saving	The baby description matches the actual item very well! The quality is awesome! Will be back to patronize again in the future!	Jingdong prefecture level city in Shandong
Power Saving King Shop	King of Power Saving	Ran the price is not high try to buy the attitude, did not expect the effect is really good, good!	Jingdong prefecture level city in Shandong
Power Saving King Shop	King of Power Saving	Power Saving King power is quite large, the quality of the leaf is quite good, the price of buying a very good deal, must be praised!	Jingdong prefecture level city in Shandong
Power Saving King Shop	King of Power Saving	Good product quality is the king of power-saving products trustworthy as always good to recommend!	Jingdong prefecture level city in Shandong

Power Saving King Shop	King of Power Saving	Works great, very affordable, shipped pretty fast, comfortable to use	Jingdong prefecture level city in Shandong
Power Saving King Shop	King of Power Saving	Shipping was pretty fast and affordable, not bad 👍	Jingdong prefecture level city in Shandong
Power Saving King Shop	King of Power Saving	Digital voltage display, voltage at a glance, plug and play, safe and simple!	Jingdong prefecture level city in Shandong
Power Saving King Shop	King of Power Saving	Quality assurance, very good, affordable, logistics is also very fast, I'm very satisfied with the	Jingdong prefecture level city in Shandong
Power Saving King Shop	King of Power Saving	The quality is very good, the first time I received the goods to open the first time to see, it is very good, very satisfied	Jingdong prefecture level city in Shandong
Power Saving King Shop	King of Power Saving	Power Saving King used, the effect is good, the price is affordable, very good, is the genuine article	Jingdong prefecture level city in Shandong
Power Saving King Shop	King of Power Saving	Good quality, fine workmanship, good appearance, time is very accurate, satisfied.	Jingdong prefecture level city in Shandong
Power Saving King Shop	King of Power Saving	I purposely used it for two days before commenting, which is responsible for myself and everyone else!	Jingdong prefecture level city in Shandong
Power Saving King Shop	King of Power Saving	Easy to install, easy to operate, helped our family save a lot of electricity this summer	Jingdong prefecture level city in Shandong
Power Saving King Shop	King of Power Saving	Bringing power savings into millions of homes. Still a great gadget. Pretty good.	Jingdong prefecture level city in Shandong
Power Saving King Shop	King of Power Saving	Digital liquid crystal display to see the clear intuitive, very formal workmanship is not rough	Jingdong prefecture level city in Shandong
Power Saving King Shop	King of Power Saving	Received. Great quality, fast shipping from the store and not too expensive!	Jingdong prefecture level city in Shandong
Power Saving King Shop	King of Power Saving	Bought myself a knuckle regulator and it was easy to use and the price was right.	Jingdong prefecture level city in Shandong
Power Saving King Shop	King of Power Saving	Received, good, powerful, good, very full of a shopping	Jingdong prefecture level city in Shandong
Power Saving King Shop	King of Power Saving	High power, consistent with the seller's description, powerful, cost-effective	Jingdong prefecture level city in Shandong
Power Saving King Shop	King of Power Saving	It works very well and also saves power at an affordable price a great deal.	Jingdong prefecture level city in Shandong
Power Saving King Shop	King of Power Saving	Good quality, good can recommend to friends to buy.	Jingdong prefecture level city in Shandong
Power Saving King Shop	King of Power Saving	Will repurchase, quality is guaranteed, safe and secure purchase.	Jingdong prefecture level city in Shandong
Power Saving King Shop	King of Power Saving	The logistics is very fast, the thing is also good to use, energy saving power rely on it!	Jingdong prefecture level city in Shandong
Power Saving King Shop	King of Power Saving	Power Saver is good quality and works very well, worth buying!	Jingdong prefecture level city in Shandong
Power Saving King Shop	King of Power Saving	The quality is very good is genuine, cost-effective recommended	Jingdong prefecture level city in Shandong
Power Saving King Shop	King of Power Saving	Quality Assurance. Favorable price. Very practical and durable	Jingdong prefecture level city in Shandong
Power Saving King Shop	King of Power Saving	Good value for money. Conscientious businessman. Recommended to everyone	Jingdong prefecture level city in Shandong
Power Saving King Shop	King of Power Saving	Buy it for your rental apartment and never worry about your landlord charging you too much.	Jingdong prefecture level city in Shandong
Power Saving King Shop	King of Power Saving	Nice and affordable, will repurchase next time	Jingdong prefecture level city in Shandong
Power Saving King Shop	King of Power Saving	It's very energy efficient. I've been using it for a few days now.	Jingdong prefecture level city in Shandong
Power Saving King Shop	King of Power Saving	Good quality, high power easy to use	Jingdong prefecture level city in Shandong
Power Saving King Shop	King of Power Saving	Power saving little helper. Deserves a favorable review.	Jingdong prefecture level city in Shandong
Power Saving King Shop	King of Power Saving	Great value for money, great value for money.	Jingdong prefecture level city in Shandong
Power Saving King Shop	King of Power Saving	Works well and as described by the seller	Jingdong prefecture level city in Shandong
Power Saving King Shop	King of Power Saving	Power saving is proper! It's good.	Jingdong prefecture level city in Shandong
Power Saving King Shop	King of Power Saving	Power saving. Works well. Not bad.	Jingdong prefecture level city in Shandong
Power Saving King Shop	King of Power Saving	Satisfied with power saving	Jingdong prefecture level city in Shandong
Power Saving King Shop	King of Power Saving	Received intact, open a super good look beautiful and generous, ~ bit of high quality is recommended to buy, the need for friends rest assured that the next order	Jingdong prefecture level city in Shandong
Power Saving King Shop	King of Power Saving	Very good to use, friends introduced, save electricity and money, long-lasting products cost-effective, large factory production, good quality, fine workmanship cost-effective!	Jingdong prefecture level city in Shandong
Power Saving King Shop	King of Power Saving	The power saving effect is very obvious, trying to pay 30% less electricity bill in a month than before, hahaha	Jingdong prefecture level city in Shandong
Power Saving King Shop	King of Power Saving	It's such a power saver to use this, it saves money on kids snacks for a month. Kudos!	Jingdong prefecture level city in Shandong
Power Saving King Shop	King of Power Saving	Plug and play, stable voltage, the screen has a display when working, this winter home appliances, need more electricity, just buy to use, save electricity and save money, with instructions, not too big and does not take up space, the quality is very good, and the seller describes exactly the same!	Jingdong prefecture level city in Shandong
Power Saving King Shop	King of Power Saving	A must have for opening a store. Save over a thousand dollars a month on your electric bill	Jingdong prefecture level city in Shandong
Power Saving King Shop	King of Power Saving	Received the goods, the first time to unpack, the quality is very good, with the seller's description, completely exceeded my expectations, the packaging is very careful, very strict, in general this is a very satisfied with the shopping	Jingdong prefecture level city in Shandong
Power Saving King Shop	King of Power Saving	As always, good quality, a very favorite product, great! o(*￣︶￣*)o The price is very affordable, cost-effective, it's considered affordable good, will repurchase!	Jingdong prefecture level city in Shandong
Power Saving King Shop	King of Power Saving	Very satisfied with a shopping, good business service, fast shipping, logistics is also very powerful, received very good, buy assured, use peace of mind!	Jingdong prefecture level city in Shandong
Power Saving King Shop	King of Power Saving	The first time to buy online, but also look at the evaluation before buying, the quality is not bad, but also very cheap, the need for friends can rest assured to buy!	Jingdong prefecture level city in Shandong
Power Saving King Shop	King of Power Saving	Better than expected, feel value for money, very satisfied with a shopping, need next time will come back!	Jingdong prefecture level city in Shandong
Power Saving King Shop	King of Power Saving	Really super like, very supportive, very good quality, exactly as described by the seller, very satisfied!	Jingdong prefecture level city in Shandong
Power Saving King Shop	King of Power Saving	It is genuine, effective, really saves power, affordable, tightly packed, fast shipping, very satisfied.	Jingdong prefecture level city in Shandong
Power Saving King Shop	King of Power Saving	Good quality, fine workmanship, is genuine and business description of the same, good value for money, fast logistics, very satisfied	Jingdong prefecture level city in Shandong
Power Saving King Shop	King of Power Saving	Saver received, very good, installation set up very well, very good, the effect is very good, recommend that the pro buy	Jingdong prefecture level city in Shandong
Power Saving King Shop	King of Power Saving	Electromagnetic good, high power convenient, enough force, the price is right it, quality can be 😊 😊 😊 😊 😊	Jingdong prefecture level city in Shandong
Power Saving King Shop	King of Power Saving	First-class quality, packaging is also good, logistics is very fast, and the price is reasonable, very affordable, I like it!	Jingdong prefecture level city in Shandong

Power Saving King Shop	King of Power Saving	It's great, it's the kind of style you want, and it arrived quickly, thank you boss, 5 stars for the service!	Jingdong prefecture level city in Shandong
Power Saving King Shop	King of Power Saving	Packaging is very good, the use of the effect is quite good, the store has a good service attitude, trustworthy	Jingdong prefecture level city in Shandong
Power Saving King Shop	King of Power Saving	The quality is very good, it works well, very satisfied, the price is affordable, it's so great value, it's worth recommending.	Jingdong prefecture level city in Shandong
Power Saving King Shop	King of Power Saving	The baby description matches the actual item very well! The quality is awesome! Will be back to patronize again in the future!	Jingdong prefecture level city in Shandong
Power Saving King Shop	King of Power Saving	Ran the price is not high try to buy the attitude, did not expect the effect is really good, good!	Jingdong prefecture level city in Shandong
Power Saving King Shop	King of Power Saving	Power Saving King power is quite large, the quality of the leaf is quite good, the price of buying a very good deal, must be praised!	Jingdong prefecture level city in Shandong
Power Saving King Shop	King of Power Saving	Good product quality is the king of power-saving products trustworthy as always good to recommend!	Jingdong prefecture level city in Shandong
Power Saving King Shop	King of Power Saving	Works great, very affordable, shipped pretty fast, comfortable to use	Jingdong prefecture level city in Shandong
Power Saving King Shop	King of Power Saving	Shipping was pretty fast and affordable, not bad 👍	Jingdong prefecture level city in Shandong
Power Saving King Shop	King of Power Saving	Digital voltage display, voltage at a glance, plug and play, safe and simple!	Jingdong prefecture level city in Shandong
Power Saving King Shop	King of Power Saving	Quality assurance, very good, affordable, logistics is also very fast, I'm very satisfied with the	Jingdong prefecture level city in Shandong
Power Saving King Shop	King of Power Saving	The quality is very good, the first time I received the goods to open the first time to see, it is very good, very satisfied	Jingdong prefecture level city in Shandong
Power Saving King Shop	King of Power Saving	Power Saving King used, the effect is good, the price is affordable, very good, is the genuine article	Jingdong prefecture level city in Shandong
Power Saving King Shop	King of Power Saving	Good quality, fine workmanship, good appearance, time is very accurate, satisfied.	Jingdong prefecture level city in Shandong
Power Saving King Shop	King of Power Saving	I purposely used it for two days before commenting, which is responsible for myself and everyone else!	Jingdong prefecture level city in Shandong
Power Saving King Shop	King of Power Saving	Easy to install, easy to operate, helped our family save a lot of electricity this summer	Jingdong prefecture level city in Shandong
Power Saving King Shop	King of Power Saving	Bringing power savings into millions of homes. Still a great gadget. Pretty good.	Jingdong prefecture level city in Shandong
Power Saving King Shop	King of Power Saving	Digital liquid crystal display to see the clear intuitive, very formal workmanship is not rough	Jingdong prefecture level city in Shandong
Power Saving King Shop	King of Power Saving	Received. Great quality, fast shipping from the store and not too expensive!	Jingdong prefecture level city in Shandong
Power Saving King Shop	King of Power Saving	Bought myself a knuckle regulator and it was easy to use and the price was right.	Jingdong prefecture level city in Shandong
Power Saving King Shop	King of Power Saving	Received, good, powerful, good, very full of a shopping	Jingdong prefecture level city in Shandong
Power Saving King Shop	King of Power Saving	High power, consistent with the seller's description, powerful, cost-effective	Jingdong prefecture level city in Shandong
Power Saving King Shop	King of Power Saving	It works very well and also saves power at an affordable price a great deal.	Jingdong prefecture level city in Shandong
Power Saving King Shop	King of Power Saving	Good quality, good can recommend to friends to buy.	Jingdong prefecture level city in Shandong
Power Saving King Shop	King of Power Saving	Will repurchase, quality is guaranteed, safe and secure purchase.	Jingdong prefecture level city in Shandong
Power Saving King Shop	King of Power Saving	The logistics is very fast, the thing is also good to use, energy saving power rely on it!	Jingdong prefecture level city in Shandong
Power Saving King Shop	King of Power Saving	Power Saver is good quality and works very well, worth buying!	Jingdong prefecture level city in Shandong
Power Saving King Shop	King of Power Saving	The quality is very good is genuine, cost-effective recommended	Jingdong prefecture level city in Shandong
Power Saving King Shop	King of Power Saving	Quality Assurance. Favorable price. Very practical and durable	Jingdong prefecture level city in Shandong
Power Saving King Shop	King of Power Saving	Good value for money. Conscientious businessman. Recommended to everyone	Jingdong prefecture level city in Shandong
Power Saving King Shop	King of Power Saving	Buy it for your rental apartment and never worry about your landlord charging you too much.	Jingdong prefecture level city in Shandong
Power Saving King Shop	King of Power Saving	Nice and affordable, will repurchase next time	Jingdong prefecture level city in Shandong
Power Saving King Shop	King of Power Saving	It's very energy efficient. I've been using it for a few days now.	Jingdong prefecture level city in Shandong
Power Saving King Shop	King of Power Saving	Good quality, high power easy to use	Jingdong prefecture level city in Shandong
Power Saving King Shop	King of Power Saving	Power saving little helper. Deserves a favorable review.	Jingdong prefecture level city in Shandong
Power Saving King Shop	King of Power Saving	Great value for money, great value for money.	Jingdong prefecture level city in Shandong
Power Saving King Shop	King of Power Saving	Works well and as described by the seller	Jingdong prefecture level city in Shandong
Power Saving King Shop	King of Power Saving	Power saving is proper! It's good.	Jingdong prefecture level city in Shandong
Power Saving King Shop	King of Power Saving	Power saving. Works well. Not bad.	Jingdong prefecture level city in Shandong
Power Saving King Shop	King of Power Saving	Satisfied with power saving	Jingdong prefecture level city in Shandong
Power Saving King Shop	King of Power Saving	Received intact, open a super good look beautiful and generous, ~ bit of high quality is recommended to buy, the need for friends rest assured that the next order	Jingdong prefecture level city in Shandong
Power Saving King Shop	King of Power Saving	Very good to use, friends introduced, save electricity and money, long-lasting products cost-effective, large factory production, good quality, fine workmanship cost-effective!	Jingdong prefecture level city in Shandong
Power Saving King Shop	King of Power Saving	The power saving effect is very obvious, trying to pay 30% less electricity bill in a month than before, hahaha	Jingdong prefecture level city in Shandong
Power Saving King Shop	King of Power Saving	It's such a power saver to use this, it saves money on kids snacks for a month. Kudos!	Jingdong prefecture level city in Shandong
Power Saving King Shop	King of Power Saving	Plug and play, stable voltage, the screen has a display when working, this winter home appliances, need more electricity, just buy to use, save electricity and save money, with instructions, not too big and does not take up space, the quality is very good, and the seller describes exactly the same!	Jingdong prefecture level city in Shandong
Power Saving King Shop	King of Power Saving	A must have for opening a store. Save over a thousand dollars a month on your electric bill	Jingdong prefecture level city in Shandong
Power Saving King Shop	King of Power Saving	Received the goods, the first time to unpack, the quality is very good, with the seller's description, completely exceeded my expectations, the packaging is very careful, very strict, in general this is a very satisfied with the shopping	Jingdong prefecture level city in Shandong
Power Saving King Shop	King of Power Saving	As always, good quality, a very favorite product, great! o(*￣︶￣*)o The price is very affordable, cost-effective, it's considered affordable good, will repurchase!	Jingdong prefecture level city in Shandong
Power Saving King Shop	King of Power Saving	Very satisfied with a shopping, good business service, fast shipping, logistics is also very powerful, received very good, buy assured, use peace of mind!	Jingdong prefecture level city in Shandong
Power Saving King Shop	King of Power Saving	The first time to buy online, but also look at the evaluation before buying, the quality is not bad, but also very cheap, the need for friends can rest assured to buy!	Jingdong prefecture level city in Shandong

Power Saving King Shop	King of Power Saving	Better than expected, feel value for money, very satisfied with a shopping, need next time will come back!	Jingdong prefecture level city in Shandong
Power Saving King Shop	King of Power Saving	Really super like, very supportive, very good quality, exactly as described by the seller, very satisfied!	Jingdong prefecture level city in Shandong
Power Saving King Shop	King of Power Saving	It is genuine, effective, really saves power, affordable, tightly packed, fast shipping, very satisfied.	Jingdong prefecture level city in Shandong
Power Saving King Shop	King of Power Saving	Good quality, fine workmanship, is genuine and business description of the same, good value for money, fast logistics, very satisfied	Jingdong prefecture level city in Shandong
Power Saving King Shop	King of Power Saving	Saver received, very good, installation set up very well, very good, the effect is very good, recommend that the pro buy	Jingdong prefecture level city in Shandong
Power Saving King Shop	King of Power Saving	Electromagnetic good, high power convenient, enough force, the price is right it, quality can be 😀 😀 😀 😀 😀	Jingdong prefecture level city in Shandong
Power Saving King Shop	King of Power Saving	First-class quality, packaging is also good, logistics is very fast, and the price is reasonable, very affordable, I like it!	Jingdong prefecture level city in Shandong
Power Saving King Shop	King of Power Saving	It's great, it's the kind of style you want, and it arrived quickly, thank you boss, 5 stars for the service!	Jingdong prefecture level city in Shandong
Power Saving King Shop	King of Power Saving	Packaging is very good, the use of the effect is quite good, the store has a good service attitude, trustworthy	Jingdong prefecture level city in Shandong
Power Saving King Shop	King of Power Saving	The quality is very good, it works well, very satisfied, the price is affordable, it's so great value, it's worth recommending.	Jingdong prefecture level city in Shandong
Power Saving King Shop	King of Power Saving	The baby description matches the actual item very well! The quality is awesome! Will be back to patronize again in the future!	Jingdong prefecture level city in Shandong
Power Saving King Shop	King of Power Saving	Ran the price is not high try to buy the attitude, did not expect the effect is really good, good!	Jingdong prefecture level city in Shandong
Power Saving King Shop	King of Power Saving	Power Saving King power is quite large, the quality of the leaf is quite good, the price of buying a very good deal, must be praised!	Jingdong prefecture level city in Shandong
Power Saving King Shop	King of Power Saving	Good product quality is the king of power-saving products trustworthy as always good to recommend!	Jingdong prefecture level city in Shandong
Power Saving King Shop	King of Power Saving	Works great, very affordable, shipped pretty fast, comfortable to use	Jingdong prefecture level city in Shandong
Power Saving King Shop	King of Power Saving	Shipping was pretty fast and affordable, not bad 👍	Jingdong prefecture level city in Shandong
Power Saving King Shop	King of Power Saving	Digital voltage display, voltage at a glance, plug and play, safe and simple!	Jingdong prefecture level city in Shandong
Power Saving King Shop	King of Power Saving	Quality assurance, very good, affordable, logistics is also very fast, I'm very satisfied with the	Jingdong prefecture level city in Shandong
Power Saving King Shop	King of Power Saving	The quality is very good, the first time I received the goods to open the first time to see, it is very good, very satisfied	Jingdong prefecture level city in Shandong
Power Saving King Shop	King of Power Saving	Power Saving King used, the effect is good, the price is affordable, very good, is the genuine article	Jingdong prefecture level city in Shandong
Power Saving King Shop	King of Power Saving	Good quality, fine workmanship, good appearance, time is very accurate, satisfied.	Jingdong prefecture level city in Shandong
Power Saving King Shop	King of Power Saving	I purposely used it for two days before commenting, which is responsible for myself and everyone else!	Jingdong prefecture level city in Shandong
Power Saving King Shop	King of Power Saving	Easy to install, easy to operate, helped our family save a lot of electricity this summer	Jingdong prefecture level city in Shandong
Power Saving King Shop	King of Power Saving	Bringing power savings into millions of homes. Still a great gadget. Pretty good.	Jingdong prefecture level city in Shandong
Power Saving King Shop	King of Power Saving	Digital liquid crystal display to see the clear intuitive, very formal workmanship is not rough	Jingdong prefecture level city in Shandong
Power Saving King Shop	King of Power Saving	Received. Great quality, fast shipping from the store and not too expensive!	Jingdong prefecture level city in Shandong
Power Saving King Shop	King of Power Saving	Bought myself a knuckle regulator and it was easy to use and the price was right.	Jingdong prefecture level city in Shandong
Power Saving King Shop	King of Power Saving	Received, good, powerful, good, very full of a shopping	Jingdong prefecture level city in Shandong
Power Saving King Shop	King of Power Saving	High power, consistent with the seller's description, powerful, cost-effective	Jingdong prefecture level city in Shandong
Power Saving King Shop	King of Power Saving	It works very well and also saves power at an affordable price a great deal.	Jingdong prefecture level city in Shandong
Power Saving King Shop	King of Power Saving	Good quality, good can recommend to friends to buy.	Jingdong prefecture level city in Shandong
Power Saving King Shop	King of Power Saving	Will repurchase, quality is guaranteed, safe and secure purchase.	Jingdong prefecture level city in Shandong
Power Saving King Shop	King of Power Saving	The logistics is very fast, the thing is also good to use, energy saving power rely on it!	Jingdong prefecture level city in Shandong
Power Saving King Shop	King of Power Saving	Power Saver is good quality and works very well, worth buying!	Jingdong prefecture level city in Shandong
Power Saving King Shop	King of Power Saving	The quality is very good is genuine, cost-effective recommended	Jingdong prefecture level city in Shandong
Power Saving King Shop	King of Power Saving	Quality Assurance. Favorable price. Very practical and durable	Jingdong prefecture level city in Shandong
Power Saving King Shop	King of Power Saving	Good value for money. Conscientious businessman. Recommended to everyone	Jingdong prefecture level city in Shandong
Power Saving King Shop	King of Power Saving	Buy it for your rental apartment and never worry about your landlord charging you too much.	Jingdong prefecture level city in Shandong
Power Saving King Shop	King of Power Saving	Nice and affordable, will repurchase next time	Jingdong prefecture level city in Shandong
Power Saving King Shop	King of Power Saving	It's very energy efficient. I've been using it for a few days now.	Jingdong prefecture level city in Shandong
Power Saving King Shop	King of Power Saving	Good quality, high power easy to use	Jingdong prefecture level city in Shandong
Power Saving King Shop	King of Power Saving	Power saving little helper. Deserves a favorable review.	Jingdong prefecture level city in Shandong
Power Saving King Shop	King of Power Saving	Great value for money, great value for money.	Jingdong prefecture level city in Shandong
Power Saving King Shop	King of Power Saving	Works well and as described by the seller	Jingdong prefecture level city in Shandong
Power Saving King Shop	King of Power Saving	Power saving is proper! It's good.	Jingdong prefecture level city in Shandong
Power Saving King Shop	King of Power Saving	Power saving. Works well. Not bad.	Jingdong prefecture level city in Shandong
Power Saving King Shop	King of Power Saving	Satisfied with power saving	Jingdong prefecture level city in Shandong
Power Saving King Shop	King of Power Saving	Received intact, open a super good look beautiful and generous, ~ bit of high quality is recommended to buy, the need for friends rest assured that the next order	Jingdong prefecture level city in Shandong
Power Saving King Shop	King of Power Saving	Very good to use, friends introduced, save electricity and money, long-lasting products cost-effective, large factory production, good quality, fine workmanship cost-effective!	Jingdong prefecture level city in Shandong
Power Saving King Shop	King of Power Saving	The power saving effect is very obvious, trying to pay 30% less electricity bill in a month than before, hahaha	Jingdong prefecture level city in Shandong
Power Saving King Shop	King of Power Saving	It's such a power saver to use this, it saves money on kids snacks for a month. Kudos!	Jingdong prefecture level city in Shandong

Power Saving King Shop	King of Power Saving	Plug and play, stable voltage, the screen has a display when working, this winter home appliances, need more electricity, just buy to use, save electricity and save money, with instructions, not too big and does not take up space, the quality is very good, and the seller describes exactly the same!	Jingdong prefecture level city in Shandong
Power Saving King Shop	King of Power Saving	A must have for opening a store. Save over a thousand dollars a month on your electric bill	Jingdong prefecture level city in Shandong
Power Saving King Shop	King of Power Saving	Received the goods, the first time to unpack, the quality is very good, with the seller's description, completely exceeded my expectations, the packaging is very careful, very strict, in general this is a very satisfied with the shopping	Jingdong prefecture level city in Shandong
Power Saving King Shop	King of Power Saving	As always, good quality, a very favorite product, great! o(*￣︶￣*)o The price is very affordable, cost-effective, it's considered affordable good, will repurchase!	Jingdong prefecture level city in Shandong
Power Saving King Shop	King of Power Saving	Very satisfied with a shopping, good business service, fast shipping, logistics is also very powerful, received very good, buy assured, use peace of mind!	Jingdong prefecture level city in Shandong
Power Saving King Shop	King of Power Saving	The first time to buy online, but also look at the evaluation before buying, the quality is not bad, but also very cheap, the need for friends can rest assured to buy!	Jingdong prefecture level city in Shandong
Power Saving King Shop	King of Power Saving	Better than expected, feel value for money, very satisfied with a shopping, need next time will come back!	Jingdong prefecture level city in Shandong
Power Saving King Shop	King of Power Saving	Really super like, very supportive, very good quality, exactly as described by the seller, very satisfied!	Jingdong prefecture level city in Shandong
Power Saving King Shop	King of Power Saving	It is genuine, effective, really saves power, affordable, tightly packed, fast shipping, very satisfied.	Jingdong prefecture level city in Shandong
Power Saving King Shop	King of Power Saving	Good quality, fine workmanship, is genuine and business description of the same, good value for money, fast logistics, very satisfied	Jingdong prefecture level city in Shandong
Power Saving King Shop	King of Power Saving	Saver received, very good, installation set up very well, very good, the effect is very good, recommend that the pro buy	Jingdong prefecture level city in Shandong
Power Saving King Shop	King of Power Saving	Electromagnetic good, high power convenient, enough force, the price is right it, quality can be 😃😃😃😃😃	Jingdong prefecture level city in Shandong
Power Saving King Shop	King of Power Saving	First-class quality, packaging is also good, logistics is very fast, and the price is reasonable, very affordable, I like it!	Jingdong prefecture level city in Shandong
Power Saving King Shop	King of Power Saving	It's great, it's the kind of style you want, and it arrived quickly, thank you boss, 5 stars for the service!	Jingdong prefecture level city in Shandong
Power Saving King Shop	King of Power Saving	Packaging is very good, the use of the effect is quite good, the store has a good service attitude, trustworthy	Jingdong prefecture level city in Shandong
Power Saving King Shop	King of Power Saving	The quality is very good, it works well, very satisfied, the price is affordable, it's so great value, it's worth recommending.	Jingdong prefecture level city in Shandong
Power Saving King Shop	King of Power Saving	The baby description matches the actual item very well! The quality is awesome! Will be back to patronize again in the future!	Jingdong prefecture level city in Shandong
Power Saving King Shop	King of Power Saving	Ran the price is not high try to buy the attitude, did not expect the effect is really good, good!	Jingdong prefecture level city in Shandong
Power Saving King Shop	King of Power Saving	Power Saving King power is quite large, the quality of the leaf is quite good, the price of buying a very good deal, must be praised!	Jingdong prefecture level city in Shandong
Power Saving King Shop	King of Power Saving	Good product quality is the king of power-saving products trustworthy as always good to recommend!	Jingdong prefecture level city in Shandong
Power Saving King Shop	King of Power Saving	Works great, very affordable, shipped pretty fast, comfortable to use	Jingdong prefecture level city in Shandong
Power Saving King Shop	King of Power Saving	Shipping was pretty fast and affordable, not bad 👍	Jingdong prefecture level city in Shandong
Power Saving King Shop	King of Power Saving	Digital voltage display, voltage at a glance, plug and play, safe and simple!	Jingdong prefecture level city in Shandong
Power Saving King Shop	King of Power Saving	Quality assurance, very good, affordable, logistics is also very fast, I'm very satisfied with the	Jingdong prefecture level city in Shandong
Power Saving King Shop	King of Power Saving	The quality is very good, the first time I received the goods to open the first time to see, it is very good, very satisfied	Jingdong prefecture level city in Shandong
Power Saving King Shop	King of Power Saving	Power Saving King used, the effect is good, the price is affordable, very good, is the genuine article	Jingdong prefecture level city in Shandong
Power Saving King Shop	King of Power Saving	Good quality, fine workmanship, good appearance, time is very accurate, satisfied.	Jingdong prefecture level city in Shandong
Power Saving King Shop	King of Power Saving	I purposely used it for two days before commenting, which is responsible for myself and everyone else!	Jingdong prefecture level city in Shandong
Power Saving King Shop	King of Power Saving	Easy to install, easy to operate, helped our family save a lot of electricity this summer	Jingdong prefecture level city in Shandong
Power Saving King Shop	King of Power Saving	Bringing power savings into millions of homes. Still a great gadget. Pretty good.	Jingdong prefecture level city in Shandong
Power Saving King Shop	King of Power Saving	Digital liquid crystal display to see the clear intuitive, very formal workmanship is not rough	Jingdong prefecture level city in Shandong
Power Saving King Shop	King of Power Saving	Received. Great quality, fast shipping from the store and not too expensive!	Jingdong prefecture level city in Shandong
Power Saving King Shop	King of Power Saving	Bought myself a knuckle regulator and it was easy to use and the price was right.	Jingdong prefecture level city in Shandong
Power Saving King Shop	King of Power Saving	Received, good, powerful, good, very full of a shopping	Jingdong prefecture level city in Shandong
Power Saving King Shop	King of Power Saving	High power, consistent with the seller's description, powerful, cost-effective	Jingdong prefecture level city in Shandong
Power Saving King Shop	King of Power Saving	It works very well and also saves power at an affordable price a great deal.	Jingdong prefecture level city in Shandong
Power Saving King Shop	King of Power Saving	Good quality, good can recommend to friends to buy.	Jingdong prefecture level city in Shandong
Power Saving King Shop	King of Power Saving	Will repurchase, quality is guaranteed, safe and secure purchase.	Jingdong prefecture level city in Shandong
Power Saving King Shop	King of Power Saving	The logistics is very fast, the thing is also good to use, energy saving power rely on it!	Jingdong prefecture level city in Shandong
Power Saving King Shop	King of Power Saving	Power Saver is good quality and works very well, worth buying!	Jingdong prefecture level city in Shandong
Power Saving King Shop	King of Power Saving	The quality is very good is genuine, cost-effective recommended	Jingdong prefecture level city in Shandong
Power Saving King Shop	King of Power Saving	Quality Assurance. Favorable price. Very practical and durable	Jingdong prefecture level city in Shandong
Power Saving King Shop	King of Power Saving	Good value for money. Conscientious businessman. Recommended to everyone	Jingdong prefecture level city in Shandong
Power Saving King Shop	King of Power Saving	Buy it for your rental apartment and never worry about your landlord charging you too much.	Jingdong prefecture level city in Shandong
Power Saving King Shop	King of Power Saving	Nice and affordable, will repurchase next time	Jingdong prefecture level city in Shandong
Power Saving King Shop	King of Power Saving	It's very energy efficient. I've been using it for a few days now.	Jingdong prefecture level city in Shandong
Power Saving King Shop	King of Power Saving	Good quality, high power easy to use	Jingdong prefecture level city in Shandong
Power Saving King Shop	King of Power Saving	Power saving little helper. Deserves a favorable review.	Jingdong prefecture level city in Shandong
Power Saving King Shop	King of Power Saving	Great value for money, great value for money.	Jingdong prefecture level city in Shandong
Power Saving King Shop	King of Power Saving	Works well and as described by the seller	Jingdong prefecture level city in Shandong

Power Saving King Shop	King of Power Saving	Power saving is proper! It's good.	Jingdong prefecture level city in Shandong
Power Saving King Shop	King of Power Saving	Power saving. Works well. Not bad.	Jingdong prefecture level city in Shandong
Power Saving King Shop	King of Power Saving	Satisfied with power saving	Jingdong prefecture level city in Shandong
Power Saving King Shop	King of Power Saving	Received intact, open a super good look beautiful and generous, ~ bit of high quality is recommended to buy, the need for friends rest assured that the next order	Jingdong prefecture level city in Shandong
Power Saving King Shop	King of Power Saving	Very good to use, friends introduced, save electricity and money, long-lasting products cost-effective, large factory production, good quality, fine workmanship cost-effective!	Jingdong prefecture level city in Shandong
Power Saving King Shop	King of Power Saving	The power saving effect is very obvious, trying to pay 30% less electricity bill in a month than before, hahaha	Jingdong prefecture level city in Shandong
Power Saving King Shop	King of Power Saving	It's such a power saver to use this, it saves money on kids snacks for a month. Kudos!	Jingdong prefecture level city in Shandong
Power Saving King Shop	King of Power Saving	Plug and play, stable voltage, the screen has a display when working, this winter home appliances, need more electricity, just buy to use, save electricity and save money, with instructions, not too big and does not take up space, the quality is very good, and the seller describes exactly the same!	Jingdong prefecture level city in Shandong
Power Saving King Shop	King of Power Saving	A must have for opening a store. Save over a thousand dollars a month on your electric bill	Jingdong prefecture level city in Shandong
Power Saving King Shop	King of Power Saving	Received the goods, the first time to unpack, the quality is very good, with the seller's description, completely exceeded my expectations, the packaging is very careful, very strict, in general this is a very satisfied with the shopping	Jingdong prefecture level city in Shandong
Power Saving King Shop	King of Power Saving	As always, good quality, a very favorite product, great! o(*￣︶￣*)o The price is very affordable, cost-effective, it's considered affordable good, will repurchase!	Jingdong prefecture level city in Shandong
Power Saving King Shop	King of Power Saving	Very satisfied with a shopping, good business service, fast shipping, logistics is also very powerful, received very good, buy assured, use peace of mind!	Jingdong prefecture level city in Shandong
Power Saving King Shop	King of Power Saving	The first time to buy online, but also look at the evaluation before buying, the quality is not bad, but also very cheap, the need for friends can rest assured to buy!	Jingdong prefecture level city in Shandong
Power Saving King Shop	King of Power Saving	Better than expected, feel value for money, very satisfied with a shopping, need next time will come back!	Jingdong prefecture level city in Shandong
Power Saving King Shop	King of Power Saving	Really super like, very supportive, very good quality, exactly as described by the seller, very satisfied!	Jingdong prefecture level city in Shandong
Power Saving King Shop	King of Power Saving	It is genuine, effective, really saves power, affordable, tightly packed, fast shipping, very satisfied.	Jingdong prefecture level city in Shandong
Power Saving King Shop	King of Power Saving	Good quality, fine workmanship, is genuine and business description of the same, good value for money, fast logistics, very satisfied	Jingdong prefecture level city in Shandong
Power Saving King Shop	King of Power Saving	Saver received, very good, installation set up very well, very good, the effect is very good, recommend that the pro buy	Jingdong prefecture level city in Shandong
Power Saving King Shop	King of Power Saving	Electromagnetic good, high power convenient, enough force, the price is right it, quality can be ☺ ☺ ☺ ☺ ☺	Jingdong prefecture level city in Shandong
Power Saving King Shop	King of Power Saving	First-class quality, packaging is also good, logistics is very fast, and the price is reasonable, very affordable, I like it!	Jingdong prefecture level city in Shandong
Power Saving King Shop	King of Power Saving	It's great, it's the kind of style you want, and it arrived quickly, thank you boss, 5 stars for the service!	Jingdong prefecture level city in Shandong
Power Saving King Shop	King of Power Saving	Packaging is very good, the use of the effect is quite good, the store has a good service attitude, trustworthy	Jingdong prefecture level city in Shandong
Power Saving King Shop	King of Power Saving	The quality is very good, it works well, very satisfied, the price is affordable, it's so great value, it's worth recommending.	Jingdong prefecture level city in Shandong
Power Saving King Shop	King of Power Saving	The baby description matches the actual item very well! The quality is awesome! Will be back to patronize again in the future!	Jingdong prefecture level city in Shandong
Power Saving King Shop	King of Power Saving	Ran the price is not high try to buy the attitude, did not expect the effect is really good, good!	Jingdong prefecture level city in Shandong
Power Saving King Shop	King of Power Saving	Power Saving King power is quite large, the quality of the leaf is quite good, the price of buying a very good deal, must be praised!	Jingdong prefecture level city in Shandong
Power Saving King Shop	King of Power Saving	Good product quality is the king of power-saving products trustworthy as always good to recommend!	Jingdong prefecture level city in Shandong
Power Saving King Shop	King of Power Saving	Works great, very affordable, shipped pretty fast, comfortable to use	Jingdong prefecture level city in Shandong
Power Saving King Shop	King of Power Saving	Shipping was pretty fast and affordable, not bad 👍	Jingdong prefecture level city in Shandong
Power Saving King Shop	King of Power Saving	Digital voltage display, voltage at a glance, plug and play, safe and simple!	Jingdong prefecture level city in Shandong
Power Saving King Shop	King of Power Saving	Quality assurance, very good, affordable, logistics is also very fast, I'm very satisfied with the	Jingdong prefecture level city in Shandong
Power Saving King Shop	King of Power Saving	The quality is very good, the first time I received the goods to open the first time to see, it is very good, very satisfied	Jingdong prefecture level city in Shandong
Power Saving King Shop	King of Power Saving	Power Saving King used, the effect is good, the price is affordable, very good, is the genuine article	Jingdong prefecture level city in Shandong
Power Saving King Shop	King of Power Saving	Good quality, fine workmanship, good appearance, time is very accurate, satisfied.	Jingdong prefecture level city in Shandong
Power Saving King Shop	King of Power Saving	I purposely used it for two days before commenting, which is responsible for myself and everyone else!	Jingdong prefecture level city in Shandong
Power Saving King Shop	King of Power Saving	Easy to install, easy to operate, helped our family save a lot of electricity this summer	Jingdong prefecture level city in Shandong
Power Saving King Shop	King of Power Saving	Bringing power savings into millions of homes. Still a great gadget. Pretty good.	Jingdong prefecture level city in Shandong
Power Saving King Shop	King of Power Saving	Digital liquid crystal display to see the clear intuitive, very formal workmanship is not rough	Jingdong prefecture level city in Shandong
Power Saving King Shop	King of Power Saving	Received. Great quality, fast shipping from the store and not too expensive!	Jingdong prefecture level city in Shandong
Power Saving King Shop	King of Power Saving	Bought myself a knuckle regulator and it was easy to use and the price was right.	Jingdong prefecture level city in Shandong
Power Saving King Shop	King of Power Saving	Received, good, powerful, good, very full of a shopping	Jingdong prefecture level city in Shandong
Power Saving King Shop	King of Power Saving	High power, consistent with the seller's description, powerful, cost-effective	Jingdong prefecture level city in Shandong
Power Saving King Shop	King of Power Saving	It works very well and also saves power at an affordable price a great deal.	Jingdong prefecture level city in Shandong
Power Saving King Shop	King of Power Saving	Good quality, good can recommend to friends to buy.	Jingdong prefecture level city in Shandong
Power Saving King Shop	King of Power Saving	Will repurchase, quality is guaranteed, safe and secure purchase.	Jingdong prefecture level city in Shandong
Power Saving King Shop	King of Power Saving	The logistics is very fast, the thing is also good to use, energy saving power rely on it!	Jingdong prefecture level city in Shandong
Power Saving King Shop	King of Power Saving	Power Saver is good quality and works very well, worth buying!	Jingdong prefecture level city in Shandong
Power Saving King Shop	King of Power Saving	The quality is very good is genuine, cost-effective recommended	Jingdong prefecture level city in Shandong
Power Saving King Shop	King of Power Saving	Quality Assurance. Favorable price. Very practical and durable	Jingdong prefecture level city in Shandong
Power Saving King Shop	King of Power Saving	Good value for money. Conscientious businessman. Recommended to everyone	Jingdong prefecture level city in Shandong

Power Saving King Shop	King of Power Saving	Buy it for your rental apartment and never worry about your landlord charging you too much.	Jingdong prefecture level city in Shandong
Power Saving King Shop	King of Power Saving	Nice and affordable, will repurchase next time	Jingdong prefecture level city in Shandong
Power Saving King Shop	King of Power Saving	It's very energy efficient. I've been using it for a few days now.	Jingdong prefecture level city in Shandong
Power Saving King Shop	King of Power Saving	Good quality, high power easy to use	Jingdong prefecture level city in Shandong
Power Saving King Shop	King of Power Saving	Power saving little helper. Deserves a favorable review.	Jingdong prefecture level city in Shandong
Power Saving King Shop	King of Power Saving	Great value for money, great value for money.	Jingdong prefecture level city in Shandong
Power Saving King Shop	King of Power Saving	Works well and as described by the seller	Jingdong prefecture level city in Shandong
Power Saving King Shop	King of Power Saving	Power saving is proper! It's good.	Jingdong prefecture level city in Shandong
Power Saving King Shop	King of Power Saving	Power saving. Works well. Not bad.	Jingdong prefecture level city in Shandong
Power Saving King Shop	King of Power Saving	Satisfied with power saving	Jingdong prefecture level city in Shandong
Power Saving King Shop	King of Power Saving	Received intact, open a super good look beautiful and generous, ~ bit of high quality is recommended to buy, the need for friends rest assured that the next order	Jingdong prefecture level city in Shandong
Power Saving King Shop	King of Power Saving	Very good to use, friends introduced, save electricity and money, long-lasting products cost-effective, large factory production, good quality, fine workmanship cost-effective!	Jingdong prefecture level city in Shandong
Power Saving King Shop	King of Power Saving	The power saving effect is very obvious, trying to pay 30% less electricity bill in a month than before, hahaha	Jingdong prefecture level city in Shandong
Power Saving King Shop	King of Power Saving	It's such a power saver to use this, it saves money on kids snacks for a month. Kudos!	Jingdong prefecture level city in Shandong
Power Saving King Shop	King of Power Saving	Plug and play, stable voltage, the screen has a display when working, this winter home appliances, need more electricity, just buy to use, save electricity and save money, with instructions, not too big and does not take up space, the quality is very good, and the seller describes exactly the same!	Jingdong prefecture level city in Shandong
Power Saving King Shop	King of Power Saving	A must have for opening a store. Save over a thousand dollars a month on your electric bill	Jingdong prefecture level city in Shandong
Power Saving King Shop	King of Power Saving	Received the goods, the first time to unpack, the quality is very good, with the seller's description, completely exceeded my expectations, the packaging is very careful, very strict, in general this is a very satisfied with the shopping	Jingdong prefecture level city in Shandong
Power Saving King Shop	King of Power Saving	As always, good quality, a very favorite product, great! o(*￣︶￣*)o The price is very affordable, cost-effective, it's considered affordable good, will repurchase!	Jingdong prefecture level city in Shandong
Power Saving King Shop	King of Power Saving	Very satisfied with a shopping, good business service, fast shipping, logistics is also very powerful, received very good, buy assured, use peace of mind!	Jingdong prefecture level city in Shandong
Power Saving King Shop	King of Power Saving	The first time to buy online, but also look at the evaluation before buying, the quality is not bad, but also very cheap, the need for friends can rest assured to buy!	Jingdong prefecture level city in Shandong
Power Saving King Shop	King of Power Saving	Better than expected, feel value for money, very satisfied with a shopping, need next time will come back!	Jingdong prefecture level city in Shandong
Power Saving King Shop	King of Power Saving	Really super like, very supportive, very good quality, exactly as described by the seller, very satisfied!	Jingdong prefecture level city in Shandong
Power Saving King Shop	King of Power Saving	It is genuine, effective, really saves power, affordable, tightly packed, fast shipping, very satisfied.	Jingdong prefecture level city in Shandong
Power Saving King Shop	King of Power Saving	Good quality, fine workmanship, is genuine and business description of the same, good value for money, fast logistics, very satisfied	Jingdong prefecture level city in Shandong
Power Saving King Shop	King of Power Saving	Saver received, very good, installation set up very well, very good, the effect is very good, recommend that the pro buy	Jingdong prefecture level city in Shandong
Power Saving King Shop	King of Power Saving	Electromagnetic good, high power convenient, enough force, the price is right it, quality can be 😀 😀 😀 😀 😀	Jingdong prefecture level city in Shandong
Power Saving King Shop	King of Power Saving	First-class quality, packaging is also good, logistics is very fast, and the price is reasonable, very affordable, I like it!	Jingdong prefecture level city in Shandong
Power Saving King Shop	King of Power Saving	It's great, it's the kind of style you want, and it arrived quickly, thank you boss, 5 stars for the service!	Jingdong prefecture level city in Shandong
Power Saving King Shop	King of Power Saving	Packaging is very good, the use of the effect is quite good, the store has a good service attitude, trustworthy	Jingdong prefecture level city in Shandong
Power Saving King Shop	King of Power Saving	The quality is very good, it works well, very satisfied, the price is affordable, it's so great value, it's worth recommending.	Jingdong prefecture level city in Shandong
Power Saving King Shop	King of Power Saving	The baby description matches the actual item very well! The quality is awesome! Will be back to patronize again in the future!	Jingdong prefecture level city in Shandong
Power Saving King Shop	King of Power Saving	Ran the price is not high try to buy the attitude, did not expect the effect is really good, good!	Jingdong prefecture level city in Shandong
Power Saving King Shop	King of Power Saving	Power Saving King power is quite large, the quality of the leaf is quite good, the price of buying a very good deal, must be praised!	Jingdong prefecture level city in Shandong
Power Saving King Shop	King of Power Saving	Good product quality is the king of power-saving products trustworthy as always good to recommend!	Jingdong prefecture level city in Shandong
Power Saving King Shop	King of Power Saving	Works great, very affordable, shipped pretty fast, comfortable to use	Jingdong prefecture level city in Shandong
Power Saving King Shop	King of Power Saving	Shipping was pretty fast and affordable, not bad 👍	Jingdong prefecture level city in Shandong
Power Saving King Shop	King of Power Saving	Digital voltage display, voltage at a glance, plug and play, safe and simple!	Jingdong prefecture level city in Shandong
Power Saving King Shop	King of Power Saving	Quality assurance, very good, affordable, logistics is also very fast, I'm very satisfied with the	Jingdong prefecture level city in Shandong
Power Saving King Shop	King of Power Saving	The quality is very good, the first time I received the goods to open the first time to see, it is very good, very satisfied	Jingdong prefecture level city in Shandong
Power Saving King Shop	King of Power Saving	Power Saving King used, the effect is good, the price is affordable, very good, is the genuine article	Jingdong prefecture level city in Shandong
Power Saving King Shop	King of Power Saving	Good quality, fine workmanship, good appearance, time is very accurate, satisfied.	Jingdong prefecture level city in Shandong
Power Saving King Shop	King of Power Saving	I purposely used it for two days before commenting, which is responsible for myself and everyone else!	Jingdong prefecture level city in Shandong
Power Saving King Shop	King of Power Saving	Easy to install, easy to operate, helped our family save a lot of electricity this summer	Jingdong prefecture level city in Shandong
Power Saving King Shop	King of Power Saving	Bringing power savings into millions of homes. Still a great gadget. Pretty good.	Jingdong prefecture level city in Shandong
Power Saving King Shop	King of Power Saving	Digital liquid crystal display to see the clear intuitive, very formal workmanship is not rough	Jingdong prefecture level city in Shandong
Power Saving King Shop	King of Power Saving	Received. Great quality, fast shipping from the store and not too expensive!	Jingdong prefecture level city in Shandong
Power Saving King Shop	King of Power Saving	Bought myself a knuckle regulator and it was easy to use and the price was right.	Jingdong prefecture level city in Shandong
Power Saving King Shop	King of Power Saving	Received, good, powerful, good, very full of a shopping	Jingdong prefecture level city in Shandong
Power Saving King Shop	King of Power Saving	High power, consistent with the seller's description, powerful, cost-effective	Jingdong prefecture level city in Shandong
Power Saving King Shop	King of Power Saving	It works very well and also saves power at an affordable price a great deal.	Jingdong prefecture level city in Shandong

Power Saving King Shop	King of Power Saving	Good quality, good can recommend to friends to buy.	Jingdong prefecture level city in Shandong
Power Saving King Shop	King of Power Saving	Will repurchase, quality is guaranteed, safe and secure purchase.	Jingdong prefecture level city in Shandong
Power Saving King Shop	King of Power Saving	The logistics is very fast, the thing is also good to use, energy saving power rely on it!	Jingdong prefecture level city in Shandong
Power Saving King Shop	King of Power Saving	Power Saver is good quality and works very well, worth buying!	Jingdong prefecture level city in Shandong
Power Saving King Shop	King of Power Saving	The quality is very good is genuine, cost-effective recommended	Jingdong prefecture level city in Shandong
Power Saving King Shop	King of Power Saving	Quality Assurance. Favorable price. Very practical and durable	Jingdong prefecture level city in Shandong
Power Saving King Shop	King of Power Saving	Good value for money. Conscientious businessman. Recommended to everyone	Jingdong prefecture level city in Shandong
Power Saving King Shop	King of Power Saving	Buy it for your rental apartment and never worry about your landlord charging you too much.	Jingdong prefecture level city in Shandong
Power Saving King Shop	King of Power Saving	Nice and affordable, will repurchase next time	Jingdong prefecture level city in Shandong
Power Saving King Shop	King of Power Saving	It's very energy efficient. I've been using it for a few days now.	Jingdong prefecture level city in Shandong
Power Saving King Shop	King of Power Saving	Good quality, high power easy to use	Jingdong prefecture level city in Shandong
Power Saving King Shop	King of Power Saving	Power saving little helper. Deserves a favorable review.	Jingdong prefecture level city in Shandong
Power Saving King Shop	King of Power Saving	Great value for money, great value for money.	Jingdong prefecture level city in Shandong
Power Saving King Shop	King of Power Saving	Works well and as described by the seller	Jingdong prefecture level city in Shandong
Power Saving King Shop	King of Power Saving	Power saving is proper! It's good.	Jingdong prefecture level city in Shandong
Power Saving King Shop	King of Power Saving	Power saving. Works well. Not bad.	Jingdong prefecture level city in Shandong
Power Saving King Shop	King of Power Saving	Satisfied with power saving	Jingdong prefecture level city in Shandong
Power Saving King Shop	King of Power Saving	Received intact, open a super good look beautiful and generous, ~ bit of high quality is recommended to buy, the need for friends rest assured that the next order	Jingdong prefecture level city in Shandong
Power Saving King Shop	King of Power Saving	Very good to use, friends introduced, save electricity and money, long-lasting products cost-effective, large factory production, good quality, fine workmanship cost-effective!	Jingdong prefecture level city in Shandong
Power Saving King Shop	King of Power Saving	The power saving effect is very obvious, trying to pay 30% less electricity bill in a month than before, hahaha	Jingdong prefecture level city in Shandong
Power Saving King Shop	King of Power Saving	It's such a power saver to use this, it saves money on kids snacks for a month. Kudos!	Jingdong prefecture level city in Shandong
Power Saving King Shop	King of Power Saving	Plug and play, stable voltage, the screen has a display when working, this winter home appliances, need more electricity, just buy to use, save electricity and save money, with instructions, not too big and does not take up space, the quality is very good, and the seller describes exactly the same!	Jingdong prefecture level city in Shandong
Power Saving King Shop	King of Power Saving	A must have for opening a store. Save over a thousand dollars a month on your electric bill	Jingdong prefecture level city in Shandong
Power Saving King Shop	King of Power Saving	Received the goods, the first time to unpack, the quality is very good, with the seller's description, completely exceeded my expectations, the packaging is very careful, very strict, in general this is a very satisfied with the shopping	Jingdong prefecture level city in Shandong
Power Saving King Shop	King of Power Saving	As always, good quality, a very favorite product, great! o(*￣︶￣*)o The price is very affordable, cost-effective, it's considered affordable good, will repurchase!	Jingdong prefecture level city in Shandong
Power Saving King Shop	King of Power Saving	Very satisfied with a shopping, good business service, fast shipping, logistics is also very powerful, received very good, buy assured, use peace of mind!	Jingdong prefecture level city in Shandong
Power Saving King Shop	King of Power Saving	The first time to buy online, but also look at the evaluation before buying, the quality is not bad, but also very cheap, the need for friends can rest assured to buy!	Jingdong prefecture level city in Shandong
Power Saving King Shop	King of Power Saving	Better than expected, feel value for money, very satisfied with a shopping, need next time will come back!	Jingdong prefecture level city in Shandong
Power Saving King Shop	King of Power Saving	Really super like, very supportive, very good quality, exactly as described by the seller, very satisfied!	Jingdong prefecture level city in Shandong
Power Saving King Shop	King of Power Saving	It is genuine, effective, really saves power, affordable, tightly packed, fast shipping, very satisfied.	Jingdong prefecture level city in Shandong
Power Saving King Shop	King of Power Saving	Good quality, fine workmanship, is genuine and business description of the same, good value for money, fast logistics, very satisfied	Jingdong prefecture level city in Shandong
Power Saving King Shop	King of Power Saving	Saver received, very good, installation set up very well, very good, the effect is very good, recommend that the pro buy	Jingdong prefecture level city in Shandong
Power Saving King Shop	King of Power Saving	Electromagnetic good, high power convenient, enough force, the price is right it, quality can be 😊 😊 😊 😊 😊	Jingdong prefecture level city in Shandong
Power Saving King Shop	King of Power Saving	First-class quality, packaging is also good, logistics is very fast, and the price is reasonable, very affordable, I like it!	Jingdong prefecture level city in Shandong
Power Saving King Shop	King of Power Saving	It's great, it's the kind of style you want, and it arrived quickly, thank you boss, 5 stars for the service!	Jingdong prefecture level city in Shandong
Power Saving King Shop	King of Power Saving	Packaging is very good, the use of the effect is quite good, the store has a good service attitude, trustworthy	Jingdong prefecture level city in Shandong
Power Saving King Shop	King of Power Saving	The quality is very good, it works well, very satisfied, the price is affordable, it's so great value, it's worth recommending.	Jingdong prefecture level city in Shandong
Power Saving King Shop	King of Power Saving	The baby description matches the actual item very well! The quality is awesome! Will be back to patronize again in the future!	Jingdong prefecture level city in Shandong
Power Saving King Shop	King of Power Saving	Ran the price is not high try to buy the attitude, did not expect the effect is really good, good!	Jingdong prefecture level city in Shandong
Power Saving King Shop	King of Power Saving	Power Saving King power is quite large, the quality of the leaf is quite good, the price of buying a very good deal, must be praised!	Jingdong prefecture level city in Shandong
Power Saving King Shop	King of Power Saving	Good product quality is the king of power-saving products trustworthy as always good to recommend!	Jingdong prefecture level city in Shandong
Power Saving King Shop	King of Power Saving	Works great, very affordable, shipped pretty fast, comfortable to use	Jingdong prefecture level city in Shandong
Power Saving King Shop	King of Power Saving	Shipping was pretty fast and affordable, not bad 👍	Jingdong prefecture level city in Shandong
Power Saving King Shop	King of Power Saving	Digital voltage display, voltage at a glance, plug and play, safe and simple!	Jingdong prefecture level city in Shandong
Power Saving King Shop	King of Power Saving	Quality assurance, very good, affordable, logistics is also very fast, I'm very satisfied with the	Jingdong prefecture level city in Shandong
Power Saving King Shop	King of Power Saving	The quality is very good, the first time I received the goods to open the first time to see, it is very good, very satisfied	Jingdong prefecture level city in Shandong
Power Saving King Shop	King of Power Saving	Power Saving King used, the effect is good, the price is affordable, very good, is the genuine article	Jingdong prefecture level city in Shandong
Power Saving King Shop	King of Power Saving	Good quality, fine workmanship, good appearance, time is very accurate, satisfied.	Jingdong prefecture level city in Shandong
Power Saving King Shop	King of Power Saving	I purposely used it for two days before commenting, which is responsible for myself and everyone else!	Jingdong prefecture level city in Shandong
Power Saving King Shop	King of Power Saving	Easy to install, easy to operate, helped our family save a lot of electricity this summer	Jingdong prefecture level city in Shandong

Power Saving King Shop	King of Power Saving	Bringing power savings into millions of homes. Still a great gadget. Pretty good.	Jingdong prefecture level city in Shandong
Power Saving King Shop	King of Power Saving	Digital liquid crystal display to see the clear intuitive, very formal workmanship is not rough	Jingdong prefecture level city in Shandong
Power Saving King Shop	King of Power Saving	Received. Great quality, fast shipping from the store and not too expensive!	Jingdong prefecture level city in Shandong
Power Saving King Shop	King of Power Saving	Bought myself a knuckle regulator and it was easy to use and the price was right.	Jingdong prefecture level city in Shandong
Power Saving King Shop	King of Power Saving	Received, good, powerful, good, very full of a shopping	Jingdong prefecture level city in Shandong
Power Saving King Shop	King of Power Saving	High power, consistent with the seller's description, powerful, cost-effective	Jingdong prefecture level city in Shandong
Power Saving King Shop	King of Power Saving	It works very well and also saves power at an affordable price a great deal.	Jingdong prefecture level city in Shandong
Power Saving King Shop	King of Power Saving	Good quality, good can recommend to friends to buy.	Jingdong prefecture level city in Shandong
Power Saving King Shop	King of Power Saving	Will repurchase, quality is guaranteed, safe and secure purchase.	Jingdong prefecture level city in Shandong
Power Saving King Shop	King of Power Saving	The logistics is very fast, the thing is also good to use, energy saving power rely on it!	Jingdong prefecture level city in Shandong
Power Saving King Shop	King of Power Saving	Power Saver is good quality and works very well, worth buying!	Jingdong prefecture level city in Shandong
Power Saving King Shop	King of Power Saving	The quality is very good is genuine, cost-effective recommended	Jingdong prefecture level city in Shandong
Power Saving King Shop	King of Power Saving	Quality Assurance. Favorable price. Very practical and durable	Jingdong prefecture level city in Shandong
Power Saving King Shop	King of Power Saving	Good value for money. Conscientious businessman. Recommended to everyone	Jingdong prefecture level city in Shandong
Power Saving King Shop	King of Power Saving	Buy it for your rental apartment and never worry about your landlord charging you too much.	Jingdong prefecture level city in Shandong
Power Saving King Shop	King of Power Saving	Nice and affordable, will repurchase next time	Jingdong prefecture level city in Shandong
Power Saving King Shop	King of Power Saving	It's very energy efficient. I've been using it for a few days now.	Jingdong prefecture level city in Shandong
Power Saving King Shop	King of Power Saving	Good quality, high power easy to use	Jingdong prefecture level city in Shandong
Power Saving King Shop	King of Power Saving	Power saving little helper. Deserves a favorable review.	Jingdong prefecture level city in Shandong
Power Saving King Shop	King of Power Saving	Great value for money, great value for money.	Jingdong prefecture level city in Shandong
Power Saving King Shop	King of Power Saving	Works well and as described by the seller	Jingdong prefecture level city in Shandong
Power Saving King Shop	King of Power Saving	Power saving is proper! It's good.	Jingdong prefecture level city in Shandong
Power Saving King Shop	King of Power Saving	Power saving. Works well. Not bad.	Jingdong prefecture level city in Shandong
Power Saving King Shop	King of Power Saving	Satisfied with power saving	Jingdong prefecture level city in Shandong
Power Saving King Shop	King of Power Saving	Received intact, open a super good look beautiful and generous, ~ bit of high quality is recommended to buy, the need for friends rest assured that the next order	Jingdong prefecture level city in Shandong
Power Saving King Shop	King of Power Saving	Very good to use, friends introduced, save electricity and money, long-lasting products cost-effective, large factory production, good quality, fine workmanship cost-effective!	Jingdong prefecture level city in Shandong
Power Saving King Shop	King of Power Saving	The power saving effect is very obvious, trying to pay 30% less electricity bill in a month than before, hahaha	Jingdong prefecture level city in Shandong
Power Saving King Shop	King of Power Saving	It's such a power saver to use this, it saves money on kids snacks for a month. Kudos!	Jingdong prefecture level city in Shandong
Power Saving King Shop	King of Power Saving	Plug and play, stable voltage, the screen has a display when working, this winter home appliances, need more electricity, just buy to use, save electricity and save money, with instructions, not too big and does not take up space, the quality is very good, and the seller describes exactly the same!	Jingdong prefecture level city in Shandong
Power Saving King Shop	King of Power Saving	A must have for opening a store. Save over a thousand dollars a month on your electric bill	Jingdong prefecture level city in Shandong
Power Saving King Shop	King of Power Saving	Received the goods, the first time to unpack, the quality is very good, with the seller's description, completely exceeded my expectations, the packaging is very careful, very strict, in general this is a very satisfied with the shopping	Jingdong prefecture level city in Shandong
Power Saving King Shop	King of Power Saving	As always, good quality, a very favorite product, great! o(*￣︶￣*)o The price is very affordable, cost-effective, it's considered affordable good, will repurchase!	Jingdong prefecture level city in Shandong
Power Saving King Shop	King of Power Saving	Very satisfied with a shopping, good business service, fast shipping, logistics is also very powerful, received very good, buy assured, use peace of mind!	Jingdong prefecture level city in Shandong
Power Saving King Shop	King of Power Saving	The first time to buy online, but also look at the evaluation before buying, the quality is not bad, but also very cheap, the need for friends can rest assured to buy!	Jingdong prefecture level city in Shandong
Power Saving King Shop	King of Power Saving	Better than expected, feel value for money, very satisfied with a shopping, need next time will come back!	Jingdong prefecture level city in Shandong
Power Saving King Shop	King of Power Saving	Really super like, very supportive, very good quality, exactly as described by the seller, very satisfied!	Jingdong prefecture level city in Shandong
Power Saving King Shop	King of Power Saving	It is genuine, effective, really saves power, affordable, tightly packed, fast shipping, very satisfied.	Jingdong prefecture level city in Shandong
Power Saving King Shop	King of Power Saving	Good quality, fine workmanship, is genuine and business description of the same, good value for money, fast logistics, very satisfied	Jingdong prefecture level city in Shandong
Power Saving King Shop	King of Power Saving	Saver received, very good, installation set up very well, very good, the effect is very good, recommend that the pro buy	Jingdong prefecture level city in Shandong
Power Saving King Shop	King of Power Saving	Electromagnetic good, high power convenient, enough force, the price is right it, quality can be 😃 😃 😃 😃 😃	Jingdong prefecture level city in Shandong
Power Saving King Shop	King of Power Saving	First-class quality, packaging is also good, logistics is very fast, and the price is reasonable, very affordable, I like it!	Jingdong prefecture level city in Shandong
Power Saving King Shop	King of Power Saving	It's great, it's the kind of style you want, and it arrived quickly, thank you boss, 5 stars for the service!	Jingdong prefecture level city in Shandong
Power Saving King Shop	King of Power Saving	Packaging is very good, the use of the effect is quite good, the store has a good service attitude, trustworthy	Jingdong prefecture level city in Shandong
Power Saving King Shop	King of Power Saving	The quality is very good, it works well, very satisfied, the price is affordable, it's so great value, it's worth recommending.	Jingdong prefecture level city in Shandong
Power Saving King Shop	King of Power Saving	The baby description matches the actual item very well! The quality is awesome! Will be back to patronize again in the future!	Jingdong prefecture level city in Shandong
Power Saving King Shop	King of Power Saving	Ran the price is not high try to buy the attitude, did not expect the effect is really good, good!	Jingdong prefecture level city in Shandong
Power Saving King Shop	King of Power Saving	Power Saving King power is quite large, the quality of the leaf is quite good, the price of buying a very good deal, must be praised!	Jingdong prefecture level city in Shandong
Power Saving King Shop	King of Power Saving	Good product quality is the king of power-saving products trustworthy as always good to recommend!	Jingdong prefecture level city in Shandong
Power Saving King Shop	King of Power Saving	Works great, very affordable, shipped pretty fast, comfortable to use	Jingdong prefecture level city in Shandong
Power Saving King Shop	King of Power Saving	Shipping was pretty fast and affordable, not bad 👍	Jingdong prefecture level city in Shandong

Power Saving King Shop	King of Power Saving	Digital voltage display, voltage at a glance, plug and play, safe and simple!	Jingdong prefecture level city in Shandong
Power Saving King Shop	King of Power Saving	Quality assurance, very good, affordable, logistics is also very fast, I'm very satisfied with the	Jingdong prefecture level city in Shandong
Power Saving King Shop	King of Power Saving	The quality is very good, the first time I received the goods to open the first time to see, it is very good, very satisfied	Jingdong prefecture level city in Shandong
Power Saving King Shop	King of Power Saving	Power Saving King used, the effect is good, the price is affordable, very good, is the genuine article	Jingdong prefecture level city in Shandong
Power Saving King Shop	King of Power Saving	Good quality, fine workmanship, good appearance, time is very accurate, satisfied.	Jingdong prefecture level city in Shandong
Power Saving King Shop	King of Power Saving	I purposely used it for two days before commenting, which is responsible for myself and everyone else!	Jingdong prefecture level city in Shandong
Power Saving King Shop	King of Power Saving	Easy to install, easy to operate, helped our family save a lot of electricity this summer	Jingdong prefecture level city in Shandong
Power Saving King Shop	King of Power Saving	Bringing power savings into millions of homes. Still a great gadget. Pretty good.	Jingdong prefecture level city in Shandong
Power Saving King Shop	King of Power Saving	Digital liquid crystal display to see the clear intuitive, very formal workmanship is not rough	Jingdong prefecture level city in Shandong
Power Saving King Shop	King of Power Saving	Received. Great quality, fast shipping from the store and not too expensive!	Jingdong prefecture level city in Shandong
Power Saving King Shop	King of Power Saving	Bought myself a knuckle regulator and it was easy to use and the price was right.	Jingdong prefecture level city in Shandong
Power Saving King Shop	King of Power Saving	Received, good, powerful, good, very full of a shopping	Jingdong prefecture level city in Shandong
Power Saving King Shop	King of Power Saving	High power, consistent with the seller's description, powerful, cost-effective	Jingdong prefecture level city in Shandong
Power Saving King Shop	King of Power Saving	It works very well and also saves power at an affordable price a great deal.	Jingdong prefecture level city in Shandong
Power Saving King Shop	King of Power Saving	Good quality, good can recommend to friends to buy.	Jingdong prefecture level city in Shandong
Power Saving King Shop	King of Power Saving	Will repurchase, quality is guaranteed, safe and secure purchase.	Jingdong prefecture level city in Shandong
Power Saving King Shop	King of Power Saving	The logistics is very fast, the thing is also good to use, energy saving power rely on it!	Jingdong prefecture level city in Shandong
Power Saving King Shop	King of Power Saving	Power Saver is good quality and works very well, worth buying!	Jingdong prefecture level city in Shandong
Power Saving King Shop	King of Power Saving	The quality is very good is genuine, cost-effective recommended	Jingdong prefecture level city in Shandong
Power Saving King Shop	King of Power Saving	Quality Assurance. Favorable price. Very practical and durable	Jingdong prefecture level city in Shandong
Power Saving King Shop	King of Power Saving	Good value for money. Conscientious businessman. Recommended to everyone	Jingdong prefecture level city in Shandong
Power Saving King Shop	King of Power Saving	Buy it for your rental apartment and never worry about your landlord charging you too much.	Jingdong prefecture level city in Shandong
Power Saving King Shop	King of Power Saving	Nice and affordable, will repurchase next time	Jingdong prefecture level city in Shandong
Power Saving King Shop	King of Power Saving	It's very energy efficient. I've been using it for a few days now.	Jingdong prefecture level city in Shandong
Power Saving King Shop	King of Power Saving	Good quality, high power easy to use	Jingdong prefecture level city in Shandong
Power Saving King Shop	King of Power Saving	Power saving little helper. Deserves a favorable review.	Jingdong prefecture level city in Shandong
Power Saving King Shop	King of Power Saving	Great value for money, great value for money.	Jingdong prefecture level city in Shandong
Power Saving King Shop	King of Power Saving	Works well and as described by the seller	Jingdong prefecture level city in Shandong
Power Saving King Shop	King of Power Saving	Power saving is proper! It's good.	Jingdong prefecture level city in Shandong
Power Saving King Shop	King of Power Saving	Power saving. Works well. Not bad.	Jingdong prefecture level city in Shandong
Power Saving King Shop	King of Power Saving	Satisfied with power saving	Jingdong prefecture level city in Shandong
Power Saving King Shop	King of Power Saving	Received intact, open a super good look beautiful and generous, ~ bit of high quality is recommended to buy, the need for friends rest assured that the next order	Jingdong prefecture level city in Shandong
Power Saving King Shop	King of Power Saving	Very good to use, friends introduced, save electricity and money, long-lasting products cost-effective, large factory production, good quality, fine workmanship cost-effective!	Jingdong prefecture level city in Shandong
Power Saving King Shop	King of Power Saving	The power saving effect is very obvious, trying to pay 30% less electricity bill in a month than before, hahaha	Jingdong prefecture level city in Shandong
Power Saving King Shop	King of Power Saving	It's such a power saver to use this, it saves money on kids snacks for a month. Kudos!	Jingdong prefecture level city in Shandong
Power Saving King Shop	King of Power Saving	Plug and play, stable voltage, the screen has a display when working, this winter home appliances, need more electricity, just buy to use, save electricity and save money, with instructions, not too big and does not take up space, the quality is very good, and the seller describes exactly the same!	Jingdong prefecture level city in Shandong
Power Saving King Shop	King of Power Saving	A must have for opening a store. Save over a thousand dollars a month on your electric bill	Jingdong prefecture level city in Shandong
Power Saving King Shop	King of Power Saving	Received the goods, the first time to unpack, the quality is very good, with the seller's description, completely exceeded my expectations, the packaging is very careful, very strict, in general this is a very satisfied with the shopping	Jingdong prefecture level city in Shandong
Power Saving King Shop	King of Power Saving	As always, good quality, a very favorite product, great! o(*￣︶￣*)o The price is very affordable, cost-effective, it's considered affordable good, will repurchase!	Jingdong prefecture level city in Shandong
Power Saving King Shop	King of Power Saving	Very satisfied with a shopping, good business service, fast shipping, logistics is also very powerful, received very good, buy assured, use peace of mind!	Jingdong prefecture level city in Shandong
Power Saving King Shop	King of Power Saving	The first time to buy online, but also look at the evaluation before buying, the quality is not bad, but also very cheap, the need for friends can rest assured to buy!	Jingdong prefecture level city in Shandong
Power Saving King Shop	King of Power Saving	Better than expected, feel value for money, very satisfied with a shopping, need next time will come back!	Jingdong prefecture level city in Shandong
Power Saving King Shop	King of Power Saving	Really super like, very supportive, very good quality, exactly as described by the seller, very satisfied!	Jingdong prefecture level city in Shandong
Power Saving King Shop	King of Power Saving	It is genuine, effective, really saves power, affordable, tightly packed, fast shipping, very satisfied.	Jingdong prefecture level city in Shandong
Power Saving King Shop	King of Power Saving	Good quality, fine workmanship, is genuine and business description of the same, good value for money, fast logistics, very satisfied	Jingdong prefecture level city in Shandong
Power Saving King Shop	King of Power Saving	Saver received, very good, installation set up very well, very good, the effect is very good, recommend that the pro buy	Jingdong prefecture level city in Shandong
Power Saving King Shop	King of Power Saving	Electromagnetic good, high power convenient, enough force, the price is right it, quality can be ☺ ☺ ☺ ☺ ☺	Jingdong prefecture level city in Shandong
Power Saving King Shop	King of Power Saving	First-class quality, packaging is also good, logistics is very fast, and the price is reasonable, very affordable, I like it!	Jingdong prefecture level city in Shandong
Power Saving King Shop	King of Power Saving	It's great, it's the kind of style you want, and it arrived quickly, thank you boss, 5 stars for the service!	Jingdong prefecture level city in Shandong
Power Saving King Shop	King of Power Saving	Packaging is very good, the use of the effect is quite good, the store has a good service attitude, trustworthy	Jingdong prefecture level city in Shandong

Power Saving King Shop	King of Power Saving	The quality is very good, it works well, very satisfied, the price is affordable, it's so great value, it's worth recommending.	Jingdong prefecture level city in Shandong
Power Saving King Shop	King of Power Saving	The baby description matches the actual item very well! The quality is awesome! Will be back to patronize again in the future!	Jingdong prefecture level city in Shandong
Power Saving King Shop	King of Power Saving	Ran the price is not high try to buy the attitude, did not expect the effect is really good, good!	Jingdong prefecture level city in Shandong
Power Saving King Shop	King of Power Saving	Power Saving King power is quite large, the quality of the leaf is quite good, the price of buying a very good deal, must be praised!	Jingdong prefecture level city in Shandong
Power Saving King Shop	King of Power Saving	Good product quality is the king of power-saving products trustworthy as always good to recommend!	Jingdong prefecture level city in Shandong
Power Saving King Shop	King of Power Saving	Works great, very affordable, shipped pretty fast, comfortable to use	Jingdong prefecture level city in Shandong
Power Saving King Shop	King of Power Saving	Shipping was pretty fast and affordable, not bad 👍	Jingdong prefecture level city in Shandong
Power Saving King Shop	King of Power Saving	Digital voltage display, voltage at a glance, plug and play, safe and simple!	Jingdong prefecture level city in Shandong
Power Saving King Shop	King of Power Saving	Quality assurance, very good, affordable, logistics is also very fast, I'm very satisfied with the	Jingdong prefecture level city in Shandong
Power Saving King Shop	King of Power Saving	The quality is very good, the first time I received the goods to open the first time to see, it is very good, very satisfied	Jingdong prefecture level city in Shandong
Power Saving King Shop	King of Power Saving	Power Saving King used, the effect is good, the price is affordable, very good, is the genuine article	Jingdong prefecture level city in Shandong
Power Saving King Shop	King of Power Saving	Good quality, fine workmanship, good appearance, time is very accurate, satisfied.	Jingdong prefecture level city in Shandong
Power Saving King Shop	King of Power Saving	I purposely used it for two days before commenting, which is responsible for myself and everyone else!	Jingdong prefecture level city in Shandong
Power Saving King Shop	King of Power Saving	Easy to install, easy to operate, helped our family save a lot of electricity this summer	Jingdong prefecture level city in Shandong
Power Saving King Shop	King of Power Saving	Bringing power savings into millions of homes. Still a great gadget. Pretty good.	Jingdong prefecture level city in Shandong
Power Saving King Shop	King of Power Saving	Digital liquid crystal display to see the clear intuitive, very formal workmanship is not rough	Jingdong prefecture level city in Shandong
Power Saving King Shop	King of Power Saving	Received. Great quality, fast shipping from the store and not too expensive!	Jingdong prefecture level city in Shandong
Power Saving King Shop	King of Power Saving	Bought myself a knuckle regulator and it was easy to use and the price was right.	Jingdong prefecture level city in Shandong
Power Saving King Shop	King of Power Saving	Received, good, powerful, good, very full of a shopping	Jingdong prefecture level city in Shandong
Power Saving King Shop	King of Power Saving	High power, consistent with the seller's description, powerful, cost-effective	Jingdong prefecture level city in Shandong
Power Saving King Shop	King of Power Saving	It works very well and also saves power at an affordable price a great deal.	Jingdong prefecture level city in Shandong
Power Saving King Shop	King of Power Saving	Good quality, good can recommend to friends to buy.	Jingdong prefecture level city in Shandong
Power Saving King Shop	King of Power Saving	Will repurchase, quality is guaranteed, safe and secure purchase.	Jingdong prefecture level city in Shandong
Power Saving King Shop	King of Power Saving	The logistics is very fast, the thing is also good to use, energy saving power rely on it!	Jingdong prefecture level city in Shandong
Power Saving King Shop	King of Power Saving	Power Saver is good quality and works very well, worth buying!	Jingdong prefecture level city in Shandong
Power Saving King Shop	King of Power Saving	The quality is very good is genuine, cost-effective recommended	Jingdong prefecture level city in Shandong
Power Saving King Shop	King of Power Saving	Quality Assurance. Favorable price. Very practical and durable	Jingdong prefecture level city in Shandong
Power Saving King Shop	King of Power Saving	Good value for money. Conscientious businessman. Recommended to everyone	Jingdong prefecture level city in Shandong
Power Saving King Shop	King of Power Saving	Buy it for your rental apartment and never worry about your landlord charging you too much.	Jingdong prefecture level city in Shandong
Power Saving King Shop	King of Power Saving	Nice and affordable, will repurchase next time	Jingdong prefecture level city in Shandong
Power Saving King Shop	King of Power Saving	It's very energy efficient. I've been using it for a few days now.	Jingdong prefecture level city in Shandong
Power Saving King Shop	King of Power Saving	Good quality, high power easy to use	Jingdong prefecture level city in Shandong
Power Saving King Shop	King of Power Saving	Power saving little helper. Deserves a favorable review.	Jingdong prefecture level city in Shandong
Power Saving King Shop	King of Power Saving	Great value for money, great value for money.	Jingdong prefecture level city in Shandong
Power Saving King Shop	King of Power Saving	Works well and as described by the seller	Jingdong prefecture level city in Shandong
Power Saving King Shop	King of Power Saving	Power saving is proper! It's good.	Jingdong prefecture level city in Shandong
Power Saving King Shop	King of Power Saving	Power saving. Works well. Not bad.	Jingdong prefecture level city in Shandong
Power Saving King Shop	King of Power Saving	Satisfied with power saving	Jingdong prefecture level city in Shandong
Power Saving King Shop	King of Power Saving	Received intact, open a super good look beautiful and generous, ~ bit of high quality is recommended to buy, the need for friends rest assured that the next order	Jingdong prefecture level city in Shandong
Power Saving King Shop	King of Power Saving	Very good to use, friends introduced, save electricity and money, long-lasting products cost-effective, large factory production, good quality, fine workmanship cost-effective!	Jingdong prefecture level city in Shandong
Power Saving King Shop	King of Power Saving	The power saving effect is very obvious, trying to pay 30% less electricity bill in a month than before, hahaha	Jingdong prefecture level city in Shandong
Power Saving King Shop	King of Power Saving	It's such a power saver to use this, it saves money on kids snacks for a month. Kudos!	Jingdong prefecture level city in Shandong
Power Saving King Shop	King of Power Saving	Plug and play, stable voltage, the screen has a display when working, this winter home appliances, need more electricity, just buy to use, save electricity and save money, with instructions, not too big and does not take up space, the quality is very good, and the seller describes exactly the same!	Jingdong prefecture level city in Shandong
Power Saving King Shop	King of Power Saving	A must have for opening a store. Save over a thousand dollars a month on your electric bill	Jingdong prefecture level city in Shandong
Power Saving King Shop	King of Power Saving	Received the goods, the first time to unpack, the quality is very good, with the seller's description, completely exceeded my expectations, the packaging is very careful, very strict, in general this is a very satisfied with the shopping	Jingdong prefecture level city in Shandong
Power Saving King Shop	King of Power Saving	As always, good quality, a very favorite product, great! o(*￣︶￣*)o The price is very affordable, cost-effective, it's considered affordable good, will repurchase!	Jingdong prefecture level city in Shandong
Power Saving King Shop	King of Power Saving	Very satisfied with a shopping, good business service, fast shipping, logistics is also very powerful, received very good, buy assured, use peace of mind!	Jingdong prefecture level city in Shandong
Power Saving King Shop	King of Power Saving	The first time to buy online, but also look at the evaluation before buying, the quality is not bad, but also very cheap, the need for friends can rest assured to buy!	Jingdong prefecture level city in Shandong
Power Saving King Shop	King of Power Saving	Better than expected, feel value for money, very satisfied with a shopping, need next time will come back!	Jingdong prefecture level city in Shandong
Power Saving King Shop	King of Power Saving	Really super like, very supportive, very good quality, exactly as described by the seller, very satisfied!	Jingdong prefecture level city in Shandong

Power Saving King Shop	King of Power Saving	It is genuine, effective, really saves power, affordable, tightly packed, fast shipping, very satisfied.	Jingdong prefecture level city in Shandong
Power Saving King Shop	King of Power Saving	Good quality, fine workmanship, is genuine and business description of the same, good value for money, fast logistics, very satisfied	Jingdong prefecture level city in Shandong
Power Saving King Shop	King of Power Saving	Saver received, very good, installation set up very well, very good, the effect is very good, recommend that the pro buy	Jingdong prefecture level city in Shandong
Power Saving King Shop	King of Power Saving	Electromagnetic good, high power convenient, enough force, the price is right it, quality can be 😃 😃 😃 😃 😃	Jingdong prefecture level city in Shandong
Power Saving King Shop	King of Power Saving	First-class quality, packaging is also good, logistics is very fast, and the price is reasonable, very affordable, I like it!	Jingdong prefecture level city in Shandong
Power Saving King Shop	King of Power Saving	It's great, it's the kind of style you want, and it arrived quickly, thank you boss, 5 stars for the service!	Jingdong prefecture level city in Shandong
Power Saving King Shop	King of Power Saving	Packaging is very good, the use of the effect is quite good, the store has a good service attitude, trustworthy	Jingdong prefecture level city in Shandong
Power Saving King Shop	King of Power Saving	The quality is very good, it works well, very satisfied, the price is affordable, it's so great value, it's worth recommending.	Jingdong prefecture level city in Shandong
Power Saving King Shop	King of Power Saving	The baby description matches the actual item very well! The quality is awesome! Will be back to patronize again in the future!	Jingdong prefecture level city in Shandong
Power Saving King Shop	King of Power Saving	Ran the price is not high try to buy the attitude, did not expect the effect is really good, good!	Jingdong prefecture level city in Shandong
Power Saving King Shop	King of Power Saving	Power Saving King power is quite large, the quality of the leaf is quite good, the price of buying a very good deal, must be praised!	Jingdong prefecture level city in Shandong
Power Saving King Shop	King of Power Saving	Good product quality is the king of power-saving products trustworthy as always good to recommend!	Jingdong prefecture level city in Shandong
Power Saving King Shop	King of Power Saving	Works great, very affordable, shipped pretty fast, comfortable to use	Jingdong prefecture level city in Shandong
Power Saving King Shop	King of Power Saving	Shipping was pretty fast and affordable, not bad 👍	Jingdong prefecture level city in Shandong
Power Saving King Shop	King of Power Saving	Digital voltage display, voltage at a glance, plug and play, safe and simple!	Jingdong prefecture level city in Shandong
Power Saving King Shop	King of Power Saving	Quality assurance, very good, affordable, logistics is also very fast, I'm very satisfied with the	Jingdong prefecture level city in Shandong
Power Saving King Shop	King of Power Saving	The quality is very good, the first time I received the goods to open the first time to see, it is very good, very satisfied	Jingdong prefecture level city in Shandong
Power Saving King Shop	King of Power Saving	Power Saving King used, the effect is good, the price is affordable, very good, is the genuine article	Jingdong prefecture level city in Shandong
Power Saving King Shop	King of Power Saving	Good quality, fine workmanship, good appearance, time is very accurate, satisfied.	Jingdong prefecture level city in Shandong
Power Saving King Shop	King of Power Saving	I purposely used it for two days before commenting, which is responsible for myself and everyone else!	Jingdong prefecture level city in Shandong
Power Saving King Shop	King of Power Saving	Easy to install, easy to operate, helped our family save a lot of electricity this summer	Jingdong prefecture level city in Shandong
Power Saving King Shop	King of Power Saving	Bringing power savings into millions of homes. Still a great gadget. Pretty good.	Jingdong prefecture level city in Shandong
Power Saving King Shop	King of Power Saving	Digital liquid crystal display to see the clear intuitive, very formal workmanship is not rough	Jingdong prefecture level city in Shandong
Power Saving King Shop	King of Power Saving	Received. Great quality, fast shipping from the store and not too expensive!	Jingdong prefecture level city in Shandong
Power Saving King Shop	King of Power Saving	Bought myself a knuckle regulator and it was easy to use and the price was right.	Jingdong prefecture level city in Shandong
Power Saving King Shop	King of Power Saving	Received, good, powerful, good, very full of a shopping	Jingdong prefecture level city in Shandong
Power Saving King Shop	King of Power Saving	High power, consistent with the seller's description, powerful, cost-effective	Jingdong prefecture level city in Shandong
Power Saving King Shop	King of Power Saving	It works very well and also saves power at an affordable price a great deal.	Jingdong prefecture level city in Shandong
Power Saving King Shop	King of Power Saving	Good quality, good can recommend to friends to buy.	Jingdong prefecture level city in Shandong
Power Saving King Shop	King of Power Saving	Will repurchase, quality is guaranteed, safe and secure purchase.	Jingdong prefecture level city in Shandong
Power Saving King Shop	King of Power Saving	The logistics is very fast, the thing is also good to use, energy saving power rely on it!	Jingdong prefecture level city in Shandong
Power Saving King Shop	King of Power Saving	Power Saver is good quality and works very well, worth buying!	Jingdong prefecture level city in Shandong
Power Saving King Shop	King of Power Saving	The quality is very good is genuine, cost-effective recommended	Jingdong prefecture level city in Shandong
Power Saving King Shop	King of Power Saving	Quality Assurance. Favorable price. Very practical and durable	Jingdong prefecture level city in Shandong
Power Saving King Shop	King of Power Saving	Good value for money. Conscientious businessman. Recommended to everyone	Jingdong prefecture level city in Shandong
Power Saving King Shop	King of Power Saving	Buy it for your rental apartment and never worry about your landlord charging you too much.	Jingdong prefecture level city in Shandong
Power Saving King Shop	King of Power Saving	Nice and affordable, will repurchase next time	Jingdong prefecture level city in Shandong
Power Saving King Shop	King of Power Saving	It's very energy efficient. I've been using it for a few days now.	Jingdong prefecture level city in Shandong
Power Saving King Shop	King of Power Saving	Good quality, high power easy to use	Jingdong prefecture level city in Shandong
Power Saving King Shop	King of Power Saving	Power saving little helper. Deserves a favorable review.	Jingdong prefecture level city in Shandong
Power Saving King Shop	King of Power Saving	Great value for money, great value for money.	Jingdong prefecture level city in Shandong
Power Saving King Shop	King of Power Saving	Works well and as described by the seller	Jingdong prefecture level city in Shandong
Power Saving King Shop	King of Power Saving	Power saving is proper! It's good.	Jingdong prefecture level city in Shandong
Power Saving King Shop	King of Power Saving	Power saving. Works well. Not bad.	Jingdong prefecture level city in Shandong
Power Saving King Shop	King of Power Saving	Satisfied with power saving	Jingdong prefecture level city in Shandong
Power Saving King Shop	King of Power Saving	Received intact, open a super good look beautiful and generous, ~ bit of high quality is recommended to buy, the need for friends rest assured that the next order	Jingdong prefecture level city in Shandong
Power Saving King Shop	King of Power Saving	Very good to use, friends introduced, save electricity and money, long-lasting products cost-effective, large factory production, good quality, fine workmanship cost-effective!	Jingdong prefecture level city in Shandong
Power Saving King Shop	King of Power Saving	The power saving effect is very obvious, trying to pay 30% less electricity bill in a month than before, hahaha	Jingdong prefecture level city in Shandong
Power Saving King Shop	King of Power Saving	It's such a power saver to use this, it saves money on kids snacks for a month. Kudos!	Jingdong prefecture level city in Shandong
Power Saving King Shop	King of Power Saving	Plug and play, stable voltage, the screen has a display when working, this winter home appliances, need more electricity, just buy to use, save electricity and save money, with instructions, not too big and does not take up space, the quality is very good, and the seller describes exactly the same!	Jingdong prefecture level city in Shandong
Power Saving King Shop	King of Power Saving	A must have for opening a store. Save over a thousand dollars a month on your electric bill	Jingdong prefecture level city in Shandong

Power Saving King Shop	King of Power Saving	Received the goods, the first time to unpack, the quality is very good, with the seller's description, completely exceeded my expectations, the packaging is very careful, very strict, in general this is a very satisfied with the shopping	Jingdong prefecture level city in Shandong
Power Saving King Shop	King of Power Saving	As always, good quality, a very favorite product, great! o(*￣︶￣*)o The price is very affordable, cost-effective, it's considered affordable good, will repurchase!	Jingdong prefecture level city in Shandong
Power Saving King Shop	King of Power Saving	Very satisfied with a shopping, good business service, fast shipping, logistics is also very powerful, received very good, buy assured, use peace of mind!	Jingdong prefecture level city in Shandong
Power Saving King Shop	King of Power Saving	The first time to buy online, but also look at the evaluation before buying, the quality is not bad, but also very cheap, the need for friends can rest assured to buy!	Jingdong prefecture level city in Shandong
Power Saving King Shop	King of Power Saving	Better than expected, feel value for money, very satisfied with a shopping, need next time will come back!	Jingdong prefecture level city in Shandong
Power Saving King Shop	King of Power Saving	Really super like, very supportive, very good quality, exactly as described by the seller, very satisfied!	Jingdong prefecture level city in Shandong
Power Saving King Shop	King of Power Saving	It is genuine, effective, really saves power, affordable, tightly packed, fast shipping, very satisfied.	Jingdong prefecture level city in Shandong
Power Saving King Shop	King of Power Saving	Good quality, fine workmanship, is genuine and business description of the same, good value for money, fast logistics, very satisfied	Jingdong prefecture level city in Shandong
Power Saving King Shop	King of Power Saving	Saver received, very good, installation set up very well, very good, the effect is very good, recommend that the pro buy	Jingdong prefecture level city in Shandong
Power Saving King Shop	King of Power Saving	Electromagnetic good, high power convenient, enough force, the price is right it, quality can be 😀 😀 😀 😀 😀	Jingdong prefecture level city in Shandong
Power Saving King Shop	King of Power Saving	First-class quality, packaging is also good, logistics is very fast, and the price is reasonable, very affordable, I like it!	Jingdong prefecture level city in Shandong
Power Saving King Shop	King of Power Saving	It's great, it's the kind of style you want, and it arrived quickly, thank you boss, 5 stars for the service!	Jingdong prefecture level city in Shandong
Power Saving King Shop	King of Power Saving	Packaging is very good, the use of the effect is quite good, the store has a good service attitude, trustworthy	Jingdong prefecture level city in Shandong
Power Saving King Shop	King of Power Saving	The quality is very good, it works well, very satisfied, the price is affordable, it's so great value, it's worth recommending.	Jingdong prefecture level city in Shandong
Power Saving King Shop	King of Power Saving	The baby description matches the actual item very well! The quality is awesome! Will be back to patronize again in the future!	Jingdong prefecture level city in Shandong
Power Saving King Shop	King of Power Saving	Ran the price is not high try to buy the attitude, did not expect the effect is really good, good!	Jingdong prefecture level city in Shandong
Power Saving King Shop	King of Power Saving	Power Saving King power is quite large, the quality of the leaf is quite good, the price of buying a very good deal, must be praised!	Jingdong prefecture level city in Shandong
Power Saving King Shop	King of Power Saving	Good product quality is the king of power-saving products trustworthy as always good to recommend!	Jingdong prefecture level city in Shandong
Power Saving King Shop	King of Power Saving	Works great, very affordable, shipped pretty fast, comfortable to use	Jingdong prefecture level city in Shandong
Power Saving King Shop	King of Power Saving	Shipping was pretty fast and affordable, not bad 👍	Jingdong prefecture level city in Shandong
Power Saving King Shop	King of Power Saving	Digital voltage display, voltage at a glance, plug and play, safe and simple!	Jingdong prefecture level city in Shandong
Power Saving King Shop	King of Power Saving	Quality assurance, very good, affordable, logistics is also very fast, I'm very satisfied with the	Jingdong prefecture level city in Shandong
Power Saving King Shop	King of Power Saving	The quality is very good, the first time I received the goods to open the first time to see, it is very good, very satisfied	Jingdong prefecture level city in Shandong
Power Saving King Shop	King of Power Saving	Power Saving King used, the effect is good, the price is affordable, very good, is the genuine article	Jingdong prefecture level city in Shandong
Power Saving King Shop	King of Power Saving	Good quality, fine workmanship, good appearance, time is very accurate, satisfied.	Jingdong prefecture level city in Shandong
Power Saving King Shop	King of Power Saving	I purposely used it for two days before commenting, which is responsible for myself and everyone else!	Jingdong prefecture level city in Shandong
Power Saving King Shop	King of Power Saving	Easy to install, easy to operate, helped our family save a lot of electricity this summer	Jingdong prefecture level city in Shandong
Power Saving King Shop	King of Power Saving	Bringing power savings into millions of homes. Still a great gadget. Pretty good.	Jingdong prefecture level city in Shandong
Power Saving King Shop	King of Power Saving	Digital liquid crystal display to see the clear intuitive, very formal workmanship is not rough	Jingdong prefecture level city in Shandong
Power Saving King Shop	King of Power Saving	Received. Great quality, fast shipping from the store and not too expensive!	Jingdong prefecture level city in Shandong
Power Saving King Shop	King of Power Saving	Bought myself a knuckle regulator and it was easy to use and the price was right.	Jingdong prefecture level city in Shandong
Power Saving King Shop	King of Power Saving	Received, good, powerful, good, very full of a shopping	Jingdong prefecture level city in Shandong
Power Saving King Shop	King of Power Saving	High power, consistent with the seller's description, powerful, cost-effective	Jingdong prefecture level city in Shandong
Power Saving King Shop	King of Power Saving	It works very well and also saves power at an affordable price a great deal.	Jingdong prefecture level city in Shandong
Power Saving King Shop	King of Power Saving	Good quality, good can recommend to friends to buy.	Jingdong prefecture level city in Shandong
Power Saving King Shop	King of Power Saving	Will repurchase, quality is guaranteed, safe and secure purchase.	Jingdong prefecture level city in Shandong
Power Saving King Shop	King of Power Saving	The logistics is very fast, the thing is also good to use, energy saving power rely on it!	Jingdong prefecture level city in Shandong
Power Saving King Shop	King of Power Saving	Power Saver is good quality and works very well, worth buying!	Jingdong prefecture level city in Shandong
Power Saving King Shop	King of Power Saving	The quality is very good is genuine, cost-effective recommended	Jingdong prefecture level city in Shandong
Power Saving King Shop	King of Power Saving	Quality Assurance. Favorable price. Very practical and durable	Jingdong prefecture level city in Shandong
Power Saving King Shop	King of Power Saving	Good value for money. Conscientious businessman. Recommended to everyone	Jingdong prefecture level city in Shandong
Power Saving King Shop	King of Power Saving	Buy it for your rental apartment and never worry about your landlord charging you too much.	Jingdong prefecture level city in Shandong
Power Saving King Shop	King of Power Saving	Nice and affordable, will repurchase next time	Jingdong prefecture level city in Shandong
Power Saving King Shop	King of Power Saving	It's very energy efficient. I've been using it for a few days now.	Jingdong prefecture level city in Shandong
Power Saving King Shop	King of Power Saving	Good quality, high power easy to use	Jingdong prefecture level city in Shandong
Power Saving King Shop	King of Power Saving	Power saving little helper. Deserves a favorable review.	Jingdong prefecture level city in Shandong
Power Saving King Shop	King of Power Saving	Great value for money, great value for money.	Jingdong prefecture level city in Shandong
Power Saving King Shop	King of Power Saving	Works well and as described by the seller	Jingdong prefecture level city in Shandong
Power Saving King Shop	King of Power Saving	Power saving is proper! It's good.	Jingdong prefecture level city in Shandong
Power Saving King Shop	King of Power Saving	Power saving. Works well. Not bad.	Jingdong prefecture level city in Shandong
Power Saving King Shop	King of Power Saving	Satisfied with power saving	Jingdong prefecture level city in Shandong

Power Saving King Shop	King of Power Saving	Received intact, open a super good look beautiful and generous, ~ bit of high quality is recommended to buy, the need for friends rest assured that the next order	Jingdong prefecture level city in Shandong
Power Saving King Shop	King of Power Saving	Very good to use, friends introduced, save electricity and money, long-lasting products cost-effective, large factory production, good quality, fine workmanship cost-effective!	Jingdong prefecture level city in Shandong
Power Saving King Shop	King of Power Saving	The power saving effect is very obvious, trying to pay 30% less electricity bill in a month than before, hahaha	Jingdong prefecture level city in Shandong
Power Saving King Shop	King of Power Saving	It's such a power saver to use this, it saves money on kids snacks for a month. Kudos!	Jingdong prefecture level city in Shandong
Power Saving King Shop	King of Power Saving	Plug and play, stable voltage, the screen has a display when working, this winter home appliances, need more electricity, just buy to use, save electricity and save money, with instructions, not too big and does not take up space, the quality is very good, and the seller describes exactly the same!	Jingdong prefecture level city in Shandong
Power Saving King Shop	King of Power Saving	A must have for opening a store. Save over a thousand dollars a month on your electric bill	Jingdong prefecture level city in Shandong
Power Saving King Shop	King of Power Saving	Received the goods, the first time to unpack, the quality is very good, with the seller's description, completely exceeded my expectations, the packaging is very careful, very strict, in general this is a very satisfied with the shopping	Jingdong prefecture level city in Shandong
Power Saving King Shop	King of Power Saving	As always, good quality, a very favorite product, great! o(*￣︶￣*)o The price is very affordable, cost-effective, it's considered affordable good, will repurchase!	Jingdong prefecture level city in Shandong
Power Saving King Shop	King of Power Saving	Very satisfied with a shopping, good business service, fast shipping, logistics is also very powerful, received very good, buy assured, use peace of mind!	Jingdong prefecture level city in Shandong
Power Saving King Shop	King of Power Saving	The first time to buy online, but also look at the evaluation before buying, the quality is not bad, but also very cheap, the need for friends can rest assured to buy!	Jingdong prefecture level city in Shandong
Power Saving King Shop	King of Power Saving	Better than expected, feel value for money, very satisfied with a shopping, need next time will come back!	Jingdong prefecture level city in Shandong
Power Saving King Shop	King of Power Saving	Really super like, very supportive, very good quality, exactly as described by the seller, very satisfied!	Jingdong prefecture level city in Shandong
Power Saving King Shop	King of Power Saving	It is genuine, effective, really saves power, affordable, tightly packed, fast shipping, very satisfied.	Jingdong prefecture level city in Shandong
Power Saving King Shop	King of Power Saving	Good quality, fine workmanship, is genuine and business description of the same, good value for money, fast logistics, very satisfied	Jingdong prefecture level city in Shandong
Power Saving King Shop	King of Power Saving	Saver received, very good, installation set up very well, very good, the effect is very good, recommend that the pro buy	Jingdong prefecture level city in Shandong
Power Saving King Shop	King of Power Saving	Electromagnetic good, high power convenient, enough force, the price is right it, quality can be 😃 😃 😃 😃 😃	Jingdong prefecture level city in Shandong
Power Saving King Shop	King of Power Saving	First-class quality, packaging is also good, logistics is very fast, and the price is reasonable, very affordable, I like it!	Jingdong prefecture level city in Shandong
Power Saving King Shop	King of Power Saving	It's great, it's the kind of style you want, and it arrived quickly, thank you boss, 5 stars for the service!	Jingdong prefecture level city in Shandong
Power Saving King Shop	King of Power Saving	Packaging is very good, the use of the effect is quite good, the store has a good service attitude, trustworthy	Jingdong prefecture level city in Shandong
Power Saving King Shop	King of Power Saving	The quality is very good, it works well, very satisfied, the price is affordable, it's so great value, it's worth recommending.	Jingdong prefecture level city in Shandong
Power Saving King Shop	King of Power Saving	The baby description matches the actual item very well! The quality is awesome! Will be back to patronize again in the future!	Jingdong prefecture level city in Shandong
Power Saving King Shop	King of Power Saving	Ran the price is not high try to buy the attitude, did not expect the effect is really good, good!	Jingdong prefecture level city in Shandong
Power Saving King Shop	King of Power Saving	Power Saving King power is quite large, the quality of the leaf is quite good, the price of buying a very good deal, must be praised!	Jingdong prefecture level city in Shandong
Power Saving King Shop	King of Power Saving	Good product quality is the king of power-saving products trustworthy as always good to recommend!	Jingdong prefecture level city in Shandong
Power Saving King Shop	King of Power Saving	Works great, very affordable, shipped pretty fast, comfortable to use	Jingdong prefecture level city in Shandong
Power Saving King Shop	King of Power Saving	Shipping was pretty fast and affordable, not bad 👍	Jingdong prefecture level city in Shandong
Power Saving King Shop	King of Power Saving	Digital voltage display, voltage at a glance, plug and play, safe and simple!	Jingdong prefecture level city in Shandong
Power Saving King Shop	King of Power Saving	Quality assurance, very good, affordable, logistics is also very fast, I'm very satisfied with the	Jingdong prefecture level city in Shandong
Power Saving King Shop	King of Power Saving	The quality is very good, the first time I received the goods to open the first time to see, it is very good, very satisfied	Jingdong prefecture level city in Shandong
Power Saving King Shop	King of Power Saving	Power Saving King used, the effect is good, the price is affordable, very good, is the genuine article	Jingdong prefecture level city in Shandong
Power Saving King Shop	King of Power Saving	Good quality, fine workmanship, good appearance, time is very accurate, satisfied.	Jingdong prefecture level city in Shandong
Power Saving King Shop	King of Power Saving	I purposely used it for two days before commenting, which is responsible for myself and everyone else!	Jingdong prefecture level city in Shandong
Power Saving King Shop	King of Power Saving	Easy to install, easy to operate, helped our family save a lot of electricity this summer	Jingdong prefecture level city in Shandong
Power Saving King Shop	King of Power Saving	Bringing power savings into millions of homes. Still a great gadget. Pretty good.	Jingdong prefecture level city in Shandong
Power Saving King Shop	King of Power Saving	Digital liquid crystal display to see the clear intuitive, very formal workmanship is not rough	Jingdong prefecture level city in Shandong
Power Saving King Shop	King of Power Saving	Received. Great quality, fast shipping from the store and not too expensive!	Jingdong prefecture level city in Shandong
Power Saving King Shop	King of Power Saving	Bought myself a knuckle regulator and it was easy to use and the price was right.	Jingdong prefecture level city in Shandong
Power Saving King Shop	King of Power Saving	Received, good, powerful, good, very full of a shopping	Jingdong prefecture level city in Shandong
Power Saving King Shop	King of Power Saving	High power, consistent with the seller's description, powerful, cost-effective	Jingdong prefecture level city in Shandong
Power Saving King Shop	King of Power Saving	It works very well and also saves power at an affordable price a great deal.	Jingdong prefecture level city in Shandong
Power Saving King Shop	King of Power Saving	Good quality, good can recommend to friends to buy.	Jingdong prefecture level city in Shandong
Power Saving King Shop	King of Power Saving	Will repurchase, quality is guaranteed, safe and secure purchase.	Jingdong prefecture level city in Shandong
Power Saving King Shop	King of Power Saving	The logistics is very fast, the thing is also good to use, energy saving power rely on it!	Jingdong prefecture level city in Shandong
Power Saving King Shop	King of Power Saving	Power Saver is good quality and works very well, worth buying!	Jingdong prefecture level city in Shandong
Power Saving King Shop	King of Power Saving	The quality is very good is genuine, cost-effective recommended	Jingdong prefecture level city in Shandong
Power Saving King Shop	King of Power Saving	Quality Assurance. Favorable price. Very practical and durable	Jingdong prefecture level city in Shandong
Power Saving King Shop	King of Power Saving	Good value for money. Conscientious businessman. Recommended to everyone	Jingdong prefecture level city in Shandong
Power Saving King Shop	King of Power Saving	Buy it for your rental apartment and never worry about your landlord charging you too much.	Jingdong prefecture level city in Shandong
Power Saving King Shop	King of Power Saving	Nice and affordable, will repurchase next time	Jingdong prefecture level city in Shandong
Power Saving King Shop	King of Power Saving	It's very energy efficient. I've been using it for a few days now.	Jingdong prefecture level city in Shandong

Power Saving King Shop	King of Power Saving	Good quality, high power easy to use	Jingdong prefecture level city in Shandong
Power Saving King Shop	King of Power Saving	Power saving little helper. Deserves a favorable review.	Jingdong prefecture level city in Shandong
Power Saving King Shop	King of Power Saving	Great value for money, great value for money.	Jingdong prefecture level city in Shandong
Power Saving King Shop	King of Power Saving	Works well and as described by the seller	Jingdong prefecture level city in Shandong
Power Saving King Shop	King of Power Saving	Power saving is proper! It's good.	Jingdong prefecture level city in Shandong
Power Saving King Shop	King of Power Saving	Power saving. Works well. Not bad.	Jingdong prefecture level city in Shandong
Power Saving King Shop	King of Power Saving	Satisfied with power saving	Jingdong prefecture level city in Shandong
Power Saving King Shop	King of Power Saving	It works. Just graduated out of college and can't afford to pay my electric bill, having this is amazing and will upgrade me from poor farmer to middle farmer. Great positive feedback!	Jingdong prefecture level city in Shandong
Power Saving King Shop	King of Power Saving	Received a few days ago, specifically to test. Still some effect, a day to save a few degrees of electricity. For us just out of work, renting a good practical. This feel is also very good. This all have anti-counterfeiting labels. Safe and secure. This quality is okay.	Jingdong prefecture level city in Shandong
Power Saving King Shop	power saver	The quality is very good, the description is consistent, the service attitude is very good, the logistics is very fast, satisfied, praise.	Jingdong prefecture level city in Shandong
Power Saving King Shop	power saver	The effect is great, genuine, and match the description, the logistics is fast, the price is reasonable 😍	Jingdong prefecture level city in Shandong
Power Saving King Shop	power saver	The seller also shipped very quickly, the quality is particularly good, quite practical, need to come back to buy again!	Jingdong prefecture level city in Shandong
Power Saving King Shop	power saver	It's genuine, works well, easy to use, plugs in and works, really saves power	Jingdong prefecture level city in Shandong
Power Saving King Shop	power saver	Good quality logistics quickly, easy to install, very satisfied with a shopping	Jingdong prefecture level city in Shandong
Power Saving King Shop	power saver	Received the quality is very good to use the effect is very satisfied with a shopping!	Jingdong prefecture level city in Shandong
Power Saving King Shop	power saver	Much cheaper than the physical store, customer service is very good, especially patient!	Jingdong prefecture level city in Shandong
Power Saving King Shop	power saver	Solid workmanship, easy to use, safe and recommendable	Jingdong prefecture level city in Shandong
Power Saving King Shop	power saver	Fine workmanship material is good, powerful, the effect is the same as described	Jingdong prefecture level city in Shandong
Power Saving King Shop	power saver	Easy to install, really effective, saves a lot of electricity than before	Jingdong prefecture level city in Shandong
Power Saving King Shop	power saver	Tested it and it's good quality, it does work well, recommended	Jingdong prefecture level city in Shandong
Power Saving King Shop	power saver	The quality of the product is quite good, fast shipping, satisfied.	Jingdong prefecture level city in Shandong
Power Saving King Shop	power saver	The logistics is very fast and satisfactory	Jingdong prefecture level city in Shandong
Power Saving King Shop	power saver	Fine workmanship material is good, powerful, the effect is the same as described	Jingdong prefecture level city in Shandong
Power Saving King Shop	power saver	Easy to install, really effective, saves a lot of electricity than before	Jingdong prefecture level city in Shandong
Power Saving King Shop	power saver	Tested it and it's good quality, it does work well, recommended	Jingdong prefecture level city in Shandong
Power Saving King Shop	power saver	The quality of the product is quite good, fast shipping, satisfied.	Jingdong prefecture level city in Shandong
Power Saving King Shop	power saver	The logistics is very fast and satisfactory	Jingdong prefecture level city in Shandong
Power Saving King Shop	power saver	Fine workmanship material is good, powerful, the effect is the same as described	Jingdong prefecture level city in Shandong
Power Saving King Shop	power saver	Easy to install, really effective, saves a lot of electricity than before	Jingdong prefecture level city in Shandong
Power Saving King Shop	power saver	Tested it and it's good quality, it does work well, recommended	Jingdong prefecture level city in Shandong
Power Saving King Shop	power saver	The quality of the product is quite good, fast shipping, satisfied.	Jingdong prefecture level city in Shandong
Power Saving King Shop	power saver	The logistics is very fast and satisfactory	Jingdong prefecture level city in Shandong
Power Saving King Shop	power saver	Fine workmanship material is good, powerful, the effect is the same as described	Jingdong prefecture level city in Shandong
Power Saving King Shop	power saver	Easy to install, really effective, saves a lot of electricity than before	Jingdong prefecture level city in Shandong
Power Saving King Shop	power saver	Tested it and it's good quality, it does work well, recommended	Jingdong prefecture level city in Shandong
Power Saving King Shop	power saver	The quality of the product is quite good, fast shipping, satisfied.	Jingdong prefecture level city in Shandong
Power Saving King Shop	power saver	The logistics is very fast and satisfactory	Jingdong prefecture level city in Shandong
Power Saving King Shop	power saver	Fine workmanship material is good, powerful, the effect is the same as described	Jingdong prefecture level city in Shandong
Power Saving King Shop	power saver	Easy to install, really effective, saves a lot of electricity than before	Jingdong prefecture level city in Shandong
Power Saving King Shop	power saver	Tested it and it's good quality, it does work well, recommended	Jingdong prefecture level city in Shandong
Power Saving King Shop	power saver	The quality of the product is quite good, fast shipping, satisfied.	Jingdong prefecture level city in Shandong
Power Saving King Shop	power saver	The logistics is very fast and satisfactory	Jingdong prefecture level city in Shandong
Power Saving King Shop	power saver	Fine workmanship material is good, powerful, the effect is the same as described	Jingdong prefecture level city in Shandong
Power Saving King Shop	power saver	Easy to install, really effective, saves a lot of electricity than before	Jingdong prefecture level city in Shandong
Power Saving King Shop	power saver	Tested it and it's good quality, it does work well, recommended	Jingdong prefecture level city in Shandong
Power Saving King Shop	power saver	The quality of the product is quite good, fast shipping, satisfied.	Jingdong prefecture level city in Shandong
Power Saving King Shop	power saver	The logistics is very fast and satisfactory	Jingdong prefecture level city in Shandong
Power Saving King Shop	power saver	Fine workmanship material is good, powerful, the effect is the same as described	Jingdong prefecture level city in Shandong
Power Saving King Shop	power saver	Easy to install, really effective, saves a lot of electricity than before	Jingdong prefecture level city in Shandong
Power Saving King Shop	power saver	Tested it and it's good quality, it does work well, recommended	Jingdong prefecture level city in Shandong
Power Saving King Shop	power saver	The quality of the product is quite good, fast shipping, satisfied.	Jingdong prefecture level city in Shandong

Power Saving King Shop	power saver	The logistics is very fast and satisfactory	Jingdong prefecture level city in Shandong
Power Saving King Shop	power saver	Fine workmanship material is good, powerful, the effect is the same as described	Jingdong prefecture level city in Shandong
Power Saving King Shop	power saver	Easy to install, really effective, saves a lot of electricity than before	Jingdong prefecture level city in Shandong
Power Saving King Shop	power saver	Tested it and it's good quality, it does work well, recommended	Jingdong prefecture level city in Shandong
Power Saving King Shop	power saver	The quality of the product is quite good, fast shipping, satisfied.	Jingdong prefecture level city in Shandong
Power Saving King Shop	power saver	The logistics is very fast and satisfactory	Jingdong prefecture level city in Shandong
Power Saving King Shop	power saver	Fine workmanship material is good, powerful, the effect is the same as described	Jingdong prefecture level city in Shandong
Power Saving King Shop	power saver	Easy to install, really effective, saves a lot of electricity than before	Jingdong prefecture level city in Shandong
Power Saving King Shop	power saver	Tested it and it's good quality, it does work well, recommended	Jingdong prefecture level city in Shandong
Power Saving King Shop	power saver	The quality of the product is quite good, fast shipping, satisfied.	Jingdong prefecture level city in Shandong
Power Saving King Shop	power saver	The logistics is very fast and satisfactory	Jingdong prefecture level city in Shandong
Power Saving King Shop	power saver	Fine workmanship material is good, powerful, the effect is the same as described	Jingdong prefecture level city in Shandong
Power Saving King Shop	power saver	Easy to install, really effective, saves a lot of electricity than before	Jingdong prefecture level city in Shandong
Power Saving King Shop	power saver	Tested it and it's good quality, it does work well, recommended	Jingdong prefecture level city in Shandong
Power Saving King Shop	power saver	The quality of the product is quite good, fast shipping, satisfied.	Jingdong prefecture level city in Shandong
Power Saving King Shop	power saver	The logistics is very fast and satisfactory	Jingdong prefecture level city in Shandong
Power Saving King Shop	power saver	Fine workmanship material is good, powerful, the effect is the same as described	Jingdong prefecture level city in Shandong
Power Saving King Shop	power saver	Easy to install, really effective, saves a lot of electricity than before	Jingdong prefecture level city in Shandong
Power Saving King Shop	power saver	Tested it and it's good quality, it does work well, recommended	Jingdong prefecture level city in Shandong
Power Saving King Shop	power saver	The quality of the product is quite good, fast shipping, satisfied.	Jingdong prefecture level city in Shandong
Power Saving King Shop	power saver	The logistics is very fast and satisfactory	Jingdong prefecture level city in Shandong
Power Saving King Shop	power saver	Fine workmanship material is good, powerful, the effect is the same as described	Jingdong prefecture level city in Shandong
Power Saving King Shop	power saver	Easy to install, really effective, saves a lot of electricity than before	Jingdong prefecture level city in Shandong
Power Saving King Shop	power saver	Tested it and it's good quality, it does work well, recommended	Jingdong prefecture level city in Shandong
Power Saving King Shop	power saver	The quality of the product is quite good, fast shipping, satisfied.	Jingdong prefecture level city in Shandong
Power Saving King Shop	power saver	The logistics is very fast and satisfactory	Jingdong prefecture level city in Shandong
Power Saving King Shop	power saver	Fine workmanship material is good, powerful, the effect is the same as described	Jingdong prefecture level city in Shandong
Power Saving King Shop	power saver	Easy to install, really effective, saves a lot of electricity than before	Jingdong prefecture level city in Shandong
Power Saving King Shop	power saver	Tested it and it's good quality, it does work well, recommended	Jingdong prefecture level city in Shandong
Power Saving King Shop	power saver	The quality of the product is quite good, fast shipping, satisfied.	Jingdong prefecture level city in Shandong
Power Saving King Shop	power saver	The logistics is very fast and satisfactory	Jingdong prefecture level city in Shandong
Power Saving King Shop	power saver	Fine workmanship material is good, powerful, the effect is the same as described	Jingdong prefecture level city in Shandong
Power Saving King Shop	power saver	Easy to install, really effective, saves a lot of electricity than before	Jingdong prefecture level city in Shandong
Power Saving King Shop	power saver	Tested it and it's good quality, it does work well, recommended	Jingdong prefecture level city in Shandong
Power Saving King Shop	power saver	The quality of the product is quite good, fast shipping, satisfied.	Jingdong prefecture level city in Shandong
Power Saving King Shop	power saver	The logistics is very fast and satisfactory	Jingdong prefecture level city in Shandong
Power Saving King Shop	power saver	Fine workmanship material is good, powerful, the effect is the same as described	Jingdong prefecture level city in Shandong
Power Saving King Shop	power saver	Easy to install, really effective, saves a lot of electricity than before	Jingdong prefecture level city in Shandong
Power Saving King Shop	power saver	Tested it and it's good quality, it does work well, recommended	Jingdong prefecture level city in Shandong
Power Saving King Shop	power saver	The quality of the product is quite good, fast shipping, satisfied.	Jingdong prefecture level city in Shandong
Power Saving King Shop	power saver	The logistics is very fast and satisfactory	Jingdong prefecture level city in Shandong
Power Saving King Shop	power saver	Fine workmanship material is good, powerful, the effect is the same as described	Jingdong prefecture level city in Shandong
Power Saving King Shop	power saver	Easy to install, really effective, saves a lot of electricity than before	Jingdong prefecture level city in Shandong
Power Saving King Shop	power saver	Tested it and it's good quality, it does work well, recommended	Jingdong prefecture level city in Shandong
Power Saving King Shop	power saver	The quality of the product is quite good, fast shipping, satisfied.	Jingdong prefecture level city in Shandong
Power Saving King Shop	power saver	The logistics is very fast and satisfactory	Jingdong prefecture level city in Shandong
Power Saving King Shop	power saver	Fine workmanship material is good, powerful, the effect is the same as described	Jingdong prefecture level city in Shandong
Power Saving King Shop	power saver	Easy to install, really effective, saves a lot of electricity than before	Jingdong prefecture level city in Shandong
Power Saving King Shop	power saver	Tested it and it's good quality, it does work well, recommended	Jingdong prefecture level city in Shandong
Power Saving King Shop	power saver	The quality of the product is quite good, fast shipping, satisfied.	Jingdong prefecture level city in Shandong
Power Saving King Shop	power saver	The logistics is very fast and satisfactory	Jingdong prefecture level city in Shandong

Power Saving King Shop	power saver	Fine workmanship material is good, powerful, the effect is the same as described	Jingdong prefecture level city in Shandong
Power Saving King Shop	power saver	Easy to install, really effective, saves a lot of electricity than before	Jingdong prefecture level city in Shandong
Power Saving King Shop	power saver	Tested it and it's good quality, it does work well, recommended	Jingdong prefecture level city in Shandong
Power Saving King Shop	power saver	The quality of the product is quite good, fast shipping, satisfied.	Jingdong prefecture level city in Shandong
Power Saving King Shop	power saver	The logistics is very fast and satisfactory	Jingdong prefecture level city in Shandong
Power Saving King Shop	power saver	Fine workmanship material is good, powerful, the effect is the same as described	Jingdong prefecture level city in Shandong
Power Saving King Shop	power saver	Easy to install, really effective, saves a lot of electricity than before	Jingdong prefecture level city in Shandong
Power Saving King Shop	power saver	Tested it and it's good quality, it does work well, recommended	Jingdong prefecture level city in Shandong
Power Saving King Shop	power saver	The quality of the product is quite good, fast shipping, satisfied.	Jingdong prefecture level city in Shandong
Power Saving King Shop	power saver	The logistics is very fast and satisfactory	Jingdong prefecture level city in Shandong
Power Saving King Shop	power saver	Fine workmanship material is good, powerful, the effect is the same as described	Jingdong prefecture level city in Shandong
Power Saving King Shop	power saver	Easy to install, really effective, saves a lot of electricity than before	Jingdong prefecture level city in Shandong
Power Saving King Shop	power saver	Tested it and it's good quality, it does work well, recommended	Jingdong prefecture level city in Shandong
Power Saving King Shop	power saver	The quality of the product is quite good, fast shipping, satisfied.	Jingdong prefecture level city in Shandong
Power Saving King Shop	power saver	The logistics is very fast and satisfactory	Jingdong prefecture level city in Shandong
Power Saving King Shop	power saver	Fine workmanship material is good, powerful, the effect is the same as described	Jingdong prefecture level city in Shandong
Power Saving King Shop	power saver	Easy to install, really effective, saves a lot of electricity than before	Jingdong prefecture level city in Shandong
Power Saving King Shop	power saver	Tested it and it's good quality, it does work well, recommended	Jingdong prefecture level city in Shandong
Power Saving King Shop	power saver	The quality of the product is quite good, fast shipping, satisfied.	Jingdong prefecture level city in Shandong
Power Saving King Shop	power saver	The logistics is very fast and satisfactory	Jingdong prefecture level city in Shandong
Power Saving King Shop	power saver	Fine workmanship material is good, powerful, the effect is the same as described	Jingdong prefecture level city in Shandong
Power Saving King Shop	power saver	Easy to install, really effective, saves a lot of electricity than before	Jingdong prefecture level city in Shandong
Power Saving King Shop	power saver	Tested it and it's good quality, it does work well, recommended	Jingdong prefecture level city in Shandong
Power Saving King Shop	power saver	The quality of the product is quite good, fast shipping, satisfied.	Jingdong prefecture level city in Shandong
Power Saving King Shop	power saver	The logistics is very fast and satisfactory	Jingdong prefecture level city in Shandong
Power Saving King Shop	power saver	Fine workmanship material is good, powerful, the effect is the same as described	Jingdong prefecture level city in Shandong
Power Saving King Shop	power saver	Easy to install, really effective, saves a lot of electricity than before	Jingdong prefecture level city in Shandong
Power Saving King Shop	power saver	Tested it and it's good quality, it does work well, recommended	Jingdong prefecture level city in Shandong
Power Saving King Shop	power saver	The quality of the product is quite good, fast shipping, satisfied.	Jingdong prefecture level city in Shandong
Power Saving King Shop	power saver	The logistics is very fast and satisfactory	Jingdong prefecture level city in Shandong
Power Saving King Shop	power saver	Fine workmanship material is good, powerful, the effect is the same as described	Jingdong prefecture level city in Shandong
Power Saving King Shop	power saver	Easy to install, really effective, saves a lot of electricity than before	Jingdong prefecture level city in Shandong
Power Saving King Shop	power saver	Tested it and it's good quality, it does work well, recommended	Jingdong prefecture level city in Shandong
Power Saving King Shop	power saver	The quality of the product is quite good, fast shipping, satisfied.	Jingdong prefecture level city in Shandong
Power Saving King Shop	power saver	The logistics is very fast and satisfactory	Jingdong prefecture level city in Shandong
Power Saving King Shop	power saver	Fine workmanship material is good, powerful, the effect is the same as described	Jingdong prefecture level city in Shandong
Power Saving King Shop	power saver	Easy to install, really effective, saves a lot of electricity than before	Jingdong prefecture level city in Shandong
Power Saving King Shop	power saver	Tested it and it's good quality, it does work well, recommended	Jingdong prefecture level city in Shandong
Power Saving King Shop	power saver	The quality of the product is quite good, fast shipping, satisfied.	Jingdong prefecture level city in Shandong
Power Saving King Shop	power saver	The logistics is very fast and satisfactory	Jingdong prefecture level city in Shandong
Power Saving King Shop	power saver	Fine workmanship material is good, powerful, the effect is the same as described	Jingdong prefecture level city in Shandong
Power Saving King Shop	power saver	Easy to install, really effective, saves a lot of electricity than before	Jingdong prefecture level city in Shandong
Power Saving King Shop	power saver	Tested it and it's good quality, it does work well, recommended	Jingdong prefecture level city in Shandong
Power Saving King Shop	power saver	The quality of the product is quite good, fast shipping, satisfied.	Jingdong prefecture level city in Shandong
Power Saving King Shop	power saver	The logistics is very fast and satisfactory	Jingdong prefecture level city in Shandong
Power Saving King Shop	power saver	Fine workmanship material is good, powerful, the effect is the same as described	Jingdong prefecture level city in Shandong
Power Saving King Shop	power saver	Easy to install, really effective, saves a lot of electricity than before	Jingdong prefecture level city in Shandong
Power Saving King Shop	power saver	Tested it and it's good quality, it does work well, recommended	Jingdong prefecture level city in Shandong
Power Saving King Shop	power saver	The quality of the product is quite good, fast shipping, satisfied.	Jingdong prefecture level city in Shandong
Power Saving King Shop	power saver	The logistics is very fast and satisfactory	Jingdong prefecture level city in Shandong
Power Saving King Shop	power saver	Fine workmanship material is good, powerful, the effect is the same as described	Jingdong prefecture level city in Shandong

Power Saving King Shop	power saver	Easy to install, really effective, saves a lot of electricity than before	Jingdong prefecture level city in Shandong
Power Saving King Shop	power saver	Tested it and it's good quality, it does work well, recommended	Jingdong prefecture level city in Shandong
Power Saving King Shop	power saver	The quality of the product is quite good, fast shipping, satisfied.	Jingdong prefecture level city in Shandong
Power Saving King Shop	power saver	The logistics is very fast and satisfactory	Jingdong prefecture level city in Shandong
Power Saving King Shop	power saver	Fine workmanship material is good, powerful, the effect is the same as described	Jingdong prefecture level city in Shandong
Power Saving King Shop	power saver	Easy to install, really effective, saves a lot of electricity than before	Jingdong prefecture level city in Shandong
Power Saving King Shop	power saver	Tested it and it's good quality, it does work well, recommended	Jingdong prefecture level city in Shandong
Power Saving King Shop	power saver	The quality of the product is quite good, fast shipping, satisfied.	Jingdong prefecture level city in Shandong
Power Saving King Shop	power saver	The logistics is very fast and satisfactory	Jingdong prefecture level city in Shandong
Power Saving King Shop	power saver	Fine workmanship material is good, powerful, the effect is the same as described	Jingdong prefecture level city in Shandong
Power Saving King Shop	power saver	Easy to install, really effective, saves a lot of electricity than before	Jingdong prefecture level city in Shandong
Power Saving King Shop	power saver	Tested it and it's good quality, it does work well, recommended	Jingdong prefecture level city in Shandong
Power Saving King Shop	power saver	The quality of the product is quite good, fast shipping, satisfied.	Jingdong prefecture level city in Shandong
Power Saving King Shop	power saver	The logistics is very fast and satisfactory	Jingdong prefecture level city in Shandong
Power Saving King Shop	power saver	Fine workmanship material is good, powerful, the effect is the same as described	Jingdong prefecture level city in Shandong
Power Saving King Shop	power saver	Easy to install, really effective, saves a lot of electricity than before	Jingdong prefecture level city in Shandong
Power Saving King Shop	power saver	Tested it and it's good quality, it does work well, recommended	Jingdong prefecture level city in Shandong
Power Saving King Shop	power saver	The quality of the product is quite good, fast shipping, satisfied.	Jingdong prefecture level city in Shandong
Power Saving King Shop	power saver	The logistics is very fast and satisfactory	Jingdong prefecture level city in Shandong
Power Saving King Shop	power saver	Fine workmanship material is good, powerful, the effect is the same as described	Jingdong prefecture level city in Shandong
Power Saving King Shop	power saver	Easy to install, really effective, saves a lot of electricity than before	Jingdong prefecture level city in Shandong
Power Saving King Shop	power saver	Tested it and it's good quality, it does work well, recommended	Jingdong prefecture level city in Shandong
Power Saving King Shop	power saver	The quality of the product is quite good, fast shipping, satisfied.	Jingdong prefecture level city in Shandong
Power Saving King Shop	power saver	The logistics is very fast and satisfactory	Jingdong prefecture level city in Shandong
Power Saving King Shop	power saver	Fine workmanship material is good, powerful, the effect is the same as described	Jingdong prefecture level city in Shandong
Power Saving King Shop	power saver	Easy to install, really effective, saves a lot of electricity than before	Jingdong prefecture level city in Shandong
Power Saving King Shop	power saver	Tested it and it's good quality, it does work well, recommended	Jingdong prefecture level city in Shandong
Power Saving King Shop	power saver	The quality of the product is quite good, fast shipping, satisfied.	Jingdong prefecture level city in Shandong
Power Saving King Shop	power saver	The logistics is very fast and satisfactory	Jingdong prefecture level city in Shandong
Power Saving King Shop	power saver	Fine workmanship material is good, powerful, the effect is the same as described	Jingdong prefecture level city in Shandong
Power Saving King Shop	power saver	Easy to install, really effective, saves a lot of electricity than before	Jingdong prefecture level city in Shandong
Power Saving King Shop	power saver	Tested it and it's good quality, it does work well, recommended	Jingdong prefecture level city in Shandong
Power Saving King Shop	power saver	The quality of the product is quite good, fast shipping, satisfied.	Jingdong prefecture level city in Shandong
Power Saving King Shop	power saver	The logistics is very fast and satisfactory	Jingdong prefecture level city in Shandong
Power Saving King Shop	power saver	Fine workmanship material is good, powerful, the effect is the same as described	Jingdong prefecture level city in Shandong
Power Saving King Shop	power saver	Easy to install, really effective, saves a lot of electricity than before	Jingdong prefecture level city in Shandong
Power Saving King Shop	power saver	Tested it and it's good quality, it does work well, recommended	Jingdong prefecture level city in Shandong
Power Saving King Shop	power saver	The quality of the product is quite good, fast shipping, satisfied.	Jingdong prefecture level city in Shandong
Power Saving King Shop	power saver	The logistics is very fast and satisfactory	Jingdong prefecture level city in Shandong
Power Saving King Shop	power saver	Fine workmanship material is good, powerful, the effect is the same as described	Jingdong prefecture level city in Shandong
Power Saving King Shop	power saver	Tested it and it's good quality, it does work well, recommended	Jingdong prefecture level city in Shandong
Power Saving King Shop	power saver	Easy to install, really effective, saves a lot of electricity than before	Jingdong prefecture level city in Shandong
Power Saving King Shop	power saver	The quality of the product is quite good, fast shipping, satisfied.	Jingdong prefecture level city in Shandong
Power Saving King Shop	power saver	The logistics is very fast and satisfactory	Jingdong prefecture level city in Shandong
Power Saving King Shop	power saver	Fine workmanship material is good, powerful, the effect is the same as described	Jingdong prefecture level city in Shandong
Power Saving King Shop	power saver	Easy to install, really effective, saves a lot of electricity than before	Jingdong prefecture level city in Shandong
Power Saving King Shop	power saver	Tested it and it's good quality, it does work well, recommended	Jingdong prefecture level city in Shandong
Power Saving King Shop	power saver	The quality of the product is quite good, fast shipping, satisfied.	Jingdong prefecture level city in Shandong
Power Saving King Shop	power saver	The logistics is very fast and satisfactory	Jingdong prefecture level city in Shandong
Power Saving King Shop	power saver	Fine workmanship material is good, powerful, the effect is the same as described	Jingdong prefecture level city in Shandong
Power Saving King Shop	power saver	Easy to install, really effective, saves a lot of electricity than before	Jingdong prefecture level city in Shandong

Power Saving King Shop	power saver	Tested it and it's good quality, it does work well, recommended	Jingdong prefecture level city in Shandong
Power Saving King Shop	power saver	The quality of the product is quite good, fast shipping, satisfied.	Jingdong prefecture level city in Shandong
Power Saving King Shop	power saver	The logistics is very fast and satisfactory	Jingdong prefecture level city in Shandong
Power Saving King Shop	power saver	Fine workmanship material is good, powerful, the effect is the same as described	Jingdong prefecture level city in Shandong
Power Saving King Shop	power saver	Easy to install, really effective, saves a lot of electricity than before	Jingdong prefecture level city in Shandong
Power Saving King Shop	power saver	Tested it and it's good quality, it does work well, recommended	Jingdong prefecture level city in Shandong
Power Saving King Shop	power saver	The quality of the product is quite good, fast shipping, satisfied.	Jingdong prefecture level city in Shandong
Power Saving King Shop	power saver	The logistics is very fast and satisfactory	Jingdong prefecture level city in Shandong
Power Saving King Shop	power saver	Fine workmanship material is good, powerful, the effect is the same as described	Jingdong prefecture level city in Shandong
Power Saving King Shop	power saver	Easy to install, really effective, saves a lot of electricity than before	Jingdong prefecture level city in Shandong
Power Saving King Shop	power saver	Tested it and it's good quality, it does work well, recommended	Jingdong prefecture level city in Shandong
Power Saving King Shop	power saver	The quality of the product is quite good, fast shipping, satisfied.	Jingdong prefecture level city in Shandong
Power Saving King Shop	power saver	The logistics is very fast and satisfactory	Jingdong prefecture level city in Shandong
Power Saving King Shop	power saver	Fine workmanship material is good, powerful, the effect is the same as described	Jingdong prefecture level city in Shandong
Power Saving King Shop	power saver	Easy to install, really effective, saves a lot of electricity than before	Jingdong prefecture level city in Shandong
Power Saving King Shop	power saver	Tested it and it's good quality, it does work well, recommended	Jingdong prefecture level city in Shandong
Power Saving King Shop	power saver	The quality of the product is quite good, fast shipping, satisfied.	Jingdong prefecture level city in Shandong
Power Saving King Shop	power saver	The logistics is very fast and satisfactory	Jingdong prefecture level city in Shandong
Power Saving King Shop	power saver	Fine workmanship material is good, powerful, the effect is the same as described	Jingdong prefecture level city in Shandong
Power Saving King Shop	power saver	Easy to install, really effective, saves a lot of electricity than before	Jingdong prefecture level city in Shandong
Power Saving King Shop	power saver	Tested it and it's good quality, it does work well, recommended	Jingdong prefecture level city in Shandong
Power Saving King Shop	power saver	The quality of the product is quite good, fast shipping, satisfied.	Jingdong prefecture level city in Shandong
Power Saving King Shop	power saver	The logistics is very fast and satisfactory	Jingdong prefecture level city in Shandong
Power Saving King Shop	power saver	Fine workmanship material is good, powerful, the effect is the same as described	Jingdong prefecture level city in Shandong
Power Saving King Shop	power saver	Easy to install, really effective, saves a lot of electricity than before	Jingdong prefecture level city in Shandong
Power Saving King Shop	power saver	Tested it and it's good quality, it does work well, recommended	Jingdong prefecture level city in Shandong
Power Saving King Shop	power saver	The quality of the product is quite good, fast shipping, satisfied.	Jingdong prefecture level city in Shandong
Power Saving King Shop	power saver	The logistics is very fast and satisfactory	Jingdong prefecture level city in Shandong
Power Saving King Shop	power saver	Fine workmanship material is good, powerful, the effect is the same as described	Jingdong prefecture level city in Shandong
Power Saving King Shop	power saver	Easy to install, really effective, saves a lot of electricity than before	Jingdong prefecture level city in Shandong
Power Saving King Shop	power saver	Tested it and it's good quality, it does work well, recommended	Jingdong prefecture level city in Shandong
Power Saving King Shop	power saver	The quality of the product is quite good, fast shipping, satisfied.	Jingdong prefecture level city in Shandong
Power Saving King Shop	power saver	The logistics is very fast and satisfactory	Jingdong prefecture level city in Shandong
Power Saving King Shop	power saver	Fine workmanship material is good, powerful, the effect is the same as described	Jingdong prefecture level city in Shandong
Power Saving King Shop	power saver	Easy to install, really effective, saves a lot of electricity than before	Jingdong prefecture level city in Shandong
Power Saving King Shop	power saver	Tested it and it's good quality, it does work well, recommended	Jingdong prefecture level city in Shandong
Power Saving King Shop	power saver	The quality of the product is quite good, fast shipping, satisfied.	Jingdong prefecture level city in Shandong
Power Saving King Shop	power saver	The logistics is very fast and satisfactory	Jingdong prefecture level city in Shandong
Power Saving King Shop	power saver	Fine workmanship material is good, powerful, the effect is the same as described	Jingdong prefecture level city in Shandong
Power Saving King Shop	power saver	Easy to install, really effective, saves a lot of electricity than before	Jingdong prefecture level city in Shandong
Power Saving King Shop	power saver	Tested it and it's good quality, it does work well, recommended	Jingdong prefecture level city in Shandong
Power Saving King Shop	power saver	The quality of the product is quite good, fast shipping, satisfied.	Jingdong prefecture level city in Shandong
Power Saving King Shop	power saver	The logistics is very fast and satisfactory	Jingdong prefecture level city in Shandong
Power Saving King Shop	power saver	Fine workmanship material is good, powerful, the effect is the same as described	Jingdong prefecture level city in Shandong
Power Saving King Shop	power saver	Easy to install, really effective, saves a lot of electricity than before	Jingdong prefecture level city in Shandong
Power Saving King Shop	power saver	Tested it and it's good quality, it does work well, recommended	Jingdong prefecture level city in Shandong
Power Saving King Shop	power saver	The quality of the product is quite good, fast shipping, satisfied.	Jingdong prefecture level city in Shandong
Power Saving King Shop	power saver	The logistics is very fast and satisfactory	Jingdong prefecture level city in Shandong
Power Saving King Shop	power saver	Fine workmanship material is good, powerful, the effect is the same as described	Jingdong prefecture level city in Shandong
Power Saving King Shop	power saver	Easy to install, really effective, saves a lot of electricity than before	Jingdong prefecture level city in Shandong
Power Saving King Shop	power saver	Tested it and it's good quality, it does work well, recommended	Jingdong prefecture level city in Shandong

Power Saving King Shop	power saver	The quality of the product is quite good, fast shipping, satisfied.	Jingdong prefecture level city in Shandong
Power Saving King Shop	power saver	The logistics is very fast and satisfactory	Jingdong prefecture level city in Shandong
Power Saving King Shop	power saver	Fine workmanship material is good, powerful, the effect is the same as described	Jingdong prefecture level city in Shandong
Power Saving King Shop	power saver	Easy to install, really effective, saves a lot of electricity than before	Jingdong prefecture level city in Shandong
Power Saving King Shop	power saver	Tested it and it's good quality, it does work well, recommended	Jingdong prefecture level city in Shandong
Power Saving King Shop	power saver	The quality of the product is quite good, fast shipping, satisfied.	Jingdong prefecture level city in Shandong
Power Saving King Shop	power saver	The logistics is very fast and satisfactory	Jingdong prefecture level city in Shandong
Power Saving King Shop	power saver	Fine workmanship material is good, powerful, the effect is the same as described	Jingdong prefecture level city in Shandong
Power Saving King Shop	power saver	Easy to install, really effective, saves a lot of electricity than before	Jingdong prefecture level city in Shandong
Power Saving King Shop	power saver	Tested it and it's good quality, it does work well, recommended	Jingdong prefecture level city in Shandong
Power Saving King Shop	power saver	The quality of the product is quite good, fast shipping, satisfied.	Jingdong prefecture level city in Shandong
Power Saving King Shop	power saver	The logistics is very fast and satisfactory	Jingdong prefecture level city in Shandong
Power Saving King Shop	power saver	Fine workmanship material is good, powerful, the effect is the same as described	Jingdong prefecture level city in Shandong
Power Saving King Shop	power saver	Easy to install, really effective, saves a lot of electricity than before	Jingdong prefecture level city in Shandong
Power Saving King Shop	power saver	Tested it and it's good quality, it does work well, recommended	Jingdong prefecture level city in Shandong
Power Saving King Shop	power saver	The quality of the product is quite good, fast shipping, satisfied.	Jingdong prefecture level city in Shandong
Power Saving King Shop	power saver	The logistics is very fast and satisfactory	Jingdong prefecture level city in Shandong
Power Saving King Shop	power saver	Fine workmanship material is good, powerful, the effect is the same as described	Jingdong prefecture level city in Shandong
Power Saving King Shop	power saver	Easy to install, really effective, saves a lot of electricity than before	Jingdong prefecture level city in Shandong
Power Saving King Shop	power saver	Tested it and it's good quality, it does work well, recommended	Jingdong prefecture level city in Shandong
Power Saving King Shop	power saver	The quality of the product is quite good, fast shipping, satisfied.	Jingdong prefecture level city in Shandong
Power Saving King Shop	power saver	The logistics is very fast and satisfactory	Jingdong prefecture level city in Shandong
Power Saving King Shop	power saver	Fine workmanship material is good, powerful, the effect is the same as described	Jingdong prefecture level city in Shandong
Power Saving King Shop	power saver	Easy to install, really effective, saves a lot of electricity than before	Jingdong prefecture level city in Shandong
Power Saving King Shop	power saver	Tested it and it's good quality, it does work well, recommended	Jingdong prefecture level city in Shandong
Power Saving King Shop	power saver	The quality of the product is quite good, fast shipping, satisfied.	Jingdong prefecture level city in Shandong
Power Saving King Shop	power saver	The logistics is very fast and satisfactory	Jingdong prefecture level city in Shandong
Power Saving King Shop	power saver	Fine workmanship material is good, powerful, the effect is the same as described	Jingdong prefecture level city in Shandong
Power Saving King Shop	power saver	Easy to install, really effective, saves a lot of electricity than before	Jingdong prefecture level city in Shandong
Power Saving King Shop	power saver	Tested it and it's good quality, it does work well, recommended	Jingdong prefecture level city in Shandong
Power Saving King Shop	power saver	The quality of the product is quite good, fast shipping, satisfied.	Jingdong prefecture level city in Shandong
Power Saving King Shop	power saver	The logistics is very fast and satisfactory	Jingdong prefecture level city in Shandong
Power Saving King Shop	power saver	Fine workmanship material is good, powerful, the effect is the same as described	Jingdong prefecture level city in Shandong
Power Saving King Shop	power saver	Easy to install, really effective, saves a lot of electricity than before	Jingdong prefecture level city in Shandong
Power Saving King Shop	power saver	Tested it and it's good quality, it does work well, recommended	Jingdong prefecture level city in Shandong
Power Saving King Shop	power saver	The quality of the product is quite good, fast shipping, satisfied.	Jingdong prefecture level city in Shandong
Power Saving King Shop	power saver	The logistics is very fast and satisfactory	Jingdong prefecture level city in Shandong
Power Saving King Shop	power saver	Fine workmanship material is good, powerful, the effect is the same as described	Jingdong prefecture level city in Shandong
Power Saving King Shop	power saver	Easy to install, really effective, saves a lot of electricity than before	Jingdong prefecture level city in Shandong
Power Saving King Shop	power saver	Tested it and it's good quality, it does work well, recommended	Jingdong prefecture level city in Shandong
Power Saving King Shop	power saver	The quality of the product is quite good, fast shipping, satisfied.	Jingdong prefecture level city in Shandong
Power Saving King Shop	power saver	The logistics is very fast and satisfactory	Jingdong prefecture level city in Shandong
Power Saving King Shop	power saver	Fine workmanship material is good, powerful, the effect is the same as described	Jingdong prefecture level city in Shandong
Power Saving King Shop	power saver	Easy to install, really effective, saves a lot of electricity than before	Jingdong prefecture level city in Shandong
Power Saving King Shop	power saver	Tested it and it's good quality, it does work well, recommended	Jingdong prefecture level city in Shandong
Power Saving King Shop	power saver	The quality of the product is quite good, fast shipping, satisfied.	Jingdong prefecture level city in Shandong
Power Saving King Shop	power saver	The logistics is very fast and satisfactory	Jingdong prefecture level city in Shandong
Power Saving King Shop	power saver	Fine workmanship material is good, powerful, the effect is the same as described	Jingdong prefecture level city in Shandong
Power Saving King Shop	power saver	Easy to install, really effective, saves a lot of electricity than before	Jingdong prefecture level city in Shandong
Power Saving King Shop	power saver	Tested it and it's good quality, it does work well, recommended	Jingdong prefecture level city in Shandong
Power Saving King Shop	power saver	The quality of the product is quite good, fast shipping, satisfied.	Jingdong prefecture level city in Shandong

Power Saving King Shop	power saver	The logistics is very fast and satisfactory	Jingdong prefecture level city in Shandong
Power Saving King Shop	power saver	Fine workmanship material is good, powerful, the effect is the same as described	Jingdong prefecture level city in Shandong
Power Saving King Shop	power saver	Easy to install, really effective, saves a lot of electricity than before	Jingdong prefecture level city in Shandong
Power Saving King Shop	power saver	Tested it and it's good quality, it does work well, recommended	Jingdong prefecture level city in Shandong
Power Saving King Shop	power saver	The quality of the product is quite good, fast shipping, satisfied.	Jingdong prefecture level city in Shandong
Power Saving King Shop	power saver	The logistics is very fast and satisfactory	Jingdong prefecture level city in Shandong
Power Saving King Shop	power saver	Fine workmanship material is good, powerful, the effect is the same as described	Jingdong prefecture level city in Shandong
Power Saving King Shop	power saver	Easy to install, really effective, saves a lot of electricity than before	Jingdong prefecture level city in Shandong
Power Saving King Shop	power saver	Tested it and it's good quality, it does work well, recommended	Jingdong prefecture level city in Shandong
Power Saving King Shop	power saver	The quality of the product is quite good, fast shipping, satisfied.	Jingdong prefecture level city in Shandong
Power Saving King Shop	power saver	The logistics is very fast and satisfactory	Jingdong prefecture level city in Shandong
Power Saving King Shop	power saver	Fine workmanship material is good, powerful, the effect is the same as described	Jingdong prefecture level city in Shandong
Power Saving King Shop	power saver	Easy to install, really effective, saves a lot of electricity than before	Jingdong prefecture level city in Shandong
Power Saving King Shop	power saver	Tested it and it's good quality, it does work well, recommended	Jingdong prefecture level city in Shandong
Power Saving King Shop	power saver	The quality of the product is quite good, fast shipping, satisfied.	Jingdong prefecture level city in Shandong
Power Saving King Shop	power saver	The logistics is very fast and satisfactory	Jingdong prefecture level city in Shandong
Power Saving King Shop	power saver	Fine workmanship material is good, powerful, the effect is the same as described	Jingdong prefecture level city in Shandong
Power Saving King Shop	power saver	Easy to install, really effective, saves a lot of electricity than before	Jingdong prefecture level city in Shandong
Power Saving King Shop	power saver	Tested it and it's good quality, it does work well, recommended	Jingdong prefecture level city in Shandong
Power Saving King Shop	power saver	The quality of the product is quite good, fast shipping, satisfied.	Jingdong prefecture level city in Shandong
Power Saving King Shop	power saver	The logistics is very fast and satisfactory	Jingdong prefecture level city in Shandong
Power Saving King Shop	power saver	Fine workmanship material is good, powerful, the effect is the same as described	Jingdong prefecture level city in Shandong
Power Saving King Shop	power saver	Easy to install, really effective, saves a lot of electricity than before	Jingdong prefecture level city in Shandong
Power Saving King Shop	power saver	Tested it and it's good quality, it does work well, recommended	Jingdong prefecture level city in Shandong
Power Saving King Shop	power saver	The quality of the product is quite good, fast shipping, satisfied.	Jingdong prefecture level city in Shandong
Power Saving King Shop	power saver	The logistics is very fast and satisfactory	Jingdong prefecture level city in Shandong
Power Saving King Shop	power saver	Fine workmanship material is good, powerful, the effect is the same as described	Jingdong prefecture level city in Shandong
Power Saving King Shop	power saver	Easy to install, really effective, saves a lot of electricity than before	Jingdong prefecture level city in Shandong
Power Saving King Shop	power saver	Tested it and it's good quality, it does work well, recommended	Jingdong prefecture level city in Shandong
Power Saving King Shop	power saver	The quality of the product is quite good, fast shipping, satisfied.	Jingdong prefecture level city in Shandong
Power Saving King Shop	power saver	The logistics is very fast and satisfactory	Jingdong prefecture level city in Shandong
Power Saving King Shop	power saver	Fine workmanship material is good, powerful, the effect is the same as described	Jingdong prefecture level city in Shandong
Power Saving King Shop	power saver	Easy to install, really effective, saves a lot of electricity than before	Jingdong prefecture level city in Shandong
Power Saving King Shop	power saver	Tested it and it's good quality, it does work well, recommended	Jingdong prefecture level city in Shandong
Power Saving King Shop	power saver	The quality of the product is quite good, fast shipping, satisfied.	Jingdong prefecture level city in Shandong
Power Saving King Shop	power saver	The logistics is very fast and satisfactory	Jingdong prefecture level city in Shandong
Power Saving King Shop	power saver	Fine workmanship material is good, powerful, the effect is the same as described	Jingdong prefecture level city in Shandong
Power Saving King Shop	power saver	Easy to install, really effective, saves a lot of electricity than before	Jingdong prefecture level city in Shandong
Power Saving King Shop	power saver	Tested it and it's good quality, it does work well, recommended	Jingdong prefecture level city in Shandong
Power Saving King Shop	power saver	The quality of the product is quite good, fast shipping, satisfied.	Jingdong prefecture level city in Shandong
Power Saving King Shop	power saver	The logistics is very fast and satisfactory	Jingdong prefecture level city in Shandong
Power Saving King Shop	power saver	Fine workmanship material is good, powerful, the effect is the same as described	Jingdong prefecture level city in Shandong
Power Saving King Shop	power saver	Easy to install, really effective, saves a lot of electricity than before	Jingdong prefecture level city in Shandong
Power Saving King Shop	power saver	Tested it and it's good quality, it does work well, recommended	Jingdong prefecture level city in Shandong
Power Saving King Shop	power saver	The quality of the product is quite good, fast shipping, satisfied.	Jingdong prefecture level city in Shandong
Power Saving King Shop	power saver	The logistics is very fast and satisfactory	Jingdong prefecture level city in Shandong
Power Saving King Shop	power saver	Fine workmanship material is good, powerful, the effect is the same as described	Jingdong prefecture level city in Shandong
Power Saving King Shop	power saver	Easy to install, really effective, saves a lot of electricity than before	Jingdong prefecture level city in Shandong
Power Saving King Shop	power saver	Tested it and it's good quality, it does work well, recommended	Jingdong prefecture level city in Shandong
Power Saving King Shop	power saver	The quality of the product is quite good, fast shipping, satisfied.	Jingdong prefecture level city in Shandong
Power Saving King Shop	power saver	The logistics is very fast and satisfactory	Jingdong prefecture level city in Shandong

Power Saving King Shop	power saver	Fine workmanship material is good, powerful, the effect is the same as described	Jingdong prefecture level city in Shandong
Power Saving King Shop	power saver	Easy to install, really effective, saves a lot of electricity than before	Jingdong prefecture level city in Shandong
Power Saving King Shop	power saver	Tested it and it’s good quality, it does work well, recommended	Jingdong prefecture level city in Shandong
Power Saving King Shop	power saver	The quality of the product is quite good, fast shipping, satisfied.	Jingdong prefecture level city in Shandong
Power Saving King Shop	power saver	The logistics is very fast and satisfactory	Jingdong prefecture level city in Shandong
Power Saving King Shop	power saver	Fine workmanship material is good, powerful, the effect is the same as described	Jingdong prefecture level city in Shandong
Power Saving King Shop	power saver	Easy to install, really effective, saves a lot of electricity than before	Jingdong prefecture level city in Shandong
Power Saving King Shop	power saver	Tested it and it’s good quality, it does work well, recommended	Jingdong prefecture level city in Shandong
Power Saving King Shop	power saver	The quality of the product is quite good, fast shipping, satisfied.	Jingdong prefecture level city in Shandong
Power Saving King Shop	power saver	The logistics is very fast and satisfactory	Jingdong prefecture level city in Shandong
Power Saving King Shop	power saver	Fine workmanship material is good, powerful, the effect is the same as described	Jingdong prefecture level city in Shandong
Power Saving King Shop	power saver	Easy to install, really effective, saves a lot of electricity than before	Jingdong prefecture level city in Shandong
Power Saving King Shop	power saver	Tested it and it’s good quality, it does work well, recommended	Jingdong prefecture level city in Shandong
Power Saving King Shop	power saver	The quality of the product is quite good, fast shipping, satisfied.	Jingdong prefecture level city in Shandong
Power Saving King Shop	power saver	The logistics is very fast and satisfactory	Jingdong prefecture level city in Shandong
Power Saving King Shop	power saver	Fine workmanship material is good, powerful, the effect is the same as described	Jingdong prefecture level city in Shandong
Power Saving King Shop	power saver	Easy to install, really effective, saves a lot of electricity than before	Jingdong prefecture level city in Shandong
Power Saving King Shop	power saver	Tested it and it’s good quality, it does work well, recommended	Jingdong prefecture level city in Shandong
Power Saving King Shop	power saver	The quality of the product is quite good, fast shipping, satisfied.	Jingdong prefecture level city in Shandong
Power Saving King Shop	power saver	The logistics is very fast and satisfactory	Jingdong prefecture level city in Shandong
Power Saving King Shop	power saver	Fine workmanship material is good, powerful, the effect is the same as described	Jingdong prefecture level city in Shandong
Power Saving King Shop	power saver	Easy to install, really effective, saves a lot of electricity than before	Jingdong prefecture level city in Shandong
Power Saving King Shop	power saver	Tested it and it’s good quality, it does work well, recommended	Jingdong prefecture level city in Shandong
Power Saving King Shop	power saver	The quality of the product is quite good, fast shipping, satisfied.	Jingdong prefecture level city in Shandong
Power Saving King Shop	power saver	The logistics is very fast and satisfactory	Jingdong prefecture level city in Shandong
Power Saving King Shop	power saver	Fine workmanship material is good, powerful, the effect is the same as described	Jingdong prefecture level city in Shandong
Power Saving King Shop	power saver	Easy to install, really effective, saves a lot of electricity than before	Jingdong prefecture level city in Shandong
Power Saving King Shop	power saver	Tested it and it’s good quality, it does work well, recommended	Jingdong prefecture level city in Shandong
Power Saving King Shop	power saver	The quality of the product is quite good, fast shipping, satisfied.	Jingdong prefecture level city in Shandong
Power Saving King Shop	power saver	The logistics is very fast and satisfactory	Jingdong prefecture level city in Shandong
Power Saving King Shop	power saver	Fine workmanship material is good, powerful, the effect is the same as described	Jingdong prefecture level city in Shandong
Power Saving King Shop	power saver	Easy to install, really effective, saves a lot of electricity than before	Jingdong prefecture level city in Shandong
Power Saving King Shop	power saver	Tested it and it’s good quality, it does work well, recommended	Jingdong prefecture level city in Shandong
Power Saving King Shop	power saver	The quality of the product is quite good, fast shipping, satisfied.	Jingdong prefecture level city in Shandong
Power Saving King Shop	power saver	The logistics is very fast and satisfactory	Jingdong prefecture level city in Shandong
Power Saving King Shop	power saver	Fine workmanship material is good, powerful, the effect is the same as described	Jingdong prefecture level city in Shandong
Power Saving King Shop	power saver	Easy to install, really effective, saves a lot of electricity than before	Jingdong prefecture level city in Shandong
Power Saving King Shop	power saver	Tested it and it’s good quality, it does work well, recommended	Jingdong prefecture level city in Shandong
Power Saving King Shop	power saver	The quality of the product is quite good, fast shipping, satisfied.	Jingdong prefecture level city in Shandong
Power Saving King Shop	power saver	The logistics is very fast and satisfactory	Jingdong prefecture level city in Shandong
Power Saving King Shop	power saver	Fine workmanship material is good, powerful, the effect is the same as described	Jingdong prefecture level city in Shandong
Power Saving King Shop	power saver	Easy to install, really effective, saves a lot of electricity than before	Jingdong prefecture level city in Shandong
Power Saving King Shop	power saver	Tested it and it’s good quality, it does work well, recommended	Jingdong prefecture level city in Shandong
Power Saving King Shop	power saver	The quality of the product is quite good, fast shipping, satisfied.	Jingdong prefecture level city in Shandong
Power Saving King Shop	power saver	The logistics is very fast and satisfactory	Jingdong prefecture level city in Shandong
Power Saving King Shop	power saver	Fine workmanship material is good, powerful, the effect is the same as described	Jingdong prefecture level city in Shandong
Power Saving King Shop	power saver	Easy to install, really effective, saves a lot of electricity than before	Jingdong prefecture level city in Shandong
Power Saving King Shop	power saver	Tested it and it’s good quality, it does work well, recommended	Jingdong prefecture level city in Shandong
Power Saving King Shop	power saver	The quality of the product is quite good, fast shipping, satisfied.	Jingdong prefecture level city in Shandong
Power Saving King Shop	power saver	The logistics is very fast and satisfactory	Jingdong prefecture level city in Shandong
Power Saving King Shop	power saver	Fine workmanship material is good, powerful, the effect is the same as described	Jingdong prefecture level city in Shandong

Power Saving King Shop	power saver	Easy to install, really effective, saves a lot of electricity than before	Jingdong prefecture level city in Shandong
Power Saving King Shop	power saver	Tested it and it's good quality, it does work well, recommended	Jingdong prefecture level city in Shandong
Power Saving King Shop	power saver	The quality of the product is quite good, fast shipping, satisfied.	Jingdong prefecture level city in Shandong
Power Saving King Shop	power saver	The logistics is very fast and satisfactory	Jingdong prefecture level city in Shandong
Power Saving King Shop	power saver	Fine workmanship material is good, powerful, the effect is the same as described	Jingdong prefecture level city in Shandong
Power Saving King Shop	power saver	Easy to install, really effective, saves a lot of electricity than before	Jingdong prefecture level city in Shandong
Power Saving King Shop	power saver	Tested it and it's good quality, it does work well, recommended	Jingdong prefecture level city in Shandong
Power Saving King Shop	power saver	The quality of the product is quite good, fast shipping, satisfied.	Jingdong prefecture level city in Shandong
Power Saving King Shop	power saver	The logistics is very fast and satisfactory	Jingdong prefecture level city in Shandong
Power Saving King Shop	power saver	Fine workmanship material is good, powerful, the effect is the same as described	Jingdong prefecture level city in Shandong
Power Saving King Shop	power saver	Easy to install, really effective, saves a lot of electricity than before	Jingdong prefecture level city in Shandong
Power Saving King Shop	power saver	Tested it and it's good quality, it does work well, recommended	Jingdong prefecture level city in Shandong
Power Saving King Shop	power saver	The quality of the product is quite good, fast shipping, satisfied.	Jingdong prefecture level city in Shandong
Power Saving King Shop	power saver	The logistics is very fast and satisfactory	Jingdong prefecture level city in Shandong
Power Saving King Shop	power saver	Fine workmanship material is good, powerful, the effect is the same as described	Jingdong prefecture level city in Shandong
Power Saving King Shop	power saver	Easy to install, really effective, saves a lot of electricity than before	Jingdong prefecture level city in Shandong
Power Saving King Shop	power saver	Tested it and it's good quality, it does work well, recommended	Jingdong prefecture level city in Shandong
Power Saving King Shop	power saver	The quality of the product is quite good, fast shipping, satisfied.	Jingdong prefecture level city in Shandong
Power Saving King Shop	power saver	The logistics is very fast and satisfactory	Jingdong prefecture level city in Shandong
Power Saving King Shop	power saver	Fine workmanship material is good, powerful, the effect is the same as described	Jingdong prefecture level city in Shandong
Power Saving King Shop	power saver	Easy to install, really effective, saves a lot of electricity than before	Jingdong prefecture level city in Shandong
Power Saving King Shop	power saver	Tested it and it's good quality, it does work well, recommended	Jingdong prefecture level city in Shandong
Power Saving King Shop	power saver	The quality of the product is quite good, fast shipping, satisfied.	Jingdong prefecture level city in Shandong
Power Saving King Shop	power saver	The logistics is very fast and satisfactory	Jingdong prefecture level city in Shandong
Power Saving King Shop	power saver	Fine workmanship material is good, powerful, the effect is the same as described	Jingdong prefecture level city in Shandong
Power Saving King Shop	power saver	Easy to install, really effective, saves a lot of electricity than before	Jingdong prefecture level city in Shandong
Power Saving King Shop	power saver	Tested it and it's good quality, it does work well, recommended	Jingdong prefecture level city in Shandong
Power Saving King Shop	power saver	The quality of the product is quite good, fast shipping, satisfied.	Jingdong prefecture level city in Shandong
Power Saving King Shop	power saver	The logistics is very fast and satisfactory	Jingdong prefecture level city in Shandong
Power Saving King Shop	power saver	Fine workmanship material is good, powerful, the effect is the same as described	Jingdong prefecture level city in Shandong
Power Saving King Shop	power saver	Easy to install, really effective, saves a lot of electricity than before	Jingdong prefecture level city in Shandong
Power Saving King Shop	power saver	Tested it and it's good quality, it does work well, recommended	Jingdong prefecture level city in Shandong
Power Saving King Shop	power saver	The quality of the product is quite good, fast shipping, satisfied.	Jingdong prefecture level city in Shandong
Power Saving King Shop	power saver	The logistics is very fast and satisfactory	Jingdong prefecture level city in Shandong
Power Saving King Shop	power saver	Fine workmanship material is good, powerful, the effect is the same as described	Jingdong prefecture level city in Shandong
Power Saving King Shop	power saver	Easy to install, really effective, saves a lot of electricity than before	Jingdong prefecture level city in Shandong
Power Saving King Shop	power saver	Tested it and it's good quality, it does work well, recommended	Jingdong prefecture level city in Shandong
Power Saving King Shop	power saver	The quality of the product is quite good, fast shipping, satisfied.	Jingdong prefecture level city in Shandong
Power Saving King Shop	power saver	The logistics is very fast and satisfactory	Jingdong prefecture level city in Shandong
Power Saving King Shop	power saver	Fine workmanship material is good, powerful, the effect is the same as described	Jingdong prefecture level city in Shandong
Power Saving King Shop	power saver	Easy to install, really effective, saves a lot of electricity than before	Jingdong prefecture level city in Shandong
Power Saving King Shop	power saver	Tested it and it's good quality, it does work well, recommended	Jingdong prefecture level city in Shandong
Power Saving King Shop	power saver	The quality of the product is quite good, fast shipping, satisfied.	Jingdong prefecture level city in Shandong
Power Saving King Shop	power saver	The logistics is very fast and satisfactory	Jingdong prefecture level city in Shandong
Power Saving King Shop	power saver	Fine workmanship material is good, powerful, the effect is the same as described	Jingdong prefecture level city in Shandong
Power Saving King Shop	power saver	Easy to install, really effective, saves a lot of electricity than before	Jingdong prefecture level city in Shandong
Power Saving King Shop	power saver	Tested it and it's good quality, it does work well, recommended	Jingdong prefecture level city in Shandong
Power Saving King Shop	power saver	The quality of the product is quite good, fast shipping, satisfied.	Jingdong prefecture level city in Shandong
Power Saving King Shop	power saver	The logistics is very fast and satisfactory	Jingdong prefecture level city in Shandong
Power Saving King Shop	power saver	Fine workmanship material is good, powerful, the effect is the same as described	Jingdong prefecture level city in Shandong
Power Saving King Shop	power saver	Easy to install, really effective, saves a lot of electricity than before	Jingdong prefecture level city in Shandong

Power Saving King Shop	power saver	Tested it and it's good quality, it does work well, recommended	Jingdong prefecture level city in Shandong
Power Saving King Shop	power saver	The quality of the product is quite good, fast shipping, satisfied.	Jingdong prefecture level city in Shandong
Power Saving King Shop	power saver	The logistics is very fast and satisfactory	Jingdong prefecture level city in Shandong
Power Saving King Shop	power saver	Fine workmanship material is good, powerful, the effect is the same as described	Jingdong prefecture level city in Shandong
Power Saving King Shop	power saver	Easy to install, really effective, saves a lot of electricity than before	Jingdong prefecture level city in Shandong
Power Saving King Shop	power saver	Tested it and it's good quality, it does work well, recommended	Jingdong prefecture level city in Shandong
Power Saving King Shop	power saver	The quality of the product is quite good, fast shipping, satisfied.	Jingdong prefecture level city in Shandong
Power Saving King Shop	power saver	The logistics is very fast and satisfactory	Jingdong prefecture level city in Shandong
Power Saving King Shop	power saver	Fine workmanship material is good, powerful, the effect is the same as described	Jingdong prefecture level city in Shandong
Power Saving King Shop	power saver	Easy to install, really effective, saves a lot of electricity than before	Jingdong prefecture level city in Shandong
Power Saving King Shop	power saver	Tested it and it's good quality, it does work well, recommended	Jingdong prefecture level city in Shandong
Power Saving King Shop	power saver	The quality of the product is quite good, fast shipping, satisfied.	Jingdong prefecture level city in Shandong
Power Saving King Shop	power saver	The logistics is very fast and satisfactory	Jingdong prefecture level city in Shandong
Power Saving King Shop	power saver	Fine workmanship material is good, powerful, the effect is the same as described	Jingdong prefecture level city in Shandong
Power Saving King Shop	power saver	Easy to install, really effective, saves a lot of electricity than before	Jingdong prefecture level city in Shandong
Power Saving King Shop	power saver	Tested it and it's good quality, it does work well, recommended	Jingdong prefecture level city in Shandong
Power Saving King Shop	power saver	The quality of the product is quite good, fast shipping, satisfied.	Jingdong prefecture level city in Shandong
Power Saving King Shop	power saver	The logistics is very fast and satisfactory	Jingdong prefecture level city in Shandong
Power Saving King Shop	power saver	Fine workmanship material is good, powerful, the effect is the same as described	Jingdong prefecture level city in Shandong
Power Saving King Shop	power saver	Easy to install, really effective, saves a lot of electricity than before	Jingdong prefecture level city in Shandong
Power Saving King Shop	power saver	Tested it and it's good quality, it does work well, recommended	Jingdong prefecture level city in Shandong
Power Saving King Shop	power saver	The quality of the product is quite good, fast shipping, satisfied.	Jingdong prefecture level city in Shandong
Power Saving King Shop	power saver	The logistics is very fast and satisfactory	Jingdong prefecture level city in Shandong
Power Saving King Shop	power saver	Fine workmanship material is good, powerful, the effect is the same as described	Jingdong prefecture level city in Shandong
Power Saving King Shop	power saver	Easy to install, really effective, saves a lot of electricity than before	Jingdong prefecture level city in Shandong
Power Saving King Shop	power saver	Tested it and it's good quality, it does work well, recommended	Jingdong prefecture level city in Shandong
Power Saving King Shop	power saver	The quality of the product is quite good, fast shipping, satisfied.	Jingdong prefecture level city in Shandong
Power Saving King Shop	power saver	The logistics is very fast and satisfactory	Jingdong prefecture level city in Shandong
Power Saving King Shop	power saver	Fine workmanship material is good, powerful, the effect is the same as described	Jingdong prefecture level city in Shandong
Power Saving King Shop	power saver	Easy to install, really effective, saves a lot of electricity than before	Jingdong prefecture level city in Shandong
Power Saving King Shop	power saver	Tested it and it's good quality, it does work well, recommended	Jingdong prefecture level city in Shandong
Power Saving King Shop	power saver	The quality of the product is quite good, fast shipping, satisfied.	Jingdong prefecture level city in Shandong
Power Saving King Shop	power saver	The logistics is very fast and satisfactory	Jingdong prefecture level city in Shandong
Power Saving King Shop	power saver	Fine workmanship material is good, powerful, the effect is the same as described	Jingdong prefecture level city in Shandong
Power Saving King Shop	power saver	Easy to install, really effective, saves a lot of electricity than before	Jingdong prefecture level city in Shandong
Power Saving King Shop	power saver	Tested it and it's good quality, it does work well, recommended	Jingdong prefecture level city in Shandong
Power Saving King Shop	power saver	The quality of the product is quite good, fast shipping, satisfied.	Jingdong prefecture level city in Shandong
Power Saving King Shop	power saver	The logistics is very fast and satisfactory	Jingdong prefecture level city in Shandong
Power Saving King Shop	power saver	Fine workmanship material is good, powerful, the effect is the same as described	Jingdong prefecture level city in Shandong
Power Saving King Shop	power saver	Easy to install, really effective, saves a lot of electricity than before	Jingdong prefecture level city in Shandong
Power Saving King Shop	power saver	Tested it and it's good quality, it does work well, recommended	Jingdong prefecture level city in Shandong
Power Saving King Shop	power saver	The quality of the product is quite good, fast shipping, satisfied.	Jingdong prefecture level city in Shandong
Power Saving King Shop	power saver	The logistics is very fast and satisfactory	Jingdong prefecture level city in Shandong
Power Saving King Shop	power saver	Fine workmanship material is good, powerful, the effect is the same as described	Jingdong prefecture level city in Shandong
Power Saving King Shop	power saver	Easy to install, really effective, saves a lot of electricity than before	Jingdong prefecture level city in Shandong
Power Saving King Shop	power saver	Tested it and it's good quality, it does work well, recommended	Jingdong prefecture level city in Shandong
Power Saving King Shop	power saver	The quality of the product is quite good, fast shipping, satisfied.	Jingdong prefecture level city in Shandong
Power Saving King Shop	power saver	The logistics is very fast and satisfactory	Jingdong prefecture level city in Shandong
Power Saving King Shop	power saver	Fine workmanship material is good, powerful, the effect is the same as described	Jingdong prefecture level city in Shandong
Power Saving King Shop	power saver	Easy to install, really effective, saves a lot of electricity than before	Jingdong prefecture level city in Shandong
Power Saving King Shop	power saver	Tested it and it's good quality, it does work well, recommended	Jingdong prefecture level city in Shandong

Power Saving King Shop	power saver	The quality of the product is quite good, fast shipping, satisfied.	Jingdong prefecture level city in Shandong
Power Saving King Shop	power saver	The logistics is very fast and satisfactory	Jingdong prefecture level city in Shandong
Power Saving King Shop	power saver	Very good, very satisfied with the quality, the price is also cost-effective, the next time will come back, merchants shipping speed, very suitable, my family with, satisfied, packaging is very good.	Jingdong prefecture level city in Shandong
Power Saving King Shop	power saver	Received very quickly. Quality is very good. Cheap price logistics very quickly good value for money worth recommending the purchase	Jingdong prefecture level city in Shandong
Power Saving King Shop	Power Saving Specialist	Power Saving King is very good quality, new style, power saving very good, cheap price, worth buying	Jingdong prefecture level city in Shandong
Power Saving King Shop	Power Saving Specialist	It's authentic, affordable, very good quality, and worth buying at this price!	Jingdong prefecture level city in Shandong
Power Saving King Shop	Power Saving Specialist	It works exceptionally well and is a good value for money	Jingdong prefecture level city in Shandong
Power Saving King Shop	Intelligent power saving	not have	Jingdong prefecture level city in Shandong
Power Saving King Shop	High Power Super Saver	not have	Jingdong prefecture level city in Shandong
Energy Saving Technology Shop	Green Intelligent Power Saver	Arrived, effective, logistics is very fast, the quality is very good, really save electricity, I have recommended friends have bought to use, especially the use of large amounts of electricity friends, the effect is more obvious. My home is a mahjong room, not using the power saver inches monthly electricity costs about 300, after using the power saver up to more than 200 yuan a month. The power saver is a real power saver! There is a power saver quality assurance genuine, friends can rest assured that the purchase	Jingdong prefecture level city in Shandong
Energy Saving Technology Shop	Green Intelligent Power Saver	Still their products are reassuringly authentic and safe to use They do save electricity!	Jingdong prefecture level city in Shandong
Energy Saving Technology Shop	Green Intelligent Power Saver	In the summer the family runs two air conditioners, a refrigerator plus some appliances, saving more than thirty percent of the electricity bill a month, and there is no breakdown.	Jingdong prefecture level city in Shandong
Energy Saving Technology Shop	Green Intelligent Power Saver	I used to have a high electricity bill, but since I've been using this power saver, I've been able to save a lot of money on my electricity bill.	Jingdong prefecture level city in Shandong
Energy Saving Technology Shop	Green Intelligent Power Saver	I just got it back and used it right away, hopefully it can help me save some money on my electricity bill, my mom's family bought one and it worked well, so I got one for myself.	Jingdong prefecture level city in Shandong
Energy Saving Technology Shop	White Intelligent Power Saver	Arrived, effective, logistics is very fast, the quality is very good, really save electricity, I have recommended friends have bought to use, especially the use of large amounts of electricity friends, the effect is more obvious. My home is a mahjong room, not using the power saver inches monthly electricity costs about 300, after using the power saver up to more than 200 yuan a month. The power saver is a real power saver! There is a power saver quality assurance genuine, friends can rest assured that the purchase	Jingdong prefecture level city in Shandong
Energy Saving Technology Shop	White Intelligent Power Saver	The previous year bought a, almost every month can save a 70 dollars or so, with a year of no problem at all, look like new, this time to move to a new home decisively buy back 2 units, very believe this store!	Jingdong prefecture level city in Shandong
Energy Saving Technology Shop	White Intelligent Power Saver	I actually purchased one of these babies online about a year ago, and based on usage observations, it does save electricity. It's just that the original one accidentally fell in the water when I was doing sanitation, so I shot one right away. This baby is genuine, beautiful appearance, with product certification, warranty card, genuine logo and other certificates, trustworthy.	Jingdong prefecture level city in Shandong
Energy Saving Technology Shop	White Intelligent Power Saver	It's been installed for some time now, and it's still saving electricity steadily, more than 600 less than last month's electricity bill.	Jingdong prefecture level city in Shandong
Energy Saving Technology Shop	In-situ compensated economizer	Hubby said he paid less for electricity this time, he said this would save electricity still	Jingdong prefecture level city in Shandong
Energy Saving Technology Shop	In-situ compensated economizer	This stuff is pretty reliable, I'm a physics and science student, I can not know? I took the schematic and studied it, and when I looked at it, I saw that it was the right thing to do, and I bought three of them and sent them back home for my parents to use.	Jingdong prefecture level city in Shandong
Energy Saving Technology Shop	In-situ compensated economizer	Arrived, effective, logistics is very fast, the quality is very good, really save electricity, I have recommended friends have bought to use, especially the use of large amounts of electricity friends, the effect is more obvious. My home is a mahjong room, not using the power saver inches monthly electricity costs about 300, after using the power saver up to more than 200 yuan a month. The power saver is a real power saver! There is a power saver quality assurance genuine, friends can rest assured that the purchase	Jingdong prefecture level city in Shandong
Energy Saving Technology Shop	In-situ compensated economizer	Usually more than 900 degrees of electricity a month, now 500 degrees, 78 months is hotter, I use the air conditioning more, is not to buy the wrong, the product is good, the effect is really good	Jingdong prefecture level city in Shandong
Energy Saving Technology Shop	In-situ compensated economizer	Baby has received, feel the quality is very good, this time not afraid of air conditioning power, can air conditioning air conditioning air conditioning free, good, worth everyone to buy!	Jingdong prefecture level city in Shandong
Energy Saving Technology Shop	In-situ compensated economizer	Is a friend's home to buy, go to do guests when the grass to come, listen to friends say really save electricity, buy back to try it, very small one, lazy people money-saving artifacts!	Jingdong prefecture level city in Shandong
Energy Saving Technology Shop	In-situ compensated economizer	Saving power received, LCD display business, this feature is really thoughtful, this is a monthly power consumption within 500 degrees of electricity. Merchants recommended, the store also inner explanation I encountered do not understand the problem, now has been plugged in the use of the observation of a few days does have the effect, at present every day can be less than a 3 degrees or so, such as the experience of 2 months to the time to follow up the evaluation!	Jingdong prefecture level city in Shandong
Energy Saving Technology Shop	In-situ compensated economizer	Simply black science and technology, we opened the hotel, before a month of electricity costs more than 7,000, installed nine power saver, this month's electricity costs only more than 5,000, the effect is too cattle it! Business is not good in these years, open source and cut back on expenditure, which saves a year down the food money is enough, really good!	Jingdong prefecture level city in Shandong
Energy Saving Technology Shop	In-situ compensated economizer	Bought two energy-saving appliances for the family, the kitchen and bathroom water heater is too expensive, the water heater to be turned on every day, the monthly electricity bill is a lot of use, with this, no longer afraid of the electricity bill is more than one month down the electricity bill can be less than half of the previous month it, the effect is particularly good!	Jingdong prefecture level city in Shandong
Energy Saving Technology Shop	In-situ compensated economizer	Saving power is really good to use, the last time I bought 3, the family used , before the family monthly electricity bills are more than 300 fast, after using this are more than 200, are several months, this time to my store is also arranged, it is really good for the people good things.	Jingdong prefecture level city in Shandong
Energy Saving Technology Shop	In-situ compensated economizer	The previous year bought a, almost every month can save a 70 dollars or so, with a year of no problem at all, look like new, this time to move to a new home decisively buy back 2 units, very believe this store!	Jingdong prefecture level city in Shandong
Energy Saving Technology Shop	In-situ compensated economizer	Power-saving effect: used a week to comment, before and now record the meter comparison, my one rental room can save twenty degrees of electricity per day. Cost-effective. Quite worth it.	Jingdong prefecture level city in Shandong
Energy Saving Technology Shop	In-situ compensated economizer	This power saver is really powerful, used to spend 300 electricity bill every month, now it's about 100	Jingdong prefecture level city in Shandong
Energy Saving Technology Shop	In-situ compensated economizer	After two days of testing. The effect is good! About the first day of power saving for 1/4. the second day for 1/3. and not "steal electricity", is through the intelligent integrated circuit, automatically reduce the loss of electrical appliances to achieve the purpose of saving electricity! Recommended	Jingdong prefecture level city in Shandong
Energy Saving Technology Shop	In-situ compensated economizer	Power Saving King has been received, really good, thank you boss, I wish the boss business prosperity, prosperity, next time to come back to patronize!	Jingdong prefecture level city in Shandong
Energy Saving Technology Shop	In-situ compensated economizer	I've been using it for a while. Than to A little less electricity than before. I'll buy it again next time.	Jingdong prefecture level city in Shandong
Energy Saving Technology Shop	In-situ compensated economizer	Used to be a couple hundred bucks a month. With him, it's a big savings. It's great.	Jingdong prefecture level city in Shandong
Energy Saving Technology Shop	In-situ compensated economizer	Previously bought a plug in the kitchen, used for a period of time after the personal feeling is still quite effective, can save a little electricity, so this time without hesitation and bought two, has been inserted in the home next to the appliances that use a lot of electricity!	Jingdong prefecture level city in Shandong
Energy Saving Technology Shop	In-situ compensated economizer	I've used it before and it does have a power saving effect	Jingdong prefecture level city in Shandong
Energy Saving Technology Shop	In-situ compensated economizer	This high-power is not bad Oh, the first few months are 1000 degrees so, I casually brush in Taobao to this product, I bought back to try, I used a month after this month left 736 degrees, scared me, I also called to the grid to confirm, really is 736 degrees, the effect is very good, and today bought a go back to the old family to use	Jingdong prefecture level city in Shandong
Energy Saving Technology Shop	In-situ compensated economizer	High-tech products, really useful, in previous years the high cost of electricity, with this product, the electricity bill came down. Praise a, hope that the research and development of this product of science and technology to continue to work hard, research out better products to.	Jingdong prefecture level city in Shandong

Energy Saving Technology Shop	In-situ compensated economizer	Used for a period of time, you do not say can really save electricity, really is a small power-saving helper, it is worth buying!	Jingdong prefecture level city in Shandong
Energy Saving Technology Shop	In-situ compensated economizer	There are many rooms and it takes a lot of electricity to run the air conditioner when the house is cold. Bought 4 inserted half a month to save me more than 300 dollars. The effect is really core good! Quality is also very good, the seller service is very good and patient.	Jingdong prefecture level city in Shandong
Energy Saving Technology Shop	In-situ compensated economizer	Recommended by the owner of the store next door, looked at his home does have to save electricity, decided to get. Indeed quite can, the voltage is much more stable, there is a reduction in electricity	Jingdong prefecture level city in Shandong
Energy Saving Technology Shop	In-situ compensated economizer	It does save electricity and works pretty well. I feel that in a few days, I will be able to use the money that I bought to save money, it's really good!	Jingdong prefecture level city in Shandong
Energy Saving Technology Shop	In-situ compensated economizer	Small store service attitude is very good Product quality is very good Suggested purchase	Jingdong prefecture level city in Shandong
Energy Saving Technology Shop	In-situ compensated economizer	Bought two energy-saving appliances for the family, the kitchen and bathroom water heater is too expensive, the water heater to be turned on every day, the monthly electricity bill is a lot of use, with this, no longer afraid of the electricity bill is more than one month down the electricity bill can be less than half of the previous month it, the effect is particularly good!	Jingdong prefecture level city in Shandong
Energy Saving Technology Shop	In-situ compensated economizer	Saving power is really good to use, the last time I bought 3, the family used , before the family monthly electricity bills are more than 300 fast, after using this are more than 200, are several months, this time to my store is also arranged, it is really good for the people good things.	Jingdong prefecture level city in Shandong
Energy Saving Technology Shop	In-situ compensated economizer	The previous year bought a, almost every month can save a 70 dollars or so, with a year of no problem at all, look like new, this time to move to a new home decisively buy back 2 units, very believe this store!	Jingdong prefecture level city in Shandong
Energy Saving Technology Shop	In-situ compensated economizer	Power-saving effect: used a week to comment, before and now record the meter comparison, my one rental room can save twenty degrees of electricity per day. Cost-effective. Quite worth it.	Jingdong prefecture level city in Shandong
Energy Saving Technology Shop	In-situ compensated economizer	This power saver is really powerful, used to spend 300 electricity bill every month, now it's about 100	Jingdong prefecture level city in Shandong
Energy Saving Technology Shop	In-situ compensated economizer	After two days of testing. The effect is good! About the first day of power saving for 1/4. the second day for 1/3. and not "steal electricity", is through the intelligent integrated circuit, automatically reduce the loss of electrical appliances to achieve the purpose of saving electricity! Recommended	Jingdong prefecture level city in Shandong
Energy Saving Technology Shop	In-situ compensated economizer	Power Saving King has been received, really good, thank you boss, I wish the boss business prosperity, prosperity, next time to come back to patronize!	Jingdong prefecture level city in Shandong
Energy Saving Technology Shop	In-situ compensated economizer	I've been using it for a while. Than to A little less electricity than before. I'll buy it again next time.	Jingdong prefecture level city in Shandong
Energy Saving Technology Shop	In-situ compensated economizer	Used to be a couple hundred bucks a month. With him, it's a big savings. It's great.	Jingdong prefecture level city in Shandong
Energy Saving Technology Shop	In-situ compensated economizer	Previously bought a plug in the kitchen, used for a period of time after the personal feeling is still quite effective, can save a little electricity, so this time without hesitation and bought two, has been inserted in the home next to the appliances that use a lot of electricity!	Jingdong prefecture level city in Shandong
Energy Saving Technology Shop	In-situ compensated economizer	I've used it before and it does have a power saving effect	Jingdong prefecture level city in Shandong
Energy Saving Technology Shop	In-situ compensated economizer	High-tech products, really useful, in previous years the high cost of electricity, with this product, the electricity bill came down. Praise a, hope that the research and development of this product of science and technology to continue to work hard, research out better products to.	Jingdong prefecture level city in Shandong
Energy Saving Technology Shop	In-situ compensated economizer	This high-power is not bad Oh, the first few months are 1000 degrees so, I casually brush in Taobao to this product, I bought back to try, I used a month after this month left 736 degrees, scared me, I also called to the grid to confirm, really is 736 degrees, the effect is very good, and today bought a go back to the old family to use	Jingdong prefecture level city in Shandong
Energy Saving Technology Shop	In-situ compensated economizer	Used for a period of time, you do not say can really save electricity, really is a small power-saving helper, it is worth buying!	Jingdong prefecture level city in Shandong
Energy Saving Technology Shop	In-situ compensated economizer	There are many rooms and it takes a lot of electricity to run the air conditioner when the house is cold. Bought 4 inserted half a month to save me more than 300 dollars. The effect is really core good! Quality is also very good, the seller service is very good and patient.	Jingdong prefecture level city in Shandong
Energy Saving Technology Shop	In-situ compensated economizer	Recommended by the owner of the store next door, looked at his home does have to save electricity, decided to get. Indeed quite can, the voltage is much more stable, there is a reduction in electricity	Jingdong prefecture level city in Shandong
Energy Saving Technology Shop	In-situ compensated economizer	It does save electricity and works pretty well. I feel that in a few days, I will be able to use the money that I bought to save money, it's really good!	Jingdong prefecture level city in Shandong
Energy Saving Technology Shop	In-situ compensated economizer	Small store service attitude is very good Product quality is very good Suggested purchase	Jingdong prefecture level city in Shandong
Energy Saving Technology Shop	In-situ compensated economizer	Bought two energy-saving appliances for the family, the kitchen and bathroom water heater is too expensive, the water heater to be turned on every day, the monthly electricity bill is a lot of use, with this, no longer afraid of the electricity bill is more than one month down the electricity bill can be less than half of the previous month it, the effect is particularly good!	Jingdong prefecture level city in Shandong
Energy Saving Technology Shop	In-situ compensated economizer	Saving power is really good to use, the last time I bought 3, the family used , before the family monthly electricity bills are more than 300 fast, after using this are more than 200, are several months, this time to my store is also arranged, it is really good for the people good things.	Jingdong prefecture level city in Shandong
Energy Saving Technology Shop	In-situ compensated economizer	The previous year bought a, almost every month can save a 70 dollars or so, with a year of no problem at all, look like new, this time to move to a new home decisively buy back 2 units, very believe this store!	Jingdong prefecture level city in Shandong
Energy Saving Technology Shop	In-situ compensated economizer	Power-saving effect: used a week to comment, before and now record the meter comparison, my one rental room can save twenty degrees of electricity per day. Cost-effective. Quite worth it.	Jingdong prefecture level city in Shandong
Energy Saving Technology Shop	In-situ compensated economizer	This power saver is really powerful, used to spend 300 electricity bill every month, now it's about 100	Jingdong prefecture level city in Shandong
Energy Saving Technology Shop	In-situ compensated economizer	After two days of testing. The effect is good! About the first day of power saving for 1/4. the second day for 1/3. and not "steal electricity", is through the intelligent integrated circuit, automatically reduce the loss of electrical appliances to achieve the purpose of saving electricity! Recommended	Jingdong prefecture level city in Shandong
Energy Saving Technology Shop	In-situ compensated economizer	Power Saving King has been received, really good, thank you boss, I wish the boss business prosperity, prosperity, next time to come back to patronize!	Jingdong prefecture level city in Shandong
Energy Saving Technology Shop	In-situ compensated economizer	I've been using it for a while. Than to A little less electricity than before. I'll buy it again next time.	Jingdong prefecture level city in Shandong
Energy Saving Technology Shop	In-situ compensated economizer	Used to be a couple hundred bucks a month. With him, it's a big savings. It's great.	Jingdong prefecture level city in Shandong
Energy Saving Technology Shop	In-situ compensated economizer	Previously bought a plug in the kitchen, used for a period of time after the personal feeling is still quite effective, can save a little electricity, so this time without hesitation and bought two, has been inserted in the home next to the appliances that use a lot of electricity!	Jingdong prefecture level city in Shandong
Energy Saving Technology Shop	In-situ compensated economizer	I've used it before and it does have a power saving effect	Jingdong prefecture level city in Shandong
Energy Saving Technology Shop	In-situ compensated economizer	High-tech products, really useful, in previous years the high cost of electricity, with this product, the electricity bill came down. Praise a, hope that the research and development of this product of science and technology to continue to work hard, research out better products to.	Jingdong prefecture level city in Shandong
Energy Saving Technology Shop	In-situ compensated economizer	This high-power is not bad Oh, the first few months are 1000 degrees so, I casually brush in Taobao to this product, I bought back to try, I used a month after this month left 736 degrees, scared me, I also called to the grid to confirm, really is 736 degrees, the effect is very good, and today bought a go back to the old family to use	Jingdong prefecture level city in Shandong
Energy Saving Technology Shop	In-situ compensated economizer	Used for a period of time, you do not say can really save electricity, really is a small power-saving helper, it is worth buying!	Jingdong prefecture level city in Shandong
Energy Saving Technology Shop	In-situ compensated economizer	There are many rooms and it takes a lot of electricity to run the air conditioner when the house is cold. Bought 4 inserted half a month to save me more than 300 dollars. The effect is really core good! Quality is also very good, the seller service is very good and patient.	Jingdong prefecture level city in Shandong
Energy Saving Technology Shop	In-situ compensated economizer	Recommended by the owner of the store next door, looked at his home does have to save electricity, decided to get. Indeed quite can, the voltage is much more stable, there is a reduction in electricity	Jingdong prefecture level city in Shandong
Energy Saving Technology Shop	In-situ compensated economizer	It does save electricity and works pretty well. I feel that in a few days, I will be able to use the money that I bought to save money, it's really good!	Jingdong prefecture level city in Shandong
Energy Saving Technology Shop	In-situ compensated economizer	Small store service attitude is very good Product quality is very good Suggested purchase	Jingdong prefecture level city in Shandong

Energy Saving Technology Shop	In-situ compensated economizer	Bought two energy-saving appliances for the family, the kitchen and bathroom water heater is too expensive, the water heater to be turned on every day, the monthly electricity bill is a lot of use, with this, no longer afraid of the electricity bill is more than one month down the electricity bill can be less than half of the previous month it, the effect is particularly good!	Jingdong prefecture level city in Shandong
Energy Saving Technology Shop	In-situ compensated economizer	Saving power is really good to use, the last time I bought 3, the family used , before the family monthly electricity bills are more than 300 fast, after using this are more than 200, are several months, this time to my store is also arranged, it is really good for the people good things.	Jingdong prefecture level city in Shandong
Energy Saving Technology Shop	In-situ compensated economizer	The previous year bought a, almost every month can save a 70 dollars or so, with a year of no problem at all, look like new, this time to move to a new home decisively buy back 2 units, very believe this store!	Jingdong prefecture level city in Shandong
Energy Saving Technology Shop	In-situ compensated economizer	Power-saving effect: used a week to comment, before and now record the meter comparison, my one rental room can save twenty degrees of electricity per day. Cost-effective. Quite worth it.	Jingdong prefecture level city in Shandong
Energy Saving Technology Shop	In-situ compensated economizer	This power saver is really powerful, used to spend 300 electricity bill every month, now it's about 100	Jingdong prefecture level city in Shandong
Energy Saving Technology Shop	In-situ compensated economizer	After two days of testing. The effect is good! About the first day of power saving for 1/4. the second day for 1/3. and not "steal electricity", is through the intelligent integrated circuit, automatically reduce the loss of electrical appliances to achieve the purpose of saving electricity! Recommended	Jingdong prefecture level city in Shandong
Energy Saving Technology Shop	In-situ compensated economizer	Power Saving King has been received, really good, thank you boss, I wish the boss business prosperity, prosperity, next time to come back to patronize!	Jingdong prefecture level city in Shandong
Energy Saving Technology Shop	In-situ compensated economizer	I've been using it for a while. Than to A little less electricity than before. I'll buy it again next time.	Jingdong prefecture level city in Shandong
Energy Saving Technology Shop	In-situ compensated economizer	Used to be a couple hundred bucks a month. With him, it's a big savings. It's great.	Jingdong prefecture level city in Shandong
Energy Saving Technology Shop	In-situ compensated economizer	Previously bought a plug in the kitchen, used for a period of time after the personal feeling is still quite effective, can save a little electricity, so this time without hesitation and bought two, has been inserted in the home next to the appliances that use a lot of electricity!	Jingdong prefecture level city in Shandong
Energy Saving Technology Shop	In-situ compensated economizer	I've used it before and it does have a power saving effect	Jingdong prefecture level city in Shandong
Energy Saving Technology Shop	In-situ compensated economizer	High-tech products, really useful, in previous years the high cost of electricity, with this product, the electricity bill came down. Praise a, hope that the research and development of this product of science and technology to continue to work hard, research out better products to.	Jingdong prefecture level city in Shandong
Energy Saving Technology Shop	In-situ compensated economizer	This high-power is not bad Oh, the first few months are 1000 degrees so, I casually brush in Taobao to this product, I bought back to try, I used a month after this month left 736 degrees, scared me, I also called to the grid to confirm, really is 736 degrees, the effect is very good, and today bought a go back to the old family to use	Jingdong prefecture level city in Shandong
Energy Saving Technology Shop	In-situ compensated economizer	Used for a period of time, you do not say can really save electricity, really is a small power-saving helper, it is worth buying!	Jingdong prefecture level city in Shandong
Energy Saving Technology Shop	In-situ compensated economizer	There are many rooms and it takes a lot of electricity to run the air conditioner when the house is cold. Bought 4 inserted half a month to save me more than 300 dollars. The effect is really core good! Quality is also very good, the seller service is very good and patient.	Jingdong prefecture level city in Shandong
Energy Saving Technology Shop	In-situ compensated economizer	Recommended by the owner of the store next door, looked at his home does have to save electricity, decided to get. Indeed quite can, the voltage is much more stable, there is a reduction in electricity	Jingdong prefecture level city in Shandong
Energy Saving Technology Shop	In-situ compensated economizer	It does save electricity and works pretty well. I feel that in a few days, I will be able to use the money that I bought to save money, it's really good!	Jingdong prefecture level city in Shandong
Energy Saving Technology Shop	In-situ compensated economizer	Small store service attitude is very good Product quality is very good Suggested purchase	Jingdong prefecture level city in Shandong
Energy Saving Technology Shop	In-situ compensated economizer	Bought two energy-saving appliances for the family, the kitchen and bathroom water heater is too expensive, the water heater to be turned on every day, the monthly electricity bill is a lot of use, with this, no longer afraid of the electricity bill is more than one month down the electricity bill can be less than half of the previous month it, the effect is particularly good!	Jingdong prefecture level city in Shandong
Energy Saving Technology Shop	In-situ compensated economizer	Saving power is really good to use, the last time I bought 3, the family used , before the family monthly electricity bills are more than 300 fast, after using this are more than 200, are several months, this time to my store is also arranged, it is really good for the people good things.	Jingdong prefecture level city in Shandong
Energy Saving Technology Shop	In-situ compensated economizer	The previous year bought a, almost every month can save a 70 dollars or so, with a year of no problem at all, look like new, this time to move to a new home decisively buy back 2 units, very believe this store!	Jingdong prefecture level city in Shandong
Energy Saving Technology Shop	In-situ compensated economizer	Power-saving effect: used a week to comment, before and now record the meter comparison, my one rental room can save twenty degrees of electricity per day. Cost-effective. Quite worth it.	Jingdong prefecture level city in Shandong
Energy Saving Technology Shop	In-situ compensated economizer	This power saver is really powerful, used to spend 300 electricity bill every month, now it's about 100	Jingdong prefecture level city in Shandong
Energy Saving Technology Shop	In-situ compensated economizer	After two days of testing. The effect is good! About the first day of power saving for 1/4. the second day for 1/3. and not "steal electricity", is through the intelligent integrated circuit, automatically reduce the loss of electrical appliances to achieve the purpose of saving electricity! Recommended	Jingdong prefecture level city in Shandong
Energy Saving Technology Shop	In-situ compensated economizer	Power Saving King has been received, really good, thank you boss, I wish the boss business prosperity, prosperity, next time to come back to patronize!	Jingdong prefecture level city in Shandong
Energy Saving Technology Shop	In-situ compensated economizer	I've been using it for a while. Than to A little less electricity than before. I'll buy it again next time.	Jingdong prefecture level city in Shandong
Energy Saving Technology Shop	In-situ compensated economizer	Used to be a couple hundred bucks a month. With him, it's a big savings. It's great.	Jingdong prefecture level city in Shandong
Energy Saving Technology Shop	In-situ compensated economizer	Previously bought a plug in the kitchen, used for a period of time after the personal feeling is still quite effective, can save a little electricity, so this time without hesitation and bought two, has been inserted in the home next to the appliances that use a lot of electricity!	Jingdong prefecture level city in Shandong
Energy Saving Technology Shop	In-situ compensated economizer	I've used it before and it does have a power saving effect	Jingdong prefecture level city in Shandong
Energy Saving Technology Shop	In-situ compensated economizer	High-tech products, really useful, in previous years the high cost of electricity, with this product, the electricity bill came down. Praise a, hope that the research and development of this product of science and technology to continue to work hard, research out better products to.	Jingdong prefecture level city in Shandong
Energy Saving Technology Shop	In-situ compensated economizer	This high-power is not bad Oh, the first few months are 1000 degrees so, I casually brush in Taobao to this product, I bought back to try, I used a month after this month left 736 degrees, scared me, I also called to the grid to confirm, really is 736 degrees, the effect is very good, and today bought a go back to the old family to use	Jingdong prefecture level city in Shandong
Energy Saving Technology Shop	In-situ compensated economizer	Used for a period of time, you do not say can really save electricity, really is a small power-saving helper, it is worth buying!	Jingdong prefecture level city in Shandong
Energy Saving Technology Shop	In-situ compensated economizer	There are many rooms and it takes a lot of electricity to run the air conditioner when the house is cold. Bought 4 inserted half a month to save me more than 300 dollars. The effect is really core good! Quality is also very good, the seller service is very good and patient.	Jingdong prefecture level city in Shandong
Energy Saving Technology Shop	In-situ compensated economizer	Recommended by the owner of the store next door, looked at his home does have to save electricity, decided to get. Indeed quite can, the voltage is much more stable, there is a reduction in electricity	Jingdong prefecture level city in Shandong
Energy Saving Technology Shop	In-situ compensated economizer	It does save electricity and works pretty well. I feel that in a few days, I will be able to use the money that I bought to save money, it's really good!	Jingdong prefecture level city in Shandong
Energy Saving Technology Shop	In-situ compensated economizer	Small store service attitude is very good Product quality is very good Suggested purchase	Jingdong prefecture level city in Shandong
Energy Saving Technology Shop	In-situ compensated economizer	Bought two energy-saving appliances for the family, the kitchen and bathroom water heater is too expensive, the water heater to be turned on every day, the monthly electricity bill is a lot of use, with this, no longer afraid of the electricity bill is more than one month down the electricity bill can be less than half of the previous month it, the effect is particularly good!	Jingdong prefecture level city in Shandong
Energy Saving Technology Shop	In-situ compensated economizer	Saving power is really good to use, the last time I bought 3, the family used , before the family monthly electricity bills are more than 300 fast, after using this are more than 200, are several months, this time to my store is also arranged, it is really good for the people good things.	Jingdong prefecture level city in Shandong
Energy Saving Technology Shop	In-situ compensated economizer	The previous year bought a, almost every month can save a 70 dollars or so, with a year of no problem at all, look like new, this time to move to a new home decisively buy back 2 units, very believe this store!	Jingdong prefecture level city in Shandong
Energy Saving Technology Shop	In-situ compensated economizer	Power-saving effect: used a week to comment, before and now record the meter comparison, my one rental room can save twenty degrees of electricity per day. Cost-effective. Quite worth it.	Jingdong prefecture level city in Shandong
Energy Saving Technology Shop	In-situ compensated economizer	This power saver is really powerful, used to spend 300 electricity bill every month, now it's about 100	Jingdong prefecture level city in Shandong

Energy Saving Technology Shop	In-situ compensated economizer	After two days of testing. The effect is good! About the first day of power saving for 1/4. the second day for 1/3. and not "steal electricity", is through the intelligent integrated circuit, automatically reduce the loss of electrical appliances to achieve the purpose of saving electricity! Recommended	Jingdong prefecture level city in Shandong
Energy Saving Technology Shop	In-situ compensated economizer	Power Saving King has been received, really good, thank you boss, I wish the boss business prosperity, prosperity, next time to come back to patronize!	Jingdong prefecture level city in Shandong
Energy Saving Technology Shop	In-situ compensated economizer	I've been using it for a while. Than to A little less electricity than before. I'll buy it again next time.	Jingdong prefecture level city in Shandong
Energy Saving Technology Shop	In-situ compensated economizer	Used to be a couple hundred bucks a month. With him, it's a big savings. It's great.	Jingdong prefecture level city in Shandong
Energy Saving Technology Shop	In-situ compensated economizer	Previously bought a plug in the kitchen, used for a period of time after the personal feeling is still quite effective, can save a little electricity, so this time without hesitation and bought two, has been inserted in the home next to the appliances that use a lot of electricity!	Jingdong prefecture level city in Shandong
Energy Saving Technology Shop	In-situ compensated economizer	I've used it before and it does have a power saving effect	Jingdong prefecture level city in Shandong
Energy Saving Technology Shop	In-situ compensated economizer	High-tech products, really useful, in previous years the high cost of electricity, with this product, the electricity bill came down. Praise a, hope that the research and development of this product of science and technology to continue to work hard, research out better products to.	Jingdong prefecture level city in Shandong
Energy Saving Technology Shop	In-situ compensated economizer	This high-power is not bad Oh, the first few months are 1000 degrees so, I casually brush in Taobao to this product, I bought back to try, I used a month after this month left 736 degrees, scared me, I also called to the grid to confirm, really is 736 degrees, the effect is very good, and today bought a go back to the old family to use	Jingdong prefecture level city in Shandong
Energy Saving Technology Shop	In-situ compensated economizer	Used for a period of time, you do not say can really save electricity, really is a small power-saving helper, it is worth buying!	Jingdong prefecture level city in Shandong
Energy Saving Technology Shop	In-situ compensated economizer	There are many rooms and it takes a lot of electricity to run the air conditioner when the house is cold. Bought 4 inserted half a month to save me more than 300 dollars. The effect is really core good! Quality is also very good, the seller service is very good and patient.	Jingdong prefecture level city in Shandong
Energy Saving Technology Shop	In-situ compensated economizer	Recommended by the owner of the store next door, looked at his home does have to save electricity, decided to get. Indeed quite can, the voltage is much more stable, there is a reduction in electricity	Jingdong prefecture level city in Shandong
Energy Saving Technology Shop	In-situ compensated economizer	It does save electricity and works pretty well. I feel that in a few days, I will be able to use the money that I bought to save money, it's really good!	Jingdong prefecture level city in Shandong
Energy Saving Technology Shop	In-situ compensated economizer	Small store service attitude is very good Product quality is very good Suggested purchase	Jingdong prefecture level city in Shandong
Energy Saving Technology Shop	In-situ compensated economizer	Bought two energy-saving appliances for the family, the kitchen and bathroom water heater is too expensive, the water heater to be turned on every day, the monthly electricity bill is a lot of use, with this, no longer afraid of the electricity bill is more than one month down the electricity bill can be less than half of the previous month it, the effect is particularly good!	Jingdong prefecture level city in Shandong
Energy Saving Technology Shop	In-situ compensated economizer	Saving power is really good to use, the last time I bought 3, the family used , before the family monthly electricity bills are more than 300 fast, after using this are more than 200, are several months, this time to my store is also arranged, it is really good for the people good things.	Jingdong prefecture level city in Shandong
Energy Saving Technology Shop	In-situ compensated economizer	The previous year bought a, almost every month can save a 70 dollars or so, with a year of no problem at all, look like new, this time to move to a new home decisively buy back 2 units, very believe this store!	Jingdong prefecture level city in Shandong
Energy Saving Technology Shop	In-situ compensated economizer	Power-saving effect: used a week to comment, before and now record the meter comparison, my one rental room can save twenty degrees of electricity per day. Cost-effective. Quite worth it.	Jingdong prefecture level city in Shandong
Energy Saving Technology Shop	In-situ compensated economizer	This power saver is really powerful, used to spend 300 electricity bill every month, now it's about 100	Jingdong prefecture level city in Shandong
Energy Saving Technology Shop	In-situ compensated economizer	After two days of testing. The effect is good! About the first day of power saving for 1/4. the second day for 1/3. and not "steal electricity", is through the intelligent integrated circuit, automatically reduce the loss of electrical appliances to achieve the purpose of saving electricity! Recommended	Jingdong prefecture level city in Shandong
Energy Saving Technology Shop	In-situ compensated economizer	Power Saving King has been received, really good, thank you boss, I wish the boss business prosperity, prosperity, next time to come back to patronize!	Jingdong prefecture level city in Shandong
Energy Saving Technology Shop	In-situ compensated economizer	I've been using it for a while. Than to A little less electricity than before. I'll buy it again next time.	Jingdong prefecture level city in Shandong
Energy Saving Technology Shop	In-situ compensated economizer	Used to be a couple hundred bucks a month. With him, it's a big savings. It's great.	Jingdong prefecture level city in Shandong
Energy Saving Technology Shop	In-situ compensated economizer	Previously bought a plug in the kitchen, used for a period of time after the personal feeling is still quite effective, can save a little electricity, so this time without hesitation and bought two, has been inserted in the home next to the appliances that use a lot of electricity!	Jingdong prefecture level city in Shandong
Energy Saving Technology Shop	In-situ compensated economizer	I've used it before and it does have a power saving effect	Jingdong prefecture level city in Shandong
Energy Saving Technology Shop	In-situ compensated economizer	High-tech products, really useful, in previous years the high cost of electricity, with this product, the electricity bill came down. Praise a, hope that the research and development of this product of science and technology to continue to work hard, research out better products to.	Jingdong prefecture level city in Shandong
Energy Saving Technology Shop	In-situ compensated economizer	This high-power is not bad Oh, the first few months are 1000 degrees so, I casually brush in Taobao to this product, I bought back to try, I used a month after this month left 736 degrees, scared me, I also called to the grid to confirm, really is 736 degrees, the effect is very good, and today bought a go back to the old family to use	Jingdong prefecture level city in Shandong
Energy Saving Technology Shop	In-situ compensated economizer	Used for a period of time, you do not say can really save electricity, really is a small power-saving helper, it is worth buying!	Jingdong prefecture level city in Shandong
Energy Saving Technology Shop	In-situ compensated economizer	There are many rooms and it takes a lot of electricity to run the air conditioner when the house is cold. Bought 4 inserted half a month to save me more than 300 dollars. The effect is really core good! Quality is also very good, the seller service is very good and patient.	Jingdong prefecture level city in Shandong
Energy Saving Technology Shop	In-situ compensated economizer	Recommended by the owner of the store next door, looked at his home does have to save electricity, decided to get. Indeed quite can, the voltage is much more stable, there is a reduction in electricity	Jingdong prefecture level city in Shandong
Energy Saving Technology Shop	In-situ compensated economizer	It does save electricity and works pretty well. I feel that in a few days, I will be able to use the money that I bought to save money, it's really good!	Jingdong prefecture level city in Shandong
Energy Saving Technology Shop	In-situ compensated economizer	Small store service attitude is very good Product quality is very good Suggested purchase	Jingdong prefecture level city in Shandong
Energy Saving Technology Shop	In-situ compensated economizer	Bought two energy-saving appliances for the family, the kitchen and bathroom water heater is too expensive, the water heater to be turned on every day, the monthly electricity bill is a lot of use, with this, no longer afraid of the electricity bill is more than one month down the electricity bill can be less than half of the previous month it, the effect is particularly good!	Jingdong prefecture level city in Shandong
Energy Saving Technology Shop	In-situ compensated economizer	Saving power is really good to use, the last time I bought 3, the family used , before the family monthly electricity bills are more than 300 fast, after using this are more than 200, are several months, this time to my store is also arranged, it is really good for the people good things.	Jingdong prefecture level city in Shandong
Energy Saving Technology Shop	In-situ compensated economizer	The previous year bought a, almost every month can save a 70 dollars or so, with a year of no problem at all, look like new, this time to move to a new home decisively buy back 2 units, very believe this store!	Jingdong prefecture level city in Shandong
Energy Saving Technology Shop	In-situ compensated economizer	Power-saving effect: used a week to comment, before and now record the meter comparison, my one rental room can save twenty degrees of electricity per day. Cost-effective. Quite worth it.	Jingdong prefecture level city in Shandong
Energy Saving Technology Shop	In-situ compensated economizer	This power saver is really powerful, used to spend 300 electricity bill every month, now it's about 100	Jingdong prefecture level city in Shandong
Energy Saving Technology Shop	In-situ compensated economizer	After two days of testing. The effect is good! About the first day of power saving for 1/4. the second day for 1/3. and not "steal electricity", is through the intelligent integrated circuit, automatically reduce the loss of electrical appliances to achieve the purpose of saving electricity! Recommended	Jingdong prefecture level city in Shandong
Energy Saving Technology Shop	In-situ compensated economizer	Power Saving King has been received, really good, thank you boss, I wish the boss business prosperity, prosperity, next time to come back to patronize!	Jingdong prefecture level city in Shandong
Energy Saving Technology Shop	In-situ compensated economizer	I've been using it for a while. Than to A little less electricity than before. I'll buy it again next time.	Jingdong prefecture level city in Shandong
Energy Saving Technology Shop	In-situ compensated economizer	Used to be a couple hundred bucks a month. With him, it's a big savings. It's great.	Jingdong prefecture level city in Shandong
Energy Saving Technology Shop	In-situ compensated economizer	Previously bought a plug in the kitchen, used for a period of time after the personal feeling is still quite effective, can save a little electricity, so this time without hesitation and bought two, has been inserted in the home next to the appliances that use a lot of electricity!	Jingdong prefecture level city in Shandong

Energy Saving Technology Shop	In-situ compensated economizer	I've used it before and it does have a power saving effect	Jingdong prefecture level city in Shandong
Energy Saving Technology Shop	In-situ compensated economizer	High-tech products, really useful, in previous years the high cost of electricity, with this product, the electricity bill came down. Praise a, hope that the research and development of this product of science and technology to continue to work hard, research out better products to.	Jingdong prefecture level city in Shandong
Energy Saving Technology Shop	In-situ compensated economizer	This high-power is not bad Oh, the first few months are 1000 degrees so, I casually brush in Taobao to this product, I bought back to try, I used a month after this month left 736 degrees, scared me, I also called to the grid to confirm, really is 736 degrees, the effect is very good, and today bought a go back to the old family to use	Jingdong prefecture level city in Shandong
Energy Saving Technology Shop	In-situ compensated economizer	Used for a period of time, you do not say can really save electricity, really is a small power-saving helper, it is worth buying!	Jingdong prefecture level city in Shandong
Energy Saving Technology Shop	In-situ compensated economizer	There are many rooms and it takes a lot of electricity to run the air conditioner when the house is cold. Bought 4 inserted half a month to save me more than 300 dollars. The effect is really core good! Quality is also very good, the seller service is very good and patient.	Jingdong prefecture level city in Shandong
Energy Saving Technology Shop	In-situ compensated economizer	Recommended by the owner of the store next door, looked at his home does have to save electricity, decided to get. Indeed quite can, the voltage is much more stable, there is a reduction in electricity	Jingdong prefecture level city in Shandong
Energy Saving Technology Shop	In-situ compensated economizer	It does save electricity and works pretty well. I feel that in a few days, I will be able to use the money that I bought to save money, it's really good!	Jingdong prefecture level city in Shandong
Energy Saving Technology Shop	In-situ compensated economizer	Small store service attitude is very good Product quality is very good Suggested purchase	Jingdong prefecture level city in Shandong
Energy Saving Technology Shop	In-situ compensated economizer	Bought two energy-saving appliances for the family, the kitchen and bathroom water heater is too expensive, the water heater to be turned on every day, the monthly electricity bill is a lot of use, with this, no longer afraid of the electricity bill is more than one month down the electricity bill can be less than half of the previous month it, the effect is particularly good!	Jingdong prefecture level city in Shandong
Energy Saving Technology Shop	In-situ compensated economizer	Saving power is really good to use, the last time I bought 3, the family used , before the family monthly electricity bills are more than 300 fast, after using this are more than 200, are several months, this time to my store is also arranged, it is really good for the people good things.	Jingdong prefecture level city in Shandong
Energy Saving Technology Shop	In-situ compensated economizer	The previous year bought a, almost every month can save a 70 dollars or so, with a year of no problem at all, look like new, this time to move to a new home decisively buy back 2 units, very believe this store!	Jingdong prefecture level city in Shandong
Energy Saving Technology Shop	In-situ compensated economizer	Power-saving effect: used a week to comment, before and now record the meter comparison, my one rental room can save twenty degrees of electricity per day. Cost-effective. Quite worth it.	Jingdong prefecture level city in Shandong
Energy Saving Technology Shop	In-situ compensated economizer	This power saver is really powerful, used to spend 300 electricity bill every month, now it's about 100	Jingdong prefecture level city in Shandong
Energy Saving Technology Shop	In-situ compensated economizer	After two days of testing. The effect is good! About the first day of power saving for 1/4. the second day for 1/3. and not "steal electricity", is through the intelligent integrated circuit, automatically reduce the loss of electrical appliances to achieve the purpose of saving electricity! Recommended	Jingdong prefecture level city in Shandong
Energy Saving Technology Shop	In-situ compensated economizer	Power Saving King has been received, really good, thank you boss, I wish the boss business prosperity, prosperity, next time to come back to patronize!	Jingdong prefecture level city in Shandong
Energy Saving Technology Shop	In-situ compensated economizer	I've been using it for a while. Than to A little less electricity than before. I'll buy it again next time.	Jingdong prefecture level city in Shandong
Energy Saving Technology Shop	In-situ compensated economizer	Used to be a couple hundred bucks a month. With him, it's a big savings. It's great.	Jingdong prefecture level city in Shandong
Energy Saving Technology Shop	In-situ compensated economizer	Previously bought a plug in the kitchen, used for a period of time after the personal feeling is still quite effective, can save a little electricity, so this time without hesitation and bought two, has been inserted in the home next to the appliances that use a lot of electricity!	Jingdong prefecture level city in Shandong
Energy Saving Technology Shop	In-situ compensated economizer	I've used it before and it does have a power saving effect	Jingdong prefecture level city in Shandong
Energy Saving Technology Shop	In-situ compensated economizer	High-tech products, really useful, in previous years the high cost of electricity, with this product, the electricity bill came down. Praise a, hope that the research and development of this product of science and technology to continue to work hard, research out better products to.	Jingdong prefecture level city in Shandong
Energy Saving Technology Shop	In-situ compensated economizer	This high-power is not bad Oh, the first few months are 1000 degrees so, I casually brush in Taobao to this product, I bought back to try, I used a month after this month left 736 degrees, scared me, I also called to the grid to confirm, really is 736 degrees, the effect is very good, and today bought a go back to the old family to use	Jingdong prefecture level city in Shandong
Energy Saving Technology Shop	In-situ compensated economizer	Used for a period of time, you do not say can really save electricity, really is a small power-saving helper, it is worth buying!	Jingdong prefecture level city in Shandong
Energy Saving Technology Shop	In-situ compensated economizer	There are many rooms and it takes a lot of electricity to run the air conditioner when the house is cold. Bought 4 inserted half a month to save me more than 300 dollars. The effect is really core good! Quality is also very good, the seller service is very good and patient.	Jingdong prefecture level city in Shandong
Energy Saving Technology Shop	In-situ compensated economizer	Recommended by the owner of the store next door, looked at his home does have to save electricity, decided to get. Indeed quite can, the voltage is much more stable, there is a reduction in electricity	Jingdong prefecture level city in Shandong
Energy Saving Technology Shop	In-situ compensated economizer	It does save electricity and works pretty well. I feel that in a few days, I will be able to use the money that I bought to save money, it's really good!	Jingdong prefecture level city in Shandong
Energy Saving Technology Shop	In-situ compensated economizer	Small store service attitude is very good Product quality is very good Suggested purchase	Jingdong prefecture level city in Shandong
Energy Saving Technology Shop	In-situ compensated economizer	Bought two energy-saving appliances for the family, the kitchen and bathroom water heater is too expensive, the water heater to be turned on every day, the monthly electricity bill is a lot of use, with this, no longer afraid of the electricity bill is more than one month down the electricity bill can be less than half of the previous month it, the effect is particularly good!	Jingdong prefecture level city in Shandong
Energy Saving Technology Shop	In-situ compensated economizer	Saving power is really good to use, the last time I bought 3, the family used , before the family monthly electricity bills are more than 300 fast, after using this are more than 200, are several months, this time to my store is also arranged, it is really good for the people good things.	Jingdong prefecture level city in Shandong
Energy Saving Technology Shop	In-situ compensated economizer	The previous year bought a, almost every month can save a 70 dollars or so, with a year of no problem at all, look like new, this time to move to a new home decisively buy back 2 units, very believe this store!	Jingdong prefecture level city in Shandong
Energy Saving Technology Shop	In-situ compensated economizer	Power-saving effect: used a week to comment, before and now record the meter comparison, my one rental room can save twenty degrees of electricity per day. Cost-effective. Quite worth it.	Jingdong prefecture level city in Shandong
Energy Saving Technology Shop	In-situ compensated economizer	This power saver is really powerful, used to spend 300 electricity bill every month, now it's about 100	Jingdong prefecture level city in Shandong
Energy Saving Technology Shop	In-situ compensated economizer	After two days of testing. The effect is good! About the first day of power saving for 1/4. the second day for 1/3. and not "steal electricity", is through the intelligent integrated circuit, automatically reduce the loss of electrical appliances to achieve the purpose of saving electricity! Recommended	Jingdong prefecture level city in Shandong
Energy Saving Technology Shop	In-situ compensated economizer	Power Saving King has been received, really good, thank you boss, I wish the boss business prosperity, prosperity, next time to come back to patronize!	Jingdong prefecture level city in Shandong
Energy Saving Technology Shop	In-situ compensated economizer	I've been using it for a while. Than to A little less electricity than before. I'll buy it again next time.	Jingdong prefecture level city in Shandong
Energy Saving Technology Shop	In-situ compensated economizer	Used to be a couple hundred bucks a month. With him, it's a big savings. It's great.	Jingdong prefecture level city in Shandong
Energy Saving Technology Shop	In-situ compensated economizer	Previously bought a plug in the kitchen, used for a period of time after the personal feeling is still quite effective, can save a little electricity, so this time without hesitation and bought two, has been inserted in the home next to the appliances that use a lot of electricity!	Jingdong prefecture level city in Shandong
Energy Saving Technology Shop	In-situ compensated economizer	I've used it before and it does have a power saving effect	Jingdong prefecture level city in Shandong
Energy Saving Technology Shop	In-situ compensated economizer	High-tech products, really useful, in previous years the high cost of electricity, with this product, the electricity bill came down. Praise a, hope that the research and development of this product of science and technology to continue to work hard, research out better products to.	Jingdong prefecture level city in Shandong
Energy Saving Technology Shop	In-situ compensated economizer	Used for a period of time, you do not say can really save electricity, really is a small power-saving helper, it is worth buying!	Jingdong prefecture level city in Shandong
Energy Saving Technology Shop	In-situ compensated economizer	This high-power is not bad Oh, the first few months are 1000 degrees so, I casually brush in Taobao to this product, I bought back to try, I used a month after this month left 736 degrees, scared me, I also called to the grid to confirm, really is 736 degrees, the effect is very good, and today bought a go back to the old family to use	Jingdong prefecture level city in Shandong

Energy Saving Technology Shop	In-situ compensated economizer	There are many rooms and it takes a lot of electricity to run the air conditioner when the house is cold. Bought 4 inserted half a month to save me more than 300 dollars. The effect is really core good! Quality is also very good, the seller service is very good and patient.	Jingdong prefecture level city in Shandong
Energy Saving Technology Shop	In-situ compensated economizer	Recommended by the owner of the store next door, looked at his home does have to save electricity, decided to get. Indeed quite can, the voltage is much more stable, there is a reduction in electricity	Jingdong prefecture level city in Shandong
Energy Saving Technology Shop	In-situ compensated economizer	It does save electricity and works pretty well. I feel that in a few days, I will be able to use the money that I bought to save money, it's really good!	Jingdong prefecture level city in Shandong
Energy Saving Technology Shop	In-situ compensated economizer	Small store service attitude is very good Product quality is very good Suggested purchase	Jingdong prefecture level city in Shandong
Energy Saving Technology Shop	In-situ compensated economizer	Bought two energy-saving appliances for the family, the kitchen and bathroom water heater is too expensive, the water heater to be turned on every day, the monthly electricity bill is a lot of use, with this, no longer afraid of the electricity bill is more than one month down the electricity bill can be less than half of the previous month it, the effect is particularly good!	Jingdong prefecture level city in Shandong
Energy Saving Technology Shop	In-situ compensated economizer	Saving power is really good to use, the last time I bought 3, the family used , before the family monthly electricity bills are more than 300 fast, after using this are more than 200, are several months, this time to my store is also arranged, it is really good for the people good things.	Jingdong prefecture level city in Shandong
Energy Saving Technology Shop	In-situ compensated economizer	The previous year bought a, almost every month can save a 70 dollars or so, with a year of no problem at all, look like new, this time to move to a new home decisively buy back 2 units, very believe this store!	Jingdong prefecture level city in Shandong
Energy Saving Technology Shop	In-situ compensated economizer	Power-saving effect: used a week to comment, before and now record the meter comparison, my one rental room can save twenty degrees of electricity per day. Cost-effective. Quite worth it.	Jingdong prefecture level city in Shandong
Energy Saving Technology Shop	In-situ compensated economizer	This power saver is really powerful, used to spend 300 electricity bill every month, now it's about 100	Jingdong prefecture level city in Shandong
Energy Saving Technology Shop	In-situ compensated economizer	After two days of testing. The effect is good! About the first day of power saving for 1/4. the second day for 1/3. and not "steal electricity", is through the intelligent integrated circuit, automatically reduce the loss of electrical appliances to achieve the purpose of saving electricity! Recommended	Jingdong prefecture level city in Shandong
Energy Saving Technology Shop	In-situ compensated economizer	Power Saving King has been received, really good, thank you boss, I wish the boss business prosperity, prosperity, next time to come back to patronize!	Jingdong prefecture level city in Shandong
Energy Saving Technology Shop	In-situ compensated economizer	I've been using it for a while. Than to A little less electricity than before. I'll buy it again next time.	Jingdong prefecture level city in Shandong
Energy Saving Technology Shop	In-situ compensated economizer	Used to be a couple hundred bucks a month. With him, it's a big savings. It's great.	Jingdong prefecture level city in Shandong
Energy Saving Technology Shop	In-situ compensated economizer	Previously bought a plug in the kitchen, used for a period of time after the personal feeling is still quite effective, can save a little electricity, so this time without hesitation and bought two, has been inserted in the home next to the appliances that use a lot of electricity!	Jingdong prefecture level city in Shandong
Energy Saving Technology Shop	In-situ compensated economizer	I've used it before and it does have a power saving effect	Jingdong prefecture level city in Shandong
Energy Saving Technology Shop	In-situ compensated economizer	High-tech products, really useful, in previous years the high cost of electricity, with this product, the electricity bill came down. Praise a, hope that the research and development of this product of science and technology to continue to work hard, research out better products to.	Jingdong prefecture level city in Shandong
Energy Saving Technology Shop	In-situ compensated economizer	This high-power is not bad Oh, the first few months are 1000 degrees so, I casually brush in Taobao to this product, I bought back to try, I used a month after this month left 736 degrees, scared me, I also called to the grid to confirm, really is 736 degrees, the effect is very good, and today bought a go back to the old family to use	Jingdong prefecture level city in Shandong
Energy Saving Technology Shop	In-situ compensated economizer	Used for a period of time, you do not say can really save electricity, really is a small power-saving helper, it is worth buying!	Jingdong prefecture level city in Shandong
Energy Saving Technology Shop	In-situ compensated economizer	There are many rooms and it takes a lot of electricity to run the air conditioner when the house is cold. Bought 4 inserted half a month to save me more than 300 dollars. The effect is really core good! Quality is also very good, the seller service is very good and patient.	Jingdong prefecture level city in Shandong
Energy Saving Technology Shop	In-situ compensated economizer	Recommended by the owner of the store next door, looked at his home does have to save electricity, decided to get. Indeed quite can, the voltage is much more stable, there is a reduction in electricity	Jingdong prefecture level city in Shandong
Energy Saving Technology Shop	In-situ compensated economizer	It does save electricity and works pretty well. I feel that in a few days, I will be able to use the money that I bought to save money, it's really good!	Jingdong prefecture level city in Shandong
Energy Saving Technology Shop	In-situ compensated economizer	Small store service attitude is very good Product quality is very good Suggested purchase	Jingdong prefecture level city in Shandong
Energy Saving Technology Shop	In-situ compensated economizer	Bought two energy-saving appliances for the family, the kitchen and bathroom water heater is too expensive, the water heater to be turned on every day, the monthly electricity bill is a lot of use, with this, no longer afraid of the electricity bill is more than one month down the electricity bill can be less than half of the previous month it, the effect is particularly good!	Jingdong prefecture level city in Shandong
Energy Saving Technology Shop	In-situ compensated economizer	Saving power is really good to use, the last time I bought 3, the family used , before the family monthly electricity bills are more than 300 fast, after using this are more than 200, are several months, this time to my store is also arranged, it is really good for the people good things.	Jingdong prefecture level city in Shandong
Energy Saving Technology Shop	In-situ compensated economizer	The previous year bought a, almost every month can save a 70 dollars or so, with a year of no problem at all, look like new, this time to move to a new home decisively buy back 2 units, very believe this store!	Jingdong prefecture level city in Shandong
Energy Saving Technology Shop	In-situ compensated economizer	Power-saving effect: used a week to comment, before and now record the meter comparison, my one rental room can save twenty degrees of electricity per day. Cost-effective. Quite worth it.	Jingdong prefecture level city in Shandong
Energy Saving Technology Shop	In-situ compensated economizer	This power saver is really powerful, used to spend 300 electricity bill every month, now it's about 100	Jingdong prefecture level city in Shandong
Energy Saving Technology Shop	In-situ compensated economizer	After two days of testing. The effect is good! About the first day of power saving for 1/4. the second day for 1/3. and not "steal electricity", is through the intelligent integrated circuit, automatically reduce the loss of electrical appliances to achieve the purpose of saving electricity! Recommended	Jingdong prefecture level city in Shandong
Energy Saving Technology Shop	In-situ compensated economizer	Power Saving King has been received, really good, thank you boss, I wish the boss business prosperity, prosperity, next time to come back to patronize!	Jingdong prefecture level city in Shandong
Energy Saving Technology Shop	In-situ compensated economizer	I've been using it for a while. Than to A little less electricity than before. I'll buy it again next time.	Jingdong prefecture level city in Shandong
Energy Saving Technology Shop	In-situ compensated economizer	Used to be a couple hundred bucks a month. With him, it's a big savings. It's great.	Jingdong prefecture level city in Shandong
Energy Saving Technology Shop	In-situ compensated economizer	Previously bought a plug in the kitchen, used for a period of time after the personal feeling is still quite effective, can save a little electricity, so this time without hesitation and bought two, has been inserted in the home next to the appliances that use a lot of electricity!	Jingdong prefecture level city in Shandong
Energy Saving Technology Shop	In-situ compensated economizer	I've used it before and it does have a power saving effect	Jingdong prefecture level city in Shandong
Energy Saving Technology Shop	In-situ compensated economizer	High-tech products, really useful, in previous years the high cost of electricity, with this product, the electricity bill came down. Praise a, hope that the research and development of this product of science and technology to continue to work hard, research out better products to.	Jingdong prefecture level city in Shandong
Energy Saving Technology Shop	In-situ compensated economizer	This high-power is not bad Oh, the first few months are 1000 degrees so, I casually brush in Taobao to this product, I bought back to try, I used a month after this month left 736 degrees, scared me, I also called to the grid to confirm, really is 736 degrees, the effect is very good, and today bought a go back to the old family to use	Jingdong prefecture level city in Shandong
Energy Saving Technology Shop	In-situ compensated economizer	Used for a period of time, you do not say can really save electricity, really is a small power-saving helper, it is worth buying!	Jingdong prefecture level city in Shandong
Energy Saving Technology Shop	In-situ compensated economizer	There are many rooms and it takes a lot of electricity to run the air conditioner when the house is cold. Bought 4 inserted half a month to save me more than 300 dollars. The effect is really core good! Quality is also very good, the seller service is very good and patient.	Jingdong prefecture level city in Shandong
Energy Saving Technology Shop	In-situ compensated economizer	Recommended by the owner of the store next door, looked at his home does have to save electricity, decided to get. Indeed quite can, the voltage is much more stable, there is a reduction in electricity	Jingdong prefecture level city in Shandong
Energy Saving Technology Shop	In-situ compensated economizer	It does save electricity and works pretty well. I feel that in a few days, I will be able to use the money that I bought to save money, it's really good!	Jingdong prefecture level city in Shandong
Energy Saving Technology Shop	In-situ compensated economizer	Small store service attitude is very good Product quality is very good Suggested purchase	Jingdong prefecture level city in Shandong
Energy Saving Technology Shop	In-situ compensated economizer	Bought two energy-saving appliances for the family, the kitchen and bathroom water heater is too expensive, the water heater to be turned on every day, the monthly electricity bill is a lot of use, with this, no longer afraid of the electricity bill is more than one month down the electricity bill can be less than half of the previous month it, the effect is particularly good!	Jingdong prefecture level city in Shandong

Energy Saving Technology Shop	In-situ compensated economizer	Saving power is really good to use, the last time I bought 3, the family used , before the family monthly electricity bills are more than 300 fast, after using this are more than 200, are several months, this time to my store is also arranged, it is really good for the people good things.	Jingdong prefecture level city in Shandong
Energy Saving Technology Shop	In-situ compensated economizer	The previous year bought a, almost every month can save a 70 dollars or so, with a year of no problem at all, look like new, this time to move to a new home decisively buy back 2 units, very believe this store!	Jingdong prefecture level city in Shandong
Energy Saving Technology Shop	In-situ compensated economizer	Power-saving effect: used a week to comment, before and now record the meter comparison, my one rental room can save twenty degrees of electricity per day. Cost-effective. Quite worth it.	Jingdong prefecture level city in Shandong
Energy Saving Technology Shop	In-situ compensated economizer	This power saver is really powerful, used to spend 300 electricity bill every month, now it's about 100	Jingdong prefecture level city in Shandong
Energy Saving Technology Shop	In-situ compensated economizer	After two days of testing. The effect is good! About the first day of power saving for 1/4. the second day for 1/3. and not "steal electricity", is through the intelligent integrated circuit, automatically reduce the loss of electrical appliances to achieve the purpose of saving electricity! Recommended	Jingdong prefecture level city in Shandong
Energy Saving Technology Shop	In-situ compensated economizer	Power Saving King has been received, really good, thank you boss, I wish the boss business prosperity, prosperity, next time to come back to patronize!	Jingdong prefecture level city in Shandong
Energy Saving Technology Shop	In-situ compensated economizer	I've been using it for a while. Than to A little less electricity than before. I'll buy it again next time.	Jingdong prefecture level city in Shandong
Energy Saving Technology Shop	In-situ compensated economizer	Used to be a couple hundred bucks a month. With him, it's a big savings. It's great.	Jingdong prefecture level city in Shandong
Energy Saving Technology Shop	In-situ compensated economizer	Previously bought a plug in the kitchen, used for a period of time after the personal feeling is still quite effective, can save a little electricity, so this time without hesitation and bought two, has been inserted in the home next to the appliances that use a lot of electricity!	Jingdong prefecture level city in Shandong
Energy Saving Technology Shop	In-situ compensated economizer	I've used it before and it does have a power saving effect	Jingdong prefecture level city in Shandong
Energy Saving Technology Shop	In-situ compensated economizer	High-tech products, really useful, in previous years the high cost of electricity, with this product, the electricity bill came down. Praise a, hope that the research and development of this product of science and technology to continue to work hard, research out better products to.	Jingdong prefecture level city in Shandong
Energy Saving Technology Shop	In-situ compensated economizer	This high-power is not bad Oh, the first few months are 1000 degrees so, I casually brush in Taobao to this product, I bought back to try, I used a month after this month left 736 degrees, scared me, I also called to the grid to confirm, really is 736 degrees, the effect is very good, and today bought a go back to the old family to use	Jingdong prefecture level city in Shandong
Energy Saving Technology Shop	In-situ compensated economizer	Used for a period of time, you do not say can really save electricity, really is a small power-saving helper, it is worth buying!	Jingdong prefecture level city in Shandong
Energy Saving Technology Shop	In-situ compensated economizer	There are many rooms and it takes a lot of electricity to run the air conditioner when the house is cold. Bought 4 inserted half a month to save me more than 300 dollars. The effect is really core good! Quality is also very good, the seller service is very good and patient.	Jingdong prefecture level city in Shandong
Energy Saving Technology Shop	In-situ compensated economizer	Recommended by the owner of the store next door, looked at his home does have to save electricity, decided to get. Indeed quite can, the voltage is much more stable, there is a reduction in electricity	Jingdong prefecture level city in Shandong
Energy Saving Technology Shop	In-situ compensated economizer	It does save electricity and works pretty well. I feel that in a few days, I will be able to use the money that I bought to save money, it's really good!	Jingdong prefecture level city in Shandong
Energy Saving Technology Shop	In-situ compensated economizer	Small store service attitude is very good Product quality is very good Suggested purchase	Jingdong prefecture level city in Shandong
Energy Saving Technology Shop	In-situ compensated economizer	Bought two energy-saving appliances for the family, the kitchen and bathroom water heater is too expensive, the water heater to be turned on every day, the monthly electricity bill is a lot of use, with this, no longer afraid of the electricity bill is more than one month down the electricity bill can be less than half of the previous month it, the effect is particularly good!	Jingdong prefecture level city in Shandong
Energy Saving Technology Shop	In-situ compensated economizer	Saving power is really good to use, the last time I bought 3, the family used , before the family monthly electricity bills are more than 300 fast, after using this are more than 200, are several months, this time to my store is also arranged, it is really good for the people good things.	Jingdong prefecture level city in Shandong
Energy Saving Technology Shop	In-situ compensated economizer	The previous year bought a, almost every month can save a 70 dollars or so, with a year of no problem at all, look like new, this time to move to a new home decisively buy back 2 units, very believe this store!	Jingdong prefecture level city in Shandong
Energy Saving Technology Shop	In-situ compensated economizer	Power-saving effect: used a week to comment, before and now record the meter comparison, my one rental room can save twenty degrees of electricity per day. Cost-effective. Quite worth it.	Jingdong prefecture level city in Shandong
Energy Saving Technology Shop	In-situ compensated economizer	This power saver is really powerful, used to spend 300 electricity bill every month, now it's about 100	Jingdong prefecture level city in Shandong
Energy Saving Technology Shop	In-situ compensated economizer	After two days of testing. The effect is good! About the first day of power saving for 1/4. the second day for 1/3. and not "steal electricity", is through the intelligent integrated circuit, automatically reduce the loss of electrical appliances to achieve the purpose of saving electricity! Recommended	Jingdong prefecture level city in Shandong
Energy Saving Technology Shop	In-situ compensated economizer	Power Saving King has been received, really good, thank you boss, I wish the boss business prosperity, prosperity, next time to come back to patronize!	Jingdong prefecture level city in Shandong
Energy Saving Technology Shop	In-situ compensated economizer	I've been using it for a while. Than to A little less electricity than before. I'll buy it again next time.	Jingdong prefecture level city in Shandong
Energy Saving Technology Shop	In-situ compensated economizer	Used to be a couple hundred bucks a month. With him, it's a big savings. It's great.	Jingdong prefecture level city in Shandong
Energy Saving Technology Shop	In-situ compensated economizer	Previously bought a plug in the kitchen, used for a period of time after the personal feeling is still quite effective, can save a little electricity, so this time without hesitation and bought two, has been inserted in the home next to the appliances that use a lot of electricity!	Jingdong prefecture level city in Shandong
Energy Saving Technology Shop	In-situ compensated economizer	I've used it before and it does have a power saving effect	Jingdong prefecture level city in Shandong
Energy Saving Technology Shop	In-situ compensated economizer	High-tech products, really useful, in previous years the high cost of electricity, with this product, the electricity bill came down. Praise a, hope that the research and development of this product of science and technology to continue to work hard, research out better products to.	Jingdong prefecture level city in Shandong
Energy Saving Technology Shop	In-situ compensated economizer	This high-power is not bad Oh, the first few months are 1000 degrees so, I casually brush in Taobao to this product, I bought back to try, I used a month after this month left 736 degrees, scared me, I also called to the grid to confirm, really is 736 degrees, the effect is very good, and today bought a go back to the old family to use	Jingdong prefecture level city in Shandong
Energy Saving Technology Shop	In-situ compensated economizer	Used for a period of time, you do not say can really save electricity, really is a small power-saving helper, it is worth buying!	Jingdong prefecture level city in Shandong
Energy Saving Technology Shop	In-situ compensated economizer	There are many rooms and it takes a lot of electricity to run the air conditioner when the house is cold. Bought 4 inserted half a month to save me more than 300 dollars. The effect is really core good! Quality is also very good, the seller service is very good and patient.	Jingdong prefecture level city in Shandong
Energy Saving Technology Shop	In-situ compensated economizer	Recommended by the owner of the store next door, looked at his home does have to save electricity, decided to get. Indeed quite can, the voltage is much more stable, there is a reduction in electricity	Jingdong prefecture level city in Shandong
Energy Saving Technology Shop	In-situ compensated economizer	It does save electricity and works pretty well. I feel that in a few days, I will be able to use the money that I bought to save money, it's really good!	Jingdong prefecture level city in Shandong
Energy Saving Technology Shop	In-situ compensated economizer	Small store service attitude is very good Product quality is very good Suggested purchase	Jingdong prefecture level city in Shandong
Energy Saving Technology Shop	In-situ compensated economizer	Bought two energy-saving appliances for the family, the kitchen and bathroom water heater is too expensive, the water heater to be turned on every day, the monthly electricity bill is a lot of use, with this, no longer afraid of the electricity bill is more than one month down the electricity bill can be less than half of the previous month it, the effect is particularly good!	Jingdong prefecture level city in Shandong
Energy Saving Technology Shop	In-situ compensated economizer	Saving power is really good to use, the last time I bought 3, the family used , before the family monthly electricity bills are more than 300 fast, after using this are more than 200, are several months, this time to my store is also arranged, it is really good for the people good things.	Jingdong prefecture level city in Shandong
Energy Saving Technology Shop	In-situ compensated economizer	The previous year bought a, almost every month can save a 70 dollars or so, with a year of no problem at all, look like new, this time to move to a new home decisively buy back 2 units, very believe this store!	Jingdong prefecture level city in Shandong
Energy Saving Technology Shop	In-situ compensated economizer	Power-saving effect: used a week to comment, before and now record the meter comparison, my one rental room can save twenty degrees of electricity per day. Cost-effective. Quite worth it.	Jingdong prefecture level city in Shandong
Energy Saving Technology Shop	In-situ compensated economizer	This power saver is really powerful, used to spend 300 electricity bill every month, now it's about 100	Jingdong prefecture level city in Shandong
Energy Saving Technology Shop	In-situ compensated economizer	After two days of testing. The effect is good! About the first day of power saving for 1/4. the second day for 1/3. and not "steal electricity", is through the intelligent integrated circuit, automatically reduce the loss of electrical appliances to achieve the purpose of saving electricity! Recommended	Jingdong prefecture level city in Shandong

Energy Saving Technology Shop	In-situ compensated economizer	Power Saving King has been received, really good, thank you boss, I wish the boss business prosperity, prosperity, next time to come back to patronize!	Jingdong prefecture level city in Shandong
Energy Saving Technology Shop	In-situ compensated economizer	I've been using it for a while. Than to A little less electricity than before. I'll buy it again next time.	Jingdong prefecture level city in Shandong
Energy Saving Technology Shop	In-situ compensated economizer	Used to be a couple hundred bucks a month. With him, it's a big savings. It's great.	Jingdong prefecture level city in Shandong
Energy Saving Technology Shop	In-situ compensated economizer	Previously bought a plug in the kitchen, used for a period of time after the personal feeling is still quite effective, can save a little electricity, so this time without hesitation and bought two, has been inserted in the home next to the appliances that use a lot of electricity!	Jingdong prefecture level city in Shandong
Energy Saving Technology Shop	In-situ compensated economizer	I've used it before and it does have a power saving effect	Jingdong prefecture level city in Shandong
Energy Saving Technology Shop	In-situ compensated economizer	High-tech products, really useful, in previous years the high cost of electricity, with this product, the electricity bill came down. Praise a, hope that the research and development of this product of science and technology to continue to work hard, research out better products to.	Jingdong prefecture level city in Shandong
Energy Saving Technology Shop	In-situ compensated economizer	This high-power is not bad Oh, the first few months are 1000 degrees so, I casually brush in Taobao to this product, I bought back to try, I used a month after this month left 736 degrees, scared me, I also called to the grid to confirm, really is 736 degrees, the effect is very good, and today bought a go back to the old family to use	Jingdong prefecture level city in Shandong
Energy Saving Technology Shop	In-situ compensated economizer	Used for a period of time, you do not say can really save electricity, really is a small power-saving helper, it is worth buying!	Jingdong prefecture level city in Shandong
Energy Saving Technology Shop	In-situ compensated economizer	There are many rooms and it takes a lot of electricity to run the air conditioner when the house is cold. Bought 4 inserted half a month to save me more than 300 dollars. The effect is really core good! Quality is also very good, the seller service is very good and patient.	Jingdong prefecture level city in Shandong
Energy Saving Technology Shop	In-situ compensated economizer	Recommended by the owner of the store next door, looked at his home does have to save electricity, decided to get. Indeed quite can, the voltage is much more stable, there is a reduction in electricity	Jingdong prefecture level city in Shandong
Energy Saving Technology Shop	In-situ compensated economizer	It does save electricity and works pretty well. I feel that in a few days, I will be able to use the money that I bought to save money, it's really good!	Jingdong prefecture level city in Shandong
Energy Saving Technology Shop	In-situ compensated economizer	Small store service attitude is very good Product quality is very good Suggested purchase	Jingdong prefecture level city in Shandong
Energy Saving Technology Shop	In-situ compensated economizer	Bought two energy-saving appliances for the family, the kitchen and bathroom water heater is too expensive, the water heater to be turned on every day, the monthly electricity bill is a lot of use, with this, no longer afraid of the electricity bill is more than one month down the electricity bill can be less than half of the previous month it, the effect is particularly good!	Jingdong prefecture level city in Shandong
Energy Saving Technology Shop	In-situ compensated economizer	Saving power is really good to use, the last time I bought 3, the family used , before the family monthly electricity bills are more than 300 fast, after using this are more than 200, are several months, this time to my store is also arranged, it is really good for the people good things.	Jingdong prefecture level city in Shandong
Energy Saving Technology Shop	In-situ compensated economizer	The previous year bought a, almost every month can save a 70 dollars or so, with a year of no problem at all, look like new, this time to move to a new home decisively buy back 2 units, very believe this store!	Jingdong prefecture level city in Shandong
Energy Saving Technology Shop	In-situ compensated economizer	Power-saving effect: used a week to comment, before and now record the meter comparison, my one rental room can save twenty degrees of electricity per day. Cost-effective. Quite worth it.	Jingdong prefecture level city in Shandong
Energy Saving Technology Shop	In-situ compensated economizer	This power saver is really powerful, used to spend 300 electricity bill every month, now it's about 100	Jingdong prefecture level city in Shandong
Energy Saving Technology Shop	In-situ compensated economizer	After two days of testing. The effect is good! About the first day of power saving for 1/4. the second day for 1/3. and not "steal electricity", is through the intelligent integrated circuit, automatically reduce the loss of electrical appliances to achieve the purpose of saving electricity! Recommended	Jingdong prefecture level city in Shandong
Energy Saving Technology Shop	In-situ compensated economizer	Power Saving King has been received, really good, thank you boss, I wish the boss business prosperity, prosperity, next time to come back to patronize!	Jingdong prefecture level city in Shandong
Energy Saving Technology Shop	In-situ compensated economizer	I've been using it for a while. Than to A little less electricity than before. I'll buy it again next time.	Jingdong prefecture level city in Shandong
Energy Saving Technology Shop	In-situ compensated economizer	Used to be a couple hundred bucks a month. With him, it's a big savings. It's great.	Jingdong prefecture level city in Shandong
Energy Saving Technology Shop	In-situ compensated economizer	Previously bought a plug in the kitchen, used for a period of time after the personal feeling is still quite effective, can save a little electricity, so this time without hesitation and bought two, has been inserted in the home next to the appliances that use a lot of electricity!	Jingdong prefecture level city in Shandong
Energy Saving Technology Shop	In-situ compensated economizer	I've used it before and it does have a power saving effect	Jingdong prefecture level city in Shandong
Energy Saving Technology Shop	In-situ compensated economizer	High-tech products, really useful, in previous years the high cost of electricity, with this product, the electricity bill came down. Praise a, hope that the research and development of this product of science and technology to continue to work hard, research out better products to.	Jingdong prefecture level city in Shandong
Energy Saving Technology Shop	In-situ compensated economizer	This high-power is not bad Oh, the first few months are 1000 degrees so, I casually brush in Taobao to this product, I bought back to try, I used a month after this month left 736 degrees, scared me, I also called to the grid to confirm, really is 736 degrees, the effect is very good, and today bought a go back to the old family to use	Jingdong prefecture level city in Shandong
Energy Saving Technology Shop	In-situ compensated economizer	Used for a period of time, you do not say can really save electricity, really is a small power-saving helper, it is worth buying!	Jingdong prefecture level city in Shandong
Energy Saving Technology Shop	In-situ compensated economizer	There are many rooms and it takes a lot of electricity to run the air conditioner when the house is cold. Bought 4 inserted half a month to save me more than 300 dollars. The effect is really core good! Quality is also very good, the seller service is very good and patient.	Jingdong prefecture level city in Shandong
Energy Saving Technology Shop	In-situ compensated economizer	Recommended by the owner of the store next door, looked at his home does have to save electricity, decided to get. Indeed quite can, the voltage is much more stable, there is a reduction in electricity	Jingdong prefecture level city in Shandong
Energy Saving Technology Shop	In-situ compensated economizer	It does save electricity and works pretty well. I feel that in a few days, I will be able to use the money that I bought to save money, it's really good!	Jingdong prefecture level city in Shandong
Energy Saving Technology Shop	In-situ compensated economizer	Small store service attitude is very good Product quality is very good Suggested purchase	Jingdong prefecture level city in Shandong
Energy Saving Technology Shop	In-situ compensated economizer	Bought two energy-saving appliances for the family, the kitchen and bathroom water heater is too expensive, the water heater to be turned on every day, the monthly electricity bill is a lot of use, with this, no longer afraid of the electricity bill is more than one month down the electricity bill can be less than half of the previous month it, the effect is particularly good!	Jingdong prefecture level city in Shandong
Energy Saving Technology Shop	In-situ compensated economizer	Saving power is really good to use, the last time I bought 3, the family used , before the family monthly electricity bills are more than 300 fast, after using this are more than 200, are several months, this time to my store is also arranged, it is really good for the people good things.	Jingdong prefecture level city in Shandong
Energy Saving Technology Shop	In-situ compensated economizer	The previous year bought a, almost every month can save a 70 dollars or so, with a year of no problem at all, look like new, this time to move to a new home decisively buy back 2 units, very believe this store!	Jingdong prefecture level city in Shandong
Energy Saving Technology Shop	In-situ compensated economizer	Power-saving effect: used a week to comment, before and now record the meter comparison, my one rental room can save twenty degrees of electricity per day. Cost-effective. Quite worth it.	Jingdong prefecture level city in Shandong
Energy Saving Technology Shop	In-situ compensated economizer	This power saver is really powerful, used to spend 300 electricity bill every month, now it's about 100	Jingdong prefecture level city in Shandong
Energy Saving Technology Shop	In-situ compensated economizer	Power Saving King has been received, really good, thank you boss, I wish the boss business prosperity, prosperity, next time to come back to patronize!	Jingdong prefecture level city in Shandong
Energy Saving Technology Shop	In-situ compensated economizer	After two days of testing. The effect is good! About the first day of power saving for 1/4. the second day for 1/3. and not "steal electricity", is through the intelligent integrated circuit, automatically reduce the loss of electrical appliances to achieve the purpose of saving electricity! Recommended	Jingdong prefecture level city in Shandong
Energy Saving Technology Shop	In-situ compensated economizer	I've been using it for a while. Than to A little less electricity than before. I'll buy it again next time.	Jingdong prefecture level city in Shandong
Energy Saving Technology Shop	In-situ compensated economizer	Used to be a couple hundred bucks a month. With him, it's a big savings. It's great.	Jingdong prefecture level city in Shandong
Energy Saving Technology Shop	In-situ compensated economizer	Previously bought a plug in the kitchen, used for a period of time after the personal feeling is still quite effective, can save a little electricity, so this time without hesitation and bought two, has been inserted in the home next to the appliances that use a lot of electricity!	Jingdong prefecture level city in Shandong
Energy Saving Technology Shop	In-situ compensated economizer	I've used it before and it does have a power saving effect	Jingdong prefecture level city in Shandong

Energy Saving Technology Shop	In-situ compensated economizer	High-tech products, really useful, in previous years the high cost of electricity, with this product, the electricity bill came down. Praise a, hope that the research and development of this product of science and technology to continue to work hard, research out better products to.	Jingdong prefecture level city in Shandong
Energy Saving Technology Shop	In-situ compensated economizer	This high-power is not bad Oh, the first few months are 1000 degrees so, I casually brush in Taobao to this product, I bought back to try, I used a month after this month left 736 degrees, scared me, I also called to the grid to confirm, really is 736 degrees, the effect is very good, and today bought a go back to the old family to use	Jingdong prefecture level city in Shandong
Energy Saving Technology Shop	In-situ compensated economizer	Used for a period of time, you do not say can really save electricity, really is a small power-saving helper, it is worth buying!	Jingdong prefecture level city in Shandong
Energy Saving Technology Shop	In-situ compensated economizer	There are many rooms and it takes a lot of electricity to run the air conditioner when the house is cold. Bought 4 inserted half a month to save me more than 300 dollars. The effect is really core good! Quality is also very good, the seller service is very good and patient.	Jingdong prefecture level city in Shandong
Energy Saving Technology Shop	In-situ compensated economizer	Recommended by the owner of the store next door, looked at his home does have to save electricity, decided to get. Indeed quite can, the voltage is much more stable, there is a reduction in electricity	Jingdong prefecture level city in Shandong
Energy Saving Technology Shop	In-situ compensated economizer	It does save electricity and works pretty well. I feel that in a few days, I will be able to use the money that I bought to save money, it's really good!	Jingdong prefecture level city in Shandong
Energy Saving Technology Shop	In-situ compensated economizer	Small store service attitude is very good Product quality is very good Suggested purchase	Jingdong prefecture level city in Shandong
Energy Saving Technology Shop	In-situ compensated economizer	Bought two energy-saving appliances for the family, the kitchen and bathroom water heater is too expensive, the water heater to be turned on every day, the monthly electricity bill is a lot of use, with this, no longer afraid of the electricity bill is more than one month down the electricity bill can be less than half of the previous month it, the effect is particularly good!	Jingdong prefecture level city in Shandong
Energy Saving Technology Shop	In-situ compensated economizer	Saving power is really good to use, the last time I bought 3, the family used , before the family monthly electricity bills are more than 300 fast, after using this are more than 200, are several months, this time to my store is also arranged, it is really good for the people good things.	Jingdong prefecture level city in Shandong
Energy Saving Technology Shop	In-situ compensated economizer	Power-saving effect: used a week to comment, before and now record the meter comparison, my one rental room can save twenty degrees of electricity per day. Cost-effective. Quite worth it.	Jingdong prefecture level city in Shandong
Energy Saving Technology Shop	In-situ compensated economizer	The previous year bought a, almost every month can save a 70 dollars or so, with a year of no problem at all, look like new, this time to move to a new home decisively buy back 2 units, very believe this store!	Jingdong prefecture level city in Shandong
Energy Saving Technology Shop	In-situ compensated economizer	This power saver is really powerful, used to spend 300 electricity bill every month, now it's about 100	Jingdong prefecture level city in Shandong
Energy Saving Technology Shop	In-situ compensated economizer	After two days of testing. The effect is good! About the first day of power saving for 1/4. the second day for 1/3. and not "steal electricity", is through the intelligent integrated circuit, automatically reduce the loss of electrical appliances to achieve the purpose of saving electricity! Recommended	Jingdong prefecture level city in Shandong
Energy Saving Technology Shop	In-situ compensated economizer	Power Saving King has been received, really good, thank you boss, I wish the boss business prosperity, prosperity, next time to come back to patronize!	Jingdong prefecture level city in Shandong
Energy Saving Technology Shop	In-situ compensated economizer	I've been using it for a while. Than to A little less electricity than before. I'll buy it again next time.	Jingdong prefecture level city in Shandong
Energy Saving Technology Shop	In-situ compensated economizer	Used to be a couple hundred bucks a month. With him, it's a big savings. It's great.	Jingdong prefecture level city in Shandong
Energy Saving Technology Shop	In-situ compensated economizer	Previously bought a plug in the kitchen, used for a period of time after the personal feeling is still quite effective, can save a little electricity, so this time without hesitation and bought two, has been inserted in the home next to the appliances that use a lot of electricity!	Jingdong prefecture level city in Shandong
Energy Saving Technology Shop	In-situ compensated economizer	I've used it before and it does have a power saving effect	Jingdong prefecture level city in Shandong
Energy Saving Technology Shop	In-situ compensated economizer	High-tech products, really useful, in previous years the high cost of electricity, with this product, the electricity bill came down. Praise a, hope that the research and development of this product of science and technology to continue to work hard, research out better products to.	Jingdong prefecture level city in Shandong
Energy Saving Technology Shop	In-situ compensated economizer	This high-power is not bad Oh, the first few months are 1000 degrees so, I casually brush in Taobao to this product, I bought back to try, I used a month after this month left 736 degrees, scared me, I also called to the grid to confirm, really is 736 degrees, the effect is very good, and today bought a go back to the old family to use	Jingdong prefecture level city in Shandong
Energy Saving Technology Shop	In-situ compensated economizer	Used for a period of time, you do not say can really save electricity, really is a small power-saving helper, it is worth buying!	Jingdong prefecture level city in Shandong
Energy Saving Technology Shop	In-situ compensated economizer	There are many rooms and it takes a lot of electricity to run the air conditioner when the house is cold. Bought 4 inserted half a month to save me more than 300 dollars. The effect is really core good! Quality is also very good, the seller service is very good and patient.	Jingdong prefecture level city in Shandong
Energy Saving Technology Shop	In-situ compensated economizer	Recommended by the owner of the store next door, looked at his home does have to save electricity, decided to get. Indeed quite can, the voltage is much more stable, there is a reduction in electricity	Jingdong prefecture level city in Shandong
Energy Saving Technology Shop	In-situ compensated economizer	It does save electricity and works pretty well. I feel that in a few days, I will be able to use the money that I bought to save money, it's really good!	Jingdong prefecture level city in Shandong
Energy Saving Technology Shop	In-situ compensated economizer	Small store service attitude is very good Product quality is very good Suggested purchase	Jingdong prefecture level city in Shandong
Energy Saving Technology Shop	In-situ compensated economizer	Bought two energy-saving appliances for the family, the kitchen and bathroom water heater is too expensive, the water heater to be turned on every day, the monthly electricity bill is a lot of use, with this, no longer afraid of the electricity bill is more than one month down the electricity bill can be less than half of the previous month it, the effect is particularly good!	Jingdong prefecture level city in Shandong
Energy Saving Technology Shop	In-situ compensated economizer	Saving power is really good to use, the last time I bought 3, the family used , before the family monthly electricity bills are more than 300 fast, after using this are more than 200, are several months, this time to my store is also arranged, it is really good for the people good things.	Jingdong prefecture level city in Shandong
Energy Saving Technology Shop	In-situ compensated economizer	The previous year bought a, almost every month can save a 70 dollars or so, with a year of no problem at all, look like new, this time to move to a new home decisively buy back 2 units, very believe this store!	Jingdong prefecture level city in Shandong
Energy Saving Technology Shop	In-situ compensated economizer	Power-saving effect: used a week to comment, before and now record the meter comparison, my one rental room can save twenty degrees of electricity per day. Cost-effective. Quite worth it.	Jingdong prefecture level city in Shandong
Energy Saving Technology Shop	In-situ compensated economizer	This power saver is really powerful, used to spend 300 electricity bill every month, now it's about 100	Jingdong prefecture level city in Shandong
Energy Saving Technology Shop	In-situ compensated economizer	After two days of testing. The effect is good! About the first day of power saving for 1/4. the second day for 1/3. and not "steal electricity", is through the intelligent integrated circuit, automatically reduce the loss of electrical appliances to achieve the purpose of saving electricity! Recommended	Jingdong prefecture level city in Shandong
Energy Saving Technology Shop	In-situ compensated economizer	Power Saving King has been received, really good, thank you boss, I wish the boss business prosperity, prosperity, next time to come back to patronize!	Jingdong prefecture level city in Shandong
Energy Saving Technology Shop	In-situ compensated economizer	I've been using it for a while. Than to A little less electricity than before. I'll buy it again next time.	Jingdong prefecture level city in Shandong
Energy Saving Technology Shop	In-situ compensated economizer	Used to be a couple hundred bucks a month. With him, it's a big savings. It's great.	Jingdong prefecture level city in Shandong
Energy Saving Technology Shop	In-situ compensated economizer	Previously bought a plug in the kitchen, used for a period of time after the personal feeling is still quite effective, can save a little electricity, so this time without hesitation and bought two, has been inserted in the home next to the appliances that use a lot of electricity!	Jingdong prefecture level city in Shandong
Energy Saving Technology Shop	In-situ compensated economizer	I've used it before and it does have a power saving effect	Jingdong prefecture level city in Shandong
Energy Saving Technology Shop	In-situ compensated economizer	High-tech products, really useful, in previous years the high cost of electricity, with this product, the electricity bill came down. Praise a, hope that the research and development of this product of science and technology to continue to work hard, research out better products to.	Jingdong prefecture level city in Shandong
Energy Saving Technology Shop	In-situ compensated economizer	This high-power is not bad Oh, the first few months are 1000 degrees so, I casually brush in Taobao to this product, I bought back to try, I used a month after this month left 736 degrees, scared me, I also called to the grid to confirm, really is 736 degrees, the effect is very good, and today bought a go back to the old family to use	Jingdong prefecture level city in Shandong
Energy Saving Technology Shop	In-situ compensated economizer	Used for a period of time, you do not say can really save electricity, really is a small power-saving helper, it is worth buying!	Jingdong prefecture level city in Shandong
Energy Saving Technology Shop	In-situ compensated economizer	There are many rooms and it takes a lot of electricity to run the air conditioner when the house is cold. Bought 4 inserted half a month to save me more than 300 dollars. The effect is really core good! Quality is also very good, the seller service is very good and patient.	Jingdong prefecture level city in Shandong

Energy Saving Technology Shop	In-situ compensated economizer	Recommended by the owner of the store next door, looked at his home does have to save electricity, decided to get. Indeed quite can, the voltage is much more stable, there is a reduction in electricity	Jingdong prefecture level city in Shandong
Energy Saving Technology Shop	In-situ compensated economizer	It does save electricity and works pretty well. I feel that in a few days, I will be able to use the money that I bought to save money, it's really good!	Jingdong prefecture level city in Shandong
Energy Saving Technology Shop	In-situ compensated economizer	Small store service attitude is very good Product quality is very good Suggested purchase	Jingdong prefecture level city in Shandong
Energy Saving Technology Shop	In-situ compensated economizer	Bought two energy-saving appliances for the family, the kitchen and bathroom water heater is too expensive, the water heater to be turned on every day, the monthly electricity bill is a lot of use, with this, no longer afraid of the electricity bill is more than one month down the electricity bill can be less than half of the previous month it, the effect is particularly good!	Jingdong prefecture level city in Shandong
Energy Saving Technology Shop	In-situ compensated economizer	Saving power is really good to use, the last time I bought 3, the family used , before the family monthly electricity bills are more than 300 fast, after using this are more than 200, are several months, this time to my store is also arranged, it is really good for the people good things.	Jingdong prefecture level city in Shandong
Energy Saving Technology Shop	In-situ compensated economizer	The previous year bought a, almost every month can save a 70 dollars or so, with a year of no problem at all, look like new, this time to move to a new home decisively buy back 2 units, very believe this store!	Jingdong prefecture level city in Shandong
Energy Saving Technology Shop	In-situ compensated economizer	Power-saving effect: used a week to comment, before and now record the meter comparison, my one rental room can save twenty degrees of electricity per day. Cost-effective. Quite worth it.	Jingdong prefecture level city in Shandong
Energy Saving Technology Shop	In-situ compensated economizer	This power saver is really powerful, used to spend 300 electricity bill every month, now it's about 100	Jingdong prefecture level city in Shandong
Energy Saving Technology Shop	In-situ compensated economizer	After two days of testing. The effect is good! About the first day of power saving for 1/4. the second day for 1/3. and not "steal electricity", is through the intelligent integrated circuit, automatically reduce the loss of electrical appliances to achieve the purpose of saving electricity! Recommended	Jingdong prefecture level city in Shandong
Energy Saving Technology Shop	In-situ compensated economizer	Power Saving King has been received, really good, thank you boss, I wish the boss business prosperity, prosperity, next time to come back to patronize!	Jingdong prefecture level city in Shandong
Energy Saving Technology Shop	In-situ compensated economizer	I've been using it for a while. Than to A little less electricity than before. I'll buy it again next time.	Jingdong prefecture level city in Shandong
Energy Saving Technology Shop	In-situ compensated economizer	Used to be a couple hundred bucks a month. With him, it's a big savings. It's great.	Jingdong prefecture level city in Shandong
Energy Saving Technology Shop	In-situ compensated economizer	Previously bought a plug in the kitchen, used for a period of time after the personal feeling is still quite effective, can save a little electricity, so this time without hesitation and bought two, has been inserted in the home next to the appliances that use a lot of electricity!	Jingdong prefecture level city in Shandong
Energy Saving Technology Shop	In-situ compensated economizer	I've used it before and it does have a power saving effect	Jingdong prefecture level city in Shandong
Energy Saving Technology Shop	In-situ compensated economizer	High-tech products, really useful, in previous years the high cost of electricity, with this product, the electricity bill came down. Praise a, hope that the research and development of this product of science and technology to continue to work hard, research out better products to.	Jingdong prefecture level city in Shandong
Energy Saving Technology Shop	In-situ compensated economizer	This high-power is not bad Oh, the first few months are 1000 degrees so, I casually brush in Taobao to this product, I bought back to try, I used a month after this month left 736 degrees, scared me, I also called to the grid to confirm, really is 736 degrees, the effect is very good, and today bought a go back to the old family to use	Jingdong prefecture level city in Shandong
Energy Saving Technology Shop	In-situ compensated economizer	There are many rooms and it takes a lot of electricity to run the air conditioner when the house is cold. Bought 4 inserted half a month to save me more than 300 dollars. The effect is really core good! Quality is also very good, the seller service is very good and patient.	Jingdong prefecture level city in Shandong
Energy Saving Technology Shop	In-situ compensated economizer	Used for a period of time, you do not say can really save electricity, really is a small power-saving helper, it is worth buying!	Jingdong prefecture level city in Shandong
Energy Saving Technology Shop	In-situ compensated economizer	Recommended by the owner of the store next door, looked at his home does have to save electricity, decided to get. Indeed quite can, the voltage is much more stable, there is a reduction in electricity	Jingdong prefecture level city in Shandong
Energy Saving Technology Shop	In-situ compensated economizer	It does save electricity and works pretty well. I feel that in a few days, I will be able to use the money that I bought to save money, it's really good!	Jingdong prefecture level city in Shandong
Energy Saving Technology Shop	In-situ compensated economizer	Small store service attitude is very good Product quality is very good Suggested purchase	Jingdong prefecture level city in Shandong
Energy Saving Technology Shop	In-situ compensated economizer	Bought two energy-saving appliances for the family, the kitchen and bathroom water heater is too expensive, the water heater to be turned on every day, the monthly electricity bill is a lot of use, with this, no longer afraid of the electricity bill is more than one month down the electricity bill can be less than half of the previous month it, the effect is particularly good!	Jingdong prefecture level city in Shandong
Energy Saving Technology Shop	In-situ compensated economizer	Saving power is really good to use, the last time I bought 3, the family used , before the family monthly electricity bills are more than 300 fast, after using this are more than 200, are several months, this time to my store is also arranged, it is really good for the people good things.	Jingdong prefecture level city in Shandong
Energy Saving Technology Shop	In-situ compensated economizer	The previous year bought a, almost every month can save a 70 dollars or so, with a year of no problem at all, look like new, this time to move to a new home decisively buy back 2 units, very believe this store!	Jingdong prefecture level city in Shandong
Energy Saving Technology Shop	In-situ compensated economizer	Power-saving effect: used a week to comment, before and now record the meter comparison, my one rental room can save twenty degrees of electricity per day. Cost-effective. Quite worth it.	Jingdong prefecture level city in Shandong
Energy Saving Technology Shop	In-situ compensated economizer	This power saver is really powerful, used to spend 300 electricity bill every month, now it's about 100	Jingdong prefecture level city in Shandong
Energy Saving Technology Shop	In-situ compensated economizer	After two days of testing. The effect is good! About the first day of power saving for 1/4. the second day for 1/3. and not "steal electricity", is through the intelligent integrated circuit, automatically reduce the loss of electrical appliances to achieve the purpose of saving electricity! Recommended	Jingdong prefecture level city in Shandong
Energy Saving Technology Shop	In-situ compensated economizer	Power Saving King has been received, really good, thank you boss, I wish the boss business prosperity, prosperity, next time to come back to patronize!	Jingdong prefecture level city in Shandong
Energy Saving Technology Shop	In-situ compensated economizer	I've been using it for a while. Than to A little less electricity than before. I'll buy it again next time.	Jingdong prefecture level city in Shandong
Energy Saving Technology Shop	In-situ compensated economizer	Used to be a couple hundred bucks a month. With him, it's a big savings. It's great.	Jingdong prefecture level city in Shandong
Energy Saving Technology Shop	In-situ compensated economizer	Previously bought a plug in the kitchen, used for a period of time after the personal feeling is still quite effective, can save a little electricity, so this time without hesitation and bought two, has been inserted in the home next to the appliances that use a lot of electricity!	Jingdong prefecture level city in Shandong
Energy Saving Technology Shop	In-situ compensated economizer	I've used it before and it does have a power saving effect	Jingdong prefecture level city in Shandong
Energy Saving Technology Shop	In-situ compensated economizer	High-tech products, really useful, in previous years the high cost of electricity, with this product, the electricity bill came down. Praise a, hope that the research and development of this product of science and technology to continue to work hard, research out better products to.	Jingdong prefecture level city in Shandong
Energy Saving Technology Shop	In-situ compensated economizer	This high-power is not bad Oh, the first few months are 1000 degrees so, I casually brush in Taobao to this product, I bought back to try, I used a month after this month left 736 degrees, scared me, I also called to the grid to confirm, really is 736 degrees, the effect is very good, and today bought a go back to the old family to use	Jingdong prefecture level city in Shandong
Energy Saving Technology Shop	In-situ compensated economizer	Used for a period of time, you do not say can really save electricity, really is a small power-saving helper, it is worth buying!	Jingdong prefecture level city in Shandong
Energy Saving Technology Shop	In-situ compensated economizer	There are many rooms and it takes a lot of electricity to run the air conditioner when the house is cold. Bought 4 inserted half a month to save me more than 300 dollars. The effect is really core good! Quality is also very good, the seller service is very good and patient.	Jingdong prefecture level city in Shandong
Energy Saving Technology Shop	In-situ compensated economizer	Recommended by the owner of the store next door, looked at his home does have to save electricity, decided to get. Indeed quite can, the voltage is much more stable, there is a reduction in electricity	Jingdong prefecture level city in Shandong
Energy Saving Technology Shop	In-situ compensated economizer	It does save electricity and works pretty well. I feel that in a few days, I will be able to use the money that I bought to save money, it's really good!	Jingdong prefecture level city in Shandong
Energy Saving Technology Shop	In-situ compensated economizer	Small store service attitude is very good Product quality is very good Suggested purchase	Jingdong prefecture level city in Shandong
Energy Saving Technology Shop	In-situ compensated economizer	Bought two energy-saving appliances for the family, the kitchen and bathroom water heater is too expensive, the water heater to be turned on every day, the monthly electricity bill is a lot of use, with this, no longer afraid of the electricity bill is more than one month down the electricity bill can be less than half of the previous month it, the effect is particularly good!	Jingdong prefecture level city in Shandong
Energy Saving Technology Shop	In-situ compensated economizer	Saving power is really good to use, the last time I bought 3, the family used , before the family monthly electricity bills are more than 300 fast, after using this are more than 200, are several months, this time to my store is also arranged, it is really good for the people good things.	Jingdong prefecture level city in Shandong

Energy Saving Technology Shop	In-situ compensated economizer	The previous year bought a, almost every month can save a 70 dollars or so, with a year of no problem at all, look like new, this time to move to a new home decisively buy back 2 units, very believe this store!	Jingdong prefecture level city in Shandong
Energy Saving Technology Shop	In-situ compensated economizer	Power-saving effect: used a week to comment, before and now record the meter comparison, my one rental room can save twenty degrees of electricity per day. Cost-effective. Quite worth it.	Jingdong prefecture level city in Shandong
Energy Saving Technology Shop	In-situ compensated economizer	This power saver is really powerful, used to spend 300 electricity bill every month, now it's about 100	Jingdong prefecture level city in Shandong
Energy Saving Technology Shop	In-situ compensated economizer	After two days of testing. The effect is good! About the first day of power saving for 1/4. the second day for 1/3. and not "steal electricity", is through the intelligent integrated circuit, automatically reduce the loss of electrical appliances to achieve the purpose of saving electricity! Recommended	Jingdong prefecture level city in Shandong
Energy Saving Technology Shop	In-situ compensated economizer	Power Saving King has been received, really good, thank you boss, I wish the boss business prosperity, prosperity, next time to come back to patronize!	Jingdong prefecture level city in Shandong
Energy Saving Technology Shop	In-situ compensated economizer	I've been using it for a while. Than to A little less electricity than before. I'll buy it again next time.	Jingdong prefecture level city in Shandong
Energy Saving Technology Shop	In-situ compensated economizer	Used to be a couple hundred bucks a month. With him, it's a big savings. It's great.	Jingdong prefecture level city in Shandong
Energy Saving Technology Shop	In-situ compensated economizer	Previously bought a plug in the kitchen, used for a period of time after the personal feeling is still quite effective, can save a little electricity, so this time without hesitation and bought two, has been inserted in the home next to the appliances that use a lot of electricity!	Jingdong prefecture level city in Shandong
Energy Saving Technology Shop	In-situ compensated economizer	I've used it before and it does have a power saving effect	Jingdong prefecture level city in Shandong
Energy Saving Technology Shop	In-situ compensated economizer	High-tech products, really useful, in previous years the high cost of electricity, with this product, the electricity bill came down. Praise a, hope that the research and development of this product of science and technology to continue to work hard, research out better products to.	Jingdong prefecture level city in Shandong
Energy Saving Technology Shop	In-situ compensated economizer	This high-power is not bad Oh, the first few months are 1000 degrees so, I casually brush in Taobao to this product, I bought back to try, I used a month after this month left 736 degrees, scared me, I also called to the grid to confirm, really is 736 degrees, the effect is very good, and today bought a go back to the old family to use	Jingdong prefecture level city in Shandong
Energy Saving Technology Shop	In-situ compensated economizer	Used for a period of time, you do not say can really save electricity, really is a small power-saving helper, it is worth buying!	Jingdong prefecture level city in Shandong
Energy Saving Technology Shop	In-situ compensated economizer	There are many rooms and it takes a lot of electricity to run the air conditioner when the house is cold. Bought 4 inserted half a month to save me more than 300 dollars. The effect is really core good! Quality is also very good, the seller service is very good and patient.	Jingdong prefecture level city in Shandong
Energy Saving Technology Shop	In-situ compensated economizer	Recommended by the owner of the store next door, looked at his home does have to save electricity, decided to get. Indeed quite can, the voltage is much more stable, there is a reduction in electricity	Jingdong prefecture level city in Shandong
Energy Saving Technology Shop	In-situ compensated economizer	It does save electricity and works pretty well. I feel that in a few days, I will be able to use the money that I bought to save money, it's really good!	Jingdong prefecture level city in Shandong
Energy Saving Technology Shop	In-situ compensated economizer	Small store service attitude is very good Product quality is very good Suggested purchase	Jingdong prefecture level city in Shandong
Energy Saving Technology Shop	In-situ compensated economizer	Bought two energy-saving appliances for the family, the kitchen and bathroom water heater is too expensive, the water heater to be turned on every day, the monthly electricity bill is a lot of use, with this, no longer afraid of the electricity bill is more than one month down the electricity bill can be less than half of the previous month it, the effect is particularly good!	Jingdong prefecture level city in Shandong
Energy Saving Technology Shop	In-situ compensated economizer	Saving power is really good to use, the last time I bought 3, the family used , before the family monthly electricity bills are more than 300 fast, after using this are more than 200, are several months, this time to my store is also arranged, it is really good for the people good things.	Jingdong prefecture level city in Shandong
Energy Saving Technology Shop	In-situ compensated economizer	The previous year bought a, almost every month can save a 70 dollars or so, with a year of no problem at all, look like new, this time to move to a new home decisively buy back 2 units, very believe this store!	Jingdong prefecture level city in Shandong
Energy Saving Technology Shop	In-situ compensated economizer	Power-saving effect: used a week to comment, before and now record the meter comparison, my one rental room can save twenty degrees of electricity per day. Cost-effective. Quite worth it.	Jingdong prefecture level city in Shandong
Energy Saving Technology Shop	In-situ compensated economizer	This power saver is really powerful, used to spend 300 electricity bill every month, now it's about 100	Jingdong prefecture level city in Shandong
Energy Saving Technology Shop	In-situ compensated economizer	After two days of testing. The effect is good! About the first day of power saving for 1/4. the second day for 1/3. and not "steal electricity", is through the intelligent integrated circuit, automatically reduce the loss of electrical appliances to achieve the purpose of saving electricity! Recommended	Jingdong prefecture level city in Shandong
Energy Saving Technology Shop	In-situ compensated economizer	Power Saving King has been received, really good, thank you boss, I wish the boss business prosperity, prosperity, next time to come back to patronize!	Jingdong prefecture level city in Shandong
Energy Saving Technology Shop	In-situ compensated economizer	I've been using it for a while. Than to A little less electricity than before. I'll buy it again next time.	Jingdong prefecture level city in Shandong
Energy Saving Technology Shop	In-situ compensated economizer	Used to be a couple hundred bucks a month. With him, it's a big savings. It's great.	Jingdong prefecture level city in Shandong
Energy Saving Technology Shop	In-situ compensated economizer	Previously bought a plug in the kitchen, used for a period of time after the personal feeling is still quite effective, can save a little electricity, so this time without hesitation and bought two, has been inserted in the home next to the appliances that use a lot of electricity!	Jingdong prefecture level city in Shandong
Energy Saving Technology Shop	In-situ compensated economizer	I've used it before and it does have a power saving effect	Jingdong prefecture level city in Shandong
Energy Saving Technology Shop	In-situ compensated economizer	High-tech products, really useful, in previous years the high cost of electricity, with this product, the electricity bill came down. Praise a, hope that the research and development of this product of science and technology to continue to work hard, research out better products to.	Jingdong prefecture level city in Shandong
Energy Saving Technology Shop	In-situ compensated economizer	This high-power is not bad Oh, the first few months are 1000 degrees so, I casually brush in Taobao to this product, I bought back to try, I used a month after this month left 736 degrees, scared me, I also called to the grid to confirm, really is 736 degrees, the effect is very good, and today bought a go back to the old family to use	Jingdong prefecture level city in Shandong
Energy Saving Technology Shop	In-situ compensated economizer	Used for a period of time, you do not say can really save electricity, really is a small power-saving helper, it is worth buying!	Jingdong prefecture level city in Shandong
Energy Saving Technology Shop	In-situ compensated economizer	There are many rooms and it takes a lot of electricity to run the air conditioner when the house is cold. Bought 4 inserted half a month to save me more than 300 dollars. The effect is really core good! Quality is also very good, the seller service is very good and patient.	Jingdong prefecture level city in Shandong
Energy Saving Technology Shop	In-situ compensated economizer	Recommended by the owner of the store next door, looked at his home does have to save electricity, decided to get. Indeed quite can, the voltage is much more stable, there is a reduction in electricity	Jingdong prefecture level city in Shandong
Energy Saving Technology Shop	In-situ compensated economizer	It does save electricity and works pretty well. I feel that in a few days, I will be able to use the money that I bought to save money, it's really good!	Jingdong prefecture level city in Shandong
Energy Saving Technology Shop	In-situ compensated economizer	Small store service attitude is very good Product quality is very good Suggested purchase	Jingdong prefecture level city in Shandong
Energy Saving Technology Shop	In-situ compensated economizer	Bought two energy-saving appliances for the family, the kitchen and bathroom water heater is too expensive, the water heater to be turned on every day, the monthly electricity bill is a lot of use, with this, no longer afraid of the electricity bill is more than one month down the electricity bill can be less than half of the previous month it, the effect is particularly good!	Jingdong prefecture level city in Shandong
Energy Saving Technology Shop	In-situ compensated economizer	Saving power is really good to use, the last time I bought 3, the family used , before the family monthly electricity bills are more than 300 fast, after using this are more than 200, are several months, this time to my store is also arranged, it is really good for the people good things.	Jingdong prefecture level city in Shandong
Energy Saving Technology Shop	In-situ compensated economizer	The previous year bought a, almost every month can save a 70 dollars or so, with a year of no problem at all, look like new, this time to move to a new home decisively buy back 2 units, very believe this store!	Jingdong prefecture level city in Shandong
Energy Saving Technology Shop	In-situ compensated economizer	Power-saving effect: used a week to comment, before and now record the meter comparison, my one rental room can save twenty degrees of electricity per day. Cost-effective. Quite worth it.	Jingdong prefecture level city in Shandong
Energy Saving Technology Shop	In-situ compensated economizer	This power saver is really powerful, used to spend 300 electricity bill every month, now it's about 100	Jingdong prefecture level city in Shandong
Energy Saving Technology Shop	In-situ compensated economizer	After two days of testing. The effect is good! About the first day of power saving for 1/4. the second day for 1/3. and not "steal electricity", is through the intelligent integrated circuit, automatically reduce the loss of electrical appliances to achieve the purpose of saving electricity! Recommended	Jingdong prefecture level city in Shandong
Energy Saving Technology Shop	In-situ compensated economizer	Power Saving King has been received, really good, thank you boss, I wish the boss business prosperity, prosperity, next time to come back to patronize!	Jingdong prefecture level city in Shandong
Energy Saving Technology Shop	In-situ compensated economizer	I've been using it for a while. Than to A little less electricity than before. I'll buy it again next time.	Jingdong prefecture level city in Shandong

Energy Saving Technology Shop	In-situ compensated economizer	Used to be a couple hundred bucks a month. With him, it's a big savings. It's great.	Jingdong prefecture level city in Shandong
Energy Saving Technology Shop	In-situ compensated economizer	Previously bought a plug in the kitchen, used for a period of time after the personal feeling is still quite effective, can save a little electricity, so this time without hesitation and bought two, has been inserted in the home next to the appliances that use a lot of electricity!	Jingdong prefecture level city in Shandong
Energy Saving Technology Shop	In-situ compensated economizer	I've used it before and it does have a power saving effect	Jingdong prefecture level city in Shandong
Energy Saving Technology Shop	In-situ compensated economizer	High-tech products, really useful, in previous years the high cost of electricity, with this product, the electricity bill came down. Praise a, hope that the research and development of this product of science and technology to continue to work hard, research out better products to.	Jingdong prefecture level city in Shandong
Energy Saving Technology Shop	In-situ compensated economizer	This high-power is not bad Oh, the first few months are 1000 degrees so, I casually brush in Taobao to this product, I bought back to try, I used a month after this month left 736 degrees, scared me, I also called to the grid to confirm, really is 736 degrees, the effect is very good, and today bought a go back to the old family to use	Jingdong prefecture level city in Shandong
Energy Saving Technology Shop	In-situ compensated economizer	Used for a period of time, you do not say can really save electricity, really is a small power-saving helper, it is worth buying!	Jingdong prefecture level city in Shandong
Energy Saving Technology Shop	In-situ compensated economizer	There are many rooms and it takes a lot of electricity to run the air conditioner when the house is cold. Bought 4 inserted half a month to save me more than 300 dollars. The effect is really core good! Quality is also very good, the seller service is very good and patient.	Jingdong prefecture level city in Shandong
Energy Saving Technology Shop	In-situ compensated economizer	Recommended by the owner of the store next door, looked at his home does have to save electricity, decided to get. Indeed quite can, the voltage is much more stable, there is a reduction in electricity	Jingdong prefecture level city in Shandong
Energy Saving Technology Shop	In-situ compensated economizer	It does save electricity and works pretty well. I feel that in a few days, I will be able to use the money that I bought to save money, it's really good!	Jingdong prefecture level city in Shandong
Energy Saving Technology Shop	In-situ compensated economizer	Small store service attitude is very good Product quality is very good Suggested purchase	Jingdong prefecture level city in Shandong
Energy Saving Technology Shop	In-situ compensated economizer	Bought two energy-saving appliances for the family, the kitchen and bathroom water heater is too expensive, the water heater to be turned on every day, the monthly electricity bill is a lot of use, with this, no longer afraid of the electricity bill is more than one month down the electricity bill can be less than half of the previous month it, the effect is particularly good!	Jingdong prefecture level city in Shandong
Energy Saving Technology Shop	In-situ compensated economizer	Saving power is really good to use, the last time I bought 3, the family used , before the family monthly electricity bills are more than 300 fast, after using this are more than 200, are several months, this time to my store is also arranged, it is really good for the people good things.	Jingdong prefecture level city in Shandong
Energy Saving Technology Shop	In-situ compensated economizer	The previous year bought a, almost every month can save a 70 dollars or so, with a year of no problem at all, look like new, this time to move to a new home decisively buy back 2 units, very believe this store!	Jingdong prefecture level city in Shandong
Energy Saving Technology Shop	In-situ compensated economizer	Power-saving effect: used a week to comment, before and now record the meter comparison, my one rental room can save twenty degrees of electricity per day. Cost-effective. Quite worth it.	Jingdong prefecture level city in Shandong
Energy Saving Technology Shop	In-situ compensated economizer	This power saver is really powerful, used to spend 300 electricity bill every month, now it's about 100	Jingdong prefecture level city in Shandong
Energy Saving Technology Shop	In-situ compensated economizer	After two days of testing. The effect is good! About the first day of power saving for 1/4. the second day for 1/3. and not "steal electricity", is through the intelligent integrated circuit, automatically reduce the loss of electrical appliances to achieve the purpose of saving electricity! Recommended	Jingdong prefecture level city in Shandong
Energy Saving Technology Shop	In-situ compensated economizer	Power Saving King has been received, really good, thank you boss, I wish the boss business prosperity, prosperity, next time to come back to patronize!	Jingdong prefecture level city in Shandong
Energy Saving Technology Shop	In-situ compensated economizer	I've been using it for a while. Than to A little less electricity than before. I'll buy it again next time.	Jingdong prefecture level city in Shandong
Energy Saving Technology Shop	In-situ compensated economizer	Used to be a couple hundred bucks a month. With him, it's a big savings. It's great.	Jingdong prefecture level city in Shandong
Energy Saving Technology Shop	In-situ compensated economizer	Previously bought a plug in the kitchen, used for a period of time after the personal feeling is still quite effective, can save a little electricity, so this time without hesitation and bought two, has been inserted in the home next to the appliances that use a lot of electricity!	Jingdong prefecture level city in Shandong
Energy Saving Technology Shop	In-situ compensated economizer	I've used it before and it does have a power saving effect	Jingdong prefecture level city in Shandong
Energy Saving Technology Shop	In-situ compensated economizer	High-tech products, really useful, in previous years the high cost of electricity, with this product, the electricity bill came down. Praise a, hope that the research and development of this product of science and technology to continue to work hard, research out better products to.	Jingdong prefecture level city in Shandong
Energy Saving Technology Shop	In-situ compensated economizer	This high-power is not bad Oh, the first few months are 1000 degrees so, I casually brush in Taobao to this product, I bought back to try, I used a month after this month left 736 degrees, scared me, I also called to the grid to confirm, really is 736 degrees, the effect is very good, and today bought a go back to the old family to use	Jingdong prefecture level city in Shandong
Energy Saving Technology Shop	In-situ compensated economizer	Used for a period of time, you do not say can really save electricity, really is a small power-saving helper, it is worth buying!	Jingdong prefecture level city in Shandong
Energy Saving Technology Shop	In-situ compensated economizer	There are many rooms and it takes a lot of electricity to run the air conditioner when the house is cold. Bought 4 inserted half a month to save me more than 300 dollars. The effect is really core good! Quality is also very good, the seller service is very good and patient.	Jingdong prefecture level city in Shandong
Energy Saving Technology Shop	In-situ compensated economizer	Recommended by the owner of the store next door, looked at his home does have to save electricity, decided to get. Indeed quite can, the voltage is much more stable, there is a reduction in electricity	Jingdong prefecture level city in Shandong
Energy Saving Technology Shop	In-situ compensated economizer	It does save electricity and works pretty well. I feel that in a few days, I will be able to use the money that I bought to save money, it's really good!	Jingdong prefecture level city in Shandong
Energy Saving Technology Shop	In-situ compensated economizer	Small store service attitude is very good Product quality is very good Suggested purchase	Jingdong prefecture level city in Shandong
Energy Saving Technology Shop	In-situ compensated economizer	Bought two energy-saving appliances for the family, the kitchen and bathroom water heater is too expensive, the water heater to be turned on every day, the monthly electricity bill is a lot of use, with this, no longer afraid of the electricity bill is more than one month down the electricity bill can be less than half of the previous month it, the effect is particularly good!	Jingdong prefecture level city in Shandong
Energy Saving Technology Shop	In-situ compensated economizer	Saving power is really good to use, the last time I bought 3, the family used , before the family monthly electricity bills are more than 300 fast, after using this are more than 200, are several months, this time to my store is also arranged, it is really good for the people good things.	Jingdong prefecture level city in Shandong
Energy Saving Technology Shop	In-situ compensated economizer	The previous year bought a, almost every month can save a 70 dollars or so, with a year of no problem at all, look like new, this time to move to a new home decisively buy back 2 units, very believe this store!	Jingdong prefecture level city in Shandong
Energy Saving Technology Shop	In-situ compensated economizer	Power-saving effect: used a week to comment, before and now record the meter comparison, my one rental room can save twenty degrees of electricity per day. Cost-effective. Quite worth it.	Jingdong prefecture level city in Shandong
Energy Saving Technology Shop	In-situ compensated economizer	This power saver is really powerful, used to spend 300 electricity bill every month, now it's about 100	Jingdong prefecture level city in Shandong
Energy Saving Technology Shop	In-situ compensated economizer	After two days of testing. The effect is good! About the first day of power saving for 1/4. the second day for 1/3. and not "steal electricity", is through the intelligent integrated circuit, automatically reduce the loss of electrical appliances to achieve the purpose of saving electricity! Recommended	Jingdong prefecture level city in Shandong
Energy Saving Technology Shop	In-situ compensated economizer	Power Saving King has been received, really good, thank you boss, I wish the boss business prosperity, prosperity, next time to come back to patronize!	Jingdong prefecture level city in Shandong
Energy Saving Technology Shop	In-situ compensated economizer	I've been using it for a while. Than to A little less electricity than before. I'll buy it again next time.	Jingdong prefecture level city in Shandong
Energy Saving Technology Shop	In-situ compensated economizer	Used to be a couple hundred bucks a month. With him, it's a big savings. It's great.	Jingdong prefecture level city in Shandong
Energy Saving Technology Shop	In-situ compensated economizer	Previously bought a plug in the kitchen, used for a period of time after the personal feeling is still quite effective, can save a little electricity, so this time without hesitation and bought two, has been inserted in the home next to the appliances that use a lot of electricity!	Jingdong prefecture level city in Shandong
Energy Saving Technology Shop	In-situ compensated economizer	I've used it before and it does have a power saving effect	Jingdong prefecture level city in Shandong
Energy Saving Technology Shop	In-situ compensated economizer	High-tech products, really useful, in previous years the high cost of electricity, with this product, the electricity bill came down. Praise a, hope that the research and development of this product of science and technology to continue to work hard, research out better products to.	Jingdong prefecture level city in Shandong

Energy Saving Technology Shop	In-situ compensated economizer	This high-power is not bad Oh, the first few months are 1000 degrees so, I casually brush in Taobao to this product, I bought back to try, I used a month after this month left 736 degrees, scared me, I also called to the grid to confirm, really is 736 degrees, the effect is very good, and today bought a go back to the old family to use	Jingdong prefecture level city in Shandong
Energy Saving Technology Shop	In-situ compensated economizer	Used for a period of time, you do not say can really save electricity, really is a small power-saving helper, it is worth buying!	Jingdong prefecture level city in Shandong
Energy Saving Technology Shop	In-situ compensated economizer	There are many rooms and it takes a lot of electricity to run the air conditioner when the house is cold. Bought 4 inserted half a month to save me more than 300 dollars. The effect is really core good! Quality is also very good, the seller service is very good and patient.	Jingdong prefecture level city in Shandong
Energy Saving Technology Shop	In-situ compensated economizer	Recommended by the owner of the store next door, looked at his home does have to save electricity, decided to get. Indeed quite can, the voltage is much more stable, there is a reduction in electricity	Jingdong prefecture level city in Shandong
Energy Saving Technology Shop	In-situ compensated economizer	It does save electricity and works pretty well. I feel that in a few days, I will be able to use the money that I bought to save money, it's really good!	Jingdong prefecture level city in Shandong
Energy Saving Technology Shop	In-situ compensated economizer	Small store service attitude is very good Product quality is very good Suggested purchase	Jingdong prefecture level city in Shandong
Energy Saving Technology Shop	In-situ compensated economizer	Bought two energy-saving appliances for the family, the kitchen and bathroom water heater is too expensive, the water heater to be turned on every day, the monthly electricity bill is a lot of use, with this, no longer afraid of the electricity bill is more than one month down the electricity bill can be less than half of the previous month it, the effect is particularly good!	Jingdong prefecture level city in Shandong
Energy Saving Technology Shop	In-situ compensated economizer	Saving power is really good to use, the last time I bought 3, the family used , before the family monthly electricity bills are more than 300 fast, after using this are more than 200, are several months, this time to my store is also arranged, it is really good for the people good things.	Jingdong prefecture level city in Shandong
Energy Saving Technology Shop	In-situ compensated economizer	The previous year bought a, almost every month can save a 70 dollars or so, with a year of no problem at all, look like new, this time to move to a new home decisively buy back 2 units, very believe this store!	Jingdong prefecture level city in Shandong
Energy Saving Technology Shop	In-situ compensated economizer	Power-saving effect: used a week to comment, before and now record the meter comparison, my one rental room can save twenty degrees of electricity per day. Cost-effective. Quite worth it.	Jingdong prefecture level city in Shandong
Energy Saving Technology Shop	In-situ compensated economizer	This power saver is really powerful, used to spend 300 electricity bill every month, now it's about 100	Jingdong prefecture level city in Shandong
Energy Saving Technology Shop	In-situ compensated economizer	After two days of testing. The effect is good! About the first day of power saving for 1/4. the second day for 1/3. and not "steal electricity", is through the intelligent integrated circuit, automatically reduce the loss of electrical appliances to achieve the purpose of saving electricity! Recommended	Jingdong prefecture level city in Shandong
Energy Saving Technology Shop	In-situ compensated economizer	Power Saving King has been received, really good, thank you boss, I wish the boss business prosperity, prosperity, next time to come back to patronize!	Jingdong prefecture level city in Shandong
Energy Saving Technology Shop	In-situ compensated economizer	I've been using it for a while. Than to A little less electricity than before. I'll buy it again next time.	Jingdong prefecture level city in Shandong
Energy Saving Technology Shop	In-situ compensated economizer	Used to be a couple hundred bucks a month. With him, it's a big savings. It's great.	Jingdong prefecture level city in Shandong
Energy Saving Technology Shop	In-situ compensated economizer	Previously bought a plug in the kitchen, used for a period of time after the personal feeling is still quite effective, can save a little electricity, so this time without hesitation and bought two, has been inserted in the home next to the appliances that use a lot of electricity!	Jingdong prefecture level city in Shandong
Energy Saving Technology Shop	In-situ compensated economizer	I've used it before and it does have a power saving effect	Jingdong prefecture level city in Shandong
Energy Saving Technology Shop	In-situ compensated economizer	High-tech products, really useful, in previous years the high cost of electricity, with this product, the electricity bill came down. Praise a, hope that the research and development of this product of science and technology to continue to work hard, research out better products to.	Jingdong prefecture level city in Shandong
Energy Saving Technology Shop	In-situ compensated economizer	This high-power is not bad Oh, the first few months are 1000 degrees so, I casually brush in Taobao to this product, I bought back to try, I used a month after this month left 736 degrees, scared me, I also called to the grid to confirm, really is 736 degrees, the effect is very good, and today bought a go back to the old family to use	Jingdong prefecture level city in Shandong
Energy Saving Technology Shop	In-situ compensated economizer	Used for a period of time, you do not say can really save electricity, really is a small power-saving helper, it is worth buying!	Jingdong prefecture level city in Shandong
Energy Saving Technology Shop	In-situ compensated economizer	There are many rooms and it takes a lot of electricity to run the air conditioner when the house is cold. Bought 4 inserted half a month to save me more than 300 dollars. The effect is really core good! Quality is also very good, the seller service is very good and patient.	Jingdong prefecture level city in Shandong
Energy Saving Technology Shop	In-situ compensated economizer	Recommended by the owner of the store next door, looked at his home does have to save electricity, decided to get. Indeed quite can, the voltage is much more stable, there is a reduction in electricity	Jingdong prefecture level city in Shandong
Energy Saving Technology Shop	In-situ compensated economizer	It does save electricity and works pretty well. I feel that in a few days, I will be able to use the money that I bought to save money, it's really good!	Jingdong prefecture level city in Shandong
Energy Saving Technology Shop	In-situ compensated economizer	Small store service attitude is very good Product quality is very good Suggested purchase	Jingdong prefecture level city in Shandong
Energy Saving Technology Shop	In-situ compensated economizer	Bought two energy-saving appliances for the family, the kitchen and bathroom water heater is too expensive, the water heater to be turned on every day, the monthly electricity bill is a lot of use, with this, no longer afraid of the electricity bill is more than one month down the electricity bill can be less than half of the previous month it, the effect is particularly good!	Jingdong prefecture level city in Shandong
Energy Saving Technology Shop	In-situ compensated economizer	Saving power is really good to use, the last time I bought 3, the family used , before the family monthly electricity bills are more than 300 fast, after using this are more than 200, are several months, this time to my store is also arranged, it is really good for the people good things.	Jingdong prefecture level city in Shandong
Energy Saving Technology Shop	In-situ compensated economizer	The previous year bought a, almost every month can save a 70 dollars or so, with a year of no problem at all, look like new, this time to move to a new home decisively buy back 2 units, very believe this store!	Jingdong prefecture level city in Shandong
Energy Saving Technology Shop	In-situ compensated economizer	Power-saving effect: used a week to comment, before and now record the meter comparison, my one rental room can save twenty degrees of electricity per day. Cost-effective. Quite worth it.	Jingdong prefecture level city in Shandong
Energy Saving Technology Shop	In-situ compensated economizer	This power saver is really powerful, used to spend 300 electricity bill every month, now it's about 100	Jingdong prefecture level city in Shandong
Energy Saving Technology Shop	In-situ compensated economizer	After two days of testing. The effect is good! About the first day of power saving for 1/4. the second day for 1/3. and not "steal electricity", is through the intelligent integrated circuit, automatically reduce the loss of electrical appliances to achieve the purpose of saving electricity! Recommended	Jingdong prefecture level city in Shandong
Energy Saving Technology Shop	In-situ compensated economizer	Power Saving King has been received, really good, thank you boss, I wish the boss business prosperity, prosperity, next time to come back to patronize!	Jingdong prefecture level city in Shandong
Energy Saving Technology Shop	In-situ compensated economizer	I've been using it for a while. Than to A little less electricity than before. I'll buy it again next time.	Jingdong prefecture level city in Shandong
Energy Saving Technology Shop	In-situ compensated economizer	Used to be a couple hundred bucks a month. With him, it's a big savings. It's great.	Jingdong prefecture level city in Shandong
Energy Saving Technology Shop	In-situ compensated economizer	Previously bought a plug in the kitchen, used for a period of time after the personal feeling is still quite effective, can save a little electricity, so this time without hesitation and bought two, has been inserted in the home next to the appliances that use a lot of electricity!	Jingdong prefecture level city in Shandong
Energy Saving Technology Shop	In-situ compensated economizer	I've used it before and it does have a power saving effect	Jingdong prefecture level city in Shandong
Energy Saving Technology Shop	In-situ compensated economizer	High-tech products, really useful, in previous years the high cost of electricity, with this product, the electricity bill came down. Praise a, hope that the research and development of this product of science and technology to continue to work hard, research out better products to.	Jingdong prefecture level city in Shandong
Energy Saving Technology Shop	In-situ compensated economizer	This high-power is not bad Oh, the first few months are 1000 degrees so, I casually brush in Taobao to this product, I bought back to try, I used a month after this month left 736 degrees, scared me, I also called to the grid to confirm, really is 736 degrees, the effect is very good, and today bought a go back to the old family to use	Jingdong prefecture level city in Shandong
Energy Saving Technology Shop	In-situ compensated economizer	Used for a period of time, you do not say can really save electricity, really is a small power-saving helper, it is worth buying!	Jingdong prefecture level city in Shandong
Energy Saving Technology Shop	In-situ compensated economizer	There are many rooms and it takes a lot of electricity to run the air conditioner when the house is cold. Bought 4 inserted half a month to save me more than 300 dollars. The effect is really core good! Quality is also very good, the seller service is very good and patient.	Jingdong prefecture level city in Shandong
Energy Saving Technology Shop	In-situ compensated economizer	Recommended by the owner of the store next door, looked at his home does have to save electricity, decided to get. Indeed quite can, the voltage is much more stable, there is a reduction in electricity	Jingdong prefecture level city in Shandong
Energy Saving Technology Shop	In-situ compensated economizer	It does save electricity and works pretty well. I feel that in a few days, I will be able to use the money that I bought to save money, it's really good!	Jingdong prefecture level city in Shandong

Energy Saving Technology Shop	In-situ compensated economizer	Small store service attitude is very good Product quality is very good Suggested purchase	Jingdong prefecture level city in Shandong
Energy Saving Technology Shop	In-situ compensated economizer	Bought two energy-saving appliances for the family, the kitchen and bathroom water heater is too expensive, the water heater to be turned on every day, the monthly electricity bill is a lot of use, with this, no longer afraid of the electricity bill is more than one month down the electricity bill can be less than half of the previous month it, the effect is particularly good!	Jingdong prefecture level city in Shandong
Energy Saving Technology Shop	In-situ compensated economizer	Saving power is really good to use, the last time I bought 3, the family used , before the family monthly electricity bills are more than 300 fast, after using this are more than 200, are several months, this time to my store is also arranged, it is really good for the people good things.	Jingdong prefecture level city in Shandong
Energy Saving Technology Shop	In-situ compensated economizer	The previous year bought a, almost every month can save a 70 dollars or so, with a year of no problem at all, look like new, this time to move to a new home decisively buy back 2 units, very believe this store!	Jingdong prefecture level city in Shandong
Energy Saving Technology Shop	In-situ compensated economizer	Power-saving effect: used a week to comment, before and now record the meter comparison, my one rental room can save twenty degrees of electricity per day. Cost-effective. Quite worth it.	Jingdong prefecture level city in Shandong
Energy Saving Technology Shop	In-situ compensated economizer	This power saver is really powerful, used to spend 300 electricity bill every month, now it's about 100	Jingdong prefecture level city in Shandong
Energy Saving Technology Shop	In-situ compensated economizer	After two days of testing. The effect is good! About the first day of power saving for 1/4. the second day for 1/3. and not "steal electricity", is through the intelligent integrated circuit, automatically reduce the loss of electrical appliances to achieve the purpose of saving electricity! Recommended	Jingdong prefecture level city in Shandong
Energy Saving Technology Shop	In-situ compensated economizer	Power Saving King has been received, really good, thank you boss, I wish the boss business prosperity, prosperity, next time to come back to patronize!	Jingdong prefecture level city in Shandong
Energy Saving Technology Shop	In-situ compensated economizer	I've been using it for a while. Than to A little less electricity than before. I'll buy it again next time.	Jingdong prefecture level city in Shandong
Energy Saving Technology Shop	In-situ compensated economizer	Used to be a couple hundred bucks a month. With him, it's a big savings. It's great.	Jingdong prefecture level city in Shandong
Energy Saving Technology Shop	In-situ compensated economizer	Previously bought a plug in the kitchen, used for a period of time after the personal feeling is still quite effective, can save a little electricity, so this time without hesitation and bought two, has been inserted in the home next to the appliances that use a lot of electricity!	Jingdong prefecture level city in Shandong
Energy Saving Technology Shop	In-situ compensated economizer	I've used it before and it does have a power saving effect	Jingdong prefecture level city in Shandong
Energy Saving Technology Shop	In-situ compensated economizer	High-tech products, really useful, in previous years the high cost of electricity, with this product, the electricity bill came down. Praise a, hope that the research and development of this product of science and technology to continue to work hard, research out better products to.	Jingdong prefecture level city in Shandong
Energy Saving Technology Shop	In-situ compensated economizer	This high-power is not bad Oh, the first few months are 1000 degrees so, I casually brush in Taobao to this product, I bought back to try, I used a month after this month left 736 degrees, scared me, I also called to the grid to confirm, really is 736 degrees, the effect is very good, and today bought a go back to the old family to use	Jingdong prefecture level city in Shandong
Energy Saving Technology Shop	In-situ compensated economizer	Used for a period of time, you do not say can really save electricity, really is a small power-saving helper, it is worth buying!	Jingdong prefecture level city in Shandong
Energy Saving Technology Shop	In-situ compensated economizer	There are many rooms and it takes a lot of electricity to run the air conditioner when the house is cold. Bought 4 inserted half a month to save me more than 300 dollars. The effect is really core good! Quality is also very good, the seller service is very good and patient.	Jingdong prefecture level city in Shandong
Energy Saving Technology Shop	In-situ compensated economizer	Recommended by the owner of the store next door, looked at his home does have to save electricity, decided to get. Indeed quite can, the voltage is much more stable, there is a reduction in electricity	Jingdong prefecture level city in Shandong
Energy Saving Technology Shop	In-situ compensated economizer	It does save electricity and works pretty well. I feel that in a few days, I will be able to use the money that I bought to save money, it's really good!	Jingdong prefecture level city in Shandong
Energy Saving Technology Shop	In-situ compensated economizer	Small store service attitude is very good Product quality is very good Suggested purchase	Jingdong prefecture level city in Shandong
Energy Saving Technology Shop	In-situ compensated economizer	Bought two energy-saving appliances for the family, the kitchen and bathroom water heater is too expensive, the water heater to be turned on every day, the monthly electricity bill is a lot of use, with this, no longer afraid of the electricity bill is more than one month down the electricity bill can be less than half of the previous month it, the effect is particularly good!	Jingdong prefecture level city in Shandong
Energy Saving Technology Shop	In-situ compensated economizer	Saving power is really good to use, the last time I bought 3, the family used , before the family monthly electricity bills are more than 300 fast, after using this are more than 200, are several months, this time to my store is also arranged, it is really good for the people good things.	Jingdong prefecture level city in Shandong
Energy Saving Technology Shop	In-situ compensated economizer	The previous year bought a, almost every month can save a 70 dollars or so, with a year of no problem at all, look like new, this time to move to a new home decisively buy back 2 units, very believe this store!	Jingdong prefecture level city in Shandong
Energy Saving Technology Shop	In-situ compensated economizer	Power-saving effect: used a week to comment, before and now record the meter comparison, my one rental room can save twenty degrees of electricity per day. Cost-effective. Quite worth it.	Jingdong prefecture level city in Shandong
Energy Saving Technology Shop	In-situ compensated economizer	This power saver is really powerful, used to spend 300 electricity bill every month, now it's about 100	Jingdong prefecture level city in Shandong
Energy Saving Technology Shop	In-situ compensated economizer	After two days of testing. The effect is good! About the first day of power saving for 1/4. the second day for 1/3. and not "steal electricity", is through the intelligent integrated circuit, automatically reduce the loss of electrical appliances to achieve the purpose of saving electricity! Recommended	Jingdong prefecture level city in Shandong
Energy Saving Technology Shop	In-situ compensated economizer	Power Saving King has been received, really good, thank you boss, I wish the boss business prosperity, prosperity, next time to come back to patronize!	Jingdong prefecture level city in Shandong
Energy Saving Technology Shop	In-situ compensated economizer	I've been using it for a while. Than to A little less electricity than before. I'll buy it again next time.	Jingdong prefecture level city in Shandong
Energy Saving Technology Shop	In-situ compensated economizer	Used to be a couple hundred bucks a month. With him, it's a big savings. It's great.	Jingdong prefecture level city in Shandong
Energy Saving Technology Shop	In-situ compensated economizer	Previously bought a plug in the kitchen, used for a period of time after the personal feeling is still quite effective, can save a little electricity, so this time without hesitation and bought two, has been inserted in the home next to the appliances that use a lot of electricity!	Jingdong prefecture level city in Shandong
Energy Saving Technology Shop	In-situ compensated economizer	I've used it before and it does have a power saving effect	Jingdong prefecture level city in Shandong
Energy Saving Technology Shop	In-situ compensated economizer	High-tech products, really useful, in previous years the high cost of electricity, with this product, the electricity bill came down. Praise a, hope that the research and development of this product of science and technology to continue to work hard, research out better products to.	Jingdong prefecture level city in Shandong
Energy Saving Technology Shop	In-situ compensated economizer	This high-power is not bad Oh, the first few months are 1000 degrees so, I casually brush in Taobao to this product, I bought back to try, I used a month after this month left 736 degrees, scared me, I also called to the grid to confirm, really is 736 degrees, the effect is very good, and today bought a go back to the old family to use	Jingdong prefecture level city in Shandong
Energy Saving Technology Shop	In-situ compensated economizer	Used for a period of time, you do not say can really save electricity, really is a small power-saving helper, it is worth buying!	Jingdong prefecture level city in Shandong
Energy Saving Technology Shop	In-situ compensated economizer	There are many rooms and it takes a lot of electricity to run the air conditioner when the house is cold. Bought 4 inserted half a month to save me more than 300 dollars. The effect is really core good! Quality is also very good, the seller service is very good and patient.	Jingdong prefecture level city in Shandong
Energy Saving Technology Shop	In-situ compensated economizer	Recommended by the owner of the store next door, looked at his home does have to save electricity, decided to get. Indeed quite can, the voltage is much more stable, there is a reduction in electricity	Jingdong prefecture level city in Shandong
Energy Saving Technology Shop	In-situ compensated economizer	It does save electricity and works pretty well. I feel that in a few days, I will be able to use the money that I bought to save money, it's really good!	Jingdong prefecture level city in Shandong
Energy Saving Technology Shop	In-situ compensated economizer	Small store service attitude is very good Product quality is very good Suggested purchase	Jingdong prefecture level city in Shandong
Energy Saving Technology Shop	In-situ compensated economizer	Bought two energy-saving appliances for the family, the kitchen and bathroom water heater is too expensive, the water heater to be turned on every day, the monthly electricity bill is a lot of use, with this, no longer afraid of the electricity bill is more than one month down the electricity bill can be less than half of the previous month it, the effect is particularly good!	Jingdong prefecture level city in Shandong
Energy Saving Technology Shop	In-situ compensated economizer	Saving power is really good to use, the last time I bought 3, the family used , before the family monthly electricity bills are more than 300 fast, after using this are more than 200, are several months, this time to my store is also arranged, it is really good for the people good things.	Jingdong prefecture level city in Shandong
Energy Saving Technology Shop	In-situ compensated economizer	The previous year bought a, almost every month can save a 70 dollars or so, with a year of no problem at all, look like new, this time to move to a new home decisively buy back 2 units, very believe this store!	Jingdong prefecture level city in Shandong
Energy Saving Technology Shop	In-situ compensated economizer	Power-saving effect: used a week to comment, before and now record the meter comparison, my one rental room can save twenty degrees of electricity per day. Cost-effective. Quite worth it.	Jingdong prefecture level city in Shandong

Energy Saving Technology Shop	In-situ compensated economizer	This power saver is really powerful, used to spend 300 electricity bill every month, now it's about 100	Jingdong prefecture level city in Shandong
Energy Saving Technology Shop	In-situ compensated economizer	After two days of testing. The effect is good! About the first day of power saving for 1/4. the second day for 1/3. and not "steal electricity", is through the intelligent integrated circuit, automatically reduce the loss of electrical appliances to achieve the purpose of saving electricity! Recommended	Jingdong prefecture level city in Shandong
Energy Saving Technology Shop	In-situ compensated economizer	Power Saving King has been received, really good, thank you boss, I wish the boss business prosperity, prosperity, next time to come back to patronize!	Jingdong prefecture level city in Shandong
Energy Saving Technology Shop	In-situ compensated economizer	I've been using it for a while. Than to A little less electricity than before. I'll buy it again next time.	Jingdong prefecture level city in Shandong
Energy Saving Technology Shop	In-situ compensated economizer	Used to be a couple hundred bucks a month. With him, it's a big savings. It's great.	Jingdong prefecture level city in Shandong
Energy Saving Technology Shop	In-situ compensated economizer	Previously bought a plug in the kitchen, used for a period of time after the personal feeling is still quite effective, can save a little electricity, so this time without hesitation and bought two, has been inserted in the home next to the appliances that use a lot of electricity!	Jingdong prefecture level city in Shandong
Energy Saving Technology Shop	In-situ compensated economizer	I've used it before and it does have a power saving effect	Jingdong prefecture level city in Shandong
Energy Saving Technology Shop	In-situ compensated economizer	High-tech products, really useful, in previous years the high cost of electricity, with this product, the electricity bill came down. Praise a, hope that the research and development of this product of science and technology to continue to work hard, research out better products to.	Jingdong prefecture level city in Shandong
Energy Saving Technology Shop	In-situ compensated economizer	This high-power is not bad Oh, the first few months are 1000 degrees so, I casually brush in Taobao to this product, I bought back to try, I used a month after this month left 736 degrees, scared me, I also called to the grid to confirm, really is 736 degrees, the effect is very good, and today bought a go back to the old family to use	Jingdong prefecture level city in Shandong
Energy Saving Technology Shop	In-situ compensated economizer	Used for a period of time, you do not say can really save electricity, really is a small power-saving helper, it is worth buying!	Jingdong prefecture level city in Shandong
Energy Saving Technology Shop	In-situ compensated economizer	There are many rooms and it takes a lot of electricity to run the air conditioner when the house is cold. Bought 4 inserted half a month to save me more than 300 dollars. The effect is really core good! Quality is also very good, the seller service is very good and patient.	Jingdong prefecture level city in Shandong
Energy Saving Technology Shop	In-situ compensated economizer	Recommended by the owner of the store next door, looked at his home does have to save electricity, decided to get. Indeed quite can, the voltage is much more stable, there is a reduction in electricity	Jingdong prefecture level city in Shandong
Energy Saving Technology Shop	In-situ compensated economizer	It does save electricity and works pretty well. I feel that in a few days, I will be able to use the money that I bought to save money, it's really good!	Jingdong prefecture level city in Shandong
Energy Saving Technology Shop	In-situ compensated economizer	Small store service attitude is very good Product quality is very good Suggested purchase	Jingdong prefecture level city in Shandong
Energy Saving Technology Shop	In-situ compensated economizer	Bought two energy-saving appliances for the family, the kitchen and bathroom water heater is too expensive, the water heater to be turned on every day, the monthly electricity bill is a lot of use, with this, no longer afraid of the electricity bill is more than one month down the electricity bill can be less than half of the previous month it, the effect is particularly good!	Jingdong prefecture level city in Shandong
Energy Saving Technology Shop	In-situ compensated economizer	Saving power is really good to use, the last time I bought 3, the family used , before the family monthly electricity bills are more than 300 fast, after using this are more than 200, are several months, this time to my store is also arranged, it is really good for the people good things.	Jingdong prefecture level city in Shandong
Energy Saving Technology Shop	In-situ compensated economizer	The previous year bought a, almost every month can save a 70 dollars or so, with a year of no problem at all, look like new, this time to move to a new home decisively buy back 2 units, very believe this store!	Jingdong prefecture level city in Shandong
Energy Saving Technology Shop	In-situ compensated economizer	Power-saving effect: used a week to comment, before and now record the meter comparison, my one rental room can save twenty degrees of electricity per day. Cost-effective. Quite worth it.	Jingdong prefecture level city in Shandong
Energy Saving Technology Shop	In-situ compensated economizer	This power saver is really powerful, used to spend 300 electricity bill every month, now it's about 100	Jingdong prefecture level city in Shandong
Energy Saving Technology Shop	In-situ compensated economizer	After two days of testing. The effect is good! About the first day of power saving for 1/4. the second day for 1/3. and not "steal electricity", is through the intelligent integrated circuit, automatically reduce the loss of electrical appliances to achieve the purpose of saving electricity! Recommended	Jingdong prefecture level city in Shandong
Energy Saving Technology Shop	In-situ compensated economizer	Power Saving King has been received, really good, thank you boss, I wish the boss business prosperity, prosperity, next time to come back to patronize!	Jingdong prefecture level city in Shandong
Energy Saving Technology Shop	In-situ compensated economizer	I've been using it for a while. Than to A little less electricity than before. I'll buy it again next time.	Jingdong prefecture level city in Shandong
Energy Saving Technology Shop	In-situ compensated economizer	Used to be a couple hundred bucks a month. With him, it's a big savings. It's great.	Jingdong prefecture level city in Shandong
Energy Saving Technology Shop	In-situ compensated economizer	Previously bought a plug in the kitchen, used for a period of time after the personal feeling is still quite effective, can save a little electricity, so this time without hesitation and bought two, has been inserted in the home next to the appliances that use a lot of electricity!	Jingdong prefecture level city in Shandong
Energy Saving Technology Shop	In-situ compensated economizer	I've used it before and it does have a power saving effect	Jingdong prefecture level city in Shandong
Energy Saving Technology Shop	In-situ compensated economizer	High-tech products, really useful, in previous years the high cost of electricity, with this product, the electricity bill came down. Praise a, hope that the research and development of this product of science and technology to continue to work hard, research out better products to.	Jingdong prefecture level city in Shandong
Energy Saving Technology Shop	In-situ compensated economizer	This high-power is not bad Oh, the first few months are 1000 degrees so, I casually brush in Taobao to this product, I bought back to try, I used a month after this month left 736 degrees, scared me, I also called to the grid to confirm, really is 736 degrees, the effect is very good, and today bought a go back to the old family to use	Jingdong prefecture level city in Shandong
Energy Saving Technology Shop	In-situ compensated economizer	Used for a period of time, you do not say can really save electricity, really is a small power-saving helper, it is worth buying!	Jingdong prefecture level city in Shandong
Energy Saving Technology Shop	In-situ compensated economizer	There are many rooms and it takes a lot of electricity to run the air conditioner when the house is cold. Bought 4 inserted half a month to save me more than 300 dollars. The effect is really core good! Quality is also very good, the seller service is very good and patient.	Jingdong prefecture level city in Shandong
Energy Saving Technology Shop	In-situ compensated economizer	Recommended by the owner of the store next door, looked at his home does have to save electricity, decided to get. Indeed quite can, the voltage is much more stable, there is a reduction in electricity	Jingdong prefecture level city in Shandong
Energy Saving Technology Shop	In-situ compensated economizer	It does save electricity and works pretty well. I feel that in a few days, I will be able to use the money that I bought to save money, it's really good!	Jingdong prefecture level city in Shandong
Energy Saving Technology Shop	In-situ compensated economizer	Small store service attitude is very good Product quality is very good Suggested purchase	Jingdong prefecture level city in Shandong
Energy Saving Technology Shop	In-situ compensated economizer	Bought two energy-saving appliances for the family, the kitchen and bathroom water heater is too expensive, the water heater to be turned on every day, the monthly electricity bill is a lot of use, with this, no longer afraid of the electricity bill is more than one month down the electricity bill can be less than half of the previous month it, the effect is particularly good!	Jingdong prefecture level city in Shandong
Energy Saving Technology Shop	In-situ compensated economizer	Saving power is really good to use, the last time I bought 3, the family used , before the family monthly electricity bills are more than 300 fast, after using this are more than 200, are several months, this time to my store is also arranged, it is really good for the people good things.	Jingdong prefecture level city in Shandong
Energy Saving Technology Shop	In-situ compensated economizer	The previous year bought a, almost every month can save a 70 dollars or so, with a year of no problem at all, look like new, this time to move to a new home decisively buy back 2 units, very believe this store!	Jingdong prefecture level city in Shandong
Energy Saving Technology Shop	In-situ compensated economizer	Power-saving effect: used a week to comment, before and now record the meter comparison, my one rental room can save twenty degrees of electricity per day. Cost-effective. Quite worth it.	Jingdong prefecture level city in Shandong
Energy Saving Technology Shop	In-situ compensated economizer	This power saver is really powerful, used to spend 300 electricity bill every month, now it's about 100	Jingdong prefecture level city in Shandong
Energy Saving Technology Shop	In-situ compensated economizer	After two days of testing. The effect is good! About the first day of power saving for 1/4. the second day for 1/3. and not "steal electricity", is through the intelligent integrated circuit, automatically reduce the loss of electrical appliances to achieve the purpose of saving electricity! Recommended	Jingdong prefecture level city in Shandong
Energy Saving Technology Shop	In-situ compensated economizer	Power Saving King has been received, really good, thank you boss, I wish the boss business prosperity, prosperity, next time to come back to patronize!	Jingdong prefecture level city in Shandong
Energy Saving Technology Shop	In-situ compensated economizer	I've been using it for a while. Than to A little less electricity than before. I'll buy it again next time.	Jingdong prefecture level city in Shandong
Energy Saving Technology Shop	In-situ compensated economizer	Used to be a couple hundred bucks a month. With him, it's a big savings. It's great.	Jingdong prefecture level city in Shandong

Energy Saving Technology Shop	In-situ compensated economizer	Previously bought a plug in the kitchen, used for a period of time after the personal feeling is still quite effective, can save a little electricity, so this time without hesitation and bought two, has been inserted in the home next to the appliances that use a lot of electricity!	Jingdong prefecture level city in Shandong
Energy Saving Technology Shop	In-situ compensated economizer	I've used it before and it does have a power saving effect	Jingdong prefecture level city in Shandong
Energy Saving Technology Shop	In-situ compensated economizer	High-tech products, really useful, in previous years the high cost of electricity, with this product, the electricity bill came down. Praise a, hope that the research and development of this product of science and technology to continue to work hard, research out better products to.	Jingdong prefecture level city in Shandong
Energy Saving Technology Shop	In-situ compensated economizer	This high-power is not bad Oh, the first few months are 1000 degrees so, I casually brush in Taobao to this product, I bought back to try, I used a month after this month left 736 degrees, scared me, I also called to the grid to confirm, really is 736 degrees, the effect is very good, and today bought a go back to the old family to use	Jingdong prefecture level city in Shandong
Energy Saving Technology Shop	In-situ compensated economizer	Used for a period of time, you do not say can really save electricity, really is a small power-saving helper, it is worth buying!	Jingdong prefecture level city in Shandong
Energy Saving Technology Shop	In-situ compensated economizer	There are many rooms and it takes a lot of electricity to run the air conditioner when the house is cold. Bought 4 inserted half a month to save me more than 300 dollars. The effect is really core good! Quality is also very good, the seller service is very good and patient.	Jingdong prefecture level city in Shandong
Energy Saving Technology Shop	In-situ compensated economizer	Recommended by the owner of the store next door, looked at his home does have to save electricity, decided to get. Indeed quite can, the voltage is much more stable, there is a reduction in electricity	Jingdong prefecture level city in Shandong
Energy Saving Technology Shop	In-situ compensated economizer	It does save electricity and works pretty well. I feel that in a few days, I will be able to use the money that I bought to save money, it's really good!	Jingdong prefecture level city in Shandong
Energy Saving Technology Shop	In-situ compensated economizer	Small store service attitude is very good Product quality is very good Suggested purchase	Jingdong prefecture level city in Shandong
Energy Saving Technology Shop	In-situ compensated economizer	Bought two energy-saving appliances for the family, the kitchen and bathroom water heater is too expensive, the water heater to be turned on every day, the monthly electricity bill is a lot of use, with this, no longer afraid of the electricity bill is more than one month down the electricity bill can be less than half of the previous month it, the effect is particularly good!	Jingdong prefecture level city in Shandong
Energy Saving Technology Shop	In-situ compensated economizer	Saving power is really good to use, the last time I bought 3, the family used , before the family monthly electricity bills are more than 300 fast, after using this are more than 200, are several months, this time to my store is also arranged, it is really good for the people good things.	Jingdong prefecture level city in Shandong
Energy Saving Technology Shop	In-situ compensated economizer	The previous year bought a, almost every month can save a 70 dollars or so, with a year of no problem at all, look like new, this time to move to a new home decisively buy back 2 units, very believe this store!	Jingdong prefecture level city in Shandong
Energy Saving Technology Shop	In-situ compensated economizer	Power-saving effect: used a week to comment, before and now record the meter comparison, my one rental room can save twenty degrees of electricity per day. Cost-effective. Quite worth it.	Jingdong prefecture level city in Shandong
Energy Saving Technology Shop	In-situ compensated economizer	This power saver is really powerful, used to spend 300 electricity bill every month, now it's about 100	Jingdong prefecture level city in Shandong
Energy Saving Technology Shop	In-situ compensated economizer	After two days of testing. The effect is good! About the first day of power saving for 1/4. the second day for 1/3. and not "steal electricity", is through the intelligent integrated circuit, automatically reduce the loss of electrical appliances to achieve the purpose of saving electricity! Recommended	Jingdong prefecture level city in Shandong
Energy Saving Technology Shop	In-situ compensated economizer	Power Saving King has been received, really good, thank you boss, I wish the boss business prosperity, prosperity, next time to come back to patronize!	Jingdong prefecture level city in Shandong
Energy Saving Technology Shop	In-situ compensated economizer	I've been using it for a while. Than to A little less electricity than before. I'll buy it again next time.	Jingdong prefecture level city in Shandong
Energy Saving Technology Shop	In-situ compensated economizer	Used to be a couple hundred bucks a month. With him, it's a big savings. It's great.	Jingdong prefecture level city in Shandong
Energy Saving Technology Shop	In-situ compensated economizer	Previously bought a plug in the kitchen, used for a period of time after the personal feeling is still quite effective, can save a little electricity, so this time without hesitation and bought two, has been inserted in the home next to the appliances that use a lot of electricity!	Jingdong prefecture level city in Shandong
Energy Saving Technology Shop	In-situ compensated economizer	I've used it before and it does have a power saving effect	Jingdong prefecture level city in Shandong
Energy Saving Technology Shop	In-situ compensated economizer	High-tech products, really useful, in previous years the high cost of electricity, with this product, the electricity bill came down. Praise a, hope that the research and development of this product of science and technology to continue to work hard, research out better products to.	Jingdong prefecture level city in Shandong
Energy Saving Technology Shop	In-situ compensated economizer	This high-power is not bad Oh, the first few months are 1000 degrees so, I casually brush in Taobao to this product, I bought back to try, I used a month after this month left 736 degrees, scared me, I also called to the grid to confirm, really is 736 degrees, the effect is very good, and today bought a go back to the old family to use	Jingdong prefecture level city in Shandong
Energy Saving Technology Shop	In-situ compensated economizer	Used for a period of time, you do not say can really save electricity, really is a small power-saving helper, it is worth buying!	Jingdong prefecture level city in Shandong
Energy Saving Technology Shop	In-situ compensated economizer	There are many rooms and it takes a lot of electricity to run the air conditioner when the house is cold. Bought 4 inserted half a month to save me more than 300 dollars. The effect is really core good! Quality is also very good, the seller service is very good and patient.	Jingdong prefecture level city in Shandong
Energy Saving Technology Shop	In-situ compensated economizer	Recommended by the owner of the store next door, looked at his home does have to save electricity, decided to get. Indeed quite can, the voltage is much more stable, there is a reduction in electricity	Jingdong prefecture level city in Shandong
Energy Saving Technology Shop	In-situ compensated economizer	It does save electricity and works pretty well. I feel that in a few days, I will be able to use the money that I bought to save money, it's really good!	Jingdong prefecture level city in Shandong
Energy Saving Technology Shop	In-situ compensated economizer	Small store service attitude is very good Product quality is very good Suggested purchase	Jingdong prefecture level city in Shandong
Energy Saving Technology Shop	In-situ compensated economizer	Bought two energy-saving appliances for the family, the kitchen and bathroom water heater is too expensive, the water heater to be turned on every day, the monthly electricity bill is a lot of use, with this, no longer afraid of the electricity bill is more than one month down the electricity bill can be less than half of the previous month it, the effect is particularly good!	Jingdong prefecture level city in Shandong
Energy Saving Technology Shop	In-situ compensated economizer	Saving power is really good to use, the last time I bought 3, the family used , before the family monthly electricity bills are more than 300 fast, after using this are more than 200, are several months, this time to my store is also arranged, it is really good for the people good things.	Jingdong prefecture level city in Shandong
Energy Saving Technology Shop	In-situ compensated economizer	The previous year bought a, almost every month can save a 70 dollars or so, with a year of no problem at all, look like new, this time to move to a new home decisively buy back 2 units, very believe this store!	Jingdong prefecture level city in Shandong
Energy Saving Technology Shop	In-situ compensated economizer	Power-saving effect: used a week to comment, before and now record the meter comparison, my one rental room can save twenty degrees of electricity per day. Cost-effective. Quite worth it.	Jingdong prefecture level city in Shandong
Energy Saving Technology Shop	In-situ compensated economizer	This power saver is really powerful, used to spend 300 electricity bill every month, now it's about 100	Jingdong prefecture level city in Shandong
Energy Saving Technology Shop	In-situ compensated economizer	After two days of testing. The effect is good! About the first day of power saving for 1/4. the second day for 1/3. and not "steal electricity", is through the intelligent integrated circuit, automatically reduce the loss of electrical appliances to achieve the purpose of saving electricity! Recommended	Jingdong prefecture level city in Shandong
Energy Saving Technology Shop	In-situ compensated economizer	Power Saving King has been received, really good, thank you boss, I wish the boss business prosperity, prosperity, next time to come back to patronize!	Jingdong prefecture level city in Shandong
Energy Saving Technology Shop	In-situ compensated economizer	I've been using it for a while. Than to A little less electricity than before. I'll buy it again next time.	Jingdong prefecture level city in Shandong
Energy Saving Technology Shop	In-situ compensated economizer	Used to be a couple hundred bucks a month. With him, it's a big savings. It's great.	Jingdong prefecture level city in Shandong
Energy Saving Technology Shop	In-situ compensated economizer	Previously bought a plug in the kitchen, used for a period of time after the personal feeling is still quite effective, can save a little electricity, so this time without hesitation and bought two, has been inserted in the home next to the appliances that use a lot of electricity!	Jingdong prefecture level city in Shandong
Energy Saving Technology Shop	In-situ compensated economizer	I've used it before and it does have a power saving effect	Jingdong prefecture level city in Shandong
Energy Saving Technology Shop	In-situ compensated economizer	High-tech products, really useful, in previous years the high cost of electricity, with this product, the electricity bill came down. Praise a, hope that the research and development of this product of science and technology to continue to work hard, research out better products to.	Jingdong prefecture level city in Shandong
Energy Saving Technology Shop	In-situ compensated economizer	This high-power is not bad Oh, the first few months are 1000 degrees so, I casually brush in Taobao to this product, I bought back to try, I used a month after this month left 736 degrees, scared me, I also called to the grid to confirm, really is 736 degrees, the effect is very good, and today bought a go back to the old family to use	Jingdong prefecture level city in Shandong

Energy Saving Technology Shop	In-situ compensated economizer	Used for a period of time, you do not say can really save electricity, really is a small power-saving helper, it is worth buying!	Jingdong prefecture level city in Shandong
Energy Saving Technology Shop	In-situ compensated economizer	There are many rooms and it takes a lot of electricity to run the air conditioner when the house is cold. Bought 4 inserted half a month to save me more than 300 dollars. The effect is really core good! Quality is also very good, the seller service is very good and patient.	Jingdong prefecture level city in Shandong
Energy Saving Technology Shop	In-situ compensated economizer	Recommended by the owner of the store next door, looked at his home does have to save electricity, decided to get. Indeed quite can, the voltage is much more stable, there is a reduction in electricity	Jingdong prefecture level city in Shandong
Energy Saving Technology Shop	In-situ compensated economizer	It does save electricity and works pretty well. I feel that in a few days, I will be able to use the money that I bought to save money, it's really good!	Jingdong prefecture level city in Shandong
Energy Saving Technology Shop	In-situ compensated economizer	Small store service attitude is very good Product quality is very good Suggested purchase	Jingdong prefecture level city in Shandong
Energy Saving Technology Shop	In-situ compensated economizer	Bought two energy-saving appliances for the family, the kitchen and bathroom water heater is too expensive, the water heater to be turned on every day, the monthly electricity bill is a lot of use, with this, no longer afraid of the electricity bill is more than one month down the electricity bill can be less than half of the previous month it, the effect is particularly good!	Jingdong prefecture level city in Shandong
Energy Saving Technology Shop	In-situ compensated economizer	Saving power is really good to use, the last time I bought 3, the family used , before the family monthly electricity bills are more than 300 fast, after using this are more than 200, are several months, this time to my store is also arranged, it is really good for the people good things.	Jingdong prefecture level city in Shandong
Energy Saving Technology Shop	In-situ compensated economizer	The previous year bought a, almost every month can save a 70 dollars or so, with a year of no problem at all, look like new, this time to move to a new home decisively buy back 2 units, very believe this store!	Jingdong prefecture level city in Shandong
Energy Saving Technology Shop	In-situ compensated economizer	Power-saving effect: used a week to comment, before and now record the meter comparison, my one rental room can save twenty degrees of electricity per day. Cost-effective. Quite worth it.	Jingdong prefecture level city in Shandong
Energy Saving Technology Shop	In-situ compensated economizer	This power saver is really powerful, used to spend 300 electricity bill every month, now it's about 100	Jingdong prefecture level city in Shandong
Energy Saving Technology Shop	In-situ compensated economizer	After two days of testing. The effect is good! About the first day of power saving for 1/4. the second day for 1/3. and not "steal electricity", is through the intelligent integrated circuit, automatically reduce the loss of electrical appliances to achieve the purpose of saving electricity! Recommended	Jingdong prefecture level city in Shandong
Energy Saving Technology Shop	In-situ compensated economizer	Power Saving King has been received, really good, thank you boss, I wish the boss business prosperity, prosperity, next time to come back to patronize!	Jingdong prefecture level city in Shandong
Energy Saving Technology Shop	In-situ compensated economizer	I've been using it for a while. Than to A little less electricity than before. I'll buy it again next time.	Jingdong prefecture level city in Shandong
Energy Saving Technology Shop	In-situ compensated economizer	Used to be a couple hundred bucks a month. With him, it's a big savings. It's great.	Jingdong prefecture level city in Shandong
Energy Saving Technology Shop	In-situ compensated economizer	Previously bought a plug in the kitchen, used for a period of time after the personal feeling is still quite effective, can save a little electricity, so this time without hesitation and bought two, has been inserted in the home next to the appliances that use a lot of electricity!	Jingdong prefecture level city in Shandong
Energy Saving Technology Shop	In-situ compensated economizer	I've used it before and it does have a power saving effect	Jingdong prefecture level city in Shandong
Energy Saving Technology Shop	In-situ compensated economizer	High-tech products, really useful, in previous years the high cost of electricity, with this product, the electricity bill came down. Praise a, hope that the research and development of this product of science and technology to continue to work hard, research out better products to.	Jingdong prefecture level city in Shandong
Energy Saving Technology Shop	In-situ compensated economizer	This high-power is not bad Oh, the first few months are 1000 degrees so, I casually brush in Taobao to this product, I bought back to try, I used a month after this month left 736 degrees, scared me, I also called to the grid to confirm, really is 736 degrees, the effect is very good, and today bought a go back to the old family to use	Jingdong prefecture level city in Shandong
Energy Saving Technology Shop	In-situ compensated economizer	Used for a period of time, you do not say can really save electricity, really is a small power-saving helper, it is worth buying!	Jingdong prefecture level city in Shandong
Energy Saving Technology Shop	In-situ compensated economizer	There are many rooms and it takes a lot of electricity to run the air conditioner when the house is cold. Bought 4 inserted half a month to save me more than 300 dollars. The effect is really core good! Quality is also very good, the seller service is very good and patient.	Jingdong prefecture level city in Shandong
Energy Saving Technology Shop	In-situ compensated economizer	Recommended by the owner of the store next door, looked at his home does have to save electricity, decided to get. Indeed quite can, the voltage is much more stable, there is a reduction in electricity	Jingdong prefecture level city in Shandong
Energy Saving Technology Shop	In-situ compensated economizer	It does save electricity and works pretty well. I feel that in a few days, I will be able to use the money that I bought to save money, it's really good!	Jingdong prefecture level city in Shandong
Energy Saving Technology Shop	In-situ compensated economizer	Small store service attitude is very good Product quality is very good Suggested purchase	Jingdong prefecture level city in Shandong
Energy Saving Technology Shop	In-situ compensated economizer	Bought two energy-saving appliances for the family, the kitchen and bathroom water heater is too expensive, the water heater to be turned on every day, the monthly electricity bill is a lot of use, with this, no longer afraid of the electricity bill is more than one month down the electricity bill can be less than half of the previous month it, the effect is particularly good!	Jingdong prefecture level city in Shandong
Energy Saving Technology Shop	In-situ compensated economizer	Saving power is really good to use, the last time I bought 3, the family used , before the family monthly electricity bills are more than 300 fast, after using this are more than 200, are several months, this time to my store is also arranged, it is really good for the people good things.	Jingdong prefecture level city in Shandong
Energy Saving Technology Shop	In-situ compensated economizer	The previous year bought a, almost every month can save a 70 dollars or so, with a year of no problem at all, look like new, this time to move to a new home decisively buy back 2 units, very believe this store!	Jingdong prefecture level city in Shandong
Energy Saving Technology Shop	In-situ compensated economizer	Power-saving effect: used a week to comment, before and now record the meter comparison, my one rental room can save twenty degrees of electricity per day. Cost-effective. Quite worth it.	Jingdong prefecture level city in Shandong
Energy Saving Technology Shop	In-situ compensated economizer	This power saver is really powerful, used to spend 300 electricity bill every month, now it's about 100	Jingdong prefecture level city in Shandong
Energy Saving Technology Shop	In-situ compensated economizer	After two days of testing. The effect is good! About the first day of power saving for 1/4. the second day for 1/3. and not "steal electricity", is through the intelligent integrated circuit, automatically reduce the loss of electrical appliances to achieve the purpose of saving electricity! Recommended	Jingdong prefecture level city in Shandong
Energy Saving Technology Shop	In-situ compensated economizer	Power Saving King has been received, really good, thank you boss, I wish the boss business prosperity, prosperity, next time to come back to patronize!	Jingdong prefecture level city in Shandong
Energy Saving Technology Shop	In-situ compensated economizer	I've been using it for a while. Than to A little less electricity than before. I'll buy it again next time.	Jingdong prefecture level city in Shandong
Energy Saving Technology Shop	In-situ compensated economizer	Used to be a couple hundred bucks a month. With him, it's a big savings. It's great.	Jingdong prefecture level city in Shandong
Energy Saving Technology Shop	In-situ compensated economizer	Previously bought a plug in the kitchen, used for a period of time after the personal feeling is still quite effective, can save a little electricity, so this time without hesitation and bought two, has been inserted in the home next to the appliances that use a lot of electricity!	Jingdong prefecture level city in Shandong
Energy Saving Technology Shop	In-situ compensated economizer	I've used it before and it does have a power saving effect	Jingdong prefecture level city in Shandong
Energy Saving Technology Shop	In-situ compensated economizer	High-tech products, really useful, in previous years the high cost of electricity, with this product, the electricity bill came down. Praise a, hope that the research and development of this product of science and technology to continue to work hard, research out better products to.	Jingdong prefecture level city in Shandong
Energy Saving Technology Shop	In-situ compensated economizer	This high-power is not bad Oh, the first few months are 1000 degrees so, I casually brush in Taobao to this product, I bought back to try, I used a month after this month left 736 degrees, scared me, I also called to the grid to confirm, really is 736 degrees, the effect is very good, and today bought a go back to the old family to use	Jingdong prefecture level city in Shandong
Energy Saving Technology Shop	In-situ compensated economizer	Used for a period of time, you do not say can really save electricity, really is a small power-saving helper, it is worth buying!	Jingdong prefecture level city in Shandong
Energy Saving Technology Shop	In-situ compensated economizer	There are many rooms and it takes a lot of electricity to run the air conditioner when the house is cold. Bought 4 inserted half a month to save me more than 300 dollars. The effect is really core good! Quality is also very good, the seller service is very good and patient.	Jingdong prefecture level city in Shandong
Energy Saving Technology Shop	In-situ compensated economizer	Recommended by the owner of the store next door, looked at his home does have to save electricity, decided to get. Indeed quite can, the voltage is much more stable, there is a reduction in electricity	Jingdong prefecture level city in Shandong
Energy Saving Technology Shop	In-situ compensated economizer	It does save electricity and works pretty well. I feel that in a few days, I will be able to use the money that I bought to save money, it's really good!	Jingdong prefecture level city in Shandong
Energy Saving Technology Shop	In-situ compensated economizer	Small store service attitude is very good Product quality is very good Suggested purchase	Jingdong prefecture level city in Shandong

Energy Saving Technology Shop	In-situ compensated economizer	Bought two energy-saving appliances for the family, the kitchen and bathroom water heater is too expensive, the water heater to be turned on every day, the monthly electricity bill is a lot of use, with this, no longer afraid of the electricity bill is more than one month down the electricity bill can be less than half of the previous month it, the effect is particularly good!	Jingdong prefecture level city in Shandong
Energy Saving Technology Shop	In-situ compensated economizer	Saving power is really good to use, the last time I bought 3, the family used , before the family monthly electricity bills are more than 300 fast, after using this are more than 200, are several months, this time to my store is also arranged, it is really good for the people good things.	Jingdong prefecture level city in Shandong
Energy Saving Technology Shop	In-situ compensated economizer	The previous year bought a, almost every month can save a 70 dollars or so, with a year of no problem at all, look like new, this time to move to a new home decisively buy back 2 units, very believe this store!	Jingdong prefecture level city in Shandong
Energy Saving Technology Shop	In-situ compensated economizer	Power-saving effect: used a week to comment, before and now record the meter comparison, my one rental room can save twenty degrees of electricity per day. Cost-effective. Quite worth it.	Jingdong prefecture level city in Shandong
Energy Saving Technology Shop	In-situ compensated economizer	This power saver is really powerful, used to spend 300 electricity bill every month, now it's about 100	Jingdong prefecture level city in Shandong
Energy Saving Technology Shop	In-situ compensated economizer	After two days of testing. The effect is good! About the first day of power saving for 1/4. the second day for 1/3. and not "steal electricity", is through the intelligent integrated circuit, automatically reduce the loss of electrical appliances to achieve the purpose of saving electricity! Recommended	Jingdong prefecture level city in Shandong
Energy Saving Technology Shop	In-situ compensated economizer	Power Saving King has been received, really good, thank you boss, I wish the boss business prosperity, prosperity, next time to come back to patronize!	Jingdong prefecture level city in Shandong
Energy Saving Technology Shop	In-situ compensated economizer	I've been using it for a while. Than to A little less electricity than before. I'll buy it again next time.	Jingdong prefecture level city in Shandong
Energy Saving Technology Shop	In-situ compensated economizer	Used to be a couple hundred bucks a month. With him, it's a big savings. It's great.	Jingdong prefecture level city in Shandong
Energy Saving Technology Shop	In-situ compensated economizer	Previously bought a plug in the kitchen, used for a period of time after the personal feeling is still quite effective, can save a little electricity, so this time without hesitation and bought two, has been inserted in the home next to the appliances that use a lot of electricity!	Jingdong prefecture level city in Shandong
Energy Saving Technology Shop	In-situ compensated economizer	I've used it before and it does have a power saving effect	Jingdong prefecture level city in Shandong
Energy Saving Technology Shop	In-situ compensated economizer	High-tech products, really useful, in previous years the high cost of electricity, with this product, the electricity bill came down. Praise a, hope that the research and development of this product of science and technology to continue to work hard, research out better products to.	Jingdong prefecture level city in Shandong
Energy Saving Technology Shop	In-situ compensated economizer	This high-power is not bad Oh, the first few months are 1000 degrees so, I casually brush in Taobao to this product, I bought back to try, I used a month after this month left 736 degrees, scared me, I also called to the grid to confirm, really is 736 degrees, the effect is very good, and today bought a go back to the old family to use	Jingdong prefecture level city in Shandong
Energy Saving Technology Shop	In-situ compensated economizer	Used for a period of time, you do not say can really save electricity, really is a small power-saving helper, it is worth buying!	Jingdong prefecture level city in Shandong
Energy Saving Technology Shop	In-situ compensated economizer	There are many rooms and it takes a lot of electricity to run the air conditioner when the house is cold. Bought 4 inserted half a month to save me more than 300 dollars. The effect is really core good! Quality is also very good, the seller service is very good and patient.	Jingdong prefecture level city in Shandong
Energy Saving Technology Shop	In-situ compensated economizer	Recommended by the owner of the store next door, looked at his home does have to save electricity, decided to get. Indeed quite can, the voltage is much more stable, there is a reduction in electricity	Jingdong prefecture level city in Shandong
Energy Saving Technology Shop	In-situ compensated economizer	It does save electricity and works pretty well. I feel that in a few days, I will be able to use the money that I bought to save money, it's really good!	Jingdong prefecture level city in Shandong
Energy Saving Technology Shop	In-situ compensated economizer	Small store service attitude is very good Product quality is very good Suggested purchase	Jingdong prefecture level city in Shandong
Energy Saving Technology Shop	In-situ compensated economizer	Bought two energy-saving appliances for the family, the kitchen and bathroom water heater is too expensive, the water heater to be turned on every day, the monthly electricity bill is a lot of use, with this, no longer afraid of the electricity bill is more than one month down the electricity bill can be less than half of the previous month it, the effect is particularly good!	Jingdong prefecture level city in Shandong
Energy Saving Technology Shop	In-situ compensated economizer	Saving power is really good to use, the last time I bought 3, the family used , before the family monthly electricity bills are more than 300 fast, after using this are more than 200, are several months, this time to my store is also arranged, it is really good for the people good things.	Jingdong prefecture level city in Shandong
Energy Saving Technology Shop	In-situ compensated economizer	The previous year bought a, almost every month can save a 70 dollars or so, with a year of no problem at all, look like new, this time to move to a new home decisively buy back 2 units, very believe this store!	Jingdong prefecture level city in Shandong
Energy Saving Technology Shop	In-situ compensated economizer	Power-saving effect: used a week to comment, before and now record the meter comparison, my one rental room can save twenty degrees of electricity per day. Cost-effective. Quite worth it.	Jingdong prefecture level city in Shandong
Energy Saving Technology Shop	In-situ compensated economizer	This power saver is really powerful, used to spend 300 electricity bill every month, now it's about 100	Jingdong prefecture level city in Shandong
Energy Saving Technology Shop	In-situ compensated economizer	After two days of testing. The effect is good! About the first day of power saving for 1/4. the second day for 1/3. and not "steal electricity", is through the intelligent integrated circuit, automatically reduce the loss of electrical appliances to achieve the purpose of saving electricity! Recommended	Jingdong prefecture level city in Shandong
Energy Saving Technology Shop	In-situ compensated economizer	Power Saving King has been received, really good, thank you boss, I wish the boss business prosperity, prosperity, next time to come back to patronize!	Jingdong prefecture level city in Shandong
Energy Saving Technology Shop	In-situ compensated economizer	I've been using it for a while. Than to A little less electricity than before. I'll buy it again next time.	Jingdong prefecture level city in Shandong
Energy Saving Technology Shop	In-situ compensated economizer	Used to be a couple hundred bucks a month. With him, it's a big savings. It's great.	Jingdong prefecture level city in Shandong
Energy Saving Technology Shop	In-situ compensated economizer	Previously bought a plug in the kitchen, used for a period of time after the personal feeling is still quite effective, can save a little electricity, so this time without hesitation and bought two, has been inserted in the home next to the appliances that use a lot of electricity!	Jingdong prefecture level city in Shandong
Energy Saving Technology Shop	In-situ compensated economizer	I've used it before and it does have a power saving effect	Jingdong prefecture level city in Shandong
Energy Saving Technology Shop	In-situ compensated economizer	High-tech products, really useful, in previous years the high cost of electricity, with this product, the electricity bill came down. Praise a, hope that the research and development of this product of science and technology to continue to work hard, research out better products to.	Jingdong prefecture level city in Shandong
Energy Saving Technology Shop	In-situ compensated economizer	This high-power is not bad Oh, the first few months are 1000 degrees so, I casually brush in Taobao to this product, I bought back to try, I used a month after this month left 736 degrees, scared me, I also called to the grid to confirm, really is 736 degrees, the effect is very good, and today bought a go back to the old family to use	Jingdong prefecture level city in Shandong
Energy Saving Technology Shop	In-situ compensated economizer	Used for a period of time, you do not say can really save electricity, really is a small power-saving helper, it is worth buying!	Jingdong prefecture level city in Shandong
Energy Saving Technology Shop	In-situ compensated economizer	There are many rooms and it takes a lot of electricity to run the air conditioner when the house is cold. Bought 4 inserted half a month to save me more than 300 dollars. The effect is really core good! Quality is also very good, the seller service is very good and patient.	Jingdong prefecture level city in Shandong
Energy Saving Technology Shop	In-situ compensated economizer	Recommended by the owner of the store next door, looked at his home does have to save electricity, decided to get. Indeed quite can, the voltage is much more stable, there is a reduction in electricity	Jingdong prefecture level city in Shandong
Energy Saving Technology Shop	In-situ compensated economizer	It does save electricity and works pretty well. I feel that in a few days, I will be able to use the money that I bought to save money, it's really good!	Jingdong prefecture level city in Shandong
Energy Saving Technology Shop	In-situ compensated economizer	Small store service attitude is very good Product quality is very good Suggested purchase	Jingdong prefecture level city in Shandong
Energy Saving Technology Shop	In-situ compensated economizer	Bought two energy-saving appliances for the family, the kitchen and bathroom water heater is too expensive, the water heater to be turned on every day, the monthly electricity bill is a lot of use, with this, no longer afraid of the electricity bill is more than one month down the electricity bill can be less than half of the previous month it, the effect is particularly good!	Jingdong prefecture level city in Shandong
Energy Saving Technology Shop	In-situ compensated economizer	Saving power is really good to use, the last time I bought 3, the family used , before the family monthly electricity bills are more than 300 fast, after using this are more than 200, are several months, this time to my store is also arranged, it is really good for the people good things.	Jingdong prefecture level city in Shandong
Energy Saving Technology Shop	In-situ compensated economizer	The previous year bought a, almost every month can save a 70 dollars or so, with a year of no problem at all, look like new, this time to move to a new home decisively buy back 2 units, very believe this store!	Jingdong prefecture level city in Shandong
Energy Saving Technology Shop	In-situ compensated economizer	Power-saving effect: used a week to comment, before and now record the meter comparison, my one rental room can save twenty degrees of electricity per day. Cost-effective. Quite worth it.	Jingdong prefecture level city in Shandong
Energy Saving Technology Shop	In-situ compensated economizer	This power saver is really powerful, used to spend 300 electricity bill every month, now it's about 100	Jingdong prefecture level city in Shandong

Energy Saving Technology Shop	In-situ compensated economizer	After two days of testing. The effect is good! About the first day of power saving for 1/4. the second day for 1/3. and not "steal electricity", is through the intelligent integrated circuit, automatically reduce the loss of electrical appliances to achieve the purpose of saving electricity! Recommended	Jingdong prefecture level city in Shandong
Energy Saving Technology Shop	In-situ compensated economizer	Power Saving King has been received, really good, thank you boss, I wish the boss business prosperity, prosperity, next time to come back to patronize!	Jingdong prefecture level city in Shandong
Energy Saving Technology Shop	In-situ compensated economizer	I've been using it for a while. Than to A little less electricity than before. I'll buy it again next time.	Jingdong prefecture level city in Shandong
Energy Saving Technology Shop	In-situ compensated economizer	Used to be a couple hundred bucks a month. With him, it's a big savings. It's great.	Jingdong prefecture level city in Shandong
Energy Saving Technology Shop	In-situ compensated economizer	Previously bought a plug in the kitchen, used for a period of time after the personal feeling is still quite effective, can save a little electricity, so this time without hesitation and bought two, has been inserted in the home next to the appliances that use a lot of electricity!	Jingdong prefecture level city in Shandong
Energy Saving Technology Shop	In-situ compensated economizer	I've used it before and it does have a power saving effect	Jingdong prefecture level city in Shandong
Energy Saving Technology Shop	In-situ compensated economizer	High-tech products, really useful, in previous years the high cost of electricity, with this product, the electricity bill came down. Praise a, hope that the research and development of this product of science and technology to continue to work hard, research out better products to.	Jingdong prefecture level city in Shandong
Energy Saving Technology Shop	In-situ compensated economizer	This high-power is not bad Oh, the first few months are 1000 degrees so, I casually brush in Taobao to this product, I bought back to try, I used a month after this month left 736 degrees, scared me, I also called to the grid to confirm, really is 736 degrees, the effect is very good, and today bought a go back to the old family to use	Jingdong prefecture level city in Shandong
Energy Saving Technology Shop	In-situ compensated economizer	Used for a period of time, you do not say can really save electricity, really is a small power-saving helper, it is worth buying!	Jingdong prefecture level city in Shandong
Energy Saving Technology Shop	In-situ compensated economizer	There are many rooms and it takes a lot of electricity to run the air conditioner when the house is cold. Bought 4 inserted half a month to save me more than 300 dollars. The effect is really core good! Quality is also very good, the seller service is very good and patient.	Jingdong prefecture level city in Shandong
Energy Saving Technology Shop	In-situ compensated economizer	Recommended by the owner of the store next door, looked at his home does have to save electricity, decided to get. Indeed quite can, the voltage is much more stable, there is a reduction in electricity	Jingdong prefecture level city in Shandong
Energy Saving Technology Shop	In-situ compensated economizer	It does save electricity and works pretty well. I feel that in a few days, I will be able to use the money that I bought to save money, it's really good!	Jingdong prefecture level city in Shandong
Energy Saving Technology Shop	In-situ compensated economizer	Small store service attitude is very good Product quality is very good Suggested purchase	Jingdong prefecture level city in Shandong
Energy Saving Technology Shop	In-situ compensated economizer	Bought two energy-saving appliances for the family, the kitchen and bathroom water heater is too expensive, the water heater to be turned on every day, the monthly electricity bill is a lot of use, with this, no longer afraid of the electricity bill is more than one month down the electricity bill can be less than half of the previous month it, the effect is particularly good!	Jingdong prefecture level city in Shandong
Energy Saving Technology Shop	In-situ compensated economizer	Saving power is really good to use, the last time I bought 3, the family used , before the family monthly electricity bills are more than 300 fast, after using this are more than 200, are several months, this time to my store is also arranged, it is really good for the people good things.	Jingdong prefecture level city in Shandong
Energy Saving Technology Shop	In-situ compensated economizer	The previous year bought a, almost every month can save a 70 dollars or so, with a year of no problem at all, look like new, this time to move to a new home decisively buy back 2 units, very believe this store!	Jingdong prefecture level city in Shandong
Energy Saving Technology Shop	In-situ compensated economizer	Power-saving effect: used a week to comment, before and now record the meter comparison, my one rental room can save twenty degrees of electricity per day. Cost-effective. Quite worth it.	Jingdong prefecture level city in Shandong
Energy Saving Technology Shop	In-situ compensated economizer	This power saver is really powerful, used to spend 300 electricity bill every month, now it's about 100	Jingdong prefecture level city in Shandong
Energy Saving Technology Shop	In-situ compensated economizer	After two days of testing. The effect is good! About the first day of power saving for 1/4. the second day for 1/3. and not "steal electricity", is through the intelligent integrated circuit, automatically reduce the loss of electrical appliances to achieve the purpose of saving electricity! Recommended	Jingdong prefecture level city in Shandong
Energy Saving Technology Shop	In-situ compensated economizer	Power Saving King has been received, really good, thank you boss, I wish the boss business prosperity, prosperity, next time to come back to patronize!	Jingdong prefecture level city in Shandong
Energy Saving Technology Shop	In-situ compensated economizer	I've been using it for a while. Than to A little less electricity than before. I'll buy it again next time.	Jingdong prefecture level city in Shandong
Energy Saving Technology Shop	In-situ compensated economizer	Used to be a couple hundred bucks a month. With him, it's a big savings. It's great.	Jingdong prefecture level city in Shandong
Energy Saving Technology Shop	In-situ compensated economizer	Previously bought a plug in the kitchen, used for a period of time after the personal feeling is still quite effective, can save a little electricity, so this time without hesitation and bought two, has been inserted in the home next to the appliances that use a lot of electricity!	Jingdong prefecture level city in Shandong
Energy Saving Technology Shop	In-situ compensated economizer	I've used it before and it does have a power saving effect	Jingdong prefecture level city in Shandong
Energy Saving Technology Shop	In-situ compensated economizer	High-tech products, really useful, in previous years the high cost of electricity, with this product, the electricity bill came down. Praise a, hope that the research and development of this product of science and technology to continue to work hard, research out better products to.	Jingdong prefecture level city in Shandong
Energy Saving Technology Shop	In-situ compensated economizer	This high-power is not bad Oh, the first few months are 1000 degrees so, I casually brush in Taobao to this product, I bought back to try, I used a month after this month left 736 degrees, scared me, I also called to the grid to confirm, really is 736 degrees, the effect is very good, and today bought a go back to the old family to use	Jingdong prefecture level city in Shandong
Energy Saving Technology Shop	In-situ compensated economizer	Used for a period of time, you do not say can really save electricity, really is a small power-saving helper, it is worth buying!	Jingdong prefecture level city in Shandong
Energy Saving Technology Shop	In-situ compensated economizer	There are many rooms and it takes a lot of electricity to run the air conditioner when the house is cold. Bought 4 inserted half a month to save me more than 300 dollars. The effect is really core good! Quality is also very good, the seller service is very good and patient.	Jingdong prefecture level city in Shandong
Energy Saving Technology Shop	In-situ compensated economizer	Recommended by the owner of the store next door, looked at his home does have to save electricity, decided to get. Indeed quite can, the voltage is much more stable, there is a reduction in electricity	Jingdong prefecture level city in Shandong
Energy Saving Technology Shop	In-situ compensated economizer	It does save electricity and works pretty well. I feel that in a few days, I will be able to use the money that I bought to save money, it's really good!	Jingdong prefecture level city in Shandong
Energy Saving Technology Shop	In-situ compensated economizer	Small store service attitude is very good Product quality is very good Suggested purchase	Jingdong prefecture level city in Shandong
Energy Saving Technology Shop	In-situ compensated economizer	Bought two energy-saving appliances for the family, the kitchen and bathroom water heater is too expensive, the water heater to be turned on every day, the monthly electricity bill is a lot of use, with this, no longer afraid of the electricity bill is more than one month down the electricity bill can be less than half of the previous month it, the effect is particularly good!	Jingdong prefecture level city in Shandong
Energy Saving Technology Shop	In-situ compensated economizer	Saving power is really good to use, the last time I bought 3, the family used , before the family monthly electricity bills are more than 300 fast, after using this are more than 200, are several months, this time to my store is also arranged, it is really good for the people good things.	Jingdong prefecture level city in Shandong
Energy Saving Technology Shop	In-situ compensated economizer	The previous year bought a, almost every month can save a 70 dollars or so, with a year of no problem at all, look like new, this time to move to a new home decisively buy back 2 units, very believe this store!	Jingdong prefecture level city in Shandong
Energy Saving Technology Shop	In-situ compensated economizer	Power-saving effect: used a week to comment, before and now record the meter comparison, my one rental room can save twenty degrees of electricity per day. Cost-effective. Quite worth it.	Jingdong prefecture level city in Shandong
Energy Saving Technology Shop	In-situ compensated economizer	This power saver is really powerful, used to spend 300 electricity bill every month, now it's about 100	Jingdong prefecture level city in Shandong
Energy Saving Technology Shop	In-situ compensated economizer	After two days of testing. The effect is good! About the first day of power saving for 1/4. the second day for 1/3. and not "steal electricity", is through the intelligent integrated circuit, automatically reduce the loss of electrical appliances to achieve the purpose of saving electricity! Recommended	Jingdong prefecture level city in Shandong
Energy Saving Technology Shop	In-situ compensated economizer	Power Saving King has been received, really good, thank you boss, I wish the boss business prosperity, prosperity, next time to come back to patronize!	Jingdong prefecture level city in Shandong
Energy Saving Technology Shop	In-situ compensated economizer	I've been using it for a while. Than to A little less electricity than before. I'll buy it again next time.	Jingdong prefecture level city in Shandong
Energy Saving Technology Shop	In-situ compensated economizer	Used to be a couple hundred bucks a month. With him, it's a big savings. It's great.	Jingdong prefecture level city in Shandong
Energy Saving Technology Shop	In-situ compensated economizer	Previously bought a plug in the kitchen, used for a period of time after the personal feeling is still quite effective, can save a little electricity, so this time without hesitation and bought two, has been inserted in the home next to the appliances that use a lot of electricity!	Jingdong prefecture level city in Shandong

Energy Saving Technology Shop	In-situ compensated economizer	I've used it before and it does have a power saving effect	Jingdong prefecture level city in Shandong
Energy Saving Technology Shop	In-situ compensated economizer	High-tech products, really useful, in previous years the high cost of electricity, with this product, the electricity bill came down. Praise a, hope that the research and development of this product of science and technology to continue to work hard, research out better products to.	Jingdong prefecture level city in Shandong
Energy Saving Technology Shop	In-situ compensated economizer	This high-power is not bad Oh, the first few months are 1000 degrees so, I casually brush in Taobao to this product, I bought back to try, I used a month after this month left 736 degrees, scared me, I also called to the grid to confirm, really is 736 degrees, the effect is very good, and today bought a go back to the old family to use	Jingdong prefecture level city in Shandong
Energy Saving Technology Shop	In-situ compensated economizer	Used for a period of time, you do not say can really save electricity, really is a small power-saving helper, it is worth buying!	Jingdong prefecture level city in Shandong
Energy Saving Technology Shop	In-situ compensated economizer	There are many rooms and it takes a lot of electricity to run the air conditioner when the house is cold. Bought 4 inserted half a month to save me more than 300 dollars. The effect is really core good! Quality is also very good, the seller service is very good and patient.	Jingdong prefecture level city in Shandong
Energy Saving Technology Shop	In-situ compensated economizer	Recommended by the owner of the store next door, looked at his home does have to save electricity, decided to get. Indeed quite can, the voltage is much more stable, there is a reduction in electricity	Jingdong prefecture level city in Shandong
Energy Saving Technology Shop	In-situ compensated economizer	It does save electricity and works pretty well. I feel that in a few days, I will be able to use the money that I bought to save money, it's really good!	Jingdong prefecture level city in Shandong
Energy Saving Technology Shop	In-situ compensated economizer	Small store service attitude is very good Product quality is very good Suggested purchase	Jingdong prefecture level city in Shandong
Energy Saving Technology Shop	In-situ compensated economizer	Bought two energy-saving appliances for the family, the kitchen and bathroom water heater is too expensive, the water heater to be turned on every day, the monthly electricity bill is a lot of use, with this, no longer afraid of the electricity bill is more than one month down the electricity bill can be less than half of the previous month it, the effect is particularly good!	Jingdong prefecture level city in Shandong
Energy Saving Technology Shop	In-situ compensated economizer	Saving power is really good to use, the last time I bought 3, the family used , before the family monthly electricity bills are more than 300 fast, after using this are more than 200, are several months, this time to my store is also arranged, it is really good for the people good things.	Jingdong prefecture level city in Shandong
Energy Saving Technology Shop	In-situ compensated economizer	The previous year bought a, almost every month can save a 70 dollars or so, with a year of no problem at all, look like new, this time to move to a new home decisively buy back 2 units, very believe this store!	Jingdong prefecture level city in Shandong
Energy Saving Technology Shop	In-situ compensated economizer	Power-saving effect: used a week to comment, before and now record the meter comparison, my one rental room can save twenty degrees of electricity per day. Cost-effective. Quite worth it.	Jingdong prefecture level city in Shandong
Energy Saving Technology Shop	In-situ compensated economizer	This power saver is really powerful, used to spend 300 electricity bill every month, now it's about 100	Jingdong prefecture level city in Shandong
Energy Saving Technology Shop	In-situ compensated economizer	After two days of testing. The effect is good! About the first day of power saving for 1/4. the second day for 1/3. and not "steal electricity", is through the intelligent integrated circuit, automatically reduce the loss of electrical appliances to achieve the purpose of saving electricity! Recommended	Jingdong prefecture level city in Shandong
Energy Saving Technology Shop	In-situ compensated economizer	Power Saving King has been received, really good, thank you boss, I wish the boss business prosperity, prosperity, next time to come back to patronize!	Jingdong prefecture level city in Shandong
Energy Saving Technology Shop	In-situ compensated economizer	I've been using it for a while. Than to A little less electricity than before. I'll buy it again next time.	Jingdong prefecture level city in Shandong
Energy Saving Technology Shop	In-situ compensated economizer	Used to be a couple hundred bucks a month. With him, it's a big savings. It's great.	Jingdong prefecture level city in Shandong
Energy Saving Technology Shop	In-situ compensated economizer	Previously bought a plug in the kitchen, used for a period of time after the personal feeling is still quite effective, can save a little electricity, so this time without hesitation and bought two, has been inserted in the home next to the appliances that use a lot of electricity!	Jingdong prefecture level city in Shandong
Energy Saving Technology Shop	In-situ compensated economizer	I've used it before and it does have a power saving effect	Jingdong prefecture level city in Shandong
Energy Saving Technology Shop	In-situ compensated economizer	High-tech products, really useful, in previous years the high cost of electricity, with this product, the electricity bill came down. Praise a, hope that the research and development of this product of science and technology to continue to work hard, research out better products to.	Jingdong prefecture level city in Shandong
Energy Saving Technology Shop	In-situ compensated economizer	This high-power is not bad Oh, the first few months are 1000 degrees so, I casually brush in Taobao to this product, I bought back to try, I used a month after this month left 736 degrees, scared me, I also called to the grid to confirm, really is 736 degrees, the effect is very good, and today bought a go back to the old family to use	Jingdong prefecture level city in Shandong
Energy Saving Technology Shop	In-situ compensated economizer	Used for a period of time, you do not say can really save electricity, really is a small power-saving helper, it is worth buying!	Jingdong prefecture level city in Shandong
Energy Saving Technology Shop	In-situ compensated economizer	There are many rooms and it takes a lot of electricity to run the air conditioner when the house is cold. Bought 4 inserted half a month to save me more than 300 dollars. The effect is really core good! Quality is also very good, the seller service is very good and patient.	Jingdong prefecture level city in Shandong
Energy Saving Technology Shop	In-situ compensated economizer	Recommended by the owner of the store next door, looked at his home does have to save electricity, decided to get. Indeed quite can, the voltage is much more stable, there is a reduction in electricity	Jingdong prefecture level city in Shandong
Energy Saving Technology Shop	In-situ compensated economizer	It does save electricity and works pretty well. I feel that in a few days, I will be able to use the money that I bought to save money, it's really good!	Jingdong prefecture level city in Shandong
Energy Saving Technology Shop	In-situ compensated economizer	Small store service attitude is very good Product quality is very good Suggested purchase	Jingdong prefecture level city in Shandong
Energy Saving Technology Shop	In-situ compensated economizer	Bought two energy-saving appliances for the family, the kitchen and bathroom water heater is too expensive, the water heater to be turned on every day, the monthly electricity bill is a lot of use, with this, no longer afraid of the electricity bill is more than one month down the electricity bill can be less than half of the previous month it, the effect is particularly good!	Jingdong prefecture level city in Shandong
Energy Saving Technology Shop	In-situ compensated economizer	Saving power is really good to use, the last time I bought 3, the family used , before the family monthly electricity bills are more than 300 fast, after using this are more than 200, are several months, this time to my store is also arranged, it is really good for the people good things.	Jingdong prefecture level city in Shandong
Energy Saving Technology Shop	In-situ compensated economizer	The previous year bought a, almost every month can save a 70 dollars or so, with a year of no problem at all, look like new, this time to move to a new home decisively buy back 2 units, very believe this store!	Jingdong prefecture level city in Shandong
Energy Saving Technology Shop	In-situ compensated economizer	Power-saving effect: used a week to comment, before and now record the meter comparison, my one rental room can save twenty degrees of electricity per day. Cost-effective. Quite worth it.	Jingdong prefecture level city in Shandong
Energy Saving Technology Shop	In-situ compensated economizer	This power saver is really powerful, used to spend 300 electricity bill every month, now it's about 100	Jingdong prefecture level city in Shandong
Energy Saving Technology Shop	In-situ compensated economizer	After two days of testing. The effect is good! About the first day of power saving for 1/4. the second day for 1/3. and not "steal electricity", is through the intelligent integrated circuit, automatically reduce the loss of electrical appliances to achieve the purpose of saving electricity! Recommended	Jingdong prefecture level city in Shandong
Energy Saving Technology Shop	In-situ compensated economizer	Power Saving King has been received, really good, thank you boss, I wish the boss business prosperity, prosperity, next time to come back to patronize!	Jingdong prefecture level city in Shandong
Energy Saving Technology Shop	In-situ compensated economizer	I've been using it for a while. Than to A little less electricity than before. I'll buy it again next time.	Jingdong prefecture level city in Shandong
Energy Saving Technology Shop	In-situ compensated economizer	Used to be a couple hundred bucks a month. With him, it's a big savings. It's great.	Jingdong prefecture level city in Shandong
Energy Saving Technology Shop	In-situ compensated economizer	Previously bought a plug in the kitchen, used for a period of time after the personal feeling is still quite effective, can save a little electricity, so this time without hesitation and bought two, has been inserted in the home next to the appliances that use a lot of electricity!	Jingdong prefecture level city in Shandong
Energy Saving Technology Shop	In-situ compensated economizer	I've used it before and it does have a power saving effect	Jingdong prefecture level city in Shandong
Energy Saving Technology Shop	In-situ compensated economizer	High-tech products, really useful, in previous years the high cost of electricity, with this product, the electricity bill came down. Praise a, hope that the research and development of this product of science and technology to continue to work hard, research out better products to.	Jingdong prefecture level city in Shandong
Energy Saving Technology Shop	In-situ compensated economizer	This high-power is not bad Oh, the first few months are 1000 degrees so, I casually brush in Taobao to this product, I bought back to try, I used a month after this month left 736 degrees, scared me, I also called to the grid to confirm, really is 736 degrees, the effect is very good, and today bought a go back to the old family to use	Jingdong prefecture level city in Shandong
Energy Saving Technology Shop	In-situ compensated economizer	Used for a period of time, you do not say can really save electricity, really is a small power-saving helper, it is worth buying!	Jingdong prefecture level city in Shandong

Energy Saving Technology Shop	In-situ compensated economizer	There are many rooms and it takes a lot of electricity to run the air conditioner when the house is cold. Bought 4 inserted half a month to save me more than 300 dollars. The effect is really core good! Quality is also very good, the seller service is very good and patient.	Jingdong prefecture level city in Shandong
Energy Saving Technology Shop	In-situ compensated economizer	Recommended by the owner of the store next door, looked at his home does have to save electricity, decided to get. Indeed quite can, the voltage is much more stable, there is a reduction in electricity	Jingdong prefecture level city in Shandong
Energy Saving Technology Shop	In-situ compensated economizer	It does save electricity and works pretty well. I feel that in a few days, I will be able to use the money that I bought to save money, it's really good!	Jingdong prefecture level city in Shandong
Energy Saving Technology Shop	In-situ compensated economizer	Small store service attitude is very good Product quality is very good Suggested purchase	Jingdong prefecture level city in Shandong
Energy Saving Technology Shop	In-situ compensated economizer	Bought two energy-saving appliances for the family, the kitchen and bathroom water heater is too expensive, the water heater to be turned on every day, the monthly electricity bill is a lot of use, with this, no longer afraid of the electricity bill is more than one month down the electricity bill can be less than half of the previous month it, the effect is particularly good!	Jingdong prefecture level city in Shandong
Energy Saving Technology Shop	In-situ compensated economizer	Saving power is really good to use, the last time I bought 3, the family used , before the family monthly electricity bills are more than 300 fast, after using this are more than 200, are several months, this time to my store is also arranged, it is really good for the people good things.	Jingdong prefecture level city in Shandong
Energy Saving Technology Shop	In-situ compensated economizer	The previous year bought a, almost every month can save a 70 dollars or so, with a year of no problem at all, look like new, this time to move to a new home decisively buy back 2 units, very believe this store!	Jingdong prefecture level city in Shandong
Energy Saving Technology Shop	In-situ compensated economizer	Power-saving effect: used a week to comment, before and now record the meter comparison, my one rental room can save twenty degrees of electricity per day. Cost-effective. Quite worth it.	Jingdong prefecture level city in Shandong
Energy Saving Technology Shop	In-situ compensated economizer	This power saver is really powerful, used to spend 300 electricity bill every month, now it's about 100	Jingdong prefecture level city in Shandong
Energy Saving Technology Shop	In-situ compensated economizer	After two days of testing. The effect is good! About the first day of power saving for 1/4. the second day for 1/3. and not "steal electricity", is through the intelligent integrated circuit, automatically reduce the loss of electrical appliances to achieve the purpose of saving electricity! Recommended	Jingdong prefecture level city in Shandong
Energy Saving Technology Shop	In-situ compensated economizer	Power Saving King has been received, really good, thank you boss, I wish the boss business prosperity, prosperity, next time to come back to patronize!	Jingdong prefecture level city in Shandong
Energy Saving Technology Shop	In-situ compensated economizer	I've been using it for a while. Than to A little less electricity than before. I'll buy it again next time.	Jingdong prefecture level city in Shandong
Energy Saving Technology Shop	In-situ compensated economizer	Used to be a couple hundred bucks a month. With him, it's a big savings. It's great.	Jingdong prefecture level city in Shandong
Energy Saving Technology Shop	In-situ compensated economizer	Previously bought a plug in the kitchen, used for a period of time after the personal feeling is still quite effective, can save a little electricity, so this time without hesitation and bought two, has been inserted in the home next to the appliances that use a lot of electricity!	Jingdong prefecture level city in Shandong
Energy Saving Technology Shop	In-situ compensated economizer	I've used it before and it does have a power saving effect	Jingdong prefecture level city in Shandong
Energy Saving Technology Shop	In-situ compensated economizer	High-tech products, really useful, in previous years the high cost of electricity, with this product, the electricity bill came down. Praise a, hope that the research and development of this product of science and technology to continue to work hard, research out better products to.	Jingdong prefecture level city in Shandong
Energy Saving Technology Shop	In-situ compensated economizer	This high-power is not bad Oh, the first few months are 1000 degrees so, I casually brush in Taobao to this product, I bought back to try, I used a month after this month left 736 degrees, scared me, I also called to the grid to confirm, really is 736 degrees, the effect is very good, and today bought a go back to the old family to use	Jingdong prefecture level city in Shandong
Energy Saving Technology Shop	In-situ compensated economizer	Used for a period of time, you do not say can really save electricity, really is a small power-saving helper, it is worth buying!	Jingdong prefecture level city in Shandong
Energy Saving Technology Shop	In-situ compensated economizer	There are many rooms and it takes a lot of electricity to run the air conditioner when the house is cold. Bought 4 inserted half a month to save me more than 300 dollars. The effect is really core good! Quality is also very good, the seller service is very good and patient.	Jingdong prefecture level city in Shandong
Energy Saving Technology Shop	In-situ compensated economizer	Recommended by the owner of the store next door, looked at his home does have to save electricity, decided to get. Indeed quite can, the voltage is much more stable, there is a reduction in electricity	Jingdong prefecture level city in Shandong
Energy Saving Technology Shop	In-situ compensated economizer	It does save electricity and works pretty well. I feel that in a few days, I will be able to use the money that I bought to save money, it's really good!	Jingdong prefecture level city in Shandong
Energy Saving Technology Shop	In-situ compensated economizer	Small store service attitude is very good Product quality is very good Suggested purchase	Jingdong prefecture level city in Shandong
Energy Saving Technology Shop	In-situ compensated economizer	Bought two energy-saving appliances for the family, the kitchen and bathroom water heater is too expensive, the water heater to be turned on every day, the monthly electricity bill is a lot of use, with this, no longer afraid of the electricity bill is more than one month down the electricity bill can be less than half of the previous month it, the effect is particularly good!	Jingdong prefecture level city in Shandong
Energy Saving Technology Shop	In-situ compensated economizer	Saving power is really good to use, the last time I bought 3, the family used , before the family monthly electricity bills are more than 300 fast, after using this are more than 200, are several months, this time to my store is also arranged, it is really good for the people good things.	Jingdong prefecture level city in Shandong
Energy Saving Technology Shop	In-situ compensated economizer	The previous year bought a, almost every month can save a 70 dollars or so, with a year of no problem at all, look like new, this time to move to a new home decisively buy back 2 units, very believe this store!	Jingdong prefecture level city in Shandong
Energy Saving Technology Shop	In-situ compensated economizer	Power-saving effect: used a week to comment, before and now record the meter comparison, my one rental room can save twenty degrees of electricity per day. Cost-effective. Quite worth it.	Jingdong prefecture level city in Shandong
Energy Saving Technology Shop	In-situ compensated economizer	This power saver is really powerful, used to spend 300 electricity bill every month, now it's about 100	Jingdong prefecture level city in Shandong
Energy Saving Technology Shop	In-situ compensated economizer	After two days of testing. The effect is good! About the first day of power saving for 1/4. the second day for 1/3. and not "steal electricity", is through the intelligent integrated circuit, automatically reduce the loss of electrical appliances to achieve the purpose of saving electricity! Recommended	Jingdong prefecture level city in Shandong
Energy Saving Technology Shop	In-situ compensated economizer	Power Saving King has been received, really good, thank you boss, I wish the boss business prosperity, prosperity, next time to come back to patronize!	Jingdong prefecture level city in Shandong
Energy Saving Technology Shop	In-situ compensated economizer	I've been using it for a while. Than to A little less electricity than before. I'll buy it again next time.	Jingdong prefecture level city in Shandong
Energy Saving Technology Shop	In-situ compensated economizer	Used to be a couple hundred bucks a month. With him, it's a big savings. It's great.	Jingdong prefecture level city in Shandong
Energy Saving Technology Shop	In-situ compensated economizer	Previously bought a plug in the kitchen, used for a period of time after the personal feeling is still quite effective, can save a little electricity, so this time without hesitation and bought two, has been inserted in the home next to the appliances that use a lot of electricity!	Jingdong prefecture level city in Shandong
Energy Saving Technology Shop	In-situ compensated economizer	I've used it before and it does have a power saving effect	Jingdong prefecture level city in Shandong
Energy Saving Technology Shop	In-situ compensated economizer	High-tech products, really useful, in previous years the high cost of electricity, with this product, the electricity bill came down. Praise a, hope that the research and development of this product of science and technology to continue to work hard, research out better products to.	Jingdong prefecture level city in Shandong
Energy Saving Technology Shop	In-situ compensated economizer	This high-power is not bad Oh, the first few months are 1000 degrees so, I casually brush in Taobao to this product, I bought back to try, I used a month after this month left 736 degrees, scared me, I also called to the grid to confirm, really is 736 degrees, the effect is very good, and today bought a go back to the old family to use	Jingdong prefecture level city in Shandong
Energy Saving Technology Shop	In-situ compensated economizer	Used for a period of time, you do not say can really save electricity, really is a small power-saving helper, it is worth buying!	Jingdong prefecture level city in Shandong
Energy Saving Technology Shop	In-situ compensated economizer	There are many rooms and it takes a lot of electricity to run the air conditioner when the house is cold. Bought 4 inserted half a month to save me more than 300 dollars. The effect is really core good! Quality is also very good, the seller service is very good and patient.	Jingdong prefecture level city in Shandong
Energy Saving Technology Shop	In-situ compensated economizer	Recommended by the owner of the store next door, looked at his home does have to save electricity, decided to get. Indeed quite can, the voltage is much more stable, there is a reduction in electricity	Jingdong prefecture level city in Shandong
Energy Saving Technology Shop	In-situ compensated economizer	It does save electricity and works pretty well. I feel that in a few days, I will be able to use the money that I bought to save money, it's really good!	Jingdong prefecture level city in Shandong
Energy Saving Technology Shop	In-situ compensated economizer	Small store service attitude is very good Product quality is very good Suggested purchase	Jingdong prefecture level city in Shandong
Energy Saving Technology Shop	In-situ compensated economizer	Bought two energy-saving appliances for the family, the kitchen and bathroom water heater is too expensive, the water heater to be turned on every day, the monthly electricity bill is a lot of use, with this, no longer afraid of the electricity bill is more than one month down the electricity bill can be less than half of the previous month it, the effect is particularly good!	Jingdong prefecture level city in Shandong

Energy Saving Technology Shop	In-situ compensated economizer	Saving power is really good to use, the last time I bought 3, the family used , before the family monthly electricity bills are more than 300 fast, after using this are more than 200, are several months, this time to my store is also arranged, it is really good for the people good things.	Jingdong prefecture level city in Shandong
Energy Saving Technology Shop	In-situ compensated economizer	The previous year bought a, almost every month can save a 70 dollars or so, with a year of no problem at all, look like new, this time to move to a new home decisively buy back 2 units, very believe this store!	Jingdong prefecture level city in Shandong
Energy Saving Technology Shop	In-situ compensated economizer	Power-saving effect: used a week to comment, before and now record the meter comparison, my one rental room can save twenty degrees of electricity per day. Cost-effective. Quite worth it.	Jingdong prefecture level city in Shandong
Energy Saving Technology Shop	In-situ compensated economizer	This power saver is really powerful, used to spend 300 electricity bill every month, now it's about 100	Jingdong prefecture level city in Shandong
Energy Saving Technology Shop	In-situ compensated economizer	After two days of testing. The effect is good! About the first day of power saving for 1/4. the second day for 1/3. and not "steal electricity", is through the intelligent integrated circuit, automatically reduce the loss of electrical appliances to achieve the purpose of saving electricity! Recommended	Jingdong prefecture level city in Shandong
Energy Saving Technology Shop	In-situ compensated economizer	Power Saving King has been received, really good, thank you boss, I wish the boss business prosperity, prosperity, next time to come back to patronize!	Jingdong prefecture level city in Shandong
Energy Saving Technology Shop	In-situ compensated economizer	I've been using it for a while. Than to A little less electricity than before. I'll buy it again next time.	Jingdong prefecture level city in Shandong
Energy Saving Technology Shop	In-situ compensated economizer	Used to be a couple hundred bucks a month. With him, it's a big savings. It's great.	Jingdong prefecture level city in Shandong
Energy Saving Technology Shop	In-situ compensated economizer	Previously bought a plug in the kitchen, used for a period of time after the personal feeling is still quite effective, can save a little electricity, so this time without hesitation and bought two, has been inserted in the home next to the appliances that use a lot of electricity!	Jingdong prefecture level city in Shandong
Energy Saving Technology Shop	In-situ compensated economizer	I've used it before and it does have a power saving effect	Jingdong prefecture level city in Shandong
Energy Saving Technology Shop	In-situ compensated economizer	High-tech products, really useful, in previous years the high cost of electricity, with this product, the electricity bill came down. Praise a, hope that the research and development of this product of science and technology to continue to work hard, research out better products to.	Jingdong prefecture level city in Shandong
Energy Saving Technology Shop	In-situ compensated economizer	This high-power is not bad Oh, the first few months are 1000 degrees so, I casually brush in Taobao to this product, I bought back to try, I used a month after this month left 736 degrees, scared me, I also called to the grid to confirm, really is 736 degrees, the effect is very good, and today bought a go back to the old family to use	Jingdong prefecture level city in Shandong
Energy Saving Technology Shop	In-situ compensated economizer	Used for a period of time, you do not say can really save electricity, really is a small power-saving helper, it is worth buying!	Jingdong prefecture level city in Shandong
Energy Saving Technology Shop	In-situ compensated economizer	There are many rooms and it takes a lot of electricity to run the air conditioner when the house is cold. Bought 4 inserted half a month to save me more than 300 dollars. The effect is really core good! Quality is also very good, the seller service is very good and patient.	Jingdong prefecture level city in Shandong
Energy Saving Technology Shop	In-situ compensated economizer	Recommended by the owner of the store next door, looked at his home does have to save electricity, decided to get. Indeed quite can, the voltage is much more stable, there is a reduction in electricity	Jingdong prefecture level city in Shandong
Energy Saving Technology Shop	In-situ compensated economizer	It does save electricity and works pretty well. I feel that in a few days, I will be able to use the money that I bought to save money, it's really good!	Jingdong prefecture level city in Shandong
Energy Saving Technology Shop	In-situ compensated economizer	Small store service attitude is very good Product quality is very good Suggested purchase	Jingdong prefecture level city in Shandong
Energy Saving Technology Shop	In-situ compensated economizer	Bought two energy-saving appliances for the family, the kitchen and bathroom water heater is too expensive, the water heater to be turned on every day, the monthly electricity bill is a lot of use, with this, no longer afraid of the electricity bill is more than one month down the electricity bill can be less than half of the previous month it, the effect is particularly good!	Jingdong prefecture level city in Shandong
Energy Saving Technology Shop	In-situ compensated economizer	Saving power is really good to use, the last time I bought 3, the family used , before the family monthly electricity bills are more than 300 fast, after using this are more than 200, are several months, this time to my store is also arranged, it is really good for the people good things.	Jingdong prefecture level city in Shandong
Energy Saving Technology Shop	In-situ compensated economizer	The previous year bought a, almost every month can save a 70 dollars or so, with a year of no problem at all, look like new, this time to move to a new home decisively buy back 2 units, very believe this store!	Jingdong prefecture level city in Shandong
Energy Saving Technology Shop	In-situ compensated economizer	Power-saving effect: used a week to comment, before and now record the meter comparison, my one rental room can save twenty degrees of electricity per day. Cost-effective. Quite worth it.	Jingdong prefecture level city in Shandong
Energy Saving Technology Shop	In-situ compensated economizer	This power saver is really powerful, used to spend 300 electricity bill every month, now it's about 100	Jingdong prefecture level city in Shandong
Energy Saving Technology Shop	In-situ compensated economizer	After two days of testing. The effect is good! About the first day of power saving for 1/4. the second day for 1/3. and not "steal electricity", is through the intelligent integrated circuit, automatically reduce the loss of electrical appliances to achieve the purpose of saving electricity! Recommended	Jingdong prefecture level city in Shandong
Energy Saving Technology Shop	In-situ compensated economizer	Power Saving King has been received, really good, thank you boss, I wish the boss business prosperity, prosperity, next time to come back to patronize!	Jingdong prefecture level city in Shandong
Energy Saving Technology Shop	In-situ compensated economizer	I've been using it for a while. Than to A little less electricity than before. I'll buy it again next time.	Jingdong prefecture level city in Shandong
Energy Saving Technology Shop	In-situ compensated economizer	Used to be a couple hundred bucks a month. With him, it's a big savings. It's great.	Jingdong prefecture level city in Shandong
Energy Saving Technology Shop	In-situ compensated economizer	Previously bought a plug in the kitchen, used for a period of time after the personal feeling is still quite effective, can save a little electricity, so this time without hesitation and bought two, has been inserted in the home next to the appliances that use a lot of electricity!	Jingdong prefecture level city in Shandong
Energy Saving Technology Shop	In-situ compensated economizer	I've used it before and it does have a power saving effect	Jingdong prefecture level city in Shandong
Energy Saving Technology Shop	In-situ compensated economizer	High-tech products, really useful, in previous years the high cost of electricity, with this product, the electricity bill came down. Praise a, hope that the research and development of this product of science and technology to continue to work hard, research out better products to.	Jingdong prefecture level city in Shandong
Energy Saving Technology Shop	In-situ compensated economizer	This high-power is not bad Oh, the first few months are 1000 degrees so, I casually brush in Taobao to this product, I bought back to try, I used a month after this month left 736 degrees, scared me, I also called to the grid to confirm, really is 736 degrees, the effect is very good, and today bought a go back to the old family to use	Jingdong prefecture level city in Shandong
Energy Saving Technology Shop	In-situ compensated economizer	Used for a period of time, you do not say can really save electricity, really is a small power-saving helper, it is worth buying!	Jingdong prefecture level city in Shandong
Energy Saving Technology Shop	In-situ compensated economizer	There are many rooms and it takes a lot of electricity to run the air conditioner when the house is cold. Bought 4 inserted half a month to save me more than 300 dollars. The effect is really core good! Quality is also very good, the seller service is very good and patient.	Jingdong prefecture level city in Shandong
Energy Saving Technology Shop	In-situ compensated economizer	Recommended by the owner of the store next door, looked at his home does have to save electricity, decided to get. Indeed quite can, the voltage is much more stable, there is a reduction in electricity	Jingdong prefecture level city in Shandong
Energy Saving Technology Shop	In-situ compensated economizer	It does save electricity and works pretty well. I feel that in a few days, I will be able to use the money that I bought to save money, it's really good!	Jingdong prefecture level city in Shandong
Energy Saving Technology Shop	In-situ compensated economizer	Small store service attitude is very good Product quality is very good Suggested purchase	Jingdong prefecture level city in Shandong
Energy Saving Technology Shop	In-situ compensated economizer	Bought two energy-saving appliances for the family, the kitchen and bathroom water heater is too expensive, the water heater to be turned on every day, the monthly electricity bill is a lot of use, with this, no longer afraid of the electricity bill is more than one month down the electricity bill can be less than half of the previous month it, the effect is particularly good!	Jingdong prefecture level city in Shandong
Energy Saving Technology Shop	In-situ compensated economizer	Saving power is really good to use, the last time I bought 3, the family used , before the family monthly electricity bills are more than 300 fast, after using this are more than 200, are several months, this time to my store is also arranged, it is really good for the people good things.	Jingdong prefecture level city in Shandong
Energy Saving Technology Shop	In-situ compensated economizer	The previous year bought a, almost every month can save a 70 dollars or so, with a year of no problem at all, look like new, this time to move to a new home decisively buy back 2 units, very believe this store!	Jingdong prefecture level city in Shandong
Energy Saving Technology Shop	In-situ compensated economizer	Power-saving effect: used a week to comment, before and now record the meter comparison, my one rental room can save twenty degrees of electricity per day. Cost-effective. Quite worth it.	Jingdong prefecture level city in Shandong
Energy Saving Technology Shop	In-situ compensated economizer	This power saver is really powerful, used to spend 300 electricity bill every month, now it's about 100	Jingdong prefecture level city in Shandong
Energy Saving Technology Shop	In-situ compensated economizer	After two days of testing. The effect is good! About the first day of power saving for 1/4. the second day for 1/3. and not "steal electricity", is through the intelligent integrated circuit, automatically reduce the loss of electrical appliances to achieve the purpose of saving electricity! Recommended	Jingdong prefecture level city in Shandong

Energy Saving Technology Shop	In-situ compensated economizer	Power Saving King has been received, really good, thank you boss, I wish the boss business prosperity, prosperity, next time to come back to patronize!	Jingdong prefecture level city in Shandong
Energy Saving Technology Shop	In-situ compensated economizer	I've been using it for a while. Than to A little less electricity than before. I'll buy it again next time.	Jingdong prefecture level city in Shandong
Energy Saving Technology Shop	In-situ compensated economizer	Used to be a couple hundred bucks a month. With him, it's a big savings. It's great.	Jingdong prefecture level city in Shandong
Energy Saving Technology Shop	In-situ compensated economizer	Previously bought a plug in the kitchen, used for a period of time after the personal feeling is still quite effective, can save a little electricity, so this time without hesitation and bought two, has been inserted in the home next to the appliances that use a lot of electricity!	Jingdong prefecture level city in Shandong
Energy Saving Technology Shop	In-situ compensated economizer	I've used it before and it does have a power saving effect	Jingdong prefecture level city in Shandong
Energy Saving Technology Shop	In-situ compensated economizer	High-tech products, really useful, in previous years the high cost of electricity, with this product, the electricity bill came down. Praise a, hope that the research and development of this product of science and technology to continue to work hard, research out better products to.	Jingdong prefecture level city in Shandong
Energy Saving Technology Shop	In-situ compensated economizer	This high-power is not bad Oh, the first few months are 1000 degrees so, I casually brush in Taobao to this product, I bought back to try, I used a month after this month left 736 degrees, scared me, I also called to the grid to confirm, really is 736 degrees, the effect is very good, and today bought a go back to the old family to use	Jingdong prefecture level city in Shandong
Energy Saving Technology Shop	In-situ compensated economizer	Used for a period of time, you do not say can really save electricity, really is a small power-saving helper, it is worth buying!	Jingdong prefecture level city in Shandong
Energy Saving Technology Shop	In-situ compensated economizer	There are many rooms and it takes a lot of electricity to run the air conditioner when the house is cold. Bought 4 inserted half a month to save me more than 300 dollars. The effect is really core good! Quality is also very good, the seller service is very good and patient.	Jingdong prefecture level city in Shandong
Energy Saving Technology Shop	In-situ compensated economizer	Recommended by the owner of the store next door, looked at his home does have to save electricity, decided to get. Indeed quite can, the voltage is much more stable, there is a reduction in electricity	Jingdong prefecture level city in Shandong
Energy Saving Technology Shop	In-situ compensated economizer	It does save electricity and works pretty well. I feel that in a few days, I will be able to use the money that I bought to save money, it's really good!	Jingdong prefecture level city in Shandong
Energy Saving Technology Shop	In-situ compensated economizer	Small store service attitude is very good Product quality is very good Suggested purchase	Jingdong prefecture level city in Shandong
Energy Saving Technology Shop	In-situ compensated economizer	Bought two energy-saving appliances for the family, the kitchen and bathroom water heater is too expensive, the water heater to be turned on every day, the monthly electricity bill is a lot of use, with this, no longer afraid of the electricity bill is more than one month down the electricity bill can be less than half of the previous month it, the effect is particularly good!	Jingdong prefecture level city in Shandong
Energy Saving Technology Shop	In-situ compensated economizer	Saving power is really good to use, the last time I bought 3, the family used , before the family monthly electricity bills are more than 300 fast, after using this are more than 200, are several months, this time to my store is also arranged, it is really good for the people good things.	Jingdong prefecture level city in Shandong
Energy Saving Technology Shop	In-situ compensated economizer	The previous year bought a, almost every month can save a 70 dollars or so, with a year of no problem at all, look like new, this time to move to a new home decisively buy back 2 units, very believe this store!	Jingdong prefecture level city in Shandong
Energy Saving Technology Shop	In-situ compensated economizer	Power-saving effect: used a week to comment, before and now record the meter comparison, my one rental room can save twenty degrees of electricity per day. Cost-effective. Quite worth it.	Jingdong prefecture level city in Shandong
Energy Saving Technology Shop	In-situ compensated economizer	This power saver is really powerful, used to spend 300 electricity bill every month, now it's about 100	Jingdong prefecture level city in Shandong
Energy Saving Technology Shop	In-situ compensated economizer	After two days of testing. The effect is good! About the first day of power saving for 1/4. the second day for 1/3. and not "steal electricity", is through the intelligent integrated circuit, automatically reduce the loss of electrical appliances to achieve the purpose of saving electricity! Recommended	Jingdong prefecture level city in Shandong
Energy Saving Technology Shop	In-situ compensated economizer	Power Saving King has been received, really good, thank you boss, I wish the boss business prosperity, prosperity, next time to come back to patronize!	Jingdong prefecture level city in Shandong
Energy Saving Technology Shop	In-situ compensated economizer	I've been using it for a while. Than to A little less electricity than before. I'll buy it again next time.	Jingdong prefecture level city in Shandong
Energy Saving Technology Shop	In-situ compensated economizer	Used to be a couple hundred bucks a month. With him, it's a big savings. It's great.	Jingdong prefecture level city in Shandong
Energy Saving Technology Shop	In-situ compensated economizer	Previously bought a plug in the kitchen, used for a period of time after the personal feeling is still quite effective, can save a little electricity, so this time without hesitation and bought two, has been inserted in the home next to the appliances that use a lot of electricity!	Jingdong prefecture level city in Shandong
Energy Saving Technology Shop	In-situ compensated economizer	I've used it before and it does have a power saving effect	Jingdong prefecture level city in Shandong
Energy Saving Technology Shop	In-situ compensated economizer	High-tech products, really useful, in previous years the high cost of electricity, with this product, the electricity bill came down. Praise a, hope that the research and development of this product of science and technology to continue to work hard, research out better products to.	Jingdong prefecture level city in Shandong
Energy Saving Technology Shop	In-situ compensated economizer	This high-power is not bad Oh, the first few months are 1000 degrees so, I casually brush in Taobao to this product, I bought back to try, I used a month after this month left 736 degrees, scared me, I also called to the grid to confirm, really is 736 degrees, the effect is very good, and today bought a go back to the old family to use	Jingdong prefecture level city in Shandong
Energy Saving Technology Shop	In-situ compensated economizer	Used for a period of time, you do not say can really save electricity, really is a small power-saving helper, it is worth buying!	Jingdong prefecture level city in Shandong
Energy Saving Technology Shop	In-situ compensated economizer	There are many rooms and it takes a lot of electricity to run the air conditioner when the house is cold. Bought 4 inserted half a month to save me more than 300 dollars. The effect is really core good! Quality is also very good, the seller service is very good and patient.	Jingdong prefecture level city in Shandong
Energy Saving Technology Shop	In-situ compensated economizer	Recommended by the owner of the store next door, looked at his home does have to save electricity, decided to get. Indeed quite can, the voltage is much more stable, there is a reduction in electricity	Jingdong prefecture level city in Shandong
Energy Saving Technology Shop	In-situ compensated economizer	It does save electricity and works pretty well. I feel that in a few days, I will be able to use the money that I bought to save money, it's really good!	Jingdong prefecture level city in Shandong
Energy Saving Technology Shop	In-situ compensated economizer	Small store service attitude is very good Product quality is very good Suggested purchase	Jingdong prefecture level city in Shandong
Energy Saving Technology Shop	In-situ compensated economizer	Bought two energy-saving appliances for the family, the kitchen and bathroom water heater is too expensive, the water heater to be turned on every day, the monthly electricity bill is a lot of use, with this, no longer afraid of the electricity bill is more than one month down the electricity bill can be less than half of the previous month it, the effect is particularly good!	Jingdong prefecture level city in Shandong
Energy Saving Technology Shop	In-situ compensated economizer	Saving power is really good to use, the last time I bought 3, the family used , before the family monthly electricity bills are more than 300 fast, after using this are more than 200, are several months, this time to my store is also arranged, it is really good for the people good things.	Jingdong prefecture level city in Shandong
Energy Saving Technology Shop	In-situ compensated economizer	The previous year bought a, almost every month can save a 70 dollars or so, with a year of no problem at all, look like new, this time to move to a new home decisively buy back 2 units, very believe this store!	Jingdong prefecture level city in Shandong
Energy Saving Technology Shop	In-situ compensated economizer	Power-saving effect: used a week to comment, before and now record the meter comparison, my one rental room can save twenty degrees of electricity per day. Cost-effective. Quite worth it.	Jingdong prefecture level city in Shandong
Energy Saving Technology Shop	In-situ compensated economizer	This power saver is really powerful, used to spend 300 electricity bill every month, now it's about 100	Jingdong prefecture level city in Shandong
Energy Saving Technology Shop	In-situ compensated economizer	After two days of testing. The effect is good! About the first day of power saving for 1/4. the second day for 1/3. and not "steal electricity", is through the intelligent integrated circuit, automatically reduce the loss of electrical appliances to achieve the purpose of saving electricity! Recommended	Jingdong prefecture level city in Shandong
Energy Saving Technology Shop	In-situ compensated economizer	Power Saving King has been received, really good, thank you boss, I wish the boss business prosperity, prosperity, next time to come back to patronize!	Jingdong prefecture level city in Shandong
Energy Saving Technology Shop	In-situ compensated economizer	I've been using it for a while. Than to A little less electricity than before. I'll buy it again next time.	Jingdong prefecture level city in Shandong
Energy Saving Technology Shop	In-situ compensated economizer	Used to be a couple hundred bucks a month. With him, it's a big savings. It's great.	Jingdong prefecture level city in Shandong
Energy Saving Technology Shop	In-situ compensated economizer	Previously bought a plug in the kitchen, used for a period of time after the personal feeling is still quite effective, can save a little electricity, so this time without hesitation and bought two, has been inserted in the home next to the appliances that use a lot of electricity!	Jingdong prefecture level city in Shandong
Energy Saving Technology Shop	In-situ compensated economizer	I've used it before and it does have a power saving effect	Jingdong prefecture level city in Shandong

Energy Saving Technology Shop	In-situ compensated economizer	High-tech products, really useful, in previous years the high cost of electricity, with this product, the electricity bill came down. Praise a, hope that the research and development of this product of science and technology to continue to work hard, research out better products to.	Jingdong prefecture level city in Shandong
Energy Saving Technology Shop	In-situ compensated economizer	This high-power is not bad Oh, the first few months are 1000 degrees so, I casually brush in Taobao to this product, I bought back to try, I used a month after this month left 736 degrees, scared me, I also called to the grid to confirm, really is 736 degrees, the effect is very good, and today bought a go back to the old family to use	Jingdong prefecture level city in Shandong
Energy Saving Technology Shop	In-situ compensated economizer	Used for a period of time, you do not say can really save electricity, really is a small power-saving helper, it is worth buying!	Jingdong prefecture level city in Shandong
Energy Saving Technology Shop	In-situ compensated economizer	There are many rooms and it takes a lot of electricity to run the air conditioner when the house is cold. Bought 4 inserted half a month to save me more than 300 dollars. The effect is really core good! Quality is also very good, the seller service is very good and patient.	Jingdong prefecture level city in Shandong
Energy Saving Technology Shop	In-situ compensated economizer	Recommended by the owner of the store next door, looked at his home does have to save electricity, decided to get. Indeed quite can, the voltage is much more stable, there is a reduction in electricity	Jingdong prefecture level city in Shandong
Energy Saving Technology Shop	In-situ compensated economizer	It does save electricity and works pretty well. I feel that in a few days, I will be able to use the money that I bought to save money, it's really good!	Jingdong prefecture level city in Shandong
Energy Saving Technology Shop	In-situ compensated economizer	Small store service attitude is very good Product quality is very good Suggested purchase	Jingdong prefecture level city in Shandong
Energy Saving Technology Shop	In-situ compensated economizer	Bought two energy-saving appliances for the family, the kitchen and bathroom water heater is too expensive, the water heater to be turned on every day, the monthly electricity bill is a lot of use, with this, no longer afraid of the electricity bill is more than one month down the electricity bill can be less than half of the previous month it, the effect is particularly good!	Jingdong prefecture level city in Shandong
Energy Saving Technology Shop	In-situ compensated economizer	Saving power is really good to use, the last time I bought 3, the family used , before the family monthly electricity bills are more than 300 fast, after using this are more than 200, are several months, this time to my store is also arranged, it is really good for the people good things.	Jingdong prefecture level city in Shandong
Energy Saving Technology Shop	In-situ compensated economizer	The previous year bought a, almost every month can save a 70 dollars or so, with a year of no problem at all, look like new, this time to move to a new home decisively buy back 2 units, very believe this store!	Jingdong prefecture level city in Shandong
Energy Saving Technology Shop	In-situ compensated economizer	Power-saving effect: used a week to comment, before and now record the meter comparison, my one rental room can save twenty degrees of electricity per day. Cost-effective. Quite worth it.	Jingdong prefecture level city in Shandong
Energy Saving Technology Shop	In-situ compensated economizer	This power saver is really powerful, used to spend 300 electricity bill every month, now it's about 100	Jingdong prefecture level city in Shandong
Energy Saving Technology Shop	In-situ compensated economizer	After two days of testing. The effect is good! About the first day of power saving for 1/4. the second day for 1/3. and not "steal electricity", is through the intelligent integrated circuit, automatically reduce the loss of electrical appliances to achieve the purpose of saving electricity! Recommended	Jingdong prefecture level city in Shandong
Energy Saving Technology Shop	In-situ compensated economizer	Power Saving King has been received, really good, thank you boss, I wish the boss business prosperity, prosperity, next time to come back to patronize!	Jingdong prefecture level city in Shandong
Energy Saving Technology Shop	In-situ compensated economizer	I've been using it for a while. Than to A little less electricity than before. I'll buy it again next time.	Jingdong prefecture level city in Shandong
Energy Saving Technology Shop	In-situ compensated economizer	Used to be a couple hundred bucks a month. With him, it's a big savings. It's great.	Jingdong prefecture level city in Shandong
Energy Saving Technology Shop	In-situ compensated economizer	Previously bought a plug in the kitchen, used for a period of time after the personal feeling is still quite effective, can save a little electricity, so this time without hesitation and bought two, has been inserted in the home next to the appliances that use a lot of electricity!	Jingdong prefecture level city in Shandong
Energy Saving Technology Shop	In-situ compensated economizer	I've used it before and it does have a power saving effect	Jingdong prefecture level city in Shandong
Energy Saving Technology Shop	In-situ compensated economizer	High-tech products, really useful, in previous years the high cost of electricity, with this product, the electricity bill came down. Praise a, hope that the research and development of this product of science and technology to continue to work hard, research out better products to.	Jingdong prefecture level city in Shandong
Energy Saving Technology Shop	In-situ compensated economizer	This high-power is not bad Oh, the first few months are 1000 degrees so, I casually brush in Taobao to this product, I bought back to try, I used a month after this month left 736 degrees, scared me, I also called to the grid to confirm, really is 736 degrees, the effect is very good, and today bought a go back to the old family to use	Jingdong prefecture level city in Shandong
Energy Saving Technology Shop	In-situ compensated economizer	Used for a period of time, you do not say can really save electricity, really is a small power-saving helper, it is worth buying!	Jingdong prefecture level city in Shandong
Energy Saving Technology Shop	In-situ compensated economizer	There are many rooms and it takes a lot of electricity to run the air conditioner when the house is cold. Bought 4 inserted half a month to save me more than 300 dollars. The effect is really core good! Quality is also very good, the seller service is very good and patient.	Jingdong prefecture level city in Shandong
Energy Saving Technology Shop	In-situ compensated economizer	Recommended by the owner of the store next door, looked at his home does have to save electricity, decided to get. Indeed quite can, the voltage is much more stable, there is a reduction in electricity	Jingdong prefecture level city in Shandong
Energy Saving Technology Shop	In-situ compensated economizer	It does save electricity and works pretty well. I feel that in a few days, I will be able to use the money that I bought to save money, it's really good!	Jingdong prefecture level city in Shandong
Energy Saving Technology Shop	In-situ compensated economizer	Small store service attitude is very good Product quality is very good Suggested purchase	Jingdong prefecture level city in Shandong
Energy Saving Technology Shop	In-situ compensated economizer	Bought two energy-saving appliances for the family, the kitchen and bathroom water heater is too expensive, the water heater to be turned on every day, the monthly electricity bill is a lot of use, with this, no longer afraid of the electricity bill is more than one month down the electricity bill can be less than half of the previous month it, the effect is particularly good!	Jingdong prefecture level city in Shandong
Energy Saving Technology Shop	In-situ compensated economizer	Saving power is really good to use, the last time I bought 3, the family used , before the family monthly electricity bills are more than 300 fast, after using this are more than 200, are several months, this time to my store is also arranged, it is really good for the people good things.	Jingdong prefecture level city in Shandong
Energy Saving Technology Shop	In-situ compensated economizer	The previous year bought a, almost every month can save a 70 dollars or so, with a year of no problem at all, look like new, this time to move to a new home decisively buy back 2 units, very believe this store!	Jingdong prefecture level city in Shandong
Energy Saving Technology Shop	In-situ compensated economizer	Power-saving effect: used a week to comment, before and now record the meter comparison, my one rental room can save twenty degrees of electricity per day. Cost-effective. Quite worth it.	Jingdong prefecture level city in Shandong
Energy Saving Technology Shop	In-situ compensated economizer	This power saver is really powerful, used to spend 300 electricity bill every month, now it's about 100	Jingdong prefecture level city in Shandong
Energy Saving Technology Shop	In-situ compensated economizer	After two days of testing. The effect is good! About the first day of power saving for 1/4. the second day for 1/3. and not "steal electricity", is through the intelligent integrated circuit, automatically reduce the loss of electrical appliances to achieve the purpose of saving electricity! Recommended	Jingdong prefecture level city in Shandong
Energy Saving Technology Shop	In-situ compensated economizer	Power Saving King has been received, really good, thank you boss, I wish the boss business prosperity, prosperity, next time to come back to patronize!	Jingdong prefecture level city in Shandong
Energy Saving Technology Shop	In-situ compensated economizer	I've been using it for a while. Than to A little less electricity than before. I'll buy it again next time.	Jingdong prefecture level city in Shandong
Energy Saving Technology Shop	In-situ compensated economizer	Used to be a couple hundred bucks a month. With him, it's a big savings. It's great.	Jingdong prefecture level city in Shandong
Energy Saving Technology Shop	In-situ compensated economizer	Previously bought a plug in the kitchen, used for a period of time after the personal feeling is still quite effective, can save a little electricity, so this time without hesitation and bought two, has been inserted in the home next to the appliances that use a lot of electricity!	Jingdong prefecture level city in Shandong
Energy Saving Technology Shop	In-situ compensated economizer	I've used it before and it does have a power saving effect	Jingdong prefecture level city in Shandong
Energy Saving Technology Shop	In-situ compensated economizer	High-tech products, really useful, in previous years the high cost of electricity, with this product, the electricity bill came down. Praise a, hope that the research and development of this product of science and technology to continue to work hard, research out better products to.	Jingdong prefecture level city in Shandong
Energy Saving Technology Shop	In-situ compensated economizer	This high-power is not bad Oh, the first few months are 1000 degrees so, I casually brush in Taobao to this product, I bought back to try, I used a month after this month left 736 degrees, scared me, I also called to the grid to confirm, really is 736 degrees, the effect is very good, and today bought a go back to the old family to use	Jingdong prefecture level city in Shandong
Energy Saving Technology Shop	In-situ compensated economizer	Used for a period of time, you do not say can really save electricity, really is a small power-saving helper, it is worth buying!	Jingdong prefecture level city in Shandong
Energy Saving Technology Shop	In-situ compensated economizer	There are many rooms and it takes a lot of electricity to run the air conditioner when the house is cold. Bought 4 inserted half a month to save me more than 300 dollars. The effect is really core good! Quality is also very good, the seller service is very good and patient.	Jingdong prefecture level city in Shandong

Energy Saving Technology Shop	In-situ compensated economizer	Recommended by the owner of the store next door, looked at his home does have to save electricity, decided to get. Indeed quite can, the voltage is much more stable, there is a reduction in electricity	Jingdong prefecture level city in Shandong
Energy Saving Technology Shop	In-situ compensated economizer	It does save electricity and works pretty well. I feel that in a few days, I will be able to use the money that I bought to save money, it's really good!	Jingdong prefecture level city in Shandong
Energy Saving Technology Shop	In-situ compensated economizer	Small store service attitude is very good Product quality is very good Suggested purchase	Jingdong prefecture level city in Shandong
Energy Saving Technology Shop	In-situ compensated economizer	Bought two energy-saving appliances for the family, the kitchen and bathroom water heater is too expensive, the water heater to be turned on every day, the monthly electricity bill is a lot of use, with this, no longer afraid of the electricity bill is more than one month down the electricity bill can be less than half of the previous month it, the effect is particularly good!	Jingdong prefecture level city in Shandong
Energy Saving Technology Shop	In-situ compensated economizer	Saving power is really good to use, the last time I bought 3, the family used , before the family monthly electricity bills are more than 300 fast, after using this are more than 200, are several months, this time to my store is also arranged, it is really good for the people good things.	Jingdong prefecture level city in Shandong
Energy Saving Technology Shop	In-situ compensated economizer	The previous year bought a, almost every month can save a 70 dollars or so, with a year of no problem at all, look like new, this time to move to a new home decisively buy back 2 units, very believe this store!	Jingdong prefecture level city in Shandong
Energy Saving Technology Shop	In-situ compensated economizer	Power-saving effect: used a week to comment, before and now record the meter comparison, my one rental room can save twenty degrees of electricity per day. Cost-effective. Quite worth it.	Jingdong prefecture level city in Shandong
Energy Saving Technology Shop	In-situ compensated economizer	This power saver is really powerful, used to spend 300 electricity bill every month, now it's about 100	Jingdong prefecture level city in Shandong
Energy Saving Technology Shop	In-situ compensated economizer	After two days of testing. The effect is good! About the first day of power saving for 1/4. the second day for 1/3. and not "steal electricity", is through the intelligent integrated circuit, automatically reduce the loss of electrical appliances to achieve the purpose of saving electricity! Recommended	Jingdong prefecture level city in Shandong
Energy Saving Technology Shop	In-situ compensated economizer	Power Saving King has been received, really good, thank you boss, I wish the boss business prosperity, prosperity, next time to come back to patronize!	Jingdong prefecture level city in Shandong
Energy Saving Technology Shop	In-situ compensated economizer	I've been using it for a while. Than to A little less electricity than before. I'll buy it again next time.	Jingdong prefecture level city in Shandong
Energy Saving Technology Shop	In-situ compensated economizer	Used to be a couple hundred bucks a month. With him, it's a big savings. It's great.	Jingdong prefecture level city in Shandong
Energy Saving Technology Shop	In-situ compensated economizer	Previously bought a plug in the kitchen, used for a period of time after the personal feeling is still quite effective, can save a little electricity, so this time without hesitation and bought two, has been inserted in the home next to the appliances that use a lot of electricity!	Jingdong prefecture level city in Shandong
Energy Saving Technology Shop	In-situ compensated economizer	I've used it before and it does have a power saving effect	Jingdong prefecture level city in Shandong
Energy Saving Technology Shop	In-situ compensated economizer	High-tech products, really useful, in previous years the high cost of electricity, with this product, the electricity bill came down. Praise a, hope that the research and development of this product of science and technology to continue to work hard, research out better products to.	Jingdong prefecture level city in Shandong
Energy Saving Technology Shop	In-situ compensated economizer	This high-power is not bad Oh, the first few months are 1000 degrees so, I casually brush in Taobao to this product, I bought back to try, I used a month after this month left 736 degrees, scared me, I also called to the grid to confirm, really is 736 degrees, the effect is very good, and today bought a go back to the old family to use	Jingdong prefecture level city in Shandong
Energy Saving Technology Shop	In-situ compensated economizer	Used for a period of time, you do not say can really save electricity, really is a small power-saving helper, it is worth buying!	Jingdong prefecture level city in Shandong
Energy Saving Technology Shop	In-situ compensated economizer	There are many rooms and it takes a lot of electricity to run the air conditioner when the house is cold. Bought 4 inserted half a month to save me more than 300 dollars. The effect is really core good! Quality is also very good, the seller service is very good and patient.	Jingdong prefecture level city in Shandong
Energy Saving Technology Shop	In-situ compensated economizer	Recommended by the owner of the store next door, looked at his home does have to save electricity, decided to get. Indeed quite can, the voltage is much more stable, there is a reduction in electricity	Jingdong prefecture level city in Shandong
Energy Saving Technology Shop	In-situ compensated economizer	It does save electricity and works pretty well. I feel that in a few days, I will be able to use the money that I bought to save money, it's really good!	Jingdong prefecture level city in Shandong
Energy Saving Technology Shop	In-situ compensated economizer	Small store service attitude is very good Product quality is very good Suggested purchase	Jingdong prefecture level city in Shandong
Energy Saving Technology Shop	In-situ compensated economizer	Bought two energy-saving appliances for the family, the kitchen and bathroom water heater is too expensive, the water heater to be turned on every day, the monthly electricity bill is a lot of use, with this, no longer afraid of the electricity bill is more than one month down the electricity bill can be less than half of the previous month it, the effect is particularly good!	Jingdong prefecture level city in Shandong
Energy Saving Technology Shop	In-situ compensated economizer	Saving power is really good to use, the last time I bought 3, the family used , before the family monthly electricity bills are more than 300 fast, after using this are more than 200, are several months, this time to my store is also arranged, it is really good for the people good things.	Jingdong prefecture level city in Shandong
Energy Saving Technology Shop	In-situ compensated economizer	The previous year bought a, almost every month can save a 70 dollars or so, with a year of no problem at all, look like new, this time to move to a new home decisively buy back 2 units, very believe this store!	Jingdong prefecture level city in Shandong
Energy Saving Technology Shop	In-situ compensated economizer	Power-saving effect: used a week to comment, before and now record the meter comparison, my one rental room can save twenty degrees of electricity per day. Cost-effective. Quite worth it.	Jingdong prefecture level city in Shandong
Energy Saving Technology Shop	In-situ compensated economizer	This power saver is really powerful, used to spend 300 electricity bill every month, now it's about 100	Jingdong prefecture level city in Shandong
Energy Saving Technology Shop	In-situ compensated economizer	After two days of testing. The effect is good! About the first day of power saving for 1/4. the second day for 1/3. and not "steal electricity", is through the intelligent integrated circuit, automatically reduce the loss of electrical appliances to achieve the purpose of saving electricity! Recommended	Jingdong prefecture level city in Shandong
Energy Saving Technology Shop	In-situ compensated economizer	Power Saving King has been received, really good, thank you boss, I wish the boss business prosperity, prosperity, next time to come back to patronize!	Jingdong prefecture level city in Shandong
Energy Saving Technology Shop	In-situ compensated economizer	I've been using it for a while. Than to A little less electricity than before. I'll buy it again next time.	Jingdong prefecture level city in Shandong
Energy Saving Technology Shop	In-situ compensated economizer	Used to be a couple hundred bucks a month. With him, it's a big savings. It's great.	Jingdong prefecture level city in Shandong
Energy Saving Technology Shop	In-situ compensated economizer	Previously bought a plug in the kitchen, used for a period of time after the personal feeling is still quite effective, can save a little electricity, so this time without hesitation and bought two, has been inserted in the home next to the appliances that use a lot of electricity!	Jingdong prefecture level city in Shandong
Energy Saving Technology Shop	In-situ compensated economizer	I've used it before and it does have a power saving effect	Jingdong prefecture level city in Shandong
Energy Saving Technology Shop	In-situ compensated economizer	High-tech products, really useful, in previous years the high cost of electricity, with this product, the electricity bill came down. Praise a, hope that the research and development of this product of science and technology to continue to work hard, research out better products to.	Jingdong prefecture level city in Shandong
Energy Saving Technology Shop	In-situ compensated economizer	This high-power is not bad Oh, the first few months are 1000 degrees so, I casually brush in Taobao to this product, I bought back to try, I used a month after this month left 736 degrees, scared me, I also called to the grid to confirm, really is 736 degrees, the effect is very good, and today bought a go back to the old family to use	Jingdong prefecture level city in Shandong
Energy Saving Technology Shop	In-situ compensated economizer	Used for a period of time, you do not say can really save electricity, really is a small power-saving helper, it is worth buying!	Jingdong prefecture level city in Shandong
Energy Saving Technology Shop	In-situ compensated economizer	There are many rooms and it takes a lot of electricity to run the air conditioner when the house is cold. Bought 4 inserted half a month to save me more than 300 dollars. The effect is really core good! Quality is also very good, the seller service is very good and patient.	Jingdong prefecture level city in Shandong
Energy Saving Technology Shop	In-situ compensated economizer	Recommended by the owner of the store next door, looked at his home does have to save electricity, decided to get. Indeed quite can, the voltage is much more stable, there is a reduction in electricity	Jingdong prefecture level city in Shandong
Energy Saving Technology Shop	In-situ compensated economizer	It does save electricity and works pretty well. I feel that in a few days, I will be able to use the money that I bought to save money, it's really good!	Jingdong prefecture level city in Shandong
Energy Saving Technology Shop	In-situ compensated economizer	Small store service attitude is very good Product quality is very good Suggested purchase	Jingdong prefecture level city in Shandong
Energy Saving Technology Shop	In-situ compensated economizer	Bought two energy-saving appliances for the family, the kitchen and bathroom water heater is too expensive, the water heater to be turned on every day, the monthly electricity bill is a lot of use, with this, no longer afraid of the electricity bill is more than one month down the electricity bill can be less than half of the previous month it, the effect is particularly good!	Jingdong prefecture level city in Shandong
Energy Saving Technology Shop	In-situ compensated economizer	Saving power is really good to use, the last time I bought 3, the family used , before the family monthly electricity bills are more than 300 fast, after using this are more than 200, are several months, this time to my store is also arranged, it is really good for the people good things.	Jingdong prefecture level city in Shandong

Energy Saving Technology Shop	In-situ compensated economizer	The previous year bought a, almost every month can save a 70 dollars or so, with a year of no problem at all, look like new, this time to move to a new home decisively buy back 2 units, very believe this store!	Jingdong prefecture level city in Shandong
Energy Saving Technology Shop	In-situ compensated economizer	Power-saving effect: used a week to comment, before and now record the meter comparison, my one rental room can save twenty degrees of electricity per day. Cost-effective. Quite worth it.	Jingdong prefecture level city in Shandong
Energy Saving Technology Shop	In-situ compensated economizer	This power saver is really powerful, used to spend 300 electricity bill every month, now it's about 100	Jingdong prefecture level city in Shandong
Energy Saving Technology Shop	In-situ compensated economizer	After two days of testing. The effect is good! About the first day of power saving for 1/4. the second day for 1/3. and not "steal electricity", is through the intelligent integrated circuit, automatically reduce the loss of electrical appliances to achieve the purpose of saving electricity! Recommended	Jingdong prefecture level city in Shandong
Energy Saving Technology Shop	In-situ compensated economizer	Power Saving King has been received, really good, thank you boss, I wish the boss business prosperity, prosperity, next time to come back to patronize!	Jingdong prefecture level city in Shandong
Energy Saving Technology Shop	In-situ compensated economizer	I've been using it for a while. Than to A little less electricity than before. I'll buy it again next time.	Jingdong prefecture level city in Shandong
Energy Saving Technology Shop	In-situ compensated economizer	Used to be a couple hundred bucks a month. With him, it's a big savings. It's great.	Jingdong prefecture level city in Shandong
Energy Saving Technology Shop	In-situ compensated economizer	Previously bought a plug in the kitchen, used for a period of time after the personal feeling is still quite effective, can save a little electricity, so this time without hesitation and bought two, has been inserted in the home next to the appliances that use a lot of electricity!	Jingdong prefecture level city in Shandong
Energy Saving Technology Shop	In-situ compensated economizer	I've used it before and it does have a power saving effect	Jingdong prefecture level city in Shandong
Energy Saving Technology Shop	In-situ compensated economizer	High-tech products, really useful, in previous years the high cost of electricity, with this product, the electricity bill came down. Praise a, hope that the research and development of this product of science and technology to continue to work hard, research out better products to.	Jingdong prefecture level city in Shandong
Energy Saving Technology Shop	In-situ compensated economizer	This high-power is not bad Oh, the first few months are 1000 degrees so, I casually brush in Taobao to this product, I bought back to try, I used a month after this month left 736 degrees, scared me, I also called to the grid to confirm, really is 736 degrees, the effect is very good, and today bought a go back to the old family to use	Jingdong prefecture level city in Shandong
Energy Saving Technology Shop	In-situ compensated economizer	Used for a period of time, you do not say can really save electricity, really is a small power-saving helper, it is worth buying!	Jingdong prefecture level city in Shandong
Energy Saving Technology Shop	In-situ compensated economizer	There are many rooms and it takes a lot of electricity to run the air conditioner when the house is cold. Bought 4 inserted half a month to save me more than 300 dollars. The effect is really core good! Quality is also very good, the seller service is very good and patient.	Jingdong prefecture level city in Shandong
Energy Saving Technology Shop	In-situ compensated economizer	Recommended by the owner of the store next door, looked at his home does have to save electricity, decided to get. Indeed quite can, the voltage is much more stable, there is a reduction in electricity	Jingdong prefecture level city in Shandong
Energy Saving Technology Shop	In-situ compensated economizer	It does save electricity and works pretty well. I feel that in a few days, I will be able to use the money that I bought to save money, it's really good!	Jingdong prefecture level city in Shandong
Energy Saving Technology Shop	In-situ compensated economizer	Small store service attitude is very good Product quality is very good Suggested purchase	Jingdong prefecture level city in Shandong
Energy Saving Technology Shop	In-situ compensated economizer	Bought two energy-saving appliances for the family, the kitchen and bathroom water heater is too expensive, the water heater to be turned on every day, the monthly electricity bill is a lot of use, with this, no longer afraid of the electricity bill is more than one month down the electricity bill can be less than half of the previous month it, the effect is particularly good!	Jingdong prefecture level city in Shandong
Energy Saving Technology Shop	In-situ compensated economizer	Saving power is really good to use, the last time I bought 3, the family used , before the family monthly electricity bills are more than 300 fast, after using this are more than 200, are several months, this time to my store is also arranged, it is really good for the people good things.	Jingdong prefecture level city in Shandong
Energy Saving Technology Shop	In-situ compensated economizer	The previous year bought a, almost every month can save a 70 dollars or so, with a year of no problem at all, look like new, this time to move to a new home decisively buy back 2 units, very believe this store!	Jingdong prefecture level city in Shandong
Energy Saving Technology Shop	In-situ compensated economizer	Power-saving effect: used a week to comment, before and now record the meter comparison, my one rental room can save twenty degrees of electricity per day. Cost-effective. Quite worth it.	Jingdong prefecture level city in Shandong
Energy Saving Technology Shop	In-situ compensated economizer	This power saver is really powerful, used to spend 300 electricity bill every month, now it's about 100	Jingdong prefecture level city in Shandong
Energy Saving Technology Shop	In-situ compensated economizer	After two days of testing. The effect is good! About the first day of power saving for 1/4. the second day for 1/3. and not "steal electricity", is through the intelligent integrated circuit, automatically reduce the loss of electrical appliances to achieve the purpose of saving electricity! Recommended	Jingdong prefecture level city in Shandong
Energy Saving Technology Shop	In-situ compensated economizer	Power Saving King has been received, really good, thank you boss, I wish the boss business prosperity, prosperity, next time to come back to patronize!	Jingdong prefecture level city in Shandong
Energy Saving Technology Shop	In-situ compensated economizer	I've been using it for a while. Than to A little less electricity than before. I'll buy it again next time.	Jingdong prefecture level city in Shandong
Energy Saving Technology Shop	In-situ compensated economizer	Used to be a couple hundred bucks a month. With him, it's a big savings. It's great.	Jingdong prefecture level city in Shandong
Energy Saving Technology Shop	In-situ compensated economizer	Previously bought a plug in the kitchen, used for a period of time after the personal feeling is still quite effective, can save a little electricity, so this time without hesitation and bought two, has been inserted in the home next to the appliances that use a lot of electricity!	Jingdong prefecture level city in Shandong
Energy Saving Technology Shop	In-situ compensated economizer	I've used it before and it does have a power saving effect	Jingdong prefecture level city in Shandong
Energy Saving Technology Shop	In-situ compensated economizer	High-tech products, really useful, in previous years the high cost of electricity, with this product, the electricity bill came down. Praise a, hope that the research and development of this product of science and technology to continue to work hard, research out better products to.	Jingdong prefecture level city in Shandong
Energy Saving Technology Shop	In-situ compensated economizer	This high-power is not bad Oh, the first few months are 1000 degrees so, I casually brush in Taobao to this product, I bought back to try, I used a month after this month left 736 degrees, scared me, I also called to the grid to confirm, really is 736 degrees, the effect is very good, and today bought a go back to the old family to use	Jingdong prefecture level city in Shandong
Energy Saving Technology Shop	In-situ compensated economizer	Used for a period of time, you do not say can really save electricity, really is a small power-saving helper, it is worth buying!	Jingdong prefecture level city in Shandong
Energy Saving Technology Shop	In-situ compensated economizer	There are many rooms and it takes a lot of electricity to run the air conditioner when the house is cold. Bought 4 inserted half a month to save me more than 300 dollars. The effect is really core good! Quality is also very good, the seller service is very good and patient.	Jingdong prefecture level city in Shandong
Energy Saving Technology Shop	In-situ compensated economizer	Recommended by the owner of the store next door, looked at his home does have to save electricity, decided to get. Indeed quite can, the voltage is much more stable, there is a reduction in electricity	Jingdong prefecture level city in Shandong
Energy Saving Technology Shop	In-situ compensated economizer	It does save electricity and works pretty well. I feel that in a few days, I will be able to use the money that I bought to save money, it's really good!	Jingdong prefecture level city in Shandong
Energy Saving Technology Shop	In-situ compensated economizer	Small store service attitude is very good Product quality is very good Suggested purchase	Jingdong prefecture level city in Shandong
Energy Saving Technology Shop	In-situ compensated economizer	Bought two energy-saving appliances for the family, the kitchen and bathroom water heater is too expensive, the water heater to be turned on every day, the monthly electricity bill is a lot of use, with this, no longer afraid of the electricity bill is more than one month down the electricity bill can be less than half of the previous month it, the effect is particularly good!	Jingdong prefecture level city in Shandong
Energy Saving Technology Shop	In-situ compensated economizer	Saving power is really good to use, the last time I bought 3, the family used , before the family monthly electricity bills are more than 300 fast, after using this are more than 200, are several months, this time to my store is also arranged, it is really good for the people good things.	Jingdong prefecture level city in Shandong
Energy Saving Technology Shop	In-situ compensated economizer	The previous year bought a, almost every month can save a 70 dollars or so, with a year of no problem at all, look like new, this time to move to a new home decisively buy back 2 units, very believe this store!	Jingdong prefecture level city in Shandong
Energy Saving Technology Shop	In-situ compensated economizer	Power-saving effect: used a week to comment, before and now record the meter comparison, my one rental room can save twenty degrees of electricity per day. Cost-effective. Quite worth it.	Jingdong prefecture level city in Shandong
Energy Saving Technology Shop	In-situ compensated economizer	This power saver is really powerful, used to spend 300 electricity bill every month, now it's about 100	Jingdong prefecture level city in Shandong
Energy Saving Technology Shop	In-situ compensated economizer	After two days of testing. The effect is good! About the first day of power saving for 1/4. the second day for 1/3. and not "steal electricity", is through the intelligent integrated circuit, automatically reduce the loss of electrical appliances to achieve the purpose of saving electricity! Recommended	Jingdong prefecture level city in Shandong
Energy Saving Technology Shop	In-situ compensated economizer	Power Saving King has been received, really good, thank you boss, I wish the boss business prosperity, prosperity, next time to come back to patronize!	Jingdong prefecture level city in Shandong
Energy Saving Technology Shop	In-situ compensated economizer	I've been using it for a while. Than to A little less electricity than before. I'll buy it again next time.	Jingdong prefecture level city in Shandong

Energy Saving Technology Shop	In-situ compensated economizer	Used to be a couple hundred bucks a month. With him, it's a big savings. It's great.	Jingdong prefecture level city in Shandong
Energy Saving Technology Shop	In-situ compensated economizer	Previously bought a plug in the kitchen, used for a period of time after the personal feeling is still quite effective, can save a little electricity, so this time without hesitation and bought two, has been inserted in the home next to the appliances that use a lot of electricity!	Jingdong prefecture level city in Shandong
Energy Saving Technology Shop	In-situ compensated economizer	I've used it before and it does have a power saving effect	Jingdong prefecture level city in Shandong
Energy Saving Technology Shop	In-situ compensated economizer	High-tech products, really useful, in previous years the high cost of electricity, with this product, the electricity bill came down. Praise a, hope that the research and development of this product of science and technology to continue to work hard, research out better products to.	Jingdong prefecture level city in Shandong
Energy Saving Technology Shop	In-situ compensated economizer	This high-power is not bad Oh, the first few months are 1000 degrees so, I casually brush in Taobao to this product, I bought back to try, I used a month after this month left 736 degrees, scared me, I also called to the grid to confirm, really is 736 degrees, the effect is very good, and today bought a go back to the old family to use	Jingdong prefecture level city in Shandong
Energy Saving Technology Shop	In-situ compensated economizer	Used for a period of time, you do not say can really save electricity, really is a small power-saving helper, it is worth buying!	Jingdong prefecture level city in Shandong
Energy Saving Technology Shop	In-situ compensated economizer	There are many rooms and it takes a lot of electricity to run the air conditioner when the house is cold. Bought 4 inserted half a month to save me more than 300 dollars. The effect is really core good! Quality is also very good, the seller service is very good and patient.	Jingdong prefecture level city in Shandong
Energy Saving Technology Shop	In-situ compensated economizer	Recommended by the owner of the store next door, looked at his home does have to save electricity, decided to get. Indeed quite can, the voltage is much more stable, there is a reduction in electricity	Jingdong prefecture level city in Shandong
Energy Saving Technology Shop	In-situ compensated economizer	It does save electricity and works pretty well. I feel that in a few days, I will be able to use the money that I bought to save money, it's really good!	Jingdong prefecture level city in Shandong
Energy Saving Technology Shop	In-situ compensated economizer	Small store service attitude is very good Product quality is very good Suggested purchase	Jingdong prefecture level city in Shandong
Energy Saving Technology Shop	In-situ compensated economizer	Bought two energy-saving appliances for the family, the kitchen and bathroom water heater is too expensive, the water heater to be turned on every day, the monthly electricity bill is a lot of use, with this, no longer afraid of the electricity bill is more than one month down the electricity bill can be less than half of the previous month it, the effect is particularly good!	Jingdong prefecture level city in Shandong
Energy Saving Technology Shop	In-situ compensated economizer	Saving power is really good to use, the last time I bought 3, the family used , before the family monthly electricity bills are more than 300 fast, after using this are more than 200, are several months, this time to my store is also arranged, it is really good for the people good things.	Jingdong prefecture level city in Shandong
Energy Saving Technology Shop	In-situ compensated economizer	The previous year bought a, almost every month can save a 70 dollars or so, with a year of no problem at all, look like new, this time to move to a new home decisively buy back 2 units, very believe this store!	Jingdong prefecture level city in Shandong
Energy Saving Technology Shop	In-situ compensated economizer	Power-saving effect: used a week to comment, before and now record the meter comparison, my one rental room can save twenty degrees of electricity per day. Cost-effective. Quite worth it.	Jingdong prefecture level city in Shandong
Energy Saving Technology Shop	In-situ compensated economizer	This power saver is really powerful, used to spend 300 electricity bill every month, now it's about 100	Jingdong prefecture level city in Shandong
Energy Saving Technology Shop	In-situ compensated economizer	After two days of testing. The effect is good! About the first day of power saving for 1/4. the second day for 1/3. and not "steal electricity", is through the intelligent integrated circuit, automatically reduce the loss of electrical appliances to achieve the purpose of saving electricity! Recommended	Jingdong prefecture level city in Shandong
Energy Saving Technology Shop	In-situ compensated economizer	Power Saving King has been received, really good, thank you boss, I wish the boss business prosperity, prosperity, next time to come back to patronize!	Jingdong prefecture level city in Shandong
Energy Saving Technology Shop	In-situ compensated economizer	I've been using it for a while. Than to A little less electricity than before. I'll buy it again next time.	Jingdong prefecture level city in Shandong
Energy Saving Technology Shop	In-situ compensated economizer	Used to be a couple hundred bucks a month. With him, it's a big savings. It's great.	Jingdong prefecture level city in Shandong
Energy Saving Technology Shop	In-situ compensated economizer	Previously bought a plug in the kitchen, used for a period of time after the personal feeling is still quite effective, can save a little electricity, so this time without hesitation and bought two, has been inserted in the home next to the appliances that use a lot of electricity!	Jingdong prefecture level city in Shandong
Energy Saving Technology Shop	In-situ compensated economizer	I've used it before and it does have a power saving effect	Jingdong prefecture level city in Shandong
Energy Saving Technology Shop	In-situ compensated economizer	High-tech products, really useful, in previous years the high cost of electricity, with this product, the electricity bill came down. Praise a, hope that the research and development of this product of science and technology to continue to work hard, research out better products to.	Jingdong prefecture level city in Shandong
Energy Saving Technology Shop	In-situ compensated economizer	This high-power is not bad Oh, the first few months are 1000 degrees so, I casually brush in Taobao to this product, I bought back to try, I used a month after this month left 736 degrees, scared me, I also called to the grid to confirm, really is 736 degrees, the effect is very good, and today bought a go back to the old family to use	Jingdong prefecture level city in Shandong
Energy Saving Technology Shop	In-situ compensated economizer	Used for a period of time, you do not say can really save electricity, really is a small power-saving helper, it is worth buying!	Jingdong prefecture level city in Shandong
Energy Saving Technology Shop	In-situ compensated economizer	There are many rooms and it takes a lot of electricity to run the air conditioner when the house is cold. Bought 4 inserted half a month to save me more than 300 dollars. The effect is really core good! Quality is also very good, the seller service is very good and patient.	Jingdong prefecture level city in Shandong
Energy Saving Technology Shop	In-situ compensated economizer	Recommended by the owner of the store next door, looked at his home does have to save electricity, decided to get. Indeed quite can, the voltage is much more stable, there is a reduction in electricity	Jingdong prefecture level city in Shandong
Energy Saving Technology Shop	In-situ compensated economizer	It does save electricity and works pretty well. I feel that in a few days, I will be able to use the money that I bought to save money, it's really good!	Jingdong prefecture level city in Shandong
Energy Saving Technology Shop	In-situ compensated economizer	Small store service attitude is very good Product quality is very good Suggested purchase	Jingdong prefecture level city in Shandong
Energy Saving Technology Shop	In-situ compensated economizer	Bought two energy-saving appliances for the family, the kitchen and bathroom water heater is too expensive, the water heater to be turned on every day, the monthly electricity bill is a lot of use, with this, no longer afraid of the electricity bill is more than one month down the electricity bill can be less than half of the previous month it, the effect is particularly good!	Jingdong prefecture level city in Shandong
Energy Saving Technology Shop	In-situ compensated economizer	Saving power is really good to use, the last time I bought 3, the family used , before the family monthly electricity bills are more than 300 fast, after using this are more than 200, are several months, this time to my store is also arranged, it is really good for the people good things.	Jingdong prefecture level city in Shandong
Energy Saving Technology Shop	In-situ compensated economizer	The previous year bought a, almost every month can save a 70 dollars or so, with a year of no problem at all, look like new, this time to move to a new home decisively buy back 2 units, very believe this store!	Jingdong prefecture level city in Shandong
Energy Saving Technology Shop	In-situ compensated economizer	Power-saving effect: used a week to comment, before and now record the meter comparison, my one rental room can save twenty degrees of electricity per day. Cost-effective. Quite worth it.	Jingdong prefecture level city in Shandong
Energy Saving Technology Shop	In-situ compensated economizer	This power saver is really powerful, used to spend 300 electricity bill every month, now it's about 100	Jingdong prefecture level city in Shandong
Energy Saving Technology Shop	In-situ compensated economizer	After two days of testing. The effect is good! About the first day of power saving for 1/4. the second day for 1/3. and not "steal electricity", is through the intelligent integrated circuit, automatically reduce the loss of electrical appliances to achieve the purpose of saving electricity! Recommended	Jingdong prefecture level city in Shandong
Energy Saving Technology Shop	In-situ compensated economizer	Power Saving King has been received, really good, thank you boss, I wish the boss business prosperity, prosperity, next time to come back to patronize!	Jingdong prefecture level city in Shandong
Energy Saving Technology Shop	In-situ compensated economizer	I've been using it for a while. Than to A little less electricity than before. I'll buy it again next time.	Jingdong prefecture level city in Shandong
Energy Saving Technology Shop	In-situ compensated economizer	Used to be a couple hundred bucks a month. With him, it's a big savings. It's great.	Jingdong prefecture level city in Shandong
Energy Saving Technology Shop	In-situ compensated economizer	Previously bought a plug in the kitchen, used for a period of time after the personal feeling is still quite effective, can save a little electricity, so this time without hesitation and bought two, has been inserted in the home next to the appliances that use a lot of electricity!	Jingdong prefecture level city in Shandong
Energy Saving Technology Shop	In-situ compensated economizer	I've used it before and it does have a power saving effect	Jingdong prefecture level city in Shandong
Energy Saving Technology Shop	In-situ compensated economizer	High-tech products, really useful, in previous years the high cost of electricity, with this product, the electricity bill came down. Praise a, hope that the research and development of this product of science and technology to continue to work hard, research out better products to.	Jingdong prefecture level city in Shandong

Energy Saving Technology Shop	In-situ compensated economizer	This high-power is not bad Oh, the first few months are 1000 degrees so, I casually brush in Taobao to this product, I bought back to try, I used a month after this month left 736 degrees, scared me, I also called to the grid to confirm, really is 736 degrees, the effect is very good, and today bought a go back to the old family to use	Jingdong prefecture level city in Shandong
Energy Saving Technology Shop	In-situ compensated economizer	Used for a period of time, you do not say can really save electricity, really is a small power-saving helper, it is worth buying!	Jingdong prefecture level city in Shandong
Energy Saving Technology Shop	In-situ compensated economizer	There are many rooms and it takes a lot of electricity to run the air conditioner when the house is cold. Bought 4 inserted half a month to save me more than 300 dollars. The effect is really core good! Quality is also very good, the seller service is very good and patient.	Jingdong prefecture level city in Shandong
Energy Saving Technology Shop	In-situ compensated economizer	Recommended by the owner of the store next door, looked at his home does have to save electricity, decided to get. Indeed quite can, the voltage is much more stable, there is a reduction in electricity	Jingdong prefecture level city in Shandong
Energy Saving Technology Shop	In-situ compensated economizer	It does save electricity and works pretty well. I feel that in a few days, I will be able to use the money that I bought to save money, it's really good!	Jingdong prefecture level city in Shandong
Energy Saving Technology Shop	In-situ compensated economizer	Small store service attitude is very good Product quality is very good Suggested purchase	Jingdong prefecture level city in Shandong
Energy Saving Technology Shop	In-situ compensated economizer	Bought two energy-saving appliances for the family, the kitchen and bathroom water heater is too expensive, the water heater to be turned on every day, the monthly electricity bill is a lot of use, with this, no longer afraid of the electricity bill is more than one month down the electricity bill can be less than half of the previous month it, the effect is particularly good!	Jingdong prefecture level city in Shandong
Energy Saving Technology Shop	In-situ compensated economizer	Saving power is really good to use, the last time I bought 3, the family used , before the family monthly electricity bills are more than 300 fast, after using this are more than 200, are several months, this time to my store is also arranged, it is really good for the people good things.	Jingdong prefecture level city in Shandong
Energy Saving Technology Shop	In-situ compensated economizer	The previous year bought a, almost every month can save a 70 dollars or so, with a year of no problem at all, look like new, this time to move to a new home decisively buy back 2 units, very believe this store!	Jingdong prefecture level city in Shandong
Energy Saving Technology Shop	In-situ compensated economizer	Power-saving effect: used a week to comment, before and now record the meter comparison, my one rental room can save twenty degrees of electricity per day. Cost-effective. Quite worth it.	Jingdong prefecture level city in Shandong
Energy Saving Technology Shop	In-situ compensated economizer	This power saver is really powerful, used to spend 300 electricity bill every month, now it's about 100	Jingdong prefecture level city in Shandong
Energy Saving Technology Shop	In-situ compensated economizer	After two days of testing. The effect is good! About the first day of power saving for 1/4. the second day for 1/3. and not "steal electricity", is through the intelligent integrated circuit, automatically reduce the loss of electrical appliances to achieve the purpose of saving electricity! Recommended	Jingdong prefecture level city in Shandong
Energy Saving Technology Shop	In-situ compensated economizer	Power Saving King has been received, really good, thank you boss, I wish the boss business prosperity, prosperity, next time to come back to patronize!	Jingdong prefecture level city in Shandong
Energy Saving Technology Shop	In-situ compensated economizer	I've been using it for a while. Than to A little less electricity than before. I'll buy it again next time.	Jingdong prefecture level city in Shandong
Energy Saving Technology Shop	In-situ compensated economizer	Used to be a couple hundred bucks a month. With him, it's a big savings. It's great.	Jingdong prefecture level city in Shandong
Energy Saving Technology Shop	In-situ compensated economizer	Previously bought a plug in the kitchen, used for a period of time after the personal feeling is still quite effective, can save a little electricity, so this time without hesitation and bought two, has been inserted in the home next to the appliances that use a lot of electricity!	Jingdong prefecture level city in Shandong
Energy Saving Technology Shop	In-situ compensated economizer	I've used it before and it does have a power saving effect	Jingdong prefecture level city in Shandong
Energy Saving Technology Shop	In-situ compensated economizer	High-tech products, really useful, in previous years the high cost of electricity, with this product, the electricity bill came down. Praise a, hope that the research and development of this product of science and technology to continue to work hard, research out better products to.	Jingdong prefecture level city in Shandong
Energy Saving Technology Shop	In-situ compensated economizer	This high-power is not bad Oh, the first few months are 1000 degrees so, I casually brush in Taobao to this product, I bought back to try, I used a month after this month left 736 degrees, scared me, I also called to the grid to confirm, really is 736 degrees, the effect is very good, and today bought a go back to the old family to use	Jingdong prefecture level city in Shandong
Energy Saving Technology Shop	In-situ compensated economizer	Used for a period of time, you do not say can really save electricity, really is a small power-saving helper, it is worth buying!	Jingdong prefecture level city in Shandong
Energy Saving Technology Shop	In-situ compensated economizer	There are many rooms and it takes a lot of electricity to run the air conditioner when the house is cold. Bought 4 inserted half a month to save me more than 300 dollars. The effect is really core good! Quality is also very good, the seller service is very good and patient.	Jingdong prefecture level city in Shandong
Energy Saving Technology Shop	In-situ compensated economizer	Recommended by the owner of the store next door, looked at his home does have to save electricity, decided to get. Indeed quite can, the voltage is much more stable, there is a reduction in electricity	Jingdong prefecture level city in Shandong
Energy Saving Technology Shop	In-situ compensated economizer	It does save electricity and works pretty well. I feel that in a few days, I will be able to use the money that I bought to save money, it's really good!	Jingdong prefecture level city in Shandong
Energy Saving Technology Shop	In-situ compensated economizer	Small store service attitude is very good Product quality is very good Suggested purchase	Jingdong prefecture level city in Shandong
Energy Saving Technology Shop	In-situ compensated economizer	Bought two energy-saving appliances for the family, the kitchen and bathroom water heater is too expensive, the water heater to be turned on every day, the monthly electricity bill is a lot of use, with this, no longer afraid of the electricity bill is more than one month down the electricity bill can be less than half of the previous month it, the effect is particularly good!	Jingdong prefecture level city in Shandong
Energy Saving Technology Shop	In-situ compensated economizer	Saving power is really good to use, the last time I bought 3, the family used , before the family monthly electricity bills are more than 300 fast, after using this are more than 200, are several months, this time to my store is also arranged, it is really good for the people good things.	Jingdong prefecture level city in Shandong
Energy Saving Technology Shop	In-situ compensated economizer	The previous year bought a, almost every month can save a 70 dollars or so, with a year of no problem at all, look like new, this time to move to a new home decisively buy back 2 units, very believe this store!	Jingdong prefecture level city in Shandong
Energy Saving Technology Shop	In-situ compensated economizer	Power-saving effect: used a week to comment, before and now record the meter comparison, my one rental room can save twenty degrees of electricity per day. Cost-effective. Quite worth it.	Jingdong prefecture level city in Shandong
Energy Saving Technology Shop	In-situ compensated economizer	This power saver is really powerful, used to spend 300 electricity bill every month, now it's about 100	Jingdong prefecture level city in Shandong
Energy Saving Technology Shop	In-situ compensated economizer	After two days of testing. The effect is good! About the first day of power saving for 1/4. the second day for 1/3. and not "steal electricity", is through the intelligent integrated circuit, automatically reduce the loss of electrical appliances to achieve the purpose of saving electricity! Recommended	Jingdong prefecture level city in Shandong
Energy Saving Technology Shop	In-situ compensated economizer	Power Saving King has been received, really good, thank you boss, I wish the boss business prosperity, prosperity, next time to come back to patronize!	Jingdong prefecture level city in Shandong
Energy Saving Technology Shop	In-situ compensated economizer	I've been using it for a while. Than to A little less electricity than before. I'll buy it again next time.	Jingdong prefecture level city in Shandong
Energy Saving Technology Shop	In-situ compensated economizer	Used to be a couple hundred bucks a month. With him, it's a big savings. It's great.	Jingdong prefecture level city in Shandong
Energy Saving Technology Shop	In-situ compensated economizer	Previously bought a plug in the kitchen, used for a period of time after the personal feeling is still quite effective, can save a little electricity, so this time without hesitation and bought two, has been inserted in the home next to the appliances that use a lot of electricity!	Jingdong prefecture level city in Shandong
Energy Saving Technology Shop	In-situ compensated economizer	I've used it before and it does have a power saving effect	Jingdong prefecture level city in Shandong
Energy Saving Technology Shop	In-situ compensated economizer	High-tech products, really useful, in previous years the high cost of electricity, with this product, the electricity bill came down. Praise a, hope that the research and development of this product of science and technology to continue to work hard, research out better products to.	Jingdong prefecture level city in Shandong
Energy Saving Technology Shop	In-situ compensated economizer	This high-power is not bad Oh, the first few months are 1000 degrees so, I casually brush in Taobao to this product, I bought back to try, I used a month after this month left 736 degrees, scared me, I also called to the grid to confirm, really is 736 degrees, the effect is very good, and today bought a go back to the old family to use	Jingdong prefecture level city in Shandong
Energy Saving Technology Shop	In-situ compensated economizer	Used for a period of time, you do not say can really save electricity, really is a small power-saving helper, it is worth buying!	Jingdong prefecture level city in Shandong
Energy Saving Technology Shop	In-situ compensated economizer	There are many rooms and it takes a lot of electricity to run the air conditioner when the house is cold. Bought 4 inserted half a month to save me more than 300 dollars. The effect is really core good! Quality is also very good, the seller service is very good and patient.	Jingdong prefecture level city in Shandong
Energy Saving Technology Shop	In-situ compensated economizer	Recommended by the owner of the store next door, looked at his home does have to save electricity, decided to get. Indeed quite can, the voltage is much more stable, there is a reduction in electricity	Jingdong prefecture level city in Shandong
Energy Saving Technology Shop	In-situ compensated economizer	It does save electricity and works pretty well. I feel that in a few days, I will be able to use the money that I bought to save money, it's really good!	Jingdong prefecture level city in Shandong

Energy Saving Technology Shop	In-situ compensated economizer	Small store service attitude is very good Product quality is very good Suggested purchase	Jingdong prefecture level city in Shandong
Energy Saving Technology Shop	In-situ compensated economizer	Bought two energy-saving appliances for the family, the kitchen and bathroom water heater is too expensive, the water heater to be turned on every day, the monthly electricity bill is a lot of use, with this, no longer afraid of the electricity bill is more than one month down the electricity bill can be less than half of the previous month it, the effect is particularly good!	Jingdong prefecture level city in Shandong
Energy Saving Technology Shop	In-situ compensated economizer	Saving power is really good to use, the last time I bought 3, the family used , before the family monthly electricity bills are more than 300 fast, after using this are more than 200, are several months, this time to my store is also arranged, it is really good for the people good things.	Jingdong prefecture level city in Shandong
Energy Saving Technology Shop	In-situ compensated economizer	The previous year bought a, almost every month can save a 70 dollars or so, with a year of no problem at all, look like new, this time to move to a new home decisively buy back 2 units, very believe this store!	Jingdong prefecture level city in Shandong
Energy Saving Technology Shop	In-situ compensated economizer	Power-saving effect: used a week to comment, before and now record the meter comparison, my one rental room can save twenty degrees of electricity per day. Cost-effective. Quite worth it.	Jingdong prefecture level city in Shandong
Energy Saving Technology Shop	In-situ compensated economizer	This power saver is really powerful, used to spend 300 electricity bill every month, now it's about 100	Jingdong prefecture level city in Shandong
Energy Saving Technology Shop	In-situ compensated economizer	After two days of testing. The effect is good! About the first day of power saving for 1/4. the second day for 1/3. and not "steal electricity", is through the intelligent integrated circuit, automatically reduce the loss of electrical appliances to achieve the purpose of saving electricity! Recommended	Jingdong prefecture level city in Shandong
Energy Saving Technology Shop	In-situ compensated economizer	Power Saving King has been received, really good, thank you boss, I wish the boss business prosperity, prosperity, next time to come back to patronize!	Jingdong prefecture level city in Shandong
Energy Saving Technology Shop	In-situ compensated economizer	I've been using it for a while. Than to A little less electricity than before. I'll buy it again next time.	Jingdong prefecture level city in Shandong
Energy Saving Technology Shop	In-situ compensated economizer	Used to be a couple hundred bucks a month. With him, it's a big savings. It's great.	Jingdong prefecture level city in Shandong
Energy Saving Technology Shop	In-situ compensated economizer	Previously bought a plug in the kitchen, used for a period of time after the personal feeling is still quite effective, can save a little electricity, so this time without hesitation and bought two, has been inserted in the home next to the appliances that use a lot of electricity!	Jingdong prefecture level city in Shandong
Energy Saving Technology Shop	In-situ compensated economizer	I've used it before and it does have a power saving effect	Jingdong prefecture level city in Shandong
Energy Saving Technology Shop	In-situ compensated economizer	High-tech products, really useful, in previous years the high cost of electricity, with this product, the electricity bill came down. Praise a, hope that the research and development of this product of science and technology to continue to work hard, research out better products to.	Jingdong prefecture level city in Shandong
Energy Saving Technology Shop	In-situ compensated economizer	This high-power is not bad Oh, the first few months are 1000 degrees so, I casually brush in Taobao to this product, I bought back to try, I used a month after this month left 736 degrees, scared me, I also called to the grid to confirm, really is 736 degrees, the effect is very good, and today bought a go back to the old family to use	Jingdong prefecture level city in Shandong
Energy Saving Technology Shop	In-situ compensated economizer	Used for a period of time, you do not say can really save electricity, really is a small power-saving helper, it is worth buying!	Jingdong prefecture level city in Shandong
Energy Saving Technology Shop	In-situ compensated economizer	There are many rooms and it takes a lot of electricity to run the air conditioner when the house is cold. Bought 4 inserted half a month to save me more than 300 dollars. The effect is really core good! Quality is also very good, the seller service is very good and patient.	Jingdong prefecture level city in Shandong
Energy Saving Technology Shop	In-situ compensated economizer	Recommended by the owner of the store next door, looked at his home does have to save electricity, decided to get. Indeed quite can, the voltage is much more stable, there is a reduction in electricity	Jingdong prefecture level city in Shandong
Energy Saving Technology Shop	In-situ compensated economizer	It does save electricity and works pretty well. I feel that in a few days, I will be able to use the money that I bought to save money, it's really good!	Jingdong prefecture level city in Shandong
Energy Saving Technology Shop	In-situ compensated economizer	Small store service attitude is very good Product quality is very good Suggested purchase	Jingdong prefecture level city in Shandong
Energy Saving Technology Shop	In-situ compensated economizer	Bought two energy-saving appliances for the family, the kitchen and bathroom water heater is too expensive, the water heater to be turned on every day, the monthly electricity bill is a lot of use, with this, no longer afraid of the electricity bill is more than one month down the electricity bill can be less than half of the previous month it, the effect is particularly good!	Jingdong prefecture level city in Shandong
Energy Saving Technology Shop	In-situ compensated economizer	Saving power is really good to use, the last time I bought 3, the family used , before the family monthly electricity bills are more than 300 fast, after using this are more than 200, are several months, this time to my store is also arranged, it is really good for the people good things.	Jingdong prefecture level city in Shandong
Energy Saving Technology Shop	In-situ compensated economizer	The previous year bought a, almost every month can save a 70 dollars or so, with a year of no problem at all, look like new, this time to move to a new home decisively buy back 2 units, very believe this store!	Jingdong prefecture level city in Shandong
Energy Saving Technology Shop	In-situ compensated economizer	Power-saving effect: used a week to comment, before and now record the meter comparison, my one rental room can save twenty degrees of electricity per day. Cost-effective. Quite worth it.	Jingdong prefecture level city in Shandong
Energy Saving Technology Shop	In-situ compensated economizer	This power saver is really powerful, used to spend 300 electricity bill every month, now it's about 100	Jingdong prefecture level city in Shandong
Energy Saving Technology Shop	In-situ compensated economizer	After two days of testing. The effect is good! About the first day of power saving for 1/4. the second day for 1/3. and not "steal electricity", is through the intelligent integrated circuit, automatically reduce the loss of electrical appliances to achieve the purpose of saving electricity! Recommended	Jingdong prefecture level city in Shandong
Energy Saving Technology Shop	In-situ compensated economizer	Power Saving King has been received, really good, thank you boss, I wish the boss business prosperity, prosperity, next time to come back to patronize!	Jingdong prefecture level city in Shandong
Energy Saving Technology Shop	In-situ compensated economizer	I've been using it for a while. Than to A little less electricity than before. I'll buy it again next time.	Jingdong prefecture level city in Shandong
Energy Saving Technology Shop	In-situ compensated economizer	Used to be a couple hundred bucks a month. With him, it's a big savings. It's great.	Jingdong prefecture level city in Shandong
Energy Saving Technology Shop	In-situ compensated economizer	Previously bought a plug in the kitchen, used for a period of time after the personal feeling is still quite effective, can save a little electricity, so this time without hesitation and bought two, has been inserted in the home next to the appliances that use a lot of electricity!	Jingdong prefecture level city in Shandong
Energy Saving Technology Shop	In-situ compensated economizer	I've used it before and it does have a power saving effect	Jingdong prefecture level city in Shandong
Energy Saving Technology Shop	In-situ compensated economizer	High-tech products, really useful, in previous years the high cost of electricity, with this product, the electricity bill came down. Praise a, hope that the research and development of this product of science and technology to continue to work hard, research out better products to.	Jingdong prefecture level city in Shandong
Energy Saving Technology Shop	In-situ compensated economizer	This high-power is not bad Oh, the first few months are 1000 degrees so, I casually brush in Taobao to this product, I bought back to try, I used a month after this month left 736 degrees, scared me, I also called to the grid to confirm, really is 736 degrees, the effect is very good, and today bought a go back to the old family to use	Jingdong prefecture level city in Shandong
Energy Saving Technology Shop	In-situ compensated economizer	Used for a period of time, you do not say can really save electricity, really is a small power-saving helper, it is worth buying!	Jingdong prefecture level city in Shandong
Energy Saving Technology Shop	In-situ compensated economizer	There are many rooms and it takes a lot of electricity to run the air conditioner when the house is cold. Bought 4 inserted half a month to save me more than 300 dollars. The effect is really core good! Quality is also very good, the seller service is very good and patient.	Jingdong prefecture level city in Shandong
Energy Saving Technology Shop	In-situ compensated economizer	Recommended by the owner of the store next door, looked at his home does have to save electricity, decided to get. Indeed quite can, the voltage is much more stable, there is a reduction in electricity	Jingdong prefecture level city in Shandong
Energy Saving Technology Shop	In-situ compensated economizer	It does save electricity and works pretty well. I feel that in a few days, I will be able to use the money that I bought to save money, it's really good!	Jingdong prefecture level city in Shandong
Energy Saving Technology Shop	In-situ compensated economizer	Small store service attitude is very good Product quality is very good Suggested purchase	Jingdong prefecture level city in Shandong
Energy Saving Technology Shop	In-situ compensated economizer	Bought two energy-saving appliances for the family, the kitchen and bathroom water heater is too expensive, the water heater to be turned on every day, the monthly electricity bill is a lot of use, with this, no longer afraid of the electricity bill is more than one month down the electricity bill can be less than half of the previous month it, the effect is particularly good!	Jingdong prefecture level city in Shandong
Energy Saving Technology Shop	In-situ compensated economizer	Saving power is really good to use, the last time I bought 3, the family used , before the family monthly electricity bills are more than 300 fast, after using this are more than 200, are several months, this time to my store is also arranged, it is really good for the people good things.	Jingdong prefecture level city in Shandong
Energy Saving Technology Shop	In-situ compensated economizer	The previous year bought a, almost every month can save a 70 dollars or so, with a year of no problem at all, look like new, this time to move to a new home decisively buy back 2 units, very believe this store!	Jingdong prefecture level city in Shandong
Energy Saving Technology Shop	In-situ compensated economizer	Power-saving effect: used a week to comment, before and now record the meter comparison, my one rental room can save twenty degrees of electricity per day. Cost-effective. Quite worth it.	Jingdong prefecture level city in Shandong

Energy Saving Technology Shop	In-situ compensated economizer	This power saver is really powerful, used to spend 300 electricity bill every month, now it's about 100	Jingdong prefecture level city in Shandong
Energy Saving Technology Shop	In-situ compensated economizer	After two days of testing. The effect is good! About the first day of power saving for 1/4. the second day for 1/3. and not "steal electricity", is through the intelligent integrated circuit, automatically reduce the loss of electrical appliances to achieve the purpose of saving electricity! Recommended	Jingdong prefecture level city in Shandong
Energy Saving Technology Shop	In-situ compensated economizer	Power Saving King has been received, really good, thank you boss, I wish the boss business prosperity, prosperity, next time to come back to patronize!	Jingdong prefecture level city in Shandong
Energy Saving Technology Shop	In-situ compensated economizer	I've been using it for a while. Than to A little less electricity than before. I'll buy it again next time.	Jingdong prefecture level city in Shandong
Energy Saving Technology Shop	In-situ compensated economizer	Used to be a couple hundred bucks a month. With him, it's a big savings. It's great.	Jingdong prefecture level city in Shandong
Energy Saving Technology Shop	In-situ compensated economizer	Previously bought a plug in the kitchen, used for a period of time after the personal feeling is still quite effective, can save a little electricity, so this time without hesitation and bought two, has been inserted in the home next to the appliances that use a lot of electricity!	Jingdong prefecture level city in Shandong
Energy Saving Technology Shop	In-situ compensated economizer	I've used it before and it does have a power saving effect	Jingdong prefecture level city in Shandong
Energy Saving Technology Shop	In-situ compensated economizer	High-tech products, really useful, in previous years the high cost of electricity, with this product, the electricity bill came down. Praise a, hope that the research and development of this product of science and technology to continue to work hard, research out better products to.	Jingdong prefecture level city in Shandong
Energy Saving Technology Shop	In-situ compensated economizer	This high-power is not bad Oh, the first few months are 1000 degrees so, I casually brush in Taobao to this product, I bought back to try, I used a month after this month left 736 degrees, scared me, I also called to the grid to confirm, really is 736 degrees, the effect is very good, and today bought a go back to the old family to use	Jingdong prefecture level city in Shandong
Energy Saving Technology Shop	In-situ compensated economizer	Used for a period of time, you do not say can really save electricity, really is a small power-saving helper, it is worth buying!	Jingdong prefecture level city in Shandong
Energy Saving Technology Shop	In-situ compensated economizer	There are many rooms and it takes a lot of electricity to run the air conditioner when the house is cold. Bought 4 inserted half a month to save me more than 300 dollars. The effect is really core good! Quality is also very good, the seller service is very good and patient.	Jingdong prefecture level city in Shandong
Energy Saving Technology Shop	In-situ compensated economizer	Recommended by the owner of the store next door, looked at his home does have to save electricity, decided to get. Indeed quite can, the voltage is much more stable, there is a reduction in electricity	Jingdong prefecture level city in Shandong
Energy Saving Technology Shop	In-situ compensated economizer	It does save electricity and works pretty well. I feel that in a few days, I will be able to use the money that I bought to save money, it's really good!	Jingdong prefecture level city in Shandong
Energy Saving Technology Shop	In-situ compensated economizer	Small store service attitude is very good Product quality is very good Suggested purchase	Jingdong prefecture level city in Shandong
Energy Saving Technology Shop	In-situ compensated economizer	Bought two energy-saving appliances for the family, the kitchen and bathroom water heater is too expensive, the water heater to be turned on every day, the monthly electricity bill is a lot of use, with this, no longer afraid of the electricity bill is more than one month down the electricity bill can be less than half of the previous month it, the effect is particularly good!	Jingdong prefecture level city in Shandong
Energy Saving Technology Shop	In-situ compensated economizer	Saving power is really good to use, the last time I bought 3, the family used , before the family monthly electricity bills are more than 300 fast, after using this are more than 200, are several months, this time to my store is also arranged, it is really good for the people good things.	Jingdong prefecture level city in Shandong
Energy Saving Technology Shop	In-situ compensated economizer	The previous year bought a, almost every month can save a 70 dollars or so, with a year of no problem at all, look like new, this time to move to a new home decisively buy back 2 units, very believe this store!	Jingdong prefecture level city in Shandong
Energy Saving Technology Shop	In-situ compensated economizer	Power-saving effect: used a week to comment, before and now record the meter comparison, my one rental room can save twenty degrees of electricity per day. Cost-effective. Quite worth it.	Jingdong prefecture level city in Shandong
Energy Saving Technology Shop	In-situ compensated economizer	This power saver is really powerful, used to spend 300 electricity bill every month, now it's about 100	Jingdong prefecture level city in Shandong
Energy Saving Technology Shop	In-situ compensated economizer	After two days of testing. The effect is good! About the first day of power saving for 1/4. the second day for 1/3. and not "steal electricity", is through the intelligent integrated circuit, automatically reduce the loss of electrical appliances to achieve the purpose of saving electricity! Recommended	Jingdong prefecture level city in Shandong
Energy Saving Technology Shop	In-situ compensated economizer	Power Saving King has been received, really good, thank you boss, I wish the boss business prosperity, prosperity, next time to come back to patronize!	Jingdong prefecture level city in Shandong
Energy Saving Technology Shop	In-situ compensated economizer	I've been using it for a while. Than to A little less electricity than before. I'll buy it again next time.	Jingdong prefecture level city in Shandong
Energy Saving Technology Shop	In-situ compensated economizer	Used to be a couple hundred bucks a month. With him, it's a big savings. It's great.	Jingdong prefecture level city in Shandong
Energy Saving Technology Shop	In-situ compensated economizer	Previously bought a plug in the kitchen, used for a period of time after the personal feeling is still quite effective, can save a little electricity, so this time without hesitation and bought two, has been inserted in the home next to the appliances that use a lot of electricity!	Jingdong prefecture level city in Shandong
Energy Saving Technology Shop	In-situ compensated economizer	I've used it before and it does have a power saving effect	Jingdong prefecture level city in Shandong
Energy Saving Technology Shop	In-situ compensated economizer	High-tech products, really useful, in previous years the high cost of electricity, with this product, the electricity bill came down. Praise a, hope that the research and development of this product of science and technology to continue to work hard, research out better products to.	Jingdong prefecture level city in Shandong
Energy Saving Technology Shop	In-situ compensated economizer	This high-power is not bad Oh, the first few months are 1000 degrees so, I casually brush in Taobao to this product, I bought back to try, I used a month after this month left 736 degrees, scared me, I also called to the grid to confirm, really is 736 degrees, the effect is very good, and today bought a go back to the old family to use	Jingdong prefecture level city in Shandong
Energy Saving Technology Shop	In-situ compensated economizer	Used for a period of time, you do not say can really save electricity, really is a small power-saving helper, it is worth buying!	Jingdong prefecture level city in Shandong
Energy Saving Technology Shop	In-situ compensated economizer	There are many rooms and it takes a lot of electricity to run the air conditioner when the house is cold. Bought 4 inserted half a month to save me more than 300 dollars. The effect is really core good! Quality is also very good, the seller service is very good and patient.	Jingdong prefecture level city in Shandong
Energy Saving Technology Shop	In-situ compensated economizer	Recommended by the owner of the store next door, looked at his home does have to save electricity, decided to get. Indeed quite can, the voltage is much more stable, there is a reduction in electricity	Jingdong prefecture level city in Shandong
Energy Saving Technology Shop	In-situ compensated economizer	It does save electricity and works pretty well. I feel that in a few days, I will be able to use the money that I bought to save money, it's really good!	Jingdong prefecture level city in Shandong
Energy Saving Technology Shop	In-situ compensated economizer	Small store service attitude is very good Product quality is very good Suggested purchase	Jingdong prefecture level city in Shandong
Energy Saving Technology Shop	In-situ compensated economizer	Bought two energy-saving appliances for the family, the kitchen and bathroom water heater is too expensive, the water heater to be turned on every day, the monthly electricity bill is a lot of use, with this, no longer afraid of the electricity bill is more than one month down the electricity bill can be less than half of the previous month it, the effect is particularly good!	Jingdong prefecture level city in Shandong
Energy Saving Technology Shop	In-situ compensated economizer	Saving power is really good to use, the last time I bought 3, the family used , before the family monthly electricity bills are more than 300 fast, after using this are more than 200, are several months, this time to my store is also arranged, it is really good for the people good things.	Jingdong prefecture level city in Shandong
Energy Saving Technology Shop	In-situ compensated economizer	The previous year bought a, almost every month can save a 70 dollars or so, with a year of no problem at all, look like new, this time to move to a new home decisively buy back 2 units, very believe this store!	Jingdong prefecture level city in Shandong
Energy Saving Technology Shop	In-situ compensated economizer	Power-saving effect: used a week to comment, before and now record the meter comparison, my one rental room can save twenty degrees of electricity per day. Cost-effective. Quite worth it.	Jingdong prefecture level city in Shandong
Energy Saving Technology Shop	In-situ compensated economizer	This power saver is really powerful, used to spend 300 electricity bill every month, now it's about 100	Jingdong prefecture level city in Shandong
Energy Saving Technology Shop	In-situ compensated economizer	After two days of testing. The effect is good! About the first day of power saving for 1/4. the second day for 1/3. and not "steal electricity", is through the intelligent integrated circuit, automatically reduce the loss of electrical appliances to achieve the purpose of saving electricity! Recommended	Jingdong prefecture level city in Shandong
Energy Saving Technology Shop	In-situ compensated economizer	Power Saving King has been received, really good, thank you boss, I wish the boss business prosperity, prosperity, next time to come back to patronize!	Jingdong prefecture level city in Shandong
Energy Saving Technology Shop	In-situ compensated economizer	I've been using it for a while. Than to A little less electricity than before. I'll buy it again next time.	Jingdong prefecture level city in Shandong
Energy Saving Technology Shop	In-situ compensated economizer	Used to be a couple hundred bucks a month. With him, it's a big savings. It's great.	Jingdong prefecture level city in Shandong

Energy Saving Technology Shop	In-situ compensated economizer	Previously bought a plug in the kitchen, used for a period of time after the personal feeling is still quite effective, can save a little electricity, so this time without hesitation and bought two, has been inserted in the home next to the appliances that use a lot of electricity!	Jingdong prefecture level city in Shandong
Energy Saving Technology Shop	In-situ compensated economizer	I've used it before and it does have a power saving effect	Jingdong prefecture level city in Shandong
Energy Saving Technology Shop	In-situ compensated economizer	High-tech products, really useful, in previous years the high cost of electricity, with this product, the electricity bill came down. Praise a, hope that the research and development of this product of science and technology to continue to work hard, research out better products to.	Jingdong prefecture level city in Shandong
Energy Saving Technology Shop	In-situ compensated economizer	This high-power is not bad Oh, the first few months are 1000 degrees so, I casually brush in Taobao to this product, I bought back to try, I used a month after this month left 736 degrees, scared me, I also called to the grid to confirm, really is 736 degrees, the effect is very good, and today bought a go back to the old family to use	Jingdong prefecture level city in Shandong
Energy Saving Technology Shop	In-situ compensated economizer	Used for a period of time, you do not say can really save electricity, really is a small power-saving helper, it is worth buying!	Jingdong prefecture level city in Shandong
Energy Saving Technology Shop	In-situ compensated economizer	There are many rooms and it takes a lot of electricity to run the air conditioner when the house is cold. Bought 4 inserted half a month to save me more than 300 dollars. The effect is really core good! Quality is also very good, the seller service is very good and patient.	Jingdong prefecture level city in Shandong
Energy Saving Technology Shop	In-situ compensated economizer	Recommended by the owner of the store next door, looked at his home does have to save electricity, decided to get. Indeed quite can, the voltage is much more stable, there is a reduction in electricity	Jingdong prefecture level city in Shandong
Energy Saving Technology Shop	In-situ compensated economizer	It does save electricity and works pretty well. I feel that in a few days, I will be able to use the money that I bought to save money, it's really good!	Jingdong prefecture level city in Shandong
Energy Saving Technology Shop	In-situ compensated economizer	Small store service attitude is very good Product quality is very good Suggested purchase	Jingdong prefecture level city in Shandong
Energy Saving Technology Shop	In-situ compensated economizer	Bought two energy-saving appliances for the family, the kitchen and bathroom water heater is too expensive, the water heater to be turned on every day, the monthly electricity bill is a lot of use, with this, no longer afraid of the electricity bill is more than one month down the electricity bill can be less than half of the previous month it, the effect is particularly good!	Jingdong prefecture level city in Shandong
Energy Saving Technology Shop	In-situ compensated economizer	Saving power is really good to use, the last time I bought 3, the family used , before the family monthly electricity bills are more than 300 fast, after using this are more than 200, are several months, this time to my store is also arranged, it is really good for the people good things.	Jingdong prefecture level city in Shandong
Energy Saving Technology Shop	In-situ compensated economizer	The previous year bought a, almost every month can save a 70 dollars or so, with a year of no problem at all, look like new, this time to move to a new home decisively buy back 2 units, very believe this store!	Jingdong prefecture level city in Shandong
Energy Saving Technology Shop	In-situ compensated economizer	Power-saving effect: used a week to comment, before and now record the meter comparison, my one rental room can save twenty degrees of electricity per day. Cost-effective. Quite worth it.	Jingdong prefecture level city in Shandong
Energy Saving Technology Shop	In-situ compensated economizer	This power saver is really powerful, used to spend 300 electricity bill every month, now it's about 100	Jingdong prefecture level city in Shandong
Energy Saving Technology Shop	In-situ compensated economizer	After two days of testing. The effect is good! About the first day of power saving for 1/4. the second day for 1/3. and not "steal electricity", is through the intelligent integrated circuit, automatically reduce the loss of electrical appliances to achieve the purpose of saving electricity! Recommended	Jingdong prefecture level city in Shandong
Energy Saving Technology Shop	In-situ compensated economizer	Power Saving King has been received, really good, thank you boss, I wish the boss business prosperity, prosperity, next time to come back to patronize!	Jingdong prefecture level city in Shandong
Energy Saving Technology Shop	In-situ compensated economizer	I've been using it for a while. Than to A little less electricity than before. I'll buy it again next time.	Jingdong prefecture level city in Shandong
Energy Saving Technology Shop	In-situ compensated economizer	Used to be a couple hundred bucks a month. With him, it's a big savings. It's great.	Jingdong prefecture level city in Shandong
Energy Saving Technology Shop	In-situ compensated economizer	Previously bought a plug in the kitchen, used for a period of time after the personal feeling is still quite effective, can save a little electricity, so this time without hesitation and bought two, has been inserted in the home next to the appliances that use a lot of electricity!	Jingdong prefecture level city in Shandong
Energy Saving Technology Shop	In-situ compensated economizer	I've used it before and it does have a power saving effect	Jingdong prefecture level city in Shandong
Energy Saving Technology Shop	In-situ compensated economizer	High-tech products, really useful, in previous years the high cost of electricity, with this product, the electricity bill came down. Praise a, hope that the research and development of this product of science and technology to continue to work hard, research out better products to.	Jingdong prefecture level city in Shandong
Energy Saving Technology Shop	In-situ compensated economizer	This high-power is not bad Oh, the first few months are 1000 degrees so, I casually brush in Taobao to this product, I bought back to try, I used a month after this month left 736 degrees, scared me, I also called to the grid to confirm, really is 736 degrees, the effect is very good, and today bought a go back to the old family to use	Jingdong prefecture level city in Shandong
Energy Saving Technology Shop	In-situ compensated economizer	Used for a period of time, you do not say can really save electricity, really is a small power-saving helper, it is worth buying!	Jingdong prefecture level city in Shandong
Energy Saving Technology Shop	In-situ compensated economizer	There are many rooms and it takes a lot of electricity to run the air conditioner when the house is cold. Bought 4 inserted half a month to save me more than 300 dollars. The effect is really core good! Quality is also very good, the seller service is very good and patient.	Jingdong prefecture level city in Shandong
Energy Saving Technology Shop	In-situ compensated economizer	Recommended by the owner of the store next door, looked at his home does have to save electricity, decided to get. Indeed quite can, the voltage is much more stable, there is a reduction in electricity	Jingdong prefecture level city in Shandong
Energy Saving Technology Shop	In-situ compensated economizer	It does save electricity and works pretty well. I feel that in a few days, I will be able to use the money that I bought to save money, it's really good!	Jingdong prefecture level city in Shandong
Energy Saving Technology Shop	In-situ compensated economizer	Small store service attitude is very good Product quality is very good Suggested purchase	Jingdong prefecture level city in Shandong
Energy Saving Technology Shop	In-situ compensated economizer	Bought two energy-saving appliances for the family, the kitchen and bathroom water heater is too expensive, the water heater to be turned on every day, the monthly electricity bill is a lot of use, with this, no longer afraid of the electricity bill is more than one month down the electricity bill can be less than half of the previous month it, the effect is particularly good!	Jingdong prefecture level city in Shandong
Energy Saving Technology Shop	In-situ compensated economizer	Saving power is really good to use, the last time I bought 3, the family used , before the family monthly electricity bills are more than 300 fast, after using this are more than 200, are several months, this time to my store is also arranged, it is really good for the people good things.	Jingdong prefecture level city in Shandong
Energy Saving Technology Shop	In-situ compensated economizer	The previous year bought a, almost every month can save a 70 dollars or so, with a year of no problem at all, look like new, this time to move to a new home decisively buy back 2 units, very believe this store!	Jingdong prefecture level city in Shandong
Energy Saving Technology Shop	In-situ compensated economizer	Power-saving effect: used a week to comment, before and now record the meter comparison, my one rental room can save twenty degrees of electricity per day. Cost-effective. Quite worth it.	Jingdong prefecture level city in Shandong
Energy Saving Technology Shop	In-situ compensated economizer	This power saver is really powerful, used to spend 300 electricity bill every month, now it's about 100	Jingdong prefecture level city in Shandong
Energy Saving Technology Shop	In-situ compensated economizer	After two days of testing. The effect is good! About the first day of power saving for 1/4. the second day for 1/3. and not "steal electricity", is through the intelligent integrated circuit, automatically reduce the loss of electrical appliances to achieve the purpose of saving electricity! Recommended	Jingdong prefecture level city in Shandong
Energy Saving Technology Shop	In-situ compensated economizer	Power Saving King has been received, really good, thank you boss, I wish the boss business prosperity, prosperity, next time to come back to patronize!	Jingdong prefecture level city in Shandong
Energy Saving Technology Shop	In-situ compensated economizer	I've been using it for a while. Than to A little less electricity than before. I'll buy it again next time.	Jingdong prefecture level city in Shandong
Energy Saving Technology Shop	In-situ compensated economizer	Used to be a couple hundred bucks a month. With him, it's a big savings. It's great.	Jingdong prefecture level city in Shandong
Energy Saving Technology Shop	In-situ compensated economizer	Previously bought a plug in the kitchen, used for a period of time after the personal feeling is still quite effective, can save a little electricity, so this time without hesitation and bought two, has been inserted in the home next to the appliances that use a lot of electricity!	Jingdong prefecture level city in Shandong
Energy Saving Technology Shop	In-situ compensated economizer	I've used it before and it does have a power saving effect	Jingdong prefecture level city in Shandong
Energy Saving Technology Shop	In-situ compensated economizer	High-tech products, really useful, in previous years the high cost of electricity, with this product, the electricity bill came down. Praise a, hope that the research and development of this product of science and technology to continue to work hard, research out better products to.	Jingdong prefecture level city in Shandong
Energy Saving Technology Shop	In-situ compensated economizer	This high-power is not bad Oh, the first few months are 1000 degrees so, I casually brush in Taobao to this product, I bought back to try, I used a month after this month left 736 degrees, scared me, I also called to the grid to confirm, really is 736 degrees, the effect is very good, and today bought a go back to the old family to use	Jingdong prefecture level city in Shandong

Energy Saving Technology Shop	In-situ compensated economizer	Used for a period of time, you do not say can really save electricity, really is a small power-saving helper, it is worth buying!	Jingdong prefecture level city in Shandong
Energy Saving Technology Shop	In-situ compensated economizer	There are many rooms and it takes a lot of electricity to run the air conditioner when the house is cold. Bought 4 inserted half a month to save me more than 300 dollars. The effect is really core good! Quality is also very good, the seller service is very good and patient.	Jingdong prefecture level city in Shandong
Energy Saving Technology Shop	In-situ compensated economizer	Recommended by the owner of the store next door, looked at his home does have to save electricity, decided to get. Indeed quite can, the voltage is much more stable, there is a reduction in electricity	Jingdong prefecture level city in Shandong
Energy Saving Technology Shop	In-situ compensated economizer	It does save electricity and works pretty well. I feel that in a few days, I will be able to use the money that I bought to save money, it's really good!	Jingdong prefecture level city in Shandong
Energy Saving Technology Shop	In-situ compensated economizer	Small store service attitude is very good Product quality is very good Suggested purchase	Jingdong prefecture level city in Shandong
Energy Saving Technology Shop	In-situ compensated economizer	Bought two energy-saving appliances for the family, the kitchen and bathroom water heater is too expensive, the water heater to be turned on every day, the monthly electricity bill is a lot of use, with this, no longer afraid of the electricity bill is more than one month down the electricity bill can be less than half of the previous month it, the effect is particularly good!	Jingdong prefecture level city in Shandong
Energy Saving Technology Shop	In-situ compensated economizer	Saving power is really good to use, the last time I bought 3, the family used , before the family monthly electricity bills are more than 300 fast, after using this are more than 200, are several months, this time to my store is also arranged, it is really good for the people good things.	Jingdong prefecture level city in Shandong
Energy Saving Technology Shop	In-situ compensated economizer	The previous year bought a, almost every month can save a 70 dollars or so, with a year of no problem at all, look like new, this time to move to a new home decisively buy back 2 units, very believe this store!	Jingdong prefecture level city in Shandong
Energy Saving Technology Shop	In-situ compensated economizer	Power-saving effect: used a week to comment, before and now record the meter comparison, my one rental room can save twenty degrees of electricity per day. Cost-effective. Quite worth it.	Jingdong prefecture level city in Shandong
Energy Saving Technology Shop	In-situ compensated economizer	This power saver is really powerful, used to spend 300 electricity bill every month, now it's about 100	Jingdong prefecture level city in Shandong
Energy Saving Technology Shop	In-situ compensated economizer	After two days of testing. The effect is good! About the first day of power saving for 1/4. the second day for 1/3. and not "steal electricity", is through the intelligent integrated circuit, automatically reduce the loss of electrical appliances to achieve the purpose of saving electricity! Recommended	Jingdong prefecture level city in Shandong
Energy Saving Technology Shop	In-situ compensated economizer	Power Saving King has been received, really good, thank you boss, I wish the boss business prosperity, prosperity, next time to come back to patronize!	Jingdong prefecture level city in Shandong
Energy Saving Technology Shop	In-situ compensated economizer	I've been using it for a while. Than to A little less electricity than before. I'll buy it again next time.	Jingdong prefecture level city in Shandong
Energy Saving Technology Shop	In-situ compensated economizer	Used to be a couple hundred bucks a month. With him, it's a big savings. It's great.	Jingdong prefecture level city in Shandong
Energy Saving Technology Shop	In-situ compensated economizer	Previously bought a plug in the kitchen, used for a period of time after the personal feeling is still quite effective, can save a little electricity, so this time without hesitation and bought two, has been inserted in the home next to the appliances that use a lot of electricity!	Jingdong prefecture level city in Shandong
Energy Saving Technology Shop	In-situ compensated economizer	I've used it before and it does have a power saving effect	Jingdong prefecture level city in Shandong
Energy Saving Technology Shop	In-situ compensated economizer	High-tech products, really useful, in previous years the high cost of electricity, with this product, the electricity bill came down. Praise a, hope that the research and development of this product of science and technology to continue to work hard, research out better products to.	Jingdong prefecture level city in Shandong
Energy Saving Technology Shop	In-situ compensated economizer	This high-power is not bad Oh, the first few months are 1000 degrees so, I casually brush in Taobao to this product, I bought back to try, I used a month after this month left 736 degrees, scared me, I also called to the grid to confirm, really is 736 degrees, the effect is very good, and today bought a go back to the old family to use	Jingdong prefecture level city in Shandong
Energy Saving Technology Shop	In-situ compensated economizer	Used for a period of time, you do not say can really save electricity, really is a small power-saving helper, it is worth buying!	Jingdong prefecture level city in Shandong
Energy Saving Technology Shop	In-situ compensated economizer	There are many rooms and it takes a lot of electricity to run the air conditioner when the house is cold. Bought 4 inserted half a month to save me more than 300 dollars. The effect is really core good! Quality is also very good, the seller service is very good and patient.	Jingdong prefecture level city in Shandong
Energy Saving Technology Shop	In-situ compensated economizer	Recommended by the owner of the store next door, looked at his home does have to save electricity, decided to get. Indeed quite can, the voltage is much more stable, there is a reduction in electricity	Jingdong prefecture level city in Shandong
Energy Saving Technology Shop	In-situ compensated economizer	It does save electricity and works pretty well. I feel that in a few days, I will be able to use the money that I bought to save money, it's really good!	Jingdong prefecture level city in Shandong
Energy Saving Technology Shop	In-situ compensated economizer	Small store service attitude is very good Product quality is very good Suggested purchase	Jingdong prefecture level city in Shandong
Energy Saving Technology Shop	In-situ compensated economizer	Bought two energy-saving appliances for the family, the kitchen and bathroom water heater is too expensive, the water heater to be turned on every day, the monthly electricity bill is a lot of use, with this, no longer afraid of the electricity bill is more than one month down the electricity bill can be less than half of the previous month it, the effect is particularly good!	Jingdong prefecture level city in Shandong
Energy Saving Technology Shop	In-situ compensated economizer	Saving power is really good to use, the last time I bought 3, the family used , before the family monthly electricity bills are more than 300 fast, after using this are more than 200, are several months, this time to my store is also arranged, it is really good for the people good things.	Jingdong prefecture level city in Shandong
Energy Saving Technology Shop	In-situ compensated economizer	The previous year bought a, almost every month can save a 70 dollars or so, with a year of no problem at all, look like new, this time to move to a new home decisively buy back 2 units, very believe this store!	Jingdong prefecture level city in Shandong
Energy Saving Technology Shop	In-situ compensated economizer	Power-saving effect: used a week to comment, before and now record the meter comparison, my one rental room can save twenty degrees of electricity per day. Cost-effective. Quite worth it.	Jingdong prefecture level city in Shandong
Energy Saving Technology Shop	In-situ compensated economizer	This power saver is really powerful, used to spend 300 electricity bill every month, now it's about 100	Jingdong prefecture level city in Shandong
Energy Saving Technology Shop	In-situ compensated economizer	After two days of testing. The effect is good! About the first day of power saving for 1/4. the second day for 1/3. and not "steal electricity", is through the intelligent integrated circuit, automatically reduce the loss of electrical appliances to achieve the purpose of saving electricity! Recommended	Jingdong prefecture level city in Shandong
Energy Saving Technology Shop	In-situ compensated economizer	Power Saving King has been received, really good, thank you boss, I wish the boss business prosperity, prosperity, next time to come back to patronize!	Jingdong prefecture level city in Shandong
Energy Saving Technology Shop	In-situ compensated economizer	I've been using it for a while. Than to A little less electricity than before. I'll buy it again next time.	Jingdong prefecture level city in Shandong
Energy Saving Technology Shop	In-situ compensated economizer	Used to be a couple hundred bucks a month. With him, it's a big savings. It's great.	Jingdong prefecture level city in Shandong
Energy Saving Technology Shop	In-situ compensated economizer	Previously bought a plug in the kitchen, used for a period of time after the personal feeling is still quite effective, can save a little electricity, so this time without hesitation and bought two, has been inserted in the home next to the appliances that use a lot of electricity!	Jingdong prefecture level city in Shandong
Energy Saving Technology Shop	In-situ compensated economizer	I've used it before and it does have a power saving effect	Jingdong prefecture level city in Shandong
Energy Saving Technology Shop	In-situ compensated economizer	High-tech products, really useful, in previous years the high cost of electricity, with this product, the electricity bill came down. Praise a, hope that the research and development of this product of science and technology to continue to work hard, research out better products to.	Jingdong prefecture level city in Shandong
Energy Saving Technology Shop	In-situ compensated economizer	This high-power is not bad Oh, the first few months are 1000 degrees so, I casually brush in Taobao to this product, I bought back to try, I used a month after this month left 736 degrees, scared me, I also called to the grid to confirm, really is 736 degrees, the effect is very good, and today bought a go back to the old family to use	Jingdong prefecture level city in Shandong
Energy Saving Technology Shop	In-situ compensated economizer	Used for a period of time, you do not say can really save electricity, really is a small power-saving helper, it is worth buying!	Jingdong prefecture level city in Shandong
Energy Saving Technology Shop	In-situ compensated economizer	There are many rooms and it takes a lot of electricity to run the air conditioner when the house is cold. Bought 4 inserted half a month to save me more than 300 dollars. The effect is really core good! Quality is also very good, the seller service is very good and patient.	Jingdong prefecture level city in Shandong
Energy Saving Technology Shop	In-situ compensated economizer	Recommended by the owner of the store next door, looked at his home does have to save electricity, decided to get. Indeed quite can, the voltage is much more stable, there is a reduction in electricity	Jingdong prefecture level city in Shandong
Energy Saving Technology Shop	In-situ compensated economizer	It does save electricity and works pretty well. I feel that in a few days, I will be able to use the money that I bought to save money, it's really good!	Jingdong prefecture level city in Shandong
Energy Saving Technology Shop	In-situ compensated economizer	Small store service attitude is very good Product quality is very good Suggested purchase	Jingdong prefecture level city in Shandong

Energy Saving Technology Shop	In-situ compensated economizer	Bought two energy-saving appliances for the family, the kitchen and bathroom water heater is too expensive, the water heater to be turned on every day, the monthly electricity bill is a lot of use, with this, no longer afraid of the electricity bill is more than one month down the electricity bill can be less than half of the previous month it, the effect is particularly good!	Jingdong prefecture level city in Shandong
Energy Saving Technology Shop	In-situ compensated economizer	Saving power is really good to use, the last time I bought 3, the family used , before the family monthly electricity bills are more than 300 fast, after using this are more than 200, are several months, this time to my store is also arranged, it is really good for the people good things.	Jingdong prefecture level city in Shandong
Energy Saving Technology Shop	In-situ compensated economizer	The previous year bought a, almost every month can save a 70 dollars or so, with a year of no problem at all, look like new, this time to move to a new home decisively buy back 2 units, very believe this store!	Jingdong prefecture level city in Shandong
Energy Saving Technology Shop	In-situ compensated economizer	Power-saving effect: used a week to comment, before and now record the meter comparison, my one rental room can save twenty degrees of electricity per day. Cost-effective. Quite worth it.	Jingdong prefecture level city in Shandong
Energy Saving Technology Shop	In-situ compensated economizer	This power saver is really powerful, used to spend 300 electricity bill every month, now it's about 100	Jingdong prefecture level city in Shandong
Energy Saving Technology Shop	In-situ compensated economizer	After two days of testing. The effect is good! About the first day of power saving for 1/4. the second day for 1/3. and not "steal electricity", is through the intelligent integrated circuit, automatically reduce the loss of electrical appliances to achieve the purpose of saving electricity! Recommended	Jingdong prefecture level city in Shandong
Energy Saving Technology Shop	In-situ compensated economizer	Power Saving King has been received, really good, thank you boss, I wish the boss business prosperity, prosperity, next time to come back to patronize!	Jingdong prefecture level city in Shandong
Energy Saving Technology Shop	In-situ compensated economizer	I've been using it for a while. Than to A little less electricity than before. I'll buy it again next time.	Jingdong prefecture level city in Shandong
Energy Saving Technology Shop	In-situ compensated economizer	Used to be a couple hundred bucks a month. With him, it's a big savings. It's great.	Jingdong prefecture level city in Shandong
Energy Saving Technology Shop	In-situ compensated economizer	Previously bought a plug in the kitchen, used for a period of time after the personal feeling is still quite effective, can save a little electricity, so this time without hesitation and bought two, has been inserted in the home next to the appliances that use a lot of electricity!	Jingdong prefecture level city in Shandong
Energy Saving Technology Shop	In-situ compensated economizer	I've used it before and it does have a power saving effect	Jingdong prefecture level city in Shandong
Energy Saving Technology Shop	In-situ compensated economizer	High-tech products, really useful, in previous years the high cost of electricity, with this product, the electricity bill came down. Praise a, hope that the research and development of this product of science and technology to continue to work hard, research out better products to.	Jingdong prefecture level city in Shandong
Energy Saving Technology Shop	In-situ compensated economizer	This high-power is not bad Oh, the first few months are 1000 degrees so, I casually brush in Taobao to this product, I bought back to try, I used a month after this month left 736 degrees, scared me, I also called to the grid to confirm, really is 736 degrees, the effect is very good, and today bought a go back to the old family to use	Jingdong prefecture level city in Shandong
Energy Saving Technology Shop	In-situ compensated economizer	Used for a period of time, you do not say can really save electricity, really is a small power-saving helper, it is worth buying!	Jingdong prefecture level city in Shandong
Energy Saving Technology Shop	In-situ compensated economizer	There are many rooms and it takes a lot of electricity to run the air conditioner when the house is cold. Bought 4 inserted half a month to save me more than 300 dollars. The effect is really core good! Quality is also very good, the seller service is very good and patient.	Jingdong prefecture level city in Shandong
Energy Saving Technology Shop	In-situ compensated economizer	Recommended by the owner of the store next door, looked at his home does have to save electricity, decided to get. Indeed quite can, the voltage is much more stable, there is a reduction in electricity	Jingdong prefecture level city in Shandong
Energy Saving Technology Shop	In-situ compensated economizer	It does save electricity and works pretty well. I feel that in a few days, I will be able to use the money that I bought to save money, it's really good!	Jingdong prefecture level city in Shandong
Energy Saving Technology Shop	In-situ compensated economizer	Small store service attitude is very good Product quality is very good Suggested purchase	Jingdong prefecture level city in Shandong
Energy Saving Technology Shop	In-situ compensated economizer	Bought two energy-saving appliances for the family, the kitchen and bathroom water heater is too expensive, the water heater to be turned on every day, the monthly electricity bill is a lot of use, with this, no longer afraid of the electricity bill is more than one month down the electricity bill can be less than half of the previous month it, the effect is particularly good!	Jingdong prefecture level city in Shandong
Energy Saving Technology Shop	In-situ compensated economizer	Saving power is really good to use, the last time I bought 3, the family used , before the family monthly electricity bills are more than 300 fast, after using this are more than 200, are several months, this time to my store is also arranged, it is really good for the people good things.	Jingdong prefecture level city in Shandong
Energy Saving Technology Shop	In-situ compensated economizer	The previous year bought a, almost every month can save a 70 dollars or so, with a year of no problem at all, look like new, this time to move to a new home decisively buy back 2 units, very believe this store!	Jingdong prefecture level city in Shandong
Energy Saving Technology Shop	In-situ compensated economizer	Power-saving effect: used a week to comment, before and now record the meter comparison, my one rental room can save twenty degrees of electricity per day. Cost-effective. Quite worth it.	Jingdong prefecture level city in Shandong
Energy Saving Technology Shop	In-situ compensated economizer	This power saver is really powerful, used to spend 300 electricity bill every month, now it's about 100	Jingdong prefecture level city in Shandong
Energy Saving Technology Shop	In-situ compensated economizer	After two days of testing. The effect is good! About the first day of power saving for 1/4. the second day for 1/3. and not "steal electricity", is through the intelligent integrated circuit, automatically reduce the loss of electrical appliances to achieve the purpose of saving electricity! Recommended	Jingdong prefecture level city in Shandong
Energy Saving Technology Shop	In-situ compensated economizer	Power Saving King has been received, really good, thank you boss, I wish the boss business prosperity, prosperity, next time to come back to patronize!	Jingdong prefecture level city in Shandong
Energy Saving Technology Shop	In-situ compensated economizer	I've been using it for a while. Than to A little less electricity than before. I'll buy it again next time.	Jingdong prefecture level city in Shandong
Energy Saving Technology Shop	In-situ compensated economizer	Used to be a couple hundred bucks a month. With him, it's a big savings. It's great.	Jingdong prefecture level city in Shandong
Energy Saving Technology Shop	In-situ compensated economizer	Previously bought a plug in the kitchen, used for a period of time after the personal feeling is still quite effective, can save a little electricity, so this time without hesitation and bought two, has been inserted in the home next to the appliances that use a lot of electricity!	Jingdong prefecture level city in Shandong
Energy Saving Technology Shop	In-situ compensated economizer	I've used it before and it does have a power saving effect	Jingdong prefecture level city in Shandong
Energy Saving Technology Shop	In-situ compensated economizer	High-tech products, really useful, in previous years the high cost of electricity, with this product, the electricity bill came down. Praise a, hope that the research and development of this product of science and technology to continue to work hard, research out better products to.	Jingdong prefecture level city in Shandong
Energy Saving Technology Shop	In-situ compensated economizer	This high-power is not bad Oh, the first few months are 1000 degrees so, I casually brush in Taobao to this product, I bought back to try, I used a month after this month left 736 degrees, scared me, I also called to the grid to confirm, really is 736 degrees, the effect is very good, and today bought a go back to the old family to use	Jingdong prefecture level city in Shandong
Energy Saving Technology Shop	In-situ compensated economizer	Used for a period of time, you do not say can really save electricity, really is a small power-saving helper, it is worth buying!	Jingdong prefecture level city in Shandong
Energy Saving Technology Shop	In-situ compensated economizer	There are many rooms and it takes a lot of electricity to run the air conditioner when the house is cold. Bought 4 inserted half a month to save me more than 300 dollars. The effect is really core good! Quality is also very good, the seller service is very good and patient.	Jingdong prefecture level city in Shandong
Energy Saving Technology Shop	In-situ compensated economizer	Recommended by the owner of the store next door, looked at his home does have to save electricity, decided to get. Indeed quite can, the voltage is much more stable, there is a reduction in electricity	Jingdong prefecture level city in Shandong
Energy Saving Technology Shop	In-situ compensated economizer	It does save electricity and works pretty well. I feel that in a few days, I will be able to use the money that I bought to save money, it's really good!	Jingdong prefecture level city in Shandong
Energy Saving Technology Shop	In-situ compensated economizer	Small store service attitude is very good Product quality is very good Suggested purchase	Jingdong prefecture level city in Shandong
Energy Saving Technology Shop	In-situ compensated economizer	Bought two energy-saving appliances for the family, the kitchen and bathroom water heater is too expensive, the water heater to be turned on every day, the monthly electricity bill is a lot of use, with this, no longer afraid of the electricity bill is more than one month down the electricity bill can be less than half of the previous month it, the effect is particularly good!	Jingdong prefecture level city in Shandong
Energy Saving Technology Shop	In-situ compensated economizer	Saving power is really good to use, the last time I bought 3, the family used , before the family monthly electricity bills are more than 300 fast, after using this are more than 200, are several months, this time to my store is also arranged, it is really good for the people good things.	Jingdong prefecture level city in Shandong
Energy Saving Technology Shop	In-situ compensated economizer	The previous year bought a, almost every month can save a 70 dollars or so, with a year of no problem at all, look like new, this time to move to a new home decisively buy back 2 units, very believe this store!	Jingdong prefecture level city in Shandong
Energy Saving Technology Shop	In-situ compensated economizer	Power-saving effect: used a week to comment, before and now record the meter comparison, my one rental room can save twenty degrees of electricity per day. Cost-effective. Quite worth it.	Jingdong prefecture level city in Shandong
Energy Saving Technology Shop	In-situ compensated economizer	This power saver is really powerful, used to spend 300 electricity bill every month, now it's about 100	Jingdong prefecture level city in Shandong

Energy Saving Technology Shop	In-situ compensated economizer	After two days of testing. The effect is good! About the first day of power saving for 1/4. the second day for 1/3. and not "steal electricity", is through the intelligent integrated circuit, automatically reduce the loss of electrical appliances to achieve the purpose of saving electricity! Recommended	Jingdong prefecture level city in Shandong
Energy Saving Technology Shop	In-situ compensated economizer	Power Saving King has been received, really good, thank you boss, I wish the boss business prosperity, prosperity, next time to come back to patronize!	Jingdong prefecture level city in Shandong
Energy Saving Technology Shop	In-situ compensated economizer	I've been using it for a while. Than to A little less electricity than before. I'll buy it again next time.	Jingdong prefecture level city in Shandong
Energy Saving Technology Shop	In-situ compensated economizer	Used to be a couple hundred bucks a month. With him, it's a big savings. It's great.	Jingdong prefecture level city in Shandong
Energy Saving Technology Shop	In-situ compensated economizer	Previously bought a plug in the kitchen, used for a period of time after the personal feeling is still quite effective, can save a little electricity, so this time without hesitation and bought two, has been inserted in the home next to the appliances that use a lot of electricity!	Jingdong prefecture level city in Shandong
Energy Saving Technology Shop	In-situ compensated economizer	I've used it before and it does have a power saving effect	Jingdong prefecture level city in Shandong
Energy Saving Technology Shop	In-situ compensated economizer	High-tech products, really useful, in previous years the high cost of electricity, with this product, the electricity bill came down. Praise a, hope that the research and development of this product of science and technology to continue to work hard, research out better products to.	Jingdong prefecture level city in Shandong
Energy Saving Technology Shop	In-situ compensated economizer	This high-power is not bad Oh, the first few months are 1000 degrees so, I casually brush in Taobao to this product, I bought back to try, I used a month after this month left 736 degrees, scared me, I also called to the grid to confirm, really is 736 degrees, the effect is very good, and today bought a go back to the old family to use	Jingdong prefecture level city in Shandong
Energy Saving Technology Shop	In-situ compensated economizer	Used for a period of time, you do not say can really save electricity, really is a small power-saving helper, it is worth buying!	Jingdong prefecture level city in Shandong
Energy Saving Technology Shop	In-situ compensated economizer	There are many rooms and it takes a lot of electricity to run the air conditioner when the house is cold. Bought 4 inserted half a month to save me more than 300 dollars. The effect is really core good! Quality is also very good, the seller service is very good and patient.	Jingdong prefecture level city in Shandong
Energy Saving Technology Shop	In-situ compensated economizer	Recommended by the owner of the store next door, looked at his home does have to save electricity, decided to get. Indeed quite can, the voltage is much more stable, there is a reduction in electricity	Jingdong prefecture level city in Shandong
Energy Saving Technology Shop	In-situ compensated economizer	It does save electricity and works pretty well. I feel that in a few days, I will be able to use the money that I bought to save money, it's really good!	Jingdong prefecture level city in Shandong
Energy Saving Technology Shop	In-situ compensated economizer	Small store service attitude is very good Product quality is very good Suggested purchase	Jingdong prefecture level city in Shandong
Energy Saving Technology Shop	In-situ compensated economizer	Bought two energy-saving appliances for the family, the kitchen and bathroom water heater is too expensive, the water heater to be turned on every day, the monthly electricity bill is a lot of use, with this, no longer afraid of the electricity bill is more than one month down the electricity bill can be less than half of the previous month it, the effect is particularly good!	Jingdong prefecture level city in Shandong
Energy Saving Technology Shop	In-situ compensated economizer	Saving power is really good to use, the last time I bought 3, the family used , before the family monthly electricity bills are more than 300 fast, after using this are more than 200, are several months, this time to my store is also arranged, it is really good for the people good things.	Jingdong prefecture level city in Shandong
Energy Saving Technology Shop	In-situ compensated economizer	The previous year bought a, almost every month can save a 70 dollars or so, with a year of no problem at all, look like new, this time to move to a new home decisively buy back 2 units, very believe this store!	Jingdong prefecture level city in Shandong
Energy Saving Technology Shop	In-situ compensated economizer	Power-saving effect: used a week to comment, before and now record the meter comparison, my one rental room can save twenty degrees of electricity per day. Cost-effective. Quite worth it.	Jingdong prefecture level city in Shandong
Energy Saving Technology Shop	In-situ compensated economizer	This power saver is really powerful, used to spend 300 electricity bill every month, now it's about 100	Jingdong prefecture level city in Shandong
Energy Saving Technology Shop	In-situ compensated economizer	After two days of testing. The effect is good! About the first day of power saving for 1/4. the second day for 1/3. and not "steal electricity", is through the intelligent integrated circuit, automatically reduce the loss of electrical appliances to achieve the purpose of saving electricity! Recommended	Jingdong prefecture level city in Shandong
Energy Saving Technology Shop	In-situ compensated economizer	Power Saving King has been received, really good, thank you boss, I wish the boss business prosperity, prosperity, next time to come back to patronize!	Jingdong prefecture level city in Shandong
Energy Saving Technology Shop	In-situ compensated economizer	I've been using it for a while. Than to A little less electricity than before. I'll buy it again next time.	Jingdong prefecture level city in Shandong
Energy Saving Technology Shop	In-situ compensated economizer	Used to be a couple hundred bucks a month. With him, it's a big savings. It's great.	Jingdong prefecture level city in Shandong
Energy Saving Technology Shop	In-situ compensated economizer	Previously bought a plug in the kitchen, used for a period of time after the personal feeling is still quite effective, can save a little electricity, so this time without hesitation and bought two, has been inserted in the home next to the appliances that use a lot of electricity!	Jingdong prefecture level city in Shandong
Energy Saving Technology Shop	In-situ compensated economizer	I've used it before and it does have a power saving effect	Jingdong prefecture level city in Shandong
Energy Saving Technology Shop	In-situ compensated economizer	High-tech products, really useful, in previous years the high cost of electricity, with this product, the electricity bill came down. Praise a, hope that the research and development of this product of science and technology to continue to work hard, research out better products to.	Jingdong prefecture level city in Shandong
Energy Saving Technology Shop	In-situ compensated economizer	This high-power is not bad Oh, the first few months are 1000 degrees so, I casually brush in Taobao to this product, I bought back to try, I used a month after this month left 736 degrees, scared me, I also called to the grid to confirm, really is 736 degrees, the effect is very good, and today bought a go back to the old family to use	Jingdong prefecture level city in Shandong
Energy Saving Technology Shop	In-situ compensated economizer	Used for a period of time, you do not say can really save electricity, really is a small power-saving helper, it is worth buying!	Jingdong prefecture level city in Shandong
Energy Saving Technology Shop	In-situ compensated economizer	There are many rooms and it takes a lot of electricity to run the air conditioner when the house is cold. Bought 4 inserted half a month to save me more than 300 dollars. The effect is really core good! Quality is also very good, the seller service is very good and patient.	Jingdong prefecture level city in Shandong
Energy Saving Technology Shop	In-situ compensated economizer	Recommended by the owner of the store next door, looked at his home does have to save electricity, decided to get. Indeed quite can, the voltage is much more stable, there is a reduction in electricity	Jingdong prefecture level city in Shandong
Energy Saving Technology Shop	In-situ compensated economizer	It does save electricity and works pretty well. I feel that in a few days, I will be able to use the money that I bought to save money, it's really good!	Jingdong prefecture level city in Shandong
Energy Saving Technology Shop	In-situ compensated economizer	Small store service attitude is very good Product quality is very good Suggested purchase	Jingdong prefecture level city in Shandong
Energy Saving Technology Shop	In-situ compensated economizer	Bought two energy-saving appliances for the family, the kitchen and bathroom water heater is too expensive, the water heater to be turned on every day, the monthly electricity bill is a lot of use, with this, no longer afraid of the electricity bill is more than one month down the electricity bill can be less than half of the previous month it, the effect is particularly good!	Jingdong prefecture level city in Shandong
Energy Saving Technology Shop	In-situ compensated economizer	Saving power is really good to use, the last time I bought 3, the family used , before the family monthly electricity bills are more than 300 fast, after using this are more than 200, are several months, this time to my store is also arranged, it is really good for the people good things.	Jingdong prefecture level city in Shandong
Energy Saving Technology Shop	In-situ compensated economizer	The previous year bought a, almost every month can save a 70 dollars or so, with a year of no problem at all, look like new, this time to move to a new home decisively buy back 2 units, very believe this store!	Jingdong prefecture level city in Shandong
Energy Saving Technology Shop	In-situ compensated economizer	Power-saving effect: used a week to comment, before and now record the meter comparison, my one rental room can save twenty degrees of electricity per day. Cost-effective. Quite worth it.	Jingdong prefecture level city in Shandong
Energy Saving Technology Shop	In-situ compensated economizer	This power saver is really powerful, used to spend 300 electricity bill every month, now it's about 100	Jingdong prefecture level city in Shandong
Energy Saving Technology Shop	In-situ compensated economizer	After two days of testing. The effect is good! About the first day of power saving for 1/4. the second day for 1/3. and not "steal electricity", is through the intelligent integrated circuit, automatically reduce the loss of electrical appliances to achieve the purpose of saving electricity! Recommended	Jingdong prefecture level city in Shandong
Energy Saving Technology Shop	In-situ compensated economizer	Power Saving King has been received, really good, thank you boss, I wish the boss business prosperity, prosperity, next time to come back to patronize!	Jingdong prefecture level city in Shandong
Energy Saving Technology Shop	In-situ compensated economizer	I've been using it for a while. Than to A little less electricity than before. I'll buy it again next time.	Jingdong prefecture level city in Shandong
Energy Saving Technology Shop	In-situ compensated economizer	Used to be a couple hundred bucks a month. With him, it's a big savings. It's great.	Jingdong prefecture level city in Shandong
Energy Saving Technology Shop	In-situ compensated economizer	Previously bought a plug in the kitchen, used for a period of time after the personal feeling is still quite effective, can save a little electricity, so this time without hesitation and bought two, has been inserted in the home next to the appliances that use a lot of electricity!	Jingdong prefecture level city in Shandong

Energy Saving Technology Shop	In-situ compensated economizer	I've used it before and it does have a power saving effect	Jingdong prefecture level city in Shandong
Energy Saving Technology Shop	In-situ compensated economizer	High-tech products, really useful, in previous years the high cost of electricity, with this product, the electricity bill came down. Praise a, hope that the research and development of this product of science and technology to continue to work hard, research out better products to.	Jingdong prefecture level city in Shandong
Energy Saving Technology Shop	In-situ compensated economizer	This high-power is not bad Oh, the first few months are 1000 degrees so, I casually brush in Taobao to this product, I bought back to try, I used a month after this month left 736 degrees, scared me, I also called to the grid to confirm, really is 736 degrees, the effect is very good, and today bought a go back to the old family to use	Jingdong prefecture level city in Shandong
Energy Saving Technology Shop	In-situ compensated economizer	Used for a period of time, you do not say can really save electricity, really is a small power-saving helper, it is worth buying!	Jingdong prefecture level city in Shandong
Energy Saving Technology Shop	In-situ compensated economizer	There are many rooms and it takes a lot of electricity to run the air conditioner when the house is cold. Bought 4 inserted half a month to save me more than 300 dollars. The effect is really core good! Quality is also very good, the seller service is very good and patient.	Jingdong prefecture level city in Shandong
Energy Saving Technology Shop	In-situ compensated economizer	Recommended by the owner of the store next door, looked at his home does have to save electricity, decided to get. Indeed quite can, the voltage is much more stable, there is a reduction in electricity	Jingdong prefecture level city in Shandong
Energy Saving Technology Shop	In-situ compensated economizer	It does save electricity and works pretty well. I feel that in a few days, I will be able to use the money that I bought to save money, it's really good!	Jingdong prefecture level city in Shandong
Energy Saving Technology Shop	In-situ compensated economizer	Small store service attitude is very good Product quality is very good Suggested purchase	Jingdong prefecture level city in Shandong
Energy Saving Technology Shop	In-situ compensated economizer	Bought two energy-saving appliances for the family, the kitchen and bathroom water heater is too expensive, the water heater to be turned on every day, the monthly electricity bill is a lot of use, with this, no longer afraid of the electricity bill is more than one month down the electricity bill can be less than half of the previous month it, the effect is particularly good!	Jingdong prefecture level city in Shandong
Energy Saving Technology Shop	In-situ compensated economizer	Saving power is really good to use, the last time I bought 3, the family used , before the family monthly electricity bills are more than 300 fast, after using this are more than 200, are several months, this time to my store is also arranged, it is really good for the people good things.	Jingdong prefecture level city in Shandong
Energy Saving Technology Shop	In-situ compensated economizer	The previous year bought a, almost every month can save a 70 dollars or so, with a year of no problem at all, look like new, this time to move to a new home decisively buy back 2 units, very believe this store!	Jingdong prefecture level city in Shandong
Energy Saving Technology Shop	In-situ compensated economizer	Power-saving effect: used a week to comment, before and now record the meter comparison, my one rental room can save twenty degrees of electricity per day. Cost-effective. Quite worth it.	Jingdong prefecture level city in Shandong
Energy Saving Technology Shop	In-situ compensated economizer	This power saver is really powerful, used to spend 300 electricity bill every month, now it's about 100	Jingdong prefecture level city in Shandong
Energy Saving Technology Shop	In-situ compensated economizer	After two days of testing. The effect is good! About the first day of power saving for 1/4. the second day for 1/3. and not "steal electricity", is through the intelligent integrated circuit, automatically reduce the loss of electrical appliances to achieve the purpose of saving electricity! Recommended	Jingdong prefecture level city in Shandong
Energy Saving Technology Shop	In-situ compensated economizer	Power Saving King has been received, really good, thank you boss, I wish the boss business prosperity, prosperity, next time to come back to patronize!	Jingdong prefecture level city in Shandong
Energy Saving Technology Shop	In-situ compensated economizer	I've been using it for a while. Than to A little less electricity than before. I'll buy it again next time.	Jingdong prefecture level city in Shandong
Energy Saving Technology Shop	In-situ compensated economizer	Used to be a couple hundred bucks a month. With him, it's a big savings. It's great.	Jingdong prefecture level city in Shandong
Energy Saving Technology Shop	In-situ compensated economizer	Previously bought a plug in the kitchen, used for a period of time after the personal feeling is still quite effective, can save a little electricity, so this time without hesitation and bought two, has been inserted in the home next to the appliances that use a lot of electricity!	Jingdong prefecture level city in Shandong
Energy Saving Technology Shop	In-situ compensated economizer	I've used it before and it does have a power saving effect	Jingdong prefecture level city in Shandong
Energy Saving Technology Shop	In-situ compensated economizer	High-tech products, really useful, in previous years the high cost of electricity, with this product, the electricity bill came down. Praise a, hope that the research and development of this product of science and technology to continue to work hard, research out better products to.	Jingdong prefecture level city in Shandong
Energy Saving Technology Shop	In-situ compensated economizer	This high-power is not bad Oh, the first few months are 1000 degrees so, I casually brush in Taobao to this product, I bought back to try, I used a month after this month left 736 degrees, scared me, I also called to the grid to confirm, really is 736 degrees, the effect is very good, and today bought a go back to the old family to use	Jingdong prefecture level city in Shandong
Energy Saving Technology Shop	In-situ compensated economizer	Used for a period of time, you do not say can really save electricity, really is a small power-saving helper, it is worth buying!	Jingdong prefecture level city in Shandong
Energy Saving Technology Shop	In-situ compensated economizer	There are many rooms and it takes a lot of electricity to run the air conditioner when the house is cold. Bought 4 inserted half a month to save me more than 300 dollars. The effect is really core good! Quality is also very good, the seller service is very good and patient.	Jingdong prefecture level city in Shandong
Energy Saving Technology Shop	In-situ compensated economizer	Recommended by the owner of the store next door, looked at his home does have to save electricity, decided to get. Indeed quite can, the voltage is much more stable, there is a reduction in electricity	Jingdong prefecture level city in Shandong
Energy Saving Technology Shop	In-situ compensated economizer	It does save electricity and works pretty well. I feel that in a few days, I will be able to use the money that I bought to save money, it's really good!	Jingdong prefecture level city in Shandong
Energy Saving Technology Shop	In-situ compensated economizer	Small store service attitude is very good Product quality is very good Suggested purchase	Jingdong prefecture level city in Shandong
Energy Saving Technology Shop	In-situ compensated economizer	Bought two energy-saving appliances for the family, the kitchen and bathroom water heater is too expensive, the water heater to be turned on every day, the monthly electricity bill is a lot of use, with this, no longer afraid of the electricity bill is more than one month down the electricity bill can be less than half of the previous month it, the effect is particularly good!	Jingdong prefecture level city in Shandong
Energy Saving Technology Shop	In-situ compensated economizer	Saving power is really good to use, the last time I bought 3, the family used , before the family monthly electricity bills are more than 300 fast, after using this are more than 200, are several months, this time to my store is also arranged, it is really good for the people good things.	Jingdong prefecture level city in Shandong
Energy Saving Technology Shop	In-situ compensated economizer	The previous year bought a, almost every month can save a 70 dollars or so, with a year of no problem at all, look like new, this time to move to a new home decisively buy back 2 units, very believe this store!	Jingdong prefecture level city in Shandong
Energy Saving Technology Shop	In-situ compensated economizer	Power-saving effect: used a week to comment, before and now record the meter comparison, my one rental room can save twenty degrees of electricity per day. Cost-effective. Quite worth it.	Jingdong prefecture level city in Shandong
Energy Saving Technology Shop	In-situ compensated economizer	This power saver is really powerful, used to spend 300 electricity bill every month, now it's about 100	Jingdong prefecture level city in Shandong
Energy Saving Technology Shop	In-situ compensated economizer	After two days of testing. The effect is good! About the first day of power saving for 1/4. the second day for 1/3. and not "steal electricity", is through the intelligent integrated circuit, automatically reduce the loss of electrical appliances to achieve the purpose of saving electricity! Recommended	Jingdong prefecture level city in Shandong
Energy Saving Technology Shop	In-situ compensated economizer	Power Saving King has been received, really good, thank you boss, I wish the boss business prosperity, prosperity, next time to come back to patronize!	Jingdong prefecture level city in Shandong
Energy Saving Technology Shop	In-situ compensated economizer	I've been using it for a while. Than to A little less electricity than before. I'll buy it again next time.	Jingdong prefecture level city in Shandong
Energy Saving Technology Shop	In-situ compensated economizer	Used to be a couple hundred bucks a month. With him, it's a big savings. It's great.	Jingdong prefecture level city in Shandong
Energy Saving Technology Shop	In-situ compensated economizer	Previously bought a plug in the kitchen, used for a period of time after the personal feeling is still quite effective, can save a little electricity, so this time without hesitation and bought two, has been inserted in the home next to the appliances that use a lot of electricity!	Jingdong prefecture level city in Shandong
Energy Saving Technology Shop	In-situ compensated economizer	I've used it before and it does have a power saving effect	Jingdong prefecture level city in Shandong
Energy Saving Technology Shop	In-situ compensated economizer	High-tech products, really useful, in previous years the high cost of electricity, with this product, the electricity bill came down. Praise a, hope that the research and development of this product of science and technology to continue to work hard, research out better products to.	Jingdong prefecture level city in Shandong
Energy Saving Technology Shop	In-situ compensated economizer	This high-power is not bad Oh, the first few months are 1000 degrees so, I casually brush in Taobao to this product, I bought back to try, I used a month after this month left 736 degrees, scared me, I also called to the grid to confirm, really is 736 degrees, the effect is very good, and today bought a go back to the old family to use	Jingdong prefecture level city in Shandong
Energy Saving Technology Shop	In-situ compensated economizer	Used for a period of time, you do not say can really save electricity, really is a small power-saving helper, it is worth buying!	Jingdong prefecture level city in Shandong

Energy Saving Technology Shop	In-situ compensated economizer	There are many rooms and it takes a lot of electricity to run the air conditioner when the house is cold. Bought 4 inserted half a month to save me more than 300 dollars. The effect is really core good! Quality is also very good, the seller service is very good and patient.	Jingdong prefecture level city in Shandong
Energy Saving Technology Shop	In-situ compensated economizer	Recommended by the owner of the store next door, looked at his home does have to save electricity, decided to get. Indeed quite can, the voltage is much more stable, there is a reduction in electricity	Jingdong prefecture level city in Shandong
Energy Saving Technology Shop	In-situ compensated economizer	It does save electricity and works pretty well. I feel that in a few days, I will be able to use the money that I bought to save money, it's really good!	Jingdong prefecture level city in Shandong
Energy Saving Technology Shop	In-situ compensated economizer	Small store service attitude is very good Product quality is very good Suggested purchase	Jingdong prefecture level city in Shandong
Energy Saving Technology Shop	In-situ compensated economizer	Bought two energy-saving appliances for the family, the kitchen and bathroom water heater is too expensive, the water heater to be turned on every day, the monthly electricity bill is a lot of use, with this, no longer afraid of the electricity bill is more than one month down the electricity bill can be less than half of the previous month it, the effect is particularly good!	Jingdong prefecture level city in Shandong
Energy Saving Technology Shop	In-situ compensated economizer	Saving power is really good to use, the last time I bought 3, the family used , before the family monthly electricity bills are more than 300 fast, after using this are more than 200, are several months, this time to my store is also arranged, it is really good for the people good things.	Jingdong prefecture level city in Shandong
Energy Saving Technology Shop	In-situ compensated economizer	The previous year bought a, almost every month can save a 70 dollars or so, with a year of no problem at all, look like new, this time to move to a new home decisively buy back 2 units, very believe this store!	Jingdong prefecture level city in Shandong
Energy Saving Technology Shop	In-situ compensated economizer	Power-saving effect: used a week to comment, before and now record the meter comparison, my one rental room can save twenty degrees of electricity per day. Cost-effective. Quite worth it.	Jingdong prefecture level city in Shandong
Energy Saving Technology Shop	In-situ compensated economizer	This power saver is really powerful, used to spend 300 electricity bill every month, now it's about 100	Jingdong prefecture level city in Shandong
Energy Saving Technology Shop	In-situ compensated economizer	After two days of testing. The effect is good! About the first day of power saving for 1/4. the second day for 1/3. and not "steal electricity", is through the intelligent integrated circuit, automatically reduce the loss of electrical appliances to achieve the purpose of saving electricity! Recommended	Jingdong prefecture level city in Shandong
Energy Saving Technology Shop	In-situ compensated economizer	Power Saving King has been received, really good, thank you boss, I wish the boss business prosperity, prosperity, next time to come back to patronize!	Jingdong prefecture level city in Shandong
Energy Saving Technology Shop	In-situ compensated economizer	I've been using it for a while. Than to A little less electricity than before. I'll buy it again next time.	Jingdong prefecture level city in Shandong
Energy Saving Technology Shop	In-situ compensated economizer	Used to be a couple hundred bucks a month. With him, it's a big savings. It's great.	Jingdong prefecture level city in Shandong
Energy Saving Technology Shop	In-situ compensated economizer	Previously bought a plug in the kitchen, used for a period of time after the personal feeling is still quite effective, can save a little electricity, so this time without hesitation and bought two, has been inserted in the home next to the appliances that use a lot of electricity!	Jingdong prefecture level city in Shandong
Energy Saving Technology Shop	In-situ compensated economizer	I've used it before and it does have a power saving effect	Jingdong prefecture level city in Shandong
Energy Saving Technology Shop	In-situ compensated economizer	High-tech products, really useful, in previous years the high cost of electricity, with this product, the electricity bill came down. Praise a, hope that the research and development of this product of science and technology to continue to work hard, research out better products to.	Jingdong prefecture level city in Shandong
Energy Saving Technology Shop	In-situ compensated economizer	This high-power is not bad Oh, the first few months are 1000 degrees so, I casually brush in Taobao to this product, I bought back to try, I used a month after this month left 736 degrees, scared me, I also called to the grid to confirm, really is 736 degrees, the effect is very good, and today bought a go back to the old family to use	Jingdong prefecture level city in Shandong
Energy Saving Technology Shop	In-situ compensated economizer	Used for a period of time, you do not say can really save electricity, really is a small power-saving helper, it is worth buying!	Jingdong prefecture level city in Shandong
Energy Saving Technology Shop	In-situ compensated economizer	There are many rooms and it takes a lot of electricity to run the air conditioner when the house is cold. Bought 4 inserted half a month to save me more than 300 dollars. The effect is really core good! Quality is also very good, the seller service is very good and patient.	Jingdong prefecture level city in Shandong
Energy Saving Technology Shop	In-situ compensated economizer	Recommended by the owner of the store next door, looked at his home does have to save electricity, decided to get. Indeed quite can, the voltage is much more stable, there is a reduction in electricity	Jingdong prefecture level city in Shandong
Energy Saving Technology Shop	In-situ compensated economizer	It does save electricity and works pretty well. I feel that in a few days, I will be able to use the money that I bought to save money, it's really good!	Jingdong prefecture level city in Shandong
Energy Saving Technology Shop	In-situ compensated economizer	Small store service attitude is very good Product quality is very good Suggested purchase	Jingdong prefecture level city in Shandong
Energy Saving Technology Shop	In-situ compensated economizer	Bought two energy-saving appliances for the family, the kitchen and bathroom water heater is too expensive, the water heater to be turned on every day, the monthly electricity bill is a lot of use, with this, no longer afraid of the electricity bill is more than one month down the electricity bill can be less than half of the previous month it, the effect is particularly good!	Jingdong prefecture level city in Shandong
Energy Saving Technology Shop	In-situ compensated economizer	Saving power is really good to use, the last time I bought 3, the family used , before the family monthly electricity bills are more than 300 fast, after using this are more than 200, are several months, this time to my store is also arranged, it is really good for the people good things.	Jingdong prefecture level city in Shandong
Energy Saving Technology Shop	In-situ compensated economizer	The previous year bought a, almost every month can save a 70 dollars or so, with a year of no problem at all, look like new, this time to move to a new home decisively buy back 2 units, very believe this store!	Jingdong prefecture level city in Shandong
Energy Saving Technology Shop	In-situ compensated economizer	Power-saving effect: used a week to comment, before and now record the meter comparison, my one rental room can save twenty degrees of electricity per day. Cost-effective. Quite worth it.	Jingdong prefecture level city in Shandong
Energy Saving Technology Shop	In-situ compensated economizer	This power saver is really powerful, used to spend 300 electricity bill every month, now it's about 100	Jingdong prefecture level city in Shandong
Energy Saving Technology Shop	In-situ compensated economizer	After two days of testing. The effect is good! About the first day of power saving for 1/4. the second day for 1/3. and not "steal electricity", is through the intelligent integrated circuit, automatically reduce the loss of electrical appliances to achieve the purpose of saving electricity! Recommended	Jingdong prefecture level city in Shandong
Energy Saving Technology Shop	In-situ compensated economizer	Power Saving King has been received, really good, thank you boss, I wish the boss business prosperity, prosperity, next time to come back to patronize!	Jingdong prefecture level city in Shandong
Energy Saving Technology Shop	In-situ compensated economizer	I've been using it for a while. Than to A little less electricity than before. I'll buy it again next time.	Jingdong prefecture level city in Shandong
Energy Saving Technology Shop	In-situ compensated economizer	Used to be a couple hundred bucks a month. With him, it's a big savings. It's great.	Jingdong prefecture level city in Shandong
Energy Saving Technology Shop	In-situ compensated economizer	Previously bought a plug in the kitchen, used for a period of time after the personal feeling is still quite effective, can save a little electricity, so this time without hesitation and bought two, has been inserted in the home next to the appliances that use a lot of electricity!	Jingdong prefecture level city in Shandong
Energy Saving Technology Shop	In-situ compensated economizer	I've used it before and it does have a power saving effect	Jingdong prefecture level city in Shandong
Energy Saving Technology Shop	In-situ compensated economizer	High-tech products, really useful, in previous years the high cost of electricity, with this product, the electricity bill came down. Praise a, hope that the research and development of this product of science and technology to continue to work hard, research out better products to.	Jingdong prefecture level city in Shandong
Energy Saving Technology Shop	In-situ compensated economizer	This high-power is not bad Oh, the first few months are 1000 degrees so, I casually brush in Taobao to this product, I bought back to try, I used a month after this month left 736 degrees, scared me, I also called to the grid to confirm, really is 736 degrees, the effect is very good, and today bought a go back to the old family to use	Jingdong prefecture level city in Shandong
Energy Saving Technology Shop	In-situ compensated economizer	Used for a period of time, you do not say can really save electricity, really is a small power-saving helper, it is worth buying!	Jingdong prefecture level city in Shandong
Energy Saving Technology Shop	In-situ compensated economizer	There are many rooms and it takes a lot of electricity to run the air conditioner when the house is cold. Bought 4 inserted half a month to save me more than 300 dollars. The effect is really core good! Quality is also very good, the seller service is very good and patient.	Jingdong prefecture level city in Shandong
Energy Saving Technology Shop	In-situ compensated economizer	Recommended by the owner of the store next door, looked at his home does have to save electricity, decided to get. Indeed quite can, the voltage is much more stable, there is a reduction in electricity	Jingdong prefecture level city in Shandong
Energy Saving Technology Shop	In-situ compensated economizer	It does save electricity and works pretty well. I feel that in a few days, I will be able to use the money that I bought to save money, it's really good!	Jingdong prefecture level city in Shandong
Energy Saving Technology Shop	In-situ compensated economizer	Small store service attitude is very good Product quality is very good Suggested purchase	Jingdong prefecture level city in Shandong
Energy Saving Technology Shop	In-situ compensated economizer	Bought two energy-saving appliances for the family, the kitchen and bathroom water heater is too expensive, the water heater to be turned on every day, the monthly electricity bill is a lot of use, with this, no longer afraid of the electricity bill is more than one month down the electricity bill can be less than half of the previous month it, the effect is particularly good!	Jingdong prefecture level city in Shandong

Energy Saving Technology Shop	In-situ compensated economizer	Saving power is really good to use, the last time I bought 3, the family used , before the family monthly electricity bills are more than 300 fast, after using this are more than 200, are several months, this time to my store is also arranged, it is really good for the people good things.	Jingdong prefecture level city in Shandong
Energy Saving Technology Shop	In-situ compensated economizer	The previous year bought a, almost every month can save a 70 dollars or so, with a year of no problem at all, look like new, this time to move to a new home decisively buy back 2 units, very believe this store!	Jingdong prefecture level city in Shandong
Energy Saving Technology Shop	In-situ compensated economizer	Power-saving effect: used a week to comment, before and now record the meter comparison, my one rental room can save twenty degrees of electricity per day. Cost-effective. Quite worth it.	Jingdong prefecture level city in Shandong
Energy Saving Technology Shop	In-situ compensated economizer	This power saver is really powerful, used to spend 300 electricity bill every month, now it's about 100	Jingdong prefecture level city in Shandong
Energy Saving Technology Shop	In-situ compensated economizer	After two days of testing. The effect is good! About the first day of power saving for 1/4. the second day for 1/3. and not "steal electricity", is through the intelligent integrated circuit, automatically reduce the loss of electrical appliances to achieve the purpose of saving electricity! Recommended	Jingdong prefecture level city in Shandong
Energy Saving Technology Shop	In-situ compensated economizer	Power Saving King has been received, really good, thank you boss, I wish the boss business prosperity, prosperity, next time to come back to patronize!	Jingdong prefecture level city in Shandong
Energy Saving Technology Shop	In-situ compensated economizer	I've been using it for a while. Than to A little less electricity than before. I'll buy it again next time.	Jingdong prefecture level city in Shandong
Energy Saving Technology Shop	In-situ compensated economizer	Used to be a couple hundred bucks a month. With him, it's a big savings. It's great.	Jingdong prefecture level city in Shandong
Energy Saving Technology Shop	In-situ compensated economizer	Previously bought a plug in the kitchen, used for a period of time after the personal feeling is still quite effective, can save a little electricity, so this time without hesitation and bought two, has been inserted in the home next to the appliances that use a lot of electricity!	Jingdong prefecture level city in Shandong
Energy Saving Technology Shop	In-situ compensated economizer	I've used it before and it does have a power saving effect	Jingdong prefecture level city in Shandong
Energy Saving Technology Shop	In-situ compensated economizer	High-tech products, really useful, in previous years the high cost of electricity, with this product, the electricity bill came down. Praise a, hope that the research and development of this product of science and technology to continue to work hard, research out better products to.	Jingdong prefecture level city in Shandong
Energy Saving Technology Shop	In-situ compensated economizer	This high-power is not bad Oh, the first few months are 1000 degrees so, I casually brush in Taobao to this product, I bought back to try, I used a month after this month left 736 degrees, scared me, I also called to the grid to confirm, really is 736 degrees, the effect is very good, and today bought a go back to the old family to use	Jingdong prefecture level city in Shandong
Energy Saving Technology Shop	In-situ compensated economizer	Used for a period of time, you do not say can really save electricity, really is a small power-saving helper, it is worth buying!	Jingdong prefecture level city in Shandong
Energy Saving Technology Shop	In-situ compensated economizer	There are many rooms and it takes a lot of electricity to run the air conditioner when the house is cold. Bought 4 inserted half a month to save me more than 300 dollars. The effect is really core good! Quality is also very good, the seller service is very good and patient.	Jingdong prefecture level city in Shandong
Energy Saving Technology Shop	In-situ compensated economizer	Recommended by the owner of the store next door, looked at his home does have to save electricity, decided to get. Indeed quite can, the voltage is much more stable, there is a reduction in electricity	Jingdong prefecture level city in Shandong
Energy Saving Technology Shop	In-situ compensated economizer	It does save electricity and works pretty well. I feel that in a few days, I will be able to use the money that I bought to save money, it's really good!	Jingdong prefecture level city in Shandong
Energy Saving Technology Shop	In-situ compensated economizer	Small store service attitude is very good Product quality is very good Suggested purchase	Jingdong prefecture level city in Shandong
Energy Saving Technology Shop	In-situ compensated economizer	Bought two energy-saving appliances for the family, the kitchen and bathroom water heater is too expensive, the water heater to be turned on every day, the monthly electricity bill is a lot of use, with this, no longer afraid of the electricity bill is more than one month down the electricity bill can be less than half of the previous month it, the effect is particularly good!	Jingdong prefecture level city in Shandong
Energy Saving Technology Shop	In-situ compensated economizer	Saving power is really good to use, the last time I bought 3, the family used , before the family monthly electricity bills are more than 300 fast, after using this are more than 200, are several months, this time to my store is also arranged, it is really good for the people good things.	Jingdong prefecture level city in Shandong
Energy Saving Technology Shop	In-situ compensated economizer	The previous year bought a, almost every month can save a 70 dollars or so, with a year of no problem at all, look like new, this time to move to a new home decisively buy back 2 units, very believe this store!	Jingdong prefecture level city in Shandong
Energy Saving Technology Shop	In-situ compensated economizer	Power-saving effect: used a week to comment, before and now record the meter comparison, my one rental room can save twenty degrees of electricity per day. Cost-effective. Quite worth it.	Jingdong prefecture level city in Shandong
Energy Saving Technology Shop	In-situ compensated economizer	This power saver is really powerful, used to spend 300 electricity bill every month, now it's about 100	Jingdong prefecture level city in Shandong
Energy Saving Technology Shop	In-situ compensated economizer	After two days of testing. The effect is good! About the first day of power saving for 1/4. the second day for 1/3. and not "steal electricity", is through the intelligent integrated circuit, automatically reduce the loss of electrical appliances to achieve the purpose of saving electricity! Recommended	Jingdong prefecture level city in Shandong
Energy Saving Technology Shop	In-situ compensated economizer	Power Saving King has been received, really good, thank you boss, I wish the boss business prosperity, prosperity, next time to come back to patronize!	Jingdong prefecture level city in Shandong
Energy Saving Technology Shop	In-situ compensated economizer	I've been using it for a while. Than to A little less electricity than before. I'll buy it again next time.	Jingdong prefecture level city in Shandong
Energy Saving Technology Shop	In-situ compensated economizer	Used to be a couple hundred bucks a month. With him, it's a big savings. It's great.	Jingdong prefecture level city in Shandong
Energy Saving Technology Shop	In-situ compensated economizer	Previously bought a plug in the kitchen, used for a period of time after the personal feeling is still quite effective, can save a little electricity, so this time without hesitation and bought two, has been inserted in the home next to the appliances that use a lot of electricity!	Jingdong prefecture level city in Shandong
Energy Saving Technology Shop	In-situ compensated economizer	I've used it before and it does have a power saving effect	Jingdong prefecture level city in Shandong
Energy Saving Technology Shop	In-situ compensated economizer	High-tech products, really useful, in previous years the high cost of electricity, with this product, the electricity bill came down. Praise a, hope that the research and development of this product of science and technology to continue to work hard, research out better products to.	Jingdong prefecture level city in Shandong
Energy Saving Technology Shop	In-situ compensated economizer	This high-power is not bad Oh, the first few months are 1000 degrees so, I casually brush in Taobao to this product, I bought back to try, I used a month after this month left 736 degrees, scared me, I also called to the grid to confirm, really is 736 degrees, the effect is very good, and today bought a go back to the old family to use	Jingdong prefecture level city in Shandong
Energy Saving Technology Shop	In-situ compensated economizer	Used for a period of time, you do not say can really save electricity, really is a small power-saving helper, it is worth buying!	Jingdong prefecture level city in Shandong
Energy Saving Technology Shop	In-situ compensated economizer	There are many rooms and it takes a lot of electricity to run the air conditioner when the house is cold. Bought 4 inserted half a month to save me more than 300 dollars. The effect is really core good! Quality is also very good, the seller service is very good and patient.	Jingdong prefecture level city in Shandong
Energy Saving Technology Shop	In-situ compensated economizer	Recommended by the owner of the store next door, looked at his home does have to save electricity, decided to get. Indeed quite can, the voltage is much more stable, there is a reduction in electricity	Jingdong prefecture level city in Shandong
Energy Saving Technology Shop	In-situ compensated economizer	It does save electricity and works pretty well. I feel that in a few days, I will be able to use the money that I bought to save money, it's really good!	Jingdong prefecture level city in Shandong
Energy Saving Technology Shop	In-situ compensated economizer	Small store service attitude is very good Product quality is very good Suggested purchase	Jingdong prefecture level city in Shandong
Energy Saving Technology Shop	In-situ compensated economizer	Bought two energy-saving appliances for the family, the kitchen and bathroom water heater is too expensive, the water heater to be turned on every day, the monthly electricity bill is a lot of use, with this, no longer afraid of the electricity bill is more than one month down the electricity bill can be less than half of the previous month it, the effect is particularly good!	Jingdong prefecture level city in Shandong
Energy Saving Technology Shop	In-situ compensated economizer	Saving power is really good to use, the last time I bought 3, the family used , before the family monthly electricity bills are more than 300 fast, after using this are more than 200, are several months, this time to my store is also arranged, it is really good for the people good things.	Jingdong prefecture level city in Shandong
Energy Saving Technology Shop	In-situ compensated economizer	The previous year bought a, almost every month can save a 70 dollars or so, with a year of no problem at all, look like new, this time to move to a new home decisively buy back 2 units, very believe this store!	Jingdong prefecture level city in Shandong
Energy Saving Technology Shop	In-situ compensated economizer	Power-saving effect: used a week to comment, before and now record the meter comparison, my one rental room can save twenty degrees of electricity per day. Cost-effective. Quite worth it.	Jingdong prefecture level city in Shandong
Energy Saving Technology Shop	In-situ compensated economizer	This power saver is really powerful, used to spend 300 electricity bill every month, now it's about 100	Jingdong prefecture level city in Shandong
Energy Saving Technology Shop	In-situ compensated economizer	After two days of testing. The effect is good! About the first day of power saving for 1/4. the second day for 1/3. and not "steal electricity", is through the intelligent integrated circuit, automatically reduce the loss of electrical appliances to achieve the purpose of saving electricity! Recommended	Jingdong prefecture level city in Shandong

Energy Saving Technology Shop	In-situ compensated economizer	Power Saving King has been received, really good, thank you boss, I wish the boss business prosperity, prosperity, next time to come back to patronize!	Jingdong prefecture level city in Shandong
Energy Saving Technology Shop	In-situ compensated economizer	I've been using it for a while. Than to A little less electricity than before. I'll buy it again next time.	Jingdong prefecture level city in Shandong
Energy Saving Technology Shop	In-situ compensated economizer	Used to be a couple hundred bucks a month. With him, it's a big savings. It's great.	Jingdong prefecture level city in Shandong
Energy Saving Technology Shop	In-situ compensated economizer	Previously bought a plug in the kitchen, used for a period of time after the personal feeling is still quite effective, can save a little electricity, so this time without hesitation and bought two, has been inserted in the home next to the appliances that use a lot of electricity!	Jingdong prefecture level city in Shandong
Energy Saving Technology Shop	In-situ compensated economizer	I've used it before and it does have a power saving effect	Jingdong prefecture level city in Shandong
Energy Saving Technology Shop	In-situ compensated economizer	High-tech products, really useful, in previous years the high cost of electricity, with this product, the electricity bill came down. Praise a, hope that the research and development of this product of science and technology to continue to work hard, research out better products to.	Jingdong prefecture level city in Shandong
Energy Saving Technology Shop	In-situ compensated economizer	This high-power is not bad Oh, the first few months are 1000 degrees so, I casually brush in Taobao to this product, I bought back to try, I used a month after this month left 736 degrees, scared me, I also called to the grid to confirm, really is 736 degrees, the effect is very good, and today bought a go back to the old family to use	Jingdong prefecture level city in Shandong
Energy Saving Technology Shop	In-situ compensated economizer	Used for a period of time, you do not say can really save electricity, really is a small power-saving helper, it is worth buying!	Jingdong prefecture level city in Shandong
Energy Saving Technology Shop	In-situ compensated economizer	There are many rooms and it takes a lot of electricity to run the air conditioner when the house is cold. Bought 4 inserted half a month to save me more than 300 dollars. The effect is really core good! Quality is also very good, the seller service is very good and patient.	Jingdong prefecture level city in Shandong
Energy Saving Technology Shop	In-situ compensated economizer	Recommended by the owner of the store next door, looked at his home does have to save electricity, decided to get. Indeed quite can, the voltage is much more stable, there is a reduction in electricity	Jingdong prefecture level city in Shandong
Energy Saving Technology Shop	In-situ compensated economizer	It does save electricity and works pretty well. I feel that in a few days, I will be able to use the money that I bought to save money, it's really good!	Jingdong prefecture level city in Shandong
Energy Saving Technology Shop	In-situ compensated economizer	Small store service attitude is very good Product quality is very good Suggested purchase	Jingdong prefecture level city in Shandong
Energy Saving Technology Shop	In-situ compensated economizer	Bought two energy-saving appliances for the family, the kitchen and bathroom water heater is too expensive, the water heater to be turned on every day, the monthly electricity bill is a lot of use, with this, no longer afraid of the electricity bill is more than one month down the electricity bill can be less than half of the previous month it, the effect is particularly good!	Jingdong prefecture level city in Shandong
Energy Saving Technology Shop	In-situ compensated economizer	Saving power is really good to use, the last time I bought 3, the family used , before the family monthly electricity bills are more than 300 fast, after using this are more than 200, are several months, this time to my store is also arranged, it is really good for the people good things.	Jingdong prefecture level city in Shandong
Energy Saving Technology Shop	In-situ compensated economizer	The previous year bought a, almost every month can save a 70 dollars or so, with a year of no problem at all, look like new, this time to move to a new home decisively buy back 2 units, very believe this store!	Jingdong prefecture level city in Shandong
Energy Saving Technology Shop	In-situ compensated economizer	Power-saving effect: used a week to comment, before and now record the meter comparison, my one rental room can save twenty degrees of electricity per day. Cost-effective. Quite worth it.	Jingdong prefecture level city in Shandong
Energy Saving Technology Shop	In-situ compensated economizer	This power saver is really powerful, used to spend 300 electricity bill every month, now it's about 100	Jingdong prefecture level city in Shandong
Energy Saving Technology Shop	In-situ compensated economizer	After two days of testing. The effect is good! About the first day of power saving for 1/4. the second day for 1/3. and not "steal electricity", is through the intelligent integrated circuit, automatically reduce the loss of electrical appliances to achieve the purpose of saving electricity! Recommended	Jingdong prefecture level city in Shandong
Energy Saving Technology Shop	In-situ compensated economizer	Power Saving King has been received, really good, thank you boss, I wish the boss business prosperity, prosperity, next time to come back to patronize!	Jingdong prefecture level city in Shandong
Energy Saving Technology Shop	In-situ compensated economizer	I've been using it for a while. Than to A little less electricity than before. I'll buy it again next time.	Jingdong prefecture level city in Shandong
Energy Saving Technology Shop	In-situ compensated economizer	Used to be a couple hundred bucks a month. With him, it's a big savings. It's great.	Jingdong prefecture level city in Shandong
Energy Saving Technology Shop	In-situ compensated economizer	Previously bought a plug in the kitchen, used for a period of time after the personal feeling is still quite effective, can save a little electricity, so this time without hesitation and bought two, has been inserted in the home next to the appliances that use a lot of electricity!	Jingdong prefecture level city in Shandong
Energy Saving Technology Shop	In-situ compensated economizer	I've used it before and it does have a power saving effect	Jingdong prefecture level city in Shandong
Energy Saving Technology Shop	In-situ compensated economizer	High-tech products, really useful, in previous years the high cost of electricity, with this product, the electricity bill came down. Praise a, hope that the research and development of this product of science and technology to continue to work hard, research out better products to.	Jingdong prefecture level city in Shandong
Energy Saving Technology Shop	In-situ compensated economizer	This high-power is not bad Oh, the first few months are 1000 degrees so, I casually brush in Taobao to this product, I bought back to try, I used a month after this month left 736 degrees, scared me, I also called to the grid to confirm, really is 736 degrees, the effect is very good, and today bought a go back to the old family to use	Jingdong prefecture level city in Shandong
Energy Saving Technology Shop	In-situ compensated economizer	Used for a period of time, you do not say can really save electricity, really is a small power-saving helper, it is worth buying!	Jingdong prefecture level city in Shandong
Energy Saving Technology Shop	In-situ compensated economizer	There are many rooms and it takes a lot of electricity to run the air conditioner when the house is cold. Bought 4 inserted half a month to save me more than 300 dollars. The effect is really core good! Quality is also very good, the seller service is very good and patient.	Jingdong prefecture level city in Shandong
Energy Saving Technology Shop	In-situ compensated economizer	Recommended by the owner of the store next door, looked at his home does have to save electricity, decided to get. Indeed quite can, the voltage is much more stable, there is a reduction in electricity	Jingdong prefecture level city in Shandong
Energy Saving Technology Shop	In-situ compensated economizer	It does save electricity and works pretty well. I feel that in a few days, I will be able to use the money that I bought to save money, it's really good!	Jingdong prefecture level city in Shandong
Energy Saving Technology Shop	In-situ compensated economizer	Small store service attitude is very good Product quality is very good Suggested purchase	Jingdong prefecture level city in Shandong
Energy Saving Technology Shop	In-situ compensated economizer	Bought two energy-saving appliances for the family, the kitchen and bathroom water heater is too expensive, the water heater to be turned on every day, the monthly electricity bill is a lot of use, with this, no longer afraid of the electricity bill is more than one month down the electricity bill can be less than half of the previous month it, the effect is particularly good!	Jingdong prefecture level city in Shandong
Energy Saving Technology Shop	In-situ compensated economizer	Saving power is really good to use, the last time I bought 3, the family used , before the family monthly electricity bills are more than 300 fast, after using this are more than 200, are several months, this time to my store is also arranged, it is really good for the people good things.	Jingdong prefecture level city in Shandong
Energy Saving Technology Shop	In-situ compensated economizer	The previous year bought a, almost every month can save a 70 dollars or so, with a year of no problem at all, look like new, this time to move to a new home decisively buy back 2 units, very believe this store!	Jingdong prefecture level city in Shandong
Energy Saving Technology Shop	In-situ compensated economizer	Power-saving effect: used a week to comment, before and now record the meter comparison, my one rental room can save twenty degrees of electricity per day. Cost-effective. Quite worth it.	Jingdong prefecture level city in Shandong
Energy Saving Technology Shop	In-situ compensated economizer	This power saver is really powerful, used to spend 300 electricity bill every month, now it's about 100	Jingdong prefecture level city in Shandong
Energy Saving Technology Shop	In-situ compensated economizer	After two days of testing. The effect is good! About the first day of power saving for 1/4. the second day for 1/3. and not "steal electricity", is through the intelligent integrated circuit, automatically reduce the loss of electrical appliances to achieve the purpose of saving electricity! Recommended	Jingdong prefecture level city in Shandong
Energy Saving Technology Shop	In-situ compensated economizer	Power Saving King has been received, really good, thank you boss, I wish the boss business prosperity, prosperity, next time to come back to patronize!	Jingdong prefecture level city in Shandong
Energy Saving Technology Shop	In-situ compensated economizer	I've been using it for a while. Than to A little less electricity than before. I'll buy it again next time.	Jingdong prefecture level city in Shandong
Energy Saving Technology Shop	In-situ compensated economizer	Used to be a couple hundred bucks a month. With him, it's a big savings. It's great.	Jingdong prefecture level city in Shandong
Energy Saving Technology Shop	In-situ compensated economizer	Previously bought a plug in the kitchen, used for a period of time after the personal feeling is still quite effective, can save a little electricity, so this time without hesitation and bought two, has been inserted in the home next to the appliances that use a lot of electricity!	Jingdong prefecture level city in Shandong
Energy Saving Technology Shop	In-situ compensated economizer	I've used it before and it does have a power saving effect	Jingdong prefecture level city in Shandong

Energy Saving Technology Shop	In-situ compensated economizer	High-tech products, really useful, in previous years the high cost of electricity, with this product, the electricity bill came down. Praise a, hope that the research and development of this product of science and technology to continue to work hard, research out better products to.	Jingdong prefecture level city in Shandong
Energy Saving Technology Shop	In-situ compensated economizer	This high-power is not bad Oh, the first few months are 1000 degrees so, I casually brush in Taobao to this product, I bought back to try, I used a month after this month left 736 degrees, scared me, I also called to the grid to confirm, really is 736 degrees, the effect is very good, and today bought a go back to the old family to use	Jingdong prefecture level city in Shandong
Energy Saving Technology Shop	In-situ compensated economizer	Used for a period of time, you do not say can really save electricity, really is a small power-saving helper, it is worth buying!	Jingdong prefecture level city in Shandong
Energy Saving Technology Shop	In-situ compensated economizer	There are many rooms and it takes a lot of electricity to run the air conditioner when the house is cold. Bought 4 inserted half a month to save me more than 300 dollars. The effect is really core good! Quality is also very good, the seller service is very good and patient.	Jingdong prefecture level city in Shandong
Energy Saving Technology Shop	In-situ compensated economizer	Recommended by the owner of the store next door, looked at his home does have to save electricity, decided to get. Indeed quite can, the voltage is much more stable, there is a reduction in electricity	Jingdong prefecture level city in Shandong
Energy Saving Technology Shop	In-situ compensated economizer	It does save electricity and works pretty well. I feel that in a few days, I will be able to use the money that I bought to save money, it's really good!	Jingdong prefecture level city in Shandong
Energy Saving Technology Shop	In-situ compensated economizer	Small store service attitude is very good Product quality is very good Suggested purchase	Jingdong prefecture level city in Shandong
Energy Saving Technology Shop	In-situ compensated economizer	Bought two energy-saving appliances for the family, the kitchen and bathroom water heater is too expensive, the water heater to be turned on every day, the monthly electricity bill is a lot of use, with this, no longer afraid of the electricity bill is more than one month down the electricity bill can be less than half of the previous month it, the effect is particularly good!	Jingdong prefecture level city in Shandong
Energy Saving Technology Shop	In-situ compensated economizer	Saving power is really good to use, the last time I bought 3, the family used , before the family monthly electricity bills are more than 300 fast, after using this are more than 200, are several months, this time to my store is also arranged, it is really good for the people good things.	Jingdong prefecture level city in Shandong
Energy Saving Technology Shop	In-situ compensated economizer	The previous year bought a, almost every month can save a 70 dollars or so, with a year of no problem at all, look like new, this time to move to a new home decisively buy back 2 units, very believe this store!	Jingdong prefecture level city in Shandong
Energy Saving Technology Shop	In-situ compensated economizer	Power-saving effect: used a week to comment, before and now record the meter comparison, my one rental room can save twenty degrees of electricity per day. Cost-effective. Quite worth it.	Jingdong prefecture level city in Shandong
Energy Saving Technology Shop	In-situ compensated economizer	This power saver is really powerful, used to spend 300 electricity bill every month, now it's about 100	Jingdong prefecture level city in Shandong
Energy Saving Technology Shop	In-situ compensated economizer	After two days of testing. The effect is good! About the first day of power saving for 1/4. the second day for 1/3. and not "steal electricity", is through the intelligent integrated circuit, automatically reduce the loss of electrical appliances to achieve the purpose of saving electricity! Recommended	Jingdong prefecture level city in Shandong
Energy Saving Technology Shop	In-situ compensated economizer	Power Saving King has been received, really good, thank you boss, I wish the boss business prosperity, prosperity, next time to come back to patronize!	Jingdong prefecture level city in Shandong
Energy Saving Technology Shop	In-situ compensated economizer	I've been using it for a while. Than to A little less electricity than before. I'll buy it again next time.	Jingdong prefecture level city in Shandong
Energy Saving Technology Shop	In-situ compensated economizer	Used to be a couple hundred bucks a month. With him, it's a big savings. It's great.	Jingdong prefecture level city in Shandong
Energy Saving Technology Shop	In-situ compensated economizer	Previously bought a plug in the kitchen, used for a period of time after the personal feeling is still quite effective, can save a little electricity, so this time without hesitation and bought two, has been inserted in the home next to the appliances that use a lot of electricity!	Jingdong prefecture level city in Shandong
Energy Saving Technology Shop	In-situ compensated economizer	I've used it before and it does have a power saving effect	Jingdong prefecture level city in Shandong
Energy Saving Technology Shop	In-situ compensated economizer	High-tech products, really useful, in previous years the high cost of electricity, with this product, the electricity bill came down. Praise a, hope that the research and development of this product of science and technology to continue to work hard, research out better products to.	Jingdong prefecture level city in Shandong
Energy Saving Technology Shop	In-situ compensated economizer	This high-power is not bad Oh, the first few months are 1000 degrees so, I casually brush in Taobao to this product, I bought back to try, I used a month after this month left 736 degrees, scared me, I also called to the grid to confirm, really is 736 degrees, the effect is very good, and today bought a go back to the old family to use	Jingdong prefecture level city in Shandong
Energy Saving Technology Shop	In-situ compensated economizer	Used for a period of time, you do not say can really save electricity, really is a small power-saving helper, it is worth buying!	Jingdong prefecture level city in Shandong
Energy Saving Technology Shop	In-situ compensated economizer	There are many rooms and it takes a lot of electricity to run the air conditioner when the house is cold. Bought 4 inserted half a month to save me more than 300 dollars. The effect is really core good! Quality is also very good, the seller service is very good and patient.	Jingdong prefecture level city in Shandong
Energy Saving Technology Shop	In-situ compensated economizer	Recommended by the owner of the store next door, looked at his home does have to save electricity, decided to get. Indeed quite can, the voltage is much more stable, there is a reduction in electricity	Jingdong prefecture level city in Shandong
Energy Saving Technology Shop	In-situ compensated economizer	It does save electricity and works pretty well. I feel that in a few days, I will be able to use the money that I bought to save money, it's really good!	Jingdong prefecture level city in Shandong
Energy Saving Technology Shop	In-situ compensated economizer	Small store service attitude is very good Product quality is very good Suggested purchase	Jingdong prefecture level city in Shandong
Energy Saving Technology Shop	In-situ compensated economizer	Bought two energy-saving appliances for the family, the kitchen and bathroom water heater is too expensive, the water heater to be turned on every day, the monthly electricity bill is a lot of use, with this, no longer afraid of the electricity bill is more than one month down the electricity bill can be less than half of the previous month it, the effect is particularly good!	Jingdong prefecture level city in Shandong
Energy Saving Technology Shop	In-situ compensated economizer	Saving power is really good to use, the last time I bought 3, the family used , before the family monthly electricity bills are more than 300 fast, after using this are more than 200, are several months, this time to my store is also arranged, it is really good for the people good things.	Jingdong prefecture level city in Shandong
Energy Saving Technology Shop	In-situ compensated economizer	The previous year bought a, almost every month can save a 70 dollars or so, with a year of no problem at all, look like new, this time to move to a new home decisively buy back 2 units, very believe this store!	Jingdong prefecture level city in Shandong
Energy Saving Technology Shop	In-situ compensated economizer	Power-saving effect: used a week to comment, before and now record the meter comparison, my one rental room can save twenty degrees of electricity per day. Cost-effective. Quite worth it.	Jingdong prefecture level city in Shandong
Energy Saving Technology Shop	In-situ compensated economizer	This power saver is really powerful, used to spend 300 electricity bill every month, now it's about 100	Jingdong prefecture level city in Shandong
Energy Saving Technology Shop	In-situ compensated economizer	After two days of testing. The effect is good! About the first day of power saving for 1/4. the second day for 1/3. and not "steal electricity", is through the intelligent integrated circuit, automatically reduce the loss of electrical appliances to achieve the purpose of saving electricity! Recommended	Jingdong prefecture level city in Shandong
Energy Saving Technology Shop	In-situ compensated economizer	Power Saving King has been received, really good, thank you boss, I wish the boss business prosperity, prosperity, next time to come back to patronize!	Jingdong prefecture level city in Shandong
Energy Saving Technology Shop	In-situ compensated economizer	I've been using it for a while. Than to A little less electricity than before. I'll buy it again next time.	Jingdong prefecture level city in Shandong
Energy Saving Technology Shop	In-situ compensated economizer	Used to be a couple hundred bucks a month. With him, it's a big savings. It's great.	Jingdong prefecture level city in Shandong
Energy Saving Technology Shop	In-situ compensated economizer	Previously bought a plug in the kitchen, used for a period of time after the personal feeling is still quite effective, can save a little electricity, so this time without hesitation and bought two, has been inserted in the home next to the appliances that use a lot of electricity!	Jingdong prefecture level city in Shandong
Energy Saving Technology Shop	In-situ compensated economizer	I've used it before and it does have a power saving effect	Jingdong prefecture level city in Shandong
Energy Saving Technology Shop	In-situ compensated economizer	High-tech products, really useful, in previous years the high cost of electricity, with this product, the electricity bill came down. Praise a, hope that the research and development of this product of science and technology to continue to work hard, research out better products to.	Jingdong prefecture level city in Shandong
Energy Saving Technology Shop	In-situ compensated economizer	This high-power is not bad Oh, the first few months are 1000 degrees so, I casually brush in Taobao to this product, I bought back to try, I used a month after this month left 736 degrees, scared me, I also called to the grid to confirm, really is 736 degrees, the effect is very good, and today bought a go back to the old family to use	Jingdong prefecture level city in Shandong
Energy Saving Technology Shop	In-situ compensated economizer	Used for a period of time, you do not say can really save electricity, really is a small power-saving helper, it is worth buying!	Jingdong prefecture level city in Shandong
Energy Saving Technology Shop	In-situ compensated economizer	There are many rooms and it takes a lot of electricity to run the air conditioner when the house is cold. Bought 4 inserted half a month to save me more than 300 dollars. The effect is really core good! Quality is also very good, the seller service is very good and patient.	Jingdong prefecture level city in Shandong

Energy Saving Technology Shop	In-situ compensated economizer	Recommended by the owner of the store next door, looked at his home does have to save electricity, decided to get. Indeed quite can, the voltage is much more stable, there is a reduction in electricity	Jingdong prefecture level city in Shandong
Energy Saving Technology Shop	In-situ compensated economizer	It does save electricity and works pretty well. I feel that in a few days, I will be able to use the money that I bought to save money, it's really good!	Jingdong prefecture level city in Shandong
Energy Saving Technology Shop	In-situ compensated economizer	Small store service attitude is very good Product quality is very good Suggested purchase	Jingdong prefecture level city in Shandong
Energy Saving Technology Shop	In-situ compensated economizer	Bought two energy-saving appliances for the family, the kitchen and bathroom water heater is too expensive, the water heater to be turned on every day, the monthly electricity bill is a lot of use, with this, no longer afraid of the electricity bill is more than one month down the electricity bill can be less than half of the previous month it, the effect is particularly good!	Jingdong prefecture level city in Shandong
Energy Saving Technology Shop	In-situ compensated economizer	Saving power is really good to use, the last time I bought 3, the family used , before the family monthly electricity bills are more than 300 fast, after using this are more than 200, are several months, this time to my store is also arranged, it is really good for the people good things.	Jingdong prefecture level city in Shandong
Energy Saving Technology Shop	In-situ compensated economizer	The previous year bought a, almost every month can save a 70 dollars or so, with a year of no problem at all, look like new, this time to move to a new home decisively buy back 2 units, very believe this store!	Jingdong prefecture level city in Shandong
Energy Saving Technology Shop	In-situ compensated economizer	Power-saving effect: used a week to comment, before and now record the meter comparison, my one rental room can save twenty degrees of electricity per day. Cost-effective. Quite worth it.	Jingdong prefecture level city in Shandong
Energy Saving Technology Shop	In-situ compensated economizer	This power saver is really powerful, used to spend 300 electricity bill every month, now it's about 100	Jingdong prefecture level city in Shandong
Energy Saving Technology Shop	In-situ compensated economizer	After two days of testing. The effect is good! About the first day of power saving for 1/4. the second day for 1/3. and not "steal electricity", is through the intelligent integrated circuit, automatically reduce the loss of electrical appliances to achieve the purpose of saving electricity! Recommended	Jingdong prefecture level city in Shandong
Energy Saving Technology Shop	In-situ compensated economizer	Power Saving King has been received, really good, thank you boss, I wish the boss business prosperity, prosperity, next time to come back to patronize!	Jingdong prefecture level city in Shandong
Energy Saving Technology Shop	In-situ compensated economizer	I've been using it for a while. Than to A little less electricity than before. I'll buy it again next time.	Jingdong prefecture level city in Shandong
Energy Saving Technology Shop	In-situ compensated economizer	Used to be a couple hundred bucks a month. With him, it's a big savings. It's great.	Jingdong prefecture level city in Shandong
Energy Saving Technology Shop	In-situ compensated economizer	Previously bought a plug in the kitchen, used for a period of time after the personal feeling is still quite effective, can save a little electricity, so this time without hesitation and bought two, has been inserted in the home next to the appliances that use a lot of electricity!	Jingdong prefecture level city in Shandong
Energy Saving Technology Shop	In-situ compensated economizer	I've used it before and it does have a power saving effect	Jingdong prefecture level city in Shandong
Energy Saving Technology Shop	In-situ compensated economizer	High-tech products, really useful, in previous years the high cost of electricity, with this product, the electricity bill came down. Praise a, hope that the research and development of this product of science and technology to continue to work hard, research out better products to.	Jingdong prefecture level city in Shandong
Energy Saving Technology Shop	In-situ compensated economizer	This high-power is not bad Oh, the first few months are 1000 degrees so, I casually brush in Taobao to this product, I bought back to try, I used a month after this month left 736 degrees, scared me, I also called to the grid to confirm, really is 736 degrees, the effect is very good, and today bought a go back to the old family to use	Jingdong prefecture level city in Shandong
Energy Saving Technology Shop	In-situ compensated economizer	Used for a period of time, you do not say can really save electricity, really is a small power-saving helper, it is worth buying!	Jingdong prefecture level city in Shandong
Energy Saving Technology Shop	In-situ compensated economizer	There are many rooms and it takes a lot of electricity to run the air conditioner when the house is cold. Bought 4 inserted half a month to save me more than 300 dollars. The effect is really core good! Quality is also very good, the seller service is very good and patient.	Jingdong prefecture level city in Shandong
Energy Saving Technology Shop	In-situ compensated economizer	Recommended by the owner of the store next door, looked at his home does have to save electricity, decided to get. Indeed quite can, the voltage is much more stable, there is a reduction in electricity	Jingdong prefecture level city in Shandong
Energy Saving Technology Shop	In-situ compensated economizer	It does save electricity and works pretty well. I feel that in a few days, I will be able to use the money that I bought to save money, it's really good!	Jingdong prefecture level city in Shandong
Energy Saving Technology Shop	In-situ compensated economizer	Small store service attitude is very good Product quality is very good Suggested purchase	Jingdong prefecture level city in Shandong
Energy Saving Technology Shop	In-situ compensated economizer	Bought two energy-saving appliances for the family, the kitchen and bathroom water heater is too expensive, the water heater to be turned on every day, the monthly electricity bill is a lot of use, with this, no longer afraid of the electricity bill is more than one month down the electricity bill can be less than half of the previous month it, the effect is particularly good!	Jingdong prefecture level city in Shandong
Energy Saving Technology Shop	In-situ compensated economizer	Saving power is really good to use, the last time I bought 3, the family used , before the family monthly electricity bills are more than 300 fast, after using this are more than 200, are several months, this time to my store is also arranged, it is really good for the people good things.	Jingdong prefecture level city in Shandong
Energy Saving Technology Shop	In-situ compensated economizer	The previous year bought a, almost every month can save a 70 dollars or so, with a year of no problem at all, look like new, this time to move to a new home decisively buy back 2 units, very believe this store!	Jingdong prefecture level city in Shandong
Energy Saving Technology Shop	In-situ compensated economizer	Power-saving effect: used a week to comment, before and now record the meter comparison, my one rental room can save twenty degrees of electricity per day. Cost-effective. Quite worth it.	Jingdong prefecture level city in Shandong
Energy Saving Technology Shop	In-situ compensated economizer	This power saver is really powerful, used to spend 300 electricity bill every month, now it's about 100	Jingdong prefecture level city in Shandong
Energy Saving Technology Shop	In-situ compensated economizer	After two days of testing. The effect is good! About the first day of power saving for 1/4. the second day for 1/3. and not "steal electricity", is through the intelligent integrated circuit, automatically reduce the loss of electrical appliances to achieve the purpose of saving electricity! Recommended	Jingdong prefecture level city in Shandong
Energy Saving Technology Shop	In-situ compensated economizer	Power Saving King has been received, really good, thank you boss, I wish the boss business prosperity, prosperity, next time to come back to patronize!	Jingdong prefecture level city in Shandong
Energy Saving Technology Shop	In-situ compensated economizer	I've been using it for a while. Than to A little less electricity than before. I'll buy it again next time.	Jingdong prefecture level city in Shandong
Energy Saving Technology Shop	In-situ compensated economizer	Used to be a couple hundred bucks a month. With him, it's a big savings. It's great.	Jingdong prefecture level city in Shandong
Energy Saving Technology Shop	In-situ compensated economizer	Previously bought a plug in the kitchen, used for a period of time after the personal feeling is still quite effective, can save a little electricity, so this time without hesitation and bought two, has been inserted in the home next to the appliances that use a lot of electricity!	Jingdong prefecture level city in Shandong
Energy Saving Technology Shop	In-situ compensated economizer	I've used it before and it does have a power saving effect	Jingdong prefecture level city in Shandong
Energy Saving Technology Shop	In-situ compensated economizer	High-tech products, really useful, in previous years the high cost of electricity, with this product, the electricity bill came down. Praise a, hope that the research and development of this product of science and technology to continue to work hard, research out better products to.	Jingdong prefecture level city in Shandong
Energy Saving Technology Shop	In-situ compensated economizer	This high-power is not bad Oh, the first few months are 1000 degrees so, I casually brush in Taobao to this product, I bought back to try, I used a month after this month left 736 degrees, scared me, I also called to the grid to confirm, really is 736 degrees, the effect is very good, and today bought a go back to the old family to use	Jingdong prefecture level city in Shandong
Energy Saving Technology Shop	In-situ compensated economizer	Used for a period of time, you do not say can really save electricity, really is a small power-saving helper, it is worth buying!	Jingdong prefecture level city in Shandong
Energy Saving Technology Shop	In-situ compensated economizer	There are many rooms and it takes a lot of electricity to run the air conditioner when the house is cold. Bought 4 inserted half a month to save me more than 300 dollars. The effect is really core good! Quality is also very good, the seller service is very good and patient.	Jingdong prefecture level city in Shandong
Energy Saving Technology Shop	In-situ compensated economizer	Recommended by the owner of the store next door, looked at his home does have to save electricity, decided to get. Indeed quite can, the voltage is much more stable, there is a reduction in electricity	Jingdong prefecture level city in Shandong
Energy Saving Technology Shop	In-situ compensated economizer	It does save electricity and works pretty well. I feel that in a few days, I will be able to use the money that I bought to save money, it's really good!	Jingdong prefecture level city in Shandong
Energy Saving Technology Shop	In-situ compensated economizer	Small store service attitude is very good Product quality is very good Suggested purchase	Jingdong prefecture level city in Shandong
Energy Saving Technology Shop	In-situ compensated economizer	Bought two energy-saving appliances for the family, the kitchen and bathroom water heater is too expensive, the water heater to be turned on every day, the monthly electricity bill is a lot of use, with this, no longer afraid of the electricity bill is more than one month down the electricity bill can be less than half of the previous month it, the effect is particularly good!	Jingdong prefecture level city in Shandong
Energy Saving Technology Shop	In-situ compensated economizer	Saving power is really good to use, the last time I bought 3, the family used , before the family monthly electricity bills are more than 300 fast, after using this are more than 200, are several months, this time to my store is also arranged, it is really good for the people good things.	Jingdong prefecture level city in Shandong

Energy Saving Technology Shop	In-situ compensated economizer	The previous year bought a, almost every month can save a 70 dollars or so, with a year of no problem at all, look like new, this time to move to a new home decisively buy back 2 units, very believe this store!	Jingdong prefecture level city in Shandong
Energy Saving Technology Shop	In-situ compensated economizer	Power-saving effect: used a week to comment, before and now record the meter comparison, my one rental room can save twenty degrees of electricity per day. Cost-effective. Quite worth it.	Jingdong prefecture level city in Shandong
Energy Saving Technology Shop	In-situ compensated economizer	This power saver is really powerful, used to spend 300 electricity bill every month, now it's about 100	Jingdong prefecture level city in Shandong
Energy Saving Technology Shop	In-situ compensated economizer	After two days of testing. The effect is good! About the first day of power saving for 1/4. the second day for 1/3. and not "steal electricity", is through the intelligent integrated circuit, automatically reduce the loss of electrical appliances to achieve the purpose of saving electricity! Recommended	Jingdong prefecture level city in Shandong
Energy Saving Technology Shop	In-situ compensated economizer	Power Saving King has been received, really good, thank you boss, I wish the boss business prosperity, prosperity, next time to come back to patronize!	Jingdong prefecture level city in Shandong
Energy Saving Technology Shop	In-situ compensated economizer	I've been using it for a while. Than to A little less electricity than before. I'll buy it again next time.	Jingdong prefecture level city in Shandong
Energy Saving Technology Shop	In-situ compensated economizer	Used to be a couple hundred bucks a month. With him, it's a big savings. It's great.	Jingdong prefecture level city in Shandong
Energy Saving Technology Shop	In-situ compensated economizer	Previously bought a plug in the kitchen, used for a period of time after the personal feeling is still quite effective, can save a little electricity, so this time without hesitation and bought two, has been inserted in the home next to the appliances that use a lot of electricity!	Jingdong prefecture level city in Shandong
Energy Saving Technology Shop	In-situ compensated economizer	I've used it before and it does have a power saving effect	Jingdong prefecture level city in Shandong
Energy Saving Technology Shop	In-situ compensated economizer	High-tech products, really useful, in previous years the high cost of electricity, with this product, the electricity bill came down. Praise a, hope that the research and development of this product of science and technology to continue to work hard, research out better products to.	Jingdong prefecture level city in Shandong
Energy Saving Technology Shop	In-situ compensated economizer	This high-power is not bad Oh, the first few months are 1000 degrees so, I casually brush in Taobao to this product, I bought back to try, I used a month after this month left 736 degrees, scared me, I also called to the grid to confirm, really is 736 degrees, the effect is very good, and today bought a go back to the old family to use	Jingdong prefecture level city in Shandong
Energy Saving Technology Shop	In-situ compensated economizer	Used for a period of time, you do not say can really save electricity, really is a small power-saving helper, it is worth buying!	Jingdong prefecture level city in Shandong
Energy Saving Technology Shop	In-situ compensated economizer	There are many rooms and it takes a lot of electricity to run the air conditioner when the house is cold. Bought 4 inserted half a month to save me more than 300 dollars. The effect is really core good! Quality is also very good, the seller service is very good and patient.	Jingdong prefecture level city in Shandong
Energy Saving Technology Shop	In-situ compensated economizer	Recommended by the owner of the store next door, looked at his home does have to save electricity, decided to get. Indeed quite can, the voltage is much more stable, there is a reduction in electricity	Jingdong prefecture level city in Shandong
Energy Saving Technology Shop	In-situ compensated economizer	It does save electricity and works pretty well. I feel that in a few days, I will be able to use the money that I bought to save money, it's really good!	Jingdong prefecture level city in Shandong
Energy Saving Technology Shop	In-situ compensated economizer	Small store service attitude is very good Product quality is very good Suggested purchase	Jingdong prefecture level city in Shandong
Energy Saving Technology Shop	In-situ compensated economizer	Bought two energy-saving appliances for the family, the kitchen and bathroom water heater is too expensive, the water heater to be turned on every day, the monthly electricity bill is a lot of use, with this, no longer afraid of the electricity bill is more than one month down the electricity bill can be less than half of the previous month it, the effect is particularly good!	Jingdong prefecture level city in Shandong
Energy Saving Technology Shop	In-situ compensated economizer	Saving power is really good to use, the last time I bought 3, the family used , before the family monthly electricity bills are more than 300 fast, after using this are more than 200, are several months, this time to my store is also arranged, it is really good for the people good things.	Jingdong prefecture level city in Shandong
Energy Saving Technology Shop	In-situ compensated economizer	The previous year bought a, almost every month can save a 70 dollars or so, with a year of no problem at all, look like new, this time to move to a new home decisively buy back 2 units, very believe this store!	Jingdong prefecture level city in Shandong
Energy Saving Technology Shop	In-situ compensated economizer	Power-saving effect: used a week to comment, before and now record the meter comparison, my one rental room can save twenty degrees of electricity per day. Cost-effective. Quite worth it.	Jingdong prefecture level city in Shandong
Energy Saving Technology Shop	In-situ compensated economizer	This power saver is really powerful, used to spend 300 electricity bill every month, now it's about 100	Jingdong prefecture level city in Shandong
Energy Saving Technology Shop	In-situ compensated economizer	After two days of testing. The effect is good! About the first day of power saving for 1/4. the second day for 1/3. and not "steal electricity", is through the intelligent integrated circuit, automatically reduce the loss of electrical appliances to achieve the purpose of saving electricity! Recommended	Jingdong prefecture level city in Shandong
Energy Saving Technology Shop	In-situ compensated economizer	Power Saving King has been received, really good, thank you boss, I wish the boss business prosperity, prosperity, next time to come back to patronize!	Jingdong prefecture level city in Shandong
Energy Saving Technology Shop	In-situ compensated economizer	I've been using it for a while. Than to A little less electricity than before. I'll buy it again next time.	Jingdong prefecture level city in Shandong
Energy Saving Technology Shop	In-situ compensated economizer	Used to be a couple hundred bucks a month. With him, it's a big savings. It's great.	Jingdong prefecture level city in Shandong
Energy Saving Technology Shop	In-situ compensated economizer	Previously bought a plug in the kitchen, used for a period of time after the personal feeling is still quite effective, can save a little electricity, so this time without hesitation and bought two, has been inserted in the home next to the appliances that use a lot of electricity!	Jingdong prefecture level city in Shandong
Energy Saving Technology Shop	In-situ compensated economizer	I've used it before and it does have a power saving effect	Jingdong prefecture level city in Shandong
Energy Saving Technology Shop	In-situ compensated economizer	High-tech products, really useful, in previous years the high cost of electricity, with this product, the electricity bill came down. Praise a, hope that the research and development of this product of science and technology to continue to work hard, research out better products to.	Jingdong prefecture level city in Shandong
Energy Saving Technology Shop	In-situ compensated economizer	This high-power is not bad Oh, the first few months are 1000 degrees so, I casually brush in Taobao to this product, I bought back to try, I used a month after this month left 736 degrees, scared me, I also called to the grid to confirm, really is 736 degrees, the effect is very good, and today bought a go back to the old family to use	Jingdong prefecture level city in Shandong
Energy Saving Technology Shop	In-situ compensated economizer	Used for a period of time, you do not say can really save electricity, really is a small power-saving helper, it is worth buying!	Jingdong prefecture level city in Shandong
Energy Saving Technology Shop	In-situ compensated economizer	There are many rooms and it takes a lot of electricity to run the air conditioner when the house is cold. Bought 4 inserted half a month to save me more than 300 dollars. The effect is really core good! Quality is also very good, the seller service is very good and patient.	Jingdong prefecture level city in Shandong
Energy Saving Technology Shop	In-situ compensated economizer	Recommended by the owner of the store next door, looked at his home does have to save electricity, decided to get. Indeed quite can, the voltage is much more stable, there is a reduction in electricity	Jingdong prefecture level city in Shandong
Energy Saving Technology Shop	In-situ compensated economizer	It does save electricity and works pretty well. I feel that in a few days, I will be able to use the money that I bought to save money, it's really good!	Jingdong prefecture level city in Shandong
Energy Saving Technology Shop	In-situ compensated economizer	Small store service attitude is very good Product quality is very good Suggested purchase	Jingdong prefecture level city in Shandong
Energy Saving Technology Shop	In-situ compensated economizer	Bought two energy-saving appliances for the family, the kitchen and bathroom water heater is too expensive, the water heater to be turned on every day, the monthly electricity bill is a lot of use, with this, no longer afraid of the electricity bill is more than one month down the electricity bill can be less than half of the previous month it, the effect is particularly good!	Jingdong prefecture level city in Shandong
Energy Saving Technology Shop	In-situ compensated economizer	Saving power is really good to use, the last time I bought 3, the family used , before the family monthly electricity bills are more than 300 fast, after using this are more than 200, are several months, this time to my store is also arranged, it is really good for the people good things.	Jingdong prefecture level city in Shandong
Energy Saving Technology Shop	In-situ compensated economizer	The previous year bought a, almost every month can save a 70 dollars or so, with a year of no problem at all, look like new, this time to move to a new home decisively buy back 2 units, very believe this store!	Jingdong prefecture level city in Shandong
Energy Saving Technology Shop	In-situ compensated economizer	Power-saving effect: used a week to comment, before and now record the meter comparison, my one rental room can save twenty degrees of electricity per day. Cost-effective. Quite worth it.	Jingdong prefecture level city in Shandong
Energy Saving Technology Shop	In-situ compensated economizer	This power saver is really powerful, used to spend 300 electricity bill every month, now it's about 100	Jingdong prefecture level city in Shandong
Energy Saving Technology Shop	In-situ compensated economizer	After two days of testing. The effect is good! About the first day of power saving for 1/4. the second day for 1/3. and not "steal electricity", is through the intelligent integrated circuit, automatically reduce the loss of electrical appliances to achieve the purpose of saving electricity! Recommended	Jingdong prefecture level city in Shandong
Energy Saving Technology Shop	In-situ compensated economizer	Power Saving King has been received, really good, thank you boss, I wish the boss business prosperity, prosperity, next time to come back to patronize!	Jingdong prefecture level city in Shandong
Energy Saving Technology Shop	In-situ compensated economizer	I've been using it for a while. Than to A little less electricity than before. I'll buy it again next time.	Jingdong prefecture level city in Shandong

Energy Saving Technology Shop	In-situ compensated economizer	Used to be a couple hundred bucks a month. With him, it's a big savings. It's great.	Jingdong prefecture level city in Shandong
Energy Saving Technology Shop	In-situ compensated economizer	Previously bought a plug in the kitchen, used for a period of time after the personal feeling is still quite effective, can save a little electricity, so this time without hesitation and bought two, has been inserted in the home next to the appliances that use a lot of electricity!	Jingdong prefecture level city in Shandong
Energy Saving Technology Shop	In-situ compensated economizer	I've used it before and it does have a power saving effect	Jingdong prefecture level city in Shandong
Energy Saving Technology Shop	In-situ compensated economizer	High-tech products, really useful, in previous years the high cost of electricity, with this product, the electricity bill came down. Praise a, hope that the research and development of this product of science and technology to continue to work hard, research out better products to.	Jingdong prefecture level city in Shandong
Energy Saving Technology Shop	In-situ compensated economizer	This high-power is not bad Oh, the first few months are 1000 degrees so, I casually brush in Taobao to this product, I bought back to try, I used a month after this month left 736 degrees, scared me, I also called to the grid to confirm, really is 736 degrees, the effect is very good, and today bought a go back to the old family to use	Jingdong prefecture level city in Shandong
Energy Saving Technology Shop	In-situ compensated economizer	Used for a period of time, you do not say can really save electricity, really is a small power-saving helper, it is worth buying!	Jingdong prefecture level city in Shandong
Energy Saving Technology Shop	In-situ compensated economizer	There are many rooms and it takes a lot of electricity to run the air conditioner when the house is cold. Bought 4 inserted half a month to save me more than 300 dollars. The effect is really core good! Quality is also very good, the seller service is very good and patient.	Jingdong prefecture level city in Shandong
Energy Saving Technology Shop	In-situ compensated economizer	Recommended by the owner of the store next door, looked at his home does have to save electricity, decided to get. Indeed quite can, the voltage is much more stable, there is a reduction in electricity	Jingdong prefecture level city in Shandong
Energy Saving Technology Shop	In-situ compensated economizer	It does save electricity and works pretty well. I feel that in a few days, I will be able to use the money that I bought to save money, it's really good!	Jingdong prefecture level city in Shandong
Energy Saving Technology Shop	In-situ compensated economizer	Small store service attitude is very good Product quality is very good Suggested purchase	Jingdong prefecture level city in Shandong
Energy Saving Technology Shop	In-situ compensated economizer	Bought two energy-saving appliances for the family, the kitchen and bathroom water heater is too expensive, the water heater to be turned on every day, the monthly electricity bill is a lot of use, with this, no longer afraid of the electricity bill is more than one month down the electricity bill can be less than half of the previous month it, the effect is particularly good!	Jingdong prefecture level city in Shandong
Energy Saving Technology Shop	In-situ compensated economizer	Saving power is really good to use, the last time I bought 3, the family used , before the family monthly electricity bills are more than 300 fast, after using this are more than 200, are several months, this time to my store is also arranged, it is really good for the people good things.	Jingdong prefecture level city in Shandong
Energy Saving Technology Shop	In-situ compensated economizer	The previous year bought a, almost every month can save a 70 dollars or so, with a year of no problem at all, look like new, this time to move to a new home decisively buy back 2 units, very believe this store!	Jingdong prefecture level city in Shandong
Energy Saving Technology Shop	In-situ compensated economizer	Power-saving effect: used a week to comment, before and now record the meter comparison, my one rental room can save twenty degrees of electricity per day. Cost-effective. Quite worth it.	Jingdong prefecture level city in Shandong
Energy Saving Technology Shop	In-situ compensated economizer	This power saver is really powerful, used to spend 300 electricity bill every month, now it's about 100	Jingdong prefecture level city in Shandong
Energy Saving Technology Shop	In-situ compensated economizer	After two days of testing. The effect is good! About the first day of power saving for 1/4. the second day for 1/3. and not "steal electricity", is through the intelligent integrated circuit, automatically reduce the loss of electrical appliances to achieve the purpose of saving electricity! Recommended	Jingdong prefecture level city in Shandong
Energy Saving Technology Shop	In-situ compensated economizer	Power Saving King has been received, really good, thank you boss, I wish the boss business prosperity, prosperity, next time to come back to patronize!	Jingdong prefecture level city in Shandong
Energy Saving Technology Shop	In-situ compensated economizer	I've been using it for a while. Than to A little less electricity than before. I'll buy it again next time.	Jingdong prefecture level city in Shandong
Energy Saving Technology Shop	In-situ compensated economizer	Used to be a couple hundred bucks a month. With him, it's a big savings. It's great.	Jingdong prefecture level city in Shandong
Energy Saving Technology Shop	In-situ compensated economizer	Previously bought a plug in the kitchen, used for a period of time after the personal feeling is still quite effective, can save a little electricity, so this time without hesitation and bought two, has been inserted in the home next to the appliances that use a lot of electricity!	Jingdong prefecture level city in Shandong
Energy Saving Technology Shop	In-situ compensated economizer	I've used it before and it does have a power saving effect	Jingdong prefecture level city in Shandong
Energy Saving Technology Shop	In-situ compensated economizer	High-tech products, really useful, in previous years the high cost of electricity, with this product, the electricity bill came down. Praise a, hope that the research and development of this product of science and technology to continue to work hard, research out better products to.	Jingdong prefecture level city in Shandong
Energy Saving Technology Shop	In-situ compensated economizer	This high-power is not bad Oh, the first few months are 1000 degrees so, I casually brush in Taobao to this product, I bought back to try, I used a month after this month left 736 degrees, scared me, I also called to the grid to confirm, really is 736 degrees, the effect is very good, and today bought a go back to the old family to use	Jingdong prefecture level city in Shandong
Energy Saving Technology Shop	In-situ compensated economizer	Used for a period of time, you do not say can really save electricity, really is a small power-saving helper, it is worth buying!	Jingdong prefecture level city in Shandong
Energy Saving Technology Shop	In-situ compensated economizer	There are many rooms and it takes a lot of electricity to run the air conditioner when the house is cold. Bought 4 inserted half a month to save me more than 300 dollars. The effect is really core good! Quality is also very good, the seller service is very good and patient.	Jingdong prefecture level city in Shandong
Energy Saving Technology Shop	In-situ compensated economizer	Recommended by the owner of the store next door, looked at his home does have to save electricity, decided to get. Indeed quite can, the voltage is much more stable, there is a reduction in electricity	Jingdong prefecture level city in Shandong
Energy Saving Technology Shop	In-situ compensated economizer	It does save electricity and works pretty well. I feel that in a few days, I will be able to use the money that I bought to save money, it's really good!	Jingdong prefecture level city in Shandong
Energy Saving Technology Shop	In-situ compensated economizer	Small store service attitude is very good Product quality is very good Suggested purchase	Jingdong prefecture level city in Shandong
Energy Saving Technology Shop	In-situ compensated economizer	Bought two energy-saving appliances for the family, the kitchen and bathroom water heater is too expensive, the water heater to be turned on every day, the monthly electricity bill is a lot of use, with this, no longer afraid of the electricity bill is more than one month down the electricity bill can be less than half of the previous month it, the effect is particularly good!	Jingdong prefecture level city in Shandong
Energy Saving Technology Shop	In-situ compensated economizer	Saving power is really good to use, the last time I bought 3, the family used , before the family monthly electricity bills are more than 300 fast, after using this are more than 200, are several months, this time to my store is also arranged, it is really good for the people good things.	Jingdong prefecture level city in Shandong
Energy Saving Technology Shop	In-situ compensated economizer	The previous year bought a, almost every month can save a 70 dollars or so, with a year of no problem at all, look like new, this time to move to a new home decisively buy back 2 units, very believe this store!	Jingdong prefecture level city in Shandong
Energy Saving Technology Shop	In-situ compensated economizer	Power-saving effect: used a week to comment, before and now record the meter comparison, my one rental room can save twenty degrees of electricity per day. Cost-effective. Quite worth it.	Jingdong prefecture level city in Shandong
Energy Saving Technology Shop	In-situ compensated economizer	This power saver is really powerful, used to spend 300 electricity bill every month, now it's about 100	Jingdong prefecture level city in Shandong
Energy Saving Technology Shop	In-situ compensated economizer	After two days of testing. The effect is good! About the first day of power saving for 1/4. the second day for 1/3. and not "steal electricity", is through the intelligent integrated circuit, automatically reduce the loss of electrical appliances to achieve the purpose of saving electricity! Recommended	Jingdong prefecture level city in Shandong
Energy Saving Technology Shop	In-situ compensated economizer	Power Saving King has been received, really good, thank you boss, I wish the boss business prosperity, prosperity, next time to come back to patronize!	Jingdong prefecture level city in Shandong
Energy Saving Technology Shop	In-situ compensated economizer	I've been using it for a while. Than to A little less electricity than before. I'll buy it again next time.	Jingdong prefecture level city in Shandong
Energy Saving Technology Shop	In-situ compensated economizer	Used to be a couple hundred bucks a month. With him, it's a big savings. It's great.	Jingdong prefecture level city in Shandong
Energy Saving Technology Shop	In-situ compensated economizer	Previously bought a plug in the kitchen, used for a period of time after the personal feeling is still quite effective, can save a little electricity, so this time without hesitation and bought two, has been inserted in the home next to the appliances that use a lot of electricity!	Jingdong prefecture level city in Shandong
Energy Saving Technology Shop	In-situ compensated economizer	I've used it before and it does have a power saving effect	Jingdong prefecture level city in Shandong
Energy Saving Technology Shop	In-situ compensated economizer	High-tech products, really useful, in previous years the high cost of electricity, with this product, the electricity bill came down. Praise a, hope that the research and development of this product of science and technology to continue to work hard, research out better products to.	Jingdong prefecture level city in Shandong

Energy Saving Technology Shop	In-situ compensated economizer	This high-power is not bad Oh, the first few months are 1000 degrees so, I casually brush in Taobao to this product, I bought back to try, I used a month after this month left 736 degrees, scared me, I also called to the grid to confirm, really is 736 degrees, the effect is very good, and today bought a go back to the old family to use	Jingdong prefecture level city in Shandong
Energy Saving Technology Shop	In-situ compensated economizer	Used for a period of time, you do not say can really save electricity, really is a small power-saving helper, it is worth buying!	Jingdong prefecture level city in Shandong
Energy Saving Technology Shop	In-situ compensated economizer	There are many rooms and it takes a lot of electricity to run the air conditioner when the house is cold. Bought 4 inserted half a month to save me more than 300 dollars. The effect is really core good! Quality is also very good, the seller service is very good and patient.	Jingdong prefecture level city in Shandong
Energy Saving Technology Shop	In-situ compensated economizer	Recommended by the owner of the store next door, looked at his home does have to save electricity, decided to get. Indeed quite can, the voltage is much more stable, there is a reduction in electricity	Jingdong prefecture level city in Shandong
Energy Saving Technology Shop	In-situ compensated economizer	It does save electricity and works pretty well. I feel that in a few days, I will be able to use the money that I bought to save money, it's really good!	Jingdong prefecture level city in Shandong
Energy Saving Technology Shop	In-situ compensated economizer	Small store service attitude is very good Product quality is very good Suggested purchase	Jingdong prefecture level city in Shandong
Energy Saving Technology Shop	In-situ compensated economizer	Bought two energy-saving appliances for the family, the kitchen and bathroom water heater is too expensive, the water heater to be turned on every day, the monthly electricity bill is a lot of use, with this, no longer afraid of the electricity bill is more than one month down the electricity bill can be less than half of the previous month it, the effect is particularly good!	Jingdong prefecture level city in Shandong
Energy Saving Technology Shop	In-situ compensated economizer	Saving power is really good to use, the last time I bought 3, the family used , before the family monthly electricity bills are more than 300 fast, after using this are more than 200, are several months, this time to my store is also arranged, it is really good for the people good things.	Jingdong prefecture level city in Shandong
Energy Saving Technology Shop	In-situ compensated economizer	The previous year bought a, almost every month can save a 70 dollars or so, with a year of no problem at all, look like new, this time to move to a new home decisively buy back 2 units, very believe this store!	Jingdong prefecture level city in Shandong
Energy Saving Technology Shop	In-situ compensated economizer	Power-saving effect: used a week to comment, before and now record the meter comparison, my one rental room can save twenty degrees of electricity per day. Cost-effective. Quite worth it.	Jingdong prefecture level city in Shandong
Energy Saving Technology Shop	In-situ compensated economizer	After two days of testing. The effect is good! About the first day of power saving for 1/4. the second day for 1/3. and not "steal electricity", is through the intelligent integrated circuit, automatically reduce the loss of electrical appliances to achieve the purpose of saving electricity! Recommended	Jingdong prefecture level city in Shandong
Energy Saving Technology Shop	In-situ compensated economizer	This power saver is really powerful, used to spend 300 electricity bill every month, now it's about 100	Jingdong prefecture level city in Shandong
Energy Saving Technology Shop	In-situ compensated economizer	Power Saving King has been received, really good, thank you boss, I wish the boss business prosperity, prosperity, next time to come back to patronize!	Jingdong prefecture level city in Shandong
Energy Saving Technology Shop	In-situ compensated economizer	I've been using it for a while. Than to A little less electricity than before. I'll buy it again next time.	Jingdong prefecture level city in Shandong
Energy Saving Technology Shop	In-situ compensated economizer	Used to be a couple hundred bucks a month. With him, it's a big savings. It's great.	Jingdong prefecture level city in Shandong
Energy Saving Technology Shop	In-situ compensated economizer	Previously bought a plug in the kitchen, used for a period of time after the personal feeling is still quite effective, can save a little electricity, so this time without hesitation and bought two, has been inserted in the home next to the appliances that use a lot of electricity!	Jingdong prefecture level city in Shandong
Energy Saving Technology Shop	In-situ compensated economizer	I've used it before and it does have a power saving effect	Jingdong prefecture level city in Shandong
Energy Saving Technology Shop	In-situ compensated economizer	High-tech products, really useful, in previous years the high cost of electricity, with this product, the electricity bill came down. Praise a, hope that the research and development of this product of science and technology to continue to work hard, research out better products to.	Jingdong prefecture level city in Shandong
Energy Saving Technology Shop	In-situ compensated economizer	This high-power is not bad Oh, the first few months are 1000 degrees so, I casually brush in Taobao to this product, I bought back to try, I used a month after this month left 736 degrees, scared me, I also called to the grid to confirm, really is 736 degrees, the effect is very good, and today bought a go back to the old family to use	Jingdong prefecture level city in Shandong
Energy Saving Technology Shop	In-situ compensated economizer	Used for a period of time, you do not say can really save electricity, really is a small power-saving helper, it is worth buying!	Jingdong prefecture level city in Shandong
Energy Saving Technology Shop	In-situ compensated economizer	There are many rooms and it takes a lot of electricity to run the air conditioner when the house is cold. Bought 4 inserted half a month to save me more than 300 dollars. The effect is really core good! Quality is also very good, the seller service is very good and patient.	Jingdong prefecture level city in Shandong
Energy Saving Technology Shop	In-situ compensated economizer	Recommended by the owner of the store next door, looked at his home does have to save electricity, decided to get. Indeed quite can, the voltage is much more stable, there is a reduction in electricity	Jingdong prefecture level city in Shandong
Energy Saving Technology Shop	In-situ compensated economizer	It does save electricity and works pretty well. I feel that in a few days, I will be able to use the money that I bought to save money, it's really good!	Jingdong prefecture level city in Shandong
Energy Saving Technology Shop	In-situ compensated economizer	Small store service attitude is very good Product quality is very good Suggested purchase	Jingdong prefecture level city in Shandong
Energy Saving Technology Shop	In-situ compensated economizer	Bought two energy-saving appliances for the family, the kitchen and bathroom water heater is too expensive, the water heater to be turned on every day, the monthly electricity bill is a lot of use, with this, no longer afraid of the electricity bill is more than one month down the electricity bill can be less than half of the previous month it, the effect is particularly good!	Jingdong prefecture level city in Shandong
Energy Saving Technology Shop	In-situ compensated economizer	Saving power is really good to use, the last time I bought 3, the family used , before the family monthly electricity bills are more than 300 fast, after using this are more than 200, are several months, this time to my store is also arranged, it is really good for the people good things.	Jingdong prefecture level city in Shandong
Energy Saving Technology Shop	In-situ compensated economizer	The previous year bought a, almost every month can save a 70 dollars or so, with a year of no problem at all, look like new, this time to move to a new home decisively buy back 2 units, very believe this store!	Jingdong prefecture level city in Shandong
Energy Saving Technology Shop	In-situ compensated economizer	Power-saving effect: used a week to comment, before and now record the meter comparison, my one rental room can save twenty degrees of electricity per day. Cost-effective. Quite worth it.	Jingdong prefecture level city in Shandong
Energy Saving Technology Shop	In-situ compensated economizer	This power saver is really powerful, used to spend 300 electricity bill every month, now it's about 100	Jingdong prefecture level city in Shandong
Energy Saving Technology Shop	In-situ compensated economizer	After two days of testing. The effect is good! About the first day of power saving for 1/4. the second day for 1/3. and not "steal electricity", is through the intelligent integrated circuit, automatically reduce the loss of electrical appliances to achieve the purpose of saving electricity! Recommended	Jingdong prefecture level city in Shandong
Energy Saving Technology Shop	In-situ compensated economizer	Power Saving King has been received, really good, thank you boss, I wish the boss business prosperity, prosperity, next time to come back to patronize!	Jingdong prefecture level city in Shandong
Energy Saving Technology Shop	In-situ compensated economizer	I've been using it for a while. Than to A little less electricity than before. I'll buy it again next time.	Jingdong prefecture level city in Shandong
Energy Saving Technology Shop	In-situ compensated economizer	Used to be a couple hundred bucks a month. With him, it's a big savings. It's great.	Jingdong prefecture level city in Shandong
Energy Saving Technology Shop	In-situ compensated economizer	Previously bought a plug in the kitchen, used for a period of time after the personal feeling is still quite effective, can save a little electricity, so this time without hesitation and bought two, has been inserted in the home next to the appliances that use a lot of electricity!	Jingdong prefecture level city in Shandong
Energy Saving Technology Shop	In-situ compensated economizer	I've used it before and it does have a power saving effect	Jingdong prefecture level city in Shandong
Energy Saving Technology Shop	In-situ compensated economizer	High-tech products, really useful, in previous years the high cost of electricity, with this product, the electricity bill came down. Praise a, hope that the research and development of this product of science and technology to continue to work hard, research out better products to.	Jingdong prefecture level city in Shandong
Energy Saving Technology Shop	In-situ compensated economizer	This high-power is not bad Oh, the first few months are 1000 degrees so, I casually brush in Taobao to this product, I bought back to try, I used a month after this month left 736 degrees, scared me, I also called to the grid to confirm, really is 736 degrees, the effect is very good, and today bought a go back to the old family to use	Jingdong prefecture level city in Shandong
Energy Saving Technology Shop	In-situ compensated economizer	Used for a period of time, you do not say can really save electricity, really is a small power-saving helper, it is worth buying!	Jingdong prefecture level city in Shandong
Energy Saving Technology Shop	In-situ compensated economizer	There are many rooms and it takes a lot of electricity to run the air conditioner when the house is cold. Bought 4 inserted half a month to save me more than 300 dollars. The effect is really core good! Quality is also very good, the seller service is very good and patient.	Jingdong prefecture level city in Shandong
Energy Saving Technology Shop	In-situ compensated economizer	Recommended by the owner of the store next door, looked at his home does have to save electricity, decided to get. Indeed quite can, the voltage is much more stable, there is a reduction in electricity	Jingdong prefecture level city in Shandong
Energy Saving Technology Shop	In-situ compensated economizer	It does save electricity and works pretty well. I feel that in a few days, I will be able to use the money that I bought to save money, it's really good!	Jingdong prefecture level city in Shandong

Energy Saving Technology Shop	In-situ compensated economizer	Small store service attitude is very good Product quality is very good Suggested purchase	Jingdong prefecture level city in Shandong
Energy Saving Technology Shop	In-situ compensated economizer	Bought two energy-saving appliances for the family, the kitchen and bathroom water heater is too expensive, the water heater to be turned on every day, the monthly electricity bill is a lot of use, with this, no longer afraid of the electricity bill is more than one month down the electricity bill can be less than half of the previous month it, the effect is particularly good!	Jingdong prefecture level city in Shandong
Energy Saving Technology Shop	In-situ compensated economizer	Saving power is really good to use, the last time I bought 3, the family used , before the family monthly electricity bills are more than 300 fast, after using this are more than 200, are several months, this time to my store is also arranged, it is really good for the people good things.	Jingdong prefecture level city in Shandong
Energy Saving Technology Shop	In-situ compensated economizer	The previous year bought a, almost every month can save a 70 dollars or so, with a year of no problem at all, look like new, this time to move to a new home decisively buy back 2 units, very believe this store!	Jingdong prefecture level city in Shandong
Energy Saving Technology Shop	In-situ compensated economizer	Power-saving effect: used a week to comment, before and now record the meter comparison, my one rental room can save twenty degrees of electricity per day. Cost-effective. Quite worth it.	Jingdong prefecture level city in Shandong
Energy Saving Technology Shop	In-situ compensated economizer	This power saver is really powerful, used to spend 300 electricity bill every month, now it's about 100	Jingdong prefecture level city in Shandong
Energy Saving Technology Shop	In-situ compensated economizer	After two days of testing. The effect is good! About the first day of power saving for 1/4. the second day for 1/3. and not "steal electricity", is through the intelligent integrated circuit, automatically reduce the loss of electrical appliances to achieve the purpose of saving electricity! Recommended	Jingdong prefecture level city in Shandong
Energy Saving Technology Shop	In-situ compensated economizer	Power Saving King has been received, really good, thank you boss, I wish the boss business prosperity, prosperity, next time to come back to patronize!	Jingdong prefecture level city in Shandong
Energy Saving Technology Shop	In-situ compensated economizer	I've been using it for a while. Than to A little less electricity than before. I'll buy it again next time.	Jingdong prefecture level city in Shandong
Energy Saving Technology Shop	In-situ compensated economizer	Used to be a couple hundred bucks a month. With him, it's a big savings. It's great.	Jingdong prefecture level city in Shandong
Energy Saving Technology Shop	In-situ compensated economizer	Previously bought a plug in the kitchen, used for a period of time after the personal feeling is still quite effective, can save a little electricity, so this time without hesitation and bought two, has been inserted in the home next to the appliances that use a lot of electricity!	Jingdong prefecture level city in Shandong
Energy Saving Technology Shop	In-situ compensated economizer	I've used it before and it does have a power saving effect	Jingdong prefecture level city in Shandong
Energy Saving Technology Shop	In-situ compensated economizer	High-tech products, really useful, in previous years the high cost of electricity, with this product, the electricity bill came down. Praise a, hope that the research and development of this product of science and technology to continue to work hard, research out better products to.	Jingdong prefecture level city in Shandong
Energy Saving Technology Shop	In-situ compensated economizer	This high-power is not bad Oh, the first few months are 1000 degrees so, I casually brush in Taobao to this product, I bought back to try, I used a month after this month left 736 degrees, scared me, I also called to the grid to confirm, really is 736 degrees, the effect is very good, and today bought a go back to the old family to use	Jingdong prefecture level city in Shandong
Energy Saving Technology Shop	In-situ compensated economizer	Used for a period of time, you do not say can really save electricity, really is a small power-saving helper, it is worth buying!	Jingdong prefecture level city in Shandong
Energy Saving Technology Shop	In-situ compensated economizer	There are many rooms and it takes a lot of electricity to run the air conditioner when the house is cold. Bought 4 inserted half a month to save me more than 300 dollars. The effect is really core good! Quality is also very good, the seller service is very good and patient.	Jingdong prefecture level city in Shandong
Energy Saving Technology Shop	In-situ compensated economizer	Recommended by the owner of the store next door, looked at his home does have to save electricity, decided to get. Indeed quite can, the voltage is much more stable, there is a reduction in electricity	Jingdong prefecture level city in Shandong
Energy Saving Technology Shop	In-situ compensated economizer	It does save electricity and works pretty well. I feel that in a few days, I will be able to use the money that I bought to save money, it's really good!	Jingdong prefecture level city in Shandong
Energy Saving Technology Shop	In-situ compensated economizer	Small store service attitude is very good Product quality is very good Suggested purchase	Jingdong prefecture level city in Shandong
Energy Saving Technology Shop	In-situ compensated economizer	Bought two energy-saving appliances for the family, the kitchen and bathroom water heater is too expensive, the water heater to be turned on every day, the monthly electricity bill is a lot of use, with this, no longer afraid of the electricity bill is more than one month down the electricity bill can be less than half of the previous month it, the effect is particularly good!	Jingdong prefecture level city in Shandong
Energy Saving Technology Shop	In-situ compensated economizer	Saving power is really good to use, the last time I bought 3, the family used , before the family monthly electricity bills are more than 300 fast, after using this are more than 200, are several months, this time to my store is also arranged, it is really good for the people good things.	Jingdong prefecture level city in Shandong
Energy Saving Technology Shop	In-situ compensated economizer	The previous year bought a, almost every month can save a 70 dollars or so, with a year of no problem at all, look like new, this time to move to a new home decisively buy back 2 units, very believe this store!	Jingdong prefecture level city in Shandong
Energy Saving Technology Shop	In-situ compensated economizer	Power-saving effect: used a week to comment, before and now record the meter comparison, my one rental room can save twenty degrees of electricity per day. Cost-effective. Quite worth it.	Jingdong prefecture level city in Shandong
Energy Saving Technology Shop	In-situ compensated economizer	This power saver is really powerful, used to spend 300 electricity bill every month, now it's about 100	Jingdong prefecture level city in Shandong
Energy Saving Technology Shop	In-situ compensated economizer	After two days of testing. The effect is good! About the first day of power saving for 1/4. the second day for 1/3. and not "steal electricity", is through the intelligent integrated circuit, automatically reduce the loss of electrical appliances to achieve the purpose of saving electricity! Recommended	Jingdong prefecture level city in Shandong
Energy Saving Technology Shop	In-situ compensated economizer	Power Saving King has been received, really good, thank you boss, I wish the boss business prosperity, prosperity, next time to come back to patronize!	Jingdong prefecture level city in Shandong
Energy Saving Technology Shop	In-situ compensated economizer	I've been using it for a while. Than to A little less electricity than before. I'll buy it again next time.	Jingdong prefecture level city in Shandong
Energy Saving Technology Shop	In-situ compensated economizer	Used to be a couple hundred bucks a month. With him, it's a big savings. It's great.	Jingdong prefecture level city in Shandong
Energy Saving Technology Shop	In-situ compensated economizer	Previously bought a plug in the kitchen, used for a period of time after the personal feeling is still quite effective, can save a little electricity, so this time without hesitation and bought two, has been inserted in the home next to the appliances that use a lot of electricity!	Jingdong prefecture level city in Shandong
Energy Saving Technology Shop	In-situ compensated economizer	I've used it before and it does have a power saving effect	Jingdong prefecture level city in Shandong
Energy Saving Technology Shop	In-situ compensated economizer	High-tech products, really useful, in previous years the high cost of electricity, with this product, the electricity bill came down. Praise a, hope that the research and development of this product of science and technology to continue to work hard, research out better products to.	Jingdong prefecture level city in Shandong
Energy Saving Technology Shop	In-situ compensated economizer	This high-power is not bad Oh, the first few months are 1000 degrees so, I casually brush in Taobao to this product, I bought back to try, I used a month after this month left 736 degrees, scared me, I also called to the grid to confirm, really is 736 degrees, the effect is very good, and today bought a go back to the old family to use	Jingdong prefecture level city in Shandong
Energy Saving Technology Shop	In-situ compensated economizer	Used for a period of time, you do not say can really save electricity, really is a small power-saving helper, it is worth buying!	Jingdong prefecture level city in Shandong
Energy Saving Technology Shop	In-situ compensated economizer	There are many rooms and it takes a lot of electricity to run the air conditioner when the house is cold. Bought 4 inserted half a month to save me more than 300 dollars. The effect is really core good! Quality is also very good, the seller service is very good and patient.	Jingdong prefecture level city in Shandong
Energy Saving Technology Shop	In-situ compensated economizer	Recommended by the owner of the store next door, looked at his home does have to save electricity, decided to get. Indeed quite can, the voltage is much more stable, there is a reduction in electricity	Jingdong prefecture level city in Shandong
Energy Saving Technology Shop	In-situ compensated economizer	It does save electricity and works pretty well. I feel that in a few days, I will be able to use the money that I bought to save money, it's really good!	Jingdong prefecture level city in Shandong
Energy Saving Technology Shop	In-situ compensated economizer	Small store service attitude is very good Product quality is very good Suggested purchase	Jingdong prefecture level city in Shandong
Energy Saving Technology Shop	In-situ compensated economizer	Bought two energy-saving appliances for the family, the kitchen and bathroom water heater is too expensive, the water heater to be turned on every day, the monthly electricity bill is a lot of use, with this, no longer afraid of the electricity bill is more than one month down the electricity bill can be less than half of the previous month it, the effect is particularly good!	Jingdong prefecture level city in Shandong
Energy Saving Technology Shop	In-situ compensated economizer	Saving power is really good to use, the last time I bought 3, the family used , before the family monthly electricity bills are more than 300 fast, after using this are more than 200, are several months, this time to my store is also arranged, it is really good for the people good things.	Jingdong prefecture level city in Shandong
Energy Saving Technology Shop	In-situ compensated economizer	The previous year bought a, almost every month can save a 70 dollars or so, with a year of no problem at all, look like new, this time to move to a new home decisively buy back 2 units, very believe this store!	Jingdong prefecture level city in Shandong
Energy Saving Technology Shop	In-situ compensated economizer	Power-saving effect: used a week to comment, before and now record the meter comparison, my one rental room can save twenty degrees of electricity per day. Cost-effective. Quite worth it.	Jingdong prefecture level city in Shandong

Energy Saving Technology Shop	In-situ compensated economizer	This power saver is really powerful, used to spend 300 electricity bill every month, now it's about 100	Jingdong prefecture level city in Shandong
Energy Saving Technology Shop	In-situ compensated economizer	After two days of testing. The effect is good! About the first day of power saving for 1/4. the second day for 1/3. and not "steal electricity", is through the intelligent integrated circuit, automatically reduce the loss of electrical appliances to achieve the purpose of saving electricity! Recommended	Jingdong prefecture level city in Shandong
Energy Saving Technology Shop	In-situ compensated economizer	Power Saving King has been received, really good, thank you boss, I wish the boss business prosperity, prosperity, next time to come back to patronize!	Jingdong prefecture level city in Shandong
Energy Saving Technology Shop	In-situ compensated economizer	I've been using it for a while. Than to A little less electricity than before. I'll buy it again next time.	Jingdong prefecture level city in Shandong
Energy Saving Technology Shop	In-situ compensated economizer	Used to be a couple hundred bucks a month. With him, it's a big savings. It's great.	Jingdong prefecture level city in Shandong
Energy Saving Technology Shop	In-situ compensated economizer	Previously bought a plug in the kitchen, used for a period of time after the personal feeling is still quite effective, can save a little electricity, so this time without hesitation and bought two, has been inserted in the home next to the appliances that use a lot of electricity!	Jingdong prefecture level city in Shandong
Energy Saving Technology Shop	In-situ compensated economizer	I've used it before and it does have a power saving effect	Jingdong prefecture level city in Shandong
Energy Saving Technology Shop	In-situ compensated economizer	High-tech products, really useful, in previous years the high cost of electricity, with this product, the electricity bill came down. Praise a, hope that the research and development of this product of science and technology to continue to work hard, research out better products to.	Jingdong prefecture level city in Shandong
Energy Saving Technology Shop	In-situ compensated economizer	This high-power is not bad Oh, the first few months are 1000 degrees so, I casually brush in Taobao to this product, I bought back to try, I used a month after this month left 736 degrees, scared me, I also called to the grid to confirm, really is 736 degrees, the effect is very good, and today bought a go back to the old family to use	Jingdong prefecture level city in Shandong
Energy Saving Technology Shop	In-situ compensated economizer	Used for a period of time, you do not say can really save electricity, really is a small power-saving helper, it is worth buying!	Jingdong prefecture level city in Shandong
Energy Saving Technology Shop	In-situ compensated economizer	There are many rooms and it takes a lot of electricity to run the air conditioner when the house is cold. Bought 4 inserted half a month to save me more than 300 dollars. The effect is really core good! Quality is also very good, the seller service is very good and patient.	Jingdong prefecture level city in Shandong
Energy Saving Technology Shop	In-situ compensated economizer	Recommended by the owner of the store next door, looked at his home does have to save electricity, decided to get. Indeed quite can, the voltage is much more stable, there is a reduction in electricity	Jingdong prefecture level city in Shandong
Energy Saving Technology Shop	In-situ compensated economizer	It does save electricity and works pretty well. I feel that in a few days, I will be able to use the money that I bought to save money, it's really good!	Jingdong prefecture level city in Shandong
Energy Saving Technology Shop	In-situ compensated economizer	Small store service attitude is very good Product quality is very good Suggested purchase	Jingdong prefecture level city in Shandong
Energy Saving Technology Shop	In-situ compensated economizer	Currently using almost two months has brought me about two hundred oceans! Energy saving can't be too small!	Jingdong prefecture level city in Shandong
Energy Saving Technology Shop	In-situ compensated economizer	The family's monthly electricity bill is more than 200, with this power saver, you can save a sum of money, especially convenient and simple, really everyone can try Believe me! If it doesn't work, I'm not	Jingdong prefecture level city in Shandong
Energy Saving Technology Shop	King of Power Saving	Arrived, effective, logistics is very fast, the quality is very good, really save electricity, I have recommended friends have bought to use, especially the use of large amounts of electricity friends, the effect is more obvious. My home is a mahjong room, not using the power saver inches monthly electricity costs about 300, after using the power saver up to more than 200 yuan a month. The power saver is a real power saver! There is a power saver quality assurance genuine, friends can rest assured that the purchase	Jingdong prefecture level city in Shandong
Energy Saving Technology Shop	King of Power Saving	Still their products are reassuringly authentic and safe to use They do save electricity!	Jingdong prefecture level city in Shandong
Energy Saving Technology Shop	King of Power Saving	In the summer the family runs two air conditioners, a refrigerator plus some appliances, saving more than thirty percent of the electricity bill a month, and there is no breakdown.	Jingdong prefecture level city in Shandong
Energy Saving Technology Shop	King of Power Saving	I used to have a high electricity bill, but since I've been using this power saver, I've been able to save a lot of money on my electricity bill.	Jingdong prefecture level city in Shandong
Energy Saving Technology Shop	King of Power Saving	I just got it back and used it right away, hopefully it can help me save some money on my electricity bill, my mom's family bought one and it worked well, so I got one for myself.	Jingdong prefecture level city in Shandong
Happy Fan Shop	Green Intelligent Power Saver	Bought two at a time, bought by the dad laughed, said it was useless, gave him a trial sticker, two months later took the initiative to find me to buy two more, and now the home summer power peak is almost the same as usual, it is indeed more practical.	Jingdong prefecture level city in Shandong
Happy Fan Shop	Green Intelligent Power Saver	Logistics quickly, the power saver received, business service is also good On the spot on the plug, waiting for the results of the trial, the appearance, the packaging is very delicate, very satisfied, I hope to achieve satisfactory results, the business service is attentive, I wish business is better!	Jingdong prefecture level city in Shandong
Happy Fan Shop	Green Intelligent Power Saver	Air conditioning adapter high-tech ah, and the business description is consistent too cattle is very surprised, this thing can really save electricity. Before a month more than 200 electricity bill, now can save 50 dollars a month, if the home air conditioning power consumption, you can rest assured to get!	Jingdong prefecture level city in Shandong
Happy Fan Shop	Green Intelligent Power Saver	Received specially used 3 months to evaluate, in fact, these days at home with electricity or growth, with the power saver instead of more power than before, the power saving rate is not bad, almost a month to save more than 200 dollars, measured effect!	Jingdong prefecture level city in Shandong
Happy Fan Shop	Green Intelligent Power Saver	On after saving me a lot of electricity, really and this baby hate each other ah, power-saving effect is really great, the appearance of the atmosphere is stylish, the use of experience is perfect, does not take up space, the next time you need will buy again. Customer service is very enthusiastic, great!	Jingdong prefecture level city in Shandong
Happy Fan Shop	Green Intelligent Power Saver	This energy-saving treasure is really, use very put point, satisfied	Jingdong prefecture level city in Shandong
Happy Fan Shop	Green Intelligent Power Saver	Has been the second time to buy, this power saving device is really good power, before every month to spend 300 electricity, now are about 200, the last time that 6, this device buy 4 get 2, this time to help lead the family to buy 6, the	Jingdong prefecture level city in Shandong
Happy Fan Shop	Green Intelligent Power Saver	Plugged in can be used easily and conveniently, every month to help my family save about 50% of the electricity bill! My home appliances are not a lot, the main air conditioning water heater power consumption, home every month to save a little bit of electricity makes me very happy, have friends need	Jingdong prefecture level city in Shandong
Happy Fan Shop	Green Intelligent Power Saver	The previous year bought a, almost every month can save a 70 dollars or so, used a year, no problem at all, look very new like, this time to move to a new home decisively repurchase 2 units, very believe this	Jingdong prefecture level city in Shandong
Happy Fan Shop	Green Intelligent Power Saver	Has been the second time to buy, this power saving device is really good power, before every month to spend 300 electricity, now are about 200, last time that 6, this time to help lead the family to buy 6,	Jingdong prefecture level city in Shandong
Happy Fan Shop	Green Intelligent Power Saver	In the spirit of trying to experience the new product, the use of down compared to the usual power consumption, with the addition of the quarter, is indeed a lot less electricity, originally more than 450	Jingdong prefecture level city in Shandong
Happy Fan Shop	Green Intelligent Power Saver	Has not been the first time to buy back, good use, power saving device is really powerful, before the monthly electricity bill of more than 500 Now more than 300, this time to help neighbors to buy several	Jingdong prefecture level city in Shandong
Happy Fan Shop	Green Intelligent Power Saver	Home appliances are very many, the circuit is also very complex, at first it is to save 9% of the electricity, and then used more than half a month, found that the power saving rate is as high as more	Jingdong prefecture level city in Shandong
Happy Fan Shop	Green Intelligent Power Saver	My friend bought it last year and saved hundreds of dollars on his electricity bill in one summer Appearance and style: it looks better than the one I bought before Quality is also good with a light.	Jingdong prefecture level city in Shandong
Happy Fan Shop	Green Intelligent Power Saver	Saver received, on the spot on the plug with the feeling of immediate power savings, appearance, packaging is very delicate, very satisfied, I hope to achieve satisfactory results!	Jingdong prefecture level city in Shandong
Happy Fan Shop	Green Intelligent Power Saver	The power saving effect is very good, is considered an old customer, also bought before, this time is to buy two for mom and dad, customer service attitude is good, praise!	Jingdong prefecture level city in Shandong
Happy Fan Shop	Green Intelligent Power Saver	After observing for a while, it really saves a lot of money I didn't realize it was still really reliable, so I'll give you a good review.	Jingdong prefecture level city in Shandong
Happy Fan Shop	Green Intelligent Power Saver	My family a month with this monthly almost save more than 200, power-saving effect: have bought a second time, really effective Cost-effective: not bad Appearance style: roughly the same A few months to the home electricity is indeed less, this time it is a re-buy three, send a friend two, they stay a	Jingdong prefecture level city in Shandong
Happy Fan Shop	Green Intelligent Power Saver	A month almost save more than 150 dollars in electricity, home appliances more, the main this can also protect high-power appliances, after using this device, these two months have not burned, things are very good, it is said that this is the latest model, the overall price is cheap and beautiful!	Jingdong prefecture level city in Shandong
Happy Fan Shop	Green Intelligent Power Saver	Received, powerful, great quality, love the style😍	Jingdong prefecture level city in Shandong
Happy Fan Shop	Green Intelligent Power Saver	This intelligent energy saver is especially good to use and save electricity hope everyone will buy it!	Jingdong prefecture level city in Shandong

Happy Fan Shop	Green Intelligent Power Saver	Air conditioning adapter effect is particularly great, the use of very simple, plug into the socket on the summer air conditioning power is too large, electricity is also expensive, with this product directly on the electricity bill to save half, has been purchased a few more, the effect is too awesome!	Jingdong prefecture level city in Shandong
Happy Fan Shop	Green Intelligent Power Saver	The family plugged in 5, monthly electricity savings of about 300 yuan. My family before a month of electricity costs more than 800, this time with this saver after the electricity bill is actually only more than 500, amazing, thanks to the store, will recommend other friends to buy!	Jingdong prefecture level city in Shandong
Happy Fan Shop	Green Intelligent Power Saver	It was recommended to me by a neighbor's family and it's nice to use it to save power.	Jingdong prefecture level city in Shandong
Happy Fan Shop	Green Intelligent Power Saver	Second time I've purchased it and the power saving is effective!	Jingdong prefecture level city in Shandong
Happy Fan Shop	Green Intelligent Power Saver	The effect is really good, before a month of more than 300 electricity, now every month every day with the air conditioning, it is more than 100 electricity! This thing is really amazing, one after another to buy and 5.6, home installed 3!	Jingdong prefecture level city in Shandong
Happy Fan Shop	Green Intelligent Power Saver	Received this product, began to use, small things look very wrong, can save a little electricity, the monthly electricity bill is too high, after plugging in did not pull, or save a lot of electricity, point	Jingdong prefecture level city in Shandong
Happy Fan Shop	Green Intelligent Power Saver	Simple and convenient, just that plug in, you can save electricity. A month can save dozens of dollars, a year down to save a lot of, very good product cost-effective, everyone can afford to buy and use!	Jingdong prefecture level city in Shandong
Happy Fan Shop	Green Intelligent Power Saver	Electricity bill from more than 200 yuan per month, down to more than 150 yuan, the original bought home, used two months, really have power-saving effect, this time and sold two, business service attitude is	Jingdong prefecture level city in Shandong
Happy Fan Shop	Green Intelligent Power Saver	Like to watch some movies at home and so on, sometimes at night until two or three o'clock, and recently the air conditioning is not turned off for a night, rather wasteful, I have to say that the effect is very good, quite like it!	Jingdong prefecture level city in Shandong
Happy Fan Shop	Green Intelligent Power Saver	Power-saving effect: the effect is very good, a month to save more than 200 dollars Cost-effective: good value for money Appearance and style: small and lovely Workmanship material: okay Experience: good	Jingdong prefecture level city in Shandong
Happy Fan Shop	Green Intelligent Power Saver	It's okay I quite like it, the stuff is very good. Come back in the future. You can rest assured to buy. Cost-effective all. Much cheaper than the physical store	Jingdong prefecture level city in Shandong
Happy Fan Shop	Green Intelligent Power Saver	More than 150 less, last month's electricity bill is almost 400, this month is only about 200, really save electricity, I am home to buy three, I did not expect a month to pay back	Jingdong prefecture level city in Shandong
Happy Fan Shop	Green Intelligent Power Saver	I bought three of these first and tried them for two months and they still felt useful, so now I've bought another one. Good and recommended!	Jingdong prefecture level city in Shandong
Happy Fan Shop	Green Intelligent Power Saver	This energy saver is very good quality and inexpensive next time I will buy it again!	Jingdong prefecture level city in Shandong
Happy Fan Shop	Green Intelligent Power Saver	Merchant service attitude is not good, the quality does not match, the business does not give the package return	Jingdong prefecture level city in Shandong
Happy Fan Shop	Green Intelligent Power Saver	The goods are very small do not know how effective after a few days to follow up the review	Jingdong prefecture level city in Shandong
Happy Fan Shop	Green Intelligent Power Saver	Not used before more than 200 a month, after using about 100 a month, my family used three, customer service said I use electricity recommended 2-3, I think more than one effect is better on the purchase of 4, the effect is very obvious, recommended to buy!	Jingdong prefecture level city in Shandong
Happy Fan Shop	Green Intelligent Power Saver	It's okay. I'll buy it again today.	Jingdong prefecture level city in Shandong
Happy Fan Shop	Green Intelligent Power Saver	Finally received the goods, very, very much like,, has been a lot of people praise, really too happy, has been his family's loyal fans, will continue to buy buy buy!	Jingdong prefecture level city in Shandong
Happy Fan Shop	Green Intelligent Power Saver	After using this, my monthly electricity bill was 1,200 pounds, now it's only 180 bucks a month, how good is it?	Jingdong prefecture level city in Shandong
Happy Fan Shop	Green Intelligent Power Saver	The item is a scam return to marry a dozen dollars in shipping fees. Buy terrific role is purely a scam.	Jingdong prefecture level city in Shandong
Happy Fan Shop	Green Intelligent Power Saver	It's really good, cost-effective and good quality, and the logistics are fast! It arrived in one day!	Jingdong prefecture level city in Shandong
Happy Fan Shop	Green Intelligent Power Saver	Great brand! Trustworthy! Works super well! Expensive for a reason! Great merchandise!	Jingdong prefecture level city in Shandong
Happy Fan Shop	Green Intelligent Power Saver	The baby is really great, I like it very much, my friends also say good to come, recommended to buy!	Jingdong prefecture level city in Shandong
Happy Fan Shop	Green Intelligent Power Saver	Fake couldn't be more fake. Bought one plugged in still so much electricity.	Jingdong prefecture level city in Shandong
Happy Fan Shop	Green Intelligent Power Saver	This appliance works great. Let's see how it works. We'll see later.	Jingdong prefecture level city in Shandong
Happy Fan Shop	Green Intelligent Power Saver	Used it before commenting, very happy with it	Jingdong prefecture level city in Shandong
Happy Fan Shop	Green Intelligent Power Saver	Goods have been received well-packed, logistics is very fast and good quality	Jingdong prefecture level city in Shandong
Happy Fan Shop	Green Intelligent Power Saver	This baby's quality is okay.	Jingdong prefecture level city in Shandong
Happy Fan Shop	Green Intelligent Power Saver	Counterfeit goods.	Jingdong prefecture level city in Shandong
Happy Fan Shop	Green Intelligent Power Saver	The effect is not very obvious	Jingdong prefecture level city in Shandong
Happy Fan Shop	Green Intelligent Power Saver	It's not the same thing.	Jingdong prefecture level city in Shandong
Happy Fan Shop	Green Intelligent Power Saver	There is no load how much w seems false, nothing works.	Jingdong prefecture level city in Shandong
Happy Fan Shop	Green Intelligent Power Saver	I received the item yesterday, got it back and plugged it in, and it used a little more power yesterday than I had before	Jingdong prefecture level city in Shandong
Happy Fan Shop	Green Intelligent Power Saver	Haven't seen the power saving mode yet	Jingdong prefecture level city in Shandong
Happy Fan Shop	Green Intelligent Power Saver	This product has no known effect	Jingdong prefecture level city in Shandong
Happy Fan Shop	Green Intelligent Power Saver	Refund Returns $16	Jingdong prefecture level city in Shandong
Happy Fan Shop	Green Intelligent Power Saver	The previous year bought a, almost every month can save a 70 dollars or so, used a year, no problem at all, look very new like, this time to move to a new home decisively repurchase 2 units, very believe this	Jingdong prefecture level city in Shandong
Happy Fan Shop	Green Intelligent Power Saver	Has been the second time to buy, this power saving device is really good power, before every month to spend 300 electricity, now are about 200, last time that 6, this time to help lead the family to buy 6,	Jingdong prefecture level city in Shandong
Happy Fan Shop	Green Intelligent Power Saver	In the spirit of trying to experience the new product, the use of down compared to the usual power consumption, with the addition of the quarter, is indeed a lot less electricity, originally more than 450	Jingdong prefecture level city in Shandong
Happy Fan Shop	Green Intelligent Power Saver	Has not been the first time to buy back, good use, power saving device is really powerful, before the monthly electricity bill of more than 500 Now more than 300, this time to help neighbors to buy several	Jingdong prefecture level city in Shandong
Happy Fan Shop	Green Intelligent Power Saver	Home appliances are very many, the circuit is also very complex, at first it is to save 9% of the electricity, and then used more than half a month, found that the power saving rate is as high as more	Jingdong prefecture level city in Shandong
Happy Fan Shop	Green Intelligent Power Saver	My friend bought it last year and saved hundreds of dollars on his electricity bill in one summer Appearance and style: it looks better than the one I bought before Quality is also good with a light.	Jingdong prefecture level city in Shandong
Happy Fan Shop	Green Intelligent Power Saver	Saver received, on the spot on the plug with the feeling of immediate power savings, appearance, packaging is very delicate, very satisfied, I hope to achieve satisfactory results!	Jingdong prefecture level city in Shandong
Happy Fan Shop	Green Intelligent Power Saver	The power saving effect is very good, is considered an old customer, also bought before, this time is to buy two for mom and dad, customer service attitude is good, praise!	Jingdong prefecture level city in Shandong
Happy Fan Shop	Green Intelligent Power Saver	After observing for a while, it really saves a lot of money I didn't realize it was still really reliable, so I'll give you a good review.	Jingdong prefecture level city in Shandong
Happy Fan Shop	Green Intelligent Power Saver	My family a month with this monthly almost save more than 200, power-saving effect: have bought a second time, really effective Cost-effective: not bad Appearance style: roughly the same A few months to the home electricity is indeed less, this time it is a re-buy three, send a friend two, they stay a	Jingdong prefecture level city in Shandong
Happy Fan Shop	Green Intelligent Power Saver	A month almost save more than 150 dollars in electricity, home appliances more, the main this can also protect high-power appliances, after using this device, these two months have not burned, things are very good, it is said that this is the latest model, the overall price is cheap and beautiful!	Jingdong prefecture level city in Shandong

Happy Fan Shop	Green Intelligent Power Saver	Received, powerful, great quality, love the style😍	Jingdong prefecture level city in Shandong
Happy Fan Shop	Green Intelligent Power Saver	This intelligent energy saver is especially good to use and save electricity hope everyone will buy it!	Jingdong prefecture level city in Shandong
Happy Fan Shop	Green Intelligent Power Saver	Air conditioning adapter effect is particularly great, the use of very simple, plug into the socket on the summer air conditioning power is too large, electricity is also expensive, with this product directly on the electricity bill to save half, has been purchased a few more, the effect is too awesome!	Jingdong prefecture level city in Shandong
Happy Fan Shop	Green Intelligent Power Saver	The family plugged in 5, monthly electricity savings of about 300 yuan. My family before a month of electricity costs more than 800, this time with this saver after the electricity bill is actually only more than 500, amazing, thanks to the store, will recommend other friends to buy!	Jingdong prefecture level city in Shandong
Happy Fan Shop	Green Intelligent Power Saver	It was recommended to me by a neighbor's family and it's nice to use it to save power.	Jingdong prefecture level city in Shandong
Happy Fan Shop	Green Intelligent Power Saver	Second time I've purchased it and the power saving is effective!	Jingdong prefecture level city in Shandong
Happy Fan Shop	Green Intelligent Power Saver	The effect is really good, before a month of more than 300 electricity, now every month every day with the air conditioning, it is more than 100 electricity! This thing is really amazing, one after another to buy and 5.6, home installed 3!	Jingdong prefecture level city in Shandong
Happy Fan Shop	Green Intelligent Power Saver	Received this product, began to use, small things look very wrong, can save a little electricity, the monthly electricity bill is too high, after plugging in did not pull, or save a lot of electricity, point	Jingdong prefecture level city in Shandong
Happy Fan Shop	Green Intelligent Power Saver	Simple and convenient, just that plug in, you can save electricity. A month can save dozens of dollars, a year down to save a lot of, very good product cost-effective, everyone can afford to buy and use!	Jingdong prefecture level city in Shandong
Happy Fan Shop	Green Intelligent Power Saver	Electricity bill from more than 200 yuan per month, down to more than 150 yuan, the original bought home, used two months, really have power-saving effect, this time and sold two, business service attitude is	Jingdong prefecture level city in Shandong
Happy Fan Shop	Green Intelligent Power Saver	Like to watch some movies at home and so on, sometimes at night until two or three o'clock, and recently the air conditioning is not turned off for a night, rather wasteful, I have to say that the effect is very good, quite like it!	Jingdong prefecture level city in Shandong
Happy Fan Shop	Green Intelligent Power Saver	Power-saving effect: the effect is very good, a month to save more than 200 dollars Cost-effective: good value for money Appearance and style: small and lovely Workmanship material: okay Experience: good	Jingdong prefecture level city in Shandong
Happy Fan Shop	Green Intelligent Power Saver	It's okay I quite like it, the stuff is very good. Come back in the future. You can rest assured to buy. Cost-effective all. Much cheaper than the physical store	Jingdong prefecture level city in Shandong
Happy Fan Shop	Green Intelligent Power Saver	More than 150 less, last month's electricity bill is almost 400, this month is only about 200, really save electricity, I am home to buy three, I did not expect a month to pay back	Jingdong prefecture level city in Shandong
Happy Fan Shop	Green Intelligent Power Saver	I bought three of these first and tried them for two months and they still felt useful, so now I've bought another one. Good and recommended!	Jingdong prefecture level city in Shandong
Happy Fan Shop	Green Intelligent Power Saver	This energy saver is very good quality and inexpensive next time I will buy it again!	Jingdong prefecture level city in Shandong
Happy Fan Shop	Green Intelligent Power Saver	Merchant service attitude is not good, the quality does not match, the business does not give the package return	Jingdong prefecture level city in Shandong
Happy Fan Shop	Green Intelligent Power Saver	The goods are very small do not know how effective after a few days to follow up the review	Jingdong prefecture level city in Shandong
Happy Fan Shop	Green Intelligent Power Saver	Not used before more than 200 a month, after using about 100 a month, my family used three, customer service said I use electricity recommended 2-3, I think more than one effect is better on the purchase of 4, the effect is very obvious, recommended to buy!	Jingdong prefecture level city in Shandong
Happy Fan Shop	Green Intelligent Power Saver	It's okay. I'll buy it again today.	Jingdong prefecture level city in Shandong
Happy Fan Shop	Green Intelligent Power Saver	Finally received the goods, very, very much like,, has been a lot of people praise, really too happy, has been his family's loyal fans, will continue to buy buy buy buy!	Jingdong prefecture level city in Shandong
Happy Fan Shop	Green Intelligent Power Saver	After using this, my monthly electricity bill was 1,200 pounds, now it's only 180 bucks a month, how good is it?	Jingdong prefecture level city in Shandong
Happy Fan Shop	Green Intelligent Power Saver	The item is a scam return to marry a dozen dollars in shipping fees. Buy terrific role is purely a scam.	Jingdong prefecture level city in Shandong
Happy Fan Shop	Green Intelligent Power Saver	It's really good, cost-effective and good quality, and the logistics are fast! It arrived in one day!	Jingdong prefecture level city in Shandong
Happy Fan Shop	Green Intelligent Power Saver	Great brand! Trustworthy! Works super well! Expensive for a reason! Great merchandise!	Jingdong prefecture level city in Shandong
Happy Fan Shop	Green Intelligent Power Saver	The baby is really great, I like it very much, my friends also say good to come, recommended to buy!	Jingdong prefecture level city in Shandong
Happy Fan Shop	Green Intelligent Power Saver	Fake couldn't be more fake. Bought one plugged in still so much electricity.	Jingdong prefecture level city in Shandong
Happy Fan Shop	Green Intelligent Power Saver	This appliance works great. Let's see how it works. We'll see later.	Jingdong prefecture level city in Shandong
Happy Fan Shop	Green Intelligent Power Saver	Used it before commenting, very happy with it	Jingdong prefecture level city in Shandong
Happy Fan Shop	Green Intelligent Power Saver	Goods have been received well-packed, logistics is very fast and good quality	Jingdong prefecture level city in Shandong
Happy Fan Shop	Green Intelligent Power Saver	This baby's quality is okay.	Jingdong prefecture level city in Shandong
Happy Fan Shop	Green Intelligent Power Saver	Counterfeit goods.	Jingdong prefecture level city in Shandong
Happy Fan Shop	Green Intelligent Power Saver	The effect is not very obvious	Jingdong prefecture level city in Shandong
Happy Fan Shop	Green Intelligent Power Saver	It's not the same thing.	Jingdong prefecture level city in Shandong
Happy Fan Shop	Green Intelligent Power Saver	There is no load how much w seems false, nothing works.	Jingdong prefecture level city in Shandong
Happy Fan Shop	Green Intelligent Power Saver	I received the item yesterday, got it back and plugged it in, and it used a little more power yesterday than I had before	Jingdong prefecture level city in Shandong
Happy Fan Shop	Green Intelligent Power Saver	Haven't seen the power saving mode yet	Jingdong prefecture level city in Shandong
Happy Fan Shop	Green Intelligent Power Saver	This product has no known effect	Jingdong prefecture level city in Shandong
Happy Fan Shop	Green Intelligent Power Saver	Refund Returns $16	Jingdong prefecture level city in Shandong
Happy Fan Shop	Green Intelligent Power Saver	The previous year bought a, almost every month can save a 70 dollars or so, used a year, no problem at all, look very new like, this time to move to a new home decisively repurchase 2 units, very believe this	Jingdong prefecture level city in Shandong
Happy Fan Shop	Green Intelligent Power Saver	Has been the second time to buy, this power saving device is really good power, before every month to spend 300 electricity, now are about 200, last time that 6, this time to help lead the family to buy 6,	Jingdong prefecture level city in Shandong
Happy Fan Shop	Green Intelligent Power Saver	In the spirit of trying to experience the new product, the use of down compared to the usual power consumption, with the addition of the quarter, is indeed a lot less electricity, originally more than 450	Jingdong prefecture level city in Shandong
Happy Fan Shop	Green Intelligent Power Saver	Has not been the first time to buy back, good use, power saving device is really powerful, before the monthly electricity bill of more than 500 Now more than 300, this time to help neighbors to buy several	Jingdong prefecture level city in Shandong
Happy Fan Shop	Green Intelligent Power Saver	Home appliances are very many, the circuit is also very complex, at first it is to save 9% of the electricity, and then used more than half a month, found that the power saving rate is as high as more	Jingdong prefecture level city in Shandong
Happy Fan Shop	Green Intelligent Power Saver	My friend bought it last year and saved hundreds of dollars on his electricity bill in one summer Appearance and style: it looks better than the one I bought before Quality is also good with a light.	Jingdong prefecture level city in Shandong
Happy Fan Shop	Green Intelligent Power Saver	Saver received, on the spot on the plug with the feeling of immediate power savings, appearance, packaging is very delicate, very satisfied, I hope to achieve satisfactory results!	Jingdong prefecture level city in Shandong
Happy Fan Shop	Green Intelligent Power Saver	The power saving effect is very good, is considered an old customer, also bought before, this time is to buy two for mom and dad, customer service attitude is good, praise!	Jingdong prefecture level city in Shandong
Happy Fan Shop	Green Intelligent Power Saver	After observing for a while, it really saves a lot of money I didn't realize it was still really reliable, so I'll give you a good review.	Jingdong prefecture level city in Shandong

Happy Fan Shop	Green Intelligent Power Saver	My family a month with this monthly almost save more than 200, power-saving effect: have bought a second time, really effective Cost-effective: not bad Appearance style: roughly the same A few months to the home electricity is indeed less, this time it is a re-buy three, send a friend two, they stay a	Jingdong prefecture level city in Shandong
Happy Fan Shop	Green Intelligent Power Saver	A month almost save more than 150 dollars in electricity, home appliances more, the main this can also protect high-power appliances, after using this device, these two months have not burned, things are very good, it is said that this is the latest model, the overall price is cheap and beautiful!	Jingdong prefecture level city in Shandong
Happy Fan Shop	Green Intelligent Power Saver	Received, powerful, great quality, love the style😍	Jingdong prefecture level city in Shandong
Happy Fan Shop	Green Intelligent Power Saver	This intelligent energy saver is especially good to use and save electricity hope everyone will buy it!	Jingdong prefecture level city in Shandong
Happy Fan Shop	Green Intelligent Power Saver	Air conditioning adapter effect is particularly great, the use of very simple, plug into the socket on the summer air conditioning power is too large, electricity is also expensive, with this product directly on the electricity bill to save half, has been purchased a few more, the effect is too awesome!	Jingdong prefecture level city in Shandong
Happy Fan Shop	Green Intelligent Power Saver	The family plugged in 5, monthly electricity savings of about 300 yuan. My family before a month of electricity costs more than 800, this time with this saver after the electricity bill is actually only more than 500, amazing, thanks to the store, will recommend other friends to buy!	Jingdong prefecture level city in Shandong
Happy Fan Shop	Green Intelligent Power Saver	It was recommended to me by a neighbor's family and it's nice to use it to save power.	Jingdong prefecture level city in Shandong
Happy Fan Shop	Green Intelligent Power Saver	Second time I've purchased it and the power saving is effective!	Jingdong prefecture level city in Shandong
Happy Fan Shop	Green Intelligent Power Saver	The effect is really good, before a month of more than 300 electricity, now every month every day with the air conditioning, it is more than 100 electricity! This thing is really amazing, one after another to buy and 5.6, home installed 3!	Jingdong prefecture level city in Shandong
Happy Fan Shop	Green Intelligent Power Saver	Received this product, began to use, small things look very wrong, can save a little electricity, the monthly electricity bill is too high, after plugging in did not pull, or save a lot of electricity, point	Jingdong prefecture level city in Shandong
Happy Fan Shop	Green Intelligent Power Saver	Simple and convenient, just that plug in, you can save electricity. A month can save dozens of dollars, a year down to save a lot of, very good product cost-effective, everyone can afford to buy and use!	Jingdong prefecture level city in Shandong
Happy Fan Shop	Green Intelligent Power Saver	Electricity bill from more than 200 yuan per month, down to more than 150 yuan, the original bought home, used two months, really have power-saving effect, this time and sold two, business service attitude is	Jingdong prefecture level city in Shandong
Happy Fan Shop	Green Intelligent Power Saver	Like to watch some movies at home and so on, sometimes at night until two or three o'clock, and recently the air conditioning is not turned off for a night, rather wasteful, I have to say that the effect is very good, quite like it!	Jingdong prefecture level city in Shandong
Happy Fan Shop	Green Intelligent Power Saver	Power-saving effect: the effect is very good, a month to save more than 200 dollars Cost-effective: good value for money Appearance and style: small and lovely Workmanship material: okay Experience: good	Jingdong prefecture level city in Shandong
Happy Fan Shop	Green Intelligent Power Saver	It's okay I quite like it, the stuff is very good. Come back in the future. You can rest assured to buy. Cost-effective all. Much cheaper than the physical store	Jingdong prefecture level city in Shandong
Happy Fan Shop	Green Intelligent Power Saver	More than 150 less, last month's electricity bill is almost 400, this month is only about 200, really save electricity, I am home to buy three, I did not expect a month to pay back	Jingdong prefecture level city in Shandong
Happy Fan Shop	Green Intelligent Power Saver	I bought three of these first and tried them for two months and they still felt useful, so now I've bought another one. Good and recommended!	Jingdong prefecture level city in Shandong
Happy Fan Shop	Green Intelligent Power Saver	This energy saver is very good quality and inexpensive next time I will buy it again!	Jingdong prefecture level city in Shandong
Happy Fan Shop	Green Intelligent Power Saver	Merchant service attitude is not good, the quality does not match, the business does not give the package return	Jingdong prefecture level city in Shandong
Happy Fan Shop	Green Intelligent Power Saver	The goods are very small do not know how effective after a few days to follow up the review	Jingdong prefecture level city in Shandong
Happy Fan Shop	Green Intelligent Power Saver	Not used before more than 200 a month, after using about 100 a month, my family used three, customer service said I use electricity recommended 2-3, I think more than one effect is better on the purchase of 4, the effect is very obvious, recommended to buy!	Jingdong prefecture level city in Shandong
Happy Fan Shop	Green Intelligent Power Saver	It's okay. I'll buy it again today.	Jingdong prefecture level city in Shandong
Happy Fan Shop	Green Intelligent Power Saver	Finally received the goods, very, very much like,, has been a lot of people praise, really too happy, has been his family's loyal fans, will continue to buy buy buy buy!	Jingdong prefecture level city in Shandong
Happy Fan Shop	Green Intelligent Power Saver	After using this, my monthly electricity bill was 1,200 pounds, now it's only 180 bucks a month, how good is it?	Jingdong prefecture level city in Shandong
Happy Fan Shop	Green Intelligent Power Saver	The item is a scam return to marry a dozen dollars in shipping fees. Buy terrific role is purely a scam.	Jingdong prefecture level city in Shandong
Happy Fan Shop	Green Intelligent Power Saver	It's really good, cost-effective and good quality, and the logistics are fast! It arrived in one day!	Jingdong prefecture level city in Shandong
Happy Fan Shop	Green Intelligent Power Saver	Great brand! Trustworthy! Works super well! Expensive for a reason! Great merchandise!	Jingdong prefecture level city in Shandong
Happy Fan Shop	Green Intelligent Power Saver	The baby is really great, I like it very much, my friends also say good to come, recommended to buy!	Jingdong prefecture level city in Shandong
Happy Fan Shop	Green Intelligent Power Saver	Fake couldn't be more fake. Bought one plugged in still so much electricity.	Jingdong prefecture level city in Shandong
Happy Fan Shop	Green Intelligent Power Saver	This appliance works great. Let's see how it works. We'll see later.	Jingdong prefecture level city in Shandong
Happy Fan Shop	Green Intelligent Power Saver	Used it before commenting, very happy with it	Jingdong prefecture level city in Shandong
Happy Fan Shop	Green Intelligent Power Saver	Goods have been received well-packed, logistics is very fast and good quality	Jingdong prefecture level city in Shandong
Happy Fan Shop	Green Intelligent Power Saver	This baby's quality is okay.	Jingdong prefecture level city in Shandong
Happy Fan Shop	Green Intelligent Power Saver	Counterfeit goods.	Jingdong prefecture level city in Shandong
Happy Fan Shop	Green Intelligent Power Saver	The effect is not very obvious	Jingdong prefecture level city in Shandong
Happy Fan Shop	Green Intelligent Power Saver	It's not the same thing.	Jingdong prefecture level city in Shandong
Happy Fan Shop	Green Intelligent Power Saver	There is no load how much w seems false, nothing works.	Jingdong prefecture level city in Shandong
Happy Fan Shop	Green Intelligent Power Saver	I received the item yesterday, got it back and plugged it in, and it used a little more power yesterday than I had before	Jingdong prefecture level city in Shandong
Happy Fan Shop	Green Intelligent Power Saver	Haven't seen the power saving mode yet	Jingdong prefecture level city in Shandong
Happy Fan Shop	Green Intelligent Power Saver	This product has no known effect	Jingdong prefecture level city in Shandong
Happy Fan Shop	Green Intelligent Power Saver	Refund Returns $16	Jingdong prefecture level city in Shandong
Happy Fan Shop	Green Intelligent Power Saver	The previous year bought a, almost every month can save a 70 dollars or so, used a year, no problem at all, look very new like, this time to move to a new home decisively repurchase 2 units, very believe this	Jingdong prefecture level city in Shandong
Happy Fan Shop	Green Intelligent Power Saver	Has been the second time to buy, this power saving device is really good power, before every month to spend 300 electricity, now are about 200, last time that 6, this time to help lead the family to buy 6,	Jingdong prefecture level city in Shandong
Happy Fan Shop	Green Intelligent Power Saver	In the spirit of trying to experience the new product, the use of down compared to the usual power consumption, with the addition of the quarter, is indeed a lot less electricity, originally more than 450	Jingdong prefecture level city in Shandong
Happy Fan Shop	Green Intelligent Power Saver	Has not been the first time to buy back, good use, power saving device is really powerful, before the monthly electricity bill of more than 500 Now more than 300, this time to help neighbors to buy several	Jingdong prefecture level city in Shandong
Happy Fan Shop	Green Intelligent Power Saver	Home appliances are very many, the circuit is also very complex, at first it is to save 9% of the electricity, and then used more than half a month, found that the power saving rate is as high as more	Jingdong prefecture level city in Shandong
Happy Fan Shop	Green Intelligent Power Saver	My friend bought it last year and saved hundreds of dollars on his electricity bill in one summer Appearance and style: it looks better than the one I bought before Quality is also good with a light.	Jingdong prefecture level city in Shandong
Happy Fan Shop	Green Intelligent Power Saver	Saver received, on the spot on the plug with the feeling of immediate power savings, appearance, packaging is very delicate, very satisfied, I hope to achieve satisfactory results!	Jingdong prefecture level city in Shandong

Happy Fan Shop	Green Intelligent Power Saver	The power saving effect is very good, is considered an old customer, also bought before, this time is to buy two for mom and dad, customer service attitude is good, praise!	Jingdong prefecture level city in Shandong
Happy Fan Shop	Green Intelligent Power Saver	After observing for a while, it really saves a lot of money I didn't realize it was still really reliable, so I'll give you a good review.	Jingdong prefecture level city in Shandong
Happy Fan Shop	Green Intelligent Power Saver	My family a month with this monthly almost save more than 200, power-saving effect: have bought a second time, really effective Cost-effective: not bad Appearance style: roughly the same A few months to the home electricity is indeed less, this time it is a re-buy three, send a friend two, they stay a	Jingdong prefecture level city in Shandong
Happy Fan Shop	Green Intelligent Power Saver	A month almost save more than 150 dollars in electricity, home appliances more, the main this can also protect high-power appliances, after using this device, these two months have not burned, things are very good, it is said that this is the latest model, the overall price is cheap and beautiful!	Jingdong prefecture level city in Shandong
Happy Fan Shop	Green Intelligent Power Saver	Received, powerful, great quality, love the style😍	Jingdong prefecture level city in Shandong
Happy Fan Shop	Green Intelligent Power Saver	This intelligent energy saver is especially good to use and save electricity hope everyone will buy it!	Jingdong prefecture level city in Shandong
Happy Fan Shop	Green Intelligent Power Saver	Air conditioning adapter effect is particularly great, the use of very simple, plug into the socket on the summer air conditioning power is too large, electricity is also expensive, with this product directly on the electricity bill to save half, has been purchased a few more, the effect is too awesome!	Jingdong prefecture level city in Shandong
Happy Fan Shop	Green Intelligent Power Saver	The family plugged in 5, monthly electricity savings of about 300 yuan. My family before a month of electricity costs more than 800, this time with this saver after the electricity bill is actually only more than 500, amazing, thanks to the store, will recommend other friends to buy!	Jingdong prefecture level city in Shandong
Happy Fan Shop	Green Intelligent Power Saver	It was recommended to me by a neighbor's family and it's nice to use it to save power.	Jingdong prefecture level city in Shandong
Happy Fan Shop	Green Intelligent Power Saver	Second time I've purchased it and the power saving is effective!	Jingdong prefecture level city in Shandong
Happy Fan Shop	Green Intelligent Power Saver	The effect is really good, before a month of more than 300 electricity, now every month every day with the air conditioning, it is more than 100 electricity! This thing is really amazing, one after another to buy and 5.6, home installed 3!	Jingdong prefecture level city in Shandong
Happy Fan Shop	Green Intelligent Power Saver	Received this product, began to use, small things look very wrong, can save a little electricity, the monthly electricity bill is too high, after plugging in did not pull, or save a lot of electricity, point	Jingdong prefecture level city in Shandong
Happy Fan Shop	Green Intelligent Power Saver	Simple and convenient, just that plug in, you can save electricity. A month can save dozens of dollars, a year down to save a lot of, very good product cost-effective, everyone can afford to buy and use!	Jingdong prefecture level city in Shandong
Happy Fan Shop	Green Intelligent Power Saver	Electricity bill from more than 200 yuan per month, down to more than 150 yuan, the original bought home, used two months, really have power-saving effect, this time and sold two, business service attitude is	Jingdong prefecture level city in Shandong
Happy Fan Shop	Green Intelligent Power Saver	Like to watch some movies at home and so on, sometimes at night until two or three o'clock, and recently the air conditioning is not turned off for a night, rather wasteful, I have to say that the effect is very good, quite like it!	Jingdong prefecture level city in Shandong
Happy Fan Shop	Green Intelligent Power Saver	Power-saving effect: the effect is very good, a month to save more than 200 dollars Cost-effective: good value for money Appearance and style: small and lovely Workmanship material: okay Experience: good	Jingdong prefecture level city in Shandong
Happy Fan Shop	Green Intelligent Power Saver	It's okay I quite like it, the stuff is very good. Come back in the future. You can rest assured to buy. Cost-effective all. Much cheaper than the physical store	Jingdong prefecture level city in Shandong
Happy Fan Shop	Green Intelligent Power Saver	More than 150 less, last month's electricity bill is almost 400, this month is only about 200, really save electricity, I am home to buy three, I did not expect a month to pay back	Jingdong prefecture level city in Shandong
Happy Fan Shop	Green Intelligent Power Saver	I bought three of these first and tried them for two months and they still felt useful, so now I've bought another one. Good and recommended!	Jingdong prefecture level city in Shandong
Happy Fan Shop	Green Intelligent Power Saver	This energy saver is very good quality and inexpensive next time I will buy it again!	Jingdong prefecture level city in Shandong
Happy Fan Shop	Green Intelligent Power Saver	Merchant service attitude is not good, the quality does not match, the business does not give the package return	Jingdong prefecture level city in Shandong
Happy Fan Shop	Green Intelligent Power Saver	The goods are very small do not know how effective after a few days to follow up the review	Jingdong prefecture level city in Shandong
Happy Fan Shop	Green Intelligent Power Saver	Not used before more than 200 a month, after using about 100 a month, my family used three, customer service said I use electricity recommended 2-3, I think more than one effect is better on the purchase of 4, the effect is very obvious, recommended to buy!	Jingdong prefecture level city in Shandong
Happy Fan Shop	Green Intelligent Power Saver	It's okay. I'll buy it again today.	Jingdong prefecture level city in Shandong
Happy Fan Shop	Green Intelligent Power Saver	Finally received the goods, very, very much like,, has been a lot of people praise, really too happy, has been his family's loyal fans, will continue to buy buy buy!	Jingdong prefecture level city in Shandong
Happy Fan Shop	Green Intelligent Power Saver	After using this, my monthly electricity bill was 1,200 pounds, now it's only 180 bucks a month, how good is it?	Jingdong prefecture level city in Shandong
Happy Fan Shop	Green Intelligent Power Saver	The item is a scam return to marry a dozen dollars in shipping fees. Buy terrific role is purely a scam.	Jingdong prefecture level city in Shandong
Happy Fan Shop	Green Intelligent Power Saver	It's really good, cost-effective and good quality, and the logistics are fast! It arrived in one day!	Jingdong prefecture level city in Shandong
Happy Fan Shop	Green Intelligent Power Saver	Great brand! Trustworthy! Works super well! Expensive for a reason! Great merchandise!	Jingdong prefecture level city in Shandong
Happy Fan Shop	Green Intelligent Power Saver	The baby is really great, I like it very much, my friends also say good to come, recommended to buy!	Jingdong prefecture level city in Shandong
Happy Fan Shop	Green Intelligent Power Saver	Fake couldn't be more fake. Bought one plugged in still so much electricity.	Jingdong prefecture level city in Shandong
Happy Fan Shop	Green Intelligent Power Saver	This appliance works great. Let's see how it works. We'll see later.	Jingdong prefecture level city in Shandong
Happy Fan Shop	Green Intelligent Power Saver	Used it before commenting, very happy with it	Jingdong prefecture level city in Shandong
Happy Fan Shop	Green Intelligent Power Saver	Goods have been received well-packed, logistics is very fast and good quality	Jingdong prefecture level city in Shandong
Happy Fan Shop	Green Intelligent Power Saver	This baby's quality is okay.	Jingdong prefecture level city in Shandong
Happy Fan Shop	Green Intelligent Power Saver	Counterfeit goods.	Jingdong prefecture level city in Shandong
Happy Fan Shop	Green Intelligent Power Saver	The effect is not very obvious	Jingdong prefecture level city in Shandong
Happy Fan Shop	Green Intelligent Power Saver	It's not the same thing.	Jingdong prefecture level city in Shandong
Happy Fan Shop	Green Intelligent Power Saver	There is no load how much w seems false, nothing works.	Jingdong prefecture level city in Shandong
Happy Fan Shop	Green Intelligent Power Saver	I received the item yesterday, got it back and plugged it in, and it used a little more power yesterday than I had before	Jingdong prefecture level city in Shandong
Happy Fan Shop	Green Intelligent Power Saver	Haven't seen the power saving mode yet	Jingdong prefecture level city in Shandong
Happy Fan Shop	Green Intelligent Power Saver	This product has no known effect	Jingdong prefecture level city in Shandong
Happy Fan Shop	Green Intelligent Power Saver	Refund Returns $16	Jingdong prefecture level city in Shandong
Happy Fan Shop	Green Intelligent Power Saver	The previous year bought a, almost every month can save a 70 dollars or so, used a year, no problem at all, look very new like, this time to move to a new home decisively repurchase 2 units, very believe this	Jingdong prefecture level city in Shandong
Happy Fan Shop	Green Intelligent Power Saver	Has been the second time to buy, this power saving device is really good power, before every month to spend 300 electricity, now are about 200, last time that 6, this time to help lead the family to buy 6,	Jingdong prefecture level city in Shandong
Happy Fan Shop	Green Intelligent Power Saver	In the spirit of trying to experience the new product, the use of down compared to the usual power consumption, with the addition of the quarter, is indeed a lot less electricity, originally more than 450	Jingdong prefecture level city in Shandong
Happy Fan Shop	Green Intelligent Power Saver	Has not been the first time to buy back, good use, power saving device is really powerful, before the monthly electricity bill of more than 500 Now more than 300, this time to help neighbors to buy several	Jingdong prefecture level city in Shandong
Happy Fan Shop	Green Intelligent Power Saver	Home appliances are very many, the circuit is also very complex, at first it is to save 9% of the electricity, and then used more than half a month, found that the power saving rate is as high as more	Jingdong prefecture level city in Shandong

Happy Fan Shop	Green Intelligent Power Saver	My friend bought it last year and saved hundreds of dollars on his electricity bill in one summer Appearance and style: it looks better than the one I bought before Quality is also good with a light.	Jingdong prefecture level city in Shandong
Happy Fan Shop	Green Intelligent Power Saver	Saver received, on the spot on the plug with the feeling of immediate power savings, appearance, packaging is very delicate, very satisfied, I hope to achieve satisfactory results!	Jingdong prefecture level city in Shandong
Happy Fan Shop	Green Intelligent Power Saver	The power saving effect is very good, is considered an old customer, also bought before, this time is to buy two for mom and dad, customer service attitude is good, praise!	Jingdong prefecture level city in Shandong
Happy Fan Shop	Green Intelligent Power Saver	After observing for a while, it really saves a lot of money I didn't realize it was still really reliable, so I'll give you a good review.	Jingdong prefecture level city in Shandong
Happy Fan Shop	Green Intelligent Power Saver	My family a month with this monthly almost save more than 200, power-saving effect: have bought a second time, really effective Cost-effective: not bad Appearance style: roughly the same A few months to the home electricity is indeed less, this time it is a re-buy three, send a friend two, they stay a	Jingdong prefecture level city in Shandong
Happy Fan Shop	Green Intelligent Power Saver	A month almost save more than 150 dollars in electricity, home appliances more, the main this can also protect high-power appliances, after using this device, these two months have not burned, things are very good, it is said that this is the latest model, the overall price is cheap and beautiful!	Jingdong prefecture level city in Shandong
Happy Fan Shop	Green Intelligent Power Saver	Received, powerful, great quality, love the style😍	Jingdong prefecture level city in Shandong
Happy Fan Shop	Green Intelligent Power Saver	This intelligent energy saver is especially good to use and save electricity hope everyone will buy it!	Jingdong prefecture level city in Shandong
Happy Fan Shop	Green Intelligent Power Saver	Air conditioning adapter effect is particularly great, the use of very simple, plug into the socket on the summer air conditioning power is too large, electricity is also expensive, with this product directly on the electricity bill to save half, has been purchased a few more, the effect is too awesome!	Jingdong prefecture level city in Shandong
Happy Fan Shop	Green Intelligent Power Saver	The family plugged in 5, monthly electricity savings of about 300 yuan. My family before a month of electricity costs more than 800, this time with this saver after the electricity bill is actually only more than 500, amazing, thanks to the store, will recommend other friends to buy!	Jingdong prefecture level city in Shandong
Happy Fan Shop	Green Intelligent Power Saver	It was recommended to me by a neighbor's family and it's nice to use it to save power.	Jingdong prefecture level city in Shandong
Happy Fan Shop	Green Intelligent Power Saver	Second time I've purchased it and the power saving is effective!	Jingdong prefecture level city in Shandong
Happy Fan Shop	Green Intelligent Power Saver	The effect is really good, before a month of more than 300 electricity, now every month every day with the air conditioning, it is more than 100 electricity! This thing is really amazing, one after another to buy and 5.6, home installed 3!	Jingdong prefecture level city in Shandong
Happy Fan Shop	Green Intelligent Power Saver	Received this product, began to use, small things look very wrong, can save a little electricity, the monthly electricity bill is too high, after plugging in did not pull, or save a lot of electricity, point	Jingdong prefecture level city in Shandong
Happy Fan Shop	Green Intelligent Power Saver	Simple and convenient, just that plug in, you can save electricity. A month can save dozens of dollars, a year down to save a lot of, very good product cost-effective, everyone can afford to buy and use!	Jingdong prefecture level city in Shandong
Happy Fan Shop	Green Intelligent Power Saver	Electricity bill from more than 200 yuan per month, down to more than 150 yuan, the original bought home, used two months, really have power-saving effect, this time and sold two, business service attitude is	Jingdong prefecture level city in Shandong
Happy Fan Shop	Green Intelligent Power Saver	Like to watch some movies at home and so on, sometimes at night until two or three o'clock, and recently the air conditioning is not turned off for a night, rather wasteful, I have to say that the effect is very good, quite like it!	Jingdong prefecture level city in Shandong
Happy Fan Shop	Green Intelligent Power Saver	Power-saving effect: the effect is very good, a month to save more than 200 dollars Cost-effective: good value for money Appearance and style: small and lovely Workmanship material: okay Experience: good	Jingdong prefecture level city in Shandong
Happy Fan Shop	Green Intelligent Power Saver	It's okay I quite like it, the stuff is very good. Come back in the future. You can rest assured to buy. Cost-effective all. Much cheaper than the physical store	Jingdong prefecture level city in Shandong
Happy Fan Shop	Green Intelligent Power Saver	More than 150 less, last month's electricity bill is almost 400, this month is only about 200, really save electricity, I am home to buy three, I did not expect a month to pay back	Jingdong prefecture level city in Shandong
Happy Fan Shop	Green Intelligent Power Saver	I bought three of these first and tried them for two months and they still felt useful, so now I've bought another one. Good and recommended!	Jingdong prefecture level city in Shandong
Happy Fan Shop	Green Intelligent Power Saver	This energy saver is very good quality and inexpensive next time I will buy it again!	Jingdong prefecture level city in Shandong
Happy Fan Shop	Green Intelligent Power Saver	Merchant service attitude is not good, the quality does not match, the business does not give the package return	Jingdong prefecture level city in Shandong
Happy Fan Shop	Green Intelligent Power Saver	The goods are very small do not know how effective after a few days to follow up the review	Jingdong prefecture level city in Shandong
Happy Fan Shop	Green Intelligent Power Saver	Not used before more than 200 a month, after using about 100 a month, my family used three, customer service said I use electricity recommended 2-3, I think more than one effect is better on the purchase of 4, the effect is very obvious, recommended to buy!	Jingdong prefecture level city in Shandong
Happy Fan Shop	Green Intelligent Power Saver	It's okay. I'll buy it again today.	Jingdong prefecture level city in Shandong
Happy Fan Shop	Green Intelligent Power Saver	Finally received the goods, very, very much like,, has been a lot of people praise, really too happy, has been his family's loyal fans, will continue to buy buy buy buy!	Jingdong prefecture level city in Shandong
Happy Fan Shop	Green Intelligent Power Saver	After using this, my monthly electricity bill was 1,200 pounds, now it's only 180 bucks a month, how good is it?	Jingdong prefecture level city in Shandong
Happy Fan Shop	Green Intelligent Power Saver	The item is a scam return to marry a dozen dollars in shipping fees. Buy terrific role is purely a scam.	Jingdong prefecture level city in Shandong
Happy Fan Shop	Green Intelligent Power Saver	It's really good, cost-effective and good quality, and the logistics are fast! It arrived in one day!	Jingdong prefecture level city in Shandong
Happy Fan Shop	Green Intelligent Power Saver	Great brand! Trustworthy! Works super well! Expensive for a reason! Great merchandise!	Jingdong prefecture level city in Shandong
Happy Fan Shop	Green Intelligent Power Saver	The baby is really great, I like it very much, my friends also say good to come, recommended to buy!	Jingdong prefecture level city in Shandong
Happy Fan Shop	Green Intelligent Power Saver	Fake couldn't be more fake. Bought one plugged in still so much electricity.	Jingdong prefecture level city in Shandong
Happy Fan Shop	Green Intelligent Power Saver	This appliance works great. Let's see how it works. We'll see later.	Jingdong prefecture level city in Shandong
Happy Fan Shop	Green Intelligent Power Saver	Used it before commenting, very happy with it	Jingdong prefecture level city in Shandong
Happy Fan Shop	Green Intelligent Power Saver	Goods have been received well-packed, logistics is very fast and good quality	Jingdong prefecture level city in Shandong
Happy Fan Shop	Green Intelligent Power Saver	This baby's quality is okay.	Jingdong prefecture level city in Shandong
Happy Fan Shop	Green Intelligent Power Saver	Counterfeit goods.	Jingdong prefecture level city in Shandong
Happy Fan Shop	Green Intelligent Power Saver	The effect is not very obvious	Jingdong prefecture level city in Shandong
Happy Fan Shop	Green Intelligent Power Saver	There is no load how much w seems false, nothing works.	Jingdong prefecture level city in Shandong
Happy Fan Shop	Green Intelligent Power Saver	It's not the same thing.	Jingdong prefecture level city in Shandong
Happy Fan Shop	Green Intelligent Power Saver	I received the item yesterday, got it back and plugged it in, and it used a little more power yesterday than I had before	Jingdong prefecture level city in Shandong
Happy Fan Shop	Green Intelligent Power Saver	Haven't seen the power saving mode yet	Jingdong prefecture level city in Shandong
Happy Fan Shop	Green Intelligent Power Saver	This product has no known effect	Jingdong prefecture level city in Shandong
Happy Fan Shop	Green Intelligent Power Saver	Refund Returns $16	Jingdong prefecture level city in Shandong
Happy Fan Shop	Green Intelligent Power Saver	The previous year bought a, almost every month can save a 70 dollars or so, used a year, no problem at all, look very new like, this time to move to a new home decisively repurchase 2 units, very believe this	Jingdong prefecture level city in Shandong
Happy Fan Shop	Green Intelligent Power Saver	Has been the second time to buy, this power saving device is really good power, before every month to spend 300 electricity, now are about 200, last time that 6, this time to help lead the family to buy 6,	Jingdong prefecture level city in Shandong
Happy Fan Shop	Green Intelligent Power Saver	In the spirit of trying to experience the new product, the use of down compared to the usual power consumption, with the addition of the quarter, is indeed a lot less electricity, originally more than 450	Jingdong prefecture level city in Shandong

Happy Fan Shop	Green Intelligent Power Saver	Has not been the first time to buy back, good use, power saving device is really powerful, before the monthly electricity bill of more than 500 Now more than 300, this time to help neighbors to buy several	Jingdong prefecture level city in Shandong
Happy Fan Shop	Green Intelligent Power Saver	Home appliances are very many, the circuit is also very complex, at first it is to save 9% of the electricity, and then used more than half a month, found that the power saving rate is as high as more	Jingdong prefecture level city in Shandong
Happy Fan Shop	Green Intelligent Power Saver	My friend bought it last year and saved hundreds of dollars on his electricity bill in one summer Appearance and style: it looks better than the one I bought before Quality is also good with a light.	Jingdong prefecture level city in Shandong
Happy Fan Shop	Green Intelligent Power Saver	Saver received, on the spot on the plug with the feeling of immediate power savings, appearance, packaging is very delicate, very satisfied, I hope to achieve satisfactory results!	Jingdong prefecture level city in Shandong
Happy Fan Shop	Green Intelligent Power Saver	The power saving effect is very good, is considered an old customer, also bought before, this time is to buy two for mom and dad, customer service attitude is good, praise!	Jingdong prefecture level city in Shandong
Happy Fan Shop	Green Intelligent Power Saver	After observing for a while, it really saves a lot of money I didn't realize it was still really reliable, so I'll give you a good review.	Jingdong prefecture level city in Shandong
Happy Fan Shop	Green Intelligent Power Saver	My family a month with this monthly almost save more than 200, power-saving effect: have bought a second time, really effective Cost-effective: not bad Appearance style: roughly the same A few months to the home electricity is indeed less, this time it is a re-buy three, send a friend two, they stay a	Jingdong prefecture level city in Shandong
Happy Fan Shop	Green Intelligent Power Saver	A month almost save more than 150 dollars in electricity, home appliances more, the main this can also protect high-power appliances, after using this device, these two months have not burned, things are very good, it is said that this is the latest model, the overall price is cheap and beautiful!	Jingdong prefecture level city in Shandong
Happy Fan Shop	Green Intelligent Power Saver	Received, powerful, great quality, love the style😍	Jingdong prefecture level city in Shandong
Happy Fan Shop	Green Intelligent Power Saver	This intelligent energy saver is especially good to use and save electricity hope everyone will buy it!	Jingdong prefecture level city in Shandong
Happy Fan Shop	Green Intelligent Power Saver	Air conditioning adapter effect is particularly great, the use of very simple, plug into the socket on the summer air conditioning power is too large, electricity is also expensive, with this product directly on the electricity bill to save half, has been purchased a few more, the effect is too awesome!	Jingdong prefecture level city in Shandong
Happy Fan Shop	Green Intelligent Power Saver	The family plugged in 5, monthly electricity savings of about 300 yuan. My family before a month of electricity costs more than 800, this time with this saver after the electricity bill is actually only more than 500, amazing, thanks to the store, will recommend other friends to buy!	Jingdong prefecture level city in Shandong
Happy Fan Shop	Green Intelligent Power Saver	It was recommended to me by a neighbor's family and it's nice to use it to save power.	Jingdong prefecture level city in Shandong
Happy Fan Shop	Green Intelligent Power Saver	Second time I've purchased it and the power saving is effective!	Jingdong prefecture level city in Shandong
Happy Fan Shop	Green Intelligent Power Saver	The effect is really good, before a month of more than 300 electricity, now every month every day with the air conditioning, it is more than 100 electricity! This thing is really amazing, one after another to buy and 5.6, home installed 3!	Jingdong prefecture level city in Shandong
Happy Fan Shop	Green Intelligent Power Saver	Received this product, began to use, small things look very wrong, can save a little electricity, the monthly electricity bill is too high, after plugging in did not pull, or save a lot of electricity, point	Jingdong prefecture level city in Shandong
Happy Fan Shop	Green Intelligent Power Saver	Simple and convenient, just that plug in, you can save electricity. A month can save dozens of dollars, a year down to save a lot of, very good product cost-effective, everyone can afford to buy and use!	Jingdong prefecture level city in Shandong
Happy Fan Shop	Green Intelligent Power Saver	Electricity bill from more than 200 yuan per month, down to more than 150 yuan, the original bought home, used two months, really have power-saving effect, this time and sold two, business service attitude is	Jingdong prefecture level city in Shandong
Happy Fan Shop	Green Intelligent Power Saver	Like to watch some movies at home and so on, sometimes at night until two or three o'clock, and recently the air conditioning is not turned off for a night, rather wasteful, I have to say that the effect is very good, quite like it!	Jingdong prefecture level city in Shandong
Happy Fan Shop	Green Intelligent Power Saver	Power-saving effect: the effect is very good, a month to save more than 200 dollars Cost-effective: good value for money Appearance and style: small and lovely Workmanship material: okay Experience: good	Jingdong prefecture level city in Shandong
Happy Fan Shop	Green Intelligent Power Saver	It's okay I quite like it, the stuff is very good. Come back in the future. You can rest assured to buy. Cost-effective all. Much cheaper than the physical store	Jingdong prefecture level city in Shandong
Happy Fan Shop	Green Intelligent Power Saver	More than 150 less, last month's electricity bill is almost 400, this month is only about 200, really save electricity, I am home to buy three, I did not expect a month to pay back	Jingdong prefecture level city in Shandong
Happy Fan Shop	Green Intelligent Power Saver	I bought three of these first and tried them for two months and they still felt useful, so now I've bought another one. Good and recommended!	Jingdong prefecture level city in Shandong
Happy Fan Shop	Green Intelligent Power Saver	This energy saver is very good quality and inexpensive next time I will buy it again!	Jingdong prefecture level city in Shandong
Happy Fan Shop	Green Intelligent Power Saver	Merchant service attitude is not good, the quality does not match, the business does not give the package return	Jingdong prefecture level city in Shandong
Happy Fan Shop	Green Intelligent Power Saver	The goods are very small do not know how effective after a few days to follow up the review	Jingdong prefecture level city in Shandong
Happy Fan Shop	Green Intelligent Power Saver	Not used before more than 200 a month, after using about 100 a month, my family used three, customer service said I use electricity recommended 2-3, I think more than one effect is better on the purchase of 4, the effect is very obvious, recommended to buy!	Jingdong prefecture level city in Shandong
Happy Fan Shop	Green Intelligent Power Saver	It's okay. I'll buy it again today.	Jingdong prefecture level city in Shandong
Happy Fan Shop	Green Intelligent Power Saver	Finally received the goods, very, very much like,, has been a lot of people praise, really too happy, has been his family's loyal fans, will continue to buy buy buy!	Jingdong prefecture level city in Shandong
Happy Fan Shop	Green Intelligent Power Saver	After using this, my monthly electricity bill was 1,200 pounds, now it's only 180 bucks a month, how good is it?	Jingdong prefecture level city in Shandong
Happy Fan Shop	Green Intelligent Power Saver	The item is a scam return to marry a dozen dollars in shipping fees. Buy terrific role is purely a scam.	Jingdong prefecture level city in Shandong
Happy Fan Shop	Green Intelligent Power Saver	It's really good, cost-effective and good quality, and the logistics are fast! It arrived in one day!	Jingdong prefecture level city in Shandong
Happy Fan Shop	Green Intelligent Power Saver	Great brand! Trustworthy! Works super well! Expensive for a reason! Great merchandise!	Jingdong prefecture level city in Shandong
Happy Fan Shop	Green Intelligent Power Saver	The baby is really great, I like it very much, my friends also say good to come, recommended to buy!	Jingdong prefecture level city in Shandong
Happy Fan Shop	Green Intelligent Power Saver	Fake couldn't be more fake. Bought one plugged in still so much electricity.	Jingdong prefecture level city in Shandong
Happy Fan Shop	Green Intelligent Power Saver	This appliance works great. Let's see how it works. We'll see later.	Jingdong prefecture level city in Shandong
Happy Fan Shop	Green Intelligent Power Saver	Used it before commenting, very happy with it	Jingdong prefecture level city in Shandong
Happy Fan Shop	Green Intelligent Power Saver	Goods have been received well-packed, logistics is very fast and good quality	Jingdong prefecture level city in Shandong
Happy Fan Shop	Green Intelligent Power Saver	This baby's quality is okay.	Jingdong prefecture level city in Shandong
Happy Fan Shop	Green Intelligent Power Saver	Counterfeit goods.	Jingdong prefecture level city in Shandong
Happy Fan Shop	Green Intelligent Power Saver	The effect is not very obvious	Jingdong prefecture level city in Shandong
Happy Fan Shop	Green Intelligent Power Saver	It's not the same thing.	Jingdong prefecture level city in Shandong
Happy Fan Shop	Green Intelligent Power Saver	There is no load how much w seems false, nothing works.	Jingdong prefecture level city in Shandong
Happy Fan Shop	Green Intelligent Power Saver	I received the item yesterday, got it back and plugged it in, and it used a little more power yesterday than I had before	Jingdong prefecture level city in Shandong
Happy Fan Shop	Green Intelligent Power Saver	Haven't seen the power saving mode yet	Jingdong prefecture level city in Shandong
Happy Fan Shop	Green Intelligent Power Saver	This product has no known effect	Jingdong prefecture level city in Shandong
Happy Fan Shop	Green Intelligent Power Saver	Refund Returns $16	Jingdong prefecture level city in Shandong
Happy Fan Shop	Green Intelligent Power Saver	The previous year bought a, almost every month can save a 70 dollars or so, used a year, no problem at all, look very new like, this time to move to a new home decisively repurchase 2 units, very believe this	Jingdong prefecture level city in Shandong

Happy Fan Shop	Green Intelligent Power Saver	Has been the second time to buy, this power saving device is really good power, before every month to spend 300 electricity, now are about 200, last time that 6, this time to help lead the family to buy 6,	Jingdong prefecture level city in Shandong
Happy Fan Shop	Green Intelligent Power Saver	In the spirit of trying to experience the new product, the use of down compared to the usual power consumption, with the addition of the quarter, is indeed a lot less electricity, originally more than 450	Jingdong prefecture level city in Shandong
Happy Fan Shop	Green Intelligent Power Saver	Has not been the first time to buy back, good use, power saving device is really powerful, before the monthly electricity bill of more than 500 Now more than 300, this time to help neighbors to buy several	Jingdong prefecture level city in Shandong
Happy Fan Shop	Green Intelligent Power Saver	Home appliances are very many, the circuit is also very complex, at first it is to save 9% of the electricity, and then used more than half a month, found that the power saving rate is as high as more	Jingdong prefecture level city in Shandong
Happy Fan Shop	Green Intelligent Power Saver	My friend bought it last year and saved hundreds of dollars on his electricity bill in one summer Appearance and style: it looks better than the one I bought before Quality is also good with a light.	Jingdong prefecture level city in Shandong
Happy Fan Shop	Green Intelligent Power Saver	Saver received, on the spot on the plug with the feeling of immediate power savings, appearance, packaging is very delicate, very satisfied, I hope to achieve satisfactory results!	Jingdong prefecture level city in Shandong
Happy Fan Shop	Green Intelligent Power Saver	The power saving effect is very good, is considered an old customer, also bought before, this time is to buy two for mom and dad, customer service attitude is good, praise!	Jingdong prefecture level city in Shandong
Happy Fan Shop	Green Intelligent Power Saver	After observing for a while, it really saves a lot of money I didn't realize it was still really reliable, so I'll give you a good review.	Jingdong prefecture level city in Shandong
Happy Fan Shop	Green Intelligent Power Saver	My family a month with this monthly almost save more than 200, power-saving effect: have bought a second time, really effective Cost-effective: not bad Appearance style: roughly the same A few months to the home electricity is indeed less, this time it is a re-buy three, send a friend two, they stay a	Jingdong prefecture level city in Shandong
Happy Fan Shop	Green Intelligent Power Saver	A month almost save more than 150 dollars in electricity, home appliances more, the main this can also protect high-power appliances, after using this device, these two months have not burned, things are very good, it is said that this is the latest model, the overall price is cheap and beautiful!	Jingdong prefecture level city in Shandong
Happy Fan Shop	Green Intelligent Power Saver	Received, powerful, great quality, love the style😍	Jingdong prefecture level city in Shandong
Happy Fan Shop	Green Intelligent Power Saver	This intelligent energy saver is especially good to use and save electricity hope everyone will buy it!	Jingdong prefecture level city in Shandong
Happy Fan Shop	Green Intelligent Power Saver	Air conditioning adapter effect is particularly great, the use of very simple, plug into the socket on the summer air conditioning power is too large, electricity is also expensive, with this product directly on the electricity bill to save half, has been purchased a few more, the effect is too awesome!	Jingdong prefecture level city in Shandong
Happy Fan Shop	Green Intelligent Power Saver	The family plugged in 5, monthly electricity savings of about 300 yuan. My family before a month of electricity costs more than 800, this time with this saver after the electricity bill is actually only more than 500, amazing, thanks to the store, will recommend other friends to buy!	Jingdong prefecture level city in Shandong
Happy Fan Shop	Green Intelligent Power Saver	It was recommended to me by a neighbor's family and it's nice to use it to save power.	Jingdong prefecture level city in Shandong
Happy Fan Shop	Green Intelligent Power Saver	Second time I've purchased it and the power saving is effective!	Jingdong prefecture level city in Shandong
Happy Fan Shop	Green Intelligent Power Saver	The effect is really good, before a month of more than 300 electricity, now every month every day with the air conditioning, it is more than 100 electricity! This thing is really amazing, one after another to buy and 5.6, home installed 3!	Jingdong prefecture level city in Shandong
Happy Fan Shop	Green Intelligent Power Saver	Received this product, began to use, small things look very wrong, can save a little electricity, the monthly electricity bill is too high, after plugging in did not pull, or save a lot of electricity, point	Jingdong prefecture level city in Shandong
Happy Fan Shop	Green Intelligent Power Saver	Simple and convenient, just that plug in, you can save electricity. A month can save dozens of dollars, a year down to save a lot of, very good product cost-effective, everyone can afford to buy and use!	Jingdong prefecture level city in Shandong
Happy Fan Shop	Green Intelligent Power Saver	Electricity bill from more than 200 yuan per month, down to more than 150 yuan, the original bought home, used two months, really have power-saving effect, this time and sold two, business service attitude is	Jingdong prefecture level city in Shandong
Happy Fan Shop	Green Intelligent Power Saver	Like to watch some movies at home and so on, sometimes at night until two or three o'clock, and recently the air conditioning is not turned off for a night, rather wasteful, I have to say that the effect is very good, quite like it!	Jingdong prefecture level city in Shandong
Happy Fan Shop	Green Intelligent Power Saver	Power-saving effect: the effect is very good, a month to save more than 200 dollars Cost-effective: good value for money Appearance and style: small and lovely Workmanship material: okay Experience: good	Jingdong prefecture level city in Shandong
Happy Fan Shop	Green Intelligent Power Saver	It's okay I quite like it, the stuff is very good. Come back in the future. You can rest assured to buy. Cost-effective all. Much cheaper than the physical store	Jingdong prefecture level city in Shandong
Happy Fan Shop	Green Intelligent Power Saver	More than 150 less, last month's electricity bill is almost 400, this month is only about 200, really save electricity, I am home to buy three, I did not expect a month to pay back	Jingdong prefecture level city in Shandong
Happy Fan Shop	Green Intelligent Power Saver	I bought three of these first and tried them for two months and they still felt useful, so now I've bought another one. Good and recommended!	Jingdong prefecture level city in Shandong
Happy Fan Shop	Green Intelligent Power Saver	This energy saver is very good quality and inexpensive next time I will buy it again!	Jingdong prefecture level city in Shandong
Happy Fan Shop	Green Intelligent Power Saver	Merchant service attitude is not good, the quality does not match, the business does not give the package return	Jingdong prefecture level city in Shandong
Happy Fan Shop	Green Intelligent Power Saver	The goods are very small do not know how effective after a few days to follow up the review	Jingdong prefecture level city in Shandong
Happy Fan Shop	Green Intelligent Power Saver	Not used before more than 200 a month, after using about 100 a month, my family used three, customer service said I use electricity recommended 2-3, I think more than one effect is better on the purchase of 4, the effect is very obvious, recommended to buy!	Jingdong prefecture level city in Shandong
Happy Fan Shop	Green Intelligent Power Saver	It's okay. I'll buy it again today.	Jingdong prefecture level city in Shandong
Happy Fan Shop	Green Intelligent Power Saver	Finally received the goods, very, very much like,, has been a lot of people praise, really too happy, has been his family's loyal fans, will continue to buy buy buy buy!	Jingdong prefecture level city in Shandong
Happy Fan Shop	Green Intelligent Power Saver	After using this, my monthly electricity bill was 1,200 pounds, now it's only 180 bucks a month, how good is it?	Jingdong prefecture level city in Shandong
Happy Fan Shop	Green Intelligent Power Saver	The item is a scam return to marry a dozen dollars in shipping fees. Buy terrific role is purely a scam.	Jingdong prefecture level city in Shandong
Happy Fan Shop	Green Intelligent Power Saver	It's really good, cost-effective and good quality, and the logistics are fast! It arrived in one day!	Jingdong prefecture level city in Shandong
Happy Fan Shop	Green Intelligent Power Saver	Great brand! Trustworthy! Works super well! Expensive for a reason! Great merchandise!	Jingdong prefecture level city in Shandong
Happy Fan Shop	Green Intelligent Power Saver	The baby is really great, I like it very much, my friends also say good to come, recommended to buy!	Jingdong prefecture level city in Shandong
Happy Fan Shop	Green Intelligent Power Saver	Fake couldn't be more fake. Bought one plugged in still so much electricity.	Jingdong prefecture level city in Shandong
Happy Fan Shop	Green Intelligent Power Saver	This appliance works great. Let's see how it works. We'll see later.	Jingdong prefecture level city in Shandong
Happy Fan Shop	Green Intelligent Power Saver	Used it before commenting, very happy with it	Jingdong prefecture level city in Shandong
Happy Fan Shop	Green Intelligent Power Saver	Goods have been received well-packed, logistics is very fast and good quality	Jingdong prefecture level city in Shandong
Happy Fan Shop	Green Intelligent Power Saver	This baby's quality is okay.	Jingdong prefecture level city in Shandong
Happy Fan Shop	Green Intelligent Power Saver	Counterfeit goods.	Jingdong prefecture level city in Shandong
Happy Fan Shop	Green Intelligent Power Saver	The effect is not very obvious	Jingdong prefecture level city in Shandong
Happy Fan Shop	Green Intelligent Power Saver	It's not the same thing.	Jingdong prefecture level city in Shandong
Happy Fan Shop	Green Intelligent Power Saver	There is no load how much w seems false, nothing works.	Jingdong prefecture level city in Shandong
Happy Fan Shop	Green Intelligent Power Saver	I received the item yesterday, got it back and plugged it in, and it used a little more power yesterday than I had before	Jingdong prefecture level city in Shandong
Happy Fan Shop	Green Intelligent Power Saver	Haven't seen the power saving mode yet	Jingdong prefecture level city in Shandong
Happy Fan Shop	Green Intelligent Power Saver	This product has no known effect	Jingdong prefecture level city in Shandong

Happy Fan Shop	Green Intelligent Power Saver	Refund Returns $16	Jingdong prefecture level city in Shandong
Happy Fan Shop	Green Intelligent Power Saver	The previous year bought a, almost every month can save a 70 dollars or so, used a year, no problem at all, look very new like, this time to move to a new home decisively repurchase 2 units, very believe this	Jingdong prefecture level city in Shandong
Happy Fan Shop	Green Intelligent Power Saver	Has been the second time to buy, this power saving device is really good power, before every month to spend 300 electricity, now are about 200, last time that 6, this time to help lead the family to buy 6,	Jingdong prefecture level city in Shandong
Happy Fan Shop	Green Intelligent Power Saver	In the spirit of trying to experience the new product, the use of down compared to the usual power consumption, with the addition of the quarter, is indeed a lot less electricity, originally more than 450	Jingdong prefecture level city in Shandong
Happy Fan Shop	Green Intelligent Power Saver	Has not been the first time to buy back, good use, power saving device is really powerful, before the monthly electricity bill of more than 500 Now more than 300, this time to help neighbors to buy several	Jingdong prefecture level city in Shandong
Happy Fan Shop	Green Intelligent Power Saver	Home appliances are very many, the circuit is also very complex, at first it is to save 9% of the electricity, and then used more than half a month, found that the power saving rate is as high as more	Jingdong prefecture level city in Shandong
Happy Fan Shop	Green Intelligent Power Saver	My friend bought it last year and saved hundreds of dollars on his electricity bill in one summer Appearance and style: it looks better than the one I bought before Quality is also good with a light.	Jingdong prefecture level city in Shandong
Happy Fan Shop	Green Intelligent Power Saver	Saver received, on the spot on the plug with the feeling of immediate power savings, appearance, packaging is very delicate, very satisfied, I hope to achieve satisfactory results!	Jingdong prefecture level city in Shandong
Happy Fan Shop	Green Intelligent Power Saver	The power saving effect is very good, is considered an old customer, also bought before, this time is to buy two for mom and dad, customer service attitude is good, praise!	Jingdong prefecture level city in Shandong
Happy Fan Shop	Green Intelligent Power Saver	After observing for a while, it really saves a lot of money I didn't realize it was still really reliable, so I'll give you a good review.	Jingdong prefecture level city in Shandong
Happy Fan Shop	Green Intelligent Power Saver	My family a month with this monthly almost save more than 200, power-saving effect: have bought a second time, really effective Cost-effective: not bad Appearance style: roughly the same A few months to the home electricity is indeed less, this time it is a re-buy three, send a friend two, they stay a	Jingdong prefecture level city in Shandong
Happy Fan Shop	Green Intelligent Power Saver	A month almost save more than 150 dollars in electricity, home appliances more, the main this can also protect high-power appliances, after using this device, these two months have not burned, things are very good, it is said that this is the latest model, the overall price is cheap and beautiful!	Jingdong prefecture level city in Shandong
Happy Fan Shop	Green Intelligent Power Saver	Received, powerful, great quality, love the style😍	Jingdong prefecture level city in Shandong
Happy Fan Shop	Green Intelligent Power Saver	This intelligent energy saver is especially good to use and save electricity hope everyone will buy it!	Jingdong prefecture level city in Shandong
Happy Fan Shop	Green Intelligent Power Saver	Air conditioning adapter effect is particularly great, the use of very simple, plug into the socket on the summer air conditioning power is too large, electricity is also expensive, with this product directly on the electricity bill to save half, has been purchased a few more, the effect is too awesome!	Jingdong prefecture level city in Shandong
Happy Fan Shop	Green Intelligent Power Saver	The family plugged in 5, monthly electricity savings of about 300 yuan. My family before a month of electricity costs more than 800, this time with this saver after the electricity bill is actually only more than 500, amazing, thanks to the store, will recommend other friends to buy!	Jingdong prefecture level city in Shandong
Happy Fan Shop	Green Intelligent Power Saver	It was recommended to me by a neighbor's family and it's nice to use it to save power.	Jingdong prefecture level city in Shandong
Happy Fan Shop	Green Intelligent Power Saver	Second time I've purchased it and the power saving is effective!	Jingdong prefecture level city in Shandong
Happy Fan Shop	Green Intelligent Power Saver	The effect is really good, before a month of more than 300 electricity, now every month every day with the air conditioning, it is more than 100 electricity! This thing is really amazing, one after another to buy and 5.6, home installed 3!	Jingdong prefecture level city in Shandong
Happy Fan Shop	Green Intelligent Power Saver	Received this product, began to use, small things look very wrong, can save a little electricity, the monthly electricity bill is too high, after plugging in did not pull, or save a lot of electricity, point	Jingdong prefecture level city in Shandong
Happy Fan Shop	Green Intelligent Power Saver	Simple and convenient, just that plug in, you can save electricity. A month can save dozens of dollars, a year down to save a lot of, very good product cost-effective, everyone can afford to buy and use!	Jingdong prefecture level city in Shandong
Happy Fan Shop	Green Intelligent Power Saver	Electricity bill from more than 200 yuan per month, down to more than 150 yuan, the original bought home, used two months, really have power-saving effect, this time and sold two, business service attitude is	Jingdong prefecture level city in Shandong
Happy Fan Shop	Green Intelligent Power Saver	Like to watch some movies at home and so on, sometimes at night until two or three o'clock, and recently the air conditioning is not turned off for a night, rather wasteful, I have to say that the effect is very good, quite like it!	Jingdong prefecture level city in Shandong
Happy Fan Shop	Green Intelligent Power Saver	Power-saving effect: the effect is very good, a month to save more than 200 dollars Cost-effective: good value for money Appearance and style: small and lovely Workmanship material: okay Experience: good	Jingdong prefecture level city in Shandong
Happy Fan Shop	Green Intelligent Power Saver	It's okay I quite like it, the stuff is very good. Come back in the future. You can rest assured to buy. Cost-effective all. Much cheaper than the physical store	Jingdong prefecture level city in Shandong
Happy Fan Shop	Green Intelligent Power Saver	More than 150 less, last month's electricity bill is almost 400, this month is only about 200, really save electricity, I am home to buy three, I did not expect a month to pay back	Jingdong prefecture level city in Shandong
Happy Fan Shop	Green Intelligent Power Saver	I bought three of these first and tried them for two months and they still felt useful, so now I've bought another one. Good and recommended!	Jingdong prefecture level city in Shandong
Happy Fan Shop	Green Intelligent Power Saver	This energy saver is very good quality and inexpensive next time I will buy it again!	Jingdong prefecture level city in Shandong
Happy Fan Shop	Green Intelligent Power Saver	Merchant service attitude is not good, the quality does not match, the business does not give the package return	Jingdong prefecture level city in Shandong
Happy Fan Shop	Green Intelligent Power Saver	The goods are very small do not know how effective after a few days to follow up the review	Jingdong prefecture level city in Shandong
Happy Fan Shop	Green Intelligent Power Saver	Not used before more than 200 a month, after using about 100 a month, my family used three, customer service said I use electricity recommended 2-3, I think more than one effect is better on the purchase of 4, the effect is very obvious, recommended to buy!	Jingdong prefecture level city in Shandong
Happy Fan Shop	Green Intelligent Power Saver	It's okay. I'll buy it again today.	Jingdong prefecture level city in Shandong
Happy Fan Shop	Green Intelligent Power Saver	Finally received the goods, very, very much like,, has been a lot of people praise, really too happy, has been his family's loyal fans, will continue to buy buy buy buy!	Jingdong prefecture level city in Shandong
Happy Fan Shop	Green Intelligent Power Saver	After using this, my monthly electricity bill was 1,200 pounds, now it's only 180 bucks a month, how good is it?	Jingdong prefecture level city in Shandong
Happy Fan Shop	Green Intelligent Power Saver	The item is a scam return to marry a dozen dollars in shipping fees. Buy terrific role is purely a scam.	Jingdong prefecture level city in Shandong
Happy Fan Shop	Green Intelligent Power Saver	It's really good, cost-effective and good quality, and the logistics are fast! It arrived in one day!	Jingdong prefecture level city in Shandong
Happy Fan Shop	Green Intelligent Power Saver	Great brand! Trustworthy! Works super well! Expensive for a reason! Great merchandise!	Jingdong prefecture level city in Shandong
Happy Fan Shop	Green Intelligent Power Saver	The baby is really great, I like it very much, my friends also say good to come, recommended to buy!	Jingdong prefecture level city in Shandong
Happy Fan Shop	Green Intelligent Power Saver	Fake couldn't be more fake. Bought one plugged in still so much electricity.	Jingdong prefecture level city in Shandong
Happy Fan Shop	Green Intelligent Power Saver	This appliance works great. Let's see how it works. We'll see later.	Jingdong prefecture level city in Shandong
Happy Fan Shop	Green Intelligent Power Saver	Used it before commenting, very happy with it	Jingdong prefecture level city in Shandong
Happy Fan Shop	Green Intelligent Power Saver	Goods have been received well-packed, logistics is very fast and good quality	Jingdong prefecture level city in Shandong
Happy Fan Shop	Green Intelligent Power Saver	This baby's quality is okay.	Jingdong prefecture level city in Shandong
Happy Fan Shop	Green Intelligent Power Saver	Counterfeit goods.	Jingdong prefecture level city in Shandong
Happy Fan Shop	Green Intelligent Power Saver	The effect is not very obvious	Jingdong prefecture level city in Shandong
Happy Fan Shop	Green Intelligent Power Saver	It's not the same thing.	Jingdong prefecture level city in Shandong
Happy Fan Shop	Green Intelligent Power Saver	There is no load how much w seems false, nothing works.	Jingdong prefecture level city in Shandong
Happy Fan Shop	Green Intelligent Power Saver	I received the item yesterday, got it back and plugged it in, and it used a little more power yesterday than I had before	Jingdong prefecture level city in Shandong

Happy Fan Shop	Green Intelligent Power Saver	Haven't seen the power saving mode yet	Jingdong prefecture level city in Shandong
Happy Fan Shop	Green Intelligent Power Saver	This product has no known effect	Jingdong prefecture level city in Shandong
Happy Fan Shop	Green Intelligent Power Saver	Refund Returns $16	Jingdong prefecture level city in Shandong
Happy Fan Shop	Green Intelligent Power Saver	The previous year bought a, almost every month can save a 70 dollars or so, used a year, no problem at all, look very new like, this time to move to a new home decisively repurchase 2 units, very believe this	Jingdong prefecture level city in Shandong
Happy Fan Shop	Green Intelligent Power Saver	Has been the second time to buy, this power saving device is really good power, before every month to spend 300 electricity, now are about 200, last time that 6, this time to help lead the family to buy 6,	Jingdong prefecture level city in Shandong
Happy Fan Shop	Green Intelligent Power Saver	In the spirit of trying to experience the new product, the use of down compared to the usual power consumption, with the addition of the quarter, is indeed a lot less electricity, originally more than 450	Jingdong prefecture level city in Shandong
Happy Fan Shop	Green Intelligent Power Saver	Has not been the first time to buy back, good use, power saving device is really powerful, before the monthly electricity bill of more than 500 Now more than 300, this time to help neighbors to buy several	Jingdong prefecture level city in Shandong
Happy Fan Shop	Green Intelligent Power Saver	Home appliances are very many, the circuit is also very complex, at first it is to save 9% of the electricity, and then used more than half a month, found that the power saving rate is as high as more	Jingdong prefecture level city in Shandong
Happy Fan Shop	Green Intelligent Power Saver	My friend bought it last year and saved hundreds of dollars on his electricity bill in one summer Appearance and style: it looks better than the one I bought before Quality is also good with a light.	Jingdong prefecture level city in Shandong
Happy Fan Shop	Green Intelligent Power Saver	Saver received, on the spot on the plug with the feeling of immediate power savings, appearance, packaging is very delicate, very satisfied, I hope to achieve satisfactory results!	Jingdong prefecture level city in Shandong
Happy Fan Shop	Green Intelligent Power Saver	The power saving effect is very good, is considered an old customer, also bought before, this time is to buy two for mom and dad, customer service attitude is good, praise!	Jingdong prefecture level city in Shandong
Happy Fan Shop	Green Intelligent Power Saver	After observing for a while, it really saves a lot of money I didn't realize it was still really reliable, so I'll give you a good review.	Jingdong prefecture level city in Shandong
Happy Fan Shop	Green Intelligent Power Saver	My family a month with this monthly almost save more than 200, power-saving effect: have bought a second time, really effective Cost-effective: not bad Appearance style: roughly the same A few months to the home electricity is indeed less, this time it is a re-buy three, send a friend two, they stay a	Jingdong prefecture level city in Shandong
Happy Fan Shop	Green Intelligent Power Saver	A month almost save more than 150 dollars in electricity, home appliances more, the main this can also protect high-power appliances, after using this device, these two months have not burned, things are very good, it is said that this is the latest model, the overall price is cheap and beautiful!	Jingdong prefecture level city in Shandong
Happy Fan Shop	Green Intelligent Power Saver	Received, powerful, great quality, love the style😍	Jingdong prefecture level city in Shandong
Happy Fan Shop	Green Intelligent Power Saver	This intelligent energy saver is especially good to use and save electricity hope everyone will buy it!	Jingdong prefecture level city in Shandong
Happy Fan Shop	Green Intelligent Power Saver	Air conditioning adapter effect is particularly great, the use of very simple, plug into the socket on the summer air conditioning power is too large, electricity is also expensive, with this product directly on the electricity bill to save half, has been purchased a few more, the effect is too awesome!	Jingdong prefecture level city in Shandong
Happy Fan Shop	Green Intelligent Power Saver	The family plugged in 5, monthly electricity savings of about 300 yuan. My family before a month of electricity costs more than 800, this time with this saver after the electricity bill is actually only more than 500, amazing, thanks to the store, will recommend other friends to buy!	Jingdong prefecture level city in Shandong
Happy Fan Shop	Green Intelligent Power Saver	It was recommended to me by a neighbor's family and it's nice to use it to save power.	Jingdong prefecture level city in Shandong
Happy Fan Shop	Green Intelligent Power Saver	Second time I've purchased it and the power saving is effective!	Jingdong prefecture level city in Shandong
Happy Fan Shop	Green Intelligent Power Saver	The effect is really good, before a month of more than 300 electricity, now every month every day with the air conditioning, it is more than 100 electricity! This thing is really amazing, one after another to buy and 5.6, home installed 3!	Jingdong prefecture level city in Shandong
Happy Fan Shop	Green Intelligent Power Saver	Received this product, began to use, small things look very wrong, can save a little electricity, the monthly electricity bill is too high, after plugging in did not pull, or save a lot of electricity, point	Jingdong prefecture level city in Shandong
Happy Fan Shop	Green Intelligent Power Saver	Simple and convenient, just that plug in, you can save electricity. A month can save dozens of dollars, a year down to save a lot of, very good product cost-effective, everyone can afford to buy and use!	Jingdong prefecture level city in Shandong
Happy Fan Shop	Green Intelligent Power Saver	Electricity bill from more than 200 yuan per month, down to more than 150 yuan, the original bought home, used two months, really have power-saving effect, this time and sold two, business service attitude is	Jingdong prefecture level city in Shandong
Happy Fan Shop	Green Intelligent Power Saver	Like to watch some movies at home and so on, sometimes at night until two or three o'clock, and recently the air conditioning is not turned off for a night, rather wasteful, I have to say that the effect is very good, quite like it!	Jingdong prefecture level city in Shandong
Happy Fan Shop	Green Intelligent Power Saver	Power-saving effect: the effect is very good, a month to save more than 200 dollars Cost-effective: good value for money Appearance and style: small and lovely Workmanship material: okay Experience: good	Jingdong prefecture level city in Shandong
Happy Fan Shop	Green Intelligent Power Saver	It's okay I quite like it, the stuff is very good. Come back in the future. You can rest assured to buy. Cost-effective all. Much cheaper than the physical store	Jingdong prefecture level city in Shandong
Happy Fan Shop	Green Intelligent Power Saver	More than 150 less, last month's electricity bill is almost 400, this month is only about 200, really save electricity, I am home to buy three, I did not expect a month to pay back	Jingdong prefecture level city in Shandong
Happy Fan Shop	Green Intelligent Power Saver	I bought three of these first and tried them for two months and they still felt useful, so now I've bought another one. Good and recommended!	Jingdong prefecture level city in Shandong
Happy Fan Shop	Green Intelligent Power Saver	This energy saver is very good quality and inexpensive next time I will buy it again!	Jingdong prefecture level city in Shandong
Happy Fan Shop	Green Intelligent Power Saver	Merchant service attitude is not good, the quality does not match, the business does not give the package return	Jingdong prefecture level city in Shandong
Happy Fan Shop	Green Intelligent Power Saver	The goods are very small do not know how effective after a few days to follow up the review	Jingdong prefecture level city in Shandong
Happy Fan Shop	Green Intelligent Power Saver	Not used before more than 200 a month, after using about 100 a month, my family used three, customer service said I use electricity recommended 2-3, I think more than one effect is better on the purchase of 4, the effect is very obvious, recommended to buy!	Jingdong prefecture level city in Shandong
Happy Fan Shop	Green Intelligent Power Saver	It's okay. I'll buy it again today.	Jingdong prefecture level city in Shandong
Happy Fan Shop	Green Intelligent Power Saver	Finally received the goods, very, very much like,, has been a lot of people praise, really too happy, has been his family's loyal fans, will continue to buy buy buy!	Jingdong prefecture level city in Shandong
Happy Fan Shop	Green Intelligent Power Saver	After using this, my monthly electricity bill was 1,200 pounds, now it's only 180 bucks a month, how good is it?	Jingdong prefecture level city in Shandong
Happy Fan Shop	Green Intelligent Power Saver	The item is a scam return to marry a dozen dollars in shipping fees. Buy terrific role is purely a scam.	Jingdong prefecture level city in Shandong
Happy Fan Shop	Green Intelligent Power Saver	It's really good, cost-effective and good quality, and the logistics are fast! It arrived in one day!	Jingdong prefecture level city in Shandong
Happy Fan Shop	Green Intelligent Power Saver	Great brand! Trustworthy! Works super well! Expensive for a reason! Great merchandise!	Jingdong prefecture level city in Shandong
Happy Fan Shop	Green Intelligent Power Saver	The baby is really great, I like it very much, my friends also say good to come, recommended to buy!	Jingdong prefecture level city in Shandong
Happy Fan Shop	Green Intelligent Power Saver	Fake couldn't be more fake. Bought one plugged in still so much electricity.	Jingdong prefecture level city in Shandong
Happy Fan Shop	Green Intelligent Power Saver	This appliance works great. Let's see how it works. We'll see later.	Jingdong prefecture level city in Shandong
Happy Fan Shop	Green Intelligent Power Saver	Used it before commenting, very happy with it	Jingdong prefecture level city in Shandong
Happy Fan Shop	Green Intelligent Power Saver	Goods have been received well-packed, logistics is very fast and good quality	Jingdong prefecture level city in Shandong
Happy Fan Shop	Green Intelligent Power Saver	This baby's quality is okay.	Jingdong prefecture level city in Shandong
Happy Fan Shop	Green Intelligent Power Saver	Counterfeit goods.	Jingdong prefecture level city in Shandong
Happy Fan Shop	Green Intelligent Power Saver	The effect is not very obvious	Jingdong prefecture level city in Shandong
Happy Fan Shop	Green Intelligent Power Saver	It's not the same thing.	Jingdong prefecture level city in Shandong

Happy Fan Shop	Green Intelligent Power Saver	There is no load how much w seems false, nothing works.	Jingdong prefecture level city in Shandong
Happy Fan Shop	Green Intelligent Power Saver	I received the item yesterday, got it back and plugged it in, and it used a little more power yesterday than I had before	Jingdong prefecture level city in Shandong
Happy Fan Shop	Green Intelligent Power Saver	Haven't seen the power saving mode yet	Jingdong prefecture level city in Shandong
Happy Fan Shop	Green Intelligent Power Saver	This product has no known effect	Jingdong prefecture level city in Shandong
Happy Fan Shop	Green Intelligent Power Saver	Refund Returns $16	Jingdong prefecture level city in Shandong
Happy Fan Shop	Green Intelligent Power Saver	The previous year bought a, almost every month can save a 70 dollars or so, used a year, no problem at all, look very new like, this time to move to a new home decisively repurchase 2 units, very believe this	Jingdong prefecture level city in Shandong
Happy Fan Shop	Green Intelligent Power Saver	Has been the second time to buy, this power saving device is really good power, before every month to spend 300 electricity, now are about 200, last time that 6, this time to help lead the family to buy 6,	Jingdong prefecture level city in Shandong
Happy Fan Shop	Green Intelligent Power Saver	In the spirit of trying to experience the new product, the use of down compared to the usual power consumption, with the addition of the quarter, is indeed a lot less electricity, originally more than 450	Jingdong prefecture level city in Shandong
Happy Fan Shop	Green Intelligent Power Saver	Has not been the first time to buy back, good use, power saving device is really powerful, before the monthly electricity bill of more than 500 Now more than 300, this time to help neighbors to buy several	Jingdong prefecture level city in Shandong
Happy Fan Shop	Green Intelligent Power Saver	Home appliances are very many, the circuit is also very complex, at first it is to save 9% of the electricity, and then used more than half a month, found that the power saving rate is as high as more	Jingdong prefecture level city in Shandong
Happy Fan Shop	Green Intelligent Power Saver	My friend bought it last year and saved hundreds of dollars on his electricity bill in one summer Appearance and style: it looks better than the one I bought before Quality is also good with a light.	Jingdong prefecture level city in Shandong
Happy Fan Shop	Green Intelligent Power Saver	Saver received, on the spot on the plug with the feeling of immediate power savings, appearance, packaging is very delicate, very satisfied, I hope to achieve satisfactory results!	Jingdong prefecture level city in Shandong
Happy Fan Shop	Green Intelligent Power Saver	The power saving effect is very good, is considered an old customer, also bought before, this time is to buy two for mom and dad, customer service attitude is good, praise!	Jingdong prefecture level city in Shandong
Happy Fan Shop	Green Intelligent Power Saver	After observing for a while, it really saves a lot of money I didn't realize it was still really reliable, so I'll give you a good review.	Jingdong prefecture level city in Shandong
Happy Fan Shop	Green Intelligent Power Saver	My family a month with this monthly almost save more than 200, power-saving effect: have bought a second time, really effective Cost-effective: not bad Appearance style: roughly the same A few months to the home electricity is indeed less, this time it is a re-buy three, send a friend two, they stay a	Jingdong prefecture level city in Shandong
Happy Fan Shop	Green Intelligent Power Saver	A month almost save more than 150 dollars in electricity, home appliances more, the main this can also protect high-power appliances, after using this device, these two months have not burned, things are very good, it is said that this is the latest model, the overall price is cheap and beautiful!	Jingdong prefecture level city in Shandong
Happy Fan Shop	Green Intelligent Power Saver	Received, powerful, great quality, love the style😍	Jingdong prefecture level city in Shandong
Happy Fan Shop	Green Intelligent Power Saver	This intelligent energy saver is especially good to use and save electricity hope everyone will buy it!	Jingdong prefecture level city in Shandong
Happy Fan Shop	Green Intelligent Power Saver	Air conditioning adapter effect is particularly great, the use of very simple, plug into the socket on the summer air conditioning power is too large, electricity is also expensive, with this product directly on the electricity bill to save half, has been purchased a few more, the effect is too awesome!	Jingdong prefecture level city in Shandong
Happy Fan Shop	Green Intelligent Power Saver	The family plugged in 5, monthly electricity savings of about 300 yuan. My family before a month of electricity costs more than 800, this time with this saver after the electricity bill is actually only more than 500, amazing, thanks to the store, will recommend other friends to buy!	Jingdong prefecture level city in Shandong
Happy Fan Shop	Green Intelligent Power Saver	It was recommended to me by a neighbor's family and it's nice to use it to save power.	Jingdong prefecture level city in Shandong
Happy Fan Shop	Green Intelligent Power Saver	Second time I've purchased it and the power saving is effective!	Jingdong prefecture level city in Shandong
Happy Fan Shop	Green Intelligent Power Saver	The effect is really good, before a month of more than 300 electricity, now every month every day with the air conditioning, it is more than 100 electricity! This thing is really amazing, one after another to buy and 5.6, home installed 3!	Jingdong prefecture level city in Shandong
Happy Fan Shop	Green Intelligent Power Saver	Received this product, began to use, small things look very wrong, can save a little electricity, the monthly electricity bill is too high, after plugging in did not pull, or save a lot of electricity, point	Jingdong prefecture level city in Shandong
Happy Fan Shop	Green Intelligent Power Saver	Simple and convenient, just that plug in, you can save electricity. A month can save dozens of dollars, a year down to save a lot of, very good product cost-effective, everyone can afford to buy and use!	Jingdong prefecture level city in Shandong
Happy Fan Shop	Green Intelligent Power Saver	Electricity bill from more than 200 yuan per month, down to more than 150 yuan, the original bought home, used two months, really have power-saving effect, this time and sold two, business service attitude is	Jingdong prefecture level city in Shandong
Happy Fan Shop	Green Intelligent Power Saver	Like to watch some movies at home and so on, sometimes at night until two or three o'clock, and recently the air conditioning is not turned off for a night, rather wasteful, I have to say that the effect is very good, quite like it!	Jingdong prefecture level city in Shandong
Happy Fan Shop	Green Intelligent Power Saver	Power-saving effect: the effect is very good, a month to save more than 200 dollars Cost-effective: good value for money Appearance and style: small and lovely Workmanship material: okay Experience: good	Jingdong prefecture level city in Shandong
Happy Fan Shop	Green Intelligent Power Saver	It's okay I quite like it, the stuff is very good. Come back in the future. You can rest assured to buy. Cost-effective all. Much cheaper than the physical store	Jingdong prefecture level city in Shandong
Happy Fan Shop	Green Intelligent Power Saver	More than 150 less, last month's electricity bill is almost 400, this month is only about 200, really save electricity, I am home to buy three, I did not expect a month to pay back	Jingdong prefecture level city in Shandong
Happy Fan Shop	Green Intelligent Power Saver	I bought three of these first and tried them for two months and they still felt useful, so now I've bought another one. Good and recommended!	Jingdong prefecture level city in Shandong
Happy Fan Shop	Green Intelligent Power Saver	This energy saver is very good quality and inexpensive next time I will buy it again!	Jingdong prefecture level city in Shandong
Happy Fan Shop	Green Intelligent Power Saver	Merchant service attitude is not good, the quality does not match, the business does not give the package return	Jingdong prefecture level city in Shandong
Happy Fan Shop	Green Intelligent Power Saver	The goods are very small do not know how effective after a few days to follow up the review	Jingdong prefecture level city in Shandong
Happy Fan Shop	Green Intelligent Power Saver	Not used before more than 200 a month, after using about 100 a month, my family used three, customer service said I use electricity recommended 2-3, I think more than one effect is better on the purchase of 4, the effect is very obvious, recommended to buy!	Jingdong prefecture level city in Shandong
Happy Fan Shop	Green Intelligent Power Saver	It's okay. I'll buy it again today.	Jingdong prefecture level city in Shandong
Happy Fan Shop	Green Intelligent Power Saver	Finally received the goods, very, very much like,, has been a lot of people praise, really too happy, has been his family's loyal fans, will continue to buy buy buy buy!	Jingdong prefecture level city in Shandong
Happy Fan Shop	Green Intelligent Power Saver	After using this, my monthly electricity bill was 1,200 pounds, now it's only 180 bucks a month, how good is it?	Jingdong prefecture level city in Shandong
Happy Fan Shop	Green Intelligent Power Saver	The item is a scam return to marry a dozen dollars in shipping fees. Buy terrific role is purely a scam.	Jingdong prefecture level city in Shandong
Happy Fan Shop	Green Intelligent Power Saver	It's really good, cost-effective and good quality, and the logistics are fast! It arrived in one day!	Jingdong prefecture level city in Shandong
Happy Fan Shop	Green Intelligent Power Saver	Great brand! Trustworthy! Works super well! Expensive for a reason! Great merchandise!	Jingdong prefecture level city in Shandong
Happy Fan Shop	Green Intelligent Power Saver	The baby is really great, I like it very much, my friends also say good to come, recommended to buy!	Jingdong prefecture level city in Shandong
Happy Fan Shop	Green Intelligent Power Saver	Fake couldn't be more fake. Bought one plugged in still so much electricity.	Jingdong prefecture level city in Shandong
Happy Fan Shop	Green Intelligent Power Saver	This appliance works great. Let's see how it works. We'll see later.	Jingdong prefecture level city in Shandong
Happy Fan Shop	Green Intelligent Power Saver	Used it before commenting, very happy with it	Jingdong prefecture level city in Shandong
Happy Fan Shop	Green Intelligent Power Saver	Goods have been received well-packed, logistics is very fast and good quality	Jingdong prefecture level city in Shandong
Happy Fan Shop	Green Intelligent Power Saver	This baby's quality is okay.	Jingdong prefecture level city in Shandong
Happy Fan Shop	Green Intelligent Power Saver	Counterfeit goods.	Jingdong prefecture level city in Shandong

Happy Fan Shop	Green Intelligent Power Saver	The effect is not very obvious	Jingdong prefecture level city in Shandong
Happy Fan Shop	Green Intelligent Power Saver	It's not the same thing.	Jingdong prefecture level city in Shandong
Happy Fan Shop	Green Intelligent Power Saver	There is no load how much w seems false, nothing works.	Jingdong prefecture level city in Shandong
Happy Fan Shop	Green Intelligent Power Saver	I received the item yesterday, got it back and plugged it in, and it used a little more power yesterday than I had before	Jingdong prefecture level city in Shandong
Happy Fan Shop	Green Intelligent Power Saver	Haven't seen the power saving mode yet	Jingdong prefecture level city in Shandong
Happy Fan Shop	Green Intelligent Power Saver	This product has no known effect	Jingdong prefecture level city in Shandong
Happy Fan Shop	Green Intelligent Power Saver	Refund Returns $16	Jingdong prefecture level city in Shandong
Happy Fan Shop	Green Intelligent Power Saver	The previous year bought a, almost every month can save a 70 dollars or so, used a year, no problem at all, look very new like, this time to move to a new home decisively repurchase 2 units, very believe this	Jingdong prefecture level city in Shandong
Happy Fan Shop	Green Intelligent Power Saver	Has been the second time to buy, this power saving device is really good power, before every month to spend 300 electricity, now are about 200, last time that 6, this time to help lead the family to buy 6,	Jingdong prefecture level city in Shandong
Happy Fan Shop	Green Intelligent Power Saver	In the spirit of trying to experience the new product, the use of down compared to the usual power consumption, with the addition of the quarter, is indeed a lot less electricity, originally more than 450	Jingdong prefecture level city in Shandong
Happy Fan Shop	Green Intelligent Power Saver	Has not been the first time to buy back, good use, power saving device is really powerful, before the monthly electricity bill of more than 500 Now more than 300, this time to help neighbors to buy several	Jingdong prefecture level city in Shandong
Happy Fan Shop	Green Intelligent Power Saver	Home appliances are very many, the circuit is also very complex, at first it is to save 9% of the electricity, and then used more than half a month, found that the power saving rate is as high as more	Jingdong prefecture level city in Shandong
Happy Fan Shop	Green Intelligent Power Saver	My friend bought it last year and saved hundreds of dollars on his electricity bill in one summer Appearance and style: it looks better than the one I bought before Quality is also good with a light.	Jingdong prefecture level city in Shandong
Happy Fan Shop	Green Intelligent Power Saver	Saver received, on the spot on the plug with the feeling of immediate power savings, appearance, packaging is very delicate, very satisfied, I hope to achieve satisfactory results!	Jingdong prefecture level city in Shandong
Happy Fan Shop	Green Intelligent Power Saver	The power saving effect is very good, is considered an old customer, also bought before, this time is to buy two for mom and dad, customer service attitude is good, praise!	Jingdong prefecture level city in Shandong
Happy Fan Shop	Green Intelligent Power Saver	After observing for a while, it really saves a lot of money I didn't realize it was still really reliable, so I'll give you a good review.	Jingdong prefecture level city in Shandong
Happy Fan Shop	Green Intelligent Power Saver	My family a month with this monthly almost save more than 200, power-saving effect: have bought a second time, really effective Cost-effective: not bad Appearance style: roughly the same A few months to the home electricity is indeed less, this time it is a re-buy three, send a friend two, they stay a	Jingdong prefecture level city in Shandong
Happy Fan Shop	Green Intelligent Power Saver	A month almost save more than 150 dollars in electricity, home appliances more, the main this can also protect high-power appliances, after using this device, these two months have not burned, things are very good, it is said that this is the latest model, the overall price is cheap and beautiful!	Jingdong prefecture level city in Shandong
Happy Fan Shop	Green Intelligent Power Saver	Received, powerful, great quality, love the style😍	Jingdong prefecture level city in Shandong
Happy Fan Shop	Green Intelligent Power Saver	This intelligent energy saver is especially good to use and save electricity hope everyone will buy it!	Jingdong prefecture level city in Shandong
Happy Fan Shop	Green Intelligent Power Saver	Air conditioning adapter effect is particularly great, the use of very simple, plug into the socket on the summer air conditioning power is too large, electricity is also expensive, with this product directly on the electricity bill to save half, has been purchased a few more, the effect is too awesome!	Jingdong prefecture level city in Shandong
Happy Fan Shop	Green Intelligent Power Saver	The family plugged in 5, monthly electricity savings of about 300 yuan. My family before a month of electricity costs more than 800, this time with this saver after the electricity bill is actually only more than 500, amazing, thanks to the store, will recommend other friends to buy!	Jingdong prefecture level city in Shandong
Happy Fan Shop	Green Intelligent Power Saver	It was recommended to me by a neighbor's family and it's nice to use it to save power.	Jingdong prefecture level city in Shandong
Happy Fan Shop	Green Intelligent Power Saver	Second time I've purchased it and the power saving is effective!	Jingdong prefecture level city in Shandong
Happy Fan Shop	Green Intelligent Power Saver	The effect is really good, before a month of more than 300 electricity, now every month every day with the air conditioning, it is more than 100 electricity! This thing is really amazing, one after another to buy and 5.6, home installed 3!	Jingdong prefecture level city in Shandong
Happy Fan Shop	Green Intelligent Power Saver	Received this product, began to use, small things look very wrong, can save a little electricity, the monthly electricity bill is too high, after plugging in did not pull, or save a lot of electricity, point	Jingdong prefecture level city in Shandong
Happy Fan Shop	Green Intelligent Power Saver	Simple and convenient, just that plug in, you can save electricity. A month can save dozens of dollars, a year down to save a lot of, very good product cost-effective, everyone can afford to buy and use!	Jingdong prefecture level city in Shandong
Happy Fan Shop	Green Intelligent Power Saver	Electricity bill from more than 200 yuan per month, down to more than 150 yuan, the original bought home, used two months, really have power-saving effect, this time and sold two, business service attitude is	Jingdong prefecture level city in Shandong
Happy Fan Shop	Green Intelligent Power Saver	Like to watch some movies at home and so on, sometimes at night until two or three o'clock, and recently the air conditioning is not turned off for a night, rather wasteful, I have to say that the effect is very good, quite like it!	Jingdong prefecture level city in Shandong
Happy Fan Shop	Green Intelligent Power Saver	Power-saving effect: the effect is very good, a month to save more than 200 dollars Cost-effective: good value for money Appearance and style: small and lovely Workmanship material: okay Experience: good	Jingdong prefecture level city in Shandong
Happy Fan Shop	Green Intelligent Power Saver	It's okay I quite like it, the stuff is very good. Come back in the future. You can rest assured to buy. Cost-effective all. Much cheaper than the physical store	Jingdong prefecture level city in Shandong
Happy Fan Shop	Green Intelligent Power Saver	More than 150 less, last month's electricity bill is almost 400, this month is only about 200, really save electricity, I am home to buy three, I did not expect a month to pay back	Jingdong prefecture level city in Shandong
Happy Fan Shop	Green Intelligent Power Saver	I bought three of these first and tried them for two months and they still felt useful, so now I've bought another one. Good and recommended!	Jingdong prefecture level city in Shandong
Happy Fan Shop	Green Intelligent Power Saver	This energy saver is very good quality and inexpensive next time I will buy it again!	Jingdong prefecture level city in Shandong
Happy Fan Shop	Green Intelligent Power Saver	Merchant service attitude is not good, the quality does not match, the business does not give the package return	Jingdong prefecture level city in Shandong
Happy Fan Shop	Green Intelligent Power Saver	The goods are very small do not know how effective after a few days to follow up the review	Jingdong prefecture level city in Shandong
Happy Fan Shop	Green Intelligent Power Saver	Not used before more than 200 a month, after using about 100 a month, my family used three, customer service said I use electricity recommended 2-3, I think more than one effect is better on the purchase of 4, the effect is very obvious, recommended to buy!	Jingdong prefecture level city in Shandong
Happy Fan Shop	Green Intelligent Power Saver	It's okay. I'll buy it again today.	Jingdong prefecture level city in Shandong
Happy Fan Shop	Green Intelligent Power Saver	Finally received the goods, very, very much like,, has been a lot of people praise, really too happy, has been his family's loyal fans, will continue to buy buy buy!	Jingdong prefecture level city in Shandong
Happy Fan Shop	Green Intelligent Power Saver	After using this, my monthly electricity bill was 1,200 pounds, now it's only 180 bucks a month, how good is it?	Jingdong prefecture level city in Shandong
Happy Fan Shop	Green Intelligent Power Saver	The item is a scam return to marry a dozen dollars in shipping fees. Buy terrific role is purely a scam.	Jingdong prefecture level city in Shandong
Happy Fan Shop	Green Intelligent Power Saver	It's really good, cost-effective and good quality, and the logistics are fast! It arrived in one day!	Jingdong prefecture level city in Shandong
Happy Fan Shop	Green Intelligent Power Saver	Great brand! Trustworthy! Works super well! Expensive for a reason! Great merchandise!	Jingdong prefecture level city in Shandong
Happy Fan Shop	Green Intelligent Power Saver	The baby is really great, I like it very much, my friends also say good to come, recommended to buy!	Jingdong prefecture level city in Shandong
Happy Fan Shop	Green Intelligent Power Saver	Fake couldn't be more fake. Bought one plugged in still so much electricity.	Jingdong prefecture level city in Shandong
Happy Fan Shop	Green Intelligent Power Saver	This appliance works great. Let's see how it works. We'll see later.	Jingdong prefecture level city in Shandong
Happy Fan Shop	Green Intelligent Power Saver	Used it before commenting, very happy with it	Jingdong prefecture level city in Shandong
Happy Fan Shop	Green Intelligent Power Saver	Goods have been received well-packed, logistics is very fast and good quality	Jingdong prefecture level city in Shandong

Happy Fan Shop	Green Intelligent Power Saver	This baby's quality is okay.	Jingdong prefecture level city in Shandong
Happy Fan Shop	Green Intelligent Power Saver	Counterfeit goods.	Jingdong prefecture level city in Shandong
Happy Fan Shop	Green Intelligent Power Saver	The effect is not very obvious	Jingdong prefecture level city in Shandong
Happy Fan Shop	Green Intelligent Power Saver	It's not the same thing.	Jingdong prefecture level city in Shandong
Happy Fan Shop	Green Intelligent Power Saver	There is no load how much w seems false, nothing works.	Jingdong prefecture level city in Shandong
Happy Fan Shop	Green Intelligent Power Saver	I received the item yesterday, got it back and plugged it in, and it used a little more power yesterday than I had before	Jingdong prefecture level city in Shandong
Happy Fan Shop	Green Intelligent Power Saver	This product has no known effect	Jingdong prefecture level city in Shandong
Happy Fan Shop	Green Intelligent Power Saver	Haven't seen the power saving mode yet	Jingdong prefecture level city in Shandong
Happy Fan Shop	Green Intelligent Power Saver	Refund Returns $16	Jingdong prefecture level city in Shandong
Happy Fan Shop	Green Intelligent Power Saver	The previous year bought a, almost every month can save a 70 dollars or so, used a year, no problem at all, look very new like, this time to move to a new home decisively repurchase 2 units, very believe this	Jingdong prefecture level city in Shandong
Happy Fan Shop	Green Intelligent Power Saver	Has been the second time to buy, this power saving device is really good power, before every month to spend 300 electricity, now are about 200, last time that 6, this time to help lead the family to buy 6,	Jingdong prefecture level city in Shandong
Happy Fan Shop	Green Intelligent Power Saver	In the spirit of trying to experience the new product, the use of down compared to the usual power consumption, with the addition of the quarter, is indeed a lot less electricity, originally more than 450	Jingdong prefecture level city in Shandong
Happy Fan Shop	Green Intelligent Power Saver	Has not been the first time to buy back, good use, power saving device is really powerful, before the monthly electricity bill of more than 500 Now more than 300, this time to help neighbors to buy several	Jingdong prefecture level city in Shandong
Happy Fan Shop	Green Intelligent Power Saver	Home appliances are very many, the circuit is also very complex, at first it is to save 9% of the electricity, and then used more than half a month, found that the power saving rate is as high as more	Jingdong prefecture level city in Shandong
Happy Fan Shop	Green Intelligent Power Saver	My friend bought it last year and saved hundreds of dollars on his electricity bill in one summer Appearance and style: it looks better than the one I bought before Quality is also good with a light.	Jingdong prefecture level city in Shandong
Happy Fan Shop	Green Intelligent Power Saver	Saver received, on the spot on the plug with the feeling of immediate power savings, appearance, packaging is very delicate, very satisfied, I hope to achieve satisfactory results!	Jingdong prefecture level city in Shandong
Happy Fan Shop	Green Intelligent Power Saver	The power saving effect is very good, is considered an old customer, also bought before, this time is to buy two for mom and dad, customer service attitude is good, praise!	Jingdong prefecture level city in Shandong
Happy Fan Shop	Green Intelligent Power Saver	After observing for a while, it really saves a lot of money I didn't realize it was still really reliable, so I'll give you a good review.	Jingdong prefecture level city in Shandong
Happy Fan Shop	Green Intelligent Power Saver	My family a month with this monthly almost save more than 200, power-saving effect: have bought a second time, really effective Cost-effective: not bad Appearance style: roughly the same A few months to the home electricity is indeed less, this time it is a re-buy three, send a friend two, they stay a	Jingdong prefecture level city in Shandong
Happy Fan Shop	Green Intelligent Power Saver	A month almost save more than 150 dollars in electricity, home appliances more, the main this can also protect high-power appliances, after using this device, these two months have not burned, things are very good, it is said that this is the latest model, the overall price is cheap and beautiful!	Jingdong prefecture level city in Shandong
Happy Fan Shop	Green Intelligent Power Saver	Received, powerful, great quality, love the style😍	Jingdong prefecture level city in Shandong
Happy Fan Shop	Green Intelligent Power Saver	This intelligent energy saver is especially good to use and save electricity hope everyone will buy it!	Jingdong prefecture level city in Shandong
Happy Fan Shop	Green Intelligent Power Saver	Air conditioning adapter effect is particularly great, the use of very simple, plug into the socket on the summer air conditioning power is too large, electricity is also expensive, with this product directly on the electricity bill to save half, has been purchased a few more, the effect is too awesome!	Jingdong prefecture level city in Shandong
Happy Fan Shop	Green Intelligent Power Saver	The family plugged in 5, monthly electricity savings of about 300 yuan. My family before a month of electricity costs more than 800, this time with this saver after the electricity bill is actually only more than 500, amazing, thanks to the store, will recommend other friends to buy!	Jingdong prefecture level city in Shandong
Happy Fan Shop	Green Intelligent Power Saver	It was recommended to me by a neighbor's family and it's nice to use it to save power.	Jingdong prefecture level city in Shandong
Happy Fan Shop	Green Intelligent Power Saver	Second time I've purchased it and the power saving is effective!	Jingdong prefecture level city in Shandong
Happy Fan Shop	Green Intelligent Power Saver	The effect is really good, before a month of more than 300 electricity, now every month every day with the air conditioning, it is more than 100 electricity! This thing is really amazing, one after another to buy and 5.6, home installed 3!	Jingdong prefecture level city in Shandong
Happy Fan Shop	Green Intelligent Power Saver	Received this product, began to use, small things look very wrong, can save a little electricity, the monthly electricity bill is too high, after plugging in did not pull, or save a lot of electricity, point	Jingdong prefecture level city in Shandong
Happy Fan Shop	Green Intelligent Power Saver	Simple and convenient, just that plug in, you can save electricity. A month can save dozens of dollars, a year down to save a lot of, very good product cost-effective, everyone can afford to buy and use!	Jingdong prefecture level city in Shandong
Happy Fan Shop	Green Intelligent Power Saver	Electricity bill from more than 200 yuan per month, down to more than 150 yuan, the original bought home, used two months, really have power-saving effect, this time and sold two, business service attitude is	Jingdong prefecture level city in Shandong
Happy Fan Shop	Green Intelligent Power Saver	Like to watch some movies at home and so on, sometimes at night until two or three o'clock, and recently the air conditioning is not turned off for a night, rather wasteful, I have to say that the effect is very good, quite like it!	Jingdong prefecture level city in Shandong
Happy Fan Shop	Green Intelligent Power Saver	Power-saving effect: the effect is very good, a month to save more than 200 dollars Cost-effective: good value for money Appearance and style: small and lovely Workmanship material: okay Experience: good	Jingdong prefecture level city in Shandong
Happy Fan Shop	Green Intelligent Power Saver	It's okay I quite like it, the stuff is very good. Come back in the future. You can rest assured to buy. Cost-effective all. Much cheaper than the physical store	Jingdong prefecture level city in Shandong
Happy Fan Shop	Green Intelligent Power Saver	More than 150 less, last month's electricity bill is almost 400, this month is only about 200, really save electricity, I am home to buy three, I did not expect a month to pay back	Jingdong prefecture level city in Shandong
Happy Fan Shop	Green Intelligent Power Saver	I bought three of these first and tried them for two months and they still felt useful, so now I've bought another one. Good and recommended!	Jingdong prefecture level city in Shandong
Happy Fan Shop	Green Intelligent Power Saver	This energy saver is very good quality and inexpensive next time I will buy it again!	Jingdong prefecture level city in Shandong
Happy Fan Shop	Green Intelligent Power Saver	Merchant service attitude is not good, the quality does not match, the business does not give the package return	Jingdong prefecture level city in Shandong
Happy Fan Shop	Green Intelligent Power Saver	The goods are very small do not know how effective after a few days to follow up the review	Jingdong prefecture level city in Shandong
Happy Fan Shop	Green Intelligent Power Saver	Not used before more than 200 a month, after using about 100 a month, my family used three, customer service said I use electricity recommended 2-3, I think more than one effect is better on the purchase of 4, the effect is very obvious, recommended to buy!	Jingdong prefecture level city in Shandong
Happy Fan Shop	Green Intelligent Power Saver	It's okay. I'll buy it again today.	Jingdong prefecture level city in Shandong
Happy Fan Shop	Green Intelligent Power Saver	Finally received the goods, very, very much like,, has been a lot of people praise, really too happy, has been his family's loyal fans, will continue to buy buy buy!	Jingdong prefecture level city in Shandong
Happy Fan Shop	Green Intelligent Power Saver	After using this, my monthly electricity bill was 1,200 pounds, now it's only 180 bucks a month, how good is it?	Jingdong prefecture level city in Shandong
Happy Fan Shop	Green Intelligent Power Saver	The item is a scam return to marry a dozen dollars in shipping fees. Buy terrific role is purely a scam.	Jingdong prefecture level city in Shandong
Happy Fan Shop	Green Intelligent Power Saver	It's really good, cost-effective and good quality, and the logistics are fast! It arrived in one day!	Jingdong prefecture level city in Shandong
Happy Fan Shop	Green Intelligent Power Saver	Great brand! Trustworthy! Works super well! Expensive for a reason! Great merchandise!	Jingdong prefecture level city in Shandong
Happy Fan Shop	Green Intelligent Power Saver	The baby is really great, I like it very much, my friends also say good to come, recommended to buy!	Jingdong prefecture level city in Shandong
Happy Fan Shop	Green Intelligent Power Saver	Fake couldn't be more fake. Bought one plugged in still so much electricity.	Jingdong prefecture level city in Shandong
Happy Fan Shop	Green Intelligent Power Saver	This appliance works great. Let's see how it works. We'll see later.	Jingdong prefecture level city in Shandong

Happy Fan Shop	Green Intelligent Power Saver	Used it before commenting, very happy with it	Jingdong prefecture level city in Shandong
Happy Fan Shop	Green Intelligent Power Saver	Goods have been received well-packed, logistics is very fast and good quality	Jingdong prefecture level city in Shandong
Happy Fan Shop	Green Intelligent Power Saver	This baby's quality is okay.	Jingdong prefecture level city in Shandong
Happy Fan Shop	Green Intelligent Power Saver	Counterfeit goods.	Jingdong prefecture level city in Shandong
Happy Fan Shop	Green Intelligent Power Saver	The effect is not very obvious	Jingdong prefecture level city in Shandong
Happy Fan Shop	Green Intelligent Power Saver	It's not the same thing.	Jingdong prefecture level city in Shandong
Happy Fan Shop	Green Intelligent Power Saver	There is no load how much w seems false, nothing works.	Jingdong prefecture level city in Shandong
Happy Fan Shop	Green Intelligent Power Saver	I received the item yesterday, got it back and plugged it in, and it used a little more power yesterday than I had before	Jingdong prefecture level city in Shandong
Happy Fan Shop	Green Intelligent Power Saver	Haven't seen the power saving mode yet	Jingdong prefecture level city in Shandong
Happy Fan Shop	Green Intelligent Power Saver	This product has no known effect	Jingdong prefecture level city in Shandong
Happy Fan Shop	Green Intelligent Power Saver	Refund Returns $16	Jingdong prefecture level city in Shandong
Happy Fan Shop	Green Intelligent Power Saver	The previous year bought a, almost every month can save a 70 dollars or so, used a year, no problem at all, look very new like, this time to move to a new home decisively repurchase 2 units, very believe this	Jingdong prefecture level city in Shandong
Happy Fan Shop	Green Intelligent Power Saver	Has been the second time to buy, this power saving device is really good power, before every month to spend 300 electricity, now are about 200, last time that 6, this time to help lead the family to buy 6,	Jingdong prefecture level city in Shandong
Happy Fan Shop	Green Intelligent Power Saver	In the spirit of trying to experience the new product, the use of down compared to the usual power consumption, with the addition of the quarter, is indeed a lot less electricity, originally more than 450	Jingdong prefecture level city in Shandong
Happy Fan Shop	Green Intelligent Power Saver	Has not been the first time to buy back, good use, power saving device is really powerful, before the monthly electricity bill of more than 500 Now more than 300, this time to help neighbors to buy several	Jingdong prefecture level city in Shandong
Happy Fan Shop	Green Intelligent Power Saver	Home appliances are very many, the circuit is also very complex, at first it is to save 9% of the electricity, and then used more than half a month, found that the power saving rate is as high as more	Jingdong prefecture level city in Shandong
Happy Fan Shop	Green Intelligent Power Saver	My friend bought it last year and saved hundreds of dollars on his electricity bill in one summer Appearance and style: it looks better than the one I bought before Quality is also good with a light,	Jingdong prefecture level city in Shandong
Happy Fan Shop	Green Intelligent Power Saver	Saver received, on the spot on the plug with the feeling of immediate power savings, appearance, packaging is very delicate, very satisfied, I hope to achieve satisfactory results!	Jingdong prefecture level city in Shandong
Happy Fan Shop	Green Intelligent Power Saver	The power saving effect is very good, is considered an old customer, also bought before, this time is to buy two for mom and dad, customer service attitude is good, praise!	Jingdong prefecture level city in Shandong
Happy Fan Shop	Green Intelligent Power Saver	After observing for a while, it really saves a lot of money I didn't realize it was still really reliable, so I'll give you a good review.	Jingdong prefecture level city in Shandong
Happy Fan Shop	Green Intelligent Power Saver	My family a month with this monthly almost save more than 200, power-saving effect: have bought a second time, really effective Cost-effective: not bad Appearance style: roughly the same A few months to the home electricity is indeed less, this time it is a re-buy three, send a friend two, they stay a	Jingdong prefecture level city in Shandong
Happy Fan Shop	Green Intelligent Power Saver	A month almost save more than 150 dollars in electricity, home appliances more, the main this can also protect high-power appliances, after using this device, these two months have not burned, things are very good, it is said that this is the latest model, the overall price is cheap and beautiful!	Jingdong prefecture level city in Shandong
Happy Fan Shop	Green Intelligent Power Saver	Received, powerful, great quality, love the style😍	Jingdong prefecture level city in Shandong
Happy Fan Shop	Green Intelligent Power Saver	This intelligent energy saver is especially good to use and save electricity hope everyone will buy it!	Jingdong prefecture level city in Shandong
Happy Fan Shop	Green Intelligent Power Saver	Air conditioning adapter effect is particularly great, the use of very simple, plug into the socket on the summer air conditioning power is too large, electricity is also expensive, with this product directly on the electricity bill to save half, has been purchased a few more, the effect is too awesome!	Jingdong prefecture level city in Shandong
Happy Fan Shop	Green Intelligent Power Saver	The family plugged in 5, monthly electricity savings of about 300 yuan. My family before a month of electricity costs more than 800, this time with this saver after the electricity bill is actually only more than 500, amazing, thanks to the store, will recommend other friends to buy!	Jingdong prefecture level city in Shandong
Happy Fan Shop	Green Intelligent Power Saver	It was recommended to me by a neighbor's family and it's nice to use it to save power.	Jingdong prefecture level city in Shandong
Happy Fan Shop	Green Intelligent Power Saver	Second time I've purchased it and the power saving is effective!	Jingdong prefecture level city in Shandong
Happy Fan Shop	Green Intelligent Power Saver	The effect is really good, before a month of more than 300 electricity, now every month every day with the air conditioning, it is more than 100 electricity! This thing is really amazing, one after another to buy and 5.6, home installed 3!	Jingdong prefecture level city in Shandong
Happy Fan Shop	Green Intelligent Power Saver	Received this product, began to use, small things look very wrong, can save a little electricity, the monthly electricity bill is too high, after plugging in did not pull, or save a lot of electricity, point	Jingdong prefecture level city in Shandong
Happy Fan Shop	Green Intelligent Power Saver	Simple and convenient, just that plug in, you can save electricity. A month can save dozens of dollars, a year down to save a lot of, very good product cost-effective, everyone can afford to buy and use!	Jingdong prefecture level city in Shandong
Happy Fan Shop	Green Intelligent Power Saver	Electricity bill from more than 200 yuan per month, down to more than 150 yuan, the original bought home, used two months, really have power-saving effect, this time and sold two, business service attitude is	Jingdong prefecture level city in Shandong
Happy Fan Shop	Green Intelligent Power Saver	Like to watch some movies at home and so on, sometimes at night until two or three o'clock, and recently the air conditioning is not turned off for a night, rather wasteful, I have to say that the effect is very good, quite like it!	Jingdong prefecture level city in Shandong
Happy Fan Shop	Green Intelligent Power Saver	Power-saving effect: the effect is very good, a month to save more than 200 dollars Cost-effective: good value for money Appearance and style: small and lovely Workmanship material: okay Experience: good	Jingdong prefecture level city in Shandong
Happy Fan Shop	Green Intelligent Power Saver	It's okay I quite like it, the stuff is very good. Come back in the future. You can rest assured to buy. Cost-effective all. Much cheaper than the physical store	Jingdong prefecture level city in Shandong
Happy Fan Shop	Green Intelligent Power Saver	More than 150 less, last month's electricity bill is almost 400, this month is only about 200, really save electricity, I am home to buy three, I did not expect a month to pay back	Jingdong prefecture level city in Shandong
Happy Fan Shop	Green Intelligent Power Saver	I bought three of these first and tried them for two months and they still felt useful, so now I've bought another one. Good and recommended!	Jingdong prefecture level city in Shandong
Happy Fan Shop	Green Intelligent Power Saver	This energy saver is very good quality and inexpensive next time I will buy it again!	Jingdong prefecture level city in Shandong
Happy Fan Shop	Green Intelligent Power Saver	Merchant service attitude is not good, the quality does not match, the business does not give the package return	Jingdong prefecture level city in Shandong
Happy Fan Shop	Green Intelligent Power Saver	The goods are very small do not know how effective after a few days to follow up the review	Jingdong prefecture level city in Shandong
Happy Fan Shop	Green Intelligent Power Saver	Not used before more than 200 a month, after using about 100 a month, my family used three, customer service said I use electricity recommended 2-3, I think more than one effect is better on the purchase of 4, the effect is very obvious, recommended to buy!	Jingdong prefecture level city in Shandong
Happy Fan Shop	Green Intelligent Power Saver	It's okay. I'll buy it again today.	Jingdong prefecture level city in Shandong
Happy Fan Shop	Green Intelligent Power Saver	Finally received the goods, very, very much like,, has been a lot of people praise, really too happy, has been his family's loyal fans, will continue to buy buy buy buy!	Jingdong prefecture level city in Shandong
Happy Fan Shop	Green Intelligent Power Saver	After using this, my monthly electricity bill was 1,200 pounds, now it's only 180 bucks a month, how good is it?	Jingdong prefecture level city in Shandong
Happy Fan Shop	Green Intelligent Power Saver	The item is a scam return to marry a dozen dollars in shipping fees. Buy terrific role is purely a scam.	Jingdong prefecture level city in Shandong
Happy Fan Shop	Green Intelligent Power Saver	It's really good, cost-effective and good quality, and the logistics are fast! It arrived in one day!	Jingdong prefecture level city in Shandong
Happy Fan Shop	Green Intelligent Power Saver	Great brand! Trustworthy! Works super well! Expensive for a reason! Great merchandise!	Jingdong prefecture level city in Shandong
Happy Fan Shop	Green Intelligent Power Saver	The baby is really great, I like it very much, my friends also say good to come, recommended to buy!	Jingdong prefecture level city in Shandong

Happy Fan Shop	Green Intelligent Power Saver	Fake couldn't be more fake. Bought one plugged in still so much electricity.	Jingdong prefecture level city in Shandong
Happy Fan Shop	Green Intelligent Power Saver	This appliance works great. Let's see how it works. We'll see later.	Jingdong prefecture level city in Shandong
Happy Fan Shop	Green Intelligent Power Saver	Used it before commenting, very happy with it	Jingdong prefecture level city in Shandong
Happy Fan Shop	Green Intelligent Power Saver	Goods have been received well-packed, logistics is very fast and good quality	Jingdong prefecture level city in Shandong
Happy Fan Shop	Green Intelligent Power Saver	This baby's quality is okay.	Jingdong prefecture level city in Shandong
Happy Fan Shop	Green Intelligent Power Saver	Counterfeit goods.	Jingdong prefecture level city in Shandong
Happy Fan Shop	Green Intelligent Power Saver	The effect is not very obvious	Jingdong prefecture level city in Shandong
Happy Fan Shop	Green Intelligent Power Saver	It's not the same thing.	Jingdong prefecture level city in Shandong
Happy Fan Shop	Green Intelligent Power Saver	There is no load how much w seems false, nothing works.	Jingdong prefecture level city in Shandong
Happy Fan Shop	Green Intelligent Power Saver	I received the item yesterday, got it back and plugged it in, and it used a little more power yesterday than I had before	Jingdong prefecture level city in Shandong
Happy Fan Shop	Green Intelligent Power Saver	Haven't seen the power saving mode yet	Jingdong prefecture level city in Shandong
Happy Fan Shop	Green Intelligent Power Saver	This product has no known effect	Jingdong prefecture level city in Shandong
Happy Fan Shop	Green Intelligent Power Saver	Refund Returns $16	Jingdong prefecture level city in Shandong
Happy Fan Shop	Green Intelligent Power Saver	The previous year bought a, almost every month can save a 70 dollars or so, used a year, no problem at all, look very new like, this time to move to a new home decisively repurchase 2 units, very believe this	Jingdong prefecture level city in Shandong
Happy Fan Shop	Green Intelligent Power Saver	Has been the second time to buy, this power saving device is really good power, before every month to spend 300 electricity, now are about 200, last time that 6, this time to help lead the family to buy 6,	Jingdong prefecture level city in Shandong
Happy Fan Shop	Green Intelligent Power Saver	In the spirit of trying to experience the new product, the use of down compared to the usual power consumption, with the addition of the quarter, is indeed a lot less electricity, originally more than 450	Jingdong prefecture level city in Shandong
Happy Fan Shop	Green Intelligent Power Saver	Has not been the first time to buy back, good use, power saving device is really powerful, before the monthly electricity bill of more than 500 Now more than 300, this time to help neighbors to buy several	Jingdong prefecture level city in Shandong
Happy Fan Shop	Green Intelligent Power Saver	Home appliances are very many, the circuit is also very complex, at first it is to save 9% of the electricity, and then used more than half a month, found that the power saving rate is as high as more	Jingdong prefecture level city in Shandong
Happy Fan Shop	Green Intelligent Power Saver	My friend bought it last year and saved hundreds of dollars on his electricity bill in one summer Appearance and style: it looks better than the one I bought before Quality is also good with a light.	Jingdong prefecture level city in Shandong
Happy Fan Shop	Green Intelligent Power Saver	Saver received, on the spot on the plug with the feeling of immediate power savings, appearance, packaging is very delicate, very satisfied, I hope to achieve satisfactory results!	Jingdong prefecture level city in Shandong
Happy Fan Shop	Green Intelligent Power Saver	The power saving effect is very good, is considered an old customer, also bought before, this time is to buy two for mom and dad, customer service attitude is good, praise!	Jingdong prefecture level city in Shandong
Happy Fan Shop	Green Intelligent Power Saver	After observing for a while, it really saves a lot of money I didn't realize it was still really reliable, so I'll give you a good review.	Jingdong prefecture level city in Shandong
Happy Fan Shop	Green Intelligent Power Saver	My family a month with this monthly almost save more than 200, power-saving effect: have bought a second time, really effective Cost-effective: not bad Appearance style: roughly the same A few months to the home electricity is indeed less, this time it is a re-buy three, send a friend two, they stay a	Jingdong prefecture level city in Shandong
Happy Fan Shop	Green Intelligent Power Saver	A month almost save more than 150 dollars in electricity, home appliances more, the main this can also protect high-power appliances, after using this device, these two months have not burned, things are very good, it is said that this is the latest model, the overall price is cheap and beautiful!	Jingdong prefecture level city in Shandong
Happy Fan Shop	Green Intelligent Power Saver	Received, powerful, great quality, love the style😍	Jingdong prefecture level city in Shandong
Happy Fan Shop	Green Intelligent Power Saver	This intelligent energy saver is especially good to use and save electricity hope everyone will buy it!	Jingdong prefecture level city in Shandong
Happy Fan Shop	Green Intelligent Power Saver	Air conditioning adapter effect is particularly great, the use of very simple, plug into the socket on the summer air conditioning power is too large, electricity is also expensive, with this product directly on the electricity bill to save half, has been purchased a few more, the effect is too awesome!	Jingdong prefecture level city in Shandong
Happy Fan Shop	Green Intelligent Power Saver	The family plugged in 5, monthly electricity savings of about 300 yuan. My family before a month of electricity costs more than 800, this time with this saver after the electricity bill is actually only more than 500, amazing, thanks to the store, will recommend other friends to buy!	Jingdong prefecture level city in Shandong
Happy Fan Shop	Green Intelligent Power Saver	It was recommended to me by a neighbor's family and it's nice to use it to save power.	Jingdong prefecture level city in Shandong
Happy Fan Shop	Green Intelligent Power Saver	Second time I've purchased it and the power saving is effective!	Jingdong prefecture level city in Shandong
Happy Fan Shop	Green Intelligent Power Saver	The effect is really good, before a month of more than 300 electricity, now every month every day with the air conditioning, it is more than 100 electricity! This thing is really amazing, one after another to buy and 5.6, home installed 3!	Jingdong prefecture level city in Shandong
Happy Fan Shop	Green Intelligent Power Saver	Received this product, began to use, small things look very wrong, can save a little electricity, the monthly electricity bill is too high, after plugging in did not pull, or save a lot of electricity, point	Jingdong prefecture level city in Shandong
Happy Fan Shop	Green Intelligent Power Saver	Simple and convenient, just that plug in, you can save electricity. A month can save dozens of dollars, a year down to save a lot of, very good product cost-effective, everyone can afford to buy and use!	Jingdong prefecture level city in Shandong
Happy Fan Shop	Green Intelligent Power Saver	Electricity bill from more than 200 yuan per month, down to more than 150 yuan, the original bought home, used two months, really have power-saving effect, this time and sold two, business service attitude is	Jingdong prefecture level city in Shandong
Happy Fan Shop	Green Intelligent Power Saver	Like to watch some movies at home and so on, sometimes at night until two or three o'clock, and recently the air conditioning is not turned off for a night, rather wasteful, I have to say that the effect is very good, quite like it!	Jingdong prefecture level city in Shandong
Happy Fan Shop	Green Intelligent Power Saver	Power-saving effect: the effect is very good, a month to save more than 200 dollars Cost-effective: good value for money Appearance and style: small and lovely Workmanship material: okay Experience: good	Jingdong prefecture level city in Shandong
Happy Fan Shop	Green Intelligent Power Saver	It's okay I quite like it, the stuff is very good. Come back in the future. You can rest assured to buy. Cost-effective all. Much cheaper than the physical store	Jingdong prefecture level city in Shandong
Happy Fan Shop	Green Intelligent Power Saver	More than 150 less, last month's electricity bill is almost 400, this month is only about 200, really save electricity, I am home to buy three, I did not expect a month to pay back	Jingdong prefecture level city in Shandong
Happy Fan Shop	Green Intelligent Power Saver	I bought three of these first and tried them for two months and they still felt useful, so now I've bought another one. Good and recommended!	Jingdong prefecture level city in Shandong
Happy Fan Shop	Green Intelligent Power Saver	This energy saver is very good quality and inexpensive next time I will buy it again!	Jingdong prefecture level city in Shandong
Happy Fan Shop	Green Intelligent Power Saver	Merchant service attitude is not good, the quality does not match, the business does not give the package return	Jingdong prefecture level city in Shandong
Happy Fan Shop	Green Intelligent Power Saver	The goods are very small do not know how effective after a few days to follow up the review	Jingdong prefecture level city in Shandong
Happy Fan Shop	Green Intelligent Power Saver	Not used before more than 200 a month, after using about 100 a month, my family used three, customer service said I use electricity recommended 2-3, I think more than one effect is better on the purchase of 4, the effect is very obvious, recommended to buy!	Jingdong prefecture level city in Shandong
Happy Fan Shop	Green Intelligent Power Saver	It's okay. I'll buy it again today.	Jingdong prefecture level city in Shandong
Happy Fan Shop	Green Intelligent Power Saver	Finally received the goods, very, very much like,, has been a lot of people praise, really too happy, has been his family's loyal fans, will continue to buy buy buy!	Jingdong prefecture level city in Shandong
Happy Fan Shop	Green Intelligent Power Saver	After using this, my monthly electricity bill was 1,200 pounds, now it's only 180 bucks a month, how good is it?	Jingdong prefecture level city in Shandong
Happy Fan Shop	Green Intelligent Power Saver	The item is a scam return to marry a dozen dollars in shipping fees. Buy terrific role is purely a scam.	Jingdong prefecture level city in Shandong
Happy Fan Shop	Green Intelligent Power Saver	It's really good, cost-effective and good quality, and the logistics are fast! It arrived in one day!	Jingdong prefecture level city in Shandong

Happy Fan Shop	Green Intelligent Power Saver	Great brand! Trustworthy! Works super well! Expensive for a reason! Great merchandise!	Jingdong prefecture level city in Shandong
Happy Fan Shop	Green Intelligent Power Saver	The baby is really great, I like it very much, my friends also say good to come, recommended to buy!	Jingdong prefecture level city in Shandong
Happy Fan Shop	Green Intelligent Power Saver	Fake couldn't be more fake. Bought one plugged in still so much electricity.	Jingdong prefecture level city in Shandong
Happy Fan Shop	Green Intelligent Power Saver	This appliance works great. Let's see how it works. We'll see later.	Jingdong prefecture level city in Shandong
Happy Fan Shop	Green Intelligent Power Saver	Used it before commenting, very happy with it	Jingdong prefecture level city in Shandong
Happy Fan Shop	Green Intelligent Power Saver	Goods have been received well-packed, logistics is very fast and good quality	Jingdong prefecture level city in Shandong
Happy Fan Shop	Green Intelligent Power Saver	This baby's quality is okay.	Jingdong prefecture level city in Shandong
Happy Fan Shop	Green Intelligent Power Saver	Counterfeit goods.	Jingdong prefecture level city in Shandong
Happy Fan Shop	Green Intelligent Power Saver	The effect is not very obvious	Jingdong prefecture level city in Shandong
Happy Fan Shop	Green Intelligent Power Saver	It's not the same thing.	Jingdong prefecture level city in Shandong
Happy Fan Shop	Green Intelligent Power Saver	There is no load how much w seems false, nothing works.	Jingdong prefecture level city in Shandong
Happy Fan Shop	Green Intelligent Power Saver	I received the item yesterday, got it back and plugged it in, and it used a little more power yesterday than I had before	Jingdong prefecture level city in Shandong
Happy Fan Shop	Green Intelligent Power Saver	Haven't seen the power saving mode yet	Jingdong prefecture level city in Shandong
Happy Fan Shop	Green Intelligent Power Saver	This product has no known effect	Jingdong prefecture level city in Shandong
Happy Fan Shop	Green Intelligent Power Saver	Refund Returns $16	Jingdong prefecture level city in Shandong
Happy Fan Shop	Green Intelligent Power Saver	The previous year bought a, almost every month can save a 70 dollars or so, used a year, no problem at all, look very new like, this time to move to a new home decisively repurchase 2 units, very believe this	Jingdong prefecture level city in Shandong
Happy Fan Shop	Green Intelligent Power Saver	Has been the second time to buy, this power saving device is really good power, before every month to spend 300 electricity, now are about 200, last time that 6, this time to help lead the family to buy 6,	Jingdong prefecture level city in Shandong
Happy Fan Shop	Green Intelligent Power Saver	In the spirit of trying to experience the new product, the use of down compared to the usual power consumption, with the addition of the quarter, is indeed a lot less electricity, originally more than 450	Jingdong prefecture level city in Shandong
Happy Fan Shop	Green Intelligent Power Saver	Has not been the first time to buy back, good use, power saving device is really powerful, before the monthly electricity bill of more than 500 Now more than 300, this time to help neighbors to buy several	Jingdong prefecture level city in Shandong
Happy Fan Shop	Green Intelligent Power Saver	Home appliances are very many, the circuit is also very complex, at first it is to save 9% of the electricity, and then used more than half a month, found that the power saving rate is as high as more	Jingdong prefecture level city in Shandong
Happy Fan Shop	Green Intelligent Power Saver	My friend bought it last year and saved hundreds of dollars on his electricity bill in one summer Appearance and style: it looks better than the one I bought before Quality is also good with a light.	Jingdong prefecture level city in Shandong
Happy Fan Shop	Green Intelligent Power Saver	Saver received, on the spot on the plug with the feeling of immediate power savings, appearance, packaging is very delicate, very satisfied, I hope to achieve satisfactory results!	Jingdong prefecture level city in Shandong
Happy Fan Shop	Green Intelligent Power Saver	The power saving effect is very good, is considered an old customer, also bought before, this time is to buy two for mom and dad, customer service attitude is good, praise!	Jingdong prefecture level city in Shandong
Happy Fan Shop	Green Intelligent Power Saver	After observing for a while, it really saves a lot of money I didn't realize it was still really reliable, so I'll give you a good review.	Jingdong prefecture level city in Shandong
Happy Fan Shop	Green Intelligent Power Saver	My family a month with this monthly almost save more than 200, power-saving effect: have bought a second time, really effective Cost-effective: not bad Appearance style: roughly the same A few months to the home electricity is indeed less, this time it is a re-buy three, send a friend two, they stay a	Jingdong prefecture level city in Shandong
Happy Fan Shop	Green Intelligent Power Saver	A month almost save more than 150 dollars in electricity, home appliances more, the main this can also protect high-power appliances, after using this device, these two months have not burned, things are very good, it is said that this is the latest model, the overall price is cheap and beautiful!	Jingdong prefecture level city in Shandong
Happy Fan Shop	Green Intelligent Power Saver	Received, powerful, great quality, love the style😍	Jingdong prefecture level city in Shandong
Happy Fan Shop	Green Intelligent Power Saver	This intelligent energy saver is especially good to use and save electricity hope everyone will buy it!	Jingdong prefecture level city in Shandong
Happy Fan Shop	Green Intelligent Power Saver	Air conditioning adapter effect is particularly great, the use of very simple, plug into the socket on the summer air conditioning power is too large, electricity is also expensive, with this product directly on the electricity bill to save half, has been purchased a few more, the effect is too awesome!	Jingdong prefecture level city in Shandong
Happy Fan Shop	Green Intelligent Power Saver	The family plugged in 5, monthly electricity savings of about 300 yuan. My family before a month of electricity costs more than 800, this time with this saver after the electricity bill is actually only more than 500, amazing, thanks to the store, will recommend other friends to buy!	Jingdong prefecture level city in Shandong
Happy Fan Shop	Green Intelligent Power Saver	It was recommended to me by a neighbor's family and it's nice to use it to save power.	Jingdong prefecture level city in Shandong
Happy Fan Shop	Green Intelligent Power Saver	Second time I've purchased it and the power saving is effective!	Jingdong prefecture level city in Shandong
Happy Fan Shop	Green Intelligent Power Saver	The effect is really good, before a month of more than 300 electricity, now every month every day with the air conditioning, it is more than 100 electricity! This thing is really amazing, one after another to buy and 5.6, home installed 3!	Jingdong prefecture level city in Shandong
Happy Fan Shop	Green Intelligent Power Saver	Received this product, began to use, small things look very wrong, can save a little electricity, the monthly electricity bill is too high, after plugging in did not pull, or save a lot of electricity, point	Jingdong prefecture level city in Shandong
Happy Fan Shop	Green Intelligent Power Saver	Simple and convenient, just that plug in, you can save electricity. A month can save dozens of dollars, a year down to save a lot of, very good product cost-effective, everyone can afford to buy and use!	Jingdong prefecture level city in Shandong
Happy Fan Shop	Green Intelligent Power Saver	Electricity bill from more than 200 yuan per month, down to more than 150 yuan, the original bought home, used two months, really have power-saving effect, this time and sold two, business service attitude is	Jingdong prefecture level city in Shandong
Happy Fan Shop	Green Intelligent Power Saver	Like to watch some movies at home and so on, sometimes at night until two or three o'clock, and recently the air conditioning is not turned off for a night, rather wasteful, I have to say that the effect is very good, quite like it!	Jingdong prefecture level city in Shandong
Happy Fan Shop	Green Intelligent Power Saver	Power-saving effect: the effect is very good, a month to save more than 200 dollars Cost-effective: good value for money Appearance and style: small and lovely Workmanship material: okay Experience: good	Jingdong prefecture level city in Shandong
Happy Fan Shop	Green Intelligent Power Saver	It's okay I quite like it, the stuff is very good. Come back in the future. You can rest assured to buy. Cost-effective all. Much cheaper than the physical store	Jingdong prefecture level city in Shandong
Happy Fan Shop	Green Intelligent Power Saver	More than 150 less, last month's electricity bill is almost 400, this month is only about 200, really save electricity, I am home to buy three, I did not expect a month to pay back	Jingdong prefecture level city in Shandong
Happy Fan Shop	Green Intelligent Power Saver	I bought three of these first and tried them for two months and they still felt useful, so now I've bought another one. Good and recommended!	Jingdong prefecture level city in Shandong
Happy Fan Shop	Green Intelligent Power Saver	This energy saver is very good quality and inexpensive next time I will buy it again!	Jingdong prefecture level city in Shandong
Happy Fan Shop	Green Intelligent Power Saver	Merchant service attitude is not good, the quality does not match, the business does not give the package return	Jingdong prefecture level city in Shandong
Happy Fan Shop	Green Intelligent Power Saver	The goods are very small do not know how effective after a few days to follow up the review	Jingdong prefecture level city in Shandong
Happy Fan Shop	Green Intelligent Power Saver	Not used before more than 200 a month, after using about 100 a month, my family used three, customer service said I use electricity recommended 2-3, I think more than one effect is better on the purchase of 4, the effect is very obvious, recommended to buy!	Jingdong prefecture level city in Shandong
Happy Fan Shop	Green Intelligent Power Saver	It's okay. I'll buy it again today.	Jingdong prefecture level city in Shandong
Happy Fan Shop	Green Intelligent Power Saver	Finally received the goods, very, very much like,, has been a lot of people praise, really too happy, has been his family's loyal fans, will continue to buy buy buy buy!	Jingdong prefecture level city in Shandong
Happy Fan Shop	Green Intelligent Power Saver	After using this, my monthly electricity bill was 1,200 pounds, now it's only 180 bucks a month, how good is it?	Jingdong prefecture level city in Shandong

Happy Fan Shop	Green Intelligent Power Saver	The item is a scam return to marry a dozen dollars in shipping fees. Buy terrific role is purely a scam.	Jingdong prefecture level city in Shandong
Happy Fan Shop	Green Intelligent Power Saver	It's really good, cost-effective and good quality, and the logistics are fast! It arrived in one day!	Jingdong prefecture level city in Shandong
Happy Fan Shop	Green Intelligent Power Saver	Great brand! Trustworthy! Works super well! Expensive for a reason! Great merchandise!	Jingdong prefecture level city in Shandong
Happy Fan Shop	Green Intelligent Power Saver	The baby is really great, I like it very much, my friends also say good to come, recommended to buy!	Jingdong prefecture level city in Shandong
Happy Fan Shop	Green Intelligent Power Saver	Fake couldn't be more fake. Bought one plugged in still so much electricity.	Jingdong prefecture level city in Shandong
Happy Fan Shop	Green Intelligent Power Saver	This appliance works great. Let's see how it works. We'll see later.	Jingdong prefecture level city in Shandong
Happy Fan Shop	Green Intelligent Power Saver	Used it before commenting, very happy with it	Jingdong prefecture level city in Shandong
Happy Fan Shop	Green Intelligent Power Saver	Goods have been received well-packed, logistics is very fast and good quality	Jingdong prefecture level city in Shandong
Happy Fan Shop	Green Intelligent Power Saver	This baby's quality is okay.	Jingdong prefecture level city in Shandong
Happy Fan Shop	Green Intelligent Power Saver	Counterfeit goods.	Jingdong prefecture level city in Shandong
Happy Fan Shop	Green Intelligent Power Saver	The effect is not very obvious	Jingdong prefecture level city in Shandong
Happy Fan Shop	Green Intelligent Power Saver	It's not the same thing.	Jingdong prefecture level city in Shandong
Happy Fan Shop	Green Intelligent Power Saver	There is no load how much w seems false, nothing works.	Jingdong prefecture level city in Shandong
Happy Fan Shop	Green Intelligent Power Saver	I received the item yesterday, got it back and plugged it in, and it used a little more power yesterday than I had before	Jingdong prefecture level city in Shandong
Happy Fan Shop	Green Intelligent Power Saver	Haven't seen the power saving mode yet	Jingdong prefecture level city in Shandong
Happy Fan Shop	Green Intelligent Power Saver	This product has no known effect	Jingdong prefecture level city in Shandong
Happy Fan Shop	Green Intelligent Power Saver	Refund Returns $16	Jingdong prefecture level city in Shandong
Happy Fan Shop	Green Intelligent Power Saver	The previous year bought a, almost every month can save a 70 dollars or so, used a year, no problem at all, look very new like, this time to move to a new home decisively repurchase 2 units, very believe this	Jingdong prefecture level city in Shandong
Happy Fan Shop	Green Intelligent Power Saver	Has been the second time to buy, this power saving device is really good power, before every month to spend 300 electricity, now are about 200, last time that 6, this time to help lead the family to buy 6,	Jingdong prefecture level city in Shandong
Happy Fan Shop	Green Intelligent Power Saver	In the spirit of trying to experience the new product, the use of down compared to the usual power consumption, with the addition of the quarter, is indeed a lot less electricity, originally more than 450	Jingdong prefecture level city in Shandong
Happy Fan Shop	Green Intelligent Power Saver	Has not been the first time to buy back, good use, power saving device is really powerful, before the monthly electricity bill of more than 500 Now more than 300, this time to help neighbors to buy several	Jingdong prefecture level city in Shandong
Happy Fan Shop	Green Intelligent Power Saver	Home appliances are very many, the circuit is also very complex, at first it is to save 9% of the electricity, and then used more than half a month, found that the power saving rate is as high as more	Jingdong prefecture level city in Shandong
Happy Fan Shop	Green Intelligent Power Saver	My friend bought it last year and saved hundreds of dollars on his electricity bill in one summer Appearance and style: it looks better than the one I bought before Quality is also good with a light.	Jingdong prefecture level city in Shandong
Happy Fan Shop	Green Intelligent Power Saver	Saver received, on the spot on the plug with the feeling of immediate power savings, appearance, packaging is very delicate, very satisfied, I hope to achieve satisfactory results!	Jingdong prefecture level city in Shandong
Happy Fan Shop	Green Intelligent Power Saver	The power saving effect is very good, is considered an old customer, also bought before, this time is to buy two for mom and dad, customer service attitude is good, praise!	Jingdong prefecture level city in Shandong
Happy Fan Shop	Green Intelligent Power Saver	After observing for a while, it really saves a lot of money I didn't realize it was still really reliable, so I'll give you a good review.	Jingdong prefecture level city in Shandong
Happy Fan Shop	Green Intelligent Power Saver	My family a month with this monthly almost save more than 200, power-saving effect: have bought a second time, really effective Cost-effective: not bad Appearance style: roughly the same A few months to the home electricity is indeed less, this time it is a re-buy three, send a friend two, they stay a	Jingdong prefecture level city in Shandong
Happy Fan Shop	Green Intelligent Power Saver	A month almost save more than 150 dollars in electricity, home appliances more, the main this can also protect high-power appliances, after using this device, these two months have not burned, things are very good, it is said that this is the latest model, the overall price is cheap and beautiful!	Jingdong prefecture level city in Shandong
Happy Fan Shop	Green Intelligent Power Saver	Received, powerful, great quality, love the style😍	Jingdong prefecture level city in Shandong
Happy Fan Shop	Green Intelligent Power Saver	This intelligent energy saver is especially good to use and save electricity hope everyone will buy it!	Jingdong prefecture level city in Shandong
Happy Fan Shop	Green Intelligent Power Saver	Air conditioning adapter effect is particularly great, the use of very simple, plug into the socket on the summer air conditioning power is too large, electricity is also expensive, with this product directly on the electricity bill to save half, has been purchased a few more, the effect is too awesome!	Jingdong prefecture level city in Shandong
Happy Fan Shop	Green Intelligent Power Saver	The family plugged in 5, monthly electricity savings of about 300 yuan. My family before a month of electricity costs more than 800, this time with this saver after the electricity bill is actually only more than 500, amazing, thanks to the store, will recommend other friends to buy!	Jingdong prefecture level city in Shandong
Happy Fan Shop	Green Intelligent Power Saver	It was recommended to me by a neighbor's family and it's nice to use it to save power.	Jingdong prefecture level city in Shandong
Happy Fan Shop	Green Intelligent Power Saver	Second time I've purchased it and the power saving is effective!	Jingdong prefecture level city in Shandong
Happy Fan Shop	Green Intelligent Power Saver	The effect is really good, before a month of more than 300 electricity, now every month every day with the air conditioning, it is more than 100 electricity! This thing is really amazing, one after another to buy and 5.6, home installed 3!	Jingdong prefecture level city in Shandong
Happy Fan Shop	Green Intelligent Power Saver	Received this product, began to use, small things look very wrong, can save a little electricity, the monthly electricity bill is too high, after plugging in did not pull, or save a lot of electricity, point	Jingdong prefecture level city in Shandong
Happy Fan Shop	Green Intelligent Power Saver	Simple and convenient, just that plug in, you can save electricity. A month can save dozens of dollars, a year down to save a lot of, very good product cost-effective, everyone can afford to buy and use!	Jingdong prefecture level city in Shandong
Happy Fan Shop	Green Intelligent Power Saver	Electricity bill from more than 200 yuan per month, down to more than 150 yuan, the original bought home, used two months, really have power-saving effect, this time and sold two, business service attitude is	Jingdong prefecture level city in Shandong
Happy Fan Shop	Green Intelligent Power Saver	Like to watch some movies at home and so on, sometimes at night until two or three o'clock, and recently the air conditioning is not turned off for a night, rather wasteful, I have to say that the effect is very good, quite like it!	Jingdong prefecture level city in Shandong
Happy Fan Shop	Green Intelligent Power Saver	Power-saving effect: the effect is very good, a month to save more than 200 dollars Cost-effective: good value for money Appearance and style: small and lovely Workmanship material: okay Experience: good	Jingdong prefecture level city in Shandong
Happy Fan Shop	Green Intelligent Power Saver	It's okay I quite like it, the stuff is very good. Come back in the future. You can rest assured to buy. Cost-effective all. Much cheaper than the physical store	Jingdong prefecture level city in Shandong
Happy Fan Shop	Green Intelligent Power Saver	More than 150 less, last month's electricity bill is almost 400, this month is only about 200, really save electricity, I am home to buy three, I did not expect a month to pay back	Jingdong prefecture level city in Shandong
Happy Fan Shop	Green Intelligent Power Saver	I bought three of these first and tried them for two months and they still felt useful, so now I've bought another one. Good and recommended!	Jingdong prefecture level city in Shandong
Happy Fan Shop	Green Intelligent Power Saver	This energy saver is very good quality and inexpensive next time I will buy it again!	Jingdong prefecture level city in Shandong
Happy Fan Shop	Green Intelligent Power Saver	Merchant service attitude is not good, the quality does not match, the business does not give the package return	Jingdong prefecture level city in Shandong
Happy Fan Shop	Green Intelligent Power Saver	The goods are very small do not know how effective after a few days to follow up the review	Jingdong prefecture level city in Shandong
Happy Fan Shop	Green Intelligent Power Saver	Not used before more than 200 a month, after using about 100 a month, my family used three, customer service said I use electricity recommended 2-3, I think more than one effect is better on the purchase of 4, the effect is very obvious, recommended to buy!	Jingdong prefecture level city in Shandong
Happy Fan Shop	Green Intelligent Power Saver	It's okay. I'll buy it again today.	Jingdong prefecture level city in Shandong

Happy Fan Shop	Green Intelligent Power Saver	Finally received the goods, very, very much like,, has been a lot of people praise, really too happy, has been his family's loyal fans, will continue to buy buy buy buy!	Jingdong prefecture level city in Shandong
Happy Fan Shop	Green Intelligent Power Saver	After using this, my monthly electricity bill was 1,200 pounds, now it's only 180 bucks a month, how good is it?	Jingdong prefecture level city in Shandong
Happy Fan Shop	Green Intelligent Power Saver	The item is a scam return to marry a dozen dollars in shipping fees. Buy terrific role is purely a scam.	Jingdong prefecture level city in Shandong
Happy Fan Shop	Green Intelligent Power Saver	It's really good, cost-effective and good quality, and the logistics are fast! It arrived in one day!	Jingdong prefecture level city in Shandong
Happy Fan Shop	Green Intelligent Power Saver	Great brand! Trustworthy! Works super well! Expensive for a reason! Great merchandise!	Jingdong prefecture level city in Shandong
Happy Fan Shop	Green Intelligent Power Saver	The baby is really great, I like it very much, my friends also say good to come, recommended to buy!	Jingdong prefecture level city in Shandong
Happy Fan Shop	Green Intelligent Power Saver	Fake couldn't be more fake. Bought one plugged in still so much electricity.	Jingdong prefecture level city in Shandong
Happy Fan Shop	Green Intelligent Power Saver	This appliance works great. Let's see how it works. We'll see later.	Jingdong prefecture level city in Shandong
Happy Fan Shop	Green Intelligent Power Saver	Used it before commenting, very happy with it	Jingdong prefecture level city in Shandong
Happy Fan Shop	Green Intelligent Power Saver	Goods have been received well-packed, logistics is very fast and good quality	Jingdong prefecture level city in Shandong
Happy Fan Shop	Green Intelligent Power Saver	This baby's quality is okay.	Jingdong prefecture level city in Shandong
Happy Fan Shop	Green Intelligent Power Saver	Counterfeit goods.	Jingdong prefecture level city in Shandong
Happy Fan Shop	Green Intelligent Power Saver	The effect is not very obvious	Jingdong prefecture level city in Shandong
Happy Fan Shop	Green Intelligent Power Saver	It's not the same thing.	Jingdong prefecture level city in Shandong
Happy Fan Shop	Green Intelligent Power Saver	There is no load how much w seems false, nothing works.	Jingdong prefecture level city in Shandong
Happy Fan Shop	Green Intelligent Power Saver	I received the item yesterday, got it back and plugged it in, and it used a little more power yesterday than I had before	Jingdong prefecture level city in Shandong
Happy Fan Shop	Green Intelligent Power Saver	Haven't seen the power saving mode yet	Jingdong prefecture level city in Shandong
Happy Fan Shop	Green Intelligent Power Saver	This product has no known effect	Jingdong prefecture level city in Shandong
Happy Fan Shop	Green Intelligent Power Saver	Refund Returns $16	Jingdong prefecture level city in Shandong
Happy Fan Shop	Green Intelligent Power Saver	The previous year bought a, almost every month can save a 70 dollars or so, used a year, no problem at all, look very new like, this time to move to a new home decisively repurchase 2 units, very believe this	Jingdong prefecture level city in Shandong
Happy Fan Shop	Green Intelligent Power Saver	Has been the second time to buy, this power saving device is really good power, before every month to spend 300 electricity, now are about 200, last time that 6, this time to help lead the family to buy 6,	Jingdong prefecture level city in Shandong
Happy Fan Shop	Green Intelligent Power Saver	In the spirit of trying to experience the new product, the use of down compared to the usual power consumption, with the addition of the quarter, is indeed a lot less electricity, originally more than 450	Jingdong prefecture level city in Shandong
Happy Fan Shop	Green Intelligent Power Saver	Has not been the first time to buy back, good use, power saving device is really powerful, before the monthly electricity bill of more than 500 Now more than 300, this time to help neighbors to buy several	Jingdong prefecture level city in Shandong
Happy Fan Shop	Green Intelligent Power Saver	Home appliances are very many, the circuit is also very complex, at first it is to save 9% of the electricity, and then used more than half a month, found that the power saving rate is as high as more	Jingdong prefecture level city in Shandong
Happy Fan Shop	Green Intelligent Power Saver	My friend bought it last year and saved hundreds of dollars on his electricity bill in one summer Appearance and style: it looks better than the one I bought before Quality is also good with a light.	Jingdong prefecture level city in Shandong
Happy Fan Shop	Green Intelligent Power Saver	Saver received, on the spot on the plug with the feeling of immediate power savings, appearance, packaging is very delicate, very satisfied, I hope to achieve satisfactory results!	Jingdong prefecture level city in Shandong
Happy Fan Shop	Green Intelligent Power Saver	The power saving effect is very good, is considered an old customer, also bought before, this time is to buy two for mom and dad, customer service attitude is good, praise!	Jingdong prefecture level city in Shandong
Happy Fan Shop	Green Intelligent Power Saver	After observing for a while, it really saves a lot of money I didn't realize it was still really reliable, so I'll give you a good review.	Jingdong prefecture level city in Shandong
Happy Fan Shop	Green Intelligent Power Saver	My family a month with this monthly almost save more than 200, power-saving effect: have bought a second time, really effective Cost-effective: not bad Appearance style: roughly the same A few months to the home electricity is indeed less, this time it is a re-buy three, send a friend two, they stay a	Jingdong prefecture level city in Shandong
Happy Fan Shop	Green Intelligent Power Saver	A month almost save more than 150 dollars in electricity, home appliances more, the main this can also protect high-power appliances, after using this device, these two months have not burned, things are very good, it is said that this is the latest model, the overall price is cheap and beautiful!	Jingdong prefecture level city in Shandong
Happy Fan Shop	Green Intelligent Power Saver	Received, powerful, great quality, love the style😍	Jingdong prefecture level city in Shandong
Happy Fan Shop	Green Intelligent Power Saver	This intelligent energy saver is especially good to use and save electricity hope everyone will buy it!	Jingdong prefecture level city in Shandong
Happy Fan Shop	Green Intelligent Power Saver	Air conditioning adapter effect is particularly great, the use of very simple, plug into the socket on the summer air conditioning power is too large, electricity is also expensive, with this product directly on the electricity bill to save half, has been purchased a few more, the effect is too awesome!	Jingdong prefecture level city in Shandong
Happy Fan Shop	Green Intelligent Power Saver	The family plugged in 5, monthly electricity savings of about 300 yuan. My family before a month of electricity costs more than 800, this time with this saver after the electricity bill is actually only more than 500, amazing, thanks to the store, will recommend other friends to buy!	Jingdong prefecture level city in Shandong
Happy Fan Shop	Green Intelligent Power Saver	It was recommended to me by a neighbor's family and it's nice to use it to save power.	Jingdong prefecture level city in Shandong
Happy Fan Shop	Green Intelligent Power Saver	Second time I've purchased it and the power saving is effective!	Jingdong prefecture level city in Shandong
Happy Fan Shop	Green Intelligent Power Saver	The effect is really good, before a month of more than 300 electricity, now every month every day with the air conditioning, it is more than 100 electricity! This thing is really amazing, one after another to buy and 5.6, home installed 3!	Jingdong prefecture level city in Shandong
Happy Fan Shop	Green Intelligent Power Saver	Received this product, began to use, small things look very wrong, can save a little electricity, the monthly electricity bill is too high, after plugging in did not pull, or save a lot of electricity, point	Jingdong prefecture level city in Shandong
Happy Fan Shop	Green Intelligent Power Saver	Simple and convenient, just that plug in, you can save electricity. A month can save dozens of dollars, a year down to save a lot of, very good product cost-effective, everyone can afford to buy and use!	Jingdong prefecture level city in Shandong
Happy Fan Shop	Green Intelligent Power Saver	Electricity bill from more than 200 yuan per month, down to more than 150 yuan, the original bought home, used two months, really have power-saving effect, this time and sold two, business service attitude is	Jingdong prefecture level city in Shandong
Happy Fan Shop	Green Intelligent Power Saver	Like to watch some movies at home and so on, sometimes at night until two or three o'clock, and recently the air conditioning is not turned off for a night, rather wasteful, I have to say that the effect is very good, quite like it!	Jingdong prefecture level city in Shandong
Happy Fan Shop	Green Intelligent Power Saver	Power-saving effect: the effect is very good, a month to save more than 200 dollars Cost-effective: good value for money Appearance and style: small and lovely Workmanship material: okay Experience: good	Jingdong prefecture level city in Shandong
Happy Fan Shop	Green Intelligent Power Saver	It's okay I quite like it, the stuff is very good. Come back in the future. You can rest assured to buy. Cost-effective all. Much cheaper than the physical store	Jingdong prefecture level city in Shandong
Happy Fan Shop	Green Intelligent Power Saver	More than 150 less, last month's electricity bill is almost 400, this month is only about 200, really save electricity, I am home to buy three, I did not expect a month to pay back	Jingdong prefecture level city in Shandong
Happy Fan Shop	Green Intelligent Power Saver	I bought three of these first and tried them for two months and they still felt useful, so now I've bought another one. Good and recommended!	Jingdong prefecture level city in Shandong
Happy Fan Shop	Green Intelligent Power Saver	This energy saver is very good quality and inexpensive next time I will buy it again!	Jingdong prefecture level city in Shandong
Happy Fan Shop	Green Intelligent Power Saver	Merchant service attitude is not good, the quality does not match, the business does not give the package return	Jingdong prefecture level city in Shandong
Happy Fan Shop	Green Intelligent Power Saver	The goods are very small do not know how effective after a few days to follow up the review	Jingdong prefecture level city in Shandong

Happy Fan Shop	Green Intelligent Power Saver	Not used before more than 200 a month, after using about 100 a month, my family used three, customer service said I use electricity recommended 2-3, I think more than one effect is better on the purchase of 4, the effect is very obvious, recommended to buy!	Jingdong prefecture level city in Shandong
Happy Fan Shop	Green Intelligent Power Saver	It's okay. I'll buy it again today.	Jingdong prefecture level city in Shandong
Happy Fan Shop	Green Intelligent Power Saver	Finally received the goods, very, very much like,, has been a lot of people praise, really too happy, has been his family's loyal fans, will continue to buy buy buy buy!	Jingdong prefecture level city in Shandong
Happy Fan Shop	Green Intelligent Power Saver	After using this, my monthly electricity bill was 1,200 pounds, now it's only 180 bucks a month, how good is it?	Jingdong prefecture level city in Shandong
Happy Fan Shop	Green Intelligent Power Saver	The item is a scam return to marry a dozen dollars in shipping fees. Buy terrific role is purely a scam.	Jingdong prefecture level city in Shandong
Happy Fan Shop	Green Intelligent Power Saver	It's really good, cost-effective and good quality, and the logistics are fast! It arrived in one day!	Jingdong prefecture level city in Shandong
Happy Fan Shop	Green Intelligent Power Saver	Great brand! Trustworthy! Works super well! Expensive for a reason! Great merchandise!	Jingdong prefecture level city in Shandong
Happy Fan Shop	Green Intelligent Power Saver	The baby is really great, I like it very much, my friends also say good to come, recommended to buy!	Jingdong prefecture level city in Shandong
Happy Fan Shop	Green Intelligent Power Saver	Fake couldn't be more fake. Bought one plugged in still so much electricity.	Jingdong prefecture level city in Shandong
Happy Fan Shop	Green Intelligent Power Saver	This appliance works great. Let's see how it works. We'll see later.	Jingdong prefecture level city in Shandong
Happy Fan Shop	Green Intelligent Power Saver	Used it before commenting, very happy with it	Jingdong prefecture level city in Shandong
Happy Fan Shop	Green Intelligent Power Saver	Goods have been received well-packed, logistics is very fast and good quality	Jingdong prefecture level city in Shandong
Happy Fan Shop	Green Intelligent Power Saver	This baby's quality is okay.	Jingdong prefecture level city in Shandong
Happy Fan Shop	Green Intelligent Power Saver	Counterfeit goods.	Jingdong prefecture level city in Shandong
Happy Fan Shop	Green Intelligent Power Saver	The effect is not very obvious	Jingdong prefecture level city in Shandong
Happy Fan Shop	Green Intelligent Power Saver	It's not the same thing.	Jingdong prefecture level city in Shandong
Happy Fan Shop	Green Intelligent Power Saver	There is no load how much w seems false, nothing works.	Jingdong prefecture level city in Shandong
Happy Fan Shop	Green Intelligent Power Saver	I received the item yesterday, got it back and plugged it in, and it used a little more power yesterday than I had before	Jingdong prefecture level city in Shandong
Happy Fan Shop	Green Intelligent Power Saver	Haven't seen the power saving mode yet	Jingdong prefecture level city in Shandong
Happy Fan Shop	Green Intelligent Power Saver	This product has no known effect	Jingdong prefecture level city in Shandong
Happy Fan Shop	Green Intelligent Power Saver	Refund Returns $16	Jingdong prefecture level city in Shandong
Happy Fan Shop	Green Intelligent Power Saver	The previous year bought a, almost every month can save a 70 dollars or so, used a year, no problem at all, look very new like, this time to move to a new home decisively repurchase 2 units, very believe this	Jingdong prefecture level city in Shandong
Happy Fan Shop	Green Intelligent Power Saver	Has been the second time to buy, this power saving device is really good power, before every month to spend 300 electricity, now are about 200, last time that 6, this time to help lead the family to buy 6,	Jingdong prefecture level city in Shandong
Happy Fan Shop	Green Intelligent Power Saver	In the spirit of trying to experience the new product, the use of down compared to the usual power consumption, with the addition of the quarter, is indeed a lot less electricity, originally more than 450	Jingdong prefecture level city in Shandong
Happy Fan Shop	Green Intelligent Power Saver	Has not been the first time to buy back, good use, power saving device is really powerful, before the monthly electricity bill of more than 500 Now more than 300, this time to help neighbors to buy several	Jingdong prefecture level city in Shandong
Happy Fan Shop	Green Intelligent Power Saver	Home appliances are very many, the circuit is also very complex, at first it is to save 9% of the electricity, and then used more than half a month, found that the power saving rate is as high as more	Jingdong prefecture level city in Shandong
Happy Fan Shop	Green Intelligent Power Saver	My friend bought it last year and saved hundreds of dollars on his electricity bill in one summer Appearance and style: it looks better than the one I bought before Quality is also good with a light.	Jingdong prefecture level city in Shandong
Happy Fan Shop	Green Intelligent Power Saver	Saver received, on the spot on the plug with the feeling of immediate power savings, appearance, packaging is very delicate, very satisfied, I hope to achieve satisfactory results!	Jingdong prefecture level city in Shandong
Happy Fan Shop	Green Intelligent Power Saver	The power saving effect is very good, is considered an old customer, also bought before, this time is to buy two for mom and dad, customer service attitude is good, praise!	Jingdong prefecture level city in Shandong
Happy Fan Shop	Green Intelligent Power Saver	After observing for a while, it really saves a lot of money I didn't realize it was still really reliable, so I'll give you a good review.	Jingdong prefecture level city in Shandong
Happy Fan Shop	Green Intelligent Power Saver	My family a month with this monthly almost save more than 200, power-saving effect: have bought a second time, really effective Cost-effective: not bad Appearance style: roughly the same A few months to the home electricity is indeed less, this time it is a re-buy three, send a friend two, they stay a	Jingdong prefecture level city in Shandong
Happy Fan Shop	Green Intelligent Power Saver	A month almost save more than 150 dollars in electricity, home appliances more, the main this can also protect high-power appliances, after using this device, these two months have not burned, things are very good, it is said that this is the latest model, the overall price is cheap and beautiful!	Jingdong prefecture level city in Shandong
Happy Fan Shop	Green Intelligent Power Saver	Received, powerful, great quality, love the style😍	Jingdong prefecture level city in Shandong
Happy Fan Shop	Green Intelligent Power Saver	This intelligent energy saver is especially good to use and save electricity hope everyone will buy it!	Jingdong prefecture level city in Shandong
Happy Fan Shop	Green Intelligent Power Saver	Air conditioning adapter effect is particularly great, the use of very simple, plug into the socket on the summer air conditioning power is too large, electricity is also expensive, with this product directly on the electricity bill to save half, has been purchased a few more, the effect is too awesome!	Jingdong prefecture level city in Shandong
Happy Fan Shop	Green Intelligent Power Saver	The family plugged in 5, monthly electricity savings of about 300 yuan. My family before a month of electricity costs more than 800, this time with this saver after the electricity bill is actually only more than 500, amazing, thanks to the store, will recommend other friends to buy!	Jingdong prefecture level city in Shandong
Happy Fan Shop	Green Intelligent Power Saver	It was recommended to me by a neighbor's family and it's nice to use it to save power.	Jingdong prefecture level city in Shandong
Happy Fan Shop	Green Intelligent Power Saver	Second time I've purchased it and the power saving is effective!	Jingdong prefecture level city in Shandong
Happy Fan Shop	Green Intelligent Power Saver	The effect is really good, before a month of more than 300 electricity, now every month every day with the air conditioning, it is more than 100 electricity! This thing is really amazing, one after another to buy and 5.6, home installed 3!	Jingdong prefecture level city in Shandong
Happy Fan Shop	Green Intelligent Power Saver	Received this product, began to use, small things look very wrong, can save a little electricity, the monthly electricity bill is too high, after plugging in did not pull, or save a lot of electricity, point	Jingdong prefecture level city in Shandong
Happy Fan Shop	Green Intelligent Power Saver	Simple and convenient, just that plug in, you can save electricity. A month can save dozens of dollars, a year down to save a lot of, very good product cost-effective, everyone can afford to buy and use!	Jingdong prefecture level city in Shandong
Happy Fan Shop	Green Intelligent Power Saver	Electricity bill from more than 200 yuan per month, down to more than 150 yuan, the original bought home, used two months, really have power-saving effect, this time and sold two, business service attitude is	Jingdong prefecture level city in Shandong
Happy Fan Shop	Green Intelligent Power Saver	Like to watch some movies at home and so on, sometimes at night until two or three o'clock, and recently the air conditioning is not turned off for a night, rather wasteful, I have to say that the effect is very good, quite like it!	Jingdong prefecture level city in Shandong
Happy Fan Shop	Green Intelligent Power Saver	Power-saving effect: the effect is very good, a month to save more than 200 dollars Cost-effective: good value for money Appearance and style: small and lovely Workmanship material: okay Experience: good	Jingdong prefecture level city in Shandong
Happy Fan Shop	Green Intelligent Power Saver	It's okay I quite like it, the stuff is very good. Come back in the future. You can rest assured to buy. Cost-effective all. Much cheaper than the physical store	Jingdong prefecture level city in Shandong
Happy Fan Shop	Green Intelligent Power Saver	More than 150 less, last month's electricity bill is almost 400, this month is only about 200, really save electricity, I am home to buy three, I did not expect a month to pay back	Jingdong prefecture level city in Shandong
Happy Fan Shop	Green Intelligent Power Saver	I bought three of these first and tried them for two months and they still felt useful, so now I've bought another one. Good and recommended!	Jingdong prefecture level city in Shandong
Happy Fan Shop	Green Intelligent Power Saver	This energy saver is very good quality and inexpensive next time I will buy it again!	Jingdong prefecture level city in Shandong

Happy Fan Shop	Green Intelligent Power Saver	Merchant service attitude is not good, the quality does not match, the business does not give the package return	Jingdong prefecture level city in Shandong
Happy Fan Shop	Green Intelligent Power Saver	The goods are very small do not know how effective after a few days to follow up the review	Jingdong prefecture level city in Shandong
Happy Fan Shop	Green Intelligent Power Saver	Not used before more than 200 a month, after using about 100 a month, my family used three, customer service said I use electricity recommended 2-3, I think more than one effect is better on the purchase of 4, the effect is very obvious, recommended to buy!	Jingdong prefecture level city in Shandong
Happy Fan Shop	Green Intelligent Power Saver	It's okay. I'll buy it again today.	Jingdong prefecture level city in Shandong
Happy Fan Shop	Green Intelligent Power Saver	Finally received the goods, very, very much like,, has been a lot of people praise, really too happy, has been his family's loyal fans, will continue to buy buy buy buy!	Jingdong prefecture level city in Shandong
Happy Fan Shop	Green Intelligent Power Saver	After using this, my monthly electricity bill was 1,200 pounds, now it's only 180 bucks a month, how good is it?	Jingdong prefecture level city in Shandong
Happy Fan Shop	Green Intelligent Power Saver	The item is a scam return to marry a dozen dollars in shipping fees. Buy terrific role is purely a scam.	Jingdong prefecture level city in Shandong
Happy Fan Shop	Green Intelligent Power Saver	It's really good, cost-effective and good quality, and the logistics are fast! It arrived in one day!	Jingdong prefecture level city in Shandong
Happy Fan Shop	Green Intelligent Power Saver	Great brand! Trustworthy! Works super well! Expensive for a reason! Great merchandise!	Jingdong prefecture level city in Shandong
Happy Fan Shop	Green Intelligent Power Saver	The baby is really great, I like it very much, my friends also say good to come, recommended to buy!	Jingdong prefecture level city in Shandong
Happy Fan Shop	Green Intelligent Power Saver	Fake couldn't be more fake. Bought one plugged in still so much electricity.	Jingdong prefecture level city in Shandong
Happy Fan Shop	Green Intelligent Power Saver	This appliance works great. Let's see how it works. We'll see later.	Jingdong prefecture level city in Shandong
Happy Fan Shop	Green Intelligent Power Saver	Used it before commenting, very happy with it	Jingdong prefecture level city in Shandong
Happy Fan Shop	Green Intelligent Power Saver	Goods have been received well-packed, logistics is very fast and good quality	Jingdong prefecture level city in Shandong
Happy Fan Shop	Green Intelligent Power Saver	This baby's quality is okay.	Jingdong prefecture level city in Shandong
Happy Fan Shop	Green Intelligent Power Saver	Counterfeit goods.	Jingdong prefecture level city in Shandong
Happy Fan Shop	Green Intelligent Power Saver	The effect is not very obvious	Jingdong prefecture level city in Shandong
Happy Fan Shop	Green Intelligent Power Saver	It's not the same thing.	Jingdong prefecture level city in Shandong
Happy Fan Shop	Green Intelligent Power Saver	There is no load how much w seems false, nothing works.	Jingdong prefecture level city in Shandong
Happy Fan Shop	Green Intelligent Power Saver	I received the item yesterday, got it back and plugged it in, and it used a little more power yesterday than I had before	Jingdong prefecture level city in Shandong
Happy Fan Shop	Green Intelligent Power Saver	Haven't seen the power saving mode yet	Jingdong prefecture level city in Shandong
Happy Fan Shop	Green Intelligent Power Saver	This product has no known effect	Jingdong prefecture level city in Shandong
Happy Fan Shop	Green Intelligent Power Saver	Refund Returns $16	Jingdong prefecture level city in Shandong
Happy Fan Shop	Green Intelligent Power Saver	The previous year bought a, almost every month can save a 70 dollars or so, used a year, no problem at all, look very new like, this time to move to a new home decisively repurchase 2 units, very believe this	Jingdong prefecture level city in Shandong
Happy Fan Shop	Green Intelligent Power Saver	Has been the second time to buy, this power saving device is really good power, before every month to spend 300 electricity, now are about 200, last time that 6, this time to help lead the family to buy 6,	Jingdong prefecture level city in Shandong
Happy Fan Shop	Green Intelligent Power Saver	In the spirit of trying to experience the new product, the use of down compared to the usual power consumption, with the addition of the quarter, is indeed a lot less electricity, originally more than 450	Jingdong prefecture level city in Shandong
Happy Fan Shop	Green Intelligent Power Saver	Has not been the first time to buy back, good use, power saving device is really powerful, before the monthly electricity bill of more than 500 Now more than 300, this time to help neighbors to buy several	Jingdong prefecture level city in Shandong
Happy Fan Shop	Green Intelligent Power Saver	Home appliances are very many, the circuit is also very complex, at first it is to save 9% of the electricity, and then used more than half a month, found that the power saving rate is as high as more	Jingdong prefecture level city in Shandong
Happy Fan Shop	Green Intelligent Power Saver	My friend bought it last year and saved hundreds of dollars on his electricity bill in one summer Appearance and style: it looks better than the one I bought before Quality is also good with a light.	Jingdong prefecture level city in Shandong
Happy Fan Shop	Green Intelligent Power Saver	Saver received, on the spot on the plug with the feeling of immediate power savings, appearance, packaging is very delicate, very satisfied, I hope to achieve satisfactory results!	Jingdong prefecture level city in Shandong
Happy Fan Shop	Green Intelligent Power Saver	The power saving effect is very good, is considered an old customer, also bought before, this time is to buy two for mom and dad, customer service attitude is good, praise!	Jingdong prefecture level city in Shandong
Happy Fan Shop	Green Intelligent Power Saver	After observing for a while, it really saves a lot of money I didn't realize it was still really reliable, so I'll give you a good review.	Jingdong prefecture level city in Shandong
Happy Fan Shop	Green Intelligent Power Saver	My family a month with this monthly almost save more than 200, power-saving effect: have bought a second time, really effective Cost-effective: not bad Appearance style: roughly the same A few months to the home electricity is indeed less, this time it is a re-buy three, send a friend two, they stay a	Jingdong prefecture level city in Shandong
Happy Fan Shop	Green Intelligent Power Saver	A month almost save more than 150 dollars in electricity, home appliances more, the main this can also protect high-power appliances, after using this device, these two months have not burned, things are very good, it is said that this is the latest model, the overall price is cheap and beautiful!	Jingdong prefecture level city in Shandong
Happy Fan Shop	Green Intelligent Power Saver	Received, powerful, great quality, love the style😍	Jingdong prefecture level city in Shandong
Happy Fan Shop	Green Intelligent Power Saver	This intelligent energy saver is especially good to use and save electricity hope everyone will buy it!	Jingdong prefecture level city in Shandong
Happy Fan Shop	Green Intelligent Power Saver	Air conditioning adapter effect is particularly great, the use of very simple, plug into the socket on the summer air conditioning power is too large, electricity is also expensive, with this product directly on the electricity bill to save half, has been purchased a few more, the effect is too awesome!	Jingdong prefecture level city in Shandong
Happy Fan Shop	Green Intelligent Power Saver	The family plugged in 5, monthly electricity savings of about 300 yuan. My family before a month of electricity costs more than 800, this time with this saver after the electricity bill is actually only more than 500, amazing, thanks to the store, will recommend other friends to buy!	Jingdong prefecture level city in Shandong
Happy Fan Shop	Green Intelligent Power Saver	It was recommended to me by a neighbor's family and it's nice to use it to save power.	Jingdong prefecture level city in Shandong
Happy Fan Shop	Green Intelligent Power Saver	Second time I've purchased it and the power saving is effective!	Jingdong prefecture level city in Shandong
Happy Fan Shop	Green Intelligent Power Saver	The effect is really good, before a month of more than 300 electricity, now every month every day with the air conditioning, it is more than 100 electricity! This thing is really amazing, one after another to buy and 5.6, home installed 3!	Jingdong prefecture level city in Shandong
Happy Fan Shop	Green Intelligent Power Saver	Received this product, began to use, small things look very wrong, can save a little electricity, the monthly electricity bill is too high, after plugging in did not pull, or save a lot of electricity, point	Jingdong prefecture level city in Shandong
Happy Fan Shop	Green Intelligent Power Saver	Simple and convenient, just that plug in, you can save electricity. A month can save dozens of dollars, a year down to save a lot of, very good product cost-effective, everyone can afford to buy and use!	Jingdong prefecture level city in Shandong
Happy Fan Shop	Green Intelligent Power Saver	Electricity bill from more than 200 yuan per month, down to more than 150 yuan, the original bought home, used two months, really have power-saving effect, this time and sold two, business service attitude is	Jingdong prefecture level city in Shandong
Happy Fan Shop	Green Intelligent Power Saver	Like to watch some movies at home and so on, sometimes at night until two or three o'clock, and recently the air conditioning is not turned off for a night, rather wasteful, I have to say that the effect is very good, quite like it!	Jingdong prefecture level city in Shandong
Happy Fan Shop	Green Intelligent Power Saver	Power-saving effect: the effect is very good, a month to save more than 200 dollars Cost-effective: good value for money Appearance and style: small and lovely Workmanship material: okay Experience: good	Jingdong prefecture level city in Shandong
Happy Fan Shop	Green Intelligent Power Saver	It's okay I quite like it, the stuff is very good. Come back in the future. You can rest assured to buy. Cost-effective all. Much cheaper than the physical store	Jingdong prefecture level city in Shandong
Happy Fan Shop	Green Intelligent Power Saver	More than 150 less, last month's electricity bill is almost 400, this month is only about 200, really save electricity, I am home to buy three, I did not expect a month to pay back	Jingdong prefecture level city in Shandong

Happy Fan Shop	Green Intelligent Power Saver	I bought three of these first and tried them for two months and they still felt useful, so now I've bought another one. Good and recommended!	Jingdong prefecture level city in Shandong
Happy Fan Shop	Green Intelligent Power Saver	This energy saver is very good quality and inexpensive next time I will buy it again!	Jingdong prefecture level city in Shandong
Happy Fan Shop	Green Intelligent Power Saver	Merchant service attitude is not good, the quality does not match, the business does not give the package return	Jingdong prefecture level city in Shandong
Happy Fan Shop	Green Intelligent Power Saver	The goods are very small do not know how effective after a few days to follow up the review	Jingdong prefecture level city in Shandong
Happy Fan Shop	Green Intelligent Power Saver	Not used before more than 200 a month, after using about 100 a month, my family used three, customer service said I use electricity recommended 2-3, I think more than one effect is better on the purchase of 4, the effect is very obvious, recommended to buy!	Jingdong prefecture level city in Shandong
Happy Fan Shop	Green Intelligent Power Saver	It's okay. I'll buy it again today.	Jingdong prefecture level city in Shandong
Happy Fan Shop	Green Intelligent Power Saver	Finally received the goods, very, very much like,, has been a lot of people praise, really too happy, has been his family's loyal fans, will continue to buy buy buy buy!	Jingdong prefecture level city in Shandong
Happy Fan Shop	Green Intelligent Power Saver	After using this, my monthly electricity bill was 1,200 pounds, now it's only 180 bucks a month, how good is it?	Jingdong prefecture level city in Shandong
Happy Fan Shop	Green Intelligent Power Saver	The item is a scam return to marry a dozen dollars in shipping fees. Buy terrific role is purely a scam.	Jingdong prefecture level city in Shandong
Happy Fan Shop	Green Intelligent Power Saver	It's really good, cost-effective and good quality, and the logistics are fast! It arrived in one day!	Jingdong prefecture level city in Shandong
Happy Fan Shop	Green Intelligent Power Saver	Great brand! Trustworthy! Works super well! Expensive for a reason! Great merchandise!	Jingdong prefecture level city in Shandong
Happy Fan Shop	Green Intelligent Power Saver	The baby is really great, I like it very much, my friends also say good to come, recommended to buy!	Jingdong prefecture level city in Shandong
Happy Fan Shop	Green Intelligent Power Saver	Fake couldn't be more fake. Bought one plugged in still so much electricity.	Jingdong prefecture level city in Shandong
Happy Fan Shop	Green Intelligent Power Saver	This appliance works great. Let's see how it works. We'll see later.	Jingdong prefecture level city in Shandong
Happy Fan Shop	Green Intelligent Power Saver	Used it before commenting, very happy with it	Jingdong prefecture level city in Shandong
Happy Fan Shop	Green Intelligent Power Saver	Goods have been received well-packed, logistics is very fast and good quality	Jingdong prefecture level city in Shandong
Happy Fan Shop	Green Intelligent Power Saver	This baby's quality is okay.	Jingdong prefecture level city in Shandong
Happy Fan Shop	Green Intelligent Power Saver	Counterfeit goods.	Jingdong prefecture level city in Shandong
Happy Fan Shop	Green Intelligent Power Saver	The effect is not very obvious	Jingdong prefecture level city in Shandong
Happy Fan Shop	Green Intelligent Power Saver	It's not the same thing.	Jingdong prefecture level city in Shandong
Happy Fan Shop	Green Intelligent Power Saver	There is no load how much w seems false, nothing works.	Jingdong prefecture level city in Shandong
Happy Fan Shop	Green Intelligent Power Saver	I received the item yesterday, got it back and plugged it in, and it used a little more power yesterday than I had before	Jingdong prefecture level city in Shandong
Happy Fan Shop	Green Intelligent Power Saver	Haven't seen the power saving mode yet	Jingdong prefecture level city in Shandong
Happy Fan Shop	Green Intelligent Power Saver	This product has no known effect	Jingdong prefecture level city in Shandong
Happy Fan Shop	Green Intelligent Power Saver	Refund Returns $16	Jingdong prefecture level city in Shandong
Happy Fan Shop	Green Intelligent Power Saver	The previous year bought a, almost every month can save a 70 dollars or so, used a year, no problem at all, look very new like, this time to move to a new home decisively repurchase 2 units, very believe this	Jingdong prefecture level city in Shandong
Happy Fan Shop	Green Intelligent Power Saver	Has been the second time to buy, this power saving device is really good power, before every month to spend 300 electricity, now are about 200, last time that 6, this time to help lead the family to buy 6,	Jingdong prefecture level city in Shandong
Happy Fan Shop	Green Intelligent Power Saver	In the spirit of trying to experience the new product, the use of down compared to the usual power consumption, with the addition of the quarter, is indeed a lot less electricity, originally more than 450	Jingdong prefecture level city in Shandong
Happy Fan Shop	Green Intelligent Power Saver	Has not been the first time to buy back, good use, power saving device is really powerful, before the monthly electricity bill of more than 500 Now more than 300, this time to help neighbors to buy several	Jingdong prefecture level city in Shandong
Happy Fan Shop	Green Intelligent Power Saver	Home appliances are very many, the circuit is also very complex, at first it is to save 9% of the electricity, and then used more than half a month, found that the power saving rate is as high as more	Jingdong prefecture level city in Shandong
Happy Fan Shop	Green Intelligent Power Saver	My friend bought it last year and saved hundreds of dollars on his electricity bill in one summer Appearance and style: it looks better than the one I bought before Quality is also good with a light.	Jingdong prefecture level city in Shandong
Happy Fan Shop	Green Intelligent Power Saver	Saver received, on the spot on the plug with the feeling of immediate power savings, appearance, packaging is very delicate, very satisfied, I hope to achieve satisfactory results!	Jingdong prefecture level city in Shandong
Happy Fan Shop	Green Intelligent Power Saver	The power saving effect is very good, is considered an old customer, also bought before, this time is to buy two for mom and dad, customer service attitude is good, praise!	Jingdong prefecture level city in Shandong
Happy Fan Shop	Green Intelligent Power Saver	After observing for a while, it really saves a lot of money I didn't realize it was still really reliable, so I'll give you a good review.	Jingdong prefecture level city in Shandong
Happy Fan Shop	Green Intelligent Power Saver	My family a month with this monthly almost save more than 200, power-saving effect: have bought a second time, really effective Cost-effective: not bad Appearance style: roughly the same A few months to the home electricity is indeed less, this time it is a re-buy three, send a friend two, they stay a	Jingdong prefecture level city in Shandong
Happy Fan Shop	Green Intelligent Power Saver	A month almost save more than 150 dollars in electricity, home appliances more, the main this can also protect high-power appliances, after using this device, these two months have not burned, things are very good, it is said that this is the latest model, the overall price is cheap and beautiful!	Jingdong prefecture level city in Shandong
Happy Fan Shop	Green Intelligent Power Saver	Received, powerful, great quality, love the style😍	Jingdong prefecture level city in Shandong
Happy Fan Shop	Green Intelligent Power Saver	This intelligent energy saver is especially good to use and save electricity hope everyone will buy it!	Jingdong prefecture level city in Shandong
Happy Fan Shop	Green Intelligent Power Saver	Air conditioning adapter effect is particularly great, the use of very simple, plug into the socket on the summer air conditioning power is too large, electricity is also expensive, with this product directly on the electricity bill to save half, has been purchased a few more, the effect is too awesome!	Jingdong prefecture level city in Shandong
Happy Fan Shop	Green Intelligent Power Saver	The family plugged in 5, monthly electricity savings of about 300 yuan. My family before a month of electricity costs more than 800, this time with this saver after the electricity bill is actually only more than 500, amazing, thanks to the store, will recommend other friends to buy!	Jingdong prefecture level city in Shandong
Happy Fan Shop	Green Intelligent Power Saver	It was recommended to me by a neighbor's family and it's nice to use it to save power.	Jingdong prefecture level city in Shandong
Happy Fan Shop	Green Intelligent Power Saver	Second time I've purchased it and the power saving is effective!	Jingdong prefecture level city in Shandong
Happy Fan Shop	Green Intelligent Power Saver	The effect is really good, before a month of more than 300 electricity, now every month every day with the air conditioning, it is more than 100 electricity! This thing is really amazing, one after another to buy and 5.6, home installed 3!	Jingdong prefecture level city in Shandong
Happy Fan Shop	Green Intelligent Power Saver	Received this product, began to use, small things look very wrong, can save a little electricity, the monthly electricity bill is too high, after plugging in did not pull, or save a lot of electricity, point	Jingdong prefecture level city in Shandong
Happy Fan Shop	Green Intelligent Power Saver	Simple and convenient, just that plug in, you can save electricity. A month can save dozens of dollars, a year down to save a lot of, very good product cost-effective, everyone can afford to buy and use!	Jingdong prefecture level city in Shandong
Happy Fan Shop	Green Intelligent Power Saver	Electricity bill from more than 200 yuan per month, down to more than 150 yuan, the original bought home, used two months, really have power-saving effect, this time and sold two, business service attitude is	Jingdong prefecture level city in Shandong
Happy Fan Shop	Green Intelligent Power Saver	Like to watch some movies at home and so on, sometimes at night until two or three o'clock, and recently the air conditioning is not turned off for a night, rather wasteful, I have to say that the effect is very good, quite like it!	Jingdong prefecture level city in Shandong
Happy Fan Shop	Green Intelligent Power Saver	Power-saving effect: the effect is very good, a month to save more than 200 dollars Cost-effective: good value for money Appearance and style: small and lovely Workmanship material: okay Experience: good	Jingdong prefecture level city in Shandong

Happy Fan Shop	Green Intelligent Power Saver	It's okay I quite like it, the stuff is very good. Come back in the future. You can rest assured to buy. Cost-effective all. Much cheaper than the physical store	Jingdong prefecture level city in Shandong
Happy Fan Shop	Green Intelligent Power Saver	More than 150 less, last month's electricity bill is almost 400, this month is only about 200, really save electricity, I am home to buy three, I did not expect a month to pay back	Jingdong prefecture level city in Shandong
Happy Fan Shop	Green Intelligent Power Saver	I bought three of these first and tried them for two months and they still felt useful, so now I've bought another one. Good and recommended!	Jingdong prefecture level city in Shandong
Happy Fan Shop	Green Intelligent Power Saver	This energy saver is very good quality and inexpensive next time I will buy it again!	Jingdong prefecture level city in Shandong
Happy Fan Shop	Green Intelligent Power Saver	Merchant service attitude is not good, the quality does not match, the business does not give the package return	Jingdong prefecture level city in Shandong
Happy Fan Shop	Green Intelligent Power Saver	The goods are very small do not know how effective after a few days to follow up the review	Jingdong prefecture level city in Shandong
Happy Fan Shop	Green Intelligent Power Saver	Not used before more than 200 a month, after using about 100 a month, my family used three, customer service said I use electricity recommended 2-3, I think more than one effect is better on the purchase of 4, the effect is very obvious, recommended to buy!	Jingdong prefecture level city in Shandong
Happy Fan Shop	Green Intelligent Power Saver	It's okay. I'll buy it again today.	Jingdong prefecture level city in Shandong
Happy Fan Shop	Green Intelligent Power Saver	Finally received the goods, very, very much like,, has been a lot of people praise, really too happy, has been his family's loyal fans, will continue to buy buy buy!	Jingdong prefecture level city in Shandong
Happy Fan Shop	Green Intelligent Power Saver	After using this, my monthly electricity bill was 1,200 pounds, now it's only 180 bucks a month, how good is it?	Jingdong prefecture level city in Shandong
Happy Fan Shop	Green Intelligent Power Saver	The item is a scam return to marry a dozen dollars in shipping fees. Buy terrific role is purely a scam.	Jingdong prefecture level city in Shandong
Happy Fan Shop	Green Intelligent Power Saver	It's really good, cost-effective and good quality, and the logistics are fast! It arrived in one day!	Jingdong prefecture level city in Shandong
Happy Fan Shop	Green Intelligent Power Saver	Great brand! Trustworthy! Works super well! Expensive for a reason! Great merchandise!	Jingdong prefecture level city in Shandong
Happy Fan Shop	Green Intelligent Power Saver	The baby is really great, I like it very much, my friends also say good to come, recommended to buy!	Jingdong prefecture level city in Shandong
Happy Fan Shop	Green Intelligent Power Saver	Fake couldn't be more fake. Bought one plugged in still so much electricity.	Jingdong prefecture level city in Shandong
Happy Fan Shop	Green Intelligent Power Saver	This appliance works great. Let's see how it works. We'll see later.	Jingdong prefecture level city in Shandong
Happy Fan Shop	Green Intelligent Power Saver	Used it before commenting, very happy with it	Jingdong prefecture level city in Shandong
Happy Fan Shop	Green Intelligent Power Saver	Goods have been received well-packed, logistics is very fast and good quality	Jingdong prefecture level city in Shandong
Happy Fan Shop	Green Intelligent Power Saver	This baby's quality is okay.	Jingdong prefecture level city in Shandong
Happy Fan Shop	Green Intelligent Power Saver	Counterfeit goods.	Jingdong prefecture level city in Shandong
Happy Fan Shop	Green Intelligent Power Saver	The effect is not very obvious	Jingdong prefecture level city in Shandong
Happy Fan Shop	Green Intelligent Power Saver	It's not the same thing.	Jingdong prefecture level city in Shandong
Happy Fan Shop	Green Intelligent Power Saver	There is no load how much w seems false, nothing works.	Jingdong prefecture level city in Shandong
Happy Fan Shop	Green Intelligent Power Saver	I received the item yesterday, got it back and plugged it in, and it used a little more power yesterday than I had before	Jingdong prefecture level city in Shandong
Happy Fan Shop	Green Intelligent Power Saver	Haven't seen the power saving mode yet	Jingdong prefecture level city in Shandong
Happy Fan Shop	Green Intelligent Power Saver	This product has no known effect	Jingdong prefecture level city in Shandong
Happy Fan Shop	Green Intelligent Power Saver	Refund Returns $16	Jingdong prefecture level city in Shandong
Happy Fan Shop	Green Intelligent Power Saver	The previous year bought a, almost every month can save a 70 dollars or so, used a year, no problem at all, look very new like, this time to move to a new home decisively repurchase 2 units, very believe this	Jingdong prefecture level city in Shandong
Happy Fan Shop	Green Intelligent Power Saver	Has been the second time to buy, this power saving device is really good power, before every month to spend 300 electricity, now are about 200, last time that 6, this time to help lead the family to buy 6,	Jingdong prefecture level city in Shandong
Happy Fan Shop	Green Intelligent Power Saver	In the spirit of trying to experience the new product, the use of down compared to the usual power consumption, with the addition of the quarter, is indeed a lot less electricity, originally more than 450	Jingdong prefecture level city in Shandong
Happy Fan Shop	Green Intelligent Power Saver	Has not been the first time to buy back, good use, power saving device is really powerful, before the monthly electricity bill of more than 500 Now more than 300, this time to help neighbors to buy several	Jingdong prefecture level city in Shandong
Happy Fan Shop	Green Intelligent Power Saver	Home appliances are very many, the circuit is also very complex, at first it is to save 9% of the electricity, and then used more than half a month, found that the power saving rate is as high as more	Jingdong prefecture level city in Shandong
Happy Fan Shop	Green Intelligent Power Saver	My friend bought it last year and saved hundreds of dollars on his electricity bill in one summer Appearance and style: it looks better than the one I bought before Quality is also good with a light.	Jingdong prefecture level city in Shandong
Happy Fan Shop	Green Intelligent Power Saver	Saver received, on the spot on the plug with the feeling of immediate power savings, appearance, packaging is very delicate, very satisfied, I hope to achieve satisfactory results!	Jingdong prefecture level city in Shandong
Happy Fan Shop	Green Intelligent Power Saver	The power saving effect is very good, is considered an old customer, also bought before, this time is to buy two for mom and dad, customer service attitude is good, praise!	Jingdong prefecture level city in Shandong
Happy Fan Shop	Green Intelligent Power Saver	After observing for a while, it really saves a lot of money I didn't realize it was still really reliable, so I'll give you a good review.	Jingdong prefecture level city in Shandong
Happy Fan Shop	Green Intelligent Power Saver	My family a month with this monthly almost save more than 200, power-saving effect: have bought a second time, really effective Cost-effective: not bad Appearance style: roughly the same A few months to the home electricity is indeed less, this time it is a re-buy three, send a friend two, they stay a	Jingdong prefecture level city in Shandong
Happy Fan Shop	Green Intelligent Power Saver	A month almost save more than 150 dollars in electricity, home appliances more, the main this can also protect high-power appliances, after using this device, these two months have not burned, things are very good, it is said that this is the latest model, the overall price is cheap and beautiful!	Jingdong prefecture level city in Shandong
Happy Fan Shop	Green Intelligent Power Saver	Received, powerful, great quality, love the style😍	Jingdong prefecture level city in Shandong
Happy Fan Shop	Green Intelligent Power Saver	This intelligent energy saver is especially good to use and save electricity hope everyone will buy it!	Jingdong prefecture level city in Shandong
Happy Fan Shop	Green Intelligent Power Saver	Air conditioning adapter effect is particularly great, the use of very simple, plug into the socket on the summer air conditioning power is too large, electricity is also expensive, with this product directly on the electricity bill to save half, has been purchased a few more, the effect is too awesome!	Jingdong prefecture level city in Shandong
Happy Fan Shop	Green Intelligent Power Saver	The family plugged in 5, monthly electricity savings of about 300 yuan. My family before a month of electricity costs more than 800, this time with this saver after the electricity bill is actually only more than 500, amazing, thanks to the store, will recommend other friends to buy!	Jingdong prefecture level city in Shandong
Happy Fan Shop	Green Intelligent Power Saver	It was recommended to me by a neighbor's family and it's nice to use it to save power.	Jingdong prefecture level city in Shandong
Happy Fan Shop	Green Intelligent Power Saver	Second time I've purchased it and the power saving is effective!	Jingdong prefecture level city in Shandong
Happy Fan Shop	Green Intelligent Power Saver	The effect is really good, before a month of more than 300 electricity, now every month every day with the air conditioning, it is more than 100 electricity! This thing is really amazing, one after another to buy and 5.6, home installed 3!	Jingdong prefecture level city in Shandong
Happy Fan Shop	Green Intelligent Power Saver	Received this product, began to use, small things look very wrong, can save a little electricity, the monthly electricity bill is too high, after plugging in did not pull, or save a lot of electricity, point	Jingdong prefecture level city in Shandong
Happy Fan Shop	Green Intelligent Power Saver	Simple and convenient, just that plug in, you can save electricity. A month can save dozens of dollars, a year down to save a lot of, very good product cost-effective, everyone can afford to buy and use!	Jingdong prefecture level city in Shandong
Happy Fan Shop	Green Intelligent Power Saver	Electricity bill from more than 200 yuan per month, down to more than 150 yuan, the original bought home, used two months, really have power-saving effect, this time and sold two, business service attitude is	Jingdong prefecture level city in Shandong

Happy Fan Shop	Green Intelligent Power Saver	Like to watch some movies at home and so on, sometimes at night until two or three o'clock, and recently the air conditioning is not turned off for a night, rather wasteful, I have to say that the effect is very good, quite like it!	Jingdong prefecture level city in Shandong
Happy Fan Shop	Green Intelligent Power Saver	Power-saving effect: the effect is very good, a month to save more than 200 dollars Cost-effective: good value for money Appearance and style: small and lovely Workmanship material: okay Experience: good	Jingdong prefecture level city in Shandong
Happy Fan Shop	Green Intelligent Power Saver	It's okay I quite like it, the stuff is very good. Come back in the future. You can rest assured to buy. Cost-effective all. Much cheaper than the physical store	Jingdong prefecture level city in Shandong
Happy Fan Shop	Green Intelligent Power Saver	More than 150 less, last month's electricity bill is almost 400, this month is only about 200, really save electricity, I am home to buy three, I did not expect a month to pay back	Jingdong prefecture level city in Shandong
Happy Fan Shop	Green Intelligent Power Saver	I bought three of these first and tried them for two months and they still felt useful, so now I've bought another one. Good and recommended!	Jingdong prefecture level city in Shandong
Happy Fan Shop	Green Intelligent Power Saver	This energy saver is very good quality and inexpensive next time I will buy it again!	Jingdong prefecture level city in Shandong
Happy Fan Shop	Green Intelligent Power Saver	Merchant service attitude is not good, the quality does not match, the business does not give the package return	Jingdong prefecture level city in Shandong
Happy Fan Shop	Green Intelligent Power Saver	The goods are very small do not know how effective after a few days to follow up the review	Jingdong prefecture level city in Shandong
Happy Fan Shop	Green Intelligent Power Saver	Not used before more than 200 a month, after using about 100 a month, my family used three, customer service said I use electricity recommended 2-3, I think more than one effect is better on the purchase of 4, the effect is very obvious, recommended to buy!	Jingdong prefecture level city in Shandong
Happy Fan Shop	Green Intelligent Power Saver	It's okay. I'll buy it again today.	Jingdong prefecture level city in Shandong
Happy Fan Shop	Green Intelligent Power Saver	Finally received the goods, very, very much like,, has been a lot of people praise, really too happy, has been his family's loyal fans, will continue to buy buy buy buy!	Jingdong prefecture level city in Shandong
Happy Fan Shop	Green Intelligent Power Saver	After using this, my monthly electricity bill was 1,200 pounds, now it's only 180 bucks a month, how good is it?	Jingdong prefecture level city in Shandong
Happy Fan Shop	Green Intelligent Power Saver	The item is a scam return to marry a dozen dollars in shipping fees. Buy terrific role is purely a scam.	Jingdong prefecture level city in Shandong
Happy Fan Shop	Green Intelligent Power Saver	It's really good, cost-effective and good quality, and the logistics are fast! It arrived in one day!	Jingdong prefecture level city in Shandong
Happy Fan Shop	Green Intelligent Power Saver	Great brand! Trustworthy! Works super well! Expensive for a reason! Great merchandise!	Jingdong prefecture level city in Shandong
Happy Fan Shop	Green Intelligent Power Saver	The baby is really great, I like it very much, my friends also say good to come, recommended to buy!	Jingdong prefecture level city in Shandong
Happy Fan Shop	Green Intelligent Power Saver	Fake couldn't be more fake. Bought one plugged in still so much electricity.	Jingdong prefecture level city in Shandong
Happy Fan Shop	Green Intelligent Power Saver	This appliance works great. Let's see how it works. We'll see later.	Jingdong prefecture level city in Shandong
Happy Fan Shop	Green Intelligent Power Saver	Used it before commenting, very happy with it	Jingdong prefecture level city in Shandong
Happy Fan Shop	Green Intelligent Power Saver	Goods have been received well-packed, logistics is very fast and good quality	Jingdong prefecture level city in Shandong
Happy Fan Shop	Green Intelligent Power Saver	This baby's quality is okay.	Jingdong prefecture level city in Shandong
Happy Fan Shop	Green Intelligent Power Saver	Counterfeit goods.	Jingdong prefecture level city in Shandong
Happy Fan Shop	Green Intelligent Power Saver	The effect is not very obvious	Jingdong prefecture level city in Shandong
Happy Fan Shop	Green Intelligent Power Saver	It's not the same thing.	Jingdong prefecture level city in Shandong
Happy Fan Shop	Green Intelligent Power Saver	There is no load how much w seems false, nothing works.	Jingdong prefecture level city in Shandong
Happy Fan Shop	Green Intelligent Power Saver	I received the item yesterday, got it back and plugged it in, and it used a little more power yesterday than I had before	Jingdong prefecture level city in Shandong
Happy Fan Shop	Green Intelligent Power Saver	Haven't seen the power saving mode yet	Jingdong prefecture level city in Shandong
Happy Fan Shop	Green Intelligent Power Saver	This product has no known effect	Jingdong prefecture level city in Shandong
Happy Fan Shop	Green Intelligent Power Saver	Refund Returns $16	Jingdong prefecture level city in Shandong
Happy Fan Shop	Green Intelligent Power Saver	The previous year bought a, almost every month can save a 70 dollars or so, used a year, no problem at all, look very new like, this time to move to a new home decisively repurchase 2 units, very believe this	Jingdong prefecture level city in Shandong
Happy Fan Shop	Green Intelligent Power Saver	Has been the second time to buy, this power saving device is really good power, before every month to spend 300 electricity, now are about 200, last time that 6, this time to help lead the family to buy 6,	Jingdong prefecture level city in Shandong
Happy Fan Shop	Green Intelligent Power Saver	In the spirit of trying to experience the new product, the use of down compared to the usual power consumption, with the addition of the quarter, is indeed a lot less electricity, originally more than 450	Jingdong prefecture level city in Shandong
Happy Fan Shop	Green Intelligent Power Saver	Has not been the first time to buy back, good use, power saving device is really powerful, before the monthly electricity bill of more than 500 Now more than 300, this time to help neighbors to buy several	Jingdong prefecture level city in Shandong
Happy Fan Shop	Green Intelligent Power Saver	Home appliances are very many, the circuit is also very complex, at first it is to save 9% of the electricity, and then used more than half a month, found that the power saving rate is as high as more	Jingdong prefecture level city in Shandong
Happy Fan Shop	Green Intelligent Power Saver	My friend bought it last year and saved hundreds of dollars on his electricity bill in one summer Appearance and style: it looks better than the one I bought before Quality is also good with a light.	Jingdong prefecture level city in Shandong
Happy Fan Shop	Green Intelligent Power Saver	Saver received, on the spot on the plug with the feeling of immediate power savings, appearance, packaging is very delicate, very satisfied, I hope to achieve satisfactory results!	Jingdong prefecture level city in Shandong
Happy Fan Shop	Green Intelligent Power Saver	The power saving effect is very good, is considered an old customer, also bought before, this time is to buy two for mom and dad, customer service attitude is good, praise!	Jingdong prefecture level city in Shandong
Happy Fan Shop	Green Intelligent Power Saver	After observing for a while, it really saves a lot of money I didn't realize it was still really reliable, so I'll give you a good review.	Jingdong prefecture level city in Shandong
Happy Fan Shop	Green Intelligent Power Saver	My family a month with this monthly almost save more than 200, power-saving effect: have bought a second time, really effective Cost-effective: not bad Appearance style: roughly the same A few months to the home electricity is indeed less, this time it is a re-buy three, send a friend two, they stay a	Jingdong prefecture level city in Shandong
Happy Fan Shop	Green Intelligent Power Saver	A month almost save more than 150 dollars in electricity, home appliances more, the main this can also protect high-power appliances, after using this device, these two months have not burned, things are very good, it is said that this is the latest model, the overall price is cheap and beautiful!	Jingdong prefecture level city in Shandong
Happy Fan Shop	Green Intelligent Power Saver	Received, powerful, great quality, love the style😍	Jingdong prefecture level city in Shandong
Happy Fan Shop	Green Intelligent Power Saver	This intelligent energy saver is especially good to use and save electricity hope everyone will buy it!	Jingdong prefecture level city in Shandong
Happy Fan Shop	Green Intelligent Power Saver	Air conditioning adapter effect is particularly great, the use of very simple, plug into the socket on the summer air conditioning power is too large, electricity is also expensive, with this product directly on the electricity bill to save half, has been purchased a few more, the effect is too awesome!	Jingdong prefecture level city in Shandong
Happy Fan Shop	Green Intelligent Power Saver	The family plugged in 5, monthly electricity savings of about 300 yuan. My family before a month of electricity costs more than 800, this time with this saver after the electricity bill is actually only more than 500, amazing, thanks to the store, will recommend other friends to buy!	Jingdong prefecture level city in Shandong
Happy Fan Shop	Green Intelligent Power Saver	It was recommended to me by a neighbor's family and it's nice to use it to save power.	Jingdong prefecture level city in Shandong
Happy Fan Shop	Green Intelligent Power Saver	Second time I've purchased it and the power saving is effective!	Jingdong prefecture level city in Shandong
Happy Fan Shop	Green Intelligent Power Saver	The effect is really good, before a month of more than 300 electricity, now every month every day with the air conditioning, it is more than 100 electricity! This thing is really amazing, one after another to buy and 5.6, home installed 3!	Jingdong prefecture level city in Shandong
Happy Fan Shop	Green Intelligent Power Saver	Received this product, began to use, small things look very wrong, can save a little electricity, the monthly electricity bill is too high, after plugging in did not pull, or save a lot of electricity, point	Jingdong prefecture level city in Shandong

Happy Fan Shop	Green Intelligent Power Saver	Simple and convenient, just that plug in, you can save electricity. A month can save dozens of dollars, a year down to save a lot of, very good product cost-effective, everyone can afford to buy and use!	Jingdong prefecture level city in Shandong
Happy Fan Shop	Green Intelligent Power Saver	Electricity bill from more than 200 yuan per month, down to more than 150 yuan, the original bought home, used two months, really have power-saving effect, this time and sold two, business service attitude is	Jingdong prefecture level city in Shandong
Happy Fan Shop	Green Intelligent Power Saver	Like to watch some movies at home and so on, sometimes at night until two or three o'clock, and recently the air conditioning is not turned off for a night, rather wasteful, I have to say that the effect is very good, quite like it!	Jingdong prefecture level city in Shandong
Happy Fan Shop	Green Intelligent Power Saver	Power-saving effect: the effect is very good, a month to save more than 200 dollars Cost-effective: good value for money Appearance and style: small and lovely Workmanship material: okay Experience: good	Jingdong prefecture level city in Shandong
Happy Fan Shop	Green Intelligent Power Saver	It's okay I quite like it, the stuff is very good. Come back in the future. You can rest assured to buy. Cost-effective all. Much cheaper than the physical store	Jingdong prefecture level city in Shandong
Happy Fan Shop	Green Intelligent Power Saver	More than 150 less, last month's electricity bill is almost 400, this month is only about 200, really save electricity, I am home to buy three, I did not expect a month to pay back	Jingdong prefecture level city in Shandong
Happy Fan Shop	Green Intelligent Power Saver	I bought three of these first and tried them for two months and they still felt useful, so now I've bought another one. Good and recommended!	Jingdong prefecture level city in Shandong
Happy Fan Shop	Green Intelligent Power Saver	This energy saver is very good quality and inexpensive next time I will buy it again!	Jingdong prefecture level city in Shandong
Happy Fan Shop	Green Intelligent Power Saver	Merchant service attitude is not good, the quality does not match, the business does not give the package return	Jingdong prefecture level city in Shandong
Happy Fan Shop	Green Intelligent Power Saver	The goods are very small do not know how effective after a few days to follow up the review	Jingdong prefecture level city in Shandong
Happy Fan Shop	Green Intelligent Power Saver	Not used before more than 200 a month, after using about 100 a month, my family used three, customer service said I use electricity recommended 2-3, I think more than one effect is better on the purchase of 4, the effect is very obvious, recommended to buy!	Jingdong prefecture level city in Shandong
Happy Fan Shop	Green Intelligent Power Saver	It's okay. I'll buy it again today.	Jingdong prefecture level city in Shandong
Happy Fan Shop	Green Intelligent Power Saver	Finally received the goods, very, very much like,, has been a lot of people praise, really too happy, has been his family's loyal fans, will continue to buy buy buy buy!	Jingdong prefecture level city in Shandong
Happy Fan Shop	Green Intelligent Power Saver	After using this, my monthly electricity bill was 1,200 pounds, now it's only 180 bucks a month, how good is it?	Jingdong prefecture level city in Shandong
Happy Fan Shop	Green Intelligent Power Saver	The item is a scam return to marry a dozen dollars in shipping fees. Buy terrific role is purely a scam.	Jingdong prefecture level city in Shandong
Happy Fan Shop	Green Intelligent Power Saver	It's really good, cost-effective and good quality, and the logistics are fast! It arrived in one day!	Jingdong prefecture level city in Shandong
Happy Fan Shop	Green Intelligent Power Saver	Great brand! Trustworthy! Works super well! Expensive for a reason! Great merchandise!	Jingdong prefecture level city in Shandong
Happy Fan Shop	Green Intelligent Power Saver	The baby is really great, I like it very much, my friends also say good to come, recommended to buy!	Jingdong prefecture level city in Shandong
Happy Fan Shop	Green Intelligent Power Saver	Fake couldn't be more fake. Bought one plugged in still so much electricity.	Jingdong prefecture level city in Shandong
Happy Fan Shop	Green Intelligent Power Saver	This appliance works great. Let's see how it works. We'll see later.	Jingdong prefecture level city in Shandong
Happy Fan Shop	Green Intelligent Power Saver	Used it before commenting, very happy with it	Jingdong prefecture level city in Shandong
Happy Fan Shop	Green Intelligent Power Saver	Goods have been received well-packed, logistics is very fast and good quality	Jingdong prefecture level city in Shandong
Happy Fan Shop	Green Intelligent Power Saver	This baby's quality is okay.	Jingdong prefecture level city in Shandong
Happy Fan Shop	Green Intelligent Power Saver	Counterfeit goods.	Jingdong prefecture level city in Shandong
Happy Fan Shop	Green Intelligent Power Saver	The effect is not very obvious	Jingdong prefecture level city in Shandong
Happy Fan Shop	Green Intelligent Power Saver	It's not the same thing.	Jingdong prefecture level city in Shandong
Happy Fan Shop	Green Intelligent Power Saver	There is no load how much w seems false, nothing works.	Jingdong prefecture level city in Shandong
Happy Fan Shop	Green Intelligent Power Saver	I received the item yesterday, got it back and plugged it in, and it used a little more power yesterday than I had before	Jingdong prefecture level city in Shandong
Happy Fan Shop	Green Intelligent Power Saver	Haven't seen the power saving mode yet	Jingdong prefecture level city in Shandong
Happy Fan Shop	Green Intelligent Power Saver	This product has no known effect	Jingdong prefecture level city in Shandong
Happy Fan Shop	Green Intelligent Power Saver	Refund Returns $16	Jingdong prefecture level city in Shandong
Happy Fan Shop	Green Intelligent Power Saver	The previous year bought a, almost every month can save a 70 dollars or so, used a year, no problem at all, look very new like, this time to move to a new home decisively repurchase 2 units, very believe this	Jingdong prefecture level city in Shandong
Happy Fan Shop	Green Intelligent Power Saver	Has been the second time to buy, this power saving device is really good power, before every month to spend 300 electricity, now are about 200, last time that 6, this time to help lead the family to buy 6,	Jingdong prefecture level city in Shandong
Happy Fan Shop	Green Intelligent Power Saver	In the spirit of trying to experience the new product, the use of down compared to the usual power consumption, with the addition of the quarter, is indeed a lot less electricity, originally more than 450	Jingdong prefecture level city in Shandong
Happy Fan Shop	Green Intelligent Power Saver	Has not been the first time to buy back, good use, power saving device is really powerful, before the monthly electricity bill of more than 500 Now more than 300, this time to help neighbors to buy several	Jingdong prefecture level city in Shandong
Happy Fan Shop	Green Intelligent Power Saver	Home appliances are very many, the circuit is also very complex, at first it is to save 9% of the electricity, and then used more than half a month, found that the power saving rate is as high as more	Jingdong prefecture level city in Shandong
Happy Fan Shop	Green Intelligent Power Saver	My friend bought it last year and saved hundreds of dollars on his electricity bill in one summer Appearance and style: it looks better than the one I bought before Quality is also good with a light.	Jingdong prefecture level city in Shandong
Happy Fan Shop	Green Intelligent Power Saver	Saver received, on the spot on the plug with the feeling of immediate power savings, appearance, packaging is very delicate, very satisfied, I hope to achieve satisfactory results!	Jingdong prefecture level city in Shandong
Happy Fan Shop	Green Intelligent Power Saver	The power saving effect is very good, is considered an old customer, also bought before, this time is to buy two for mom and dad, customer service attitude is good, praise!	Jingdong prefecture level city in Shandong
Happy Fan Shop	Green Intelligent Power Saver	After observing for a while, it really saves a lot of money I didn't realize it was still really reliable, so I'll give you a good review.	Jingdong prefecture level city in Shandong
Happy Fan Shop	Green Intelligent Power Saver	My family a month with this monthly almost save more than 200, power-saving effect: have bought a second time, really effective Cost-effective: not bad Appearance style: roughly the same A few months to the home electricity is indeed less, this time it is a re-buy three, send a friend two, they stay a	Jingdong prefecture level city in Shandong
Happy Fan Shop	Green Intelligent Power Saver	A month almost save more than 150 dollars in electricity, home appliances more, the main this can also protect high-power appliances, after using this device, these two months have not burned, things are very good, it is said that this is the latest model, the overall price is cheap and beautiful!	Jingdong prefecture level city in Shandong
Happy Fan Shop	Green Intelligent Power Saver	Received, powerful, great quality, love the style😍	Jingdong prefecture level city in Shandong
Happy Fan Shop	Green Intelligent Power Saver	This intelligent energy saver is especially good to use and save electricity hope everyone will buy it!	Jingdong prefecture level city in Shandong
Happy Fan Shop	Green Intelligent Power Saver	Air conditioning adapter effect is particularly great, the use of very simple, plug into the socket on the summer air conditioning power is too large, electricity is also expensive, with this product directly on the electricity bill to save half, has been purchased a few more, the effect is too awesome!	Jingdong prefecture level city in Shandong
Happy Fan Shop	Green Intelligent Power Saver	The family plugged in 5, monthly electricity savings of about 300 yuan. My family before a month of electricity costs more than 800, this time with this saver after the electricity bill is actually only more than 500, amazing, thanks to the store, will recommend other friends to buy!	Jingdong prefecture level city in Shandong
Happy Fan Shop	Green Intelligent Power Saver	It was recommended to me by a neighbor's family and it's nice to use it to save power.	Jingdong prefecture level city in Shandong
Happy Fan Shop	Green Intelligent Power Saver	Second time I've purchased it and the power saving is effective!	Jingdong prefecture level city in Shandong

Happy Fan Shop	Green Intelligent Power Saver	The effect is really good, before a month of more than 300 electricity, now every month every day with the air conditioning, it is more than 100 electricity! This thing is really amazing, one after another to buy and 5.6, home installed 3!	Jingdong prefecture level city in Shandong
Happy Fan Shop	Green Intelligent Power Saver	Received this product, began to use, small things look very wrong, can save a little electricity, the monthly electricity bill is too high, after plugging in did not pull, or save a lot of electricity, point	Jingdong prefecture level city in Shandong
Happy Fan Shop	Green Intelligent Power Saver	Simple and convenient, just that plug in, you can save electricity. A month can save dozens of dollars, a year down to save a lot of, very good product cost-effective, everyone can afford to buy and use!	Jingdong prefecture level city in Shandong
Happy Fan Shop	Green Intelligent Power Saver	Electricity bill from more than 200 yuan per month, down to more than 150 yuan, the original bought home, used two months, really have power-saving effect, this time and sold two, business service attitude is	Jingdong prefecture level city in Shandong
Happy Fan Shop	Green Intelligent Power Saver	Like to watch some movies at home and so on, sometimes at night until two or three o'clock, and recently the air conditioning is not turned off for a night, rather wasteful, I have to say that the effect is very good, quite like it!	Jingdong prefecture level city in Shandong
Happy Fan Shop	Green Intelligent Power Saver	Power-saving effect: the effect is very good, a month to save more than 200 dollars Cost-effective: good value for money Appearance and style: small and lovely Workmanship material: okay Experience: good	Jingdong prefecture level city in Shandong
Happy Fan Shop	Green Intelligent Power Saver	It's okay I quite like it, the stuff is very good. Come back in the future. You can rest assured to buy. Cost-effective all. Much cheaper than the physical store	Jingdong prefecture level city in Shandong
Happy Fan Shop	Green Intelligent Power Saver	More than 150 less, last month's electricity bill is almost 400, this month is only about 200, really save electricity, I am home to buy three, I did not expect a month to pay back	Jingdong prefecture level city in Shandong
Happy Fan Shop	Green Intelligent Power Saver	I bought three of these first and tried them for two months and they still felt useful, so now I've bought another one. Good and recommended!	Jingdong prefecture level city in Shandong
Happy Fan Shop	Green Intelligent Power Saver	This energy saver is very good quality and inexpensive next time I will buy it again!	Jingdong prefecture level city in Shandong
Happy Fan Shop	Green Intelligent Power Saver	Merchant service attitude is not good, the quality does not match, the business does not give the package return	Jingdong prefecture level city in Shandong
Happy Fan Shop	Green Intelligent Power Saver	The goods are very small do not know how effective after a few days to follow up the review	Jingdong prefecture level city in Shandong
Happy Fan Shop	Green Intelligent Power Saver	Not used before more than 200 a month, after using about 100 a month, my family used three, customer service said I use electricity recommended 2-3, I think more than one effect is better on the purchase of 4, the effect is very obvious, recommended to buy!	Jingdong prefecture level city in Shandong
Happy Fan Shop	Green Intelligent Power Saver	It's okay. I'll buy it again today.	Jingdong prefecture level city in Shandong
Happy Fan Shop	Green Intelligent Power Saver	Finally received the goods, very, very much like,, has been a lot of people praise, really too happy, has been his family's loyal fans, will continue to buy buy buy buy!	Jingdong prefecture level city in Shandong
Happy Fan Shop	Green Intelligent Power Saver	After using this, my monthly electricity bill was 1,200 pounds, now it's only 180 bucks a month, how good is it?	Jingdong prefecture level city in Shandong
Happy Fan Shop	Green Intelligent Power Saver	The item is a scam return to marry a dozen dollars in shipping fees. Buy terrific role is purely a scam.	Jingdong prefecture level city in Shandong
Happy Fan Shop	Green Intelligent Power Saver	It's really good, cost-effective and good quality, and the logistics are fast! It arrived in one day!	Jingdong prefecture level city in Shandong
Happy Fan Shop	Green Intelligent Power Saver	Great brand! Trustworthy! Works super well! Expensive for a reason! Great merchandise!	Jingdong prefecture level city in Shandong
Happy Fan Shop	Green Intelligent Power Saver	The baby is really great, I like it very much, my friends also say good to come, recommended to buy!	Jingdong prefecture level city in Shandong
Happy Fan Shop	Green Intelligent Power Saver	Fake couldn't be more fake. Bought one plugged in still so much electricity.	Jingdong prefecture level city in Shandong
Happy Fan Shop	Green Intelligent Power Saver	This appliance works great. Let's see how it works. We'll see later.	Jingdong prefecture level city in Shandong
Happy Fan Shop	Green Intelligent Power Saver	Used it before commenting, very happy with it	Jingdong prefecture level city in Shandong
Happy Fan Shop	Green Intelligent Power Saver	Goods have been received well-packed, logistics is very fast and good quality	Jingdong prefecture level city in Shandong
Happy Fan Shop	Green Intelligent Power Saver	This baby's quality is okay.	Jingdong prefecture level city in Shandong
Happy Fan Shop	Green Intelligent Power Saver	Counterfeit goods.	Jingdong prefecture level city in Shandong
Happy Fan Shop	Green Intelligent Power Saver	The effect is not very obvious	Jingdong prefecture level city in Shandong
Happy Fan Shop	Green Intelligent Power Saver	It's not the same thing.	Jingdong prefecture level city in Shandong
Happy Fan Shop	Green Intelligent Power Saver	There is no load how much w seems false, nothing works.	Jingdong prefecture level city in Shandong
Happy Fan Shop	Green Intelligent Power Saver	I received the item yesterday, got it back and plugged it in, and it used a little more power yesterday than I had before	Jingdong prefecture level city in Shandong
Happy Fan Shop	Green Intelligent Power Saver	Haven't seen the power saving mode yet	Jingdong prefecture level city in Shandong
Happy Fan Shop	Green Intelligent Power Saver	This product has no known effect	Jingdong prefecture level city in Shandong
Happy Fan Shop	Green Intelligent Power Saver	Refund Returns $16	Jingdong prefecture level city in Shandong
Happy Fan Shop	Green Intelligent Power Saver	The previous year bought a, almost every month can save a 70 dollars or so, used a year, no problem at all, look very new like, this time to move to a new home decisively repurchase 2 units, very believe this	Jingdong prefecture level city in Shandong
Happy Fan Shop	Green Intelligent Power Saver	Has been the second time to buy, this power saving device is really good power, before every month to spend 300 electricity, now are about 200, last time that 6, this time to help lead the family to buy 6,	Jingdong prefecture level city in Shandong
Happy Fan Shop	Green Intelligent Power Saver	In the spirit of trying to experience the new product, the use of down compared to the usual power consumption, with the addition of the quarter, is indeed a lot less electricity, originally more than 450	Jingdong prefecture level city in Shandong
Happy Fan Shop	Green Intelligent Power Saver	Has not been the first time to buy back, good use, power saving device is really powerful, before the monthly electricity bill of more than 500 Now more than 300, this time to help neighbors to buy several	Jingdong prefecture level city in Shandong
Happy Fan Shop	Green Intelligent Power Saver	Home appliances are very many, the circuit is also very complex, at first it is to save 9% of the electricity, and then used more than half a month, found that the power saving rate is as high as more	Jingdong prefecture level city in Shandong
Happy Fan Shop	Green Intelligent Power Saver	My friend bought it last year and saved hundreds of dollars on his electricity bill in one summer Appearance and style: it looks better than the one I bought before Quality is also good with a light.	Jingdong prefecture level city in Shandong
Happy Fan Shop	Green Intelligent Power Saver	Saver received, on the spot on the plug with the feeling of immediate power savings, appearance, packaging is very delicate, very satisfied, I hope to achieve satisfactory results!	Jingdong prefecture level city in Shandong
Happy Fan Shop	Green Intelligent Power Saver	The power saving effect is very good, is considered an old customer, also bought before, this time is to buy two for mom and dad, customer service attitude is good, praise!	Jingdong prefecture level city in Shandong
Happy Fan Shop	Green Intelligent Power Saver	After observing for a while, it really saves a lot of money I didn't realize it was still really reliable, so I'll give you a good review.	Jingdong prefecture level city in Shandong
Happy Fan Shop	Green Intelligent Power Saver	My family a month with this monthly almost save more than 200, power-saving effect: have bought a second time, really effective Cost-effective: not bad Appearance style: roughly the same A few months to the home electricity is indeed less, this time it is a re-buy three, send a friend two, they stay a	Jingdong prefecture level city in Shandong
Happy Fan Shop	Green Intelligent Power Saver	A month almost save more than 150 dollars in electricity, home appliances more, the main this can also protect high-power appliances, after using this device, these two months have not burned, things are very good, it is said that this is the latest model, the overall price is cheap and beautiful!	Jingdong prefecture level city in Shandong
Happy Fan Shop	Green Intelligent Power Saver	Received, powerful, great quality, love the style😍	Jingdong prefecture level city in Shandong
Happy Fan Shop	Green Intelligent Power Saver	This intelligent energy saver is especially good to use and save electricity hope everyone will buy it!	Jingdong prefecture level city in Shandong
Happy Fan Shop	Green Intelligent Power Saver	Air conditioning adapter effect is particularly great, the use of very simple, plug into the socket on the summer air conditioning power is too large, electricity is also expensive, with this product directly on the electricity bill to save half, has been purchased a few more, the effect is too awesome!	Jingdong prefecture level city in Shandong
Happy Fan Shop	Green Intelligent Power Saver	The family plugged in 5, monthly electricity savings of about 300 yuan. My family before a month of electricity costs more than 800, this time with this saver after the electricity bill is actually only more than 500, amazing, thanks to the store, will recommend other friends to buy!	Jingdong prefecture level city in Shandong

Happy Fan Shop	Green Intelligent Power Saver	It was recommended to me by a neighbor's family and it's nice to use it to save power.	Jingdong prefecture level city in Shandong
Happy Fan Shop	Green Intelligent Power Saver	Second time I've purchased it and the power saving is effective!	Jingdong prefecture level city in Shandong
Happy Fan Shop	Green Intelligent Power Saver	The effect is really good, before a month of more than 300 electricity, now every month every day with the air conditioning, it is more than 100 electricity! This thing is really amazing, one after another to buy and 5.6, home installed 3!	Jingdong prefecture level city in Shandong
Happy Fan Shop	Green Intelligent Power Saver	Received this product, began to use, small things look very wrong, can save a little electricity, the monthly electricity bill is too high, after plugging in did not pull, or save a lot of electricity, point	Jingdong prefecture level city in Shandong
Happy Fan Shop	Green Intelligent Power Saver	Simple and convenient, just that plug in, you can save electricity. A month can save dozens of dollars, a year down to save a lot of, very good product cost-effective, everyone can afford to buy and use!	Jingdong prefecture level city in Shandong
Happy Fan Shop	Green Intelligent Power Saver	Electricity bill from more than 200 yuan per month, down to more than 150 yuan, the original bought home, used two months, really have power-saving effect, this time and sold two, business service attitude is	Jingdong prefecture level city in Shandong
Happy Fan Shop	Green Intelligent Power Saver	Like to watch some movies at home and so on, sometimes at night until two or three o'clock, and recently the air conditioning is not turned off for a night, rather wasteful, I have to say that the effect is very good, quite like it!	Jingdong prefecture level city in Shandong
Happy Fan Shop	Green Intelligent Power Saver	Power-saving effect: the effect is very good, a month to save more than 200 dollars Cost-effective: good value for money Appearance and style: small and lovely Workmanship material: okay Experience: good	Jingdong prefecture level city in Shandong
Happy Fan Shop	Green Intelligent Power Saver	It's okay I quite like it, the stuff is very good. Come back in the future. You can rest assured to buy. Cost-effective all. Much cheaper than the physical store	Jingdong prefecture level city in Shandong
Happy Fan Shop	Green Intelligent Power Saver	More than 150 less, last month's electricity bill is almost 400, this month is only about 200, really save electricity, I am home to buy three, I did not expect a month to pay back	Jingdong prefecture level city in Shandong
Happy Fan Shop	Green Intelligent Power Saver	I bought three of these first and tried them for two months and they still felt useful, so now I've bought another one. Good and recommended!	Jingdong prefecture level city in Shandong
Happy Fan Shop	Green Intelligent Power Saver	This energy saver is very good quality and inexpensive next time I will buy it again!	Jingdong prefecture level city in Shandong
Happy Fan Shop	Green Intelligent Power Saver	Merchant service attitude is not good, the quality does not match, the business does not give the package return	Jingdong prefecture level city in Shandong
Happy Fan Shop	Green Intelligent Power Saver	The goods are very small do not know how effective after a few days to follow up the review	Jingdong prefecture level city in Shandong
Happy Fan Shop	Green Intelligent Power Saver	Not used before more than 200 a month, after using about 100 a month, my family used three, customer service said I use electricity recommended 2-3, I think more than one effect is better on the purchase of 4, the effect is very obvious, recommended to buy!	Jingdong prefecture level city in Shandong
Happy Fan Shop	Green Intelligent Power Saver	It's okay. I'll buy it again today.	Jingdong prefecture level city in Shandong
Happy Fan Shop	Green Intelligent Power Saver	Finally received the goods, very, very much like,, has been a lot of people praise, really too happy, has been his family's loyal fans, will continue to buy buy buy buy!	Jingdong prefecture level city in Shandong
Happy Fan Shop	Green Intelligent Power Saver	After using this, my monthly electricity bill was 1,200 pounds, now it's only 180 bucks a month, how good is it?	Jingdong prefecture level city in Shandong
Happy Fan Shop	Green Intelligent Power Saver	The item is a scam return to marry a dozen dollars in shipping fees. Buy terrific role is purely a scam.	Jingdong prefecture level city in Shandong
Happy Fan Shop	Green Intelligent Power Saver	It's really good, cost-effective and good quality, and the logistics are fast! It arrived in one day!	Jingdong prefecture level city in Shandong
Happy Fan Shop	Green Intelligent Power Saver	Great brand! Trustworthy! Works super well! Expensive for a reason! Great merchandise!	Jingdong prefecture level city in Shandong
Happy Fan Shop	Green Intelligent Power Saver	The baby is really great, I like it very much, my friends also say good to come, recommended to buy!	Jingdong prefecture level city in Shandong
Happy Fan Shop	Green Intelligent Power Saver	Fake couldn't be more fake. Bought one plugged in still so much electricity.	Jingdong prefecture level city in Shandong
Happy Fan Shop	Green Intelligent Power Saver	This appliance works great. Let's see how it works. We'll see later.	Jingdong prefecture level city in Shandong
Happy Fan Shop	Green Intelligent Power Saver	Used it before commenting, very happy with it	Jingdong prefecture level city in Shandong
Happy Fan Shop	Green Intelligent Power Saver	Goods have been received well-packed, logistics is very fast and good quality	Jingdong prefecture level city in Shandong
Happy Fan Shop	Green Intelligent Power Saver	This baby's quality is okay.	Jingdong prefecture level city in Shandong
Happy Fan Shop	Green Intelligent Power Saver	Counterfeit goods.	Jingdong prefecture level city in Shandong
Happy Fan Shop	Green Intelligent Power Saver	The effect is not very obvious	Jingdong prefecture level city in Shandong
Happy Fan Shop	Green Intelligent Power Saver	It's not the same thing.	Jingdong prefecture level city in Shandong
Happy Fan Shop	Green Intelligent Power Saver	There is no load how much w seems false, nothing works.	Jingdong prefecture level city in Shandong
Happy Fan Shop	Green Intelligent Power Saver	I received the item yesterday, got it back and plugged it in, and it used a little more power yesterday than I had before	Jingdong prefecture level city in Shandong
Happy Fan Shop	Green Intelligent Power Saver	Haven't seen the power saving mode yet	Jingdong prefecture level city in Shandong
Happy Fan Shop	Green Intelligent Power Saver	This product has no known effect	Jingdong prefecture level city in Shandong
Happy Fan Shop	Green Intelligent Power Saver	Refund Returns $16	Jingdong prefecture level city in Shandong
Happy Fan Shop	Green Intelligent Power Saver	The previous year bought a, almost every month can save a 70 dollars or so, used a year, no problem at all, look very new like, this time to move to a new home decisively repurchase 2 units, very believe this	Jingdong prefecture level city in Shandong
Happy Fan Shop	Green Intelligent Power Saver	Has been the second time to buy, this power saving device is really good power, before every month to spend 300 electricity, now are about 200, last time that 6, this time to help lead the family to buy 6,	Jingdong prefecture level city in Shandong
Happy Fan Shop	Green Intelligent Power Saver	In the spirit of trying to experience the new product, the use of down compared to the usual power consumption, with the addition of the quarter, is indeed a lot less electricity, originally more than 450	Jingdong prefecture level city in Shandong
Happy Fan Shop	Green Intelligent Power Saver	Has not been the first time to buy back, good use, power saving device is really powerful, before the monthly electricity bill of more than 500 Now more than 300, this time to help neighbors to buy several	Jingdong prefecture level city in Shandong
Happy Fan Shop	Green Intelligent Power Saver	Home appliances are very many, the circuit is also very complex, at first it is to save 9% of the electricity, and then used more than half a month, found that the power saving rate is as high as more	Jingdong prefecture level city in Shandong
Happy Fan Shop	Green Intelligent Power Saver	My friend bought it last year and saved hundreds of dollars on his electricity bill in one summer Appearance and style: it looks better than the one I bought before Quality is also good with a light.	Jingdong prefecture level city in Shandong
Happy Fan Shop	Green Intelligent Power Saver	Saver received, on the spot on the plug with the feeling of immediate power savings, appearance, packaging is very delicate, very satisfied, I hope to achieve satisfactory results!	Jingdong prefecture level city in Shandong
Happy Fan Shop	Green Intelligent Power Saver	The power saving effect is very good, is considered an old customer, also bought before, this time is to buy two for mom and dad, customer service attitude is good, praise!	Jingdong prefecture level city in Shandong
Happy Fan Shop	Green Intelligent Power Saver	After observing for a while, it really saves a lot of money I didn't realize it was still really reliable, so I'll give you a good review.	Jingdong prefecture level city in Shandong
Happy Fan Shop	Green Intelligent Power Saver	My family a month with this monthly almost save more than 200, power-saving effect: have bought a second time, really effective Cost-effective: not bad Appearance style: roughly the same A few months to the home electricity is indeed less, this time it is a re-buy three, send a friend two, they stay a	Jingdong prefecture level city in Shandong
Happy Fan Shop	Green Intelligent Power Saver	A month almost save more than 150 dollars in electricity, home appliances more, the main this can also protect high-power appliances, after using this device, these two months have not burned, things are very good, it is said that this is the latest model, the overall price is cheap and beautiful!	Jingdong prefecture level city in Shandong
Happy Fan Shop	Green Intelligent Power Saver	Received, powerful, great quality, love the style😍	Jingdong prefecture level city in Shandong
Happy Fan Shop	Green Intelligent Power Saver	This intelligent energy saver is especially good to use and save electricity hope everyone will buy it!	Jingdong prefecture level city in Shandong

Happy Fan Shop	Green Intelligent Power Saver	Air conditioning adapter effect is particularly great, the use of very simple, plug into the socket on the summer air conditioning power is too large, electricity is also expensive, with this product directly on the electricity bill to save half, has been purchased a few more, the effect is too awesome!	Jingdong prefecture level city in Shandong
Happy Fan Shop	Green Intelligent Power Saver	The family plugged in 5, monthly electricity savings of about 300 yuan. My family before a month of electricity costs more than 800, this time with this saver after the electricity bill is actually only more than 500, amazing, thanks to the store, will recommend other friends to buy!	Jingdong prefecture level city in Shandong
Happy Fan Shop	Green Intelligent Power Saver	It was recommended to me by a neighbor's family and it's nice to use it to save power.	Jingdong prefecture level city in Shandong
Happy Fan Shop	Green Intelligent Power Saver	Second time I've purchased it and the power saving is effective!	Jingdong prefecture level city in Shandong
Happy Fan Shop	Green Intelligent Power Saver	The effect is really good, before a month of more than 300 electricity, now every month every day with the air conditioning, it is more than 100 electricity! This thing is really amazing, one after another to buy and 5.6, home installed 3!	Jingdong prefecture level city in Shandong
Happy Fan Shop	Green Intelligent Power Saver	Received this product, began to use, small things look very wrong, can save a little electricity, the monthly electricity bill is too high, after plugging in did not pull, or save a lot of electricity, point	Jingdong prefecture level city in Shandong
Happy Fan Shop	Green Intelligent Power Saver	Simple and convenient, just that plug in, you can save electricity. A month can save dozens of dollars, a year down to save a lot of, very good product cost-effective, everyone can afford to buy and use!	Jingdong prefecture level city in Shandong
Happy Fan Shop	Green Intelligent Power Saver	Electricity bill from more than 200 yuan per month, down to more than 150 yuan, the original bought home, used two months, really have power-saving effect, this time and sold two, business service attitude is	Jingdong prefecture level city in Shandong
Happy Fan Shop	Green Intelligent Power Saver	Like to watch some movies at home and so on, sometimes at night until two or three o'clock, and recently the air conditioning is not turned off for a night, rather wasteful, I have to say that the effect is very good, quite like it!	Jingdong prefecture level city in Shandong
Happy Fan Shop	Green Intelligent Power Saver	Power-saving effect: the effect is very good, a month to save more than 200 dollars Cost-effective: good value for money Appearance and style: small and lovely Workmanship material: okay Experience: good	Jingdong prefecture level city in Shandong
Happy Fan Shop	Green Intelligent Power Saver	It's okay I quite like it, the stuff is very good. Come back in the future. You can rest assured to buy. Cost-effective all. Much cheaper than the physical store	Jingdong prefecture level city in Shandong
Happy Fan Shop	Green Intelligent Power Saver	More than 150 less, last month's electricity bill is almost 400, this month is only about 200, really save electricity, I am home to buy three, I did not expect a month to pay back	Jingdong prefecture level city in Shandong
Happy Fan Shop	Green Intelligent Power Saver	I bought three of these first and tried them for two months and they still felt useful, so now I've bought another one. Good and recommended!	Jingdong prefecture level city in Shandong
Happy Fan Shop	Green Intelligent Power Saver	This energy saver is very good quality and inexpensive next time I will buy it again!	Jingdong prefecture level city in Shandong
Happy Fan Shop	Green Intelligent Power Saver	Merchant service attitude is not good, the quality does not match, the business does not give the package return	Jingdong prefecture level city in Shandong
Happy Fan Shop	Green Intelligent Power Saver	The goods are very small do not know how effective after a few days to follow up the review	Jingdong prefecture level city in Shandong
Happy Fan Shop	Green Intelligent Power Saver	Not used before more than 200 a month, after using about 100 a month, my family used three, customer service said I use electricity recommended 2-3, I think more than one effect is better on the purchase of 4, the effect is very obvious, recommended to buy!	Jingdong prefecture level city in Shandong
Happy Fan Shop	Green Intelligent Power Saver	It's okay. I'll buy it again today.	Jingdong prefecture level city in Shandong
Happy Fan Shop	Green Intelligent Power Saver	Finally received the goods, very, very much like,, has been a lot of people praise, really too happy, has been his family's loyal fans, will continue to buy buy buy buy!	Jingdong prefecture level city in Shandong
Happy Fan Shop	Green Intelligent Power Saver	After using this, my monthly electricity bill was 1,200 pounds, now it's only 180 bucks a month, how good is it?	Jingdong prefecture level city in Shandong
Happy Fan Shop	Green Intelligent Power Saver	The item is a scam return to marry a dozen dollars in shipping fees. Buy terrific role is purely a scam.	Jingdong prefecture level city in Shandong
Happy Fan Shop	Green Intelligent Power Saver	It's really good, cost-effective and good quality, and the logistics are fast! It arrived in one day!	Jingdong prefecture level city in Shandong
Happy Fan Shop	Green Intelligent Power Saver	Great brand! Trustworthy! Works super well! Expensive for a reason! Great merchandise!	Jingdong prefecture level city in Shandong
Happy Fan Shop	Green Intelligent Power Saver	The baby is really great, I like it very much, my friends also say good to come, recommended to buy!	Jingdong prefecture level city in Shandong
Happy Fan Shop	Green Intelligent Power Saver	Fake couldn't be more fake. Bought one plugged in still so much electricity.	Jingdong prefecture level city in Shandong
Happy Fan Shop	Green Intelligent Power Saver	This appliance works great. Let's see how it works. We'll see later.	Jingdong prefecture level city in Shandong
Happy Fan Shop	Green Intelligent Power Saver	Used it before commenting, very happy with it	Jingdong prefecture level city in Shandong
Happy Fan Shop	Green Intelligent Power Saver	Goods have been received well-packed, logistics is very fast and good quality	Jingdong prefecture level city in Shandong
Happy Fan Shop	Green Intelligent Power Saver	This baby's quality is okay.	Jingdong prefecture level city in Shandong
Happy Fan Shop	Green Intelligent Power Saver	Counterfeit goods.	Jingdong prefecture level city in Shandong
Happy Fan Shop	Green Intelligent Power Saver	The effect is not very obvious	Jingdong prefecture level city in Shandong
Happy Fan Shop	Green Intelligent Power Saver	It's not the same thing.	Jingdong prefecture level city in Shandong
Happy Fan Shop	Green Intelligent Power Saver	There is no load how much w seems false, nothing works.	Jingdong prefecture level city in Shandong
Happy Fan Shop	Green Intelligent Power Saver	I received the item yesterday, got it back and plugged it in, and it used a little more power yesterday than I had before	Jingdong prefecture level city in Shandong
Happy Fan Shop	Green Intelligent Power Saver	Haven't seen the power saving mode yet	Jingdong prefecture level city in Shandong
Happy Fan Shop	Green Intelligent Power Saver	This product has no known effect	Jingdong prefecture level city in Shandong
Happy Fan Shop	Green Intelligent Power Saver	Refund Returns $16	Jingdong prefecture level city in Shandong
Happy Fan Shop	Green Intelligent Power Saver	The previous year bought a, almost every month can save a 70 dollars or so, used a year, no problem at all, look very new like, this time to move to a new home decisively repurchase 2 units, very believe this	Jingdong prefecture level city in Shandong
Happy Fan Shop	Green Intelligent Power Saver	Has been the second time to buy, this power saving device is really good power, before every month to spend 300 electricity, now are about 200, last time that 6, this time to help lead the family to buy 6,	Jingdong prefecture level city in Shandong
Happy Fan Shop	Green Intelligent Power Saver	In the spirit of trying to experience the new product, the use of down compared to the usual power consumption, with the addition of the quarter, is indeed a lot less electricity, originally more than 450	Jingdong prefecture level city in Shandong
Happy Fan Shop	Green Intelligent Power Saver	Has not been the first time to buy back, good use, power saving device is really powerful, before the monthly electricity bill of more than 500 Now more than 300, this time to help neighbors to buy several	Jingdong prefecture level city in Shandong
Happy Fan Shop	Green Intelligent Power Saver	Home appliances are very many, the circuit is also very complex, at first it is to save 9% of the electricity, and then used more than half a month, found that the power saving rate is as high as more	Jingdong prefecture level city in Shandong
Happy Fan Shop	Green Intelligent Power Saver	My friend bought it last year and saved hundreds of dollars on his electricity bill in one summer Appearance and style: it looks better than the one I bought before Quality is also good with a light.	Jingdong prefecture level city in Shandong
Happy Fan Shop	Green Intelligent Power Saver	Saver received, on the spot on the plug with the feeling of immediate power savings, appearance, packaging is very delicate, very satisfied, I hope to achieve satisfactory results!	Jingdong prefecture level city in Shandong
Happy Fan Shop	Green Intelligent Power Saver	The power saving effect is very good, is considered an old customer, also bought before, this time is to buy two for mom and dad, customer service attitude is good, praise!	Jingdong prefecture level city in Shandong
Happy Fan Shop	Green Intelligent Power Saver	After observing for a while, it really saves a lot of money I didn't realize it was still really reliable, so I'll give you a good review.	Jingdong prefecture level city in Shandong
Happy Fan Shop	Green Intelligent Power Saver	My family a month with this monthly almost save more than 200, power-saving effect: have bought a second time, really effective Cost-effective: not bad Appearance style: roughly the same A few months to the home electricity is indeed less, this time it is a re-buy three, send a friend two, they stay a	Jingdong prefecture level city in Shandong
Happy Fan Shop	Green Intelligent Power Saver	A month almost save more than 150 dollars in electricity, home appliances more, the main this can also protect high-power appliances, after using this device, these two months have not burned, things are very good, it is said that this is the latest model, the overall price is cheap and beautiful!	Jingdong prefecture level city in Shandong

Happy Fan Shop	Green Intelligent Power Saver	Received, powerful, great quality, love the style😍	Jingdong prefecture level city in Shandong
Happy Fan Shop	Green Intelligent Power Saver	This intelligent energy saver is especially good to use and save electricity hope everyone will buy it!	Jingdong prefecture level city in Shandong
Happy Fan Shop	Green Intelligent Power Saver	Air conditioning adapter effect is particularly great, the use of very simple, plug into the socket on the summer air conditioning power is too large, electricity is also expensive, with this product directly on the electricity bill to save half, has been purchased a few more, the effect is too awesome!	Jingdong prefecture level city in Shandong
Happy Fan Shop	Green Intelligent Power Saver	The family plugged in 5, monthly electricity savings of about 300 yuan. My family before a month of electricity costs more than 800, this time with this saver after the electricity bill is actually only more than 500, amazing, thanks to the store, will recommend other friends to buy!	Jingdong prefecture level city in Shandong
Happy Fan Shop	Green Intelligent Power Saver	It was recommended to me by a neighbor's family and it's nice to use it to save power.	Jingdong prefecture level city in Shandong
Happy Fan Shop	Green Intelligent Power Saver	Second time I've purchased it and the power saving is effective!	Jingdong prefecture level city in Shandong
Happy Fan Shop	Green Intelligent Power Saver	The effect is really good, before a month of more than 300 electricity, now every month every day with the air conditioning, it is more than 100 electricity! This thing is really amazing, one after another to buy and 5.6, home installed 3!	Jingdong prefecture level city in Shandong
Happy Fan Shop	Green Intelligent Power Saver	Received this product, began to use, small things look very wrong, can save a little electricity, the monthly electricity bill is too high, after plugging in did not pull, or save a lot of electricity, point	Jingdong prefecture level city in Shandong
Happy Fan Shop	Green Intelligent Power Saver	Simple and convenient, just that plug in, you can save electricity. A month can save dozens of dollars, a year down to save a lot of, very good product cost-effective, everyone can afford to buy and use!	Jingdong prefecture level city in Shandong
Happy Fan Shop	Green Intelligent Power Saver	Electricity bill from more than 200 yuan per month, down to more than 150 yuan, the original bought home, used two months, really have power-saving effect, this time and sold two, business service attitude is	Jingdong prefecture level city in Shandong
Happy Fan Shop	Green Intelligent Power Saver	Like to watch some movies at home and so on, sometimes at night until two or three o'clock, and recently the air conditioning is not turned off for a night, rather wasteful, I have to say that the effect is very good, quite like it!	Jingdong prefecture level city in Shandong
Happy Fan Shop	Green Intelligent Power Saver	Power-saving effect: the effect is very good, a month to save more than 200 dollars Cost-effective: good value for money Appearance and style: small and lovely Workmanship material: okay Experience: good	Jingdong prefecture level city in Shandong
Happy Fan Shop	Green Intelligent Power Saver	It's okay I quite like it, the stuff is very good. Come back in the future. You can rest assured to buy. Cost-effective all. Much cheaper than the physical store	Jingdong prefecture level city in Shandong
Happy Fan Shop	Green Intelligent Power Saver	More than 150 less, last month's electricity bill is almost 400, this month is only about 200, really save electricity, I am home to buy three, I did not expect a month to pay back	Jingdong prefecture level city in Shandong
Happy Fan Shop	Green Intelligent Power Saver	I bought three of these first and tried them for two months and they still felt useful, so now I've bought another one. Good and recommended!	Jingdong prefecture level city in Shandong
Happy Fan Shop	Green Intelligent Power Saver	This energy saver is very good quality and inexpensive next time I will buy it again!	Jingdong prefecture level city in Shandong
Happy Fan Shop	Green Intelligent Power Saver	Merchant service attitude is not good, the quality does not match, the business does not give the package return	Jingdong prefecture level city in Shandong
Happy Fan Shop	Green Intelligent Power Saver	The goods are very small do not know how effective after a few days to follow up the review	Jingdong prefecture level city in Shandong
Happy Fan Shop	Green Intelligent Power Saver	Not used before more than 200 a month, after using about 100 a month, my family used three, customer service said I use electricity recommended 2-3, I think more than one effect is better on the purchase of 4, the effect is very obvious, recommended to buy!	Jingdong prefecture level city in Shandong
Happy Fan Shop	Green Intelligent Power Saver	It's okay. I'll buy it again today.	Jingdong prefecture level city in Shandong
Happy Fan Shop	Green Intelligent Power Saver	Finally received the goods, very, very much like,, has been a lot of people praise, really too happy, has been his family's loyal fans, will continue to buy buy buy buy!	Jingdong prefecture level city in Shandong
Happy Fan Shop	Green Intelligent Power Saver	After using this, my monthly electricity bill was 1,200 pounds, now it's only 180 bucks a month, how good is it?	Jingdong prefecture level city in Shandong
Happy Fan Shop	Green Intelligent Power Saver	The item is a scam return to marry a dozen dollars in shipping fees. Buy terrific role is purely a scam.	Jingdong prefecture level city in Shandong
Happy Fan Shop	Green Intelligent Power Saver	It's really good, cost-effective and good quality, and the logistics are fast! It arrived in one day!	Jingdong prefecture level city in Shandong
Happy Fan Shop	Green Intelligent Power Saver	Great brand! Trustworthy! Works super well! Expensive for a reason! Great merchandise!	Jingdong prefecture level city in Shandong
Happy Fan Shop	Green Intelligent Power Saver	The baby is really great, I like it very much, my friends also say good to come, recommended to buy!	Jingdong prefecture level city in Shandong
Happy Fan Shop	Green Intelligent Power Saver	Fake couldn't be more fake. Bought one plugged in still so much electricity.	Jingdong prefecture level city in Shandong
Happy Fan Shop	Green Intelligent Power Saver	This appliance works great. Let's see how it works. We'll see later.	Jingdong prefecture level city in Shandong
Happy Fan Shop	Green Intelligent Power Saver	Used it before commenting, very happy with it	Jingdong prefecture level city in Shandong
Happy Fan Shop	Green Intelligent Power Saver	Goods have been received well-packed, logistics is very fast and good quality	Jingdong prefecture level city in Shandong
Happy Fan Shop	Green Intelligent Power Saver	This baby's quality is okay.	Jingdong prefecture level city in Shandong
Happy Fan Shop	Green Intelligent Power Saver	Counterfeit goods.	Jingdong prefecture level city in Shandong
Happy Fan Shop	Green Intelligent Power Saver	The effect is not very obvious	Jingdong prefecture level city in Shandong
Happy Fan Shop	Green Intelligent Power Saver	It's not the same thing.	Jingdong prefecture level city in Shandong
Happy Fan Shop	Green Intelligent Power Saver	There is no load how much w seems false, nothing works.	Jingdong prefecture level city in Shandong
Happy Fan Shop	Green Intelligent Power Saver	I received the item yesterday, got it back and plugged it in, and it used a little more power yesterday than I had before	Jingdong prefecture level city in Shandong
Happy Fan Shop	Green Intelligent Power Saver	Haven't seen the power saving mode yet	Jingdong prefecture level city in Shandong
Happy Fan Shop	Green Intelligent Power Saver	This product has no known effect	Jingdong prefecture level city in Shandong
Happy Fan Shop	Green Intelligent Power Saver	Refund Returns $16	Jingdong prefecture level city in Shandong
Happy Fan Shop	Green Intelligent Power Saver	The previous year bought a, almost every month can save a 70 dollars or so, used a year, no problem at all, look very new like, this time to move to a new home decisively repurchase 2 units, very believe this	Jingdong prefecture level city in Shandong
Happy Fan Shop	Green Intelligent Power Saver	Has been the second time to buy, this power saving device is really good power, before every month to spend 300 electricity, now are about 200, last time that 6, this time to help lead the family to buy 6,	Jingdong prefecture level city in Shandong
Happy Fan Shop	Green Intelligent Power Saver	In the spirit of trying to experience the new product, the use of down compared to the usual power consumption, with the addition of the quarter, is indeed a lot less electricity, originally more than 450	Jingdong prefecture level city in Shandong
Happy Fan Shop	Green Intelligent Power Saver	Has not been the first time to buy back, good use, power saving device is really powerful, before the monthly electricity bill of more than 500 Now more than 300, this time to help neighbors to buy several	Jingdong prefecture level city in Shandong
Happy Fan Shop	Green Intelligent Power Saver	Home appliances are very many, the circuit is also very complex, at first it is to save 9% of the electricity, and then used more than half a month, found that the power saving rate is as high as more	Jingdong prefecture level city in Shandong
Happy Fan Shop	Green Intelligent Power Saver	My friend bought it last year and saved hundreds of dollars on his electricity bill in one summer Appearance and style: it looks better than the one I bought before Quality is also good with a light.	Jingdong prefecture level city in Shandong
Happy Fan Shop	Green Intelligent Power Saver	Saver received, on the spot on the plug with the feeling of immediate power savings, appearance, packaging is very delicate, very satisfied, I hope to achieve satisfactory results!	Jingdong prefecture level city in Shandong
Happy Fan Shop	Green Intelligent Power Saver	The power saving effect is very good, is considered an old customer, also bought before, this time is to buy two for mom and dad, customer service attitude is good, praise!	Jingdong prefecture level city in Shandong
Happy Fan Shop	Green Intelligent Power Saver	After observing for a while, it really saves a lot of money I didn't realize it was still really reliable, so I'll give you a good review.	Jingdong prefecture level city in Shandong

Happy Fan Shop	Green Intelligent Power Saver	My family a month with this monthly almost save more than 200, power-saving effect: have bought a second time, really effective Cost-effective: not bad Appearance style: roughly the same A few months to the home electricity is indeed less, this time it is a re-buy three, send a friend two, they stay a	Jingdong prefecture level city in Shandong
Happy Fan Shop	Green Intelligent Power Saver	A month almost save more than 150 dollars in electricity, home appliances more, the main this can also protect high-power appliances, after using this device, these two months have not burned, things are very good, it is said that this is the latest model, the overall price is cheap and beautiful!	Jingdong prefecture level city in Shandong
Happy Fan Shop	Green Intelligent Power Saver	Received, powerful, great quality, love the style😍	Jingdong prefecture level city in Shandong
Happy Fan Shop	Green Intelligent Power Saver	This intelligent energy saver is especially good to use and save electricity hope everyone will buy it!	Jingdong prefecture level city in Shandong
Happy Fan Shop	Green Intelligent Power Saver	Air conditioning adapter effect is particularly great, the use of very simple, plug into the socket on the summer air conditioning power is too large, electricity is also expensive, with this product directly on the electricity bill to save half, has been purchased a few more, the effect is too awesome!	Jingdong prefecture level city in Shandong
Happy Fan Shop	Green Intelligent Power Saver	The family plugged in 5, monthly electricity savings of about 300 yuan. My family before a month of electricity costs more than 800, this time with this saver after the electricity bill is actually only more than 500, amazing, thanks to the store, will recommend other friends to buy!	Jingdong prefecture level city in Shandong
Happy Fan Shop	Green Intelligent Power Saver	It was recommended to me by a neighbor's family and it's nice to use it to save power.	Jingdong prefecture level city in Shandong
Happy Fan Shop	Green Intelligent Power Saver	Second time I've purchased it and the power saving is effective!	Jingdong prefecture level city in Shandong
Happy Fan Shop	Green Intelligent Power Saver	The effect is really good, before a month of more than 300 electricity, now every month every day with the air conditioning, it is more than 100 electricity! This thing is really amazing, one after another to buy and 5.6, home installed 3!	Jingdong prefecture level city in Shandong
Happy Fan Shop	Green Intelligent Power Saver	Received this product, began to use, small things look very wrong, can save a little electricity, the monthly electricity bill is too high, after plugging in did not pull, or save a lot of electricity, point	Jingdong prefecture level city in Shandong
Happy Fan Shop	Green Intelligent Power Saver	Simple and convenient, just that plug in, you can save electricity. A month can save dozens of dollars, a year down to save a lot of, very good product cost-effective, everyone can afford to buy and use!	Jingdong prefecture level city in Shandong
Happy Fan Shop	Green Intelligent Power Saver	Electricity bill from more than 200 yuan per month, down to more than 150 yuan, the original bought home, used two months, really have power-saving effect, this time and sold two, business service attitude is	Jingdong prefecture level city in Shandong
Happy Fan Shop	Green Intelligent Power Saver	Like to watch some movies at home and so on, sometimes at night until two or three o'clock, and recently the air conditioning is not turned off for a night, rather wasteful, I have to say that the effect is very good, quite like it!	Jingdong prefecture level city in Shandong
Happy Fan Shop	Green Intelligent Power Saver	Power-saving effect: the effect is very good, a month to save more than 200 dollars Cost-effective: good value for money Appearance and style: small and lovely Workmanship material: okay Experience: good	Jingdong prefecture level city in Shandong
Happy Fan Shop	Green Intelligent Power Saver	It's okay I quite like it, the stuff is very good. Come back in the future. You can rest assured to buy. Cost-effective all. Much cheaper than the physical store	Jingdong prefecture level city in Shandong
Happy Fan Shop	Green Intelligent Power Saver	More than 150 less, last month's electricity bill is almost 400, this month is only about 200, really save electricity, I am home to buy three, I did not expect a month to pay back	Jingdong prefecture level city in Shandong
Happy Fan Shop	Green Intelligent Power Saver	I bought three of these first and tried them for two months and they still felt useful, so now I've bought another one. Good and recommended!	Jingdong prefecture level city in Shandong
Happy Fan Shop	Green Intelligent Power Saver	This energy saver is very good quality and inexpensive next time I will buy it again!	Jingdong prefecture level city in Shandong
Happy Fan Shop	Green Intelligent Power Saver	Merchant service attitude is not good, the quality does not match, the business does not give the package return	Jingdong prefecture level city in Shandong
Happy Fan Shop	Green Intelligent Power Saver	The goods are very small do not know how effective after a few days to follow up the review	Jingdong prefecture level city in Shandong
Happy Fan Shop	Green Intelligent Power Saver	Not used before more than 200 a month, after using about 100 a month, my family used three, customer service said I use electricity recommended 2-3, I think more than one effect is better on the purchase of 4, the effect is very obvious, recommended to buy!	Jingdong prefecture level city in Shandong
Happy Fan Shop	Green Intelligent Power Saver	It's okay. I'll buy it again today.	Jingdong prefecture level city in Shandong
Happy Fan Shop	Green Intelligent Power Saver	Finally received the goods, very, very much like,, has been a lot of people praise, really too happy, has been his family's loyal fans, will continue to buy buy buy buy!	Jingdong prefecture level city in Shandong
Happy Fan Shop	Green Intelligent Power Saver	After using this, my monthly electricity bill was 1,200 pounds, now it's only 180 bucks a month, how good is it?	Jingdong prefecture level city in Shandong
Happy Fan Shop	Green Intelligent Power Saver	The item is a scam return to marry a dozen dollars in shipping fees. Buy terrific role is purely a scam.	Jingdong prefecture level city in Shandong
Happy Fan Shop	Green Intelligent Power Saver	It's really good, cost-effective and good quality, and the logistics are fast! It arrived in one day!	Jingdong prefecture level city in Shandong
Happy Fan Shop	Green Intelligent Power Saver	Great brand! Trustworthy! Works super well! Expensive for a reason! Great merchandise!	Jingdong prefecture level city in Shandong
Happy Fan Shop	Green Intelligent Power Saver	The baby is really great, I like it very much, my friends also say good to come, recommended to buy!	Jingdong prefecture level city in Shandong
Happy Fan Shop	Green Intelligent Power Saver	Fake couldn't be more fake. Bought one plugged in still so much electricity.	Jingdong prefecture level city in Shandong
Happy Fan Shop	Green Intelligent Power Saver	This appliance works great. Let's see how it works. We'll see later.	Jingdong prefecture level city in Shandong
Happy Fan Shop	Green Intelligent Power Saver	Used it before commenting, very happy with it	Jingdong prefecture level city in Shandong
Happy Fan Shop	Green Intelligent Power Saver	Goods have been received well-packed, logistics is very fast and good quality	Jingdong prefecture level city in Shandong
Happy Fan Shop	Green Intelligent Power Saver	This baby's quality is okay.	Jingdong prefecture level city in Shandong
Happy Fan Shop	Green Intelligent Power Saver	Counterfeit goods.	Jingdong prefecture level city in Shandong
Happy Fan Shop	Green Intelligent Power Saver	The effect is not very obvious	Jingdong prefecture level city in Shandong
Happy Fan Shop	Green Intelligent Power Saver	It's not the same thing.	Jingdong prefecture level city in Shandong
Happy Fan Shop	Green Intelligent Power Saver	There is no load how much w seems false, nothing works.	Jingdong prefecture level city in Shandong
Happy Fan Shop	Green Intelligent Power Saver	I received the item yesterday, got it back and plugged it in, and it used a little more power yesterday than I had before	Jingdong prefecture level city in Shandong
Happy Fan Shop	Green Intelligent Power Saver	Haven't seen the power saving mode yet	Jingdong prefecture level city in Shandong
Happy Fan Shop	Green Intelligent Power Saver	This product has no known effect	Jingdong prefecture level city in Shandong
Happy Fan Shop	Green Intelligent Power Saver	Refund Returns $16	Jingdong prefecture level city in Shandong
Happy Fan Shop	Green Intelligent Power Saver	The previous year bought a, almost every month can save a 70 dollars or so, used a year, no problem at all, look very new like, this time to move to a new home decisively repurchase 2 units, very believe this	Jingdong prefecture level city in Shandong
Happy Fan Shop	Green Intelligent Power Saver	Has been the second time to buy, this power saving device is really good power, before every month to spend 300 electricity, now are about 200, last time that 6, this time to help lead the family to buy 6,	Jingdong prefecture level city in Shandong
Happy Fan Shop	Green Intelligent Power Saver	In the spirit of trying to experience the new product, the use of down compared to the usual power consumption, with the addition of the quarter, is indeed a lot less electricity, originally more than 450	Jingdong prefecture level city in Shandong
Happy Fan Shop	Green Intelligent Power Saver	Has not been the first time to buy back, good use, power saving device is really powerful, before the monthly electricity bill of more than 500 Now more than 300, this time to help neighbors to buy several	Jingdong prefecture level city in Shandong
Happy Fan Shop	Green Intelligent Power Saver	Home appliances are very many, the circuit is also very complex, at first it is to save 9% of the electricity, and then used more than half a month, found that the power saving rate is as high as more	Jingdong prefecture level city in Shandong
Happy Fan Shop	Green Intelligent Power Saver	My friend bought it last year and saved hundreds of dollars on his electricity bill in one summer Appearance and style: it looks better than the one I bought before Quality is also good with a light.	Jingdong prefecture level city in Shandong
Happy Fan Shop	Green Intelligent Power Saver	Saver received, on the spot on the plug with the feeling of immediate power savings, appearance, packaging is very delicate, very satisfied, I hope to achieve satisfactory results!	Jingdong prefecture level city in Shandong

Happy Fan Shop	Green Intelligent Power Saver	The power saving effect is very good, is considered an old customer, also bought before, this time is to buy two for mom and dad, customer service attitude is good, praise!	Jingdong prefecture level city in Shandong
Happy Fan Shop	Green Intelligent Power Saver	After observing for a while, it really saves a lot of money I didn't realize it was still really reliable, so I'll give you a good review.	Jingdong prefecture level city in Shandong
Happy Fan Shop	Green Intelligent Power Saver	My family a month with this monthly almost save more than 200, power-saving effect: have bought a second time, really effective Cost-effective: not bad Appearance style: roughly the same A few months to the home electricity is indeed less, this time it is a re-buy three, send a friend two, they stay a	Jingdong prefecture level city in Shandong
Happy Fan Shop	Green Intelligent Power Saver	A month almost save more than 150 dollars in electricity, home appliances more, the main this can also protect high-power appliances, after using this device, these two months have not burned, things are very good, it is said that this is the latest model, the overall price is cheap and beautiful!	Jingdong prefecture level city in Shandong
Happy Fan Shop	Green Intelligent Power Saver	Received, powerful, great quality, love the style😍	Jingdong prefecture level city in Shandong
Happy Fan Shop	Green Intelligent Power Saver	This intelligent energy saver is especially good to use and save electricity hope everyone will buy it!	Jingdong prefecture level city in Shandong
Happy Fan Shop	Green Intelligent Power Saver	Air conditioning adapter effect is particularly great, the use of very simple, plug into the socket on the summer air conditioning power is too large, electricity is also expensive, with this product directly on the electricity bill to save half, has been purchased a few more, the effect is too awesome!	Jingdong prefecture level city in Shandong
Happy Fan Shop	Green Intelligent Power Saver	The family plugged in 5, monthly electricity savings of about 300 yuan. My family before a month of electricity costs more than 800, this time with this saver after the electricity bill is actually only more than 500, amazing, thanks to the store, will recommend other friends to buy!	Jingdong prefecture level city in Shandong
Happy Fan Shop	Green Intelligent Power Saver	It was recommended to me by a neighbor's family and it's nice to use it to save power.	Jingdong prefecture level city in Shandong
Happy Fan Shop	Green Intelligent Power Saver	Second time I've purchased it and the power saving is effective!	Jingdong prefecture level city in Shandong
Happy Fan Shop	Green Intelligent Power Saver	The effect is really good, before a month of more than 300 electricity, now every month every day with the air conditioning, it is more than 100 electricity! This thing is really amazing, one after another to buy and 5.6, home installed 3!	Jingdong prefecture level city in Shandong
Happy Fan Shop	Green Intelligent Power Saver	Received this product, began to use, small things look very wrong, can save a little electricity, the monthly electricity bill is too high, after plugging in did not pull, or save a lot of electricity, point	Jingdong prefecture level city in Shandong
Happy Fan Shop	Green Intelligent Power Saver	Simple and convenient, just that plug in, you can save electricity. A month can save dozens of dollars, a year down to save a lot of, very good product cost-effective, everyone can afford to buy and use!	Jingdong prefecture level city in Shandong
Happy Fan Shop	Green Intelligent Power Saver	Electricity bill from more than 200 yuan per month, down to more than 150 yuan, the original bought home, used two months, really have power-saving effect, this time and sold two, business service attitude is	Jingdong prefecture level city in Shandong
Happy Fan Shop	Green Intelligent Power Saver	Like to watch some movies at home and so on, sometimes at night until two or three o'clock, and recently the air conditioning is not turned off for a night, rather wasteful, I have to say that the effect is very good, quite like it!	Jingdong prefecture level city in Shandong
Happy Fan Shop	Green Intelligent Power Saver	Power-saving effect: the effect is very good, a month to save more than 200 dollars Cost-effective: good value for money Appearance and style: small and lovely Workmanship material: okay Experience: good	Jingdong prefecture level city in Shandong
Happy Fan Shop	Green Intelligent Power Saver	It's okay I quite like it, the stuff is very good. Come back in the future. You can rest assured to buy. Cost-effective all. Much cheaper than the physical store	Jingdong prefecture level city in Shandong
Happy Fan Shop	Green Intelligent Power Saver	More than 150 less, last month's electricity bill is almost 400, this month is only about 200, really save electricity, I am home to buy three, I did not expect a month to pay back	Jingdong prefecture level city in Shandong
Happy Fan Shop	Green Intelligent Power Saver	I bought three of these first and tried them for two months and they still felt useful, so now I've bought another one. Good and recommended!	Jingdong prefecture level city in Shandong
Happy Fan Shop	Green Intelligent Power Saver	This energy saver is very good quality and inexpensive next time I will buy it again!	Jingdong prefecture level city in Shandong
Happy Fan Shop	Green Intelligent Power Saver	Merchant service attitude is not good, the quality does not match, the business does not give the package return	Jingdong prefecture level city in Shandong
Happy Fan Shop	Green Intelligent Power Saver	The goods are very small do not know how effective after a few days to follow up the review	Jingdong prefecture level city in Shandong
Happy Fan Shop	Green Intelligent Power Saver	Not used before more than 200 a month, after using about 100 a month, my family used three, customer service said I use electricity recommended 2-3, I think more than one effect is better on the purchase of 4, the effect is very obvious, recommended to buy!	Jingdong prefecture level city in Shandong
Happy Fan Shop	Green Intelligent Power Saver	It's okay. I'll buy it again today.	Jingdong prefecture level city in Shandong
Happy Fan Shop	Green Intelligent Power Saver	Finally received the goods, very, very much like,, has been a lot of people praise, really too happy, has been his family's loyal fans, will continue to buy buy buy!	Jingdong prefecture level city in Shandong
Happy Fan Shop	Green Intelligent Power Saver	After using this, my monthly electricity bill was 1,200 pounds, now it's only 180 bucks a month, how good is it?	Jingdong prefecture level city in Shandong
Happy Fan Shop	Green Intelligent Power Saver	The item is a scam return to marry a dozen dollars in shipping fees. Buy terrific role is purely a scam.	Jingdong prefecture level city in Shandong
Happy Fan Shop	Green Intelligent Power Saver	It's really good, cost-effective and good quality, and the logistics are fast! It arrived in one day!	Jingdong prefecture level city in Shandong
Happy Fan Shop	Green Intelligent Power Saver	Great brand! Trustworthy! Works super well! Expensive for a reason! Great merchandise!	Jingdong prefecture level city in Shandong
Happy Fan Shop	Green Intelligent Power Saver	The baby is really great, I like it very much, my friends also say good to come, recommended to buy!	Jingdong prefecture level city in Shandong
Happy Fan Shop	Green Intelligent Power Saver	Fake couldn't be more fake. Bought one plugged in still so much electricity.	Jingdong prefecture level city in Shandong
Happy Fan Shop	Green Intelligent Power Saver	This appliance works great. Let's see how it works. We'll see later.	Jingdong prefecture level city in Shandong
Happy Fan Shop	Green Intelligent Power Saver	Used it before commenting, very happy with it	Jingdong prefecture level city in Shandong
Happy Fan Shop	Green Intelligent Power Saver	Goods have been received well-packed, logistics is very fast and good quality	Jingdong prefecture level city in Shandong
Happy Fan Shop	Green Intelligent Power Saver	This baby's quality is okay.	Jingdong prefecture level city in Shandong
Happy Fan Shop	Green Intelligent Power Saver	Counterfeit goods.	Jingdong prefecture level city in Shandong
Happy Fan Shop	Green Intelligent Power Saver	The effect is not very obvious	Jingdong prefecture level city in Shandong
Happy Fan Shop	Green Intelligent Power Saver	It's not the same thing.	Jingdong prefecture level city in Shandong
Happy Fan Shop	Green Intelligent Power Saver	There is no load how much w seems false, nothing works.	Jingdong prefecture level city in Shandong
Happy Fan Shop	Green Intelligent Power Saver	I received the item yesterday, got it back and plugged it in, and it used a little more power yesterday than I had before	Jingdong prefecture level city in Shandong
Happy Fan Shop	Green Intelligent Power Saver	Haven't seen the power saving mode yet	Jingdong prefecture level city in Shandong
Happy Fan Shop	Green Intelligent Power Saver	This product has no known effect	Jingdong prefecture level city in Shandong
Happy Fan Shop	Green Intelligent Power Saver	Refund Returns $16	Jingdong prefecture level city in Shandong
Happy Fan Shop	Green Intelligent Power Saver	The previous year bought a, almost every month can save a 70 dollars or so, used a year, no problem at all, look very new like, this time to move to a new home decisively repurchase 2 units, very believe this	Jingdong prefecture level city in Shandong
Happy Fan Shop	Green Intelligent Power Saver	Has been the second time to buy, this power saving device is really good power, before every month to spend 300 electricity, now are about 200, last time that 6, this time to help lead the family to buy 6,	Jingdong prefecture level city in Shandong
Happy Fan Shop	Green Intelligent Power Saver	In the spirit of trying to experience the new product, the use of down compared to the usual power consumption, with the addition of the quarter, is indeed a lot less electricity, originally more than 450	Jingdong prefecture level city in Shandong
Happy Fan Shop	Green Intelligent Power Saver	Has not been the first time to buy back, good use, power saving device is really powerful, before the monthly electricity bill of more than 500 Now more than 300, this time to help neighbors to buy several	Jingdong prefecture level city in Shandong
Happy Fan Shop	Green Intelligent Power Saver	Home appliances are very many, the circuit is also very complex, at first it is to save 9% of the electricity, and then used more than half a month, found that the power saving rate is as high as more	Jingdong prefecture level city in Shandong

Happy Fan Shop	Green Intelligent Power Saver	My friend bought it last year and saved hundreds of dollars on his electricity bill in one summer Appearance and style: it looks better than the one I bought before Quality is also good with a light.	Jingdong prefecture level city in Shandong
Happy Fan Shop	Green Intelligent Power Saver	Saver received, on the spot on the plug with the feeling of immediate power savings, appearance, packaging is very delicate, very satisfied, I hope to achieve satisfactory results!	Jingdong prefecture level city in Shandong
Happy Fan Shop	Green Intelligent Power Saver	The power saving effect is very good, is considered an old customer, also bought before, this time is to buy two for mom and dad, customer service attitude is good, praise!	Jingdong prefecture level city in Shandong
Happy Fan Shop	Green Intelligent Power Saver	After observing for a while, it really saves a lot of money I didn't realize it was still really reliable, so I'll give you a good review.	Jingdong prefecture level city in Shandong
Happy Fan Shop	Green Intelligent Power Saver	My family a month with this monthly almost save more than 200, power-saving effect: have bought a second time, really effective Cost-effective: not bad Appearance style: roughly the same A few months to the home electricity is indeed less, this time it is a re-buy three, send a friend two, they stay a	Jingdong prefecture level city in Shandong
Happy Fan Shop	Green Intelligent Power Saver	A month almost save more than 150 dollars in electricity, home appliances more, the main this can also protect high-power appliances, after using this device, these two months have not burned, things are very good, it is said that this is the latest model, the overall price is cheap and beautiful!	Jingdong prefecture level city in Shandong
Happy Fan Shop	Green Intelligent Power Saver	Received, powerful, great quality, love the style😍	Jingdong prefecture level city in Shandong
Happy Fan Shop	Green Intelligent Power Saver	This intelligent energy saver is especially good to use and save electricity hope everyone will buy it!	Jingdong prefecture level city in Shandong
Happy Fan Shop	Green Intelligent Power Saver	Air conditioning adapter effect is particularly great, the use of very simple, plug into the socket on the summer air conditioning power is too large, electricity is also expensive, with this product directly on the electricity bill to save half, has been purchased a few more, the effect is too awesome!	Jingdong prefecture level city in Shandong
Happy Fan Shop	Green Intelligent Power Saver	The family plugged in 5, monthly electricity savings of about 300 yuan. My family before a month of electricity costs more than 800, this time with this saver after the electricity bill is actually only more than 500, amazing, thanks to the store, will recommend other friends to buy!	Jingdong prefecture level city in Shandong
Happy Fan Shop	Green Intelligent Power Saver	It was recommended to me by a neighbor's family and it's nice to use it to save power.	Jingdong prefecture level city in Shandong
Happy Fan Shop	Green Intelligent Power Saver	Second time I've purchased it and the power saving is effective!	Jingdong prefecture level city in Shandong
Happy Fan Shop	Green Intelligent Power Saver	The effect is really good, before a month of more than 300 electricity, now every month every day with the air conditioning, it is more than 100 electricity! This thing is really amazing, one after another to buy and 5.6, home installed 3!	Jingdong prefecture level city in Shandong
Happy Fan Shop	Green Intelligent Power Saver	Received this product, began to use, small things look very wrong, can save a little electricity, the monthly electricity bill is too high, after plugging in did not pull, or save a lot of electricity, point	Jingdong prefecture level city in Shandong
Happy Fan Shop	Green Intelligent Power Saver	Simple and convenient, just that plug in, you can save electricity. A month can save dozens of dollars, a year down to save a lot of, very good product cost-effective, everyone can afford to buy and use!	Jingdong prefecture level city in Shandong
Happy Fan Shop	Green Intelligent Power Saver	Electricity bill from more than 200 yuan per month, down to more than 150 yuan, the original bought home, used two months, really have power-saving effect, this time and sold two, business service attitude is	Jingdong prefecture level city in Shandong
Happy Fan Shop	Green Intelligent Power Saver	Like to watch some movies at home and so on, sometimes at night until two or three o'clock, and recently the air conditioning is not turned off for a night, rather wasteful, I have to say that the effect is very good, quite like it!	Jingdong prefecture level city in Shandong
Happy Fan Shop	Green Intelligent Power Saver	Power-saving effect: the effect is very good, a month to save more than 200 dollars Cost-effective: good value for money Appearance and style: small and lovely Workmanship material: okay Experience: good	Jingdong prefecture level city in Shandong
Happy Fan Shop	Green Intelligent Power Saver	It's okay I quite like it, the stuff is very good. Come back in the future. You can rest assured to buy. Cost-effective all. Much cheaper than the physical store	Jingdong prefecture level city in Shandong
Happy Fan Shop	Green Intelligent Power Saver	More than 150 less, last month's electricity bill is almost 400, this month is only about 200, really save electricity, I am home to buy three, I did not expect a month to pay back	Jingdong prefecture level city in Shandong
Happy Fan Shop	Green Intelligent Power Saver	I bought three of these first and tried them for two months and they still felt useful, so now I've bought another one. Good and recommended!	Jingdong prefecture level city in Shandong
Happy Fan Shop	Green Intelligent Power Saver	This energy saver is very good quality and inexpensive next time I will buy it again!	Jingdong prefecture level city in Shandong
Happy Fan Shop	Green Intelligent Power Saver	Merchant service attitude is not good, the quality does not match, the business does not give the package return	Jingdong prefecture level city in Shandong
Happy Fan Shop	Green Intelligent Power Saver	The goods are very small do not know how effective after a few days to follow up the review	Jingdong prefecture level city in Shandong
Happy Fan Shop	Green Intelligent Power Saver	Not used before more than 200 a month, after using about 100 a month, my family used three, customer service said I use electricity recommended 2-3, I think more than one effect is better on the purchase of 4, the effect is very obvious, recommended to buy!	Jingdong prefecture level city in Shandong
Happy Fan Shop	Green Intelligent Power Saver	It's okay. I'll buy it again today.	Jingdong prefecture level city in Shandong
Happy Fan Shop	Green Intelligent Power Saver	Finally received the goods, very, very much like,, has been a lot of people praise, really too happy, has been his family's loyal fans, will continue to buy buy buy buy!	Jingdong prefecture level city in Shandong
Happy Fan Shop	Green Intelligent Power Saver	After using this, my monthly electricity bill was 1,200 pounds, now it's only 180 bucks a month, how good is it?	Jingdong prefecture level city in Shandong
Happy Fan Shop	Green Intelligent Power Saver	The item is a scam return to marry a dozen dollars in shipping fees. Buy terrific role is purely a scam.	Jingdong prefecture level city in Shandong
Happy Fan Shop	Green Intelligent Power Saver	It's really good, cost-effective and good quality, and the logistics are fast! It arrived in one day!	Jingdong prefecture level city in Shandong
Happy Fan Shop	Green Intelligent Power Saver	Great brand! Trustworthy! Works super well! Expensive for a reason! Great merchandise!	Jingdong prefecture level city in Shandong
Happy Fan Shop	Green Intelligent Power Saver	The baby is really great, I like it very much, my friends also say good to come, recommended to buy!	Jingdong prefecture level city in Shandong
Happy Fan Shop	Green Intelligent Power Saver	Fake couldn't be more fake. Bought one plugged in still so much electricity.	Jingdong prefecture level city in Shandong
Happy Fan Shop	Green Intelligent Power Saver	This appliance works great. Let's see how it works. We'll see later.	Jingdong prefecture level city in Shandong
Happy Fan Shop	Green Intelligent Power Saver	Used it before commenting, very happy with it	Jingdong prefecture level city in Shandong
Happy Fan Shop	Green Intelligent Power Saver	Goods have been received well-packed, logistics is very fast and good quality	Jingdong prefecture level city in Shandong
Happy Fan Shop	Green Intelligent Power Saver	This baby's quality is okay.	Jingdong prefecture level city in Shandong
Happy Fan Shop	Green Intelligent Power Saver	Counterfeit goods.	Jingdong prefecture level city in Shandong
Happy Fan Shop	Green Intelligent Power Saver	The effect is not very obvious	Jingdong prefecture level city in Shandong
Happy Fan Shop	Green Intelligent Power Saver	It's not the same thing.	Jingdong prefecture level city in Shandong
Happy Fan Shop	Green Intelligent Power Saver	There is no load how much w seems false, nothing works.	Jingdong prefecture level city in Shandong
Happy Fan Shop	Green Intelligent Power Saver	I received the item yesterday, got it back and plugged it in, and it used a little more power yesterday than I had before	Jingdong prefecture level city in Shandong
Happy Fan Shop	Green Intelligent Power Saver	Haven't seen the power saving mode yet	Jingdong prefecture level city in Shandong
Happy Fan Shop	Green Intelligent Power Saver	This product has no known effect	Jingdong prefecture level city in Shandong
Happy Fan Shop	Green Intelligent Power Saver	Refund Returns $16	Jingdong prefecture level city in Shandong
Happy Fan Shop	Green Intelligent Power Saver	The previous year bought a, almost every month can save a 70 dollars or so, used a year, no problem at all, look very new like, this time to move to a new home decisively repurchase 2 units, very believe this	Jingdong prefecture level city in Shandong
Happy Fan Shop	Green Intelligent Power Saver	Has been the second time to buy, this power saving device is really good power, before every month to spend 300 electricity, now are about 200, last time that 6, this time to help lead the family to buy 6,	Jingdong prefecture level city in Shandong
Happy Fan Shop	Green Intelligent Power Saver	In the spirit of trying to experience the new product, the use of down compared to the usual power consumption, with the addition of the quarter, is indeed a lot less electricity, originally more than 450	Jingdong prefecture level city in Shandong

Happy Fan Shop	Green Intelligent Power Saver	Has not been the first time to buy back, good use, power saving device is really powerful, before the monthly electricity bill of more than 500 Now more than 300, this time to help neighbors to buy several	Jingdong prefecture level city in Shandong
Happy Fan Shop	Green Intelligent Power Saver	Home appliances are very many, the circuit is also very complex, at first it is to save 9% of the electricity, and then used more than half a month, found that the power saving rate is as high as more	Jingdong prefecture level city in Shandong
Happy Fan Shop	Green Intelligent Power Saver	My friend bought it last year and saved hundreds of dollars on his electricity bill in one summer Appearance and style: it looks better than the one I bought before Quality is also good with a light.	Jingdong prefecture level city in Shandong
Happy Fan Shop	Green Intelligent Power Saver	Saver received, on the spot on the plug with the feeling of immediate power savings, appearance, packaging is very delicate, very satisfied, I hope to achieve satisfactory results!	Jingdong prefecture level city in Shandong
Happy Fan Shop	Green Intelligent Power Saver	The power saving effect is very good, is considered an old customer, also bought before, this time is to buy two for mom and dad, customer service attitude is good, praise!	Jingdong prefecture level city in Shandong
Happy Fan Shop	Green Intelligent Power Saver	After observing for a while, it really saves a lot of money I didn't realize it was still really reliable, so I'll give you a good review.	Jingdong prefecture level city in Shandong
Happy Fan Shop	Green Intelligent Power Saver	My family a month with this monthly almost save more than 200, power-saving effect: have bought a second time, really effective Cost-effective: not bad Appearance style: roughly the same A few months to the home electricity is indeed less, this time it is a re-buy three, send a friend two, they stay a	Jingdong prefecture level city in Shandong
Happy Fan Shop	Green Intelligent Power Saver	A month almost save more than 150 dollars in electricity, home appliances more, the main this can also protect high-power appliances, after using this device, these two months have not burned, things are very good, it is said that this is the latest model, the overall price is cheap and beautiful!	Jingdong prefecture level city in Shandong
Happy Fan Shop	Green Intelligent Power Saver	Received, powerful, great quality, love the style😍	Jingdong prefecture level city in Shandong
Happy Fan Shop	Green Intelligent Power Saver	This intelligent energy saver is especially good to use and save electricity hope everyone will buy it!	Jingdong prefecture level city in Shandong
Happy Fan Shop	Green Intelligent Power Saver	Air conditioning adapter effect is particularly great, the use of very simple, plug into the socket on the summer air conditioning power is too large, electricity is also expensive, with this product directly on the electricity bill to save half, has been purchased a few more, the effect is too awesome!	Jingdong prefecture level city in Shandong
Happy Fan Shop	Green Intelligent Power Saver	The family plugged in 5, monthly electricity savings of about 300 yuan. My family before a month of electricity costs more than 800, this time with this saver after the electricity bill is actually only more than 500, amazing, thanks to the store, will recommend other friends to buy!	Jingdong prefecture level city in Shandong
Happy Fan Shop	Green Intelligent Power Saver	It was recommended to me by a neighbor's family and it's nice to use it to save power.	Jingdong prefecture level city in Shandong
Happy Fan Shop	Green Intelligent Power Saver	Second time I've purchased it and the power saving is effective!	Jingdong prefecture level city in Shandong
Happy Fan Shop	Green Intelligent Power Saver	The effect is really good, before a month of more than 300 electricity, now every month every day with the air conditioning, it is more than 100 electricity! This thing is really amazing, one after another to buy and 5.6, home installed 3!	Jingdong prefecture level city in Shandong
Happy Fan Shop	Green Intelligent Power Saver	Received this product, began to use, small things look very wrong, can save a little electricity, the monthly electricity bill is too high, after plugging in did not pull, or save a lot of electricity, point	Jingdong prefecture level city in Shandong
Happy Fan Shop	Green Intelligent Power Saver	Simple and convenient, just that plug in, you can save electricity. A month can save dozens of dollars, a year down to save a lot of, very good product cost-effective, everyone can afford to buy and use!	Jingdong prefecture level city in Shandong
Happy Fan Shop	Green Intelligent Power Saver	Electricity bill from more than 200 yuan per month, down to more than 150 yuan, the original bought home, used two months, really have power-saving effect, this time and sold two, business service attitude is	Jingdong prefecture level city in Shandong
Happy Fan Shop	Green Intelligent Power Saver	Like to watch some movies at home and so on, sometimes at night until two or three o'clock, and recently the air conditioning is not turned off for a night, rather wasteful, I have to say that the effect is very good, quite like it!	Jingdong prefecture level city in Shandong
Happy Fan Shop	Green Intelligent Power Saver	Power-saving effect: the effect is very good, a month to save more than 200 dollars Cost-effective: good value for money Appearance and style: small and lovely Workmanship material: okay Experience: good	Jingdong prefecture level city in Shandong
Happy Fan Shop	Green Intelligent Power Saver	It's okay I quite like it, the stuff is very good. Come back in the future. You can rest assured to buy. Cost-effective all. Much cheaper than the physical store	Jingdong prefecture level city in Shandong
Happy Fan Shop	Green Intelligent Power Saver	More than 150 less, last month's electricity bill is almost 400, this month is only about 200, really save electricity, I am home to buy three, I did not expect a month to pay back	Jingdong prefecture level city in Shandong
Happy Fan Shop	Green Intelligent Power Saver	I bought three of these first and tried them for two months and they still felt useful, so now I've bought another one. Good and recommended!	Jingdong prefecture level city in Shandong
Happy Fan Shop	Green Intelligent Power Saver	This energy saver is very good quality and inexpensive next time I will buy it again!	Jingdong prefecture level city in Shandong
Happy Fan Shop	Green Intelligent Power Saver	Merchant service attitude is not good, the quality does not match, the business does not give the package return	Jingdong prefecture level city in Shandong
Happy Fan Shop	Green Intelligent Power Saver	The goods are very small do not know how effective after a few days to follow up the review	Jingdong prefecture level city in Shandong
Happy Fan Shop	Green Intelligent Power Saver	Not used before more than 200 a month, after using about 100 a month, my family used three, customer service said I use electricity recommended 2-3, I think more than one effect is better on the purchase of 4, the effect is very obvious, recommended to buy!	Jingdong prefecture level city in Shandong
Happy Fan Shop	Green Intelligent Power Saver	It's okay. I'll buy it again today.	Jingdong prefecture level city in Shandong
Happy Fan Shop	Green Intelligent Power Saver	Finally received the goods, very, very much like,, has been a lot of people praise, really too happy, has been his family's loyal fans, will continue to buy buy buy!	Jingdong prefecture level city in Shandong
Happy Fan Shop	Green Intelligent Power Saver	After using this, my monthly electricity bill was 1,200 pounds, now it's only 180 bucks a month, how good is it?	Jingdong prefecture level city in Shandong
Happy Fan Shop	Green Intelligent Power Saver	The item is a scam return to marry a dozen dollars in shipping fees. Buy terrific role is purely a scam.	Jingdong prefecture level city in Shandong
Happy Fan Shop	Green Intelligent Power Saver	It's really good, cost-effective and good quality, and the logistics are fast! It arrived in one day!	Jingdong prefecture level city in Shandong
Happy Fan Shop	Green Intelligent Power Saver	Great brand! Trustworthy! Works super well! Expensive for a reason! Great merchandise!	Jingdong prefecture level city in Shandong
Happy Fan Shop	Green Intelligent Power Saver	The baby is really great, I like it very much, my friends also say good to come, recommended to buy!	Jingdong prefecture level city in Shandong
Happy Fan Shop	Green Intelligent Power Saver	Fake couldn't be more fake. Bought one plugged in still so much electricity.	Jingdong prefecture level city in Shandong
Happy Fan Shop	Green Intelligent Power Saver	This appliance works great. Let's see how it works. We'll see later.	Jingdong prefecture level city in Shandong
Happy Fan Shop	Green Intelligent Power Saver	Used it before commenting, very happy with it	Jingdong prefecture level city in Shandong
Happy Fan Shop	Green Intelligent Power Saver	Goods have been received well-packed, logistics is very fast and good quality	Jingdong prefecture level city in Shandong
Happy Fan Shop	Green Intelligent Power Saver	This baby's quality is okay.	Jingdong prefecture level city in Shandong
Happy Fan Shop	Green Intelligent Power Saver	Counterfeit goods.	Jingdong prefecture level city in Shandong
Happy Fan Shop	Green Intelligent Power Saver	The effect is not very obvious	Jingdong prefecture level city in Shandong
Happy Fan Shop	Green Intelligent Power Saver	It's not the same thing.	Jingdong prefecture level city in Shandong
Happy Fan Shop	Green Intelligent Power Saver	There is no load how much w seems false, nothing works.	Jingdong prefecture level city in Shandong
Happy Fan Shop	Green Intelligent Power Saver	I received the item yesterday, got it back and plugged it in, and it used a little more power yesterday than I had before	Jingdong prefecture level city in Shandong
Happy Fan Shop	Green Intelligent Power Saver	Haven't seen the power saving mode yet	Jingdong prefecture level city in Shandong
Happy Fan Shop	Green Intelligent Power Saver	This product has no known effect	Jingdong prefecture level city in Shandong
Happy Fan Shop	Green Intelligent Power Saver	Refund Returns $16	Jingdong prefecture level city in Shandong
Happy Fan Shop	Green Intelligent Power Saver	The previous year bought a, almost every month can save a 70 dollars or so, used a year, no problem at all, look very new like, this time to move to a new home decisively repurchase 2 units, very believe this	Jingdong prefecture level city in Shandong

Happy Fan Shop	Green Intelligent Power Saver	Has been the second time to buy, this power saving device is really good power, before every month to spend 300 electricity, now are about 200, last time that 6, this time to help lead the family to buy 6,	Jingdong prefecture level city in Shandong
Happy Fan Shop	Green Intelligent Power Saver	In the spirit of trying to experience the new product, the use of down compared to the usual power consumption, with the addition of the quarter, is indeed a lot less electricity, originally more than 450	Jingdong prefecture level city in Shandong
Happy Fan Shop	Green Intelligent Power Saver	Has not been the first time to buy back, good use, power saving device is really powerful, before the monthly electricity bill of more than 500 Now more than 300, this time to help neighbors to buy several	Jingdong prefecture level city in Shandong
Happy Fan Shop	Green Intelligent Power Saver	Home appliances are very many, the circuit is also very complex, at first it is to save 9% of the electricity, and then used more than half a month, found that the power saving rate is as high as more	Jingdong prefecture level city in Shandong
Happy Fan Shop	Green Intelligent Power Saver	My friend bought it last year and saved hundreds of dollars on his electricity bill in one summer Appearance and style: it looks better than the one I bought before Quality is also good with a light.	Jingdong prefecture level city in Shandong
Happy Fan Shop	Green Intelligent Power Saver	Saver received, on the spot on the plug with the feeling of immediate power savings, appearance, packaging is very delicate, very satisfied, I hope to achieve satisfactory results!	Jingdong prefecture level city in Shandong
Happy Fan Shop	Green Intelligent Power Saver	The power saving effect is very good, is considered an old customer, also bought before, this time is to buy two for mom and dad, customer service attitude is good, praise!	Jingdong prefecture level city in Shandong
Happy Fan Shop	Green Intelligent Power Saver	After observing for a while, it really saves a lot of money I didn't realize it was still really reliable, so I'll give you a good review.	Jingdong prefecture level city in Shandong
Happy Fan Shop	Green Intelligent Power Saver	My family a month with this monthly almost save more than 200, power-saving effect: have bought a second time, really effective Cost-effective: not bad Appearance style: roughly the same A few months to the home electricity is indeed less, this time it is a re-buy three, send a friend two, they stay a	Jingdong prefecture level city in Shandong
Happy Fan Shop	Green Intelligent Power Saver	A month almost save more than 150 dollars in electricity, home appliances more, the main this can also protect high-power appliances, after using this device, these two months have not burned, things are very good, it is said that this is the latest model, the overall price is cheap and beautiful!	Jingdong prefecture level city in Shandong
Happy Fan Shop	Green Intelligent Power Saver	Received, powerful, great quality, love the style😍	Jingdong prefecture level city in Shandong
Happy Fan Shop	Green Intelligent Power Saver	This intelligent energy saver is especially good to use and save electricity hope everyone will buy it!	Jingdong prefecture level city in Shandong
Happy Fan Shop	Green Intelligent Power Saver	Air conditioning adapter effect is particularly great, the use of very simple, plug into the socket on the summer air conditioning power is too large, electricity is also expensive, with this product directly on the electricity bill to save half, has been purchased a few more, the effect is too awesome!	Jingdong prefecture level city in Shandong
Happy Fan Shop	Green Intelligent Power Saver	The family plugged in 5, monthly electricity savings of about 300 yuan. My family before a month of electricity costs more than 800, this time with this saver after the electricity bill is actually only more than 500, amazing, thanks to the store, will recommend other friends to buy!	Jingdong prefecture level city in Shandong
Happy Fan Shop	Green Intelligent Power Saver	It was recommended to me by a neighbor's family and it's nice to use it to save power.	Jingdong prefecture level city in Shandong
Happy Fan Shop	Green Intelligent Power Saver	Second time I've purchased it and the power saving is effective!	Jingdong prefecture level city in Shandong
Happy Fan Shop	Green Intelligent Power Saver	The effect is really good, before a month of more than 300 electricity, now every month every day with the air conditioning, it is more than 100 electricity! This thing is really amazing, one after another to buy and 5.6, home installed 3!	Jingdong prefecture level city in Shandong
Happy Fan Shop	Green Intelligent Power Saver	Received this product, began to use, small things look very wrong, can save a little electricity, the monthly electricity bill is too high, after plugging in did not pull, or save a lot of electricity, point	Jingdong prefecture level city in Shandong
Happy Fan Shop	Green Intelligent Power Saver	Simple and convenient, just that plug in, you can save electricity. A month can save dozens of dollars, a year down to save a lot of, very good product cost-effective, everyone can afford to buy and use!	Jingdong prefecture level city in Shandong
Happy Fan Shop	Green Intelligent Power Saver	Electricity bill from more than 200 yuan per month, down to more than 150 yuan, the original bought home, used two months, really have power-saving effect, this time and sold two, business service attitude is	Jingdong prefecture level city in Shandong
Happy Fan Shop	Green Intelligent Power Saver	Like to watch some movies at home and so on, sometimes at night until two or three o'clock, and recently the air conditioning is not turned off for a night, rather wasteful, I have to say that the effect is very good, quite like it!	Jingdong prefecture level city in Shandong
Happy Fan Shop	Green Intelligent Power Saver	Power-saving effect: the effect is very good, a month to save more than 200 dollars Cost-effective: good value for money Appearance and style: small and lovely Workmanship material: okay Experience: good	Jingdong prefecture level city in Shandong
Happy Fan Shop	Green Intelligent Power Saver	It's okay I quite like it, the stuff is very good. Come back in the future. You can rest assured to buy. Cost-effective all. Much cheaper than the physical store	Jingdong prefecture level city in Shandong
Happy Fan Shop	Green Intelligent Power Saver	More than 150 less, last month's electricity bill is almost 400, this month is only about 200, really save electricity, I am home to buy three, I did not expect a month to pay back	Jingdong prefecture level city in Shandong
Happy Fan Shop	Green Intelligent Power Saver	I bought three of these first and tried them for two months and they still felt useful, so now I've bought another one. Good and recommended!	Jingdong prefecture level city in Shandong
Happy Fan Shop	Green Intelligent Power Saver	This energy saver is very good quality and inexpensive next time I will buy it again!	Jingdong prefecture level city in Shandong
Happy Fan Shop	Green Intelligent Power Saver	Merchant service attitude is not good, the quality does not match, the business does not give the package return	Jingdong prefecture level city in Shandong
Happy Fan Shop	Green Intelligent Power Saver	The goods are very small do not know how effective after a few days to follow up the review	Jingdong prefecture level city in Shandong
Happy Fan Shop	Green Intelligent Power Saver	Not used before more than 200 a month, after using about 100 a month, my family used three, customer service said I use electricity recommended 2-3, I think more than one effect is better on the purchase of 4, the effect is very obvious, recommended to buy!	Jingdong prefecture level city in Shandong
Happy Fan Shop	Green Intelligent Power Saver	It's okay. I'll buy it again today.	Jingdong prefecture level city in Shandong
Happy Fan Shop	Green Intelligent Power Saver	Finally received the goods, very, very much like,, has been a lot of people praise, really too happy, has been his family's loyal fans, will continue to buy buy buy buy!	Jingdong prefecture level city in Shandong
Happy Fan Shop	Green Intelligent Power Saver	After using this, my monthly electricity bill was 1,200 pounds, now it's only 180 bucks a month, how good is it?	Jingdong prefecture level city in Shandong
Happy Fan Shop	Green Intelligent Power Saver	The item is a scam return to marry a dozen dollars in shipping fees. Buy terrific role is purely a scam.	Jingdong prefecture level city in Shandong
Happy Fan Shop	Green Intelligent Power Saver	It's really good, cost-effective and good quality, and the logistics are fast! It arrived in one day!	Jingdong prefecture level city in Shandong
Happy Fan Shop	Green Intelligent Power Saver	Great brand! Trustworthy! Works super well! Expensive for a reason! Great merchandise!	Jingdong prefecture level city in Shandong
Happy Fan Shop	Green Intelligent Power Saver	The baby is really great, I like it very much, my friends also say good to come, recommended to buy!	Jingdong prefecture level city in Shandong
Happy Fan Shop	Green Intelligent Power Saver	Fake couldn't be more fake. Bought one plugged in still so much electricity.	Jingdong prefecture level city in Shandong
Happy Fan Shop	Green Intelligent Power Saver	This appliance works great. Let's see how it works. We'll see later.	Jingdong prefecture level city in Shandong
Happy Fan Shop	Green Intelligent Power Saver	Used it before commenting, very happy with it	Jingdong prefecture level city in Shandong
Happy Fan Shop	Green Intelligent Power Saver	Goods have been received well-packed, logistics is very fast and good quality	Jingdong prefecture level city in Shandong
Happy Fan Shop	Green Intelligent Power Saver	This baby's quality is okay.	Jingdong prefecture level city in Shandong
Happy Fan Shop	Green Intelligent Power Saver	Counterfeit goods.	Jingdong prefecture level city in Shandong
Happy Fan Shop	Green Intelligent Power Saver	The effect is not very obvious	Jingdong prefecture level city in Shandong
Happy Fan Shop	Green Intelligent Power Saver	It's not the same thing.	Jingdong prefecture level city in Shandong
Happy Fan Shop	Green Intelligent Power Saver	There is no load how much w seems false, nothing works.	Jingdong prefecture level city in Shandong
Happy Fan Shop	Green Intelligent Power Saver	I received the item yesterday, got it back and plugged it in, and it used a little more power yesterday than I had before	Jingdong prefecture level city in Shandong
Happy Fan Shop	Green Intelligent Power Saver	Haven't seen the power saving mode yet	Jingdong prefecture level city in Shandong
Happy Fan Shop	Green Intelligent Power Saver	This product has no known effect	Jingdong prefecture level city in Shandong

Happy Fan Shop	Green Intelligent Power Saver	Refund Returns $16	Jingdong prefecture level city in Shandong
Happy Fan Shop	Green Intelligent Power Saver	The previous year bought a, almost every month can save a 70 dollars or so, used a year, no problem at all, look very new like, this time to move to a new home decisively repurchase 2 units, very believe this	Jingdong prefecture level city in Shandong
Happy Fan Shop	Green Intelligent Power Saver	Has been the second time to buy, this power saving device is really good power, before every month to spend 300 electricity, now are about 200, last time that 6, this time to help lead the family to buy 6,	Jingdong prefecture level city in Shandong
Happy Fan Shop	Green Intelligent Power Saver	In the spirit of trying to experience the new product, the use of down compared to the usual power consumption, with the addition of the quarter, is indeed a lot less electricity, originally more than 450	Jingdong prefecture level city in Shandong
Happy Fan Shop	Green Intelligent Power Saver	Has not been the first time to buy back, good use, power saving device is really powerful, before the monthly electricity bill of more than 500 Now more than 300, this time to help neighbors to buy several	Jingdong prefecture level city in Shandong
Happy Fan Shop	Green Intelligent Power Saver	Home appliances are very many, the circuit is also very complex, at first it is to save 9% of the electricity, and then used more than half a month, found that the power saving rate is as high as more	Jingdong prefecture level city in Shandong
Happy Fan Shop	Green Intelligent Power Saver	My friend bought it last year and saved hundreds of dollars on his electricity bill in one summer Appearance and style: it looks better than the one I bought before Quality is also good with a light.	Jingdong prefecture level city in Shandong
Happy Fan Shop	Green Intelligent Power Saver	Saver received, on the spot on the plug with the feeling of immediate power savings, appearance, packaging is very delicate, very satisfied, I hope to achieve satisfactory results!	Jingdong prefecture level city in Shandong
Happy Fan Shop	Green Intelligent Power Saver	The power saving effect is very good, is considered an old customer, also bought before, this time is to buy two for mom and dad, customer service attitude is good, praise!	Jingdong prefecture level city in Shandong
Happy Fan Shop	Green Intelligent Power Saver	After observing for a while, it really saves a lot of money I didn't realize it was still really reliable, so I'll give you a good review.	Jingdong prefecture level city in Shandong
Happy Fan Shop	Green Intelligent Power Saver	My family a month with this monthly almost save more than 200, power-saving effect: have bought a second time, really effective Cost-effective: not bad Appearance style: roughly the same A few months to the home electricity is indeed less, this time it is a re-buy three, send a friend two, they stay a	Jingdong prefecture level city in Shandong
Happy Fan Shop	Green Intelligent Power Saver	A month almost save more than 150 dollars in electricity, home appliances more, the main this can also protect high-power appliances, after using this device, these two months have not burned, things are very good, it is said that this is the latest model, the overall price is cheap and beautiful!	Jingdong prefecture level city in Shandong
Happy Fan Shop	Green Intelligent Power Saver	Received, powerful, great quality, love the style😍	Jingdong prefecture level city in Shandong
Happy Fan Shop	Green Intelligent Power Saver	This intelligent energy saver is especially good to use and save electricity hope everyone will buy it!	Jingdong prefecture level city in Shandong
Happy Fan Shop	Green Intelligent Power Saver	Air conditioning adapter effect is particularly great, the use of very simple, plug into the socket on the summer air conditioning power is too large, electricity is also expensive, with this product directly on the electricity bill to save half, has been purchased a few more, the effect is too awesome!	Jingdong prefecture level city in Shandong
Happy Fan Shop	Green Intelligent Power Saver	The family plugged in 5, monthly electricity savings of about 300 yuan. My family before a month of electricity costs more than 800, this time with this saver after the electricity bill is actually only more than 500, amazing, thanks to the store, will recommend other friends to buy!	Jingdong prefecture level city in Shandong
Happy Fan Shop	Green Intelligent Power Saver	It was recommended to me by a neighbor's family and it's nice to use it to save power.	Jingdong prefecture level city in Shandong
Happy Fan Shop	Green Intelligent Power Saver	Second time I've purchased it and the power saving is effective!	Jingdong prefecture level city in Shandong
Happy Fan Shop	Green Intelligent Power Saver	The effect is really good, before a month of more than 300 electricity, now every month every day with the air conditioning, it is more than 100 electricity! This thing is really amazing, one after another to buy and 5.6, home installed 3!	Jingdong prefecture level city in Shandong
Happy Fan Shop	Green Intelligent Power Saver	Received this product, began to use, small things look very wrong, can save a little electricity, the monthly electricity bill is too high, after plugging in did not pull, or save a lot of electricity, point	Jingdong prefecture level city in Shandong
Happy Fan Shop	Green Intelligent Power Saver	Simple and convenient, just that plug in, you can save electricity. A month can save dozens of dollars, a year down to save a lot of, very good product cost-effective, everyone can afford to buy and use!	Jingdong prefecture level city in Shandong
Happy Fan Shop	Green Intelligent Power Saver	Electricity bill from more than 200 yuan per month, down to more than 150 yuan, the original bought home, used two months, really have power-saving effect, this time and sold two, business service attitude is	Jingdong prefecture level city in Shandong
Happy Fan Shop	Green Intelligent Power Saver	Like to watch some movies at home and so on, sometimes at night until two or three o'clock, and recently the air conditioning is not turned off for a night, rather wasteful, I have to say that the effect is very good, quite like it!	Jingdong prefecture level city in Shandong
Happy Fan Shop	Green Intelligent Power Saver	Power-saving effect: the effect is very good, a month to save more than 200 dollars Cost-effective: good value for money Appearance and style: small and lovely Workmanship material: okay Experience: good	Jingdong prefecture level city in Shandong
Happy Fan Shop	Green Intelligent Power Saver	It's okay I quite like it, the stuff is very good. Come back in the future. You can rest assured to buy. Cost-effective all. Much cheaper than the physical store	Jingdong prefecture level city in Shandong
Happy Fan Shop	Green Intelligent Power Saver	More than 150 less, last month's electricity bill is almost 400, this month is only about 200, really save electricity, I am home to buy three, I did not expect a month to pay back	Jingdong prefecture level city in Shandong
Happy Fan Shop	Green Intelligent Power Saver	I bought three of these first and tried them for two months and they still felt useful, so now I've bought another one. Good and recommended!	Jingdong prefecture level city in Shandong
Happy Fan Shop	Green Intelligent Power Saver	This energy saver is very good quality and inexpensive next time I will buy it again!	Jingdong prefecture level city in Shandong
Happy Fan Shop	Green Intelligent Power Saver	Merchant service attitude is not good, the quality does not match, the business does not give the package return	Jingdong prefecture level city in Shandong
Happy Fan Shop	Green Intelligent Power Saver	The goods are very small do not know how effective after a few days to follow up the review	Jingdong prefecture level city in Shandong
Happy Fan Shop	Green Intelligent Power Saver	Not used before more than 200 a month, after using about 100 a month, my family used three, customer service said I use electricity recommended 2-3, I think more than one effect is better on the purchase of 4, the effect is very obvious, recommended to buy!	Jingdong prefecture level city in Shandong
Happy Fan Shop	Green Intelligent Power Saver	It's okay. I'll buy it again today.	Jingdong prefecture level city in Shandong
Happy Fan Shop	Green Intelligent Power Saver	Finally received the goods, very, very much like,, has been a lot of people praise, really too happy, has been his family's loyal fans, will continue to buy buy buy buy!	Jingdong prefecture level city in Shandong
Happy Fan Shop	Green Intelligent Power Saver	After using this, my monthly electricity bill was 1,200 pounds, now it's only 180 bucks a month, how good is it?	Jingdong prefecture level city in Shandong
Happy Fan Shop	Green Intelligent Power Saver	The item is a scam return to marry a dozen dollars in shipping fees. Buy terrific role is purely a scam.	Jingdong prefecture level city in Shandong
Happy Fan Shop	Green Intelligent Power Saver	It's really good, cost-effective and good quality, and the logistics are fast! It arrived in one day!	Jingdong prefecture level city in Shandong
Happy Fan Shop	Green Intelligent Power Saver	Great brand! Trustworthy! Works super well! Expensive for a reason! Great merchandise!	Jingdong prefecture level city in Shandong
Happy Fan Shop	Green Intelligent Power Saver	The baby is really great, I like it very much, my friends also say good to come, recommended to buy!	Jingdong prefecture level city in Shandong
Happy Fan Shop	Green Intelligent Power Saver	Fake couldn't be more fake. Bought one plugged in still so much electricity.	Jingdong prefecture level city in Shandong
Happy Fan Shop	Green Intelligent Power Saver	This appliance works great. Let's see how it works. We'll see later.	Jingdong prefecture level city in Shandong
Happy Fan Shop	Green Intelligent Power Saver	Used it before commenting, very happy with it	Jingdong prefecture level city in Shandong
Happy Fan Shop	Green Intelligent Power Saver	Goods have been received well-packed, logistics is very fast and good quality	Jingdong prefecture level city in Shandong
Happy Fan Shop	Green Intelligent Power Saver	This baby's quality is okay.	Jingdong prefecture level city in Shandong
Happy Fan Shop	Green Intelligent Power Saver	Counterfeit goods.	Jingdong prefecture level city in Shandong
Happy Fan Shop	Green Intelligent Power Saver	The effect is not very obvious	Jingdong prefecture level city in Shandong
Happy Fan Shop	Green Intelligent Power Saver	It's not the same thing.	Jingdong prefecture level city in Shandong
Happy Fan Shop	Green Intelligent Power Saver	There is no load how much w seems false, nothing works.	Jingdong prefecture level city in Shandong
Happy Fan Shop	Green Intelligent Power Saver	I received the item yesterday, got it back and plugged it in, and it used a little more power yesterday than I had before	Jingdong prefecture level city in Shandong

Happy Fan Shop	Green Intelligent Power Saver	Haven't seen the power saving mode yet	Jingdong prefecture level city in Shandong
Happy Fan Shop	Green Intelligent Power Saver	This product has no known effect	Jingdong prefecture level city in Shandong
Happy Fan Shop	Green Intelligent Power Saver	Refund Returns $16	Jingdong prefecture level city in Shandong
Happy Fan Shop	Green Intelligent Power Saver	The previous year bought a, almost every month can save a 70 dollars or so, used a year, no problem at all, look very new like, this time to move to a new home decisively repurchase 2 units, very believe this	Jingdong prefecture level city in Shandong
Happy Fan Shop	Green Intelligent Power Saver	Has been the second time to buy, this power saving device is really good power, before every month to spend 300 electricity, now are about 200, last time that 6, this time to help lead the family to buy 6,	Jingdong prefecture level city in Shandong
Happy Fan Shop	Green Intelligent Power Saver	In the spirit of trying to experience the new product, the use of down compared to the usual power consumption, with the addition of the quarter, is indeed a lot less electricity, originally more than 450	Jingdong prefecture level city in Shandong
Happy Fan Shop	Green Intelligent Power Saver	Has not been the first time to buy back, good use, power saving device is really powerful, before the monthly electricity bill of more than 500 Now more than 300, this time to help neighbors to buy several	Jingdong prefecture level city in Shandong
Happy Fan Shop	Green Intelligent Power Saver	Home appliances are very many, the circuit is also very complex, at first it is to save 9% of the electricity, and then used more than half a month, found that the power saving rate is as high as more	Jingdong prefecture level city in Shandong
Happy Fan Shop	Green Intelligent Power Saver	My friend bought it last year and saved hundreds of dollars on his electricity bill in one summer Appearance and style: it looks better than the one I bought before Quality is also good with a light.	Jingdong prefecture level city in Shandong
Happy Fan Shop	Green Intelligent Power Saver	Saver received, on the spot on the plug with the feeling of immediate power savings, appearance, packaging is very delicate, very satisfied, I hope to achieve satisfactory results!	Jingdong prefecture level city in Shandong
Happy Fan Shop	Green Intelligent Power Saver	The power saving effect is very good, is considered an old customer, also bought before, this time is to buy two for mom and dad, customer service attitude is good, praise!	Jingdong prefecture level city in Shandong
Happy Fan Shop	Green Intelligent Power Saver	After observing for a while, it really saves a lot of money I didn't realize it was still really reliable, so I'll give you a good review.	Jingdong prefecture level city in Shandong
Happy Fan Shop	Green Intelligent Power Saver	My family a month with this monthly almost save more than 200, power-saving effect: have bought a second time, really effective Cost-effective: not bad Appearance style: roughly the same A few months to the home electricity is indeed less, this time it is a re-buy three, send a friend two, they stay a	Jingdong prefecture level city in Shandong
Happy Fan Shop	Green Intelligent Power Saver	A month almost save more than 150 dollars in electricity, home appliances more, the main this can also protect high-power appliances, after using this device, these two months have not burned, things are very good, it is said that this is the latest model, the overall price is cheap and beautiful!	Jingdong prefecture level city in Shandong
Happy Fan Shop	Green Intelligent Power Saver	Received, powerful, great quality, love the style😍	Jingdong prefecture level city in Shandong
Happy Fan Shop	Green Intelligent Power Saver	This intelligent energy saver is especially good to use and save electricity hope everyone will buy it!	Jingdong prefecture level city in Shandong
Happy Fan Shop	Green Intelligent Power Saver	Air conditioning adapter effect is particularly great, the use of very simple, plug into the socket on the summer air conditioning power is too large, electricity is also expensive, with this product directly on the electricity bill to save half, has been purchased a few more, the effect is too awesome!	Jingdong prefecture level city in Shandong
Happy Fan Shop	Green Intelligent Power Saver	The family plugged in 5, monthly electricity savings of about 300 yuan. My family before a month of electricity costs more than 800, this time with this saver after the electricity bill is actually only more than 500, amazing, thanks to the store, will recommend other friends to buy!	Jingdong prefecture level city in Shandong
Happy Fan Shop	Green Intelligent Power Saver	It was recommended to me by a neighbor's family and it's nice to use it to save power.	Jingdong prefecture level city in Shandong
Happy Fan Shop	Green Intelligent Power Saver	Second time I've purchased it and the power saving is effective!	Jingdong prefecture level city in Shandong
Happy Fan Shop	Green Intelligent Power Saver	The effect is really good, before a month of more than 300 electricity, now every month every day with the air conditioning, it is more than 100 electricity! This thing is really amazing, one after another to buy and 5.6, home installed 3!	Jingdong prefecture level city in Shandong
Happy Fan Shop	Green Intelligent Power Saver	Received this product, began to use, small things look very wrong, can save a little electricity, the monthly electricity bill is too high, after plugging in did not pull, or save a lot of electricity, point	Jingdong prefecture level city in Shandong
Happy Fan Shop	Green Intelligent Power Saver	Simple and convenient, just that plug in, you can save electricity. A month can save dozens of dollars, a year down to save a lot of, very good product cost-effective, everyone can afford to buy and use!	Jingdong prefecture level city in Shandong
Happy Fan Shop	Green Intelligent Power Saver	Electricity bill from more than 200 yuan per month, down to more than 150 yuan, the original bought home, used two months, really have power-saving effect, this time and sold two, business service attitude is	Jingdong prefecture level city in Shandong
Happy Fan Shop	Green Intelligent Power Saver	Like to watch some movies at home and so on, sometimes at night until two or three o'clock, and recently the air conditioning is not turned off for a night, rather wasteful, I have to say that the effect is very good, quite like it!	Jingdong prefecture level city in Shandong
Happy Fan Shop	Green Intelligent Power Saver	Power-saving effect: the effect is very good, a month to save more than 200 dollars Cost-effective: good value for money Appearance and style: small and lovely Workmanship material: okay Experience: good	Jingdong prefecture level city in Shandong
Happy Fan Shop	Green Intelligent Power Saver	It's okay I quite like it, the stuff is very good. Come back in the future. You can rest assured to buy. Cost-effective all. Much cheaper than the physical store	Jingdong prefecture level city in Shandong
Happy Fan Shop	Green Intelligent Power Saver	More than 150 less, last month's electricity bill is almost 400, this month is only about 200, really save electricity, I am home to buy three, I did not expect a month to pay back	Jingdong prefecture level city in Shandong
Happy Fan Shop	Green Intelligent Power Saver	I bought three of these first and tried them for two months and they still felt useful, so now I've bought another one. Good and recommended!	Jingdong prefecture level city in Shandong
Happy Fan Shop	Green Intelligent Power Saver	This energy saver is very good quality and inexpensive next time I will buy it again!	Jingdong prefecture level city in Shandong
Happy Fan Shop	Green Intelligent Power Saver	Merchant service attitude is not good, the quality does not match, the business does not give the package return	Jingdong prefecture level city in Shandong
Happy Fan Shop	Green Intelligent Power Saver	The goods are very small do not know how effective after a few days to follow up the review	Jingdong prefecture level city in Shandong
Happy Fan Shop	Green Intelligent Power Saver	Not used before more than 200 a month, after using about 100 a month, my family used three, customer service said I use electricity recommended 2-3, I think more than one effect is better on the purchase of 4, the effect is very obvious, recommended to buy!	Jingdong prefecture level city in Shandong
Happy Fan Shop	Green Intelligent Power Saver	It's okay. I'll buy it again today.	Jingdong prefecture level city in Shandong
Happy Fan Shop	Green Intelligent Power Saver	Finally received the goods, very, very much like,, has been a lot of people praise, really too happy, has been his family's loyal fans, will continue to buy buy buy!	Jingdong prefecture level city in Shandong
Happy Fan Shop	Green Intelligent Power Saver	After using this, my monthly electricity bill was 1,200 pounds, now it's only 180 bucks a month, how good is it?	Jingdong prefecture level city in Shandong
Happy Fan Shop	Green Intelligent Power Saver	The item is a scam return to marry a dozen dollars in shipping fees. Buy terrific role is purely a scam.	Jingdong prefecture level city in Shandong
Happy Fan Shop	Green Intelligent Power Saver	It's really good, cost-effective and good quality, and the logistics are fast! It arrived in one day!	Jingdong prefecture level city in Shandong
Happy Fan Shop	Green Intelligent Power Saver	Great brand! Trustworthy! Works super well! Expensive for a reason! Great merchandise!	Jingdong prefecture level city in Shandong
Happy Fan Shop	Green Intelligent Power Saver	The baby is really great, I like it very much, my friends also say good to come, recommended to buy!	Jingdong prefecture level city in Shandong
Happy Fan Shop	Green Intelligent Power Saver	Fake couldn't be more fake. Bought one plugged in still so much electricity.	Jingdong prefecture level city in Shandong
Happy Fan Shop	Green Intelligent Power Saver	This appliance works great. Let's see how it works. We'll see later.	Jingdong prefecture level city in Shandong
Happy Fan Shop	Green Intelligent Power Saver	Used it before commenting, very happy with it	Jingdong prefecture level city in Shandong
Happy Fan Shop	Green Intelligent Power Saver	Goods have been received well-packed, logistics is very fast and good quality	Jingdong prefecture level city in Shandong
Happy Fan Shop	Green Intelligent Power Saver	This baby's quality is okay.	Jingdong prefecture level city in Shandong
Happy Fan Shop	Green Intelligent Power Saver	Counterfeit goods.	Jingdong prefecture level city in Shandong
Happy Fan Shop	Green Intelligent Power Saver	The effect is not very obvious	Jingdong prefecture level city in Shandong
Happy Fan Shop	Green Intelligent Power Saver	It's not the same thing.	Jingdong prefecture level city in Shandong

Happy Fan Shop	Green Intelligent Power Saver	There is no load how much w seems false, nothing works.	Jingdong prefecture level city in Shandong
Happy Fan Shop	Green Intelligent Power Saver	I received the item yesterday, got it back and plugged it in, and it used a little more power yesterday than I had before	Jingdong prefecture level city in Shandong
Happy Fan Shop	Green Intelligent Power Saver	Haven't seen the power saving mode yet	Jingdong prefecture level city in Shandong
Happy Fan Shop	Green Intelligent Power Saver	This product has no known effect	Jingdong prefecture level city in Shandong
Happy Fan Shop	Green Intelligent Power Saver	Refund Returns $16	Jingdong prefecture level city in Shandong
Happy Fan Shop	Green Intelligent Power Saver	The previous year bought a, almost every month can save a 70 dollars or so, used a year, no problem at all, look very new like, this time to move to a new home decisively repurchase 2 units, very believe this	Jingdong prefecture level city in Shandong
Happy Fan Shop	Green Intelligent Power Saver	Has been the second time to buy, this power saving device is really good power, before every month to spend 300 electricity, now are about 200, last time that 6, this time to help lead the family to buy 6,	Jingdong prefecture level city in Shandong
Happy Fan Shop	Green Intelligent Power Saver	In the spirit of trying to experience the new product, the use of down compared to the usual power consumption, with the addition of the quarter, is indeed a lot less electricity, originally more than 450	Jingdong prefecture level city in Shandong
Happy Fan Shop	Green Intelligent Power Saver	Has not been the first time to buy back, good use, power saving device is really powerful, before the monthly electricity bill of more than 500 Now more than 300, this time to help neighbors to buy several	Jingdong prefecture level city in Shandong
Happy Fan Shop	Green Intelligent Power Saver	Home appliances are very many, the circuit is also very complex, at first it is to save 9% of the electricity, and then used more than half a month, found that the power saving rate is as high as more	Jingdong prefecture level city in Shandong
Happy Fan Shop	Green Intelligent Power Saver	My friend bought it last year and saved hundreds of dollars on his electricity bill in one summer Appearance and style: it looks better than the one I bought before Quality is also good with a light.	Jingdong prefecture level city in Shandong
Happy Fan Shop	Green Intelligent Power Saver	Saver received, on the spot on the plug with the feeling of immediate power savings, appearance, packaging is very delicate, very satisfied, I hope to achieve satisfactory results!	Jingdong prefecture level city in Shandong
Happy Fan Shop	Green Intelligent Power Saver	The power saving effect is very good, is considered an old customer, also bought before, this time is to buy two for mom and dad, customer service attitude is good, praise!	Jingdong prefecture level city in Shandong
Happy Fan Shop	Green Intelligent Power Saver	After observing for a while, it really saves a lot of money I didn't realize it was still really reliable, so I'll give you a good review.	Jingdong prefecture level city in Shandong
Happy Fan Shop	Green Intelligent Power Saver	My family a month with this monthly almost save more than 200, power-saving effect: have bought a second time, really effective Cost-effective: not bad Appearance style: roughly the same A few months to the home electricity is indeed less, this time it is a re-buy three, send a friend two, they stay a	Jingdong prefecture level city in Shandong
Happy Fan Shop	Green Intelligent Power Saver	A month almost save more than 150 dollars in electricity, home appliances more, the main this can also protect high-power appliances, after using this device, these two months have not burned, things are very good, it is said that this is the latest model, the overall price is cheap and beautiful!	Jingdong prefecture level city in Shandong
Happy Fan Shop	Green Intelligent Power Saver	Received, powerful, great quality, love the style😍	Jingdong prefecture level city in Shandong
Happy Fan Shop	Green Intelligent Power Saver	This intelligent energy saver is especially good to use and save electricity hope everyone will buy it!	Jingdong prefecture level city in Shandong
Happy Fan Shop	Green Intelligent Power Saver	Air conditioning adapter effect is particularly great, the use of very simple, plug into the socket on the summer air conditioning power is too large, electricity is also expensive, with this product directly on the electricity bill to save half, has been purchased a few more, the effect is too awesome!	Jingdong prefecture level city in Shandong
Happy Fan Shop	Green Intelligent Power Saver	The family plugged in 5, monthly electricity savings of about 300 yuan. My family before a month of electricity costs more than 800, this time with this saver after the electricity bill is actually only more than 500, amazing, thanks to the store, will recommend other friends to buy!	Jingdong prefecture level city in Shandong
Happy Fan Shop	Green Intelligent Power Saver	It was recommended to me by a neighbor's family and it's nice to use it to save power.	Jingdong prefecture level city in Shandong
Happy Fan Shop	Green Intelligent Power Saver	Second time I've purchased it and the power saving is effective!	Jingdong prefecture level city in Shandong
Happy Fan Shop	Green Intelligent Power Saver	The effect is really good, before a month of more than 300 electricity, now every month every day with the air conditioning, it is more than 100 electricity! This thing is really amazing, one after another to buy and 5.6, home installed 3!	Jingdong prefecture level city in Shandong
Happy Fan Shop	Green Intelligent Power Saver	Received this product, began to use, small things look very wrong, can save a little electricity, the monthly electricity bill is too high, after plugging in did not pull, or save a lot of electricity, point	Jingdong prefecture level city in Shandong
Happy Fan Shop	Green Intelligent Power Saver	Simple and convenient, just that plug in, you can save electricity. A month can save dozens of dollars, a year down to save a lot of, very good product cost-effective, everyone can afford to buy and use!	Jingdong prefecture level city in Shandong
Happy Fan Shop	Green Intelligent Power Saver	Electricity bill from more than 200 yuan per month, down to more than 150 yuan, the original bought home, used two months, really have power-saving effect, this time and sold two, business service attitude is	Jingdong prefecture level city in Shandong
Happy Fan Shop	Green Intelligent Power Saver	Like to watch some movies at home and so on, sometimes at night until two or three o'clock, and recently the air conditioning is not turned off for a night, rather wasteful, I have to say that the effect is very good, quite like it!	Jingdong prefecture level city in Shandong
Happy Fan Shop	Green Intelligent Power Saver	Power-saving effect: the effect is very good, a month to save more than 200 dollars Cost-effective: good value for money Appearance and style: small and lovely Workmanship material: okay Experience: good	Jingdong prefecture level city in Shandong
Happy Fan Shop	Green Intelligent Power Saver	It's okay I quite like it, the stuff is very good. Come back in the future. You can rest assured to buy. Cost-effective all. Much cheaper than the physical store	Jingdong prefecture level city in Shandong
Happy Fan Shop	Green Intelligent Power Saver	More than 150 less, last month's electricity bill is almost 400, this month is only about 200, really save electricity, I am home to buy three, I did not expect a month to pay back	Jingdong prefecture level city in Shandong
Happy Fan Shop	Green Intelligent Power Saver	I bought three of these first and tried them for two months and they still felt useful, so now I've bought another one. Good and recommended!	Jingdong prefecture level city in Shandong
Happy Fan Shop	Green Intelligent Power Saver	This energy saver is very good quality and inexpensive next time I will buy it again!	Jingdong prefecture level city in Shandong
Happy Fan Shop	Green Intelligent Power Saver	Merchant service attitude is not good, the quality does not match, the business does not give the package return	Jingdong prefecture level city in Shandong
Happy Fan Shop	Green Intelligent Power Saver	The goods are very small do not know how effective after a few days to follow up the review	Jingdong prefecture level city in Shandong
Happy Fan Shop	Green Intelligent Power Saver	Not used before more than 200 a month, after using about 100 a month, my family used three, customer service said I use electricity recommended 2-3, I think more than one effect is better on the purchase of 4, the effect is very obvious, recommended to buy!	Jingdong prefecture level city in Shandong
Happy Fan Shop	Green Intelligent Power Saver	It's okay. I'll buy it again today.	Jingdong prefecture level city in Shandong
Happy Fan Shop	Green Intelligent Power Saver	Finally received the goods, very, very much like,, has been a lot of people praise, really too happy, has been his family's loyal fans, will continue to buy buy buy buy!	Jingdong prefecture level city in Shandong
Happy Fan Shop	Green Intelligent Power Saver	After using this, my monthly electricity bill was 1,200 pounds, now it's only 180 bucks a month, how good is it?	Jingdong prefecture level city in Shandong
Happy Fan Shop	Green Intelligent Power Saver	The item is a scam return to marry a dozen dollars in shipping fees. Buy terrific role is purely a scam.	Jingdong prefecture level city in Shandong
Happy Fan Shop	Green Intelligent Power Saver	It's really good, cost-effective and good quality, and the logistics are fast! It arrived in one day!	Jingdong prefecture level city in Shandong
Happy Fan Shop	Green Intelligent Power Saver	Great brand! Trustworthy! Works super well! Expensive for a reason! Great merchandise!	Jingdong prefecture level city in Shandong
Happy Fan Shop	Green Intelligent Power Saver	The baby is really great, I like it very much, my friends also say good to come, recommended to buy!	Jingdong prefecture level city in Shandong
Happy Fan Shop	Green Intelligent Power Saver	Fake couldn't be more fake. Bought one plugged in still so much electricity.	Jingdong prefecture level city in Shandong
Happy Fan Shop	Green Intelligent Power Saver	This appliance works great. Let's see how it works. We'll see later.	Jingdong prefecture level city in Shandong
Happy Fan Shop	Green Intelligent Power Saver	Used it before commenting, very happy with it	Jingdong prefecture level city in Shandong
Happy Fan Shop	Green Intelligent Power Saver	Goods have been received well-packed, logistics is very fast and good quality	Jingdong prefecture level city in Shandong
Happy Fan Shop	Green Intelligent Power Saver	This baby's quality is okay.	Jingdong prefecture level city in Shandong
Happy Fan Shop	Green Intelligent Power Saver	Counterfeit goods.	Jingdong prefecture level city in Shandong

Happy Fan Shop	Green Intelligent Power Saver	The effect is not very obvious	Jingdong prefecture level city in Shandong
Happy Fan Shop	Green Intelligent Power Saver	It's not the same thing.	Jingdong prefecture level city in Shandong
Happy Fan Shop	Green Intelligent Power Saver	There is no load how much w seems false, nothing works.	Jingdong prefecture level city in Shandong
Happy Fan Shop	Green Intelligent Power Saver	I received the item yesterday, got it back and plugged it in, and it used a little more power yesterday than I had before	Jingdong prefecture level city in Shandong
Happy Fan Shop	Green Intelligent Power Saver	Haven't seen the power saving mode yet	Jingdong prefecture level city in Shandong
Happy Fan Shop	Green Intelligent Power Saver	This product has no known effect	Jingdong prefecture level city in Shandong
Happy Fan Shop	Green Intelligent Power Saver	Refund Returns $16	Jingdong prefecture level city in Shandong
Happy Fan Shop	Green Intelligent Power Saver	The previous year bought a, almost every month can save a 70 dollars or so, used a year, no problem at all, look very new like, this time to move to a new home decisively repurchase 2 units, very believe this	Jingdong prefecture level city in Shandong
Happy Fan Shop	Green Intelligent Power Saver	Has been the second time to buy, this power saving device is really good power, before every month to spend 300 electricity, now are about 200, last time that 6, this time to help lead the family to buy 6,	Jingdong prefecture level city in Shandong
Happy Fan Shop	Green Intelligent Power Saver	In the spirit of trying to experience the new product, the use of down compared to the usual power consumption, with the addition of the quarter, is indeed a lot less electricity, originally more than 450	Jingdong prefecture level city in Shandong
Happy Fan Shop	Green Intelligent Power Saver	Has not been the first time to buy back, good use, power saving device is really powerful, before the monthly electricity bill of more than 500 Now more than 300, this time to help neighbors to buy several	Jingdong prefecture level city in Shandong
Happy Fan Shop	Green Intelligent Power Saver	Home appliances are very many, the circuit is also very complex, at first it is to save 9% of the electricity, and then used more than half a month, found that the power saving rate is as high as more	Jingdong prefecture level city in Shandong
Happy Fan Shop	Green Intelligent Power Saver	My friend bought it last year and saved hundreds of dollars on his electricity bill in one summer Appearance and style: it looks better than the one I bought before Quality is also good with a light.	Jingdong prefecture level city in Shandong
Happy Fan Shop	Green Intelligent Power Saver	Saver received, on the spot on the plug with the feeling of immediate power savings, appearance, packaging is very delicate, very satisfied, I hope to achieve satisfactory results!	Jingdong prefecture level city in Shandong
Happy Fan Shop	Green Intelligent Power Saver	The power saving effect is very good, is considered an old customer, also bought before, this time is to buy two for mom and dad, customer service attitude is good, praise!	Jingdong prefecture level city in Shandong
Happy Fan Shop	Green Intelligent Power Saver	After observing for a while, it really saves a lot of money I didn't realize it was still really reliable, so I'll give you a good review.	Jingdong prefecture level city in Shandong
Happy Fan Shop	Green Intelligent Power Saver	My family a month with this monthly almost save more than 200, power-saving effect: have bought a second time, really effective Cost-effective: not bad Appearance style: roughly the same A few months to the home electricity is indeed less, this time it is a re-buy three, send a friend two, they stay a	Jingdong prefecture level city in Shandong
Happy Fan Shop	Green Intelligent Power Saver	A month almost save more than 150 dollars in electricity, home appliances more, the main this can also protect high-power appliances, after using this device, these two months have not burned, things are very good, it is said that this is the latest model, the overall price is cheap and beautiful!	Jingdong prefecture level city in Shandong
Happy Fan Shop	Green Intelligent Power Saver	Received, powerful, great quality, love the style😍	Jingdong prefecture level city in Shandong
Happy Fan Shop	Green Intelligent Power Saver	This intelligent energy saver is especially good to use and save electricity hope everyone will buy it!	Jingdong prefecture level city in Shandong
Happy Fan Shop	Green Intelligent Power Saver	Air conditioning adapter effect is particularly great, the use of very simple, plug into the socket on the summer air conditioning power is too large, electricity is also expensive, with this product directly on the electricity bill to save half, has been purchased a few more, the effect is too awesome!	Jingdong prefecture level city in Shandong
Happy Fan Shop	Green Intelligent Power Saver	The family plugged in 5, monthly electricity savings of about 300 yuan. My family before a month of electricity costs more than 800, this time with this saver after the electricity bill is actually only more than 500, amazing, thanks to the store, will recommend other friends to buy!	Jingdong prefecture level city in Shandong
Happy Fan Shop	Green Intelligent Power Saver	It was recommended to me by a neighbor's family and it's nice to use it to save power.	Jingdong prefecture level city in Shandong
Happy Fan Shop	Green Intelligent Power Saver	Second time I've purchased it and the power saving is effective!	Jingdong prefecture level city in Shandong
Happy Fan Shop	Green Intelligent Power Saver	The effect is really good, before a month of more than 300 electricity, now every month every day with the air conditioning, it is more than 100 electricity! This thing is really amazing, one after another to buy and 5.6, home installed 3!	Jingdong prefecture level city in Shandong
Happy Fan Shop	Green Intelligent Power Saver	Received this product, began to use, small things look very wrong, can save a little electricity, the monthly electricity bill is too high, after plugging in did not pull, or save a lot of electricity, point	Jingdong prefecture level city in Shandong
Happy Fan Shop	Green Intelligent Power Saver	Simple and convenient, just that plug in, you can save electricity. A month can save dozens of dollars, a year down to save a lot of, very good product cost-effective, everyone can afford to buy and use!	Jingdong prefecture level city in Shandong
Happy Fan Shop	Green Intelligent Power Saver	Electricity bill from more than 200 yuan per month, down to more than 150 yuan, the original bought home, used two months, really have power-saving effect, this time and sold two, business service attitude is	Jingdong prefecture level city in Shandong
Happy Fan Shop	Green Intelligent Power Saver	Like to watch some movies at home and so on, sometimes at night until two or three o'clock, and recently the air conditioning is not turned off for a night, rather wasteful, I have to say that the effect is very good, quite like it!	Jingdong prefecture level city in Shandong
Happy Fan Shop	Green Intelligent Power Saver	Power-saving effect: the effect is very good, a month to save more than 200 dollars Cost-effective: good value for money Appearance and style: small and lovely Workmanship material: okay Experience: good	Jingdong prefecture level city in Shandong
Happy Fan Shop	Green Intelligent Power Saver	It's okay I quite like it, the stuff is very good. Come back in the future. You can rest assured to buy. Cost-effective all. Much cheaper than the physical store	Jingdong prefecture level city in Shandong
Happy Fan Shop	Green Intelligent Power Saver	More than 150 less, last month's electricity bill is almost 400, this month is only about 200, really save electricity, I am home to buy three, I did not expect a month to pay back	Jingdong prefecture level city in Shandong
Happy Fan Shop	Green Intelligent Power Saver	I bought three of these first and tried them for two months and they still felt useful, so now I've bought another one. Good and recommended!	Jingdong prefecture level city in Shandong
Happy Fan Shop	Green Intelligent Power Saver	This energy saver is very good quality and inexpensive next time I will buy it again!	Jingdong prefecture level city in Shandong
Happy Fan Shop	Green Intelligent Power Saver	Merchant service attitude is not good, the quality does not match, the business does not give the package return	Jingdong prefecture level city in Shandong
Happy Fan Shop	Green Intelligent Power Saver	The goods are very small do not know how effective after a few days to follow up the review	Jingdong prefecture level city in Shandong
Happy Fan Shop	Green Intelligent Power Saver	Not used before more than 200 a month, after using about 100 a month, my family used three, customer service said I use electricity recommended 2-3, I think more than one effect is better on the purchase of 4, the effect is very obvious, recommended to buy!	Jingdong prefecture level city in Shandong
Happy Fan Shop	Green Intelligent Power Saver	It's okay. I'll buy it again today.	Jingdong prefecture level city in Shandong
Happy Fan Shop	Green Intelligent Power Saver	Finally received the goods, very, very much like,, has been a lot of people praise, really too happy, has been his family's loyal fans, will continue to buy buy buy buy!	Jingdong prefecture level city in Shandong
Happy Fan Shop	Green Intelligent Power Saver	After using this, my monthly electricity bill was 1,200 pounds, now it's only 180 bucks a month, how good is it?	Jingdong prefecture level city in Shandong
Happy Fan Shop	Green Intelligent Power Saver	The item is a scam return to marry a dozen dollars in shipping fees. Buy terrific role is purely a scam.	Jingdong prefecture level city in Shandong
Happy Fan Shop	Green Intelligent Power Saver	It's really good, cost-effective and good quality, and the logistics are fast! It arrived in one day!	Jingdong prefecture level city in Shandong
Happy Fan Shop	Green Intelligent Power Saver	Great brand! Trustworthy! Works super well! Expensive for a reason! Great merchandise!	Jingdong prefecture level city in Shandong
Happy Fan Shop	Green Intelligent Power Saver	The baby is really great, I like it very much, my friends also say good to come, recommended to buy!	Jingdong prefecture level city in Shandong
Happy Fan Shop	Green Intelligent Power Saver	Fake couldn't be more fake. Bought one plugged in still so much electricity.	Jingdong prefecture level city in Shandong
Happy Fan Shop	Green Intelligent Power Saver	This appliance works great. Let's see how it works. We'll see later.	Jingdong prefecture level city in Shandong
Happy Fan Shop	Green Intelligent Power Saver	Used it before commenting, very happy with it	Jingdong prefecture level city in Shandong
Happy Fan Shop	Green Intelligent Power Saver	Goods have been received well-packed, logistics is very fast and good quality	Jingdong prefecture level city in Shandong

Happy Fan Shop	Green Intelligent Power Saver	This baby's quality is okay.	Jingdong prefecture level city in Shandong
Happy Fan Shop	Green Intelligent Power Saver	Counterfeit goods.	Jingdong prefecture level city in Shandong
Happy Fan Shop	Green Intelligent Power Saver	The effect is not very obvious	Jingdong prefecture level city in Shandong
Happy Fan Shop	Green Intelligent Power Saver	It's not the same thing.	Jingdong prefecture level city in Shandong
Happy Fan Shop	Green Intelligent Power Saver	There is no load how much w seems false, nothing works.	Jingdong prefecture level city in Shandong
Happy Fan Shop	Green Intelligent Power Saver	I received the item yesterday, got it back and plugged it in, and it used a little more power yesterday than I had before	Jingdong prefecture level city in Shandong
Happy Fan Shop	Green Intelligent Power Saver	Haven't seen the power saving mode yet	Jingdong prefecture level city in Shandong
Happy Fan Shop	Green Intelligent Power Saver	This product has no known effect	Jingdong prefecture level city in Shandong
Happy Fan Shop	Green Intelligent Power Saver	Refund Returns $16	Jingdong prefecture level city in Shandong
Happy Fan Shop	Green Intelligent Power Saver	The previous year bought a, almost every month can save a 70 dollars or so, used a year, no problem at all, look very new like, this time to move to a new home decisively repurchase 2 units, very believe this	Jingdong prefecture level city in Shandong
Happy Fan Shop	Green Intelligent Power Saver	Has been the second time to buy, this power saving device is really good power, before every month to spend 300 electricity, now are about 200, last time that 6, this time to help lead the family to buy 6,	Jingdong prefecture level city in Shandong
Happy Fan Shop	Green Intelligent Power Saver	In the spirit of trying to experience the new product, the use of down compared to the usual power consumption, with the addition of the quarter, is indeed a lot less electricity, originally more than 450	Jingdong prefecture level city in Shandong
Happy Fan Shop	Green Intelligent Power Saver	Has not been the first time to buy back, good use, power saving device is really powerful, before the monthly electricity bill of more than 500 Now more than 300, this time to help neighbors to buy several	Jingdong prefecture level city in Shandong
Happy Fan Shop	Green Intelligent Power Saver	Home appliances are very many, the circuit is also very complex, at first it is to save 9% of the electricity, and then used more than half a month, found that the power saving rate is as high as more	Jingdong prefecture level city in Shandong
Happy Fan Shop	Green Intelligent Power Saver	My friend bought it last year and saved hundreds of dollars on his electricity bill in one summer Appearance and style: it looks better than the one I bought before Quality is also good with a light.	Jingdong prefecture level city in Shandong
Happy Fan Shop	Green Intelligent Power Saver	Saver received, on the spot on the plug with the feeling of immediate power savings, appearance, packaging is very delicate, very satisfied, I hope to achieve satisfactory results!	Jingdong prefecture level city in Shandong
Happy Fan Shop	Green Intelligent Power Saver	The power saving effect is very good, is considered an old customer, also bought before, this time is to buy two for mom and dad, customer service attitude is good, praise!	Jingdong prefecture level city in Shandong
Happy Fan Shop	Green Intelligent Power Saver	After observing for a while, it really saves a lot of money I didn't realize it was still really reliable, so I'll give you a good review.	Jingdong prefecture level city in Shandong
Happy Fan Shop	Green Intelligent Power Saver	My family a month with this monthly almost save more than 200, power-saving effect: have bought a second time, really effective Cost-effective: not bad Appearance style: roughly the same A few months to the home electricity is indeed less, this time it is a re-buy three, send a friend two, they stay a	Jingdong prefecture level city in Shandong
Happy Fan Shop	Green Intelligent Power Saver	A month almost save more than 150 dollars in electricity, home appliances more, the main this can also protect high-power appliances, after using this device, these two months have not burned, things are very good, it is said that this is the latest model, the overall price is cheap and beautiful!	Jingdong prefecture level city in Shandong
Happy Fan Shop	Green Intelligent Power Saver	Received, powerful, great quality, love the style😍	Jingdong prefecture level city in Shandong
Happy Fan Shop	Green Intelligent Power Saver	This intelligent energy saver is especially good to use and save electricity hope everyone will buy it!	Jingdong prefecture level city in Shandong
Happy Fan Shop	Green Intelligent Power Saver	Air conditioning adapter effect is particularly great, the use of very simple, plug into the socket on the summer air conditioning power is too large, electricity is also expensive, with this product directly on the electricity bill to save half, has been purchased a few more, the effect is too awesome!	Jingdong prefecture level city in Shandong
Happy Fan Shop	Green Intelligent Power Saver	The family plugged in 5, monthly electricity savings of about 300 yuan. My family before a month of electricity costs more than 800, this time with this saver after the electricity bill is actually only more than 500, amazing, thanks to the store, will recommend other friends to buy!	Jingdong prefecture level city in Shandong
Happy Fan Shop	Green Intelligent Power Saver	It was recommended to me by a neighbor's family and it's nice to use it to save power.	Jingdong prefecture level city in Shandong
Happy Fan Shop	Green Intelligent Power Saver	Second time I've purchased it and the power saving is effective!	Jingdong prefecture level city in Shandong
Happy Fan Shop	Green Intelligent Power Saver	The effect is really good, before a month of more than 300 electricity, now every month every day with the air conditioning, it is more than 100 electricity! This thing is really amazing, one after another to buy and 5.6, home installed 3!	Jingdong prefecture level city in Shandong
Happy Fan Shop	Green Intelligent Power Saver	Received this product, began to use, small things look very wrong, can save a little electricity, the monthly electricity bill is too high, after plugging in did not pull, or save a lot of electricity, point	Jingdong prefecture level city in Shandong
Happy Fan Shop	Green Intelligent Power Saver	Simple and convenient, just that plug in, you can save electricity. A month can save dozens of dollars, a year down to save a lot of, very good product cost-effective, everyone can afford to buy and use!	Jingdong prefecture level city in Shandong
Happy Fan Shop	Green Intelligent Power Saver	Electricity bill from more than 200 yuan per month, down to more than 150 yuan, the original bought home, used two months, really have power-saving effect, this time and sold two, business service attitude is	Jingdong prefecture level city in Shandong
Happy Fan Shop	Green Intelligent Power Saver	Like to watch some movies at home and so on, sometimes at night until two or three o'clock, and recently the air conditioning is not turned off for a night, rather wasteful, I have to say that the effect is very good, quite like it!	Jingdong prefecture level city in Shandong
Happy Fan Shop	Green Intelligent Power Saver	Power-saving effect: the effect is very good, a month to save more than 200 dollars Cost-effective: good value for money Appearance and style: small and lovely Workmanship material: okay Experience: good	Jingdong prefecture level city in Shandong
Happy Fan Shop	Green Intelligent Power Saver	It's okay I quite like it, the stuff is very good. Come back in the future. You can rest assured to buy. Cost-effective all. Much cheaper than the physical store	Jingdong prefecture level city in Shandong
Happy Fan Shop	Green Intelligent Power Saver	More than 150 less, last month's electricity bill is almost 400, this month is only about 200, really save electricity, I am home to buy three, I did not expect a month to pay back	Jingdong prefecture level city in Shandong
Happy Fan Shop	Green Intelligent Power Saver	I bought three of these first and tried them for two months and they still felt useful, so now I've bought another one. Good and recommended!	Jingdong prefecture level city in Shandong
Happy Fan Shop	Green Intelligent Power Saver	This energy saver is very good quality and inexpensive next time I will buy it again!	Jingdong prefecture level city in Shandong
Happy Fan Shop	Green Intelligent Power Saver	Merchant service attitude is not good, the quality does not match, the business does not give the package return	Jingdong prefecture level city in Shandong
Happy Fan Shop	Green Intelligent Power Saver	The goods are very small do not know how effective after a few days to follow up the review	Jingdong prefecture level city in Shandong
Happy Fan Shop	Green Intelligent Power Saver	Not used before more than 200 a month, after using about 100 a month, my family used three, customer service said I use electricity recommended 2-3, I think more than one effect is better on the purchase of 4, the effect is very obvious, recommended to buy!	Jingdong prefecture level city in Shandong
Happy Fan Shop	Green Intelligent Power Saver	It's okay. I'll buy it again today.	Jingdong prefecture level city in Shandong
Happy Fan Shop	Green Intelligent Power Saver	Finally received the goods, very, very much like,, has been a lot of people praise, really too happy, has been his family's loyal fans, will continue to buy buy buy!	Jingdong prefecture level city in Shandong
Happy Fan Shop	Green Intelligent Power Saver	After using this, my monthly electricity bill was 1,200 pounds, now it's only 180 bucks a month, how good is it?	Jingdong prefecture level city in Shandong
Happy Fan Shop	Green Intelligent Power Saver	The item is a scam return to marry a dozen dollars in shipping fees. Buy terrific role is purely a scam.	Jingdong prefecture level city in Shandong
Happy Fan Shop	Green Intelligent Power Saver	It's really good, cost-effective and good quality, and the logistics are fast! It arrived in one day!	Jingdong prefecture level city in Shandong
Happy Fan Shop	Green Intelligent Power Saver	Great brand! Trustworthy! Works super well! Expensive for a reason! Great merchandise!	Jingdong prefecture level city in Shandong
Happy Fan Shop	Green Intelligent Power Saver	The baby is really great, I like it very much, my friends also say good to come, recommended to buy!	Jingdong prefecture level city in Shandong
Happy Fan Shop	Green Intelligent Power Saver	Fake couldn't be more fake. Bought one plugged in still so much electricity.	Jingdong prefecture level city in Shandong
Happy Fan Shop	Green Intelligent Power Saver	This appliance works great. Let's see how it works. We'll see later.	Jingdong prefecture level city in Shandong

Happy Fan Shop	Green Intelligent Power Saver	Used it before commenting, very happy with it	Jingdong prefecture level city in Shandong
Happy Fan Shop	Green Intelligent Power Saver	Goods have been received well-packed, logistics is very fast and good quality	Jingdong prefecture level city in Shandong
Happy Fan Shop	Green Intelligent Power Saver	This baby's quality is okay.	Jingdong prefecture level city in Shandong
Happy Fan Shop	Green Intelligent Power Saver	Counterfeit goods.	Jingdong prefecture level city in Shandong
Happy Fan Shop	Green Intelligent Power Saver	The effect is not very obvious	Jingdong prefecture level city in Shandong
Happy Fan Shop	Green Intelligent Power Saver	It's not the same thing.	Jingdong prefecture level city in Shandong
Happy Fan Shop	Green Intelligent Power Saver	There is no load how much w seems false, nothing works.	Jingdong prefecture level city in Shandong
Happy Fan Shop	Green Intelligent Power Saver	I received the item yesterday, got it back and plugged it in, and it used a little more power yesterday than I had before	Jingdong prefecture level city in Shandong
Happy Fan Shop	Green Intelligent Power Saver	Haven't seen the power saving mode yet	Jingdong prefecture level city in Shandong
Happy Fan Shop	Green Intelligent Power Saver	This product has no known effect	Jingdong prefecture level city in Shandong
Happy Fan Shop	Green Intelligent Power Saver	Refund Returns $16	Jingdong prefecture level city in Shandong
Happy Fan Shop	Green Intelligent Power Saver	The previous year bought a, almost every month can save a 70 dollars or so, used a year, no problem at all, look very new like, this time to move to a new home decisively repurchase 2 units, very believe this	Jingdong prefecture level city in Shandong
Happy Fan Shop	Green Intelligent Power Saver	Has been the second time to buy, this power saving device is really good power, before every month to spend 300 electricity, now are about 200, last time that 6, this time to help lead the family to buy 6,	Jingdong prefecture level city in Shandong
Happy Fan Shop	Green Intelligent Power Saver	In the spirit of trying to experience the new product, the use of down compared to the usual power consumption, with the addition of the quarter, is indeed a lot less electricity, originally more than 450	Jingdong prefecture level city in Shandong
Happy Fan Shop	Green Intelligent Power Saver	Has not been the first time to buy back, good use, power saving device is really powerful, before the monthly electricity bill of more than 500 Now more than 300, this time to help neighbors to buy several	Jingdong prefecture level city in Shandong
Happy Fan Shop	Green Intelligent Power Saver	Home appliances are very many, the circuit is also very complex, at first it is to save 9% of the electricity, and then used more than half a month, found that the power saving rate is as high as more	Jingdong prefecture level city in Shandong
Happy Fan Shop	Green Intelligent Power Saver	My friend bought it last year and saved hundreds of dollars on his electricity bill in one summer Appearance and style: it looks better than the one I bought before Quality is also good with a light.	Jingdong prefecture level city in Shandong
Happy Fan Shop	Green Intelligent Power Saver	Saver received, on the spot on the plug with the feeling of immediate power savings, appearance, packaging is very delicate, very satisfied, I hope to achieve satisfactory results!	Jingdong prefecture level city in Shandong
Happy Fan Shop	Green Intelligent Power Saver	The power saving effect is very good, is considered an old customer, also bought before, this time is to buy two for mom and dad, customer service attitude is good, praise!	Jingdong prefecture level city in Shandong
Happy Fan Shop	Green Intelligent Power Saver	After observing for a while, it really saves a lot of money I didn't realize it was still really reliable, so I'll give you a good review.	Jingdong prefecture level city in Shandong
Happy Fan Shop	Green Intelligent Power Saver	My family a month with this monthly almost save more than 200, power-saving effect: have bought a second time, really effective Cost-effective: not bad Appearance style: roughly the same A few months to the home electricity is indeed less, this time it is a re-buy three, send a friend two, they stay a	Jingdong prefecture level city in Shandong
Happy Fan Shop	Green Intelligent Power Saver	A month almost save more than 150 dollars in electricity, home appliances more, the main this can also protect high-power appliances, after using this device, these two months have not burned, things are very good, it is said that this is the latest model, the overall price is cheap and beautiful!	Jingdong prefecture level city in Shandong
Happy Fan Shop	Green Intelligent Power Saver	Received, powerful, great quality, love the style😍	Jingdong prefecture level city in Shandong
Happy Fan Shop	Green Intelligent Power Saver	This intelligent energy saver is especially good to use and save electricity hope everyone will buy it!	Jingdong prefecture level city in Shandong
Happy Fan Shop	Green Intelligent Power Saver	Air conditioning adapter effect is particularly great, the use of very simple, plug into the socket on the summer air conditioning power is too large, electricity is also expensive, with this product directly on the electricity bill to save half, has been purchased a few more, the effect is too awesome!	Jingdong prefecture level city in Shandong
Happy Fan Shop	Green Intelligent Power Saver	The family plugged in 5, monthly electricity savings of about 300 yuan. My family before a month of electricity costs more than 800, this time with this saver after the electricity bill is actually only more than 500, amazing, thanks to the store, will recommend other friends to buy!	Jingdong prefecture level city in Shandong
Happy Fan Shop	Green Intelligent Power Saver	It was recommended to me by a neighbor's family and it's nice to use it to save power.	Jingdong prefecture level city in Shandong
Happy Fan Shop	Green Intelligent Power Saver	Second time I've purchased it and the power saving is effective!	Jingdong prefecture level city in Shandong
Happy Fan Shop	Green Intelligent Power Saver	The effect is really good, before a month of more than 300 electricity, now every month every day with the air conditioning, it is more than 100 electricity! This thing is really amazing, one after another to buy and 5.6, home installed 3!	Jingdong prefecture level city in Shandong
Happy Fan Shop	Green Intelligent Power Saver	Received this product, began to use, small things look very wrong, can save a little electricity, the monthly electricity bill is too high, after plugging in did not pull, or save a lot of electricity, point	Jingdong prefecture level city in Shandong
Happy Fan Shop	Green Intelligent Power Saver	Simple and convenient, just that plug in, you can save electricity. A month can save dozens of dollars, a year down to save a lot of, very good product cost-effective, everyone can afford to buy and use!	Jingdong prefecture level city in Shandong
Happy Fan Shop	Green Intelligent Power Saver	Electricity bill from more than 200 yuan per month, down to more than 150 yuan, the original bought home, used two months, really have power-saving effect, this time and sold two, business service attitude is	Jingdong prefecture level city in Shandong
Happy Fan Shop	Green Intelligent Power Saver	Like to watch some movies at home and so on, sometimes at night until two or three o'clock, and recently the air conditioning is not turned off for a night, rather wasteful, I have to say that the effect is very good, quite like it!	Jingdong prefecture level city in Shandong
Happy Fan Shop	Green Intelligent Power Saver	Power-saving effect: the effect is very good, a month to save more than 200 dollars Cost-effective: good value for money Appearance and style: small and lovely Workmanship material: okay Experience: good	Jingdong prefecture level city in Shandong
Happy Fan Shop	Green Intelligent Power Saver	It's okay I quite like it, the stuff is very good. Come back in the future. You can rest assured to buy. Cost-effective all. Much cheaper than the physical store	Jingdong prefecture level city in Shandong
Happy Fan Shop	Green Intelligent Power Saver	More than 150 less, last month's electricity bill is almost 400, this month is only about 200, really save electricity, I am home to buy three, I did not expect a month to pay back	Jingdong prefecture level city in Shandong
Happy Fan Shop	Green Intelligent Power Saver	I bought three of these first and tried them for two months and they still felt useful, so now I've bought another one. Good and recommended!	Jingdong prefecture level city in Shandong
Happy Fan Shop	Green Intelligent Power Saver	This energy saver is very good quality and inexpensive next time I will buy it again!	Jingdong prefecture level city in Shandong
Happy Fan Shop	Green Intelligent Power Saver	Merchant service attitude is not good, the quality does not match, the business does not give the package return	Jingdong prefecture level city in Shandong
Happy Fan Shop	Green Intelligent Power Saver	The goods are very small do not know how effective after a few days to follow up the review	Jingdong prefecture level city in Shandong
Happy Fan Shop	Green Intelligent Power Saver	Not used before more than 200 a month, after using about 100 a month, my family used three, customer service said I use electricity recommended 2-3, I think more than one effect is better on the purchase of 4, the effect is very obvious, recommended to buy!	Jingdong prefecture level city in Shandong
Happy Fan Shop	Green Intelligent Power Saver	It's okay. I'll buy it again today.	Jingdong prefecture level city in Shandong
Happy Fan Shop	Green Intelligent Power Saver	Finally received the goods, very, very much like,, has been a lot of people praise, really too happy, has been his family's loyal fans, will continue to buy buy buy buy!	Jingdong prefecture level city in Shandong
Happy Fan Shop	Green Intelligent Power Saver	After using this, my monthly electricity bill was 1,200 pounds, now it's only 180 bucks a month, how good is it?	Jingdong prefecture level city in Shandong
Happy Fan Shop	Green Intelligent Power Saver	The item is a scam return to marry a dozen dollars in shipping fees. Buy terrific role is purely a scam.	Jingdong prefecture level city in Shandong
Happy Fan Shop	Green Intelligent Power Saver	It's really good, cost-effective and good quality, and the logistics are fast! It arrived in one day!	Jingdong prefecture level city in Shandong
Happy Fan Shop	Green Intelligent Power Saver	Great brand! Trustworthy! Works super well! Expensive for a reason! Great merchandise!	Jingdong prefecture level city in Shandong
Happy Fan Shop	Green Intelligent Power Saver	The baby is really great, I like it very much, my friends also say good to come, recommended to buy!	Jingdong prefecture level city in Shandong

Happy Fan Shop	Green Intelligent Power Saver	Fake couldn't be more fake. Bought one plugged in still so much electricity.	Jingdong prefecture level city in Shandong
Happy Fan Shop	Green Intelligent Power Saver	This appliance works great. Let's see how it works. We'll see later.	Jingdong prefecture level city in Shandong
Happy Fan Shop	Green Intelligent Power Saver	Used it before commenting, very happy with it	Jingdong prefecture level city in Shandong
Happy Fan Shop	Green Intelligent Power Saver	Goods have been received well-packed, logistics is very fast and good quality	Jingdong prefecture level city in Shandong
Happy Fan Shop	Green Intelligent Power Saver	This baby's quality is okay.	Jingdong prefecture level city in Shandong
Happy Fan Shop	Green Intelligent Power Saver	Counterfeit goods.	Jingdong prefecture level city in Shandong
Happy Fan Shop	Green Intelligent Power Saver	The effect is not very obvious	Jingdong prefecture level city in Shandong
Happy Fan Shop	Green Intelligent Power Saver	It's not the same thing.	Jingdong prefecture level city in Shandong
Happy Fan Shop	Green Intelligent Power Saver	There is no load how much w seems false, nothing works.	Jingdong prefecture level city in Shandong
Happy Fan Shop	Green Intelligent Power Saver	I received the item yesterday, got it back and plugged it in, and it used a little more power yesterday than I had before	Jingdong prefecture level city in Shandong
Happy Fan Shop	Green Intelligent Power Saver	Haven't seen the power saving mode yet	Jingdong prefecture level city in Shandong
Happy Fan Shop	Green Intelligent Power Saver	This product has no known effect	Jingdong prefecture level city in Shandong
Happy Fan Shop	Green Intelligent Power Saver	Refund Returns $16	Jingdong prefecture level city in Shandong
Happy Fan Shop	Green Intelligent Power Saver	The previous year bought a, almost every month can save a 70 dollars or so, used a year, no problem at all, look very new like, this time to move to a new home decisively repurchase 2 units, very believe this	Jingdong prefecture level city in Shandong
Happy Fan Shop	Green Intelligent Power Saver	Has been the second time to buy, this power saving device is really good power, before every month to spend 300 electricity, now are about 200, last time that 6, this time to help lead the family to buy 6,	Jingdong prefecture level city in Shandong
Happy Fan Shop	Green Intelligent Power Saver	In the spirit of trying to experience the new product, the use of down compared to the usual power consumption, with the addition of the quarter, is indeed a lot less electricity, originally more than 450	Jingdong prefecture level city in Shandong
Happy Fan Shop	Green Intelligent Power Saver	Has not been the first time to buy back, good use, power saving device is really powerful, before the monthly electricity bill of more than 500 Now more than 300, this time to help neighbors to buy several	Jingdong prefecture level city in Shandong
Happy Fan Shop	Green Intelligent Power Saver	Home appliances are very many, the circuit is also very complex, at first it is to save 9% of the electricity, and then used more than half a month, found that the power saving rate is as high as more	Jingdong prefecture level city in Shandong
Happy Fan Shop	Green Intelligent Power Saver	My friend bought it last year and saved hundreds of dollars on his electricity bill in one summer Appearance and style: it looks better than the one I bought before Quality is also good with a light.	Jingdong prefecture level city in Shandong
Happy Fan Shop	Green Intelligent Power Saver	Saver received, on the spot on the plug with the feeling of immediate power savings, appearance, packaging is very delicate, very satisfied, I hope to achieve satisfactory results!	Jingdong prefecture level city in Shandong
Happy Fan Shop	Green Intelligent Power Saver	The power saving effect is very good, is considered an old customer, also bought before, this time is to buy two for mom and dad, customer service attitude is good, praise!	Jingdong prefecture level city in Shandong
Happy Fan Shop	Green Intelligent Power Saver	After observing for a while, it really saves a lot of money I didn't realize it was still really reliable, so I'll give you a good review.	Jingdong prefecture level city in Shandong
Happy Fan Shop	Green Intelligent Power Saver	My family a month with this monthly almost save more than 200, power-saving effect: have bought a second time, really effective Cost-effective: not bad Appearance style: roughly the same A few months to the home electricity is indeed less, this time it is a re-buy three, send a friend two, they stay a	Jingdong prefecture level city in Shandong
Happy Fan Shop	Green Intelligent Power Saver	A month almost save more than 150 dollars in electricity, home appliances more, the main this can also protect high-power appliances, after using this device, these two months have not burned, things are very good, it is said that this is the latest model, the overall price is cheap and beautiful!	Jingdong prefecture level city in Shandong
Happy Fan Shop	Green Intelligent Power Saver	Received, powerful, great quality, love the style😍	Jingdong prefecture level city in Shandong
Happy Fan Shop	Green Intelligent Power Saver	This intelligent energy saver is especially good to use and save electricity hope everyone will buy it!	Jingdong prefecture level city in Shandong
Happy Fan Shop	Green Intelligent Power Saver	Air conditioning adapter effect is particularly great, the use of very simple, plug into the socket on the summer air conditioning power is too large, electricity is also expensive, with this product directly on the electricity bill to save half, has been purchased a few more, the effect is too awesome!	Jingdong prefecture level city in Shandong
Happy Fan Shop	Green Intelligent Power Saver	The family plugged in 5, monthly electricity savings of about 300 yuan. My family before a month of electricity costs more than 800, this time with this saver after the electricity bill is actually only more than 500, amazing, thanks to the store, will recommend other friends to buy!	Jingdong prefecture level city in Shandong
Happy Fan Shop	Green Intelligent Power Saver	It was recommended to me by a neighbor's family and it's nice to use it to save power.	Jingdong prefecture level city in Shandong
Happy Fan Shop	Green Intelligent Power Saver	Second time I've purchased it and the power saving is effective!	Jingdong prefecture level city in Shandong
Happy Fan Shop	Green Intelligent Power Saver	The effect is really good, before a month of more than 300 electricity, now every month every day with the air conditioning, it is more than 100 electricity! This thing is really amazing, one after another to buy and 5.6, home installed 3!	Jingdong prefecture level city in Shandong
Happy Fan Shop	Green Intelligent Power Saver	Received this product, began to use, small things look very wrong, can save a little electricity, the monthly electricity bill is too high, after plugging in did not pull, or save a lot of electricity, point	Jingdong prefecture level city in Shandong
Happy Fan Shop	Green Intelligent Power Saver	Simple and convenient, just that plug in, you can save electricity. A month can save dozens of dollars, a year down to save a lot of, very good product cost-effective, everyone can afford to buy and use!	Jingdong prefecture level city in Shandong
Happy Fan Shop	Green Intelligent Power Saver	Electricity bill from more than 200 yuan per month, down to more than 150 yuan, the original bought home, used two months, really have power-saving effect, this time and sold two, business service attitude is	Jingdong prefecture level city in Shandong
Happy Fan Shop	Green Intelligent Power Saver	Like to watch some movies at home and so on, sometimes at night until two or three o'clock, and recently the air conditioning is not turned off for a night, rather wasteful, I have to say that the effect is very good, quite like it!	Jingdong prefecture level city in Shandong
Happy Fan Shop	Green Intelligent Power Saver	Power-saving effect: the effect is very good, a month to save more than 200 dollars Cost-effective: good value for money Appearance and style: small and lovely Workmanship material: okay Experience: good	Jingdong prefecture level city in Shandong
Happy Fan Shop	Green Intelligent Power Saver	It's okay I quite like it, the stuff is very good. Come back in the future. You can rest assured to buy. Cost-effective all. Much cheaper than the physical store	Jingdong prefecture level city in Shandong
Happy Fan Shop	Green Intelligent Power Saver	More than 150 less, last month's electricity bill is almost 400, this month is only about 200, really save electricity, I am home to buy three, I did not expect a month to pay back	Jingdong prefecture level city in Shandong
Happy Fan Shop	Green Intelligent Power Saver	I bought three of these first and tried them for two months and they still felt useful, so now I've bought another one. Good and recommended!	Jingdong prefecture level city in Shandong
Happy Fan Shop	Green Intelligent Power Saver	This energy saver is very good quality and inexpensive next time I will buy it again!	Jingdong prefecture level city in Shandong
Happy Fan Shop	Green Intelligent Power Saver	Merchant service attitude is not good, the quality does not match, the business does not give the package return	Jingdong prefecture level city in Shandong
Happy Fan Shop	Green Intelligent Power Saver	The goods are very small do not know how effective after a few days to follow up the review	Jingdong prefecture level city in Shandong
Happy Fan Shop	Green Intelligent Power Saver	Not used before more than 200 a month, after using about 100 a month, my family used three, customer service said I use electricity recommended 2-3, I think more than one effect is better on the purchase of 4, the effect is very obvious, recommended to buy!	Jingdong prefecture level city in Shandong
Happy Fan Shop	Green Intelligent Power Saver	It's okay. I'll buy it again today.	Jingdong prefecture level city in Shandong
Happy Fan Shop	Green Intelligent Power Saver	Finally received the goods, very, very much like,, has been a lot of people praise, really too happy, has been his family's loyal fans, will continue to buy buy buy!	Jingdong prefecture level city in Shandong
Happy Fan Shop	Green Intelligent Power Saver	After using this, my monthly electricity bill was 1,200 pounds, now it's only 180 bucks a month, how good is it?	Jingdong prefecture level city in Shandong
Happy Fan Shop	Green Intelligent Power Saver	The item is a scam return to marry a dozen dollars in shipping fees. Buy terrific role is purely a scam.	Jingdong prefecture level city in Shandong
Happy Fan Shop	Green Intelligent Power Saver	It's really good, cost-effective and good quality, and the logistics are fast! It arrived in one day!	Jingdong prefecture level city in Shandong

Happy Fan Shop	Green Intelligent Power Saver	Great brand! Trustworthy! Works super well! Expensive for a reason! Great merchandise!	Jingdong prefecture level city in Shandong
Happy Fan Shop	Green Intelligent Power Saver	The baby is really great, I like it very much, my friends also say good to come, recommended to buy!	Jingdong prefecture level city in Shandong
Happy Fan Shop	Green Intelligent Power Saver	Fake couldn't be more fake. Bought one plugged in still so much electricity.	Jingdong prefecture level city in Shandong
Happy Fan Shop	Green Intelligent Power Saver	This appliance works great. Let's see how it works. We'll see later.	Jingdong prefecture level city in Shandong
Happy Fan Shop	Green Intelligent Power Saver	Used it before commenting, very happy with it	Jingdong prefecture level city in Shandong
Happy Fan Shop	Green Intelligent Power Saver	Goods have been received well-packed, logistics is very fast and good quality	Jingdong prefecture level city in Shandong
Happy Fan Shop	Green Intelligent Power Saver	This baby's quality is okay.	Jingdong prefecture level city in Shandong
Happy Fan Shop	Green Intelligent Power Saver	Counterfeit goods.	Jingdong prefecture level city in Shandong
Happy Fan Shop	Green Intelligent Power Saver	The effect is not very obvious	Jingdong prefecture level city in Shandong
Happy Fan Shop	Green Intelligent Power Saver	It's not the same thing.	Jingdong prefecture level city in Shandong
Happy Fan Shop	Green Intelligent Power Saver	There is no load how much w seems false, nothing works.	Jingdong prefecture level city in Shandong
Happy Fan Shop	Green Intelligent Power Saver	I received the item yesterday, got it back and plugged it in, and it used a little more power yesterday than I had before	Jingdong prefecture level city in Shandong
Happy Fan Shop	Green Intelligent Power Saver	Haven't seen the power saving mode yet	Jingdong prefecture level city in Shandong
Happy Fan Shop	Green Intelligent Power Saver	This product has no known effect	Jingdong prefecture level city in Shandong
Happy Fan Shop	Green Intelligent Power Saver	Refund Returns $16	Jingdong prefecture level city in Shandong
Happy Fan Shop	Green Intelligent Power Saver	The previous year bought a, almost every month can save a 70 dollars or so, used a year, no problem at all, look very new like, this time to move to a new home decisively repurchase 2 units, very believe this	Jingdong prefecture level city in Shandong
Happy Fan Shop	Green Intelligent Power Saver	Has been the second time to buy, this power saving device is really good power, before every month to spend 300 electricity, now are about 200, last time that 6, this time to help lead the family to buy 6,	Jingdong prefecture level city in Shandong
Happy Fan Shop	Green Intelligent Power Saver	In the spirit of trying to experience the new product, the use of down compared to the usual power consumption, with the addition of the quarter, is indeed a lot less electricity, originally more than 450	Jingdong prefecture level city in Shandong
Happy Fan Shop	Green Intelligent Power Saver	Has not been the first time to buy back, good use, power saving device is really powerful, before the monthly electricity bill of more than 500 Now more than 300, this time to help neighbors to buy several	Jingdong prefecture level city in Shandong
Happy Fan Shop	Green Intelligent Power Saver	Home appliances are very many, the circuit is also very complex, at first it is to save 9% of the electricity, and then used more than half a month, found that the power saving rate is as high as more	Jingdong prefecture level city in Shandong
Happy Fan Shop	Green Intelligent Power Saver	My friend bought it last year and saved hundreds of dollars on his electricity bill in one summer Appearance and style: it looks better than the one I bought before Quality is also good with a light.	Jingdong prefecture level city in Shandong
Happy Fan Shop	Green Intelligent Power Saver	Saver received, on the spot on the plug with the feeling of immediate power savings, appearance, packaging is very delicate, very satisfied, I hope to achieve satisfactory results!	Jingdong prefecture level city in Shandong
Happy Fan Shop	Green Intelligent Power Saver	The power saving effect is very good, is considered an old customer, also bought before, this time is to buy two for mom and dad, customer service attitude is good, praise!	Jingdong prefecture level city in Shandong
Happy Fan Shop	Green Intelligent Power Saver	After observing for a while, it really saves a lot of money I didn't realize it was still really reliable, so I'll give you a good review.	Jingdong prefecture level city in Shandong
Happy Fan Shop	Green Intelligent Power Saver	My family a month with this monthly almost save more than 200, power-saving effect: have bought a second time, really effective Cost-effective: not bad Appearance style: roughly the same A few months to the home electricity is indeed less, this time it is a re-buy three, send a friend two, they stay a	Jingdong prefecture level city in Shandong
Happy Fan Shop	Green Intelligent Power Saver	A month almost save more than 150 dollars in electricity, home appliances more, the main this can also protect high-power appliances, after using this device, these two months have not burned, things are very good, it is said that this is the latest model, the overall price is cheap and beautiful!	Jingdong prefecture level city in Shandong
Happy Fan Shop	Green Intelligent Power Saver	Received, powerful, great quality, love the style😍	Jingdong prefecture level city in Shandong
Happy Fan Shop	Green Intelligent Power Saver	This intelligent energy saver is especially good to use and save electricity hope everyone will buy it!	Jingdong prefecture level city in Shandong
Happy Fan Shop	Green Intelligent Power Saver	Air conditioning adapter effect is particularly great, the use of very simple, plug into the socket on the summer air conditioning power is too large, electricity is also expensive, with this product directly on the electricity bill to save half, has been purchased a few more, the effect is too awesome!	Jingdong prefecture level city in Shandong
Happy Fan Shop	Green Intelligent Power Saver	The family plugged in 5, monthly electricity savings of about 300 yuan. My family before a month of electricity costs more than 800, this time with this saver after the electricity bill is actually only more than 500, amazing, thanks to the store, will recommend other friends to buy!	Jingdong prefecture level city in Shandong
Happy Fan Shop	Green Intelligent Power Saver	It was recommended to me by a neighbor's family and it's nice to use it to save power.	Jingdong prefecture level city in Shandong
Happy Fan Shop	Green Intelligent Power Saver	Second time I've purchased it and the power saving is effective!	Jingdong prefecture level city in Shandong
Happy Fan Shop	Green Intelligent Power Saver	The effect is really good, before a month of more than 300 electricity, now every month every day with the air conditioning, it is more than 100 electricity! This thing is really amazing, one after another to buy and 5.6, home installed 3!	Jingdong prefecture level city in Shandong
Happy Fan Shop	Green Intelligent Power Saver	Received this product, began to use, small things look very wrong, can save a little electricity, the monthly electricity bill is too high, after plugging in did not pull, or save a lot of electricity, point	Jingdong prefecture level city in Shandong
Happy Fan Shop	Green Intelligent Power Saver	Simple and convenient, just that plug in, you can save electricity. A month can save dozens of dollars, a year down to save a lot of, very good product cost-effective, everyone can afford to buy and use!	Jingdong prefecture level city in Shandong
Happy Fan Shop	Green Intelligent Power Saver	Electricity bill from more than 200 yuan per month, down to more than 150 yuan, the original bought home, used two months, really have power-saving effect, this time and sold two, business service attitude is	Jingdong prefecture level city in Shandong
Happy Fan Shop	Green Intelligent Power Saver	Like to watch some movies at home and so on, sometimes at night until two or three o'clock, and recently the air conditioning is not turned off for a night, rather wasteful, I have to say that the effect is very good, quite like it!	Jingdong prefecture level city in Shandong
Happy Fan Shop	Green Intelligent Power Saver	Power-saving effect: the effect is very good, a month to save more than 200 dollars Cost-effective: good value for money Appearance and style: small and lovely Workmanship material: okay Experience: good	Jingdong prefecture level city in Shandong
Happy Fan Shop	Green Intelligent Power Saver	It's okay I quite like it, the stuff is very good. Come back in the future. You can rest assured to buy. Cost-effective all. Much cheaper than the physical store	Jingdong prefecture level city in Shandong
Happy Fan Shop	Green Intelligent Power Saver	More than 150 less, last month's electricity bill is almost 400, this month is only about 200, really save electricity, I am home to buy three, I did not expect a month to pay back	Jingdong prefecture level city in Shandong
Happy Fan Shop	Green Intelligent Power Saver	I bought three of these first and tried them for two months and they still felt useful, so now I've bought another one. Good and recommended!	Jingdong prefecture level city in Shandong
Happy Fan Shop	Green Intelligent Power Saver	This energy saver is very good quality and inexpensive next time I will buy it again!	Jingdong prefecture level city in Shandong
Happy Fan Shop	Green Intelligent Power Saver	Merchant service attitude is not good, the quality does not match, the business does not give the package return	Jingdong prefecture level city in Shandong
Happy Fan Shop	Green Intelligent Power Saver	The goods are very small do not know how effective after a few days to follow up the review	Jingdong prefecture level city in Shandong
Happy Fan Shop	Green Intelligent Power Saver	Not used before more than 200 a month, after using about 100 a month, my family used three, customer service said I use electricity recommended 2-3, I think more than one effect is better on the purchase of 4, the effect is very obvious, recommended to buy!	Jingdong prefecture level city in Shandong
Happy Fan Shop	Green Intelligent Power Saver	It's okay. I'll buy it again today.	Jingdong prefecture level city in Shandong
Happy Fan Shop	Green Intelligent Power Saver	Finally received the goods, very, very much like,, has been a lot of people praise, really too happy, has been his family's loyal fans, will continue to buy buy buy buy!	Jingdong prefecture level city in Shandong
Happy Fan Shop	Green Intelligent Power Saver	After using this, my monthly electricity bill was 1,200 pounds, now it's only 180 bucks a month, how good is it?	Jingdong prefecture level city in Shandong

Happy Fan Shop	Green Intelligent Power Saver	The item is a scam return to marry a dozen dollars in shipping fees. Buy terrific role is purely a scam.	Jingdong prefecture level city in Shandong
Happy Fan Shop	Green Intelligent Power Saver	It's really good, cost-effective and good quality, and the logistics are fast! It arrived in one day!	Jingdong prefecture level city in Shandong
Happy Fan Shop	Green Intelligent Power Saver	Great brand! Trustworthy! Works super well! Expensive for a reason! Great merchandise!	Jingdong prefecture level city in Shandong
Happy Fan Shop	Green Intelligent Power Saver	The baby is really great, I like it very much, my friends also say good to come, recommended to buy!	Jingdong prefecture level city in Shandong
Happy Fan Shop	Green Intelligent Power Saver	Fake couldn't be more fake. Bought one plugged in still so much electricity.	Jingdong prefecture level city in Shandong
Happy Fan Shop	Green Intelligent Power Saver	This appliance works great. Let's see how it works. We'll see later.	Jingdong prefecture level city in Shandong
Happy Fan Shop	Green Intelligent Power Saver	Used it before commenting, very happy with it	Jingdong prefecture level city in Shandong
Happy Fan Shop	Green Intelligent Power Saver	Goods have been received well-packed, logistics is very fast and good quality	Jingdong prefecture level city in Shandong
Happy Fan Shop	Green Intelligent Power Saver	This baby's quality is okay.	Jingdong prefecture level city in Shandong
Happy Fan Shop	Green Intelligent Power Saver	Counterfeit goods.	Jingdong prefecture level city in Shandong
Happy Fan Shop	Green Intelligent Power Saver	The effect is not very obvious	Jingdong prefecture level city in Shandong
Happy Fan Shop	Green Intelligent Power Saver	It's not the same thing.	Jingdong prefecture level city in Shandong
Happy Fan Shop	Green Intelligent Power Saver	There is no load how much w seems false, nothing works.	Jingdong prefecture level city in Shandong
Happy Fan Shop	Green Intelligent Power Saver	I received the item yesterday, got it back and plugged it in, and it used a little more power yesterday than I had before	Jingdong prefecture level city in Shandong
Happy Fan Shop	Green Intelligent Power Saver	Haven't seen the power saving mode yet	Jingdong prefecture level city in Shandong
Happy Fan Shop	Green Intelligent Power Saver	This product has no known effect	Jingdong prefecture level city in Shandong
Happy Fan Shop	Green Intelligent Power Saver	Refund Returns $16	Jingdong prefecture level city in Shandong
Happy Fan Shop	Green Intelligent Power Saver	The previous year bought a, almost every month can save a 70 dollars or so, used a year, no problem at all, look very new like, this time to move to a new home decisively repurchase 2 units, very believe this	Jingdong prefecture level city in Shandong
Happy Fan Shop	Green Intelligent Power Saver	Has been the second time to buy, this power saving device is really good power, before every month to spend 300 electricity, now are about 200, last time that 6, this time to help lead the family to buy 6,	Jingdong prefecture level city in Shandong
Happy Fan Shop	Green Intelligent Power Saver	In the spirit of trying to experience the new product, the use of down compared to the usual power consumption, with the addition of the quarter, is indeed a lot less electricity, originally more than 450	Jingdong prefecture level city in Shandong
Happy Fan Shop	Green Intelligent Power Saver	Has not been the first time to buy back, good use, power saving device is really powerful, before the monthly electricity bill of more than 500 Now more than 300, this time to help neighbors to buy several	Jingdong prefecture level city in Shandong
Happy Fan Shop	Green Intelligent Power Saver	Home appliances are very many, the circuit is also very complex, at first it is to save 9% of the electricity, and then used more than half a month, found that the power saving rate is as high as more	Jingdong prefecture level city in Shandong
Happy Fan Shop	Green Intelligent Power Saver	My friend bought it last year and saved hundreds of dollars on his electricity bill in one summer Appearance and style: it looks better than the one I bought before Quality is also good with a light.	Jingdong prefecture level city in Shandong
Happy Fan Shop	Green Intelligent Power Saver	Saver received, on the spot on the plug with the feeling of immediate power savings, appearance, packaging is very delicate, very satisfied, I hope to achieve satisfactory results!	Jingdong prefecture level city in Shandong
Happy Fan Shop	Green Intelligent Power Saver	The power saving effect is very good, is considered an old customer, also bought before, this time is to buy two for mom and dad, customer service attitude is good, praise!	Jingdong prefecture level city in Shandong
Happy Fan Shop	Green Intelligent Power Saver	After observing for a while, it really saves a lot of money I didn't realize it was still really reliable, so I'll give you a good review.	Jingdong prefecture level city in Shandong
Happy Fan Shop	Green Intelligent Power Saver	My family a month with this monthly almost save more than 200, power-saving effect: have bought a second time, really effective Cost-effective: not bad Appearance style: roughly the same A few months to the home electricity is indeed less, this time it is a re-buy three, send a friend two, they stay a	Jingdong prefecture level city in Shandong
Happy Fan Shop	Green Intelligent Power Saver	A month almost save more than 150 dollars in electricity, home appliances more, the main this can also protect high-power appliances, after using this device, these two months have not burned, things are very good, it is said that this is the latest model, the overall price is cheap and beautiful!	Jingdong prefecture level city in Shandong
Happy Fan Shop	Green Intelligent Power Saver	Received, powerful, great quality, love the style😍	Jingdong prefecture level city in Shandong
Happy Fan Shop	Green Intelligent Power Saver	This intelligent energy saver is especially good to use and save electricity hope everyone will buy it!	Jingdong prefecture level city in Shandong
Happy Fan Shop	Green Intelligent Power Saver	Air conditioning adapter effect is particularly great, the use of very simple, plug into the socket on the summer air conditioning power is too large, electricity is also expensive, with this product directly on the electricity bill to save half, has been purchased a few more, the effect is too awesome!	Jingdong prefecture level city in Shandong
Happy Fan Shop	Green Intelligent Power Saver	The family plugged in 5, monthly electricity savings of about 300 yuan. My family before a month of electricity costs more than 800, this time with this saver after the electricity bill is actually only more than 500, amazing, thanks to the store, will recommend other friends to buy!	Jingdong prefecture level city in Shandong
Happy Fan Shop	Green Intelligent Power Saver	It was recommended to me by a neighbor's family and it's nice to use it to save power.	Jingdong prefecture level city in Shandong
Happy Fan Shop	Green Intelligent Power Saver	Second time I've purchased it and the power saving is effective!	Jingdong prefecture level city in Shandong
Happy Fan Shop	Green Intelligent Power Saver	The effect is really good, before a month of more than 300 electricity, now every month every day with the air conditioning, it is more than 100 electricity! This thing is really amazing, one after another to buy and 5.6, home installed 3!	Jingdong prefecture level city in Shandong
Happy Fan Shop	Green Intelligent Power Saver	Received this product, began to use, small things look very wrong, can save a little electricity, the monthly electricity bill is too high, after plugging in did not pull, or save a lot of electricity, point	Jingdong prefecture level city in Shandong
Happy Fan Shop	Green Intelligent Power Saver	Simple and convenient, just that plug in, you can save electricity. A month can save dozens of dollars, a year down to save a lot of, very good product cost-effective, everyone can afford to buy and use!	Jingdong prefecture level city in Shandong
Happy Fan Shop	Green Intelligent Power Saver	Electricity bill from more than 200 yuan per month, down to more than 150 yuan, the original bought home, used two months, really have power-saving effect, this time and sold two, business service attitude is	Jingdong prefecture level city in Shandong
Happy Fan Shop	Green Intelligent Power Saver	Like to watch some movies at home and so on, sometimes at night until two or three o'clock, and recently the air conditioning is not turned off for a night, rather wasteful, I have to say that the effect is very good, quite like it!	Jingdong prefecture level city in Shandong
Happy Fan Shop	Green Intelligent Power Saver	Power-saving effect: the effect is very good, a month to save more than 200 dollars Cost-effective: good value for money Appearance and style: small and lovely Workmanship material: okay Experience: good	Jingdong prefecture level city in Shandong
Happy Fan Shop	Green Intelligent Power Saver	It's okay I quite like it, the stuff is very good. Come back in the future. You can rest assured to buy. Cost-effective all. Much cheaper than the physical store	Jingdong prefecture level city in Shandong
Happy Fan Shop	Green Intelligent Power Saver	More than 150 less, last month's electricity bill is almost 400, this month is only about 200, really save electricity, I am home to buy three, I did not expect a month to pay back	Jingdong prefecture level city in Shandong
Happy Fan Shop	Green Intelligent Power Saver	I bought three of these first and tried them for two months and they still felt useful, so now I've bought another one. Good and recommended!	Jingdong prefecture level city in Shandong
Happy Fan Shop	Green Intelligent Power Saver	This energy saver is very good quality and inexpensive next time I will buy it again!	Jingdong prefecture level city in Shandong
Happy Fan Shop	Green Intelligent Power Saver	Merchant service attitude is not good, the quality does not match, the business does not give the package return	Jingdong prefecture level city in Shandong
Happy Fan Shop	Green Intelligent Power Saver	The goods are very small do not know how effective after a few days to follow up the review	Jingdong prefecture level city in Shandong
Happy Fan Shop	Green Intelligent Power Saver	Not used before more than 200 a month, after using about 100 a month, my family used three, customer service said I use electricity recommended 2-3, I think more than one effect is better on the purchase of 4, the effect is very obvious, recommended to buy!	Jingdong prefecture level city in Shandong
Happy Fan Shop	Green Intelligent Power Saver	It's okay. I'll buy it again today.	Jingdong prefecture level city in Shandong

Happy Fan Shop	Green Intelligent Power Saver	Finally received the goods, very, very much like,, has been a lot of people praise, really too happy, has been his family's loyal fans, will continue to buy buy buy buy!	Jingdong prefecture level city in Shandong
Happy Fan Shop	Green Intelligent Power Saver	After using this, my monthly electricity bill was 1,200 pounds, now it's only 180 bucks a month, how good is it?	Jingdong prefecture level city in Shandong
Happy Fan Shop	Green Intelligent Power Saver	The item is a scam return to marry a dozen dollars in shipping fees. Buy terrific role is purely a scam.	Jingdong prefecture level city in Shandong
Happy Fan Shop	Green Intelligent Power Saver	It's really good, cost-effective and good quality, and the logistics are fast! It arrived in one day!	Jingdong prefecture level city in Shandong
Happy Fan Shop	Green Intelligent Power Saver	Great brand! Trustworthy! Works super well! Expensive for a reason! Great merchandise!	Jingdong prefecture level city in Shandong
Happy Fan Shop	Green Intelligent Power Saver	The baby is really great, I like it very much, my friends also say good to come, recommended to buy!	Jingdong prefecture level city in Shandong
Happy Fan Shop	Green Intelligent Power Saver	Fake couldn't be more fake. Bought one plugged in still so much electricity.	Jingdong prefecture level city in Shandong
Happy Fan Shop	Green Intelligent Power Saver	This appliance works great. Let's see how it works. We'll see later.	Jingdong prefecture level city in Shandong
Happy Fan Shop	Green Intelligent Power Saver	Used it before commenting, very happy with it	Jingdong prefecture level city in Shandong
Happy Fan Shop	Green Intelligent Power Saver	Goods have been received well-packed, logistics is very fast and good quality	Jingdong prefecture level city in Shandong
Happy Fan Shop	Green Intelligent Power Saver	This baby's quality is okay.	Jingdong prefecture level city in Shandong
Happy Fan Shop	Green Intelligent Power Saver	Counterfeit goods.	Jingdong prefecture level city in Shandong
Happy Fan Shop	Green Intelligent Power Saver	The effect is not very obvious	Jingdong prefecture level city in Shandong
Happy Fan Shop	Green Intelligent Power Saver	It's not the same thing.	Jingdong prefecture level city in Shandong
Happy Fan Shop	Green Intelligent Power Saver	There is no load how much w seems false, nothing works.	Jingdong prefecture level city in Shandong
Happy Fan Shop	Green Intelligent Power Saver	I received the item yesterday, got it back and plugged it in, and it used a little more power yesterday than I had before	Jingdong prefecture level city in Shandong
Happy Fan Shop	Green Intelligent Power Saver	Haven't seen the power saving mode yet	Jingdong prefecture level city in Shandong
Happy Fan Shop	Green Intelligent Power Saver	This product has no known effect	Jingdong prefecture level city in Shandong
Happy Fan Shop	Green Intelligent Power Saver	Refund Returns $16	Jingdong prefecture level city in Shandong
Happy Fan Shop	Green Intelligent Power Saver	The previous year bought a, almost every month can save a 70 dollars or so, used a year, no problem at all, look very new like, this time to move to a new home decisively repurchase 2 units, very believe this	Jingdong prefecture level city in Shandong
Happy Fan Shop	Green Intelligent Power Saver	Has been the second time to buy, this power saving device is really good power, before every month to spend 300 electricity, now are about 200, last time that 6, this time to help lead the family to buy 6,	Jingdong prefecture level city in Shandong
Happy Fan Shop	Green Intelligent Power Saver	In the spirit of trying to experience the new product, the use of down compared to the usual power consumption, with the addition of the quarter, is indeed a lot less electricity, originally more than 450	Jingdong prefecture level city in Shandong
Happy Fan Shop	Green Intelligent Power Saver	Has not been the first time to buy back, good use, power saving device is really powerful, before the monthly electricity bill of more than 500 Now more than 300, this time to help neighbors to buy several	Jingdong prefecture level city in Shandong
Happy Fan Shop	Green Intelligent Power Saver	Home appliances are very many, the circuit is also very complex, at first it is to save 9% of the electricity, and then used more than half a month, found that the power saving rate is as high as more	Jingdong prefecture level city in Shandong
Happy Fan Shop	Green Intelligent Power Saver	My friend bought it last year and saved hundreds of dollars on his electricity bill in one summer Appearance and style: it looks better than the one I bought before Quality is also good with a light.	Jingdong prefecture level city in Shandong
Happy Fan Shop	Green Intelligent Power Saver	Saver received, on the spot on the plug with the feeling of immediate power savings, appearance, packaging is very delicate, very satisfied, I hope to achieve satisfactory results!	Jingdong prefecture level city in Shandong
Happy Fan Shop	Green Intelligent Power Saver	The power saving effect is very good, is considered an old customer, also bought before, this time is to buy two for mom and dad, customer service attitude is good, praise!	Jingdong prefecture level city in Shandong
Happy Fan Shop	Green Intelligent Power Saver	After observing for a while, it really saves a lot of money I didn't realize it was still really reliable, so I'll give you a good review.	Jingdong prefecture level city in Shandong
Happy Fan Shop	Green Intelligent Power Saver	My family a month with this month almost save more than 200, power-saving effect: have bought a second time, really effective Cost-effective: not bad Appearance style: roughly the same A few months to the home electricity is indeed less, this time it is a re-buy three, send a friend two, they stay a	Jingdong prefecture level city in Shandong
Happy Fan Shop	Green Intelligent Power Saver	A month almost save more than 150 dollars in electricity, home appliances more, the main this can also protect high-power appliances, after using this device, these two months have not burned, things are very good, it is said that this is the latest model, the overall price is cheap and beautiful!	Jingdong prefecture level city in Shandong
Happy Fan Shop	Green Intelligent Power Saver	Received, powerful, great quality, love the style😍	Jingdong prefecture level city in Shandong
Happy Fan Shop	Green Intelligent Power Saver	This intelligent energy saver is especially good to use and save electricity hope everyone will buy it!	Jingdong prefecture level city in Shandong
Happy Fan Shop	Green Intelligent Power Saver	Air conditioning adapter effect is particularly great, the use of very simple, plug into the socket on the summer air conditioning power is too large, electricity is also expensive, with this product directly on the electricity bill to save half, has been purchased a few more, the effect is too awesome!	Jingdong prefecture level city in Shandong
Happy Fan Shop	Green Intelligent Power Saver	The family plugged in 5, monthly electricity savings of about 300 yuan. My family before a month of electricity costs more than 800, this time with this saver after the electricity bill is actually only more than 500, amazing, thanks to the store, will recommend other friends to buy!	Jingdong prefecture level city in Shandong
Happy Fan Shop	Green Intelligent Power Saver	It was recommended to me by a neighbor's family and it's nice to use it to save power.	Jingdong prefecture level city in Shandong
Happy Fan Shop	Green Intelligent Power Saver	Second time I've purchased it and the power saving is effective!	Jingdong prefecture level city in Shandong
Happy Fan Shop	Green Intelligent Power Saver	The effect is really good, before a month of more than 300 electricity, now every month every day with the air conditioning, it is more than 100 electricity! This thing is really amazing, one after another to buy and 5.6, home installed 3!	Jingdong prefecture level city in Shandong
Happy Fan Shop	Green Intelligent Power Saver	Received this product, began to use, small things look very wrong, can save a little electricity, the monthly electricity bill is too high, after plugging in did not pull, or save a lot of electricity, point	Jingdong prefecture level city in Shandong
Happy Fan Shop	Green Intelligent Power Saver	Simple and convenient, just that plug in, you can save electricity. A month can save dozens of dollars, a year down to save a lot of, very good product cost-effective, everyone can afford to buy and use!	Jingdong prefecture level city in Shandong
Happy Fan Shop	Green Intelligent Power Saver	Electricity bill from more than 200 yuan per month, down to more than 150 yuan, the original bought home, used two months, really have power-saving effect, this time and sold two, business service attitude is	Jingdong prefecture level city in Shandong
Happy Fan Shop	Green Intelligent Power Saver	Like to watch some movies at home and so on, sometimes at night until two or three o'clock, and recently the air conditioning is not turned off for a night, rather wasteful, I have to say that the effect is very good, quite like it!	Jingdong prefecture level city in Shandong
Happy Fan Shop	Green Intelligent Power Saver	Power-saving effect: the effect is very good, a month to save more than 200 dollars Cost-effective: good value for money Appearance and style: small and lovely Workmanship material: okay Experience: good	Jingdong prefecture level city in Shandong
Happy Fan Shop	Green Intelligent Power Saver	It's okay I quite like it, the stuff is very good. Come back in the future. You can rest assured to buy. Cost-effective all. Much cheaper than the physical store	Jingdong prefecture level city in Shandong
Happy Fan Shop	Green Intelligent Power Saver	More than 150 less, last month's electricity bill is almost 400, this month is only about 200, really save electricity, I am home to buy three, I did not expect a month to pay back	Jingdong prefecture level city in Shandong
Happy Fan Shop	Green Intelligent Power Saver	I bought three of these first and tried them for two months and they still felt useful, so now I've bought another one. Good and recommended!	Jingdong prefecture level city in Shandong
Happy Fan Shop	Green Intelligent Power Saver	This energy saver is very good quality and inexpensive next time I will buy it again!	Jingdong prefecture level city in Shandong
Happy Fan Shop	Green Intelligent Power Saver	Merchant service attitude is not good, the quality does not match, the business does not give the package return	Jingdong prefecture level city in Shandong
Happy Fan Shop	Green Intelligent Power Saver	The goods are very small do not know how effective after a few days to follow up the review	Jingdong prefecture level city in Shandong

Happy Fan Shop	Green Intelligent Power Saver	Not used before more than 200 a month, after using about 100 a month, my family used three, customer service said I use electricity recommended 2-3, I think more than one effect is better on the purchase of 4, the effect is very obvious, recommended to buy!	Jingdong prefecture level city in Shandong
Happy Fan Shop	Green Intelligent Power Saver	It's okay. I'll buy it again today.	Jingdong prefecture level city in Shandong
Happy Fan Shop	Green Intelligent Power Saver	Finally received the goods, very, very much like,, has been a lot of people praise, really too happy, has been his family's loyal fans, will continue to buy buy buy buy!	Jingdong prefecture level city in Shandong
Happy Fan Shop	Green Intelligent Power Saver	After using this, my monthly electricity bill was 1,200 pounds, now it's only 180 bucks a month, how good is it?	Jingdong prefecture level city in Shandong
Happy Fan Shop	Green Intelligent Power Saver	The item is a scam return to marry a dozen dollars in shipping fees. Buy terrific role is purely a scam.	Jingdong prefecture level city in Shandong
Happy Fan Shop	Green Intelligent Power Saver	It's really good, cost-effective and good quality, and the logistics are fast! It arrived in one day!	Jingdong prefecture level city in Shandong
Happy Fan Shop	Green Intelligent Power Saver	Great brand! Trustworthy! Works super well! Expensive for a reason! Great merchandise!	Jingdong prefecture level city in Shandong
Happy Fan Shop	Green Intelligent Power Saver	The baby is really great, I like it very much, my friends also say good to come, recommended to buy!	Jingdong prefecture level city in Shandong
Happy Fan Shop	Green Intelligent Power Saver	Fake couldn't be more fake. Bought one plugged in still so much electricity.	Jingdong prefecture level city in Shandong
Happy Fan Shop	Green Intelligent Power Saver	This appliance works great. Let's see how it works. We'll see later.	Jingdong prefecture level city in Shandong
Happy Fan Shop	Green Intelligent Power Saver	Used it before commenting, very happy with it	Jingdong prefecture level city in Shandong
Happy Fan Shop	Green Intelligent Power Saver	Goods have been received well-packed, logistics is very fast and good quality	Jingdong prefecture level city in Shandong
Happy Fan Shop	Green Intelligent Power Saver	This baby's quality is okay.	Jingdong prefecture level city in Shandong
Happy Fan Shop	Green Intelligent Power Saver	Counterfeit goods.	Jingdong prefecture level city in Shandong
Happy Fan Shop	Green Intelligent Power Saver	The effect is not very obvious	Jingdong prefecture level city in Shandong
Happy Fan Shop	Green Intelligent Power Saver	It's not the same thing.	Jingdong prefecture level city in Shandong
Happy Fan Shop	Green Intelligent Power Saver	There is no load how much w seems false, nothing works.	Jingdong prefecture level city in Shandong
Happy Fan Shop	Green Intelligent Power Saver	I received the item yesterday, got it back and plugged it in, and it used a little more power yesterday than I had before	Jingdong prefecture level city in Shandong
Happy Fan Shop	Green Intelligent Power Saver	Haven't seen the power saving mode yet	Jingdong prefecture level city in Shandong
Happy Fan Shop	Green Intelligent Power Saver	This product has no known effect	Jingdong prefecture level city in Shandong
Happy Fan Shop	Green Intelligent Power Saver	Refund Returns $16	Jingdong prefecture level city in Shandong
Happy Fan Shop	Green Intelligent Power Saver	The previous year bought a, almost every month can save a 70 dollars or so, used a year, no problem at all, look very new like, this time to move to a new home decisively repurchase 2 units, very believe this	Jingdong prefecture level city in Shandong
Happy Fan Shop	Green Intelligent Power Saver	Has been the second time to buy, this power saving device is really good power, before every month to spend 300 electricity, now are about 200, last time that 6, this time to help lead the family to buy 6,	Jingdong prefecture level city in Shandong
Happy Fan Shop	Green Intelligent Power Saver	In the spirit of trying to experience the new product, the use of down compared to the usual power consumption, with the addition of the quarter, is indeed a lot less electricity, originally more than 450	Jingdong prefecture level city in Shandong
Happy Fan Shop	Green Intelligent Power Saver	Has not been the first time to buy back, good use, power saving device is really powerful, before the monthly electricity bill of more than 500 Now more than 300, this time to help neighbors to buy several	Jingdong prefecture level city in Shandong
Happy Fan Shop	Green Intelligent Power Saver	Home appliances are very many, the circuit is also very complex, at first it is to save 9% of the electricity, and then used more than half a month, found that the power saving rate is as high as more	Jingdong prefecture level city in Shandong
Happy Fan Shop	Green Intelligent Power Saver	My friend bought it last year and saved hundreds of dollars on his electricity bill in one summer Appearance and style: it looks better than the one I bought before Quality is also good with a light.	Jingdong prefecture level city in Shandong
Happy Fan Shop	Green Intelligent Power Saver	Saver received, on the spot on the plug with the feeling of immediate power savings, appearance, packaging is very delicate, very satisfied, I hope to achieve satisfactory results!	Jingdong prefecture level city in Shandong
Happy Fan Shop	Green Intelligent Power Saver	The power saving effect is very good, is considered an old customer, also bought before, this time is to buy two for mom and dad, customer service attitude is good, praise!	Jingdong prefecture level city in Shandong
Happy Fan Shop	Green Intelligent Power Saver	After observing for a while, it really saves a lot of money I didn't realize it was still really reliable, so I'll give you a good review.	Jingdong prefecture level city in Shandong
Happy Fan Shop	Green Intelligent Power Saver	My family a month with this monthly almost save more than 200, power-saving effect: have bought a second time, really effective Cost-effective: not bad Appearance style: roughly the same A few months to the home electricity is indeed less, this time it is a re-buy three, send a friend two, they stay a	Jingdong prefecture level city in Shandong
Happy Fan Shop	Green Intelligent Power Saver	A month almost save more than 150 dollars in electricity, home appliances more, the main this can also protect high-power appliances, after using this device, these two months have not burned, things are very good, it is said that this is the latest model, the overall price is cheap and beautiful!	Jingdong prefecture level city in Shandong
Happy Fan Shop	Green Intelligent Power Saver	Received, powerful, great quality, love the style😍	Jingdong prefecture level city in Shandong
Happy Fan Shop	Green Intelligent Power Saver	This intelligent energy saver is especially good to use and save electricity hope everyone will buy it!	Jingdong prefecture level city in Shandong
Happy Fan Shop	Green Intelligent Power Saver	Air conditioning adapter effect is particularly great, the use of very simple, plug into the socket on the summer air conditioning power is too large, electricity is also expensive, with this product directly on the electricity bill to save half, has been purchased a few more, the effect is too awesome!	Jingdong prefecture level city in Shandong
Happy Fan Shop	Green Intelligent Power Saver	The family plugged in 5, monthly electricity savings of about 300 yuan. My family before a month of electricity costs more than 800, this time with this saver after the electricity bill is actually only more than 500, amazing, thanks to the store, will recommend other friends to buy!	Jingdong prefecture level city in Shandong
Happy Fan Shop	Green Intelligent Power Saver	It was recommended to me by a neighbor's family and it's nice to use it to save power.	Jingdong prefecture level city in Shandong
Happy Fan Shop	Green Intelligent Power Saver	Second time I've purchased it and the power saving is effective!	Jingdong prefecture level city in Shandong
Happy Fan Shop	Green Intelligent Power Saver	The effect is really good, before a month of more than 300 electricity, now every month every day with the air conditioning, it is more than 100 electricity! This thing is really amazing, one after another to buy and 5.6, home installed 3!	Jingdong prefecture level city in Shandong
Happy Fan Shop	Green Intelligent Power Saver	Received this product, began to use, small things look very wrong, can save a little electricity, the monthly electricity bill is too high, after plugging in did not pull, or save a lot of electricity, point	Jingdong prefecture level city in Shandong
Happy Fan Shop	Green Intelligent Power Saver	Simple and convenient, just that plug in, you can save electricity. A month can save dozens of dollars, a year down to save a lot of, very good product cost-effective, everyone can afford to buy and use!	Jingdong prefecture level city in Shandong
Happy Fan Shop	Green Intelligent Power Saver	Electricity bill from more than 200 yuan per month, down to more than 150 yuan, the original bought home, used two months, really have power-saving effect, this time and sold two, business service attitude is	Jingdong prefecture level city in Shandong
Happy Fan Shop	Green Intelligent Power Saver	Like to watch some movies at home and so on, sometimes at night until two or three o'clock, and recently the air conditioning is not turned off for a night, rather wasteful, I have to say that the effect is very good, quite like it!	Jingdong prefecture level city in Shandong
Happy Fan Shop	Green Intelligent Power Saver	Power-saving effect: the effect is very good, a month to save more than 200 dollars Cost-effective: good value for money Appearance and style: small and lovely Workmanship material: okay Experience: good	Jingdong prefecture level city in Shandong
Happy Fan Shop	Green Intelligent Power Saver	It's okay I quite like it, the stuff is very good. Come back in the future. You can rest assured to buy. Cost-effective all. Much cheaper than the physical store	Jingdong prefecture level city in Shandong
Happy Fan Shop	Green Intelligent Power Saver	More than 150 less, last month's electricity bill is almost 400, this month is only about 200, really save electricity, I am home to buy three, I did not expect a month to pay back	Jingdong prefecture level city in Shandong
Happy Fan Shop	Green Intelligent Power Saver	I bought three of these first and tried them for two months and they still felt useful, so now I've bought another one. Good and recommended!	Jingdong prefecture level city in Shandong
Happy Fan Shop	Green Intelligent Power Saver	This energy saver is very good quality and inexpensive next time I will buy it again!	Jingdong prefecture level city in Shandong

Happy Fan Shop	Green Intelligent Power Saver	Merchant service attitude is not good, the quality does not match, the business does not give the package return	Jingdong prefecture level city in Shandong
Happy Fan Shop	Green Intelligent Power Saver	The goods are very small do not know how effective after a few days to follow up the review	Jingdong prefecture level city in Shandong
Happy Fan Shop	Green Intelligent Power Saver	Not used before more than 200 a month, after using about 100 a month, my family used three, customer service said I use electricity recommended 2-3, I think more than one effect is better on the purchase of 4, the effect is very obvious, recommended to buy!	Jingdong prefecture level city in Shandong
Happy Fan Shop	Green Intelligent Power Saver	It's okay. I'll buy it again today.	Jingdong prefecture level city in Shandong
Happy Fan Shop	Green Intelligent Power Saver	Finally received the goods, very, very much like,, has been a lot of people praise, really too happy, has been his family's loyal fans, will continue to buy buy buy buy!	Jingdong prefecture level city in Shandong
Happy Fan Shop	Green Intelligent Power Saver	After using this, my monthly electricity bill was 1,200 pounds, now it's only 180 bucks a month, how good is it?	Jingdong prefecture level city in Shandong
Happy Fan Shop	Green Intelligent Power Saver	The item is a scam return to marry a dozen dollars in shipping fees. Buy terrific role is purely a scam.	Jingdong prefecture level city in Shandong
Happy Fan Shop	Green Intelligent Power Saver	It's really good, cost-effective and good quality, and the logistics are fast! It arrived in one day!	Jingdong prefecture level city in Shandong
Happy Fan Shop	Green Intelligent Power Saver	Great brand! Trustworthy! Works super well! Expensive for a reason! Great merchandise!	Jingdong prefecture level city in Shandong
Happy Fan Shop	Green Intelligent Power Saver	The baby is really great, I like it very much, my friends also say good to come, recommended to buy!	Jingdong prefecture level city in Shandong
Happy Fan Shop	Green Intelligent Power Saver	Fake couldn't be more fake. Bought one plugged in still so much electricity.	Jingdong prefecture level city in Shandong
Happy Fan Shop	Green Intelligent Power Saver	This appliance works great. Let's see how it works. We'll see later.	Jingdong prefecture level city in Shandong
Happy Fan Shop	Green Intelligent Power Saver	Used it before commenting, very happy with it	Jingdong prefecture level city in Shandong
Happy Fan Shop	Green Intelligent Power Saver	Goods have been received well-packed, logistics is very fast and good quality	Jingdong prefecture level city in Shandong
Happy Fan Shop	Green Intelligent Power Saver	This baby's quality is okay.	Jingdong prefecture level city in Shandong
Happy Fan Shop	Green Intelligent Power Saver	Counterfeit goods.	Jingdong prefecture level city in Shandong
Happy Fan Shop	Green Intelligent Power Saver	The effect is not very obvious	Jingdong prefecture level city in Shandong
Happy Fan Shop	Green Intelligent Power Saver	It's not the same thing.	Jingdong prefecture level city in Shandong
Happy Fan Shop	Green Intelligent Power Saver	There is no load how much w seems false, nothing works.	Jingdong prefecture level city in Shandong
Happy Fan Shop	Green Intelligent Power Saver	I received the item yesterday, got it back and plugged it in, and it used a little more power yesterday than I had before	Jingdong prefecture level city in Shandong
Happy Fan Shop	Green Intelligent Power Saver	Haven't seen the power saving mode yet	Jingdong prefecture level city in Shandong
Happy Fan Shop	Green Intelligent Power Saver	This product has no known effect	Jingdong prefecture level city in Shandong
Happy Fan Shop	Green Intelligent Power Saver	Refund Returns $16	Jingdong prefecture level city in Shandong
Happy Fan Shop	Green Intelligent Power Saver	The previous year bought a, almost every month can save a 70 dollars or so, used a year, no problem at all, look very new like, this time to move to a new home decisively repurchase 2 units, very believe this	Jingdong prefecture level city in Shandong
Happy Fan Shop	Green Intelligent Power Saver	Has been the second time to buy, this power saving device is really good power, before every month to spend 300 electricity, now are about 200, last time that 6, this time to help lead the family to buy 6,	Jingdong prefecture level city in Shandong
Happy Fan Shop	Green Intelligent Power Saver	In the spirit of trying to experience the new product, the use of down compared to the usual power consumption, with the addition of the quarter, is indeed a lot less electricity, originally more than 450	Jingdong prefecture level city in Shandong
Happy Fan Shop	Green Intelligent Power Saver	Has not been the first time to buy back, good use, power saving device is really powerful, before the monthly electricity bill of more than 500 Now more than 300, this time to help neighbors to buy several	Jingdong prefecture level city in Shandong
Happy Fan Shop	Green Intelligent Power Saver	Home appliances are very many, the circuit is also very complex, at first it is to save 9% of the electricity, and then used more than half a month, found that the power saving rate is as high as more	Jingdong prefecture level city in Shandong
Happy Fan Shop	Green Intelligent Power Saver	My friend bought it last year and saved hundreds of dollars on his electricity bill in one summer Appearance and style: it looks better than the one I bought before Quality is also good with a light.	Jingdong prefecture level city in Shandong
Happy Fan Shop	Green Intelligent Power Saver	Saver received, on the spot on the plug with the feeling of immediate power savings, appearance, packaging is very delicate, very satisfied, I hope to achieve satisfactory results!	Jingdong prefecture level city in Shandong
Happy Fan Shop	Green Intelligent Power Saver	The power saving effect is very good, is considered an old customer, also bought before, this time is to buy two for mom and dad, customer service attitude is good, praise!	Jingdong prefecture level city in Shandong
Happy Fan Shop	Green Intelligent Power Saver	After observing for a while, it really saves a lot of money I didn't realize it was still really reliable, so I'll give you a good review.	Jingdong prefecture level city in Shandong
Happy Fan Shop	Green Intelligent Power Saver	My family a month with this monthly almost save more than 200, power-saving effect: have bought a second time, really effective Cost-effective: not bad Appearance style: roughly the same A few months to the home electricity is indeed less, this time it is a re-buy three, send a friend two, they stay a	Jingdong prefecture level city in Shandong
Happy Fan Shop	Green Intelligent Power Saver	A month almost save more than 150 dollars in electricity, home appliances more, the main this can also protect high-power appliances, after using this device, these two months have not burned, things are very good, it is said that this is the latest model, the overall price is cheap and beautiful!	Jingdong prefecture level city in Shandong
Happy Fan Shop	Green Intelligent Power Saver	Received, powerful, great quality, love the style😍	Jingdong prefecture level city in Shandong
Happy Fan Shop	Green Intelligent Power Saver	This intelligent energy saver is especially good to use and save electricity hope everyone will buy it!	Jingdong prefecture level city in Shandong
Happy Fan Shop	Green Intelligent Power Saver	Air conditioning adapter effect is particularly great, the use of very simple, plug into the socket on the summer air conditioning power is too large, electricity is also expensive, with this product directly on the electricity bill to save half, has been purchased a few more, the effect is too awesome!	Jingdong prefecture level city in Shandong
Happy Fan Shop	Green Intelligent Power Saver	The family plugged in 5, monthly electricity savings of about 300 yuan. My family before a month of electricity costs more than 800, this time with this saver after the electricity bill is actually only more than 500, amazing, thanks to the store, will recommend other friends to buy!	Jingdong prefecture level city in Shandong
Happy Fan Shop	Green Intelligent Power Saver	It was recommended to me by a neighbor's family and it's nice to use it to save power.	Jingdong prefecture level city in Shandong
Happy Fan Shop	Green Intelligent Power Saver	Second time I've purchased it and the power saving is effective!	Jingdong prefecture level city in Shandong
Happy Fan Shop	Green Intelligent Power Saver	The effect is really good, before a month of more than 300 electricity, now every month every day with the air conditioning, it is more than 100 electricity! This thing is really amazing, one after another to buy and 5.6, home installed 3!	Jingdong prefecture level city in Shandong
Happy Fan Shop	Green Intelligent Power Saver	Received this product, began to use, small things look very wrong, can save a little electricity, the monthly electricity bill is too high, after plugging in did not pull, or save a lot of electricity, point	Jingdong prefecture level city in Shandong
Happy Fan Shop	Green Intelligent Power Saver	Simple and convenient, just that plug in, you can save electricity. A month can save dozens of dollars, a year down to save a lot of, very good product cost-effective, everyone can afford to buy and use!	Jingdong prefecture level city in Shandong
Happy Fan Shop	Green Intelligent Power Saver	Electricity bill from more than 200 yuan per month, down to more than 150 yuan, the original bought home, used two months, really have power-saving effect, this time and sold two, business service attitude is	Jingdong prefecture level city in Shandong
Happy Fan Shop	Green Intelligent Power Saver	Like to watch some movies at home and so on, sometimes at night until two or three o'clock, and recently the air conditioning is not turned off for a night, rather wasteful, I have to say that the effect is very good, quite like it!	Jingdong prefecture level city in Shandong
Happy Fan Shop	Green Intelligent Power Saver	Power-saving effect: the effect is very good, a month to save more than 200 dollars Cost-effective: good value for money Appearance and style: small and lovely Workmanship material: okay Experience: good	Jingdong prefecture level city in Shandong
Happy Fan Shop	Green Intelligent Power Saver	It's okay I quite like it, the stuff is very good. Come back in the future. You can rest assured to buy. Cost-effective all. Much cheaper than the physical store	Jingdong prefecture level city in Shandong
Happy Fan Shop	Green Intelligent Power Saver	More than 150 less, last month's electricity bill is almost 400, this month is only about 200, really save electricity, I am home to buy three, I did not expect a month to pay back	Jingdong prefecture level city in Shandong

Happy Fan Shop	Green Intelligent Power Saver	I bought three of these first and tried them for two months and they still felt useful, so now I've bought another one. Good and recommended!	Jingdong prefecture level city in Shandong
Happy Fan Shop	Green Intelligent Power Saver	This energy saver is very good quality and inexpensive next time I will buy it again!	Jingdong prefecture level city in Shandong
Happy Fan Shop	Green Intelligent Power Saver	Merchant service attitude is not good, the quality does not match, the business does not give the package return	Jingdong prefecture level city in Shandong
Happy Fan Shop	Green Intelligent Power Saver	The goods are very small do not know how effective after a few days to follow up the review	Jingdong prefecture level city in Shandong
Happy Fan Shop	Green Intelligent Power Saver	Not used before more than 200 a month, after using about 100 a month, my family used three, customer service said I use electricity recommended 2-3, I think more than one effect is better on the purchase of 4, the effect is very obvious, recommended to buy!	Jingdong prefecture level city in Shandong
Happy Fan Shop	Green Intelligent Power Saver	It's okay. I'll buy it again today.	Jingdong prefecture level city in Shandong
Happy Fan Shop	Green Intelligent Power Saver	Finally received the goods, very, very much like,, has been a lot of people praise, really too happy, has been his family's loyal fans, will continue to buy buy buy buy!	Jingdong prefecture level city in Shandong
Happy Fan Shop	Green Intelligent Power Saver	After using this, my monthly electricity bill was 1,200 pounds, now it's only 180 bucks a month, how good is it?	Jingdong prefecture level city in Shandong
Happy Fan Shop	Green Intelligent Power Saver	The item is a scam return to marry a dozen dollars in shipping fees. Buy terrific role is purely a scam.	Jingdong prefecture level city in Shandong
Happy Fan Shop	Green Intelligent Power Saver	It's really good, cost-effective and good quality, and the logistics are fast! It arrived in one day!	Jingdong prefecture level city in Shandong
Happy Fan Shop	Green Intelligent Power Saver	Great brand! Trustworthy! Works super well! Expensive for a reason! Great merchandise!	Jingdong prefecture level city in Shandong
Happy Fan Shop	Green Intelligent Power Saver	The baby is really great, I like it very much, my friends also say good to come, recommended to buy!	Jingdong prefecture level city in Shandong
Happy Fan Shop	Green Intelligent Power Saver	Fake couldn't be more fake. Bought one plugged in still so much electricity.	Jingdong prefecture level city in Shandong
Happy Fan Shop	Green Intelligent Power Saver	This appliance works great. Let's see how it works. We'll see later.	Jingdong prefecture level city in Shandong
Happy Fan Shop	Green Intelligent Power Saver	Used it before commenting, very happy with it	Jingdong prefecture level city in Shandong
Happy Fan Shop	Green Intelligent Power Saver	Goods have been received well-packed, logistics is very fast and good quality	Jingdong prefecture level city in Shandong
Happy Fan Shop	Green Intelligent Power Saver	This baby's quality is okay.	Jingdong prefecture level city in Shandong
Happy Fan Shop	Green Intelligent Power Saver	Counterfeit goods.	Jingdong prefecture level city in Shandong
Happy Fan Shop	Green Intelligent Power Saver	The effect is not very obvious	Jingdong prefecture level city in Shandong
Happy Fan Shop	Green Intelligent Power Saver	It's not the same thing.	Jingdong prefecture level city in Shandong
Happy Fan Shop	Green Intelligent Power Saver	There is no load how much w seems false, nothing works.	Jingdong prefecture level city in Shandong
Happy Fan Shop	Green Intelligent Power Saver	I received the item yesterday, got it back and plugged it in, and it used a little more power yesterday than I had before	Jingdong prefecture level city in Shandong
Happy Fan Shop	Green Intelligent Power Saver	Haven't seen the power saving mode yet	Jingdong prefecture level city in Shandong
Happy Fan Shop	Green Intelligent Power Saver	This product has no known effect	Jingdong prefecture level city in Shandong
Happy Fan Shop	Green Intelligent Power Saver	Refund Returns $16	Jingdong prefecture level city in Shandong
Happy Fan Shop	Green Intelligent Power Saver	The previous year bought a, almost every month can save a 70 dollars or so, used a year, no problem at all, look very new like, this time to move to a new home decisively repurchase 2 units, very believe this	Jingdong prefecture level city in Shandong
Happy Fan Shop	Green Intelligent Power Saver	Has been the second time to buy, this power saving device is really good power, before every month to spend 300 electricity, now are about 200, last time that 6, this time to help lead the family to buy 6,	Jingdong prefecture level city in Shandong
Happy Fan Shop	Green Intelligent Power Saver	In the spirit of trying to experience the new product, the use of down compared to the usual power consumption, with the addition of the quarter, is indeed a lot less electricity, originally more than 450	Jingdong prefecture level city in Shandong
Happy Fan Shop	Green Intelligent Power Saver	Has not been the first time to buy back, good use, power saving device is really powerful, before the monthly electricity bill of more than 500 Now more than 300, this time to help neighbors to buy several	Jingdong prefecture level city in Shandong
Happy Fan Shop	Green Intelligent Power Saver	Home appliances are very many, the circuit is also very complex, at first it is to save 9% of the electricity, and then used more than half a month, found that the power saving rate is as high as more	Jingdong prefecture level city in Shandong
Happy Fan Shop	Green Intelligent Power Saver	My friend bought it last year and saved hundreds of dollars on his electricity bill in one summer Appearance and style: it looks better than the one I bought before Quality is also good with a light.	Jingdong prefecture level city in Shandong
Happy Fan Shop	Green Intelligent Power Saver	Saver received, on the spot on the plug with the feeling of immediate power savings, appearance, packaging is very delicate, very satisfied, I hope to achieve satisfactory results!	Jingdong prefecture level city in Shandong
Happy Fan Shop	Green Intelligent Power Saver	The power saving effect is very good, is considered an old customer, also bought before, this time is to buy two for mom and dad, customer service attitude is good, praise!	Jingdong prefecture level city in Shandong
Happy Fan Shop	Green Intelligent Power Saver	After observing for a while, it really saves a lot of money I didn't realize it was still really reliable, so I'll give you a good review.	Jingdong prefecture level city in Shandong
Happy Fan Shop	Green Intelligent Power Saver	My family a month with this monthly almost save more than 200, power-saving effect: have bought a second time, really effective Cost-effective: not bad Appearance style: roughly the same A few months to the home electricity is indeed less, this time it is a re-buy three, send a friend two, they stay a	Jingdong prefecture level city in Shandong
Happy Fan Shop	Green Intelligent Power Saver	A month almost save more than 150 dollars in electricity, home appliances more, the main this can also protect high-power appliances, after using this device, these two months have not burned, things are very good, it is said that this is the latest model, the overall price is cheap and beautiful!	Jingdong prefecture level city in Shandong
Happy Fan Shop	Green Intelligent Power Saver	Received, powerful, great quality, love the style😍	Jingdong prefecture level city in Shandong
Happy Fan Shop	Green Intelligent Power Saver	This intelligent energy saver is especially good to use and save electricity hope everyone will buy it!	Jingdong prefecture level city in Shandong
Happy Fan Shop	Green Intelligent Power Saver	Air conditioning adapter effect is particularly great, the use of very simple, plug into the socket on the summer air conditioning power is too large, electricity is also expensive, with this product directly on the electricity bill to save half, has been purchased a few more, the effect is too awesome!	Jingdong prefecture level city in Shandong
Happy Fan Shop	Green Intelligent Power Saver	The family plugged in 5, monthly electricity savings of about 300 yuan. My family before a month of electricity costs more than 800, this time with this saver after the electricity bill is actually only more than 500, amazing, thanks to the store, will recommend other friends to buy!	Jingdong prefecture level city in Shandong
Happy Fan Shop	Green Intelligent Power Saver	It was recommended to me by a neighbor's family and it's nice to use it to save power.	Jingdong prefecture level city in Shandong
Happy Fan Shop	Green Intelligent Power Saver	Second time I've purchased it and the power saving is effective!	Jingdong prefecture level city in Shandong
Happy Fan Shop	Green Intelligent Power Saver	The effect is really good, before a month of more than 300 electricity, now every month every day with the air conditioning, it is more than 100 electricity! This thing is really amazing, one after another to buy and 5.6, home installed 3!	Jingdong prefecture level city in Shandong
Happy Fan Shop	Green Intelligent Power Saver	Received this product, began to use, small things look very wrong, can save a little electricity, the monthly electricity bill is too high, after plugging in did not pull, or save a lot of electricity, point	Jingdong prefecture level city in Shandong
Happy Fan Shop	Green Intelligent Power Saver	Simple and convenient, just that plug in, you can save electricity. A month can save dozens of dollars, a year down to save a lot of, very good product cost-effective, everyone can afford to buy and use!	Jingdong prefecture level city in Shandong
Happy Fan Shop	Green Intelligent Power Saver	Electricity bill from more than 200 yuan per month, down to more than 150 yuan, the original bought home, used two months, really have power-saving effect, this time and sold two, business service attitude is	Jingdong prefecture level city in Shandong
Happy Fan Shop	Green Intelligent Power Saver	Like to watch some movies at home and so on, sometimes at night until two or three o'clock, and recently the air conditioning is not turned off for a night, rather wasteful, I have to say that the effect is very good, quite like it!	Jingdong prefecture level city in Shandong
Happy Fan Shop	Green Intelligent Power Saver	Power-saving effect: the effect is very good, a month to save more than 200 dollars Cost-effective: good value for money Appearance and style: small and lovely Workmanship material: okay Experience: good	Jingdong prefecture level city in Shandong

Happy Fan Shop	Green Intelligent Power Saver	It's okay I quite like it, the stuff is very good. Come back in the future. You can rest assured to buy. Cost-effective all. Much cheaper than the physical store	Jingdong prefecture level city in Shandong
Happy Fan Shop	Green Intelligent Power Saver	More than 150 less, last month's electricity bill is almost 400, this month is only about 200, really save electricity, I am home to buy three, I did not expect a month to pay back	Jingdong prefecture level city in Shandong
Happy Fan Shop	Green Intelligent Power Saver	I bought three of these first and tried them for two months and they still felt useful, so now I've bought another one. Good and recommended!	Jingdong prefecture level city in Shandong
Happy Fan Shop	Green Intelligent Power Saver	This energy saver is very good quality and inexpensive next time I will buy it again!	Jingdong prefecture level city in Shandong
Happy Fan Shop	Green Intelligent Power Saver	Merchant service attitude is not good, the quality does not match, the business does not give the package return	Jingdong prefecture level city in Shandong
Happy Fan Shop	Green Intelligent Power Saver	The goods are very small do not know how effective after a few days to follow up the review	Jingdong prefecture level city in Shandong
Happy Fan Shop	Green Intelligent Power Saver	Not used before more than 200 a month, after using about 100 a month, my family used three, customer service said I use electricity recommended 2-3, I think more than one effect is better on the purchase of 4, the effect is very obvious, recommended to buy!	Jingdong prefecture level city in Shandong
Happy Fan Shop	Green Intelligent Power Saver	It's okay. I'll buy it again today.	Jingdong prefecture level city in Shandong
Happy Fan Shop	Green Intelligent Power Saver	Finally received the goods, very, very much like,, has been a lot of people praise, really too happy, has been his family's loyal fans, will continue to buy buy buy!	Jingdong prefecture level city in Shandong
Happy Fan Shop	Green Intelligent Power Saver	After using this, my monthly electricity bill was 1,200 pounds, now it's only 180 bucks a month, how good is it?	Jingdong prefecture level city in Shandong
Happy Fan Shop	Green Intelligent Power Saver	The item is a scam return to marry a dozen dollars in shipping fees. Buy terrific role is purely a scam.	Jingdong prefecture level city in Shandong
Happy Fan Shop	Green Intelligent Power Saver	It's really good, cost-effective and good quality, and the logistics are fast! It arrived in one day!	Jingdong prefecture level city in Shandong
Happy Fan Shop	Green Intelligent Power Saver	Great brand! Trustworthy! Works super well! Expensive for a reason! Great merchandise!	Jingdong prefecture level city in Shandong
Happy Fan Shop	Green Intelligent Power Saver	The baby is really great, I like it very much, my friends also say good to come, recommended to buy!	Jingdong prefecture level city in Shandong
Happy Fan Shop	Green Intelligent Power Saver	Fake couldn't be more fake. Bought one plugged in still so much electricity.	Jingdong prefecture level city in Shandong
Happy Fan Shop	Green Intelligent Power Saver	This appliance works great. Let's see how it works. We'll see later.	Jingdong prefecture level city in Shandong
Happy Fan Shop	Green Intelligent Power Saver	Used it before commenting, very happy with it	Jingdong prefecture level city in Shandong
Happy Fan Shop	Green Intelligent Power Saver	Goods have been received well-packed, logistics is very fast and good quality	Jingdong prefecture level city in Shandong
Happy Fan Shop	Green Intelligent Power Saver	This baby's quality is okay.	Jingdong prefecture level city in Shandong
Happy Fan Shop	Green Intelligent Power Saver	Counterfeit goods.	Jingdong prefecture level city in Shandong
Happy Fan Shop	Green Intelligent Power Saver	The effect is not very obvious	Jingdong prefecture level city in Shandong
Happy Fan Shop	Green Intelligent Power Saver	It's not the same thing.	Jingdong prefecture level city in Shandong
Happy Fan Shop	Green Intelligent Power Saver	There is no load how much w seems false, nothing works.	Jingdong prefecture level city in Shandong
Happy Fan Shop	Green Intelligent Power Saver	I received the item yesterday, got it back and plugged it in, and it used a little more power yesterday than I had before	Jingdong prefecture level city in Shandong
Happy Fan Shop	Green Intelligent Power Saver	Haven't seen the power saving mode yet	Jingdong prefecture level city in Shandong
Happy Fan Shop	Green Intelligent Power Saver	This product has no known effect	Jingdong prefecture level city in Shandong
Happy Fan Shop	Green Intelligent Power Saver	Refund Returns $16	Jingdong prefecture level city in Shandong
Happy Fan Shop	Green Intelligent Power Saver	The previous year bought a, almost every month can save a 70 dollars or so, used a year, no problem at all, look very new like, this time to move to a new home decisively repurchase 2 units, very believe this	Jingdong prefecture level city in Shandong
Happy Fan Shop	Green Intelligent Power Saver	Has been the second time to buy, this power saving device is really good power, before every month to spend 300 electricity, now are about 200, last time that 6, this time to help lead the family to buy 6,	Jingdong prefecture level city in Shandong
Happy Fan Shop	Green Intelligent Power Saver	In the spirit of trying to experience the new product, the use of down compared to the usual power consumption, with the addition of the quarter, is indeed a lot less electricity, originally more than 450	Jingdong prefecture level city in Shandong
Happy Fan Shop	Green Intelligent Power Saver	Has not been the first time to buy back, good use, power saving device is really powerful, before the monthly electricity bill of more than 500 Now more than 300, this time to help neighbors to buy several	Jingdong prefecture level city in Shandong
Happy Fan Shop	Green Intelligent Power Saver	Home appliances are very many, the circuit is also very complex, at first it is to save 9% of the electricity, and then used more than half a month, found that the power saving rate is as high as more	Jingdong prefecture level city in Shandong
Happy Fan Shop	Green Intelligent Power Saver	My friend bought it last year and saved hundreds of dollars on his electricity bill in one summer Appearance and style: it looks better than the one I bought before Quality is also good with a light.	Jingdong prefecture level city in Shandong
Happy Fan Shop	Green Intelligent Power Saver	Saver received, on the spot on the plug with the feeling of immediate power savings, appearance, packaging is very delicate, very satisfied, I hope to achieve satisfactory results!	Jingdong prefecture level city in Shandong
Happy Fan Shop	Green Intelligent Power Saver	The power saving effect is very good, is considered an old customer, also bought before, this time is to buy two for mom and dad, customer service attitude is good, praise!	Jingdong prefecture level city in Shandong
Happy Fan Shop	Green Intelligent Power Saver	After observing for a while, it really saves a lot of money I didn't realize it was still really reliable, so I'll give you a good review.	Jingdong prefecture level city in Shandong
Happy Fan Shop	Green Intelligent Power Saver	My family a month with this monthly almost save more than 200, power-saving effect: have bought a second time, really effective Cost-effective: not bad Appearance style: roughly the same A few months to the home electricity is indeed less, this time it is a re-buy three, send a friend two, they stay a	Jingdong prefecture level city in Shandong
Happy Fan Shop	Green Intelligent Power Saver	A month almost save more than 150 dollars in electricity, home appliances more, the main this can also protect high-power appliances, after using this device, these two months have not burned, things are very good, it is said that this is the latest model, the overall price is cheap and beautiful!	Jingdong prefecture level city in Shandong
Happy Fan Shop	Green Intelligent Power Saver	Received, powerful, great quality, love the style😍	Jingdong prefecture level city in Shandong
Happy Fan Shop	Green Intelligent Power Saver	This intelligent energy saver is especially good to use and save electricity hope everyone will buy it!	Jingdong prefecture level city in Shandong
Happy Fan Shop	Green Intelligent Power Saver	Air conditioning adapter effect is particularly great, the use of very simple, plug into the socket on the summer air conditioning power is too large, electricity is also expensive, with this product directly on the electricity bill to save half, has been purchased a few more, the effect is too awesome!	Jingdong prefecture level city in Shandong
Happy Fan Shop	Green Intelligent Power Saver	The family plugged in 5, monthly electricity savings of about 300 yuan. My family before a month of electricity costs more than 800, this time with this saver after the electricity bill is actually only more than 500, amazing, thanks to the store, will recommend other friends to buy!	Jingdong prefecture level city in Shandong
Happy Fan Shop	Green Intelligent Power Saver	It was recommended to me by a neighbor's family and it's nice to use it to save power.	Jingdong prefecture level city in Shandong
Happy Fan Shop	Green Intelligent Power Saver	Second time I've purchased it and the power saving is effective!	Jingdong prefecture level city in Shandong
Happy Fan Shop	Green Intelligent Power Saver	The effect is really good, before a month of more than 300 electricity, now every month every day with the air conditioning, it is more than 100 electricity! This thing is really amazing, one after another to buy and 5.6, home installed 3!	Jingdong prefecture level city in Shandong
Happy Fan Shop	Green Intelligent Power Saver	Received this product, began to use, small things look very wrong, can save a little electricity, the monthly electricity bill is too high, after plugging in did not pull, or save a lot of electricity, point	Jingdong prefecture level city in Shandong
Happy Fan Shop	Green Intelligent Power Saver	Simple and convenient, just that plug in, you can save electricity. A month can save dozens of dollars, a year down to save a lot of, very good product cost-effective, everyone can afford to buy and use!	Jingdong prefecture level city in Shandong
Happy Fan Shop	Green Intelligent Power Saver	Electricity bill from more than 200 yuan per month, down to more than 150 yuan, the original bought home, used two months, really have power-saving effect, this time and sold two, business service attitude is	Jingdong prefecture level city in Shandong

Happy Fan Shop	Green Intelligent Power Saver	Like to watch some movies at home and so on, sometimes at night until two or three o'clock, and recently the air conditioning is not turned off for a night, rather wasteful, I have to say that the effect is very good, quite like it!	Jingdong prefecture level city in Shandong
Happy Fan Shop	Green Intelligent Power Saver	Power-saving effect: the effect is very good, a month to save more than 200 dollars Cost-effective: good value for money Appearance and style: small and lovely Workmanship material: okay Experience: good	Jingdong prefecture level city in Shandong
Happy Fan Shop	Green Intelligent Power Saver	It's okay I quite like it, the stuff is very good. Come back in the future. You can rest assured to buy. Cost-effective all. Much cheaper than the physical store	Jingdong prefecture level city in Shandong
Happy Fan Shop	Green Intelligent Power Saver	More than 150 less, last month's electricity bill is almost 400, this month is only about 200, really save electricity, I am home to buy three, I did not expect a month to pay back	Jingdong prefecture level city in Shandong
Happy Fan Shop	Green Intelligent Power Saver	I bought three of these first and tried them for two months and they still felt useful, so now I've bought another one. Good and recommended!	Jingdong prefecture level city in Shandong
Happy Fan Shop	Green Intelligent Power Saver	This energy saver is very good quality and inexpensive next time I will buy it again!	Jingdong prefecture level city in Shandong
Happy Fan Shop	Green Intelligent Power Saver	Merchant service attitude is not good, the quality does not match, the business does not give the package return	Jingdong prefecture level city in Shandong
Happy Fan Shop	Green Intelligent Power Saver	The goods are very small do not know how effective after a few days to follow up the review	Jingdong prefecture level city in Shandong
Happy Fan Shop	Green Intelligent Power Saver	Not used before more than 200 a month, after using about 100 a month, my family used three, customer service said I use electricity recommended 2-3, I think more than one effect is better on the purchase of 4, the effect is very obvious, recommended to buy!	Jingdong prefecture level city in Shandong
Happy Fan Shop	Green Intelligent Power Saver	It's okay. I'll buy it again today.	Jingdong prefecture level city in Shandong
Happy Fan Shop	Green Intelligent Power Saver	Finally received the goods, very, very much like,, has been a lot of people praise, really too happy, has been his family's loyal fans, will continue to buy buy buy buy!	Jingdong prefecture level city in Shandong
Happy Fan Shop	Green Intelligent Power Saver	After using this, my monthly electricity bill was 1,200 pounds, now it's only 180 bucks a month, how good is it?	Jingdong prefecture level city in Shandong
Happy Fan Shop	Green Intelligent Power Saver	The item is a scam return to marry a dozen dollars in shipping fees. Buy terrific role is purely a scam.	Jingdong prefecture level city in Shandong
Happy Fan Shop	Green Intelligent Power Saver	It's really good, cost-effective and good quality, and the logistics are fast! It arrived in one day!	Jingdong prefecture level city in Shandong
Happy Fan Shop	Green Intelligent Power Saver	Great brand! Trustworthy! Works super well! Expensive for a reason! Great merchandise!	Jingdong prefecture level city in Shandong
Happy Fan Shop	Green Intelligent Power Saver	The baby is really great, I like it very much, my friends also say good to come, recommended to buy!	Jingdong prefecture level city in Shandong
Happy Fan Shop	Green Intelligent Power Saver	Fake couldn't be more fake. Bought one plugged in still so much electricity.	Jingdong prefecture level city in Shandong
Happy Fan Shop	Green Intelligent Power Saver	This appliance works great. Let's see how it works. We'll see later.	Jingdong prefecture level city in Shandong
Happy Fan Shop	Green Intelligent Power Saver	Used it before commenting, very happy with it	Jingdong prefecture level city in Shandong
Happy Fan Shop	Green Intelligent Power Saver	Goods have been received well-packed, logistics is very fast and good quality	Jingdong prefecture level city in Shandong
Happy Fan Shop	Green Intelligent Power Saver	This baby's quality is okay.	Jingdong prefecture level city in Shandong
Happy Fan Shop	Green Intelligent Power Saver	Counterfeit goods.	Jingdong prefecture level city in Shandong
Happy Fan Shop	Green Intelligent Power Saver	The effect is not very obvious	Jingdong prefecture level city in Shandong
Happy Fan Shop	Green Intelligent Power Saver	It's not the same thing.	Jingdong prefecture level city in Shandong
Happy Fan Shop	Green Intelligent Power Saver	There is no load how much w seems false, nothing works.	Jingdong prefecture level city in Shandong
Happy Fan Shop	Green Intelligent Power Saver	I received the item yesterday, got it back and plugged it in, and it used a little more power yesterday than I had before	Jingdong prefecture level city in Shandong
Happy Fan Shop	Green Intelligent Power Saver	Haven't seen the power saving mode yet	Jingdong prefecture level city in Shandong
Happy Fan Shop	Green Intelligent Power Saver	This product has no known effect	Jingdong prefecture level city in Shandong
Happy Fan Shop	Green Intelligent Power Saver	Refund Returns $16	Jingdong prefecture level city in Shandong
Happy Fan Shop	Green Intelligent Power Saver	The previous year bought a, almost every month can save a 70 dollars or so, used a year, no problem at all, look very new like, this time to move to a new home decisively repurchase 2 units, very believe this	Jingdong prefecture level city in Shandong
Happy Fan Shop	Green Intelligent Power Saver	Has been the second time to buy, this power saving device is really good power, before every month to spend 300 electricity, now are about 200, last time that 6, this time to help lead the family to buy 6,	Jingdong prefecture level city in Shandong
Happy Fan Shop	Green Intelligent Power Saver	In the spirit of trying to experience the new product, the use of down compared to the usual power consumption, with the addition of the quarter, is indeed a lot less electricity, originally more than 450	Jingdong prefecture level city in Shandong
Happy Fan Shop	Green Intelligent Power Saver	Has not been the first time to buy back, good use, power saving device is really powerful, before the monthly electricity bill of more than 500 Now more than 300, this time to help neighbors to buy several	Jingdong prefecture level city in Shandong
Happy Fan Shop	Green Intelligent Power Saver	Home appliances are very many, the circuit is also very complex, at first it is to save 9% of the electricity, and then used more than half a month, found that the power saving rate is as high as more	Jingdong prefecture level city in Shandong
Happy Fan Shop	Green Intelligent Power Saver	My friend bought it last year and saved hundreds of dollars on his electricity bill in one summer Appearance and style: it looks better than the one I bought before Quality is also good with a light.	Jingdong prefecture level city in Shandong
Happy Fan Shop	Green Intelligent Power Saver	Saver received, on the spot on the plug with the feeling of immediate power savings, appearance, packaging is very delicate, very satisfied, I hope to achieve satisfactory results!	Jingdong prefecture level city in Shandong
Happy Fan Shop	Green Intelligent Power Saver	The power saving effect is very good, is considered an old customer, also bought before, this time is to buy two for mom and dad, customer service attitude is good, praise!	Jingdong prefecture level city in Shandong
Happy Fan Shop	Green Intelligent Power Saver	After observing for a while, it really saves a lot of money I didn't realize it was still really reliable, so I'll give you a good review.	Jingdong prefecture level city in Shandong
Happy Fan Shop	Green Intelligent Power Saver	My family a month with this monthly almost save more than 200, power-saving effect: have bought a second time, really effective Cost-effective: not bad Appearance style: roughly the same A few months to the home electricity is indeed less, this time it is a re-buy three, send a friend two, they stay a	Jingdong prefecture level city in Shandong
Happy Fan Shop	Green Intelligent Power Saver	A month almost save more than 150 dollars in electricity, home appliances more, the main this can also protect high-power appliances, after using this device, these two months have not burned, things are very good, it is said that this is the latest model, the overall price is cheap and beautiful!	Jingdong prefecture level city in Shandong
Happy Fan Shop	Green Intelligent Power Saver	Received, powerful, great quality, love the style😍	Jingdong prefecture level city in Shandong
Happy Fan Shop	Green Intelligent Power Saver	This intelligent energy saver is especially good to use and save electricity hope everyone will buy it!	Jingdong prefecture level city in Shandong
Happy Fan Shop	Green Intelligent Power Saver	Air conditioning adapter effect is particularly great, the use of very simple, plug into the socket on the summer air conditioning power is too large, electricity is also expensive, with this product directly on the electricity bill to save half, has been purchased a few more, the effect is too awesome!	Jingdong prefecture level city in Shandong
Happy Fan Shop	Green Intelligent Power Saver	The family plugged in 5, monthly electricity savings of about 300 yuan. My family before a month of electricity costs more than 800, this time with this saver after the electricity bill is actually only more than 500, amazing, thanks to the store, will recommend other friends to buy!	Jingdong prefecture level city in Shandong
Happy Fan Shop	Green Intelligent Power Saver	It was recommended to me by a neighbor's family and it's nice to use it to save power.	Jingdong prefecture level city in Shandong
Happy Fan Shop	Green Intelligent Power Saver	Second time I've purchased it and the power saving is effective!	Jingdong prefecture level city in Shandong
Happy Fan Shop	Green Intelligent Power Saver	The effect is really good, before a month of more than 300 electricity, now every month every day with the air conditioning, it is more than 100 electricity! This thing is really amazing, one after another to buy and 5.6, home installed 3!	Jingdong prefecture level city in Shandong
Happy Fan Shop	Green Intelligent Power Saver	Received this product, began to use, small things look very wrong, can save a little electricity, the monthly electricity bill is too high, after plugging in did not pull, or save a lot of electricity, point	Jingdong prefecture level city in Shandong

Happy Fan Shop	Green Intelligent Power Saver	Simple and convenient, just that plug in, you can save electricity. A month can save dozens of dollars, a year down to save a lot of, very good product cost-effective, everyone can afford to buy and use!	Jingdong prefecture level city in Shandong
Happy Fan Shop	Green Intelligent Power Saver	Electricity bill from more than 200 yuan per month, down to more than 150 yuan, the original bought home, used two months, really have power-saving effect, this time and sold two, business service attitude is	Jingdong prefecture level city in Shandong
Happy Fan Shop	Green Intelligent Power Saver	Like to watch some movies at home and so on, sometimes at night until two or three o'clock, and recently the air conditioning is not turned off for a night, rather wasteful, I have to say that the effect is very good, quite like it!	Jingdong prefecture level city in Shandong
Happy Fan Shop	Green Intelligent Power Saver	Power-saving effect: the effect is very good, a month to save more than 200 dollars Cost-effective: good value for money Appearance and style: small and lovely Workmanship material: okay Experience: good	Jingdong prefecture level city in Shandong
Happy Fan Shop	Green Intelligent Power Saver	It's okay I quite like it, the stuff is very good. Come back in the future. You can rest assured to buy. Cost-effective all. Much cheaper than the physical store	Jingdong prefecture level city in Shandong
Happy Fan Shop	Green Intelligent Power Saver	More than 150 less, last month's electricity bill is almost 400, this month is only about 200, really save electricity, I am home to buy three, I did not expect a month to pay back	Jingdong prefecture level city in Shandong
Happy Fan Shop	Green Intelligent Power Saver	I bought three of these first and tried them for two months and they still felt useful, so now I've bought another one. Good and recommended!	Jingdong prefecture level city in Shandong
Happy Fan Shop	Green Intelligent Power Saver	This energy saver is very good quality and inexpensive next time I will buy it again!	Jingdong prefecture level city in Shandong
Happy Fan Shop	Green Intelligent Power Saver	Merchant service attitude is not good, the quality does not match, the business does not give the package return	Jingdong prefecture level city in Shandong
Happy Fan Shop	Green Intelligent Power Saver	The goods are very small do not know how effective after a few days to follow up the review	Jingdong prefecture level city in Shandong
Happy Fan Shop	Green Intelligent Power Saver	Not used before more than 200 a month, after using about 100 a month, my family used three, customer service said I use electricity recommended 2-3, I think more than one effect is better on the purchase of 4, the effect is very obvious, recommended to buy!	Jingdong prefecture level city in Shandong
Happy Fan Shop	Green Intelligent Power Saver	It's okay. I'll buy it again today.	Jingdong prefecture level city in Shandong
Happy Fan Shop	Green Intelligent Power Saver	Finally received the goods, very, very much like,, has been a lot of people praise, really too happy, has been his family's loyal fans, will continue to buy buy buy buy!	Jingdong prefecture level city in Shandong
Happy Fan Shop	Green Intelligent Power Saver	After using this, my monthly electricity bill was 1,200 pounds, now it's only 180 bucks a month, how good is it?	Jingdong prefecture level city in Shandong
Happy Fan Shop	Green Intelligent Power Saver	The item is a scam return to marry a dozen dollars in shipping fees. Buy terrific role is purely a scam.	Jingdong prefecture level city in Shandong
Happy Fan Shop	Green Intelligent Power Saver	It's really good, cost-effective and good quality, and the logistics are fast! It arrived in one day!	Jingdong prefecture level city in Shandong
Happy Fan Shop	Green Intelligent Power Saver	Great brand! Trustworthy! Works super well! Expensive for a reason! Great merchandise!	Jingdong prefecture level city in Shandong
Happy Fan Shop	Green Intelligent Power Saver	The baby is really great, I like it very much, my friends also say good to come, recommended to buy!	Jingdong prefecture level city in Shandong
Happy Fan Shop	Green Intelligent Power Saver	Fake couldn't be more fake. Bought one plugged in still so much electricity.	Jingdong prefecture level city in Shandong
Happy Fan Shop	Green Intelligent Power Saver	This appliance works great. Let's see how it works. We'll see later.	Jingdong prefecture level city in Shandong
Happy Fan Shop	Green Intelligent Power Saver	Used it before commenting, very happy with it	Jingdong prefecture level city in Shandong
Happy Fan Shop	Green Intelligent Power Saver	Goods have been received well-packed, logistics is very fast and good quality	Jingdong prefecture level city in Shandong
Happy Fan Shop	Green Intelligent Power Saver	This baby's quality is okay.	Jingdong prefecture level city in Shandong
Happy Fan Shop	Green Intelligent Power Saver	Counterfeit goods.	Jingdong prefecture level city in Shandong
Happy Fan Shop	Green Intelligent Power Saver	The effect is not very obvious	Jingdong prefecture level city in Shandong
Happy Fan Shop	Green Intelligent Power Saver	It's not the same thing.	Jingdong prefecture level city in Shandong
Happy Fan Shop	Green Intelligent Power Saver	There is no load how much w seems false, nothing works.	Jingdong prefecture level city in Shandong
Happy Fan Shop	Green Intelligent Power Saver	I received the item yesterday, got it back and plugged it in, and it used a little more power yesterday than I had before	Jingdong prefecture level city in Shandong
Happy Fan Shop	Green Intelligent Power Saver	Haven't seen the power saving mode yet	Jingdong prefecture level city in Shandong
Happy Fan Shop	Green Intelligent Power Saver	This product has no known effect	Jingdong prefecture level city in Shandong
Happy Fan Shop	Green Intelligent Power Saver	Refund Returns $16	Jingdong prefecture level city in Shandong
Happy Fan Shop	Green Intelligent Power Saver	The previous year bought a, almost every month can save a 70 dollars or so, used a year, no problem at all, look very new like, this time to move to a new home decisively repurchase 2 units, very believe this	Jingdong prefecture level city in Shandong
Happy Fan Shop	Green Intelligent Power Saver	Has been the second time to buy, this power saving device is really good power, before every month to spend 300 electricity, now are about 200, last time that 6, this time to help lead the family to buy 6,	Jingdong prefecture level city in Shandong
Happy Fan Shop	Green Intelligent Power Saver	In the spirit of trying to experience the new product, the use of down compared to the usual power consumption, with the addition of the quarter, is indeed a lot less electricity, originally more than 450	Jingdong prefecture level city in Shandong
Happy Fan Shop	Green Intelligent Power Saver	Has not been the first time to buy back, good use, power saving device is really powerful, before the monthly electricity bill of more than 500 Now more than 300, this time to help neighbors to buy several	Jingdong prefecture level city in Shandong
Happy Fan Shop	Green Intelligent Power Saver	Home appliances are very many, the circuit is also very complex, at first it is to save 9% of the electricity, and then used more than half a month, found that the power saving rate is as high as more	Jingdong prefecture level city in Shandong
Happy Fan Shop	Green Intelligent Power Saver	My friend bought it last year and saved hundreds of dollars on his electricity bill in one summer Appearance and style: it looks better than the one I bought before Quality is also good with a light.	Jingdong prefecture level city in Shandong
Happy Fan Shop	Green Intelligent Power Saver	Saver received, on the spot on the plug with the feeling of immediate power savings, appearance, packaging is very delicate, very satisfied, I hope to achieve satisfactory results!	Jingdong prefecture level city in Shandong
Happy Fan Shop	Green Intelligent Power Saver	The power saving effect is very good, is considered an old customer, also bought before, this time is to buy two for mom and dad, customer service attitude is good, praise!	Jingdong prefecture level city in Shandong
Happy Fan Shop	Green Intelligent Power Saver	After observing for a while, it really saves a lot of money I didn't realize it was still really reliable, so I'll give you a good review.	Jingdong prefecture level city in Shandong
Happy Fan Shop	Green Intelligent Power Saver	My family a month with this monthly almost save more than 200, power-saving effect: have bought a second time, really effective Cost-effective: not bad Appearance style: roughly the same A few months to the home electricity is indeed less, this time it is a re-buy three, send a friend two, they stay a	Jingdong prefecture level city in Shandong
Happy Fan Shop	Green Intelligent Power Saver	A month almost save more than 150 dollars in electricity, home appliances more, the main this can also protect high-power appliances, after using this device, these two months have not burned, things are very good, it is said that this is the latest model, the overall price is cheap and beautiful!	Jingdong prefecture level city in Shandong
Happy Fan Shop	Green Intelligent Power Saver	Received, powerful, great quality, love the style😍	Jingdong prefecture level city in Shandong
Happy Fan Shop	Green Intelligent Power Saver	This intelligent energy saver is especially good to use and save electricity hope everyone will buy it!	Jingdong prefecture level city in Shandong
Happy Fan Shop	Green Intelligent Power Saver	Air conditioning adapter effect is particularly great, the use of very simple, plug into the socket on the summer air conditioning power is too large, electricity is also expensive, with this product directly on the electricity bill to save half, has been purchased a few more, the effect is too awesome!	Jingdong prefecture level city in Shandong
Happy Fan Shop	Green Intelligent Power Saver	The family plugged in 5, monthly electricity savings of about 300 yuan. My family before a month of electricity costs more than 800, this time with this saver after the electricity bill is actually only more than 500, amazing, thanks to the store, will recommend other friends to buy!	Jingdong prefecture level city in Shandong
Happy Fan Shop	Green Intelligent Power Saver	It was recommended to me by a neighbor's family and it's nice to use it to save power.	Jingdong prefecture level city in Shandong
Happy Fan Shop	Green Intelligent Power Saver	Second time I've purchased it and the power saving is effective!	Jingdong prefecture level city in Shandong

Happy Fan Shop	Green Intelligent Power Saver	The effect is really good, before a month of more than 300 electricity, now every month every day with the air conditioning, it is more than 100 electricity! This thing is really amazing, one after another to buy and 5.6, home installed 3!	Jingdong prefecture level city in Shandong
Happy Fan Shop	Green Intelligent Power Saver	Received this product, began to use, small things look very wrong, can save a little electricity, the monthly electricity bill is too high, after plugging in did not pull, or save a lot of electricity, point	Jingdong prefecture level city in Shandong
Happy Fan Shop	Green Intelligent Power Saver	Simple and convenient, just that plug in, you can save electricity. A month can save dozens of dollars, a year down to save a lot of, very good product cost-effective, everyone can afford to buy and use!	Jingdong prefecture level city in Shandong
Happy Fan Shop	Green Intelligent Power Saver	Electricity bill from more than 200 yuan per month, down to more than 150 yuan, the original bought home, used two months, really have power-saving effect, this time and sold two, business service attitude is	Jingdong prefecture level city in Shandong
Happy Fan Shop	Green Intelligent Power Saver	Like to watch some movies at home and so on, sometimes at night until two or three o'clock, and recently the air conditioning is not turned off for a night, rather wasteful, I have to say that the effect is very good, quite like it!	Jingdong prefecture level city in Shandong
Happy Fan Shop	Green Intelligent Power Saver	Power-saving effect: the effect is very good, a month to save more than 200 dollars Cost-effective: good value for money Appearance and style: small and lovely Workmanship material: okay Experience: good	Jingdong prefecture level city in Shandong
Happy Fan Shop	Green Intelligent Power Saver	It's okay I quite like it, the stuff is very good. Come back in the future. You can rest assured to buy. Cost-effective all. Much cheaper than the physical store	Jingdong prefecture level city in Shandong
Happy Fan Shop	Green Intelligent Power Saver	More than 150 less, last month's electricity bill is almost 400, this month is only about 200, really save electricity, I am home to buy three, I did not expect a month to pay back	Jingdong prefecture level city in Shandong
Happy Fan Shop	Green Intelligent Power Saver	I bought three of these first and tried them for two months and they still felt useful, so now I've bought another one. Good and recommended!	Jingdong prefecture level city in Shandong
Happy Fan Shop	Green Intelligent Power Saver	This energy saver is very good quality and inexpensive next time I will buy it again!	Jingdong prefecture level city in Shandong
Happy Fan Shop	Green Intelligent Power Saver	Merchant service attitude is not good, the quality does not match, the business does not give the package return	Jingdong prefecture level city in Shandong
Happy Fan Shop	Green Intelligent Power Saver	The goods are very small do not know how effective after a few days to follow up the review	Jingdong prefecture level city in Shandong
Happy Fan Shop	Green Intelligent Power Saver	Not used before more than 200 a month, after using about 100 a month, my family used three, customer service said I use electricity recommended 2-3, I think more than one effect is better on the purchase of 4, the effect is very obvious, recommended to buy!	Jingdong prefecture level city in Shandong
Happy Fan Shop	Green Intelligent Power Saver	It's okay. I'll buy it again today.	Jingdong prefecture level city in Shandong
Happy Fan Shop	Green Intelligent Power Saver	Finally received the goods, very, very much like,, has been a lot of people praise, really too happy, has been his family's loyal fans, will continue to buy buy buy buy!	Jingdong prefecture level city in Shandong
Happy Fan Shop	Green Intelligent Power Saver	After using this, my monthly electricity bill was 1,200 pounds, now it's only 180 bucks a month, how good is it?	Jingdong prefecture level city in Shandong
Happy Fan Shop	Green Intelligent Power Saver	The item is a scam return to marry a dozen dollars in shipping fees. Buy terrific role is purely a scam.	Jingdong prefecture level city in Shandong
Happy Fan Shop	Green Intelligent Power Saver	It's really good, cost-effective and good quality, and the logistics are fast! It arrived in one day!	Jingdong prefecture level city in Shandong
Happy Fan Shop	Green Intelligent Power Saver	Great brand! Trustworthy! Works super well! Expensive for a reason! Great merchandise!	Jingdong prefecture level city in Shandong
Happy Fan Shop	Green Intelligent Power Saver	The baby is really great, I like it very much, my friends also say good to come, recommended to buy!	Jingdong prefecture level city in Shandong
Happy Fan Shop	Green Intelligent Power Saver	Fake couldn't be more fake. Bought one plugged in still so much electricity.	Jingdong prefecture level city in Shandong
Happy Fan Shop	Green Intelligent Power Saver	This appliance works great. Let's see how it works. We'll see later.	Jingdong prefecture level city in Shandong
Happy Fan Shop	Green Intelligent Power Saver	Used it before commenting, very happy with it	Jingdong prefecture level city in Shandong
Happy Fan Shop	Green Intelligent Power Saver	Goods have been received well-packed, logistics is very fast and good quality	Jingdong prefecture level city in Shandong
Happy Fan Shop	Green Intelligent Power Saver	This baby's quality is okay.	Jingdong prefecture level city in Shandong
Happy Fan Shop	Green Intelligent Power Saver	Counterfeit goods.	Jingdong prefecture level city in Shandong
Happy Fan Shop	Green Intelligent Power Saver	The effect is not very obvious	Jingdong prefecture level city in Shandong
Happy Fan Shop	Green Intelligent Power Saver	It's not the same thing.	Jingdong prefecture level city in Shandong
Happy Fan Shop	Green Intelligent Power Saver	There is no load how much w seems false, nothing works.	Jingdong prefecture level city in Shandong
Happy Fan Shop	Green Intelligent Power Saver	I received the item yesterday, got it back and plugged it in, and it used a little more power yesterday than I had before	Jingdong prefecture level city in Shandong
Happy Fan Shop	Green Intelligent Power Saver	Haven't seen the power saving mode yet	Jingdong prefecture level city in Shandong
Happy Fan Shop	Green Intelligent Power Saver	This product has no known effect	Jingdong prefecture level city in Shandong
Happy Fan Shop	Green Intelligent Power Saver	Refund Returns $16	Jingdong prefecture level city in Shandong
Happy Fan Shop	Green Intelligent Power Saver	The previous year bought a, almost every month can save a 70 dollars or so, used a year, no problem at all, look very new like, this time to move to a new home decisively repurchase 2 units, very believe this	Jingdong prefecture level city in Shandong
Happy Fan Shop	Green Intelligent Power Saver	Has been the second time to buy, this power saving device is really good power, before every month to spend 300 electricity, now are about 200, last time that 6, this time to help lead the family to buy 6,	Jingdong prefecture level city in Shandong
Happy Fan Shop	Green Intelligent Power Saver	In the spirit of trying to experience the new product, the use of down compared to the usual power consumption, with the addition of the quarter, is indeed a lot less electricity, originally more than 450	Jingdong prefecture level city in Shandong
Happy Fan Shop	Green Intelligent Power Saver	Has not been the first time to buy back, good use, power saving device is really powerful, before the monthly electricity bill of more than 500 Now more than 300, this time to help neighbors to buy several	Jingdong prefecture level city in Shandong
Happy Fan Shop	Green Intelligent Power Saver	Home appliances are very many, the circuit is also very complex, at first it is to save 9% of the electricity, and then used more than half a month, found that the power saving rate is as high as more	Jingdong prefecture level city in Shandong
Happy Fan Shop	Green Intelligent Power Saver	My friend bought it last year and saved hundreds of dollars on his electricity bill in one summer Appearance and style: it looks better than the one I bought before Quality is also good with a light.	Jingdong prefecture level city in Shandong
Happy Fan Shop	Green Intelligent Power Saver	Saver received, on the spot on the plug with the feeling of immediate power savings, appearance, packaging is very delicate, very satisfied, I hope to achieve satisfactory results!	Jingdong prefecture level city in Shandong
Happy Fan Shop	Green Intelligent Power Saver	The power saving effect is very good, is considered an old customer, also bought before, this time is to buy two for mom and dad, customer service attitude is good, praise!	Jingdong prefecture level city in Shandong
Happy Fan Shop	Green Intelligent Power Saver	After observing for a while, it really saves a lot of money I didn't realize it was still really reliable, so I'll give you a good review.	Jingdong prefecture level city in Shandong
Happy Fan Shop	Green Intelligent Power Saver	My family a month with this monthly almost save more than 200, power-saving effect: have bought a second time, really effective Cost-effective: not bad Appearance style: roughly the same A few months to the home electricity is indeed less, this time it is a re-buy three, send a friend two, they stay a	Jingdong prefecture level city in Shandong
Happy Fan Shop	Green Intelligent Power Saver	A month almost save more than 150 dollars in electricity, home appliances more, the main this can also protect high-power appliances, after using this device, these two months have not burned, things are very good, it is said that this is the latest model, the overall price is cheap and beautiful!	Jingdong prefecture level city in Shandong
Happy Fan Shop	Green Intelligent Power Saver	Received, powerful, great quality, love the style😍	Jingdong prefecture level city in Shandong
Happy Fan Shop	Green Intelligent Power Saver	This intelligent energy saver is especially good to use and save electricity hope everyone will buy it!	Jingdong prefecture level city in Shandong
Happy Fan Shop	Green Intelligent Power Saver	Air conditioning adapter effect is particularly great, the use of very simple, plug into the socket on the summer air conditioning power is too large, electricity is also expensive, with this product directly on the electricity bill to save half, has been purchased a few more, the effect is too awesome!	Jingdong prefecture level city in Shandong
Happy Fan Shop	Green Intelligent Power Saver	The family plugged in 5, monthly electricity savings of about 300 yuan. My family before a month of electricity costs more than 800, this time with this saver after the electricity bill is actually only more than 500, amazing, thanks to the store, will recommend other friends to buy!	Jingdong prefecture level city in Shandong

Happy Fan Shop	Green Intelligent Power Saver	It was recommended to me by a neighbor's family and it's nice to use it to save power.	Jingdong prefecture level city in Shandong
Happy Fan Shop	Green Intelligent Power Saver	Second time I've purchased it and the power saving is effective!	Jingdong prefecture level city in Shandong
Happy Fan Shop	Green Intelligent Power Saver	The effect is really good, before a month of more than 300 electricity, now every month every day with the air conditioning, it is more than 100 electricity! This thing is really amazing, one after another to buy and 5.6, home installed 3!	Jingdong prefecture level city in Shandong
Happy Fan Shop	Green Intelligent Power Saver	Received this product, began to use, small things look very wrong, can save a little electricity, the monthly electricity bill is too high, after plugging in did not pull, or save a lot of electricity, point	Jingdong prefecture level city in Shandong
Happy Fan Shop	Green Intelligent Power Saver	Simple and convenient, just that plug in, you can save electricity. A month can save dozens of dollars, a year down to save a lot of, very good product cost-effective, everyone can afford to buy and use!	Jingdong prefecture level city in Shandong
Happy Fan Shop	Green Intelligent Power Saver	Electricity bill from more than 200 yuan per month, down to more than 150 yuan, the original bought home, used two months, really have power-saving effect, this time and sold two, business service attitude is	Jingdong prefecture level city in Shandong
Happy Fan Shop	Green Intelligent Power Saver	Like to watch some movies at home and so on, sometimes at night until two or three o'clock, and recently the air conditioning is not turned off for a night, rather wasteful, I have to say that the effect is very good, quite like it!	Jingdong prefecture level city in Shandong
Happy Fan Shop	Green Intelligent Power Saver	Power-saving effect: the effect is very good, a month to save more than 200 dollars Cost-effective: good value for money Appearance and style: small and lovely Workmanship material: okay Experience: good	Jingdong prefecture level city in Shandong
Happy Fan Shop	Green Intelligent Power Saver	It's okay I quite like it, the stuff is very good. Come back in the future. You can rest assured to buy. Cost-effective all. Much cheaper than the physical store	Jingdong prefecture level city in Shandong
Happy Fan Shop	Green Intelligent Power Saver	More than 150 less, last month's electricity bill is almost 400, this month is only about 200, really save electricity, I am home to buy three, I did not expect a month to pay back	Jingdong prefecture level city in Shandong
Happy Fan Shop	Green Intelligent Power Saver	I bought three of these first and tried them for two months and they still felt useful, so now I've bought another one. Good and recommended!	Jingdong prefecture level city in Shandong
Happy Fan Shop	Green Intelligent Power Saver	This energy saver is very good quality and inexpensive next time I will buy it again!	Jingdong prefecture level city in Shandong
Happy Fan Shop	Green Intelligent Power Saver	Merchant service attitude is not good, the quality does not match, the business does not give the package return	Jingdong prefecture level city in Shandong
Happy Fan Shop	Green Intelligent Power Saver	The goods are very small do not know how effective after a few days to follow up the review	Jingdong prefecture level city in Shandong
Happy Fan Shop	Green Intelligent Power Saver	Not used before more than 200 a month, after using about 100 a month, my family used three, customer service said I use electricity recommended 2-3, I think more than one effect is better on the purchase of 4, the effect is very obvious, recommended to buy!	Jingdong prefecture level city in Shandong
Happy Fan Shop	Green Intelligent Power Saver	It's okay. I'll buy it again today.	Jingdong prefecture level city in Shandong
Happy Fan Shop	Green Intelligent Power Saver	Finally received the goods, very, very much like,, has been a lot of people praise, really too happy, has been his family's loyal fans, will continue to buy buy buy buy!	Jingdong prefecture level city in Shandong
Happy Fan Shop	Green Intelligent Power Saver	After using this, my monthly electricity bill was 1,200 pounds, now it's only 180 bucks a month, how good is it?	Jingdong prefecture level city in Shandong
Happy Fan Shop	Green Intelligent Power Saver	The item is a scam return to marry a dozen dollars in shipping fees. Buy terrific role is purely a scam.	Jingdong prefecture level city in Shandong
Happy Fan Shop	Green Intelligent Power Saver	It's really good, cost-effective and good quality, and the logistics are fast! It arrived in one day!	Jingdong prefecture level city in Shandong
Happy Fan Shop	Green Intelligent Power Saver	Great brand! Trustworthy! Works super well! Expensive for a reason! Great merchandise!	Jingdong prefecture level city in Shandong
Happy Fan Shop	Green Intelligent Power Saver	The baby is really great, I like it very much, my friends also say good to come, recommended to buy!	Jingdong prefecture level city in Shandong
Happy Fan Shop	Green Intelligent Power Saver	Fake couldn't be more fake. Bought one plugged in still so much electricity.	Jingdong prefecture level city in Shandong
Happy Fan Shop	Green Intelligent Power Saver	This appliance works great. Let's see how it works. We'll see later.	Jingdong prefecture level city in Shandong
Happy Fan Shop	Green Intelligent Power Saver	Used it before commenting, very happy with it	Jingdong prefecture level city in Shandong
Happy Fan Shop	Green Intelligent Power Saver	Goods have been received well-packed, logistics is very fast and good quality	Jingdong prefecture level city in Shandong
Happy Fan Shop	Green Intelligent Power Saver	This baby's quality is okay.	Jingdong prefecture level city in Shandong
Happy Fan Shop	Green Intelligent Power Saver	Counterfeit goods.	Jingdong prefecture level city in Shandong
Happy Fan Shop	Green Intelligent Power Saver	The effect is not very obvious	Jingdong prefecture level city in Shandong
Happy Fan Shop	Green Intelligent Power Saver	It's not the same thing.	Jingdong prefecture level city in Shandong
Happy Fan Shop	Green Intelligent Power Saver	There is no load how much w seems false, nothing works.	Jingdong prefecture level city in Shandong
Happy Fan Shop	Green Intelligent Power Saver	I received the item yesterday, got it back and plugged it in, and it used a little more power yesterday than I had before	Jingdong prefecture level city in Shandong
Happy Fan Shop	Green Intelligent Power Saver	Haven't seen the power saving mode yet	Jingdong prefecture level city in Shandong
Happy Fan Shop	Green Intelligent Power Saver	This product has no known effect	Jingdong prefecture level city in Shandong
Happy Fan Shop	Green Intelligent Power Saver	Refund Returns $16	Jingdong prefecture level city in Shandong
Happy Fan Shop	Green Intelligent Power Saver	The previous year bought a, almost every month can save a 70 dollars or so, used a year, no problem at all, look very new like, this time to move to a new home decisively repurchase 2 units, very believe this	Jingdong prefecture level city in Shandong
Happy Fan Shop	Green Intelligent Power Saver	Has been the second time to buy, this power saving device is really good power, before every month to spend 300 electricity, now are about 200, last time that 6, this time to help lead the family to buy 6,	Jingdong prefecture level city in Shandong
Happy Fan Shop	Green Intelligent Power Saver	In the spirit of trying to experience the new product, the use of down compared to the usual power consumption, with the addition of the quarter, is indeed a lot less electricity, originally more than 450	Jingdong prefecture level city in Shandong
Happy Fan Shop	Green Intelligent Power Saver	Has not been the first time to buy back, good use, power saving device is really powerful, before the monthly electricity bill of more than 500 Now more than 300, this time to help neighbors to buy several	Jingdong prefecture level city in Shandong
Happy Fan Shop	Green Intelligent Power Saver	Home appliances are very many, the circuit is also very complex, at first it is to save 9% of the electricity, and then used more than half a month, found that the power saving rate is as high as more	Jingdong prefecture level city in Shandong
Happy Fan Shop	Green Intelligent Power Saver	My friend bought it last year and saved hundreds of dollars on his electricity bill in one summer Appearance and style: it looks better than the one I bought before Quality is also good with a light.	Jingdong prefecture level city in Shandong
Happy Fan Shop	Green Intelligent Power Saver	Saver received, on the spot on the plug with the feeling of immediate power savings, appearance, packaging is very delicate, very satisfied, I hope to achieve satisfactory results!	Jingdong prefecture level city in Shandong
Happy Fan Shop	Green Intelligent Power Saver	The power saving effect is very good, is considered an old customer, also bought before, this time is to buy two for mom and dad, customer service attitude is good, praise!	Jingdong prefecture level city in Shandong
Happy Fan Shop	Green Intelligent Power Saver	After observing for a while, it really saves a lot of money I didn't realize it was still really reliable, so I'll give you a good review.	Jingdong prefecture level city in Shandong
Happy Fan Shop	Green Intelligent Power Saver	My family a month with this monthly almost save more than 200, power-saving effect: have bought a second time, really effective Cost-effective: not bad Appearance style: roughly the same A few months to the home electricity is indeed less, this time it is a re-buy three, send a friend two, they stay a	Jingdong prefecture level city in Shandong
Happy Fan Shop	Green Intelligent Power Saver	A month almost save more than 150 dollars in electricity, home appliances more, the main this can also protect high-power appliances, after using this device, these two months have not burned, things are very good, it is said that this is the latest model, the overall price is cheap and beautiful!	Jingdong prefecture level city in Shandong
Happy Fan Shop	Green Intelligent Power Saver	Received, powerful, great quality, love the style😍	Jingdong prefecture level city in Shandong
Happy Fan Shop	Green Intelligent Power Saver	This intelligent energy saver is especially good to use and save electricity hope everyone will buy it!	Jingdong prefecture level city in Shandong

Happy Fan Shop	Green Intelligent Power Saver	Air conditioning adapter effect is particularly great, the use of very simple, plug into the socket on the summer air conditioning power is too large, electricity is also expensive, with this product directly on the electricity bill to save half, has been purchased a few more, the effect is too awesome!	Jingdong prefecture level city in Shandong
Happy Fan Shop	Green Intelligent Power Saver	The family plugged in 5, monthly electricity savings of about 300 yuan. My family before a month of electricity costs more than 800, this time with this saver after the electricity bill is actually only more than 500, amazing, thanks to the store, will recommend other friends to buy!	Jingdong prefecture level city in Shandong
Happy Fan Shop	Green Intelligent Power Saver	It was recommended to me by a neighbor's family and it's nice to use it to save power.	Jingdong prefecture level city in Shandong
Happy Fan Shop	Green Intelligent Power Saver	Second time I've purchased it and the power saving is effective!	Jingdong prefecture level city in Shandong
Happy Fan Shop	Green Intelligent Power Saver	The effect is really good, before a month of more than 300 electricity, now every month every day with the air conditioning, it is more than 100 electricity! This thing is really amazing, one after another to buy and 5.6, home installed 3!	Jingdong prefecture level city in Shandong
Happy Fan Shop	Green Intelligent Power Saver	Received this product, began to use, small things look very wrong, can save a little electricity, the monthly electricity bill is too high, after plugging in did not pull, or save a lot of electricity, point	Jingdong prefecture level city in Shandong
Happy Fan Shop	Green Intelligent Power Saver	Simple and convenient, just that plug in, you can save electricity. A month can save dozens of dollars, a year down to save a lot of, very good product cost-effective, everyone can afford to buy and use!	Jingdong prefecture level city in Shandong
Happy Fan Shop	Green Intelligent Power Saver	Electricity bill from more than 200 yuan per month, down to more than 150 yuan, the original bought home, used two months, really have power-saving effect, this time and sold two, business service attitude is	Jingdong prefecture level city in Shandong
Happy Fan Shop	Green Intelligent Power Saver	Like to watch some movies at home and so on, sometimes at night until two or three o'clock, and recently the air conditioning is not turned off for a night, rather wasteful, I have to say that the effect is very good, quite like it!	Jingdong prefecture level city in Shandong
Happy Fan Shop	Green Intelligent Power Saver	Power-saving effect: the effect is very good, a month to save more than 200 dollars Cost-effective: good value for money Appearance and style: small and lovely Workmanship material: okay Experience: good	Jingdong prefecture level city in Shandong
Happy Fan Shop	Green Intelligent Power Saver	It's okay I quite like it, the stuff is very good. Come back in the future. You can rest assured to buy. Cost-effective all. Much cheaper than the physical store	Jingdong prefecture level city in Shandong
Happy Fan Shop	Green Intelligent Power Saver	More than 150 less, last month's electricity bill is almost 400, this month is only about 200, really save electricity, I am home to buy three, I did not expect a month to pay back	Jingdong prefecture level city in Shandong
Happy Fan Shop	Green Intelligent Power Saver	I bought three of these first and tried them for two months and they still felt useful, so now I've bought another one. Good and recommended!	Jingdong prefecture level city in Shandong
Happy Fan Shop	Green Intelligent Power Saver	This energy saver is very good quality and inexpensive next time I will buy it again!	Jingdong prefecture level city in Shandong
Happy Fan Shop	Green Intelligent Power Saver	Merchant service attitude is not good, the quality does not match, the business does not give the package return	Jingdong prefecture level city in Shandong
Happy Fan Shop	Green Intelligent Power Saver	The goods are very small do not know how effective after a few days to follow up the review	Jingdong prefecture level city in Shandong
Happy Fan Shop	Green Intelligent Power Saver	Not used before more than 200 a month, after using about 100 a month, my family used three, customer service said I use electricity recommended 2-3, I think more than one effect is better on the purchase of 4, the effect is very obvious, recommended to buy!	Jingdong prefecture level city in Shandong
Happy Fan Shop	Green Intelligent Power Saver	It's okay. I'll buy it again today.	Jingdong prefecture level city in Shandong
Happy Fan Shop	Green Intelligent Power Saver	Finally received the goods, very, very much like,, has been a lot of people praise, really too happy, has been his family's loyal fans, will continue to buy buy buy!	Jingdong prefecture level city in Shandong
Happy Fan Shop	Green Intelligent Power Saver	After using this, my monthly electricity bill was 1,200 pounds, now it's only 180 bucks a month, how good is it?	Jingdong prefecture level city in Shandong
Happy Fan Shop	Green Intelligent Power Saver	The item is a scam return to marry a dozen dollars in shipping fees. Buy terrific role is purely a scam.	Jingdong prefecture level city in Shandong
Happy Fan Shop	Green Intelligent Power Saver	It's really good, cost-effective and good quality, and the logistics are fast! It arrived in one day!	Jingdong prefecture level city in Shandong
Happy Fan Shop	Green Intelligent Power Saver	Great brand! Trustworthy! Works super well! Expensive for a reason! Great merchandise!	Jingdong prefecture level city in Shandong
Happy Fan Shop	Green Intelligent Power Saver	The baby is really great, I like it very much, my friends also say good to come, recommended to buy!	Jingdong prefecture level city in Shandong
Happy Fan Shop	Green Intelligent Power Saver	Fake couldn't be more fake. Bought one plugged in still so much electricity.	Jingdong prefecture level city in Shandong
Happy Fan Shop	Green Intelligent Power Saver	This appliance works great. Let's see how it works. We'll see later.	Jingdong prefecture level city in Shandong
Happy Fan Shop	Green Intelligent Power Saver	Used it before commenting, very happy with it	Jingdong prefecture level city in Shandong
Happy Fan Shop	Green Intelligent Power Saver	Goods have been received well-packed, logistics is very fast and good quality	Jingdong prefecture level city in Shandong
Happy Fan Shop	Green Intelligent Power Saver	This baby's quality is okay.	Jingdong prefecture level city in Shandong
Happy Fan Shop	Green Intelligent Power Saver	Counterfeit goods.	Jingdong prefecture level city in Shandong
Happy Fan Shop	Green Intelligent Power Saver	The effect is not very obvious	Jingdong prefecture level city in Shandong
Happy Fan Shop	Green Intelligent Power Saver	It's not the same thing.	Jingdong prefecture level city in Shandong
Happy Fan Shop	Green Intelligent Power Saver	There is no load how much w seems false, nothing works.	Jingdong prefecture level city in Shandong
Happy Fan Shop	Green Intelligent Power Saver	I received the item yesterday, got it back and plugged it in, and it used a little more power yesterday than I had before	Jingdong prefecture level city in Shandong
Happy Fan Shop	Green Intelligent Power Saver	Haven't seen the power saving mode yet	Jingdong prefecture level city in Shandong
Happy Fan Shop	Green Intelligent Power Saver	This product has no known effect	Jingdong prefecture level city in Shandong
Happy Fan Shop	Green Intelligent Power Saver	Refund Returns $16	Jingdong prefecture level city in Shandong
Happy Fan Shop	Green Intelligent Power Saver	The previous year bought a, almost every month can save a 70 dollars or so, used a year, no problem at all, look very new like, this time to move to a new home decisively repurchase 2 units, very believe this	Jingdong prefecture level city in Shandong
Happy Fan Shop	Green Intelligent Power Saver	Has been the second time to buy, this power saving device is really good power, before every month to spend 300 electricity, now are about 200, last time that 6, this time to help lead the family to buy 6,	Jingdong prefecture level city in Shandong
Happy Fan Shop	Green Intelligent Power Saver	In the spirit of trying to experience the new product, the use of down compared to the usual power consumption, with the addition of the quarter, is indeed a lot less electricity, originally more than 450	Jingdong prefecture level city in Shandong
Happy Fan Shop	Green Intelligent Power Saver	Has not been the first time to buy back, good use, power saving device is really powerful, before the monthly electricity bill of more than 500 Now more than 300, this time to help neighbors to buy several	Jingdong prefecture level city in Shandong
Happy Fan Shop	Green Intelligent Power Saver	Home appliances are very many, the circuit is also very complex, at first it is to save 9% of the electricity, and then used more than half a month, found that the power saving rate is as high as more	Jingdong prefecture level city in Shandong
Happy Fan Shop	Green Intelligent Power Saver	My friend bought it last year and saved hundreds of dollars on his electricity bill in one summer Appearance and style: it looks better than the one I bought before Quality is also good with a light.	Jingdong prefecture level city in Shandong
Happy Fan Shop	Green Intelligent Power Saver	Saver received, on the spot on the plug with the feeling of immediate power savings, appearance, packaging is very delicate, very satisfied, I hope to achieve satisfactory results!	Jingdong prefecture level city in Shandong
Happy Fan Shop	Green Intelligent Power Saver	The power saving effect is very good, is considered an old customer, also bought before, this time is to buy two for mom and dad, customer service attitude is good, praise!	Jingdong prefecture level city in Shandong
Happy Fan Shop	Green Intelligent Power Saver	After observing for a while, it really saves a lot of money I didn't realize it was still really reliable, so I'll give you a good review.	Jingdong prefecture level city in Shandong
Happy Fan Shop	Green Intelligent Power Saver	My family a month with this monthly almost save more than 200, power-saving effect: have bought a second time, really effective Cost-effective: not bad Appearance style: roughly the same A few months to the home electricity is indeed less, this time it is a re-buy three, send a friend two, they stay a	Jingdong prefecture level city in Shandong
Happy Fan Shop	Green Intelligent Power Saver	A month almost save more than 150 dollars in electricity, home appliances more, the main this can also protect high-power appliances, after using this device, these two months have not burned, things are very good, it is said that this is the latest model, the overall price is cheap and beautiful!	Jingdong prefecture level city in Shandong

Happy Fan Shop	Green Intelligent Power Saver	Received, powerful, great quality, love the style😍	Jingdong prefecture level city in Shandong
Happy Fan Shop	Green Intelligent Power Saver	This intelligent energy saver is especially good to use and save electricity hope everyone will buy it!	Jingdong prefecture level city in Shandong
Happy Fan Shop	Green Intelligent Power Saver	Air conditioning adapter effect is particularly great, the use of very simple, plug into the socket on the summer air conditioning power is too large, electricity is also expensive, with this product directly on the electricity bill to save half, has been purchased a few more, the effect is too awesome!	Jingdong prefecture level city in Shandong
Happy Fan Shop	Green Intelligent Power Saver	The family plugged in 5, monthly electricity savings of about 300 yuan. My family before a month of electricity costs more than 800, this time with this saver after the electricity bill is actually only more than 500, amazing, thanks to the store, will recommend other friends to buy!	Jingdong prefecture level city in Shandong
Happy Fan Shop	Green Intelligent Power Saver	It was recommended to me by a neighbor's family and it's nice to use it to save power.	Jingdong prefecture level city in Shandong
Happy Fan Shop	Green Intelligent Power Saver	Second time I've purchased it and the power saving is effective!	Jingdong prefecture level city in Shandong
Happy Fan Shop	Green Intelligent Power Saver	The effect is really good, before a month of more than 300 electricity, now every month every day with the air conditioning, it is more than 100 electricity! This thing is really amazing, one after another to buy and 5.6, home installed 3!	Jingdong prefecture level city in Shandong
Happy Fan Shop	Green Intelligent Power Saver	Received this product, began to use, small things look very wrong, can save a little electricity, the monthly electricity bill is too high, after plugging in did not pull, or save a lot of electricity, point	Jingdong prefecture level city in Shandong
Happy Fan Shop	Green Intelligent Power Saver	Simple and convenient, just that plug in, you can save electricity. A month can save dozens of dollars, a year down to save a lot of, very good product cost-effective, everyone can afford to buy and use!	Jingdong prefecture level city in Shandong
Happy Fan Shop	Green Intelligent Power Saver	Electricity bill from more than 200 yuan per month, down to more than 150 yuan, the original bought home, used two months, really have power-saving effect, this time and sold two, business service attitude is	Jingdong prefecture level city in Shandong
Happy Fan Shop	Green Intelligent Power Saver	Like to watch some movies at home and so on, sometimes at night until two or three o'clock, and recently the air conditioning is not turned off for a night, rather wasteful, I have to say that the effect is very good, quite like it!	Jingdong prefecture level city in Shandong
Happy Fan Shop	Green Intelligent Power Saver	Power-saving effect: the effect is very good, a month to save more than 200 dollars Cost-effective: good value for money Appearance and style: small and lovely Workmanship material: okay Experience: good	Jingdong prefecture level city in Shandong
Happy Fan Shop	Green Intelligent Power Saver	It's okay I quite like it, the stuff is very good. Come back in the future. You can rest assured to buy. Cost-effective all. Much cheaper than the physical store	Jingdong prefecture level city in Shandong
Happy Fan Shop	Green Intelligent Power Saver	More than 150 less, last month's electricity bill is almost 400, this month is only about 200, really save electricity, I am home to buy three, I did not expect a month to pay back	Jingdong prefecture level city in Shandong
Happy Fan Shop	Green Intelligent Power Saver	I bought three of these first and tried them for two months and they still felt useful, so now I've bought another one. Good and recommended!	Jingdong prefecture level city in Shandong
Happy Fan Shop	Green Intelligent Power Saver	This energy saver is very good quality and inexpensive next time I will buy it again!	Jingdong prefecture level city in Shandong
Happy Fan Shop	Green Intelligent Power Saver	Merchant service attitude is not good, the quality does not match, the business does not give the package return	Jingdong prefecture level city in Shandong
Happy Fan Shop	Green Intelligent Power Saver	The goods are very small do not know how effective after a few days to follow up the review	Jingdong prefecture level city in Shandong
Happy Fan Shop	Green Intelligent Power Saver	Not used before more than 200 a month, after using about 100 a month, my family used three, customer service said I use electricity recommended 2-3, I think more than one effect is better on the purchase of 4, the effect is very obvious, recommended to buy!	Jingdong prefecture level city in Shandong
Happy Fan Shop	Green Intelligent Power Saver	It's okay. I'll buy it again today.	Jingdong prefecture level city in Shandong
Happy Fan Shop	Green Intelligent Power Saver	Finally received the goods, very, very much like,, has been a lot of people praise, really too happy, has been his family's loyal fans, will continue to buy buy buy buy!	Jingdong prefecture level city in Shandong
Happy Fan Shop	Green Intelligent Power Saver	After using this, my monthly electricity bill was 1,200 pounds, now it's only 180 bucks a month, how good is it?	Jingdong prefecture level city in Shandong
Happy Fan Shop	Green Intelligent Power Saver	The item is a scam return to marry a dozen dollars in shipping fees. Buy terrific role is purely a scam.	Jingdong prefecture level city in Shandong
Happy Fan Shop	Green Intelligent Power Saver	It's really good, cost-effective and good quality, and the logistics are fast! It arrived in one day!	Jingdong prefecture level city in Shandong
Happy Fan Shop	Green Intelligent Power Saver	Great brand! Trustworthy! Works super well! Expensive for a reason! Great merchandise!	Jingdong prefecture level city in Shandong
Happy Fan Shop	Green Intelligent Power Saver	The baby is really great, I like it very much, my friends also say good to come, recommended to buy!	Jingdong prefecture level city in Shandong
Happy Fan Shop	Green Intelligent Power Saver	Fake couldn't be more fake. Bought one plugged in still so much electricity.	Jingdong prefecture level city in Shandong
Happy Fan Shop	Green Intelligent Power Saver	This appliance works great. Let's see how it works. We'll see later.	Jingdong prefecture level city in Shandong
Happy Fan Shop	Green Intelligent Power Saver	Used it before commenting, very happy with it	Jingdong prefecture level city in Shandong
Happy Fan Shop	Green Intelligent Power Saver	Goods have been received well-packed, logistics is very fast and good quality	Jingdong prefecture level city in Shandong
Happy Fan Shop	Green Intelligent Power Saver	This baby's quality is okay.	Jingdong prefecture level city in Shandong
Happy Fan Shop	Green Intelligent Power Saver	Counterfeit goods.	Jingdong prefecture level city in Shandong
Happy Fan Shop	Green Intelligent Power Saver	The effect is not very obvious	Jingdong prefecture level city in Shandong
Happy Fan Shop	Green Intelligent Power Saver	It's not the same thing.	Jingdong prefecture level city in Shandong
Happy Fan Shop	Green Intelligent Power Saver	There is no load how much w seems false, nothing works.	Jingdong prefecture level city in Shandong
Happy Fan Shop	Green Intelligent Power Saver	I received the item yesterday, got it back and plugged it in, and it used a little more power yesterday than I had before	Jingdong prefecture level city in Shandong
Happy Fan Shop	Green Intelligent Power Saver	Haven't seen the power saving mode yet	Jingdong prefecture level city in Shandong
Happy Fan Shop	Green Intelligent Power Saver	This product has no known effect	Jingdong prefecture level city in Shandong
Happy Fan Shop	Green Intelligent Power Saver	Refund Returns $16	Jingdong prefecture level city in Shandong
Happy Fan Shop	Green Intelligent Power Saver	The previous year bought a, almost every month can save a 70 dollars or so, used a year, no problem at all, look very new like, this time to move to a new home decisively repurchase 2 units, very believe this	Jingdong prefecture level city in Shandong
Happy Fan Shop	Green Intelligent Power Saver	Has been the second time to buy, this power saving device is really good power, before every month to spend 300 electricity, now are about 200, last time that 6, this time to help lead the family to buy 6,	Jingdong prefecture level city in Shandong
Happy Fan Shop	Green Intelligent Power Saver	In the spirit of trying to experience the new product, the use of down compared to the usual power consumption, with the addition of the quarter, is indeed a lot less electricity, originally more than 450	Jingdong prefecture level city in Shandong
Happy Fan Shop	Green Intelligent Power Saver	Has not been the first time to buy back, good use, power saving device is really powerful, before the monthly electricity bill of more than 500 Now more than 300, this time to help neighbors to buy several	Jingdong prefecture level city in Shandong
Happy Fan Shop	Green Intelligent Power Saver	Home appliances are very many, the circuit is also very complex, at first it is to save 9% of the electricity, and then used more than half a month, found that the power saving rate is as high as more	Jingdong prefecture level city in Shandong
Happy Fan Shop	Green Intelligent Power Saver	My friend bought it last year and saved hundreds of dollars on his electricity bill in one summer Appearance and style: it looks better than the one I bought before Quality is also good with a light.	Jingdong prefecture level city in Shandong
Happy Fan Shop	Green Intelligent Power Saver	Saver received, on the spot on the plug with the feeling of immediate power savings, appearance, packaging is very delicate, very satisfied, I hope to achieve satisfactory results!	Jingdong prefecture level city in Shandong
Happy Fan Shop	Green Intelligent Power Saver	The power saving effect is very good, is considered an old customer, also bought before, this time is to buy two for mom and dad, customer service attitude is good, praise!	Jingdong prefecture level city in Shandong
Happy Fan Shop	Green Intelligent Power Saver	After observing for a while, it really saves a lot of money I didn't realize it was still really reliable, so I'll give you a good review.	Jingdong prefecture level city in Shandong

Happy Fan Shop	Green Intelligent Power Saver	My family a month with this monthly almost save more than 200, power-saving effect: have bought a second time, really effective Cost-effective: not bad Appearance style: roughly the same A few months to the home electricity is indeed less, this time it is a re-buy three, send a friend two, they stay a	Jingdong prefecture level city in Shandong
Happy Fan Shop	Green Intelligent Power Saver	A month almost save more than 150 dollars in electricity, home appliances more, the main this can also protect high-power appliances, after using this device, these two months have not burned, things are very good, it is said that this is the latest model, the overall price is cheap and beautiful!	Jingdong prefecture level city in Shandong
Happy Fan Shop	Green Intelligent Power Saver	Received, powerful, great quality, love the style😍	Jingdong prefecture level city in Shandong
Happy Fan Shop	Green Intelligent Power Saver	This intelligent energy saver is especially good to use and save electricity hope everyone will buy it!	Jingdong prefecture level city in Shandong
Happy Fan Shop	Green Intelligent Power Saver	Air conditioning adapter effect is particularly great, the use of very simple, plug into the socket on the summer air conditioning power is too large, electricity is also expensive, with this product directly on the electricity bill to save half, has been purchased a few more, the effect is too awesome!	Jingdong prefecture level city in Shandong
Happy Fan Shop	Green Intelligent Power Saver	The family plugged in 5, monthly electricity savings of about 300 yuan. My family before a month of electricity costs more than 800, this time with this saver after the electricity bill is actually only more than 500, amazing, thanks to the store, will recommend other friends to buy!	Jingdong prefecture level city in Shandong
Happy Fan Shop	Green Intelligent Power Saver	It was recommended to me by a neighbor's family and it's nice to use it to save power.	Jingdong prefecture level city in Shandong
Happy Fan Shop	Green Intelligent Power Saver	Second time I've purchased it and the power saving is effective!	Jingdong prefecture level city in Shandong
Happy Fan Shop	Green Intelligent Power Saver	The effect is really good, before a month of more than 300 electricity, now every month every day with the air conditioning, it is more than 100 electricity! This thing is really amazing, one after another to buy and 5.6, home installed 3!	Jingdong prefecture level city in Shandong
Happy Fan Shop	Green Intelligent Power Saver	Received this product, began to use, small things look very wrong, can save a little electricity, the monthly electricity bill is too high, after plugging in did not pull, or save a lot of electricity, point	Jingdong prefecture level city in Shandong
Happy Fan Shop	Green Intelligent Power Saver	Simple and convenient, just that plug in, you can save electricity. A month can save dozens of dollars, a year down to save a lot of, very good product cost-effective, everyone can afford to buy and use!	Jingdong prefecture level city in Shandong
Happy Fan Shop	Green Intelligent Power Saver	Electricity bill from more than 200 yuan per month, down to more than 150 yuan, the original bought home, used two months, really have power-saving effect, this time and sold two, business service attitude is	Jingdong prefecture level city in Shandong
Happy Fan Shop	Green Intelligent Power Saver	Like to watch some movies at home and so on, sometimes at night until two or three o'clock, and recently the air conditioning is not turned off for a night, rather wasteful, I have to say that the effect is very good, quite like it!	Jingdong prefecture level city in Shandong
Happy Fan Shop	Green Intelligent Power Saver	Power-saving effect: the effect is very good, a month to save more than 200 dollars Cost-effective: good value for money Appearance and style: small and lovely Workmanship material: okay Experience: good	Jingdong prefecture level city in Shandong
Happy Fan Shop	Green Intelligent Power Saver	It's okay I quite like it, the stuff is very good. Come back in the future. You can rest assured to buy. Cost-effective all. Much cheaper than the physical store	Jingdong prefecture level city in Shandong
Happy Fan Shop	Green Intelligent Power Saver	More than 150 less, last month's electricity bill is almost 400, this month is only about 200, really save electricity, I am home to buy three, I did not expect a month to pay back	Jingdong prefecture level city in Shandong
Happy Fan Shop	Green Intelligent Power Saver	I bought three of these first and tried them for two months and they still felt useful, so now I've bought another one. Good and recommended!	Jingdong prefecture level city in Shandong
Happy Fan Shop	Green Intelligent Power Saver	This energy saver is very good quality and inexpensive next time I will buy it again!	Jingdong prefecture level city in Shandong
Happy Fan Shop	Green Intelligent Power Saver	Merchant service attitude is not good, the quality does not match, the business does not give the package return	Jingdong prefecture level city in Shandong
Happy Fan Shop	Green Intelligent Power Saver	The goods are very small do not know how effective after a few days to follow up the review	Jingdong prefecture level city in Shandong
Happy Fan Shop	Green Intelligent Power Saver	Not used before more than 200 a month, after using about 100 a month, my family used three, customer service said I use electricity recommended 2-3, I think more than one effect is better on the purchase of 4, the effect is very obvious, recommended to buy!	Jingdong prefecture level city in Shandong
Happy Fan Shop	Green Intelligent Power Saver	It's okay. I'll buy it again today.	Jingdong prefecture level city in Shandong
Happy Fan Shop	Green Intelligent Power Saver	Finally received the goods, very, very much like,, has been a lot of people praise, really too happy, has been his family's loyal fans, will continue to buy buy buy buy!	Jingdong prefecture level city in Shandong
Happy Fan Shop	Green Intelligent Power Saver	After using this, my monthly electricity bill was 1,200 pounds, now it's only 180 bucks a month, how good is it?	Jingdong prefecture level city in Shandong
Happy Fan Shop	Green Intelligent Power Saver	The item is a scam return to marry a dozen dollars in shipping fees. Buy terrific role is purely a scam.	Jingdong prefecture level city in Shandong
Happy Fan Shop	Green Intelligent Power Saver	It's really good, cost-effective and good quality, and the logistics are fast! It arrived in one day!	Jingdong prefecture level city in Shandong
Happy Fan Shop	Green Intelligent Power Saver	Great brand! Trustworthy! Works super well! Expensive for a reason! Great merchandise!	Jingdong prefecture level city in Shandong
Happy Fan Shop	Green Intelligent Power Saver	The baby is really great, I like it very much, my friends also say good to come, recommended to buy!	Jingdong prefecture level city in Shandong
Happy Fan Shop	Green Intelligent Power Saver	Fake couldn't be more fake. Bought one plugged in still so much electricity.	Jingdong prefecture level city in Shandong
Happy Fan Shop	Green Intelligent Power Saver	This appliance works great. Let's see how it works. We'll see later.	Jingdong prefecture level city in Shandong
Happy Fan Shop	Green Intelligent Power Saver	Used it before commenting, very happy with it	Jingdong prefecture level city in Shandong
Happy Fan Shop	Green Intelligent Power Saver	Goods have been received well-packed, logistics is very fast and good quality	Jingdong prefecture level city in Shandong
Happy Fan Shop	Green Intelligent Power Saver	This baby's quality is okay.	Jingdong prefecture level city in Shandong
Happy Fan Shop	Green Intelligent Power Saver	Counterfeit goods.	Jingdong prefecture level city in Shandong
Happy Fan Shop	Green Intelligent Power Saver	The effect is not very obvious	Jingdong prefecture level city in Shandong
Happy Fan Shop	Green Intelligent Power Saver	It's not the same thing.	Jingdong prefecture level city in Shandong
Happy Fan Shop	Green Intelligent Power Saver	There is no load how much w seems false, nothing works.	Jingdong prefecture level city in Shandong
Happy Fan Shop	Green Intelligent Power Saver	I received the item yesterday, got it back and plugged it in, and it used a little more power yesterday than I had before	Jingdong prefecture level city in Shandong
Happy Fan Shop	Green Intelligent Power Saver	Haven't seen the power saving mode yet	Jingdong prefecture level city in Shandong
Happy Fan Shop	Green Intelligent Power Saver	This product has no known effect	Jingdong prefecture level city in Shandong
Happy Fan Shop	Green Intelligent Power Saver	Refund Returns $16	Jingdong prefecture level city in Shandong
Happy Fan Shop	Green Intelligent Power Saver	The previous year bought a, almost every month can save a 70 dollars or so, used a year, no problem at all, look very new like, this time to move to a new home decisively repurchase 2 units, very believe this	Jingdong prefecture level city in Shandong
Happy Fan Shop	Green Intelligent Power Saver	Has been the second time to buy, this power saving device is really good power, before every month to spend 300 electricity, now are about 200, last time that 6, this time to help lead the family to buy 6,	Jingdong prefecture level city in Shandong
Happy Fan Shop	Green Intelligent Power Saver	In the spirit of trying to experience the new product, the use of down compared to the usual power consumption, with the addition of the quarter, is indeed a lot less electricity, originally more than 450	Jingdong prefecture level city in Shandong
Happy Fan Shop	Green Intelligent Power Saver	Has not been the first time to buy back, good use, power saving device is really powerful, before the monthly electricity bill of more than 500 Now more than 300, this time to help neighbors to buy several	Jingdong prefecture level city in Shandong
Happy Fan Shop	Green Intelligent Power Saver	Home appliances are very many, the circuit is also very complex, at first it is to save 9% of the electricity, and then used more than half a month, found that the power saving rate is as high as more	Jingdong prefecture level city in Shandong
Happy Fan Shop	Green Intelligent Power Saver	My friend bought it last year and saved hundreds of dollars on his electricity bill in one summer Appearance and style: it looks better than the one I bought before Quality is also good with a light.	Jingdong prefecture level city in Shandong
Happy Fan Shop	Green Intelligent Power Saver	Saver received, on the spot on the plug with the feeling of immediate power savings, appearance, packaging is very delicate, very satisfied, I hope to achieve satisfactory results!	Jingdong prefecture level city in Shandong

Happy Fan Shop	Green Intelligent Power Saver	The power saving effect is very good, is considered an old customer, also bought before, this time is to buy two for mom and dad, customer service attitude is good, praise!	Jingdong prefecture level city in Shandong
Happy Fan Shop	Green Intelligent Power Saver	After observing for a while, it really saves a lot of money I didn't realize it was still really reliable, so I'll give you a good review.	Jingdong prefecture level city in Shandong
Happy Fan Shop	Green Intelligent Power Saver	My family a month with this monthly almost save more than 200, power-saving effect: have bought a second time, really effective Cost-effective: not bad Appearance style: roughly the same A few months to the home electricity is indeed less, this time it is a re-buy three, send a friend two, they stay a	Jingdong prefecture level city in Shandong
Happy Fan Shop	Green Intelligent Power Saver	A month almost save more than 150 dollars in electricity, home appliances more, the main this can also protect high-power appliances, after using this device, these two months have not burned, things are very good, it is said that this is the latest model, the overall price is cheap and beautiful!	Jingdong prefecture level city in Shandong
Happy Fan Shop	Green Intelligent Power Saver	Received, powerful, great quality, love the style😍	Jingdong prefecture level city in Shandong
Happy Fan Shop	Green Intelligent Power Saver	This intelligent energy saver is especially good to use and save electricity hope everyone will buy it!	Jingdong prefecture level city in Shandong
Happy Fan Shop	Green Intelligent Power Saver	Air conditioning adapter effect is particularly great, the use of very simple, plug into the socket on the summer air conditioning power is too large, electricity is also expensive, with this product directly on the electricity bill to save half, has been purchased a few more, the effect is too awesome!	Jingdong prefecture level city in Shandong
Happy Fan Shop	Green Intelligent Power Saver	The family plugged in 5, monthly electricity savings of about 300 yuan. My family before a month of electricity costs more than 800, this time with this saver after the electricity bill is actually only more than 500, amazing, thanks to the store, will recommend other friends to buy!	Jingdong prefecture level city in Shandong
Happy Fan Shop	Green Intelligent Power Saver	It was recommended to me by a neighbor's family and it's nice to use it to save power.	Jingdong prefecture level city in Shandong
Happy Fan Shop	Green Intelligent Power Saver	Second time I've purchased it and the power saving is effective!	Jingdong prefecture level city in Shandong
Happy Fan Shop	Green Intelligent Power Saver	The effect is really good, before a month of more than 300 electricity, now every month every day with the air conditioning, it is more than 100 electricity! This thing is really amazing, one after another to buy and 5.6, home installed 3!	Jingdong prefecture level city in Shandong
Happy Fan Shop	Green Intelligent Power Saver	Received this product, began to use, small things look very wrong, can save a little electricity, the monthly electricity bill is too high, after plugging in did not pull, or save a lot of electricity, point	Jingdong prefecture level city in Shandong
Happy Fan Shop	Green Intelligent Power Saver	Simple and convenient, just that plug in, you can save electricity. A month can save dozens of dollars, a year down to save a lot of, very good product cost-effective, everyone can afford to buy and use!	Jingdong prefecture level city in Shandong
Happy Fan Shop	Green Intelligent Power Saver	Electricity bill from more than 200 yuan per month, down to more than 150 yuan, the original bought home, used two months, really have power-saving effect, this time and sold two, business service attitude is	Jingdong prefecture level city in Shandong
Happy Fan Shop	Green Intelligent Power Saver	Like to watch some movies at home and so on, sometimes at night until two or three o'clock, and recently the air conditioning is not turned off for a night, rather wasteful, I have to say that the effect is very good, quite like it!	Jingdong prefecture level city in Shandong
Happy Fan Shop	Green Intelligent Power Saver	Power-saving effect: the effect is very good, a month to save more than 200 dollars Cost-effective: good value for money Appearance and style: small and lovely Workmanship material: okay Experience: good	Jingdong prefecture level city in Shandong
Happy Fan Shop	Green Intelligent Power Saver	It's okay I quite like it, the stuff is very good. Come back in the future. You can rest assured to buy. Cost-effective all. Much cheaper than the physical store	Jingdong prefecture level city in Shandong
Happy Fan Shop	Green Intelligent Power Saver	More than 150 less, last month's electricity bill is almost 400, this month is only about 200, really save electricity, I am home to buy three, I did not expect a month to pay back	Jingdong prefecture level city in Shandong
Happy Fan Shop	Green Intelligent Power Saver	I bought three of these first and tried them for two months and they still felt useful, so now I've bought another one. Good and recommended!	Jingdong prefecture level city in Shandong
Happy Fan Shop	Green Intelligent Power Saver	This energy saver is very good quality and inexpensive next time I will buy it again!	Jingdong prefecture level city in Shandong
Happy Fan Shop	Green Intelligent Power Saver	Merchant service attitude is not good, the quality does not match, the business does not give the package return	Jingdong prefecture level city in Shandong
Happy Fan Shop	Green Intelligent Power Saver	The goods are very small do not know how effective after a few days to follow up the review	Jingdong prefecture level city in Shandong
Happy Fan Shop	Green Intelligent Power Saver	Not used before more than 200 a month, after using about 100 a month, my family used three, customer service said I use electricity recommended 2-3, I think more than one effect is better on the purchase of 4, the effect is very obvious, recommended to buy!	Jingdong prefecture level city in Shandong
Happy Fan Shop	Green Intelligent Power Saver	It's okay. I'll buy it again today.	Jingdong prefecture level city in Shandong
Happy Fan Shop	Green Intelligent Power Saver	Finally received the goods, very, very much like,, has been a lot of people praise, really too happy, has been his family's loyal fans, will continue to buy buy buy!	Jingdong prefecture level city in Shandong
Happy Fan Shop	Green Intelligent Power Saver	After using this, my monthly electricity bill was 1,200 pounds, now it's only 180 bucks a month, how good is it?	Jingdong prefecture level city in Shandong
Happy Fan Shop	Green Intelligent Power Saver	The item is a scam return to marry a dozen dollars in shipping fees. Buy terrific role is purely a scam.	Jingdong prefecture level city in Shandong
Happy Fan Shop	Green Intelligent Power Saver	It's really good, cost-effective and good quality, and the logistics are fast! It arrived in one day!	Jingdong prefecture level city in Shandong
Happy Fan Shop	Green Intelligent Power Saver	Great brand! Trustworthy! Works super well! Expensive for a reason! Great merchandise!	Jingdong prefecture level city in Shandong
Happy Fan Shop	Green Intelligent Power Saver	The baby is really great, I like it very much, my friends also say good to come, recommended to buy!	Jingdong prefecture level city in Shandong
Happy Fan Shop	Green Intelligent Power Saver	Fake couldn't be more fake. Bought one plugged in still so much electricity.	Jingdong prefecture level city in Shandong
Happy Fan Shop	Green Intelligent Power Saver	This appliance works great. Let's see how it works. We'll see later.	Jingdong prefecture level city in Shandong
Happy Fan Shop	Green Intelligent Power Saver	Used it before commenting, very happy with it	Jingdong prefecture level city in Shandong
Happy Fan Shop	Green Intelligent Power Saver	Goods have been received well-packed, logistics is very fast and good quality	Jingdong prefecture level city in Shandong
Happy Fan Shop	Green Intelligent Power Saver	This baby's quality is okay.	Jingdong prefecture level city in Shandong
Happy Fan Shop	Green Intelligent Power Saver	Counterfeit goods.	Jingdong prefecture level city in Shandong
Happy Fan Shop	Green Intelligent Power Saver	The effect is not very obvious	Jingdong prefecture level city in Shandong
Happy Fan Shop	Green Intelligent Power Saver	It's not the same thing.	Jingdong prefecture level city in Shandong
Happy Fan Shop	Green Intelligent Power Saver	There is no load how much w seems false, nothing works.	Jingdong prefecture level city in Shandong
Happy Fan Shop	Green Intelligent Power Saver	I received the item yesterday, got it back and plugged it in, and it used a little more power yesterday than I had before	Jingdong prefecture level city in Shandong
Happy Fan Shop	Green Intelligent Power Saver	Haven't seen the power saving mode yet	Jingdong prefecture level city in Shandong
Happy Fan Shop	Green Intelligent Power Saver	This product has no known effect	Jingdong prefecture level city in Shandong
Happy Fan Shop	Green Intelligent Power Saver	Refund Returns $16	Jingdong prefecture level city in Shandong
Happy Fan Shop	Green Intelligent Power Saver	The previous year bought a, almost every month can save a 70 dollars or so, used a year, no problem at all, look very new like, this time to move to a new home decisively repurchase 2 units, very believe this	Jingdong prefecture level city in Shandong
Happy Fan Shop	Green Intelligent Power Saver	Has been the second time to buy, this power saving device is really good power, before every month to spend 300 electricity, now are about 200, last time that 6, this time to help lead the family to buy 6,	Jingdong prefecture level city in Shandong
Happy Fan Shop	Green Intelligent Power Saver	In the spirit of trying to experience the new product, the use of down compared to the usual power consumption, with the addition of the quarter, is indeed a lot less electricity, originally more than 450	Jingdong prefecture level city in Shandong
Happy Fan Shop	Green Intelligent Power Saver	Has not been the first time to buy back, good use, power saving device is really powerful, before the monthly electricity bill of more than 500 Now more than 300, this time to help neighbors to buy several	Jingdong prefecture level city in Shandong
Happy Fan Shop	Green Intelligent Power Saver	Home appliances are very many, the circuit is also very complex, at first it is to save 9% of the electricity, and then used more than half a month, found that the power saving rate is as high as more	Jingdong prefecture level city in Shandong

Happy Fan Shop	Green Intelligent Power Saver	My friend bought it last year and saved hundreds of dollars on his electricity bill in one summer Appearance and style: it looks better than the one I bought before Quality is also good with a light.	Jingdong prefecture level city in Shandong
Happy Fan Shop	Green Intelligent Power Saver	Saver received, on the spot on the plug with the feeling of immediate power savings, appearance, packaging is very delicate, very satisfied, I hope to achieve satisfactory results!	Jingdong prefecture level city in Shandong
Happy Fan Shop	Green Intelligent Power Saver	The power saving effect is very good, is considered an old customer, also bought before, this time is to buy two for mom and dad, customer service attitude is good, praise!	Jingdong prefecture level city in Shandong
Happy Fan Shop	Green Intelligent Power Saver	After observing for a while, it really saves a lot of money I didn't realize it was still really reliable, so I'll give you a good review.	Jingdong prefecture level city in Shandong
Happy Fan Shop	Green Intelligent Power Saver	My family a month with this monthly almost save more than 200, power-saving effect: have bought a second time, really effective Cost-effective: not bad Appearance style: roughly the same A few months to the home electricity is indeed less, this time it is a re-buy three, send a friend two, they stay a	Jingdong prefecture level city in Shandong
Happy Fan Shop	Green Intelligent Power Saver	A month almost save more than 150 dollars in electricity, home appliances more, the main this can also protect high-power appliances, after using this device, these two months have not burned, things are very good, it is said that this is the latest model, the overall price is cheap and beautiful!	Jingdong prefecture level city in Shandong
Happy Fan Shop	Green Intelligent Power Saver	Received, powerful, great quality, love the style😍	Jingdong prefecture level city in Shandong
Happy Fan Shop	Green Intelligent Power Saver	This intelligent energy saver is especially good to use and save electricity hope everyone will buy it!	Jingdong prefecture level city in Shandong
Happy Fan Shop	Green Intelligent Power Saver	Air conditioning adapter effect is particularly great, the use of very simple, plug into the socket on the summer air conditioning power is too large, electricity is also expensive, with this product directly on the electricity bill to save half, has been purchased a few more, the effect is too awesome!	Jingdong prefecture level city in Shandong
Happy Fan Shop	Green Intelligent Power Saver	The family plugged in 5, monthly electricity savings of about 300 yuan. My family before a month of electricity costs more than 800, this time with this saver after the electricity bill is actually only more than 500, amazing, thanks to the store, will recommend other friends to buy!	Jingdong prefecture level city in Shandong
Happy Fan Shop	Green Intelligent Power Saver	It was recommended to me by a neighbor's family and it's nice to use it to save power.	Jingdong prefecture level city in Shandong
Happy Fan Shop	Green Intelligent Power Saver	Second time I've purchased it and the power saving is effective!	Jingdong prefecture level city in Shandong
Happy Fan Shop	Green Intelligent Power Saver	The effect is really good, before a month of more than 300 electricity, now every month every day with the air conditioning, it is more than 100 electricity! This thing is really amazing, one after another to buy and 5.6, home installed 3!	Jingdong prefecture level city in Shandong
Happy Fan Shop	Green Intelligent Power Saver	Received this product, began to use, small things look very wrong, can save a little electricity, the monthly electricity bill is too high, after plugging in did not pull, or save a lot of electricity, point	Jingdong prefecture level city in Shandong
Happy Fan Shop	Green Intelligent Power Saver	Simple and convenient, just that plug in, you can save electricity. A month can save dozens of dollars, a year down to save a lot of, very good product cost-effective, everyone can afford to buy and use!	Jingdong prefecture level city in Shandong
Happy Fan Shop	Green Intelligent Power Saver	Electricity bill from more than 200 yuan per month, down to more than 150 yuan, the original bought home, used two months, really have power-saving effect, this time and sold two, business service attitude is	Jingdong prefecture level city in Shandong
Happy Fan Shop	Green Intelligent Power Saver	Like to watch some movies at home and so on, sometimes at night until two or three o'clock, and recently the air conditioning is not turned off for a night, rather wasteful, I have to say that the effect is very good, quite like it!	Jingdong prefecture level city in Shandong
Happy Fan Shop	Green Intelligent Power Saver	Power-saving effect: the effect is very good, a month to save more than 200 dollars Cost-effective: good value for money Appearance and style: small and lovely Workmanship material: okay Experience: good	Jingdong prefecture level city in Shandong
Happy Fan Shop	Green Intelligent Power Saver	It's okay I quite like it, the stuff is very good. Come back in the future. You can rest assured to buy. Cost-effective all. Much cheaper than the physical store	Jingdong prefecture level city in Shandong
Happy Fan Shop	Green Intelligent Power Saver	More than 150 less, last month's electricity bill is almost 400, this month is only about 200, really save electricity, I am home to buy three, I did not expect a month to pay back	Jingdong prefecture level city in Shandong
Happy Fan Shop	Green Intelligent Power Saver	I bought three of these first and tried them for two months and they still felt useful, so now I've bought another one. Good and recommended!	Jingdong prefecture level city in Shandong
Happy Fan Shop	Green Intelligent Power Saver	This energy saver is very good quality and inexpensive next time I will buy it again!	Jingdong prefecture level city in Shandong
Happy Fan Shop	Green Intelligent Power Saver	Merchant service attitude is not good, the quality does not match, the business does not give the package return	Jingdong prefecture level city in Shandong
Happy Fan Shop	Green Intelligent Power Saver	The goods are very small do not know how effective after a few days to follow up the review	Jingdong prefecture level city in Shandong
Happy Fan Shop	Green Intelligent Power Saver	Not used before more than 200 a month, after using about 100 a month, my family used three, customer service said I use electricity recommended 2-3, I think more than one effect is better on the purchase of 4, the effect is very obvious, recommended to buy!	Jingdong prefecture level city in Shandong
Happy Fan Shop	Green Intelligent Power Saver	It's okay. I'll buy it again today.	Jingdong prefecture level city in Shandong
Happy Fan Shop	Green Intelligent Power Saver	Finally received the goods, very, very much like,, has been a lot of people praise, really too happy, has been his family's loyal fans, will continue to buy buy buy buy!	Jingdong prefecture level city in Shandong
Happy Fan Shop	Green Intelligent Power Saver	After using this, my monthly electricity bill was 1,200 pounds, now it's only 180 bucks a month, how good is it?	Jingdong prefecture level city in Shandong
Happy Fan Shop	Green Intelligent Power Saver	The item is a scam return to marry a dozen dollars in shipping fees. Buy terrific role is purely a scam.	Jingdong prefecture level city in Shandong
Happy Fan Shop	Green Intelligent Power Saver	It's really good, cost-effective and good quality, and the logistics are fast! It arrived in one day!	Jingdong prefecture level city in Shandong
Happy Fan Shop	Green Intelligent Power Saver	Great brand! Trustworthy! Works super well! Expensive for a reason! Great merchandise!	Jingdong prefecture level city in Shandong
Happy Fan Shop	Green Intelligent Power Saver	The baby is really great, I like it very much, my friends also say good to come, recommended to buy!	Jingdong prefecture level city in Shandong
Happy Fan Shop	Green Intelligent Power Saver	Fake couldn't be more fake. Bought one plugged in still so much electricity.	Jingdong prefecture level city in Shandong
Happy Fan Shop	Green Intelligent Power Saver	This appliance works great. Let's see how it works. We'll see later.	Jingdong prefecture level city in Shandong
Happy Fan Shop	Green Intelligent Power Saver	Used it before commenting, very happy with it	Jingdong prefecture level city in Shandong
Happy Fan Shop	Green Intelligent Power Saver	Goods have been received well-packed, logistics is very fast and good quality	Jingdong prefecture level city in Shandong
Happy Fan Shop	Green Intelligent Power Saver	This baby's quality is okay.	Jingdong prefecture level city in Shandong
Happy Fan Shop	Green Intelligent Power Saver	Counterfeit goods.	Jingdong prefecture level city in Shandong
Happy Fan Shop	Green Intelligent Power Saver	The effect is not very obvious	Jingdong prefecture level city in Shandong
Happy Fan Shop	Green Intelligent Power Saver	It's not the same thing.	Jingdong prefecture level city in Shandong
Happy Fan Shop	Green Intelligent Power Saver	There is no load how much w seems false, nothing works.	Jingdong prefecture level city in Shandong
Happy Fan Shop	Green Intelligent Power Saver	I received the item yesterday, got it back and plugged it in, and it used a little more power yesterday than I had before	Jingdong prefecture level city in Shandong
Happy Fan Shop	Green Intelligent Power Saver	Haven't seen the power saving mode yet	Jingdong prefecture level city in Shandong
Happy Fan Shop	Green Intelligent Power Saver	This product has no known effect	Jingdong prefecture level city in Shandong
Happy Fan Shop	Green Intelligent Power Saver	Refund Returns $16	Jingdong prefecture level city in Shandong
Happy Fan Shop	Green Intelligent Power Saver	The previous year bought a, almost every month can save a 70 dollars or so, used a year, no problem at all, look very new like, this time to move to a new home decisively repurchase 2 units, very believe this	Jingdong prefecture level city in Shandong
Happy Fan Shop	Green Intelligent Power Saver	Has been the second time to buy, this power saving device is really good power, before every month to spend 300 electricity, now are about 200, last time that 6, this time to help lead the family to buy 6,	Jingdong prefecture level city in Shandong
Happy Fan Shop	Green Intelligent Power Saver	In the spirit of trying to experience the new product, the use of down compared to the usual power consumption, with the addition of the quarter, is indeed a lot less electricity, originally more than 450	Jingdong prefecture level city in Shandong

Happy Fan Shop	Green Intelligent Power Saver	Has not been the first time to buy back, good use, power saving device is really powerful, before the monthly electricity bill of more than 500 Now more than 300, this time to help neighbors to buy several	Jingdong prefecture level city in Shandong
Happy Fan Shop	Green Intelligent Power Saver	Home appliances are very many, the circuit is also very complex, at first it is to save 9% of the electricity, and then used more than half a month, found that the power saving rate is as high as more	Jingdong prefecture level city in Shandong
Happy Fan Shop	Green Intelligent Power Saver	My friend bought it last year and saved hundreds of dollars on his electricity bill in one summer Appearance and style: it looks better than the one I bought before Quality is also good with a light.	Jingdong prefecture level city in Shandong
Happy Fan Shop	Green Intelligent Power Saver	Saver received, on the spot on the plug with the feeling of immediate power savings, appearance, packaging is very delicate, very satisfied, I hope to achieve satisfactory results!	Jingdong prefecture level city in Shandong
Happy Fan Shop	Green Intelligent Power Saver	The power saving effect is very good, is considered an old customer, also bought before, this time is to buy two for mom and dad, customer service attitude is good, praise!	Jingdong prefecture level city in Shandong
Happy Fan Shop	Green Intelligent Power Saver	After observing for a while, it really saves a lot of money I didn't realize it was still really reliable, so I'll give you a good review.	Jingdong prefecture level city in Shandong
Happy Fan Shop	Green Intelligent Power Saver	My family a month with this monthly almost save more than 200, power-saving effect: have bought a second time, really effective Cost-effective: not bad Appearance style: roughly the same A few months to the home electricity is indeed less, this time it is a re-buy three, send a friend two, they stay a	Jingdong prefecture level city in Shandong
Happy Fan Shop	Green Intelligent Power Saver	A month almost save more than 150 dollars in electricity, home appliances more, the main this can also protect high-power appliances, after using this device, these two months have not burned, things are very good, it is said that this is the latest model, the overall price is cheap and beautiful!	Jingdong prefecture level city in Shandong
Happy Fan Shop	Green Intelligent Power Saver	Received, powerful, great quality, love the style😍	Jingdong prefecture level city in Shandong
Happy Fan Shop	Green Intelligent Power Saver	This intelligent energy saver is especially good to use and save electricity hope everyone will buy it!	Jingdong prefecture level city in Shandong
Happy Fan Shop	Green Intelligent Power Saver	Air conditioning adapter effect is particularly great, the use of very simple, plug into the socket on the summer air conditioning power is too large, electricity is also expensive, with this product directly on the electricity bill to save half, has been purchased a few more, the effect is too awesome!	Jingdong prefecture level city in Shandong
Happy Fan Shop	Green Intelligent Power Saver	The family plugged in 5, monthly electricity savings of about 300 yuan. My family before a month of electricity costs more than 800, this time with this saver after the electricity bill is actually only more than 500, amazing, thanks to the store, will recommend other friends to buy!	Jingdong prefecture level city in Shandong
Happy Fan Shop	Green Intelligent Power Saver	It was recommended to me by a neighbor's family and it's nice to use it to save power.	Jingdong prefecture level city in Shandong
Happy Fan Shop	Green Intelligent Power Saver	Second time I've purchased it and the power saving is effective!	Jingdong prefecture level city in Shandong
Happy Fan Shop	Green Intelligent Power Saver	The effect is really good, before a month of more than 300 electricity, now every month every day with the air conditioning, it is more than 100 electricity! This thing is really amazing, one after another to buy and 5.6, home installed 3!	Jingdong prefecture level city in Shandong
Happy Fan Shop	Green Intelligent Power Saver	Received this product, began to use, small things look very wrong, can save a little electricity, the monthly electricity bill is too high, after plugging in did not pull, or save a lot of electricity, point	Jingdong prefecture level city in Shandong
Happy Fan Shop	Green Intelligent Power Saver	Simple and convenient, just that plug in, you can save electricity. A month can save dozens of dollars, a year down to save a lot of, very good product cost-effective, everyone can afford to buy and use!	Jingdong prefecture level city in Shandong
Happy Fan Shop	Green Intelligent Power Saver	Electricity bill from more than 200 yuan per month, down to more than 150 yuan, the original bought home, used two months, really have power-saving effect, this time and sold two, business service attitude is	Jingdong prefecture level city in Shandong
Happy Fan Shop	Green Intelligent Power Saver	Like to watch some movies at home and so on, sometimes at night until two or three o'clock, and recently the air conditioning is not turned off for a night, rather wasteful, I have to say that the effect is very good, quite like it!	Jingdong prefecture level city in Shandong
Happy Fan Shop	Green Intelligent Power Saver	Power-saving effect: the effect is very good, a month to save more than 200 dollars Cost-effective: good value for money Appearance and style: small and lovely Workmanship material: okay Experience: good	Jingdong prefecture level city in Shandong
Happy Fan Shop	Green Intelligent Power Saver	It's okay I quite like it, the stuff is very good. Come back in the future. You can rest assured to buy. Cost-effective all. Much cheaper than the physical store	Jingdong prefecture level city in Shandong
Happy Fan Shop	Green Intelligent Power Saver	More than 150 less, last month's electricity bill is almost 400, this month is only about 200, really save electricity, I am home to buy three, I did not expect a month to pay back	Jingdong prefecture level city in Shandong
Happy Fan Shop	Green Intelligent Power Saver	I bought three of these first and tried them for two months and they still felt useful, so now I've bought another one. Good and recommended!	Jingdong prefecture level city in Shandong
Happy Fan Shop	Green Intelligent Power Saver	This energy saver is very good quality and inexpensive next time I will buy it again!	Jingdong prefecture level city in Shandong
Happy Fan Shop	Green Intelligent Power Saver	Merchant service attitude is not good, the quality does not match, the business does not give the package return	Jingdong prefecture level city in Shandong
Happy Fan Shop	Green Intelligent Power Saver	The goods are very small do not know how effective after a few days to follow up the review	Jingdong prefecture level city in Shandong
Happy Fan Shop	Green Intelligent Power Saver	Not used before more than 200 a month, after using about 100 a month, my family used three, customer service said I use electricity recommended 2-3, I think more than one effect is better on the purchase of 4, the effect is very obvious, recommended to buy!	Jingdong prefecture level city in Shandong
Happy Fan Shop	Green Intelligent Power Saver	It's okay. I'll buy it again today.	Jingdong prefecture level city in Shandong
Happy Fan Shop	Green Intelligent Power Saver	Finally received the goods, very, very much like,, has been a lot of people praise, really too happy, has been his family's loyal fans, will continue to buy buy buy!	Jingdong prefecture level city in Shandong
Happy Fan Shop	Green Intelligent Power Saver	After using this, my monthly electricity bill was 1,200 pounds, now it's only 180 bucks a month, how good is it?	Jingdong prefecture level city in Shandong
Happy Fan Shop	Green Intelligent Power Saver	The item is a scam return to marry a dozen dollars in shipping fees. Buy terrific role is purely a scam.	Jingdong prefecture level city in Shandong
Happy Fan Shop	Green Intelligent Power Saver	It's really good, cost-effective and good quality, and the logistics are fast! It arrived in one day!	Jingdong prefecture level city in Shandong
Happy Fan Shop	Green Intelligent Power Saver	Great brand! Trustworthy! Works super well! Expensive for a reason! Great merchandise!	Jingdong prefecture level city in Shandong
Happy Fan Shop	Green Intelligent Power Saver	The baby is really great, I like it very much, my friends also say good to come, recommended to buy!	Jingdong prefecture level city in Shandong
Happy Fan Shop	Green Intelligent Power Saver	Fake couldn't be more fake. Bought one plugged in still so much electricity.	Jingdong prefecture level city in Shandong
Happy Fan Shop	Green Intelligent Power Saver	This appliance works great. Let's see how it works. We'll see later.	Jingdong prefecture level city in Shandong
Happy Fan Shop	Green Intelligent Power Saver	Used it before commenting, very happy with it	Jingdong prefecture level city in Shandong
Happy Fan Shop	Green Intelligent Power Saver	Goods have been received well-packed, logistics is very fast and good quality	Jingdong prefecture level city in Shandong
Happy Fan Shop	Green Intelligent Power Saver	This baby's quality is okay.	Jingdong prefecture level city in Shandong
Happy Fan Shop	Green Intelligent Power Saver	Counterfeit goods.	Jingdong prefecture level city in Shandong
Happy Fan Shop	Green Intelligent Power Saver	The effect is not very obvious	Jingdong prefecture level city in Shandong
Happy Fan Shop	Green Intelligent Power Saver	It's not the same thing.	Jingdong prefecture level city in Shandong
Happy Fan Shop	Green Intelligent Power Saver	There is no load how much w seems false, nothing works.	Jingdong prefecture level city in Shandong
Happy Fan Shop	Green Intelligent Power Saver	I received the item yesterday, got it back and plugged it in, and it used a little more power yesterday than I had before	Jingdong prefecture level city in Shandong
Happy Fan Shop	Green Intelligent Power Saver	Haven't seen the power saving mode yet	Jingdong prefecture level city in Shandong
Happy Fan Shop	Green Intelligent Power Saver	This product has no known effect	Jingdong prefecture level city in Shandong
Happy Fan Shop	Green Intelligent Power Saver	Refund Returns $16	Jingdong prefecture level city in Shandong
Happy Fan Shop	Green Intelligent Power Saver	The previous year bought a, almost every month can save a 70 dollars or so, used a year, no problem at all, look very new like, this time to move to a new home decisively repurchase 2 units, very believe this	Jingdong prefecture level city in Shandong

Happy Fan Shop	Green Intelligent Power Saver	Has been the second time to buy, this power saving device is really good power, before every month to spend 300 electricity, now are about 200, last time that 6, this time to help lead the family to buy 6,	Jingdong prefecture level city in Shandong
Happy Fan Shop	Green Intelligent Power Saver	In the spirit of trying to experience the new product, the use of down compared to the usual power consumption, with the addition of the quarter, is indeed a lot less electricity, originally more than 450	Jingdong prefecture level city in Shandong
Happy Fan Shop	Green Intelligent Power Saver	Has not been the first time to buy back, good use, power saving device is really powerful, before the monthly electricity bill of more than 500 Now more than 300, this time to help neighbors to buy several	Jingdong prefecture level city in Shandong
Happy Fan Shop	Green Intelligent Power Saver	Home appliances are very many, the circuit is also very complex, at first it is to save 9% of the electricity, and then used more than half a month, found that the power saving rate is as high as more	Jingdong prefecture level city in Shandong
Happy Fan Shop	Green Intelligent Power Saver	My friend bought it last year and saved hundreds of dollars on his electricity bill in one summer Appearance and style: it looks better than the one I bought before Quality is also good with a light.	Jingdong prefecture level city in Shandong
Happy Fan Shop	Green Intelligent Power Saver	Saver received, on the spot on the plug with the feeling of immediate power savings, appearance, packaging is very delicate, very satisfied, I hope to achieve satisfactory results!	Jingdong prefecture level city in Shandong
Happy Fan Shop	Green Intelligent Power Saver	The power saving effect is very good, is considered an old customer, also bought before, this time is to buy two for mom and dad, customer service attitude is good, praise!	Jingdong prefecture level city in Shandong
Happy Fan Shop	Green Intelligent Power Saver	After observing for a while, it really saves a lot of money I didn't realize it was still really reliable, so I'll give you a good review.	Jingdong prefecture level city in Shandong
Happy Fan Shop	Green Intelligent Power Saver	My family a month with this monthly almost save more than 200, power-saving effect: have bought a second time, really effective Cost-effective: not bad Appearance style: roughly the same A few months to the home electricity is indeed less, this time it is a re-buy three, send a friend two, they stay a	Jingdong prefecture level city in Shandong
Happy Fan Shop	Green Intelligent Power Saver	A month almost save more than 150 dollars in electricity, home appliances more, the main this can also protect high-power appliances, after using this device, these two months have not burned, things are very good, it is said that this is the latest model, the overall price is cheap and beautiful!	Jingdong prefecture level city in Shandong
Happy Fan Shop	Green Intelligent Power Saver	Received, powerful, great quality, love the style😍	Jingdong prefecture level city in Shandong
Happy Fan Shop	Green Intelligent Power Saver	This intelligent energy saver is especially good to use and save electricity hope everyone will buy it!	Jingdong prefecture level city in Shandong
Happy Fan Shop	Green Intelligent Power Saver	Air conditioning adapter effect is particularly great, the use of very simple, plug into the socket on the summer air conditioning power is too large, electricity is also expensive, with this product directly on the electricity bill to save half, has been purchased a few more, the effect is too awesome!	Jingdong prefecture level city in Shandong
Happy Fan Shop	Green Intelligent Power Saver	The family plugged in 5, monthly electricity savings of about 300 yuan. My family before a month of electricity costs more than 800, this time with this saver after the electricity bill is actually only more than 500, amazing, thanks to the store, will recommend other friends to buy!	Jingdong prefecture level city in Shandong
Happy Fan Shop	Green Intelligent Power Saver	It was recommended to me by a neighbor's family and it's nice to use it to save power.	Jingdong prefecture level city in Shandong
Happy Fan Shop	Green Intelligent Power Saver	Second time I've purchased it and the power saving is effective!	Jingdong prefecture level city in Shandong
Happy Fan Shop	Green Intelligent Power Saver	The effect is really good, before a month of more than 300 electricity, now every month every day with the air conditioning, it is more than 100 electricity! This thing is really amazing, one after another to buy and 5.6, home installed 3!	Jingdong prefecture level city in Shandong
Happy Fan Shop	Green Intelligent Power Saver	Received this product, began to use, small things look very wrong, can save a little electricity, the monthly electricity bill is too high, after plugging in did not pull, or save a lot of electricity, point	Jingdong prefecture level city in Shandong
Happy Fan Shop	Green Intelligent Power Saver	Simple and convenient, just that plug in, you can save electricity. A month can save dozens of dollars, a year down to save a lot of, very good product cost-effective, everyone can afford to buy and use!	Jingdong prefecture level city in Shandong
Happy Fan Shop	Green Intelligent Power Saver	Electricity bill from more than 200 yuan per month, down to more than 150 yuan, the original bought home, used two months, really have power-saving effect, this time and sold two, business service attitude is	Jingdong prefecture level city in Shandong
Happy Fan Shop	Green Intelligent Power Saver	Like to watch some movies at home and so on, sometimes at night until two or three o'clock, and recently the air conditioning is not turned off for a night, rather wasteful, I have to say that the effect is very good, quite like it!	Jingdong prefecture level city in Shandong
Happy Fan Shop	Green Intelligent Power Saver	Power-saving effect: the effect is very good, a month to save more than 200 dollars Cost-effective: good value for money Appearance and style: small and lovely Workmanship material: okay Experience: good	Jingdong prefecture level city in Shandong
Happy Fan Shop	Green Intelligent Power Saver	It's okay I quite like it, the stuff is very good. Come back in the future. You can rest assured to buy. Cost-effective all. Much cheaper than the physical store	Jingdong prefecture level city in Shandong
Happy Fan Shop	Green Intelligent Power Saver	More than 150 less, last month's electricity bill is almost 400, this month is only about 200, really save electricity, I am home to buy three, I did not expect a month to pay back	Jingdong prefecture level city in Shandong
Happy Fan Shop	Green Intelligent Power Saver	I bought three of these first and tried them for two months and they still felt useful, so now I've bought another one. Good and recommended!	Jingdong prefecture level city in Shandong
Happy Fan Shop	Green Intelligent Power Saver	This energy saver is very good quality and inexpensive next time I will buy it again!	Jingdong prefecture level city in Shandong
Happy Fan Shop	Green Intelligent Power Saver	Merchant service attitude is not good, the quality does not match, the business does not give the package return	Jingdong prefecture level city in Shandong
Happy Fan Shop	Green Intelligent Power Saver	The goods are very small do not know how effective after a few days to follow up the review	Jingdong prefecture level city in Shandong
Happy Fan Shop	Green Intelligent Power Saver	Not used before more than 200 a month, after using about 100 a month, my family used three, customer service said I use electricity recommended 2-3, I think more than one effect is better on the purchase of 4, the effect is very obvious, recommended to buy!	Jingdong prefecture level city in Shandong
Happy Fan Shop	Green Intelligent Power Saver	It's okay. I'll buy it again today.	Jingdong prefecture level city in Shandong
Happy Fan Shop	Green Intelligent Power Saver	Finally received the goods, very, very much like,, has been a lot of people praise, really too happy, has been his family's loyal fans, will continue to buy buy buy buy!	Jingdong prefecture level city in Shandong
Happy Fan Shop	Green Intelligent Power Saver	After using this, my monthly electricity bill was 1,200 pounds, now it's only 180 bucks a month, how good is it?	Jingdong prefecture level city in Shandong
Happy Fan Shop	Green Intelligent Power Saver	The item is a scam return to marry a dozen dollars in shipping fees. Buy terrific role is purely a scam.	Jingdong prefecture level city in Shandong
Happy Fan Shop	Green Intelligent Power Saver	It's really good, cost-effective and good quality, and the logistics are fast! It arrived in one day!	Jingdong prefecture level city in Shandong
Happy Fan Shop	Green Intelligent Power Saver	Great brand! Trustworthy! Works super well! Expensive for a reason! Great merchandise!	Jingdong prefecture level city in Shandong
Happy Fan Shop	Green Intelligent Power Saver	The baby is really great, I like it very much, my friends also say good to come, recommended to buy!	Jingdong prefecture level city in Shandong
Happy Fan Shop	Green Intelligent Power Saver	Fake couldn't be more fake. Bought one plugged in still so much electricity.	Jingdong prefecture level city in Shandong
Happy Fan Shop	Green Intelligent Power Saver	This appliance works great. Let's see how it works. We'll see later.	Jingdong prefecture level city in Shandong
Happy Fan Shop	Green Intelligent Power Saver	Used it before commenting, very happy with it	Jingdong prefecture level city in Shandong
Happy Fan Shop	Green Intelligent Power Saver	Goods have been received well-packed, logistics is very fast and good quality	Jingdong prefecture level city in Shandong
Happy Fan Shop	Green Intelligent Power Saver	This baby's quality is okay.	Jingdong prefecture level city in Shandong
Happy Fan Shop	Green Intelligent Power Saver	Counterfeit goods.	Jingdong prefecture level city in Shandong
Happy Fan Shop	Green Intelligent Power Saver	The effect is not very obvious	Jingdong prefecture level city in Shandong
Happy Fan Shop	Green Intelligent Power Saver	It's not the same thing.	Jingdong prefecture level city in Shandong
Happy Fan Shop	Green Intelligent Power Saver	There is no load how much w seems false, nothing works.	Jingdong prefecture level city in Shandong
Happy Fan Shop	Green Intelligent Power Saver	I received the item yesterday, got it back and plugged it in, and it used a little more power yesterday than I had before	Jingdong prefecture level city in Shandong
Happy Fan Shop	Green Intelligent Power Saver	Haven't seen the power saving mode yet	Jingdong prefecture level city in Shandong
Happy Fan Shop	Green Intelligent Power Saver	This product has no known effect	Jingdong prefecture level city in Shandong

Happy Fan Shop	Green Intelligent Power Saver	Refund Returns $16	Jingdong prefecture level city in Shandong
Happy Fan Shop	Green Intelligent Power Saver	The previous year bought a, almost every month can save a 70 dollars or so, used a year, no problem at all, look very new like, this time to move to a new home decisively repurchase 2 units, very believe this	Jingdong prefecture level city in Shandong
Happy Fan Shop	Green Intelligent Power Saver	Has been the second time to buy, this power saving device is really good power, before every month to spend 300 electricity, now are about 200, last time that 6, this time to help lead the family to buy 6,	Jingdong prefecture level city in Shandong
Happy Fan Shop	Green Intelligent Power Saver	In the spirit of trying to experience the new product, the use of down compared to the usual power consumption, with the addition of the quarter, is indeed a lot less electricity, originally more than 450	Jingdong prefecture level city in Shandong
Happy Fan Shop	Green Intelligent Power Saver	Has not been the first time to buy back, good use, power saving device is really powerful, before the monthly electricity bill of more than 500 Now more than 300, this time to help neighbors to buy several	Jingdong prefecture level city in Shandong
Happy Fan Shop	Green Intelligent Power Saver	Home appliances are very many, the circuit is also very complex, at first it is to save 9% of the electricity, and then used more than half a month, found that the power saving rate is as high as more	Jingdong prefecture level city in Shandong
Happy Fan Shop	Green Intelligent Power Saver	My friend bought it last year and saved hundreds of dollars on his electricity bill in one summer Appearance and style: it looks better than the one I bought before Quality is also good with a light.	Jingdong prefecture level city in Shandong
Happy Fan Shop	Green Intelligent Power Saver	Saver received, on the spot on the plug with the feeling of immediate power savings, appearance, packaging is very delicate, very satisfied, I hope to achieve satisfactory results!	Jingdong prefecture level city in Shandong
Happy Fan Shop	Green Intelligent Power Saver	The power saving effect is very good, is considered an old customer, also bought before, this time is to buy two for mom and dad, customer service attitude is good, praise!	Jingdong prefecture level city in Shandong
Happy Fan Shop	Green Intelligent Power Saver	After observing for a while, it really saves a lot of money I didn't realize it was still really reliable, so I'll give you a good review.	Jingdong prefecture level city in Shandong
Happy Fan Shop	Green Intelligent Power Saver	My family a month with this monthly almost save more than 200, power-saving effect: have bought a second time, really effective Cost-effective: not bad Appearance style: roughly the same A few months to the home electricity is indeed less, this time it is a re-buy three, send a friend two, they stay a	Jingdong prefecture level city in Shandong
Happy Fan Shop	Green Intelligent Power Saver	A month almost save more than 150 dollars in electricity, home appliances more, the main this can also protect high-power appliances, after using this device, these two months have not burned, things are very good, it is said that this is the latest model, the overall price is cheap and beautiful!	Jingdong prefecture level city in Shandong
Happy Fan Shop	Green Intelligent Power Saver	Received, powerful, great quality, love the style😍	Jingdong prefecture level city in Shandong
Happy Fan Shop	Green Intelligent Power Saver	This intelligent energy saver is especially good to use and save electricity hope everyone will buy it!	Jingdong prefecture level city in Shandong
Happy Fan Shop	Green Intelligent Power Saver	Air conditioning adapter effect is particularly great, the use of very simple, plug into the socket on the summer air conditioning power is too large, electricity is also expensive, with this product directly on the electricity bill to save half, has been purchased a few more, the effect is too awesome!	Jingdong prefecture level city in Shandong
Happy Fan Shop	Green Intelligent Power Saver	The family plugged in 5, monthly electricity savings of about 300 yuan. My family before a month of electricity costs more than 800, this time with this saver after the electricity bill is actually only more than 500, amazing, thanks to the store, will recommend other friends to buy!	Jingdong prefecture level city in Shandong
Happy Fan Shop	Green Intelligent Power Saver	It was recommended to me by a neighbor's family and it's nice to use it to save power.	Jingdong prefecture level city in Shandong
Happy Fan Shop	Green Intelligent Power Saver	Second time I've purchased it and the power saving is effective!	Jingdong prefecture level city in Shandong
Happy Fan Shop	Green Intelligent Power Saver	The effect is really good, before a month of more than 300 electricity, now every month every day with the air conditioning, it is more than 100 electricity! This thing is really amazing, one after another to buy and 5.6, home installed 3!	Jingdong prefecture level city in Shandong
Happy Fan Shop	Green Intelligent Power Saver	Received this product, began to use, small things look very wrong, can save a little electricity, the monthly electricity bill is too high, after plugging in did not pull, or save a lot of electricity, point	Jingdong prefecture level city in Shandong
Happy Fan Shop	Green Intelligent Power Saver	Simple and convenient, just that plug in, you can save electricity. A month can save dozens of dollars, a year down to save a lot of, very good product cost-effective, everyone can afford to buy and use!	Jingdong prefecture level city in Shandong
Happy Fan Shop	Green Intelligent Power Saver	Electricity bill from more than 200 yuan per month, down to more than 150 yuan, the original bought home, used two months, really have power-saving effect, this time and sold two, business service attitude is	Jingdong prefecture level city in Shandong
Happy Fan Shop	Green Intelligent Power Saver	Like to watch some movies at home and so on, sometimes at night until two or three o'clock, and recently the air conditioning is not turned off for a night, rather wasteful, I have to say that the effect is very good, quite like it!	Jingdong prefecture level city in Shandong
Happy Fan Shop	Green Intelligent Power Saver	Power-saving effect: the effect is very good, a month to save more than 200 dollars Cost-effective: good value for money Appearance and style: small and lovely Workmanship material: okay Experience: good	Jingdong prefecture level city in Shandong
Happy Fan Shop	Green Intelligent Power Saver	It's okay I quite like it, the stuff is very good. Come back in the future. You can rest assured to buy. Cost-effective all. Much cheaper than the physical store	Jingdong prefecture level city in Shandong
Happy Fan Shop	Green Intelligent Power Saver	More than 150 less, last month's electricity bill is almost 400, this month is only about 200, really save electricity, I am home to buy three, I did not expect a month to pay back	Jingdong prefecture level city in Shandong
Happy Fan Shop	Green Intelligent Power Saver	I bought three of these first and tried them for two months and they still felt useful, so now I've bought another one. Good and recommended!	Jingdong prefecture level city in Shandong
Happy Fan Shop	Green Intelligent Power Saver	This energy saver is very good quality and inexpensive next time I will buy it again!	Jingdong prefecture level city in Shandong
Happy Fan Shop	Green Intelligent Power Saver	Merchant service attitude is not good, the quality does not match, the business does not give the package return	Jingdong prefecture level city in Shandong
Happy Fan Shop	Green Intelligent Power Saver	The goods are very small do not know how effective after a few days to follow up the review	Jingdong prefecture level city in Shandong
Happy Fan Shop	Green Intelligent Power Saver	Not used before more than 200 a month, after using about 100 a month, my family used three, customer service said I use electricity recommended 2-3, I think more than one effect is better on the purchase of 4, the effect is very obvious, recommended to buy!	Jingdong prefecture level city in Shandong
Happy Fan Shop	Green Intelligent Power Saver	It's okay. I'll buy it again today.	Jingdong prefecture level city in Shandong
Happy Fan Shop	Green Intelligent Power Saver	Finally received the goods, very, very much like,, has been a lot of people praise, really too happy, has been his family's loyal fans, will continue to buy buy buy buy!	Jingdong prefecture level city in Shandong
Happy Fan Shop	Green Intelligent Power Saver	After using this, my monthly electricity bill was 1,200 pounds, now it's only 180 bucks a month, how good is it?	Jingdong prefecture level city in Shandong
Happy Fan Shop	Green Intelligent Power Saver	The item is a scam return to marry a dozen dollars in shipping fees. Buy terrific role is purely a scam.	Jingdong prefecture level city in Shandong
Happy Fan Shop	Green Intelligent Power Saver	It's really good, cost-effective and good quality, and the logistics are fast! It arrived in one day!	Jingdong prefecture level city in Shandong
Happy Fan Shop	Green Intelligent Power Saver	Great brand! Trustworthy! Works super well! Expensive for a reason! Great merchandise!	Jingdong prefecture level city in Shandong
Happy Fan Shop	Green Intelligent Power Saver	The baby is really great, I like it very much, my friends also say good to come, recommended to buy!	Jingdong prefecture level city in Shandong
Happy Fan Shop	Green Intelligent Power Saver	Fake couldn't be more fake. Bought one plugged in still so much electricity.	Jingdong prefecture level city in Shandong
Happy Fan Shop	Green Intelligent Power Saver	This appliance works great. Let's see how it works. We'll see later.	Jingdong prefecture level city in Shandong
Happy Fan Shop	Green Intelligent Power Saver	Used it before commenting, very happy with it	Jingdong prefecture level city in Shandong
Happy Fan Shop	Green Intelligent Power Saver	Goods have been received well-packed, logistics is very fast and good quality	Jingdong prefecture level city in Shandong
Happy Fan Shop	Green Intelligent Power Saver	This baby's quality is okay.	Jingdong prefecture level city in Shandong
Happy Fan Shop	Green Intelligent Power Saver	Counterfeit goods.	Jingdong prefecture level city in Shandong
Happy Fan Shop	Green Intelligent Power Saver	The effect is not very obvious	Jingdong prefecture level city in Shandong
Happy Fan Shop	Green Intelligent Power Saver	It's not the same thing.	Jingdong prefecture level city in Shandong
Happy Fan Shop	Green Intelligent Power Saver	There is no load how much w seems false, nothing works.	Jingdong prefecture level city in Shandong
Happy Fan Shop	Green Intelligent Power Saver	I received the item yesterday, got it back and plugged it in, and it used a little more power yesterday than I had before	Jingdong prefecture level city in Shandong

Happy Fan Shop	Green Intelligent Power Saver	Haven't seen the power saving mode yet	Jingdong prefecture level city in Shandong
Happy Fan Shop	Green Intelligent Power Saver	This product has no known effect	Jingdong prefecture level city in Shandong
Happy Fan Shop	Green Intelligent Power Saver	Refund Returns $16	Jingdong prefecture level city in Shandong
Happy Fan Shop	Green Intelligent Power Saver	The previous year bought a, almost every month can save a 70 dollars or so, used a year, no problem at all, look very new like, this time to move to a new home decisively repurchase 2 units, very believe this	Jingdong prefecture level city in Shandong
Happy Fan Shop	Green Intelligent Power Saver	Has been the second time to buy, this power saving device is really good power, before every month to spend 300 electricity, now are about 200, last time that 6, this time to help lead the family to buy 6,	Jingdong prefecture level city in Shandong
Happy Fan Shop	Green Intelligent Power Saver	In the spirit of trying to experience the new product, the use of down compared to the usual power consumption, with the addition of the quarter, is indeed a lot less electricity, originally more than 450	Jingdong prefecture level city in Shandong
Happy Fan Shop	Green Intelligent Power Saver	Has not been the first time to buy back, good use, power saving device is really powerful, before the monthly electricity bill of more than 500 Now more than 300, this time to help neighbors to buy several	Jingdong prefecture level city in Shandong
Happy Fan Shop	Green Intelligent Power Saver	Home appliances are very many, the circuit is also very complex, at first it is to save 9% of the electricity, and then used more than half a month, found that the power saving rate is as high as more	Jingdong prefecture level city in Shandong
Happy Fan Shop	Green Intelligent Power Saver	My friend bought it last year and saved hundreds of dollars on his electricity bill in one summer Appearance and style: it looks better than the one I bought before Quality is also good with a light.	Jingdong prefecture level city in Shandong
Happy Fan Shop	Green Intelligent Power Saver	Saver received, on the spot on the plug with the feeling of immediate power savings, appearance, packaging is very delicate, very satisfied, I hope to achieve satisfactory results!	Jingdong prefecture level city in Shandong
Happy Fan Shop	Green Intelligent Power Saver	The power saving effect is very good, is considered an old customer, also bought before, this time is to buy two for mom and dad, customer service attitude is good, praise!	Jingdong prefecture level city in Shandong
Happy Fan Shop	Green Intelligent Power Saver	After observing for a while, it really saves a lot of money I didn't realize it was still really reliable, so I'll give you a good review.	Jingdong prefecture level city in Shandong
Happy Fan Shop	Green Intelligent Power Saver	My family a month with this monthly almost save more than 200, power-saving effect: have bought a second time, really effective Cost-effective: not bad Appearance style: roughly the same A few months to the home electricity is indeed less, this time it is a re-buy three, send a friend two, they stay a	Jingdong prefecture level city in Shandong
Happy Fan Shop	Green Intelligent Power Saver	A month almost save more than 150 dollars in electricity, home appliances more, the main this can also protect high-power appliances, after using this device, these two months have not burned, things are very good, it is said that this is the latest model, the overall price is cheap and beautiful!	Jingdong prefecture level city in Shandong
Happy Fan Shop	Green Intelligent Power Saver	Received, powerful, great quality, love the style😍	Jingdong prefecture level city in Shandong
Happy Fan Shop	Green Intelligent Power Saver	This intelligent energy saver is especially good to use and save electricity hope everyone will buy it!	Jingdong prefecture level city in Shandong
Happy Fan Shop	Green Intelligent Power Saver	Air conditioning adapter effect is particularly great, the use of very simple, plug into the socket on the summer air conditioning power is too large, electricity is also expensive, with this product directly on the electricity bill to save half, has been purchased a few more, the effect is too awesome!	Jingdong prefecture level city in Shandong
Happy Fan Shop	Green Intelligent Power Saver	The family plugged in 5, monthly electricity savings of about 300 yuan. My family before a month of electricity costs more than 800, this time with this saver after the electricity bill is actually only more than 500, amazing, thanks to the store, will recommend other friends to buy!	Jingdong prefecture level city in Shandong
Happy Fan Shop	Green Intelligent Power Saver	It was recommended to me by a neighbor's family and it's nice to use it to save power.	Jingdong prefecture level city in Shandong
Happy Fan Shop	Green Intelligent Power Saver	Second time I've purchased it and the power saving is effective!	Jingdong prefecture level city in Shandong
Happy Fan Shop	Green Intelligent Power Saver	The effect is really good, before a month of more than 300 electricity, now every month every day with the air conditioning, it is more than 100 electricity! This thing is really amazing, one after another to buy and 5.6, home installed 3!	Jingdong prefecture level city in Shandong
Happy Fan Shop	Green Intelligent Power Saver	Received this product, began to use, small things look very wrong, can save a little electricity, the monthly electricity bill is too high, after plugging in did not pull, or save a lot of electricity, point	Jingdong prefecture level city in Shandong
Happy Fan Shop	Green Intelligent Power Saver	Simple and convenient, just that plug in, you can save electricity. A month can save dozens of dollars, a year down to save a lot of, very good product cost-effective, everyone can afford to buy and use!	Jingdong prefecture level city in Shandong
Happy Fan Shop	Green Intelligent Power Saver	Electricity bill from more than 200 yuan per month, down to more than 150 yuan, the original bought home, used two months, really have power-saving effect, this time and sold two, business service attitude is	Jingdong prefecture level city in Shandong
Happy Fan Shop	Green Intelligent Power Saver	Like to watch some movies at home and so on, sometimes at night until two or three o'clock, and recently the air conditioning is not turned off for a night, rather wasteful, I have to say that the effect is very good, quite like it!	Jingdong prefecture level city in Shandong
Happy Fan Shop	Green Intelligent Power Saver	Power-saving effect: the effect is very good, a month to save more than 200 dollars Cost-effective: good value for money Appearance and style: small and lovely Workmanship material: okay Experience: good	Jingdong prefecture level city in Shandong
Happy Fan Shop	Green Intelligent Power Saver	It's okay I quite like it, the stuff is very good. Come back in the future. You can rest assured to buy. Cost-effective all. Much cheaper than the physical store	Jingdong prefecture level city in Shandong
Happy Fan Shop	Green Intelligent Power Saver	More than 150 less, last month's electricity bill is almost 400, this month is only about 200, really save electricity, I am home to buy three, I did not expect a month to pay back	Jingdong prefecture level city in Shandong
Happy Fan Shop	Green Intelligent Power Saver	I bought three of these first and tried them for two months and they still felt useful, so now I've bought another one. Good and recommended!	Jingdong prefecture level city in Shandong
Happy Fan Shop	Green Intelligent Power Saver	This energy saver is very good quality and inexpensive next time I will buy it again!	Jingdong prefecture level city in Shandong
Happy Fan Shop	Green Intelligent Power Saver	Merchant service attitude is not good, the quality does not match, the business does not give the package return	Jingdong prefecture level city in Shandong
Happy Fan Shop	Green Intelligent Power Saver	The goods are very small do not know how effective after a few days to follow up the review	Jingdong prefecture level city in Shandong
Happy Fan Shop	Green Intelligent Power Saver	Not used before more than 200 a month, after using about 100 a month, my family used three, customer service said I use electricity recommended 2-3, I think more than one effect is better on the purchase of 4, the effect is very obvious, recommended to buy!	Jingdong prefecture level city in Shandong
Happy Fan Shop	Green Intelligent Power Saver	It's okay. I'll buy it again today.	Jingdong prefecture level city in Shandong
Happy Fan Shop	Green Intelligent Power Saver	Finally received the goods, very, very much like,, has been a lot of people praise, really too happy, has been his family's loyal fans, will continue to buy buy buy!	Jingdong prefecture level city in Shandong
Happy Fan Shop	Green Intelligent Power Saver	After using this, my monthly electricity bill was 1,200 pounds, now it's only 180 bucks a month, how good is it?	Jingdong prefecture level city in Shandong
Happy Fan Shop	Green Intelligent Power Saver	The item is a scam return to marry a dozen dollars in shipping fees. Buy terrific role is purely a scam.	Jingdong prefecture level city in Shandong
Happy Fan Shop	Green Intelligent Power Saver	It's really good, cost-effective and good quality, and the logistics are fast! It arrived in one day!	Jingdong prefecture level city in Shandong
Happy Fan Shop	Green Intelligent Power Saver	Great brand! Trustworthy! Works super well! Expensive for a reason! Great merchandise!	Jingdong prefecture level city in Shandong
Happy Fan Shop	Green Intelligent Power Saver	The baby is really great, I like it very much, my friends also say good to come, recommended to buy!	Jingdong prefecture level city in Shandong
Happy Fan Shop	Green Intelligent Power Saver	Fake couldn't be more fake. Bought one plugged in still so much electricity.	Jingdong prefecture level city in Shandong
Happy Fan Shop	Green Intelligent Power Saver	This appliance works great. Let's see how it works. We'll see later.	Jingdong prefecture level city in Shandong
Happy Fan Shop	Green Intelligent Power Saver	Used it before commenting, very happy with it	Jingdong prefecture level city in Shandong
Happy Fan Shop	Green Intelligent Power Saver	Goods have been received well-packed, logistics is very fast and good quality	Jingdong prefecture level city in Shandong
Happy Fan Shop	Green Intelligent Power Saver	This baby's quality is okay.	Jingdong prefecture level city in Shandong
Happy Fan Shop	Green Intelligent Power Saver	Counterfeit goods.	Jingdong prefecture level city in Shandong
Happy Fan Shop	Green Intelligent Power Saver	The effect is not very obvious	Jingdong prefecture level city in Shandong
Happy Fan Shop	Green Intelligent Power Saver	It's not the same thing.	Jingdong prefecture level city in Shandong

Happy Fan Shop	Green Intelligent Power Saver	There is no load how much w seems false, nothing works.	Jingdong prefecture level city in Shandong
Happy Fan Shop	Green Intelligent Power Saver	I received the item yesterday, got it back and plugged it in, and it used a little more power yesterday than I had before	Jingdong prefecture level city in Shandong
Happy Fan Shop	Green Intelligent Power Saver	Haven't seen the power saving mode yet	Jingdong prefecture level city in Shandong
Happy Fan Shop	Green Intelligent Power Saver	This product has no known effect	Jingdong prefecture level city in Shandong
Happy Fan Shop	Green Intelligent Power Saver	Refund Returns $16	Jingdong prefecture level city in Shandong
Happy Fan Shop	Green Intelligent Power Saver	The previous year bought a, almost every month can save a 70 dollars or so, used a year, no problem at all, look very new like, this time to move to a new home decisively repurchase 2 units, very believe this	Jingdong prefecture level city in Shandong
Happy Fan Shop	Green Intelligent Power Saver	Has been the second time to buy, this power saving device is really good power, before every month to spend 300 electricity, now are about 200, last time that 6, this time to help lead the family to buy 6,	Jingdong prefecture level city in Shandong
Happy Fan Shop	Green Intelligent Power Saver	In the spirit of trying to experience the new product, the use of down compared to the usual power consumption, with the addition of the quarter, is indeed a lot less electricity, originally more than 450	Jingdong prefecture level city in Shandong
Happy Fan Shop	Green Intelligent Power Saver	Has not been the first time to buy back, good use, power saving device is really powerful, before the monthly electricity bill of more than 500 Now more than 300, this time to help neighbors to buy several	Jingdong prefecture level city in Shandong
Happy Fan Shop	Green Intelligent Power Saver	Home appliances are very many, the circuit is also very complex, at first it is to save 9% of the electricity, and then used more than half a month, found that the power saving rate is as high as more	Jingdong prefecture level city in Shandong
Happy Fan Shop	Green Intelligent Power Saver	My friend bought it last year and saved hundreds of dollars on his electricity bill in one summer Appearance and style: it looks better than the one I bought before Quality is also good with a light.	Jingdong prefecture level city in Shandong
Happy Fan Shop	Green Intelligent Power Saver	Saver received, on the spot on the plug with the feeling of immediate power savings, appearance, packaging is very delicate, very satisfied, I hope to achieve satisfactory results!	Jingdong prefecture level city in Shandong
Happy Fan Shop	Green Intelligent Power Saver	The power saving effect is very good, is considered an old customer, also bought before, this time is to buy two for mom and dad, customer service attitude is good, praise!	Jingdong prefecture level city in Shandong
Happy Fan Shop	Green Intelligent Power Saver	After observing for a while, it really saves a lot of money I didn't realize it was still really reliable, so I'll give you a good review.	Jingdong prefecture level city in Shandong
Happy Fan Shop	Green Intelligent Power Saver	My family a month with this monthly almost save more than 200, power-saving effect: have bought a second time, really effective Cost-effective: not bad Appearance style: roughly the same A few months to the home electricity is indeed less, this time it is a re-buy three, send a friend two, they stay a	Jingdong prefecture level city in Shandong
Happy Fan Shop	Green Intelligent Power Saver	A month almost save more than 150 dollars in electricity, home appliances more, the main this can also protect high-power appliances, after using this device, these two months have not burned, things are very good, it is said that this is the latest model, the overall price is cheap and beautiful!	Jingdong prefecture level city in Shandong
Happy Fan Shop	Green Intelligent Power Saver	Received, powerful, great quality, love the style😍	Jingdong prefecture level city in Shandong
Happy Fan Shop	Green Intelligent Power Saver	This intelligent energy saver is especially good to use and save electricity hope everyone will buy it!	Jingdong prefecture level city in Shandong
Happy Fan Shop	Green Intelligent Power Saver	Air conditioning adapter effect is particularly great, the use of very simple, plug into the socket on the summer air conditioning power is too large, electricity is also expensive, with this product directly on the electricity bill to save half, has been purchased a few more, the effect is too awesome!	Jingdong prefecture level city in Shandong
Happy Fan Shop	Green Intelligent Power Saver	The family plugged in 5, monthly electricity savings of about 300 yuan. My family before a month of electricity costs more than 800, this time with this saver after the electricity bill is actually only more than 500, amazing, thanks to the store, will recommend other friends to buy!	Jingdong prefecture level city in Shandong
Happy Fan Shop	Green Intelligent Power Saver	It was recommended to me by a neighbor's family and it's nice to use it to save power.	Jingdong prefecture level city in Shandong
Happy Fan Shop	Green Intelligent Power Saver	Second time I've purchased it and the power saving is effective!	Jingdong prefecture level city in Shandong
Happy Fan Shop	Green Intelligent Power Saver	The effect is really good, before a month of more than 300 electricity, now every month every day with the air conditioning, it is more than 100 electricity! This thing is really amazing, one after another to buy and 5.6, home installed 3!	Jingdong prefecture level city in Shandong
Happy Fan Shop	Green Intelligent Power Saver	Received this product, began to use, small things look very wrong, can save a little electricity, the monthly electricity bill is too high, after plugging in did not pull, or save a lot of electricity, point	Jingdong prefecture level city in Shandong
Happy Fan Shop	Green Intelligent Power Saver	Simple and convenient, just that plug in, you can save electricity. A month can save dozens of dollars, a year down to save a lot of, very good product cost-effective, everyone can afford to buy and use!	Jingdong prefecture level city in Shandong
Happy Fan Shop	Green Intelligent Power Saver	Electricity bill from more than 200 yuan per month, down to more than 150 yuan, the original bought home, used two months, really have power-saving effect, this time and sold two, business service attitude is	Jingdong prefecture level city in Shandong
Happy Fan Shop	Green Intelligent Power Saver	Like to watch some movies at home and so on, sometimes at night until two or three o'clock, and recently the air conditioning is not turned off for a night, rather wasteful, I have to say that the effect is very good, quite like it!	Jingdong prefecture level city in Shandong
Happy Fan Shop	Green Intelligent Power Saver	Power-saving effect: the effect is very good, a month to save more than 200 dollars Cost-effective: good value for money Appearance and style: small and lovely Workmanship material: okay Experience: good	Jingdong prefecture level city in Shandong
Happy Fan Shop	Green Intelligent Power Saver	It's okay I quite like it, the stuff is very good. Come back in the future. You can rest assured to buy. Cost-effective all. Much cheaper than the physical store	Jingdong prefecture level city in Shandong
Happy Fan Shop	Green Intelligent Power Saver	More than 150 less, last month's electricity bill is almost 400, this month is only about 200, really save electricity, I am home to buy three, I did not expect a month to pay back	Jingdong prefecture level city in Shandong
Happy Fan Shop	Green Intelligent Power Saver	I bought three of these first and tried them for two months and they still felt useful, so now I've bought another one. Good and recommended!	Jingdong prefecture level city in Shandong
Happy Fan Shop	Green Intelligent Power Saver	This energy saver is very good quality and inexpensive next time I will buy it again!	Jingdong prefecture level city in Shandong
Happy Fan Shop	Green Intelligent Power Saver	Merchant service attitude is not good, the quality does not match, the business does not give the package return	Jingdong prefecture level city in Shandong
Happy Fan Shop	Green Intelligent Power Saver	The goods are very small do not know how effective after a few days to follow up the review	Jingdong prefecture level city in Shandong
Happy Fan Shop	Green Intelligent Power Saver	Not used before more than 200 a month, after using about 100 a month, my family used three, customer service said I use electricity recommended 2-3, I think more than one effect is better on the purchase of 4, the effect is very obvious, recommended to buy!	Jingdong prefecture level city in Shandong
Happy Fan Shop	Green Intelligent Power Saver	It's okay. I'll buy it again today.	Jingdong prefecture level city in Shandong
Happy Fan Shop	Green Intelligent Power Saver	Finally received the goods, very, very much like,, has been a lot of people praise, really too happy, has been his family's loyal fans, will continue to buy buy buy buy!	Jingdong prefecture level city in Shandong
Happy Fan Shop	Green Intelligent Power Saver	After using this, my monthly electricity bill was 1,200 pounds, now it's only 180 bucks a month, how good is it?	Jingdong prefecture level city in Shandong
Happy Fan Shop	Green Intelligent Power Saver	The item is a scam return to marry a dozen dollars in shipping fees. Buy terrific role is purely a scam.	Jingdong prefecture level city in Shandong
Happy Fan Shop	Green Intelligent Power Saver	It's really good, cost-effective and good quality, and the logistics are fast! It arrived in one day!	Jingdong prefecture level city in Shandong
Happy Fan Shop	Green Intelligent Power Saver	Great brand! Trustworthy! Works super well! Expensive for a reason! Great merchandise!	Jingdong prefecture level city in Shandong
Happy Fan Shop	Green Intelligent Power Saver	The baby is really great, I like it very much, my friends also say good to come, recommended to buy!	Jingdong prefecture level city in Shandong
Happy Fan Shop	Green Intelligent Power Saver	Fake couldn't be more fake. Bought one plugged in still so much electricity.	Jingdong prefecture level city in Shandong
Happy Fan Shop	Green Intelligent Power Saver	This appliance works great. Let's see how it works. We'll see later.	Jingdong prefecture level city in Shandong
Happy Fan Shop	Green Intelligent Power Saver	Used it before commenting, very happy with it	Jingdong prefecture level city in Shandong
Happy Fan Shop	Green Intelligent Power Saver	Goods have been received well-packed, logistics is very fast and good quality	Jingdong prefecture level city in Shandong
Happy Fan Shop	Green Intelligent Power Saver	This baby's quality is okay.	Jingdong prefecture level city in Shandong
Happy Fan Shop	Green Intelligent Power Saver	Counterfeit goods.	Jingdong prefecture level city in Shandong

Happy Fan Shop	Green Intelligent Power Saver	The effect is not very obvious	Jingdong prefecture level city in Shandong
Happy Fan Shop	Green Intelligent Power Saver	It's not the same thing.	Jingdong prefecture level city in Shandong
Happy Fan Shop	Green Intelligent Power Saver	There is no load how much w seems false, nothing works.	Jingdong prefecture level city in Shandong
Happy Fan Shop	Green Intelligent Power Saver	I received the item yesterday, got it back and plugged it in, and it used a little more power yesterday than I had before	Jingdong prefecture level city in Shandong
Happy Fan Shop	Green Intelligent Power Saver	Haven't seen the power saving mode yet	Jingdong prefecture level city in Shandong
Happy Fan Shop	Green Intelligent Power Saver	This product has no known effect	Jingdong prefecture level city in Shandong
Happy Fan Shop	Green Intelligent Power Saver	Refund Returns $16	Jingdong prefecture level city in Shandong
Happy Fan Shop	Green Intelligent Power Saver	The previous year bought a, almost every month can save a 70 dollars or so, used a year, no problem at all, look very new like, this time to move to a new home decisively repurchase 2 units, very believe this	Jingdong prefecture level city in Shandong
Happy Fan Shop	Green Intelligent Power Saver	Has been the second time to buy, this power saving device is really good power, before every month to spend 300 electricity, now are about 200, last time that 6, this time to help lead the family to buy 6,	Jingdong prefecture level city in Shandong
Happy Fan Shop	Green Intelligent Power Saver	In the spirit of trying to experience the new product, the use of down compared to the usual power consumption, with the addition of the quarter, is indeed a lot less electricity, originally more than 450	Jingdong prefecture level city in Shandong
Happy Fan Shop	Green Intelligent Power Saver	Has not been the first time to buy back, good use, power saving device is really powerful, before the monthly electricity bill of more than 500 Now more than 300, this time to help neighbors to buy several	Jingdong prefecture level city in Shandong
Happy Fan Shop	Green Intelligent Power Saver	Home appliances are very many, the circuit is also very complex, at first it is to save 9% of the electricity, and then used more than half a month, found that the power saving rate is as high as more	Jingdong prefecture level city in Shandong
Happy Fan Shop	Green Intelligent Power Saver	My friend bought it last year and saved hundreds of dollars on his electricity bill in one summer Appearance and style: it looks better than the one I bought before Quality is also good with a light.	Jingdong prefecture level city in Shandong
Happy Fan Shop	Green Intelligent Power Saver	Saver received, on the spot on the plug with the feeling of immediate power savings, appearance, packaging is very delicate, very satisfied, I hope to achieve satisfactory results!	Jingdong prefecture level city in Shandong
Happy Fan Shop	Green Intelligent Power Saver	The power saving effect is very good, is considered an old customer, also bought before, this time is to buy two for mom and dad, customer service attitude is good, praise!	Jingdong prefecture level city in Shandong
Happy Fan Shop	Green Intelligent Power Saver	After observing for a while, it really saves a lot of money I didn't realize it was still really reliable, so I'll give you a good review.	Jingdong prefecture level city in Shandong
Happy Fan Shop	Green Intelligent Power Saver	My family a month with this monthly almost save more than 200, power-saving effect: have bought a second time, really effective Cost-effective: not bad Appearance style: roughly the same A few months to the home electricity is indeed less, this time it is a re-buy three, send a friend two, they stay a	Jingdong prefecture level city in Shandong
Happy Fan Shop	Green Intelligent Power Saver	A month almost save more than 150 dollars in electricity, home appliances more, the main this can also protect high-power appliances, after using this device, these two months have not burned, things are very good, it is said that this is the latest model, the overall price is cheap and beautiful!	Jingdong prefecture level city in Shandong
Happy Fan Shop	Green Intelligent Power Saver	Received, powerful, great quality, love the style😍	Jingdong prefecture level city in Shandong
Happy Fan Shop	Green Intelligent Power Saver	This intelligent energy saver is especially good to use and save electricity hope everyone will buy it!	Jingdong prefecture level city in Shandong
Happy Fan Shop	Green Intelligent Power Saver	Air conditioning adapter effect is particularly great, the use of very simple, plug into the socket on the summer air conditioning power is too large, electricity is also expensive, with this product directly on the electricity bill to save half, has been purchased a few more, the effect is too awesome!	Jingdong prefecture level city in Shandong
Happy Fan Shop	Green Intelligent Power Saver	The family plugged in 5, monthly electricity savings of about 300 yuan. My family before a month of electricity costs more than 800, this time with this saver after the electricity bill is actually only more than 500, amazing, thanks to the store, will recommend other friends to buy!	Jingdong prefecture level city in Shandong
Happy Fan Shop	Green Intelligent Power Saver	It was recommended to me by a neighbor's family and it's nice to use it to save power.	Jingdong prefecture level city in Shandong
Happy Fan Shop	Green Intelligent Power Saver	Second time I've purchased it and the power saving is effective!	Jingdong prefecture level city in Shandong
Happy Fan Shop	Green Intelligent Power Saver	The effect is really good, before a month of more than 300 electricity, now every month every day with the air conditioning, it is more than 100 electricity! This thing is really amazing, one after another to buy and 5.6, home installed 3!	Jingdong prefecture level city in Shandong
Happy Fan Shop	Green Intelligent Power Saver	Received this product, began to use, small things look very wrong, can save a little electricity, the monthly electricity bill is too high, after plugging in did not pull, or save a lot of electricity, point	Jingdong prefecture level city in Shandong
Happy Fan Shop	Green Intelligent Power Saver	Simple and convenient, just that plug in, you can save electricity. A month can save dozens of dollars, a year down to save a lot of, very good product cost-effective, everyone can afford to buy and use!	Jingdong prefecture level city in Shandong
Happy Fan Shop	Green Intelligent Power Saver	Electricity bill from more than 200 yuan per month, down to more than 150 yuan, the original bought home, used two months, really have power-saving effect, this time and sold two, business service attitude is	Jingdong prefecture level city in Shandong
Happy Fan Shop	Green Intelligent Power Saver	Like to watch some movies at home and so on, sometimes at night until two or three o'clock, and recently the air conditioning is not turned off for a night, rather wasteful, I have to say that the effect is very good, quite like it!	Jingdong prefecture level city in Shandong
Happy Fan Shop	Green Intelligent Power Saver	Power-saving effect: the effect is very good, a month to save more than 200 dollars Cost-effective: good value for money Appearance and style: small and lovely Workmanship material: okay Experience: good	Jingdong prefecture level city in Shandong
Happy Fan Shop	Green Intelligent Power Saver	It's okay I quite like it, the stuff is very good. Come back in the future. You can rest assured to buy. Cost-effective all. Much cheaper than the physical store	Jingdong prefecture level city in Shandong
Happy Fan Shop	Green Intelligent Power Saver	More than 150 less, last month's electricity bill is almost 400, this month is only about 200, really save electricity, I am home to buy three, I did not expect a month to pay back	Jingdong prefecture level city in Shandong
Happy Fan Shop	Green Intelligent Power Saver	I bought three of these first and tried them for two months and they still felt useful, so now I've bought another one. Good and recommended!	Jingdong prefecture level city in Shandong
Happy Fan Shop	Green Intelligent Power Saver	This energy saver is very good quality and inexpensive next time I will buy it again!	Jingdong prefecture level city in Shandong
Happy Fan Shop	Green Intelligent Power Saver	Merchant service attitude is not good, the quality does not match, the business does not give the package return	Jingdong prefecture level city in Shandong
Happy Fan Shop	Green Intelligent Power Saver	The goods are very small do not know how effective after a few days to follow up the review	Jingdong prefecture level city in Shandong
Happy Fan Shop	Green Intelligent Power Saver	Not used before more than 200 a month, after using about 100 a month, my family used three, customer service said I use electricity recommended 2-3, I think more than one effect is better on the purchase of 4, the effect is very obvious, recommended to buy!	Jingdong prefecture level city in Shandong
Happy Fan Shop	Green Intelligent Power Saver	It's okay. I'll buy it again today.	Jingdong prefecture level city in Shandong
Happy Fan Shop	Green Intelligent Power Saver	Finally received the goods, very, very much like,, has been a lot of people praise, really too happy, has been his family's loyal fans, will continue to buy buy buy!	Jingdong prefecture level city in Shandong
Happy Fan Shop	Green Intelligent Power Saver	After using this, my monthly electricity bill was 1,200 pounds, now it's only 180 bucks a month, how good is it?	Jingdong prefecture level city in Shandong
Happy Fan Shop	Green Intelligent Power Saver	The item is a scam return to marry a dozen dollars in shipping fees. Buy terrific role is purely a scam.	Jingdong prefecture level city in Shandong
Happy Fan Shop	Green Intelligent Power Saver	It's really good, cost-effective and good quality, and the logistics are fast! It arrived in one day!	Jingdong prefecture level city in Shandong
Happy Fan Shop	Green Intelligent Power Saver	Great brand! Trustworthy! Works super well! Expensive for a reason! Great merchandise!	Jingdong prefecture level city in Shandong
Happy Fan Shop	Green Intelligent Power Saver	The baby is really great, I like it very much, my friends also say good to come, recommended to buy!	Jingdong prefecture level city in Shandong
Happy Fan Shop	Green Intelligent Power Saver	Fake couldn't be more fake. Bought one plugged in still so much electricity.	Jingdong prefecture level city in Shandong
Happy Fan Shop	Green Intelligent Power Saver	This appliance works great. Let's see how it works. We'll see later.	Jingdong prefecture level city in Shandong
Happy Fan Shop	Green Intelligent Power Saver	Used it before commenting, very happy with it	Jingdong prefecture level city in Shandong
Happy Fan Shop	Green Intelligent Power Saver	Goods have been received well-packed, logistics is very fast and good quality	Jingdong prefecture level city in Shandong

Happy Fan Shop	Green Intelligent Power Saver	This baby's quality is okay.	Jingdong prefecture level city in Shandong
Happy Fan Shop	Green Intelligent Power Saver	Counterfeit goods.	Jingdong prefecture level city in Shandong
Happy Fan Shop	Green Intelligent Power Saver	The effect is not very obvious	Jingdong prefecture level city in Shandong
Happy Fan Shop	Green Intelligent Power Saver	It's not the same thing.	Jingdong prefecture level city in Shandong
Happy Fan Shop	Green Intelligent Power Saver	There is no load how much w seems false, nothing works.	Jingdong prefecture level city in Shandong
Happy Fan Shop	Green Intelligent Power Saver	I received the item yesterday, got it back and plugged it in, and it used a little more power yesterday than I had before	Jingdong prefecture level city in Shandong
Happy Fan Shop	Green Intelligent Power Saver	Haven't seen the power saving mode yet	Jingdong prefecture level city in Shandong
Happy Fan Shop	Green Intelligent Power Saver	This product has no known effect	Jingdong prefecture level city in Shandong
Happy Fan Shop	Green Intelligent Power Saver	Refund Returns $16	Jingdong prefecture level city in Shandong
Happy Fan Shop	Green Intelligent Power Saver	The previous year bought a, almost every month can save a 70 dollars or so, used a year, no problem at all, look very new like, this time to move to a new home decisively repurchase 2 units, very believe this	Jingdong prefecture level city in Shandong
Happy Fan Shop	Green Intelligent Power Saver	Has been the second time to buy, this power saving device is really good power, before every month to spend 300 electricity, now are about 200, last time that 6, this time to help lead the family to buy 6,	Jingdong prefecture level city in Shandong
Happy Fan Shop	Green Intelligent Power Saver	In the spirit of trying to experience the new product, the use of down compared to the usual power consumption, with the addition of the quarter, is indeed a lot less electricity, originally more than 450	Jingdong prefecture level city in Shandong
Happy Fan Shop	Green Intelligent Power Saver	Has not been the first time to buy back, good use, power saving device is really powerful, before the monthly electricity bill of more than 500 Now more than 300, this time to help neighbors to buy several	Jingdong prefecture level city in Shandong
Happy Fan Shop	Green Intelligent Power Saver	Home appliances are very many, the circuit is also very complex, at first it is to save 9% of the electricity, and then used more than half a month, found that the power saving rate is as high as more	Jingdong prefecture level city in Shandong
Happy Fan Shop	Green Intelligent Power Saver	My friend bought it last year and saved hundreds of dollars on his electricity bill in one summer Appearance and style: it looks better than the one I bought before Quality is also good with a light.	Jingdong prefecture level city in Shandong
Happy Fan Shop	Green Intelligent Power Saver	Saver received, on the spot on the plug with the feeling of immediate power savings, appearance, packaging is very delicate, very satisfied, I hope to achieve satisfactory results!	Jingdong prefecture level city in Shandong
Happy Fan Shop	Green Intelligent Power Saver	The power saving effect is very good, is considered an old customer, also bought before, this time is to buy two for mom and dad, customer service attitude is good, praise!	Jingdong prefecture level city in Shandong
Happy Fan Shop	Green Intelligent Power Saver	After observing for a while, it really saves a lot of money I didn't realize it was still really reliable, so I'll give you a good review.	Jingdong prefecture level city in Shandong
Happy Fan Shop	Green Intelligent Power Saver	My family a month with this monthly almost save more than 200, power-saving effect: have bought a second time, really effective Cost-effective: not bad Appearance style: roughly the same A few months to the home electricity is indeed less, this time it is a re-buy three, send a friend two, they stay a	Jingdong prefecture level city in Shandong
Happy Fan Shop	Green Intelligent Power Saver	A month almost save more than 150 dollars in electricity, home appliances more, the main this can also protect high-power appliances, after using this device, these two months have not burned, things are very good, it is said that this is the latest model, the overall price is cheap and beautiful!	Jingdong prefecture level city in Shandong
Happy Fan Shop	Green Intelligent Power Saver	Received, powerful, great quality, love the style😍	Jingdong prefecture level city in Shandong
Happy Fan Shop	Green Intelligent Power Saver	This intelligent energy saver is especially good to use and save electricity hope everyone will buy it!	Jingdong prefecture level city in Shandong
Happy Fan Shop	Green Intelligent Power Saver	Air conditioning adapter effect is particularly great, the use of very simple, plug into the socket on the summer air conditioning power is too large, electricity is also expensive, with this product directly on the electricity bill to save half, has been purchased a few more, the effect is too awesome!	Jingdong prefecture level city in Shandong
Happy Fan Shop	Green Intelligent Power Saver	The family plugged in 5, monthly electricity savings of about 300 yuan. My family before a month of electricity costs more than 800, this time with this saver after the electricity bill is actually only more than 500, amazing, thanks to the store, will recommend other friends to buy!	Jingdong prefecture level city in Shandong
Happy Fan Shop	Green Intelligent Power Saver	It was recommended to me by a neighbor's family and it's nice to use it to save power.	Jingdong prefecture level city in Shandong
Happy Fan Shop	Green Intelligent Power Saver	Second time I've purchased it and the power saving is effective!	Jingdong prefecture level city in Shandong
Happy Fan Shop	Green Intelligent Power Saver	The effect is really good, before a month of more than 300 electricity, now every month every day with the air conditioning, it is more than 100 electricity! This thing is really amazing, one after another to buy and 5.6, home installed 3!	Jingdong prefecture level city in Shandong
Happy Fan Shop	Green Intelligent Power Saver	Received this product, began to use, small things look very wrong, can save a little electricity, the monthly electricity bill is too high, after plugging in did not pull, or save a lot of electricity, point	Jingdong prefecture level city in Shandong
Happy Fan Shop	Green Intelligent Power Saver	Simple and convenient, just that plug in, you can save electricity. A month can save dozens of dollars, a year down to save a lot of, very good product cost-effective, everyone can afford to buy and use!	Jingdong prefecture level city in Shandong
Happy Fan Shop	Green Intelligent Power Saver	Electricity bill from more than 200 yuan per month, down to more than 150 yuan, the original bought home, used two months, really have power-saving effect, this time and sold two, business service attitude is	Jingdong prefecture level city in Shandong
Happy Fan Shop	Green Intelligent Power Saver	Like to watch some movies at home and so on, sometimes at night until two or three o'clock, and recently the air conditioning is not turned off for a night, rather wasteful, I have to say that the effect is very good, quite like it!	Jingdong prefecture level city in Shandong
Happy Fan Shop	Green Intelligent Power Saver	Power-saving effect: the effect is very good, a month to save more than 200 dollars Cost-effective: good value for money Appearance and style: small and lovely Workmanship material: okay Experience: good	Jingdong prefecture level city in Shandong
Happy Fan Shop	Green Intelligent Power Saver	It's okay I quite like it, the stuff is very good. Come back in the future. You can rest assured to buy. Cost-effective all. Much cheaper than the physical store	Jingdong prefecture level city in Shandong
Happy Fan Shop	Green Intelligent Power Saver	More than 150 less, last month's electricity bill is almost 400, this month is only about 200, really save electricity, I am home to buy three, I did not expect a month to pay back	Jingdong prefecture level city in Shandong
Happy Fan Shop	Green Intelligent Power Saver	I bought three of these first and tried them for two months and they still felt useful, so now I've bought another one. Good and recommended!	Jingdong prefecture level city in Shandong
Happy Fan Shop	Green Intelligent Power Saver	This energy saver is very good quality and inexpensive next time I will buy it again!	Jingdong prefecture level city in Shandong
Happy Fan Shop	Green Intelligent Power Saver	Merchant service attitude is not good, the quality does not match, the business does not give the package return	Jingdong prefecture level city in Shandong
Happy Fan Shop	Green Intelligent Power Saver	The goods are very small do not know how effective after a few days to follow up the review	Jingdong prefecture level city in Shandong
Happy Fan Shop	Green Intelligent Power Saver	Not used before more than 200 a month, after using about 100 a month, my family used three, customer service said I use electricity recommended 2-3, I think more than one effect is better on the purchase of 4, the effect is very obvious, recommended to buy!	Jingdong prefecture level city in Shandong
Happy Fan Shop	Green Intelligent Power Saver	It's okay. I'll buy it again today.	Jingdong prefecture level city in Shandong
Happy Fan Shop	Green Intelligent Power Saver	Finally received the goods, very, very much like,, has been a lot of people praise, really too happy, has been his family's loyal fans, will continue to buy buy buy!	Jingdong prefecture level city in Shandong
Happy Fan Shop	Green Intelligent Power Saver	After using this, my monthly electricity bill was 1,200 pounds, now it's only 180 bucks a month, how good is it?	Jingdong prefecture level city in Shandong
Happy Fan Shop	Green Intelligent Power Saver	The item is a scam return to marry a dozen dollars in shipping fees. Buy terrific role is purely a scam.	Jingdong prefecture level city in Shandong
Happy Fan Shop	Green Intelligent Power Saver	It's really good, cost-effective and good quality, and the logistics are fast! It arrived in one day!	Jingdong prefecture level city in Shandong
Happy Fan Shop	Green Intelligent Power Saver	Great brand! Trustworthy! Works super well! Expensive for a reason! Great merchandise!	Jingdong prefecture level city in Shandong
Happy Fan Shop	Green Intelligent Power Saver	The baby is really great, I like it very much, my friends also say good to come, recommended to buy!	Jingdong prefecture level city in Shandong
Happy Fan Shop	Green Intelligent Power Saver	Fake couldn't be more fake. Bought one plugged in still so much electricity.	Jingdong prefecture level city in Shandong
Happy Fan Shop	Green Intelligent Power Saver	This appliance works great. Let's see how it works. We'll see later.	Jingdong prefecture level city in Shandong

Happy Fan Shop	Green Intelligent Power Saver	Used it before commenting, very happy with it	Jingdong prefecture level city in Shandong
Happy Fan Shop	Green Intelligent Power Saver	Goods have been received well-packed, logistics is very fast and good quality	Jingdong prefecture level city in Shandong
Happy Fan Shop	Green Intelligent Power Saver	This baby's quality is okay.	Jingdong prefecture level city in Shandong
Happy Fan Shop	Green Intelligent Power Saver	Counterfeit goods.	Jingdong prefecture level city in Shandong
Happy Fan Shop	Green Intelligent Power Saver	The effect is not very obvious	Jingdong prefecture level city in Shandong
Happy Fan Shop	Green Intelligent Power Saver	It's not the same thing.	Jingdong prefecture level city in Shandong
Happy Fan Shop	Green Intelligent Power Saver	There is no load how much w seems false, nothing works.	Jingdong prefecture level city in Shandong
Happy Fan Shop	Green Intelligent Power Saver	I received the item yesterday, got it back and plugged it in, and it used a little more power yesterday than I had before	Jingdong prefecture level city in Shandong
Happy Fan Shop	Green Intelligent Power Saver	Haven't seen the power saving mode yet	Jingdong prefecture level city in Shandong
Happy Fan Shop	Green Intelligent Power Saver	This product has no known effect	Jingdong prefecture level city in Shandong
Happy Fan Shop	Green Intelligent Power Saver	Refund Returns $16	Jingdong prefecture level city in Shandong
Happy Fan Shop	Green Intelligent Power Saver	The previous year bought a, almost every month can save a 70 dollars or so, used a year, no problem at all, look very new like, this time to move to a new home decisively repurchase 2 units, very believe this	Jingdong prefecture level city in Shandong
Happy Fan Shop	Green Intelligent Power Saver	Has been the second time to buy, this power saving device is really good power, before every month to spend 300 electricity, now are about 200, last time that 6, this time to help lead the family to buy 6,	Jingdong prefecture level city in Shandong
Happy Fan Shop	Green Intelligent Power Saver	In the spirit of trying to experience the new product, the use of down compared to the usual power consumption, with the addition of the quarter, is indeed a lot less electricity, originally more than 450	Jingdong prefecture level city in Shandong
Happy Fan Shop	Green Intelligent Power Saver	Has not been the first time to buy back, good use, power saving device is really powerful, before the monthly electricity bill of more than 500 Now more than 300, this time to help neighbors to buy several	Jingdong prefecture level city in Shandong
Happy Fan Shop	Green Intelligent Power Saver	Home appliances are very many, the circuit is also very complex, at first it is to save 9% of the electricity, and then used more than half a month, found that the power saving rate is as high as more	Jingdong prefecture level city in Shandong
Happy Fan Shop	Green Intelligent Power Saver	My friend bought it last year and saved hundreds of dollars on his electricity bill in one summer Appearance and style: it looks better than the one I bought before Quality is also good with a light,	Jingdong prefecture level city in Shandong
Happy Fan Shop	Green Intelligent Power Saver	Saver received, on the spot on the plug with the feeling of immediate power savings, appearance, packaging is very delicate, very satisfied, I hope to achieve satisfactory results!	Jingdong prefecture level city in Shandong
Happy Fan Shop	Green Intelligent Power Saver	The power saving effect is very good, is considered an old customer, also bought before, this time is to buy two for mom and dad, customer service attitude is good, praise!	Jingdong prefecture level city in Shandong
Happy Fan Shop	Green Intelligent Power Saver	After observing for a while, it really saves a lot of money I didn't realize it was still really reliable, so I'll give you a good review.	Jingdong prefecture level city in Shandong
Happy Fan Shop	Green Intelligent Power Saver	My family a month with this monthly almost save more than 200, power-saving effect: have bought a second time, really effective Cost-effective: not bad Appearance style: roughly the same A few months to the home electricity is indeed less, this time it is a re-buy three, send a friend two, they stay a	Jingdong prefecture level city in Shandong
Happy Fan Shop	Green Intelligent Power Saver	A month almost save more than 150 dollars in electricity, home appliances more, the main this can also protect high-power appliances, after using this device, these two months have not burned, things are very good, it is said that this is the latest model, the overall price is cheap and beautiful!	Jingdong prefecture level city in Shandong
Happy Fan Shop	Green Intelligent Power Saver	Received, powerful, great quality, love the style😍	Jingdong prefecture level city in Shandong
Happy Fan Shop	Green Intelligent Power Saver	This intelligent energy saver is especially good to use and save electricity hope everyone will buy it!	Jingdong prefecture level city in Shandong
Happy Fan Shop	Green Intelligent Power Saver	Air conditioning adapter effect is particularly great, the use of very simple, plug into the socket on the summer air conditioning power is too large, electricity is also expensive, with this product directly on the electricity bill to save half, has been purchased a few more, the effect is too awesome!	Jingdong prefecture level city in Shandong
Happy Fan Shop	Green Intelligent Power Saver	The family plugged in 5, monthly electricity savings of about 300 yuan. My family before a month of electricity costs more than 800, this time with this saver after the electricity bill is actually only more than 500, amazing, thanks to the store, will recommend other friends to buy!	Jingdong prefecture level city in Shandong
Happy Fan Shop	Green Intelligent Power Saver	It was recommended to me by a neighbor's family and it's nice to use it to save power.	Jingdong prefecture level city in Shandong
Happy Fan Shop	Green Intelligent Power Saver	Second time I've purchased it and the power saving is effective!	Jingdong prefecture level city in Shandong
Happy Fan Shop	Green Intelligent Power Saver	The effect is really good, before a month of more than 300 electricity, now every month every day with the air conditioning, it is more than 100 electricity! This thing is really amazing, one after another to buy and 5.6, home installed 3!	Jingdong prefecture level city in Shandong
Happy Fan Shop	Green Intelligent Power Saver	Received this product, began to use, small things look very wrong, can save a little electricity, the monthly electricity bill is too high, after plugging in did not pull, or save a lot of electricity, point	Jingdong prefecture level city in Shandong
Happy Fan Shop	Green Intelligent Power Saver	Simple and convenient, just that plug in, you can save electricity. A month can save dozens of dollars, a year down to save a lot of, very good product cost-effective, everyone can afford to buy and use!	Jingdong prefecture level city in Shandong
Happy Fan Shop	Green Intelligent Power Saver	Electricity bill from more than 200 yuan per month, down to more than 150 yuan, the original bought home, used two months, really have power-saving effect, this time and sold two, business service attitude is	Jingdong prefecture level city in Shandong
Happy Fan Shop	Green Intelligent Power Saver	Like to watch some movies at home and so on, sometimes at night until two or three o'clock, and recently the air conditioning is not turned off for a night, rather wasteful, I have to say that the effect is very good, quite like it!	Jingdong prefecture level city in Shandong
Happy Fan Shop	Green Intelligent Power Saver	Power-saving effect: the effect is very good, a month to save more than 200 dollars Cost-effective: good value for money Appearance and style: small and lovely Workmanship material: okay Experience: good	Jingdong prefecture level city in Shandong
Happy Fan Shop	Green Intelligent Power Saver	It's okay I quite like it, the stuff is very good. Come back in the future. You can rest assured to buy. Cost-effective all. Much cheaper than the physical store	Jingdong prefecture level city in Shandong
Happy Fan Shop	Green Intelligent Power Saver	More than 150 less, last month's electricity bill is almost 400, this month is only about 200, really save electricity, I am home to buy three, I did not expect a month to pay back	Jingdong prefecture level city in Shandong
Happy Fan Shop	Green Intelligent Power Saver	I bought three of these first and tried them for two months and they still felt useful, so now I've bought another one. Good and recommended!	Jingdong prefecture level city in Shandong
Happy Fan Shop	Green Intelligent Power Saver	This energy saver is very good quality and inexpensive next time I will buy it again!	Jingdong prefecture level city in Shandong
Happy Fan Shop	Green Intelligent Power Saver	Merchant service attitude is not good, the quality does not match, the business does not give the package return	Jingdong prefecture level city in Shandong
Happy Fan Shop	Green Intelligent Power Saver	The goods are very small do not know how effective after a few days to follow up the review	Jingdong prefecture level city in Shandong
Happy Fan Shop	Green Intelligent Power Saver	Not used before more than 200 a month, after using about 100 a month, my family used three, customer service said I use electricity recommended 2-3, I think more than one effect is better on the purchase of 4, the effect is very obvious, recommended to buy!	Jingdong prefecture level city in Shandong
Happy Fan Shop	Green Intelligent Power Saver	It's okay. I'll buy it again today.	Jingdong prefecture level city in Shandong
Happy Fan Shop	Green Intelligent Power Saver	Finally received the goods, very, very much like,, has been a lot of people praise, really too happy, has been his family's loyal fans, will continue to buy buy buy buy!	Jingdong prefecture level city in Shandong
Happy Fan Shop	Green Intelligent Power Saver	After using this, my monthly electricity bill was 1,200 pounds, now it's only 180 bucks a month, how good is it?	Jingdong prefecture level city in Shandong
Happy Fan Shop	Green Intelligent Power Saver	The item is a scam return to marry a dozen dollars in shipping fees. Buy terrific role is purely a scam.	Jingdong prefecture level city in Shandong
Happy Fan Shop	Green Intelligent Power Saver	It's really good, cost-effective and good quality, and the logistics are fast! It arrived in one day!	Jingdong prefecture level city in Shandong
Happy Fan Shop	Green Intelligent Power Saver	Great brand! Trustworthy! Works super well! Expensive for a reason! Great merchandise!	Jingdong prefecture level city in Shandong
Happy Fan Shop	Green Intelligent Power Saver	The baby is really great, I like it very much, my friends also say good to come, recommended to buy!	Jingdong prefecture level city in Shandong

Happy Fan Shop	Green Intelligent Power Saver	Fake couldn't be more fake. Bought one plugged in still so much electricity.	Jingdong prefecture level city in Shandong
Happy Fan Shop	Green Intelligent Power Saver	This appliance works great. Let's see how it works. We'll see later.	Jingdong prefecture level city in Shandong
Happy Fan Shop	Green Intelligent Power Saver	Used it before commenting, very happy with it	Jingdong prefecture level city in Shandong
Happy Fan Shop	Green Intelligent Power Saver	Goods have been received well-packed, logistics is very fast and good quality	Jingdong prefecture level city in Shandong
Happy Fan Shop	Green Intelligent Power Saver	This baby's quality is okay.	Jingdong prefecture level city in Shandong
Happy Fan Shop	Green Intelligent Power Saver	Counterfeit goods.	Jingdong prefecture level city in Shandong
Happy Fan Shop	Green Intelligent Power Saver	The effect is not very obvious	Jingdong prefecture level city in Shandong
Happy Fan Shop	Green Intelligent Power Saver	It's not the same thing.	Jingdong prefecture level city in Shandong
Happy Fan Shop	Green Intelligent Power Saver	There is no load how much w seems false, nothing works.	Jingdong prefecture level city in Shandong
Happy Fan Shop	Green Intelligent Power Saver	I received the item yesterday, got it back and plugged it in, and it used a little more power yesterday than I had before	Jingdong prefecture level city in Shandong
Happy Fan Shop	Green Intelligent Power Saver	Haven't seen the power saving mode yet	Jingdong prefecture level city in Shandong
Happy Fan Shop	Green Intelligent Power Saver	This product has no known effect	Jingdong prefecture level city in Shandong
Happy Fan Shop	Green Intelligent Power Saver	Refund Returns $16	Jingdong prefecture level city in Shandong
Happy Fan Shop	Green Intelligent Power Saver	The previous year bought a, almost every month can save a 70 dollars or so, used a year, no problem at all, look very new like, this time to move to a new home decisively repurchase 2 units, very believe this	Jingdong prefecture level city in Shandong
Happy Fan Shop	Green Intelligent Power Saver	Has been the second time to buy, this power saving device is really good power, before every month to spend 300 electricity, now are about 200, last time that 6, this time to help lead the family to buy 6,	Jingdong prefecture level city in Shandong
Happy Fan Shop	Green Intelligent Power Saver	In the spirit of trying to experience the new product, the use of down compared to the usual power consumption, with the addition of the quarter, is indeed a lot less electricity, originally more than 450	Jingdong prefecture level city in Shandong
Happy Fan Shop	Green Intelligent Power Saver	Has not been the first time to buy back, good use, power saving device is really powerful, before the monthly electricity bill of more than 500 Now more than 300, this time to help neighbors to buy several	Jingdong prefecture level city in Shandong
Happy Fan Shop	Green Intelligent Power Saver	Home appliances are very many, the circuit is also very complex, at first it is to save 9% of the electricity, and then used more than half a month, found that the power saving rate is as high as more	Jingdong prefecture level city in Shandong
Happy Fan Shop	Green Intelligent Power Saver	My friend bought it last year and saved hundreds of dollars on his electricity bill in one summer Appearance and style: it looks better than the one I bought before Quality is also good with a light.	Jingdong prefecture level city in Shandong
Happy Fan Shop	Green Intelligent Power Saver	Saver received, on the spot on the plug with the feeling of immediate power savings, appearance, packaging is very delicate, very satisfied, I hope to achieve satisfactory results!	Jingdong prefecture level city in Shandong
Happy Fan Shop	Green Intelligent Power Saver	The power saving effect is very good, is considered an old customer, also bought before, this time is to buy two for mom and dad, customer service attitude is good, praise!	Jingdong prefecture level city in Shandong
Happy Fan Shop	Green Intelligent Power Saver	After observing for a while, it really saves a lot of money I didn't realize it was still really reliable, so I'll give you a good review.	Jingdong prefecture level city in Shandong
Happy Fan Shop	Green Intelligent Power Saver	My family a month with this monthly almost save more than 200, power-saving effect: have bought a second time, really effective Cost-effective: not bad Appearance style: roughly the same A few months to the home electricity is indeed less, this time it is a re-buy three, send a friend two, they stay a	Jingdong prefecture level city in Shandong
Happy Fan Shop	Green Intelligent Power Saver	A month almost save more than 150 dollars in electricity, home appliances more, the main this can also protect high-power appliances, after using this device, these two months have not burned, things are very good, it is said that this is the latest model, the overall price is cheap and beautiful!	Jingdong prefecture level city in Shandong
Happy Fan Shop	Green Intelligent Power Saver	Received, powerful, great quality, love the style😍	Jingdong prefecture level city in Shandong
Happy Fan Shop	Green Intelligent Power Saver	This intelligent energy saver is especially good to use and save electricity hope everyone will buy it!	Jingdong prefecture level city in Shandong
Happy Fan Shop	Green Intelligent Power Saver	Air conditioning adapter effect is particularly great, the use of very simple, plug into the socket on the summer air conditioning power is too large, electricity is also expensive, with this product directly on the electricity bill to save half, has been purchased a few more, the effect is too awesome!	Jingdong prefecture level city in Shandong
Happy Fan Shop	Green Intelligent Power Saver	The family plugged in 5, monthly electricity savings of about 300 yuan. My family before a month of electricity costs more than 800, this time with this saver after the electricity bill is actually only more than 500, amazing, thanks to the store, will recommend other friends to buy!	Jingdong prefecture level city in Shandong
Happy Fan Shop	Green Intelligent Power Saver	It was recommended to me by a neighbor's family and it's nice to use it to save power.	Jingdong prefecture level city in Shandong
Happy Fan Shop	Green Intelligent Power Saver	Second time I've purchased it and the power saving is effective!	Jingdong prefecture level city in Shandong
Happy Fan Shop	Green Intelligent Power Saver	The effect is really good, before a month of more than 300 electricity, now every month every day with the air conditioning, it is more than 100 electricity! This thing is really amazing, one after another to buy and 5.6, home installed 3!	Jingdong prefecture level city in Shandong
Happy Fan Shop	Green Intelligent Power Saver	Received this product, began to use, small things look very wrong, can save a little electricity, the monthly electricity bill is too high, after plugging in did not pull, or save a lot of electricity, point	Jingdong prefecture level city in Shandong
Happy Fan Shop	Green Intelligent Power Saver	Simple and convenient, just that plug in, you can save electricity. A month can save dozens of dollars, a year down to save a lot of, very good product cost-effective, everyone can afford to buy and use!	Jingdong prefecture level city in Shandong
Happy Fan Shop	Green Intelligent Power Saver	Electricity bill from more than 200 yuan per month, down to more than 150 yuan, the original bought home, used two months, really have power-saving effect, this time and sold two, business service attitude is	Jingdong prefecture level city in Shandong
Happy Fan Shop	Green Intelligent Power Saver	Like to watch some movies at home and so on, sometimes at night until two or three o'clock, and recently the air conditioning is not turned off for a night, rather wasteful, I have to say that the effect is very good, quite like it!	Jingdong prefecture level city in Shandong
Happy Fan Shop	Green Intelligent Power Saver	Power-saving effect: the effect is very good, a month to save more than 200 dollars Cost-effective: good value for money Appearance and style: small and lovely Workmanship material: okay Experience: good	Jingdong prefecture level city in Shandong
Happy Fan Shop	Green Intelligent Power Saver	It's okay I quite like it, the stuff is very good. Come back in the future. You can rest assured to buy. Cost-effective all. Much cheaper than the physical store	Jingdong prefecture level city in Shandong
Happy Fan Shop	Green Intelligent Power Saver	More than 150 less, last month's electricity bill is almost 400, this month is only about 200, really save electricity, I am home to buy three, I did not expect a month to pay back	Jingdong prefecture level city in Shandong
Happy Fan Shop	Green Intelligent Power Saver	I bought three of these first and tried them for two months and they still felt useful, so now I've bought another one. Good and recommended!	Jingdong prefecture level city in Shandong
Happy Fan Shop	Green Intelligent Power Saver	This energy saver is very good quality and inexpensive next time I will buy it again!	Jingdong prefecture level city in Shandong
Happy Fan Shop	Green Intelligent Power Saver	Merchant service attitude is not good, the quality does not match, the business does not give the package return	Jingdong prefecture level city in Shandong
Happy Fan Shop	Green Intelligent Power Saver	The goods are very small do not know how effective after a few days to follow up the review	Jingdong prefecture level city in Shandong
Happy Fan Shop	Green Intelligent Power Saver	Not used before more than 200 a month, after using about 100 a month, my family used three, customer service said I use electricity recommended 2-3, I think more than one effect is better on the purchase of 4, the effect is very obvious, recommended to buy!	Jingdong prefecture level city in Shandong
Happy Fan Shop	Green Intelligent Power Saver	It's okay. I'll buy it again today.	Jingdong prefecture level city in Shandong
Happy Fan Shop	Green Intelligent Power Saver	Finally received the goods, very, very much like,, has been a lot of people praise, really too happy, has been his family's loyal fans, will continue to buy buy buy!	Jingdong prefecture level city in Shandong
Happy Fan Shop	Green Intelligent Power Saver	After using this, my monthly electricity bill was 1,200 pounds, now it's only 180 bucks a month, how good is it?	Jingdong prefecture level city in Shandong
Happy Fan Shop	Green Intelligent Power Saver	The item is a scam return to marry a dozen dollars in shipping fees. Buy terrific role is purely a scam.	Jingdong prefecture level city in Shandong
Happy Fan Shop	Green Intelligent Power Saver	It's really good, cost-effective and good quality, and the logistics are fast! It arrived in one day!	Jingdong prefecture level city in Shandong

Happy Fan Shop	Green Intelligent Power Saver	Great brand! Trustworthy! Works super well! Expensive for a reason! Great merchandise!	Jingdong prefecture level city in Shandong
Happy Fan Shop	Green Intelligent Power Saver	The baby is really great, I like it very much, my friends also say good to come, recommended to buy!	Jingdong prefecture level city in Shandong
Happy Fan Shop	Green Intelligent Power Saver	Fake couldn't be more fake. Bought one plugged in still so much electricity.	Jingdong prefecture level city in Shandong
Happy Fan Shop	Green Intelligent Power Saver	This appliance works great. Let's see how it works. We'll see later.	Jingdong prefecture level city in Shandong
Happy Fan Shop	Green Intelligent Power Saver	Used it before commenting, very happy with it	Jingdong prefecture level city in Shandong
Happy Fan Shop	Green Intelligent Power Saver	Goods have been received well-packed, logistics is very fast and good quality	Jingdong prefecture level city in Shandong
Happy Fan Shop	Green Intelligent Power Saver	This baby's quality is okay.	Jingdong prefecture level city in Shandong
Happy Fan Shop	Green Intelligent Power Saver	Counterfeit goods.	Jingdong prefecture level city in Shandong
Happy Fan Shop	Green Intelligent Power Saver	The effect is not very obvious	Jingdong prefecture level city in Shandong
Happy Fan Shop	Green Intelligent Power Saver	It's not the same thing.	Jingdong prefecture level city in Shandong
Happy Fan Shop	Green Intelligent Power Saver	There is no load how much w seems false, nothing works.	Jingdong prefecture level city in Shandong
Happy Fan Shop	Green Intelligent Power Saver	I received the item yesterday, got it back and plugged it in, and it used a little more power yesterday than I had before	Jingdong prefecture level city in Shandong
Happy Fan Shop	Green Intelligent Power Saver	Haven't seen the power saving mode yet	Jingdong prefecture level city in Shandong
Happy Fan Shop	Green Intelligent Power Saver	This product has no known effect	Jingdong prefecture level city in Shandong
Happy Fan Shop	Green Intelligent Power Saver	Refund Returns $16	Jingdong prefecture level city in Shandong
Happy Fan Shop	Green Intelligent Power Saver	The previous year bought a, almost every month can save a 70 dollars or so, used a year, no problem at all, look very new like, this time to move to a new home decisively repurchase 2 units, very believe this	Jingdong prefecture level city in Shandong
Happy Fan Shop	Green Intelligent Power Saver	Has been the second time to buy, this power saving device is really good power, before every month to spend 300 electricity, now are about 200, last time that 6, this time to help lead the family to buy 6,	Jingdong prefecture level city in Shandong
Happy Fan Shop	Green Intelligent Power Saver	In the spirit of trying to experience the new product, the use of down compared to the usual power consumption, with the addition of the quarter, is indeed a lot less electricity, originally more than 450	Jingdong prefecture level city in Shandong
Happy Fan Shop	Green Intelligent Power Saver	Has not been the first time to buy back, good use, power saving device is really powerful, before the monthly electricity bill of more than 500 Now more than 300, this time to help neighbors to buy several	Jingdong prefecture level city in Shandong
Happy Fan Shop	Green Intelligent Power Saver	Home appliances are very many, the circuit is also very complex, at first it is to save 9% of the electricity, and then used more than half a month, found that the power saving rate is as high as more	Jingdong prefecture level city in Shandong
Happy Fan Shop	Green Intelligent Power Saver	My friend bought it last year and saved hundreds of dollars on his electricity bill in one summer Appearance and style: it looks better than the one I bought before Quality is also good with a light.	Jingdong prefecture level city in Shandong
Happy Fan Shop	Green Intelligent Power Saver	Saver received, on the spot on the plug with the feeling of immediate power savings, appearance, packaging is very delicate, very satisfied, I hope to achieve satisfactory results!	Jingdong prefecture level city in Shandong
Happy Fan Shop	Green Intelligent Power Saver	The power saving effect is very good, is considered an old customer, also bought before, this time is to buy two for mom and dad, customer service attitude is good, praise!	Jingdong prefecture level city in Shandong
Happy Fan Shop	Green Intelligent Power Saver	After observing for a while, it really saves a lot of money I didn't realize it was still really reliable, so I'll give you a good review.	Jingdong prefecture level city in Shandong
Happy Fan Shop	Green Intelligent Power Saver	My family a month with this monthly almost save more than 200, power-saving effect: have bought a second time, really effective Cost-effective: not bad Appearance style: roughly the same A few months to the home electricity is indeed less, this time it is a re-buy three, send a friend two, they stay a	Jingdong prefecture level city in Shandong
Happy Fan Shop	Green Intelligent Power Saver	A month almost save more than 150 dollars in electricity, home appliances more, the main this can also protect high-power appliances, after using this device, these two months have not burned, things are very good, it is said that this is the latest model, the overall price is cheap and beautiful!	Jingdong prefecture level city in Shandong
Happy Fan Shop	Green Intelligent Power Saver	Received, powerful, great quality, love the style😍	Jingdong prefecture level city in Shandong
Happy Fan Shop	Green Intelligent Power Saver	This intelligent energy saver is especially good to use and save electricity hope everyone will buy it!	Jingdong prefecture level city in Shandong
Happy Fan Shop	Green Intelligent Power Saver	Air conditioning adapter effect is particularly great, the use of very simple, plug into the socket on the summer air conditioning power is too large, electricity is also expensive, with this product directly on the electricity bill to save half, has been purchased a few more, the effect is too awesome!	Jingdong prefecture level city in Shandong
Happy Fan Shop	Green Intelligent Power Saver	The family plugged in 5, monthly electricity savings of about 300 yuan. My family before a month of electricity costs more than 800, this time with this saver after the electricity bill is actually only more than 500, amazing, thanks to the store, will recommend other friends to buy!	Jingdong prefecture level city in Shandong
Happy Fan Shop	Green Intelligent Power Saver	It was recommended to me by a neighbor's family and it's nice to use it to save power.	Jingdong prefecture level city in Shandong
Happy Fan Shop	Green Intelligent Power Saver	Second time I've purchased it and the power saving is effective!	Jingdong prefecture level city in Shandong
Happy Fan Shop	Green Intelligent Power Saver	The effect is really good, before a month of more than 300 electricity, now every month every day with the air conditioning, it is more than 100 electricity! This thing is really amazing, one after another to buy and 5.6, home installed 3!	Jingdong prefecture level city in Shandong
Happy Fan Shop	Green Intelligent Power Saver	Received this product, began to use, small things look very wrong, can save a little electricity, the monthly electricity bill is too high, after plugging in did not pull, or save a lot of electricity, point	Jingdong prefecture level city in Shandong
Happy Fan Shop	Green Intelligent Power Saver	Simple and convenient, just that plug in, you can save electricity. A month can save dozens of dollars, a year down to save a lot of, very good product cost-effective, everyone can afford to buy and use!	Jingdong prefecture level city in Shandong
Happy Fan Shop	Green Intelligent Power Saver	Electricity bill from more than 200 yuan per month, down to more than 150 yuan, the original bought home, used two months, really have power-saving effect, this time and sold two, business service attitude is	Jingdong prefecture level city in Shandong
Happy Fan Shop	Green Intelligent Power Saver	Like to watch some movies at home and so on, sometimes at night until two or three o'clock, and recently the air conditioning is not turned off for a night, rather wasteful, I have to say that the effect is very good, quite like it!	Jingdong prefecture level city in Shandong
Happy Fan Shop	Green Intelligent Power Saver	Power-saving effect: the effect is very good, a month to save more than 200 dollars Cost-effective: good value for money Appearance and style: small and lovely Workmanship material: okay Experience: good	Jingdong prefecture level city in Shandong
Happy Fan Shop	Green Intelligent Power Saver	It's okay I quite like it, the stuff is very good. Come back in the future. You can rest assured to buy. Cost-effective all. Much cheaper than the physical store	Jingdong prefecture level city in Shandong
Happy Fan Shop	Green Intelligent Power Saver	More than 150 less, last month's electricity bill is almost 400, this month is only about 200, really save electricity, I am home to buy three, I did not expect a month to pay back	Jingdong prefecture level city in Shandong
Happy Fan Shop	Green Intelligent Power Saver	I bought three of these first and tried them for two months and they still felt useful, so now I've bought another one. Good and recommended!	Jingdong prefecture level city in Shandong
Happy Fan Shop	Green Intelligent Power Saver	This energy saver is very good quality and inexpensive next time I will buy it again!	Jingdong prefecture level city in Shandong
Happy Fan Shop	Green Intelligent Power Saver	Merchant service attitude is not good, the quality does not match, the business does not give the package return	Jingdong prefecture level city in Shandong
Happy Fan Shop	Green Intelligent Power Saver	The goods are very small do not know how effective after a few days to follow up the review	Jingdong prefecture level city in Shandong
Happy Fan Shop	Green Intelligent Power Saver	Not used before more than 200 a month, after using about 100 a month, my family used three, customer service said I use electricity recommended 2-3, I think more than one effect is better on the purchase of 4, the effect is very obvious, recommended to buy!	Jingdong prefecture level city in Shandong
Happy Fan Shop	Green Intelligent Power Saver	It's okay. I'll buy it again today.	Jingdong prefecture level city in Shandong
Happy Fan Shop	Green Intelligent Power Saver	Finally received the goods, very, very much like,, has been a lot of people praise, really too happy, has been his family's loyal fans, will continue to buy buy buy buy!	Jingdong prefecture level city in Shandong
Happy Fan Shop	Green Intelligent Power Saver	After using this, my monthly electricity bill was 1,200 pounds, now it's only 180 bucks a month, how good is it?	Jingdong prefecture level city in Shandong

Happy Fan Shop	Green Intelligent Power Saver	The item is a scam return to marry a dozen dollars in shipping fees. Buy terrific role is purely a scam.	Jingdong prefecture level city in Shandong
Happy Fan Shop	Green Intelligent Power Saver	It's really good, cost-effective and good quality, and the logistics are fast! It arrived in one day!	Jingdong prefecture level city in Shandong
Happy Fan Shop	Green Intelligent Power Saver	Great brand! Trustworthy! Works super well! Expensive for a reason! Great merchandise!	Jingdong prefecture level city in Shandong
Happy Fan Shop	Green Intelligent Power Saver	The baby is really great, I like it very much, my friends also say good to come, recommended to buy!	Jingdong prefecture level city in Shandong
Happy Fan Shop	Green Intelligent Power Saver	Fake couldn't be more fake. Bought one plugged in still so much electricity.	Jingdong prefecture level city in Shandong
Happy Fan Shop	Green Intelligent Power Saver	This appliance works great. Let's see how it works. We'll see later.	Jingdong prefecture level city in Shandong
Happy Fan Shop	Green Intelligent Power Saver	Used it before commenting, very happy with it	Jingdong prefecture level city in Shandong
Happy Fan Shop	Green Intelligent Power Saver	Goods have been received well-packed, logistics is very fast and good quality	Jingdong prefecture level city in Shandong
Happy Fan Shop	Green Intelligent Power Saver	This baby's quality is okay.	Jingdong prefecture level city in Shandong
Happy Fan Shop	Green Intelligent Power Saver	Counterfeit goods.	Jingdong prefecture level city in Shandong
Happy Fan Shop	Green Intelligent Power Saver	The effect is not very obvious	Jingdong prefecture level city in Shandong
Happy Fan Shop	Green Intelligent Power Saver	It's not the same thing.	Jingdong prefecture level city in Shandong
Happy Fan Shop	Green Intelligent Power Saver	There is no load how much w seems false, nothing works.	Jingdong prefecture level city in Shandong
Happy Fan Shop	Green Intelligent Power Saver	I received the item yesterday, got it back and plugged it in, and it used a little more power yesterday than I had before	Jingdong prefecture level city in Shandong
Happy Fan Shop	Green Intelligent Power Saver	Haven't seen the power saving mode yet	Jingdong prefecture level city in Shandong
Happy Fan Shop	Green Intelligent Power Saver	This product has no known effect	Jingdong prefecture level city in Shandong
Happy Fan Shop	Green Intelligent Power Saver	Refund Returns $16	Jingdong prefecture level city in Shandong
Happy Fan Shop	Green Intelligent Power Saver	The previous year bought a, almost every month can save a 70 dollars or so, used a year, no problem at all, look very new like, this time to move to a new home decisively repurchase 2 units, very believe this	Jingdong prefecture level city in Shandong
Happy Fan Shop	Green Intelligent Power Saver	Has been the second time to buy, this power saving device is really good power, before every month to spend 300 electricity, now are about 200, last time that 6, this time to help lead the family to buy 6,	Jingdong prefecture level city in Shandong
Happy Fan Shop	Green Intelligent Power Saver	In the spirit of trying to experience the new product, the use of down compared to the usual power consumption, with the addition of the quarter, is indeed a lot less electricity, originally more than 450	Jingdong prefecture level city in Shandong
Happy Fan Shop	Green Intelligent Power Saver	Has not been the first time to buy back, good use, power saving device is really powerful, before the monthly electricity bill of more than 500 Now more than 300, this time to help neighbors to buy several	Jingdong prefecture level city in Shandong
Happy Fan Shop	Green Intelligent Power Saver	Home appliances are very many, the circuit is also very complex, at first it is to save 9% of the electricity, and then used more than half a month, found that the power saving rate is as high as more	Jingdong prefecture level city in Shandong
Happy Fan Shop	Green Intelligent Power Saver	My friend bought it last year and saved hundreds of dollars on his electricity bill in one summer Appearance and style: it looks better than the one I bought before Quality is also good with a light.	Jingdong prefecture level city in Shandong
Happy Fan Shop	Green Intelligent Power Saver	Saver received, on the spot on the plug with the feeling of immediate power savings, appearance, packaging is very delicate, very satisfied, I hope to achieve satisfactory results!	Jingdong prefecture level city in Shandong
Happy Fan Shop	Green Intelligent Power Saver	The power saving effect is very good, is considered an old customer, also bought before, this time is to buy two for mom and dad, customer service attitude is good, praise!	Jingdong prefecture level city in Shandong
Happy Fan Shop	Green Intelligent Power Saver	After observing for a while, it really saves a lot of money I didn't realize it was still really reliable, so I'll give you a good review.	Jingdong prefecture level city in Shandong
Happy Fan Shop	Green Intelligent Power Saver	My family a month with this monthly almost save more than 200, power-saving effect: have bought a second time, really effective Cost-effective: not bad Appearance style: roughly the same A few months to the home electricity is indeed less, this time it is a re-buy three, send a friend two, they stay a	Jingdong prefecture level city in Shandong
Happy Fan Shop	Green Intelligent Power Saver	A month almost save more than 150 dollars in electricity, home appliances more, the main this can also protect high-power appliances, after using this device, these two months have not burned, things are very good, it is said that this is the latest model, the overall price is cheap and beautiful!	Jingdong prefecture level city in Shandong
Happy Fan Shop	Green Intelligent Power Saver	Received, powerful, great quality, love the style😍	Jingdong prefecture level city in Shandong
Happy Fan Shop	Green Intelligent Power Saver	This intelligent energy saver is especially good to use and save electricity hope everyone will buy it!	Jingdong prefecture level city in Shandong
Happy Fan Shop	Green Intelligent Power Saver	Air conditioning adapter effect is particularly great, the use of very simple, plug into the socket on the summer air conditioning power is too large, electricity is also expensive, with this product directly on the electricity bill to save half, has been purchased a few more, the effect is too awesome!	Jingdong prefecture level city in Shandong
Happy Fan Shop	Green Intelligent Power Saver	The family plugged in 5, monthly electricity savings of about 300 yuan. My family before a month of electricity costs more than 800, this time with this saver after the electricity bill is actually only more than 500, amazing, thanks to the store, will recommend other friends to buy!	Jingdong prefecture level city in Shandong
Happy Fan Shop	Green Intelligent Power Saver	It was recommended to me by a neighbor's family and it's nice to use it to save power.	Jingdong prefecture level city in Shandong
Happy Fan Shop	Green Intelligent Power Saver	Second time I've purchased it and the power saving is effective!	Jingdong prefecture level city in Shandong
Happy Fan Shop	Green Intelligent Power Saver	The effect is really good, before a month of more than 300 electricity, now every month every day with the air conditioning, it is more than 100 electricity! This thing is really amazing, one after another to buy and 5.6, home installed 3!	Jingdong prefecture level city in Shandong
Happy Fan Shop	Green Intelligent Power Saver	Received this product, began to use, small things look very wrong, can save a little electricity, the monthly electricity bill is too high, after plugging in did not pull, or save a lot of electricity, point	Jingdong prefecture level city in Shandong
Happy Fan Shop	Green Intelligent Power Saver	Simple and convenient, just that plug in, you can save electricity. A month can save dozens of dollars, a year down to save a lot of, very good product cost-effective, everyone can afford to buy and use!	Jingdong prefecture level city in Shandong
Happy Fan Shop	Green Intelligent Power Saver	Electricity bill from more than 200 yuan per month, down to more than 150 yuan, the original bought home, used two months, really have power-saving effect, this time and sold two, business service attitude is	Jingdong prefecture level city in Shandong
Happy Fan Shop	Green Intelligent Power Saver	Like to watch some movies at home and so on, sometimes at night until two or three o'clock, and recently the air conditioning is not turned off for a night, rather wasteful, I have to say that the effect is very good, quite like it!	Jingdong prefecture level city in Shandong
Happy Fan Shop	Green Intelligent Power Saver	Power-saving effect: the effect is very good, a month to save more than 200 dollars Cost-effective: good value for money Appearance and style: small and lovely Workmanship material: okay Experience: good	Jingdong prefecture level city in Shandong
Happy Fan Shop	Green Intelligent Power Saver	It's okay I quite like it, the stuff is very good. Come back in the future. You can rest assured to buy. Cost-effective all. Much cheaper than the physical store	Jingdong prefecture level city in Shandong
Happy Fan Shop	Green Intelligent Power Saver	More than 150 less, last month's electricity bill is almost 400, this month is only about 200, really save electricity, I am home to buy three, I did not expect a month to pay back	Jingdong prefecture level city in Shandong
Happy Fan Shop	Green Intelligent Power Saver	I bought three of these first and tried them for two months and they still felt useful, so now I've bought another one. Good and recommended!	Jingdong prefecture level city in Shandong
Happy Fan Shop	Green Intelligent Power Saver	This energy saver is very good quality and inexpensive next time I will buy it again!	Jingdong prefecture level city in Shandong
Happy Fan Shop	Green Intelligent Power Saver	Merchant service attitude is not good, the quality does not match, the business does not give the package return	Jingdong prefecture level city in Shandong
Happy Fan Shop	Green Intelligent Power Saver	The goods are very small do not know how effective after a few days to follow up the review	Jingdong prefecture level city in Shandong
Happy Fan Shop	Green Intelligent Power Saver	Not used before more than 200 a month, after using about 100 a month, my family used three, customer service said I use electricity recommended 2-3, I think more than one effect is better on the purchase of 4, the effect is very obvious, recommended to buy!	Jingdong prefecture level city in Shandong
Happy Fan Shop	Green Intelligent Power Saver	It's okay. I'll buy it again today.	Jingdong prefecture level city in Shandong

Happy Fan Shop	Green Intelligent Power Saver	Finally received the goods, very, very much like,, has been a lot of people praise, really too happy, has been his family's loyal fans, will continue to buy buy buy buy!	Jingdong prefecture level city in Shandong
Happy Fan Shop	Green Intelligent Power Saver	After using this, my monthly electricity bill was 1,200 pounds, now it's only 180 bucks a month, how good is it?	Jingdong prefecture level city in Shandong
Happy Fan Shop	Green Intelligent Power Saver	The item is a scam return to marry a dozen dollars in shipping fees. Buy terrific role is purely a scam.	Jingdong prefecture level city in Shandong
Happy Fan Shop	Green Intelligent Power Saver	It's really good, cost-effective and good quality, and the logistics are fast! It arrived in one day!	Jingdong prefecture level city in Shandong
Happy Fan Shop	Green Intelligent Power Saver	Great brand! Trustworthy! Works super well! Expensive for a reason! Great merchandise!	Jingdong prefecture level city in Shandong
Happy Fan Shop	Green Intelligent Power Saver	The baby is really great, I like it very much, my friends also say good to come, recommended to buy!	Jingdong prefecture level city in Shandong
Happy Fan Shop	Green Intelligent Power Saver	Fake couldn't be more fake. Bought one plugged in still so much electricity.	Jingdong prefecture level city in Shandong
Happy Fan Shop	Green Intelligent Power Saver	This appliance works great. Let's see how it works. We'll see later.	Jingdong prefecture level city in Shandong
Happy Fan Shop	Green Intelligent Power Saver	Used it before commenting, very happy with it	Jingdong prefecture level city in Shandong
Happy Fan Shop	Green Intelligent Power Saver	Goods have been received well-packed, logistics is very fast and good quality	Jingdong prefecture level city in Shandong
Happy Fan Shop	Green Intelligent Power Saver	This baby's quality is okay.	Jingdong prefecture level city in Shandong
Happy Fan Shop	Green Intelligent Power Saver	Counterfeit goods.	Jingdong prefecture level city in Shandong
Happy Fan Shop	Green Intelligent Power Saver	The effect is not very obvious	Jingdong prefecture level city in Shandong
Happy Fan Shop	Green Intelligent Power Saver	It's not the same thing.	Jingdong prefecture level city in Shandong
Happy Fan Shop	Green Intelligent Power Saver	There is no load how much w seems false, nothing works.	Jingdong prefecture level city in Shandong
Happy Fan Shop	Green Intelligent Power Saver	I received the item yesterday, got it back and plugged it in, and it used a little more power yesterday than I had before	Jingdong prefecture level city in Shandong
Happy Fan Shop	Green Intelligent Power Saver	Haven't seen the power saving mode yet	Jingdong prefecture level city in Shandong
Happy Fan Shop	Green Intelligent Power Saver	This product has no known effect	Jingdong prefecture level city in Shandong
Happy Fan Shop	Green Intelligent Power Saver	Refund Returns $16	Jingdong prefecture level city in Shandong
Happy Fan Shop	Green Intelligent Power Saver	The previous year bought a, almost every month can save a 70 dollars or so, used a year, no problem at all, look very new like, this time to move to a new home decisively repurchase 2 units, very believe this	Jingdong prefecture level city in Shandong
Happy Fan Shop	Green Intelligent Power Saver	Has been the second time to buy, this power saving device is really good power, before every month to spend 300 electricity, now are about 200, last time that 6, this time to help lead the family to buy 6,	Jingdong prefecture level city in Shandong
Happy Fan Shop	Green Intelligent Power Saver	In the spirit of trying to experience the new product, the use of down compared to the usual power consumption, with the addition of the quarter, is indeed a lot less electricity, originally more than 450	Jingdong prefecture level city in Shandong
Happy Fan Shop	Green Intelligent Power Saver	Has not been the first time to buy back, good use, power saving device is really powerful, before the monthly electricity bill of more than 500 Now more than 300, this time to help neighbors to buy several	Jingdong prefecture level city in Shandong
Happy Fan Shop	Green Intelligent Power Saver	Home appliances are very many, the circuit is also very complex, at first it is to save 9% of the electricity, and then used more than half a month, found that the power saving rate is as high as more	Jingdong prefecture level city in Shandong
Happy Fan Shop	Green Intelligent Power Saver	My friend bought it last year and saved hundreds of dollars on his electricity bill in one summer Appearance and style: it looks better than the one I bought before Quality is also good with a light.	Jingdong prefecture level city in Shandong
Happy Fan Shop	Green Intelligent Power Saver	Saver received, on the spot on the plug with the feeling of immediate power savings, appearance, packaging is very delicate, very satisfied, I hope to achieve satisfactory results!	Jingdong prefecture level city in Shandong
Happy Fan Shop	Green Intelligent Power Saver	The power saving effect is very good, is considered an old customer, also bought before, this time is to buy two for mom and dad, customer service attitude is good, praise!	Jingdong prefecture level city in Shandong
Happy Fan Shop	Green Intelligent Power Saver	After observing for a while, it really saves a lot of money I didn't realize it was still really reliable, so I'll give you a good review.	Jingdong prefecture level city in Shandong
Happy Fan Shop	Green Intelligent Power Saver	My family a month with this monthly almost save more than 200, power-saving effect: have bought a second time, really effective Cost-effective: not bad Appearance style: roughly the same A few months to the home electricity is indeed less, this time it is a re-buy three, send a friend two, they stay a	Jingdong prefecture level city in Shandong
Happy Fan Shop	Green Intelligent Power Saver	A month almost save more than 150 dollars in electricity, home appliances more, the main this can also protect high-power appliances, after using this device, these two months have not burned, things are very good, it is said that this is the latest model, the overall price is cheap and beautiful!	Jingdong prefecture level city in Shandong
Happy Fan Shop	Green Intelligent Power Saver	Received, powerful, great quality, love the style😍	Jingdong prefecture level city in Shandong
Happy Fan Shop	Green Intelligent Power Saver	This intelligent energy saver is especially good to use and save electricity hope everyone will buy it!	Jingdong prefecture level city in Shandong
Happy Fan Shop	Green Intelligent Power Saver	Air conditioning adapter effect is particularly great, the use of very simple, plug into the socket on the summer air conditioning power is too large, electricity is also expensive, with this product directly on the electricity bill to save half, has been purchased a few more, the effect is too awesome!	Jingdong prefecture level city in Shandong
Happy Fan Shop	Green Intelligent Power Saver	The family plugged in 5, monthly electricity savings of about 300 yuan. My family before a month of electricity costs more than 800, this time with this saver after the electricity bill is actually only more than 500, amazing, thanks to the store, will recommend other friends to buy!	Jingdong prefecture level city in Shandong
Happy Fan Shop	Green Intelligent Power Saver	It was recommended to me by a neighbor's family and it's nice to use it to save power.	Jingdong prefecture level city in Shandong
Happy Fan Shop	Green Intelligent Power Saver	Second time I've purchased it and the power saving is effective!	Jingdong prefecture level city in Shandong
Happy Fan Shop	Green Intelligent Power Saver	The effect is really good, before a month of more than 300 electricity, now every month every day with the air conditioning, it is more than 100 electricity! This thing is really amazing, one after another to buy and 5.6, home installed 3!	Jingdong prefecture level city in Shandong
Happy Fan Shop	Green Intelligent Power Saver	Received this product, began to use, small things look very wrong, can save a little electricity, the monthly electricity bill is too high, after plugging in did not pull, or save a lot of electricity, point	Jingdong prefecture level city in Shandong
Happy Fan Shop	Green Intelligent Power Saver	Simple and convenient, just that plug in, you can save electricity. A month can save dozens of dollars, a year down to save a lot of, very good product cost-effective, everyone can afford to buy and use!	Jingdong prefecture level city in Shandong
Happy Fan Shop	Green Intelligent Power Saver	Electricity bill from more than 200 yuan per month, down to more than 150 yuan, the original bought home, used two months, really have power-saving effect, this time and sold two, business service attitude is	Jingdong prefecture level city in Shandong
Happy Fan Shop	Green Intelligent Power Saver	Like to watch some movies at home and so on, sometimes at night until two or three o'clock, and recently the air conditioning is not turned off for a night, rather wasteful, I have to say that the effect is very good, quite like it!	Jingdong prefecture level city in Shandong
Happy Fan Shop	Green Intelligent Power Saver	Power-saving effect: the effect is very good, a month to save more than 200 dollars Cost-effective: good value for money Appearance and style: small and lovely Workmanship material: okay Experience: good	Jingdong prefecture level city in Shandong
Happy Fan Shop	Green Intelligent Power Saver	It's okay I quite like it, the stuff is very good. Come back in the future. You can rest assured to buy. Cost-effective all. Much cheaper than the physical store	Jingdong prefecture level city in Shandong
Happy Fan Shop	Green Intelligent Power Saver	More than 150 less, last month's electricity bill is almost 400, this month is only about 200, really save electricity, I am home to buy three, I did not expect a month to pay back	Jingdong prefecture level city in Shandong
Happy Fan Shop	Green Intelligent Power Saver	I bought three of these first and tried them for two months and they still felt useful, so now I've bought another one. Good and recommended!	Jingdong prefecture level city in Shandong
Happy Fan Shop	Green Intelligent Power Saver	This energy saver is very good quality and inexpensive next time I will buy it again!	Jingdong prefecture level city in Shandong
Happy Fan Shop	Green Intelligent Power Saver	Merchant service attitude is not good, the quality does not match, the business does not give the package return	Jingdong prefecture level city in Shandong
Happy Fan Shop	Green Intelligent Power Saver	The goods are very small do not know how effective after a few days to follow up the review	Jingdong prefecture level city in Shandong

Happy Fan Shop	Green Intelligent Power Saver	Not used before more than 200 a month, after using about 100 a month, my family used three, customer service said I use electricity recommended 2-3, I think more than one effect is better on the purchase of 4, the effect is very obvious, recommended to buy!	Jingdong prefecture level city in Shandong
Happy Fan Shop	Green Intelligent Power Saver	It's okay. I'll buy it again today.	Jingdong prefecture level city in Shandong
Happy Fan Shop	Green Intelligent Power Saver	Finally received the goods, very, very much like,, has been a lot of people praise, really too happy, has been his family's loyal fans, will continue to buy buy buy buy!	Jingdong prefecture level city in Shandong
Happy Fan Shop	Green Intelligent Power Saver	After using this, my monthly electricity bill was 1,200 pounds, now it's only 180 bucks a month, how good is it?	Jingdong prefecture level city in Shandong
Happy Fan Shop	Green Intelligent Power Saver	The item is a scam return to marry a dozen dollars in shipping fees. Buy terrific role is purely a scam.	Jingdong prefecture level city in Shandong
Happy Fan Shop	Green Intelligent Power Saver	It's really good, cost-effective and good quality, and the logistics are fast! It arrived in one day!	Jingdong prefecture level city in Shandong
Happy Fan Shop	Green Intelligent Power Saver	Great brand! Trustworthy! Works super well! Expensive for a reason! Great merchandise!	Jingdong prefecture level city in Shandong
Happy Fan Shop	Green Intelligent Power Saver	The baby is really great, I like it very much, my friends also say good to come, recommended to buy!	Jingdong prefecture level city in Shandong
Happy Fan Shop	Green Intelligent Power Saver	Fake couldn't be more fake. Bought one plugged in still so much electricity.	Jingdong prefecture level city in Shandong
Happy Fan Shop	Green Intelligent Power Saver	This appliance works great. Let's see how it works. We'll see later.	Jingdong prefecture level city in Shandong
Happy Fan Shop	Green Intelligent Power Saver	Used it before commenting, very happy with it	Jingdong prefecture level city in Shandong
Happy Fan Shop	Green Intelligent Power Saver	Goods have been received well-packed, logistics is very fast and good quality	Jingdong prefecture level city in Shandong
Happy Fan Shop	Green Intelligent Power Saver	This baby's quality is okay.	Jingdong prefecture level city in Shandong
Happy Fan Shop	Green Intelligent Power Saver	Counterfeit goods.	Jingdong prefecture level city in Shandong
Happy Fan Shop	Green Intelligent Power Saver	The effect is not very obvious	Jingdong prefecture level city in Shandong
Happy Fan Shop	Green Intelligent Power Saver	It's not the same thing.	Jingdong prefecture level city in Shandong
Happy Fan Shop	Green Intelligent Power Saver	There is no load how much w seems false, nothing works.	Jingdong prefecture level city in Shandong
Happy Fan Shop	Green Intelligent Power Saver	I received the item yesterday, got it back and plugged it in, and it used a little more power yesterday than I had before	Jingdong prefecture level city in Shandong
Happy Fan Shop	Green Intelligent Power Saver	Haven't seen the power saving mode yet	Jingdong prefecture level city in Shandong
Happy Fan Shop	Green Intelligent Power Saver	This product has no known effect	Jingdong prefecture level city in Shandong
Happy Fan Shop	Green Intelligent Power Saver	Refund Returns $16	Jingdong prefecture level city in Shandong
Happy Fan Shop	Green Intelligent Power Saver	The previous year bought a, almost every month can save a 70 dollars or so, used a year, no problem at all, look very new like, this time to move to a new home decisively repurchase 2 units, very believe this	Jingdong prefecture level city in Shandong
Happy Fan Shop	Green Intelligent Power Saver	Has been the second time to buy, this power saving device is really good power, before every month to spend 300 electricity, now are about 200, last time that 6, this time to help lead the family to buy 6,	Jingdong prefecture level city in Shandong
Happy Fan Shop	Green Intelligent Power Saver	In the spirit of trying to experience the new product, the use of down compared to the usual power consumption, with the addition of the quarter, is indeed a lot less electricity, originally more than 450	Jingdong prefecture level city in Shandong
Happy Fan Shop	Green Intelligent Power Saver	Has not been the first time to buy back, good use, power saving device is really powerful, before the monthly electricity bill of more than 500 Now more than 300, this time to help neighbors to buy several	Jingdong prefecture level city in Shandong
Happy Fan Shop	Green Intelligent Power Saver	Home appliances are very many, the circuit is also very complex, at first it is to save 9% of the electricity, and then used more than half a month, found that the power saving rate is as high as more	Jingdong prefecture level city in Shandong
Happy Fan Shop	Green Intelligent Power Saver	My friend bought it last year and saved hundreds of dollars on his electricity bill in one summer Appearance and style: it looks better than the one I bought before Quality is also good with a light.	Jingdong prefecture level city in Shandong
Happy Fan Shop	Green Intelligent Power Saver	Saver received, on the spot on the plug with the feeling of immediate power savings, appearance, packaging is very delicate, very satisfied, I hope to achieve satisfactory results!	Jingdong prefecture level city in Shandong
Happy Fan Shop	Green Intelligent Power Saver	The power saving effect is very good, is considered an old customer, also bought before, this time is to buy two for mom and dad, customer service attitude is good, praise!	Jingdong prefecture level city in Shandong
Happy Fan Shop	Green Intelligent Power Saver	After observing for a while, it really saves a lot of money I didn't realize it was still really reliable, so I'll give you a good review.	Jingdong prefecture level city in Shandong
Happy Fan Shop	Green Intelligent Power Saver	My family a month with this monthly almost save more than 200, power-saving effect: have bought a second time, really effective Cost-effective: not bad Appearance style: roughly the same A few months to the home electricity is indeed less, this time it is a re-buy three, send a friend two, they stay a	Jingdong prefecture level city in Shandong
Happy Fan Shop	Green Intelligent Power Saver	A month almost save more than 150 dollars in electricity, home appliances more, the main this can also protect high-power appliances, after using this device, these two months have not burned, things are very good, it is said that this is the latest model, the overall price is cheap and beautiful!	Jingdong prefecture level city in Shandong
Happy Fan Shop	Green Intelligent Power Saver	Received, powerful, great quality, love the style😍	Jingdong prefecture level city in Shandong
Happy Fan Shop	Green Intelligent Power Saver	This intelligent energy saver is especially good to use and save electricity hope everyone will buy it!	Jingdong prefecture level city in Shandong
Happy Fan Shop	Green Intelligent Power Saver	Air conditioning adapter effect is particularly great, the use of very simple, plug into the socket on the summer air conditioning power is too large, electricity is also expensive, with this product directly on the electricity bill to save half, has been purchased a few more, the effect is too awesome!	Jingdong prefecture level city in Shandong
Happy Fan Shop	Green Intelligent Power Saver	The family plugged in 5, monthly electricity savings of about 300 yuan. My family before a month of electricity costs more than 800, this time with this saver after the electricity bill is actually only more than 500, amazing, thanks to the store, will recommend other friends to buy!	Jingdong prefecture level city in Shandong
Happy Fan Shop	Green Intelligent Power Saver	It was recommended to me by a neighbor's family and it's nice to use it to save power.	Jingdong prefecture level city in Shandong
Happy Fan Shop	Green Intelligent Power Saver	Second time I've purchased it and the power saving is effective!	Jingdong prefecture level city in Shandong
Happy Fan Shop	Green Intelligent Power Saver	The effect is really good, before a month of more than 300 electricity, now every month every day with the air conditioning, it is more than 100 electricity! This thing is really amazing, one after another to buy and 5.6, home installed 3!	Jingdong prefecture level city in Shandong
Happy Fan Shop	Green Intelligent Power Saver	Received this product, began to use, small things look very wrong, can save a little electricity, the monthly electricity bill is too high, after plugging in did not pull, or save a lot of electricity, point	Jingdong prefecture level city in Shandong
Happy Fan Shop	Green Intelligent Power Saver	Simple and convenient, just that plug in, you can save electricity. A month can save dozens of dollars, a year down to save a lot of, very good product cost-effective, everyone can afford to buy and use!	Jingdong prefecture level city in Shandong
Happy Fan Shop	Green Intelligent Power Saver	Electricity bill from more than 200 yuan per month, down to more than 150 yuan, the original bought home, used two months, really have power-saving effect, this time and sold two, business service attitude is	Jingdong prefecture level city in Shandong
Happy Fan Shop	Green Intelligent Power Saver	Like to watch some movies at home and so on, sometimes at night until two or three o'clock, and recently the air conditioning is not turned off for a night, rather wasteful, I have to say that the effect is very good, quite like it!	Jingdong prefecture level city in Shandong
Happy Fan Shop	Green Intelligent Power Saver	Power-saving effect: the effect is very good, a month to save more than 200 dollars Cost-effective: good value for money Appearance and style: small and lovely Workmanship material: okay Experience: good	Jingdong prefecture level city in Shandong
Happy Fan Shop	Green Intelligent Power Saver	It's okay I quite like it, the stuff is very good. Come back in the future. You can rest assured to buy. Cost-effective all. Much cheaper than the physical store	Jingdong prefecture level city in Shandong
Happy Fan Shop	Green Intelligent Power Saver	More than 150 less, last month's electricity bill is almost 400, this month is only about 200, really save electricity, I am home to buy three, I did not expect a month to pay back	Jingdong prefecture level city in Shandong
Happy Fan Shop	Green Intelligent Power Saver	I bought three of these first and tried them for two months and they still felt useful, so now I've bought another one. Good and recommended!	Jingdong prefecture level city in Shandong
Happy Fan Shop	Green Intelligent Power Saver	This energy saver is very good quality and inexpensive next time I will buy it again!	Jingdong prefecture level city in Shandong

Happy Fan Shop	Green Intelligent Power Saver	Merchant service attitude is not good, the quality does not match, the business does not give the package return	Jingdong prefecture level city in Shandong
Happy Fan Shop	Green Intelligent Power Saver	The goods are very small do not know how effective after a few days to follow up the review	Jingdong prefecture level city in Shandong
Happy Fan Shop	Green Intelligent Power Saver	Not used before more than 200 a month, after using about 100 a month, my family used three, customer service said I use electricity recommended 2-3, I think more than one effect is better on the purchase of 4, the effect is very obvious, recommended to buy!	Jingdong prefecture level city in Shandong
Happy Fan Shop	Green Intelligent Power Saver	It's okay. I'll buy it again today.	Jingdong prefecture level city in Shandong
Happy Fan Shop	Green Intelligent Power Saver	Finally received the goods, very, very much like,, has been a lot of people praise, really too happy, has been his family's loyal fans, will continue to buy buy buy buy!	Jingdong prefecture level city in Shandong
Happy Fan Shop	Green Intelligent Power Saver	After using this, my monthly electricity bill was 1,200 pounds, now it's only 180 bucks a month, how good is it?	Jingdong prefecture level city in Shandong
Happy Fan Shop	Green Intelligent Power Saver	The item is a scam return to marry a dozen dollars in shipping fees. Buy terrific role is purely a scam.	Jingdong prefecture level city in Shandong
Happy Fan Shop	Green Intelligent Power Saver	It's really good, cost-effective and good quality, and the logistics are fast! It arrived in one day!	Jingdong prefecture level city in Shandong
Happy Fan Shop	Green Intelligent Power Saver	Great brand! Trustworthy! Works super well! Expensive for a reason! Great merchandise!	Jingdong prefecture level city in Shandong
Happy Fan Shop	Green Intelligent Power Saver	The baby is really great, I like it very much, my friends also say good to come, recommended to buy!	Jingdong prefecture level city in Shandong
Happy Fan Shop	Green Intelligent Power Saver	Fake couldn't be more fake. Bought one plugged in still so much electricity.	Jingdong prefecture level city in Shandong
Happy Fan Shop	Green Intelligent Power Saver	This appliance works great. Let's see how it works. We'll see later.	Jingdong prefecture level city in Shandong
Happy Fan Shop	Green Intelligent Power Saver	Used it before commenting, very happy with it	Jingdong prefecture level city in Shandong
Happy Fan Shop	Green Intelligent Power Saver	Goods have been received well-packed, logistics is very fast and good quality	Jingdong prefecture level city in Shandong
Happy Fan Shop	Green Intelligent Power Saver	This baby's quality is okay.	Jingdong prefecture level city in Shandong
Happy Fan Shop	Green Intelligent Power Saver	Counterfeit goods.	Jingdong prefecture level city in Shandong
Happy Fan Shop	Green Intelligent Power Saver	The effect is not very obvious	Jingdong prefecture level city in Shandong
Happy Fan Shop	Green Intelligent Power Saver	It's not the same thing.	Jingdong prefecture level city in Shandong
Happy Fan Shop	Green Intelligent Power Saver	There is no load how much w seems false, nothing works.	Jingdong prefecture level city in Shandong
Happy Fan Shop	Green Intelligent Power Saver	I received the item yesterday, got it back and plugged it in, and it used a little more power yesterday than I had before	Jingdong prefecture level city in Shandong
Happy Fan Shop	Green Intelligent Power Saver	Haven't seen the power saving mode yet	Jingdong prefecture level city in Shandong
Happy Fan Shop	Green Intelligent Power Saver	This product has no known effect	Jingdong prefecture level city in Shandong
Happy Fan Shop	Green Intelligent Power Saver	Refund Returns $16	Jingdong prefecture level city in Shandong
Happy Fan Shop	Green Intelligent Power Saver	The previous year bought a, almost every month can save a 70 dollars or so, used a year, no problem at all, look very new like, this time to move to a new home decisively repurchase 2 units, very believe this	Jingdong prefecture level city in Shandong
Happy Fan Shop	Green Intelligent Power Saver	Has been the second time to buy, this power saving device is really good power, before every month to spend 300 electricity, now are about 200, last time that 6, this time to help lead the family to buy 6,	Jingdong prefecture level city in Shandong
Happy Fan Shop	Green Intelligent Power Saver	In the spirit of trying to experience the new product, the use of down compared to the usual power consumption, with the addition of the quarter, is indeed a lot less electricity, originally more than 450	Jingdong prefecture level city in Shandong
Happy Fan Shop	Green Intelligent Power Saver	Has not been the first time to buy back, good use, power saving device is really powerful, before the monthly electricity bill of more than 500 Now more than 300, this time to help neighbors to buy several	Jingdong prefecture level city in Shandong
Happy Fan Shop	Green Intelligent Power Saver	Home appliances are very many, the circuit is also very complex, at first it is to save 9% of the electricity, and then used more than half a month, found that the power saving rate is as high as more	Jingdong prefecture level city in Shandong
Happy Fan Shop	Green Intelligent Power Saver	My friend bought it last year and saved hundreds of dollars on his electricity bill in one summer Appearance and style: it looks better than the one I bought before Quality is also good with a light.	Jingdong prefecture level city in Shandong
Happy Fan Shop	Green Intelligent Power Saver	Saver received, on the spot on the plug with the feeling of immediate power savings, appearance, packaging is very delicate, very satisfied, I hope to achieve satisfactory results!	Jingdong prefecture level city in Shandong
Happy Fan Shop	Green Intelligent Power Saver	The power saving effect is very good, is considered an old customer, also bought before, this time is to buy two for mom and dad, customer service attitude is good, praise!	Jingdong prefecture level city in Shandong
Happy Fan Shop	Green Intelligent Power Saver	After observing for a while, it really saves a lot of money I didn't realize it was still really reliable, so I'll give you a good review.	Jingdong prefecture level city in Shandong
Happy Fan Shop	Green Intelligent Power Saver	My family a month with this monthly almost save more than 200, power-saving effect: have bought a second time, really effective Cost-effective: not bad Appearance style: roughly the same A few months to the home electricity is indeed less, this time it is a re-buy three, send a friend two, they stay a	Jingdong prefecture level city in Shandong
Happy Fan Shop	Green Intelligent Power Saver	A month almost save more than 150 dollars in electricity, home appliances more, the main this can also protect high-power appliances, after using this device, these two months have not burned, things are very good, it is said that this is the latest model, the overall price is cheap and beautiful!	Jingdong prefecture level city in Shandong
Happy Fan Shop	Green Intelligent Power Saver	Received, powerful, great quality, love the style😍	Jingdong prefecture level city in Shandong
Happy Fan Shop	Green Intelligent Power Saver	This intelligent energy saver is especially good to use and save electricity hope everyone will buy it!	Jingdong prefecture level city in Shandong
Happy Fan Shop	Green Intelligent Power Saver	Air conditioning adapter effect is particularly great, the use of very simple, plug into the socket on the summer air conditioning power is too large, electricity is also expensive, with this product directly on the electricity bill to save half, has been purchased a few more, the effect is too awesome!	Jingdong prefecture level city in Shandong
Happy Fan Shop	Green Intelligent Power Saver	The family plugged in 5, monthly electricity savings of about 300 yuan. My family before a month of electricity costs more than 800, this time with this saver after the electricity bill is actually only more than 500, amazing, thanks to the store, will recommend other friends to buy!	Jingdong prefecture level city in Shandong
Happy Fan Shop	Green Intelligent Power Saver	It was recommended to me by a neighbor's family and it's nice to use it to save power.	Jingdong prefecture level city in Shandong
Happy Fan Shop	Green Intelligent Power Saver	Second time I've purchased it and the power saving is effective!	Jingdong prefecture level city in Shandong
Happy Fan Shop	Green Intelligent Power Saver	The effect is really good, before a month of more than 300 electricity, now every month every day with the air conditioning, it is more than 100 electricity! This thing is really amazing, one after another to buy and 5.6, home installed 3!	Jingdong prefecture level city in Shandong
Happy Fan Shop	Green Intelligent Power Saver	Received this product, began to use, small things look very wrong, can save a little electricity, the monthly electricity bill is too high, after plugging in did not pull, or save a lot of electricity, point	Jingdong prefecture level city in Shandong
Happy Fan Shop	Green Intelligent Power Saver	Simple and convenient, just that plug in, you can save electricity. A month can save dozens of dollars, a year down to save a lot of, very good product cost-effective, everyone can afford to buy and use!	Jingdong prefecture level city in Shandong
Happy Fan Shop	Green Intelligent Power Saver	Electricity bill from more than 200 yuan per month, down to more than 150 yuan, the original bought home, used two months, really have power-saving effect, this time and sold two, business service attitude is	Jingdong prefecture level city in Shandong
Happy Fan Shop	Green Intelligent Power Saver	Like to watch some movies at home and so on, sometimes at night until two or three o'clock, and recently the air conditioning is not turned off for a night, rather wasteful, I have to say that the effect is very good, quite like it!	Jingdong prefecture level city in Shandong
Happy Fan Shop	Green Intelligent Power Saver	Power-saving effect: the effect is very good, a month to save more than 200 dollars Cost-effective: good value for money Appearance and style: small and lovely Workmanship material: okay Experience: good	Jingdong prefecture level city in Shandong
Happy Fan Shop	Green Intelligent Power Saver	It's okay I quite like it, the stuff is very good. Come back in the future. You can rest assured to buy. Cost-effective all. Much cheaper than the physical store	Jingdong prefecture level city in Shandong
Happy Fan Shop	Green Intelligent Power Saver	More than 150 less, last month's electricity bill is almost 400, this month is only about 200, really save electricity, I am home to buy three, I did not expect a month to pay back	Jingdong prefecture level city in Shandong

Happy Fan Shop	Green Intelligent Power Saver	I bought three of these first and tried them for two months and they still felt useful, so now I've bought another one. Good and recommended!	Jingdong prefecture level city in Shandong
Happy Fan Shop	Green Intelligent Power Saver	This energy saver is very good quality and inexpensive next time I will buy it again!	Jingdong prefecture level city in Shandong
Happy Fan Shop	Green Intelligent Power Saver	Merchant service attitude is not good, the quality does not match, the business does not give the package return	Jingdong prefecture level city in Shandong
Happy Fan Shop	Green Intelligent Power Saver	The goods are very small do not know how effective after a few days to follow up the review	Jingdong prefecture level city in Shandong
Happy Fan Shop	Green Intelligent Power Saver	Not used before more than 200 a month, after using about 100 a month, my family used three, customer service said I use electricity recommended 2-3, I think more than one effect is better on the purchase of 4, the effect is very obvious, recommended to buy!	Jingdong prefecture level city in Shandong
Happy Fan Shop	Green Intelligent Power Saver	It's okay. I'll buy it again today.	Jingdong prefecture level city in Shandong
Happy Fan Shop	Green Intelligent Power Saver	Finally received the goods, very, very much like,, has been a lot of people praise, really too happy, has been his family's loyal fans, will continue to buy buy buy buy!	Jingdong prefecture level city in Shandong
Happy Fan Shop	Green Intelligent Power Saver	After using this, my monthly electricity bill was 1,200 pounds, now it's only 180 bucks a month, how good is it?	Jingdong prefecture level city in Shandong
Happy Fan Shop	Green Intelligent Power Saver	The item is a scam return to marry a dozen dollars in shipping fees. Buy terrific role is purely a scam.	Jingdong prefecture level city in Shandong
Happy Fan Shop	Green Intelligent Power Saver	It's really good, cost-effective and good quality, and the logistics are fast! It arrived in one day!	Jingdong prefecture level city in Shandong
Happy Fan Shop	Green Intelligent Power Saver	Great brand! Trustworthy! Works super well! Expensive for a reason! Great merchandise!	Jingdong prefecture level city in Shandong
Happy Fan Shop	Green Intelligent Power Saver	The baby is really great, I like it very much, my friends also say good to come, recommended to buy!	Jingdong prefecture level city in Shandong
Happy Fan Shop	Green Intelligent Power Saver	Fake couldn't be more fake. Bought one plugged in still so much electricity.	Jingdong prefecture level city in Shandong
Happy Fan Shop	Green Intelligent Power Saver	This appliance works great. Let's see how it works. We'll see later.	Jingdong prefecture level city in Shandong
Happy Fan Shop	Green Intelligent Power Saver	Used it before commenting, very happy with it	Jingdong prefecture level city in Shandong
Happy Fan Shop	Green Intelligent Power Saver	Goods have been received well-packed, logistics is very fast and good quality	Jingdong prefecture level city in Shandong
Happy Fan Shop	Green Intelligent Power Saver	This baby's quality is okay.	Jingdong prefecture level city in Shandong
Happy Fan Shop	Green Intelligent Power Saver	Counterfeit goods.	Jingdong prefecture level city in Shandong
Happy Fan Shop	Green Intelligent Power Saver	The effect is not very obvious	Jingdong prefecture level city in Shandong
Happy Fan Shop	Green Intelligent Power Saver	It's not the same thing.	Jingdong prefecture level city in Shandong
Happy Fan Shop	Green Intelligent Power Saver	There is no load how much w seems false, nothing works.	Jingdong prefecture level city in Shandong
Happy Fan Shop	Green Intelligent Power Saver	I received the item yesterday, got it back and plugged it in, and it used a little more power yesterday than I had before	Jingdong prefecture level city in Shandong
Happy Fan Shop	Green Intelligent Power Saver	Haven't seen the power saving mode yet	Jingdong prefecture level city in Shandong
Happy Fan Shop	Green Intelligent Power Saver	This product has no known effect	Jingdong prefecture level city in Shandong
Happy Fan Shop	Green Intelligent Power Saver	Refund Returns $16	Jingdong prefecture level city in Shandong
Happy Fan Shop	Green Intelligent Power Saver	The previous year bought a, almost every month can save a 70 dollars or so, used a year, no problem at all, look very new like, this time to move to a new home decisively repurchase 2 units, very believe this	Jingdong prefecture level city in Shandong
Happy Fan Shop	Green Intelligent Power Saver	Has been the second time to buy, this power saving device is really good power, before every month to spend 300 electricity, now are about 200, last time that 6, this time to help lead the family to buy 6,	Jingdong prefecture level city in Shandong
Happy Fan Shop	Green Intelligent Power Saver	In the spirit of trying to experience the new product, the use of down compared to the usual power consumption, with the addition of the quarter, is indeed a lot less electricity, originally more than 450	Jingdong prefecture level city in Shandong
Happy Fan Shop	Green Intelligent Power Saver	Has not been the first time to buy back, good use, power saving device is really powerful, before the monthly electricity bill of more than 500 Now more than 300, this time to help neighbors to buy several	Jingdong prefecture level city in Shandong
Happy Fan Shop	Green Intelligent Power Saver	Home appliances are very many, the circuit is also very complex, at first it is to save 9% of the electricity, and then used more than half a month, found that the power saving rate is as high as more	Jingdong prefecture level city in Shandong
Happy Fan Shop	Green Intelligent Power Saver	My friend bought it last year and saved hundreds of dollars on his electricity bill in one summer Appearance and style: it looks better than the one I bought before Quality is also good with a light.	Jingdong prefecture level city in Shandong
Happy Fan Shop	Green Intelligent Power Saver	Saver received, on the spot on the plug with the feeling of immediate power savings, appearance, packaging is very delicate, very satisfied, I hope to achieve satisfactory results!	Jingdong prefecture level city in Shandong
Happy Fan Shop	Green Intelligent Power Saver	The power saving effect is very good, is considered an old customer, also bought before, this time is to buy two for mom and dad, customer service attitude is good, praise!	Jingdong prefecture level city in Shandong
Happy Fan Shop	Green Intelligent Power Saver	After observing for a while, it really saves a lot of money I didn't realize it was still really reliable, so I'll give you a good review.	Jingdong prefecture level city in Shandong
Happy Fan Shop	Green Intelligent Power Saver	My family a month with this monthly almost save more than 200, power-saving effect: have bought a second time, really effective Cost-effective: not bad Appearance style: roughly the same A few months to the home electricity is indeed less, this time it is a re-buy three, send a friend two, they stay a	Jingdong prefecture level city in Shandong
Happy Fan Shop	Green Intelligent Power Saver	A month almost save more than 150 dollars in electricity, home appliances more, the main this can also protect high-power appliances, after using this device, these two months have not burned, things are very good, it is said that this is the latest model, the overall price is cheap and beautiful!	Jingdong prefecture level city in Shandong
Happy Fan Shop	Green Intelligent Power Saver	Received, powerful, great quality, love the style😍	Jingdong prefecture level city in Shandong
Happy Fan Shop	Green Intelligent Power Saver	This intelligent energy saver is especially good to use and save electricity hope everyone will buy it!	Jingdong prefecture level city in Shandong
Happy Fan Shop	Green Intelligent Power Saver	Air conditioning adapter effect is particularly great, the use of very simple, plug into the socket on the summer air conditioning power is too large, electricity is also expensive, with this product directly on the electricity bill to save half, has been purchased a few more, the effect is too awesome!	Jingdong prefecture level city in Shandong
Happy Fan Shop	Green Intelligent Power Saver	The family plugged in 5, monthly electricity savings of about 300 yuan. My family before a month of electricity costs more than 800, this time with this saver after the electricity bill is actually only more than 500, amazing, thanks to the store, will recommend other friends to buy!	Jingdong prefecture level city in Shandong
Happy Fan Shop	Green Intelligent Power Saver	It was recommended to me by a neighbor's family and it's nice to use it to save power.	Jingdong prefecture level city in Shandong
Happy Fan Shop	Green Intelligent Power Saver	Second time I've purchased it and the power saving is effective!	Jingdong prefecture level city in Shandong
Happy Fan Shop	Green Intelligent Power Saver	The effect is really good, before a month of more than 300 electricity, now every month every day with the air conditioning, it is more than 100 electricity! This thing is really amazing, one after another to buy and 5.6, home installed 3!	Jingdong prefecture level city in Shandong
Happy Fan Shop	Green Intelligent Power Saver	Received this product, began to use, small things look very wrong, can save a little electricity, the monthly electricity bill is too high, after plugging in did not pull, or save a lot of electricity, point	Jingdong prefecture level city in Shandong
Happy Fan Shop	Green Intelligent Power Saver	Simple and convenient, just that plug in, you can save electricity. A month can save dozens of dollars, a year down to save a lot of, very good product cost-effective, everyone can afford to buy and use!	Jingdong prefecture level city in Shandong
Happy Fan Shop	Green Intelligent Power Saver	Electricity bill from more than 200 yuan per month, down to more than 150 yuan, the original bought home, used two months, really have power-saving effect, this time and sold two, business service attitude is	Jingdong prefecture level city in Shandong
Happy Fan Shop	Green Intelligent Power Saver	Like to watch some movies at home and so on, sometimes at night until two or three o'clock, and recently the air conditioning is not turned off for a night, rather wasteful, I have to say that the effect is very good, quite like it!	Jingdong prefecture level city in Shandong
Happy Fan Shop	Green Intelligent Power Saver	Power-saving effect: the effect is very good, a month to save more than 200 dollars Cost-effective: good value for money Appearance and style: small and lovely Workmanship material: okay Experience: good	Jingdong prefecture level city in Shandong

Happy Fan Shop	Green Intelligent Power Saver	It's okay I quite like it, the stuff is very good. Come back in the future. You can rest assured to buy. Cost-effective all. Much cheaper than the physical store	Jingdong prefecture level city in Shandong
Happy Fan Shop	Green Intelligent Power Saver	More than 150 less, last month's electricity bill is almost 400, this month is only about 200, really save electricity, I am home to buy three, I did not expect a month to pay back	Jingdong prefecture level city in Shandong
Happy Fan Shop	Green Intelligent Power Saver	I bought three of these first and tried them for two months and they still felt useful, so now I've bought another one. Good and recommended!	Jingdong prefecture level city in Shandong
Happy Fan Shop	Green Intelligent Power Saver	This energy saver is very good quality and inexpensive next time I will buy it again!	Jingdong prefecture level city in Shandong
Happy Fan Shop	Green Intelligent Power Saver	Merchant service attitude is not good, the quality does not match, the business does not give the package return	Jingdong prefecture level city in Shandong
Happy Fan Shop	Green Intelligent Power Saver	The goods are very small do not know how effective after a few days to follow up the review	Jingdong prefecture level city in Shandong
Happy Fan Shop	Green Intelligent Power Saver	Not used before more than 200 a month, after using about 100 a month, my family used three, customer service said I use electricity recommended 2-3, I think more than one effect is better on the purchase of 4, the effect is very obvious, recommended to buy!	Jingdong prefecture level city in Shandong
Happy Fan Shop	Green Intelligent Power Saver	It's okay. I'll buy it again today.	Jingdong prefecture level city in Shandong
Happy Fan Shop	Green Intelligent Power Saver	Finally received the goods, very, very much like,, has been a lot of people praise, really too happy, has been his family's loyal fans, will continue to buy buy buy!	Jingdong prefecture level city in Shandong
Happy Fan Shop	Green Intelligent Power Saver	After using this, my monthly electricity bill was 1,200 pounds, now it's only 180 bucks a month, how good is it?	Jingdong prefecture level city in Shandong
Happy Fan Shop	Green Intelligent Power Saver	The item is a scam return to marry a dozen dollars in shipping fees. Buy terrific role is purely a scam.	Jingdong prefecture level city in Shandong
Happy Fan Shop	Green Intelligent Power Saver	It's really good, cost-effective and good quality, and the logistics are fast! It arrived in one day!	Jingdong prefecture level city in Shandong
Happy Fan Shop	Green Intelligent Power Saver	Great brand! Trustworthy! Works super well! Expensive for a reason! Great merchandise!	Jingdong prefecture level city in Shandong
Happy Fan Shop	Green Intelligent Power Saver	The baby is really great, I like it very much, my friends also say good to come, recommended to buy!	Jingdong prefecture level city in Shandong
Happy Fan Shop	Green Intelligent Power Saver	Fake couldn't be more fake. Bought one plugged in still so much electricity.	Jingdong prefecture level city in Shandong
Happy Fan Shop	Green Intelligent Power Saver	This appliance works great. Let's see how it works. We'll see later.	Jingdong prefecture level city in Shandong
Happy Fan Shop	Green Intelligent Power Saver	Used it before commenting, very happy with it	Jingdong prefecture level city in Shandong
Happy Fan Shop	Green Intelligent Power Saver	Goods have been received well-packed, logistics is very fast and good quality	Jingdong prefecture level city in Shandong
Happy Fan Shop	Green Intelligent Power Saver	This baby's quality is okay.	Jingdong prefecture level city in Shandong
Happy Fan Shop	Green Intelligent Power Saver	Counterfeit goods.	Jingdong prefecture level city in Shandong
Happy Fan Shop	Green Intelligent Power Saver	The effect is not very obvious	Jingdong prefecture level city in Shandong
Happy Fan Shop	Green Intelligent Power Saver	It's not the same thing.	Jingdong prefecture level city in Shandong
Happy Fan Shop	Green Intelligent Power Saver	There is no load how much w seems false, nothing works.	Jingdong prefecture level city in Shandong
Happy Fan Shop	Green Intelligent Power Saver	I received the item yesterday, got it back and plugged it in, and it used a little more power yesterday than I had before	Jingdong prefecture level city in Shandong
Happy Fan Shop	Green Intelligent Power Saver	Haven't seen the power saving mode yet	Jingdong prefecture level city in Shandong
Happy Fan Shop	Green Intelligent Power Saver	This product has no known effect	Jingdong prefecture level city in Shandong
Happy Fan Shop	Green Intelligent Power Saver	Refund Returns $16	Jingdong prefecture level city in Shandong
Happy Fan Shop	Green Intelligent Power Saver	The previous year bought a, almost every month can save a 70 dollars or so, used a year, no problem at all, look very new like, this time to move to a new home decisively repurchase 2 units, very believe this	Jingdong prefecture level city in Shandong
Happy Fan Shop	Green Intelligent Power Saver	Has been the second time to buy, this power saving device is really good power, before every month to spend 300 electricity, now are about 200, last time that 6, this time to help lead the family to buy 6,	Jingdong prefecture level city in Shandong
Happy Fan Shop	Green Intelligent Power Saver	In the spirit of trying to experience the new product, the use of down compared to the usual power consumption, with the addition of the quarter, is indeed a lot less electricity, originally more than 450	Jingdong prefecture level city in Shandong
Happy Fan Shop	Green Intelligent Power Saver	Has not been the first time to buy back, good use, power saving device is really powerful, before the monthly electricity bill of more than 500 Now more than 300, this time to help neighbors to buy several	Jingdong prefecture level city in Shandong
Happy Fan Shop	Green Intelligent Power Saver	Home appliances are very many, the circuit is also very complex, at first it is to save 9% of the electricity, and then used more than half a month, found that the power saving rate is as high as more	Jingdong prefecture level city in Shandong
Happy Fan Shop	Green Intelligent Power Saver	My friend bought it last year and saved hundreds of dollars on his electricity bill in one summer Appearance and style: it looks better than the one I bought before Quality is also good with a light.	Jingdong prefecture level city in Shandong
Happy Fan Shop	Green Intelligent Power Saver	Saver received, on the spot on the plug with the feeling of immediate power savings, appearance, packaging is very delicate, very satisfied, I hope to achieve satisfactory results!	Jingdong prefecture level city in Shandong
Happy Fan Shop	Green Intelligent Power Saver	The power saving effect is very good, is considered an old customer, also bought before, this time is to buy two for mom and dad, customer service attitude is good, praise!	Jingdong prefecture level city in Shandong
Happy Fan Shop	Green Intelligent Power Saver	After observing for a while, it really saves a lot of money I didn't realize it was still really reliable, so I'll give you a good review.	Jingdong prefecture level city in Shandong
Happy Fan Shop	Green Intelligent Power Saver	My family a month with this monthly almost save more than 200, power-saving effect: have bought a second time, really effective Cost-effective: not bad Appearance style: roughly the same A few months to the home electricity is indeed less, this time it is a re-buy three, send a friend two, they stay a	Jingdong prefecture level city in Shandong
Happy Fan Shop	Green Intelligent Power Saver	A month almost save more than 150 dollars in electricity, home appliances more, the main this can also protect high-power appliances, after using this device, these two months have not burned, things are very good, it is said that this is the latest model, the overall price is cheap and beautiful!	Jingdong prefecture level city in Shandong
Happy Fan Shop	Green Intelligent Power Saver	Received, powerful, great quality, love the style😍	Jingdong prefecture level city in Shandong
Happy Fan Shop	Green Intelligent Power Saver	This intelligent energy saver is especially good to use and save electricity hope everyone will buy it!	Jingdong prefecture level city in Shandong
Happy Fan Shop	Green Intelligent Power Saver	Air conditioning adapter effect is particularly great, the use of very simple, plug into the socket on the summer air conditioning power is too large, electricity is also expensive, with this product directly on the electricity bill to save half, has been purchased a few more, the effect is too awesome!	Jingdong prefecture level city in Shandong
Happy Fan Shop	Green Intelligent Power Saver	The family plugged in 5, monthly electricity savings of about 300 yuan. My family before a month of electricity costs more than 800, this time with this saver after the electricity bill is actually only more than 500, amazing, thanks to the store, will recommend other friends to buy!	Jingdong prefecture level city in Shandong
Happy Fan Shop	Green Intelligent Power Saver	It was recommended to me by a neighbor's family and it's nice to use it to save power.	Jingdong prefecture level city in Shandong
Happy Fan Shop	Green Intelligent Power Saver	Second time I've purchased it and the power saving is effective!	Jingdong prefecture level city in Shandong
Happy Fan Shop	Green Intelligent Power Saver	The effect is really good, before a month of more than 300 electricity, now every month every day with the air conditioning, it is more than 100 electricity! This thing is really amazing, one after another to buy and 5.6, home installed 3!	Jingdong prefecture level city in Shandong
Happy Fan Shop	Green Intelligent Power Saver	Received this product, began to use, small things look very wrong, can save a little electricity, the monthly electricity bill is too high, after plugging in did not pull, or save a lot of electricity, point	Jingdong prefecture level city in Shandong
Happy Fan Shop	Green Intelligent Power Saver	Simple and convenient, just that plug in, you can save electricity. A month can save dozens of dollars, a year down to save a lot of, very good product cost-effective, everyone can afford to buy and use!	Jingdong prefecture level city in Shandong
Happy Fan Shop	Green Intelligent Power Saver	Electricity bill from more than 200 yuan per month, down to more than 150 yuan, the original bought home, used two months, really have power-saving effect, this time and sold two, business service attitude is	Jingdong prefecture level city in Shandong

Happy Fan Shop	Green Intelligent Power Saver	Like to watch some movies at home and so on, sometimes at night until two or three o'clock, and recently the air conditioning is not turned off for a night, rather wasteful, I have to say that the effect is very good, quite like it!	Jingdong prefecture level city in Shandong
Happy Fan Shop	Green Intelligent Power Saver	Power-saving effect: the effect is very good, a month to save more than 200 dollars Cost-effective: good value for money Appearance and style: small and lovely Workmanship material: okay Experience: good	Jingdong prefecture level city in Shandong
Happy Fan Shop	Green Intelligent Power Saver	It's okay I quite like it, the stuff is very good. Come back in the future. You can rest assured to buy. Cost-effective all. Much cheaper than the physical store	Jingdong prefecture level city in Shandong
Happy Fan Shop	Green Intelligent Power Saver	More than 150 less, last month's electricity bill is almost 400, this month is only about 200, really save electricity, I am home to buy three, I did not expect a month to pay back	Jingdong prefecture level city in Shandong
Happy Fan Shop	Green Intelligent Power Saver	I bought three of these first and tried them for two months and they still felt useful, so now I've bought another one. Good and recommended!	Jingdong prefecture level city in Shandong
Happy Fan Shop	Green Intelligent Power Saver	This energy saver is very good quality and inexpensive next time I will buy it again!	Jingdong prefecture level city in Shandong
Happy Fan Shop	Green Intelligent Power Saver	Merchant service attitude is not good, the quality does not match, the business does not give the package return	Jingdong prefecture level city in Shandong
Happy Fan Shop	Green Intelligent Power Saver	The goods are very small do not know how effective after a few days to follow up the review	Jingdong prefecture level city in Shandong
Happy Fan Shop	Green Intelligent Power Saver	Not used before more than 200 a month, after using about 100 a month, my family used three, customer service said I use electricity recommended 2-3, I think more than one effect is better on the purchase of 4, the effect is very obvious, recommended to buy!	Jingdong prefecture level city in Shandong
Happy Fan Shop	Green Intelligent Power Saver	It's okay. I'll buy it again today.	Jingdong prefecture level city in Shandong
Happy Fan Shop	Green Intelligent Power Saver	Finally received the goods, very, very much like,, has been a lot of people praise, really too happy, has been his family's loyal fans, will continue to buy buy buy buy!	Jingdong prefecture level city in Shandong
Happy Fan Shop	Green Intelligent Power Saver	After using this, my monthly electricity bill was 1,200 pounds, now it's only 180 bucks a month, how good is it?	Jingdong prefecture level city in Shandong
Happy Fan Shop	Green Intelligent Power Saver	The item is a scam return to marry a dozen dollars in shipping fees. Buy terrific role is purely a scam.	Jingdong prefecture level city in Shandong
Happy Fan Shop	Green Intelligent Power Saver	It's really good, cost-effective and good quality, and the logistics are fast! It arrived in one day!	Jingdong prefecture level city in Shandong
Happy Fan Shop	Green Intelligent Power Saver	Great brand! Trustworthy! Works super well! Expensive for a reason! Great merchandise!	Jingdong prefecture level city in Shandong
Happy Fan Shop	Green Intelligent Power Saver	The baby is really great, I like it very much, my friends also say good to come, recommended to buy!	Jingdong prefecture level city in Shandong
Happy Fan Shop	Green Intelligent Power Saver	Fake couldn't be more fake. Bought one plugged in still so much electricity.	Jingdong prefecture level city in Shandong
Happy Fan Shop	Green Intelligent Power Saver	This appliance works great. Let's see how it works. We'll see later.	Jingdong prefecture level city in Shandong
Happy Fan Shop	Green Intelligent Power Saver	Used it before commenting, very happy with it	Jingdong prefecture level city in Shandong
Happy Fan Shop	Green Intelligent Power Saver	Goods have been received well-packed, logistics is very fast and good quality	Jingdong prefecture level city in Shandong
Happy Fan Shop	Green Intelligent Power Saver	This baby's quality is okay.	Jingdong prefecture level city in Shandong
Happy Fan Shop	Green Intelligent Power Saver	Counterfeit goods.	Jingdong prefecture level city in Shandong
Happy Fan Shop	Green Intelligent Power Saver	The effect is not very obvious	Jingdong prefecture level city in Shandong
Happy Fan Shop	Green Intelligent Power Saver	It's not the same thing.	Jingdong prefecture level city in Shandong
Happy Fan Shop	Green Intelligent Power Saver	There is no load how much w seems false, nothing works.	Jingdong prefecture level city in Shandong
Happy Fan Shop	Green Intelligent Power Saver	I received the item yesterday, got it back and plugged it in, and it used a little more power yesterday than I had before	Jingdong prefecture level city in Shandong
Happy Fan Shop	Green Intelligent Power Saver	Haven't seen the power saving mode yet	Jingdong prefecture level city in Shandong
Happy Fan Shop	Green Intelligent Power Saver	This product has no known effect	Jingdong prefecture level city in Shandong
Happy Fan Shop	Green Intelligent Power Saver	Refund Returns $16	Jingdong prefecture level city in Shandong
Happy Fan Shop	Green Intelligent Power Saver	The previous year bought a, almost every month can save a 70 dollars or so, used a year, no problem at all, look very new like, this time to move to a new home decisively repurchase 2 units, very believe this	Jingdong prefecture level city in Shandong
Happy Fan Shop	Green Intelligent Power Saver	Has been the second time to buy, this power saving device is really good power, before every month to spend 300 electricity, now are about 200, last time that 6, this time to help lead the family to buy 6,	Jingdong prefecture level city in Shandong
Happy Fan Shop	Green Intelligent Power Saver	In the spirit of trying to experience the new product, the use of down compared to the usual power consumption, with the addition of the quarter, is indeed a lot less electricity, originally more than 450	Jingdong prefecture level city in Shandong
Happy Fan Shop	Green Intelligent Power Saver	Has not been the first time to buy back, good use, power saving device is really powerful, before the monthly electricity bill of more than 500 Now more than 300, this time to help neighbors to buy several	Jingdong prefecture level city in Shandong
Happy Fan Shop	Green Intelligent Power Saver	Home appliances are very many, the circuit is also very complex, at first it is to save 9% of the electricity, and then used more than half a month, found that the power saving rate is as high as more	Jingdong prefecture level city in Shandong
Happy Fan Shop	Green Intelligent Power Saver	My friend bought it last year and saved hundreds of dollars on his electricity bill in one summer Appearance and style: it looks better than the one I bought before Quality is also good with a light.	Jingdong prefecture level city in Shandong
Happy Fan Shop	Green Intelligent Power Saver	Saver received, on the spot on the plug with the feeling of immediate power savings, appearance, packaging is very delicate, very satisfied, I hope to achieve satisfactory results!	Jingdong prefecture level city in Shandong
Happy Fan Shop	Green Intelligent Power Saver	The power saving effect is very good, is considered an old customer, also bought before, this time is to buy two for mom and dad, customer service attitude is good, praise!	Jingdong prefecture level city in Shandong
Happy Fan Shop	Green Intelligent Power Saver	After observing for a while, it really saves a lot of money I didn't realize it was still really reliable, so I'll give you a good review.	Jingdong prefecture level city in Shandong
Happy Fan Shop	Green Intelligent Power Saver	My family a month with this monthly almost save more than 200, power-saving effect: have bought a second time, really effective Cost-effective: not bad Appearance style: roughly the same A few months to the home electricity is indeed less, this time it is a re-buy three, send a friend two, they stay a	Jingdong prefecture level city in Shandong
Happy Fan Shop	Green Intelligent Power Saver	A month almost save more than 150 dollars in electricity, home appliances more, the main this can also protect high-power appliances, after using this device, these two months have not burned, things are very good, it is said that this is the latest model, the overall price is cheap and beautiful!	Jingdong prefecture level city in Shandong
Happy Fan Shop	Green Intelligent Power Saver	Received, powerful, great quality, love the style😍	Jingdong prefecture level city in Shandong
Happy Fan Shop	Green Intelligent Power Saver	This intelligent energy saver is especially good to use and save electricity hope everyone will buy it!	Jingdong prefecture level city in Shandong
Happy Fan Shop	Green Intelligent Power Saver	Air conditioning adapter effect is particularly great, the use of very simple, plug into the socket on the summer air conditioning power is too large, electricity is also expensive, with this product directly on the electricity bill to save half, has been purchased a few more, the effect is too awesome!	Jingdong prefecture level city in Shandong
Happy Fan Shop	Green Intelligent Power Saver	The family plugged in 5, monthly electricity savings of about 300 yuan. My family before a month of electricity costs more than 800, this time with this saver after the electricity bill is actually only more than 500, amazing, thanks to the store, will recommend other friends to buy!	Jingdong prefecture level city in Shandong
Happy Fan Shop	Green Intelligent Power Saver	It was recommended to me by a neighbor's family and it's nice to use it to save power.	Jingdong prefecture level city in Shandong
Happy Fan Shop	Green Intelligent Power Saver	Second time I've purchased it and the power saving is effective!	Jingdong prefecture level city in Shandong
Happy Fan Shop	Green Intelligent Power Saver	The effect is really good, before a month of more than 300 electricity, now every month every day with the air conditioning, it is more than 100 electricity! This thing is really amazing, one after another to buy and 5.6, home installed 3!	Jingdong prefecture level city in Shandong
Happy Fan Shop	Green Intelligent Power Saver	Received this product, began to use, small things look very wrong, can save a little electricity, the monthly electricity bill is too high, after plugging in did not pull, or save a lot of electricity, point	Jingdong prefecture level city in Shandong

Happy Fan Shop	Green Intelligent Power Saver	Simple and convenient, just that plug in, you can save electricity. A month can save dozens of dollars, a year down to save a lot of, very good product cost-effective, everyone can afford to buy and use!	Jingdong prefecture level city in Shandong
Happy Fan Shop	Green Intelligent Power Saver	Electricity bill from more than 200 yuan per month, down to more than 150 yuan, the original bought home, used two months, really have power-saving effect, this time and sold two, business service attitude is	Jingdong prefecture level city in Shandong
Happy Fan Shop	Green Intelligent Power Saver	Like to watch some movies at home and so on, sometimes at night until two or three o'clock, and recently the air conditioning is not turned off for a night, rather wasteful, I have to say that the effect is very good, quite like it!	Jingdong prefecture level city in Shandong
Happy Fan Shop	Green Intelligent Power Saver	Power-saving effect: the effect is very good, a month to save more than 200 dollars Cost-effective: good value for money Appearance and style: small and lovely Workmanship material: okay Experience: good	Jingdong prefecture level city in Shandong
Happy Fan Shop	Green Intelligent Power Saver	It's okay I quite like it, the stuff is very good. Come back in the future. You can rest assured to buy. Cost-effective all. Much cheaper than the physical store	Jingdong prefecture level city in Shandong
Happy Fan Shop	Green Intelligent Power Saver	More than 150 less, last month's electricity bill is almost 400, this month is only about 200, really save electricity, I am home to buy three, I did not expect a month to pay back	Jingdong prefecture level city in Shandong
Happy Fan Shop	Green Intelligent Power Saver	I bought three of these first and tried them for two months and they still felt useful, so now I've bought another one. Good and recommended!	Jingdong prefecture level city in Shandong
Happy Fan Shop	Green Intelligent Power Saver	This energy saver is very good quality and inexpensive next time I will buy it again!	Jingdong prefecture level city in Shandong
Happy Fan Shop	Green Intelligent Power Saver	Merchant service attitude is not good, the quality does not match, the business does not give the package return	Jingdong prefecture level city in Shandong
Happy Fan Shop	Green Intelligent Power Saver	The goods are very small do not know how effective after a few days to follow up the review	Jingdong prefecture level city in Shandong
Happy Fan Shop	Green Intelligent Power Saver	Not used before more than 200 a month, after using about 100 a month, my family used three, customer service said I use electricity recommended 2-3, I think more than one effect is better on the purchase of 4, the effect is very obvious, recommended to buy!	Jingdong prefecture level city in Shandong
Happy Fan Shop	Green Intelligent Power Saver	It's okay. I'll buy it again today.	Jingdong prefecture level city in Shandong
Happy Fan Shop	Green Intelligent Power Saver	Finally received the goods, very, very much like,, has been a lot of people praise, really too happy, has been his family's loyal fans, will continue to buy buy buy buy!	Jingdong prefecture level city in Shandong
Happy Fan Shop	Green Intelligent Power Saver	After using this, my monthly electricity bill was 1,200 pounds, now it's only 180 bucks a month, how good is it?	Jingdong prefecture level city in Shandong
Happy Fan Shop	Green Intelligent Power Saver	The item is a scam return to marry a dozen dollars in shipping fees. Buy terrific role is purely a scam.	Jingdong prefecture level city in Shandong
Happy Fan Shop	Green Intelligent Power Saver	It's really good, cost-effective and good quality, and the logistics are fast! It arrived in one day!	Jingdong prefecture level city in Shandong
Happy Fan Shop	Green Intelligent Power Saver	Great brand! Trustworthy! Works super well! Expensive for a reason! Great merchandise!	Jingdong prefecture level city in Shandong
Happy Fan Shop	Green Intelligent Power Saver	The baby is really great, I like it very much, my friends also say good to come, recommended to buy!	Jingdong prefecture level city in Shandong
Happy Fan Shop	Green Intelligent Power Saver	Fake couldn't be more fake. Bought one plugged in still so much electricity.	Jingdong prefecture level city in Shandong
Happy Fan Shop	Green Intelligent Power Saver	This appliance works great. Let's see how it works. We'll see later.	Jingdong prefecture level city in Shandong
Happy Fan Shop	Green Intelligent Power Saver	Used it before commenting, very happy with it	Jingdong prefecture level city in Shandong
Happy Fan Shop	Green Intelligent Power Saver	Goods have been received well-packed, logistics is very fast and good quality	Jingdong prefecture level city in Shandong
Happy Fan Shop	Green Intelligent Power Saver	This baby's quality is okay.	Jingdong prefecture level city in Shandong
Happy Fan Shop	Green Intelligent Power Saver	Counterfeit goods.	Jingdong prefecture level city in Shandong
Happy Fan Shop	Green Intelligent Power Saver	The effect is not very obvious	Jingdong prefecture level city in Shandong
Happy Fan Shop	Green Intelligent Power Saver	It's not the same thing.	Jingdong prefecture level city in Shandong
Happy Fan Shop	Green Intelligent Power Saver	There is no load how much w seems false, nothing works.	Jingdong prefecture level city in Shandong
Happy Fan Shop	Green Intelligent Power Saver	I received the item yesterday, got it back and plugged it in, and it used a little more power yesterday than I had before	Jingdong prefecture level city in Shandong
Happy Fan Shop	Green Intelligent Power Saver	Haven't seen the power saving mode yet	Jingdong prefecture level city in Shandong
Happy Fan Shop	Green Intelligent Power Saver	This product has no known effect	Jingdong prefecture level city in Shandong
Happy Fan Shop	Green Intelligent Power Saver	Refund Returns $16	Jingdong prefecture level city in Shandong
Happy Fan Shop	Green Intelligent Power Saver	The previous year bought a, almost every month can save a 70 dollars or so, used a year, no problem at all, look very new like, this time to move to a new home decisively repurchase 2 units, very believe this	Jingdong prefecture level city in Shandong
Happy Fan Shop	Green Intelligent Power Saver	Has been the second time to buy, this power saving device is really good power, before every month to spend 300 electricity, now are about 200, last time that 6, this time to help lead the family to buy 6,	Jingdong prefecture level city in Shandong
Happy Fan Shop	Green Intelligent Power Saver	In the spirit of trying to experience the new product, the use of down compared to the usual power consumption, with the addition of the quarter, is indeed a lot less electricity, originally more than 450	Jingdong prefecture level city in Shandong
Happy Fan Shop	Green Intelligent Power Saver	Has not been the first time to buy back, good use, power saving device is really powerful, before the monthly electricity bill of more than 500 Now more than 300, this time to help neighbors to buy several	Jingdong prefecture level city in Shandong
Happy Fan Shop	Green Intelligent Power Saver	Home appliances are very many, the circuit is also very complex, at first it is to save 9% of the electricity, and then used more than half a month, found that the power saving rate is as high as more	Jingdong prefecture level city in Shandong
Happy Fan Shop	Green Intelligent Power Saver	My friend bought it last year and saved hundreds of dollars on his electricity bill in one summer Appearance and style: it looks better than the one I bought before Quality is also good with a light.	Jingdong prefecture level city in Shandong
Happy Fan Shop	Green Intelligent Power Saver	Saver received, on the spot on the plug with the feeling of immediate power savings, appearance, packaging is very delicate, very satisfied, I hope to achieve satisfactory results!	Jingdong prefecture level city in Shandong
Happy Fan Shop	Green Intelligent Power Saver	The power saving effect is very good, is considered an old customer, also bought before, this time is to buy two for mom and dad, customer service attitude is good, praise!	Jingdong prefecture level city in Shandong
Happy Fan Shop	Green Intelligent Power Saver	After observing for a while, it really saves a lot of money I didn't realize it was still really reliable, so I'll give you a good review.	Jingdong prefecture level city in Shandong
Happy Fan Shop	Green Intelligent Power Saver	My family a month with this monthly almost save more than 200, power-saving effect: have bought a second time, really effective Cost-effective: not bad Appearance style: roughly the same A few months to the home electricity is indeed less, this time it is a re-buy three, send a friend two, they stay a	Jingdong prefecture level city in Shandong
Happy Fan Shop	Green Intelligent Power Saver	A month almost save more than 150 dollars in electricity, home appliances more, the main this can also protect high-power appliances, after using this device, these two months have not burned, things are very good, it is said that this is the latest model, the overall price is cheap and beautiful!	Jingdong prefecture level city in Shandong
Happy Fan Shop	Green Intelligent Power Saver	Received, powerful, great quality, love the style😍	Jingdong prefecture level city in Shandong
Happy Fan Shop	Green Intelligent Power Saver	This intelligent energy saver is especially good to use and save electricity hope everyone will buy it!	Jingdong prefecture level city in Shandong
Happy Fan Shop	Green Intelligent Power Saver	Air conditioning adapter effect is particularly great, the use of very simple, plug into the socket on the summer air conditioning power is too large, electricity is also expensive, with this product directly on the electricity bill to save half, has been purchased a few more, the effect is too awesome!	Jingdong prefecture level city in Shandong
Happy Fan Shop	Green Intelligent Power Saver	The family plugged in 5, monthly electricity savings of about 300 yuan. My family before a month of electricity costs more than 800, this time with this saver after the electricity bill is actually only more than 500, amazing, thanks to the store, will recommend other friends to buy!	Jingdong prefecture level city in Shandong
Happy Fan Shop	Green Intelligent Power Saver	It was recommended to me by a neighbor's family and it's nice to use it to save power.	Jingdong prefecture level city in Shandong
Happy Fan Shop	Green Intelligent Power Saver	Second time I've purchased it and the power saving is effective!	Jingdong prefecture level city in Shandong

Happy Fan Shop	Green Intelligent Power Saver	The effect is really good, before a month of more than 300 electricity, now every month every day with the air conditioning, it is more than 100 electricity! This thing is really amazing, one after another to buy and 5.6, home installed 3!	Jingdong prefecture level city in Shandong
Happy Fan Shop	Green Intelligent Power Saver	Received this product, began to use, small things look very wrong, can save a little electricity, the monthly electricity bill is too high, after plugging in did not pull, or save a lot of electricity, point	Jingdong prefecture level city in Shandong
Happy Fan Shop	Green Intelligent Power Saver	Simple and convenient, just that plug in, you can save electricity. A month can save dozens of dollars, a year down to save a lot of, very good product cost-effective, everyone can afford to buy and use!	Jingdong prefecture level city in Shandong
Happy Fan Shop	Green Intelligent Power Saver	Electricity bill from more than 200 yuan per month, down to more than 150 yuan, the original bought home, used two months, really have power-saving effect, this time and sold two, business service attitude is	Jingdong prefecture level city in Shandong
Happy Fan Shop	Green Intelligent Power Saver	Like to watch some movies at home and so on, sometimes at night until two or three o'clock, and recently the air conditioning is not turned off for a night, rather wasteful, I have to say that the effect is very good, quite like it!	Jingdong prefecture level city in Shandong
Happy Fan Shop	Green Intelligent Power Saver	Power-saving effect: the effect is very good, a month to save more than 200 dollars Cost-effective: good value for money Appearance and style: small and lovely Workmanship material: okay Experience: good	Jingdong prefecture level city in Shandong
Happy Fan Shop	Green Intelligent Power Saver	It's okay I quite like it, the stuff is very good. Come back in the future. You can rest assured to buy. Cost-effective all. Much cheaper than the physical store	Jingdong prefecture level city in Shandong
Happy Fan Shop	Green Intelligent Power Saver	More than 150 less, last month's electricity bill is almost 400, this month is only about 200, really save electricity, I am home to buy three, I did not expect a month to pay back	Jingdong prefecture level city in Shandong
Happy Fan Shop	Green Intelligent Power Saver	I bought three of these first and tried them for two months and they still felt useful, so now I've bought another one. Good and recommended!	Jingdong prefecture level city in Shandong
Happy Fan Shop	Green Intelligent Power Saver	This energy saver is very good quality and inexpensive next time I will buy it again!	Jingdong prefecture level city in Shandong
Happy Fan Shop	Green Intelligent Power Saver	Merchant service attitude is not good, the quality does not match, the business does not give the package return	Jingdong prefecture level city in Shandong
Happy Fan Shop	Green Intelligent Power Saver	The goods are very small do not know how effective after a few days to follow up the review	Jingdong prefecture level city in Shandong
Happy Fan Shop	Green Intelligent Power Saver	Not used before more than 200 a month, after using about 100 a month, my family used three, customer service said I use electricity recommended 2-3, I think more than one effect is better on the purchase of 4, the effect is very obvious, recommended to buy!	Jingdong prefecture level city in Shandong
Happy Fan Shop	Green Intelligent Power Saver	It's okay. I'll buy it again today.	Jingdong prefecture level city in Shandong
Happy Fan Shop	Green Intelligent Power Saver	Finally received the goods, very, very much like,, has been a lot of people praise, really too happy, has been his family's loyal fans, will continue to buy buy buy buy!	Jingdong prefecture level city in Shandong
Happy Fan Shop	Green Intelligent Power Saver	After using this, my monthly electricity bill was 1,200 pounds, now it's only 180 bucks a month, how good is it?	Jingdong prefecture level city in Shandong
Happy Fan Shop	Green Intelligent Power Saver	The item is a scam return to marry a dozen dollars in shipping fees. Buy terrific role is purely a scam.	Jingdong prefecture level city in Shandong
Happy Fan Shop	Green Intelligent Power Saver	It's really good, cost-effective and good quality, and the logistics are fast! It arrived in one day!	Jingdong prefecture level city in Shandong
Happy Fan Shop	Green Intelligent Power Saver	Great brand! Trustworthy! Works super well! Expensive for a reason! Great merchandise!	Jingdong prefecture level city in Shandong
Happy Fan Shop	Green Intelligent Power Saver	The baby is really great, I like it very much, my friends also say good to come, recommended to buy!	Jingdong prefecture level city in Shandong
Happy Fan Shop	Green Intelligent Power Saver	Fake couldn't be more fake. Bought one plugged in still so much electricity.	Jingdong prefecture level city in Shandong
Happy Fan Shop	Green Intelligent Power Saver	This appliance works great. Let's see how it works. We'll see later.	Jingdong prefecture level city in Shandong
Happy Fan Shop	Green Intelligent Power Saver	Used it before commenting, very happy with it	Jingdong prefecture level city in Shandong
Happy Fan Shop	Green Intelligent Power Saver	Goods have been received well-packed, logistics is very fast and good quality	Jingdong prefecture level city in Shandong
Happy Fan Shop	Green Intelligent Power Saver	This baby's quality is okay.	Jingdong prefecture level city in Shandong
Happy Fan Shop	Green Intelligent Power Saver	Counterfeit goods.	Jingdong prefecture level city in Shandong
Happy Fan Shop	Green Intelligent Power Saver	The effect is not very obvious	Jingdong prefecture level city in Shandong
Happy Fan Shop	Green Intelligent Power Saver	It's not the same thing.	Jingdong prefecture level city in Shandong
Happy Fan Shop	Green Intelligent Power Saver	There is no load how much w seems false, nothing works.	Jingdong prefecture level city in Shandong
Happy Fan Shop	Green Intelligent Power Saver	I received the item yesterday, got it back and plugged it in, and it used a little more power yesterday than I had before	Jingdong prefecture level city in Shandong
Happy Fan Shop	Green Intelligent Power Saver	Haven't seen the power saving mode yet	Jingdong prefecture level city in Shandong
Happy Fan Shop	Green Intelligent Power Saver	This product has no known effect	Jingdong prefecture level city in Shandong
Happy Fan Shop	Green Intelligent Power Saver	Refund Returns $16	Jingdong prefecture level city in Shandong
Happy Fan Shop	Green Intelligent Power Saver	The previous year bought a, almost every month can save a 70 dollars or so, used a year, no problem at all, look very new like, this time to move to a new home decisively repurchase 2 units, very believe this	Jingdong prefecture level city in Shandong
Happy Fan Shop	Green Intelligent Power Saver	Has been the second time to buy, this power saving device is really good power, before every month to spend 300 electricity, now are about 200, last time that 6, this time to help lead the family to buy 6,	Jingdong prefecture level city in Shandong
Happy Fan Shop	Green Intelligent Power Saver	In the spirit of trying to experience the new product, the use of down compared to the usual power consumption, with the addition of the quarter, is indeed a lot less electricity, originally more than 450	Jingdong prefecture level city in Shandong
Happy Fan Shop	Green Intelligent Power Saver	Has not been the first time to buy back, good use, power saving device is really powerful, before the monthly electricity bill of more than 500 Now more than 300, this time to help neighbors to buy several	Jingdong prefecture level city in Shandong
Happy Fan Shop	Green Intelligent Power Saver	Home appliances are very many, the circuit is also very complex, at first it is to save 9% of the electricity, and then used more than half a month, found that the power saving rate is as high as more	Jingdong prefecture level city in Shandong
Happy Fan Shop	Green Intelligent Power Saver	My friend bought it last year and saved hundreds of dollars on his electricity bill in one summer Appearance and style: it looks better than the one I bought before Quality is also good with a light.	Jingdong prefecture level city in Shandong
Happy Fan Shop	Green Intelligent Power Saver	Saver received, on the spot on the plug with the feeling of immediate power savings, appearance, packaging is very delicate, very satisfied, I hope to achieve satisfactory results!	Jingdong prefecture level city in Shandong
Happy Fan Shop	Green Intelligent Power Saver	The power saving effect is very good, is considered an old customer, also bought before, this time is to buy two for mom and dad, customer service attitude is good, praise!	Jingdong prefecture level city in Shandong
Happy Fan Shop	Green Intelligent Power Saver	After observing for a while, it really saves a lot of money I didn't realize it was still really reliable, so I'll give you a good review.	Jingdong prefecture level city in Shandong
Happy Fan Shop	Green Intelligent Power Saver	My family a month with this monthly almost save more than 200, power-saving effect: have bought a second time, really effective Cost-effective: not bad Appearance style: roughly the same A few months to the home electricity is indeed less, this time it is a re-buy three, send a friend two, they stay a	Jingdong prefecture level city in Shandong
Happy Fan Shop	Green Intelligent Power Saver	A month almost save more than 150 dollars in electricity, home appliances more, the main this can also protect high-power appliances, after using this device, these two months have not burned, things are very good, it is said that this is the latest model, the overall price is cheap and beautiful!	Jingdong prefecture level city in Shandong
Happy Fan Shop	Green Intelligent Power Saver	Received, powerful, great quality, love the style😍	Jingdong prefecture level city in Shandong
Happy Fan Shop	Green Intelligent Power Saver	This intelligent energy saver is especially good to use and save electricity hope everyone will buy it!	Jingdong prefecture level city in Shandong
Happy Fan Shop	Green Intelligent Power Saver	Air conditioning adapter effect is particularly great, the use of very simple, plug into the socket on the summer air conditioning power is too large, electricity is also expensive, with this product directly on the electricity bill to save half, has been purchased a few more, the effect is too awesome!	Jingdong prefecture level city in Shandong
Happy Fan Shop	Green Intelligent Power Saver	The family plugged in 5, monthly electricity savings of about 300 yuan. My family before a month of electricity costs more than 800, this time with this saver after the electricity bill is actually only more than 500, amazing, thanks to the store, will recommend other friends to buy!	Jingdong prefecture level city in Shandong

Happy Fan Shop	Green Intelligent Power Saver	It was recommended to me by a neighbor's family and it's nice to use it to save power.	Jingdong prefecture level city in Shandong
Happy Fan Shop	Green Intelligent Power Saver	Second time I've purchased it and the power saving is effective!	Jingdong prefecture level city in Shandong
Happy Fan Shop	Green Intelligent Power Saver	The effect is really good, before a month of more than 300 electricity, now every month every day with the air conditioning, it is more than 100 electricity! This thing is really amazing, one after another to buy and 5.6, home installed 3!	Jingdong prefecture level city in Shandong
Happy Fan Shop	Green Intelligent Power Saver	Received this product, began to use, small things look very wrong, can save a little electricity, the monthly electricity bill is too high, after plugging in did not pull, or save a lot of electricity, point	Jingdong prefecture level city in Shandong
Happy Fan Shop	Green Intelligent Power Saver	Simple and convenient, just that plug in, you can save electricity. A month can save dozens of dollars, a year down to save a lot of, very good product cost-effective, everyone can afford to buy and use!	Jingdong prefecture level city in Shandong
Happy Fan Shop	Green Intelligent Power Saver	Electricity bill from more than 200 yuan per month, down to more than 150 yuan, the original bought home, used two months, really have power-saving effect, this time and sold two, business service attitude is	Jingdong prefecture level city in Shandong
Happy Fan Shop	Green Intelligent Power Saver	Like to watch some movies at home and so on, sometimes at night until two or three o'clock, and recently the air conditioning is not turned off for a night, rather wasteful, I have to say that the effect is very good, quite like it!	Jingdong prefecture level city in Shandong
Happy Fan Shop	Green Intelligent Power Saver	Power-saving effect: the effect is very good, a month to save more than 200 dollars Cost-effective: good value for money Appearance and style: small and lovely Workmanship material: okay Experience: good	Jingdong prefecture level city in Shandong
Happy Fan Shop	Green Intelligent Power Saver	It's okay I quite like it, the stuff is very good. Come back in the future. You can rest assured to buy. Cost-effective all. Much cheaper than the physical store	Jingdong prefecture level city in Shandong
Happy Fan Shop	Green Intelligent Power Saver	More than 150 less, last month's electricity bill is almost 400, this month is only about 200, really save electricity, I am home to buy three, I did not expect a month to pay back	Jingdong prefecture level city in Shandong
Happy Fan Shop	Green Intelligent Power Saver	I bought three of these first and tried them for two months and they still felt useful, so now I've bought another one. Good and recommended!	Jingdong prefecture level city in Shandong
Happy Fan Shop	Green Intelligent Power Saver	This energy saver is very good quality and inexpensive next time I will buy it again!	Jingdong prefecture level city in Shandong
Happy Fan Shop	Green Intelligent Power Saver	Merchant service attitude is not good, the quality does not match, the business does not give the package return	Jingdong prefecture level city in Shandong
Happy Fan Shop	Green Intelligent Power Saver	The goods are very small do not know how effective after a few days to follow up the review	Jingdong prefecture level city in Shandong
Happy Fan Shop	Green Intelligent Power Saver	Not used before more than 200 a month, after using about 100 a month, my family used three, customer service said I use electricity recommended 2-3, I think more than one effect is better on the purchase of 4, the effect is very obvious, recommended to buy!	Jingdong prefecture level city in Shandong
Happy Fan Shop	Green Intelligent Power Saver	It's okay. I'll buy it again today.	Jingdong prefecture level city in Shandong
Happy Fan Shop	Green Intelligent Power Saver	Finally received the goods, very, very much like,, has been a lot of people praise, really too happy, has been his family's loyal fans, will continue to buy buy buy buy!	Jingdong prefecture level city in Shandong
Happy Fan Shop	Green Intelligent Power Saver	After using this, my monthly electricity bill was 1,200 pounds, now it's only 180 bucks a month, how good is it?	Jingdong prefecture level city in Shandong
Happy Fan Shop	Green Intelligent Power Saver	The item is a scam return to marry a dozen dollars in shipping fees. Buy terrific role is purely a scam.	Jingdong prefecture level city in Shandong
Happy Fan Shop	Green Intelligent Power Saver	It's really good, cost-effective and good quality, and the logistics are fast! It arrived in one day!	Jingdong prefecture level city in Shandong
Happy Fan Shop	Green Intelligent Power Saver	Great brand! Trustworthy! Works super well! Expensive for a reason! Great merchandise!	Jingdong prefecture level city in Shandong
Happy Fan Shop	Green Intelligent Power Saver	The baby is really great, I like it very much, my friends also say good to come, recommended to buy!	Jingdong prefecture level city in Shandong
Happy Fan Shop	Green Intelligent Power Saver	Fake couldn't be more fake. Bought one plugged in still so much electricity.	Jingdong prefecture level city in Shandong
Happy Fan Shop	Green Intelligent Power Saver	This appliance works great. Let's see how it works. We'll see later.	Jingdong prefecture level city in Shandong
Happy Fan Shop	Green Intelligent Power Saver	Used it before commenting, very happy with it	Jingdong prefecture level city in Shandong
Happy Fan Shop	Green Intelligent Power Saver	Goods have been received well-packed, logistics is very fast and good quality	Jingdong prefecture level city in Shandong
Happy Fan Shop	Green Intelligent Power Saver	This baby's quality is okay.	Jingdong prefecture level city in Shandong
Happy Fan Shop	Green Intelligent Power Saver	Counterfeit goods.	Jingdong prefecture level city in Shandong
Happy Fan Shop	Green Intelligent Power Saver	The effect is not very obvious	Jingdong prefecture level city in Shandong
Happy Fan Shop	Green Intelligent Power Saver	It's not the same thing.	Jingdong prefecture level city in Shandong
Happy Fan Shop	Green Intelligent Power Saver	There is no load how much w seems false, nothing works.	Jingdong prefecture level city in Shandong
Happy Fan Shop	Green Intelligent Power Saver	I received the item yesterday, got it back and plugged it in, and it used a little more power yesterday than I had before	Jingdong prefecture level city in Shandong
Happy Fan Shop	Green Intelligent Power Saver	Haven't seen the power saving mode yet	Jingdong prefecture level city in Shandong
Happy Fan Shop	Green Intelligent Power Saver	This product has no known effect	Jingdong prefecture level city in Shandong
Happy Fan Shop	Green Intelligent Power Saver	Refund Returns $16	Jingdong prefecture level city in Shandong
Happy Fan Shop	Green Intelligent Power Saver	The previous year bought a, almost every month can save a 70 dollars or so, used a year, no problem at all, look very new like, this time to move to a new home decisively repurchase 2 units, very believe this	Jingdong prefecture level city in Shandong
Happy Fan Shop	Green Intelligent Power Saver	Has been the second time to buy, this power saving device is really good power, before every month to spend 300 electricity, now are about 200, last time that 6, this time to help lead the family to buy 6,	Jingdong prefecture level city in Shandong
Happy Fan Shop	Green Intelligent Power Saver	In the spirit of trying to experience the new product, the use of down compared to the usual power consumption, with the addition of the quarter, is indeed a lot less electricity, originally more than 450	Jingdong prefecture level city in Shandong
Happy Fan Shop	Green Intelligent Power Saver	Has not been the first time to buy back, good use, power saving device is really powerful, before the monthly electricity bill of more than 500 Now more than 300, this time to help neighbors to buy several	Jingdong prefecture level city in Shandong
Happy Fan Shop	Green Intelligent Power Saver	Home appliances are very many, the circuit is also very complex, at first it is to save 9% of the electricity, and then used more than half a month, found that the power saving rate is as high as more	Jingdong prefecture level city in Shandong
Happy Fan Shop	Green Intelligent Power Saver	My friend bought it last year and saved hundreds of dollars on his electricity bill in one summer Appearance and style: it looks better than the one I bought before Quality is also good with a light.	Jingdong prefecture level city in Shandong
Happy Fan Shop	Green Intelligent Power Saver	Saver received, on the spot on the plug with the feeling of immediate power savings, appearance, packaging is very delicate, very satisfied, I hope to achieve satisfactory results!	Jingdong prefecture level city in Shandong
Happy Fan Shop	Green Intelligent Power Saver	The power saving effect is very good, is considered an old customer, also bought before, this time is to buy two for mom and dad, customer service attitude is good, praise!	Jingdong prefecture level city in Shandong
Happy Fan Shop	Green Intelligent Power Saver	After observing for a while, it really saves a lot of money I didn't realize it was still really reliable, so I'll give you a good review.	Jingdong prefecture level city in Shandong
Happy Fan Shop	Green Intelligent Power Saver	My family a month with this monthly almost save more than 200, power-saving effect: have bought a second time, really effective Cost-effective: not bad Appearance style: roughly the same A few months to the home electricity is indeed less, this time it is a re-buy three, send a friend two, they stay a	Jingdong prefecture level city in Shandong
Happy Fan Shop	Green Intelligent Power Saver	A month almost save more than 150 dollars in electricity, home appliances more, the main this can also protect high-power appliances, after using this device, these two months have not burned, things are very good, it is said that this is the latest model, the overall price is cheap and beautiful!	Jingdong prefecture level city in Shandong
Happy Fan Shop	Green Intelligent Power Saver	Received, powerful, great quality, love the style😍	Jingdong prefecture level city in Shandong
Happy Fan Shop	Green Intelligent Power Saver	This intelligent energy saver is especially good to use and save electricity hope everyone will buy it!	Jingdong prefecture level city in Shandong

Happy Fan Shop	Green Intelligent Power Saver	Air conditioning adapter effect is particularly great, the use of very simple, plug into the socket on the summer air conditioning power is too large, electricity is also expensive, with this product directly on the electricity bill to save half, has been purchased a few more, the effect is too awesome!	Jingdong prefecture level city in Shandong
Happy Fan Shop	Green Intelligent Power Saver	The family plugged in 5, monthly electricity savings of about 300 yuan. My family before a month of electricity costs more than 800, this time with this saver after the electricity bill is actually only more than 500, amazing, thanks to the store, will recommend other friends to buy!	Jingdong prefecture level city in Shandong
Happy Fan Shop	Green Intelligent Power Saver	It was recommended to me by a neighbor's family and it's nice to use it to save power.	Jingdong prefecture level city in Shandong
Happy Fan Shop	Green Intelligent Power Saver	Second time I've purchased it and the power saving is effective!	Jingdong prefecture level city in Shandong
Happy Fan Shop	Green Intelligent Power Saver	The effect is really good, before a month of more than 300 electricity, now every month every day with the air conditioning, it is more than 100 electricity! This thing is really amazing, one after another to buy and 5.6, home installed 3!	Jingdong prefecture level city in Shandong
Happy Fan Shop	Green Intelligent Power Saver	Received this product, began to use, small things look very wrong, can save a little electricity, the monthly electricity bill is too high, after plugging in did not pull, or save a lot of electricity, point	Jingdong prefecture level city in Shandong
Happy Fan Shop	Green Intelligent Power Saver	Simple and convenient, just that plug in, you can save electricity. A month can save dozens of dollars, a year down to save a lot of, very good product cost-effective, everyone can afford to buy and use!	Jingdong prefecture level city in Shandong
Happy Fan Shop	Green Intelligent Power Saver	Electricity bill from more than 200 yuan per month, down to more than 150 yuan, the original bought home, used two months, really have power-saving effect, this time and sold two, business service attitude is	Jingdong prefecture level city in Shandong
Happy Fan Shop	Green Intelligent Power Saver	Like to watch some movies at home and so on, sometimes at night until two or three o'clock, and recently the air conditioning is not turned off for a night, rather wasteful, I have to say that the effect is very good, quite like it!	Jingdong prefecture level city in Shandong
Happy Fan Shop	Green Intelligent Power Saver	Power-saving effect: the effect is very good, a month to save more than 200 dollars Cost-effective: good value for money Appearance and style: small and lovely Workmanship material: okay Experience: good	Jingdong prefecture level city in Shandong
Happy Fan Shop	Green Intelligent Power Saver	It's okay I quite like it, the stuff is very good. Come back in the future. You can rest assured to buy. Cost-effective all. Much cheaper than the physical store	Jingdong prefecture level city in Shandong
Happy Fan Shop	Green Intelligent Power Saver	More than 150 less, last month's electricity bill is almost 400, this month is only about 200, really save electricity, I am home to buy three, I did not expect a month to pay back	Jingdong prefecture level city in Shandong
Happy Fan Shop	Green Intelligent Power Saver	I bought three of these first and tried them for two months and they still felt useful, so now I've bought another one. Good and recommended!	Jingdong prefecture level city in Shandong
Happy Fan Shop	Green Intelligent Power Saver	This energy saver is very good quality and inexpensive next time I will buy it again!	Jingdong prefecture level city in Shandong
Happy Fan Shop	Green Intelligent Power Saver	Merchant service attitude is not good, the quality does not match, the business does not give the package return	Jingdong prefecture level city in Shandong
Happy Fan Shop	Green Intelligent Power Saver	The goods are very small do not know how effective after a few days to follow up the review	Jingdong prefecture level city in Shandong
Happy Fan Shop	Green Intelligent Power Saver	Not used before more than 200 a month, after using about 100 a month, my family used three, customer service said I use electricity recommended 2-3, I think more than one effect is better on the purchase of 4, the effect is very obvious, recommended to buy!	Jingdong prefecture level city in Shandong
Happy Fan Shop	Green Intelligent Power Saver	It's okay. I'll buy it again today.	Jingdong prefecture level city in Shandong
Happy Fan Shop	Green Intelligent Power Saver	Finally received the goods, very, very much like,, has been a lot of people praise, really too happy, has been his family's loyal fans, will continue to buy buy buy!	Jingdong prefecture level city in Shandong
Happy Fan Shop	Green Intelligent Power Saver	After using this, my monthly electricity bill was 1,200 pounds, now it's only 180 bucks a month, how good is it?	Jingdong prefecture level city in Shandong
Happy Fan Shop	Green Intelligent Power Saver	The item is a scam return to marry a dozen dollars in shipping fees. Buy terrific role is purely a scam.	Jingdong prefecture level city in Shandong
Happy Fan Shop	Green Intelligent Power Saver	It's really good, cost-effective and good quality, and the logistics are fast! It arrived in one day!	Jingdong prefecture level city in Shandong
Happy Fan Shop	Green Intelligent Power Saver	Great brand! Trustworthy! Works super well! Expensive for a reason! Great merchandise!	Jingdong prefecture level city in Shandong
Happy Fan Shop	Green Intelligent Power Saver	The baby is really great, I like it very much, my friends also say good to come, recommended to buy!	Jingdong prefecture level city in Shandong
Happy Fan Shop	Green Intelligent Power Saver	Fake couldn't be more fake. Bought one plugged in still so much electricity.	Jingdong prefecture level city in Shandong
Happy Fan Shop	Green Intelligent Power Saver	This appliance works great. Let's see how it works. We'll see later.	Jingdong prefecture level city in Shandong
Happy Fan Shop	Green Intelligent Power Saver	Used it before commenting, very happy with it	Jingdong prefecture level city in Shandong
Happy Fan Shop	Green Intelligent Power Saver	Goods have been received well-packed, logistics is very fast and good quality	Jingdong prefecture level city in Shandong
Happy Fan Shop	Green Intelligent Power Saver	This baby's quality is okay.	Jingdong prefecture level city in Shandong
Happy Fan Shop	Green Intelligent Power Saver	Counterfeit goods.	Jingdong prefecture level city in Shandong
Happy Fan Shop	Green Intelligent Power Saver	The effect is not very obvious	Jingdong prefecture level city in Shandong
Happy Fan Shop	Green Intelligent Power Saver	It's not the same thing.	Jingdong prefecture level city in Shandong
Happy Fan Shop	Green Intelligent Power Saver	There is no load how much w seems false, nothing works.	Jingdong prefecture level city in Shandong
Happy Fan Shop	Green Intelligent Power Saver	I received the item yesterday, got it back and plugged it in, and it used a little more power yesterday than I had before	Jingdong prefecture level city in Shandong
Happy Fan Shop	Green Intelligent Power Saver	Haven't seen the power saving mode yet	Jingdong prefecture level city in Shandong
Happy Fan Shop	Green Intelligent Power Saver	This product has no known effect	Jingdong prefecture level city in Shandong
Happy Fan Shop	Green Intelligent Power Saver	Refund Returns $16	Jingdong prefecture level city in Shandong
Happy Fan Shop	Green Intelligent Power Saver	The previous year bought a, almost every month can save a 70 dollars or so, used a year, no problem at all, look very new like, this time to move to a new home decisively repurchase 2 units, very believe this	Jingdong prefecture level city in Shandong
Happy Fan Shop	Green Intelligent Power Saver	Has been the second time to buy, this power saving device is really good power, before every month to spend 300 electricity, now are about 200, last time that 6, this time to help lead the family to buy 6,	Jingdong prefecture level city in Shandong
Happy Fan Shop	Green Intelligent Power Saver	In the spirit of trying to experience the new product, the use of down compared to the usual power consumption, with the addition of the quarter, is indeed a lot less electricity, originally more than 450	Jingdong prefecture level city in Shandong
Happy Fan Shop	Green Intelligent Power Saver	Has not been the first time to buy back, good use, power saving device is really powerful, before the monthly electricity bill of more than 500 Now more than 300, this time to help neighbors to buy several	Jingdong prefecture level city in Shandong
Happy Fan Shop	Green Intelligent Power Saver	Home appliances are very many, the circuit is also very complex, at first it is to save 9% of the electricity, and then used more than half a month, found that the power saving rate is as high as more	Jingdong prefecture level city in Shandong
Happy Fan Shop	Green Intelligent Power Saver	My friend bought it last year and saved hundreds of dollars on his electricity bill in one summer Appearance and style: it looks better than the one I bought before Quality is also good with a light.	Jingdong prefecture level city in Shandong
Happy Fan Shop	Green Intelligent Power Saver	Saver received, on the spot on the plug with the feeling of immediate power savings, appearance, packaging is very delicate, very satisfied, I hope to achieve satisfactory results!	Jingdong prefecture level city in Shandong
Happy Fan Shop	Green Intelligent Power Saver	The power saving effect is very good, is considered an old customer, also bought before, this time is to buy two for mom and dad, customer service attitude is good, praise!	Jingdong prefecture level city in Shandong
Happy Fan Shop	Green Intelligent Power Saver	After observing for a while, it really saves a lot of money I didn't realize it was still really reliable, so I'll give you a good review.	Jingdong prefecture level city in Shandong
Happy Fan Shop	Green Intelligent Power Saver	My family a month with this monthly almost save more than 200, power-saving effect: have bought a second time, really effective Cost-effective: not bad Appearance style: roughly the same A few months to the home electricity is indeed less, this time it is a re-buy three, send a friend two, they stay a	Jingdong prefecture level city in Shandong
Happy Fan Shop	Green Intelligent Power Saver	A month almost save more than 150 dollars in electricity, home appliances more, the main this can also protect high-power appliances, after using this device, these two months have not burned, things are very good, it is said that this is the latest model, the overall price is cheap and beautiful!	Jingdong prefecture level city in Shandong

Happy Fan Shop	Green Intelligent Power Saver	Received, powerful, great quality, love the style😍	Jingdong prefecture level city in Shandong
Happy Fan Shop	Green Intelligent Power Saver	This intelligent energy saver is especially good to use and save electricity hope everyone will buy it!	Jingdong prefecture level city in Shandong
Happy Fan Shop	Green Intelligent Power Saver	Air conditioning adapter effect is particularly great, the use of very simple, plug into the socket on the summer air conditioning power is too large, electricity is also expensive, with this product directly on the electricity bill to save half, has been purchased a few more, the effect is too awesome!	Jingdong prefecture level city in Shandong
Happy Fan Shop	Green Intelligent Power Saver	The family plugged in 5, monthly electricity savings of about 300 yuan. My family before a month of electricity costs more than 800, this time with this saver after the electricity bill is actually only more than 500, amazing, thanks to the store, will recommend other friends to buy!	Jingdong prefecture level city in Shandong
Happy Fan Shop	Green Intelligent Power Saver	It was recommended to me by a neighbor's family and it's nice to use it to save power.	Jingdong prefecture level city in Shandong
Happy Fan Shop	Green Intelligent Power Saver	Second time I've purchased it and the power saving is effective!	Jingdong prefecture level city in Shandong
Happy Fan Shop	Green Intelligent Power Saver	The effect is really good, before a month of more than 300 electricity, now every month every day with the air conditioning, it is more than 100 electricity! This thing is really amazing, one after another to buy and 5.6, home installed 3!	Jingdong prefecture level city in Shandong
Happy Fan Shop	Green Intelligent Power Saver	Received this product, began to use, small things look very wrong, can save a little electricity, the monthly electricity bill is too high, after plugging in did not pull, or save a lot of electricity, point	Jingdong prefecture level city in Shandong
Happy Fan Shop	Green Intelligent Power Saver	Simple and convenient, just that plug in, you can save electricity. A month can save dozens of dollars, a year down to save a lot of, very good product cost-effective, everyone can afford to buy and use!	Jingdong prefecture level city in Shandong
Happy Fan Shop	Green Intelligent Power Saver	Electricity bill from more than 200 yuan per month, down to more than 150 yuan, the original bought home, used two months, really have power-saving effect, this time and sold two, business service attitude is	Jingdong prefecture level city in Shandong
Happy Fan Shop	Green Intelligent Power Saver	Like to watch some movies at home and so on, sometimes at night until two or three o'clock, and recently the air conditioning is not turned off for a night, rather wasteful, I have to say that the effect is very good, quite like it!	Jingdong prefecture level city in Shandong
Happy Fan Shop	Green Intelligent Power Saver	Power-saving effect: the effect is very good, a month to save more than 200 dollars Cost-effective: good value for money Appearance and style: small and lovely Workmanship material: okay Experience: good	Jingdong prefecture level city in Shandong
Happy Fan Shop	Green Intelligent Power Saver	It's okay I quite like it, the stuff is very good. Come back in the future. You can rest assured to buy. Cost-effective all. Much cheaper than the physical store	Jingdong prefecture level city in Shandong
Happy Fan Shop	Green Intelligent Power Saver	More than 150 less, last month's electricity bill is almost 400, this month is only about 200, really save electricity, I am home to buy three, I did not expect a month to pay back	Jingdong prefecture level city in Shandong
Happy Fan Shop	Green Intelligent Power Saver	I bought three of these first and tried them for two months and they still felt useful, so now I've bought another one. Good and recommended!	Jingdong prefecture level city in Shandong
Happy Fan Shop	Green Intelligent Power Saver	This energy saver is very good quality and inexpensive next time I will buy it again!	Jingdong prefecture level city in Shandong
Happy Fan Shop	Green Intelligent Power Saver	Merchant service attitude is not good, the quality does not match, the business does not give the package return	Jingdong prefecture level city in Shandong
Happy Fan Shop	Green Intelligent Power Saver	The goods are very small do not know how effective after a few days to follow up the review	Jingdong prefecture level city in Shandong
Happy Fan Shop	Green Intelligent Power Saver	Not used before more than 200 a month, after using about 100 a month, my family used three, customer service said I use electricity recommended 2-3, I think more than one effect is better on the purchase of 4, the effect is very obvious, recommended to buy!	Jingdong prefecture level city in Shandong
Happy Fan Shop	Green Intelligent Power Saver	It's okay. I'll buy it again today.	Jingdong prefecture level city in Shandong
Happy Fan Shop	Green Intelligent Power Saver	Finally received the goods, very, very much like,, has been a lot of people praise, really too happy, has been his family's loyal fans, will continue to buy buy buy buy!	Jingdong prefecture level city in Shandong
Happy Fan Shop	Green Intelligent Power Saver	After using this, my monthly electricity bill was 1,200 pounds, now it's only 180 bucks a month, how good is it?	Jingdong prefecture level city in Shandong
Happy Fan Shop	Green Intelligent Power Saver	The item is a scam return to marry a dozen dollars in shipping fees. Buy terrific role is purely a scam.	Jingdong prefecture level city in Shandong
Happy Fan Shop	Green Intelligent Power Saver	It's really good, cost-effective and good quality, and the logistics are fast! It arrived in one day!	Jingdong prefecture level city in Shandong
Happy Fan Shop	Green Intelligent Power Saver	Great brand! Trustworthy! Works super well! Expensive for a reason! Great merchandise!	Jingdong prefecture level city in Shandong
Happy Fan Shop	Green Intelligent Power Saver	The baby is really great, I like it very much, my friends also say good to come, recommended to buy!	Jingdong prefecture level city in Shandong
Happy Fan Shop	Green Intelligent Power Saver	Fake couldn't be more fake. Bought one plugged in still so much electricity.	Jingdong prefecture level city in Shandong
Happy Fan Shop	Green Intelligent Power Saver	This appliance works great. Let's see how it works. We'll see later.	Jingdong prefecture level city in Shandong
Happy Fan Shop	Green Intelligent Power Saver	Used it before commenting, very happy with it	Jingdong prefecture level city in Shandong
Happy Fan Shop	Green Intelligent Power Saver	Goods have been received well-packed, logistics is very fast and good quality	Jingdong prefecture level city in Shandong
Happy Fan Shop	Green Intelligent Power Saver	This baby's quality is okay.	Jingdong prefecture level city in Shandong
Happy Fan Shop	Green Intelligent Power Saver	Counterfeit goods.	Jingdong prefecture level city in Shandong
Happy Fan Shop	Green Intelligent Power Saver	The effect is not very obvious	Jingdong prefecture level city in Shandong
Happy Fan Shop	Green Intelligent Power Saver	It's not the same thing.	Jingdong prefecture level city in Shandong
Happy Fan Shop	Green Intelligent Power Saver	There is no load how much w seems false, nothing works.	Jingdong prefecture level city in Shandong
Happy Fan Shop	Green Intelligent Power Saver	I received the item yesterday, got it back and plugged it in, and it used a little more power yesterday than I had before	Jingdong prefecture level city in Shandong
Happy Fan Shop	Green Intelligent Power Saver	Haven't seen the power saving mode yet	Jingdong prefecture level city in Shandong
Happy Fan Shop	Green Intelligent Power Saver	This product has no known effect	Jingdong prefecture level city in Shandong
Happy Fan Shop	Green Intelligent Power Saver	Refund Returns $16	Jingdong prefecture level city in Shandong
Happy Fan Shop	Green Intelligent Power Saver	The previous year bought a, almost every month can save a 70 dollars or so, used a year, no problem at all, look very new like, this time to move to a new home decisively repurchase 2 units, very believe this	Jingdong prefecture level city in Shandong
Happy Fan Shop	Green Intelligent Power Saver	Has been the second time to buy, this power saving device is really good power, before every month to spend 300 electricity, now are about 200, last time that 6, this time to help lead the family to buy 6,	Jingdong prefecture level city in Shandong
Happy Fan Shop	Green Intelligent Power Saver	In the spirit of trying to experience the new product, the use of down compared to the usual power consumption, with the addition of the quarter, is indeed a lot less electricity, originally more than 450	Jingdong prefecture level city in Shandong
Happy Fan Shop	Green Intelligent Power Saver	Has not been the first time to buy back, good use, power saving device is really powerful, before the monthly electricity bill of more than 500 Now more than 300, this time to help neighbors to buy several	Jingdong prefecture level city in Shandong
Happy Fan Shop	Green Intelligent Power Saver	Home appliances are very many, the circuit is also very complex, at first it is to save 9% of the electricity, and then used more than half a month, found that the power saving rate is as high as more	Jingdong prefecture level city in Shandong
Happy Fan Shop	Green Intelligent Power Saver	My friend bought it last year and saved hundreds of dollars on his electricity bill in one summer Appearance and style: it looks better than the one I bought before Quality is also good with a light.	Jingdong prefecture level city in Shandong
Happy Fan Shop	Green Intelligent Power Saver	Saver received, on the spot on the plug with the feeling of immediate power savings, appearance, packaging is very delicate, very satisfied, I hope to achieve satisfactory results!	Jingdong prefecture level city in Shandong
Happy Fan Shop	Green Intelligent Power Saver	The power saving effect is very good, is considered an old customer, also bought before, this time is to buy two for mom and dad, customer service attitude is good, praise!	Jingdong prefecture level city in Shandong
Happy Fan Shop	Green Intelligent Power Saver	After observing for a while, it really saves a lot of money I didn't realize it was still really reliable, so I'll give you a good review.	Jingdong prefecture level city in Shandong

Happy Fan Shop	Green Intelligent Power Saver	My family a month with this monthly almost save more than 200, power-saving effect: have bought a second time, really effective Cost-effective: not bad Appearance style: roughly the same A few months to the home electricity is indeed less, this time it is a re-buy three, send a friend two, they stay a	Jingdong prefecture level city in Shandong
Happy Fan Shop	Green Intelligent Power Saver	A month almost save more than 150 dollars in electricity, home appliances more, the main this can also protect high-power appliances, after using this device, these two months have not burned, things are very good, it is said that this is the latest model, the overall price is cheap and beautiful!	Jingdong prefecture level city in Shandong
Happy Fan Shop	Green Intelligent Power Saver	Received, powerful, great quality, love the style😍	Jingdong prefecture level city in Shandong
Happy Fan Shop	Green Intelligent Power Saver	This intelligent energy saver is especially good to use and save electricity hope everyone will buy it!	Jingdong prefecture level city in Shandong
Happy Fan Shop	Green Intelligent Power Saver	Air conditioning adapter effect is particularly great, the use of very simple, plug into the socket on the summer air conditioning power is too large, electricity is also expensive, with this product directly on the electricity bill to save half, has been purchased a few more, the effect is too awesome!	Jingdong prefecture level city in Shandong
Happy Fan Shop	Green Intelligent Power Saver	The family plugged in 5, monthly electricity savings of about 300 yuan. My family before a month of electricity costs more than 800, this time with this saver after the electricity bill is actually only more than 500, amazing, thanks to the store, will recommend other friends to buy!	Jingdong prefecture level city in Shandong
Happy Fan Shop	Green Intelligent Power Saver	It was recommended to me by a neighbor's family and it's nice to use it to save power.	Jingdong prefecture level city in Shandong
Happy Fan Shop	Green Intelligent Power Saver	Second time I've purchased it and the power saving is effective!	Jingdong prefecture level city in Shandong
Happy Fan Shop	Green Intelligent Power Saver	The effect is really good, before a month of more than 300 electricity, now every month every day with the air conditioning, it is more than 100 electricity! This thing is really amazing, one after another to buy and 5.6, home installed 3!	Jingdong prefecture level city in Shandong
Happy Fan Shop	Green Intelligent Power Saver	Received this product, began to use, small things look very wrong, can save a little electricity, the monthly electricity bill is too high, after plugging in did not pull, or save a lot of electricity, point	Jingdong prefecture level city in Shandong
Happy Fan Shop	Green Intelligent Power Saver	Simple and convenient, just that plug in, you can save electricity. A month can save dozens of dollars, a year down to save a lot of, very good product cost-effective, everyone can afford to buy and use!	Jingdong prefecture level city in Shandong
Happy Fan Shop	Green Intelligent Power Saver	Electricity bill from more than 200 yuan per month, down to more than 150 yuan, the original bought home, used two months, really have power-saving effect, this time and sold two, business service attitude is	Jingdong prefecture level city in Shandong
Happy Fan Shop	Green Intelligent Power Saver	Like to watch some movies at home and so on, sometimes at night until two or three o'clock, and recently the air conditioning is not turned off for a night, rather wasteful, I have to say that the effect is very good, quite like it!	Jingdong prefecture level city in Shandong
Happy Fan Shop	Green Intelligent Power Saver	Power-saving effect: the effect is very good, a month to save more than 200 dollars Cost-effective: good value for money Appearance and style: small and lovely Workmanship material: okay Experience: good	Jingdong prefecture level city in Shandong
Happy Fan Shop	Green Intelligent Power Saver	It's okay I quite like it, the stuff is very good. Come back in the future. You can rest assured to buy. Cost-effective all. Much cheaper than the physical store	Jingdong prefecture level city in Shandong
Happy Fan Shop	Green Intelligent Power Saver	More than 150 less, last month's electricity bill is almost 400, this month is only about 200, really save electricity, I am home to buy three, I did not expect a month to pay back	Jingdong prefecture level city in Shandong
Happy Fan Shop	Green Intelligent Power Saver	I bought three of these first and tried them for two months and they still felt useful, so now I've bought another one. Good and recommended!	Jingdong prefecture level city in Shandong
Happy Fan Shop	Green Intelligent Power Saver	This energy saver is very good quality and inexpensive next time I will buy it again!	Jingdong prefecture level city in Shandong
Happy Fan Shop	Green Intelligent Power Saver	Merchant service attitude is not good, the quality does not match, the business does not give the package return	Jingdong prefecture level city in Shandong
Happy Fan Shop	Green Intelligent Power Saver	The goods are very small do not know how effective after a few days to follow up the review	Jingdong prefecture level city in Shandong
Happy Fan Shop	Green Intelligent Power Saver	Not used before more than 200 a month, after using about 100 a month, my family used three, customer service said I use electricity recommended 2-3, I think more than one effect is better on the purchase of 4, the effect is very obvious, recommended to buy!	Jingdong prefecture level city in Shandong
Happy Fan Shop	Green Intelligent Power Saver	It's okay. I'll buy it again today.	Jingdong prefecture level city in Shandong
Happy Fan Shop	Green Intelligent Power Saver	Finally received the goods, very, very much like,, has been a lot of people praise, really too happy, has been his family's loyal fans, will continue to buy buy buy buy!	Jingdong prefecture level city in Shandong
Happy Fan Shop	Green Intelligent Power Saver	After using this, my monthly electricity bill was 1,200 pounds, now it's only 180 bucks a month, how good is it?	Jingdong prefecture level city in Shandong
Happy Fan Shop	Green Intelligent Power Saver	The item is a scam return to marry a dozen dollars in shipping fees. Buy terrific role is purely a scam.	Jingdong prefecture level city in Shandong
Happy Fan Shop	Green Intelligent Power Saver	It's really good, cost-effective and good quality, and the logistics are fast! It arrived in one day!	Jingdong prefecture level city in Shandong
Happy Fan Shop	Green Intelligent Power Saver	Great brand! Trustworthy! Works super well! Expensive for a reason! Great merchandise!	Jingdong prefecture level city in Shandong
Happy Fan Shop	Green Intelligent Power Saver	The baby is really great, I like it very much, my friends also say good to come, recommended to buy!	Jingdong prefecture level city in Shandong
Happy Fan Shop	Green Intelligent Power Saver	Fake couldn't be more fake. Bought one plugged in still so much electricity.	Jingdong prefecture level city in Shandong
Happy Fan Shop	Green Intelligent Power Saver	This appliance works great. Let's see how it works. We'll see later.	Jingdong prefecture level city in Shandong
Happy Fan Shop	Green Intelligent Power Saver	Used it before commenting, very happy with it	Jingdong prefecture level city in Shandong
Happy Fan Shop	Green Intelligent Power Saver	Goods have been received well-packed, logistics is very fast and good quality	Jingdong prefecture level city in Shandong
Happy Fan Shop	Green Intelligent Power Saver	This baby's quality is okay.	Jingdong prefecture level city in Shandong
Happy Fan Shop	Green Intelligent Power Saver	Counterfeit goods.	Jingdong prefecture level city in Shandong
Happy Fan Shop	Green Intelligent Power Saver	The effect is not very obvious	Jingdong prefecture level city in Shandong
Happy Fan Shop	Green Intelligent Power Saver	It's not the same thing.	Jingdong prefecture level city in Shandong
Happy Fan Shop	Green Intelligent Power Saver	There is no load how much w seems false, nothing works.	Jingdong prefecture level city in Shandong
Happy Fan Shop	Green Intelligent Power Saver	I received the item yesterday, got it back and plugged it in, and it used a little more power yesterday than I had before	Jingdong prefecture level city in Shandong
Happy Fan Shop	Green Intelligent Power Saver	Haven't seen the power saving mode yet	Jingdong prefecture level city in Shandong
Happy Fan Shop	Green Intelligent Power Saver	This product has no known effect	Jingdong prefecture level city in Shandong
Happy Fan Shop	Green Intelligent Power Saver	Refund Returns $16	Jingdong prefecture level city in Shandong
Happy Fan Shop	Green Intelligent Power Saver	The previous year bought a, almost every month can save a 70 dollars or so, used a year, no problem at all, look very new like, this time to move to a new home decisively repurchase 2 units, very believe this	Jingdong prefecture level city in Shandong
Happy Fan Shop	Green Intelligent Power Saver	Has been the second time to buy, this power saving device is really good power, before every month to spend 300 electricity, now are about 200, last time that 6, this time to help lead the family to buy 6,	Jingdong prefecture level city in Shandong
Happy Fan Shop	Green Intelligent Power Saver	In the spirit of trying to experience the new product, the use of down compared to the usual power consumption, with the addition of the quarter, is indeed a lot less electricity, originally more than 450	Jingdong prefecture level city in Shandong
Happy Fan Shop	Green Intelligent Power Saver	Has not been the first time to buy back, good use, power saving device is really powerful, before the monthly electricity bill of more than 500 Now more than 300, this time to help neighbors to buy several	Jingdong prefecture level city in Shandong
Happy Fan Shop	Green Intelligent Power Saver	Home appliances are very many, the circuit is also very complex, at first it is to save 9% of the electricity, and then used more than half a month, found that the power saving rate is as high as more	Jingdong prefecture level city in Shandong
Happy Fan Shop	Green Intelligent Power Saver	My friend bought it last year and saved hundreds of dollars on his electricity bill in one summer Appearance and style: it looks better than the one I bought before Quality is also good with a light.	Jingdong prefecture level city in Shandong
Happy Fan Shop	Green Intelligent Power Saver	Saver received, on the spot on the plug with the feeling of immediate power savings, appearance, packaging is very delicate, very satisfied, I hope to achieve satisfactory results!	Jingdong prefecture level city in Shandong

Happy Fan Shop	Green Intelligent Power Saver	The power saving effect is very good, is considered an old customer, also bought before, this time is to buy two for mom and dad, customer service attitude is good, praise!	Jingdong prefecture level city in Shandong
Happy Fan Shop	Green Intelligent Power Saver	After observing for a while, it really saves a lot of money I didn't realize it was still really reliable, so I'll give you a good review.	Jingdong prefecture level city in Shandong
Happy Fan Shop	Green Intelligent Power Saver	My family a month with this monthly almost save more than 200, power-saving effect: have bought a second time, really effective Cost-effective: not bad Appearance style: roughly the same A few months to the home electricity is indeed less, this time it is a re-buy three, send a friend two, they stay a	Jingdong prefecture level city in Shandong
Happy Fan Shop	Green Intelligent Power Saver	A month almost save more than 150 dollars in electricity, home appliances more, the main this can also protect high-power appliances, after using this device, these two months have not burned, things are very good, it is said that this is the latest model, the overall price is cheap and beautiful!	Jingdong prefecture level city in Shandong
Happy Fan Shop	Green Intelligent Power Saver	Received, powerful, great quality, love the style😍	Jingdong prefecture level city in Shandong
Happy Fan Shop	Green Intelligent Power Saver	This intelligent energy saver is especially good to use and save electricity hope everyone will buy it!	Jingdong prefecture level city in Shandong
Happy Fan Shop	Green Intelligent Power Saver	Air conditioning adapter effect is particularly great, the use of very simple, plug into the socket on the summer air conditioning power is too large, electricity is also expensive, with this product directly on the electricity bill to save half, has been purchased a few more, the effect is too awesome!	Jingdong prefecture level city in Shandong
Happy Fan Shop	Green Intelligent Power Saver	The family plugged in 5, monthly electricity savings of about 300 yuan. My family before a month of electricity costs more than 800, this time with this saver after the electricity bill is actually only more than 500, amazing, thanks to the store, will recommend other friends to buy!	Jingdong prefecture level city in Shandong
Happy Fan Shop	Green Intelligent Power Saver	It was recommended to me by a neighbor's family and it's nice to use it to save power.	Jingdong prefecture level city in Shandong
Happy Fan Shop	Green Intelligent Power Saver	Second time I've purchased it and the power saving is effective!	Jingdong prefecture level city in Shandong
Happy Fan Shop	Green Intelligent Power Saver	The effect is really good, before a month of more than 300 electricity, now every month every day with the air conditioning, it is more than 100 electricity! This thing is really amazing, one after another to buy and 5.6, home installed 3!	Jingdong prefecture level city in Shandong
Happy Fan Shop	Green Intelligent Power Saver	Received this product, began to use, small things look very wrong, can save a little electricity, the monthly electricity bill is too high, after plugging in did not pull, or save a lot of electricity, point	Jingdong prefecture level city in Shandong
Happy Fan Shop	Green Intelligent Power Saver	Simple and convenient, just that plug in, you can save electricity. A month can save dozens of dollars, a year down to save a lot of, very good product cost-effective, everyone can afford to buy and use!	Jingdong prefecture level city in Shandong
Happy Fan Shop	Green Intelligent Power Saver	Electricity bill from more than 200 yuan per month, down to more than 150 yuan, the original bought home, used two months, really have power-saving effect, this time and sold two, business service attitude is	Jingdong prefecture level city in Shandong
Happy Fan Shop	Green Intelligent Power Saver	Like to watch some movies at home and so on, sometimes at night until two or three o'clock, and recently the air conditioning is not turned off for a night, rather wasteful, I have to say that the effect is very good, quite like it!	Jingdong prefecture level city in Shandong
Happy Fan Shop	Green Intelligent Power Saver	Power-saving effect: the effect is very good, a month to save more than 200 dollars Cost-effective: good value for money Appearance and style: small and lovely Workmanship material: okay Experience: good	Jingdong prefecture level city in Shandong
Happy Fan Shop	Green Intelligent Power Saver	It's okay I quite like it, the stuff is very good. Come back in the future. You can rest assured to buy. Cost-effective all. Much cheaper than the physical store	Jingdong prefecture level city in Shandong
Happy Fan Shop	Green Intelligent Power Saver	More than 150 less, last month's electricity bill is almost 400, this month is only about 200, really save electricity, I am home to buy three, I did not expect a month to pay back	Jingdong prefecture level city in Shandong
Happy Fan Shop	Green Intelligent Power Saver	I bought three of these first and tried them for two months and they still felt useful, so now I've bought another one. Good and recommended!	Jingdong prefecture level city in Shandong
Happy Fan Shop	Green Intelligent Power Saver	This energy saver is very good quality and inexpensive next time I will buy it again!	Jingdong prefecture level city in Shandong
Happy Fan Shop	Green Intelligent Power Saver	Merchant service attitude is not good, the quality does not match, the business does not give the package return	Jingdong prefecture level city in Shandong
Happy Fan Shop	Green Intelligent Power Saver	The goods are very small do not know how effective after a few days to follow up the review	Jingdong prefecture level city in Shandong
Happy Fan Shop	Green Intelligent Power Saver	Not used before more than 200 a month, after using about 100 a month, my family used three, customer service said I use electricity recommended 2-3, I think more than one effect is better on the purchase of 4, the effect is very obvious, recommended to buy!	Jingdong prefecture level city in Shandong
Happy Fan Shop	Green Intelligent Power Saver	It's okay. I'll buy it again today.	Jingdong prefecture level city in Shandong
Happy Fan Shop	Green Intelligent Power Saver	Finally received the goods, very, very much like,, has been a lot of people praise, really too happy, has been his family's loyal fans, will continue to buy buy buy!	Jingdong prefecture level city in Shandong
Happy Fan Shop	Green Intelligent Power Saver	After using this, my monthly electricity bill was 1,200 pounds, now it's only 180 bucks a month, how good is it?	Jingdong prefecture level city in Shandong
Happy Fan Shop	Green Intelligent Power Saver	The item is a scam return to marry a dozen dollars in shipping fees. Buy terrific role is purely a scam.	Jingdong prefecture level city in Shandong
Happy Fan Shop	Green Intelligent Power Saver	It's really good, cost-effective and good quality, and the logistics are fast! It arrived in one day!	Jingdong prefecture level city in Shandong
Happy Fan Shop	Green Intelligent Power Saver	Great brand! Trustworthy! Works super well! Expensive for a reason! Great merchandise!	Jingdong prefecture level city in Shandong
Happy Fan Shop	Green Intelligent Power Saver	The baby is really great, I like it very much, my friends also say good to come, recommended to buy!	Jingdong prefecture level city in Shandong
Happy Fan Shop	Green Intelligent Power Saver	Fake couldn't be more fake. Bought one plugged in still so much electricity.	Jingdong prefecture level city in Shandong
Happy Fan Shop	Green Intelligent Power Saver	This appliance works great. Let's see how it works. We'll see later.	Jingdong prefecture level city in Shandong
Happy Fan Shop	Green Intelligent Power Saver	Used it before commenting, very happy with it	Jingdong prefecture level city in Shandong
Happy Fan Shop	Green Intelligent Power Saver	Goods have been received well-packed, logistics is very fast and good quality	Jingdong prefecture level city in Shandong
Happy Fan Shop	Green Intelligent Power Saver	This baby's quality is okay.	Jingdong prefecture level city in Shandong
Happy Fan Shop	Green Intelligent Power Saver	Counterfeit goods.	Jingdong prefecture level city in Shandong
Happy Fan Shop	Green Intelligent Power Saver	The effect is not very obvious	Jingdong prefecture level city in Shandong
Happy Fan Shop	Green Intelligent Power Saver	It's not the same thing.	Jingdong prefecture level city in Shandong
Happy Fan Shop	Green Intelligent Power Saver	There is no load how much w seems false, nothing works.	Jingdong prefecture level city in Shandong
Happy Fan Shop	Green Intelligent Power Saver	I received the item yesterday, got it back and plugged it in, and it used a little more power yesterday than I had before	Jingdong prefecture level city in Shandong
Happy Fan Shop	Green Intelligent Power Saver	Haven't seen the power saving mode yet	Jingdong prefecture level city in Shandong
Happy Fan Shop	Green Intelligent Power Saver	This product has no known effect	Jingdong prefecture level city in Shandong
Happy Fan Shop	Green Intelligent Power Saver	Refund Returns $16	Jingdong prefecture level city in Shandong
Happy Fan Shop	Green Intelligent Power Saver	The previous year bought a, almost every month can save a 70 dollars or so, used a year, no problem at all, look very new like, this time to move to a new home decisively repurchase 2 units, very believe this	Jingdong prefecture level city in Shandong
Happy Fan Shop	Green Intelligent Power Saver	Has been the second time to buy, this power saving device is really good power, before every month to spend 300 electricity, now are about 200, last time that 6, this time to help lead the family to buy 6,	Jingdong prefecture level city in Shandong
Happy Fan Shop	Green Intelligent Power Saver	In the spirit of trying to experience the new product, the use of down compared to the usual power consumption, with the addition of the quarter, is indeed a lot less electricity, originally more than 450	Jingdong prefecture level city in Shandong
Happy Fan Shop	Green Intelligent Power Saver	Has not been the first time to buy back, good use, power saving device is really powerful, before the monthly electricity bill of more than 500 Now more than 300, this time to help neighbors to buy several	Jingdong prefecture level city in Shandong
Happy Fan Shop	Green Intelligent Power Saver	Home appliances are very many, the circuit is also very complex, at first it is to save 9% of the electricity, and then used more than half a month, found that the power saving rate is as high as more	Jingdong prefecture level city in Shandong

Happy Fan Shop	Green Intelligent Power Saver	My friend bought it last year and saved hundreds of dollars on his electricity bill in one summer Appearance and style: it looks better than the one I bought before Quality is also good with a light.	Jingdong prefecture level city in Shandong
Happy Fan Shop	Green Intelligent Power Saver	Saver received, on the spot on the plug with the feeling of immediate power savings, appearance, packaging is very delicate, very satisfied, I hope to achieve satisfactory results!	Jingdong prefecture level city in Shandong
Happy Fan Shop	Green Intelligent Power Saver	The power saving effect is very good, is considered an old customer, also bought before, this time is to buy two for mom and dad, customer service attitude is good, praise!	Jingdong prefecture level city in Shandong
Happy Fan Shop	Green Intelligent Power Saver	After observing for a while, it really saves a lot of money I didn't realize it was still really reliable, so I'll give you a good review.	Jingdong prefecture level city in Shandong
Happy Fan Shop	Green Intelligent Power Saver	My family a month with this monthly almost save more than 200, power-saving effect: have bought a second time, really effective Cost-effective: not bad Appearance style: roughly the same A few months to the home electricity is indeed less, this time it is a re-buy three, send a friend two, they stay a	Jingdong prefecture level city in Shandong
Happy Fan Shop	Green Intelligent Power Saver	A month almost save more than 150 dollars in electricity, home appliances more, the main this can also protect high-power appliances, after using this device, these two months have not burned, things are very good, it is said that this is the latest model, the overall price is cheap and beautiful!	Jingdong prefecture level city in Shandong
Happy Fan Shop	Green Intelligent Power Saver	Received, powerful, great quality, love the style😍	Jingdong prefecture level city in Shandong
Happy Fan Shop	Green Intelligent Power Saver	This intelligent energy saver is especially good to use and save electricity hope everyone will buy it!	Jingdong prefecture level city in Shandong
Happy Fan Shop	Green Intelligent Power Saver	Air conditioning adapter effect is particularly great, the use of very simple, plug into the socket on the summer air conditioning power is too large, electricity is also expensive, with this product directly on the electricity bill to save half, has been purchased a few more, the effect is too awesome!	Jingdong prefecture level city in Shandong
Happy Fan Shop	Green Intelligent Power Saver	The family plugged in 5, monthly electricity savings of about 300 yuan. My family before a month of electricity costs more than 800, this time with this saver after the electricity bill is actually only more than 500, amazing, thanks to the store, will recommend other friends to buy!	Jingdong prefecture level city in Shandong
Happy Fan Shop	Green Intelligent Power Saver	It was recommended to me by a neighbor's family and it's nice to use it to save power.	Jingdong prefecture level city in Shandong
Happy Fan Shop	Green Intelligent Power Saver	Second time I've purchased it and the power saving is effective!	Jingdong prefecture level city in Shandong
Happy Fan Shop	Green Intelligent Power Saver	The effect is really good, before a month of more than 300 electricity, now every month every day with the air conditioning, it is more than 100 electricity! This thing is really amazing, one after another to buy and 5.6, home installed 3!	Jingdong prefecture level city in Shandong
Happy Fan Shop	Green Intelligent Power Saver	Received this product, began to use, small things look very wrong, can save a little electricity, the monthly electricity bill is too high, after plugging in did not pull, or save a lot of electricity, point	Jingdong prefecture level city in Shandong
Happy Fan Shop	Green Intelligent Power Saver	Simple and convenient, just that plug in, you can save electricity. A month can save dozens of dollars, a year down to save a lot of, very good product cost-effective, everyone can afford to buy and use!	Jingdong prefecture level city in Shandong
Happy Fan Shop	Green Intelligent Power Saver	Electricity bill from more than 200 yuan per month, down to more than 150 yuan, the original bought home, used two months, really have power-saving effect, this time and sold two, business service attitude is	Jingdong prefecture level city in Shandong
Happy Fan Shop	Green Intelligent Power Saver	Like to watch some movies at home and so on, sometimes at night until two or three o'clock, and recently the air conditioning is not turned off for a night, rather wasteful, I have to say that the effect is very good, quite like it!	Jingdong prefecture level city in Shandong
Happy Fan Shop	Green Intelligent Power Saver	Power-saving effect: the effect is very good, a month to save more than 200 dollars Cost-effective: good value for money Appearance and style: small and lovely Workmanship material: okay Experience: good	Jingdong prefecture level city in Shandong
Happy Fan Shop	Green Intelligent Power Saver	It's okay I quite like it, the stuff is very good. Come back in the future. You can rest assured to buy. Cost-effective all. Much cheaper than the physical store	Jingdong prefecture level city in Shandong
Happy Fan Shop	Green Intelligent Power Saver	More than 150 less, last month's electricity bill is almost 400, this month is only about 200, really save electricity, I am home to buy three, I did not expect a month to pay back	Jingdong prefecture level city in Shandong
Happy Fan Shop	Green Intelligent Power Saver	I bought three of these first and tried them for two months and they still felt useful, so now I've bought another one. Good and recommended!	Jingdong prefecture level city in Shandong
Happy Fan Shop	Green Intelligent Power Saver	This energy saver is very good quality and inexpensive next time I will buy it again!	Jingdong prefecture level city in Shandong
Happy Fan Shop	Green Intelligent Power Saver	Merchant service attitude is not good, the quality does not match, the business does not give the package return	Jingdong prefecture level city in Shandong
Happy Fan Shop	Green Intelligent Power Saver	The goods are very small do not know how effective after a few days to follow up the review	Jingdong prefecture level city in Shandong
Happy Fan Shop	Green Intelligent Power Saver	Not used before more than 200 a month, after using about 100 a month, my family used three, customer service said I use electricity recommended 2-3, I think more than one effect is better on the purchase of 4, the effect is very obvious, recommended to buy!	Jingdong prefecture level city in Shandong
Happy Fan Shop	Green Intelligent Power Saver	It's okay. I'll buy it again today.	Jingdong prefecture level city in Shandong
Happy Fan Shop	Green Intelligent Power Saver	Finally received the goods, very, very much like,, has been a lot of people praise, really too happy, has been his family's loyal fans, will continue to buy buy buy buy!	Jingdong prefecture level city in Shandong
Happy Fan Shop	Green Intelligent Power Saver	After using this, my monthly electricity bill was 1,200 pounds, now it's only 180 bucks a month, how good is it?	Jingdong prefecture level city in Shandong
Happy Fan Shop	Green Intelligent Power Saver	The item is a scam return to marry a dozen dollars in shipping fees. Buy terrific role is purely a scam.	Jingdong prefecture level city in Shandong
Happy Fan Shop	Green Intelligent Power Saver	It's really good, cost-effective and good quality, and the logistics are fast! It arrived in one day!	Jingdong prefecture level city in Shandong
Happy Fan Shop	Green Intelligent Power Saver	Great brand! Trustworthy! Works super well! Expensive for a reason! Great merchandise!	Jingdong prefecture level city in Shandong
Happy Fan Shop	Green Intelligent Power Saver	The baby is really great, I like it very much, my friends also say good to come, recommended to buy!	Jingdong prefecture level city in Shandong
Happy Fan Shop	Green Intelligent Power Saver	Fake couldn't be more fake. Bought one plugged in still so much electricity.	Jingdong prefecture level city in Shandong
Happy Fan Shop	Green Intelligent Power Saver	This appliance works great. Let's see how it works. We'll see later.	Jingdong prefecture level city in Shandong
Happy Fan Shop	Green Intelligent Power Saver	Used it before commenting, very happy with it	Jingdong prefecture level city in Shandong
Happy Fan Shop	Green Intelligent Power Saver	Goods have been received well-packed, logistics is very fast and good quality	Jingdong prefecture level city in Shandong
Happy Fan Shop	Green Intelligent Power Saver	This baby's quality is okay.	Jingdong prefecture level city in Shandong
Happy Fan Shop	Green Intelligent Power Saver	Counterfeit goods.	Jingdong prefecture level city in Shandong
Happy Fan Shop	Green Intelligent Power Saver	The effect is not very obvious	Jingdong prefecture level city in Shandong
Happy Fan Shop	Green Intelligent Power Saver	It's not the same thing.	Jingdong prefecture level city in Shandong
Happy Fan Shop	Green Intelligent Power Saver	There is no load how much w seems false, nothing works.	Jingdong prefecture level city in Shandong
Happy Fan Shop	Green Intelligent Power Saver	I received the item yesterday, got it back and plugged it in, and it used a little more power yesterday than I had before	Jingdong prefecture level city in Shandong
Happy Fan Shop	Green Intelligent Power Saver	Haven't seen the power saving mode yet	Jingdong prefecture level city in Shandong
Happy Fan Shop	Green Intelligent Power Saver	This product has no known effect	Jingdong prefecture level city in Shandong
Happy Fan Shop	Green Intelligent Power Saver	Refund Returns $16	Jingdong prefecture level city in Shandong
Happy Fan Shop	Green Intelligent Power Saver	The previous year bought a, almost every month can save a 70 dollars or so, used a year, no problem at all, look very new like, this time to move to a new home decisively repurchase 2 units, very believe this	Jingdong prefecture level city in Shandong
Happy Fan Shop	Green Intelligent Power Saver	Has been the second time to buy, this power saving device is really good power, before every month to spend 300 electricity, now are about 200, last time that 6, this time to help lead the family to buy 6,	Jingdong prefecture level city in Shandong
Happy Fan Shop	Green Intelligent Power Saver	In the spirit of trying to experience the new product, the use of down compared to the usual power consumption, with the addition of the quarter, is indeed a lot less electricity, originally more than 450	Jingdong prefecture level city in Shandong

Happy Fan Shop	Green Intelligent Power Saver	Has not been the first time to buy back, good use, power saving device is really powerful, before the monthly electricity bill of more than 500 Now more than 300, this time to help neighbors to buy several	Jingdong prefecture level city in Shandong
Happy Fan Shop	Green Intelligent Power Saver	Home appliances are very many, the circuit is also very complex, at first it is to save 9% of the electricity, and then used more than half a month, found that the power saving rate is as high as more	Jingdong prefecture level city in Shandong
Happy Fan Shop	Green Intelligent Power Saver	My friend bought it last year and saved hundreds of dollars on his electricity bill in one summer Appearance and style: it looks better than the one I bought before Quality is also good with a light.	Jingdong prefecture level city in Shandong
Happy Fan Shop	Green Intelligent Power Saver	Saver received, on the spot on the plug with the feeling of immediate power savings, appearance, packaging is very delicate, very satisfied, I hope to achieve satisfactory results!	Jingdong prefecture level city in Shandong
Happy Fan Shop	Green Intelligent Power Saver	The power saving effect is very good, is considered an old customer, also bought before, this time is to buy two for mom and dad, customer service attitude is good, praise!	Jingdong prefecture level city in Shandong
Happy Fan Shop	Green Intelligent Power Saver	After observing for a while, it really saves a lot of money I didn't realize it was still really reliable, so I'll give you a good review.	Jingdong prefecture level city in Shandong
Happy Fan Shop	Green Intelligent Power Saver	My family a month with this monthly almost save more than 200, power-saving effect: have bought a second time, really effective Cost-effective: not bad Appearance style: roughly the same A few months to the home electricity is indeed less, this time it is a re-buy three, send a friend two, they stay a	Jingdong prefecture level city in Shandong
Happy Fan Shop	Green Intelligent Power Saver	A month almost save more than 150 dollars in electricity, home appliances more, the main this can also protect high-power appliances, after using this device, these two months have not burned, things are very good, it is said that this is the latest model, the overall price is cheap and beautiful!	Jingdong prefecture level city in Shandong
Happy Fan Shop	Green Intelligent Power Saver	Received, powerful, great quality, love the style😍	Jingdong prefecture level city in Shandong
Happy Fan Shop	Green Intelligent Power Saver	This intelligent energy saver is especially good to use and save electricity hope everyone will buy it!	Jingdong prefecture level city in Shandong
Happy Fan Shop	Green Intelligent Power Saver	Air conditioning adapter effect is particularly great, the use of very simple, plug into the socket on the summer air conditioning power is too large, electricity is also expensive, with this product directly on the electricity bill to save half, has been purchased a few more, the effect is too awesome!	Jingdong prefecture level city in Shandong
Happy Fan Shop	Green Intelligent Power Saver	The family plugged in 5, monthly electricity savings of about 300 yuan. My family before a month of electricity costs more than 800, this time with this saver after the electricity bill is actually only more than 500, amazing, thanks to the store, will recommend other friends to buy!	Jingdong prefecture level city in Shandong
Happy Fan Shop	Green Intelligent Power Saver	It was recommended to me by a neighbor's family and it's nice to use it to save power.	Jingdong prefecture level city in Shandong
Happy Fan Shop	Green Intelligent Power Saver	Second time I've purchased it and the power saving is effective!	Jingdong prefecture level city in Shandong
Happy Fan Shop	Green Intelligent Power Saver	The effect is really good, before a month of more than 300 electricity, now every month every day with the air conditioning, it is more than 100 electricity! This thing is really amazing, one after another to buy and 5.6, home installed 3!	Jingdong prefecture level city in Shandong
Happy Fan Shop	Green Intelligent Power Saver	Received this product, began to use, small things look very wrong, can save a little electricity, the monthly electricity bill is too high, after plugging in did not pull, or save a lot of electricity, point	Jingdong prefecture level city in Shandong
Happy Fan Shop	Green Intelligent Power Saver	Simple and convenient, just that plug in, you can save electricity. A month can save dozens of dollars, a year down to save a lot of, very good product cost-effective, everyone can afford to buy and use!	Jingdong prefecture level city in Shandong
Happy Fan Shop	Green Intelligent Power Saver	Electricity bill from more than 200 yuan per month, down to more than 150 yuan, the original bought home, used two months, really have power-saving effect, this time and sold two, business service attitude is	Jingdong prefecture level city in Shandong
Happy Fan Shop	Green Intelligent Power Saver	Like to watch some movies at home and so on, sometimes at night until two or three o'clock, and recently the air conditioning is not turned off for a night, rather wasteful, I have to say that the effect is very good, quite like it!	Jingdong prefecture level city in Shandong
Happy Fan Shop	Green Intelligent Power Saver	Power-saving effect: the effect is very good, a month to save more than 200 dollars Cost-effective: good value for money Appearance and style: small and lovely Workmanship material: okay Experience: good	Jingdong prefecture level city in Shandong
Happy Fan Shop	Green Intelligent Power Saver	It's okay I quite like it, the stuff is very good. Come back in the future. You can rest assured to buy. Cost-effective all. Much cheaper than the physical store	Jingdong prefecture level city in Shandong
Happy Fan Shop	Green Intelligent Power Saver	More than 150 less, last month's electricity bill is almost 400, this month is only about 200, really save electricity, I am home to buy three, I did not expect a month to pay back	Jingdong prefecture level city in Shandong
Happy Fan Shop	Green Intelligent Power Saver	I bought three of these first and tried them for two months and they still felt useful, so now I've bought another one. Good and recommended!	Jingdong prefecture level city in Shandong
Happy Fan Shop	Green Intelligent Power Saver	This energy saver is very good quality and inexpensive next time I will buy it again!	Jingdong prefecture level city in Shandong
Happy Fan Shop	Green Intelligent Power Saver	Merchant service attitude is not good, the quality does not match, the business does not give the package return	Jingdong prefecture level city in Shandong
Happy Fan Shop	Green Intelligent Power Saver	The goods are very small do not know how effective after a few days to follow up the review	Jingdong prefecture level city in Shandong
Happy Fan Shop	Green Intelligent Power Saver	Not used before more than 200 a month, after using about 100 a month, my family used three, customer service said I use electricity recommended 2-3, I think more than one effect is better on the purchase of 4, the effect is very obvious, recommended to buy!	Jingdong prefecture level city in Shandong
Happy Fan Shop	Green Intelligent Power Saver	It's okay. I'll buy it again today.	Jingdong prefecture level city in Shandong
Happy Fan Shop	Green Intelligent Power Saver	Finally received the goods, very, very much like,, has been a lot of people praise, really too happy, has been his family's loyal fans, will continue to buy buy buy!	Jingdong prefecture level city in Shandong
Happy Fan Shop	Green Intelligent Power Saver	After using this, my monthly electricity bill was 1,200 pounds, now it's only 180 bucks a month, how good is it?	Jingdong prefecture level city in Shandong
Happy Fan Shop	Green Intelligent Power Saver	The item is a scam return to marry a dozen dollars in shipping fees. Buy terrific role is purely a scam.	Jingdong prefecture level city in Shandong
Happy Fan Shop	Green Intelligent Power Saver	It's really good, cost-effective and good quality, and the logistics are fast! It arrived in one day!	Jingdong prefecture level city in Shandong
Happy Fan Shop	Green Intelligent Power Saver	Great brand! Trustworthy! Works super well! Expensive for a reason! Great merchandise!	Jingdong prefecture level city in Shandong
Happy Fan Shop	Green Intelligent Power Saver	The baby is really great, I like it very much, my friends also say good to come, recommended to buy!	Jingdong prefecture level city in Shandong
Happy Fan Shop	Green Intelligent Power Saver	Fake couldn't be more fake. Bought one plugged in still so much electricity.	Jingdong prefecture level city in Shandong
Happy Fan Shop	Green Intelligent Power Saver	This appliance works great. Let's see how it works. We'll see later.	Jingdong prefecture level city in Shandong
Happy Fan Shop	Green Intelligent Power Saver	Used it before commenting, very happy with it	Jingdong prefecture level city in Shandong
Happy Fan Shop	Green Intelligent Power Saver	Goods have been received well-packed, logistics is very fast and good quality	Jingdong prefecture level city in Shandong
Happy Fan Shop	Green Intelligent Power Saver	This baby's quality is okay.	Jingdong prefecture level city in Shandong
Happy Fan Shop	Green Intelligent Power Saver	Counterfeit goods.	Jingdong prefecture level city in Shandong
Happy Fan Shop	Green Intelligent Power Saver	The effect is not very obvious	Jingdong prefecture level city in Shandong
Happy Fan Shop	Green Intelligent Power Saver	It's not the same thing.	Jingdong prefecture level city in Shandong
Happy Fan Shop	Green Intelligent Power Saver	There is no load how much w seems false, nothing works.	Jingdong prefecture level city in Shandong
Happy Fan Shop	Green Intelligent Power Saver	I received the item yesterday, got it back and plugged it in, and it used a little more power yesterday than I had before	Jingdong prefecture level city in Shandong
Happy Fan Shop	Green Intelligent Power Saver	Haven't seen the power saving mode yet	Jingdong prefecture level city in Shandong
Happy Fan Shop	Green Intelligent Power Saver	This product has no known effect	Jingdong prefecture level city in Shandong
Happy Fan Shop	Green Intelligent Power Saver	Refund Returns $16	Jingdong prefecture level city in Shandong
Happy Fan Shop	Green Intelligent Power Saver	The previous year bought a, almost every month can save a 70 dollars or so, used a year, no problem at all, look very new like, this time to move to a new home decisively repurchase 2 units, very believe this	Jingdong prefecture level city in Shandong

Happy Fan Shop	Green Intelligent Power Saver	Has been the second time to buy, this power saving device is really good power, before every month to spend 300 electricity, now are about 200, last time that 6, this time to help lead the family to buy 6,	Jingdong prefecture level city in Shandong
Happy Fan Shop	Green Intelligent Power Saver	In the spirit of trying to experience the new product, the use of down compared to the usual power consumption, with the addition of the quarter, is indeed a lot less electricity, originally more than 450	Jingdong prefecture level city in Shandong
Happy Fan Shop	Green Intelligent Power Saver	Has not been the first time to buy back, good use, power saving device is really powerful, before the monthly electricity bill of more than 500 Now more than 300, this time to help neighbors to buy several	Jingdong prefecture level city in Shandong
Happy Fan Shop	Green Intelligent Power Saver	Home appliances are very many, the circuit is also very complex, at first it is to save 9% of the electricity, and then used more than half a month, found that the power saving rate is as high as more	Jingdong prefecture level city in Shandong
Happy Fan Shop	Green Intelligent Power Saver	My friend bought it last year and saved hundreds of dollars on his electricity bill in one summer Appearance and style: it looks better than the one I bought before Quality is also good with a light.	Jingdong prefecture level city in Shandong
Happy Fan Shop	Green Intelligent Power Saver	Saver received, on the spot on the plug with the feeling of immediate power savings, appearance, packaging is very delicate, very satisfied, I hope to achieve satisfactory results!	Jingdong prefecture level city in Shandong
Happy Fan Shop	Green Intelligent Power Saver	The power saving effect is very good, is considered an old customer, also bought before, this time is to buy two for mom and dad, customer service attitude is good, praise!	Jingdong prefecture level city in Shandong
Happy Fan Shop	Green Intelligent Power Saver	After observing for a while, it really saves a lot of money I didn't realize it was still really reliable, so I'll give you a good review.	Jingdong prefecture level city in Shandong
Happy Fan Shop	Green Intelligent Power Saver	My family a month with this monthly almost save more than 200, power-saving effect: have bought a second time, really effective Cost-effective: not bad Appearance style: roughly the same A few months to the home electricity is indeed less, this time it is a re-buy three, send a friend two, they stay a	Jingdong prefecture level city in Shandong
Happy Fan Shop	Green Intelligent Power Saver	A month almost save more than 150 dollars in electricity, home appliances more, the main this can also protect high-power appliances, after using this device, these two months have not burned, things are very good, it is said that this is the latest model, the overall price is cheap and beautiful!	Jingdong prefecture level city in Shandong
Happy Fan Shop	Green Intelligent Power Saver	Received, powerful, great quality, love the style😍	Jingdong prefecture level city in Shandong
Happy Fan Shop	Green Intelligent Power Saver	This intelligent energy saver is especially good to use and save electricity hope everyone will buy it!	Jingdong prefecture level city in Shandong
Happy Fan Shop	Green Intelligent Power Saver	Air conditioning adapter effect is particularly great, the use of very simple, plug into the socket on the summer air conditioning power is too large, electricity is also expensive, with this product directly on the electricity bill to save half, has been purchased a few more, the effect is too awesome!	Jingdong prefecture level city in Shandong
Happy Fan Shop	Green Intelligent Power Saver	The family plugged in 5, monthly electricity savings of about 300 yuan. My family before a month of electricity costs more than 800, this time with this saver after the electricity bill is actually only more than 500, amazing, thanks to the store, will recommend other friends to buy!	Jingdong prefecture level city in Shandong
Happy Fan Shop	Green Intelligent Power Saver	It was recommended to me by a neighbor's family and it's nice to use it to save power.	Jingdong prefecture level city in Shandong
Happy Fan Shop	Green Intelligent Power Saver	Second time I've purchased it and the power saving is effective!	Jingdong prefecture level city in Shandong
Happy Fan Shop	Green Intelligent Power Saver	The effect is really good, before a month of more than 300 electricity, now every month every day with the air conditioning, it is more than 100 electricity! This thing is really amazing, one after another to buy and 5.6, home installed 3!	Jingdong prefecture level city in Shandong
Happy Fan Shop	Green Intelligent Power Saver	Received this product, began to use, small things look very wrong, can save a little electricity, the monthly electricity bill is too high, after plugging in did not pull, or save a lot of electricity, point	Jingdong prefecture level city in Shandong
Happy Fan Shop	Green Intelligent Power Saver	Simple and convenient, just that plug in, you can save electricity. A month can save dozens of dollars, a year down to save a lot of, very good product cost-effective, everyone can afford to buy and use!	Jingdong prefecture level city in Shandong
Happy Fan Shop	Green Intelligent Power Saver	Electricity bill from more than 200 yuan per month, down to more than 150 yuan, the original bought home, used two months, really have power-saving effect, this time and sold two, business service attitude is	Jingdong prefecture level city in Shandong
Happy Fan Shop	Green Intelligent Power Saver	Like to watch some movies at home and so on, sometimes at night until two or three o'clock, and recently the air conditioning is not turned off for a night, rather wasteful, I have to say that the effect is very good, quite like it!	Jingdong prefecture level city in Shandong
Happy Fan Shop	Green Intelligent Power Saver	Power-saving effect: the effect is very good, a month to save more than 200 dollars Cost-effective: good value for money Appearance and style: small and lovely Workmanship material: okay Experience: good	Jingdong prefecture level city in Shandong
Happy Fan Shop	Green Intelligent Power Saver	It's okay I quite like it, the stuff is very good. Come back in the future. You can rest assured to buy. Cost-effective all. Much cheaper than the physical store	Jingdong prefecture level city in Shandong
Happy Fan Shop	Green Intelligent Power Saver	More than 150 less, last month's electricity bill is almost 400, this month is only about 200, really save electricity, I am home to buy three, I did not expect a month to pay back	Jingdong prefecture level city in Shandong
Happy Fan Shop	Green Intelligent Power Saver	I bought three of these first and tried them for two months and they still felt useful, so now I've bought another one. Good and recommended!	Jingdong prefecture level city in Shandong
Happy Fan Shop	Green Intelligent Power Saver	This energy saver is very good quality and inexpensive next time I will buy it again!	Jingdong prefecture level city in Shandong
Happy Fan Shop	Green Intelligent Power Saver	Merchant service attitude is not good, the quality does not match, the business does not give the package return	Jingdong prefecture level city in Shandong
Happy Fan Shop	Green Intelligent Power Saver	The goods are very small do not know how effective after a few days to follow up the review	Jingdong prefecture level city in Shandong
Happy Fan Shop	Green Intelligent Power Saver	Not used before more than 200 a month, after using about 100 a month, my family used three, customer service said I use electricity recommended 2-3, I think more than one effect is better on the purchase of 4, the effect is very obvious, recommended to buy!	Jingdong prefecture level city in Shandong
Happy Fan Shop	Green Intelligent Power Saver	It's okay. I'll buy it again today.	Jingdong prefecture level city in Shandong
Happy Fan Shop	Green Intelligent Power Saver	Finally received the goods, very, very much like,, has been a lot of people praise, really too happy, has been his family's loyal fans, will continue to buy buy buy buy!	Jingdong prefecture level city in Shandong
Happy Fan Shop	Green Intelligent Power Saver	After using this, my monthly electricity bill was 1,200 pounds, now it's only 180 bucks a month, how good is it?	Jingdong prefecture level city in Shandong
Happy Fan Shop	Green Intelligent Power Saver	The item is a scam return to marry a dozen dollars in shipping fees. Buy terrific role is purely a scam.	Jingdong prefecture level city in Shandong
Happy Fan Shop	Green Intelligent Power Saver	It's really good, cost-effective and good quality, and the logistics are fast! It arrived in one day!	Jingdong prefecture level city in Shandong
Happy Fan Shop	Green Intelligent Power Saver	Great brand! Trustworthy! Works super well! Expensive for a reason! Great merchandise!	Jingdong prefecture level city in Shandong
Happy Fan Shop	Green Intelligent Power Saver	The baby is really great, I like it very much, my friends also say good to come, recommended to buy!	Jingdong prefecture level city in Shandong
Happy Fan Shop	Green Intelligent Power Saver	Fake couldn't be more fake. Bought one plugged in still so much electricity.	Jingdong prefecture level city in Shandong
Happy Fan Shop	Green Intelligent Power Saver	This appliance works great. Let's see how it works. We'll see later.	Jingdong prefecture level city in Shandong
Happy Fan Shop	Green Intelligent Power Saver	Used it before commenting, very happy with it	Jingdong prefecture level city in Shandong
Happy Fan Shop	Green Intelligent Power Saver	Goods have been received well-packed, logistics is very fast and good quality	Jingdong prefecture level city in Shandong
Happy Fan Shop	Green Intelligent Power Saver	This baby's quality is okay.	Jingdong prefecture level city in Shandong
Happy Fan Shop	Green Intelligent Power Saver	Counterfeit goods.	Jingdong prefecture level city in Shandong
Happy Fan Shop	Green Intelligent Power Saver	The effect is not very obvious	Jingdong prefecture level city in Shandong
Happy Fan Shop	Green Intelligent Power Saver	It's not the same thing.	Jingdong prefecture level city in Shandong
Happy Fan Shop	Green Intelligent Power Saver	There is no load how much w seems false, nothing works.	Jingdong prefecture level city in Shandong
Happy Fan Shop	Green Intelligent Power Saver	I received the item yesterday, got it back and plugged it in, and it used a little more power yesterday than I had before	Jingdong prefecture level city in Shandong
Happy Fan Shop	Green Intelligent Power Saver	Haven't seen the power saving mode yet	Jingdong prefecture level city in Shandong
Happy Fan Shop	Green Intelligent Power Saver	This product has no known effect	Jingdong prefecture level city in Shandong

Happy Fan Shop	Green Intelligent Power Saver	Refund Returns $16	Jingdong prefecture level city in Shandong
Happy Fan Shop	Green Intelligent Power Saver	The previous year bought a, almost every month can save a 70 dollars or so, used a year, no problem at all, look very new like, this time to move to a new home decisively repurchase 2 units, very believe this	Jingdong prefecture level city in Shandong
Happy Fan Shop	Green Intelligent Power Saver	Has been the second time to buy, this power saving device is really good power, before every month to spend 300 electricity, now are about 200, last time that 6, this time to help lead the family to buy 6,	Jingdong prefecture level city in Shandong
Happy Fan Shop	Green Intelligent Power Saver	In the spirit of trying to experience the new product, the use of down compared to the usual power consumption, with the addition of the quarter, is indeed a lot less electricity, originally more than 450	Jingdong prefecture level city in Shandong
Happy Fan Shop	Green Intelligent Power Saver	Has not been the first time to buy back, good use, power saving device is really powerful, before the monthly electricity bill of more than 500 Now more than 300, this time to help neighbors to buy several	Jingdong prefecture level city in Shandong
Happy Fan Shop	Green Intelligent Power Saver	Home appliances are very many, the circuit is also very complex, at first it is to save 9% of the electricity, and then used more than half a month, found that the power saving rate is as high as more	Jingdong prefecture level city in Shandong
Happy Fan Shop	Green Intelligent Power Saver	My friend bought it last year and saved hundreds of dollars on his electricity bill in one summer Appearance and style: it looks better than the one I bought before Quality is also good with a light.	Jingdong prefecture level city in Shandong
Happy Fan Shop	Green Intelligent Power Saver	Saver received, on the spot on the plug with the feeling of immediate power savings, appearance, packaging is very delicate, very satisfied, I hope to achieve satisfactory results!	Jingdong prefecture level city in Shandong
Happy Fan Shop	Green Intelligent Power Saver	The power saving effect is very good, is considered an old customer, also bought before, this time is to buy two for mom and dad, customer service attitude is good, praise!	Jingdong prefecture level city in Shandong
Happy Fan Shop	Green Intelligent Power Saver	After observing for a while, it really saves a lot of money I didn't realize it was still really reliable, so I'll give you a good review.	Jingdong prefecture level city in Shandong
Happy Fan Shop	Green Intelligent Power Saver	My family a month with this monthly almost save more than 200, power-saving effect: have bought a second time, really effective Cost-effective: not bad Appearance style: roughly the same A few months to the home electricity is indeed less, this time it is a re-buy three, send a friend two, they stay a	Jingdong prefecture level city in Shandong
Happy Fan Shop	Green Intelligent Power Saver	A month almost save more than 150 dollars in electricity, home appliances more, the main this can also protect high-power appliances, after using this device, these two months have not burned, things are very good, it is said that this is the latest model, the overall price is cheap and beautiful!	Jingdong prefecture level city in Shandong
Happy Fan Shop	Green Intelligent Power Saver	Received, powerful, great quality, love the style😍	Jingdong prefecture level city in Shandong
Happy Fan Shop	Green Intelligent Power Saver	This intelligent energy saver is especially good to use and save electricity hope everyone will buy it!	Jingdong prefecture level city in Shandong
Happy Fan Shop	Green Intelligent Power Saver	Air conditioning adapter effect is particularly great, the use of very simple, plug into the socket on the summer air conditioning power is too large, electricity is also expensive, with this product directly on the electricity bill to save half, has been purchased a few more, the effect is too awesome!	Jingdong prefecture level city in Shandong
Happy Fan Shop	Green Intelligent Power Saver	The family plugged in 5, monthly electricity savings of about 300 yuan. My family before a month of electricity costs more than 800, this time with this saver after the electricity bill is actually only more than 500, amazing, thanks to the store, will recommend other friends to buy!	Jingdong prefecture level city in Shandong
Happy Fan Shop	Green Intelligent Power Saver	It was recommended to me by a neighbor's family and it's nice to use it to save power.	Jingdong prefecture level city in Shandong
Happy Fan Shop	Green Intelligent Power Saver	Second time I've purchased it and the power saving is effective!	Jingdong prefecture level city in Shandong
Happy Fan Shop	Green Intelligent Power Saver	The effect is really good, before a month of more than 300 electricity, now every month every day with the air conditioning, it is more than 100 electricity! This thing is really amazing, one after another to buy and 5.6, home installed 3!	Jingdong prefecture level city in Shandong
Happy Fan Shop	Green Intelligent Power Saver	Received this product, began to use, small things look very wrong, can save a little electricity, the monthly electricity bill is too high, after plugging in did not pull, or save a lot of electricity, point	Jingdong prefecture level city in Shandong
Happy Fan Shop	Green Intelligent Power Saver	Simple and convenient, just that plug in, you can save electricity. A month can save dozens of dollars, a year down to save a lot of, very good product cost-effective, everyone can afford to buy and use!	Jingdong prefecture level city in Shandong
Happy Fan Shop	Green Intelligent Power Saver	Electricity bill from more than 200 yuan per month, down to more than 150 yuan, the original bought home, used two months, really have power-saving effect, this time and sold two, business service attitude is	Jingdong prefecture level city in Shandong
Happy Fan Shop	Green Intelligent Power Saver	Like to watch some movies at home and so on, sometimes at night until two or three o'clock, and recently the air conditioning is not turned off for a night, rather wasteful, I have to say that the effect is very good, quite like it!	Jingdong prefecture level city in Shandong
Happy Fan Shop	Green Intelligent Power Saver	Power-saving effect: the effect is very good, a month to save more than 200 dollars Cost-effective: good value for money Appearance and style: small and lovely Workmanship material: okay Experience: good	Jingdong prefecture level city in Shandong
Happy Fan Shop	Green Intelligent Power Saver	It's okay I quite like it, the stuff is very good. Come back in the future. You can rest assured to buy. Cost-effective all. Much cheaper than the physical store	Jingdong prefecture level city in Shandong
Happy Fan Shop	Green Intelligent Power Saver	More than 150 less, last month's electricity bill is almost 400, this month is only about 200, really save electricity, I am home to buy three, I did not expect a month to pay back	Jingdong prefecture level city in Shandong
Happy Fan Shop	Green Intelligent Power Saver	I bought three of these first and tried them for two months and they still felt useful, so now I've bought another one. Good and recommended!	Jingdong prefecture level city in Shandong
Happy Fan Shop	Green Intelligent Power Saver	This energy saver is very good quality and inexpensive next time I will buy it again!	Jingdong prefecture level city in Shandong
Happy Fan Shop	Green Intelligent Power Saver	Merchant service attitude is not good, the quality does not match, the business does not give the package return	Jingdong prefecture level city in Shandong
Happy Fan Shop	Green Intelligent Power Saver	The goods are very small do not know how effective after a few days to follow up the review	Jingdong prefecture level city in Shandong
Happy Fan Shop	Green Intelligent Power Saver	Not used before more than 200 a month, after using about 100 a month, my family used three, customer service said I use electricity recommended 2-3, I think more than one effect is better on the purchase of 4, the effect is very obvious, recommended to buy!	Jingdong prefecture level city in Shandong
Happy Fan Shop	Green Intelligent Power Saver	It's okay. I'll buy it again today.	Jingdong prefecture level city in Shandong
Happy Fan Shop	Green Intelligent Power Saver	Finally received the goods, very, very much like,, has been a lot of people praise, really too happy, has been his family's loyal fans, will continue to buy buy buy buy!	Jingdong prefecture level city in Shandong
Happy Fan Shop	Green Intelligent Power Saver	After using this, my monthly electricity bill was 1,200 pounds, now it's only 180 bucks a month, how good is it?	Jingdong prefecture level city in Shandong
Happy Fan Shop	Green Intelligent Power Saver	The item is a scam return to marry a dozen dollars in shipping fees. Buy terrific role is purely a scam.	Jingdong prefecture level city in Shandong
Happy Fan Shop	Green Intelligent Power Saver	It's really good, cost-effective and good quality, and the logistics are fast! It arrived in one day!	Jingdong prefecture level city in Shandong
Happy Fan Shop	Green Intelligent Power Saver	Great brand! Trustworthy! Works super well! Expensive for a reason! Great merchandise!	Jingdong prefecture level city in Shandong
Happy Fan Shop	Green Intelligent Power Saver	The baby is really great, I like it very much, my friends also say good to come, recommended to buy!	Jingdong prefecture level city in Shandong
Happy Fan Shop	Green Intelligent Power Saver	Fake couldn't be more fake. Bought one plugged in still so much electricity.	Jingdong prefecture level city in Shandong
Happy Fan Shop	Green Intelligent Power Saver	This appliance works great. Let's see how it works. We'll see later.	Jingdong prefecture level city in Shandong
Happy Fan Shop	Green Intelligent Power Saver	Used it before commenting, very happy with it	Jingdong prefecture level city in Shandong
Happy Fan Shop	Green Intelligent Power Saver	Goods have been received well-packed, logistics is very fast and good quality	Jingdong prefecture level city in Shandong
Happy Fan Shop	Green Intelligent Power Saver	This baby's quality is okay.	Jingdong prefecture level city in Shandong
Happy Fan Shop	Green Intelligent Power Saver	Counterfeit goods.	Jingdong prefecture level city in Shandong
Happy Fan Shop	Green Intelligent Power Saver	The effect is not very obvious	Jingdong prefecture level city in Shandong
Happy Fan Shop	Green Intelligent Power Saver	It's not the same thing.	Jingdong prefecture level city in Shandong
Happy Fan Shop	Green Intelligent Power Saver	There is no load how much w seems false, nothing works.	Jingdong prefecture level city in Shandong
Happy Fan Shop	Green Intelligent Power Saver	I received the item yesterday, got it back and plugged it in, and it used a little more power yesterday than I had before	Jingdong prefecture level city in Shandong

Happy Fan Shop	Green Intelligent Power Saver	Haven't seen the power saving mode yet	Jingdong prefecture level city in Shandong
Happy Fan Shop	Green Intelligent Power Saver	This product has no known effect	Jingdong prefecture level city in Shandong
Happy Fan Shop	Green Intelligent Power Saver	Refund Returns $16	Jingdong prefecture level city in Shandong
Happy Fan Shop	Green Intelligent Power Saver	The previous year bought a, almost every month can save a 70 dollars or so, used a year, no problem at all, look very new like, this time to move to a new home decisively repurchase 2 units, very believe this	Jingdong prefecture level city in Shandong
Happy Fan Shop	Green Intelligent Power Saver	Has been the second time to buy, this power saving device is really good power, before every month to spend 300 electricity, now are about 200, last time that 6, this time to help lead the family to buy 6,	Jingdong prefecture level city in Shandong
Happy Fan Shop	Green Intelligent Power Saver	In the spirit of trying to experience the new product, the use of down compared to the usual power consumption, with the addition of the quarter, is indeed a lot less electricity, originally more than 450	Jingdong prefecture level city in Shandong
Happy Fan Shop	Green Intelligent Power Saver	Has not been the first time to buy back, good use, power saving device is really powerful, before the monthly electricity bill of more than 500 Now more than 300, this time to help neighbors to buy several	Jingdong prefecture level city in Shandong
Happy Fan Shop	Green Intelligent Power Saver	Home appliances are very many, the circuit is also very complex, at first it is to save 9% of the electricity, and then used more than half a month, found that the power saving rate is as high as more	Jingdong prefecture level city in Shandong
Happy Fan Shop	Green Intelligent Power Saver	My friend bought it last year and saved hundreds of dollars on his electricity bill in one summer Appearance and style: it looks better than the one I bought before Quality is also good with a light.	Jingdong prefecture level city in Shandong
Happy Fan Shop	Green Intelligent Power Saver	Saver received, on the spot on the plug with the feeling of immediate power savings, appearance, packaging is very delicate, very satisfied, I hope to achieve satisfactory results!	Jingdong prefecture level city in Shandong
Happy Fan Shop	Green Intelligent Power Saver	The power saving effect is very good, is considered an old customer, also bought before, this time is to buy two for mom and dad, customer service attitude is good, praise!	Jingdong prefecture level city in Shandong
Happy Fan Shop	Green Intelligent Power Saver	After observing for a while, it really saves a lot of money I didn't realize it was still really reliable, so I'll give you a good review.	Jingdong prefecture level city in Shandong
Happy Fan Shop	Green Intelligent Power Saver	My family a month with this monthly almost save more than 200, power-saving effect: have bought a second time, really effective Cost-effective: not bad Appearance style: roughly the same A few months to the home electricity is indeed less, this time it is a re-buy three, send a friend two, they stay a	Jingdong prefecture level city in Shandong
Happy Fan Shop	Green Intelligent Power Saver	A month almost save more than 150 dollars in electricity, home appliances more, the main this can also protect high-power appliances, after using this device, these two months have not burned, things are very good, it is said that this is the latest model, the overall price is cheap and beautiful!	Jingdong prefecture level city in Shandong
Happy Fan Shop	Green Intelligent Power Saver	Received, powerful, great quality, love the style😍	Jingdong prefecture level city in Shandong
Happy Fan Shop	Green Intelligent Power Saver	This intelligent energy saver is especially good to use and save electricity hope everyone will buy it!	Jingdong prefecture level city in Shandong
Happy Fan Shop	Green Intelligent Power Saver	Air conditioning adapter effect is particularly great, the use of very simple, plug into the socket on the summer air conditioning power is too large, electricity is also expensive, with this product directly on the electricity bill to save half, has been purchased a few more, the effect is too awesome!	Jingdong prefecture level city in Shandong
Happy Fan Shop	Green Intelligent Power Saver	The family plugged in 5, monthly electricity savings of about 300 yuan. My family before a month of electricity costs more than 800, this time with this saver after the electricity bill is actually only more than 500, amazing, thanks to the store, will recommend other friends to buy!	Jingdong prefecture level city in Shandong
Happy Fan Shop	Green Intelligent Power Saver	It was recommended to me by a neighbor's family and it's nice to use it to save power.	Jingdong prefecture level city in Shandong
Happy Fan Shop	Green Intelligent Power Saver	Second time I've purchased it and the power saving is effective!	Jingdong prefecture level city in Shandong
Happy Fan Shop	Green Intelligent Power Saver	The effect is really good, before a month of more than 300 electricity, now every month every day with the air conditioning, it is more than 100 electricity! This thing is really amazing, one after another to buy and 5.6, home installed 3!	Jingdong prefecture level city in Shandong
Happy Fan Shop	Green Intelligent Power Saver	Received this product, began to use, small things look very wrong, can save a little electricity, the monthly electricity bill is too high, after plugging in did not pull, or save a lot of electricity, point	Jingdong prefecture level city in Shandong
Happy Fan Shop	Green Intelligent Power Saver	Simple and convenient, just that plug in, you can save electricity. A month can save dozens of dollars, a year down to save a lot of, very good product cost-effective, everyone can afford to buy and use!	Jingdong prefecture level city in Shandong
Happy Fan Shop	Green Intelligent Power Saver	Electricity bill from more than 200 yuan per month, down to more than 150 yuan, the original bought home, used two months, really have power-saving effect, this time and sold two, business service attitude is	Jingdong prefecture level city in Shandong
Happy Fan Shop	Green Intelligent Power Saver	Like to watch some movies at home and so on, sometimes at night until two or three o'clock, and recently the air conditioning is not turned off for a night, rather wasteful, I have to say that the effect is very good, quite like it!	Jingdong prefecture level city in Shandong
Happy Fan Shop	Green Intelligent Power Saver	Power-saving effect: the effect is very good, a month to save more than 200 dollars Cost-effective: good value for money Appearance and style: small and lovely Workmanship material: okay Experience: good	Jingdong prefecture level city in Shandong
Happy Fan Shop	Green Intelligent Power Saver	It's okay I quite like it, the stuff is very good. Come back in the future. You can rest assured to buy. Cost-effective all. Much cheaper than the physical store	Jingdong prefecture level city in Shandong
Happy Fan Shop	Green Intelligent Power Saver	More than 150 less, last month's electricity bill is almost 400, this month is only about 200, really save electricity, I am home to buy three, I did not expect a month to pay back	Jingdong prefecture level city in Shandong
Happy Fan Shop	Green Intelligent Power Saver	I bought three of these first and tried them for two months and they still felt useful, so now I've bought another one. Good and recommended!	Jingdong prefecture level city in Shandong
Happy Fan Shop	Green Intelligent Power Saver	This energy saver is very good quality and inexpensive next time I will buy it again!	Jingdong prefecture level city in Shandong
Happy Fan Shop	Green Intelligent Power Saver	Merchant service attitude is not good, the quality does not match, the business does not give the package return	Jingdong prefecture level city in Shandong
Happy Fan Shop	Green Intelligent Power Saver	The goods are very small do not know how effective after a few days to follow up the review	Jingdong prefecture level city in Shandong
Happy Fan Shop	Green Intelligent Power Saver	Not used before more than 200 a month, after using about 100 a month, my family used three, customer service said I use electricity recommended 2-3, I think more than one effect is better on the purchase of 4, the effect is very obvious, recommended to buy!	Jingdong prefecture level city in Shandong
Happy Fan Shop	Green Intelligent Power Saver	It's okay. I'll buy it again today.	Jingdong prefecture level city in Shandong
Happy Fan Shop	Green Intelligent Power Saver	Finally received the goods, very, very much like,, has been a lot of people praise, really too happy, has been his family's loyal fans, will continue to buy buy buy!	Jingdong prefecture level city in Shandong
Happy Fan Shop	Green Intelligent Power Saver	After using this, my monthly electricity bill was 1,200 pounds, now it's only 180 bucks a month, how good is it?	Jingdong prefecture level city in Shandong
Happy Fan Shop	Green Intelligent Power Saver	The item is a scam return to marry a dozen dollars in shipping fees. Buy terrific role is purely a scam.	Jingdong prefecture level city in Shandong
Happy Fan Shop	Green Intelligent Power Saver	It's really good, cost-effective and good quality, and the logistics are fast! It arrived in one day!	Jingdong prefecture level city in Shandong
Happy Fan Shop	Green Intelligent Power Saver	Great brand! Trustworthy! Works super well! Expensive for a reason! Great merchandise!	Jingdong prefecture level city in Shandong
Happy Fan Shop	Green Intelligent Power Saver	The baby is really great, I like it very much, my friends also say good to come, recommended to buy!	Jingdong prefecture level city in Shandong
Happy Fan Shop	Green Intelligent Power Saver	Fake couldn't be more fake. Bought one plugged in still so much electricity.	Jingdong prefecture level city in Shandong
Happy Fan Shop	Green Intelligent Power Saver	This appliance works great. Let's see how it works. We'll see later.	Jingdong prefecture level city in Shandong
Happy Fan Shop	Green Intelligent Power Saver	Used it before commenting, very happy with it	Jingdong prefecture level city in Shandong
Happy Fan Shop	Green Intelligent Power Saver	Goods have been received well-packed, logistics is very fast and good quality	Jingdong prefecture level city in Shandong
Happy Fan Shop	Green Intelligent Power Saver	This baby's quality is okay.	Jingdong prefecture level city in Shandong
Happy Fan Shop	Green Intelligent Power Saver	Counterfeit goods.	Jingdong prefecture level city in Shandong
Happy Fan Shop	Green Intelligent Power Saver	The effect is not very obvious	Jingdong prefecture level city in Shandong
Happy Fan Shop	Green Intelligent Power Saver	It's not the same thing.	Jingdong prefecture level city in Shandong

Happy Fan Shop	Green Intelligent Power Saver	There is no load how much w seems false, nothing works.	Jingdong prefecture level city in Shandong
Happy Fan Shop	Green Intelligent Power Saver	I received the item yesterday, got it back and plugged it in, and it used a little more power yesterday than I had before	Jingdong prefecture level city in Shandong
Happy Fan Shop	Green Intelligent Power Saver	Haven't seen the power saving mode yet	Jingdong prefecture level city in Shandong
Happy Fan Shop	Green Intelligent Power Saver	This product has no known effect	Jingdong prefecture level city in Shandong
Happy Fan Shop	Green Intelligent Power Saver	Refund Returns $16	Jingdong prefecture level city in Shandong
Happy Fan Shop	Green Intelligent Power Saver	The previous year bought a, almost every month can save a 70 dollars or so, used a year, no problem at all, look very new like, this time to move to a new home decisively repurchase 2 units, very believe this	Jingdong prefecture level city in Shandong
Happy Fan Shop	Green Intelligent Power Saver	Has been the second time to buy, this power saving device is really good power, before every month to spend 300 electricity, now are about 200, last time that 6, this time to help lead the family to buy 6,	Jingdong prefecture level city in Shandong
Happy Fan Shop	Green Intelligent Power Saver	In the spirit of trying to experience the new product, the use of down compared to the usual power consumption, with the addition of the quarter, is indeed a lot less electricity, originally more than 450	Jingdong prefecture level city in Shandong
Happy Fan Shop	Green Intelligent Power Saver	Has not been the first time to buy back, good use, power saving device is really powerful, before the monthly electricity bill of more than 500 Now more than 300, this time to help neighbors to buy several	Jingdong prefecture level city in Shandong
Happy Fan Shop	Green Intelligent Power Saver	Home appliances are very many, the circuit is also very complex, at first it is to save 9% of the electricity, and then used more than half a month, found that the power saving rate is as high as more	Jingdong prefecture level city in Shandong
Happy Fan Shop	Green Intelligent Power Saver	My friend bought it last year and saved hundreds of dollars on his electricity bill in one summer Appearance and style: it looks better than the one I bought before Quality is also good with a light.	Jingdong prefecture level city in Shandong
Happy Fan Shop	Green Intelligent Power Saver	Saver received, on the spot on the plug with the feeling of immediate power savings, appearance, packaging is very delicate, very satisfied, I hope to achieve satisfactory results!	Jingdong prefecture level city in Shandong
Happy Fan Shop	Green Intelligent Power Saver	The power saving effect is very good, is considered an old customer, also bought before, this time is to buy two for mom and dad, customer service attitude is good, praise!	Jingdong prefecture level city in Shandong
Happy Fan Shop	Green Intelligent Power Saver	After observing for a while, it really saves a lot of money I didn't realize it was still really reliable, so I'll give you a good review.	Jingdong prefecture level city in Shandong
Happy Fan Shop	Green Intelligent Power Saver	My family a month with this monthly almost save more than 200, power-saving effect: have bought a second time, really effective Cost-effective: not bad Appearance style: roughly the same A few months to the home electricity is indeed less, this time it is a re-buy three, send a friend two, they stay a	Jingdong prefecture level city in Shandong
Happy Fan Shop	Green Intelligent Power Saver	A month almost save more than 150 dollars in electricity, home appliances more, the main this can also protect high-power appliances, after using this device, these two months have not burned, things are very good, it is said that this is the latest model, the overall price is cheap and beautiful!	Jingdong prefecture level city in Shandong
Happy Fan Shop	Green Intelligent Power Saver	Received, powerful, great quality, love the style😍	Jingdong prefecture level city in Shandong
Happy Fan Shop	Green Intelligent Power Saver	This intelligent energy saver is especially good to use and save electricity hope everyone will buy it!	Jingdong prefecture level city in Shandong
Happy Fan Shop	Green Intelligent Power Saver	Air conditioning adapter effect is particularly great, the use of very simple, plug into the socket on the summer air conditioning power is too large, electricity is also expensive, with this product directly on the electricity bill to save half, has been purchased a few more, the effect is too awesome!	Jingdong prefecture level city in Shandong
Happy Fan Shop	Green Intelligent Power Saver	The family plugged in 5, monthly electricity savings of about 300 yuan. My family before a month of electricity costs more than 800, this time with this saver after the electricity bill is actually only more than 500, amazing, thanks to the store, will recommend other friends to buy!	Jingdong prefecture level city in Shandong
Happy Fan Shop	Green Intelligent Power Saver	It was recommended to me by a neighbor's family and it's nice to use it to save power.	Jingdong prefecture level city in Shandong
Happy Fan Shop	Green Intelligent Power Saver	Second time I've purchased it and the power saving is effective!	Jingdong prefecture level city in Shandong
Happy Fan Shop	Green Intelligent Power Saver	The effect is really good, before a month of more than 300 electricity, now every month every day with the air conditioning, it is more than 100 electricity! This thing is really amazing, one after another to buy and 5.6, home installed 3!	Jingdong prefecture level city in Shandong
Happy Fan Shop	Green Intelligent Power Saver	Received this product, began to use, small things look very wrong, can save a little electricity, the monthly electricity bill is too high, after plugging in did not pull, or save a lot of electricity, point	Jingdong prefecture level city in Shandong
Happy Fan Shop	Green Intelligent Power Saver	Simple and convenient, just that plug in, you can save electricity. A month can save dozens of dollars, a year down to save a lot of, very good product cost-effective, everyone can afford to buy and use!	Jingdong prefecture level city in Shandong
Happy Fan Shop	Green Intelligent Power Saver	Electricity bill from more than 200 yuan per month, down to more than 150 yuan, the original bought home, used two months, really have power-saving effect, this time and sold two, business service attitude is	Jingdong prefecture level city in Shandong
Happy Fan Shop	Green Intelligent Power Saver	Like to watch some movies at home and so on, sometimes at night until two or three o'clock, and recently the air conditioning is not turned off for a night, rather wasteful, I have to say that the effect is very good, quite like it!	Jingdong prefecture level city in Shandong
Happy Fan Shop	Green Intelligent Power Saver	Power-saving effect: the effect is very good, a month to save more than 200 dollars Cost-effective: good value for money Appearance and style: small and lovely Workmanship material: okay Experience: good	Jingdong prefecture level city in Shandong
Happy Fan Shop	Green Intelligent Power Saver	It's okay I quite like it, the stuff is very good. Come back in the future. You can rest assured to buy. Cost-effective all. Much cheaper than the physical store	Jingdong prefecture level city in Shandong
Happy Fan Shop	Green Intelligent Power Saver	More than 150 less, last month's electricity bill is almost 400, this month is only about 200, really save electricity, I am home to buy three, I did not expect a month to pay back	Jingdong prefecture level city in Shandong
Happy Fan Shop	Green Intelligent Power Saver	I bought three of these first and tried them for two months and they still felt useful, so now I've bought another one. Good and recommended!	Jingdong prefecture level city in Shandong
Happy Fan Shop	Green Intelligent Power Saver	This energy saver is very good quality and inexpensive next time I will buy it again!	Jingdong prefecture level city in Shandong
Happy Fan Shop	Green Intelligent Power Saver	Merchant service attitude is not good, the quality does not match, the business does not give the package return	Jingdong prefecture level city in Shandong
Happy Fan Shop	Green Intelligent Power Saver	The goods are very small do not know how effective after a few days to follow up the review	Jingdong prefecture level city in Shandong
Happy Fan Shop	Green Intelligent Power Saver	Not used before more than 200 a month, after using about 100 a month, my family used three, customer service said I use electricity recommended 2-3, I think more than one effect is better on the purchase of 4, the effect is very obvious, recommended to buy!	Jingdong prefecture level city in Shandong
Happy Fan Shop	Green Intelligent Power Saver	It's okay. I'll buy it again today.	Jingdong prefecture level city in Shandong
Happy Fan Shop	Green Intelligent Power Saver	Finally received the goods, very, very much like,, has been a lot of people praise, really too happy, has been his family's loyal fans, will continue to buy buy buy buy!	Jingdong prefecture level city in Shandong
Happy Fan Shop	Green Intelligent Power Saver	After using this, my monthly electricity bill was 1,200 pounds, now it's only 180 bucks a month, how good is it?	Jingdong prefecture level city in Shandong
Happy Fan Shop	Green Intelligent Power Saver	The item is a scam return to marry a dozen dollars in shipping fees. Buy terrific role is purely a scam.	Jingdong prefecture level city in Shandong
Happy Fan Shop	Green Intelligent Power Saver	It's really good, cost-effective and good quality, and the logistics are fast! It arrived in one day!	Jingdong prefecture level city in Shandong
Happy Fan Shop	Green Intelligent Power Saver	Great brand! Trustworthy! Works super well! Expensive for a reason! Great merchandise!	Jingdong prefecture level city in Shandong
Happy Fan Shop	Green Intelligent Power Saver	The baby is really great, I like it very much, my friends also say good to come, recommended to buy!	Jingdong prefecture level city in Shandong
Happy Fan Shop	Green Intelligent Power Saver	Fake couldn't be more fake. Bought one plugged in still so much electricity.	Jingdong prefecture level city in Shandong
Happy Fan Shop	Green Intelligent Power Saver	This appliance works great. Let's see how it works. We'll see later.	Jingdong prefecture level city in Shandong
Happy Fan Shop	Green Intelligent Power Saver	Used it before commenting, very happy with it	Jingdong prefecture level city in Shandong
Happy Fan Shop	Green Intelligent Power Saver	Goods have been received well-packed, logistics is very fast and good quality	Jingdong prefecture level city in Shandong
Happy Fan Shop	Green Intelligent Power Saver	This baby's quality is okay.	Jingdong prefecture level city in Shandong
Happy Fan Shop	Green Intelligent Power Saver	Counterfeit goods.	Jingdong prefecture level city in Shandong

Happy Fan Shop	Green Intelligent Power Saver	The effect is not very obvious	Jingdong prefecture level city in Shandong
Happy Fan Shop	Green Intelligent Power Saver	It's not the same thing.	Jingdong prefecture level city in Shandong
Happy Fan Shop	Green Intelligent Power Saver	There is no load how much w seems false, nothing works.	Jingdong prefecture level city in Shandong
Happy Fan Shop	Green Intelligent Power Saver	I received the item yesterday, got it back and plugged it in, and it used a little more power yesterday than I had before	Jingdong prefecture level city in Shandong
Happy Fan Shop	Green Intelligent Power Saver	Haven't seen the power saving mode yet	Jingdong prefecture level city in Shandong
Happy Fan Shop	Green Intelligent Power Saver	This product has no known effect	Jingdong prefecture level city in Shandong
Happy Fan Shop	Green Intelligent Power Saver	Refund Returns $16	Jingdong prefecture level city in Shandong
Happy Fan Shop	Green Intelligent Power Saver	The previous year bought a, almost every month can save a 70 dollars or so, used a year, no problem at all, look very new like, this time to move to a new home decisively repurchase 2 units, very believe this	Jingdong prefecture level city in Shandong
Happy Fan Shop	Green Intelligent Power Saver	Has been the second time to buy, this power saving device is really good power, before every month to spend 300 electricity, now are about 200, last time that 6, this time to help lead the family to buy 6,	Jingdong prefecture level city in Shandong
Happy Fan Shop	Green Intelligent Power Saver	In the spirit of trying to experience the new product, the use of down compared to the usual power consumption, with the addition of the quarter, is indeed a lot less electricity, originally more than 450	Jingdong prefecture level city in Shandong
Happy Fan Shop	Green Intelligent Power Saver	Has not been the first time to buy back, good use, power saving device is really powerful, before the monthly electricity bill of more than 500 Now more than 300, this time to help neighbors to buy several	Jingdong prefecture level city in Shandong
Happy Fan Shop	Green Intelligent Power Saver	Home appliances are very many, the circuit is also very complex, at first it is to save 9% of the electricity, and then used more than half a month, found that the power saving rate is as high as more	Jingdong prefecture level city in Shandong
Happy Fan Shop	Green Intelligent Power Saver	My friend bought it last year and saved hundreds of dollars on his electricity bill in one summer Appearance and style: it looks better than the one I bought before Quality is also good with a light.	Jingdong prefecture level city in Shandong
Happy Fan Shop	Green Intelligent Power Saver	Saver received, on the spot on the plug with the feeling of immediate power savings, appearance, packaging is very delicate, very satisfied, I hope to achieve satisfactory results!	Jingdong prefecture level city in Shandong
Happy Fan Shop	Green Intelligent Power Saver	The power saving effect is very good, is considered an old customer, also bought before, this time is to buy two for mom and dad, customer service attitude is good, praise!	Jingdong prefecture level city in Shandong
Happy Fan Shop	Green Intelligent Power Saver	After observing for a while, it really saves a lot of money I didn't realize it was still really reliable, so I'll give you a good review.	Jingdong prefecture level city in Shandong
Happy Fan Shop	Green Intelligent Power Saver	My family a month with this monthly almost save more than 200, power-saving effect: have bought a second time, really effective Cost-effective: not bad Appearance style: roughly the same A few months to the home electricity is indeed less, this time it is a re-buy three, send a friend two, they stay a	Jingdong prefecture level city in Shandong
Happy Fan Shop	Green Intelligent Power Saver	A month almost save more than 150 dollars in electricity, home appliances more, the main this can also protect high-power appliances, after using this device, these two months have not burned, things are very good, it is said that this is the latest model, the overall price is cheap and beautiful!	Jingdong prefecture level city in Shandong
Happy Fan Shop	Green Intelligent Power Saver	Received, powerful, great quality, love the style😍	Jingdong prefecture level city in Shandong
Happy Fan Shop	Green Intelligent Power Saver	This intelligent energy saver is especially good to use and save electricity hope everyone will buy it!	Jingdong prefecture level city in Shandong
Happy Fan Shop	Green Intelligent Power Saver	Air conditioning adapter effect is particularly great, the use of very simple, plug into the socket on the summer air conditioning power is too large, electricity is also expensive, with this product directly on the electricity bill to save half, has been purchased a few more, the effect is too awesome!	Jingdong prefecture level city in Shandong
Happy Fan Shop	Green Intelligent Power Saver	The family plugged in 5, monthly electricity savings of about 300 yuan. My family before a month of electricity costs more than 800, this time with this saver after the electricity bill is actually only more than 500, amazing, thanks to the store, will recommend other friends to buy!	Jingdong prefecture level city in Shandong
Happy Fan Shop	Green Intelligent Power Saver	It was recommended to me by a neighbor's family and it's nice to use it to save power.	Jingdong prefecture level city in Shandong
Happy Fan Shop	Green Intelligent Power Saver	Second time I've purchased it and the power saving is effective!	Jingdong prefecture level city in Shandong
Happy Fan Shop	Green Intelligent Power Saver	The effect is really good, before a month of more than 300 electricity, now every month every day with the air conditioning, it is more than 100 electricity! This thing is really amazing, one after another to buy and 5.6, home installed 3!	Jingdong prefecture level city in Shandong
Happy Fan Shop	Green Intelligent Power Saver	Received this product, began to use, small things look very wrong, can save a little electricity, the monthly electricity bill is too high, after plugging in did not pull, or save a lot of electricity, point	Jingdong prefecture level city in Shandong
Happy Fan Shop	Green Intelligent Power Saver	Simple and convenient, just that plug in, you can save electricity. A month can save dozens of dollars, a year down to save a lot of, very good product cost-effective, everyone can afford to buy and use!	Jingdong prefecture level city in Shandong
Happy Fan Shop	Green Intelligent Power Saver	Electricity bill from more than 200 yuan per month, down to more than 150 yuan, the original bought home, used two months, really have power-saving effect, this time and sold two, business service attitude is	Jingdong prefecture level city in Shandong
Happy Fan Shop	Green Intelligent Power Saver	Like to watch some movies at home and so on, sometimes at night until two or three o'clock, and recently the air conditioning is not turned off for a night, rather wasteful, I have to say that the effect is very good, quite like it!	Jingdong prefecture level city in Shandong
Happy Fan Shop	Green Intelligent Power Saver	Power-saving effect: the effect is very good, a month to save more than 200 dollars Cost-effective: good value for money Appearance and style: small and lovely Workmanship material: okay Experience: good	Jingdong prefecture level city in Shandong
Happy Fan Shop	Green Intelligent Power Saver	It's okay I quite like it, the stuff is very good. Come back in the future. You can rest assured to buy. Cost-effective all. Much cheaper than the physical store	Jingdong prefecture level city in Shandong
Happy Fan Shop	Green Intelligent Power Saver	More than 150 less, last month's electricity bill is almost 400, this month is only about 200, really save electricity, I am home to buy three, I did not expect a month to pay back	Jingdong prefecture level city in Shandong
Happy Fan Shop	Green Intelligent Power Saver	I bought three of these first and tried them for two months and they still felt useful, so now I've bought another one. Good and recommended!	Jingdong prefecture level city in Shandong
Happy Fan Shop	Green Intelligent Power Saver	This energy saver is very good quality and inexpensive next time I will buy it again!	Jingdong prefecture level city in Shandong
Happy Fan Shop	Green Intelligent Power Saver	Merchant service attitude is not good, the quality does not match, the business does not give the package return	Jingdong prefecture level city in Shandong
Happy Fan Shop	Green Intelligent Power Saver	The goods are very small do not know how effective after a few days to follow up the review	Jingdong prefecture level city in Shandong
Happy Fan Shop	Green Intelligent Power Saver	Not used before more than 200 a month, after using about 100 a month, my family used three, customer service said I use electricity recommended 2-3, I think more than one effect is better on the purchase of 4, the effect is very obvious, recommended to buy!	Jingdong prefecture level city in Shandong
Happy Fan Shop	Green Intelligent Power Saver	It's okay. I'll buy it again today.	Jingdong prefecture level city in Shandong
Happy Fan Shop	Green Intelligent Power Saver	Finally received the goods, very, very much like,, has been a lot of people praise, really too happy, has been his family's loyal fans, will continue to buy buy buy buy!	Jingdong prefecture level city in Shandong
Happy Fan Shop	Green Intelligent Power Saver	After using this, my monthly electricity bill was 1,200 pounds, now it's only 180 bucks a month, how good is it?	Jingdong prefecture level city in Shandong
Happy Fan Shop	Green Intelligent Power Saver	The item is a scam return to marry a dozen dollars in shipping fees. Buy terrific role is purely a scam.	Jingdong prefecture level city in Shandong
Happy Fan Shop	Green Intelligent Power Saver	It's really good, cost-effective and good quality, and the logistics are fast! It arrived in one day!	Jingdong prefecture level city in Shandong
Happy Fan Shop	Green Intelligent Power Saver	Great brand! Trustworthy! Works super well! Expensive for a reason! Great merchandise!	Jingdong prefecture level city in Shandong
Happy Fan Shop	Green Intelligent Power Saver	The baby is really great, I like it very much, my friends also say good to come, recommended to buy!	Jingdong prefecture level city in Shandong
Happy Fan Shop	Green Intelligent Power Saver	Fake couldn't be more fake. Bought one plugged in still so much electricity.	Jingdong prefecture level city in Shandong
Happy Fan Shop	Green Intelligent Power Saver	This appliance works great. Let's see how it works. We'll see later.	Jingdong prefecture level city in Shandong
Happy Fan Shop	Green Intelligent Power Saver	Used it before commenting, very happy with it	Jingdong prefecture level city in Shandong
Happy Fan Shop	Green Intelligent Power Saver	Goods have been received well-packed, logistics is very fast and good quality	Jingdong prefecture level city in Shandong

Happy Fan Shop	Green Intelligent Power Saver	This baby's quality is okay.	Jingdong prefecture level city in Shandong
Happy Fan Shop	Green Intelligent Power Saver	Counterfeit goods.	Jingdong prefecture level city in Shandong
Happy Fan Shop	Green Intelligent Power Saver	The effect is not very obvious	Jingdong prefecture level city in Shandong
Happy Fan Shop	Green Intelligent Power Saver	It's not the same thing.	Jingdong prefecture level city in Shandong
Happy Fan Shop	Green Intelligent Power Saver	There is no load how much w seems false, nothing works.	Jingdong prefecture level city in Shandong
Happy Fan Shop	Green Intelligent Power Saver	I received the item yesterday, got it back and plugged it in, and it used a little more power yesterday than I had before	Jingdong prefecture level city in Shandong
Happy Fan Shop	Green Intelligent Power Saver	Haven't seen the power saving mode yet	Jingdong prefecture level city in Shandong
Happy Fan Shop	Green Intelligent Power Saver	This product has no known effect	Jingdong prefecture level city in Shandong
Happy Fan Shop	Green Intelligent Power Saver	Refund Returns $16	Jingdong prefecture level city in Shandong
Happy Fan Shop	Green Intelligent Power Saver	The previous year bought a, almost every month can save a 70 dollars or so, used a year, no problem at all, look very new like, this time to move to a new home decisively repurchase 2 units, very believe this	Jingdong prefecture level city in Shandong
Happy Fan Shop	Green Intelligent Power Saver	Has been the second time to buy, this power saving device is really good power, before every month to spend 300 electricity, now are about 200, last time that 6, this time to help lead the family to buy 6,	Jingdong prefecture level city in Shandong
Happy Fan Shop	Green Intelligent Power Saver	In the spirit of trying to experience the new product, the use of down compared to the usual power consumption, with the addition of the quarter, is indeed a lot less electricity, originally more than 450	Jingdong prefecture level city in Shandong
Happy Fan Shop	Green Intelligent Power Saver	Has not been the first time to buy back, good use, power saving device is really power, before the monthly electricity bill of more than 500 Now more than 300, this time to help neighbors to buy several	Jingdong prefecture level city in Shandong
Happy Fan Shop	Green Intelligent Power Saver	Home appliances are very many, the circuit is also very complex, at first it is to save 9% of the electricity, and then used more than half a month, found that the power saving rate is as high as more	Jingdong prefecture level city in Shandong
Happy Fan Shop	Green Intelligent Power Saver	My friend bought it last year and saved hundreds of dollars on his electricity bill in one summer Appearance and style: it looks better than the one I bought before Quality is also good with a light.	Jingdong prefecture level city in Shandong
Happy Fan Shop	Green Intelligent Power Saver	Saver received, on the spot on the plug with the feeling of immediate power savings, appearance, packaging is very delicate, very satisfied, I hope to achieve satisfactory results!	Jingdong prefecture level city in Shandong
Happy Fan Shop	Green Intelligent Power Saver	The power saving effect is very good, is considered an old customer, also bought before, this time is to buy two for mom and dad, customer service attitude is good, praise!	Jingdong prefecture level city in Shandong
Happy Fan Shop	Green Intelligent Power Saver	After observing for a while, it really saves a lot of money I didn't realize it was still really reliable, so I'll give you a good review.	Jingdong prefecture level city in Shandong
Happy Fan Shop	Green Intelligent Power Saver	My family a month with this monthly almost save more than 200, power-saving effect: have bought a second time, really effective Cost-effective: not bad Appearance style: roughly the same A few months to the home electricity is indeed less, this time it is a re-buy three, send a friend two, they stay a	Jingdong prefecture level city in Shandong
Happy Fan Shop	Green Intelligent Power Saver	A month almost save more than 150 dollars in electricity, home appliances more, the main this can also protect high-power appliances, after using this device, these two months have not burned, things are very good, it is said that this is the latest model, the overall price is cheap and beautiful!	Jingdong prefecture level city in Shandong
Happy Fan Shop	Green Intelligent Power Saver	Received, powerful, great quality, love the style😍	Jingdong prefecture level city in Shandong
Happy Fan Shop	Green Intelligent Power Saver	This intelligent energy saver is especially good to use and save electricity hope everyone will buy it!	Jingdong prefecture level city in Shandong
Happy Fan Shop	Green Intelligent Power Saver	Air conditioning adapter effect is particularly great, the use of very simple, plug into the socket on the summer air conditioning power is too large, electricity is also expensive, with this product directly on the electricity bill to save half, has been purchased a few more, the effect is too awesome!	Jingdong prefecture level city in Shandong
Happy Fan Shop	Green Intelligent Power Saver	The family plugged in 5, monthly electricity savings of about 300 yuan. My family before a month of electricity costs more than 800, this time with this saver after the electricity bill is actually only more than 500, amazing, thanks to the store, will recommend other friends to buy!	Jingdong prefecture level city in Shandong
Happy Fan Shop	Green Intelligent Power Saver	It was recommended to me by a neighbor's family and it's nice to use it to save power.	Jingdong prefecture level city in Shandong
Happy Fan Shop	Green Intelligent Power Saver	Second time I've purchased it and the power saving is effective!	Jingdong prefecture level city in Shandong
Happy Fan Shop	Green Intelligent Power Saver	The effect is really good, before a month of more than 300 electricity, now every month every day with the air conditioning, it is more than 100 electricity! This thing is really amazing, one after another to buy and 5.6, home installed 3!	Jingdong prefecture level city in Shandong
Happy Fan Shop	Green Intelligent Power Saver	Received this product, began to use, small things look very wrong, can save a little electricity, the monthly electricity bill is too high, after plugging in did not pull, or save a lot of electricity, point	Jingdong prefecture level city in Shandong
Happy Fan Shop	Green Intelligent Power Saver	Simple and convenient, just that plug in, you can save electricity. A month can save dozens of dollars, a year down to save a lot of, very good product cost-effective, everyone can afford to buy and use!	Jingdong prefecture level city in Shandong
Happy Fan Shop	Green Intelligent Power Saver	Electricity bill from more than 200 yuan per month, down to more than 150 yuan, the original bought home, used two months, really have power-saving effect, this time and sold two, business service attitude is	Jingdong prefecture level city in Shandong
Happy Fan Shop	Green Intelligent Power Saver	Like to watch some movies at home and so on, sometimes at night until two or three o'clock, and recently the air conditioning is not turned off for a night, rather wasteful, I have to say that the effect is very good, quite like it!	Jingdong prefecture level city in Shandong
Happy Fan Shop	Green Intelligent Power Saver	Power-saving effect: the effect is very good, a month to save more than 200 dollars Cost-effective: good value for money Appearance and style: small and lovely Workmanship material: okay Experience: good	Jingdong prefecture level city in Shandong
Happy Fan Shop	Green Intelligent Power Saver	It's okay I quite like it, the stuff is very good. Come back in the future. You can rest assured to buy. Cost-effective all. Much cheaper than the physical store	Jingdong prefecture level city in Shandong
Happy Fan Shop	Green Intelligent Power Saver	More than 150 less, last month's electricity bill is almost 400, this month is only about 200, really save electricity, I am home to buy three, I did not expect a month to pay back	Jingdong prefecture level city in Shandong
Happy Fan Shop	Green Intelligent Power Saver	I bought three of these first and tried them for two months and they still felt useful, so now I've bought another one. Good and recommended!	Jingdong prefecture level city in Shandong
Happy Fan Shop	Green Intelligent Power Saver	This energy saver is very good quality and inexpensive next time I will buy it again!	Jingdong prefecture level city in Shandong
Happy Fan Shop	Green Intelligent Power Saver	Merchant service attitude is not good, the quality does not match, the business does not give the package return	Jingdong prefecture level city in Shandong
Happy Fan Shop	Green Intelligent Power Saver	The goods are very small do not know how effective after a few days to follow up the review	Jingdong prefecture level city in Shandong
Happy Fan Shop	Green Intelligent Power Saver	Not used before more than 200 a month, after using about 100 a month, my family used three, customer service said I use electricity recommended 2-3, I think more than one effect is better on the purchase of 4, the effect is very obvious, recommended to buy!	Jingdong prefecture level city in Shandong
Happy Fan Shop	Green Intelligent Power Saver	It's okay. I'll buy it again today.	Jingdong prefecture level city in Shandong
Happy Fan Shop	Green Intelligent Power Saver	Finally received the goods, very, very much like,, has been a lot of people praise, really too happy, has been his family's loyal fans, will continue to buy buy buy!	Jingdong prefecture level city in Shandong
Happy Fan Shop	Green Intelligent Power Saver	After using this, my monthly electricity bill was 1,200 pounds, now it's only 180 bucks a month, how good is it?	Jingdong prefecture level city in Shandong
Happy Fan Shop	Green Intelligent Power Saver	The item is a scam return to marry a dozen dollars in shipping fees. Buy terrific role is purely a scam.	Jingdong prefecture level city in Shandong
Happy Fan Shop	Green Intelligent Power Saver	It's really good, cost-effective and good quality, and the logistics are fast! It arrived in one day!	Jingdong prefecture level city in Shandong
Happy Fan Shop	Green Intelligent Power Saver	Great brand! Trustworthy! Works super well! Expensive for a reason! Great merchandise!	Jingdong prefecture level city in Shandong
Happy Fan Shop	Green Intelligent Power Saver	The baby is really great, I like it very much, my friends also say good to come, recommended to buy!	Jingdong prefecture level city in Shandong
Happy Fan Shop	Green Intelligent Power Saver	Fake couldn't be more fake. Bought one plugged in still so much electricity.	Jingdong prefecture level city in Shandong
Happy Fan Shop	Green Intelligent Power Saver	This appliance works great. Let's see how it works. We'll see later.	Jingdong prefecture level city in Shandong

Happy Fan Shop	Green Intelligent Power Saver	Used it before commenting, very happy with it	Jingdong prefecture level city in Shandong
Happy Fan Shop	Green Intelligent Power Saver	Goods have been received well-packed, logistics is very fast and good quality	Jingdong prefecture level city in Shandong
Happy Fan Shop	Green Intelligent Power Saver	This baby's quality is okay.	Jingdong prefecture level city in Shandong
Happy Fan Shop	Green Intelligent Power Saver	Counterfeit goods.	Jingdong prefecture level city in Shandong
Happy Fan Shop	Green Intelligent Power Saver	The effect is not very obvious	Jingdong prefecture level city in Shandong
Happy Fan Shop	Green Intelligent Power Saver	It's not the same thing.	Jingdong prefecture level city in Shandong
Happy Fan Shop	Green Intelligent Power Saver	There is no load how much w seems false, nothing works.	Jingdong prefecture level city in Shandong
Happy Fan Shop	Green Intelligent Power Saver	I received the item yesterday, got it back and plugged it in, and it used a little more power yesterday than I had before	Jingdong prefecture level city in Shandong
Happy Fan Shop	Green Intelligent Power Saver	Haven't seen the power saving mode yet	Jingdong prefecture level city in Shandong
Happy Fan Shop	Green Intelligent Power Saver	This product has no known effect	Jingdong prefecture level city in Shandong
Happy Fan Shop	Green Intelligent Power Saver	Refund Returns $16	Jingdong prefecture level city in Shandong
Happy Fan Shop	Green Intelligent Power Saver	The previous year bought a, almost every month can save a 70 dollars or so, used a year, no problem at all, look very new like, this time to move to a new home decisively repurchase 2 units, very believe this	Jingdong prefecture level city in Shandong
Happy Fan Shop	Green Intelligent Power Saver	Has been the second time to buy, this power saving device is really good power, before every month to spend 300 electricity, now are about 200, last time that 6, this time to help lead the family to buy 6,	Jingdong prefecture level city in Shandong
Happy Fan Shop	Green Intelligent Power Saver	In the spirit of trying to experience the new product, the use of down compared to the usual power consumption, with the addition of the quarter, is indeed a lot less electricity, originally more than 450	Jingdong prefecture level city in Shandong
Happy Fan Shop	Green Intelligent Power Saver	Has not been the first time to buy back, good use, power saving device is really powerful, before the monthly electricity bill of more than 500 Now more than 300, this time to help neighbors to buy several	Jingdong prefecture level city in Shandong
Happy Fan Shop	Green Intelligent Power Saver	Home appliances are very many, the circuit is also very complex, at first it is to save 9% of the electricity, and then used more than half a month, found that the power saving rate is as high as more	Jingdong prefecture level city in Shandong
Happy Fan Shop	Green Intelligent Power Saver	My friend bought it last year and saved hundreds of dollars on his electricity bill in one summer Appearance and style: it looks better than the one I bought before Quality is also good with a light.	Jingdong prefecture level city in Shandong
Happy Fan Shop	Green Intelligent Power Saver	Saver received, on the spot on the plug with the feeling of immediate power savings, appearance, packaging is very delicate, very satisfied, I hope to achieve satisfactory results!	Jingdong prefecture level city in Shandong
Happy Fan Shop	Green Intelligent Power Saver	The power saving effect is very good, is considered an old customer, also bought before, this time is to buy two for mom and dad, customer service attitude is good, praise!	Jingdong prefecture level city in Shandong
Happy Fan Shop	Green Intelligent Power Saver	After observing for a while, it really saves a lot of money I didn't realize it was still really reliable, so I'll give you a good review.	Jingdong prefecture level city in Shandong
Happy Fan Shop	Green Intelligent Power Saver	My family a month with this monthly almost save more than 200, power-saving effect: have bought a second time, really effective Cost-effective: not bad Appearance style: roughly the same A few months to the home electricity is indeed less, this time it is a re-buy three, send a friend two, they stay a	Jingdong prefecture level city in Shandong
Happy Fan Shop	Green Intelligent Power Saver	A month almost save more than 150 dollars in electricity, home appliances more, the main this can also protect high-power appliances, after using this device, these two months have not burned, things are very good, it is said that this is the latest model, the overall price is cheap and beautiful!	Jingdong prefecture level city in Shandong
Happy Fan Shop	Green Intelligent Power Saver	Received, powerful, great quality, love the style😍	Jingdong prefecture level city in Shandong
Happy Fan Shop	Green Intelligent Power Saver	This intelligent energy saver is especially good to use and save electricity hope everyone will buy it!	Jingdong prefecture level city in Shandong
Happy Fan Shop	Green Intelligent Power Saver	Air conditioning adapter effect is particularly great, the use of very simple, plug into the socket on the summer air conditioning power is too large, electricity is also expensive, with this product directly on the electricity bill to save half, has been purchased a few more, the effect is too awesome!	Jingdong prefecture level city in Shandong
Happy Fan Shop	Green Intelligent Power Saver	The family plugged in 5, monthly electricity savings of about 300 yuan. My family before a month of electricity costs more than 800, this time with this saver after the electricity bill is actually only more than 500, amazing, thanks to the store, will recommend other friends to buy!	Jingdong prefecture level city in Shandong
Happy Fan Shop	Green Intelligent Power Saver	It was recommended to me by a neighbor's family and it's nice to use it to save power.	Jingdong prefecture level city in Shandong
Happy Fan Shop	Green Intelligent Power Saver	Second time I've purchased it and the power saving is effective!	Jingdong prefecture level city in Shandong
Happy Fan Shop	Green Intelligent Power Saver	The effect is really good, before a month of more than 300 electricity, now every month every day with the air conditioning, it is more than 100 electricity! This thing is really amazing, one after another to buy and 5.6, home installed 3!	Jingdong prefecture level city in Shandong
Happy Fan Shop	Green Intelligent Power Saver	Received this product, began to use, small things look very wrong, can save a little electricity, the monthly electricity bill is too high, after plugging in did not pull, or save a lot of electricity, point	Jingdong prefecture level city in Shandong
Happy Fan Shop	Green Intelligent Power Saver	Simple and convenient, just that plug in, you can save electricity. A month can save dozens of dollars, a year down to save a lot of, very good product cost-effective, everyone can afford to buy and use!	Jingdong prefecture level city in Shandong
Happy Fan Shop	Green Intelligent Power Saver	Electricity bill from more than 200 yuan per month, down to more than 150 yuan, the original bought home, used two months, really have power-saving effect, this time and sold two, business service attitude is	Jingdong prefecture level city in Shandong
Happy Fan Shop	Green Intelligent Power Saver	Like to watch some movies at home and so on, sometimes at night until two or three o'clock, and recently the air conditioning is not turned off for a night, rather wasteful, I have to say that the effect is very good, quite like it!	Jingdong prefecture level city in Shandong
Happy Fan Shop	Green Intelligent Power Saver	Power-saving effect: the effect is very good, a month to save more than 200 dollars Cost-effective: good value for money Appearance and style: small and lovely Workmanship material: okay Experience: good	Jingdong prefecture level city in Shandong
Happy Fan Shop	Green Intelligent Power Saver	It's okay I quite like it, the stuff is very good. Come back in the future. You can rest assured to buy. Cost-effective all. Much cheaper than the physical store	Jingdong prefecture level city in Shandong
Happy Fan Shop	Green Intelligent Power Saver	More than 150 less, last month's electricity bill is almost 400, this month is only about 200, really save electricity, I am home to buy three, I did not expect a month to pay back	Jingdong prefecture level city in Shandong
Happy Fan Shop	Green Intelligent Power Saver	I bought three of these first and tried them for two months and they still felt useful, so now I've bought another one. Good and recommended!	Jingdong prefecture level city in Shandong
Happy Fan Shop	Green Intelligent Power Saver	This energy saver is very good quality and inexpensive next time I will buy it again!	Jingdong prefecture level city in Shandong
Happy Fan Shop	Green Intelligent Power Saver	Merchant service attitude is not good, the quality does not match, the business does not give the package return	Jingdong prefecture level city in Shandong
Happy Fan Shop	Green Intelligent Power Saver	The goods are very small do not know how effective after a few days to follow up the review	Jingdong prefecture level city in Shandong
Happy Fan Shop	Green Intelligent Power Saver	Not used before more than 200 a month, after using about 100 a month, my family used three, customer service said I use electricity recommended 2-3, I think more than one effect is better on the purchase of 4, the effect is very obvious, recommended to buy!	Jingdong prefecture level city in Shandong
Happy Fan Shop	Green Intelligent Power Saver	It's okay. I'll buy it again today.	Jingdong prefecture level city in Shandong
Happy Fan Shop	Green Intelligent Power Saver	Finally received the goods, very, very much like,, has been a lot of people praise, really too happy, has been his family's loyal fans, will continue to buy buy buy buy!	Jingdong prefecture level city in Shandong
Happy Fan Shop	Green Intelligent Power Saver	After using this, my monthly electricity bill was 1,200 pounds, now it's only 180 bucks a month, how good is it?	Jingdong prefecture level city in Shandong
Happy Fan Shop	Green Intelligent Power Saver	The item is a scam return to marry a dozen dollars in shipping fees. Buy terrific role is purely a scam.	Jingdong prefecture level city in Shandong
Happy Fan Shop	Green Intelligent Power Saver	It's really good, cost-effective and good quality, and the logistics are fast! It arrived in one day!	Jingdong prefecture level city in Shandong
Happy Fan Shop	Green Intelligent Power Saver	Great brand! Trustworthy! Works super well! Expensive for a reason! Great merchandise!	Jingdong prefecture level city in Shandong
Happy Fan Shop	Green Intelligent Power Saver	The baby is really great, I like it very much, my friends also say good to come, recommended to buy!	Jingdong prefecture level city in Shandong

Happy Fan Shop	Green Intelligent Power Saver	Fake couldn't be more fake. Bought one plugged in still so much electricity.	Jingdong prefecture level city in Shandong
Happy Fan Shop	Green Intelligent Power Saver	This appliance works great. Let's see how it works. We'll see later.	Jingdong prefecture level city in Shandong
Happy Fan Shop	Green Intelligent Power Saver	Used it before commenting, very happy with it	Jingdong prefecture level city in Shandong
Happy Fan Shop	Green Intelligent Power Saver	Goods have been received well-packed, logistics is very fast and good quality	Jingdong prefecture level city in Shandong
Happy Fan Shop	Green Intelligent Power Saver	This baby's quality is okay.	Jingdong prefecture level city in Shandong
Happy Fan Shop	Green Intelligent Power Saver	Counterfeit goods.	Jingdong prefecture level city in Shandong
Happy Fan Shop	Green Intelligent Power Saver	The effect is not very obvious	Jingdong prefecture level city in Shandong
Happy Fan Shop	Green Intelligent Power Saver	It's not the same thing.	Jingdong prefecture level city in Shandong
Happy Fan Shop	Green Intelligent Power Saver	There is no load how much w seems false, nothing works.	Jingdong prefecture level city in Shandong
Happy Fan Shop	Green Intelligent Power Saver	I received the item yesterday, got it back and plugged it in, and it used a little more power yesterday than I had before	Jingdong prefecture level city in Shandong
Happy Fan Shop	Green Intelligent Power Saver	Haven't seen the power saving mode yet	Jingdong prefecture level city in Shandong
Happy Fan Shop	Green Intelligent Power Saver	This product has no known effect	Jingdong prefecture level city in Shandong
Happy Fan Shop	Green Intelligent Power Saver	Refund Returns $16	Jingdong prefecture level city in Shandong
Happy Fan Shop	Green Intelligent Power Saver	The previous year bought a, almost every month can save a 70 dollars or so, used a year, no problem at all, look very new like, this time to move to a new home decisively repurchase 2 units, very believe this	Jingdong prefecture level city in Shandong
Happy Fan Shop	Green Intelligent Power Saver	Has been the second time to buy, this power saving device is really good power, before every month to spend 300 electricity, now are about 200, last time that 6, this time to help lead the family to buy 6,	Jingdong prefecture level city in Shandong
Happy Fan Shop	Green Intelligent Power Saver	In the spirit of trying to experience the new product, the use of down compared to the usual power consumption, with the addition of the quarter, is indeed a lot less electricity, originally more than 450	Jingdong prefecture level city in Shandong
Happy Fan Shop	Green Intelligent Power Saver	Has not been the first time to buy back, good use, power saving device is really powerful, before the monthly electricity bill of more than 500 Now more than 300, this time to help neighbors to buy several	Jingdong prefecture level city in Shandong
Happy Fan Shop	Green Intelligent Power Saver	Home appliances are very many, the circuit is also very complex, at first it is to save 9% of the electricity, and then used more than half a month, found that the power saving rate is as high as more	Jingdong prefecture level city in Shandong
Happy Fan Shop	Green Intelligent Power Saver	My friend bought it last year and saved hundreds of dollars on his electricity bill in one summer Appearance and style: it looks better than the one I bought before Quality is also good with a light.	Jingdong prefecture level city in Shandong
Happy Fan Shop	Green Intelligent Power Saver	Saver received, on the spot on the plug with the feeling of immediate power savings, appearance, packaging is very delicate, very satisfied, I hope to achieve satisfactory results!	Jingdong prefecture level city in Shandong
Happy Fan Shop	Green Intelligent Power Saver	The power saving effect is very good, is considered an old customer, also bought before, this time is to buy two for mom and dad, customer service attitude is good, praise!	Jingdong prefecture level city in Shandong
Happy Fan Shop	Green Intelligent Power Saver	After observing for a while, it really saves a lot of money I didn't realize it was still really reliable, so I'll give you a good review.	Jingdong prefecture level city in Shandong
Happy Fan Shop	Green Intelligent Power Saver	My family a month with this monthly almost save more than 200, power-saving effect: have bought a second time, really effective Cost-effective: not bad Appearance style: roughly the same A few months to the home electricity is indeed less, this time it is a re-buy three, send a friend two, they stay a	Jingdong prefecture level city in Shandong
Happy Fan Shop	Green Intelligent Power Saver	A month almost save more than 150 dollars in electricity, home appliances more, the main this can also protect high-power appliances, after using this device, these two months have not burned, things are very good, it is said that this is the latest model, the overall price is cheap and beautiful!	Jingdong prefecture level city in Shandong
Happy Fan Shop	Green Intelligent Power Saver	Received, powerful, great quality, love the style😍	Jingdong prefecture level city in Shandong
Happy Fan Shop	Green Intelligent Power Saver	This intelligent energy saver is especially good to use and save electricity hope everyone will buy it!	Jingdong prefecture level city in Shandong
Happy Fan Shop	Green Intelligent Power Saver	Air conditioning adapter effect is particularly great, the use of very simple, plug into the socket on the summer air conditioning power is too large, electricity is also expensive, with this product directly on the electricity bill to save half, has been purchased a few more, the effect is too awesome!	Jingdong prefecture level city in Shandong
Happy Fan Shop	Green Intelligent Power Saver	The family plugged in 5, monthly electricity savings of about 300 yuan. My family before a month of electricity costs more than 800, this time with this saver after the electricity bill is actually only more than 500, amazing, thanks to the store, will recommend other friends to buy!	Jingdong prefecture level city in Shandong
Happy Fan Shop	Green Intelligent Power Saver	It was recommended to me by a neighbor's family and it's nice to use it to save power.	Jingdong prefecture level city in Shandong
Happy Fan Shop	Green Intelligent Power Saver	Second time I've purchased it and the power saving is effective!	Jingdong prefecture level city in Shandong
Happy Fan Shop	Green Intelligent Power Saver	The effect is really good, before a month of more than 300 electricity, now every month every day with the air conditioning, it is more than 100 electricity! This thing is really amazing, one after another to buy and 5.6, home installed 3!	Jingdong prefecture level city in Shandong
Happy Fan Shop	Green Intelligent Power Saver	Received this product, began to use, small things look very wrong, can save a little electricity, the monthly electricity bill is too high, after plugging in did not pull, or save a lot of electricity, point	Jingdong prefecture level city in Shandong
Happy Fan Shop	Green Intelligent Power Saver	Simple and convenient, just that plug in, you can save electricity. A month can save dozens of dollars, a year down to save a lot of, very good product cost-effective, everyone can afford to buy and use!	Jingdong prefecture level city in Shandong
Happy Fan Shop	Green Intelligent Power Saver	Electricity bill from more than 200 yuan per month, down to more than 150 yuan, the original bought home, used two months, really have power-saving effect, this time and sold two, business service attitude is	Jingdong prefecture level city in Shandong
Happy Fan Shop	Green Intelligent Power Saver	Like to watch some movies at home and so on, sometimes at night until two or three o'clock, and recently the air conditioning is not turned off for a night, rather wasteful, I have to say that the effect is very good, quite like it!	Jingdong prefecture level city in Shandong
Happy Fan Shop	Green Intelligent Power Saver	Power-saving effect: the effect is very good, a month to save more than 200 dollars Cost-effective: good value for money Appearance and style: small and lovely Workmanship material: okay Experience: good	Jingdong prefecture level city in Shandong
Happy Fan Shop	Green Intelligent Power Saver	It's okay I quite like it, the stuff is very good. Come back in the future. You can rest assured to buy. Cost-effective all. Much cheaper than the physical store	Jingdong prefecture level city in Shandong
Happy Fan Shop	Green Intelligent Power Saver	More than 150 less, last month's electricity bill is almost 400, this month is only about 200, really save electricity, I am home to buy three, I did not expect a month to pay back	Jingdong prefecture level city in Shandong
Happy Fan Shop	Green Intelligent Power Saver	I bought three of these first and tried them for two months and they still felt useful, so now I've bought another one. Good and recommended!	Jingdong prefecture level city in Shandong
Happy Fan Shop	Green Intelligent Power Saver	This energy saver is very good quality and inexpensive next time I will buy it again!	Jingdong prefecture level city in Shandong
Happy Fan Shop	Green Intelligent Power Saver	Merchant service attitude is not good, the quality does not match, the business does not give the package return	Jingdong prefecture level city in Shandong
Happy Fan Shop	Green Intelligent Power Saver	The goods are very small do not know how effective after a few days to follow up the review	Jingdong prefecture level city in Shandong
Happy Fan Shop	Green Intelligent Power Saver	Not used before more than 200 a month, after using about 100 a month, my family used three, customer service said I use electricity recommended 2-3, I think more than one effect is better on the purchase of 4, the effect is very obvious, recommended to buy!	Jingdong prefecture level city in Shandong
Happy Fan Shop	Green Intelligent Power Saver	It's okay. I'll buy it again today.	Jingdong prefecture level city in Shandong
Happy Fan Shop	Green Intelligent Power Saver	Finally received the goods, very, very much like,, has been a lot of people praise, really too happy, has been his family's loyal fans, will continue to buy buy buy!	Jingdong prefecture level city in Shandong
Happy Fan Shop	Green Intelligent Power Saver	After using this, my monthly electricity bill was 1,200 pounds, now it's only 180 bucks a month, how good is it?	Jingdong prefecture level city in Shandong
Happy Fan Shop	Green Intelligent Power Saver	The item is a scam return to marry a dozen dollars in shipping fees. Buy terrific role is purely a scam.	Jingdong prefecture level city in Shandong
Happy Fan Shop	Green Intelligent Power Saver	It's really good, cost-effective and good quality, and the logistics are fast! It arrived in one day!	Jingdong prefecture level city in Shandong

Happy Fan Shop	Green Intelligent Power Saver	Great brand! Trustworthy! Works super well! Expensive for a reason! Great merchandise!	Jingdong prefecture level city in Shandong
Happy Fan Shop	Green Intelligent Power Saver	The baby is really great, I like it very much, my friends also say good to come, recommended to buy!	Jingdong prefecture level city in Shandong
Happy Fan Shop	Green Intelligent Power Saver	Fake couldn't be more fake. Bought one plugged in still so much electricity.	Jingdong prefecture level city in Shandong
Happy Fan Shop	Green Intelligent Power Saver	This appliance works great. Let's see how it works. We'll see later.	Jingdong prefecture level city in Shandong
Happy Fan Shop	Green Intelligent Power Saver	Used it before commenting, very happy with it	Jingdong prefecture level city in Shandong
Happy Fan Shop	Green Intelligent Power Saver	Goods have been received well-packed, logistics is very fast and good quality	Jingdong prefecture level city in Shandong
Happy Fan Shop	Green Intelligent Power Saver	This baby's quality is okay.	Jingdong prefecture level city in Shandong
Happy Fan Shop	Green Intelligent Power Saver	Counterfeit goods.	Jingdong prefecture level city in Shandong
Happy Fan Shop	Green Intelligent Power Saver	The effect is not very obvious	Jingdong prefecture level city in Shandong
Happy Fan Shop	Green Intelligent Power Saver	It's not the same thing.	Jingdong prefecture level city in Shandong
Happy Fan Shop	Green Intelligent Power Saver	There is no load how much w seems false, nothing works.	Jingdong prefecture level city in Shandong
Happy Fan Shop	Green Intelligent Power Saver	I received the item yesterday, got it back and plugged it in, and it used a little more power yesterday than I had before	Jingdong prefecture level city in Shandong
Happy Fan Shop	Green Intelligent Power Saver	Haven't seen the power saving mode yet	Jingdong prefecture level city in Shandong
Happy Fan Shop	Green Intelligent Power Saver	This product has no known effect	Jingdong prefecture level city in Shandong
Happy Fan Shop	Green Intelligent Power Saver	Refund Returns $16	Jingdong prefecture level city in Shandong
Happy Fan Shop	Green Intelligent Power Saver	The previous year bought a, almost every month can save a 70 dollars or so, used a year, no problem at all, look very new like, this time to move to a new home decisively repurchase 2 units, very believe this	Jingdong prefecture level city in Shandong
Happy Fan Shop	Green Intelligent Power Saver	Has been the second time to buy, this power saving device is really good power, before every month to spend 300 electricity, now are about 200, last time that 6, this time to help lead the family to buy 6,	Jingdong prefecture level city in Shandong
Happy Fan Shop	Green Intelligent Power Saver	In the spirit of trying to experience the new product, the use of down compared to the usual power consumption, with the addition of the quarter, is indeed a lot less electricity, originally more than 450	Jingdong prefecture level city in Shandong
Happy Fan Shop	Green Intelligent Power Saver	Has not been the first time to buy back, good use, power saving device is really powerful, before the monthly electricity bill of more than 500 Now more than 300, this time to help neighbors to buy several	Jingdong prefecture level city in Shandong
Happy Fan Shop	Green Intelligent Power Saver	Home appliances are very many, the circuit is also very complex, at first it is to save 9% of the electricity, and then used more than half a month, found that the power saving rate is as high as more	Jingdong prefecture level city in Shandong
Happy Fan Shop	Green Intelligent Power Saver	My friend bought it last year and saved hundreds of dollars on his electricity bill in one summer Appearance and style: it looks better than the one I bought before Quality is also good with a light.	Jingdong prefecture level city in Shandong
Happy Fan Shop	Green Intelligent Power Saver	Saver received, on the spot on the plug with the feeling of immediate power savings, appearance, packaging is very delicate, very satisfied, I hope to achieve satisfactory results!	Jingdong prefecture level city in Shandong
Happy Fan Shop	Green Intelligent Power Saver	The power saving effect is very good, is considered an old customer, also bought before, this time is to buy two for mom and dad, customer service attitude is good, praise!	Jingdong prefecture level city in Shandong
Happy Fan Shop	Green Intelligent Power Saver	After observing for a while, it really saves a lot of money I didn't realize it was still really reliable, so I'll give you a good review.	Jingdong prefecture level city in Shandong
Happy Fan Shop	Green Intelligent Power Saver	My family a month with this monthly almost save more than 200, power-saving effect: have bought a second time, really effective Cost-effective: not bad Appearance style: roughly the same A few months to the home electricity is indeed less, this time it is a re-buy three, send a friend two, they stay a	Jingdong prefecture level city in Shandong
Happy Fan Shop	Green Intelligent Power Saver	A month almost save more than 150 dollars in electricity, home appliances more, the main this can also protect high-power appliances, after using this device, these two months have not burned, things are very good, it is said that this is the latest model, the overall price is cheap and beautiful!	Jingdong prefecture level city in Shandong
Happy Fan Shop	Green Intelligent Power Saver	Received, powerful, great quality, love the style😍	Jingdong prefecture level city in Shandong
Happy Fan Shop	Green Intelligent Power Saver	This intelligent energy saver is especially good to use and save electricity hope everyone will buy it!	Jingdong prefecture level city in Shandong
Happy Fan Shop	Green Intelligent Power Saver	Air conditioning adapter effect is particularly great, the use of very simple, plug into the socket on the summer air conditioning power is too large, electricity is also expensive, with this product directly on the electricity bill to save half, has been purchased a few more, the effect is too awesome!	Jingdong prefecture level city in Shandong
Happy Fan Shop	Green Intelligent Power Saver	The family plugged in 5, monthly electricity savings of about 300 yuan. My family before a month of electricity costs more than 800, this time with this saver after the electricity bill is actually only more than 500, amazing, thanks to the store, will recommend other friends to buy!	Jingdong prefecture level city in Shandong
Happy Fan Shop	Green Intelligent Power Saver	It was recommended to me by a neighbor's family and it's nice to use it to save power.	Jingdong prefecture level city in Shandong
Happy Fan Shop	Green Intelligent Power Saver	Second time I've purchased it and the power saving is effective!	Jingdong prefecture level city in Shandong
Happy Fan Shop	Green Intelligent Power Saver	The effect is really good, before a month of more than 300 electricity, now every month every day with the air conditioning, it is more than 100 electricity! This thing is really amazing, one after another to buy and 5.6, home installed 3!	Jingdong prefecture level city in Shandong
Happy Fan Shop	Green Intelligent Power Saver	Received this product, began to use, small things look very wrong, can save a little electricity, the monthly electricity bill is too high, after plugging in did not pull, or save a lot of electricity, point	Jingdong prefecture level city in Shandong
Happy Fan Shop	Green Intelligent Power Saver	Simple and convenient, just that plug in, you can save electricity. A month can save dozens of dollars, a year down to save a lot of, very good product cost-effective, everyone can afford to buy and use!	Jingdong prefecture level city in Shandong
Happy Fan Shop	Green Intelligent Power Saver	Electricity bill from more than 200 yuan per month, down to more than 150 yuan, the original bought home, used two months, really have power-saving effect, this time and sold two, business service attitude is	Jingdong prefecture level city in Shandong
Happy Fan Shop	Green Intelligent Power Saver	Like to watch some movies at home and so on, sometimes at night until two or three o'clock, and recently the air conditioning is not turned off for a night, rather wasteful, I have to say that the effect is very good, quite like it!	Jingdong prefecture level city in Shandong
Happy Fan Shop	Green Intelligent Power Saver	Power-saving effect: the effect is very good, a month to save more than 200 dollars Cost-effective: good value for money Appearance and style: small and lovely Workmanship material: okay Experience: good	Jingdong prefecture level city in Shandong
Happy Fan Shop	Green Intelligent Power Saver	It's okay I quite like it, the stuff is very good. Come back in the future. You can rest assured to buy. Cost-effective all. Much cheaper than the physical store	Jingdong prefecture level city in Shandong
Happy Fan Shop	Green Intelligent Power Saver	More than 150 less, last month's electricity bill is almost 400, this month is only about 200, really save electricity, I am home to buy three, I did not expect a month to pay back	Jingdong prefecture level city in Shandong
Happy Fan Shop	Green Intelligent Power Saver	I bought three of these first and tried them for two months and they still felt useful, so now I've bought another one. Good and recommended!	Jingdong prefecture level city in Shandong
Happy Fan Shop	Green Intelligent Power Saver	This energy saver is very good quality and inexpensive next time I will buy it again!	Jingdong prefecture level city in Shandong
Happy Fan Shop	Green Intelligent Power Saver	Merchant service attitude is not good, the quality does not match, the business does not give the package return	Jingdong prefecture level city in Shandong
Happy Fan Shop	Green Intelligent Power Saver	The goods are very small do not know how effective after a few days to follow up the review	Jingdong prefecture level city in Shandong
Happy Fan Shop	Green Intelligent Power Saver	Not used before more than 200 a month, after using about 100 a month, my family used three, customer service said I use electricity recommended 2-3, I think more than one effect is better on the purchase of 4, the effect is very obvious, recommended to buy!	Jingdong prefecture level city in Shandong
Happy Fan Shop	Green Intelligent Power Saver	It's okay. I'll buy it again today.	Jingdong prefecture level city in Shandong
Happy Fan Shop	Green Intelligent Power Saver	Finally received the goods, very, very much like,, has been a lot of people praise, really too happy, has been his family's loyal fans, will continue to buy buy buy buy!	Jingdong prefecture level city in Shandong
Happy Fan Shop	Green Intelligent Power Saver	After using this, my monthly electricity bill was 1,200 pounds, now it's only 180 bucks a month, how good is it?	Jingdong prefecture level city in Shandong

Happy Fan Shop	Green Intelligent Power Saver	The item is a scam return to marry a dozen dollars in shipping fees. Buy terrific role is purely a scam.	Jingdong prefecture level city in Shandong
Happy Fan Shop	Green Intelligent Power Saver	It's really good, cost-effective and good quality, and the logistics are fast! It arrived in one day!	Jingdong prefecture level city in Shandong
Happy Fan Shop	Green Intelligent Power Saver	Great brand! Trustworthy! Works super well! Expensive for a reason! Great merchandise!	Jingdong prefecture level city in Shandong
Happy Fan Shop	Green Intelligent Power Saver	The baby is really great, I like it very much, my friends also say good to come, recommended to buy!	Jingdong prefecture level city in Shandong
Happy Fan Shop	Green Intelligent Power Saver	Fake couldn't be more fake. Bought one plugged in still so much electricity.	Jingdong prefecture level city in Shandong
Happy Fan Shop	Green Intelligent Power Saver	This appliance works great. Let's see how it works. We'll see later.	Jingdong prefecture level city in Shandong
Happy Fan Shop	Green Intelligent Power Saver	Used it before commenting, very happy with it	Jingdong prefecture level city in Shandong
Happy Fan Shop	Green Intelligent Power Saver	Goods have been received well-packed, logistics is very fast and good quality	Jingdong prefecture level city in Shandong
Happy Fan Shop	Green Intelligent Power Saver	This baby's quality is okay.	Jingdong prefecture level city in Shandong
Happy Fan Shop	Green Intelligent Power Saver	Counterfeit goods.	Jingdong prefecture level city in Shandong
Happy Fan Shop	Green Intelligent Power Saver	The effect is not very obvious	Jingdong prefecture level city in Shandong
Happy Fan Shop	Green Intelligent Power Saver	It's not the same thing.	Jingdong prefecture level city in Shandong
Happy Fan Shop	Green Intelligent Power Saver	There is no load how much w seems false, nothing works.	Jingdong prefecture level city in Shandong
Happy Fan Shop	Green Intelligent Power Saver	I received the item yesterday, got it back and plugged it in, and it used a little more power yesterday than I had before	Jingdong prefecture level city in Shandong
Happy Fan Shop	Green Intelligent Power Saver	Haven't seen the power saving mode yet	Jingdong prefecture level city in Shandong
Happy Fan Shop	Green Intelligent Power Saver	This product has no known effect	Jingdong prefecture level city in Shandong
Happy Fan Shop	Green Intelligent Power Saver	Refund Returns $16	Jingdong prefecture level city in Shandong
Happy Fan Shop	Green Intelligent Power Saver	The previous year bought a, almost every month can save a 70 dollars or so, used a year, no problem at all, look very new like, this time to move to a new home decisively repurchase 2 units, very believe this	Jingdong prefecture level city in Shandong
Happy Fan Shop	Green Intelligent Power Saver	Has been the second time to buy, this power saving device is really good power, before every month to spend 300 electricity, now are about 200, last time that 6, this time to help lead the family to buy 6,	Jingdong prefecture level city in Shandong
Happy Fan Shop	Green Intelligent Power Saver	In the spirit of trying to experience the new product, the use of down compared to the usual power consumption, with the addition of the quarter, is indeed a lot less electricity, originally more than 450	Jingdong prefecture level city in Shandong
Happy Fan Shop	Green Intelligent Power Saver	Has not been the first time to buy back, good use, power saving device is really powerful, before the monthly electricity bill of more than 500 Now more than 300, this time to help neighbors to buy several	Jingdong prefecture level city in Shandong
Happy Fan Shop	Green Intelligent Power Saver	Home appliances are very many, the circuit is also very complex, at first it is to save 9% of the electricity, and then used more than half a month, found that the power saving rate is as high as more	Jingdong prefecture level city in Shandong
Happy Fan Shop	Green Intelligent Power Saver	My friend bought it last year and saved hundreds of dollars on his electricity bill in one summer Appearance and style: it looks better than the one I bought before Quality is also good with a light.	Jingdong prefecture level city in Shandong
Happy Fan Shop	Green Intelligent Power Saver	Saver received, on the spot on the plug with the feeling of immediate power savings, appearance, packaging is very delicate, very satisfied, I hope to achieve satisfactory results!	Jingdong prefecture level city in Shandong
Happy Fan Shop	Green Intelligent Power Saver	The power saving effect is very good, is considered an old customer, also bought before, this time is to buy two for mom and dad, customer service attitude is good, praise!	Jingdong prefecture level city in Shandong
Happy Fan Shop	Green Intelligent Power Saver	After observing for a while, it really saves a lot of money I didn't realize it was still really reliable, so I'll give you a good review.	Jingdong prefecture level city in Shandong
Happy Fan Shop	Green Intelligent Power Saver	My family a month with this monthly almost save more than 200, power-saving effect: have bought a second time, really effective Cost-effective: not bad Appearance style: roughly the same A few months to the home electricity is indeed less, this time it is a re-buy three, send a friend two, they stay a	Jingdong prefecture level city in Shandong
Happy Fan Shop	Green Intelligent Power Saver	A month almost save more than 150 dollars in electricity, home appliances more, the main this can also protect high-power appliances, after using this device, these two months have not burned, things are very good, it is said that this is the latest model, the overall price is cheap and beautiful!	Jingdong prefecture level city in Shandong
Happy Fan Shop	Green Intelligent Power Saver	Received, powerful, great quality, love the style😍	Jingdong prefecture level city in Shandong
Happy Fan Shop	Green Intelligent Power Saver	This intelligent energy saver is especially good to use and save electricity hope everyone will buy it!	Jingdong prefecture level city in Shandong
Happy Fan Shop	Green Intelligent Power Saver	Air conditioning adapter effect is particularly great, the use of very simple, plug into the socket on the summer air conditioning power is too large, electricity is also expensive, with this product directly on the electricity bill to save half, has been purchased a few more, the effect is too awesome!	Jingdong prefecture level city in Shandong
Happy Fan Shop	Green Intelligent Power Saver	The family plugged in 5, monthly electricity savings of about 300 yuan. My family before a month of electricity costs more than 800, this time with this saver after the electricity bill is actually only more than 500, amazing, thanks to the store, will recommend other friends to buy!	Jingdong prefecture level city in Shandong
Happy Fan Shop	Green Intelligent Power Saver	It was recommended to me by a neighbor's family and it's nice to use it to save power.	Jingdong prefecture level city in Shandong
Happy Fan Shop	Green Intelligent Power Saver	Second time I've purchased it and the power saving is effective!	Jingdong prefecture level city in Shandong
Happy Fan Shop	Green Intelligent Power Saver	The effect is really good, before a month of more than 300 electricity, now every month every day with the air conditioning, it is more than 100 electricity! This thing is really amazing, one after another to buy and 5.6, home installed 3!	Jingdong prefecture level city in Shandong
Happy Fan Shop	Green Intelligent Power Saver	Received this product, began to use, small things look very wrong, can save a little electricity, the monthly electricity bill is too high, after plugging in did not pull, or save a lot of electricity, point	Jingdong prefecture level city in Shandong
Happy Fan Shop	Green Intelligent Power Saver	Simple and convenient, just that plug in, you can save electricity. A month can save dozens of dollars, a year down to save a lot of, very good product cost-effective, everyone can afford to buy and use!	Jingdong prefecture level city in Shandong
Happy Fan Shop	Green Intelligent Power Saver	Electricity bill from more than 200 yuan per month, down to more than 150 yuan, the original bought home, used two months, really have power-saving effect, this time and sold two, business service attitude is	Jingdong prefecture level city in Shandong
Happy Fan Shop	Green Intelligent Power Saver	Like to watch some movies at home and so on, sometimes at night until two or three o'clock, and recently the air conditioning is not turned off for a night, rather wasteful, I have to say that the effect is very good, quite like it!	Jingdong prefecture level city in Shandong
Happy Fan Shop	Green Intelligent Power Saver	Power-saving effect: the effect is very good, a month to save more than 200 dollars Cost-effective: good value for money Appearance and style: small and lovely Workmanship material: okay Experience: good	Jingdong prefecture level city in Shandong
Happy Fan Shop	Green Intelligent Power Saver	It's okay I quite like it, the stuff is very good. Come back in the future. You can rest assured to buy. Cost-effective all. Much cheaper than the physical store	Jingdong prefecture level city in Shandong
Happy Fan Shop	Green Intelligent Power Saver	More than 150 less, last month's electricity bill is almost 400, this month is only about 200, really save electricity, I am home to buy three, I did not expect a month to pay back	Jingdong prefecture level city in Shandong
Happy Fan Shop	Green Intelligent Power Saver	I bought three of these first and tried them for two months and they still felt useful, so now I've bought another one. Good and recommended!	Jingdong prefecture level city in Shandong
Happy Fan Shop	Green Intelligent Power Saver	This energy saver is very good quality and inexpensive next time I will buy it again!	Jingdong prefecture level city in Shandong
Happy Fan Shop	Green Intelligent Power Saver	Merchant service attitude is not good, the quality does not match, the business does not give the package return	Jingdong prefecture level city in Shandong
Happy Fan Shop	Green Intelligent Power Saver	The goods are very small do not know how effective after a few days to follow up the review	Jingdong prefecture level city in Shandong
Happy Fan Shop	Green Intelligent Power Saver	Not used before more than 200 a month, after using about 100 a month, my family used three, customer service said I use electricity recommended 2-3, I think more than one effect is better on the purchase of 4, the effect is very obvious, recommended to buy!	Jingdong prefecture level city in Shandong
Happy Fan Shop	Green Intelligent Power Saver	It's okay. I'll buy it again today.	Jingdong prefecture level city in Shandong

Happy Fan Shop	Green Intelligent Power Saver	Finally received the goods, very, very much like,, has been a lot of people praise, really too happy, has been his family's loyal fans, will continue to buy buy buy buy!	Jingdong prefecture level city in Shandong
Happy Fan Shop	Green Intelligent Power Saver	After using this, my monthly electricity bill was 1,200 pounds, now it's only 180 bucks a month, how good is it?	Jingdong prefecture level city in Shandong
Happy Fan Shop	Green Intelligent Power Saver	The item is a scam return to marry a dozen dollars in shipping fees. Buy terrific role is purely a scam.	Jingdong prefecture level city in Shandong
Happy Fan Shop	Green Intelligent Power Saver	It's really good, cost-effective and good quality, and the logistics are fast! It arrived in one day!	Jingdong prefecture level city in Shandong
Happy Fan Shop	Green Intelligent Power Saver	Great brand! Trustworthy! Works super well! Expensive for a reason! Great merchandise!	Jingdong prefecture level city in Shandong
Happy Fan Shop	Green Intelligent Power Saver	The baby is really great, I like it very much, my friends also say good to come, recommended to buy!	Jingdong prefecture level city in Shandong
Happy Fan Shop	Green Intelligent Power Saver	Fake couldn't be more fake. Bought one plugged in still so much electricity.	Jingdong prefecture level city in Shandong
Happy Fan Shop	Green Intelligent Power Saver	This appliance works great. Let's see how it works. We'll see later.	Jingdong prefecture level city in Shandong
Happy Fan Shop	Green Intelligent Power Saver	Used it before commenting, very happy with it	Jingdong prefecture level city in Shandong
Happy Fan Shop	Green Intelligent Power Saver	Goods have been received well-packed, logistics is very fast and good quality	Jingdong prefecture level city in Shandong
Happy Fan Shop	Green Intelligent Power Saver	This baby's quality is okay.	Jingdong prefecture level city in Shandong
Happy Fan Shop	Green Intelligent Power Saver	Counterfeit goods.	Jingdong prefecture level city in Shandong
Happy Fan Shop	Green Intelligent Power Saver	The effect is not very obvious	Jingdong prefecture level city in Shandong
Happy Fan Shop	Green Intelligent Power Saver	It's not the same thing.	Jingdong prefecture level city in Shandong
Happy Fan Shop	Green Intelligent Power Saver	There is no load how much w seems false, nothing works.	Jingdong prefecture level city in Shandong
Happy Fan Shop	Green Intelligent Power Saver	I received the item yesterday, got it back and plugged it in, and it used a little more power yesterday than I had before	Jingdong prefecture level city in Shandong
Happy Fan Shop	Green Intelligent Power Saver	Haven't seen the power saving mode yet	Jingdong prefecture level city in Shandong
Happy Fan Shop	Green Intelligent Power Saver	This product has no known effect	Jingdong prefecture level city in Shandong
Happy Fan Shop	Green Intelligent Power Saver	Refund Returns $16	Jingdong prefecture level city in Shandong
Happy Fan Shop	Green Intelligent Power Saver	The previous year bought a, almost every month can save a 70 dollars or so, used a year, no problem at all, look very new like, this time to move to a new home decisively repurchase 2 units, very believe this	Jingdong prefecture level city in Shandong
Happy Fan Shop	Green Intelligent Power Saver	Has been the second time to buy, this power saving device is really good power, before every month to spend 300 electricity, now are about 200, last time that 6, this time to help lead the family to buy 6,	Jingdong prefecture level city in Shandong
Happy Fan Shop	Green Intelligent Power Saver	In the spirit of trying to experience the new product, the use of down compared to the usual power consumption, with the addition of the quarter, is indeed a lot less electricity, originally more than 450	Jingdong prefecture level city in Shandong
Happy Fan Shop	Green Intelligent Power Saver	Has not been the first time to buy back, good use, power saving device is really powerful, before the monthly electricity bill of more than 500 Now more than 300, this time to help neighbors to buy several	Jingdong prefecture level city in Shandong
Happy Fan Shop	Green Intelligent Power Saver	Home appliances are very many, the circuit is also very complex, at first it is to save 9% of the electricity, and then used more than half a month, found that the power saving rate is as high as more	Jingdong prefecture level city in Shandong
Happy Fan Shop	Green Intelligent Power Saver	My friend bought it last year and saved hundreds of dollars on his electricity bill in one summer Appearance and style: it looks better than the one I bought before Quality is also good with a light.	Jingdong prefecture level city in Shandong
Happy Fan Shop	Green Intelligent Power Saver	Saver received, on the spot on the plug with the feeling of immediate power savings, appearance, packaging is very delicate, very satisfied, I hope to achieve satisfactory results!	Jingdong prefecture level city in Shandong
Happy Fan Shop	Green Intelligent Power Saver	The power saving effect is very good, is considered an old customer, also bought before, this time is to buy two for mom and dad, customer service attitude is good, praise!	Jingdong prefecture level city in Shandong
Happy Fan Shop	Green Intelligent Power Saver	After observing for a while, it really saves a lot of money I didn't realize it was still really reliable, so I'll give you a good review.	Jingdong prefecture level city in Shandong
Happy Fan Shop	Green Intelligent Power Saver	My family a month with this monthly almost save more than 200, power-saving effect: have bought a second time, really effective Cost-effective: not bad Appearance style: roughly the same A few months to the home electricity is indeed less, this time it is a re-buy three, send a friend two, they stay a	Jingdong prefecture level city in Shandong
Happy Fan Shop	Green Intelligent Power Saver	A month almost save more than 150 dollars in electricity, home appliances more, the main this can also protect high-power appliances, after using this device, these two months have not burned, things are very good, it is said that this is the latest model, the overall price is cheap and beautiful!	Jingdong prefecture level city in Shandong
Happy Fan Shop	Green Intelligent Power Saver	Received, powerful, great quality, love the style😍	Jingdong prefecture level city in Shandong
Happy Fan Shop	Green Intelligent Power Saver	This intelligent energy saver is especially good to use and save electricity hope everyone will buy it!	Jingdong prefecture level city in Shandong
Happy Fan Shop	Green Intelligent Power Saver	Air conditioning adapter effect is particularly great, the use of very simple, plug into the socket on the summer air conditioning power is too large, electricity is also expensive, with this product directly on the electricity bill to save half, has been purchased a few more, the effect is too awesome!	Jingdong prefecture level city in Shandong
Happy Fan Shop	Green Intelligent Power Saver	The family plugged in 5, monthly electricity savings of about 300 yuan. My family before a month of electricity costs more than 800, this time with this saver after the electricity bill is actually only more than 500, amazing, thanks to the store, will recommend other friends to buy!	Jingdong prefecture level city in Shandong
Happy Fan Shop	Green Intelligent Power Saver	It was recommended to me by a neighbor's family and it's nice to use it to save power.	Jingdong prefecture level city in Shandong
Happy Fan Shop	Green Intelligent Power Saver	Second time I've purchased it and the power saving is effective!	Jingdong prefecture level city in Shandong
Happy Fan Shop	Green Intelligent Power Saver	The effect is really good, before a month of more than 300 electricity, now every month every day with the air conditioning, it is more than 100 electricity! This thing is really amazing, one after another to buy and 5.6, home installed 3!	Jingdong prefecture level city in Shandong
Happy Fan Shop	Green Intelligent Power Saver	Received this product, began to use, small things look very wrong, can save a little electricity, the monthly electricity bill is too high, after plugging in did not pull, or save a lot of electricity, point	Jingdong prefecture level city in Shandong
Happy Fan Shop	Green Intelligent Power Saver	Simple and convenient, just that plug in, you can save electricity. A month can save dozens of dollars, a year down to save a lot of, very good product cost-effective, everyone can afford to buy and use!	Jingdong prefecture level city in Shandong
Happy Fan Shop	Green Intelligent Power Saver	Electricity bill from more than 200 yuan per month, down to more than 150 yuan, the original bought home, used two months, really have power-saving effect, this time and sold two, business service attitude is	Jingdong prefecture level city in Shandong
Happy Fan Shop	Green Intelligent Power Saver	Like to watch some movies at home and so on, sometimes at night until two or three o'clock, and recently the air conditioning is not turned off for a night, rather wasteful, I have to say that the effect is very good, quite like it!	Jingdong prefecture level city in Shandong
Happy Fan Shop	Green Intelligent Power Saver	Power-saving effect: the effect is very good, a month to save more than 200 dollars Cost-effective: good value for money Appearance and style: small and lovely Workmanship material: okay Experience: good	Jingdong prefecture level city in Shandong
Happy Fan Shop	Green Intelligent Power Saver	It's okay I quite like it, the stuff is very good. Come back in the future. You can rest assured to buy. Cost-effective all. Much cheaper than the physical store	Jingdong prefecture level city in Shandong
Happy Fan Shop	Green Intelligent Power Saver	More than 150 less, last month's electricity bill is almost 400, this month is only about 200, really save electricity, I am home to buy three, I did not expect a month to pay back	Jingdong prefecture level city in Shandong
Happy Fan Shop	Green Intelligent Power Saver	I bought three of these first and tried them for two months and they still felt useful, so now I've bought another one. Good and recommended!	Jingdong prefecture level city in Shandong
Happy Fan Shop	Green Intelligent Power Saver	This energy saver is very good quality and inexpensive next time I will buy it again!	Jingdong prefecture level city in Shandong
Happy Fan Shop	Green Intelligent Power Saver	Merchant service attitude is not good, the quality does not match, the business does not give the package return	Jingdong prefecture level city in Shandong
Happy Fan Shop	Green Intelligent Power Saver	The goods are very small do not know how effective after a few days to follow up the review	Jingdong prefecture level city in Shandong

Happy Fan Shop	Green Intelligent Power Saver	Not used before more than 200 a month, after using about 100 a month, my family used three, customer service said I use electricity recommended 2-3, I think more than one effect is better on the purchase of 4, the effect is very obvious, recommended to buy!	Jingdong prefecture level city in Shandong
Happy Fan Shop	Green Intelligent Power Saver	It's okay. I'll buy it again today.	Jingdong prefecture level city in Shandong
Happy Fan Shop	Green Intelligent Power Saver	Finally received the goods, very, very much like,, has been a lot of people praise, really too happy, has been his family's loyal fans, will continue to buy buy buy buy!	Jingdong prefecture level city in Shandong
Happy Fan Shop	Green Intelligent Power Saver	After using this, my monthly electricity bill was 1,200 pounds, now it's only 180 bucks a month, how good is it?	Jingdong prefecture level city in Shandong
Happy Fan Shop	Green Intelligent Power Saver	The item is a scam return to marry a dozen dollars in shipping fees. Buy terrific role is purely a scam.	Jingdong prefecture level city in Shandong
Happy Fan Shop	Green Intelligent Power Saver	It's really good, cost-effective and good quality, and the logistics are fast! It arrived in one day!	Jingdong prefecture level city in Shandong
Happy Fan Shop	Green Intelligent Power Saver	Great brand! Trustworthy! Works super well! Expensive for a reason! Great merchandise!	Jingdong prefecture level city in Shandong
Happy Fan Shop	Green Intelligent Power Saver	The baby is really great, I like it very much, my friends also say good to come, recommended to buy!	Jingdong prefecture level city in Shandong
Happy Fan Shop	Green Intelligent Power Saver	Fake couldn't be more fake. Bought one plugged in still so much electricity.	Jingdong prefecture level city in Shandong
Happy Fan Shop	Green Intelligent Power Saver	This appliance works great. Let's see how it works. We'll see later.	Jingdong prefecture level city in Shandong
Happy Fan Shop	Green Intelligent Power Saver	Used it before commenting, very happy with it	Jingdong prefecture level city in Shandong
Happy Fan Shop	Green Intelligent Power Saver	Goods have been received well-packed, logistics is very fast and good quality	Jingdong prefecture level city in Shandong
Happy Fan Shop	Green Intelligent Power Saver	This baby's quality is okay.	Jingdong prefecture level city in Shandong
Happy Fan Shop	Green Intelligent Power Saver	Counterfeit goods.	Jingdong prefecture level city in Shandong
Happy Fan Shop	Green Intelligent Power Saver	The effect is not very obvious	Jingdong prefecture level city in Shandong
Happy Fan Shop	Green Intelligent Power Saver	It's not the same thing.	Jingdong prefecture level city in Shandong
Happy Fan Shop	Green Intelligent Power Saver	There is no load how much w seems false, nothing works.	Jingdong prefecture level city in Shandong
Happy Fan Shop	Green Intelligent Power Saver	I received the item yesterday, got it back and plugged it in, and it used a little more power yesterday than I had before	Jingdong prefecture level city in Shandong
Happy Fan Shop	Green Intelligent Power Saver	Haven't seen the power saving mode yet	Jingdong prefecture level city in Shandong
Happy Fan Shop	Green Intelligent Power Saver	This product has no known effect	Jingdong prefecture level city in Shandong
Happy Fan Shop	Green Intelligent Power Saver	Refund Returns $16	Jingdong prefecture level city in Shandong
Happy Fan Shop	Green Intelligent Power Saver	The previous year bought a, almost every month can save a 70 dollars or so, used a year, no problem at all, look very new like, this time to move to a new home decisively repurchase 2 units, very believe this	Jingdong prefecture level city in Shandong
Happy Fan Shop	Green Intelligent Power Saver	Has been the second time to buy, this power saving device is really good power, before every month to spend 300 electricity, now are about 200, last time that 6, this time to help lead the family to buy 6,	Jingdong prefecture level city in Shandong
Happy Fan Shop	Green Intelligent Power Saver	In the spirit of trying to experience the new product, the use of down compared to the usual power consumption, with the addition of the quarter, is indeed a lot less electricity, originally more than 450	Jingdong prefecture level city in Shandong
Happy Fan Shop	Green Intelligent Power Saver	Has not been the first time to buy back, good use, power saving device is really powerful, before the monthly electricity bill of more than 500 Now more than 300, this time to help neighbors to buy several	Jingdong prefecture level city in Shandong
Happy Fan Shop	Green Intelligent Power Saver	Home appliances are very many, the circuit is also very complex, at first it is to save 9% of the electricity, and then used more than half a month, found that the power saving rate is as high as more	Jingdong prefecture level city in Shandong
Happy Fan Shop	Green Intelligent Power Saver	My friend bought it last year and saved hundreds of dollars on his electricity bill in one summer Appearance and style: it looks better than the one I bought before Quality is also good with a light.	Jingdong prefecture level city in Shandong
Happy Fan Shop	Green Intelligent Power Saver	Saver received, on the spot on the plug with the feeling of immediate power savings, appearance, packaging is very delicate, very satisfied, I hope to achieve satisfactory results!	Jingdong prefecture level city in Shandong
Happy Fan Shop	Green Intelligent Power Saver	The power saving effect is very good, is considered an old customer, also bought before, this time is to buy two for mom and dad, customer service attitude is good, praise!	Jingdong prefecture level city in Shandong
Happy Fan Shop	Green Intelligent Power Saver	After observing for a while, it really saves a lot of money I didn't realize it was still really reliable, so I'll give you a good review.	Jingdong prefecture level city in Shandong
Happy Fan Shop	Green Intelligent Power Saver	My family a month with this monthly almost save more than 200, power-saving effect: have bought a second time, really effective Cost-effective: not bad Appearance style: roughly the same A few months to the home electricity is indeed less, this time it is a re-buy three, send a friend two, they stay a	Jingdong prefecture level city in Shandong
Happy Fan Shop	Green Intelligent Power Saver	A month almost save more than 150 dollars in electricity, home appliances more, the main this can also protect high-power appliances, after using this device, these two months have not burned, things are very good, it is said that this is the latest model, the overall price is cheap and beautiful!	Jingdong prefecture level city in Shandong
Happy Fan Shop	Green Intelligent Power Saver	Received, powerful, great quality, love the style😍	Jingdong prefecture level city in Shandong
Happy Fan Shop	Green Intelligent Power Saver	This intelligent energy saver is especially good to use and save electricity hope everyone will buy it!	Jingdong prefecture level city in Shandong
Happy Fan Shop	Green Intelligent Power Saver	Air conditioning adapter effect is particularly great, the use of very simple, plug into the socket on the summer air conditioning power is too large, electricity is also expensive, with this product directly on the electricity bill to save half, has been purchased a few more, the effect is too awesome!	Jingdong prefecture level city in Shandong
Happy Fan Shop	Green Intelligent Power Saver	The family plugged in 5, monthly electricity savings of about 300 yuan. My family before a month of electricity costs more than 800, this time with this saver after the electricity bill is actually only more than 500, amazing, thanks to the store, will recommend other friends to buy!	Jingdong prefecture level city in Shandong
Happy Fan Shop	Green Intelligent Power Saver	It was recommended to me by a neighbor's family and it's nice to use it to save power.	Jingdong prefecture level city in Shandong
Happy Fan Shop	Green Intelligent Power Saver	Second time I've purchased it and the power saving is effective!	Jingdong prefecture level city in Shandong
Happy Fan Shop	Green Intelligent Power Saver	The effect is really good, before a month of more than 300 electricity, now every month every day with the air conditioning, it is more than 100 electricity! This thing is really amazing, one after another to buy and 5.6, home installed 3!	Jingdong prefecture level city in Shandong
Happy Fan Shop	Green Intelligent Power Saver	Received this product, began to use, small things look very wrong, can save a little electricity, the monthly electricity bill is too high, after plugging in did not pull, or save a lot of electricity, point	Jingdong prefecture level city in Shandong
Happy Fan Shop	Green Intelligent Power Saver	Simple and convenient, just that plug in, you can save electricity. A month can save dozens of dollars, a year down to save a lot of, very good product cost-effective, everyone can afford to buy and use!	Jingdong prefecture level city in Shandong
Happy Fan Shop	Green Intelligent Power Saver	Electricity bill from more than 200 yuan per month, down to more than 150 yuan, the original bought home, used two months, really have power-saving effect, this time and sold two, business service attitude is	Jingdong prefecture level city in Shandong
Happy Fan Shop	Green Intelligent Power Saver	Like to watch some movies at home and so on, sometimes at night until two or three o'clock, and recently the air conditioning is not turned off for a night, rather wasteful, I have to say that the effect is very good, quite like it!	Jingdong prefecture level city in Shandong
Happy Fan Shop	Green Intelligent Power Saver	Power-saving effect: the effect is very good, a month to save more than 200 dollars Cost-effective: good value for money Appearance and style: small and lovely Workmanship material: okay Experience: good	Jingdong prefecture level city in Shandong
Happy Fan Shop	Green Intelligent Power Saver	It's okay I quite like it, the stuff is very good. Come back in the future. You can rest assured to buy. Cost-effective all. Much cheaper than the physical store	Jingdong prefecture level city in Shandong
Happy Fan Shop	Green Intelligent Power Saver	More than 150 less, last month's electricity bill is almost 400, this month is only about 200, really save electricity, I am home to buy three, I did not expect a month to pay back	Jingdong prefecture level city in Shandong
Happy Fan Shop	Green Intelligent Power Saver	I bought three of these first and tried them for two months and they still felt useful, so now I've bought another one. Good and recommended!	Jingdong prefecture level city in Shandong
Happy Fan Shop	Green Intelligent Power Saver	This energy saver is very good quality and inexpensive next time I will buy it again!	Jingdong prefecture level city in Shandong

Happy Fan Shop	Green Intelligent Power Saver	Merchant service attitude is not good, the quality does not match, the business does not give the package return	Jingdong prefecture level city in Shandong
Happy Fan Shop	Green Intelligent Power Saver	The goods are very small do not know how effective after a few days to follow up the review	Jingdong prefecture level city in Shandong
Happy Fan Shop	Green Intelligent Power Saver	Not used before more than 200 a month, after using about 100 a month, my family used three, customer service said I use electricity recommended 2-3, I think more than one effect is better on the purchase of 4, the effect is very obvious, recommended to buy!	Jingdong prefecture level city in Shandong
Happy Fan Shop	Green Intelligent Power Saver	It's okay. I'll buy it again today.	Jingdong prefecture level city in Shandong
Happy Fan Shop	Green Intelligent Power Saver	Finally received the goods, very, very much like,, has been a lot of people praise, really too happy, has been his family's loyal fans, will continue to buy buy buy buy!	Jingdong prefecture level city in Shandong
Happy Fan Shop	Green Intelligent Power Saver	After using this, my monthly electricity bill was 1,200 pounds, now it's only 180 bucks a month, how good is it?	Jingdong prefecture level city in Shandong
Happy Fan Shop	Green Intelligent Power Saver	The item is a scam return to marry a dozen dollars in shipping fees. Buy terrific role is purely a scam.	Jingdong prefecture level city in Shandong
Happy Fan Shop	Green Intelligent Power Saver	It's really good, cost-effective and good quality, and the logistics are fast! It arrived in one day!	Jingdong prefecture level city in Shandong
Happy Fan Shop	Green Intelligent Power Saver	Great brand! Trustworthy! Works super well! Expensive for a reason! Great merchandise!	Jingdong prefecture level city in Shandong
Happy Fan Shop	Green Intelligent Power Saver	The baby is really great, I like it very much, my friends also say good to come, recommended to buy!	Jingdong prefecture level city in Shandong
Happy Fan Shop	Green Intelligent Power Saver	Fake couldn't be more fake. Bought one plugged in still so much electricity.	Jingdong prefecture level city in Shandong
Happy Fan Shop	Green Intelligent Power Saver	Used it before commenting, very happy with it	Jingdong prefecture level city in Shandong
Happy Fan Shop	Green Intelligent Power Saver	This appliance works great. Let's see how it works. We'll see later.	Jingdong prefecture level city in Shandong
Happy Fan Shop	Green Intelligent Power Saver	Goods have been received well-packed, logistics is very fast and good quality	Jingdong prefecture level city in Shandong
Happy Fan Shop	Green Intelligent Power Saver	Counterfeit goods.	Jingdong prefecture level city in Shandong
Happy Fan Shop	Green Intelligent Power Saver	This baby's quality is okay.	Jingdong prefecture level city in Shandong
Happy Fan Shop	Green Intelligent Power Saver	The effect is not very obvious	Jingdong prefecture level city in Shandong
Happy Fan Shop	Green Intelligent Power Saver	It's not the same thing.	Jingdong prefecture level city in Shandong
Happy Fan Shop	Green Intelligent Power Saver	There is no load how much w seems false, nothing works.	Jingdong prefecture level city in Shandong
Happy Fan Shop	Green Intelligent Power Saver	I received the item yesterday, got it back and plugged it in, and it used a little more power yesterday than I had before	Jingdong prefecture level city in Shandong
Happy Fan Shop	Green Intelligent Power Saver	Haven't seen the power saving mode yet	Jingdong prefecture level city in Shandong
Happy Fan Shop	Green Intelligent Power Saver	This product has no known effect	Jingdong prefecture level city in Shandong
Happy Fan Shop	Green Intelligent Power Saver	Refund Returns $16	Jingdong prefecture level city in Shandong
Happy Fan Shop	Green Intelligent Power Saver	The previous year bought a, almost every month can save a 70 dollars or so, used a year, no problem at all, look very new like, this time to move to a new home decisively repurchase 2 units, very believe this	Jingdong prefecture level city in Shandong
Happy Fan Shop	Green Intelligent Power Saver	Has been the second time to buy, this power saving device is really good power, before every month to spend 300 electricity, now are about 200, last time that 6, this time to help lead the family to buy 6,	Jingdong prefecture level city in Shandong
Happy Fan Shop	Green Intelligent Power Saver	In the spirit of trying to experience the new product, the use of down compared to the usual power consumption, with the addition of the quarter, is indeed a lot less electricity, originally more than 450	Jingdong prefecture level city in Shandong
Happy Fan Shop	Green Intelligent Power Saver	Has not been the first time to buy back, good use, power saving device is really powerful, before the monthly electricity bill of more than 500 Now more than 300, this time to help neighbors to buy several	Jingdong prefecture level city in Shandong
Happy Fan Shop	Green Intelligent Power Saver	Home appliances are very many, the circuit is also very complex, at first it is to save 9% of the electricity, and then used more than half a month, found that the power saving rate is as high as more	Jingdong prefecture level city in Shandong
Happy Fan Shop	Green Intelligent Power Saver	My friend bought it last year and saved hundreds of dollars on his electricity bill in one summer Appearance and style: it looks better than the one I bought before Quality is also good with a light.	Jingdong prefecture level city in Shandong
Happy Fan Shop	Green Intelligent Power Saver	Saver received, on the spot on the plug with the feeling of immediate power savings, appearance, packaging is very delicate, very satisfied, I hope to achieve satisfactory results!	Jingdong prefecture level city in Shandong
Happy Fan Shop	Green Intelligent Power Saver	The power saving effect is very good, is considered an old customer, also bought before, this time is to buy two for mom and dad, customer service attitude is good, praise!	Jingdong prefecture level city in Shandong
Happy Fan Shop	Green Intelligent Power Saver	After observing for a while, it really saves a lot of money I didn't realize it was still really reliable, so I'll give you a good review.	Jingdong prefecture level city in Shandong
Happy Fan Shop	Green Intelligent Power Saver	My family a month with this monthly almost save more than 200, power-saving effect: have bought a second time, really effective Cost-effective: not bad Appearance style: roughly the same A few months to the home electricity is indeed less, this time it is a re-buy three, send a friend two, they stay a	Jingdong prefecture level city in Shandong
Happy Fan Shop	Green Intelligent Power Saver	A month almost save more than 150 dollars in electricity, home appliances more, the main this can also protect high-power appliances, after using this device, these two months have not burned, things are very good, it is said that this is the latest model, the overall price is cheap and beautiful!	Jingdong prefecture level city in Shandong
Happy Fan Shop	Green Intelligent Power Saver	Received, powerful, great quality, love the style😍	Jingdong prefecture level city in Shandong
Happy Fan Shop	Green Intelligent Power Saver	This intelligent energy saver is especially good to use and save electricity hope everyone will buy it!	Jingdong prefecture level city in Shandong
Happy Fan Shop	Green Intelligent Power Saver	Air conditioning adapter effect is particularly great, the use of very simple, plug into the socket on the summer air conditioning power is too large, electricity is also expensive, with this product directly on the electricity bill to save half, has been purchased a few more, the effect is too awesome!	Jingdong prefecture level city in Shandong
Happy Fan Shop	Green Intelligent Power Saver	The family plugged in 5, monthly electricity savings of about 300 yuan. My family before a month of electricity costs more than 800, this time with this saver after the electricity bill is actually only more than 500, amazing, thanks to the store, will recommend other friends to buy!	Jingdong prefecture level city in Shandong
Happy Fan Shop	Green Intelligent Power Saver	It was recommended to me by a neighbor's family and it's nice to use it to save power.	Jingdong prefecture level city in Shandong
Happy Fan Shop	Green Intelligent Power Saver	Second time I've purchased it and the power saving is effective!	Jingdong prefecture level city in Shandong
Happy Fan Shop	Green Intelligent Power Saver	The effect is really good, before a month of more than 300 electricity, now every month every day with the air conditioning, it is more than 100 electricity! This thing is really amazing, one after another to buy and 5.6, home installed 3!	Jingdong prefecture level city in Shandong
Happy Fan Shop	Green Intelligent Power Saver	Received this product, began to use, small things look very wrong, can save a little electricity, the monthly electricity bill is too high, after plugging in did not pull, or save a lot of electricity, point	Jingdong prefecture level city in Shandong
Happy Fan Shop	Green Intelligent Power Saver	Simple and convenient, just that plug in, you can save electricity. A month can save dozens of dollars, a year down to save a lot of, very good product cost-effective, everyone can afford to buy and use!	Jingdong prefecture level city in Shandong
Happy Fan Shop	Green Intelligent Power Saver	Electricity bill from more than 200 yuan per month, down to more than 150 yuan, the original bought home, used two months, really have power-saving effect, this time and sold two, business service attitude is	Jingdong prefecture level city in Shandong
Happy Fan Shop	Green Intelligent Power Saver	Like to watch some movies at home and so on, sometimes at night until two or three o'clock, and recently the air conditioning is not turned off for a night, rather wasteful, I have to say that the effect is very good, quite like it!	Jingdong prefecture level city in Shandong
Happy Fan Shop	Green Intelligent Power Saver	Power-saving effect: the effect is very good, a month to save more than 200 dollars Cost-effective: good value for money Appearance and style: small and lovely Workmanship material: okay Experience: good	Jingdong prefecture level city in Shandong
Happy Fan Shop	Green Intelligent Power Saver	It's okay I quite like it, the stuff is very good. Come back in the future. You can rest assured to buy. Cost-effective all. Much cheaper than the physical store	Jingdong prefecture level city in Shandong
Happy Fan Shop	Green Intelligent Power Saver	More than 150 less, last month's electricity bill is almost 400, this month is only about 200, really save electricity, I am home to buy three, I did not expect a month to pay back	Jingdong prefecture level city in Shandong

Happy Fan Shop	Green Intelligent Power Saver	I bought three of these first and tried them for two months and they still felt useful, so now I've bought another one. Good and recommended!	Jingdong prefecture level city in Shandong
Happy Fan Shop	Green Intelligent Power Saver	This energy saver is very good quality and inexpensive next time I will buy it again!	Jingdong prefecture level city in Shandong
Happy Fan Shop	Green Intelligent Power Saver	Merchant service attitude is not good, the quality does not match, the business does not give the package return	Jingdong prefecture level city in Shandong
Happy Fan Shop	Green Intelligent Power Saver	The goods are very small do not know how effective after a few days to follow up the review	Jingdong prefecture level city in Shandong
Happy Fan Shop	Green Intelligent Power Saver	Not used before more than 200 a month, after using about 100 a month, my family used three, customer service said I use electricity recommended 2-3, I think more than one effect is better on the purchase of 4, the effect is very obvious, recommended to buy!	Jingdong prefecture level city in Shandong
Happy Fan Shop	Green Intelligent Power Saver	It's okay. I'll buy it again today.	Jingdong prefecture level city in Shandong
Happy Fan Shop	Green Intelligent Power Saver	Finally received the goods, very, very much like,, has been a lot of people praise, really too happy, has been his family's loyal fans, will continue to buy buy buy buy!	Jingdong prefecture level city in Shandong
Happy Fan Shop	Green Intelligent Power Saver	After using this, my monthly electricity bill was 1,200 pounds, now it's only 180 bucks a month, how good is it?	Jingdong prefecture level city in Shandong
Happy Fan Shop	Green Intelligent Power Saver	The item is a scam return to marry a dozen dollars in shipping fees. Buy terrific role is purely a scam.	Jingdong prefecture level city in Shandong
Happy Fan Shop	Green Intelligent Power Saver	It's really good, cost-effective and good quality, and the logistics are fast! It arrived in one day!	Jingdong prefecture level city in Shandong
Happy Fan Shop	Green Intelligent Power Saver	Great brand! Trustworthy! Works super well! Expensive for a reason! Great merchandise!	Jingdong prefecture level city in Shandong
Happy Fan Shop	Green Intelligent Power Saver	The baby is really great, I like it very much, my friends also say good to come, recommended to buy!	Jingdong prefecture level city in Shandong
Happy Fan Shop	Green Intelligent Power Saver	Fake couldn't be more fake. Bought one plugged in still so much electricity.	Jingdong prefecture level city in Shandong
Happy Fan Shop	Green Intelligent Power Saver	This appliance works great. Let's see how it works. We'll see later.	Jingdong prefecture level city in Shandong
Happy Fan Shop	Green Intelligent Power Saver	Used it before commenting, very happy with it	Jingdong prefecture level city in Shandong
Happy Fan Shop	Green Intelligent Power Saver	Goods have been received well-packed, logistics is very fast and good quality	Jingdong prefecture level city in Shandong
Happy Fan Shop	Green Intelligent Power Saver	This baby's quality is okay.	Jingdong prefecture level city in Shandong
Happy Fan Shop	Green Intelligent Power Saver	Counterfeit goods.	Jingdong prefecture level city in Shandong
Happy Fan Shop	Green Intelligent Power Saver	The effect is not very obvious	Jingdong prefecture level city in Shandong
Happy Fan Shop	Green Intelligent Power Saver	It's not the same thing.	Jingdong prefecture level city in Shandong
Happy Fan Shop	Green Intelligent Power Saver	There is no load how much w seems false, nothing works.	Jingdong prefecture level city in Shandong
Happy Fan Shop	Green Intelligent Power Saver	I received the item yesterday, got it back and plugged it in, and it used a little more power yesterday than I had before	Jingdong prefecture level city in Shandong
Happy Fan Shop	Green Intelligent Power Saver	Haven't seen the power saving mode yet	Jingdong prefecture level city in Shandong
Happy Fan Shop	Green Intelligent Power Saver	This product has no known effect	Jingdong prefecture level city in Shandong
Happy Fan Shop	Green Intelligent Power Saver	Refund Returns $16	Jingdong prefecture level city in Shandong
Happy Fan Shop	Green Intelligent Power Saver	The previous year bought a, almost every month can save a 70 dollars or so, used a year, no problem at all, look very new like, this time to move to a new home decisively repurchase 2 units, very believe this	Jingdong prefecture level city in Shandong
Happy Fan Shop	Green Intelligent Power Saver	Has been the second time to buy, this power saving device is really good power, before every month to spend 300 electricity, now are about 200, last time that 6, this time to help lead the family to buy 6,	Jingdong prefecture level city in Shandong
Happy Fan Shop	Green Intelligent Power Saver	In the spirit of trying to experience the new product, the use of down compared to the usual power consumption, with the addition of the quarter, is indeed a lot less electricity, originally more than 450	Jingdong prefecture level city in Shandong
Happy Fan Shop	Green Intelligent Power Saver	Has not been the first time to buy back, good use, power saving device is really powerful, before the monthly electricity bill of more than 500 Now more than 300, this time to help neighbors to buy several	Jingdong prefecture level city in Shandong
Happy Fan Shop	Green Intelligent Power Saver	Home appliances are very many, the circuit is also very complex, at first it is to save 9% of the electricity, and then used more than half a month, found that the power saving rate is as high as more	Jingdong prefecture level city in Shandong
Happy Fan Shop	Green Intelligent Power Saver	My friend bought it last year and saved hundreds of dollars on his electricity bill in one summer Appearance and style: it looks better than the one I bought before Quality is also good with a light.	Jingdong prefecture level city in Shandong
Happy Fan Shop	Green Intelligent Power Saver	Saver received, on the spot on the plug with the feeling of immediate power savings, appearance, packaging is very delicate, very satisfied, I hope to achieve satisfactory results!	Jingdong prefecture level city in Shandong
Happy Fan Shop	Green Intelligent Power Saver	The power saving effect is very good, is considered an old customer, also bought before, this time is to buy two for mom and dad, customer service attitude is good, praise!	Jingdong prefecture level city in Shandong
Happy Fan Shop	Green Intelligent Power Saver	After observing for a while, it really saves a lot of money I didn't realize it was still really reliable, so I'll give you a good review.	Jingdong prefecture level city in Shandong
Happy Fan Shop	Green Intelligent Power Saver	My family a month with this monthly almost save more than 200, power-saving effect: have bought a second time, really effective Cost-effective: not bad Appearance style: roughly the same A few months to the home electricity is indeed less, this time it is a re-buy three, send a friend two, they stay a	Jingdong prefecture level city in Shandong
Happy Fan Shop	Green Intelligent Power Saver	A month almost save more than 150 dollars in electricity, home appliances more, the main this can also protect high-power appliances, after using this device, these two months have not burned, things are very good, it is said that this is the latest model, the overall price is cheap and beautiful!	Jingdong prefecture level city in Shandong
Happy Fan Shop	Green Intelligent Power Saver	Received, powerful, great quality, love the style😍	Jingdong prefecture level city in Shandong
Happy Fan Shop	Green Intelligent Power Saver	This intelligent energy saver is especially good to use and save electricity hope everyone will buy it!	Jingdong prefecture level city in Shandong
Happy Fan Shop	Green Intelligent Power Saver	Air conditioning adapter effect is particularly great, the use of very simple, plug into the socket on the summer air conditioning power is too large, electricity is also expensive, with this product directly on the electricity bill to save half, has been purchased a few more, the effect is too awesome!	Jingdong prefecture level city in Shandong
Happy Fan Shop	Green Intelligent Power Saver	The family plugged in 5, monthly electricity savings of about 300 yuan. My family before a month of electricity costs more than 800, this time with this saver after the electricity bill is actually only more than 500, amazing, thanks to the store, will recommend other friends to buy!	Jingdong prefecture level city in Shandong
Happy Fan Shop	Green Intelligent Power Saver	It was recommended to me by a neighbor's family and it's nice to use it to save power.	Jingdong prefecture level city in Shandong
Happy Fan Shop	Green Intelligent Power Saver	Second time I've purchased it and the power saving is effective!	Jingdong prefecture level city in Shandong
Happy Fan Shop	Green Intelligent Power Saver	The effect is really good, before a month of more than 300 electricity, now every month every day with the air conditioning, it is more than 100 electricity! This thing is really amazing, one after another to buy and 5.6, home installed 3!	Jingdong prefecture level city in Shandong
Happy Fan Shop	Green Intelligent Power Saver	Received this product, began to use, small things look very wrong, can save a little electricity, the monthly electricity bill is too high, after plugging in did not pull, or save a lot of electricity, point	Jingdong prefecture level city in Shandong
Happy Fan Shop	Green Intelligent Power Saver	Simple and convenient, just that plug in, you can save electricity. A month can save dozens of dollars, a year down to save a lot of, very good product cost-effective, everyone can afford to buy and use!	Jingdong prefecture level city in Shandong
Happy Fan Shop	Green Intelligent Power Saver	Electricity bill from more than 200 yuan per month, down to more than 150 yuan, the original bought home, used two months, really have power-saving effect, this time and sold two, business service attitude is	Jingdong prefecture level city in Shandong
Happy Fan Shop	Green Intelligent Power Saver	Like to watch some movies at home and so on, sometimes at night until two or three o'clock, and recently the air conditioning is not turned off for a night, rather wasteful, I have to say that the effect is very good, quite like it!	Jingdong prefecture level city in Shandong
Happy Fan Shop	Green Intelligent Power Saver	Power-saving effect: the effect is very good, a month to save more than 200 dollars Cost-effective: good value for money Appearance and style: small and lovely Workmanship material: okay Experience: good	Jingdong prefecture level city in Shandong

Happy Fan Shop	Green Intelligent Power Saver	It's okay I quite like it, the stuff is very good. Come back in the future. You can rest assured to buy. Cost-effective all. Much cheaper than the physical store	Jingdong prefecture level city in Shandong
Happy Fan Shop	Green Intelligent Power Saver	More than 150 less, last month's electricity bill is almost 400, this month is only about 200, really save electricity, I am home to buy three, I did not expect a month to pay back	Jingdong prefecture level city in Shandong
Happy Fan Shop	Green Intelligent Power Saver	I bought three of these first and tried them for two months and they still felt useful, so now I've bought another one. Good and recommended!	Jingdong prefecture level city in Shandong
Happy Fan Shop	Green Intelligent Power Saver	This energy saver is very good quality and inexpensive next time I will buy it again!	Jingdong prefecture level city in Shandong
Happy Fan Shop	Green Intelligent Power Saver	Merchant service attitude is not good, the quality does not match, the business does not give the package return	Jingdong prefecture level city in Shandong
Happy Fan Shop	Green Intelligent Power Saver	The goods are very small do not know how effective after a few days to follow up the review	Jingdong prefecture level city in Shandong
Happy Fan Shop	Green Intelligent Power Saver	Not used before more than 200 a month, after using about 100 a month, my family used three, customer service said I use electricity recommended 2-3, I think more than one effect is better on the purchase of 4, the effect is very obvious, recommended to buy!	Jingdong prefecture level city in Shandong
Happy Fan Shop	Green Intelligent Power Saver	It's okay. I'll buy it again today.	Jingdong prefecture level city in Shandong
Happy Fan Shop	Green Intelligent Power Saver	Finally received the goods, very, very much like,, has been a lot of people praise, really too happy, has been his family's loyal fans, will continue to buy buy buy!	Jingdong prefecture level city in Shandong
Happy Fan Shop	Green Intelligent Power Saver	After using this, my monthly electricity bill was 1,200 pounds, now it's only 180 bucks a month, how good is it?	Jingdong prefecture level city in Shandong
Happy Fan Shop	Green Intelligent Power Saver	The item is a scam return to marry a dozen dollars in shipping fees. Buy terrific role is purely a scam.	Jingdong prefecture level city in Shandong
Happy Fan Shop	Green Intelligent Power Saver	It's really good, cost-effective and good quality, and the logistics are fast! It arrived in one day!	Jingdong prefecture level city in Shandong
Happy Fan Shop	Green Intelligent Power Saver	Great brand! Trustworthy! Works super well! Expensive for a reason! Great merchandise!	Jingdong prefecture level city in Shandong
Happy Fan Shop	Green Intelligent Power Saver	The baby is really great, I like it very much, my friends also say good to come, recommended to buy!	Jingdong prefecture level city in Shandong
Happy Fan Shop	Green Intelligent Power Saver	Fake couldn't be more fake. Bought one plugged in still so much electricity.	Jingdong prefecture level city in Shandong
Happy Fan Shop	Green Intelligent Power Saver	This appliance works great. Let's see how it works. We'll see later.	Jingdong prefecture level city in Shandong
Happy Fan Shop	Green Intelligent Power Saver	Used it before commenting, very happy with it	Jingdong prefecture level city in Shandong
Happy Fan Shop	Green Intelligent Power Saver	Goods have been received well-packed, logistics is very fast and good quality	Jingdong prefecture level city in Shandong
Happy Fan Shop	Green Intelligent Power Saver	This baby's quality is okay.	Jingdong prefecture level city in Shandong
Happy Fan Shop	Green Intelligent Power Saver	Counterfeit goods.	Jingdong prefecture level city in Shandong
Happy Fan Shop	Green Intelligent Power Saver	The effect is not very obvious	Jingdong prefecture level city in Shandong
Happy Fan Shop	Green Intelligent Power Saver	It's not the same thing.	Jingdong prefecture level city in Shandong
Happy Fan Shop	Green Intelligent Power Saver	There is no load how much w seems false, nothing works.	Jingdong prefecture level city in Shandong
Happy Fan Shop	Green Intelligent Power Saver	I received the item yesterday, got it back and plugged it in, and it used a little more power yesterday than I had before	Jingdong prefecture level city in Shandong
Happy Fan Shop	Green Intelligent Power Saver	Haven't seen the power saving mode yet	Jingdong prefecture level city in Shandong
Happy Fan Shop	Green Intelligent Power Saver	This product has no known effect	Jingdong prefecture level city in Shandong
Happy Fan Shop	Green Intelligent Power Saver	Refund Returns $16	Jingdong prefecture level city in Shandong
Happy Fan Shop	Green Intelligent Power Saver	The previous year bought a, almost every month can save a 70 dollars or so, used a year, no problem at all, look very new like, this time to move to a new home decisively repurchase 2 units, very believe this	Jingdong prefecture level city in Shandong
Happy Fan Shop	Green Intelligent Power Saver	Has been the second time to buy, this power saving device is really good power, before every month to spend 300 electricity, now are about 200, last time that 6, this time to help lead the family to buy 6,	Jingdong prefecture level city in Shandong
Happy Fan Shop	Green Intelligent Power Saver	In the spirit of trying to experience the new product, the use of down compared to the usual power consumption, with the addition of the quarter, is indeed a lot less electricity, originally more than 450	Jingdong prefecture level city in Shandong
Happy Fan Shop	Green Intelligent Power Saver	Has not been the first time to buy back, good use, power saving device is really powerful, before the monthly electricity bill of more than 500 Now more than 300, this time to help neighbors to buy several	Jingdong prefecture level city in Shandong
Happy Fan Shop	Green Intelligent Power Saver	Home appliances are very many, the circuit is also very complex, at first it is to save 9% of the electricity, and then used more than half a month, found that the power saving rate is as high as more	Jingdong prefecture level city in Shandong
Happy Fan Shop	Green Intelligent Power Saver	My friend bought it last year and saved hundreds of dollars on his electricity bill in one summer Appearance and style: it looks better than the one I bought before Quality is also good with a light.	Jingdong prefecture level city in Shandong
Happy Fan Shop	Green Intelligent Power Saver	Saver received, on the spot on the plug with the feeling of immediate power savings, appearance, packaging is very delicate, very satisfied, I hope to achieve satisfactory results!	Jingdong prefecture level city in Shandong
Happy Fan Shop	Green Intelligent Power Saver	The power saving effect is very good, is considered an old customer, also bought before, this time is to buy two for mom and dad, customer service attitude is good, praise!	Jingdong prefecture level city in Shandong
Happy Fan Shop	Green Intelligent Power Saver	After observing for a while, it really saves a lot of money I didn't realize it was still really reliable, so I'll give you a good review.	Jingdong prefecture level city in Shandong
Happy Fan Shop	Green Intelligent Power Saver	My family a month with this monthly almost save more than 200, power-saving effect: have bought a second time, really effective Cost-effective: not bad Appearance style: roughly the same A few months to the home electricity is indeed less, this time it is a re-buy three, send a friend two, they stay a	Jingdong prefecture level city in Shandong
Happy Fan Shop	Green Intelligent Power Saver	A month almost save more than 150 dollars in electricity, home appliances more, the main this can also protect high-power appliances, after using this device, these two months have not burned, things are very good, it is said that this is the latest model, the overall price is cheap and beautiful!	Jingdong prefecture level city in Shandong
Happy Fan Shop	Green Intelligent Power Saver	Received, powerful, great quality, love the style😍	Jingdong prefecture level city in Shandong
Happy Fan Shop	Green Intelligent Power Saver	This intelligent energy saver is especially good to use and save electricity hope everyone will buy it!	Jingdong prefecture level city in Shandong
Happy Fan Shop	Green Intelligent Power Saver	Air conditioning adapter effect is particularly great, the use of very simple, plug into the socket on the summer air conditioning power is too large, electricity is also expensive, with this product directly on the electricity bill to save half, has been purchased a few more, the effect is too awesome!	Jingdong prefecture level city in Shandong
Happy Fan Shop	Green Intelligent Power Saver	The family plugged in 5, monthly electricity savings of about 300 yuan. My family before a month of electricity costs more than 800, this time with this saver after the electricity bill is actually only more than 500, amazing, thanks to the store, will recommend other friends to buy!	Jingdong prefecture level city in Shandong
Happy Fan Shop	Green Intelligent Power Saver	It was recommended to me by a neighbor's family and it's nice to use it to save power.	Jingdong prefecture level city in Shandong
Happy Fan Shop	Green Intelligent Power Saver	Second time I've purchased it and the power saving is effective!	Jingdong prefecture level city in Shandong
Happy Fan Shop	Green Intelligent Power Saver	The effect is really good, before a month of more than 300 electricity, now every month every day with the air conditioning, it is more than 100 electricity! This thing is really amazing, one after another to buy and 5.6, home installed 3!	Jingdong prefecture level city in Shandong
Happy Fan Shop	Green Intelligent Power Saver	Received this product, began to use, small things look very wrong, can save a little electricity, the monthly electricity bill is too high, after plugging in did not pull, or save a lot of electricity, point	Jingdong prefecture level city in Shandong
Happy Fan Shop	Green Intelligent Power Saver	Simple and convenient, just that plug in, you can save electricity. A month can save dozens of dollars, a year down to save a lot of, very good product cost-effective, everyone can afford to buy and use!	Jingdong prefecture level city in Shandong
Happy Fan Shop	Green Intelligent Power Saver	Electricity bill from more than 200 yuan per month, down to more than 150 yuan, the original bought home, used two months, really have power-saving effect, this time and sold two, business service attitude is	Jingdong prefecture level city in Shandong

Happy Fan Shop	Green Intelligent Power Saver	Like to watch some movies at home and so on, sometimes at night until two or three o'clock, and recently the air conditioning is not turned off for a night, rather wasteful, I have to say that the effect is very good, quite like it!	Jingdong prefecture level city in Shandong
Happy Fan Shop	Green Intelligent Power Saver	Power-saving effect: the effect is very good, a month to save more than 200 dollars Cost-effective: good value for money Appearance and style: small and lovely Workmanship material: okay Experience: good	Jingdong prefecture level city in Shandong
Happy Fan Shop	Green Intelligent Power Saver	It's okay I quite like it, the stuff is very good. Come back in the future. You can rest assured to buy. Cost-effective all. Much cheaper than the physical store	Jingdong prefecture level city in Shandong
Happy Fan Shop	Green Intelligent Power Saver	More than 150 less, last month's electricity bill is almost 400, this month is only about 200, really save electricity, I am home to buy three, I did not expect a month to pay back	Jingdong prefecture level city in Shandong
Happy Fan Shop	Green Intelligent Power Saver	I bought three of these first and tried them for two months and they still felt useful, so now I've bought another one. Good and recommended!	Jingdong prefecture level city in Shandong
Happy Fan Shop	Green Intelligent Power Saver	This energy saver is very good quality and inexpensive next time I will buy it again!	Jingdong prefecture level city in Shandong
Happy Fan Shop	Green Intelligent Power Saver	Merchant service attitude is not good, the quality does not match, the business does not give the package return	Jingdong prefecture level city in Shandong
Happy Fan Shop	Green Intelligent Power Saver	The goods are very small do not know how effective after a few days to follow up the review	Jingdong prefecture level city in Shandong
Happy Fan Shop	Green Intelligent Power Saver	Not used before more than 200 a month, after using about 100 a month, my family used three, customer service said I use electricity recommended 2-3, I think more than one effect is better on the purchase of 4, the effect is very obvious, recommended to buy!	Jingdong prefecture level city in Shandong
Happy Fan Shop	Green Intelligent Power Saver	It's okay. I'll buy it again today.	Jingdong prefecture level city in Shandong
Happy Fan Shop	Green Intelligent Power Saver	Finally received the goods, very, very much like,, has been a lot of people praise, really too happy, has been his family's loyal fans, will continue to buy buy buy buy!	Jingdong prefecture level city in Shandong
Happy Fan Shop	Green Intelligent Power Saver	After using this, my monthly electricity bill was 1,200 pounds, now it's only 180 bucks a month, how good is it?	Jingdong prefecture level city in Shandong
Happy Fan Shop	Green Intelligent Power Saver	The item is a scam return to marry a dozen dollars in shipping fees. Buy terrific role is purely a scam.	Jingdong prefecture level city in Shandong
Happy Fan Shop	Green Intelligent Power Saver	It's really good, cost-effective and good quality, and the logistics are fast! It arrived in one day!	Jingdong prefecture level city in Shandong
Happy Fan Shop	Green Intelligent Power Saver	Great brand! Trustworthy! Works super well! Expensive for a reason! Great merchandise!	Jingdong prefecture level city in Shandong
Happy Fan Shop	Green Intelligent Power Saver	The baby is really great, I like it very much, my friends also say good to come, recommended to buy!	Jingdong prefecture level city in Shandong
Happy Fan Shop	Green Intelligent Power Saver	Fake couldn't be more fake. Bought one plugged in still so much electricity.	Jingdong prefecture level city in Shandong
Happy Fan Shop	Green Intelligent Power Saver	This appliance works great. Let's see how it works. We'll see later.	Jingdong prefecture level city in Shandong
Happy Fan Shop	Green Intelligent Power Saver	Used it before commenting, very happy with it	Jingdong prefecture level city in Shandong
Happy Fan Shop	Green Intelligent Power Saver	Goods have been received well-packed, logistics is very fast and good quality	Jingdong prefecture level city in Shandong
Happy Fan Shop	Green Intelligent Power Saver	This baby's quality is okay.	Jingdong prefecture level city in Shandong
Happy Fan Shop	Green Intelligent Power Saver	Counterfeit goods.	Jingdong prefecture level city in Shandong
Happy Fan Shop	Green Intelligent Power Saver	The effect is not very obvious	Jingdong prefecture level city in Shandong
Happy Fan Shop	Green Intelligent Power Saver	It's not the same thing.	Jingdong prefecture level city in Shandong
Happy Fan Shop	Green Intelligent Power Saver	There is no load how much w seems false, nothing works.	Jingdong prefecture level city in Shandong
Happy Fan Shop	Green Intelligent Power Saver	I received the item yesterday, got it back and plugged it in, and it used a little more power yesterday than I had before	Jingdong prefecture level city in Shandong
Happy Fan Shop	Green Intelligent Power Saver	Haven't seen the power saving mode yet	Jingdong prefecture level city in Shandong
Happy Fan Shop	Green Intelligent Power Saver	This product has no known effect	Jingdong prefecture level city in Shandong
Happy Fan Shop	Green Intelligent Power Saver	Refund Returns $16	Jingdong prefecture level city in Shandong
Happy Fan Shop	Green Intelligent Power Saver	The previous year bought a, almost every month can save a 70 dollars or so, used a year, no problem at all, look very new like, this time to move to a new home decisively repurchase 2 units, very believe this	Jingdong prefecture level city in Shandong
Happy Fan Shop	Green Intelligent Power Saver	Has been the second time to buy, this power saving device is really good power, before every month to spend 300 electricity, now are about 200, last time that 6, this time to help lead the family to buy 6,	Jingdong prefecture level city in Shandong
Happy Fan Shop	Green Intelligent Power Saver	In the spirit of trying to experience the new product, the use of down compared to the usual power consumption, with the addition of the quarter, is indeed a lot less electricity, originally more than 450	Jingdong prefecture level city in Shandong
Happy Fan Shop	Green Intelligent Power Saver	Has not been the first time to buy back, good use, power saving device is really powerful, before the monthly electricity bill of more than 500 Now more than 300, this time to help neighbors to buy several	Jingdong prefecture level city in Shandong
Happy Fan Shop	Green Intelligent Power Saver	Home appliances are very many, the circuit is also very complex, at first it is to save 9% of the electricity, and then used more than half a month, found that the power saving rate is as high as more	Jingdong prefecture level city in Shandong
Happy Fan Shop	Green Intelligent Power Saver	My friend bought it last year and saved hundreds of dollars on his electricity bill in one summer Appearance and style: it looks better than the one I bought before Quality is also good with a light.	Jingdong prefecture level city in Shandong
Happy Fan Shop	Green Intelligent Power Saver	Saver received, on the spot on the plug with the feeling of immediate power savings, appearance, packaging is very delicate, very satisfied, I hope to achieve satisfactory results!	Jingdong prefecture level city in Shandong
Happy Fan Shop	Green Intelligent Power Saver	The power saving effect is very good, is considered an old customer, also bought before, this time is to buy two for mom and dad, customer service attitude is good, praise!	Jingdong prefecture level city in Shandong
Happy Fan Shop	Green Intelligent Power Saver	After observing for a while, it really saves a lot of money I didn't realize it was still really reliable, so I'll give you a good review.	Jingdong prefecture level city in Shandong
Happy Fan Shop	Green Intelligent Power Saver	My family a month with this monthly almost save more than 200, power-saving effect: have bought a second time, really effective Cost-effective: not bad Appearance style: roughly the same A few months to the home electricity is indeed less, this time it is a re-buy three, send a friend two, they stay a	Jingdong prefecture level city in Shandong
Happy Fan Shop	Green Intelligent Power Saver	A month almost save more than 150 dollars in electricity, home appliances more, the main this can also protect high-power appliances, after using this device, these two months have not burned, things are very good, it is said that this is the latest model, the overall price is cheap and beautiful!	Jingdong prefecture level city in Shandong
Happy Fan Shop	Green Intelligent Power Saver	Received, powerful, great quality, love the style😍	Jingdong prefecture level city in Shandong
Happy Fan Shop	Green Intelligent Power Saver	This intelligent energy saver is especially good to use and save electricity hope everyone will buy it!	Jingdong prefecture level city in Shandong
Happy Fan Shop	Green Intelligent Power Saver	Air conditioning adapter effect is particularly great, the use of very simple, plug into the socket on the summer air conditioning power is too large, electricity is also expensive, with this product directly on the electricity bill to save half, has been purchased a few more, the effect is too awesome!	Jingdong prefecture level city in Shandong
Happy Fan Shop	Green Intelligent Power Saver	The family plugged in 5, monthly electricity savings of about 300 yuan. My family before a month of electricity costs more than 800, this time with this saver after the electricity bill is actually only more than 500, amazing, thanks to the store, will recommend other friends to buy!	Jingdong prefecture level city in Shandong
Happy Fan Shop	Green Intelligent Power Saver	It was recommended to me by a neighbor's family and it's nice to use it to save power.	Jingdong prefecture level city in Shandong
Happy Fan Shop	Green Intelligent Power Saver	Second time I've purchased it and the power saving is effective!	Jingdong prefecture level city in Shandong
Happy Fan Shop	Green Intelligent Power Saver	The effect is really good, before a month of more than 300 electricity, now every month every day with the air conditioning, it is more than 100 electricity! This thing is really amazing, one after another to buy and 5.6, home installed 3!	Jingdong prefecture level city in Shandong
Happy Fan Shop	Green Intelligent Power Saver	Received this product, began to use, small things look very wrong, can save a little electricity, the monthly electricity bill is too high, after plugging in did not pull, or save a lot of electricity, point	Jingdong prefecture level city in Shandong

Happy Fan Shop	Green Intelligent Power Saver	Simple and convenient, just that plug in, you can save electricity. A month can save dozens of dollars, a year down to save a lot of, very good product cost-effective, everyone can afford to buy and use!	Jingdong prefecture level city in Shandong
Happy Fan Shop	Green Intelligent Power Saver	Electricity bill from more than 200 yuan per month, down to more than 150 yuan, the original bought home, used two months, really have power-saving effect, this time and sold two, business service attitude is	Jingdong prefecture level city in Shandong
Happy Fan Shop	Green Intelligent Power Saver	Like to watch some movies at home and so on, sometimes at night until two or three o'clock, and recently the air conditioning is not turned off for a night, rather wasteful, I have to say that the effect is very good, quite like it!	Jingdong prefecture level city in Shandong
Happy Fan Shop	Green Intelligent Power Saver	Power-saving effect: the effect is very good, a month to save more than 200 dollars Cost-effective: good value for money Appearance and style: small and lovely Workmanship material: okay Experience: good	Jingdong prefecture level city in Shandong
Happy Fan Shop	Green Intelligent Power Saver	It's okay I quite like it, the stuff is very good. Come back in the future. You can rest assured to buy. Cost-effective all. Much cheaper than the physical store	Jingdong prefecture level city in Shandong
Happy Fan Shop	Green Intelligent Power Saver	More than 150 less, last month's electricity bill is almost 400, this month is only about 200, really save electricity, I am home to buy three, I did not expect a month to pay back	Jingdong prefecture level city in Shandong
Happy Fan Shop	Green Intelligent Power Saver	I bought three of these first and tried them for two months and they still felt useful, so now I've bought another one. Good and recommended!	Jingdong prefecture level city in Shandong
Happy Fan Shop	Green Intelligent Power Saver	This energy saver is very good quality and inexpensive next time I will buy it again!	Jingdong prefecture level city in Shandong
Happy Fan Shop	Green Intelligent Power Saver	Merchant service attitude is not good, the quality does not match, the business does not give the package return	Jingdong prefecture level city in Shandong
Happy Fan Shop	Green Intelligent Power Saver	The goods are very small do not know how effective after a few days to follow up the review	Jingdong prefecture level city in Shandong
Happy Fan Shop	Green Intelligent Power Saver	Not used before more than 200 a month, after using about 100 a month, my family used three, customer service said I use electricity recommended 2-3, I think more than one effect is better on the purchase of 4, the effect is very obvious, recommended to buy!	Jingdong prefecture level city in Shandong
Happy Fan Shop	Green Intelligent Power Saver	It's okay. I'll buy it again today.	Jingdong prefecture level city in Shandong
Happy Fan Shop	Green Intelligent Power Saver	Finally received the goods, very, very much like,, has been a lot of people praise, really too happy, has been his family's loyal fans, will continue to buy buy buy buy!	Jingdong prefecture level city in Shandong
Happy Fan Shop	Green Intelligent Power Saver	After using this, my monthly electricity bill was 1,200 pounds, now it's only 180 bucks a month, how good is it?	Jingdong prefecture level city in Shandong
Happy Fan Shop	Green Intelligent Power Saver	The item is a scam return to marry a dozen dollars in shipping fees. Buy terrific role is purely a scam.	Jingdong prefecture level city in Shandong
Happy Fan Shop	Green Intelligent Power Saver	It's really good, cost-effective and good quality, and the logistics are fast! It arrived in one day!	Jingdong prefecture level city in Shandong
Happy Fan Shop	Green Intelligent Power Saver	Great brand! Trustworthy! Works super well! Expensive for a reason! Great merchandise!	Jingdong prefecture level city in Shandong
Happy Fan Shop	Green Intelligent Power Saver	The baby is really great, I like it very much, my friends also say good to come, recommended to buy!	Jingdong prefecture level city in Shandong
Happy Fan Shop	Green Intelligent Power Saver	Fake couldn't be more fake. Bought one plugged in still so much electricity.	Jingdong prefecture level city in Shandong
Happy Fan Shop	Green Intelligent Power Saver	This appliance works great. Let's see how it works. We'll see later.	Jingdong prefecture level city in Shandong
Happy Fan Shop	Green Intelligent Power Saver	Used it before commenting, very happy with it	Jingdong prefecture level city in Shandong
Happy Fan Shop	Green Intelligent Power Saver	Goods have been received well-packed, logistics is very fast and good quality	Jingdong prefecture level city in Shandong
Happy Fan Shop	Green Intelligent Power Saver	This baby's quality is okay.	Jingdong prefecture level city in Shandong
Happy Fan Shop	Green Intelligent Power Saver	Counterfeit goods.	Jingdong prefecture level city in Shandong
Happy Fan Shop	Green Intelligent Power Saver	The effect is not very obvious	Jingdong prefecture level city in Shandong
Happy Fan Shop	Green Intelligent Power Saver	It's not the same thing.	Jingdong prefecture level city in Shandong
Happy Fan Shop	Green Intelligent Power Saver	There is no load how much w seems false, nothing works.	Jingdong prefecture level city in Shandong
Happy Fan Shop	Green Intelligent Power Saver	I received the item yesterday, got it back and plugged it in, and it used a little more power yesterday than I had before	Jingdong prefecture level city in Shandong
Happy Fan Shop	Green Intelligent Power Saver	Haven't seen the power saving mode yet	Jingdong prefecture level city in Shandong
Happy Fan Shop	Green Intelligent Power Saver	This product has no known effect	Jingdong prefecture level city in Shandong
Happy Fan Shop	Green Intelligent Power Saver	Refund Returns $16	Jingdong prefecture level city in Shandong
Happy Fan Shop	Green Intelligent Power Saver	The previous year bought a, almost every month can save a 70 dollars or so, used a year, no problem at all, look very new like, this time to move to a new home decisively repurchase 2 units, very believe this	Jingdong prefecture level city in Shandong
Happy Fan Shop	Green Intelligent Power Saver	Has been the second time to buy, this power saving device is really good power, before every month to spend 300 electricity, now are about 200, last time that 6, this time to help lead the family to buy 6,	Jingdong prefecture level city in Shandong
Happy Fan Shop	Green Intelligent Power Saver	In the spirit of trying to experience the new product, the use of down compared to the usual power consumption, with the addition of the quarter, is indeed a lot less electricity, originally more than 450	Jingdong prefecture level city in Shandong
Happy Fan Shop	Green Intelligent Power Saver	Has not been the first time to buy back, good use, power saving device is really powerful, before the monthly electricity bill of more than 500 Now more than 300, this time to help neighbors to buy several	Jingdong prefecture level city in Shandong
Happy Fan Shop	Green Intelligent Power Saver	Home appliances are very many, the circuit is also very complex, at first it is to save 9% of the electricity, and then used more than half a month, found that the power saving rate is as high as more	Jingdong prefecture level city in Shandong
Happy Fan Shop	Green Intelligent Power Saver	My friend bought it last year and saved hundreds of dollars on his electricity bill in one summer Appearance and style: it looks better than the one I bought before Quality is also good with a light.	Jingdong prefecture level city in Shandong
Happy Fan Shop	Green Intelligent Power Saver	Saver received, on the spot on the plug with the feeling of immediate power savings, appearance, packaging is very delicate, very satisfied, I hope to achieve satisfactory results!	Jingdong prefecture level city in Shandong
Happy Fan Shop	Green Intelligent Power Saver	The power saving effect is very good, is considered an old customer, also bought before, this time is to buy two for mom and dad, customer service attitude is good, praise!	Jingdong prefecture level city in Shandong
Happy Fan Shop	Green Intelligent Power Saver	After observing for a while, it really saves a lot of money I didn't realize it was still really reliable, so I'll give you a good review.	Jingdong prefecture level city in Shandong
Happy Fan Shop	Green Intelligent Power Saver	My family a month with this monthly almost save more than 200, power-saving effect: have bought a second time, really effective Cost-effective: not bad Appearance style: roughly the same A few months to the home electricity is indeed less, this time it is a re-buy three, send a friend two, they stay a	Jingdong prefecture level city in Shandong
Happy Fan Shop	Green Intelligent Power Saver	A month almost save more than 150 dollars in electricity, home appliances more, the main this can also protect high-power appliances, after using this device, these two months have not burned, things are very good, it is said that this is the latest model, the overall price is cheap and beautiful!	Jingdong prefecture level city in Shandong
Happy Fan Shop	Green Intelligent Power Saver	Received, powerful, great quality, love the style😍	Jingdong prefecture level city in Shandong
Happy Fan Shop	Green Intelligent Power Saver	This intelligent energy saver is especially good to use and save electricity hope everyone will buy it!	Jingdong prefecture level city in Shandong
Happy Fan Shop	Green Intelligent Power Saver	Air conditioning adapter effect is particularly great, the use of very simple, plug into the socket on the summer air conditioning power is too large, electricity is also expensive, with this product directly on the electricity bill to save half, has been purchased a few more, the effect is too awesome!	Jingdong prefecture level city in Shandong
Happy Fan Shop	Green Intelligent Power Saver	The family plugged in 5, monthly electricity savings of about 300 yuan. My family before a month of electricity costs more than 800, this time with this saver after the electricity bill is actually only more than 500, amazing, thanks to the store, will recommend other friends to buy!	Jingdong prefecture level city in Shandong
Happy Fan Shop	Green Intelligent Power Saver	It was recommended to me by a neighbor's family and it's nice to use it to save power.	Jingdong prefecture level city in Shandong
Happy Fan Shop	Green Intelligent Power Saver	Second time I've purchased it and the power saving is effective!	Jingdong prefecture level city in Shandong

Happy Fan Shop	Green Intelligent Power Saver	The effect is really good, before a month of more than 300 electricity, now every month every day with the air conditioning, it is more than 100 electricity! This thing is really amazing, one after another to buy and 5.6, home installed 3!	Jingdong prefecture level city in Shandong
Happy Fan Shop	Green Intelligent Power Saver	Received this product, began to use, small things look very wrong, can save a little electricity, the monthly electricity bill is too high, after plugging in did not pull, or save a lot of electricity, point	Jingdong prefecture level city in Shandong
Happy Fan Shop	Green Intelligent Power Saver	Simple and convenient, just that plug in, you can save electricity. A month can save dozens of dollars, a year down to save a lot of, very good product cost-effective, everyone can afford to buy and use!	Jingdong prefecture level city in Shandong
Happy Fan Shop	Green Intelligent Power Saver	Electricity bill from more than 200 yuan per month, down to more than 150 yuan, the original bought home, used two months, really have power-saving effect, this time and sold two, business service attitude is	Jingdong prefecture level city in Shandong
Happy Fan Shop	Green Intelligent Power Saver	Like to watch some movies at home and so on, sometimes at night until two or three o'clock, and recently the air conditioning is not turned off for a night, rather wasteful, I have to say that the effect is very good, quite like it!	Jingdong prefecture level city in Shandong
Happy Fan Shop	Green Intelligent Power Saver	Power-saving effect: the effect is very good, a month to save more than 200 dollars Cost-effective: good value for money Appearance and style: small and lovely Workmanship material: okay Experience: good	Jingdong prefecture level city in Shandong
Happy Fan Shop	Green Intelligent Power Saver	It's okay I quite like it, the stuff is very good. Come back in the future. You can rest assured to buy. Cost-effective all. Much cheaper than the physical store	Jingdong prefecture level city in Shandong
Happy Fan Shop	Green Intelligent Power Saver	More than 150 less, last month's electricity bill is almost 400, this month is only about 200, really save electricity, I am home to buy three, I did not expect a month to pay back	Jingdong prefecture level city in Shandong
Happy Fan Shop	Green Intelligent Power Saver	I bought three of these first and tried them for two months and they still felt useful, so now I've bought another one. Good and recommended!	Jingdong prefecture level city in Shandong
Happy Fan Shop	Green Intelligent Power Saver	This energy saver is very good quality and inexpensive next time I will buy it again!	Jingdong prefecture level city in Shandong
Happy Fan Shop	Green Intelligent Power Saver	Merchant service attitude is not good, the quality does not match, the business does not give the package return	Jingdong prefecture level city in Shandong
Happy Fan Shop	Green Intelligent Power Saver	The goods are very small do not know how effective after a few days to follow up the review	Jingdong prefecture level city in Shandong
Happy Fan Shop	Green Intelligent Power Saver	Not used before more than 200 a month, after using about 100 a month, my family used three, customer service said I use electricity recommended 2-3, I think more than one effect is better on the purchase of 4, the effect is very obvious, recommended to buy!	Jingdong prefecture level city in Shandong
Happy Fan Shop	Green Intelligent Power Saver	It's okay. I'll buy it again today.	Jingdong prefecture level city in Shandong
Happy Fan Shop	Green Intelligent Power Saver	Finally received the goods, very, very much like,, has been a lot of people praise, really too happy, has been his family's loyal fans, will continue to buy buy buy buy!	Jingdong prefecture level city in Shandong
Happy Fan Shop	Green Intelligent Power Saver	After using this, my monthly electricity bill was 1,200 pounds, now it's only 180 bucks a month, how good is it?	Jingdong prefecture level city in Shandong
Happy Fan Shop	Green Intelligent Power Saver	The item is a scam return to marry a dozen dollars in shipping fees. Buy terrific role is purely a scam.	Jingdong prefecture level city in Shandong
Happy Fan Shop	Green Intelligent Power Saver	It's really good, cost-effective and good quality, and the logistics are fast! It arrived in one day!	Jingdong prefecture level city in Shandong
Happy Fan Shop	Green Intelligent Power Saver	Great brand! Trustworthy! Works super well! Expensive for a reason! Great merchandise!	Jingdong prefecture level city in Shandong
Happy Fan Shop	Green Intelligent Power Saver	The baby is really great, I like it very much, my friends also say good to come, recommended to buy!	Jingdong prefecture level city in Shandong
Happy Fan Shop	Green Intelligent Power Saver	Fake couldn't be more fake. Bought one plugged in still so much electricity.	Jingdong prefecture level city in Shandong
Happy Fan Shop	Green Intelligent Power Saver	This appliance works great. Let's see how it works. We'll see later.	Jingdong prefecture level city in Shandong
Happy Fan Shop	Green Intelligent Power Saver	Used it before commenting, very happy with it	Jingdong prefecture level city in Shandong
Happy Fan Shop	Green Intelligent Power Saver	Goods have been received well-packed, logistics is very fast and good quality	Jingdong prefecture level city in Shandong
Happy Fan Shop	Green Intelligent Power Saver	This baby's quality is okay.	Jingdong prefecture level city in Shandong
Happy Fan Shop	Green Intelligent Power Saver	Counterfeit goods.	Jingdong prefecture level city in Shandong
Happy Fan Shop	Green Intelligent Power Saver	The effect is not very obvious	Jingdong prefecture level city in Shandong
Happy Fan Shop	Green Intelligent Power Saver	It's not the same thing.	Jingdong prefecture level city in Shandong
Happy Fan Shop	Green Intelligent Power Saver	There is no load how much w seems false, nothing works.	Jingdong prefecture level city in Shandong
Happy Fan Shop	Green Intelligent Power Saver	I received the item yesterday, got it back and plugged it in, and it used a little more power yesterday than I had before	Jingdong prefecture level city in Shandong
Happy Fan Shop	Green Intelligent Power Saver	Haven't seen the power saving mode yet	Jingdong prefecture level city in Shandong
Happy Fan Shop	Green Intelligent Power Saver	This product has no known effect	Jingdong prefecture level city in Shandong
Happy Fan Shop	Green Intelligent Power Saver	Refund Returns $16	Jingdong prefecture level city in Shandong
Happy Fan Shop	Green Intelligent Power Saver	The previous year bought a, almost every month can save a 70 dollars or so, used a year, no problem at all, look very new like, this time to move to a new home decisively repurchase 2 units, very believe this	Jingdong prefecture level city in Shandong
Happy Fan Shop	Green Intelligent Power Saver	Has been the second time to buy, this power saving device is really good power, before every month to spend 300 electricity, now are about 200, last time that 6, this time to help lead the family to buy 6,	Jingdong prefecture level city in Shandong
Happy Fan Shop	Green Intelligent Power Saver	In the spirit of trying to experience the new product, the use of down compared to the usual power consumption, with the addition of the quarter, is indeed a lot less electricity, originally more than 450	Jingdong prefecture level city in Shandong
Happy Fan Shop	Green Intelligent Power Saver	Has not been the first time to buy back, good use, power saving device is really powerful, before the monthly electricity bill of more than 500 Now more than 300, this time to help neighbors to buy several	Jingdong prefecture level city in Shandong
Happy Fan Shop	Green Intelligent Power Saver	Home appliances are very many, the circuit is also very complex, at first it is to save 9% of the electricity, and then used more than half a month, found that the power saving rate is as high as more	Jingdong prefecture level city in Shandong
Happy Fan Shop	Green Intelligent Power Saver	My friend bought it last year and saved hundreds of dollars on his electricity bill in one summer Appearance and style: it looks better than the one I bought before Quality is also good with a light.	Jingdong prefecture level city in Shandong
Happy Fan Shop	Green Intelligent Power Saver	Saver received, on the spot on the plug with the feeling of immediate power savings, appearance, packaging is very delicate, very satisfied, I hope to achieve satisfactory results!	Jingdong prefecture level city in Shandong
Happy Fan Shop	Green Intelligent Power Saver	The power saving effect is very good, is considered an old customer, also bought before, this time is to buy two for mom and dad, customer service attitude is good, praise!	Jingdong prefecture level city in Shandong
Happy Fan Shop	Green Intelligent Power Saver	After observing for a while, it really saves a lot of money I didn't realize it was still really reliable, so I'll give you a good review.	Jingdong prefecture level city in Shandong
Happy Fan Shop	Green Intelligent Power Saver	My family a month with this monthly almost save more than 200, power-saving effect: have bought a second time, really effective Cost-effective: not bad Appearance style: roughly the same A few months to the home electricity is indeed less, this time it is a re-buy three, send a friend two, they stay a	Jingdong prefecture level city in Shandong
Happy Fan Shop	Green Intelligent Power Saver	A month almost save more than 150 dollars in electricity, home appliances more, the main this can also protect high-power appliances, after using this device, these two months have not burned, things are very good, it is said that this is the latest model, the overall price is cheap and beautiful!	Jingdong prefecture level city in Shandong
Happy Fan Shop	Green Intelligent Power Saver	Received, powerful, great quality, love the style😍	Jingdong prefecture level city in Shandong
Happy Fan Shop	Green Intelligent Power Saver	This intelligent energy saver is especially good to use and save electricity hope everyone will buy it!	Jingdong prefecture level city in Shandong
Happy Fan Shop	Green Intelligent Power Saver	Air conditioning adapter effect is particularly great, the use of very simple, plug into the socket on the summer air conditioning power is too large, electricity is also expensive, with this product directly on the electricity bill to save half, has been purchased a few more, the effect is too awesome!	Jingdong prefecture level city in Shandong
Happy Fan Shop	Green Intelligent Power Saver	The family plugged in 5, monthly electricity savings of about 300 yuan. My family before a month of electricity costs more than 800, this time with this saver after the electricity bill is actually only more than 500, amazing, thanks to the store, will recommend other friends to buy!	Jingdong prefecture level city in Shandong

Happy Fan Shop	Green Intelligent Power Saver	It was recommended to me by a neighbor's family and it's nice to use it to save power.	Jingdong prefecture level city in Shandong
Happy Fan Shop	Green Intelligent Power Saver	Second time I've purchased it and the power saving is effective!	Jingdong prefecture level city in Shandong
Happy Fan Shop	Green Intelligent Power Saver	The effect is really good, before a month of more than 300 electricity, now every month every day with the air conditioning, it is more than 100 electricity! This thing is really amazing, one after another to buy and 5.6, home installed 3!	Jingdong prefecture level city in Shandong
Happy Fan Shop	Green Intelligent Power Saver	Received this product, began to use, small things look very wrong, can save a little electricity, the monthly electricity bill is too high, after plugging in did not pull, or save a lot of electricity, point	Jingdong prefecture level city in Shandong
Happy Fan Shop	Green Intelligent Power Saver	Simple and convenient, just that plug in, you can save electricity. A month can save dozens of dollars, a year down to save a lot of, very good product cost-effective, everyone can afford to buy and use!	Jingdong prefecture level city in Shandong
Happy Fan Shop	Green Intelligent Power Saver	Electricity bill from more than 200 yuan per month, down to more than 150 yuan, the original bought home, used two months, really have power-saving effect, this time and sold two, business service attitude is	Jingdong prefecture level city in Shandong
Happy Fan Shop	Green Intelligent Power Saver	Like to watch some movies at home and so on, sometimes at night until two or three o'clock, and recently the air conditioning is not turned off for a night, rather wasteful, I have to say that the effect is very good, quite like it!	Jingdong prefecture level city in Shandong
Happy Fan Shop	Green Intelligent Power Saver	Power-saving effect: the effect is very good, a month to save more than 200 dollars Cost-effective: good value for money Appearance and style: small and lovely Workmanship material: okay Experience: good	Jingdong prefecture level city in Shandong
Happy Fan Shop	Green Intelligent Power Saver	It's okay I quite like it, the stuff is very good. Come back in the future. You can rest assured to buy. Cost-effective all. Much cheaper than the physical store	Jingdong prefecture level city in Shandong
Happy Fan Shop	Green Intelligent Power Saver	More than 150 less, last month's electricity bill is almost 400, this month is only about 200, really save electricity, I am home to buy three, I did not expect a month to pay back	Jingdong prefecture level city in Shandong
Happy Fan Shop	Green Intelligent Power Saver	I bought three of these first and tried them for two months and they still felt useful, so now I've bought another one. Good and recommended!	Jingdong prefecture level city in Shandong
Happy Fan Shop	Green Intelligent Power Saver	This energy saver is very good quality and inexpensive next time I will buy it again!	Jingdong prefecture level city in Shandong
Happy Fan Shop	Green Intelligent Power Saver	Merchant service attitude is not good, the quality does not match, the business does not give the package return	Jingdong prefecture level city in Shandong
Happy Fan Shop	Green Intelligent Power Saver	The goods are very small do not know how effective after a few days to follow up the review	Jingdong prefecture level city in Shandong
Happy Fan Shop	Green Intelligent Power Saver	Not used before more than 200 a month, after using about 100 a month, my family used three, customer service said I use electricity recommended 2-3, I think more than one effect is better on the purchase of 4, the effect is very obvious, recommended to buy!	Jingdong prefecture level city in Shandong
Happy Fan Shop	Green Intelligent Power Saver	It's okay. I'll buy it again today.	Jingdong prefecture level city in Shandong
Happy Fan Shop	Green Intelligent Power Saver	Finally received the goods, very, very much like,, has been a lot of people praise, really too happy, has been his family's loyal fans, will continue to buy buy buy buy!	Jingdong prefecture level city in Shandong
Happy Fan Shop	Green Intelligent Power Saver	After using this, my monthly electricity bill was 1,200 pounds, now it's only 180 bucks a month, how good is it?	Jingdong prefecture level city in Shandong
Happy Fan Shop	Green Intelligent Power Saver	The item is a scam return to marry a dozen dollars in shipping fees. Buy terrific role is purely a scam.	Jingdong prefecture level city in Shandong
Happy Fan Shop	Green Intelligent Power Saver	It's really good, cost-effective and good quality, and the logistics are fast! It arrived in one day!	Jingdong prefecture level city in Shandong
Happy Fan Shop	Green Intelligent Power Saver	Great brand! Trustworthy! Works super well! Expensive for a reason! Great merchandise!	Jingdong prefecture level city in Shandong
Happy Fan Shop	Green Intelligent Power Saver	The baby is really great, I like it very much, my friends also say good to come, recommended to buy!	Jingdong prefecture level city in Shandong
Happy Fan Shop	Green Intelligent Power Saver	Fake couldn't be more fake. Bought one plugged in still so much electricity.	Jingdong prefecture level city in Shandong
Happy Fan Shop	Green Intelligent Power Saver	This appliance works great. Let's see how it works. We'll see later.	Jingdong prefecture level city in Shandong
Happy Fan Shop	Green Intelligent Power Saver	Used it before commenting, very happy with it	Jingdong prefecture level city in Shandong
Happy Fan Shop	Green Intelligent Power Saver	Goods have been received well-packed, logistics is very fast and good quality	Jingdong prefecture level city in Shandong
Happy Fan Shop	Green Intelligent Power Saver	This baby's quality is okay.	Jingdong prefecture level city in Shandong
Happy Fan Shop	Green Intelligent Power Saver	Counterfeit goods.	Jingdong prefecture level city in Shandong
Happy Fan Shop	Green Intelligent Power Saver	The effect is not very obvious	Jingdong prefecture level city in Shandong
Happy Fan Shop	Green Intelligent Power Saver	It's not the same thing.	Jingdong prefecture level city in Shandong
Happy Fan Shop	Green Intelligent Power Saver	There is no load how much w seems false, nothing works.	Jingdong prefecture level city in Shandong
Happy Fan Shop	Green Intelligent Power Saver	I received the item yesterday, got it back and plugged it in, and it used a little more power yesterday than I had before	Jingdong prefecture level city in Shandong
Happy Fan Shop	Green Intelligent Power Saver	Haven't seen the power saving mode yet	Jingdong prefecture level city in Shandong
Happy Fan Shop	Green Intelligent Power Saver	This product has no known effect	Jingdong prefecture level city in Shandong
Happy Fan Shop	Green Intelligent Power Saver	Refund Returns $16	Jingdong prefecture level city in Shandong
Happy Fan Shop	Green Intelligent Power Saver	The previous year bought a, almost every month can save a 70 dollars or so, used a year, no problem at all, look very new like, this time to move to a new home decisively repurchase 2 units, very believe this	Jingdong prefecture level city in Shandong
Happy Fan Shop	Green Intelligent Power Saver	Has been the second time to buy, this power saving device is really good power, before every month to spend 300 electricity, now are about 200, last time that 6, this time to help lead the family to buy 6,	Jingdong prefecture level city in Shandong
Happy Fan Shop	Green Intelligent Power Saver	In the spirit of trying to experience the new product, the use of down compared to the usual power consumption, with the addition of the quarter, is indeed a lot less electricity, originally more than 450	Jingdong prefecture level city in Shandong
Happy Fan Shop	Green Intelligent Power Saver	Has not been the first time to buy back, good use, power saving device is really powerful, before the monthly electricity bill of more than 500 Now more than 300, this time to help neighbors to buy several	Jingdong prefecture level city in Shandong
Happy Fan Shop	Green Intelligent Power Saver	Home appliances are very many, the circuit is also very complex, at first it is to save 9% of the electricity, and then used more than half a month, found that the power saving rate is as high as more	Jingdong prefecture level city in Shandong
Happy Fan Shop	Green Intelligent Power Saver	My friend bought it last year and saved hundreds of dollars on his electricity bill in one summer Appearance and style: it looks better than the one I bought before Quality is also good with a light.	Jingdong prefecture level city in Shandong
Happy Fan Shop	Green Intelligent Power Saver	Saver received, on the spot on the plug with the feeling of immediate power savings, appearance, packaging is very delicate, very satisfied, I hope to achieve satisfactory results!	Jingdong prefecture level city in Shandong
Happy Fan Shop	Green Intelligent Power Saver	The power saving effect is very good, is considered an old customer, also bought before, this time is to buy two for mom and dad, customer service attitude is good, praise!	Jingdong prefecture level city in Shandong
Happy Fan Shop	Green Intelligent Power Saver	After observing for a while, it really saves a lot of money I didn't realize it was still really reliable, so I'll give you a good review.	Jingdong prefecture level city in Shandong
Happy Fan Shop	Green Intelligent Power Saver	My family a month with this monthly almost save more than 200, power-saving effect: have bought a second time, really effective Cost-effective: not bad Appearance style: roughly the same A few months to the home electricity is indeed less, this time it is a re-buy three, send a friend two, they stay a	Jingdong prefecture level city in Shandong
Happy Fan Shop	Green Intelligent Power Saver	A month almost save more than 150 dollars in electricity, home appliances more, the main this can also protect high-power appliances, after using this device, these two months have not burned, things are very good, it is said that this is the latest model, the overall price is cheap and beautiful!	Jingdong prefecture level city in Shandong
Happy Fan Shop	Green Intelligent Power Saver	Received, powerful, great quality, love the style😍	Jingdong prefecture level city in Shandong
Happy Fan Shop	Green Intelligent Power Saver	This intelligent energy saver is especially good to use and save electricity hope everyone will buy it!	Jingdong prefecture level city in Shandong

Happy Fan Shop	Green Intelligent Power Saver	Air conditioning adapter effect is particularly great, the use of very simple, plug into the socket on the summer air conditioning power is too large, electricity is also expensive, with this product directly on the electricity bill to save half, has been purchased a few more, the effect is too awesome!	Jingdong prefecture level city in Shandong
Happy Fan Shop	Green Intelligent Power Saver	The family plugged in 5, monthly electricity savings of about 300 yuan. My family before a month of electricity costs more than 800, this time with this saver after the electricity bill is actually only more than 500, amazing, thanks to the store, will recommend other friends to buy!	Jingdong prefecture level city in Shandong
Happy Fan Shop	Green Intelligent Power Saver	It was recommended to me by a neighbor's family and it's nice to use it to save power.	Jingdong prefecture level city in Shandong
Happy Fan Shop	Green Intelligent Power Saver	Second time I've purchased it and the power saving is effective!	Jingdong prefecture level city in Shandong
Happy Fan Shop	Green Intelligent Power Saver	The effect is really good, before a month of more than 300 electricity, now every month every day with the air conditioning, it is more than 100 electricity! This thing is really amazing, one after another to buy and 5.6, home installed 3!	Jingdong prefecture level city in Shandong
Happy Fan Shop	Green Intelligent Power Saver	Received this product, began to use, small things look very wrong, can save a little electricity, the monthly electricity bill is too high, after plugging in did not pull, or save a lot of electricity, point	Jingdong prefecture level city in Shandong
Happy Fan Shop	Green Intelligent Power Saver	Simple and convenient, just that plug in, you can save electricity. A month can save dozens of dollars, a year down to save a lot of, very good product cost-effective, everyone can afford to buy and use!	Jingdong prefecture level city in Shandong
Happy Fan Shop	Green Intelligent Power Saver	Electricity bill from more than 200 yuan per month, down to more than 150 yuan, the original bought home, used two months, really have power-saving effect, this time and sold two, business service attitude is	Jingdong prefecture level city in Shandong
Happy Fan Shop	Green Intelligent Power Saver	Like to watch some movies at home and so on, sometimes at night until two or three o'clock, and recently the air conditioning is not turned off for a night, rather wasteful, I have to say that the effect is very good, quite like it!	Jingdong prefecture level city in Shandong
Happy Fan Shop	Green Intelligent Power Saver	Power-saving effect: the effect is very good, a month to save more than 200 dollars Cost-effective: good value for money Appearance and style: small and lovely Workmanship material: okay Experience: good	Jingdong prefecture level city in Shandong
Happy Fan Shop	Green Intelligent Power Saver	It's okay I quite like it, the stuff is very good. Come back in the future. You can rest assured to buy. Cost-effective all. Much cheaper than the physical store	Jingdong prefecture level city in Shandong
Happy Fan Shop	Green Intelligent Power Saver	More than 150 less, last month's electricity bill is almost 400, this month is only about 200, really save electricity, I am home to buy three, I did not expect a month to pay back	Jingdong prefecture level city in Shandong
Happy Fan Shop	Green Intelligent Power Saver	I bought three of these first and tried them for two months and they still felt useful, so now I've bought another one. Good and recommended!	Jingdong prefecture level city in Shandong
Happy Fan Shop	Green Intelligent Power Saver	This energy saver is very good quality and inexpensive next time I will buy it again!	Jingdong prefecture level city in Shandong
Happy Fan Shop	Green Intelligent Power Saver	Merchant service attitude is not good, the quality does not match, the business does not give the package return	Jingdong prefecture level city in Shandong
Happy Fan Shop	Green Intelligent Power Saver	The goods are very small do not know how effective after a few days to follow up the review	Jingdong prefecture level city in Shandong
Happy Fan Shop	Green Intelligent Power Saver	Not used before more than 200 a month, after using about 100 a month, my family used three, customer service said I use electricity recommended 2-3, I think more than one effect is better on the purchase of 4, the effect is very obvious, recommended to buy!	Jingdong prefecture level city in Shandong
Happy Fan Shop	Green Intelligent Power Saver	It's okay. I'll buy it again today.	Jingdong prefecture level city in Shandong
Happy Fan Shop	Green Intelligent Power Saver	Finally received the goods, very, very much like,, has been a lot of people praise, really too happy, has been his family's loyal fans, will continue to buy buy buy!	Jingdong prefecture level city in Shandong
Happy Fan Shop	Green Intelligent Power Saver	After using this, my monthly electricity bill was 1,200 pounds, now it's only 180 bucks a month, how good is it?	Jingdong prefecture level city in Shandong
Happy Fan Shop	Green Intelligent Power Saver	The item is a scam return to marry a dozen dollars in shipping fees. Buy terrific role is purely a scam.	Jingdong prefecture level city in Shandong
Happy Fan Shop	Green Intelligent Power Saver	It's really good, cost-effective and good quality, and the logistics are fast! It arrived in one day!	Jingdong prefecture level city in Shandong
Happy Fan Shop	Green Intelligent Power Saver	Great brand! Trustworthy! Works super well! Expensive for a reason! Great merchandise!	Jingdong prefecture level city in Shandong
Happy Fan Shop	Green Intelligent Power Saver	The baby is really great, I like it very much, my friends also say good to come, recommended to buy!	Jingdong prefecture level city in Shandong
Happy Fan Shop	Green Intelligent Power Saver	Fake couldn't be more fake. Bought one plugged in still so much electricity.	Jingdong prefecture level city in Shandong
Happy Fan Shop	Green Intelligent Power Saver	This appliance works great. Let's see how it works. We'll see later.	Jingdong prefecture level city in Shandong
Happy Fan Shop	Green Intelligent Power Saver	Used it before commenting, very happy with it	Jingdong prefecture level city in Shandong
Happy Fan Shop	Green Intelligent Power Saver	Goods have been received well-packed, logistics is very fast and good quality	Jingdong prefecture level city in Shandong
Happy Fan Shop	Green Intelligent Power Saver	This baby's quality is okay.	Jingdong prefecture level city in Shandong
Happy Fan Shop	Green Intelligent Power Saver	Counterfeit goods.	Jingdong prefecture level city in Shandong
Happy Fan Shop	Green Intelligent Power Saver	The effect is not very obvious	Jingdong prefecture level city in Shandong
Happy Fan Shop	Green Intelligent Power Saver	It's not the same thing.	Jingdong prefecture level city in Shandong
Happy Fan Shop	Green Intelligent Power Saver	There is no load how much w seems false, nothing works.	Jingdong prefecture level city in Shandong
Happy Fan Shop	Green Intelligent Power Saver	I received the item yesterday, got it back and plugged it in, and it used a little more power yesterday than I had before	Jingdong prefecture level city in Shandong
Happy Fan Shop	Green Intelligent Power Saver	Haven't seen the power saving mode yet	Jingdong prefecture level city in Shandong
Happy Fan Shop	Green Intelligent Power Saver	This product has no known effect	Jingdong prefecture level city in Shandong
Happy Fan Shop	Green Intelligent Power Saver	Refund Returns $16	Jingdong prefecture level city in Shandong
Happy Fan Shop	Green Intelligent Power Saver	The previous year bought a, almost every month can save a 70 dollars or so, used a year, no problem at all, look very new like, this time to move to a new home decisively repurchase 2 units, very believe this	Jingdong prefecture level city in Shandong
Happy Fan Shop	Green Intelligent Power Saver	Has been the second time to buy, this power saving device is really good power, before every month to spend 300 electricity, now are about 200, last time that 6, this time to help lead the family to buy 6,	Jingdong prefecture level city in Shandong
Happy Fan Shop	Green Intelligent Power Saver	In the spirit of trying to experience the new product, the use of down compared to the usual power consumption, with the addition of the quarter, is indeed a lot less electricity, originally more than 450	Jingdong prefecture level city in Shandong
Happy Fan Shop	Green Intelligent Power Saver	Has not been the first time to buy back, good use, power saving device is really powerful, before the monthly electricity bill of more than 500 Now more than 300, this time to help neighbors to buy several	Jingdong prefecture level city in Shandong
Happy Fan Shop	Green Intelligent Power Saver	Home appliances are very many, the circuit is also very complex, at first it is to save 9% of the electricity, and then used more than half a month, found that the power saving rate is as high as more	Jingdong prefecture level city in Shandong
Happy Fan Shop	Green Intelligent Power Saver	My friend bought it last year and saved hundreds of dollars on his electricity bill in one summer Appearance and style: it looks better than the one I bought before Quality is also good with a light.	Jingdong prefecture level city in Shandong
Happy Fan Shop	Green Intelligent Power Saver	Saver received, on the spot on the plug with the feeling of immediate power savings, appearance, packaging is very delicate, very satisfied, I hope to achieve satisfactory results!	Jingdong prefecture level city in Shandong
Happy Fan Shop	Green Intelligent Power Saver	The power saving effect is very good, is considered an old customer, also bought before, this time is to buy two for mom and dad, customer service attitude is good, praise!	Jingdong prefecture level city in Shandong
Happy Fan Shop	Green Intelligent Power Saver	After observing for a while, it really saves a lot of money I didn't realize it was still really reliable, so I'll give you a good review.	Jingdong prefecture level city in Shandong
Happy Fan Shop	Green Intelligent Power Saver	My family a month with this monthly almost save more than 200, power-saving effect: have bought a second time, really effective Cost-effective: not bad Appearance style: roughly the same A few months to the home electricity is indeed less, this time it is a re-buy three, send a friend two, they stay a	Jingdong prefecture level city in Shandong
Happy Fan Shop	Green Intelligent Power Saver	A month almost save more than 150 dollars in electricity, home appliances more, the main this can also protect high-power appliances, after using this device, these two months have not burned, things are very good, it is said that this is the latest model, the overall price is cheap and beautiful!	Jingdong prefecture level city in Shandong

Happy Fan Shop	Green Intelligent Power Saver	Received, powerful, great quality, love the style😍	Jingdong prefecture level city in Shandong
Happy Fan Shop	Green Intelligent Power Saver	This intelligent energy saver is especially good to use and save electricity hope everyone will buy it!	Jingdong prefecture level city in Shandong
Happy Fan Shop	Green Intelligent Power Saver	Air conditioning adapter effect is particularly great, the use of very simple, plug into the socket on the summer air conditioning power is too large, electricity is also expensive, with this product directly on the electricity bill to save half, has been purchased a few more, the effect is too awesome!	Jingdong prefecture level city in Shandong
Happy Fan Shop	Green Intelligent Power Saver	The family plugged in 5, monthly electricity savings of about 300 yuan. My family before a month of electricity costs more than 800, this time with this saver after the electricity bill is actually only more than 500, amazing, thanks to the store, will recommend other friends to buy!	Jingdong prefecture level city in Shandong
Happy Fan Shop	Green Intelligent Power Saver	It was recommended to me by a neighbor's family and it's nice to use it to save power.	Jingdong prefecture level city in Shandong
Happy Fan Shop	Green Intelligent Power Saver	Second time I've purchased it and the power saving is effective!	Jingdong prefecture level city in Shandong
Happy Fan Shop	Green Intelligent Power Saver	The effect is really good, before a month of more than 300 electricity, now every month every day with the air conditioning, it is more than 100 electricity! This thing is really amazing, one after another to buy and 5.6, home installed 3!	Jingdong prefecture level city in Shandong
Happy Fan Shop	Green Intelligent Power Saver	Received this product, began to use, small things look very wrong, can save a little electricity, the monthly electricity bill is too high, after plugging in did not pull, or save a lot of electricity, point	Jingdong prefecture level city in Shandong
Happy Fan Shop	Green Intelligent Power Saver	Simple and convenient, just that plug in, you can save electricity. A month can save dozens of dollars, a year down to save a lot of, very good product cost-effective, everyone can afford to buy and use!	Jingdong prefecture level city in Shandong
Happy Fan Shop	Green Intelligent Power Saver	Electricity bill from more than 200 yuan per month, down to more than 150 yuan, the original bought home, used two months, really have power-saving effect, this time and sold two, business service attitude is	Jingdong prefecture level city in Shandong
Happy Fan Shop	Green Intelligent Power Saver	Like to watch some movies at home and so on, sometimes at night until two or three o'clock, and recently the air conditioning is not turned off for a night, rather wasteful, I have to say that the effect is very good, quite like it!	Jingdong prefecture level city in Shandong
Happy Fan Shop	Green Intelligent Power Saver	Power-saving effect: the effect is very good, a month to save more than 200 dollars Cost-effective: good value for money Appearance and style: small and lovely Workmanship material: okay Experience: good	Jingdong prefecture level city in Shandong
Happy Fan Shop	Green Intelligent Power Saver	It's okay I quite like it, the stuff is very good. Come back in the future. You can rest assured to buy. Cost-effective all. Much cheaper than the physical store	Jingdong prefecture level city in Shandong
Happy Fan Shop	Green Intelligent Power Saver	More than 150 less, last month's electricity bill is almost 400, this month is only about 200, really save electricity, I am home to buy three, I did not expect a month to pay back	Jingdong prefecture level city in Shandong
Happy Fan Shop	Green Intelligent Power Saver	I bought three of these first and tried them for two months and they still felt useful, so now I've bought another one. Good and recommended!	Jingdong prefecture level city in Shandong
Happy Fan Shop	Green Intelligent Power Saver	This energy saver is very good quality and inexpensive next time I will buy it again!	Jingdong prefecture level city in Shandong
Happy Fan Shop	Green Intelligent Power Saver	Merchant service attitude is not good, the quality does not match, the business does not give the package return	Jingdong prefecture level city in Shandong
Happy Fan Shop	Green Intelligent Power Saver	The goods are very small do not know how effective after a few days to follow up the review	Jingdong prefecture level city in Shandong
Happy Fan Shop	Green Intelligent Power Saver	Not used before more than 200 a month, after using about 100 a month, my family used three, customer service said I use electricity recommended 2-3, I think more than one effect is better on the purchase of 4, the effect is very obvious, recommended to buy!	Jingdong prefecture level city in Shandong
Happy Fan Shop	Green Intelligent Power Saver	It's okay. I'll buy it again today.	Jingdong prefecture level city in Shandong
Happy Fan Shop	Green Intelligent Power Saver	Finally received the goods, very, very much like,, has been a lot of people praise, really too happy, has been his family's loyal fans, will continue to buy buy buy buy!	Jingdong prefecture level city in Shandong
Happy Fan Shop	Green Intelligent Power Saver	After using this, my monthly electricity bill was 1,200 pounds, now it's only 180 bucks a month, how good is it?	Jingdong prefecture level city in Shandong
Happy Fan Shop	Green Intelligent Power Saver	The item is a scam return to marry a dozen dollars in shipping fees. Buy terrific role is purely a scam.	Jingdong prefecture level city in Shandong
Happy Fan Shop	Green Intelligent Power Saver	It's really good, cost-effective and good quality, and the logistics are fast! It arrived in one day!	Jingdong prefecture level city in Shandong
Happy Fan Shop	Green Intelligent Power Saver	Great brand! Trustworthy! Works super well! Expensive for a reason! Great merchandise!	Jingdong prefecture level city in Shandong
Happy Fan Shop	Green Intelligent Power Saver	The baby is really great, I like it very much, my friends also say good to come, recommended to buy!	Jingdong prefecture level city in Shandong
Happy Fan Shop	Green Intelligent Power Saver	Fake couldn't be more fake. Bought one plugged in still so much electricity.	Jingdong prefecture level city in Shandong
Happy Fan Shop	Green Intelligent Power Saver	This appliance works great. Let's see how it works. We'll see later.	Jingdong prefecture level city in Shandong
Happy Fan Shop	Green Intelligent Power Saver	Used it before commenting, very happy with it	Jingdong prefecture level city in Shandong
Happy Fan Shop	Green Intelligent Power Saver	Goods have been received well-packed, logistics is very fast and good quality	Jingdong prefecture level city in Shandong
Happy Fan Shop	Green Intelligent Power Saver	This baby's quality is okay.	Jingdong prefecture level city in Shandong
Happy Fan Shop	Green Intelligent Power Saver	Counterfeit goods.	Jingdong prefecture level city in Shandong
Happy Fan Shop	Green Intelligent Power Saver	The effect is not very obvious	Jingdong prefecture level city in Shandong
Happy Fan Shop	Green Intelligent Power Saver	It's not the same thing.	Jingdong prefecture level city in Shandong
Happy Fan Shop	Green Intelligent Power Saver	There is no load how much w seems false, nothing works.	Jingdong prefecture level city in Shandong
Happy Fan Shop	Green Intelligent Power Saver	I received the item yesterday, got it back and plugged it in, and it used a little more power yesterday than I had before	Jingdong prefecture level city in Shandong
Happy Fan Shop	Green Intelligent Power Saver	Haven't seen the power saving mode yet	Jingdong prefecture level city in Shandong
Happy Fan Shop	Green Intelligent Power Saver	This product has no known effect	Jingdong prefecture level city in Shandong
Happy Fan Shop	Green Intelligent Power Saver	Refund Returns $16	Jingdong prefecture level city in Shandong
Happy Fan Shop	Green Intelligent Power Saver	The previous year bought a, almost every month can save a 70 dollars or so, used a year, no problem at all, look very new like, this time to move to a new home decisively repurchase 2 units, very believe this	Jingdong prefecture level city in Shandong
Happy Fan Shop	Green Intelligent Power Saver	Has been the second time to buy, this power saving device is really good power, before every month to spend 300 electricity, now are about 200, last time that 6, this time to help lead the family to buy 6,	Jingdong prefecture level city in Shandong
Happy Fan Shop	Green Intelligent Power Saver	In the spirit of trying to experience the new product, the use of down compared to the usual power consumption, with the addition of the quarter, is indeed a lot less electricity, originally more than 450	Jingdong prefecture level city in Shandong
Happy Fan Shop	Green Intelligent Power Saver	Has not been the first time to buy back, good use, power saving device is really powerful, before the monthly electricity bill of more than 500 Now more than 300, this time to help neighbors to buy several	Jingdong prefecture level city in Shandong
Happy Fan Shop	Green Intelligent Power Saver	Home appliances are very many, the circuit is also very complex, at first it is to save 9% of the electricity, and then used more than half a month, found that the power saving rate is as high as more	Jingdong prefecture level city in Shandong
Happy Fan Shop	Green Intelligent Power Saver	My friend bought it last year and saved hundreds of dollars on his electricity bill in one summer Appearance and style: it looks better than the one I bought before Quality is also good with a light.	Jingdong prefecture level city in Shandong
Happy Fan Shop	Green Intelligent Power Saver	Saver received, on the spot on the plug with the feeling of immediate power savings, appearance, packaging is very delicate, very satisfied, I hope to achieve satisfactory results!	Jingdong prefecture level city in Shandong
Happy Fan Shop	Green Intelligent Power Saver	The power saving effect is very good, is considered an old customer, also bought before, this time is to buy two for mom and dad, customer service attitude is good, praise!	Jingdong prefecture level city in Shandong
Happy Fan Shop	Green Intelligent Power Saver	After observing for a while, it really saves a lot of money I didn't realize it was still really reliable, so I'll give you a good review.	Jingdong prefecture level city in Shandong

Happy Fan Shop	Green Intelligent Power Saver	My family a month with this monthly almost save more than 200, power-saving effect: have bought a second time, really effective Cost-effective: not bad Appearance style: roughly the same A few months to the home electricity is indeed less, this time it is a re-buy three, send a friend two, they stay a	Jingdong prefecture level city in Shandong
Happy Fan Shop	Green Intelligent Power Saver	A month almost save more than 150 dollars in electricity, home appliances more, the main this can also protect high-power appliances, after using this device, these two months have not burned, things are very good, it is said that this is the latest model, the overall price is cheap and beautiful!	Jingdong prefecture level city in Shandong
Happy Fan Shop	Green Intelligent Power Saver	Received, powerful, great quality, love the style😍	Jingdong prefecture level city in Shandong
Happy Fan Shop	Green Intelligent Power Saver	This intelligent energy saver is especially good to use and save electricity hope everyone will buy it!	Jingdong prefecture level city in Shandong
Happy Fan Shop	Green Intelligent Power Saver	Air conditioning adapter effect is particularly great, the use of very simple, plug into the socket on the summer air conditioning power is too large, electricity is also expensive, with this product directly on the electricity bill to save half, has been purchased a few more, the effect is too awesome!	Jingdong prefecture level city in Shandong
Happy Fan Shop	Green Intelligent Power Saver	The family plugged in 5, monthly electricity savings of about 300 yuan. My family before a month of electricity costs more than 800, this time with this saver after the electricity bill is actually only more than 500, amazing, thanks to the store, will recommend other friends to buy!	Jingdong prefecture level city in Shandong
Happy Fan Shop	Green Intelligent Power Saver	It was recommended to me by a neighbor's family and it's nice to use it to save power.	Jingdong prefecture level city in Shandong
Happy Fan Shop	Green Intelligent Power Saver	Second time I've purchased it and the power saving is effective!	Jingdong prefecture level city in Shandong
Happy Fan Shop	Green Intelligent Power Saver	The effect is really good, before a month of more than 300 electricity, now every month every day with the air conditioning, it is more than 100 electricity! This thing is really amazing, one after another to buy and 5.6, home installed 3!	Jingdong prefecture level city in Shandong
Happy Fan Shop	Green Intelligent Power Saver	Received this product, began to use, small things look very wrong, can save a little electricity, the monthly electricity bill is too high, after plugging in did not pull, or save a lot of electricity, point	Jingdong prefecture level city in Shandong
Happy Fan Shop	Green Intelligent Power Saver	Simple and convenient, just that plug in, you can save electricity. A month can save dozens of dollars, a year down to save a lot of, very good product cost-effective, everyone can afford to buy and use!	Jingdong prefecture level city in Shandong
Happy Fan Shop	Green Intelligent Power Saver	Electricity bill from more than 200 yuan per month, down to more than 150 yuan, the original bought home, used two months, really have power-saving effect, this time and sold two, business service attitude is	Jingdong prefecture level city in Shandong
Happy Fan Shop	Green Intelligent Power Saver	Like to watch some movies at home and so on, sometimes at night until two or three o'clock, and recently the air conditioning is not turned off for a night, rather wasteful, I have to say that the effect is very good, quite like it!	Jingdong prefecture level city in Shandong
Happy Fan Shop	Green Intelligent Power Saver	Power-saving effect: the effect is very good, a month to save more than 200 dollars Cost-effective: good value for money Appearance and style: small and lovely Workmanship material: okay Experience: good	Jingdong prefecture level city in Shandong
Happy Fan Shop	Green Intelligent Power Saver	It's okay I quite like it, the stuff is very good. Come back in the future. You can rest assured to buy. Cost-effective all. Much cheaper than the physical store	Jingdong prefecture level city in Shandong
Happy Fan Shop	Green Intelligent Power Saver	More than 150 less, last month's electricity bill is almost 400, this month is only about 200, really save electricity, I am home to buy three, I did not expect a month to pay back	Jingdong prefecture level city in Shandong
Happy Fan Shop	Green Intelligent Power Saver	I bought three of these first and tried them for two months and they still felt useful, so now I've bought another one. Good and recommended!	Jingdong prefecture level city in Shandong
Happy Fan Shop	Green Intelligent Power Saver	This energy saver is very good quality and inexpensive next time I will buy it again!	Jingdong prefecture level city in Shandong
Happy Fan Shop	Green Intelligent Power Saver	Merchant service attitude is not good, the quality does not match, the business does not give the package return	Jingdong prefecture level city in Shandong
Happy Fan Shop	Green Intelligent Power Saver	The goods are very small do not know how effective after a few days to follow up the review	Jingdong prefecture level city in Shandong
Happy Fan Shop	Green Intelligent Power Saver	Not used before more than 200 a month, after using about 100 a month, my family used three, customer service said I use electricity recommended 2-3, I think more than one effect is better on the purchase of 4, the effect is very obvious, recommended to buy!	Jingdong prefecture level city in Shandong
Happy Fan Shop	Green Intelligent Power Saver	It's okay. I'll buy it again today.	Jingdong prefecture level city in Shandong
Happy Fan Shop	Green Intelligent Power Saver	Finally received the goods, very, very much like,, has been a lot of people praise, really too happy, has been his family's loyal fans, will continue to buy buy buy buy!	Jingdong prefecture level city in Shandong
Happy Fan Shop	Green Intelligent Power Saver	After using this, my monthly electricity bill was 1,200 pounds, now it's only 180 bucks a month, how good is it?	Jingdong prefecture level city in Shandong
Happy Fan Shop	Green Intelligent Power Saver	The item is a scam return to marry a dozen dollars in shipping fees. Buy terrific role is purely a scam.	Jingdong prefecture level city in Shandong
Happy Fan Shop	Green Intelligent Power Saver	It's really good, cost-effective and good quality, and the logistics are fast! It arrived in one day!	Jingdong prefecture level city in Shandong
Happy Fan Shop	Green Intelligent Power Saver	Great brand! Trustworthy! Works super well! Expensive for a reason! Great merchandise!	Jingdong prefecture level city in Shandong
Happy Fan Shop	Green Intelligent Power Saver	The baby is really great, I like it very much, my friends also say good to come, recommended to buy!	Jingdong prefecture level city in Shandong
Happy Fan Shop	Green Intelligent Power Saver	Fake couldn't be more fake. Bought one plugged in still so much electricity.	Jingdong prefecture level city in Shandong
Happy Fan Shop	Green Intelligent Power Saver	This appliance works great. Let's see how it works. We'll see later.	Jingdong prefecture level city in Shandong
Happy Fan Shop	Green Intelligent Power Saver	Used it before commenting, very happy with it	Jingdong prefecture level city in Shandong
Happy Fan Shop	Green Intelligent Power Saver	Goods have been received well-packed, logistics is very fast and good quality	Jingdong prefecture level city in Shandong
Happy Fan Shop	Green Intelligent Power Saver	This baby's quality is okay.	Jingdong prefecture level city in Shandong
Happy Fan Shop	Green Intelligent Power Saver	Counterfeit goods.	Jingdong prefecture level city in Shandong
Happy Fan Shop	Green Intelligent Power Saver	The effect is not very obvious	Jingdong prefecture level city in Shandong
Happy Fan Shop	Green Intelligent Power Saver	It's not the same thing.	Jingdong prefecture level city in Shandong
Happy Fan Shop	Green Intelligent Power Saver	There is no load how much w seems false, nothing works.	Jingdong prefecture level city in Shandong
Happy Fan Shop	Green Intelligent Power Saver	I received the item yesterday, got it back and plugged it in, and it used a little more power yesterday than I had before	Jingdong prefecture level city in Shandong
Happy Fan Shop	Green Intelligent Power Saver	Haven't seen the power saving mode yet	Jingdong prefecture level city in Shandong
Happy Fan Shop	Green Intelligent Power Saver	This product has no known effect	Jingdong prefecture level city in Shandong
Happy Fan Shop	Green Intelligent Power Saver	Refund Returns $16	Jingdong prefecture level city in Shandong
Happy Fan Shop	Green Intelligent Power Saver	I don't understand how it saves power, but after experimenting and comparing, it does save power, kudos!	Jingdong prefecture level city in Shandong
Happy Fan Shop	Green Intelligent Power Saver	Used half a month does save electricity, after plugging in to save me a lot of electricity, really and this baby hate each other ah, power-saving effect is really great, the appearance of the atmosphere is stylish, the use of the experience is perfect, does not take up space, the next time you need will buy again. Customer service is very enthusiastic, great!	Jingdong prefecture level city in Shandong
Happy Fan Shop	King of Power Saving	Commodities have arrived, the design is unique power powerful, save electricity more than forty percent, after the test, the effect is very good is the economic benefits, good faith business service attitude is good, worthy of praise.	Jingdong prefecture level city in Shandong
Happy Fan Shop	King of Power Saving	Last month's electricity cost 310, after using this this month's electricity cost 198, this saver is quite good, summer open air conditioning electricity fast, praise, will recommend to the side with.	Jingdong prefecture level city in Shandong
Happy Fan Shop	King of Power Saving	Originally bought for home use, used for two months, the electricity bill from more than 200 yuan per month, down to more than 140 yuan, there is indeed a power-saving effect, this time and sold two, the unit with. Merchant service attitude is really good.	Jingdong prefecture level city in Shandong

Happy Fan Shop	King of Power Saving	And back to buy it, my electricity bill is relatively high, before the customer service recommended that I buy 2-3, I bought a try, the result is really effective quite obvious, hurry back to buy a, this is not yet received the goods, first to give a favorable comment, can save electricity really too happy! Thank	Jingdong prefecture level city in Shandong
Happy Fan Shop	King of Power Saving	This brand has been used for many years, and this time I saw this upgraded version of the product and got it again. The purpose of this product is to reduce the loss of electricity for a long time, so as to achieve the purpose of saving electricity.	Jingdong prefecture level city in Shandong
Happy Fan Shop	King of Power Saving	Concerned about the praise collection, believe him, friends recommended decisively out, once to buy a few, very good, inexpensive, brand-name is good, the price is pro, buy a few or can be, collection of	Jingdong prefecture level city in Shandong
Happy Fan Shop	King of Power Saving	Always buy this brand, check is genuine, a variety of anti-counterfeiting mark and Pacific insurance mark, the quality is also good, but also with LCD display, the price is not expensive, my family uses a lot of electricity, half a month to save a hundred dollars, summer open air conditioning is more, has	Jingdong prefecture level city in Shandong
Happy Fan Shop	King of Power Saving	275267324498 jd_5222cc992fe7d<br><br>Indeed very effective, because now are cell phone **** payment,, summer air conditioning with a month of 300, we use this energy-saving products are about 28 days less than 200 yuan of electricity, it is indeed save a lot, this time and to the old man bought two. Also recommended their relatives do business with a	Jingdong prefecture level city in Shandong
Happy Fan Shop	King of Power Saving	Things are more perfect than I imagined, very powerful, it is worth getting Packaging is very strong, things are well protected, it is worth the praise things are very satisfied Oh, beautiful and stylish atmosphere, customer service is super patient, service is meticulous and attentive, the big brand is not	Jingdong prefecture level city in Shandong
Happy Fan Shop	King of Power Saving	The effect is too good, the family has now several units, back to buy a number of times, the power saver received, power-saving effect is very good,, fine workmanship, long warranty, but also purify the air, before buying a used for a period of time, with the okay, and then bought a, and only now come to the	Jingdong prefecture level city in Shandong
Happy Fan Shop	King of Power Saving	Quite good, and imagine the same, casual ride are very good, satisfied! Baby received it 👌, really great ah! 🌈 quality is really nothing to say, very satisfied! 🆗 happy happy 😃 genuine, great, the price is very good, in short, quite good, will buy again a very good shopping experience, attentive service, fast logistics, distribution is also thoughtful, the attitude is also very good, value for money, ah,	Jingdong prefecture level city in Shandong
Happy Fan Shop	King of Power Saving	Power-saving effect: strong Cost-effective: quite good Appearance style: beautiful Workmanship material: meticulous Experience: looking forward to power-saving effect	Jingdong prefecture level city in Shandong
Happy Fan Shop	King of Power Saving	Cost-effective is okay, the appearance of the style of meticulous workmanship, looking forward to energy-saving effect.	Jingdong prefecture level city in Shandong
Happy Fan Shop	King of Power Saving	Very Good Very Power Saving This Power Saver is really power saving As soon as the power is turned on, the Power Saver starts the power saving function for you.	Jingdong prefecture level city in Shandong
Happy Fan Shop	King of Power Saving	Logistics is very fast, the price is favorable, friends introduced that it is very good to use, bought 2 at once, looking forward to the effect, used after the follow-up comment.	Jingdong prefecture level city in Shandong
Happy Fan Shop	King of Power Saving	It's all fake. It doesn't save any electricity.	Jingdong prefecture level city in Shandong
Happy Fan Shop	King of Power Saving	This Power Saver is so much better than the one I bought before, not only the effect is good, the quality is very good in all aspects, the customer service attitude is also very good, recommended to buy!	Jingdong prefecture level city in Shandong
Happy Fan Shop	King of Power Saving	Logistics is very fast, buy for parents, good quality, once bought 2, power-saving effect is good to buy 2 more, look forward to using the effect, satisfied will recommend to friends.	Jingdong prefecture level city in Shandong
Happy Fan Shop	King of Power Saving	With the description is the same, very high-grade, good value, to buy friends can order Oh!	Jingdong prefecture level city in Shandong
Happy Fan Shop	King of Power Saving	Not using much electricity in the winter saving over 100 per month! Ready to buy 2 more units in the summer ！！！！！ Good quality, good price, power saving effect is obvious, cost-effective, worth buying, can be recommended!	Jingdong prefecture level city in Shandong
Happy Fan Shop	King of Power Saving	Purchased a power saver received on the put into use, rent a room with many electrical appliances, power consumption is very large, we have a piece of electricity or 1.2 dollars a time, with this power saver is much better, every day can save a lot of money, very convenient and practical.	Jingdong prefecture level city in Shandong
Happy Fan Shop	King of Power Saving	The power saving effect is very good: my supermarket monthly electricity bill is more than 7000 yuan. On May 1st someone recommended buying a couple of power savers, and I bought 5 of them first. The electricity bill was about 2000 less in June. This time, I bought 5 more. The seller said 800 degrees of electricity with one. I feel it's basically enough. A total of 10 units	Jingdong prefecture level city in Shandong
Happy Fan Shop	King of Power Saving	I never thought I could get such a good baby at such an affordable price, it's very good value for money, I love it!	Jingdong prefecture level city in Shandong
Happy Fan Shop	King of Power Saving	Previously bought other brands of power savers, but found no effect at home, this time to see how the products on the Jingdong, inserted in the home socket, the LED display is blue, the night light is very nice to see, look at the instructions that have to be half a year in order to achieve the best results, try it, I believe that the quality of the Jingdong, it should not be wrong.	Jingdong prefecture level city in Shandong
Happy Fan Shop	King of Power Saving	Superb, inexpensive, saves a lot of points, been using it for a long time!	Jingdong prefecture level city in Shandong
Happy Fan Shop	King of Power Saving	There are a lot of appliances in the house, the summer air conditioning is very costly, I bought this in front of the air conditioning effect is very good, I explain that I bought three in one breath, I use more electricity a month down to 200 or so, very satisfied.	Jingdong prefecture level city in Shandong
Happy Fan Shop	King of Power Saving	A month can save more than 200, home monthly electricity bill of more than 600 dollars, inserted 3 or so, an amount of the balance of the average can save more than 200. Product is very good, has been buying this one, has given a lot of relatives recommended, the quality is also very good, before buying a look with this same, who knows to buy home has not been used for more than a week on the bad, the quality of this is really good with more than two years!	Jingdong prefecture level city in Shandong
Happy Fan Shop	King of Power Saving	Whole house power saving king, can really save electricity, friends recommended, high power saving appliances, only replacement, no repair, after-sales worry-free!	Jingdong prefecture level city in Shandong
Happy Fan Shop	King of Power Saving	Great item love it, great customer service, great shopping experience!	Jingdong prefecture level city in Shandong
Happy Fan Shop	King of Power Saving	Power saving, very good use, much cheaper than the physical store, satisfaction	Jingdong prefecture level city in Shandong
Happy Fan Shop	King of Power Saving	In use, very well packaged and fast delivery of credit!	Jingdong prefecture level city in Shandong
Happy Fan Shop	King of Power Saving	Product quality, customer service attitude is particularly good, like, praise!	Jingdong prefecture level city in Shandong
Happy Fan Shop	King of Power Saving	The logistics is very fast, the packaging is not broken, the quality is very good, I like it very much!	Jingdong prefecture level city in Shandong
Happy Fan Shop	King of Power Saving	The saver was well packaged and was as described when plugged in	Jingdong prefecture level city in Shandong
Happy Fan Shop	King of Power Saving	Energy efficient and very satisfied	Jingdong prefecture level city in Shandong
Happy Fan Shop	King of Power Saving	Great quality for the price and fast shipping.	Jingdong prefecture level city in Shandong
Happy Fan Shop	King of Power Saving	It's great, just as described. Perfect.	Jingdong prefecture level city in Shandong
Happy Fan Shop	King of Power Saving	There is no power saving effect, but rather ring up an increase in electricity costs, because this machine is on continuously all day long, uninterruptedly, its own electricity and increased costs, plus the cost of purchasing the machine, so I feel that the use of this machine instead of increasing the	Jingdong prefecture level city in Shandong
Happy Fan Shop	King of Power Saving	Recommended by the owner of the store next door, looked at his home does have to save electricity, decided to get. Indeed quite can, the voltage is much more stable, there is a reduction in electricity	Jingdong prefecture level city in Shandong
Happy Fan Shop	King of Power Saving	Goods have been received value for money, affordable and reasonable price, and the description of the same, power-saving safe and reliable!	Jingdong prefecture level city in Shandong
Happy Fan Shop	King of Power Saving	Listen to friends introduced to buy, tried to save a lot of electricity is good, quite good, worth buying.	Jingdong prefecture level city in Shandong
Happy Fan Shop	King of Power Saving	The product is easy to use, safe and environmentally friendly, only used for a short time, to the next month, after the electricity bill status display to know how the performance of the power saver efficacy.	Jingdong prefecture level city in Shandong
Happy Fan Shop	King of Power Saving	Last month's electricity bill is 178 yuan, with this power saver is 92 yuan, ready to buy two more back home, I was introduced to buy a friend, just started to not believe that I did not expect the effect!	Jingdong prefecture level city in Shandong
Happy Fan Shop	King of Power Saving	It looks nice, but after using it for a few days I can't see how it saves power?	Jingdong prefecture level city in Shandong

Happy Fan Shop	King of Power Saving	Recommended by a friend, it is genuine, as described by the merchant, cost-effective	Jingdong prefecture level city in Shandong
Happy Fan Shop	King of Power Saving	Works well, affordable and saves money.	Jingdong prefecture level city in Shandong
Happy Fan Shop	King of Power Saving	Feels good haven't compared it yet, the air conditioning has been on a lot in the past two months	Jingdong prefecture level city in Shandong
Happy Fan Shop	King of Power Saving	Saving about 5 kWh per day is a good thing	Jingdong prefecture level city in Shandong
Happy Fan Shop	King of Power Saving	Not bad, affordable, best value for money	Jingdong prefecture level city in Shandong
Happy Fan Shop	King of Power Saving	The goods are genuine and the logistics are faster.	Jingdong prefecture level city in Shandong
Happy Fan Shop	King of Power Saving	Goods have been received today, has been plugged into the outlet, looking forward to saving electricity, after a period of time to see how the effect in the follow-up comment!	Jingdong prefecture level city in Shandong
Happy Fan Shop	King of Power Saving	I just got it home and haven't used it for long, so I'll use it for a couple months and see what happens. Seeing that most people have good reviews, it should be fine.	Jingdong prefecture level city in Shandong
Happy Fan Shop	King of Power Saving	I'm looking forward to the results, so I'll give you a good review out of habit! Follow up review after a while	Jingdong prefecture level city in Shandong
Happy Fan Shop	King of Power Saving	Use a dish first to know if it will help save energy	Jingdong prefecture level city in Shandong
Happy Fan Shop	King of Power Saving	Whether it saves electricity or not, we don't know yet.	Jingdong prefecture level city in Shandong
Happy Fan Shop	King of Power Saving	Goods have been received, use a period of time to know how oh. The courier did not deliver to the door ah! Sent to the station ah! The heat is inconvenient ah!	Jingdong prefecture level city in Shandong
Happy Fan Shop	King of Power Saving	A little effect, before an hour a power, now an hour 0.76 degrees, a dollar a power = save 24 cents, although not a lot, first use it!	Jingdong prefecture level city in Shandong
Happy Fan Shop	King of Power Saving	Just received the goods, use a month to evaluate	Jingdong prefecture level city in Shandong
Happy Fan Shop	King of Power Saving	Home every month with a lot of electricity, inadvertently found this good thing online that can save the beginning of a skeptical attitude to consult customer service, customer service replied very professional, said 120 days can be refunded that first try it!	Jingdong prefecture level city in Shandong
Happy Fan Shop	King of Power Saving	After receiving the goods this afternoon, immediately installed in the bathroom Smith energy storage electric water heater on the power outlet (instead of connecting with a plug board), when used, found that the "Power Saving King" LCD display, is the value of the voltage, not the store's video said so	Jingdong prefecture level city in Shandong
Happy Fan Shop	King of Power Saving	Has made many days, did not feel the power saving, the effect is not obvious.	Jingdong prefecture level city in Shandong
Happy Fan Shop	King of Power Saving	Energy savers do not save energy at all, but rather consume a lot of electricity.	Jingdong prefecture level city in Shandong
Happy Fan Shop	King of Power Saving	It's useless. It's a waste of money.	Jingdong prefecture level city in Shandong
Happy Fan Shop	King of Power Saving	Last month saved more than 500, which came back to buy, the store bought three, and then buy 1 home use, my family is dry beauty salon, more instruments, especially electricity, a month almost 1000. or so, with this did not expect to save so much, great, five-star praise!	Jingdong prefecture level city in Shandong
Happy Fan Shop	King of Power Saving	It rained a couple days ago and I just went to get the item back today , not bad, hey I looked at the meter and that red display light takes several seconds to jump, did not make a mistake buying it. After a period of time to evaluate.	Jingdong prefecture level city in Shandong
Happy Fan Shop	King of Power Saving	Without further ado, the electricity bill came down, this month less 102 yuan, has been repurchased, I also recommended to my friends, all said that there are effects, the product is okay, the effect is quite	Jingdong prefecture level city in Shandong
Happy Fan Shop	King of Power Saving	After buying and using two months really see the effect, so only to evaluate! Originally held the mindset of trying, I did not expect really effective, worth having!	Jingdong prefecture level city in Shandong
Happy Fan Shop	King of Power Saving	In the use of this energy saver before the monthly electricity bill is more than 150 yuan or so, now save thirty percent, every day to work, six or seven o'clock in the afternoon to go home, I do not know why the electricity bill is so expensive, energy-saving appliances really have the effect of recommending	Jingdong prefecture level city in Shandong
Happy Fan Shop	King of Power Saving	It saves about 100 bucks a month. A friend recommended it to me, and he's been using it for a while, and the effect is so obvious that I bought one too.	Jingdong prefecture level city in Shandong
Happy Fan Shop	King of Power Saving	My dad asked me to buy one, but after I paid the bill, I asked and he said it was a lot less this month, so he asked me to buy another one.	Jingdong prefecture level city in Shandong
Happy Fan Shop	King of Power Saving	The specific effect will have to be tested for at least two months to compare whether there is a power saving effect or not.	Jingdong prefecture level city in Shandong
Happy Fan Shop	King of Power Saving	'The goods have been received, immediately go to use, please ask the boss you instructions do not allow to withdraw, there are two fuses installed there!	Jingdong prefecture level city in Shandong
Happy Fan Shop	King of Power Saving	First praise is using, after using if good then follow up, now because I have not yet seen the results	Jingdong prefecture level city in Shandong
Happy Fan Shop	King of Power Saving	I've just been using it, and I haven't seen if it has a power-saving effect yet, so I'll follow up after 10 days and half a month.	Jingdong prefecture level city in Shandong
Happy Fan Shop	King of Power Saving	Just received the intelligent power saving meter, immediately plugged in to use, see it is not bad, and order to sell the second time	Jingdong prefecture level city in Shandong
Happy Fan Shop	King of Power Saving	This power saver is a bit effective, used for half a month, the effect is still visible.	Jingdong prefecture level city in Shandong
Happy Fan Shop	King of Power Saving	I received the baby and it looks great, I'm trying it out now and will keep an eye on it afterward.	Jingdong prefecture level city in Shandong
Happy Fan Shop	King of Power Saving	I've just received it, but I'm still trying to see how it works, so I'll come back in a little while.	Jingdong prefecture level city in Shandong
Happy Fan Shop	King of Power Saving	Just used it wait to use it for a while and then comment thanks, all first five stars.	Jingdong prefecture level city in Shandong
Happy Fan Shop	King of Power Saving	Very trustworthy brand, quality is guaranteed, the price is still affordable, quite satisfied!	Jingdong prefecture level city in Shandong
Happy Fan Shop	King of Power Saving	Things received, I hope that the platform of Jingdong do not sell fake goods are available	Jingdong prefecture level city in Shandong
Happy Fan Shop	King of Power Saving	Energy saver received, the use of the effect is good and the business description of the same	Jingdong prefecture level city in Shandong
Happy Fan Shop	King of Power Saving	The surface looks beautiful, the actual effect is not known, after using additional evaluation.	Jingdong prefecture level city in Shandong
Happy Fan Shop	King of Power Saving	Tried it for a few months, it really saves electricity, and I bought it for a friend.	Jingdong prefecture level city in Shandong
Happy Fan Shop	King of Power Saving	It didn't work and wanted to return it, it really didn't work at all.	Jingdong prefecture level city in Shandong
Happy Fan Shop	King of Power Saving	Just used it, don't know how effective it is, looking forward to usefulness	Jingdong prefecture level city in Shandong
Happy Fan Shop	King of Power Saving	After two months before the effect I do not know if it is true!	Jingdong prefecture level city in Shandong
Happy Fan Shop	King of Power Saving	So fast! Ordered yesterday and received today 👍👍👍👍👍👍👍👍👍 🌹🌹🌹🌹🌹🌹🌹🌹🌹	Jingdong prefecture level city in Shandong
Happy Fan Shop	King of Power Saving	I've started using it. We'll see how it works.	Jingdong prefecture level city in Shandong
Happy Fan Shop	King of Power Saving	I just received the goods, I won't know the effect until after two.	Jingdong prefecture level city in Shandong
Happy Fan Shop	King of Power Saving	Just received the goods, the specific effect will be evaluated later	Jingdong prefecture level city in Shandong
Happy Fan Shop	King of Power Saving	I've received the goods, I don't know if it's any good yet	Jingdong prefecture level city in Shandong
Happy Fan Shop	King of Power Saving	Try it for a month and then come back with a concrete evaluation	Jingdong prefecture level city in Shandong

Happy Fan Shop	King of Power Saving	In use, after two months to see the effect of power saving	Jingdong prefecture level city in Shandong
Happy Fan Shop	King of Power Saving	Power Saver. It's authentic. It's black. It works.	Jingdong prefecture level city in Shandong
Happy Fan Shop	King of Power Saving	I've been using it for a week now and it's not working.	Jingdong prefecture level city in Shandong
Happy Fan Shop	King of Power Saving	I'm just using it. I don't know how it's going to work.	Jingdong prefecture level city in Shandong
Happy Fan Shop	King of Power Saving	Products are better looking out for power savings	Jingdong prefecture level city in Shandong
Happy Fan Shop	King of Power Saving	I received it and I've kept it on. I hope it works.	Jingdong prefecture level city in Shandong
Happy Fan Shop	King of Power Saving	It's really good! Worth recommending to buy!	Jingdong prefecture level city in Shandong
Happy Fan Shop	King of Power Saving	Try it for a few days and see if it doesn't work, then return it.	Jingdong prefecture level city in Shandong
Happy Fan Shop	King of Power Saving	It's in use and I hope to be pleasantly surprised.	Jingdong prefecture level city in Shandong
Happy Fan Shop	King of Power Saving	We'll see how it works when we use it	Jingdong prefecture level city in Shandong
Happy Fan Shop	King of Power Saving	Let's see if it works.	Jingdong prefecture level city in Shandong
Happy Fan Shop	King of Power Saving	I'm using it and will follow up with a review	Jingdong prefecture level city in Shandong
Happy Fan Shop	King of Power Saving	It really works. It saves energy.	Jingdong prefecture level city in Shandong
Happy Fan Shop	King of Power Saving	It does have some power savings!	Jingdong prefecture level city in Shandong
Happy Fan Shop	King of Power Saving	Jingdong shopping speed is convenient and timely	Jingdong prefecture level city in Shandong
Happy Fan Shop	King of Power Saving	Second time I've bought it. Everything's fine.	Jingdong prefecture level city in Shandong
Happy Fan Shop	King of Power Saving	I don't know if it works yet.	Jingdong prefecture level city in Shandong
Happy Fan Shop	King of Power Saving	Not used, don't know how well it works.	Jingdong prefecture level city in Shandong
Happy Fan Shop	King of Power Saving	No visible results so far	Jingdong prefecture level city in Shandong
Happy Fan Shop	King of Power Saving	It doesn't feel like it's working.	Jingdong prefecture level city in Shandong
Happy Fan Shop	King of Power Saving	Where's the effect? Where are the power savings?	Jingdong prefecture level city in Shandong
Happy Fan Shop	King of Power Saving	Let's see how it works in practice!	Jingdong prefecture level city in Shandong
Happy Fan Shop	King of Power Saving	I'll wait until I use it to give you a good review	Jingdong prefecture level city in Shandong
Happy Fan Shop	King of Power Saving	I'll follow up after I've used it	Jingdong prefecture level city in Shandong
Happy Fan Shop	King of Power Saving	Well, that's good for saving power.	Jingdong prefecture level city in Shandong
Happy Fan Shop	King of Power Saving	Received. Used in the know.	Jingdong prefecture level city in Shandong
Happy Fan Shop	King of Power Saving	It doesn't do much good.	Jingdong prefecture level city in Shandong
Happy Fan Shop	King of Power Saving	It's not working.	Jingdong prefecture level city in Shandong
Happy Fan Shop	King of Power Saving	I don't know. I just used it.	Jingdong prefecture level city in Shandong
Happy Fan Shop	King of Power Saving	Use it and come back to evaluate.	Jingdong prefecture level city in Shandong
Happy Fan Shop	King of Power Saving	Two words: useless.	Jingdong prefecture level city in Shandong
Happy Fan Shop	King of Power Saving	To be used and then evaluated	Jingdong prefecture level city in Shandong
Happy Fan Shop	King of Power Saving	No shipment.	Jingdong prefecture level city in Shandong
Happy Fan Shop	King of Power Saving	Fake. Speechless.	Jingdong prefecture level city in Shandong
Happy Fan Shop	King of Power Saving	fantastic (idiom); awesome	Jingdong prefecture level city in Shandong
Happy Fan Shop	King of Power Saving	an excellent	Jingdong prefecture level city in Shandong
Happy Fan Shop	King of Power Saving	an excellent	Jingdong prefecture level city in Shandong
Happy Fan Shop	King of Power Saving	Want to buy! Recommended not to buy! No effect	Jingdong prefecture level city in Shandong
Happy Fan Shop	King of Power Saving	That's all there is to it. I can't figure out how to save electricity.	Jingdong prefecture level city in Shandong
Happy Fan Shop	King of Power Saving	It's no use.	Jingdong prefecture level city in Shandong
Happy Fan Shop	King of Power Saving	Things are more perfect than I imagined, very powerful, it is worth getting Packaging is very strong, things are well protected, it is worth the praise things are very satisfied Oh, beautiful and stylish atmosphere, customer service is super patient, service is meticulous and attentive, the big brand is not	Jingdong prefecture level city in Shandong
Happy Fan Shop	King of Power Saving	The effect is too good, the family has now several units, back to buy a number of times, the power saver received, power-saving effect is very good,, fine workmanship, long warranty, but also purify the air, before buying a used for a period of time, with the okay, and then bought a, and only now come to the	Jingdong prefecture level city in Shandong
Happy Fan Shop	King of Power Saving	Quite good, and imagine the same, casual ride are very good, satisfied! Baby received it 👌, really great ah! 🌈 quality is really nothing to say, very satisfied! 🆗 happy happy 😃 genuine, great, the price is very good, in short, quite good, will buy again a very good shopping experience, attentive service, fast logistics, distribution is also thoughtful, the attitude is also very good, value for money, ah,	Jingdong prefecture level city in Shandong
Happy Fan Shop	King of Power Saving	Power-saving effect: strong Cost-effective: quite good Appearance style: beautiful Workmanship material: meticulous Experience: looking forward to power-saving effect	Jingdong prefecture level city in Shandong
Happy Fan Shop	King of Power Saving	Cost-effective is okay, the appearance of the style of meticulous workmanship, looking forward to energy-saving effect.	Jingdong prefecture level city in Shandong
Happy Fan Shop	King of Power Saving	Very Good Very Power Saving This Power Saver is really power saving As soon as the power is turned on, the Power Saver starts the power saving function for you.	Jingdong prefecture level city in Shandong
Happy Fan Shop	King of Power Saving	Logistics is very fast, the price is favorable, friends introduced that it is very good to use, bought 2 at once, looking forward to the effect, used after the follow-up comment.	Jingdong prefecture level city in Shandong
Happy Fan Shop	King of Power Saving	It's all fake. It doesn't save any electricity.	Jingdong prefecture level city in Shandong
Happy Fan Shop	King of Power Saving	This Power Saver is so much better than the one I bought before, not only the effect is good, the quality is very good in all aspects, the customer service attitude is also very good, recommended to buy!	Jingdong prefecture level city in Shandong

Happy Fan Shop	King of Power Saving	Logistics is very fast, buy for parents, good quality, once bought 2, power-saving effect is good to buy 2 more, look forward to using the effect, satisfied will recommend to friends.	Jingdong prefecture level city in Shandong
Happy Fan Shop	King of Power Saving	With the description is the same, very high-grade, good value, to buy friends can order Oh!	Jingdong prefecture level city in Shandong
Happy Fan Shop	King of Power Saving	Not using much electricity in the winter saving over 100 per month! Ready to buy 2 more units in the summer ! ! ! ! ! Good quality, good price, power saving effect is obvious, cost-effective, worth buying, can be recommended!	Jingdong prefecture level city in Shandong
Happy Fan Shop	King of Power Saving	Purchased a power saver received on the put into use, rent a room with many electrical appliances, power consumption is very large, we have a piece of electricity or 1.2 dollars a time, with this power saver is much better, every day can save a lot of money, very convenient and practical.	Jingdong prefecture level city in Shandong
Happy Fan Shop	King of Power Saving	The power saving effect is very good: my supermarket monthly electricity bill is more than 7000 yuan. On May 1st someone recommended buying a couple of power savers, and I bought 5 of them first. The electricity bill was about 2000 less in June. This time, I bought 5 more. The seller said 800 degrees of electricity with one. I feel it's basically enough. A total of 10 units	Jingdong prefecture level city in Shandong
Happy Fan Shop	King of Power Saving	I never thought I could get such a good baby at such an affordable price, it's very good value for money, I love it!	Jingdong prefecture level city in Shandong
Happy Fan Shop	King of Power Saving	Previously bought other brands of power savers, but found no effect at home, this time to see how the products on the Jingdong, inserted in the home socket, the LED display is blue, the night light is very nice to see, look at the instructions that have to be half a year in order to achieve the best results, try it, I believe that the quality of the Jingdong, it should not be wrong.	Jingdong prefecture level city in Shandong
Happy Fan Shop	King of Power Saving	Superb, inexpensive, saves a lot of points, been using it for a long time!	Jingdong prefecture level city in Shandong
Happy Fan Shop	King of Power Saving	There are a lot of appliances in the house, the summer air conditioning is very costly, I bought this in front of the air conditioning effect is very good, I explain that I bought three in one breath, I use more electricity a month down to 200 or so, very satisfied.	Jingdong prefecture level city in Shandong
Happy Fan Shop	King of Power Saving	A month can save more than 200, home monthly electricity bill of more than 600 dollars, inserted 3 or so, an amount of the balance of the average can save more than 200. Product is very good, has been buying this one, has given a lot of relatives recommended, the quality is also very good, before buying a look with this same, who knows to buy home has not been used for more than a week on the bad, the quality of this is really good with more than two years!	Jingdong prefecture level city in Shandong
Happy Fan Shop	King of Power Saving	Whole house power saving king, can really save electricity, friends recommended, high power saving appliances, only replacement, no repair, after-sales worry-free!	Jingdong prefecture level city in Shandong
Happy Fan Shop	King of Power Saving	Great item love it, great customer service, great shopping experience!	Jingdong prefecture level city in Shandong
Happy Fan Shop	King of Power Saving	Power saving, very good use, much cheaper than the physical store, satisfaction	Jingdong prefecture level city in Shandong
Happy Fan Shop	King of Power Saving	In use, very well packaged and fast delivery of credit!	Jingdong prefecture level city in Shandong
Happy Fan Shop	King of Power Saving	Product quality, customer service attitude is particularly good, like, praise!	Jingdong prefecture level city in Shandong
Happy Fan Shop	King of Power Saving	The logistics is very fast, the packaging is not broken, the quality is very good, I like it very much!	Jingdong prefecture level city in Shandong
Happy Fan Shop	King of Power Saving	The saver was well packaged and was as described when plugged in	Jingdong prefecture level city in Shandong
Happy Fan Shop	King of Power Saving	Energy efficient and very satisfied	Jingdong prefecture level city in Shandong
Happy Fan Shop	King of Power Saving	Great quality for the price and fast shipping.	Jingdong prefecture level city in Shandong
Happy Fan Shop	King of Power Saving	It's great, just as described. Perfect.	Jingdong prefecture level city in Shandong
Happy Fan Shop	King of Power Saving	There is no power saving effect, but rather ring up an increase in electricity costs, because this machine is on continuously all day long, uninterruptedly, its own electricity and increased costs, plus the cost of purchasing the machine, so I feel that the use of this machine instead of increasing the	Jingdong prefecture level city in Shandong
Happy Fan Shop	King of Power Saving	Recommended by the owner of the store next door, looked at his home does have to save electricity, decided to get. Indeed quite can, the voltage is much more stable, there is a reduction in electricity	Jingdong prefecture level city in Shandong
Happy Fan Shop	King of Power Saving	Goods have been received value for money, affordable and reasonable price, and the description of the same, power-saving safe and reliable!	Jingdong prefecture level city in Shandong
Happy Fan Shop	King of Power Saving	Listen to friends introduced to buy, tried to save a lot of electricity is good, quite good, worth buying.	Jingdong prefecture level city in Shandong
Happy Fan Shop	King of Power Saving	The product is easy to use, safe and environmentally friendly, only used for a short time, to the next month, after the electricity bill status display to know how the performance of the power saver efficacy.	Jingdong prefecture level city in Shandong
Happy Fan Shop	King of Power Saving	Last month's electricity bill is 178 yuan, with this power saver is 92 yuan, ready to buy two more back home, I was introduced to buy a friend, just started to not believe that I did not expect the effect!	Jingdong prefecture level city in Shandong
Happy Fan Shop	King of Power Saving	It looks nice, but after using it for a few days I can't see how it saves power?	Jingdong prefecture level city in Shandong
Happy Fan Shop	King of Power Saving	Recommended by a friend, it is genuine, as described by the merchant, cost-effective	Jingdong prefecture level city in Shandong
Happy Fan Shop	King of Power Saving	Works well, affordable and saves money.	Jingdong prefecture level city in Shandong
Happy Fan Shop	King of Power Saving	Feels good haven't compared it yet, the air conditioning has been on a lot in the past two months	Jingdong prefecture level city in Shandong
Happy Fan Shop	King of Power Saving	Saving about 5 kWh per day is a good thing	Jingdong prefecture level city in Shandong
Happy Fan Shop	King of Power Saving	Not bad, affordable, best value for money	Jingdong prefecture level city in Shandong
Happy Fan Shop	King of Power Saving	The goods are genuine and the logistics are faster.	Jingdong prefecture level city in Shandong
Happy Fan Shop	King of Power Saving	Goods have been received today, has been plugged into the outlet, looking forward to saving electricity, after a period of time to see how the effect in the follow-up comment!	Jingdong prefecture level city in Shandong
Happy Fan Shop	King of Power Saving	I just got it home and haven't used it for long, so I'll use it for a couple months and see what happens. Seeing that most people have good reviews, it should be fine.	Jingdong prefecture level city in Shandong
Happy Fan Shop	King of Power Saving	I'm looking forward to the results, so I'll give you a good review out of habit! Follow up review after a while	Jingdong prefecture level city in Shandong
Happy Fan Shop	King of Power Saving	Use a dish first to know if it will help save energy	Jingdong prefecture level city in Shandong
Happy Fan Shop	King of Power Saving	Whether it saves electricity or not, we don't know yet.	Jingdong prefecture level city in Shandong
Happy Fan Shop	King of Power Saving	Goods have been received, use a period of time to know how oh. The courier did not deliver to the door ah! Sent to the station ah! The heat is inconvenient ah!	Jingdong prefecture level city in Shandong
Happy Fan Shop	King of Power Saving	A little effect, before an hour a power, now an hour 0.76 degrees, a dollar a power = save 24 cents, although not a lot, first use it!	Jingdong prefecture level city in Shandong
Happy Fan Shop	King of Power Saving	Just received the goods, use a month to evaluate	Jingdong prefecture level city in Shandong
Happy Fan Shop	King of Power Saving	Home every month with a lot of electricity, inadvertently found this good thing online that can save the beginning of a skeptical attitude to consult customer service, customer service replied very professional, said 120 days can be refunded that first try it!	Jingdong prefecture level city in Shandong
Happy Fan Shop	King of Power Saving	After receiving the goods this afternoon, immediately installed in the bathroom Smith energy storage electric water heater on the power outlet (instead of connecting with a plug board), when used, found that the "Power Saving King" LCD display, is the value of the voltage, not the store's video said so	Jingdong prefecture level city in Shandong
Happy Fan Shop	King of Power Saving	Has made many days, did not feel the power saving, the effect is not obvious.	Jingdong prefecture level city in Shandong
Happy Fan Shop	King of Power Saving	Energy savers do not save energy at all, but rather consume a lot of electricity.	Jingdong prefecture level city in Shandong

Happy Fan Shop	King of Power Saving	It's useless. It's a waste of money.	Jingdong prefecture level city in Shandong
Happy Fan Shop	King of Power Saving	Last month saved more than 500, which came back to buy, the store bought three, and then buy 1 home use, my family is dry beauty salon, more instruments, especially electricity, a month almost 1000. or so, with this did not expect to save so much, great, five-star praise!	Jingdong prefecture level city in Shandong
Happy Fan Shop	King of Power Saving	It rained a couple days ago and I just went to get the item back today , not bad, hey I looked at the meter and that red display light takes several seconds to jump, did not make a mistake buying it. After a period of time to evaluate.	Jingdong prefecture level city in Shandong
Happy Fan Shop	King of Power Saving	Without further ado, the electricity bill came down, this month less 102 yuan, has been repurchased, I also recommended to my friends, all said that there are effects, the product is okay, the effect is quite	Jingdong prefecture level city in Shandong
Happy Fan Shop	King of Power Saving	After buying and using two months really see the effect, so only to evaluate! Originally held the mindset of trying, I did not expect really effective, worth having!	Jingdong prefecture level city in Shandong
Happy Fan Shop	King of Power Saving	In the use of this energy saver before the monthly electricity bill is more than 150 yuan or so, now save thirty percent, every day to work, six or seven o'clock in the afternoon to go home, I do not know why the electricity bill is so expensive, energy-saving appliances really have the effect of recommending	Jingdong prefecture level city in Shandong
Happy Fan Shop	King of Power Saving	It saves about 100 bucks a month. A friend recommended it to me, and he's been using it for a while, and the effect is so obvious that I bought one too.	Jingdong prefecture level city in Shandong
Happy Fan Shop	King of Power Saving	My dad asked me to buy one, but after I paid the bill, I asked and he said it was a lot less this month, so he asked me to buy another one.	Jingdong prefecture level city in Shandong
Happy Fan Shop	King of Power Saving	The specific effect will have to be tested for at least two months to compare whether there is a power saving effect or not.	Jingdong prefecture level city in Shandong
Happy Fan Shop	King of Power Saving	'The goods have been received, immediately go to use, please ask the boss you instructions do not allow to withdraw, there are two fuses installed there!	Jingdong prefecture level city in Shandong
Happy Fan Shop	King of Power Saving	First praise is using, after using if good then follow up, now because I have not yet seen the results	Jingdong prefecture level city in Shandong
Happy Fan Shop	King of Power Saving	I've just been using it, and I haven't seen if it has a power-saving effect yet, so I'll follow up after 10 days and half a month.	Jingdong prefecture level city in Shandong
Happy Fan Shop	King of Power Saving	Just received the intelligent power saving meter, immediately plugged in to use, see it is not bad, and order to sell the second time	Jingdong prefecture level city in Shandong
Happy Fan Shop	King of Power Saving	This power saver is a bit effective, used for half a month, the effect is still visible.	Jingdong prefecture level city in Shandong
Happy Fan Shop	King of Power Saving	I received the baby and it looks great, I'm trying it out now and will keep an eye on it afterward.	Jingdong prefecture level city in Shandong
Happy Fan Shop	King of Power Saving	I've just received it, but I'm still trying to see how it works, so I'll come back in a little while.	Jingdong prefecture level city in Shandong
Happy Fan Shop	King of Power Saving	Just used it wait to use it for a while and then comment thanks, all first five stars.	Jingdong prefecture level city in Shandong
Happy Fan Shop	King of Power Saving	Very trustworthy brand, quality is guaranteed, the price is still affordable, quite satisfied!	Jingdong prefecture level city in Shandong
Happy Fan Shop	King of Power Saving	Things received, I hope that the platform of Jingdong do not sell fake goods are available	Jingdong prefecture level city in Shandong
Happy Fan Shop	King of Power Saving	Energy saver received, the use of the effect is good and the business description of the same	Jingdong prefecture level city in Shandong
Happy Fan Shop	King of Power Saving	The surface looks beautiful, the actual effect is not known, after using additional evaluation.	Jingdong prefecture level city in Shandong
Happy Fan Shop	King of Power Saving	Tried it for a few months, it really saves electricity, and I bought it for a friend.	Jingdong prefecture level city in Shandong
Happy Fan Shop	King of Power Saving	It didn't work and wanted to return it, it really didn't work at all.	Jingdong prefecture level city in Shandong
Happy Fan Shop	King of Power Saving	Just used it, don't know how effective it is, looking forward to usefulness	Jingdong prefecture level city in Shandong
Happy Fan Shop	King of Power Saving	After two months before the effect I do not know if it is true!	Jingdong prefecture level city in Shandong
Happy Fan Shop	King of Power Saving	So fast! Ordered yesterday and received today 👍👍👍👍👍👍👍👍👍 🌹🌹🌹🌹🌹🌹🌹🌹🌹	Jingdong prefecture level city in Shandong
Happy Fan Shop	King of Power Saving	I've started using it. We'll see how it works.	Jingdong prefecture level city in Shandong
Happy Fan Shop	King of Power Saving	I just received the goods, I won't know the effect until after two.	Jingdong prefecture level city in Shandong
Happy Fan Shop	King of Power Saving	Just received the goods, the specific effect will be evaluated later	Jingdong prefecture level city in Shandong
Happy Fan Shop	King of Power Saving	I've received the goods, I don't know if it's any good yet	Jingdong prefecture level city in Shandong
Happy Fan Shop	King of Power Saving	Try it for a month and then come back with a concrete evaluation	Jingdong prefecture level city in Shandong
Happy Fan Shop	King of Power Saving	In use, after two months to see the effect of power saving	Jingdong prefecture level city in Shandong
Happy Fan Shop	King of Power Saving	Power Saver. It's authentic. It's black. It works.	Jingdong prefecture level city in Shandong
Happy Fan Shop	King of Power Saving	I've been using it for a week now and it's not working.	Jingdong prefecture level city in Shandong
Happy Fan Shop	King of Power Saving	I'm just using it. I don't know how it's going to work.	Jingdong prefecture level city in Shandong
Happy Fan Shop	King of Power Saving	Products are better looking out for power savings	Jingdong prefecture level city in Shandong
Happy Fan Shop	King of Power Saving	I received it and I've kept it on. I hope it works.	Jingdong prefecture level city in Shandong
Happy Fan Shop	King of Power Saving	It's really good! Worth recommending to buy!	Jingdong prefecture level city in Shandong
Happy Fan Shop	King of Power Saving	Try it for a few days and see if it doesn't work, then return it.	Jingdong prefecture level city in Shandong
Happy Fan Shop	King of Power Saving	It's in use and I hope to be pleasantly surprised.	Jingdong prefecture level city in Shandong
Happy Fan Shop	King of Power Saving	We'll see how it works when we use it	Jingdong prefecture level city in Shandong
Happy Fan Shop	King of Power Saving	Let's see if it works.	Jingdong prefecture level city in Shandong
Happy Fan Shop	King of Power Saving	I'm using it and will follow up with a review	Jingdong prefecture level city in Shandong
Happy Fan Shop	King of Power Saving	It really works. It saves energy.	Jingdong prefecture level city in Shandong
Happy Fan Shop	King of Power Saving	It does have some power savings!	Jingdong prefecture level city in Shandong
Happy Fan Shop	King of Power Saving	Jingdong shopping speed is convenient and timely	Jingdong prefecture level city in Shandong
Happy Fan Shop	King of Power Saving	Second time I've bought it. Everything's fine.	Jingdong prefecture level city in Shandong
Happy Fan Shop	King of Power Saving	I don't know if it works yet.	Jingdong prefecture level city in Shandong
Happy Fan Shop	King of Power Saving	Not used, don't know how well it works.	Jingdong prefecture level city in Shandong
Happy Fan Shop	King of Power Saving	No visible results so far	Jingdong prefecture level city in Shandong

Happy Fan Shop	King of Power Saving	It doesn't feel like it's working.	Jingdong prefecture level city in Shandong
Happy Fan Shop	King of Power Saving	Where's the effect? Where are the power savings?	Jingdong prefecture level city in Shandong
Happy Fan Shop	King of Power Saving	I'll wait until I use it to give you a good review	Jingdong prefecture level city in Shandong
Happy Fan Shop	King of Power Saving	Let's see how it works in practice!	Jingdong prefecture level city in Shandong
Happy Fan Shop	King of Power Saving	I'll follow up after I've used it	Jingdong prefecture level city in Shandong
Happy Fan Shop	King of Power Saving	Well, that's good for saving power.	Jingdong prefecture level city in Shandong
Happy Fan Shop	King of Power Saving	Received. Used in the know.	Jingdong prefecture level city in Shandong
Happy Fan Shop	King of Power Saving	It doesn't do much good.	Jingdong prefecture level city in Shandong
Happy Fan Shop	King of Power Saving	It's not working.	Jingdong prefecture level city in Shandong
Happy Fan Shop	King of Power Saving	I don't know. I just used it.	Jingdong prefecture level city in Shandong
Happy Fan Shop	King of Power Saving	Use it and come back to evaluate.	Jingdong prefecture level city in Shandong
Happy Fan Shop	King of Power Saving	Two words: useless.	Jingdong prefecture level city in Shandong
Happy Fan Shop	King of Power Saving	To be used and then evaluated	Jingdong prefecture level city in Shandong
Happy Fan Shop	King of Power Saving	No shipment.	Jingdong prefecture level city in Shandong
Happy Fan Shop	King of Power Saving	Fake. Speechless.	Jingdong prefecture level city in Shandong
Happy Fan Shop	King of Power Saving	fantastic (idiom); awesome	Jingdong prefecture level city in Shandong
Happy Fan Shop	King of Power Saving	an excellent	Jingdong prefecture level city in Shandong
Happy Fan Shop	King of Power Saving	an excellent	Jingdong prefecture level city in Shandong
Happy Fan Shop	King of Power Saving	Want to buy! Recommended not to buy! No effect	Jingdong prefecture level city in Shandong
Happy Fan Shop	King of Power Saving	That's all there is to it. I can't figure out how to save electricity.	Jingdong prefecture level city in Shandong
Happy Fan Shop	King of Power Saving	It's no use.	Jingdong prefecture level city in Shandong
Happy Fan Shop	King of Power Saving	Things are more perfect than I imagined, very powerful, it is worth getting Packaging is very strong, things are well protected, it is worth the praise things are very satisfied Oh, beautiful and stylish atmosphere, customer service is super patient, service is meticulous and attentive, the big brand is not	Jingdong prefecture level city in Shandong
Happy Fan Shop	King of Power Saving	The effect is too good, the family has now several units, back to buy a number of times, the power saver received, power-saving effect is very good,, fine workmanship, long warranty, but also purify the air, before buying a used for a period of time, with the okay, and then bought a, and only now come to the	Jingdong prefecture level city in Shandong
Happy Fan Shop	King of Power Saving	Quite good, and imagine the same, casual ride are very good, satisfied! Baby received it 👌, really great ah! 🌈 quality is really nothing to say, very satisfied! 🆗 happy happy 😃 genuine, great, the price is very good, in short, quite good, will buy again a very good shopping experience, attentive service, fast logistics, distribution is also thoughtful, the attitude is also very good, value for money, ah,	Jingdong prefecture level city in Shandong
Happy Fan Shop	King of Power Saving	Power-saving effect: strong Cost-effective: quite good Appearance style: beautiful Workmanship material: meticulous Experience: looking forward to power-saving effect	Jingdong prefecture level city in Shandong
Happy Fan Shop	King of Power Saving	Cost-effective is okay, the appearance of the style of meticulous workmanship, looking forward to energy-saving effect.	Jingdong prefecture level city in Shandong
Happy Fan Shop	King of Power Saving	Very Good Very Power Saving This Power Saver is really power saving As soon as the power is turned on, the Power Saver starts the power saving function for you.	Jingdong prefecture level city in Shandong
Happy Fan Shop	King of Power Saving	Logistics is very fast, the price is favorable, friends introduced that it is very good to use, bought 2 at once, looking forward to the effect, used after the follow-up comment.	Jingdong prefecture level city in Shandong
Happy Fan Shop	King of Power Saving	It's all fake. It doesn't save any electricity.	Jingdong prefecture level city in Shandong
Happy Fan Shop	King of Power Saving	This Power Saver is so much better than the one I bought before, not only the effect is good, the quality is very good in all aspects, the customer service attitude is also very good, recommended to buy!	Jingdong prefecture level city in Shandong
Happy Fan Shop	King of Power Saving	Logistics is very fast, buy for parents, good quality, once bought 2, power-saving effect is good to buy 2 more, look forward to using the effect, satisfied will recommend to friends.	Jingdong prefecture level city in Shandong
Happy Fan Shop	King of Power Saving	With the description is the same, very high-grade, good value, to buy friends can order Oh!	Jingdong prefecture level city in Shandong
Happy Fan Shop	King of Power Saving	Not using much electricity in the winter saving over 100 per month! Ready to buy 2 more units in the summer ！！！！！ Good quality, good price, power saving effect is obvious, cost-effective, worth buying, can be recommended!	Jingdong prefecture level city in Shandong
Happy Fan Shop	King of Power Saving	Purchased a power saver received on the put into use, rent a room with many electrical appliances, power consumption is very large, we have a piece of electricity or 1.2 dollars a time, with this power saver is much better, every day can save a lot of money, very convenient and practical.	Jingdong prefecture level city in Shandong
Happy Fan Shop	King of Power Saving	The power saving effect is very good: my supermarket monthly electricity bill is more than 7000 yuan. On May 1st someone recommended buying a couple of power savers, and I bought 5 of them first. The electricity bill was about 2000 less in June. This time, I bought 5 more. The seller said 800 degrees of electricity with one. I feel it's basically enough. A total of 10 units	Jingdong prefecture level city in Shandong
Happy Fan Shop	King of Power Saving	I never thought I could get such a good baby at such an affordable price, it's very good value for money, I love it!	Jingdong prefecture level city in Shandong
Happy Fan Shop	King of Power Saving	Previously bought other brands of power savers, but found no effect at home, this time to see how the products on the Jingdong, inserted in the home socket, the LED display is blue, the night light is very nice to see, look at the instructions that have to be half a year in order to achieve the best results, try it, I believe that the quality of the Jingdong, it should not be wrong.	Jingdong prefecture level city in Shandong
Happy Fan Shop	King of Power Saving	Superb, inexpensive, saves a lot of points, been using it for a long time!	Jingdong prefecture level city in Shandong
Happy Fan Shop	King of Power Saving	There are a lot of appliances in the house, the summer air conditioning is very costly, I bought this in front of the air conditioning effect is very good, I explain that I bought three in one breath, I use more electricity a month down to 200 or so, very satisfied.	Jingdong prefecture level city in Shandong
Happy Fan Shop	King of Power Saving	A month can save more than 200, home monthly electricity bill of more than 600 dollars, inserted 3 or so, an amount of the balance of the average can save more than 200. Product is very good, has been buying this one, has given a lot of relatives recommended, the quality is also very good, before buying a look with this same, who knows to buy home has not been used for more than a week on the bad, the quality of this is really good with more than two years!	Jingdong prefecture level city in Shandong
Happy Fan Shop	King of Power Saving	Whole house power saving king, can really save electricity, friends recommended, high power saving appliances, only replacement, no repair, after-sales worry-free!	Jingdong prefecture level city in Shandong
Happy Fan Shop	King of Power Saving	Great item love it, great customer service, great shopping experience!	Jingdong prefecture level city in Shandong
Happy Fan Shop	King of Power Saving	Power saving, very good use, much cheaper than the physical store, satisfaction	Jingdong prefecture level city in Shandong
Happy Fan Shop	King of Power Saving	In use, very well packaged and fast delivery of credit!	Jingdong prefecture level city in Shandong

Happy Fan Shop	King of Power Saving	Product quality, customer service attitude is particularly good, like, praise!	Jingdong prefecture level city in Shandong
Happy Fan Shop	King of Power Saving	The logistics is very fast, the packaging is not broken, the quality is very good, I like it very much!	Jingdong prefecture level city in Shandong
Happy Fan Shop	King of Power Saving	The saver was well packaged and was as described when plugged in	Jingdong prefecture level city in Shandong
Happy Fan Shop	King of Power Saving	Energy efficient and very satisfied	Jingdong prefecture level city in Shandong
Happy Fan Shop	King of Power Saving	Great quality for the price and fast shipping.	Jingdong prefecture level city in Shandong
Happy Fan Shop	King of Power Saving	It's great, just as described. Perfect.	Jingdong prefecture level city in Shandong
Happy Fan Shop	King of Power Saving	There is no power saving effect, but rather ring up an increase in electricity costs, because this machine is on continuously all day long, uninterruptedly, its own electricity and increased costs, plus the cost of purchasing the machine, so I feel that the use of this machine instead of increasing the	Jingdong prefecture level city in Shandong
Happy Fan Shop	King of Power Saving	Recommended by the owner of the store next door, looked at his home does have to save electricity, decided to get. Indeed quite can, the voltage is much more stable, there is a reduction in electricity	Jingdong prefecture level city in Shandong
Happy Fan Shop	King of Power Saving	Goods have been received value for money, affordable and reasonable price, and the description of the same, power-saving safe and reliable!	Jingdong prefecture level city in Shandong
Happy Fan Shop	King of Power Saving	Listen to friends introduced to buy, tried to save a lot of electricity is good, quite good, worth buying.	Jingdong prefecture level city in Shandong
Happy Fan Shop	King of Power Saving	The product is easy to use, safe and environmentally friendly, only used for a short time, to the next month, after the electricity bill status display to know how the performance of the power saver efficacy.	Jingdong prefecture level city in Shandong
Happy Fan Shop	King of Power Saving	Last month's electricity bill is 178 yuan, with this power saver is 92 yuan, ready to buy two more back home, I was introduced to buy a friend, just started to not believe that I did not expect the effect!	Jingdong prefecture level city in Shandong
Happy Fan Shop	King of Power Saving	It looks nice, but after using it for a few days I can't see how it saves power?	Jingdong prefecture level city in Shandong
Happy Fan Shop	King of Power Saving	Recommended by a friend, it is genuine, as described by the merchant, cost-effective	Jingdong prefecture level city in Shandong
Happy Fan Shop	King of Power Saving	Works well, affordable and saves money.	Jingdong prefecture level city in Shandong
Happy Fan Shop	King of Power Saving	Feels good haven't compared it yet, the air conditioning has been on a lot in the past two months	Jingdong prefecture level city in Shandong
Happy Fan Shop	King of Power Saving	Saving about 5 kWh per day is a good thing	Jingdong prefecture level city in Shandong
Happy Fan Shop	King of Power Saving	Not bad, affordable, best value for money	Jingdong prefecture level city in Shandong
Happy Fan Shop	King of Power Saving	The goods are genuine and the logistics are faster.	Jingdong prefecture level city in Shandong
Happy Fan Shop	King of Power Saving	Goods have been received today, has been plugged into the outlet, looking forward to saving electricity, after a period of time to see how the effect in the follow-up comment!	Jingdong prefecture level city in Shandong
Happy Fan Shop	King of Power Saving	I just got it home and haven't used it for long, so I'll use it for a couple months and see what happens. Seeing that most people have good reviews, it should be fine.	Jingdong prefecture level city in Shandong
Happy Fan Shop	King of Power Saving	I'm looking forward to the results, so I'll give you a good review out of habit! Follow up review after a while	Jingdong prefecture level city in Shandong
Happy Fan Shop	King of Power Saving	Use a dish first to know if it will help save energy	Jingdong prefecture level city in Shandong
Happy Fan Shop	King of Power Saving	Whether it saves electricity or not, we don't know yet.	Jingdong prefecture level city in Shandong
Happy Fan Shop	King of Power Saving	Goods have been received, use a period of time to know how oh. The courier did not deliver to the door ah! Sent to the station ah! The heat is inconvenient ah!	Jingdong prefecture level city in Shandong
Happy Fan Shop	King of Power Saving	A little effect, before an hour a power, now an hour 0.76 degrees, a dollar a power = save 24 cents, although not a lot, first use it!	Jingdong prefecture level city in Shandong
Happy Fan Shop	King of Power Saving	Just received the goods, use a month to evaluate	Jingdong prefecture level city in Shandong
Happy Fan Shop	King of Power Saving	Home every month with a lot of electricity, inadvertently found this good thing online that can save the beginning of a skeptical attitude to consult customer service, customer service replied very professional, said 120 days can be refunded that first try it!	Jingdong prefecture level city in Shandong
Happy Fan Shop	King of Power Saving	After receiving the goods this afternoon, immediately installed in the bathroom Smith energy storage electric water heater on the power outlet (instead of connecting with a plug board), when used, found that the "Power Saving King" LCD display, is the value of the voltage, not the store's video said so	Jingdong prefecture level city in Shandong
Happy Fan Shop	King of Power Saving	Has made many days, did not feel the power saving, the effect is not obvious.	Jingdong prefecture level city in Shandong
Happy Fan Shop	King of Power Saving	Energy savers do not save energy at all, but rather consume a lot of electricity.	Jingdong prefecture level city in Shandong
Happy Fan Shop	King of Power Saving	It's useless. It's a waste of money.	Jingdong prefecture level city in Shandong
Happy Fan Shop	King of Power Saving	Last month saved more than 500, which came back to buy, the store bought three, and then buy 1 home use, my family is dry beauty salon, more instruments, especially electricity, a month almost 1000. or so, with this did not expect to save so much, great, five-star praise!	Jingdong prefecture level city in Shandong
Happy Fan Shop	King of Power Saving	It rained a couple days ago and I just went to get the item back today , not bad, hey I looked at the meter and that red display light takes several seconds to jump, did not make a mistake buying it. After a period of time to evaluate.	Jingdong prefecture level city in Shandong
Happy Fan Shop	King of Power Saving	Without further ado, the electricity bill came down, this month less 102 yuan, has been repurchased, I also recommended to my friends, all said that there are effects, the product is okay, the effect is quite	Jingdong prefecture level city in Shandong
Happy Fan Shop	King of Power Saving	After buying and using two months really see the effect, so only to evaluate! Originally held the mindset of trying, I did not expect really effective, worth having!	Jingdong prefecture level city in Shandong
Happy Fan Shop	King of Power Saving	In the use of this energy saver before the monthly electricity bill is more than 150 yuan or so, now save thirty percent, every day to work, six or seven o'clock in the afternoon to go home, I do not know why the electricity bill is so expensive, energy-saving appliances really have the effect of recommending	Jingdong prefecture level city in Shandong
Happy Fan Shop	King of Power Saving	It saves about 100 bucks a month. A friend recommended it to me, and he's been using it for a while, and the effect is so obvious that I bought one too.	Jingdong prefecture level city in Shandong
Happy Fan Shop	King of Power Saving	My dad asked me to buy one, but after I paid the bill, I asked and he said it was a lot less this month, so he asked me to buy another one.	Jingdong prefecture level city in Shandong
Happy Fan Shop	King of Power Saving	The specific effect will have to be tested for at least two months to compare whether there is a power saving effect or not.	Jingdong prefecture level city in Shandong
Happy Fan Shop	King of Power Saving	'The goods have been received, immediately go to use, please ask the boss you instructions do not allow to withdraw, there are two fuses installed there!	Jingdong prefecture level city in Shandong
Happy Fan Shop	King of Power Saving	First praise is using, after using if good then follow up, now because I have not yet seen the results	Jingdong prefecture level city in Shandong
Happy Fan Shop	King of Power Saving	I've just been using it, and I haven't seen if it has a power-saving effect yet, so I'll follow up after 10 days and half a month.	Jingdong prefecture level city in Shandong
Happy Fan Shop	King of Power Saving	Just received the intelligent power saving meter, immediately plugged in to use, see it is not bad, and order to sell the second time	Jingdong prefecture level city in Shandong
Happy Fan Shop	King of Power Saving	This power saver is a bit effective, used for half a month, the effect is still visible.	Jingdong prefecture level city in Shandong
Happy Fan Shop	King of Power Saving	I received the baby and it looks great, I'm trying it out now and will keep an eye on it afterward.	Jingdong prefecture level city in Shandong
Happy Fan Shop	King of Power Saving	I've just received it, but I'm still trying to see how it works, so I'll come back in a little while.	Jingdong prefecture level city in Shandong
Happy Fan Shop	King of Power Saving	Just used it wait to use it for a while and then comment thanks, all first five stars.	Jingdong prefecture level city in Shandong

Happy Fan Shop	King of Power Saving	Very trustworthy brand, quality is guaranteed, the price is still affordable, quite satisfied!	Jingdong prefecture level city in Shandong
Happy Fan Shop	King of Power Saving	Things received, I hope that the platform of Jingdong do not sell fake goods are available	Jingdong prefecture level city in Shandong
Happy Fan Shop	King of Power Saving	Energy saver received, the use of the effect is good and the business description of the same	Jingdong prefecture level city in Shandong
Happy Fan Shop	King of Power Saving	The surface looks beautiful, the actual effect is not known, after using additional evaluation.	Jingdong prefecture level city in Shandong
Happy Fan Shop	King of Power Saving	Tried it for a few months, it really saves electricity, and I bought it for a friend.	Jingdong prefecture level city in Shandong
Happy Fan Shop	King of Power Saving	It didn't work and wanted to return it, it really didn't work at all.	Jingdong prefecture level city in Shandong
Happy Fan Shop	King of Power Saving	Just used it, don't know how effective it is, looking forward to usefulness	Jingdong prefecture level city in Shandong
Happy Fan Shop	King of Power Saving	After two months before the effect I do not know if it is true!	Jingdong prefecture level city in Shandong
Happy Fan Shop	King of Power Saving	So fast! Ordered yesterday and received today 👍👍👍👍👍👍👍👍👍 🌹🌹🌹🌹🌹🌹🌹🌹🌹	Jingdong prefecture level city in Shandong
Happy Fan Shop	King of Power Saving	I've started using it. We'll see how it works.	Jingdong prefecture level city in Shandong
Happy Fan Shop	King of Power Saving	I just received the goods, I won't know the effect until after two.	Jingdong prefecture level city in Shandong
Happy Fan Shop	King of Power Saving	Just received the goods, the specific effect will be evaluated later	Jingdong prefecture level city in Shandong
Happy Fan Shop	King of Power Saving	I've received the goods, I don't know if it's any good yet	Jingdong prefecture level city in Shandong
Happy Fan Shop	King of Power Saving	Try it for a month and then come back with a concrete evaluation	Jingdong prefecture level city in Shandong
Happy Fan Shop	King of Power Saving	In use, after two months to see the effect of power saving	Jingdong prefecture level city in Shandong
Happy Fan Shop	King of Power Saving	Power Saver. It's authentic. It's black. It works.	Jingdong prefecture level city in Shandong
Happy Fan Shop	King of Power Saving	I've been using it for a week now and it's not working.	Jingdong prefecture level city in Shandong
Happy Fan Shop	King of Power Saving	I'm just using it. I don't know how it's going to work.	Jingdong prefecture level city in Shandong
Happy Fan Shop	King of Power Saving	Products are better looking out for power savings	Jingdong prefecture level city in Shandong
Happy Fan Shop	King of Power Saving	I received it and I've kept it on. I hope it works.	Jingdong prefecture level city in Shandong
Happy Fan Shop	King of Power Saving	It's really good! Worth recommending to buy!	Jingdong prefecture level city in Shandong
Happy Fan Shop	King of Power Saving	Try it for a few days and see if it doesn't work, then return it.	Jingdong prefecture level city in Shandong
Happy Fan Shop	King of Power Saving	It's in use and I hope to be pleasantly surprised.	Jingdong prefecture level city in Shandong
Happy Fan Shop	King of Power Saving	We'll see how it works when we use it	Jingdong prefecture level city in Shandong
Happy Fan Shop	King of Power Saving	Let's see if it works.	Jingdong prefecture level city in Shandong
Happy Fan Shop	King of Power Saving	I'm using it and will follow up with a review	Jingdong prefecture level city in Shandong
Happy Fan Shop	King of Power Saving	It really works. It saves energy.	Jingdong prefecture level city in Shandong
Happy Fan Shop	King of Power Saving	It does have some power savings!	Jingdong prefecture level city in Shandong
Happy Fan Shop	King of Power Saving	Jingdong shopping speed is convenient and timely	Jingdong prefecture level city in Shandong
Happy Fan Shop	King of Power Saving	Second time I've bought it. Everything's fine.	Jingdong prefecture level city in Shandong
Happy Fan Shop	King of Power Saving	I don't know if it works yet.	Jingdong prefecture level city in Shandong
Happy Fan Shop	King of Power Saving	Not used, don't know how well it works.	Jingdong prefecture level city in Shandong
Happy Fan Shop	King of Power Saving	No visible results so far	Jingdong prefecture level city in Shandong
Happy Fan Shop	King of Power Saving	It doesn't feel like it's working.	Jingdong prefecture level city in Shandong
Happy Fan Shop	King of Power Saving	Where's the effect? Where are the power savings?	Jingdong prefecture level city in Shandong
Happy Fan Shop	King of Power Saving	Let's see how it works in practice!	Jingdong prefecture level city in Shandong
Happy Fan Shop	King of Power Saving	I'll wait until I use it to give you a good review	Jingdong prefecture level city in Shandong
Happy Fan Shop	King of Power Saving	I'll follow up after I've used it	Jingdong prefecture level city in Shandong
Happy Fan Shop	King of Power Saving	Well, that's good for saving power.	Jingdong prefecture level city in Shandong
Happy Fan Shop	King of Power Saving	Received. Used in the know.	Jingdong prefecture level city in Shandong
Happy Fan Shop	King of Power Saving	It doesn't do much good.	Jingdong prefecture level city in Shandong
Happy Fan Shop	King of Power Saving	It's not working.	Jingdong prefecture level city in Shandong
Happy Fan Shop	King of Power Saving	I don't know. I just used it.	Jingdong prefecture level city in Shandong
Happy Fan Shop	King of Power Saving	Use it and come back to evaluate.	Jingdong prefecture level city in Shandong
Happy Fan Shop	King of Power Saving	Two words: useless.	Jingdong prefecture level city in Shandong
Happy Fan Shop	King of Power Saving	To be used and then evaluated	Jingdong prefecture level city in Shandong
Happy Fan Shop	King of Power Saving	No shipment.	Jingdong prefecture level city in Shandong
Happy Fan Shop	King of Power Saving	Fake. Speechless.	Jingdong prefecture level city in Shandong
Happy Fan Shop	King of Power Saving	fantastic (idiom); awesome	Jingdong prefecture level city in Shandong
Happy Fan Shop	King of Power Saving	an excellent	Jingdong prefecture level city in Shandong
Happy Fan Shop	King of Power Saving	an excellent	Jingdong prefecture level city in Shandong

Happy Fan Shop	King of Power Saving	Want to buy! Recommended not to buy! No effect	Jingdong prefecture level city in Shandong
Happy Fan Shop	King of Power Saving	That's all there is to it. I can't figure out how to save electricity.	Jingdong prefecture level city in Shandong
Happy Fan Shop	King of Power Saving	It's no use.	Jingdong prefecture level city in Shandong
Happy Fan Shop	King of Power Saving	Things are more perfect than I imagined, very powerful, it is worth getting Packaging is very strong, things are well protected, it is worth the praise things are very satisfied Oh, beautiful and stylish atmosphere, customer service is super patient, service is meticulous and attentive, the big brand is not	Jingdong prefecture level city in Shandong
Happy Fan Shop	King of Power Saving	The effect is too good, the family has now several units, back to buy a number of times, the power saver received, power-saving effect is very good,, fine workmanship, long warranty, but also purify the air, before buying a used for a period of time, with the okay, and then bought a, and only now come to the	Jingdong prefecture level city in Shandong
Happy Fan Shop	King of Power Saving	Quite good, and imagine the same, casual ride are very good, satisfied! Baby received it 👌, really great ah! 🌈 quality is really nothing to say, very satisfied! 🆗 happy happy 😃 genuine, great, the price is very good, in short, quite good, will buy again a very good shopping experience, attentive service, fast logistics, distribution is also thoughtful, the attitude is also very good, value for money, ah,	Jingdong prefecture level city in Shandong
Happy Fan Shop	King of Power Saving	Power-saving effect: strong Cost-effective: quite good Appearance style: beautiful Workmanship material: meticulous Experience: looking forward to power-saving effect	Jingdong prefecture level city in Shandong
Happy Fan Shop	King of Power Saving	Cost-effective is okay, the appearance of the style of meticulous workmanship, looking forward to energy-saving effect.	Jingdong prefecture level city in Shandong
Happy Fan Shop	King of Power Saving	Very Good Very Power Saving This Power Saver is really power saving As soon as the power is turned on, the Power Saver starts the power saving function for you.	Jingdong prefecture level city in Shandong
Happy Fan Shop	King of Power Saving	Logistics is very fast, the price is favorable, friends introduced that it is very good to use, bought 2 at once, looking forward to the effect, used after the follow-up comment.	Jingdong prefecture level city in Shandong
Happy Fan Shop	King of Power Saving	It's all fake. It doesn't save any electricity.	Jingdong prefecture level city in Shandong
Happy Fan Shop	King of Power Saving	This Power Saver is so much better than the one I bought before, not only the effect is good, the quality is very good in all aspects, the customer service attitude is also very good, recommended to buy!	Jingdong prefecture level city in Shandong
Happy Fan Shop	King of Power Saving	Logistics is very fast, buy for parents, good quality, once bought 2, power-saving effect is good to buy 2 more, look forward to using the effect, satisfied will recommend to friends.	Jingdong prefecture level city in Shandong
Happy Fan Shop	King of Power Saving	With the description is the same, very high-grade, good value, to buy friends can order Oh!	Jingdong prefecture level city in Shandong
Happy Fan Shop	King of Power Saving	Not using much electricity in the winter saving over 100 per month! Ready to buy 2 more units in the summer ! ! ! ! ! Good quality, good price, power saving effect is obvious, cost-effective, worth buying, can be recommended!	Jingdong prefecture level city in Shandong
Happy Fan Shop	King of Power Saving	Purchased a power saver received on the put into use, rent a room with many electrical appliances, power consumption is very large, we have a piece of electricity or 1.2 dollars a time, with this power saver is much better, every day can save a lot of money, very convenient and practical.	Jingdong prefecture level city in Shandong
Happy Fan Shop	King of Power Saving	The power saving effect is very good: my supermarket monthly electricity bill is more than 7000 yuan. On May 1st someone recommended buying a couple of power savers, and I bought 5 of them first. The electricity bill was about 2000 less in June. This time, I bought 5 more. The seller said 800 degrees of electricity with one. I feel it's basically enough. A total of 10 units	Jingdong prefecture level city in Shandong
Happy Fan Shop	King of Power Saving	I never thought I could get such a good baby at such an affordable price, it's very good value for money, I love it!	Jingdong prefecture level city in Shandong
Happy Fan Shop	King of Power Saving	Previously bought other brands of power savers, but found no effect at home, this time to see how the products on the Jingdong, inserted in the home socket, the LED display is blue, the night light is very nice to see, look at the instructions that have to be half a year in order to achieve the best results, try it, I believe that the quality of the Jingdong, it should not be wrong.	Jingdong prefecture level city in Shandong
Happy Fan Shop	King of Power Saving	Superb, inexpensive, saves a lot of points, been using it for a long time!	Jingdong prefecture level city in Shandong
Happy Fan Shop	King of Power Saving	There are a lot of appliances in the house, the summer air conditioning is very costly, I bought this in front of the air conditioning effect is very good, I explain that I bought three in one breath, I use more electricity a month down to 200 or so, very satisfied.	Jingdong prefecture level city in Shandong
Happy Fan Shop	King of Power Saving	A month can save more than 200, home monthly electricity bill of more than 600 dollars, inserted 3 or so, an amount of the balance of the average can save more than 200. Product is very good, has been buying this one, has given a lot of relatives recommended, the quality is also very good, before buying a look with this same, who knows to buy home has not been used for more than a week on the bad, the quality of this is really good with more than two years!	Jingdong prefecture level city in Shandong
Happy Fan Shop	King of Power Saving	Whole house power saving king, can really save electricity, friends recommended, high power saving appliances, only replacement, no repair, after-sales worry-free!	Jingdong prefecture level city in Shandong
Happy Fan Shop	King of Power Saving	Great item love it, great customer service, great shopping experience!	Jingdong prefecture level city in Shandong
Happy Fan Shop	King of Power Saving	Power saving, very good use, much cheaper than the physical store, satisfaction	Jingdong prefecture level city in Shandong
Happy Fan Shop	King of Power Saving	In use, very well packaged and fast delivery of credit!	Jingdong prefecture level city in Shandong
Happy Fan Shop	King of Power Saving	Product quality, customer service attitude is particularly good, like, praise!	Jingdong prefecture level city in Shandong
Happy Fan Shop	King of Power Saving	The logistics is very fast, the packaging is not broken, the quality is very good, I like it very much!	Jingdong prefecture level city in Shandong
Happy Fan Shop	King of Power Saving	The saver was well packaged and was as described when plugged in	Jingdong prefecture level city in Shandong
Happy Fan Shop	King of Power Saving	Energy efficient and very satisfied	Jingdong prefecture level city in Shandong
Happy Fan Shop	King of Power Saving	Great quality for the price and fast shipping.	Jingdong prefecture level city in Shandong
Happy Fan Shop	King of Power Saving	It's great, just as described. Perfect.	Jingdong prefecture level city in Shandong
Happy Fan Shop	King of Power Saving	There is no power saving effect, but rather ring up an increase in electricity costs, because this machine is on continuously all day long, uninterruptedly, its own electricity and increased costs, plus the cost of purchasing the machine, so I feel that the use of this machine instead of increasing the	Jingdong prefecture level city in Shandong
Happy Fan Shop	King of Power Saving	Recommended by the owner of the store next door, looked at his home does have to save electricity, decided to get. Indeed quite can, the voltage is much more stable, there is a reduction in electricity	Jingdong prefecture level city in Shandong
Happy Fan Shop	King of Power Saving	Goods have been received value for money, affordable and reasonable price, and the description of the same, power-saving safe and reliable!	Jingdong prefecture level city in Shandong
Happy Fan Shop	King of Power Saving	Listen to friends introduced to buy, tried to save a lot of electricity is good, quite good, worth buying.	Jingdong prefecture level city in Shandong
Happy Fan Shop	King of Power Saving	The product is easy to use, safe and environmentally friendly, only used for a short time, to the next month, after the electricity bill status display to know how the performance of the power saver efficacy.	Jingdong prefecture level city in Shandong
Happy Fan Shop	King of Power Saving	Last month's electricity bill is 178 yuan, with this power saver is 92 yuan, ready to buy two more back home, I was introduced to buy a friend, just started to not believe that I did not expect the effect!	Jingdong prefecture level city in Shandong
Happy Fan Shop	King of Power Saving	It looks nice, but after using it for a few days I can't see how it saves power?	Jingdong prefecture level city in Shandong
Happy Fan Shop	King of Power Saving	Recommended by a friend, it is genuine, as described by the merchant, cost-effective	Jingdong prefecture level city in Shandong
Happy Fan Shop	King of Power Saving	Works well, affordable and saves money.	Jingdong prefecture level city in Shandong
Happy Fan Shop	King of Power Saving	Feels good haven't compared it yet, the air conditioning has been on a lot in the past two months	Jingdong prefecture level city in Shandong
Happy Fan Shop	King of Power Saving	Saving about 5 kWh per day is a good thing	Jingdong prefecture level city in Shandong
Happy Fan Shop	King of Power Saving	Not bad, affordable, best value for money	Jingdong prefecture level city in Shandong

Happy Fan Shop	King of Power Saving	The goods are genuine and the logistics are faster.	Jingdong prefecture level city in Shandong
Happy Fan Shop	King of Power Saving	Goods have been received today, has been plugged into the outlet, looking forward to saving electricity, after a period of time to see how the effect in the follow-up comment!	Jingdong prefecture level city in Shandong
Happy Fan Shop	King of Power Saving	I just got it home and haven't used it for long, so I'll use it for a couple months and see what happens. Seeing that most people have good reviews, it should be fine.	Jingdong prefecture level city in Shandong
Happy Fan Shop	King of Power Saving	I'm looking forward to the results, so I'll give you a good review out of habit! Follow up review after a while	Jingdong prefecture level city in Shandong
Happy Fan Shop	King of Power Saving	Use a dish first to know if it will help save energy	Jingdong prefecture level city in Shandong
Happy Fan Shop	King of Power Saving	Whether it saves electricity or not, we don't know yet.	Jingdong prefecture level city in Shandong
Happy Fan Shop	King of Power Saving	Goods have been received, use a period of time to know how oh. The courier did not deliver to the door ah! Sent to the station ah! The heat is inconvenient ah!	Jingdong prefecture level city in Shandong
Happy Fan Shop	King of Power Saving	A little effect, before an hour a power, now an hour 0.76 degrees, a dollar a power = save 24 cents, although not a lot, first use it!	Jingdong prefecture level city in Shandong
Happy Fan Shop	King of Power Saving	Just received the goods, use a month to evaluate	Jingdong prefecture level city in Shandong
Happy Fan Shop	King of Power Saving	Home every month with a lot of electricity, inadvertently found this good thing online that can save the beginning of a skeptical attitude to consult customer service, customer service replied very professional, said 120 days can be refunded that first try it!	Jingdong prefecture level city in Shandong
Happy Fan Shop	King of Power Saving	After receiving the goods this afternoon, immediately installed in the bathroom Smith energy storage electric water heater on the power outlet (instead of connecting with a plug board), when used, found that the "Power Saving King" LCD display, is the value of the voltage, not the store's video said so	Jingdong prefecture level city in Shandong
Happy Fan Shop	King of Power Saving	Has made many days, did not feel the power saving, the effect is not obvious.	Jingdong prefecture level city in Shandong
Happy Fan Shop	King of Power Saving	Energy savers do not save energy at all, but rather consume a lot of electricity.	Jingdong prefecture level city in Shandong
Happy Fan Shop	King of Power Saving	It's useless. It's a waste of money.	Jingdong prefecture level city in Shandong
Happy Fan Shop	King of Power Saving	Last month saved more than 500, which came back to buy, the store bought three, and then buy 1 home use, my family is dry beauty salon, more instruments, especially electricity, a month almost 1000. or so, with this did not expect to save so much, great, five-star praise!	Jingdong prefecture level city in Shandong
Happy Fan Shop	King of Power Saving	It rained a couple days ago and I just went to get the item back today , not bad, hey I looked at the meter and that red display light takes several seconds to jump, did not make a mistake buying it. After a period of time to evaluate.	Jingdong prefecture level city in Shandong
Happy Fan Shop	King of Power Saving	Without further ado, the electricity bill came down, this month less 102 yuan, has been repurchased, I also recommended to my friends, all said that there are effects, the product is okay, the effect is quite	Jingdong prefecture level city in Shandong
Happy Fan Shop	King of Power Saving	After buying and using two months really see the effect, so only to evaluate! Originally held the mindset of trying, I did not expect really effective, worth having!	Jingdong prefecture level city in Shandong
Happy Fan Shop	King of Power Saving	In the use of this energy saver before the monthly electricity bill is more than 150 yuan or so, now save thirty percent, every day to work, six or seven o'clock in the afternoon to go home, I do not know why the electricity bill is so expensive, energy-saving appliances really have the effect of recommending	Jingdong prefecture level city in Shandong
Happy Fan Shop	King of Power Saving	It saves about 100 bucks a month. A friend recommended it to me, and he's been using it for a while, and the effect is so obvious that I bought one too.	Jingdong prefecture level city in Shandong
Happy Fan Shop	King of Power Saving	My dad asked me to buy one, but after I paid the bill, I asked and he said it was a lot less this month, so he asked me to buy another one.	Jingdong prefecture level city in Shandong
Happy Fan Shop	King of Power Saving	The specific effect will have to be tested for at least two months to compare whether there is a power saving effect or not.	Jingdong prefecture level city in Shandong
Happy Fan Shop	King of Power Saving	'The goods have been received, immediately go to use, please ask the boss you instructions do not allow to withdraw, there are two fuses installed there!	Jingdong prefecture level city in Shandong
Happy Fan Shop	King of Power Saving	First praise is using, after using if good then follow up, now because I have not yet seen the results	Jingdong prefecture level city in Shandong
Happy Fan Shop	King of Power Saving	I've just been using it, and I haven't seen if it has a power-saving effect yet, so I'll follow up after 10 days and half a month.	Jingdong prefecture level city in Shandong
Happy Fan Shop	King of Power Saving	Just received the intelligent power saving meter, immediately plugged in to use, see it is not bad, and order to sell the second time	Jingdong prefecture level city in Shandong
Happy Fan Shop	King of Power Saving	This power saver is a bit effective, used for half a month, the effect is still visible.	Jingdong prefecture level city in Shandong
Happy Fan Shop	King of Power Saving	I received the baby and it looks great, I'm trying it out now and will keep an eye on it afterward.	Jingdong prefecture level city in Shandong
Happy Fan Shop	King of Power Saving	I've just received it, but I'm still trying to see how it works, so I'll come back in a little while.	Jingdong prefecture level city in Shandong
Happy Fan Shop	King of Power Saving	Just used it wait to use it for a while and then comment thanks, all first five stars.	Jingdong prefecture level city in Shandong
Happy Fan Shop	King of Power Saving	Very trustworthy brand, quality is guaranteed, the price is still affordable, quite satisfied!	Jingdong prefecture level city in Shandong
Happy Fan Shop	King of Power Saving	Things received, I hope that the platform of Jingdong do not sell fake goods are available	Jingdong prefecture level city in Shandong
Happy Fan Shop	King of Power Saving	Energy saver received, the use of the effect is good and the business description of the same	Jingdong prefecture level city in Shandong
Happy Fan Shop	King of Power Saving	The surface looks beautiful, the actual effect is not known, after using additional evaluation.	Jingdong prefecture level city in Shandong
Happy Fan Shop	King of Power Saving	Tried it for a few months, it really saves electricity, and I bought it for a friend.	Jingdong prefecture level city in Shandong
Happy Fan Shop	King of Power Saving	It didn't work and wanted to return it, it really didn't work at all.	Jingdong prefecture level city in Shandong
Happy Fan Shop	King of Power Saving	Just used it, don't know how effective it is, looking forward to usefulness	Jingdong prefecture level city in Shandong
Happy Fan Shop	King of Power Saving	After two months before the effect I do not know if it is true!	Jingdong prefecture level city in Shandong
Happy Fan Shop	King of Power Saving	So fast! Ordered yesterday and received today 👍👍👍👍👍👍👍👍👍 🌹🌹🌹🌹🌹🌹🌹🌹🌹	Jingdong prefecture level city in Shandong
Happy Fan Shop	King of Power Saving	I've started using it. We'll see how it works.	Jingdong prefecture level city in Shandong
Happy Fan Shop	King of Power Saving	I just received the goods, I won't know the effect until after two.	Jingdong prefecture level city in Shandong
Happy Fan Shop	King of Power Saving	Just received the goods, the specific effect will be evaluated later	Jingdong prefecture level city in Shandong
Happy Fan Shop	King of Power Saving	I've received the goods, I don't know if it's any good yet	Jingdong prefecture level city in Shandong
Happy Fan Shop	King of Power Saving	Try it for a month and then come back with a concrete evaluation	Jingdong prefecture level city in Shandong
Happy Fan Shop	King of Power Saving	In use, after two months to see the effect of power saving	Jingdong prefecture level city in Shandong
Happy Fan Shop	King of Power Saving	Power Saver. It's authentic. It's black. It works.	Jingdong prefecture level city in Shandong
Happy Fan Shop	King of Power Saving	I've been using it for a week now and it's not working.	Jingdong prefecture level city in Shandong
Happy Fan Shop	King of Power Saving	I'm just using it. I don't know how it's going to work.	Jingdong prefecture level city in Shandong
Happy Fan Shop	King of Power Saving	Products are better looking out for power savings	Jingdong prefecture level city in Shandong

Happy Fan Shop	King of Power Saving	I received it and I've kept it on. I hope it works.	Jingdong prefecture level city in Shandong
Happy Fan Shop	King of Power Saving	It's really good! Worth recommending to buy!	Jingdong prefecture level city in Shandong
Happy Fan Shop	King of Power Saving	Try it for a few days and see if it doesn't work, then return it.	Jingdong prefecture level city in Shandong
Happy Fan Shop	King of Power Saving	It's in use and I hope to be pleasantly surprised.	Jingdong prefecture level city in Shandong
Happy Fan Shop	King of Power Saving	We'll see how it works when we use it	Jingdong prefecture level city in Shandong
Happy Fan Shop	King of Power Saving	Let's see if it works.	Jingdong prefecture level city in Shandong
Happy Fan Shop	King of Power Saving	I'm using it and will follow up with a review	Jingdong prefecture level city in Shandong
Happy Fan Shop	King of Power Saving	It really works. It saves energy.	Jingdong prefecture level city in Shandong
Happy Fan Shop	King of Power Saving	It does have some power savings!	Jingdong prefecture level city in Shandong
Happy Fan Shop	King of Power Saving	Jingdong shopping speed is convenient and timely	Jingdong prefecture level city in Shandong
Happy Fan Shop	King of Power Saving	Second time I've bought it. Everything's fine.	Jingdong prefecture level city in Shandong
Happy Fan Shop	King of Power Saving	I don't know if it works yet.	Jingdong prefecture level city in Shandong
Happy Fan Shop	King of Power Saving	Not used, don't know how well it works.	Jingdong prefecture level city in Shandong
Happy Fan Shop	King of Power Saving	No visible results so far	Jingdong prefecture level city in Shandong
Happy Fan Shop	King of Power Saving	It doesn't feel like it's working.	Jingdong prefecture level city in Shandong
Happy Fan Shop	King of Power Saving	Where's the effect? Where are the power savings?	Jingdong prefecture level city in Shandong
Happy Fan Shop	King of Power Saving	Let's see how it works in practice!	Jingdong prefecture level city in Shandong
Happy Fan Shop	King of Power Saving	I'll wait until I use it to give you a good review	Jingdong prefecture level city in Shandong
Happy Fan Shop	King of Power Saving	I'll follow up after I've used it	Jingdong prefecture level city in Shandong
Happy Fan Shop	King of Power Saving	Well, that's good for saving power.	Jingdong prefecture level city in Shandong
Happy Fan Shop	King of Power Saving	Received. Used in the know.	Jingdong prefecture level city in Shandong
Happy Fan Shop	King of Power Saving	It doesn't do much good.	Jingdong prefecture level city in Shandong
Happy Fan Shop	King of Power Saving	It's not working.	Jingdong prefecture level city in Shandong
Happy Fan Shop	King of Power Saving	I don't know. I just used it.	Jingdong prefecture level city in Shandong
Happy Fan Shop	King of Power Saving	Use it and come back to evaluate.	Jingdong prefecture level city in Shandong
Happy Fan Shop	King of Power Saving	Two words: useless.	Jingdong prefecture level city in Shandong
Happy Fan Shop	King of Power Saving	To be used and then evaluated	Jingdong prefecture level city in Shandong
Happy Fan Shop	King of Power Saving	No shipment.	Jingdong prefecture level city in Shandong
Happy Fan Shop	King of Power Saving	Fake. Speechless.	Jingdong prefecture level city in Shandong
Happy Fan Shop	King of Power Saving	fantastic (idiom); awesome	Jingdong prefecture level city in Shandong
Happy Fan Shop	King of Power Saving	an excellent	Jingdong prefecture level city in Shandong
Happy Fan Shop	King of Power Saving	an excellent	Jingdong prefecture level city in Shandong
Happy Fan Shop	King of Power Saving	Want to buy! Recommended not to buy! No effect	Jingdong prefecture level city in Shandong
Happy Fan Shop	King of Power Saving	That's all there is to it. I can't figure out how to save electricity.	Jingdong prefecture level city in Shandong
Happy Fan Shop	King of Power Saving	It's no use.	Jingdong prefecture level city in Shandong
Happy Fan Shop	King of Power Saving	Things are more perfect than I imagined, very powerful, it is worth getting Packaging is very strong, things are well protected, it is worth the praise things are very satisfied Oh, beautiful and stylish atmosphere, customer service is super patient, service is meticulous and attentive, the big brand is not	Jingdong prefecture level city in Shandong
Happy Fan Shop	King of Power Saving	The effect is too good, the family has now several units, back to buy a number of times, the power saver received, power-saving effect is very good,, fine workmanship, long warranty, but also purify the air, before buying a used for a period of time, with the okay, and then bought a, and only now come to the	Jingdong prefecture level city in Shandong
Happy Fan Shop	King of Power Saving	Quite good, and imagine the same, casual ride are very good, satisfied! Baby received it 👌, really great ah! 🌈 quality is really nothing to say, very satisfied! 🆗 happy happy 😃 genuine, great, the price is very good, in short, quite good, will buy again a very good shopping experience, attentive service, fast logistics, distribution is also thoughtful, the attitude is also very good, value for money, ah,	Jingdong prefecture level city in Shandong
Happy Fan Shop	King of Power Saving	Power-saving effect: strong Cost-effective: quite good Appearance style: beautiful Workmanship material: meticulous Experience: looking forward to power-saving effect	Jingdong prefecture level city in Shandong
Happy Fan Shop	King of Power Saving	Cost-effective is okay, the appearance of the style of meticulous workmanship, looking forward to energy-saving effect.	Jingdong prefecture level city in Shandong
Happy Fan Shop	King of Power Saving	Very Good Very Power Saving This Power Saver is really power saving As soon as the power is turned on, the Power Saver starts the power saving function for you.	Jingdong prefecture level city in Shandong
Happy Fan Shop	King of Power Saving	Logistics is very fast, the price is favorable, friends introduced that it is very good to use, bought 2 at once, looking forward to the effect, used after the follow-up comment.	Jingdong prefecture level city in Shandong
Happy Fan Shop	King of Power Saving	It's all fake. It doesn't save any electricity.	Jingdong prefecture level city in Shandong
Happy Fan Shop	King of Power Saving	This Power Saver is so much better than the one I bought before, not only the effect is good, the quality is very good in all aspects, the customer service attitude is also very good, recommended to buy!	Jingdong prefecture level city in Shandong
Happy Fan Shop	King of Power Saving	Logistics is very fast, buy for parents, good quality, once bought 2, power-saving effect is good to buy 2 more, look forward to using the effect, satisfied will recommend to friends.	Jingdong prefecture level city in Shandong
Happy Fan Shop	King of Power Saving	With the description is the same, very high-grade, good value, to buy friends can order Oh!	Jingdong prefecture level city in Shandong
Happy Fan Shop	King of Power Saving	Not using much electricity in the winter saving over 100 per month! Ready to buy 2 more units in the summer ！！！！！ Good quality, good price, power saving effect is obvious, cost-effective, worth buying, can be recommended!	Jingdong prefecture level city in Shandong
Happy Fan Shop	King of Power Saving	Purchased a power saver received on the put into use, rent a room with many electrical appliances, power consumption is very large, we have a piece of electricity or 1.2 dollars a time, with this power saver is much better, every day can save a lot of money, very convenient and practical.	Jingdong prefecture level city in Shandong

Happy Fan Shop	King of Power Saving	The power saving effect is very good: my supermarket monthly electricity bill is more than 7000 yuan. On May 1st someone recommended buying a couple of power savers, and I bought 5 of them first. The electricity bill was about 2000 less in June. This time, I bought 5 more. The seller said 800 degrees of electricity with one. I feel it's basically enough. A total of 10 units	Jingdong prefecture level city in Shandong
Happy Fan Shop	King of Power Saving	I never thought I could get such a good baby at such an affordable price, it's very good value for money, I love it!	Jingdong prefecture level city in Shandong
Happy Fan Shop	King of Power Saving	Previously bought other brands of power savers, but found no effect at home, this time to see how the products on the Jingdong, inserted in the home socket, the LED display is blue, the night light is very nice to see, look at the instructions that have to be half a year in order to achieve the best results, try it, I believe that the quality of the Jingdong, it should not be wrong.	Jingdong prefecture level city in Shandong
Happy Fan Shop	King of Power Saving	Superb, inexpensive, saves a lot of points, been using it for a long time!	Jingdong prefecture level city in Shandong
Happy Fan Shop	King of Power Saving	There are a lot of appliances in the house, the summer air conditioning is very costly, I bought this in front of the air conditioning effect is very good, I explain that I bought three in one breath, I use more electricity a month down to 200 or so, very satisfied.	Jingdong prefecture level city in Shandong
Happy Fan Shop	King of Power Saving	A month can save more than 200, home monthly electricity bill of more than 600 dollars, inserted 3 or so, an amount of the balance of the average can save more than 200. Product is very good, has been buying this one, has given a lot of relatives recommended, the quality is also very good, before buying a look with this same, who knows to buy home has not been used for more than a week on the bad, the quality of this is really good with more than two years!	Jingdong prefecture level city in Shandong
Happy Fan Shop	King of Power Saving	Whole house power saving king, can really save electricity, friends recommended, high power saving appliances, only replacement, no repair, after-sales worry-free!	Jingdong prefecture level city in Shandong
Happy Fan Shop	King of Power Saving	Great item love it, great customer service, great shopping experience!	Jingdong prefecture level city in Shandong
Happy Fan Shop	King of Power Saving	Power saving, very good use, much cheaper than the physical store, satisfaction	Jingdong prefecture level city in Shandong
Happy Fan Shop	King of Power Saving	In use, very well packaged and fast delivery of credit!	Jingdong prefecture level city in Shandong
Happy Fan Shop	King of Power Saving	Product quality, customer service attitude is particularly good, like, praise!	Jingdong prefecture level city in Shandong
Happy Fan Shop	King of Power Saving	The logistics is very fast, the packaging is not broken, the quality is very good, I like it very much!	Jingdong prefecture level city in Shandong
Happy Fan Shop	King of Power Saving	The saver was well packaged and was as described when plugged in	Jingdong prefecture level city in Shandong
Happy Fan Shop	King of Power Saving	Energy efficient and very satisfied	Jingdong prefecture level city in Shandong
Happy Fan Shop	King of Power Saving	Great quality for the price and fast shipping.	Jingdong prefecture level city in Shandong
Happy Fan Shop	King of Power Saving	It's great, just as described. Perfect.	Jingdong prefecture level city in Shandong
Happy Fan Shop	King of Power Saving	There is no power saving effect, but rather ring up an increase in electricity costs, because this machine is on continuously all day long, uninterruptedly, its own electricity and increased costs, plus the cost of purchasing the machine, so I feel that the use of this machine instead of increasing the	Jingdong prefecture level city in Shandong
Happy Fan Shop	King of Power Saving	Recommended by the owner of the store next door, looked at his home does have to save electricity, decided to get. Indeed quite can, the voltage is much more stable, there is a reduction in electricity	Jingdong prefecture level city in Shandong
Happy Fan Shop	King of Power Saving	Goods have been received value for money, affordable and reasonable price, and the description of the same, power-saving safe and reliable!	Jingdong prefecture level city in Shandong
Happy Fan Shop	King of Power Saving	Listen to friends introduced to buy, tried to save a lot of electricity is good, quite good, worth buying.	Jingdong prefecture level city in Shandong
Happy Fan Shop	King of Power Saving	The product is easy to use, safe and environmentally friendly, only used for a short time, to the next month, after the electricity bill status display to know how the performance of the power saver efficacy.	Jingdong prefecture level city in Shandong
Happy Fan Shop	King of Power Saving	Last month's electricity bill is 178 yuan, with this power saver is 92 yuan, ready to buy two more back home, I was introduced to buy a friend, just started to not believe that I did not expect the effect!	Jingdong prefecture level city in Shandong
Happy Fan Shop	King of Power Saving	It looks nice, but after using it for a few days I can't see how it saves power?	Jingdong prefecture level city in Shandong
Happy Fan Shop	King of Power Saving	Recommended by a friend, it is genuine, as described by the merchant, cost-effective	Jingdong prefecture level city in Shandong
Happy Fan Shop	King of Power Saving	Works well, affordable and saves money.	Jingdong prefecture level city in Shandong
Happy Fan Shop	King of Power Saving	Feels good haven't compared it yet, the air conditioning has been on a lot in the past two months	Jingdong prefecture level city in Shandong
Happy Fan Shop	King of Power Saving	Saving about 5 kWh per day is a good thing	Jingdong prefecture level city in Shandong
Happy Fan Shop	King of Power Saving	Not bad, affordable, best value for money	Jingdong prefecture level city in Shandong
Happy Fan Shop	King of Power Saving	The goods are genuine and the logistics are faster.	Jingdong prefecture level city in Shandong
Happy Fan Shop	King of Power Saving	Goods have been received today, has been plugged into the outlet, looking forward to saving electricity, after a period of time to see how the effect in the follow-up comment!	Jingdong prefecture level city in Shandong
Happy Fan Shop	King of Power Saving	I just got it home and haven't used it for long, so I'll use it for a couple months and see what happens. Seeing that most people have good reviews, it should be fine.	Jingdong prefecture level city in Shandong
Happy Fan Shop	King of Power Saving	I'm looking forward to the results, so I'll give you a good review out of habit! Follow up review after a while	Jingdong prefecture level city in Shandong
Happy Fan Shop	King of Power Saving	Use a dish first to know if it will help save energy	Jingdong prefecture level city in Shandong
Happy Fan Shop	King of Power Saving	Whether it saves electricity or not, we don't know yet.	Jingdong prefecture level city in Shandong
Happy Fan Shop	King of Power Saving	Goods have been received, use a period of time to know how oh. The courier did not deliver to the door ah! Sent to the station ah! The heat is inconvenient ah!	Jingdong prefecture level city in Shandong
Happy Fan Shop	King of Power Saving	A little effect, before an hour a power, now an hour 0.76 degrees, a dollar a power = save 24 cents, although not a lot, first use it!	Jingdong prefecture level city in Shandong
Happy Fan Shop	King of Power Saving	Just received the goods, use a month to evaluate	Jingdong prefecture level city in Shandong
Happy Fan Shop	King of Power Saving	Home every month with a lot of electricity, inadvertently found this good thing online that can save the beginning of a skeptical attitude to consult customer service, customer service replied very professional, said 120 days can be refunded that first try it!	Jingdong prefecture level city in Shandong
Happy Fan Shop	King of Power Saving	After receiving the goods this afternoon, immediately installed in the bathroom Smith energy storage electric water heater on the power outlet (instead of connecting with a plug board), when used, found that the "Power Saving King" LCD display, is the value of the voltage, not the store's video said so	Jingdong prefecture level city in Shandong
Happy Fan Shop	King of Power Saving	Has made many days, did not feel the power saving, the effect is not obvious.	Jingdong prefecture level city in Shandong
Happy Fan Shop	King of Power Saving	Energy savers do not save energy at all, but rather consume a lot of electricity.	Jingdong prefecture level city in Shandong
Happy Fan Shop	King of Power Saving	It's useless. It's a waste of money.	Jingdong prefecture level city in Shandong
Happy Fan Shop	King of Power Saving	Last month saved more than 500, which came back to buy, the store bought three, and then buy 1 home use, my family is dry beauty salon, more instruments, especially electricity, a month almost 1000. or so, with this did not expect to save so much, great, five-star praise!	Jingdong prefecture level city in Shandong
Happy Fan Shop	King of Power Saving	It rained a couple days ago and I just went to get the item back today , not bad, hey I looked at the meter and that red display light takes several seconds to jump, did not make a mistake buying it. After a period of time to evaluate.	Jingdong prefecture level city in Shandong
Happy Fan Shop	King of Power Saving	Without further ado, the electricity bill came down, this month less 102 yuan, has been repurchased, I also recommended to my friends, all said that there are effects, the product is okay, the effect is quite	Jingdong prefecture level city in Shandong

Happy Fan Shop	King of Power Saving	After buying and using two months really see the effect, so only to evaluate! Originally held the mindset of trying, I did not expect really effective, worth having!	Jingdong prefecture level city in Shandong
Happy Fan Shop	King of Power Saving	In the use of this energy saver before the monthly electricity bill is more than 150 yuan or so, now save thirty percent, every day to work, six or seven o'clock in the afternoon to go home, I do not know why the electricity bill is so expensive, energy-saving appliances really have the effect of recommending	Jingdong prefecture level city in Shandong
Happy Fan Shop	King of Power Saving	It saves about 100 bucks a month. A friend recommended it to me, and he's been using it for a while, and the effect is so obvious that I bought one too.	Jingdong prefecture level city in Shandong
Happy Fan Shop	King of Power Saving	My dad asked me to buy one, but after I paid the bill, I asked and he said it was a lot less this month, so he asked me to buy another one.	Jingdong prefecture level city in Shandong
Happy Fan Shop	King of Power Saving	The specific effect will have to be tested for at least two months to compare whether there is a power saving effect or not.	Jingdong prefecture level city in Shandong
Happy Fan Shop	King of Power Saving	'The goods have been received, immediately go to use, please ask the boss you instructions do not allow to withdraw, there are two fuses installed there!	Jingdong prefecture level city in Shandong
Happy Fan Shop	King of Power Saving	First praise is using, after using if good then follow up, now because I have not yet seen the results	Jingdong prefecture level city in Shandong
Happy Fan Shop	King of Power Saving	I've just been using it, and I haven't seen if it has a power-saving effect yet, so I'll follow up after 10 days and half a month.	Jingdong prefecture level city in Shandong
Happy Fan Shop	King of Power Saving	Just received the intelligent power saving meter, immediately plugged in to use, see it is not bad, and order to sell the second time	Jingdong prefecture level city in Shandong
Happy Fan Shop	King of Power Saving	This power saver is a bit effective, used for half a month, the effect is still visible.	Jingdong prefecture level city in Shandong
Happy Fan Shop	King of Power Saving	I received the baby and it looks great, I'm trying it out now and will keep an eye on it afterward.	Jingdong prefecture level city in Shandong
Happy Fan Shop	King of Power Saving	I've just received it, but I'm still trying to see how it works, so I'll come back in a little while.	Jingdong prefecture level city in Shandong
Happy Fan Shop	King of Power Saving	Just used it wait to use it for a while and then comment thanks, all first five stars.	Jingdong prefecture level city in Shandong
Happy Fan Shop	King of Power Saving	Very trustworthy brand, quality is guaranteed, the price is still affordable, quite satisfied!	Jingdong prefecture level city in Shandong
Happy Fan Shop	King of Power Saving	Things received, I hope that the platform of Jingdong do not sell fake goods are available	Jingdong prefecture level city in Shandong
Happy Fan Shop	King of Power Saving	Energy saver received, the use of the effect is good and the business description of the same	Jingdong prefecture level city in Shandong
Happy Fan Shop	King of Power Saving	The surface looks beautiful, the actual effect is not known, after using additional evaluation.	Jingdong prefecture level city in Shandong
Happy Fan Shop	King of Power Saving	Tried it for a few months, it really saves electricity, and I bought it for a friend.	Jingdong prefecture level city in Shandong
Happy Fan Shop	King of Power Saving	It didn't work and wanted to return it, it really didn't work at all.	Jingdong prefecture level city in Shandong
Happy Fan Shop	King of Power Saving	Just used it, don't know how effective it is, looking forward to usefulness	Jingdong prefecture level city in Shandong
Happy Fan Shop	King of Power Saving	After two months before the effect I do not know if it is true!	Jingdong prefecture level city in Shandong
Happy Fan Shop	King of Power Saving	So fast! Ordered yesterday and received today 👍👍👍👍👍👍👍👍👍 🌹🌹🌹🌹🌹🌹🌹🌹🌹	Jingdong prefecture level city in Shandong
Happy Fan Shop	King of Power Saving	I've started using it. We'll see how it works.	Jingdong prefecture level city in Shandong
Happy Fan Shop	King of Power Saving	I just received the goods, I won't know the effect until after two.	Jingdong prefecture level city in Shandong
Happy Fan Shop	King of Power Saving	Just received the goods, the specific effect will be evaluated later	Jingdong prefecture level city in Shandong
Happy Fan Shop	King of Power Saving	I've received the goods, I don't know if it's any good yet	Jingdong prefecture level city in Shandong
Happy Fan Shop	King of Power Saving	Try it for a month and then come back with a concrete evaluation	Jingdong prefecture level city in Shandong
Happy Fan Shop	King of Power Saving	In use, after two months to see the effect of power saving	Jingdong prefecture level city in Shandong
Happy Fan Shop	King of Power Saving	Power Saver. It's authentic. It's black. It works.	Jingdong prefecture level city in Shandong
Happy Fan Shop	King of Power Saving	I've been using it for a week now and it's not working.	Jingdong prefecture level city in Shandong
Happy Fan Shop	King of Power Saving	I'm just using it. I don't know how it's going to work.	Jingdong prefecture level city in Shandong
Happy Fan Shop	King of Power Saving	Products are better looking out for power savings	Jingdong prefecture level city in Shandong
Happy Fan Shop	King of Power Saving	I received it and I've kept it on. I hope it works.	Jingdong prefecture level city in Shandong
Happy Fan Shop	King of Power Saving	It's really good! Worth recommending to buy!	Jingdong prefecture level city in Shandong
Happy Fan Shop	King of Power Saving	Try it for a few days and see if it doesn't work, then return it.	Jingdong prefecture level city in Shandong
Happy Fan Shop	King of Power Saving	It's in use and I hope to be pleasantly surprised.	Jingdong prefecture level city in Shandong
Happy Fan Shop	King of Power Saving	We'll see how it works when we use it	Jingdong prefecture level city in Shandong
Happy Fan Shop	King of Power Saving	Let's see if it works.	Jingdong prefecture level city in Shandong
Happy Fan Shop	King of Power Saving	I'm using it and will follow up with a review	Jingdong prefecture level city in Shandong
Happy Fan Shop	King of Power Saving	It really works. It saves energy.	Jingdong prefecture level city in Shandong
Happy Fan Shop	King of Power Saving	It does have some power savings!	Jingdong prefecture level city in Shandong
Happy Fan Shop	King of Power Saving	Jingdong shopping speed is convenient and timely	Jingdong prefecture level city in Shandong
Happy Fan Shop	King of Power Saving	Second time I've bought it. Everything's fine.	Jingdong prefecture level city in Shandong
Happy Fan Shop	King of Power Saving	I don't know if it works yet.	Jingdong prefecture level city in Shandong
Happy Fan Shop	King of Power Saving	Not used, don't know how well it works.	Jingdong prefecture level city in Shandong
Happy Fan Shop	King of Power Saving	No visible results so far	Jingdong prefecture level city in Shandong
Happy Fan Shop	King of Power Saving	It doesn't feel like it's working.	Jingdong prefecture level city in Shandong
Happy Fan Shop	King of Power Saving	Where's the effect? Where are the power savings?	Jingdong prefecture level city in Shandong
Happy Fan Shop	King of Power Saving	Let's see how it works in practice!	Jingdong prefecture level city in Shandong
Happy Fan Shop	King of Power Saving	I'll wait until I use it to give you a good review	Jingdong prefecture level city in Shandong
Happy Fan Shop	King of Power Saving	I'll follow up after I've used it	Jingdong prefecture level city in Shandong

Happy Fan Shop	King of Power Saving	Well, that's good for saving power.	Jingdong prefecture level city in Shandong
Happy Fan Shop	King of Power Saving	Received. Used in the know.	Jingdong prefecture level city in Shandong
Happy Fan Shop	King of Power Saving	It doesn't do much good.	Jingdong prefecture level city in Shandong
Happy Fan Shop	King of Power Saving	It's not working.	Jingdong prefecture level city in Shandong
Happy Fan Shop	King of Power Saving	I don't know. I just used it.	Jingdong prefecture level city in Shandong
Happy Fan Shop	King of Power Saving	Use it and come back to evaluate.	Jingdong prefecture level city in Shandong
Happy Fan Shop	King of Power Saving	Two words: useless.	Jingdong prefecture level city in Shandong
Happy Fan Shop	King of Power Saving	To be used and then evaluated	Jingdong prefecture level city in Shandong
Happy Fan Shop	King of Power Saving	No shipment.	Jingdong prefecture level city in Shandong
Happy Fan Shop	King of Power Saving	Fake. Speechless.	Jingdong prefecture level city in Shandong
Happy Fan Shop	King of Power Saving	fantastic (idiom); awesome	Jingdong prefecture level city in Shandong
Happy Fan Shop	King of Power Saving	an excellent	Jingdong prefecture level city in Shandong
Happy Fan Shop	King of Power Saving	an excellent	Jingdong prefecture level city in Shandong
Happy Fan Shop	King of Power Saving	Want to buy! Recommended not to buy! No effect	Jingdong prefecture level city in Shandong
Happy Fan Shop	King of Power Saving	That's all there is to it. I can't figure out how to save electricity.	Jingdong prefecture level city in Shandong
Happy Fan Shop	King of Power Saving	It's no use.	Jingdong prefecture level city in Shandong
Happy Fan Shop	King of Power Saving	Things are more perfect than I imagined, very powerful, it is worth getting Packaging is very strong, things are well protected, it is worth the praise things are very satisfied Oh, beautiful and stylish atmosphere, customer service is super patient, service is meticulous and attentive, the big brand is not	Jingdong prefecture level city in Shandong
Happy Fan Shop	King of Power Saving	The effect is too good, the family has now several units, back to buy a number of times, the power saver received, power-saving effect is very good,, fine workmanship, long warranty, but also purify the air, before buying a used for a period of time, with the okay, and then bought a, and only now come to the	Jingdong prefecture level city in Shandong
Happy Fan Shop	King of Power Saving	Quite good, and imagine the same, casual ride are very good, satisfied! Baby received it 👌, really great ah! 🌈 quality is really nothing to say, very satisfied! 🆗 happy happy 😃 genuine, great, the price is very good, in short, quite good, will buy again a very good shopping experience, attentive service, fast logistics, distribution is also thoughtful, the attitude is also very good, value for money, ah,	Jingdong prefecture level city in Shandong
Happy Fan Shop	King of Power Saving	Power-saving effect: strong Cost-effective: quite good Appearance style: beautiful Workmanship material: meticulous Experience: looking forward to power-saving effect	Jingdong prefecture level city in Shandong
Happy Fan Shop	King of Power Saving	Cost-effective is okay, the appearance of the style of meticulous workmanship, looking forward to energy-saving effect.	Jingdong prefecture level city in Shandong
Happy Fan Shop	King of Power Saving	Very Good Very Power Saving This Power Saver is really power saving As soon as the power is turned on, the Power Saver starts the power saving function for you.	Jingdong prefecture level city in Shandong
Happy Fan Shop	King of Power Saving	Logistics is very fast, the price is favorable, friends introduced that it is very good to use, bought 2 at once, looking forward to the effect, used after the follow-up comment.	Jingdong prefecture level city in Shandong
Happy Fan Shop	King of Power Saving	It's all fake. It doesn't save any electricity.	Jingdong prefecture level city in Shandong
Happy Fan Shop	King of Power Saving	This Power Saver is so much better than the one I bought before, not only the effect is good, the quality is very good in all aspects, the customer service attitude is also very good, recommended to buy!	Jingdong prefecture level city in Shandong
Happy Fan Shop	King of Power Saving	Logistics is very fast, buy for parents, good quality, once bought 2, power-saving effect is good to buy 2 more, look forward to using the effect, satisfied will recommend to friends.	Jingdong prefecture level city in Shandong
Happy Fan Shop	King of Power Saving	With the description is the same, very high-grade, good value, to buy friends can order Oh!	Jingdong prefecture level city in Shandong
Happy Fan Shop	King of Power Saving	Not using much electricity in the winter saving over 100 per month! Ready to buy 2 more units in the summer ! ! ! ! ! Good quality, good price, power saving effect is obvious, cost-effective, worth buying, can be recommended!	Jingdong prefecture level city in Shandong
Happy Fan Shop	King of Power Saving	Purchased a power saver received on the put into use, rent a room with many electrical appliances, power consumption is very large, we have a piece of electricity or 1.2 dollars a time, with this power saver is much better, every day can save a lot of money, very convenient and practical.	Jingdong prefecture level city in Shandong
Happy Fan Shop	King of Power Saving	The power saving effect is very good: my supermarket monthly electricity bill is more than 7000 yuan. On May 1st someone recommended buying a couple of power savers, and I bought 5 of them first. The electricity bill was about 2000 less in June. This time, I bought 5 more. The seller said 800 degrees of electricity with one. I feel it's basically enough. A total of 10 units	Jingdong prefecture level city in Shandong
Happy Fan Shop	King of Power Saving	I never thought I could get such a good baby at such an affordable price, it's very good value for money, I love it!	Jingdong prefecture level city in Shandong
Happy Fan Shop	King of Power Saving	Previously bought other brands of power savers, but found no effect at home, this time to see how the products on the Jingdong, inserted in the home socket, the LED display is blue, the night light is very nice to see, look at the instructions that have to be half a year in order to achieve the best results, try it, I believe that the quality of the Jingdong, it should not be wrong.	Jingdong prefecture level city in Shandong
Happy Fan Shop	King of Power Saving	Superb, inexpensive, saves a lot of points, been using it for a long time!	Jingdong prefecture level city in Shandong
Happy Fan Shop	King of Power Saving	There are a lot of appliances in the house, the summer air conditioning is very costly, I bought this in front of the air conditioning effect is very good, I explain that I bought three in one breath, I use more electricity a month down to 200 or so, very satisfied.	Jingdong prefecture level city in Shandong
Happy Fan Shop	King of Power Saving	A month can save more than 200, home monthly electricity bill of more than 600 dollars, inserted 3 or so, an amount of the balance of the average can save more than 200. Product is very good, has been buying this one, has given a lot of relatives recommended, the quality is also very good, before buying a look with this same, who knows to buy home has not been used for more than a week on the bad, the quality of this is really good with more than two years!	Jingdong prefecture level city in Shandong
Happy Fan Shop	King of Power Saving	Whole house power saving king, can really save electricity, friends recommended, high power saving appliances, only replacement, no repair, after-sales worry-free!	Jingdong prefecture level city in Shandong
Happy Fan Shop	King of Power Saving	Great item love it, great customer service, great shopping experience!	Jingdong prefecture level city in Shandong
Happy Fan Shop	King of Power Saving	Power saving, very good use, much cheaper than the physical store, satisfaction	Jingdong prefecture level city in Shandong
Happy Fan Shop	King of Power Saving	In use, very well packaged and fast delivery of credit!	Jingdong prefecture level city in Shandong
Happy Fan Shop	King of Power Saving	Product quality, customer service attitude is particularly good, like, praise!	Jingdong prefecture level city in Shandong
Happy Fan Shop	King of Power Saving	The logistics is very fast, the packaging is not broken, the quality is very good, I like it very much!	Jingdong prefecture level city in Shandong
Happy Fan Shop	King of Power Saving	The saver was well packaged and was as described when plugged in	Jingdong prefecture level city in Shandong
Happy Fan Shop	King of Power Saving	Energy efficient and very satisfied	Jingdong prefecture level city in Shandong
Happy Fan Shop	King of Power Saving	Great quality for the price and fast shipping.	Jingdong prefecture level city in Shandong

Happy Fan Shop	King of Power Saving	It's great, just as described. Perfect.	Jingdong prefecture level city in Shandong
Happy Fan Shop	King of Power Saving	There is no power saving effect, but rather ring up an increase in electricity costs, because this machine is on continuously all day long, uninterruptedly, its own electricity and increased costs, plus the cost of purchasing the machine, so I feel that the use of this machine instead of increasing the	Jingdong prefecture level city in Shandong
Happy Fan Shop	King of Power Saving	Recommended by the owner of the store next door, looked at his home does have to save electricity, decided to get. Indeed quite can, the voltage is much more stable, there is a reduction in electricity	Jingdong prefecture level city in Shandong
Happy Fan Shop	King of Power Saving	Goods have been received value for money, affordable and reasonable price, and the description of the same, power-saving safe and reliable!	Jingdong prefecture level city in Shandong
Happy Fan Shop	King of Power Saving	Listen to friends introduced to buy, tried to save a lot of electricity is good, quite good, worth buying.	Jingdong prefecture level city in Shandong
Happy Fan Shop	King of Power Saving	The product is easy to use, safe and environmentally friendly, only used for a short time, to the next month, after the electricity bill status display to know how the performance of the power saver efficacy.	Jingdong prefecture level city in Shandong
Happy Fan Shop	King of Power Saving	Last month's electricity bill is 178 yuan, with this power saver is 92 yuan, ready to buy two more back home, I was introduced to buy a friend, just started to not believe that I did not expect the effect!	Jingdong prefecture level city in Shandong
Happy Fan Shop	King of Power Saving	It looks nice, but after using it for a few days I can't see how it saves power?	Jingdong prefecture level city in Shandong
Happy Fan Shop	King of Power Saving	Recommended by a friend, it is genuine, as described by the merchant, cost-effective	Jingdong prefecture level city in Shandong
Happy Fan Shop	King of Power Saving	Works well, affordable and saves money.	Jingdong prefecture level city in Shandong
Happy Fan Shop	King of Power Saving	Feels good haven't compared it yet, the air conditioning has been on a lot in the past two months	Jingdong prefecture level city in Shandong
Happy Fan Shop	King of Power Saving	Saving about 5 kWh per day is a good thing	Jingdong prefecture level city in Shandong
Happy Fan Shop	King of Power Saving	Not bad, affordable, best value for money	Jingdong prefecture level city in Shandong
Happy Fan Shop	King of Power Saving	The goods are genuine and the logistics are faster.	Jingdong prefecture level city in Shandong
Happy Fan Shop	King of Power Saving	Goods have been received today, has been plugged into the outlet, looking forward to saving electricity, after a period of time to see how the effect in the follow-up comment!	Jingdong prefecture level city in Shandong
Happy Fan Shop	King of Power Saving	I just got it home and haven't used it for long, so I'll use it for a couple months and see what happens. Seeing that most people have good reviews, it should be fine.	Jingdong prefecture level city in Shandong
Happy Fan Shop	King of Power Saving	I'm looking forward to the results, so I'll give you a good review out of habit! Follow up review after a while	Jingdong prefecture level city in Shandong
Happy Fan Shop	King of Power Saving	Use a dish first to know if it will help save energy	Jingdong prefecture level city in Shandong
Happy Fan Shop	King of Power Saving	Whether it saves electricity or not, we don't know yet.	Jingdong prefecture level city in Shandong
Happy Fan Shop	King of Power Saving	Goods have been received, use a period of time to know how oh. The courier did not deliver to the door ah! Sent to the station ah! The heat is inconvenient ah!	Jingdong prefecture level city in Shandong
Happy Fan Shop	King of Power Saving	A little effect, before an hour a power, now an hour 0.76 degrees, a dollar a power = save 24 cents, although not a lot, first use it!	Jingdong prefecture level city in Shandong
Happy Fan Shop	King of Power Saving	Just received the goods, use a month to evaluate	Jingdong prefecture level city in Shandong
Happy Fan Shop	King of Power Saving	Home every month with a lot of electricity, inadvertently found this good thing online that can save the beginning of a skeptical attitude to consult customer service, customer service replied very professional, said 120 days can be refunded that first try it!	Jingdong prefecture level city in Shandong
Happy Fan Shop	King of Power Saving	After receiving the goods this afternoon, immediately installed in the bathroom Smith energy storage electric water heater on the power outlet (instead of connecting with a plug board), when used, found that the "Power Saving King" LCD display, is the value of the voltage, not the store's video said so	Jingdong prefecture level city in Shandong
Happy Fan Shop	King of Power Saving	Has made many days, did not feel the power saving, the effect is not obvious.	Jingdong prefecture level city in Shandong
Happy Fan Shop	King of Power Saving	Energy savers do not save energy at all, but rather consume a lot of electricity.	Jingdong prefecture level city in Shandong
Happy Fan Shop	King of Power Saving	It's useless. It's a waste of money.	Jingdong prefecture level city in Shandong
Happy Fan Shop	King of Power Saving	Last month saved more than 500, which came back to buy, the store bought three, and then buy 1 home use, my family is dry beauty salon, more instruments, especially electricity, a month almost 1000. or so, with this did not expect to save so much, great, five-star praise!	Jingdong prefecture level city in Shandong
Happy Fan Shop	King of Power Saving	It rained a couple days ago and I just went to get the item back today , not bad, hey I looked at the meter and that red display light takes several seconds to jump, did not make a mistake buying it. After a period of time to evaluate.	Jingdong prefecture level city in Shandong
Happy Fan Shop	King of Power Saving	Without further ado, the electricity bill came down, this month less 102 yuan, has been repurchased, I also recommended to my friends, all said that there are effects, the product is okay, the effect is quite	Jingdong prefecture level city in Shandong
Happy Fan Shop	King of Power Saving	After buying and using two months really see the effect, so only to evaluate! Originally held the mindset of trying, I did not expect really effective, worth having!	Jingdong prefecture level city in Shandong
Happy Fan Shop	King of Power Saving	In the use of this energy saver before the monthly electricity bill is more than 150 yuan or so, now save thirty percent, every day to work, six or seven o'clock in the afternoon to go home, I do not know why the electricity bill is so expensive, energy-saving appliances really have the effect of recommending	Jingdong prefecture level city in Shandong
Happy Fan Shop	King of Power Saving	It saves about 100 bucks a month. A friend recommended it to me, and he's been using it for a while, and the effect is so obvious that I bought one too.	Jingdong prefecture level city in Shandong
Happy Fan Shop	King of Power Saving	My dad asked me to buy one, but after I paid the bill, I asked and he said it was a lot less this month, so he asked me to buy another one.	Jingdong prefecture level city in Shandong
Happy Fan Shop	King of Power Saving	The specific effect will have to be tested for at least two months to compare whether there is a power saving effect or not.	Jingdong prefecture level city in Shandong
Happy Fan Shop	King of Power Saving	'The goods have been received, immediately go to use, please ask the boss you instructions do not allow to withdraw, there are two fuses installed there!	Jingdong prefecture level city in Shandong
Happy Fan Shop	King of Power Saving	First praise is using, after using if good then follow up, now because I have not yet seen the results	Jingdong prefecture level city in Shandong
Happy Fan Shop	King of Power Saving	I've just been using it, and I haven't seen if it has a power-saving effect yet, so I'll follow up after 10 days and half a month.	Jingdong prefecture level city in Shandong
Happy Fan Shop	King of Power Saving	Just received the intelligent power saving meter, immediately plugged in to use, see it is not bad, and order to sell the second time	Jingdong prefecture level city in Shandong
Happy Fan Shop	King of Power Saving	This power saver is a bit effective, used for half a month, the effect is still visible.	Jingdong prefecture level city in Shandong
Happy Fan Shop	King of Power Saving	I received the baby and it looks great, I'm trying it out now and will keep an eye on it afterward.	Jingdong prefecture level city in Shandong
Happy Fan Shop	King of Power Saving	I've just received it, but I'm still trying to see how it works, so I'll come back in a little while.	Jingdong prefecture level city in Shandong
Happy Fan Shop	King of Power Saving	Just used it wait to use it for a while and then comment thanks, all first five stars.	Jingdong prefecture level city in Shandong
Happy Fan Shop	King of Power Saving	Very trustworthy brand, quality is guaranteed, the price is still affordable, quite satisfied!	Jingdong prefecture level city in Shandong
Happy Fan Shop	King of Power Saving	Things received, I hope that the platform of Jingdong do not sell fake goods are available	Jingdong prefecture level city in Shandong
Happy Fan Shop	King of Power Saving	Energy saver received, the use of the effect is good and the business description of the same	Jingdong prefecture level city in Shandong
Happy Fan Shop	King of Power Saving	The surface looks beautiful, the actual effect is not known, after using additional evaluation.	Jingdong prefecture level city in Shandong
Happy Fan Shop	King of Power Saving	Tried it for a few months, it really saves electricity, and I bought it for a friend.	Jingdong prefecture level city in Shandong

Happy Fan Shop	King of Power Saving	It didn't work and wanted to return it, it really didn't work at all.	Jingdong prefecture level city in Shandong
Happy Fan Shop	King of Power Saving	Just used it, don't know how effective it is, looking forward to usefulness	Jingdong prefecture level city in Shandong
Happy Fan Shop	King of Power Saving	After two months before the effect I do not know if it is true!	Jingdong prefecture level city in Shandong
Happy Fan Shop	King of Power Saving	So fast! Ordered yesterday and received today 👍👍👍👍👍👍👍👍👍 🌹🌹🌹🌹🌹🌹🌹🌹🌹	Jingdong prefecture level city in Shandong
Happy Fan Shop	King of Power Saving	I've started using it. We'll see how it works.	Jingdong prefecture level city in Shandong
Happy Fan Shop	King of Power Saving	I just received the goods, I won't know the effect until after two.	Jingdong prefecture level city in Shandong
Happy Fan Shop	King of Power Saving	Just received the goods, the specific effect will be evaluated later	Jingdong prefecture level city in Shandong
Happy Fan Shop	King of Power Saving	I've received the goods, I don't know if it's any good yet	Jingdong prefecture level city in Shandong
Happy Fan Shop	King of Power Saving	Try it for a month and then come back with a concrete evaluation	Jingdong prefecture level city in Shandong
Happy Fan Shop	King of Power Saving	In use, after two months to see the effect of power saving	Jingdong prefecture level city in Shandong
Happy Fan Shop	King of Power Saving	Power Saver. It's authentic. It's black. It works.	Jingdong prefecture level city in Shandong
Happy Fan Shop	King of Power Saving	I've been using it for a week now and it's not working.	Jingdong prefecture level city in Shandong
Happy Fan Shop	King of Power Saving	I'm just using it. I don't know how it's going to work.	Jingdong prefecture level city in Shandong
Happy Fan Shop	King of Power Saving	Products are better looking out for power savings	Jingdong prefecture level city in Shandong
Happy Fan Shop	King of Power Saving	I received it and I've kept it on. I hope it works.	Jingdong prefecture level city in Shandong
Happy Fan Shop	King of Power Saving	It's really good! Worth recommending to buy!	Jingdong prefecture level city in Shandong
Happy Fan Shop	King of Power Saving	Try it for a few days and see if it doesn't work, then return it.	Jingdong prefecture level city in Shandong
Happy Fan Shop	King of Power Saving	It's in use and I hope to be pleasantly surprised.	Jingdong prefecture level city in Shandong
Happy Fan Shop	King of Power Saving	We'll see how it works when we use it	Jingdong prefecture level city in Shandong
Happy Fan Shop	King of Power Saving	Let's see if it works.	Jingdong prefecture level city in Shandong
Happy Fan Shop	King of Power Saving	I'm using it and will follow up with a review	Jingdong prefecture level city in Shandong
Happy Fan Shop	King of Power Saving	It really works. It saves energy.	Jingdong prefecture level city in Shandong
Happy Fan Shop	King of Power Saving	It does have some power savings!	Jingdong prefecture level city in Shandong
Happy Fan Shop	King of Power Saving	Jingdong shopping speed is convenient and timely	Jingdong prefecture level city in Shandong
Happy Fan Shop	King of Power Saving	Second time I've bought it. Everything's fine.	Jingdong prefecture level city in Shandong
Happy Fan Shop	King of Power Saving	I don't know if it works yet.	Jingdong prefecture level city in Shandong
Happy Fan Shop	King of Power Saving	Not used, don't know how well it works.	Jingdong prefecture level city in Shandong
Happy Fan Shop	King of Power Saving	No visible results so far	Jingdong prefecture level city in Shandong
Happy Fan Shop	King of Power Saving	It doesn't feel like it's working.	Jingdong prefecture level city in Shandong
Happy Fan Shop	King of Power Saving	Where's the effect? Where are the power savings?	Jingdong prefecture level city in Shandong
Happy Fan Shop	King of Power Saving	Let's see how it works in practice!	Jingdong prefecture level city in Shandong
Happy Fan Shop	King of Power Saving	I'll wait until I use it to give you a good review	Jingdong prefecture level city in Shandong
Happy Fan Shop	King of Power Saving	I'll follow up after I've used it	Jingdong prefecture level city in Shandong
Happy Fan Shop	King of Power Saving	Well, that's good for saving power.	Jingdong prefecture level city in Shandong
Happy Fan Shop	King of Power Saving	Received. Used in the know.	Jingdong prefecture level city in Shandong
Happy Fan Shop	King of Power Saving	It doesn't do much good.	Jingdong prefecture level city in Shandong
Happy Fan Shop	King of Power Saving	It's not working.	Jingdong prefecture level city in Shandong
Happy Fan Shop	King of Power Saving	I don't know. I just used it.	Jingdong prefecture level city in Shandong
Happy Fan Shop	King of Power Saving	Use it and come back to evaluate.	Jingdong prefecture level city in Shandong
Happy Fan Shop	King of Power Saving	Two words: useless.	Jingdong prefecture level city in Shandong
Happy Fan Shop	King of Power Saving	To be used and then evaluated	Jingdong prefecture level city in Shandong
Happy Fan Shop	King of Power Saving	No shipment.	Jingdong prefecture level city in Shandong
Happy Fan Shop	King of Power Saving	Fake. Speechless.	Jingdong prefecture level city in Shandong
Happy Fan Shop	King of Power Saving	fantastic (idiom); awesome	Jingdong prefecture level city in Shandong
Happy Fan Shop	King of Power Saving	an excellent	Jingdong prefecture level city in Shandong
Happy Fan Shop	King of Power Saving	an excellent	Jingdong prefecture level city in Shandong
Happy Fan Shop	King of Power Saving	Want to buy! Recommended not to buy! No effect	Jingdong prefecture level city in Shandong
Happy Fan Shop	King of Power Saving	That's all there is to it. I can't figure out how to save electricity.	Jingdong prefecture level city in Shandong
Happy Fan Shop	King of Power Saving	It's no use.	Jingdong prefecture level city in Shandong
Happy Fan Shop	King of Power Saving	Things are more perfect than I imagined, very powerful, it is worth getting Packaging is very strong, things are well protected, it is worth the praise things are very satisfied Oh, beautiful and stylish atmosphere, customer service is super patient, service is meticulous and attentive, the big brand is not	Jingdong prefecture level city in Shandong

Happy Fan Shop	King of Power Saving	The effect is too good, the family has now several units, back to buy a number of times, the power saver received, power-saving effect is very good,, fine workmanship, long warranty, but also purify the air, before buying a used for a period of time, with the okay, and then bought a, and only now come to the	Jingdong prefecture level city in Shandong
Happy Fan Shop	King of Power Saving	Quite good, and imagine the same, casual ride are very good, satisfied! Baby received it 👌, really great ah! 🌈 quality is really nothing to say, very satisfied! 🆗 happy happy 😃 genuine, great, the price is very good, in short, quite good, will buy again a very good shopping experience, attentive service, fast logistics, distribution is also thoughtful, the attitude is also very good, value for money, ah,	Jingdong prefecture level city in Shandong
Happy Fan Shop	King of Power Saving	Power-saving effect: strong Cost-effective: quite good Appearance style: beautiful Workmanship material: meticulous Experience: looking forward to power-saving effect	Jingdong prefecture level city in Shandong
Happy Fan Shop	King of Power Saving	Cost-effective is okay, the appearance of the style of meticulous workmanship, looking forward to energy-saving effect.	Jingdong prefecture level city in Shandong
Happy Fan Shop	King of Power Saving	Very Good Very Power Saving This Power Saver is really power saving As soon as the power is turned on, the Power Saver starts the power saving function for you.	Jingdong prefecture level city in Shandong
Happy Fan Shop	King of Power Saving	Logistics is very fast, the price is favorable, friends introduced that it is very good to use, bought 2 at once, looking forward to the effect, used after the follow-up comment.	Jingdong prefecture level city in Shandong
Happy Fan Shop	King of Power Saving	It's all fake. It doesn't save any electricity.	Jingdong prefecture level city in Shandong
Happy Fan Shop	King of Power Saving	This Power Saver is so much better than the one I bought before, not only the effect is good, the quality is very good in all aspects, the customer service attitude is also very good, recommended to buy!	Jingdong prefecture level city in Shandong
Happy Fan Shop	King of Power Saving	Logistics is very fast, buy for parents, good quality, once bought 2, power-saving effect is good to buy 2 more, look forward to using the effect, satisfied will recommend to friends.	Jingdong prefecture level city in Shandong
Happy Fan Shop	King of Power Saving	With the description is the same, very high-grade, good value, to buy friends can order Oh!	Jingdong prefecture level city in Shandong
Happy Fan Shop	King of Power Saving	Not using much electricity in the winter saving over 100 per month! Ready to buy 2 more units in the summer ! ! ! ! ! Good quality, good price, power saving effect is obvious, cost-effective, worth buying, can be recommended!	Jingdong prefecture level city in Shandong
Happy Fan Shop	King of Power Saving	Purchased a power saver received on the put into use, rent a room with many electrical appliances, power consumption is very large, we have a piece of electricity or 1.2 dollars a time, with this power saver is much better, every day can save a lot of money, very convenient and practical.	Jingdong prefecture level city in Shandong
Happy Fan Shop	King of Power Saving	The power saving effect is very good: my supermarket monthly electricity bill is more than 7000 yuan. On May 1st someone recommended buying a couple of power savers, and I bought 5 of them first. The electricity bill was about 2000 less in June. This time, I bought 5 more. The seller said 800 degrees of electricity with one. I feel it's basically enough. A total of 10 units	Jingdong prefecture level city in Shandong
Happy Fan Shop	King of Power Saving	I never thought I could get such a good baby at such an affordable price, it's very good value for money, I love it!	Jingdong prefecture level city in Shandong
Happy Fan Shop	King of Power Saving	Previously bought other brands of power savers, but found no effect at home, this time to see how the products on the Jingdong, inserted in the home socket, the LED display is blue, the night light is very nice to see, look at the instructions that have to be half a year in order to achieve the best results, try it, I believe that the quality of the Jingdong, it should not be wrong.	Jingdong prefecture level city in Shandong
Happy Fan Shop	King of Power Saving	Superb, inexpensive, saves a lot of points, been using it for a long time!	Jingdong prefecture level city in Shandong
Happy Fan Shop	King of Power Saving	There are a lot of appliances in the house, the summer air conditioning is very costly, I bought this in front of the air conditioning effect is very good, I explain that I bought three in one breath, I use more electricity a month down to 200 or so, very satisfied.	Jingdong prefecture level city in Shandong
Happy Fan Shop	King of Power Saving	A month can save more than 200, home monthly electricity bill of more than 600 dollars, inserted 3 or so, an amount of the balance of the average can save more than 200. Product is very good, has been buying this one, has given a lot of relatives recommended, the quality is also very good, before buying a look with this same, who knows to buy home has not been used for more than a week on the bad, the quality of this is really good with more than two years!	Jingdong prefecture level city in Shandong
Happy Fan Shop	King of Power Saving	Whole house power saving king, can really save electricity, friends recommended, high power saving appliances, only replacement, no repair, after-sales worry-free!	Jingdong prefecture level city in Shandong
Happy Fan Shop	King of Power Saving	Great item love it, great customer service, great shopping experience!	Jingdong prefecture level city in Shandong
Happy Fan Shop	King of Power Saving	Power saving, very good use, much cheaper than the physical store, satisfaction	Jingdong prefecture level city in Shandong
Happy Fan Shop	King of Power Saving	In use, very well packaged and fast delivery of credit!	Jingdong prefecture level city in Shandong
Happy Fan Shop	King of Power Saving	Product quality, customer service attitude is particularly good, like, praise!	Jingdong prefecture level city in Shandong
Happy Fan Shop	King of Power Saving	The logistics is very fast, the packaging is not broken, the quality is very good, I like it very much!	Jingdong prefecture level city in Shandong
Happy Fan Shop	King of Power Saving	The saver was well packaged and was as described when plugged in	Jingdong prefecture level city in Shandong
Happy Fan Shop	King of Power Saving	Energy efficient and very satisfied	Jingdong prefecture level city in Shandong
Happy Fan Shop	King of Power Saving	Great quality for the price and fast shipping.	Jingdong prefecture level city in Shandong
Happy Fan Shop	King of Power Saving	It's great, just as described. Perfect.	Jingdong prefecture level city in Shandong
Happy Fan Shop	King of Power Saving	There is no power saving effect, but rather ring up an increase in electricity costs, because this machine is on continuously all day long, uninterruptedly, its own electricity and increased costs, plus the cost of purchasing the machine, so I feel that the use of this machine instead of increasing the	Jingdong prefecture level city in Shandong
Happy Fan Shop	King of Power Saving	Recommended by the owner of the store next door, looked at his home does have to save electricity, decided to get. Indeed quite can, the voltage is much more stable, there is a reduction in electricity	Jingdong prefecture level city in Shandong
Happy Fan Shop	King of Power Saving	Goods have been received value for money, affordable and reasonable price, and the description of the same, power-saving safe and reliable!	Jingdong prefecture level city in Shandong
Happy Fan Shop	King of Power Saving	Listen to friends introduced to buy, tried to save a lot of electricity is good, quite good, worth buying.	Jingdong prefecture level city in Shandong
Happy Fan Shop	King of Power Saving	The product is easy to use, safe and environmentally friendly, only used for a short time, to the next month, after the electricity bill status display to know how the performance of the power saver efficacy.	Jingdong prefecture level city in Shandong
Happy Fan Shop	King of Power Saving	Last month's electricity bill is 178 yuan, with this power saver is 92 yuan, ready to buy two more back home, I was introduced to buy a friend, just started to not believe that I did not expect the effect!	Jingdong prefecture level city in Shandong
Happy Fan Shop	King of Power Saving	It looks nice, but after using it for a few days I can't see how it saves power?	Jingdong prefecture level city in Shandong
Happy Fan Shop	King of Power Saving	Recommended by a friend, it is genuine, as described by the merchant, cost-effective	Jingdong prefecture level city in Shandong
Happy Fan Shop	King of Power Saving	Works well, affordable and saves money.	Jingdong prefecture level city in Shandong
Happy Fan Shop	King of Power Saving	Feels good haven't compared it yet, the air conditioning has been on a lot in the past two months	Jingdong prefecture level city in Shandong
Happy Fan Shop	King of Power Saving	Saving about 5 kWh per day is a good thing	Jingdong prefecture level city in Shandong
Happy Fan Shop	King of Power Saving	Not bad, affordable, best value for money	Jingdong prefecture level city in Shandong
Happy Fan Shop	King of Power Saving	The goods are genuine and the logistics are faster.	Jingdong prefecture level city in Shandong
Happy Fan Shop	King of Power Saving	Goods have been received today, has been plugged into the outlet, looking forward to saving electricity, after a period of time to see how the effect in the follow-up comment!	Jingdong prefecture level city in Shandong
Happy Fan Shop	King of Power Saving	I just got it home and haven't used it for long, so I'll use it for a couple months and see what happens. Seeing that most people have good reviews, it should be fine.	Jingdong prefecture level city in Shandong
Happy Fan Shop	King of Power Saving	I'm looking forward to the results, so I'll give you a good review out of habit! Follow up review after a while	Jingdong prefecture level city in Shandong

Happy Fan Shop	King of Power Saving	Use a dish first to know if it will help save energy	Jingdong prefecture level city in Shandong
Happy Fan Shop	King of Power Saving	Whether it saves electricity or not, we don't know yet.	Jingdong prefecture level city in Shandong
Happy Fan Shop	King of Power Saving	Goods have been received, use a period of time to know how oh. The courier did not deliver to the door ah! Sent to the station ah! The heat is inconvenient ah!	Jingdong prefecture level city in Shandong
Happy Fan Shop	King of Power Saving	A little effect, before an hour a power, now an hour 0.76 degrees, a dollar a power = save 24 cents, although not a lot, first use it!	Jingdong prefecture level city in Shandong
Happy Fan Shop	King of Power Saving	Just received the goods, use a month to evaluate	Jingdong prefecture level city in Shandong
Happy Fan Shop	King of Power Saving	Home every month with a lot of electricity, inadvertently found this good thing online that can save the beginning of a skeptical attitude to consult customer service, customer service replied very professional, said 120 days can be refunded that first try it!	Jingdong prefecture level city in Shandong
Happy Fan Shop	King of Power Saving	After receiving the goods this afternoon, immediately installed in the bathroom Smith energy storage electric water heater on the power outlet (instead of connecting with a plug board), when used, found that the "Power Saving King" LCD display, is the value of the voltage, not the store's video said so	Jingdong prefecture level city in Shandong
Happy Fan Shop	King of Power Saving	Has made many days, did not feel the power saving, the effect is not obvious.	Jingdong prefecture level city in Shandong
Happy Fan Shop	King of Power Saving	Energy savers do not save energy at all, but rather consume a lot of electricity.	Jingdong prefecture level city in Shandong
Happy Fan Shop	King of Power Saving	It's useless. It's a waste of money.	Jingdong prefecture level city in Shandong
Happy Fan Shop	King of Power Saving	Last month saved more than 500, which came back to buy, the store bought three, and then buy 1 home use, my family is dry beauty salon, more instruments, especially electricity, a month almost 1000. or so, with this did not expect to save so much, great, five-star praise!	Jingdong prefecture level city in Shandong
Happy Fan Shop	King of Power Saving	It rained a couple days ago and I just went to get the item back today , not bad, hey I looked at the meter and that red display light takes several seconds to jump, did not make a mistake buying it. After a period of time to evaluate.	Jingdong prefecture level city in Shandong
Happy Fan Shop	King of Power Saving	Without further ado, the electricity bill came down, this month less 102 yuan, has been repurchased, I also recommended to my friends, all said that there are effects, the product is okay, the effect is quite	Jingdong prefecture level city in Shandong
Happy Fan Shop	King of Power Saving	After buying and using two months really see the effect, so only to evaluate! Originally held the mindset of trying, I did not expect really effective, worth having!	Jingdong prefecture level city in Shandong
Happy Fan Shop	King of Power Saving	In the use of this energy saver before the monthly electricity bill is more than 150 yuan or so, now save thirty percent, every day to work, six or seven o'clock in the afternoon to go home, I do not know why the electricity bill is so expensive, energy-saving appliances really have the effect of recommending	Jingdong prefecture level city in Shandong
Happy Fan Shop	King of Power Saving	It saves about 100 bucks a month. A friend recommended it to me, and he's been using it for a while, and the effect is so obvious that I bought one too.	Jingdong prefecture level city in Shandong
Happy Fan Shop	King of Power Saving	My dad asked me to buy one, but after I paid the bill, I asked and he said it was a lot less this month, so he asked me to buy another one.	Jingdong prefecture level city in Shandong
Happy Fan Shop	King of Power Saving	The specific effect will have to be tested for at least two months to compare whether there is a power saving effect or not.	Jingdong prefecture level city in Shandong
Happy Fan Shop	King of Power Saving	'The goods have been received, immediately go to use, please ask the boss you instructions do not allow to withdraw, there are two fuses installed there!	Jingdong prefecture level city in Shandong
Happy Fan Shop	King of Power Saving	First praise is using, after using if good then follow up, now because I have not yet seen the results	Jingdong prefecture level city in Shandong
Happy Fan Shop	King of Power Saving	I've just been using it, and I haven't seen if it has a power-saving effect yet, so I'll follow up after 10 days and half a month.	Jingdong prefecture level city in Shandong
Happy Fan Shop	King of Power Saving	Just received the intelligent power saving meter, immediately plugged in to use, see it is not bad, and order to sell the second time	Jingdong prefecture level city in Shandong
Happy Fan Shop	King of Power Saving	This power saver is a bit effective, used for half a month, the effect is still visible.	Jingdong prefecture level city in Shandong
Happy Fan Shop	King of Power Saving	I received the baby and it looks great, I'm trying it out now and will keep an eye on it afterward.	Jingdong prefecture level city in Shandong
Happy Fan Shop	King of Power Saving	I've just received it, but I'm still trying to see how it works, so I'll come back in a little while.	Jingdong prefecture level city in Shandong
Happy Fan Shop	King of Power Saving	Just used it wait to use it for a while and then comment thanks, all first five stars.	Jingdong prefecture level city in Shandong
Happy Fan Shop	King of Power Saving	Very trustworthy brand, quality is guaranteed, the price is still affordable, quite satisfied!	Jingdong prefecture level city in Shandong
Happy Fan Shop	King of Power Saving	Things received, I hope that the platform of Jingdong do not sell fake goods are available	Jingdong prefecture level city in Shandong
Happy Fan Shop	King of Power Saving	Energy saver received, the use of the effect is good and the business description of the same	Jingdong prefecture level city in Shandong
Happy Fan Shop	King of Power Saving	The surface looks beautiful, the actual effect is not known, after using additional evaluation.	Jingdong prefecture level city in Shandong
Happy Fan Shop	King of Power Saving	Tried it for a few months, it really saves electricity, and I bought it for a friend.	Jingdong prefecture level city in Shandong
Happy Fan Shop	King of Power Saving	It didn't work and wanted to return it, it really didn't work at all.	Jingdong prefecture level city in Shandong
Happy Fan Shop	King of Power Saving	Just used it, don't know how effective it is, looking forward to usefulness	Jingdong prefecture level city in Shandong
Happy Fan Shop	King of Power Saving	After two months before the effect I do not know if it is true!	Jingdong prefecture level city in Shandong
Happy Fan Shop	King of Power Saving	So fast! Ordered yesterday and received today 👍👍👍👍👍👍👍👍👍 🌹🌹🌹🌹🌹🌹🌹🌹🌹	Jingdong prefecture level city in Shandong
Happy Fan Shop	King of Power Saving	I've started using it. We'll see how it works.	Jingdong prefecture level city in Shandong
Happy Fan Shop	King of Power Saving	I just received the goods, I won't know the effect until after two.	Jingdong prefecture level city in Shandong
Happy Fan Shop	King of Power Saving	Just received the goods, the specific effect will be evaluated later	Jingdong prefecture level city in Shandong
Happy Fan Shop	King of Power Saving	I've received the goods, I don't know if it's any good yet	Jingdong prefecture level city in Shandong
Happy Fan Shop	King of Power Saving	Try it for a month and then come back with a concrete evaluation	Jingdong prefecture level city in Shandong
Happy Fan Shop	King of Power Saving	In use, after two months to see the effect of power saving	Jingdong prefecture level city in Shandong
Happy Fan Shop	King of Power Saving	Power Saver. It's authentic. It's black. It works.	Jingdong prefecture level city in Shandong
Happy Fan Shop	King of Power Saving	I've been using it for a week now and it's not working.	Jingdong prefecture level city in Shandong
Happy Fan Shop	King of Power Saving	I'm just using it. I don't know how it's going to work.	Jingdong prefecture level city in Shandong
Happy Fan Shop	King of Power Saving	Products are better looking out for power savings	Jingdong prefecture level city in Shandong
Happy Fan Shop	King of Power Saving	I received it and I've kept it on. I hope it works.	Jingdong prefecture level city in Shandong
Happy Fan Shop	King of Power Saving	It's really good! Worth recommending to buy!	Jingdong prefecture level city in Shandong
Happy Fan Shop	King of Power Saving	Try it for a few days and see if it doesn't work, then return it.	Jingdong prefecture level city in Shandong
Happy Fan Shop	King of Power Saving	It's in use and I hope to be pleasantly surprised.	Jingdong prefecture level city in Shandong

Happy Fan Shop	King of Power Saving	We'll see how it works when we use it	Jingdong prefecture level city in Shandong
Happy Fan Shop	King of Power Saving	Let's see if it works.	Jingdong prefecture level city in Shandong
Happy Fan Shop	King of Power Saving	I'm using it and will follow up with a review	Jingdong prefecture level city in Shandong
Happy Fan Shop	King of Power Saving	It really works. It saves energy.	Jingdong prefecture level city in Shandong
Happy Fan Shop	King of Power Saving	It does have some power savings!	Jingdong prefecture level city in Shandong
Happy Fan Shop	King of Power Saving	Jingdong shopping speed is convenient and timely	Jingdong prefecture level city in Shandong
Happy Fan Shop	King of Power Saving	Second time I've bought it. Everything's fine.	Jingdong prefecture level city in Shandong
Happy Fan Shop	King of Power Saving	I don't know if it works yet.	Jingdong prefecture level city in Shandong
Happy Fan Shop	King of Power Saving	Not used, don't know how well it works.	Jingdong prefecture level city in Shandong
Happy Fan Shop	King of Power Saving	No visible results so far	Jingdong prefecture level city in Shandong
Happy Fan Shop	King of Power Saving	It doesn't feel like it's working.	Jingdong prefecture level city in Shandong
Happy Fan Shop	King of Power Saving	Where's the effect? Where are the power savings?	Jingdong prefecture level city in Shandong
Happy Fan Shop	King of Power Saving	Let's see how it works in practice!	Jingdong prefecture level city in Shandong
Happy Fan Shop	King of Power Saving	I'll wait until I use it to give you a good review	Jingdong prefecture level city in Shandong
Happy Fan Shop	King of Power Saving	I'll follow up after I've used it	Jingdong prefecture level city in Shandong
Happy Fan Shop	King of Power Saving	Well, that's good for saving power.	Jingdong prefecture level city in Shandong
Happy Fan Shop	King of Power Saving	Received. Used in the know.	Jingdong prefecture level city in Shandong
Happy Fan Shop	King of Power Saving	It doesn't do much good.	Jingdong prefecture level city in Shandong
Happy Fan Shop	King of Power Saving	It's not working.	Jingdong prefecture level city in Shandong
Happy Fan Shop	King of Power Saving	I don't know. I just used it.	Jingdong prefecture level city in Shandong
Happy Fan Shop	King of Power Saving	Use it and come back to evaluate.	Jingdong prefecture level city in Shandong
Happy Fan Shop	King of Power Saving	Two words: useless.	Jingdong prefecture level city in Shandong
Happy Fan Shop	King of Power Saving	To be used and then evaluated	Jingdong prefecture level city in Shandong
Happy Fan Shop	King of Power Saving	No shipment.	Jingdong prefecture level city in Shandong
Happy Fan Shop	King of Power Saving	Fake. Speechless.	Jingdong prefecture level city in Shandong
Happy Fan Shop	King of Power Saving	fantastic (idiom); awesome	Jingdong prefecture level city in Shandong
Happy Fan Shop	King of Power Saving	an excellent	Jingdong prefecture level city in Shandong
Happy Fan Shop	King of Power Saving	an excellent	Jingdong prefecture level city in Shandong
Happy Fan Shop	King of Power Saving	Want to buy! Recommended not to buy! No effect	Jingdong prefecture level city in Shandong
Happy Fan Shop	King of Power Saving	That's all there is to it. I can't figure out how to save electricity.	Jingdong prefecture level city in Shandong
Happy Fan Shop	King of Power Saving	It's no use.	Jingdong prefecture level city in Shandong
Happy Fan Shop	King of Power Saving	Things are more perfect than I imagined, very powerful, it is worth getting Packaging is very strong, things are well protected, it is worth the praise things are very satisfied Oh, beautiful and stylish atmosphere, customer service is super patient, service is meticulous and attentive, the big brand is not	Jingdong prefecture level city in Shandong
Happy Fan Shop	King of Power Saving	The effect is too good, the family has now several units, back to buy a number of times, the power saver received, power-saving effect is very good,, fine workmanship, long warranty, but also purify the air, before buying a used for a period of time, with the okay, and then bought a, and only now come to the	Jingdong prefecture level city in Shandong
Happy Fan Shop	King of Power Saving	Quite good, and imagine the same, casual ride are very good, satisfied! Baby received it 👌, really great ah! 🌈 quality is really nothing to say, very satisfied! 🆗 happy happy 😃 genuine, great, the price is very good, in short, quite good, will buy again a very good shopping experience, attentive service, fast logistics, distribution is also thoughtful, the attitude is also very good, value for money, ah,	Jingdong prefecture level city in Shandong
Happy Fan Shop	King of Power Saving	Power-saving effect: strong Cost-effective: quite good Appearance style: beautiful Workmanship material: meticulous Experience: looking forward to power-saving effect	Jingdong prefecture level city in Shandong
Happy Fan Shop	King of Power Saving	Cost-effective is okay, the appearance of the style of meticulous workmanship, looking forward to energy-saving effect.	Jingdong prefecture level city in Shandong
Happy Fan Shop	King of Power Saving	Very Good Very Power Saving This Power Saver is really power saving As soon as the power is turned on, the Power Saver starts the power saving function for you.	Jingdong prefecture level city in Shandong
Happy Fan Shop	King of Power Saving	Logistics is very fast, the price is favorable, friends introduced that it is very good to use, bought 2 at once, looking forward to the effect, used after the follow-up comment.	Jingdong prefecture level city in Shandong
Happy Fan Shop	King of Power Saving	It's all fake. It doesn't save any electricity.	Jingdong prefecture level city in Shandong
Happy Fan Shop	King of Power Saving	This Power Saver is so much better than the one I bought before, not only the effect is good, the quality is very good in all aspects, the customer service attitude is also very good, recommended to buy!	Jingdong prefecture level city in Shandong
Happy Fan Shop	King of Power Saving	Logistics is very fast, buy for parents, good quality, once bought 2, power-saving effect is good to buy 2 more, look forward to using the effect, satisfied will recommend to friends.	Jingdong prefecture level city in Shandong
Happy Fan Shop	King of Power Saving	With the description is the same, very high-grade, good value, to buy friends can order Oh!	Jingdong prefecture level city in Shandong
Happy Fan Shop	King of Power Saving	Not using much electricity in the winter saving over 100 per month! Ready to buy 2 more units in the summer ！！！！！ Good quality, good price, power saving effect is obvious, cost-effective, worth buying, can be recommended!	Jingdong prefecture level city in Shandong
Happy Fan Shop	King of Power Saving	Purchased a power saver received on the put into use, rent a room with many electrical appliances, power consumption is very large, we have a piece of electricity or 1.2 dollars a time, with this power saver is much better, every day can save a lot of money, very convenient and practical.	Jingdong prefecture level city in Shandong
Happy Fan Shop	King of Power Saving	The power saving effect is very good: my supermarket monthly electricity bill is more than 7000 yuan. On May 1st someone recommended buying a couple of power savers, and I bought 5 of them first. The electricity bill was about 2000 less in June. This time, I bought 5 more. The seller said 800 degrees of electricity with one. I feel it's basically enough. A total of 10 units	Jingdong prefecture level city in Shandong
Happy Fan Shop	King of Power Saving	I never thought I could get such a good baby at such an affordable price, it's very good value for money, I love it!	Jingdong prefecture level city in Shandong

Happy Fan Shop	King of Power Saving	Previously bought other brands of power savers, but found no effect at home, this time to see how the products on the Jingdong, inserted in the home socket, the LED display is blue, the night light is very nice to see, look at the instructions that have to be half a year in order to achieve the best results, try it, I believe that the quality of the Jingdong, it should not be wrong.	Jingdong prefecture level city in Shandong
Happy Fan Shop	King of Power Saving	Superb, inexpensive, saves a lot of points, been using it for a long time!	Jingdong prefecture level city in Shandong
Happy Fan Shop	King of Power Saving	There are a lot of appliances in the house, the summer air conditioning is very costly, I bought this in front of the air conditioning effect is very good, I explain that I bought three in one breath, I use more electricity a month down to 200 or so, very satisfied.	Jingdong prefecture level city in Shandong
Happy Fan Shop	King of Power Saving	A month can save more than 200, home monthly electricity bill of more than 600 dollars, inserted 3 or so, an amount of the balance of the average can save more than 200. Product is very good, has been buying this one, has given a lot of relatives recommended, the quality is also very good, before buying a look with this same, who knows to buy home has not been used for more than a week on the bad, the quality of this is really good with more than two years!	Jingdong prefecture level city in Shandong
Happy Fan Shop	King of Power Saving	Whole house power saving king, can really save electricity, friends recommended, high power saving appliances, only replacement, no repair, after-sales worry-free!	Jingdong prefecture level city in Shandong
Happy Fan Shop	King of Power Saving	Great item love it, great customer service, great shopping experience!	Jingdong prefecture level city in Shandong
Happy Fan Shop	King of Power Saving	Power saving, very good use, much cheaper than the physical store, satisfaction	Jingdong prefecture level city in Shandong
Happy Fan Shop	King of Power Saving	In use, very well packaged and fast delivery of credit!	Jingdong prefecture level city in Shandong
Happy Fan Shop	King of Power Saving	Product quality, customer service attitude is particularly good, like, praise!	Jingdong prefecture level city in Shandong
Happy Fan Shop	King of Power Saving	The logistics is very fast, the packaging is not broken, the quality is very good, I like it very much!	Jingdong prefecture level city in Shandong
Happy Fan Shop	King of Power Saving	The saver was well packaged and was as described when plugged in	Jingdong prefecture level city in Shandong
Happy Fan Shop	King of Power Saving	Energy efficient and very satisfied	Jingdong prefecture level city in Shandong
Happy Fan Shop	King of Power Saving	Great quality for the price and fast shipping.	Jingdong prefecture level city in Shandong
Happy Fan Shop	King of Power Saving	It's great, just as described. Perfect.	Jingdong prefecture level city in Shandong
Happy Fan Shop	King of Power Saving	There is no power saving effect, but rather ring up an increase in electricity costs, because this machine is on continuously all day long, uninterruptedly, its own electricity and increased costs, plus the cost of purchasing the machine, so I feel that the use of this machine instead of increasing the	Jingdong prefecture level city in Shandong
Happy Fan Shop	King of Power Saving	Recommended by the owner of the store next door, looked at his home does have to save electricity, decided to get. Indeed quite can, the voltage is much more stable, there is a reduction in electricity	Jingdong prefecture level city in Shandong
Happy Fan Shop	King of Power Saving	Goods have been received value for money, affordable and reasonable price, and the description of the same, power-saving safe and reliable!	Jingdong prefecture level city in Shandong
Happy Fan Shop	King of Power Saving	Listen to friends introduced to buy, tried to save a lot of electricity is good, quite good, worth buying.	Jingdong prefecture level city in Shandong
Happy Fan Shop	King of Power Saving	The product is easy to use, safe and environmentally friendly, only used for a short time, to the next month, after the electricity bill status display to know how the performance of the power saver efficacy.	Jingdong prefecture level city in Shandong
Happy Fan Shop	King of Power Saving	Last month's electricity bill is 178 yuan, with this power saver is 92 yuan, ready to buy two more back home, I was introduced to buy a friend, just started to not believe that I did not expect the effect!	Jingdong prefecture level city in Shandong
Happy Fan Shop	King of Power Saving	It looks nice, but after using it for a few days I can't see how it saves power?	Jingdong prefecture level city in Shandong
Happy Fan Shop	King of Power Saving	Recommended by a friend, it is genuine, as described by the merchant, cost-effective	Jingdong prefecture level city in Shandong
Happy Fan Shop	King of Power Saving	Works well, affordable and saves money.	Jingdong prefecture level city in Shandong
Happy Fan Shop	King of Power Saving	Feels good haven't compared it yet, the air conditioning has been on a lot in the past two months	Jingdong prefecture level city in Shandong
Happy Fan Shop	King of Power Saving	Saving about 5 kWh per day is a good thing	Jingdong prefecture level city in Shandong
Happy Fan Shop	King of Power Saving	Not bad, affordable, best value for money	Jingdong prefecture level city in Shandong
Happy Fan Shop	King of Power Saving	The goods are genuine and the logistics are faster.	Jingdong prefecture level city in Shandong
Happy Fan Shop	King of Power Saving	Goods have been received today, has been plugged into the outlet, looking forward to saving electricity, after a period of time to see how the effect in the follow-up comment!	Jingdong prefecture level city in Shandong
Happy Fan Shop	King of Power Saving	I just got it home and haven't used it for long, so I'll use it for a couple months and see what happens. Seeing that most people have good reviews, it should be fine.	Jingdong prefecture level city in Shandong
Happy Fan Shop	King of Power Saving	I'm looking forward to the results, so I'll give you a good review out of habit! Follow up review after a while	Jingdong prefecture level city in Shandong
Happy Fan Shop	King of Power Saving	Use a dish first to know if it will help save energy	Jingdong prefecture level city in Shandong
Happy Fan Shop	King of Power Saving	Whether it saves electricity or not, we don't know yet.	Jingdong prefecture level city in Shandong
Happy Fan Shop	King of Power Saving	Goods have been received, use a period of time to know how oh. The courier did not deliver to the door ah! Sent to the station ah! The heat is inconvenient ah!	Jingdong prefecture level city in Shandong
Happy Fan Shop	King of Power Saving	A little effect, before an hour a power, now an hour 0.76 degrees, a dollar a power = save 24 cents, although not a lot, first use it!	Jingdong prefecture level city in Shandong
Happy Fan Shop	King of Power Saving	Just received the goods, use a month to evaluate	Jingdong prefecture level city in Shandong
Happy Fan Shop	King of Power Saving	Home every month with a lot of electricity, inadvertently found this good thing online that can save the beginning of a skeptical attitude to consult customer service, customer service replied very professional, said 120 days can be refunded that first try it!	Jingdong prefecture level city in Shandong
Happy Fan Shop	King of Power Saving	After receiving the goods this afternoon, immediately installed in the bathroom Smith energy storage electric water heater on the power outlet (instead of connecting with a plug board), when used, found that the "Power Saving King" LCD display, is the value of the voltage, not the store's video said so	Jingdong prefecture level city in Shandong
Happy Fan Shop	King of Power Saving	Has made many days, did not feel the power saving, the effect is not obvious.	Jingdong prefecture level city in Shandong
Happy Fan Shop	King of Power Saving	Energy savers do not save energy at all, but rather consume a lot of electricity.	Jingdong prefecture level city in Shandong
Happy Fan Shop	King of Power Saving	It's useless. It's a waste of money.	Jingdong prefecture level city in Shandong
Happy Fan Shop	King of Power Saving	Last month saved more than 500, which came back to buy, the store bought three, and then buy 1 home use, my family is dry beauty salon, more instruments, especially electricity, a month almost 1000. or so, with this did not expect to save so much, great, five-star praise!	Jingdong prefecture level city in Shandong
Happy Fan Shop	King of Power Saving	It rained a couple days ago and I just went to get the item back today , not bad, hey I looked at the meter and that red display light takes several seconds to jump, did not make a mistake buying it. After a period of time to evaluate.	Jingdong prefecture level city in Shandong
Happy Fan Shop	King of Power Saving	Without further ado, the electricity bill came down, this month less 102 yuan, has been repurchased, I also recommended to my friends, all said that there are effects, the product is okay, the effect is quite	Jingdong prefecture level city in Shandong
Happy Fan Shop	King of Power Saving	After buying and using two months really see the effect, so only to evaluate! Originally held the mindset of trying, I did not expect really effective, worth having!	Jingdong prefecture level city in Shandong
Happy Fan Shop	King of Power Saving	In the use of this energy saver before the monthly electricity bill is more than 150 yuan or so, now save thirty percent, every day to work, six or seven o'clock in the afternoon to go home, I do not know why the electricity bill is so expensive, energy-saving appliances really have the effect of recommending	Jingdong prefecture level city in Shandong

Happy Fan Shop	King of Power Saving	It saves about 100 bucks a month. A friend recommended it to me, and he's been using it for a while, and the effect is so obvious that I bought one too.	Jingdong prefecture level city in Shandong
Happy Fan Shop	King of Power Saving	My dad asked me to buy one, but after I paid the bill, I asked and he said it was a lot less this month, so he asked me to buy another one.	Jingdong prefecture level city in Shandong
Happy Fan Shop	King of Power Saving	The specific effect will have to be tested for at least two months to compare whether there is a power saving effect or not.	Jingdong prefecture level city in Shandong
Happy Fan Shop	King of Power Saving	'The goods have been received, immediately go to use, please ask the boss you instructions do not allow to withdraw, there are two fuses installed there!	Jingdong prefecture level city in Shandong
Happy Fan Shop	King of Power Saving	First praise is using, after using if good then follow up, now because I have not yet seen the results	Jingdong prefecture level city in Shandong
Happy Fan Shop	King of Power Saving	I've just been using it, and I haven't seen if it has a power-saving effect yet, so I'll follow up after 10 days and half a month.	Jingdong prefecture level city in Shandong
Happy Fan Shop	King of Power Saving	Just received the intelligent power saving meter, immediately plugged in to use, see it is not bad, and order to sell the second time	Jingdong prefecture level city in Shandong
Happy Fan Shop	King of Power Saving	This power saver is a bit effective, used for half a month, the effect is still visible.	Jingdong prefecture level city in Shandong
Happy Fan Shop	King of Power Saving	I received the baby and it looks great, I'm trying it out now and will keep an eye on it afterward.	Jingdong prefecture level city in Shandong
Happy Fan Shop	King of Power Saving	I've just received it, but I'm still trying to see how it works, so I'll come back in a little while.	Jingdong prefecture level city in Shandong
Happy Fan Shop	King of Power Saving	Just used it wait to use it for a while and then comment thanks, all first five stars.	Jingdong prefecture level city in Shandong
Happy Fan Shop	King of Power Saving	Very trustworthy brand, quality is guaranteed, the price is still affordable, quite satisfied!	Jingdong prefecture level city in Shandong
Happy Fan Shop	King of Power Saving	Things received, I hope that the platform of Jingdong do not sell fake goods are available	Jingdong prefecture level city in Shandong
Happy Fan Shop	King of Power Saving	Energy saver received, the use of the effect is good and the business description of the same	Jingdong prefecture level city in Shandong
Happy Fan Shop	King of Power Saving	The surface looks beautiful, the actual effect is not known, after using additional evaluation.	Jingdong prefecture level city in Shandong
Happy Fan Shop	King of Power Saving	Tried it for a few months, it really saves electricity, and I bought it for a friend.	Jingdong prefecture level city in Shandong
Happy Fan Shop	King of Power Saving	It didn't work and wanted to return it, it really didn't work at all.	Jingdong prefecture level city in Shandong
Happy Fan Shop	King of Power Saving	Just used it, don't know how effective it is, looking forward to usefulness	Jingdong prefecture level city in Shandong
Happy Fan Shop	King of Power Saving	After two months before the effect I do not know if it is true!	Jingdong prefecture level city in Shandong
Happy Fan Shop	King of Power Saving	So fast! Ordered yesterday and received today 👍👍👍👍👍👍👍👍👍 🌹🌹🌹🌹🌹🌹🌹🌹🌹	Jingdong prefecture level city in Shandong
Happy Fan Shop	King of Power Saving	I've started using it. We'll see how it works.	Jingdong prefecture level city in Shandong
Happy Fan Shop	King of Power Saving	I just received the goods, I won't know the effect until after two.	Jingdong prefecture level city in Shandong
Happy Fan Shop	King of Power Saving	Just received the goods, the specific effect will be evaluated later	Jingdong prefecture level city in Shandong
Happy Fan Shop	King of Power Saving	I've received the goods, I don't know if it's any good yet	Jingdong prefecture level city in Shandong
Happy Fan Shop	King of Power Saving	Try it for a month and then come back with a concrete evaluation	Jingdong prefecture level city in Shandong
Happy Fan Shop	King of Power Saving	In use, after two months to see the effect of power saving	Jingdong prefecture level city in Shandong
Happy Fan Shop	King of Power Saving	Power Saver. It's authentic. It's black. It works.	Jingdong prefecture level city in Shandong
Happy Fan Shop	King of Power Saving	I've been using it for a week now and it's not working.	Jingdong prefecture level city in Shandong
Happy Fan Shop	King of Power Saving	I'm just using it. I don't know how it's going to work.	Jingdong prefecture level city in Shandong
Happy Fan Shop	King of Power Saving	Products are better looking out for power savings	Jingdong prefecture level city in Shandong
Happy Fan Shop	King of Power Saving	I received it and I've kept it on. I hope it works.	Jingdong prefecture level city in Shandong
Happy Fan Shop	King of Power Saving	It's really good! Worth recommending to buy!	Jingdong prefecture level city in Shandong
Happy Fan Shop	King of Power Saving	Try it for a few days and see if it doesn't work, then return it.	Jingdong prefecture level city in Shandong
Happy Fan Shop	King of Power Saving	It's in use and I hope to be pleasantly surprised.	Jingdong prefecture level city in Shandong
Happy Fan Shop	King of Power Saving	We'll see how it works when we use it	Jingdong prefecture level city in Shandong
Happy Fan Shop	King of Power Saving	Let's see if it works.	Jingdong prefecture level city in Shandong
Happy Fan Shop	King of Power Saving	I'm using it and will follow up with a review	Jingdong prefecture level city in Shandong
Happy Fan Shop	King of Power Saving	It really works. It saves energy.	Jingdong prefecture level city in Shandong
Happy Fan Shop	King of Power Saving	It does have some power savings!	Jingdong prefecture level city in Shandong
Happy Fan Shop	King of Power Saving	Jingdong shopping speed is convenient and timely	Jingdong prefecture level city in Shandong
Happy Fan Shop	King of Power Saving	Second time I've bought it. Everything's fine.	Jingdong prefecture level city in Shandong
Happy Fan Shop	King of Power Saving	I don't know if it works yet.	Jingdong prefecture level city in Shandong
Happy Fan Shop	King of Power Saving	Not used, don't know how well it works.	Jingdong prefecture level city in Shandong
Happy Fan Shop	King of Power Saving	No visible results so far	Jingdong prefecture level city in Shandong
Happy Fan Shop	King of Power Saving	It doesn't feel like it's working.	Jingdong prefecture level city in Shandong
Happy Fan Shop	King of Power Saving	Where's the effect? Where are the power savings?	Jingdong prefecture level city in Shandong
Happy Fan Shop	King of Power Saving	Let's see how it works in practice!	Jingdong prefecture level city in Shandong
Happy Fan Shop	King of Power Saving	I'll wait until I use it to give you a good review	Jingdong prefecture level city in Shandong
Happy Fan Shop	King of Power Saving	I'll follow up after I've used it	Jingdong prefecture level city in Shandong
Happy Fan Shop	King of Power Saving	Well, that's good for saving power.	Jingdong prefecture level city in Shandong
Happy Fan Shop	King of Power Saving	Received. Used in the know.	Jingdong prefecture level city in Shandong

Happy Fan Shop	King of Power Saving	It doesn't do much good.	Jingdong prefecture level city in Shandong
Happy Fan Shop	King of Power Saving	It's not working.	Jingdong prefecture level city in Shandong
Happy Fan Shop	King of Power Saving	I don't know. I just used it.	Jingdong prefecture level city in Shandong
Happy Fan Shop	King of Power Saving	Use it and come back to evaluate.	Jingdong prefecture level city in Shandong
Happy Fan Shop	King of Power Saving	Two words: useless.	Jingdong prefecture level city in Shandong
Happy Fan Shop	King of Power Saving	To be used and then evaluated	Jingdong prefecture level city in Shandong
Happy Fan Shop	King of Power Saving	No shipment.	Jingdong prefecture level city in Shandong
Happy Fan Shop	King of Power Saving	Fake. Speechless.	Jingdong prefecture level city in Shandong
Happy Fan Shop	King of Power Saving	fantastic (idiom); awesome	Jingdong prefecture level city in Shandong
Happy Fan Shop	King of Power Saving	an excellent	Jingdong prefecture level city in Shandong
Happy Fan Shop	King of Power Saving	an excellent	Jingdong prefecture level city in Shandong
Happy Fan Shop	King of Power Saving	Want to buy! Recommended not to buy! No effect	Jingdong prefecture level city in Shandong
Happy Fan Shop	King of Power Saving	That's all there is to it. I can't figure out how to save electricity.	Jingdong prefecture level city in Shandong
Happy Fan Shop	King of Power Saving	It's no use.	Jingdong prefecture level city in Shandong
Happy Fan Shop	King of Power Saving	Things are more perfect than I imagined, very powerful, it is worth getting Packaging is very strong, things are well protected, it is worth the praise things are very satisfied Oh, beautiful and stylish atmosphere, customer service is super patient, service is meticulous and attentive, the big brand is not	Jingdong prefecture level city in Shandong
Happy Fan Shop	King of Power Saving	The effect is too good, the family has now several units, back to buy a number of times, the power saver received, power-saving effect is very good,, fine workmanship, long warranty, but also purify the air, before buying a used for a period of time, with the okay, and then bought a, and only now come to the	Jingdong prefecture level city in Shandong
Happy Fan Shop	King of Power Saving	Quite good, and imagine the same, casual ride are very good, satisfied! Baby received it 👌, really great ah! 🌈 quality is really nothing to say, very satisfied! 🆗 happy happy 😀 genuine, great, the price is very good, in short, quite good, will buy again a very good shopping experience, attentive service, fast logistics, distribution is also thoughtful, the attitude is also very good, value for money, ah,	Jingdong prefecture level city in Shandong
Happy Fan Shop	King of Power Saving	Power-saving effect: strong Cost-effective: quite good Appearance style: beautiful Workmanship material: meticulous Experience: looking forward to power-saving effect	Jingdong prefecture level city in Shandong
Happy Fan Shop	King of Power Saving	Cost-effective is okay, the appearance of the style of meticulous workmanship, looking forward to energy-saving effect.	Jingdong prefecture level city in Shandong
Happy Fan Shop	King of Power Saving	Very Good Very Power Saving This Power Saver is really power saving As soon as the power is turned on, the Power Saver starts the power saving function for you.	Jingdong prefecture level city in Shandong
Happy Fan Shop	King of Power Saving	Logistics is very fast, the price is favorable, friends introduced that it is very good to use, bought 2 at once, looking forward to the effect, used after the follow-up comment.	Jingdong prefecture level city in Shandong
Happy Fan Shop	King of Power Saving	It's all fake. It doesn't save any electricity.	Jingdong prefecture level city in Shandong
Happy Fan Shop	King of Power Saving	This Power Saver is so much better than the one I bought before, not only the effect is good, the quality is very good in all aspects, the customer service attitude is also very good, recommended to buy!	Jingdong prefecture level city in Shandong
Happy Fan Shop	King of Power Saving	Logistics is very fast, buy for parents, good quality, once bought 2, power-saving effect is good to buy 2 more, look forward to using the effect, satisfied will recommend to friends.	Jingdong prefecture level city in Shandong
Happy Fan Shop	King of Power Saving	With the description is the same, very high-grade, good value, to buy friends can order Oh!	Jingdong prefecture level city in Shandong
Happy Fan Shop	King of Power Saving	Not using much electricity in the winter saving over 100 per month! Ready to buy 2 more units in the summer ！！！！！ Good quality, good price, power saving effect is obvious, cost-effective, worth buying, can be recommended!	Jingdong prefecture level city in Shandong
Happy Fan Shop	King of Power Saving	Purchased a power saver received on the put into use, rent a room with many electrical appliances, power consumption is very large, we have a piece of electricity or 1.2 dollars a time, with this power saver is much better, every day can save a lot of money, very convenient and practical.	Jingdong prefecture level city in Shandong
Happy Fan Shop	King of Power Saving	The power saving effect is very good: my supermarket monthly electricity bill is more than 7000 yuan. On May 1st someone recommended buying a couple of power savers, and I bought 5 of them first. The electricity bill was about 2000 less in June. This time, I bought 5 more. The seller said 800 degrees of electricity with one. I feel it's basically enough. A total of 10 units	Jingdong prefecture level city in Shandong
Happy Fan Shop	King of Power Saving	I never thought I could get such a good baby at such an affordable price, it's very good value for money, I love it!	Jingdong prefecture level city in Shandong
Happy Fan Shop	King of Power Saving	Previously bought other brands of power savers, but found no effect at home, this time to see how the products on the Jingdong, inserted in the home socket, the LED display is blue, the night light is very nice to see, look at the instructions that have to be half a year in order to achieve the best results, try it, I believe that the quality of the Jingdong, it should not be wrong.	Jingdong prefecture level city in Shandong
Happy Fan Shop	King of Power Saving	Superb, inexpensive, saves a lot of points, been using it for a long time!	Jingdong prefecture level city in Shandong
Happy Fan Shop	King of Power Saving	There are a lot of appliances in the house, the summer air conditioning is very costly, I bought this in front of the air conditioning effect is very good, I explain that I bought three in one breath, I use more electricity a month down to 200 or so, very satisfied.	Jingdong prefecture level city in Shandong
Happy Fan Shop	King of Power Saving	A month can save more than 200, home monthly electricity bill of more than 600 dollars, inserted 3 or so, an amount of the balance of the average can save more than 200. Product is very good, has been buying this one, has given a lot of relatives recommended, the quality is also very good, before buying a look with this same, who knows to buy home has not been used for more than a week on the bad, the quality of this is really good with more than two years!	Jingdong prefecture level city in Shandong
Happy Fan Shop	King of Power Saving	Whole house power saving king, can really save electricity, friends recommended, high power saving appliances, only replacement, no repair, after-sales worry-free!	Jingdong prefecture level city in Shandong
Happy Fan Shop	King of Power Saving	Great item love it, great customer service, great shopping experience!	Jingdong prefecture level city in Shandong
Happy Fan Shop	King of Power Saving	Power saving, very good use, much cheaper than the physical store, satisfaction	Jingdong prefecture level city in Shandong
Happy Fan Shop	King of Power Saving	In use, very well packaged and fast delivery of credit!	Jingdong prefecture level city in Shandong
Happy Fan Shop	King of Power Saving	Product quality, customer service attitude is particularly good, like, praise!	Jingdong prefecture level city in Shandong
Happy Fan Shop	King of Power Saving	The logistics is very fast, the packaging is not broken, the quality is very good, I like it very much!	Jingdong prefecture level city in Shandong
Happy Fan Shop	King of Power Saving	The saver was well packaged and was as described when plugged in	Jingdong prefecture level city in Shandong
Happy Fan Shop	King of Power Saving	Energy efficient and very satisfied	Jingdong prefecture level city in Shandong
Happy Fan Shop	King of Power Saving	Great quality for the price and fast shipping.	Jingdong prefecture level city in Shandong
Happy Fan Shop	King of Power Saving	It's great, just as described. Perfect.	Jingdong prefecture level city in Shandong
Happy Fan Shop	King of Power Saving	There is no power saving effect, but rather ring up an increase in electricity costs, because this machine is on continuously all day long, uninterruptedly, its own electricity and increased costs, plus the cost of purchasing the machine, so I feel that the use of this machine instead of increasing the	Jingdong prefecture level city in Shandong

Happy Fan Shop	King of Power Saving	Recommended by the owner of the store next door, looked at his home does have to save electricity, decided to get. Indeed quite can, the voltage is much more stable, there is a reduction in electricity	Jingdong prefecture level city in Shandong
Happy Fan Shop	King of Power Saving	Goods have been received value for money, affordable and reasonable price, and the description of the same, power-saving safe and reliable!	Jingdong prefecture level city in Shandong
Happy Fan Shop	King of Power Saving	Listen to friends introduced to buy, tried to save a lot of electricity is good, quite good, worth buying.	Jingdong prefecture level city in Shandong
Happy Fan Shop	King of Power Saving	The product is easy to use, safe and environmentally friendly, only used for a short time, to the next month, after the electricity bill status display to know how the performance of the power saver efficacy.	Jingdong prefecture level city in Shandong
Happy Fan Shop	King of Power Saving	Last month's electricity bill is 178 yuan, with this power saver is 92 yuan, ready to buy two more back home, I was introduced to buy a friend, just started to not believe that I did not expect the effect!	Jingdong prefecture level city in Shandong
Happy Fan Shop	King of Power Saving	It looks nice, but after using it for a few days I can't see how it saves power?	Jingdong prefecture level city in Shandong
Happy Fan Shop	King of Power Saving	Recommended by a friend, it is genuine, as described by the merchant, cost-effective	Jingdong prefecture level city in Shandong
Happy Fan Shop	King of Power Saving	Works well, affordable and saves money.	Jingdong prefecture level city in Shandong
Happy Fan Shop	King of Power Saving	Feels good haven't compared it yet, the air conditioning has been on a lot in the past two months	Jingdong prefecture level city in Shandong
Happy Fan Shop	King of Power Saving	Saving about 5 kWh per day is a good thing	Jingdong prefecture level city in Shandong
Happy Fan Shop	King of Power Saving	Not bad, affordable, best value for money	Jingdong prefecture level city in Shandong
Happy Fan Shop	King of Power Saving	The goods are genuine and the logistics are faster.	Jingdong prefecture level city in Shandong
Happy Fan Shop	King of Power Saving	Goods have been received today, has been plugged into the outlet, looking forward to saving electricity, after a period of time to see how the effect in the follow-up comment!	Jingdong prefecture level city in Shandong
Happy Fan Shop	King of Power Saving	I just got it home and haven't used it for long, so I'll use it for a couple months and see what happens. Seeing that most people have good reviews, it should be fine.	Jingdong prefecture level city in Shandong
Happy Fan Shop	King of Power Saving	I'm looking forward to the results, so I'll give you a good review out of habit! Follow up review after a while	Jingdong prefecture level city in Shandong
Happy Fan Shop	King of Power Saving	Use a dish first to know if it will help save energy	Jingdong prefecture level city in Shandong
Happy Fan Shop	King of Power Saving	Whether it saves electricity or not, we don't know yet.	Jingdong prefecture level city in Shandong
Happy Fan Shop	King of Power Saving	Goods have been received, use a period of time to know how oh. The courier did not deliver to the door ah! Sent to the station ah! The heat is inconvenient ah!	Jingdong prefecture level city in Shandong
Happy Fan Shop	King of Power Saving	A little effect, before an hour a power, now an hour 0.76 degrees, a dollar a power = save 24 cents, although not a lot, first use it!	Jingdong prefecture level city in Shandong
Happy Fan Shop	King of Power Saving	Just received the goods, use a month to evaluate	Jingdong prefecture level city in Shandong
Happy Fan Shop	King of Power Saving	Home every month with a lot of electricity, inadvertently found this good thing online that can save the beginning of a skeptical attitude to consult customer service, customer service replied very professional, said 120 days can be refunded that first try it!	Jingdong prefecture level city in Shandong
Happy Fan Shop	King of Power Saving	After receiving the goods this afternoon, immediately installed in the bathroom Smith energy storage electric water heater on the power outlet (instead of connecting with a plug board), when used, found that the "Power Saving King" LCD display, is the value of the voltage, not the store's video said so	Jingdong prefecture level city in Shandong
Happy Fan Shop	King of Power Saving	Has made many days, did not feel the power saving, the effect is not obvious.	Jingdong prefecture level city in Shandong
Happy Fan Shop	King of Power Saving	Energy savers do not save energy at all, but rather consume a lot of electricity.	Jingdong prefecture level city in Shandong
Happy Fan Shop	King of Power Saving	It's useless. It's a waste of money.	Jingdong prefecture level city in Shandong
Happy Fan Shop	King of Power Saving	Last month saved more than 500, which came back to buy, the store bought three, and then buy 1 home use, my family is dry beauty salon, more instruments, especially electricity, a month almost 1000. or so, with this did not expect to save so much, great, five-star praise!	Jingdong prefecture level city in Shandong
Happy Fan Shop	King of Power Saving	It rained a couple days ago and I just went to get the item back today , not bad, hey I looked at the meter and that red display light takes several seconds to jump, did not make a mistake buying it. After a period of time to evaluate.	Jingdong prefecture level city in Shandong
Happy Fan Shop	King of Power Saving	Without further ado, the electricity bill came down, this month less 102 yuan, has been repurchased, I also recommended to my friends, all said that there are effects, the product is okay, the effect is quite	Jingdong prefecture level city in Shandong
Happy Fan Shop	King of Power Saving	After buying and using two months really see the effect, so only to evaluate! Originally held the mindset of trying, I did not expect really effective, worth having!	Jingdong prefecture level city in Shandong
Happy Fan Shop	King of Power Saving	In the use of this energy saver before the monthly electricity bill is more than 150 yuan or so, now save thirty percent, every day to work, six or seven o'clock in the afternoon to go home, I do not know why the electricity bill is so expensive, energy-saving appliances really have the effect of recommending	Jingdong prefecture level city in Shandong
Happy Fan Shop	King of Power Saving	It saves about 100 bucks a month. A friend recommended it to me, and he's been using it for a while, and the effect is so obvious that I bought one too.	Jingdong prefecture level city in Shandong
Happy Fan Shop	King of Power Saving	My dad asked me to buy one, but after I paid the bill, I asked and he said it was a lot less this month, so he asked me to buy another one.	Jingdong prefecture level city in Shandong
Happy Fan Shop	King of Power Saving	The specific effect will have to be tested for at least two months to compare whether there is a power saving effect or not.	Jingdong prefecture level city in Shandong
Happy Fan Shop	King of Power Saving	'The goods have been received, immediately go to use, please ask the boss you instructions do not allow to withdraw, there are two fuses installed there!	Jingdong prefecture level city in Shandong
Happy Fan Shop	King of Power Saving	First praise is using, after using if good then follow up, now because I have not yet seen the results	Jingdong prefecture level city in Shandong
Happy Fan Shop	King of Power Saving	I've just been using it, and I haven't seen if it has a power-saving effect yet, so I'll follow up after 10 days and half a month.	Jingdong prefecture level city in Shandong
Happy Fan Shop	King of Power Saving	Just received the intelligent power saving meter, immediately plugged in to use, see it is not bad, and order to sell the second time	Jingdong prefecture level city in Shandong
Happy Fan Shop	King of Power Saving	This power saver is a bit effective, used for half a month, the effect is still visible.	Jingdong prefecture level city in Shandong
Happy Fan Shop	King of Power Saving	I received the baby and it looks great, I'm trying it out now and will keep an eye on it afterward.	Jingdong prefecture level city in Shandong
Happy Fan Shop	King of Power Saving	I've just received it, but I'm still trying to see how it works, so I'll come back in a little while.	Jingdong prefecture level city in Shandong
Happy Fan Shop	King of Power Saving	Just used it wait to use it for a while and then comment thanks, all first five stars.	Jingdong prefecture level city in Shandong
Happy Fan Shop	King of Power Saving	Very trustworthy brand, quality is guaranteed, the price is still affordable, quite satisfied!	Jingdong prefecture level city in Shandong
Happy Fan Shop	King of Power Saving	Things received, I hope that the platform of Jingdong do not sell fake goods are available	Jingdong prefecture level city in Shandong
Happy Fan Shop	King of Power Saving	Energy saver received, the use of the effect is good and the business description of the same	Jingdong prefecture level city in Shandong
Happy Fan Shop	King of Power Saving	The surface looks beautiful, the actual effect is not known, after using additional evaluation.	Jingdong prefecture level city in Shandong
Happy Fan Shop	King of Power Saving	Tried it for a few months, it really saves electricity, and I bought it for a friend.	Jingdong prefecture level city in Shandong
Happy Fan Shop	King of Power Saving	It didn't work and wanted to return it, it really didn't work at all.	Jingdong prefecture level city in Shandong
Happy Fan Shop	King of Power Saving	Just used it, don't know how effective it is, looking forward to usefulness	Jingdong prefecture level city in Shandong
Happy Fan Shop	King of Power Saving	After two months before the effect I do not know if it is true!	Jingdong prefecture level city in Shandong

Happy Fan Shop	King of Power Saving	So fast! Ordered yesterday and received today 👍👍👍👍👍👍👍👍👍 🌹🌹🌹🌹🌹🌹🌹🌹🌹	Jingdong prefecture level city in Shandong
Happy Fan Shop	King of Power Saving	I’ve started using it. We’ll see how it works.	Jingdong prefecture level city in Shandong
Happy Fan Shop	King of Power Saving	I just received the goods, I won't know the effect until after two.	Jingdong prefecture level city in Shandong
Happy Fan Shop	King of Power Saving	Just received the goods, the specific effect will be evaluated later	Jingdong prefecture level city in Shandong
Happy Fan Shop	King of Power Saving	I've received the goods, I don't know if it's any good yet	Jingdong prefecture level city in Shandong
Happy Fan Shop	King of Power Saving	Try it for a month and then come back with a concrete evaluation	Jingdong prefecture level city in Shandong
Happy Fan Shop	King of Power Saving	In use, after two months to see the effect of power saving	Jingdong prefecture level city in Shandong
Happy Fan Shop	King of Power Saving	Power Saver. It's authentic. It's black. It works.	Jingdong prefecture level city in Shandong
Happy Fan Shop	King of Power Saving	I've been using it for a week now and it's not working.	Jingdong prefecture level city in Shandong
Happy Fan Shop	King of Power Saving	I'm just using it. I don't know how it's going to work.	Jingdong prefecture level city in Shandong
Happy Fan Shop	King of Power Saving	Products are better looking out for power savings	Jingdong prefecture level city in Shandong
Happy Fan Shop	King of Power Saving	I received it and I've kept it on. I hope it works.	Jingdong prefecture level city in Shandong
Happy Fan Shop	King of Power Saving	It's really good! Worth recommending to buy!	Jingdong prefecture level city in Shandong
Happy Fan Shop	King of Power Saving	Try it for a few days and see if it doesn't work, then return it.	Jingdong prefecture level city in Shandong
Happy Fan Shop	King of Power Saving	It's in use and I hope to be pleasantly surprised.	Jingdong prefecture level city in Shandong
Happy Fan Shop	King of Power Saving	We'll see how it works when we use it	Jingdong prefecture level city in Shandong
Happy Fan Shop	King of Power Saving	Let's see if it works.	Jingdong prefecture level city in Shandong
Happy Fan Shop	King of Power Saving	I'm using it and will follow up with a review	Jingdong prefecture level city in Shandong
Happy Fan Shop	King of Power Saving	It really works. It saves energy.	Jingdong prefecture level city in Shandong
Happy Fan Shop	King of Power Saving	It does have some power savings!	Jingdong prefecture level city in Shandong
Happy Fan Shop	King of Power Saving	Jingdong shopping speed is convenient and timely	Jingdong prefecture level city in Shandong
Happy Fan Shop	King of Power Saving	Second time I've bought it. Everything's fine.	Jingdong prefecture level city in Shandong
Happy Fan Shop	King of Power Saving	I don't know if it works yet.	Jingdong prefecture level city in Shandong
Happy Fan Shop	King of Power Saving	Not used, don't know how well it works.	Jingdong prefecture level city in Shandong
Happy Fan Shop	King of Power Saving	No visible results so far	Jingdong prefecture level city in Shandong
Happy Fan Shop	King of Power Saving	It doesn't feel like it's working.	Jingdong prefecture level city in Shandong
Happy Fan Shop	King of Power Saving	Where's the effect? Where are the power savings?	Jingdong prefecture level city in Shandong
Happy Fan Shop	King of Power Saving	Let's see how it works in practice!	Jingdong prefecture level city in Shandong
Happy Fan Shop	King of Power Saving	I'll wait until I use it to give you a good review	Jingdong prefecture level city in Shandong
Happy Fan Shop	King of Power Saving	I'll follow up after I've used it	Jingdong prefecture level city in Shandong
Happy Fan Shop	King of Power Saving	Well, that's good for saving power.	Jingdong prefecture level city in Shandong
Happy Fan Shop	King of Power Saving	Received. Used in the know.	Jingdong prefecture level city in Shandong
Happy Fan Shop	King of Power Saving	It doesn't do much good.	Jingdong prefecture level city in Shandong
Happy Fan Shop	King of Power Saving	It's not working.	Jingdong prefecture level city in Shandong
Happy Fan Shop	King of Power Saving	I don't know. I just used it.	Jingdong prefecture level city in Shandong
Happy Fan Shop	King of Power Saving	Use it and come back to evaluate.	Jingdong prefecture level city in Shandong
Happy Fan Shop	King of Power Saving	Two words: useless.	Jingdong prefecture level city in Shandong
Happy Fan Shop	King of Power Saving	To be used and then evaluated	Jingdong prefecture level city in Shandong
Happy Fan Shop	King of Power Saving	No shipment.	Jingdong prefecture level city in Shandong
Happy Fan Shop	King of Power Saving	Fake. Speechless.	Jingdong prefecture level city in Shandong
Happy Fan Shop	King of Power Saving	fantastic (idiom); awesome	Jingdong prefecture level city in Shandong
Happy Fan Shop	King of Power Saving	an excellent	Jingdong prefecture level city in Shandong
Happy Fan Shop	King of Power Saving	an excellent	Jingdong prefecture level city in Shandong
Happy Fan Shop	King of Power Saving	Want to buy! Recommended not to buy! No effect	Jingdong prefecture level city in Shandong
Happy Fan Shop	King of Power Saving	That's all there is to it. I can't figure out how to save electricity.	Jingdong prefecture level city in Shandong
Happy Fan Shop	King of Power Saving	It's no use.	Jingdong prefecture level city in Shandong
Happy Fan Shop	King of Power Saving	Things are more perfect than I imagined, very powerful, it is worth getting Packaging is very strong, things are well protected, it is worth the praise things are very satisfied Oh, beautiful and stylish atmosphere, customer service is super patient, service is meticulous and attentive, the big brand is not	Jingdong prefecture level city in Shandong
Happy Fan Shop	King of Power Saving	The effect is too good, the family has now several units, back to buy a number of times, the power saver received, power-saving effect is very good,, fine workmanship, long warranty, but also purify the air, before buying a used for a period of time, with the okay, and then bought a, and only now come to the	Jingdong prefecture level city in Shandong
Happy Fan Shop	King of Power Saving	Quite good, and imagine the same, casual ride are very good, satisfied! Baby received it 👌, really great ah! 🌈 quality is really nothing to say, very satisfied! 🆗 happy happy 😃 genuine, great, the price is very good, in short, quite good, will buy again a very good shopping experience, attentive service, fast logistics, distribution is also thoughtful, the attitude is also very good, value for money, ah,	Jingdong prefecture level city in Shandong

Happy Fan Shop	King of Power Saving	Power-saving effect: strong Cost-effective: quite good Appearance style: beautiful Workmanship material: meticulous Experience: looking forward to power-saving effect	Jingdong prefecture level city in Shandong
Happy Fan Shop	King of Power Saving	Cost-effective is okay, the appearance of the style of meticulous workmanship, looking forward to energy-saving effect.	Jingdong prefecture level city in Shandong
Happy Fan Shop	King of Power Saving	Very Good Very Power Saving This Power Saver is really power saving As soon as the power is turned on, the Power Saver starts the power saving function for you.	Jingdong prefecture level city in Shandong
Happy Fan Shop	King of Power Saving	Logistics is very fast, the price is favorable, friends introduced that it is very good to use, bought 2 at once, looking forward to the effect, used after the follow-up comment.	Jingdong prefecture level city in Shandong
Happy Fan Shop	King of Power Saving	It's all fake. It doesn't save any electricity.	Jingdong prefecture level city in Shandong
Happy Fan Shop	King of Power Saving	This Power Saver is so much better than the one I bought before, not only the effect is good, the quality is very good in all aspects, the customer service attitude is also very good, recommended to buy!	Jingdong prefecture level city in Shandong
Happy Fan Shop	King of Power Saving	Logistics is very fast, buy for parents, good quality, once bought 2, power-saving effect is good to buy 2 more, look forward to using the effect, satisfied will recommend to friends.	Jingdong prefecture level city in Shandong
Happy Fan Shop	King of Power Saving	With the description is the same, very high-grade, good value, to buy friends can order Oh!	Jingdong prefecture level city in Shandong
Happy Fan Shop	King of Power Saving	Not using much electricity in the winter saving over 100 per month! Ready to buy 2 more units in the summer ! ! ! ! ! Good quality, good price, power saving effect is obvious, cost-effective, worth buying, can be recommended!	Jingdong prefecture level city in Shandong
Happy Fan Shop	King of Power Saving	Purchased a power saver received on the put into use, rent a room with many electrical appliances, power consumption is very large, we have a piece of electricity or 1.2 dollars a time, with this power saver is much better, every day can save a lot of money, very convenient and practical.	Jingdong prefecture level city in Shandong
Happy Fan Shop	King of Power Saving	The power saving effect is very good: my supermarket monthly electricity bill is more than 7000 yuan. On May 1st someone recommended buying a couple of power savers, and I bought 5 of them first. The electricity bill was about 2000 less in June. This time, I bought 5 more. The seller said 800 degrees of electricity with one. I feel it's basically enough. A total of 10 units	Jingdong prefecture level city in Shandong
Happy Fan Shop	King of Power Saving	I never thought I could get such a good baby at such an affordable price, it's very good value for money, I love it!	Jingdong prefecture level city in Shandong
Happy Fan Shop	King of Power Saving	Previously bought other brands of power savers, but found no effect at home, this time to see how the products on the Jingdong, inserted in the home socket, the LED display is blue, the night light is very nice to see, look at the instructions that have to be half a year in order to achieve the best results, try it, I believe that the quality of the Jingdong, it should not be wrong.	Jingdong prefecture level city in Shandong
Happy Fan Shop	King of Power Saving	Superb, inexpensive, saves a lot of points, been using it for a long time!	Jingdong prefecture level city in Shandong
Happy Fan Shop	King of Power Saving	There are a lot of appliances in the house, the summer air conditioning is very costly, I bought this in front of the air conditioning effect is very good, I explain that I bought three in one breath, I use more electricity a month down to 200 or so, very satisfied.	Jingdong prefecture level city in Shandong
Happy Fan Shop	King of Power Saving	A month can save more than 200, home monthly electricity bill of more than 600 dollars, inserted 3 or so, an amount of the balance of the average can save more than 200. Product is very good, has been buying this one, has given a lot of relatives recommended, the quality is also very good, before buying a look with this same, who knows to buy home has not been used for more than a week on the bad, the quality of this is really good with more than two years!	Jingdong prefecture level city in Shandong
Happy Fan Shop	King of Power Saving	Whole house power saving king, can really save electricity, friends recommended, high power saving appliances, only replacement, no repair, after-sales worry-free!	Jingdong prefecture level city in Shandong
Happy Fan Shop	King of Power Saving	Great item love it, great customer service, great shopping experience!	Jingdong prefecture level city in Shandong
Happy Fan Shop	King of Power Saving	Power saving, very good use, much cheaper than the physical store, satisfaction	Jingdong prefecture level city in Shandong
Happy Fan Shop	King of Power Saving	In use, very well packaged and fast delivery of credit!	Jingdong prefecture level city in Shandong
Happy Fan Shop	King of Power Saving	Product quality, customer service attitude is particularly good, like, praise!	Jingdong prefecture level city in Shandong
Happy Fan Shop	King of Power Saving	The logistics is very fast, the packaging is not broken, the quality is very good, I like it very much!	Jingdong prefecture level city in Shandong
Happy Fan Shop	King of Power Saving	The saver was well packaged and was as described when plugged in	Jingdong prefecture level city in Shandong
Happy Fan Shop	King of Power Saving	Energy efficient and very satisfied	Jingdong prefecture level city in Shandong
Happy Fan Shop	King of Power Saving	Great quality for the price and fast shipping.	Jingdong prefecture level city in Shandong
Happy Fan Shop	King of Power Saving	It's great, just as described. Perfect.	Jingdong prefecture level city in Shandong
Happy Fan Shop	King of Power Saving	There is no power saving effect, but rather ring up an increase in electricity costs, because this machine is on continuously all day long, uninterruptedly, its own electricity and increased costs, plus the cost of purchasing the machine, so I feel that the use of this machine instead of increasing the	Jingdong prefecture level city in Shandong
Happy Fan Shop	King of Power Saving	Recommended by the owner of the store next door, looked at his home does have to save electricity, decided to get. Indeed quite can, the voltage is much more stable, there is a reduction in electricity	Jingdong prefecture level city in Shandong
Happy Fan Shop	King of Power Saving	Goods have been received value for money, affordable and reasonable price, and the description of the same, power-saving safe and reliable!	Jingdong prefecture level city in Shandong
Happy Fan Shop	King of Power Saving	Listen to friends introduced to buy, tried to save a lot of electricity is good, quite good, worth buying.	Jingdong prefecture level city in Shandong
Happy Fan Shop	King of Power Saving	The product is easy to use, safe and environmentally friendly, only used for a short time, to the next month, after the electricity bill status display to know how the performance of the power saver efficacy.	Jingdong prefecture level city in Shandong
Happy Fan Shop	King of Power Saving	Last month's electricity bill is 178 yuan, with this power saver is 92 yuan, ready to buy two more back home, I was introduced to buy a friend, just started to not believe that I did not expect the effect!	Jingdong prefecture level city in Shandong
Happy Fan Shop	King of Power Saving	It looks nice, but after using it for a few days I can't see how it saves power?	Jingdong prefecture level city in Shandong
Happy Fan Shop	King of Power Saving	Recommended by a friend, it is genuine, as described by the merchant, cost-effective	Jingdong prefecture level city in Shandong
Happy Fan Shop	King of Power Saving	Works well, affordable and saves money.	Jingdong prefecture level city in Shandong
Happy Fan Shop	King of Power Saving	Feels good haven't compared it yet, the air conditioning has been on a lot in the past two months	Jingdong prefecture level city in Shandong
Happy Fan Shop	King of Power Saving	Saving about 5 kWh per day is a good thing	Jingdong prefecture level city in Shandong
Happy Fan Shop	King of Power Saving	Not bad, affordable, best value for money	Jingdong prefecture level city in Shandong
Happy Fan Shop	King of Power Saving	The goods are genuine and the logistics are faster.	Jingdong prefecture level city in Shandong
Happy Fan Shop	King of Power Saving	Goods have been received today, has been plugged into the outlet, looking forward to saving electricity, after a period of time to see how the effect in the follow-up comment!	Jingdong prefecture level city in Shandong
Happy Fan Shop	King of Power Saving	I just got it home and haven't used it for long, so I'll use it for a couple months and see what happens. Seeing that most people have good reviews, it should be fine.	Jingdong prefecture level city in Shandong
Happy Fan Shop	King of Power Saving	I'm looking forward to the results, so I'll give you a good review out of habit! Follow up review after a while	Jingdong prefecture level city in Shandong
Happy Fan Shop	King of Power Saving	Use a dish first to know if it will help save energy	Jingdong prefecture level city in Shandong
Happy Fan Shop	King of Power Saving	Whether it saves electricity or not, we don't know yet.	Jingdong prefecture level city in Shandong
Happy Fan Shop	King of Power Saving	Goods have been received, use a period of time to know how oh. The courier did not deliver to the door ah! Sent to the station ah! The heat is inconvenient ah!	Jingdong prefecture level city in Shandong
Happy Fan Shop	King of Power Saving	A little effect, before an hour a power, now an hour 0.76 degrees, a dollar a power = save 24 cents, although not a lot, first use it!	Jingdong prefecture level city in Shandong

Happy Fan Shop	King of Power Saving	Just received the goods, use a month to evaluate	Jingdong prefecture level city in Shandong
Happy Fan Shop	King of Power Saving	Home every month with a lot of electricity, inadvertently found this good thing online that can save the beginning of a skeptical attitude to consult customer service, customer service replied very professional, said 120 days can be refunded that first try it!	Jingdong prefecture level city in Shandong
Happy Fan Shop	King of Power Saving	After receiving the goods this afternoon, immediately installed in the bathroom Smith energy storage electric water heater on the power outlet (instead of connecting with a plug board), when used, found that the "Power Saving King" LCD display, is the value of the voltage, not the store's video said so	Jingdong prefecture level city in Shandong
Happy Fan Shop	King of Power Saving	Has made many days, did not feel the power saving, the effect is not obvious.	Jingdong prefecture level city in Shandong
Happy Fan Shop	King of Power Saving	Energy savers do not save energy at all, but rather consume a lot of electricity.	Jingdong prefecture level city in Shandong
Happy Fan Shop	King of Power Saving	It's useless. It's a waste of money.	Jingdong prefecture level city in Shandong
Happy Fan Shop	King of Power Saving	Last month saved more than 500, which came back to buy, the store bought three, and then buy 1 home use, my family is dry beauty salon, more instruments, especially electricity, a month almost 1000. or so, with this did not expect to save so much, great, five-star praise!	Jingdong prefecture level city in Shandong
Happy Fan Shop	King of Power Saving	It rained a couple days ago and I just went to get the item back today , not bad, hey I looked at the meter and that red display light takes several seconds to jump, did not make a mistake buying it. After a period of time to evaluate.	Jingdong prefecture level city in Shandong
Happy Fan Shop	King of Power Saving	Without further ado, the electricity bill came down, this month less 102 yuan, has been repurchased, I also recommended to my friends, all said that there are effects, the product is okay, the effect is quite	Jingdong prefecture level city in Shandong
Happy Fan Shop	King of Power Saving	After buying and using two months really see the effect, so only to evaluate! Originally held the mindset of trying, I did not expect really effective, worth having!	Jingdong prefecture level city in Shandong
Happy Fan Shop	King of Power Saving	In the use of this energy saver before the monthly electricity bill is more than 150 yuan or so, now save thirty percent, every day to work, six or seven o'clock in the afternoon to go home, I do not know why the electricity bill is so expensive, energy-saving appliances really have the effect of recommending	Jingdong prefecture level city in Shandong
Happy Fan Shop	King of Power Saving	It saves about 100 bucks a month. A friend recommended it to me, and he's been using it for a while, and the effect is so obvious that I bought one too.	Jingdong prefecture level city in Shandong
Happy Fan Shop	King of Power Saving	My dad asked me to buy one, but after I paid the bill, I asked and he said it was a lot less this month, so he asked me to buy another one.	Jingdong prefecture level city in Shandong
Happy Fan Shop	King of Power Saving	The specific effect will have to be tested for at least two months to compare whether there is a power saving effect or not.	Jingdong prefecture level city in Shandong
Happy Fan Shop	King of Power Saving	'The goods have been received, immediately go to use, please ask the boss you instructions do not allow to withdraw, there are two fuses installed there!	Jingdong prefecture level city in Shandong
Happy Fan Shop	King of Power Saving	First praise is using, after using if good then follow up, now because I have not yet seen the results	Jingdong prefecture level city in Shandong
Happy Fan Shop	King of Power Saving	I've just been using it, and I haven't seen if it has a power-saving effect yet, so I'll follow up after 10 days and half a month.	Jingdong prefecture level city in Shandong
Happy Fan Shop	King of Power Saving	Just received the intelligent power saving meter, immediately plugged in to use, see it is not bad, and order to sell the second time	Jingdong prefecture level city in Shandong
Happy Fan Shop	King of Power Saving	This power saver is a bit effective, used for half a month, the effect is still visible.	Jingdong prefecture level city in Shandong
Happy Fan Shop	King of Power Saving	I received the baby and it looks great, I'm trying it out now and will keep an eye on it afterward.	Jingdong prefecture level city in Shandong
Happy Fan Shop	King of Power Saving	I've just received it, but I'm still trying to see how it works, so I'll come back in a little while.	Jingdong prefecture level city in Shandong
Happy Fan Shop	King of Power Saving	Just used it wait to use it for a while and then comment thanks, all first five stars.	Jingdong prefecture level city in Shandong
Happy Fan Shop	King of Power Saving	Very trustworthy brand, quality is guaranteed, the price is still affordable, quite satisfied!	Jingdong prefecture level city in Shandong
Happy Fan Shop	King of Power Saving	Things received, I hope that the platform of Jingdong do not sell fake goods are available	Jingdong prefecture level city in Shandong
Happy Fan Shop	King of Power Saving	Energy saver received, the use of the effect is good and the business description of the same	Jingdong prefecture level city in Shandong
Happy Fan Shop	King of Power Saving	The surface looks beautiful, the actual effect is not known, after using additional evaluation.	Jingdong prefecture level city in Shandong
Happy Fan Shop	King of Power Saving	Tried it for a few months, it really saves electricity, and I bought it for a friend.	Jingdong prefecture level city in Shandong
Happy Fan Shop	King of Power Saving	It didn't work and wanted to return it, it really didn't work at all.	Jingdong prefecture level city in Shandong
Happy Fan Shop	King of Power Saving	Just used it, don't know how effective it is, looking forward to usefulness	Jingdong prefecture level city in Shandong
Happy Fan Shop	King of Power Saving	After two months before the effect I do not know if it is true!	Jingdong prefecture level city in Shandong
Happy Fan Shop	King of Power Saving	So fast! Ordered yesterday and received today 👍👍👍👍👍👍👍👍👍 🌹🌹🌹🌹🌹🌹🌹🌹🌹	Jingdong prefecture level city in Shandong
Happy Fan Shop	King of Power Saving	I've started using it. We'll see how it works.	Jingdong prefecture level city in Shandong
Happy Fan Shop	King of Power Saving	I just received the goods, I won't know the effect until after two.	Jingdong prefecture level city in Shandong
Happy Fan Shop	King of Power Saving	Just received the goods, the specific effect will be evaluated later	Jingdong prefecture level city in Shandong
Happy Fan Shop	King of Power Saving	I've received the goods, I don't know if it's any good yet	Jingdong prefecture level city in Shandong
Happy Fan Shop	King of Power Saving	Try it for a month and then come back with a concrete evaluation	Jingdong prefecture level city in Shandong
Happy Fan Shop	King of Power Saving	In use, after two months to see the effect of power saving	Jingdong prefecture level city in Shandong
Happy Fan Shop	King of Power Saving	Power Saver. It's authentic. It's black. It works.	Jingdong prefecture level city in Shandong
Happy Fan Shop	King of Power Saving	I've been using it for a week now and it's not working.	Jingdong prefecture level city in Shandong
Happy Fan Shop	King of Power Saving	I'm just using it. I don't know how it's going to work.	Jingdong prefecture level city in Shandong
Happy Fan Shop	King of Power Saving	Products are better looking out for power savings	Jingdong prefecture level city in Shandong
Happy Fan Shop	King of Power Saving	I received it and I've kept it on. I hope it works.	Jingdong prefecture level city in Shandong
Happy Fan Shop	King of Power Saving	It's really good! Worth recommending to buy!	Jingdong prefecture level city in Shandong
Happy Fan Shop	King of Power Saving	Try it for a few days and see if it doesn't work, then return it.	Jingdong prefecture level city in Shandong
Happy Fan Shop	King of Power Saving	It's in use and I hope to be pleasantly surprised.	Jingdong prefecture level city in Shandong
Happy Fan Shop	King of Power Saving	We'll see how it works when we use it	Jingdong prefecture level city in Shandong
Happy Fan Shop	King of Power Saving	Let's see if it works.	Jingdong prefecture level city in Shandong
Happy Fan Shop	King of Power Saving	I'm using it and will follow up with a review	Jingdong prefecture level city in Shandong
Happy Fan Shop	King of Power Saving	It really works. It saves energy.	Jingdong prefecture level city in Shandong

Happy Fan Shop	King of Power Saving	It does have some power savings!	Jingdong prefecture level city in Shandong
Happy Fan Shop	King of Power Saving	Jingdong shopping speed is convenient and timely	Jingdong prefecture level city in Shandong
Happy Fan Shop	King of Power Saving	Second time I've bought it. Everything's fine.	Jingdong prefecture level city in Shandong
Happy Fan Shop	King of Power Saving	I don't know if it works yet.	Jingdong prefecture level city in Shandong
Happy Fan Shop	King of Power Saving	Not used, don't know how well it works.	Jingdong prefecture level city in Shandong
Happy Fan Shop	King of Power Saving	No visible results so far	Jingdong prefecture level city in Shandong
Happy Fan Shop	King of Power Saving	It doesn't feel like it's working.	Jingdong prefecture level city in Shandong
Happy Fan Shop	King of Power Saving	Where's the effect? Where are the power savings?	Jingdong prefecture level city in Shandong
Happy Fan Shop	King of Power Saving	Let's see how it works in practice!	Jingdong prefecture level city in Shandong
Happy Fan Shop	King of Power Saving	I'll wait until I use it to give you a good review	Jingdong prefecture level city in Shandong
Happy Fan Shop	King of Power Saving	I'll follow up after I've used it	Jingdong prefecture level city in Shandong
Happy Fan Shop	King of Power Saving	Well, that's good for saving power.	Jingdong prefecture level city in Shandong
Happy Fan Shop	King of Power Saving	Received. Used in the know.	Jingdong prefecture level city in Shandong
Happy Fan Shop	King of Power Saving	It doesn't do much good.	Jingdong prefecture level city in Shandong
Happy Fan Shop	King of Power Saving	It's not working.	Jingdong prefecture level city in Shandong
Happy Fan Shop	King of Power Saving	I don't know. I just used it.	Jingdong prefecture level city in Shandong
Happy Fan Shop	King of Power Saving	Use it and come back to evaluate.	Jingdong prefecture level city in Shandong
Happy Fan Shop	King of Power Saving	Two words: useless.	Jingdong prefecture level city in Shandong
Happy Fan Shop	King of Power Saving	To be used and then evaluated	Jingdong prefecture level city in Shandong
Happy Fan Shop	King of Power Saving	No shipment.	Jingdong prefecture level city in Shandong
Happy Fan Shop	King of Power Saving	Fake. Speechless.	Jingdong prefecture level city in Shandong
Happy Fan Shop	King of Power Saving	fantastic (idiom); awesome	Jingdong prefecture level city in Shandong
Happy Fan Shop	King of Power Saving	an excellent	Jingdong prefecture level city in Shandong
Happy Fan Shop	King of Power Saving	an excellent	Jingdong prefecture level city in Shandong
Happy Fan Shop	King of Power Saving	Want to buy! Recommended not to buy! No effect	Jingdong prefecture level city in Shandong
Happy Fan Shop	King of Power Saving	That's all there is to it. I can't figure out how to save electricity.	Jingdong prefecture level city in Shandong
Happy Fan Shop	King of Power Saving	It's no use.	Jingdong prefecture level city in Shandong
Happy Fan Shop	King of Power Saving	Things are more perfect than I imagined, very powerful, it is worth getting Packaging is very strong, things are well protected, it is worth the praise things are very satisfied Oh, beautiful and stylish atmosphere, customer service is super patient, service is meticulous and attentive, the big brand is not	Jingdong prefecture level city in Shandong
Happy Fan Shop	King of Power Saving	The effect is too good, the family has now several units, back to buy a number of times, the power saver received, power-saving effect is very good,, fine workmanship, long warranty, but also purify the air, before buying a used for a period of time, with the okay, and then bought a, and only now come to the	Jingdong prefecture level city in Shandong
Happy Fan Shop	King of Power Saving	Quite good, and imagine the same, casual ride are very good, satisfied! Baby received it 👌, really great ah! 🌈 quality is really nothing to say, very satisfied! 🆗 happy happy 😃 genuine, great, the price is very good, in short, quite good, will buy again a very good shopping experience, attentive service, fast logistics, distribution is also thoughtful, the attitude is also very good, value for money, ah,	Jingdong prefecture level city in Shandong
Happy Fan Shop	King of Power Saving	Power-saving effect: strong Cost-effective: quite good Appearance style: beautiful Workmanship material: meticulous Experience: looking forward to power-saving effect	Jingdong prefecture level city in Shandong
Happy Fan Shop	King of Power Saving	Cost-effective is okay, the appearance of the style of meticulous workmanship, looking forward to energy-saving effect.	Jingdong prefecture level city in Shandong
Happy Fan Shop	King of Power Saving	Very Good Very Power Saving This Power Saver is really power saving As soon as the power is turned on, the Power Saver starts the power saving function for you.	Jingdong prefecture level city in Shandong
Happy Fan Shop	King of Power Saving	Logistics is very fast, the price is favorable, friends introduced that it is very good to use, bought 2 at once, looking forward to the effect, used after the follow-up comment.	Jingdong prefecture level city in Shandong
Happy Fan Shop	King of Power Saving	It's all fake. It doesn't save any electricity.	Jingdong prefecture level city in Shandong
Happy Fan Shop	King of Power Saving	This Power Saver is so much better than the one I bought before, not only the effect is good, the quality is very good in all aspects, the customer service attitude is also very good, recommended to buy!	Jingdong prefecture level city in Shandong
Happy Fan Shop	King of Power Saving	Logistics is very fast, buy for parents, good quality, once bought 2, power-saving effect is good to buy 2 more, look forward to using the effect, satisfied will recommend to friends.	Jingdong prefecture level city in Shandong
Happy Fan Shop	King of Power Saving	With the description is the same, very high-grade, good value, to buy friends can order Oh!	Jingdong prefecture level city in Shandong
Happy Fan Shop	King of Power Saving	Not using much electricity in the winter saving over 100 per month! Ready to buy 2 more units in the summer ！！！！！ Good quality, good price, power saving effect is obvious, cost-effective, worth buying, can be recommended!	Jingdong prefecture level city in Shandong
Happy Fan Shop	King of Power Saving	Purchased a power saver received on the put into use, rent a room with many electrical appliances, power consumption is very large, we have a piece of electricity or 1.2 dollars a time, with this power saver is much better, every day can save a lot of money, very convenient and practical.	Jingdong prefecture level city in Shandong
Happy Fan Shop	King of Power Saving	The power saving effect is very good: my supermarket monthly electricity bill is more than 7000 yuan. On May 1st someone recommended buying a couple of power savers, and I bought 5 of them first. The electricity bill was about 2000 less in June. This time, I bought 5 more. The seller said 800 degrees of electricity with one. I feel it's basically enough. A total of 10 units	Jingdong prefecture level city in Shandong
Happy Fan Shop	King of Power Saving	I never thought I could get such a good baby at such an affordable price, it's very good value for money, I love it!	Jingdong prefecture level city in Shandong
Happy Fan Shop	King of Power Saving	Previously bought other brands of power savers, but found no effect at home, this time to see how the products on the Jingdong, inserted in the home socket, the LED display is blue, the night light is very nice to see, look at the instructions that have to be half a year in order to achieve the best results, try it, I believe that the quality of the Jingdong, it should not be wrong.	Jingdong prefecture level city in Shandong
Happy Fan Shop	King of Power Saving	Superb, inexpensive, saves a lot of points, been using it for a long time!	Jingdong prefecture level city in Shandong
Happy Fan Shop	King of Power Saving	There are a lot of appliances in the house, the summer air conditioning is very costly, I bought this in front of the air conditioning effect is very good, I explain that I bought three in one breath, I use more electricity a month down to 200 or so, very satisfied.	Jingdong prefecture level city in Shandong

Happy Fan Shop	King of Power Saving	A month can save more than 200, home monthly electricity bill of more than 600 dollars, inserted 3 or so, an amount of the balance of the average can save more than 200. Product is very good, has been buying this one, has given a lot of relatives recommended, the quality is also very good, before buying a look with this same, who knows to buy home has not been used for more than a week on the bad, the quality of this is really good with more than two years!	Jingdong prefecture level city in Shandong
Happy Fan Shop	King of Power Saving	Whole house power saving king, can really save electricity, friends recommended, high power saving appliances, only replacement, no repair, after-sales worry-free!	Jingdong prefecture level city in Shandong
Happy Fan Shop	King of Power Saving	Great item love it, great customer service, great shopping experience!	Jingdong prefecture level city in Shandong
Happy Fan Shop	King of Power Saving	Power saving, very good use, much cheaper than the physical store, satisfaction	Jingdong prefecture level city in Shandong
Happy Fan Shop	King of Power Saving	In use, very well packaged and fast delivery of credit!	Jingdong prefecture level city in Shandong
Happy Fan Shop	King of Power Saving	Product quality, customer service attitude is particularly good, like, praise!	Jingdong prefecture level city in Shandong
Happy Fan Shop	King of Power Saving	The logistics is very fast, the packaging is not broken, the quality is very good, I like it very much!	Jingdong prefecture level city in Shandong
Happy Fan Shop	King of Power Saving	The saver was well packaged and was as described when plugged in	Jingdong prefecture level city in Shandong
Happy Fan Shop	King of Power Saving	Energy efficient and very satisfied	Jingdong prefecture level city in Shandong
Happy Fan Shop	King of Power Saving	Great quality for the price and fast shipping.	Jingdong prefecture level city in Shandong
Happy Fan Shop	King of Power Saving	It's great, just as described. Perfect.	Jingdong prefecture level city in Shandong
Happy Fan Shop	King of Power Saving	There is no power saving effect, but rather ring up an increase in electricity costs, because this machine is on continuously all day long, uninterruptedly, its own electricity and increased costs, plus the cost of purchasing the machine, so I feel that the use of this machine instead of increasing the	Jingdong prefecture level city in Shandong
Happy Fan Shop	King of Power Saving	Recommended by the owner of the store next door, looked at his home does have to save electricity, decided to get. Indeed quite can, the voltage is much more stable, there is a reduction in electricity	Jingdong prefecture level city in Shandong
Happy Fan Shop	King of Power Saving	Goods have been received value for money, affordable and reasonable price, and the description of the same, power-saving safe and reliable!	Jingdong prefecture level city in Shandong
Happy Fan Shop	King of Power Saving	Listen to friends introduced to buy, tried to save a lot of electricity is good, quite good, worth buying.	Jingdong prefecture level city in Shandong
Happy Fan Shop	King of Power Saving	The product is easy to use, safe and environmentally friendly, only used for a short time, to the next month, after the electricity bill status display to know how the performance of the power saver efficacy.	Jingdong prefecture level city in Shandong
Happy Fan Shop	King of Power Saving	Last month's electricity bill is 178 yuan, with this power saver is 92 yuan, ready to buy two more back home, I was introduced to buy a friend, just started to not believe that I did not expect the effect!	Jingdong prefecture level city in Shandong
Happy Fan Shop	King of Power Saving	It looks nice, but after using it for a few days I can't see how it saves power?	Jingdong prefecture level city in Shandong
Happy Fan Shop	King of Power Saving	Recommended by a friend, it is genuine, as described by the merchant, cost-effective	Jingdong prefecture level city in Shandong
Happy Fan Shop	King of Power Saving	Works well, affordable and saves money.	Jingdong prefecture level city in Shandong
Happy Fan Shop	King of Power Saving	Feels good haven't compared it yet, the air conditioning has been on a lot in the past two months	Jingdong prefecture level city in Shandong
Happy Fan Shop	King of Power Saving	Saving about 5 kWh per day is a good thing	Jingdong prefecture level city in Shandong
Happy Fan Shop	King of Power Saving	Not bad, affordable, best value for money	Jingdong prefecture level city in Shandong
Happy Fan Shop	King of Power Saving	The goods are genuine and the logistics are faster.	Jingdong prefecture level city in Shandong
Happy Fan Shop	King of Power Saving	Goods have been received today, has been plugged into the outlet, looking forward to saving electricity, after a period of time to see how the effect in the follow-up comment!	Jingdong prefecture level city in Shandong
Happy Fan Shop	King of Power Saving	I just got it home and haven't used it for long, so I'll use it for a couple months and see what happens. Seeing that most people have good reviews, it should be fine.	Jingdong prefecture level city in Shandong
Happy Fan Shop	King of Power Saving	I'm looking forward to the results, so I'll give you a good review out of habit! Follow up review after a while	Jingdong prefecture level city in Shandong
Happy Fan Shop	King of Power Saving	Use a dish first to know if it will help save energy	Jingdong prefecture level city in Shandong
Happy Fan Shop	King of Power Saving	Whether it saves electricity or not, we don't know yet.	Jingdong prefecture level city in Shandong
Happy Fan Shop	King of Power Saving	Goods have been received, use a period of time to know how oh. The courier did not deliver to the door ah! Sent to the station ah! The heat is inconvenient ah!	Jingdong prefecture level city in Shandong
Happy Fan Shop	King of Power Saving	A little effect, before an hour a power, now an hour 0.76 degrees, a dollar a power = save 24 cents, although not a lot, first use it!	Jingdong prefecture level city in Shandong
Happy Fan Shop	King of Power Saving	Just received the goods, use a month to evaluate	Jingdong prefecture level city in Shandong
Happy Fan Shop	King of Power Saving	Home every month with a lot of electricity, inadvertently found this good thing online that can save the beginning of a skeptical attitude to consult customer service, customer service replied very professional, said 120 days can be refunded that first try it!	Jingdong prefecture level city in Shandong
Happy Fan Shop	King of Power Saving	After receiving the goods this afternoon, immediately installed in the bathroom Smith energy storage electric water heater on the power outlet (instead of connecting with a plug board), when used, found that the "Power Saving King" LCD display, is the value of the voltage, not the store's video said so	Jingdong prefecture level city in Shandong
Happy Fan Shop	King of Power Saving	Has made many days, did not feel the power saving, the effect is not obvious.	Jingdong prefecture level city in Shandong
Happy Fan Shop	King of Power Saving	Energy savers do not save energy at all, but rather consume a lot of electricity.	Jingdong prefecture level city in Shandong
Happy Fan Shop	King of Power Saving	It's useless. It's a waste of money.	Jingdong prefecture level city in Shandong
Happy Fan Shop	King of Power Saving	Last month saved more than 500, which came back to buy, the store bought three, and then buy 1 home use, my family is dry beauty salon, more instruments, especially electricity, a month almost 1000. or so, with this did not expect to save so much, great, five-star praise!	Jingdong prefecture level city in Shandong
Happy Fan Shop	King of Power Saving	It rained a couple days ago and I just went to get the item back today , not bad, hey I looked at the meter and that red display light takes several seconds to jump, did not make a mistake buying it. After a period of time to evaluate.	Jingdong prefecture level city in Shandong
Happy Fan Shop	King of Power Saving	Without further ado, the electricity bill came down, this month less 102 yuan, has been repurchased, I also recommended to my friends, all said that there are effects, the product is okay, the effect is quite	Jingdong prefecture level city in Shandong
Happy Fan Shop	King of Power Saving	After buying and using two months really see the effect, so only to evaluate! Originally held the mindset of trying, I did not expect really effective, worth having!	Jingdong prefecture level city in Shandong
Happy Fan Shop	King of Power Saving	In the use of this energy saver before the monthly electricity bill is more than 150 yuan or so, now save thirty percent, every day to work, six or seven o'clock in the afternoon to go home, I do not know why the electricity bill is so expensive, energy-saving appliances really have the effect of recommending	Jingdong prefecture level city in Shandong
Happy Fan Shop	King of Power Saving	It saves about 100 bucks a month. A friend recommended it to me, and he's been using it for a while, and the effect is so obvious that I bought one too.	Jingdong prefecture level city in Shandong
Happy Fan Shop	King of Power Saving	My dad asked me to buy one, but after I paid the bill, I asked and he said it was a lot less this month, so he asked me to buy another one.	Jingdong prefecture level city in Shandong
Happy Fan Shop	King of Power Saving	The specific effect will have to be tested for at least two months to compare whether there is a power saving effect or not.	Jingdong prefecture level city in Shandong
Happy Fan Shop	King of Power Saving	'The goods have been received, immediately go to use, please ask the boss you instructions do not allow to withdraw, there are two fuses installed there!	Jingdong prefecture level city in Shandong
Happy Fan Shop	King of Power Saving	First praise is using, after using if good then follow up, now because I have not yet seen the results	Jingdong prefecture level city in Shandong

Happy Fan Shop	King of Power Saving	I've just been using it, and I haven't seen if it has a power-saving effect yet, so I'll follow up after 10 days and half a month.	Jingdong prefecture level city in Shandong
Happy Fan Shop	King of Power Saving	Just received the intelligent power saving meter, immediately plugged in to use, see it is not bad, and order to sell the second time	Jingdong prefecture level city in Shandong
Happy Fan Shop	King of Power Saving	This power saver is a bit effective, used for half a month, the effect is still visible.	Jingdong prefecture level city in Shandong
Happy Fan Shop	King of Power Saving	I received the baby and it looks great, I'm trying it out now and will keep an eye on it afterward.	Jingdong prefecture level city in Shandong
Happy Fan Shop	King of Power Saving	I've just received it, but I'm still trying to see how it works, so I'll come back in a little while.	Jingdong prefecture level city in Shandong
Happy Fan Shop	King of Power Saving	Just used it wait to use it for a while and then comment thanks, all first five stars.	Jingdong prefecture level city in Shandong
Happy Fan Shop	King of Power Saving	Very trustworthy brand, quality is guaranteed, the price is still affordable, quite satisfied!	Jingdong prefecture level city in Shandong
Happy Fan Shop	King of Power Saving	Things received, I hope that the platform of Jingdong do not sell fake goods are available	Jingdong prefecture level city in Shandong
Happy Fan Shop	King of Power Saving	Energy saver received, the use of the effect is good and the business description of the same	Jingdong prefecture level city in Shandong
Happy Fan Shop	King of Power Saving	The surface looks beautiful, the actual effect is not known, after using additional evaluation.	Jingdong prefecture level city in Shandong
Happy Fan Shop	King of Power Saving	Tried it for a few months, it really saves electricity, and I bought it for a friend.	Jingdong prefecture level city in Shandong
Happy Fan Shop	King of Power Saving	It didn't work and wanted to return it, it really didn't work at all.	Jingdong prefecture level city in Shandong
Happy Fan Shop	King of Power Saving	Just used it, don't know how effective it is, looking forward to usefulness	Jingdong prefecture level city in Shandong
Happy Fan Shop	King of Power Saving	After two months before the effect I do not know if it is true!	Jingdong prefecture level city in Shandong
Happy Fan Shop	King of Power Saving	So fast! Ordered yesterday and received today 👍👍👍👍👍👍👍👍👍 🌹🌹🌹🌹🌹🌹🌹🌹🌹	Jingdong prefecture level city in Shandong
Happy Fan Shop	King of Power Saving	I've started using it. We'll see how it works.	Jingdong prefecture level city in Shandong
Happy Fan Shop	King of Power Saving	I just received the goods, I won't know the effect until after two.	Jingdong prefecture level city in Shandong
Happy Fan Shop	King of Power Saving	Just received the goods, the specific effect will be evaluated later	Jingdong prefecture level city in Shandong
Happy Fan Shop	King of Power Saving	I've received the goods, I don't know if it's any good yet	Jingdong prefecture level city in Shandong
Happy Fan Shop	King of Power Saving	Try it for a month and then come back with a concrete evaluation	Jingdong prefecture level city in Shandong
Happy Fan Shop	King of Power Saving	In use, after two months to see the effect of power saving	Jingdong prefecture level city in Shandong
Happy Fan Shop	King of Power Saving	Power Saver. It's authentic. It's black. It works.	Jingdong prefecture level city in Shandong
Happy Fan Shop	King of Power Saving	I've been using it for a week now and it's not working.	Jingdong prefecture level city in Shandong
Happy Fan Shop	King of Power Saving	I'm just using it. I don't know how it's going to work.	Jingdong prefecture level city in Shandong
Happy Fan Shop	King of Power Saving	Products are better looking out for power savings	Jingdong prefecture level city in Shandong
Happy Fan Shop	King of Power Saving	I received it and I've kept it on. I hope it works.	Jingdong prefecture level city in Shandong
Happy Fan Shop	King of Power Saving	It's really good! Worth recommending to buy!	Jingdong prefecture level city in Shandong
Happy Fan Shop	King of Power Saving	Try it for a few days and see if it doesn't work, then return it.	Jingdong prefecture level city in Shandong
Happy Fan Shop	King of Power Saving	It's in use and I hope to be pleasantly surprised.	Jingdong prefecture level city in Shandong
Happy Fan Shop	King of Power Saving	We'll see how it works when we use it	Jingdong prefecture level city in Shandong
Happy Fan Shop	King of Power Saving	Let's see if it works.	Jingdong prefecture level city in Shandong
Happy Fan Shop	King of Power Saving	I'm using it and will follow up with a review	Jingdong prefecture level city in Shandong
Happy Fan Shop	King of Power Saving	It really works. It saves energy.	Jingdong prefecture level city in Shandong
Happy Fan Shop	King of Power Saving	It does have some power savings!	Jingdong prefecture level city in Shandong
Happy Fan Shop	King of Power Saving	Jingdong shopping speed is convenient and timely	Jingdong prefecture level city in Shandong
Happy Fan Shop	King of Power Saving	Second time I've bought it. Everything's fine.	Jingdong prefecture level city in Shandong
Happy Fan Shop	King of Power Saving	I don't know if it works yet.	Jingdong prefecture level city in Shandong
Happy Fan Shop	King of Power Saving	Not used, don't know how well it works.	Jingdong prefecture level city in Shandong
Happy Fan Shop	King of Power Saving	No visible results so far	Jingdong prefecture level city in Shandong
Happy Fan Shop	King of Power Saving	It doesn't feel like it's working.	Jingdong prefecture level city in Shandong
Happy Fan Shop	King of Power Saving	Where's the effect? Where are the power savings?	Jingdong prefecture level city in Shandong
Happy Fan Shop	King of Power Saving	Let's see how it works in practice!	Jingdong prefecture level city in Shandong
Happy Fan Shop	King of Power Saving	I'll wait until I use it to give you a good review	Jingdong prefecture level city in Shandong
Happy Fan Shop	King of Power Saving	I'll follow up after I've used it	Jingdong prefecture level city in Shandong
Happy Fan Shop	King of Power Saving	Well, that's good for saving power.	Jingdong prefecture level city in Shandong
Happy Fan Shop	King of Power Saving	Received. Used in the know.	Jingdong prefecture level city in Shandong
Happy Fan Shop	King of Power Saving	It doesn't do much good.	Jingdong prefecture level city in Shandong
Happy Fan Shop	King of Power Saving	It's not working.	Jingdong prefecture level city in Shandong
Happy Fan Shop	King of Power Saving	I don't know. I just used it.	Jingdong prefecture level city in Shandong
Happy Fan Shop	King of Power Saving	Use it and come back to evaluate.	Jingdong prefecture level city in Shandong
Happy Fan Shop	King of Power Saving	Two words: useless.	Jingdong prefecture level city in Shandong

Happy Fan Shop	King of Power Saving	To be used and then evaluated	Jingdong prefecture level city in Shandong
Happy Fan Shop	King of Power Saving	No shipment.	Jingdong prefecture level city in Shandong
Happy Fan Shop	King of Power Saving	Fake. Speechless.	Jingdong prefecture level city in Shandong
Happy Fan Shop	King of Power Saving	fantastic (idiom); awesome	Jingdong prefecture level city in Shandong
Happy Fan Shop	King of Power Saving	an excellent	Jingdong prefecture level city in Shandong
Happy Fan Shop	King of Power Saving	an excellent	Jingdong prefecture level city in Shandong
Happy Fan Shop	King of Power Saving	Want to buy! Recommended not to buy! No effect	Jingdong prefecture level city in Shandong
Happy Fan Shop	King of Power Saving	That's all there is to it. I can't figure out how to save electricity.	Jingdong prefecture level city in Shandong
Happy Fan Shop	King of Power Saving	It's no use.	Jingdong prefecture level city in Shandong
Happy Fan Shop	King of Power Saving	Things are more perfect than I imagined, very powerful, it is worth getting Packaging is very strong, things are well protected, it is worth the praise things are very satisfied Oh, beautiful and stylish atmosphere, customer service is super patient, service is meticulous and attentive, the big brand is not	Jingdong prefecture level city in Shandong
Happy Fan Shop	King of Power Saving	The effect is too good, the family has now several units, back to buy a number of times, the power saver received, power-saving effect is very good,, fine workmanship, long warranty, but also purify the air, before buying a used for a period of time, with the okay, and then bought a, and only now come to the	Jingdong prefecture level city in Shandong
Happy Fan Shop	King of Power Saving	Quite good, and imagine the same, casual ride are very good, satisfied! Baby received it 👌, really great ah! 🌈 quality is really nothing to say, very satisfied! 🆗 happy happy 😃 genuine, great, the price is very good, in short, quite good, will buy again a very good shopping experience, attentive service, fast logistics, distribution is also thoughtful, the attitude is also very good, value for money, ah,	Jingdong prefecture level city in Shandong
Happy Fan Shop	King of Power Saving	Power-saving effect: strong Cost-effective: quite good Appearance style: beautiful Workmanship material: meticulous Experience: looking forward to power-saving effect	Jingdong prefecture level city in Shandong
Happy Fan Shop	King of Power Saving	Cost-effective is okay, the appearance of the style of meticulous workmanship, looking forward to energy-saving effect.	Jingdong prefecture level city in Shandong
Happy Fan Shop	King of Power Saving	Very Good Very Power Saving This Power Saver is really power saving As soon as the power is turned on, the Power Saver starts the power saving function for you.	Jingdong prefecture level city in Shandong
Happy Fan Shop	King of Power Saving	Logistics is very fast, the price is favorable, friends introduced that it is very good to use, bought 2 at once, looking forward to the effect, used after the follow-up comment.	Jingdong prefecture level city in Shandong
Happy Fan Shop	King of Power Saving	It's all fake. It doesn't save any electricity.	Jingdong prefecture level city in Shandong
Happy Fan Shop	King of Power Saving	This Power Saver is so much better than the one I bought before, not only the effect is good, the quality is very good in all aspects, the customer service attitude is also very good, recommended to buy!	Jingdong prefecture level city in Shandong
Happy Fan Shop	King of Power Saving	Logistics is very fast, buy for parents, good quality, once bought 2, power-saving effect is good to buy 2 more, look forward to using the effect, satisfied will recommend to friends.	Jingdong prefecture level city in Shandong
Happy Fan Shop	King of Power Saving	With the description is the same, very high-grade, good value, to buy friends can order Oh!	Jingdong prefecture level city in Shandong
Happy Fan Shop	King of Power Saving	Not using much electricity in the winter saving over 100 per month! Ready to buy 2 more units in the summer ！！！！！ Good quality, good price, power saving effect is obvious, cost-effective, worth buying, can be recommended!	Jingdong prefecture level city in Shandong
Happy Fan Shop	King of Power Saving	Purchased a power saver received on the put into use, rent a room with many electrical appliances, power consumption is very large, we have a piece of electricity or 1.2 dollars a time, with this power saver is much better, every day can save a lot of money, very convenient and practical.	Jingdong prefecture level city in Shandong
Happy Fan Shop	King of Power Saving	The power saving effect is very good: my supermarket monthly electricity bill is more than 7000 yuan. On May 1st someone recommended buying a couple of power savers, and I bought 5 of them first. The electricity bill was about 2000 less in June. This time, I bought 5 more. The seller said 800 degrees of electricity with one. I feel it's basically enough. A total of 10 units	Jingdong prefecture level city in Shandong
Happy Fan Shop	King of Power Saving	I never thought I could get such a good baby at such an affordable price, it's very good value for money, I love it!	Jingdong prefecture level city in Shandong
Happy Fan Shop	King of Power Saving	Previously bought other brands of power savers, but found no effect at home, this time to see how the products on the Jingdong, inserted in the home socket, the LED display is blue, the night light is very nice to see, look at the instructions that have to be half a year in order to achieve the best results, try it, I believe that the quality of the Jingdong, it should not be wrong.	Jingdong prefecture level city in Shandong
Happy Fan Shop	King of Power Saving	Superb, inexpensive, saves a lot of points, been using it for a long time!	Jingdong prefecture level city in Shandong
Happy Fan Shop	King of Power Saving	There are a lot of appliances in the house, the summer air conditioning is very costly, I bought this in front of the air conditioning effect is very good, I explain that I bought three in one breath, I use more electricity a month down to 200 or so, very satisfied.	Jingdong prefecture level city in Shandong
Happy Fan Shop	King of Power Saving	A month can save more than 200, home monthly electricity bill of more than 600 dollars, inserted 3 or so, an amount of the balance of the average can save more than 200. Product is very good, has been buying this one, has given a lot of relatives recommended, the quality is also very good, before buying a look with this same, who knows to buy home has not been used for more than a week on the bad, the quality of this is really good with more than two years!	Jingdong prefecture level city in Shandong
Happy Fan Shop	King of Power Saving	Whole house power saving king, can really save electricity, friends recommended, high power saving appliances, only replacement, no repair, after-sales worry-free!	Jingdong prefecture level city in Shandong
Happy Fan Shop	King of Power Saving	Great item love it, great customer service, great shopping experience!	Jingdong prefecture level city in Shandong
Happy Fan Shop	King of Power Saving	Power saving, very good use, much cheaper than the physical store, satisfaction	Jingdong prefecture level city in Shandong
Happy Fan Shop	King of Power Saving	In use, very well packaged and fast delivery of credit!	Jingdong prefecture level city in Shandong
Happy Fan Shop	King of Power Saving	Product quality, customer service attitude is particularly good, like, praise!	Jingdong prefecture level city in Shandong
Happy Fan Shop	King of Power Saving	The logistics is very fast, the packaging is not broken, the quality is very good, I like it very much!	Jingdong prefecture level city in Shandong
Happy Fan Shop	King of Power Saving	The saver was well packaged and was as described when plugged in	Jingdong prefecture level city in Shandong
Happy Fan Shop	King of Power Saving	Energy efficient and very satisfied	Jingdong prefecture level city in Shandong
Happy Fan Shop	King of Power Saving	Great quality for the price and fast shipping.	Jingdong prefecture level city in Shandong
Happy Fan Shop	King of Power Saving	It's great, just as described. Perfect.	Jingdong prefecture level city in Shandong
Happy Fan Shop	King of Power Saving	There is no power saving effect, but rather ring up an increase in electricity costs, because this machine is on continuously all day long, uninterruptedly, its own electricity and increased costs, plus the cost of purchasing the machine, so I feel that the use of this machine instead of increasing the	Jingdong prefecture level city in Shandong
Happy Fan Shop	King of Power Saving	Recommended by the owner of the store next door, looked at his home does have to save electricity, decided to get. Indeed quite can, the voltage is much more stable, there is a reduction in electricity	Jingdong prefecture level city in Shandong
Happy Fan Shop	King of Power Saving	Goods have been received value for money, affordable and reasonable price, and the description of the same, power-saving safe and reliable!	Jingdong prefecture level city in Shandong
Happy Fan Shop	King of Power Saving	Listen to friends introduced to buy, tried to save a lot of electricity is good, quite good, worth buying.	Jingdong prefecture level city in Shandong
Happy Fan Shop	King of Power Saving	The product is easy to use, safe and environmentally friendly, only used for a short time, to the next month, after the electricity bill status display to know how the performance of the power saver efficacy.	Jingdong prefecture level city in Shandong
Happy Fan Shop	King of Power Saving	Last month's electricity bill is 178 yuan, with this power saver is 92 yuan, ready to buy two more back home, I was introduced to buy a friend, just started to not believe that I did not expect the effect!	Jingdong prefecture level city in Shandong

Happy Fan Shop	King of Power Saving	It looks nice, but after using it for a few days I can't see how it saves power?	Jingdong prefecture level city in Shandong
Happy Fan Shop	King of Power Saving	Recommended by a friend, it is genuine, as described by the merchant, cost-effective	Jingdong prefecture level city in Shandong
Happy Fan Shop	King of Power Saving	Works well, affordable and saves money.	Jingdong prefecture level city in Shandong
Happy Fan Shop	King of Power Saving	Feels good haven't compared it yet, the air conditioning has been on a lot in the past two months	Jingdong prefecture level city in Shandong
Happy Fan Shop	King of Power Saving	Saving about 5 kWh per day is a good thing	Jingdong prefecture level city in Shandong
Happy Fan Shop	King of Power Saving	Not bad, affordable, best value for money	Jingdong prefecture level city in Shandong
Happy Fan Shop	King of Power Saving	The goods are genuine and the logistics are faster.	Jingdong prefecture level city in Shandong
Happy Fan Shop	King of Power Saving	Goods have been received today, has been plugged into the outlet, looking forward to saving electricity, after a period of time to see how the effect in the follow-up comment!	Jingdong prefecture level city in Shandong
Happy Fan Shop	King of Power Saving	I just got it home and haven't used it for long, so I'll use it for a couple months and see what happens. Seeing that most people have good reviews, it should be fine.	Jingdong prefecture level city in Shandong
Happy Fan Shop	King of Power Saving	I'm looking forward to the results, so I'll give you a good review out of habit! Follow up review after a while	Jingdong prefecture level city in Shandong
Happy Fan Shop	King of Power Saving	Use a dish first to know if it will help save energy	Jingdong prefecture level city in Shandong
Happy Fan Shop	King of Power Saving	Whether it saves electricity or not, we don't know yet.	Jingdong prefecture level city in Shandong
Happy Fan Shop	King of Power Saving	Goods have been received, use a period of time to know how oh. The courier did not deliver to the door ah! Sent to the station ah! The heat is inconvenient ah!	Jingdong prefecture level city in Shandong
Happy Fan Shop	King of Power Saving	A little effect, before an hour a power, now an hour 0.76 degrees, a dollar a power = save 24 cents, although not a lot, first use it!	Jingdong prefecture level city in Shandong
Happy Fan Shop	King of Power Saving	Just received the goods, use a month to evaluate	Jingdong prefecture level city in Shandong
Happy Fan Shop	King of Power Saving	Home every month with a lot of electricity, inadvertently found this good thing online that can save the beginning of a skeptical attitude to consult customer service, customer service replied very professional, said 120 days can be refunded that first try it!	Jingdong prefecture level city in Shandong
Happy Fan Shop	King of Power Saving	After receiving the goods this afternoon, immediately installed in the bathroom Smith energy storage electric water heater on the power outlet (instead of connecting with a plug board), when used, found that the "Power Saving King" LCD display, is the value of the voltage, not the store's video said so	Jingdong prefecture level city in Shandong
Happy Fan Shop	King of Power Saving	Has made many days, did not feel the power saving, the effect is not obvious.	Jingdong prefecture level city in Shandong
Happy Fan Shop	King of Power Saving	Energy savers do not save energy at all, but rather consume a lot of electricity.	Jingdong prefecture level city in Shandong
Happy Fan Shop	King of Power Saving	It's useless. It's a waste of money.	Jingdong prefecture level city in Shandong
Happy Fan Shop	King of Power Saving	Last month saved more than 500, which came back to buy, the store bought three, and then buy 1 home use, my family is dry beauty salon, more instruments, especially electricity, a month almost 1000. or so, with this did not expect to save so much, great, five-star praise!	Jingdong prefecture level city in Shandong
Happy Fan Shop	King of Power Saving	It rained a couple days ago and I just went to get the item back today , not bad, hey I looked at the meter and that red display light takes several seconds to jump, did not make a mistake buying it. After a period of time to evaluate.	Jingdong prefecture level city in Shandong
Happy Fan Shop	King of Power Saving	Without further ado, the electricity bill came down, this month less 102 yuan, has been repurchased, I also recommended to my friends, all said that there are effects, the product is okay, the effect is quite	Jingdong prefecture level city in Shandong
Happy Fan Shop	King of Power Saving	After buying and using two months really see the effect, so only to evaluate! Originally held the mindset of trying, I did not expect really effective, worth having!	Jingdong prefecture level city in Shandong
Happy Fan Shop	King of Power Saving	In the use of this energy saver before the monthly electricity bill is more than 150 yuan or so, now save thirty percent, every day to work, six or seven o'clock in the afternoon to go home, I do not know why the electricity bill is so expensive, energy-saving appliances really have the effect of recommending	Jingdong prefecture level city in Shandong
Happy Fan Shop	King of Power Saving	It saves about 100 bucks a month. A friend recommended it to me, and he's been using it for a while, and the effect is so obvious that I bought one too.	Jingdong prefecture level city in Shandong
Happy Fan Shop	King of Power Saving	My dad asked me to buy one, but after I paid the bill, I asked and he said it was a lot less this month, so he asked me to buy another one.	Jingdong prefecture level city in Shandong
Happy Fan Shop	King of Power Saving	The specific effect will have to be tested for at least two months to compare whether there is a power saving effect or not.	Jingdong prefecture level city in Shandong
Happy Fan Shop	King of Power Saving	'The goods have been received, immediately go to use, please ask the boss you instructions do not allow to withdraw, there are two fuses installed there!	Jingdong prefecture level city in Shandong
Happy Fan Shop	King of Power Saving	First praise is using, after using if good then follow up, now because I have not yet seen the results	Jingdong prefecture level city in Shandong
Happy Fan Shop	King of Power Saving	I've just been using it, and I haven't seen if it has a power-saving effect yet, so I'll follow up after 10 days and half a month.	Jingdong prefecture level city in Shandong
Happy Fan Shop	King of Power Saving	Just received the intelligent power saving meter, immediately plugged in to use, see it is not bad, and order to sell the second time	Jingdong prefecture level city in Shandong
Happy Fan Shop	King of Power Saving	This power saver is a bit effective, used for half a month, the effect is still visible.	Jingdong prefecture level city in Shandong
Happy Fan Shop	King of Power Saving	I received the baby and it looks great, I'm trying it out now and will keep an eye on it afterward.	Jingdong prefecture level city in Shandong
Happy Fan Shop	King of Power Saving	I've just received it, but I'm still trying to see how it works, so I'll come back in a little while.	Jingdong prefecture level city in Shandong
Happy Fan Shop	King of Power Saving	Just used it wait to use it for a while and then comment thanks, all first five stars.	Jingdong prefecture level city in Shandong
Happy Fan Shop	King of Power Saving	Very trustworthy brand, quality is guaranteed, the price is still affordable, quite satisfied!	Jingdong prefecture level city in Shandong
Happy Fan Shop	King of Power Saving	Things received, I hope that the platform of Jingdong do not sell fake goods are available	Jingdong prefecture level city in Shandong
Happy Fan Shop	King of Power Saving	Energy saver received, the use of the effect is good and the business description of the same	Jingdong prefecture level city in Shandong
Happy Fan Shop	King of Power Saving	The surface looks beautiful, the actual effect is not known, after using additional evaluation.	Jingdong prefecture level city in Shandong
Happy Fan Shop	King of Power Saving	Tried it for a few months, it really saves electricity, and I bought it for a friend.	Jingdong prefecture level city in Shandong
Happy Fan Shop	King of Power Saving	It didn't work and wanted to return it, it really didn't work at all.	Jingdong prefecture level city in Shandong
Happy Fan Shop	King of Power Saving	Just used it, don't know how effective it is, looking forward to usefulness	Jingdong prefecture level city in Shandong
Happy Fan Shop	King of Power Saving	After two months before the effect I do not know if it is true!	Jingdong prefecture level city in Shandong
Happy Fan Shop	King of Power Saving	So fast! Ordered yesterday and received today 👍👍👍👍👍👍👍👍👍 🌹🌹🌹🌹🌹🌹🌹🌹🌹	Jingdong prefecture level city in Shandong
Happy Fan Shop	King of Power Saving	I've started using it. We'll see how it works.	Jingdong prefecture level city in Shandong
Happy Fan Shop	King of Power Saving	I just received the goods, I won't know the effect until after two.	Jingdong prefecture level city in Shandong
Happy Fan Shop	King of Power Saving	Just received the goods, the specific effect will be evaluated later	Jingdong prefecture level city in Shandong
Happy Fan Shop	King of Power Saving	I've received the goods, I don't know if it's any good yet	Jingdong prefecture level city in Shandong

Happy Fan Shop	King of Power Saving	Try it for a month and then come back with a concrete evaluation	Jingdong prefecture level city in Shandong
Happy Fan Shop	King of Power Saving	In use, after two months to see the effect of power saving	Jingdong prefecture level city in Shandong
Happy Fan Shop	King of Power Saving	Power Saver. It's authentic. It's black. It works.	Jingdong prefecture level city in Shandong
Happy Fan Shop	King of Power Saving	I've been using it for a week now and it's not working.	Jingdong prefecture level city in Shandong
Happy Fan Shop	King of Power Saving	I'm just using it. I don't know how it's going to work.	Jingdong prefecture level city in Shandong
Happy Fan Shop	King of Power Saving	Products are better looking out for power savings	Jingdong prefecture level city in Shandong
Happy Fan Shop	King of Power Saving	I received it and I've kept it on. I hope it works.	Jingdong prefecture level city in Shandong
Happy Fan Shop	King of Power Saving	It's really good! Worth recommending to buy!	Jingdong prefecture level city in Shandong
Happy Fan Shop	King of Power Saving	Try it for a few days and see if it doesn't work, then return it.	Jingdong prefecture level city in Shandong
Happy Fan Shop	King of Power Saving	It's in use and I hope to be pleasantly surprised.	Jingdong prefecture level city in Shandong
Happy Fan Shop	King of Power Saving	We'll see how it works when we use it	Jingdong prefecture level city in Shandong
Happy Fan Shop	King of Power Saving	Let's see if it works.	Jingdong prefecture level city in Shandong
Happy Fan Shop	King of Power Saving	I'm using it and will follow up with a review	Jingdong prefecture level city in Shandong
Happy Fan Shop	King of Power Saving	It really works. It saves energy.	Jingdong prefecture level city in Shandong
Happy Fan Shop	King of Power Saving	It does have some power savings!	Jingdong prefecture level city in Shandong
Happy Fan Shop	King of Power Saving	Jingdong shopping speed is convenient and timely	Jingdong prefecture level city in Shandong
Happy Fan Shop	King of Power Saving	Second time I've bought it. Everything's fine.	Jingdong prefecture level city in Shandong
Happy Fan Shop	King of Power Saving	I don't know if it works yet.	Jingdong prefecture level city in Shandong
Happy Fan Shop	King of Power Saving	Not used, don't know how well it works.	Jingdong prefecture level city in Shandong
Happy Fan Shop	King of Power Saving	No visible results so far	Jingdong prefecture level city in Shandong
Happy Fan Shop	King of Power Saving	It doesn't feel like it's working.	Jingdong prefecture level city in Shandong
Happy Fan Shop	King of Power Saving	Where's the effect? Where are the power savings?	Jingdong prefecture level city in Shandong
Happy Fan Shop	King of Power Saving	Let's see how it works in practice!	Jingdong prefecture level city in Shandong
Happy Fan Shop	King of Power Saving	I'll wait until I use it to give you a good review	Jingdong prefecture level city in Shandong
Happy Fan Shop	King of Power Saving	I'll follow up after I've used it	Jingdong prefecture level city in Shandong
Happy Fan Shop	King of Power Saving	Well, that's good for saving power.	Jingdong prefecture level city in Shandong
Happy Fan Shop	King of Power Saving	Received. Used in the know.	Jingdong prefecture level city in Shandong
Happy Fan Shop	King of Power Saving	It doesn't do much good.	Jingdong prefecture level city in Shandong
Happy Fan Shop	King of Power Saving	It's not working.	Jingdong prefecture level city in Shandong
Happy Fan Shop	King of Power Saving	I don't know. I just used it.	Jingdong prefecture level city in Shandong
Happy Fan Shop	King of Power Saving	Use it and come back to evaluate.	Jingdong prefecture level city in Shandong
Happy Fan Shop	King of Power Saving	Two words: useless.	Jingdong prefecture level city in Shandong
Happy Fan Shop	King of Power Saving	To be used and then evaluated	Jingdong prefecture level city in Shandong
Happy Fan Shop	King of Power Saving	No shipment.	Jingdong prefecture level city in Shandong
Happy Fan Shop	King of Power Saving	Fake. Speechless.	Jingdong prefecture level city in Shandong
Happy Fan Shop	King of Power Saving	fantastic (idiom); awesome	Jingdong prefecture level city in Shandong
Happy Fan Shop	King of Power Saving	an excellent	Jingdong prefecture level city in Shandong
Happy Fan Shop	King of Power Saving	an excellent	Jingdong prefecture level city in Shandong
Happy Fan Shop	King of Power Saving	Want to buy! Recommended not to buy! No effect	Jingdong prefecture level city in Shandong
Happy Fan Shop	King of Power Saving	That's all there is to it. I can't figure out how to save electricity.	Jingdong prefecture level city in Shandong
Happy Fan Shop	King of Power Saving	It's no use.	Jingdong prefecture level city in Shandong
Happy Fan Shop	King of Power Saving	Things are more perfect than I imagined, very powerful, it is worth getting Packaging is very strong, things are well protected, it is worth the praise things are very satisfied Oh, beautiful and stylish atmosphere, customer service is super patient, service is meticulous and attentive, the big brand is not	Jingdong prefecture level city in Shandong
Happy Fan Shop	King of Power Saving	The effect is too good, the family has now several units, back to buy a number of times, the power saver received, power-saving effect is very good,, fine workmanship, long warranty, but also purify the air, before buying a used for a period of time, with the okay, and then bought a, and only now come to the	Jingdong prefecture level city in Shandong
Happy Fan Shop	King of Power Saving	Quite good, and imagine the same, casual ride are very good, satisfied! Baby received it 👌, really great ah! 🌈 quality is really nothing to say, very satisfied! 🆗 happy happy 😃 genuine, great, the price is very good, in short, quite good, will buy again a very good shopping experience, attentive service, fast logistics, distribution is also thoughtful, the attitude is also very good, value for money, ah,	Jingdong prefecture level city in Shandong
Happy Fan Shop	King of Power Saving	Power-saving effect: strong Cost-effective: quite good Appearance style: beautiful Workmanship material: meticulous Experience: looking forward to power-saving effect	Jingdong prefecture level city in Shandong
Happy Fan Shop	King of Power Saving	Cost-effective is okay, the appearance of the style of meticulous workmanship, looking forward to energy-saving effect.	Jingdong prefecture level city in Shandong
Happy Fan Shop	King of Power Saving	Very Good Very Power Saving This Power Saver is really power saving As soon as the power is turned on, the Power Saver starts the power saving function for you.	Jingdong prefecture level city in Shandong
Happy Fan Shop	King of Power Saving	Logistics is very fast, the price is favorable, friends introduced that it is very good to use, bought 2 at once, looking forward to the effect, used after the follow-up comment.	Jingdong prefecture level city in Shandong
Happy Fan Shop	King of Power Saving	It's all fake. It doesn't save any electricity.	Jingdong prefecture level city in Shandong

Happy Fan Shop	King of Power Saving	This Power Saver is so much better than the one I bought before, not only the effect is good, the quality is very good in all aspects, the customer service attitude is also very good, recommended to buy!	Jingdong prefecture level city in Shandong
Happy Fan Shop	King of Power Saving	Logistics is very fast, buy for parents, good quality, once bought 2, power-saving effect is good to buy 2 more, look forward to using the effect, satisfied will recommend to friends.	Jingdong prefecture level city in Shandong
Happy Fan Shop	King of Power Saving	With the description is the same, very high-grade, good value, to buy friends can order Oh!	Jingdong prefecture level city in Shandong
Happy Fan Shop	King of Power Saving	Not using much electricity in the winter saving over 100 per month! Ready to buy 2 more units in the summer ! ! ! ! ! Good quality, good price, power saving effect is obvious, cost-effective, worth buying, can be recommended!	Jingdong prefecture level city in Shandong
Happy Fan Shop	King of Power Saving	Purchased a power saver received on the put into use, rent a room with many electrical appliances, power consumption is very large, we have a piece of electricity or 1.2 dollars a time, with this power saver is much better, every day can save a lot of money, very convenient and practical.	Jingdong prefecture level city in Shandong
Happy Fan Shop	King of Power Saving	The power saving effect is very good: my supermarket monthly electricity bill is more than 7000 yuan. On May 1st someone recommended buying a couple of power savers, and I bought 5 of them first. The electricity bill was about 2000 less in June. This time, I bought 5 more. The seller said 800 degrees of electricity with one. I feel it's basically enough. A total of 10 units	Jingdong prefecture level city in Shandong
Happy Fan Shop	King of Power Saving	I never thought I could get such a good baby at such an affordable price, it's very good value for money, I love it!	Jingdong prefecture level city in Shandong
Happy Fan Shop	King of Power Saving	Previously bought other brands of power savers, but found no effect at home, this time to see how the products on the Jingdong, inserted in the home socket, the LED display is blue, the night light is very nice to see, look at the instructions that have to be half a year in order to achieve the best results, try it, I believe that the quality of the Jingdong, it should not be wrong.	Jingdong prefecture level city in Shandong
Happy Fan Shop	King of Power Saving	Superb, inexpensive, saves a lot of points, been using it for a long time!	Jingdong prefecture level city in Shandong
Happy Fan Shop	King of Power Saving	There are a lot of appliances in the house, the summer air conditioning is very costly, I bought this in front of the air conditioning effect is very good, I explain that I bought three in one breath, I use more electricity a month down to 200 or so, very satisfied.	Jingdong prefecture level city in Shandong
Happy Fan Shop	King of Power Saving	A month can save more than 200, home monthly electricity bill of more than 600 dollars, inserted 3 or so, an amount of the balance of the average can save more than 200. Product is very good, has been buying this one, has given a lot of relatives recommended, the quality is also very good, before buying a look with this same, who knows to buy home has not been used for more than a week on the bad, the quality of this is really good with more than two years!	Jingdong prefecture level city in Shandong
Happy Fan Shop	King of Power Saving	Whole house power saving king, can really save electricity, friends recommended, high power saving appliances, only replacement, no repair, after-sales worry-free!	Jingdong prefecture level city in Shandong
Happy Fan Shop	King of Power Saving	Great item love it, great customer service, great shopping experience!	Jingdong prefecture level city in Shandong
Happy Fan Shop	King of Power Saving	Power saving, very good use, much cheaper than the physical store, satisfaction	Jingdong prefecture level city in Shandong
Happy Fan Shop	King of Power Saving	In use, very well packaged and fast delivery of credit!	Jingdong prefecture level city in Shandong
Happy Fan Shop	King of Power Saving	Product quality, customer service attitude is particularly good, like, praise!	Jingdong prefecture level city in Shandong
Happy Fan Shop	King of Power Saving	The logistics is very fast, the packaging is not broken, the quality is very good, I like it very much!	Jingdong prefecture level city in Shandong
Happy Fan Shop	King of Power Saving	The saver was well packaged and was as described when plugged in	Jingdong prefecture level city in Shandong
Happy Fan Shop	King of Power Saving	Energy efficient and very satisfied	Jingdong prefecture level city in Shandong
Happy Fan Shop	King of Power Saving	Great quality for the price and fast shipping.	Jingdong prefecture level city in Shandong
Happy Fan Shop	King of Power Saving	It's great, just as described. Perfect.	Jingdong prefecture level city in Shandong
Happy Fan Shop	King of Power Saving	There is no power saving effect, but rather ring up an increase in electricity costs, because this machine is on continuously all day long, uninterruptedly, its own electricity and increased costs, plus the cost of purchasing the machine, so I feel that the use of this machine instead of increasing the	Jingdong prefecture level city in Shandong
Happy Fan Shop	King of Power Saving	Recommended by the owner of the store next door, looked at his home does have to save electricity, decided to get. Indeed quite can, the voltage is much more stable, there is a reduction in electricity	Jingdong prefecture level city in Shandong
Happy Fan Shop	King of Power Saving	Goods have been received value for money, affordable and reasonable price, and the description of the same, power-saving safe and reliable!	Jingdong prefecture level city in Shandong
Happy Fan Shop	King of Power Saving	Listen to friends introduced to buy, tried to save a lot of electricity is good, quite good, worth buying.	Jingdong prefecture level city in Shandong
Happy Fan Shop	King of Power Saving	The product is easy to use, safe and environmentally friendly, only used for a short time, to the next month, after the electricity bill status display to know how the performance of the power saver efficacy.	Jingdong prefecture level city in Shandong
Happy Fan Shop	King of Power Saving	Last month's electricity bill is 178 yuan, with this power saver is 92 yuan, ready to buy two more back home, I was introduced to buy a friend, just started to not believe that I did not expect the effect!	Jingdong prefecture level city in Shandong
Happy Fan Shop	King of Power Saving	It looks nice, but after using it for a few days I can't see how it saves power?	Jingdong prefecture level city in Shandong
Happy Fan Shop	King of Power Saving	Recommended by a friend, it is genuine, as described by the merchant, cost-effective	Jingdong prefecture level city in Shandong
Happy Fan Shop	King of Power Saving	Works well, affordable and saves money.	Jingdong prefecture level city in Shandong
Happy Fan Shop	King of Power Saving	Feels good haven't compared it yet, the air conditioning has been on a lot in the past two months	Jingdong prefecture level city in Shandong
Happy Fan Shop	King of Power Saving	Saving about 5 kWh per day is a good thing	Jingdong prefecture level city in Shandong
Happy Fan Shop	King of Power Saving	Not bad, affordable, best value for money	Jingdong prefecture level city in Shandong
Happy Fan Shop	King of Power Saving	The goods are genuine and the logistics are faster.	Jingdong prefecture level city in Shandong
Happy Fan Shop	King of Power Saving	Goods have been received today, has been plugged into the outlet, looking forward to saving electricity, after a period of time to see how the effect in the follow-up comment!	Jingdong prefecture level city in Shandong
Happy Fan Shop	King of Power Saving	I just got it home and haven't used it for long, so I'll use it for a couple months and see what happens. Seeing that most people have good reviews, it should be fine.	Jingdong prefecture level city in Shandong
Happy Fan Shop	King of Power Saving	I'm looking forward to the results, so I'll give you a good review out of habit! Follow up review after a while	Jingdong prefecture level city in Shandong
Happy Fan Shop	King of Power Saving	Use a dish first to know if it will help save energy	Jingdong prefecture level city in Shandong
Happy Fan Shop	King of Power Saving	Whether it saves electricity or not, we don't know yet.	Jingdong prefecture level city in Shandong
Happy Fan Shop	King of Power Saving	Goods have been received, use a period of time to know how oh. The courier did not deliver to the door ah! Sent to the station ah! The heat is inconvenient ah!	Jingdong prefecture level city in Shandong
Happy Fan Shop	King of Power Saving	A little effect, before an hour a power, now an hour 0.76 degrees, a dollar a power = save 24 cents, although not a lot, first use it!	Jingdong prefecture level city in Shandong
Happy Fan Shop	King of Power Saving	Just received the goods, use a month to evaluate	Jingdong prefecture level city in Shandong
Happy Fan Shop	King of Power Saving	Home every month with a lot of electricity, inadvertently found this good thing online that can save the beginning of a skeptical attitude to consult customer service, customer service replied very professional, said 120 days can be refunded that first try it!	Jingdong prefecture level city in Shandong
Happy Fan Shop	King of Power Saving	After receiving the goods this afternoon, immediately installed in the bathroom Smith energy storage electric water heater on the power outlet (instead of connecting with a plug board), when used, found that the "Power Saving King" LCD display, is the value of the voltage, not the store's video said so	Jingdong prefecture level city in Shandong
Happy Fan Shop	King of Power Saving	Has made many days, did not feel the power saving, the effect is not obvious.	Jingdong prefecture level city in Shandong

Happy Fan Shop	King of Power Saving	Energy savers do not save energy at all, but rather consume a lot of electricity.	Jingdong prefecture level city in Shandong
Happy Fan Shop	King of Power Saving	It's useless. It's a waste of money.	Jingdong prefecture level city in Shandong
Happy Fan Shop	King of Power Saving	Last month saved more than 500, which came back to buy, the store bought three, and then buy 1 home use, my family is dry beauty salon, more instruments, especially electricity, a month almost 1000. or so, with this did not expect to save so much, great, five-star praise!	Jingdong prefecture level city in Shandong
Happy Fan Shop	King of Power Saving	It rained a couple days ago and I just went to get the item back today , not bad, hey I looked at the meter and that red display light takes several seconds to jump, did not make a mistake buying it. After a period of time to evaluate.	Jingdong prefecture level city in Shandong
Happy Fan Shop	King of Power Saving	Without further ado, the electricity bill came down, this month less 102 yuan, has been repurchased, I also recommended to my friends, all said that there are effects, the product is okay, the effect is quite	Jingdong prefecture level city in Shandong
Happy Fan Shop	King of Power Saving	After buying and using two months really see the effect, so only to evaluate! Originally held the mindset of trying, I did not expect really effective, worth having!	Jingdong prefecture level city in Shandong
Happy Fan Shop	King of Power Saving	In the use of this energy saver before the monthly electricity bill is more than 150 yuan or so, now save thirty percent, every day to work, six or seven o'clock in the afternoon to go home, I do not know why the electricity bill is so expensive, energy-saving appliances really have the effect of recommending	Jingdong prefecture level city in Shandong
Happy Fan Shop	King of Power Saving	It saves about 100 bucks a month. A friend recommended it to me, and he's been using it for a while, and the effect is so obvious that I bought one too.	Jingdong prefecture level city in Shandong
Happy Fan Shop	King of Power Saving	My dad asked me to buy one, but after I paid the bill, I asked and he said it was a lot less this month, so he asked me to buy another one.	Jingdong prefecture level city in Shandong
Happy Fan Shop	King of Power Saving	The specific effect will have to be tested for at least two months to compare whether there is a power saving effect or not.	Jingdong prefecture level city in Shandong
Happy Fan Shop	King of Power Saving	'The goods have been received, immediately go to use, please ask the boss you instructions do not allow to withdraw, there are two fuses installed there!	Jingdong prefecture level city in Shandong
Happy Fan Shop	King of Power Saving	First praise is using, after using if good then follow up, now because I have not yet seen the results	Jingdong prefecture level city in Shandong
Happy Fan Shop	King of Power Saving	I've just been using it, and I haven't seen if it has a power-saving effect yet, so I'll follow up after 10 days and half a month.	Jingdong prefecture level city in Shandong
Happy Fan Shop	King of Power Saving	Just received the intelligent power saving meter, immediately plugged in to use, see it is not bad, and order to sell the second time	Jingdong prefecture level city in Shandong
Happy Fan Shop	King of Power Saving	This power saver is a bit effective, used for half a month, the effect is still visible.	Jingdong prefecture level city in Shandong
Happy Fan Shop	King of Power Saving	I received the baby and it looks great, I'm trying it out now and will keep an eye on it afterward.	Jingdong prefecture level city in Shandong
Happy Fan Shop	King of Power Saving	I've just received it, but I'm still trying to see how it works, so I'll come back in a little while.	Jingdong prefecture level city in Shandong
Happy Fan Shop	King of Power Saving	Just used it wait to use it for a while and then comment thanks, all first five stars.	Jingdong prefecture level city in Shandong
Happy Fan Shop	King of Power Saving	Very trustworthy brand, quality is guaranteed, the price is still affordable, quite satisfied!	Jingdong prefecture level city in Shandong
Happy Fan Shop	King of Power Saving	Things received, I hope that the platform of Jingdong do not sell fake goods are available	Jingdong prefecture level city in Shandong
Happy Fan Shop	King of Power Saving	Energy saver received, the use of the effect is good and the business description of the same	Jingdong prefecture level city in Shandong
Happy Fan Shop	King of Power Saving	The surface looks beautiful, the actual effect is not known, after using additional evaluation.	Jingdong prefecture level city in Shandong
Happy Fan Shop	King of Power Saving	Tried it for a few months, it really saves electricity, and I bought it for a friend.	Jingdong prefecture level city in Shandong
Happy Fan Shop	King of Power Saving	It didn't work and wanted to return it, it really didn't work at all.	Jingdong prefecture level city in Shandong
Happy Fan Shop	King of Power Saving	Just used it, don't know how effective it is, looking forward to usefulness	Jingdong prefecture level city in Shandong
Happy Fan Shop	King of Power Saving	After two months before the effect I do not know if it is true!	Jingdong prefecture level city in Shandong
Happy Fan Shop	King of Power Saving	So fast! Ordered yesterday and received today 👍👍👍👍👍👍👍👍👍 🌹🌹🌹🌹🌹🌹🌹🌹🌹	Jingdong prefecture level city in Shandong
Happy Fan Shop	King of Power Saving	I've started using it. We'll see how it works.	Jingdong prefecture level city in Shandong
Happy Fan Shop	King of Power Saving	I just received the goods, I won't know the effect until after two.	Jingdong prefecture level city in Shandong
Happy Fan Shop	King of Power Saving	Just received the goods, the specific effect will be evaluated later	Jingdong prefecture level city in Shandong
Happy Fan Shop	King of Power Saving	I've received the goods, I don't know if it's any good yet	Jingdong prefecture level city in Shandong
Happy Fan Shop	King of Power Saving	Try it for a month and then come back with a concrete evaluation	Jingdong prefecture level city in Shandong
Happy Fan Shop	King of Power Saving	In use, after two months to see the effect of power saving	Jingdong prefecture level city in Shandong
Happy Fan Shop	King of Power Saving	Power Saver. It's authentic. It's black. It works.	Jingdong prefecture level city in Shandong
Happy Fan Shop	King of Power Saving	I've been using it for a week now and it's not working.	Jingdong prefecture level city in Shandong
Happy Fan Shop	King of Power Saving	I'm just using it. I don't know how it's going to work.	Jingdong prefecture level city in Shandong
Happy Fan Shop	King of Power Saving	Products are better looking out for power savings	Jingdong prefecture level city in Shandong
Happy Fan Shop	King of Power Saving	I received it and I've kept it on. I hope it works.	Jingdong prefecture level city in Shandong
Happy Fan Shop	King of Power Saving	It's really good! Worth recommending to buy!	Jingdong prefecture level city in Shandong
Happy Fan Shop	King of Power Saving	Try it for a few days and see if it doesn't work, then return it.	Jingdong prefecture level city in Shandong
Happy Fan Shop	King of Power Saving	It's in use and I hope to be pleasantly surprised.	Jingdong prefecture level city in Shandong
Happy Fan Shop	King of Power Saving	We'll see how it works when we use it	Jingdong prefecture level city in Shandong
Happy Fan Shop	King of Power Saving	Let's see if it works.	Jingdong prefecture level city in Shandong
Happy Fan Shop	King of Power Saving	I'm using it and will follow up with a review	Jingdong prefecture level city in Shandong
Happy Fan Shop	King of Power Saving	It really works. It saves energy.	Jingdong prefecture level city in Shandong
Happy Fan Shop	King of Power Saving	It does have some power savings!	Jingdong prefecture level city in Shandong
Happy Fan Shop	King of Power Saving	Jingdong shopping speed is convenient and timely	Jingdong prefecture level city in Shandong
Happy Fan Shop	King of Power Saving	Second time I've bought it. Everything's fine.	Jingdong prefecture level city in Shandong
Happy Fan Shop	King of Power Saving	I don't know if it works yet.	Jingdong prefecture level city in Shandong
Happy Fan Shop	King of Power Saving	Not used, don't know how well it works.	Jingdong prefecture level city in Shandong

Happy Fan Shop	King of Power Saving	No visible results so far	Jingdong prefecture level city in Shandong
Happy Fan Shop	King of Power Saving	It doesn't feel like it's working.	Jingdong prefecture level city in Shandong
Happy Fan Shop	King of Power Saving	Where's the effect? Where are the power savings?	Jingdong prefecture level city in Shandong
Happy Fan Shop	King of Power Saving	Let's see how it works in practice!	Jingdong prefecture level city in Shandong
Happy Fan Shop	King of Power Saving	I'll wait until I use it to give you a good review	Jingdong prefecture level city in Shandong
Happy Fan Shop	King of Power Saving	I'll follow up after I've used it	Jingdong prefecture level city in Shandong
Happy Fan Shop	King of Power Saving	Well, that's good for saving power.	Jingdong prefecture level city in Shandong
Happy Fan Shop	King of Power Saving	Received. Used in the know.	Jingdong prefecture level city in Shandong
Happy Fan Shop	King of Power Saving	It doesn't do much good.	Jingdong prefecture level city in Shandong
Happy Fan Shop	King of Power Saving	It's not working.	Jingdong prefecture level city in Shandong
Happy Fan Shop	King of Power Saving	I don't know. I just used it.	Jingdong prefecture level city in Shandong
Happy Fan Shop	King of Power Saving	Use it and come back to evaluate.	Jingdong prefecture level city in Shandong
Happy Fan Shop	King of Power Saving	Two words: useless.	Jingdong prefecture level city in Shandong
Happy Fan Shop	King of Power Saving	To be used and then evaluated	Jingdong prefecture level city in Shandong
Happy Fan Shop	King of Power Saving	No shipment.	Jingdong prefecture level city in Shandong
Happy Fan Shop	King of Power Saving	Fake. Speechless.	Jingdong prefecture level city in Shandong
Happy Fan Shop	King of Power Saving	fantastic (idiom); awesome	Jingdong prefecture level city in Shandong
Happy Fan Shop	King of Power Saving	an excellent	Jingdong prefecture level city in Shandong
Happy Fan Shop	King of Power Saving	an excellent	Jingdong prefecture level city in Shandong
Happy Fan Shop	King of Power Saving	Want to buy! Recommended not to buy! No effect	Jingdong prefecture level city in Shandong
Happy Fan Shop	King of Power Saving	That's all there is to it. I can't figure out how to save electricity.	Jingdong prefecture level city in Shandong
Happy Fan Shop	King of Power Saving	It's no use.	Jingdong prefecture level city in Shandong
Happy Fan Shop	King of Power Saving	Things are more perfect than I imagined, very powerful, it is worth getting Packaging is very strong, things are well protected, it is worth the praise things are very satisfied Oh, beautiful and stylish atmosphere, customer service is super patient, service is meticulous and attentive, the big brand is not	Jingdong prefecture level city in Shandong
Happy Fan Shop	King of Power Saving	The effect is too good, the family has now several units, back to buy a number of times, the power saver received, power-saving effect is very good,, fine workmanship, long warranty, but also purify the air, before buying a used for a period of time, with the okay, and then bought a, and only now come to the	Jingdong prefecture level city in Shandong
Happy Fan Shop	King of Power Saving	Quite good, and imagine the same, casual ride are very good, satisfied! Baby received it 👌, really great ah! 🌈 quality is really nothing to say, very satisfied! 🆗 happy happy 😀 genuine, great, the price is very good, in short, quite good, will buy again a very good shopping experience, attentive service, fast logistics, distribution is also thoughtful, the attitude is also very good, value for money, ah,	Jingdong prefecture level city in Shandong
Happy Fan Shop	King of Power Saving	Power-saving effect: strong Cost-effective: quite good Appearance style: beautiful Workmanship material: meticulous Experience: looking forward to power-saving effect	Jingdong prefecture level city in Shandong
Happy Fan Shop	King of Power Saving	Cost-effective is okay, the appearance of the style of meticulous workmanship, looking forward to energy-saving effect.	Jingdong prefecture level city in Shandong
Happy Fan Shop	King of Power Saving	Very Good Very Power Saving This Power Saver is really power saving As soon as the power is turned on, the Power Saver starts the power saving function for you.	Jingdong prefecture level city in Shandong
Happy Fan Shop	King of Power Saving	Logistics is very fast, the price is favorable, friends introduced that it is very good to use, bought 2 at once, looking forward to the effect, used after the follow-up comment.	Jingdong prefecture level city in Shandong
Happy Fan Shop	King of Power Saving	It's all fake. It doesn't save any electricity.	Jingdong prefecture level city in Shandong
Happy Fan Shop	King of Power Saving	This Power Saver is so much better than the one I bought before, not only the effect is good, the quality is very good in all aspects, the customer service attitude is also very good, recommended to buy!	Jingdong prefecture level city in Shandong
Happy Fan Shop	King of Power Saving	Logistics is very fast, buy for parents, good quality, once bought 2, power-saving effect is good to buy 2 more, look forward to using the effect, satisfied will recommend to friends.	Jingdong prefecture level city in Shandong
Happy Fan Shop	King of Power Saving	With the description is the same, very high-grade, good value, to buy friends can order Oh!	Jingdong prefecture level city in Shandong
Happy Fan Shop	King of Power Saving	Not using much electricity in the winter saving over 100 per month! Ready to buy 2 more units in the summer ! ! ! ! ! Good quality, good price, power saving effect is obvious, cost-effective, worth buying, can be recommended!	Jingdong prefecture level city in Shandong
Happy Fan Shop	King of Power Saving	Purchased a power saver received on the put into use, rent a room with many electrical appliances, power consumption is very large, we have a piece of electricity or 1.2 dollars a time, with this power saver is much better, every day can save a lot of money, very convenient and practical.	Jingdong prefecture level city in Shandong
Happy Fan Shop	King of Power Saving	The power saving effect is very good: my supermarket monthly electricity bill is more than 7000 yuan. On May 1st someone recommended buying a couple of power savers, and I bought 5 of them first. The electricity bill was about 2000 less in June. This time, I bought 5 more. The seller said 800 degrees of electricity with one. I feel it's basically enough. A total of 10 units	Jingdong prefecture level city in Shandong
Happy Fan Shop	King of Power Saving	I never thought I could get such a good baby at such an affordable price, it's very good value for money, I love it!	Jingdong prefecture level city in Shandong
Happy Fan Shop	King of Power Saving	Previously bought other brands of power savers, but found no effect at home, this time to see how the products on the Jingdong, inserted in the home socket, the LED display is blue, the night light is very nice to see, look at the instructions that have to be half a year in order to achieve the best results, try it, I believe that the quality of the Jingdong, it should not be wrong.	Jingdong prefecture level city in Shandong
Happy Fan Shop	King of Power Saving	Superb, inexpensive, saves a lot of points, been using it for a long time!	Jingdong prefecture level city in Shandong
Happy Fan Shop	King of Power Saving	There are a lot of appliances in the house, the summer air conditioning is very costly, I bought this in front of the air conditioning effect is very good, I explain that I bought three in one breath, I use more electricity a month down to 200 or so, very satisfied.	Jingdong prefecture level city in Shandong
Happy Fan Shop	King of Power Saving	A month can save more than 200, home monthly electricity bill of more than 600 dollars, inserted 3 or so, an amount of the balance of the average can save more than 200. Product is very good, has been buying this one, has given a lot of relatives recommended, the quality is also very good, before buying a look with this same, who knows to buy home has not been used for more than a week on the bad, the quality of this is really good with more than two years!	Jingdong prefecture level city in Shandong
Happy Fan Shop	King of Power Saving	Whole house power saving king, can really save electricity, friends recommended, high power saving appliances, only replacement, no repair, after-sales worry-free!	Jingdong prefecture level city in Shandong
Happy Fan Shop	King of Power Saving	Great item love it, great customer service, great shopping experience!	Jingdong prefecture level city in Shandong
Happy Fan Shop	King of Power Saving	Power saving, very good use, much cheaper than the physical store, satisfaction	Jingdong prefecture level city in Shandong

Happy Fan Shop	King of Power Saving	In use, very well packaged and fast delivery of credit!	Jingdong prefecture level city in Shandong
Happy Fan Shop	King of Power Saving	Product quality, customer service attitude is particularly good, like, praise!	Jingdong prefecture level city in Shandong
Happy Fan Shop	King of Power Saving	The logistics is very fast, the packaging is not broken, the quality is very good, I like it very much!	Jingdong prefecture level city in Shandong
Happy Fan Shop	King of Power Saving	The saver was well packaged and was as described when plugged in	Jingdong prefecture level city in Shandong
Happy Fan Shop	King of Power Saving	Energy efficient and very satisfied	Jingdong prefecture level city in Shandong
Happy Fan Shop	King of Power Saving	Great quality for the price and fast shipping.	Jingdong prefecture level city in Shandong
Happy Fan Shop	King of Power Saving	It's great, just as described. Perfect.	Jingdong prefecture level city in Shandong
Happy Fan Shop	King of Power Saving	There is no power saving effect, but rather ring up an increase in electricity costs, because this machine is on continuously all day long, uninterruptedly, its own electricity and increased costs, plus the cost of purchasing the machine, so I feel that the use of this machine instead of increasing the	Jingdong prefecture level city in Shandong
Happy Fan Shop	King of Power Saving	Recommended by the owner of the store next door, looked at his home does have to save electricity, decided to get. Indeed quite can, the voltage is much more stable, there is a reduction in electricity	Jingdong prefecture level city in Shandong
Happy Fan Shop	King of Power Saving	Goods have been received value for money, affordable and reasonable price, and the description of the same, power-saving safe and reliable!	Jingdong prefecture level city in Shandong
Happy Fan Shop	King of Power Saving	Listen to friends introduced to buy, tried to save a lot of electricity is good, quite good, worth buying.	Jingdong prefecture level city in Shandong
Happy Fan Shop	King of Power Saving	The product is easy to use, safe and environmentally friendly, only used for a short time, to the next month, after the electricity bill status display to know how the performance of the power saver efficacy.	Jingdong prefecture level city in Shandong
Happy Fan Shop	King of Power Saving	Last month's electricity bill is 178 yuan, with this power saver is 92 yuan, ready to buy two more back home, I was introduced to buy a friend, just started to not believe that I did not expect the effect!	Jingdong prefecture level city in Shandong
Happy Fan Shop	King of Power Saving	It looks nice, but after using it for a few days I can't see how it saves power?	Jingdong prefecture level city in Shandong
Happy Fan Shop	King of Power Saving	Recommended by a friend, it is genuine, as described by the merchant, cost-effective	Jingdong prefecture level city in Shandong
Happy Fan Shop	King of Power Saving	Works well, affordable and saves money.	Jingdong prefecture level city in Shandong
Happy Fan Shop	King of Power Saving	Feels good haven't compared it yet, the air conditioning has been on a lot in the past two months	Jingdong prefecture level city in Shandong
Happy Fan Shop	King of Power Saving	Saving about 5 kWh per day is a good thing	Jingdong prefecture level city in Shandong
Happy Fan Shop	King of Power Saving	Not bad, affordable, best value for money	Jingdong prefecture level city in Shandong
Happy Fan Shop	King of Power Saving	The goods are genuine and the logistics are faster.	Jingdong prefecture level city in Shandong
Happy Fan Shop	King of Power Saving	Goods have been received today, has been plugged into the outlet, looking forward to saving electricity, after a period of time to see how the effect in the follow-up comment!	Jingdong prefecture level city in Shandong
Happy Fan Shop	King of Power Saving	I just got it home and haven't used it for long, so I'll use it for a couple months and see what happens. Seeing that most people have good reviews, it should be fine.	Jingdong prefecture level city in Shandong
Happy Fan Shop	King of Power Saving	I'm looking forward to the results, so I'll give you a good review out of habit! Follow up review after a while	Jingdong prefecture level city in Shandong
Happy Fan Shop	King of Power Saving	Use a dish first to know if it will help save energy	Jingdong prefecture level city in Shandong
Happy Fan Shop	King of Power Saving	Whether it saves electricity or not, we don't know yet.	Jingdong prefecture level city in Shandong
Happy Fan Shop	King of Power Saving	Goods have been received, use a period of time to know how oh. The courier did not deliver to the door ah! Sent to the station ah! The heat is inconvenient ah!	Jingdong prefecture level city in Shandong
Happy Fan Shop	King of Power Saving	A little effect, before an hour a power, now an hour 0.76 degrees, a dollar a power = save 24 cents, although not a lot, first use it!	Jingdong prefecture level city in Shandong
Happy Fan Shop	King of Power Saving	Just received the goods, use a month to evaluate	Jingdong prefecture level city in Shandong
Happy Fan Shop	King of Power Saving	Home every month with a lot of electricity, inadvertently found this good thing online that can save the beginning of a skeptical attitude to consult customer service, customer service replied very professional, said 120 days can be refunded that first try it!	Jingdong prefecture level city in Shandong
Happy Fan Shop	King of Power Saving	After receiving the goods this afternoon, immediately installed in the bathroom Smith energy storage electric water heater on the power outlet (instead of connecting with a plug board), when used, found that the "Power Saving King" LCD display, is the value of the voltage, not the store's video said so	Jingdong prefecture level city in Shandong
Happy Fan Shop	King of Power Saving	Has made many days, did not feel the power saving, the effect is not obvious.	Jingdong prefecture level city in Shandong
Happy Fan Shop	King of Power Saving	Energy savers do not save energy at all, but rather consume a lot of electricity.	Jingdong prefecture level city in Shandong
Happy Fan Shop	King of Power Saving	It's useless. It's a waste of money.	Jingdong prefecture level city in Shandong
Happy Fan Shop	King of Power Saving	Last month saved more than 500, which came back to buy, the store bought three, and then buy 1 home use, my family is dry beauty salon, more instruments, especially electricity, a month almost 1000. or so, with this did not expect to save so much, great, five-star praise!	Jingdong prefecture level city in Shandong
Happy Fan Shop	King of Power Saving	It rained a couple days ago and I just went to get the item back today , not bad, hey I looked at the meter and that red display light takes several seconds to jump, did not make a mistake buying it. After a period of time to evaluate.	Jingdong prefecture level city in Shandong
Happy Fan Shop	King of Power Saving	Without further ado, the electricity bill came down, this month less 102 yuan, has been repurchased, I also recommended to my friends, all said that there are effects, the product is okay, the effect is quite	Jingdong prefecture level city in Shandong
Happy Fan Shop	King of Power Saving	After buying and using two months really see the effect, so only to evaluate! Originally held the mindset of trying, I did not expect really effective, worth having!	Jingdong prefecture level city in Shandong
Happy Fan Shop	King of Power Saving	In the use of this energy saver before the monthly electricity bill is more than 150 yuan or so, now save thirty percent, every day to work, six or seven o'clock in the afternoon to go home, I do not know why the electricity bill is so expensive, energy-saving appliances really have the effect of recommending	Jingdong prefecture level city in Shandong
Happy Fan Shop	King of Power Saving	It saves about 100 bucks a month. A friend recommended it to me, and he's been using it for a while, and the effect is so obvious that I bought one too.	Jingdong prefecture level city in Shandong
Happy Fan Shop	King of Power Saving	My dad asked me to buy one, but after I paid the bill, I asked and he said it was a lot less this month, so he asked me to buy another one.	Jingdong prefecture level city in Shandong
Happy Fan Shop	King of Power Saving	The specific effect will have to be tested for at least two months to compare whether there is a power saving effect or not.	Jingdong prefecture level city in Shandong
Happy Fan Shop	King of Power Saving	'The goods have been received, immediately go to use, please ask the boss you instructions do not allow to withdraw, there are two fuses installed there!	Jingdong prefecture level city in Shandong
Happy Fan Shop	King of Power Saving	First praise is using, after using if good then follow up, now because I have not yet seen the results	Jingdong prefecture level city in Shandong
Happy Fan Shop	King of Power Saving	I've just been using it, and I haven't seen if it has a power-saving effect yet, so I'll follow up after 10 days and half a month.	Jingdong prefecture level city in Shandong
Happy Fan Shop	King of Power Saving	Just received the intelligent power saving meter, immediately plugged in to use, see it is not bad, and order to sell the second time	Jingdong prefecture level city in Shandong
Happy Fan Shop	King of Power Saving	This power saver is a bit effective, used for half a month, the effect is still visible.	Jingdong prefecture level city in Shandong
Happy Fan Shop	King of Power Saving	I received the baby and it looks great, I'm trying it out now and will keep an eye on it afterward.	Jingdong prefecture level city in Shandong
Happy Fan Shop	King of Power Saving	I've just received it, but I'm still trying to see how it works, so I'll come back in a little while.	Jingdong prefecture level city in Shandong

Happy Fan Shop	King of Power Saving	Just used it wait to use it for a while and then comment thanks, all first five stars.	Jingdong prefecture level city in Shandong
Happy Fan Shop	King of Power Saving	Very trustworthy brand, quality is guaranteed, the price is still affordable, quite satisfied!	Jingdong prefecture level city in Shandong
Happy Fan Shop	King of Power Saving	Things received, I hope that the platform of Jingdong do not sell fake goods are available	Jingdong prefecture level city in Shandong
Happy Fan Shop	King of Power Saving	Energy saver received, the use of the effect is good and the business description of the same	Jingdong prefecture level city in Shandong
Happy Fan Shop	King of Power Saving	The surface looks beautiful, the actual effect is not known, after using additional evaluation.	Jingdong prefecture level city in Shandong
Happy Fan Shop	King of Power Saving	Tried it for a few months, it really saves electricity, and I bought it for a friend.	Jingdong prefecture level city in Shandong
Happy Fan Shop	King of Power Saving	It didn't work and wanted to return it, it really didn't work at all.	Jingdong prefecture level city in Shandong
Happy Fan Shop	King of Power Saving	Just used it, don't know how effective it is, looking forward to usefulness	Jingdong prefecture level city in Shandong
Happy Fan Shop	King of Power Saving	After two months before the effect I do not know if it is true!	Jingdong prefecture level city in Shandong
Happy Fan Shop	King of Power Saving	So fast! Ordered yesterday and received today 👍👍👍👍👍👍👍👍👍 🌹🌹🌹🌹🌹🌹🌹🌹🌹	Jingdong prefecture level city in Shandong
Happy Fan Shop	King of Power Saving	I've started using it. We'll see how it works.	Jingdong prefecture level city in Shandong
Happy Fan Shop	King of Power Saving	I just received the goods, I won't know the effect until after two.	Jingdong prefecture level city in Shandong
Happy Fan Shop	King of Power Saving	Just received the goods, the specific effect will be evaluated later	Jingdong prefecture level city in Shandong
Happy Fan Shop	King of Power Saving	I've received the goods, I don't know if it's any good yet	Jingdong prefecture level city in Shandong
Happy Fan Shop	King of Power Saving	Try it for a month and then come back with a concrete evaluation	Jingdong prefecture level city in Shandong
Happy Fan Shop	King of Power Saving	In use, after two months to see the effect of power saving	Jingdong prefecture level city in Shandong
Happy Fan Shop	King of Power Saving	Power Saver. It's authentic. It's black. It works.	Jingdong prefecture level city in Shandong
Happy Fan Shop	King of Power Saving	I've been using it for a week now and it's not working.	Jingdong prefecture level city in Shandong
Happy Fan Shop	King of Power Saving	I'm just using it. I don't know how it's going to work.	Jingdong prefecture level city in Shandong
Happy Fan Shop	King of Power Saving	Products are better looking out for power savings	Jingdong prefecture level city in Shandong
Happy Fan Shop	King of Power Saving	I received it and I've kept it on. I hope it works.	Jingdong prefecture level city in Shandong
Happy Fan Shop	King of Power Saving	It's really good! Worth recommending to buy!	Jingdong prefecture level city in Shandong
Happy Fan Shop	King of Power Saving	Try it for a few days and see if it doesn't work, then return it.	Jingdong prefecture level city in Shandong
Happy Fan Shop	King of Power Saving	It's in use and I hope to be pleasantly surprised.	Jingdong prefecture level city in Shandong
Happy Fan Shop	King of Power Saving	We'll see how it works when we use it	Jingdong prefecture level city in Shandong
Happy Fan Shop	King of Power Saving	Let's see if it works.	Jingdong prefecture level city in Shandong
Happy Fan Shop	King of Power Saving	I'm using it and will follow up with a review	Jingdong prefecture level city in Shandong
Happy Fan Shop	King of Power Saving	It really works. It saves energy.	Jingdong prefecture level city in Shandong
Happy Fan Shop	King of Power Saving	It does have some power savings!	Jingdong prefecture level city in Shandong
Happy Fan Shop	King of Power Saving	Jingdong shopping speed is convenient and timely	Jingdong prefecture level city in Shandong
Happy Fan Shop	King of Power Saving	Second time I've bought it. Everything's fine.	Jingdong prefecture level city in Shandong
Happy Fan Shop	King of Power Saving	I don't know if it works yet.	Jingdong prefecture level city in Shandong
Happy Fan Shop	King of Power Saving	Not used, don't know how well it works.	Jingdong prefecture level city in Shandong
Happy Fan Shop	King of Power Saving	No visible results so far	Jingdong prefecture level city in Shandong
Happy Fan Shop	King of Power Saving	It doesn't feel like it's working.	Jingdong prefecture level city in Shandong
Happy Fan Shop	King of Power Saving	Where's the effect? Where are the power savings?	Jingdong prefecture level city in Shandong
Happy Fan Shop	King of Power Saving	Let's see how it works in practice!	Jingdong prefecture level city in Shandong
Happy Fan Shop	King of Power Saving	I'll wait until I use it to give you a good review	Jingdong prefecture level city in Shandong
Happy Fan Shop	King of Power Saving	I'll follow up after I've used it	Jingdong prefecture level city in Shandong
Happy Fan Shop	King of Power Saving	Well, that's good for saving power.	Jingdong prefecture level city in Shandong
Happy Fan Shop	King of Power Saving	Received. Used in the know.	Jingdong prefecture level city in Shandong
Happy Fan Shop	King of Power Saving	It doesn't do much good.	Jingdong prefecture level city in Shandong
Happy Fan Shop	King of Power Saving	It's not working.	Jingdong prefecture level city in Shandong
Happy Fan Shop	King of Power Saving	I don't know. I just used it.	Jingdong prefecture level city in Shandong
Happy Fan Shop	King of Power Saving	Use it and come back to evaluate.	Jingdong prefecture level city in Shandong
Happy Fan Shop	King of Power Saving	Two words: useless.	Jingdong prefecture level city in Shandong
Happy Fan Shop	King of Power Saving	To be used and then evaluated	Jingdong prefecture level city in Shandong
Happy Fan Shop	King of Power Saving	No shipment.	Jingdong prefecture level city in Shandong
Happy Fan Shop	King of Power Saving	Fake. Speechless.	Jingdong prefecture level city in Shandong
Happy Fan Shop	King of Power Saving	fantastic (idiom); awesome	Jingdong prefecture level city in Shandong
Happy Fan Shop	King of Power Saving	an excellent	Jingdong prefecture level city in Shandong

Happy Fan Shop	King of Power Saving	an excellent	Jingdong prefecture level city in Shandong
Happy Fan Shop	King of Power Saving	Want to buy! Recommended not to buy! No effect	Jingdong prefecture level city in Shandong
Happy Fan Shop	King of Power Saving	That's all there is to it. I can't figure out how to save electricity.	Jingdong prefecture level city in Shandong
Happy Fan Shop	King of Power Saving	It's no use.	Jingdong prefecture level city in Shandong
Happy Fan Shop	King of Power Saving	Things are more perfect than I imagined, very powerful, it is worth getting Packaging is very strong, things are well protected, it is worth the praise things are very satisfied Oh, beautiful and stylish atmosphere, customer service is super patient, service is meticulous and attentive, the big brand is not	Jingdong prefecture level city in Shandong
Happy Fan Shop	King of Power Saving	The effect is too good, the family has now several units, back to buy a number of times, the power saver received, power-saving effect is very good,, fine workmanship, long warranty, but also purify the air, before buying a used for a period of time, with the okay, and then bought a, and only now come to the	Jingdong prefecture level city in Shandong
Happy Fan Shop	King of Power Saving	Quite good, and imagine the same, casual ride are very good, satisfied! Baby received it 👌, really great ah! 🌈 quality is really nothing to say, very satisfied! 🆗 happy happy 😃 genuine, great, the price is very good, in short, quite good, will buy again a very good shopping experience, attentive service, fast logistics, distribution is also thoughtful, the attitude is also very good, value for money, ah,	Jingdong prefecture level city in Shandong
Happy Fan Shop	King of Power Saving	Power-saving effect: strong Cost-effective: quite good Appearance style: beautiful Workmanship material: meticulous Experience: looking forward to power-saving effect	Jingdong prefecture level city in Shandong
Happy Fan Shop	King of Power Saving	Cost-effective is okay, the appearance of the style of meticulous workmanship, looking forward to energy-saving effect.	Jingdong prefecture level city in Shandong
Happy Fan Shop	King of Power Saving	Very Good Very Power Saving This Power Saver is really power saving As soon as the power is turned on, the Power Saver starts the power saving function for you.	Jingdong prefecture level city in Shandong
Happy Fan Shop	King of Power Saving	Logistics is very fast, the price is favorable, friends introduced that it is very good to use, bought 2 at once, looking forward to the effect, used after the follow-up comment.	Jingdong prefecture level city in Shandong
Happy Fan Shop	King of Power Saving	It's all fake. It doesn't save any electricity.	Jingdong prefecture level city in Shandong
Happy Fan Shop	King of Power Saving	This Power Saver is so much better than the one I bought before, not only the effect is good, the quality is very good in all aspects, the customer service attitude is also very good, recommended to buy!	Jingdong prefecture level city in Shandong
Happy Fan Shop	King of Power Saving	Logistics is very fast, buy for parents, good quality, once bought 2, power-saving effect is good to buy 2 more, look forward to using the effect, satisfied will recommend to friends.	Jingdong prefecture level city in Shandong
Happy Fan Shop	King of Power Saving	With the description is the same, very high-grade, good value, to buy friends can order Oh!	Jingdong prefecture level city in Shandong
Happy Fan Shop	King of Power Saving	Not using much electricity in the winter saving over 100 per month! Ready to buy 2 more units in the summer ！！！！！ Good quality, good price, power saving effect is obvious, cost-effective, worth buying, can be recommended!	Jingdong prefecture level city in Shandong
Happy Fan Shop	King of Power Saving	Purchased a power saver received on the put into use, rent a room with many electrical appliances, power consumption is very large, we have a piece of electricity or 1.2 dollars a time, with this power saver is much better, every day can save a lot of money, very convenient and practical.	Jingdong prefecture level city in Shandong
Happy Fan Shop	King of Power Saving	The power saving effect is very good: my supermarket monthly electricity bill is more than 7000 yuan. On May 1st someone recommended buying a couple of power savers, and I bought 5 of them first. The electricity bill was about 2000 less in June. This time, I bought 5 more. The seller said 800 degrees of electricity with one. I feel it's basically enough. A total of 10 units	Jingdong prefecture level city in Shandong
Happy Fan Shop	King of Power Saving	I never thought I could get such a good baby at such an affordable price, it's very good value for money, I love it!	Jingdong prefecture level city in Shandong
Happy Fan Shop	King of Power Saving	Previously bought other brands of power savers, but found no effect at home, this time to see how the products on the Jingdong, inserted in the home socket, the LED display is blue, the night light is very nice to see, look at the instructions that have to be half a year in order to achieve the best results, try it, I believe that the quality of the Jingdong, it should not be wrong.	Jingdong prefecture level city in Shandong
Happy Fan Shop	King of Power Saving	Superb, inexpensive, saves a lot of points, been using it for a long time!	Jingdong prefecture level city in Shandong
Happy Fan Shop	King of Power Saving	There are a lot of appliances in the house, the summer air conditioning is very costly, I bought this in front of the air conditioning effect is very good, I explain that I bought three in one breath, I use more electricity a month down to 200 or so, very satisfied.	Jingdong prefecture level city in Shandong
Happy Fan Shop	King of Power Saving	A month can save more than 200, home monthly electricity bill of more than 600 dollars, inserted 3 or so, an amount of the balance of the average can save more than 200. Product is very good, has been buying this one, has given a lot of relatives recommended, the quality is also very good, before buying a look with this same, who knows to buy home has not been used for more than a week on the bad, the quality of this is really good with more than two years!	Jingdong prefecture level city in Shandong
Happy Fan Shop	King of Power Saving	Whole house power saving king, can really save electricity, friends recommended, high power saving appliances, only replacement, no repair, after-sales worry-free!	Jingdong prefecture level city in Shandong
Happy Fan Shop	King of Power Saving	Great item love it, great customer service, great shopping experience!	Jingdong prefecture level city in Shandong
Happy Fan Shop	King of Power Saving	Power saving, very good use, much cheaper than the physical store, satisfaction	Jingdong prefecture level city in Shandong
Happy Fan Shop	King of Power Saving	In use, very well packaged and fast delivery of credit!	Jingdong prefecture level city in Shandong
Happy Fan Shop	King of Power Saving	Product quality, customer service attitude is particularly good, like, praise!	Jingdong prefecture level city in Shandong
Happy Fan Shop	King of Power Saving	The logistics is very fast, the packaging is not broken, the quality is very good, I like it very much!	Jingdong prefecture level city in Shandong
Happy Fan Shop	King of Power Saving	The saver was well packaged and was as described when plugged in	Jingdong prefecture level city in Shandong
Happy Fan Shop	King of Power Saving	Energy efficient and very satisfied	Jingdong prefecture level city in Shandong
Happy Fan Shop	King of Power Saving	Great quality for the price and fast shipping.	Jingdong prefecture level city in Shandong
Happy Fan Shop	King of Power Saving	It's great, just as described. Perfect.	Jingdong prefecture level city in Shandong
Happy Fan Shop	King of Power Saving	There is no power saving effect, but rather ring up an increase in electricity costs, because this machine is on continuously all day long, uninterruptedly, its own electricity and increased costs, plus the cost of purchasing the machine, so I feel that the use of this machine instead of increasing the	Jingdong prefecture level city in Shandong
Happy Fan Shop	King of Power Saving	Recommended by the owner of the store next door, looked at his home does have to save electricity, decided to get. Indeed quite can, the voltage is much more stable, there is a reduction in electricity	Jingdong prefecture level city in Shandong
Happy Fan Shop	King of Power Saving	Goods have been received value for money, affordable and reasonable price, and the description of the same, power-saving safe and reliable!	Jingdong prefecture level city in Shandong
Happy Fan Shop	King of Power Saving	Listen to friends introduced to buy, tried to save a lot of electricity is good, quite good, worth buying.	Jingdong prefecture level city in Shandong
Happy Fan Shop	King of Power Saving	The product is easy to use, safe and environmentally friendly, only used for a short time, to the next month, after the electricity bill status display to know how the performance of the power saver efficacy.	Jingdong prefecture level city in Shandong
Happy Fan Shop	King of Power Saving	Last month's electricity bill is 178 yuan, with this power saver is 92 yuan, ready to buy two more back home, I was introduced to buy a friend, just started to not believe that I did not expect the effect!	Jingdong prefecture level city in Shandong
Happy Fan Shop	King of Power Saving	It looks nice, but after using it for a few days I can't see how it saves power?	Jingdong prefecture level city in Shandong
Happy Fan Shop	King of Power Saving	Recommended by a friend, it is genuine, as described by the merchant, cost-effective	Jingdong prefecture level city in Shandong
Happy Fan Shop	King of Power Saving	Works well, affordable and saves money.	Jingdong prefecture level city in Shandong
Happy Fan Shop	King of Power Saving	Feels good haven't compared it yet, the air conditioning has been on a lot in the past two months	Jingdong prefecture level city in Shandong
Happy Fan Shop	King of Power Saving	Saving about 5 kWh per day is a good thing	Jingdong prefecture level city in Shandong

Happy Fan Shop	King of Power Saving	Not bad, affordable, best value for money	Jingdong prefecture level city in Shandong
Happy Fan Shop	King of Power Saving	The goods are genuine and the logistics are faster.	Jingdong prefecture level city in Shandong
Happy Fan Shop	King of Power Saving	Goods have been received today, has been plugged into the outlet, looking forward to saving electricity, after a period of time to see how the effect in the follow-up comment!	Jingdong prefecture level city in Shandong
Happy Fan Shop	King of Power Saving	I just got it home and haven't used it for long, so I'll use it for a couple months and see what happens. Seeing that most people have good reviews, it should be fine.	Jingdong prefecture level city in Shandong
Happy Fan Shop	King of Power Saving	I'm looking forward to the results, so I'll give you a good review out of habit! Follow up review after a while	Jingdong prefecture level city in Shandong
Happy Fan Shop	King of Power Saving	Use a dish first to know if it will help save energy	Jingdong prefecture level city in Shandong
Happy Fan Shop	King of Power Saving	Whether it saves electricity or not, we don't know yet.	Jingdong prefecture level city in Shandong
Happy Fan Shop	King of Power Saving	Goods have been received, use a period of time to know how oh. The courier did not deliver to the door ah! Sent to the station ah! The heat is inconvenient ah!	Jingdong prefecture level city in Shandong
Happy Fan Shop	King of Power Saving	A little effect, before an hour a power, now an hour 0.76 degrees, a dollar a power = save 24 cents, although not a lot, first use it!	Jingdong prefecture level city in Shandong
Happy Fan Shop	King of Power Saving	Just received the goods, use a month to evaluate	Jingdong prefecture level city in Shandong
Happy Fan Shop	King of Power Saving	Home every month with a lot of electricity, inadvertently found this good thing online that can save the beginning of a skeptical attitude to consult customer service, customer service replied very professional, said 120 days can be refunded that first try it!	Jingdong prefecture level city in Shandong
Happy Fan Shop	King of Power Saving	After receiving the goods this afternoon, immediately installed in the bathroom Smith energy storage electric water heater on the power outlet (instead of connecting with a plug board), when used, found that the "Power Saving King" LCD display, is the value of the voltage, not the store's video said so	Jingdong prefecture level city in Shandong
Happy Fan Shop	King of Power Saving	Has made many days, did not feel the power saving, the effect is not obvious.	Jingdong prefecture level city in Shandong
Happy Fan Shop	King of Power Saving	Energy savers do not save energy at all, but rather consume a lot of electricity.	Jingdong prefecture level city in Shandong
Happy Fan Shop	King of Power Saving	It's useless. It's a waste of money.	Jingdong prefecture level city in Shandong
Happy Fan Shop	King of Power Saving	Last month saved more than 500, which came back to buy, the store bought three, and then buy 1 home use, my family is dry beauty salon, more instruments, especially electricity, a month almost 1000. or so, with this did not expect to save so much, great, five-star praise!	Jingdong prefecture level city in Shandong
Happy Fan Shop	King of Power Saving	It rained a couple days ago and I just went to get the item back today , not bad, hey I looked at the meter and that red display light takes several seconds to jump, did not make a mistake buying it. After a period of time to evaluate.	Jingdong prefecture level city in Shandong
Happy Fan Shop	King of Power Saving	Without further ado, the electricity bill came down, this month less 102 yuan, has been repurchased, I also recommended to my friends, all said that there are effects, the product is okay, the effect is quite	Jingdong prefecture level city in Shandong
Happy Fan Shop	King of Power Saving	After buying and using two months really see the effect, so only to evaluate! Originally held the mindset of trying, I did not expect really effective, worth having!	Jingdong prefecture level city in Shandong
Happy Fan Shop	King of Power Saving	In the use of this energy saver before the monthly electricity bill is more than 150 yuan or so, now save thirty percent, every day to work, six or seven o'clock in the afternoon to go home, I do not know why the electricity bill is so expensive, energy-saving appliances really have the effect of recommending	Jingdong prefecture level city in Shandong
Happy Fan Shop	King of Power Saving	It saves about 100 bucks a month. A friend recommended it to me, and he's been using it for a while, and the effect is so obvious that I bought one too.	Jingdong prefecture level city in Shandong
Happy Fan Shop	King of Power Saving	My dad asked me to buy one, but after I paid the bill, I asked and he said it was a lot less this month, so he asked me to buy another one.	Jingdong prefecture level city in Shandong
Happy Fan Shop	King of Power Saving	The specific effect will have to be tested for at least two months to compare whether there is a power saving effect or not.	Jingdong prefecture level city in Shandong
Happy Fan Shop	King of Power Saving	'The goods have been received, immediately go to use, please ask the boss you instructions do not allow to withdraw, there are two fuses installed there!	Jingdong prefecture level city in Shandong
Happy Fan Shop	King of Power Saving	First praise is using, after using if good then follow up, now because I have not yet seen the results	Jingdong prefecture level city in Shandong
Happy Fan Shop	King of Power Saving	I've just been using it, and I haven't seen if it has a power-saving effect yet, so I'll follow up after 10 days and half a month.	Jingdong prefecture level city in Shandong
Happy Fan Shop	King of Power Saving	Just received the intelligent power saving meter, immediately plugged in to use, see it is not bad, and order to sell the second time	Jingdong prefecture level city in Shandong
Happy Fan Shop	King of Power Saving	This power saver is a bit effective, used for half a month, the effect is still visible.	Jingdong prefecture level city in Shandong
Happy Fan Shop	King of Power Saving	I received the baby and it looks great, I'm trying it out now and will keep an eye on it afterward.	Jingdong prefecture level city in Shandong
Happy Fan Shop	King of Power Saving	I've just received it, but I'm still trying to see how it works, so I'll come back in a little while.	Jingdong prefecture level city in Shandong
Happy Fan Shop	King of Power Saving	Just used it wait to use it for a while and then comment thanks, all first five stars.	Jingdong prefecture level city in Shandong
Happy Fan Shop	King of Power Saving	Very trustworthy brand, quality is guaranteed, the price is still affordable, quite satisfied!	Jingdong prefecture level city in Shandong
Happy Fan Shop	King of Power Saving	Things received, I hope that the platform of Jingdong do not sell fake goods are available	Jingdong prefecture level city in Shandong
Happy Fan Shop	King of Power Saving	Energy saver received, the use of the effect is good and the business description of the same	Jingdong prefecture level city in Shandong
Happy Fan Shop	King of Power Saving	The surface looks beautiful, the actual effect is not known, after using additional evaluation.	Jingdong prefecture level city in Shandong
Happy Fan Shop	King of Power Saving	Tried it for a few months, it really saves electricity, and I bought it for a friend.	Jingdong prefecture level city in Shandong
Happy Fan Shop	King of Power Saving	It didn't work and wanted to return it, it really didn't work at all.	Jingdong prefecture level city in Shandong
Happy Fan Shop	King of Power Saving	Just used it, don't know how effective it is, looking forward to usefulness	Jingdong prefecture level city in Shandong
Happy Fan Shop	King of Power Saving	After two months before the effect I do not know if it is true!	Jingdong prefecture level city in Shandong
Happy Fan Shop	King of Power Saving	So fast! Ordered yesterday and received today 👍👍👍👍👍👍👍👍👍 🌹🌹🌹🌹🌹🌹🌹🌹🌹	Jingdong prefecture level city in Shandong
Happy Fan Shop	King of Power Saving	I've started using it. We'll see how it works.	Jingdong prefecture level city in Shandong
Happy Fan Shop	King of Power Saving	I just received the goods, I won't know the effect until after two.	Jingdong prefecture level city in Shandong
Happy Fan Shop	King of Power Saving	Just received the goods, the specific effect will be evaluated later	Jingdong prefecture level city in Shandong
Happy Fan Shop	King of Power Saving	I've received the goods, I don't know if it's any good yet	Jingdong prefecture level city in Shandong
Happy Fan Shop	King of Power Saving	Try it for a month and then come back with a concrete evaluation	Jingdong prefecture level city in Shandong
Happy Fan Shop	King of Power Saving	In use, after two months to see the effect of power saving	Jingdong prefecture level city in Shandong
Happy Fan Shop	King of Power Saving	Power Saver. It's authentic. It's black. It works.	Jingdong prefecture level city in Shandong
Happy Fan Shop	King of Power Saving	I've been using it for a week now and it's not working.	Jingdong prefecture level city in Shandong
Happy Fan Shop	King of Power Saving	I'm just using it. I don't know how it's going to work.	Jingdong prefecture level city in Shandong

Happy Fan Shop	King of Power Saving	Products are better looking out for power savings	Jingdong prefecture level city in Shandong
Happy Fan Shop	King of Power Saving	I received it and I've kept it on. I hope it works.	Jingdong prefecture level city in Shandong
Happy Fan Shop	King of Power Saving	It's really good! Worth recommending to buy!	Jingdong prefecture level city in Shandong
Happy Fan Shop	King of Power Saving	Try it for a few days and see if it doesn't work, then return it.	Jingdong prefecture level city in Shandong
Happy Fan Shop	King of Power Saving	It's in use and I hope to be pleasantly surprised.	Jingdong prefecture level city in Shandong
Happy Fan Shop	King of Power Saving	We'll see how it works when we use it	Jingdong prefecture level city in Shandong
Happy Fan Shop	King of Power Saving	Let's see if it works.	Jingdong prefecture level city in Shandong
Happy Fan Shop	King of Power Saving	I'm using it and will follow up with a review	Jingdong prefecture level city in Shandong
Happy Fan Shop	King of Power Saving	It really works. It saves energy.	Jingdong prefecture level city in Shandong
Happy Fan Shop	King of Power Saving	It does have some power savings!	Jingdong prefecture level city in Shandong
Happy Fan Shop	King of Power Saving	Jingdong shopping speed is convenient and timely	Jingdong prefecture level city in Shandong
Happy Fan Shop	King of Power Saving	Second time I've bought it. Everything's fine.	Jingdong prefecture level city in Shandong
Happy Fan Shop	King of Power Saving	I don't know if it works yet.	Jingdong prefecture level city in Shandong
Happy Fan Shop	King of Power Saving	Not used, don't know how well it works.	Jingdong prefecture level city in Shandong
Happy Fan Shop	King of Power Saving	No visible results so far	Jingdong prefecture level city in Shandong
Happy Fan Shop	King of Power Saving	It doesn't feel like it's working.	Jingdong prefecture level city in Shandong
Happy Fan Shop	King of Power Saving	Where's the effect? Where are the power savings?	Jingdong prefecture level city in Shandong
Happy Fan Shop	King of Power Saving	Let's see how it works in practice!	Jingdong prefecture level city in Shandong
Happy Fan Shop	King of Power Saving	I'll wait until I use it to give you a good review	Jingdong prefecture level city in Shandong
Happy Fan Shop	King of Power Saving	I'll follow up after I've used it	Jingdong prefecture level city in Shandong
Happy Fan Shop	King of Power Saving	Well, that's good for saving power.	Jingdong prefecture level city in Shandong
Happy Fan Shop	King of Power Saving	Received. Used in the know.	Jingdong prefecture level city in Shandong
Happy Fan Shop	King of Power Saving	It doesn't do much good.	Jingdong prefecture level city in Shandong
Happy Fan Shop	King of Power Saving	It's not working.	Jingdong prefecture level city in Shandong
Happy Fan Shop	King of Power Saving	I don't know. I just used it.	Jingdong prefecture level city in Shandong
Happy Fan Shop	King of Power Saving	Use it and come back to evaluate.	Jingdong prefecture level city in Shandong
Happy Fan Shop	King of Power Saving	Two words: useless.	Jingdong prefecture level city in Shandong
Happy Fan Shop	King of Power Saving	To be used and then evaluated	Jingdong prefecture level city in Shandong
Happy Fan Shop	King of Power Saving	No shipment.	Jingdong prefecture level city in Shandong
Happy Fan Shop	King of Power Saving	Fake. Speechless.	Jingdong prefecture level city in Shandong
Happy Fan Shop	King of Power Saving	fantastic (idiom); awesome	Jingdong prefecture level city in Shandong
Happy Fan Shop	King of Power Saving	an excellent	Jingdong prefecture level city in Shandong
Happy Fan Shop	King of Power Saving	an excellent	Jingdong prefecture level city in Shandong
Happy Fan Shop	King of Power Saving	Want to buy! Recommended not to buy! No effect	Jingdong prefecture level city in Shandong
Happy Fan Shop	King of Power Saving	That's all there is to it. I can't figure out how to save electricity.	Jingdong prefecture level city in Shandong
Happy Fan Shop	King of Power Saving	It's no use.	Jingdong prefecture level city in Shandong
Happy Fan Shop	King of Power Saving	Things are more perfect than I imagined, very powerful, it is worth getting Packaging is very strong, things are well protected, it is worth the praise things are very satisfied Oh, beautiful and stylish atmosphere, customer service is super patient, service is meticulous and attentive, the big brand is not	Jingdong prefecture level city in Shandong
Happy Fan Shop	King of Power Saving	The effect is too good, the family has now several units, back to buy a number of times, the power saver received, power-saving effect is very good,, fine workmanship, long warranty, but also purify the air, before buying a used for a period of time, with the okay, and then bought a, and only now come to the	Jingdong prefecture level city in Shandong
Happy Fan Shop	King of Power Saving	Quite good, and imagine the same, casual ride are very good, satisfied! Baby received it 👌, really great ah! 🌈 quality is really nothing to say, very satisfied! 🆗 happy happy 😃 genuine, great, the price is very good, in short, quite good, will buy again a very good shopping experience, attentive service, fast logistics, distribution is also thoughtful, the attitude is also very good, value for money, ah,	Jingdong prefecture level city in Shandong
Happy Fan Shop	King of Power Saving	Power-saving effect: strong Cost-effective: quite good Appearance style: beautiful Workmanship material: meticulous Experience: looking forward to power-saving effect	Jingdong prefecture level city in Shandong
Happy Fan Shop	King of Power Saving	Cost-effective is okay, the appearance of the style of meticulous workmanship, looking forward to energy-saving effect.	Jingdong prefecture level city in Shandong
Happy Fan Shop	King of Power Saving	Very Good Very Power Saving This Power Saver is really power saving As soon as the power is turned on, the Power Saver starts the power saving function for you.	Jingdong prefecture level city in Shandong
Happy Fan Shop	King of Power Saving	Logistics is very fast, the price is favorable, friends introduced that it is very good to use, bought 2 at once, looking forward to the effect, used after the follow-up comment.	Jingdong prefecture level city in Shandong
Happy Fan Shop	King of Power Saving	It's all fake. It doesn't save any electricity.	Jingdong prefecture level city in Shandong
Happy Fan Shop	King of Power Saving	This Power Saver is so much better than the one I bought before, not only the effect is good, the quality is very good in all aspects, the customer service attitude is also very good, recommended to buy!	Jingdong prefecture level city in Shandong
Happy Fan Shop	King of Power Saving	Logistics is very fast, buy for parents, good quality, once bought 2, power-saving effect is good to buy 2 more, look forward to using the effect, satisfied will recommend to friends.	Jingdong prefecture level city in Shandong
Happy Fan Shop	King of Power Saving	With the description is the same, very high-grade, good value, to buy friends can order Oh!	Jingdong prefecture level city in Shandong
Happy Fan Shop	King of Power Saving	Not using much electricity in the winter saving over 100 per month! Ready to buy 2 more units in the summer ！！！！！ Good quality, good price, power saving effect is obvious, cost-effective, worth buying, can be recommended!	Jingdong prefecture level city in Shandong

Happy Fan Shop	King of Power Saving	Purchased a power saver received on the put into use, rent a room with many electrical appliances, power consumption is very large, we have a piece of electricity or 1.2 dollars a time, with this power saver is much better, every day can save a lot of money, very convenient and practical.	Jingdong prefecture level city in Shandong
Happy Fan Shop	King of Power Saving	The power saving effect is very good: my supermarket monthly electricity bill is more than 7000 yuan. On May 1st someone recommended buying a couple of power savers, and I bought 5 of them first. The electricity bill was about 2000 less in June. This time, I bought 5 more. The seller said 800 degrees of electricity with one. I feel it's basically enough. A total of 10 units	Jingdong prefecture level city in Shandong
Happy Fan Shop	King of Power Saving	I never thought I could get such a good baby at such an affordable price, it's very good value for money, I love it!	Jingdong prefecture level city in Shandong
Happy Fan Shop	King of Power Saving	Previously bought other brands of power savers, but found no effect at home, this time to see how the products on the Jingdong, inserted in the home socket, the LED display is blue, the night light is very nice to see, look at the instructions that have to be half a year in order to achieve the best results, try it, I believe that the quality of the Jingdong, it should not be wrong.	Jingdong prefecture level city in Shandong
Happy Fan Shop	King of Power Saving	Superb, inexpensive, saves a lot of points, been using it for a long time!	Jingdong prefecture level city in Shandong
Happy Fan Shop	King of Power Saving	There are a lot of appliances in the house, the summer air conditioning is very costly, I bought this in front of the air conditioning effect is very good, I explain that I bought three in one breath, I use more electricity a month down to 200 or so, very satisfied.	Jingdong prefecture level city in Shandong
Happy Fan Shop	King of Power Saving	A month can save more than 200, home monthly electricity bill of more than 600 dollars, inserted 3 or so, an amount of the balance of the average can save more than 200. Product is very good, has been buying this one, has given a lot of relatives recommended, the quality is also very good, before buying a look with this same, who knows to buy home has not been used for more than a week on the bad, the quality of this is really good with more than two years!	Jingdong prefecture level city in Shandong
Happy Fan Shop	King of Power Saving	Whole house power saving king, can really save electricity, friends recommended, high power saving appliances, only replacement, no repair, after-sales worry-free!	Jingdong prefecture level city in Shandong
Happy Fan Shop	King of Power Saving	Great item love it, great customer service, great shopping experience!	Jingdong prefecture level city in Shandong
Happy Fan Shop	King of Power Saving	Power saving, very good use, much cheaper than the physical store, satisfaction	Jingdong prefecture level city in Shandong
Happy Fan Shop	King of Power Saving	In use, very well packaged and fast delivery of credit!	Jingdong prefecture level city in Shandong
Happy Fan Shop	King of Power Saving	Product quality, customer service attitude is particularly good, like, praise!	Jingdong prefecture level city in Shandong
Happy Fan Shop	King of Power Saving	The logistics is very fast, the packaging is not broken, the quality is very good, I like it very much!	Jingdong prefecture level city in Shandong
Happy Fan Shop	King of Power Saving	The saver was well packaged and was as described when plugged in	Jingdong prefecture level city in Shandong
Happy Fan Shop	King of Power Saving	Energy efficient and very satisfied	Jingdong prefecture level city in Shandong
Happy Fan Shop	King of Power Saving	Great quality for the price and fast shipping.	Jingdong prefecture level city in Shandong
Happy Fan Shop	King of Power Saving	It's great, just as described. Perfect.	Jingdong prefecture level city in Shandong
Happy Fan Shop	King of Power Saving	There is no power saving effect, but rather ring up an increase in electricity costs, because this machine is on continuously all day long, uninterruptedly, its own electricity and increased costs, plus the cost of purchasing the machine, so I feel that the use of this machine instead of increasing the	Jingdong prefecture level city in Shandong
Happy Fan Shop	King of Power Saving	Recommended by the owner of the store next door, looked at his home does have to save electricity, decided to get. Indeed quite can, the voltage is much more stable, there is a reduction in electricity	Jingdong prefecture level city in Shandong
Happy Fan Shop	King of Power Saving	Goods have been received value for money, affordable and reasonable price, and the description of the same, power-saving safe and reliable!	Jingdong prefecture level city in Shandong
Happy Fan Shop	King of Power Saving	Listen to friends introduced to buy, tried to save a lot of electricity is good, quite good, worth buying.	Jingdong prefecture level city in Shandong
Happy Fan Shop	King of Power Saving	The product is easy to use, safe and environmentally friendly, only used for a short time, to the next month, after the electricity bill status display to know how the performance of the power saver efficacy.	Jingdong prefecture level city in Shandong
Happy Fan Shop	King of Power Saving	Last month's electricity bill is 178 yuan, with this power saver is 92 yuan, ready to buy two more back home, I was introduced to buy a friend, just started to not believe that I did not expect the effect!	Jingdong prefecture level city in Shandong
Happy Fan Shop	King of Power Saving	It looks nice, but after using it for a few days I can't see how it saves power?	Jingdong prefecture level city in Shandong
Happy Fan Shop	King of Power Saving	Recommended by a friend, it is genuine, as described by the merchant, cost-effective	Jingdong prefecture level city in Shandong
Happy Fan Shop	King of Power Saving	Works well, affordable and saves money.	Jingdong prefecture level city in Shandong
Happy Fan Shop	King of Power Saving	Feels good haven't compared it yet, the air conditioning has been on a lot in the past two months	Jingdong prefecture level city in Shandong
Happy Fan Shop	King of Power Saving	Saving about 5 kWh per day is a good thing	Jingdong prefecture level city in Shandong
Happy Fan Shop	King of Power Saving	Not bad, affordable, best value for money	Jingdong prefecture level city in Shandong
Happy Fan Shop	King of Power Saving	The goods are genuine and the logistics are faster.	Jingdong prefecture level city in Shandong
Happy Fan Shop	King of Power Saving	Goods have been received today, has been plugged into the outlet, looking forward to saving electricity, after a period of time to see how the effect in the follow-up comment!	Jingdong prefecture level city in Shandong
Happy Fan Shop	King of Power Saving	I just got it home and haven't used it for long, so I'll use it for a couple months and see what happens. Seeing that most people have good reviews, it should be fine.	Jingdong prefecture level city in Shandong
Happy Fan Shop	King of Power Saving	I'm looking forward to the results, so I'll give you a good review out of habit! Follow up review after a while	Jingdong prefecture level city in Shandong
Happy Fan Shop	King of Power Saving	Use a dish first to know if it will help save energy	Jingdong prefecture level city in Shandong
Happy Fan Shop	King of Power Saving	Whether it saves electricity or not, we don't know yet.	Jingdong prefecture level city in Shandong
Happy Fan Shop	King of Power Saving	Goods have been received, use a period of time to know how oh. The courier did not deliver to the door ah! Sent to the station ah! The heat is inconvenient ah!	Jingdong prefecture level city in Shandong
Happy Fan Shop	King of Power Saving	A little effect, before an hour a power, now an hour 0.76 degrees, a dollar a power = save 24 cents, although not a lot, first use it!	Jingdong prefecture level city in Shandong
Happy Fan Shop	King of Power Saving	Just received the goods, use a month to evaluate	Jingdong prefecture level city in Shandong
Happy Fan Shop	King of Power Saving	Home every month with a lot of electricity, inadvertently found this good thing online that can save the beginning of a skeptical attitude to consult customer service, customer service replied very professional, said 120 days can be refunded that first try it!	Jingdong prefecture level city in Shandong
Happy Fan Shop	King of Power Saving	After receiving the goods this afternoon, immediately installed in the bathroom Smith energy storage electric water heater on the power outlet (instead of connecting with a plug board), when used, found that the "Power Saving King" LCD display, is the value of the voltage, not the store's video said so	Jingdong prefecture level city in Shandong
Happy Fan Shop	King of Power Saving	Has made many days, did not feel the power saving, the effect is not obvious.	Jingdong prefecture level city in Shandong
Happy Fan Shop	King of Power Saving	Energy savers do not save energy at all, but rather consume a lot of electricity.	Jingdong prefecture level city in Shandong
Happy Fan Shop	King of Power Saving	It's useless. It's a waste of money.	Jingdong prefecture level city in Shandong
Happy Fan Shop	King of Power Saving	Last month saved more than 500, which came back to buy, the store bought three, and then buy 1 home use, my family is dry beauty salon, more instruments, especially electricity, a month almost 1000. or so, with this did not expect to save so much, great, five-star praise!	Jingdong prefecture level city in Shandong

Happy Fan Shop	King of Power Saving	It rained a couple days ago and I just went to get the item back today , not bad, hey I looked at the meter and that red display light takes several seconds to jump, did not make a mistake buying it. After a period of time to evaluate.	Jingdong prefecture level city in Shandong
Happy Fan Shop	King of Power Saving	Without further ado, the electricity bill came down, this month less 102 yuan, has been repurchased, I also recommended to my friends, all said that there are effects, the product is okay, the effect is quite	Jingdong prefecture level city in Shandong
Happy Fan Shop	King of Power Saving	After buying and using two months really see the effect, so only to evaluate! Originally held the mindset of trying, I did not expect really effective, worth having!	Jingdong prefecture level city in Shandong
Happy Fan Shop	King of Power Saving	In the use of this energy saver before the monthly electricity bill is more than 150 yuan or so, now save thirty percent, every day to work, six or seven o'clock in the afternoon to go home, I do not know why the electricity bill is so expensive, energy-saving appliances really have the effect of recommending	Jingdong prefecture level city in Shandong
Happy Fan Shop	King of Power Saving	It saves about 100 bucks a month. A friend recommended it to me, and he's been using it for a while, and the effect is so obvious that I bought one too.	Jingdong prefecture level city in Shandong
Happy Fan Shop	King of Power Saving	My dad asked me to buy one, but after I paid the bill, I asked and he said it was a lot less this month, so he asked me to buy another one.	Jingdong prefecture level city in Shandong
Happy Fan Shop	King of Power Saving	The specific effect will have to be tested for at least two months to compare whether there is a power saving effect or not.	Jingdong prefecture level city in Shandong
Happy Fan Shop	King of Power Saving	'The goods have been received, immediately go to use, please ask the boss you instructions do not allow to withdraw, there are two fuses installed there!	Jingdong prefecture level city in Shandong
Happy Fan Shop	King of Power Saving	First praise is using, after using if good then follow up, now because I have not yet seen the results	Jingdong prefecture level city in Shandong
Happy Fan Shop	King of Power Saving	I've just been using it, and I haven't seen if it has a power-saving effect yet, so I'll follow up after 10 days and half a month.	Jingdong prefecture level city in Shandong
Happy Fan Shop	King of Power Saving	Just received the intelligent power saving meter, immediately plugged in to use, see it is not bad, and order to sell the second time	Jingdong prefecture level city in Shandong
Happy Fan Shop	King of Power Saving	This power saver is a bit effective, used for half a month, the effect is still visible.	Jingdong prefecture level city in Shandong
Happy Fan Shop	King of Power Saving	I received the baby and it looks great, I'm trying it out now and will keep an eye on it afterward.	Jingdong prefecture level city in Shandong
Happy Fan Shop	King of Power Saving	I've just received it, but I'm still trying to see how it works, so I'll come back in a little while.	Jingdong prefecture level city in Shandong
Happy Fan Shop	King of Power Saving	Just used it wait to use it for a while and then comment thanks, all first five stars.	Jingdong prefecture level city in Shandong
Happy Fan Shop	King of Power Saving	Very trustworthy brand, quality is guaranteed, the price is still affordable, quite satisfied!	Jingdong prefecture level city in Shandong
Happy Fan Shop	King of Power Saving	Things received, I hope that the platform of Jingdong do not sell fake goods are available	Jingdong prefecture level city in Shandong
Happy Fan Shop	King of Power Saving	Energy saver received, the use of the effect is good and the business description of the same	Jingdong prefecture level city in Shandong
Happy Fan Shop	King of Power Saving	The surface looks beautiful, the actual effect is not known, after using additional evaluation.	Jingdong prefecture level city in Shandong
Happy Fan Shop	King of Power Saving	Tried it for a few months, it really saves electricity, and I bought it for a friend.	Jingdong prefecture level city in Shandong
Happy Fan Shop	King of Power Saving	It didn't work and wanted to return it, it really didn't work at all.	Jingdong prefecture level city in Shandong
Happy Fan Shop	King of Power Saving	Just used it, don't know how effective it is, looking forward to usefulness	Jingdong prefecture level city in Shandong
Happy Fan Shop	King of Power Saving	After two months before the effect I do not know if it is true!	Jingdong prefecture level city in Shandong
Happy Fan Shop	King of Power Saving	So fast! Ordered yesterday and received today 👍👍👍👍👍👍👍👍👍 🌹🌹🌹🌹🌹🌹🌹🌹🌹	Jingdong prefecture level city in Shandong
Happy Fan Shop	King of Power Saving	I've started using it. We'll see how it works.	Jingdong prefecture level city in Shandong
Happy Fan Shop	King of Power Saving	I just received the goods, I won't know the effect until after two.	Jingdong prefecture level city in Shandong
Happy Fan Shop	King of Power Saving	Just received the goods, the specific effect will be evaluated later	Jingdong prefecture level city in Shandong
Happy Fan Shop	King of Power Saving	I've received the goods, I don't know if it's any good yet	Jingdong prefecture level city in Shandong
Happy Fan Shop	King of Power Saving	Try it for a month and then come back with a concrete evaluation	Jingdong prefecture level city in Shandong
Happy Fan Shop	King of Power Saving	In use, after two months to see the effect of power saving	Jingdong prefecture level city in Shandong
Happy Fan Shop	King of Power Saving	Power Saver. It's authentic. It's black. It works.	Jingdong prefecture level city in Shandong
Happy Fan Shop	King of Power Saving	I've been using it for a week now and it's not working.	Jingdong prefecture level city in Shandong
Happy Fan Shop	King of Power Saving	I'm just using it. I don't know how it's going to work.	Jingdong prefecture level city in Shandong
Happy Fan Shop	King of Power Saving	Products are better looking out for power savings	Jingdong prefecture level city in Shandong
Happy Fan Shop	King of Power Saving	I received it and I've kept it on. I hope it works.	Jingdong prefecture level city in Shandong
Happy Fan Shop	King of Power Saving	It's really good! Worth recommending to buy!	Jingdong prefecture level city in Shandong
Happy Fan Shop	King of Power Saving	Try it for a few days and see if it doesn't work, then return it.	Jingdong prefecture level city in Shandong
Happy Fan Shop	King of Power Saving	It's in use and I hope to be pleasantly surprised.	Jingdong prefecture level city in Shandong
Happy Fan Shop	King of Power Saving	We'll see how it works when we use it	Jingdong prefecture level city in Shandong
Happy Fan Shop	King of Power Saving	Let's see if it works.	Jingdong prefecture level city in Shandong
Happy Fan Shop	King of Power Saving	I'm using it and will follow up with a review	Jingdong prefecture level city in Shandong
Happy Fan Shop	King of Power Saving	It really works. It saves energy.	Jingdong prefecture level city in Shandong
Happy Fan Shop	King of Power Saving	It does have some power savings!	Jingdong prefecture level city in Shandong
Happy Fan Shop	King of Power Saving	Jingdong shopping speed is convenient and timely	Jingdong prefecture level city in Shandong
Happy Fan Shop	King of Power Saving	Second time I've bought it. Everything's fine.	Jingdong prefecture level city in Shandong
Happy Fan Shop	King of Power Saving	I don't know if it works yet.	Jingdong prefecture level city in Shandong
Happy Fan Shop	King of Power Saving	Not used, don't know how well it works.	Jingdong prefecture level city in Shandong
Happy Fan Shop	King of Power Saving	No visible results so far	Jingdong prefecture level city in Shandong
Happy Fan Shop	King of Power Saving	It doesn't feel like it's working.	Jingdong prefecture level city in Shandong
Happy Fan Shop	King of Power Saving	Where's the effect? Where are the power savings?	Jingdong prefecture level city in Shandong

Happy Fan Shop	King of Power Saving	Let's see how it works in practice!	Jingdong prefecture level city in Shandong
Happy Fan Shop	King of Power Saving	I'll wait until I use it to give you a good review	Jingdong prefecture level city in Shandong
Happy Fan Shop	King of Power Saving	I'll follow up after I've used it	Jingdong prefecture level city in Shandong
Happy Fan Shop	King of Power Saving	Well, that's good for saving power.	Jingdong prefecture level city in Shandong
Happy Fan Shop	King of Power Saving	Received. Used in the know.	Jingdong prefecture level city in Shandong
Happy Fan Shop	King of Power Saving	It doesn't do much good.	Jingdong prefecture level city in Shandong
Happy Fan Shop	King of Power Saving	It's not working.	Jingdong prefecture level city in Shandong
Happy Fan Shop	King of Power Saving	I don't know. I just used it.	Jingdong prefecture level city in Shandong
Happy Fan Shop	King of Power Saving	Use it and come back to evaluate.	Jingdong prefecture level city in Shandong
Happy Fan Shop	King of Power Saving	Two words: useless.	Jingdong prefecture level city in Shandong
Happy Fan Shop	King of Power Saving	To be used and then evaluated	Jingdong prefecture level city in Shandong
Happy Fan Shop	King of Power Saving	No shipment.	Jingdong prefecture level city in Shandong
Happy Fan Shop	King of Power Saving	Fake. Speechless.	Jingdong prefecture level city in Shandong
Happy Fan Shop	King of Power Saving	fantastic (idiom); awesome	Jingdong prefecture level city in Shandong
Happy Fan Shop	King of Power Saving	an excellent	Jingdong prefecture level city in Shandong
Happy Fan Shop	King of Power Saving	an excellent	Jingdong prefecture level city in Shandong
Happy Fan Shop	King of Power Saving	Want to buy! Recommended not to buy! No effect	Jingdong prefecture level city in Shandong
Happy Fan Shop	King of Power Saving	That's all there is to it. I can't figure out how to save electricity.	Jingdong prefecture level city in Shandong
Happy Fan Shop	King of Power Saving	It's no use.	Jingdong prefecture level city in Shandong
Happy Fan Shop	King of Power Saving	Things are more perfect than I imagined, very powerful, it is worth getting Packaging is very strong, things are well protected, it is worth the praise things are very satisfied Oh, beautiful and stylish atmosphere, customer service is super patient, service is meticulous and attentive, the big brand is not	Jingdong prefecture level city in Shandong
Happy Fan Shop	King of Power Saving	The effect is too good, the family has now several units, back to buy a number of times, the power saver received, power-saving effect is very good,, fine workmanship, long warranty, but also purify the air, before buying a used for a period of time, with the okay, and then bought a, and only now come to the	Jingdong prefecture level city in Shandong
Happy Fan Shop	King of Power Saving	Quite good, and imagine the same, casual ride are very good, satisfied! Baby received it 👌, really great ah! 🌈 quality is really nothing to say, very satisfied! 🆗 happy happy 😃 genuine, great, the price is very good, in short, quite good, will buy again a very good shopping experience, attentive service, fast logistics, distribution is also thoughtful, the attitude is also very good, value for money, ah,	Jingdong prefecture level city in Shandong
Happy Fan Shop	King of Power Saving	Power-saving effect: strong Cost-effective: quite good Appearance style: beautiful Workmanship material: meticulous Experience: looking forward to power-saving effect	Jingdong prefecture level city in Shandong
Happy Fan Shop	King of Power Saving	Cost-effective is okay, the appearance of the style of meticulous workmanship, looking forward to energy-saving effect.	Jingdong prefecture level city in Shandong
Happy Fan Shop	King of Power Saving	Very Good Very Power Saving This Power Saver is really power saving As soon as the power is turned on, the Power Saver starts the power saving function for you.	Jingdong prefecture level city in Shandong
Happy Fan Shop	King of Power Saving	Logistics is very fast, the price is favorable, friends introduced that it is very good to use, bought 2 at once, looking forward to the effect, used after the follow-up comment.	Jingdong prefecture level city in Shandong
Happy Fan Shop	King of Power Saving	It's all fake. It doesn't save any electricity.	Jingdong prefecture level city in Shandong
Happy Fan Shop	King of Power Saving	This Power Saver is so much better than the one I bought before, not only the effect is good, the quality is very good in all aspects, the customer service attitude is also very good, recommended to buy!	Jingdong prefecture level city in Shandong
Happy Fan Shop	King of Power Saving	Logistics is very fast, buy for parents, good quality, once bought 2, power-saving effect is good to buy 2 more, look forward to using the effect, satisfied will recommend to friends.	Jingdong prefecture level city in Shandong
Happy Fan Shop	King of Power Saving	With the description is the same, very high-grade, good value, to buy friends can order Oh!	Jingdong prefecture level city in Shandong
Happy Fan Shop	King of Power Saving	Not using much electricity in the winter saving over 100 per month! Ready to buy 2 more units in the summer ！！！！！ Good quality, good price, power saving effect is obvious, cost-effective, worth buying, can be recommended!	Jingdong prefecture level city in Shandong
Happy Fan Shop	King of Power Saving	Purchased a power saver received on the put into use, rent a room with many electrical appliances, power consumption is very large, we have a piece of electricity or 1.2 dollars a time, with this power saver is much better, every day can save a lot of money, very convenient and practical.	Jingdong prefecture level city in Shandong
Happy Fan Shop	King of Power Saving	The power saving effect is very good: my supermarket monthly electricity bill is more than 7000 yuan. On May 1st someone recommended buying a couple of power savers, and I bought 5 of them first. The electricity bill was about 2000 less in June. This time, I bought 5 more. The seller said 800 degrees of electricity with one. I feel it's basically enough. A total of 10 units	Jingdong prefecture level city in Shandong
Happy Fan Shop	King of Power Saving	I never thought I could get such a good baby at such an affordable price, it's very good value for money, I love it!	Jingdong prefecture level city in Shandong
Happy Fan Shop	King of Power Saving	Previously bought other brands of power savers, but found no effect at home, this time to see how the products on the Jingdong, inserted in the home socket, the LED display is blue, the night light is very nice to see, look at the instructions that have to be half a year in order to achieve the best results, try it, I believe that the quality of the Jingdong, it should not be wrong.	Jingdong prefecture level city in Shandong
Happy Fan Shop	King of Power Saving	Superb, inexpensive, saves a lot of points, been using it for a long time!	Jingdong prefecture level city in Shandong
Happy Fan Shop	King of Power Saving	There are a lot of appliances in the house, the summer air conditioning is very costly, I bought this in front of the air conditioning effect is very good, I explain that I bought three in one breath, I use more electricity a month down to 200 or so, very satisfied.	Jingdong prefecture level city in Shandong
Happy Fan Shop	King of Power Saving	A month can save more than 200, home monthly electricity bill of more than 600 dollars, inserted 3 or so, an amount of the balance of the average can save more than 200. Product is very good, has been buying this one, has given a lot of relatives recommended, the quality is also very good, before buying a look with this same, who knows to buy home has not been used for more than a week on the bad, the quality of this is really good with more than two years!	Jingdong prefecture level city in Shandong
Happy Fan Shop	King of Power Saving	Whole house power saving king, can really save electricity, friends recommended, high power saving appliances, only replacement, no repair, after-sales worry-free!	Jingdong prefecture level city in Shandong
Happy Fan Shop	King of Power Saving	Great item love it, great customer service, great shopping experience!	Jingdong prefecture level city in Shandong
Happy Fan Shop	King of Power Saving	Power saving, very good use, much cheaper than the physical store, satisfaction	Jingdong prefecture level city in Shandong
Happy Fan Shop	King of Power Saving	In use, very well packaged and fast delivery of credit!	Jingdong prefecture level city in Shandong
Happy Fan Shop	King of Power Saving	Product quality, customer service attitude is particularly good, like, praise!	Jingdong prefecture level city in Shandong
Happy Fan Shop	King of Power Saving	The logistics is very fast, the packaging is not broken, the quality is very good, I like it very much!	Jingdong prefecture level city in Shandong

Happy Fan Shop	King of Power Saving	The saver was well packaged and was as described when plugged in	Jingdong prefecture level city in Shandong
Happy Fan Shop	King of Power Saving	Energy efficient and very satisfied	Jingdong prefecture level city in Shandong
Happy Fan Shop	King of Power Saving	Great quality for the price and fast shipping.	Jingdong prefecture level city in Shandong
Happy Fan Shop	King of Power Saving	It's great, just as described. Perfect.	Jingdong prefecture level city in Shandong
Happy Fan Shop	King of Power Saving	There is no power saving effect, but rather ring up an increase in electricity costs, because this machine is on continuously all day long, uninterruptedly, its own electricity and increased costs, plus the cost of purchasing the machine, so I feel that the use of this machine instead of increasing the	Jingdong prefecture level city in Shandong
Happy Fan Shop	King of Power Saving	Recommended by the owner of the store next door, looked at his home does have to save electricity, decided to get. Indeed quite can, the voltage is much more stable, there is a reduction in electricity	Jingdong prefecture level city in Shandong
Happy Fan Shop	King of Power Saving	Goods have been received value for money, affordable and reasonable price, and the description of the same, power-saving safe and reliable!	Jingdong prefecture level city in Shandong
Happy Fan Shop	King of Power Saving	Listen to friends introduced to buy, tried to save a lot of electricity is good, quite good, worth buying.	Jingdong prefecture level city in Shandong
Happy Fan Shop	King of Power Saving	The product is easy to use, safe and environmentally friendly, only used for a short time, to the next month, after the electricity bill status display to know how the performance of the power saver efficacy.	Jingdong prefecture level city in Shandong
Happy Fan Shop	King of Power Saving	Last month's electricity bill is 178 yuan, with this power saver is 92 yuan, ready to buy two more back home, I was introduced to buy a friend, just started to not believe that I did not expect the effect!	Jingdong prefecture level city in Shandong
Happy Fan Shop	King of Power Saving	It looks nice, but after using it for a few days I can't see how it saves power?	Jingdong prefecture level city in Shandong
Happy Fan Shop	King of Power Saving	Recommended by a friend, it is genuine, as described by the merchant, cost-effective	Jingdong prefecture level city in Shandong
Happy Fan Shop	King of Power Saving	Works well, affordable and saves money.	Jingdong prefecture level city in Shandong
Happy Fan Shop	King of Power Saving	Feels good haven't compared it yet, the air conditioning has been on a lot in the past two months	Jingdong prefecture level city in Shandong
Happy Fan Shop	King of Power Saving	Saving about 5 kWh per day is a good thing	Jingdong prefecture level city in Shandong
Happy Fan Shop	King of Power Saving	Not bad, affordable, best value for money	Jingdong prefecture level city in Shandong
Happy Fan Shop	King of Power Saving	The goods are genuine and the logistics are faster.	Jingdong prefecture level city in Shandong
Happy Fan Shop	King of Power Saving	Goods have been received today, has been plugged into the outlet, looking forward to saving electricity, after a period of time to see how the effect in the follow-up comment!	Jingdong prefecture level city in Shandong
Happy Fan Shop	King of Power Saving	I just got it home and haven't used it for long, so I'll use it for a couple months and see what happens. Seeing that most people have good reviews, it should be fine.	Jingdong prefecture level city in Shandong
Happy Fan Shop	King of Power Saving	I'm looking forward to the results, so I'll give you a good review out of habit! Follow up review after a while	Jingdong prefecture level city in Shandong
Happy Fan Shop	King of Power Saving	Use a dish first to know if it will help save energy	Jingdong prefecture level city in Shandong
Happy Fan Shop	King of Power Saving	Whether it saves electricity or not, we don't know yet.	Jingdong prefecture level city in Shandong
Happy Fan Shop	King of Power Saving	Goods have been received, use a period of time to know how oh. The courier did not deliver to the door ah! Sent to the station ah! The heat is inconvenient ah!	Jingdong prefecture level city in Shandong
Happy Fan Shop	King of Power Saving	A little effect, before an hour a power, now an hour 0.76 degrees, a dollar a power = save 24 cents, although not a lot, first use it!	Jingdong prefecture level city in Shandong
Happy Fan Shop	King of Power Saving	Just received the goods, use a month to evaluate	Jingdong prefecture level city in Shandong
Happy Fan Shop	King of Power Saving	Home every month with a lot of electricity, inadvertently found this good thing online that can save the beginning of a skeptical attitude to consult customer service, customer service replied very professional, said 120 days can be refunded that first try it!	Jingdong prefecture level city in Shandong
Happy Fan Shop	King of Power Saving	After receiving the goods this afternoon, immediately installed in the bathroom Smith energy storage electric water heater on the power outlet (instead of connecting with a plug board), when used, found that the "Power Saving King" LCD display, is the value of the voltage, not the store's video said so	Jingdong prefecture level city in Shandong
Happy Fan Shop	King of Power Saving	Has made many days, did not feel the power saving, the effect is not obvious.	Jingdong prefecture level city in Shandong
Happy Fan Shop	King of Power Saving	Energy savers do not save energy at all, but rather consume a lot of electricity.	Jingdong prefecture level city in Shandong
Happy Fan Shop	King of Power Saving	It's useless. It's a waste of money.	Jingdong prefecture level city in Shandong
Happy Fan Shop	King of Power Saving	Last month saved more than 500, which came back to buy, the store bought three, and then buy 1 home use, my family is dry beauty salon, more instruments, especially electricity, a month almost 1000. or so, with this did not expect to save so much, great, five-star praise!	Jingdong prefecture level city in Shandong
Happy Fan Shop	King of Power Saving	It rained a couple days ago and I just went to get the item back today , not bad, hey I looked at the meter and that red display light takes several seconds to jump, did not make a mistake buying it. After a period of time to evaluate.	Jingdong prefecture level city in Shandong
Happy Fan Shop	King of Power Saving	Without further ado, the electricity bill came down, this month less 102 yuan, has been repurchased, I also recommended to my friends, all said that there are effects, the product is okay, the effect is quite	Jingdong prefecture level city in Shandong
Happy Fan Shop	King of Power Saving	After buying and using two months really see the effect, so only to evaluate! Originally held the mindset of trying, I did not expect really effective, worth having!	Jingdong prefecture level city in Shandong
Happy Fan Shop	King of Power Saving	In the use of this energy saver before the monthly electricity bill is more than 150 yuan or so, now save thirty percent, every day to work, six or seven o'clock in the afternoon to go home, I do not know why the electricity bill is so expensive, energy-saving appliances really have the effect of recommending	Jingdong prefecture level city in Shandong
Happy Fan Shop	King of Power Saving	It saves about 100 bucks a month. A friend recommended it to me, and he's been using it for a while, and the effect is so obvious that I bought one too.	Jingdong prefecture level city in Shandong
Happy Fan Shop	King of Power Saving	My dad asked me to buy one, but after I paid the bill, I asked and he said it was a lot less this month, so he asked me to buy another one.	Jingdong prefecture level city in Shandong
Happy Fan Shop	King of Power Saving	The specific effect will have to be tested for at least two months to compare whether there is a power saving effect or not.	Jingdong prefecture level city in Shandong
Happy Fan Shop	King of Power Saving	'The goods have been received, immediately go to use, please ask the boss you instructions do not allow to withdraw, there are two fuses installed there!	Jingdong prefecture level city in Shandong
Happy Fan Shop	King of Power Saving	First praise is using, after using if good then follow up, now because I have not yet seen the results	Jingdong prefecture level city in Shandong
Happy Fan Shop	King of Power Saving	I've just been using it, and I haven't seen if it has a power-saving effect yet, so I'll follow up after 10 days and half a month.	Jingdong prefecture level city in Shandong
Happy Fan Shop	King of Power Saving	Just received the intelligent power saving meter, immediately plugged in to use, see it is not bad, and order to sell the second time	Jingdong prefecture level city in Shandong
Happy Fan Shop	King of Power Saving	This power saver is a bit effective, used for half a month, the effect is still visible.	Jingdong prefecture level city in Shandong
Happy Fan Shop	King of Power Saving	I received the baby and it looks great, I'm trying it out now and will keep an eye on it afterward.	Jingdong prefecture level city in Shandong
Happy Fan Shop	King of Power Saving	I've just received it, but I'm still trying to see how it works, so I'll come back in a little while.	Jingdong prefecture level city in Shandong
Happy Fan Shop	King of Power Saving	Just used it wait to use it for a while and then comment thanks, all first five stars.	Jingdong prefecture level city in Shandong
Happy Fan Shop	King of Power Saving	Very trustworthy brand, quality is guaranteed, the price is still affordable, quite satisfied!	Jingdong prefecture level city in Shandong
Happy Fan Shop	King of Power Saving	Things received, I hope that the platform of Jingdong do not sell fake goods are available	Jingdong prefecture level city in Shandong

Happy Fan Shop	King of Power Saving	Energy saver received, the use of the effect is good and the business description of the same	Jingdong prefecture level city in Shandong
Happy Fan Shop	King of Power Saving	The surface looks beautiful, the actual effect is not known, after using additional evaluation.	Jingdong prefecture level city in Shandong
Happy Fan Shop	King of Power Saving	Tried it for a few months, it really saves electricity, and I bought it for a friend.	Jingdong prefecture level city in Shandong
Happy Fan Shop	King of Power Saving	It didn't work and wanted to return it, it really didn't work at all.	Jingdong prefecture level city in Shandong
Happy Fan Shop	King of Power Saving	Just used it, don't know how effective it is, looking forward to usefulness	Jingdong prefecture level city in Shandong
Happy Fan Shop	King of Power Saving	After two months before the effect I do not know if it is true!	Jingdong prefecture level city in Shandong
Happy Fan Shop	King of Power Saving	So fast! Ordered yesterday and received today 👍👍👍👍👍👍👍👍👍 🌹🌹🌹🌹🌹🌹🌹🌹🌹	Jingdong prefecture level city in Shandong
Happy Fan Shop	King of Power Saving	I've started using it. We'll see how it works.	Jingdong prefecture level city in Shandong
Happy Fan Shop	King of Power Saving	I just received the goods, I won't know the effect until after two.	Jingdong prefecture level city in Shandong
Happy Fan Shop	King of Power Saving	Just received the goods, the specific effect will be evaluated later	Jingdong prefecture level city in Shandong
Happy Fan Shop	King of Power Saving	I've received the goods, I don't know if it's any good yet	Jingdong prefecture level city in Shandong
Happy Fan Shop	King of Power Saving	Try it for a month and then come back with a concrete evaluation	Jingdong prefecture level city in Shandong
Happy Fan Shop	King of Power Saving	In use, after two months to see the effect of power saving	Jingdong prefecture level city in Shandong
Happy Fan Shop	King of Power Saving	Power Saver. It's authentic. It's black. It works.	Jingdong prefecture level city in Shandong
Happy Fan Shop	King of Power Saving	I've been using it for a week now and it's not working.	Jingdong prefecture level city in Shandong
Happy Fan Shop	King of Power Saving	I'm just using it. I don't know how it's going to work.	Jingdong prefecture level city in Shandong
Happy Fan Shop	King of Power Saving	Products are better looking out for power savings	Jingdong prefecture level city in Shandong
Happy Fan Shop	King of Power Saving	I received it and I've kept it on. I hope it works.	Jingdong prefecture level city in Shandong
Happy Fan Shop	King of Power Saving	It's really good! Worth recommending to buy!	Jingdong prefecture level city in Shandong
Happy Fan Shop	King of Power Saving	Try it for a few days and see if it doesn't work, then return it.	Jingdong prefecture level city in Shandong
Happy Fan Shop	King of Power Saving	It's in use and I hope to be pleasantly surprised.	Jingdong prefecture level city in Shandong
Happy Fan Shop	King of Power Saving	We'll see how it works when we use it	Jingdong prefecture level city in Shandong
Happy Fan Shop	King of Power Saving	Let's see if it works.	Jingdong prefecture level city in Shandong
Happy Fan Shop	King of Power Saving	I'm using it and will follow up with a review	Jingdong prefecture level city in Shandong
Happy Fan Shop	King of Power Saving	It really works. It saves energy.	Jingdong prefecture level city in Shandong
Happy Fan Shop	King of Power Saving	It does have some power savings!	Jingdong prefecture level city in Shandong
Happy Fan Shop	King of Power Saving	Jingdong shopping speed is convenient and timely	Jingdong prefecture level city in Shandong
Happy Fan Shop	King of Power Saving	Second time I've bought it. Everything's fine.	Jingdong prefecture level city in Shandong
Happy Fan Shop	King of Power Saving	I don't know if it works yet.	Jingdong prefecture level city in Shandong
Happy Fan Shop	King of Power Saving	Not used, don't know how well it works.	Jingdong prefecture level city in Shandong
Happy Fan Shop	King of Power Saving	No visible results so far	Jingdong prefecture level city in Shandong
Happy Fan Shop	King of Power Saving	It doesn't feel like it's working.	Jingdong prefecture level city in Shandong
Happy Fan Shop	King of Power Saving	Where's the effect? Where are the power savings?	Jingdong prefecture level city in Shandong
Happy Fan Shop	King of Power Saving	Let's see how it works in practice!	Jingdong prefecture level city in Shandong
Happy Fan Shop	King of Power Saving	I'll wait until I use it to give you a good review	Jingdong prefecture level city in Shandong
Happy Fan Shop	King of Power Saving	I'll follow up after I've used it	Jingdong prefecture level city in Shandong
Happy Fan Shop	King of Power Saving	Well, that's good for saving power.	Jingdong prefecture level city in Shandong
Happy Fan Shop	King of Power Saving	Received. Used in the know.	Jingdong prefecture level city in Shandong
Happy Fan Shop	King of Power Saving	It doesn't do much good.	Jingdong prefecture level city in Shandong
Happy Fan Shop	King of Power Saving	It's not working.	Jingdong prefecture level city in Shandong
Happy Fan Shop	King of Power Saving	I don't know. I just used it.	Jingdong prefecture level city in Shandong
Happy Fan Shop	King of Power Saving	Use it and come back to evaluate.	Jingdong prefecture level city in Shandong
Happy Fan Shop	King of Power Saving	Two words: useless.	Jingdong prefecture level city in Shandong
Happy Fan Shop	King of Power Saving	To be used and then evaluated	Jingdong prefecture level city in Shandong
Happy Fan Shop	King of Power Saving	No shipment.	Jingdong prefecture level city in Shandong
Happy Fan Shop	King of Power Saving	Fake. Speechless.	Jingdong prefecture level city in Shandong
Happy Fan Shop	King of Power Saving	fantastic (idiom); awesome	Jingdong prefecture level city in Shandong
Happy Fan Shop	King of Power Saving	an excellent	Jingdong prefecture level city in Shandong
Happy Fan Shop	King of Power Saving	an excellent	Jingdong prefecture level city in Shandong
Happy Fan Shop	King of Power Saving	Want to buy! Recommended not to buy! No effect	Jingdong prefecture level city in Shandong
Happy Fan Shop	King of Power Saving	That's all there is to it. I can't figure out how to save electricity.	Jingdong prefecture level city in Shandong

Happy Fan Shop	King of Power Saving	It's no use.	Jingdong prefecture level city in Shandong
Happy Fan Shop	King of Power Saving	Things are more perfect than I imagined, very powerful, it is worth getting Packaging is very strong, things are well protected, it is worth the praise things are very satisfied Oh, beautiful and stylish atmosphere, customer service is super patient, service is meticulous and attentive, the big brand is not	Jingdong prefecture level city in Shandong
Happy Fan Shop	King of Power Saving	The effect is too good, the family has now several units, back to buy a number of times, the power saver received, power-saving effect is very good,, fine workmanship, long warranty, but also purify the air, before buying a used for a period of time, with the okay, and then bought a, and only now come to the	Jingdong prefecture level city in Shandong
Happy Fan Shop	King of Power Saving	Quite good, and imagine the same, casual ride are very good, satisfied! Baby received it 👌, really great ah! 🌈 quality is really nothing to say, very satisfied! 🆗 happy happy 😃 genuine, great, the price is very good, in short, quite good, will buy again a very good shopping experience, attentive service, fast logistics, distribution is also thoughtful, the attitude is also very good, value for money, ah,	Jingdong prefecture level city in Shandong
Happy Fan Shop	King of Power Saving	Power-saving effect: strong Cost-effective: quite good Appearance style: beautiful Workmanship material: meticulous Experience: looking forward to power-saving effect	Jingdong prefecture level city in Shandong
Happy Fan Shop	King of Power Saving	Cost-effective is okay, the appearance of the style of meticulous workmanship, looking forward to energy-saving effect.	Jingdong prefecture level city in Shandong
Happy Fan Shop	King of Power Saving	Very Good Very Power Saving This Power Saver is really power saving As soon as the power is turned on, the Power Saver starts the power saving function for you.	Jingdong prefecture level city in Shandong
Happy Fan Shop	King of Power Saving	Logistics is very fast, the price is favorable, friends introduced that it is very good to use, bought 2 at once, looking forward to the effect, used after the follow-up comment.	Jingdong prefecture level city in Shandong
Happy Fan Shop	King of Power Saving	It's all fake. It doesn't save any electricity.	Jingdong prefecture level city in Shandong
Happy Fan Shop	King of Power Saving	This Power Saver is so much better than the one I bought before, not only the effect is good, the quality is very good in all aspects, the customer service attitude is also very good, recommended to buy!	Jingdong prefecture level city in Shandong
Happy Fan Shop	King of Power Saving	Logistics is very fast, buy for parents, good quality, once bought 2, power-saving effect is good to buy 2 more, look forward to using the effect, satisfied will recommend to friends.	Jingdong prefecture level city in Shandong
Happy Fan Shop	King of Power Saving	With the description is the same, very high-grade, good value, to buy friends can order Oh!	Jingdong prefecture level city in Shandong
Happy Fan Shop	King of Power Saving	Not using much electricity in the winter saving over 100 per month! Ready to buy 2 more units in the summer ! ! ! ! ! Good quality, good price, power saving effect is obvious, cost-effective, worth buying, can be recommended!	Jingdong prefecture level city in Shandong
Happy Fan Shop	King of Power Saving	Purchased a power saver received on the put into use, rent a room with many electrical appliances, power consumption is very large, we have a piece of electricity or 1.2 dollars a time, with this power saver is much better, every day can save a lot of money, very convenient and practical.	Jingdong prefecture level city in Shandong
Happy Fan Shop	King of Power Saving	The power saving effect is very good: my supermarket monthly electricity bill is more than 7000 yuan. On May 1st someone recommended buying a couple of power savers, and I bought 5 of them first. The electricity bill was about 2000 less in June. This time, I bought 5 more. The seller said 800 degrees of electricity with one. I feel it's basically enough. A total of 10 units	Jingdong prefecture level city in Shandong
Happy Fan Shop	King of Power Saving	I never thought I could get such a good baby at such an affordable price, it's very good value for money, I love it!	Jingdong prefecture level city in Shandong
Happy Fan Shop	King of Power Saving	Previously bought other brands of power savers, but found no effect at home, this time to see how the products on the Jingdong, inserted in the home socket, the LED display is blue, the night light is very nice to see, look at the instructions that have to be half a year in order to achieve the best results, try it, I believe that the quality of the Jingdong, it should not be wrong.	Jingdong prefecture level city in Shandong
Happy Fan Shop	King of Power Saving	Superb, inexpensive, saves a lot of points, been using it for a long time!	Jingdong prefecture level city in Shandong
Happy Fan Shop	King of Power Saving	There are a lot of appliances in the house, the summer air conditioning is very costly, I bought this in front of the air conditioning effect is very good, I explain that I bought three in one breath, I use more electricity a month down to 200 or so, very satisfied.	Jingdong prefecture level city in Shandong
Happy Fan Shop	King of Power Saving	A month can save more than 200, home monthly electricity bill of more than 600 dollars, inserted 3 or so, an amount of the balance of the average can save more than 200. Product is very good, has been buying this one, has given a lot of relatives recommended, the quality is also very good, before buying a look with this same, who knows to buy home has not been used for more than a week on the bad, the quality of this is really good with more than two years!	Jingdong prefecture level city in Shandong
Happy Fan Shop	King of Power Saving	Whole house power saving king, can really save electricity, friends recommended, high power saving appliances, only replacement, no repair, after-sales worry-free!	Jingdong prefecture level city in Shandong
Happy Fan Shop	King of Power Saving	Great item love it, great customer service, great shopping experience!	Jingdong prefecture level city in Shandong
Happy Fan Shop	King of Power Saving	Power saving, very good use, much cheaper than the physical store, satisfaction	Jingdong prefecture level city in Shandong
Happy Fan Shop	King of Power Saving	In use, very well packaged and fast delivery of credit!	Jingdong prefecture level city in Shandong
Happy Fan Shop	King of Power Saving	Product quality, customer service attitude is particularly good, like, praise!	Jingdong prefecture level city in Shandong
Happy Fan Shop	King of Power Saving	The logistics is very fast, the packaging is not broken, the quality is very good, I like it very much!	Jingdong prefecture level city in Shandong
Happy Fan Shop	King of Power Saving	The saver was well packaged and was as described when plugged in	Jingdong prefecture level city in Shandong
Happy Fan Shop	King of Power Saving	Energy efficient and very satisfied	Jingdong prefecture level city in Shandong
Happy Fan Shop	King of Power Saving	Great quality for the price and fast shipping.	Jingdong prefecture level city in Shandong
Happy Fan Shop	King of Power Saving	It's great, just as described. Perfect.	Jingdong prefecture level city in Shandong
Happy Fan Shop	King of Power Saving	There is no power saving effect, but rather ring up an increase in electricity costs, because this machine is on continuously all day long, uninterruptedly, its own electricity and increased costs, plus the cost of purchasing the machine, so I feel that the use of this machine instead of increasing the	Jingdong prefecture level city in Shandong
Happy Fan Shop	King of Power Saving	Recommended by the owner of the store next door, looked at his home does have to save electricity, decided to get. Indeed quite can, the voltage is much more stable, there is a reduction in electricity	Jingdong prefecture level city in Shandong
Happy Fan Shop	King of Power Saving	Goods have been received value for money, affordable and reasonable price, and the description of the same, power-saving safe and reliable!	Jingdong prefecture level city in Shandong
Happy Fan Shop	King of Power Saving	Listen to friends introduced to buy, tried to save a lot of electricity is good, quite good, worth buying.	Jingdong prefecture level city in Shandong
Happy Fan Shop	King of Power Saving	The product is easy to use, safe and environmentally friendly, only used for a short time, to the next month, after the electricity bill status display to know how the performance of the power saver efficacy.	Jingdong prefecture level city in Shandong
Happy Fan Shop	King of Power Saving	Last month's electricity bill is 178 yuan, with this power saver is 92 yuan, ready to buy two more back home, I was introduced to buy a friend, just started to not believe that I did not expect the effect!	Jingdong prefecture level city in Shandong
Happy Fan Shop	King of Power Saving	It looks nice, but after using it for a few days I can't see how it saves power?	Jingdong prefecture level city in Shandong
Happy Fan Shop	King of Power Saving	Recommended by a friend, it is genuine, as described by the merchant, cost-effective	Jingdong prefecture level city in Shandong
Happy Fan Shop	King of Power Saving	Works well, affordable and saves money.	Jingdong prefecture level city in Shandong
Happy Fan Shop	King of Power Saving	Feels good haven't compared it yet, the air conditioning has been on a lot in the past two months	Jingdong prefecture level city in Shandong
Happy Fan Shop	King of Power Saving	Saving about 5 kWh per day is a good thing	Jingdong prefecture level city in Shandong
Happy Fan Shop	King of Power Saving	Not bad, affordable, best value for money	Jingdong prefecture level city in Shandong
Happy Fan Shop	King of Power Saving	The goods are genuine and the logistics are faster.	Jingdong prefecture level city in Shandong
Happy Fan Shop	King of Power Saving	Goods have been received today, has been plugged into the outlet, looking forward to saving electricity, after a period of time to see how the effect in the follow-up comment!	Jingdong prefecture level city in Shandong

Happy Fan Shop	King of Power Saving	I just got it home and haven't used it for long, so I'll use it for a couple months and see what happens. Seeing that most people have good reviews, it should be fine.	Jingdong prefecture level city in Shandong
Happy Fan Shop	King of Power Saving	I'm looking forward to the results, so I'll give you a good review out of habit! Follow up review after a while	Jingdong prefecture level city in Shandong
Happy Fan Shop	King of Power Saving	Use a dish first to know if it will help save energy	Jingdong prefecture level city in Shandong
Happy Fan Shop	King of Power Saving	Whether it saves electricity or not, we don't know yet.	Jingdong prefecture level city in Shandong
Happy Fan Shop	King of Power Saving	Goods have been received, use a period of time to know how oh. The courier did not deliver to the door ah! Sent to the station ah! The heat is inconvenient ah!	Jingdong prefecture level city in Shandong
Happy Fan Shop	King of Power Saving	A little effect, before an hour a power, now an hour 0.76 degrees, a dollar a power = save 24 cents, although not a lot, first use it!	Jingdong prefecture level city in Shandong
Happy Fan Shop	King of Power Saving	Just received the goods, use a month to evaluate	Jingdong prefecture level city in Shandong
Happy Fan Shop	King of Power Saving	Home every month with a lot of electricity, inadvertently found this good thing online that can save the beginning of a skeptical attitude to consult customer service, customer service replied very professional, said 120 days can be refunded that first try it!	Jingdong prefecture level city in Shandong
Happy Fan Shop	King of Power Saving	After receiving the goods this afternoon, immediately installed in the bathroom Smith energy storage electric water heater on the power outlet (instead of connecting with a plug board), when used, found that the "Power Saving King" LCD display, is the value of the voltage, not the store's video said so	Jingdong prefecture level city in Shandong
Happy Fan Shop	King of Power Saving	Has made many days, did not feel the power saving, the effect is not obvious.	Jingdong prefecture level city in Shandong
Happy Fan Shop	King of Power Saving	Energy savers do not save energy at all, but rather consume a lot of electricity.	Jingdong prefecture level city in Shandong
Happy Fan Shop	King of Power Saving	It's useless. It's a waste of money.	Jingdong prefecture level city in Shandong
Happy Fan Shop	King of Power Saving	Last month saved more than 500, which came back to buy, the store bought three, and then buy 1 home use, my family is dry beauty salon, more instruments, especially electricity, a month almost 1000. or so, with this did not expect to save so much, great, five-star praise!	Jingdong prefecture level city in Shandong
Happy Fan Shop	King of Power Saving	It rained a couple days ago and I just went to get the item back today , not bad, hey I looked at the meter and that red display light takes several seconds to jump, did not make a mistake buying it. After a period of time to evaluate.	Jingdong prefecture level city in Shandong
Happy Fan Shop	King of Power Saving	Without further ado, the electricity bill came down, this month less 102 yuan, has been repurchased, I also recommended to my friends, all said that there are effects, the product is okay, the effect is quite	Jingdong prefecture level city in Shandong
Happy Fan Shop	King of Power Saving	After buying and using two months really see the effect, so only to evaluate! Originally held the mindset of trying, I did not expect really effective, worth having!	Jingdong prefecture level city in Shandong
Happy Fan Shop	King of Power Saving	In the use of this energy saver before the monthly electricity bill is more than 150 yuan or so, now save thirty percent, every day to work, six or seven o'clock in the afternoon to go home, I do not know why the electricity bill is so expensive, energy-saving appliances really have the effect of recommending	Jingdong prefecture level city in Shandong
Happy Fan Shop	King of Power Saving	It saves about 100 bucks a month. A friend recommended it to me, and he's been using it for a while, and the effect is so obvious that I bought one too.	Jingdong prefecture level city in Shandong
Happy Fan Shop	King of Power Saving	My dad asked me to buy one, but after I paid the bill, I asked and he said it was a lot less this month, so he asked me to buy another one.	Jingdong prefecture level city in Shandong
Happy Fan Shop	King of Power Saving	The specific effect will have to be tested for at least two months to compare whether there is a power saving effect or not.	Jingdong prefecture level city in Shandong
Happy Fan Shop	King of Power Saving	'The goods have been received, immediately go to use, please ask the boss you instructions do not allow to withdraw, there are two fuses installed there!	Jingdong prefecture level city in Shandong
Happy Fan Shop	King of Power Saving	First praise is using, after using if good then follow up, now because I have not yet seen the results	Jingdong prefecture level city in Shandong
Happy Fan Shop	King of Power Saving	I've just been using it, and I haven't seen if it has a power-saving effect yet, so I'll follow up after 10 days and half a month.	Jingdong prefecture level city in Shandong
Happy Fan Shop	King of Power Saving	Just received the intelligent power saving meter, immediately plugged in to use, see it is not bad, and order to sell the second time	Jingdong prefecture level city in Shandong
Happy Fan Shop	King of Power Saving	This power saver is a bit effective, used for half a month, the effect is still visible.	Jingdong prefecture level city in Shandong
Happy Fan Shop	King of Power Saving	I received the baby and it looks great, I'm trying it out now and will keep an eye on it afterward.	Jingdong prefecture level city in Shandong
Happy Fan Shop	King of Power Saving	I've just received it, but I'm still trying to see how it works, so I'll come back in a little while.	Jingdong prefecture level city in Shandong
Happy Fan Shop	King of Power Saving	Just used it wait to use it for a while and then comment thanks, all first five stars.	Jingdong prefecture level city in Shandong
Happy Fan Shop	King of Power Saving	Very trustworthy brand, quality is guaranteed, the price is still affordable, quite satisfied!	Jingdong prefecture level city in Shandong
Happy Fan Shop	King of Power Saving	Things received, I hope that the platform of Jingdong do not sell fake goods are available	Jingdong prefecture level city in Shandong
Happy Fan Shop	King of Power Saving	Energy saver received, the use of the effect is good and the business description of the same	Jingdong prefecture level city in Shandong
Happy Fan Shop	King of Power Saving	The surface looks beautiful, the actual effect is not known, after using additional evaluation.	Jingdong prefecture level city in Shandong
Happy Fan Shop	King of Power Saving	Tried it for a few months, it really saves electricity, and I bought it for a friend.	Jingdong prefecture level city in Shandong
Happy Fan Shop	King of Power Saving	It didn't work and wanted to return it, it really didn't work at all.	Jingdong prefecture level city in Shandong
Happy Fan Shop	King of Power Saving	Just used it, don't know how effective it is, looking forward to usefulness	Jingdong prefecture level city in Shandong
Happy Fan Shop	King of Power Saving	After two months before the effect I do not know if it is true!	Jingdong prefecture level city in Shandong
Happy Fan Shop	King of Power Saving	So fast! Ordered yesterday and received today 👍👍👍👍👍👍👍👍👍 🌹🌹🌹🌹🌹🌹🌹🌹🌹	Jingdong prefecture level city in Shandong
Happy Fan Shop	King of Power Saving	I've started using it. We'll see how it works.	Jingdong prefecture level city in Shandong
Happy Fan Shop	King of Power Saving	I just received the goods, I won't know the effect until after two.	Jingdong prefecture level city in Shandong
Happy Fan Shop	King of Power Saving	Just received the goods, the specific effect will be evaluated later	Jingdong prefecture level city in Shandong
Happy Fan Shop	King of Power Saving	I've received the goods, I don't know if it's any good yet	Jingdong prefecture level city in Shandong
Happy Fan Shop	King of Power Saving	Try it for a month and then come back with a concrete evaluation	Jingdong prefecture level city in Shandong
Happy Fan Shop	King of Power Saving	In use, after two months to see the effect of power saving	Jingdong prefecture level city in Shandong
Happy Fan Shop	King of Power Saving	Power Saver. It's authentic. It's black. It works.	Jingdong prefecture level city in Shandong
Happy Fan Shop	King of Power Saving	I've been using it for a week now and it's not working.	Jingdong prefecture level city in Shandong
Happy Fan Shop	King of Power Saving	I'm just using it. I don't know how it's going to work.	Jingdong prefecture level city in Shandong
Happy Fan Shop	King of Power Saving	Products are better looking out for power savings	Jingdong prefecture level city in Shandong
Happy Fan Shop	King of Power Saving	I received it and I've kept it on. I hope it works.	Jingdong prefecture level city in Shandong
Happy Fan Shop	King of Power Saving	It's really good! Worth recommending to buy!	Jingdong prefecture level city in Shandong

Happy Fan Shop	King of Power Saving	Try it for a few days and see if it doesn't work, then return it.	Jingdong prefecture level city in Shandong
Happy Fan Shop	King of Power Saving	It's in use and I hope to be pleasantly surprised.	Jingdong prefecture level city in Shandong
Happy Fan Shop	King of Power Saving	We'll see how it works when we use it	Jingdong prefecture level city in Shandong
Happy Fan Shop	King of Power Saving	Let's see if it works.	Jingdong prefecture level city in Shandong
Happy Fan Shop	King of Power Saving	I'm using it and will follow up with a review	Jingdong prefecture level city in Shandong
Happy Fan Shop	King of Power Saving	It really works. It saves energy.	Jingdong prefecture level city in Shandong
Happy Fan Shop	King of Power Saving	It does have some power savings!	Jingdong prefecture level city in Shandong
Happy Fan Shop	King of Power Saving	Jingdong shopping speed is convenient and timely	Jingdong prefecture level city in Shandong
Happy Fan Shop	King of Power Saving	Second time I've bought it. Everything's fine.	Jingdong prefecture level city in Shandong
Happy Fan Shop	King of Power Saving	I don't know if it works yet.	Jingdong prefecture level city in Shandong
Happy Fan Shop	King of Power Saving	Not used, don't know how well it works.	Jingdong prefecture level city in Shandong
Happy Fan Shop	King of Power Saving	No visible results so far	Jingdong prefecture level city in Shandong
Happy Fan Shop	King of Power Saving	It doesn't feel like it's working.	Jingdong prefecture level city in Shandong
Happy Fan Shop	King of Power Saving	Where's the effect? Where are the power savings?	Jingdong prefecture level city in Shandong
Happy Fan Shop	King of Power Saving	Let's see how it works in practice!	Jingdong prefecture level city in Shandong
Happy Fan Shop	King of Power Saving	I'll wait until I use it to give you a good review	Jingdong prefecture level city in Shandong
Happy Fan Shop	King of Power Saving	I'll follow up after I've used it	Jingdong prefecture level city in Shandong
Happy Fan Shop	King of Power Saving	Well, that's good for saving power.	Jingdong prefecture level city in Shandong
Happy Fan Shop	King of Power Saving	Received. Used in the know.	Jingdong prefecture level city in Shandong
Happy Fan Shop	King of Power Saving	It doesn't do much good.	Jingdong prefecture level city in Shandong
Happy Fan Shop	King of Power Saving	It's not working.	Jingdong prefecture level city in Shandong
Happy Fan Shop	King of Power Saving	I don't know. I just used it.	Jingdong prefecture level city in Shandong
Happy Fan Shop	King of Power Saving	Use it and come back to evaluate.	Jingdong prefecture level city in Shandong
Happy Fan Shop	King of Power Saving	Two words: useless.	Jingdong prefecture level city in Shandong
Happy Fan Shop	King of Power Saving	To be used and then evaluated	Jingdong prefecture level city in Shandong
Happy Fan Shop	King of Power Saving	No shipment.	Jingdong prefecture level city in Shandong
Happy Fan Shop	King of Power Saving	Fake. Speechless.	Jingdong prefecture level city in Shandong
Happy Fan Shop	King of Power Saving	fantastic (idiom); awesome	Jingdong prefecture level city in Shandong
Happy Fan Shop	King of Power Saving	an excellent	Jingdong prefecture level city in Shandong
Happy Fan Shop	King of Power Saving	an excellent	Jingdong prefecture level city in Shandong
Happy Fan Shop	King of Power Saving	Want to buy! Recommended not to buy! No effect	Jingdong prefecture level city in Shandong
Happy Fan Shop	King of Power Saving	That's all there is to it. I can't figure out how to save electricity.	Jingdong prefecture level city in Shandong
Happy Fan Shop	King of Power Saving	It's no use.	Jingdong prefecture level city in Shandong
Happy Fan Shop	King of Power Saving	Things are more perfect than I imagined, very powerful, it is worth getting Packaging is very strong, things are well protected, it is worth the praise things are very satisfied Oh, beautiful and stylish atmosphere, customer service is super patient, service is meticulous and attentive, the big brand is not	Jingdong prefecture level city in Shandong
Happy Fan Shop	King of Power Saving	The effect is too good, the family has now several units, back to buy a number of times, the power saver received, power-saving effect is very good,, fine workmanship, long warranty, but also purify the air, before buying a used for a period of time, with the okay, and then bought a, and only now come to the	Jingdong prefecture level city in Shandong
Happy Fan Shop	King of Power Saving	Quite good, and imagine the same, casual ride are very good, satisfied! Baby received it 👌, really great ah! 🌈 quality is really nothing to say, very satisfied! 🆗 happy happy 😃 genuine, great, the price is very good, in short, quite good, will buy again a very good shopping experience, attentive service, fast logistics, distribution is also thoughtful, the attitude is also very good, value for money, ah,	Jingdong prefecture level city in Shandong
Happy Fan Shop	King of Power Saving	Power-saving effect: strong Cost-effective: quite good Appearance style: beautiful Workmanship material: meticulous Experience: looking forward to power-saving effect	Jingdong prefecture level city in Shandong
Happy Fan Shop	King of Power Saving	Cost-effective is okay, the appearance of the style of meticulous workmanship, looking forward to energy-saving effect.	Jingdong prefecture level city in Shandong
Happy Fan Shop	King of Power Saving	Very Good Very Power Saving This Power Saver is really power saving As soon as the power is turned on, the Power Saver starts the power saving function for you.	Jingdong prefecture level city in Shandong
Happy Fan Shop	King of Power Saving	Logistics is very fast, the price is favorable, friends introduced that it is very good to use, bought 2 at once, looking forward to the effect, used after the follow-up comment.	Jingdong prefecture level city in Shandong
Happy Fan Shop	King of Power Saving	It's all fake. It doesn't save any electricity.	Jingdong prefecture level city in Shandong
Happy Fan Shop	King of Power Saving	This Power Saver is so much better than the one I bought before, not only the effect is good, the quality is very good in all aspects, the customer service attitude is also very good, recommended to buy!	Jingdong prefecture level city in Shandong
Happy Fan Shop	King of Power Saving	Logistics is very fast, buy for parents, good quality, once bought 2, power-saving effect is good to buy 2 more, look forward to using the effect, satisfied will recommend to friends.	Jingdong prefecture level city in Shandong
Happy Fan Shop	King of Power Saving	With the description is the same, very high-grade, good value, to buy friends can order Oh!	Jingdong prefecture level city in Shandong
Happy Fan Shop	King of Power Saving	Not using much electricity in the winter saving over 100 per month! Ready to buy 2 more units in the summer ！！！！！ Good quality, good price, power saving effect is obvious, cost-effective, worth buying, can be recommended!	Jingdong prefecture level city in Shandong
Happy Fan Shop	King of Power Saving	Purchased a power saver received on the put into use, rent a room with many electrical appliances, power consumption is very large, we have a piece of electricity or 1.2 dollars a time, with this power saver is much better, every day can save a lot of money, very convenient and practical.	Jingdong prefecture level city in Shandong
Happy Fan Shop	King of Power Saving	The power saving effect is very good: my supermarket monthly electricity bill is more than 7000 yuan. On May 1st someone recommended buying a couple of power savers, and I bought 5 of them first. The electricity bill was about 2000 less in June. This time, I bought 5 more. The seller said 800 degrees of electricity with one. I feel it's basically enough. A total of 10 units	Jingdong prefecture level city in Shandong

Happy Fan Shop	King of Power Saving	I never thought I could get such a good baby at such an affordable price, it's very good value for money, I love it!	Jingdong prefecture level city in Shandong
Happy Fan Shop	King of Power Saving	Previously bought other brands of power savers, but found no effect at home, this time to see how the products on the Jingdong, inserted in the home socket, the LED display is blue, the night light is very nice to see, look at the instructions that have to be half a year in order to achieve the best results, try it, I believe that the quality of the Jingdong, it should not be wrong.	Jingdong prefecture level city in Shandong
Happy Fan Shop	King of Power Saving	Superb, inexpensive, saves a lot of points, been using it for a long time!	Jingdong prefecture level city in Shandong
Happy Fan Shop	King of Power Saving	There are a lot of appliances in the house, the summer air conditioning is very costly, I bought this in front of the air conditioning effect is very good, I explain that I bought three in one breath, I use more electricity a month down to 200 or so, very satisfied.	Jingdong prefecture level city in Shandong
Happy Fan Shop	King of Power Saving	A month can save more than 200, home monthly electricity bill of more than 600 dollars, inserted 3 or so, an amount of the balance of the average can save more than 200. Product is very good, has been buying this one, has given a lot of relatives recommended, the quality is also very good, before buying a look with this same, who knows to buy home has not been used for more than a week on the bad, the quality of this is really good with more than two years!	Jingdong prefecture level city in Shandong
Happy Fan Shop	King of Power Saving	Whole house power saving king, can really save electricity, friends recommended, high power saving appliances, only replacement, no repair, after-sales worry-free!	Jingdong prefecture level city in Shandong
Happy Fan Shop	King of Power Saving	Great item love it, great customer service, great shopping experience!	Jingdong prefecture level city in Shandong
Happy Fan Shop	King of Power Saving	Power saving, very good use, much cheaper than the physical store, satisfaction	Jingdong prefecture level city in Shandong
Happy Fan Shop	King of Power Saving	In use, very well packaged and fast delivery of credit!	Jingdong prefecture level city in Shandong
Happy Fan Shop	King of Power Saving	Product quality, customer service attitude is particularly good, like, praise!	Jingdong prefecture level city in Shandong
Happy Fan Shop	King of Power Saving	The logistics is very fast, the packaging is not broken, the quality is very good, I like it very much!	Jingdong prefecture level city in Shandong
Happy Fan Shop	King of Power Saving	The saver was well packaged and was as described when plugged in	Jingdong prefecture level city in Shandong
Happy Fan Shop	King of Power Saving	Energy efficient and very satisfied	Jingdong prefecture level city in Shandong
Happy Fan Shop	King of Power Saving	Great quality for the price and fast shipping.	Jingdong prefecture level city in Shandong
Happy Fan Shop	King of Power Saving	It's great, just as described. Perfect.	Jingdong prefecture level city in Shandong
Happy Fan Shop	King of Power Saving	There is no power saving effect, but rather ring up an increase in electricity costs, because this machine is on continuously all day long, uninterruptedly, its own electricity and increased costs, plus the cost of purchasing the machine, so I feel that the use of this machine instead of increasing the	Jingdong prefecture level city in Shandong
Happy Fan Shop	King of Power Saving	Recommended by the owner of the store next door, looked at his home does have to save electricity, decided to get. Indeed quite can, the voltage is much more stable, there is a reduction in electricity	Jingdong prefecture level city in Shandong
Happy Fan Shop	King of Power Saving	Goods have been received value for money, affordable and reasonable price, and the description of the same, power-saving safe and reliable!	Jingdong prefecture level city in Shandong
Happy Fan Shop	King of Power Saving	Listen to friends introduced to buy, tried to save a lot of electricity is good, quite good, worth buying.	Jingdong prefecture level city in Shandong
Happy Fan Shop	King of Power Saving	The product is easy to use, safe and environmentally friendly, only used for a short time, to the next month, after the electricity bill status display to know how the performance of the power saver efficacy.	Jingdong prefecture level city in Shandong
Happy Fan Shop	King of Power Saving	Last month's electricity bill is 178 yuan, with this power saver is 92 yuan, ready to buy two more back home, I was introduced to buy a friend, just started to not believe that I did not expect the effect!	Jingdong prefecture level city in Shandong
Happy Fan Shop	King of Power Saving	It looks nice, but after using it for a few days I can't see how it saves power?	Jingdong prefecture level city in Shandong
Happy Fan Shop	King of Power Saving	Recommended by a friend, it is genuine, as described by the merchant, cost-effective	Jingdong prefecture level city in Shandong
Happy Fan Shop	King of Power Saving	Works well, affordable and saves money.	Jingdong prefecture level city in Shandong
Happy Fan Shop	King of Power Saving	Feels good haven't compared it yet, the air conditioning has been on a lot in the past two months	Jingdong prefecture level city in Shandong
Happy Fan Shop	King of Power Saving	Saving about 5 kWh per day is a good thing	Jingdong prefecture level city in Shandong
Happy Fan Shop	King of Power Saving	Not bad, affordable, best value for money	Jingdong prefecture level city in Shandong
Happy Fan Shop	King of Power Saving	The goods are genuine and the logistics are faster.	Jingdong prefecture level city in Shandong
Happy Fan Shop	King of Power Saving	Goods have been received today, has been plugged into the outlet, looking forward to saving electricity, after a period of time to see how the effect in the follow-up comment!	Jingdong prefecture level city in Shandong
Happy Fan Shop	King of Power Saving	I just got it home and haven't used it for long, so I'll use it for a couple months and see what happens. Seeing that most people have good reviews, it should be fine.	Jingdong prefecture level city in Shandong
Happy Fan Shop	King of Power Saving	I'm looking forward to the results, so I'll give you a good review out of habit! Follow up review after a while	Jingdong prefecture level city in Shandong
Happy Fan Shop	King of Power Saving	Use a dish first to know if it will help save energy	Jingdong prefecture level city in Shandong
Happy Fan Shop	King of Power Saving	Whether it saves electricity or not, we don't know yet.	Jingdong prefecture level city in Shandong
Happy Fan Shop	King of Power Saving	Goods have been received, use a period of time to know how oh. The courier did not deliver to the door ah! Sent to the station ah! The heat is inconvenient ah!	Jingdong prefecture level city in Shandong
Happy Fan Shop	King of Power Saving	A little effect, before an hour a power, now an hour 0.76 degrees, a dollar a power = save 24 cents, although not a lot, first use it!	Jingdong prefecture level city in Shandong
Happy Fan Shop	King of Power Saving	Just received the goods, use a month to evaluate	Jingdong prefecture level city in Shandong
Happy Fan Shop	King of Power Saving	Home every month with a lot of electricity, inadvertently found this good thing online that can save the beginning of a skeptical attitude to consult customer service, customer service replied very professional, said 120 days can be refunded that first try it!	Jingdong prefecture level city in Shandong
Happy Fan Shop	King of Power Saving	After receiving the goods this afternoon, immediately installed in the bathroom Smith energy storage electric water heater on the power outlet (instead of connecting with a plug board), when used, found that the "Power Saving King" LCD display, is the value of the voltage, not the store's video said so	Jingdong prefecture level city in Shandong
Happy Fan Shop	King of Power Saving	Has made many days, did not feel the power saving, the effect is not obvious.	Jingdong prefecture level city in Shandong
Happy Fan Shop	King of Power Saving	Energy savers do not save energy at all, but rather consume a lot of electricity.	Jingdong prefecture level city in Shandong
Happy Fan Shop	King of Power Saving	It's useless. It's a waste of money.	Jingdong prefecture level city in Shandong
Happy Fan Shop	King of Power Saving	Last month saved more than 500, which came back to buy, the store bought three, and then buy 1 home use, my family is dry beauty salon, more instruments, especially electricity, a month almost 1000. or so, with this did not expect to save so much, great, five-star praise!	Jingdong prefecture level city in Shandong
Happy Fan Shop	King of Power Saving	It rained a couple days ago and I just went to get the item back today , not bad, hey I looked at the meter and that red display light takes several seconds to jump, did not make a mistake buying it. After a period of time to evaluate.	Jingdong prefecture level city in Shandong
Happy Fan Shop	King of Power Saving	Without further ado, the electricity bill came down, this month less 102 yuan, has been repurchased, I also recommended to my friends, all said that there are effects, the product is okay, the effect is quite	Jingdong prefecture level city in Shandong
Happy Fan Shop	King of Power Saving	After buying and using two months really see the effect, so only to evaluate! Originally held the mindset of trying, I did not expect really effective, worth having!	Jingdong prefecture level city in Shandong

Happy Fan Shop	King of Power Saving	In the use of this energy saver before the monthly electricity bill is more than 150 yuan or so, now save thirty percent, every day to work, six or seven o'clock in the afternoon to go home, I do not know why the electricity bill is so expensive, energy-saving appliances really have the effect of recommending	Jingdong prefecture level city in Shandong
Happy Fan Shop	King of Power Saving	It saves about 100 bucks a month. A friend recommended it to me, and he's been using it for a while, and the effect is so obvious that I bought one too.	Jingdong prefecture level city in Shandong
Happy Fan Shop	King of Power Saving	My dad asked me to buy one, but after I paid the bill, I asked and he said it was a lot less this month, so he asked me to buy another one.	Jingdong prefecture level city in Shandong
Happy Fan Shop	King of Power Saving	The specific effect will have to be tested for at least two months to compare whether there is a power saving effect or not.	Jingdong prefecture level city in Shandong
Happy Fan Shop	King of Power Saving	'The goods have been received, immediately go to use, please ask the boss you instructions do not allow to withdraw, there are two fuses installed there!	Jingdong prefecture level city in Shandong
Happy Fan Shop	King of Power Saving	First praise is using, after using if good then follow up, now because I have not yet seen the results	Jingdong prefecture level city in Shandong
Happy Fan Shop	King of Power Saving	I've just been using it, and I haven't seen if it has a power-saving effect yet, so I'll follow up after 10 days and half a month.	Jingdong prefecture level city in Shandong
Happy Fan Shop	King of Power Saving	Just received the intelligent power saving meter, immediately plugged in to use, see it is not bad, and order to sell the second time	Jingdong prefecture level city in Shandong
Happy Fan Shop	King of Power Saving	This power saver is a bit effective, used for half a month, the effect is still visible.	Jingdong prefecture level city in Shandong
Happy Fan Shop	King of Power Saving	I received the baby and it looks great, I'm trying it out now and will keep an eye on it afterward.	Jingdong prefecture level city in Shandong
Happy Fan Shop	King of Power Saving	I've just received it, but I'm still trying to see how it works, so I'll come back in a little while.	Jingdong prefecture level city in Shandong
Happy Fan Shop	King of Power Saving	Just used it wait to use it for a while and then comment thanks, all first five stars.	Jingdong prefecture level city in Shandong
Happy Fan Shop	King of Power Saving	Very trustworthy brand, quality is guaranteed, the price is still affordable, quite satisfied!	Jingdong prefecture level city in Shandong
Happy Fan Shop	King of Power Saving	Things received, I hope that the platform of Jingdong do not sell fake goods are available	Jingdong prefecture level city in Shandong
Happy Fan Shop	King of Power Saving	Energy saver received, the use of the effect is good and the business description of the same	Jingdong prefecture level city in Shandong
Happy Fan Shop	King of Power Saving	The surface looks beautiful, the actual effect is not known, after using additional evaluation.	Jingdong prefecture level city in Shandong
Happy Fan Shop	King of Power Saving	Tried it for a few months, it really saves electricity, and I bought it for a friend.	Jingdong prefecture level city in Shandong
Happy Fan Shop	King of Power Saving	It didn't work and wanted to return it, it really didn't work at all.	Jingdong prefecture level city in Shandong
Happy Fan Shop	King of Power Saving	Just used it, don't know how effective it is, looking forward to usefulness	Jingdong prefecture level city in Shandong
Happy Fan Shop	King of Power Saving	After two months before the effect I do not know if it is true!	Jingdong prefecture level city in Shandong
Happy Fan Shop	King of Power Saving	So fast! Ordered yesterday and received today 👍👍👍👍👍👍👍👍👍 🌹🌹🌹🌹🌹🌹🌹🌹🌹	Jingdong prefecture level city in Shandong
Happy Fan Shop	King of Power Saving	I've started using it. We'll see how it works.	Jingdong prefecture level city in Shandong
Happy Fan Shop	King of Power Saving	I just received the goods, I won't know the effect until after two.	Jingdong prefecture level city in Shandong
Happy Fan Shop	King of Power Saving	Just received the goods, the specific effect will be evaluated later	Jingdong prefecture level city in Shandong
Happy Fan Shop	King of Power Saving	I've received the goods, I don't know if it's any good yet	Jingdong prefecture level city in Shandong
Happy Fan Shop	King of Power Saving	Try it for a month and then come back with a concrete evaluation	Jingdong prefecture level city in Shandong
Happy Fan Shop	King of Power Saving	In use, after two months to see the effect of power saving	Jingdong prefecture level city in Shandong
Happy Fan Shop	King of Power Saving	Power Saver. It's authentic. It's black. It works.	Jingdong prefecture level city in Shandong
Happy Fan Shop	King of Power Saving	I've been using it for a week now and it's not working.	Jingdong prefecture level city in Shandong
Happy Fan Shop	King of Power Saving	I'm just using it. I don't know how it's going to work.	Jingdong prefecture level city in Shandong
Happy Fan Shop	King of Power Saving	Products are better looking out for power savings	Jingdong prefecture level city in Shandong
Happy Fan Shop	King of Power Saving	I received it and I've kept it on. I hope it works.	Jingdong prefecture level city in Shandong
Happy Fan Shop	King of Power Saving	It's really good! Worth recommending to buy!	Jingdong prefecture level city in Shandong
Happy Fan Shop	King of Power Saving	Try it for a few days and see if it doesn't work, then return it.	Jingdong prefecture level city in Shandong
Happy Fan Shop	King of Power Saving	It's in use and I hope to be pleasantly surprised.	Jingdong prefecture level city in Shandong
Happy Fan Shop	King of Power Saving	We'll see how it works when we use it	Jingdong prefecture level city in Shandong
Happy Fan Shop	King of Power Saving	Let's see if it works.	Jingdong prefecture level city in Shandong
Happy Fan Shop	King of Power Saving	I'm using it and will follow up with a review	Jingdong prefecture level city in Shandong
Happy Fan Shop	King of Power Saving	It really works. It saves energy.	Jingdong prefecture level city in Shandong
Happy Fan Shop	King of Power Saving	It does have some power savings!	Jingdong prefecture level city in Shandong
Happy Fan Shop	King of Power Saving	Jingdong shopping speed is convenient and timely	Jingdong prefecture level city in Shandong
Happy Fan Shop	King of Power Saving	Second time I've bought it. Everything's fine.	Jingdong prefecture level city in Shandong
Happy Fan Shop	King of Power Saving	I don't know if it works yet.	Jingdong prefecture level city in Shandong
Happy Fan Shop	King of Power Saving	Not used, don't know how well it works.	Jingdong prefecture level city in Shandong
Happy Fan Shop	King of Power Saving	No visible results so far	Jingdong prefecture level city in Shandong
Happy Fan Shop	King of Power Saving	It doesn't feel like it's working.	Jingdong prefecture level city in Shandong
Happy Fan Shop	King of Power Saving	Where's the effect? Where are the power savings?	Jingdong prefecture level city in Shandong
Happy Fan Shop	King of Power Saving	Let's see how it works in practice!	Jingdong prefecture level city in Shandong
Happy Fan Shop	King of Power Saving	I'll wait until I use it to give you a good review	Jingdong prefecture level city in Shandong
Happy Fan Shop	King of Power Saving	I'll follow up after I've used it	Jingdong prefecture level city in Shandong
Happy Fan Shop	King of Power Saving	Well, that's good for saving power.	Jingdong prefecture level city in Shandong

Happy Fan Shop	King of Power Saving	Received. Used in the know.	Jingdong prefecture level city in Shandong
Happy Fan Shop	King of Power Saving	It doesn't do much good.	Jingdong prefecture level city in Shandong
Happy Fan Shop	King of Power Saving	It's not working.	Jingdong prefecture level city in Shandong
Happy Fan Shop	King of Power Saving	I don't know. I just used it.	Jingdong prefecture level city in Shandong
Happy Fan Shop	King of Power Saving	Use it and come back to evaluate.	Jingdong prefecture level city in Shandong
Happy Fan Shop	King of Power Saving	Two words: useless.	Jingdong prefecture level city in Shandong
Happy Fan Shop	King of Power Saving	To be used and then evaluated	Jingdong prefecture level city in Shandong
Happy Fan Shop	King of Power Saving	No shipment.	Jingdong prefecture level city in Shandong
Happy Fan Shop	King of Power Saving	Fake. Speechless.	Jingdong prefecture level city in Shandong
Happy Fan Shop	King of Power Saving	fantastic (idiom); awesome	Jingdong prefecture level city in Shandong
Happy Fan Shop	King of Power Saving	an excellent	Jingdong prefecture level city in Shandong
Happy Fan Shop	King of Power Saving	an excellent	Jingdong prefecture level city in Shandong
Happy Fan Shop	King of Power Saving	Want to buy! Recommended not to buy! No effect	Jingdong prefecture level city in Shandong
Happy Fan Shop	King of Power Saving	That's all there is to it. I can't figure out how to save electricity.	Jingdong prefecture level city in Shandong
Happy Fan Shop	King of Power Saving	It's no use.	Jingdong prefecture level city in Shandong
Happy Fan Shop	King of Power Saving	Things are more perfect than I imagined, very powerful, it is worth getting Packaging is very strong, things are well protected, it is worth the praise things are very satisfied Oh, beautiful and stylish atmosphere, customer service is super patient, service is meticulous and attentive, the big brand is not	Jingdong prefecture level city in Shandong
Happy Fan Shop	King of Power Saving	The effect is too good, the family has now several units, back to buy a number of times, the power saver received, power-saving effect is very good,, fine workmanship, long warranty, but also purify the air, before buying a used for a period of time, with the okay, and then bought a, and only now come to the	Jingdong prefecture level city in Shandong
Happy Fan Shop	King of Power Saving	Quite good, and imagine the same, casual ride are very good, satisfied! Baby received it 👌, really great ah! 🌈 quality is really nothing to say, very satisfied! 🆗 happy happy 😃 genuine, great, the price is very good, in short, quite good, will buy again a very good shopping experience, attentive service, fast logistics, distribution is also thoughtful, the attitude is also very good, value for money, ah,	Jingdong prefecture level city in Shandong
Happy Fan Shop	King of Power Saving	Power-saving effect: strong Cost-effective: quite good Appearance style: beautiful Workmanship material: meticulous Experience: looking forward to power-saving effect	Jingdong prefecture level city in Shandong
Happy Fan Shop	King of Power Saving	Cost-effective is okay, the appearance of the style of meticulous workmanship, looking forward to energy-saving effect.	Jingdong prefecture level city in Shandong
Happy Fan Shop	King of Power Saving	Very Good Very Power Saving This Power Saver is really power saving As soon as the power is turned on, the Power Saver starts the power saving function for you.	Jingdong prefecture level city in Shandong
Happy Fan Shop	King of Power Saving	Logistics is very fast, the price is favorable, friends introduced that it is very good to use, bought 2 at once, looking forward to the effect, used after the follow-up comment.	Jingdong prefecture level city in Shandong
Happy Fan Shop	King of Power Saving	It's all fake. It doesn't save any electricity.	Jingdong prefecture level city in Shandong
Happy Fan Shop	King of Power Saving	This Power Saver is so much better than the one I bought before, not only the effect is good, the quality is very good in all aspects, the customer service attitude is also very good, recommended to buy!	Jingdong prefecture level city in Shandong
Happy Fan Shop	King of Power Saving	Logistics is very fast, buy for parents, good quality, once bought 2, power-saving effect is good to buy 2 more, look forward to using the effect, satisfied will recommend to friends.	Jingdong prefecture level city in Shandong
Happy Fan Shop	King of Power Saving	With the description is the same, very high-grade, good value, to buy friends can order Oh!	Jingdong prefecture level city in Shandong
Happy Fan Shop	King of Power Saving	Not using much electricity in the winter saving over 100 per month! Ready to buy 2 more units in the summer ！！！！！ Good quality, good price, power saving effect is obvious, cost-effective, worth buying, can be recommended!	Jingdong prefecture level city in Shandong
Happy Fan Shop	King of Power Saving	Purchased a power saver received on the put into use, rent a room with many electrical appliances, power consumption is very large, we have a piece of electricity or 1.2 dollars a time, with this power saver is much better, every day can save a lot of money, very convenient and practical.	Jingdong prefecture level city in Shandong
Happy Fan Shop	King of Power Saving	The power saving effect is very good: my supermarket monthly electricity bill is more than 7000 yuan. On May 1st someone recommended buying a couple of power savers, and I bought 5 of them first. The electricity bill was about 2000 less in June. This time, I bought 5 more. The seller said 800 degrees of electricity with one. I feel it's basically enough. A total of 10 units	Jingdong prefecture level city in Shandong
Happy Fan Shop	King of Power Saving	I never thought I could get such a good baby at such an affordable price, it's very good value for money, I love it!	Jingdong prefecture level city in Shandong
Happy Fan Shop	King of Power Saving	Previously bought other brands of power savers, but found no effect at home, this time to see how the products on the Jingdong, inserted in the home socket, the LED display is blue, the night light is very nice to see, look at the instructions that have to be half a year in order to achieve the best results, try it, I believe that the quality of the Jingdong, it should not be wrong.	Jingdong prefecture level city in Shandong
Happy Fan Shop	King of Power Saving	Superb, inexpensive, saves a lot of points, been using it for a long time!	Jingdong prefecture level city in Shandong
Happy Fan Shop	King of Power Saving	There are a lot of appliances in the house, the summer air conditioning is very costly, I bought this in front of the air conditioning effect is very good, I explain that I bought three in one breath, I use more electricity a month down to 200 or so, very satisfied.	Jingdong prefecture level city in Shandong
Happy Fan Shop	King of Power Saving	A month can save more than 200, home monthly electricity bill of more than 600 dollars, inserted 3 or so, an amount of the balance of the average can save more than 200. Product is very good, has been buying this one, has given a lot of relatives recommended, the quality is also very good, before buying a look with this same, who knows to buy home has not been used for more than a week on the bad, the quality of this is really good with more than two years!	Jingdong prefecture level city in Shandong
Happy Fan Shop	King of Power Saving	Whole house power saving king, can really save electricity, friends recommended, high power saving appliances, only replacement, no repair, after-sales worry-free!	Jingdong prefecture level city in Shandong
Happy Fan Shop	King of Power Saving	Great item love it, great customer service, great shopping experience!	Jingdong prefecture level city in Shandong
Happy Fan Shop	King of Power Saving	Power saving, very good use, much cheaper than the physical store, satisfaction	Jingdong prefecture level city in Shandong
Happy Fan Shop	King of Power Saving	In use, very well packaged and fast delivery of credit!	Jingdong prefecture level city in Shandong
Happy Fan Shop	King of Power Saving	Product quality, customer service attitude is particularly good, like, praise!	Jingdong prefecture level city in Shandong
Happy Fan Shop	King of Power Saving	The logistics is very fast, the packaging is not broken, the quality is very good, I like it very much!	Jingdong prefecture level city in Shandong
Happy Fan Shop	King of Power Saving	The saver was well packaged and was as described when plugged in	Jingdong prefecture level city in Shandong
Happy Fan Shop	King of Power Saving	Energy efficient and very satisfied	Jingdong prefecture level city in Shandong
Happy Fan Shop	King of Power Saving	Great quality for the price and fast shipping.	Jingdong prefecture level city in Shandong
Happy Fan Shop	King of Power Saving	It's great, just as described. Perfect.	Jingdong prefecture level city in Shandong

Happy Fan Shop	King of Power Saving	There is no power saving effect, but rather ring up an increase in electricity costs, because this machine is on continuously all day long, uninterruptedly, its own electricity and increased costs, plus the cost of purchasing the machine, so I feel that the use of this machine instead of increasing the	Jingdong prefecture level city in Shandong
Happy Fan Shop	King of Power Saving	Recommended by the owner of the store next door, looked at his home does have to save electricity, decided to get. Indeed quite can, the voltage is much more stable, there is a reduction in electricity	Jingdong prefecture level city in Shandong
Happy Fan Shop	King of Power Saving	Goods have been received value for money, affordable and reasonable price, and the description of the same, power-saving safe and reliable!	Jingdong prefecture level city in Shandong
Happy Fan Shop	King of Power Saving	Listen to friends introduced to buy, tried to save a lot of electricity is good, quite good, worth buying.	Jingdong prefecture level city in Shandong
Happy Fan Shop	King of Power Saving	The product is easy to use, safe and environmentally friendly, only used for a short time, to the next month, after the electricity bill status display to know how the performance of the power saver efficacy.	Jingdong prefecture level city in Shandong
Happy Fan Shop	King of Power Saving	Last month's electricity bill is 178 yuan, with this power saver is 92 yuan, ready to buy two more back home, I was introduced to buy a friend, just started to not believe that I did not expect the effect!	Jingdong prefecture level city in Shandong
Happy Fan Shop	King of Power Saving	It looks nice, but after using it for a few days I can't see how it saves power?	Jingdong prefecture level city in Shandong
Happy Fan Shop	King of Power Saving	Recommended by a friend, it is genuine, as described by the merchant, cost-effective	Jingdong prefecture level city in Shandong
Happy Fan Shop	King of Power Saving	Works well, affordable and saves money.	Jingdong prefecture level city in Shandong
Happy Fan Shop	King of Power Saving	Feels good haven't compared it yet, the air conditioning has been on a lot in the past two months	Jingdong prefecture level city in Shandong
Happy Fan Shop	King of Power Saving	Saving about 5 kWh per day is a good thing	Jingdong prefecture level city in Shandong
Happy Fan Shop	King of Power Saving	Not bad, affordable, best value for money	Jingdong prefecture level city in Shandong
Happy Fan Shop	King of Power Saving	The goods are genuine and the logistics are faster.	Jingdong prefecture level city in Shandong
Happy Fan Shop	King of Power Saving	Goods have been received today, has been plugged into the outlet, looking forward to saving electricity, after a period of time to see how the effect in the follow-up comment!	Jingdong prefecture level city in Shandong
Happy Fan Shop	King of Power Saving	I just got it home and haven't used it for long, so I'll use it for a couple months and see what happens. Seeing that most people have good reviews, it should be fine.	Jingdong prefecture level city in Shandong
Happy Fan Shop	King of Power Saving	I'm looking forward to the results, so I'll give you a good review out of habit! Follow up review after a while	Jingdong prefecture level city in Shandong
Happy Fan Shop	King of Power Saving	Use a dish first to know if it will help save energy	Jingdong prefecture level city in Shandong
Happy Fan Shop	King of Power Saving	Whether it saves electricity or not, we don't know yet.	Jingdong prefecture level city in Shandong
Happy Fan Shop	King of Power Saving	Goods have been received, use a period of time to know how oh. The courier did not deliver to the door ah! Sent to the station ah! The heat is inconvenient ah!	Jingdong prefecture level city in Shandong
Happy Fan Shop	King of Power Saving	A little effect, before an hour a power, now an hour 0.76 degrees, a dollar a power = save 24 cents, although not a lot, first use it!	Jingdong prefecture level city in Shandong
Happy Fan Shop	King of Power Saving	Just received the goods, use a month to evaluate	Jingdong prefecture level city in Shandong
Happy Fan Shop	King of Power Saving	Home every month with a lot of electricity, inadvertently found this good thing online that can save the beginning of a skeptical attitude to consult customer service, customer service replied very professional, said 120 days can be refunded that first try it!	Jingdong prefecture level city in Shandong
Happy Fan Shop	King of Power Saving	After receiving the goods this afternoon, immediately installed in the bathroom Smith energy storage electric water heater on the power outlet (instead of connecting with a plug board), when used, found that the "Power Saving King" LCD display, is the value of the voltage, not the store's video said so	Jingdong prefecture level city in Shandong
Happy Fan Shop	King of Power Saving	Has made many days, did not feel the power saving, the effect is not obvious.	Jingdong prefecture level city in Shandong
Happy Fan Shop	King of Power Saving	Energy savers do not save energy at all, but rather consume a lot of electricity.	Jingdong prefecture level city in Shandong
Happy Fan Shop	King of Power Saving	It's useless. It's a waste of money.	Jingdong prefecture level city in Shandong
Happy Fan Shop	King of Power Saving	Last month saved more than 500, which came back to buy, the store bought three, and then buy 1 home use, my family is dry beauty salon, more instruments, especially electricity, a month almost 1000. or so, with this did not expect to save so much, great, five-star praise!	Jingdong prefecture level city in Shandong
Happy Fan Shop	King of Power Saving	It rained a couple days ago and I just went to get the item back today , not bad, hey I looked at the meter and that red display light takes several seconds to jump, did not make a mistake buying it. After a period of time to evaluate.	Jingdong prefecture level city in Shandong
Happy Fan Shop	King of Power Saving	Without further ado, the electricity bill came down, this month less 102 yuan, has been repurchased, I also recommended to my friends, all said that there are effects, the product is okay, the effect is quite	Jingdong prefecture level city in Shandong
Happy Fan Shop	King of Power Saving	After buying and using two months really see the effect, so only to evaluate! Originally held the mindset of trying, I did not expect really effective, worth having!	Jingdong prefecture level city in Shandong
Happy Fan Shop	King of Power Saving	In the use of this energy saver before the monthly electricity bill is more than 150 yuan or so, now save thirty percent, every day to work, six or seven o'clock in the afternoon to go home, I do not know why the electricity bill is so expensive, energy-saving appliances really have the effect of recommending	Jingdong prefecture level city in Shandong
Happy Fan Shop	King of Power Saving	It saves about 100 bucks a month. A friend recommended it to me, and he's been using it for a while, and the effect is so obvious that I bought one too.	Jingdong prefecture level city in Shandong
Happy Fan Shop	King of Power Saving	The specific effect will have to be tested for at least two months to compare whether there is a power saving effect or not.	Jingdong prefecture level city in Shandong
Happy Fan Shop	King of Power Saving	My dad asked me to buy one, but after I paid the bill, I asked and he said it was a lot less this month, so he asked me to buy another one.	Jingdong prefecture level city in Shandong
Happy Fan Shop	King of Power Saving	'The goods have been received, immediately go to use, please ask the boss you instructions do not allow to withdraw, there are two fuses installed there!	Jingdong prefecture level city in Shandong
Happy Fan Shop	King of Power Saving	First praise is using, after using if good then follow up, now because I have not yet seen the results	Jingdong prefecture level city in Shandong
Happy Fan Shop	King of Power Saving	I've just been using it, and I haven't seen if it has a power-saving effect yet, so I'll follow up after 10 days and half a month.	Jingdong prefecture level city in Shandong
Happy Fan Shop	King of Power Saving	Just received the intelligent power saving meter, immediately plugged in to use, see it is not bad, and order to sell the second time	Jingdong prefecture level city in Shandong
Happy Fan Shop	King of Power Saving	This power saver is a bit effective, used for half a month, the effect is still visible.	Jingdong prefecture level city in Shandong
Happy Fan Shop	King of Power Saving	I received the baby and it looks great, I'm trying it out now and will keep an eye on it afterward.	Jingdong prefecture level city in Shandong
Happy Fan Shop	King of Power Saving	I've just received it, but I'm still trying to see how it works, so I'll come back in a little while.	Jingdong prefecture level city in Shandong
Happy Fan Shop	King of Power Saving	Just used it wait to use it for a while and then comment thanks, all first five stars.	Jingdong prefecture level city in Shandong
Happy Fan Shop	King of Power Saving	Very trustworthy brand, quality is guaranteed, the price is still affordable, quite satisfied!	Jingdong prefecture level city in Shandong
Happy Fan Shop	King of Power Saving	Things received, I hope that the platform of Jingdong do not sell fake goods are available	Jingdong prefecture level city in Shandong
Happy Fan Shop	King of Power Saving	Energy saver received, the use of the effect is good and the business description of the same	Jingdong prefecture level city in Shandong
Happy Fan Shop	King of Power Saving	The surface looks beautiful, the actual effect is not known, after using additional evaluation.	Jingdong prefecture level city in Shandong
Happy Fan Shop	King of Power Saving	Tried it for a few months, it really saves electricity, and I bought it for a friend.	Jingdong prefecture level city in Shandong
Happy Fan Shop	King of Power Saving	It didn't work and wanted to return it, it really didn't work at all.	Jingdong prefecture level city in Shandong

Happy Fan Shop	King of Power Saving	Just used it, don't know how effective it is, looking forward to usefulness	Jingdong prefecture level city in Shandong
Happy Fan Shop	King of Power Saving	After two months before the effect I do not know if it is true!	Jingdong prefecture level city in Shandong
Happy Fan Shop	King of Power Saving	So fast! Ordered yesterday and received today 👍👍👍👍👍👍👍👍👍 🌹🌹🌹🌹🌹🌹🌹🌹🌹	Jingdong prefecture level city in Shandong
Happy Fan Shop	King of Power Saving	I've started using it. We'll see how it works.	Jingdong prefecture level city in Shandong
Happy Fan Shop	King of Power Saving	I just received the goods, I won't know the effect until after two.	Jingdong prefecture level city in Shandong
Happy Fan Shop	King of Power Saving	Just received the goods, the specific effect will be evaluated later	Jingdong prefecture level city in Shandong
Happy Fan Shop	King of Power Saving	I've received the goods, I don't know if it's any good yet	Jingdong prefecture level city in Shandong
Happy Fan Shop	King of Power Saving	Try it for a month and then come back with a concrete evaluation	Jingdong prefecture level city in Shandong
Happy Fan Shop	King of Power Saving	In use, after two months to see the effect of power saving	Jingdong prefecture level city in Shandong
Happy Fan Shop	King of Power Saving	Power Saver. It's authentic. It's black. It works.	Jingdong prefecture level city in Shandong
Happy Fan Shop	King of Power Saving	I've been using it for a week now and it's not working.	Jingdong prefecture level city in Shandong
Happy Fan Shop	King of Power Saving	I'm just using it. I don't know how it's going to work.	Jingdong prefecture level city in Shandong
Happy Fan Shop	King of Power Saving	Products are better looking out for power savings	Jingdong prefecture level city in Shandong
Happy Fan Shop	King of Power Saving	I received it and I've kept it on. I hope it works.	Jingdong prefecture level city in Shandong
Happy Fan Shop	King of Power Saving	It's really good! Worth recommending to buy!	Jingdong prefecture level city in Shandong
Happy Fan Shop	King of Power Saving	Try it for a few days and see if it doesn't work, then return it.	Jingdong prefecture level city in Shandong
Happy Fan Shop	King of Power Saving	It's in use and I hope to be pleasantly surprised.	Jingdong prefecture level city in Shandong
Happy Fan Shop	King of Power Saving	We'll see how it works when we use it	Jingdong prefecture level city in Shandong
Happy Fan Shop	King of Power Saving	Let's see if it works.	Jingdong prefecture level city in Shandong
Happy Fan Shop	King of Power Saving	I'm using it and will follow up with a review	Jingdong prefecture level city in Shandong
Happy Fan Shop	King of Power Saving	It really works. It saves energy.	Jingdong prefecture level city in Shandong
Happy Fan Shop	King of Power Saving	It does have some power savings!	Jingdong prefecture level city in Shandong
Happy Fan Shop	King of Power Saving	Jingdong shopping speed is convenient and timely	Jingdong prefecture level city in Shandong
Happy Fan Shop	King of Power Saving	Second time I've bought it. Everything's fine.	Jingdong prefecture level city in Shandong
Happy Fan Shop	King of Power Saving	I don't know if it works yet.	Jingdong prefecture level city in Shandong
Happy Fan Shop	King of Power Saving	Not used, don't know how well it works.	Jingdong prefecture level city in Shandong
Happy Fan Shop	King of Power Saving	No visible results so far	Jingdong prefecture level city in Shandong
Happy Fan Shop	King of Power Saving	It doesn't feel like it's working.	Jingdong prefecture level city in Shandong
Happy Fan Shop	King of Power Saving	Where's the effect? Where are the power savings?	Jingdong prefecture level city in Shandong
Happy Fan Shop	King of Power Saving	Let's see how it works in practice!	Jingdong prefecture level city in Shandong
Happy Fan Shop	King of Power Saving	I'll wait until I use it to give you a good review	Jingdong prefecture level city in Shandong
Happy Fan Shop	King of Power Saving	I'll follow up after I've used it	Jingdong prefecture level city in Shandong
Happy Fan Shop	King of Power Saving	Well, that's good for saving power.	Jingdong prefecture level city in Shandong
Happy Fan Shop	King of Power Saving	Received. Used in the know.	Jingdong prefecture level city in Shandong
Happy Fan Shop	King of Power Saving	It doesn't do much good.	Jingdong prefecture level city in Shandong
Happy Fan Shop	King of Power Saving	It's not working.	Jingdong prefecture level city in Shandong
Happy Fan Shop	King of Power Saving	I don't know. I just used it.	Jingdong prefecture level city in Shandong
Happy Fan Shop	King of Power Saving	Use it and come back to evaluate.	Jingdong prefecture level city in Shandong
Happy Fan Shop	King of Power Saving	Two words: useless.	Jingdong prefecture level city in Shandong
Happy Fan Shop	King of Power Saving	To be used and then evaluated	Jingdong prefecture level city in Shandong
Happy Fan Shop	King of Power Saving	No shipment.	Jingdong prefecture level city in Shandong
Happy Fan Shop	King of Power Saving	Fake. Speechless.	Jingdong prefecture level city in Shandong
Happy Fan Shop	King of Power Saving	fantastic (idiom); awesome	Jingdong prefecture level city in Shandong
Happy Fan Shop	King of Power Saving	an excellent	Jingdong prefecture level city in Shandong
Happy Fan Shop	King of Power Saving	an excellent	Jingdong prefecture level city in Shandong
Happy Fan Shop	King of Power Saving	Want to buy! Recommended not to buy! No effect	Jingdong prefecture level city in Shandong
Happy Fan Shop	King of Power Saving	That's all there is to it. I can't figure out how to save electricity.	Jingdong prefecture level city in Shandong
Happy Fan Shop	King of Power Saving	It's no use.	Jingdong prefecture level city in Shandong
Happy Fan Shop	King of Power Saving	Things are more perfect than I imagined, very powerful, it is worth getting Packaging is very strong, things are well protected, it is worth the praise things are very satisfied Oh, beautiful and stylish atmosphere, customer service is super patient, service is meticulous and attentive, the big brand is not	Jingdong prefecture level city in Shandong
Happy Fan Shop	King of Power Saving	The effect is too good, the family has now several units, back to buy a number of times, the power saver received, power-saving effect is very good,, fine workmanship, long warranty, but also purify the air, before buying a used for a period of time, with the okay, and then bought a, and only now come to the	Jingdong prefecture level city in Shandong

Happy Fan Shop	King of Power Saving	Quite good, and imagine the same, casual ride are very good, satisfied! Baby received it 👌, really great ah! 🌈 quality is really nothing to say, very satisfied! 🆗 happy happy 😃 genuine, great, the price is very good, in short, quite good, will buy again a very good shopping experience, attentive service, fast logistics, distribution is also thoughtful, the attitude is also very good, value for money, ah,	Jingdong prefecture level city in Shandong
Happy Fan Shop	King of Power Saving	Power-saving effect: strong Cost-effective: quite good Appearance style: beautiful Workmanship material: meticulous Experience: looking forward to power-saving effect	Jingdong prefecture level city in Shandong
Happy Fan Shop	King of Power Saving	Cost-effective is okay, the appearance of the style of meticulous workmanship, looking forward to energy-saving effect.	Jingdong prefecture level city in Shandong
Happy Fan Shop	King of Power Saving	Very Good Very Power Saving This Power Saver is really power saving As soon as the power is turned on, the Power Saver starts the power saving function for you.	Jingdong prefecture level city in Shandong
Happy Fan Shop	King of Power Saving	Logistics is very fast, the price is favorable, friends introduced that it is very good to use, bought 2 at once, looking forward to the effect, used after the follow-up comment.	Jingdong prefecture level city in Shandong
Happy Fan Shop	King of Power Saving	It's all fake. It doesn't save any electricity.	Jingdong prefecture level city in Shandong
Happy Fan Shop	King of Power Saving	This Power Saver is so much better than the one I bought before, not only the effect is good, the quality is very good in all aspects, the customer service attitude is also very good, recommended to buy!	Jingdong prefecture level city in Shandong
Happy Fan Shop	King of Power Saving	Logistics is very fast, buy for parents, good quality, once bought 2, power-saving effect is good to buy 2 more, look forward to using the effect, satisfied will recommend to friends.	Jingdong prefecture level city in Shandong
Happy Fan Shop	King of Power Saving	With the description is the same, very high-grade, good value, to buy friends can order Oh!	Jingdong prefecture level city in Shandong
Happy Fan Shop	King of Power Saving	Not using much electricity in the winter saving over 100 per month! Ready to buy 2 more units in the summer ！！！！！ Good quality, good price, power saving effect is obvious, cost-effective, worth buying, can be recommended!	Jingdong prefecture level city in Shandong
Happy Fan Shop	King of Power Saving	Purchased a power saver received on the put into use, rent a room with many electrical appliances, power consumption is very large, we have a piece of electricity or 1.2 dollars a time, with this power saver is much better, every day can save a lot of money, very convenient and practical.	Jingdong prefecture level city in Shandong
Happy Fan Shop	King of Power Saving	The power saving effect is very good: my supermarket monthly electricity bill is more than 7000 yuan. On May 1st someone recommended buying a couple of power savers, and I bought 5 of them first. The electricity bill was about 2000 less in June. This time, I bought 5 more. The seller said 800 degrees of electricity with one. I feel it's basically enough. A total of 10 units	Jingdong prefecture level city in Shandong
Happy Fan Shop	King of Power Saving	I never thought I could get such a good baby at such an affordable price, it's very good value for money, I love it!	Jingdong prefecture level city in Shandong
Happy Fan Shop	King of Power Saving	Previously bought other brands of power savers, but found no effect at home, this time to see how the products on the Jingdong, inserted in the home socket, the LED display is blue, the night light is very nice to see, look at the instructions that have to be half a year in order to achieve the best results, try it, I believe that the quality of the Jingdong, it should not be wrong.	Jingdong prefecture level city in Shandong
Happy Fan Shop	King of Power Saving	Superb, inexpensive, saves a lot of points, been using it for a long time!	Jingdong prefecture level city in Shandong
Happy Fan Shop	King of Power Saving	There are a lot of appliances in the house, the summer air conditioning is very costly, I bought this in front of the air conditioning effect is very good, I explain that I bought three in one breath, I use more electricity a month down to 200 or so, very satisfied.	Jingdong prefecture level city in Shandong
Happy Fan Shop	King of Power Saving	A month can save more than 200, home monthly electricity bill of more than 600 dollars, inserted 3 or so, an amount of the balance of the average can save more than 200. Product is very good, has been buying this one, has given a lot of relatives recommended, the quality is also very good, before buying a look with this same, who knows to buy home has not been used for more than a week on the bad, the quality of this is really good with more than two years!	Jingdong prefecture level city in Shandong
Happy Fan Shop	King of Power Saving	Whole house power saving king, can really save electricity, friends recommended, high power saving appliances, only replacement, no repair, after-sales worry-free!	Jingdong prefecture level city in Shandong
Happy Fan Shop	King of Power Saving	Great item love it, great customer service, great shopping experience!	Jingdong prefecture level city in Shandong
Happy Fan Shop	King of Power Saving	Power saving, very good use, much cheaper than the physical store, satisfaction	Jingdong prefecture level city in Shandong
Happy Fan Shop	King of Power Saving	In use, very well packaged and fast delivery of credit!	Jingdong prefecture level city in Shandong
Happy Fan Shop	King of Power Saving	Product quality, customer service attitude is particularly good, like, praise!	Jingdong prefecture level city in Shandong
Happy Fan Shop	King of Power Saving	The logistics is very fast, the packaging is not broken, the quality is very good, I like it very much!	Jingdong prefecture level city in Shandong
Happy Fan Shop	King of Power Saving	The saver was well packaged and was as described when plugged in	Jingdong prefecture level city in Shandong
Happy Fan Shop	King of Power Saving	Energy efficient and very satisfied	Jingdong prefecture level city in Shandong
Happy Fan Shop	King of Power Saving	Great quality for the price and fast shipping.	Jingdong prefecture level city in Shandong
Happy Fan Shop	King of Power Saving	It's great, just as described. Perfect.	Jingdong prefecture level city in Shandong
Happy Fan Shop	King of Power Saving	There is no power saving effect, but rather ring up an increase in electricity costs, because this machine is on continuously all day long, uninterruptedly, its own electricity and increased costs, plus the cost of purchasing the machine, so I feel that the use of this machine instead of increasing the	Jingdong prefecture level city in Shandong
Happy Fan Shop	King of Power Saving	Recommended by the owner of the store next door, looked at his home does have to save electricity, decided to get. Indeed quite can, the voltage is much more stable, there is a reduction in electricity	Jingdong prefecture level city in Shandong
Happy Fan Shop	King of Power Saving	Goods have been received value for money, affordable and reasonable price, and the description of the same, power-saving safe and reliable!	Jingdong prefecture level city in Shandong
Happy Fan Shop	King of Power Saving	Listen to friends introduced to buy, tried to save a lot of electricity is good, quite good, worth buying.	Jingdong prefecture level city in Shandong
Happy Fan Shop	King of Power Saving	The product is easy to use, safe and environmentally friendly, only used for a short time, to the next month, after the electricity bill status display to know how the performance of the power saver efficacy.	Jingdong prefecture level city in Shandong
Happy Fan Shop	King of Power Saving	Last month's electricity bill is 178 yuan, with this power saver is 92 yuan, ready to buy two more back home, I was introduced to buy a friend, just started to not believe that I did not expect the effect!	Jingdong prefecture level city in Shandong
Happy Fan Shop	King of Power Saving	It looks nice, but after using it for a few days I can't see how it saves power?	Jingdong prefecture level city in Shandong
Happy Fan Shop	King of Power Saving	Recommended by a friend, it is genuine, as described by the merchant, cost-effective	Jingdong prefecture level city in Shandong
Happy Fan Shop	King of Power Saving	Works well, affordable and saves money.	Jingdong prefecture level city in Shandong
Happy Fan Shop	King of Power Saving	Feels good haven't compared it yet, the air conditioning has been on a lot in the past two months	Jingdong prefecture level city in Shandong
Happy Fan Shop	King of Power Saving	Saving about 5 kWh per day is a good thing	Jingdong prefecture level city in Shandong
Happy Fan Shop	King of Power Saving	Not bad, affordable, best value for money	Jingdong prefecture level city in Shandong
Happy Fan Shop	King of Power Saving	The goods are genuine and the logistics are faster.	Jingdong prefecture level city in Shandong
Happy Fan Shop	King of Power Saving	Goods have been received today, has been plugged into the outlet, looking forward to saving electricity, after a period of time to see how the effect in the follow-up comment!	Jingdong prefecture level city in Shandong
Happy Fan Shop	King of Power Saving	I just got it home and haven't used it for long, so I'll use it for a couple months and see what happens. Seeing that most people have good reviews, it should be fine.	Jingdong prefecture level city in Shandong
Happy Fan Shop	King of Power Saving	I'm looking forward to the results, so I'll give you a good review out of habit! Follow up review after a while	Jingdong prefecture level city in Shandong
Happy Fan Shop	King of Power Saving	Use a dish first to know if it will help save energy	Jingdong prefecture level city in Shandong
Happy Fan Shop	King of Power Saving	Whether it saves electricity or not, we don't know yet.	Jingdong prefecture level city in Shandong

Happy Fan Shop	King of Power Saving	Goods have been received, use a period of time to know how oh. The courier did not deliver to the door ah! Sent to the station ah! The heat is inconvenient ah!	Jingdong prefecture level city in Shandong
Happy Fan Shop	King of Power Saving	A little effect, before an hour a power, now an hour 0.76 degrees, a dollar a power = save 24 cents, although not a lot, first use it!	Jingdong prefecture level city in Shandong
Happy Fan Shop	King of Power Saving	Just received the goods, use a month to evaluate	Jingdong prefecture level city in Shandong
Happy Fan Shop	King of Power Saving	Home every month with a lot of electricity, inadvertently found this good thing online that can save the beginning of a skeptical attitude to consult customer service, customer service replied very professional, said 120 days can be refunded that first try it!	Jingdong prefecture level city in Shandong
Happy Fan Shop	King of Power Saving	After receiving the goods this afternoon, immediately installed in the bathroom Smith energy storage electric water heater on the power outlet (instead of connecting with a plug board), when used, found that the "Power Saving King" LCD display, is the value of the voltage, not the store's video said so	Jingdong prefecture level city in Shandong
Happy Fan Shop	King of Power Saving	Has made many days, did not feel the power saving, the effect is not obvious.	Jingdong prefecture level city in Shandong
Happy Fan Shop	King of Power Saving	Energy savers do not save energy at all, but rather consume a lot of electricity.	Jingdong prefecture level city in Shandong
Happy Fan Shop	King of Power Saving	It's useless. It's a waste of money.	Jingdong prefecture level city in Shandong
Happy Fan Shop	King of Power Saving	Last month saved more than 500, which came back to buy, the store bought three, and then buy 1 home use, my family is dry beauty salon, more instruments, especially electricity, a month almost 1000. or so, with this did not expect to save so much, great, five-star praise!	Jingdong prefecture level city in Shandong
Happy Fan Shop	King of Power Saving	It rained a couple days ago and I just went to get the item back today , not bad, hey I looked at the meter and that red display light takes several seconds to jump, did not make a mistake buying it. After a period of time to evaluate.	Jingdong prefecture level city in Shandong
Happy Fan Shop	King of Power Saving	Without further ado, the electricity bill came down, this month less 102 yuan, has been repurchased, I also recommended to my friends, all said that there are effects, the product is okay, the effect is quite	Jingdong prefecture level city in Shandong
Happy Fan Shop	King of Power Saving	After buying and using two months really see the effect, so only to evaluate! Originally held the mindset of trying, I did not expect really effective, worth having!	Jingdong prefecture level city in Shandong
Happy Fan Shop	King of Power Saving	In the use of this energy saver before the monthly electricity bill is more than 150 yuan or so, now save thirty percent, every day to work, six or seven o'clock in the afternoon to go home, I do not know why the electricity bill is so expensive, energy-saving appliances really have the effect of recommending	Jingdong prefecture level city in Shandong
Happy Fan Shop	King of Power Saving	It saves about 100 bucks a month. A friend recommended it to me, and he's been using it for a while, and the effect is so obvious that I bought one too.	Jingdong prefecture level city in Shandong
Happy Fan Shop	King of Power Saving	My dad asked me to buy one, but after I paid the bill, I asked and he said it was a lot less this month, so he asked me to buy another one.	Jingdong prefecture level city in Shandong
Happy Fan Shop	King of Power Saving	The specific effect will have to be tested for at least two months to compare whether there is a power saving effect or not.	Jingdong prefecture level city in Shandong
Happy Fan Shop	King of Power Saving	'The goods have been received, immediately go to use, please ask the boss you instructions do not allow to withdraw, there are two fuses installed there!	Jingdong prefecture level city in Shandong
Happy Fan Shop	King of Power Saving	First praise is using, after using if good then follow up, now because I have not yet seen the results	Jingdong prefecture level city in Shandong
Happy Fan Shop	King of Power Saving	I've just been using it, and I haven't seen if it has a power-saving effect yet, so I'll follow up after 10 days and half a month.	Jingdong prefecture level city in Shandong
Happy Fan Shop	King of Power Saving	Just received the intelligent power saving meter, immediately plugged in to use, see it is not bad, and order to sell the second time	Jingdong prefecture level city in Shandong
Happy Fan Shop	King of Power Saving	This power saver is a bit effective, used for half a month, the effect is still visible.	Jingdong prefecture level city in Shandong
Happy Fan Shop	King of Power Saving	I received the baby and it looks great, I'm trying it out now and will keep an eye on it afterward.	Jingdong prefecture level city in Shandong
Happy Fan Shop	King of Power Saving	I've just received it, but I'm still trying to see how it works, so I'll come back in a little while.	Jingdong prefecture level city in Shandong
Happy Fan Shop	King of Power Saving	Just used it wait to use it for a while and then comment thanks, all first five stars.	Jingdong prefecture level city in Shandong
Happy Fan Shop	King of Power Saving	Very trustworthy brand, quality is guaranteed, the price is still affordable, quite satisfied!	Jingdong prefecture level city in Shandong
Happy Fan Shop	King of Power Saving	Things received, I hope that the platform of Jingdong do not sell fake goods are available	Jingdong prefecture level city in Shandong
Happy Fan Shop	King of Power Saving	Energy saver received, the use of the effect is good and the business description of the same	Jingdong prefecture level city in Shandong
Happy Fan Shop	King of Power Saving	The surface looks beautiful, the actual effect is not known, after using additional evaluation.	Jingdong prefecture level city in Shandong
Happy Fan Shop	King of Power Saving	Tried it for a few months, it really saves electricity, and I bought it for a friend.	Jingdong prefecture level city in Shandong
Happy Fan Shop	King of Power Saving	It didn't work and wanted to return it, it really didn't work at all.	Jingdong prefecture level city in Shandong
Happy Fan Shop	King of Power Saving	Just used it, don't know how effective it is, looking forward to usefulness	Jingdong prefecture level city in Shandong
Happy Fan Shop	King of Power Saving	After two months before the effect I do not know if it is true!	Jingdong prefecture level city in Shandong
Happy Fan Shop	King of Power Saving	So fast! Ordered yesterday and received today 👍👍👍👍👍👍👍👍👍 🌹🌹🌹🌹🌹🌹🌹🌹🌹	Jingdong prefecture level city in Shandong
Happy Fan Shop	King of Power Saving	I've started using it. We'll see how it works.	Jingdong prefecture level city in Shandong
Happy Fan Shop	King of Power Saving	I just received the goods, I won't know the effect until after two.	Jingdong prefecture level city in Shandong
Happy Fan Shop	King of Power Saving	Just received the goods, the specific effect will be evaluated later	Jingdong prefecture level city in Shandong
Happy Fan Shop	King of Power Saving	I've received the goods, I don't know if it's any good yet	Jingdong prefecture level city in Shandong
Happy Fan Shop	King of Power Saving	Try it for a month and then come back with a concrete evaluation	Jingdong prefecture level city in Shandong
Happy Fan Shop	King of Power Saving	In use, after two months to see the effect of power saving	Jingdong prefecture level city in Shandong
Happy Fan Shop	King of Power Saving	Power Saver. It's authentic. It's black. It works.	Jingdong prefecture level city in Shandong
Happy Fan Shop	King of Power Saving	I've been using it for a week now and it's not working.	Jingdong prefecture level city in Shandong
Happy Fan Shop	King of Power Saving	I'm just using it. I don't know how it's going to work.	Jingdong prefecture level city in Shandong
Happy Fan Shop	King of Power Saving	Products are better looking out for power savings	Jingdong prefecture level city in Shandong
Happy Fan Shop	King of Power Saving	I received it and I've kept it on. I hope it works.	Jingdong prefecture level city in Shandong
Happy Fan Shop	King of Power Saving	It's really good! Worth recommending to buy!	Jingdong prefecture level city in Shandong
Happy Fan Shop	King of Power Saving	Try it for a few days and see if it doesn't work, then return it.	Jingdong prefecture level city in Shandong
Happy Fan Shop	King of Power Saving	It's in use and I hope to be pleasantly surprised.	Jingdong prefecture level city in Shandong
Happy Fan Shop	King of Power Saving	We'll see how it works when we use it	Jingdong prefecture level city in Shandong
Happy Fan Shop	King of Power Saving	Let's see if it works.	Jingdong prefecture level city in Shandong

Happy Fan Shop	King of Power Saving	I'm using it and will follow up with a review	Jingdong prefecture level city in Shandong
Happy Fan Shop	King of Power Saving	It really works. It saves energy.	Jingdong prefecture level city in Shandong
Happy Fan Shop	King of Power Saving	It does have some power savings!	Jingdong prefecture level city in Shandong
Happy Fan Shop	King of Power Saving	Jingdong shopping speed is convenient and timely	Jingdong prefecture level city in Shandong
Happy Fan Shop	King of Power Saving	Second time I've bought it. Everything's fine.	Jingdong prefecture level city in Shandong
Happy Fan Shop	King of Power Saving	I don't know if it works yet.	Jingdong prefecture level city in Shandong
Happy Fan Shop	King of Power Saving	Not used, don't know how well it works.	Jingdong prefecture level city in Shandong
Happy Fan Shop	King of Power Saving	No visible results so far	Jingdong prefecture level city in Shandong
Happy Fan Shop	King of Power Saving	It doesn't feel like it's working.	Jingdong prefecture level city in Shandong
Happy Fan Shop	King of Power Saving	Where's the effect? Where are the power savings?	Jingdong prefecture level city in Shandong
Happy Fan Shop	King of Power Saving	Let's see how it works in practice!	Jingdong prefecture level city in Shandong
Happy Fan Shop	King of Power Saving	I'll wait until I use it to give you a good review	Jingdong prefecture level city in Shandong
Happy Fan Shop	King of Power Saving	I'll follow up after I've used it	Jingdong prefecture level city in Shandong
Happy Fan Shop	King of Power Saving	Well, that's good for saving power.	Jingdong prefecture level city in Shandong
Happy Fan Shop	King of Power Saving	Received. Used in the know.	Jingdong prefecture level city in Shandong
Happy Fan Shop	King of Power Saving	It doesn't do much good.	Jingdong prefecture level city in Shandong
Happy Fan Shop	King of Power Saving	It's not working.	Jingdong prefecture level city in Shandong
Happy Fan Shop	King of Power Saving	I don't know. I just used it.	Jingdong prefecture level city in Shandong
Happy Fan Shop	King of Power Saving	Use it and come back to evaluate.	Jingdong prefecture level city in Shandong
Happy Fan Shop	King of Power Saving	Two words: useless.	Jingdong prefecture level city in Shandong
Happy Fan Shop	King of Power Saving	To be used and then evaluated	Jingdong prefecture level city in Shandong
Happy Fan Shop	King of Power Saving	No shipment.	Jingdong prefecture level city in Shandong
Happy Fan Shop	King of Power Saving	Fake. Speechless.	Jingdong prefecture level city in Shandong
Happy Fan Shop	King of Power Saving	fantastic (idiom); awesome	Jingdong prefecture level city in Shandong
Happy Fan Shop	King of Power Saving	an excellent	Jingdong prefecture level city in Shandong
Happy Fan Shop	King of Power Saving	an excellent	Jingdong prefecture level city in Shandong
Happy Fan Shop	King of Power Saving	Want to buy! Recommended not to buy! No effect	Jingdong prefecture level city in Shandong
Happy Fan Shop	King of Power Saving	That's all there is to it. I can't figure out how to save electricity.	Jingdong prefecture level city in Shandong
Happy Fan Shop	King of Power Saving	It's no use.	Jingdong prefecture level city in Shandong
Happy Fan Shop	King of Power Saving	Things are more perfect than I imagined, very powerful, it is worth getting Packaging is very strong, things are well protected, it is worth the praise things are very satisfied Oh, beautiful and stylish atmosphere, customer service is super patient, service is meticulous and attentive, the big brand is not	Jingdong prefecture level city in Shandong
Happy Fan Shop	King of Power Saving	The effect is too good, the family has now several units, back to buy a number of times, the power saver received, power-saving effect is very good,, fine workmanship, long warranty, but also purify the air, before buying a used for a period of time, with the okay, and then bought a, and only now come to the	Jingdong prefecture level city in Shandong
Happy Fan Shop	King of Power Saving	Quite good, and imagine the same, casual ride are very good, satisfied! Baby received it 👌, really great ah! 🌈 quality is really nothing to say, very satisfied! 🆗 happy happy 😃 genuine, great, the price is very good, in short, quite good, will buy again a very good shopping experience, attentive service, fast logistics, distribution is also thoughtful, the attitude is also very good, value for money, ah,	Jingdong prefecture level city in Shandong
Happy Fan Shop	King of Power Saving	Power-saving effect: strong Cost-effective: quite good Appearance style: beautiful Workmanship material: meticulous Experience: looking forward to power-saving effect	Jingdong prefecture level city in Shandong
Happy Fan Shop	King of Power Saving	Cost-effective is okay, the appearance of the style of meticulous workmanship, looking forward to energy-saving effect.	Jingdong prefecture level city in Shandong
Happy Fan Shop	King of Power Saving	Very Good Very Power Saving This Power Saver is really power saving As soon as the power is turned on, the Power Saver starts the power saving function for you.	Jingdong prefecture level city in Shandong
Happy Fan Shop	King of Power Saving	Logistics is very fast, the price is favorable, friends introduced that it is very good to use, bought 2 at once, looking forward to the effect, used after the follow-up comment.	Jingdong prefecture level city in Shandong
Happy Fan Shop	King of Power Saving	It's all fake. It doesn't save any electricity.	Jingdong prefecture level city in Shandong
Happy Fan Shop	King of Power Saving	This Power Saver is so much better than the one I bought before, not only the effect is good, the quality is very good in all aspects, the customer service attitude is also very good, recommended to buy!	Jingdong prefecture level city in Shandong
Happy Fan Shop	King of Power Saving	Logistics is very fast, buy for parents, good quality, once bought 2, power-saving effect is good to buy 2 more, look forward to using the effect, satisfied will recommend to friends.	Jingdong prefecture level city in Shandong
Happy Fan Shop	King of Power Saving	With the description is the same, very high-grade, good value, to buy friends can order Oh!	Jingdong prefecture level city in Shandong
Happy Fan Shop	King of Power Saving	Not using much electricity in the winter saving over 100 per month! Ready to buy 2 more units in the summer ！！！！ Good quality, good price, power saving effect is obvious, cost-effective, worth buying, can be recommended!	Jingdong prefecture level city in Shandong
Happy Fan Shop	King of Power Saving	Purchased a power saver received on the put into use, rent a room with many electrical appliances, power consumption is very large, we have a piece of electricity or 1.2 dollars a time, with this power saver is much better, every day can save a lot of money, very convenient and practical.	Jingdong prefecture level city in Shandong
Happy Fan Shop	King of Power Saving	The power saving effect is very good: my supermarket monthly electricity bill is more than 7000 yuan. On May 1st someone recommended buying a couple of power savers, and I bought 5 of them first. The electricity bill was about 2000 less in June. This time, I bought 5 more. The seller said 800 degrees of electricity with one. I feel it's basically enough. A total of 10 units	Jingdong prefecture level city in Shandong
Happy Fan Shop	King of Power Saving	I never thought I could get such a good baby at such an affordable price, it's very good value for money, I love it!	Jingdong prefecture level city in Shandong
Happy Fan Shop	King of Power Saving	Previously bought other brands of power savers, but found no effect at home, this time to see how the products on the Jingdong, inserted in the home socket, the LED display is blue, the night light is very nice to see, look at the instructions that have to be half a year in order to achieve the best results, try it, I believe that the quality of the Jingdong, it should not be wrong.	Jingdong prefecture level city in Shandong
Happy Fan Shop	King of Power Saving	Superb, inexpensive, saves a lot of points, been using it for a long time!	Jingdong prefecture level city in Shandong

Happy Fan Shop	King of Power Saving	There are a lot of appliances in the house, the summer air conditioning is very costly, I bought this in front of the air conditioning effect is very good, I explain that I bought three in one breath, I use more electricity a month down to 200 or so, very satisfied.	Jingdong prefecture level city in Shandong
Happy Fan Shop	King of Power Saving	A month can save more than 200, home monthly electricity bill of more than 600 dollars, inserted 3 or so, an amount of the balance of the average can save more than 200. Product is very good, has been buying this one, has given a lot of relatives recommended, the quality is also very good, before buying a look with this same, who knows to buy home has not been used for more than a week on the bad, the quality of this is really good with more than two years!	Jingdong prefecture level city in Shandong
Happy Fan Shop	King of Power Saving	Whole house power saving king, can really save electricity, friends recommended, high power saving appliances, only replacement, no repair, after-sales worry-free!	Jingdong prefecture level city in Shandong
Happy Fan Shop	King of Power Saving	Great item love it, great customer service, great shopping experience!	Jingdong prefecture level city in Shandong
Happy Fan Shop	King of Power Saving	Power saving, very good use, much cheaper than the physical store, satisfaction	Jingdong prefecture level city in Shandong
Happy Fan Shop	King of Power Saving	In use, very well packaged and fast delivery of credit!	Jingdong prefecture level city in Shandong
Happy Fan Shop	King of Power Saving	Product quality, customer service attitude is particularly good, like, praise!	Jingdong prefecture level city in Shandong
Happy Fan Shop	King of Power Saving	The logistics is very fast, the packaging is not broken, the quality is very good, I like it very much!	Jingdong prefecture level city in Shandong
Happy Fan Shop	King of Power Saving	The saver was well packaged and was as described when plugged in	Jingdong prefecture level city in Shandong
Happy Fan Shop	King of Power Saving	Energy efficient and very satisfied	Jingdong prefecture level city in Shandong
Happy Fan Shop	King of Power Saving	Great quality for the price and fast shipping.	Jingdong prefecture level city in Shandong
Happy Fan Shop	King of Power Saving	It's great, just as described. Perfect.	Jingdong prefecture level city in Shandong
Happy Fan Shop	King of Power Saving	There is no power saving effect, but rather ring up an increase in electricity costs, because this machine is on continuously all day long, uninterruptedly, its own electricity and increased costs, plus the cost of purchasing the machine, so I feel that the use of this machine instead of increasing the	Jingdong prefecture level city in Shandong
Happy Fan Shop	King of Power Saving	Recommended by the owner of the store next door, looked at his home does have to save electricity, decided to get. Indeed quite can, the voltage is much more stable, there is a reduction in electricity	Jingdong prefecture level city in Shandong
Happy Fan Shop	King of Power Saving	Goods have been received value for money, affordable and reasonable price, and the description of the same, power-saving safe and reliable!	Jingdong prefecture level city in Shandong
Happy Fan Shop	King of Power Saving	Listen to friends introduced to buy, tried to save a lot of electricity is good, quite good, worth buying.	Jingdong prefecture level city in Shandong
Happy Fan Shop	King of Power Saving	The product is easy to use, safe and environmentally friendly, only used for a short time, to the next month, after the electricity bill status display to know how the performance of the power saver efficacy.	Jingdong prefecture level city in Shandong
Happy Fan Shop	King of Power Saving	Last month's electricity bill is 178 yuan, with this power saver is 92 yuan, ready to buy two more back home, I was introduced to buy a friend, just started to not believe that I did not expect the effect!	Jingdong prefecture level city in Shandong
Happy Fan Shop	King of Power Saving	It looks nice, but after using it for a few days I can't see how it saves power?	Jingdong prefecture level city in Shandong
Happy Fan Shop	King of Power Saving	Recommended by a friend, it is genuine, as described by the merchant, cost-effective	Jingdong prefecture level city in Shandong
Happy Fan Shop	King of Power Saving	Works well, affordable and saves money.	Jingdong prefecture level city in Shandong
Happy Fan Shop	King of Power Saving	Feels good haven't compared it yet, the air conditioning has been on a lot in the past two months	Jingdong prefecture level city in Shandong
Happy Fan Shop	King of Power Saving	Saving about 5 kWh per day is a good thing	Jingdong prefecture level city in Shandong
Happy Fan Shop	King of Power Saving	Not bad, affordable, best value for money	Jingdong prefecture level city in Shandong
Happy Fan Shop	King of Power Saving	The goods are genuine and the logistics are faster.	Jingdong prefecture level city in Shandong
Happy Fan Shop	King of Power Saving	Goods have been received today, has been plugged into the outlet, looking forward to saving electricity, after a period of time to see how the effect in the follow-up comment!	Jingdong prefecture level city in Shandong
Happy Fan Shop	King of Power Saving	I just got it home and haven't used it for long, so I'll use it for a couple months and see what happens. Seeing that most people have good reviews, it should be fine.	Jingdong prefecture level city in Shandong
Happy Fan Shop	King of Power Saving	I'm looking forward to the results, so I'll give you a good review out of habit! Follow up review after a while	Jingdong prefecture level city in Shandong
Happy Fan Shop	King of Power Saving	Use a dish first to know if it will help save energy	Jingdong prefecture level city in Shandong
Happy Fan Shop	King of Power Saving	Whether it saves electricity or not, we don't know yet.	Jingdong prefecture level city in Shandong
Happy Fan Shop	King of Power Saving	Goods have been received, use a period of time to know how oh. The courier did not deliver to the door ah! Sent to the station ah! The heat is inconvenient ah!	Jingdong prefecture level city in Shandong
Happy Fan Shop	King of Power Saving	A little effect, before an hour a power, now an hour 0.76 degrees, a dollar a power = save 24 cents, although not a lot, first use it!	Jingdong prefecture level city in Shandong
Happy Fan Shop	King of Power Saving	Just received the goods, use a month to evaluate	Jingdong prefecture level city in Shandong
Happy Fan Shop	King of Power Saving	Home every month with a lot of electricity, inadvertently found this good thing online that can save the beginning of a skeptical attitude to consult customer service, customer service replied very professional, said 120 days can be refunded that first try it!	Jingdong prefecture level city in Shandong
Happy Fan Shop	King of Power Saving	After receiving the goods this afternoon, immediately installed in the bathroom Smith energy storage electric water heater on the power outlet (instead of connecting with a plug board), when used, found that the "Power Saving King" LCD display, is the value of the voltage, not the store's video said so	Jingdong prefecture level city in Shandong
Happy Fan Shop	King of Power Saving	Has made many days, did not feel the power saving, the effect is not obvious.	Jingdong prefecture level city in Shandong
Happy Fan Shop	King of Power Saving	Energy savers do not save energy at all, but rather consume a lot of electricity.	Jingdong prefecture level city in Shandong
Happy Fan Shop	King of Power Saving	It's useless. It's a waste of money.	Jingdong prefecture level city in Shandong
Happy Fan Shop	King of Power Saving	Last month saved more than 500, which came back to buy, the store bought three, and then buy 1 home use, my family is dry beauty salon, more instruments, especially electricity, a month almost 1000. or so, with this did not expect to save so much, great, five-star praise!	Jingdong prefecture level city in Shandong
Happy Fan Shop	King of Power Saving	It rained a couple days ago and I just went to get the item back today , not bad, hey I looked at the meter and that red display light takes several seconds to jump, did not make a mistake buying it. After a period of time to evaluate.	Jingdong prefecture level city in Shandong
Happy Fan Shop	King of Power Saving	Without further ado, the electricity bill came down, this month less 102 yuan, has been repurchased, I also recommended to my friends, all said that there are effects, the product is okay, the effect is quite	Jingdong prefecture level city in Shandong
Happy Fan Shop	King of Power Saving	After buying and using two months really see the effect, so only to evaluate! Originally held the mindset of trying, I did not expect really effective, worth having!	Jingdong prefecture level city in Shandong
Happy Fan Shop	King of Power Saving	In the use of this energy saver before the monthly electricity bill is more than 150 yuan or so, now save thirty percent, every day to work, six or seven o'clock in the afternoon to go home, I do not know why the electricity bill is so expensive, energy-saving appliances really have the effect of recommending	Jingdong prefecture level city in Shandong
Happy Fan Shop	King of Power Saving	It saves about 100 bucks a month. A friend recommended it to me, and he's been using it for a while, and the effect is so obvious that I bought one too.	Jingdong prefecture level city in Shandong
Happy Fan Shop	King of Power Saving	My dad asked me to buy one, but after I paid the bill, I asked and he said it was a lot less this month, so he asked me to buy another one.	Jingdong prefecture level city in Shandong
Happy Fan Shop	King of Power Saving	The specific effect will have to be tested for at least two months to compare whether there is a power saving effect or not.	Jingdong prefecture level city in Shandong

Happy Fan Shop	King of Power Saving	'The goods have been received, immediately go to use, please ask the boss you instructions do not allow to withdraw, there are two fuses installed there!	Jingdong prefecture level city in Shandong
Happy Fan Shop	King of Power Saving	First praise is using, after using if good then follow up, now because I have not yet seen the results	Jingdong prefecture level city in Shandong
Happy Fan Shop	King of Power Saving	I've just been using it, and I haven't seen if it has a power-saving effect yet, so I'll follow up after 10 days and half a month.	Jingdong prefecture level city in Shandong
Happy Fan Shop	King of Power Saving	Just received the intelligent power saving meter, immediately plugged in to use, see it is not bad, and order to sell the second time	Jingdong prefecture level city in Shandong
Happy Fan Shop	King of Power Saving	This power saver is a bit effective, used for half a month, the effect is still visible.	Jingdong prefecture level city in Shandong
Happy Fan Shop	King of Power Saving	I received the baby and it looks great, I'm trying it out now and will keep an eye on it afterward.	Jingdong prefecture level city in Shandong
Happy Fan Shop	King of Power Saving	I've just received it, but I'm still trying to see how it works, so I'll come back in a little while.	Jingdong prefecture level city in Shandong
Happy Fan Shop	King of Power Saving	Just used it wait to use it for a while and then comment thanks, all first five stars.	Jingdong prefecture level city in Shandong
Happy Fan Shop	King of Power Saving	Very trustworthy brand, quality is guaranteed, the price is still affordable, quite satisfied!	Jingdong prefecture level city in Shandong
Happy Fan Shop	King of Power Saving	Things received, I hope that the platform of Jingdong do not sell fake goods are available	Jingdong prefecture level city in Shandong
Happy Fan Shop	King of Power Saving	Energy saver received, the use of the effect is good and the business description of the same	Jingdong prefecture level city in Shandong
Happy Fan Shop	King of Power Saving	The surface looks beautiful, the actual effect is not known, after using additional evaluation.	Jingdong prefecture level city in Shandong
Happy Fan Shop	King of Power Saving	Tried it for a few months, it really saves electricity, and I bought it for a friend.	Jingdong prefecture level city in Shandong
Happy Fan Shop	King of Power Saving	It didn't work and wanted to return it, it really didn't work at all.	Jingdong prefecture level city in Shandong
Happy Fan Shop	King of Power Saving	Just used it, don't know how effective it is, looking forward to usefulness	Jingdong prefecture level city in Shandong
Happy Fan Shop	King of Power Saving	After two months before the effect I do not know if it is true!	Jingdong prefecture level city in Shandong
Happy Fan Shop	King of Power Saving	So fast! Ordered yesterday and received today 👍👍👍👍👍👍👍👍👍 🌹🌹🌹🌹🌹🌹🌹🌹🌹	Jingdong prefecture level city in Shandong
Happy Fan Shop	King of Power Saving	I've started using it. We'll see how it works.	Jingdong prefecture level city in Shandong
Happy Fan Shop	King of Power Saving	I just received the goods, I won't know the effect until after two.	Jingdong prefecture level city in Shandong
Happy Fan Shop	King of Power Saving	Just received the goods, the specific effect will be evaluated later	Jingdong prefecture level city in Shandong
Happy Fan Shop	King of Power Saving	I've received the goods, I don't know if it's any good yet	Jingdong prefecture level city in Shandong
Happy Fan Shop	King of Power Saving	Try it for a month and then come back with a concrete evaluation	Jingdong prefecture level city in Shandong
Happy Fan Shop	King of Power Saving	In use, after two months to see the effect of power saving	Jingdong prefecture level city in Shandong
Happy Fan Shop	King of Power Saving	Power Saver. It's authentic. It's black. It works.	Jingdong prefecture level city in Shandong
Happy Fan Shop	King of Power Saving	I've been using it for a week now and it's not working.	Jingdong prefecture level city in Shandong
Happy Fan Shop	King of Power Saving	I'm just using it. I don't know how it's going to work.	Jingdong prefecture level city in Shandong
Happy Fan Shop	King of Power Saving	Products are better looking out for power savings	Jingdong prefecture level city in Shandong
Happy Fan Shop	King of Power Saving	I received it and I've kept it on. I hope it works.	Jingdong prefecture level city in Shandong
Happy Fan Shop	King of Power Saving	It's really good! Worth recommending to buy!	Jingdong prefecture level city in Shandong
Happy Fan Shop	King of Power Saving	Try it for a few days and see if it doesn't work, then return it.	Jingdong prefecture level city in Shandong
Happy Fan Shop	King of Power Saving	It's in use and I hope to be pleasantly surprised.	Jingdong prefecture level city in Shandong
Happy Fan Shop	King of Power Saving	We'll see how it works when we use it	Jingdong prefecture level city in Shandong
Happy Fan Shop	King of Power Saving	Let's see if it works.	Jingdong prefecture level city in Shandong
Happy Fan Shop	King of Power Saving	I'm using it and will follow up with a review	Jingdong prefecture level city in Shandong
Happy Fan Shop	King of Power Saving	It really works. It saves energy.	Jingdong prefecture level city in Shandong
Happy Fan Shop	King of Power Saving	It does have some power savings!	Jingdong prefecture level city in Shandong
Happy Fan Shop	King of Power Saving	Jingdong shopping speed is convenient and timely	Jingdong prefecture level city in Shandong
Happy Fan Shop	King of Power Saving	Second time I've bought it. Everything's fine.	Jingdong prefecture level city in Shandong
Happy Fan Shop	King of Power Saving	I don't know if it works yet.	Jingdong prefecture level city in Shandong
Happy Fan Shop	King of Power Saving	Not used, don't know how well it works.	Jingdong prefecture level city in Shandong
Happy Fan Shop	King of Power Saving	No visible results so far	Jingdong prefecture level city in Shandong
Happy Fan Shop	King of Power Saving	It doesn't feel like it's working.	Jingdong prefecture level city in Shandong
Happy Fan Shop	King of Power Saving	Where's the effect? Where are the power savings?	Jingdong prefecture level city in Shandong
Happy Fan Shop	King of Power Saving	Let's see how it works in practice!	Jingdong prefecture level city in Shandong
Happy Fan Shop	King of Power Saving	I'll wait until I use it to give you a good review	Jingdong prefecture level city in Shandong
Happy Fan Shop	King of Power Saving	I'll follow up after I've used it	Jingdong prefecture level city in Shandong
Happy Fan Shop	King of Power Saving	Well, that's good for saving power.	Jingdong prefecture level city in Shandong
Happy Fan Shop	King of Power Saving	Received. Used in the know.	Jingdong prefecture level city in Shandong
Happy Fan Shop	King of Power Saving	It doesn't do much good.	Jingdong prefecture level city in Shandong
Happy Fan Shop	King of Power Saving	It's not working.	Jingdong prefecture level city in Shandong
Happy Fan Shop	King of Power Saving	I don't know. I just used it.	Jingdong prefecture level city in Shandong

Happy Fan Shop	King of Power Saving	Use it and come back to evaluate.	Jingdong prefecture level city in Shandong
Happy Fan Shop	King of Power Saving	Two words: useless.	Jingdong prefecture level city in Shandong
Happy Fan Shop	King of Power Saving	To be used and then evaluated	Jingdong prefecture level city in Shandong
Happy Fan Shop	King of Power Saving	No shipment.	Jingdong prefecture level city in Shandong
Happy Fan Shop	King of Power Saving	Fake. Speechless.	Jingdong prefecture level city in Shandong
Happy Fan Shop	King of Power Saving	fantastic (idiom); awesome	Jingdong prefecture level city in Shandong
Happy Fan Shop	King of Power Saving	an excellent	Jingdong prefecture level city in Shandong
Happy Fan Shop	King of Power Saving	an excellent	Jingdong prefecture level city in Shandong
Happy Fan Shop	King of Power Saving	Want to buy! Recommended not to buy! No effect	Jingdong prefecture level city in Shandong
Happy Fan Shop	King of Power Saving	That's all there is to it. I can't figure out how to save electricity.	Jingdong prefecture level city in Shandong
Happy Fan Shop	King of Power Saving	It's no use.	Jingdong prefecture level city in Shandong
Happy Fan Shop	King of Power Saving	Things are more perfect than I imagined, very powerful, it is worth getting Packaging is very strong, things are well protected, it is worth the praise things are very satisfied Oh, beautiful and stylish atmosphere, customer service is super patient, service is meticulous and attentive, the big brand is not	Jingdong prefecture level city in Shandong
Happy Fan Shop	King of Power Saving	The effect is too good, the family has now several units, back to buy a number of times, the power saver received, power-saving effect is very good,, fine workmanship, long warranty, but also purify the air, before buying a used for a period of time, with the okay, and then bought a, and only now come to the	Jingdong prefecture level city in Shandong
Happy Fan Shop	King of Power Saving	Quite good, and imagine the same, casual ride are very good, satisfied! Baby received it 👌, really great ah! 🌈 quality is really nothing to say, very satisfied! 🆗 happy happy 😃 genuine, great, the price is very good, in short, quite good, will buy again a very good shopping experience, attentive service, fast logistics, distribution is also thoughtful, the attitude is also very good, value for money, ah,	Jingdong prefecture level city in Shandong
Happy Fan Shop	King of Power Saving	Power-saving effect: strong Cost-effective: quite good Appearance style: beautiful Workmanship material: meticulous Experience: looking forward to power-saving effect	Jingdong prefecture level city in Shandong
Happy Fan Shop	King of Power Saving	Cost-effective is okay, the appearance of the style of meticulous workmanship, looking forward to energy-saving effect.	Jingdong prefecture level city in Shandong
Happy Fan Shop	King of Power Saving	Very Good Very Power Saving This Power Saver is really power saving As soon as the power is turned on, the Power Saver starts the power saving function for you.	Jingdong prefecture level city in Shandong
Happy Fan Shop	King of Power Saving	Logistics is very fast, the price is favorable, friends introduced that it is very good to use, bought 2 at once, looking forward to the effect, used after the follow-up comment.	Jingdong prefecture level city in Shandong
Happy Fan Shop	King of Power Saving	It's all fake. It doesn't save any electricity.	Jingdong prefecture level city in Shandong
Happy Fan Shop	King of Power Saving	This Power Saver is so much better than the one I bought before, not only the effect is good, the quality is very good in all aspects, the customer service attitude is also very good, recommended to buy!	Jingdong prefecture level city in Shandong
Happy Fan Shop	King of Power Saving	Logistics is very fast, buy for parents, good quality, once bought 2, power-saving effect is good to buy 2 more, look forward to using the effect, satisfied will recommend to friends.	Jingdong prefecture level city in Shandong
Happy Fan Shop	King of Power Saving	With the description is the same, very high-grade, good value, to buy friends can order Oh!	Jingdong prefecture level city in Shandong
Happy Fan Shop	King of Power Saving	Not using much electricity in the winter saving over 100 per month! Ready to buy 2 more units in the summer ！！！！！ Good quality, good price, power saving effect is obvious, cost-effective, worth buying, can be recommended!	Jingdong prefecture level city in Shandong
Happy Fan Shop	King of Power Saving	Purchased a power saver received on the put into use, rent a room with many electrical appliances, power consumption is very large, we have a piece of electricity or 1.2 dollars a time, with this power saver is much better, every day can save a lot of money, very convenient and practical.	Jingdong prefecture level city in Shandong
Happy Fan Shop	King of Power Saving	The power saving effect is very good: my supermarket monthly electricity bill is more than 7000 yuan. On May 1st someone recommended buying a couple of power savers, and I bought 5 of them first. The electricity bill was about 2000 less in June. This time, I bought 5 more. The seller said 800 degrees of electricity with one. I feel it's basically enough. A total of 10 units	Jingdong prefecture level city in Shandong
Happy Fan Shop	King of Power Saving	I never thought I could get such a good baby at such an affordable price, it's very good value for money, I love it!	Jingdong prefecture level city in Shandong
Happy Fan Shop	King of Power Saving	Previously bought other brands of power savers, but found no effect at home, this time to see how the products on the Jingdong, inserted in the home socket, the LED display is blue, the night light is very nice to see, look at the instructions that have to be half a year in order to achieve the best results, try it, I believe that the quality of the Jingdong, it should not be wrong.	Jingdong prefecture level city in Shandong
Happy Fan Shop	King of Power Saving	Superb, inexpensive, saves a lot of points, been using it for a long time!	Jingdong prefecture level city in Shandong
Happy Fan Shop	King of Power Saving	There are a lot of appliances in the house, the summer air conditioning is very costly, I bought this in front of the air conditioning effect is very good, I explain that I bought three in one breath, I use more electricity a month down to 200 or so, very satisfied.	Jingdong prefecture level city in Shandong
Happy Fan Shop	King of Power Saving	A month can save more than 200, home monthly electricity bill of more than 600 dollars, inserted 3 or so, an amount of the balance of the average can save more than 200. Product is very good, has been buying this one, has given a lot of relatives recommended, the quality is also very good, before buying a look with this same, who knows to buy home has not been used for more than a week on the bad, the quality of this is really good with more than two years!	Jingdong prefecture level city in Shandong
Happy Fan Shop	King of Power Saving	Whole house power saving king, can really save electricity, friends recommended, high power saving appliances, only replacement, no repair, after-sales worry-free!	Jingdong prefecture level city in Shandong
Happy Fan Shop	King of Power Saving	Great item love it, great customer service, great shopping experience!	Jingdong prefecture level city in Shandong
Happy Fan Shop	King of Power Saving	Power saving, very good use, much cheaper than the physical store, satisfaction	Jingdong prefecture level city in Shandong
Happy Fan Shop	King of Power Saving	In use, very well packaged and fast delivery of credit!	Jingdong prefecture level city in Shandong
Happy Fan Shop	King of Power Saving	Product quality, customer service attitude is particularly good, like, praise!	Jingdong prefecture level city in Shandong
Happy Fan Shop	King of Power Saving	The logistics is very fast, the packaging is not broken, the quality is very good, I like it very much!	Jingdong prefecture level city in Shandong
Happy Fan Shop	King of Power Saving	The saver was well packaged and was as described when plugged in	Jingdong prefecture level city in Shandong
Happy Fan Shop	King of Power Saving	Energy efficient and very satisfied	Jingdong prefecture level city in Shandong
Happy Fan Shop	King of Power Saving	Great quality for the price and fast shipping.	Jingdong prefecture level city in Shandong
Happy Fan Shop	King of Power Saving	It's great, just as described. Perfect.	Jingdong prefecture level city in Shandong
Happy Fan Shop	King of Power Saving	There is no power saving effect, but rather ring up an increase in electricity costs, because this machine is on continuously all day long, uninterruptedly, its own electricity and increased costs, plus the cost of purchasing the machine, so I feel that the use of this machine instead of increasing the	Jingdong prefecture level city in Shandong
Happy Fan Shop	King of Power Saving	Recommended by the owner of the store next door, looked at his home does have to save electricity, decided to get. Indeed quite can, the voltage is much more stable, there is a reduction in electricity	Jingdong prefecture level city in Shandong
Happy Fan Shop	King of Power Saving	Goods have been received value for money, affordable and reasonable price, and the description of the same, power-saving safe and reliable!	Jingdong prefecture level city in Shandong
Happy Fan Shop	King of Power Saving	Listen to friends introduced to buy, tried to save a lot of electricity is good, quite good, worth buying.	Jingdong prefecture level city in Shandong

Happy Fan Shop	King of Power Saving	The product is easy to use, safe and environmentally friendly, only used for a short time, to the next month, after the electricity bill status display to know how the performance of the power saver efficacy.	Jingdong prefecture level city in Shandong
Happy Fan Shop	King of Power Saving	Last month's electricity bill is 178 yuan, with this power saver is 92 yuan, ready to buy two more back home, I was introduced to buy a friend, just started to not believe that I did not expect the effect!	Jingdong prefecture level city in Shandong
Happy Fan Shop	King of Power Saving	It looks nice, but after using it for a few days I can't see how it saves power?	Jingdong prefecture level city in Shandong
Happy Fan Shop	King of Power Saving	Recommended by a friend, it is genuine, as described by the merchant, cost-effective	Jingdong prefecture level city in Shandong
Happy Fan Shop	King of Power Saving	Works well, affordable and saves money.	Jingdong prefecture level city in Shandong
Happy Fan Shop	King of Power Saving	Feels good haven't compared it yet, the air conditioning has been on a lot in the past two months	Jingdong prefecture level city in Shandong
Happy Fan Shop	King of Power Saving	Saving about 5 kWh per day is a good thing	Jingdong prefecture level city in Shandong
Happy Fan Shop	King of Power Saving	Not bad, affordable, best value for money	Jingdong prefecture level city in Shandong
Happy Fan Shop	King of Power Saving	The goods are genuine and the logistics are faster.	Jingdong prefecture level city in Shandong
Happy Fan Shop	King of Power Saving	Goods have been received today, has been plugged into the outlet, looking forward to saving electricity, after a period of time to see how the effect in the follow-up comment!	Jingdong prefecture level city in Shandong
Happy Fan Shop	King of Power Saving	I just got it home and haven't used it for long, so I'll use it for a couple months and see what happens. Seeing that most people have good reviews, it should be fine.	Jingdong prefecture level city in Shandong
Happy Fan Shop	King of Power Saving	I'm looking forward to the results, so I'll give you a good review out of habit! Follow up review after a while	Jingdong prefecture level city in Shandong
Happy Fan Shop	King of Power Saving	Use a dish first to know if it will help save energy	Jingdong prefecture level city in Shandong
Happy Fan Shop	King of Power Saving	Whether it saves electricity or not, we don't know yet.	Jingdong prefecture level city in Shandong
Happy Fan Shop	King of Power Saving	Goods have been received, use a period of time to know how oh. The courier did not deliver to the door ah! Sent to the station ah! The heat is inconvenient ah!	Jingdong prefecture level city in Shandong
Happy Fan Shop	King of Power Saving	A little effect, before an hour a power, now an hour 0.76 degrees, a dollar a power = save 24 cents, although not a lot, first use it!	Jingdong prefecture level city in Shandong
Happy Fan Shop	King of Power Saving	Just received the goods, use a month to evaluate	Jingdong prefecture level city in Shandong
Happy Fan Shop	King of Power Saving	Home every month with a lot of electricity, inadvertently found this good thing online that can save the beginning of a skeptical attitude to consult customer service, customer service replied very professional, said 120 days can be refunded that first try it!	Jingdong prefecture level city in Shandong
Happy Fan Shop	King of Power Saving	After receiving the goods this afternoon, immediately installed in the bathroom Smith energy storage electric water heater on the power outlet (instead of connecting with a plug board), when used, found that the "Power Saving King" LCD display, is the value of the voltage, not the store's video said so	Jingdong prefecture level city in Shandong
Happy Fan Shop	King of Power Saving	Has made many days, did not feel the power saving, the effect is not obvious.	Jingdong prefecture level city in Shandong
Happy Fan Shop	King of Power Saving	Energy savers do not save energy at all, but rather consume a lot of electricity.	Jingdong prefecture level city in Shandong
Happy Fan Shop	King of Power Saving	It's useless. It's a waste of money.	Jingdong prefecture level city in Shandong
Happy Fan Shop	King of Power Saving	Last month saved more than 500, which came back to buy, the store bought three, and then buy 1 home use, my family is dry beauty salon, more instruments, especially electricity, a month almost 1000. or so, with this did not expect to save so much, great, five-star praise!	Jingdong prefecture level city in Shandong
Happy Fan Shop	King of Power Saving	It rained a couple days ago and I just went to get the item back today , not bad, hey I looked at the meter and that red display light takes several seconds to jump, did not make a mistake buying it. After a period of time to evaluate.	Jingdong prefecture level city in Shandong
Happy Fan Shop	King of Power Saving	Without further ado, the electricity bill came down, this month less 102 yuan, has been repurchased, I also recommended to my friends, all said that there are effects, the product is okay, the effect is quite	Jingdong prefecture level city in Shandong
Happy Fan Shop	King of Power Saving	After buying and using two months really see the effect, so only to evaluate! Originally held the mindset of trying, I did not expect really effective, worth having!	Jingdong prefecture level city in Shandong
Happy Fan Shop	King of Power Saving	In the use of this energy saver before the monthly electricity bill is more than 150 yuan or so, now save thirty percent, every day to work, six or seven o'clock in the afternoon to go home, I do not know why the electricity bill is so expensive, energy-saving appliances really have the effect of recommending	Jingdong prefecture level city in Shandong
Happy Fan Shop	King of Power Saving	It saves about 100 bucks a month. A friend recommended it to me, and he's been using it for a while, and the effect is so obvious that I bought one too.	Jingdong prefecture level city in Shandong
Happy Fan Shop	King of Power Saving	My dad asked me to buy one, but after I paid the bill, I asked and he said it was a lot less this month, so he asked me to buy another one.	Jingdong prefecture level city in Shandong
Happy Fan Shop	King of Power Saving	The specific effect will have to be tested for at least two months to compare whether there is a power saving effect or not.	Jingdong prefecture level city in Shandong
Happy Fan Shop	King of Power Saving	'The goods have been received, immediately go to use, please ask the boss you instructions do not allow to withdraw, there are two fuses installed there!	Jingdong prefecture level city in Shandong
Happy Fan Shop	King of Power Saving	First praise is using, after using if good then follow up, now because I have not yet seen the results	Jingdong prefecture level city in Shandong
Happy Fan Shop	King of Power Saving	I've just been using it, and I haven't seen if it has a power-saving effect yet, so I'll follow up after 10 days and half a month.	Jingdong prefecture level city in Shandong
Happy Fan Shop	King of Power Saving	Just received the intelligent power saving meter, immediately plugged in to use, see it is not bad, and order to sell the second time	Jingdong prefecture level city in Shandong
Happy Fan Shop	King of Power Saving	This power saver is a bit effective, used for half a month, the effect is still visible.	Jingdong prefecture level city in Shandong
Happy Fan Shop	King of Power Saving	I received the baby and it looks great, I'm trying it out now and will keep an eye on it afterward.	Jingdong prefecture level city in Shandong
Happy Fan Shop	King of Power Saving	I've just received it, but I'm still trying to see how it works, so I'll come back in a little while.	Jingdong prefecture level city in Shandong
Happy Fan Shop	King of Power Saving	Just used it wait to use it for a while and then comment thanks, all first five stars.	Jingdong prefecture level city in Shandong
Happy Fan Shop	King of Power Saving	Very trustworthy brand, quality is guaranteed, the price is still affordable, quite satisfied!	Jingdong prefecture level city in Shandong
Happy Fan Shop	King of Power Saving	Things received, I hope that the platform of Jingdong do not sell fake goods are available	Jingdong prefecture level city in Shandong
Happy Fan Shop	King of Power Saving	Energy saver received, the use of the effect is good and the business description of the same	Jingdong prefecture level city in Shandong
Happy Fan Shop	King of Power Saving	The surface looks beautiful, the actual effect is not known, after using additional evaluation.	Jingdong prefecture level city in Shandong
Happy Fan Shop	King of Power Saving	Tried it for a few months, it really saves electricity, and I bought it for a friend.	Jingdong prefecture level city in Shandong
Happy Fan Shop	King of Power Saving	It didn't work and wanted to return it, it really didn't work at all.	Jingdong prefecture level city in Shandong
Happy Fan Shop	King of Power Saving	Just used it, don't know how effective it is, looking forward to usefulness	Jingdong prefecture level city in Shandong
Happy Fan Shop	King of Power Saving	After two months before the effect I do not know if it is true!	Jingdong prefecture level city in Shandong
Happy Fan Shop	King of Power Saving	So fast! Ordered yesterday and received today 👍👍👍👍👍👍👍👍👍 🌹🌹🌹🌹🌹🌹🌹🌹🌹	Jingdong prefecture level city in Shandong
Happy Fan Shop	King of Power Saving	I've started using it. We'll see how it works.	Jingdong prefecture level city in Shandong
Happy Fan Shop	King of Power Saving	I just received the goods, I won't know the effect until after two.	Jingdong prefecture level city in Shandong

Happy Fan Shop	King of Power Saving	Just received the goods, the specific effect will be evaluated later	Jingdong prefecture level city in Shandong
Happy Fan Shop	King of Power Saving	I've received the goods, I don't know if it's any good yet	Jingdong prefecture level city in Shandong
Happy Fan Shop	King of Power Saving	Try it for a month and then come back with a concrete evaluation	Jingdong prefecture level city in Shandong
Happy Fan Shop	King of Power Saving	In use, after two months to see the effect of power saving	Jingdong prefecture level city in Shandong
Happy Fan Shop	King of Power Saving	Power Saver. It's authentic. It's black. It works.	Jingdong prefecture level city in Shandong
Happy Fan Shop	King of Power Saving	I've been using it for a week now and it's not working.	Jingdong prefecture level city in Shandong
Happy Fan Shop	King of Power Saving	I'm just using it. I don't know how it's going to work.	Jingdong prefecture level city in Shandong
Happy Fan Shop	King of Power Saving	Products are better looking out for power savings	Jingdong prefecture level city in Shandong
Happy Fan Shop	King of Power Saving	I received it and I've kept it on. I hope it works.	Jingdong prefecture level city in Shandong
Happy Fan Shop	King of Power Saving	It's really good! Worth recommending to buy!	Jingdong prefecture level city in Shandong
Happy Fan Shop	King of Power Saving	Try it for a few days and see if it doesn't work, then return it.	Jingdong prefecture level city in Shandong
Happy Fan Shop	King of Power Saving	It's in use and I hope to be pleasantly surprised.	Jingdong prefecture level city in Shandong
Happy Fan Shop	King of Power Saving	We'll see how it works when we use it	Jingdong prefecture level city in Shandong
Happy Fan Shop	King of Power Saving	Let's see if it works.	Jingdong prefecture level city in Shandong
Happy Fan Shop	King of Power Saving	I'm using it and will follow up with a review	Jingdong prefecture level city in Shandong
Happy Fan Shop	King of Power Saving	It really works. It saves energy.	Jingdong prefecture level city in Shandong
Happy Fan Shop	King of Power Saving	It does have some power savings!	Jingdong prefecture level city in Shandong
Happy Fan Shop	King of Power Saving	Jingdong shopping speed is convenient and timely	Jingdong prefecture level city in Shandong
Happy Fan Shop	King of Power Saving	Second time I've bought it. Everything's fine.	Jingdong prefecture level city in Shandong
Happy Fan Shop	King of Power Saving	I don't know if it works yet.	Jingdong prefecture level city in Shandong
Happy Fan Shop	King of Power Saving	Not used, don't know how well it works.	Jingdong prefecture level city in Shandong
Happy Fan Shop	King of Power Saving	No visible results so far	Jingdong prefecture level city in Shandong
Happy Fan Shop	King of Power Saving	It doesn't feel like it's working.	Jingdong prefecture level city in Shandong
Happy Fan Shop	King of Power Saving	Where's the effect? Where are the power savings?	Jingdong prefecture level city in Shandong
Happy Fan Shop	King of Power Saving	Let's see how it works in practice!	Jingdong prefecture level city in Shandong
Happy Fan Shop	King of Power Saving	I'll wait until I use it to give you a good review	Jingdong prefecture level city in Shandong
Happy Fan Shop	King of Power Saving	I'll follow up after I've used it	Jingdong prefecture level city in Shandong
Happy Fan Shop	King of Power Saving	Well, that's good for saving power.	Jingdong prefecture level city in Shandong
Happy Fan Shop	King of Power Saving	Received. Used in the know.	Jingdong prefecture level city in Shandong
Happy Fan Shop	King of Power Saving	It doesn't do much good.	Jingdong prefecture level city in Shandong
Happy Fan Shop	King of Power Saving	It's not working.	Jingdong prefecture level city in Shandong
Happy Fan Shop	King of Power Saving	I don't know. I just used it.	Jingdong prefecture level city in Shandong
Happy Fan Shop	King of Power Saving	Use it and come back to evaluate.	Jingdong prefecture level city in Shandong
Happy Fan Shop	King of Power Saving	Two words: useless.	Jingdong prefecture level city in Shandong
Happy Fan Shop	King of Power Saving	To be used and then evaluated	Jingdong prefecture level city in Shandong
Happy Fan Shop	King of Power Saving	No shipment.	Jingdong prefecture level city in Shandong
Happy Fan Shop	King of Power Saving	Fake. Speechless.	Jingdong prefecture level city in Shandong
Happy Fan Shop	King of Power Saving	fantastic (idiom); awesome	Jingdong prefecture level city in Shandong
Happy Fan Shop	King of Power Saving	an excellent	Jingdong prefecture level city in Shandong
Happy Fan Shop	King of Power Saving	an excellent	Jingdong prefecture level city in Shandong
Happy Fan Shop	King of Power Saving	Want to buy! Recommended not to buy! No effect	Jingdong prefecture level city in Shandong
Happy Fan Shop	King of Power Saving	That's all there is to it. I can't figure out how to save electricity.	Jingdong prefecture level city in Shandong
Happy Fan Shop	King of Power Saving	It's no use.	Jingdong prefecture level city in Shandong
Happy Fan Shop	King of Power Saving	Things are more perfect than I imagined, very powerful, it is worth getting Packaging is very strong, things are well protected, it is worth the praise things are very satisfied Oh, beautiful and stylish atmosphere, customer service is super patient, service is meticulous and attentive, the big brand is not	Jingdong prefecture level city in Shandong
Happy Fan Shop	King of Power Saving	The effect is too good, the family has now several units, back to buy a number of times, the power saver received, power-saving effect is very good,, fine workmanship, long warranty, but also purify the air, before buying a used for a period of time, with the okay, and then bought a, and only now come to the	Jingdong prefecture level city in Shandong
Happy Fan Shop	King of Power Saving	Quite good, and imagine the same, casual ride are very good, satisfied! Baby received it 👌, really great ah! 🌈 quality is really nothing to say, very satisfied! 🆗 happy happy 😃 genuine, great, the price is very good, in short, quite good, will buy again a very good shopping experience, attentive service, fast logistics, distribution is also thoughtful, the attitude is also very good, value for money, ah,	Jingdong prefecture level city in Shandong
Happy Fan Shop	King of Power Saving	Power-saving effect: strong Cost-effective: quite good Appearance style: beautiful Workmanship material: meticulous Experience: looking forward to power-saving effect	Jingdong prefecture level city in Shandong
Happy Fan Shop	King of Power Saving	Cost-effective is okay, the appearance of the style of meticulous workmanship, looking forward to energy-saving effect.	Jingdong prefecture level city in Shandong
Happy Fan Shop	King of Power Saving	Very Good Very Power Saving This Power Saver is really power saving As soon as the power is turned on, the Power Saver starts the power saving function for you.	Jingdong prefecture level city in Shandong

Happy Fan Shop	King of Power Saving	Logistics is very fast, the price is favorable, friends introduced that it is very good to use, bought 2 at once, looking forward to the effect, used after the follow-up comment.	Jingdong prefecture level city in Shandong
Happy Fan Shop	King of Power Saving	It's all fake. It doesn't save any electricity.	Jingdong prefecture level city in Shandong
Happy Fan Shop	King of Power Saving	This Power Saver is so much better than the one I bought before, not only the effect is good, the quality is very good in all aspects, the customer service attitude is also very good, recommended to buy!	Jingdong prefecture level city in Shandong
Happy Fan Shop	King of Power Saving	Logistics is very fast, buy for parents, good quality, once bought 2, power-saving effect is good to buy 2 more, look forward to using the effect, satisfied will recommend to friends.	Jingdong prefecture level city in Shandong
Happy Fan Shop	King of Power Saving	With the description is the same, very high-grade, good value, to buy friends can order Oh!	Jingdong prefecture level city in Shandong
Happy Fan Shop	King of Power Saving	Not using much electricity in the winter saving over 100 per month! Ready to buy 2 more units in the summer ! ! ! ! ! Good quality, good price, power saving effect is obvious, cost-effective, worth buying, can be recommended!	Jingdong prefecture level city in Shandong
Happy Fan Shop	King of Power Saving	Purchased a power saver received on the put into use, rent a room with many electrical appliances, power consumption is very large, we have a piece of electricity or 1.2 dollars a time, with this power saver is much better, every day can save a lot of money, very convenient and practical.	Jingdong prefecture level city in Shandong
Happy Fan Shop	King of Power Saving	The power saving effect is very good: my supermarket monthly electricity bill is more than 7000 yuan. On May 1st someone recommended buying a couple of power savers, and I bought 5 of them first. The electricity bill was about 2000 less in June. This time, I bought 5 more. The seller said 800 degrees of electricity with one. I feel it's basically enough. A total of 10 units	Jingdong prefecture level city in Shandong
Happy Fan Shop	King of Power Saving	I never thought I could get such a good baby at such an affordable price, it's very good value for money, I love it!	Jingdong prefecture level city in Shandong
Happy Fan Shop	King of Power Saving	Previously bought other brands of power savers, but found no effect at home, this time to see how the products on the Jingdong, inserted in the home socket, the LED display is blue, the night light is very nice to see, look at the instructions that have to be half a year in order to achieve the best results, try it, I believe that the quality of the Jingdong, it should not be wrong.	Jingdong prefecture level city in Shandong
Happy Fan Shop	King of Power Saving	Superb, inexpensive, saves a lot of points, been using it for a long time!	Jingdong prefecture level city in Shandong
Happy Fan Shop	King of Power Saving	There are a lot of appliances in the house, the summer air conditioning is very costly, I bought this in front of the air conditioning effect is very good, I explain that I bought three in one breath, I use more electricity a month down to 200 or so, very satisfied.	Jingdong prefecture level city in Shandong
Happy Fan Shop	King of Power Saving	A month can save more than 200, home monthly electricity bill of more than 600 dollars, inserted 3 or so, an amount of the balance of the average can save more than 200. Product is very good, has been buying this one, has given a lot of relatives recommended, the quality is also very good, before buying a look with this same, who knows to buy home has not been used for more than a week on the bad, the quality of this is really good with more than two years!	Jingdong prefecture level city in Shandong
Happy Fan Shop	King of Power Saving	Whole house power saving king, can really save electricity, friends recommended, high power saving appliances, only replacement, no repair, after-sales worry-free!	Jingdong prefecture level city in Shandong
Happy Fan Shop	King of Power Saving	Great item love it, great customer service, great shopping experience!	Jingdong prefecture level city in Shandong
Happy Fan Shop	King of Power Saving	Power saving, very good use, much cheaper than the physical store, satisfaction	Jingdong prefecture level city in Shandong
Happy Fan Shop	King of Power Saving	In use, very well packaged and fast delivery of credit!	Jingdong prefecture level city in Shandong
Happy Fan Shop	King of Power Saving	Product quality, customer service attitude is particularly good, like, praise!	Jingdong prefecture level city in Shandong
Happy Fan Shop	King of Power Saving	The logistics is very fast, the packaging is not broken, the quality is very good, I like it very much!	Jingdong prefecture level city in Shandong
Happy Fan Shop	King of Power Saving	The saver was well packaged and was as described when plugged in	Jingdong prefecture level city in Shandong
Happy Fan Shop	King of Power Saving	Energy efficient and very satisfied	Jingdong prefecture level city in Shandong
Happy Fan Shop	King of Power Saving	Great quality for the price and fast shipping.	Jingdong prefecture level city in Shandong
Happy Fan Shop	King of Power Saving	It's great, just as described. Perfect.	Jingdong prefecture level city in Shandong
Happy Fan Shop	King of Power Saving	There is no power saving effect, but rather ring up an increase in electricity costs, because this machine is on continuously all day long, uninterruptedly, its own electricity and increased costs, plus the cost of purchasing the machine, so I feel that the use of this machine instead of increasing the	Jingdong prefecture level city in Shandong
Happy Fan Shop	King of Power Saving	Recommended by the owner of the store next door, looked at his home does have to save electricity, decided to get. Indeed quite can, the voltage is much more stable, there is a reduction in electricity	Jingdong prefecture level city in Shandong
Happy Fan Shop	King of Power Saving	Goods have been received value for money, affordable and reasonable price, and the description of the same, power-saving safe and reliable!	Jingdong prefecture level city in Shandong
Happy Fan Shop	King of Power Saving	Listen to friends introduced to buy, tried to save a lot of electricity is good, quite good, worth buying.	Jingdong prefecture level city in Shandong
Happy Fan Shop	King of Power Saving	The product is easy to use, safe and environmentally friendly, only used for a short time, to the next month, after the electricity bill status display to know how the performance of the power saver efficacy.	Jingdong prefecture level city in Shandong
Happy Fan Shop	King of Power Saving	Last month's electricity bill is 178 yuan, with this power saver is 92 yuan, ready to buy two more back home, I was introduced to buy a friend, just started to not believe that I did not expect the effect!	Jingdong prefecture level city in Shandong
Happy Fan Shop	King of Power Saving	It looks nice, but after using it for a few days I can't see how it saves power?	Jingdong prefecture level city in Shandong
Happy Fan Shop	King of Power Saving	Recommended by a friend, it is genuine, as described by the merchant, cost-effective	Jingdong prefecture level city in Shandong
Happy Fan Shop	King of Power Saving	Works well, affordable and saves money.	Jingdong prefecture level city in Shandong
Happy Fan Shop	King of Power Saving	Feels good haven't compared it yet, the air conditioning has been on a lot in the past two months	Jingdong prefecture level city in Shandong
Happy Fan Shop	King of Power Saving	Saving about 5 kWh per day is a good thing	Jingdong prefecture level city in Shandong
Happy Fan Shop	King of Power Saving	Not bad, affordable, best value for money	Jingdong prefecture level city in Shandong
Happy Fan Shop	King of Power Saving	The goods are genuine and the logistics are faster.	Jingdong prefecture level city in Shandong
Happy Fan Shop	King of Power Saving	Goods have been received today, has been plugged into the outlet, looking forward to saving electricity, after a period of time to see how the effect in the follow-up comment!	Jingdong prefecture level city in Shandong
Happy Fan Shop	King of Power Saving	I just got it home and haven't used it for long, so I'll use it for a couple months and see what happens. Seeing that most people have good reviews, it should be fine.	Jingdong prefecture level city in Shandong
Happy Fan Shop	King of Power Saving	I'm looking forward to the results, so I'll give you a good review out of habit! Follow up review after a while	Jingdong prefecture level city in Shandong
Happy Fan Shop	King of Power Saving	Use a dish first to know if it will help save energy	Jingdong prefecture level city in Shandong
Happy Fan Shop	King of Power Saving	Whether it saves electricity or not, we don't know yet.	Jingdong prefecture level city in Shandong
Happy Fan Shop	King of Power Saving	Goods have been received, use a period of time to know how oh. The courier did not deliver to the door ah! Sent to the station ah! The heat is inconvenient ah!	Jingdong prefecture level city in Shandong
Happy Fan Shop	King of Power Saving	A little effect, before an hour a power, now an hour 0.76 degrees, a dollar a power = save 24 cents, although not a lot, first use it!	Jingdong prefecture level city in Shandong
Happy Fan Shop	King of Power Saving	Just received the goods, use a month to evaluate	Jingdong prefecture level city in Shandong
Happy Fan Shop	King of Power Saving	Home every month with a lot of electricity, inadvertently found this good thing online that can save the beginning of a skeptical attitude to consult customer service, customer service replied very professional, said 120 days can be refunded that first try it!	Jingdong prefecture level city in Shandong

Happy Fan Shop	King of Power Saving	After receiving the goods this afternoon, immediately installed in the bathroom Smith energy storage electric water heater on the power outlet (instead of connecting with a plug board), when used, found that the "Power Saving King" LCD display, is the value of the voltage, not the store's video said so	Jingdong prefecture level city in Shandong
Happy Fan Shop	King of Power Saving	Has made many days, did not feel the power saving, the effect is not obvious.	Jingdong prefecture level city in Shandong
Happy Fan Shop	King of Power Saving	Energy savers do not save energy at all, but rather consume a lot of electricity.	Jingdong prefecture level city in Shandong
Happy Fan Shop	King of Power Saving	It's useless. It's a waste of money.	Jingdong prefecture level city in Shandong
Happy Fan Shop	King of Power Saving	Last month saved more than 500, which came back to buy, the store bought three, and then buy 1 home use, my family is dry beauty salon, more instruments, especially electricity, a month almost 1000. or so, with this did not expect to save so much, great, five-star praise!	Jingdong prefecture level city in Shandong
Happy Fan Shop	King of Power Saving	It rained a couple days ago and I just went to get the item back today , not bad, hey I looked at the meter and that red display light takes several seconds to jump, did not make a mistake buying it. After a period of time to evaluate.	Jingdong prefecture level city in Shandong
Happy Fan Shop	King of Power Saving	Without further ado, the electricity bill came down, this month less 102 yuan, has been repurchased, I also recommended to my friends, all said that there are effects, the product is okay, the effect is quite	Jingdong prefecture level city in Shandong
Happy Fan Shop	King of Power Saving	After buying and using two months really see the effect, so only to evaluate! Originally held the mindset of trying, I did not expect really effective, worth having!	Jingdong prefecture level city in Shandong
Happy Fan Shop	King of Power Saving	In the use of this energy saver before the monthly electricity bill is more than 150 yuan or so, now save thirty percent, every day to work, six or seven o'clock in the afternoon to go home, I do not know why the electricity bill is so expensive, energy-saving appliances really have the effect of recommending	Jingdong prefecture level city in Shandong
Happy Fan Shop	King of Power Saving	It saves about 100 bucks a month. A friend recommended it to me, and he's been using it for a while, and the effect is so obvious that I bought one too.	Jingdong prefecture level city in Shandong
Happy Fan Shop	King of Power Saving	My dad asked me to buy one, but after I paid the bill, I asked and he said it was a lot less this month, so he asked me to buy another one.	Jingdong prefecture level city in Shandong
Happy Fan Shop	King of Power Saving	The specific effect will have to be tested for at least two months to compare whether there is a power saving effect or not.	Jingdong prefecture level city in Shandong
Happy Fan Shop	King of Power Saving	'The goods have been received, immediately go to use, please ask the boss you instructions do not allow to withdraw, there are two fuses installed there!	Jingdong prefecture level city in Shandong
Happy Fan Shop	King of Power Saving	First praise is using, after using if good then follow up, now because I have not yet seen the results	Jingdong prefecture level city in Shandong
Happy Fan Shop	King of Power Saving	I've just been using it, and I haven't seen if it has a power-saving effect yet, so I'll follow up after 10 days and half a month.	Jingdong prefecture level city in Shandong
Happy Fan Shop	King of Power Saving	Just received the intelligent power saving meter, immediately plugged in to use, see it is not bad, and order to sell the second time	Jingdong prefecture level city in Shandong
Happy Fan Shop	King of Power Saving	This power saver is a bit effective, used for half a month, the effect is still visible.	Jingdong prefecture level city in Shandong
Happy Fan Shop	King of Power Saving	I received the baby and it looks great, I'm trying it out now and will keep an eye on it afterward.	Jingdong prefecture level city in Shandong
Happy Fan Shop	King of Power Saving	I've just received it, but I'm still trying to see how it works, so I'll come back in a little while.	Jingdong prefecture level city in Shandong
Happy Fan Shop	King of Power Saving	Just used it wait to use it for a while and then comment thanks, all first five stars.	Jingdong prefecture level city in Shandong
Happy Fan Shop	King of Power Saving	Very trustworthy brand, quality is guaranteed, the price is still affordable, quite satisfied!	Jingdong prefecture level city in Shandong
Happy Fan Shop	King of Power Saving	Things received, I hope that the platform of Jingdong do not sell fake goods are available	Jingdong prefecture level city in Shandong
Happy Fan Shop	King of Power Saving	Energy saver received, the use of the effect is good and the business description of the same	Jingdong prefecture level city in Shandong
Happy Fan Shop	King of Power Saving	The surface looks beautiful, the actual effect is not known, after using additional evaluation.	Jingdong prefecture level city in Shandong
Happy Fan Shop	King of Power Saving	Tried it for a few months, it really saves electricity, and I bought it for a friend.	Jingdong prefecture level city in Shandong
Happy Fan Shop	King of Power Saving	It didn't work and wanted to return it, it really didn't work at all.	Jingdong prefecture level city in Shandong
Happy Fan Shop	King of Power Saving	Just used it, don't know how effective it is, looking forward to usefulness	Jingdong prefecture level city in Shandong
Happy Fan Shop	King of Power Saving	After two months before the effect I do not know if it is true!	Jingdong prefecture level city in Shandong
Happy Fan Shop	King of Power Saving	So fast! Ordered yesterday and received today 👍👍👍👍👍👍👍👍👍 🌹🌹🌹🌹🌹🌹🌹🌹🌹	Jingdong prefecture level city in Shandong
Happy Fan Shop	King of Power Saving	I've started using it. We'll see how it works.	Jingdong prefecture level city in Shandong
Happy Fan Shop	King of Power Saving	I just received the goods, I won't know the effect until after two.	Jingdong prefecture level city in Shandong
Happy Fan Shop	King of Power Saving	Just received the goods, the specific effect will be evaluated later	Jingdong prefecture level city in Shandong
Happy Fan Shop	King of Power Saving	I've received the goods, I don't know if it's any good yet	Jingdong prefecture level city in Shandong
Happy Fan Shop	King of Power Saving	Try it for a month and then come back with a concrete evaluation	Jingdong prefecture level city in Shandong
Happy Fan Shop	King of Power Saving	In use, after two months to see the effect of power saving	Jingdong prefecture level city in Shandong
Happy Fan Shop	King of Power Saving	Power Saver. It's authentic. It's black. It works.	Jingdong prefecture level city in Shandong
Happy Fan Shop	King of Power Saving	I've been using it for a week now and it's not working.	Jingdong prefecture level city in Shandong
Happy Fan Shop	King of Power Saving	I'm just using it. I don't know how it's going to work.	Jingdong prefecture level city in Shandong
Happy Fan Shop	King of Power Saving	Products are better looking out for power savings	Jingdong prefecture level city in Shandong
Happy Fan Shop	King of Power Saving	I received it and I've kept it on. I hope it works.	Jingdong prefecture level city in Shandong
Happy Fan Shop	King of Power Saving	It's really good! Worth recommending to buy!	Jingdong prefecture level city in Shandong
Happy Fan Shop	King of Power Saving	Try it for a few days and see if it doesn't work, then return it.	Jingdong prefecture level city in Shandong
Happy Fan Shop	King of Power Saving	It's in use and I hope to be pleasantly surprised.	Jingdong prefecture level city in Shandong
Happy Fan Shop	King of Power Saving	We'll see how it works when we use it	Jingdong prefecture level city in Shandong
Happy Fan Shop	King of Power Saving	Let's see if it works.	Jingdong prefecture level city in Shandong
Happy Fan Shop	King of Power Saving	I'm using it and will follow up with a review	Jingdong prefecture level city in Shandong
Happy Fan Shop	King of Power Saving	It really works. It saves energy.	Jingdong prefecture level city in Shandong
Happy Fan Shop	King of Power Saving	It does have some power savings!	Jingdong prefecture level city in Shandong
Happy Fan Shop	King of Power Saving	Jingdong shopping speed is convenient and timely	Jingdong prefecture level city in Shandong

Happy Fan Shop	King of Power Saving	Second time I've bought it. Everything's fine.	Jingdong prefecture level city in Shandong
Happy Fan Shop	King of Power Saving	I don't know if it works yet.	Jingdong prefecture level city in Shandong
Happy Fan Shop	King of Power Saving	Not used, don't know how well it works.	Jingdong prefecture level city in Shandong
Happy Fan Shop	King of Power Saving	No visible results so far	Jingdong prefecture level city in Shandong
Happy Fan Shop	King of Power Saving	It doesn't feel like it's working.	Jingdong prefecture level city in Shandong
Happy Fan Shop	King of Power Saving	Where's the effect? Where are the power savings?	Jingdong prefecture level city in Shandong
Happy Fan Shop	King of Power Saving	Let's see how it works in practice!	Jingdong prefecture level city in Shandong
Happy Fan Shop	King of Power Saving	I'll wait until I use it to give you a good review	Jingdong prefecture level city in Shandong
Happy Fan Shop	King of Power Saving	I'll follow up after I've used it	Jingdong prefecture level city in Shandong
Happy Fan Shop	King of Power Saving	Well, that's good for saving power.	Jingdong prefecture level city in Shandong
Happy Fan Shop	King of Power Saving	Received. Used in the know.	Jingdong prefecture level city in Shandong
Happy Fan Shop	King of Power Saving	It doesn't do much good.	Jingdong prefecture level city in Shandong
Happy Fan Shop	King of Power Saving	It's not working.	Jingdong prefecture level city in Shandong
Happy Fan Shop	King of Power Saving	I don't know. I just used it.	Jingdong prefecture level city in Shandong
Happy Fan Shop	King of Power Saving	Use it and come back to evaluate.	Jingdong prefecture level city in Shandong
Happy Fan Shop	King of Power Saving	Two words: useless.	Jingdong prefecture level city in Shandong
Happy Fan Shop	King of Power Saving	To be used and then evaluated	Jingdong prefecture level city in Shandong
Happy Fan Shop	King of Power Saving	No shipment.	Jingdong prefecture level city in Shandong
Happy Fan Shop	King of Power Saving	Fake. Speechless.	Jingdong prefecture level city in Shandong
Happy Fan Shop	King of Power Saving	fantastic (idiom); awesome	Jingdong prefecture level city in Shandong
Happy Fan Shop	King of Power Saving	an excellent	Jingdong prefecture level city in Shandong
Happy Fan Shop	King of Power Saving	an excellent	Jingdong prefecture level city in Shandong
Happy Fan Shop	King of Power Saving	Want to buy! Recommended not to buy! No effect	Jingdong prefecture level city in Shandong
Happy Fan Shop	King of Power Saving	That's all there is to it. I can't figure out how to save electricity.	Jingdong prefecture level city in Shandong
Happy Fan Shop	King of Power Saving	It's no use.	Jingdong prefecture level city in Shandong
Happy Fan Shop	King of Power Saving	Things are more perfect than I imagined, very powerful, it is worth getting Packaging is very strong, things are well protected, it is worth the praise things are very satisfied Oh, beautiful and stylish atmosphere, customer service is super patient, service is meticulous and attentive, the big brand is not	Jingdong prefecture level city in Shandong
Happy Fan Shop	King of Power Saving	The effect is too good, the family has now several units, back to buy a number of times, the power saver received, power-saving effect is very good,, fine workmanship, long warranty, but also purify the air, before buying a used for a period of time, with the okay, and then bought a, and only now come to the	Jingdong prefecture level city in Shandong
Happy Fan Shop	King of Power Saving	Quite good, and imagine the same, casual ride are very good, satisfied! Baby received it 👌, really great ah! 🌈 quality is really nothing to say, very satisfied! 🆗 happy happy 😀 genuine, great, the price is very good, in short, quite good, will buy again a very good shopping experience, attentive service, fast logistics, distribution is also thoughtful, the attitude is also very good, value for money, ah,	Jingdong prefecture level city in Shandong
Happy Fan Shop	King of Power Saving	Power-saving effect: strong Cost-effective: quite good Appearance style: beautiful Workmanship material: meticulous Experience: looking forward to power-saving effect	Jingdong prefecture level city in Shandong
Happy Fan Shop	King of Power Saving	Cost-effective is okay, the appearance of the style of meticulous workmanship, looking forward to energy-saving effect.	Jingdong prefecture level city in Shandong
Happy Fan Shop	King of Power Saving	Very Good Very Power Saving This Power Saver is really power saving As soon as the power is turned on, the Power Saver starts the power saving function for you.	Jingdong prefecture level city in Shandong
Happy Fan Shop	King of Power Saving	Logistics is very fast, the price is favorable, friends introduced that it is very good to use, bought 2 at once, looking forward to the effect, used after the follow-up comment.	Jingdong prefecture level city in Shandong
Happy Fan Shop	King of Power Saving	It's all fake. It doesn't save any electricity.	Jingdong prefecture level city in Shandong
Happy Fan Shop	King of Power Saving	This Power Saver is so much better than the one I bought before, not only the effect is good, the quality is very good in all aspects, the customer service attitude is also very good, recommended to buy!	Jingdong prefecture level city in Shandong
Happy Fan Shop	King of Power Saving	Logistics is very fast, buy for parents, good quality, once bought 2, power-saving effect is good to buy 2 more, look forward to using the effect, satisfied will recommend to friends.	Jingdong prefecture level city in Shandong
Happy Fan Shop	King of Power Saving	With the description is the same, very high-grade, good value, to buy friends can order Oh!	Jingdong prefecture level city in Shandong
Happy Fan Shop	King of Power Saving	Not using much electricity in the winter saving over 100 per month! Ready to buy 2 more units in the summer ！！！！！ Good quality, good price, power saving effect is obvious, cost-effective, worth buying, can be recommended!	Jingdong prefecture level city in Shandong
Happy Fan Shop	King of Power Saving	Purchased a power saver received on the put into use, rent a room with many electrical appliances, power consumption is very large, we have a piece of electricity or 1.2 dollars a time, with this power saver is much better, every day can save a lot of money, very convenient and practical.	Jingdong prefecture level city in Shandong
Happy Fan Shop	King of Power Saving	The power saving effect is very good: my supermarket monthly electricity bill is more than 7000 yuan. On May 1st someone recommended buying a couple of power savers, and I bought 5 of them first. The electricity bill was about 2000 less in June. This time, I bought 5 more. The seller said 800 degrees of electricity with one. I feel it's basically enough. A total of 10 units	Jingdong prefecture level city in Shandong
Happy Fan Shop	King of Power Saving	I never thought I could get such a good baby at such an affordable price, it's very good value for money, I love it!	Jingdong prefecture level city in Shandong
Happy Fan Shop	King of Power Saving	Previously bought other brands of power savers, but found no effect at home, this time to see how the products on the Jingdong, inserted in the home socket, the LED display is blue, the night light is very nice to see, look at the instructions that have to be half a year in order to achieve the best results, try it, I believe that the quality of the Jingdong, it should not be wrong.	Jingdong prefecture level city in Shandong
Happy Fan Shop	King of Power Saving	Superb, inexpensive, saves a lot of points, been using it for a long time!	Jingdong prefecture level city in Shandong
Happy Fan Shop	King of Power Saving	There are a lot of appliances in the house, the summer air conditioning is very costly, I bought this in front of the air conditioning effect is very good, I explain that I bought three in one breath, I use more electricity a month down to 200 or so, very satisfied.	Jingdong prefecture level city in Shandong
Happy Fan Shop	King of Power Saving	A month can save more than 200, home monthly electricity bill of more than 600 dollars, inserted 3 or so, an amount of the balance of the average can save more than 200. Product is very good, has been buying this one, has given a lot of relatives recommended, the quality is also very good, before buying a look with this same, who knows to buy home has not been used for more than a week on the bad, the quality of this is really good with more than two years!	Jingdong prefecture level city in Shandong

Happy Fan Shop	King of Power Saving	Whole house power saving king, can really save electricity, friends recommended, high power saving appliances, only replacement, no repair, after-sales worry-free!	Jingdong prefecture level city in Shandong
Happy Fan Shop	King of Power Saving	Great item love it, great customer service, great shopping experience!	Jingdong prefecture level city in Shandong
Happy Fan Shop	King of Power Saving	Power saving, very good use, much cheaper than the physical store, satisfaction	Jingdong prefecture level city in Shandong
Happy Fan Shop	King of Power Saving	In use, very well packaged and fast delivery of credit!	Jingdong prefecture level city in Shandong
Happy Fan Shop	King of Power Saving	Product quality, customer service attitude is particularly good, like, praise!	Jingdong prefecture level city in Shandong
Happy Fan Shop	King of Power Saving	The logistics is very fast, the packaging is not broken, the quality is very good, I like it very much!	Jingdong prefecture level city in Shandong
Happy Fan Shop	King of Power Saving	The saver was well packaged and was as described when plugged in	Jingdong prefecture level city in Shandong
Happy Fan Shop	King of Power Saving	Energy efficient and very satisfied	Jingdong prefecture level city in Shandong
Happy Fan Shop	King of Power Saving	Great quality for the price and fast shipping.	Jingdong prefecture level city in Shandong
Happy Fan Shop	King of Power Saving	It's great, just as described. Perfect.	Jingdong prefecture level city in Shandong
Happy Fan Shop	King of Power Saving	There is no power saving effect, but rather ring up an increase in electricity costs, because this machine is on continuously all day long, uninterruptedly, its own electricity and increased costs, plus the cost of purchasing the machine, so I feel that the use of this machine instead of increasing the	Jingdong prefecture level city in Shandong
Happy Fan Shop	King of Power Saving	Recommended by the owner of the store next door, looked at his home does have to save electricity, decided to get. Indeed quite can, the voltage is much more stable, there is a reduction in electricity	Jingdong prefecture level city in Shandong
Happy Fan Shop	King of Power Saving	Goods have been received value for money, affordable and reasonable price, and the description of the same, power-saving safe and reliable!	Jingdong prefecture level city in Shandong
Happy Fan Shop	King of Power Saving	Listen to friends introduced to buy, tried to save a lot of electricity is good, quite good, worth buying.	Jingdong prefecture level city in Shandong
Happy Fan Shop	King of Power Saving	The product is easy to use, safe and environmentally friendly, only used for a short time, to the next month, after the electricity bill status display to know how the performance of the power saver efficacy.	Jingdong prefecture level city in Shandong
Happy Fan Shop	King of Power Saving	Last month's electricity bill is 178 yuan, with this power saver is 92 yuan, ready to buy two more back home, I was introduced to buy a friend, just started to not believe that I did not expect the effect!	Jingdong prefecture level city in Shandong
Happy Fan Shop	King of Power Saving	It looks nice, but after using it for a few days I can't see how it saves power?	Jingdong prefecture level city in Shandong
Happy Fan Shop	King of Power Saving	Recommended by a friend, it is genuine, as described by the merchant, cost-effective	Jingdong prefecture level city in Shandong
Happy Fan Shop	King of Power Saving	Works well, affordable and saves money.	Jingdong prefecture level city in Shandong
Happy Fan Shop	King of Power Saving	Feels good haven't compared it yet, the air conditioning has been on a lot in the past two months	Jingdong prefecture level city in Shandong
Happy Fan Shop	King of Power Saving	Saving about 5 kWh per day is a good thing	Jingdong prefecture level city in Shandong
Happy Fan Shop	King of Power Saving	Not bad, affordable, best value for money	Jingdong prefecture level city in Shandong
Happy Fan Shop	King of Power Saving	The goods are genuine and the logistics are faster.	Jingdong prefecture level city in Shandong
Happy Fan Shop	King of Power Saving	Goods have been received today, has been plugged into the outlet, looking forward to saving electricity, after a period of time to see how the effect in the follow-up comment!	Jingdong prefecture level city in Shandong
Happy Fan Shop	King of Power Saving	I just got it home and haven't used it for long, so I'll use it for a couple months and see what happens. Seeing that most people have good reviews, it should be fine.	Jingdong prefecture level city in Shandong
Happy Fan Shop	King of Power Saving	I'm looking forward to the results, so I'll give you a good review out of habit! Follow up review after a while	Jingdong prefecture level city in Shandong
Happy Fan Shop	King of Power Saving	Use a dish first to know if it will help save energy	Jingdong prefecture level city in Shandong
Happy Fan Shop	King of Power Saving	Whether it saves electricity or not, we don't know yet.	Jingdong prefecture level city in Shandong
Happy Fan Shop	King of Power Saving	Goods have been received, use a period of time to know how oh. The courier did not deliver to the door ah! Sent to the station ah! The heat is inconvenient ah!	Jingdong prefecture level city in Shandong
Happy Fan Shop	King of Power Saving	A little effect, before an hour a power, now an hour 0.76 degrees, a dollar a power = save 24 cents, although not a lot, first use it!	Jingdong prefecture level city in Shandong
Happy Fan Shop	King of Power Saving	Just received the goods, use a month to evaluate	Jingdong prefecture level city in Shandong
Happy Fan Shop	King of Power Saving	Home every month with a lot of electricity, inadvertently found this good thing online that can save the beginning of a skeptical attitude to consult customer service, customer service replied very professional, said 120 days can be refunded that first try it!	Jingdong prefecture level city in Shandong
Happy Fan Shop	King of Power Saving	After receiving the goods this afternoon, immediately installed in the bathroom Smith energy storage electric water heater on the power outlet (instead of connecting with a plug board), when used, found that the "Power Saving King" LCD display, is the value of the voltage, not the store's video said so	Jingdong prefecture level city in Shandong
Happy Fan Shop	King of Power Saving	Has made many days, did not feel the power saving, the effect is not obvious.	Jingdong prefecture level city in Shandong
Happy Fan Shop	King of Power Saving	Energy savers do not save energy at all, but rather consume a lot of electricity.	Jingdong prefecture level city in Shandong
Happy Fan Shop	King of Power Saving	It's useless. It's a waste of money.	Jingdong prefecture level city in Shandong
Happy Fan Shop	King of Power Saving	Last month saved more than 500, which came back to buy, the store bought three, and then buy 1 home use, my family is dry beauty salon, more instruments, especially electricity, a month almost 1000. or so, with this did not expect to save so much, great, five-star praise!	Jingdong prefecture level city in Shandong
Happy Fan Shop	King of Power Saving	It rained a couple days ago and I just went to get the item back today , not bad, hey I looked at the meter and that red display light takes several seconds to jump, did not make a mistake buying it. After a period of time to evaluate.	Jingdong prefecture level city in Shandong
Happy Fan Shop	King of Power Saving	Without further ado, the electricity bill came down, this month less 102 yuan, has been repurchased, I also recommended to my friends, all said that there are effects, the product is okay, the effect is quite	Jingdong prefecture level city in Shandong
Happy Fan Shop	King of Power Saving	After buying and using two months really see the effect, so only to evaluate! Originally held the mindset of trying, I did not expect really effective, worth having!	Jingdong prefecture level city in Shandong
Happy Fan Shop	King of Power Saving	In the use of this energy saver before the monthly electricity bill is more than 150 yuan or so, now save thirty percent, every day to work, six or seven o'clock in the afternoon to go home, I do not know why the electricity bill is so expensive, energy-saving appliances really have the effect of recommending	Jingdong prefecture level city in Shandong
Happy Fan Shop	King of Power Saving	It saves about 100 bucks a month. A friend recommended it to me, and he's been using it for a while, and the effect is so obvious that I bought one too.	Jingdong prefecture level city in Shandong
Happy Fan Shop	King of Power Saving	My dad asked me to buy one, but after I paid the bill, I asked and he said it was a lot less this month, so he asked me to buy another one.	Jingdong prefecture level city in Shandong
Happy Fan Shop	King of Power Saving	The specific effect will have to be tested for at least two months to compare whether there is a power saving effect or not.	Jingdong prefecture level city in Shandong
Happy Fan Shop	King of Power Saving	'The goods have been received, immediately go to use, please ask the boss you instructions do not allow to withdraw, there are two fuses installed there!	Jingdong prefecture level city in Shandong
Happy Fan Shop	King of Power Saving	First praise is using, after using if good then follow up, now because I have not yet seen the results	Jingdong prefecture level city in Shandong
Happy Fan Shop	King of Power Saving	I've just been using it, and I haven't seen if it has a power-saving effect yet, so I'll follow up after 10 days and half a month.	Jingdong prefecture level city in Shandong
Happy Fan Shop	King of Power Saving	Just received the intelligent power saving meter, immediately plugged in to use, see it is not bad, and order to sell the second time	Jingdong prefecture level city in Shandong

Happy Fan Shop	King of Power Saving	This power saver is a bit effective, used for half a month, the effect is still visible.	Jingdong prefecture level city in Shandong
Happy Fan Shop	King of Power Saving	I received the baby and it looks great, I'm trying it out now and will keep an eye on it afterward.	Jingdong prefecture level city in Shandong
Happy Fan Shop	King of Power Saving	I've just received it, but I'm still trying to see how it works, so I'll come back in a little while.	Jingdong prefecture level city in Shandong
Happy Fan Shop	King of Power Saving	Just used it wait to use it for a while and then comment thanks, all first five stars.	Jingdong prefecture level city in Shandong
Happy Fan Shop	King of Power Saving	Very trustworthy brand, quality is guaranteed, the price is still affordable, quite satisfied!	Jingdong prefecture level city in Shandong
Happy Fan Shop	King of Power Saving	Things received, I hope that the platform of Jingdong do not sell fake goods are available	Jingdong prefecture level city in Shandong
Happy Fan Shop	King of Power Saving	Energy saver received, the use of the effect is good and the business description of the same	Jingdong prefecture level city in Shandong
Happy Fan Shop	King of Power Saving	The surface looks beautiful, the actual effect is not known, after using additional evaluation.	Jingdong prefecture level city in Shandong
Happy Fan Shop	King of Power Saving	Tried it for a few months, it really saves electricity, and I bought it for a friend.	Jingdong prefecture level city in Shandong
Happy Fan Shop	King of Power Saving	It didn't work and wanted to return it, it really didn't work at all.	Jingdong prefecture level city in Shandong
Happy Fan Shop	King of Power Saving	Just used it, don't know how effective it is, looking forward to usefulness	Jingdong prefecture level city in Shandong
Happy Fan Shop	King of Power Saving	After two months before the effect I do not know if it is true!	Jingdong prefecture level city in Shandong
Happy Fan Shop	King of Power Saving	So fast! Ordered yesterday and received today 👍👍👍👍👍👍👍👍👍 🌹🌹🌹🌹🌹🌹🌹🌹🌹	Jingdong prefecture level city in Shandong
Happy Fan Shop	King of Power Saving	I've started using it. We'll see how it works.	Jingdong prefecture level city in Shandong
Happy Fan Shop	King of Power Saving	I just received the goods, I won't know the effect until after two.	Jingdong prefecture level city in Shandong
Happy Fan Shop	King of Power Saving	Just received the goods, the specific effect will be evaluated later	Jingdong prefecture level city in Shandong
Happy Fan Shop	King of Power Saving	I've received the goods, I don't know if it's any good yet	Jingdong prefecture level city in Shandong
Happy Fan Shop	King of Power Saving	Try it for a month and then come back with a concrete evaluation	Jingdong prefecture level city in Shandong
Happy Fan Shop	King of Power Saving	In use, after two months to see the effect of power saving	Jingdong prefecture level city in Shandong
Happy Fan Shop	King of Power Saving	Power Saver. It's authentic. It's black. It works.	Jingdong prefecture level city in Shandong
Happy Fan Shop	King of Power Saving	I've been using it for a week now and it's not working.	Jingdong prefecture level city in Shandong
Happy Fan Shop	King of Power Saving	I'm just using it. I don't know how it's going to work.	Jingdong prefecture level city in Shandong
Happy Fan Shop	King of Power Saving	Products are better looking out for power savings	Jingdong prefecture level city in Shandong
Happy Fan Shop	King of Power Saving	I received it and I've kept it on. I hope it works.	Jingdong prefecture level city in Shandong
Happy Fan Shop	King of Power Saving	It's really good! Worth recommending to buy!	Jingdong prefecture level city in Shandong
Happy Fan Shop	King of Power Saving	Try it for a few days and see if it doesn't work, then return it.	Jingdong prefecture level city in Shandong
Happy Fan Shop	King of Power Saving	It's in use and I hope to be pleasantly surprised.	Jingdong prefecture level city in Shandong
Happy Fan Shop	King of Power Saving	We'll see how it works when we use it	Jingdong prefecture level city in Shandong
Happy Fan Shop	King of Power Saving	Let's see if it works.	Jingdong prefecture level city in Shandong
Happy Fan Shop	King of Power Saving	I'm using it and will follow up with a review	Jingdong prefecture level city in Shandong
Happy Fan Shop	King of Power Saving	It really works. It saves energy.	Jingdong prefecture level city in Shandong
Happy Fan Shop	King of Power Saving	It does have some power savings!	Jingdong prefecture level city in Shandong
Happy Fan Shop	King of Power Saving	Jingdong shopping speed is convenient and timely	Jingdong prefecture level city in Shandong
Happy Fan Shop	King of Power Saving	Second time I've bought it. Everything's fine.	Jingdong prefecture level city in Shandong
Happy Fan Shop	King of Power Saving	I don't know if it works yet.	Jingdong prefecture level city in Shandong
Happy Fan Shop	King of Power Saving	Not used, don't know how well it works.	Jingdong prefecture level city in Shandong
Happy Fan Shop	King of Power Saving	No visible results so far	Jingdong prefecture level city in Shandong
Happy Fan Shop	King of Power Saving	It doesn't feel like it's working.	Jingdong prefecture level city in Shandong
Happy Fan Shop	King of Power Saving	Where's the effect? Where are the power savings?	Jingdong prefecture level city in Shandong
Happy Fan Shop	King of Power Saving	Let's see how it works in practice!	Jingdong prefecture level city in Shandong
Happy Fan Shop	King of Power Saving	I'll wait until I use it to give you a good review	Jingdong prefecture level city in Shandong
Happy Fan Shop	King of Power Saving	I'll follow up after I've used it	Jingdong prefecture level city in Shandong
Happy Fan Shop	King of Power Saving	Well, that's good for saving power.	Jingdong prefecture level city in Shandong
Happy Fan Shop	King of Power Saving	Received. Used in the know.	Jingdong prefecture level city in Shandong
Happy Fan Shop	King of Power Saving	It doesn't do much good.	Jingdong prefecture level city in Shandong
Happy Fan Shop	King of Power Saving	It's not working.	Jingdong prefecture level city in Shandong
Happy Fan Shop	King of Power Saving	I don't know. I just used it.	Jingdong prefecture level city in Shandong
Happy Fan Shop	King of Power Saving	Use it and come back to evaluate.	Jingdong prefecture level city in Shandong
Happy Fan Shop	King of Power Saving	Two words: useless.	Jingdong prefecture level city in Shandong
Happy Fan Shop	King of Power Saving	To be used and then evaluated	Jingdong prefecture level city in Shandong
Happy Fan Shop	King of Power Saving	No shipment.	Jingdong prefecture level city in Shandong

Happy Fan Shop	King of Power Saving	Fake. Speechless.	Jingdong prefecture level city in Shandong
Happy Fan Shop	King of Power Saving	fantastic (idiom); awesome	Jingdong prefecture level city in Shandong
Happy Fan Shop	King of Power Saving	an excellent	Jingdong prefecture level city in Shandong
Happy Fan Shop	King of Power Saving	an excellent	Jingdong prefecture level city in Shandong
Happy Fan Shop	King of Power Saving	Want to buy! Recommended not to buy! No effect	Jingdong prefecture level city in Shandong
Happy Fan Shop	King of Power Saving	That's all there is to it. I can't figure out how to save electricity.	Jingdong prefecture level city in Shandong
Happy Fan Shop	King of Power Saving	It's no use.	Jingdong prefecture level city in Shandong
Happy Fan Shop	King of Power Saving	Things are more perfect than I imagined, very powerful, it is worth getting Packaging is very strong, things are well protected, it is worth the praise things are very satisfied Oh, beautiful and stylish atmosphere, customer service is super patient, service is meticulous and attentive, the big brand is not	Jingdong prefecture level city in Shandong
Happy Fan Shop	King of Power Saving	The effect is too good, the family has now several units, back to buy a number of times, the power saver received, power-saving effect is very good,, fine workmanship, long warranty, but also purify the air, before buying a used for a period of time, with the okay, and then bought a, and only now come to the	Jingdong prefecture level city in Shandong
Happy Fan Shop	King of Power Saving	Quite good, and imagine the same, casual ride are very good, satisfied! Baby received it 👌, really great ah! 🌈 quality is really nothing to say, very satisfied! 🆗 happy happy 😃 genuine, great, the price is very good, in short, quite good, will buy again a very good shopping experience, attentive service, fast logistics, distribution is also thoughtful, the attitude is also very good, value for money, ah,	Jingdong prefecture level city in Shandong
Happy Fan Shop	King of Power Saving	Power-saving effect: strong Cost-effective: quite good Appearance style: beautiful Workmanship material: meticulous Experience: looking forward to power-saving effect	Jingdong prefecture level city in Shandong
Happy Fan Shop	King of Power Saving	Cost-effective is okay, the appearance of the style of meticulous workmanship, looking forward to energy-saving effect.	Jingdong prefecture level city in Shandong
Happy Fan Shop	King of Power Saving	Very Good Very Power Saving This Power Saver is really power saving As soon as the power is turned on, the Power Saver starts the power saving function for you.	Jingdong prefecture level city in Shandong
Happy Fan Shop	King of Power Saving	Logistics is very fast, the price is favorable, friends introduced that it is very good to use, bought 2 at once, looking forward to the effect, used after the follow-up comment.	Jingdong prefecture level city in Shandong
Happy Fan Shop	King of Power Saving	It's all fake. It doesn't save any electricity.	Jingdong prefecture level city in Shandong
Happy Fan Shop	King of Power Saving	This Power Saver is so much better than the one I bought before, not only the effect is good, the quality is very good in all aspects, the customer service attitude is also very good, recommended to buy!	Jingdong prefecture level city in Shandong
Happy Fan Shop	King of Power Saving	Logistics is very fast, buy for parents, good quality, once bought 2, power-saving effect is good to buy 2 more, look forward to using the effect, satisfied will recommend to friends.	Jingdong prefecture level city in Shandong
Happy Fan Shop	King of Power Saving	With the description is the same, very high-grade, good value, to buy friends can order Oh!	Jingdong prefecture level city in Shandong
Happy Fan Shop	King of Power Saving	Not using much electricity in the winter saving over 100 per month! Ready to buy 2 more units in the summer ！！！！！ Good quality, good price, power saving effect is obvious, cost-effective, worth buying, can be recommended!	Jingdong prefecture level city in Shandong
Happy Fan Shop	King of Power Saving	Purchased a power saver received on the put into use, rent a room with many electrical appliances, power consumption is very large, we have a piece of electricity or 1.2 dollars a time, with this power saver is much better, every day can save a lot of money, very convenient and practical.	Jingdong prefecture level city in Shandong
Happy Fan Shop	King of Power Saving	The power saving effect is very good: my supermarket monthly electricity bill is more than 7000 yuan. On May 1st someone recommended buying a couple of power savers, and I bought 5 of them first. The electricity bill was about 2000 less in June. This time, I bought 5 more. The seller said 800 degrees of electricity with one. I feel it's basically enough. A total of 10 units	Jingdong prefecture level city in Shandong
Happy Fan Shop	King of Power Saving	I never thought I could get such a good baby at such an affordable price, it's very good value for money, I love it!	Jingdong prefecture level city in Shandong
Happy Fan Shop	King of Power Saving	Previously bought other brands of power savers, but found no effect at home, this time to see how the products on the Jingdong, inserted in the home socket, the LED display is blue, the night light is very nice to see, look at the instructions that have to be half a year in order to achieve the best results, try it, I believe that the quality of the Jingdong, it should not be wrong.	Jingdong prefecture level city in Shandong
Happy Fan Shop	King of Power Saving	Superb, inexpensive, saves a lot of points, been using it for a long time!	Jingdong prefecture level city in Shandong
Happy Fan Shop	King of Power Saving	There are a lot of appliances in the house, the summer air conditioning is very costly, I bought this in front of the air conditioning effect is very good, I explain that I bought three in one breath, I use more electricity a month down to 200 or so, very satisfied.	Jingdong prefecture level city in Shandong
Happy Fan Shop	King of Power Saving	A month can save more than 200, home monthly electricity bill of more than 600 dollars, inserted 3 or so, an amount of the balance of the average can save more than 200. Product is very good, has been buying this one, has given a lot of relatives recommended, the quality is also very good, before buying a look with this same, who knows to buy home has not been used for more than a week on the bad, the quality of this is really good with more than two years!	Jingdong prefecture level city in Shandong
Happy Fan Shop	King of Power Saving	Whole house power saving king, can really save electricity, friends recommended, high power saving appliances, only replacement, no repair, after-sales worry-free!	Jingdong prefecture level city in Shandong
Happy Fan Shop	King of Power Saving	Great item love it, great customer service, great shopping experience!	Jingdong prefecture level city in Shandong
Happy Fan Shop	King of Power Saving	Power saving, very good use, much cheaper than the physical store, satisfaction	Jingdong prefecture level city in Shandong
Happy Fan Shop	King of Power Saving	In use, very well packaged and fast delivery of credit!	Jingdong prefecture level city in Shandong
Happy Fan Shop	King of Power Saving	Product quality, customer service attitude is particularly good, like, praise!	Jingdong prefecture level city in Shandong
Happy Fan Shop	King of Power Saving	The logistics is very fast, the packaging is not broken, the quality is very good, I like it very much!	Jingdong prefecture level city in Shandong
Happy Fan Shop	King of Power Saving	The saver was well packaged and was as described when plugged in	Jingdong prefecture level city in Shandong
Happy Fan Shop	King of Power Saving	Energy efficient and very satisfied	Jingdong prefecture level city in Shandong
Happy Fan Shop	King of Power Saving	Great quality for the price and fast shipping.	Jingdong prefecture level city in Shandong
Happy Fan Shop	King of Power Saving	It's great, just as described. Perfect.	Jingdong prefecture level city in Shandong
Happy Fan Shop	King of Power Saving	There is no power saving effect, but rather ring up an increase in electricity costs, because this machine is on continuously all day long, uninterruptedly, its own electricity and increased costs, plus the cost of purchasing the machine, so I feel that the use of this machine instead of increasing the	Jingdong prefecture level city in Shandong
Happy Fan Shop	King of Power Saving	Recommended by the owner of the store next door, looked at his home does have to save electricity, decided to get. Indeed quite can, the voltage is much more stable, there is a reduction in electricity	Jingdong prefecture level city in Shandong
Happy Fan Shop	King of Power Saving	Goods have been received value for money, affordable and reasonable price, and the description of the same, power-saving safe and reliable!	Jingdong prefecture level city in Shandong
Happy Fan Shop	King of Power Saving	Listen to friends introduced to buy, tried to save a lot of electricity is good, quite good, worth buying.	Jingdong prefecture level city in Shandong
Happy Fan Shop	King of Power Saving	The product is easy to use, safe and environmentally friendly, only used for a short time, to the next month, after the electricity bill status display to know how the performance of the power saver efficacy.	Jingdong prefecture level city in Shandong
Happy Fan Shop	King of Power Saving	Last month's electricity bill is 178 yuan, with this power saver is 92 yuan, ready to buy two more back home, I was introduced to buy a friend, just started to not believe that I did not expect the effect!	Jingdong prefecture level city in Shandong
Happy Fan Shop	King of Power Saving	It looks nice, but after using it for a few days I can't see how it saves power?	Jingdong prefecture level city in Shandong
Happy Fan Shop	King of Power Saving	Recommended by a friend, it is genuine, as described by the merchant, cost-effective	Jingdong prefecture level city in Shandong

Happy Fan Shop	King of Power Saving	Works well, affordable and saves money.	Jingdong prefecture level city in Shandong
Happy Fan Shop	King of Power Saving	Feels good haven't compared it yet, the air conditioning has been on a lot in the past two months	Jingdong prefecture level city in Shandong
Happy Fan Shop	King of Power Saving	Saving about 5 kWh per day is a good thing	Jingdong prefecture level city in Shandong
Happy Fan Shop	King of Power Saving	Not bad, affordable, best value for money	Jingdong prefecture level city in Shandong
Happy Fan Shop	King of Power Saving	The goods are genuine and the logistics are faster.	Jingdong prefecture level city in Shandong
Happy Fan Shop	King of Power Saving	Goods have been received today, has been plugged into the outlet, looking forward to saving electricity, after a period of time to see how the effect in the follow-up comment!	Jingdong prefecture level city in Shandong
Happy Fan Shop	King of Power Saving	I just got it home and haven't used it for long, so I'll use it for a couple months and see what happens. Seeing that most people have good reviews, it should be fine.	Jingdong prefecture level city in Shandong
Happy Fan Shop	King of Power Saving	I'm looking forward to the results, so I'll give you a good review out of habit! Follow up review after a while	Jingdong prefecture level city in Shandong
Happy Fan Shop	King of Power Saving	Use a dish first to know if it will help save energy	Jingdong prefecture level city in Shandong
Happy Fan Shop	King of Power Saving	Whether it saves electricity or not, we don't know yet.	Jingdong prefecture level city in Shandong
Happy Fan Shop	King of Power Saving	Goods have been received, use a period of time to know how oh. The courier did not deliver to the door ah! Sent to the station ah! The heat is inconvenient ah!	Jingdong prefecture level city in Shandong
Happy Fan Shop	King of Power Saving	A little effect, before an hour a power, now an hour 0.76 degrees, a dollar a power = save 24 cents, although not a lot, first use it!	Jingdong prefecture level city in Shandong
Happy Fan Shop	King of Power Saving	Just received the goods, use a month to evaluate	Jingdong prefecture level city in Shandong
Happy Fan Shop	King of Power Saving	Home every month with a lot of electricity, inadvertently found this good thing online that can save the beginning of a skeptical attitude to consult customer service, customer service replied very professional, said 120 days can be refunded that first try it!	Jingdong prefecture level city in Shandong
Happy Fan Shop	King of Power Saving	After receiving the goods this afternoon, immediately installed in the bathroom Smith energy storage electric water heater on the power outlet (instead of connecting with a plug board), when used, found that the "Power Saving King" LCD display, is the value of the voltage, not the store's video said so	Jingdong prefecture level city in Shandong
Happy Fan Shop	King of Power Saving	Has made many days, did not feel the power saving, the effect is not obvious.	Jingdong prefecture level city in Shandong
Happy Fan Shop	King of Power Saving	Energy savers do not save energy at all, but rather consume a lot of electricity.	Jingdong prefecture level city in Shandong
Happy Fan Shop	King of Power Saving	It's useless. It's a waste of money.	Jingdong prefecture level city in Shandong
Happy Fan Shop	King of Power Saving	Last month saved more than 500, which came back to buy, the store bought three, and then buy 1 home use, my family is dry beauty salon, more instruments, especially electricity, a month almost 1000. or so, with this did not expect to save so much, great, five-star praise!	Jingdong prefecture level city in Shandong
Happy Fan Shop	King of Power Saving	It rained a couple days ago and I just went to get the item back today , not bad, hey I looked at the meter and that red display light takes several seconds to jump, did not make a mistake buying it. After a period of time to evaluate.	Jingdong prefecture level city in Shandong
Happy Fan Shop	King of Power Saving	Without further ado, the electricity bill came down, this month less 102 yuan, has been repurchased, I also recommended to my friends, all said that there are effects, the product is okay, the effect is quite	Jingdong prefecture level city in Shandong
Happy Fan Shop	King of Power Saving	After buying and using two months really see the effect, so only to evaluate! Originally held the mindset of trying, I did not expect really effective, worth having!	Jingdong prefecture level city in Shandong
Happy Fan Shop	King of Power Saving	In the use of this energy saver before the monthly electricity bill is more than 150 yuan or so, now save thirty percent, every day to work, six or seven o'clock in the afternoon to go home, I do not know why the electricity bill is so expensive, energy-saving appliances really have the effect of recommending	Jingdong prefecture level city in Shandong
Happy Fan Shop	King of Power Saving	It saves about 100 bucks a month. A friend recommended it to me, and he's been using it for a while, and the effect is so obvious that I bought one too.	Jingdong prefecture level city in Shandong
Happy Fan Shop	King of Power Saving	My dad asked me to buy one, but after I paid the bill, I asked and he said it was a lot less this month, so he asked me to buy another one.	Jingdong prefecture level city in Shandong
Happy Fan Shop	King of Power Saving	The specific effect will have to be tested for at least two months to compare whether there is a power saving effect or not.	Jingdong prefecture level city in Shandong
Happy Fan Shop	King of Power Saving	'The goods have been received, immediately go to use, please ask the boss you instructions do not allow to withdraw, there are two fuses installed there!	Jingdong prefecture level city in Shandong
Happy Fan Shop	King of Power Saving	First praise is using, after using if good then follow up, now because I have not yet seen the results	Jingdong prefecture level city in Shandong
Happy Fan Shop	King of Power Saving	I've just been using it, and I haven't seen if it has a power-saving effect yet, so I'll follow up after 10 days and half a month.	Jingdong prefecture level city in Shandong
Happy Fan Shop	King of Power Saving	Just received the intelligent power saving meter, immediately plugged in to use, see it is not bad, and order to sell the second time	Jingdong prefecture level city in Shandong
Happy Fan Shop	King of Power Saving	This power saver is a bit effective, used for half a month, the effect is still visible.	Jingdong prefecture level city in Shandong
Happy Fan Shop	King of Power Saving	I received the baby and it looks great, I'm trying it out now and will keep an eye on it afterward.	Jingdong prefecture level city in Shandong
Happy Fan Shop	King of Power Saving	I've just received it, but I'm still trying to see how it works, so I'll come back in a little while.	Jingdong prefecture level city in Shandong
Happy Fan Shop	King of Power Saving	Just used it wait to use it for a while and then comment thanks, all first five stars.	Jingdong prefecture level city in Shandong
Happy Fan Shop	King of Power Saving	Very trustworthy brand, quality is guaranteed, the price is still affordable, quite satisfied!	Jingdong prefecture level city in Shandong
Happy Fan Shop	King of Power Saving	Things received, I hope that the platform of Jingdong do not sell fake goods are available	Jingdong prefecture level city in Shandong
Happy Fan Shop	King of Power Saving	Energy saver received, the use of the effect is good and the business description of the same	Jingdong prefecture level city in Shandong
Happy Fan Shop	King of Power Saving	The surface looks beautiful, the actual effect is not known, after using additional evaluation.	Jingdong prefecture level city in Shandong
Happy Fan Shop	King of Power Saving	Tried it for a few months, it really saves electricity, and I bought it for a friend.	Jingdong prefecture level city in Shandong
Happy Fan Shop	King of Power Saving	It didn't work and wanted to return it, it really didn't work at all.	Jingdong prefecture level city in Shandong
Happy Fan Shop	King of Power Saving	Just used it, don't know how effective it is, looking forward to usefulness	Jingdong prefecture level city in Shandong
Happy Fan Shop	King of Power Saving	After two months before the effect I do not know if it is true!	Jingdong prefecture level city in Shandong
Happy Fan Shop	King of Power Saving	So fast! Ordered yesterday and received today 👍👍👍👍👍👍👍👍👍 🌹🌹🌹🌹🌹🌹🌹🌹🌹	Jingdong prefecture level city in Shandong
Happy Fan Shop	King of Power Saving	I've started using it. We'll see how it works.	Jingdong prefecture level city in Shandong
Happy Fan Shop	King of Power Saving	I just received the goods, I won't know the effect until after two.	Jingdong prefecture level city in Shandong
Happy Fan Shop	King of Power Saving	Just received the goods, the specific effect will be evaluated later	Jingdong prefecture level city in Shandong
Happy Fan Shop	King of Power Saving	I've received the goods, I don't know if it's any good yet	Jingdong prefecture level city in Shandong
Happy Fan Shop	King of Power Saving	Try it for a month and then come back with a concrete evaluation	Jingdong prefecture level city in Shandong
Happy Fan Shop	King of Power Saving	In use, after two months to see the effect of power saving	Jingdong prefecture level city in Shandong

Happy Fan Shop	King of Power Saving	Power Saver. It's authentic. It's black. It works.	Jingdong prefecture level city in Shandong
Happy Fan Shop	King of Power Saving	I've been using it for a week now and it's not working.	Jingdong prefecture level city in Shandong
Happy Fan Shop	King of Power Saving	I'm just using it. I don't know how it's going to work.	Jingdong prefecture level city in Shandong
Happy Fan Shop	King of Power Saving	Products are better looking out for power savings	Jingdong prefecture level city in Shandong
Happy Fan Shop	King of Power Saving	I received it and I've kept it on. I hope it works.	Jingdong prefecture level city in Shandong
Happy Fan Shop	King of Power Saving	It's really good! Worth recommending to buy!	Jingdong prefecture level city in Shandong
Happy Fan Shop	King of Power Saving	Try it for a few days and see if it doesn't work, then return it.	Jingdong prefecture level city in Shandong
Happy Fan Shop	King of Power Saving	It's in use and I hope to be pleasantly surprised.	Jingdong prefecture level city in Shandong
Happy Fan Shop	King of Power Saving	We'll see how it works when we use it	Jingdong prefecture level city in Shandong
Happy Fan Shop	King of Power Saving	Let's see if it works.	Jingdong prefecture level city in Shandong
Happy Fan Shop	King of Power Saving	I'm using it and will follow up with a review	Jingdong prefecture level city in Shandong
Happy Fan Shop	King of Power Saving	It really works. It saves energy.	Jingdong prefecture level city in Shandong
Happy Fan Shop	King of Power Saving	It does have some power savings!	Jingdong prefecture level city in Shandong
Happy Fan Shop	King of Power Saving	Jingdong shopping speed is convenient and timely	Jingdong prefecture level city in Shandong
Happy Fan Shop	King of Power Saving	Second time I've bought it. Everything's fine.	Jingdong prefecture level city in Shandong
Happy Fan Shop	King of Power Saving	I don't know if it works yet.	Jingdong prefecture level city in Shandong
Happy Fan Shop	King of Power Saving	Not used, don't know how well it works.	Jingdong prefecture level city in Shandong
Happy Fan Shop	King of Power Saving	No visible results so far	Jingdong prefecture level city in Shandong
Happy Fan Shop	King of Power Saving	It doesn't feel like it's working.	Jingdong prefecture level city in Shandong
Happy Fan Shop	King of Power Saving	Where's the effect? Where are the power savings?	Jingdong prefecture level city in Shandong
Happy Fan Shop	King of Power Saving	Let's see how it works in practice!	Jingdong prefecture level city in Shandong
Happy Fan Shop	King of Power Saving	I'll wait until I use it to give you a good review	Jingdong prefecture level city in Shandong
Happy Fan Shop	King of Power Saving	I'll follow up after I've used it	Jingdong prefecture level city in Shandong
Happy Fan Shop	King of Power Saving	Well, that's good for saving power.	Jingdong prefecture level city in Shandong
Happy Fan Shop	King of Power Saving	Received. Used in the know.	Jingdong prefecture level city in Shandong
Happy Fan Shop	King of Power Saving	It doesn't do much good.	Jingdong prefecture level city in Shandong
Happy Fan Shop	King of Power Saving	It's not working.	Jingdong prefecture level city in Shandong
Happy Fan Shop	King of Power Saving	I don't know. I just used it.	Jingdong prefecture level city in Shandong
Happy Fan Shop	King of Power Saving	Use it and come back to evaluate.	Jingdong prefecture level city in Shandong
Happy Fan Shop	King of Power Saving	Two words: useless.	Jingdong prefecture level city in Shandong
Happy Fan Shop	King of Power Saving	To be used and then evaluated	Jingdong prefecture level city in Shandong
Happy Fan Shop	King of Power Saving	No shipment.	Jingdong prefecture level city in Shandong
Happy Fan Shop	King of Power Saving	Fake. Speechless.	Jingdong prefecture level city in Shandong
Happy Fan Shop	King of Power Saving	fantastic (idiom); awesome	Jingdong prefecture level city in Shandong
Happy Fan Shop	King of Power Saving	an excellent	Jingdong prefecture level city in Shandong
Happy Fan Shop	King of Power Saving	an excellent	Jingdong prefecture level city in Shandong
Happy Fan Shop	King of Power Saving	Want to buy! Recommended not to buy! No effect	Jingdong prefecture level city in Shandong
Happy Fan Shop	King of Power Saving	That's all there is to it. I can't figure out how to save electricity.	Jingdong prefecture level city in Shandong
Happy Fan Shop	King of Power Saving	It's no use.	Jingdong prefecture level city in Shandong
Happy Fan Shop	King of Power Saving	Things are more perfect than I imagined, very powerful, it is worth getting Packaging is very strong, things are well protected, it is worth the praise things are very satisfied Oh, beautiful and stylish atmosphere, customer service is super patient, service is meticulous and attentive, the big brand is not	Jingdong prefecture level city in Shandong
Happy Fan Shop	King of Power Saving	The effect is too good, the family has now several units, back to buy a number of times, the power saver received, power-saving effect is very good,, fine workmanship, long warranty, but also purify the air, before buying a used for a period of time, with the okay, and then bought a, and only now come to the	Jingdong prefecture level city in Shandong
Happy Fan Shop	King of Power Saving	Quite good, and imagine the same, casual ride are very good, satisfied! Baby received it 👌, really great ah! 🌈 quality is really nothing to say, very satisfied! 🆗 happy happy 😃 genuine, great, the price is very good, in short, quite good, will buy again a very good shopping experience, attentive service, fast logistics, distribution is also thoughtful, the attitude is also very good, value for money, ah,	Jingdong prefecture level city in Shandong
Happy Fan Shop	King of Power Saving	Power-saving effect: strong Cost-effective: quite good Appearance style: beautiful Workmanship material: meticulous Experience: looking forward to power-saving effect	Jingdong prefecture level city in Shandong
Happy Fan Shop	King of Power Saving	Cost-effective is okay, the appearance of the style of meticulous workmanship, looking forward to energy-saving effect.	Jingdong prefecture level city in Shandong
Happy Fan Shop	King of Power Saving	Very Good Very Power Saving This Power Saver is really power saving As soon as the power is turned on, the Power Saver starts the power saving function for you.	Jingdong prefecture level city in Shandong
Happy Fan Shop	King of Power Saving	Logistics is very fast, the price is favorable, friends introduced that it is very good to use, bought 2 at once, looking forward to the effect, used after the follow-up comment.	Jingdong prefecture level city in Shandong
Happy Fan Shop	King of Power Saving	It's all fake. It doesn't save any electricity.	Jingdong prefecture level city in Shandong
Happy Fan Shop	King of Power Saving	This Power Saver is so much better than the one I bought before, not only the effect is good, the quality is very good in all aspects, the customer service attitude is also very good, recommended to buy!	Jingdong prefecture level city in Shandong
Happy Fan Shop	King of Power Saving	Logistics is very fast, buy for parents, good quality, once bought 2, power-saving effect is good to buy 2 more, look forward to using the effect, satisfied will recommend to friends.	Jingdong prefecture level city in Shandong

Happy Fan Shop	King of Power Saving	With the description is the same, very high-grade, good value, to buy friends can order Oh!	Jingdong prefecture level city in Shandong
Happy Fan Shop	King of Power Saving	Not using much electricity in the winter saving over 100 per month! Ready to buy 2 more units in the summer ！！！！！ Good quality, good price, power saving effect is obvious, cost-effective, worth buying, can be recommended!	Jingdong prefecture level city in Shandong
Happy Fan Shop	King of Power Saving	Purchased a power saver received on the put into use, rent a room with many electrical appliances, power consumption is very large, we have a piece of electricity or 1.2 dollars a time, with this power saver is much better, every day can save a lot of money, very convenient and practical.	Jingdong prefecture level city in Shandong
Happy Fan Shop	King of Power Saving	The power saving effect is very good: my supermarket monthly electricity bill is more than 7000 yuan. On May 1st someone recommended buying a couple of power savers, and I bought 5 of them first. The electricity bill was about 2000 less in June. This time, I bought 5 more. The seller said 800 degrees of electricity with one. I feel it's basically enough. A total of 10 units	Jingdong prefecture level city in Shandong
Happy Fan Shop	King of Power Saving	I never thought I could get such a good baby at such an affordable price, it's very good value for money, I love it!	Jingdong prefecture level city in Shandong
Happy Fan Shop	King of Power Saving	Previously bought other brands of power savers, but found no effect at home, this time to see how the products on the Jingdong, inserted in the home socket, the LED display is blue, the night light is very nice to see, look at the instructions that have to be half a year in order to achieve the best results, try it, I believe that the quality of the Jingdong, it should not be wrong.	Jingdong prefecture level city in Shandong
Happy Fan Shop	King of Power Saving	Superb, inexpensive, saves a lot of points, been using it for a long time!	Jingdong prefecture level city in Shandong
Happy Fan Shop	King of Power Saving	There are a lot of appliances in the house, the summer air conditioning is very costly, I bought this in front of the air conditioning effect is very good, I explain that I bought three in one breath, I use more electricity a month down to 200 or so, very satisfied.	Jingdong prefecture level city in Shandong
Happy Fan Shop	King of Power Saving	A month can save more than 200, home monthly electricity bill of more than 600 dollars, inserted 3 or so, an amount of the balance of the average can save more than 200. Product is very good, has been buying this one, has given a lot of relatives recommended, the quality is also very good, before buying a look with this same, who knows to buy home has not been used for more than a week on the bad, the quality of this is really good with more than two years!	Jingdong prefecture level city in Shandong
Happy Fan Shop	King of Power Saving	Whole house power saving king, can really save electricity, friends recommended, high power saving appliances, only replacement, no repair, after-sales worry-free!	Jingdong prefecture level city in Shandong
Happy Fan Shop	King of Power Saving	Great item love it, great customer service, great shopping experience!	Jingdong prefecture level city in Shandong
Happy Fan Shop	King of Power Saving	Power saving, very good use, much cheaper than the physical store, satisfaction	Jingdong prefecture level city in Shandong
Happy Fan Shop	King of Power Saving	In use, very well packaged and fast delivery of credit!	Jingdong prefecture level city in Shandong
Happy Fan Shop	King of Power Saving	Product quality, customer service attitude is particularly good, like, praise!	Jingdong prefecture level city in Shandong
Happy Fan Shop	King of Power Saving	The logistics is very fast, the packaging is not broken, the quality is very good, I like it very much!	Jingdong prefecture level city in Shandong
Happy Fan Shop	King of Power Saving	The saver was well packaged and was as described when plugged in	Jingdong prefecture level city in Shandong
Happy Fan Shop	King of Power Saving	Energy efficient and very satisfied	Jingdong prefecture level city in Shandong
Happy Fan Shop	King of Power Saving	Great quality for the price and fast shipping.	Jingdong prefecture level city in Shandong
Happy Fan Shop	King of Power Saving	It's great, just as described. Perfect.	Jingdong prefecture level city in Shandong
Happy Fan Shop	King of Power Saving	There is no power saving effect, but rather ring up an increase in electricity costs, because this machine is on continuously all day long, uninterruptedly, its own electricity and increased costs, plus the cost of purchasing the machine, so I feel that the use of this machine instead of increasing the	Jingdong prefecture level city in Shandong
Happy Fan Shop	King of Power Saving	Recommended by the owner of the store next door, looked at his home does have to save electricity, decided to get. Indeed quite can, the voltage is much more stable, there is a reduction in electricity	Jingdong prefecture level city in Shandong
Happy Fan Shop	King of Power Saving	Goods have been received value for money, affordable and reasonable price, and the description of the same, power-saving safe and reliable!	Jingdong prefecture level city in Shandong
Happy Fan Shop	King of Power Saving	Listen to friends introduced to buy, tried to save a lot of electricity is good, quite good, worth buying.	Jingdong prefecture level city in Shandong
Happy Fan Shop	King of Power Saving	The product is easy to use, safe and environmentally friendly, only used for a short time, to the next month, after the electricity bill status display to know how the performance of the power saver efficacy.	Jingdong prefecture level city in Shandong
Happy Fan Shop	King of Power Saving	Last month's electricity bill is 178 yuan, with this power saver is 92 yuan, ready to buy two more back home, I was introduced to buy a friend, just started to not believe that I did not expect the effect!	Jingdong prefecture level city in Shandong
Happy Fan Shop	King of Power Saving	It looks nice, but after using it for a few days I can't see how it saves power?	Jingdong prefecture level city in Shandong
Happy Fan Shop	King of Power Saving	Recommended by a friend, it is genuine, as described by the merchant, cost-effective	Jingdong prefecture level city in Shandong
Happy Fan Shop	King of Power Saving	Works well, affordable and saves money.	Jingdong prefecture level city in Shandong
Happy Fan Shop	King of Power Saving	Feels good haven't compared it yet, the air conditioning has been on a lot in the past two months	Jingdong prefecture level city in Shandong
Happy Fan Shop	King of Power Saving	Saving about 5 kWh per day is a good thing	Jingdong prefecture level city in Shandong
Happy Fan Shop	King of Power Saving	Not bad, affordable, best value for money	Jingdong prefecture level city in Shandong
Happy Fan Shop	King of Power Saving	The goods are genuine and the logistics are faster.	Jingdong prefecture level city in Shandong
Happy Fan Shop	King of Power Saving	Goods have been received today, has been plugged into the outlet, looking forward to saving electricity, after a period of time to see how the effect in the follow-up comment!	Jingdong prefecture level city in Shandong
Happy Fan Shop	King of Power Saving	I just got it home and haven't used it for long, so I'll use it for a couple months and see what happens. Seeing that most people have good reviews, it should be fine.	Jingdong prefecture level city in Shandong
Happy Fan Shop	King of Power Saving	I'm looking forward to the results, so I'll give you a good review out of habit! Follow up review after a while	Jingdong prefecture level city in Shandong
Happy Fan Shop	King of Power Saving	Use a dish first to know if it will help save energy	Jingdong prefecture level city in Shandong
Happy Fan Shop	King of Power Saving	Whether it saves electricity or not, we don't know yet.	Jingdong prefecture level city in Shandong
Happy Fan Shop	King of Power Saving	Goods have been received, use a period of time to know how oh. The courier did not deliver to the door ah! Sent to the station ah! The heat is inconvenient ah!	Jingdong prefecture level city in Shandong
Happy Fan Shop	King of Power Saving	A little effect, before an hour a power, now an hour 0.76 degrees, a dollar a power = save 24 cents, although not a lot, first use it!	Jingdong prefecture level city in Shandong
Happy Fan Shop	King of Power Saving	Just received the goods, use a month to evaluate	Jingdong prefecture level city in Shandong
Happy Fan Shop	King of Power Saving	Home every month with a lot of electricity, inadvertently found this good thing online that can save the beginning of a skeptical attitude to consult customer service, customer service replied very professional, said 120 days can be refunded that first try it!	Jingdong prefecture level city in Shandong
Happy Fan Shop	King of Power Saving	After receiving the goods this afternoon, immediately installed in the bathroom Smith energy storage electric water heater on the power outlet (instead of connecting with a plug board), when used, found that the "Power Saving King" LCD display, is the value of the voltage, not the store's video said so	Jingdong prefecture level city in Shandong
Happy Fan Shop	King of Power Saving	Has made many days, did not feel the power saving, the effect is not obvious.	Jingdong prefecture level city in Shandong
Happy Fan Shop	King of Power Saving	Energy savers do not save energy at all, but rather consume a lot of electricity.	Jingdong prefecture level city in Shandong
Happy Fan Shop	King of Power Saving	It's useless. It's a waste of money.	Jingdong prefecture level city in Shandong

Happy Fan Shop	King of Power Saving	Last month saved more than 500, which came back to buy, the store bought three, and then buy 1 home use, my family is dry beauty salon, more instruments, especially electricity, a month almost 1000. or so, with this did not expect to save so much, great, five-star praise!	Jingdong prefecture level city in Shandong
Happy Fan Shop	King of Power Saving	It rained a couple days ago and I just went to get the item back today , not bad, hey I looked at the meter and that red display light takes several seconds to jump, did not make a mistake buying it. After a period of time to evaluate.	Jingdong prefecture level city in Shandong
Happy Fan Shop	King of Power Saving	Without further ado, the electricity bill came down, this month less 102 yuan, has been repurchased, I also recommended to my friends, all said that there are effects, the product is okay, the effect is quite	Jingdong prefecture level city in Shandong
Happy Fan Shop	King of Power Saving	After buying and using two months really see the effect, so only to evaluate! Originally held the mindset of trying, I did not expect really effective, worth having!	Jingdong prefecture level city in Shandong
Happy Fan Shop	King of Power Saving	In the use of this energy saver before the monthly electricity bill is more than 150 yuan or so, now save thirty percent, every day to work, six or seven o'clock in the afternoon to go home, I do not know why the electricity bill is so expensive, energy-saving appliances really have the effect of recommending	Jingdong prefecture level city in Shandong
Happy Fan Shop	King of Power Saving	It saves about 100 bucks a month. A friend recommended it to me, and he's been using it for a while, and the effect is so obvious that I bought one too.	Jingdong prefecture level city in Shandong
Happy Fan Shop	King of Power Saving	My dad asked me to buy one, but after I paid the bill, I asked and he said it was a lot less this month, so he asked me to buy another one.	Jingdong prefecture level city in Shandong
Happy Fan Shop	King of Power Saving	The specific effect will have to be tested for at least two months to compare whether there is a power saving effect or not.	Jingdong prefecture level city in Shandong
Happy Fan Shop	King of Power Saving	'The goods have been received, immediately go to use, please ask the boss you instructions do not allow to withdraw, there are two fuses installed there!	Jingdong prefecture level city in Shandong
Happy Fan Shop	King of Power Saving	First praise is using, after using if good then follow up, now because I have not yet seen the results	Jingdong prefecture level city in Shandong
Happy Fan Shop	King of Power Saving	I've just been using it, and I haven't seen if it has a power-saving effect yet, so I'll follow up after 10 days and half a month.	Jingdong prefecture level city in Shandong
Happy Fan Shop	King of Power Saving	Just received the intelligent power saving meter, immediately plugged in to use, see it is not bad, and order to sell the second time	Jingdong prefecture level city in Shandong
Happy Fan Shop	King of Power Saving	This power saver is a bit effective, used for half a month, the effect is still visible.	Jingdong prefecture level city in Shandong
Happy Fan Shop	King of Power Saving	I received the baby and it looks great, I'm trying it out now and will keep an eye on it afterward.	Jingdong prefecture level city in Shandong
Happy Fan Shop	King of Power Saving	I've just received it, but I'm still trying to see how it works, so I'll come back in a little while.	Jingdong prefecture level city in Shandong
Happy Fan Shop	King of Power Saving	Just used it wait to use it for a while and then comment thanks, all first five stars.	Jingdong prefecture level city in Shandong
Happy Fan Shop	King of Power Saving	Very trustworthy brand, quality is guaranteed, the price is still affordable, quite satisfied!	Jingdong prefecture level city in Shandong
Happy Fan Shop	King of Power Saving	Things received, I hope that the platform of Jingdong do not sell fake goods are available	Jingdong prefecture level city in Shandong
Happy Fan Shop	King of Power Saving	Energy saver received, the use of the effect is good and the business description of the same	Jingdong prefecture level city in Shandong
Happy Fan Shop	King of Power Saving	The surface looks beautiful, the actual effect is not known, after using additional evaluation.	Jingdong prefecture level city in Shandong
Happy Fan Shop	King of Power Saving	Tried it for a few months, it really saves electricity, and I bought it for a friend.	Jingdong prefecture level city in Shandong
Happy Fan Shop	King of Power Saving	It didn't work and wanted to return it, it really didn't work at all.	Jingdong prefecture level city in Shandong
Happy Fan Shop	King of Power Saving	Just used it, don't know how effective it is, looking forward to usefulness	Jingdong prefecture level city in Shandong
Happy Fan Shop	King of Power Saving	After two months before the effect I do not know if it is true!	Jingdong prefecture level city in Shandong
Happy Fan Shop	King of Power Saving	So fast! Ordered yesterday and received today 👍👍👍👍👍👍👍👍👍 🌹🌹🌹🌹🌹🌹🌹🌹🌹	Jingdong prefecture level city in Shandong
Happy Fan Shop	King of Power Saving	I've started using it. We'll see how it works.	Jingdong prefecture level city in Shandong
Happy Fan Shop	King of Power Saving	I just received the goods, I won't know the effect until after two.	Jingdong prefecture level city in Shandong
Happy Fan Shop	King of Power Saving	Just received the goods, the specific effect will be evaluated later	Jingdong prefecture level city in Shandong
Happy Fan Shop	King of Power Saving	I've received the goods, I don't know if it's any good yet	Jingdong prefecture level city in Shandong
Happy Fan Shop	King of Power Saving	Try it for a month and then come back with a concrete evaluation	Jingdong prefecture level city in Shandong
Happy Fan Shop	King of Power Saving	In use, after two months to see the effect of power saving	Jingdong prefecture level city in Shandong
Happy Fan Shop	King of Power Saving	Power Saver. It's authentic. It's black. It works.	Jingdong prefecture level city in Shandong
Happy Fan Shop	King of Power Saving	I've been using it for a week now and it's not working.	Jingdong prefecture level city in Shandong
Happy Fan Shop	King of Power Saving	I'm just using it. I don't know how it's going to work.	Jingdong prefecture level city in Shandong
Happy Fan Shop	King of Power Saving	Products are better looking out for power savings	Jingdong prefecture level city in Shandong
Happy Fan Shop	King of Power Saving	I received it and I've kept it on. I hope it works.	Jingdong prefecture level city in Shandong
Happy Fan Shop	King of Power Saving	It's really good! Worth recommending to buy!	Jingdong prefecture level city in Shandong
Happy Fan Shop	King of Power Saving	Try it for a few days and see if it doesn't work, then return it.	Jingdong prefecture level city in Shandong
Happy Fan Shop	King of Power Saving	It's in use and I hope to be pleasantly surprised.	Jingdong prefecture level city in Shandong
Happy Fan Shop	King of Power Saving	We'll see how it works when we use it	Jingdong prefecture level city in Shandong
Happy Fan Shop	King of Power Saving	Let's see if it works.	Jingdong prefecture level city in Shandong
Happy Fan Shop	King of Power Saving	I'm using it and will follow up with a review	Jingdong prefecture level city in Shandong
Happy Fan Shop	King of Power Saving	It really works. It saves energy.	Jingdong prefecture level city in Shandong
Happy Fan Shop	King of Power Saving	It does have some power savings!	Jingdong prefecture level city in Shandong
Happy Fan Shop	King of Power Saving	Jingdong shopping speed is convenient and timely	Jingdong prefecture level city in Shandong
Happy Fan Shop	King of Power Saving	Second time I've bought it. Everything's fine.	Jingdong prefecture level city in Shandong
Happy Fan Shop	King of Power Saving	I don't know if it works yet.	Jingdong prefecture level city in Shandong
Happy Fan Shop	King of Power Saving	Not used, don't know how well it works.	Jingdong prefecture level city in Shandong
Happy Fan Shop	King of Power Saving	No visible results so far	Jingdong prefecture level city in Shandong
Happy Fan Shop	King of Power Saving	It doesn't feel like it's working.	Jingdong prefecture level city in Shandong

Happy Fan Shop	King of Power Saving	Where's the effect? Where are the power savings?	Jingdong prefecture level city in Shandong
Happy Fan Shop	King of Power Saving	Let's see how it works in practice!	Jingdong prefecture level city in Shandong
Happy Fan Shop	King of Power Saving	I'll wait until I use it to give you a good review	Jingdong prefecture level city in Shandong
Happy Fan Shop	King of Power Saving	I'll follow up after I've used it	Jingdong prefecture level city in Shandong
Happy Fan Shop	King of Power Saving	Well, that's good for saving power.	Jingdong prefecture level city in Shandong
Happy Fan Shop	King of Power Saving	Received. Used in the know.	Jingdong prefecture level city in Shandong
Happy Fan Shop	King of Power Saving	It doesn't do much good.	Jingdong prefecture level city in Shandong
Happy Fan Shop	King of Power Saving	It's not working.	Jingdong prefecture level city in Shandong
Happy Fan Shop	King of Power Saving	I don't know. I just used it.	Jingdong prefecture level city in Shandong
Happy Fan Shop	King of Power Saving	Use it and come back to evaluate.	Jingdong prefecture level city in Shandong
Happy Fan Shop	King of Power Saving	Two words: useless.	Jingdong prefecture level city in Shandong
Happy Fan Shop	King of Power Saving	To be used and then evaluated	Jingdong prefecture level city in Shandong
Happy Fan Shop	King of Power Saving	No shipment.	Jingdong prefecture level city in Shandong
Happy Fan Shop	King of Power Saving	Fake. Speechless.	Jingdong prefecture level city in Shandong
Happy Fan Shop	King of Power Saving	fantastic (idiom); awesome	Jingdong prefecture level city in Shandong
Happy Fan Shop	King of Power Saving	an excellent	Jingdong prefecture level city in Shandong
Happy Fan Shop	King of Power Saving	an excellent	Jingdong prefecture level city in Shandong
Happy Fan Shop	King of Power Saving	Want to buy! Recommended not to buy! No effect	Jingdong prefecture level city in Shandong
Happy Fan Shop	King of Power Saving	That's all there is to it. I can't figure out how to save electricity.	Jingdong prefecture level city in Shandong
Happy Fan Shop	King of Power Saving	It's no use.	Jingdong prefecture level city in Shandong
Happy Fan Shop	King of Power Saving	Things are more perfect than I imagined, very powerful, it is worth getting Packaging is very strong, things are well protected, it is worth the praise things are very satisfied Oh, beautiful and stylish atmosphere, customer service is super patient, service is meticulous and attentive, the big brand is not	Jingdong prefecture level city in Shandong
Happy Fan Shop	King of Power Saving	The effect is too good, the family has now several units, back to buy a number of times, the power saver received, power-saving effect is very good,, fine workmanship, long warranty, but also purify the air, before buying a used for a period of time, with the okay, and then bought a, and only now come to the	Jingdong prefecture level city in Shandong
Happy Fan Shop	King of Power Saving	Quite good, and imagine the same, casual ride are very good, satisfied! Baby received it 👌, really great ah! 🌈 quality is really nothing to say, very satisfied! 🆗 happy happy 😃 genuine, great, the price is very good, in short, quite good, will buy again a very good shopping experience, attentive service, fast logistics, distribution is also thoughtful, the attitude is also very good, value for money, ah,	Jingdong prefecture level city in Shandong
Happy Fan Shop	King of Power Saving	Power-saving effect: strong Cost-effective: quite good Appearance style: beautiful Workmanship material: meticulous Experience: looking forward to power-saving effect	Jingdong prefecture level city in Shandong
Happy Fan Shop	King of Power Saving	Cost-effective is okay, the appearance of the style of meticulous workmanship, looking forward to energy-saving effect.	Jingdong prefecture level city in Shandong
Happy Fan Shop	King of Power Saving	Very Good Very Power Saving This Power Saver is really power saving As soon as the power is turned on, the Power Saver starts the power saving function for you.	Jingdong prefecture level city in Shandong
Happy Fan Shop	King of Power Saving	Logistics is very fast, the price is favorable, friends introduced that it is very good to use, bought 2 at once, looking forward to the effect, used after the follow-up comment.	Jingdong prefecture level city in Shandong
Happy Fan Shop	King of Power Saving	It's all fake. It doesn't save any electricity.	Jingdong prefecture level city in Shandong
Happy Fan Shop	King of Power Saving	This Power Saver is so much better than the one I bought before, not only the effect is good, the quality is very good in all aspects, the customer service attitude is also very good, recommended to buy!	Jingdong prefecture level city in Shandong
Happy Fan Shop	King of Power Saving	Logistics is very fast, buy for parents, good quality, once bought 2, power-saving effect is good to buy 2 more, look forward to using the effect, satisfied will recommend to friends.	Jingdong prefecture level city in Shandong
Happy Fan Shop	King of Power Saving	With the description is the same, very high-grade, good value, to buy friends can order Oh!	Jingdong prefecture level city in Shandong
Happy Fan Shop	King of Power Saving	Not using much electricity in the winter saving over 100 per month! Ready to buy 2 more units in the summer ！！！！！ Good quality, good price, power saving effect is obvious, cost-effective, worth buying, can be recommended!	Jingdong prefecture level city in Shandong
Happy Fan Shop	King of Power Saving	Purchased a power saver received on the put into use, rent a room with many electrical appliances, power consumption is very large, we have a piece of electricity or 1.2 dollars a time, with this power saver is much better, every day can save a lot of money, very convenient and practical.	Jingdong prefecture level city in Shandong
Happy Fan Shop	King of Power Saving	The power saving effect is very good: my supermarket monthly electricity bill is more than 7000 yuan. On May 1st someone recommended buying a couple of power savers, and I bought 5 of them first. The electricity bill was about 2000 less in June. This time, I bought 5 more. The seller said 800 degrees of electricity with one. I feel it's basically enough. A total of 10 units	Jingdong prefecture level city in Shandong
Happy Fan Shop	King of Power Saving	I never thought I could get such a good baby at such an affordable price, it's very good value for money, I love it!	Jingdong prefecture level city in Shandong
Happy Fan Shop	King of Power Saving	Previously bought other brands of power savers, but found no effect at home, this time to see how the products on the Jingdong, inserted in the home socket, the LED display is blue, the night light is very nice to see, look at the instructions that have to be half a year in order to achieve the best results, try it, I believe that the quality of the Jingdong, it should not be wrong.	Jingdong prefecture level city in Shandong
Happy Fan Shop	King of Power Saving	Superb, inexpensive, saves a lot of points, been using it for a long time!	Jingdong prefecture level city in Shandong
Happy Fan Shop	King of Power Saving	There are a lot of appliances in the house, the summer air conditioning is very costly, I bought this in front of the air conditioning effect is very good, I explain that I bought three in one breath, I use more electricity a month down to 200 or so, very satisfied.	Jingdong prefecture level city in Shandong
Happy Fan Shop	King of Power Saving	A month can save more than 200, home monthly electricity bill of more than 600 dollars, inserted 3 or so, an amount of the balance of the average can save more than 200. Product is very good, has been buying this one, has given a lot of relatives recommended, the quality is also very good, before buying a look with this same, who knows to buy home has not been used for more than a week on the bad, the quality of this is really good with more than two years!	Jingdong prefecture level city in Shandong
Happy Fan Shop	King of Power Saving	Whole house power saving king, can really save electricity, friends recommended, high power saving appliances, only replacement, no repair, after-sales worry-free!	Jingdong prefecture level city in Shandong
Happy Fan Shop	King of Power Saving	Great item love it, great customer service, great shopping experience!	Jingdong prefecture level city in Shandong
Happy Fan Shop	King of Power Saving	Power saving, very good use, much cheaper than the physical store, satisfaction	Jingdong prefecture level city in Shandong
Happy Fan Shop	King of Power Saving	In use, very well packaged and fast delivery of credit!	Jingdong prefecture level city in Shandong
Happy Fan Shop	King of Power Saving	Product quality, customer service attitude is particularly good, like, praise!	Jingdong prefecture level city in Shandong

Happy Fan Shop	King of Power Saving	The logistics is very fast, the packaging is not broken, the quality is very good, I like it very much!	Jingdong prefecture level city in Shandong
Happy Fan Shop	King of Power Saving	The saver was well packaged and was as described when plugged in	Jingdong prefecture level city in Shandong
Happy Fan Shop	King of Power Saving	Energy efficient and very satisfied	Jingdong prefecture level city in Shandong
Happy Fan Shop	King of Power Saving	Great quality for the price and fast shipping.	Jingdong prefecture level city in Shandong
Happy Fan Shop	King of Power Saving	It's great, just as described. Perfect.	Jingdong prefecture level city in Shandong
Happy Fan Shop	King of Power Saving	There is no power saving effect, but rather ring up an increase in electricity costs, because this machine is on continuously all day long, uninterruptedly, its own electricity and increased costs, plus the cost of purchasing the machine, so I feel that the use of this machine instead of increasing the	Jingdong prefecture level city in Shandong
Happy Fan Shop	King of Power Saving	Recommended by the owner of the store next door, looked at his home does have to save electricity, decided to get. Indeed quite can, the voltage is much more stable, there is a reduction in electricity	Jingdong prefecture level city in Shandong
Happy Fan Shop	King of Power Saving	Goods have been received value for money, affordable and reasonable price, and the description of the same, power-saving safe and reliable!	Jingdong prefecture level city in Shandong
Happy Fan Shop	King of Power Saving	Listen to friends introduced to buy, tried to save a lot of electricity is good, quite good, worth buying.	Jingdong prefecture level city in Shandong
Happy Fan Shop	King of Power Saving	The product is easy to use, safe and environmentally friendly, only used for a short time, to the next month, after the electricity bill status display to know how the performance of the power saver efficacy.	Jingdong prefecture level city in Shandong
Happy Fan Shop	King of Power Saving	Last month's electricity bill is 178 yuan, with this power saver is 92 yuan, ready to buy two more back home, I was introduced to buy a friend, just started to not believe that I did not expect the effect!	Jingdong prefecture level city in Shandong
Happy Fan Shop	King of Power Saving	It looks nice, but after using it for a few days I can't see how it saves power?	Jingdong prefecture level city in Shandong
Happy Fan Shop	King of Power Saving	Recommended by a friend, it is genuine, as described by the merchant, cost-effective	Jingdong prefecture level city in Shandong
Happy Fan Shop	King of Power Saving	Works well, affordable and saves money.	Jingdong prefecture level city in Shandong
Happy Fan Shop	King of Power Saving	Feels good haven't compared it yet, the air conditioning has been on a lot in the past two months	Jingdong prefecture level city in Shandong
Happy Fan Shop	King of Power Saving	Saving about 5 kWh per day is a good thing	Jingdong prefecture level city in Shandong
Happy Fan Shop	King of Power Saving	Not bad, affordable, best value for money	Jingdong prefecture level city in Shandong
Happy Fan Shop	King of Power Saving	The goods are genuine and the logistics are faster.	Jingdong prefecture level city in Shandong
Happy Fan Shop	King of Power Saving	Goods have been received today, has been plugged into the outlet, looking forward to saving electricity, after a period of time to see how the effect in the follow-up comment!	Jingdong prefecture level city in Shandong
Happy Fan Shop	King of Power Saving	I just got it home and haven't used it for long, so I'll use it for a couple months and see what happens. Seeing that most people have good reviews, it should be fine.	Jingdong prefecture level city in Shandong
Happy Fan Shop	King of Power Saving	I'm looking forward to the results, so I'll give you a good review out of habit! Follow up review after a while	Jingdong prefecture level city in Shandong
Happy Fan Shop	King of Power Saving	Use a dish first to know if it will help save energy	Jingdong prefecture level city in Shandong
Happy Fan Shop	King of Power Saving	Whether it saves electricity or not, we don't know yet.	Jingdong prefecture level city in Shandong
Happy Fan Shop	King of Power Saving	Goods have been received, use a period of time to know how oh. The courier did not deliver to the door ah! Sent to the station ah! The heat is inconvenient ah!	Jingdong prefecture level city in Shandong
Happy Fan Shop	King of Power Saving	A little effect, before an hour a power, now an hour 0.76 degrees, a dollar a power = save 24 cents, although not a lot, first use it!	Jingdong prefecture level city in Shandong
Happy Fan Shop	King of Power Saving	Just received the goods, use a month to evaluate	Jingdong prefecture level city in Shandong
Happy Fan Shop	King of Power Saving	Home every month with a lot of electricity, inadvertently found this good thing online that can save the beginning of a skeptical attitude to consult customer service, customer service replied very professional, said 120 days can be refunded that first try it!	Jingdong prefecture level city in Shandong
Happy Fan Shop	King of Power Saving	After receiving the goods this afternoon, immediately installed in the bathroom Smith energy storage electric water heater on the power outlet (instead of connecting with a plug board), when used, found that the "Power Saving King" LCD display, is the value of the voltage, not the store's video said so	Jingdong prefecture level city in Shandong
Happy Fan Shop	King of Power Saving	Has made many days, did not feel the power saving, the effect is not obvious.	Jingdong prefecture level city in Shandong
Happy Fan Shop	King of Power Saving	Energy savers do not save energy at all, but rather consume a lot of electricity.	Jingdong prefecture level city in Shandong
Happy Fan Shop	King of Power Saving	It's useless. It's a waste of money.	Jingdong prefecture level city in Shandong
Happy Fan Shop	King of Power Saving	Last month saved more than 500, which came back to buy, the store bought three, and then buy 1 home use, my family is dry beauty salon, more instruments, especially electricity, a month almost 1000. or so, with this did not expect to save so much, great, five-star praise!	Jingdong prefecture level city in Shandong
Happy Fan Shop	King of Power Saving	It rained a couple days ago and I just went to get the item back today , not bad, hey I looked at the meter and that red display light takes several seconds to jump, did not make a mistake buying it. After a period of time to evaluate.	Jingdong prefecture level city in Shandong
Happy Fan Shop	King of Power Saving	Without further ado, the electricity bill came down, this month less 102 yuan, has been repurchased, I also recommended to my friends, all said that there are effects, the product is okay, the effect is quite	Jingdong prefecture level city in Shandong
Happy Fan Shop	King of Power Saving	After buying and using two months really see the effect, so only to evaluate! Originally held the mindset of trying, I did not expect really effective, worth having!	Jingdong prefecture level city in Shandong
Happy Fan Shop	King of Power Saving	In the use of this energy saver before the monthly electricity bill is more than 150 yuan or so, now save thirty percent, every day to work, six or seven o'clock in the afternoon to go home, I do not know why the electricity bill is so expensive, energy-saving appliances really have the effect of recommending	Jingdong prefecture level city in Shandong
Happy Fan Shop	King of Power Saving	It saves about 100 bucks a month. A friend recommended it to me, and he's been using it for a while, and the effect is so obvious that I bought one too.	Jingdong prefecture level city in Shandong
Happy Fan Shop	King of Power Saving	My dad asked me to buy one, but after I paid the bill, I asked and he said it was a lot less this month, so he asked me to buy another one.	Jingdong prefecture level city in Shandong
Happy Fan Shop	King of Power Saving	The specific effect will have to be tested for at least two months to compare whether there is a power saving effect or not.	Jingdong prefecture level city in Shandong
Happy Fan Shop	King of Power Saving	'The goods have been received, immediately go to use, please ask the boss you instructions do not allow to withdraw, there are two fuses installed there!	Jingdong prefecture level city in Shandong
Happy Fan Shop	King of Power Saving	First praise is using, after using if good then follow up, now because I have not yet seen the results	Jingdong prefecture level city in Shandong
Happy Fan Shop	King of Power Saving	I've just been using it, and I haven't seen if it has a power-saving effect yet, so I'll follow up after 10 days and half a month.	Jingdong prefecture level city in Shandong
Happy Fan Shop	King of Power Saving	Just received the intelligent power saving meter, immediately plugged in to use, see it is not bad, and order to sell the second time	Jingdong prefecture level city in Shandong
Happy Fan Shop	King of Power Saving	This power saver is a bit effective, used for half a month, the effect is still visible.	Jingdong prefecture level city in Shandong
Happy Fan Shop	King of Power Saving	I received the baby and it looks great, I'm trying it out now and will keep an eye on it afterward.	Jingdong prefecture level city in Shandong
Happy Fan Shop	King of Power Saving	I've just received it, but I'm still trying to see how it works, so I'll come back in a little while.	Jingdong prefecture level city in Shandong
Happy Fan Shop	King of Power Saving	Just used it wait to use it for a while and then comment thanks, all first five stars.	Jingdong prefecture level city in Shandong
Happy Fan Shop	King of Power Saving	Very trustworthy brand, quality is guaranteed, the price is still affordable, quite satisfied!	Jingdong prefecture level city in Shandong

Happy Fan Shop	King of Power Saving	Things received, I hope that the platform of Jingdong do not sell fake goods are available	Jingdong prefecture level city in Shandong
Happy Fan Shop	King of Power Saving	Energy saver received, the use of the effect is good and the business description of the same	Jingdong prefecture level city in Shandong
Happy Fan Shop	King of Power Saving	The surface looks beautiful, the actual effect is not known, after using additional evaluation.	Jingdong prefecture level city in Shandong
Happy Fan Shop	King of Power Saving	Tried it for a few months, it really saves electricity, and I bought it for a friend.	Jingdong prefecture level city in Shandong
Happy Fan Shop	King of Power Saving	It didn't work and wanted to return it, it really didn't work at all.	Jingdong prefecture level city in Shandong
Happy Fan Shop	King of Power Saving	Just used it, don't know how effective it is, looking forward to usefulness	Jingdong prefecture level city in Shandong
Happy Fan Shop	King of Power Saving	After two months before the effect I do not know if it is true!	Jingdong prefecture level city in Shandong
Happy Fan Shop	King of Power Saving	So fast! Ordered yesterday and received today 👍👍👍👍👍👍👍👍👍 🌹🌹🌹🌹🌹🌹🌹🌹🌹	Jingdong prefecture level city in Shandong
Happy Fan Shop	King of Power Saving	I've started using it. We'll see how it works.	Jingdong prefecture level city in Shandong
Happy Fan Shop	King of Power Saving	I just received the goods, I won't know the effect until after two.	Jingdong prefecture level city in Shandong
Happy Fan Shop	King of Power Saving	Just received the goods, the specific effect will be evaluated later	Jingdong prefecture level city in Shandong
Happy Fan Shop	King of Power Saving	I've received the goods, I don't know if it's any good yet	Jingdong prefecture level city in Shandong
Happy Fan Shop	King of Power Saving	Try it for a month and then come back with a concrete evaluation	Jingdong prefecture level city in Shandong
Happy Fan Shop	King of Power Saving	In use, after two months to see the effect of power saving	Jingdong prefecture level city in Shandong
Happy Fan Shop	King of Power Saving	Power Saver. It's authentic. It's black. It works.	Jingdong prefecture level city in Shandong
Happy Fan Shop	King of Power Saving	I've been using it for a week now and it's not working.	Jingdong prefecture level city in Shandong
Happy Fan Shop	King of Power Saving	I'm just using it. I don't know how it's going to work.	Jingdong prefecture level city in Shandong
Happy Fan Shop	King of Power Saving	Products are better looking out for power savings	Jingdong prefecture level city in Shandong
Happy Fan Shop	King of Power Saving	I received it and I've kept it on. I hope it works.	Jingdong prefecture level city in Shandong
Happy Fan Shop	King of Power Saving	It's really good! Worth recommending to buy!	Jingdong prefecture level city in Shandong
Happy Fan Shop	King of Power Saving	Try it for a few days and see if it doesn't work, then return it.	Jingdong prefecture level city in Shandong
Happy Fan Shop	King of Power Saving	It's in use and I hope to be pleasantly surprised.	Jingdong prefecture level city in Shandong
Happy Fan Shop	King of Power Saving	We'll see how it works when we use it	Jingdong prefecture level city in Shandong
Happy Fan Shop	King of Power Saving	Let's see if it works.	Jingdong prefecture level city in Shandong
Happy Fan Shop	King of Power Saving	I'm using it and will follow up with a review	Jingdong prefecture level city in Shandong
Happy Fan Shop	King of Power Saving	It really works. It saves energy.	Jingdong prefecture level city in Shandong
Happy Fan Shop	King of Power Saving	It does have some power savings!	Jingdong prefecture level city in Shandong
Happy Fan Shop	King of Power Saving	Jingdong shopping speed is convenient and timely	Jingdong prefecture level city in Shandong
Happy Fan Shop	King of Power Saving	Second time I've bought it. Everything's fine.	Jingdong prefecture level city in Shandong
Happy Fan Shop	King of Power Saving	I don't know if it works yet.	Jingdong prefecture level city in Shandong
Happy Fan Shop	King of Power Saving	Not used, don't know how well it works.	Jingdong prefecture level city in Shandong
Happy Fan Shop	King of Power Saving	No visible results so far	Jingdong prefecture level city in Shandong
Happy Fan Shop	King of Power Saving	It doesn't feel like it's working.	Jingdong prefecture level city in Shandong
Happy Fan Shop	King of Power Saving	Where's the effect? Where are the power savings?	Jingdong prefecture level city in Shandong
Happy Fan Shop	King of Power Saving	Let's see how it works in practice!	Jingdong prefecture level city in Shandong
Happy Fan Shop	King of Power Saving	I'll wait until I use it to give you a good review	Jingdong prefecture level city in Shandong
Happy Fan Shop	King of Power Saving	I'll follow up after I've used it	Jingdong prefecture level city in Shandong
Happy Fan Shop	King of Power Saving	Well, that's good for saving power.	Jingdong prefecture level city in Shandong
Happy Fan Shop	King of Power Saving	Received. Used in the know.	Jingdong prefecture level city in Shandong
Happy Fan Shop	King of Power Saving	It doesn't do much good.	Jingdong prefecture level city in Shandong
Happy Fan Shop	King of Power Saving	It's not working.	Jingdong prefecture level city in Shandong
Happy Fan Shop	King of Power Saving	I don't know. I just used it.	Jingdong prefecture level city in Shandong
Happy Fan Shop	King of Power Saving	Use it and come back to evaluate.	Jingdong prefecture level city in Shandong
Happy Fan Shop	King of Power Saving	Two words: useless.	Jingdong prefecture level city in Shandong
Happy Fan Shop	King of Power Saving	To be used and then evaluated	Jingdong prefecture level city in Shandong
Happy Fan Shop	King of Power Saving	No shipment.	Jingdong prefecture level city in Shandong
Happy Fan Shop	King of Power Saving	Fake. Speechless.	Jingdong prefecture level city in Shandong
Happy Fan Shop	King of Power Saving	fantastic (idiom); awesome	Jingdong prefecture level city in Shandong
Happy Fan Shop	King of Power Saving	an excellent	Jingdong prefecture level city in Shandong
Happy Fan Shop	King of Power Saving	an excellent	Jingdong prefecture level city in Shandong
Happy Fan Shop	King of Power Saving	Want to buy! Recommended not to buy! No effect	Jingdong prefecture level city in Shandong

Happy Fan Shop	King of Power Saving	That's all there is to it. I can't figure out how to save electricity.	Jingdong prefecture level city in Shandong
Happy Fan Shop	King of Power Saving	It's no use.	Jingdong prefecture level city in Shandong
Happy Fan Shop	King of Power Saving	Things are more perfect than I imagined, very powerful, it is worth getting Packaging is very strong, things are well protected, it is worth the praise things are very satisfied Oh, beautiful and stylish atmosphere, customer service is super patient, service is meticulous and attentive, the big brand is not	Jingdong prefecture level city in Shandong
Happy Fan Shop	King of Power Saving	The effect is too good, the family has now several units, back to buy a number of times, the power saver received, power-saving effect is very good,, fine workmanship, long warranty, but also purify the air, before buying a used for a period of time, with the okay, and then bought a, and only now come to the	Jingdong prefecture level city in Shandong
Happy Fan Shop	King of Power Saving	Quite good, and imagine the same, casual ride are very good, satisfied! Baby received it 👌, really great ah! 🌈 quality is really nothing to say, very satisfied! 🆗 happy happy 😃 genuine, great, the price is very good, in short, quite good, will buy again a very good shopping experience, attentive service, fast logistics, distribution is also thoughtful, the attitude is also very good, value for money, ah,	Jingdong prefecture level city in Shandong
Happy Fan Shop	King of Power Saving	Power-saving effect: strong Cost-effective: quite good Appearance style: beautiful Workmanship material: meticulous Experience: looking forward to power-saving effect	Jingdong prefecture level city in Shandong
Happy Fan Shop	King of Power Saving	Cost-effective is okay, the appearance of the style of meticulous workmanship, looking forward to energy-saving effect.	Jingdong prefecture level city in Shandong
Happy Fan Shop	King of Power Saving	Very Good Very Power Saving This Power Saver is really power saving As soon as the power is turned on, the Power Saver starts the power saving function for you.	Jingdong prefecture level city in Shandong
Happy Fan Shop	King of Power Saving	Logistics is very fast, the price is favorable, friends introduced that it is very good to use, bought 2 at once, looking forward to the effect, used after the follow-up comment.	Jingdong prefecture level city in Shandong
Happy Fan Shop	King of Power Saving	It's all fake. It doesn't save any electricity.	Jingdong prefecture level city in Shandong
Happy Fan Shop	King of Power Saving	This Power Saver is so much better than the one I bought before, not only the effect is good, the quality is very good in all aspects, the customer service attitude is also very good, recommended to buy!	Jingdong prefecture level city in Shandong
Happy Fan Shop	King of Power Saving	Logistics is very fast, buy for parents, good quality, once bought 2, power-saving effect is good to buy 2 more, look forward to using the effect, satisfied will recommend to friends.	Jingdong prefecture level city in Shandong
Happy Fan Shop	King of Power Saving	With the description is the same, very high-grade, good value, to buy friends can order Oh!	Jingdong prefecture level city in Shandong
Happy Fan Shop	King of Power Saving	Not using much electricity in the winter saving over 100 per month! Ready to buy 2 more units in the summer ！！！！！ Good quality, good price, power saving effect is obvious, cost-effective, worth buying, can be recommended!	Jingdong prefecture level city in Shandong
Happy Fan Shop	King of Power Saving	Purchased a power saver received on the put into use, rent a room with many electrical appliances, power consumption is very large, we have a piece of electricity or 1.2 dollars a time, with this power saver is much better, every day can save a lot of money, very convenient and practical.	Jingdong prefecture level city in Shandong
Happy Fan Shop	King of Power Saving	The power saving effect is very good: my supermarket monthly electricity bill is more than 7000 yuan. On May 1st someone recommended buying a couple of power savers, and I bought 5 of them first. The electricity bill was about 2000 less in June. This time, I bought 5 more. The seller said 800 degrees of electricity with one. I feel it's basically enough. A total of 10 units	Jingdong prefecture level city in Shandong
Happy Fan Shop	King of Power Saving	I never thought I could get such a good baby at such an affordable price, it's very good value for money, I love it!	Jingdong prefecture level city in Shandong
Happy Fan Shop	King of Power Saving	Previously bought other brands of power savers, but found no effect at home, this time to see how the products on the Jingdong, inserted in the home socket, the LED display is blue, the night light is very nice to see, look at the instructions that have to be half a year in order to achieve the best results, try it, I believe that the quality of the Jingdong, it should not be wrong.	Jingdong prefecture level city in Shandong
Happy Fan Shop	King of Power Saving	Superb, inexpensive, saves a lot of points, been using it for a long time!	Jingdong prefecture level city in Shandong
Happy Fan Shop	King of Power Saving	There are a lot of appliances in the house, the summer air conditioning is very costly, I bought this in front of the air conditioning effect is very good, I explain that I bought three in one breath, I use more electricity a month down to 200 or so, very satisfied.	Jingdong prefecture level city in Shandong
Happy Fan Shop	King of Power Saving	A month can save more than 200, home monthly electricity bill of more than 600 dollars, inserted 3 or so, an amount of the balance of the average can save more than 200. Product is very good, has been buying this one, has given a lot of relatives recommended, the quality is also very good, before buying a look with this same, who knows to buy home has not been used for more than a week on the bad, the quality of this is really good with more than two years!	Jingdong prefecture level city in Shandong
Happy Fan Shop	King of Power Saving	Whole house power saving king, can really save electricity, friends recommended, high power saving appliances, only replacement, no repair, after-sales worry-free!	Jingdong prefecture level city in Shandong
Happy Fan Shop	King of Power Saving	Great item love it, great customer service, great shopping experience!	Jingdong prefecture level city in Shandong
Happy Fan Shop	King of Power Saving	Power saving, very good use, much cheaper than the physical store, satisfaction	Jingdong prefecture level city in Shandong
Happy Fan Shop	King of Power Saving	In use, very well packaged and fast delivery of credit!	Jingdong prefecture level city in Shandong
Happy Fan Shop	King of Power Saving	Product quality, customer service attitude is particularly good, like, praise!	Jingdong prefecture level city in Shandong
Happy Fan Shop	King of Power Saving	The logistics is very fast, the packaging is not broken, the quality is very good, I like it very much!	Jingdong prefecture level city in Shandong
Happy Fan Shop	King of Power Saving	The saver was well packaged and was as described when plugged in	Jingdong prefecture level city in Shandong
Happy Fan Shop	King of Power Saving	Energy efficient and very satisfied	Jingdong prefecture level city in Shandong
Happy Fan Shop	King of Power Saving	Great quality for the price and fast shipping.	Jingdong prefecture level city in Shandong
Happy Fan Shop	King of Power Saving	It's great, just as described. Perfect.	Jingdong prefecture level city in Shandong
Happy Fan Shop	King of Power Saving	There is no power saving effect, but rather ring up an increase in electricity costs, because this machine is on continuously all day long, uninterruptedly, its own electricity and increased costs, plus the cost of purchasing the machine, so I feel that the use of this machine instead of increasing the	Jingdong prefecture level city in Shandong
Happy Fan Shop	King of Power Saving	Recommended by the owner of the store next door, looked at his home does have to save electricity, decided to get. Indeed quite can, the voltage is much more stable, there is a reduction in electricity	Jingdong prefecture level city in Shandong
Happy Fan Shop	King of Power Saving	Goods have been received value for money, affordable and reasonable price, and the description of the same, power-saving safe and reliable!	Jingdong prefecture level city in Shandong
Happy Fan Shop	King of Power Saving	Listen to friends introduced to buy, tried to save a lot of electricity is good, quite good, worth buying.	Jingdong prefecture level city in Shandong
Happy Fan Shop	King of Power Saving	The product is easy to use, safe and environmentally friendly, only used for a short time, to the next month, after the electricity bill status display to know how the performance of the power saver efficacy.	Jingdong prefecture level city in Shandong
Happy Fan Shop	King of Power Saving	Last month's electricity bill is 178 yuan, with this power saver is 92 yuan, ready to buy two more back home, I was introduced to buy a friend, just started to not believe that I did not expect the effect!	Jingdong prefecture level city in Shandong
Happy Fan Shop	King of Power Saving	It looks nice, but after using it for a few days I can't see how it saves power?	Jingdong prefecture level city in Shandong
Happy Fan Shop	King of Power Saving	Recommended by a friend, it is genuine, as described by the merchant, cost-effective	Jingdong prefecture level city in Shandong
Happy Fan Shop	King of Power Saving	Works well, affordable and saves money.	Jingdong prefecture level city in Shandong
Happy Fan Shop	King of Power Saving	Feels good haven't compared it yet, the air conditioning has been on a lot in the past two months	Jingdong prefecture level city in Shandong
Happy Fan Shop	King of Power Saving	Saving about 5 kWh per day is a good thing	Jingdong prefecture level city in Shandong
Happy Fan Shop	King of Power Saving	Not bad, affordable, best value for money	Jingdong prefecture level city in Shandong
Happy Fan Shop	King of Power Saving	The goods are genuine and the logistics are faster.	Jingdong prefecture level city in Shandong

Happy Fan Shop	King of Power Saving	Goods have been received today, has been plugged into the outlet, looking forward to saving electricity, after a period of time to see how the effect in the follow-up comment!	Jingdong prefecture level city in Shandong
Happy Fan Shop	King of Power Saving	I just got it home and haven't used it for long, so I'll use it for a couple months and see what happens. Seeing that most people have good reviews, it should be fine.	Jingdong prefecture level city in Shandong
Happy Fan Shop	King of Power Saving	I'm looking forward to the results, so I'll give you a good review out of habit! Follow up review after a while	Jingdong prefecture level city in Shandong
Happy Fan Shop	King of Power Saving	Use a dish first to know if it will help save energy	Jingdong prefecture level city in Shandong
Happy Fan Shop	King of Power Saving	Whether it saves electricity or not, we don't know yet.	Jingdong prefecture level city in Shandong
Happy Fan Shop	King of Power Saving	Goods have been received, use a period of time to know how oh. The courier did not deliver to the door ah! Sent to the station ah! The heat is inconvenient ah!	Jingdong prefecture level city in Shandong
Happy Fan Shop	King of Power Saving	A little effect, before an hour a power, now an hour 0.76 degrees, a dollar a power = save 24 cents, although not a lot, first use it!	Jingdong prefecture level city in Shandong
Happy Fan Shop	King of Power Saving	Just received the goods, use a month to evaluate	Jingdong prefecture level city in Shandong
Happy Fan Shop	King of Power Saving	Home every month with a lot of electricity, inadvertently found this good thing online that can save the beginning of a skeptical attitude to consult customer service, customer service replied very professional, said 120 days can be refunded that first try it!	Jingdong prefecture level city in Shandong
Happy Fan Shop	King of Power Saving	After receiving the goods this afternoon, immediately installed in the bathroom Smith energy storage electric water heater on the power outlet (instead of connecting with a plug board), when used, found that the "Power Saving King" LCD display, is the value of the voltage, not the store's video said so	Jingdong prefecture level city in Shandong
Happy Fan Shop	King of Power Saving	Has made many days, did not feel the power saving, the effect is not obvious.	Jingdong prefecture level city in Shandong
Happy Fan Shop	King of Power Saving	Energy savers do not save energy at all, but rather consume a lot of electricity.	Jingdong prefecture level city in Shandong
Happy Fan Shop	King of Power Saving	It's useless. It's a waste of money.	Jingdong prefecture level city in Shandong
Happy Fan Shop	King of Power Saving	Last month saved more than 500, which came back to buy, the store bought three, and then buy 1 home use, my family is dry beauty salon, more instruments, especially electricity, a month almost 1000. or so, with this did not expect to save so much, great, five-star praise!	Jingdong prefecture level city in Shandong
Happy Fan Shop	King of Power Saving	It rained a couple days ago and I just went to get the item back today , not bad, hey I looked at the meter and that red display light takes several seconds to jump, did not make a mistake buying it. After a period of time to evaluate.	Jingdong prefecture level city in Shandong
Happy Fan Shop	King of Power Saving	Without further ado, the electricity bill came down, this month less 102 yuan, has been repurchased, I also recommended to my friends, all said that there are effects, the product is okay, the effect is quite	Jingdong prefecture level city in Shandong
Happy Fan Shop	King of Power Saving	After buying and using two months really see the effect, so only to evaluate! Originally held the mindset of trying, I did not expect really effective, worth having!	Jingdong prefecture level city in Shandong
Happy Fan Shop	King of Power Saving	In the use of this energy saver before the monthly electricity bill is more than 150 yuan or so, now save thirty percent, every day to work, six or seven o'clock in the afternoon to go home, I do not know why the electricity bill is so expensive, energy-saving appliances really have the effect of recommending	Jingdong prefecture level city in Shandong
Happy Fan Shop	King of Power Saving	It saves about 100 bucks a month. A friend recommended it to me, and he's been using it for a while, and the effect is so obvious that I bought one too.	Jingdong prefecture level city in Shandong
Happy Fan Shop	King of Power Saving	My dad asked me to buy one, but after I paid the bill, I asked and he said it was a lot less this month, so he asked me to buy another one.	Jingdong prefecture level city in Shandong
Happy Fan Shop	King of Power Saving	The specific effect will have to be tested for at least two months to compare whether there is a power saving effect or not.	Jingdong prefecture level city in Shandong
Happy Fan Shop	King of Power Saving	'The goods have been received, immediately go to use, please ask the boss you instructions do not allow to withdraw, there are two fuses installed there!	Jingdong prefecture level city in Shandong
Happy Fan Shop	King of Power Saving	First praise is using, after using if good then follow up, now because I have not yet seen the results	Jingdong prefecture level city in Shandong
Happy Fan Shop	King of Power Saving	I've just been using it, and I haven't seen if it has a power-saving effect yet, so I'll follow up after 10 days and half a month.	Jingdong prefecture level city in Shandong
Happy Fan Shop	King of Power Saving	Just received the intelligent power saving meter, immediately plugged in to use, see it is not bad, and order to sell the second time	Jingdong prefecture level city in Shandong
Happy Fan Shop	King of Power Saving	This power saver is a bit effective, used for half a month, the effect is still visible.	Jingdong prefecture level city in Shandong
Happy Fan Shop	King of Power Saving	I received the baby and it looks great, I'm trying it out now and will keep an eye on it afterward.	Jingdong prefecture level city in Shandong
Happy Fan Shop	King of Power Saving	I've just received it, but I'm still trying to see how it works, so I'll come back in a little while.	Jingdong prefecture level city in Shandong
Happy Fan Shop	King of Power Saving	Just used it wait to use it for a while and then comment thanks, all first five stars.	Jingdong prefecture level city in Shandong
Happy Fan Shop	King of Power Saving	Very trustworthy brand, quality is guaranteed, the price is still affordable, quite satisfied!	Jingdong prefecture level city in Shandong
Happy Fan Shop	King of Power Saving	Things received, I hope that the platform of Jingdong do not sell fake goods are available	Jingdong prefecture level city in Shandong
Happy Fan Shop	King of Power Saving	Energy saver received, the use of the effect is good and the business description of the same	Jingdong prefecture level city in Shandong
Happy Fan Shop	King of Power Saving	The surface looks beautiful, the actual effect is not known, after using additional evaluation.	Jingdong prefecture level city in Shandong
Happy Fan Shop	King of Power Saving	Tried it for a few months, it really saves electricity, and I bought it for a friend.	Jingdong prefecture level city in Shandong
Happy Fan Shop	King of Power Saving	It didn't work and wanted to return it, it really didn't work at all.	Jingdong prefecture level city in Shandong
Happy Fan Shop	King of Power Saving	Just used it, don't know how effective it is, looking forward to usefulness	Jingdong prefecture level city in Shandong
Happy Fan Shop	King of Power Saving	After two months before the effect I do not know if it is true!	Jingdong prefecture level city in Shandong
Happy Fan Shop	King of Power Saving	So fast! Ordered yesterday and received today 👍👍👍👍👍👍👍👍👍 🌹🌹🌹🌹🌹🌹🌹🌹🌹	Jingdong prefecture level city in Shandong
Happy Fan Shop	King of Power Saving	I've started using it. We'll see how it works.	Jingdong prefecture level city in Shandong
Happy Fan Shop	King of Power Saving	I just received the goods, I won't know the effect until after two.	Jingdong prefecture level city in Shandong
Happy Fan Shop	King of Power Saving	Just received the goods, the specific effect will be evaluated later	Jingdong prefecture level city in Shandong
Happy Fan Shop	King of Power Saving	I've received the goods, I don't know if it's any good yet	Jingdong prefecture level city in Shandong
Happy Fan Shop	King of Power Saving	Try it for a month and then come back with a concrete evaluation	Jingdong prefecture level city in Shandong
Happy Fan Shop	King of Power Saving	In use, after two months to see the effect of power saving	Jingdong prefecture level city in Shandong
Happy Fan Shop	King of Power Saving	Power Saver. It's authentic. It's black. It works.	Jingdong prefecture level city in Shandong
Happy Fan Shop	King of Power Saving	I've been using it for a week now and it's not working.	Jingdong prefecture level city in Shandong
Happy Fan Shop	King of Power Saving	I'm just using it. I don't know how it's going to work.	Jingdong prefecture level city in Shandong
Happy Fan Shop	King of Power Saving	Products are better looking out for power savings	Jingdong prefecture level city in Shandong
Happy Fan Shop	King of Power Saving	I received it and I've kept it on. I hope it works.	Jingdong prefecture level city in Shandong

Happy Fan Shop	King of Power Saving	It's really good! Worth recommending to buy!	Jingdong prefecture level city in Shandong
Happy Fan Shop	King of Power Saving	Try it for a few days and see if it doesn't work, then return it.	Jingdong prefecture level city in Shandong
Happy Fan Shop	King of Power Saving	It's in use and I hope to be pleasantly surprised.	Jingdong prefecture level city in Shandong
Happy Fan Shop	King of Power Saving	We'll see how it works when we use it	Jingdong prefecture level city in Shandong
Happy Fan Shop	King of Power Saving	Let's see if it works.	Jingdong prefecture level city in Shandong
Happy Fan Shop	King of Power Saving	I'm using it and will follow up with a review	Jingdong prefecture level city in Shandong
Happy Fan Shop	King of Power Saving	It really works. It saves energy.	Jingdong prefecture level city in Shandong
Happy Fan Shop	King of Power Saving	It does have some power savings!	Jingdong prefecture level city in Shandong
Happy Fan Shop	King of Power Saving	Jingdong shopping speed is convenient and timely	Jingdong prefecture level city in Shandong
Happy Fan Shop	King of Power Saving	Second time I've bought it. Everything's fine.	Jingdong prefecture level city in Shandong
Happy Fan Shop	King of Power Saving	I don't know if it works yet.	Jingdong prefecture level city in Shandong
Happy Fan Shop	King of Power Saving	Not used, don't know how well it works.	Jingdong prefecture level city in Shandong
Happy Fan Shop	King of Power Saving	No visible results so far	Jingdong prefecture level city in Shandong
Happy Fan Shop	King of Power Saving	It doesn't feel like it's working.	Jingdong prefecture level city in Shandong
Happy Fan Shop	King of Power Saving	Where's the effect? Where are the power savings?	Jingdong prefecture level city in Shandong
Happy Fan Shop	King of Power Saving	Let's see how it works in practice!	Jingdong prefecture level city in Shandong
Happy Fan Shop	King of Power Saving	I'll wait until I use it to give you a good review	Jingdong prefecture level city in Shandong
Happy Fan Shop	King of Power Saving	I'll follow up after I've used it	Jingdong prefecture level city in Shandong
Happy Fan Shop	King of Power Saving	Well, that's good for saving power.	Jingdong prefecture level city in Shandong
Happy Fan Shop	King of Power Saving	Received. Used in the know.	Jingdong prefecture level city in Shandong
Happy Fan Shop	King of Power Saving	It doesn't do much good.	Jingdong prefecture level city in Shandong
Happy Fan Shop	King of Power Saving	It's not working.	Jingdong prefecture level city in Shandong
Happy Fan Shop	King of Power Saving	I don't know. I just used it.	Jingdong prefecture level city in Shandong
Happy Fan Shop	King of Power Saving	Use it and come back to evaluate.	Jingdong prefecture level city in Shandong
Happy Fan Shop	King of Power Saving	Two words: useless.	Jingdong prefecture level city in Shandong
Happy Fan Shop	King of Power Saving	To be used and then evaluated	Jingdong prefecture level city in Shandong
Happy Fan Shop	King of Power Saving	No shipment.	Jingdong prefecture level city in Shandong
Happy Fan Shop	King of Power Saving	Fake. Speechless.	Jingdong prefecture level city in Shandong
Happy Fan Shop	King of Power Saving	fantastic (idiom); awesome	Jingdong prefecture level city in Shandong
Happy Fan Shop	King of Power Saving	an excellent	Jingdong prefecture level city in Shandong
Happy Fan Shop	King of Power Saving	an excellent	Jingdong prefecture level city in Shandong
Happy Fan Shop	King of Power Saving	Want to buy! Recommended not to buy! No effect	Jingdong prefecture level city in Shandong
Happy Fan Shop	King of Power Saving	That's all there is to it. I can't figure out how to save electricity.	Jingdong prefecture level city in Shandong
Happy Fan Shop	King of Power Saving	It's no use.	Jingdong prefecture level city in Shandong
Happy Fan Shop	King of Power Saving	Things are more perfect than I imagined, very powerful, it is worth getting Packaging is very strong, things are well protected, it is worth the praise things are very satisfied Oh, beautiful and stylish atmosphere, customer service is super patient, service is meticulous and attentive, the big brand is not	Jingdong prefecture level city in Shandong
Happy Fan Shop	King of Power Saving	The effect is too good, the family has now several units, back to buy a number of times, the power saver received, power-saving effect is very good,, fine workmanship, long warranty, but also purify the air, before buying a used for a period of time, with the okay, and then bought a, and only now come to the	Jingdong prefecture level city in Shandong
Happy Fan Shop	King of Power Saving	Quite good, and imagine the same, casual ride are very good, satisfied! Baby received it 👌, really great ah! 🌈 quality is really nothing to say, very satisfied! 🆗 happy happy 😃 genuine, great, the price is very good, in short, quite good, will buy again a very good shopping experience, attentive service, fast logistics, distribution is also thoughtful, the attitude is also very good, value for money, ah,	Jingdong prefecture level city in Shandong
Happy Fan Shop	King of Power Saving	Power-saving effect: strong Cost-effective: quite good Appearance style: beautiful Workmanship material: meticulous Experience: looking forward to power-saving effect	Jingdong prefecture level city in Shandong
Happy Fan Shop	King of Power Saving	Cost-effective is okay, the appearance of the style of meticulous workmanship, looking forward to energy-saving effect.	Jingdong prefecture level city in Shandong
Happy Fan Shop	King of Power Saving	Very Good Very Power Saving This Power Saver is really power saving As soon as the power is turned on, the Power Saver starts the power saving function for you.	Jingdong prefecture level city in Shandong
Happy Fan Shop	King of Power Saving	Logistics is very fast, the price is favorable, friends introduced that it is very good to use, bought 2 at once, looking forward to the effect, used after the follow-up comment.	Jingdong prefecture level city in Shandong
Happy Fan Shop	King of Power Saving	It's all fake. It doesn't save any electricity.	Jingdong prefecture level city in Shandong
Happy Fan Shop	King of Power Saving	This Power Saver is so much better than the one I bought before, not only the effect is good, the quality is very good in all aspects, the customer service attitude is also very good, recommended to buy!	Jingdong prefecture level city in Shandong
Happy Fan Shop	King of Power Saving	Logistics is very fast, buy for parents, good quality, once bought 2, power-saving effect is good to buy 2 more, look forward to using the effect, satisfied will recommend to friends.	Jingdong prefecture level city in Shandong
Happy Fan Shop	King of Power Saving	With the description is the same, very high-grade, good value, to buy friends can order Oh!	Jingdong prefecture level city in Shandong
Happy Fan Shop	King of Power Saving	Not using much electricity in the winter saving over 100 per month! Ready to buy 2 more units in the summer ！！！！！ Good quality, good price, power saving effect is obvious, cost-effective, worth buying, can be recommended!	Jingdong prefecture level city in Shandong
Happy Fan Shop	King of Power Saving	Purchased a power saver received on the put into use, rent a room with many electrical appliances, power consumption is very large, we have a piece of electricity or 1.2 dollars a time, with this power saver is much better, every day can save a lot of money, very convenient and practical.	Jingdong prefecture level city in Shandong

Happy Fan Shop	King of Power Saving	The power saving effect is very good: my supermarket monthly electricity bill is more than 7000 yuan. On May 1st someone recommended buying a couple of power savers, and I bought 5 of them first. The electricity bill was about 2000 less in June. This time, I bought 5 more. The seller said 800 degrees of electricity with one. I feel it's basically enough. A total of 10 units	Jingdong prefecture level city in Shandong
Happy Fan Shop	King of Power Saving	I never thought I could get such a good baby at such an affordable price, it's very good value for money, I love it!	Jingdong prefecture level city in Shandong
Happy Fan Shop	King of Power Saving	Previously bought other brands of power savers, but found no effect at home, this time to see how the products on the Jingdong, inserted in the home socket, the LED display is blue, the night light is very nice to see, look at the instructions that have to be half a year in order to achieve the best results, try it, I believe that the quality of the Jingdong, it should not be wrong.	Jingdong prefecture level city in Shandong
Happy Fan Shop	King of Power Saving	Superb, inexpensive, saves a lot of points, been using it for a long time!	Jingdong prefecture level city in Shandong
Happy Fan Shop	King of Power Saving	There are a lot of appliances in the house, the summer air conditioning is very costly, I bought this in front of the air conditioning effect is very good, I explain that I bought three in one breath, I use more electricity a month down to 200 or so, very satisfied.	Jingdong prefecture level city in Shandong
Happy Fan Shop	King of Power Saving	A month can save more than 200, home monthly electricity bill of more than 600 dollars, inserted 3 or so, an amount of the balance of the average can save more than 200. Product is very good, has been buying this one, has given a lot of relatives recommended, the quality is also very good, before buying a look with this same, who knows to buy home has not been used for more than a week on the bad, the quality of this is really good with more than two years!	Jingdong prefecture level city in Shandong
Happy Fan Shop	King of Power Saving	Whole house power saving king, can really save electricity, friends recommended, high power saving appliances, only replacement, no repair, after-sales worry-free!	Jingdong prefecture level city in Shandong
Happy Fan Shop	King of Power Saving	Great item love it, great customer service, great shopping experience!	Jingdong prefecture level city in Shandong
Happy Fan Shop	King of Power Saving	Power saving, very good use, much cheaper than the physical store, satisfaction	Jingdong prefecture level city in Shandong
Happy Fan Shop	King of Power Saving	In use, very well packaged and fast delivery of credit!	Jingdong prefecture level city in Shandong
Happy Fan Shop	King of Power Saving	Product quality, customer service attitude is particularly good, like, praise!	Jingdong prefecture level city in Shandong
Happy Fan Shop	King of Power Saving	The logistics is very fast, the packaging is not broken, the quality is very good, I like it very much!	Jingdong prefecture level city in Shandong
Happy Fan Shop	King of Power Saving	The saver was well packaged and was as described when plugged in	Jingdong prefecture level city in Shandong
Happy Fan Shop	King of Power Saving	Energy efficient and very satisfied	Jingdong prefecture level city in Shandong
Happy Fan Shop	King of Power Saving	Great quality for the price and fast shipping.	Jingdong prefecture level city in Shandong
Happy Fan Shop	King of Power Saving	It's great, just as described. Perfect.	Jingdong prefecture level city in Shandong
Happy Fan Shop	King of Power Saving	There is no power saving effect, but rather ring up an increase in electricity costs, because this machine is on continuously all day long, uninterruptedly, its own electricity and increased costs, plus the cost of purchasing the machine, so I feel that the use of this machine instead of increasing the	Jingdong prefecture level city in Shandong
Happy Fan Shop	King of Power Saving	Recommended by the owner of the store next door, looked at his home does have to save electricity, decided to get. Indeed quite can, the voltage is much more stable, there is a reduction in electricity	Jingdong prefecture level city in Shandong
Happy Fan Shop	King of Power Saving	Goods have been received value for money, affordable and reasonable price, and the description of the same, power-saving safe and reliable!	Jingdong prefecture level city in Shandong
Happy Fan Shop	King of Power Saving	Listen to friends introduced to buy, tried to save a lot of electricity is good, quite good, worth buying.	Jingdong prefecture level city in Shandong
Happy Fan Shop	King of Power Saving	The product is easy to use, safe and environmentally friendly, only used for a short time, to the next month, after the electricity bill status display to know how the performance of the power saver efficacy.	Jingdong prefecture level city in Shandong
Happy Fan Shop	King of Power Saving	Last month's electricity bill is 178 yuan, with this power saver is 92 yuan, ready to buy two more back home, I was introduced to buy a friend, just started to not believe that I did not expect the effect!	Jingdong prefecture level city in Shandong
Happy Fan Shop	King of Power Saving	It looks nice, but after using it for a few days I can't see how it saves power?	Jingdong prefecture level city in Shandong
Happy Fan Shop	King of Power Saving	Recommended by a friend, it is genuine, as described by the merchant, cost-effective	Jingdong prefecture level city in Shandong
Happy Fan Shop	King of Power Saving	Works well, affordable and saves money.	Jingdong prefecture level city in Shandong
Happy Fan Shop	King of Power Saving	Feels good haven't compared it yet, the air conditioning has been on a lot in the past two months	Jingdong prefecture level city in Shandong
Happy Fan Shop	King of Power Saving	Saving about 5 kWh per day is a good thing	Jingdong prefecture level city in Shandong
Happy Fan Shop	King of Power Saving	Not bad, affordable, best value for money	Jingdong prefecture level city in Shandong
Happy Fan Shop	King of Power Saving	The goods are genuine and the logistics are faster.	Jingdong prefecture level city in Shandong
Happy Fan Shop	King of Power Saving	Goods have been received today, has been plugged into the outlet, looking forward to saving electricity, after a period of time to see how the effect in the follow-up comment!	Jingdong prefecture level city in Shandong
Happy Fan Shop	King of Power Saving	I just got it home and haven't used it for long, so I'll use it for a couple months and see what happens. Seeing that most people have good reviews, it should be fine.	Jingdong prefecture level city in Shandong
Happy Fan Shop	King of Power Saving	I'm looking forward to the results, so I'll give you a good review out of habit! Follow up review after a while	Jingdong prefecture level city in Shandong
Happy Fan Shop	King of Power Saving	Use a dish first to know if it will help save energy	Jingdong prefecture level city in Shandong
Happy Fan Shop	King of Power Saving	Whether it saves electricity or not, we don't know yet.	Jingdong prefecture level city in Shandong
Happy Fan Shop	King of Power Saving	Goods have been received, use a period of time to know how oh. The courier did not deliver to the door ah! Sent to the station ah! The heat is inconvenient ah!	Jingdong prefecture level city in Shandong
Happy Fan Shop	King of Power Saving	A little effect, before an hour a power, now an hour 0.76 degrees, a dollar a power = save 24 cents, although not a lot, first use it!	Jingdong prefecture level city in Shandong
Happy Fan Shop	King of Power Saving	Just received the goods, use a month to evaluate	Jingdong prefecture level city in Shandong
Happy Fan Shop	King of Power Saving	Home every month with a lot of electricity, inadvertently found this good thing online that can save the beginning of a skeptical attitude to consult customer service, customer service replied very professional, said 120 days can be refunded that first try it!	Jingdong prefecture level city in Shandong
Happy Fan Shop	King of Power Saving	After receiving the goods this afternoon, immediately installed in the bathroom Smith energy storage electric water heater on the power outlet (instead of connecting with a plug board), when used, found that the "Power Saving King" LCD display, is the value of the voltage, not the store's video said so	Jingdong prefecture level city in Shandong
Happy Fan Shop	King of Power Saving	Has made many days, did not feel the power saving, the effect is not obvious.	Jingdong prefecture level city in Shandong
Happy Fan Shop	King of Power Saving	Energy savers do not save energy at all, but rather consume a lot of electricity.	Jingdong prefecture level city in Shandong
Happy Fan Shop	King of Power Saving	It's useless. It's a waste of money.	Jingdong prefecture level city in Shandong
Happy Fan Shop	King of Power Saving	Last month saved more than 500, which came back to buy, the store bought three, and then buy 1 home use, my family is dry beauty salon, more instruments, especially electricity, a month almost 1000. or so, with this did not expect to save so much, great, five-star praise!	Jingdong prefecture level city in Shandong
Happy Fan Shop	King of Power Saving	It rained a couple days ago and I just went to get the item back today , not bad, hey I looked at the meter and that red display light takes several seconds to jump, did not make a mistake buying it. After a period of time to evaluate.	Jingdong prefecture level city in Shandong
Happy Fan Shop	King of Power Saving	Without further ado, the electricity bill came down, this month less 102 yuan, has been repurchased, I also recommended to my friends, all said that there are effects, the product is okay, the effect is quite	Jingdong prefecture level city in Shandong

Happy Fan Shop	King of Power Saving	After buying and using two months really see the effect, so only to evaluate! Originally held the mindset of trying, I did not expect really effective, worth having!	Jingdong prefecture level city in Shandong
Happy Fan Shop	King of Power Saving	In the use of this energy saver before the monthly electricity bill is more than 150 yuan or so, now save thirty percent, every day to work, six or seven o'clock in the afternoon to go home, I do not know why the electricity bill is so expensive, energy-saving appliances really have the effect of recommending	Jingdong prefecture level city in Shandong
Happy Fan Shop	King of Power Saving	It saves about 100 bucks a month. A friend recommended it to me, and he's been using it for a while, and the effect is so obvious that I bought one too.	Jingdong prefecture level city in Shandong
Happy Fan Shop	King of Power Saving	My dad asked me to buy one, but after I paid the bill, I asked and he said it was a lot less this month, so he asked me to buy another one.	Jingdong prefecture level city in Shandong
Happy Fan Shop	King of Power Saving	The specific effect will have to be tested for at least two months to compare whether there is a power saving effect or not.	Jingdong prefecture level city in Shandong
Happy Fan Shop	King of Power Saving	'The goods have been received, immediately go to use, please ask the boss you instructions do not allow to withdraw, there are two fuses installed there!	Jingdong prefecture level city in Shandong
Happy Fan Shop	King of Power Saving	First praise is using, after using if good then follow up, now because I have not yet seen the results	Jingdong prefecture level city in Shandong
Happy Fan Shop	King of Power Saving	I've just been using it, and I haven't seen if it has a power-saving effect yet, so I'll follow up after 10 days and half a month.	Jingdong prefecture level city in Shandong
Happy Fan Shop	King of Power Saving	Just received the intelligent power saving meter, immediately plugged in to use, see it is not bad, and order to sell the second time	Jingdong prefecture level city in Shandong
Happy Fan Shop	King of Power Saving	This power saver is a bit effective, used for half a month, the effect is still visible.	Jingdong prefecture level city in Shandong
Happy Fan Shop	King of Power Saving	I received the baby and it looks great, I'm trying it out now and will keep an eye on it afterward.	Jingdong prefecture level city in Shandong
Happy Fan Shop	King of Power Saving	I've just received it, but I'm still trying to see how it works, so I'll come back in a little while.	Jingdong prefecture level city in Shandong
Happy Fan Shop	King of Power Saving	Just used it wait to use it for a while and then comment thanks, all first five stars.	Jingdong prefecture level city in Shandong
Happy Fan Shop	King of Power Saving	Very trustworthy brand, quality is guaranteed, the price is still affordable, quite satisfied!	Jingdong prefecture level city in Shandong
Happy Fan Shop	King of Power Saving	Things received, I hope that the platform of Jingdong do not sell fake goods are available	Jingdong prefecture level city in Shandong
Happy Fan Shop	King of Power Saving	Energy saver received, the use of the effect is good and the business description of the same	Jingdong prefecture level city in Shandong
Happy Fan Shop	King of Power Saving	The surface looks beautiful, the actual effect is not known, after using additional evaluation.	Jingdong prefecture level city in Shandong
Happy Fan Shop	King of Power Saving	Tried it for a few months, it really saves electricity, and I bought it for a friend.	Jingdong prefecture level city in Shandong
Happy Fan Shop	King of Power Saving	It didn't work and wanted to return it, it really didn't work at all.	Jingdong prefecture level city in Shandong
Happy Fan Shop	King of Power Saving	Just used it, don't know how effective it is, looking forward to usefulness	Jingdong prefecture level city in Shandong
Happy Fan Shop	King of Power Saving	After two months before the effect I do not know if it is true!	Jingdong prefecture level city in Shandong
Happy Fan Shop	King of Power Saving	So fast! Ordered yesterday and received today 👍👍👍👍👍👍👍👍👍 🌹🌹🌹🌹🌹🌹🌹🌹🌹	Jingdong prefecture level city in Shandong
Happy Fan Shop	King of Power Saving	I've started using it. We'll see how it works.	Jingdong prefecture level city in Shandong
Happy Fan Shop	King of Power Saving	I just received the goods, I won't know the effect until after two.	Jingdong prefecture level city in Shandong
Happy Fan Shop	King of Power Saving	Just received the goods, the specific effect will be evaluated later	Jingdong prefecture level city in Shandong
Happy Fan Shop	King of Power Saving	I've received the goods, I don't know if it's any good yet	Jingdong prefecture level city in Shandong
Happy Fan Shop	King of Power Saving	Try it for a month and then come back with a concrete evaluation	Jingdong prefecture level city in Shandong
Happy Fan Shop	King of Power Saving	In use, after two months to see the effect of power saving	Jingdong prefecture level city in Shandong
Happy Fan Shop	King of Power Saving	Power Saver. It's authentic. It's black. It works.	Jingdong prefecture level city in Shandong
Happy Fan Shop	King of Power Saving	I've been using it for a week now and it's not working.	Jingdong prefecture level city in Shandong
Happy Fan Shop	King of Power Saving	I'm just using it. I don't know how it's going to work.	Jingdong prefecture level city in Shandong
Happy Fan Shop	King of Power Saving	Products are better looking out for power savings	Jingdong prefecture level city in Shandong
Happy Fan Shop	King of Power Saving	I received it and I've kept it on. I hope it works.	Jingdong prefecture level city in Shandong
Happy Fan Shop	King of Power Saving	It's really good! Worth recommending to buy!	Jingdong prefecture level city in Shandong
Happy Fan Shop	King of Power Saving	Try it for a few days and see if it doesn't work, then return it.	Jingdong prefecture level city in Shandong
Happy Fan Shop	King of Power Saving	It's in use and I hope to be pleasantly surprised.	Jingdong prefecture level city in Shandong
Happy Fan Shop	King of Power Saving	We'll see how it works when we use it	Jingdong prefecture level city in Shandong
Happy Fan Shop	King of Power Saving	Let's see if it works.	Jingdong prefecture level city in Shandong
Happy Fan Shop	King of Power Saving	I'm using it and will follow up with a review	Jingdong prefecture level city in Shandong
Happy Fan Shop	King of Power Saving	It really works. It saves energy.	Jingdong prefecture level city in Shandong
Happy Fan Shop	King of Power Saving	It does have some power savings!	Jingdong prefecture level city in Shandong
Happy Fan Shop	King of Power Saving	Jingdong shopping speed is convenient and timely	Jingdong prefecture level city in Shandong
Happy Fan Shop	King of Power Saving	Second time I've bought it. Everything's fine.	Jingdong prefecture level city in Shandong
Happy Fan Shop	King of Power Saving	I don't know if it works yet.	Jingdong prefecture level city in Shandong
Happy Fan Shop	King of Power Saving	Not used, don't know how well it works.	Jingdong prefecture level city in Shandong
Happy Fan Shop	King of Power Saving	No visible results so far	Jingdong prefecture level city in Shandong
Happy Fan Shop	King of Power Saving	It doesn't feel like it's working.	Jingdong prefecture level city in Shandong
Happy Fan Shop	King of Power Saving	Where's the effect? Where are the power savings?	Jingdong prefecture level city in Shandong
Happy Fan Shop	King of Power Saving	Let's see how it works in practice!	Jingdong prefecture level city in Shandong
Happy Fan Shop	King of Power Saving	I'll wait until I use it to give you a good review	Jingdong prefecture level city in Shandong
Happy Fan Shop	King of Power Saving	I'll follow up after I've used it	Jingdong prefecture level city in Shandong

Happy Fan Shop	King of Power Saving	Well, that's good for saving power.	Jingdong prefecture level city in Shandong
Happy Fan Shop	King of Power Saving	Received. Used in the know.	Jingdong prefecture level city in Shandong
Happy Fan Shop	King of Power Saving	It doesn't do much good.	Jingdong prefecture level city in Shandong
Happy Fan Shop	King of Power Saving	It's not working.	Jingdong prefecture level city in Shandong
Happy Fan Shop	King of Power Saving	I don't know. I just used it.	Jingdong prefecture level city in Shandong
Happy Fan Shop	King of Power Saving	Use it and come back to evaluate.	Jingdong prefecture level city in Shandong
Happy Fan Shop	King of Power Saving	Two words: useless.	Jingdong prefecture level city in Shandong
Happy Fan Shop	King of Power Saving	To be used and then evaluated	Jingdong prefecture level city in Shandong
Happy Fan Shop	King of Power Saving	No shipment.	Jingdong prefecture level city in Shandong
Happy Fan Shop	King of Power Saving	Fake. Speechless.	Jingdong prefecture level city in Shandong
Happy Fan Shop	King of Power Saving	fantastic (idiom); awesome	Jingdong prefecture level city in Shandong
Happy Fan Shop	King of Power Saving	an excellent	Jingdong prefecture level city in Shandong
Happy Fan Shop	King of Power Saving	an excellent	Jingdong prefecture level city in Shandong
Happy Fan Shop	King of Power Saving	Want to buy! Recommended not to buy! No effect	Jingdong prefecture level city in Shandong
Happy Fan Shop	King of Power Saving	That's all there is to it. I can't figure out how to save electricity.	Jingdong prefecture level city in Shandong
Happy Fan Shop	King of Power Saving	It's no use.	Jingdong prefecture level city in Shandong
Happy Fan Shop	King of Power Saving	Things are more perfect than I imagined, very powerful, it is worth getting Packaging is very strong, things are well protected, it is worth the praise things are very satisfied Oh, beautiful and stylish atmosphere, customer service is super patient, service is meticulous and attentive, the big brand is not	Jingdong prefecture level city in Shandong
Happy Fan Shop	King of Power Saving	The effect is too good, the family has now several units, back to buy a number of times, the power saver received, power-saving effect is very good,, fine workmanship, long warranty, but also purify the air, before buying a used for a period of time, with the okay, and then bought a, and only now come to the	Jingdong prefecture level city in Shandong
Happy Fan Shop	King of Power Saving	Quite good, and imagine the same, casual ride are very good, satisfied! Baby received it 👌, really great ah! 🌈 quality is really nothing to say, very satisfied! 🆗 happy happy 😃 genuine, great, the price is very good, in short, quite good, will buy again a very good shopping experience, attentive service, fast logistics, distribution is also thoughtful, the attitude is also very good, value for money, ah,	Jingdong prefecture level city in Shandong
Happy Fan Shop	King of Power Saving	Power-saving effect: strong Cost-effective: quite good Appearance style: beautiful Workmanship material: meticulous Experience: looking forward to power-saving effect	Jingdong prefecture level city in Shandong
Happy Fan Shop	King of Power Saving	Cost-effective is okay, the appearance of the style of meticulous workmanship, looking forward to energy-saving effect.	Jingdong prefecture level city in Shandong
Happy Fan Shop	King of Power Saving	Very Good Very Power Saving This Power Saver is really power saving As soon as the power is turned on, the Power Saver starts the power saving function for you.	Jingdong prefecture level city in Shandong
Happy Fan Shop	King of Power Saving	Logistics is very fast, the price is favorable, friends introduced that it is very good to use, bought 2 at once, looking forward to the effect, used after the follow-up comment.	Jingdong prefecture level city in Shandong
Happy Fan Shop	King of Power Saving	It's all fake. It doesn't save any electricity.	Jingdong prefecture level city in Shandong
Happy Fan Shop	King of Power Saving	This Power Saver is so much better than the one I bought before, not only the effect is good, the quality is very good in all aspects, the customer service attitude is also very good, recommended to buy!	Jingdong prefecture level city in Shandong
Happy Fan Shop	King of Power Saving	Logistics is very fast, buy for parents, good quality, once bought 2, power-saving effect is good to buy 2 more, look forward to using the effect, satisfied will recommend to friends.	Jingdong prefecture level city in Shandong
Happy Fan Shop	King of Power Saving	With the description is the same, very high-grade, good value, to buy friends can order Oh!	Jingdong prefecture level city in Shandong
Happy Fan Shop	King of Power Saving	Not using much electricity in the winter saving over 100 per month! Ready to buy 2 more units in the summer !!!!! Good quality, good price, power saving effect is obvious, cost-effective, worth buying, can be recommended!	Jingdong prefecture level city in Shandong
Happy Fan Shop	King of Power Saving	Purchased a power saver received on the put into use, rent a room with many electrical appliances, power consumption is very large, we have a piece of electricity or 1.2 dollars a time, with this power saver is much better, every day can save a lot of money, very convenient and practical.	Jingdong prefecture level city in Shandong
Happy Fan Shop	King of Power Saving	The power saving effect is very good: my supermarket monthly electricity bill is more than 7000 yuan. On May 1st someone recommended buying a couple of power savers, and I bought 5 of them first. The electricity bill was about 2000 less in June. This time, I bought 5 more. The seller said 800 degrees of electricity with one. I feel it's basically enough. A total of 10 units	Jingdong prefecture level city in Shandong
Happy Fan Shop	King of Power Saving	I never thought I could get such a good baby at such an affordable price, it's very good value for money, I love it!	Jingdong prefecture level city in Shandong
Happy Fan Shop	King of Power Saving	Previously bought other brands of power savers, but found no effect at home, this time to see how the products on the Jingdong, inserted in the home socket, the LED display is blue, the night light is very nice to see, look at the instructions that have to be half a year in order to achieve the best results, try it, I believe that the quality of the Jingdong, it should not be wrong.	Jingdong prefecture level city in Shandong
Happy Fan Shop	King of Power Saving	Superb, inexpensive, saves a lot of points, been using it for a long time!	Jingdong prefecture level city in Shandong
Happy Fan Shop	King of Power Saving	There are a lot of appliances in the house, the summer air conditioning is very costly, I bought this in front of the air conditioning effect is very good, I explain that I bought three in one breath, I use more electricity a month down to 200 or so, very satisfied.	Jingdong prefecture level city in Shandong
Happy Fan Shop	King of Power Saving	A month can save more than 200, home monthly electricity bill of more than 600 dollars, inserted 3 or so, an amount of the balance of the average can save more than 200. Product is very good, has been buying this one, has given a lot of relatives recommended, the quality is also very good, before buying a look with this same, who knows to buy home has not been used for more than a week on the bad, the quality of this is really good with more than two years!	Jingdong prefecture level city in Shandong
Happy Fan Shop	King of Power Saving	Whole house power saving king, can really save electricity, friends recommended, high power saving appliances, only replacement, no repair, after-sales worry-free!	Jingdong prefecture level city in Shandong
Happy Fan Shop	King of Power Saving	Great item love it, great customer service, great shopping experience!	Jingdong prefecture level city in Shandong
Happy Fan Shop	King of Power Saving	Power saving, very good use, much cheaper than the physical store, satisfaction	Jingdong prefecture level city in Shandong
Happy Fan Shop	King of Power Saving	In use, very well packaged and fast delivery of credit!	Jingdong prefecture level city in Shandong
Happy Fan Shop	King of Power Saving	Product quality, customer service attitude is particularly good, like, praise!	Jingdong prefecture level city in Shandong
Happy Fan Shop	King of Power Saving	The logistics is very fast, the packaging is not broken, the quality is very good, I like it very much!	Jingdong prefecture level city in Shandong
Happy Fan Shop	King of Power Saving	The saver was well packaged and was as described when plugged in	Jingdong prefecture level city in Shandong
Happy Fan Shop	King of Power Saving	Energy efficient and very satisfied	Jingdong prefecture level city in Shandong
Happy Fan Shop	King of Power Saving	Great quality for the price and fast shipping.	Jingdong prefecture level city in Shandong

Happy Fan Shop	King of Power Saving	It's great, just as described. Perfect.	Jingdong prefecture level city in Shandong
Happy Fan Shop	King of Power Saving	There is no power saving effect, but rather ring up an increase in electricity costs, because this machine is on continuously all day long, uninterruptedly, its own electricity and increased costs, plus the cost of purchasing the machine, so I feel that the use of this machine instead of increasing the	Jingdong prefecture level city in Shandong
Happy Fan Shop	King of Power Saving	Recommended by the owner of the store next door, looked at his home does have to save electricity, decided to get. Indeed quite can, the voltage is much more stable, there is a reduction in electricity	Jingdong prefecture level city in Shandong
Happy Fan Shop	King of Power Saving	Goods have been received value for money, affordable and reasonable price, and the description of the same, power-saving safe and reliable!	Jingdong prefecture level city in Shandong
Happy Fan Shop	King of Power Saving	Listen to friends introduced to buy, tried to save a lot of electricity is good, quite good, worth buying.	Jingdong prefecture level city in Shandong
Happy Fan Shop	King of Power Saving	The product is easy to use, safe and environmentally friendly, only used for a short time, to the next month, after the electricity bill status display to know how the performance of the power saver efficacy.	Jingdong prefecture level city in Shandong
Happy Fan Shop	King of Power Saving	Last month's electricity bill is 178 yuan, with this power saver is 92 yuan, ready to buy two more back home, I was introduced to buy a friend, just started to not believe that I did not expect the effect!	Jingdong prefecture level city in Shandong
Happy Fan Shop	King of Power Saving	It looks nice, but after using it for a few days I can't see how it saves power?	Jingdong prefecture level city in Shandong
Happy Fan Shop	King of Power Saving	Recommended by a friend, it is genuine, as described by the merchant, cost-effective	Jingdong prefecture level city in Shandong
Happy Fan Shop	King of Power Saving	Works well, affordable and saves money.	Jingdong prefecture level city in Shandong
Happy Fan Shop	King of Power Saving	Feels good haven't compared it yet, the air conditioning has been on a lot in the past two months	Jingdong prefecture level city in Shandong
Happy Fan Shop	King of Power Saving	Saving about 5 kWh per day is a good thing	Jingdong prefecture level city in Shandong
Happy Fan Shop	King of Power Saving	Not bad, affordable, best value for money	Jingdong prefecture level city in Shandong
Happy Fan Shop	King of Power Saving	The goods are genuine and the logistics are faster.	Jingdong prefecture level city in Shandong
Happy Fan Shop	King of Power Saving	Goods have been received today, has been plugged into the outlet, looking forward to saving electricity, after a period of time to see how the effect in the follow-up comment!	Jingdong prefecture level city in Shandong
Happy Fan Shop	King of Power Saving	I just got it home and haven't used it for long, so I'll use it for a couple months and see what happens. Seeing that most people have good reviews, it should be fine.	Jingdong prefecture level city in Shandong
Happy Fan Shop	King of Power Saving	I'm looking forward to the results, so I'll give you a good review out of habit! Follow up review after a while	Jingdong prefecture level city in Shandong
Happy Fan Shop	King of Power Saving	Use a dish first to know if it will help save energy	Jingdong prefecture level city in Shandong
Happy Fan Shop	King of Power Saving	Whether it saves electricity or not, we don't know yet.	Jingdong prefecture level city in Shandong
Happy Fan Shop	King of Power Saving	Goods have been received, use a period of time to know how oh. The courier did not deliver to the door ah! Sent to the station ah! The heat is inconvenient ah!	Jingdong prefecture level city in Shandong
Happy Fan Shop	King of Power Saving	A little effect, before an hour a power, now an hour 0.76 degrees, a dollar a power = save 24 cents, although not a lot, first use it!	Jingdong prefecture level city in Shandong
Happy Fan Shop	King of Power Saving	Just received the goods, use a month to evaluate	Jingdong prefecture level city in Shandong
Happy Fan Shop	King of Power Saving	Home every month with a lot of electricity, inadvertently found this good thing online that can save the beginning of a skeptical attitude to consult customer service, customer service replied very professional, said 120 days can be refunded that first try it!	Jingdong prefecture level city in Shandong
Happy Fan Shop	King of Power Saving	After receiving the goods this afternoon, immediately installed in the bathroom Smith energy storage electric water heater on the power outlet (instead of connecting with a plug board), when used, found that the "Power Saving King" LCD display, is the value of the voltage, not the store's video said so	Jingdong prefecture level city in Shandong
Happy Fan Shop	King of Power Saving	Has made many days, did not feel the power saving, the effect is not obvious.	Jingdong prefecture level city in Shandong
Happy Fan Shop	King of Power Saving	Energy savers do not save energy at all, but rather consume a lot of electricity.	Jingdong prefecture level city in Shandong
Happy Fan Shop	King of Power Saving	It's useless. It's a waste of money.	Jingdong prefecture level city in Shandong
Happy Fan Shop	King of Power Saving	Last month saved more than 500, which came back to buy, the store bought three, and then buy 1 home use, my family is dry beauty salon, more instruments, especially electricity, a month almost 1000. or so, with this did not expect to save so much, great, five-star praise!	Jingdong prefecture level city in Shandong
Happy Fan Shop	King of Power Saving	It rained a couple days ago and I just went to get the item back today , not bad, hey I looked at the meter and that red display light takes several seconds to jump, did not make a mistake buying it. After a period of time to evaluate.	Jingdong prefecture level city in Shandong
Happy Fan Shop	King of Power Saving	Without further ado, the electricity bill came down, this month less 102 yuan, has been repurchased, I also recommended to my friends, all said that there are effects, the product is okay, the effect is quite	Jingdong prefecture level city in Shandong
Happy Fan Shop	King of Power Saving	After buying and using two months really see the effect, so only to evaluate! Originally held the mindset of trying, I did not expect really effective, worth having!	Jingdong prefecture level city in Shandong
Happy Fan Shop	King of Power Saving	In the use of this energy saver before the monthly electricity bill is more than 150 yuan or so, now save thirty percent, every day to work, six or seven o'clock in the afternoon to go home, I do not know why the electricity bill is so expensive, energy-saving appliances really have the effect of recommending	Jingdong prefecture level city in Shandong
Happy Fan Shop	King of Power Saving	It saves about 100 bucks a month. A friend recommended it to me, and he's been using it for a while, and the effect is so obvious that I bought one too.	Jingdong prefecture level city in Shandong
Happy Fan Shop	King of Power Saving	My dad asked me to buy one, but after I paid the bill, I asked and he said it was a lot less this month, so he asked me to buy another one.	Jingdong prefecture level city in Shandong
Happy Fan Shop	King of Power Saving	The specific effect will have to be tested for at least two months to compare whether there is a power saving effect or not.	Jingdong prefecture level city in Shandong
Happy Fan Shop	King of Power Saving	'The goods have been received, immediately go to use, please ask the boss you instructions do not allow to withdraw, there are two fuses installed there!	Jingdong prefecture level city in Shandong
Happy Fan Shop	King of Power Saving	First praise is using, after using if good then follow up, now because I have not yet seen the results	Jingdong prefecture level city in Shandong
Happy Fan Shop	King of Power Saving	I've just been using it, and I haven't seen if it has a power-saving effect yet, so I'll follow up after 10 days and half a month.	Jingdong prefecture level city in Shandong
Happy Fan Shop	King of Power Saving	Just received the intelligent power saving meter, immediately plugged in to use, see it is not bad, and order to sell the second time	Jingdong prefecture level city in Shandong
Happy Fan Shop	King of Power Saving	This power saver is a bit effective, used for half a month, the effect is still visible.	Jingdong prefecture level city in Shandong
Happy Fan Shop	King of Power Saving	I received the baby and it looks great, I'm trying it out now and will keep an eye on it afterward.	Jingdong prefecture level city in Shandong
Happy Fan Shop	King of Power Saving	I've just received it, but I'm still trying to see how it works, so I'll come back in a little while.	Jingdong prefecture level city in Shandong
Happy Fan Shop	King of Power Saving	Just used it wait to use it for a while and then comment thanks, all first five stars.	Jingdong prefecture level city in Shandong
Happy Fan Shop	King of Power Saving	Very trustworthy brand, quality is guaranteed, the price is still affordable, quite satisfied!	Jingdong prefecture level city in Shandong
Happy Fan Shop	King of Power Saving	Things received, I hope that the platform of Jingdong do not sell fake goods are available	Jingdong prefecture level city in Shandong
Happy Fan Shop	King of Power Saving	Energy saver received, the use of the effect is good and the business description of the same	Jingdong prefecture level city in Shandong
Happy Fan Shop	King of Power Saving	The surface looks beautiful, the actual effect is not known, after using additional evaluation.	Jingdong prefecture level city in Shandong
Happy Fan Shop	King of Power Saving	Tried it for a few months, it really saves electricity, and I bought it for a friend.	Jingdong prefecture level city in Shandong

Happy Fan Shop	King of Power Saving	It didn't work and wanted to return it, it really didn't work at all.	Jingdong prefecture level city in Shandong
Happy Fan Shop	King of Power Saving	Just used it, don't know how effective it is, looking forward to usefulness	Jingdong prefecture level city in Shandong
Happy Fan Shop	King of Power Saving	After two months before the effect I do not know if it is true!	Jingdong prefecture level city in Shandong
Happy Fan Shop	King of Power Saving	So fast! Ordered yesterday and received today 👍👍👍👍👍👍👍👍👍 🌹🌹🌹🌹🌹🌹🌹🌹🌹	Jingdong prefecture level city in Shandong
Happy Fan Shop	King of Power Saving	I've started using it. We'll see how it works.	Jingdong prefecture level city in Shandong
Happy Fan Shop	King of Power Saving	I just received the goods, I won't know the effect until after two.	Jingdong prefecture level city in Shandong
Happy Fan Shop	King of Power Saving	Just received the goods, the specific effect will be evaluated later	Jingdong prefecture level city in Shandong
Happy Fan Shop	King of Power Saving	I've received the goods, I don't know if it's any good yet	Jingdong prefecture level city in Shandong
Happy Fan Shop	King of Power Saving	Try it for a month and then come back with a concrete evaluation	Jingdong prefecture level city in Shandong
Happy Fan Shop	King of Power Saving	In use, after two months to see the effect of power saving	Jingdong prefecture level city in Shandong
Happy Fan Shop	King of Power Saving	Power Saver. It's authentic. It's black. It works.	Jingdong prefecture level city in Shandong
Happy Fan Shop	King of Power Saving	I've been using it for a week now and it's not working.	Jingdong prefecture level city in Shandong
Happy Fan Shop	King of Power Saving	I'm just using it. I don't know how it's going to work.	Jingdong prefecture level city in Shandong
Happy Fan Shop	King of Power Saving	Products are better looking out for power savings	Jingdong prefecture level city in Shandong
Happy Fan Shop	King of Power Saving	I received it and I've kept it on. I hope it works.	Jingdong prefecture level city in Shandong
Happy Fan Shop	King of Power Saving	It's really good! Worth recommending to buy!	Jingdong prefecture level city in Shandong
Happy Fan Shop	King of Power Saving	Try it for a few days and see if it doesn't work, then return it.	Jingdong prefecture level city in Shandong
Happy Fan Shop	King of Power Saving	It's in use and I hope to be pleasantly surprised.	Jingdong prefecture level city in Shandong
Happy Fan Shop	King of Power Saving	We'll see how it works when we use it	Jingdong prefecture level city in Shandong
Happy Fan Shop	King of Power Saving	Let's see if it works.	Jingdong prefecture level city in Shandong
Happy Fan Shop	King of Power Saving	I'm using it and will follow up with a review	Jingdong prefecture level city in Shandong
Happy Fan Shop	King of Power Saving	It really works. It saves energy.	Jingdong prefecture level city in Shandong
Happy Fan Shop	King of Power Saving	It does have some power savings!	Jingdong prefecture level city in Shandong
Happy Fan Shop	King of Power Saving	Jingdong shopping speed is convenient and timely	Jingdong prefecture level city in Shandong
Happy Fan Shop	King of Power Saving	Second time I've bought it. Everything's fine.	Jingdong prefecture level city in Shandong
Happy Fan Shop	King of Power Saving	I don't know if it works yet.	Jingdong prefecture level city in Shandong
Happy Fan Shop	King of Power Saving	Not used, don't know how well it works.	Jingdong prefecture level city in Shandong
Happy Fan Shop	King of Power Saving	No visible results so far	Jingdong prefecture level city in Shandong
Happy Fan Shop	King of Power Saving	It doesn't feel like it's working.	Jingdong prefecture level city in Shandong
Happy Fan Shop	King of Power Saving	Where's the effect? Where are the power savings?	Jingdong prefecture level city in Shandong
Happy Fan Shop	King of Power Saving	Let's see how it works in practice!	Jingdong prefecture level city in Shandong
Happy Fan Shop	King of Power Saving	I'll wait until I use it to give you a good review	Jingdong prefecture level city in Shandong
Happy Fan Shop	King of Power Saving	I'll follow up after I've used it	Jingdong prefecture level city in Shandong
Happy Fan Shop	King of Power Saving	Well, that's good for saving power.	Jingdong prefecture level city in Shandong
Happy Fan Shop	King of Power Saving	Received. Used in the know.	Jingdong prefecture level city in Shandong
Happy Fan Shop	King of Power Saving	It doesn't do much good.	Jingdong prefecture level city in Shandong
Happy Fan Shop	King of Power Saving	It's not working.	Jingdong prefecture level city in Shandong
Happy Fan Shop	King of Power Saving	I don't know. I just used it.	Jingdong prefecture level city in Shandong
Happy Fan Shop	King of Power Saving	Use it and come back to evaluate.	Jingdong prefecture level city in Shandong
Happy Fan Shop	King of Power Saving	Two words: useless.	Jingdong prefecture level city in Shandong
Happy Fan Shop	King of Power Saving	To be used and then evaluated	Jingdong prefecture level city in Shandong
Happy Fan Shop	King of Power Saving	No shipment.	Jingdong prefecture level city in Shandong
Happy Fan Shop	King of Power Saving	Fake. Speechless.	Jingdong prefecture level city in Shandong
Happy Fan Shop	King of Power Saving	fantastic (idiom); awesome	Jingdong prefecture level city in Shandong
Happy Fan Shop	King of Power Saving	an excellent	Jingdong prefecture level city in Shandong
Happy Fan Shop	King of Power Saving	an excellent	Jingdong prefecture level city in Shandong
Happy Fan Shop	King of Power Saving	Want to buy! Recommended not to buy! No effect	Jingdong prefecture level city in Shandong
Happy Fan Shop	King of Power Saving	That's all there is to it. I can't figure out how to save electricity.	Jingdong prefecture level city in Shandong
Happy Fan Shop	King of Power Saving	It's no use.	Jingdong prefecture level city in Shandong
Happy Fan Shop	King of Power Saving	Things are more perfect than I imagined, very powerful, it is worth getting Packaging is very strong, things are well protected, it is worth the praise things are very satisfied Oh, beautiful and stylish atmosphere, customer service is super patient, service is meticulous and attentive, the big brand is not	Jingdong prefecture level city in Shandong

Happy Fan Shop	King of Power Saving	The effect is too good, the family has now several units, back to buy a number of times, the power saver received, power-saving effect is very good,, fine workmanship, long warranty, but also purify the air, before buying a used for a period of time, with the okay, and then bought a, and only now come to the	Jingdong prefecture level city in Shandong
Happy Fan Shop	King of Power Saving	Quite good, and imagine the same, casual ride are very good, satisfied! Baby received it, really great ah! quality is really nothing to say, very satisfied! OK happy happy genuine, great, the price is very good, in short, quite good, will buy again a very good shopping experience, attentive service, fast logistics, distribution is also thoughtful, the attitude is also very good, value for money, ah,	Jingdong prefecture level city in Shandong
Happy Fan Shop	King of Power Saving	Power-saving effect: strong Cost-effective: quite good Appearance style: beautiful Workmanship material: meticulous Experience: looking forward to power-saving effect	Jingdong prefecture level city in Shandong
Happy Fan Shop	King of Power Saving	Cost-effective is okay, the appearance of the style of meticulous workmanship, looking forward to energy-saving effect.	Jingdong prefecture level city in Shandong
Happy Fan Shop	King of Power Saving	Very Good Very Power Saving This Power Saver is really power saving As soon as the power is turned on, the Power Saver starts the power saving function for you.	Jingdong prefecture level city in Shandong
Happy Fan Shop	King of Power Saving	Logistics is very fast, the price is favorable, friends introduced that it is very good to use, bought 2 at once, looking forward to the effect, used after the follow-up comment.	Jingdong prefecture level city in Shandong
Happy Fan Shop	King of Power Saving	It's all fake. It doesn't save any electricity.	Jingdong prefecture level city in Shandong
Happy Fan Shop	King of Power Saving	This Power Saver is so much better than the one I bought before, not only the effect is good, the quality is very good in all aspects, the customer service attitude is also very good, recommended to buy!	Jingdong prefecture level city in Shandong
Happy Fan Shop	King of Power Saving	Logistics is very fast, buy for parents, good quality, once bought 2, power-saving effect is good to buy 2 more, look forward to using the effect, satisfied will recommend to friends.	Jingdong prefecture level city in Shandong
Happy Fan Shop	King of Power Saving	With the description is the same, very high-grade, good value, to buy friends can order Oh!	Jingdong prefecture level city in Shandong
Happy Fan Shop	King of Power Saving	Not using much electricity in the winter saving over 100 per month! Ready to buy 2 more units in the summer ! ! ! ! ! Good quality, good price, power saving effect is obvious, cost-effective, worth buying, can be recommended!	Jingdong prefecture level city in Shandong
Happy Fan Shop	King of Power Saving	Purchased a power saver received on the put into use, rent a room with many electrical appliances, power consumption is very large, we have a piece of electricity or 1.2 dollars a time, with this power saver is much better, every day can save a lot of money, very convenient and practical.	Jingdong prefecture level city in Shandong
Happy Fan Shop	King of Power Saving	The power saving effect is very good: my supermarket monthly electricity bill is more than 7000 yuan. On May 1st someone recommended buying a couple of power savers, and I bought 5 of them first. The electricity bill was about 2000 less in June. This time, I bought 5 more. The seller said 800 degrees of electricity with one. I feel it's basically enough. A total of 10 units	Jingdong prefecture level city in Shandong
Happy Fan Shop	King of Power Saving	I never thought I could get such a good baby at such an affordable price, it's very good value for money, I love it!	Jingdong prefecture level city in Shandong
Happy Fan Shop	King of Power Saving	Previously bought other brands of power savers, but found no effect at home, this time to see how the products on the Jingdong, inserted in the home socket, the LED display is blue, the night light is very nice to see, look at the instructions that have to be half a year in order to achieve the best results, try it, I believe that the quality of the Jingdong, it should not be wrong.	Jingdong prefecture level city in Shandong
Happy Fan Shop	King of Power Saving	Superb, inexpensive, saves a lot of points, been using it for a long time!	Jingdong prefecture level city in Shandong
Happy Fan Shop	King of Power Saving	There are a lot of appliances in the house, the summer air conditioning is very costly, I bought this in front of the air conditioning effect is very good, I explain that I bought three in one breath, I use more electricity a month down to 200 or so, very satisfied.	Jingdong prefecture level city in Shandong
Happy Fan Shop	King of Power Saving	A month can save more than 200, home monthly electricity bill of more than 600 dollars, inserted 3 or so, an amount of the balance of the average can save more than 200. Product is very good, has been buying this one, has given a lot of relatives recommended, the quality is also very good, before buying a look with this same, who knows to buy home has not been used for more than a week on the bad, the quality of this is really good with more than two years!	Jingdong prefecture level city in Shandong
Happy Fan Shop	King of Power Saving	Whole house power saving king, can really save electricity, friends recommended, high power saving appliances, only replacement, no repair, after-sales worry-free!	Jingdong prefecture level city in Shandong
Happy Fan Shop	King of Power Saving	Great item love it, great customer service, great shopping experience!	Jingdong prefecture level city in Shandong
Happy Fan Shop	King of Power Saving	Power saving, very good use, much cheaper than the physical store, satisfaction	Jingdong prefecture level city in Shandong
Happy Fan Shop	King of Power Saving	In use, very well packaged and fast delivery of credit!	Jingdong prefecture level city in Shandong
Happy Fan Shop	King of Power Saving	Product quality, customer service attitude is particularly good, like, praise!	Jingdong prefecture level city in Shandong
Happy Fan Shop	King of Power Saving	The logistics is very fast, the packaging is not broken, the quality is very good, I like it very much!	Jingdong prefecture level city in Shandong
Happy Fan Shop	King of Power Saving	The saver was well packaged and was as described when plugged in	Jingdong prefecture level city in Shandong
Happy Fan Shop	King of Power Saving	Energy efficient and very satisfied	Jingdong prefecture level city in Shandong
Happy Fan Shop	King of Power Saving	Great quality for the price and fast shipping.	Jingdong prefecture level city in Shandong
Happy Fan Shop	King of Power Saving	It's great, just as described. Perfect.	Jingdong prefecture level city in Shandong
Happy Fan Shop	King of Power Saving	There is no power saving effect, but rather ring up an increase in electricity costs, because this machine is on continuously all day long, uninterruptedly, its own electricity and increased costs, plus the cost of purchasing the machine, so I feel that the use of this machine instead of increasing the	Jingdong prefecture level city in Shandong
Happy Fan Shop	King of Power Saving	Recommended by the owner of the store next door, looked at his home does have to save electricity, decided to get. Indeed quite can, the voltage is much more stable, there is a reduction in electricity	Jingdong prefecture level city in Shandong
Happy Fan Shop	King of Power Saving	Goods have been received value for money, affordable and reasonable price, and the description of the same, power-saving safe and reliable!	Jingdong prefecture level city in Shandong
Happy Fan Shop	King of Power Saving	Listen to friends introduced to buy, tried to save a lot of electricity is good, quite good, worth buying.	Jingdong prefecture level city in Shandong
Happy Fan Shop	King of Power Saving	The product is easy to use, safe and environmentally friendly, only used for a short time, to the next month, after the electricity bill status display to know how the performance of the power saver efficacy.	Jingdong prefecture level city in Shandong
Happy Fan Shop	King of Power Saving	Last month's electricity bill is 178 yuan, with this power saver is 92 yuan, ready to buy two more back home, I was introduced to buy a friend, just started to not believe that I did not expect the effect!	Jingdong prefecture level city in Shandong
Happy Fan Shop	King of Power Saving	It looks nice, but after using it for a few days I can't see how it saves power?	Jingdong prefecture level city in Shandong
Happy Fan Shop	King of Power Saving	Recommended by a friend, it is genuine, as described by the merchant, cost-effective	Jingdong prefecture level city in Shandong
Happy Fan Shop	King of Power Saving	Works well, affordable and saves money.	Jingdong prefecture level city in Shandong
Happy Fan Shop	King of Power Saving	Feels good haven't compared it yet, the air conditioning has been on a lot in the past two months	Jingdong prefecture level city in Shandong
Happy Fan Shop	King of Power Saving	Saving about 5 kWh per day is a good thing	Jingdong prefecture level city in Shandong
Happy Fan Shop	King of Power Saving	Not bad, affordable, best value for money	Jingdong prefecture level city in Shandong
Happy Fan Shop	King of Power Saving	The goods are genuine and the logistics are faster.	Jingdong prefecture level city in Shandong
Happy Fan Shop	King of Power Saving	Goods have been received today, has been plugged into the outlet, looking forward to saving electricity, after a period of time to see how the effect in the follow-up comment!	Jingdong prefecture level city in Shandong
Happy Fan Shop	King of Power Saving	I just got it home and haven't used it for long, so I'll use it for a couple months and see what happens. Seeing that most people have good reviews, it should be fine.	Jingdong prefecture level city in Shandong
Happy Fan Shop	King of Power Saving	I'm looking forward to the results, so I'll give you a good review out of habit! Follow up review after a while	Jingdong prefecture level city in Shandong

Happy Fan Shop	King of Power Saving	Use a dish first to know if it will help save energy	Jingdong prefecture level city in Shandong
Happy Fan Shop	King of Power Saving	Whether it saves electricity or not, we don't know yet.	Jingdong prefecture level city in Shandong
Happy Fan Shop	King of Power Saving	Goods have been received, use a period of time to know how oh. The courier did not deliver to the door ah! Sent to the station ah! The heat is inconvenient ah!	Jingdong prefecture level city in Shandong
Happy Fan Shop	King of Power Saving	A little effect, before an hour a power, now an hour 0.76 degrees, a dollar a power = save 24 cents, although not a lot, first use it!	Jingdong prefecture level city in Shandong
Happy Fan Shop	King of Power Saving	Just received the goods, use a month to evaluate	Jingdong prefecture level city in Shandong
Happy Fan Shop	King of Power Saving	Home every month with a lot of electricity, inadvertently found this good thing online that can save the beginning of a skeptical attitude to consult customer service, customer service replied very professional, said 120 days can be refunded that first try it!	Jingdong prefecture level city in Shandong
Happy Fan Shop	King of Power Saving	After receiving the goods this afternoon, immediately installed in the bathroom Smith energy storage electric water heater on the power outlet (instead of connecting with a plug board), when used, found that the "Power Saving King" LCD display, is the value of the voltage, not the store's video said so	Jingdong prefecture level city in Shandong
Happy Fan Shop	King of Power Saving	Has made many days, did not feel the power saving, the effect is not obvious.	Jingdong prefecture level city in Shandong
Happy Fan Shop	King of Power Saving	Energy savers do not save energy at all, but rather consume a lot of electricity.	Jingdong prefecture level city in Shandong
Happy Fan Shop	King of Power Saving	It's useless. It's a waste of money.	Jingdong prefecture level city in Shandong
Happy Fan Shop	King of Power Saving	Last month saved more than 500, which came back to buy, the store bought three, and then buy 1 home use, my family is dry beauty salon, more instruments, especially electricity, a month almost 1000. or so, with this did not expect to save so much, great, five-star praise!	Jingdong prefecture level city in Shandong
Happy Fan Shop	King of Power Saving	It rained a couple days ago and I just went to get the item back today , not bad, hey I looked at the meter and that red display light takes several seconds to jump, did not make a mistake buying it. After a period of time to evaluate.	Jingdong prefecture level city in Shandong
Happy Fan Shop	King of Power Saving	Without further ado, the electricity bill came down, this month less 102 yuan, has been repurchased, I also recommended to my friends, all said that there are effects, the product is okay, the effect is quite	Jingdong prefecture level city in Shandong
Happy Fan Shop	King of Power Saving	After buying and using two months really see the effect, so only to evaluate! Originally held the mindset of trying, I did not expect really effective, worth having!	Jingdong prefecture level city in Shandong
Happy Fan Shop	King of Power Saving	In the use of this energy saver before the monthly electricity bill is more than 150 yuan or so, now save thirty percent, every day to work, six or seven o'clock in the afternoon to go home, I do not know why the electricity bill is so expensive, energy-saving appliances really have the effect of recommending	Jingdong prefecture level city in Shandong
Happy Fan Shop	King of Power Saving	It saves about 100 bucks a month. A friend recommended it to me, and he's been using it for a while, and the effect is so obvious that I bought one too.	Jingdong prefecture level city in Shandong
Happy Fan Shop	King of Power Saving	My dad asked me to buy one, but after I paid the bill, I asked and he said it was a lot less this month, so he asked me to buy another one.	Jingdong prefecture level city in Shandong
Happy Fan Shop	King of Power Saving	The specific effect will have to be tested for at least two months to compare whether there is a power saving effect or not.	Jingdong prefecture level city in Shandong
Happy Fan Shop	King of Power Saving	'The goods have been received, immediately go to use, please ask the boss you instructions do not allow to withdraw, there are two fuses installed there!	Jingdong prefecture level city in Shandong
Happy Fan Shop	King of Power Saving	First praise is using, after using if good then follow up, now because I have not yet seen the results	Jingdong prefecture level city in Shandong
Happy Fan Shop	King of Power Saving	I've just been using it, and I haven't seen if it has a power-saving effect yet, so I'll follow up after 10 days and half a month.	Jingdong prefecture level city in Shandong
Happy Fan Shop	King of Power Saving	Just received the intelligent power saving meter, immediately plugged in to use, see it is not bad, and order to sell the second time	Jingdong prefecture level city in Shandong
Happy Fan Shop	King of Power Saving	This power saver is a bit effective, used for half a month, the effect is still visible.	Jingdong prefecture level city in Shandong
Happy Fan Shop	King of Power Saving	I received the baby and it looks great, I'm trying it out now and will keep an eye on it afterward.	Jingdong prefecture level city in Shandong
Happy Fan Shop	King of Power Saving	I've just received it, but I'm still trying to see how it works, so I'll come back in a little while.	Jingdong prefecture level city in Shandong
Happy Fan Shop	King of Power Saving	Just used it wait to use it for a while and then comment thanks, all first five stars.	Jingdong prefecture level city in Shandong
Happy Fan Shop	King of Power Saving	Very trustworthy brand, quality is guaranteed, the price is still affordable, quite satisfied!	Jingdong prefecture level city in Shandong
Happy Fan Shop	King of Power Saving	Things received, I hope that the platform of Jingdong do not sell fake goods are available	Jingdong prefecture level city in Shandong
Happy Fan Shop	King of Power Saving	Energy saver received, the use of the effect is good and the business description of the same	Jingdong prefecture level city in Shandong
Happy Fan Shop	King of Power Saving	The surface looks beautiful, the actual effect is not known, after using additional evaluation.	Jingdong prefecture level city in Shandong
Happy Fan Shop	King of Power Saving	Tried it for a few months, it really saves electricity, and I bought it for a friend.	Jingdong prefecture level city in Shandong
Happy Fan Shop	King of Power Saving	It didn't work and wanted to return it, it really didn't work at all.	Jingdong prefecture level city in Shandong
Happy Fan Shop	King of Power Saving	Just used it, don't know how effective it is, looking forward to usefulness	Jingdong prefecture level city in Shandong
Happy Fan Shop	King of Power Saving	After two months before the effect I do not know if it is true!	Jingdong prefecture level city in Shandong
Happy Fan Shop	King of Power Saving	So fast! Ordered yesterday and received today 👍👍👍👍👍👍👍👍👍 🌹🌹🌹🌹🌹🌹🌹🌹🌹	Jingdong prefecture level city in Shandong
Happy Fan Shop	King of Power Saving	I've started using it. We'll see how it works.	Jingdong prefecture level city in Shandong
Happy Fan Shop	King of Power Saving	I just received the goods, I won't know the effect until after two.	Jingdong prefecture level city in Shandong
Happy Fan Shop	King of Power Saving	Just received the goods, the specific effect will be evaluated later	Jingdong prefecture level city in Shandong
Happy Fan Shop	King of Power Saving	I've received the goods, I don't know if it's any good yet	Jingdong prefecture level city in Shandong
Happy Fan Shop	King of Power Saving	Try it for a month and then come back with a concrete evaluation	Jingdong prefecture level city in Shandong
Happy Fan Shop	King of Power Saving	In use, after two months to see the effect of power saving	Jingdong prefecture level city in Shandong
Happy Fan Shop	King of Power Saving	Power Saver. It's authentic. It's black. It works.	Jingdong prefecture level city in Shandong
Happy Fan Shop	King of Power Saving	I've been using it for a week now and it's not working.	Jingdong prefecture level city in Shandong
Happy Fan Shop	King of Power Saving	I'm just using it. I don't know how it's going to work.	Jingdong prefecture level city in Shandong
Happy Fan Shop	King of Power Saving	Products are better looking out for power savings	Jingdong prefecture level city in Shandong
Happy Fan Shop	King of Power Saving	I received it and I've kept it on. I hope it works.	Jingdong prefecture level city in Shandong
Happy Fan Shop	King of Power Saving	It's really good! Worth recommending to buy!	Jingdong prefecture level city in Shandong
Happy Fan Shop	King of Power Saving	Try it for a few days and see if it doesn't work, then return it.	Jingdong prefecture level city in Shandong
Happy Fan Shop	King of Power Saving	It's in use and I hope to be pleasantly surprised.	Jingdong prefecture level city in Shandong

Happy Fan Shop	King of Power Saving	We'll see how it works when we use it	Jingdong prefecture level city in Shandong
Happy Fan Shop	King of Power Saving	Let's see if it works.	Jingdong prefecture level city in Shandong
Happy Fan Shop	King of Power Saving	I'm using it and will follow up with a review	Jingdong prefecture level city in Shandong
Happy Fan Shop	King of Power Saving	It really works. It saves energy.	Jingdong prefecture level city in Shandong
Happy Fan Shop	King of Power Saving	It does have some power savings!	Jingdong prefecture level city in Shandong
Happy Fan Shop	King of Power Saving	Jingdong shopping speed is convenient and timely	Jingdong prefecture level city in Shandong
Happy Fan Shop	King of Power Saving	Second time I've bought it. Everything's fine.	Jingdong prefecture level city in Shandong
Happy Fan Shop	King of Power Saving	I don't know if it works yet.	Jingdong prefecture level city in Shandong
Happy Fan Shop	King of Power Saving	Not used, don't know how well it works.	Jingdong prefecture level city in Shandong
Happy Fan Shop	King of Power Saving	No visible results so far	Jingdong prefecture level city in Shandong
Happy Fan Shop	King of Power Saving	It doesn't feel like it's working.	Jingdong prefecture level city in Shandong
Happy Fan Shop	King of Power Saving	Where's the effect? Where are the power savings?	Jingdong prefecture level city in Shandong
Happy Fan Shop	King of Power Saving	Let's see how it works in practice!	Jingdong prefecture level city in Shandong
Happy Fan Shop	King of Power Saving	I'll wait until I use it to give you a good review	Jingdong prefecture level city in Shandong
Happy Fan Shop	King of Power Saving	I'll follow up after I've used it	Jingdong prefecture level city in Shandong
Happy Fan Shop	King of Power Saving	Well, that's good for saving power.	Jingdong prefecture level city in Shandong
Happy Fan Shop	King of Power Saving	Received. Used in the know.	Jingdong prefecture level city in Shandong
Happy Fan Shop	King of Power Saving	It doesn't do much good.	Jingdong prefecture level city in Shandong
Happy Fan Shop	King of Power Saving	It's not working.	Jingdong prefecture level city in Shandong
Happy Fan Shop	King of Power Saving	I don't know. I just used it.	Jingdong prefecture level city in Shandong
Happy Fan Shop	King of Power Saving	Use it and come back to evaluate.	Jingdong prefecture level city in Shandong
Happy Fan Shop	King of Power Saving	Two words: useless.	Jingdong prefecture level city in Shandong
Happy Fan Shop	King of Power Saving	To be used and then evaluated	Jingdong prefecture level city in Shandong
Happy Fan Shop	King of Power Saving	No shipment.	Jingdong prefecture level city in Shandong
Happy Fan Shop	King of Power Saving	Fake. Speechless.	Jingdong prefecture level city in Shandong
Happy Fan Shop	King of Power Saving	fantastic (idiom); awesome	Jingdong prefecture level city in Shandong
Happy Fan Shop	King of Power Saving	an excellent	Jingdong prefecture level city in Shandong
Happy Fan Shop	King of Power Saving	an excellent	Jingdong prefecture level city in Shandong
Happy Fan Shop	King of Power Saving	Want to buy! Recommended not to buy! No effect	Jingdong prefecture level city in Shandong
Happy Fan Shop	King of Power Saving	That's all there is to it. I can't figure out how to save electricity.	Jingdong prefecture level city in Shandong
Happy Fan Shop	King of Power Saving	It's no use.	Jingdong prefecture level city in Shandong
Happy Fan Shop	King of Power Saving	Things are more perfect than I imagined, very powerful, it is worth getting Packaging is very strong, things are well protected, it is worth the praise things are very satisfied Oh, beautiful and stylish atmosphere, customer service is super patient, service is meticulous and attentive, the big brand is not	Jingdong prefecture level city in Shandong
Happy Fan Shop	King of Power Saving	The effect is too good, the family has now several units, back to buy a number of times, the power saver received, power-saving effect is very good,, fine workmanship, long warranty, but also purify the air, before buying a used for a period of time, with the okay, and then bought a, and only now come to the	Jingdong prefecture level city in Shandong
Happy Fan Shop	King of Power Saving	Quite good, and imagine the same, casual ride are very good, satisfied! Baby received it 👌, really great ah! 🌈 quality is really nothing to say, very satisfied! 🆗 happy happy 😃 genuine, great, the price is very good, in short, quite good, will buy again a very good shopping experience, attentive service, fast logistics, distribution is also thoughtful, the attitude is also very good, value for money, ah,	Jingdong prefecture level city in Shandong
Happy Fan Shop	King of Power Saving	Power-saving effect: strong Cost-effective: quite good Appearance style: beautiful Workmanship material: meticulous Experience: looking forward to power-saving effect	Jingdong prefecture level city in Shandong
Happy Fan Shop	King of Power Saving	Cost-effective is okay, the appearance of the style of meticulous workmanship, looking forward to energy-saving effect.	Jingdong prefecture level city in Shandong
Happy Fan Shop	King of Power Saving	Very Good Very Power Saving This Power Saver is really power saving As soon as the power is turned on, the Power Saver starts the power saving function for you.	Jingdong prefecture level city in Shandong
Happy Fan Shop	King of Power Saving	Logistics is very fast, the price is favorable, friends introduced that it is very good to use, bought 2 at once, looking forward to the effect, used after the follow-up comment.	Jingdong prefecture level city in Shandong
Happy Fan Shop	King of Power Saving	It's all fake. It doesn't save any electricity.	Jingdong prefecture level city in Shandong
Happy Fan Shop	King of Power Saving	This Power Saver is so much better than the one I bought before, not only the effect is good, the quality is very good in all aspects, the customer service attitude is also very good, recommended to buy!	Jingdong prefecture level city in Shandong
Happy Fan Shop	King of Power Saving	Logistics is very fast, buy for parents, good quality, once bought 2, power-saving effect is good to buy 2 more, look forward to using the effect, satisfied will recommend to friends.	Jingdong prefecture level city in Shandong
Happy Fan Shop	King of Power Saving	With the description is the same, very high-grade, good value, to buy friends can order Oh!	Jingdong prefecture level city in Shandong
Happy Fan Shop	King of Power Saving	Not using much electricity in the winter saving over 100 per month! Ready to buy 2 more units in the summer !!!!! Good quality, good price, power saving effect is obvious, cost-effective, worth buying, can be recommended!	Jingdong prefecture level city in Shandong
Happy Fan Shop	King of Power Saving	Purchased a power saver received on the put into use, rent a room with many electrical appliances, power consumption is very large, we have a piece of electricity or 1.2 dollars a time, with this power saver is much better, every day can save a lot of money, very convenient and practical.	Jingdong prefecture level city in Shandong
Happy Fan Shop	King of Power Saving	The power saving effect is very good: my supermarket monthly electricity bill is more than 7000 yuan. On May 1st someone recommended buying a couple of power savers, and I bought 5 of them first. The electricity bill was about 2000 less in June. This time, I bought 5 more. The seller said 800 degrees of electricity with one. I feel it's basically enough. A total of 10 units	Jingdong prefecture level city in Shandong
Happy Fan Shop	King of Power Saving	I never thought I could get such a good baby at such an affordable price, it's very good value for money, I love it!	Jingdong prefecture level city in Shandong

Happy Fan Shop	King of Power Saving	Previously bought other brands of power savers, but found no effect at home, this time to see how the products on the Jingdong, inserted in the home socket, the LED display is blue, the night light is very nice to see, look at the instructions that have to be half a year in order to achieve the best results, try it, I believe that the quality of the Jingdong, it should not be wrong.	Jingdong prefecture level city in Shandong
Happy Fan Shop	King of Power Saving	Superb, inexpensive, saves a lot of points, been using it for a long time!	Jingdong prefecture level city in Shandong
Happy Fan Shop	King of Power Saving	There are a lot of appliances in the house, the summer air conditioning is very costly, I bought this in front of the air conditioning effect is very good, I explain that I bought three in one breath, I use more electricity a month down to 200 or so, very satisfied.	Jingdong prefecture level city in Shandong
Happy Fan Shop	King of Power Saving	A month can save more than 200, home monthly electricity bill of more than 600 dollars, inserted 3 or so, an amount of the balance of the average can save more than 200. Product is very good, has been buying this one, has given a lot of relatives recommended, the quality is also very good, before buying a look with this same, who knows to buy home has not been used for more than a week on the bad, the quality of this is really good with more than two years!	Jingdong prefecture level city in Shandong
Happy Fan Shop	King of Power Saving	Whole house power saving king, can really save electricity, friends recommended, high power saving appliances, only replacement, no repair, after-sales worry-free!	Jingdong prefecture level city in Shandong
Happy Fan Shop	King of Power Saving	Great item love it, great customer service, great shopping experience!	Jingdong prefecture level city in Shandong
Happy Fan Shop	King of Power Saving	Power saving, very good use, much cheaper than the physical store, satisfaction	Jingdong prefecture level city in Shandong
Happy Fan Shop	King of Power Saving	In use, very well packaged and fast delivery of credit!	Jingdong prefecture level city in Shandong
Happy Fan Shop	King of Power Saving	Product quality, customer service attitude is particularly good, like, praise!	Jingdong prefecture level city in Shandong
Happy Fan Shop	King of Power Saving	The logistics is very fast, the packaging is not broken, the quality is very good, I like it very much!	Jingdong prefecture level city in Shandong
Happy Fan Shop	King of Power Saving	The saver was well packaged and was as described when plugged in	Jingdong prefecture level city in Shandong
Happy Fan Shop	King of Power Saving	Energy efficient and very satisfied	Jingdong prefecture level city in Shandong
Happy Fan Shop	King of Power Saving	Great quality for the price and fast shipping.	Jingdong prefecture level city in Shandong
Happy Fan Shop	King of Power Saving	It's great, just as described. Perfect.	Jingdong prefecture level city in Shandong
Happy Fan Shop	King of Power Saving	There is no power saving effect, but rather ring up an increase in electricity costs, because this machine is on continuously all day long, uninterruptedly, its own electricity and increased costs, plus the cost of purchasing the machine, so I feel that the use of this machine instead of increasing the	Jingdong prefecture level city in Shandong
Happy Fan Shop	King of Power Saving	Recommended by the owner of the store next door, looked at his home does have to save electricity, decided to get. Indeed quite can, the voltage is much more stable, there is a reduction in electricity	Jingdong prefecture level city in Shandong
Happy Fan Shop	King of Power Saving	Goods have been received value for money, affordable and reasonable price, and the description of the same, power-saving safe and reliable!	Jingdong prefecture level city in Shandong
Happy Fan Shop	King of Power Saving	Listen to friends introduced to buy, tried to save a lot of electricity is good, quite good, worth buying.	Jingdong prefecture level city in Shandong
Happy Fan Shop	King of Power Saving	The product is easy to use, safe and environmentally friendly, only used for a short time, to the next month, after the electricity bill status display to know how the performance of the power saver efficacy.	Jingdong prefecture level city in Shandong
Happy Fan Shop	King of Power Saving	Last month's electricity bill is 178 yuan, with this power saver is 92 yuan, ready to buy two more back home, I was introduced to buy a friend, just started to not believe that I did not expect the effect!	Jingdong prefecture level city in Shandong
Happy Fan Shop	King of Power Saving	It looks nice, but after using it for a few days I can't see how it saves power?	Jingdong prefecture level city in Shandong
Happy Fan Shop	King of Power Saving	Recommended by a friend, it is genuine, as described by the merchant, cost-effective	Jingdong prefecture level city in Shandong
Happy Fan Shop	King of Power Saving	Works well, affordable and saves money.	Jingdong prefecture level city in Shandong
Happy Fan Shop	King of Power Saving	Feels good haven't compared it yet, the air conditioning has been on a lot in the past two months	Jingdong prefecture level city in Shandong
Happy Fan Shop	King of Power Saving	Saving about 5 kWh per day is a good thing	Jingdong prefecture level city in Shandong
Happy Fan Shop	King of Power Saving	Not bad, affordable, best value for money	Jingdong prefecture level city in Shandong
Happy Fan Shop	King of Power Saving	The goods are genuine and the logistics are faster.	Jingdong prefecture level city in Shandong
Happy Fan Shop	King of Power Saving	Goods have been received today, has been plugged into the outlet, looking forward to saving electricity, after a period of time to see how the effect in the follow-up comment!	Jingdong prefecture level city in Shandong
Happy Fan Shop	King of Power Saving	I just got it home and haven't used it for long, so I'll use it for a couple months and see what happens. Seeing that most people have good reviews, it should be fine.	Jingdong prefecture level city in Shandong
Happy Fan Shop	King of Power Saving	I'm looking forward to the results, so I'll give you a good review out of habit! Follow up review after a while	Jingdong prefecture level city in Shandong
Happy Fan Shop	King of Power Saving	Use a dish first to know if it will help save energy	Jingdong prefecture level city in Shandong
Happy Fan Shop	King of Power Saving	Whether it saves electricity or not, we don't know yet.	Jingdong prefecture level city in Shandong
Happy Fan Shop	King of Power Saving	Goods have been received, use a period of time to know how oh. The courier did not deliver to the door ah! Sent to the station ah! The heat is inconvenient ah!	Jingdong prefecture level city in Shandong
Happy Fan Shop	King of Power Saving	A little effect, before an hour a power, now an hour 0.76 degrees, a dollar a power = save 24 cents, although not a lot, first use it!	Jingdong prefecture level city in Shandong
Happy Fan Shop	King of Power Saving	Just received the goods, use a month to evaluate	Jingdong prefecture level city in Shandong
Happy Fan Shop	King of Power Saving	Home every month with a lot of electricity, inadvertently found this good thing online that can save the beginning of a skeptical attitude to consult customer service, customer service replied very professional, said 120 days can be refunded that first try it!	Jingdong prefecture level city in Shandong
Happy Fan Shop	King of Power Saving	After receiving the goods this afternoon, immediately installed in the bathroom Smith energy storage electric water heater on the power outlet (instead of connecting with a plug board), when used, found that the "Power Saving King" LCD display, is the value of the voltage, not the store's video said so	Jingdong prefecture level city in Shandong
Happy Fan Shop	King of Power Saving	Has made many days, did not feel the power saving, the effect is not obvious.	Jingdong prefecture level city in Shandong
Happy Fan Shop	King of Power Saving	Energy savers do not save energy at all, but rather consume a lot of electricity.	Jingdong prefecture level city in Shandong
Happy Fan Shop	King of Power Saving	It's useless. It's a waste of money.	Jingdong prefecture level city in Shandong
Happy Fan Shop	King of Power Saving	Last month saved more than 500, which came back to buy, the store bought three, and then buy 1 home use, my family is dry beauty salon, more instruments, especially electricity, a month almost 1000. or so, with this did not expect to save so much, great, five-star praise!	Jingdong prefecture level city in Shandong
Happy Fan Shop	King of Power Saving	It rained a couple days ago and I just went to get the item back today , not bad, hey I looked at the meter and that red display light takes several seconds to jump, did not make a mistake buying it. After a period of time to evaluate.	Jingdong prefecture level city in Shandong
Happy Fan Shop	King of Power Saving	Without further ado, the electricity bill came down, this month less 102 yuan, has been repurchased, I also recommended to my friends, all said that there are effects, the product is okay, the effect is quite	Jingdong prefecture level city in Shandong
Happy Fan Shop	King of Power Saving	After buying and using two months really see the effect, so only to evaluate! Originally held the mindset of trying, I did not expect really effective, worth having!	Jingdong prefecture level city in Shandong
Happy Fan Shop	King of Power Saving	In the use of this energy saver before the monthly electricity bill is more than 150 yuan or so, now save thirty percent, every day to work, six or seven o'clock in the afternoon to go home, I do not know why the electricity bill is so expensive, energy-saving appliances really have the effect of recommending	Jingdong prefecture level city in Shandong

Happy Fan Shop	King of Power Saving	It saves about 100 bucks a month. A friend recommended it to me, and he's been using it for a while, and the effect is so obvious that I bought one too.	Jingdong prefecture level city in Shandong
Happy Fan Shop	King of Power Saving	My dad asked me to buy one, but after I paid the bill, I asked and he said it was a lot less this month, so he asked me to buy another one.	Jingdong prefecture level city in Shandong
Happy Fan Shop	King of Power Saving	The specific effect will have to be tested for at least two months to compare whether there is a power saving effect or not.	Jingdong prefecture level city in Shandong
Happy Fan Shop	King of Power Saving	'The goods have been received, immediately go to use, please ask the boss you instructions do not allow to withdraw, there are two fuses installed there!	Jingdong prefecture level city in Shandong
Happy Fan Shop	King of Power Saving	First praise is using, after using if good then follow up, now because I have not yet seen the results	Jingdong prefecture level city in Shandong
Happy Fan Shop	King of Power Saving	I've just been using it, and I haven't seen if it has a power-saving effect yet, so I'll follow up after 10 days and half a month.	Jingdong prefecture level city in Shandong
Happy Fan Shop	King of Power Saving	Just received the intelligent power saving meter, immediately plugged in to use, see it is not bad, and order to sell the second time	Jingdong prefecture level city in Shandong
Happy Fan Shop	King of Power Saving	This power saver is a bit effective, used for half a month, the effect is still visible.	Jingdong prefecture level city in Shandong
Happy Fan Shop	King of Power Saving	I received the baby and it looks great, I'm trying it out now and will keep an eye on it afterward.	Jingdong prefecture level city in Shandong
Happy Fan Shop	King of Power Saving	I've just received it, but I'm still trying to see how it works, so I'll come back in a little while.	Jingdong prefecture level city in Shandong
Happy Fan Shop	King of Power Saving	Just used it wait to use it for a while and then comment thanks, all first five stars.	Jingdong prefecture level city in Shandong
Happy Fan Shop	King of Power Saving	Very trustworthy brand, quality is guaranteed, the price is still affordable, quite satisfied!	Jingdong prefecture level city in Shandong
Happy Fan Shop	King of Power Saving	Things received, I hope that the platform of Jingdong do not sell fake goods are available	Jingdong prefecture level city in Shandong
Happy Fan Shop	King of Power Saving	Energy saver received, the use of the effect is good and the business description of the same	Jingdong prefecture level city in Shandong
Happy Fan Shop	King of Power Saving	The surface looks beautiful, the actual effect is not known, after using additional evaluation.	Jingdong prefecture level city in Shandong
Happy Fan Shop	King of Power Saving	Tried it for a few months, it really saves electricity, and I bought it for a friend.	Jingdong prefecture level city in Shandong
Happy Fan Shop	King of Power Saving	It didn't work and wanted to return it, it really didn't work at all.	Jingdong prefecture level city in Shandong
Happy Fan Shop	King of Power Saving	Just used it, don't know how effective it is, looking forward to usefulness	Jingdong prefecture level city in Shandong
Happy Fan Shop	King of Power Saving	After two months before the effect I do not know if it is true!	Jingdong prefecture level city in Shandong
Happy Fan Shop	King of Power Saving	So fast! Ordered yesterday and received today 👍👍👍👍👍👍👍👍👍 🌹🌹🌹🌹🌹🌹🌹🌹🌹	Jingdong prefecture level city in Shandong
Happy Fan Shop	King of Power Saving	I've started using it. We'll see how it works.	Jingdong prefecture level city in Shandong
Happy Fan Shop	King of Power Saving	I just received the goods, I won't know the effect until after two.	Jingdong prefecture level city in Shandong
Happy Fan Shop	King of Power Saving	Just received the goods, the specific effect will be evaluated later	Jingdong prefecture level city in Shandong
Happy Fan Shop	King of Power Saving	I've received the goods, I don't know if it's any good yet	Jingdong prefecture level city in Shandong
Happy Fan Shop	King of Power Saving	Try it for a month and then come back with a concrete evaluation	Jingdong prefecture level city in Shandong
Happy Fan Shop	King of Power Saving	In use, after two months to see the effect of power saving	Jingdong prefecture level city in Shandong
Happy Fan Shop	King of Power Saving	Power Saver. It's authentic. It's black. It works.	Jingdong prefecture level city in Shandong
Happy Fan Shop	King of Power Saving	I've been using it for a week now and it's not working.	Jingdong prefecture level city in Shandong
Happy Fan Shop	King of Power Saving	I'm just using it. I don't know how it's going to work.	Jingdong prefecture level city in Shandong
Happy Fan Shop	King of Power Saving	Products are better looking out for power savings	Jingdong prefecture level city in Shandong
Happy Fan Shop	King of Power Saving	I received it and I've kept it on. I hope it works.	Jingdong prefecture level city in Shandong
Happy Fan Shop	King of Power Saving	It's really good! Worth recommending to buy!	Jingdong prefecture level city in Shandong
Happy Fan Shop	King of Power Saving	Try it for a few days and see if it doesn't work, then return it.	Jingdong prefecture level city in Shandong
Happy Fan Shop	King of Power Saving	It's in use and I hope to be pleasantly surprised.	Jingdong prefecture level city in Shandong
Happy Fan Shop	King of Power Saving	We'll see how it works when we use it	Jingdong prefecture level city in Shandong
Happy Fan Shop	King of Power Saving	Let's see if it works.	Jingdong prefecture level city in Shandong
Happy Fan Shop	King of Power Saving	I'm using it and will follow up with a review	Jingdong prefecture level city in Shandong
Happy Fan Shop	King of Power Saving	It really works. It saves energy.	Jingdong prefecture level city in Shandong
Happy Fan Shop	King of Power Saving	It does have some power savings!	Jingdong prefecture level city in Shandong
Happy Fan Shop	King of Power Saving	Jingdong shopping speed is convenient and timely	Jingdong prefecture level city in Shandong
Happy Fan Shop	King of Power Saving	Second time I've bought it. Everything's fine.	Jingdong prefecture level city in Shandong
Happy Fan Shop	King of Power Saving	I don't know if it works yet.	Jingdong prefecture level city in Shandong
Happy Fan Shop	King of Power Saving	Not used, don't know how well it works.	Jingdong prefecture level city in Shandong
Happy Fan Shop	King of Power Saving	No visible results so far	Jingdong prefecture level city in Shandong
Happy Fan Shop	King of Power Saving	It doesn't feel like it's working.	Jingdong prefecture level city in Shandong
Happy Fan Shop	King of Power Saving	Where's the effect? Where are the power savings?	Jingdong prefecture level city in Shandong
Happy Fan Shop	King of Power Saving	Let's see how it works in practice!	Jingdong prefecture level city in Shandong
Happy Fan Shop	King of Power Saving	I'll wait until I use it to give you a good review	Jingdong prefecture level city in Shandong
Happy Fan Shop	King of Power Saving	I'll follow up after I've used it	Jingdong prefecture level city in Shandong
Happy Fan Shop	King of Power Saving	Well, that's good for saving power.	Jingdong prefecture level city in Shandong
Happy Fan Shop	King of Power Saving	Received. Used in the know.	Jingdong prefecture level city in Shandong

Happy Fan Shop	King of Power Saving	It doesn't do much good.	Jingdong prefecture level city in Shandong
Happy Fan Shop	King of Power Saving	It's not working.	Jingdong prefecture level city in Shandong
Happy Fan Shop	King of Power Saving	I don't know. I just used it.	Jingdong prefecture level city in Shandong
Happy Fan Shop	King of Power Saving	Use it and come back to evaluate.	Jingdong prefecture level city in Shandong
Happy Fan Shop	King of Power Saving	Two words: useless.	Jingdong prefecture level city in Shandong
Happy Fan Shop	King of Power Saving	To be used and then evaluated	Jingdong prefecture level city in Shandong
Happy Fan Shop	King of Power Saving	No shipment.	Jingdong prefecture level city in Shandong
Happy Fan Shop	King of Power Saving	Fake. Speechless.	Jingdong prefecture level city in Shandong
Happy Fan Shop	King of Power Saving	fantastic (idiom); awesome	Jingdong prefecture level city in Shandong
Happy Fan Shop	King of Power Saving	an excellent	Jingdong prefecture level city in Shandong
Happy Fan Shop	King of Power Saving	an excellent	Jingdong prefecture level city in Shandong
Happy Fan Shop	King of Power Saving	Want to buy! Recommended not to buy! No effect	Jingdong prefecture level city in Shandong
Happy Fan Shop	King of Power Saving	That's all there is to it. I can't figure out how to save electricity.	Jingdong prefecture level city in Shandong
Happy Fan Shop	King of Power Saving	It's no use.	Jingdong prefecture level city in Shandong
Happy Fan Shop	King of Power Saving	Things are more perfect than I imagined, very powerful, it is worth getting Packaging is very strong, things are well protected, it is worth the praise things are very satisfied Oh, beautiful and stylish atmosphere, customer service is super patient, service is meticulous and attentive, the big brand is not	Jingdong prefecture level city in Shandong
Happy Fan Shop	King of Power Saving	The effect is too good, the family has now several units, back to buy a number of times, the power saver received, power-saving effect is very good,, fine workmanship, long warranty, but also purify the air, before buying a used for a period of time, with the okay, and then bought a, and only now come to the	Jingdong prefecture level city in Shandong
Happy Fan Shop	King of Power Saving	Quite good, and imagine the same, casual ride are very good, satisfied! Baby received it 👌, really great ah! 🌈 quality is really nothing to say, very satisfied! 🆗 happy happy 😃 genuine, great, the price is very good, in short, quite good, will buy again a very good shopping experience, attentive service, fast logistics, distribution is also thoughtful, the attitude is also very good, value for money, ah,	Jingdong prefecture level city in Shandong
Happy Fan Shop	King of Power Saving	Power-saving effect: strong Cost-effective: quite good Appearance style: beautiful Workmanship material: meticulous Experience: looking forward to power-saving effect	Jingdong prefecture level city in Shandong
Happy Fan Shop	King of Power Saving	Cost-effective is okay, the appearance of the style of meticulous workmanship, looking forward to energy-saving effect.	Jingdong prefecture level city in Shandong
Happy Fan Shop	King of Power Saving	Very Good Very Power Saving This Power Saver is really power saving As soon as the power is turned on, the Power Saver starts the power saving function for you.	Jingdong prefecture level city in Shandong
Happy Fan Shop	King of Power Saving	Logistics is very fast, the price is favorable, friends introduced that it is very good to use, bought 2 at once, looking forward to the effect, used after the follow-up comment.	Jingdong prefecture level city in Shandong
Happy Fan Shop	King of Power Saving	It's all fake. It doesn't save any electricity.	Jingdong prefecture level city in Shandong
Happy Fan Shop	King of Power Saving	This Power Saver is so much better than the one I bought before, not only the effect is good, the quality is very good in all aspects, the customer service attitude is also very good, recommended to buy!	Jingdong prefecture level city in Shandong
Happy Fan Shop	King of Power Saving	Logistics is very fast, buy for parents, good quality, once bought 2, power-saving effect is good to buy 2 more, look forward to using the effect, satisfied will recommend to friends.	Jingdong prefecture level city in Shandong
Happy Fan Shop	King of Power Saving	With the description is the same, very high-grade, good value, to buy friends can order Oh!	Jingdong prefecture level city in Shandong
Happy Fan Shop	King of Power Saving	Not using much electricity in the winter saving over 100 per month! Ready to buy 2 more units in the summer ! ! ! ! ! Good quality, good price, power saving effect is obvious, cost-effective, worth buying, can be recommended!	Jingdong prefecture level city in Shandong
Happy Fan Shop	King of Power Saving	Purchased a power saver received on the put into use, rent a room with many electrical appliances, power consumption is very large, we have a piece of electricity or 1.2 dollars a time, with this power saver is much better, every day can save a lot of money, very convenient and practical.	Jingdong prefecture level city in Shandong
Happy Fan Shop	King of Power Saving	The power saving effect is very good: my supermarket monthly electricity bill is more than 7000 yuan. On May 1st someone recommended buying a couple of power savers, and I bought 5 of them first. The electricity bill was about 2000 less in June. This time, I bought 5 more. The seller said 800 degrees of electricity with one. I feel it's basically enough. A total of 10 units	Jingdong prefecture level city in Shandong
Happy Fan Shop	King of Power Saving	I never thought I could get such a good baby at such an affordable price, it's very good value for money, I love it!	Jingdong prefecture level city in Shandong
Happy Fan Shop	King of Power Saving	Previously bought other brands of power savers, but found no effect at home, this time to see how the products on the Jingdong, inserted in the home socket, the LED display is blue, the night light is very nice to see, look at the instructions that have to be half a year in order to achieve the best results, try it, I believe that the quality of the Jingdong, it should not be wrong.	Jingdong prefecture level city in Shandong
Happy Fan Shop	King of Power Saving	Superb, inexpensive, saves a lot of points, been using it for a long time!	Jingdong prefecture level city in Shandong
Happy Fan Shop	King of Power Saving	There are a lot of appliances in the house, the summer air conditioning is very costly, I bought this in front of the air conditioning effect is very good, I explain that I bought three in one breath, I use more electricity a month down to 200 or so, very satisfied.	Jingdong prefecture level city in Shandong
Happy Fan Shop	King of Power Saving	A month can save more than 200, home monthly electricity bill of more than 600 dollars, inserted 3 or so, an amount of the balance of the average can save more than 200. Product is very good, has been buying this one, has given a lot of relatives recommended, the quality is also very good, before buying a look with this same, who knows to buy home has not been used for more than a week on the bad, the quality of this is really good with more than two years!	Jingdong prefecture level city in Shandong
Happy Fan Shop	King of Power Saving	Whole house power saving king, can really save electricity, friends recommended, high power saving appliances, only replacement, no repair, after-sales worry-free!	Jingdong prefecture level city in Shandong
Happy Fan Shop	King of Power Saving	Great item love it, great customer service, great shopping experience!	Jingdong prefecture level city in Shandong
Happy Fan Shop	King of Power Saving	Power saving, very good use, much cheaper than the physical store, satisfaction	Jingdong prefecture level city in Shandong
Happy Fan Shop	King of Power Saving	In use, very well packaged and fast delivery of credit!	Jingdong prefecture level city in Shandong
Happy Fan Shop	King of Power Saving	Product quality, customer service attitude is particularly good, like, praise!	Jingdong prefecture level city in Shandong
Happy Fan Shop	King of Power Saving	The logistics is very fast, the packaging is not broken, the quality is very good, I like it very much!	Jingdong prefecture level city in Shandong
Happy Fan Shop	King of Power Saving	The saver was well packaged and was as described when plugged in	Jingdong prefecture level city in Shandong
Happy Fan Shop	King of Power Saving	Energy efficient and very satisfied	Jingdong prefecture level city in Shandong
Happy Fan Shop	King of Power Saving	Great quality for the price and fast shipping.	Jingdong prefecture level city in Shandong
Happy Fan Shop	King of Power Saving	It's great, just as described. Perfect.	Jingdong prefecture level city in Shandong
Happy Fan Shop	King of Power Saving	There is no power saving effect, but rather ring up an increase in electricity costs, because this machine is on continuously all day long, uninterruptedly, its own electricity and increased costs, plus the cost of purchasing the machine, so I feel that the use of this machine instead of increasing the	Jingdong prefecture level city in Shandong

Happy Fan Shop	King of Power Saving	Recommended by the owner of the store next door, looked at his home does have to save electricity, decided to get. Indeed quite can, the voltage is much more stable, there is a reduction in electricity	Jingdong prefecture level city in Shandong
Happy Fan Shop	King of Power Saving	Goods have been received value for money, affordable and reasonable price, and the description of the same, power-saving safe and reliable!	Jingdong prefecture level city in Shandong
Happy Fan Shop	King of Power Saving	Listen to friends introduced to buy, tried to save a lot of electricity is good, quite good, worth buying.	Jingdong prefecture level city in Shandong
Happy Fan Shop	King of Power Saving	The product is easy to use, safe and environmentally friendly, only used for a short time, to the next month, after the electricity bill status display to know how the performance of the power saver efficacy.	Jingdong prefecture level city in Shandong
Happy Fan Shop	King of Power Saving	Last month's electricity bill is 178 yuan, with this power saver is 92 yuan, ready to buy two more back home, I was introduced to buy a friend, just started to not believe that I did not expect the effect!	Jingdong prefecture level city in Shandong
Happy Fan Shop	King of Power Saving	It looks nice, but after using it for a few days I can't see how it saves power?	Jingdong prefecture level city in Shandong
Happy Fan Shop	King of Power Saving	Recommended by a friend, it is genuine, as described by the merchant, cost-effective	Jingdong prefecture level city in Shandong
Happy Fan Shop	King of Power Saving	Works well, affordable and saves money.	Jingdong prefecture level city in Shandong
Happy Fan Shop	King of Power Saving	Feels good haven't compared it yet, the air conditioning has been on a lot in the past two months	Jingdong prefecture level city in Shandong
Happy Fan Shop	King of Power Saving	Saving about 5 kWh per day is a good thing	Jingdong prefecture level city in Shandong
Happy Fan Shop	King of Power Saving	Not bad, affordable, best value for money	Jingdong prefecture level city in Shandong
Happy Fan Shop	King of Power Saving	The goods are genuine and the logistics are faster.	Jingdong prefecture level city in Shandong
Happy Fan Shop	King of Power Saving	Goods have been received today, has been plugged into the outlet, looking forward to saving electricity, after a period of time to see how the effect in the follow-up comment!	Jingdong prefecture level city in Shandong
Happy Fan Shop	King of Power Saving	I just got it home and haven't used it for long, so I'll use it for a couple months and see what happens. Seeing that most people have good reviews, it should be fine.	Jingdong prefecture level city in Shandong
Happy Fan Shop	King of Power Saving	I'm looking forward to the results, so I'll give you a good review out of habit! Follow up review after a while	Jingdong prefecture level city in Shandong
Happy Fan Shop	King of Power Saving	Use a dish first to know if it will help save energy	Jingdong prefecture level city in Shandong
Happy Fan Shop	King of Power Saving	Whether it saves electricity or not, we don't know yet.	Jingdong prefecture level city in Shandong
Happy Fan Shop	King of Power Saving	Goods have been received, use a period of time to know how oh. The courier did not deliver to the door ah! Sent to the station ah! The heat is inconvenient ah!	Jingdong prefecture level city in Shandong
Happy Fan Shop	King of Power Saving	A little effect, before an hour a power, now an hour 0.76 degrees, a dollar a power = save 24 cents, although not a lot, first use it!	Jingdong prefecture level city in Shandong
Happy Fan Shop	King of Power Saving	Just received the goods, use a month to evaluate	Jingdong prefecture level city in Shandong
Happy Fan Shop	King of Power Saving	Home every month with a lot of electricity, inadvertently found this good thing online that can save the beginning of a skeptical attitude to consult customer service, customer service replied very professional, said 120 days can be refunded that first try it!	Jingdong prefecture level city in Shandong
Happy Fan Shop	King of Power Saving	After receiving the goods this afternoon, immediately installed in the bathroom Smith energy storage electric water heater on the power outlet (instead of connecting with a plug board), when used, found that the "Power Saving King" LCD display, is the value of the voltage, not the store's video said so	Jingdong prefecture level city in Shandong
Happy Fan Shop	King of Power Saving	Has made many days, did not feel the power saving, the effect is not obvious.	Jingdong prefecture level city in Shandong
Happy Fan Shop	King of Power Saving	Energy savers do not save energy at all, but rather consume a lot of electricity.	Jingdong prefecture level city in Shandong
Happy Fan Shop	King of Power Saving	It's useless. It's a waste of money.	Jingdong prefecture level city in Shandong
Happy Fan Shop	King of Power Saving	Last month saved more than 500, which came back to buy, the store bought three, and then buy 1 home use, my family is dry beauty salon, more instruments, especially electricity, a month almost 1000. or so, with this did not expect to save so much, great, five-star praise!	Jingdong prefecture level city in Shandong
Happy Fan Shop	King of Power Saving	It rained a couple days ago and I just went to get the item back today , not bad, hey I looked at the meter and that red display light takes several seconds to jump, did not make a mistake buying it. After a period of time to evaluate.	Jingdong prefecture level city in Shandong
Happy Fan Shop	King of Power Saving	Without further ado, the electricity bill came down, this month less 102 yuan, has been repurchased, I also recommended to my friends, all said that there are effects, the product is okay, the effect is quite	Jingdong prefecture level city in Shandong
Happy Fan Shop	King of Power Saving	After buying and using two months really see the effect, so only to evaluate! Originally held the mindset of trying, I did not expect really effective, worth having!	Jingdong prefecture level city in Shandong
Happy Fan Shop	King of Power Saving	In the use of this energy saver before the monthly electricity bill is more than 150 yuan or so, now save thirty percent, every day to work, six or seven o'clock in the afternoon to go home, I do not know why the electricity bill is so expensive, energy-saving appliances really have the effect of recommending	Jingdong prefecture level city in Shandong
Happy Fan Shop	King of Power Saving	It saves about 100 bucks a month. A friend recommended it to me, and he's been using it for a while, and the effect is so obvious that I bought one too.	Jingdong prefecture level city in Shandong
Happy Fan Shop	King of Power Saving	My dad asked me to buy one, but after I paid the bill, I asked and he said it was a lot less this month, so he asked me to buy another one.	Jingdong prefecture level city in Shandong
Happy Fan Shop	King of Power Saving	The specific effect will have to be tested for at least two months to compare whether there is a power saving effect or not.	Jingdong prefecture level city in Shandong
Happy Fan Shop	King of Power Saving	'The goods have been received, immediately go to use, please ask the boss you instructions do not allow to withdraw, there are two fuses installed there!	Jingdong prefecture level city in Shandong
Happy Fan Shop	King of Power Saving	First praise is using, after using if good then follow up, now because I have not yet seen the results	Jingdong prefecture level city in Shandong
Happy Fan Shop	King of Power Saving	I've just been using it, and I haven't seen if it has a power-saving effect yet, so I'll follow up after 10 days and half a month.	Jingdong prefecture level city in Shandong
Happy Fan Shop	King of Power Saving	Just received the intelligent power saving meter, immediately plugged in to use, see it is not bad, and order to sell the second time	Jingdong prefecture level city in Shandong
Happy Fan Shop	King of Power Saving	This power saver is a bit effective, used for half a month, the effect is still visible.	Jingdong prefecture level city in Shandong
Happy Fan Shop	King of Power Saving	I received the baby and it looks great, I'm trying it out now and will keep an eye on it afterward.	Jingdong prefecture level city in Shandong
Happy Fan Shop	King of Power Saving	I've just received it, but I'm still trying to see how it works, so I'll come back in a little while.	Jingdong prefecture level city in Shandong
Happy Fan Shop	King of Power Saving	Just used it wait to use it for a while and then comment thanks, all first five stars.	Jingdong prefecture level city in Shandong
Happy Fan Shop	King of Power Saving	Very trustworthy brand, quality is guaranteed, the price is still affordable, quite satisfied!	Jingdong prefecture level city in Shandong
Happy Fan Shop	King of Power Saving	Things received, I hope that the platform of Jingdong do not sell fake goods are available	Jingdong prefecture level city in Shandong
Happy Fan Shop	King of Power Saving	Energy saver received, the use of the effect is good and the business description of the same	Jingdong prefecture level city in Shandong
Happy Fan Shop	King of Power Saving	The surface looks beautiful, the actual effect is not known, after using additional evaluation.	Jingdong prefecture level city in Shandong
Happy Fan Shop	King of Power Saving	Tried it for a few months, it really saves electricity, and I bought it for a friend.	Jingdong prefecture level city in Shandong
Happy Fan Shop	King of Power Saving	It didn't work and wanted to return it, it really didn't work at all.	Jingdong prefecture level city in Shandong
Happy Fan Shop	King of Power Saving	Just used it, don't know how effective it is, looking forward to usefulness	Jingdong prefecture level city in Shandong
Happy Fan Shop	King of Power Saving	After two months before the effect I do not know if it is true!	Jingdong prefecture level city in Shandong

Happy Fan Shop	King of Power Saving	So fast! Ordered yesterday and received today 👍👍👍👍👍👍👍👍👍 🌹🌹🌹🌹🌹🌹🌹🌹🌹	Jingdong prefecture level city in Shandong
Happy Fan Shop	King of Power Saving	I've started using it. We'll see how it works.	Jingdong prefecture level city in Shandong
Happy Fan Shop	King of Power Saving	I just received the goods, I won't know the effect until after two.	Jingdong prefecture level city in Shandong
Happy Fan Shop	King of Power Saving	Just received the goods, the specific effect will be evaluated later	Jingdong prefecture level city in Shandong
Happy Fan Shop	King of Power Saving	I've received the goods, I don't know if it's any good yet	Jingdong prefecture level city in Shandong
Happy Fan Shop	King of Power Saving	Try it for a month and then come back with a concrete evaluation	Jingdong prefecture level city in Shandong
Happy Fan Shop	King of Power Saving	In use, after two months to see the effect of power saving	Jingdong prefecture level city in Shandong
Happy Fan Shop	King of Power Saving	Power Saver. It's authentic. It's black. It works.	Jingdong prefecture level city in Shandong
Happy Fan Shop	King of Power Saving	I've been using it for a week now and it's not working.	Jingdong prefecture level city in Shandong
Happy Fan Shop	King of Power Saving	I'm just using it. I don't know how it's going to work.	Jingdong prefecture level city in Shandong
Happy Fan Shop	King of Power Saving	Products are better looking out for power savings	Jingdong prefecture level city in Shandong
Happy Fan Shop	King of Power Saving	I received it and I've kept it on. I hope it works.	Jingdong prefecture level city in Shandong
Happy Fan Shop	King of Power Saving	It's really good! Worth recommending to buy!	Jingdong prefecture level city in Shandong
Happy Fan Shop	King of Power Saving	Try it for a few days and see if it doesn't work, then return it.	Jingdong prefecture level city in Shandong
Happy Fan Shop	King of Power Saving	It's in use and I hope to be pleasantly surprised.	Jingdong prefecture level city in Shandong
Happy Fan Shop	King of Power Saving	We'll see how it works when we use it	Jingdong prefecture level city in Shandong
Happy Fan Shop	King of Power Saving	Let's see if it works.	Jingdong prefecture level city in Shandong
Happy Fan Shop	King of Power Saving	I'm using it and will follow up with a review	Jingdong prefecture level city in Shandong
Happy Fan Shop	King of Power Saving	It really works. It saves energy.	Jingdong prefecture level city in Shandong
Happy Fan Shop	King of Power Saving	It does have some power savings!	Jingdong prefecture level city in Shandong
Happy Fan Shop	King of Power Saving	Jingdong shopping speed is convenient and timely	Jingdong prefecture level city in Shandong
Happy Fan Shop	King of Power Saving	Second time I've bought it. Everything's fine.	Jingdong prefecture level city in Shandong
Happy Fan Shop	King of Power Saving	I don't know if it works yet.	Jingdong prefecture level city in Shandong
Happy Fan Shop	King of Power Saving	Not used, don't know how well it works.	Jingdong prefecture level city in Shandong
Happy Fan Shop	King of Power Saving	No visible results so far	Jingdong prefecture level city in Shandong
Happy Fan Shop	King of Power Saving	It doesn't feel like it's working.	Jingdong prefecture level city in Shandong
Happy Fan Shop	King of Power Saving	Where's the effect? Where are the power savings?	Jingdong prefecture level city in Shandong
Happy Fan Shop	King of Power Saving	Let's see how it works in practice!	Jingdong prefecture level city in Shandong
Happy Fan Shop	King of Power Saving	I'll wait until I use it to give you a good review	Jingdong prefecture level city in Shandong
Happy Fan Shop	King of Power Saving	I'll follow up after I've used it	Jingdong prefecture level city in Shandong
Happy Fan Shop	King of Power Saving	Well, that's good for saving power.	Jingdong prefecture level city in Shandong
Happy Fan Shop	King of Power Saving	Received. Used in the know.	Jingdong prefecture level city in Shandong
Happy Fan Shop	King of Power Saving	It doesn't do much good.	Jingdong prefecture level city in Shandong
Happy Fan Shop	King of Power Saving	It's not working.	Jingdong prefecture level city in Shandong
Happy Fan Shop	King of Power Saving	I don't know. I just used it.	Jingdong prefecture level city in Shandong
Happy Fan Shop	King of Power Saving	Use it and come back to evaluate.	Jingdong prefecture level city in Shandong
Happy Fan Shop	King of Power Saving	Two words: useless.	Jingdong prefecture level city in Shandong
Happy Fan Shop	King of Power Saving	To be used and then evaluated	Jingdong prefecture level city in Shandong
Happy Fan Shop	King of Power Saving	No shipment.	Jingdong prefecture level city in Shandong
Happy Fan Shop	King of Power Saving	Fake. Speechless.	Jingdong prefecture level city in Shandong
Happy Fan Shop	King of Power Saving	fantastic (idiom); awesome	Jingdong prefecture level city in Shandong
Happy Fan Shop	King of Power Saving	an excellent	Jingdong prefecture level city in Shandong
Happy Fan Shop	King of Power Saving	an excellent	Jingdong prefecture level city in Shandong
Happy Fan Shop	King of Power Saving	Want to buy! Recommended not to buy! No effect	Jingdong prefecture level city in Shandong
Happy Fan Shop	King of Power Saving	That's all there is to it. I can't figure out how to save electricity.	Jingdong prefecture level city in Shandong
Happy Fan Shop	King of Power Saving	It's no use.	Jingdong prefecture level city in Shandong
Happy Fan Shop	King of Power Saving	Things are more perfect than I imagined, very powerful, it is worth getting Packaging is very strong, things are well protected, it is worth the praise things are very satisfied Oh, beautiful and stylish atmosphere, customer service is super patient, service is meticulous and attentive, the big brand is not	Jingdong prefecture level city in Shandong
Happy Fan Shop	King of Power Saving	The effect is too good, the family has now several units, back to buy a number of times, the power saver received, power-saving effect is very good,, fine workmanship, long warranty, but also purify the air, before buying a used for a period of time, with the okay, and then bought a, and only now come to the	Jingdong prefecture level city in Shandong
Happy Fan Shop	King of Power Saving	Quite good, and imagine the same, casual ride are very good, satisfied! Baby received it 👌, really great ah! 🌈 quality is really nothing to say, very satisfied! 🆗 happy happy 😃 genuine, great, the price is very good, in short, quite good, will buy again a very good shopping experience, attentive service, fast logistics, distribution is also thoughtful, the attitude is also very good, value for money, ah,	Jingdong prefecture level city in Shandong

Happy Fan Shop	King of Power Saving	Power-saving effect: strong Cost-effective: quite good Appearance style: beautiful Workmanship material: meticulous Experience: looking forward to power-saving effect	Jingdong prefecture level city in Shandong
Happy Fan Shop	King of Power Saving	Cost-effective is okay, the appearance of the style of meticulous workmanship, looking forward to energy-saving effect.	Jingdong prefecture level city in Shandong
Happy Fan Shop	King of Power Saving	Very Good Very Power Saving This Power Saver is really power saving As soon as the power is turned on, the Power Saver starts the power saving function for you.	Jingdong prefecture level city in Shandong
Happy Fan Shop	King of Power Saving	Logistics is very fast, the price is favorable, friends introduced that it is very good to use, bought 2 at once, looking forward to the effect, used after the follow-up comment.	Jingdong prefecture level city in Shandong
Happy Fan Shop	King of Power Saving	It's all fake. It doesn't save any electricity.	Jingdong prefecture level city in Shandong
Happy Fan Shop	King of Power Saving	This Power Saver is so much better than the one I bought before, not only the effect is good, the quality is very good in all aspects, the customer service attitude is also very good, recommended to buy!	Jingdong prefecture level city in Shandong
Happy Fan Shop	King of Power Saving	Logistics is very fast, buy for parents, good quality, once bought 2, power-saving effect is good to buy 2 more, look forward to using the effect, satisfied will recommend to friends.	Jingdong prefecture level city in Shandong
Happy Fan Shop	King of Power Saving	With the description is the same, very high-grade, good value, to buy friends can order Oh!	Jingdong prefecture level city in Shandong
Happy Fan Shop	King of Power Saving	Not using much electricity in the winter saving over 100 per month! Ready to buy 2 more units in the summer ! ! ! ! ! Good quality, good price, power saving effect is obvious, cost-effective, worth buying, can be recommended!	Jingdong prefecture level city in Shandong
Happy Fan Shop	King of Power Saving	Purchased a power saver received on the put into use, rent a room with many electrical appliances, power consumption is very large, we have a piece of electricity or 1.2 dollars a time, with this power saver is much better, every day can save a lot of money, very convenient and practical.	Jingdong prefecture level city in Shandong
Happy Fan Shop	King of Power Saving	The power saving effect is very good: my supermarket monthly electricity bill is more than 7000 yuan. On May 1st someone recommended buying a couple of power savers, and I bought 5 of them first. The electricity bill was about 2000 less in June. This time, I bought 5 more. The seller said 800 degrees of electricity with one. I feel it's basically enough. A total of 10 units	Jingdong prefecture level city in Shandong
Happy Fan Shop	King of Power Saving	I never thought I could get such a good baby at such an affordable price, it's very good value for money, I love it!	Jingdong prefecture level city in Shandong
Happy Fan Shop	King of Power Saving	Previously bought other brands of power savers, but found no effect at home, this time to see how the products on the Jingdong, inserted in the home socket, the LED display is blue, the night light is very nice to see, look at the instructions that have to be half a year in order to achieve the best results, try it, I believe that the quality of the Jingdong, it should not be wrong.	Jingdong prefecture level city in Shandong
Happy Fan Shop	King of Power Saving	Superb, inexpensive, saves a lot of points, been using it for a long time!	Jingdong prefecture level city in Shandong
Happy Fan Shop	King of Power Saving	There are a lot of appliances in the house, the summer air conditioning is very costly, I bought this in front of the air conditioning effect is very good, I explain that I bought three in one breath, I use more electricity a month down to 200 or so, very satisfied.	Jingdong prefecture level city in Shandong
Happy Fan Shop	King of Power Saving	A month can save more than 200, home monthly electricity bill of more than 600 dollars, inserted 3 or so, an amount of the balance of the average can save more than 200. Product is very good, has been buying this one, has given a lot of relatives recommended, the quality is also very good, before buying a look with this same, who knows to buy home has not been used for more than a week on the bad, the quality of this is really good with more than two years!	Jingdong prefecture level city in Shandong
Happy Fan Shop	King of Power Saving	Whole house power saving king, can really save electricity, friends recommended, high power saving appliances, only replacement, no repair, after-sales worry-free!	Jingdong prefecture level city in Shandong
Happy Fan Shop	King of Power Saving	Great item love it, great customer service, great shopping experience!	Jingdong prefecture level city in Shandong
Happy Fan Shop	King of Power Saving	Power saving, very good use, much cheaper than the physical store, satisfaction	Jingdong prefecture level city in Shandong
Happy Fan Shop	King of Power Saving	In use, very well packaged and fast delivery of credit!	Jingdong prefecture level city in Shandong
Happy Fan Shop	King of Power Saving	Product quality, customer service attitude is particularly good, like, praise!	Jingdong prefecture level city in Shandong
Happy Fan Shop	King of Power Saving	The logistics is very fast, the packaging is not broken, the quality is very good, I like it very much!	Jingdong prefecture level city in Shandong
Happy Fan Shop	King of Power Saving	The saver was well packaged and was as described when plugged in	Jingdong prefecture level city in Shandong
Happy Fan Shop	King of Power Saving	Energy efficient and very satisfied	Jingdong prefecture level city in Shandong
Happy Fan Shop	King of Power Saving	Great quality for the price and fast shipping.	Jingdong prefecture level city in Shandong
Happy Fan Shop	King of Power Saving	It's great, just as described. Perfect.	Jingdong prefecture level city in Shandong
Happy Fan Shop	King of Power Saving	There is no power saving effect, but rather ring up an increase in electricity costs, because this machine is on continuously all day long, uninterruptedly, its own electricity and increased costs, plus the cost of purchasing the machine, so I feel that the use of this machine instead of increasing the	Jingdong prefecture level city in Shandong
Happy Fan Shop	King of Power Saving	Recommended by the owner of the store next door, looked at his home does have to save electricity, decided to get. Indeed quite can, the voltage is much more stable, there is a reduction in electricity	Jingdong prefecture level city in Shandong
Happy Fan Shop	King of Power Saving	Goods have been received value for money, affordable and reasonable price, and the description of the same, power-saving safe and reliable!	Jingdong prefecture level city in Shandong
Happy Fan Shop	King of Power Saving	Listen to friends introduced to buy, tried to save a lot of electricity is good, quite good, worth buying.	Jingdong prefecture level city in Shandong
Happy Fan Shop	King of Power Saving	The product is easy to use, safe and environmentally friendly, only used for a short time, to the next month, after the electricity bill status display to know how the performance of the power saver efficacy.	Jingdong prefecture level city in Shandong
Happy Fan Shop	King of Power Saving	Last month's electricity bill is 178 yuan, with this power saver is 92 yuan, ready to buy two more back home, I was introduced to buy a friend, just started to not believe that I did not expect the effect!	Jingdong prefecture level city in Shandong
Happy Fan Shop	King of Power Saving	It looks nice, but after using it for a few days I can't see how it saves power?	Jingdong prefecture level city in Shandong
Happy Fan Shop	King of Power Saving	Recommended by a friend, it is genuine, as described by the merchant, cost-effective	Jingdong prefecture level city in Shandong
Happy Fan Shop	King of Power Saving	Works well, affordable and saves money.	Jingdong prefecture level city in Shandong
Happy Fan Shop	King of Power Saving	Feels good haven't compared it yet, the air conditioning has been on a lot in the past two months	Jingdong prefecture level city in Shandong
Happy Fan Shop	King of Power Saving	Saving about 5 kWh per day is a good thing	Jingdong prefecture level city in Shandong
Happy Fan Shop	King of Power Saving	Not bad, affordable, best value for money	Jingdong prefecture level city in Shandong
Happy Fan Shop	King of Power Saving	The goods are genuine and the logistics are faster.	Jingdong prefecture level city in Shandong
Happy Fan Shop	King of Power Saving	Goods have been received today, has been plugged into the outlet, looking forward to saving electricity, after a period of time to see how the effect in the follow-up comment!	Jingdong prefecture level city in Shandong
Happy Fan Shop	King of Power Saving	I just got it home and haven't used it for long, so I'll use it for a couple months and see what happens. Seeing that most people have good reviews, it should be fine.	Jingdong prefecture level city in Shandong
Happy Fan Shop	King of Power Saving	I'm looking forward to the results, so I'll give you a good review out of habit! Follow up review after a while	Jingdong prefecture level city in Shandong
Happy Fan Shop	King of Power Saving	Use a dish first to know if it will help save energy	Jingdong prefecture level city in Shandong
Happy Fan Shop	King of Power Saving	Whether it saves electricity or not, we don't know yet.	Jingdong prefecture level city in Shandong
Happy Fan Shop	King of Power Saving	Goods have been received, use a period of time to know how oh. The courier did not deliver to the door ah! Sent to the station ah! The heat is inconvenient ah!	Jingdong prefecture level city in Shandong
Happy Fan Shop	King of Power Saving	A little effect, before an hour a power, now an hour 0.76 degrees, a dollar a power = save 24 cents, although not a lot, first use it!	Jingdong prefecture level city in Shandong

Happy Fan Shop	King of Power Saving	Just received the goods, use a month to evaluate	Jingdong prefecture level city in Shandong
Happy Fan Shop	King of Power Saving	Home every month with a lot of electricity, inadvertently found this good thing online that can save the beginning of a skeptical attitude to consult customer service, customer service replied very professional, said 120 days can be refunded that first try it!	Jingdong prefecture level city in Shandong
Happy Fan Shop	King of Power Saving	After receiving the goods this afternoon, immediately installed in the bathroom Smith energy storage electric water heater on the power outlet (instead of connecting with a plug board), when used, found that the "Power Saving King" LCD display, is the value of the voltage, not the store's video said so	Jingdong prefecture level city in Shandong
Happy Fan Shop	King of Power Saving	Has made many days, did not feel the power saving, the effect is not obvious.	Jingdong prefecture level city in Shandong
Happy Fan Shop	King of Power Saving	Energy savers do not save energy at all, but rather consume a lot of electricity.	Jingdong prefecture level city in Shandong
Happy Fan Shop	King of Power Saving	It's useless. It's a waste of money.	Jingdong prefecture level city in Shandong
Happy Fan Shop	King of Power Saving	Last month saved more than 500, which came back to buy, the store bought three, and then buy 1 home use, my family is dry beauty salon, more instruments, especially electricity, a month almost 1000. or so, with this did not expect to save so much, great, five-star praise!	Jingdong prefecture level city in Shandong
Happy Fan Shop	King of Power Saving	It rained a couple days ago and I just went to get the item back today , not bad, hey I looked at the meter and that red display light takes several seconds to jump, did not make a mistake buying it. After a period of time to evaluate.	Jingdong prefecture level city in Shandong
Happy Fan Shop	King of Power Saving	Without further ado, the electricity bill came down, this month less 102 yuan, has been repurchased, I also recommended to my friends, all said that there are effects, the product is okay, the effect is quite	Jingdong prefecture level city in Shandong
Happy Fan Shop	King of Power Saving	After buying and using two months really see the effect, so only to evaluate! Originally held the mindset of trying, I did not expect really effective, worth having!	Jingdong prefecture level city in Shandong
Happy Fan Shop	King of Power Saving	In the use of this energy saver before the monthly electricity bill is more than 150 yuan or so, now save thirty percent, every day to work, six or seven o'clock in the afternoon to go home, I do not know why the electricity bill is so expensive, energy-saving appliances really have the effect of recommending	Jingdong prefecture level city in Shandong
Happy Fan Shop	King of Power Saving	It saves about 100 bucks a month. A friend recommended it to me, and he's been using it for a while, and the effect is so obvious that I bought one too.	Jingdong prefecture level city in Shandong
Happy Fan Shop	King of Power Saving	My dad asked me to buy one, but after I paid the bill, I asked and he said it was a lot less this month, so he asked me to buy another one.	Jingdong prefecture level city in Shandong
Happy Fan Shop	King of Power Saving	The specific effect will have to be tested for at least two months to compare whether there is a power saving effect or not.	Jingdong prefecture level city in Shandong
Happy Fan Shop	King of Power Saving	'The goods have been received, immediately go to use, please ask the boss you instructions do not allow to withdraw, there are two fuses installed there!	Jingdong prefecture level city in Shandong
Happy Fan Shop	King of Power Saving	First praise is using, after using if good then follow up, now because I have not yet seen the results	Jingdong prefecture level city in Shandong
Happy Fan Shop	King of Power Saving	I've just been using it, and I haven't seen if it has a power-saving effect yet, so I'll follow up after 10 days and half a month.	Jingdong prefecture level city in Shandong
Happy Fan Shop	King of Power Saving	Just received the intelligent power saving meter, immediately plugged in to use, see it is not bad, and order to sell the second time	Jingdong prefecture level city in Shandong
Happy Fan Shop	King of Power Saving	This power saver is a bit effective, used for half a month, the effect is still visible.	Jingdong prefecture level city in Shandong
Happy Fan Shop	King of Power Saving	I received the baby and it looks great, I'm trying it out now and will keep an eye on it afterward.	Jingdong prefecture level city in Shandong
Happy Fan Shop	King of Power Saving	I've just received it, but I'm still trying to see how it works, so I'll come back in a little while.	Jingdong prefecture level city in Shandong
Happy Fan Shop	King of Power Saving	Just used it wait to use it for a while and then comment thanks, all first five stars.	Jingdong prefecture level city in Shandong
Happy Fan Shop	King of Power Saving	Very trustworthy brand, quality is guaranteed, the price is still affordable, quite satisfied!	Jingdong prefecture level city in Shandong
Happy Fan Shop	King of Power Saving	Things received, I hope that the platform of Jingdong do not sell fake goods are available	Jingdong prefecture level city in Shandong
Happy Fan Shop	King of Power Saving	Energy saver received, the use of the effect is good and the business description of the same	Jingdong prefecture level city in Shandong
Happy Fan Shop	King of Power Saving	The surface looks beautiful, the actual effect is not known, after using additional evaluation.	Jingdong prefecture level city in Shandong
Happy Fan Shop	King of Power Saving	Tried it for a few months, it really saves electricity, and I bought it for a friend.	Jingdong prefecture level city in Shandong
Happy Fan Shop	King of Power Saving	It didn't work and wanted to return it, it really didn't work at all.	Jingdong prefecture level city in Shandong
Happy Fan Shop	King of Power Saving	Just used it, don't know how effective it is, looking forward to usefulness	Jingdong prefecture level city in Shandong
Happy Fan Shop	King of Power Saving	After two months before the effect I do not know if it is true!	Jingdong prefecture level city in Shandong
Happy Fan Shop	King of Power Saving	So fast! Ordered yesterday and received today 👍👍👍👍👍👍👍👍👍 🌹🌹🌹🌹🌹🌹🌹🌹🌹	Jingdong prefecture level city in Shandong
Happy Fan Shop	King of Power Saving	I've started using it. We'll see how it works.	Jingdong prefecture level city in Shandong
Happy Fan Shop	King of Power Saving	I just received the goods, I won't know the effect until after two.	Jingdong prefecture level city in Shandong
Happy Fan Shop	King of Power Saving	Just received the goods, the specific effect will be evaluated later	Jingdong prefecture level city in Shandong
Happy Fan Shop	King of Power Saving	I've received the goods, I don't know if it's any good yet	Jingdong prefecture level city in Shandong
Happy Fan Shop	King of Power Saving	Try it for a month and then come back with a concrete evaluation	Jingdong prefecture level city in Shandong
Happy Fan Shop	King of Power Saving	In use, after two months to see the effect of power saving	Jingdong prefecture level city in Shandong
Happy Fan Shop	King of Power Saving	Power Saver. It's authentic. It's black. It works.	Jingdong prefecture level city in Shandong
Happy Fan Shop	King of Power Saving	I've been using it for a week now and it's not working.	Jingdong prefecture level city in Shandong
Happy Fan Shop	King of Power Saving	I'm just using it. I don't know how it's going to work.	Jingdong prefecture level city in Shandong
Happy Fan Shop	King of Power Saving	Products are better looking out for power savings	Jingdong prefecture level city in Shandong
Happy Fan Shop	King of Power Saving	I received it and I've kept it on. I hope it works.	Jingdong prefecture level city in Shandong
Happy Fan Shop	King of Power Saving	It's really good! Worth recommending to buy!	Jingdong prefecture level city in Shandong
Happy Fan Shop	King of Power Saving	Try it for a few days and see if it doesn't work, then return it.	Jingdong prefecture level city in Shandong
Happy Fan Shop	King of Power Saving	It's in use and I hope to be pleasantly surprised.	Jingdong prefecture level city in Shandong
Happy Fan Shop	King of Power Saving	We'll see how it works when we use it	Jingdong prefecture level city in Shandong
Happy Fan Shop	King of Power Saving	Let's see if it works.	Jingdong prefecture level city in Shandong
Happy Fan Shop	King of Power Saving	I'm using it and will follow up with a review	Jingdong prefecture level city in Shandong
Happy Fan Shop	King of Power Saving	It really works. It saves energy.	Jingdong prefecture level city in Shandong

Happy Fan Shop	King of Power Saving	It does have some power savings!	Jingdong prefecture level city in Shandong
Happy Fan Shop	King of Power Saving	Jingdong shopping speed is convenient and timely	Jingdong prefecture level city in Shandong
Happy Fan Shop	King of Power Saving	Second time I've bought it. Everything's fine.	Jingdong prefecture level city in Shandong
Happy Fan Shop	King of Power Saving	I don't know if it works yet.	Jingdong prefecture level city in Shandong
Happy Fan Shop	King of Power Saving	Not used, don't know how well it works.	Jingdong prefecture level city in Shandong
Happy Fan Shop	King of Power Saving	No visible results so far	Jingdong prefecture level city in Shandong
Happy Fan Shop	King of Power Saving	It doesn't feel like it's working.	Jingdong prefecture level city in Shandong
Happy Fan Shop	King of Power Saving	Where's the effect? Where are the power savings?	Jingdong prefecture level city in Shandong
Happy Fan Shop	King of Power Saving	Let's see how it works in practice!	Jingdong prefecture level city in Shandong
Happy Fan Shop	King of Power Saving	I'll wait until I use it to give you a good review	Jingdong prefecture level city in Shandong
Happy Fan Shop	King of Power Saving	I'll follow up after I've used it	Jingdong prefecture level city in Shandong
Happy Fan Shop	King of Power Saving	Well, that's good for saving power.	Jingdong prefecture level city in Shandong
Happy Fan Shop	King of Power Saving	Received. Used in the know.	Jingdong prefecture level city in Shandong
Happy Fan Shop	King of Power Saving	It doesn't do much good.	Jingdong prefecture level city in Shandong
Happy Fan Shop	King of Power Saving	It's not working.	Jingdong prefecture level city in Shandong
Happy Fan Shop	King of Power Saving	I don't know. I just used it.	Jingdong prefecture level city in Shandong
Happy Fan Shop	King of Power Saving	Use it and come back to evaluate.	Jingdong prefecture level city in Shandong
Happy Fan Shop	King of Power Saving	Two words: useless.	Jingdong prefecture level city in Shandong
Happy Fan Shop	King of Power Saving	To be used and then evaluated	Jingdong prefecture level city in Shandong
Happy Fan Shop	King of Power Saving	No shipment.	Jingdong prefecture level city in Shandong
Happy Fan Shop	King of Power Saving	Fake. Speechless.	Jingdong prefecture level city in Shandong
Happy Fan Shop	King of Power Saving	fantastic (idiom); awesome	Jingdong prefecture level city in Shandong
Happy Fan Shop	King of Power Saving	an excellent	Jingdong prefecture level city in Shandong
Happy Fan Shop	King of Power Saving	an excellent	Jingdong prefecture level city in Shandong
Happy Fan Shop	King of Power Saving	Want to buy! Recommended not to buy! No effect	Jingdong prefecture level city in Shandong
Happy Fan Shop	King of Power Saving	That's all there is to it. I can't figure out how to save electricity.	Jingdong prefecture level city in Shandong
Happy Fan Shop	King of Power Saving	It's no use.	Jingdong prefecture level city in Shandong
Happy Fan Shop	King of Power Saving	Things are more perfect than I imagined, very powerful, it is worth getting Packaging is very strong, things are well protected, it is worth the praise things are very satisfied Oh, beautiful and stylish atmosphere, customer service is super patient, service is meticulous and attentive, the big brand is not	Jingdong prefecture level city in Shandong
Happy Fan Shop	King of Power Saving	The effect is too good, the family has now several units, back to buy a number of times, the power saver received, power-saving effect is very good,, fine workmanship, long warranty, but also purify the air, before buying a used for a period of time, with the okay, and then bought a, and only now come to the	Jingdong prefecture level city in Shandong
Happy Fan Shop	King of Power Saving	Quite good, and imagine the same, casual ride are very good, satisfied! Baby received it 👌, really great ah! 🌈 quality is really nothing to say, very satisfied! 🆗 happy happy 😃 genuine, great, the price is very good, in short, quite good, will buy again a very good shopping experience, attentive service, fast logistics, distribution is also thoughtful, the attitude is also very good, value for money, ah,	Jingdong prefecture level city in Shandong
Happy Fan Shop	King of Power Saving	Power-saving effect: strong Cost-effective: quite good Appearance style: beautiful Workmanship material: meticulous Experience: looking forward to power-saving effect	Jingdong prefecture level city in Shandong
Happy Fan Shop	King of Power Saving	Cost-effective is okay, the appearance of the style of meticulous workmanship, looking forward to energy-saving effect.	Jingdong prefecture level city in Shandong
Happy Fan Shop	King of Power Saving	Very Good Very Power Saving This Power Saver is really power saving As soon as the power is turned on, the Power Saver starts the power saving function for you.	Jingdong prefecture level city in Shandong
Happy Fan Shop	King of Power Saving	Logistics is very fast, the price is favorable, friends introduced that it is very good to use, bought 2 at once, looking forward to the effect, used after the follow-up comment.	Jingdong prefecture level city in Shandong
Happy Fan Shop	King of Power Saving	It's all fake. It doesn't save any electricity.	Jingdong prefecture level city in Shandong
Happy Fan Shop	King of Power Saving	This Power Saver is so much better than the one I bought before, not only the effect is good, the quality is very good in all aspects, the customer service attitude is also very good, recommended to buy!	Jingdong prefecture level city in Shandong
Happy Fan Shop	King of Power Saving	Logistics is very fast, buy for parents, good quality, once bought 2, power-saving effect is good to buy 2 more, look forward to using the effect, satisfied will recommend to friends.	Jingdong prefecture level city in Shandong
Happy Fan Shop	King of Power Saving	With the description is the same, very high-grade, good value, to buy friends can order Oh!	Jingdong prefecture level city in Shandong
Happy Fan Shop	King of Power Saving	Not using much electricity in the winter saving over 100 per month! Ready to buy 2 more units in the summer ! ! ! ! ! Good quality, good price, power saving effect is obvious, cost-effective, worth buying, can be recommended!	Jingdong prefecture level city in Shandong
Happy Fan Shop	King of Power Saving	Purchased a power saver received on the put into use, rent a room with many electrical appliances, power consumption is very large, we have a piece of electricity or 1.2 dollars a time, with this power saver is much better, every day can save a lot of money, very convenient and practical.	Jingdong prefecture level city in Shandong
Happy Fan Shop	King of Power Saving	The power saving effect is very good: my supermarket monthly electricity bill is more than 7000 yuan. On May 1st someone recommended buying a couple of power savers, and I bought 5 of them first. The electricity bill was about 2000 less in June. This time, I bought 5 more. The seller said 800 degrees of electricity with one. I feel it's basically enough. A total of 10 units	Jingdong prefecture level city in Shandong
Happy Fan Shop	King of Power Saving	I never thought I could get such a good baby at such an affordable price, it's very good value for money, I love it!	Jingdong prefecture level city in Shandong
Happy Fan Shop	King of Power Saving	Previously bought other brands of power savers, but found no effect at home, this time to see how the products on the Jingdong, inserted in the home socket, the LED display is blue, the night light is very nice to see, look at the instructions that have to be half a year in order to achieve the best results, try it, I believe that the quality of the Jingdong, it should not be wrong.	Jingdong prefecture level city in Shandong
Happy Fan Shop	King of Power Saving	Superb, inexpensive, saves a lot of points, been using it for a long time!	Jingdong prefecture level city in Shandong
Happy Fan Shop	King of Power Saving	There are a lot of appliances in the house, the summer air conditioning is very costly, I bought this in front of the air conditioning effect is very good, I explain that I bought three in one breath, I use more electricity a month down to 200 or so, very satisfied.	Jingdong prefecture level city in Shandong

Happy Fan Shop	King of Power Saving	A month can save more than 200, home monthly electricity bill of more than 600 dollars, inserted 3 or so, an amount of the balance of the average can save more than 200. Product is very good, has been buying this one, has given a lot of relatives recommended, the quality is also very good, before buying a look with this same, who knows to buy home has not been used for more than a week on the bad, the quality of this is really good with more than two years!	Jingdong prefecture level city in Shandong
Happy Fan Shop	King of Power Saving	Whole house power saving king, can really save electricity, friends recommended, high power saving appliances, only replacement, no repair, after-sales worry-free!	Jingdong prefecture level city in Shandong
Happy Fan Shop	King of Power Saving	Great item love it, great customer service, great shopping experience!	Jingdong prefecture level city in Shandong
Happy Fan Shop	King of Power Saving	Power saving, very good use, much cheaper than the physical store, satisfaction	Jingdong prefecture level city in Shandong
Happy Fan Shop	King of Power Saving	In use, very well packaged and fast delivery of credit!	Jingdong prefecture level city in Shandong
Happy Fan Shop	King of Power Saving	Product quality, customer service attitude is particularly good, like, praise!	Jingdong prefecture level city in Shandong
Happy Fan Shop	King of Power Saving	The logistics is very fast, the packaging is not broken, the quality is very good, I like it very much!	Jingdong prefecture level city in Shandong
Happy Fan Shop	King of Power Saving	The saver was well packaged and was as described when plugged in	Jingdong prefecture level city in Shandong
Happy Fan Shop	King of Power Saving	Energy efficient and very satisfied	Jingdong prefecture level city in Shandong
Happy Fan Shop	King of Power Saving	Great quality for the price and fast shipping.	Jingdong prefecture level city in Shandong
Happy Fan Shop	King of Power Saving	It's great, just as described. Perfect.	Jingdong prefecture level city in Shandong
Happy Fan Shop	King of Power Saving	There is no power saving effect, but rather ring up an increase in electricity costs, because this machine is on continuously all day long, uninterruptedly, its own electricity and increased costs, plus the cost of purchasing the machine, so I feel that the use of this machine instead of increasing the	Jingdong prefecture level city in Shandong
Happy Fan Shop	King of Power Saving	Recommended by the owner of the store next door, looked at his home does have to save electricity, decided to get. Indeed quite can, the voltage is much more stable, there is a reduction in electricity	Jingdong prefecture level city in Shandong
Happy Fan Shop	King of Power Saving	Goods have been received value for money, affordable and reasonable price, and the description of the same, power-saving safe and reliable!	Jingdong prefecture level city in Shandong
Happy Fan Shop	King of Power Saving	Listen to friends introduced to buy, tried to save a lot of electricity is good, quite good, worth buying.	Jingdong prefecture level city in Shandong
Happy Fan Shop	King of Power Saving	The product is easy to use, safe and environmentally friendly, only used for a short time, to the next month, after the electricity bill status display to know how the performance of the power saver efficacy.	Jingdong prefecture level city in Shandong
Happy Fan Shop	King of Power Saving	Last month's electricity bill is 178 yuan, with this power saver is 92 yuan, ready to buy two more back home, I was introduced to buy a friend, just started to not believe that I did not expect the effect!	Jingdong prefecture level city in Shandong
Happy Fan Shop	King of Power Saving	It looks nice, but after using it for a few days I can't see how it saves power?	Jingdong prefecture level city in Shandong
Happy Fan Shop	King of Power Saving	Recommended by a friend, it is genuine, as described by the merchant, cost-effective	Jingdong prefecture level city in Shandong
Happy Fan Shop	King of Power Saving	Works well, affordable and saves money.	Jingdong prefecture level city in Shandong
Happy Fan Shop	King of Power Saving	Feels good haven't compared it yet, the air conditioning has been on a lot in the past two months	Jingdong prefecture level city in Shandong
Happy Fan Shop	King of Power Saving	Saving about 5 kWh per day is a good thing	Jingdong prefecture level city in Shandong
Happy Fan Shop	King of Power Saving	Not bad, affordable, best value for money	Jingdong prefecture level city in Shandong
Happy Fan Shop	King of Power Saving	The goods are genuine and the logistics are faster.	Jingdong prefecture level city in Shandong
Happy Fan Shop	King of Power Saving	Goods have been received today, has been plugged into the outlet, looking forward to saving electricity, after a period of time to see how the effect in the follow-up comment!	Jingdong prefecture level city in Shandong
Happy Fan Shop	King of Power Saving	I just got it home and haven't used it for long, so I'll use it for a couple months and see what happens. Seeing that most people have good reviews, it should be fine.	Jingdong prefecture level city in Shandong
Happy Fan Shop	King of Power Saving	I'm looking forward to the results, so I'll give you a good review out of habit! Follow up review after a while	Jingdong prefecture level city in Shandong
Happy Fan Shop	King of Power Saving	Use a dish first to know if it will help save energy	Jingdong prefecture level city in Shandong
Happy Fan Shop	King of Power Saving	Whether it saves electricity or not, we don't know yet.	Jingdong prefecture level city in Shandong
Happy Fan Shop	King of Power Saving	Goods have been received, use a period of time to know how oh. The courier did not deliver to the door ah! Sent to the station ah! The heat is inconvenient ah!	Jingdong prefecture level city in Shandong
Happy Fan Shop	King of Power Saving	A little effect, before an hour a power, now an hour 0.76 degrees, a dollar a power = save 24 cents, although not a lot, first use it!	Jingdong prefecture level city in Shandong
Happy Fan Shop	King of Power Saving	Just received the goods, use a month to evaluate	Jingdong prefecture level city in Shandong
Happy Fan Shop	King of Power Saving	Home every month with a lot of electricity, inadvertently found this good thing online that can save the beginning of a skeptical attitude to consult customer service, customer service replied very professional, said 120 days can be refunded that first try it!	Jingdong prefecture level city in Shandong
Happy Fan Shop	King of Power Saving	After receiving the goods this afternoon, immediately installed in the bathroom Smith energy storage electric water heater on the power outlet (instead of connecting with a plug board), when used, found that the "Power Saving King" LCD display, is the value of the voltage, not the store's video said so	Jingdong prefecture level city in Shandong
Happy Fan Shop	King of Power Saving	Has made many days, did not feel the power saving, the effect is not obvious.	Jingdong prefecture level city in Shandong
Happy Fan Shop	King of Power Saving	Energy savers do not save energy at all, but rather consume a lot of electricity.	Jingdong prefecture level city in Shandong
Happy Fan Shop	King of Power Saving	It's useless. It's a waste of money.	Jingdong prefecture level city in Shandong
Happy Fan Shop	King of Power Saving	Last month saved more than 500, which came back to buy, the store bought three, and then buy 1 home use, my family is dry beauty salon, more instruments, especially electricity, a month almost 1000. or so, with this did not expect to save so much, great, five-star praise!	Jingdong prefecture level city in Shandong
Happy Fan Shop	King of Power Saving	It rained a couple days ago and I just went to get the item back today , not bad, hey I looked at the meter and that red display light takes several seconds to jump, did not make a mistake buying it. After a period of time to evaluate.	Jingdong prefecture level city in Shandong
Happy Fan Shop	King of Power Saving	Without further ado, the electricity bill came down, this month less 102 yuan, has been repurchased, I also recommended to my friends, all said that there are effects, the product is okay, the effect is quite	Jingdong prefecture level city in Shandong
Happy Fan Shop	King of Power Saving	After buying and using two months really see the effect, so only to evaluate! Originally held the mindset of trying, I did not expect really effective, worth having!	Jingdong prefecture level city in Shandong
Happy Fan Shop	King of Power Saving	In the use of this energy saver before the monthly electricity bill is more than 150 yuan or so, now save thirty percent, every day to work, six or seven o'clock in the afternoon to go home, I do not know why the electricity bill is so expensive, energy-saving appliances really have the effect of recommending	Jingdong prefecture level city in Shandong
Happy Fan Shop	King of Power Saving	It saves about 100 bucks a month. A friend recommended it to me, and he's been using it for a while, and the effect is so obvious that I bought one too.	Jingdong prefecture level city in Shandong
Happy Fan Shop	King of Power Saving	My dad asked me to buy one, but after I paid the bill, I asked and he said it was a lot less this month, so he asked me to buy another one.	Jingdong prefecture level city in Shandong
Happy Fan Shop	King of Power Saving	The specific effect will have to be tested for at least two months to compare whether there is a power saving effect or not.	Jingdong prefecture level city in Shandong
Happy Fan Shop	King of Power Saving	'The goods have been received, immediately go to use, please ask the boss you instructions do not allow to withdraw, there are two fuses installed there!	Jingdong prefecture level city in Shandong
Happy Fan Shop	King of Power Saving	First praise is using, after using if good then follow up, now because I have not yet seen the results	Jingdong prefecture level city in Shandong

Happy Fan Shop	King of Power Saving	I've just been using it, and I haven't seen if it has a power-saving effect yet, so I'll follow up after 10 days and half a month.	Jingdong prefecture level city in Shandong
Happy Fan Shop	King of Power Saving	Just received the intelligent power saving meter, immediately plugged in to use, see it is not bad, and order to sell the second time	Jingdong prefecture level city in Shandong
Happy Fan Shop	King of Power Saving	This power saver is a bit effective, used for half a month, the effect is still visible.	Jingdong prefecture level city in Shandong
Happy Fan Shop	King of Power Saving	I received the baby and it looks great, I'm trying it out now and will keep an eye on it afterward.	Jingdong prefecture level city in Shandong
Happy Fan Shop	King of Power Saving	I've just received it, but I'm still trying to see how it works, so I'll come back in a little while.	Jingdong prefecture level city in Shandong
Happy Fan Shop	King of Power Saving	Just used it wait to use it for a while and then comment thanks, all first five stars.	Jingdong prefecture level city in Shandong
Happy Fan Shop	King of Power Saving	Very trustworthy brand, quality is guaranteed, the price is still affordable, quite satisfied!	Jingdong prefecture level city in Shandong
Happy Fan Shop	King of Power Saving	Things received, I hope that the platform of Jingdong do not sell fake goods are available	Jingdong prefecture level city in Shandong
Happy Fan Shop	King of Power Saving	Energy saver received, the use of the effect is good and the business description of the same	Jingdong prefecture level city in Shandong
Happy Fan Shop	King of Power Saving	The surface looks beautiful, the actual effect is not known, after using additional evaluation.	Jingdong prefecture level city in Shandong
Happy Fan Shop	King of Power Saving	Tried it for a few months, it really saves electricity, and I bought it for a friend.	Jingdong prefecture level city in Shandong
Happy Fan Shop	King of Power Saving	It didn't work and wanted to return it, it really didn't work at all.	Jingdong prefecture level city in Shandong
Happy Fan Shop	King of Power Saving	Just used it, don't know how effective it is, looking forward to usefulness	Jingdong prefecture level city in Shandong
Happy Fan Shop	King of Power Saving	After two months before the effect I do not know if it is true!	Jingdong prefecture level city in Shandong
Happy Fan Shop	King of Power Saving	So fast! Ordered yesterday and received today 👍👍👍👍👍👍👍👍👍 🌹🌹🌹🌹🌹🌹🌹🌹🌹	Jingdong prefecture level city in Shandong
Happy Fan Shop	King of Power Saving	I've started using it. We'll see how it works.	Jingdong prefecture level city in Shandong
Happy Fan Shop	King of Power Saving	I just received the goods, I won't know the effect until after two.	Jingdong prefecture level city in Shandong
Happy Fan Shop	King of Power Saving	Just received the goods, the specific effect will be evaluated later	Jingdong prefecture level city in Shandong
Happy Fan Shop	King of Power Saving	I've received the goods, I don't know if it's any good yet	Jingdong prefecture level city in Shandong
Happy Fan Shop	King of Power Saving	Try it for a month and then come back with a concrete evaluation	Jingdong prefecture level city in Shandong
Happy Fan Shop	King of Power Saving	In use, after two months to see the effect of power saving	Jingdong prefecture level city in Shandong
Happy Fan Shop	King of Power Saving	Power Saver. It's authentic. It's black. It works.	Jingdong prefecture level city in Shandong
Happy Fan Shop	King of Power Saving	I've been using it for a week now and it's not working.	Jingdong prefecture level city in Shandong
Happy Fan Shop	King of Power Saving	I'm just using it. I don't know how it's going to work.	Jingdong prefecture level city in Shandong
Happy Fan Shop	King of Power Saving	Products are better looking out for power savings	Jingdong prefecture level city in Shandong
Happy Fan Shop	King of Power Saving	I received it and I've kept it on. I hope it works.	Jingdong prefecture level city in Shandong
Happy Fan Shop	King of Power Saving	It's really good! Worth recommending to buy!	Jingdong prefecture level city in Shandong
Happy Fan Shop	King of Power Saving	Try it for a few days and see if it doesn't work, then return it.	Jingdong prefecture level city in Shandong
Happy Fan Shop	King of Power Saving	It's in use and I hope to be pleasantly surprised.	Jingdong prefecture level city in Shandong
Happy Fan Shop	King of Power Saving	We'll see how it works when we use it	Jingdong prefecture level city in Shandong
Happy Fan Shop	King of Power Saving	Let's see if it works.	Jingdong prefecture level city in Shandong
Happy Fan Shop	King of Power Saving	I'm using it and will follow up with a review	Jingdong prefecture level city in Shandong
Happy Fan Shop	King of Power Saving	It really works. It saves energy.	Jingdong prefecture level city in Shandong
Happy Fan Shop	King of Power Saving	It does have some power savings!	Jingdong prefecture level city in Shandong
Happy Fan Shop	King of Power Saving	Jingdong shopping speed is convenient and timely	Jingdong prefecture level city in Shandong
Happy Fan Shop	King of Power Saving	Second time I've bought it. Everything's fine.	Jingdong prefecture level city in Shandong
Happy Fan Shop	King of Power Saving	I don't know if it works yet.	Jingdong prefecture level city in Shandong
Happy Fan Shop	King of Power Saving	Not used, don't know how well it works.	Jingdong prefecture level city in Shandong
Happy Fan Shop	King of Power Saving	No visible results so far	Jingdong prefecture level city in Shandong
Happy Fan Shop	King of Power Saving	It doesn't feel like it's working.	Jingdong prefecture level city in Shandong
Happy Fan Shop	King of Power Saving	Where's the effect? Where are the power savings?	Jingdong prefecture level city in Shandong
Happy Fan Shop	King of Power Saving	Let's see how it works in practice!	Jingdong prefecture level city in Shandong
Happy Fan Shop	King of Power Saving	I'll wait until I use it to give you a good review	Jingdong prefecture level city in Shandong
Happy Fan Shop	King of Power Saving	I'll follow up after I've used it	Jingdong prefecture level city in Shandong
Happy Fan Shop	King of Power Saving	Well, that's good for saving power.	Jingdong prefecture level city in Shandong
Happy Fan Shop	King of Power Saving	Received. Used in the know.	Jingdong prefecture level city in Shandong
Happy Fan Shop	King of Power Saving	It doesn't do much good.	Jingdong prefecture level city in Shandong
Happy Fan Shop	King of Power Saving	It's not working.	Jingdong prefecture level city in Shandong
Happy Fan Shop	King of Power Saving	I don't know. I just used it.	Jingdong prefecture level city in Shandong
Happy Fan Shop	King of Power Saving	Use it and come back to evaluate.	Jingdong prefecture level city in Shandong
Happy Fan Shop	King of Power Saving	Two words: useless.	Jingdong prefecture level city in Shandong

Happy Fan Shop	King of Power Saving	To be used and then evaluated	Jingdong prefecture level city in Shandong
Happy Fan Shop	King of Power Saving	No shipment.	Jingdong prefecture level city in Shandong
Happy Fan Shop	King of Power Saving	Fake. Speechless.	Jingdong prefecture level city in Shandong
Happy Fan Shop	King of Power Saving	fantastic (idiom); awesome	Jingdong prefecture level city in Shandong
Happy Fan Shop	King of Power Saving	an excellent	Jingdong prefecture level city in Shandong
Happy Fan Shop	King of Power Saving	an excellent	Jingdong prefecture level city in Shandong
Happy Fan Shop	King of Power Saving	Want to buy! Recommended not to buy! No effect	Jingdong prefecture level city in Shandong
Happy Fan Shop	King of Power Saving	That's all there is to it. I can't figure out how to save electricity.	Jingdong prefecture level city in Shandong
Happy Fan Shop	King of Power Saving	It's no use.	Jingdong prefecture level city in Shandong
Happy Fan Shop	King of Power Saving	Things are more perfect than I imagined, very powerful, it is worth getting Packaging is very strong, things are well protected, it is worth the praise things are very satisfied Oh, beautiful and stylish atmosphere, customer service is super patient, service is meticulous and attentive, the big brand is not	Jingdong prefecture level city in Shandong
Happy Fan Shop	King of Power Saving	The effect is too good, the family has now several units, back to buy a number of times, the power saver received, power-saving effect is very good,, fine workmanship, long warranty, but also purify the air, before buying a used for a period of time, with the okay, and then bought a, and only now come to the	Jingdong prefecture level city in Shandong
Happy Fan Shop	King of Power Saving	Quite good, and imagine the same, casual ride are very good, satisfied! Baby received it 👌, really great ah! 🌈 quality is really nothing to say, very satisfied! 🆗 happy happy 😃 genuine, great, the price is very good, in short, quite good, will buy again a very good shopping experience, attentive service, fast logistics, distribution is also thoughtful, the attitude is also very good, value for money, ah,	Jingdong prefecture level city in Shandong
Happy Fan Shop	King of Power Saving	Power-saving effect: strong Cost-effective: quite good Appearance style: beautiful Workmanship material: meticulous Experience: looking forward to power-saving effect	Jingdong prefecture level city in Shandong
Happy Fan Shop	King of Power Saving	Cost-effective is okay, the appearance of the style of meticulous workmanship, looking forward to energy-saving effect.	Jingdong prefecture level city in Shandong
Happy Fan Shop	King of Power Saving	Very Good Very Power Saving This Power Saver is really power saving As soon as the power is turned on, the Power Saver starts the power saving function for you.	Jingdong prefecture level city in Shandong
Happy Fan Shop	King of Power Saving	Logistics is very fast, the price is favorable, friends introduced that it is very good to use, bought 2 at once, looking forward to the effect, used after the follow-up comment.	Jingdong prefecture level city in Shandong
Happy Fan Shop	King of Power Saving	It's all fake. It doesn't save any electricity.	Jingdong prefecture level city in Shandong
Happy Fan Shop	King of Power Saving	This Power Saver is so much better than the one I bought before, not only the effect is good, the quality is very good in all aspects, the customer service attitude is also very good, recommended to buy!	Jingdong prefecture level city in Shandong
Happy Fan Shop	King of Power Saving	Logistics is very fast, buy for parents, good quality, once bought 2, power-saving effect is good to buy 2 more, look forward to using the effect, satisfied will recommend to friends.	Jingdong prefecture level city in Shandong
Happy Fan Shop	King of Power Saving	With the description is the same, very high-grade, good value, to buy friends can order Oh!	Jingdong prefecture level city in Shandong
Happy Fan Shop	King of Power Saving	Not using much electricity in the winter saving over 100 per month! Ready to buy 2 more units in the summer ！！！！！ Good quality, good price, power saving effect is obvious, cost-effective, worth buying, can be recommended!	Jingdong prefecture level city in Shandong
Happy Fan Shop	King of Power Saving	Purchased a power saver received on the put into use, rent a room with many electrical appliances, power consumption is very large, we have a piece of electricity or 1.2 dollars a time, with this power saver is much better, every day can save a lot of money, very convenient and practical.	Jingdong prefecture level city in Shandong
Happy Fan Shop	King of Power Saving	The power saving effect is very good: my supermarket monthly electricity bill is more than 7000 yuan. On May 1st someone recommended buying a couple of power savers, and I bought 5 of them first. The electricity bill was about 2000 less in June. This time, I bought 5 more. The seller said 800 degrees of electricity with one. I feel it's basically enough. A total of 10 units	Jingdong prefecture level city in Shandong
Happy Fan Shop	King of Power Saving	I never thought I could get such a good baby at such an affordable price, it's very good value for money, I love it!	Jingdong prefecture level city in Shandong
Happy Fan Shop	King of Power Saving	Previously bought other brands of power savers, but found no effect at home, this time to see how the products on the Jingdong, inserted in the home socket, the LED display is blue, the night light is very nice to see, look at the instructions that have to be half a year in order to achieve the best results, try it, I believe that the quality of the Jingdong, it should not be wrong.	Jingdong prefecture level city in Shandong
Happy Fan Shop	King of Power Saving	Superb, inexpensive, saves a lot of points, been using it for a long time!	Jingdong prefecture level city in Shandong
Happy Fan Shop	King of Power Saving	There are a lot of appliances in the house, the summer air conditioning is very costly, I bought this in front of the air conditioning effect is very good, I explain that I bought three in one breath, I use more electricity a month down to 200 or so, very satisfied.	Jingdong prefecture level city in Shandong
Happy Fan Shop	King of Power Saving	A month can save more than 200, home monthly electricity bill of more than 600 dollars, inserted 3 or so, an amount of the balance of the average can save more than 200. Product is very good, has been buying this one, has given a lot of relatives recommended, the quality is also very good, before buying a look with this same, who knows to buy home has not been used for more than a week on the bad, the quality of this is really good with more than two years!	Jingdong prefecture level city in Shandong
Happy Fan Shop	King of Power Saving	Whole house power saving king, can really save electricity, friends recommended, high power saving appliances, only replacement, no repair, after-sales worry-free!	Jingdong prefecture level city in Shandong
Happy Fan Shop	King of Power Saving	Great item love it, great customer service, great shopping experience!	Jingdong prefecture level city in Shandong
Happy Fan Shop	King of Power Saving	Power saving, very good use, much cheaper than the physical store, satisfaction	Jingdong prefecture level city in Shandong
Happy Fan Shop	King of Power Saving	In use, very well packaged and fast delivery of credit!	Jingdong prefecture level city in Shandong
Happy Fan Shop	King of Power Saving	Product quality, customer service attitude is particularly good, like, praise!	Jingdong prefecture level city in Shandong
Happy Fan Shop	King of Power Saving	The logistics is very fast, the packaging is not broken, the quality is very good, I like it very much!	Jingdong prefecture level city in Shandong
Happy Fan Shop	King of Power Saving	The saver was well packaged and was as described when plugged in	Jingdong prefecture level city in Shandong
Happy Fan Shop	King of Power Saving	Energy efficient and very satisfied	Jingdong prefecture level city in Shandong
Happy Fan Shop	King of Power Saving	Great quality for the price and fast shipping.	Jingdong prefecture level city in Shandong
Happy Fan Shop	King of Power Saving	It's great, just as described. Perfect.	Jingdong prefecture level city in Shandong
Happy Fan Shop	King of Power Saving	There is no power saving effect, but rather ring up an increase in electricity costs, because this machine is on continuously all day long, uninterruptedly, its own electricity and increased costs, plus the cost of purchasing the machine, so I feel that the use of this machine instead of increasing the	Jingdong prefecture level city in Shandong
Happy Fan Shop	King of Power Saving	Recommended by the owner of the store next door, looked at his home does have to save electricity, decided to get. Indeed quite can, the voltage is much more stable, there is a reduction in electricity	Jingdong prefecture level city in Shandong
Happy Fan Shop	King of Power Saving	Goods have been received value for money, affordable and reasonable price, and the description of the same, power-saving safe and reliable!	Jingdong prefecture level city in Shandong
Happy Fan Shop	King of Power Saving	Listen to friends introduced to buy, tried to save a lot of electricity is good, quite good, worth buying.	Jingdong prefecture level city in Shandong
Happy Fan Shop	King of Power Saving	The product is easy to use, safe and environmentally friendly, only used for a short time, to the next month, after the electricity bill status display to know how the performance of the power saver efficacy.	Jingdong prefecture level city in Shandong
Happy Fan Shop	King of Power Saving	Last month's electricity bill is 178 yuan, with this power saver is 92 yuan, ready to buy two more back home, I was introduced to buy a friend, just started to not believe that I did not expect the effect!	Jingdong prefecture level city in Shandong

Happy Fan Shop	King of Power Saving	It looks nice, but after using it for a few days I can't see how it saves power?	Jingdong prefecture level city in Shandong
Happy Fan Shop	King of Power Saving	Recommended by a friend, it is genuine, as described by the merchant, cost-effective	Jingdong prefecture level city in Shandong
Happy Fan Shop	King of Power Saving	Works well, affordable and saves money.	Jingdong prefecture level city in Shandong
Happy Fan Shop	King of Power Saving	Feels good haven't compared it yet, the air conditioning has been on a lot in the past two months	Jingdong prefecture level city in Shandong
Happy Fan Shop	King of Power Saving	Saving about 5 kWh per day is a good thing	Jingdong prefecture level city in Shandong
Happy Fan Shop	King of Power Saving	Not bad, affordable, best value for money	Jingdong prefecture level city in Shandong
Happy Fan Shop	King of Power Saving	The goods are genuine and the logistics are faster.	Jingdong prefecture level city in Shandong
Happy Fan Shop	King of Power Saving	Goods have been received today, has been plugged into the outlet, looking forward to saving electricity, after a period of time to see how the effect in the follow-up comment!	Jingdong prefecture level city in Shandong
Happy Fan Shop	King of Power Saving	I just got it home and haven't used it for long, so I'll use it for a couple months and see what happens. Seeing that most people have good reviews, it should be fine.	Jingdong prefecture level city in Shandong
Happy Fan Shop	King of Power Saving	I'm looking forward to the results, so I'll give you a good review out of habit! Follow up review after a while	Jingdong prefecture level city in Shandong
Happy Fan Shop	King of Power Saving	Use a dish first to know if it will help save energy	Jingdong prefecture level city in Shandong
Happy Fan Shop	King of Power Saving	Whether it saves electricity or not, we don't know yet.	Jingdong prefecture level city in Shandong
Happy Fan Shop	King of Power Saving	Goods have been received, use a period of time to know how oh. The courier did not deliver to the door ah! Sent to the station ah! The heat is inconvenient ah!	Jingdong prefecture level city in Shandong
Happy Fan Shop	King of Power Saving	A little effect, before an hour a power, now an hour 0.76 degrees, a dollar a power = save 24 cents, although not a lot, first use it!	Jingdong prefecture level city in Shandong
Happy Fan Shop	King of Power Saving	Just received the goods, use a month to evaluate	Jingdong prefecture level city in Shandong
Happy Fan Shop	King of Power Saving	Home every month with a lot of electricity, inadvertently found this good thing online that can save the beginning of a skeptical attitude to consult customer service, customer service replied very professional, said 120 days can be refunded that first try it!	Jingdong prefecture level city in Shandong
Happy Fan Shop	King of Power Saving	After receiving the goods this afternoon, immediately installed in the bathroom Smith energy storage electric water heater on the power outlet (instead of connecting with a plug board), when used, found that the "Power Saving King" LCD display, is the value of the voltage, not the store's video said so	Jingdong prefecture level city in Shandong
Happy Fan Shop	King of Power Saving	Has made many days, did not feel the power saving, the effect is not obvious.	Jingdong prefecture level city in Shandong
Happy Fan Shop	King of Power Saving	Energy savers do not save energy at all, but rather consume a lot of electricity.	Jingdong prefecture level city in Shandong
Happy Fan Shop	King of Power Saving	It's useless. It's a waste of money.	Jingdong prefecture level city in Shandong
Happy Fan Shop	King of Power Saving	Last month saved more than 500, which came back to buy, the store bought three, and then buy 1 home use, my family is dry beauty salon, more instruments, especially electricity, a month almost 1000. or so, with this did not expect to save so much, great, five-star praise!	Jingdong prefecture level city in Shandong
Happy Fan Shop	King of Power Saving	It rained a couple days ago and I just went to get the item back today , not bad, hey I looked at the meter and that red display light takes several seconds to jump, did not make a mistake buying it. After a period of time to evaluate.	Jingdong prefecture level city in Shandong
Happy Fan Shop	King of Power Saving	Without further ado, the electricity bill came down, this month less 102 yuan, has been repurchased, I also recommended to my friends, all said that there are effects, the product is okay, the effect is quite	Jingdong prefecture level city in Shandong
Happy Fan Shop	King of Power Saving	After buying and using two months really see the effect, so only to evaluate! Originally held the mindset of trying, I did not expect really effective, worth having!	Jingdong prefecture level city in Shandong
Happy Fan Shop	King of Power Saving	In the use of this energy saver before the monthly electricity bill is more than 150 yuan or so, now save thirty percent, every day to work, six or seven o'clock in the afternoon to go home, I do not know why the electricity bill is so expensive, energy-saving appliances really have the effect of recommending	Jingdong prefecture level city in Shandong
Happy Fan Shop	King of Power Saving	It saves about 100 bucks a month. A friend recommended it to me, and he's been using it for a while, and the effect is so obvious that I bought one too.	Jingdong prefecture level city in Shandong
Happy Fan Shop	King of Power Saving	My dad asked me to buy one, but after I paid the bill, I asked and he said it was a lot less this month, so he asked me to buy another one.	Jingdong prefecture level city in Shandong
Happy Fan Shop	King of Power Saving	The specific effect will have to be tested for at least two months to compare whether there is a power saving effect or not.	Jingdong prefecture level city in Shandong
Happy Fan Shop	King of Power Saving	'The goods have been received, immediately go to use, please ask the boss you instructions do not allow to withdraw, there are two fuses installed there!	Jingdong prefecture level city in Shandong
Happy Fan Shop	King of Power Saving	First praise is using, after using if good then follow up, now because I have not yet seen the results	Jingdong prefecture level city in Shandong
Happy Fan Shop	King of Power Saving	I've just been using it, and I haven't seen if it has a power-saving effect yet, so I'll follow up after 10 days and half a month.	Jingdong prefecture level city in Shandong
Happy Fan Shop	King of Power Saving	Just received the intelligent power saving meter, immediately plugged in to use, see it is not bad, and order to sell the second time	Jingdong prefecture level city in Shandong
Happy Fan Shop	King of Power Saving	This power saver is a bit effective, used for half a month, the effect is still visible.	Jingdong prefecture level city in Shandong
Happy Fan Shop	King of Power Saving	I received the baby and it looks great, I'm trying it out now and will keep an eye on it afterward.	Jingdong prefecture level city in Shandong
Happy Fan Shop	King of Power Saving	I've just received it, but I'm still trying to see how it works, so I'll come back in a little while.	Jingdong prefecture level city in Shandong
Happy Fan Shop	King of Power Saving	Just used it wait to use it for a while and then comment thanks, all first five stars.	Jingdong prefecture level city in Shandong
Happy Fan Shop	King of Power Saving	Very trustworthy brand, quality is guaranteed, the price is still affordable, quite satisfied!	Jingdong prefecture level city in Shandong
Happy Fan Shop	King of Power Saving	Things received, I hope that the platform of Jingdong do not sell fake goods are available	Jingdong prefecture level city in Shandong
Happy Fan Shop	King of Power Saving	Energy saver received, the use of the effect is good and the business description of the same	Jingdong prefecture level city in Shandong
Happy Fan Shop	King of Power Saving	The surface looks beautiful, the actual effect is not known, after using additional evaluation.	Jingdong prefecture level city in Shandong
Happy Fan Shop	King of Power Saving	Tried it for a few months, it really saves electricity, and I bought it for a friend.	Jingdong prefecture level city in Shandong
Happy Fan Shop	King of Power Saving	It didn't work and wanted to return it, it really didn't work at all.	Jingdong prefecture level city in Shandong
Happy Fan Shop	King of Power Saving	Just used it, don't know how effective it is, looking forward to usefulness	Jingdong prefecture level city in Shandong
Happy Fan Shop	King of Power Saving	After two months before the effect I do not know if it is true!	Jingdong prefecture level city in Shandong
Happy Fan Shop	King of Power Saving	So fast! Ordered yesterday and received today 👍👍👍👍👍👍👍👍👍 🌹🌹🌹🌹🌹🌹🌹🌹🌹	Jingdong prefecture level city in Shandong
Happy Fan Shop	King of Power Saving	I've started using it. We'll see how it works.	Jingdong prefecture level city in Shandong
Happy Fan Shop	King of Power Saving	I just received the goods, I won't know the effect until after two.	Jingdong prefecture level city in Shandong
Happy Fan Shop	King of Power Saving	Just received the goods, the specific effect will be evaluated later	Jingdong prefecture level city in Shandong
Happy Fan Shop	King of Power Saving	I've received the goods, I don't know if it's any good yet	Jingdong prefecture level city in Shandong

Happy Fan Shop	King of Power Saving	Try it for a month and then come back with a concrete evaluation	Jingdong prefecture level city in Shandong
Happy Fan Shop	King of Power Saving	In use, after two months to see the effect of power saving	Jingdong prefecture level city in Shandong
Happy Fan Shop	King of Power Saving	Power Saver. It's authentic. It's black. It works.	Jingdong prefecture level city in Shandong
Happy Fan Shop	King of Power Saving	I've been using it for a week now and it's not working.	Jingdong prefecture level city in Shandong
Happy Fan Shop	King of Power Saving	I'm just using it. I don't know how it's going to work.	Jingdong prefecture level city in Shandong
Happy Fan Shop	King of Power Saving	Products are better looking out for power savings	Jingdong prefecture level city in Shandong
Happy Fan Shop	King of Power Saving	I received it and I've kept it on. I hope it works.	Jingdong prefecture level city in Shandong
Happy Fan Shop	King of Power Saving	It's really good! Worth recommending to buy!	Jingdong prefecture level city in Shandong
Happy Fan Shop	King of Power Saving	Try it for a few days and see if it doesn't work, then return it.	Jingdong prefecture level city in Shandong
Happy Fan Shop	King of Power Saving	It's in use and I hope to be pleasantly surprised.	Jingdong prefecture level city in Shandong
Happy Fan Shop	King of Power Saving	We'll see how it works when we use it	Jingdong prefecture level city in Shandong
Happy Fan Shop	King of Power Saving	Let's see if it works.	Jingdong prefecture level city in Shandong
Happy Fan Shop	King of Power Saving	I'm using it and will follow up with a review	Jingdong prefecture level city in Shandong
Happy Fan Shop	King of Power Saving	It really works. It saves energy.	Jingdong prefecture level city in Shandong
Happy Fan Shop	King of Power Saving	It does have some power savings!	Jingdong prefecture level city in Shandong
Happy Fan Shop	King of Power Saving	Jingdong shopping speed is convenient and timely	Jingdong prefecture level city in Shandong
Happy Fan Shop	King of Power Saving	Second time I've bought it. Everything's fine.	Jingdong prefecture level city in Shandong
Happy Fan Shop	King of Power Saving	I don't know if it works yet.	Jingdong prefecture level city in Shandong
Happy Fan Shop	King of Power Saving	Not used, don't know how well it works.	Jingdong prefecture level city in Shandong
Happy Fan Shop	King of Power Saving	No visible results so far	Jingdong prefecture level city in Shandong
Happy Fan Shop	King of Power Saving	It doesn't feel like it's working.	Jingdong prefecture level city in Shandong
Happy Fan Shop	King of Power Saving	Where's the effect? Where are the power savings?	Jingdong prefecture level city in Shandong
Happy Fan Shop	King of Power Saving	Let's see how it works in practice!	Jingdong prefecture level city in Shandong
Happy Fan Shop	King of Power Saving	I'll wait until I use it to give you a good review	Jingdong prefecture level city in Shandong
Happy Fan Shop	King of Power Saving	I'll follow up after I've used it	Jingdong prefecture level city in Shandong
Happy Fan Shop	King of Power Saving	Well, that's good for saving power.	Jingdong prefecture level city in Shandong
Happy Fan Shop	King of Power Saving	Received. Used in the know.	Jingdong prefecture level city in Shandong
Happy Fan Shop	King of Power Saving	It doesn't do much good.	Jingdong prefecture level city in Shandong
Happy Fan Shop	King of Power Saving	It's not working.	Jingdong prefecture level city in Shandong
Happy Fan Shop	King of Power Saving	I don't know. I just used it.	Jingdong prefecture level city in Shandong
Happy Fan Shop	King of Power Saving	Use it and come back to evaluate.	Jingdong prefecture level city in Shandong
Happy Fan Shop	King of Power Saving	Two words: useless.	Jingdong prefecture level city in Shandong
Happy Fan Shop	King of Power Saving	To be used and then evaluated	Jingdong prefecture level city in Shandong
Happy Fan Shop	King of Power Saving	No shipment.	Jingdong prefecture level city in Shandong
Happy Fan Shop	King of Power Saving	Fake. Speechless.	Jingdong prefecture level city in Shandong
Happy Fan Shop	King of Power Saving	fantastic (idiom); awesome	Jingdong prefecture level city in Shandong
Happy Fan Shop	King of Power Saving	an excellent	Jingdong prefecture level city in Shandong
Happy Fan Shop	King of Power Saving	an excellent	Jingdong prefecture level city in Shandong
Happy Fan Shop	King of Power Saving	Want to buy! Recommended not to buy! No effect	Jingdong prefecture level city in Shandong
Happy Fan Shop	King of Power Saving	That's all there is to it. I can't figure out how to save electricity.	Jingdong prefecture level city in Shandong
Happy Fan Shop	King of Power Saving	It's no use.	Jingdong prefecture level city in Shandong
Happy Fan Shop	King of Power Saving	Things are more perfect than I imagined, very powerful, it is worth getting Packaging is very strong, things are well protected, it is worth the praise things are very satisfied Oh, beautiful and stylish atmosphere, customer service is super patient, service is meticulous and attentive, the big brand is not	Jingdong prefecture level city in Shandong
Happy Fan Shop	King of Power Saving	The effect is too good, the family has now several units, back to buy a number of times, the power saver received, power-saving effect is very good,, fine workmanship, long warranty, but also purify the air, before buying a used for a period of time, with the okay, and then bought a, and only now come to the	Jingdong prefecture level city in Shandong
Happy Fan Shop	King of Power Saving	Quite good, and imagine the same, casual ride are very good, satisfied! Baby received it 👌, really great ah! 🌈 quality is really nothing to say, very satisfied! 🆗 happy happy 😃 genuine, great, the price is very good, in short, quite good, will buy again a very good shopping experience, attentive service, fast logistics, distribution is also thoughtful, the attitude is also very good, value for money, ah,	Jingdong prefecture level city in Shandong
Happy Fan Shop	King of Power Saving	Power-saving effect: strong Cost-effective: quite good Appearance style: beautiful Workmanship material: meticulous Experience: looking forward to power-saving effect	Jingdong prefecture level city in Shandong
Happy Fan Shop	King of Power Saving	Cost-effective is okay, the appearance of the style of meticulous workmanship, looking forward to energy-saving effect.	Jingdong prefecture level city in Shandong
Happy Fan Shop	King of Power Saving	Very Good Very Power Saving This Power Saver is really power saving As soon as the power is turned on, the Power Saver starts the power saving function for you.	Jingdong prefecture level city in Shandong
Happy Fan Shop	King of Power Saving	Logistics is very fast, the price is favorable, friends introduced that it is very good to use, bought 2 at once, looking forward to the effect, used after the follow-up comment.	Jingdong prefecture level city in Shandong
Happy Fan Shop	King of Power Saving	It's all fake. It doesn't save any electricity.	Jingdong prefecture level city in Shandong

Happy Fan Shop	King of Power Saving	This Power Saver is so much better than the one I bought before, not only the effect is good, the quality is very good in all aspects, the customer service attitude is also very good, recommended to buy!	Jingdong prefecture level city in Shandong
Happy Fan Shop	King of Power Saving	Logistics is very fast, buy for parents, good quality, once bought 2, power-saving effect is good to buy 2 more, look forward to using the effect, satisfied will recommend to friends.	Jingdong prefecture level city in Shandong
Happy Fan Shop	King of Power Saving	With the description is the same, very high-grade, good value, to buy friends can order Oh!	Jingdong prefecture level city in Shandong
Happy Fan Shop	King of Power Saving	Not using much electricity in the winter saving over 100 per month! Ready to buy 2 more units in the summer ! ! ! ! ! Good quality, good price, power saving effect is obvious, cost-effective, worth buying, can be recommended!	Jingdong prefecture level city in Shandong
Happy Fan Shop	King of Power Saving	Purchased a power saver received on the put into use, rent a room with many electrical appliances, power consumption is very large, we have a piece of electricity or 1.2 dollars a time, with this power saver is much better, every day can save a lot of money, very convenient and practical.	Jingdong prefecture level city in Shandong
Happy Fan Shop	King of Power Saving	The power saving effect is very good: my supermarket monthly electricity bill is more than 7000 yuan. On May 1st someone recommended buying a couple of power savers, and I bought 5 of them first. The electricity bill was about 2000 less in June. This time, I bought 5 more. The seller said 800 degrees of electricity with one. I feel it's basically enough. A total of 10 units	Jingdong prefecture level city in Shandong
Happy Fan Shop	King of Power Saving	I never thought I could get such a good baby at such an affordable price, it's very good value for money, I love it!	Jingdong prefecture level city in Shandong
Happy Fan Shop	King of Power Saving	Previously bought other brands of power savers, but found no effect at home, this time to see how the products on the Jingdong, inserted in the home socket, the LED display is blue, the night light is very nice to see, look at the instructions that have to be half a year in order to achieve the best results, try it, I believe that the quality of the Jingdong, it should not be wrong.	Jingdong prefecture level city in Shandong
Happy Fan Shop	King of Power Saving	Superb, inexpensive, saves a lot of points, been using it for a long time!	Jingdong prefecture level city in Shandong
Happy Fan Shop	King of Power Saving	There are a lot of appliances in the house, the summer air conditioning is very costly, I bought this in front of the air conditioning effect is very good, I explain that I bought three in one breath, I use more electricity a month down to 200 or so, very satisfied.	Jingdong prefecture level city in Shandong
Happy Fan Shop	King of Power Saving	A month can save more than 200, home monthly electricity bill of more than 600 dollars, inserted 3 or so, an amount of the balance of the average can save more than 200. Product is very good, has been buying this one, has given a lot of relatives recommended, the quality is also very good, before buying a look with this same, who knows to buy home has not been used for more than a week on the bad, the quality of this is really good with more than two years!	Jingdong prefecture level city in Shandong
Happy Fan Shop	King of Power Saving	Whole house power saving king, can really save electricity, friends recommended, high power saving appliances, only replacement, no repair, after-sales worry-free!	Jingdong prefecture level city in Shandong
Happy Fan Shop	King of Power Saving	Great item love it, great customer service, great shopping experience!	Jingdong prefecture level city in Shandong
Happy Fan Shop	King of Power Saving	Power saving, very good use, much cheaper than the physical store, satisfaction	Jingdong prefecture level city in Shandong
Happy Fan Shop	King of Power Saving	In use, very well packaged and fast delivery of credit!	Jingdong prefecture level city in Shandong
Happy Fan Shop	King of Power Saving	Product quality, customer service attitude is particularly good, like, praise!	Jingdong prefecture level city in Shandong
Happy Fan Shop	King of Power Saving	The logistics is very fast, the packaging is not broken, the quality is very good, I like it very much!	Jingdong prefecture level city in Shandong
Happy Fan Shop	King of Power Saving	The saver was well packaged and was as described when plugged in	Jingdong prefecture level city in Shandong
Happy Fan Shop	King of Power Saving	Energy efficient and very satisfied	Jingdong prefecture level city in Shandong
Happy Fan Shop	King of Power Saving	Great quality for the price and fast shipping.	Jingdong prefecture level city in Shandong
Happy Fan Shop	King of Power Saving	It's great, just as described. Perfect.	Jingdong prefecture level city in Shandong
Happy Fan Shop	King of Power Saving	There is no power saving effect, but rather ring up an increase in electricity costs, because this machine is on continuously all day long, uninterruptedly, its own electricity and increased costs, plus the cost of purchasing the machine, so I feel that the use of this machine instead of increasing the	Jingdong prefecture level city in Shandong
Happy Fan Shop	King of Power Saving	Recommended by the owner of the store next door, looked at his home does have to save electricity, decided to get. Indeed quite can, the voltage is much more stable, there is a reduction in electricity	Jingdong prefecture level city in Shandong
Happy Fan Shop	King of Power Saving	Goods have been received value for money, affordable and reasonable price, and the description of the same, power-saving safe and reliable!	Jingdong prefecture level city in Shandong
Happy Fan Shop	King of Power Saving	Listen to friends introduced to buy, tried to save a lot of electricity is good, quite good, worth buying.	Jingdong prefecture level city in Shandong
Happy Fan Shop	King of Power Saving	The product is easy to use, safe and environmentally friendly, only used for a short time, to the next month, after the electricity bill status display to know how the performance of the power saver efficacy.	Jingdong prefecture level city in Shandong
Happy Fan Shop	King of Power Saving	Last month's electricity bill is 178 yuan, with this power saver is 92 yuan, ready to buy two more back home, I was introduced to buy a friend, just started to not believe that I did not expect the effect!	Jingdong prefecture level city in Shandong
Happy Fan Shop	King of Power Saving	It looks nice, but after using it for a few days I can't see how it saves power?	Jingdong prefecture level city in Shandong
Happy Fan Shop	King of Power Saving	Recommended by a friend, it is genuine, as described by the merchant, cost-effective	Jingdong prefecture level city in Shandong
Happy Fan Shop	King of Power Saving	Works well, affordable and saves money.	Jingdong prefecture level city in Shandong
Happy Fan Shop	King of Power Saving	Feels good haven't compared it yet, the air conditioning has been on a lot in the past two months	Jingdong prefecture level city in Shandong
Happy Fan Shop	King of Power Saving	Saving about 5 kWh per day is a good thing	Jingdong prefecture level city in Shandong
Happy Fan Shop	King of Power Saving	Not bad, affordable, best value for money	Jingdong prefecture level city in Shandong
Happy Fan Shop	King of Power Saving	The goods are genuine and the logistics are faster.	Jingdong prefecture level city in Shandong
Happy Fan Shop	King of Power Saving	Goods have been received today, has been plugged into the outlet, looking forward to saving electricity, after a period of time to see how the effect in the follow-up comment!	Jingdong prefecture level city in Shandong
Happy Fan Shop	King of Power Saving	I just got it home and haven't used it for long, so I'll use it for a couple months and see what happens. Seeing that most people have good reviews, it should be fine.	Jingdong prefecture level city in Shandong
Happy Fan Shop	King of Power Saving	I'm looking forward to the results, so I'll give you a good review out of habit! Follow up review after a while	Jingdong prefecture level city in Shandong
Happy Fan Shop	King of Power Saving	Use a dish first to know if it will help save energy	Jingdong prefecture level city in Shandong
Happy Fan Shop	King of Power Saving	Whether it saves electricity or not, we don't know yet.	Jingdong prefecture level city in Shandong
Happy Fan Shop	King of Power Saving	Goods have been received, use a period of time to know how oh. The courier did not deliver to the door ah! Sent to the station ah! The heat is inconvenient ah!	Jingdong prefecture level city in Shandong
Happy Fan Shop	King of Power Saving	A little effect, before an hour a power, now an hour 0.76 degrees, a dollar a power = save 24 cents, although not a lot, first use it!	Jingdong prefecture level city in Shandong
Happy Fan Shop	King of Power Saving	Just received the goods, use a month to evaluate	Jingdong prefecture level city in Shandong
Happy Fan Shop	King of Power Saving	Home every month with a lot of electricity, inadvertently found this good thing online that can save the beginning of a skeptical attitude to consult customer service, customer service replied very professional, said 120 days can be refunded that first try it!	Jingdong prefecture level city in Shandong
Happy Fan Shop	King of Power Saving	After receiving the goods this afternoon, immediately installed in the bathroom Smith energy storage electric water heater on the power outlet (instead of connecting with a plug board), when used, found that the "Power Saving King" LCD display, is the value of the voltage, not the store's video said so	Jingdong prefecture level city in Shandong
Happy Fan Shop	King of Power Saving	Has made many days, did not feel the power saving, the effect is not obvious.	Jingdong prefecture level city in Shandong

Happy Fan Shop	King of Power Saving	Energy savers do not save energy at all, but rather consume a lot of electricity.	Jingdong prefecture level city in Shandong
Happy Fan Shop	King of Power Saving	It's useless. It's a waste of money.	Jingdong prefecture level city in Shandong
Happy Fan Shop	King of Power Saving	Last month saved more than 500, which came back to buy, the store bought three, and then buy 1 home use, my family is dry beauty salon, more instruments, especially electricity, a month almost 1000. or so, with this did not expect to save so much, great, five-star praise!	Jingdong prefecture level city in Shandong
Happy Fan Shop	King of Power Saving	It rained a couple days ago and I just went to get the item back today , not bad, hey I looked at the meter and that red display light takes several seconds to jump, did not make a mistake buying it. After a period of time to evaluate.	Jingdong prefecture level city in Shandong
Happy Fan Shop	King of Power Saving	Without further ado, the electricity bill came down, this month less 102 yuan, has been repurchased, I also recommended to my friends, all said that there are effects, the product is okay, the effect is quite	Jingdong prefecture level city in Shandong
Happy Fan Shop	King of Power Saving	After buying and using two months really see the effect, so only to evaluate! Originally held the mindset of trying, I did not expect really effective, worth having!	Jingdong prefecture level city in Shandong
Happy Fan Shop	King of Power Saving	In the use of this energy saver before the monthly electricity bill is more than 150 yuan or so, now save thirty percent, every day to work, six or seven o'clock in the afternoon to go home, I do not know why the electricity bill is so expensive, energy-saving appliances really have the effect of recommending	Jingdong prefecture level city in Shandong
Happy Fan Shop	King of Power Saving	It saves about 100 bucks a month. A friend recommended it to me, and he's been using it for a while, and the effect is so obvious that I bought one too.	Jingdong prefecture level city in Shandong
Happy Fan Shop	King of Power Saving	My dad asked me to buy one, but after I paid the bill, I asked and he said it was a lot less this month, so he asked me to buy another one.	Jingdong prefecture level city in Shandong
Happy Fan Shop	King of Power Saving	The specific effect will have to be tested for at least two months to compare whether there is a power saving effect or not.	Jingdong prefecture level city in Shandong
Happy Fan Shop	King of Power Saving	'The goods have been received, immediately go to use, please ask the boss you instructions do not allow to withdraw, there are two fuses installed there!	Jingdong prefecture level city in Shandong
Happy Fan Shop	King of Power Saving	First praise is using, after using if good then follow up, now because I have not yet seen the results	Jingdong prefecture level city in Shandong
Happy Fan Shop	King of Power Saving	I've just been using it, and I haven't seen if it has a power-saving effect yet, so I'll follow up after 10 days and half a month.	Jingdong prefecture level city in Shandong
Happy Fan Shop	King of Power Saving	Just received the intelligent power saving meter, immediately plugged in to use, see it is not bad, and order to sell the second time	Jingdong prefecture level city in Shandong
Happy Fan Shop	King of Power Saving	This power saver is a bit effective, used for half a month, the effect is still visible.	Jingdong prefecture level city in Shandong
Happy Fan Shop	King of Power Saving	I received the baby and it looks great, I'm trying it out now and will keep an eye on it afterward.	Jingdong prefecture level city in Shandong
Happy Fan Shop	King of Power Saving	I've just received it, but I'm still trying to see how it works, so I'll come back in a little while.	Jingdong prefecture level city in Shandong
Happy Fan Shop	King of Power Saving	Just used it wait to use it for a while and then comment thanks, all first five stars.	Jingdong prefecture level city in Shandong
Happy Fan Shop	King of Power Saving	Very trustworthy brand, quality is guaranteed, the price is still affordable, quite satisfied!	Jingdong prefecture level city in Shandong
Happy Fan Shop	King of Power Saving	Things received, I hope that the platform of Jingdong do not sell fake goods are available	Jingdong prefecture level city in Shandong
Happy Fan Shop	King of Power Saving	Energy saver received, the use of the effect is good and the business description of the same	Jingdong prefecture level city in Shandong
Happy Fan Shop	King of Power Saving	The surface looks beautiful, the actual effect is not known, after using additional evaluation.	Jingdong prefecture level city in Shandong
Happy Fan Shop	King of Power Saving	Tried it for a few months, it really saves electricity, and I bought it for a friend.	Jingdong prefecture level city in Shandong
Happy Fan Shop	King of Power Saving	It didn't work and wanted to return it, it really didn't work at all.	Jingdong prefecture level city in Shandong
Happy Fan Shop	King of Power Saving	Just used it, don't know how effective it is, looking forward to usefulness	Jingdong prefecture level city in Shandong
Happy Fan Shop	King of Power Saving	After two months before the effect I do not know if it is true!	Jingdong prefecture level city in Shandong
Happy Fan Shop	King of Power Saving	So fast! Ordered yesterday and received today 👍👍👍👍👍👍👍👍👍 🌹🌹🌹🌹🌹🌹🌹🌹🌹	Jingdong prefecture level city in Shandong
Happy Fan Shop	King of Power Saving	I've started using it. We'll see how it works.	Jingdong prefecture level city in Shandong
Happy Fan Shop	King of Power Saving	I just received the goods, I won't know the effect until after two.	Jingdong prefecture level city in Shandong
Happy Fan Shop	King of Power Saving	Just received the goods, the specific effect will be evaluated later	Jingdong prefecture level city in Shandong
Happy Fan Shop	King of Power Saving	I've received the goods, I don't know if it's any good yet	Jingdong prefecture level city in Shandong
Happy Fan Shop	King of Power Saving	Try it for a month and then come back with a concrete evaluation	Jingdong prefecture level city in Shandong
Happy Fan Shop	King of Power Saving	In use, after two months to see the effect of power saving	Jingdong prefecture level city in Shandong
Happy Fan Shop	King of Power Saving	Power Saver. It's authentic. It's black. It works.	Jingdong prefecture level city in Shandong
Happy Fan Shop	King of Power Saving	I've been using it for a week now and it's not working.	Jingdong prefecture level city in Shandong
Happy Fan Shop	King of Power Saving	I'm just using it. I don't know how it's going to work.	Jingdong prefecture level city in Shandong
Happy Fan Shop	King of Power Saving	Products are better looking out for power savings	Jingdong prefecture level city in Shandong
Happy Fan Shop	King of Power Saving	I received it and I've kept it on. I hope it works.	Jingdong prefecture level city in Shandong
Happy Fan Shop	King of Power Saving	It's really good! Worth recommending to buy!	Jingdong prefecture level city in Shandong
Happy Fan Shop	King of Power Saving	Try it for a few days and see if it doesn't work, then return it.	Jingdong prefecture level city in Shandong
Happy Fan Shop	King of Power Saving	It's in use and I hope to be pleasantly surprised.	Jingdong prefecture level city in Shandong
Happy Fan Shop	King of Power Saving	We'll see how it works when we use it	Jingdong prefecture level city in Shandong
Happy Fan Shop	King of Power Saving	Let's see if it works.	Jingdong prefecture level city in Shandong
Happy Fan Shop	King of Power Saving	I'm using it and will follow up with a review	Jingdong prefecture level city in Shandong
Happy Fan Shop	King of Power Saving	It really works. It saves energy.	Jingdong prefecture level city in Shandong
Happy Fan Shop	King of Power Saving	It does have some power savings!	Jingdong prefecture level city in Shandong
Happy Fan Shop	King of Power Saving	Jingdong shopping speed is convenient and timely	Jingdong prefecture level city in Shandong
Happy Fan Shop	King of Power Saving	Second time I've bought it. Everything's fine.	Jingdong prefecture level city in Shandong
Happy Fan Shop	King of Power Saving	I don't know if it works yet.	Jingdong prefecture level city in Shandong
Happy Fan Shop	King of Power Saving	Not used, don't know how well it works.	Jingdong prefecture level city in Shandong

Happy Fan Shop	King of Power Saving	No visible results so far	Jingdong prefecture level city in Shandong
Happy Fan Shop	King of Power Saving	It doesn't feel like it's working.	Jingdong prefecture level city in Shandong
Happy Fan Shop	King of Power Saving	Where's the effect? Where are the power savings?	Jingdong prefecture level city in Shandong
Happy Fan Shop	King of Power Saving	Let's see how it works in practice!	Jingdong prefecture level city in Shandong
Happy Fan Shop	King of Power Saving	I'll wait until I use it to give you a good review	Jingdong prefecture level city in Shandong
Happy Fan Shop	King of Power Saving	I'll follow up after I've used it	Jingdong prefecture level city in Shandong
Happy Fan Shop	King of Power Saving	Well, that's good for saving power.	Jingdong prefecture level city in Shandong
Happy Fan Shop	King of Power Saving	Received. Used in the know.	Jingdong prefecture level city in Shandong
Happy Fan Shop	King of Power Saving	It doesn't do much good.	Jingdong prefecture level city in Shandong
Happy Fan Shop	King of Power Saving	It's not working.	Jingdong prefecture level city in Shandong
Happy Fan Shop	King of Power Saving	I don't know. I just used it.	Jingdong prefecture level city in Shandong
Happy Fan Shop	King of Power Saving	Use it and come back to evaluate.	Jingdong prefecture level city in Shandong
Happy Fan Shop	King of Power Saving	Two words: useless.	Jingdong prefecture level city in Shandong
Happy Fan Shop	King of Power Saving	To be used and then evaluated	Jingdong prefecture level city in Shandong
Happy Fan Shop	King of Power Saving	No shipment.	Jingdong prefecture level city in Shandong
Happy Fan Shop	King of Power Saving	Fake. Speechless.	Jingdong prefecture level city in Shandong
Happy Fan Shop	King of Power Saving	fantastic (idiom); awesome	Jingdong prefecture level city in Shandong
Happy Fan Shop	King of Power Saving	an excellent	Jingdong prefecture level city in Shandong
Happy Fan Shop	King of Power Saving	an excellent	Jingdong prefecture level city in Shandong
Happy Fan Shop	King of Power Saving	Want to buy! Recommended not to buy! No effect	Jingdong prefecture level city in Shandong
Happy Fan Shop	King of Power Saving	That's all there is to it. I can't figure out how to save electricity.	Jingdong prefecture level city in Shandong
Happy Fan Shop	King of Power Saving	It's no use.	Jingdong prefecture level city in Shandong
Happy Fan Shop	King of Power Saving	Things are more perfect than I imagined, very powerful, it is worth getting Packaging is very strong, things are well protected, it is worth the praise things are very satisfied Oh, beautiful and stylish atmosphere, customer service is super patient, service is meticulous and attentive, the big brand is not	Jingdong prefecture level city in Shandong
Happy Fan Shop	King of Power Saving	The effect is too good, the family has now several units, back to buy a number of times, the power saver received, power-saving effect is very good,, fine workmanship, long warranty, but also purify the air, before buying a used for a period of time, with the okay, and then bought a, and only now come to the	Jingdong prefecture level city in Shandong
Happy Fan Shop	King of Power Saving	Quite good, and imagine the same, casual ride are very good, satisfied! Baby received it 👌, really great ah! 🌈 quality is really nothing to say, very satisfied! 🆗 happy happy 😃 genuine, great, the price is very good, in short, quite good, will buy again a very good shopping experience, attentive service, fast logistics, distribution is also thoughtful, the attitude is also very good, value for money, ah,	Jingdong prefecture level city in Shandong
Happy Fan Shop	King of Power Saving	Power-saving effect: strong Cost-effective: quite good Appearance style: beautiful Workmanship material: meticulous Experience: looking forward to power-saving effect	Jingdong prefecture level city in Shandong
Happy Fan Shop	King of Power Saving	Cost-effective is okay, the appearance of the style of meticulous workmanship, looking forward to energy-saving effect.	Jingdong prefecture level city in Shandong
Happy Fan Shop	King of Power Saving	Very Good Very Power Saving This Power Saver is really power saving As soon as the power is turned on, the Power Saver starts the power saving function for you.	Jingdong prefecture level city in Shandong
Happy Fan Shop	King of Power Saving	Logistics is very fast, the price is favorable, friends introduced that it is very good to use, bought 2 at once, looking forward to the effect, used after the follow-up comment.	Jingdong prefecture level city in Shandong
Happy Fan Shop	King of Power Saving	It's all fake. It doesn't save any electricity.	Jingdong prefecture level city in Shandong
Happy Fan Shop	King of Power Saving	This Power Saver is so much better than the one I bought before, not only the effect is good, the quality is very good in all aspects, the customer service attitude is also very good, recommended to buy!	Jingdong prefecture level city in Shandong
Happy Fan Shop	King of Power Saving	Logistics is very fast, buy for parents, good quality, once bought 2, power-saving effect is good to buy 2 more, look forward to using the effect, satisfied will recommend to friends.	Jingdong prefecture level city in Shandong
Happy Fan Shop	King of Power Saving	With the description is the same, very high-grade, good value, to buy friends can order Oh!	Jingdong prefecture level city in Shandong
Happy Fan Shop	King of Power Saving	Not using much electricity in the winter saving over 100 per month! Ready to buy 2 more units in the summer ! ! ! ! ! Good quality, good price, power saving effect is obvious, cost-effective, worth buying, can be recommended!	Jingdong prefecture level city in Shandong
Happy Fan Shop	King of Power Saving	Purchased a power saver received on the put into use, rent a room with many electrical appliances, power consumption is very large, we have a piece of electricity or 1.2 dollars a time, with this power saver is much better, every day can save a lot of money, very convenient and practical.	Jingdong prefecture level city in Shandong
Happy Fan Shop	King of Power Saving	The power saving effect is very good: my supermarket monthly electricity bill is more than 7000 yuan. On May 1st someone recommended buying a couple of power savers, and I bought 5 of them first. The electricity bill was about 2000 less in June. This time, I bought 5 more. The seller said 800 degrees of electricity with one. I feel it's basically enough. A total of 10 units	Jingdong prefecture level city in Shandong
Happy Fan Shop	King of Power Saving	I never thought I could get such a good baby at such an affordable price, it's very good value for money, I love it!	Jingdong prefecture level city in Shandong
Happy Fan Shop	King of Power Saving	Previously bought other brands of power savers, but found no effect at home, this time to see how the products on the Jingdong, inserted in the home socket, the LED display is blue, the night light is very nice to see, look at the instructions that have to be half a year in order to achieve the best results, try it, I believe that the quality of the Jingdong, it should not be wrong.	Jingdong prefecture level city in Shandong
Happy Fan Shop	King of Power Saving	Superb, inexpensive, saves a lot of points, been using it for a long time!	Jingdong prefecture level city in Shandong
Happy Fan Shop	King of Power Saving	There are a lot of appliances in the house, the summer air conditioning is very costly, I bought this in front of the air conditioning effect is very good, I explain that I bought three in one breath, I use more electricity a month down to 200 or so, very satisfied.	Jingdong prefecture level city in Shandong
Happy Fan Shop	King of Power Saving	A month can save more than 200, home monthly electricity bill of more than 600 dollars, inserted 3 or so, an amount of the balance of the average can save more than 200. Product is very good, has been buying this one, has given a lot of relatives recommended, the quality is also very good, before buying a look with this same, who knows to buy home has not been used for more than a week on the bad, the quality of this is really good with more than two years!	Jingdong prefecture level city in Shandong
Happy Fan Shop	King of Power Saving	Whole house power saving king, can really save electricity, friends recommended, high power saving appliances, only replacement, no repair, after-sales worry-free!	Jingdong prefecture level city in Shandong
Happy Fan Shop	King of Power Saving	Great item love it, great customer service, great shopping experience!	Jingdong prefecture level city in Shandong
Happy Fan Shop	King of Power Saving	Power saving, very good use, much cheaper than the physical store, satisfaction	Jingdong prefecture level city in Shandong

Happy Fan Shop	King of Power Saving	In use, very well packaged and fast delivery of credit!	Jingdong prefecture level city in Shandong
Happy Fan Shop	King of Power Saving	Product quality, customer service attitude is particularly good, like, praise!	Jingdong prefecture level city in Shandong
Happy Fan Shop	King of Power Saving	The logistics is very fast, the packaging is not broken, the quality is very good, I like it very much!	Jingdong prefecture level city in Shandong
Happy Fan Shop	King of Power Saving	The saver was well packaged and was as described when plugged in	Jingdong prefecture level city in Shandong
Happy Fan Shop	King of Power Saving	Energy efficient and very satisfied	Jingdong prefecture level city in Shandong
Happy Fan Shop	King of Power Saving	Great quality for the price and fast shipping.	Jingdong prefecture level city in Shandong
Happy Fan Shop	King of Power Saving	It's great, just as described. Perfect.	Jingdong prefecture level city in Shandong
Happy Fan Shop	King of Power Saving	There is no power saving effect, but rather ring up an increase in electricity costs, because this machine is on continuously all day long, uninterruptedly, its own electricity and increased costs, plus the cost of purchasing the machine, so I feel that the use of this machine instead of increasing the	Jingdong prefecture level city in Shandong
Happy Fan Shop	King of Power Saving	Recommended by the owner of the store next door, looked at his home does have to save electricity, decided to get. Indeed quite can, the voltage is much more stable, there is a reduction in electricity	Jingdong prefecture level city in Shandong
Happy Fan Shop	King of Power Saving	Goods have been received value for money, affordable and reasonable price, and the description of the same, power-saving safe and reliable!	Jingdong prefecture level city in Shandong
Happy Fan Shop	King of Power Saving	Listen to friends introduced to buy, tried to save a lot of electricity is good, quite good, worth buying.	Jingdong prefecture level city in Shandong
Happy Fan Shop	King of Power Saving	The product is easy to use, safe and environmentally friendly, only used for a short time, to the next month, after the electricity bill status display to know how the performance of the power saver efficacy.	Jingdong prefecture level city in Shandong
Happy Fan Shop	King of Power Saving	Last month's electricity bill is 178 yuan, with this power saver is 92 yuan, ready to buy two more back home, I was introduced to buy a friend, just started to not believe that I did not expect the effect!	Jingdong prefecture level city in Shandong
Happy Fan Shop	King of Power Saving	It looks nice, but after using it for a few days I can't see how it saves power?	Jingdong prefecture level city in Shandong
Happy Fan Shop	King of Power Saving	Recommended by a friend, it is genuine, as described by the merchant, cost-effective	Jingdong prefecture level city in Shandong
Happy Fan Shop	King of Power Saving	Works well, affordable and saves money.	Jingdong prefecture level city in Shandong
Happy Fan Shop	King of Power Saving	Feels good haven't compared it yet, the air conditioning has been on a lot in the past two months	Jingdong prefecture level city in Shandong
Happy Fan Shop	King of Power Saving	Saving about 5 kWh per day is a good thing	Jingdong prefecture level city in Shandong
Happy Fan Shop	King of Power Saving	Not bad, affordable, best value for money	Jingdong prefecture level city in Shandong
Happy Fan Shop	King of Power Saving	The goods are genuine and the logistics are faster.	Jingdong prefecture level city in Shandong
Happy Fan Shop	King of Power Saving	Goods have been received today, has been plugged into the outlet, looking forward to saving electricity, after a period of time to see how the effect in the follow-up comment!	Jingdong prefecture level city in Shandong
Happy Fan Shop	King of Power Saving	I just got it home and haven't used it for long, so I'll use it for a couple months and see what happens. Seeing that most people have good reviews, it should be fine.	Jingdong prefecture level city in Shandong
Happy Fan Shop	King of Power Saving	I'm looking forward to the results, so I'll give you a good review out of habit! Follow up review after a while	Jingdong prefecture level city in Shandong
Happy Fan Shop	King of Power Saving	Use a dish first to know if it will help save energy	Jingdong prefecture level city in Shandong
Happy Fan Shop	King of Power Saving	Whether it saves electricity or not, we don't know yet.	Jingdong prefecture level city in Shandong
Happy Fan Shop	King of Power Saving	Goods have been received, use a period of time to know how oh. The courier did not deliver to the door ah! Sent to the station ah! The heat is inconvenient ah!	Jingdong prefecture level city in Shandong
Happy Fan Shop	King of Power Saving	A little effect, before an hour a power, now an hour 0.76 degrees, a dollar a power = save 24 cents, although not a lot, first use it!	Jingdong prefecture level city in Shandong
Happy Fan Shop	King of Power Saving	Just received the goods, use a month to evaluate	Jingdong prefecture level city in Shandong
Happy Fan Shop	King of Power Saving	Home every month with a lot of electricity, inadvertently found this good thing online that can save the beginning of a skeptical attitude to consult customer service, customer service replied very professional, said 120 days can be refunded that first try it!	Jingdong prefecture level city in Shandong
Happy Fan Shop	King of Power Saving	After receiving the goods this afternoon, immediately installed in the bathroom Smith energy storage electric water heater on the power outlet (instead of connecting with a plug board), when used, found that the "Power Saving King" LCD display, is the value of the voltage, not the store's video said so	Jingdong prefecture level city in Shandong
Happy Fan Shop	King of Power Saving	Has made many days, did not feel the power saving, the effect is not obvious.	Jingdong prefecture level city in Shandong
Happy Fan Shop	King of Power Saving	Energy savers do not save energy at all, but rather consume a lot of electricity.	Jingdong prefecture level city in Shandong
Happy Fan Shop	King of Power Saving	It's useless. It's a waste of money.	Jingdong prefecture level city in Shandong
Happy Fan Shop	King of Power Saving	Last month saved more than 500, which came back to buy, the store bought three, and then buy 1 home use, my family is dry beauty salon, more instruments, especially electricity, a month almost 1000. or so, with this did not expect to save so much, great, five-star praise!	Jingdong prefecture level city in Shandong
Happy Fan Shop	King of Power Saving	It rained a couple days ago and I just went to get the item back today , not bad, hey I looked at the meter and that red display light takes several seconds to jump, did not make a mistake buying it. After a period of time to evaluate.	Jingdong prefecture level city in Shandong
Happy Fan Shop	King of Power Saving	Without further ado, the electricity bill came down, this month less 102 yuan, has been repurchased, I also recommended to my friends, all said that there are effects, the product is okay, the effect is quite	Jingdong prefecture level city in Shandong
Happy Fan Shop	King of Power Saving	After buying and using two months really see the effect, so only to evaluate! Originally held the mindset of trying, I did not expect really effective, worth having!	Jingdong prefecture level city in Shandong
Happy Fan Shop	King of Power Saving	In the use of this energy saver before the monthly electricity bill is more than 150 yuan or so, now save thirty percent, every day to work, six or seven o'clock in the afternoon to go home, I do not know why the electricity bill is so expensive, energy-saving appliances really have the effect of recommending	Jingdong prefecture level city in Shandong
Happy Fan Shop	King of Power Saving	It saves about 100 bucks a month. A friend recommended it to me, and he's been using it for a while, and the effect is so obvious that I bought one too.	Jingdong prefecture level city in Shandong
Happy Fan Shop	King of Power Saving	My dad asked me to buy one, but after I paid the bill, I asked and he said it was a lot less this month, so he asked me to buy another one.	Jingdong prefecture level city in Shandong
Happy Fan Shop	King of Power Saving	The specific effect will have to be tested for at least two months to compare whether there is a power saving effect or not.	Jingdong prefecture level city in Shandong
Happy Fan Shop	King of Power Saving	'The goods have been received, immediately go to use, please ask the boss you instructions do not allow to withdraw, there are two fuses installed there!	Jingdong prefecture level city in Shandong
Happy Fan Shop	King of Power Saving	First praise is using, after using if good then follow up, now because I have not yet seen the results	Jingdong prefecture level city in Shandong
Happy Fan Shop	King of Power Saving	I've just been using it, and I haven't seen if it has a power-saving effect yet, so I'll follow up after 10 days and half a month.	Jingdong prefecture level city in Shandong
Happy Fan Shop	King of Power Saving	Just received the intelligent power saving meter, immediately plugged in to use, see it is not bad, and order to sell the second time	Jingdong prefecture level city in Shandong
Happy Fan Shop	King of Power Saving	This power saver is a bit effective, used for half a month, the effect is still visible.	Jingdong prefecture level city in Shandong
Happy Fan Shop	King of Power Saving	I received the baby and it looks great, I'm trying it out now and will keep an eye on it afterward.	Jingdong prefecture level city in Shandong
Happy Fan Shop	King of Power Saving	I've just received it, but I'm still trying to see how it works, so I'll come back in a little while.	Jingdong prefecture level city in Shandong

Happy Fan Shop	King of Power Saving	Just used it wait to use it for a while and then comment thanks, all first five stars.	Jingdong prefecture level city in Shandong
Happy Fan Shop	King of Power Saving	Very trustworthy brand, quality is guaranteed, the price is still affordable, quite satisfied!	Jingdong prefecture level city in Shandong
Happy Fan Shop	King of Power Saving	Things received, I hope that the platform of Jingdong do not sell fake goods are available	Jingdong prefecture level city in Shandong
Happy Fan Shop	King of Power Saving	Energy saver received, the use of the effect is good and the business description of the same	Jingdong prefecture level city in Shandong
Happy Fan Shop	King of Power Saving	The surface looks beautiful, the actual effect is not known, after using additional evaluation.	Jingdong prefecture level city in Shandong
Happy Fan Shop	King of Power Saving	Tried it for a few months, it really saves electricity, and I bought it for a friend.	Jingdong prefecture level city in Shandong
Happy Fan Shop	King of Power Saving	It didn't work and wanted to return it, it really didn't work at all.	Jingdong prefecture level city in Shandong
Happy Fan Shop	King of Power Saving	Just used it, don't know how effective it is, looking forward to usefulness	Jingdong prefecture level city in Shandong
Happy Fan Shop	King of Power Saving	After two months before the effect I do not know if it is true!	Jingdong prefecture level city in Shandong
Happy Fan Shop	King of Power Saving	So fast! Ordered yesterday and received today 👍👍👍👍👍👍👍👍👍 🌹🌹🌹🌹🌹🌹🌹🌹🌹	Jingdong prefecture level city in Shandong
Happy Fan Shop	King of Power Saving	I've started using it. We'll see how it works.	Jingdong prefecture level city in Shandong
Happy Fan Shop	King of Power Saving	I just received the goods, I won't know the effect until after two.	Jingdong prefecture level city in Shandong
Happy Fan Shop	King of Power Saving	Just received the goods, the specific effect will be evaluated later	Jingdong prefecture level city in Shandong
Happy Fan Shop	King of Power Saving	I've received the goods, I don't know if it's any good yet	Jingdong prefecture level city in Shandong
Happy Fan Shop	King of Power Saving	Try it for a month and then come back with a concrete evaluation	Jingdong prefecture level city in Shandong
Happy Fan Shop	King of Power Saving	In use, after two months to see the effect of power saving	Jingdong prefecture level city in Shandong
Happy Fan Shop	King of Power Saving	Power Saver. It's authentic. It's black. It works.	Jingdong prefecture level city in Shandong
Happy Fan Shop	King of Power Saving	I've been using it for a week now and it's not working.	Jingdong prefecture level city in Shandong
Happy Fan Shop	King of Power Saving	I'm just using it. I don't know how it's going to work.	Jingdong prefecture level city in Shandong
Happy Fan Shop	King of Power Saving	Products are better looking out for power savings	Jingdong prefecture level city in Shandong
Happy Fan Shop	King of Power Saving	I received it and I've kept it on. I hope it works.	Jingdong prefecture level city in Shandong
Happy Fan Shop	King of Power Saving	It's really good! Worth recommending to buy!	Jingdong prefecture level city in Shandong
Happy Fan Shop	King of Power Saving	Try it for a few days and see if it doesn't work, then return it.	Jingdong prefecture level city in Shandong
Happy Fan Shop	King of Power Saving	It's in use and I hope to be pleasantly surprised.	Jingdong prefecture level city in Shandong
Happy Fan Shop	King of Power Saving	We'll see how it works when we use it	Jingdong prefecture level city in Shandong
Happy Fan Shop	King of Power Saving	Let's see if it works.	Jingdong prefecture level city in Shandong
Happy Fan Shop	King of Power Saving	I'm using it and will follow up with a review	Jingdong prefecture level city in Shandong
Happy Fan Shop	King of Power Saving	It really works. It saves energy.	Jingdong prefecture level city in Shandong
Happy Fan Shop	King of Power Saving	It does have some power savings!	Jingdong prefecture level city in Shandong
Happy Fan Shop	King of Power Saving	Jingdong shopping speed is convenient and timely	Jingdong prefecture level city in Shandong
Happy Fan Shop	King of Power Saving	Second time I've bought it. Everything's fine.	Jingdong prefecture level city in Shandong
Happy Fan Shop	King of Power Saving	I don't know if it works yet.	Jingdong prefecture level city in Shandong
Happy Fan Shop	King of Power Saving	Not used, don't know how well it works.	Jingdong prefecture level city in Shandong
Happy Fan Shop	King of Power Saving	No visible results so far	Jingdong prefecture level city in Shandong
Happy Fan Shop	King of Power Saving	It doesn't feel like it's working.	Jingdong prefecture level city in Shandong
Happy Fan Shop	King of Power Saving	Where's the effect? Where are the power savings?	Jingdong prefecture level city in Shandong
Happy Fan Shop	King of Power Saving	Let's see how it works in practice!	Jingdong prefecture level city in Shandong
Happy Fan Shop	King of Power Saving	I'll wait until I use it to give you a good review	Jingdong prefecture level city in Shandong
Happy Fan Shop	King of Power Saving	I'll follow up after I've used it	Jingdong prefecture level city in Shandong
Happy Fan Shop	King of Power Saving	Well, that's good for saving power.	Jingdong prefecture level city in Shandong
Happy Fan Shop	King of Power Saving	Received. Used in the know.	Jingdong prefecture level city in Shandong
Happy Fan Shop	King of Power Saving	It doesn't do much good.	Jingdong prefecture level city in Shandong
Happy Fan Shop	King of Power Saving	It's not working.	Jingdong prefecture level city in Shandong
Happy Fan Shop	King of Power Saving	I don't know. I just used it.	Jingdong prefecture level city in Shandong
Happy Fan Shop	King of Power Saving	Use it and come back to evaluate.	Jingdong prefecture level city in Shandong
Happy Fan Shop	King of Power Saving	Two words: useless.	Jingdong prefecture level city in Shandong
Happy Fan Shop	King of Power Saving	To be used and then evaluated	Jingdong prefecture level city in Shandong
Happy Fan Shop	King of Power Saving	No shipment.	Jingdong prefecture level city in Shandong
Happy Fan Shop	King of Power Saving	Fake. Speechless.	Jingdong prefecture level city in Shandong
Happy Fan Shop	King of Power Saving	fantastic (idiom); awesome	Jingdong prefecture level city in Shandong
Happy Fan Shop	King of Power Saving	an excellent	Jingdong prefecture level city in Shandong

Happy Fan Shop	King of Power Saving	an excellent	Jingdong prefecture level city in Shandong
Happy Fan Shop	King of Power Saving	Want to buy! Recommended not to buy! No effect	Jingdong prefecture level city in Shandong
Happy Fan Shop	King of Power Saving	That's all there is to it. I can't figure out how to save electricity.	Jingdong prefecture level city in Shandong
Happy Fan Shop	King of Power Saving	It's no use.	Jingdong prefecture level city in Shandong
Happy Fan Shop	King of Power Saving	Things are more perfect than I imagined, very powerful, it is worth getting Packaging is very strong, things are well protected, it is worth the praise things are very satisfied Oh, beautiful and stylish atmosphere, customer service is super patient, service is meticulous and attentive, the big brand is not	Jingdong prefecture level city in Shandong
Happy Fan Shop	King of Power Saving	The effect is too good, the family has now several units, back to buy a number of times, the power saver received, power-saving effect is very good,, fine workmanship, long warranty, but also purify the air, before buying a used for a period of time, with the okay, and then bought a, and only now come to the	Jingdong prefecture level city in Shandong
Happy Fan Shop	King of Power Saving	Quite good, and imagine the same, casual ride are very good, satisfied! Baby received it 👌, really great ah! 🌈 quality is really nothing to say, very satisfied! 🆗 happy happy 😃 genuine, great, the price is very good, in short, quite good, will buy again a very good shopping experience, attentive service, fast logistics, distribution is also thoughtful, the attitude is also very good, value for money, ah,	Jingdong prefecture level city in Shandong
Happy Fan Shop	King of Power Saving	Power-saving effect: strong Cost-effective: quite good Appearance style: beautiful Workmanship material: meticulous Experience: looking forward to power-saving effect	Jingdong prefecture level city in Shandong
Happy Fan Shop	King of Power Saving	Cost-effective is okay, the appearance of the style of meticulous workmanship, looking forward to energy-saving effect.	Jingdong prefecture level city in Shandong
Happy Fan Shop	King of Power Saving	Very Good Very Power Saving This Power Saver is really power saving As soon as the power is turned on, the Power Saver starts the power saving function for you.	Jingdong prefecture level city in Shandong
Happy Fan Shop	King of Power Saving	Logistics is very fast, the price is favorable, friends introduced that it is very good to use, bought 2 at once, looking forward to the effect, used after the follow-up comment.	Jingdong prefecture level city in Shandong
Happy Fan Shop	King of Power Saving	It's all fake. It doesn't save any electricity.	Jingdong prefecture level city in Shandong
Happy Fan Shop	King of Power Saving	This Power Saver is so much better than the one I bought before, not only the effect is good, the quality is very good in all aspects, the customer service attitude is also very good, recommended to buy!	Jingdong prefecture level city in Shandong
Happy Fan Shop	King of Power Saving	Logistics is very fast, buy for parents, good quality, once bought 2, power-saving effect is good to buy 2 more, look forward to using the effect, satisfied will recommend to friends.	Jingdong prefecture level city in Shandong
Happy Fan Shop	King of Power Saving	With the description is the same, very high-grade, good value, to buy friends can order Oh!	Jingdong prefecture level city in Shandong
Happy Fan Shop	King of Power Saving	Not using much electricity in the winter saving over 100 per month! Ready to buy 2 more units in the summer ！！！！！ Good quality, good price, power saving effect is obvious, cost-effective, worth buying, can be recommended!	Jingdong prefecture level city in Shandong
Happy Fan Shop	King of Power Saving	Purchased a power saver received on the put into use, rent a room with many electrical appliances, power consumption is very large, we have a piece of electricity or 1.2 dollars a time, with this power saver is much better, every day can save a lot of money, very convenient and practical.	Jingdong prefecture level city in Shandong
Happy Fan Shop	King of Power Saving	The power saving effect is very good: my supermarket monthly electricity bill is more than 7000 yuan. On May 1st someone recommended buying a couple of power savers, and I bought 5 of them first. The electricity bill was about 2000 less in June. This time, I bought 5 more. The seller said 800 degrees of electricity with one. I feel it's basically enough. A total of 10 units	Jingdong prefecture level city in Shandong
Happy Fan Shop	King of Power Saving	I never thought I could get such a good baby at such an affordable price, it's very good value for money, I love it!	Jingdong prefecture level city in Shandong
Happy Fan Shop	King of Power Saving	Previously bought other brands of power savers, but found no effect at home, this time to see how the products on the Jingdong, inserted in the home socket, the LED display is blue, the night light is very nice to see, look at the instructions that have to be half a year in order to achieve the best results, try it, I believe that the quality of the Jingdong, it should not be wrong.	Jingdong prefecture level city in Shandong
Happy Fan Shop	King of Power Saving	Superb, inexpensive, saves a lot of points, been using it for a long time!	Jingdong prefecture level city in Shandong
Happy Fan Shop	King of Power Saving	There are a lot of appliances in the house, the summer air conditioning is very costly, I bought this in front of the air conditioning effect is very good, I explain that I bought three in one breath, I use more electricity a month down to 200 or so, very satisfied.	Jingdong prefecture level city in Shandong
Happy Fan Shop	King of Power Saving	A month can save more than 200, home monthly electricity bill of more than 600 dollars, inserted 3 or so, an amount of the balance of the average can save more than 200. Product is very good, has been buying this one, has given a lot of relatives recommended, the quality is also very good, before buying a look with this same, who knows to buy home has not been used for more than a week on the bad, the quality of this is really good with more than two years!	Jingdong prefecture level city in Shandong
Happy Fan Shop	King of Power Saving	Whole house power saving king, can really save electricity, friends recommended, high power saving appliances, only replacement, no repair, after-sales worry-free!	Jingdong prefecture level city in Shandong
Happy Fan Shop	King of Power Saving	Great item love it, great customer service, great shopping experience!	Jingdong prefecture level city in Shandong
Happy Fan Shop	King of Power Saving	Power saving, very good use, much cheaper than the physical store, satisfaction	Jingdong prefecture level city in Shandong
Happy Fan Shop	King of Power Saving	In use, very well packaged and fast delivery of credit!	Jingdong prefecture level city in Shandong
Happy Fan Shop	King of Power Saving	Product quality, customer service attitude is particularly good, like, praise!	Jingdong prefecture level city in Shandong
Happy Fan Shop	King of Power Saving	The logistics is very fast, the packaging is not broken, the quality is very good, I like it very much!	Jingdong prefecture level city in Shandong
Happy Fan Shop	King of Power Saving	The saver was well packaged and was as described when plugged in	Jingdong prefecture level city in Shandong
Happy Fan Shop	King of Power Saving	Energy efficient and very satisfied	Jingdong prefecture level city in Shandong
Happy Fan Shop	King of Power Saving	Great quality for the price and fast shipping.	Jingdong prefecture level city in Shandong
Happy Fan Shop	King of Power Saving	It's great, just as described. Perfect.	Jingdong prefecture level city in Shandong
Happy Fan Shop	King of Power Saving	There is no power saving effect, but rather ring up an increase in electricity costs, because this machine is on continuously all day long, uninterruptedly, its own electricity and increased costs, plus the cost of purchasing the machine, so I feel that the use of this machine instead of increasing the	Jingdong prefecture level city in Shandong
Happy Fan Shop	King of Power Saving	Recommended by the owner of the store next door, looked at his home does have to save electricity, decided to get. Indeed quite can, the voltage is much more stable, there is a reduction in electricity	Jingdong prefecture level city in Shandong
Happy Fan Shop	King of Power Saving	Goods have been received value for money, affordable and reasonable price, and the description of the same, power-saving safe and reliable!	Jingdong prefecture level city in Shandong
Happy Fan Shop	King of Power Saving	Listen to friends introduced to buy, tried to save a lot of electricity is good, quite good, worth buying.	Jingdong prefecture level city in Shandong
Happy Fan Shop	King of Power Saving	The product is easy to use, safe and environmentally friendly, only used for a short time, to the next month, after the electricity bill status display to know how the performance of the power saver efficacy.	Jingdong prefecture level city in Shandong
Happy Fan Shop	King of Power Saving	Last month's electricity bill is 178 yuan, with this power saver is 92 yuan, ready to buy two more back home, I was introduced to buy a friend, just started to not believe that I did not expect the effect!	Jingdong prefecture level city in Shandong
Happy Fan Shop	King of Power Saving	It looks nice, but after using it for a few days I can't see how it saves power?	Jingdong prefecture level city in Shandong
Happy Fan Shop	King of Power Saving	Recommended by a friend, it is genuine, as described by the merchant, cost-effective	Jingdong prefecture level city in Shandong
Happy Fan Shop	King of Power Saving	Works well, affordable and saves money.	Jingdong prefecture level city in Shandong
Happy Fan Shop	King of Power Saving	Feels good haven't compared it yet, the air conditioning has been on a lot in the past two months	Jingdong prefecture level city in Shandong
Happy Fan Shop	King of Power Saving	Saving about 5 kWh per day is a good thing	Jingdong prefecture level city in Shandong

Happy Fan Shop	King of Power Saving	Not bad, affordable, best value for money	Jingdong prefecture level city in Shandong
Happy Fan Shop	King of Power Saving	The goods are genuine and the logistics are faster.	Jingdong prefecture level city in Shandong
Happy Fan Shop	King of Power Saving	Goods have been received today, has been plugged into the outlet, looking forward to saving electricity, after a period of time to see how the effect in the follow-up comment!	Jingdong prefecture level city in Shandong
Happy Fan Shop	King of Power Saving	I just got it home and haven't used it for long, so I'll use it for a couple months and see what happens. Seeing that most people have good reviews, it should be fine.	Jingdong prefecture level city in Shandong
Happy Fan Shop	King of Power Saving	I'm looking forward to the results, so I'll give you a good review out of habit! Follow up review after a while	Jingdong prefecture level city in Shandong
Happy Fan Shop	King of Power Saving	Use a dish first to know if it will help save energy	Jingdong prefecture level city in Shandong
Happy Fan Shop	King of Power Saving	Whether it saves electricity or not, we don't know yet.	Jingdong prefecture level city in Shandong
Happy Fan Shop	King of Power Saving	Goods have been received, use a period of time to know how oh. The courier did not deliver to the door ah! Sent to the station ah! The heat is inconvenient ah!	Jingdong prefecture level city in Shandong
Happy Fan Shop	King of Power Saving	A little effect, before an hour a power, now an hour 0.76 degrees, a dollar a power = save 24 cents, although not a lot, first use it!	Jingdong prefecture level city in Shandong
Happy Fan Shop	King of Power Saving	Just received the goods, use a month to evaluate	Jingdong prefecture level city in Shandong
Happy Fan Shop	King of Power Saving	Home every month with a lot of electricity, inadvertently found this good thing online that can save the beginning of a skeptical attitude to consult customer service, customer service replied very professional, said 120 days can be refunded that first try it!	Jingdong prefecture level city in Shandong
Happy Fan Shop	King of Power Saving	After receiving the goods this afternoon, immediately installed in the bathroom Smith energy storage electric water heater on the power outlet (instead of connecting with a plug board), when used, found that the "Power Saving King" LCD display, is the value of the voltage, not the store's video said so	Jingdong prefecture level city in Shandong
Happy Fan Shop	King of Power Saving	Has made many days, did not feel the power saving, the effect is not obvious.	Jingdong prefecture level city in Shandong
Happy Fan Shop	King of Power Saving	Energy savers do not save energy at all, but rather consume a lot of electricity.	Jingdong prefecture level city in Shandong
Happy Fan Shop	King of Power Saving	It's useless. It's a waste of money.	Jingdong prefecture level city in Shandong
Happy Fan Shop	King of Power Saving	Last month saved more than 500, which came back to buy, the store bought three, and then buy 1 home use, my family is dry beauty salon, more instruments, especially electricity, a month almost 1000. or so, with this did not expect to save so much, great, five-star praise!	Jingdong prefecture level city in Shandong
Happy Fan Shop	King of Power Saving	It rained a couple days ago and I just went to get the item back today , not bad, hey I looked at the meter and that red display light takes several seconds to jump, did not make a mistake buying it. After a period of time to evaluate.	Jingdong prefecture level city in Shandong
Happy Fan Shop	King of Power Saving	Without further ado, the electricity bill came down, this month less 102 yuan, has been repurchased, I also recommended to my friends, all said that there are effects, the product is okay, the effect is quite	Jingdong prefecture level city in Shandong
Happy Fan Shop	King of Power Saving	After buying and using two months really see the effect, so only to evaluate! Originally held the mindset of trying, I did not expect really effective, worth having!	Jingdong prefecture level city in Shandong
Happy Fan Shop	King of Power Saving	In the use of this energy saver before the monthly electricity bill is more than 150 yuan or so, now save thirty percent, every day to work, six or seven o'clock in the afternoon to go home, I do not know why the electricity bill is so expensive, energy-saving appliances really have the effect of recommending	Jingdong prefecture level city in Shandong
Happy Fan Shop	King of Power Saving	It saves about 100 bucks a month. A friend recommended it to me, and he's been using it for a while, and the effect is so obvious that I bought one too.	Jingdong prefecture level city in Shandong
Happy Fan Shop	King of Power Saving	My dad asked me to buy one, but after I paid the bill, I asked and he said it was a lot less this month, so he asked me to buy another one.	Jingdong prefecture level city in Shandong
Happy Fan Shop	King of Power Saving	The specific effect will have to be tested for at least two months to compare whether there is a power saving effect or not.	Jingdong prefecture level city in Shandong
Happy Fan Shop	King of Power Saving	'The goods have been received, immediately go to use, please ask the boss you instructions do not allow to withdraw, there are two fuses installed there!	Jingdong prefecture level city in Shandong
Happy Fan Shop	King of Power Saving	First praise is using, after using if good then follow up, now because I have not yet seen the results	Jingdong prefecture level city in Shandong
Happy Fan Shop	King of Power Saving	I've just been using it, and I haven't seen if it has a power-saving effect yet, so I'll follow up after 10 days and half a month.	Jingdong prefecture level city in Shandong
Happy Fan Shop	King of Power Saving	Just received the intelligent power saving meter, immediately plugged in to use, see it is not bad, and order to sell the second time	Jingdong prefecture level city in Shandong
Happy Fan Shop	King of Power Saving	This power saver is a bit effective, used for half a month, the effect is still visible.	Jingdong prefecture level city in Shandong
Happy Fan Shop	King of Power Saving	I received the baby and it looks great, I'm trying it out now and will keep an eye on it afterward.	Jingdong prefecture level city in Shandong
Happy Fan Shop	King of Power Saving	I've just received it, but I'm still trying to see how it works, so I'll come back in a little while.	Jingdong prefecture level city in Shandong
Happy Fan Shop	King of Power Saving	Just used it wait to use it for a while and then comment thanks, all first five stars.	Jingdong prefecture level city in Shandong
Happy Fan Shop	King of Power Saving	Very trustworthy brand, quality is guaranteed, the price is still affordable, quite satisfied!	Jingdong prefecture level city in Shandong
Happy Fan Shop	King of Power Saving	Things received, I hope that the platform of Jingdong do not sell fake goods are available	Jingdong prefecture level city in Shandong
Happy Fan Shop	King of Power Saving	Energy saver received, the use of the effect is good and the business description of the same	Jingdong prefecture level city in Shandong
Happy Fan Shop	King of Power Saving	The surface looks beautiful, the actual effect is not known, after using additional evaluation.	Jingdong prefecture level city in Shandong
Happy Fan Shop	King of Power Saving	Tried it for a few months, it really saves electricity, and I bought it for a friend.	Jingdong prefecture level city in Shandong
Happy Fan Shop	King of Power Saving	It didn't work and wanted to return it, it really didn't work at all.	Jingdong prefecture level city in Shandong
Happy Fan Shop	King of Power Saving	Just used it, don't know how effective it is, looking forward to usefulness	Jingdong prefecture level city in Shandong
Happy Fan Shop	King of Power Saving	After two months before the effect I do not know if it is true!	Jingdong prefecture level city in Shandong
Happy Fan Shop	King of Power Saving	So fast! Ordered yesterday and received today 👍👍👍👍👍👍👍👍👍 🌹🌹🌹🌹🌹🌹🌹🌹🌹	Jingdong prefecture level city in Shandong
Happy Fan Shop	King of Power Saving	I've started using it. We'll see how it works.	Jingdong prefecture level city in Shandong
Happy Fan Shop	King of Power Saving	I just received the goods, I won't know the effect until after two.	Jingdong prefecture level city in Shandong
Happy Fan Shop	King of Power Saving	Just received the goods, the specific effect will be evaluated later	Jingdong prefecture level city in Shandong
Happy Fan Shop	King of Power Saving	I've received the goods, I don't know if it's any good yet	Jingdong prefecture level city in Shandong
Happy Fan Shop	King of Power Saving	Try it for a month and then come back with a concrete evaluation	Jingdong prefecture level city in Shandong
Happy Fan Shop	King of Power Saving	In use, after two months to see the effect of power saving	Jingdong prefecture level city in Shandong
Happy Fan Shop	King of Power Saving	Power Saver. It's authentic. It's black. It works.	Jingdong prefecture level city in Shandong
Happy Fan Shop	King of Power Saving	I've been using it for a week now and it's not working.	Jingdong prefecture level city in Shandong
Happy Fan Shop	King of Power Saving	I'm just using it. I don't know how it's going to work.	Jingdong prefecture level city in Shandong

Happy Fan Shop	King of Power Saving	Products are better looking out for power savings	Jingdong prefecture level city in Shandong
Happy Fan Shop	King of Power Saving	I received it and I've kept it on. I hope it works.	Jingdong prefecture level city in Shandong
Happy Fan Shop	King of Power Saving	It's really good! Worth recommending to buy!	Jingdong prefecture level city in Shandong
Happy Fan Shop	King of Power Saving	Try it for a few days and see if it doesn't work, then return it.	Jingdong prefecture level city in Shandong
Happy Fan Shop	King of Power Saving	It's in use and I hope to be pleasantly surprised.	Jingdong prefecture level city in Shandong
Happy Fan Shop	King of Power Saving	We'll see how it works when we use it	Jingdong prefecture level city in Shandong
Happy Fan Shop	King of Power Saving	Let's see if it works.	Jingdong prefecture level city in Shandong
Happy Fan Shop	King of Power Saving	I'm using it and will follow up with a review	Jingdong prefecture level city in Shandong
Happy Fan Shop	King of Power Saving	It really works. It saves energy.	Jingdong prefecture level city in Shandong
Happy Fan Shop	King of Power Saving	It does have some power savings!	Jingdong prefecture level city in Shandong
Happy Fan Shop	King of Power Saving	Jingdong shopping speed is convenient and timely	Jingdong prefecture level city in Shandong
Happy Fan Shop	King of Power Saving	Second time I've bought it. Everything's fine.	Jingdong prefecture level city in Shandong
Happy Fan Shop	King of Power Saving	I don't know if it works yet.	Jingdong prefecture level city in Shandong
Happy Fan Shop	King of Power Saving	Not used, don't know how well it works.	Jingdong prefecture level city in Shandong
Happy Fan Shop	King of Power Saving	No visible results so far	Jingdong prefecture level city in Shandong
Happy Fan Shop	King of Power Saving	It doesn't feel like it's working.	Jingdong prefecture level city in Shandong
Happy Fan Shop	King of Power Saving	Where's the effect? Where are the power savings?	Jingdong prefecture level city in Shandong
Happy Fan Shop	King of Power Saving	Let's see how it works in practice!	Jingdong prefecture level city in Shandong
Happy Fan Shop	King of Power Saving	I'll wait until I use it to give you a good review	Jingdong prefecture level city in Shandong
Happy Fan Shop	King of Power Saving	I'll follow up after I've used it	Jingdong prefecture level city in Shandong
Happy Fan Shop	King of Power Saving	Well, that's good for saving power.	Jingdong prefecture level city in Shandong
Happy Fan Shop	King of Power Saving	Received. Used in the know.	Jingdong prefecture level city in Shandong
Happy Fan Shop	King of Power Saving	It doesn't do much good.	Jingdong prefecture level city in Shandong
Happy Fan Shop	King of Power Saving	It's not working.	Jingdong prefecture level city in Shandong
Happy Fan Shop	King of Power Saving	I don't know. I just used it.	Jingdong prefecture level city in Shandong
Happy Fan Shop	King of Power Saving	Use it and come back to evaluate.	Jingdong prefecture level city in Shandong
Happy Fan Shop	King of Power Saving	Two words: useless.	Jingdong prefecture level city in Shandong
Happy Fan Shop	King of Power Saving	To be used and then evaluated	Jingdong prefecture level city in Shandong
Happy Fan Shop	King of Power Saving	No shipment.	Jingdong prefecture level city in Shandong
Happy Fan Shop	King of Power Saving	Fake. Speechless.	Jingdong prefecture level city in Shandong
Happy Fan Shop	King of Power Saving	fantastic (idiom); awesome	Jingdong prefecture level city in Shandong
Happy Fan Shop	King of Power Saving	an excellent	Jingdong prefecture level city in Shandong
Happy Fan Shop	King of Power Saving	an excellent	Jingdong prefecture level city in Shandong
Happy Fan Shop	King of Power Saving	Want to buy! Recommended not to buy! No effect	Jingdong prefecture level city in Shandong
Happy Fan Shop	King of Power Saving	That's all there is to it. I can't figure out how to save electricity.	Jingdong prefecture level city in Shandong
Happy Fan Shop	King of Power Saving	It's no use.	Jingdong prefecture level city in Shandong
Happy Fan Shop	King of Power Saving	Things are more perfect than I imagined, very powerful, it is worth getting Packaging is very strong, things are well protected, it is worth the praise things are very satisfied Oh, beautiful and stylish atmosphere, customer service is super patient, service is meticulous and attentive, the big brand is not	Jingdong prefecture level city in Shandong
Happy Fan Shop	King of Power Saving	The effect is too good, the family has now several units, back to buy a number of times, the power saver received, power-saving effect is very good,, fine workmanship, long warranty, but also purify the air, before buying a used for a period of time, with the okay, and then bought a, and only now come to the	Jingdong prefecture level city in Shandong
Happy Fan Shop	King of Power Saving	Quite good, and imagine the same, casual ride are very good, satisfied! Baby received it 👌, really great ah! 🌈 quality is really nothing to say, very satisfied! 🆗 happy happy 😃 genuine, great, the price is very good, in short, quite good, will buy again a very good shopping experience, attentive service, fast logistics, distribution is also thoughtful, the attitude is also very good, value for money, ah,	Jingdong prefecture level city in Shandong
Happy Fan Shop	King of Power Saving	Power-saving effect: strong Cost-effective: quite good Appearance style: beautiful Workmanship material: meticulous Experience: looking forward to power-saving effect	Jingdong prefecture level city in Shandong
Happy Fan Shop	King of Power Saving	Cost-effective is okay, the appearance of the style of meticulous workmanship, looking forward to energy-saving effect.	Jingdong prefecture level city in Shandong
Happy Fan Shop	King of Power Saving	Very Good Very Power Saving This Power Saver is really power saving As soon as the power is turned on, the Power Saver starts the power saving function for you.	Jingdong prefecture level city in Shandong
Happy Fan Shop	King of Power Saving	Logistics is very fast, the price is favorable, friends introduced that it is very good to use, bought 2 at once, looking forward to the effect, used after the follow-up comment.	Jingdong prefecture level city in Shandong
Happy Fan Shop	King of Power Saving	It's all fake. It doesn't save any electricity.	Jingdong prefecture level city in Shandong
Happy Fan Shop	King of Power Saving	This Power Saver is so much better than the one I bought before, not only the effect is good, the quality is very good in all aspects, the customer service attitude is also very good, recommended to buy!	Jingdong prefecture level city in Shandong
Happy Fan Shop	King of Power Saving	Logistics is very fast, buy for parents, good quality, once bought 2, power-saving effect is good to buy 2 more, look forward to using the effect, satisfied will recommend to friends.	Jingdong prefecture level city in Shandong
Happy Fan Shop	King of Power Saving	With the description is the same, very high-grade, good value, to buy friends can order Oh!	Jingdong prefecture level city in Shandong
Happy Fan Shop	King of Power Saving	Not using much electricity in the winter saving over 100 per month! Ready to buy 2 more units in the summer ！！！！！ Good quality, good price, power saving effect is obvious, cost-effective, worth buying, can be recommended!	Jingdong prefecture level city in Shandong

Happy Fan Shop	King of Power Saving	Purchased a power saver received on the put into use, rent a room with many electrical appliances, power consumption is very large, we have a piece of electricity or 1.2 dollars a time, with this power saver is much better, every day can save a lot of money, very convenient and practical.	Jingdong prefecture level city in Shandong
Happy Fan Shop	King of Power Saving	The power saving effect is very good: my supermarket monthly electricity bill is more than 7000 yuan. On May 1st someone recommended buying a couple of power savers, and I bought 5 of them first. The electricity bill was about 2000 less in June. This time, I bought 5 more. The seller said 800 degrees of electricity with one. I feel it's basically enough. A total of 10 units	Jingdong prefecture level city in Shandong
Happy Fan Shop	King of Power Saving	I never thought I could get such a good baby at such an affordable price, it's very good value for money, I love it!	Jingdong prefecture level city in Shandong
Happy Fan Shop	King of Power Saving	Previously bought other brands of power savers, but found no effect at home, this time to see how the products on the Jingdong, inserted in the home socket, the LED display is blue, the night light is very nice to see, look at the instructions that have to be half a year in order to achieve the best results, try it, I believe that the quality of the Jingdong, it should not be wrong.	Jingdong prefecture level city in Shandong
Happy Fan Shop	King of Power Saving	Superb, inexpensive, saves a lot of points, been using it for a long time!	Jingdong prefecture level city in Shandong
Happy Fan Shop	King of Power Saving	There are a lot of appliances in the house, the summer air conditioning is very costly, I bought this in front of the air conditioning effect is very good, I explain that I bought three in one breath, I use more electricity a month down to 200 or so, very satisfied.	Jingdong prefecture level city in Shandong
Happy Fan Shop	King of Power Saving	A month can save more than 200, home monthly electricity bill of more than 600 dollars, inserted 3 or so, an amount of the balance of the average can save more than 200. Product is very good, has been buying this one, has given a lot of relatives recommended, the quality is also very good, before buying a look with this same, who knows to buy home has not been used for more than a week on the bad, the quality of this is really good with more than two years!	Jingdong prefecture level city in Shandong
Happy Fan Shop	King of Power Saving	Whole house power saving king, can really save electricity, friends recommended, high power saving appliances, only replacement, no repair, after-sales worry-free!	Jingdong prefecture level city in Shandong
Happy Fan Shop	King of Power Saving	Great item love it, great customer service, great shopping experience!	Jingdong prefecture level city in Shandong
Happy Fan Shop	King of Power Saving	Power saving, very good use, much cheaper than the physical store, satisfaction	Jingdong prefecture level city in Shandong
Happy Fan Shop	King of Power Saving	In use, very well packaged and fast delivery of credit!	Jingdong prefecture level city in Shandong
Happy Fan Shop	King of Power Saving	Product quality, customer service attitude is particularly good, like, praise!	Jingdong prefecture level city in Shandong
Happy Fan Shop	King of Power Saving	The logistics is very fast, the packaging is not broken, the quality is very good, I like it very much!	Jingdong prefecture level city in Shandong
Happy Fan Shop	King of Power Saving	The saver was well packaged and was as described when plugged in	Jingdong prefecture level city in Shandong
Happy Fan Shop	King of Power Saving	Energy efficient and very satisfied	Jingdong prefecture level city in Shandong
Happy Fan Shop	King of Power Saving	Great quality for the price and fast shipping.	Jingdong prefecture level city in Shandong
Happy Fan Shop	King of Power Saving	It's great, just as described. Perfect.	Jingdong prefecture level city in Shandong
Happy Fan Shop	King of Power Saving	There is no power saving effect, but rather ring up an increase in electricity costs, because this machine is on continuously all day long, uninterruptedly, its own electricity and increased costs, plus the cost of purchasing the machine, so I feel that the use of this machine instead of increasing the	Jingdong prefecture level city in Shandong
Happy Fan Shop	King of Power Saving	Recommended by the owner of the store next door, looked at his home does have to save electricity, decided to get. Indeed quite can, the voltage is much more stable, there is a reduction in electricity	Jingdong prefecture level city in Shandong
Happy Fan Shop	King of Power Saving	Goods have been received value for money, affordable and reasonable price, and the description of the same, power-saving safe and reliable!	Jingdong prefecture level city in Shandong
Happy Fan Shop	King of Power Saving	Listen to friends introduced to buy, tried to save a lot of electricity is good, quite good, worth buying.	Jingdong prefecture level city in Shandong
Happy Fan Shop	King of Power Saving	The product is easy to use, safe and environmentally friendly, only used for a short time, to the next month, after the electricity bill status display to know how the performance of the power saver efficacy.	Jingdong prefecture level city in Shandong
Happy Fan Shop	King of Power Saving	Last month's electricity bill is 178 yuan, with this power saver is 92 yuan, ready to buy two more back home, I was introduced to buy a friend, just started to not believe that I did not expect the effect!	Jingdong prefecture level city in Shandong
Happy Fan Shop	King of Power Saving	It looks nice, but after using it for a few days I can't see how it saves power?	Jingdong prefecture level city in Shandong
Happy Fan Shop	King of Power Saving	Recommended by a friend, it is genuine, as described by the merchant, cost-effective	Jingdong prefecture level city in Shandong
Happy Fan Shop	King of Power Saving	Works well, affordable and saves money.	Jingdong prefecture level city in Shandong
Happy Fan Shop	King of Power Saving	Feels good haven't compared it yet, the air conditioning has been on a lot in the past two months	Jingdong prefecture level city in Shandong
Happy Fan Shop	King of Power Saving	Saving about 5 kWh per day is a good thing	Jingdong prefecture level city in Shandong
Happy Fan Shop	King of Power Saving	Not bad, affordable, best value for money	Jingdong prefecture level city in Shandong
Happy Fan Shop	King of Power Saving	The goods are genuine and the logistics are faster.	Jingdong prefecture level city in Shandong
Happy Fan Shop	King of Power Saving	Goods have been received today, has been plugged into the outlet, looking forward to saving electricity, after a period of time to see how the effect in the follow-up comment!	Jingdong prefecture level city in Shandong
Happy Fan Shop	King of Power Saving	I just got it home and haven't used it for long, so I'll use it for a couple months and see what happens. Seeing that most people have good reviews, it should be fine.	Jingdong prefecture level city in Shandong
Happy Fan Shop	King of Power Saving	I'm looking forward to the results, so I'll give you a good review out of habit! Follow up review after a while	Jingdong prefecture level city in Shandong
Happy Fan Shop	King of Power Saving	Use a dish first to know if it will help save energy	Jingdong prefecture level city in Shandong
Happy Fan Shop	King of Power Saving	Whether it saves electricity or not, we don't know yet.	Jingdong prefecture level city in Shandong
Happy Fan Shop	King of Power Saving	Goods have been received, use a period of time to know how oh. The courier did not deliver to the door ah! Sent to the station ah! The heat is inconvenient ah!	Jingdong prefecture level city in Shandong
Happy Fan Shop	King of Power Saving	A little effect, before an hour a power, now an hour 0.76 degrees, a dollar a power = save 24 cents, although not a lot, first use it!	Jingdong prefecture level city in Shandong
Happy Fan Shop	King of Power Saving	Just received the goods, use a month to evaluate	Jingdong prefecture level city in Shandong
Happy Fan Shop	King of Power Saving	Home every month with a lot of electricity, inadvertently found this good thing online that can save the beginning of a skeptical attitude to consult customer service, customer service replied very professional, said 120 days can be refunded that first try it!	Jingdong prefecture level city in Shandong
Happy Fan Shop	King of Power Saving	After receiving the goods this afternoon, immediately installed in the bathroom Smith energy storage electric water heater on the power outlet (instead of connecting with a plug board), when used, found that the "Power Saving King" LCD display, is the value of the voltage, not the store's video said so	Jingdong prefecture level city in Shandong
Happy Fan Shop	King of Power Saving	Has made many days, did not feel the power saving, the effect is not obvious.	Jingdong prefecture level city in Shandong
Happy Fan Shop	King of Power Saving	Energy savers do not save energy at all, but rather consume a lot of electricity.	Jingdong prefecture level city in Shandong
Happy Fan Shop	King of Power Saving	It's useless. It's a waste of money.	Jingdong prefecture level city in Shandong
Happy Fan Shop	King of Power Saving	Last month saved more than 500, which came back to buy, the store bought three, and then buy 1 home use, my family is dry beauty salon, more instruments, especially electricity, a month almost 1000. or so, with this did not expect to save so much, great, five-star praise!	Jingdong prefecture level city in Shandong

Happy Fan Shop	King of Power Saving	It rained a couple days ago and I just went to get the item back today , not bad, hey I looked at the meter and that red display light takes several seconds to jump, did not make a mistake buying it. After a period of time to evaluate.	Jingdong prefecture level city in Shandong
Happy Fan Shop	King of Power Saving	Without further ado, the electricity bill came down, this month less 102 yuan, has been repurchased, I also recommended to my friends, all said that there are effects, the product is okay, the effect is quite	Jingdong prefecture level city in Shandong
Happy Fan Shop	King of Power Saving	After buying and using two months really see the effect, so only to evaluate! Originally held the mindset of trying, I did not expect really effective, worth having!	Jingdong prefecture level city in Shandong
Happy Fan Shop	King of Power Saving	In the use of this energy saver before the monthly electricity bill is more than 150 yuan or so, now save thirty percent, every day to work, six or seven o'clock in the afternoon to go home, I do not know why the electricity bill is so expensive, energy-saving appliances really have the effect of recommending	Jingdong prefecture level city in Shandong
Happy Fan Shop	King of Power Saving	It saves about 100 bucks a month. A friend recommended it to me, and he's been using it for a while, and the effect is so obvious that I bought one too.	Jingdong prefecture level city in Shandong
Happy Fan Shop	King of Power Saving	My dad asked me to buy one, but after I paid the bill, I asked and he said it was a lot less this month, so he asked me to buy another one.	Jingdong prefecture level city in Shandong
Happy Fan Shop	King of Power Saving	The specific effect will have to be tested for at least two months to compare whether there is a power saving effect or not.	Jingdong prefecture level city in Shandong
Happy Fan Shop	King of Power Saving	'The goods have been received, immediately go to use, please ask the boss you instructions do not allow to withdraw, there are two fuses installed there!	Jingdong prefecture level city in Shandong
Happy Fan Shop	King of Power Saving	First praise is using, after using if good then follow up, now because I have not yet seen the results	Jingdong prefecture level city in Shandong
Happy Fan Shop	King of Power Saving	I've just been using it, and I haven't seen if it has a power-saving effect yet, so I'll follow up after 10 days and half a month.	Jingdong prefecture level city in Shandong
Happy Fan Shop	King of Power Saving	Just received the intelligent power saving meter, immediately plugged in to use, see it is not bad, and order to sell the second time	Jingdong prefecture level city in Shandong
Happy Fan Shop	King of Power Saving	This power saver is a bit effective, used for half a month, the effect is still visible.	Jingdong prefecture level city in Shandong
Happy Fan Shop	King of Power Saving	I received the baby and it looks great, I'm trying it out now and will keep an eye on it afterward.	Jingdong prefecture level city in Shandong
Happy Fan Shop	King of Power Saving	I've just received it, but I'm still trying to see how it works, so I'll come back in a little while.	Jingdong prefecture level city in Shandong
Happy Fan Shop	King of Power Saving	Just used it wait to use it for a while and then comment thanks, all first five stars.	Jingdong prefecture level city in Shandong
Happy Fan Shop	King of Power Saving	Very trustworthy brand, quality is guaranteed, the price is still affordable, quite satisfied!	Jingdong prefecture level city in Shandong
Happy Fan Shop	King of Power Saving	Things received, I hope that the platform of Jingdong do not sell fake goods are available	Jingdong prefecture level city in Shandong
Happy Fan Shop	King of Power Saving	Energy saver received, the use of the effect is good and the business description of the same	Jingdong prefecture level city in Shandong
Happy Fan Shop	King of Power Saving	The surface looks beautiful, the actual effect is not known, after using additional evaluation.	Jingdong prefecture level city in Shandong
Happy Fan Shop	King of Power Saving	Tried it for a few months, it really saves electricity, and I bought it for a friend.	Jingdong prefecture level city in Shandong
Happy Fan Shop	King of Power Saving	It didn't work and wanted to return it, it really didn't work at all.	Jingdong prefecture level city in Shandong
Happy Fan Shop	King of Power Saving	Just used it, don't know how effective it is, looking forward to usefulness	Jingdong prefecture level city in Shandong
Happy Fan Shop	King of Power Saving	After two months before the effect I do not know if it is true!	Jingdong prefecture level city in Shandong
Happy Fan Shop	King of Power Saving	So fast! Ordered yesterday and received today 👍👍👍👍👍👍👍👍👍 🌹🌹🌹🌹🌹🌹🌹🌹🌹	Jingdong prefecture level city in Shandong
Happy Fan Shop	King of Power Saving	I've started using it. We'll see how it works.	Jingdong prefecture level city in Shandong
Happy Fan Shop	King of Power Saving	I just received the goods, I won't know the effect until after two.	Jingdong prefecture level city in Shandong
Happy Fan Shop	King of Power Saving	Just received the goods, the specific effect will be evaluated later	Jingdong prefecture level city in Shandong
Happy Fan Shop	King of Power Saving	I've received the goods, I don't know if it's any good yet	Jingdong prefecture level city in Shandong
Happy Fan Shop	King of Power Saving	Try it for a month and then come back with a concrete evaluation	Jingdong prefecture level city in Shandong
Happy Fan Shop	King of Power Saving	In use, after two months to see the effect of power saving	Jingdong prefecture level city in Shandong
Happy Fan Shop	King of Power Saving	Power Saver. It's authentic. It's black. It works.	Jingdong prefecture level city in Shandong
Happy Fan Shop	King of Power Saving	I've been using it for a week now and it's not working.	Jingdong prefecture level city in Shandong
Happy Fan Shop	King of Power Saving	I'm just using it. I don't know how it's going to work.	Jingdong prefecture level city in Shandong
Happy Fan Shop	King of Power Saving	Products are better looking out for power savings	Jingdong prefecture level city in Shandong
Happy Fan Shop	King of Power Saving	I received it and I've kept it on. I hope it works.	Jingdong prefecture level city in Shandong
Happy Fan Shop	King of Power Saving	It's really good! Worth recommending to buy!	Jingdong prefecture level city in Shandong
Happy Fan Shop	King of Power Saving	Try it for a few days and see if it doesn't work, then return it.	Jingdong prefecture level city in Shandong
Happy Fan Shop	King of Power Saving	It's in use and I hope to be pleasantly surprised.	Jingdong prefecture level city in Shandong
Happy Fan Shop	King of Power Saving	We'll see how it works when we use it	Jingdong prefecture level city in Shandong
Happy Fan Shop	King of Power Saving	Let's see if it works.	Jingdong prefecture level city in Shandong
Happy Fan Shop	King of Power Saving	I'm using it and will follow up with a review	Jingdong prefecture level city in Shandong
Happy Fan Shop	King of Power Saving	It really works. It saves energy.	Jingdong prefecture level city in Shandong
Happy Fan Shop	King of Power Saving	It does have some power savings!	Jingdong prefecture level city in Shandong
Happy Fan Shop	King of Power Saving	Jingdong shopping speed is convenient and timely	Jingdong prefecture level city in Shandong
Happy Fan Shop	King of Power Saving	Second time I've bought it. Everything's fine.	Jingdong prefecture level city in Shandong
Happy Fan Shop	King of Power Saving	I don't know if it works yet.	Jingdong prefecture level city in Shandong
Happy Fan Shop	King of Power Saving	Not used, don't know how well it works.	Jingdong prefecture level city in Shandong
Happy Fan Shop	King of Power Saving	No visible results so far	Jingdong prefecture level city in Shandong
Happy Fan Shop	King of Power Saving	It doesn't feel like it's working.	Jingdong prefecture level city in Shandong
Happy Fan Shop	King of Power Saving	Where's the effect? Where are the power savings?	Jingdong prefecture level city in Shandong

Happy Fan Shop	King of Power Saving	Let's see how it works in practice!	Jingdong prefecture level city in Shandong
Happy Fan Shop	King of Power Saving	I'll wait until I use it to give you a good review	Jingdong prefecture level city in Shandong
Happy Fan Shop	King of Power Saving	I'll follow up after I've used it	Jingdong prefecture level city in Shandong
Happy Fan Shop	King of Power Saving	Well, that's good for saving power.	Jingdong prefecture level city in Shandong
Happy Fan Shop	King of Power Saving	Received. Used in the know.	Jingdong prefecture level city in Shandong
Happy Fan Shop	King of Power Saving	It doesn't do much good.	Jingdong prefecture level city in Shandong
Happy Fan Shop	King of Power Saving	It's not working.	Jingdong prefecture level city in Shandong
Happy Fan Shop	King of Power Saving	I don't know. I just used it.	Jingdong prefecture level city in Shandong
Happy Fan Shop	King of Power Saving	Use it and come back to evaluate.	Jingdong prefecture level city in Shandong
Happy Fan Shop	King of Power Saving	Two words: useless.	Jingdong prefecture level city in Shandong
Happy Fan Shop	King of Power Saving	To be used and then evaluated	Jingdong prefecture level city in Shandong
Happy Fan Shop	King of Power Saving	No shipment.	Jingdong prefecture level city in Shandong
Happy Fan Shop	King of Power Saving	Fake. Speechless.	Jingdong prefecture level city in Shandong
Happy Fan Shop	King of Power Saving	fantastic (idiom); awesome	Jingdong prefecture level city in Shandong
Happy Fan Shop	King of Power Saving	an excellent	Jingdong prefecture level city in Shandong
Happy Fan Shop	King of Power Saving	an excellent	Jingdong prefecture level city in Shandong
Happy Fan Shop	King of Power Saving	Want to buy! Recommended not to buy! No effect	Jingdong prefecture level city in Shandong
Happy Fan Shop	King of Power Saving	That's all there is to it. I can't figure out how to save electricity.	Jingdong prefecture level city in Shandong
Happy Fan Shop	King of Power Saving	It's no use.	Jingdong prefecture level city in Shandong
Happy Fan Shop	King of Power Saving	Things are more perfect than I imagined, very powerful, it is worth getting Packaging is very strong, things are well protected, it is worth the praise things are very satisfied Oh, beautiful and stylish atmosphere, customer service is super patient, service is meticulous and attentive, the big brand is not	Jingdong prefecture level city in Shandong
Happy Fan Shop	King of Power Saving	The effect is too good, the family has now several units, back to buy a number of times, the power saver received, power-saving effect is very good,, fine workmanship, long warranty, but also purify the air, before buying a used for a period of time, with the okay, and then bought a, and only now come to the	Jingdong prefecture level city in Shandong
Happy Fan Shop	King of Power Saving	Quite good, and imagine the same, casual ride are very good, satisfied! Baby received it 👌, really great ah! 🌈 quality is really nothing to say, very satisfied! 🆗 happy happy 😃 genuine, great, the price is very good, in short, quite good, will buy again a very good shopping experience, attentive service, fast logistics, distribution is also thoughtful, the attitude is also very good, value for money, ah,	Jingdong prefecture level city in Shandong
Happy Fan Shop	King of Power Saving	Power-saving effect: strong Cost-effective: quite good Appearance style: beautiful Workmanship material: meticulous Experience: looking forward to power-saving effect	Jingdong prefecture level city in Shandong
Happy Fan Shop	King of Power Saving	Cost-effective is okay, the appearance of the style of meticulous workmanship, looking forward to energy-saving effect.	Jingdong prefecture level city in Shandong
Happy Fan Shop	King of Power Saving	Very Good Very Power Saving This Power Saver is really power saving As soon as the power is turned on, the Power Saver starts the power saving function for you.	Jingdong prefecture level city in Shandong
Happy Fan Shop	King of Power Saving	Logistics is very fast, the price is favorable, friends introduced that it is very good to use, bought 2 at once, looking forward to the effect, used after the follow-up comment.	Jingdong prefecture level city in Shandong
Happy Fan Shop	King of Power Saving	It's all fake. It doesn't save any electricity.	Jingdong prefecture level city in Shandong
Happy Fan Shop	King of Power Saving	This Power Saver is so much better than the one I bought before, not only the effect is good, the quality is very good in all aspects, the customer service attitude is also very good, recommended to buy!	Jingdong prefecture level city in Shandong
Happy Fan Shop	King of Power Saving	Logistics is very fast, buy for parents, good quality, once bought 2, power-saving effect is good to buy 2 more, look forward to using the effect, satisfied will recommend to friends.	Jingdong prefecture level city in Shandong
Happy Fan Shop	King of Power Saving	With the description is the same, very high-grade, good value, to buy friends can order Oh!	Jingdong prefecture level city in Shandong
Happy Fan Shop	King of Power Saving	Not using much electricity in the winter saving over 100 per month! Ready to buy 2 more units in the summer ！！！！！ Good quality, good price, power saving effect is obvious, cost-effective, worth buying, can be recommended!	Jingdong prefecture level city in Shandong
Happy Fan Shop	King of Power Saving	Purchased a power saver received on the put into use, rent a room with many electrical appliances, power consumption is very large, we have a piece of electricity or 1.2 dollars a time, with this power saver is much better, every day can save a lot of money, very convenient and practical.	Jingdong prefecture level city in Shandong
Happy Fan Shop	King of Power Saving	The power saving effect is very good: my supermarket monthly electricity bill is more than 7000 yuan. On May 1st someone recommended buying a couple of power savers, and I bought 5 of them first. The electricity bill was about 2000 less in June. This time, I bought 5 more. The seller said 800 degrees of electricity with one. I feel it's basically enough. A total of 10 units	Jingdong prefecture level city in Shandong
Happy Fan Shop	King of Power Saving	I never thought I could get such a good baby at such an affordable price, it's very good value for money, I love it!	Jingdong prefecture level city in Shandong
Happy Fan Shop	King of Power Saving	Previously bought other brands of power savers, but found no effect at home, this time to see how the products on the Jingdong, inserted in the home socket, the LED display is blue, the night light is very nice to see, look at the instructions that have to be half a year in order to achieve the best results, try it, I believe that the quality of the Jingdong, it should not be wrong.	Jingdong prefecture level city in Shandong
Happy Fan Shop	King of Power Saving	Superb, inexpensive, saves a lot of points, been using it for a long time!	Jingdong prefecture level city in Shandong
Happy Fan Shop	King of Power Saving	There are a lot of appliances in the house, the summer air conditioning is very costly, I bought this in front of the air conditioning effect is very good, I explain that I bought three in one breath, I use more electricity a month down to 200 or so, very satisfied.	Jingdong prefecture level city in Shandong
Happy Fan Shop	King of Power Saving	A month can save more than 200, home monthly electricity bill of more than 600 dollars, inserted 3 or so, an amount of the balance of the average can save more than 200. Product is very good, has been buying this one, has given a lot of relatives recommended, the quality is also very good, before buying a look with this same, who knows to buy home has not been used for more than a week on the bad, the quality of this is really good with more than two years!	Jingdong prefecture level city in Shandong
Happy Fan Shop	King of Power Saving	Whole house power saving king, can really save electricity, friends recommended, high power saving appliances, only replacement, no repair, after-sales worry-free!	Jingdong prefecture level city in Shandong
Happy Fan Shop	King of Power Saving	Great item love it, great customer service, great shopping experience!	Jingdong prefecture level city in Shandong
Happy Fan Shop	King of Power Saving	Power saving, very good use, much cheaper than the physical store, satisfaction	Jingdong prefecture level city in Shandong
Happy Fan Shop	King of Power Saving	In use, very well packaged and fast delivery of credit!	Jingdong prefecture level city in Shandong
Happy Fan Shop	King of Power Saving	Product quality, customer service attitude is particularly good, like, praise!	Jingdong prefecture level city in Shandong
Happy Fan Shop	King of Power Saving	The logistics is very fast, the packaging is not broken, the quality is very good, I like it very much!	Jingdong prefecture level city in Shandong

Happy Fan Shop	King of Power Saving	The saver was well packaged and was as described when plugged in	Jingdong prefecture level city in Shandong
Happy Fan Shop	King of Power Saving	Energy efficient and very satisfied	Jingdong prefecture level city in Shandong
Happy Fan Shop	King of Power Saving	Great quality for the price and fast shipping.	Jingdong prefecture level city in Shandong
Happy Fan Shop	King of Power Saving	It's great, just as described. Perfect.	Jingdong prefecture level city in Shandong
Happy Fan Shop	King of Power Saving	There is no power saving effect, but rather ring up an increase in electricity costs, because this machine is on continuously all day long, uninterruptedly, its own electricity and increased costs, plus the cost of purchasing the machine, so I feel that the use of this machine instead of increasing the	Jingdong prefecture level city in Shandong
Happy Fan Shop	King of Power Saving	Recommended by the owner of the store next door, looked at his home does have to save electricity, decided to get. Indeed quite can, the voltage is much more stable, there is a reduction in electricity	Jingdong prefecture level city in Shandong
Happy Fan Shop	King of Power Saving	Goods have been received value for money, affordable and reasonable price, and the description of the same, power-saving safe and reliable!	Jingdong prefecture level city in Shandong
Happy Fan Shop	King of Power Saving	Listen to friends introduced to buy, tried to save a lot of electricity is good, quite good, worth buying.	Jingdong prefecture level city in Shandong
Happy Fan Shop	King of Power Saving	The product is easy to use, safe and environmentally friendly, only used for a short time, to the next month, after the electricity bill status display to know how the performance of the power saver efficacy.	Jingdong prefecture level city in Shandong
Happy Fan Shop	King of Power Saving	Last month's electricity bill is 178 yuan, with this power saver is 92 yuan, ready to buy two more back home, I was introduced to buy a friend, just started to not believe that I did not expect the effect!	Jingdong prefecture level city in Shandong
Happy Fan Shop	King of Power Saving	It looks nice, but after using it for a few days I can't see how it saves power?	Jingdong prefecture level city in Shandong
Happy Fan Shop	King of Power Saving	Recommended by a friend, it is genuine, as described by the merchant, cost-effective	Jingdong prefecture level city in Shandong
Happy Fan Shop	King of Power Saving	Works well, affordable and saves money.	Jingdong prefecture level city in Shandong
Happy Fan Shop	King of Power Saving	Feels good haven't compared it yet, the air conditioning has been on a lot in the past two months	Jingdong prefecture level city in Shandong
Happy Fan Shop	King of Power Saving	Saving about 5 kWh per day is a good thing	Jingdong prefecture level city in Shandong
Happy Fan Shop	King of Power Saving	Not bad, affordable, best value for money	Jingdong prefecture level city in Shandong
Happy Fan Shop	King of Power Saving	The goods are genuine and the logistics are faster.	Jingdong prefecture level city in Shandong
Happy Fan Shop	King of Power Saving	Goods have been received today, has been plugged into the outlet, looking forward to saving electricity, after a period of time to see how the effect in the follow-up comment!	Jingdong prefecture level city in Shandong
Happy Fan Shop	King of Power Saving	I just got it home and haven't used it for long, so I'll use it for a couple months and see what happens. Seeing that most people have good reviews, it should be fine.	Jingdong prefecture level city in Shandong
Happy Fan Shop	King of Power Saving	I'm looking forward to the results, so I'll give you a good review out of habit! Follow up review after a while	Jingdong prefecture level city in Shandong
Happy Fan Shop	King of Power Saving	Use a dish first to know if it will help save energy	Jingdong prefecture level city in Shandong
Happy Fan Shop	King of Power Saving	Whether it saves electricity or not, we don't know yet.	Jingdong prefecture level city in Shandong
Happy Fan Shop	King of Power Saving	Goods have been received, use a period of time to know how oh. The courier did not deliver to the door ah! Sent to the station ah! The heat is inconvenient ah!	Jingdong prefecture level city in Shandong
Happy Fan Shop	King of Power Saving	A little effect, before an hour a power, now an hour 0.76 degrees, a dollar a power = save 24 cents, although not a lot, first use it!	Jingdong prefecture level city in Shandong
Happy Fan Shop	King of Power Saving	Just received the goods, use a month to evaluate	Jingdong prefecture level city in Shandong
Happy Fan Shop	King of Power Saving	Home every month with a lot of electricity, inadvertently found this good thing online that can save the beginning of a skeptical attitude to consult customer service, customer service replied very professional, said 120 days can be refunded that first try it!	Jingdong prefecture level city in Shandong
Happy Fan Shop	King of Power Saving	After receiving the goods this afternoon, immediately installed in the bathroom Smith energy storage electric water heater on the power outlet (instead of connecting with a plug board), when used, found that the "Power Saving King" LCD display, is the value of the voltage, not the store's video said so	Jingdong prefecture level city in Shandong
Happy Fan Shop	King of Power Saving	Has made many days, did not feel the power saving, the effect is not obvious.	Jingdong prefecture level city in Shandong
Happy Fan Shop	King of Power Saving	Energy savers do not save energy at all, but rather consume a lot of electricity.	Jingdong prefecture level city in Shandong
Happy Fan Shop	King of Power Saving	It's useless. It's a waste of money.	Jingdong prefecture level city in Shandong
Happy Fan Shop	King of Power Saving	Last month saved more than 500, which came back to buy, the store bought three, and then buy 1 home use, my family is dry beauty salon, more instruments, especially electricity, a month almost 1000. or so, with this did not expect to save so much, great, five-star praise!	Jingdong prefecture level city in Shandong
Happy Fan Shop	King of Power Saving	It rained a couple days ago and I just went to get the item back today , not bad, hey I looked at the meter and that red display light takes several seconds to jump, did not make a mistake buying it. After a period of time to evaluate.	Jingdong prefecture level city in Shandong
Happy Fan Shop	King of Power Saving	Without further ado, the electricity bill came down, this month less 102 yuan, has been repurchased, I also recommended to my friends, all said that there are effects, the product is okay, the effect is quite	Jingdong prefecture level city in Shandong
Happy Fan Shop	King of Power Saving	After buying and using two months really see the effect, so only to evaluate! Originally held the mindset of trying, I did not expect really effective, worth having!	Jingdong prefecture level city in Shandong
Happy Fan Shop	King of Power Saving	In the use of this energy saver before the monthly electricity bill is more than 150 yuan or so, now save thirty percent, every day to work, six or seven o'clock in the afternoon to go home, I do not know why the electricity bill is so expensive, energy-saving appliances really have the effect of recommending	Jingdong prefecture level city in Shandong
Happy Fan Shop	King of Power Saving	It saves about 100 bucks a month. A friend recommended it to me, and he's been using it for a while, and the effect is so obvious that I bought one too.	Jingdong prefecture level city in Shandong
Happy Fan Shop	King of Power Saving	My dad asked me to buy one, but after I paid the bill, I asked and he said it was a lot less this month, so he asked me to buy another one.	Jingdong prefecture level city in Shandong
Happy Fan Shop	King of Power Saving	The specific effect will have to be tested for at least two months to compare whether there is a power saving effect or not.	Jingdong prefecture level city in Shandong
Happy Fan Shop	King of Power Saving	'The goods have been received, immediately go to use, please ask the boss you instructions do not allow to withdraw, there are two fuses installed there!	Jingdong prefecture level city in Shandong
Happy Fan Shop	King of Power Saving	First praise is using, after using if good then follow up, now because I have not yet seen the results	Jingdong prefecture level city in Shandong
Happy Fan Shop	King of Power Saving	I've just been using it, and I haven't seen if it has a power-saving effect yet, so I'll follow up after 10 days and half a month.	Jingdong prefecture level city in Shandong
Happy Fan Shop	King of Power Saving	Just received the intelligent power saving meter, immediately plugged in to use, see it is not bad, and order to sell the second time	Jingdong prefecture level city in Shandong
Happy Fan Shop	King of Power Saving	This power saver is a bit effective, used for half a month, the effect is still visible.	Jingdong prefecture level city in Shandong
Happy Fan Shop	King of Power Saving	I received the baby and it looks great, I'm trying it out now and will keep an eye on it afterward.	Jingdong prefecture level city in Shandong
Happy Fan Shop	King of Power Saving	I've just received it, but I'm still trying to see how it works, so I'll come back in a little while.	Jingdong prefecture level city in Shandong
Happy Fan Shop	King of Power Saving	Just used it wait to use it for a while and then comment thanks, all first five stars.	Jingdong prefecture level city in Shandong
Happy Fan Shop	King of Power Saving	Very trustworthy brand, quality is guaranteed, the price is still affordable, quite satisfied!	Jingdong prefecture level city in Shandong
Happy Fan Shop	King of Power Saving	Things received, I hope that the platform of Jingdong do not sell fake goods are available	Jingdong prefecture level city in Shandong

Happy Fan Shop	King of Power Saving	Energy saver received, the use of the effect is good and the business description of the same	Jingdong prefecture level city in Shandong
Happy Fan Shop	King of Power Saving	The surface looks beautiful, the actual effect is not known, after using additional evaluation.	Jingdong prefecture level city in Shandong
Happy Fan Shop	King of Power Saving	Tried it for a few months, it really saves electricity, and I bought it for a friend.	Jingdong prefecture level city in Shandong
Happy Fan Shop	King of Power Saving	It didn't work and wanted to return it, it really didn't work at all.	Jingdong prefecture level city in Shandong
Happy Fan Shop	King of Power Saving	Just used it, don't know how effective it is, looking forward to usefulness	Jingdong prefecture level city in Shandong
Happy Fan Shop	King of Power Saving	After two months before the effect I do not know if it is true!	Jingdong prefecture level city in Shandong
Happy Fan Shop	King of Power Saving	So fast! Ordered yesterday and received today 👍👍👍👍👍👍👍👍👍 🌹🌹🌹🌹🌹🌹🌹🌹🌹	Jingdong prefecture level city in Shandong
Happy Fan Shop	King of Power Saving	I've started using it. We'll see how it works.	Jingdong prefecture level city in Shandong
Happy Fan Shop	King of Power Saving	I just received the goods, I won't know the effect until after two.	Jingdong prefecture level city in Shandong
Happy Fan Shop	King of Power Saving	Just received the goods, the specific effect will be evaluated later	Jingdong prefecture level city in Shandong
Happy Fan Shop	King of Power Saving	I've received the goods, I don't know if it's any good yet	Jingdong prefecture level city in Shandong
Happy Fan Shop	King of Power Saving	Try it for a month and then come back with a concrete evaluation	Jingdong prefecture level city in Shandong
Happy Fan Shop	King of Power Saving	In use, after two months to see the effect of power saving	Jingdong prefecture level city in Shandong
Happy Fan Shop	King of Power Saving	Power Saver. It's authentic. It's black. It works.	Jingdong prefecture level city in Shandong
Happy Fan Shop	King of Power Saving	I've been using it for a week now and it's not working.	Jingdong prefecture level city in Shandong
Happy Fan Shop	King of Power Saving	I'm just using it. I don't know how it's going to work.	Jingdong prefecture level city in Shandong
Happy Fan Shop	King of Power Saving	Products are better looking out for power savings	Jingdong prefecture level city in Shandong
Happy Fan Shop	King of Power Saving	I received it and I've kept it on. I hope it works.	Jingdong prefecture level city in Shandong
Happy Fan Shop	King of Power Saving	It's really good! Worth recommending to buy!	Jingdong prefecture level city in Shandong
Happy Fan Shop	King of Power Saving	Try it for a few days and see if it doesn't work, then return it.	Jingdong prefecture level city in Shandong
Happy Fan Shop	King of Power Saving	It's in use and I hope to be pleasantly surprised.	Jingdong prefecture level city in Shandong
Happy Fan Shop	King of Power Saving	We'll see how it works when we use it	Jingdong prefecture level city in Shandong
Happy Fan Shop	King of Power Saving	Let's see if it works.	Jingdong prefecture level city in Shandong
Happy Fan Shop	King of Power Saving	I'm using it and will follow up with a review	Jingdong prefecture level city in Shandong
Happy Fan Shop	King of Power Saving	It really works. It saves energy.	Jingdong prefecture level city in Shandong
Happy Fan Shop	King of Power Saving	It does have some power savings!	Jingdong prefecture level city in Shandong
Happy Fan Shop	King of Power Saving	Jingdong shopping speed is convenient and timely	Jingdong prefecture level city in Shandong
Happy Fan Shop	King of Power Saving	Second time I've bought it. Everything's fine.	Jingdong prefecture level city in Shandong
Happy Fan Shop	King of Power Saving	I don't know if it works yet.	Jingdong prefecture level city in Shandong
Happy Fan Shop	King of Power Saving	Not used, don't know how well it works.	Jingdong prefecture level city in Shandong
Happy Fan Shop	King of Power Saving	No visible results so far	Jingdong prefecture level city in Shandong
Happy Fan Shop	King of Power Saving	It doesn't feel like it's working.	Jingdong prefecture level city in Shandong
Happy Fan Shop	King of Power Saving	Where's the effect? Where are the power savings?	Jingdong prefecture level city in Shandong
Happy Fan Shop	King of Power Saving	Let's see how it works in practice!	Jingdong prefecture level city in Shandong
Happy Fan Shop	King of Power Saving	I'll wait until I use it to give you a good review	Jingdong prefecture level city in Shandong
Happy Fan Shop	King of Power Saving	I'll follow up after I've used it	Jingdong prefecture level city in Shandong
Happy Fan Shop	King of Power Saving	Well, that's good for saving power.	Jingdong prefecture level city in Shandong
Happy Fan Shop	King of Power Saving	Received. Used in the know.	Jingdong prefecture level city in Shandong
Happy Fan Shop	King of Power Saving	It doesn't do much good.	Jingdong prefecture level city in Shandong
Happy Fan Shop	King of Power Saving	It's not working.	Jingdong prefecture level city in Shandong
Happy Fan Shop	King of Power Saving	I don't know. I just used it.	Jingdong prefecture level city in Shandong
Happy Fan Shop	King of Power Saving	Use it and come back to evaluate.	Jingdong prefecture level city in Shandong
Happy Fan Shop	King of Power Saving	Two words: useless.	Jingdong prefecture level city in Shandong
Happy Fan Shop	King of Power Saving	To be used and then evaluated	Jingdong prefecture level city in Shandong
Happy Fan Shop	King of Power Saving	No shipment.	Jingdong prefecture level city in Shandong
Happy Fan Shop	King of Power Saving	Fake. Speechless.	Jingdong prefecture level city in Shandong
Happy Fan Shop	King of Power Saving	fantastic (idiom); awesome	Jingdong prefecture level city in Shandong
Happy Fan Shop	King of Power Saving	an excellent	Jingdong prefecture level city in Shandong
Happy Fan Shop	King of Power Saving	an excellent	Jingdong prefecture level city in Shandong
Happy Fan Shop	King of Power Saving	Want to buy! Recommended not to buy! No effect	Jingdong prefecture level city in Shandong
Happy Fan Shop	King of Power Saving	That's all there is to it. I can't figure out how to save electricity.	Jingdong prefecture level city in Shandong

Happy Fan Shop	King of Power Saving	It's no use.	Jingdong prefecture level city in Shandong
Happy Fan Shop	King of Power Saving	Things are more perfect than I imagined, very powerful, it is worth getting Packaging is very strong, things are well protected, it is worth the praise things are very satisfied Oh, beautiful and stylish atmosphere, customer service is super patient, service is meticulous and attentive, the big brand is not	Jingdong prefecture level city in Shandong
Happy Fan Shop	King of Power Saving	The effect is too good, the family has now several units, back to buy a number of times, the power saver received, power-saving effect is very good,, fine workmanship, long warranty, but also purify the air, before buying a used for a period of time, with the okay, and then bought a, and only now come to the	Jingdong prefecture level city in Shandong
Happy Fan Shop	King of Power Saving	Quite good, and imagine the same, casual ride are very good, satisfied! Baby received it 👌, really great ah! 🌈 quality is really nothing to say, very satisfied! 🆗 happy happy 😃 genuine, great, the price is very good, in short, quite good, will buy again a very good shopping experience, attentive service, fast logistics, distribution is also thoughtful, the attitude is also very good, value for money, ah,	Jingdong prefecture level city in Shandong
Happy Fan Shop	King of Power Saving	Power-saving effect: strong Cost-effective: quite good Appearance style: beautiful Workmanship material: meticulous Experience: looking forward to power-saving effect	Jingdong prefecture level city in Shandong
Happy Fan Shop	King of Power Saving	Cost-effective is okay, the appearance of the style of meticulous workmanship, looking forward to energy-saving effect.	Jingdong prefecture level city in Shandong
Happy Fan Shop	King of Power Saving	Very Good Very Power Saving This Power Saver is really power saving As soon as the power is turned on, the Power Saver starts the power saving function for you.	Jingdong prefecture level city in Shandong
Happy Fan Shop	King of Power Saving	Logistics is very fast, the price is favorable, friends introduced that it is very good to use, bought 2 at once, looking forward to the effect, used after the follow-up comment.	Jingdong prefecture level city in Shandong
Happy Fan Shop	King of Power Saving	It's all fake. It doesn't save any electricity.	Jingdong prefecture level city in Shandong
Happy Fan Shop	King of Power Saving	This Power Saver is so much better than the one I bought before, not only the effect is good, the quality is very good in all aspects, the customer service attitude is also very good, recommended to buy!	Jingdong prefecture level city in Shandong
Happy Fan Shop	King of Power Saving	Logistics is very fast, buy for parents, good quality, once bought 2, power-saving effect is good to buy 2 more, look forward to using the effect, satisfied will recommend to friends.	Jingdong prefecture level city in Shandong
Happy Fan Shop	King of Power Saving	With the description is the same, very high-grade, good value, to buy friends can order Oh!	Jingdong prefecture level city in Shandong
Happy Fan Shop	King of Power Saving	Not using much electricity in the winter saving over 100 per month! Ready to buy 2 more units in the summer ！！！！！ Good quality, good price, power saving effect is obvious, cost-effective, worth buying, can be recommended!	Jingdong prefecture level city in Shandong
Happy Fan Shop	King of Power Saving	Purchased a power saver received on the put into use, rent a room with many electrical appliances, power consumption is very large, we have a piece of electricity or 1.2 dollars a time, with this power saver is much better, every day can save a lot of money, very convenient and practical.	Jingdong prefecture level city in Shandong
Happy Fan Shop	King of Power Saving	The power saving effect is very good: my supermarket monthly electricity bill is more than 7000 yuan. On May 1st someone recommended buying a couple of power savers, and I bought 5 of them first. The electricity bill was about 2000 less in June. This time, I bought 5 more. The seller said 800 degrees of electricity with one. I feel it's basically enough. A total of 10 units	Jingdong prefecture level city in Shandong
Happy Fan Shop	King of Power Saving	I never thought I could get such a good baby at such an affordable price, it's very good value for money, I love it!	Jingdong prefecture level city in Shandong
Happy Fan Shop	King of Power Saving	Previously bought other brands of power savers, but found no effect at home, this time to see how the products on the Jingdong, inserted in the home socket, the LED display is blue, the night light is very nice to see, look at the instructions that have to be half a year in order to achieve the best results, try it, I believe that the quality of the Jingdong, it should not be wrong.	Jingdong prefecture level city in Shandong
Happy Fan Shop	King of Power Saving	Superb, inexpensive, saves a lot of points, been using it for a long time!	Jingdong prefecture level city in Shandong
Happy Fan Shop	King of Power Saving	There are a lot of appliances in the house, the summer air conditioning is very costly, I bought this in front of the air conditioning effect is very good, I explain that I bought three in one breath, I use more electricity a month down to 200 or so, very satisfied.	Jingdong prefecture level city in Shandong
Happy Fan Shop	King of Power Saving	A month can save more than 200, home monthly electricity bill of more than 600 dollars, inserted 3 or so, an amount of the balance of the average can save more than 200. Product is very good, has been buying this one, has given a lot of relatives recommended, the quality is also very good, before buying a look with this same, who knows to buy home has not been used for more than a week on the bad, the quality of this is really good with more than two years!	Jingdong prefecture level city in Shandong
Happy Fan Shop	King of Power Saving	Whole house power saving king, can really save electricity, friends recommended, high power saving appliances, only replacement, no repair, after-sales worry-free!	Jingdong prefecture level city in Shandong
Happy Fan Shop	King of Power Saving	Great item love it, great customer service, great shopping experience!	Jingdong prefecture level city in Shandong
Happy Fan Shop	King of Power Saving	Power saving, very good use, much cheaper than the physical store, satisfaction	Jingdong prefecture level city in Shandong
Happy Fan Shop	King of Power Saving	In use, very well packaged and fast delivery of credit!	Jingdong prefecture level city in Shandong
Happy Fan Shop	King of Power Saving	Product quality, customer service attitude is particularly good, like, praise!	Jingdong prefecture level city in Shandong
Happy Fan Shop	King of Power Saving	The logistics is very fast, the packaging is not broken, the quality is very good, I like it very much!	Jingdong prefecture level city in Shandong
Happy Fan Shop	King of Power Saving	The saver was well packaged and was as described when plugged in	Jingdong prefecture level city in Shandong
Happy Fan Shop	King of Power Saving	Energy efficient and very satisfied	Jingdong prefecture level city in Shandong
Happy Fan Shop	King of Power Saving	Great quality for the price and fast shipping.	Jingdong prefecture level city in Shandong
Happy Fan Shop	King of Power Saving	It's great, just as described. Perfect.	Jingdong prefecture level city in Shandong
Happy Fan Shop	King of Power Saving	There is no power saving effect, but rather ring up an increase in electricity costs, because this machine is on continuously all day long, uninterruptedly, its own electricity and increased costs, plus the cost of purchasing the machine, so I feel that the use of this machine instead of increasing the	Jingdong prefecture level city in Shandong
Happy Fan Shop	King of Power Saving	Recommended by the owner of the store next door, looked at his home does have to save electricity, decided to get. Indeed quite can, the voltage is much more stable, there is a reduction in electricity	Jingdong prefecture level city in Shandong
Happy Fan Shop	King of Power Saving	Goods have been received value for money, affordable and reasonable price, and the description of the same, power-saving safe and reliable!	Jingdong prefecture level city in Shandong
Happy Fan Shop	King of Power Saving	Listen to friends introduced to buy, tried to save a lot of electricity is good, quite good, worth buying.	Jingdong prefecture level city in Shandong
Happy Fan Shop	King of Power Saving	The product is easy to use, safe and environmentally friendly, only used for a short time, to the next month, after the electricity bill status display to know how the performance of the power saver efficacy.	Jingdong prefecture level city in Shandong
Happy Fan Shop	King of Power Saving	Last month's electricity bill is 178 yuan, with this power saver is 92 yuan, ready to buy two more back home, I was introduced to buy a friend, just started to not believe that I did not expect the effect!	Jingdong prefecture level city in Shandong
Happy Fan Shop	King of Power Saving	It looks nice, but after using it for a few days I can't see how it saves power?	Jingdong prefecture level city in Shandong
Happy Fan Shop	King of Power Saving	Recommended by a friend, it is genuine, as described by the merchant, cost-effective	Jingdong prefecture level city in Shandong
Happy Fan Shop	King of Power Saving	Works well, affordable and saves money.	Jingdong prefecture level city in Shandong
Happy Fan Shop	King of Power Saving	Feels good haven't compared it yet, the air conditioning has been on a lot in the past two months	Jingdong prefecture level city in Shandong
Happy Fan Shop	King of Power Saving	Saving about 5 kWh per day is a good thing	Jingdong prefecture level city in Shandong
Happy Fan Shop	King of Power Saving	Not bad, affordable, best value for money	Jingdong prefecture level city in Shandong
Happy Fan Shop	King of Power Saving	The goods are genuine and the logistics are faster.	Jingdong prefecture level city in Shandong
Happy Fan Shop	King of Power Saving	Goods have been received today, has been plugged into the outlet, looking forward to saving electricity, after a period of time to see how the effect in the follow-up comment!	Jingdong prefecture level city in Shandong

Happy Fan Shop	King of Power Saving	I just got it home and haven't used it for long, so I'll use it for a couple months and see what happens. Seeing that most people have good reviews, it should be fine.	Jingdong prefecture level city in Shandong
Happy Fan Shop	King of Power Saving	I'm looking forward to the results, so I'll give you a good review out of habit! Follow up review after a while	Jingdong prefecture level city in Shandong
Happy Fan Shop	King of Power Saving	Use a dish first to know if it will help save energy	Jingdong prefecture level city in Shandong
Happy Fan Shop	King of Power Saving	Whether it saves electricity or not, we don't know yet.	Jingdong prefecture level city in Shandong
Happy Fan Shop	King of Power Saving	Goods have been received, use a period of time to know how oh. The courier did not deliver to the door ah! Sent to the station ah! The heat is inconvenient ah!	Jingdong prefecture level city in Shandong
Happy Fan Shop	King of Power Saving	A little effect, before an hour a power, now an hour 0.76 degrees, a dollar a power = save 24 cents, although not a lot, first use it!	Jingdong prefecture level city in Shandong
Happy Fan Shop	King of Power Saving	Just received the goods, use a month to evaluate	Jingdong prefecture level city in Shandong
Happy Fan Shop	King of Power Saving	Home every month with a lot of electricity, inadvertently found this good thing online that can save the beginning of a skeptical attitude to consult customer service, customer service replied very professional, said 120 days can be refunded that first try it!	Jingdong prefecture level city in Shandong
Happy Fan Shop	King of Power Saving	After receiving the goods this afternoon, immediately installed in the bathroom Smith energy storage electric water heater on the power outlet (instead of connecting with a plug board), when used, found that the "Power Saving King" LCD display, is the value of the voltage, not the store's video said so	Jingdong prefecture level city in Shandong
Happy Fan Shop	King of Power Saving	Has made many days, did not feel the power saving, the effect is not obvious.	Jingdong prefecture level city in Shandong
Happy Fan Shop	King of Power Saving	Energy savers do not save energy at all, but rather consume a lot of electricity.	Jingdong prefecture level city in Shandong
Happy Fan Shop	King of Power Saving	It's useless. It's a waste of money.	Jingdong prefecture level city in Shandong
Happy Fan Shop	King of Power Saving	Last month saved more than 500, which came back to buy, the store bought three, and then buy 1 home use, my family is dry beauty salon, more instruments, especially electricity, a month almost 1000. or so, with this did not expect to save so much, great, five-star praise!	Jingdong prefecture level city in Shandong
Happy Fan Shop	King of Power Saving	It rained a couple days ago and I just went to get the item back today , not bad, hey I looked at the meter and that red display light takes several seconds to jump, did not make a mistake buying it. After a period of time to evaluate.	Jingdong prefecture level city in Shandong
Happy Fan Shop	King of Power Saving	Without further ado, the electricity bill came down, this month less 102 yuan, has been repurchased, I also recommended to my friends, all said that there are effects, the product is okay, the effect is quite	Jingdong prefecture level city in Shandong
Happy Fan Shop	King of Power Saving	After buying and using two months really see the effect, so only to evaluate! Originally held the mindset of trying, I did not expect really effective, worth having!	Jingdong prefecture level city in Shandong
Happy Fan Shop	King of Power Saving	In the use of this energy saver before the monthly electricity bill is more than 150 yuan or so, now save thirty percent, every day to work, six or seven o'clock in the afternoon to go home, I do not know why the electricity bill is so expensive, energy-saving appliances really have the effect of recommending	Jingdong prefecture level city in Shandong
Happy Fan Shop	King of Power Saving	It saves about 100 bucks a month. A friend recommended it to me, and he's been using it for a while, and the effect is so obvious that I bought one too.	Jingdong prefecture level city in Shandong
Happy Fan Shop	King of Power Saving	My dad asked me to buy one, but after I paid the bill, I asked and he said it was a lot less this month, so he asked me to buy another one.	Jingdong prefecture level city in Shandong
Happy Fan Shop	King of Power Saving	The specific effect will have to be tested for at least two months to compare whether there is a power saving effect or not.	Jingdong prefecture level city in Shandong
Happy Fan Shop	King of Power Saving	'The goods have been received, immediately go to use, please ask the boss you instructions do not allow to withdraw, there are two fuses installed there!	Jingdong prefecture level city in Shandong
Happy Fan Shop	King of Power Saving	First praise is using, after using if good then follow up, now because I have not yet seen the results	Jingdong prefecture level city in Shandong
Happy Fan Shop	King of Power Saving	I've just been using it, and I haven't seen if it has a power-saving effect yet, so I'll follow up after 10 days and half a month.	Jingdong prefecture level city in Shandong
Happy Fan Shop	King of Power Saving	Just received the intelligent power saving meter, immediately plugged in to use, see it is not bad, and order to sell the second time	Jingdong prefecture level city in Shandong
Happy Fan Shop	King of Power Saving	This power saver is a bit effective, used for half a month, the effect is still visible.	Jingdong prefecture level city in Shandong
Happy Fan Shop	King of Power Saving	I received the baby and it looks great, I'm trying it out now and will keep an eye on it afterward.	Jingdong prefecture level city in Shandong
Happy Fan Shop	King of Power Saving	I've just received it, but I'm still trying to see how it works, so I'll come back in a little while.	Jingdong prefecture level city in Shandong
Happy Fan Shop	King of Power Saving	Just used it wait to use it for a while and then comment thanks, all first five stars.	Jingdong prefecture level city in Shandong
Happy Fan Shop	King of Power Saving	Very trustworthy brand, quality is guaranteed, the price is still affordable, quite satisfied!	Jingdong prefecture level city in Shandong
Happy Fan Shop	King of Power Saving	Things received, I hope that the platform of Jingdong do not sell fake goods are available	Jingdong prefecture level city in Shandong
Happy Fan Shop	King of Power Saving	Energy saver received, the use of the effect is good and the business description of the same	Jingdong prefecture level city in Shandong
Happy Fan Shop	King of Power Saving	The surface looks beautiful, the actual effect is not known, after using additional evaluation.	Jingdong prefecture level city in Shandong
Happy Fan Shop	King of Power Saving	Tried it for a few months, it really saves electricity, and I bought it for a friend.	Jingdong prefecture level city in Shandong
Happy Fan Shop	King of Power Saving	It didn't work and wanted to return it, it really didn't work at all.	Jingdong prefecture level city in Shandong
Happy Fan Shop	King of Power Saving	Just used it, don't know how effective it is, looking forward to usefulness	Jingdong prefecture level city in Shandong
Happy Fan Shop	King of Power Saving	After two months before the effect I do not know if it is true!	Jingdong prefecture level city in Shandong
Happy Fan Shop	King of Power Saving	So fast! Ordered yesterday and received today 👍👍👍👍👍👍👍👍👍 🌹🌹🌹🌹🌹🌹🌹🌹🌹	Jingdong prefecture level city in Shandong
Happy Fan Shop	King of Power Saving	I've started using it. We'll see how it works.	Jingdong prefecture level city in Shandong
Happy Fan Shop	King of Power Saving	I just received the goods, I won't know the effect until after two.	Jingdong prefecture level city in Shandong
Happy Fan Shop	King of Power Saving	Just received the goods, the specific effect will be evaluated later	Jingdong prefecture level city in Shandong
Happy Fan Shop	King of Power Saving	I've received the goods, I don't know if it's any good yet	Jingdong prefecture level city in Shandong
Happy Fan Shop	King of Power Saving	Try it for a month and then come back with a concrete evaluation	Jingdong prefecture level city in Shandong
Happy Fan Shop	King of Power Saving	In use, after two months to see the effect of power saving	Jingdong prefecture level city in Shandong
Happy Fan Shop	King of Power Saving	Power Saver. It's authentic. It's black. It works.	Jingdong prefecture level city in Shandong
Happy Fan Shop	King of Power Saving	I've been using it for a week now and it's not working.	Jingdong prefecture level city in Shandong
Happy Fan Shop	King of Power Saving	I'm just using it. I don't know how it's going to work.	Jingdong prefecture level city in Shandong
Happy Fan Shop	King of Power Saving	Products are better looking out for power savings	Jingdong prefecture level city in Shandong
Happy Fan Shop	King of Power Saving	I received it and I've kept it on. I hope it works.	Jingdong prefecture level city in Shandong
Happy Fan Shop	King of Power Saving	It's really good! Worth recommending to buy!	Jingdong prefecture level city in Shandong

Happy Fan Shop	King of Power Saving	Try it for a few days and see if it doesn't work, then return it.	Jingdong prefecture level city in Shandong
Happy Fan Shop	King of Power Saving	It's in use and I hope to be pleasantly surprised.	Jingdong prefecture level city in Shandong
Happy Fan Shop	King of Power Saving	We'll see how it works when we use it	Jingdong prefecture level city in Shandong
Happy Fan Shop	King of Power Saving	Let's see if it works.	Jingdong prefecture level city in Shandong
Happy Fan Shop	King of Power Saving	I'm using it and will follow up with a review	Jingdong prefecture level city in Shandong
Happy Fan Shop	King of Power Saving	It really works. It saves energy.	Jingdong prefecture level city in Shandong
Happy Fan Shop	King of Power Saving	It does have some power savings!	Jingdong prefecture level city in Shandong
Happy Fan Shop	King of Power Saving	Jingdong shopping speed is convenient and timely	Jingdong prefecture level city in Shandong
Happy Fan Shop	King of Power Saving	Second time I've bought it. Everything's fine.	Jingdong prefecture level city in Shandong
Happy Fan Shop	King of Power Saving	I don't know if it works yet.	Jingdong prefecture level city in Shandong
Happy Fan Shop	King of Power Saving	Not used, don't know how well it works.	Jingdong prefecture level city in Shandong
Happy Fan Shop	King of Power Saving	No visible results so far	Jingdong prefecture level city in Shandong
Happy Fan Shop	King of Power Saving	It doesn't feel like it's working.	Jingdong prefecture level city in Shandong
Happy Fan Shop	King of Power Saving	Where's the effect? Where are the power savings?	Jingdong prefecture level city in Shandong
Happy Fan Shop	King of Power Saving	Let's see how it works in practice!	Jingdong prefecture level city in Shandong
Happy Fan Shop	King of Power Saving	I'll wait until I use it to give you a good review	Jingdong prefecture level city in Shandong
Happy Fan Shop	King of Power Saving	I'll follow up after I've used it	Jingdong prefecture level city in Shandong
Happy Fan Shop	King of Power Saving	Well, that's good for saving power.	Jingdong prefecture level city in Shandong
Happy Fan Shop	King of Power Saving	Received. Used in the know.	Jingdong prefecture level city in Shandong
Happy Fan Shop	King of Power Saving	It doesn't do much good.	Jingdong prefecture level city in Shandong
Happy Fan Shop	King of Power Saving	It's not working.	Jingdong prefecture level city in Shandong
Happy Fan Shop	King of Power Saving	I don't know. I just used it.	Jingdong prefecture level city in Shandong
Happy Fan Shop	King of Power Saving	Use it and come back to evaluate.	Jingdong prefecture level city in Shandong
Happy Fan Shop	King of Power Saving	Two words: useless.	Jingdong prefecture level city in Shandong
Happy Fan Shop	King of Power Saving	To be used and then evaluated	Jingdong prefecture level city in Shandong
Happy Fan Shop	King of Power Saving	No shipment.	Jingdong prefecture level city in Shandong
Happy Fan Shop	King of Power Saving	Fake. Speechless.	Jingdong prefecture level city in Shandong
Happy Fan Shop	King of Power Saving	fantastic (idiom); awesome	Jingdong prefecture level city in Shandong
Happy Fan Shop	King of Power Saving	an excellent	Jingdong prefecture level city in Shandong
Happy Fan Shop	King of Power Saving	an excellent	Jingdong prefecture level city in Shandong
Happy Fan Shop	King of Power Saving	Want to buy! Recommended not to buy! No effect	Jingdong prefecture level city in Shandong
Happy Fan Shop	King of Power Saving	That's all there is to it. I can't figure out how to save electricity.	Jingdong prefecture level city in Shandong
Happy Fan Shop	King of Power Saving	It's no use.	Jingdong prefecture level city in Shandong
Happy Fan Shop	King of Power Saving	Things are more perfect than I imagined, very powerful, it is worth getting Packaging is very strong, things are well protected, it is worth the praise things are very satisfied Oh, beautiful and stylish atmosphere, customer service is super patient, service is meticulous and attentive, the big brand is not	Jingdong prefecture level city in Shandong
Happy Fan Shop	King of Power Saving	The effect is too good, the family has now several units, back to buy a number of times, the power saver received, power-saving effect is very good,, fine workmanship, long warranty, but also purify the air, before buying a used for a period of time, with the okay, and then bought a, and only now come to the	Jingdong prefecture level city in Shandong
Happy Fan Shop	King of Power Saving	Quite good, and imagine the same, casual ride are very good, satisfied! Baby received it 👌, really great ah! 🌈 quality is really nothing to say, very satisfied! 🆗 happy happy 😃 genuine, great, the price is very good, in short, quite good, will buy again a very good shopping experience, attentive service, fast logistics, distribution is also thoughtful, the attitude is also very good, value for money, ah,	Jingdong prefecture level city in Shandong
Happy Fan Shop	King of Power Saving	Power-saving effect: strong Cost-effective: quite good Appearance style: beautiful Workmanship material: meticulous Experience: looking forward to power-saving effect	Jingdong prefecture level city in Shandong
Happy Fan Shop	King of Power Saving	Cost-effective is okay, the appearance of the style of meticulous workmanship, looking forward to energy-saving effect.	Jingdong prefecture level city in Shandong
Happy Fan Shop	King of Power Saving	Very Good Very Power Saving This Power Saver is really power saving As soon as the power is turned on, the Power Saver starts the power saving function for you.	Jingdong prefecture level city in Shandong
Happy Fan Shop	King of Power Saving	Logistics is very fast, the price is favorable, friends introduced that it is very good to use, bought 2 at once, looking forward to the effect, used after the follow-up comment.	Jingdong prefecture level city in Shandong
Happy Fan Shop	King of Power Saving	It's all fake. It doesn't save any electricity.	Jingdong prefecture level city in Shandong
Happy Fan Shop	King of Power Saving	This Power Saver is so much better than the one I bought before, not only the effect is good, the quality is very good in all aspects, the customer service attitude is also very good, recommended to buy!	Jingdong prefecture level city in Shandong
Happy Fan Shop	King of Power Saving	Logistics is very fast, buy for parents, good quality, once bought 2, power-saving effect is good to buy 2 more, look forward to using the effect, satisfied will recommend to friends.	Jingdong prefecture level city in Shandong
Happy Fan Shop	King of Power Saving	With the description is the same, very high-grade, good value, to buy friends can order Oh!	Jingdong prefecture level city in Shandong
Happy Fan Shop	King of Power Saving	Not using much electricity in the winter saving over 100 per month! Ready to buy 2 more units in the summer ！！！！！ Good quality, good price, power saving effect is obvious, cost-effective, worth buying, can be recommended!	Jingdong prefecture level city in Shandong
Happy Fan Shop	King of Power Saving	Purchased a power saver received on the put into use, rent a room with many electrical appliances, power consumption is very large, we have a piece of electricity or 1.2 dollars a time, with this power saver is much better, every day can save a lot of money, very convenient and practical.	Jingdong prefecture level city in Shandong
Happy Fan Shop	King of Power Saving	The power saving effect is very good: my supermarket monthly electricity bill is more than 7000 yuan. On May 1st someone recommended buying a couple of power savers, and I bought 5 of them first. The electricity bill was about 2000 less in June. This time, I bought 5 more. The seller said 800 degrees of electricity with one. I feel it's basically enough. A total of 10 units	Jingdong prefecture level city in Shandong

Happy Fan Shop	King of Power Saving	I never thought I could get such a good baby at such an affordable price, it's very good value for money, I love it!	Jingdong prefecture level city in Shandong
Happy Fan Shop	King of Power Saving	Previously bought other brands of power savers, but found no effect at home, this time to see how the products on the Jingdong, inserted in the home socket, the LED display is blue, the night light is very nice to see, look at the instructions that have to be half a year in order to achieve the best results, try it, I believe that the quality of the Jingdong, it should not be wrong.	Jingdong prefecture level city in Shandong
Happy Fan Shop	King of Power Saving	Superb, inexpensive, saves a lot of points, been using it for a long time!	Jingdong prefecture level city in Shandong
Happy Fan Shop	King of Power Saving	There are a lot of appliances in the house, the summer air conditioning is very costly, I bought this in front of the air conditioning effect is very good, I explain that I bought three in one breath, I use more electricity a month down to 200 or so, very satisfied.	Jingdong prefecture level city in Shandong
Happy Fan Shop	King of Power Saving	A month can save more than 200, home monthly electricity bill of more than 600 dollars, inserted 3 or so, an amount of the balance of the average can save more than 200. Product is very good, has been buying this one, has given a lot of relatives recommended, the quality is also very good, before buying a look with this same, who knows to buy home has not been used for more than a week on the bad, the quality of this is really good with more than two years!	Jingdong prefecture level city in Shandong
Happy Fan Shop	King of Power Saving	Whole house power saving king, can really save electricity, friends recommended, high power saving appliances, only replacement, no repair, after-sales worry-free!	Jingdong prefecture level city in Shandong
Happy Fan Shop	King of Power Saving	Great item love it, great customer service, great shopping experience!	Jingdong prefecture level city in Shandong
Happy Fan Shop	King of Power Saving	Power saving, very good use, much cheaper than the physical store, satisfaction	Jingdong prefecture level city in Shandong
Happy Fan Shop	King of Power Saving	In use, very well packaged and fast delivery of credit!	Jingdong prefecture level city in Shandong
Happy Fan Shop	King of Power Saving	Product quality, customer service attitude is particularly good, like, praise!	Jingdong prefecture level city in Shandong
Happy Fan Shop	King of Power Saving	The logistics is very fast, the packaging is not broken, the quality is very good, I like it very much!	Jingdong prefecture level city in Shandong
Happy Fan Shop	King of Power Saving	The saver was well packaged and was as described when plugged in	Jingdong prefecture level city in Shandong
Happy Fan Shop	King of Power Saving	Energy efficient and very satisfied	Jingdong prefecture level city in Shandong
Happy Fan Shop	King of Power Saving	Great quality for the price and fast shipping.	Jingdong prefecture level city in Shandong
Happy Fan Shop	King of Power Saving	It's great, just as described. Perfect.	Jingdong prefecture level city in Shandong
Happy Fan Shop	King of Power Saving	There is no power saving effect, but rather ring up an increase in electricity costs, because this machine is on continuously all day long, uninterruptedly, its own electricity and increased costs, plus the cost of purchasing the machine, so I feel that the use of this machine instead of increasing the	Jingdong prefecture level city in Shandong
Happy Fan Shop	King of Power Saving	Recommended by the owner of the store next door, looked at his home does have to save electricity, decided to get. Indeed quite can, the voltage is much more stable, there is a reduction in electricity	Jingdong prefecture level city in Shandong
Happy Fan Shop	King of Power Saving	Goods have been received value for money, affordable and reasonable price, and the description of the same, power-saving safe and reliable!	Jingdong prefecture level city in Shandong
Happy Fan Shop	King of Power Saving	Listen to friends introduced to buy, tried to save a lot of electricity is good, quite good, worth buying.	Jingdong prefecture level city in Shandong
Happy Fan Shop	King of Power Saving	The product is easy to use, safe and environmentally friendly, only used for a short time, to the next month, after the electricity bill status display to know how the performance of the power saver efficacy.	Jingdong prefecture level city in Shandong
Happy Fan Shop	King of Power Saving	Last month's electricity bill is 178 yuan, with this power saver is 92 yuan, ready to buy two more back home, I was introduced to buy a friend, just started to not believe that I did not expect the effect!	Jingdong prefecture level city in Shandong
Happy Fan Shop	King of Power Saving	It looks nice, but after using it for a few days I can't see how it saves power?	Jingdong prefecture level city in Shandong
Happy Fan Shop	King of Power Saving	Recommended by a friend, it is genuine, as described by the merchant, cost-effective	Jingdong prefecture level city in Shandong
Happy Fan Shop	King of Power Saving	Works well, affordable and saves money.	Jingdong prefecture level city in Shandong
Happy Fan Shop	King of Power Saving	Feels good haven't compared it yet, the air conditioning has been on a lot in the past two months	Jingdong prefecture level city in Shandong
Happy Fan Shop	King of Power Saving	Saving about 5 kWh per day is a good thing	Jingdong prefecture level city in Shandong
Happy Fan Shop	King of Power Saving	Not bad, affordable, best value for money	Jingdong prefecture level city in Shandong
Happy Fan Shop	King of Power Saving	The goods are genuine and the logistics are faster.	Jingdong prefecture level city in Shandong
Happy Fan Shop	King of Power Saving	Goods have been received today, has been plugged into the outlet, looking forward to saving electricity, after a period of time to see how the effect in the follow-up comment!	Jingdong prefecture level city in Shandong
Happy Fan Shop	King of Power Saving	I just got it home and haven't used it for long, so I'll use it for a couple months and see what happens. Seeing that most people have good reviews, it should be fine.	Jingdong prefecture level city in Shandong
Happy Fan Shop	King of Power Saving	I'm looking forward to the results, so I'll give you a good review out of habit! Follow up review after a while	Jingdong prefecture level city in Shandong
Happy Fan Shop	King of Power Saving	Use a dish first to know if it will help save energy	Jingdong prefecture level city in Shandong
Happy Fan Shop	King of Power Saving	Whether it saves electricity or not, we don't know yet.	Jingdong prefecture level city in Shandong
Happy Fan Shop	King of Power Saving	Goods have been received, use a period of time to know how oh. The courier did not deliver to the door ah! Sent to the station ah! The heat is inconvenient ah!	Jingdong prefecture level city in Shandong
Happy Fan Shop	King of Power Saving	A little effect, before an hour a power, now an hour 0.76 degrees, a dollar a power = save 24 cents, although not a lot, first use it!	Jingdong prefecture level city in Shandong
Happy Fan Shop	King of Power Saving	Just received the goods, use a month to evaluate	Jingdong prefecture level city in Shandong
Happy Fan Shop	King of Power Saving	Home every month with a lot of electricity, inadvertently found this good thing online that can save the beginning of a skeptical attitude to consult customer service, customer service replied very professional, said 120 days can be refunded that first try it!	Jingdong prefecture level city in Shandong
Happy Fan Shop	King of Power Saving	After receiving the goods this afternoon, immediately installed in the bathroom Smith energy storage electric water heater on the power outlet (instead of connecting with a plug board), when used, found that the "Power Saving King" LCD display, is the value of the voltage, not the store's video said so	Jingdong prefecture level city in Shandong
Happy Fan Shop	King of Power Saving	Has made many days, did not feel the power saving, the effect is not obvious.	Jingdong prefecture level city in Shandong
Happy Fan Shop	King of Power Saving	Energy savers do not save energy at all, but rather consume a lot of electricity.	Jingdong prefecture level city in Shandong
Happy Fan Shop	King of Power Saving	It's useless. It's a waste of money.	Jingdong prefecture level city in Shandong
Happy Fan Shop	King of Power Saving	Last month saved more than 500, which came back to buy, the store bought three, and then buy 1 home use, my family is dry beauty salon, more instruments, especially electricity, a month almost 1000. or so, with this did not expect to save so much, great, five-star praise!	Jingdong prefecture level city in Shandong
Happy Fan Shop	King of Power Saving	It rained a couple days ago and I just went to get the item back today , not bad, hey I looked at the meter and that red display light takes several seconds to jump, did not make a mistake buying it. After a period of time to evaluate.	Jingdong prefecture level city in Shandong
Happy Fan Shop	King of Power Saving	Without further ado, the electricity bill came down, this month less 102 yuan, has been repurchased, I also recommended to my friends, all said that there are effects, the product is okay, the effect is quite	Jingdong prefecture level city in Shandong
Happy Fan Shop	King of Power Saving	After buying and using two months really see the effect, so only to evaluate! Originally held the mindset of trying, I did not expect really effective, worth having!	Jingdong prefecture level city in Shandong

Happy Fan Shop	King of Power Saving	In the use of this energy saver before the monthly electricity bill is more than 150 yuan or so, now save thirty percent, every day to work, six or seven o'clock in the afternoon to go home, I do not know why the electricity bill is so expensive, energy-saving appliances really have the effect of recommending	Jingdong prefecture level city in Shandong
Happy Fan Shop	King of Power Saving	It saves about 100 bucks a month. A friend recommended it to me, and he's been using it for a while, and the effect is so obvious that I bought one too.	Jingdong prefecture level city in Shandong
Happy Fan Shop	King of Power Saving	My dad asked me to buy one, but after I paid the bill, I asked and he said it was a lot less this month, so he asked me to buy another one.	Jingdong prefecture level city in Shandong
Happy Fan Shop	King of Power Saving	The specific effect will have to be tested for at least two months to compare whether there is a power saving effect or not.	Jingdong prefecture level city in Shandong
Happy Fan Shop	King of Power Saving	'The goods have been received, immediately go to use, please ask the boss you instructions do not allow to withdraw, there are two fuses installed there!	Jingdong prefecture level city in Shandong
Happy Fan Shop	King of Power Saving	First praise is using, after using if good then follow up, now because I have not yet seen the results	Jingdong prefecture level city in Shandong
Happy Fan Shop	King of Power Saving	I've just been using it, and I haven't seen if it has a power-saving effect yet, so I'll follow up after 10 days and half a month.	Jingdong prefecture level city in Shandong
Happy Fan Shop	King of Power Saving	Just received the intelligent power saving meter, immediately plugged in to use, see it is not bad, and order to sell the second time	Jingdong prefecture level city in Shandong
Happy Fan Shop	King of Power Saving	This power saver is a bit effective, used for half a month, the effect is still visible.	Jingdong prefecture level city in Shandong
Happy Fan Shop	King of Power Saving	I received the baby and it looks great, I'm trying it out now and will keep an eye on it afterward.	Jingdong prefecture level city in Shandong
Happy Fan Shop	King of Power Saving	I've just received it, but I'm still trying to see how it works, so I'll come back in a little while.	Jingdong prefecture level city in Shandong
Happy Fan Shop	King of Power Saving	Just used it wait to use it for a while and then comment thanks, all first five stars.	Jingdong prefecture level city in Shandong
Happy Fan Shop	King of Power Saving	Very trustworthy brand, quality is guaranteed, the price is still affordable, quite satisfied!	Jingdong prefecture level city in Shandong
Happy Fan Shop	King of Power Saving	Things received, I hope that the platform of Jingdong do not sell fake goods are available	Jingdong prefecture level city in Shandong
Happy Fan Shop	King of Power Saving	Energy saver received, the use of the effect is good and the business description of the same	Jingdong prefecture level city in Shandong
Happy Fan Shop	King of Power Saving	The surface looks beautiful, the actual effect is not known, after using additional evaluation.	Jingdong prefecture level city in Shandong
Happy Fan Shop	King of Power Saving	Tried it for a few months, it really saves electricity, and I bought it for a friend.	Jingdong prefecture level city in Shandong
Happy Fan Shop	King of Power Saving	It didn't work and wanted to return it, it really didn't work at all.	Jingdong prefecture level city in Shandong
Happy Fan Shop	King of Power Saving	Just used it, don't know how effective it is, looking forward to usefulness	Jingdong prefecture level city in Shandong
Happy Fan Shop	King of Power Saving	After two months before the effect I do not know if it is true!	Jingdong prefecture level city in Shandong
Happy Fan Shop	King of Power Saving	So fast! Ordered yesterday and received today 👍👍👍👍👍👍👍👍👍 🌹🌹🌹🌹🌹🌹🌹🌹🌹	Jingdong prefecture level city in Shandong
Happy Fan Shop	King of Power Saving	I've started using it. We'll see how it works.	Jingdong prefecture level city in Shandong
Happy Fan Shop	King of Power Saving	I just received the goods, I won't know the effect until after two.	Jingdong prefecture level city in Shandong
Happy Fan Shop	King of Power Saving	Just received the goods, the specific effect will be evaluated later	Jingdong prefecture level city in Shandong
Happy Fan Shop	King of Power Saving	I've received the goods, I don't know if it's any good yet	Jingdong prefecture level city in Shandong
Happy Fan Shop	King of Power Saving	Try it for a month and then come back with a concrete evaluation	Jingdong prefecture level city in Shandong
Happy Fan Shop	King of Power Saving	In use, after two months to see the effect of power saving	Jingdong prefecture level city in Shandong
Happy Fan Shop	King of Power Saving	Power Saver. It's authentic. It's black. It works.	Jingdong prefecture level city in Shandong
Happy Fan Shop	King of Power Saving	I've been using it for a week now and it's not working.	Jingdong prefecture level city in Shandong
Happy Fan Shop	King of Power Saving	I'm just using it. I don't know how it's going to work.	Jingdong prefecture level city in Shandong
Happy Fan Shop	King of Power Saving	Products are better looking out for power savings	Jingdong prefecture level city in Shandong
Happy Fan Shop	King of Power Saving	I received it and I've kept it on. I hope it works.	Jingdong prefecture level city in Shandong
Happy Fan Shop	King of Power Saving	It's really good! Worth recommending to buy!	Jingdong prefecture level city in Shandong
Happy Fan Shop	King of Power Saving	Try it for a few days and see if it doesn't work, then return it.	Jingdong prefecture level city in Shandong
Happy Fan Shop	King of Power Saving	It's in use and I hope to be pleasantly surprised.	Jingdong prefecture level city in Shandong
Happy Fan Shop	King of Power Saving	We'll see how it works when we use it	Jingdong prefecture level city in Shandong
Happy Fan Shop	King of Power Saving	Let's see if it works.	Jingdong prefecture level city in Shandong
Happy Fan Shop	King of Power Saving	I'm using it and will follow up with a review	Jingdong prefecture level city in Shandong
Happy Fan Shop	King of Power Saving	It really works. It saves energy.	Jingdong prefecture level city in Shandong
Happy Fan Shop	King of Power Saving	It does have some power savings!	Jingdong prefecture level city in Shandong
Happy Fan Shop	King of Power Saving	Jingdong shopping speed is convenient and timely	Jingdong prefecture level city in Shandong
Happy Fan Shop	King of Power Saving	Second time I've bought it. Everything's fine.	Jingdong prefecture level city in Shandong
Happy Fan Shop	King of Power Saving	I don't know if it works yet.	Jingdong prefecture level city in Shandong
Happy Fan Shop	King of Power Saving	Not used, don't know how well it works.	Jingdong prefecture level city in Shandong
Happy Fan Shop	King of Power Saving	No visible results so far	Jingdong prefecture level city in Shandong
Happy Fan Shop	King of Power Saving	It doesn't feel like it's working.	Jingdong prefecture level city in Shandong
Happy Fan Shop	King of Power Saving	Where's the effect? Where are the power savings?	Jingdong prefecture level city in Shandong
Happy Fan Shop	King of Power Saving	Let's see how it works in practice!	Jingdong prefecture level city in Shandong
Happy Fan Shop	King of Power Saving	I'll wait until I use it to give you a good review	Jingdong prefecture level city in Shandong
Happy Fan Shop	King of Power Saving	I'll follow up after I've used it	Jingdong prefecture level city in Shandong
Happy Fan Shop	King of Power Saving	Well, that's good for saving power.	Jingdong prefecture level city in Shandong

Happy Fan Shop	King of Power Saving	Received. Used in the know.	Jingdong prefecture level city in Shandong
Happy Fan Shop	King of Power Saving	It doesn't do much good.	Jingdong prefecture level city in Shandong
Happy Fan Shop	King of Power Saving	It's not working.	Jingdong prefecture level city in Shandong
Happy Fan Shop	King of Power Saving	I don't know. I just used it.	Jingdong prefecture level city in Shandong
Happy Fan Shop	King of Power Saving	Use it and come back to evaluate.	Jingdong prefecture level city in Shandong
Happy Fan Shop	King of Power Saving	Two words: useless.	Jingdong prefecture level city in Shandong
Happy Fan Shop	King of Power Saving	To be used and then evaluated	Jingdong prefecture level city in Shandong
Happy Fan Shop	King of Power Saving	No shipment.	Jingdong prefecture level city in Shandong
Happy Fan Shop	King of Power Saving	Fake. Speechless.	Jingdong prefecture level city in Shandong
Happy Fan Shop	King of Power Saving	fantastic (idiom); awesome	Jingdong prefecture level city in Shandong
Happy Fan Shop	King of Power Saving	an excellent	Jingdong prefecture level city in Shandong
Happy Fan Shop	King of Power Saving	an excellent	Jingdong prefecture level city in Shandong
Happy Fan Shop	King of Power Saving	Want to buy! Recommended not to buy! No effect	Jingdong prefecture level city in Shandong
Happy Fan Shop	King of Power Saving	That's all there is to it. I can't figure out how to save electricity.	Jingdong prefecture level city in Shandong
Happy Fan Shop	King of Power Saving	It's no use.	Jingdong prefecture level city in Shandong
Happy Fan Shop	King of Power Saving	Things are more perfect than I imagined, very powerful, it is worth getting Packaging is very strong, things are well protected, it is worth the praise things are very satisfied Oh, beautiful and stylish atmosphere, customer service is super patient, service is meticulous and attentive, the big brand is not	Jingdong prefecture level city in Shandong
Happy Fan Shop	King of Power Saving	The effect is too good, the family has now several units, back to buy a number of times, the power saver received, power-saving effect is very good,, fine workmanship, long warranty, but also purify the air, before buying a used for a period of time, with the okay, and then bought a, and only now come to the	Jingdong prefecture level city in Shandong
Happy Fan Shop	King of Power Saving	Quite good, and imagine the same, casual ride are very good, satisfied! Baby received it 👌, really great ah! 🌈 quality is really nothing to say, very satisfied! 🆗 happy happy 😃 genuine, great, the price is very good, in short, quite good, will buy again a very good shopping experience, attentive service, fast logistics, distribution is also thoughtful, the attitude is also very good, value for money, ah,	Jingdong prefecture level city in Shandong
Happy Fan Shop	King of Power Saving	Power-saving effect: strong Cost-effective: quite good Appearance style: beautiful Workmanship material: meticulous Experience: looking forward to power-saving effect	Jingdong prefecture level city in Shandong
Happy Fan Shop	King of Power Saving	Cost-effective is okay, the appearance of the style of meticulous workmanship, looking forward to energy-saving effect.	Jingdong prefecture level city in Shandong
Happy Fan Shop	King of Power Saving	Very Good Very Power Saving This Power Saver is really power saving As soon as the power is turned on, the Power Saver starts the power saving function for you.	Jingdong prefecture level city in Shandong
Happy Fan Shop	King of Power Saving	Logistics is very fast, the price is favorable, friends introduced that it is very good to use, bought 2 at once, looking forward to the effect, used after the follow-up comment.	Jingdong prefecture level city in Shandong
Happy Fan Shop	King of Power Saving	It's all fake. It doesn't save any electricity.	Jingdong prefecture level city in Shandong
Happy Fan Shop	King of Power Saving	This Power Saver is so much better than the one I bought before, not only the effect is good, the quality is very good in all aspects, the customer service attitude is also very good, recommended to buy!	Jingdong prefecture level city in Shandong
Happy Fan Shop	King of Power Saving	Logistics is very fast, buy for parents, good quality, once bought 2, power-saving effect is good to buy 2 more, look forward to using the effect, satisfied will recommend to friends.	Jingdong prefecture level city in Shandong
Happy Fan Shop	King of Power Saving	With the description is the same, very high-grade, good value, to buy friends can order Oh!	Jingdong prefecture level city in Shandong
Happy Fan Shop	King of Power Saving	Not using much electricity in the winter saving over 100 per month! Ready to buy 2 more units in the summer ！！！！！ Good quality, good price, power saving effect is obvious, cost-effective, worth buying, can be recommended!	Jingdong prefecture level city in Shandong
Happy Fan Shop	King of Power Saving	Purchased a power saver received on the put into use, rent a room with many electrical appliances, power consumption is very large, we have a piece of electricity or 1.2 dollars a time, with this power saver is much better, every day can save a lot of money, very convenient and practical.	Jingdong prefecture level city in Shandong
Happy Fan Shop	King of Power Saving	The power saving effect is very good: my supermarket monthly electricity bill is more than 7000 yuan. On May 1st someone recommended buying a couple of power savers, and I bought 5 of them first. The electricity bill was about 2000 less in June. This time, I bought 5 more. The seller said 800 degrees of electricity with one. I feel it's basically enough. A total of 10 units	Jingdong prefecture level city in Shandong
Happy Fan Shop	King of Power Saving	I never thought I could get such a good baby at such an affordable price, it's very good value for money, I love it!	Jingdong prefecture level city in Shandong
Happy Fan Shop	King of Power Saving	Previously bought other brands of power savers, but found no effect at home, this time to see how the products on the Jingdong, inserted in the home socket, the LED display is blue, the night light is very nice to see, look at the instructions that have to be half a year in order to achieve the best results, try it, I believe that the quality of the Jingdong, it should not be wrong.	Jingdong prefecture level city in Shandong
Happy Fan Shop	King of Power Saving	Superb, inexpensive, saves a lot of points, been using it for a long time!	Jingdong prefecture level city in Shandong
Happy Fan Shop	King of Power Saving	There are a lot of appliances in the house, the summer air conditioning is very costly, I bought this in front of the air conditioning effect is very good, I explain that I bought three in one breath, I use more electricity a month down to 200 or so, very satisfied.	Jingdong prefecture level city in Shandong
Happy Fan Shop	King of Power Saving	A month can save more than 200, home monthly electricity bill of more than 600 dollars, inserted 3 or so, an amount of the balance of the average can save more than 200. Product is very good, has been buying this one, has given a lot of relatives recommended, the quality is also very good, before buying a look with this same, who knows to buy home has not been used for more than a week on the bad, the quality of this is really good with more than two years!	Jingdong prefecture level city in Shandong
Happy Fan Shop	King of Power Saving	Whole house power saving king, can really save electricity, friends recommended, high power saving appliances, only replacement, no repair, after-sales worry-free!	Jingdong prefecture level city in Shandong
Happy Fan Shop	King of Power Saving	Great item love it, great customer service, great shopping experience!	Jingdong prefecture level city in Shandong
Happy Fan Shop	King of Power Saving	Power saving, very good use, much cheaper than the physical store, satisfaction	Jingdong prefecture level city in Shandong
Happy Fan Shop	King of Power Saving	In use, very well packaged and fast delivery of credit!	Jingdong prefecture level city in Shandong
Happy Fan Shop	King of Power Saving	Product quality, customer service attitude is particularly good, like, praise!	Jingdong prefecture level city in Shandong
Happy Fan Shop	King of Power Saving	The logistics is very fast, the packaging is not broken, the quality is very good, I like it very much!	Jingdong prefecture level city in Shandong
Happy Fan Shop	King of Power Saving	The saver was well packaged and was as described when plugged in	Jingdong prefecture level city in Shandong
Happy Fan Shop	King of Power Saving	Energy efficient and very satisfied	Jingdong prefecture level city in Shandong
Happy Fan Shop	King of Power Saving	Great quality for the price and fast shipping.	Jingdong prefecture level city in Shandong
Happy Fan Shop	King of Power Saving	It's great, just as described. Perfect.	Jingdong prefecture level city in Shandong

Happy Fan Shop	King of Power Saving	There is no power saving effect, but rather ring up an increase in electricity costs, because this machine is on continuously all day long, uninterruptedly, its own electricity and increased costs, plus the cost of purchasing the machine, so I feel that the use of this machine instead of increasing the	Jingdong prefecture level city in Shandong
Happy Fan Shop	King of Power Saving	Recommended by the owner of the store next door, looked at his home does have to save electricity, decided to get. Indeed quite can, the voltage is much more stable, there is a reduction in electricity	Jingdong prefecture level city in Shandong
Happy Fan Shop	King of Power Saving	Goods have been received value for money, affordable and reasonable price, and the description of the same, power-saving safe and reliable!	Jingdong prefecture level city in Shandong
Happy Fan Shop	King of Power Saving	Listen to friends introduced to buy, tried to save a lot of electricity is good, quite good, worth buying.	Jingdong prefecture level city in Shandong
Happy Fan Shop	King of Power Saving	The product is easy to use, safe and environmentally friendly, only used for a short time, to the next month, after the electricity bill status display to know how the performance of the power saver efficacy.	Jingdong prefecture level city in Shandong
Happy Fan Shop	King of Power Saving	Last month's electricity bill is 178 yuan, with this power saver is 92 yuan, ready to buy two more back home, I was introduced to buy a friend, just started to not believe that I did not expect the effect!	Jingdong prefecture level city in Shandong
Happy Fan Shop	King of Power Saving	It looks nice, but after using it for a few days I can't see how it saves power?	Jingdong prefecture level city in Shandong
Happy Fan Shop	King of Power Saving	Recommended by a friend, it is genuine, as described by the merchant, cost-effective	Jingdong prefecture level city in Shandong
Happy Fan Shop	King of Power Saving	Works well, affordable and saves money.	Jingdong prefecture level city in Shandong
Happy Fan Shop	King of Power Saving	Feels good haven't compared it yet, the air conditioning has been on a lot in the past two months	Jingdong prefecture level city in Shandong
Happy Fan Shop	King of Power Saving	Saving about 5 kWh per day is a good thing	Jingdong prefecture level city in Shandong
Happy Fan Shop	King of Power Saving	Not bad, affordable, best value for money	Jingdong prefecture level city in Shandong
Happy Fan Shop	King of Power Saving	The goods are genuine and the logistics are faster.	Jingdong prefecture level city in Shandong
Happy Fan Shop	King of Power Saving	Goods have been received today, has been plugged into the outlet, looking forward to saving electricity, after a period of time to see how the effect in the follow-up comment!	Jingdong prefecture level city in Shandong
Happy Fan Shop	King of Power Saving	I just got it home and haven't used it for long, so I'll use it for a couple months and see what happens. Seeing that most people have good reviews, it should be fine.	Jingdong prefecture level city in Shandong
Happy Fan Shop	King of Power Saving	I'm looking forward to the results, so I'll give you a good review out of habit! Follow up review after a while	Jingdong prefecture level city in Shandong
Happy Fan Shop	King of Power Saving	Use a dish first to know if it will help save energy	Jingdong prefecture level city in Shandong
Happy Fan Shop	King of Power Saving	Whether it saves electricity or not, we don't know yet.	Jingdong prefecture level city in Shandong
Happy Fan Shop	King of Power Saving	Goods have been received, use a period of time to know how oh. The courier did not deliver to the door ah! Sent to the station ah! The heat is inconvenient ah!	Jingdong prefecture level city in Shandong
Happy Fan Shop	King of Power Saving	A little effect, before an hour a power, now an hour 0.76 degrees, a dollar a power = save 24 cents, although not a lot, first use it!	Jingdong prefecture level city in Shandong
Happy Fan Shop	King of Power Saving	Just received the goods, use a month to evaluate	Jingdong prefecture level city in Shandong
Happy Fan Shop	King of Power Saving	Home every month with a lot of electricity, inadvertently found this good thing online that can save the beginning of a skeptical attitude to consult customer service, customer service replied very professional, said 120 days can be refunded that first try it!	Jingdong prefecture level city in Shandong
Happy Fan Shop	King of Power Saving	After receiving the goods this afternoon, immediately installed in the bathroom Smith energy storage electric water heater on the power outlet (instead of connecting with a plug board), when used, found that the "Power Saving King" LCD display, is the value of the voltage, not the store's video said so	Jingdong prefecture level city in Shandong
Happy Fan Shop	King of Power Saving	Has made many days, did not feel the power saving, the effect is not obvious.	Jingdong prefecture level city in Shandong
Happy Fan Shop	King of Power Saving	Energy savers do not save energy at all, but rather consume a lot of electricity.	Jingdong prefecture level city in Shandong
Happy Fan Shop	King of Power Saving	It's useless. It's a waste of money.	Jingdong prefecture level city in Shandong
Happy Fan Shop	King of Power Saving	Last month saved more than 500, which came back to buy, the store bought three, and then buy 1 home use, my family is dry beauty salon, more instruments, especially electricity, a month almost 1000. or so, with this did not expect to save so much, great, five-star praise!	Jingdong prefecture level city in Shandong
Happy Fan Shop	King of Power Saving	It rained a couple days ago and I just went to get the item back today , not bad, hey I looked at the meter and that red display light takes several seconds to jump, did not make a mistake buying it. After a period of time to evaluate.	Jingdong prefecture level city in Shandong
Happy Fan Shop	King of Power Saving	Without further ado, the electricity bill came down, this month less 102 yuan, has been repurchased, I also recommended to my friends, all said that there are effects, the product is okay, the effect is quite	Jingdong prefecture level city in Shandong
Happy Fan Shop	King of Power Saving	After buying and using two months really see the effect, so only to evaluate! Originally held the mindset of trying, I did not expect really effective, worth having!	Jingdong prefecture level city in Shandong
Happy Fan Shop	King of Power Saving	In the use of this energy saver before the monthly electricity bill is more than 150 yuan or so, now save thirty percent, every day to work, six or seven o'clock in the afternoon to go home, I do not know why the electricity bill is so expensive, energy-saving appliances really have the effect of recommending	Jingdong prefecture level city in Shandong
Happy Fan Shop	King of Power Saving	It saves about 100 bucks a month. A friend recommended it to me, and he's been using it for a while, and the effect is so obvious that I bought one too.	Jingdong prefecture level city in Shandong
Happy Fan Shop	King of Power Saving	My dad asked me to buy one, but after I paid the bill, I asked and he said it was a lot less this month, so he asked me to buy another one.	Jingdong prefecture level city in Shandong
Happy Fan Shop	King of Power Saving	The specific effect will have to be tested for at least two months to compare whether there is a power saving effect or not.	Jingdong prefecture level city in Shandong
Happy Fan Shop	King of Power Saving	'The goods have been received, immediately go to use, please ask the boss you instructions do not allow to withdraw, there are two fuses installed there!	Jingdong prefecture level city in Shandong
Happy Fan Shop	King of Power Saving	First praise is using, after using if good then follow up, now because I have not yet seen the results	Jingdong prefecture level city in Shandong
Happy Fan Shop	King of Power Saving	I've just been using it, and I haven't seen if it has a power-saving effect yet, so I'll follow up after 10 days and half a month.	Jingdong prefecture level city in Shandong
Happy Fan Shop	King of Power Saving	Just received the intelligent power saving meter, immediately plugged in to use, see it is not bad, and order to sell the second time	Jingdong prefecture level city in Shandong
Happy Fan Shop	King of Power Saving	This power saver is a bit effective, used for half a month, the effect is still visible.	Jingdong prefecture level city in Shandong
Happy Fan Shop	King of Power Saving	I received the baby and it looks great, I'm trying it out now and will keep an eye on it afterward.	Jingdong prefecture level city in Shandong
Happy Fan Shop	King of Power Saving	I've just received it, but I'm still trying to see how it works, so I'll come back in a little while.	Jingdong prefecture level city in Shandong
Happy Fan Shop	King of Power Saving	Just used it wait to use it for a while and then comment thanks, all first five stars.	Jingdong prefecture level city in Shandong
Happy Fan Shop	King of Power Saving	Very trustworthy brand, quality is guaranteed, the price is still affordable, quite satisfied!	Jingdong prefecture level city in Shandong
Happy Fan Shop	King of Power Saving	Things received, I hope that the platform of Jingdong do not sell fake goods are available	Jingdong prefecture level city in Shandong
Happy Fan Shop	King of Power Saving	Energy saver received, the use of the effect is good and the business description of the same	Jingdong prefecture level city in Shandong
Happy Fan Shop	King of Power Saving	The surface looks beautiful, the actual effect is not known, after using additional evaluation.	Jingdong prefecture level city in Shandong
Happy Fan Shop	King of Power Saving	Tried it for a few months, it really saves electricity, and I bought it for a friend.	Jingdong prefecture level city in Shandong
Happy Fan Shop	King of Power Saving	It didn't work and wanted to return it, it really didn't work at all.	Jingdong prefecture level city in Shandong

Happy Fan Shop	King of Power Saving	Just used it, don't know how effective it is, looking forward to usefulness	Jingdong prefecture level city in Shandong
Happy Fan Shop	King of Power Saving	After two months before the effect I do not know if it is true!	Jingdong prefecture level city in Shandong
Happy Fan Shop	King of Power Saving	So fast! Ordered yesterday and received today 👍👍👍👍👍👍👍👍👍 🌹🌹🌹🌹🌹🌹🌹🌹🌹	Jingdong prefecture level city in Shandong
Happy Fan Shop	King of Power Saving	I've started using it. We'll see how it works.	Jingdong prefecture level city in Shandong
Happy Fan Shop	King of Power Saving	I just received the goods, I won't know the effect until after two.	Jingdong prefecture level city in Shandong
Happy Fan Shop	King of Power Saving	Just received the goods, the specific effect will be evaluated later	Jingdong prefecture level city in Shandong
Happy Fan Shop	King of Power Saving	I've received the goods, I don't know if it's any good yet	Jingdong prefecture level city in Shandong
Happy Fan Shop	King of Power Saving	Try it for a month and then come back with a concrete evaluation	Jingdong prefecture level city in Shandong
Happy Fan Shop	King of Power Saving	In use, after two months to see the effect of power saving	Jingdong prefecture level city in Shandong
Happy Fan Shop	King of Power Saving	Power Saver. It's authentic. It's black. It works.	Jingdong prefecture level city in Shandong
Happy Fan Shop	King of Power Saving	I've been using it for a week now and it's not working.	Jingdong prefecture level city in Shandong
Happy Fan Shop	King of Power Saving	I'm just using it. I don't know how it's going to work.	Jingdong prefecture level city in Shandong
Happy Fan Shop	King of Power Saving	Products are better looking out for power savings	Jingdong prefecture level city in Shandong
Happy Fan Shop	King of Power Saving	I received it and I've kept it on. I hope it works.	Jingdong prefecture level city in Shandong
Happy Fan Shop	King of Power Saving	It's really good! Worth recommending to buy!	Jingdong prefecture level city in Shandong
Happy Fan Shop	King of Power Saving	Try it for a few days and see if it doesn't work, then return it.	Jingdong prefecture level city in Shandong
Happy Fan Shop	King of Power Saving	It's in use and I hope to be pleasantly surprised.	Jingdong prefecture level city in Shandong
Happy Fan Shop	King of Power Saving	We'll see how it works when we use it	Jingdong prefecture level city in Shandong
Happy Fan Shop	King of Power Saving	Let's see if it works.	Jingdong prefecture level city in Shandong
Happy Fan Shop	King of Power Saving	I'm using it and will follow up with a review	Jingdong prefecture level city in Shandong
Happy Fan Shop	King of Power Saving	It really works. It saves energy.	Jingdong prefecture level city in Shandong
Happy Fan Shop	King of Power Saving	It does have some power savings!	Jingdong prefecture level city in Shandong
Happy Fan Shop	King of Power Saving	Jingdong shopping speed is convenient and timely	Jingdong prefecture level city in Shandong
Happy Fan Shop	King of Power Saving	Second time I've bought it. Everything's fine.	Jingdong prefecture level city in Shandong
Happy Fan Shop	King of Power Saving	I don't know if it works yet.	Jingdong prefecture level city in Shandong
Happy Fan Shop	King of Power Saving	Not used, don't know how well it works.	Jingdong prefecture level city in Shandong
Happy Fan Shop	King of Power Saving	No visible results so far	Jingdong prefecture level city in Shandong
Happy Fan Shop	King of Power Saving	It doesn't feel like it's working.	Jingdong prefecture level city in Shandong
Happy Fan Shop	King of Power Saving	Where's the effect? Where are the power savings?	Jingdong prefecture level city in Shandong
Happy Fan Shop	King of Power Saving	Let's see how it works in practice!	Jingdong prefecture level city in Shandong
Happy Fan Shop	King of Power Saving	I'll wait until I use it to give you a good review	Jingdong prefecture level city in Shandong
Happy Fan Shop	King of Power Saving	I'll follow up after I've used it	Jingdong prefecture level city in Shandong
Happy Fan Shop	King of Power Saving	Well, that's good for saving power.	Jingdong prefecture level city in Shandong
Happy Fan Shop	King of Power Saving	Received. Used in the know.	Jingdong prefecture level city in Shandong
Happy Fan Shop	King of Power Saving	It doesn't do much good.	Jingdong prefecture level city in Shandong
Happy Fan Shop	King of Power Saving	It's not working.	Jingdong prefecture level city in Shandong
Happy Fan Shop	King of Power Saving	I don't know. I just used it.	Jingdong prefecture level city in Shandong
Happy Fan Shop	King of Power Saving	Use it and come back to evaluate.	Jingdong prefecture level city in Shandong
Happy Fan Shop	King of Power Saving	Two words: useless.	Jingdong prefecture level city in Shandong
Happy Fan Shop	King of Power Saving	To be used and then evaluated	Jingdong prefecture level city in Shandong
Happy Fan Shop	King of Power Saving	No shipment.	Jingdong prefecture level city in Shandong
Happy Fan Shop	King of Power Saving	Fake. Speechless.	Jingdong prefecture level city in Shandong
Happy Fan Shop	King of Power Saving	fantastic (idiom); awesome	Jingdong prefecture level city in Shandong
Happy Fan Shop	King of Power Saving	an excellent	Jingdong prefecture level city in Shandong
Happy Fan Shop	King of Power Saving	an excellent	Jingdong prefecture level city in Shandong
Happy Fan Shop	King of Power Saving	Want to buy! Recommended not to buy! No effect	Jingdong prefecture level city in Shandong
Happy Fan Shop	King of Power Saving	That's all there is to it. I can't figure out how to save electricity.	Jingdong prefecture level city in Shandong
Happy Fan Shop	King of Power Saving	It's no use.	Jingdong prefecture level city in Shandong
Happy Fan Shop	King of Power Saving	Things are more perfect than I imagined, very powerful, it is worth getting Packaging is very strong, things are well protected, it is worth the praise things are very satisfied Oh, beautiful and stylish atmosphere, customer service is super patient, service is meticulous and attentive, the big brand is not	Jingdong prefecture level city in Shandong
Happy Fan Shop	King of Power Saving	The effect is too good, the family has now several units, back to buy a number of times, the power saver received, power-saving effect is very good,, fine workmanship, long warranty, but also purify the air, before buying a used for a period of time, with the okay, and then bought a, and only now come to the	Jingdong prefecture level city in Shandong

Happy Fan Shop	King of Power Saving	Quite good, and imagine the same, casual ride are very good, satisfied! Baby received it 👌, really great ah! 🌈 quality is really nothing to say, very satisfied! 🆗 happy happy 😃 genuine, great, the price is very good, in short, quite good, will buy again a very good shopping experience, attentive service, fast logistics, distribution is also thoughtful, the attitude is also very good, value for money, ah,	Jingdong prefecture level city in Shandong
Happy Fan Shop	King of Power Saving	Power-saving effect: strong Cost-effective: quite good Appearance style: beautiful Workmanship material: meticulous Experience: looking forward to power-saving effect	Jingdong prefecture level city in Shandong
Happy Fan Shop	King of Power Saving	Cost-effective is okay, the appearance of the style of meticulous workmanship, looking forward to energy-saving effect.	Jingdong prefecture level city in Shandong
Happy Fan Shop	King of Power Saving	Very Good Very Power Saving This Power Saver is really power saving As soon as the power is turned on, the Power Saver starts the power saving function for you.	Jingdong prefecture level city in Shandong
Happy Fan Shop	King of Power Saving	Logistics is very fast, the price is favorable, friends introduced that it is very good to use, bought 2 at once, looking forward to the effect, used after the follow-up comment.	Jingdong prefecture level city in Shandong
Happy Fan Shop	King of Power Saving	It's all fake. It doesn't save any electricity.	Jingdong prefecture level city in Shandong
Happy Fan Shop	King of Power Saving	This Power Saver is so much better than the one I bought before, not only the effect is good, the quality is very good in all aspects, the customer service attitude is also very good, recommended to buy!	Jingdong prefecture level city in Shandong
Happy Fan Shop	King of Power Saving	Logistics is very fast, buy for parents, good quality, once bought 2, power-saving effect is good to buy 2 more, look forward to using the effect, satisfied will recommend to friends.	Jingdong prefecture level city in Shandong
Happy Fan Shop	King of Power Saving	With the description is the same, very high-grade, good value, to buy friends can order Oh!	Jingdong prefecture level city in Shandong
Happy Fan Shop	King of Power Saving	Not using much electricity in the winter saving over 100 per month! Ready to buy 2 more units in the summer ！！！！！ Good quality, good price, power saving effect is obvious, cost-effective, worth buying, can be recommended!	Jingdong prefecture level city in Shandong
Happy Fan Shop	King of Power Saving	Purchased a power saver received on the put into use, rent a room with many electrical appliances, power consumption is very large, we have a piece of electricity or 1.2 dollars a time, with this power saver is much better, every day can save a lot of money, very convenient and practical.	Jingdong prefecture level city in Shandong
Happy Fan Shop	King of Power Saving	The power saving effect is very good: my supermarket monthly electricity bill is more than 7000 yuan. On May 1st someone recommended buying a couple of power savers, and I bought 5 of them first. The electricity bill was about 2000 less in June. This time, I bought 5 more. The seller said 800 degrees of electricity with one. I feel it's basically enough. A total of 10 units	Jingdong prefecture level city in Shandong
Happy Fan Shop	King of Power Saving	I never thought I could get such a good baby at such an affordable price, it's very good value for money, I love it!	Jingdong prefecture level city in Shandong
Happy Fan Shop	King of Power Saving	Previously bought other brands of power savers, but found no effect at home, this time to see how the products on the Jingdong, inserted in the home socket, the LED display is blue, the night light is very nice to see, look at the instructions that have to be half a year in order to achieve the best results, try it, I believe that the quality of the Jingdong, it should not be wrong.	Jingdong prefecture level city in Shandong
Happy Fan Shop	King of Power Saving	Superb, inexpensive, saves a lot of points, been using it for a long time!	Jingdong prefecture level city in Shandong
Happy Fan Shop	King of Power Saving	There are a lot of appliances in the house, the summer air conditioning is very costly, I bought this in front of the air conditioning effect is very good, I explain that I bought three in one breath, I use more electricity a month down to 200 or so, very satisfied.	Jingdong prefecture level city in Shandong
Happy Fan Shop	King of Power Saving	A month can save more than 200, home monthly electricity bill of more than 600 dollars, inserted 3 or so, an amount of the balance of the average can save more than 200. Product is very good, has been buying this one, has given a lot of relatives recommended, the quality is also very good, before buying a look with this same, who knows to buy home has not been used for more than a week on the bad, the quality of this is really good with more than two years!	Jingdong prefecture level city in Shandong
Happy Fan Shop	King of Power Saving	Whole house power saving king, can really save electricity, friends recommended, high power saving appliances, only replacement, no repair, after-sales worry-free!	Jingdong prefecture level city in Shandong
Happy Fan Shop	King of Power Saving	Great item love it, great customer service, great shopping experience!	Jingdong prefecture level city in Shandong
Happy Fan Shop	King of Power Saving	Power saving, very good use, much cheaper than the physical store, satisfaction	Jingdong prefecture level city in Shandong
Happy Fan Shop	King of Power Saving	In use, very well packaged and fast delivery of credit!	Jingdong prefecture level city in Shandong
Happy Fan Shop	King of Power Saving	Product quality, customer service attitude is particularly good, like, praise!	Jingdong prefecture level city in Shandong
Happy Fan Shop	King of Power Saving	The logistics is very fast, the packaging is not broken, the quality is very good, I like it very much!	Jingdong prefecture level city in Shandong
Happy Fan Shop	King of Power Saving	The saver was well packaged and was as described when plugged in	Jingdong prefecture level city in Shandong
Happy Fan Shop	King of Power Saving	Energy efficient and very satisfied	Jingdong prefecture level city in Shandong
Happy Fan Shop	King of Power Saving	Great quality for the price and fast shipping.	Jingdong prefecture level city in Shandong
Happy Fan Shop	King of Power Saving	It's great, just as described. Perfect.	Jingdong prefecture level city in Shandong
Happy Fan Shop	King of Power Saving	There is no power saving effect, but rather ring up an increase in electricity costs, because this machine is on continuously all day long, uninterruptedly, its own electricity and increased costs, plus the cost of purchasing the machine, so I feel that the use of this machine instead of increasing the	Jingdong prefecture level city in Shandong
Happy Fan Shop	King of Power Saving	Recommended by the owner of the store next door, looked at his home does have to save electricity, decided to get. Indeed quite can, the voltage is much more stable, there is a reduction in electricity	Jingdong prefecture level city in Shandong
Happy Fan Shop	King of Power Saving	Goods have been received value for money, affordable and reasonable price, and the description of the same, power-saving safe and reliable!	Jingdong prefecture level city in Shandong
Happy Fan Shop	King of Power Saving	Listen to friends introduced to buy, tried to save a lot of electricity is good, quite good, worth buying.	Jingdong prefecture level city in Shandong
Happy Fan Shop	King of Power Saving	The product is easy to use, safe and environmentally friendly, only used for a short time, to the next month, after the electricity bill status display to know how the performance of the power saver efficacy.	Jingdong prefecture level city in Shandong
Happy Fan Shop	King of Power Saving	Last month's electricity bill is 178 yuan, with this power saver is 92 yuan, ready to buy two more back home, I was introduced to buy a friend, just started to not believe that I did not expect the effect!	Jingdong prefecture level city in Shandong
Happy Fan Shop	King of Power Saving	It looks nice, but after using it for a few days I can't see how it saves power?	Jingdong prefecture level city in Shandong
Happy Fan Shop	King of Power Saving	Recommended by a friend, it is genuine, as described by the merchant, cost-effective	Jingdong prefecture level city in Shandong
Happy Fan Shop	King of Power Saving	Works well, affordable and saves money.	Jingdong prefecture level city in Shandong
Happy Fan Shop	King of Power Saving	Feels good haven't compared it yet, the air conditioning has been on a lot in the past two months	Jingdong prefecture level city in Shandong
Happy Fan Shop	King of Power Saving	Saving about 5 kWh per day is a good thing	Jingdong prefecture level city in Shandong
Happy Fan Shop	King of Power Saving	Not bad, affordable, best value for money	Jingdong prefecture level city in Shandong
Happy Fan Shop	King of Power Saving	The goods are genuine and the logistics are faster.	Jingdong prefecture level city in Shandong
Happy Fan Shop	King of Power Saving	Goods have been received today, has been plugged into the outlet, looking forward to saving electricity, after a period of time to see how the effect in the follow-up comment!	Jingdong prefecture level city in Shandong
Happy Fan Shop	King of Power Saving	I just got it home and haven't used it for long, so I'll use it for a couple months and see what happens. Seeing that most people have good reviews, it should be fine.	Jingdong prefecture level city in Shandong
Happy Fan Shop	King of Power Saving	I'm looking forward to the results, so I'll give you a good review out of habit! Follow up review after a while	Jingdong prefecture level city in Shandong
Happy Fan Shop	King of Power Saving	Use a dish first to know if it will help save energy	Jingdong prefecture level city in Shandong
Happy Fan Shop	King of Power Saving	Whether it saves electricity or not, we don't know yet.	Jingdong prefecture level city in Shandong

Happy Fan Shop	King of Power Saving	Goods have been received, use a period of time to know how oh. The courier did not deliver to the door ah! Sent to the station ah! The heat is inconvenient ah!	Jingdong prefecture level city in Shandong
Happy Fan Shop	King of Power Saving	A little effect, before an hour a power, now an hour 0.76 degrees, a dollar a power = save 24 cents, although not a lot, first use it!	Jingdong prefecture level city in Shandong
Happy Fan Shop	King of Power Saving	Just received the goods, use a month to evaluate	Jingdong prefecture level city in Shandong
Happy Fan Shop	King of Power Saving	Home every month with a lot of electricity, inadvertently found this good thing online that can save the beginning of a skeptical attitude to consult customer service, customer service replied very professional, said 120 days can be refunded that first try it!	Jingdong prefecture level city in Shandong
Happy Fan Shop	King of Power Saving	After receiving the goods this afternoon, immediately installed in the bathroom Smith energy storage electric water heater on the power outlet (instead of connecting with a plug board), when used, found that the "Power Saving King" LCD display, is the value of the voltage, not the store's video said so	Jingdong prefecture level city in Shandong
Happy Fan Shop	King of Power Saving	Has made many days, did not feel the power saving, the effect is not obvious.	Jingdong prefecture level city in Shandong
Happy Fan Shop	King of Power Saving	Energy savers do not save energy at all, but rather consume a lot of electricity.	Jingdong prefecture level city in Shandong
Happy Fan Shop	King of Power Saving	It's useless. It's a waste of money.	Jingdong prefecture level city in Shandong
Happy Fan Shop	King of Power Saving	Last month saved more than 500, which came back to buy, the store bought three, and then buy 1 home use, my family is dry beauty salon, more instruments, especially electricity, a month almost 1000. or so, with this did not expect to save so much, great, five-star praise!	Jingdong prefecture level city in Shandong
Happy Fan Shop	King of Power Saving	It rained a couple days ago and I just went to get the item back today , not bad, hey I looked at the meter and that red display light takes several seconds to jump, did not make a mistake buying it. After a period of time to evaluate.	Jingdong prefecture level city in Shandong
Happy Fan Shop	King of Power Saving	Without further ado, the electricity bill came down, this month less 102 yuan, has been repurchased, I also recommended to my friends, all said that there are effects, the product is okay, the effect is quite	Jingdong prefecture level city in Shandong
Happy Fan Shop	King of Power Saving	After buying and using two months really see the effect, so only to evaluate! Originally held the mindset of trying, I did not expect really effective, worth having!	Jingdong prefecture level city in Shandong
Happy Fan Shop	King of Power Saving	In the use of this energy saver before the monthly electricity bill is more than 150 yuan or so, now save thirty percent, every day to work, six or seven o'clock in the afternoon to go home, I do not know why the electricity bill is so expensive, energy-saving appliances really have the effect of recommending	Jingdong prefecture level city in Shandong
Happy Fan Shop	King of Power Saving	It saves about 100 bucks a month. A friend recommended it to me, and he's been using it for a while, and the effect is so obvious that I bought one too.	Jingdong prefecture level city in Shandong
Happy Fan Shop	King of Power Saving	My dad asked me to buy one, but after I paid the bill, I asked and he said it was a lot less this month, so he asked me to buy another one.	Jingdong prefecture level city in Shandong
Happy Fan Shop	King of Power Saving	The specific effect will have to be tested for at least two months to compare whether there is a power saving effect or not.	Jingdong prefecture level city in Shandong
Happy Fan Shop	King of Power Saving	'The goods have been received, immediately go to use, please ask the boss you instructions do not allow to withdraw, there are two fuses installed there!	Jingdong prefecture level city in Shandong
Happy Fan Shop	King of Power Saving	First praise is using, after using if good then follow up, now because I have not yet seen the results	Jingdong prefecture level city in Shandong
Happy Fan Shop	King of Power Saving	I've just been using it, and I haven't seen if it has a power-saving effect yet, so I'll follow up after 10 days and half a month.	Jingdong prefecture level city in Shandong
Happy Fan Shop	King of Power Saving	Just received the intelligent power saving meter, immediately plugged in to use, see it is not bad, and order to sell the second time	Jingdong prefecture level city in Shandong
Happy Fan Shop	King of Power Saving	This power saver is a bit effective, used for half a month, the effect is still visible.	Jingdong prefecture level city in Shandong
Happy Fan Shop	King of Power Saving	I received the baby and it looks great, I'm trying it out now and will keep an eye on it afterward.	Jingdong prefecture level city in Shandong
Happy Fan Shop	King of Power Saving	I've just received it, but I'm still trying to see how it works, so I'll come back in a little while.	Jingdong prefecture level city in Shandong
Happy Fan Shop	King of Power Saving	Just used it wait to use it for a while and then comment thanks, all first five stars.	Jingdong prefecture level city in Shandong
Happy Fan Shop	King of Power Saving	Very trustworthy brand, quality is guaranteed, the price is still affordable, quite satisfied!	Jingdong prefecture level city in Shandong
Happy Fan Shop	King of Power Saving	Things received, I hope that the platform of Jingdong do not sell fake goods are available	Jingdong prefecture level city in Shandong
Happy Fan Shop	King of Power Saving	Energy saver received, the use of the effect is good and the business description of the same	Jingdong prefecture level city in Shandong
Happy Fan Shop	King of Power Saving	The surface looks beautiful, the actual effect is not known, after using additional evaluation.	Jingdong prefecture level city in Shandong
Happy Fan Shop	King of Power Saving	Tried it for a few months, it really saves electricity, and I bought it for a friend.	Jingdong prefecture level city in Shandong
Happy Fan Shop	King of Power Saving	It didn't work and wanted to return it, it really didn't work at all.	Jingdong prefecture level city in Shandong
Happy Fan Shop	King of Power Saving	Just used it, don't know how effective it is, looking forward to usefulness	Jingdong prefecture level city in Shandong
Happy Fan Shop	King of Power Saving	After two months before the effect I do not know if it is true!	Jingdong prefecture level city in Shandong
Happy Fan Shop	King of Power Saving	So fast! Ordered yesterday and received today 👍👍👍👍👍👍👍👍👍 🌹🌹🌹🌹🌹🌹🌹🌹🌹	Jingdong prefecture level city in Shandong
Happy Fan Shop	King of Power Saving	I've started using it. We'll see how it works.	Jingdong prefecture level city in Shandong
Happy Fan Shop	King of Power Saving	I just received the goods, I won't know the effect until after two.	Jingdong prefecture level city in Shandong
Happy Fan Shop	King of Power Saving	Just received the goods, the specific effect will be evaluated later	Jingdong prefecture level city in Shandong
Happy Fan Shop	King of Power Saving	I've received the goods, I don't know if it's any good yet	Jingdong prefecture level city in Shandong
Happy Fan Shop	King of Power Saving	Try it for a month and then come back with a concrete evaluation	Jingdong prefecture level city in Shandong
Happy Fan Shop	King of Power Saving	In use, after two months to see the effect of power saving	Jingdong prefecture level city in Shandong
Happy Fan Shop	King of Power Saving	Power Saver. It's authentic. It's black. It works.	Jingdong prefecture level city in Shandong
Happy Fan Shop	King of Power Saving	I've been using it for a week now and it's not working.	Jingdong prefecture level city in Shandong
Happy Fan Shop	King of Power Saving	I'm just using it. I don't know how it's going to work.	Jingdong prefecture level city in Shandong
Happy Fan Shop	King of Power Saving	Products are better looking out for power savings	Jingdong prefecture level city in Shandong
Happy Fan Shop	King of Power Saving	I received it and I've kept it on. I hope it works.	Jingdong prefecture level city in Shandong
Happy Fan Shop	King of Power Saving	It's really good! Worth recommending to buy!	Jingdong prefecture level city in Shandong
Happy Fan Shop	King of Power Saving	Try it for a few days and see if it doesn't work, then return it.	Jingdong prefecture level city in Shandong
Happy Fan Shop	King of Power Saving	It's in use and I hope to be pleasantly surprised.	Jingdong prefecture level city in Shandong
Happy Fan Shop	King of Power Saving	We'll see how it works when we use it	Jingdong prefecture level city in Shandong
Happy Fan Shop	King of Power Saving	Let's see if it works.	Jingdong prefecture level city in Shandong

Happy Fan Shop	King of Power Saving	I'm using it and will follow up with a review	Jingdong prefecture level city in Shandong
Happy Fan Shop	King of Power Saving	It really works. It saves energy.	Jingdong prefecture level city in Shandong
Happy Fan Shop	King of Power Saving	It does have some power savings!	Jingdong prefecture level city in Shandong
Happy Fan Shop	King of Power Saving	Jingdong shopping speed is convenient and timely	Jingdong prefecture level city in Shandong
Happy Fan Shop	King of Power Saving	Second time I've bought it. Everything's fine.	Jingdong prefecture level city in Shandong
Happy Fan Shop	King of Power Saving	I don't know if it works yet.	Jingdong prefecture level city in Shandong
Happy Fan Shop	King of Power Saving	Not used, don't know how well it works.	Jingdong prefecture level city in Shandong
Happy Fan Shop	King of Power Saving	No visible results so far	Jingdong prefecture level city in Shandong
Happy Fan Shop	King of Power Saving	It doesn't feel like it's working.	Jingdong prefecture level city in Shandong
Happy Fan Shop	King of Power Saving	Where's the effect? Where are the power savings?	Jingdong prefecture level city in Shandong
Happy Fan Shop	King of Power Saving	Let's see how it works in practice!	Jingdong prefecture level city in Shandong
Happy Fan Shop	King of Power Saving	I'll wait until I use it to give you a good review	Jingdong prefecture level city in Shandong
Happy Fan Shop	King of Power Saving	I'll follow up after I've used it	Jingdong prefecture level city in Shandong
Happy Fan Shop	King of Power Saving	Well, that's good for saving power.	Jingdong prefecture level city in Shandong
Happy Fan Shop	King of Power Saving	Received. Used in the know.	Jingdong prefecture level city in Shandong
Happy Fan Shop	King of Power Saving	It doesn't do much good.	Jingdong prefecture level city in Shandong
Happy Fan Shop	King of Power Saving	It's not working.	Jingdong prefecture level city in Shandong
Happy Fan Shop	King of Power Saving	I don't know. I just used it.	Jingdong prefecture level city in Shandong
Happy Fan Shop	King of Power Saving	Use it and come back to evaluate.	Jingdong prefecture level city in Shandong
Happy Fan Shop	King of Power Saving	Two words: useless.	Jingdong prefecture level city in Shandong
Happy Fan Shop	King of Power Saving	To be used and then evaluated	Jingdong prefecture level city in Shandong
Happy Fan Shop	King of Power Saving	No shipment.	Jingdong prefecture level city in Shandong
Happy Fan Shop	King of Power Saving	Fake. Speechless.	Jingdong prefecture level city in Shandong
Happy Fan Shop	King of Power Saving	fantastic (idiom); awesome	Jingdong prefecture level city in Shandong
Happy Fan Shop	King of Power Saving	an excellent	Jingdong prefecture level city in Shandong
Happy Fan Shop	King of Power Saving	an excellent	Jingdong prefecture level city in Shandong
Happy Fan Shop	King of Power Saving	Want to buy! Recommended not to buy! No effect	Jingdong prefecture level city in Shandong
Happy Fan Shop	King of Power Saving	That's all there is to it. I can't figure out how to save electricity.	Jingdong prefecture level city in Shandong
Happy Fan Shop	King of Power Saving	It's no use.	Jingdong prefecture level city in Shandong
Happy Fan Shop	King of Power Saving	Things are more perfect than I imagined, very powerful, it is worth getting Packaging is very strong, things are well protected, it is worth the praise things are very satisfied Oh, beautiful and stylish atmosphere, customer service is super patient, service is meticulous and attentive, the big brand is not	Jingdong prefecture level city in Shandong
Happy Fan Shop	King of Power Saving	The effect is too good, the family has now several units, back to buy a number of times, the power saver received, power-saving effect is very good,, fine workmanship, long warranty, but also purify the air, before buying a used for a period of time, with the okay, and then bought a, and only now come to the	Jingdong prefecture level city in Shandong
Happy Fan Shop	King of Power Saving	Quite good, and imagine the same, casual ride are very good, satisfied! Baby received it 👌, really great ah! 🌈 quality is really nothing to say, very satisfied! 🆗 happy happy 😃 genuine, great, the price is very good, in short, quite good, will buy again a very good shopping experience, attentive service, fast logistics, distribution is also thoughtful, the attitude is also very good, value for money, ah,	Jingdong prefecture level city in Shandong
Happy Fan Shop	King of Power Saving	Power-saving effect: strong Cost-effective: quite good Appearance style: beautiful Workmanship material: meticulous Experience: looking forward to power-saving effect	Jingdong prefecture level city in Shandong
Happy Fan Shop	King of Power Saving	Cost-effective is okay, the appearance of the style of meticulous workmanship, looking forward to energy-saving effect.	Jingdong prefecture level city in Shandong
Happy Fan Shop	King of Power Saving	Very Good Very Power Saving This Power Saver is really power saving As soon as the power is turned on, the Power Saver starts the power saving function for you.	Jingdong prefecture level city in Shandong
Happy Fan Shop	King of Power Saving	Logistics is very fast, the price is favorable, friends introduced that it is very good to use, bought 2 at once, looking forward to the effect, used after the follow-up comment.	Jingdong prefecture level city in Shandong
Happy Fan Shop	King of Power Saving	It's all fake. It doesn't save any electricity.	Jingdong prefecture level city in Shandong
Happy Fan Shop	King of Power Saving	This Power Saver is so much better than the one I bought before, not only the effect is good, the quality is very good in all aspects, the customer service attitude is also very good, recommended to buy!	Jingdong prefecture level city in Shandong
Happy Fan Shop	King of Power Saving	Logistics is very fast, buy for parents, good quality, once bought 2, power-saving effect is good to buy 2 more, look forward to using the effect, satisfied will recommend to friends.	Jingdong prefecture level city in Shandong
Happy Fan Shop	King of Power Saving	With the description is the same, very high-grade, good value, to buy friends can order Oh!	Jingdong prefecture level city in Shandong
Happy Fan Shop	King of Power Saving	Not using much electricity in the winter saving over 100 per month! Ready to buy 2 more units in the summer ！！！！ Good quality, good price, power saving effect is obvious, cost-effective, worth buying, can be recommended!	Jingdong prefecture level city in Shandong
Happy Fan Shop	King of Power Saving	Purchased a power saver received on the put into use, rent a room with many electrical appliances, power consumption is very large, we have a piece of electricity or 1.2 dollars a time, with this power saver is much better, every day can save a lot of money, very convenient and practical.	Jingdong prefecture level city in Shandong
Happy Fan Shop	King of Power Saving	The power saving effect is very good: my supermarket monthly electricity bill is more than 7000 yuan. On May 1st someone recommended buying a couple of power savers, and I bought 5 of them first. The electricity bill was about 2000 less in June. This time, I bought 5 more. The seller said 800 degrees of electricity with one. I feel it's basically enough. A total of 10 units	Jingdong prefecture level city in Shandong
Happy Fan Shop	King of Power Saving	I never thought I could get such a good baby at such an affordable price, it's very good value for money, I love it!	Jingdong prefecture level city in Shandong
Happy Fan Shop	King of Power Saving	Previously bought other brands of power savers, but found no effect at home, this time to see how the products on the Jingdong, inserted in the home socket, the LED display is blue, the night light is very nice to see, look at the instructions that have to be half a year in order to achieve the best results, try it, I believe that the quality of the Jingdong, it should not be wrong.	Jingdong prefecture level city in Shandong
Happy Fan Shop	King of Power Saving	Superb, inexpensive, saves a lot of points, been using it for a long time!	Jingdong prefecture level city in Shandong

Happy Fan Shop	King of Power Saving	There are a lot of appliances in the house, the summer air conditioning is very costly, I bought this in front of the air conditioning effect is very good, I explain that I bought three in one breath, I use more electricity a month down to 200 or so, very satisfied.	Jingdong prefecture level city in Shandong
Happy Fan Shop	King of Power Saving	A month can save more than 200, home monthly electricity bill of more than 600 dollars, inserted 3 or so, an amount of the balance of the average can save more than 200. Product is very good, has been buying this one, has given a lot of relatives recommended, the quality is also very good, before buying a look with this same, who knows to buy home has not been used for more than a week on the bad, the quality of this is really good with more than two years!	Jingdong prefecture level city in Shandong
Happy Fan Shop	King of Power Saving	Whole house power saving king, can really save electricity, friends recommended, high power saving appliances, only replacement, no repair, after-sales worry-free!	Jingdong prefecture level city in Shandong
Happy Fan Shop	King of Power Saving	Great item love it, great customer service, great shopping experience!	Jingdong prefecture level city in Shandong
Happy Fan Shop	King of Power Saving	Power saving, very good use, much cheaper than the physical store, satisfaction	Jingdong prefecture level city in Shandong
Happy Fan Shop	King of Power Saving	In use, very well packaged and fast delivery of credit!	Jingdong prefecture level city in Shandong
Happy Fan Shop	King of Power Saving	Product quality, customer service attitude is particularly good, like, praise!	Jingdong prefecture level city in Shandong
Happy Fan Shop	King of Power Saving	The logistics is very fast, the packaging is not broken, the quality is very good, I like it very much!	Jingdong prefecture level city in Shandong
Happy Fan Shop	King of Power Saving	The saver was well packaged and was as described when plugged in	Jingdong prefecture level city in Shandong
Happy Fan Shop	King of Power Saving	Energy efficient and very satisfied	Jingdong prefecture level city in Shandong
Happy Fan Shop	King of Power Saving	Great quality for the price and fast shipping.	Jingdong prefecture level city in Shandong
Happy Fan Shop	King of Power Saving	It's great, just as described. Perfect.	Jingdong prefecture level city in Shandong
Happy Fan Shop	King of Power Saving	There is no power saving effect, but rather ring up an increase in electricity costs, because this machine is on continuously all day long, uninterruptedly, its own electricity and increased costs, plus the cost of purchasing the machine, so I feel that the use of this machine instead of increasing the	Jingdong prefecture level city in Shandong
Happy Fan Shop	King of Power Saving	Recommended by the owner of the store next door, looked at his home does have to save electricity, decided to get. Indeed quite can, the voltage is much more stable, there is a reduction in electricity	Jingdong prefecture level city in Shandong
Happy Fan Shop	King of Power Saving	Goods have been received value for money, affordable and reasonable price, and the description of the same, power-saving safe and reliable!	Jingdong prefecture level city in Shandong
Happy Fan Shop	King of Power Saving	Listen to friends introduced to buy, tried to save a lot of electricity is good, quite good, worth buying.	Jingdong prefecture level city in Shandong
Happy Fan Shop	King of Power Saving	The product is easy to use, safe and environmentally friendly, only used for a short time, to the next month, after the electricity bill status display to know how the performance of the power saver efficacy.	Jingdong prefecture level city in Shandong
Happy Fan Shop	King of Power Saving	Last month's electricity bill is 178 yuan, with this power saver is 92 yuan, ready to buy two more back home, I was introduced to buy a friend, just started to not believe that I did not expect the effect!	Jingdong prefecture level city in Shandong
Happy Fan Shop	King of Power Saving	It looks nice, but after using it for a few days I can't see how it saves power?	Jingdong prefecture level city in Shandong
Happy Fan Shop	King of Power Saving	Recommended by a friend, it is genuine, as described by the merchant, cost-effective	Jingdong prefecture level city in Shandong
Happy Fan Shop	King of Power Saving	Works well, affordable and saves money.	Jingdong prefecture level city in Shandong
Happy Fan Shop	King of Power Saving	Feels good haven't compared it yet, the air conditioning has been on a lot in the past two months	Jingdong prefecture level city in Shandong
Happy Fan Shop	King of Power Saving	Saving about 5 kWh per day is a good thing	Jingdong prefecture level city in Shandong
Happy Fan Shop	King of Power Saving	Not bad, affordable, best value for money	Jingdong prefecture level city in Shandong
Happy Fan Shop	King of Power Saving	The goods are genuine and the logistics are faster.	Jingdong prefecture level city in Shandong
Happy Fan Shop	King of Power Saving	Goods have been received today, has been plugged into the outlet, looking forward to saving electricity, after a period of time to see how the effect in the follow-up comment!	Jingdong prefecture level city in Shandong
Happy Fan Shop	King of Power Saving	I just got it home and haven't used it for long, so I'll use it for a couple months and see what happens. Seeing that most people have good reviews, it should be fine.	Jingdong prefecture level city in Shandong
Happy Fan Shop	King of Power Saving	I'm looking forward to the results, so I'll give you a good review out of habit! Follow up review after a while	Jingdong prefecture level city in Shandong
Happy Fan Shop	King of Power Saving	Use a dish first to know if it will help save energy	Jingdong prefecture level city in Shandong
Happy Fan Shop	King of Power Saving	Whether it saves electricity or not, we don't know yet.	Jingdong prefecture level city in Shandong
Happy Fan Shop	King of Power Saving	Goods have been received, use a period of time to know how oh. The courier did not deliver to the door ah! Sent to the station ah! The heat is inconvenient ah!	Jingdong prefecture level city in Shandong
Happy Fan Shop	King of Power Saving	A little effect, before an hour a power, now an hour 0.76 degrees, a dollar a power = save 24 cents, although not a lot, first use it!	Jingdong prefecture level city in Shandong
Happy Fan Shop	King of Power Saving	Just received the goods, use a month to evaluate	Jingdong prefecture level city in Shandong
Happy Fan Shop	King of Power Saving	Home every month with a lot of electricity, inadvertently found this good thing online that can save the beginning of a skeptical attitude to consult customer service, customer service replied very professional, said 120 days can be refunded that first try it!	Jingdong prefecture level city in Shandong
Happy Fan Shop	King of Power Saving	After receiving the goods this afternoon, immediately installed in the bathroom Smith energy storage electric water heater on the power outlet (instead of connecting with a plug board), when used, found that the "Power Saving King" LCD display, is the value of the voltage, not the store's video said so	Jingdong prefecture level city in Shandong
Happy Fan Shop	King of Power Saving	Has made many days, did not feel the power saving, the effect is not obvious.	Jingdong prefecture level city in Shandong
Happy Fan Shop	King of Power Saving	Energy savers do not save energy at all, but rather consume a lot of electricity.	Jingdong prefecture level city in Shandong
Happy Fan Shop	King of Power Saving	It's useless. It's a waste of money.	Jingdong prefecture level city in Shandong
Happy Fan Shop	King of Power Saving	Last month saved more than 500, which came back to buy, the store bought three, and then buy 1 home use, my family is dry beauty salon, more instruments, especially electricity, a month almost 1000. or so, with this did not expect to save so much, great, five-star praise!	Jingdong prefecture level city in Shandong
Happy Fan Shop	King of Power Saving	It rained a couple days ago and I just went to get the item back today , not bad, hey I looked at the meter and that red display light takes several seconds to jump, did not make a mistake buying it. After a period of time to evaluate.	Jingdong prefecture level city in Shandong
Happy Fan Shop	King of Power Saving	Without further ado, the electricity bill came down, this month less 102 yuan, has been repurchased, I also recommended to my friends, all said that there are effects, the product is okay, the effect is quite	Jingdong prefecture level city in Shandong
Happy Fan Shop	King of Power Saving	After buying and using two months really see the effect, so only to evaluate! Originally held the mindset of trying, I did not expect really effective, worth having!	Jingdong prefecture level city in Shandong
Happy Fan Shop	King of Power Saving	In the use of this energy saver before the monthly electricity bill is more than 150 yuan or so, now save thirty percent, every day to work, six or seven o'clock in the afternoon to go home, I do not know why the electricity bill is so expensive, energy-saving appliances really have the effect of recommending	Jingdong prefecture level city in Shandong
Happy Fan Shop	King of Power Saving	It saves about 100 bucks a month. A friend recommended it to me, and he's been using it for a while, and the effect is so obvious that I bought one too.	Jingdong prefecture level city in Shandong
Happy Fan Shop	King of Power Saving	My dad asked me to buy one, but after I paid the bill, I asked and he said it was a lot less this month, so he asked me to buy another one.	Jingdong prefecture level city in Shandong
Happy Fan Shop	King of Power Saving	The specific effect will have to be tested for at least two months to compare whether there is a power saving effect or not.	Jingdong prefecture level city in Shandong

Happy Fan Shop	King of Power Saving	'The goods have been received, immediately go to use, please ask the boss you instructions do not allow to withdraw, there are two fuses installed there!	Jingdong prefecture level city in Shandong
Happy Fan Shop	King of Power Saving	First praise is using, after using if good then follow up, now because I have not yet seen the results	Jingdong prefecture level city in Shandong
Happy Fan Shop	King of Power Saving	I've just been using it, and I haven't seen if it has a power-saving effect yet, so I'll follow up after 10 days and half a month.	Jingdong prefecture level city in Shandong
Happy Fan Shop	King of Power Saving	Just received the intelligent power saving meter, immediately plugged in to use, see it is not bad, and order to sell the second time	Jingdong prefecture level city in Shandong
Happy Fan Shop	King of Power Saving	This power saver is a bit effective, used for half a month, the effect is still visible.	Jingdong prefecture level city in Shandong
Happy Fan Shop	King of Power Saving	I received the baby and it looks great, I'm trying it out now and will keep an eye on it afterward.	Jingdong prefecture level city in Shandong
Happy Fan Shop	King of Power Saving	I've just received it, but I'm still trying to see how it works, so I'll come back in a little while.	Jingdong prefecture level city in Shandong
Happy Fan Shop	King of Power Saving	Just used it wait to use it for a while and then comment thanks, all first five stars.	Jingdong prefecture level city in Shandong
Happy Fan Shop	King of Power Saving	Very trustworthy brand, quality is guaranteed, the price is still affordable, quite satisfied!	Jingdong prefecture level city in Shandong
Happy Fan Shop	King of Power Saving	Things received, I hope that the platform of Jingdong do not sell fake goods are available	Jingdong prefecture level city in Shandong
Happy Fan Shop	King of Power Saving	Energy saver received, the use of the effect is good and the business description of the same	Jingdong prefecture level city in Shandong
Happy Fan Shop	King of Power Saving	The surface looks beautiful, the actual effect is not known, after using additional evaluation.	Jingdong prefecture level city in Shandong
Happy Fan Shop	King of Power Saving	Tried it for a few months, it really saves electricity, and I bought it for a friend.	Jingdong prefecture level city in Shandong
Happy Fan Shop	King of Power Saving	It didn't work and wanted to return it, it really didn't work at all.	Jingdong prefecture level city in Shandong
Happy Fan Shop	King of Power Saving	Just used it, don't know how effective it is, looking forward to usefulness	Jingdong prefecture level city in Shandong
Happy Fan Shop	King of Power Saving	After two months before the effect I do not know if it is true!	Jingdong prefecture level city in Shandong
Happy Fan Shop	King of Power Saving	So fast! Ordered yesterday and received today 👍👍👍👍👍👍👍👍👍 🌹🌹🌹🌹🌹🌹🌹🌹🌹	Jingdong prefecture level city in Shandong
Happy Fan Shop	King of Power Saving	I've started using it. We'll see how it works.	Jingdong prefecture level city in Shandong
Happy Fan Shop	King of Power Saving	I just received the goods, I won't know the effect until after two.	Jingdong prefecture level city in Shandong
Happy Fan Shop	King of Power Saving	Just received the goods, the specific effect will be evaluated later	Jingdong prefecture level city in Shandong
Happy Fan Shop	King of Power Saving	I've received the goods, I don't know if it's any good yet	Jingdong prefecture level city in Shandong
Happy Fan Shop	King of Power Saving	Try it for a month and then come back with a concrete evaluation	Jingdong prefecture level city in Shandong
Happy Fan Shop	King of Power Saving	In use, after two months to see the effect of power saving	Jingdong prefecture level city in Shandong
Happy Fan Shop	King of Power Saving	Power Saver. It's authentic. It's black. It works.	Jingdong prefecture level city in Shandong
Happy Fan Shop	King of Power Saving	I've been using it for a week now and it's not working.	Jingdong prefecture level city in Shandong
Happy Fan Shop	King of Power Saving	I'm just using it. I don't know how it's going to work.	Jingdong prefecture level city in Shandong
Happy Fan Shop	King of Power Saving	Products are better looking out for power savings	Jingdong prefecture level city in Shandong
Happy Fan Shop	King of Power Saving	I received it and I've kept it on. I hope it works.	Jingdong prefecture level city in Shandong
Happy Fan Shop	King of Power Saving	It's really good! Worth recommending to buy!	Jingdong prefecture level city in Shandong
Happy Fan Shop	King of Power Saving	Try it for a few days and see if it doesn't work, then return it.	Jingdong prefecture level city in Shandong
Happy Fan Shop	King of Power Saving	It's in use and I hope to be pleasantly surprised.	Jingdong prefecture level city in Shandong
Happy Fan Shop	King of Power Saving	We'll see how it works when we use it	Jingdong prefecture level city in Shandong
Happy Fan Shop	King of Power Saving	Let's see if it works.	Jingdong prefecture level city in Shandong
Happy Fan Shop	King of Power Saving	I'm using it and will follow up with a review	Jingdong prefecture level city in Shandong
Happy Fan Shop	King of Power Saving	It really works. It saves energy.	Jingdong prefecture level city in Shandong
Happy Fan Shop	King of Power Saving	It does have some power savings!	Jingdong prefecture level city in Shandong
Happy Fan Shop	King of Power Saving	Jingdong shopping speed is convenient and timely	Jingdong prefecture level city in Shandong
Happy Fan Shop	King of Power Saving	Second time I've bought it. Everything's fine.	Jingdong prefecture level city in Shandong
Happy Fan Shop	King of Power Saving	I don't know if it works yet.	Jingdong prefecture level city in Shandong
Happy Fan Shop	King of Power Saving	Not used, don't know how well it works.	Jingdong prefecture level city in Shandong
Happy Fan Shop	King of Power Saving	No visible results so far	Jingdong prefecture level city in Shandong
Happy Fan Shop	King of Power Saving	It doesn't feel like it's working.	Jingdong prefecture level city in Shandong
Happy Fan Shop	King of Power Saving	Where's the effect? Where are the power savings?	Jingdong prefecture level city in Shandong
Happy Fan Shop	King of Power Saving	Let's see how it works in practice!	Jingdong prefecture level city in Shandong
Happy Fan Shop	King of Power Saving	I'll wait until I use it to give you a good review	Jingdong prefecture level city in Shandong
Happy Fan Shop	King of Power Saving	I'll follow up after I've used it	Jingdong prefecture level city in Shandong
Happy Fan Shop	King of Power Saving	Well, that's good for saving power.	Jingdong prefecture level city in Shandong
Happy Fan Shop	King of Power Saving	Received. Used in the know.	Jingdong prefecture level city in Shandong
Happy Fan Shop	King of Power Saving	It doesn't do much good.	Jingdong prefecture level city in Shandong
Happy Fan Shop	King of Power Saving	It's not working.	Jingdong prefecture level city in Shandong
Happy Fan Shop	King of Power Saving	I don't know. I just used it.	Jingdong prefecture level city in Shandong

Happy Fan Shop	King of Power Saving	Use it and come back to evaluate.	Jingdong prefecture level city in Shandong
Happy Fan Shop	King of Power Saving	Two words: useless.	Jingdong prefecture level city in Shandong
Happy Fan Shop	King of Power Saving	To be used and then evaluated	Jingdong prefecture level city in Shandong
Happy Fan Shop	King of Power Saving	No shipment.	Jingdong prefecture level city in Shandong
Happy Fan Shop	King of Power Saving	Fake. Speechless.	Jingdong prefecture level city in Shandong
Happy Fan Shop	King of Power Saving	fantastic (idiom); awesome	Jingdong prefecture level city in Shandong
Happy Fan Shop	King of Power Saving	an excellent	Jingdong prefecture level city in Shandong
Happy Fan Shop	King of Power Saving	an excellent	Jingdong prefecture level city in Shandong
Happy Fan Shop	King of Power Saving	Want to buy! Recommended not to buy! No effect	Jingdong prefecture level city in Shandong
Happy Fan Shop	King of Power Saving	That's all there is to it. I can't figure out how to save electricity.	Jingdong prefecture level city in Shandong
Happy Fan Shop	King of Power Saving	It's no use.	Jingdong prefecture level city in Shandong
Happy Fan Shop	King of Power Saving	Things are more perfect than I imagined, very powerful, it is worth getting Packaging is very strong, things are well protected, it is worth the praise things are very satisfied Oh, beautiful and stylish atmosphere, customer service is super patient, service is meticulous and attentive, the big brand is not	Jingdong prefecture level city in Shandong
Happy Fan Shop	King of Power Saving	The effect is too good, the family has now several units, back to buy a number of times, the power saver received, power-saving effect is very good,, fine workmanship, long warranty, but also purify the air, before buying a used for a period of time, with the okay, and then bought a, and only now come to the	Jingdong prefecture level city in Shandong
Happy Fan Shop	King of Power Saving	Quite good, and imagine the same, casual ride are very good, satisfied! Baby received it, really great ah! quality is really nothing to say, very satisfied! happy happy genuine, great, the price is very good, in short, quite good, will buy again a very good shopping experience, attentive service, fast logistics, distribution is also thoughtful, the attitude is also very good, value for money, ah,	Jingdong prefecture level city in Shandong
Happy Fan Shop	King of Power Saving	Power-saving effect: strong Cost-effective: quite good Appearance style: beautiful Workmanship material: meticulous Experience: looking forward to power-saving effect	Jingdong prefecture level city in Shandong
Happy Fan Shop	King of Power Saving	Cost-effective is okay, the appearance of the style of meticulous workmanship, looking forward to energy-saving effect.	Jingdong prefecture level city in Shandong
Happy Fan Shop	King of Power Saving	Very Good Very Power Saving This Power Saver is really power saving As soon as the power is turned on, the Power Saver starts the power saving function for you.	Jingdong prefecture level city in Shandong
Happy Fan Shop	King of Power Saving	Logistics is very fast, the price is favorable, friends introduced that it is very good to use, bought 2 at once, looking forward to the effect, used after the follow-up comment.	Jingdong prefecture level city in Shandong
Happy Fan Shop	King of Power Saving	It's all fake. It doesn't save any electricity.	Jingdong prefecture level city in Shandong
Happy Fan Shop	King of Power Saving	This Power Saver is so much better than the one I bought before, not only the effect is good, the quality is very good in all aspects, the customer service attitude is also very good, recommended to buy!	Jingdong prefecture level city in Shandong
Happy Fan Shop	King of Power Saving	Logistics is very fast, buy for parents, good quality, once bought 2, power-saving effect is good to buy 2 more, look forward to using the effect, satisfied will recommend to friends.	Jingdong prefecture level city in Shandong
Happy Fan Shop	King of Power Saving	With the description is the same, very high-grade, good value, to buy friends can order Oh!	Jingdong prefecture level city in Shandong
Happy Fan Shop	King of Power Saving	Not using much electricity in the winter saving over 100 per month! Ready to buy 2 more units in the summer ！！！！！ Good quality, good price, power saving effect is obvious, cost-effective, worth buying, can be recommended!	Jingdong prefecture level city in Shandong
Happy Fan Shop	King of Power Saving	Purchased a power saver received on the put into use, rent a room with many electrical appliances, power consumption is very large, we have a piece of electricity or 1.2 dollars a time, with this power saver is much better, every day can save a lot of money, very convenient and practical.	Jingdong prefecture level city in Shandong
Happy Fan Shop	King of Power Saving	The power saving effect is very good: my supermarket monthly electricity bill is more than 7000 yuan. On May 1st someone recommended buying a couple of power savers, and I bought 5 of them first. The electricity bill was about 2000 less in June. This time, I bought 5 more. The seller said 800 degrees of electricity with one. I feel it's basically enough. A total of 10 units	Jingdong prefecture level city in Shandong
Happy Fan Shop	King of Power Saving	I never thought I could get such a good baby at such an affordable price, it's very good value for money, I love it!	Jingdong prefecture level city in Shandong
Happy Fan Shop	King of Power Saving	Previously bought other brands of power savers, but found no effect at home, this time to see how the products on the Jingdong, inserted in the home socket, the LED display is blue, the night light is very nice to see, look at the instructions that have to be half a year in order to achieve the best results, try it, I believe that the quality of the Jingdong, it should not be wrong.	Jingdong prefecture level city in Shandong
Happy Fan Shop	King of Power Saving	Superb, inexpensive, saves a lot of points, been using it for a long time!	Jingdong prefecture level city in Shandong
Happy Fan Shop	King of Power Saving	There are a lot of appliances in the house, the summer air conditioning is very costly, I bought this in front of the air conditioning effect is very good, I explain that I bought three in one breath, I use more electricity a month down to 200 or so, very satisfied.	Jingdong prefecture level city in Shandong
Happy Fan Shop	King of Power Saving	A month can save more than 200, home monthly electricity bill of more than 600 dollars, inserted 3 or so, an amount of the balance of the average can save more than 200. Product is very good, has been buying this one, has given a lot of relatives recommended, the quality is also very good, before buying a look with this same, who knows to buy home has not been used for more than a week on the bad, the quality of this is really good with more than two years!	Jingdong prefecture level city in Shandong
Happy Fan Shop	King of Power Saving	Whole house power saving king, can really save electricity, friends recommended, high power saving appliances, only replacement, no repair, after-sales worry-free!	Jingdong prefecture level city in Shandong
Happy Fan Shop	King of Power Saving	Great item love it, great customer service, great shopping experience!	Jingdong prefecture level city in Shandong
Happy Fan Shop	King of Power Saving	Power saving, very good use, much cheaper than the physical store, satisfaction	Jingdong prefecture level city in Shandong
Happy Fan Shop	King of Power Saving	In use, very well packaged and fast delivery of credit!	Jingdong prefecture level city in Shandong
Happy Fan Shop	King of Power Saving	Product quality, customer service attitude is particularly good, like, praise!	Jingdong prefecture level city in Shandong
Happy Fan Shop	King of Power Saving	The logistics is very fast, the packaging is not broken, the quality is very good, I like it very much!	Jingdong prefecture level city in Shandong
Happy Fan Shop	King of Power Saving	The saver was well packaged and was as described when plugged in	Jingdong prefecture level city in Shandong
Happy Fan Shop	King of Power Saving	Energy efficient and very satisfied	Jingdong prefecture level city in Shandong
Happy Fan Shop	King of Power Saving	Great quality for the price and fast shipping.	Jingdong prefecture level city in Shandong
Happy Fan Shop	King of Power Saving	It's great, just as described. Perfect.	Jingdong prefecture level city in Shandong
Happy Fan Shop	King of Power Saving	There is no power saving effect, but rather ring up an increase in electricity costs, because this machine is on continuously all day long, uninterruptedly, its own electricity and increased costs, plus the cost of purchasing the machine, so I feel that the use of this machine instead of increasing the	Jingdong prefecture level city in Shandong
Happy Fan Shop	King of Power Saving	Recommended by the owner of the store next door, looked at his home does have to save electricity, decided to get. Indeed quite can, the voltage is much more stable, there is a reduction in electricity	Jingdong prefecture level city in Shandong
Happy Fan Shop	King of Power Saving	Goods have been received value for money, affordable and reasonable price, and the description of the same, power-saving safe and reliable!	Jingdong prefecture level city in Shandong
Happy Fan Shop	King of Power Saving	Listen to friends introduced to buy, tried to save a lot of electricity is good, quite good, worth buying.	Jingdong prefecture level city in Shandong

Happy Fan Shop	King of Power Saving	The product is easy to use, safe and environmentally friendly, only used for a short time, to the next month, after the electricity bill status display to know how the performance of the power saver efficacy.	Jingdong prefecture level city in Shandong
Happy Fan Shop	King of Power Saving	Last month's electricity bill is 178 yuan, with this power saver is 92 yuan, ready to buy two more back home, I was introduced to buy a friend, just started to not believe that I did not expect the effect!	Jingdong prefecture level city in Shandong
Happy Fan Shop	King of Power Saving	It looks nice, but after using it for a few days I can't see how it saves power?	Jingdong prefecture level city in Shandong
Happy Fan Shop	King of Power Saving	Recommended by a friend, it is genuine, as described by the merchant, cost-effective	Jingdong prefecture level city in Shandong
Happy Fan Shop	King of Power Saving	Works well, affordable and saves money.	Jingdong prefecture level city in Shandong
Happy Fan Shop	King of Power Saving	Feels good haven't compared it yet, the air conditioning has been on a lot in the past two months	Jingdong prefecture level city in Shandong
Happy Fan Shop	King of Power Saving	Saving about 5 kWh per day is a good thing	Jingdong prefecture level city in Shandong
Happy Fan Shop	King of Power Saving	Not bad, affordable, best value for money	Jingdong prefecture level city in Shandong
Happy Fan Shop	King of Power Saving	The goods are genuine and the logistics are faster.	Jingdong prefecture level city in Shandong
Happy Fan Shop	King of Power Saving	Goods have been received today, has been plugged into the outlet, looking forward to saving electricity, after a period of time to see how the effect in the follow-up comment!	Jingdong prefecture level city in Shandong
Happy Fan Shop	King of Power Saving	I just got it home and haven't used it for long, so I'll use it for a couple months and see what happens. Seeing that most people have good reviews, it should be fine.	Jingdong prefecture level city in Shandong
Happy Fan Shop	King of Power Saving	I'm looking forward to the results, so I'll give you a good review out of habit! Follow up review after a while	Jingdong prefecture level city in Shandong
Happy Fan Shop	King of Power Saving	Use a dish first to know if it will help save energy	Jingdong prefecture level city in Shandong
Happy Fan Shop	King of Power Saving	Whether it saves electricity or not, we don't know yet.	Jingdong prefecture level city in Shandong
Happy Fan Shop	King of Power Saving	Goods have been received, use a period of time to know how oh. The courier did not deliver to the door ah! Sent to the station ah! The heat is inconvenient ah!	Jingdong prefecture level city in Shandong
Happy Fan Shop	King of Power Saving	A little effect, before an hour a power, now an hour 0.76 degrees, a dollar a power = save 24 cents, although not a lot, first use it!	Jingdong prefecture level city in Shandong
Happy Fan Shop	King of Power Saving	Just received the goods, use a month to evaluate	Jingdong prefecture level city in Shandong
Happy Fan Shop	King of Power Saving	Home every month with a lot of electricity, inadvertently found this good thing online that can save the beginning of a skeptical attitude to consult customer service, customer service replied very professional, said 120 days can be refunded that first try it!	Jingdong prefecture level city in Shandong
Happy Fan Shop	King of Power Saving	After receiving the goods this afternoon, immediately installed in the bathroom Smith energy storage electric water heater on the power outlet (instead of connecting with a plug board), when used, found that the "Power Saving King" LCD display, is the value of the voltage, not the store's video said so	Jingdong prefecture level city in Shandong
Happy Fan Shop	King of Power Saving	Has made many days, did not feel the power saving, the effect is not obvious.	Jingdong prefecture level city in Shandong
Happy Fan Shop	King of Power Saving	Energy savers do not save energy at all, but rather consume a lot of electricity.	Jingdong prefecture level city in Shandong
Happy Fan Shop	King of Power Saving	It's useless. It's a waste of money.	Jingdong prefecture level city in Shandong
Happy Fan Shop	King of Power Saving	Last month saved more than 500, which came back to buy, the store bought three, and then buy 1 home use, my family is dry beauty salon, more instruments, especially electricity, a month almost 1000. or so, with this did not expect to save so much, great, five-star praise!	Jingdong prefecture level city in Shandong
Happy Fan Shop	King of Power Saving	It rained a couple days ago and I just went to get the item back today , not bad, hey I looked at the meter and that red display light takes several seconds to jump, did not make a mistake buying it. After a period of time to evaluate.	Jingdong prefecture level city in Shandong
Happy Fan Shop	King of Power Saving	Without further ado, the electricity bill came down, this month less 102 yuan, has been repurchased, I also recommended to my friends, all said that there are effects, the product is okay, the effect is quite	Jingdong prefecture level city in Shandong
Happy Fan Shop	King of Power Saving	After buying and using two months really see the effect, so only to evaluate! Originally held the mindset of trying, I did not expect really effective, worth having!	Jingdong prefecture level city in Shandong
Happy Fan Shop	King of Power Saving	In the use of this energy saver before the monthly electricity bill is more than 150 yuan or so, now save thirty percent, every day to work, six or seven o'clock in the afternoon to go home, I do not know why the electricity bill is so expensive, energy-saving appliances really have the effect of recommending	Jingdong prefecture level city in Shandong
Happy Fan Shop	King of Power Saving	It saves about 100 bucks a month. A friend recommended it to me, and he's been using it for a while, and the effect is so obvious that I bought one too.	Jingdong prefecture level city in Shandong
Happy Fan Shop	King of Power Saving	My dad asked me to buy one, but after I paid the bill, I asked and he said it was a lot less this month, so he asked me to buy another one.	Jingdong prefecture level city in Shandong
Happy Fan Shop	King of Power Saving	The specific effect will have to be tested for at least two months to compare whether there is a power saving effect or not.	Jingdong prefecture level city in Shandong
Happy Fan Shop	King of Power Saving	'The goods have been received, immediately go to use, please ask the boss you instructions do not allow to withdraw, there are two fuses installed there!	Jingdong prefecture level city in Shandong
Happy Fan Shop	King of Power Saving	First praise is using, after using if good then follow up, now because I have not yet seen the results	Jingdong prefecture level city in Shandong
Happy Fan Shop	King of Power Saving	I've just been using it, and I haven't seen if it has a power-saving effect yet, so I'll follow up after 10 days and half a month.	Jingdong prefecture level city in Shandong
Happy Fan Shop	King of Power Saving	Just received the intelligent power saving meter, immediately plugged in to use, see it is not bad, and order to sell the second time	Jingdong prefecture level city in Shandong
Happy Fan Shop	King of Power Saving	This power saver is a bit effective, used for half a month, the effect is still visible.	Jingdong prefecture level city in Shandong
Happy Fan Shop	King of Power Saving	I received the baby and it looks great, I'm trying it out now and will keep an eye on it afterward.	Jingdong prefecture level city in Shandong
Happy Fan Shop	King of Power Saving	I've just received it, but I'm still trying to see how it works, so I'll come back in a little while.	Jingdong prefecture level city in Shandong
Happy Fan Shop	King of Power Saving	Just used it wait to use it for a while and then comment thanks, all first five stars.	Jingdong prefecture level city in Shandong
Happy Fan Shop	King of Power Saving	Very trustworthy brand, quality is guaranteed, the price is still affordable, quite satisfied!	Jingdong prefecture level city in Shandong
Happy Fan Shop	King of Power Saving	Things received, I hope that the platform of Jingdong do not sell fake goods are available	Jingdong prefecture level city in Shandong
Happy Fan Shop	King of Power Saving	Energy saver received, the use of the effect is good and the business description of the same	Jingdong prefecture level city in Shandong
Happy Fan Shop	King of Power Saving	The surface looks beautiful, the actual effect is not known, after using additional evaluation.	Jingdong prefecture level city in Shandong
Happy Fan Shop	King of Power Saving	Tried it for a few months, it really saves electricity, and I bought it for a friend.	Jingdong prefecture level city in Shandong
Happy Fan Shop	King of Power Saving	It didn't work and wanted to return it, it really didn't work at all.	Jingdong prefecture level city in Shandong
Happy Fan Shop	King of Power Saving	Just used it, don't know how effective it is, looking forward to usefulness	Jingdong prefecture level city in Shandong
Happy Fan Shop	King of Power Saving	After two months before the effect I do not know if it is true!	Jingdong prefecture level city in Shandong
Happy Fan Shop	King of Power Saving	So fast! Ordered yesterday and received today 👍👍👍👍👍👍👍👍👍 🌹🌹🌹🌹🌹🌹🌹🌹🌹	Jingdong prefecture level city in Shandong
Happy Fan Shop	King of Power Saving	I've started using it. We'll see how it works.	Jingdong prefecture level city in Shandong
Happy Fan Shop	King of Power Saving	I just received the goods, I won't know the effect until after two.	Jingdong prefecture level city in Shandong

Happy Fan Shop	King of Power Saving	Just received the goods, the specific effect will be evaluated later	Jingdong prefecture level city in Shandong
Happy Fan Shop	King of Power Saving	I've received the goods, I don't know if it's any good yet	Jingdong prefecture level city in Shandong
Happy Fan Shop	King of Power Saving	Try it for a month and then come back with a concrete evaluation	Jingdong prefecture level city in Shandong
Happy Fan Shop	King of Power Saving	In use, after two months to see the effect of power saving	Jingdong prefecture level city in Shandong
Happy Fan Shop	King of Power Saving	Power Saver. It's authentic. It's black. It works.	Jingdong prefecture level city in Shandong
Happy Fan Shop	King of Power Saving	I've been using it for a week now and it's not working.	Jingdong prefecture level city in Shandong
Happy Fan Shop	King of Power Saving	I'm just using it. I don't know how it's going to work.	Jingdong prefecture level city in Shandong
Happy Fan Shop	King of Power Saving	Products are better looking out for power savings	Jingdong prefecture level city in Shandong
Happy Fan Shop	King of Power Saving	I received it and I've kept it on. I hope it works.	Jingdong prefecture level city in Shandong
Happy Fan Shop	King of Power Saving	It's really good! Worth recommending to buy!	Jingdong prefecture level city in Shandong
Happy Fan Shop	King of Power Saving	Try it for a few days and see if it doesn't work, then return it.	Jingdong prefecture level city in Shandong
Happy Fan Shop	King of Power Saving	It's in use and I hope to be pleasantly surprised.	Jingdong prefecture level city in Shandong
Happy Fan Shop	King of Power Saving	We'll see how it works when we use it	Jingdong prefecture level city in Shandong
Happy Fan Shop	King of Power Saving	Let's see if it works.	Jingdong prefecture level city in Shandong
Happy Fan Shop	King of Power Saving	I'm using it and will follow up with a review	Jingdong prefecture level city in Shandong
Happy Fan Shop	King of Power Saving	It really works. It saves energy.	Jingdong prefecture level city in Shandong
Happy Fan Shop	King of Power Saving	It does have some power savings!	Jingdong prefecture level city in Shandong
Happy Fan Shop	King of Power Saving	Jingdong shopping speed is convenient and timely	Jingdong prefecture level city in Shandong
Happy Fan Shop	King of Power Saving	Second time I've bought it. Everything's fine.	Jingdong prefecture level city in Shandong
Happy Fan Shop	King of Power Saving	I don't know if it works yet.	Jingdong prefecture level city in Shandong
Happy Fan Shop	King of Power Saving	Not used, don't know how well it works.	Jingdong prefecture level city in Shandong
Happy Fan Shop	King of Power Saving	No visible results so far	Jingdong prefecture level city in Shandong
Happy Fan Shop	King of Power Saving	It doesn't feel like it's working.	Jingdong prefecture level city in Shandong
Happy Fan Shop	King of Power Saving	Where's the effect? Where are the power savings?	Jingdong prefecture level city in Shandong
Happy Fan Shop	King of Power Saving	Let's see how it works in practice!	Jingdong prefecture level city in Shandong
Happy Fan Shop	King of Power Saving	I'll wait until I use it to give you a good review	Jingdong prefecture level city in Shandong
Happy Fan Shop	King of Power Saving	I'll follow up after I've used it	Jingdong prefecture level city in Shandong
Happy Fan Shop	King of Power Saving	Well, that's good for saving power.	Jingdong prefecture level city in Shandong
Happy Fan Shop	King of Power Saving	Received. Used in the know.	Jingdong prefecture level city in Shandong
Happy Fan Shop	King of Power Saving	It doesn't do much good.	Jingdong prefecture level city in Shandong
Happy Fan Shop	King of Power Saving	It's not working.	Jingdong prefecture level city in Shandong
Happy Fan Shop	King of Power Saving	I don't know. I just used it.	Jingdong prefecture level city in Shandong
Happy Fan Shop	King of Power Saving	Use it and come back to evaluate.	Jingdong prefecture level city in Shandong
Happy Fan Shop	King of Power Saving	Two words: useless.	Jingdong prefecture level city in Shandong
Happy Fan Shop	King of Power Saving	To be used and then evaluated	Jingdong prefecture level city in Shandong
Happy Fan Shop	King of Power Saving	No shipment.	Jingdong prefecture level city in Shandong
Happy Fan Shop	King of Power Saving	Fake. Speechless.	Jingdong prefecture level city in Shandong
Happy Fan Shop	King of Power Saving	fantastic (idiom); awesome	Jingdong prefecture level city in Shandong
Happy Fan Shop	King of Power Saving	an excellent	Jingdong prefecture level city in Shandong
Happy Fan Shop	King of Power Saving	an excellent	Jingdong prefecture level city in Shandong
Happy Fan Shop	King of Power Saving	Want to buy! Recommended not to buy! No effect	Jingdong prefecture level city in Shandong
Happy Fan Shop	King of Power Saving	That's all there is to it. I can't figure out how to save electricity.	Jingdong prefecture level city in Shandong
Happy Fan Shop	King of Power Saving	It's no use.	Jingdong prefecture level city in Shandong
Happy Fan Shop	King of Power Saving	Things are more perfect than I imagined, very powerful, it is worth getting Packaging is very strong, things are well protected, it is worth the praise things are very satisfied Oh, beautiful and stylish atmosphere, customer service is super patient, service is meticulous and attentive, the big brand is not	Jingdong prefecture level city in Shandong
Happy Fan Shop	King of Power Saving	The effect is too good, the family has now several units, back to buy a number of times, the power saver received, power-saving effect is very good,, fine workmanship, long warranty, but also purify the air, before buying a used for a period of time, with the okay, and then bought a, and only now come to the	Jingdong prefecture level city in Shandong
Happy Fan Shop	King of Power Saving	Quite good, and imagine the same, casual ride are very good, satisfied! Baby received it 👌, really great ah! 🌈 quality is really nothing to say, very satisfied! 🆗 happy happy 😃 genuine, great, the price is very good, in short, quite good, will buy again a very good shopping experience, attentive service, fast logistics, distribution is also thoughtful, the attitude is also very good, value for money, ah,	Jingdong prefecture level city in Shandong
Happy Fan Shop	King of Power Saving	Power-saving effect: strong Cost-effective: quite good Appearance style: beautiful Workmanship material: meticulous Experience: looking forward to power-saving effect	Jingdong prefecture level city in Shandong
Happy Fan Shop	King of Power Saving	Cost-effective is okay, the appearance of the style of meticulous workmanship, looking forward to energy-saving effect.	Jingdong prefecture level city in Shandong
Happy Fan Shop	King of Power Saving	Very Good Very Power Saving This Power Saver is really power saving As soon as the power is turned on, the Power Saver starts the power saving function for you.	Jingdong prefecture level city in Shandong

Happy Fan Shop	King of Power Saving	Logistics is very fast, the price is favorable, friends introduced that it is very good to use, bought 2 at once, looking forward to the effect, used after the follow-up comment.	Jingdong prefecture level city in Shandong
Happy Fan Shop	King of Power Saving	It's all fake. It doesn't save any electricity.	Jingdong prefecture level city in Shandong
Happy Fan Shop	King of Power Saving	This Power Saver is so much better than the one I bought before, not only the effect is good, the quality is very good in all aspects, the customer service attitude is also very good, recommended to buy!	Jingdong prefecture level city in Shandong
Happy Fan Shop	King of Power Saving	Logistics is very fast, buy for parents, good quality, once bought 2, power-saving effect is good to buy 2 more, look forward to using the effect, satisfied will recommend to friends.	Jingdong prefecture level city in Shandong
Happy Fan Shop	King of Power Saving	With the description is the same, very high-grade, good value, to buy friends can order Oh!	Jingdong prefecture level city in Shandong
Happy Fan Shop	King of Power Saving	Not using much electricity in the winter saving over 100 per month! Ready to buy 2 more units in the summer ！！！！！ Good quality, good price, power saving effect is obvious, cost-effective, worth buying, can be recommended!	Jingdong prefecture level city in Shandong
Happy Fan Shop	King of Power Saving	Purchased a power saver received on the put into use, rent a room with many electrical appliances, power consumption is very large, we have a piece of electricity or 1.2 dollars a time, with this power saver is much better, every day can save a lot of money, very convenient and practical.	Jingdong prefecture level city in Shandong
Happy Fan Shop	King of Power Saving	The power saving effect is very good: my supermarket monthly electricity bill is more than 7000 yuan. On May 1st someone recommended buying a couple of power savers, and I bought 5 of them first. The electricity bill was about 2000 less in June. This time, I bought 5 more. The seller said 800 degrees of electricity with one. I feel it's basically enough. A total of 10 units	Jingdong prefecture level city in Shandong
Happy Fan Shop	King of Power Saving	I never thought I could get such a good baby at such an affordable price, it's very good value for money, I love it!	Jingdong prefecture level city in Shandong
Happy Fan Shop	King of Power Saving	Previously bought other brands of power savers, but found no effect at home, this time to see how the products on the Jingdong, inserted in the home socket, the LED display is blue, the night light is very nice to see, look at the instructions that have to be half a year in order to achieve the best results, try it, I believe that the quality of the Jingdong, it should not be wrong.	Jingdong prefecture level city in Shandong
Happy Fan Shop	King of Power Saving	Superb, inexpensive, saves a lot of points, been using it for a long time!	Jingdong prefecture level city in Shandong
Happy Fan Shop	King of Power Saving	There are a lot of appliances in the house, the summer air conditioning is very costly, I bought this in front of the air conditioning effect is very good, I explain that I bought three in one breath, I use more electricity a month down to 200 or so, very satisfied.	Jingdong prefecture level city in Shandong
Happy Fan Shop	King of Power Saving	A month can save more than 200, home monthly electricity bill of more than 600 dollars, inserted 3 or so, an amount of the balance of the average can save more than 200. Product is very good, has been buying this one, has given a lot of relatives recommended, the quality is also very good, before buying a look with this same, who knows to buy home has not been used for more than a week on the bad, the quality of this is really good with more than two years!	Jingdong prefecture level city in Shandong
Happy Fan Shop	King of Power Saving	Whole house power saving king, can really save electricity, friends recommended, high power saving appliances, only replacement, no repair, after-sales worry-free!	Jingdong prefecture level city in Shandong
Happy Fan Shop	King of Power Saving	Great item love it, great customer service, great shopping experience!	Jingdong prefecture level city in Shandong
Happy Fan Shop	King of Power Saving	Power saving, very good use, much cheaper than the physical store, satisfaction	Jingdong prefecture level city in Shandong
Happy Fan Shop	King of Power Saving	In use, very well packaged and fast delivery of credit!	Jingdong prefecture level city in Shandong
Happy Fan Shop	King of Power Saving	Product quality, customer service attitude is particularly good, like, praise!	Jingdong prefecture level city in Shandong
Happy Fan Shop	King of Power Saving	The logistics is very fast, the packaging is not broken, the quality is very good, I like it very much!	Jingdong prefecture level city in Shandong
Happy Fan Shop	King of Power Saving	The saver was well packaged and was as described when plugged in	Jingdong prefecture level city in Shandong
Happy Fan Shop	King of Power Saving	Energy efficient and very satisfied	Jingdong prefecture level city in Shandong
Happy Fan Shop	King of Power Saving	Great quality for the price and fast shipping.	Jingdong prefecture level city in Shandong
Happy Fan Shop	King of Power Saving	It's great, just as described. Perfect.	Jingdong prefecture level city in Shandong
Happy Fan Shop	King of Power Saving	There is no power saving effect, but rather ring up an increase in electricity costs, because this machine is on continuously all day long, uninterruptedly, its own electricity and increased costs, plus the cost of purchasing the machine, so I feel that the use of this machine instead of increasing the	Jingdong prefecture level city in Shandong
Happy Fan Shop	King of Power Saving	Recommended by the owner of the store next door, looked at his home does have to save electricity, decided to get. Indeed quite can, the voltage is much more stable, there is a reduction in electricity	Jingdong prefecture level city in Shandong
Happy Fan Shop	King of Power Saving	Goods have been received value for money, affordable and reasonable price, and the description of the same, power-saving safe and reliable!	Jingdong prefecture level city in Shandong
Happy Fan Shop	King of Power Saving	Listen to friends introduced to buy, tried to save a lot of electricity is good, quite good, worth buying.	Jingdong prefecture level city in Shandong
Happy Fan Shop	King of Power Saving	The product is easy to use, safe and environmentally friendly, only used for a short time, to the next month, after the electricity bill status display to know how the performance of the power saver efficacy.	Jingdong prefecture level city in Shandong
Happy Fan Shop	King of Power Saving	Last month's electricity bill is 178 yuan, with this power saver is 92 yuan, ready to buy two more back home, I was introduced to buy a friend, just started to not believe that I did not expect the effect!	Jingdong prefecture level city in Shandong
Happy Fan Shop	King of Power Saving	It looks nice, but after using it for a few days I can't see how it saves power?	Jingdong prefecture level city in Shandong
Happy Fan Shop	King of Power Saving	Recommended by a friend, it is genuine, as described by the merchant, cost-effective	Jingdong prefecture level city in Shandong
Happy Fan Shop	King of Power Saving	Works well, affordable and saves money.	Jingdong prefecture level city in Shandong
Happy Fan Shop	King of Power Saving	Feels good haven't compared it yet, the air conditioning has been on a lot in the past two months	Jingdong prefecture level city in Shandong
Happy Fan Shop	King of Power Saving	Saving about 5 kWh per day is a good thing	Jingdong prefecture level city in Shandong
Happy Fan Shop	King of Power Saving	Not bad, affordable, best value for money	Jingdong prefecture level city in Shandong
Happy Fan Shop	King of Power Saving	The goods are genuine and the logistics are faster.	Jingdong prefecture level city in Shandong
Happy Fan Shop	King of Power Saving	Goods have been received today, has been plugged into the outlet, looking forward to saving electricity, after a period of time to see how the effect in the follow-up comment!	Jingdong prefecture level city in Shandong
Happy Fan Shop	King of Power Saving	I just got it home and haven't used it for long, so I'll use it for a couple months and see what happens. Seeing that most people have good reviews, it should be fine.	Jingdong prefecture level city in Shandong
Happy Fan Shop	King of Power Saving	I'm looking forward to the results, so I'll give you a good review out of habit! Follow up review after a while	Jingdong prefecture level city in Shandong
Happy Fan Shop	King of Power Saving	Use a dish first to know if it will help save energy	Jingdong prefecture level city in Shandong
Happy Fan Shop	King of Power Saving	Whether it saves electricity or not, we don't know yet.	Jingdong prefecture level city in Shandong
Happy Fan Shop	King of Power Saving	Goods have been received, use a period of time to know how oh. The courier did not deliver to the door ah! Sent to the station ah! The heat is inconvenient ah!	Jingdong prefecture level city in Shandong
Happy Fan Shop	King of Power Saving	A little effect, before an hour a power, now an hour 0.76 degrees, a dollar a power = save 24 cents, although not a lot, first use it!	Jingdong prefecture level city in Shandong
Happy Fan Shop	King of Power Saving	Just received the goods, use a month to evaluate	Jingdong prefecture level city in Shandong
Happy Fan Shop	King of Power Saving	Home every month with a lot of electricity, inadvertently found this good thing online that can save the beginning of a skeptical attitude to consult customer service, customer service replied very professional, said 120 days can be refunded that first try it!	Jingdong prefecture level city in Shandong

Happy Fan Shop	King of Power Saving	After receiving the goods this afternoon, immediately installed in the bathroom Smith energy storage electric water heater on the power outlet (instead of connecting with a plug board), when used, found that the "Power Saving King" LCD display, is the value of the voltage, not the store's video said so	Jingdong prefecture level city in Shandong
Happy Fan Shop	King of Power Saving	Has made many days, did not feel the power saving, the effect is not obvious.	Jingdong prefecture level city in Shandong
Happy Fan Shop	King of Power Saving	Energy savers do not save energy at all, but rather consume a lot of electricity.	Jingdong prefecture level city in Shandong
Happy Fan Shop	King of Power Saving	It's useless. It's a waste of money.	Jingdong prefecture level city in Shandong
Happy Fan Shop	King of Power Saving	Last month saved more than 500, which came back to buy, the store bought three, and then buy 1 home use, my family is dry beauty salon, more instruments, especially electricity, a month almost 1000. or so, with this did not expect to save so much, great, five-star praise!	Jingdong prefecture level city in Shandong
Happy Fan Shop	King of Power Saving	It rained a couple days ago and I just went to get the item back today , not bad, hey I looked at the meter and that red display light takes several seconds to jump, did not make a mistake buying it. After a period of time to evaluate.	Jingdong prefecture level city in Shandong
Happy Fan Shop	King of Power Saving	Without further ado, the electricity bill came down, this month less 102 yuan, has been repurchased, I also recommended to my friends, all said that there are effects, the product is okay, the effect is quite	Jingdong prefecture level city in Shandong
Happy Fan Shop	King of Power Saving	After buying and using two months really see the effect, so only to evaluate! Originally held the mindset of trying, I did not expect really effective, worth having!	Jingdong prefecture level city in Shandong
Happy Fan Shop	King of Power Saving	In the use of this energy saver before the monthly electricity bill is more than 150 yuan or so, now save thirty percent, every day to work, six or seven o'clock in the afternoon to go home, I do not know why the electricity bill is so expensive, energy-saving appliances really have the effect of recommending	Jingdong prefecture level city in Shandong
Happy Fan Shop	King of Power Saving	It saves about 100 bucks a month. A friend recommended it to me, and he's been using it for a while, and the effect is so obvious that I bought one too.	Jingdong prefecture level city in Shandong
Happy Fan Shop	King of Power Saving	My dad asked me to buy one, but after I paid the bill, I asked and he said it was a lot less this month, so he asked me to buy another one.	Jingdong prefecture level city in Shandong
Happy Fan Shop	King of Power Saving	The specific effect will have to be tested for at least two months to compare whether there is a power saving effect or not.	Jingdong prefecture level city in Shandong
Happy Fan Shop	King of Power Saving	'The goods have been received, immediately go to use, please ask the boss you instructions do not allow to withdraw, there are two fuses installed there!	Jingdong prefecture level city in Shandong
Happy Fan Shop	King of Power Saving	First praise is using, after using if good then follow up, now because I have not yet seen the results	Jingdong prefecture level city in Shandong
Happy Fan Shop	King of Power Saving	I've just been using it, and I haven't seen if it has a power-saving effect yet, so I'll follow up after 10 days and half a month.	Jingdong prefecture level city in Shandong
Happy Fan Shop	King of Power Saving	Just received the intelligent power saving meter, immediately plugged in to use, see it is not bad, and order to sell the second time	Jingdong prefecture level city in Shandong
Happy Fan Shop	King of Power Saving	This power saver is a bit effective, used for half a month, the effect is still visible.	Jingdong prefecture level city in Shandong
Happy Fan Shop	King of Power Saving	I received the baby and it looks great, I'm trying it out now and will keep an eye on it afterward.	Jingdong prefecture level city in Shandong
Happy Fan Shop	King of Power Saving	I've just received it, but I'm still trying to see how it works, so I'll come back in a little while.	Jingdong prefecture level city in Shandong
Happy Fan Shop	King of Power Saving	Just used it wait to use it for a while and then comment thanks, all first five stars.	Jingdong prefecture level city in Shandong
Happy Fan Shop	King of Power Saving	Very trustworthy brand, quality is guaranteed, the price is still affordable, quite satisfied!	Jingdong prefecture level city in Shandong
Happy Fan Shop	King of Power Saving	Things received, I hope that the platform of Jingdong do not sell fake goods are available	Jingdong prefecture level city in Shandong
Happy Fan Shop	King of Power Saving	Energy saver received, the use of the effect is good and the business description of the same	Jingdong prefecture level city in Shandong
Happy Fan Shop	King of Power Saving	The surface looks beautiful, the actual effect is not known, after using additional evaluation.	Jingdong prefecture level city in Shandong
Happy Fan Shop	King of Power Saving	Tried it for a few months, it really saves electricity, and I bought it for a friend.	Jingdong prefecture level city in Shandong
Happy Fan Shop	King of Power Saving	It didn't work and wanted to return it, it really didn't work at all.	Jingdong prefecture level city in Shandong
Happy Fan Shop	King of Power Saving	Just used it, don't know how effective it is, looking forward to usefulness	Jingdong prefecture level city in Shandong
Happy Fan Shop	King of Power Saving	After two months before the effect I do not know if it is true!	Jingdong prefecture level city in Shandong
Happy Fan Shop	King of Power Saving	So fast! Ordered yesterday and received today 👍👍👍👍👍👍👍👍👍 🌹🌹🌹🌹🌹🌹🌹🌹🌹	Jingdong prefecture level city in Shandong
Happy Fan Shop	King of Power Saving	I've started using it. We'll see how it works.	Jingdong prefecture level city in Shandong
Happy Fan Shop	King of Power Saving	I just received the goods, I won't know the effect until after two.	Jingdong prefecture level city in Shandong
Happy Fan Shop	King of Power Saving	Just received the goods, the specific effect will be evaluated later	Jingdong prefecture level city in Shandong
Happy Fan Shop	King of Power Saving	I've received the goods, I don't know if it's any good yet	Jingdong prefecture level city in Shandong
Happy Fan Shop	King of Power Saving	Try it for a month and then come back with a concrete evaluation	Jingdong prefecture level city in Shandong
Happy Fan Shop	King of Power Saving	In use, after two months to see the effect of power saving	Jingdong prefecture level city in Shandong
Happy Fan Shop	King of Power Saving	Power Saver. It's authentic. It's black. It works.	Jingdong prefecture level city in Shandong
Happy Fan Shop	King of Power Saving	I've been using it for a week now and it's not working.	Jingdong prefecture level city in Shandong
Happy Fan Shop	King of Power Saving	I'm just using it. I don't know how it's going to work.	Jingdong prefecture level city in Shandong
Happy Fan Shop	King of Power Saving	Products are better looking out for power savings	Jingdong prefecture level city in Shandong
Happy Fan Shop	King of Power Saving	I received it and I've kept it on. I hope it works.	Jingdong prefecture level city in Shandong
Happy Fan Shop	King of Power Saving	It's really good! Worth recommending to buy!	Jingdong prefecture level city in Shandong
Happy Fan Shop	King of Power Saving	Try it for a few days and see if it doesn't work, then return it.	Jingdong prefecture level city in Shandong
Happy Fan Shop	King of Power Saving	It's in use and I hope to be pleasantly surprised.	Jingdong prefecture level city in Shandong
Happy Fan Shop	King of Power Saving	We'll see how it works when we use it	Jingdong prefecture level city in Shandong
Happy Fan Shop	King of Power Saving	Let's see if it works.	Jingdong prefecture level city in Shandong
Happy Fan Shop	King of Power Saving	I'm using it and will follow up with a review	Jingdong prefecture level city in Shandong
Happy Fan Shop	King of Power Saving	It really works. It saves energy.	Jingdong prefecture level city in Shandong
Happy Fan Shop	King of Power Saving	It does have some power savings!	Jingdong prefecture level city in Shandong
Happy Fan Shop	King of Power Saving	Jingdong shopping speed is convenient and timely	Jingdong prefecture level city in Shandong

Happy Fan Shop	King of Power Saving	Second time I've bought it. Everything's fine.	Jingdong prefecture level city in Shandong
Happy Fan Shop	King of Power Saving	I don't know if it works yet.	Jingdong prefecture level city in Shandong
Happy Fan Shop	King of Power Saving	Not used, don't know how well it works.	Jingdong prefecture level city in Shandong
Happy Fan Shop	King of Power Saving	No visible results so far	Jingdong prefecture level city in Shandong
Happy Fan Shop	King of Power Saving	It doesn't feel like it's working.	Jingdong prefecture level city in Shandong
Happy Fan Shop	King of Power Saving	Where's the effect? Where are the power savings?	Jingdong prefecture level city in Shandong
Happy Fan Shop	King of Power Saving	Let's see how it works in practice!	Jingdong prefecture level city in Shandong
Happy Fan Shop	King of Power Saving	I'll wait until I use it to give you a good review	Jingdong prefecture level city in Shandong
Happy Fan Shop	King of Power Saving	I'll follow up after I've used it	Jingdong prefecture level city in Shandong
Happy Fan Shop	King of Power Saving	Well, that's good for saving power.	Jingdong prefecture level city in Shandong
Happy Fan Shop	King of Power Saving	Received. Used in the know.	Jingdong prefecture level city in Shandong
Happy Fan Shop	King of Power Saving	It doesn't do much good.	Jingdong prefecture level city in Shandong
Happy Fan Shop	King of Power Saving	It's not working.	Jingdong prefecture level city in Shandong
Happy Fan Shop	King of Power Saving	I don't know. I just used it.	Jingdong prefecture level city in Shandong
Happy Fan Shop	King of Power Saving	Use it and come back to evaluate.	Jingdong prefecture level city in Shandong
Happy Fan Shop	King of Power Saving	Two words: useless.	Jingdong prefecture level city in Shandong
Happy Fan Shop	King of Power Saving	To be used and then evaluated	Jingdong prefecture level city in Shandong
Happy Fan Shop	King of Power Saving	No shipment.	Jingdong prefecture level city in Shandong
Happy Fan Shop	King of Power Saving	Fake. Speechless.	Jingdong prefecture level city in Shandong
Happy Fan Shop	King of Power Saving	fantastic (idiom); awesome	Jingdong prefecture level city in Shandong
Happy Fan Shop	King of Power Saving	an excellent	Jingdong prefecture level city in Shandong
Happy Fan Shop	King of Power Saving	an excellent	Jingdong prefecture level city in Shandong
Happy Fan Shop	King of Power Saving	Want to buy! Recommended not to buy! No effect	Jingdong prefecture level city in Shandong
Happy Fan Shop	King of Power Saving	That's all there is to it. I can't figure out how to save electricity.	Jingdong prefecture level city in Shandong
Happy Fan Shop	King of Power Saving	It's no use.	Jingdong prefecture level city in Shandong
Happy Fan Shop	King of Power Saving	Things are more perfect than I imagined, very powerful, it is worth getting Packaging is very strong, things are well protected, it is worth the praise things are very satisfied Oh, beautiful and stylish atmosphere, customer service is super patient, service is meticulous and attentive, the big brand is not	Jingdong prefecture level city in Shandong
Happy Fan Shop	King of Power Saving	The effect is too good, the family has now several units, back to buy a number of times, the power saver received, power-saving effect is very good,, fine workmanship, long warranty, but also purify the air, before buying a used for a period of time, with the okay, and then bought a, and only now come to the	Jingdong prefecture level city in Shandong
Happy Fan Shop	King of Power Saving	Quite good, and imagine the same, casual ride are very good, satisfied! Baby received it 👌, really great ah! 🌈 quality is really nothing to say, very satisfied! 🆗 happy happy 😃 genuine, great, the price is very good, in short, quite good, will buy again a very good shopping experience, attentive service, fast logistics, distribution is also thoughtful, the attitude is also very good, value for money, ah,	Jingdong prefecture level city in Shandong
Happy Fan Shop	King of Power Saving	Power-saving effect: strong Cost-effective: quite good Appearance style: beautiful Workmanship material: meticulous Experience: looking forward to power-saving effect	Jingdong prefecture level city in Shandong
Happy Fan Shop	King of Power Saving	Cost-effective is okay, the appearance of the style of meticulous workmanship, looking forward to energy-saving effect.	Jingdong prefecture level city in Shandong
Happy Fan Shop	King of Power Saving	Very Good Very Power Saving This Power Saver is really power saving As soon as the power is turned on, the Power Saver starts the power saving function for you.	Jingdong prefecture level city in Shandong
Happy Fan Shop	King of Power Saving	Logistics is very fast, the price is favorable, friends introduced that it is very good to use, bought 2 at once, looking forward to the effect, used after the follow-up comment.	Jingdong prefecture level city in Shandong
Happy Fan Shop	King of Power Saving	It's all fake. It doesn't save any electricity.	Jingdong prefecture level city in Shandong
Happy Fan Shop	King of Power Saving	This Power Saver is so much better than the one I bought before, not only the effect is good, the quality is very good in all aspects, the customer service attitude is also very good, recommended to buy!	Jingdong prefecture level city in Shandong
Happy Fan Shop	King of Power Saving	Logistics is very fast, buy for parents, good quality, once bought 2, power-saving effect is good to buy 2 more, look forward to using the effect, satisfied will recommend to friends.	Jingdong prefecture level city in Shandong
Happy Fan Shop	King of Power Saving	With the description is the same, very high-grade, good value, to buy friends can order Oh!	Jingdong prefecture level city in Shandong
Happy Fan Shop	King of Power Saving	Not using much electricity in the winter saving over 100 per month! Ready to buy 2 more units in the summer ！！！！！ Good quality, good price, power saving effect is obvious, cost-effective, worth buying, can be recommended!	Jingdong prefecture level city in Shandong
Happy Fan Shop	King of Power Saving	Purchased a power saver received on the put into use, rent a room with many electrical appliances, power consumption is very large, we have a piece of electricity or 1.2 dollars a time, with this power saver is much better, every day can save a lot of money, very convenient and practical.	Jingdong prefecture level city in Shandong
Happy Fan Shop	King of Power Saving	The power saving effect is very good: my supermarket monthly electricity bill is more than 7000 yuan. On May 1st someone recommended buying a couple of power savers, and I bought 5 of them first. The electricity bill was about 2000 less in June. This time, I bought 5 more. The seller said 800 degrees of electricity with one. I feel it's basically enough. A total of 10 units	Jingdong prefecture level city in Shandong
Happy Fan Shop	King of Power Saving	I never thought I could get such a good baby at such an affordable price, it's very good value for money, I love it!	Jingdong prefecture level city in Shandong
Happy Fan Shop	King of Power Saving	Previously bought other brands of power savers, but found no effect at home, this time to see how the products on the Jingdong, inserted in the home socket, the LED display is blue, the night light is very nice to see, look at the instructions that have to be half a year in order to achieve the best results, try it, I believe that the quality of the Jingdong, it should not be wrong.	Jingdong prefecture level city in Shandong
Happy Fan Shop	King of Power Saving	Superb, inexpensive, saves a lot of points, been using it for a long time!	Jingdong prefecture level city in Shandong
Happy Fan Shop	King of Power Saving	There are a lot of appliances in the house, the summer air conditioning is very costly, I bought this in front of the air conditioning effect is very good, I explain that I bought three in one breath, I use more electricity a month down to 200 or so, very satisfied.	Jingdong prefecture level city in Shandong
Happy Fan Shop	King of Power Saving	A month can save more than 200, home monthly electricity bill of more than 600 dollars, inserted 3 or so, an amount of the balance of the average can save more than 200. Product is very good, has been buying this one, has given a lot of relatives recommended, the quality is also very good, before buying a look with this same, who knows to buy home has not been used for more than a week on the bad, the quality of this is really good with more than two years!	Jingdong prefecture level city in Shandong

Happy Fan Shop	King of Power Saving	Whole house power saving king, can really save electricity, friends recommended, high power saving appliances, only replacement, no repair, after-sales worry-free!	Jingdong prefecture level city in Shandong
Happy Fan Shop	King of Power Saving	Great item love it, great customer service, great shopping experience!	Jingdong prefecture level city in Shandong
Happy Fan Shop	King of Power Saving	Power saving, very good use, much cheaper than the physical store, satisfaction	Jingdong prefecture level city in Shandong
Happy Fan Shop	King of Power Saving	In use, very well packaged and fast delivery of credit!	Jingdong prefecture level city in Shandong
Happy Fan Shop	King of Power Saving	Product quality, customer service attitude is particularly good, like, praise!	Jingdong prefecture level city in Shandong
Happy Fan Shop	King of Power Saving	The logistics is very fast, the packaging is not broken, the quality is very good, I like it very much!	Jingdong prefecture level city in Shandong
Happy Fan Shop	King of Power Saving	The saver was well packaged and was as described when plugged in	Jingdong prefecture level city in Shandong
Happy Fan Shop	King of Power Saving	Energy efficient and very satisfied	Jingdong prefecture level city in Shandong
Happy Fan Shop	King of Power Saving	Great quality for the price and fast shipping.	Jingdong prefecture level city in Shandong
Happy Fan Shop	King of Power Saving	It's great, just as described. Perfect.	Jingdong prefecture level city in Shandong
Happy Fan Shop	King of Power Saving	There is no power saving effect, but rather ring up an increase in electricity costs, because this machine is on continuously all day long, uninterruptedly, its own electricity and increased costs, plus the cost of purchasing the machine, so I feel that the use of this machine instead of increasing the	Jingdong prefecture level city in Shandong
Happy Fan Shop	King of Power Saving	Recommended by the owner of the store next door, looked at his home does have to save electricity, decided to get. Indeed quite can, the voltage is much more stable, there is a reduction in electricity	Jingdong prefecture level city in Shandong
Happy Fan Shop	King of Power Saving	Goods have been received value for money, affordable and reasonable price, and the description of the same, power-saving safe and reliable!	Jingdong prefecture level city in Shandong
Happy Fan Shop	King of Power Saving	Listen to friends introduced to buy, tried to save a lot of electricity is good, quite good, worth buying.	Jingdong prefecture level city in Shandong
Happy Fan Shop	King of Power Saving	The product is easy to use, safe and environmentally friendly, only used for a short time, to the next month, after the electricity bill status display to know how the performance of the power saver efficacy.	Jingdong prefecture level city in Shandong
Happy Fan Shop	King of Power Saving	Last month's electricity bill is 178 yuan, with this power saver is 92 yuan, ready to buy two more back home, I was introduced to buy a friend, just started to not believe that I did not expect the effect!	Jingdong prefecture level city in Shandong
Happy Fan Shop	King of Power Saving	It looks nice, but after using it for a few days I can't see how it saves power?	Jingdong prefecture level city in Shandong
Happy Fan Shop	King of Power Saving	Recommended by a friend, it is genuine, as described by the merchant, cost-effective	Jingdong prefecture level city in Shandong
Happy Fan Shop	King of Power Saving	Works well, affordable and saves money.	Jingdong prefecture level city in Shandong
Happy Fan Shop	King of Power Saving	Feels good haven't compared it yet, the air conditioning has been on a lot in the past two months	Jingdong prefecture level city in Shandong
Happy Fan Shop	King of Power Saving	Saving about 5 kWh per day is a good thing	Jingdong prefecture level city in Shandong
Happy Fan Shop	King of Power Saving	Not bad, affordable, best value for money	Jingdong prefecture level city in Shandong
Happy Fan Shop	King of Power Saving	The goods are genuine and the logistics are faster.	Jingdong prefecture level city in Shandong
Happy Fan Shop	King of Power Saving	Goods have been received today, has been plugged into the outlet, looking forward to saving electricity, after a period of time to see how the effect in the follow-up comment!	Jingdong prefecture level city in Shandong
Happy Fan Shop	King of Power Saving	I just got it home and haven't used it for long, so I'll use it for a couple months and see what happens. Seeing that most people have good reviews, it should be fine.	Jingdong prefecture level city in Shandong
Happy Fan Shop	King of Power Saving	I'm looking forward to the results, so I'll give you a good review out of habit! Follow up review after a while	Jingdong prefecture level city in Shandong
Happy Fan Shop	King of Power Saving	Use a dish first to know if it will help save energy	Jingdong prefecture level city in Shandong
Happy Fan Shop	King of Power Saving	Whether it saves electricity or not, we don't know yet.	Jingdong prefecture level city in Shandong
Happy Fan Shop	King of Power Saving	Goods have been received, use a period of time to know how oh. The courier did not deliver to the door ah! Sent to the station ah! The heat is inconvenient ah!	Jingdong prefecture level city in Shandong
Happy Fan Shop	King of Power Saving	A little effect, before an hour a power, now an hour 0.76 degrees, a dollar a power = save 24 cents, although not a lot, first use it!	Jingdong prefecture level city in Shandong
Happy Fan Shop	King of Power Saving	Just received the goods, use a month to evaluate	Jingdong prefecture level city in Shandong
Happy Fan Shop	King of Power Saving	Home every month with a lot of electricity, inadvertently found this good thing online that can save the beginning of a skeptical attitude to consult customer service, customer service replied very professional, said 120 days can be refunded that first try it!	Jingdong prefecture level city in Shandong
Happy Fan Shop	King of Power Saving	After receiving the goods this afternoon, immediately installed in the bathroom Smith energy storage electric water heater on the power outlet (instead of connecting with a plug board), when used, found that the "Power Saving King" LCD display, is the value of the voltage, not the store's video said so	Jingdong prefecture level city in Shandong
Happy Fan Shop	King of Power Saving	Has made many days, did not feel the power saving, the effect is not obvious.	Jingdong prefecture level city in Shandong
Happy Fan Shop	King of Power Saving	Energy savers do not save energy at all, but rather consume a lot of electricity.	Jingdong prefecture level city in Shandong
Happy Fan Shop	King of Power Saving	It's useless. It's a waste of money.	Jingdong prefecture level city in Shandong
Happy Fan Shop	King of Power Saving	Last month saved more than 500, which came back to buy, the store bought three, and then buy 1 home use, my family is dry beauty salon, more instruments, especially electricity, a month almost 1000. or so, with this did not expect to save so much, great, five-star praise!	Jingdong prefecture level city in Shandong
Happy Fan Shop	King of Power Saving	It rained a couple days ago and I just went to get the item back today , not bad, hey I looked at the meter and that red display light takes several seconds to jump, did not make a mistake buying it. After a period of time to evaluate.	Jingdong prefecture level city in Shandong
Happy Fan Shop	King of Power Saving	Without further ado, the electricity bill came down, this month less 102 yuan, has been repurchased, I also recommended to my friends, all said that there are effects, the product is okay, the effect is quite	Jingdong prefecture level city in Shandong
Happy Fan Shop	King of Power Saving	After buying and using two months really see the effect, so only to evaluate! Originally held the mindset of trying, I did not expect really effective, worth having!	Jingdong prefecture level city in Shandong
Happy Fan Shop	King of Power Saving	In the use of this energy saver before the monthly electricity bill is more than 150 yuan or so, now save thirty percent, every day to work, six or seven o'clock in the afternoon to go home, I do not know why the electricity bill is so expensive, energy-saving appliances really have the effect of recommending	Jingdong prefecture level city in Shandong
Happy Fan Shop	King of Power Saving	It saves about 100 bucks a month. A friend recommended it to me, and he's been using it for a while, and the effect is so obvious that I bought one too.	Jingdong prefecture level city in Shandong
Happy Fan Shop	King of Power Saving	My dad asked me to buy one, but after I paid the bill, I asked and he said it was a lot less this month, so he asked me to buy another one.	Jingdong prefecture level city in Shandong
Happy Fan Shop	King of Power Saving	The specific effect will have to be tested for at least two months to compare whether there is a power saving effect or not.	Jingdong prefecture level city in Shandong
Happy Fan Shop	King of Power Saving	'The goods have been received, immediately go to use, please ask the boss you instructions do not allow to withdraw, there are two fuses installed there!	Jingdong prefecture level city in Shandong
Happy Fan Shop	King of Power Saving	First praise is using, after using if good then follow up, now because I have not yet seen the results	Jingdong prefecture level city in Shandong
Happy Fan Shop	King of Power Saving	I've just been using it, and I haven't seen if it has a power-saving effect yet, so I'll follow up after 10 days and half a month.	Jingdong prefecture level city in Shandong
Happy Fan Shop	King of Power Saving	Just received the intelligent power saving meter, immediately plugged in to use, see it is not bad, and order to sell the second time	Jingdong prefecture level city in Shandong

Happy Fan Shop	King of Power Saving	This power saver is a bit effective, used for half a month, the effect is still visible.	Jingdong prefecture level city in Shandong
Happy Fan Shop	King of Power Saving	I received the baby and it looks great, I'm trying it out now and will keep an eye on it afterward.	Jingdong prefecture level city in Shandong
Happy Fan Shop	King of Power Saving	I've just received it, but I'm still trying to see how it works, so I'll come back in a little while.	Jingdong prefecture level city in Shandong
Happy Fan Shop	King of Power Saving	Just used it wait to use it for a while and then comment thanks, all first five stars.	Jingdong prefecture level city in Shandong
Happy Fan Shop	King of Power Saving	Very trustworthy brand, quality is guaranteed, the price is still affordable, quite satisfied!	Jingdong prefecture level city in Shandong
Happy Fan Shop	King of Power Saving	Things received, I hope that the platform of Jingdong do not sell fake goods are available	Jingdong prefecture level city in Shandong
Happy Fan Shop	King of Power Saving	Energy saver received, the use of the effect is good and the business description of the same	Jingdong prefecture level city in Shandong
Happy Fan Shop	King of Power Saving	The surface looks beautiful, the actual effect is not known, after using additional evaluation.	Jingdong prefecture level city in Shandong
Happy Fan Shop	King of Power Saving	Tried it for a few months, it really saves electricity, and I bought it for a friend.	Jingdong prefecture level city in Shandong
Happy Fan Shop	King of Power Saving	It didn't work and wanted to return it, it really didn't work at all.	Jingdong prefecture level city in Shandong
Happy Fan Shop	King of Power Saving	Just used it, don't know how effective it is, looking forward to usefulness	Jingdong prefecture level city in Shandong
Happy Fan Shop	King of Power Saving	After two months before the effect I do not know if it is true!	Jingdong prefecture level city in Shandong
Happy Fan Shop	King of Power Saving	So fast! Ordered yesterday and received today 👍👍👍👍👍👍👍👍👍 🌹🌹🌹🌹🌹🌹🌹🌹🌹	Jingdong prefecture level city in Shandong
Happy Fan Shop	King of Power Saving	I've started using it. We'll see how it works.	Jingdong prefecture level city in Shandong
Happy Fan Shop	King of Power Saving	I just received the goods, I won't know the effect until after two.	Jingdong prefecture level city in Shandong
Happy Fan Shop	King of Power Saving	Just received the goods, the specific effect will be evaluated later	Jingdong prefecture level city in Shandong
Happy Fan Shop	King of Power Saving	I've received the goods, I don't know if it's any good yet	Jingdong prefecture level city in Shandong
Happy Fan Shop	King of Power Saving	Try it for a month and then come back with a concrete evaluation	Jingdong prefecture level city in Shandong
Happy Fan Shop	King of Power Saving	In use, after two months to see the effect of power saving	Jingdong prefecture level city in Shandong
Happy Fan Shop	King of Power Saving	Power Saver. It's authentic. It's black. It works.	Jingdong prefecture level city in Shandong
Happy Fan Shop	King of Power Saving	I've been using it for a week now and it's not working.	Jingdong prefecture level city in Shandong
Happy Fan Shop	King of Power Saving	I'm just using it. I don't know how it's going to work.	Jingdong prefecture level city in Shandong
Happy Fan Shop	King of Power Saving	Products are better looking out for power savings	Jingdong prefecture level city in Shandong
Happy Fan Shop	King of Power Saving	I received it and I've kept it on. I hope it works.	Jingdong prefecture level city in Shandong
Happy Fan Shop	King of Power Saving	It's really good! Worth recommending to buy!	Jingdong prefecture level city in Shandong
Happy Fan Shop	King of Power Saving	Try it for a few days and see if it doesn't work, then return it.	Jingdong prefecture level city in Shandong
Happy Fan Shop	King of Power Saving	It's in use and I hope to be pleasantly surprised.	Jingdong prefecture level city in Shandong
Happy Fan Shop	King of Power Saving	We'll see how it works when we use it	Jingdong prefecture level city in Shandong
Happy Fan Shop	King of Power Saving	Let's see if it works.	Jingdong prefecture level city in Shandong
Happy Fan Shop	King of Power Saving	I'm using it and will follow up with a review	Jingdong prefecture level city in Shandong
Happy Fan Shop	King of Power Saving	It really works. It saves energy.	Jingdong prefecture level city in Shandong
Happy Fan Shop	King of Power Saving	It does have some power savings!	Jingdong prefecture level city in Shandong
Happy Fan Shop	King of Power Saving	Jingdong shopping speed is convenient and timely	Jingdong prefecture level city in Shandong
Happy Fan Shop	King of Power Saving	Second time I've bought it. Everything's fine.	Jingdong prefecture level city in Shandong
Happy Fan Shop	King of Power Saving	I don't know if it works yet.	Jingdong prefecture level city in Shandong
Happy Fan Shop	King of Power Saving	Not used, don't know how well it works.	Jingdong prefecture level city in Shandong
Happy Fan Shop	King of Power Saving	No visible results so far	Jingdong prefecture level city in Shandong
Happy Fan Shop	King of Power Saving	It doesn't feel like it's working.	Jingdong prefecture level city in Shandong
Happy Fan Shop	King of Power Saving	Where's the effect? Where are the power savings?	Jingdong prefecture level city in Shandong
Happy Fan Shop	King of Power Saving	Let's see how it works in practice!	Jingdong prefecture level city in Shandong
Happy Fan Shop	King of Power Saving	I'll wait until I use it to give you a good review	Jingdong prefecture level city in Shandong
Happy Fan Shop	King of Power Saving	I'll follow up after I've used it	Jingdong prefecture level city in Shandong
Happy Fan Shop	King of Power Saving	Well, that's good for saving power.	Jingdong prefecture level city in Shandong
Happy Fan Shop	King of Power Saving	Received. Used in the know.	Jingdong prefecture level city in Shandong
Happy Fan Shop	King of Power Saving	It doesn't do much good.	Jingdong prefecture level city in Shandong
Happy Fan Shop	King of Power Saving	It's not working.	Jingdong prefecture level city in Shandong
Happy Fan Shop	King of Power Saving	I don't know. I just used it.	Jingdong prefecture level city in Shandong
Happy Fan Shop	King of Power Saving	Use it and come back to evaluate.	Jingdong prefecture level city in Shandong
Happy Fan Shop	King of Power Saving	Two words: useless.	Jingdong prefecture level city in Shandong
Happy Fan Shop	King of Power Saving	To be used and then evaluated	Jingdong prefecture level city in Shandong
Happy Fan Shop	King of Power Saving	No shipment.	Jingdong prefecture level city in Shandong

Happy Fan Shop	King of Power Saving	Fake. Speechless.	Jingdong prefecture level city in Shandong
Happy Fan Shop	King of Power Saving	fantastic (idiom); awesome	Jingdong prefecture level city in Shandong
Happy Fan Shop	King of Power Saving	an excellent	Jingdong prefecture level city in Shandong
Happy Fan Shop	King of Power Saving	an excellent	Jingdong prefecture level city in Shandong
Happy Fan Shop	King of Power Saving	Want to buy! Recommended not to buy! No effect	Jingdong prefecture level city in Shandong
Happy Fan Shop	King of Power Saving	That's all there is to it. I can't figure out how to save electricity.	Jingdong prefecture level city in Shandong
Happy Fan Shop	King of Power Saving	It's no use.	Jingdong prefecture level city in Shandong
Happy Fan Shop	King of Power Saving	Things are more perfect than I imagined, very powerful, it is worth getting Packaging is very strong, things are well protected, it is worth the praise things are very satisfied Oh, beautiful and stylish atmosphere, customer service is super patient, service is meticulous and attentive, the big brand is not	Jingdong prefecture level city in Shandong
Happy Fan Shop	King of Power Saving	The effect is too good, the family has now several units, back to buy a number of times, the power saver received, power-saving effect is very good,, fine workmanship, long warranty, but also purify the air, before buying a used for a period of time, with the okay, and then bought a, and only now come to the	Jingdong prefecture level city in Shandong
Happy Fan Shop	King of Power Saving	Quite good, and imagine the same, casual ride are very good, satisfied! Baby received it 👌, really great ah! 🌈 quality is really nothing to say, very satisfied! 🆗 happy happy 😃 genuine, great, the price is very good, in short, quite good, will buy again a very good shopping experience, attentive service, fast logistics, distribution is also thoughtful, the attitude is also very good, value for money, ah,	Jingdong prefecture level city in Shandong
Happy Fan Shop	King of Power Saving	Power-saving effect: strong Cost-effective: quite good Appearance style: beautiful Workmanship material: meticulous Experience: looking forward to power-saving effect	Jingdong prefecture level city in Shandong
Happy Fan Shop	King of Power Saving	Cost-effective is okay, the appearance of the style of meticulous workmanship, looking forward to energy-saving effect.	Jingdong prefecture level city in Shandong
Happy Fan Shop	King of Power Saving	Very Good Very Power Saving This Power Saver is really power saving As soon as the power is turned on, the Power Saver starts the power saving function for you.	Jingdong prefecture level city in Shandong
Happy Fan Shop	King of Power Saving	Logistics is very fast, the price is favorable, friends introduced that it is very good to use, bought 2 at once, looking forward to the effect, used after the follow-up comment.	Jingdong prefecture level city in Shandong
Happy Fan Shop	King of Power Saving	It's all fake. It doesn't save any electricity.	Jingdong prefecture level city in Shandong
Happy Fan Shop	King of Power Saving	This Power Saver is so much better than the one I bought before, not only the effect is good, the quality is very good in all aspects, the customer service attitude is also very good, recommended to buy!	Jingdong prefecture level city in Shandong
Happy Fan Shop	King of Power Saving	Logistics is very fast, buy for parents, good quality, once bought 2, power-saving effect is good to buy 2 more, look forward to using the effect, satisfied will recommend to friends.	Jingdong prefecture level city in Shandong
Happy Fan Shop	King of Power Saving	With the description is the same, very high-grade, good value, to buy friends can order Oh!	Jingdong prefecture level city in Shandong
Happy Fan Shop	King of Power Saving	Not using much electricity in the winter saving over 100 per month! Ready to buy 2 more units in the summer ！！！！！ Good quality, good price, power saving effect is obvious, cost-effective, worth buying, can be recommended!	Jingdong prefecture level city in Shandong
Happy Fan Shop	King of Power Saving	Purchased a power saver received on the put into use, rent a room with many electrical appliances, power consumption is very large, we have a piece of electricity or 1.2 dollars a time, with this power saver is much better, every day can save a lot of money, very convenient and practical.	Jingdong prefecture level city in Shandong
Happy Fan Shop	King of Power Saving	The power saving effect is very good: my supermarket monthly electricity bill is more than 7000 yuan. On May 1st someone recommended buying a couple of power savers, and I bought 5 of them first. The electricity bill was about 2000 less in June. This time, I bought 5 more. The seller said 800 degrees of electricity with one. I feel it's basically enough. A total of 10 units	Jingdong prefecture level city in Shandong
Happy Fan Shop	King of Power Saving	I never thought I could get such a good baby at such an affordable price, it's very good value for money, I love it!	Jingdong prefecture level city in Shandong
Happy Fan Shop	King of Power Saving	Previously bought other brands of power savers, but found no effect at home, this time to see how the products on the Jingdong, inserted in the home socket, the LED display is blue, the night light is very nice to see, look at the instructions that have to be half a year in order to achieve the best results, try it, I believe that the quality of the Jingdong, it should not be wrong.	Jingdong prefecture level city in Shandong
Happy Fan Shop	King of Power Saving	Superb, inexpensive, saves a lot of points, been using it for a long time!	Jingdong prefecture level city in Shandong
Happy Fan Shop	King of Power Saving	There are a lot of appliances in the house, the summer air conditioning is very costly, I bought this in front of the air conditioning effect is very good, I explain that I bought three in one breath, I use more electricity a month down to 200 or so, very satisfied.	Jingdong prefecture level city in Shandong
Happy Fan Shop	King of Power Saving	A month can save more than 200, home monthly electricity bill of more than 600 dollars, inserted 3 or so, an amount of the balance of the average can save more than 200. Product is very good, has been buying this one, has given a lot of relatives recommended, the quality is also very good, before buying a look with this same, who knows to buy home has not been used for more than a week on the bad, the quality of this is really good with more than two years!	Jingdong prefecture level city in Shandong
Happy Fan Shop	King of Power Saving	Whole house power saving king, can really save electricity, friends recommended, high power saving appliances, only replacement, no repair, after-sales worry-free!	Jingdong prefecture level city in Shandong
Happy Fan Shop	King of Power Saving	Great item love it, great customer service, great shopping experience!	Jingdong prefecture level city in Shandong
Happy Fan Shop	King of Power Saving	Power saving, very good use, much cheaper than the physical store, satisfaction	Jingdong prefecture level city in Shandong
Happy Fan Shop	King of Power Saving	In use, very well packaged and fast delivery of credit!	Jingdong prefecture level city in Shandong
Happy Fan Shop	King of Power Saving	Product quality, customer service attitude is particularly good, like, praise!	Jingdong prefecture level city in Shandong
Happy Fan Shop	King of Power Saving	The logistics is very fast, the packaging is not broken, the quality is very good, I like it very much!	Jingdong prefecture level city in Shandong
Happy Fan Shop	King of Power Saving	The saver was well packaged and was as described when plugged in	Jingdong prefecture level city in Shandong
Happy Fan Shop	King of Power Saving	Energy efficient and very satisfied	Jingdong prefecture level city in Shandong
Happy Fan Shop	King of Power Saving	Great quality for the price and fast shipping.	Jingdong prefecture level city in Shandong
Happy Fan Shop	King of Power Saving	It's great, just as described. Perfect.	Jingdong prefecture level city in Shandong
Happy Fan Shop	King of Power Saving	There is no power saving effect, but rather ring up an increase in electricity costs, because this machine is on continuously all day long, uninterruptedly, its own electricity and increased costs, plus the cost of purchasing the machine, so I feel that the use of this machine instead of increasing the	Jingdong prefecture level city in Shandong
Happy Fan Shop	King of Power Saving	Recommended by the owner of the store next door, looked at his home does have to save electricity, decided to get. Indeed quite can, the voltage is much more stable, there is a reduction in electricity	Jingdong prefecture level city in Shandong
Happy Fan Shop	King of Power Saving	Goods have been received value for money, affordable and reasonable price, and the description of the same, power-saving safe and reliable!	Jingdong prefecture level city in Shandong
Happy Fan Shop	King of Power Saving	Listen to friends introduced to buy, tried to save a lot of electricity is good, quite good, worth buying.	Jingdong prefecture level city in Shandong
Happy Fan Shop	King of Power Saving	The product is easy to use, safe and environmentally friendly, only used for a short time, to the next month, after the electricity bill status display to know how the performance of the power saver efficacy.	Jingdong prefecture level city in Shandong
Happy Fan Shop	King of Power Saving	Last month's electricity bill is 178 yuan, with this power saver is 92 yuan, ready to buy two more back home, I was introduced to buy a friend, just started to not believe that I did not expect the effect!	Jingdong prefecture level city in Shandong
Happy Fan Shop	King of Power Saving	It looks nice, but after using it for a few days I can't see how it saves power?	Jingdong prefecture level city in Shandong
Happy Fan Shop	King of Power Saving	Recommended by a friend, it is genuine, as described by the merchant, cost-effective	Jingdong prefecture level city in Shandong

Happy Fan Shop	King of Power Saving	Works well, affordable and saves money.	Jingdong prefecture level city in Shandong
Happy Fan Shop	King of Power Saving	Feels good haven't compared it yet, the air conditioning has been on a lot in the past two months	Jingdong prefecture level city in Shandong
Happy Fan Shop	King of Power Saving	Saving about 5 kWh per day is a good thing	Jingdong prefecture level city in Shandong
Happy Fan Shop	King of Power Saving	Not bad, affordable, best value for money	Jingdong prefecture level city in Shandong
Happy Fan Shop	King of Power Saving	The goods are genuine and the logistics are faster.	Jingdong prefecture level city in Shandong
Happy Fan Shop	King of Power Saving	Goods have been received today, has been plugged into the outlet, looking forward to saving electricity, after a period of time to see how the effect in the follow-up comment!	Jingdong prefecture level city in Shandong
Happy Fan Shop	King of Power Saving	I just got it home and haven't used it for long, so I'll use it for a couple months and see what happens. Seeing that most people have good reviews, it should be fine.	Jingdong prefecture level city in Shandong
Happy Fan Shop	King of Power Saving	I'm looking forward to the results, so I'll give you a good review out of habit! Follow up review after a while	Jingdong prefecture level city in Shandong
Happy Fan Shop	King of Power Saving	Use a dish first to know if it will help save energy	Jingdong prefecture level city in Shandong
Happy Fan Shop	King of Power Saving	Whether it saves electricity or not, we don't know yet.	Jingdong prefecture level city in Shandong
Happy Fan Shop	King of Power Saving	Goods have been received, use a period of time to know how oh. The courier did not deliver to the door ah! Sent to the station ah! The heat is inconvenient ah!	Jingdong prefecture level city in Shandong
Happy Fan Shop	King of Power Saving	A little effect, before an hour a power, now an hour 0.76 degrees, a dollar a power = save 24 cents, although not a lot, first use it!	Jingdong prefecture level city in Shandong
Happy Fan Shop	King of Power Saving	Just received the goods, use a month to evaluate	Jingdong prefecture level city in Shandong
Happy Fan Shop	King of Power Saving	Home every month with a lot of electricity, inadvertently found this good thing online that can save the beginning of a skeptical attitude to consult customer service, customer service replied very professional, said 120 days can be refunded that first try it!	Jingdong prefecture level city in Shandong
Happy Fan Shop	King of Power Saving	After receiving the goods this afternoon, immediately installed in the bathroom Smith energy storage electric water heater on the power outlet (instead of connecting with a plug board), when used, found that the "Power Saving King" LCD display, is the value of the voltage, not the store's video said so	Jingdong prefecture level city in Shandong
Happy Fan Shop	King of Power Saving	Has made many days, did not feel the power saving, the effect is not obvious.	Jingdong prefecture level city in Shandong
Happy Fan Shop	King of Power Saving	Energy savers do not save energy at all, but rather consume a lot of electricity.	Jingdong prefecture level city in Shandong
Happy Fan Shop	King of Power Saving	It's useless. It's a waste of money.	Jingdong prefecture level city in Shandong
Happy Fan Shop	King of Power Saving	Last month saved more than 500, which came back to buy, the store bought three, and then buy 1 home use, my family is dry beauty salon, more instruments, especially electricity, a month almost 1000. or so, with this did not expect to save so much, great, five-star praise!	Jingdong prefecture level city in Shandong
Happy Fan Shop	King of Power Saving	It rained a couple days ago and I just went to get the item back today , not bad, hey I looked at the meter and that red display light takes several seconds to jump, did not make a mistake buying it. After a period of time to evaluate.	Jingdong prefecture level city in Shandong
Happy Fan Shop	King of Power Saving	Without further ado, the electricity bill came down, this month less 102 yuan, has been repurchased, I also recommended to my friends, all said that there are effects, the product is okay, the effect is quite	Jingdong prefecture level city in Shandong
Happy Fan Shop	King of Power Saving	After buying and using two months really see the effect, so only to evaluate! Originally held the mindset of trying, I did not expect really effective, worth having!	Jingdong prefecture level city in Shandong
Happy Fan Shop	King of Power Saving	In the use of this energy saver before the monthly electricity bill is more than 150 yuan or so, now save thirty percent, every day to work, six or seven o'clock in the afternoon to go home, I do not know why the electricity bill is so expensive, energy-saving appliances really have the effect of recommending	Jingdong prefecture level city in Shandong
Happy Fan Shop	King of Power Saving	It saves about 100 bucks a month. A friend recommended it to me, and he's been using it for a while, and the effect is so obvious that I bought one too.	Jingdong prefecture level city in Shandong
Happy Fan Shop	King of Power Saving	My dad asked me to buy one, but after I paid the bill, I asked and he said it was a lot less this month, so he asked me to buy another one.	Jingdong prefecture level city in Shandong
Happy Fan Shop	King of Power Saving	The specific effect will have to be tested for at least two months to compare whether there is a power saving effect or not.	Jingdong prefecture level city in Shandong
Happy Fan Shop	King of Power Saving	'The goods have been received, immediately go to use, please ask the boss you instructions do not allow to withdraw, there are two fuses installed there!	Jingdong prefecture level city in Shandong
Happy Fan Shop	King of Power Saving	First praise is using, after using if good then follow up, now because I have not yet seen the results	Jingdong prefecture level city in Shandong
Happy Fan Shop	King of Power Saving	I've just been using it, and I haven't seen if it has a power-saving effect yet, so I'll follow up after 10 days and half a month.	Jingdong prefecture level city in Shandong
Happy Fan Shop	King of Power Saving	Just received the intelligent power saving meter, immediately plugged in to use, see it is not bad, and order to sell the second time	Jingdong prefecture level city in Shandong
Happy Fan Shop	King of Power Saving	This power saver is a bit effective, used for half a month, the effect is still visible.	Jingdong prefecture level city in Shandong
Happy Fan Shop	King of Power Saving	I received the baby and it looks great, I'm trying it out now and will keep an eye on it afterward.	Jingdong prefecture level city in Shandong
Happy Fan Shop	King of Power Saving	I've just received it, but I'm still trying to see how it works, so I'll come back in a little while.	Jingdong prefecture level city in Shandong
Happy Fan Shop	King of Power Saving	Just used it wait to use it for a while and then comment thanks, all first five stars.	Jingdong prefecture level city in Shandong
Happy Fan Shop	King of Power Saving	Very trustworthy brand, quality is guaranteed, the price is still affordable, quite satisfied!	Jingdong prefecture level city in Shandong
Happy Fan Shop	King of Power Saving	Things received, I hope that the platform of Jingdong do not sell fake goods are available	Jingdong prefecture level city in Shandong
Happy Fan Shop	King of Power Saving	Energy saver received, the use of the effect is good and the business description of the same	Jingdong prefecture level city in Shandong
Happy Fan Shop	King of Power Saving	The surface looks beautiful, the actual effect is not known, after using additional evaluation.	Jingdong prefecture level city in Shandong
Happy Fan Shop	King of Power Saving	Tried it for a few months, it really saves electricity, and I bought it for a friend.	Jingdong prefecture level city in Shandong
Happy Fan Shop	King of Power Saving	It didn't work and wanted to return it, it really didn't work at all.	Jingdong prefecture level city in Shandong
Happy Fan Shop	King of Power Saving	Just used it, don't know how effective it is, looking forward to usefulness	Jingdong prefecture level city in Shandong
Happy Fan Shop	King of Power Saving	After two months before the effect I do not know if it is true!	Jingdong prefecture level city in Shandong
Happy Fan Shop	King of Power Saving	So fast! Ordered yesterday and received today 👍👍👍👍👍👍👍👍👍 🌹🌹🌹🌹🌹🌹🌹🌹🌹	Jingdong prefecture level city in Shandong
Happy Fan Shop	King of Power Saving	I've started using it. We'll see how it works.	Jingdong prefecture level city in Shandong
Happy Fan Shop	King of Power Saving	I just received the goods, I won't know the effect until after two.	Jingdong prefecture level city in Shandong
Happy Fan Shop	King of Power Saving	Just received the goods, the specific effect will be evaluated later	Jingdong prefecture level city in Shandong
Happy Fan Shop	King of Power Saving	I've received the goods, I don't know if it's any good yet	Jingdong prefecture level city in Shandong
Happy Fan Shop	King of Power Saving	Try it for a month and then come back with a concrete evaluation	Jingdong prefecture level city in Shandong
Happy Fan Shop	King of Power Saving	In use, after two months to see the effect of power saving	Jingdong prefecture level city in Shandong

Happy Fan Shop	King of Power Saving	Power Saver. It's authentic. It's black. It works.	Jingdong prefecture level city in Shandong
Happy Fan Shop	King of Power Saving	I've been using it for a week now and it's not working.	Jingdong prefecture level city in Shandong
Happy Fan Shop	King of Power Saving	I'm just using it. I don't know how it's going to work.	Jingdong prefecture level city in Shandong
Happy Fan Shop	King of Power Saving	Products are better looking out for power savings	Jingdong prefecture level city in Shandong
Happy Fan Shop	King of Power Saving	I received it and I've kept it on. I hope it works.	Jingdong prefecture level city in Shandong
Happy Fan Shop	King of Power Saving	It's really good! Worth recommending to buy!	Jingdong prefecture level city in Shandong
Happy Fan Shop	King of Power Saving	Try it for a few days and see if it doesn't work, then return it.	Jingdong prefecture level city in Shandong
Happy Fan Shop	King of Power Saving	It's in use and I hope to be pleasantly surprised.	Jingdong prefecture level city in Shandong
Happy Fan Shop	King of Power Saving	We'll see how it works when we use it	Jingdong prefecture level city in Shandong
Happy Fan Shop	King of Power Saving	Let's see if it works.	Jingdong prefecture level city in Shandong
Happy Fan Shop	King of Power Saving	I'm using it and will follow up with a review	Jingdong prefecture level city in Shandong
Happy Fan Shop	King of Power Saving	It really works. It saves energy.	Jingdong prefecture level city in Shandong
Happy Fan Shop	King of Power Saving	It does have some power savings!	Jingdong prefecture level city in Shandong
Happy Fan Shop	King of Power Saving	Jingdong shopping speed is convenient and timely	Jingdong prefecture level city in Shandong
Happy Fan Shop	King of Power Saving	Second time I've bought it. Everything's fine.	Jingdong prefecture level city in Shandong
Happy Fan Shop	King of Power Saving	I don't know if it works yet.	Jingdong prefecture level city in Shandong
Happy Fan Shop	King of Power Saving	Not used, don't know how well it works.	Jingdong prefecture level city in Shandong
Happy Fan Shop	King of Power Saving	No visible results so far	Jingdong prefecture level city in Shandong
Happy Fan Shop	King of Power Saving	It doesn't feel like it's working.	Jingdong prefecture level city in Shandong
Happy Fan Shop	King of Power Saving	Where's the effect? Where are the power savings?	Jingdong prefecture level city in Shandong
Happy Fan Shop	King of Power Saving	Let's see how it works in practice!	Jingdong prefecture level city in Shandong
Happy Fan Shop	King of Power Saving	I'll wait until I use it to give you a good review	Jingdong prefecture level city in Shandong
Happy Fan Shop	King of Power Saving	I'll follow up after I've used it	Jingdong prefecture level city in Shandong
Happy Fan Shop	King of Power Saving	Well, that's good for saving power.	Jingdong prefecture level city in Shandong
Happy Fan Shop	King of Power Saving	Received. Used in the know.	Jingdong prefecture level city in Shandong
Happy Fan Shop	King of Power Saving	It doesn't do much good.	Jingdong prefecture level city in Shandong
Happy Fan Shop	King of Power Saving	It's not working.	Jingdong prefecture level city in Shandong
Happy Fan Shop	King of Power Saving	I don't know. I just used it.	Jingdong prefecture level city in Shandong
Happy Fan Shop	King of Power Saving	Use it and come back to evaluate.	Jingdong prefecture level city in Shandong
Happy Fan Shop	King of Power Saving	Two words: useless.	Jingdong prefecture level city in Shandong
Happy Fan Shop	King of Power Saving	To be used and then evaluated	Jingdong prefecture level city in Shandong
Happy Fan Shop	King of Power Saving	No shipment.	Jingdong prefecture level city in Shandong
Happy Fan Shop	King of Power Saving	Fake. Speechless.	Jingdong prefecture level city in Shandong
Happy Fan Shop	King of Power Saving	fantastic (idiom); awesome	Jingdong prefecture level city in Shandong
Happy Fan Shop	King of Power Saving	an excellent	Jingdong prefecture level city in Shandong
Happy Fan Shop	King of Power Saving	an excellent	Jingdong prefecture level city in Shandong
Happy Fan Shop	King of Power Saving	Want to buy! Recommended not to buy! No effect	Jingdong prefecture level city in Shandong
Happy Fan Shop	King of Power Saving	That's all there is to it. I can't figure out how to save electricity.	Jingdong prefecture level city in Shandong
Happy Fan Shop	King of Power Saving	It's no use.	Jingdong prefecture level city in Shandong
Happy Fan Shop	King of Power Saving	Things are more perfect than I imagined, very powerful, it is worth getting Packaging is very strong, things are well protected, it is worth the praise things are very satisfied Oh, beautiful and stylish atmosphere, customer service is super patient, service is meticulous and attentive, the big brand is not	Jingdong prefecture level city in Shandong
Happy Fan Shop	King of Power Saving	The effect is too good, the family has now several units, back to buy a number of times, the power saver received, power-saving effect is very good,, fine workmanship, long warranty, but also purify the air, before buying a used for a period of time, with the okay, and then bought a, and only now come to the	Jingdong prefecture level city in Shandong
Happy Fan Shop	King of Power Saving	Quite good, and imagine the same, casual ride are very good, satisfied! Baby received it 👌, really great ah! 🌈 quality is really nothing to say, very satisfied! 🆗 happy happy 😃 genuine, great, the price is very good, in short, quite good, will buy again a very good shopping experience, attentive service, fast logistics, distribution is also thoughtful, the attitude is also very good, value for money, ah,	Jingdong prefecture level city in Shandong
Happy Fan Shop	King of Power Saving	Power-saving effect: strong Cost-effective: quite good Appearance style: beautiful Workmanship material: meticulous Experience: looking forward to power-saving effect	Jingdong prefecture level city in Shandong
Happy Fan Shop	King of Power Saving	Cost-effective is okay, the appearance of the style of meticulous workmanship, looking forward to energy-saving effect.	Jingdong prefecture level city in Shandong
Happy Fan Shop	King of Power Saving	Very Good Very Power Saving This Power Saver is really power saving As soon as the power is turned on, the Power Saver starts the power saving function for you.	Jingdong prefecture level city in Shandong
Happy Fan Shop	King of Power Saving	Logistics is very fast, the price is favorable, friends introduced that it is very good to use, bought 2 at once, looking forward to the effect, used after the follow-up comment.	Jingdong prefecture level city in Shandong
Happy Fan Shop	King of Power Saving	It's all fake. It doesn't save any electricity.	Jingdong prefecture level city in Shandong
Happy Fan Shop	King of Power Saving	This Power Saver is so much better than the one I bought before, not only the effect is good, the quality is very good in all aspects, the customer service attitude is also very good, recommended to buy!	Jingdong prefecture level city in Shandong
Happy Fan Shop	King of Power Saving	Logistics is very fast, buy for parents, good quality, once bought 2, power-saving effect is good to buy 2 more, look forward to using the effect, satisfied will recommend to friends.	Jingdong prefecture level city in Shandong

Happy Fan Shop	King of Power Saving	With the description is the same, very high-grade, good value, to buy friends can order Oh!	Jingdong prefecture level city in Shandong
Happy Fan Shop	King of Power Saving	Not using much electricity in the winter saving over 100 per month! Ready to buy 2 more units in the summer ！！！！！ Good quality, good price, power saving effect is obvious, cost-effective, worth buying, can be recommended!	Jingdong prefecture level city in Shandong
Happy Fan Shop	King of Power Saving	Purchased a power saver received on the put into use, rent a room with many electrical appliances, power consumption is very large, we have a piece of electricity or 1.2 dollars a time, with this power saver is much better, every day can save a lot of money, very convenient and practical.	Jingdong prefecture level city in Shandong
Happy Fan Shop	King of Power Saving	The power saving effect is very good: my supermarket monthly electricity bill is more than 7000 yuan. On May 1st someone recommended buying a couple of power savers, and I bought 5 of them first. The electricity bill was about 2000 less in June. This time, I bought 5 more. The seller said 800 degrees of electricity with one. I feel it's basically enough. A total of 10 units	Jingdong prefecture level city in Shandong
Happy Fan Shop	King of Power Saving	I never thought I could get such a good baby at such an affordable price, it's very good value for money, I love it!	Jingdong prefecture level city in Shandong
Happy Fan Shop	King of Power Saving	Previously bought other brands of power savers, but found no effect at home, this time to see how the products on the Jingdong, inserted in the home socket, the LED display is blue, the night light is very nice to see, look at the instructions that have to be half a year in order to achieve the best results, try it, I believe that the quality of the Jingdong, it should not be wrong.	Jingdong prefecture level city in Shandong
Happy Fan Shop	King of Power Saving	Superb, inexpensive, saves a lot of points, been using it for a long time!	Jingdong prefecture level city in Shandong
Happy Fan Shop	King of Power Saving	There are a lot of appliances in the house, the summer air conditioning is very costly, I bought this in front of the air conditioning effect is very good, I explain that I bought three in one breath, I use more electricity a month down to 200 or so, very satisfied.	Jingdong prefecture level city in Shandong
Happy Fan Shop	King of Power Saving	A month can save more than 200, home monthly electricity bill of more than 600 dollars, inserted 3 or so, an amount of the balance of the average can save more than 200. Product is very good, has been buying this one, has given a lot of relatives recommended, the quality is also very good, before buying a look with this same, who knows to buy home has not been used for more than a week on the bad, the quality of this is really good with more than two years!	Jingdong prefecture level city in Shandong
Happy Fan Shop	King of Power Saving	Whole house power saving king, can really save electricity, friends recommended, high power saving appliances, only replacement, no repair, after-sales worry-free!	Jingdong prefecture level city in Shandong
Happy Fan Shop	King of Power Saving	Great item love it, great customer service, great shopping experience!	Jingdong prefecture level city in Shandong
Happy Fan Shop	King of Power Saving	Power saving, very good use, much cheaper than the physical store, satisfaction	Jingdong prefecture level city in Shandong
Happy Fan Shop	King of Power Saving	In use, very well packaged and fast delivery of credit!	Jingdong prefecture level city in Shandong
Happy Fan Shop	King of Power Saving	Product quality, customer service attitude is particularly good, like, praise!	Jingdong prefecture level city in Shandong
Happy Fan Shop	King of Power Saving	The logistics is very fast, the packaging is not broken, the quality is very good, I like it very much!	Jingdong prefecture level city in Shandong
Happy Fan Shop	King of Power Saving	The saver was well packaged and was as described when plugged in	Jingdong prefecture level city in Shandong
Happy Fan Shop	King of Power Saving	Energy efficient and very satisfied	Jingdong prefecture level city in Shandong
Happy Fan Shop	King of Power Saving	Great quality for the price and fast shipping.	Jingdong prefecture level city in Shandong
Happy Fan Shop	King of Power Saving	It's great, just as described. Perfect.	Jingdong prefecture level city in Shandong
Happy Fan Shop	King of Power Saving	There is no power saving effect, but rather ring up an increase in electricity costs, because this machine is on continuously all day long, uninterruptedly, its own electricity and increased costs, plus the cost of purchasing the machine, so I feel that the use of this machine instead of increasing the	Jingdong prefecture level city in Shandong
Happy Fan Shop	King of Power Saving	Recommended by the owner of the store next door, looked at his home does have to save electricity, decided to get. Indeed quite can, the voltage is much more stable, there is a reduction in electricity	Jingdong prefecture level city in Shandong
Happy Fan Shop	King of Power Saving	Goods have been received value for money, affordable and reasonable price, and the description of the same, power-saving safe and reliable!	Jingdong prefecture level city in Shandong
Happy Fan Shop	King of Power Saving	Listen to friends introduced to buy, tried to save a lot of electricity is good, quite good, worth buying.	Jingdong prefecture level city in Shandong
Happy Fan Shop	King of Power Saving	The product is easy to use, safe and environmentally friendly, only used for a short time, to the next month, after the electricity bill status display to know how the performance of the power saver efficacy.	Jingdong prefecture level city in Shandong
Happy Fan Shop	King of Power Saving	Last month's electricity bill is 178 yuan, with this power saver is 92 yuan, ready to buy two more back home, I was introduced to buy a friend, just started to not believe that I did not expect the effect!	Jingdong prefecture level city in Shandong
Happy Fan Shop	King of Power Saving	It looks nice, but after using it for a few days I can't see how it saves power?	Jingdong prefecture level city in Shandong
Happy Fan Shop	King of Power Saving	Recommended by a friend, it is genuine, as described by the merchant, cost-effective	Jingdong prefecture level city in Shandong
Happy Fan Shop	King of Power Saving	Works well, affordable and saves money.	Jingdong prefecture level city in Shandong
Happy Fan Shop	King of Power Saving	Feels good haven't compared it yet, the air conditioning has been on a lot in the past two months	Jingdong prefecture level city in Shandong
Happy Fan Shop	King of Power Saving	Saving about 5 kWh per day is a good thing	Jingdong prefecture level city in Shandong
Happy Fan Shop	King of Power Saving	Not bad, affordable, best value for money	Jingdong prefecture level city in Shandong
Happy Fan Shop	King of Power Saving	The goods are genuine and the logistics are faster.	Jingdong prefecture level city in Shandong
Happy Fan Shop	King of Power Saving	Goods have been received today, has been plugged into the outlet, looking forward to saving electricity, after a period of time to see how the effect in the follow-up comment!	Jingdong prefecture level city in Shandong
Happy Fan Shop	King of Power Saving	I just got it home and haven't used it for long, so I'll use it for a couple months and see what happens. Seeing that most people have good reviews, it should be fine.	Jingdong prefecture level city in Shandong
Happy Fan Shop	King of Power Saving	I'm looking forward to the results, so I'll give you a good review out of habit! Follow up review after a while	Jingdong prefecture level city in Shandong
Happy Fan Shop	King of Power Saving	Use a dish first to know if it will help save energy	Jingdong prefecture level city in Shandong
Happy Fan Shop	King of Power Saving	Whether it saves electricity or not, we don't know yet.	Jingdong prefecture level city in Shandong
Happy Fan Shop	King of Power Saving	Goods have been received, use a period of time to know how oh. The courier did not deliver to the door ah! Sent to the station ah! The heat is inconvenient ah!	Jingdong prefecture level city in Shandong
Happy Fan Shop	King of Power Saving	A little effect, before an hour a power, now an hour 0.76 degrees, a dollar a power = save 24 cents, although not a lot, first use it!	Jingdong prefecture level city in Shandong
Happy Fan Shop	King of Power Saving	Just received the goods, use a month to evaluate	Jingdong prefecture level city in Shandong
Happy Fan Shop	King of Power Saving	Home every month with a lot of electricity, inadvertently found this good thing online that can save the beginning of a skeptical attitude to consult customer service, customer service replied very professional, said 120 days can be refunded that first try it!	Jingdong prefecture level city in Shandong
Happy Fan Shop	King of Power Saving	After receiving the goods this afternoon, immediately installed in the bathroom Smith energy storage electric water heater on the power outlet (instead of connecting with a plug board), when used, found that the "Power Saving King" LCD display, is the value of the voltage, not the store's video said so	Jingdong prefecture level city in Shandong
Happy Fan Shop	King of Power Saving	Has made many days, did not feel the power saving, the effect is not obvious.	Jingdong prefecture level city in Shandong
Happy Fan Shop	King of Power Saving	Energy savers do not save energy at all, but rather consume a lot of electricity.	Jingdong prefecture level city in Shandong
Happy Fan Shop	King of Power Saving	It's useless. It's a waste of money.	Jingdong prefecture level city in Shandong

Happy Fan Shop	King of Power Saving	Last month saved more than 500, which came back to buy, the store bought three, and then buy 1 home use, my family is dry beauty salon, more instruments, especially electricity, a month almost 1000. or so, with this did not expect to save so much, great, five-star praise!	Jingdong prefecture level city in Shandong
Happy Fan Shop	King of Power Saving	It rained a couple days ago and I just went to get the item back today , not bad, hey I looked at the meter and that red display light takes several seconds to jump, did not make a mistake buying it. After a period of time to evaluate.	Jingdong prefecture level city in Shandong
Happy Fan Shop	King of Power Saving	Without further ado, the electricity bill came down, this month less 102 yuan, has been repurchased, I also recommended to my friends, all said that there are effects, the product is okay, the effect is quite	Jingdong prefecture level city in Shandong
Happy Fan Shop	King of Power Saving	After buying and using two months really see the effect, so only to evaluate! Originally held the mindset of trying, I did not expect really effective, worth having!	Jingdong prefecture level city in Shandong
Happy Fan Shop	King of Power Saving	In the use of this energy saver before the monthly electricity bill is more than 150 yuan or so, now save thirty percent, every day to work, six or seven o'clock in the afternoon to go home, I do not know why the electricity bill is so expensive, energy-saving appliances really have the effect of recommending	Jingdong prefecture level city in Shandong
Happy Fan Shop	King of Power Saving	It saves about 100 bucks a month. A friend recommended it to me, and he's been using it for a while, and the effect is so obvious that I bought one too.	Jingdong prefecture level city in Shandong
Happy Fan Shop	King of Power Saving	My dad asked me to buy one, but after I paid the bill, I asked and he said it was a lot less this month, so he asked me to buy another one.	Jingdong prefecture level city in Shandong
Happy Fan Shop	King of Power Saving	The specific effect will have to be tested for at least two months to compare whether there is a power saving effect or not.	Jingdong prefecture level city in Shandong
Happy Fan Shop	King of Power Saving	'The goods have been received, immediately go to use, please ask the boss you instructions do not allow to withdraw, there are two fuses installed there!	Jingdong prefecture level city in Shandong
Happy Fan Shop	King of Power Saving	First praise is using, after using if good then follow up, now because I have not yet seen the results	Jingdong prefecture level city in Shandong
Happy Fan Shop	King of Power Saving	I've just been using it, and I haven't seen if it has a power-saving effect yet, so I'll follow up after 10 days and half a month.	Jingdong prefecture level city in Shandong
Happy Fan Shop	King of Power Saving	Just received the intelligent power saving meter, immediately plugged in to use, see it is not bad, and order to sell the second time	Jingdong prefecture level city in Shandong
Happy Fan Shop	King of Power Saving	This power saver is a bit effective, used for half a month, the effect is still visible.	Jingdong prefecture level city in Shandong
Happy Fan Shop	King of Power Saving	I received the baby and it looks great, I'm trying it out now and will keep an eye on it afterward.	Jingdong prefecture level city in Shandong
Happy Fan Shop	King of Power Saving	I've just received it, but I'm still trying to see how it works, so I'll come back in a little while.	Jingdong prefecture level city in Shandong
Happy Fan Shop	King of Power Saving	Just used it wait to use it for a while and then comment thanks, all first five stars.	Jingdong prefecture level city in Shandong
Happy Fan Shop	King of Power Saving	Very trustworthy brand, quality is guaranteed, the price is still affordable, quite satisfied!	Jingdong prefecture level city in Shandong
Happy Fan Shop	King of Power Saving	Things received, I hope that the platform of Jingdong do not sell fake goods are available	Jingdong prefecture level city in Shandong
Happy Fan Shop	King of Power Saving	Energy saver received, the use of the effect is good and the business description of the same	Jingdong prefecture level city in Shandong
Happy Fan Shop	King of Power Saving	The surface looks beautiful, the actual effect is not known, after using additional evaluation.	Jingdong prefecture level city in Shandong
Happy Fan Shop	King of Power Saving	Tried it for a few months, it really saves electricity, and I bought it for a friend.	Jingdong prefecture level city in Shandong
Happy Fan Shop	King of Power Saving	It didn't work and wanted to return it, it really didn't work at all.	Jingdong prefecture level city in Shandong
Happy Fan Shop	King of Power Saving	Just used it, don't know how effective it is, looking forward to usefulness	Jingdong prefecture level city in Shandong
Happy Fan Shop	King of Power Saving	After two months before the effect I do not know if it is true!	Jingdong prefecture level city in Shandong
Happy Fan Shop	King of Power Saving	So fast! Ordered yesterday and received today 👍👍👍👍👍👍👍👍👍 🌹🌹🌹🌹🌹🌹🌹🌹🌹	Jingdong prefecture level city in Shandong
Happy Fan Shop	King of Power Saving	I've started using it. We'll see how it works.	Jingdong prefecture level city in Shandong
Happy Fan Shop	King of Power Saving	I just received the goods, I won't know the effect until after two.	Jingdong prefecture level city in Shandong
Happy Fan Shop	King of Power Saving	Just received the goods, the specific effect will be evaluated later	Jingdong prefecture level city in Shandong
Happy Fan Shop	King of Power Saving	I've received the goods, I don't know if it's any good yet	Jingdong prefecture level city in Shandong
Happy Fan Shop	King of Power Saving	Try it for a month and then come back with a concrete evaluation	Jingdong prefecture level city in Shandong
Happy Fan Shop	King of Power Saving	In use, after two months to see the effect of power saving	Jingdong prefecture level city in Shandong
Happy Fan Shop	King of Power Saving	Power Saver. It's authentic. It's black. It works.	Jingdong prefecture level city in Shandong
Happy Fan Shop	King of Power Saving	I've been using it for a week now and it's not working.	Jingdong prefecture level city in Shandong
Happy Fan Shop	King of Power Saving	I'm just using it. I don't know how it's going to work.	Jingdong prefecture level city in Shandong
Happy Fan Shop	King of Power Saving	Products are better looking out for power savings	Jingdong prefecture level city in Shandong
Happy Fan Shop	King of Power Saving	I received it and I've kept it on. I hope it works.	Jingdong prefecture level city in Shandong
Happy Fan Shop	King of Power Saving	It's really good! Worth recommending to buy!	Jingdong prefecture level city in Shandong
Happy Fan Shop	King of Power Saving	Try it for a few days and see if it doesn't work, then return it.	Jingdong prefecture level city in Shandong
Happy Fan Shop	King of Power Saving	It's in use and I hope to be pleasantly surprised.	Jingdong prefecture level city in Shandong
Happy Fan Shop	King of Power Saving	We'll see how it works when we use it	Jingdong prefecture level city in Shandong
Happy Fan Shop	King of Power Saving	Let's see if it works.	Jingdong prefecture level city in Shandong
Happy Fan Shop	King of Power Saving	I'm using it and will follow up with a review	Jingdong prefecture level city in Shandong
Happy Fan Shop	King of Power Saving	It really works. It saves energy.	Jingdong prefecture level city in Shandong
Happy Fan Shop	King of Power Saving	It does have some power savings!	Jingdong prefecture level city in Shandong
Happy Fan Shop	King of Power Saving	Jingdong shopping speed is convenient and timely	Jingdong prefecture level city in Shandong
Happy Fan Shop	King of Power Saving	Second time I've bought it. Everything's fine.	Jingdong prefecture level city in Shandong
Happy Fan Shop	King of Power Saving	I don't know if it works yet.	Jingdong prefecture level city in Shandong
Happy Fan Shop	King of Power Saving	Not used, don't know how well it works.	Jingdong prefecture level city in Shandong
Happy Fan Shop	King of Power Saving	No visible results so far	Jingdong prefecture level city in Shandong
Happy Fan Shop	King of Power Saving	It doesn't feel like it's working.	Jingdong prefecture level city in Shandong

Happy Fan Shop	King of Power Saving	Where's the effect? Where are the power savings?	Jingdong prefecture level city in Shandong
Happy Fan Shop	King of Power Saving	Let's see how it works in practice!	Jingdong prefecture level city in Shandong
Happy Fan Shop	King of Power Saving	I'll wait until I use it to give you a good review	Jingdong prefecture level city in Shandong
Happy Fan Shop	King of Power Saving	I'll follow up after I've used it	Jingdong prefecture level city in Shandong
Happy Fan Shop	King of Power Saving	Well, that's good for saving power.	Jingdong prefecture level city in Shandong
Happy Fan Shop	King of Power Saving	Received. Used in the know.	Jingdong prefecture level city in Shandong
Happy Fan Shop	King of Power Saving	It doesn't do much good.	Jingdong prefecture level city in Shandong
Happy Fan Shop	King of Power Saving	It's not working.	Jingdong prefecture level city in Shandong
Happy Fan Shop	King of Power Saving	I don't know. I just used it.	Jingdong prefecture level city in Shandong
Happy Fan Shop	King of Power Saving	Use it and come back to evaluate.	Jingdong prefecture level city in Shandong
Happy Fan Shop	King of Power Saving	Two words: useless.	Jingdong prefecture level city in Shandong
Happy Fan Shop	King of Power Saving	To be used and then evaluated	Jingdong prefecture level city in Shandong
Happy Fan Shop	King of Power Saving	No shipment.	Jingdong prefecture level city in Shandong
Happy Fan Shop	King of Power Saving	Fake. Speechless.	Jingdong prefecture level city in Shandong
Happy Fan Shop	King of Power Saving	fantastic (idiom); awesome	Jingdong prefecture level city in Shandong
Happy Fan Shop	King of Power Saving	an excellent	Jingdong prefecture level city in Shandong
Happy Fan Shop	King of Power Saving	an excellent	Jingdong prefecture level city in Shandong
Happy Fan Shop	King of Power Saving	Want to buy! Recommended not to buy! No effect	Jingdong prefecture level city in Shandong
Happy Fan Shop	King of Power Saving	That's all there is to it. I can't figure out how to save electricity.	Jingdong prefecture level city in Shandong
Happy Fan Shop	King of Power Saving	It's no use.	Jingdong prefecture level city in Shandong
Happy Fan Shop	King of Power Saving	Things are more perfect than I imagined, very powerful, it is worth getting Packaging is very strong, things are well protected, it is worth the praise things are very satisfied Oh, beautiful and stylish atmosphere, customer service is super patient, service is meticulous and attentive, the big brand is not	Jingdong prefecture level city in Shandong
Happy Fan Shop	King of Power Saving	The effect is too good, the family has now several units, back to buy a number of times, the power saver received, power-saving effect is very good,, fine workmanship, long warranty, but also purify the air, before buying a used for a period of time, with the okay, and then bought a, and only now come to the	Jingdong prefecture level city in Shandong
Happy Fan Shop	King of Power Saving	Quite good, and imagine the same, casual ride are very good, satisfied! Baby received it 👌, really great ah! 🌈 quality is really nothing to say, very satisfied! 🆗 happy happy 😃 genuine, great, the price is very good, in short, quite good, will buy again a very good shopping experience, attentive service, fast logistics, distribution is also thoughtful, the attitude is also very good, value for money, ah,	Jingdong prefecture level city in Shandong
Happy Fan Shop	King of Power Saving	Power-saving effect: strong Cost-effective: quite good Appearance style: beautiful Workmanship material: meticulous Experience: looking forward to power-saving effect	Jingdong prefecture level city in Shandong
Happy Fan Shop	King of Power Saving	Cost-effective is okay, the appearance of the style of meticulous workmanship, looking forward to energy-saving effect.	Jingdong prefecture level city in Shandong
Happy Fan Shop	King of Power Saving	Very Good Very Power Saving This Power Saver is really power saving As soon as the power is turned on, the Power Saver starts the power saving function for you.	Jingdong prefecture level city in Shandong
Happy Fan Shop	King of Power Saving	Logistics is very fast, the price is favorable, friends introduced that it is very good to use, bought 2 at once, looking forward to the effect, used after the follow-up comment.	Jingdong prefecture level city in Shandong
Happy Fan Shop	King of Power Saving	It's all fake. It doesn't save any electricity.	Jingdong prefecture level city in Shandong
Happy Fan Shop	King of Power Saving	This Power Saver is so much better than the one I bought before, not only the effect is good, the quality is very good in all aspects, the customer service attitude is also very good, recommended to buy!	Jingdong prefecture level city in Shandong
Happy Fan Shop	King of Power Saving	Logistics is very fast, buy for parents, good quality, once bought 2, power-saving effect is good to buy 2 more, look forward to using the effect, satisfied will recommend to friends.	Jingdong prefecture level city in Shandong
Happy Fan Shop	King of Power Saving	With the description is the same, very high-grade, good value, to buy friends can order Oh!	Jingdong prefecture level city in Shandong
Happy Fan Shop	King of Power Saving	Not using much electricity in the winter saving over 100 per month! Ready to buy 2 more units in the summer !!!!! Good quality, good price, power saving effect is obvious, cost-effective, worth buying, can be recommended!	Jingdong prefecture level city in Shandong
Happy Fan Shop	King of Power Saving	Purchased a power saver received on the put into use, rent a room with many electrical appliances, power consumption is very large, we have a piece of electricity or 1.2 dollars a time, with this power saver is much better, every day can save a lot of money, very convenient and practical.	Jingdong prefecture level city in Shandong
Happy Fan Shop	King of Power Saving	The power saving effect is very good: my supermarket monthly electricity bill is more than 7000 yuan. On May 1st someone recommended buying a couple of power savers, and I bought 5 of them first. The electricity bill was about 2000 less in June. This time, I bought 5 more. The seller said 800 degrees of electricity with one. I feel it's basically enough. A total of 10 units	Jingdong prefecture level city in Shandong
Happy Fan Shop	King of Power Saving	I never thought I could get such a good baby at such an affordable price, it's very good value for money, I love it!	Jingdong prefecture level city in Shandong
Happy Fan Shop	King of Power Saving	Previously bought other brands of power savers, but found no effect at home, this time to see how the products on the Jingdong, inserted in the home socket, the LED display is blue, the night light is very nice to see, look at the instructions that have to be half a year in order to achieve the best results, try it, I believe that the quality of the Jingdong, it should not be wrong.	Jingdong prefecture level city in Shandong
Happy Fan Shop	King of Power Saving	Superb, inexpensive, saves a lot of points, been using it for a long time!	Jingdong prefecture level city in Shandong
Happy Fan Shop	King of Power Saving	There are a lot of appliances in the house, the summer air conditioning is very costly, I bought this in front of the air conditioning effect is very good, I explain that I bought three in one breath, I use more electricity a month down to 200 or so, very satisfied.	Jingdong prefecture level city in Shandong
Happy Fan Shop	King of Power Saving	A month can save more than 200, home monthly electricity bill of more than 600 dollars, inserted 3 or so, an amount of the balance of the average can save more than 200. Product is very good, has been buying this one, has given a lot of relatives recommended, the quality is also very good, before buying a look with this same, who knows to buy home has not been used for more than a week on the bad, the quality of this is really good with more than two years!	Jingdong prefecture level city in Shandong
Happy Fan Shop	King of Power Saving	Whole house power saving king, can really save electricity, friends recommended, high power saving appliances, only replacement, no repair, after-sales worry-free!	Jingdong prefecture level city in Shandong
Happy Fan Shop	King of Power Saving	Great item love it, great customer service, great shopping experience!	Jingdong prefecture level city in Shandong
Happy Fan Shop	King of Power Saving	Power saving, very good use, much cheaper than the physical store, satisfaction	Jingdong prefecture level city in Shandong
Happy Fan Shop	King of Power Saving	In use, very well packaged and fast delivery of credit!	Jingdong prefecture level city in Shandong
Happy Fan Shop	King of Power Saving	Product quality, customer service attitude is particularly good, like, praise!	Jingdong prefecture level city in Shandong

Happy Fan Shop	King of Power Saving	The logistics is very fast, the packaging is not broken, the quality is very good, I like it very much!	Jingdong prefecture level city in Shandong
Happy Fan Shop	King of Power Saving	The saver was well packaged and was as described when plugged in	Jingdong prefecture level city in Shandong
Happy Fan Shop	King of Power Saving	Energy efficient and very satisfied	Jingdong prefecture level city in Shandong
Happy Fan Shop	King of Power Saving	Great quality for the price and fast shipping.	Jingdong prefecture level city in Shandong
Happy Fan Shop	King of Power Saving	It's great, just as described. Perfect.	Jingdong prefecture level city in Shandong
Happy Fan Shop	King of Power Saving	There is no power saving effect, but rather ring up an increase in electricity costs, because this machine is on continuously all day long, uninterruptedly, its own electricity and increased costs, plus the cost of purchasing the machine, so I feel that the use of this machine instead of increasing the	Jingdong prefecture level city in Shandong
Happy Fan Shop	King of Power Saving	Recommended by the owner of the store next door, looked at his home does have to save electricity, decided to get. Indeed quite can, the voltage is much more stable, there is a reduction in electricity	Jingdong prefecture level city in Shandong
Happy Fan Shop	King of Power Saving	Goods have been received value for money, affordable and reasonable price, and the description of the same, power-saving safe and reliable!	Jingdong prefecture level city in Shandong
Happy Fan Shop	King of Power Saving	Listen to friends introduced to buy, tried to save a lot of electricity is good, quite good, worth buying.	Jingdong prefecture level city in Shandong
Happy Fan Shop	King of Power Saving	The product is easy to use, safe and environmentally friendly, only used for a short time, to the next month, after the electricity bill status display to know how the performance of the power saver efficacy.	Jingdong prefecture level city in Shandong
Happy Fan Shop	King of Power Saving	Last month's electricity bill is 178 yuan, with this power saver is 92 yuan, ready to buy two more back home, I was introduced to buy a friend, just started to not believe that I did not expect the effect!	Jingdong prefecture level city in Shandong
Happy Fan Shop	King of Power Saving	It looks nice, but after using it for a few days I can't see how it saves power?	Jingdong prefecture level city in Shandong
Happy Fan Shop	King of Power Saving	Recommended by a friend, it is genuine, as described by the merchant, cost-effective	Jingdong prefecture level city in Shandong
Happy Fan Shop	King of Power Saving	Works well, affordable and saves money.	Jingdong prefecture level city in Shandong
Happy Fan Shop	King of Power Saving	Feels good haven't compared it yet, the air conditioning has been on a lot in the past two months	Jingdong prefecture level city in Shandong
Happy Fan Shop	King of Power Saving	Saving about 5 kWh per day is a good thing	Jingdong prefecture level city in Shandong
Happy Fan Shop	King of Power Saving	Not bad, affordable, best value for money	Jingdong prefecture level city in Shandong
Happy Fan Shop	King of Power Saving	The goods are genuine and the logistics are faster.	Jingdong prefecture level city in Shandong
Happy Fan Shop	King of Power Saving	Goods have been received today, has been plugged into the outlet, looking forward to saving electricity, after a period of time to see how the effect in the follow-up comment!	Jingdong prefecture level city in Shandong
Happy Fan Shop	King of Power Saving	I just got it home and haven't used it for long, so I'll use it for a couple months and see what happens. Seeing that most people have good reviews, it should be fine.	Jingdong prefecture level city in Shandong
Happy Fan Shop	King of Power Saving	I'm looking forward to the results, so I'll give you a good review out of habit! Follow up review after a while	Jingdong prefecture level city in Shandong
Happy Fan Shop	King of Power Saving	Use a dish first to know if it will help save energy	Jingdong prefecture level city in Shandong
Happy Fan Shop	King of Power Saving	Whether it saves electricity or not, we don't know yet.	Jingdong prefecture level city in Shandong
Happy Fan Shop	King of Power Saving	Goods have been received, use a period of time to know how oh. The courier did not deliver to the door ah! Sent to the station ah! The heat is inconvenient ah!	Jingdong prefecture level city in Shandong
Happy Fan Shop	King of Power Saving	A little effect, before an hour a power, now an hour 0.76 degrees, a dollar a power = save 24 cents, although not a lot, first use it!	Jingdong prefecture level city in Shandong
Happy Fan Shop	King of Power Saving	Just received the goods, use a month to evaluate	Jingdong prefecture level city in Shandong
Happy Fan Shop	King of Power Saving	Home every month with a lot of electricity, inadvertently found this good thing online that can save the beginning of a skeptical attitude to consult customer service, customer service replied very professional, said 120 days can be refunded that first try it!	Jingdong prefecture level city in Shandong
Happy Fan Shop	King of Power Saving	After receiving the goods this afternoon, immediately installed in the bathroom Smith energy storage electric water heater on the power outlet (instead of connecting with a plug board), when used, found that the "Power Saving King" LCD display, is the value of the voltage, not the store's video said so	Jingdong prefecture level city in Shandong
Happy Fan Shop	King of Power Saving	Has made many days, did not feel the power saving, the effect is not obvious.	Jingdong prefecture level city in Shandong
Happy Fan Shop	King of Power Saving	Energy savers do not save energy at all, but rather consume a lot of electricity.	Jingdong prefecture level city in Shandong
Happy Fan Shop	King of Power Saving	It's useless. It's a waste of money.	Jingdong prefecture level city in Shandong
Happy Fan Shop	King of Power Saving	Last month saved more than 500, which came back to buy, the store bought three, and then buy 1 home use, my family is dry beauty salon, more instruments, especially electricity, a month almost 1000. or so, with this did not expect to save so much, great, five-star praise!	Jingdong prefecture level city in Shandong
Happy Fan Shop	King of Power Saving	It rained a couple days ago and I just went to get the item back today , not bad, hey I looked at the meter and that red display light takes several seconds to jump, did not make a mistake buying it. After a period of time to evaluate.	Jingdong prefecture level city in Shandong
Happy Fan Shop	King of Power Saving	Without further ado, the electricity bill came down, this month less 102 yuan, has been repurchased, I also recommended to my friends, all said that there are effects, the product is okay, the effect is quite	Jingdong prefecture level city in Shandong
Happy Fan Shop	King of Power Saving	After buying and using two months really see the effect, so only to evaluate! Originally held the mindset of trying, I did not expect really effective, worth having!	Jingdong prefecture level city in Shandong
Happy Fan Shop	King of Power Saving	In the use of this energy saver before the monthly electricity bill is more than 150 yuan or so, now save thirty percent, every day to work, six or seven o'clock in the afternoon to go home, I do not know why the electricity bill is so expensive, energy-saving appliances really have the effect of recommending	Jingdong prefecture level city in Shandong
Happy Fan Shop	King of Power Saving	It saves about 100 bucks a month. A friend recommended it to me, and he's been using it for a while, and the effect is so obvious that I bought one too.	Jingdong prefecture level city in Shandong
Happy Fan Shop	King of Power Saving	My dad asked me to buy one, but after I paid the bill, I asked and he said it was a lot less this month, so he asked me to buy another one.	Jingdong prefecture level city in Shandong
Happy Fan Shop	King of Power Saving	The specific effect will have to be tested for at least two months to compare whether there is a power saving effect or not.	Jingdong prefecture level city in Shandong
Happy Fan Shop	King of Power Saving	'The goods have been received, immediately go to use, please ask the boss you instructions do not allow to withdraw, there are two fuses installed there!	Jingdong prefecture level city in Shandong
Happy Fan Shop	King of Power Saving	First praise is using, after using if good then follow up, now because I have not yet seen the results	Jingdong prefecture level city in Shandong
Happy Fan Shop	King of Power Saving	I've just been using it, and I haven't seen if it has a power-saving effect yet, so I'll follow up after 10 days and half a month.	Jingdong prefecture level city in Shandong
Happy Fan Shop	King of Power Saving	Just received the intelligent power saving meter, immediately plugged in to use, see it is not bad, and order to sell the second time	Jingdong prefecture level city in Shandong
Happy Fan Shop	King of Power Saving	This power saver is a bit effective, used for half a month, the effect is still visible.	Jingdong prefecture level city in Shandong
Happy Fan Shop	King of Power Saving	I received the baby and it looks great, I'm trying it out now and will keep an eye on it afterward.	Jingdong prefecture level city in Shandong
Happy Fan Shop	King of Power Saving	I've just received it, but I'm still trying to see how it works, so I'll come back in a little while.	Jingdong prefecture level city in Shandong
Happy Fan Shop	King of Power Saving	Just used it wait to use it for a while and then comment thanks, all first five stars.	Jingdong prefecture level city in Shandong
Happy Fan Shop	King of Power Saving	Very trustworthy brand, quality is guaranteed, the price is still affordable, quite satisfied!	Jingdong prefecture level city in Shandong

Happy Fan Shop	King of Power Saving	Things received, I hope that the platform of Jingdong do not sell fake goods are available	Jingdong prefecture level city in Shandong
Happy Fan Shop	King of Power Saving	Energy saver received, the use of the effect is good and the business description of the same	Jingdong prefecture level city in Shandong
Happy Fan Shop	King of Power Saving	The surface looks beautiful, the actual effect is not known, after using additional evaluation.	Jingdong prefecture level city in Shandong
Happy Fan Shop	King of Power Saving	Tried it for a few months, it really saves electricity, and I bought it for a friend.	Jingdong prefecture level city in Shandong
Happy Fan Shop	King of Power Saving	It didn't work and wanted to return it, it really didn't work at all.	Jingdong prefecture level city in Shandong
Happy Fan Shop	King of Power Saving	Just used it, don't know how effective it is, looking forward to usefulness	Jingdong prefecture level city in Shandong
Happy Fan Shop	King of Power Saving	After two months before the effect I do not know if it is true!	Jingdong prefecture level city in Shandong
Happy Fan Shop	King of Power Saving	So fast! Ordered yesterday and received today 👍👍👍👍👍👍👍👍👍 🌹🌹🌹🌹🌹🌹🌹🌹🌹	Jingdong prefecture level city in Shandong
Happy Fan Shop	King of Power Saving	I've started using it. We'll see how it works.	Jingdong prefecture level city in Shandong
Happy Fan Shop	King of Power Saving	I just received the goods, I won't know the effect until after two.	Jingdong prefecture level city in Shandong
Happy Fan Shop	King of Power Saving	Just received the goods, the specific effect will be evaluated later	Jingdong prefecture level city in Shandong
Happy Fan Shop	King of Power Saving	I've received the goods, I don't know if it's any good yet	Jingdong prefecture level city in Shandong
Happy Fan Shop	King of Power Saving	Try it for a month and then come back with a concrete evaluation	Jingdong prefecture level city in Shandong
Happy Fan Shop	King of Power Saving	In use, after two months to see the effect of power saving	Jingdong prefecture level city in Shandong
Happy Fan Shop	King of Power Saving	Power Saver. It's authentic. It's black. It works.	Jingdong prefecture level city in Shandong
Happy Fan Shop	King of Power Saving	I've been using it for a week now and it's not working.	Jingdong prefecture level city in Shandong
Happy Fan Shop	King of Power Saving	I'm just using it. I don't know how it's going to work.	Jingdong prefecture level city in Shandong
Happy Fan Shop	King of Power Saving	Products are better looking out for power savings	Jingdong prefecture level city in Shandong
Happy Fan Shop	King of Power Saving	I received it and I've kept it on. I hope it works.	Jingdong prefecture level city in Shandong
Happy Fan Shop	King of Power Saving	It's really good! Worth recommending to buy!	Jingdong prefecture level city in Shandong
Happy Fan Shop	King of Power Saving	Try it for a few days and see if it doesn't work, then return it.	Jingdong prefecture level city in Shandong
Happy Fan Shop	King of Power Saving	It's in use and I hope to be pleasantly surprised.	Jingdong prefecture level city in Shandong
Happy Fan Shop	King of Power Saving	We'll see how it works when we use it	Jingdong prefecture level city in Shandong
Happy Fan Shop	King of Power Saving	Let's see if it works.	Jingdong prefecture level city in Shandong
Happy Fan Shop	King of Power Saving	I'm using it and will follow up with a review	Jingdong prefecture level city in Shandong
Happy Fan Shop	King of Power Saving	It really works. It saves energy.	Jingdong prefecture level city in Shandong
Happy Fan Shop	King of Power Saving	It does have some power savings!	Jingdong prefecture level city in Shandong
Happy Fan Shop	King of Power Saving	Jingdong shopping speed is convenient and timely	Jingdong prefecture level city in Shandong
Happy Fan Shop	King of Power Saving	Second time I've bought it. Everything's fine.	Jingdong prefecture level city in Shandong
Happy Fan Shop	King of Power Saving	I don't know if it works yet.	Jingdong prefecture level city in Shandong
Happy Fan Shop	King of Power Saving	Not used, don't know how well it works.	Jingdong prefecture level city in Shandong
Happy Fan Shop	King of Power Saving	No visible results so far	Jingdong prefecture level city in Shandong
Happy Fan Shop	King of Power Saving	It doesn't feel like it's working.	Jingdong prefecture level city in Shandong
Happy Fan Shop	King of Power Saving	Where's the effect? Where are the power savings?	Jingdong prefecture level city in Shandong
Happy Fan Shop	King of Power Saving	Let's see how it works in practice!	Jingdong prefecture level city in Shandong
Happy Fan Shop	King of Power Saving	I'll wait until I use it to give you a good review	Jingdong prefecture level city in Shandong
Happy Fan Shop	King of Power Saving	I'll follow up after I've used it	Jingdong prefecture level city in Shandong
Happy Fan Shop	King of Power Saving	Well, that's good for saving power.	Jingdong prefecture level city in Shandong
Happy Fan Shop	King of Power Saving	Received. Used in the know.	Jingdong prefecture level city in Shandong
Happy Fan Shop	King of Power Saving	It doesn't do much good.	Jingdong prefecture level city in Shandong
Happy Fan Shop	King of Power Saving	It's not working.	Jingdong prefecture level city in Shandong
Happy Fan Shop	King of Power Saving	I don't know. I just used it.	Jingdong prefecture level city in Shandong
Happy Fan Shop	King of Power Saving	Use it and come back to evaluate.	Jingdong prefecture level city in Shandong
Happy Fan Shop	King of Power Saving	Two words: useless.	Jingdong prefecture level city in Shandong
Happy Fan Shop	King of Power Saving	To be used and then evaluated	Jingdong prefecture level city in Shandong
Happy Fan Shop	King of Power Saving	No shipment.	Jingdong prefecture level city in Shandong
Happy Fan Shop	King of Power Saving	Fake. Speechless.	Jingdong prefecture level city in Shandong
Happy Fan Shop	King of Power Saving	fantastic (idiom); awesome	Jingdong prefecture level city in Shandong
Happy Fan Shop	King of Power Saving	an excellent	Jingdong prefecture level city in Shandong
Happy Fan Shop	King of Power Saving	an excellent	Jingdong prefecture level city in Shandong
Happy Fan Shop	King of Power Saving	Want to buy! Recommended not to buy! No effect	Jingdong prefecture level city in Shandong

Happy Fan Shop	King of Power Saving	That's all there is to it. I can't figure out how to save electricity.	Jingdong prefecture level city in Shandong
Happy Fan Shop	King of Power Saving	It's no use.	Jingdong prefecture level city in Shandong
Happy Fan Shop	King of Power Saving	Things are more perfect than I imagined, very powerful, it is worth getting Packaging is very strong, things are well protected, it is worth the praise things are very satisfied Oh, beautiful and stylish atmosphere, customer service is super patient, service is meticulous and attentive, the big brand is not	Jingdong prefecture level city in Shandong
Happy Fan Shop	King of Power Saving	The effect is too good, the family has now several units, back to buy a number of times, the power saver received, power-saving effect is very good,, fine workmanship, long warranty, but also purify the air, before buying a used for a period of time, with the okay, and then bought a, and only now come to the	Jingdong prefecture level city in Shandong
Happy Fan Shop	King of Power Saving	Quite good, and imagine the same, casual ride are very good, satisfied! Baby received it 👌, really great ah! 🌈 quality is really nothing to say, very satisfied! 🆗 happy happy 😃 genuine, great, the price is very good, in short, quite good, will buy again a very good shopping experience, attentive service, fast logistics, distribution is also thoughtful, the attitude is also very good, value for money, ah,	Jingdong prefecture level city in Shandong
Happy Fan Shop	King of Power Saving	Power-saving effect: strong Cost-effective: quite good Appearance style: beautiful Workmanship material: meticulous Experience: looking forward to power-saving effect	Jingdong prefecture level city in Shandong
Happy Fan Shop	King of Power Saving	Cost-effective is okay, the appearance of the style of meticulous workmanship, looking forward to energy-saving effect.	Jingdong prefecture level city in Shandong
Happy Fan Shop	King of Power Saving	Very Good Very Power Saving This Power Saver is really power saving As soon as the power is turned on, the Power Saver starts the power saving function for you.	Jingdong prefecture level city in Shandong
Happy Fan Shop	King of Power Saving	Logistics is very fast, the price is favorable, friends introduced that it is very good to use, bought 2 at once, looking forward to the effect, used after the follow-up comment.	Jingdong prefecture level city in Shandong
Happy Fan Shop	King of Power Saving	It's all fake. It doesn't save any electricity.	Jingdong prefecture level city in Shandong
Happy Fan Shop	King of Power Saving	This Power Saver is so much better than the one I bought before, not only the effect is good, the quality is very good in all aspects, the customer service attitude is also very good, recommended to buy!	Jingdong prefecture level city in Shandong
Happy Fan Shop	King of Power Saving	Logistics is very fast, buy for parents, good quality, once bought 2, power-saving effect is good to buy 2 more, look forward to using the effect, satisfied will recommend to friends.	Jingdong prefecture level city in Shandong
Happy Fan Shop	King of Power Saving	With the description is the same, very high-grade, good value, to buy friends can order Oh!	Jingdong prefecture level city in Shandong
Happy Fan Shop	King of Power Saving	Not using much electricity in the winter saving over 100 per month! Ready to buy 2 more units in the summer ！！！！！ Good quality, good price, power saving effect is obvious, cost-effective, worth buying, can be recommended!	Jingdong prefecture level city in Shandong
Happy Fan Shop	King of Power Saving	Purchased a power saver received on the put into use, rent a room with many electrical appliances, power consumption is very large, we have a piece of electricity or 1.2 dollars a time, with this power saver is much better, every day can save a lot of money, very convenient and practical.	Jingdong prefecture level city in Shandong
Happy Fan Shop	King of Power Saving	The power saving effect is very good: my supermarket monthly electricity bill is more than 7000 yuan. On May 1st someone recommended buying a couple of power savers, and I bought 5 of them first. The electricity bill was about 2000 less in June. This time, I bought 5 more. The seller said 800 degrees of electricity with one. I feel it's basically enough. A total of 10 units	Jingdong prefecture level city in Shandong
Happy Fan Shop	King of Power Saving	I never thought I could get such a good baby at such an affordable price, it's very good value for money, I love it!	Jingdong prefecture level city in Shandong
Happy Fan Shop	King of Power Saving	Previously bought other brands of power savers, but found no effect at home, this time to see how the products on the Jingdong, inserted in the home socket, the LED display is blue, the night light is very nice to see, look at the instructions that have to be half a year in order to achieve the best results, try it, I believe that the quality of the Jingdong, it should not be wrong.	Jingdong prefecture level city in Shandong
Happy Fan Shop	King of Power Saving	Superb, inexpensive, saves a lot of points, been using it for a long time!	Jingdong prefecture level city in Shandong
Happy Fan Shop	King of Power Saving	There are a lot of appliances in the house, the summer air conditioning is very costly, I bought this in front of the air conditioning effect is very good, I explain that I bought three in one breath, I use more electricity a month down to 200 or so, very satisfied.	Jingdong prefecture level city in Shandong
Happy Fan Shop	King of Power Saving	A month can save more than 200, home monthly electricity bill of more than 600 dollars, inserted 3 or so, an amount of the balance of the average can save more than 200. Product is very good, has been buying this one, has given a lot of relatives recommended, the quality is also very good, before buying a look with this same, who knows to buy home has not been used for more than a week on the bad, the quality of this is really good with more than two years!	Jingdong prefecture level city in Shandong
Happy Fan Shop	King of Power Saving	Whole house power saving king, can really save electricity, friends recommended, high power saving appliances, only replacement, no repair, after-sales worry-free!	Jingdong prefecture level city in Shandong
Happy Fan Shop	King of Power Saving	Great item love it, great customer service, great shopping experience!	Jingdong prefecture level city in Shandong
Happy Fan Shop	King of Power Saving	Power saving, very good use, much cheaper than the physical store, satisfaction	Jingdong prefecture level city in Shandong
Happy Fan Shop	King of Power Saving	In use, very well packaged and fast delivery of credit!	Jingdong prefecture level city in Shandong
Happy Fan Shop	King of Power Saving	Product quality, customer service attitude is particularly good, like, praise!	Jingdong prefecture level city in Shandong
Happy Fan Shop	King of Power Saving	The logistics is very fast, the packaging is not broken, the quality is very good, I like it very much!	Jingdong prefecture level city in Shandong
Happy Fan Shop	King of Power Saving	The saver was well packaged and was as described when plugged in	Jingdong prefecture level city in Shandong
Happy Fan Shop	King of Power Saving	Energy efficient and very satisfied	Jingdong prefecture level city in Shandong
Happy Fan Shop	King of Power Saving	Great quality for the price and fast shipping.	Jingdong prefecture level city in Shandong
Happy Fan Shop	King of Power Saving	It's great, just as described. Perfect.	Jingdong prefecture level city in Shandong
Happy Fan Shop	King of Power Saving	There is no power saving effect, but rather ring up an increase in electricity costs, because this machine is on continuously all day long, uninterruptedly, its own electricity and increased costs, plus the cost of purchasing the machine, so I feel that the use of this machine instead of increasing the	Jingdong prefecture level city in Shandong
Happy Fan Shop	King of Power Saving	Recommended by the owner of the store next door, looked at his home does have to save electricity, decided to get. Indeed quite can, the voltage is much more stable, there is a reduction in electricity	Jingdong prefecture level city in Shandong
Happy Fan Shop	King of Power Saving	Goods have been received value for money, affordable and reasonable price, and the description of the same, power-saving safe and reliable!	Jingdong prefecture level city in Shandong
Happy Fan Shop	King of Power Saving	Listen to friends introduced to buy, tried to save a lot of electricity is good, quite good, worth buying.	Jingdong prefecture level city in Shandong
Happy Fan Shop	King of Power Saving	The product is easy to use, safe and environmentally friendly, only used for a short time, to the next month, after the electricity bill status display to know how the performance of the power saver efficacy.	Jingdong prefecture level city in Shandong
Happy Fan Shop	King of Power Saving	Last month's electricity bill is 178 yuan, with this power saver is 92 yuan, ready to buy two more back home, I was introduced to buy a friend, just started to not believe that I did not expect the effect!	Jingdong prefecture level city in Shandong
Happy Fan Shop	King of Power Saving	It looks nice, but after using it for a few days I can't see how it saves power?	Jingdong prefecture level city in Shandong
Happy Fan Shop	King of Power Saving	Recommended by a friend, it is genuine, as described by the merchant, cost-effective	Jingdong prefecture level city in Shandong
Happy Fan Shop	King of Power Saving	Works well, affordable and saves money.	Jingdong prefecture level city in Shandong
Happy Fan Shop	King of Power Saving	Feels good haven't compared it yet, the air conditioning has been on a lot in the past two months	Jingdong prefecture level city in Shandong
Happy Fan Shop	King of Power Saving	Saving about 5 kWh per day is a good thing	Jingdong prefecture level city in Shandong
Happy Fan Shop	King of Power Saving	Not bad, affordable, best value for money	Jingdong prefecture level city in Shandong
Happy Fan Shop	King of Power Saving	The goods are genuine and the logistics are faster.	Jingdong prefecture level city in Shandong

Happy Fan Shop	King of Power Saving	Goods have been received today, has been plugged into the outlet, looking forward to saving electricity, after a period of time to see how the effect in the follow-up comment!	Jingdong prefecture level city in Shandong
Happy Fan Shop	King of Power Saving	I just got it home and haven't used it for long, so I'll use it for a couple months and see what happens. Seeing that most people have good reviews, it should be fine.	Jingdong prefecture level city in Shandong
Happy Fan Shop	King of Power Saving	I'm looking forward to the results, so I'll give you a good review out of habit! Follow up review after a while	Jingdong prefecture level city in Shandong
Happy Fan Shop	King of Power Saving	Use a dish first to know if it will help save energy	Jingdong prefecture level city in Shandong
Happy Fan Shop	King of Power Saving	Whether it saves electricity or not, we don't know yet.	Jingdong prefecture level city in Shandong
Happy Fan Shop	King of Power Saving	Goods have been received, use a period of time to know how oh. The courier did not deliver to the door ah! Sent to the station ah! The heat is inconvenient ah!	Jingdong prefecture level city in Shandong
Happy Fan Shop	King of Power Saving	A little effect, before an hour a power, now an hour 0.76 degrees, a dollar a power = save 24 cents, although not a lot, first use it!	Jingdong prefecture level city in Shandong
Happy Fan Shop	King of Power Saving	Just received the goods, use a month to evaluate	Jingdong prefecture level city in Shandong
Happy Fan Shop	King of Power Saving	Home every month with a lot of electricity, inadvertently found this good thing online that can save the beginning of a skeptical attitude to consult customer service, customer service replied very professional, said 120 days can be refunded that first try it!	Jingdong prefecture level city in Shandong
Happy Fan Shop	King of Power Saving	After receiving the goods this afternoon, immediately installed in the bathroom Smith energy storage electric water heater on the power outlet (instead of connecting with a plug board), when used, found that the "Power Saving King" LCD display, is the value of the voltage, not the store's video said so	Jingdong prefecture level city in Shandong
Happy Fan Shop	King of Power Saving	Has made many days, did not feel the power saving, the effect is not obvious.	Jingdong prefecture level city in Shandong
Happy Fan Shop	King of Power Saving	Energy savers do not save energy at all, but rather consume a lot of electricity.	Jingdong prefecture level city in Shandong
Happy Fan Shop	King of Power Saving	It's useless. It's a waste of money.	Jingdong prefecture level city in Shandong
Happy Fan Shop	King of Power Saving	Last month saved more than 500, which came back to buy, the store bought three, and then buy 1 home use, my family is dry beauty salon, more instruments, especially electricity, a month almost 1000. or so, with this did not expect to save so much, great, five-star praise!	Jingdong prefecture level city in Shandong
Happy Fan Shop	King of Power Saving	It rained a couple days ago and I just went to get the item back today , not bad, hey I looked at the meter and that red display light takes several seconds to jump, did not make a mistake buying it. After a period of time to evaluate.	Jingdong prefecture level city in Shandong
Happy Fan Shop	King of Power Saving	Without further ado, the electricity bill came down, this month less 102 yuan, has been repurchased, I also recommended to my friends, all said that there are effects, the product is okay, the effect is quite	Jingdong prefecture level city in Shandong
Happy Fan Shop	King of Power Saving	After buying and using two months really see the effect, so only to evaluate! Originally held the mindset of trying, I did not expect really effective, worth having!	Jingdong prefecture level city in Shandong
Happy Fan Shop	King of Power Saving	In the use of this energy saver before the monthly electricity bill is more than 150 yuan or so, now save thirty percent, every day to work, six or seven o'clock in the afternoon to go home, I do not know why the electricity bill is so expensive, energy-saving appliances really have the effect of recommending	Jingdong prefecture level city in Shandong
Happy Fan Shop	King of Power Saving	It saves about 100 bucks a month. A friend recommended it to me, and he's been using it for a while, and the effect is so obvious that I bought one too.	Jingdong prefecture level city in Shandong
Happy Fan Shop	King of Power Saving	My dad asked me to buy one, but after I paid the bill, I asked and he said it was a lot less this month, so he asked me to buy another one.	Jingdong prefecture level city in Shandong
Happy Fan Shop	King of Power Saving	The specific effect will have to be tested for at least two months to compare whether there is a power saving effect or not.	Jingdong prefecture level city in Shandong
Happy Fan Shop	King of Power Saving	'The goods have been received, immediately go to use, please ask the boss you instructions do not allow to withdraw, there are two fuses installed there!	Jingdong prefecture level city in Shandong
Happy Fan Shop	King of Power Saving	First praise is using, after using if good then follow up, now because I have not yet seen the results	Jingdong prefecture level city in Shandong
Happy Fan Shop	King of Power Saving	I've just been using it, and I haven't seen if it has a power-saving effect yet, so I'll follow up after 10 days and half a month.	Jingdong prefecture level city in Shandong
Happy Fan Shop	King of Power Saving	Just received the intelligent power saving meter, immediately plugged in to use, see it is not bad, and order to sell the second time	Jingdong prefecture level city in Shandong
Happy Fan Shop	King of Power Saving	This power saver is a bit effective, used for half a month, the effect is still visible.	Jingdong prefecture level city in Shandong
Happy Fan Shop	King of Power Saving	I received the baby and it looks great, I'm trying it out now and will keep an eye on it afterward.	Jingdong prefecture level city in Shandong
Happy Fan Shop	King of Power Saving	I've just received it, but I'm still trying to see how it works, so I'll come back in a little while.	Jingdong prefecture level city in Shandong
Happy Fan Shop	King of Power Saving	Just used it wait to use it for a while and then comment thanks, all first five stars.	Jingdong prefecture level city in Shandong
Happy Fan Shop	King of Power Saving	Very trustworthy brand, quality is guaranteed, the price is still affordable, quite satisfied!	Jingdong prefecture level city in Shandong
Happy Fan Shop	King of Power Saving	Things received, I hope that the platform of Jingdong do not sell fake goods are available	Jingdong prefecture level city in Shandong
Happy Fan Shop	King of Power Saving	Energy saver received, the use of the effect is good and the business description of the same	Jingdong prefecture level city in Shandong
Happy Fan Shop	King of Power Saving	The surface looks beautiful, the actual effect is not known, after using additional evaluation.	Jingdong prefecture level city in Shandong
Happy Fan Shop	King of Power Saving	Tried it for a few months, it really saves electricity, and I bought it for a friend.	Jingdong prefecture level city in Shandong
Happy Fan Shop	King of Power Saving	It didn't work and wanted to return it, it really didn't work at all.	Jingdong prefecture level city in Shandong
Happy Fan Shop	King of Power Saving	Just used it, don't know how effective it is, looking forward to usefulness	Jingdong prefecture level city in Shandong
Happy Fan Shop	King of Power Saving	After two months before the effect I do not know if it is true!	Jingdong prefecture level city in Shandong
Happy Fan Shop	King of Power Saving	So fast! Ordered yesterday and received today 👍👍👍👍👍👍👍👍👍 🌹🌹🌹🌹🌹🌹🌹🌹🌹	Jingdong prefecture level city in Shandong
Happy Fan Shop	King of Power Saving	I've started using it. We'll see how it works.	Jingdong prefecture level city in Shandong
Happy Fan Shop	King of Power Saving	I just received the goods, I won't know the effect until after two.	Jingdong prefecture level city in Shandong
Happy Fan Shop	King of Power Saving	Just received the goods, the specific effect will be evaluated later	Jingdong prefecture level city in Shandong
Happy Fan Shop	King of Power Saving	I've received the goods, I don't know if it's any good yet	Jingdong prefecture level city in Shandong
Happy Fan Shop	King of Power Saving	Try it for a month and then come back with a concrete evaluation	Jingdong prefecture level city in Shandong
Happy Fan Shop	King of Power Saving	In use, after two months to see the effect of power saving	Jingdong prefecture level city in Shandong
Happy Fan Shop	King of Power Saving	Power Saver. It's authentic. It's black. It works.	Jingdong prefecture level city in Shandong
Happy Fan Shop	King of Power Saving	I've been using it for a week now and it's not working.	Jingdong prefecture level city in Shandong
Happy Fan Shop	King of Power Saving	I'm just using it. I don't know how it's going to work.	Jingdong prefecture level city in Shandong
Happy Fan Shop	King of Power Saving	Products are better looking out for power savings	Jingdong prefecture level city in Shandong
Happy Fan Shop	King of Power Saving	I received it and I've kept it on. I hope it works.	Jingdong prefecture level city in Shandong

Happy Fan Shop	King of Power Saving	It's really good! Worth recommending to buy!	Jingdong prefecture level city in Shandong
Happy Fan Shop	King of Power Saving	Try it for a few days and see if it doesn't work, then return it.	Jingdong prefecture level city in Shandong
Happy Fan Shop	King of Power Saving	It's in use and I hope to be pleasantly surprised.	Jingdong prefecture level city in Shandong
Happy Fan Shop	King of Power Saving	We'll see how it works when we use it	Jingdong prefecture level city in Shandong
Happy Fan Shop	King of Power Saving	Let's see if it works.	Jingdong prefecture level city in Shandong
Happy Fan Shop	King of Power Saving	I'm using it and will follow up with a review	Jingdong prefecture level city in Shandong
Happy Fan Shop	King of Power Saving	It really works. It saves energy.	Jingdong prefecture level city in Shandong
Happy Fan Shop	King of Power Saving	It does have some power savings!	Jingdong prefecture level city in Shandong
Happy Fan Shop	King of Power Saving	Jingdong shopping speed is convenient and timely	Jingdong prefecture level city in Shandong
Happy Fan Shop	King of Power Saving	Second time I've bought it. Everything's fine.	Jingdong prefecture level city in Shandong
Happy Fan Shop	King of Power Saving	I don't know if it works yet.	Jingdong prefecture level city in Shandong
Happy Fan Shop	King of Power Saving	Not used, don't know how well it works.	Jingdong prefecture level city in Shandong
Happy Fan Shop	King of Power Saving	No visible results so far	Jingdong prefecture level city in Shandong
Happy Fan Shop	King of Power Saving	It doesn't feel like it's working.	Jingdong prefecture level city in Shandong
Happy Fan Shop	King of Power Saving	Where's the effect? Where are the power savings?	Jingdong prefecture level city in Shandong
Happy Fan Shop	King of Power Saving	Let's see how it works in practice!	Jingdong prefecture level city in Shandong
Happy Fan Shop	King of Power Saving	I'll wait until I use it to give you a good review	Jingdong prefecture level city in Shandong
Happy Fan Shop	King of Power Saving	I'll follow up after I've used it	Jingdong prefecture level city in Shandong
Happy Fan Shop	King of Power Saving	Well, that's good for saving power.	Jingdong prefecture level city in Shandong
Happy Fan Shop	King of Power Saving	Received. Used in the know.	Jingdong prefecture level city in Shandong
Happy Fan Shop	King of Power Saving	It doesn't do much good.	Jingdong prefecture level city in Shandong
Happy Fan Shop	King of Power Saving	It's not working.	Jingdong prefecture level city in Shandong
Happy Fan Shop	King of Power Saving	I don't know. I just used it.	Jingdong prefecture level city in Shandong
Happy Fan Shop	King of Power Saving	Use it and come back to evaluate.	Jingdong prefecture level city in Shandong
Happy Fan Shop	King of Power Saving	Two words: useless.	Jingdong prefecture level city in Shandong
Happy Fan Shop	King of Power Saving	To be used and then evaluated	Jingdong prefecture level city in Shandong
Happy Fan Shop	King of Power Saving	No shipment.	Jingdong prefecture level city in Shandong
Happy Fan Shop	King of Power Saving	Fake. Speechless.	Jingdong prefecture level city in Shandong
Happy Fan Shop	King of Power Saving	fantastic (idiom); awesome	Jingdong prefecture level city in Shandong
Happy Fan Shop	King of Power Saving	an excellent	Jingdong prefecture level city in Shandong
Happy Fan Shop	King of Power Saving	an excellent	Jingdong prefecture level city in Shandong
Happy Fan Shop	King of Power Saving	Want to buy! Recommended not to buy! No effect	Jingdong prefecture level city in Shandong
Happy Fan Shop	King of Power Saving	That's all there is to it. I can't figure out how to save electricity.	Jingdong prefecture level city in Shandong
Happy Fan Shop	King of Power Saving	It's no use.	Jingdong prefecture level city in Shandong
Happy Fan Shop	King of Power Saving	Things are more perfect than I imagined, very powerful, it is worth getting Packaging is very strong, things are well protected, it is worth the praise things are very satisfied Oh, beautiful and stylish atmosphere, customer service is super patient, service is meticulous and attentive, the big brand is not	Jingdong prefecture level city in Shandong
Happy Fan Shop	King of Power Saving	The effect is too good, the family has now several units, back to buy a number of times, the power saver received, power-saving effect is very good,, fine workmanship, long warranty, but also purify the air, before buying a used for a period of time, with the okay, and then bought a, and only now come to the	Jingdong prefecture level city in Shandong
Happy Fan Shop	King of Power Saving	Quite good, and imagine the same, casual ride are very good, satisfied! Baby received it 👌, really great ah! 🌈 quality is really nothing to say, very satisfied! 🆗 happy happy 😃 genuine, great, the price is very good, in short, quite good, will buy again a very good shopping experience, attentive service, fast logistics, distribution is also thoughtful, the attitude is also very good, value for money, ah,	Jingdong prefecture level city in Shandong
Happy Fan Shop	King of Power Saving	Power-saving effect: strong Cost-effective: quite good Appearance style: beautiful Workmanship material: meticulous Experience: looking forward to power-saving effect	Jingdong prefecture level city in Shandong
Happy Fan Shop	King of Power Saving	Cost-effective is okay, the appearance of the style of meticulous workmanship, looking forward to energy-saving effect.	Jingdong prefecture level city in Shandong
Happy Fan Shop	King of Power Saving	Very Good Very Power Saving This Power Saver is really power saving As soon as the power is turned on, the Power Saver starts the power saving function for you.	Jingdong prefecture level city in Shandong
Happy Fan Shop	King of Power Saving	Logistics is very fast, the price is favorable, friends introduced that it is very good to use, bought 2 at once, looking forward to the effect, used after the follow-up comment.	Jingdong prefecture level city in Shandong
Happy Fan Shop	King of Power Saving	It's all fake. It doesn't save any electricity.	Jingdong prefecture level city in Shandong
Happy Fan Shop	King of Power Saving	This Power Saver is so much better than the one I bought before, not only the effect is good, the quality is very good in all aspects, the customer service attitude is also very good, recommended to buy!	Jingdong prefecture level city in Shandong
Happy Fan Shop	King of Power Saving	Logistics is very fast, buy for parents, good quality, once bought 2, power-saving effect is good to buy 2 more, look forward to using the effect, satisfied will recommend to friends.	Jingdong prefecture level city in Shandong
Happy Fan Shop	King of Power Saving	With the description is the same, very high-grade, good value, to buy friends can order Oh!	Jingdong prefecture level city in Shandong
Happy Fan Shop	King of Power Saving	Not using much electricity in the winter saving over 100 per month! Ready to buy 2 more units in the summer ！！！！！ Good quality, good price, power saving effect is obvious, cost-effective, worth buying, can be recommended!	Jingdong prefecture level city in Shandong
Happy Fan Shop	King of Power Saving	Purchased a power saver received on the put into use, rent a room with many electrical appliances, power consumption is very large, we have a piece of electricity or 1.2 dollars a time, with this power saver is much better, every day can save a lot of money, very convenient and practical.	Jingdong prefecture level city in Shandong

Happy Fan Shop	King of Power Saving	The power saving effect is very good: my supermarket monthly electricity bill is more than 7000 yuan. On May 1st someone recommended buying a couple of power savers, and I bought 5 of them first. The electricity bill was about 2000 less in June. This time, I bought 5 more. The seller said 800 degrees of electricity with one. I feel it's basically enough. A total of 10 units	Jingdong prefecture level city in Shandong
Happy Fan Shop	King of Power Saving	I never thought I could get such a good baby at such an affordable price, it's very good value for money, I love it!	Jingdong prefecture level city in Shandong
Happy Fan Shop	King of Power Saving	Previously bought other brands of power savers, but found no effect at home, this time to see how the products on the Jingdong, inserted in the home socket, the LED display is blue, the night light is very nice to see, look at the instructions that have to be half a year in order to achieve the best results, try it, I believe that the quality of the Jingdong, it should not be wrong.	Jingdong prefecture level city in Shandong
Happy Fan Shop	King of Power Saving	Superb, inexpensive, saves a lot of points, been using it for a long time!	Jingdong prefecture level city in Shandong
Happy Fan Shop	King of Power Saving	There are a lot of appliances in the house, the summer air conditioning is very costly, I bought this in front of the air conditioning effect is very good, I explain that I bought three in one breath, I use more electricity a month down to 200 or so, very satisfied.	Jingdong prefecture level city in Shandong
Happy Fan Shop	King of Power Saving	A month can save more than 200, home monthly electricity bill of more than 600 dollars, inserted 3 or so, an amount of the balance of the average can save more than 200. Product is very good, has been buying this one, has given a lot of relatives recommended, the quality is also very good, before buying a look with this same, who knows to buy home has not been used for more than a week on the bad, the quality of this is really good with more than two years!	Jingdong prefecture level city in Shandong
Happy Fan Shop	King of Power Saving	Whole house power saving king, can really save electricity, friends recommended, high power saving appliances, only replacement, no repair, after-sales worry-free!	Jingdong prefecture level city in Shandong
Happy Fan Shop	King of Power Saving	Great item love it, great customer service, great shopping experience!	Jingdong prefecture level city in Shandong
Happy Fan Shop	King of Power Saving	Power saving, very good use, much cheaper than the physical store, satisfaction	Jingdong prefecture level city in Shandong
Happy Fan Shop	King of Power Saving	In use, very well packaged and fast delivery of credit!	Jingdong prefecture level city in Shandong
Happy Fan Shop	King of Power Saving	Product quality, customer service attitude is particularly good, like, praise!	Jingdong prefecture level city in Shandong
Happy Fan Shop	King of Power Saving	The logistics is very fast, the packaging is not broken, the quality is very good, I like it very much!	Jingdong prefecture level city in Shandong
Happy Fan Shop	King of Power Saving	The saver was well packaged and was as described when plugged in	Jingdong prefecture level city in Shandong
Happy Fan Shop	King of Power Saving	Energy efficient and very satisfied	Jingdong prefecture level city in Shandong
Happy Fan Shop	King of Power Saving	Great quality for the price and fast shipping.	Jingdong prefecture level city in Shandong
Happy Fan Shop	King of Power Saving	It's great, just as described. Perfect.	Jingdong prefecture level city in Shandong
Happy Fan Shop	King of Power Saving	There is no power saving effect, but rather ring up an increase in electricity costs, because this machine is on continuously all day long, uninterruptedly, its own electricity and increased costs, plus the cost of purchasing the machine, so I feel that the use of this machine instead of increasing the	Jingdong prefecture level city in Shandong
Happy Fan Shop	King of Power Saving	Recommended by the owner of the store next door, looked at his home does have to save electricity, decided to get. Indeed quite can, the voltage is much more stable, there is a reduction in electricity	Jingdong prefecture level city in Shandong
Happy Fan Shop	King of Power Saving	Goods have been received value for money, affordable and reasonable price, and the description of the same, power-saving safe and reliable!	Jingdong prefecture level city in Shandong
Happy Fan Shop	King of Power Saving	Listen to friends introduced to buy, tried to save a lot of electricity is good, quite good, worth buying.	Jingdong prefecture level city in Shandong
Happy Fan Shop	King of Power Saving	The product is easy to use, safe and environmentally friendly, only used for a short time, to the next month, after the electricity bill status display to know how the performance of the power saver efficacy.	Jingdong prefecture level city in Shandong
Happy Fan Shop	King of Power Saving	Last month's electricity bill is 178 yuan, with this power saver is 92 yuan, ready to buy two more back home, I was introduced to buy a friend, just started to not believe that I did not expect the effect!	Jingdong prefecture level city in Shandong
Happy Fan Shop	King of Power Saving	It looks nice, but after using it for a few days I can't see how it saves power?	Jingdong prefecture level city in Shandong
Happy Fan Shop	King of Power Saving	Recommended by a friend, it is genuine, as described by the merchant, cost-effective	Jingdong prefecture level city in Shandong
Happy Fan Shop	King of Power Saving	Works well, affordable and saves money.	Jingdong prefecture level city in Shandong
Happy Fan Shop	King of Power Saving	Feels good haven't compared it yet, the air conditioning has been on a lot in the past two months	Jingdong prefecture level city in Shandong
Happy Fan Shop	King of Power Saving	Saving about 5 kWh per day is a good thing	Jingdong prefecture level city in Shandong
Happy Fan Shop	King of Power Saving	Not bad, affordable, best value for money	Jingdong prefecture level city in Shandong
Happy Fan Shop	King of Power Saving	The goods are genuine and the logistics are faster.	Jingdong prefecture level city in Shandong
Happy Fan Shop	King of Power Saving	Goods have been received today, has been plugged into the outlet, looking forward to saving electricity, after a period of time to see how the effect in the follow-up comment!	Jingdong prefecture level city in Shandong
Happy Fan Shop	King of Power Saving	I just got it home and haven't used it for long, so I'll use it for a couple months and see what happens. Seeing that most people have good reviews, it should be fine.	Jingdong prefecture level city in Shandong
Happy Fan Shop	King of Power Saving	I'm looking forward to the results, so I'll give you a good review out of habit! Follow up review after a while	Jingdong prefecture level city in Shandong
Happy Fan Shop	King of Power Saving	Use a dish first to know if it will help save energy	Jingdong prefecture level city in Shandong
Happy Fan Shop	King of Power Saving	Whether it saves electricity or not, we don't know yet.	Jingdong prefecture level city in Shandong
Happy Fan Shop	King of Power Saving	Goods have been received, use a period of time to know how oh. The courier did not deliver to the door ah! Sent to the station ah! The heat is inconvenient ah!	Jingdong prefecture level city in Shandong
Happy Fan Shop	King of Power Saving	A little effect, before an hour a power, now an hour 0.76 degrees, a dollar a power = save 24 cents, although not a lot, first use it!	Jingdong prefecture level city in Shandong
Happy Fan Shop	King of Power Saving	Just received the goods, use a month to evaluate	Jingdong prefecture level city in Shandong
Happy Fan Shop	King of Power Saving	Home every month with a lot of electricity, inadvertently found this good thing online that can save the beginning of a skeptical attitude to consult customer service, customer service replied very professional, said 120 days can be refunded that first try it!	Jingdong prefecture level city in Shandong
Happy Fan Shop	King of Power Saving	After receiving the goods this afternoon, immediately installed in the bathroom Smith energy storage electric water heater on the power outlet (instead of connecting with a plug board), when used, found that the "Power Saving King" LCD display, is the value of the voltage, not the store's video said so	Jingdong prefecture level city in Shandong
Happy Fan Shop	King of Power Saving	Has made many days, did not feel the power saving, the effect is not obvious.	Jingdong prefecture level city in Shandong
Happy Fan Shop	King of Power Saving	Energy savers do not save energy at all, but rather consume a lot of electricity.	Jingdong prefecture level city in Shandong
Happy Fan Shop	King of Power Saving	It's useless. It's a waste of money.	Jingdong prefecture level city in Shandong
Happy Fan Shop	King of Power Saving	Last month saved more than 500, which came back to buy, the store bought three, and then buy 1 home use, my family is dry beauty salon, more instruments, especially electricity, a month almost 1000. or so, with this did not expect to save so much, great, five-star praise!	Jingdong prefecture level city in Shandong
Happy Fan Shop	King of Power Saving	It rained a couple days ago and I just went to get the item back today , not bad, hey I looked at the meter and that red display light takes several seconds to jump, did not make a mistake buying it. After a period of time to evaluate.	Jingdong prefecture level city in Shandong
Happy Fan Shop	King of Power Saving	Without further ado, the electricity bill came down, this month less 102 yuan, has been repurchased, I also recommended to my friends, all said that there are effects, the product is okay, the effect is quite	Jingdong prefecture level city in Shandong

Happy Fan Shop	King of Power Saving	After buying and using two months really see the effect, so only to evaluate! Originally held the mindset of trying, I did not expect really effective, worth having!	Jingdong prefecture level city in Shandong
Happy Fan Shop	King of Power Saving	In the use of this energy saver before the monthly electricity bill is more than 150 yuan or so, now save thirty percent, every day to work, six or seven o'clock in the afternoon to go home, I do not know why the electricity bill is so expensive, energy-saving appliances really have the effect of recommending	Jingdong prefecture level city in Shandong
Happy Fan Shop	King of Power Saving	It saves about 100 bucks a month. A friend recommended it to me, and he's been using it for a while, and the effect is so obvious that I bought one too.	Jingdong prefecture level city in Shandong
Happy Fan Shop	King of Power Saving	My dad asked me to buy one, but after I paid the bill, I asked and he said it was a lot less this month, so he asked me to buy another one.	Jingdong prefecture level city in Shandong
Happy Fan Shop	King of Power Saving	The specific effect will have to be tested for at least two months to compare whether there is a power saving effect or not.	Jingdong prefecture level city in Shandong
Happy Fan Shop	King of Power Saving	'The goods have been received, immediately go to use, please ask the boss you instructions do not allow to withdraw, there are two fuses installed there!	Jingdong prefecture level city in Shandong
Happy Fan Shop	King of Power Saving	First praise is using, after using if good then follow up, now because I have not yet seen the results	Jingdong prefecture level city in Shandong
Happy Fan Shop	King of Power Saving	I've just been using it, and I haven't seen if it has a power-saving effect yet, so I'll follow up after 10 days and half a month.	Jingdong prefecture level city in Shandong
Happy Fan Shop	King of Power Saving	Just received the intelligent power saving meter, immediately plugged in to use, see it is not bad, and order to sell the second time	Jingdong prefecture level city in Shandong
Happy Fan Shop	King of Power Saving	This power saver is a bit effective, used for half a month, the effect is still visible.	Jingdong prefecture level city in Shandong
Happy Fan Shop	King of Power Saving	I received the baby and it looks great, I'm trying it out now and will keep an eye on it afterward.	Jingdong prefecture level city in Shandong
Happy Fan Shop	King of Power Saving	I've just received it, but I'm still trying to see how it works, so I'll come back in a little while.	Jingdong prefecture level city in Shandong
Happy Fan Shop	King of Power Saving	Just used it wait to use it for a while and then comment thanks, all first five stars.	Jingdong prefecture level city in Shandong
Happy Fan Shop	King of Power Saving	Very trustworthy brand, quality is guaranteed, the price is still affordable, quite satisfied!	Jingdong prefecture level city in Shandong
Happy Fan Shop	King of Power Saving	Things received, I hope that the platform of Jingdong do not sell fake goods are available	Jingdong prefecture level city in Shandong
Happy Fan Shop	King of Power Saving	Energy saver received, the use of the effect is good and the business description of the same	Jingdong prefecture level city in Shandong
Happy Fan Shop	King of Power Saving	The surface looks beautiful, the actual effect is not known, after using additional evaluation.	Jingdong prefecture level city in Shandong
Happy Fan Shop	King of Power Saving	Tried it for a few months, it really saves electricity, and I bought it for a friend.	Jingdong prefecture level city in Shandong
Happy Fan Shop	King of Power Saving	It didn't work and wanted to return it, it really didn't work at all.	Jingdong prefecture level city in Shandong
Happy Fan Shop	King of Power Saving	Just used it, don't know how effective it is, looking forward to usefulness	Jingdong prefecture level city in Shandong
Happy Fan Shop	King of Power Saving	After two months before the effect I do not know if it is true!	Jingdong prefecture level city in Shandong
Happy Fan Shop	King of Power Saving	So fast! Ordered yesterday and received today 👍👍👍👍👍👍👍👍👍 🌹🌹🌹🌹🌹🌹🌹🌹🌹	Jingdong prefecture level city in Shandong
Happy Fan Shop	King of Power Saving	I've started using it. We'll see how it works.	Jingdong prefecture level city in Shandong
Happy Fan Shop	King of Power Saving	I just received the goods, I won't know the effect until after two.	Jingdong prefecture level city in Shandong
Happy Fan Shop	King of Power Saving	Just received the goods, the specific effect will be evaluated later	Jingdong prefecture level city in Shandong
Happy Fan Shop	King of Power Saving	I've received the goods, I don't know if it's any good yet	Jingdong prefecture level city in Shandong
Happy Fan Shop	King of Power Saving	Try it for a month and then come back with a concrete evaluation	Jingdong prefecture level city in Shandong
Happy Fan Shop	King of Power Saving	In use, after two months to see the effect of power saving	Jingdong prefecture level city in Shandong
Happy Fan Shop	King of Power Saving	Power Saver. It's authentic. It's black. It works.	Jingdong prefecture level city in Shandong
Happy Fan Shop	King of Power Saving	I've been using it for a week now and it's not working.	Jingdong prefecture level city in Shandong
Happy Fan Shop	King of Power Saving	I'm just using it. I don't know how it's going to work.	Jingdong prefecture level city in Shandong
Happy Fan Shop	King of Power Saving	Products are better looking out for power savings	Jingdong prefecture level city in Shandong
Happy Fan Shop	King of Power Saving	I received it and I've kept it on. I hope it works.	Jingdong prefecture level city in Shandong
Happy Fan Shop	King of Power Saving	It's really good! Worth recommending to buy!	Jingdong prefecture level city in Shandong
Happy Fan Shop	King of Power Saving	Try it for a few days and see if it doesn't work, then return it.	Jingdong prefecture level city in Shandong
Happy Fan Shop	King of Power Saving	It's in use and I hope to be pleasantly surprised.	Jingdong prefecture level city in Shandong
Happy Fan Shop	King of Power Saving	We'll see how it works when we use it	Jingdong prefecture level city in Shandong
Happy Fan Shop	King of Power Saving	Let's see if it works.	Jingdong prefecture level city in Shandong
Happy Fan Shop	King of Power Saving	I'm using it and will follow up with a review	Jingdong prefecture level city in Shandong
Happy Fan Shop	King of Power Saving	It really works. It saves energy.	Jingdong prefecture level city in Shandong
Happy Fan Shop	King of Power Saving	It does have some power savings!	Jingdong prefecture level city in Shandong
Happy Fan Shop	King of Power Saving	Jingdong shopping speed is convenient and timely	Jingdong prefecture level city in Shandong
Happy Fan Shop	King of Power Saving	Second time I've bought it. Everything's fine.	Jingdong prefecture level city in Shandong
Happy Fan Shop	King of Power Saving	I don't know if it works yet.	Jingdong prefecture level city in Shandong
Happy Fan Shop	King of Power Saving	Not used, don't know how well it works.	Jingdong prefecture level city in Shandong
Happy Fan Shop	King of Power Saving	No visible results so far	Jingdong prefecture level city in Shandong
Happy Fan Shop	King of Power Saving	It doesn't feel like it's working.	Jingdong prefecture level city in Shandong
Happy Fan Shop	King of Power Saving	Where's the effect? Where are the power savings?	Jingdong prefecture level city in Shandong
Happy Fan Shop	King of Power Saving	Let's see how it works in practice!	Jingdong prefecture level city in Shandong
Happy Fan Shop	King of Power Saving	I'll wait until I use it to give you a good review	Jingdong prefecture level city in Shandong
Happy Fan Shop	King of Power Saving	I'll follow up after I've used it	Jingdong prefecture level city in Shandong

Happy Fan Shop	King of Power Saving	Well, that's good for saving power.	Jingdong prefecture level city in Shandong
Happy Fan Shop	King of Power Saving	Received. Used in the know.	Jingdong prefecture level city in Shandong
Happy Fan Shop	King of Power Saving	It doesn't do much good.	Jingdong prefecture level city in Shandong
Happy Fan Shop	King of Power Saving	It's not working.	Jingdong prefecture level city in Shandong
Happy Fan Shop	King of Power Saving	I don't know. I just used it.	Jingdong prefecture level city in Shandong
Happy Fan Shop	King of Power Saving	Use it and come back to evaluate.	Jingdong prefecture level city in Shandong
Happy Fan Shop	King of Power Saving	Two words: useless.	Jingdong prefecture level city in Shandong
Happy Fan Shop	King of Power Saving	To be used and then evaluated	Jingdong prefecture level city in Shandong
Happy Fan Shop	King of Power Saving	No shipment.	Jingdong prefecture level city in Shandong
Happy Fan Shop	King of Power Saving	Fake. Speechless.	Jingdong prefecture level city in Shandong
Happy Fan Shop	King of Power Saving	fantastic (idiom); awesome	Jingdong prefecture level city in Shandong
Happy Fan Shop	King of Power Saving	an excellent	Jingdong prefecture level city in Shandong
Happy Fan Shop	King of Power Saving	an excellent	Jingdong prefecture level city in Shandong
Happy Fan Shop	King of Power Saving	Want to buy! Recommended not to buy! No effect	Jingdong prefecture level city in Shandong
Happy Fan Shop	King of Power Saving	That's all there is to it. I can't figure out how to save electricity.	Jingdong prefecture level city in Shandong
Happy Fan Shop	King of Power Saving	It's no use.	Jingdong prefecture level city in Shandong
Happy Fan Shop	King of Power Saving	Things are more perfect than I imagined, very powerful, it is worth getting Packaging is very strong, things are well protected, it is worth the praise things are very satisfied Oh, beautiful and stylish atmosphere, customer service is super patient, service is meticulous and attentive, the big brand is not	Jingdong prefecture level city in Shandong
Happy Fan Shop	King of Power Saving	The effect is too good, the family has now several units, back to buy a number of times, the power saver received, power-saving effect is very good,, fine workmanship, long warranty, but also purify the air, before buying a used for a period of time, with the okay, and then bought a, and only now come to the	Jingdong prefecture level city in Shandong
Happy Fan Shop	King of Power Saving	Quite good, and imagine the same, casual ride are very good, satisfied! Baby received it 👌, really great ah! 🌈 quality is really nothing to say, very satisfied! 🆗 happy happy 😃 genuine, great, the price is very good, in short, quite good, will buy again a very good shopping experience, attentive service, fast logistics, distribution is also thoughtful, the attitude is also very good, value for money, ah,	Jingdong prefecture level city in Shandong
Happy Fan Shop	King of Power Saving	Power-saving effect: strong Cost-effective: quite good Appearance style: beautiful Workmanship material: meticulous Experience: looking forward to power-saving effect	Jingdong prefecture level city in Shandong
Happy Fan Shop	King of Power Saving	Cost-effective is okay, the appearance of the style of meticulous workmanship, looking forward to energy-saving effect.	Jingdong prefecture level city in Shandong
Happy Fan Shop	King of Power Saving	Very Good Very Power Saving This Power Saver is really power saving As soon as the power is turned on, the Power Saver starts the power saving function for you.	Jingdong prefecture level city in Shandong
Happy Fan Shop	King of Power Saving	Logistics is very fast, the price is favorable, friends introduced that it is very good to use, bought 2 at once, looking forward to the effect, used after the follow-up comment.	Jingdong prefecture level city in Shandong
Happy Fan Shop	King of Power Saving	It's all fake. It doesn't save any electricity.	Jingdong prefecture level city in Shandong
Happy Fan Shop	King of Power Saving	This Power Saver is so much better than the one I bought before, not only the effect is good, the quality is very good in all aspects, the customer service attitude is also very good, recommended to buy!	Jingdong prefecture level city in Shandong
Happy Fan Shop	King of Power Saving	Logistics is very fast, buy for parents, good quality, once bought 2, power-saving effect is good to buy 2 more, look forward to using the effect, satisfied will recommend to friends.	Jingdong prefecture level city in Shandong
Happy Fan Shop	King of Power Saving	With the description is the same, very high-grade, good value, to buy friends can order Oh!	Jingdong prefecture level city in Shandong
Happy Fan Shop	King of Power Saving	Not using much electricity in the winter saving over 100 per month! Ready to buy 2 more units in the summer ! ! ! ! ! Good quality, good price, power saving effect is obvious, cost-effective, worth buying, can be recommended!	Jingdong prefecture level city in Shandong
Happy Fan Shop	King of Power Saving	Purchased a power saver received on the put into use, rent a room with many electrical appliances, power consumption is very large, we have a piece of electricity or 1.2 dollars a time, with this power saver is much better, every day can save a lot of money, very convenient and practical.	Jingdong prefecture level city in Shandong
Happy Fan Shop	King of Power Saving	The power saving effect is very good: my supermarket monthly electricity bill is more than 7000 yuan. On May 1st someone recommended buying a couple of power savers, and I bought 5 of them first. The electricity bill was about 2000 less in June. This time, I bought 5 more. The seller said 800 degrees of electricity with one. I feel it's basically enough. A total of 10 units	Jingdong prefecture level city in Shandong
Happy Fan Shop	King of Power Saving	I never thought I could get such a good baby at such an affordable price, it's very good value for money, I love it!	Jingdong prefecture level city in Shandong
Happy Fan Shop	King of Power Saving	Previously bought other brands of power savers, but found no effect at home, this time to see how the products on the Jingdong, inserted in the home socket, the LED display is blue, the night light is very nice to see, look at the instructions that have to be half a year in order to achieve the best results, try it, I believe that the quality of the Jingdong, it should not be wrong.	Jingdong prefecture level city in Shandong
Happy Fan Shop	King of Power Saving	Superb, inexpensive, saves a lot of points, been using it for a long time!	Jingdong prefecture level city in Shandong
Happy Fan Shop	King of Power Saving	There are a lot of appliances in the house, the summer air conditioning is very costly, I bought this in front of the air conditioning effect is very good, I explain that I bought three in one breath, I use more electricity a month down to 200 or so, very satisfied.	Jingdong prefecture level city in Shandong
Happy Fan Shop	King of Power Saving	A month can save more than 200, home monthly electricity bill of more than 600 dollars, inserted 3 or so, an amount of the balance of the average can save more than 200. Product is very good, has been buying this one, has given a lot of relatives recommended, the quality is also very good, before buying a look with this same, who knows to buy home has not been used for more than a week on the bad, the quality of this is really good with more than two years!	Jingdong prefecture level city in Shandong
Happy Fan Shop	King of Power Saving	Whole house power saving king, can really save electricity, friends recommended, high power saving appliances, only replacement, no repair, after-sales worry-free!	Jingdong prefecture level city in Shandong
Happy Fan Shop	King of Power Saving	Great item love it, great customer service, great shopping experience!	Jingdong prefecture level city in Shandong
Happy Fan Shop	King of Power Saving	Power saving, very good use, much cheaper than the physical store, satisfaction	Jingdong prefecture level city in Shandong
Happy Fan Shop	King of Power Saving	In use, very well packaged and fast delivery of credit!	Jingdong prefecture level city in Shandong
Happy Fan Shop	King of Power Saving	Product quality, customer service attitude is particularly good, like, praise!	Jingdong prefecture level city in Shandong
Happy Fan Shop	King of Power Saving	The logistics is very fast, the packaging is not broken, the quality is very good, I like it very much!	Jingdong prefecture level city in Shandong
Happy Fan Shop	King of Power Saving	The saver was well packaged and was as described when plugged in	Jingdong prefecture level city in Shandong
Happy Fan Shop	King of Power Saving	Energy efficient and very satisfied	Jingdong prefecture level city in Shandong
Happy Fan Shop	King of Power Saving	Great quality for the price and fast shipping.	Jingdong prefecture level city in Shandong

Happy Fan Shop	King of Power Saving	It's great, just as described. Perfect.	Jingdong prefecture level city in Shandong
Happy Fan Shop	King of Power Saving	There is no power saving effect, but rather ring up an increase in electricity costs, because this machine is on continuously all day long, uninterruptedly, its own electricity and increased costs, plus the cost of purchasing the machine, so I feel that the use of this machine instead of increasing the	Jingdong prefecture level city in Shandong
Happy Fan Shop	King of Power Saving	Recommended by the owner of the store next door, looked at his home does have to save electricity, decided to get. Indeed quite can, the voltage is much more stable, there is a reduction in electricity	Jingdong prefecture level city in Shandong
Happy Fan Shop	King of Power Saving	Goods have been received value for money, affordable and reasonable price, and the description of the same, power-saving safe and reliable!	Jingdong prefecture level city in Shandong
Happy Fan Shop	King of Power Saving	Listen to friends introduced to buy, tried to save a lot of electricity is good, quite good, worth buying.	Jingdong prefecture level city in Shandong
Happy Fan Shop	King of Power Saving	The product is easy to use, safe and environmentally friendly, only used for a short time, to the next month, after the electricity bill status display to know how the performance of the power saver efficacy.	Jingdong prefecture level city in Shandong
Happy Fan Shop	King of Power Saving	Last month's electricity bill is 178 yuan, with this power saver is 92 yuan, ready to buy two more back home, I was introduced to buy a friend, just started to not believe that I did not expect the effect!	Jingdong prefecture level city in Shandong
Happy Fan Shop	King of Power Saving	It looks nice, but after using it for a few days I can't see how it saves power?	Jingdong prefecture level city in Shandong
Happy Fan Shop	King of Power Saving	Recommended by a friend, it is genuine, as described by the merchant, cost-effective	Jingdong prefecture level city in Shandong
Happy Fan Shop	King of Power Saving	Works well, affordable and saves money.	Jingdong prefecture level city in Shandong
Happy Fan Shop	King of Power Saving	Feels good haven't compared it yet, the air conditioning has been on a lot in the past two months	Jingdong prefecture level city in Shandong
Happy Fan Shop	King of Power Saving	Saving about 5 kWh per day is a good thing	Jingdong prefecture level city in Shandong
Happy Fan Shop	King of Power Saving	Not bad, affordable, best value for money	Jingdong prefecture level city in Shandong
Happy Fan Shop	King of Power Saving	The goods are genuine and the logistics are faster.	Jingdong prefecture level city in Shandong
Happy Fan Shop	King of Power Saving	Goods have been received today, has been plugged into the outlet, looking forward to saving electricity, after a period of time to see how the effect in the follow-up comment!	Jingdong prefecture level city in Shandong
Happy Fan Shop	King of Power Saving	I just got it home and haven't used it for long, so I'll use it for a couple months and see what happens. Seeing that most people have good reviews, it should be fine.	Jingdong prefecture level city in Shandong
Happy Fan Shop	King of Power Saving	I'm looking forward to the results, so I'll give you a good review out of habit! Follow up review after a while	Jingdong prefecture level city in Shandong
Happy Fan Shop	King of Power Saving	Use a dish first to know if it will help save energy	Jingdong prefecture level city in Shandong
Happy Fan Shop	King of Power Saving	Whether it saves electricity or not, we don't know yet.	Jingdong prefecture level city in Shandong
Happy Fan Shop	King of Power Saving	Goods have been received, use a period of time to know how oh. The courier did not deliver to the door ah! Sent to the station ah! The heat is inconvenient ah!	Jingdong prefecture level city in Shandong
Happy Fan Shop	King of Power Saving	A little effect, before an hour a power, now an hour 0.76 degrees, a dollar a power = save 24 cents, although not a lot, first use it!	Jingdong prefecture level city in Shandong
Happy Fan Shop	King of Power Saving	Just received the goods, use a month to evaluate	Jingdong prefecture level city in Shandong
Happy Fan Shop	King of Power Saving	Home every month with a lot of electricity, inadvertently found this good thing online that can save the beginning of a skeptical attitude to consult customer service, customer service replied very professional, said 120 days can be refunded that first try it!	Jingdong prefecture level city in Shandong
Happy Fan Shop	King of Power Saving	After receiving the goods this afternoon, immediately installed in the bathroom Smith energy storage electric water heater on the power outlet (instead of connecting with a plug board), when used, found that the "Power Saving King" LCD display, is the value of the voltage, not the store's video said so	Jingdong prefecture level city in Shandong
Happy Fan Shop	King of Power Saving	Has made many days, did not feel the power saving, the effect is not obvious.	Jingdong prefecture level city in Shandong
Happy Fan Shop	King of Power Saving	Energy savers do not save energy at all, but rather consume a lot of electricity.	Jingdong prefecture level city in Shandong
Happy Fan Shop	King of Power Saving	It's useless. It's a waste of money.	Jingdong prefecture level city in Shandong
Happy Fan Shop	King of Power Saving	Last month saved more than 500, which came back to buy, the store bought three, and then buy 1 home use, my family is dry beauty salon, more instruments, especially electricity, a month almost 1000. or so, with this did not expect to save so much, great, five-star praise!	Jingdong prefecture level city in Shandong
Happy Fan Shop	King of Power Saving	It rained a couple days ago and I just went to get the item back today , not bad, hey I looked at the meter and that red display light takes several seconds to jump, did not make a mistake buying it. After a period of time to evaluate.	Jingdong prefecture level city in Shandong
Happy Fan Shop	King of Power Saving	Without further ado, the electricity bill came down, this month less 102 yuan, has been repurchased, I also recommended to my friends, all said that there are effects, the product is okay, the effect is quite	Jingdong prefecture level city in Shandong
Happy Fan Shop	King of Power Saving	After buying and using two months really see the effect, so only to evaluate! Originally held the mindset of trying, I did not expect really effective, worth having!	Jingdong prefecture level city in Shandong
Happy Fan Shop	King of Power Saving	In the use of this energy saver before the monthly electricity bill is more than 150 yuan or so, now save thirty percent, every day to work, six or seven o'clock in the afternoon to go home, I do not know why the electricity bill is so expensive, energy-saving appliances really have the effect of recommending	Jingdong prefecture level city in Shandong
Happy Fan Shop	King of Power Saving	It saves about 100 bucks a month. A friend recommended it to me, and he's been using it for a while, and the effect is so obvious that I bought one too.	Jingdong prefecture level city in Shandong
Happy Fan Shop	King of Power Saving	My dad asked me to buy one, but after I paid the bill, I asked and he said it was a lot less this month, so he asked me to buy another one.	Jingdong prefecture level city in Shandong
Happy Fan Shop	King of Power Saving	The specific effect will have to be tested for at least two months to compare whether there is a power saving effect or not.	Jingdong prefecture level city in Shandong
Happy Fan Shop	King of Power Saving	'The goods have been received, immediately go to use, please ask the boss you instructions do not allow to withdraw, there are two fuses installed there!	Jingdong prefecture level city in Shandong
Happy Fan Shop	King of Power Saving	First praise is using, after using if good then follow up, now because I have not yet seen the results	Jingdong prefecture level city in Shandong
Happy Fan Shop	King of Power Saving	I've just been using it, and I haven't seen if it has a power-saving effect yet, so I'll follow up after 10 days and half a month.	Jingdong prefecture level city in Shandong
Happy Fan Shop	King of Power Saving	Just received the intelligent power saving meter, immediately plugged in to use, see it is not bad, and order to sell the second time	Jingdong prefecture level city in Shandong
Happy Fan Shop	King of Power Saving	This power saver is a bit effective, used for half a month, the effect is still visible.	Jingdong prefecture level city in Shandong
Happy Fan Shop	King of Power Saving	I received the baby and it looks great, I'm trying it out now and will keep an eye on it afterward.	Jingdong prefecture level city in Shandong
Happy Fan Shop	King of Power Saving	I've just received it, but I'm still trying to see how it works, so I'll come back in a little while.	Jingdong prefecture level city in Shandong
Happy Fan Shop	King of Power Saving	Just used it wait to use it for a while and then comment thanks, all first five stars.	Jingdong prefecture level city in Shandong
Happy Fan Shop	King of Power Saving	Very trustworthy brand, quality is guaranteed, the price is still affordable, quite satisfied!	Jingdong prefecture level city in Shandong
Happy Fan Shop	King of Power Saving	Things received, I hope that the platform of Jingdong do not sell fake goods are available	Jingdong prefecture level city in Shandong
Happy Fan Shop	King of Power Saving	Energy saver received, the use of the effect is good and the business description of the same	Jingdong prefecture level city in Shandong
Happy Fan Shop	King of Power Saving	The surface looks beautiful, the actual effect is not known, after using additional evaluation.	Jingdong prefecture level city in Shandong
Happy Fan Shop	King of Power Saving	Tried it for a few months, it really saves electricity, and I bought it for a friend.	Jingdong prefecture level city in Shandong

Happy Fan Shop	King of Power Saving	It didn't work and wanted to return it, it really didn't work at all.	Jingdong prefecture level city in Shandong
Happy Fan Shop	King of Power Saving	Just used it, don't know how effective it is, looking forward to usefulness	Jingdong prefecture level city in Shandong
Happy Fan Shop	King of Power Saving	After two months before the effect I do not know if it is true!	Jingdong prefecture level city in Shandong
Happy Fan Shop	King of Power Saving	So fast! Ordered yesterday and received today 👍👍👍👍👍👍👍👍👍 🌹🌹🌹🌹🌹🌹🌹🌹🌹	Jingdong prefecture level city in Shandong
Happy Fan Shop	King of Power Saving	I've started using it. We'll see how it works.	Jingdong prefecture level city in Shandong
Happy Fan Shop	King of Power Saving	I just received the goods, I won't know the effect until after two.	Jingdong prefecture level city in Shandong
Happy Fan Shop	King of Power Saving	Just received the goods, the specific effect will be evaluated later	Jingdong prefecture level city in Shandong
Happy Fan Shop	King of Power Saving	I've received the goods, I don't know if it's any good yet	Jingdong prefecture level city in Shandong
Happy Fan Shop	King of Power Saving	Try it for a month and then come back with a concrete evaluation	Jingdong prefecture level city in Shandong
Happy Fan Shop	King of Power Saving	In use, after two months to see the effect of power saving	Jingdong prefecture level city in Shandong
Happy Fan Shop	King of Power Saving	Power Saver. It's authentic. It's black. It works.	Jingdong prefecture level city in Shandong
Happy Fan Shop	King of Power Saving	I've been using it for a week now and it's not working.	Jingdong prefecture level city in Shandong
Happy Fan Shop	King of Power Saving	I'm just using it. I don't know how it's going to work.	Jingdong prefecture level city in Shandong
Happy Fan Shop	King of Power Saving	Products are better looking out for power savings	Jingdong prefecture level city in Shandong
Happy Fan Shop	King of Power Saving	I received it and I've kept it on. I hope it works.	Jingdong prefecture level city in Shandong
Happy Fan Shop	King of Power Saving	It's really good! Worth recommending to buy!	Jingdong prefecture level city in Shandong
Happy Fan Shop	King of Power Saving	Try it for a few days and see if it doesn't work, then return it.	Jingdong prefecture level city in Shandong
Happy Fan Shop	King of Power Saving	It's in use and I hope to be pleasantly surprised.	Jingdong prefecture level city in Shandong
Happy Fan Shop	King of Power Saving	We'll see how it works when we use it	Jingdong prefecture level city in Shandong
Happy Fan Shop	King of Power Saving	Let's see if it works.	Jingdong prefecture level city in Shandong
Happy Fan Shop	King of Power Saving	I'm using it and will follow up with a review	Jingdong prefecture level city in Shandong
Happy Fan Shop	King of Power Saving	It really works. It saves energy.	Jingdong prefecture level city in Shandong
Happy Fan Shop	King of Power Saving	It does have some power savings!	Jingdong prefecture level city in Shandong
Happy Fan Shop	King of Power Saving	Jingdong shopping speed is convenient and timely	Jingdong prefecture level city in Shandong
Happy Fan Shop	King of Power Saving	Second time I've bought it. Everything's fine.	Jingdong prefecture level city in Shandong
Happy Fan Shop	King of Power Saving	I don't know if it works yet.	Jingdong prefecture level city in Shandong
Happy Fan Shop	King of Power Saving	Not used, don't know how well it works.	Jingdong prefecture level city in Shandong
Happy Fan Shop	King of Power Saving	No visible results so far	Jingdong prefecture level city in Shandong
Happy Fan Shop	King of Power Saving	It doesn't feel like it's working.	Jingdong prefecture level city in Shandong
Happy Fan Shop	King of Power Saving	Where's the effect? Where are the power savings?	Jingdong prefecture level city in Shandong
Happy Fan Shop	King of Power Saving	Let's see how it works in practice!	Jingdong prefecture level city in Shandong
Happy Fan Shop	King of Power Saving	I'll wait until I use it to give you a good review	Jingdong prefecture level city in Shandong
Happy Fan Shop	King of Power Saving	I'll follow up after I've used it	Jingdong prefecture level city in Shandong
Happy Fan Shop	King of Power Saving	Well, that's good for saving power.	Jingdong prefecture level city in Shandong
Happy Fan Shop	King of Power Saving	Received. Used in the know.	Jingdong prefecture level city in Shandong
Happy Fan Shop	King of Power Saving	It doesn't do much good.	Jingdong prefecture level city in Shandong
Happy Fan Shop	King of Power Saving	It's not working.	Jingdong prefecture level city in Shandong
Happy Fan Shop	King of Power Saving	I don't know. I just used it.	Jingdong prefecture level city in Shandong
Happy Fan Shop	King of Power Saving	Use it and come back to evaluate.	Jingdong prefecture level city in Shandong
Happy Fan Shop	King of Power Saving	Two words: useless.	Jingdong prefecture level city in Shandong
Happy Fan Shop	King of Power Saving	To be used and then evaluated	Jingdong prefecture level city in Shandong
Happy Fan Shop	King of Power Saving	No shipment.	Jingdong prefecture level city in Shandong
Happy Fan Shop	King of Power Saving	Fake. Speechless.	Jingdong prefecture level city in Shandong
Happy Fan Shop	King of Power Saving	fantastic (idiom); awesome	Jingdong prefecture level city in Shandong
Happy Fan Shop	King of Power Saving	an excellent	Jingdong prefecture level city in Shandong
Happy Fan Shop	King of Power Saving	an excellent	Jingdong prefecture level city in Shandong
Happy Fan Shop	King of Power Saving	Want to buy! Recommended not to buy! No effect	Jingdong prefecture level city in Shandong
Happy Fan Shop	King of Power Saving	That's all there is to it. I can't figure out how to save electricity.	Jingdong prefecture level city in Shandong
Happy Fan Shop	King of Power Saving	It's no use.	Jingdong prefecture level city in Shandong
Happy Fan Shop	King of Power Saving	Things are more perfect than I imagined, very powerful, it is worth getting Packaging is very strong, things are well protected, it is worth the praise things are very satisfied Oh, beautiful and stylish atmosphere, customer service is super patient, service is meticulous and attentive, the big brand is not	Jingdong prefecture level city in Shandong

Happy Fan Shop	King of Power Saving	The effect is too good, the family has now several units, back to buy a number of times, the power saver received, power-saving effect is very good,, fine workmanship, long warranty, but also purify the air, before buying a used for a period of time, with the okay, and then bought a, and only now come to the	Jingdong prefecture level city in Shandong
Happy Fan Shop	King of Power Saving	Quite good, and imagine the same, casual ride are very good, satisfied! Baby received it 👌, really great ah! 🌈 quality is really nothing to say, very satisfied! 🆗 happy happy 😀 genuine, great, the price is very good, in short, quite good, will buy again a very good shopping experience, attentive service, fast logistics, distribution is also thoughtful, the attitude is also very good, value for money, ah,	Jingdong prefecture level city in Shandong
Happy Fan Shop	King of Power Saving	Power-saving effect: strong Cost-effective: quite good Appearance style: beautiful Workmanship material: meticulous Experience: looking forward to power-saving effect	Jingdong prefecture level city in Shandong
Happy Fan Shop	King of Power Saving	Cost-effective is okay, the appearance of the style of meticulous workmanship, looking forward to energy-saving effect.	Jingdong prefecture level city in Shandong
Happy Fan Shop	King of Power Saving	Very Good Very Power Saving This Power Saver is really power saving As soon as the power is turned on, the Power Saver starts the power saving function for you.	Jingdong prefecture level city in Shandong
Happy Fan Shop	King of Power Saving	Logistics is very fast, the price is favorable, friends introduced that it is very good to use, bought 2 at once, looking forward to the effect, used after the follow-up comment.	Jingdong prefecture level city in Shandong
Happy Fan Shop	King of Power Saving	It's all fake. It doesn't save any electricity.	Jingdong prefecture level city in Shandong
Happy Fan Shop	King of Power Saving	This Power Saver is so much better than the one I bought before, not only the effect is good, the quality is very good in all aspects, the customer service attitude is also very good, recommended to buy!	Jingdong prefecture level city in Shandong
Happy Fan Shop	King of Power Saving	Logistics is very fast, buy for parents, good quality, once bought 2, power-saving effect is good to buy 2 more, look forward to using the effect, satisfied will recommend to friends.	Jingdong prefecture level city in Shandong
Happy Fan Shop	King of Power Saving	With the description is the same, very high-grade, good value, to buy friends can order Oh!	Jingdong prefecture level city in Shandong
Happy Fan Shop	King of Power Saving	Not using much electricity in the winter saving over 100 per month! Ready to buy 2 more units in the summer ! ! ! ! ! Good quality, good price, power saving effect is obvious, cost-effective, worth buying, can be recommended!	Jingdong prefecture level city in Shandong
Happy Fan Shop	King of Power Saving	Purchased a power saver received on the put into use, rent a room with many electrical appliances, power consumption is very large, we have a piece of electricity or 1.2 dollars a time, with this power saver is much better, every day can save a lot of money, very convenient and practical.	Jingdong prefecture level city in Shandong
Happy Fan Shop	King of Power Saving	The power saving effect is very good: my supermarket monthly electricity bill is more than 7000 yuan. On May 1st someone recommended buying a couple of power savers, and I bought 5 of them first. The electricity bill was about 2000 less in June. This time, I bought 5 more. The seller said 800 degrees of electricity with one. I feel it's basically enough. A total of 10 units	Jingdong prefecture level city in Shandong
Happy Fan Shop	King of Power Saving	I never thought I could get such a good baby at such an affordable price, it's very good value for money, I love it!	Jingdong prefecture level city in Shandong
Happy Fan Shop	King of Power Saving	Previously bought other brands of power savers, but found no effect at home, this time to see how the products on the Jingdong, inserted in the home socket, the LED display is blue, the night light is very nice to see, look at the instructions that have to be half a year in order to achieve the best results, try it, I believe that the quality of the Jingdong, it should not be wrong.	Jingdong prefecture level city in Shandong
Happy Fan Shop	King of Power Saving	Superb, inexpensive, saves a lot of points, been using it for a long time!	Jingdong prefecture level city in Shandong
Happy Fan Shop	King of Power Saving	There are a lot of appliances in the house, the summer air conditioning is very costly, I bought this in front of the air conditioning effect is very good, I explain that I bought three in one breath, I use more electricity a month down to 200 or so, very satisfied.	Jingdong prefecture level city in Shandong
Happy Fan Shop	King of Power Saving	A month can save more than 200, home monthly electricity bill of more than 600 dollars, inserted 3 or so, an amount of the balance of the average can save more than 200. Product is very good, has been buying this one, has given a lot of relatives recommended, the quality is also very good, before buying a look with this same, who knows to buy home has not been used for more than a week on the bad, the quality of this is really good with more than two years!	Jingdong prefecture level city in Shandong
Happy Fan Shop	King of Power Saving	Whole house power saving king, can really save electricity, friends recommended, high power saving appliances, only replacement, no repair, after-sales worry-free!	Jingdong prefecture level city in Shandong
Happy Fan Shop	King of Power Saving	Great item love it, great customer service, great shopping experience!	Jingdong prefecture level city in Shandong
Happy Fan Shop	King of Power Saving	Power saving, very good use, much cheaper than the physical store, satisfaction	Jingdong prefecture level city in Shandong
Happy Fan Shop	King of Power Saving	In use, very well packaged and fast delivery of credit!	Jingdong prefecture level city in Shandong
Happy Fan Shop	King of Power Saving	Product quality, customer service attitude is particularly good, like, praise!	Jingdong prefecture level city in Shandong
Happy Fan Shop	King of Power Saving	The logistics is very fast, the packaging is not broken, the quality is very good, I like it very much!	Jingdong prefecture level city in Shandong
Happy Fan Shop	King of Power Saving	The saver was well packaged and was as described when plugged in	Jingdong prefecture level city in Shandong
Happy Fan Shop	King of Power Saving	Energy efficient and very satisfied	Jingdong prefecture level city in Shandong
Happy Fan Shop	King of Power Saving	Great quality for the price and fast shipping.	Jingdong prefecture level city in Shandong
Happy Fan Shop	King of Power Saving	It's great, just as described. Perfect.	Jingdong prefecture level city in Shandong
Happy Fan Shop	King of Power Saving	There is no power saving effect, but rather ring up an increase in electricity costs, because this machine is on continuously all day long, uninterruptedly, its own electricity and increased costs, plus the cost of purchasing the machine, so I feel that the use of this machine instead of increasing the	Jingdong prefecture level city in Shandong
Happy Fan Shop	King of Power Saving	Recommended by the owner of the store next door, looked at his home does have to save electricity, decided to get. Indeed quite can, the voltage is much more stable, there is a reduction in electricity	Jingdong prefecture level city in Shandong
Happy Fan Shop	King of Power Saving	Goods have been received value for money, affordable and reasonable price, and the description of the same, power-saving safe and reliable!	Jingdong prefecture level city in Shandong
Happy Fan Shop	King of Power Saving	Listen to friends introduced to buy, tried to save a lot of electricity is good, quite good, worth buying.	Jingdong prefecture level city in Shandong
Happy Fan Shop	King of Power Saving	The product is easy to use, safe and environmentally friendly, only used for a short time, to the next month, after the electricity bill status display to know how the performance of the power saver efficacy.	Jingdong prefecture level city in Shandong
Happy Fan Shop	King of Power Saving	Last month's electricity bill is 178 yuan, with this power saver is 92 yuan, ready to buy two more back home, I was introduced to buy a friend, just started to not believe that I did not expect the effect!	Jingdong prefecture level city in Shandong
Happy Fan Shop	King of Power Saving	It looks nice, but after using it for a few days I can't see how it saves power?	Jingdong prefecture level city in Shandong
Happy Fan Shop	King of Power Saving	Recommended by a friend, it is genuine, as described by the merchant, cost-effective	Jingdong prefecture level city in Shandong
Happy Fan Shop	King of Power Saving	Works well, affordable and saves money.	Jingdong prefecture level city in Shandong
Happy Fan Shop	King of Power Saving	Feels good haven't compared it yet, the air conditioning has been on a lot in the past two months	Jingdong prefecture level city in Shandong
Happy Fan Shop	King of Power Saving	Saving about 5 kWh per day is a good thing	Jingdong prefecture level city in Shandong
Happy Fan Shop	King of Power Saving	Not bad, affordable, best value for money	Jingdong prefecture level city in Shandong
Happy Fan Shop	King of Power Saving	The goods are genuine and the logistics are faster.	Jingdong prefecture level city in Shandong
Happy Fan Shop	King of Power Saving	Goods have been received today, has been plugged into the outlet, looking forward to saving electricity, after a period of time to see how the effect in the follow-up comment!	Jingdong prefecture level city in Shandong
Happy Fan Shop	King of Power Saving	I just got it home and haven't used it for long, so I'll use it for a couple months and see what happens. Seeing that most people have good reviews, it should be fine.	Jingdong prefecture level city in Shandong
Happy Fan Shop	King of Power Saving	I'm looking forward to the results, so I'll give you a good review out of habit! Follow up review after a while	Jingdong prefecture level city in Shandong

Happy Fan Shop	King of Power Saving	Use a dish first to know if it will help save energy	Jingdong prefecture level city in Shandong
Happy Fan Shop	King of Power Saving	Whether it saves electricity or not, we don't know yet.	Jingdong prefecture level city in Shandong
Happy Fan Shop	King of Power Saving	Goods have been received, use a period of time to know how oh. The courier did not deliver to the door ah! Sent to the station ah! The heat is inconvenient ah!	Jingdong prefecture level city in Shandong
Happy Fan Shop	King of Power Saving	A little effect, before an hour a power, now an hour 0.76 degrees, a dollar a power = save 24 cents, although not a lot, first use it!	Jingdong prefecture level city in Shandong
Happy Fan Shop	King of Power Saving	Just received the goods, use a month to evaluate	Jingdong prefecture level city in Shandong
Happy Fan Shop	King of Power Saving	Home every month with a lot of electricity, inadvertently found this good thing online that can save the beginning of a skeptical attitude to consult customer service, customer service replied very professional, said 120 days can be refunded that first try it!	Jingdong prefecture level city in Shandong
Happy Fan Shop	King of Power Saving	After receiving the goods this afternoon, immediately installed in the bathroom Smith energy storage electric water heater on the power outlet (instead of connecting with a plug board), when used, found that the "Power Saving King" LCD display, is the value of the voltage, not the store's video said so	Jingdong prefecture level city in Shandong
Happy Fan Shop	King of Power Saving	Has made many days, did not feel the power saving, the effect is not obvious.	Jingdong prefecture level city in Shandong
Happy Fan Shop	King of Power Saving	Energy savers do not save energy at all, but rather consume a lot of electricity.	Jingdong prefecture level city in Shandong
Happy Fan Shop	King of Power Saving	It's useless. It's a waste of money.	Jingdong prefecture level city in Shandong
Happy Fan Shop	King of Power Saving	Last month saved more than 500, which came back to buy, the store bought three, and then buy 1 home use, my family is dry beauty salon, more instruments, especially electricity, a month almost 1000. or so, with this did not expect to save so much, great, five-star praise!	Jingdong prefecture level city in Shandong
Happy Fan Shop	King of Power Saving	It rained a couple days ago and I just went to get the item back today , not bad, hey I looked at the meter and that red display light takes several seconds to jump, did not make a mistake buying it. After a period of time to evaluate.	Jingdong prefecture level city in Shandong
Happy Fan Shop	King of Power Saving	Without further ado, the electricity bill came down, this month less 102 yuan, has been repurchased, I also recommended to my friends, all said that there are effects, the product is okay, the effect is quite	Jingdong prefecture level city in Shandong
Happy Fan Shop	King of Power Saving	After buying and using two months really see the effect, so only to evaluate! Originally held the mindset of trying, I did not expect really effective, worth having!	Jingdong prefecture level city in Shandong
Happy Fan Shop	King of Power Saving	In the use of this energy saver before the monthly electricity bill is more than 150 yuan or so, now save thirty percent, every day to work, six or seven o'clock in the afternoon to go home, I do not know why the electricity bill is so expensive, energy-saving appliances really have the effect of recommending	Jingdong prefecture level city in Shandong
Happy Fan Shop	King of Power Saving	It saves about 100 bucks a month. A friend recommended it to me, and he's been using it for a while, and the effect is so obvious that I bought one too.	Jingdong prefecture level city in Shandong
Happy Fan Shop	King of Power Saving	My dad asked me to buy one, but after I paid the bill, I asked and he said it was a lot less this month, so he asked me to buy another one.	Jingdong prefecture level city in Shandong
Happy Fan Shop	King of Power Saving	The specific effect will have to be tested for at least two months to compare whether there is a power saving effect or not.	Jingdong prefecture level city in Shandong
Happy Fan Shop	King of Power Saving	'The goods have been received, immediately go to use, please ask the boss you instructions do not allow to withdraw, there are two fuses installed there!	Jingdong prefecture level city in Shandong
Happy Fan Shop	King of Power Saving	First praise is using, after using if good then follow up, now because I have not yet seen the results	Jingdong prefecture level city in Shandong
Happy Fan Shop	King of Power Saving	I've just been using it, and I haven't seen if it has a power-saving effect yet, so I'll follow up after 10 days and half a month.	Jingdong prefecture level city in Shandong
Happy Fan Shop	King of Power Saving	Just received the intelligent power saving meter, immediately plugged in to use, see it is not bad, and order to sell the second time	Jingdong prefecture level city in Shandong
Happy Fan Shop	King of Power Saving	This power saver is a bit effective, used for half a month, the effect is still visible.	Jingdong prefecture level city in Shandong
Happy Fan Shop	King of Power Saving	I received the baby and it looks great, I'm trying it out now and will keep an eye on it afterward.	Jingdong prefecture level city in Shandong
Happy Fan Shop	King of Power Saving	I've just received it, but I'm still trying to see how it works, so I'll come back in a little while.	Jingdong prefecture level city in Shandong
Happy Fan Shop	King of Power Saving	Just used it wait to use it for a while and then comment thanks, all first five stars.	Jingdong prefecture level city in Shandong
Happy Fan Shop	King of Power Saving	Very trustworthy brand, quality is guaranteed, the price is still affordable, quite satisfied!	Jingdong prefecture level city in Shandong
Happy Fan Shop	King of Power Saving	Things received, I hope that the platform of Jingdong do not sell fake goods are available	Jingdong prefecture level city in Shandong
Happy Fan Shop	King of Power Saving	Energy saver received, the use of the effect is good and the business description of the same	Jingdong prefecture level city in Shandong
Happy Fan Shop	King of Power Saving	The surface looks beautiful, the actual effect is not known, after using additional evaluation.	Jingdong prefecture level city in Shandong
Happy Fan Shop	King of Power Saving	Tried it for a few months, it really saves electricity, and I bought it for a friend.	Jingdong prefecture level city in Shandong
Happy Fan Shop	King of Power Saving	It didn't work and wanted to return it, it really didn't work at all.	Jingdong prefecture level city in Shandong
Happy Fan Shop	King of Power Saving	Just used it, don't know how effective it is, looking forward to usefulness	Jingdong prefecture level city in Shandong
Happy Fan Shop	King of Power Saving	After two months before the effect I do not know if it is true!	Jingdong prefecture level city in Shandong
Happy Fan Shop	King of Power Saving	So fast! Ordered yesterday and received today 👍👍👍👍👍👍👍👍👍 🌹🌹🌹🌹🌹🌹🌹🌹🌹	Jingdong prefecture level city in Shandong
Happy Fan Shop	King of Power Saving	I've started using it. We'll see how it works.	Jingdong prefecture level city in Shandong
Happy Fan Shop	King of Power Saving	I just received the goods, I won't know the effect until after two.	Jingdong prefecture level city in Shandong
Happy Fan Shop	King of Power Saving	Just received the goods, the specific effect will be evaluated later	Jingdong prefecture level city in Shandong
Happy Fan Shop	King of Power Saving	I've received the goods, I don't know if it's any good yet	Jingdong prefecture level city in Shandong
Happy Fan Shop	King of Power Saving	Try it for a month and then come back with a concrete evaluation	Jingdong prefecture level city in Shandong
Happy Fan Shop	King of Power Saving	In use, after two months to see the effect of power saving	Jingdong prefecture level city in Shandong
Happy Fan Shop	King of Power Saving	Power Saver. It's authentic. It's black. It works.	Jingdong prefecture level city in Shandong
Happy Fan Shop	King of Power Saving	I've been using it for a week now and it's not working.	Jingdong prefecture level city in Shandong
Happy Fan Shop	King of Power Saving	I'm just using it. I don't know how it's going to work.	Jingdong prefecture level city in Shandong
Happy Fan Shop	King of Power Saving	Products are better looking out for power savings	Jingdong prefecture level city in Shandong
Happy Fan Shop	King of Power Saving	I received it and I've kept it on. I hope it works.	Jingdong prefecture level city in Shandong
Happy Fan Shop	King of Power Saving	It's really good! Worth recommending to buy!	Jingdong prefecture level city in Shandong
Happy Fan Shop	King of Power Saving	Try it for a few days and see if it doesn't work, then return it.	Jingdong prefecture level city in Shandong
Happy Fan Shop	King of Power Saving	It's in use and I hope to be pleasantly surprised.	Jingdong prefecture level city in Shandong

Happy Fan Shop	King of Power Saving	We'll see how it works when we use it	Jingdong prefecture level city in Shandong
Happy Fan Shop	King of Power Saving	Let's see if it works.	Jingdong prefecture level city in Shandong
Happy Fan Shop	King of Power Saving	I'm using it and will follow up with a review	Jingdong prefecture level city in Shandong
Happy Fan Shop	King of Power Saving	It really works. It saves energy.	Jingdong prefecture level city in Shandong
Happy Fan Shop	King of Power Saving	It does have some power savings!	Jingdong prefecture level city in Shandong
Happy Fan Shop	King of Power Saving	Jingdong shopping speed is convenient and timely	Jingdong prefecture level city in Shandong
Happy Fan Shop	King of Power Saving	Second time I've bought it. Everything's fine.	Jingdong prefecture level city in Shandong
Happy Fan Shop	King of Power Saving	I don't know if it works yet.	Jingdong prefecture level city in Shandong
Happy Fan Shop	King of Power Saving	Not used, don't know how well it works.	Jingdong prefecture level city in Shandong
Happy Fan Shop	King of Power Saving	No visible results so far	Jingdong prefecture level city in Shandong
Happy Fan Shop	King of Power Saving	It doesn't feel like it's working.	Jingdong prefecture level city in Shandong
Happy Fan Shop	King of Power Saving	Where's the effect? Where are the power savings?	Jingdong prefecture level city in Shandong
Happy Fan Shop	King of Power Saving	Let's see how it works in practice!	Jingdong prefecture level city in Shandong
Happy Fan Shop	King of Power Saving	I'll wait until I use it to give you a good review	Jingdong prefecture level city in Shandong
Happy Fan Shop	King of Power Saving	I'll follow up after I've used it	Jingdong prefecture level city in Shandong
Happy Fan Shop	King of Power Saving	Well, that's good for saving power.	Jingdong prefecture level city in Shandong
Happy Fan Shop	King of Power Saving	Received. Used in the know.	Jingdong prefecture level city in Shandong
Happy Fan Shop	King of Power Saving	It doesn't do much good.	Jingdong prefecture level city in Shandong
Happy Fan Shop	King of Power Saving	It's not working.	Jingdong prefecture level city in Shandong
Happy Fan Shop	King of Power Saving	I don't know. I just used it.	Jingdong prefecture level city in Shandong
Happy Fan Shop	King of Power Saving	Use it and come back to evaluate.	Jingdong prefecture level city in Shandong
Happy Fan Shop	King of Power Saving	Two words: useless.	Jingdong prefecture level city in Shandong
Happy Fan Shop	King of Power Saving	To be used and then evaluated	Jingdong prefecture level city in Shandong
Happy Fan Shop	King of Power Saving	No shipment.	Jingdong prefecture level city in Shandong
Happy Fan Shop	King of Power Saving	Fake. Speechless.	Jingdong prefecture level city in Shandong
Happy Fan Shop	King of Power Saving	fantastic (idiom); awesome	Jingdong prefecture level city in Shandong
Happy Fan Shop	King of Power Saving	an excellent	Jingdong prefecture level city in Shandong
Happy Fan Shop	King of Power Saving	an excellent	Jingdong prefecture level city in Shandong
Happy Fan Shop	King of Power Saving	Want to buy! Recommended not to buy! No effect	Jingdong prefecture level city in Shandong
Happy Fan Shop	King of Power Saving	That's all there is to it. I can't figure out how to save electricity.	Jingdong prefecture level city in Shandong
Happy Fan Shop	King of Power Saving	It's no use.	Jingdong prefecture level city in Shandong
Happy Fan Shop	King of Power Saving	Things are more perfect than I imagined, very powerful, it is worth getting Packaging is very strong, things are well protected, it is worth the praise things are very satisfied Oh, beautiful and stylish atmosphere, customer service is super patient, service is meticulous and attentive, the big brand is not	Jingdong prefecture level city in Shandong
Happy Fan Shop	King of Power Saving	The effect is too good, the family has now several units, back to buy a number of times, the power saver received, power-saving effect is very good,, fine workmanship, long warranty, but also purify the air, before buying a used for a period of time, with the okay, and then bought a, and only now come to the	Jingdong prefecture level city in Shandong
Happy Fan Shop	King of Power Saving	Quite good, and imagine the same, casual ride are very good, satisfied! Baby received it 👌, really great ah! 🌈 quality is really nothing to say, very satisfied! 🆗 happy happy 😃 genuine, great, the price is very good, in short, quite good, will buy again a very good shopping experience, attentive service, fast logistics, distribution is also thoughtful, the attitude is also very good, value for money, ah,	Jingdong prefecture level city in Shandong
Happy Fan Shop	King of Power Saving	Power-saving effect: strong Cost-effective: quite good Appearance style: beautiful Workmanship material: meticulous Experience: looking forward to power-saving effect	Jingdong prefecture level city in Shandong
Happy Fan Shop	King of Power Saving	Cost-effective is okay, the appearance of the style of meticulous workmanship, looking forward to energy-saving effect.	Jingdong prefecture level city in Shandong
Happy Fan Shop	King of Power Saving	Very Good Very Power Saving This Power Saver is really power saving As soon as the power is turned on, the Power Saver starts the power saving function for you.	Jingdong prefecture level city in Shandong
Happy Fan Shop	King of Power Saving	Logistics is very fast, the price is favorable, friends introduced that it is very good to use, bought 2 at once, looking forward to the effect, used after the follow-up comment.	Jingdong prefecture level city in Shandong
Happy Fan Shop	King of Power Saving	It's all fake. It doesn't save any electricity.	Jingdong prefecture level city in Shandong
Happy Fan Shop	King of Power Saving	This Power Saver is so much better than the one I bought before, not only the effect is good, the quality is very good in all aspects, the customer service attitude is also very good, recommended to buy!	Jingdong prefecture level city in Shandong
Happy Fan Shop	King of Power Saving	Logistics is very fast, buy for parents, good quality, once bought 2, power-saving effect is good to buy 2 more, look forward to using the effect, satisfied will recommend to friends.	Jingdong prefecture level city in Shandong
Happy Fan Shop	King of Power Saving	With the description is the same, very high-grade, good value, to buy friends can order Oh!	Jingdong prefecture level city in Shandong
Happy Fan Shop	King of Power Saving	Not using much electricity in the winter saving over 100 per month! Ready to buy 2 more units in the summer !!!!! Good quality, good price, power saving effect is obvious, cost-effective, worth buying, can be recommended!	Jingdong prefecture level city in Shandong
Happy Fan Shop	King of Power Saving	Purchased a power saver received on the put into use, rent a room with many electrical appliances, power consumption is very large, we have a piece of electricity or 1.2 dollars a time, with this power saver is much better, every day can save a lot of money, very convenient and practical.	Jingdong prefecture level city in Shandong
Happy Fan Shop	King of Power Saving	The power saving effect is very good: my supermarket monthly electricity bill is more than 7000 yuan. On May 1st someone recommended buying a couple of power savers, and I bought 5 of them first. The electricity bill was about 2000 less in June. This time, I bought 5 more. The seller said 800 degrees of electricity with one. I feel it's basically enough. A total of 10 units	Jingdong prefecture level city in Shandong
Happy Fan Shop	King of Power Saving	I never thought I could get such a good baby at such an affordable price, it's very good value for money, I love it!	Jingdong prefecture level city in Shandong

Happy Fan Shop	King of Power Saving	Previously bought other brands of power savers, but found no effect at home, this time to see how the products on the Jingdong, inserted in the home socket, the LED display is blue, the night light is very nice to see, look at the instructions that have to be half a year in order to achieve the best results, try it, I believe that the quality of the Jingdong, it should not be wrong.	Jingdong prefecture level city in Shandong
Happy Fan Shop	King of Power Saving	Superb, inexpensive, saves a lot of points, been using it for a long time!	Jingdong prefecture level city in Shandong
Happy Fan Shop	King of Power Saving	There are a lot of appliances in the house, the summer air conditioning is very costly, I bought this in front of the air conditioning effect is very good, I explain that I bought three in one breath, I use more electricity a month down to 200 or so, very satisfied.	Jingdong prefecture level city in Shandong
Happy Fan Shop	King of Power Saving	A month can save more than 200, home monthly electricity bill of more than 600 dollars, inserted 3 or so, an amount of the balance of the average can save more than 200. Product is very good, has been buying this one, has given a lot of relatives recommended, the quality is also very good, before buying a look with this same, who knows to buy home has not been used for more than a week on the bad, the quality of this is really good with more than two years!	Jingdong prefecture level city in Shandong
Happy Fan Shop	King of Power Saving	Whole house power saving king, can really save electricity, friends recommended, high power saving appliances, only replacement, no repair, after-sales worry-free!	Jingdong prefecture level city in Shandong
Happy Fan Shop	King of Power Saving	Great item love it, great customer service, great shopping experience!	Jingdong prefecture level city in Shandong
Happy Fan Shop	King of Power Saving	Power saving, very good use, much cheaper than the physical store, satisfaction	Jingdong prefecture level city in Shandong
Happy Fan Shop	King of Power Saving	In use, very well packaged and fast delivery of credit!	Jingdong prefecture level city in Shandong
Happy Fan Shop	King of Power Saving	Product quality, customer service attitude is particularly good, like, praise!	Jingdong prefecture level city in Shandong
Happy Fan Shop	King of Power Saving	The logistics is very fast, the packaging is not broken, the quality is very good, I like it very much!	Jingdong prefecture level city in Shandong
Happy Fan Shop	King of Power Saving	The saver was well packaged and was as described when plugged in	Jingdong prefecture level city in Shandong
Happy Fan Shop	King of Power Saving	Energy efficient and very satisfied	Jingdong prefecture level city in Shandong
Happy Fan Shop	King of Power Saving	Great quality for the price and fast shipping.	Jingdong prefecture level city in Shandong
Happy Fan Shop	King of Power Saving	It's great, just as described. Perfect.	Jingdong prefecture level city in Shandong
Happy Fan Shop	King of Power Saving	There is no power saving effect, but rather ring up an increase in electricity costs, because this machine is on continuously all day long, uninterruptedly, its own electricity and increased costs, plus the cost of purchasing the machine, so I feel that the use of this machine instead of increasing the	Jingdong prefecture level city in Shandong
Happy Fan Shop	King of Power Saving	Recommended by the owner of the store next door, looked at his home does have to save electricity, decided to get. Indeed quite can, the voltage is much more stable, there is a reduction in electricity	Jingdong prefecture level city in Shandong
Happy Fan Shop	King of Power Saving	Goods have been received value for money, affordable and reasonable price, and the description of the same, power-saving safe and reliable!	Jingdong prefecture level city in Shandong
Happy Fan Shop	King of Power Saving	Listen to friends introduced to buy, tried to save a lot of electricity is good, quite good, worth buying.	Jingdong prefecture level city in Shandong
Happy Fan Shop	King of Power Saving	The product is easy to use, safe and environmentally friendly, only used for a short time, to the next month, after the electricity bill status display to know how the performance of the power saver efficacy.	Jingdong prefecture level city in Shandong
Happy Fan Shop	King of Power Saving	Last month's electricity bill is 178 yuan, with this power saver is 92 yuan, ready to buy two more back home, I was introduced to buy a friend, just started to not believe that I did not expect the effect!	Jingdong prefecture level city in Shandong
Happy Fan Shop	King of Power Saving	It looks nice, but after using it for a few days I can't see how it saves power?	Jingdong prefecture level city in Shandong
Happy Fan Shop	King of Power Saving	Recommended by a friend, it is genuine, as described by the merchant, cost-effective	Jingdong prefecture level city in Shandong
Happy Fan Shop	King of Power Saving	Works well, affordable and saves money.	Jingdong prefecture level city in Shandong
Happy Fan Shop	King of Power Saving	Feels good haven't compared it yet, the air conditioning has been on a lot in the past two months	Jingdong prefecture level city in Shandong
Happy Fan Shop	King of Power Saving	Saving about 5 kWh per day is a good thing	Jingdong prefecture level city in Shandong
Happy Fan Shop	King of Power Saving	Not bad, affordable, best value for money	Jingdong prefecture level city in Shandong
Happy Fan Shop	King of Power Saving	The goods are genuine and the logistics are faster.	Jingdong prefecture level city in Shandong
Happy Fan Shop	King of Power Saving	Goods have been received today, has been plugged into the outlet, looking forward to saving electricity, after a period of time to see how the effect in the follow-up comment!	Jingdong prefecture level city in Shandong
Happy Fan Shop	King of Power Saving	I just got it home and haven't used it for long, so I'll use it for a couple months and see what happens. Seeing that most people have good reviews, it should be fine.	Jingdong prefecture level city in Shandong
Happy Fan Shop	King of Power Saving	I'm looking forward to the results, so I'll give you a good review out of habit! Follow up review after a while	Jingdong prefecture level city in Shandong
Happy Fan Shop	King of Power Saving	Use a dish first to know if it will help save energy	Jingdong prefecture level city in Shandong
Happy Fan Shop	King of Power Saving	Whether it saves electricity or not, we don't know yet.	Jingdong prefecture level city in Shandong
Happy Fan Shop	King of Power Saving	Goods have been received, use a period of time to know how oh. The courier did not deliver to the door ah! Sent to the station ah! The heat is inconvenient ah!	Jingdong prefecture level city in Shandong
Happy Fan Shop	King of Power Saving	A little effect, before an hour a power, now an hour 0.76 degrees, a dollar a power = save 24 cents, although not a lot, first use it!	Jingdong prefecture level city in Shandong
Happy Fan Shop	King of Power Saving	Just received the goods, use a month to evaluate	Jingdong prefecture level city in Shandong
Happy Fan Shop	King of Power Saving	Home every month with a lot of electricity, inadvertently found this good thing online that can save the beginning of a skeptical attitude to consult customer service, customer service replied very professional, said 120 days can be refunded that first try it!	Jingdong prefecture level city in Shandong
Happy Fan Shop	King of Power Saving	After receiving the goods this afternoon, immediately installed in the bathroom Smith energy storage electric water heater on the power outlet (instead of connecting with a plug board), when used, found that the "Power Saving King" LCD display, is the value of the voltage, not the store's video said so	Jingdong prefecture level city in Shandong
Happy Fan Shop	King of Power Saving	Has made many days, did not feel the power saving, the effect is not obvious.	Jingdong prefecture level city in Shandong
Happy Fan Shop	King of Power Saving	Energy savers do not save energy at all, but rather consume a lot of electricity.	Jingdong prefecture level city in Shandong
Happy Fan Shop	King of Power Saving	It's useless. It's a waste of money.	Jingdong prefecture level city in Shandong
Happy Fan Shop	King of Power Saving	Last month saved more than 500, which came back to buy, the store bought three, and then buy 1 home use, my family is dry beauty salon, more instruments, especially electricity, a month almost 1000. or so, with this did not expect to save so much, great, five-star praise!	Jingdong prefecture level city in Shandong
Happy Fan Shop	King of Power Saving	It rained a couple days ago and I just went to get the item back today , not bad, hey I looked at the meter and that red display light takes several seconds to jump, did not make a mistake buying it. After a period of time to evaluate.	Jingdong prefecture level city in Shandong
Happy Fan Shop	King of Power Saving	Without further ado, the electricity bill came down, this month less 102 yuan, has been repurchased, I also recommended to my friends, all said that there are effects, the product is okay, the effect is quite	Jingdong prefecture level city in Shandong
Happy Fan Shop	King of Power Saving	After buying and using two months really see the effect, so only to evaluate! Originally held the mindset of trying, I did not expect really effective, worth having!	Jingdong prefecture level city in Shandong
Happy Fan Shop	King of Power Saving	In the use of this energy saver before the monthly electricity bill is more than 150 yuan or so, now save thirty percent, every day to work, six or seven o'clock in the afternoon to go home, I do not know why the electricity bill is so expensive, energy-saving appliances really have the effect of recommending	Jingdong prefecture level city in Shandong

Happy Fan Shop	King of Power Saving	It saves about 100 bucks a month. A friend recommended it to me, and he's been using it for a while, and the effect is so obvious that I bought one too.	Jingdong prefecture level city in Shandong
Happy Fan Shop	King of Power Saving	My dad asked me to buy one, but after I paid the bill, I asked and he said it was a lot less this month, so he asked me to buy another one.	Jingdong prefecture level city in Shandong
Happy Fan Shop	King of Power Saving	The specific effect will have to be tested for at least two months to compare whether there is a power saving effect or not.	Jingdong prefecture level city in Shandong
Happy Fan Shop	King of Power Saving	'The goods have been received, immediately go to use, please ask the boss you instructions do not allow to withdraw, there are two fuses installed there!	Jingdong prefecture level city in Shandong
Happy Fan Shop	King of Power Saving	First praise is using, after using if good then follow up, now because I have not yet seen the results	Jingdong prefecture level city in Shandong
Happy Fan Shop	King of Power Saving	I've just been using it, and I haven't seen if it has a power-saving effect yet, so I'll follow up after 10 days and half a month.	Jingdong prefecture level city in Shandong
Happy Fan Shop	King of Power Saving	Just received the intelligent power saving meter, immediately plugged in to use, see it is not bad, and order to sell the second time	Jingdong prefecture level city in Shandong
Happy Fan Shop	King of Power Saving	This power saver is a bit effective, used for half a month, the effect is still visible.	Jingdong prefecture level city in Shandong
Happy Fan Shop	King of Power Saving	I received the baby and it looks great, I'm trying it out now and will keep an eye on it afterward.	Jingdong prefecture level city in Shandong
Happy Fan Shop	King of Power Saving	I've just received it, but I'm still trying to see how it works, so I'll come back in a little while.	Jingdong prefecture level city in Shandong
Happy Fan Shop	King of Power Saving	Just used it wait to use it for a while and then comment thanks, all first five stars.	Jingdong prefecture level city in Shandong
Happy Fan Shop	King of Power Saving	Very trustworthy brand, quality is guaranteed, the price is still affordable, quite satisfied!	Jingdong prefecture level city in Shandong
Happy Fan Shop	King of Power Saving	Things received, I hope that the platform of Jingdong do not sell fake goods are available	Jingdong prefecture level city in Shandong
Happy Fan Shop	King of Power Saving	Energy saver received, the use of the effect is good and the business description of the same	Jingdong prefecture level city in Shandong
Happy Fan Shop	King of Power Saving	The surface looks beautiful, the actual effect is not known, after using additional evaluation.	Jingdong prefecture level city in Shandong
Happy Fan Shop	King of Power Saving	Tried it for a few months, it really saves electricity, and I bought it for a friend.	Jingdong prefecture level city in Shandong
Happy Fan Shop	King of Power Saving	It didn't work and wanted to return it, it really didn't work at all.	Jingdong prefecture level city in Shandong
Happy Fan Shop	King of Power Saving	Just used it, don't know how effective it is, looking forward to usefulness	Jingdong prefecture level city in Shandong
Happy Fan Shop	King of Power Saving	After two months before the effect I do not know if it is true!	Jingdong prefecture level city in Shandong
Happy Fan Shop	King of Power Saving	So fast! Ordered yesterday and received today 👍👍👍👍👍👍👍👍👍 🌹🌹🌹🌹🌹🌹🌹🌹🌹	Jingdong prefecture level city in Shandong
Happy Fan Shop	King of Power Saving	I've started using it. We'll see how it works.	Jingdong prefecture level city in Shandong
Happy Fan Shop	King of Power Saving	I just received the goods, I won't know the effect until after two.	Jingdong prefecture level city in Shandong
Happy Fan Shop	King of Power Saving	Just received the goods, the specific effect will be evaluated later	Jingdong prefecture level city in Shandong
Happy Fan Shop	King of Power Saving	I've received the goods, I don't know if it's any good yet	Jingdong prefecture level city in Shandong
Happy Fan Shop	King of Power Saving	Try it for a month and then come back with a concrete evaluation	Jingdong prefecture level city in Shandong
Happy Fan Shop	King of Power Saving	In use, after two months to see the effect of power saving	Jingdong prefecture level city in Shandong
Happy Fan Shop	King of Power Saving	Power Saver. It's authentic. It's black. It works.	Jingdong prefecture level city in Shandong
Happy Fan Shop	King of Power Saving	I've been using it for a week now and it's not working.	Jingdong prefecture level city in Shandong
Happy Fan Shop	King of Power Saving	I'm just using it. I don't know how it's going to work.	Jingdong prefecture level city in Shandong
Happy Fan Shop	King of Power Saving	Products are better looking out for power savings	Jingdong prefecture level city in Shandong
Happy Fan Shop	King of Power Saving	I received it and I've kept it on. I hope it works.	Jingdong prefecture level city in Shandong
Happy Fan Shop	King of Power Saving	It's really good! Worth recommending to buy!	Jingdong prefecture level city in Shandong
Happy Fan Shop	King of Power Saving	Try it for a few days and see if it doesn't work, then return it.	Jingdong prefecture level city in Shandong
Happy Fan Shop	King of Power Saving	It's in use and I hope to be pleasantly surprised.	Jingdong prefecture level city in Shandong
Happy Fan Shop	King of Power Saving	We'll see how it works when we use it	Jingdong prefecture level city in Shandong
Happy Fan Shop	King of Power Saving	Let's see if it works.	Jingdong prefecture level city in Shandong
Happy Fan Shop	King of Power Saving	I'm using it and will follow up with a review	Jingdong prefecture level city in Shandong
Happy Fan Shop	King of Power Saving	It really works. It saves energy.	Jingdong prefecture level city in Shandong
Happy Fan Shop	King of Power Saving	It does have some power savings!	Jingdong prefecture level city in Shandong
Happy Fan Shop	King of Power Saving	Jingdong shopping speed is convenient and timely	Jingdong prefecture level city in Shandong
Happy Fan Shop	King of Power Saving	Second time I've bought it. Everything's fine.	Jingdong prefecture level city in Shandong
Happy Fan Shop	King of Power Saving	I don't know if it works yet.	Jingdong prefecture level city in Shandong
Happy Fan Shop	King of Power Saving	Not used, don't know how well it works.	Jingdong prefecture level city in Shandong
Happy Fan Shop	King of Power Saving	No visible results so far	Jingdong prefecture level city in Shandong
Happy Fan Shop	King of Power Saving	It doesn't feel like it's working.	Jingdong prefecture level city in Shandong
Happy Fan Shop	King of Power Saving	Where's the effect? Where are the power savings?	Jingdong prefecture level city in Shandong
Happy Fan Shop	King of Power Saving	Let's see how it works in practice!	Jingdong prefecture level city in Shandong
Happy Fan Shop	King of Power Saving	I'll wait until I use it to give you a good review	Jingdong prefecture level city in Shandong
Happy Fan Shop	King of Power Saving	I'll follow up after I've used it	Jingdong prefecture level city in Shandong
Happy Fan Shop	King of Power Saving	Well, that's good for saving power.	Jingdong prefecture level city in Shandong
Happy Fan Shop	King of Power Saving	Received. Used in the know.	Jingdong prefecture level city in Shandong

Happy Fan Shop	King of Power Saving	It doesn't do much good.	Jingdong prefecture level city in Shandong
Happy Fan Shop	King of Power Saving	It's not working.	Jingdong prefecture level city in Shandong
Happy Fan Shop	King of Power Saving	I don't know. I just used it.	Jingdong prefecture level city in Shandong
Happy Fan Shop	King of Power Saving	Use it and come back to evaluate.	Jingdong prefecture level city in Shandong
Happy Fan Shop	King of Power Saving	Two words: useless.	Jingdong prefecture level city in Shandong
Happy Fan Shop	King of Power Saving	To be used and then evaluated	Jingdong prefecture level city in Shandong
Happy Fan Shop	King of Power Saving	No shipment.	Jingdong prefecture level city in Shandong
Happy Fan Shop	King of Power Saving	Fake. Speechless.	Jingdong prefecture level city in Shandong
Happy Fan Shop	King of Power Saving	fantastic (idiom); awesome	Jingdong prefecture level city in Shandong
Happy Fan Shop	King of Power Saving	an excellent	Jingdong prefecture level city in Shandong
Happy Fan Shop	King of Power Saving	an excellent	Jingdong prefecture level city in Shandong
Happy Fan Shop	King of Power Saving	Want to buy! Recommended not to buy! No effect	Jingdong prefecture level city in Shandong
Happy Fan Shop	King of Power Saving	That's all there is to it. I can't figure out how to save electricity.	Jingdong prefecture level city in Shandong
Happy Fan Shop	King of Power Saving	It's no use.	Jingdong prefecture level city in Shandong
Happy Fan Shop	King of Power Saving	Things are more perfect than I imagined, very powerful, it is worth getting Packaging is very strong, things are well protected, it is worth the praise things are very satisfied Oh, beautiful and stylish atmosphere, customer service is super patient, service is meticulous and attentive, the big brand is not	Jingdong prefecture level city in Shandong
Happy Fan Shop	King of Power Saving	The effect is too good, the family has now several units, back to buy a number of times, the power saver received, power-saving effect is very good,, fine workmanship, long warranty, but also purify the air, before buying a used for a period of time, with the okay, and then bought a, and only now come to the	Jingdong prefecture level city in Shandong
Happy Fan Shop	King of Power Saving	Quite good, and imagine the same, casual ride are very good, satisfied! Baby received it 👌, really great ah! 🌈 quality is really nothing to say, very satisfied! 🆗 happy happy 😃 genuine, great, the price is very good, in short, quite good, will buy again a very good shopping experience, attentive service, fast logistics, distribution is also thoughtful, the attitude is also very good, value for money, ah,	Jingdong prefecture level city in Shandong
Happy Fan Shop	King of Power Saving	Power-saving effect: strong Cost-effective: quite good Appearance style: beautiful Workmanship material: meticulous Experience: looking forward to power-saving effect	Jingdong prefecture level city in Shandong
Happy Fan Shop	King of Power Saving	Cost-effective is okay, the appearance of the style of meticulous workmanship, looking forward to energy-saving effect.	Jingdong prefecture level city in Shandong
Happy Fan Shop	King of Power Saving	Very Good Very Power Saving This Power Saver is really power saving As soon as the power is turned on, the Power Saver starts the power saving function for you.	Jingdong prefecture level city in Shandong
Happy Fan Shop	King of Power Saving	Logistics is very fast, the price is favorable, friends introduced that it is very good to use, bought 2 at once, looking forward to the effect, used after the follow-up comment.	Jingdong prefecture level city in Shandong
Happy Fan Shop	King of Power Saving	It's all fake. It doesn't save any electricity.	Jingdong prefecture level city in Shandong
Happy Fan Shop	King of Power Saving	This Power Saver is so much better than the one I bought before, not only the effect is good, the quality is very good in all aspects, the customer service attitude is also very good, recommended to buy!	Jingdong prefecture level city in Shandong
Happy Fan Shop	King of Power Saving	Logistics is very fast, buy for parents, good quality, once bought 2, power-saving effect is good to buy 2 more, look forward to using the effect, satisfied will recommend to friends.	Jingdong prefecture level city in Shandong
Happy Fan Shop	King of Power Saving	With the description is the same, very high-grade, good value, to buy friends can order Oh!	Jingdong prefecture level city in Shandong
Happy Fan Shop	King of Power Saving	Not using much electricity in the winter saving over 100 per month! Ready to buy 2 more units in the summer ! ! ! ! ! Good quality, good price, power saving effect is obvious, cost-effective, worth buying, can be recommended!	Jingdong prefecture level city in Shandong
Happy Fan Shop	King of Power Saving	Purchased a power saver received on the put into use, rent a room with many electrical appliances, power consumption is very large, we have a piece of electricity or 1.2 dollars a time, with this power saver is much better, every day can save a lot of money, very convenient and practical.	Jingdong prefecture level city in Shandong
Happy Fan Shop	King of Power Saving	The power saving effect is very good: my supermarket monthly electricity bill is more than 7000 yuan. On May 1st someone recommended buying a couple of power savers, and I bought 5 of them first. The electricity bill was about 2000 less in June. This time, I bought 5 more. The seller said 800 degrees of electricity with one. I feel it's basically enough. A total of 10 units	Jingdong prefecture level city in Shandong
Happy Fan Shop	King of Power Saving	I never thought I could get such a good baby at such an affordable price, it's very good value for money, I love it!	Jingdong prefecture level city in Shandong
Happy Fan Shop	King of Power Saving	Previously bought other brands of power savers, but found no effect at home, this time to see how the products on the Jingdong, inserted in the home socket, the LED display is blue, the night light is very nice to see, look at the instructions that have to be half a year in order to achieve the best results, try it, I believe that the quality of the Jingdong, it should not be wrong.	Jingdong prefecture level city in Shandong
Happy Fan Shop	King of Power Saving	Superb, inexpensive, saves a lot of points, been using it for a long time!	Jingdong prefecture level city in Shandong
Happy Fan Shop	King of Power Saving	There are a lot of appliances in the house, the summer air conditioning is very costly, I bought this in front of the air conditioning effect is very good, I explain that I bought three in one breath, I use more electricity a month down to 200 or so, very satisfied.	Jingdong prefecture level city in Shandong
Happy Fan Shop	King of Power Saving	A month can save more than 200, home monthly electricity bill of more than 600 dollars, inserted 3 or so, an amount of the balance of the average can save more than 200. Product is very good, has been buying this one, has given a lot of relatives recommended, the quality is also very good, before buying a look with this same, who knows to buy home has not been used for more than a week on the bad, the quality of this is really good with more than two years!	Jingdong prefecture level city in Shandong
Happy Fan Shop	King of Power Saving	Whole house power saving king, can really save electricity, friends recommended, high power saving appliances, only replacement, no repair, after-sales worry-free!	Jingdong prefecture level city in Shandong
Happy Fan Shop	King of Power Saving	Great item love it, great customer service, great shopping experience!	Jingdong prefecture level city in Shandong
Happy Fan Shop	King of Power Saving	Power saving, very good use, much cheaper than the physical store, satisfaction	Jingdong prefecture level city in Shandong
Happy Fan Shop	King of Power Saving	In use, very well packaged and fast delivery of credit!	Jingdong prefecture level city in Shandong
Happy Fan Shop	King of Power Saving	Product quality, customer service attitude is particularly good, like, praise!	Jingdong prefecture level city in Shandong
Happy Fan Shop	King of Power Saving	The logistics is very fast, the packaging is not broken, the quality is very good, I like it very much!	Jingdong prefecture level city in Shandong
Happy Fan Shop	King of Power Saving	The saver was well packaged and was as described when plugged in	Jingdong prefecture level city in Shandong
Happy Fan Shop	King of Power Saving	Energy efficient and very satisfied	Jingdong prefecture level city in Shandong
Happy Fan Shop	King of Power Saving	Great quality for the price and fast shipping.	Jingdong prefecture level city in Shandong
Happy Fan Shop	King of Power Saving	It's great, just as described. Perfect.	Jingdong prefecture level city in Shandong
Happy Fan Shop	King of Power Saving	There is no power saving effect, but rather ring up an increase in electricity costs, because this machine is on continuously all day long, uninterruptedly, its own electricity and increased costs, plus the cost of purchasing the machine, so I feel that the use of this machine instead of increasing the	Jingdong prefecture level city in Shandong

Happy Fan Shop	King of Power Saving	Recommended by the owner of the store next door, looked at his home does have to save electricity, decided to get. Indeed quite can, the voltage is much more stable, there is a reduction in electricity	Jingdong prefecture level city in Shandong
Happy Fan Shop	King of Power Saving	Goods have been received value for money, affordable and reasonable price, and the description of the same, power-saving safe and reliable!	Jingdong prefecture level city in Shandong
Happy Fan Shop	King of Power Saving	Listen to friends introduced to buy, tried to save a lot of electricity is good, quite good, worth buying.	Jingdong prefecture level city in Shandong
Happy Fan Shop	King of Power Saving	The product is easy to use, safe and environmentally friendly, only used for a short time, to the next month, after the electricity bill status display to know how the performance of the power saver efficacy.	Jingdong prefecture level city in Shandong
Happy Fan Shop	King of Power Saving	Last month's electricity bill is 178 yuan, with this power saver is 92 yuan, ready to buy two more back home, I was introduced to buy a friend, just started to not believe that I did not expect the effect!	Jingdong prefecture level city in Shandong
Happy Fan Shop	King of Power Saving	It looks nice, but after using it for a few days I can't see how it saves power?	Jingdong prefecture level city in Shandong
Happy Fan Shop	King of Power Saving	Recommended by a friend, it is genuine, as described by the merchant, cost-effective	Jingdong prefecture level city in Shandong
Happy Fan Shop	King of Power Saving	Works well, affordable and saves money.	Jingdong prefecture level city in Shandong
Happy Fan Shop	King of Power Saving	Feels good haven't compared it yet, the air conditioning has been on a lot in the past two months	Jingdong prefecture level city in Shandong
Happy Fan Shop	King of Power Saving	Saving about 5 kWh per day is a good thing	Jingdong prefecture level city in Shandong
Happy Fan Shop	King of Power Saving	Not bad, affordable, best value for money	Jingdong prefecture level city in Shandong
Happy Fan Shop	King of Power Saving	The goods are genuine and the logistics are faster.	Jingdong prefecture level city in Shandong
Happy Fan Shop	King of Power Saving	Goods have been received today, has been plugged into the outlet, looking forward to saving electricity, after a period of time to see how the effect in the follow-up comment!	Jingdong prefecture level city in Shandong
Happy Fan Shop	King of Power Saving	I just got it home and haven't used it for long, so I'll use it for a couple months and see what happens. Seeing that most people have good reviews, it should be fine.	Jingdong prefecture level city in Shandong
Happy Fan Shop	King of Power Saving	I'm looking forward to the results, so I'll give you a good review out of habit! Follow up review after a while	Jingdong prefecture level city in Shandong
Happy Fan Shop	King of Power Saving	Use a dish first to know if it will help save energy	Jingdong prefecture level city in Shandong
Happy Fan Shop	King of Power Saving	Whether it saves electricity or not, we don't know yet.	Jingdong prefecture level city in Shandong
Happy Fan Shop	King of Power Saving	Goods have been received, use a period of time to know how oh. The courier did not deliver to the door ah! Sent to the station ah! The heat is inconvenient ah!	Jingdong prefecture level city in Shandong
Happy Fan Shop	King of Power Saving	A little effect, before an hour a power, now an hour 0.76 degrees, a dollar a power = save 24 cents, although not a lot, first use it!	Jingdong prefecture level city in Shandong
Happy Fan Shop	King of Power Saving	Just received the goods, use a month to evaluate	Jingdong prefecture level city in Shandong
Happy Fan Shop	King of Power Saving	Home every month with a lot of electricity, inadvertently found this good thing online that can save the beginning of a skeptical attitude to consult customer service, customer service replied very professional, said 120 days can be refunded that first try it!	Jingdong prefecture level city in Shandong
Happy Fan Shop	King of Power Saving	After receiving the goods this afternoon, immediately installed in the bathroom Smith energy storage electric water heater on the power outlet (instead of connecting with a plug board), when used, found that the "Power Saving King" LCD display, is the value of the voltage, not the store's video said so	Jingdong prefecture level city in Shandong
Happy Fan Shop	King of Power Saving	Has made many days, did not feel the power saving, the effect is not obvious.	Jingdong prefecture level city in Shandong
Happy Fan Shop	King of Power Saving	Energy savers do not save energy at all, but rather consume a lot of electricity.	Jingdong prefecture level city in Shandong
Happy Fan Shop	King of Power Saving	It's useless. It's a waste of money.	Jingdong prefecture level city in Shandong
Happy Fan Shop	King of Power Saving	Last month saved more than 500, which came back to buy, the store bought three, and then buy 1 home use, my family is dry beauty salon, more instruments, especially electricity, a month almost 1000. or so, with this did not expect to save so much, great, five-star praise!	Jingdong prefecture level city in Shandong
Happy Fan Shop	King of Power Saving	It rained a couple days ago and I just went to get the item back today , not bad, hey I looked at the meter and that red display light takes several seconds to jump, did not make a mistake buying it. After a period of time to evaluate.	Jingdong prefecture level city in Shandong
Happy Fan Shop	King of Power Saving	Without further ado, the electricity bill came down, this month less 102 yuan, has been repurchased, I also recommended to my friends, all said that there are effects, the product is okay, the effect is quite	Jingdong prefecture level city in Shandong
Happy Fan Shop	King of Power Saving	After buying and using two months really see the effect, so only to evaluate! Originally held the mindset of trying, I did not expect really effective, worth having!	Jingdong prefecture level city in Shandong
Happy Fan Shop	King of Power Saving	In the use of this energy saver before the monthly electricity bill is more than 150 yuan or so, now save thirty percent, every day to work, six or seven o'clock in the afternoon to go home, I do not know why the electricity bill is so expensive, energy-saving appliances really have the effect of recommending	Jingdong prefecture level city in Shandong
Happy Fan Shop	King of Power Saving	It saves about 100 bucks a month. A friend recommended it to me, and he's been using it for a while, and the effect is so obvious that I bought one too.	Jingdong prefecture level city in Shandong
Happy Fan Shop	King of Power Saving	My dad asked me to buy one, but after I paid the bill, I asked and he said it was a lot less this month, so he asked me to buy another one.	Jingdong prefecture level city in Shandong
Happy Fan Shop	King of Power Saving	The specific effect will have to be tested for at least two months to compare whether there is a power saving effect or not.	Jingdong prefecture level city in Shandong
Happy Fan Shop	King of Power Saving	'The goods have been received, immediately go to use, please ask the boss you instructions do not allow to withdraw, there are two fuses installed there!	Jingdong prefecture level city in Shandong
Happy Fan Shop	King of Power Saving	First praise is using, after using if good then follow up, now because I have not yet seen the results	Jingdong prefecture level city in Shandong
Happy Fan Shop	King of Power Saving	I've just been using it, and I haven't seen if it has a power-saving effect yet, so I'll follow up after 10 days and half a month.	Jingdong prefecture level city in Shandong
Happy Fan Shop	King of Power Saving	Just received the intelligent power saving meter, immediately plugged in to use, see it is not bad, and order to sell the second time	Jingdong prefecture level city in Shandong
Happy Fan Shop	King of Power Saving	This power saver is a bit effective, used for half a month, the effect is still visible.	Jingdong prefecture level city in Shandong
Happy Fan Shop	King of Power Saving	I received the baby and it looks great, I'm trying it out now and will keep an eye on it afterward.	Jingdong prefecture level city in Shandong
Happy Fan Shop	King of Power Saving	I've just received it, but I'm still trying to see how it works, so I'll come back in a little while.	Jingdong prefecture level city in Shandong
Happy Fan Shop	King of Power Saving	Just used it wait to use it for a while and then comment thanks, all first five stars.	Jingdong prefecture level city in Shandong
Happy Fan Shop	King of Power Saving	Very trustworthy brand, quality is guaranteed, the price is still affordable, quite satisfied!	Jingdong prefecture level city in Shandong
Happy Fan Shop	King of Power Saving	Things received, I hope that the platform of Jingdong do not sell fake goods are available	Jingdong prefecture level city in Shandong
Happy Fan Shop	King of Power Saving	Energy saver received, the use of the effect is good and the business description of the same	Jingdong prefecture level city in Shandong
Happy Fan Shop	King of Power Saving	The surface looks beautiful, the actual effect is not known, after using additional evaluation.	Jingdong prefecture level city in Shandong
Happy Fan Shop	King of Power Saving	Tried it for a few months, it really saves electricity, and I bought it for a friend.	Jingdong prefecture level city in Shandong
Happy Fan Shop	King of Power Saving	It didn't work and wanted to return it, it really didn't work at all.	Jingdong prefecture level city in Shandong
Happy Fan Shop	King of Power Saving	Just used it, don't know how effective it is, looking forward to usefulness	Jingdong prefecture level city in Shandong
Happy Fan Shop	King of Power Saving	After two months before the effect I do not know if it is true!	Jingdong prefecture level city in Shandong

Happy Fan Shop	King of Power Saving	So fast! Ordered yesterday and received today 👍👍👍👍👍👍👍👍👍 🌹🌹🌹🌹🌹🌹🌹🌹🌹	Jingdong prefecture level city in Shandong
Happy Fan Shop	King of Power Saving	I've started using it. We'll see how it works.	Jingdong prefecture level city in Shandong
Happy Fan Shop	King of Power Saving	I just received the goods, I won't know the effect until after two.	Jingdong prefecture level city in Shandong
Happy Fan Shop	King of Power Saving	Just received the goods, the specific effect will be evaluated later	Jingdong prefecture level city in Shandong
Happy Fan Shop	King of Power Saving	I've received the goods, I don't know if it's any good yet	Jingdong prefecture level city in Shandong
Happy Fan Shop	King of Power Saving	Try it for a month and then come back with a concrete evaluation	Jingdong prefecture level city in Shandong
Happy Fan Shop	King of Power Saving	In use, after two months to see the effect of power saving	Jingdong prefecture level city in Shandong
Happy Fan Shop	King of Power Saving	Power Saver. It's authentic. It's black. It works.	Jingdong prefecture level city in Shandong
Happy Fan Shop	King of Power Saving	I've been using it for a week now and it's not working.	Jingdong prefecture level city in Shandong
Happy Fan Shop	King of Power Saving	I'm just using it. I don't know how it's going to work.	Jingdong prefecture level city in Shandong
Happy Fan Shop	King of Power Saving	Products are better looking out for power savings	Jingdong prefecture level city in Shandong
Happy Fan Shop	King of Power Saving	I received it and I've kept it on. I hope it works.	Jingdong prefecture level city in Shandong
Happy Fan Shop	King of Power Saving	It's really good! Worth recommending to buy!	Jingdong prefecture level city in Shandong
Happy Fan Shop	King of Power Saving	Try it for a few days and see if it doesn't work, then return it.	Jingdong prefecture level city in Shandong
Happy Fan Shop	King of Power Saving	It's in use and I hope to be pleasantly surprised.	Jingdong prefecture level city in Shandong
Happy Fan Shop	King of Power Saving	We'll see how it works when we use it	Jingdong prefecture level city in Shandong
Happy Fan Shop	King of Power Saving	Let's see if it works.	Jingdong prefecture level city in Shandong
Happy Fan Shop	King of Power Saving	I'm using it and will follow up with a review	Jingdong prefecture level city in Shandong
Happy Fan Shop	King of Power Saving	It really works. It saves energy.	Jingdong prefecture level city in Shandong
Happy Fan Shop	King of Power Saving	It does have some power savings!	Jingdong prefecture level city in Shandong
Happy Fan Shop	King of Power Saving	Jingdong shopping speed is convenient and timely	Jingdong prefecture level city in Shandong
Happy Fan Shop	King of Power Saving	Second time I've bought it. Everything's fine.	Jingdong prefecture level city in Shandong
Happy Fan Shop	King of Power Saving	I don't know if it works yet.	Jingdong prefecture level city in Shandong
Happy Fan Shop	King of Power Saving	Not used, don't know how well it works.	Jingdong prefecture level city in Shandong
Happy Fan Shop	King of Power Saving	No visible results so far	Jingdong prefecture level city in Shandong
Happy Fan Shop	King of Power Saving	It doesn't feel like it's working.	Jingdong prefecture level city in Shandong
Happy Fan Shop	King of Power Saving	Where's the effect? Where are the power savings?	Jingdong prefecture level city in Shandong
Happy Fan Shop	King of Power Saving	Let's see how it works in practice!	Jingdong prefecture level city in Shandong
Happy Fan Shop	King of Power Saving	I'll wait until I use it to give you a good review	Jingdong prefecture level city in Shandong
Happy Fan Shop	King of Power Saving	I'll follow up after I've used it	Jingdong prefecture level city in Shandong
Happy Fan Shop	King of Power Saving	Well, that's good for saving power.	Jingdong prefecture level city in Shandong
Happy Fan Shop	King of Power Saving	Received. Used in the know.	Jingdong prefecture level city in Shandong
Happy Fan Shop	King of Power Saving	It doesn't do much good.	Jingdong prefecture level city in Shandong
Happy Fan Shop	King of Power Saving	It's not working.	Jingdong prefecture level city in Shandong
Happy Fan Shop	King of Power Saving	I don't know. I just used it.	Jingdong prefecture level city in Shandong
Happy Fan Shop	King of Power Saving	Use it and come back to evaluate.	Jingdong prefecture level city in Shandong
Happy Fan Shop	King of Power Saving	Two words: useless.	Jingdong prefecture level city in Shandong
Happy Fan Shop	King of Power Saving	To be used and then evaluated	Jingdong prefecture level city in Shandong
Happy Fan Shop	King of Power Saving	No shipment.	Jingdong prefecture level city in Shandong
Happy Fan Shop	King of Power Saving	Fake. Speechless.	Jingdong prefecture level city in Shandong
Happy Fan Shop	King of Power Saving	fantastic (idiom); awesome	Jingdong prefecture level city in Shandong
Happy Fan Shop	King of Power Saving	an excellent	Jingdong prefecture level city in Shandong
Happy Fan Shop	King of Power Saving	an excellent	Jingdong prefecture level city in Shandong
Happy Fan Shop	King of Power Saving	Want to buy! Recommended not to buy! No effect	Jingdong prefecture level city in Shandong
Happy Fan Shop	King of Power Saving	That's all there is to it. I can't figure out how to save electricity.	Jingdong prefecture level city in Shandong
Happy Fan Shop	King of Power Saving	It's no use.	Jingdong prefecture level city in Shandong
Happy Fan Shop	King of Power Saving	Things are more perfect than I imagined, very powerful, it is worth getting Packaging is very strong, things are well protected, it is worth the praise things are very satisfied Oh, beautiful and stylish atmosphere, customer service is super patient, service is meticulous and attentive, the big brand is not	Jingdong prefecture level city in Shandong
Happy Fan Shop	King of Power Saving	The effect is too good, the family has now several units, back to buy a number of times, the power saver received, power-saving effect is very good,, fine workmanship, long warranty, but also purify the air, before buying a used for a period of time, with the okay, and then bought a, and only now come to the	Jingdong prefecture level city in Shandong
Happy Fan Shop	King of Power Saving	Quite good, and imagine the same, casual ride are very good, satisfied! Baby received it 👌, really great ah! 🌈 quality is really nothing to say, very satisfied! 🆗 happy happy 😀 genuine, great, the price is very good, in short, quite good, will buy again a very good shopping experience, attentive service, fast logistics, distribution is also thoughtful, the attitude is also very good, value for money, ah,	Jingdong prefecture level city in Shandong

Happy Fan Shop	King of Power Saving	Power-saving effect: strong Cost-effective: quite good Appearance style: beautiful Workmanship material: meticulous Experience: looking forward to power-saving effect	Jingdong prefecture level city in Shandong
Happy Fan Shop	King of Power Saving	Cost-effective is okay, the appearance of the style of meticulous workmanship, looking forward to energy-saving effect.	Jingdong prefecture level city in Shandong
Happy Fan Shop	King of Power Saving	Very Good Very Power Saving This Power Saver is really power saving As soon as the power is turned on, the Power Saver starts the power saving function for you.	Jingdong prefecture level city in Shandong
Happy Fan Shop	King of Power Saving	Logistics is very fast, the price is favorable, friends introduced that it is very good to use, bought 2 at once, looking forward to the effect, used after the follow-up comment.	Jingdong prefecture level city in Shandong
Happy Fan Shop	King of Power Saving	It's all fake. It doesn't save any electricity.	Jingdong prefecture level city in Shandong
Happy Fan Shop	King of Power Saving	This Power Saver is so much better than the one I bought before, not only the effect is good, the quality is very good in all aspects, the customer service attitude is also very good, recommended to buy!	Jingdong prefecture level city in Shandong
Happy Fan Shop	King of Power Saving	Logistics is very fast, buy for parents, good quality, once bought 2, power-saving effect is good to buy 2 more, look forward to using the effect, satisfied will recommend to friends.	Jingdong prefecture level city in Shandong
Happy Fan Shop	King of Power Saving	With the description is the same, very high-grade, good value, to buy friends can order Oh!	Jingdong prefecture level city in Shandong
Happy Fan Shop	King of Power Saving	Not using much electricity in the winter saving over 100 per month! Ready to buy 2 more units in the summer ! ! ! ! ! Good quality, good price, power saving effect is obvious, cost-effective, worth buying, can be recommended!	Jingdong prefecture level city in Shandong
Happy Fan Shop	King of Power Saving	Purchased a power saver received on the put into use, rent a room with many electrical appliances, power consumption is very large, we have a piece of electricity or 1.2 dollars a time, with this power saver is much better, every day can save a lot of money, very convenient and practical.	Jingdong prefecture level city in Shandong
Happy Fan Shop	King of Power Saving	The power saving effect is very good: my supermarket monthly electricity bill is more than 7000 yuan. On May 1st someone recommended buying a couple of power savers, and I bought 5 of them first. The electricity bill was about 2000 less in June. This time, I bought 5 more. The seller said 800 degrees of electricity with one. I feel it's basically enough. A total of 10 units	Jingdong prefecture level city in Shandong
Happy Fan Shop	King of Power Saving	I never thought I could get such a good baby at such an affordable price, it's very good value for money, I love it!	Jingdong prefecture level city in Shandong
Happy Fan Shop	King of Power Saving	Previously bought other brands of power savers, but found no effect at home, this time to see how the products on the Jingdong, inserted in the home socket, the LED display is blue, the night light is very nice to see, look at the instructions that have to be half a year in order to achieve the best results, try it, I believe that the quality of the Jingdong, it should not be wrong.	Jingdong prefecture level city in Shandong
Happy Fan Shop	King of Power Saving	Superb, inexpensive, saves a lot of points, been using it for a long time!	Jingdong prefecture level city in Shandong
Happy Fan Shop	King of Power Saving	There are a lot of appliances in the house, the summer air conditioning is very costly, I bought this in front of the air conditioning effect is very good, I explain that I bought three in one breath, I use more electricity a month down to 200 or so, very satisfied.	Jingdong prefecture level city in Shandong
Happy Fan Shop	King of Power Saving	A month can save more than 200, home monthly electricity bill of more than 600 dollars, inserted 3 or so, an amount of the balance of the average can save more than 200. Product is very good, has been buying this one, has given a lot of relatives recommended, the quality is also very good, before buying a look with this same, who knows to buy home has not been used for more than a week on the bad, the quality of this is really good with more than two years!	Jingdong prefecture level city in Shandong
Happy Fan Shop	King of Power Saving	Whole house power saving king, can really save electricity, friends recommended, high power saving appliances, only replacement, no repair, after-sales worry-free!	Jingdong prefecture level city in Shandong
Happy Fan Shop	King of Power Saving	Great item love it, great customer service, great shopping experience!	Jingdong prefecture level city in Shandong
Happy Fan Shop	King of Power Saving	Power saving, very good use, much cheaper than the physical store, satisfaction	Jingdong prefecture level city in Shandong
Happy Fan Shop	King of Power Saving	In use, very well packaged and fast delivery of credit!	Jingdong prefecture level city in Shandong
Happy Fan Shop	King of Power Saving	Product quality, customer service attitude is particularly good, like, praise!	Jingdong prefecture level city in Shandong
Happy Fan Shop	King of Power Saving	The logistics is very fast, the packaging is not broken, the quality is very good, I like it very much!	Jingdong prefecture level city in Shandong
Happy Fan Shop	King of Power Saving	The saver was well packaged and was as described when plugged in	Jingdong prefecture level city in Shandong
Happy Fan Shop	King of Power Saving	Energy efficient and very satisfied	Jingdong prefecture level city in Shandong
Happy Fan Shop	King of Power Saving	Great quality for the price and fast shipping.	Jingdong prefecture level city in Shandong
Happy Fan Shop	King of Power Saving	It's great, just as described. Perfect.	Jingdong prefecture level city in Shandong
Happy Fan Shop	King of Power Saving	There is no power saving effect, but rather ring up an increase in electricity costs, because this machine is on continuously all day long, uninterruptedly, its own electricity and increased costs, plus the cost of purchasing the machine, so I feel that the use of this machine instead of increasing the	Jingdong prefecture level city in Shandong
Happy Fan Shop	King of Power Saving	Recommended by the owner of the store next door, looked at his home does have to save electricity, decided to get. Indeed quite can, the voltage is much more stable, there is a reduction in electricity	Jingdong prefecture level city in Shandong
Happy Fan Shop	King of Power Saving	Goods have been received value for money, affordable and reasonable price, and the description of the same, power-saving safe and reliable!	Jingdong prefecture level city in Shandong
Happy Fan Shop	King of Power Saving	Listen to friends introduced to buy, tried to save a lot of electricity is good, quite good, worth buying.	Jingdong prefecture level city in Shandong
Happy Fan Shop	King of Power Saving	The product is easy to use, safe and environmentally friendly, only used for a short time, to the next month, after the electricity bill status display to know how the performance of the power saver efficacy.	Jingdong prefecture level city in Shandong
Happy Fan Shop	King of Power Saving	Last month's electricity bill is 178 yuan, with this power saver is 92 yuan, ready to buy two more back home, I was introduced to buy a friend, just started to not believe that I did not expect the effect!	Jingdong prefecture level city in Shandong
Happy Fan Shop	King of Power Saving	It looks nice, but after using it for a few days I can't see how it saves power?	Jingdong prefecture level city in Shandong
Happy Fan Shop	King of Power Saving	Recommended by a friend, it is genuine, as described by the merchant, cost-effective	Jingdong prefecture level city in Shandong
Happy Fan Shop	King of Power Saving	Works well, affordable and saves money.	Jingdong prefecture level city in Shandong
Happy Fan Shop	King of Power Saving	Feels good haven't compared it yet, the air conditioning has been on a lot in the past two months	Jingdong prefecture level city in Shandong
Happy Fan Shop	King of Power Saving	Saving about 5 kWh per day is a good thing	Jingdong prefecture level city in Shandong
Happy Fan Shop	King of Power Saving	Not bad, affordable, best value for money	Jingdong prefecture level city in Shandong
Happy Fan Shop	King of Power Saving	The goods are genuine and the logistics are faster.	Jingdong prefecture level city in Shandong
Happy Fan Shop	King of Power Saving	Goods have been received today, has been plugged into the outlet, looking forward to saving electricity, after a period of time to see how the effect in the follow-up comment!	Jingdong prefecture level city in Shandong
Happy Fan Shop	King of Power Saving	I just got it home and haven't used it for long, so I'll use it for a couple months and see what happens. Seeing that most people have good reviews, it should be fine.	Jingdong prefecture level city in Shandong
Happy Fan Shop	King of Power Saving	I'm looking forward to the results, so I'll give you a good review out of habit! Follow up review after a while	Jingdong prefecture level city in Shandong
Happy Fan Shop	King of Power Saving	Use a dish first to know if it will help save energy	Jingdong prefecture level city in Shandong
Happy Fan Shop	King of Power Saving	Whether it saves electricity or not, we don't know yet.	Jingdong prefecture level city in Shandong
Happy Fan Shop	King of Power Saving	Goods have been received, use a period of time to know how oh. The courier did not deliver to the door ah! Sent to the station ah! The heat is inconvenient ah!	Jingdong prefecture level city in Shandong
Happy Fan Shop	King of Power Saving	A little effect, before an hour a power, now an hour 0.76 degrees, a dollar a power = save 24 cents, although not a lot, first use it!	Jingdong prefecture level city in Shandong

Happy Fan Shop	King of Power Saving	Just received the goods, use a month to evaluate	Jingdong prefecture level city in Shandong
Happy Fan Shop	King of Power Saving	Home every month with a lot of electricity, inadvertently found this good thing online that can save the beginning of a skeptical attitude to consult customer service, customer service replied very professional, said 120 days can be refunded that first try it!	Jingdong prefecture level city in Shandong
Happy Fan Shop	King of Power Saving	After receiving the goods this afternoon, immediately installed in the bathroom Smith energy storage electric water heater on the power outlet (instead of connecting with a plug board), when used, found that the "Power Saving King" LCD display, is the value of the voltage, not the store's video said so	Jingdong prefecture level city in Shandong
Happy Fan Shop	King of Power Saving	Has made many days, did not feel the power saving, the effect is not obvious.	Jingdong prefecture level city in Shandong
Happy Fan Shop	King of Power Saving	Energy savers do not save energy at all, but rather consume a lot of electricity.	Jingdong prefecture level city in Shandong
Happy Fan Shop	King of Power Saving	It's useless. It's a waste of money.	Jingdong prefecture level city in Shandong
Happy Fan Shop	King of Power Saving	Last month saved more than 500, which came back to buy, the store bought three, and then buy 1 home use, my family is dry beauty salon, more instruments, especially electricity, a month almost 1000. or so, with this did not expect to save so much, great, five-star praise!	Jingdong prefecture level city in Shandong
Happy Fan Shop	King of Power Saving	It rained a couple days ago and I just went to get the item back today , not bad, hey I looked at the meter and that red display light takes several seconds to jump, did not make a mistake buying it. After a period of time to evaluate.	Jingdong prefecture level city in Shandong
Happy Fan Shop	King of Power Saving	Without further ado, the electricity bill came down, this month less 102 yuan, has been repurchased, I also recommended to my friends, all said that there are effects, the product is okay, the effect is quite	Jingdong prefecture level city in Shandong
Happy Fan Shop	King of Power Saving	After buying and using two months really see the effect, so only to evaluate! Originally held the mindset of trying, I did not expect really effective, worth having!	Jingdong prefecture level city in Shandong
Happy Fan Shop	King of Power Saving	In the use of this energy saver before the monthly electricity bill is more than 150 yuan or so, now save thirty percent, every day to work, six or seven o'clock in the afternoon to go home, I do not know why the electricity bill is so expensive, energy-saving appliances really have the effect of recommending	Jingdong prefecture level city in Shandong
Happy Fan Shop	King of Power Saving	It saves about 100 bucks a month. A friend recommended it to me, and he's been using it for a while, and the effect is so obvious that I bought one too.	Jingdong prefecture level city in Shandong
Happy Fan Shop	King of Power Saving	My dad asked me to buy one, but after I paid the bill, I asked and he said it was a lot less this month, so he asked me to buy another one.	Jingdong prefecture level city in Shandong
Happy Fan Shop	King of Power Saving	The specific effect will have to be tested for at least two months to compare whether there is a power saving effect or not.	Jingdong prefecture level city in Shandong
Happy Fan Shop	King of Power Saving	'The goods have been received, immediately go to use, please ask the boss you instructions do not allow to withdraw, there are two fuses installed there!	Jingdong prefecture level city in Shandong
Happy Fan Shop	King of Power Saving	First praise is using, after using if good then follow up, now because I have not yet seen the results	Jingdong prefecture level city in Shandong
Happy Fan Shop	King of Power Saving	I've just been using it, and I haven't seen if it has a power-saving effect yet, so I'll follow up after 10 days and half a month.	Jingdong prefecture level city in Shandong
Happy Fan Shop	King of Power Saving	Just received the intelligent power saving meter, immediately plugged in to use, see it is not bad, and order to sell the second time	Jingdong prefecture level city in Shandong
Happy Fan Shop	King of Power Saving	This power saver is a bit effective, used for half a month, the effect is still visible.	Jingdong prefecture level city in Shandong
Happy Fan Shop	King of Power Saving	I received the baby and it looks great, I'm trying it out now and will keep an eye on it afterward.	Jingdong prefecture level city in Shandong
Happy Fan Shop	King of Power Saving	I've just received it, but I'm still trying to see how it works, so I'll come back in a little while.	Jingdong prefecture level city in Shandong
Happy Fan Shop	King of Power Saving	Just used it wait to use it for a while and then comment thanks, all first five stars.	Jingdong prefecture level city in Shandong
Happy Fan Shop	King of Power Saving	Very trustworthy brand, quality is guaranteed, the price is still affordable, quite satisfied!	Jingdong prefecture level city in Shandong
Happy Fan Shop	King of Power Saving	Things received, I hope that the platform of Jingdong do not sell fake goods are available	Jingdong prefecture level city in Shandong
Happy Fan Shop	King of Power Saving	Energy saver received, the use of the effect is good and the business description of the same	Jingdong prefecture level city in Shandong
Happy Fan Shop	King of Power Saving	The surface looks beautiful, the actual effect is not known, after using additional evaluation.	Jingdong prefecture level city in Shandong
Happy Fan Shop	King of Power Saving	Tried it for a few months, it really saves electricity, and I bought it for a friend.	Jingdong prefecture level city in Shandong
Happy Fan Shop	King of Power Saving	It didn't work and wanted to return it, it really didn't work at all.	Jingdong prefecture level city in Shandong
Happy Fan Shop	King of Power Saving	Just used it, don't know how effective it is, looking forward to usefulness	Jingdong prefecture level city in Shandong
Happy Fan Shop	King of Power Saving	After two months before the effect I do not know if it is true!	Jingdong prefecture level city in Shandong
Happy Fan Shop	King of Power Saving	So fast! Ordered yesterday and received today 👍👍👍👍👍👍👍👍👍 🌹🌹🌹🌹🌹🌹🌹🌹🌹	Jingdong prefecture level city in Shandong
Happy Fan Shop	King of Power Saving	I've started using it. We'll see how it works.	Jingdong prefecture level city in Shandong
Happy Fan Shop	King of Power Saving	I just received the goods, I won't know the effect until after two.	Jingdong prefecture level city in Shandong
Happy Fan Shop	King of Power Saving	Just received the goods, the specific effect will be evaluated later	Jingdong prefecture level city in Shandong
Happy Fan Shop	King of Power Saving	I've received the goods, I don't know if it's any good yet	Jingdong prefecture level city in Shandong
Happy Fan Shop	King of Power Saving	Try it for a month and then come back with a concrete evaluation	Jingdong prefecture level city in Shandong
Happy Fan Shop	King of Power Saving	In use, after two months to see the effect of power saving	Jingdong prefecture level city in Shandong
Happy Fan Shop	King of Power Saving	Power Saver. It's authentic. It's black. It works.	Jingdong prefecture level city in Shandong
Happy Fan Shop	King of Power Saving	I've been using it for a week now and it's not working.	Jingdong prefecture level city in Shandong
Happy Fan Shop	King of Power Saving	I'm just using it. I don't know how it's going to work.	Jingdong prefecture level city in Shandong
Happy Fan Shop	King of Power Saving	Products are better looking out for power savings	Jingdong prefecture level city in Shandong
Happy Fan Shop	King of Power Saving	I received it and I've kept it on. I hope it works.	Jingdong prefecture level city in Shandong
Happy Fan Shop	King of Power Saving	It's really good! Worth recommending to buy!	Jingdong prefecture level city in Shandong
Happy Fan Shop	King of Power Saving	Try it for a few days and see if it doesn't work, then return it.	Jingdong prefecture level city in Shandong
Happy Fan Shop	King of Power Saving	It's in use and I hope to be pleasantly surprised.	Jingdong prefecture level city in Shandong
Happy Fan Shop	King of Power Saving	We'll see how it works when we use it	Jingdong prefecture level city in Shandong
Happy Fan Shop	King of Power Saving	Let's see if it works.	Jingdong prefecture level city in Shandong
Happy Fan Shop	King of Power Saving	I'm using it and will follow up with a review	Jingdong prefecture level city in Shandong
Happy Fan Shop	King of Power Saving	It really works. It saves energy.	Jingdong prefecture level city in Shandong

Happy Fan Shop	King of Power Saving	It does have some power savings!	Jingdong prefecture level city in Shandong
Happy Fan Shop	King of Power Saving	Jingdong shopping speed is convenient and timely	Jingdong prefecture level city in Shandong
Happy Fan Shop	King of Power Saving	Second time I've bought it. Everything's fine.	Jingdong prefecture level city in Shandong
Happy Fan Shop	King of Power Saving	I don't know if it works yet.	Jingdong prefecture level city in Shandong
Happy Fan Shop	King of Power Saving	Not used, don't know how well it works.	Jingdong prefecture level city in Shandong
Happy Fan Shop	King of Power Saving	No visible results so far	Jingdong prefecture level city in Shandong
Happy Fan Shop	King of Power Saving	It doesn't feel like it's working.	Jingdong prefecture level city in Shandong
Happy Fan Shop	King of Power Saving	Where's the effect? Where are the power savings?	Jingdong prefecture level city in Shandong
Happy Fan Shop	King of Power Saving	Let's see how it works in practice!	Jingdong prefecture level city in Shandong
Happy Fan Shop	King of Power Saving	I'll wait until I use it to give you a good review	Jingdong prefecture level city in Shandong
Happy Fan Shop	King of Power Saving	I'll follow up after I've used it	Jingdong prefecture level city in Shandong
Happy Fan Shop	King of Power Saving	Well, that's good for saving power.	Jingdong prefecture level city in Shandong
Happy Fan Shop	King of Power Saving	Received. Used in the know.	Jingdong prefecture level city in Shandong
Happy Fan Shop	King of Power Saving	It doesn't do much good.	Jingdong prefecture level city in Shandong
Happy Fan Shop	King of Power Saving	It's not working.	Jingdong prefecture level city in Shandong
Happy Fan Shop	King of Power Saving	I don't know. I just used it.	Jingdong prefecture level city in Shandong
Happy Fan Shop	King of Power Saving	Use it and come back to evaluate.	Jingdong prefecture level city in Shandong
Happy Fan Shop	King of Power Saving	Two words: useless.	Jingdong prefecture level city in Shandong
Happy Fan Shop	King of Power Saving	To be used and then evaluated	Jingdong prefecture level city in Shandong
Happy Fan Shop	King of Power Saving	No shipment.	Jingdong prefecture level city in Shandong
Happy Fan Shop	King of Power Saving	Fake. Speechless.	Jingdong prefecture level city in Shandong
Happy Fan Shop	King of Power Saving	fantastic (idiom); awesome	Jingdong prefecture level city in Shandong
Happy Fan Shop	King of Power Saving	an excellent	Jingdong prefecture level city in Shandong
Happy Fan Shop	King of Power Saving	an excellent	Jingdong prefecture level city in Shandong
Happy Fan Shop	King of Power Saving	Want to buy! Recommended not to buy! No effect	Jingdong prefecture level city in Shandong
Happy Fan Shop	King of Power Saving	That's all there is to it. I can't figure out how to save electricity.	Jingdong prefecture level city in Shandong
Happy Fan Shop	King of Power Saving	It's no use.	Jingdong prefecture level city in Shandong
Happy Fan Shop	King of Power Saving	Things are more perfect than I imagined, very powerful, it is worth getting Packaging is very strong, things are well protected, it is worth the praise things are very satisfied Oh, beautiful and stylish atmosphere, customer service is super patient, service is meticulous and attentive, the big brand is not	Jingdong prefecture level city in Shandong
Happy Fan Shop	King of Power Saving	The effect is too good, the family has now several units, back to buy a number of times, the power saver received, power-saving effect is very good,, fine workmanship, long warranty, but also purify the air, before buying a used for a period of time, with the okay, and then bought a, and only now come to the	Jingdong prefecture level city in Shandong
Happy Fan Shop	King of Power Saving	Quite good, and imagine the same, casual ride are very good, satisfied! Baby received it 👌, really great ah! 🌈 quality is really nothing to say, very satisfied! 🆗 happy happy 😀 genuine, great, the price is very good, in short, quite good, will buy again a very good shopping experience, attentive service, fast logistics, distribution is also thoughtful, the attitude is also very good, value for money, ah,	Jingdong prefecture level city in Shandong
Happy Fan Shop	King of Power Saving	Power-saving effect: strong Cost-effective: quite good Appearance style: beautiful Workmanship material: meticulous Experience: looking forward to power-saving effect	Jingdong prefecture level city in Shandong
Happy Fan Shop	King of Power Saving	Cost-effective is okay, the appearance of the style of meticulous workmanship, looking forward to energy-saving effect.	Jingdong prefecture level city in Shandong
Happy Fan Shop	King of Power Saving	Very Good Very Power Saving This Power Saver is really power saving As soon as the power is turned on, the Power Saver starts the power saving function for you.	Jingdong prefecture level city in Shandong
Happy Fan Shop	King of Power Saving	Logistics is very fast, the price is favorable, friends introduced that it is very good to use, bought 2 at once, looking forward to the effect, used after the follow-up comment.	Jingdong prefecture level city in Shandong
Happy Fan Shop	King of Power Saving	It's all fake. It doesn't save any electricity.	Jingdong prefecture level city in Shandong
Happy Fan Shop	King of Power Saving	This Power Saver is so much better than the one I bought before, not only the effect is good, the quality is very good in all aspects, the customer service attitude is also very good, recommended to buy!	Jingdong prefecture level city in Shandong
Happy Fan Shop	King of Power Saving	Logistics is very fast, buy for parents, good quality, once bought 2, power-saving effect is good to buy 2 more, look forward to using the effect, satisfied will recommend to friends.	Jingdong prefecture level city in Shandong
Happy Fan Shop	King of Power Saving	With the description is the same, very high-grade, good value, to buy friends can order Oh!	Jingdong prefecture level city in Shandong
Happy Fan Shop	King of Power Saving	Not using much electricity in the winter saving over 100 per month! Ready to buy 2 more units in the summer ！！！！！ Good quality, good price, power saving effect is obvious, cost-effective, worth buying, can be recommended!	Jingdong prefecture level city in Shandong
Happy Fan Shop	King of Power Saving	Purchased a power saver received on the put into use, rent a room with many electrical appliances, power consumption is very large, we have a piece of electricity or 1.2 dollars a time, with this power saver is much better, every day can save a lot of money, very convenient and practical.	Jingdong prefecture level city in Shandong
Happy Fan Shop	King of Power Saving	The power saving effect is very good: my supermarket monthly electricity bill is more than 7000 yuan. On May 1st someone recommended buying a couple of power savers, and I bought 5 of them first. The electricity bill was about 2000 less in June. This time, I bought 5 more. The seller said 800 degrees of electricity with one. I feel it's basically enough. A total of 10 units	Jingdong prefecture level city in Shandong
Happy Fan Shop	King of Power Saving	I never thought I could get such a good baby at such an affordable price, it's very good value for money, I love it!	Jingdong prefecture level city in Shandong
Happy Fan Shop	King of Power Saving	Previously bought other brands of power savers, but found no effect at home, this time to see how the products on the Jingdong, inserted in the home socket, the LED display is blue, the night light is very nice to see, look at the instructions that have to be half a year in order to achieve the best results, try it, I believe that the quality of the Jingdong, it should not be wrong.	Jingdong prefecture level city in Shandong
Happy Fan Shop	King of Power Saving	Superb, inexpensive, saves a lot of points, been using it for a long time!	Jingdong prefecture level city in Shandong
Happy Fan Shop	King of Power Saving	There are a lot of appliances in the house, the summer air conditioning is very costly, I bought this in front of the air conditioning effect is very good, I explain that I bought three in one breath, I use more electricity a month down to 200 or so, very satisfied.	Jingdong prefecture level city in Shandong

Happy Fan Shop	King of Power Saving	A month can save more than 200, home monthly electricity bill of more than 600 dollars, inserted 3 or so, an amount of the balance of the average can save more than 200. Product is very good, has been buying this one, has given a lot of relatives recommended, the quality is also very good, before buying a look with this same, who knows to buy home has not been used for more than a week on the bad, the quality of this is really good with more than two years!	Jingdong prefecture level city in Shandong
Happy Fan Shop	King of Power Saving	Whole house power saving king, can really save electricity, friends recommended, high power saving appliances, only replacement, no repair, after-sales worry-free!	Jingdong prefecture level city in Shandong
Happy Fan Shop	King of Power Saving	Great item love it, great customer service, great shopping experience!	Jingdong prefecture level city in Shandong
Happy Fan Shop	King of Power Saving	Power saving, very good use, much cheaper than the physical store, satisfaction	Jingdong prefecture level city in Shandong
Happy Fan Shop	King of Power Saving	In use, very well packaged and fast delivery of credit!	Jingdong prefecture level city in Shandong
Happy Fan Shop	King of Power Saving	Product quality, customer service attitude is particularly good, like, praise!	Jingdong prefecture level city in Shandong
Happy Fan Shop	King of Power Saving	The logistics is very fast, the packaging is not broken, the quality is very good, I like it very much!	Jingdong prefecture level city in Shandong
Happy Fan Shop	King of Power Saving	The saver was well packaged and was as described when plugged in	Jingdong prefecture level city in Shandong
Happy Fan Shop	King of Power Saving	Energy efficient and very satisfied	Jingdong prefecture level city in Shandong
Happy Fan Shop	King of Power Saving	Great quality for the price and fast shipping.	Jingdong prefecture level city in Shandong
Happy Fan Shop	King of Power Saving	It's great, just as described. Perfect.	Jingdong prefecture level city in Shandong
Happy Fan Shop	King of Power Saving	There is no power saving effect, but rather ring up an increase in electricity costs, because this machine is on continuously all day long, uninterruptedly, its own electricity and increased costs, plus the cost of purchasing the machine, so I feel that the use of this machine instead of increasing the	Jingdong prefecture level city in Shandong
Happy Fan Shop	King of Power Saving	Recommended by the owner of the store next door, looked at his home does have to save electricity, decided to get. Indeed quite can, the voltage is much more stable, there is a reduction in electricity	Jingdong prefecture level city in Shandong
Happy Fan Shop	King of Power Saving	Goods have been received value for money, affordable and reasonable price, and the description of the same, power-saving safe and reliable!	Jingdong prefecture level city in Shandong
Happy Fan Shop	King of Power Saving	Listen to friends introduced to buy, tried to save a lot of electricity is good, quite good, worth buying.	Jingdong prefecture level city in Shandong
Happy Fan Shop	King of Power Saving	The product is easy to use, safe and environmentally friendly, only used for a short time, to the next month, after the electricity bill status display to know how the performance of the power saver efficacy.	Jingdong prefecture level city in Shandong
Happy Fan Shop	King of Power Saving	Last month's electricity bill is 178 yuan, with this power saver is 92 yuan, ready to buy two more back home, I was introduced to buy a friend, just started to not believe that I did not expect the effect!	Jingdong prefecture level city in Shandong
Happy Fan Shop	King of Power Saving	It looks nice, but after using it for a few days I can't see how it saves power?	Jingdong prefecture level city in Shandong
Happy Fan Shop	King of Power Saving	Recommended by a friend, it is genuine, as described by the merchant, cost-effective	Jingdong prefecture level city in Shandong
Happy Fan Shop	King of Power Saving	Works well, affordable and saves money.	Jingdong prefecture level city in Shandong
Happy Fan Shop	King of Power Saving	Feels good haven't compared it yet, the air conditioning has been on a lot in the past two months	Jingdong prefecture level city in Shandong
Happy Fan Shop	King of Power Saving	Saving about 5 kWh per day is a good thing	Jingdong prefecture level city in Shandong
Happy Fan Shop	King of Power Saving	Not bad, affordable, best value for money	Jingdong prefecture level city in Shandong
Happy Fan Shop	King of Power Saving	The goods are genuine and the logistics are faster.	Jingdong prefecture level city in Shandong
Happy Fan Shop	King of Power Saving	Goods have been received today, has been plugged into the outlet, looking forward to saving electricity, after a period of time to see how the effect in the follow-up comment!	Jingdong prefecture level city in Shandong
Happy Fan Shop	King of Power Saving	I just got it home and haven't used it for long, so I'll use it for a couple months and see what happens. Seeing that most people have good reviews, it should be fine.	Jingdong prefecture level city in Shandong
Happy Fan Shop	King of Power Saving	I'm looking forward to the results, so I'll give you a good review out of habit! Follow up review after a while	Jingdong prefecture level city in Shandong
Happy Fan Shop	King of Power Saving	Use a dish first to know if it will help save energy	Jingdong prefecture level city in Shandong
Happy Fan Shop	King of Power Saving	Whether it saves electricity or not, we don't know yet.	Jingdong prefecture level city in Shandong
Happy Fan Shop	King of Power Saving	Goods have been received, use a period of time to know how oh. The courier did not deliver to the door ah! Sent to the station ah! The heat is inconvenient ah!	Jingdong prefecture level city in Shandong
Happy Fan Shop	King of Power Saving	A little effect, before an hour a power, now an hour 0.76 degrees, a dollar a power = save 24 cents, although not a lot, first use it!	Jingdong prefecture level city in Shandong
Happy Fan Shop	King of Power Saving	Just received the goods, use a month to evaluate	Jingdong prefecture level city in Shandong
Happy Fan Shop	King of Power Saving	Home every month with a lot of electricity, inadvertently found this good thing online that can save the beginning of a skeptical attitude to consult customer service, customer service replied very professional, said 120 days can be refunded that first try it!	Jingdong prefecture level city in Shandong
Happy Fan Shop	King of Power Saving	After receiving the goods this afternoon, immediately installed in the bathroom Smith energy storage electric water heater on the power outlet (instead of connecting with a plug board), when used, found that the "Power Saving King" LCD display, is the value of the voltage, not the store's video said so	Jingdong prefecture level city in Shandong
Happy Fan Shop	King of Power Saving	Has made many days, did not feel the power saving, the effect is not obvious.	Jingdong prefecture level city in Shandong
Happy Fan Shop	King of Power Saving	Energy savers do not save energy at all, but rather consume a lot of electricity.	Jingdong prefecture level city in Shandong
Happy Fan Shop	King of Power Saving	It's useless. It's a waste of money.	Jingdong prefecture level city in Shandong
Happy Fan Shop	King of Power Saving	Last month saved more than 500, which came back to buy, the store bought three, and then buy 1 home use, my family is dry beauty salon, more instruments, especially electricity, a month almost 1000. or so, with this did not expect to save so much, great, five-star praise!	Jingdong prefecture level city in Shandong
Happy Fan Shop	King of Power Saving	It rained a couple days ago and I just went to get the item back today , not bad, hey I looked at the meter and that red display light takes several seconds to jump, did not make a mistake buying it. After a period of time to evaluate.	Jingdong prefecture level city in Shandong
Happy Fan Shop	King of Power Saving	Without further ado, the electricity bill came down, this month less 102 yuan, has been repurchased, I also recommended to my friends, all said that there are effects, the product is okay, the effect is quite	Jingdong prefecture level city in Shandong
Happy Fan Shop	King of Power Saving	After buying and using two months really see the effect, so only to evaluate! Originally held the mindset of trying, I did not expect really effective, worth having!	Jingdong prefecture level city in Shandong
Happy Fan Shop	King of Power Saving	In the use of this energy saver before the monthly electricity bill is more than 150 yuan or so, now save thirty percent, every day to work, six or seven o'clock in the afternoon to go home, I do not know why the electricity bill is so expensive, energy-saving appliances really have the effect of recommending	Jingdong prefecture level city in Shandong
Happy Fan Shop	King of Power Saving	It saves about 100 bucks a month. A friend recommended it to me, and he's been using it for a while, and the effect is so obvious that I bought one too.	Jingdong prefecture level city in Shandong
Happy Fan Shop	King of Power Saving	My dad asked me to buy one, but after I paid the bill, I asked and he said it was a lot less this month, so he asked me to buy another one.	Jingdong prefecture level city in Shandong
Happy Fan Shop	King of Power Saving	The specific effect will have to be tested for at least two months to compare whether there is a power saving effect or not.	Jingdong prefecture level city in Shandong
Happy Fan Shop	King of Power Saving	'The goods have been received, immediately go to use, please ask the boss you instructions do not allow to withdraw, there are two fuses installed there!	Jingdong prefecture level city in Shandong
Happy Fan Shop	King of Power Saving	First praise is using, after using if good then follow up, now because I have not yet seen the results	Jingdong prefecture level city in Shandong

Happy Fan Shop	King of Power Saving	I've just been using it, and I haven't seen if it has a power-saving effect yet, so I'll follow up after 10 days and half a month.	Jingdong prefecture level city in Shandong
Happy Fan Shop	King of Power Saving	Just received the intelligent power saving meter, immediately plugged in to use, see it is not bad, and order to sell the second time	Jingdong prefecture level city in Shandong
Happy Fan Shop	King of Power Saving	This power saver is a bit effective, used for half a month, the effect is still visible.	Jingdong prefecture level city in Shandong
Happy Fan Shop	King of Power Saving	I received the baby and it looks great, I'm trying it out now and will keep an eye on it afterward.	Jingdong prefecture level city in Shandong
Happy Fan Shop	King of Power Saving	I've just received it, but I'm still trying to see how it works, so I'll come back in a little while.	Jingdong prefecture level city in Shandong
Happy Fan Shop	King of Power Saving	Just used it wait to use it for a while and then comment thanks, all first five stars.	Jingdong prefecture level city in Shandong
Happy Fan Shop	King of Power Saving	Very trustworthy brand, quality is guaranteed, the price is still affordable, quite satisfied!	Jingdong prefecture level city in Shandong
Happy Fan Shop	King of Power Saving	Things received, I hope that the platform of Jingdong do not sell fake goods are available	Jingdong prefecture level city in Shandong
Happy Fan Shop	King of Power Saving	Energy saver received, the use of the effect is good and the business description of the same	Jingdong prefecture level city in Shandong
Happy Fan Shop	King of Power Saving	The surface looks beautiful, the actual effect is not known, after using additional evaluation.	Jingdong prefecture level city in Shandong
Happy Fan Shop	King of Power Saving	Tried it for a few months, it really saves electricity, and I bought it for a friend.	Jingdong prefecture level city in Shandong
Happy Fan Shop	King of Power Saving	It didn't work and wanted to return it, it really didn't work at all.	Jingdong prefecture level city in Shandong
Happy Fan Shop	King of Power Saving	Just used it, don't know how effective it is, looking forward to usefulness	Jingdong prefecture level city in Shandong
Happy Fan Shop	King of Power Saving	After two months before the effect I do not know if it is true!	Jingdong prefecture level city in Shandong
Happy Fan Shop	King of Power Saving	So fast! Ordered yesterday and received today 👍👍👍👍👍👍👍👍👍 🌹🌹🌹🌹🌹🌹🌹🌹🌹	Jingdong prefecture level city in Shandong
Happy Fan Shop	King of Power Saving	I've started using it. We'll see how it works.	Jingdong prefecture level city in Shandong
Happy Fan Shop	King of Power Saving	I just received the goods, I won't know the effect until after two.	Jingdong prefecture level city in Shandong
Happy Fan Shop	King of Power Saving	Just received the goods, the specific effect will be evaluated later	Jingdong prefecture level city in Shandong
Happy Fan Shop	King of Power Saving	I've received the goods, I don't know if it's any good yet	Jingdong prefecture level city in Shandong
Happy Fan Shop	King of Power Saving	Try it for a month and then come back with a concrete evaluation	Jingdong prefecture level city in Shandong
Happy Fan Shop	King of Power Saving	In use, after two months to see the effect of power saving	Jingdong prefecture level city in Shandong
Happy Fan Shop	King of Power Saving	Power Saver. It's authentic. It's black. It works.	Jingdong prefecture level city in Shandong
Happy Fan Shop	King of Power Saving	I've been using it for a week now and it's not working.	Jingdong prefecture level city in Shandong
Happy Fan Shop	King of Power Saving	I'm just using it. I don't know how it's going to work.	Jingdong prefecture level city in Shandong
Happy Fan Shop	King of Power Saving	Products are better looking out for power savings	Jingdong prefecture level city in Shandong
Happy Fan Shop	King of Power Saving	I received it and I've kept it on. I hope it works.	Jingdong prefecture level city in Shandong
Happy Fan Shop	King of Power Saving	It's really good! Worth recommending to buy!	Jingdong prefecture level city in Shandong
Happy Fan Shop	King of Power Saving	Try it for a few days and see if it doesn't work, then return it.	Jingdong prefecture level city in Shandong
Happy Fan Shop	King of Power Saving	It's in use and I hope to be pleasantly surprised.	Jingdong prefecture level city in Shandong
Happy Fan Shop	King of Power Saving	We'll see how it works when we use it	Jingdong prefecture level city in Shandong
Happy Fan Shop	King of Power Saving	Let's see if it works.	Jingdong prefecture level city in Shandong
Happy Fan Shop	King of Power Saving	I'm using it and will follow up with a review	Jingdong prefecture level city in Shandong
Happy Fan Shop	King of Power Saving	It really works. It saves energy.	Jingdong prefecture level city in Shandong
Happy Fan Shop	King of Power Saving	It does have some power savings!	Jingdong prefecture level city in Shandong
Happy Fan Shop	King of Power Saving	Jingdong shopping speed is convenient and timely	Jingdong prefecture level city in Shandong
Happy Fan Shop	King of Power Saving	Second time I've bought it. Everything's fine.	Jingdong prefecture level city in Shandong
Happy Fan Shop	King of Power Saving	I don't know if it works yet.	Jingdong prefecture level city in Shandong
Happy Fan Shop	King of Power Saving	Not used, don't know how well it works.	Jingdong prefecture level city in Shandong
Happy Fan Shop	King of Power Saving	No visible results so far	Jingdong prefecture level city in Shandong
Happy Fan Shop	King of Power Saving	It doesn't feel like it's working.	Jingdong prefecture level city in Shandong
Happy Fan Shop	King of Power Saving	Where's the effect? Where are the power savings?	Jingdong prefecture level city in Shandong
Happy Fan Shop	King of Power Saving	Let's see how it works in practice!	Jingdong prefecture level city in Shandong
Happy Fan Shop	King of Power Saving	I'll wait until I use it to give you a good review	Jingdong prefecture level city in Shandong
Happy Fan Shop	King of Power Saving	I'll follow up after I've used it	Jingdong prefecture level city in Shandong
Happy Fan Shop	King of Power Saving	Well, that's good for saving power.	Jingdong prefecture level city in Shandong
Happy Fan Shop	King of Power Saving	Received. Used in the know.	Jingdong prefecture level city in Shandong
Happy Fan Shop	King of Power Saving	It doesn't do much good.	Jingdong prefecture level city in Shandong
Happy Fan Shop	King of Power Saving	It's not working.	Jingdong prefecture level city in Shandong
Happy Fan Shop	King of Power Saving	I don't know. I just used it.	Jingdong prefecture level city in Shandong
Happy Fan Shop	King of Power Saving	Use it and come back to evaluate.	Jingdong prefecture level city in Shandong
Happy Fan Shop	King of Power Saving	Two words: useless.	Jingdong prefecture level city in Shandong

Happy Fan Shop	King of Power Saving	To be used and then evaluated	Jingdong prefecture level city in Shandong
Happy Fan Shop	King of Power Saving	No shipment.	Jingdong prefecture level city in Shandong
Happy Fan Shop	King of Power Saving	Fake. Speechless.	Jingdong prefecture level city in Shandong
Happy Fan Shop	King of Power Saving	fantastic (idiom); awesome	Jingdong prefecture level city in Shandong
Happy Fan Shop	King of Power Saving	an excellent	Jingdong prefecture level city in Shandong
Happy Fan Shop	King of Power Saving	an excellent	Jingdong prefecture level city in Shandong
Happy Fan Shop	King of Power Saving	Want to buy! Recommended not to buy! No effect	Jingdong prefecture level city in Shandong
Happy Fan Shop	King of Power Saving	That's all there is to it. I can't figure out how to save electricity.	Jingdong prefecture level city in Shandong
Happy Fan Shop	King of Power Saving	It's no use.	Jingdong prefecture level city in Shandong
Happy Fan Shop	King of Power Saving	Things are more perfect than I imagined, very powerful, it is worth getting Packaging is very strong, things are well protected, it is worth the praise things are very satisfied Oh, beautiful and stylish atmosphere, customer service is super patient, service is meticulous and attentive, the big brand is not	Jingdong prefecture level city in Shandong
Happy Fan Shop	King of Power Saving	The effect is too good, the family has now several units, back to buy a number of times, the power saver received, power-saving effect is very good,, fine workmanship, long warranty, but also purify the air, before buying a used for a period of time, with the okay, and then bought a, and only now come to the	Jingdong prefecture level city in Shandong
Happy Fan Shop	King of Power Saving	Quite good, and imagine the same, casual ride are very good, satisfied! Baby received it 👌, really great ah! 🌈 quality is really nothing to say, very satisfied! 🆗 happy happy 😃 genuine, great, the price is very good, in short, quite good, will buy again a very good shopping experience, attentive service, fast logistics, distribution is also thoughtful, the attitude is also very good, value for money, ah,	Jingdong prefecture level city in Shandong
Happy Fan Shop	King of Power Saving	Power-saving effect: strong Cost-effective: quite good Appearance style: beautiful Workmanship material: meticulous Experience: looking forward to power-saving effect	Jingdong prefecture level city in Shandong
Happy Fan Shop	King of Power Saving	Cost-effective is okay, the appearance of the style of meticulous workmanship, looking forward to energy-saving effect.	Jingdong prefecture level city in Shandong
Happy Fan Shop	King of Power Saving	Very Good Very Power Saving This Power Saver is really power saving As soon as the power is turned on, the Power Saver starts the power saving function for you.	Jingdong prefecture level city in Shandong
Happy Fan Shop	King of Power Saving	Logistics is very fast, the price is favorable, friends introduced that it is very good to use, bought 2 at once, looking forward to the effect, used after the follow-up comment.	Jingdong prefecture level city in Shandong
Happy Fan Shop	King of Power Saving	It's all fake. It doesn't save any electricity.	Jingdong prefecture level city in Shandong
Happy Fan Shop	King of Power Saving	This Power Saver is so much better than the one I bought before, not only the effect is good, the quality is very good in all aspects, the customer service attitude is also very good, recommended to buy!	Jingdong prefecture level city in Shandong
Happy Fan Shop	King of Power Saving	Logistics is very fast, buy for parents, good quality, once bought 2, power-saving effect is good to buy 2 more, look forward to using the effect, satisfied will recommend to friends.	Jingdong prefecture level city in Shandong
Happy Fan Shop	King of Power Saving	With the description is the same, very high-grade, good value, to buy friends can order Oh!	Jingdong prefecture level city in Shandong
Happy Fan Shop	King of Power Saving	Not using much electricity in the winter saving over 100 per month! Ready to buy 2 more units in the summer ！！！！！ Good quality, good price, power saving effect is obvious, cost-effective, worth buying, can be recommended!	Jingdong prefecture level city in Shandong
Happy Fan Shop	King of Power Saving	Purchased a power saver received on the put into use, rent a room with many electrical appliances, power consumption is very large, we have a piece of electricity or 1.2 dollars a time, with this power saver is much better, every day can save a lot of money, very convenient and practical.	Jingdong prefecture level city in Shandong
Happy Fan Shop	King of Power Saving	The power saving effect is very good: my supermarket monthly electricity bill is more than 7000 yuan. On May 1st someone recommended buying a couple of power savers, and I bought 5 of them first. The electricity bill was about 2000 less in June. This time, I bought 5 more. The seller said 800 degrees of electricity with one. I feel it's basically enough. A total of 10 units	Jingdong prefecture level city in Shandong
Happy Fan Shop	King of Power Saving	I never thought I could get such a good baby at such an affordable price, it's very good value for money, I love it!	Jingdong prefecture level city in Shandong
Happy Fan Shop	King of Power Saving	Previously bought other brands of power savers, but found no effect at home, this time to see how the products on the Jingdong, inserted in the home socket, the LED display is blue, the night light is very nice to see, look at the instructions that have to be half a year in order to achieve the best results, try it, I believe that the quality of the Jingdong, it should not be wrong.	Jingdong prefecture level city in Shandong
Happy Fan Shop	King of Power Saving	Superb, inexpensive, saves a lot of points, been using it for a long time!	Jingdong prefecture level city in Shandong
Happy Fan Shop	King of Power Saving	There are a lot of appliances in the house, the summer air conditioning is very costly, I bought this in front of the air conditioning effect is very good, I explain that I bought three in one breath, I use more electricity a month down to 200 or so, very satisfied.	Jingdong prefecture level city in Shandong
Happy Fan Shop	King of Power Saving	A month can save more than 200, home monthly electricity bill of more than 600 dollars, inserted 3 or so, an amount of the balance of the average can save more than 200. Product is very good, has been buying this one, has given a lot of relatives recommended, the quality is also very good, before buying a look with this same, who knows to buy home has not been used for more than a week on the bad, the quality of this is really good with more than two years!	Jingdong prefecture level city in Shandong
Happy Fan Shop	King of Power Saving	Whole house power saving king, can really save electricity, friends recommended, high power saving appliances, only replacement, no repair, after-sales worry-free!	Jingdong prefecture level city in Shandong
Happy Fan Shop	King of Power Saving	Great item love it, great customer service, great shopping experience!	Jingdong prefecture level city in Shandong
Happy Fan Shop	King of Power Saving	Power saving, very good use, much cheaper than the physical store, satisfaction	Jingdong prefecture level city in Shandong
Happy Fan Shop	King of Power Saving	In use, very well packaged and fast delivery of credit!	Jingdong prefecture level city in Shandong
Happy Fan Shop	King of Power Saving	Product quality, customer service attitude is particularly good, like, praise!	Jingdong prefecture level city in Shandong
Happy Fan Shop	King of Power Saving	The logistics is very fast, the packaging is not broken, the quality is very good, I like it very much!	Jingdong prefecture level city in Shandong
Happy Fan Shop	King of Power Saving	The saver was well packaged and was as described when plugged in	Jingdong prefecture level city in Shandong
Happy Fan Shop	King of Power Saving	Energy efficient and very satisfied	Jingdong prefecture level city in Shandong
Happy Fan Shop	King of Power Saving	Great quality for the price and fast shipping.	Jingdong prefecture level city in Shandong
Happy Fan Shop	King of Power Saving	It's great, just as described. Perfect.	Jingdong prefecture level city in Shandong
Happy Fan Shop	King of Power Saving	There is no power saving effect, but rather ring up an increase in electricity costs, because this machine is on continuously all day long, uninterruptedly, its own electricity and increased costs, plus the cost of purchasing the machine, so I feel that the use of this machine instead of increasing the	Jingdong prefecture level city in Shandong
Happy Fan Shop	King of Power Saving	Recommended by the owner of the store next door, looked at his home does have to save electricity, decided to get. Indeed quite can, the voltage is much more stable, there is a reduction in electricity	Jingdong prefecture level city in Shandong
Happy Fan Shop	King of Power Saving	Goods have been received value for money, affordable and reasonable price, and the description of the same, power-saving safe and reliable!	Jingdong prefecture level city in Shandong
Happy Fan Shop	King of Power Saving	Listen to friends introduced to buy, tried to save a lot of electricity is good, quite good, worth buying.	Jingdong prefecture level city in Shandong
Happy Fan Shop	King of Power Saving	The product is easy to use, safe and environmentally friendly, only used for a short time, to the next month, after the electricity bill status display to know how the performance of the power saver efficacy.	Jingdong prefecture level city in Shandong
Happy Fan Shop	King of Power Saving	Last month's electricity bill is 178 yuan, with this power saver is 92 yuan, ready to buy two more back home, I was introduced to buy a friend, just started to not believe that I did not expect the effect!	Jingdong prefecture level city in Shandong

Happy Fan Shop	King of Power Saving	It looks nice, but after using it for a few days I can't see how it saves power?	Jingdong prefecture level city in Shandong
Happy Fan Shop	King of Power Saving	Recommended by a friend, it is genuine, as described by the merchant, cost-effective	Jingdong prefecture level city in Shandong
Happy Fan Shop	King of Power Saving	Works well, affordable and saves money.	Jingdong prefecture level city in Shandong
Happy Fan Shop	King of Power Saving	Feels good haven't compared it yet, the air conditioning has been on a lot in the past two months	Jingdong prefecture level city in Shandong
Happy Fan Shop	King of Power Saving	Saving about 5 kWh per day is a good thing	Jingdong prefecture level city in Shandong
Happy Fan Shop	King of Power Saving	Not bad, affordable, best value for money	Jingdong prefecture level city in Shandong
Happy Fan Shop	King of Power Saving	The goods are genuine and the logistics are faster.	Jingdong prefecture level city in Shandong
Happy Fan Shop	King of Power Saving	Goods have been received today, has been plugged into the outlet, looking forward to saving electricity, after a period of time to see how the effect in the follow-up comment!	Jingdong prefecture level city in Shandong
Happy Fan Shop	King of Power Saving	I just got it home and haven't used it for long, so I'll use it for a couple months and see what happens. Seeing that most people have good reviews, it should be fine.	Jingdong prefecture level city in Shandong
Happy Fan Shop	King of Power Saving	I'm looking forward to the results, so I'll give you a good review out of habit! Follow up review after a while	Jingdong prefecture level city in Shandong
Happy Fan Shop	King of Power Saving	Use a dish first to know if it will help save energy	Jingdong prefecture level city in Shandong
Happy Fan Shop	King of Power Saving	Whether it saves electricity or not, we don't know yet.	Jingdong prefecture level city in Shandong
Happy Fan Shop	King of Power Saving	Goods have been received, use a period of time to know how oh. The courier did not deliver to the door ah! Sent to the station ah! The heat is inconvenient ah!	Jingdong prefecture level city in Shandong
Happy Fan Shop	King of Power Saving	A little effect, before an hour a power, now an hour 0.76 degrees, a dollar a power = save 24 cents, although not a lot, first use it!	Jingdong prefecture level city in Shandong
Happy Fan Shop	King of Power Saving	Just received the goods, use a month to evaluate	Jingdong prefecture level city in Shandong
Happy Fan Shop	King of Power Saving	Home every month with a lot of electricity, inadvertently found this good thing online that can save the beginning of a skeptical attitude to consult customer service, customer service replied very professional, said 120 days can be refunded that first try it!	Jingdong prefecture level city in Shandong
Happy Fan Shop	King of Power Saving	After receiving the goods this afternoon, immediately installed in the bathroom Smith energy storage electric water heater on the power outlet (instead of connecting with a plug board), when used, found that the "Power Saving King" LCD display, is the value of the voltage, not the store's video said so	Jingdong prefecture level city in Shandong
Happy Fan Shop	King of Power Saving	Has made many days, did not feel the power saving, the effect is not obvious.	Jingdong prefecture level city in Shandong
Happy Fan Shop	King of Power Saving	Energy savers do not save energy at all, but rather consume a lot of electricity.	Jingdong prefecture level city in Shandong
Happy Fan Shop	King of Power Saving	It's useless. It's a waste of money.	Jingdong prefecture level city in Shandong
Happy Fan Shop	King of Power Saving	Last month saved more than 500, which came back to buy, the store bought three, and then buy 1 home use, my family is dry beauty salon, more instruments, especially electricity, a month almost 1000. or so, with this did not expect to save so much, great, five-star praise!	Jingdong prefecture level city in Shandong
Happy Fan Shop	King of Power Saving	It rained a couple days ago and I just went to get the item back today , not bad, hey I looked at the meter and that red display light takes several seconds to jump, did not make a mistake buying it. After a period of time to evaluate.	Jingdong prefecture level city in Shandong
Happy Fan Shop	King of Power Saving	Without further ado, the electricity bill came down, this month less 102 yuan, has been repurchased, I also recommended to my friends, all said that there are effects, the product is okay, the effect is quite	Jingdong prefecture level city in Shandong
Happy Fan Shop	King of Power Saving	After buying and using two months really see the effect, so only to evaluate! Originally held the mindset of trying, I did not expect really effective, worth having!	Jingdong prefecture level city in Shandong
Happy Fan Shop	King of Power Saving	In the use of this energy saver before the monthly electricity bill is more than 150 yuan or so, now save thirty percent, every day to work, six or seven o'clock in the afternoon to go home, I do not know why the electricity bill is so expensive, energy-saving appliances really have the effect of recommending	Jingdong prefecture level city in Shandong
Happy Fan Shop	King of Power Saving	It saves about 100 bucks a month. A friend recommended it to me, and he's been using it for a while, and the effect is so obvious that I bought one too.	Jingdong prefecture level city in Shandong
Happy Fan Shop	King of Power Saving	My dad asked me to buy one, but after I paid the bill, I asked and he said it was a lot less this month, so he asked me to buy another one.	Jingdong prefecture level city in Shandong
Happy Fan Shop	King of Power Saving	The specific effect will have to be tested for at least two months to compare whether there is a power saving effect or not.	Jingdong prefecture level city in Shandong
Happy Fan Shop	King of Power Saving	'The goods have been received, immediately go to use, please ask the boss you instructions do not allow to withdraw, there are two fuses installed there!	Jingdong prefecture level city in Shandong
Happy Fan Shop	King of Power Saving	First praise is using, after using if good then follow up, now because I have not yet seen the results	Jingdong prefecture level city in Shandong
Happy Fan Shop	King of Power Saving	I've just been using it, and I haven't seen if it has a power-saving effect yet, so I'll follow up after 10 days and half a month.	Jingdong prefecture level city in Shandong
Happy Fan Shop	King of Power Saving	Just received the intelligent power saving meter, immediately plugged in to use, see it is not bad, and order to sell the second time	Jingdong prefecture level city in Shandong
Happy Fan Shop	King of Power Saving	This power saver is a bit effective, used for half a month, the effect is still visible.	Jingdong prefecture level city in Shandong
Happy Fan Shop	King of Power Saving	I received the baby and it looks great, I'm trying it out now and will keep an eye on it afterward.	Jingdong prefecture level city in Shandong
Happy Fan Shop	King of Power Saving	I've just received it, but I'm still trying to see how it works, so I'll come back in a little while.	Jingdong prefecture level city in Shandong
Happy Fan Shop	King of Power Saving	Just used it wait to use it for a while and then comment thanks, all first five stars.	Jingdong prefecture level city in Shandong
Happy Fan Shop	King of Power Saving	Very trustworthy brand, quality is guaranteed, the price is still affordable, quite satisfied!	Jingdong prefecture level city in Shandong
Happy Fan Shop	King of Power Saving	Things received, I hope that the platform of Jingdong do not sell fake goods are available	Jingdong prefecture level city in Shandong
Happy Fan Shop	King of Power Saving	Energy saver received, the use of the effect is good and the business description of the same	Jingdong prefecture level city in Shandong
Happy Fan Shop	King of Power Saving	The surface looks beautiful, the actual effect is not known, after using additional evaluation.	Jingdong prefecture level city in Shandong
Happy Fan Shop	King of Power Saving	Tried it for a few months, it really saves electricity, and I bought it for a friend.	Jingdong prefecture level city in Shandong
Happy Fan Shop	King of Power Saving	It didn't work and wanted to return it, it really didn't work at all.	Jingdong prefecture level city in Shandong
Happy Fan Shop	King of Power Saving	Just used it, don't know how effective it is, looking forward to usefulness	Jingdong prefecture level city in Shandong
Happy Fan Shop	King of Power Saving	After two months before the effect I do not know if it is true!	Jingdong prefecture level city in Shandong
Happy Fan Shop	King of Power Saving	So fast! Ordered yesterday and received today 👍👍👍👍👍👍👍👍👍 🌹🌹🌹🌹🌹🌹🌹🌹🌹	Jingdong prefecture level city in Shandong
Happy Fan Shop	King of Power Saving	I've started using it. We'll see how it works.	Jingdong prefecture level city in Shandong
Happy Fan Shop	King of Power Saving	I just received the goods, I won't know the effect until after two.	Jingdong prefecture level city in Shandong
Happy Fan Shop	King of Power Saving	Just received the goods, the specific effect will be evaluated later	Jingdong prefecture level city in Shandong
Happy Fan Shop	King of Power Saving	I've received the goods, I don't know if it's any good yet	Jingdong prefecture level city in Shandong

Happy Fan Shop	King of Power Saving	Try it for a month and then come back with a concrete evaluation	Jingdong prefecture level city in Shandong
Happy Fan Shop	King of Power Saving	In use, after two months to see the effect of power saving	Jingdong prefecture level city in Shandong
Happy Fan Shop	King of Power Saving	Power Saver. It's authentic. It's black. It works.	Jingdong prefecture level city in Shandong
Happy Fan Shop	King of Power Saving	I've been using it for a week now and it's not working.	Jingdong prefecture level city in Shandong
Happy Fan Shop	King of Power Saving	I'm just using it. I don't know how it's going to work.	Jingdong prefecture level city in Shandong
Happy Fan Shop	King of Power Saving	Products are better looking out for power savings	Jingdong prefecture level city in Shandong
Happy Fan Shop	King of Power Saving	I received it and I've kept it on. I hope it works.	Jingdong prefecture level city in Shandong
Happy Fan Shop	King of Power Saving	It's really good! Worth recommending to buy!	Jingdong prefecture level city in Shandong
Happy Fan Shop	King of Power Saving	Try it for a few days and see if it doesn't work, then return it.	Jingdong prefecture level city in Shandong
Happy Fan Shop	King of Power Saving	It's in use and I hope to be pleasantly surprised.	Jingdong prefecture level city in Shandong
Happy Fan Shop	King of Power Saving	We'll see how it works when we use it	Jingdong prefecture level city in Shandong
Happy Fan Shop	King of Power Saving	Let's see if it works.	Jingdong prefecture level city in Shandong
Happy Fan Shop	King of Power Saving	I'm using it and will follow up with a review	Jingdong prefecture level city in Shandong
Happy Fan Shop	King of Power Saving	It really works. It saves energy.	Jingdong prefecture level city in Shandong
Happy Fan Shop	King of Power Saving	It does have some power savings!	Jingdong prefecture level city in Shandong
Happy Fan Shop	King of Power Saving	Jingdong shopping speed is convenient and timely	Jingdong prefecture level city in Shandong
Happy Fan Shop	King of Power Saving	Second time I've bought it. Everything's fine.	Jingdong prefecture level city in Shandong
Happy Fan Shop	King of Power Saving	I don't know if it works yet.	Jingdong prefecture level city in Shandong
Happy Fan Shop	King of Power Saving	Not used, don't know how well it works.	Jingdong prefecture level city in Shandong
Happy Fan Shop	King of Power Saving	No visible results so far	Jingdong prefecture level city in Shandong
Happy Fan Shop	King of Power Saving	It doesn't feel like it's working.	Jingdong prefecture level city in Shandong
Happy Fan Shop	King of Power Saving	Where's the effect? Where are the power savings?	Jingdong prefecture level city in Shandong
Happy Fan Shop	King of Power Saving	Let's see how it works in practice!	Jingdong prefecture level city in Shandong
Happy Fan Shop	King of Power Saving	I'll wait until I use it to give you a good review	Jingdong prefecture level city in Shandong
Happy Fan Shop	King of Power Saving	I'll follow up after I've used it	Jingdong prefecture level city in Shandong
Happy Fan Shop	King of Power Saving	Well, that's good for saving power.	Jingdong prefecture level city in Shandong
Happy Fan Shop	King of Power Saving	Received. Used in the know.	Jingdong prefecture level city in Shandong
Happy Fan Shop	King of Power Saving	It doesn't do much good.	Jingdong prefecture level city in Shandong
Happy Fan Shop	King of Power Saving	It's not working.	Jingdong prefecture level city in Shandong
Happy Fan Shop	King of Power Saving	I don't know. I just used it.	Jingdong prefecture level city in Shandong
Happy Fan Shop	King of Power Saving	Use it and come back to evaluate.	Jingdong prefecture level city in Shandong
Happy Fan Shop	King of Power Saving	Two words: useless.	Jingdong prefecture level city in Shandong
Happy Fan Shop	King of Power Saving	To be used and then evaluated	Jingdong prefecture level city in Shandong
Happy Fan Shop	King of Power Saving	No shipment.	Jingdong prefecture level city in Shandong
Happy Fan Shop	King of Power Saving	Fake. Speechless.	Jingdong prefecture level city in Shandong
Happy Fan Shop	King of Power Saving	fantastic (idiom); awesome	Jingdong prefecture level city in Shandong
Happy Fan Shop	King of Power Saving	an excellent	Jingdong prefecture level city in Shandong
Happy Fan Shop	King of Power Saving	an excellent	Jingdong prefecture level city in Shandong
Happy Fan Shop	King of Power Saving	Want to buy! Recommended not to buy! No effect	Jingdong prefecture level city in Shandong
Happy Fan Shop	King of Power Saving	That's all there is to it. I can't figure out how to save electricity.	Jingdong prefecture level city in Shandong
Happy Fan Shop	King of Power Saving	It's no use.	Jingdong prefecture level city in Shandong
Happy Fan Shop	King of Power Saving	Things are more perfect than I imagined, very powerful, it is worth getting Packaging is very strong, things are well protected, it is worth the praise things are very satisfied Oh, beautiful and stylish atmosphere, customer service is super patient, service is meticulous and attentive, the big brand is not	Jingdong prefecture level city in Shandong
Happy Fan Shop	King of Power Saving	The effect is too good, the family has now several units, back to buy a number of times, the power saver received, power-saving effect is very good,, fine workmanship, long warranty, but also purify the air, before buying a used for a period of time, with the okay, and then bought a, and only now come to the	Jingdong prefecture level city in Shandong
Happy Fan Shop	King of Power Saving	Quite good, and imagine the same, casual ride are very good, satisfied! Baby received it 👌, really great ah! 🌈 quality is really nothing to say, very satisfied! 🆗 happy happy 😃 genuine, great, the price is very good, in short, quite good, will buy again a very good shopping experience, attentive service, fast logistics, distribution is also thoughtful, the attitude is also very good, value for money, ah,	Jingdong prefecture level city in Shandong
Happy Fan Shop	King of Power Saving	Power-saving effect: strong Cost-effective: quite good Appearance style: beautiful Workmanship material: meticulous Experience: looking forward to power-saving effect	Jingdong prefecture level city in Shandong
Happy Fan Shop	King of Power Saving	Cost-effective is okay, the appearance of the style of meticulous workmanship, looking forward to energy-saving effect.	Jingdong prefecture level city in Shandong
Happy Fan Shop	King of Power Saving	Very Good Very Power Saving This Power Saver is really power saving As soon as the power is turned on, the Power Saver starts the power saving function for you.	Jingdong prefecture level city in Shandong
Happy Fan Shop	King of Power Saving	Logistics is very fast, the price is favorable, friends introduced that it is very good to use, bought 2 at once, looking forward to the effect, used after the follow-up comment.	Jingdong prefecture level city in Shandong
Happy Fan Shop	King of Power Saving	It's all fake. It doesn't save any electricity.	Jingdong prefecture level city in Shandong

Happy Fan Shop	King of Power Saving	This Power Saver is so much better than the one I bought before, not only the effect is good, the quality is very good in all aspects, the customer service attitude is also very good, recommended to buy!	Jingdong prefecture level city in Shandong
Happy Fan Shop	King of Power Saving	Logistics is very fast, buy for parents, good quality, once bought 2, power-saving effect is good to buy 2 more, look forward to using the effect, satisfied will recommend to friends.	Jingdong prefecture level city in Shandong
Happy Fan Shop	King of Power Saving	With the description is the same, very high-grade, good value, to buy friends can order Oh!	Jingdong prefecture level city in Shandong
Happy Fan Shop	King of Power Saving	Not using much electricity in the winter saving over 100 per month! Ready to buy 2 more units in the summer ！！！！！ Good quality, good price, power saving effect is obvious, cost-effective, worth buying, can be recommended!	Jingdong prefecture level city in Shandong
Happy Fan Shop	King of Power Saving	Purchased a power saver received on the put into use, rent a room with many electrical appliances, power consumption is very large, we have a piece of electricity or 1.2 dollars a time, with this power saver is much better, every day can save a lot of money, very convenient and practical.	Jingdong prefecture level city in Shandong
Happy Fan Shop	King of Power Saving	The power saving effect is very good: my supermarket monthly electricity bill is more than 7000 yuan. On May 1st someone recommended buying a couple of power savers, and I bought 5 of them first. The electricity bill was about 2000 less in June. This time, I bought 5 more. The seller said 800 degrees of electricity with one. I feel it's basically enough. A total of 10 units	Jingdong prefecture level city in Shandong
Happy Fan Shop	King of Power Saving	I never thought I could get such a good baby at such an affordable price, it's very good value for money, I love it!	Jingdong prefecture level city in Shandong
Happy Fan Shop	King of Power Saving	Previously bought other brands of power savers, but found no effect at home, this time to see how the products on the Jingdong, inserted in the home socket, the LED display is blue, the night light is very nice to see, look at the instructions that have to be half a year in order to achieve the best results, try it, I believe that the quality of the Jingdong, it should not be wrong.	Jingdong prefecture level city in Shandong
Happy Fan Shop	King of Power Saving	Superb, inexpensive, saves a lot of points, been using it for a long time!	Jingdong prefecture level city in Shandong
Happy Fan Shop	King of Power Saving	There are a lot of appliances in the house, the summer air conditioning is very costly, I bought this in front of the air conditioning effect is very good, I explain that I bought three in one breath, I use more electricity a month down to 200 or so, very satisfied.	Jingdong prefecture level city in Shandong
Happy Fan Shop	King of Power Saving	A month can save more than 200, home monthly electricity bill of more than 600 dollars, inserted 3 or so, an amount of the balance of the average can save more than 200. Product is very good, has been buying this one, has given a lot of relatives recommended, the quality is also very good, before buying a look with this same, who knows to buy home has not been used for more than a week on the bad, the quality of this is really good with more than two years!	Jingdong prefecture level city in Shandong
Happy Fan Shop	King of Power Saving	Whole house power saving king, can really save electricity, friends recommended, high power saving appliances, only replacement, no repair, after-sales worry-free!	Jingdong prefecture level city in Shandong
Happy Fan Shop	King of Power Saving	Great item love it, great customer service, great shopping experience!	Jingdong prefecture level city in Shandong
Happy Fan Shop	King of Power Saving	Power saving, very good use, much cheaper than the physical store, satisfaction	Jingdong prefecture level city in Shandong
Happy Fan Shop	King of Power Saving	In use, very well packaged and fast delivery of credit!	Jingdong prefecture level city in Shandong
Happy Fan Shop	King of Power Saving	Product quality, customer service attitude is particularly good, like, praise!	Jingdong prefecture level city in Shandong
Happy Fan Shop	King of Power Saving	The logistics is very fast, the packaging is not broken, the quality is very good, I like it very much!	Jingdong prefecture level city in Shandong
Happy Fan Shop	King of Power Saving	The saver was well packaged and was as described when plugged in	Jingdong prefecture level city in Shandong
Happy Fan Shop	King of Power Saving	Energy efficient and very satisfied	Jingdong prefecture level city in Shandong
Happy Fan Shop	King of Power Saving	Great quality for the price and fast shipping.	Jingdong prefecture level city in Shandong
Happy Fan Shop	King of Power Saving	It's great, just as described. Perfect.	Jingdong prefecture level city in Shandong
Happy Fan Shop	King of Power Saving	There is no power saving effect, but rather ring up an increase in electricity costs, because this machine is on continuously all day long, uninterruptedly, its own electricity and increased costs, plus the cost of purchasing the machine, so I feel that the use of this machine instead of increasing the	Jingdong prefecture level city in Shandong
Happy Fan Shop	King of Power Saving	Recommended by the owner of the store next door, looked at his home does have to save electricity, decided to get. Indeed quite can, the voltage is much more stable, there is a reduction in electricity	Jingdong prefecture level city in Shandong
Happy Fan Shop	King of Power Saving	Goods have been received value for money, affordable and reasonable price, and the description of the same, power-saving safe and reliable!	Jingdong prefecture level city in Shandong
Happy Fan Shop	King of Power Saving	Listen to friends introduced to buy, tried to save a lot of electricity is good, quite good, worth buying.	Jingdong prefecture level city in Shandong
Happy Fan Shop	King of Power Saving	The product is easy to use, safe and environmentally friendly, only used for a short time, to the next month, after the electricity bill status display to know how the performance of the power saver efficacy.	Jingdong prefecture level city in Shandong
Happy Fan Shop	King of Power Saving	Last month's electricity bill is 178 yuan, with this power saver is 92 yuan, ready to buy two more back home, I was introduced to buy a friend, just started to not believe that I did not expect the effect!	Jingdong prefecture level city in Shandong
Happy Fan Shop	King of Power Saving	It looks nice, but after using it for a few days I can't see how it saves power?	Jingdong prefecture level city in Shandong
Happy Fan Shop	King of Power Saving	Recommended by a friend, it is genuine, as described by the merchant, cost-effective	Jingdong prefecture level city in Shandong
Happy Fan Shop	King of Power Saving	Works well, affordable and saves money.	Jingdong prefecture level city in Shandong
Happy Fan Shop	King of Power Saving	Feels good haven't compared it yet, the air conditioning has been on a lot in the past two months	Jingdong prefecture level city in Shandong
Happy Fan Shop	King of Power Saving	Saving about 5 kWh per day is a good thing	Jingdong prefecture level city in Shandong
Happy Fan Shop	King of Power Saving	Not bad, affordable, best value for money	Jingdong prefecture level city in Shandong
Happy Fan Shop	King of Power Saving	The goods are genuine and the logistics are faster.	Jingdong prefecture level city in Shandong
Happy Fan Shop	King of Power Saving	Goods have been received today, has been plugged into the outlet, looking forward to saving electricity, after a period of time to see how the effect in the follow-up comment!	Jingdong prefecture level city in Shandong
Happy Fan Shop	King of Power Saving	I just got it home and haven't used it for long, so I'll use it for a couple months and see what happens. Seeing that most people have good reviews, it should be fine.	Jingdong prefecture level city in Shandong
Happy Fan Shop	King of Power Saving	I'm looking forward to the results, so I'll give you a good review out of habit! Follow up review after a while	Jingdong prefecture level city in Shandong
Happy Fan Shop	King of Power Saving	Use a dish first to know if it will help save energy	Jingdong prefecture level city in Shandong
Happy Fan Shop	King of Power Saving	Whether it saves electricity or not, we don't know yet.	Jingdong prefecture level city in Shandong
Happy Fan Shop	King of Power Saving	Goods have been received, use a period of time to know how oh. The courier did not deliver to the door ah! Sent to the station ah! The heat is inconvenient ah!	Jingdong prefecture level city in Shandong
Happy Fan Shop	King of Power Saving	A little effect, before an hour a power, now an hour 0.76 degrees, a dollar a power = save 24 cents, although not a lot, first use it!	Jingdong prefecture level city in Shandong
Happy Fan Shop	King of Power Saving	Just received the goods, use a month to evaluate	Jingdong prefecture level city in Shandong
Happy Fan Shop	King of Power Saving	Home every month with a lot of electricity, inadvertently found this good thing online that can save the beginning of a skeptical attitude to consult customer service, customer service replied very professional, said 120 days can be refunded that first try it!	Jingdong prefecture level city in Shandong
Happy Fan Shop	King of Power Saving	After receiving the goods this afternoon, immediately installed in the bathroom Smith energy storage electric water heater on the power outlet (instead of connecting with a plug board), when used, found that the "Power Saving King" LCD display, is the value of the voltage, not the store's video said so	Jingdong prefecture level city in Shandong
Happy Fan Shop	King of Power Saving	Has made many days, did not feel the power saving, the effect is not obvious.	Jingdong prefecture level city in Shandong

Happy Fan Shop	King of Power Saving	Energy savers do not save energy at all, but rather consume a lot of electricity.	Jingdong prefecture level city in Shandong
Happy Fan Shop	King of Power Saving	It's useless. It's a waste of money.	Jingdong prefecture level city in Shandong
Happy Fan Shop	King of Power Saving	Last month saved more than 500, which came back to buy, the store bought three, and then buy 1 home use, my family is dry beauty salon, more instruments, especially electricity, a month almost 1000. or so, with this did not expect to save so much, great, five-star praise!	Jingdong prefecture level city in Shandong
Happy Fan Shop	King of Power Saving	It rained a couple days ago and I just went to get the item back today , not bad, hey I looked at the meter and that red display light takes several seconds to jump, did not make a mistake buying it. After a period of time to evaluate.	Jingdong prefecture level city in Shandong
Happy Fan Shop	King of Power Saving	Without further ado, the electricity bill came down, this month less 102 yuan, has been repurchased, I also recommended to my friends, all said that there are effects, the product is okay, the effect is quite	Jingdong prefecture level city in Shandong
Happy Fan Shop	King of Power Saving	After buying and using two months really see the effect, so only to evaluate! Originally held the mindset of trying, I did not expect really effective, worth having!	Jingdong prefecture level city in Shandong
Happy Fan Shop	King of Power Saving	In the use of this energy saver before the monthly electricity bill is more than 150 yuan or so, now save thirty percent, every day to work, six or seven o'clock in the afternoon to go home, I do not know why the electricity bill is so expensive, energy-saving appliances really have the effect of recommending	Jingdong prefecture level city in Shandong
Happy Fan Shop	King of Power Saving	It saves about 100 bucks a month. A friend recommended it to me, and he's been using it for a while, and the effect is so obvious that I bought one too.	Jingdong prefecture level city in Shandong
Happy Fan Shop	King of Power Saving	My dad asked me to buy one, but after I paid the bill, I asked and he said it was a lot less this month, so he asked me to buy another one.	Jingdong prefecture level city in Shandong
Happy Fan Shop	King of Power Saving	The specific effect will have to be tested for at least two months to compare whether there is a power saving effect or not.	Jingdong prefecture level city in Shandong
Happy Fan Shop	King of Power Saving	'The goods have been received, immediately go to use, please ask the boss you instructions do not allow to withdraw, there are two fuses installed there!	Jingdong prefecture level city in Shandong
Happy Fan Shop	King of Power Saving	First praise is using, after using if good then follow up, now because I have not yet seen the results	Jingdong prefecture level city in Shandong
Happy Fan Shop	King of Power Saving	I've just been using it, and I haven't seen if it has a power-saving effect yet, so I'll follow up after 10 days and half a month.	Jingdong prefecture level city in Shandong
Happy Fan Shop	King of Power Saving	Just received the intelligent power saving meter, immediately plugged in to use, see it is not bad, and order to sell the second time	Jingdong prefecture level city in Shandong
Happy Fan Shop	King of Power Saving	This power saver is a bit effective, used for half a month, the effect is still visible.	Jingdong prefecture level city in Shandong
Happy Fan Shop	King of Power Saving	I received the baby and it looks great, I'm trying it out now and will keep an eye on it afterward.	Jingdong prefecture level city in Shandong
Happy Fan Shop	King of Power Saving	I've just received it, but I'm still trying to see how it works, so I'll come back in a little while.	Jingdong prefecture level city in Shandong
Happy Fan Shop	King of Power Saving	Just used it wait to use it for a while and then comment thanks, all first five stars.	Jingdong prefecture level city in Shandong
Happy Fan Shop	King of Power Saving	Very trustworthy brand, quality is guaranteed, the price is still affordable, quite satisfied!	Jingdong prefecture level city in Shandong
Happy Fan Shop	King of Power Saving	Things received, I hope that the platform of Jingdong do not sell fake goods are available	Jingdong prefecture level city in Shandong
Happy Fan Shop	King of Power Saving	Energy saver received, the use of the effect is good and the business description of the same	Jingdong prefecture level city in Shandong
Happy Fan Shop	King of Power Saving	The surface looks beautiful, the actual effect is not known, after using additional evaluation.	Jingdong prefecture level city in Shandong
Happy Fan Shop	King of Power Saving	Tried it for a few months, it really saves electricity, and I bought it for a friend.	Jingdong prefecture level city in Shandong
Happy Fan Shop	King of Power Saving	It didn't work and wanted to return it, it really didn't work at all.	Jingdong prefecture level city in Shandong
Happy Fan Shop	King of Power Saving	Just used it, don't know how effective it is, looking forward to usefulness	Jingdong prefecture level city in Shandong
Happy Fan Shop	King of Power Saving	After two months before the effect I do not know if it is true!	Jingdong prefecture level city in Shandong
Happy Fan Shop	King of Power Saving	So fast! Ordered yesterday and received today 👍👍👍👍👍👍👍👍👍 🌹🌹🌹🌹🌹🌹🌹🌹🌹	Jingdong prefecture level city in Shandong
Happy Fan Shop	King of Power Saving	I've started using it. We'll see how it works.	Jingdong prefecture level city in Shandong
Happy Fan Shop	King of Power Saving	I just received the goods, I won't know the effect until after two.	Jingdong prefecture level city in Shandong
Happy Fan Shop	King of Power Saving	Just received the goods, the specific effect will be evaluated later	Jingdong prefecture level city in Shandong
Happy Fan Shop	King of Power Saving	I've received the goods, I don't know if it's any good yet	Jingdong prefecture level city in Shandong
Happy Fan Shop	King of Power Saving	Try it for a month and then come back with a concrete evaluation	Jingdong prefecture level city in Shandong
Happy Fan Shop	King of Power Saving	In use, after two months to see the effect of power saving	Jingdong prefecture level city in Shandong
Happy Fan Shop	King of Power Saving	Power Saver. It's authentic. It's black. It works.	Jingdong prefecture level city in Shandong
Happy Fan Shop	King of Power Saving	I've been using it for a week now and it's not working.	Jingdong prefecture level city in Shandong
Happy Fan Shop	King of Power Saving	I'm just using it. I don't know how it's going to work.	Jingdong prefecture level city in Shandong
Happy Fan Shop	King of Power Saving	Products are better looking out for power savings	Jingdong prefecture level city in Shandong
Happy Fan Shop	King of Power Saving	I received it and I've kept it on. I hope it works.	Jingdong prefecture level city in Shandong
Happy Fan Shop	King of Power Saving	It's really good! Worth recommending to buy!	Jingdong prefecture level city in Shandong
Happy Fan Shop	King of Power Saving	Try it for a few days and see if it doesn't work, then return it.	Jingdong prefecture level city in Shandong
Happy Fan Shop	King of Power Saving	It's in use and I hope to be pleasantly surprised.	Jingdong prefecture level city in Shandong
Happy Fan Shop	King of Power Saving	We'll see how it works when we use it	Jingdong prefecture level city in Shandong
Happy Fan Shop	King of Power Saving	Let's see if it works.	Jingdong prefecture level city in Shandong
Happy Fan Shop	King of Power Saving	I'm using it and will follow up with a review	Jingdong prefecture level city in Shandong
Happy Fan Shop	King of Power Saving	It really works. It saves energy.	Jingdong prefecture level city in Shandong
Happy Fan Shop	King of Power Saving	It does have some power savings!	Jingdong prefecture level city in Shandong
Happy Fan Shop	King of Power Saving	Jingdong shopping speed is convenient and timely	Jingdong prefecture level city in Shandong
Happy Fan Shop	King of Power Saving	Second time I've bought it. Everything's fine.	Jingdong prefecture level city in Shandong
Happy Fan Shop	King of Power Saving	I don't know if it works yet.	Jingdong prefecture level city in Shandong
Happy Fan Shop	King of Power Saving	Not used, don't know how well it works.	Jingdong prefecture level city in Shandong

Happy Fan Shop	King of Power Saving	No visible results so far	Jingdong prefecture level city in Shandong
Happy Fan Shop	King of Power Saving	It doesn't feel like it's working.	Jingdong prefecture level city in Shandong
Happy Fan Shop	King of Power Saving	Where's the effect? Where are the power savings?	Jingdong prefecture level city in Shandong
Happy Fan Shop	King of Power Saving	Let's see how it works in practice!	Jingdong prefecture level city in Shandong
Happy Fan Shop	King of Power Saving	I'll wait until I use it to give you a good review	Jingdong prefecture level city in Shandong
Happy Fan Shop	King of Power Saving	I'll follow up after I've used it	Jingdong prefecture level city in Shandong
Happy Fan Shop	King of Power Saving	Well, that's good for saving power.	Jingdong prefecture level city in Shandong
Happy Fan Shop	King of Power Saving	Received. Used in the know.	Jingdong prefecture level city in Shandong
Happy Fan Shop	King of Power Saving	It doesn't do much good.	Jingdong prefecture level city in Shandong
Happy Fan Shop	King of Power Saving	It's not working.	Jingdong prefecture level city in Shandong
Happy Fan Shop	King of Power Saving	I don't know. I just used it.	Jingdong prefecture level city in Shandong
Happy Fan Shop	King of Power Saving	Use it and come back to evaluate.	Jingdong prefecture level city in Shandong
Happy Fan Shop	King of Power Saving	Two words: useless.	Jingdong prefecture level city in Shandong
Happy Fan Shop	King of Power Saving	To be used and then evaluated	Jingdong prefecture level city in Shandong
Happy Fan Shop	King of Power Saving	No shipment.	Jingdong prefecture level city in Shandong
Happy Fan Shop	King of Power Saving	Fake. Speechless.	Jingdong prefecture level city in Shandong
Happy Fan Shop	King of Power Saving	fantastic (idiom); awesome	Jingdong prefecture level city in Shandong
Happy Fan Shop	King of Power Saving	an excellent	Jingdong prefecture level city in Shandong
Happy Fan Shop	King of Power Saving	an excellent	Jingdong prefecture level city in Shandong
Happy Fan Shop	King of Power Saving	Want to buy! Recommended not to buy! No effect	Jingdong prefecture level city in Shandong
Happy Fan Shop	King of Power Saving	That's all there is to it. I can't figure out how to save electricity.	Jingdong prefecture level city in Shandong
Happy Fan Shop	King of Power Saving	It's no use.	Jingdong prefecture level city in Shandong
Happy Fan Shop	King of Power Saving	Things are more perfect than I imagined, very powerful, it is worth getting Packaging is very strong, things are well protected, it is worth the praise things are very satisfied Oh, beautiful and stylish atmosphere, customer service is super patient, service is meticulous and attentive, the big brand is not	Jingdong prefecture level city in Shandong
Happy Fan Shop	King of Power Saving	The effect is too good, the family has now several units, back to buy a number of times, the power saver received, power-saving effect is very good,, fine workmanship, long warranty, but also purify the air, before buying a used for a period of time, with the okay, and then bought a, and only now come to the	Jingdong prefecture level city in Shandong
Happy Fan Shop	King of Power Saving	Quite good, and imagine the same, casual ride are very good, satisfied! Baby received it 👌, really great ah! 🌈 quality is really nothing to say, very satisfied! 🆗 happy happy 😃 genuine, great, the price is very good, in short, quite good, will buy again a very good shopping experience, attentive service, fast logistics, distribution is also thoughtful, the attitude is also very good, value for money, ah,	Jingdong prefecture level city in Shandong
Happy Fan Shop	King of Power Saving	Power-saving effect: strong Cost-effective: quite good Appearance style: beautiful Workmanship material: meticulous Experience: looking forward to power-saving effect	Jingdong prefecture level city in Shandong
Happy Fan Shop	King of Power Saving	Cost-effective is okay, the appearance of the style of meticulous workmanship, looking forward to energy-saving effect.	Jingdong prefecture level city in Shandong
Happy Fan Shop	King of Power Saving	Very Good Very Power Saving This Power Saver is really power saving As soon as the power is turned on, the Power Saver starts the power saving function for you.	Jingdong prefecture level city in Shandong
Happy Fan Shop	King of Power Saving	Logistics is very fast, the price is favorable, friends introduced that it is very good to use, bought 2 at once, looking forward to the effect, used after the follow-up comment.	Jingdong prefecture level city in Shandong
Happy Fan Shop	King of Power Saving	It's all fake. It doesn't save any electricity.	Jingdong prefecture level city in Shandong
Happy Fan Shop	King of Power Saving	This Power Saver is so much better than the one I bought before, not only the effect is good, the quality is very good in all aspects, the customer service attitude is also very good, recommended to buy!	Jingdong prefecture level city in Shandong
Happy Fan Shop	King of Power Saving	Logistics is very fast, buy for parents, good quality, once bought 2, power-saving effect is good to buy 2 more, look forward to using the effect, satisfied will recommend to friends.	Jingdong prefecture level city in Shandong
Happy Fan Shop	King of Power Saving	With the description is the same, very high-grade, good value, to buy friends can order Oh!	Jingdong prefecture level city in Shandong
Happy Fan Shop	King of Power Saving	Not using much electricity in the winter saving over 100 per month! Ready to buy 2 more units in the summer ! ! ! ! ! Good quality, good price, power saving effect is obvious, cost-effective, worth buying, can be recommended!	Jingdong prefecture level city in Shandong
Happy Fan Shop	King of Power Saving	Purchased a power saver received on the put into use, rent a room with many electrical appliances, power consumption is very large, we have a piece of electricity or 1.2 dollars a time, with this power saver is much better, every day can save a lot of money, very convenient and practical.	Jingdong prefecture level city in Shandong
Happy Fan Shop	King of Power Saving	The power saving effect is very good: my supermarket monthly electricity bill is more than 7000 yuan. On May 1st someone recommended buying a couple of power savers, and I bought 5 of them first. The electricity bill was about 2000 less in June. This time, I bought 5 more. The seller said 800 degrees of electricity with one. I feel it's basically enough. A total of 10 units	Jingdong prefecture level city in Shandong
Happy Fan Shop	King of Power Saving	I never thought I could get such a good baby at such an affordable price, it's very good value for money, I love it!	Jingdong prefecture level city in Shandong
Happy Fan Shop	King of Power Saving	Previously bought other brands of power savers, but found no effect at home, this time to see how the products on the Jingdong, inserted in the home socket, the LED display is blue, the night light is very nice to see, look at the instructions that have to be half a year in order to achieve the best results, try it, I believe that the quality of the Jingdong, it should not be wrong.	Jingdong prefecture level city in Shandong
Happy Fan Shop	King of Power Saving	Superb, inexpensive, saves a lot of points, been using it for a long time!	Jingdong prefecture level city in Shandong
Happy Fan Shop	King of Power Saving	There are a lot of appliances in the house, the summer air conditioning is very costly, I bought this in front of the air conditioning effect is very good, I explain that I bought three in one breath, I use more electricity a month down to 200 or so, very satisfied.	Jingdong prefecture level city in Shandong
Happy Fan Shop	King of Power Saving	A month can save more than 200, home monthly electricity bill of more than 600 dollars, inserted 3 or so, an amount of the balance of the average can save more than 200. Product is very good, has been buying this one, has given a lot of relatives recommended, the quality is also very good, before buying a look with this same, who knows to buy home has not been used for more than a week on the bad, the quality of this is really good with more than two years!	Jingdong prefecture level city in Shandong
Happy Fan Shop	King of Power Saving	Whole house power saving king, can really save electricity, friends recommended, high power saving appliances, only replacement, no repair, after-sales worry-free!	Jingdong prefecture level city in Shandong
Happy Fan Shop	King of Power Saving	Great item love it, great customer service, great shopping experience!	Jingdong prefecture level city in Shandong
Happy Fan Shop	King of Power Saving	Power saving, very good use, much cheaper than the physical store, satisfaction	Jingdong prefecture level city in Shandong

Happy Fan Shop	King of Power Saving	In use, very well packaged and fast delivery of credit!	Jingdong prefecture level city in Shandong
Happy Fan Shop	King of Power Saving	Product quality, customer service attitude is particularly good, like, praise!	Jingdong prefecture level city in Shandong
Happy Fan Shop	King of Power Saving	The logistics is very fast, the packaging is not broken, the quality is very good, I like it very much!	Jingdong prefecture level city in Shandong
Happy Fan Shop	King of Power Saving	The saver was well packaged and was as described when plugged in	Jingdong prefecture level city in Shandong
Happy Fan Shop	King of Power Saving	Energy efficient and very satisfied	Jingdong prefecture level city in Shandong
Happy Fan Shop	King of Power Saving	Great quality for the price and fast shipping.	Jingdong prefecture level city in Shandong
Happy Fan Shop	King of Power Saving	It's great, just as described. Perfect.	Jingdong prefecture level city in Shandong
Happy Fan Shop	King of Power Saving	There is no power saving effect, but rather ring up an increase in electricity costs, because this machine is on continuously all day long, uninterruptedly, its own electricity and increased costs, plus the cost of purchasing the machine, so I feel that the use of this machine instead of increasing the	Jingdong prefecture level city in Shandong
Happy Fan Shop	King of Power Saving	Recommended by the owner of the store next door, looked at his home does have to save electricity, decided to get. Indeed quite can, the voltage is much more stable, there is a reduction in electricity	Jingdong prefecture level city in Shandong
Happy Fan Shop	King of Power Saving	Goods have been received value for money, affordable and reasonable price, and the description of the same, power-saving safe and reliable!	Jingdong prefecture level city in Shandong
Happy Fan Shop	King of Power Saving	Listen to friends introduced to buy, tried to save a lot of electricity is good, quite good, worth buying.	Jingdong prefecture level city in Shandong
Happy Fan Shop	King of Power Saving	The product is easy to use, safe and environmentally friendly, only used for a short time, to the next month, after the electricity bill status display to know how the performance of the power saver efficacy.	Jingdong prefecture level city in Shandong
Happy Fan Shop	King of Power Saving	Last month's electricity bill is 178 yuan, with this power saver is 92 yuan, ready to buy two more back home, I was introduced to buy a friend, just started to not believe that I did not expect the effect!	Jingdong prefecture level city in Shandong
Happy Fan Shop	King of Power Saving	It looks nice, but after using it for a few days I can't see how it saves power?	Jingdong prefecture level city in Shandong
Happy Fan Shop	King of Power Saving	Recommended by a friend, it is genuine, as described by the merchant, cost-effective	Jingdong prefecture level city in Shandong
Happy Fan Shop	King of Power Saving	Works well, affordable and saves money.	Jingdong prefecture level city in Shandong
Happy Fan Shop	King of Power Saving	Feels good haven't compared it yet, the air conditioning has been on a lot in the past two months	Jingdong prefecture level city in Shandong
Happy Fan Shop	King of Power Saving	Saving about 5 kWh per day is a good thing	Jingdong prefecture level city in Shandong
Happy Fan Shop	King of Power Saving	Not bad, affordable, best value for money	Jingdong prefecture level city in Shandong
Happy Fan Shop	King of Power Saving	The goods are genuine and the logistics are faster.	Jingdong prefecture level city in Shandong
Happy Fan Shop	King of Power Saving	Goods have been received today, has been plugged into the outlet, looking forward to saving electricity, after a period of time to see how the effect in the follow-up comment!	Jingdong prefecture level city in Shandong
Happy Fan Shop	King of Power Saving	I just got it home and haven't used it for long, so I'll use it for a couple months and see what happens. Seeing that most people have good reviews, it should be fine.	Jingdong prefecture level city in Shandong
Happy Fan Shop	King of Power Saving	I'm looking forward to the results, so I'll give you a good review out of habit! Follow up review after a while	Jingdong prefecture level city in Shandong
Happy Fan Shop	King of Power Saving	Use a dish first to know if it will help save energy	Jingdong prefecture level city in Shandong
Happy Fan Shop	King of Power Saving	Whether it saves electricity or not, we don't know yet.	Jingdong prefecture level city in Shandong
Happy Fan Shop	King of Power Saving	Goods have been received, use a period of time to know how oh. The courier did not deliver to the door ah! Sent to the station ah! The heat is inconvenient ah!	Jingdong prefecture level city in Shandong
Happy Fan Shop	King of Power Saving	A little effect, before an hour a power, now an hour 0.76 degrees, a dollar a power = save 24 cents, although not a lot, first use it!	Jingdong prefecture level city in Shandong
Happy Fan Shop	King of Power Saving	Just received the goods, use a month to evaluate	Jingdong prefecture level city in Shandong
Happy Fan Shop	King of Power Saving	Home every month with a lot of electricity, inadvertently found this good thing online that can save the beginning of a skeptical attitude to consult customer service, customer service replied very professional, said 120 days can be refunded that first try it!	Jingdong prefecture level city in Shandong
Happy Fan Shop	King of Power Saving	After receiving the goods this afternoon, immediately installed in the bathroom Smith energy storage electric water heater on the power outlet (instead of connecting with a plug board), when used, found that the "Power Saving King" LCD display, is the value of the voltage, not the store's video said so	Jingdong prefecture level city in Shandong
Happy Fan Shop	King of Power Saving	Has made many days, did not feel the power saving, the effect is not obvious.	Jingdong prefecture level city in Shandong
Happy Fan Shop	King of Power Saving	Energy savers do not save energy at all, but rather consume a lot of electricity.	Jingdong prefecture level city in Shandong
Happy Fan Shop	King of Power Saving	It's useless. It's a waste of money.	Jingdong prefecture level city in Shandong
Happy Fan Shop	King of Power Saving	Last month saved more than 500, which came back to buy, the store bought three, and then buy 1 home use, my family is dry beauty salon, more instruments, especially electricity, a month almost 1000. or so, with this did not expect to save so much, great, five-star praise!	Jingdong prefecture level city in Shandong
Happy Fan Shop	King of Power Saving	It rained a couple days ago and I just went to get the item back today , not bad, hey I looked at the meter and that red display light takes several seconds to jump, did not make a mistake buying it. After a period of time to evaluate.	Jingdong prefecture level city in Shandong
Happy Fan Shop	King of Power Saving	Without further ado, the electricity bill came down, this month less 102 yuan, has been repurchased, I also recommended to my friends, all said that there are effects, the product is okay, the effect is quite	Jingdong prefecture level city in Shandong
Happy Fan Shop	King of Power Saving	After buying and using two months really see the effect, so only to evaluate! Originally held the mindset of trying, I did not expect really effective, worth having!	Jingdong prefecture level city in Shandong
Happy Fan Shop	King of Power Saving	In the use of this energy saver before the monthly electricity bill is more than 150 yuan or so, now save thirty percent, every day to work, six or seven o'clock in the afternoon to go home, I do not know why the electricity bill is so expensive, energy-saving appliances really have the effect of recommending	Jingdong prefecture level city in Shandong
Happy Fan Shop	King of Power Saving	It saves about 100 bucks a month. A friend recommended it to me, and he's been using it for a while, and the effect is so obvious that I bought one too.	Jingdong prefecture level city in Shandong
Happy Fan Shop	King of Power Saving	My dad asked me to buy one, but after I paid the bill, I asked and he said it was a lot less this month, so he asked me to buy another one.	Jingdong prefecture level city in Shandong
Happy Fan Shop	King of Power Saving	The specific effect will have to be tested for at least two months to compare whether there is a power saving effect or not.	Jingdong prefecture level city in Shandong
Happy Fan Shop	King of Power Saving	'The goods have been received, immediately go to use, please ask the boss you instructions do not allow to withdraw, there are two fuses installed there!	Jingdong prefecture level city in Shandong
Happy Fan Shop	King of Power Saving	First praise is using, after using if good then follow up, now because I have not yet seen the results	Jingdong prefecture level city in Shandong
Happy Fan Shop	King of Power Saving	I've just been using it, and I haven't seen if it has a power-saving effect yet, so I'll follow up after 10 days and half a month.	Jingdong prefecture level city in Shandong
Happy Fan Shop	King of Power Saving	Just received the intelligent power saving meter, immediately plugged in to use, see it is not bad, and order to sell the second time	Jingdong prefecture level city in Shandong
Happy Fan Shop	King of Power Saving	This power saver is a bit effective, used for half a month, the effect is still visible.	Jingdong prefecture level city in Shandong
Happy Fan Shop	King of Power Saving	I received the baby and it looks great, I'm trying it out now and will keep an eye on it afterward.	Jingdong prefecture level city in Shandong
Happy Fan Shop	King of Power Saving	I've just received it, but I'm still trying to see how it works, so I'll come back in a little while.	Jingdong prefecture level city in Shandong

Happy Fan Shop	King of Power Saving	Just used it wait to use it for a while and then comment thanks, all first five stars.	Jingdong prefecture level city in Shandong
Happy Fan Shop	King of Power Saving	Very trustworthy brand, quality is guaranteed, the price is still affordable, quite satisfied!	Jingdong prefecture level city in Shandong
Happy Fan Shop	King of Power Saving	Things received, I hope that the platform of Jingdong do not sell fake goods are available	Jingdong prefecture level city in Shandong
Happy Fan Shop	King of Power Saving	Energy saver received, the use of the effect is good and the business description of the same	Jingdong prefecture level city in Shandong
Happy Fan Shop	King of Power Saving	The surface looks beautiful, the actual effect is not known, after using additional evaluation.	Jingdong prefecture level city in Shandong
Happy Fan Shop	King of Power Saving	Tried it for a few months, it really saves electricity, and I bought it for a friend.	Jingdong prefecture level city in Shandong
Happy Fan Shop	King of Power Saving	It didn't work and wanted to return it, it really didn't work at all.	Jingdong prefecture level city in Shandong
Happy Fan Shop	King of Power Saving	Just used it, don't know how effective it is, looking forward to usefulness	Jingdong prefecture level city in Shandong
Happy Fan Shop	King of Power Saving	After two months before the effect I do not know if it is true!	Jingdong prefecture level city in Shandong
Happy Fan Shop	King of Power Saving	So fast! Ordered yesterday and received today 👍👍👍👍👍👍👍👍👍 🌹🌹🌹🌹🌹🌹🌹🌹🌹	Jingdong prefecture level city in Shandong
Happy Fan Shop	King of Power Saving	I've started using it. We'll see how it works.	Jingdong prefecture level city in Shandong
Happy Fan Shop	King of Power Saving	I just received the goods, I won't know the effect until after two.	Jingdong prefecture level city in Shandong
Happy Fan Shop	King of Power Saving	Just received the goods, the specific effect will be evaluated later	Jingdong prefecture level city in Shandong
Happy Fan Shop	King of Power Saving	I've received the goods, I don't know if it's any good yet	Jingdong prefecture level city in Shandong
Happy Fan Shop	King of Power Saving	Try it for a month and then come back with a concrete evaluation	Jingdong prefecture level city in Shandong
Happy Fan Shop	King of Power Saving	In use, after two months to see the effect of power saving	Jingdong prefecture level city in Shandong
Happy Fan Shop	King of Power Saving	Power Saver. It's authentic. It's black. It works.	Jingdong prefecture level city in Shandong
Happy Fan Shop	King of Power Saving	I've been using it for a week now and it's not working.	Jingdong prefecture level city in Shandong
Happy Fan Shop	King of Power Saving	I'm just using it. I don't know how it's going to work.	Jingdong prefecture level city in Shandong
Happy Fan Shop	King of Power Saving	Products are better looking out for power savings	Jingdong prefecture level city in Shandong
Happy Fan Shop	King of Power Saving	I received it and I've kept it on. I hope it works.	Jingdong prefecture level city in Shandong
Happy Fan Shop	King of Power Saving	It's really good! Worth recommending to buy!	Jingdong prefecture level city in Shandong
Happy Fan Shop	King of Power Saving	Try it for a few days and see if it doesn't work, then return it.	Jingdong prefecture level city in Shandong
Happy Fan Shop	King of Power Saving	It's in use and I hope to be pleasantly surprised.	Jingdong prefecture level city in Shandong
Happy Fan Shop	King of Power Saving	We'll see how it works when we use it	Jingdong prefecture level city in Shandong
Happy Fan Shop	King of Power Saving	Let's see if it works.	Jingdong prefecture level city in Shandong
Happy Fan Shop	King of Power Saving	I'm using it and will follow up with a review	Jingdong prefecture level city in Shandong
Happy Fan Shop	King of Power Saving	It really works. It saves energy.	Jingdong prefecture level city in Shandong
Happy Fan Shop	King of Power Saving	It does have some power savings!	Jingdong prefecture level city in Shandong
Happy Fan Shop	King of Power Saving	Jingdong shopping speed is convenient and timely	Jingdong prefecture level city in Shandong
Happy Fan Shop	King of Power Saving	Second time I've bought it. Everything's fine.	Jingdong prefecture level city in Shandong
Happy Fan Shop	King of Power Saving	I don't know if it works yet.	Jingdong prefecture level city in Shandong
Happy Fan Shop	King of Power Saving	Not used, don't know how well it works.	Jingdong prefecture level city in Shandong
Happy Fan Shop	King of Power Saving	No visible results so far	Jingdong prefecture level city in Shandong
Happy Fan Shop	King of Power Saving	It doesn't feel like it's working.	Jingdong prefecture level city in Shandong
Happy Fan Shop	King of Power Saving	Where's the effect? Where are the power savings?	Jingdong prefecture level city in Shandong
Happy Fan Shop	King of Power Saving	Let's see how it works in practice!	Jingdong prefecture level city in Shandong
Happy Fan Shop	King of Power Saving	I'll wait until I use it to give you a good review	Jingdong prefecture level city in Shandong
Happy Fan Shop	King of Power Saving	I'll follow up after I've used it	Jingdong prefecture level city in Shandong
Happy Fan Shop	King of Power Saving	Well, that's good for saving power.	Jingdong prefecture level city in Shandong
Happy Fan Shop	King of Power Saving	Received. Used in the know.	Jingdong prefecture level city in Shandong
Happy Fan Shop	King of Power Saving	It doesn't do much good.	Jingdong prefecture level city in Shandong
Happy Fan Shop	King of Power Saving	It's not working.	Jingdong prefecture level city in Shandong
Happy Fan Shop	King of Power Saving	I don't know. I just used it.	Jingdong prefecture level city in Shandong
Happy Fan Shop	King of Power Saving	Use it and come back to evaluate.	Jingdong prefecture level city in Shandong
Happy Fan Shop	King of Power Saving	Two words: useless.	Jingdong prefecture level city in Shandong
Happy Fan Shop	King of Power Saving	To be used and then evaluated	Jingdong prefecture level city in Shandong
Happy Fan Shop	King of Power Saving	No shipment.	Jingdong prefecture level city in Shandong
Happy Fan Shop	King of Power Saving	Fake. Speechless.	Jingdong prefecture level city in Shandong
Happy Fan Shop	King of Power Saving	fantastic (idiom); awesome	Jingdong prefecture level city in Shandong
Happy Fan Shop	King of Power Saving	an excellent	Jingdong prefecture level city in Shandong

Happy Fan Shop	King of Power Saving	an excellent	Jingdong prefecture level city in Shandong
Happy Fan Shop	King of Power Saving	Want to buy! Recommended not to buy! No effect	Jingdong prefecture level city in Shandong
Happy Fan Shop	King of Power Saving	That's all there is to it. I can't figure out how to save electricity.	Jingdong prefecture level city in Shandong
Happy Fan Shop	King of Power Saving	It's no use.	Jingdong prefecture level city in Shandong
Happy Fan Shop	King of Power Saving	Things are more perfect than I imagined, very powerful, it is worth getting Packaging is very strong, things are well protected, it is worth the praise things are very satisfied Oh, beautiful and stylish atmosphere, customer service is super patient, service is meticulous and attentive, the big brand is not	Jingdong prefecture level city in Shandong
Happy Fan Shop	King of Power Saving	The effect is too good, the family has now several units, back to buy a number of times, the power saver received, power-saving effect is very good,, fine workmanship, long warranty, but also purify the air, before buying a used for a period of time, with the okay, and then bought a, and only now come to the	Jingdong prefecture level city in Shandong
Happy Fan Shop	King of Power Saving	Quite good, and imagine the same, casual ride are very good, satisfied! Baby received it 👌, really great ah! 🌈 quality is really nothing to say, very satisfied! 🆗 happy happy 😃 genuine, great, the price is very good, in short, quite good, will buy again a very good shopping experience, attentive service, fast logistics, distribution is also thoughtful, the attitude is also very good, value for money, ah,	Jingdong prefecture level city in Shandong
Happy Fan Shop	King of Power Saving	Power-saving effect: strong Cost-effective: quite good Appearance style: beautiful Workmanship material: meticulous Experience: looking forward to power-saving effect	Jingdong prefecture level city in Shandong
Happy Fan Shop	King of Power Saving	Cost-effective is okay, the appearance of the style of meticulous workmanship, looking forward to energy-saving effect.	Jingdong prefecture level city in Shandong
Happy Fan Shop	King of Power Saving	Very Good Very Power Saving This Power Saver is really power saving As soon as the power is turned on, the Power Saver starts the power saving function for you.	Jingdong prefecture level city in Shandong
Happy Fan Shop	King of Power Saving	Logistics is very fast, the price is favorable, friends introduced that it is very good to use, bought 2 at once, looking forward to the effect, used after the follow-up comment.	Jingdong prefecture level city in Shandong
Happy Fan Shop	King of Power Saving	It's all fake. It doesn't save any electricity.	Jingdong prefecture level city in Shandong
Happy Fan Shop	King of Power Saving	This Power Saver is so much better than the one I bought before, not only the effect is good, the quality is very good in all aspects, the customer service attitude is also very good, recommended to buy!	Jingdong prefecture level city in Shandong
Happy Fan Shop	King of Power Saving	Logistics is very fast, buy for parents, good quality, once bought 2, power-saving effect is good to buy 2 more, look forward to using the effect, satisfied will recommend to friends.	Jingdong prefecture level city in Shandong
Happy Fan Shop	King of Power Saving	With the description is the same, very high-grade, good value, to buy friends can order Oh!	Jingdong prefecture level city in Shandong
Happy Fan Shop	King of Power Saving	Not using much electricity in the winter saving over 100 per month! Ready to buy 2 more units in the summer ！！！！！ Good quality, good price, power saving effect is obvious, cost-effective, worth buying, can be recommended!	Jingdong prefecture level city in Shandong
Happy Fan Shop	King of Power Saving	Purchased a power saver received on the put into use, rent a room with many electrical appliances, power consumption is very large, we have a piece of electricity or 1.2 dollars a time, with this power saver is much better, every day can save a lot of money, very convenient and practical.	Jingdong prefecture level city in Shandong
Happy Fan Shop	King of Power Saving	The power saving effect is very good: my supermarket monthly electricity bill is more than 7000 yuan. On May 1st someone recommended buying a couple of power savers, and I bought 5 of them first. The electricity bill was about 2000 less in June. This time, I bought 5 more. The seller said 800 degrees of electricity with one. I feel it's basically enough. A total of 10 units	Jingdong prefecture level city in Shandong
Happy Fan Shop	King of Power Saving	I never thought I could get such a good baby at such an affordable price, it's very good value for money, I love it!	Jingdong prefecture level city in Shandong
Happy Fan Shop	King of Power Saving	Previously bought other brands of power savers, but found no effect at home, this time to see how the products on the Jingdong, inserted in the home socket, the LED display is blue, the night light is very nice to see, look at the instructions that have to be half a year in order to achieve the best results, try it, I believe that the quality of the Jingdong, it should not be wrong.	Jingdong prefecture level city in Shandong
Happy Fan Shop	King of Power Saving	Superb, inexpensive, saves a lot of points, been using it for a long time!	Jingdong prefecture level city in Shandong
Happy Fan Shop	King of Power Saving	There are a lot of appliances in the house, the summer air conditioning is very costly, I bought this in front of the air conditioning effect is very good, I explain that I bought three in one breath, I use more electricity a month down to 200 or so, very satisfied.	Jingdong prefecture level city in Shandong
Happy Fan Shop	King of Power Saving	A month can save more than 200, home monthly electricity bill of more than 600 dollars, inserted 3 or so, an amount of the balance of the average can save more than 200. Product is very good, has been buying this one, has given a lot of relatives recommended, the quality is also very good, before buying a look with this same, who knows to buy home has not been used for more than a week on the bad, the quality of this is really good with more than two years!	Jingdong prefecture level city in Shandong
Happy Fan Shop	King of Power Saving	Whole house power saving king, can really save electricity, friends recommended, high power saving appliances, only replacement, no repair, after-sales worry-free!	Jingdong prefecture level city in Shandong
Happy Fan Shop	King of Power Saving	Great item love it, great customer service, great shopping experience!	Jingdong prefecture level city in Shandong
Happy Fan Shop	King of Power Saving	Power saving, very good use, much cheaper than the physical store, satisfaction	Jingdong prefecture level city in Shandong
Happy Fan Shop	King of Power Saving	In use, very well packaged and fast delivery of credit!	Jingdong prefecture level city in Shandong
Happy Fan Shop	King of Power Saving	Product quality, customer service attitude is particularly good, like, praise!	Jingdong prefecture level city in Shandong
Happy Fan Shop	King of Power Saving	The logistics is very fast, the packaging is not broken, the quality is very good, I like it very much!	Jingdong prefecture level city in Shandong
Happy Fan Shop	King of Power Saving	The saver was well packaged and was as described when plugged in	Jingdong prefecture level city in Shandong
Happy Fan Shop	King of Power Saving	Energy efficient and very satisfied	Jingdong prefecture level city in Shandong
Happy Fan Shop	King of Power Saving	Great quality for the price and fast shipping.	Jingdong prefecture level city in Shandong
Happy Fan Shop	King of Power Saving	It's great, just as described. Perfect.	Jingdong prefecture level city in Shandong
Happy Fan Shop	King of Power Saving	There is no power saving effect, but rather ring up an increase in electricity costs, because this machine is on continuously all day long, uninterruptedly, its own electricity and increased costs, plus the cost of purchasing the machine, so I feel that the use of this machine instead of increasing the	Jingdong prefecture level city in Shandong
Happy Fan Shop	King of Power Saving	Recommended by the owner of the store next door, looked at his home does have to save electricity, decided to get. Indeed quite can, the voltage is much more stable, there is a reduction in electricity	Jingdong prefecture level city in Shandong
Happy Fan Shop	King of Power Saving	Goods have been received value for money, affordable and reasonable price, and the description of the same, power-saving safe and reliable!	Jingdong prefecture level city in Shandong
Happy Fan Shop	King of Power Saving	Listen to friends introduced to buy, tried to save a lot of electricity is good, quite good, worth buying.	Jingdong prefecture level city in Shandong
Happy Fan Shop	King of Power Saving	The product is easy to use, safe and environmentally friendly, only used for a short time, to the next month, after the electricity bill status display to know how the performance of the power saver efficacy.	Jingdong prefecture level city in Shandong
Happy Fan Shop	King of Power Saving	Last month's electricity bill is 178 yuan, with this power saver is 92 yuan, ready to buy two more back home, I was introduced to buy a friend, just started to not believe that I did not expect the effect!	Jingdong prefecture level city in Shandong
Happy Fan Shop	King of Power Saving	It looks nice, but after using it for a few days I can't see how it saves power?	Jingdong prefecture level city in Shandong
Happy Fan Shop	King of Power Saving	Recommended by a friend, it is genuine, as described by the merchant, cost-effective	Jingdong prefecture level city in Shandong
Happy Fan Shop	King of Power Saving	Works well, affordable and saves money.	Jingdong prefecture level city in Shandong
Happy Fan Shop	King of Power Saving	Feels good haven't compared it yet, the air conditioning has been on a lot in the past two months	Jingdong prefecture level city in Shandong
Happy Fan Shop	King of Power Saving	Saving about 5 kWh per day is a good thing	Jingdong prefecture level city in Shandong

Happy Fan Shop	King of Power Saving	Not bad, affordable, best value for money	Jingdong prefecture level city in Shandong
Happy Fan Shop	King of Power Saving	The goods are genuine and the logistics are faster.	Jingdong prefecture level city in Shandong
Happy Fan Shop	King of Power Saving	Goods have been received today, has been plugged into the outlet, looking forward to saving electricity, after a period of time to see how the effect in the follow-up comment!	Jingdong prefecture level city in Shandong
Happy Fan Shop	King of Power Saving	I just got it home and haven't used it for long, so I'll use it for a couple months and see what happens. Seeing that most people have good reviews, it should be fine.	Jingdong prefecture level city in Shandong
Happy Fan Shop	King of Power Saving	I'm looking forward to the results, so I'll give you a good review out of habit! Follow up review after a while	Jingdong prefecture level city in Shandong
Happy Fan Shop	King of Power Saving	Use a dish first to know if it will help save energy	Jingdong prefecture level city in Shandong
Happy Fan Shop	King of Power Saving	Whether it saves electricity or not, we don't know yet.	Jingdong prefecture level city in Shandong
Happy Fan Shop	King of Power Saving	Goods have been received, use a period of time to know how oh. The courier did not deliver to the door ah! Sent to the station ah! The heat is inconvenient ah!	Jingdong prefecture level city in Shandong
Happy Fan Shop	King of Power Saving	A little effect, before an hour a power, now an hour 0.76 degrees, a dollar a power = save 24 cents, although not a lot, first use it!	Jingdong prefecture level city in Shandong
Happy Fan Shop	King of Power Saving	Just received the goods, use a month to evaluate	Jingdong prefecture level city in Shandong
Happy Fan Shop	King of Power Saving	Home every month with a lot of electricity, inadvertently found this good thing online that can save the beginning of a skeptical attitude to consult customer service, customer service replied very professional, said 120 days can be refunded that first try it!	Jingdong prefecture level city in Shandong
Happy Fan Shop	King of Power Saving	After receiving the goods this afternoon, immediately installed in the bathroom Smith energy storage electric water heater on the power outlet (instead of connecting with a plug board), when used, found that the "Power Saving King" LCD display, is the value of the voltage, not the store's video said so	Jingdong prefecture level city in Shandong
Happy Fan Shop	King of Power Saving	Has made many days, did not feel the power saving, the effect is not obvious.	Jingdong prefecture level city in Shandong
Happy Fan Shop	King of Power Saving	Energy savers do not save energy at all, but rather consume a lot of electricity.	Jingdong prefecture level city in Shandong
Happy Fan Shop	King of Power Saving	It's useless. It's a waste of money.	Jingdong prefecture level city in Shandong
Happy Fan Shop	King of Power Saving	Last month saved more than 500, which came back to buy, the store bought three, and then buy 1 home use, my family is dry beauty salon, more instruments, especially electricity, a month almost 1000. or so, with this did not expect to save so much, great, five-star praise!	Jingdong prefecture level city in Shandong
Happy Fan Shop	King of Power Saving	It rained a couple days ago and I just went to get the item back today , not bad, hey I looked at the meter and that red display light takes several seconds to jump, did not make a mistake buying it. After a period of time to evaluate.	Jingdong prefecture level city in Shandong
Happy Fan Shop	King of Power Saving	Without further ado, the electricity bill came down, this month less 102 yuan, has been repurchased, I also recommended to my friends, all said that there are effects, the product is okay, the effect is quite	Jingdong prefecture level city in Shandong
Happy Fan Shop	King of Power Saving	After buying and using two months really see the effect, so only to evaluate! Originally held the mindset of trying, I did not expect really effective, worth having!	Jingdong prefecture level city in Shandong
Happy Fan Shop	King of Power Saving	In the use of this energy saver before the monthly electricity bill is more than 150 yuan or so, now save thirty percent, every day to work, six or seven o'clock in the afternoon to go home, I do not know why the electricity bill is so expensive, energy-saving appliances really have the effect of recommending	Jingdong prefecture level city in Shandong
Happy Fan Shop	King of Power Saving	It saves about 100 bucks a month. A friend recommended it to me, and he's been using it for a while, and the effect is so obvious that I bought one too.	Jingdong prefecture level city in Shandong
Happy Fan Shop	King of Power Saving	My dad asked me to buy one, but after I paid the bill, I asked and he said it was a lot less this month, so he asked me to buy another one.	Jingdong prefecture level city in Shandong
Happy Fan Shop	King of Power Saving	The specific effect will have to be tested for at least two months to compare whether there is a power saving effect or not.	Jingdong prefecture level city in Shandong
Happy Fan Shop	King of Power Saving	'The goods have been received, immediately go to use, please ask the boss you instructions do not allow to withdraw, there are two fuses installed there!	Jingdong prefecture level city in Shandong
Happy Fan Shop	King of Power Saving	First praise is using, after using if good then follow up, now because I have not yet seen the results	Jingdong prefecture level city in Shandong
Happy Fan Shop	King of Power Saving	I've just been using it, and I haven't seen if it has a power-saving effect yet, so I'll follow up after 10 days and half a month.	Jingdong prefecture level city in Shandong
Happy Fan Shop	King of Power Saving	Just received the intelligent power saving meter, immediately plugged in to use, see it is not bad, and order to sell the second time	Jingdong prefecture level city in Shandong
Happy Fan Shop	King of Power Saving	This power saver is a bit effective, used for half a month, the effect is still visible.	Jingdong prefecture level city in Shandong
Happy Fan Shop	King of Power Saving	I received the baby and it looks great, I'm trying it out now and will keep an eye on it afterward.	Jingdong prefecture level city in Shandong
Happy Fan Shop	King of Power Saving	I've just received it, but I'm still trying to see how it works, so I'll come back in a little while.	Jingdong prefecture level city in Shandong
Happy Fan Shop	King of Power Saving	Just used it wait to use it for a while and then comment thanks, all first five stars.	Jingdong prefecture level city in Shandong
Happy Fan Shop	King of Power Saving	Very trustworthy brand, quality is guaranteed, the price is still affordable, quite satisfied!	Jingdong prefecture level city in Shandong
Happy Fan Shop	King of Power Saving	Things received, I hope that the platform of Jingdong do not sell fake goods are available	Jingdong prefecture level city in Shandong
Happy Fan Shop	King of Power Saving	Energy saver received, the use of the effect is good and the business description of the same	Jingdong prefecture level city in Shandong
Happy Fan Shop	King of Power Saving	The surface looks beautiful, the actual effect is not known, after using additional evaluation.	Jingdong prefecture level city in Shandong
Happy Fan Shop	King of Power Saving	Tried it for a few months, it really saves electricity, and I bought it for a friend.	Jingdong prefecture level city in Shandong
Happy Fan Shop	King of Power Saving	It didn't work and wanted to return it, it really didn't work at all.	Jingdong prefecture level city in Shandong
Happy Fan Shop	King of Power Saving	Just used it, don't know how effective it is, looking forward to usefulness	Jingdong prefecture level city in Shandong
Happy Fan Shop	King of Power Saving	After two months before the effect I do not know if it is true!	Jingdong prefecture level city in Shandong
Happy Fan Shop	King of Power Saving	So fast! Ordered yesterday and received today 👍👍👍👍👍👍👍👍👍 🌹🌹🌹🌹🌹🌹🌹🌹🌹	Jingdong prefecture level city in Shandong
Happy Fan Shop	King of Power Saving	I've started using it. We'll see how it works.	Jingdong prefecture level city in Shandong
Happy Fan Shop	King of Power Saving	I just received the goods, I won't know the effect until after two.	Jingdong prefecture level city in Shandong
Happy Fan Shop	King of Power Saving	Just received the goods, the specific effect will be evaluated later	Jingdong prefecture level city in Shandong
Happy Fan Shop	King of Power Saving	I've received the goods, I don't know if it's any good yet	Jingdong prefecture level city in Shandong
Happy Fan Shop	King of Power Saving	Try it for a month and then come back with a concrete evaluation	Jingdong prefecture level city in Shandong
Happy Fan Shop	King of Power Saving	In use, after two months to see the effect of power saving	Jingdong prefecture level city in Shandong
Happy Fan Shop	King of Power Saving	Power Saver. It's authentic. It's black. It works.	Jingdong prefecture level city in Shandong
Happy Fan Shop	King of Power Saving	I've been using it for a week now and it's not working.	Jingdong prefecture level city in Shandong
Happy Fan Shop	King of Power Saving	I'm just using it. I don't know how it's going to work.	Jingdong prefecture level city in Shandong

Happy Fan Shop	King of Power Saving	Products are better looking out for power savings	Jingdong prefecture level city in Shandong
Happy Fan Shop	King of Power Saving	I received it and I've kept it on. I hope it works.	Jingdong prefecture level city in Shandong
Happy Fan Shop	King of Power Saving	It's really good! Worth recommending to buy!	Jingdong prefecture level city in Shandong
Happy Fan Shop	King of Power Saving	Try it for a few days and see if it doesn't work, then return it.	Jingdong prefecture level city in Shandong
Happy Fan Shop	King of Power Saving	It's in use and I hope to be pleasantly surprised.	Jingdong prefecture level city in Shandong
Happy Fan Shop	King of Power Saving	We'll see how it works when we use it	Jingdong prefecture level city in Shandong
Happy Fan Shop	King of Power Saving	Let's see if it works.	Jingdong prefecture level city in Shandong
Happy Fan Shop	King of Power Saving	I'm using it and will follow up with a review	Jingdong prefecture level city in Shandong
Happy Fan Shop	King of Power Saving	It really works. It saves energy.	Jingdong prefecture level city in Shandong
Happy Fan Shop	King of Power Saving	It does have some power savings!	Jingdong prefecture level city in Shandong
Happy Fan Shop	King of Power Saving	Jingdong shopping speed is convenient and timely	Jingdong prefecture level city in Shandong
Happy Fan Shop	King of Power Saving	Second time I've bought it. Everything's fine.	Jingdong prefecture level city in Shandong
Happy Fan Shop	King of Power Saving	I don't know if it works yet.	Jingdong prefecture level city in Shandong
Happy Fan Shop	King of Power Saving	Not used, don't know how well it works.	Jingdong prefecture level city in Shandong
Happy Fan Shop	King of Power Saving	No visible results so far	Jingdong prefecture level city in Shandong
Happy Fan Shop	King of Power Saving	It doesn't feel like it's working.	Jingdong prefecture level city in Shandong
Happy Fan Shop	King of Power Saving	Where's the effect? Where are the power savings?	Jingdong prefecture level city in Shandong
Happy Fan Shop	King of Power Saving	Let's see how it works in practice!	Jingdong prefecture level city in Shandong
Happy Fan Shop	King of Power Saving	I'll wait until I use it to give you a good review	Jingdong prefecture level city in Shandong
Happy Fan Shop	King of Power Saving	I'll follow up after I've used it	Jingdong prefecture level city in Shandong
Happy Fan Shop	King of Power Saving	Well, that's good for saving power.	Jingdong prefecture level city in Shandong
Happy Fan Shop	King of Power Saving	Received. Used in the know.	Jingdong prefecture level city in Shandong
Happy Fan Shop	King of Power Saving	It doesn't do much good.	Jingdong prefecture level city in Shandong
Happy Fan Shop	King of Power Saving	It's not working.	Jingdong prefecture level city in Shandong
Happy Fan Shop	King of Power Saving	I don't know. I just used it.	Jingdong prefecture level city in Shandong
Happy Fan Shop	King of Power Saving	Use it and come back to evaluate.	Jingdong prefecture level city in Shandong
Happy Fan Shop	King of Power Saving	Two words: useless.	Jingdong prefecture level city in Shandong
Happy Fan Shop	King of Power Saving	To be used and then evaluated	Jingdong prefecture level city in Shandong
Happy Fan Shop	King of Power Saving	No shipment.	Jingdong prefecture level city in Shandong
Happy Fan Shop	King of Power Saving	Fake. Speechless.	Jingdong prefecture level city in Shandong
Happy Fan Shop	King of Power Saving	fantastic (idiom); awesome	Jingdong prefecture level city in Shandong
Happy Fan Shop	King of Power Saving	an excellent	Jingdong prefecture level city in Shandong
Happy Fan Shop	King of Power Saving	an excellent	Jingdong prefecture level city in Shandong
Happy Fan Shop	King of Power Saving	Want to buy! Recommended not to buy! No effect	Jingdong prefecture level city in Shandong
Happy Fan Shop	King of Power Saving	That's all there is to it. I can't figure out how to save electricity.	Jingdong prefecture level city in Shandong
Happy Fan Shop	King of Power Saving	It's no use.	Jingdong prefecture level city in Shandong
Happy Fan Shop	King of Power Saving	Things are more perfect than I imagined, very powerful, it is worth getting Packaging is very strong, things are well protected, it is worth the praise things are very satisfied Oh, beautiful and stylish atmosphere, customer service is super patient, service is meticulous and attentive, the big brand is not	Jingdong prefecture level city in Shandong
Happy Fan Shop	King of Power Saving	The effect is too good, the family has now several units, back to buy a number of times, the power saver received, power-saving effect is very good,, fine workmanship, long warranty, but also purify the air, before buying a used for a period of time, with the okay, and then bought a, and only now come to the	Jingdong prefecture level city in Shandong
Happy Fan Shop	King of Power Saving	Quite good, and imagine the same, casual ride are very good, satisfied! Baby received it 👌, really great ah! 🌈 quality is really nothing to say, very satisfied! 🆗 happy happy 😃 genuine, great, the price is very good, in short, quite good, will buy again a very good shopping experience, attentive service, fast logistics, distribution is also thoughtful, the attitude is also very good, value for money, ah,	Jingdong prefecture level city in Shandong
Happy Fan Shop	King of Power Saving	Power-saving effect: strong Cost-effective: quite good Appearance style: beautiful Workmanship material: meticulous Experience: looking forward to power-saving effect	Jingdong prefecture level city in Shandong
Happy Fan Shop	King of Power Saving	Cost-effective is okay, the appearance of the style of meticulous workmanship, looking forward to energy-saving effect.	Jingdong prefecture level city in Shandong
Happy Fan Shop	King of Power Saving	Very Good Very Power Saving This Power Saver is really power saving As soon as the power is turned on, the Power Saver starts the power saving function for you.	Jingdong prefecture level city in Shandong
Happy Fan Shop	King of Power Saving	Logistics is very fast, the price is favorable, friends introduced that it is very good to use, bought 2 at once, looking forward to the effect, used after the follow-up comment.	Jingdong prefecture level city in Shandong
Happy Fan Shop	King of Power Saving	It's all fake. It doesn't save any electricity.	Jingdong prefecture level city in Shandong
Happy Fan Shop	King of Power Saving	This Power Saver is so much better than the one I bought before, not only the effect is good, the quality is very good in all aspects, the customer service attitude is also very good, recommended to buy!	Jingdong prefecture level city in Shandong
Happy Fan Shop	King of Power Saving	Logistics is very fast, buy for parents, good quality, once bought 2, power-saving effect is good to buy 2 more, look forward to using the effect, satisfied will recommend to friends.	Jingdong prefecture level city in Shandong
Happy Fan Shop	King of Power Saving	With the description is the same, very high-grade, good value, to buy friends can order Oh!	Jingdong prefecture level city in Shandong
Happy Fan Shop	King of Power Saving	Not using much electricity in the winter saving over 100 per month! Ready to buy 2 more units in the summer !!!!! Good quality, good price, power saving effect is obvious, cost-effective, worth buying, can be recommended!	Jingdong prefecture level city in Shandong

Happy Fan Shop	King of Power Saving	Purchased a power saver received on the put into use, rent a room with many electrical appliances, power consumption is very large, we have a piece of electricity or 1.2 dollars a time, with this power saver is much better, every day can save a lot of money, very convenient and practical.	Jingdong prefecture level city in Shandong
Happy Fan Shop	King of Power Saving	The power saving effect is very good: my supermarket monthly electricity bill is more than 7000 yuan. On May 1st someone recommended buying a couple of power savers, and I bought 5 of them first. The electricity bill was about 2000 less in June. This time, I bought 5 more. The seller said 800 degrees of electricity with one. I feel it's basically enough. A total of 10 units	Jingdong prefecture level city in Shandong
Happy Fan Shop	King of Power Saving	I never thought I could get such a good baby at such an affordable price, it's very good value for money, I love it!	Jingdong prefecture level city in Shandong
Happy Fan Shop	King of Power Saving	Previously bought other brands of power savers, but found no effect at home, this time to see how the products on the Jingdong, inserted in the home socket, the LED display is blue, the night light is very nice to see, look at the instructions that have to be half a year in order to achieve the best results, try it, I believe that the quality of the Jingdong, it should not be wrong.	Jingdong prefecture level city in Shandong
Happy Fan Shop	King of Power Saving	Superb, inexpensive, saves a lot of points, been using it for a long time!	Jingdong prefecture level city in Shandong
Happy Fan Shop	King of Power Saving	There are a lot of appliances in the house, the summer air conditioning is very costly, I bought this in front of the air conditioning effect is very good, I explain that I bought three in one breath, I use more electricity a month down to 200 or so, very satisfied.	Jingdong prefecture level city in Shandong
Happy Fan Shop	King of Power Saving	A month can save more than 200, home monthly electricity bill of more than 600 dollars, inserted 3 or so, an amount of the balance of the average can save more than 200. Product is very good, has been buying this one, has given a lot of relatives recommended, the quality is also very good, before buying a look with this same, who knows to buy home has not been used for more than a week on the bad, the quality of this is really good with more than two years!	Jingdong prefecture level city in Shandong
Happy Fan Shop	King of Power Saving	Whole house power saving king, can really save electricity, friends recommended, high power saving appliances, only replacement, no repair, after-sales worry-free!	Jingdong prefecture level city in Shandong
Happy Fan Shop	King of Power Saving	Great item love it, great customer service, great shopping experience!	Jingdong prefecture level city in Shandong
Happy Fan Shop	King of Power Saving	Power saving, very good use, much cheaper than the physical store, satisfaction	Jingdong prefecture level city in Shandong
Happy Fan Shop	King of Power Saving	In use, very well packaged and fast delivery of credit!	Jingdong prefecture level city in Shandong
Happy Fan Shop	King of Power Saving	Product quality, customer service attitude is particularly good, like, praise!	Jingdong prefecture level city in Shandong
Happy Fan Shop	King of Power Saving	The logistics is very fast, the packaging is not broken, the quality is very good, I like it very much!	Jingdong prefecture level city in Shandong
Happy Fan Shop	King of Power Saving	The saver was well packaged and was as described when plugged in	Jingdong prefecture level city in Shandong
Happy Fan Shop	King of Power Saving	Energy efficient and very satisfied	Jingdong prefecture level city in Shandong
Happy Fan Shop	King of Power Saving	Great quality for the price and fast shipping.	Jingdong prefecture level city in Shandong
Happy Fan Shop	King of Power Saving	It's great, just as described. Perfect.	Jingdong prefecture level city in Shandong
Happy Fan Shop	King of Power Saving	There is no power saving effect, but rather ring up an increase in electricity costs, because this machine is on continuously all day long, uninterruptedly, its own electricity and increased costs, plus the cost of purchasing the machine, so I feel that the use of this machine instead of increasing the	Jingdong prefecture level city in Shandong
Happy Fan Shop	King of Power Saving	Recommended by the owner of the store next door, looked at his home does have to save electricity, decided to get. Indeed quite can, the voltage is much more stable, there is a reduction in electricity	Jingdong prefecture level city in Shandong
Happy Fan Shop	King of Power Saving	Goods have been received value for money, affordable and reasonable price, and the description of the same, power-saving safe and reliable!	Jingdong prefecture level city in Shandong
Happy Fan Shop	King of Power Saving	Listen to friends introduced to buy, tried to save a lot of electricity is good, quite good, worth buying.	Jingdong prefecture level city in Shandong
Happy Fan Shop	King of Power Saving	The product is easy to use, safe and environmentally friendly, only used for a short time, to the next month, after the electricity bill status display to know how the performance of the power saver efficacy.	Jingdong prefecture level city in Shandong
Happy Fan Shop	King of Power Saving	Last month's electricity bill is 178 yuan, with this power saver is 92 yuan, ready to buy two more back home, I was introduced to buy a friend, just started to not believe that I did not expect the effect!	Jingdong prefecture level city in Shandong
Happy Fan Shop	King of Power Saving	It looks nice, but after using it for a few days I can't see how it saves power?	Jingdong prefecture level city in Shandong
Happy Fan Shop	King of Power Saving	Recommended by a friend, it is genuine, as described by the merchant, cost-effective	Jingdong prefecture level city in Shandong
Happy Fan Shop	King of Power Saving	Works well, affordable and saves money.	Jingdong prefecture level city in Shandong
Happy Fan Shop	King of Power Saving	Feels good haven't compared it yet, the air conditioning has been on a lot in the past two months	Jingdong prefecture level city in Shandong
Happy Fan Shop	King of Power Saving	Saving about 5 kWh per day is a good thing	Jingdong prefecture level city in Shandong
Happy Fan Shop	King of Power Saving	Not bad, affordable, best value for money	Jingdong prefecture level city in Shandong
Happy Fan Shop	King of Power Saving	The goods are genuine and the logistics are faster.	Jingdong prefecture level city in Shandong
Happy Fan Shop	King of Power Saving	Goods have been received today, has been plugged into the outlet, looking forward to saving electricity, after a period of time to see how the effect in the follow-up comment!	Jingdong prefecture level city in Shandong
Happy Fan Shop	King of Power Saving	I just got it home and haven't used it for long, so I'll use it for a couple months and see what happens. Seeing that most people have good reviews, it should be fine.	Jingdong prefecture level city in Shandong
Happy Fan Shop	King of Power Saving	I'm looking forward to the results, so I'll give you a good review out of habit! Follow up review after a while	Jingdong prefecture level city in Shandong
Happy Fan Shop	King of Power Saving	Use a dish first to know if it will help save energy	Jingdong prefecture level city in Shandong
Happy Fan Shop	King of Power Saving	Whether it saves electricity or not, we don't know yet.	Jingdong prefecture level city in Shandong
Happy Fan Shop	King of Power Saving	Goods have been received, use a period of time to know how oh. The courier did not deliver to the door ah! Sent to the station ah! The heat is inconvenient ah!	Jingdong prefecture level city in Shandong
Happy Fan Shop	King of Power Saving	A little effect, before an hour a power, now an hour 0.76 degrees, a dollar a power = save 24 cents, although not a lot, first use it!	Jingdong prefecture level city in Shandong
Happy Fan Shop	King of Power Saving	Just received the goods, use a month to evaluate	Jingdong prefecture level city in Shandong
Happy Fan Shop	King of Power Saving	Home every month with a lot of electricity, inadvertently found this good thing online that can save the beginning of a skeptical attitude to consult customer service, customer service replied very professional, said 120 days can be refunded that first try it!	Jingdong prefecture level city in Shandong
Happy Fan Shop	King of Power Saving	After receiving the goods this afternoon, immediately installed in the bathroom Smith energy storage electric water heater on the power outlet (instead of connecting with a plug board), when used, found that the "Power Saving King" LCD display, is the value of the voltage, not the store's video said so	Jingdong prefecture level city in Shandong
Happy Fan Shop	King of Power Saving	Has made many days, did not feel the power saving, the effect is not obvious.	Jingdong prefecture level city in Shandong
Happy Fan Shop	King of Power Saving	Energy savers do not save energy at all, but rather consume a lot of electricity.	Jingdong prefecture level city in Shandong
Happy Fan Shop	King of Power Saving	It's useless. It's a waste of money.	Jingdong prefecture level city in Shandong
Happy Fan Shop	King of Power Saving	Last month saved more than 500, which came back to buy, the store bought three, and then buy 1 home use, my family is dry beauty salon, more instruments, especially electricity, a month almost 1000. or so, with this did not expect to save so much, great, five-star praise!	Jingdong prefecture level city in Shandong

Happy Fan Shop	King of Power Saving	It rained a couple days ago and I just went to get the item back today , not bad, hey I looked at the meter and that red display light takes several seconds to jump, did not make a mistake buying it. After a period of time to evaluate.	Jingdong prefecture level city in Shandong
Happy Fan Shop	King of Power Saving	Without further ado, the electricity bill came down, this month less 102 yuan, has been repurchased, I also recommended to my friends, all said that there are effects, the product is okay, the effect is quite	Jingdong prefecture level city in Shandong
Happy Fan Shop	King of Power Saving	After buying and using two months really see the effect, so only to evaluate! Originally held the mindset of trying, I did not expect really effective, worth having!	Jingdong prefecture level city in Shandong
Happy Fan Shop	King of Power Saving	In the use of this energy saver before the monthly electricity bill is more than 150 yuan or so, now save thirty percent, every day to work, six or seven o'clock in the afternoon to go home, I do not know why the electricity bill is so expensive, energy-saving appliances really have the effect of recommending	Jingdong prefecture level city in Shandong
Happy Fan Shop	King of Power Saving	It saves about 100 bucks a month. A friend recommended it to me, and he's been using it for a while, and the effect is so obvious that I bought one too.	Jingdong prefecture level city in Shandong
Happy Fan Shop	King of Power Saving	My dad asked me to buy one, but after I paid the bill, I asked and he said it was a lot less this month, so he asked me to buy another one.	Jingdong prefecture level city in Shandong
Happy Fan Shop	King of Power Saving	The specific effect will have to be tested for at least two months to compare whether there is a power saving effect or not.	Jingdong prefecture level city in Shandong
Happy Fan Shop	King of Power Saving	'The goods have been received, immediately go to use, please ask the boss you instructions do not allow to withdraw, there are two fuses installed there!	Jingdong prefecture level city in Shandong
Happy Fan Shop	King of Power Saving	First praise is using, after using if good then follow up, now because I have not yet seen the results	Jingdong prefecture level city in Shandong
Happy Fan Shop	King of Power Saving	I've just been using it, and I haven't seen if it has a power-saving effect yet, so I'll follow up after 10 days and half a month.	Jingdong prefecture level city in Shandong
Happy Fan Shop	King of Power Saving	Just received the intelligent power saving meter, immediately plugged in to use, see it is not bad, and order to sell the second time	Jingdong prefecture level city in Shandong
Happy Fan Shop	King of Power Saving	This power saver is a bit effective, used for half a month, the effect is still visible.	Jingdong prefecture level city in Shandong
Happy Fan Shop	King of Power Saving	I received the baby and it looks great, I'm trying it out now and will keep an eye on it afterward.	Jingdong prefecture level city in Shandong
Happy Fan Shop	King of Power Saving	I've just received it, but I'm still trying to see how it works, so I'll come back in a little while.	Jingdong prefecture level city in Shandong
Happy Fan Shop	King of Power Saving	Just used it wait to use it for a while and then comment thanks, all first five stars.	Jingdong prefecture level city in Shandong
Happy Fan Shop	King of Power Saving	Very trustworthy brand, quality is guaranteed, the price is still affordable, quite satisfied!	Jingdong prefecture level city in Shandong
Happy Fan Shop	King of Power Saving	Things received, I hope that the platform of Jingdong do not sell fake goods are available	Jingdong prefecture level city in Shandong
Happy Fan Shop	King of Power Saving	Energy saver received, the use of the effect is good and the business description of the same	Jingdong prefecture level city in Shandong
Happy Fan Shop	King of Power Saving	The surface looks beautiful, the actual effect is not known, after using additional evaluation.	Jingdong prefecture level city in Shandong
Happy Fan Shop	King of Power Saving	Tried it for a few months, it really saves electricity, and I bought it for a friend.	Jingdong prefecture level city in Shandong
Happy Fan Shop	King of Power Saving	It didn't work and wanted to return it, it really didn't work at all.	Jingdong prefecture level city in Shandong
Happy Fan Shop	King of Power Saving	Just used it, don't know how effective it is, looking forward to usefulness	Jingdong prefecture level city in Shandong
Happy Fan Shop	King of Power Saving	After two months before the effect I do not know if it is true!	Jingdong prefecture level city in Shandong
Happy Fan Shop	King of Power Saving	So fast! Ordered yesterday and received today 👍👍👍👍👍👍👍👍👍 🌹🌹🌹🌹🌹🌹🌹🌹🌹	Jingdong prefecture level city in Shandong
Happy Fan Shop	King of Power Saving	I've started using it. We'll see how it works.	Jingdong prefecture level city in Shandong
Happy Fan Shop	King of Power Saving	I just received the goods, I won't know the effect until after two.	Jingdong prefecture level city in Shandong
Happy Fan Shop	King of Power Saving	Just received the goods, the specific effect will be evaluated later	Jingdong prefecture level city in Shandong
Happy Fan Shop	King of Power Saving	I've received the goods, I don't know if it's any good yet	Jingdong prefecture level city in Shandong
Happy Fan Shop	King of Power Saving	Try it for a month and then come back with a concrete evaluation	Jingdong prefecture level city in Shandong
Happy Fan Shop	King of Power Saving	In use, after two months to see the effect of power saving	Jingdong prefecture level city in Shandong
Happy Fan Shop	King of Power Saving	Power Saver. It's authentic. It's black. It works.	Jingdong prefecture level city in Shandong
Happy Fan Shop	King of Power Saving	I've been using it for a week now and it's not working.	Jingdong prefecture level city in Shandong
Happy Fan Shop	King of Power Saving	I'm just using it. I don't know how it's going to work.	Jingdong prefecture level city in Shandong
Happy Fan Shop	King of Power Saving	Products are better looking out for power savings	Jingdong prefecture level city in Shandong
Happy Fan Shop	King of Power Saving	I received it and I've kept it on. I hope it works.	Jingdong prefecture level city in Shandong
Happy Fan Shop	King of Power Saving	It's really good! Worth recommending to buy!	Jingdong prefecture level city in Shandong
Happy Fan Shop	King of Power Saving	Try it for a few days and see if it doesn't work, then return it.	Jingdong prefecture level city in Shandong
Happy Fan Shop	King of Power Saving	It's in use and I hope to be pleasantly surprised.	Jingdong prefecture level city in Shandong
Happy Fan Shop	King of Power Saving	We'll see how it works when we use it	Jingdong prefecture level city in Shandong
Happy Fan Shop	King of Power Saving	Let's see if it works.	Jingdong prefecture level city in Shandong
Happy Fan Shop	King of Power Saving	I'm using it and will follow up with a review	Jingdong prefecture level city in Shandong
Happy Fan Shop	King of Power Saving	It really works. It saves energy.	Jingdong prefecture level city in Shandong
Happy Fan Shop	King of Power Saving	It does have some power savings!	Jingdong prefecture level city in Shandong
Happy Fan Shop	King of Power Saving	Jingdong shopping speed is convenient and timely	Jingdong prefecture level city in Shandong
Happy Fan Shop	King of Power Saving	Things are more perfect than I imagined, very powerful, it is worth getting Packaging is very strong, things are well protected, it is worth the praise things are very satisfied Oh, beautiful and stylish atmosphere, customer service is super patient, service is meticulous and attentive, the big brand is not	Jingdong prefecture level city in Shandong
Happy Fan Shop	King of Power Saving	The effect is too good, the family has now several units, back to buy a number of times, the power saver received, power-saving effect is very good,, fine workmanship, long warranty, but also purify the air, before buying a used for a period of time, with the okay, and then bought a, and only now come to the	Jingdong prefecture level city in Shandong
Happy Fan Shop	King of Power Saving	Quite good, and imagine the same, casual ride are very good, satisfied! Baby received it 👌, really great ah! 🌈 quality is really nothing to say, very satisfied! 🆗 happy happy 😃 genuine, great, the price is very good, in short, quite good, will buy again a very good shopping experience, attentive service, fast logistics, distribution is also thoughtful, the attitude is also very good, value for money, ah,	Jingdong prefecture level city in Shandong
Happy Fan Shop	King of Power Saving	Power-saving effect: strong Cost-effective: quite good Appearance style: beautiful Workmanship material: meticulous Experience: looking forward to power-saving effect	Jingdong prefecture level city in Shandong

Happy Fan Shop	King of Power Saving	Cost-effective is okay, the appearance of the style of meticulous workmanship, looking forward to energy-saving effect.	Jingdong prefecture level city in Shandong
Happy Fan Shop	King of Power Saving	Very Good Very Power Saving This Power Saver is really power saving As soon as the power is turned on, the Power Saver starts the power saving function for you.	Jingdong prefecture level city in Shandong
Happy Fan Shop	King of Power Saving	Logistics is very fast, the price is favorable, friends introduced that it is very good to use, bought 2 at once, looking forward to the effect, used after the follow-up comment.	Jingdong prefecture level city in Shandong
Happy Fan Shop	King of Power Saving	It's all fake. It doesn't save any electricity.	Jingdong prefecture level city in Shandong
Happy Fan Shop	King of Power Saving	This Power Saver is so much better than the one I bought before, not only the effect is good, the quality is very good in all aspects, the customer service attitude is also very good, recommended to buy!	Jingdong prefecture level city in Shandong
Happy Fan Shop	King of Power Saving	Logistics is very fast, buy for parents, good quality, once bought 2, power-saving effect is good to buy 2 more, look forward to using the effect, satisfied will recommend to friends.	Jingdong prefecture level city in Shandong
Happy Fan Shop	King of Power Saving	With the description is the same, very high-grade, good value, to buy friends can order Oh!	Jingdong prefecture level city in Shandong
Happy Fan Shop	King of Power Saving	Not using much electricity in the winter saving over 100 per month! Ready to buy 2 more units in the summer ! ! ! ! ! Good quality, good price, power saving effect is obvious, cost-effective, worth buying, can be recommended!	Jingdong prefecture level city in Shandong
Happy Fan Shop	King of Power Saving	Purchased a power saver received on the put into use, rent a room with many electrical appliances, power consumption is very large, we have a piece of electricity or 1.2 dollars a time, with this power saver is much better, every day can save a lot of money, very convenient and practical.	Jingdong prefecture level city in Shandong
Happy Fan Shop	King of Power Saving	The power saving effect is very good: my supermarket monthly electricity bill is more than 7000 yuan. On May 1st someone recommended buying a couple of power savers, and I bought 5 of them first. The electricity bill was about 2000 less in June. This time, I bought 5 more. The seller said 800 degrees of electricity with one. I feel it's basically enough. A total of 10 units	Jingdong prefecture level city in Shandong
Happy Fan Shop	King of Power Saving	I never thought I could get such a good baby at such an affordable price, it's very good value for money, I love it!	Jingdong prefecture level city in Shandong
Happy Fan Shop	King of Power Saving	Previously bought other brands of power savers, but found no effect at home, this time to see how the products on the Jingdong, inserted in the home socket, the LED display is blue, the night light is very nice to see, look at the instructions that have to be half a year in order to achieve the best results, try it, I believe that the quality of the Jingdong, it should not be wrong.	Jingdong prefecture level city in Shandong
Happy Fan Shop	King of Power Saving	Superb, inexpensive, saves a lot of points, been using it for a long time!	Jingdong prefecture level city in Shandong
Happy Fan Shop	King of Power Saving	There are a lot of appliances in the house, the summer air conditioning is very costly, I bought this in front of the air conditioning effect is very good, I explain that I bought three in one breath, I use more electricity a month down to 200 or so, very satisfied.	Jingdong prefecture level city in Shandong
Happy Fan Shop	King of Power Saving	A month can save more than 200, home monthly electricity bill of more than 600 dollars, inserted 3 or so, an amount of the balance of the average can save more than 200. Product is very good, has been buying this one, has given a lot of relatives recommended, the quality is also very good, before buying a look with this same, who knows to buy home has not been used for more than a week on the bad, the quality of this is really good with more than two years!	Jingdong prefecture level city in Shandong
Happy Fan Shop	King of Power Saving	Whole house power saving king, can really save electricity, friends recommended, high power saving appliances, only replacement, no repair, after-sales worry-free!	Jingdong prefecture level city in Shandong
Happy Fan Shop	King of Power Saving	Great item love it, great customer service, great shopping experience!	Jingdong prefecture level city in Shandong
Happy Fan Shop	King of Power Saving	Power saving, very good use, much cheaper than the physical store, satisfaction	Jingdong prefecture level city in Shandong
Happy Fan Shop	King of Power Saving	In use, very well packaged and fast delivery of credit!	Jingdong prefecture level city in Shandong
Happy Fan Shop	King of Power Saving	Product quality, customer service attitude is particularly good, like, praise!	Jingdong prefecture level city in Shandong
Happy Fan Shop	King of Power Saving	The logistics is very fast, the packaging is not broken, the quality is very good, I like it very much!	Jingdong prefecture level city in Shandong
Happy Fan Shop	King of Power Saving	The saver was well packaged and was as described when plugged in	Jingdong prefecture level city in Shandong
Happy Fan Shop	King of Power Saving	Energy efficient and very satisfied	Jingdong prefecture level city in Shandong
Happy Fan Shop	King of Power Saving	Great quality for the price and fast shipping.	Jingdong prefecture level city in Shandong
Happy Fan Shop	King of Power Saving	It's great, just as described. Perfect.	Jingdong prefecture level city in Shandong
Happy Fan Shop	King of Power Saving	There is no power saving effect, but rather ring up an increase in electricity costs, because this machine is on continuously all day long, uninterruptedly, its own electricity and increased costs, plus the cost of purchasing the machine, so I feel that the use of this machine instead of increasing the	Jingdong prefecture level city in Shandong
Happy Fan Shop	King of Power Saving	Recommended by the owner of the store next door, looked at his home does have to save electricity, decided to get. Indeed quite can, the voltage is much more stable, there is a reduction in electricity	Jingdong prefecture level city in Shandong
Happy Fan Shop	King of Power Saving	Goods have been received value for money, affordable and reasonable price, and the description of the same, power-saving safe and reliable!	Jingdong prefecture level city in Shandong
Happy Fan Shop	King of Power Saving	Listen to friends introduced to buy, tried to save a lot of electricity is good, quite good, worth buying.	Jingdong prefecture level city in Shandong
Happy Fan Shop	King of Power Saving	The product is easy to use, safe and environmentally friendly, only used for a short time, to the next month, after the electricity bill status display to know how the performance of the power saver efficacy.	Jingdong prefecture level city in Shandong
Happy Fan Shop	King of Power Saving	Last month's electricity bill is 178 yuan, with this power saver is 92 yuan, ready to buy two more back home, I was introduced to buy a friend, just started to not believe that I did not expect the effect!	Jingdong prefecture level city in Shandong
Happy Fan Shop	King of Power Saving	It looks nice, but after using it for a few days I can't see how it saves power?	Jingdong prefecture level city in Shandong
Happy Fan Shop	King of Power Saving	Recommended by a friend, it is genuine, as described by the merchant, cost-effective	Jingdong prefecture level city in Shandong
Happy Fan Shop	King of Power Saving	Works well, affordable and saves money.	Jingdong prefecture level city in Shandong
Happy Fan Shop	King of Power Saving	Feels good haven't compared it yet, the air conditioning has been on a lot in the past two months	Jingdong prefecture level city in Shandong
Happy Fan Shop	King of Power Saving	Saving about 5 kWh per day is a good thing	Jingdong prefecture level city in Shandong
Happy Fan Shop	King of Power Saving	Not bad, affordable, best value for money	Jingdong prefecture level city in Shandong
Happy Fan Shop	King of Power Saving	The goods are genuine and the logistics are faster.	Jingdong prefecture level city in Shandong
Happy Fan Shop	King of Power Saving	Goods have been received today, has been plugged into the outlet, looking forward to saving electricity, after a period of time to see how the effect in the follow-up comment!	Jingdong prefecture level city in Shandong
Happy Fan Shop	King of Power Saving	I just got it home and haven't used it for long, so I'll use it for a couple months and see what happens. Seeing that most people have good reviews, it should be fine.	Jingdong prefecture level city in Shandong
Happy Fan Shop	King of Power Saving	I'm looking forward to the results, so I'll give you a good review out of habit! Follow up review after a while	Jingdong prefecture level city in Shandong
Happy Fan Shop	King of Power Saving	Use a dish first to know if it will help save energy	Jingdong prefecture level city in Shandong
Happy Fan Shop	King of Power Saving	Whether it saves electricity or not, we don't know yet.	Jingdong prefecture level city in Shandong
Happy Fan Shop	King of Power Saving	Goods have been received, use a period of time to know how oh. The courier did not deliver to the door ah! Sent to the station ah! The heat is inconvenient ah!	Jingdong prefecture level city in Shandong
Happy Fan Shop	King of Power Saving	A little effect, before an hour a power, now an hour 0.76 degrees, a dollar a power = save 24 cents, although not a lot, first use it!	Jingdong prefecture level city in Shandong
Happy Fan Shop	King of Power Saving	Just received the goods, use a month to evaluate	Jingdong prefecture level city in Shandong

Happy Fan Shop	King of Power Saving	Home every month with a lot of electricity, inadvertently found this good thing online that can save the beginning of a skeptical attitude to consult customer service, customer service replied very professional, said 120 days can be refunded that first try it!	Jingdong prefecture level city in Shandong
Happy Fan Shop	King of Power Saving	After receiving the goods this afternoon, immediately installed in the bathroom Smith energy storage electric water heater on the power outlet (instead of connecting with a plug board), when used, found that the "Power Saving King" LCD display, is the value of the voltage, not the store's video said so	Jingdong prefecture level city in Shandong
Happy Fan Shop	King of Power Saving	Has made many days, did not feel the power saving, the effect is not obvious.	Jingdong prefecture level city in Shandong
Happy Fan Shop	King of Power Saving	Energy savers do not save energy at all, but rather consume a lot of electricity.	Jingdong prefecture level city in Shandong
Happy Fan Shop	King of Power Saving	It's useless. It's a waste of money.	Jingdong prefecture level city in Shandong
Happy Fan Shop	King of Power Saving	Last month saved more than 500, which came back to buy, the store bought three, and then buy 1 home use, my family is dry beauty salon, more instruments, especially electricity, a month almost 1000. or so, with this did not expect to save so much, great, five-star praise!	Jingdong prefecture level city in Shandong
Happy Fan Shop	King of Power Saving	It rained a couple days ago and I just went to get the item back today , not bad, hey I looked at the meter and that red display light takes several seconds to jump, did not make a mistake buying it. After a period of time to evaluate.	Jingdong prefecture level city in Shandong
Happy Fan Shop	King of Power Saving	Without further ado, the electricity bill came down, this month less 102 yuan, has been repurchased, I also recommended to my friends, all said that there are effects, the product is okay, the effect is quite	Jingdong prefecture level city in Shandong
Happy Fan Shop	King of Power Saving	After buying and using two months really see the effect, so only to evaluate! Originally held the mindset of trying, I did not expect really effective, worth having!	Jingdong prefecture level city in Shandong
Happy Fan Shop	King of Power Saving	In the use of this energy saver before the monthly electricity bill is more than 150 yuan or so, now save thirty percent, every day to work, six or seven o'clock in the afternoon to go home, I do not know why the electricity bill is so expensive, energy-saving appliances really have the effect of recommending	Jingdong prefecture level city in Shandong
Happy Fan Shop	King of Power Saving	It saves about 100 bucks a month. A friend recommended it to me, and he's been using it for a while, and the effect is so obvious that I bought one too.	Jingdong prefecture level city in Shandong
Happy Fan Shop	King of Power Saving	My dad asked me to buy one, but after I paid the bill, I asked and he said it was a lot less this month, so he asked me to buy another one.	Jingdong prefecture level city in Shandong
Happy Fan Shop	King of Power Saving	The specific effect will have to be tested for at least two months to compare whether there is a power saving effect or not.	Jingdong prefecture level city in Shandong
Happy Fan Shop	King of Power Saving	'The goods have been received, immediately go to use, please ask the boss you instructions do not allow to withdraw, there are two fuses installed there!	Jingdong prefecture level city in Shandong
Happy Fan Shop	King of Power Saving	First praise is using, after using if good then follow up, now because I have not yet seen the results	Jingdong prefecture level city in Shandong
Happy Fan Shop	King of Power Saving	I've just been using it, and I haven't seen if it has a power-saving effect yet, so I'll follow up after 10 days and half a month.	Jingdong prefecture level city in Shandong
Happy Fan Shop	King of Power Saving	Just received the intelligent power saving meter, immediately plugged in to use, see it is not bad, and order to sell the second time	Jingdong prefecture level city in Shandong
Happy Fan Shop	King of Power Saving	This power saver is a bit effective, used for half a month, the effect is still visible.	Jingdong prefecture level city in Shandong
Happy Fan Shop	King of Power Saving	I received the baby and it looks great, I'm trying it out now and will keep an eye on it afterward.	Jingdong prefecture level city in Shandong
Happy Fan Shop	King of Power Saving	I've just received it, but I'm still trying to see how it works, so I'll come back in a little while.	Jingdong prefecture level city in Shandong
Happy Fan Shop	King of Power Saving	Just used it wait to use it for a while and then comment thanks, all first five stars.	Jingdong prefecture level city in Shandong
Happy Fan Shop	King of Power Saving	Very trustworthy brand, quality is guaranteed, the price is still affordable, quite satisfied!	Jingdong prefecture level city in Shandong
Happy Fan Shop	King of Power Saving	Things received, I hope that the platform of Jingdong do not sell fake goods are available	Jingdong prefecture level city in Shandong
Happy Fan Shop	King of Power Saving	Energy saver received, the use of the effect is good and the business description of the same	Jingdong prefecture level city in Shandong
Happy Fan Shop	King of Power Saving	The surface looks beautiful, the actual effect is not known, after using additional evaluation.	Jingdong prefecture level city in Shandong
Happy Fan Shop	King of Power Saving	Tried it for a few months, it really saves electricity, and I bought it for a friend.	Jingdong prefecture level city in Shandong
Happy Fan Shop	King of Power Saving	It didn't work and wanted to return it, it really didn't work at all.	Jingdong prefecture level city in Shandong
Happy Fan Shop	King of Power Saving	Just used it, don't know how effective it is, looking forward to usefulness	Jingdong prefecture level city in Shandong
Happy Fan Shop	King of Power Saving	After two months before the effect I do not know if it is true!	Jingdong prefecture level city in Shandong
Happy Fan Shop	King of Power Saving	So fast! Ordered yesterday and received today 👍👍👍👍👍👍👍👍👍 🌹🌹🌹🌹🌹🌹🌹🌹🌹	Jingdong prefecture level city in Shandong
Happy Fan Shop	King of Power Saving	I've started using it. We'll see how it works.	Jingdong prefecture level city in Shandong
Happy Fan Shop	King of Power Saving	I just received the goods, I won't know the effect until after two.	Jingdong prefecture level city in Shandong
Happy Fan Shop	King of Power Saving	Just received the goods, the specific effect will be evaluated later	Jingdong prefecture level city in Shandong
Happy Fan Shop	King of Power Saving	I've received the goods, I don't know if it's any good yet	Jingdong prefecture level city in Shandong
Happy Fan Shop	King of Power Saving	Try it for a month and then come back with a concrete evaluation	Jingdong prefecture level city in Shandong
Happy Fan Shop	King of Power Saving	In use, after two months to see the effect of power saving	Jingdong prefecture level city in Shandong
Happy Fan Shop	King of Power Saving	Power Saver. It's authentic. It's black. It works.	Jingdong prefecture level city in Shandong
Happy Fan Shop	King of Power Saving	I've been using it for a week now and it's not working.	Jingdong prefecture level city in Shandong
Happy Fan Shop	King of Power Saving	I'm just using it. I don't know how it's going to work.	Jingdong prefecture level city in Shandong
Happy Fan Shop	King of Power Saving	Products are better looking out for power savings	Jingdong prefecture level city in Shandong
Happy Fan Shop	King of Power Saving	I received it and I've kept it on. I hope it works.	Jingdong prefecture level city in Shandong
Happy Fan Shop	King of Power Saving	It's really good! Worth recommending to buy!	Jingdong prefecture level city in Shandong
Happy Fan Shop	King of Power Saving	Try it for a few days and see if it doesn't work, then return it.	Jingdong prefecture level city in Shandong
Happy Fan Shop	King of Power Saving	It's in use and I hope to be pleasantly surprised.	Jingdong prefecture level city in Shandong
Happy Fan Shop	King of Power Saving	We'll see how it works when we use it	Jingdong prefecture level city in Shandong
Happy Fan Shop	King of Power Saving	Let's see if it works.	Jingdong prefecture level city in Shandong
Happy Fan Shop	King of Power Saving	I'm using it and will follow up with a review	Jingdong prefecture level city in Shandong
Happy Fan Shop	King of Power Saving	It really works. It saves energy.	Jingdong prefecture level city in Shandong
Happy Fan Shop	King of Power Saving	It does have some power savings!	Jingdong prefecture level city in Shandong

Happy Fan Shop	King of Power Saving	Jingdong shopping speed is convenient and timely	Jingdong prefecture level city in Shandong
Happy Fan Shop	King of Power Saving	Second time I've bought it. Everything's fine.	Jingdong prefecture level city in Shandong
Happy Fan Shop	King of Power Saving	I don't know if it works yet.	Jingdong prefecture level city in Shandong
Happy Fan Shop	King of Power Saving	Not used, don't know how well it works.	Jingdong prefecture level city in Shandong
Happy Fan Shop	King of Power Saving	No visible results so far	Jingdong prefecture level city in Shandong
Happy Fan Shop	King of Power Saving	It doesn't feel like it's working.	Jingdong prefecture level city in Shandong
Happy Fan Shop	King of Power Saving	Where's the effect? Where are the power savings?	Jingdong prefecture level city in Shandong
Happy Fan Shop	King of Power Saving	Let's see how it works in practice!	Jingdong prefecture level city in Shandong
Happy Fan Shop	King of Power Saving	I'll wait until I use it to give you a good review	Jingdong prefecture level city in Shandong
Happy Fan Shop	King of Power Saving	I'll follow up after I've used it	Jingdong prefecture level city in Shandong
Happy Fan Shop	King of Power Saving	Well, that's good for saving power.	Jingdong prefecture level city in Shandong
Happy Fan Shop	King of Power Saving	Received. Used in the know.	Jingdong prefecture level city in Shandong
Happy Fan Shop	King of Power Saving	It doesn't do much good.	Jingdong prefecture level city in Shandong
Happy Fan Shop	King of Power Saving	It's not working.	Jingdong prefecture level city in Shandong
Happy Fan Shop	King of Power Saving	I don't know. I just used it.	Jingdong prefecture level city in Shandong
Happy Fan Shop	King of Power Saving	Use it and come back to evaluate.	Jingdong prefecture level city in Shandong
Happy Fan Shop	King of Power Saving	Two words: useless.	Jingdong prefecture level city in Shandong
Happy Fan Shop	King of Power Saving	To be used and then evaluated	Jingdong prefecture level city in Shandong
Happy Fan Shop	King of Power Saving	No shipment.	Jingdong prefecture level city in Shandong
Happy Fan Shop	King of Power Saving	Fake. Speechless.	Jingdong prefecture level city in Shandong
Happy Fan Shop	King of Power Saving	fantastic (idiom); awesome	Jingdong prefecture level city in Shandong
Happy Fan Shop	King of Power Saving	an excellent	Jingdong prefecture level city in Shandong
Happy Fan Shop	King of Power Saving	an excellent	Jingdong prefecture level city in Shandong
Happy Fan Shop	King of Power Saving	Want to buy! Recommended not to buy! No effect	Jingdong prefecture level city in Shandong
Happy Fan Shop	King of Power Saving	That's all there is to it. I can't figure out how to save electricity.	Jingdong prefecture level city in Shandong
Happy Fan Shop	King of Power Saving	It's no use.	Jingdong prefecture level city in Shandong
Happy Fan Shop	King of Power Saving	Things are more perfect than I imagined, very powerful, it is worth getting Packaging is very strong, things are well protected, it is worth the praise things are very satisfied Oh, beautiful and stylish atmosphere, customer service is super patient, service is meticulous and attentive, the big brand is not	Jingdong prefecture level city in Shandong
Happy Fan Shop	King of Power Saving	The effect is too good, the family has now several units, back to buy a number of times, the power saver received, power-saving effect is very good,, fine workmanship, long warranty, but also purify the air, before buying a used for a period of time, with the okay, and then bought a, and only now come to the	Jingdong prefecture level city in Shandong
Happy Fan Shop	King of Power Saving	Quite good, and imagine the same, casual ride are very good, satisfied! Baby received it 👌, really great ah! 🌈 quality is really nothing to say, very satisfied! 🆗 happy happy 😃 genuine, great, the price is very good, in short, quite good, will buy again a very good shopping experience, attentive service, fast logistics, distribution is also thoughtful, the attitude is also very good, value for money, ah,	Jingdong prefecture level city in Shandong
Happy Fan Shop	King of Power Saving	Power-saving effect: strong Cost-effective: quite good Appearance style: beautiful Workmanship material: meticulous Experience: looking forward to power-saving effect	Jingdong prefecture level city in Shandong
Happy Fan Shop	King of Power Saving	Cost-effective is okay, the appearance of the style of meticulous workmanship, looking forward to energy-saving effect.	Jingdong prefecture level city in Shandong
Happy Fan Shop	King of Power Saving	Very Good Very Power Saving This Power Saver is really power saving As soon as the power is turned on, the Power Saver starts the power saving function for you.	Jingdong prefecture level city in Shandong
Happy Fan Shop	King of Power Saving	Logistics is very fast, the price is favorable, friends introduced that it is very good to use, bought 2 at once, looking forward to the effect, used after the follow-up comment.	Jingdong prefecture level city in Shandong
Happy Fan Shop	King of Power Saving	It's all fake. It doesn't save any electricity.	Jingdong prefecture level city in Shandong
Happy Fan Shop	King of Power Saving	This Power Saver is so much better than the one I bought before, not only the effect is good, the quality is very good in all aspects, the customer service attitude is also very good, recommended to buy!	Jingdong prefecture level city in Shandong
Happy Fan Shop	King of Power Saving	Logistics is very fast, buy for parents, good quality, once bought 2, power-saving effect is good to buy 2 more, look forward to using the effect, satisfied will recommend to friends.	Jingdong prefecture level city in Shandong
Happy Fan Shop	King of Power Saving	With the description is the same, very high-grade, good value, to buy friends can order Oh!	Jingdong prefecture level city in Shandong
Happy Fan Shop	King of Power Saving	Not using much electricity in the winter saving over 100 per month! Ready to buy 2 more units in the summer ！！！！！ Good quality, good price, power saving effect is obvious, cost-effective, worth buying, can be recommended!	Jingdong prefecture level city in Shandong
Happy Fan Shop	King of Power Saving	Purchased a power saver received on the put into use, rent a room with many electrical appliances, power consumption is very large, we have a piece of electricity or 1.2 dollars a time, with this power saver is much better, every day can save a lot of money, very convenient and practical.	Jingdong prefecture level city in Shandong
Happy Fan Shop	King of Power Saving	The power saving effect is very good: my supermarket monthly electricity bill is more than 7000 yuan. On May 1st someone recommended buying a couple of power savers, and I bought 5 of them first. The electricity bill was about 2000 less in June. This time, I bought 5 more. The seller said 800 degrees of electricity with one. I feel it's basically enough. A total of 10 units	Jingdong prefecture level city in Shandong
Happy Fan Shop	King of Power Saving	I never thought I could get such a good baby at such an affordable price, it's very good value for money, I love it!	Jingdong prefecture level city in Shandong
Happy Fan Shop	King of Power Saving	Previously bought other brands of power savers, but found no effect at home, this time to see how the products on the Jingdong, inserted in the home socket, the LED display is blue, the night light is very nice to see, look at the instructions that have to be half a year in order to achieve the best results, try it, I believe that the quality of the Jingdong, it should not be wrong.	Jingdong prefecture level city in Shandong
Happy Fan Shop	King of Power Saving	Superb, inexpensive, saves a lot of points, been using it for a long time!	Jingdong prefecture level city in Shandong
Happy Fan Shop	King of Power Saving	There are a lot of appliances in the house, the summer air conditioning is very costly, I bought this in front of the air conditioning effect is very good, I explain that I bought three in one breath, I use more electricity a month down to 200 or so, very satisfied.	Jingdong prefecture level city in Shandong

Happy Fan Shop	King of Power Saving	A month can save more than 200, home monthly electricity bill of more than 600 dollars, inserted 3 or so, an amount of the balance of the average can save more than 200. Product is very good, has been buying this one, has given a lot of relatives recommended, the quality is also very good, before buying a look with this same, who knows to buy home has not been used for more than a week on the bad, the quality of this is really good with more than two years!	Jingdong prefecture level city in Shandong
Happy Fan Shop	King of Power Saving	Whole house power saving king, can really save electricity, friends recommended, high power saving appliances, only replacement, no repair, after-sales worry-free!	Jingdong prefecture level city in Shandong
Happy Fan Shop	King of Power Saving	Great item love it, great customer service, great shopping experience!	Jingdong prefecture level city in Shandong
Happy Fan Shop	King of Power Saving	Power saving, very good use, much cheaper than the physical store, satisfaction	Jingdong prefecture level city in Shandong
Happy Fan Shop	King of Power Saving	In use, very well packaged and fast delivery of credit!	Jingdong prefecture level city in Shandong
Happy Fan Shop	King of Power Saving	Product quality, customer service attitude is particularly good, like, praise!	Jingdong prefecture level city in Shandong
Happy Fan Shop	King of Power Saving	The logistics is very fast, the packaging is not broken, the quality is very good, I like it very much!	Jingdong prefecture level city in Shandong
Happy Fan Shop	King of Power Saving	The saver was well packaged and was as described when plugged in	Jingdong prefecture level city in Shandong
Happy Fan Shop	King of Power Saving	Energy efficient and very satisfied	Jingdong prefecture level city in Shandong
Happy Fan Shop	King of Power Saving	Great quality for the price and fast shipping.	Jingdong prefecture level city in Shandong
Happy Fan Shop	King of Power Saving	It's great, just as described. Perfect.	Jingdong prefecture level city in Shandong
Happy Fan Shop	King of Power Saving	There is no power saving effect, but rather ring up an increase in electricity costs, because this machine is on continuously all day long, uninterruptedly, its own electricity and increased costs, plus the cost of purchasing the machine, so I feel that the use of this machine instead of increasing the	Jingdong prefecture level city in Shandong
Happy Fan Shop	King of Power Saving	Recommended by the owner of the store next door, looked at his home does have to save electricity, decided to get. Indeed quite can, the voltage is much more stable, there is a reduction in electricity	Jingdong prefecture level city in Shandong
Happy Fan Shop	King of Power Saving	Goods have been received value for money, affordable and reasonable price, and the description of the same, power-saving safe and reliable!	Jingdong prefecture level city in Shandong
Happy Fan Shop	King of Power Saving	Listen to friends introduced to buy, tried to save a lot of electricity is good, quite good, worth buying.	Jingdong prefecture level city in Shandong
Happy Fan Shop	King of Power Saving	The product is easy to use, safe and environmentally friendly, only used for a short time, to the next month, after the electricity bill status display to know how the performance of the power saver efficacy.	Jingdong prefecture level city in Shandong
Happy Fan Shop	King of Power Saving	Last month's electricity bill is 178 yuan, with this power saver is 92 yuan, ready to buy two more back home, I was introduced to buy a friend, just started to not believe that I did not expect the effect!	Jingdong prefecture level city in Shandong
Happy Fan Shop	King of Power Saving	It looks nice, but after using it for a few days I can't see how it saves power?	Jingdong prefecture level city in Shandong
Happy Fan Shop	King of Power Saving	Recommended by a friend, it is genuine, as described by the merchant, cost-effective	Jingdong prefecture level city in Shandong
Happy Fan Shop	King of Power Saving	Works well, affordable and saves money.	Jingdong prefecture level city in Shandong
Happy Fan Shop	King of Power Saving	Feels good haven't compared it yet, the air conditioning has been on a lot in the past two months	Jingdong prefecture level city in Shandong
Happy Fan Shop	King of Power Saving	Saving about 5 kWh per day is a good thing	Jingdong prefecture level city in Shandong
Happy Fan Shop	King of Power Saving	Not bad, affordable, best value for money	Jingdong prefecture level city in Shandong
Happy Fan Shop	King of Power Saving	The goods are genuine and the logistics are faster.	Jingdong prefecture level city in Shandong
Happy Fan Shop	King of Power Saving	Goods have been received today, has been plugged into the outlet, looking forward to saving electricity, after a period of time to see how the effect in the follow-up comment!	Jingdong prefecture level city in Shandong
Happy Fan Shop	King of Power Saving	I just got it home and haven't used it for long, so I'll use it for a couple months and see what happens. Seeing that most people have good reviews, it should be fine.	Jingdong prefecture level city in Shandong
Happy Fan Shop	King of Power Saving	I'm looking forward to the results, so I'll give you a good review out of habit! Follow up review after a while	Jingdong prefecture level city in Shandong
Happy Fan Shop	King of Power Saving	Use a dish first to know if it will help save energy	Jingdong prefecture level city in Shandong
Happy Fan Shop	King of Power Saving	Whether it saves electricity or not, we don't know yet.	Jingdong prefecture level city in Shandong
Happy Fan Shop	King of Power Saving	Goods have been received, use a period of time to know how oh. The courier did not deliver to the door ah! Sent to the station ah! The heat is inconvenient ah!	Jingdong prefecture level city in Shandong
Happy Fan Shop	King of Power Saving	A little effect, before an hour a power, now an hour 0.76 degrees, a dollar a power = save 24 cents, although not a lot, first use it!	Jingdong prefecture level city in Shandong
Happy Fan Shop	King of Power Saving	Just received the goods, use a month to evaluate	Jingdong prefecture level city in Shandong
Happy Fan Shop	King of Power Saving	Home every month with a lot of electricity, inadvertently found this good thing online that can save the beginning of a skeptical attitude to consult customer service, customer service replied very professional, said 120 days can be refunded that first try it!	Jingdong prefecture level city in Shandong
Happy Fan Shop	King of Power Saving	After receiving the goods this afternoon, immediately installed in the bathroom Smith energy storage electric water heater on the power outlet (instead of connecting with a plug board), when used, found that the "Power Saving King" LCD display, is the value of the voltage, not the store's video said so	Jingdong prefecture level city in Shandong
Happy Fan Shop	King of Power Saving	Has made many days, did not feel the power saving, the effect is not obvious.	Jingdong prefecture level city in Shandong
Happy Fan Shop	King of Power Saving	Energy savers do not save energy at all, but rather consume a lot of electricity.	Jingdong prefecture level city in Shandong
Happy Fan Shop	King of Power Saving	It's useless. It's a waste of money.	Jingdong prefecture level city in Shandong
Happy Fan Shop	King of Power Saving	Last month saved more than 500, which came back to buy, the store bought three, and then buy 1 home use, my family is dry beauty salon, more instruments, especially electricity, a month almost 1000. or so, with this did not expect to save so much, great, five-star praise!	Jingdong prefecture level city in Shandong
Happy Fan Shop	King of Power Saving	It rained a couple days ago and I just went to get the item back today , not bad, hey I looked at the meter and that red display light takes several seconds to jump, did not make a mistake buying it. After a period of time to evaluate.	Jingdong prefecture level city in Shandong
Happy Fan Shop	King of Power Saving	Without further ado, the electricity bill came down, this month less 102 yuan, has been repurchased, I also recommended to my friends, all said that there are effects, the product is okay, the effect is quite	Jingdong prefecture level city in Shandong
Happy Fan Shop	King of Power Saving	After buying and using two months really see the effect, so only to evaluate! Originally held the mindset of trying, I did not expect really effective, worth having!	Jingdong prefecture level city in Shandong
Happy Fan Shop	King of Power Saving	In the use of this energy saver before the monthly electricity bill is more than 150 yuan or so, now save thirty percent, every day to work, six or seven o'clock in the afternoon to go home, I do not know why the electricity bill is so expensive, energy-saving appliances really have the effect of recommending	Jingdong prefecture level city in Shandong
Happy Fan Shop	King of Power Saving	It saves about 100 bucks a month. A friend recommended it to me, and he's been using it for a while, and the effect is so obvious that I bought one too.	Jingdong prefecture level city in Shandong
Happy Fan Shop	King of Power Saving	My dad asked me to buy one, but after I paid the bill, I asked and he said it was a lot less this month, so he asked me to buy another one.	Jingdong prefecture level city in Shandong
Happy Fan Shop	King of Power Saving	The specific effect will have to be tested for at least two months to compare whether there is a power saving effect or not.	Jingdong prefecture level city in Shandong
Happy Fan Shop	King of Power Saving	'The goods have been received, immediately go to use, please ask the boss you instructions do not allow to withdraw, there are two fuses installed there!	Jingdong prefecture level city in Shandong
Happy Fan Shop	King of Power Saving	First praise is using, after using if good then follow up, now because I have not yet seen the results	Jingdong prefecture level city in Shandong

Happy Fan Shop	King of Power Saving	I've just been using it, and I haven't seen if it has a power-saving effect yet, so I'll follow up after 10 days and half a month.	Jingdong prefecture level city in Shandong
Happy Fan Shop	King of Power Saving	Just received the intelligent power saving meter, immediately plugged in to use, see it is not bad, and order to sell the second time	Jingdong prefecture level city in Shandong
Happy Fan Shop	King of Power Saving	This power saver is a bit effective, used for half a month, the effect is still visible.	Jingdong prefecture level city in Shandong
Happy Fan Shop	King of Power Saving	I received the baby and it looks great, I'm trying it out now and will keep an eye on it afterward.	Jingdong prefecture level city in Shandong
Happy Fan Shop	King of Power Saving	I've just received it, but I'm still trying to see how it works, so I'll come back in a little while.	Jingdong prefecture level city in Shandong
Happy Fan Shop	King of Power Saving	Just used it wait to use it for a while and then comment thanks, all first five stars.	Jingdong prefecture level city in Shandong
Happy Fan Shop	King of Power Saving	Very trustworthy brand, quality is guaranteed, the price is still affordable, quite satisfied!	Jingdong prefecture level city in Shandong
Happy Fan Shop	King of Power Saving	Things received, I hope that the platform of Jingdong do not sell fake goods are available	Jingdong prefecture level city in Shandong
Happy Fan Shop	King of Power Saving	Energy saver received, the use of the effect is good and the business description of the same	Jingdong prefecture level city in Shandong
Happy Fan Shop	King of Power Saving	The surface looks beautiful, the actual effect is not known, after using additional evaluation.	Jingdong prefecture level city in Shandong
Happy Fan Shop	King of Power Saving	Tried it for a few months, it really saves electricity, and I bought it for a friend.	Jingdong prefecture level city in Shandong
Happy Fan Shop	King of Power Saving	It didn't work and wanted to return it, it really didn't work at all.	Jingdong prefecture level city in Shandong
Happy Fan Shop	King of Power Saving	Just used it, don't know how effective it is, looking forward to usefulness	Jingdong prefecture level city in Shandong
Happy Fan Shop	King of Power Saving	After two months before the effect I do not know if it is true!	Jingdong prefecture level city in Shandong
Happy Fan Shop	King of Power Saving	So fast! Ordered yesterday and received today 👍👍👍👍👍👍👍👍👍 🌹🌹🌹🌹🌹🌹🌹🌹🌹	Jingdong prefecture level city in Shandong
Happy Fan Shop	King of Power Saving	I've started using it. We'll see how it works.	Jingdong prefecture level city in Shandong
Happy Fan Shop	King of Power Saving	I just received the goods, I won't know the effect until after two.	Jingdong prefecture level city in Shandong
Happy Fan Shop	King of Power Saving	Just received the goods, the specific effect will be evaluated later	Jingdong prefecture level city in Shandong
Happy Fan Shop	King of Power Saving	I've received the goods, I don't know if it's any good yet	Jingdong prefecture level city in Shandong
Happy Fan Shop	King of Power Saving	Try it for a month and then come back with a concrete evaluation	Jingdong prefecture level city in Shandong
Happy Fan Shop	King of Power Saving	In use, after two months to see the effect of power saving	Jingdong prefecture level city in Shandong
Happy Fan Shop	King of Power Saving	Power Saver. It's authentic. It's black. It works.	Jingdong prefecture level city in Shandong
Happy Fan Shop	King of Power Saving	I've been using it for a week now and it's not working.	Jingdong prefecture level city in Shandong
Happy Fan Shop	King of Power Saving	I'm just using it. I don't know how it's going to work.	Jingdong prefecture level city in Shandong
Happy Fan Shop	King of Power Saving	Products are better looking out for power savings	Jingdong prefecture level city in Shandong
Happy Fan Shop	King of Power Saving	I received it and I've kept it on. I hope it works.	Jingdong prefecture level city in Shandong
Happy Fan Shop	King of Power Saving	It's really good! Worth recommending to buy!	Jingdong prefecture level city in Shandong
Happy Fan Shop	King of Power Saving	Try it for a few days and see if it doesn't work, then return it.	Jingdong prefecture level city in Shandong
Happy Fan Shop	King of Power Saving	It's in use and I hope to be pleasantly surprised.	Jingdong prefecture level city in Shandong
Happy Fan Shop	King of Power Saving	We'll see how it works when we use it	Jingdong prefecture level city in Shandong
Happy Fan Shop	King of Power Saving	Let's see if it works.	Jingdong prefecture level city in Shandong
Happy Fan Shop	King of Power Saving	I'm using it and will follow up with a review	Jingdong prefecture level city in Shandong
Happy Fan Shop	King of Power Saving	It really works. It saves energy.	Jingdong prefecture level city in Shandong
Happy Fan Shop	King of Power Saving	It does have some power savings!	Jingdong prefecture level city in Shandong
Happy Fan Shop	King of Power Saving	Jingdong shopping speed is convenient and timely	Jingdong prefecture level city in Shandong
Happy Fan Shop	King of Power Saving	Second time I've bought it. Everything's fine.	Jingdong prefecture level city in Shandong
Happy Fan Shop	King of Power Saving	I don't know if it works yet.	Jingdong prefecture level city in Shandong
Happy Fan Shop	King of Power Saving	Not used, don't know how well it works.	Jingdong prefecture level city in Shandong
Happy Fan Shop	King of Power Saving	No visible results so far	Jingdong prefecture level city in Shandong
Happy Fan Shop	King of Power Saving	It doesn't feel like it's working.	Jingdong prefecture level city in Shandong
Happy Fan Shop	King of Power Saving	Where's the effect? Where are the power savings?	Jingdong prefecture level city in Shandong
Happy Fan Shop	King of Power Saving	Let's see how it works in practice!	Jingdong prefecture level city in Shandong
Happy Fan Shop	King of Power Saving	I'll wait until I use it to give you a good review	Jingdong prefecture level city in Shandong
Happy Fan Shop	King of Power Saving	I'll follow up after I've used it	Jingdong prefecture level city in Shandong
Happy Fan Shop	King of Power Saving	Well, that's good for saving power.	Jingdong prefecture level city in Shandong
Happy Fan Shop	King of Power Saving	Received. Used in the know.	Jingdong prefecture level city in Shandong
Happy Fan Shop	King of Power Saving	It doesn't do much good.	Jingdong prefecture level city in Shandong
Happy Fan Shop	King of Power Saving	It's not working.	Jingdong prefecture level city in Shandong
Happy Fan Shop	King of Power Saving	I don't know. I just used it.	Jingdong prefecture level city in Shandong
Happy Fan Shop	King of Power Saving	Use it and come back to evaluate.	Jingdong prefecture level city in Shandong
Happy Fan Shop	King of Power Saving	Two words: useless.	Jingdong prefecture level city in Shandong

Happy Fan Shop	King of Power Saving	To be used and then evaluated	Jingdong prefecture level city in Shandong
Happy Fan Shop	King of Power Saving	No shipment.	Jingdong prefecture level city in Shandong
Happy Fan Shop	King of Power Saving	Fake. Speechless.	Jingdong prefecture level city in Shandong
Happy Fan Shop	King of Power Saving	fantastic (idiom); awesome	Jingdong prefecture level city in Shandong
Happy Fan Shop	King of Power Saving	an excellent	Jingdong prefecture level city in Shandong
Happy Fan Shop	King of Power Saving	an excellent	Jingdong prefecture level city in Shandong
Happy Fan Shop	King of Power Saving	Want to buy! Recommended not to buy! No effect	Jingdong prefecture level city in Shandong
Happy Fan Shop	King of Power Saving	That's all there is to it. I can't figure out how to save electricity.	Jingdong prefecture level city in Shandong
Happy Fan Shop	King of Power Saving	It's no use.	Jingdong prefecture level city in Shandong
Happy Fan Shop	King of Power Saving	Things are more perfect than I imagined, very powerful, it is worth getting Packaging is very strong, things are well protected, it is worth the praise things are very satisfied Oh, beautiful and stylish atmosphere, customer service is super patient, service is meticulous and attentive, the big brand is not	Jingdong prefecture level city in Shandong
Happy Fan Shop	King of Power Saving	The effect is too good, the family has now several units, back to buy a number of times, the power saver received, power-saving effect is very good,, fine workmanship, long warranty, but also purify the air, before buying a used for a period of time, with the okay, and then bought a, and only now come to the	Jingdong prefecture level city in Shandong
Happy Fan Shop	King of Power Saving	Quite good, and imagine the same, casual ride are very good, satisfied! Baby received it 👌, really great ah! 🌈 quality is really nothing to say, very satisfied! 🆗 happy happy 😃 genuine, great, the price is very good, in short, quite good, will buy again a very good shopping experience, attentive service, fast logistics, distribution is also thoughtful, the attitude is also very good, value for money, ah,	Jingdong prefecture level city in Shandong
Happy Fan Shop	King of Power Saving	Power-saving effect: strong Cost-effective: quite good Appearance style: beautiful Workmanship material: meticulous Experience: looking forward to power-saving effect	Jingdong prefecture level city in Shandong
Happy Fan Shop	King of Power Saving	Cost-effective is okay, the appearance of the style of meticulous workmanship, looking forward to energy-saving effect.	Jingdong prefecture level city in Shandong
Happy Fan Shop	King of Power Saving	Very Good Very Power Saving This Power Saver is really power saving As soon as the power is turned on, the Power Saver starts the power saving function for you.	Jingdong prefecture level city in Shandong
Happy Fan Shop	King of Power Saving	Logistics is very fast, the price is favorable, friends introduced that it is very good to use, bought 2 at once, looking forward to the effect, used after the follow-up comment.	Jingdong prefecture level city in Shandong
Happy Fan Shop	King of Power Saving	It's all fake. It doesn't save any electricity.	Jingdong prefecture level city in Shandong
Happy Fan Shop	King of Power Saving	This Power Saver is so much better than the one I bought before, not only the effect is good, the quality is very good in all aspects, the customer service attitude is also very good, recommended to buy!	Jingdong prefecture level city in Shandong
Happy Fan Shop	King of Power Saving	Logistics is very fast, buy for parents, good quality, once bought 2, power-saving effect is good to buy 2 more, look forward to using the effect, satisfied will recommend to friends.	Jingdong prefecture level city in Shandong
Happy Fan Shop	King of Power Saving	With the description is the same, very high-grade, good value, to buy friends can order Oh!	Jingdong prefecture level city in Shandong
Happy Fan Shop	King of Power Saving	Not using much electricity in the winter saving over 100 per month! Ready to buy 2 more units in the summer ！！！！！ Good quality, good price, power saving effect is obvious, cost-effective, worth buying, can be recommended!	Jingdong prefecture level city in Shandong
Happy Fan Shop	King of Power Saving	Purchased a power saver received on the put into use, rent a room with many electrical appliances, power consumption is very large, we have a piece of electricity or 1.2 dollars a time, with this power saver is much better, every day can save a lot of money, very convenient and practical.	Jingdong prefecture level city in Shandong
Happy Fan Shop	King of Power Saving	The power saving effect is very good: my supermarket monthly electricity bill is more than 7000 yuan. On May 1st someone recommended buying a couple of power savers, and I bought 5 of them first. The electricity bill was about 2000 less in June. This time, I bought 5 more. The seller said 800 degrees of electricity with one. I feel it's basically enough. A total of 10 units	Jingdong prefecture level city in Shandong
Happy Fan Shop	King of Power Saving	I never thought I could get such a good baby at such an affordable price, it's very good value for money, I love it!	Jingdong prefecture level city in Shandong
Happy Fan Shop	King of Power Saving	Previously bought other brands of power savers, but found no effect at home, this time to see how the products on the Jingdong, inserted in the home socket, the LED display is blue, the night light is very nice to see, look at the instructions that have to be half a year in order to achieve the best results, try it, I believe that the quality of the Jingdong, it should not be wrong.	Jingdong prefecture level city in Shandong
Happy Fan Shop	King of Power Saving	Superb, inexpensive, saves a lot of points, been using it for a long time!	Jingdong prefecture level city in Shandong
Happy Fan Shop	King of Power Saving	There are a lot of appliances in the house, the summer air conditioning is very costly, I bought this in front of the air conditioning effect is very good, I explain that I bought three in one breath, I use more electricity a month down to 200 or so, very satisfied.	Jingdong prefecture level city in Shandong
Happy Fan Shop	King of Power Saving	A month can save more than 200, home monthly electricity bill of more than 600 dollars, inserted 3 or so, an amount of the balance of the average can save more than 200. Product is very good, has been buying this one, has given a lot of relatives recommended, the quality is also very good, before buying a look with this same, who knows to buy home has not been used for more than a week on the bad, the quality of this is really good with more than two years!	Jingdong prefecture level city in Shandong
Happy Fan Shop	King of Power Saving	Whole house power saving king, can really save electricity, friends recommended, high power saving appliances, only replacement, no repair, after-sales worry-free!	Jingdong prefecture level city in Shandong
Happy Fan Shop	King of Power Saving	Great item love it, great customer service, great shopping experience!	Jingdong prefecture level city in Shandong
Happy Fan Shop	King of Power Saving	Power saving, very good use, much cheaper than the physical store, satisfaction	Jingdong prefecture level city in Shandong
Happy Fan Shop	King of Power Saving	In use, very well packaged and fast delivery of credit!	Jingdong prefecture level city in Shandong
Happy Fan Shop	King of Power Saving	Product quality, customer service attitude is particularly good, like, praise!	Jingdong prefecture level city in Shandong
Happy Fan Shop	King of Power Saving	The logistics is very fast, the packaging is not broken, the quality is very good, I like it very much!	Jingdong prefecture level city in Shandong
Happy Fan Shop	King of Power Saving	The saver was well packaged and was as described when plugged in	Jingdong prefecture level city in Shandong
Happy Fan Shop	King of Power Saving	Energy efficient and very satisfied	Jingdong prefecture level city in Shandong
Happy Fan Shop	King of Power Saving	Great quality for the price and fast shipping.	Jingdong prefecture level city in Shandong
Happy Fan Shop	King of Power Saving	It's great, just as described. Perfect.	Jingdong prefecture level city in Shandong
Happy Fan Shop	King of Power Saving	There is no power saving effect, but rather ring up an increase in electricity costs, because this machine is on continuously all day long, uninterruptedly, its own electricity and increased costs, plus the cost of purchasing the machine, so I feel that the use of this machine instead of increasing the	Jingdong prefecture level city in Shandong
Happy Fan Shop	King of Power Saving	Recommended by the owner of the store next door, looked at his home does have to save electricity, decided to get. Indeed quite can, the voltage is much more stable, there is a reduction in electricity	Jingdong prefecture level city in Shandong
Happy Fan Shop	King of Power Saving	Goods have been received value for money, affordable and reasonable price, and the description of the same, power-saving safe and reliable!	Jingdong prefecture level city in Shandong
Happy Fan Shop	King of Power Saving	Listen to friends introduced to buy, tried to save a lot of electricity is good, quite good, worth buying.	Jingdong prefecture level city in Shandong
Happy Fan Shop	King of Power Saving	The product is easy to use, safe and environmentally friendly, only used for a short time, to the next month, after the electricity bill status display to know how the performance of the power saver efficacy.	Jingdong prefecture level city in Shandong
Happy Fan Shop	King of Power Saving	Last month's electricity bill is 178 yuan, with this power saver is 92 yuan, ready to buy two more back home, I was introduced to buy a friend, just started to not believe that I did not expect the effect!	Jingdong prefecture level city in Shandong

Happy Fan Shop	King of Power Saving	It looks nice, but after using it for a few days I can't see how it saves power?	Jingdong prefecture level city in Shandong
Happy Fan Shop	King of Power Saving	Recommended by a friend, it is genuine, as described by the merchant, cost-effective	Jingdong prefecture level city in Shandong
Happy Fan Shop	King of Power Saving	Works well, affordable and saves money.	Jingdong prefecture level city in Shandong
Happy Fan Shop	King of Power Saving	Feels good haven't compared it yet, the air conditioning has been on a lot in the past two months	Jingdong prefecture level city in Shandong
Happy Fan Shop	King of Power Saving	Saving about 5 kWh per day is a good thing	Jingdong prefecture level city in Shandong
Happy Fan Shop	King of Power Saving	Not bad, affordable, best value for money	Jingdong prefecture level city in Shandong
Happy Fan Shop	King of Power Saving	The goods are genuine and the logistics are faster.	Jingdong prefecture level city in Shandong
Happy Fan Shop	King of Power Saving	Goods have been received today, has been plugged into the outlet, looking forward to saving electricity, after a period of time to see how the effect in the follow-up comment!	Jingdong prefecture level city in Shandong
Happy Fan Shop	King of Power Saving	I just got it home and haven't used it for long, so I'll use it for a couple months and see what happens. Seeing that most people have good reviews, it should be fine.	Jingdong prefecture level city in Shandong
Happy Fan Shop	King of Power Saving	I'm looking forward to the results, so I'll give you a good review out of habit! Follow up review after a while	Jingdong prefecture level city in Shandong
Happy Fan Shop	King of Power Saving	Use a dish first to know if it will help save energy	Jingdong prefecture level city in Shandong
Happy Fan Shop	King of Power Saving	Whether it saves electricity or not, we don't know yet.	Jingdong prefecture level city in Shandong
Happy Fan Shop	King of Power Saving	Goods have been received, use a period of time to know how oh. The courier did not deliver to the door ah! Sent to the station ah! The heat is inconvenient ah!	Jingdong prefecture level city in Shandong
Happy Fan Shop	King of Power Saving	A little effect, before an hour a power, now an hour 0.76 degrees, a dollar a power = save 24 cents, although not a lot, first use it!	Jingdong prefecture level city in Shandong
Happy Fan Shop	King of Power Saving	Just received the goods, use a month to evaluate	Jingdong prefecture level city in Shandong
Happy Fan Shop	King of Power Saving	Home every month with a lot of electricity, inadvertently found this good thing online that can save the beginning of a skeptical attitude to consult customer service, customer service replied very professional, said 120 days can be refunded that first try it!	Jingdong prefecture level city in Shandong
Happy Fan Shop	King of Power Saving	After receiving the goods this afternoon, immediately installed in the bathroom Smith energy storage electric water heater on the power outlet (instead of connecting with a plug board), when used, found that the "Power Saving King" LCD display, is the value of the voltage, not the store's video said so	Jingdong prefecture level city in Shandong
Happy Fan Shop	King of Power Saving	Has made many days, did not feel the power saving, the effect is not obvious.	Jingdong prefecture level city in Shandong
Happy Fan Shop	King of Power Saving	Energy savers do not save energy at all, but rather consume a lot of electricity.	Jingdong prefecture level city in Shandong
Happy Fan Shop	King of Power Saving	It's useless. It's a waste of money.	Jingdong prefecture level city in Shandong
Happy Fan Shop	King of Power Saving	Last month saved more than 500, which came back to buy, the store bought three, and then buy 1 home use, my family is dry beauty salon, more instruments, especially electricity, a month almost 1000. or so, with this did not expect to save so much, great, five-star praise!	Jingdong prefecture level city in Shandong
Happy Fan Shop	King of Power Saving	It rained a couple days ago and I just went to get the item back today , not bad, hey I looked at the meter and that red display light takes several seconds to jump, did not make a mistake buying it. After a period of time to evaluate.	Jingdong prefecture level city in Shandong
Happy Fan Shop	King of Power Saving	Without further ado, the electricity bill came down, this month less 102 yuan, has been repurchased, I also recommended to my friends, all said that there are effects, the product is okay, the effect is quite	Jingdong prefecture level city in Shandong
Happy Fan Shop	King of Power Saving	After buying and using two months really see the effect, so only to evaluate! Originally held the mindset of trying, I did not expect really effective, worth having!	Jingdong prefecture level city in Shandong
Happy Fan Shop	King of Power Saving	In the use of this energy saver before the monthly electricity bill is more than 150 yuan or so, now save thirty percent, every day to work, six or seven o'clock in the afternoon to go home, I do not know why the electricity bill is so expensive, energy-saving appliances really have the effect of recommending	Jingdong prefecture level city in Shandong
Happy Fan Shop	King of Power Saving	It saves about 100 bucks a month. A friend recommended it to me, and he's been using it for a while, and the effect is so obvious that I bought one too.	Jingdong prefecture level city in Shandong
Happy Fan Shop	King of Power Saving	My dad asked me to buy one, but after I paid the bill, I asked and he said it was a lot less this month, so he asked me to buy another one.	Jingdong prefecture level city in Shandong
Happy Fan Shop	King of Power Saving	The specific effect will have to be tested for at least two months to compare whether there is a power saving effect or not.	Jingdong prefecture level city in Shandong
Happy Fan Shop	King of Power Saving	'The goods have been received, immediately go to use, please ask the boss you instructions do not allow to withdraw, there are two fuses installed there!	Jingdong prefecture level city in Shandong
Happy Fan Shop	King of Power Saving	First praise is using, after using if good then follow up, now because I have not yet seen the results	Jingdong prefecture level city in Shandong
Happy Fan Shop	King of Power Saving	I've just been using it, and I haven't seen if it has a power-saving effect yet, so I'll follow up after 10 days and half a month.	Jingdong prefecture level city in Shandong
Happy Fan Shop	King of Power Saving	Just received the intelligent power saving meter, immediately plugged in to use, see it is not bad, and order to sell the second time	Jingdong prefecture level city in Shandong
Happy Fan Shop	King of Power Saving	This power saver is a bit effective, used for half a month, the effect is still visible.	Jingdong prefecture level city in Shandong
Happy Fan Shop	King of Power Saving	I received the baby and it looks great, I'm trying it out now and will keep an eye on it afterward.	Jingdong prefecture level city in Shandong
Happy Fan Shop	King of Power Saving	I've just received it, but I'm still trying to see how it works, so I'll come back in a little while.	Jingdong prefecture level city in Shandong
Happy Fan Shop	King of Power Saving	Just used it wait to use it for a while and then comment thanks, all first five stars.	Jingdong prefecture level city in Shandong
Happy Fan Shop	King of Power Saving	Very trustworthy brand, quality is guaranteed, the price is still affordable, quite satisfied!	Jingdong prefecture level city in Shandong
Happy Fan Shop	King of Power Saving	Things received, I hope that the platform of Jingdong do not sell fake goods are available	Jingdong prefecture level city in Shandong
Happy Fan Shop	King of Power Saving	Energy saver received, the use of the effect is good and the business description of the same	Jingdong prefecture level city in Shandong
Happy Fan Shop	King of Power Saving	The surface looks beautiful, the actual effect is not known, after using additional evaluation.	Jingdong prefecture level city in Shandong
Happy Fan Shop	King of Power Saving	Tried it for a few months, it really saves electricity, and I bought it for a friend.	Jingdong prefecture level city in Shandong
Happy Fan Shop	King of Power Saving	It didn't work and wanted to return it, it really didn't work at all.	Jingdong prefecture level city in Shandong
Happy Fan Shop	King of Power Saving	Just used it, don't know how effective it is, looking forward to usefulness	Jingdong prefecture level city in Shandong
Happy Fan Shop	King of Power Saving	After two months before the effect I do not know if it is true!	Jingdong prefecture level city in Shandong
Happy Fan Shop	King of Power Saving	So fast! Ordered yesterday and received today 👍👍👍👍👍👍👍👍👍 🌹🌹🌹🌹🌹🌹🌹🌹🌹	Jingdong prefecture level city in Shandong
Happy Fan Shop	King of Power Saving	I've started using it. We'll see how it works.	Jingdong prefecture level city in Shandong
Happy Fan Shop	King of Power Saving	I just received the goods, I won't know the effect until after two.	Jingdong prefecture level city in Shandong
Happy Fan Shop	King of Power Saving	Just received the goods, the specific effect will be evaluated later	Jingdong prefecture level city in Shandong
Happy Fan Shop	King of Power Saving	I've received the goods, I don't know if it's any good yet	Jingdong prefecture level city in Shandong

Happy Fan Shop	King of Power Saving	Try it for a month and then come back with a concrete evaluation	Jingdong prefecture level city in Shandong
Happy Fan Shop	King of Power Saving	In use, after two months to see the effect of power saving	Jingdong prefecture level city in Shandong
Happy Fan Shop	King of Power Saving	Power Saver. It's authentic. It's black. It works.	Jingdong prefecture level city in Shandong
Happy Fan Shop	King of Power Saving	I've been using it for a week now and it's not working.	Jingdong prefecture level city in Shandong
Happy Fan Shop	King of Power Saving	I'm just using it. I don't know how it's going to work.	Jingdong prefecture level city in Shandong
Happy Fan Shop	King of Power Saving	Products are better looking out for power savings	Jingdong prefecture level city in Shandong
Happy Fan Shop	King of Power Saving	I received it and I've kept it on. I hope it works.	Jingdong prefecture level city in Shandong
Happy Fan Shop	King of Power Saving	It's really good! Worth recommending to buy!	Jingdong prefecture level city in Shandong
Happy Fan Shop	King of Power Saving	Try it for a few days and see if it doesn't work, then return it.	Jingdong prefecture level city in Shandong
Happy Fan Shop	King of Power Saving	It's in use and I hope to be pleasantly surprised.	Jingdong prefecture level city in Shandong
Happy Fan Shop	King of Power Saving	We'll see how it works when we use it	Jingdong prefecture level city in Shandong
Happy Fan Shop	King of Power Saving	Let's see if it works.	Jingdong prefecture level city in Shandong
Happy Fan Shop	King of Power Saving	I'm using it and will follow up with a review	Jingdong prefecture level city in Shandong
Happy Fan Shop	King of Power Saving	It really works. It saves energy.	Jingdong prefecture level city in Shandong
Happy Fan Shop	King of Power Saving	It does have some power savings!	Jingdong prefecture level city in Shandong
Happy Fan Shop	King of Power Saving	Jingdong shopping speed is convenient and timely	Jingdong prefecture level city in Shandong
Happy Fan Shop	King of Power Saving	Second time I've bought it. Everything's fine.	Jingdong prefecture level city in Shandong
Happy Fan Shop	King of Power Saving	I don't know if it works yet.	Jingdong prefecture level city in Shandong
Happy Fan Shop	King of Power Saving	Not used, don't know how well it works.	Jingdong prefecture level city in Shandong
Happy Fan Shop	King of Power Saving	No visible results so far	Jingdong prefecture level city in Shandong
Happy Fan Shop	King of Power Saving	It doesn't feel like it's working.	Jingdong prefecture level city in Shandong
Happy Fan Shop	King of Power Saving	Where's the effect? Where are the power savings?	Jingdong prefecture level city in Shandong
Happy Fan Shop	King of Power Saving	Let's see how it works in practice!	Jingdong prefecture level city in Shandong
Happy Fan Shop	King of Power Saving	I'll wait until I use it to give you a good review	Jingdong prefecture level city in Shandong
Happy Fan Shop	King of Power Saving	I'll follow up after I've used it	Jingdong prefecture level city in Shandong
Happy Fan Shop	King of Power Saving	Well, that's good for saving power.	Jingdong prefecture level city in Shandong
Happy Fan Shop	King of Power Saving	Received. Used in the know.	Jingdong prefecture level city in Shandong
Happy Fan Shop	King of Power Saving	It doesn't do much good.	Jingdong prefecture level city in Shandong
Happy Fan Shop	King of Power Saving	It's not working.	Jingdong prefecture level city in Shandong
Happy Fan Shop	King of Power Saving	I don't know. I just used it.	Jingdong prefecture level city in Shandong
Happy Fan Shop	King of Power Saving	Use it and come back to evaluate.	Jingdong prefecture level city in Shandong
Happy Fan Shop	King of Power Saving	Two words: useless.	Jingdong prefecture level city in Shandong
Happy Fan Shop	King of Power Saving	To be used and then evaluated	Jingdong prefecture level city in Shandong
Happy Fan Shop	King of Power Saving	No shipment.	Jingdong prefecture level city in Shandong
Happy Fan Shop	King of Power Saving	Fake. Speechless.	Jingdong prefecture level city in Shandong
Happy Fan Shop	King of Power Saving	fantastic (idiom); awesome	Jingdong prefecture level city in Shandong
Happy Fan Shop	King of Power Saving	an excellent	Jingdong prefecture level city in Shandong
Happy Fan Shop	King of Power Saving	an excellent	Jingdong prefecture level city in Shandong
Happy Fan Shop	King of Power Saving	Want to buy! Recommended not to buy! No effect	Jingdong prefecture level city in Shandong
Happy Fan Shop	King of Power Saving	That's all there is to it. I can't figure out how to save electricity.	Jingdong prefecture level city in Shandong
Happy Fan Shop	King of Power Saving	It's no use.	Jingdong prefecture level city in Shandong
Happy Fan Shop	King of Power Saving	Things are more perfect than I imagined, very powerful, it is worth getting Packaging is very strong, things are well protected, it is worth the praise things are very satisfied Oh, beautiful and stylish atmosphere, customer service is super patient, service is meticulous and attentive, the big brand is not	Jingdong prefecture level city in Shandong
Happy Fan Shop	King of Power Saving	The effect is too good, the family has now several units, back to buy a number of times, the power saver received, power-saving effect is very good,, fine workmanship, long warranty, but also purify the air, before buying a used for a period of time, with the okay, and then bought a, and only now come to the	Jingdong prefecture level city in Shandong
Happy Fan Shop	King of Power Saving	Quite good, and imagine the same, casual ride are very good, satisfied! Baby received it 👌, really great ah! 🌈 quality is really nothing to say, very satisfied! 🆗 happy happy 😃 genuine, great, the price is very good, in short, quite good, will buy again a very good shopping experience, attentive service, fast logistics, distribution is also thoughtful, the attitude is also very good, value for money, ah,	Jingdong prefecture level city in Shandong
Happy Fan Shop	King of Power Saving	Power-saving effect: strong Cost-effective: quite good Appearance style: beautiful Workmanship material: meticulous Experience: looking forward to power-saving effect	Jingdong prefecture level city in Shandong
Happy Fan Shop	King of Power Saving	Cost-effective is okay, the appearance of the style of meticulous workmanship, looking forward to energy-saving effect.	Jingdong prefecture level city in Shandong
Happy Fan Shop	King of Power Saving	Very Good Very Power Saving This Power Saver is really power saving As soon as the power is turned on, the Power Saver starts the power saving function for you.	Jingdong prefecture level city in Shandong
Happy Fan Shop	King of Power Saving	Logistics is very fast, the price is favorable, friends introduced that it is very good to use, bought 2 at once, looking forward to the effect, used after the follow-up comment.	Jingdong prefecture level city in Shandong
Happy Fan Shop	King of Power Saving	It's all fake. It doesn't save any electricity.	Jingdong prefecture level city in Shandong

Happy Fan Shop	King of Power Saving	This Power Saver is so much better than the one I bought before, not only the effect is good, the quality is very good in all aspects, the customer service attitude is also very good, recommended to buy!	Jingdong prefecture level city in Shandong
Happy Fan Shop	King of Power Saving	Logistics is very fast, buy for parents, good quality, once bought 2, power-saving effect is good to buy 2 more, look forward to using the effect, satisfied will recommend to friends.	Jingdong prefecture level city in Shandong
Happy Fan Shop	King of Power Saving	With the description is the same, very high-grade, good value, to buy friends can order Oh!	Jingdong prefecture level city in Shandong
Happy Fan Shop	King of Power Saving	Not using much electricity in the winter saving over 100 per month! Ready to buy 2 more units in the summer ! ! ! ! ! Good quality, good price, power saving effect is obvious, cost-effective, worth buying, can be recommended!	Jingdong prefecture level city in Shandong
Happy Fan Shop	King of Power Saving	Purchased a power saver received on the put into use, rent a room with many electrical appliances, power consumption is very large, we have a piece of electricity or 1.2 dollars a time, with this power saver is much better, every day can save a lot of money, very convenient and practical.	Jingdong prefecture level city in Shandong
Happy Fan Shop	King of Power Saving	The power saving effect is very good: my supermarket monthly electricity bill is more than 7000 yuan. On May 1st someone recommended buying a couple of power savers, and I bought 5 of them first. The electricity bill was about 2000 less in June. This time, I bought 5 more. The seller said 800 degrees of electricity with one. I feel it's basically enough. A total of 10 units	Jingdong prefecture level city in Shandong
Happy Fan Shop	King of Power Saving	I never thought I could get such a good baby at such an affordable price, it's very good value for money, I love it!	Jingdong prefecture level city in Shandong
Happy Fan Shop	King of Power Saving	Previously bought other brands of power savers, but found no effect at home, this time to see how the products on the Jingdong, inserted in the home socket, the LED display is blue, the night light is very nice to see, look at the instructions that have to be half a year in order to achieve the best results, try it, I believe that the quality of the Jingdong, it should not be wrong.	Jingdong prefecture level city in Shandong
Happy Fan Shop	King of Power Saving	Superb, inexpensive, saves a lot of points, been using it for a long time!	Jingdong prefecture level city in Shandong
Happy Fan Shop	King of Power Saving	There are a lot of appliances in the house, the summer air conditioning is very costly, I bought this in front of the air conditioning effect is very good, I explain that I bought three in one breath, I use more electricity a month down to 200 or so, very satisfied.	Jingdong prefecture level city in Shandong
Happy Fan Shop	King of Power Saving	A month can save more than 200, home monthly electricity bill of more than 600 dollars, inserted 3 or so, an amount of the balance of the average can save more than 200. Product is very good, has been buying this one, has given a lot of relatives recommended, the quality is also very good, before buying a look with this same, who knows to buy home has not been used for more than a week on the bad, the quality of this is really good with more than two years!	Jingdong prefecture level city in Shandong
Happy Fan Shop	King of Power Saving	Whole house power saving king, can really save electricity, friends recommended, high power saving appliances, only replacement, no repair, after-sales worry-free!	Jingdong prefecture level city in Shandong
Happy Fan Shop	King of Power Saving	Great item love it, great customer service, great shopping experience!	Jingdong prefecture level city in Shandong
Happy Fan Shop	King of Power Saving	Power saving, very good use, much cheaper than the physical store, satisfaction	Jingdong prefecture level city in Shandong
Happy Fan Shop	King of Power Saving	In use, very well packaged and fast delivery of credit!	Jingdong prefecture level city in Shandong
Happy Fan Shop	King of Power Saving	Product quality, customer service attitude is particularly good, like, praise!	Jingdong prefecture level city in Shandong
Happy Fan Shop	King of Power Saving	The logistics is very fast, the packaging is not broken, the quality is very good, I like it very much!	Jingdong prefecture level city in Shandong
Happy Fan Shop	King of Power Saving	The saver was well packaged and was as described when plugged in	Jingdong prefecture level city in Shandong
Happy Fan Shop	King of Power Saving	Energy efficient and very satisfied	Jingdong prefecture level city in Shandong
Happy Fan Shop	King of Power Saving	Great quality for the price and fast shipping.	Jingdong prefecture level city in Shandong
Happy Fan Shop	King of Power Saving	It's great, just as described. Perfect.	Jingdong prefecture level city in Shandong
Happy Fan Shop	King of Power Saving	There is no power saving effect, but rather ring up an increase in electricity costs, because this machine is on continuously all day long, uninterruptedly, its own electricity and increased costs, plus the cost of purchasing the machine, so I feel that the use of this machine instead of increasing the	Jingdong prefecture level city in Shandong
Happy Fan Shop	King of Power Saving	Recommended by the owner of the store next door, looked at his home does have to save electricity, decided to get. Indeed quite can, the voltage is much more stable, there is a reduction in electricity	Jingdong prefecture level city in Shandong
Happy Fan Shop	King of Power Saving	Goods have been received value for money, affordable and reasonable price, and the description of the same, power-saving safe and reliable!	Jingdong prefecture level city in Shandong
Happy Fan Shop	King of Power Saving	Listen to friends introduced to buy, tried to save a lot of electricity is good, quite good, worth buying.	Jingdong prefecture level city in Shandong
Happy Fan Shop	King of Power Saving	The product is easy to use, safe and environmentally friendly, only used for a short time, to the next month, after the electricity bill status display to know how the performance of the power saver efficacy.	Jingdong prefecture level city in Shandong
Happy Fan Shop	King of Power Saving	Last month's electricity bill is 178 yuan, with this power saver is 92 yuan, ready to buy two more back home, I was introduced to buy a friend, just started to not believe that I did not expect the effect!	Jingdong prefecture level city in Shandong
Happy Fan Shop	King of Power Saving	It looks nice, but after using it for a few days I can't see how it saves power?	Jingdong prefecture level city in Shandong
Happy Fan Shop	King of Power Saving	Recommended by a friend, it is genuine, as described by the merchant, cost-effective	Jingdong prefecture level city in Shandong
Happy Fan Shop	King of Power Saving	Works well, affordable and saves money.	Jingdong prefecture level city in Shandong
Happy Fan Shop	King of Power Saving	Feels good haven't compared it yet, the air conditioning has been on a lot in the past two months	Jingdong prefecture level city in Shandong
Happy Fan Shop	King of Power Saving	Saving about 5 kWh per day is a good thing	Jingdong prefecture level city in Shandong
Happy Fan Shop	King of Power Saving	Not bad, affordable, best value for money	Jingdong prefecture level city in Shandong
Happy Fan Shop	King of Power Saving	The goods are genuine and the logistics are faster.	Jingdong prefecture level city in Shandong
Happy Fan Shop	King of Power Saving	Goods have been received today, has been plugged into the outlet, looking forward to saving electricity, after a period of time to see how the effect in the follow-up comment!	Jingdong prefecture level city in Shandong
Happy Fan Shop	King of Power Saving	I just got it home and haven't used it for long, so I'll use it for a couple months and see what happens. Seeing that most people have good reviews, it should be fine.	Jingdong prefecture level city in Shandong
Happy Fan Shop	King of Power Saving	I'm looking forward to the results, so I'll give you a good review out of habit! Follow up review after a while	Jingdong prefecture level city in Shandong
Happy Fan Shop	King of Power Saving	Use a dish first to know if it will help save energy	Jingdong prefecture level city in Shandong
Happy Fan Shop	King of Power Saving	Whether it saves electricity or not, we don't know yet.	Jingdong prefecture level city in Shandong
Happy Fan Shop	King of Power Saving	Goods have been received, use a period of time to know how oh. The courier did not deliver to the door ah! Sent to the station ah! The heat is inconvenient ah!	Jingdong prefecture level city in Shandong
Happy Fan Shop	King of Power Saving	A little effect, before an hour a power, now an hour 0.76 degrees, a dollar a power = save 24 cents, although not a lot, first use it!	Jingdong prefecture level city in Shandong
Happy Fan Shop	King of Power Saving	Just received the goods, use a month to evaluate	Jingdong prefecture level city in Shandong
Happy Fan Shop	King of Power Saving	Home every month with a lot of electricity, inadvertently found this good thing online that can save the beginning of a skeptical attitude to consult customer service, customer service replied very professional, said 120 days can be refunded that first try it!	Jingdong prefecture level city in Shandong
Happy Fan Shop	King of Power Saving	After receiving the goods this afternoon, immediately installed in the bathroom Smith energy storage electric water heater on the power outlet (instead of connecting with a plug board), when used, found that the "Power Saving King" LCD display, is the value of the voltage, not the store's video said so	Jingdong prefecture level city in Shandong
Happy Fan Shop	King of Power Saving	Has made many days, did not feel the power saving, the effect is not obvious.	Jingdong prefecture level city in Shandong

Happy Fan Shop	King of Power Saving	Energy savers do not save energy at all, but rather consume a lot of electricity.	Jingdong prefecture level city in Shandong
Happy Fan Shop	King of Power Saving	It's useless. It's a waste of money.	Jingdong prefecture level city in Shandong
Happy Fan Shop	King of Power Saving	Last month saved more than 500, which came back to buy, the store bought three, and then buy 1 home use, my family is dry beauty salon, more instruments, especially electricity, a month almost 1000. or so, with this did not expect to save so much, great, five-star praise!	Jingdong prefecture level city in Shandong
Happy Fan Shop	King of Power Saving	It rained a couple days ago and I just went to get the item back today , not bad, hey I looked at the meter and that red display light takes several seconds to jump, did not make a mistake buying it. After a period of time to evaluate.	Jingdong prefecture level city in Shandong
Happy Fan Shop	King of Power Saving	Without further ado, the electricity bill came down, this month less 102 yuan, has been repurchased, I also recommended to my friends, all said that there are effects, the product is okay, the effect is quite	Jingdong prefecture level city in Shandong
Happy Fan Shop	King of Power Saving	After buying and using two months really see the effect, so only to evaluate! Originally held the mindset of trying, I did not expect really effective, worth having!	Jingdong prefecture level city in Shandong
Happy Fan Shop	King of Power Saving	In the use of this energy saver before the monthly electricity bill is more than 150 yuan or so, now save thirty percent, every day to work, six or seven o'clock in the afternoon to go home, I do not know why the electricity bill is so expensive, energy-saving appliances really have the effect of recommending	Jingdong prefecture level city in Shandong
Happy Fan Shop	King of Power Saving	It saves about 100 bucks a month. A friend recommended it to me, and he's been using it for a while, and the effect is so obvious that I bought one too.	Jingdong prefecture level city in Shandong
Happy Fan Shop	King of Power Saving	My dad asked me to buy one, but after I paid the bill, I asked and he said it was a lot less this month, so he asked me to buy another one.	Jingdong prefecture level city in Shandong
Happy Fan Shop	King of Power Saving	The specific effect will have to be tested for at least two months to compare whether there is a power saving effect or not.	Jingdong prefecture level city in Shandong
Happy Fan Shop	King of Power Saving	'The goods have been received, immediately go to use, please ask the boss you instructions do not allow to withdraw, there are two fuses installed there!	Jingdong prefecture level city in Shandong
Happy Fan Shop	King of Power Saving	First praise is using, after using if good then follow up, now because I have not yet seen the results	Jingdong prefecture level city in Shandong
Happy Fan Shop	King of Power Saving	I've just been using it, and I haven't seen if it has a power-saving effect yet, so I'll follow up after 10 days and half a month.	Jingdong prefecture level city in Shandong
Happy Fan Shop	King of Power Saving	Just received the intelligent power saving meter, immediately plugged in to use, see it is not bad, and order to sell the second time	Jingdong prefecture level city in Shandong
Happy Fan Shop	King of Power Saving	This power saver is a bit effective, used for half a month, the effect is still visible.	Jingdong prefecture level city in Shandong
Happy Fan Shop	King of Power Saving	I received the baby and it looks great, I'm trying it out now and will keep an eye on it afterward.	Jingdong prefecture level city in Shandong
Happy Fan Shop	King of Power Saving	I've just received it, but I'm still trying to see how it works, so I'll come back in a little while.	Jingdong prefecture level city in Shandong
Happy Fan Shop	King of Power Saving	Just used it wait to use it for a while and then comment thanks, all first five stars.	Jingdong prefecture level city in Shandong
Happy Fan Shop	King of Power Saving	Very trustworthy brand, quality is guaranteed, the price is still affordable, quite satisfied!	Jingdong prefecture level city in Shandong
Happy Fan Shop	King of Power Saving	Things received, I hope that the platform of Jingdong do not sell fake goods are available	Jingdong prefecture level city in Shandong
Happy Fan Shop	King of Power Saving	Energy saver received, the use of the effect is good and the business description of the same	Jingdong prefecture level city in Shandong
Happy Fan Shop	King of Power Saving	The surface looks beautiful, the actual effect is not known, after using additional evaluation.	Jingdong prefecture level city in Shandong
Happy Fan Shop	King of Power Saving	Tried it for a few months, it really saves electricity, and I bought it for a friend.	Jingdong prefecture level city in Shandong
Happy Fan Shop	King of Power Saving	It didn't work and wanted to return it, it really didn't work at all.	Jingdong prefecture level city in Shandong
Happy Fan Shop	King of Power Saving	Just used it, don't know how effective it is, looking forward to usefulness	Jingdong prefecture level city in Shandong
Happy Fan Shop	King of Power Saving	After two months before the effect I do not know if it is true!	Jingdong prefecture level city in Shandong
Happy Fan Shop	King of Power Saving	So fast! Ordered yesterday and received today 👍👍👍👍👍👍👍👍👍 🌹🌹🌹🌹🌹🌹🌹🌹🌹	Jingdong prefecture level city in Shandong
Happy Fan Shop	King of Power Saving	I've started using it. We'll see how it works.	Jingdong prefecture level city in Shandong
Happy Fan Shop	King of Power Saving	I just received the goods, I won't know the effect until after two.	Jingdong prefecture level city in Shandong
Happy Fan Shop	King of Power Saving	Just received the goods, the specific effect will be evaluated later	Jingdong prefecture level city in Shandong
Happy Fan Shop	King of Power Saving	I've received the goods, I don't know if it's any good yet	Jingdong prefecture level city in Shandong
Happy Fan Shop	King of Power Saving	Try it for a month and then come back with a concrete evaluation	Jingdong prefecture level city in Shandong
Happy Fan Shop	King of Power Saving	In use, after two months to see the effect of power saving	Jingdong prefecture level city in Shandong
Happy Fan Shop	King of Power Saving	Power Saver. It's authentic. It's black. It works.	Jingdong prefecture level city in Shandong
Happy Fan Shop	King of Power Saving	I've been using it for a week now and it's not working.	Jingdong prefecture level city in Shandong
Happy Fan Shop	King of Power Saving	I'm just using it. I don't know how it's going to work.	Jingdong prefecture level city in Shandong
Happy Fan Shop	King of Power Saving	Products are better looking out for power savings	Jingdong prefecture level city in Shandong
Happy Fan Shop	King of Power Saving	I received it and I've kept it on. I hope it works.	Jingdong prefecture level city in Shandong
Happy Fan Shop	King of Power Saving	It's really good! Worth recommending to buy!	Jingdong prefecture level city in Shandong
Happy Fan Shop	King of Power Saving	Try it for a few days and see if it doesn't work, then return it.	Jingdong prefecture level city in Shandong
Happy Fan Shop	King of Power Saving	It's in use and I hope to be pleasantly surprised.	Jingdong prefecture level city in Shandong
Happy Fan Shop	King of Power Saving	We'll see how it works when we use it	Jingdong prefecture level city in Shandong
Happy Fan Shop	King of Power Saving	Let's see if it works.	Jingdong prefecture level city in Shandong
Happy Fan Shop	King of Power Saving	I'm using it and will follow up with a review	Jingdong prefecture level city in Shandong
Happy Fan Shop	King of Power Saving	It really works. It saves energy.	Jingdong prefecture level city in Shandong
Happy Fan Shop	King of Power Saving	It does have some power savings!	Jingdong prefecture level city in Shandong
Happy Fan Shop	King of Power Saving	Jingdong shopping speed is convenient and timely	Jingdong prefecture level city in Shandong
Happy Fan Shop	King of Power Saving	Second time I've bought it. Everything's fine.	Jingdong prefecture level city in Shandong
Happy Fan Shop	King of Power Saving	I don't know if it works yet.	Jingdong prefecture level city in Shandong
Happy Fan Shop	King of Power Saving	Not used, don't know how well it works.	Jingdong prefecture level city in Shandong

Happy Fan Shop	King of Power Saving	No visible results so far	Jingdong prefecture level city in Shandong
Happy Fan Shop	King of Power Saving	It doesn't feel like it's working.	Jingdong prefecture level city in Shandong
Happy Fan Shop	King of Power Saving	Where's the effect? Where are the power savings?	Jingdong prefecture level city in Shandong
Happy Fan Shop	King of Power Saving	Let's see how it works in practice!	Jingdong prefecture level city in Shandong
Happy Fan Shop	King of Power Saving	I'll wait until I use it to give you a good review	Jingdong prefecture level city in Shandong
Happy Fan Shop	King of Power Saving	I'll follow up after I've used it	Jingdong prefecture level city in Shandong
Happy Fan Shop	King of Power Saving	Well, that's good for saving power.	Jingdong prefecture level city in Shandong
Happy Fan Shop	King of Power Saving	Received. Used in the know.	Jingdong prefecture level city in Shandong
Happy Fan Shop	King of Power Saving	It doesn't do much good.	Jingdong prefecture level city in Shandong
Happy Fan Shop	King of Power Saving	It's not working.	Jingdong prefecture level city in Shandong
Happy Fan Shop	King of Power Saving	I don't know. I just used it.	Jingdong prefecture level city in Shandong
Happy Fan Shop	King of Power Saving	Use it and come back to evaluate.	Jingdong prefecture level city in Shandong
Happy Fan Shop	King of Power Saving	Two words: useless.	Jingdong prefecture level city in Shandong
Happy Fan Shop	King of Power Saving	To be used and then evaluated	Jingdong prefecture level city in Shandong
Happy Fan Shop	King of Power Saving	No shipment.	Jingdong prefecture level city in Shandong
Happy Fan Shop	King of Power Saving	Fake. Speechless.	Jingdong prefecture level city in Shandong
Happy Fan Shop	King of Power Saving	fantastic (idiom); awesome	Jingdong prefecture level city in Shandong
Happy Fan Shop	King of Power Saving	an excellent	Jingdong prefecture level city in Shandong
Happy Fan Shop	King of Power Saving	an excellent	Jingdong prefecture level city in Shandong
Happy Fan Shop	King of Power Saving	Want to buy! Recommended not to buy! No effect	Jingdong prefecture level city in Shandong
Happy Fan Shop	King of Power Saving	That's all there is to it. I can't figure out how to save electricity.	Jingdong prefecture level city in Shandong
Happy Fan Shop	King of Power Saving	It's no use.	Jingdong prefecture level city in Shandong
Happy Fan Shop	King of Power Saving	Things are more perfect than I imagined, very powerful, it is worth getting Packaging is very strong, things are well protected, it is worth the praise things are very satisfied Oh, beautiful and stylish atmosphere, customer service is super patient, service is meticulous and attentive, the big brand is not	Jingdong prefecture level city in Shandong
Happy Fan Shop	King of Power Saving	The effect is too good, the family has now several units, back to buy a number of times, the power saver received, power-saving effect is very good,, fine workmanship, long warranty, but also purify the air, before buying a used for a period of time, with the okay, and then bought a, and only now come to the	Jingdong prefecture level city in Shandong
Happy Fan Shop	King of Power Saving	Quite good, and imagine the same, casual ride are very good, satisfied! Baby received it 👌, really great ah! 🌈 quality is really nothing to say, very satisfied! 🆗 happy happy 😃 genuine, great, the price is very good, in short, quite good, will buy again a very good shopping experience, attentive service, fast logistics, distribution is also thoughtful, the attitude is also very good, value for money, ah,	Jingdong prefecture level city in Shandong
Happy Fan Shop	King of Power Saving	Power-saving effect: strong Cost-effective: quite good Appearance style: beautiful Workmanship material: meticulous Experience: looking forward to power-saving effect	Jingdong prefecture level city in Shandong
Happy Fan Shop	King of Power Saving	Cost-effective is okay, the appearance of the style of meticulous workmanship, looking forward to energy-saving effect.	Jingdong prefecture level city in Shandong
Happy Fan Shop	King of Power Saving	Very Good Very Power Saving This Power Saver is really power saving As soon as the power is turned on, the Power Saver starts the power saving function for you.	Jingdong prefecture level city in Shandong
Happy Fan Shop	King of Power Saving	Logistics is very fast, the price is favorable, friends introduced that it is very good to use, bought 2 at once, looking forward to the effect, used after the follow-up comment.	Jingdong prefecture level city in Shandong
Happy Fan Shop	King of Power Saving	It's all fake. It doesn't save any electricity.	Jingdong prefecture level city in Shandong
Happy Fan Shop	King of Power Saving	This Power Saver is so much better than the one I bought before, not only the effect is good, the quality is very good in all aspects, the customer service attitude is also very good, recommended to buy!	Jingdong prefecture level city in Shandong
Happy Fan Shop	King of Power Saving	Logistics is very fast, buy for parents, good quality, once bought 2, power-saving effect is good to buy 2 more, look forward to using the effect, satisfied will recommend to friends.	Jingdong prefecture level city in Shandong
Happy Fan Shop	King of Power Saving	With the description is the same, very high-grade, good value, to buy friends can order Oh!	Jingdong prefecture level city in Shandong
Happy Fan Shop	King of Power Saving	Not using much electricity in the winter saving over 100 per month! Ready to buy 2 more units in the summer ! ! ! ! ! Good quality, good price, power saving effect is obvious, cost-effective, worth buying, can be recommended!	Jingdong prefecture level city in Shandong
Happy Fan Shop	King of Power Saving	Purchased a power saver received on the put into use, rent a room with many electrical appliances, power consumption is very large, we have a piece of electricity or 1.2 dollars a time, with this power saver is much better, every day can save a lot of money, very convenient and practical.	Jingdong prefecture level city in Shandong
Happy Fan Shop	King of Power Saving	The power saving effect is very good: my supermarket monthly electricity bill is more than 7000 yuan. On May 1st someone recommended buying a couple of power savers, and I bought 5 of them first. The electricity bill was about 2000 less in June. This time, I bought 5 more. The seller said 800 degrees of electricity with one. I feel it's basically enough. A total of 10 units	Jingdong prefecture level city in Shandong
Happy Fan Shop	King of Power Saving	I never thought I could get such a good baby at such an affordable price, it's very good value for money, I love it!	Jingdong prefecture level city in Shandong
Happy Fan Shop	King of Power Saving	Previously bought other brands of power savers, but found no effect at home, this time to see how the products on the Jingdong, inserted in the home socket, the LED display is blue, the night light is very nice to see, look at the instructions that have to be half a year in order to achieve the best results, try it, I believe that the quality of the Jingdong, it should not be wrong.	Jingdong prefecture level city in Shandong
Happy Fan Shop	King of Power Saving	Superb, inexpensive, saves a lot of points, been using it for a long time!	Jingdong prefecture level city in Shandong
Happy Fan Shop	King of Power Saving	There are a lot of appliances in the house, the summer air conditioning is very costly, I bought this in front of the air conditioning effect is very good, I explain that I bought three in one breath, I use more electricity a month down to 200 or so, very satisfied.	Jingdong prefecture level city in Shandong
Happy Fan Shop	King of Power Saving	A month can save more than 200, home monthly electricity bill of more than 600 dollars, inserted 3 or so, an amount of the balance of the average can save more than 200. Product is very good, has been buying this one, has given a lot of relatives recommended, the quality is also very good, before buying a look with this same, who knows to buy home has not been used for more than a week on the bad, the quality of this is really good with more than two years!	Jingdong prefecture level city in Shandong
Happy Fan Shop	King of Power Saving	Whole house power saving king, can really save electricity, friends recommended, high power saving appliances, only replacement, no repair, after-sales worry-free!	Jingdong prefecture level city in Shandong
Happy Fan Shop	King of Power Saving	Great item love it, great customer service, great shopping experience!	Jingdong prefecture level city in Shandong
Happy Fan Shop	King of Power Saving	Power saving, very good use, much cheaper than the physical store, satisfaction	Jingdong prefecture level city in Shandong

Happy Fan Shop	King of Power Saving	In use, very well packaged and fast delivery of credit!	Jingdong prefecture level city in Shandong
Happy Fan Shop	King of Power Saving	Product quality, customer service attitude is particularly good, like, praise!	Jingdong prefecture level city in Shandong
Happy Fan Shop	King of Power Saving	The logistics is very fast, the packaging is not broken, the quality is very good, I like it very much!	Jingdong prefecture level city in Shandong
Happy Fan Shop	King of Power Saving	The saver was well packaged and was as described when plugged in	Jingdong prefecture level city in Shandong
Happy Fan Shop	King of Power Saving	Energy efficient and very satisfied	Jingdong prefecture level city in Shandong
Happy Fan Shop	King of Power Saving	Great quality for the price and fast shipping.	Jingdong prefecture level city in Shandong
Happy Fan Shop	King of Power Saving	It's great, just as described. Perfect.	Jingdong prefecture level city in Shandong
Happy Fan Shop	King of Power Saving	There is no power saving effect, but rather ring up an increase in electricity costs, because this machine is on continuously all day long, uninterruptedly, its own electricity and increased costs, plus the cost of purchasing the machine, so I feel that the use of this machine instead of increasing the	Jingdong prefecture level city in Shandong
Happy Fan Shop	King of Power Saving	Recommended by the owner of the store next door, looked at his home does have to save electricity, decided to get. Indeed quite can, the voltage is much more stable, there is a reduction in electricity	Jingdong prefecture level city in Shandong
Happy Fan Shop	King of Power Saving	Goods have been received value for money, affordable and reasonable price, and the description of the same, power-saving safe and reliable!	Jingdong prefecture level city in Shandong
Happy Fan Shop	King of Power Saving	Listen to friends introduced to buy, tried to save a lot of electricity is good, quite good, worth buying.	Jingdong prefecture level city in Shandong
Happy Fan Shop	King of Power Saving	The product is easy to use, safe and environmentally friendly, only used for a short time, to the next month, after the electricity bill status display to know how the performance of the power saver efficacy.	Jingdong prefecture level city in Shandong
Happy Fan Shop	King of Power Saving	Last month's electricity bill is 178 yuan, with this power saver is 92 yuan, ready to buy two more back home, I was introduced to buy a friend, just started to not believe that I did not expect the effect!	Jingdong prefecture level city in Shandong
Happy Fan Shop	King of Power Saving	It looks nice, but after using it for a few days I can't see how it saves power?	Jingdong prefecture level city in Shandong
Happy Fan Shop	King of Power Saving	Recommended by a friend, it is genuine, as described by the merchant, cost-effective	Jingdong prefecture level city in Shandong
Happy Fan Shop	King of Power Saving	Works well, affordable and saves money.	Jingdong prefecture level city in Shandong
Happy Fan Shop	King of Power Saving	Feels good haven't compared it yet, the air conditioning has been on a lot in the past two months	Jingdong prefecture level city in Shandong
Happy Fan Shop	King of Power Saving	Saving about 5 kWh per day is a good thing	Jingdong prefecture level city in Shandong
Happy Fan Shop	King of Power Saving	Not bad, affordable, best value for money	Jingdong prefecture level city in Shandong
Happy Fan Shop	King of Power Saving	The goods are genuine and the logistics are faster.	Jingdong prefecture level city in Shandong
Happy Fan Shop	King of Power Saving	Goods have been received today, has been plugged into the outlet, looking forward to saving electricity, after a period of time to see how the effect in the follow-up comment!	Jingdong prefecture level city in Shandong
Happy Fan Shop	King of Power Saving	I just got it home and haven't used it for long, so I'll use it for a couple months and see what happens. Seeing that most people have good reviews, it should be fine.	Jingdong prefecture level city in Shandong
Happy Fan Shop	King of Power Saving	I'm looking forward to the results, so I'll give you a good review out of habit! Follow up review after a while	Jingdong prefecture level city in Shandong
Happy Fan Shop	King of Power Saving	Use a dish first to know if it will help save energy	Jingdong prefecture level city in Shandong
Happy Fan Shop	King of Power Saving	Whether it saves electricity or not, we don't know yet.	Jingdong prefecture level city in Shandong
Happy Fan Shop	King of Power Saving	Goods have been received, use a period of time to know how oh. The courier did not deliver to the door ah! Sent to the station ah! The heat is inconvenient ah!	Jingdong prefecture level city in Shandong
Happy Fan Shop	King of Power Saving	A little effect, before an hour a power, now an hour 0.76 degrees, a dollar a power = save 24 cents, although not a lot, first use it!	Jingdong prefecture level city in Shandong
Happy Fan Shop	King of Power Saving	Just received the goods, use a month to evaluate	Jingdong prefecture level city in Shandong
Happy Fan Shop	King of Power Saving	Home every month with a lot of electricity, inadvertently found this good thing online that can save the beginning of a skeptical attitude to consult customer service, customer service replied very professional, said 120 days can be refunded that first try it!	Jingdong prefecture level city in Shandong
Happy Fan Shop	King of Power Saving	After receiving the goods this afternoon, immediately installed in the bathroom Smith energy storage electric water heater on the power outlet (instead of connecting with a plug board), when used, found that the "Power Saving King" LCD display, is the value of the voltage, not the store's video said so	Jingdong prefecture level city in Shandong
Happy Fan Shop	King of Power Saving	Has made many days, did not feel the power saving, the effect is not obvious.	Jingdong prefecture level city in Shandong
Happy Fan Shop	King of Power Saving	Energy savers do not save energy at all, but rather consume a lot of electricity.	Jingdong prefecture level city in Shandong
Happy Fan Shop	King of Power Saving	It's useless. It's a waste of money.	Jingdong prefecture level city in Shandong
Happy Fan Shop	King of Power Saving	Last month saved more than 500, which came back to buy, the store bought three, and then buy 1 home use, my family is dry beauty salon, more instruments, especially electricity, a month almost 1000. or so, with this did not expect to save so much, great, five-star praise!	Jingdong prefecture level city in Shandong
Happy Fan Shop	King of Power Saving	It rained a couple days ago and I just went to get the item back today , not bad, hey I looked at the meter and that red display light takes several seconds to jump, did not make a mistake buying it. After a period of time to evaluate.	Jingdong prefecture level city in Shandong
Happy Fan Shop	King of Power Saving	Without further ado, the electricity bill came down, this month less 102 yuan, has been repurchased, I also recommended to my friends, all said that there are effects, the product is okay, the effect is quite	Jingdong prefecture level city in Shandong
Happy Fan Shop	King of Power Saving	After buying and using two months really see the effect, so only to evaluate! Originally held the mindset of trying, I did not expect really effective, worth having!	Jingdong prefecture level city in Shandong
Happy Fan Shop	King of Power Saving	In the use of this energy saver before the monthly electricity bill is more than 150 yuan or so, now save thirty percent, every day to work, six or seven o'clock in the afternoon to go home, I do not know why the electricity bill is so expensive, energy-saving appliances really have the effect of recommending	Jingdong prefecture level city in Shandong
Happy Fan Shop	King of Power Saving	It saves about 100 bucks a month. A friend recommended it to me, and he's been using it for a while, and the effect is so obvious that I bought one too.	Jingdong prefecture level city in Shandong
Happy Fan Shop	King of Power Saving	My dad asked me to buy one, but after I paid the bill, I asked and he said it was a lot less this month, so he asked me to buy another one.	Jingdong prefecture level city in Shandong
Happy Fan Shop	King of Power Saving	The specific effect will have to be tested for at least two months to compare whether there is a power saving effect or not.	Jingdong prefecture level city in Shandong
Happy Fan Shop	King of Power Saving	'The goods have been received, immediately go to use, please ask the boss you instructions do not allow to withdraw, there are two fuses installed there!	Jingdong prefecture level city in Shandong
Happy Fan Shop	King of Power Saving	First praise is using, after using if good then follow up, now because I have not yet seen the results	Jingdong prefecture level city in Shandong
Happy Fan Shop	King of Power Saving	I've just been using it, and I haven't seen if it has a power-saving effect yet, so I'll follow up after 10 days and half a month.	Jingdong prefecture level city in Shandong
Happy Fan Shop	King of Power Saving	Just received the intelligent power saving meter, immediately plugged in to use, see it is not bad, and order to sell the second time	Jingdong prefecture level city in Shandong
Happy Fan Shop	King of Power Saving	This power saver is a bit effective, used for half a month, the effect is still visible.	Jingdong prefecture level city in Shandong
Happy Fan Shop	King of Power Saving	I received the baby and it looks great, I'm trying it out now and will keep an eye on it afterward.	Jingdong prefecture level city in Shandong
Happy Fan Shop	King of Power Saving	I've just received it, but I'm still trying to see how it works, so I'll come back in a little while.	Jingdong prefecture level city in Shandong

Happy Fan Shop	King of Power Saving	Just used it wait to use it for a while and then comment thanks, all first five stars.	Jingdong prefecture level city in Shandong
Happy Fan Shop	King of Power Saving	Very trustworthy brand, quality is guaranteed, the price is still affordable, quite satisfied!	Jingdong prefecture level city in Shandong
Happy Fan Shop	King of Power Saving	Things received, I hope that the platform of Jingdong do not sell fake goods are available	Jingdong prefecture level city in Shandong
Happy Fan Shop	King of Power Saving	Energy saver received, the use of the effect is good and the business description of the same	Jingdong prefecture level city in Shandong
Happy Fan Shop	King of Power Saving	The surface looks beautiful, the actual effect is not known, after using additional evaluation.	Jingdong prefecture level city in Shandong
Happy Fan Shop	King of Power Saving	Tried it for a few months, it really saves electricity, and I bought it for a friend.	Jingdong prefecture level city in Shandong
Happy Fan Shop	King of Power Saving	It didn't work and wanted to return it, it really didn't work at all.	Jingdong prefecture level city in Shandong
Happy Fan Shop	King of Power Saving	Just used it, don't know how effective it is, looking forward to usefulness	Jingdong prefecture level city in Shandong
Happy Fan Shop	King of Power Saving	After two months before the effect I do not know if it is true!	Jingdong prefecture level city in Shandong
Happy Fan Shop	King of Power Saving	So fast! Ordered yesterday and received today 👍👍👍👍👍👍👍👍👍 🌹🌹🌹🌹🌹🌹🌹🌹🌹	Jingdong prefecture level city in Shandong
Happy Fan Shop	King of Power Saving	I've started using it. We'll see how it works.	Jingdong prefecture level city in Shandong
Happy Fan Shop	King of Power Saving	I just received the goods, I won't know the effect until after two.	Jingdong prefecture level city in Shandong
Happy Fan Shop	King of Power Saving	Just received the goods, the specific effect will be evaluated later	Jingdong prefecture level city in Shandong
Happy Fan Shop	King of Power Saving	I've received the goods, I don't know if it's any good yet	Jingdong prefecture level city in Shandong
Happy Fan Shop	King of Power Saving	Try it for a month and then come back with a concrete evaluation	Jingdong prefecture level city in Shandong
Happy Fan Shop	King of Power Saving	In use, after two months to see the effect of power saving	Jingdong prefecture level city in Shandong
Happy Fan Shop	King of Power Saving	Power Saver. It's authentic. It's black. It works.	Jingdong prefecture level city in Shandong
Happy Fan Shop	King of Power Saving	I've been using it for a week now and it's not working.	Jingdong prefecture level city in Shandong
Happy Fan Shop	King of Power Saving	I'm just using it. I don't know how it's going to work.	Jingdong prefecture level city in Shandong
Happy Fan Shop	King of Power Saving	Products are better looking out for power savings	Jingdong prefecture level city in Shandong
Happy Fan Shop	King of Power Saving	I received it and I've kept it on. I hope it works.	Jingdong prefecture level city in Shandong
Happy Fan Shop	King of Power Saving	It's really good! Worth recommending to buy!	Jingdong prefecture level city in Shandong
Happy Fan Shop	King of Power Saving	Try it for a few days and see if it doesn't work, then return it.	Jingdong prefecture level city in Shandong
Happy Fan Shop	King of Power Saving	It's in use and I hope to be pleasantly surprised.	Jingdong prefecture level city in Shandong
Happy Fan Shop	King of Power Saving	We'll see how it works when we use it	Jingdong prefecture level city in Shandong
Happy Fan Shop	King of Power Saving	Let's see if it works.	Jingdong prefecture level city in Shandong
Happy Fan Shop	King of Power Saving	I'm using it and will follow up with a review	Jingdong prefecture level city in Shandong
Happy Fan Shop	King of Power Saving	It really works. It saves energy.	Jingdong prefecture level city in Shandong
Happy Fan Shop	King of Power Saving	It does have some power savings!	Jingdong prefecture level city in Shandong
Happy Fan Shop	King of Power Saving	Jingdong shopping speed is convenient and timely	Jingdong prefecture level city in Shandong
Happy Fan Shop	King of Power Saving	Second time I've bought it. Everything's fine.	Jingdong prefecture level city in Shandong
Happy Fan Shop	King of Power Saving	I don't know if it works yet.	Jingdong prefecture level city in Shandong
Happy Fan Shop	King of Power Saving	Not used, don't know how well it works.	Jingdong prefecture level city in Shandong
Happy Fan Shop	King of Power Saving	No visible results so far	Jingdong prefecture level city in Shandong
Happy Fan Shop	King of Power Saving	It doesn't feel like it's working.	Jingdong prefecture level city in Shandong
Happy Fan Shop	King of Power Saving	Where's the effect? Where are the power savings?	Jingdong prefecture level city in Shandong
Happy Fan Shop	King of Power Saving	Let's see how it works in practice!	Jingdong prefecture level city in Shandong
Happy Fan Shop	King of Power Saving	I'll wait until I use it to give you a good review	Jingdong prefecture level city in Shandong
Happy Fan Shop	King of Power Saving	I'll follow up after I've used it	Jingdong prefecture level city in Shandong
Happy Fan Shop	King of Power Saving	Well, that's good for saving power.	Jingdong prefecture level city in Shandong
Happy Fan Shop	King of Power Saving	Received. Used in the know.	Jingdong prefecture level city in Shandong
Happy Fan Shop	King of Power Saving	It doesn't do much good.	Jingdong prefecture level city in Shandong
Happy Fan Shop	King of Power Saving	It's not working.	Jingdong prefecture level city in Shandong
Happy Fan Shop	King of Power Saving	I don't know. I just used it.	Jingdong prefecture level city in Shandong
Happy Fan Shop	King of Power Saving	Use it and come back to evaluate.	Jingdong prefecture level city in Shandong
Happy Fan Shop	King of Power Saving	Two words: useless.	Jingdong prefecture level city in Shandong
Happy Fan Shop	King of Power Saving	To be used and then evaluated	Jingdong prefecture level city in Shandong
Happy Fan Shop	King of Power Saving	No shipment.	Jingdong prefecture level city in Shandong
Happy Fan Shop	King of Power Saving	Fake. Speechless.	Jingdong prefecture level city in Shandong
Happy Fan Shop	King of Power Saving	fantastic (idiom); awesome	Jingdong prefecture level city in Shandong
Happy Fan Shop	King of Power Saving	an excellent	Jingdong prefecture level city in Shandong

Happy Fan Shop	King of Power Saving	an excellent	Jingdong prefecture level city in Shandong
Happy Fan Shop	King of Power Saving	Want to buy! Recommended not to buy! No effect	Jingdong prefecture level city in Shandong
Happy Fan Shop	King of Power Saving	That's all there is to it. I can't figure out how to save electricity.	Jingdong prefecture level city in Shandong
Happy Fan Shop	King of Power Saving	It's no use.	Jingdong prefecture level city in Shandong
Happy Fan Shop	King of Power Saving	Things are more perfect than I imagined, very powerful, it is worth getting Packaging is very strong, things are well protected, it is worth the praise things are very satisfied Oh, beautiful and stylish atmosphere, customer service is super patient, service is meticulous and attentive, the big brand is not	Jingdong prefecture level city in Shandong
Happy Fan Shop	King of Power Saving	The effect is too good, the family has now several units, back to buy a number of times, the power saver received, power-saving effect is very good,, fine workmanship, long warranty, but also purify the air, before buying a used for a period of time, with the okay, and then bought a, and only now come to the	Jingdong prefecture level city in Shandong
Happy Fan Shop	King of Power Saving	Quite good, and imagine the same, casual ride are very good, satisfied! Baby received it 👌, really great ah! 🌈 quality is really nothing to say, very satisfied! 🆗 happy happy 😃 genuine, great, the price is very good, in short, quite good, will buy again a very good shopping experience, attentive service, fast logistics, distribution is also thoughtful, the attitude is also very good, value for money, ah,	Jingdong prefecture level city in Shandong
Happy Fan Shop	King of Power Saving	Power-saving effect: strong Cost-effective: quite good Appearance style: beautiful Workmanship material: meticulous Experience: looking forward to power-saving effect	Jingdong prefecture level city in Shandong
Happy Fan Shop	King of Power Saving	Cost-effective is okay, the appearance of the style of meticulous workmanship, looking forward to energy-saving effect.	Jingdong prefecture level city in Shandong
Happy Fan Shop	King of Power Saving	Very Good Very Power Saving This Power Saver is really power saving As soon as the power is turned on, the Power Saver starts the power saving function for you.	Jingdong prefecture level city in Shandong
Happy Fan Shop	King of Power Saving	Logistics is very fast, the price is favorable, friends introduced that it is very good to use, bought 2 at once, looking forward to the effect, used after the follow-up comment.	Jingdong prefecture level city in Shandong
Happy Fan Shop	King of Power Saving	It's all fake. It doesn't save any electricity.	Jingdong prefecture level city in Shandong
Happy Fan Shop	King of Power Saving	This Power Saver is so much better than the one I bought before, not only the effect is good, the quality is very good in all aspects, the customer service attitude is also very good, recommended to buy!	Jingdong prefecture level city in Shandong
Happy Fan Shop	King of Power Saving	Logistics is very fast, buy for parents, good quality, once bought 2, power-saving effect is good to buy 2 more, look forward to using the effect, satisfied will recommend to friends.	Jingdong prefecture level city in Shandong
Happy Fan Shop	King of Power Saving	With the description is the same, very high-grade, good value, to buy friends can order Oh!	Jingdong prefecture level city in Shandong
Happy Fan Shop	King of Power Saving	Not using much electricity in the winter saving over 100 per month! Ready to buy 2 more units in the summer ！！！！！ Good quality, good price, power saving effect is obvious, cost-effective, worth buying, can be recommended!	Jingdong prefecture level city in Shandong
Happy Fan Shop	King of Power Saving	Purchased a power saver received on the put into use, rent a room with many electrical appliances, power consumption is very large, we have a piece of electricity or 1.2 dollars a time, with this power saver is much better, every day can save a lot of money, very convenient and practical.	Jingdong prefecture level city in Shandong
Happy Fan Shop	King of Power Saving	The power saving effect is very good: my supermarket monthly electricity bill is more than 7000 yuan. On May 1st someone recommended buying a couple of power savers, and I bought 5 of them first. The electricity bill was about 2000 less in June. This time, I bought 5 more. The seller said 800 degrees of electricity with one. I feel it's basically enough. A total of 10 units	Jingdong prefecture level city in Shandong
Happy Fan Shop	King of Power Saving	I never thought I could get such a good baby at such an affordable price, it's very good value for money, I love it!	Jingdong prefecture level city in Shandong
Happy Fan Shop	King of Power Saving	Previously bought other brands of power savers, but found no effect at home, this time to see how the products on the Jingdong, inserted in the home socket, the LED display is blue, the night light is very nice to see, look at the instructions that have to be half a year in order to achieve the best results, try it, I believe that the quality of the Jingdong, it should not be wrong.	Jingdong prefecture level city in Shandong
Happy Fan Shop	King of Power Saving	Superb, inexpensive, saves a lot of points, been using it for a long time!	Jingdong prefecture level city in Shandong
Happy Fan Shop	King of Power Saving	There are a lot of appliances in the house, the summer air conditioning is very costly, I bought this in front of the air conditioning effect is very good, I explain that I bought three in one breath, I use more electricity a month down to 200 or so, very satisfied.	Jingdong prefecture level city in Shandong
Happy Fan Shop	King of Power Saving	A month can save more than 200, home monthly electricity bill of more than 600 dollars, inserted 3 or so, an amount of the balance of the average can save more than 200. Product is very good, has been buying this one, has given a lot of relatives recommended, the quality is also very good, before buying a look with this same, who knows to buy home has not been used for more than a week on the bad, the quality of this is really good with more than two years!	Jingdong prefecture level city in Shandong
Happy Fan Shop	King of Power Saving	Whole house power saving king, can really save electricity, friends recommended, high power saving appliances, only replacement, no repair, after-sales worry-free!	Jingdong prefecture level city in Shandong
Happy Fan Shop	King of Power Saving	Great item love it, great customer service, great shopping experience!	Jingdong prefecture level city in Shandong
Happy Fan Shop	King of Power Saving	Power saving, very good use, much cheaper than the physical store, satisfaction	Jingdong prefecture level city in Shandong
Happy Fan Shop	King of Power Saving	In use, very well packaged and fast delivery of credit!	Jingdong prefecture level city in Shandong
Happy Fan Shop	King of Power Saving	Product quality, customer service attitude is particularly good, like, praise!	Jingdong prefecture level city in Shandong
Happy Fan Shop	King of Power Saving	The logistics is very fast, the packaging is not broken, the quality is very good, I like it very much!	Jingdong prefecture level city in Shandong
Happy Fan Shop	King of Power Saving	The saver was well packaged and was as described when plugged in	Jingdong prefecture level city in Shandong
Happy Fan Shop	King of Power Saving	Energy efficient and very satisfied	Jingdong prefecture level city in Shandong
Happy Fan Shop	King of Power Saving	Great quality for the price and fast shipping.	Jingdong prefecture level city in Shandong
Happy Fan Shop	King of Power Saving	It's great, just as described. Perfect.	Jingdong prefecture level city in Shandong
Happy Fan Shop	King of Power Saving	There is no power saving effect, but rather ring up an increase in electricity costs, because this machine is on continuously all day long, uninterruptedly, its own electricity and increased costs, plus the cost of purchasing the machine, so I feel that the use of this machine instead of increasing the	Jingdong prefecture level city in Shandong
Happy Fan Shop	King of Power Saving	Recommended by the owner of the store next door, looked at his home does have to save electricity, decided to get. Indeed quite can, the voltage is much more stable, there is a reduction in electricity	Jingdong prefecture level city in Shandong
Happy Fan Shop	King of Power Saving	Goods have been received value for money, affordable and reasonable price, and the description of the same, power-saving safe and reliable!	Jingdong prefecture level city in Shandong
Happy Fan Shop	King of Power Saving	Listen to friends introduced to buy, tried to save a lot of electricity is good, quite good, worth buying.	Jingdong prefecture level city in Shandong
Happy Fan Shop	King of Power Saving	The product is easy to use, safe and environmentally friendly, only used for a short time, to the next month, after the electricity bill status display to know how the performance of the power saver efficacy.	Jingdong prefecture level city in Shandong
Happy Fan Shop	King of Power Saving	Last month's electricity bill is 178 yuan, with this power saver is 92 yuan, ready to buy two more back home, I was introduced to buy a friend, just started to not believe that I did not expect the effect!	Jingdong prefecture level city in Shandong
Happy Fan Shop	King of Power Saving	It looks nice, but after using it for a few days I can't see how it saves power?	Jingdong prefecture level city in Shandong
Happy Fan Shop	King of Power Saving	Recommended by a friend, it is genuine, as described by the merchant, cost-effective	Jingdong prefecture level city in Shandong
Happy Fan Shop	King of Power Saving	Works well, affordable and saves money.	Jingdong prefecture level city in Shandong
Happy Fan Shop	King of Power Saving	Feels good haven't compared it yet, the air conditioning has been on a lot in the past two months	Jingdong prefecture level city in Shandong
Happy Fan Shop	King of Power Saving	Saving about 5 kWh per day is a good thing	Jingdong prefecture level city in Shandong

Happy Fan Shop	King of Power Saving	Not bad, affordable, best value for money	Jingdong prefecture level city in Shandong
Happy Fan Shop	King of Power Saving	The goods are genuine and the logistics are faster.	Jingdong prefecture level city in Shandong
Happy Fan Shop	King of Power Saving	Goods have been received today, has been plugged into the outlet, looking forward to saving electricity, after a period of time to see how the effect in the follow-up comment!	Jingdong prefecture level city in Shandong
Happy Fan Shop	King of Power Saving	I just got it home and haven't used it for long, so I'll use it for a couple months and see what happens. Seeing that most people have good reviews, it should be fine.	Jingdong prefecture level city in Shandong
Happy Fan Shop	King of Power Saving	I'm looking forward to the results, so I'll give you a good review out of habit! Follow up review after a while	Jingdong prefecture level city in Shandong
Happy Fan Shop	King of Power Saving	Use a dish first to know if it will help save energy	Jingdong prefecture level city in Shandong
Happy Fan Shop	King of Power Saving	Whether it saves electricity or not, we don't know yet.	Jingdong prefecture level city in Shandong
Happy Fan Shop	King of Power Saving	Goods have been received, use a period of time to know how oh. The courier did not deliver to the door ah! Sent to the station ah! The heat is inconvenient ah!	Jingdong prefecture level city in Shandong
Happy Fan Shop	King of Power Saving	A little effect, before an hour a power, now an hour 0.76 degrees, a dollar a power = save 24 cents, although not a lot, first use it!	Jingdong prefecture level city in Shandong
Happy Fan Shop	King of Power Saving	Just received the goods, use a month to evaluate	Jingdong prefecture level city in Shandong
Happy Fan Shop	King of Power Saving	Home every month with a lot of electricity, inadvertently found this good thing online that can save the beginning of a skeptical attitude to consult customer service, customer service replied very professional, said 120 days can be refunded that first try it!	Jingdong prefecture level city in Shandong
Happy Fan Shop	King of Power Saving	After receiving the goods this afternoon, immediately installed in the bathroom Smith energy storage electric water heater on the power outlet (instead of connecting with a plug board), when used, found that the "Power Saving King" LCD display, is the value of the voltage, not the store's video said so	Jingdong prefecture level city in Shandong
Happy Fan Shop	King of Power Saving	Has made many days, did not feel the power saving, the effect is not obvious.	Jingdong prefecture level city in Shandong
Happy Fan Shop	King of Power Saving	Energy savers do not save energy at all, but rather consume a lot of electricity.	Jingdong prefecture level city in Shandong
Happy Fan Shop	King of Power Saving	It's useless. It's a waste of money.	Jingdong prefecture level city in Shandong
Happy Fan Shop	King of Power Saving	Last month saved more than 500, which came back to buy, the store bought three, and then buy 1 home use, my family is dry beauty salon, more instruments, especially electricity, a month almost 1000. or so, with this did not expect to save so much, great, five-star praise!	Jingdong prefecture level city in Shandong
Happy Fan Shop	King of Power Saving	It rained a couple days ago and I just went to get the item back today , not bad, hey I looked at the meter and that red display light takes several seconds to jump, did not make a mistake buying it. After a period of time to evaluate.	Jingdong prefecture level city in Shandong
Happy Fan Shop	King of Power Saving	Without further ado, the electricity bill came down, this month less 102 yuan, has been repurchased, I also recommended to my friends, all said that there are effects, the product is okay, the effect is quite	Jingdong prefecture level city in Shandong
Happy Fan Shop	King of Power Saving	After buying and using two months really see the effect, so only to evaluate! Originally held the mindset of trying, I did not expect really effective, worth having!	Jingdong prefecture level city in Shandong
Happy Fan Shop	King of Power Saving	In the use of this energy saver before the monthly electricity bill is more than 150 yuan or so, now save thirty percent, every day to work, six or seven o'clock in the afternoon to go home, I do not know why the electricity bill is so expensive, energy-saving appliances really have the effect of recommending	Jingdong prefecture level city in Shandong
Happy Fan Shop	King of Power Saving	It saves about 100 bucks a month. A friend recommended it to me, and he's been using it for a while, and the effect is so obvious that I bought one too.	Jingdong prefecture level city in Shandong
Happy Fan Shop	King of Power Saving	My dad asked me to buy one, but after I paid the bill, I asked and he said it was a lot less this month, so he asked me to buy another one.	Jingdong prefecture level city in Shandong
Happy Fan Shop	King of Power Saving	The specific effect will have to be tested for at least two months to compare whether there is a power saving effect or not.	Jingdong prefecture level city in Shandong
Happy Fan Shop	King of Power Saving	'The goods have been received, immediately go to use, please ask the boss you instructions do not allow to withdraw, there are two fuses installed there!	Jingdong prefecture level city in Shandong
Happy Fan Shop	King of Power Saving	First praise is using, after using if good then follow up, now because I have not yet seen the results	Jingdong prefecture level city in Shandong
Happy Fan Shop	King of Power Saving	I've just been using it, and I haven't seen if it has a power-saving effect yet, so I'll follow up after 10 days and half a month.	Jingdong prefecture level city in Shandong
Happy Fan Shop	King of Power Saving	Just received the intelligent power saving meter, immediately plugged in to use, see it is not bad, and order to sell the second time	Jingdong prefecture level city in Shandong
Happy Fan Shop	King of Power Saving	This power saver is a bit effective, used for half a month, the effect is still visible.	Jingdong prefecture level city in Shandong
Happy Fan Shop	King of Power Saving	I received the baby and it looks great, I'm trying it out now and will keep an eye on it afterward.	Jingdong prefecture level city in Shandong
Happy Fan Shop	King of Power Saving	I've just received it, but I'm still trying to see how it works, so I'll come back in a little while.	Jingdong prefecture level city in Shandong
Happy Fan Shop	King of Power Saving	Just used it wait to use it for a while and then comment thanks, all first five stars.	Jingdong prefecture level city in Shandong
Happy Fan Shop	King of Power Saving	Very trustworthy brand, quality is guaranteed, the price is still affordable, quite satisfied!	Jingdong prefecture level city in Shandong
Happy Fan Shop	King of Power Saving	Things received, I hope that the platform of Jingdong do not sell fake goods are available	Jingdong prefecture level city in Shandong
Happy Fan Shop	King of Power Saving	Energy saver received, the use of the effect is good and the business description of the same	Jingdong prefecture level city in Shandong
Happy Fan Shop	King of Power Saving	The surface looks beautiful, the actual effect is not known, after using additional evaluation.	Jingdong prefecture level city in Shandong
Happy Fan Shop	King of Power Saving	Tried it for a few months, it really saves electricity, and I bought it for a friend.	Jingdong prefecture level city in Shandong
Happy Fan Shop	King of Power Saving	It didn't work and wanted to return it, it really didn't work at all.	Jingdong prefecture level city in Shandong
Happy Fan Shop	King of Power Saving	Just used it, don't know how effective it is, looking forward to usefulness	Jingdong prefecture level city in Shandong
Happy Fan Shop	King of Power Saving	After two months before the effect I do not know if it is true!	Jingdong prefecture level city in Shandong
Happy Fan Shop	King of Power Saving	So fast! Ordered yesterday and received today 👍👍👍👍👍👍👍👍👍 🌹🌹🌹🌹🌹🌹🌹🌹🌹	Jingdong prefecture level city in Shandong
Happy Fan Shop	King of Power Saving	I've started using it. We'll see how it works.	Jingdong prefecture level city in Shandong
Happy Fan Shop	King of Power Saving	I just received the goods, I won't know the effect until after two.	Jingdong prefecture level city in Shandong
Happy Fan Shop	King of Power Saving	Just received the goods, the specific effect will be evaluated later	Jingdong prefecture level city in Shandong
Happy Fan Shop	King of Power Saving	I've received the goods, I don't know if it's any good yet	Jingdong prefecture level city in Shandong
Happy Fan Shop	King of Power Saving	Try it for a month and then come back with a concrete evaluation	Jingdong prefecture level city in Shandong
Happy Fan Shop	King of Power Saving	In use, after two months to see the effect of power saving	Jingdong prefecture level city in Shandong
Happy Fan Shop	King of Power Saving	Power Saver. It's authentic. It's black. It works.	Jingdong prefecture level city in Shandong
Happy Fan Shop	King of Power Saving	I've been using it for a week now and it's not working.	Jingdong prefecture level city in Shandong
Happy Fan Shop	King of Power Saving	I'm just using it. I don't know how it's going to work.	Jingdong prefecture level city in Shandong

Happy Fan Shop	King of Power Saving	Products are better looking out for power savings	Jingdong prefecture level city in Shandong
Happy Fan Shop	King of Power Saving	I received it and I've kept it on. I hope it works.	Jingdong prefecture level city in Shandong
Happy Fan Shop	King of Power Saving	It's really good! Worth recommending to buy!	Jingdong prefecture level city in Shandong
Happy Fan Shop	King of Power Saving	Try it for a few days and see if it doesn't work, then return it.	Jingdong prefecture level city in Shandong
Happy Fan Shop	King of Power Saving	It's in use and I hope to be pleasantly surprised.	Jingdong prefecture level city in Shandong
Happy Fan Shop	King of Power Saving	We'll see how it works when we use it	Jingdong prefecture level city in Shandong
Happy Fan Shop	King of Power Saving	Let's see if it works.	Jingdong prefecture level city in Shandong
Happy Fan Shop	King of Power Saving	I'm using it and will follow up with a review	Jingdong prefecture level city in Shandong
Happy Fan Shop	King of Power Saving	It really works. It saves energy.	Jingdong prefecture level city in Shandong
Happy Fan Shop	King of Power Saving	It does have some power savings!	Jingdong prefecture level city in Shandong
Happy Fan Shop	King of Power Saving	Jingdong shopping speed is convenient and timely	Jingdong prefecture level city in Shandong
Happy Fan Shop	King of Power Saving	Second time I've bought it. Everything's fine.	Jingdong prefecture level city in Shandong
Happy Fan Shop	King of Power Saving	I don't know if it works yet.	Jingdong prefecture level city in Shandong
Happy Fan Shop	King of Power Saving	Not used, don't know how well it works.	Jingdong prefecture level city in Shandong
Happy Fan Shop	King of Power Saving	No visible results so far	Jingdong prefecture level city in Shandong
Happy Fan Shop	King of Power Saving	It doesn't feel like it's working.	Jingdong prefecture level city in Shandong
Happy Fan Shop	King of Power Saving	Where's the effect? Where are the power savings?	Jingdong prefecture level city in Shandong
Happy Fan Shop	King of Power Saving	Let's see how it works in practice!	Jingdong prefecture level city in Shandong
Happy Fan Shop	King of Power Saving	I'll wait until I use it to give you a good review	Jingdong prefecture level city in Shandong
Happy Fan Shop	King of Power Saving	I'll follow up after I've used it	Jingdong prefecture level city in Shandong
Happy Fan Shop	King of Power Saving	Well, that's good for saving power.	Jingdong prefecture level city in Shandong
Happy Fan Shop	King of Power Saving	Received. Used in the know.	Jingdong prefecture level city in Shandong
Happy Fan Shop	King of Power Saving	It doesn't do much good.	Jingdong prefecture level city in Shandong
Happy Fan Shop	King of Power Saving	It's not working.	Jingdong prefecture level city in Shandong
Happy Fan Shop	King of Power Saving	I don't know. I just used it.	Jingdong prefecture level city in Shandong
Happy Fan Shop	King of Power Saving	Use it and come back to evaluate.	Jingdong prefecture level city in Shandong
Happy Fan Shop	King of Power Saving	Two words: useless.	Jingdong prefecture level city in Shandong
Happy Fan Shop	King of Power Saving	To be used and then evaluated	Jingdong prefecture level city in Shandong
Happy Fan Shop	King of Power Saving	No shipment.	Jingdong prefecture level city in Shandong
Happy Fan Shop	King of Power Saving	Fake. Speechless.	Jingdong prefecture level city in Shandong
Happy Fan Shop	King of Power Saving	fantastic (idiom); awesome	Jingdong prefecture level city in Shandong
Happy Fan Shop	King of Power Saving	an excellent	Jingdong prefecture level city in Shandong
Happy Fan Shop	King of Power Saving	an excellent	Jingdong prefecture level city in Shandong
Happy Fan Shop	King of Power Saving	Want to buy! Recommended not to buy! No effect	Jingdong prefecture level city in Shandong
Happy Fan Shop	King of Power Saving	That's all there is to it. I can't figure out how to save electricity.	Jingdong prefecture level city in Shandong
Happy Fan Shop	King of Power Saving	It's no use.	Jingdong prefecture level city in Shandong
Happy Fan Shop	King of Power Saving	Things are more perfect than I imagined, very powerful, it is worth getting Packaging is very strong, things are well protected, it is worth the praise things are very satisfied Oh, beautiful and stylish atmosphere, customer service is super patient, service is meticulous and attentive, the big brand is not	Jingdong prefecture level city in Shandong
Happy Fan Shop	King of Power Saving	The effect is too good, the family has now several units, back to buy a number of times, the power saver received, power-saving effect is very good,, fine workmanship, long warranty, but also purify the air, before buying a used for a period of time, with the okay, and then bought a, and only now come to the	Jingdong prefecture level city in Shandong
Happy Fan Shop	King of Power Saving	Quite good, and imagine the same, casual ride are very good, satisfied! Baby received it 👌, really great ah! 🌈 quality is really nothing to say, very satisfied! 🆗 happy happy 😃 genuine, great, the price is very good, in short, quite good, will buy again a very good shopping experience, attentive service, fast logistics, distribution is also thoughtful, the attitude is also very good, value for money, ah,	Jingdong prefecture level city in Shandong
Happy Fan Shop	King of Power Saving	Power-saving effect: strong Cost-effective: quite good Appearance style: beautiful Workmanship material: meticulous Experience: looking forward to power-saving effect	Jingdong prefecture level city in Shandong
Happy Fan Shop	King of Power Saving	Cost-effective is okay, the appearance of the style of meticulous workmanship, looking forward to energy-saving effect.	Jingdong prefecture level city in Shandong
Happy Fan Shop	King of Power Saving	Very Good Very Power Saving This Power Saver is really power saving As soon as the power is turned on, the Power Saver starts the power saving function for you.	Jingdong prefecture level city in Shandong
Happy Fan Shop	King of Power Saving	Logistics is very fast, the price is favorable, friends introduced that it is very good to use, bought 2 at once, looking forward to the effect, used after the follow-up comment.	Jingdong prefecture level city in Shandong
Happy Fan Shop	King of Power Saving	It's all fake. It doesn't save any electricity.	Jingdong prefecture level city in Shandong
Happy Fan Shop	King of Power Saving	This Power Saver is so much better than the one I bought before, not only the effect is good, the quality is very good in all aspects, the customer service attitude is also very good, recommended to buy!	Jingdong prefecture level city in Shandong
Happy Fan Shop	King of Power Saving	Logistics is very fast, buy for parents, good quality, once bought 2, power-saving effect is good to buy 2 more, look forward to using the effect, satisfied will recommend to friends.	Jingdong prefecture level city in Shandong
Happy Fan Shop	King of Power Saving	With the description is the same, very high-grade, good value, to buy friends can order Oh!	Jingdong prefecture level city in Shandong
Happy Fan Shop	King of Power Saving	Not using much electricity in the winter saving over 100 per month! Ready to buy 2 more units in the summer ！！！！！ Good quality, good price, power saving effect is obvious, cost-effective, worth buying, can be recommended!	Jingdong prefecture level city in Shandong

Happy Fan Shop	King of Power Saving	Purchased a power saver received on the put into use, rent a room with many electrical appliances, power consumption is very large, we have a piece of electricity or 1.2 dollars a time, with this power saver is much better, every day can save a lot of money, very convenient and practical.	Jingdong prefecture level city in Shandong
Happy Fan Shop	King of Power Saving	The power saving effect is very good: my supermarket monthly electricity bill is more than 7000 yuan. On May 1st someone recommended buying a couple of power savers, and I bought 5 of them first. The electricity bill was about 2000 less in June. This time, I bought 5 more. The seller said 800 degrees of electricity with one. I feel it's basically enough. A total of 10 units	Jingdong prefecture level city in Shandong
Happy Fan Shop	King of Power Saving	I never thought I could get such a good baby at such an affordable price, it's very good value for money, I love it!	Jingdong prefecture level city in Shandong
Happy Fan Shop	King of Power Saving	Previously bought other brands of power savers, but found no effect at home, this time to see how the products on the Jingdong, inserted in the home socket, the LED display is blue, the night light is very nice to see, look at the instructions that have to be half a year in order to achieve the best results, try it, I believe that the quality of the Jingdong, it should not be wrong.	Jingdong prefecture level city in Shandong
Happy Fan Shop	King of Power Saving	Superb, inexpensive, saves a lot of points, been using it for a long time!	Jingdong prefecture level city in Shandong
Happy Fan Shop	King of Power Saving	There are a lot of appliances in the house, the summer air conditioning is very costly, I bought this in front of the air conditioning effect is very good, I explain that I bought three in one breath, I use more electricity a month down to 200 or so, very satisfied.	Jingdong prefecture level city in Shandong
Happy Fan Shop	King of Power Saving	A month can save more than 200, home monthly electricity bill of more than 600 dollars, inserted 3 or so, an amount of the balance of the average can save more than 200. Product is very good, has been buying this one, has given a lot of relatives recommended, the quality is also very good, before buying a look with this same, who knows to buy home has not been used for more than a week on the bad, the quality of this is really good with more than two years!	Jingdong prefecture level city in Shandong
Happy Fan Shop	King of Power Saving	Whole house power saving king, can really save electricity, friends recommended, high power saving appliances, only replacement, no repair, after-sales worry-free!	Jingdong prefecture level city in Shandong
Happy Fan Shop	King of Power Saving	Great item love it, great customer service, great shopping experience!	Jingdong prefecture level city in Shandong
Happy Fan Shop	King of Power Saving	Power saving, very good use, much cheaper than the physical store, satisfaction	Jingdong prefecture level city in Shandong
Happy Fan Shop	King of Power Saving	In use, very well packaged and fast delivery of credit!	Jingdong prefecture level city in Shandong
Happy Fan Shop	King of Power Saving	Product quality, customer service attitude is particularly good, like, praise!	Jingdong prefecture level city in Shandong
Happy Fan Shop	King of Power Saving	The logistics is very fast, the packaging is not broken, the quality is very good, I like it very much!	Jingdong prefecture level city in Shandong
Happy Fan Shop	King of Power Saving	The saver was well packaged and was as described when plugged in	Jingdong prefecture level city in Shandong
Happy Fan Shop	King of Power Saving	Energy efficient and very satisfied	Jingdong prefecture level city in Shandong
Happy Fan Shop	King of Power Saving	Great quality for the price and fast shipping.	Jingdong prefecture level city in Shandong
Happy Fan Shop	King of Power Saving	It's great, just as described. Perfect.	Jingdong prefecture level city in Shandong
Happy Fan Shop	King of Power Saving	There is no power saving effect, but rather ring up an increase in electricity costs, because this machine is on continuously all day long, uninterruptedly, its own electricity and increased costs, plus the cost of purchasing the machine, so I feel that the use of this machine instead of increasing the	Jingdong prefecture level city in Shandong
Happy Fan Shop	King of Power Saving	Recommended by the owner of the store next door, looked at his home does have to save electricity, decided to get. Indeed quite can, the voltage is much more stable, there is a reduction in electricity	Jingdong prefecture level city in Shandong
Happy Fan Shop	King of Power Saving	Goods have been received value for money, affordable and reasonable price, and the description of the same, power-saving safe and reliable!	Jingdong prefecture level city in Shandong
Happy Fan Shop	King of Power Saving	Listen to friends introduced to buy, tried to save a lot of electricity is good, quite good, worth buying.	Jingdong prefecture level city in Shandong
Happy Fan Shop	King of Power Saving	The product is easy to use, safe and environmentally friendly, only used for a short time, to the next month, after the electricity bill status display to know how the performance of the power saver efficacy.	Jingdong prefecture level city in Shandong
Happy Fan Shop	King of Power Saving	Last month's electricity bill is 178 yuan, with this power saver is 92 yuan, ready to buy two more back home, I was introduced to buy a friend, just started to not believe that I did not expect the effect!	Jingdong prefecture level city in Shandong
Happy Fan Shop	King of Power Saving	It looks nice, but after using it for a few days I can't see how it saves power?	Jingdong prefecture level city in Shandong
Happy Fan Shop	King of Power Saving	Recommended by a friend, it is genuine, as described by the merchant, cost-effective	Jingdong prefecture level city in Shandong
Happy Fan Shop	King of Power Saving	Works well, affordable and saves money.	Jingdong prefecture level city in Shandong
Happy Fan Shop	King of Power Saving	Feels good haven't compared it yet, the air conditioning has been on a lot in the past two months	Jingdong prefecture level city in Shandong
Happy Fan Shop	King of Power Saving	Saving about 5 kWh per day is a good thing	Jingdong prefecture level city in Shandong
Happy Fan Shop	King of Power Saving	Not bad, affordable, best value for money	Jingdong prefecture level city in Shandong
Happy Fan Shop	King of Power Saving	The goods are genuine and the logistics are faster.	Jingdong prefecture level city in Shandong
Happy Fan Shop	King of Power Saving	Goods have been received today, has been plugged into the outlet, looking forward to saving electricity, after a period of time to see how the effect in the follow-up comment!	Jingdong prefecture level city in Shandong
Happy Fan Shop	King of Power Saving	I just got it home and haven't used it for long, so I'll use it for a couple months and see what happens. Seeing that most people have good reviews, it should be fine.	Jingdong prefecture level city in Shandong
Happy Fan Shop	King of Power Saving	I'm looking forward to the results, so I'll give you a good review out of habit! Follow up review after a while	Jingdong prefecture level city in Shandong
Happy Fan Shop	King of Power Saving	Use a dish first to know if it will help save energy	Jingdong prefecture level city in Shandong
Happy Fan Shop	King of Power Saving	Whether it saves electricity or not, we don't know yet.	Jingdong prefecture level city in Shandong
Happy Fan Shop	King of Power Saving	Goods have been received, use a period of time to know how oh. The courier did not deliver to the door ah! Sent to the station ah! The heat is inconvenient ah!	Jingdong prefecture level city in Shandong
Happy Fan Shop	King of Power Saving	A little effect, before an hour a power, now an hour 0.76 degrees, a dollar a power = save 24 cents, although not a lot, first use it!	Jingdong prefecture level city in Shandong
Happy Fan Shop	King of Power Saving	Just received the goods, use a month to evaluate	Jingdong prefecture level city in Shandong
Happy Fan Shop	King of Power Saving	Home every month with a lot of electricity, inadvertently found this good thing online that can save the beginning of a skeptical attitude to consult customer service, customer service replied very professional, said 120 days can be refunded that first try it!	Jingdong prefecture level city in Shandong
Happy Fan Shop	King of Power Saving	After receiving the goods this afternoon, immediately installed in the bathroom Smith energy storage electric water heater on the power outlet (instead of connecting with a plug board), when used, found that the "Power Saving King" LCD display, is the value of the voltage, not the store's video said so	Jingdong prefecture level city in Shandong
Happy Fan Shop	King of Power Saving	Has made many days, did not feel the power saving, the effect is not obvious.	Jingdong prefecture level city in Shandong
Happy Fan Shop	King of Power Saving	Energy savers do not save energy at all, but rather consume a lot of electricity.	Jingdong prefecture level city in Shandong
Happy Fan Shop	King of Power Saving	It's useless. It's a waste of money.	Jingdong prefecture level city in Shandong
Happy Fan Shop	King of Power Saving	Last month saved more than 500, which came back to buy, the store bought three, and then buy 1 home use, my family is dry beauty salon, more instruments, especially electricity, a month almost 1000. or so, with this did not expect to save so much, great, five-star praise!	Jingdong prefecture level city in Shandong

Happy Fan Shop	King of Power Saving	It rained a couple days ago and I just went to get the item back today , not bad, hey I looked at the meter and that red display light takes several seconds to jump, did not make a mistake buying it. After a period of time to evaluate.	Jingdong prefecture level city in Shandong
Happy Fan Shop	King of Power Saving	Without further ado, the electricity bill came down, this month less 102 yuan, has been repurchased, I also recommended to my friends, all said that there are effects, the product is okay, the effect is quite	Jingdong prefecture level city in Shandong
Happy Fan Shop	King of Power Saving	After buying and using two months really see the effect, so only to evaluate! Originally held the mindset of trying, I did not expect really effective, worth having!	Jingdong prefecture level city in Shandong
Happy Fan Shop	King of Power Saving	In the use of this energy saver before the monthly electricity bill is more than 150 yuan or so, now save thirty percent, every day to work, six or seven o'clock in the afternoon to go home, I do not know why the electricity bill is so expensive, energy-saving appliances really have the effect of recommending	Jingdong prefecture level city in Shandong
Happy Fan Shop	King of Power Saving	It saves about 100 bucks a month. A friend recommended it to me, and he's been using it for a while, and the effect is so obvious that I bought one too.	Jingdong prefecture level city in Shandong
Happy Fan Shop	King of Power Saving	My dad asked me to buy one, but after I paid the bill, I asked and he said it was a lot less this month, so he asked me to buy another one.	Jingdong prefecture level city in Shandong
Happy Fan Shop	King of Power Saving	The specific effect will have to be tested for at least two months to compare whether there is a power saving effect or not.	Jingdong prefecture level city in Shandong
Happy Fan Shop	King of Power Saving	'The goods have been received, immediately go to use, please ask the boss you instructions do not allow to withdraw, there are two fuses installed there!	Jingdong prefecture level city in Shandong
Happy Fan Shop	King of Power Saving	First praise is using, after using if good then follow up, now because I have not yet seen the results	Jingdong prefecture level city in Shandong
Happy Fan Shop	King of Power Saving	I've just been using it, and I haven't seen if it has a power-saving effect yet, so I'll follow up after 10 days and half a month.	Jingdong prefecture level city in Shandong
Happy Fan Shop	King of Power Saving	Just received the intelligent power saving meter, immediately plugged in to use, see it is not bad, and order to sell the second time	Jingdong prefecture level city in Shandong
Happy Fan Shop	King of Power Saving	This power saver is a bit effective, used for half a month, the effect is still visible.	Jingdong prefecture level city in Shandong
Happy Fan Shop	King of Power Saving	I received the baby and it looks great, I'm trying it out now and will keep an eye on it afterward.	Jingdong prefecture level city in Shandong
Happy Fan Shop	King of Power Saving	I've just received it, but I'm still trying to see how it works, so I'll come back in a little while.	Jingdong prefecture level city in Shandong
Happy Fan Shop	King of Power Saving	Just used it wait to use it for a while and then comment thanks, all first five stars.	Jingdong prefecture level city in Shandong
Happy Fan Shop	King of Power Saving	Very trustworthy brand, quality is guaranteed, the price is still affordable, quite satisfied!	Jingdong prefecture level city in Shandong
Happy Fan Shop	King of Power Saving	Things received, I hope that the platform of Jingdong do not sell fake goods are available	Jingdong prefecture level city in Shandong
Happy Fan Shop	King of Power Saving	Energy saver received, the use of the effect is good and the business description of the same	Jingdong prefecture level city in Shandong
Happy Fan Shop	King of Power Saving	The surface looks beautiful, the actual effect is not known, after using additional evaluation.	Jingdong prefecture level city in Shandong
Happy Fan Shop	King of Power Saving	Tried it for a few months, it really saves electricity, and I bought it for a friend.	Jingdong prefecture level city in Shandong
Happy Fan Shop	King of Power Saving	It didn't work and wanted to return it, it really didn't work at all.	Jingdong prefecture level city in Shandong
Happy Fan Shop	King of Power Saving	Just used it, don't know how effective it is, looking forward to usefulness	Jingdong prefecture level city in Shandong
Happy Fan Shop	King of Power Saving	After two months before the effect I do not know if it is true!	Jingdong prefecture level city in Shandong
Happy Fan Shop	King of Power Saving	So fast! Ordered yesterday and received today 👍👍👍👍👍👍👍👍👍 🌹🌹🌹🌹🌹🌹🌹🌹🌹	Jingdong prefecture level city in Shandong
Happy Fan Shop	King of Power Saving	Things are more perfect than I imagined, very powerful, it is worth getting Packaging is very strong, things are well protected, it is worth the praise things are very satisfied Oh, beautiful and stylish atmosphere, customer service is super patient, service is meticulous and attentive, the big brand is not	Jingdong prefecture level city in Shandong
Happy Fan Shop	King of Power Saving	The effect is too good, the family has now several units, back to buy a number of times, the power saver received, power-saving effect is very good,, fine workmanship, long warranty, but also purify the air, before buying a used for a period of time, with the okay, and then bought a, and only now come to the	Jingdong prefecture level city in Shandong
Happy Fan Shop	King of Power Saving	Quite good, and imagine the same, casual ride are very good, satisfied! Baby received it 👌, really great ah! 🌈 quality is really nothing to say, very satisfied! 🆗 happy happy 😃 genuine, great, the price is very good, in short, quite good, will buy again a very good shopping experience, attentive service, fast logistics, distribution is also thoughtful, the attitude is also very good, value for money, ah,	Jingdong prefecture level city in Shandong
Happy Fan Shop	King of Power Saving	Power-saving effect: strong Cost-effective: quite good Appearance style: beautiful Workmanship material: meticulous Experience: looking forward to power-saving effect	Jingdong prefecture level city in Shandong
Happy Fan Shop	King of Power Saving	Cost-effective is okay, the appearance of the style of meticulous workmanship, looking forward to energy-saving effect.	Jingdong prefecture level city in Shandong
Happy Fan Shop	King of Power Saving	Very Good Very Power Saving This Power Saver is really power saving As soon as the power is turned on, the Power Saver starts the power saving function for you.	Jingdong prefecture level city in Shandong
Happy Fan Shop	King of Power Saving	Logistics is very fast, the price is favorable, friends introduced that it is very good to use, bought 2 at once, looking forward to the effect, used after the follow-up comment.	Jingdong prefecture level city in Shandong
Happy Fan Shop	King of Power Saving	It's all fake. It doesn't save any electricity.	Jingdong prefecture level city in Shandong
Happy Fan Shop	King of Power Saving	This Power Saver is so much better than the one I bought before, not only the effect is good, the quality is very good in all aspects, the customer service attitude is also very good, recommended to buy!	Jingdong prefecture level city in Shandong
Happy Fan Shop	King of Power Saving	Logistics is very fast, buy for parents, good quality, once bought 2, power-saving effect is good to buy 2 more, look forward to using the effect, satisfied will recommend to friends.	Jingdong prefecture level city in Shandong
Happy Fan Shop	King of Power Saving	With the description is the same, very high-grade, good value, to buy friends can order Oh!	Jingdong prefecture level city in Shandong
Happy Fan Shop	King of Power Saving	Not using much electricity in the winter saving over 100 per month! Ready to buy 2 more units in the summer ! ! ! ! ! Good quality, good price, power saving effect is obvious, cost-effective, worth buying, can be recommended!	Jingdong prefecture level city in Shandong
Happy Fan Shop	King of Power Saving	Purchased a power saver received on the put into use, rent a room with many electrical appliances, power consumption is very large, we have a piece of electricity or 1.2 dollars a time, with this power saver is much better, every day can save a lot of money, very convenient and practical.	Jingdong prefecture level city in Shandong
Happy Fan Shop	King of Power Saving	The power saving effect is very good: my supermarket monthly electricity bill is more than 7000 yuan. On May 1st someone recommended buying a couple of power savers, and I bought 5 of them first. The electricity bill was about 2000 less in June. This time, I bought 5 more. The seller said 800 degrees of electricity with one. I feel it's basically enough. A total of 10 units	Jingdong prefecture level city in Shandong
Happy Fan Shop	King of Power Saving	I never thought I could get such a good baby at such an affordable price, it's very good value for money, I love it!	Jingdong prefecture level city in Shandong
Happy Fan Shop	King of Power Saving	Previously bought other brands of power savers, but found no effect at home, this time to see how the products on the Jingdong, inserted in the home socket, the LED display is blue, the night light is very nice to see, look at the instructions that have to be half a year in order to achieve the best results, try it, I believe that the quality of the Jingdong, it should not be wrong.	Jingdong prefecture level city in Shandong
Happy Fan Shop	King of Power Saving	Superb, inexpensive, saves a lot of points, been using it for a long time!	Jingdong prefecture level city in Shandong
Happy Fan Shop	King of Power Saving	There are a lot of appliances in the house, the summer air conditioning is very costly, I bought this in front of the air conditioning effect is very good, I explain that I bought three in one breath, I use more electricity a month down to 200 or so, very satisfied.	Jingdong prefecture level city in Shandong
Happy Fan Shop	King of Power Saving	A month can save more than 200, home monthly electricity bill of more than 600 dollars, inserted 3 or so, an amount of the balance of the average can save more than 200. Product is very good, has been buying this one, has given a lot of relatives recommended, the quality is also very good, before buying a look with this same, who knows to buy home has not been used for more than a week on the bad, the quality of this is really good with more than two years!	Jingdong prefecture level city in Shandong

Happy Fan Shop	King of Power Saving	Whole house power saving king, can really save electricity, friends recommended, high power saving appliances, only replacement, no repair, after-sales worry-free!	Jingdong prefecture level city in Shandong
Happy Fan Shop	King of Power Saving	Great item love it, great customer service, great shopping experience!	Jingdong prefecture level city in Shandong
Happy Fan Shop	King of Power Saving	Power saving, very good use, much cheaper than the physical store, satisfaction	Jingdong prefecture level city in Shandong
Happy Fan Shop	King of Power Saving	In use, very well packaged and fast delivery of credit!	Jingdong prefecture level city in Shandong
Happy Fan Shop	King of Power Saving	Product quality, customer service attitude is particularly good, like, praise!	Jingdong prefecture level city in Shandong
Happy Fan Shop	King of Power Saving	The logistics is very fast, the packaging is not broken, the quality is very good, I like it very much!	Jingdong prefecture level city in Shandong
Happy Fan Shop	King of Power Saving	The saver was well packaged and was as described when plugged in	Jingdong prefecture level city in Shandong
Happy Fan Shop	King of Power Saving	Energy efficient and very satisfied	Jingdong prefecture level city in Shandong
Happy Fan Shop	King of Power Saving	Great quality for the price and fast shipping.	Jingdong prefecture level city in Shandong
Happy Fan Shop	King of Power Saving	It's great, just as described. Perfect.	Jingdong prefecture level city in Shandong
Happy Fan Shop	King of Power Saving	There is no power saving effect, but rather ring up an increase in electricity costs, because this machine is on continuously all day long, uninterruptedly, its own electricity and increased costs, plus the cost of purchasing the machine, so I feel that the use of this machine instead of increasing the	Jingdong prefecture level city in Shandong
Happy Fan Shop	King of Power Saving	Recommended by the owner of the store next door, looked at his home does have to save electricity, decided to get. Indeed quite can, the voltage is much more stable, there is a reduction in electricity	Jingdong prefecture level city in Shandong
Happy Fan Shop	King of Power Saving	Goods have been received value for money, affordable and reasonable price, and the description of the same, power-saving safe and reliable!	Jingdong prefecture level city in Shandong
Happy Fan Shop	King of Power Saving	Listen to friends introduced to buy, tried to save a lot of electricity is good, quite good, worth buying.	Jingdong prefecture level city in Shandong
Happy Fan Shop	King of Power Saving	The product is easy to use, safe and environmentally friendly, only used for a short time, to the next month, after the electricity bill status display to know how the performance of the power saver efficacy.	Jingdong prefecture level city in Shandong
Happy Fan Shop	King of Power Saving	Last month's electricity bill is 178 yuan, with this power saver is 92 yuan, ready to buy two more back home, I was introduced to buy a friend, just started to not believe that I did not expect the effect!	Jingdong prefecture level city in Shandong
Happy Fan Shop	King of Power Saving	It looks nice, but after using it for a few days I can't see how it saves power?	Jingdong prefecture level city in Shandong
Happy Fan Shop	King of Power Saving	Recommended by a friend, it is genuine, as described by the merchant, cost-effective	Jingdong prefecture level city in Shandong
Happy Fan Shop	King of Power Saving	Works well, affordable and saves money.	Jingdong prefecture level city in Shandong
Happy Fan Shop	King of Power Saving	Feels good haven't compared it yet, the air conditioning has been on a lot in the past two months	Jingdong prefecture level city in Shandong
Happy Fan Shop	King of Power Saving	Saving about 5 kWh per day is a good thing	Jingdong prefecture level city in Shandong
Happy Fan Shop	King of Power Saving	Not bad, affordable, best value for money	Jingdong prefecture level city in Shandong
Happy Fan Shop	King of Power Saving	The goods are genuine and the logistics are faster.	Jingdong prefecture level city in Shandong
Happy Fan Shop	King of Power Saving	Goods have been received today, has been plugged into the outlet, looking forward to saving electricity, after a period of time to see how the effect in the follow-up comment!	Jingdong prefecture level city in Shandong
Happy Fan Shop	King of Power Saving	I just got it home and haven't used it for long, so I'll use it for a couple months and see what happens. Seeing that most people have good reviews, it should be fine.	Jingdong prefecture level city in Shandong
Happy Fan Shop	King of Power Saving	I'm looking forward to the results, so I'll give you a good review out of habit! Follow up review after a while	Jingdong prefecture level city in Shandong
Happy Fan Shop	King of Power Saving	Use a dish first to know if it will help save energy	Jingdong prefecture level city in Shandong
Happy Fan Shop	King of Power Saving	Whether it saves electricity or not, we don't know yet.	Jingdong prefecture level city in Shandong
Happy Fan Shop	King of Power Saving	Goods have been received, use a period of time to know how oh. The courier did not deliver to the door ah! Sent to the station ah! The heat is inconvenient ah!	Jingdong prefecture level city in Shandong
Happy Fan Shop	King of Power Saving	A little effect, before an hour a power, now an hour 0.76 degrees, a dollar a power = save 24 cents, although not a lot, first use it!	Jingdong prefecture level city in Shandong
Happy Fan Shop	King of Power Saving	Just received the goods, use a month to evaluate	Jingdong prefecture level city in Shandong
Happy Fan Shop	King of Power Saving	Home every month with a lot of electricity, inadvertently found this good thing online that can save the beginning of a skeptical attitude to consult customer service, customer service replied very professional, said 120 days can be refunded that first try it!	Jingdong prefecture level city in Shandong
Happy Fan Shop	King of Power Saving	After receiving the goods this afternoon, immediately installed in the bathroom Smith energy storage electric water heater on the power outlet (instead of connecting with a plug board), when used, found that the "Power Saving King" LCD display, is the value of the voltage, not the store's video said so	Jingdong prefecture level city in Shandong
Happy Fan Shop	King of Power Saving	Has made many days, did not feel the power saving, the effect is not obvious.	Jingdong prefecture level city in Shandong
Happy Fan Shop	King of Power Saving	Energy savers do not save energy at all, but rather consume a lot of electricity.	Jingdong prefecture level city in Shandong
Happy Fan Shop	King of Power Saving	It's useless. It's a waste of money.	Jingdong prefecture level city in Shandong
Happy Fan Shop	King of Power Saving	Last month saved more than 500, which came back to buy, the store bought three, and then buy 1 home use, my family is dry beauty salon, more instruments, especially electricity, a month almost 1000. or so, with this did not expect to save so much, great, five-star praise!	Jingdong prefecture level city in Shandong
Happy Fan Shop	King of Power Saving	It rained a couple days ago and I just went to get the item back today , not bad, hey I looked at the meter and that red display light takes several seconds to jump, did not make a mistake buying it. After a period of time to evaluate.	Jingdong prefecture level city in Shandong
Happy Fan Shop	King of Power Saving	Without further ado, the electricity bill came down, this month less 102 yuan, has been repurchased, I also recommended to my friends, all said that there are effects, the product is okay, the effect is quite	Jingdong prefecture level city in Shandong
Happy Fan Shop	King of Power Saving	After buying and using two months really see the effect, so only to evaluate! Originally held the mindset of trying, I did not expect really effective, worth having!	Jingdong prefecture level city in Shandong
Happy Fan Shop	King of Power Saving	In the use of this energy saver before the monthly electricity bill is more than 150 yuan or so, now save thirty percent, every day to work, six or seven o'clock in the afternoon to go home, I do not know why the electricity bill is so expensive, energy-saving appliances really have the effect of recommending	Jingdong prefecture level city in Shandong
Happy Fan Shop	King of Power Saving	It saves about 100 bucks a month. A friend recommended it to me, and he's been using it for a while, and the effect is so obvious that I bought one too.	Jingdong prefecture level city in Shandong
Happy Fan Shop	King of Power Saving	My dad asked me to buy one, but after I paid the bill, I asked and he said it was a lot less this month, so he asked me to buy another one.	Jingdong prefecture level city in Shandong
Happy Fan Shop	King of Power Saving	The specific effect will have to be tested for at least two months to compare whether there is a power saving effect or not.	Jingdong prefecture level city in Shandong
Happy Fan Shop	King of Power Saving	'The goods have been received, immediately go to use, please ask the boss you instructions do not allow to withdraw, there are two fuses installed there!	Jingdong prefecture level city in Shandong
Happy Fan Shop	King of Power Saving	First praise is using, after using if good then follow up, now because I have not yet seen the results	Jingdong prefecture level city in Shandong
Happy Fan Shop	King of Power Saving	I've just been using it, and I haven't seen if it has a power-saving effect yet, so I'll follow up after 10 days and half a month.	Jingdong prefecture level city in Shandong
Happy Fan Shop	King of Power Saving	Just received the intelligent power saving meter, immediately plugged in to use, see it is not bad, and order to sell the second time	Jingdong prefecture level city in Shandong

Happy Fan Shop	King of Power Saving	This power saver is a bit effective, used for half a month, the effect is still visible.	Jingdong prefecture level city in Shandong
Happy Fan Shop	King of Power Saving	I received the baby and it looks great, I'm trying it out now and will keep an eye on it afterward.	Jingdong prefecture level city in Shandong
Happy Fan Shop	King of Power Saving	I've just received it, but I'm still trying to see how it works, so I'll come back in a little while.	Jingdong prefecture level city in Shandong
Happy Fan Shop	King of Power Saving	Just used it wait to use it for a while and then comment thanks, all first five stars.	Jingdong prefecture level city in Shandong
Happy Fan Shop	King of Power Saving	Very trustworthy brand, quality is guaranteed, the price is still affordable, quite satisfied!	Jingdong prefecture level city in Shandong
Happy Fan Shop	King of Power Saving	Things received, I hope that the platform of Jingdong do not sell fake goods are available	Jingdong prefecture level city in Shandong
Happy Fan Shop	King of Power Saving	Energy saver received, the use of the effect is good and the business description of the same	Jingdong prefecture level city in Shandong
Happy Fan Shop	King of Power Saving	The surface looks beautiful, the actual effect is not known, after using additional evaluation.	Jingdong prefecture level city in Shandong
Happy Fan Shop	King of Power Saving	Tried it for a few months, it really saves electricity, and I bought it for a friend.	Jingdong prefecture level city in Shandong
Happy Fan Shop	King of Power Saving	It didn't work and wanted to return it, it really didn't work at all.	Jingdong prefecture level city in Shandong
Happy Fan Shop	King of Power Saving	Just used it, don't know how effective it is, looking forward to usefulness	Jingdong prefecture level city in Shandong
Happy Fan Shop	King of Power Saving	After two months before the effect I do not know if it is true!	Jingdong prefecture level city in Shandong
Happy Fan Shop	King of Power Saving	So fast! Ordered yesterday and received today 👍👍👍👍👍👍👍👍👍 🌹🌹🌹🌹🌹🌹🌹🌹🌹	Jingdong prefecture level city in Shandong
Happy Fan Shop	King of Power Saving	I've started using it. We'll see how it works.	Jingdong prefecture level city in Shandong
Happy Fan Shop	King of Power Saving	I just received the goods, I won't know the effect until after two.	Jingdong prefecture level city in Shandong
Happy Fan Shop	King of Power Saving	Just received the goods, the specific effect will be evaluated later	Jingdong prefecture level city in Shandong
Happy Fan Shop	King of Power Saving	I've received the goods, I don't know if it's any good yet	Jingdong prefecture level city in Shandong
Happy Fan Shop	King of Power Saving	Try it for a month and then come back with a concrete evaluation	Jingdong prefecture level city in Shandong
Happy Fan Shop	King of Power Saving	In use, after two months to see the effect of power saving	Jingdong prefecture level city in Shandong
Happy Fan Shop	King of Power Saving	Power Saver. It's authentic. It's black. It works.	Jingdong prefecture level city in Shandong
Happy Fan Shop	King of Power Saving	I've been using it for a week now and it's not working.	Jingdong prefecture level city in Shandong
Happy Fan Shop	King of Power Saving	I'm just using it. I don't know how it's going to work.	Jingdong prefecture level city in Shandong
Happy Fan Shop	King of Power Saving	Products are better looking out for power savings	Jingdong prefecture level city in Shandong
Happy Fan Shop	King of Power Saving	I received it and I've kept it on. I hope it works.	Jingdong prefecture level city in Shandong
Happy Fan Shop	King of Power Saving	It's really good! Worth recommending to buy!	Jingdong prefecture level city in Shandong
Happy Fan Shop	King of Power Saving	Try it for a few days and see if it doesn't work, then return it.	Jingdong prefecture level city in Shandong
Happy Fan Shop	King of Power Saving	It's in use and I hope to be pleasantly surprised.	Jingdong prefecture level city in Shandong
Happy Fan Shop	King of Power Saving	We'll see how it works when we use it	Jingdong prefecture level city in Shandong
Happy Fan Shop	King of Power Saving	Let's see if it works.	Jingdong prefecture level city in Shandong
Happy Fan Shop	King of Power Saving	I'm using it and will follow up with a review	Jingdong prefecture level city in Shandong
Happy Fan Shop	King of Power Saving	It really works. It saves energy.	Jingdong prefecture level city in Shandong
Happy Fan Shop	King of Power Saving	It does have some power savings!	Jingdong prefecture level city in Shandong
Happy Fan Shop	King of Power Saving	Jingdong shopping speed is convenient and timely	Jingdong prefecture level city in Shandong
Happy Fan Shop	King of Power Saving	Second time I've bought it. Everything's fine.	Jingdong prefecture level city in Shandong
Happy Fan Shop	King of Power Saving	I don't know if it works yet.	Jingdong prefecture level city in Shandong
Happy Fan Shop	King of Power Saving	Not used, don't know how well it works.	Jingdong prefecture level city in Shandong
Happy Fan Shop	King of Power Saving	No visible results so far	Jingdong prefecture level city in Shandong
Happy Fan Shop	King of Power Saving	It doesn't feel like it's working.	Jingdong prefecture level city in Shandong
Happy Fan Shop	King of Power Saving	Where's the effect? Where are the power savings?	Jingdong prefecture level city in Shandong
Happy Fan Shop	King of Power Saving	Let's see how it works in practice!	Jingdong prefecture level city in Shandong
Happy Fan Shop	King of Power Saving	I'll wait until I use it to give you a good review	Jingdong prefecture level city in Shandong
Happy Fan Shop	King of Power Saving	I'll follow up after I've used it	Jingdong prefecture level city in Shandong
Happy Fan Shop	King of Power Saving	Well, that's good for saving power.	Jingdong prefecture level city in Shandong
Happy Fan Shop	King of Power Saving	Received. Used in the know.	Jingdong prefecture level city in Shandong
Happy Fan Shop	King of Power Saving	It doesn't do much good.	Jingdong prefecture level city in Shandong
Happy Fan Shop	King of Power Saving	It's not working.	Jingdong prefecture level city in Shandong
Happy Fan Shop	King of Power Saving	I don't know. I just used it.	Jingdong prefecture level city in Shandong
Happy Fan Shop	King of Power Saving	Use it and come back to evaluate.	Jingdong prefecture level city in Shandong
Happy Fan Shop	King of Power Saving	Two words: useless.	Jingdong prefecture level city in Shandong
Happy Fan Shop	King of Power Saving	To be used and then evaluated	Jingdong prefecture level city in Shandong
Happy Fan Shop	King of Power Saving	No shipment.	Jingdong prefecture level city in Shandong

Happy Fan Shop	King of Power Saving	Fake. Speechless.	Jingdong prefecture level city in Shandong
Happy Fan Shop	King of Power Saving	fantastic (idiom); awesome	Jingdong prefecture level city in Shandong
Happy Fan Shop	King of Power Saving	an excellent	Jingdong prefecture level city in Shandong
Happy Fan Shop	King of Power Saving	an excellent	Jingdong prefecture level city in Shandong
Happy Fan Shop	King of Power Saving	Want to buy! Recommended not to buy! No effect	Jingdong prefecture level city in Shandong
Happy Fan Shop	King of Power Saving	That's all there is to it. I can't figure out how to save electricity.	Jingdong prefecture level city in Shandong
Happy Fan Shop	King of Power Saving	It's no use.	Jingdong prefecture level city in Shandong
Happy Fan Shop	King of Power Saving	Things are more perfect than I imagined, very powerful, it is worth getting Packaging is very strong, things are well protected, it is worth the praise things are very satisfied Oh, beautiful and stylish atmosphere, customer service is super patient, service is meticulous and attentive, the big brand is not	Jingdong prefecture level city in Shandong
Happy Fan Shop	King of Power Saving	The effect is too good, the family has now several units, back to buy a number of times, the power saver received, power-saving effect is very good,, fine workmanship, long warranty, but also purify the air, before buying a used for a period of time, with the okay, and then bought a, and only now come to the	Jingdong prefecture level city in Shandong
Happy Fan Shop	King of Power Saving	Quite good, and imagine the same, casual ride are very good, satisfied! Baby received it 👌, really great ah! 🌈 quality is really nothing to say, very satisfied! 🆗 happy happy 😃 genuine, great, the price is very good, in short, quite good, will buy again a very good shopping experience, attentive service, fast logistics, distribution is also thoughtful, the attitude is also very good, value for money, ah,	Jingdong prefecture level city in Shandong
Happy Fan Shop	King of Power Saving	Power-saving effect: strong Cost-effective: quite good Appearance style: beautiful Workmanship material: meticulous Experience: looking forward to power-saving effect	Jingdong prefecture level city in Shandong
Happy Fan Shop	King of Power Saving	Cost-effective is okay, the appearance of the style of meticulous workmanship, looking forward to energy-saving effect.	Jingdong prefecture level city in Shandong
Happy Fan Shop	King of Power Saving	Very Good Very Power Saving This Power Saver is really power saving As soon as the power is turned on, the Power Saver starts the power saving function for you.	Jingdong prefecture level city in Shandong
Happy Fan Shop	King of Power Saving	Logistics is very fast, the price is favorable, friends introduced that it is very good to use, bought 2 at once, looking forward to the effect, used after the follow-up comment.	Jingdong prefecture level city in Shandong
Happy Fan Shop	King of Power Saving	It's all fake. It doesn't save any electricity.	Jingdong prefecture level city in Shandong
Happy Fan Shop	King of Power Saving	This Power Saver is so much better than the one I bought before, not only the effect is good, the quality is very good in all aspects, the customer service attitude is also very good, recommended to buy!	Jingdong prefecture level city in Shandong
Happy Fan Shop	King of Power Saving	Logistics is very fast, buy for parents, good quality, once bought 2, power-saving effect is good to buy 2 more, look forward to using the effect, satisfied will recommend to friends.	Jingdong prefecture level city in Shandong
Happy Fan Shop	King of Power Saving	With the description is the same, very high-grade, good value, to buy friends can order Oh!	Jingdong prefecture level city in Shandong
Happy Fan Shop	King of Power Saving	Not using much electricity in the winter saving over 100 per month! Ready to buy 2 more units in the summer ! ! ! ! ! Good quality, good price, power saving effect is obvious, cost-effective, worth buying, can be recommended!	Jingdong prefecture level city in Shandong
Happy Fan Shop	King of Power Saving	Purchased a power saver received on the put into use, rent a room with many electrical appliances, power consumption is very large, we have a piece of electricity or 1.2 dollars a time, with this power saver is much better, every day can save a lot of money, very convenient and practical.	Jingdong prefecture level city in Shandong
Happy Fan Shop	King of Power Saving	The power saving effect is very good: my supermarket monthly electricity bill is more than 7000 yuan. On May 1st someone recommended buying a couple of power savers, and I bought 5 of them first. The electricity bill was about 2000 less in June. This time, I bought 5 more. The seller said 800 degrees of electricity with one. I feel it's basically enough. A total of 10 units	Jingdong prefecture level city in Shandong
Happy Fan Shop	King of Power Saving	I never thought I could get such a good baby at such an affordable price, it's very good value for money, I love it!	Jingdong prefecture level city in Shandong
Happy Fan Shop	King of Power Saving	Previously bought other brands of power savers, but found no effect at home, this time to see how the products on the Jingdong, inserted in the home socket, the LED display is blue, the night light is very nice to see, look at the instructions that have to be half a year in order to achieve the best results, try it, I believe that the quality of the Jingdong, it should not be wrong.	Jingdong prefecture level city in Shandong
Happy Fan Shop	King of Power Saving	Superb, inexpensive, saves a lot of points, been using it for a long time!	Jingdong prefecture level city in Shandong
Happy Fan Shop	King of Power Saving	There are a lot of appliances in the house, the summer air conditioning is very costly, I bought this in front of the air conditioning effect is very good, I explain that I bought three in one breath, I use more electricity a month down to 200 or so, very satisfied.	Jingdong prefecture level city in Shandong
Happy Fan Shop	King of Power Saving	A month can save more than 200, home monthly electricity bill of more than 600 dollars, inserted 3 or so, an amount of the balance of the average can save more than 200. Product is very good, has been buying this one, has given a lot of relatives recommended, the quality is also very good, before buying a look with this same, who knows to buy home has not been used for more than a week on the bad, the quality of this is really good with more than two years!	Jingdong prefecture level city in Shandong
Happy Fan Shop	King of Power Saving	Whole house power saving king, can really save electricity, friends recommended, high power saving appliances, only replacement, no repair, after-sales worry-free!	Jingdong prefecture level city in Shandong
Happy Fan Shop	King of Power Saving	Great item love it, great customer service, great shopping experience!	Jingdong prefecture level city in Shandong
Happy Fan Shop	King of Power Saving	Power saving, very good use, much cheaper than the physical store, satisfaction	Jingdong prefecture level city in Shandong
Happy Fan Shop	King of Power Saving	In use, very well packaged and fast delivery of credit!	Jingdong prefecture level city in Shandong
Happy Fan Shop	King of Power Saving	Product quality, customer service attitude is particularly good, like, praise!	Jingdong prefecture level city in Shandong
Happy Fan Shop	King of Power Saving	The logistics is very fast, the packaging is not broken, the quality is very good, I like it very much!	Jingdong prefecture level city in Shandong
Happy Fan Shop	King of Power Saving	The saver was well packaged and was as described when plugged in	Jingdong prefecture level city in Shandong
Happy Fan Shop	King of Power Saving	Energy efficient and very satisfied	Jingdong prefecture level city in Shandong
Happy Fan Shop	King of Power Saving	Great quality for the price and fast shipping.	Jingdong prefecture level city in Shandong
Happy Fan Shop	King of Power Saving	It's great, just as described. Perfect.	Jingdong prefecture level city in Shandong
Happy Fan Shop	King of Power Saving	There is no power saving effect, but rather ring up an increase in electricity costs, because this machine is on continuously all day long, uninterruptedly, its own electricity and increased costs, plus the cost of purchasing the machine, so I feel that the use of this machine instead of increasing the	Jingdong prefecture level city in Shandong
Happy Fan Shop	King of Power Saving	Recommended by the owner of the store next door, looked at his home does have to save electricity, decided to get. Indeed quite can, the voltage is much more stable, there is a reduction in electricity	Jingdong prefecture level city in Shandong
Happy Fan Shop	King of Power Saving	Goods have been received value for money, affordable and reasonable price, and the description of the same, power-saving safe and reliable!	Jingdong prefecture level city in Shandong
Happy Fan Shop	King of Power Saving	Listen to friends introduced to buy, tried to save a lot of electricity is good, quite good, worth buying.	Jingdong prefecture level city in Shandong
Happy Fan Shop	King of Power Saving	The product is easy to use, safe and environmentally friendly, only used for a short time, to the next month, after the electricity bill status display to know how the performance of the power saver efficacy.	Jingdong prefecture level city in Shandong
Happy Fan Shop	King of Power Saving	Last month's electricity bill is 178 yuan, with this power saver is 92 yuan, ready to buy two more back home, I was introduced to buy a friend, just started to not believe that I did not expect the effect!	Jingdong prefecture level city in Shandong
Happy Fan Shop	King of Power Saving	It looks nice, but after using it for a few days I can't see how it saves power?	Jingdong prefecture level city in Shandong
Happy Fan Shop	King of Power Saving	Recommended by a friend, it is genuine, as described by the merchant, cost-effective	Jingdong prefecture level city in Shandong

Happy Fan Shop	King of Power Saving	Works well, affordable and saves money.	Jingdong prefecture level city in Shandong
Happy Fan Shop	King of Power Saving	Feels good haven't compared it yet, the air conditioning has been on a lot in the past two months	Jingdong prefecture level city in Shandong
Happy Fan Shop	King of Power Saving	Saving about 5 kWh per day is a good thing	Jingdong prefecture level city in Shandong
Happy Fan Shop	King of Power Saving	Not bad, affordable, best value for money	Jingdong prefecture level city in Shandong
Happy Fan Shop	King of Power Saving	The goods are genuine and the logistics are faster.	Jingdong prefecture level city in Shandong
Happy Fan Shop	King of Power Saving	Goods have been received today, has been plugged into the outlet, looking forward to saving electricity, after a period of time to see how the effect in the follow-up comment!	Jingdong prefecture level city in Shandong
Happy Fan Shop	King of Power Saving	I just got it home and haven't used it for long, so I'll use it for a couple months and see what happens. Seeing that most people have good reviews, it should be fine.	Jingdong prefecture level city in Shandong
Happy Fan Shop	King of Power Saving	I'm looking forward to the results, so I'll give you a good review out of habit! Follow up review after a while	Jingdong prefecture level city in Shandong
Happy Fan Shop	King of Power Saving	Use a dish first to know if it will help save energy	Jingdong prefecture level city in Shandong
Happy Fan Shop	King of Power Saving	Whether it saves electricity or not, we don't know yet.	Jingdong prefecture level city in Shandong
Happy Fan Shop	King of Power Saving	Goods have been received, use a period of time to know how oh. The courier did not deliver to the door ah! Sent to the station ah! The heat is inconvenient ah!	Jingdong prefecture level city in Shandong
Happy Fan Shop	King of Power Saving	A little effect, before an hour a power, now an hour 0.76 degrees, a dollar a power = save 24 cents, although not a lot, first use it!	Jingdong prefecture level city in Shandong
Happy Fan Shop	King of Power Saving	Just received the goods, use a month to evaluate	Jingdong prefecture level city in Shandong
Happy Fan Shop	King of Power Saving	Home every month with a lot of electricity, inadvertently found this good thing online that can save the beginning of a skeptical attitude to consult customer service, customer service replied very professional, said 120 days can be refunded that first try it!	Jingdong prefecture level city in Shandong
Happy Fan Shop	King of Power Saving	After receiving the goods this afternoon, immediately installed in the bathroom Smith energy storage electric water heater on the power outlet (instead of connecting with a plug board), when used, found that the "Power Saving King" LCD display, is the value of the voltage, not the store's video said so	Jingdong prefecture level city in Shandong
Happy Fan Shop	King of Power Saving	Has made many days, did not feel the power saving, the effect is not obvious.	Jingdong prefecture level city in Shandong
Happy Fan Shop	King of Power Saving	Energy savers do not save energy at all, but rather consume a lot of electricity.	Jingdong prefecture level city in Shandong
Happy Fan Shop	King of Power Saving	It's useless. It's a waste of money.	Jingdong prefecture level city in Shandong
Happy Fan Shop	King of Power Saving	Last month saved more than 500, which came back to buy, the store bought three, and then buy 1 home use, my family is dry beauty salon, more instruments, especially electricity, a month almost 1000. or so, with this did not expect to save so much, great, five-star praise!	Jingdong prefecture level city in Shandong
Happy Fan Shop	King of Power Saving	It rained a couple days ago and I just went to get the item back today , not bad, hey I looked at the meter and that red display light takes several seconds to jump, did not make a mistake buying it. After a period of time to evaluate.	Jingdong prefecture level city in Shandong
Happy Fan Shop	King of Power Saving	Without further ado, the electricity bill came down, this month less 102 yuan, has been repurchased, I also recommended to my friends, all said that there are effects, the product is okay, the effect is quite	Jingdong prefecture level city in Shandong
Happy Fan Shop	King of Power Saving	After buying and using two months really see the effect, so only to evaluate! Originally held the mindset of trying, I did not expect really effective, worth having!	Jingdong prefecture level city in Shandong
Happy Fan Shop	King of Power Saving	In the use of this energy saver before the monthly electricity bill is more than 150 yuan or so, now save thirty percent, every day to work, six or seven o'clock in the afternoon to go home, I do not know why the electricity bill is so expensive, energy-saving appliances really have the effect of recommending	Jingdong prefecture level city in Shandong
Happy Fan Shop	King of Power Saving	It saves about 100 bucks a month. A friend recommended it to me, and he's been using it for a while, and the effect is so obvious that I bought one too.	Jingdong prefecture level city in Shandong
Happy Fan Shop	King of Power Saving	My dad asked me to buy one, but after I paid the bill, I asked and he said it was a lot less this month, so he asked me to buy another one.	Jingdong prefecture level city in Shandong
Happy Fan Shop	King of Power Saving	The specific effect will have to be tested for at least two months to compare whether there is a power saving effect or not.	Jingdong prefecture level city in Shandong
Happy Fan Shop	King of Power Saving	'The goods have been received, immediately go to use, please ask the boss you instructions do not allow to withdraw, there are two fuses installed there!	Jingdong prefecture level city in Shandong
Happy Fan Shop	King of Power Saving	First praise is using, after using if good then follow up, now because I have not yet seen the results	Jingdong prefecture level city in Shandong
Happy Fan Shop	King of Power Saving	I've just been using it, and I haven't seen if it has a power-saving effect yet, so I'll follow up after 10 days and half a month.	Jingdong prefecture level city in Shandong
Happy Fan Shop	King of Power Saving	Just received the intelligent power saving meter, immediately plugged in to use, see it is not bad, and order to sell the second time	Jingdong prefecture level city in Shandong
Happy Fan Shop	King of Power Saving	This power saver is a bit effective, used for half a month, the effect is still visible.	Jingdong prefecture level city in Shandong
Happy Fan Shop	King of Power Saving	I received the baby and it looks great, I'm trying it out now and will keep an eye on it afterward.	Jingdong prefecture level city in Shandong
Happy Fan Shop	King of Power Saving	I've just received it, but I'm still trying to see how it works, so I'll come back in a little while.	Jingdong prefecture level city in Shandong
Happy Fan Shop	King of Power Saving	Just used it wait to use it for a while and then comment thanks, all first five stars.	Jingdong prefecture level city in Shandong
Happy Fan Shop	King of Power Saving	Very trustworthy brand, quality is guaranteed, the price is still affordable, quite satisfied!	Jingdong prefecture level city in Shandong
Happy Fan Shop	King of Power Saving	Things received, I hope that the platform of Jingdong do not sell fake goods are available	Jingdong prefecture level city in Shandong
Happy Fan Shop	King of Power Saving	Energy saver received, the use of the effect is good and the business description of the same	Jingdong prefecture level city in Shandong
Happy Fan Shop	King of Power Saving	The surface looks beautiful, the actual effect is not known, after using additional evaluation.	Jingdong prefecture level city in Shandong
Happy Fan Shop	King of Power Saving	Tried it for a few months, it really saves electricity, and I bought it for a friend.	Jingdong prefecture level city in Shandong
Happy Fan Shop	King of Power Saving	It didn't work and wanted to return it, it really didn't work at all.	Jingdong prefecture level city in Shandong
Happy Fan Shop	King of Power Saving	Just used it, don't know how effective it is, looking forward to usefulness	Jingdong prefecture level city in Shandong
Happy Fan Shop	King of Power Saving	After two months before the effect I do not know if it is true!	Jingdong prefecture level city in Shandong
Happy Fan Shop	King of Power Saving	So fast! Ordered yesterday and received today 👍👍👍👍👍👍👍👍👍 🌹🌹🌹🌹🌹🌹🌹🌹🌹	Jingdong prefecture level city in Shandong
Happy Fan Shop	King of Power Saving	I've started using it. We'll see how it works.	Jingdong prefecture level city in Shandong
Happy Fan Shop	King of Power Saving	I just received the goods, I won't know the effect until after two.	Jingdong prefecture level city in Shandong
Happy Fan Shop	King of Power Saving	Just received the goods, the specific effect will be evaluated later	Jingdong prefecture level city in Shandong
Happy Fan Shop	King of Power Saving	I've received the goods, I don't know if it's any good yet	Jingdong prefecture level city in Shandong
Happy Fan Shop	King of Power Saving	Try it for a month and then come back with a concrete evaluation	Jingdong prefecture level city in Shandong
Happy Fan Shop	King of Power Saving	In use, after two months to see the effect of power saving	Jingdong prefecture level city in Shandong

Happy Fan Shop	King of Power Saving	Power Saver. It's authentic. It's black. It works.	Jingdong prefecture level city in Shandong
Happy Fan Shop	King of Power Saving	I've been using it for a week now and it's not working.	Jingdong prefecture level city in Shandong
Happy Fan Shop	King of Power Saving	I'm just using it. I don't know how it's going to work.	Jingdong prefecture level city in Shandong
Happy Fan Shop	King of Power Saving	Products are better looking out for power savings	Jingdong prefecture level city in Shandong
Happy Fan Shop	King of Power Saving	I received it and I've kept it on. I hope it works.	Jingdong prefecture level city in Shandong
Happy Fan Shop	King of Power Saving	It's really good! Worth recommending to buy!	Jingdong prefecture level city in Shandong
Happy Fan Shop	King of Power Saving	Try it for a few days and see if it doesn't work, then return it.	Jingdong prefecture level city in Shandong
Happy Fan Shop	King of Power Saving	It's in use and I hope to be pleasantly surprised.	Jingdong prefecture level city in Shandong
Happy Fan Shop	King of Power Saving	We'll see how it works when we use it	Jingdong prefecture level city in Shandong
Happy Fan Shop	King of Power Saving	Let's see if it works.	Jingdong prefecture level city in Shandong
Happy Fan Shop	King of Power Saving	I'm using it and will follow up with a review	Jingdong prefecture level city in Shandong
Happy Fan Shop	King of Power Saving	It really works. It saves energy.	Jingdong prefecture level city in Shandong
Happy Fan Shop	King of Power Saving	It does have some power savings!	Jingdong prefecture level city in Shandong
Happy Fan Shop	King of Power Saving	Jingdong shopping speed is convenient and timely	Jingdong prefecture level city in Shandong
Happy Fan Shop	King of Power Saving	Second time I've bought it. Everything's fine.	Jingdong prefecture level city in Shandong
Happy Fan Shop	King of Power Saving	I don't know if it works yet.	Jingdong prefecture level city in Shandong
Happy Fan Shop	King of Power Saving	Not used, don't know how well it works.	Jingdong prefecture level city in Shandong
Happy Fan Shop	King of Power Saving	No visible results so far	Jingdong prefecture level city in Shandong
Happy Fan Shop	King of Power Saving	It doesn't feel like it's working.	Jingdong prefecture level city in Shandong
Happy Fan Shop	King of Power Saving	Where's the effect? Where are the power savings?	Jingdong prefecture level city in Shandong
Happy Fan Shop	King of Power Saving	Let's see how it works in practice!	Jingdong prefecture level city in Shandong
Happy Fan Shop	King of Power Saving	I'll wait until I use it to give you a good review	Jingdong prefecture level city in Shandong
Happy Fan Shop	King of Power Saving	I'll follow up after I've used it	Jingdong prefecture level city in Shandong
Happy Fan Shop	King of Power Saving	Well, that's good for saving power.	Jingdong prefecture level city in Shandong
Happy Fan Shop	King of Power Saving	Received. Used in the know.	Jingdong prefecture level city in Shandong
Happy Fan Shop	King of Power Saving	It doesn't do much good.	Jingdong prefecture level city in Shandong
Happy Fan Shop	King of Power Saving	It's not working.	Jingdong prefecture level city in Shandong
Happy Fan Shop	King of Power Saving	I don't know. I just used it.	Jingdong prefecture level city in Shandong
Happy Fan Shop	King of Power Saving	Use it and come back to evaluate.	Jingdong prefecture level city in Shandong
Happy Fan Shop	King of Power Saving	Two words: useless.	Jingdong prefecture level city in Shandong
Happy Fan Shop	King of Power Saving	To be used and then evaluated	Jingdong prefecture level city in Shandong
Happy Fan Shop	King of Power Saving	No shipment.	Jingdong prefecture level city in Shandong
Happy Fan Shop	King of Power Saving	Fake. Speechless.	Jingdong prefecture level city in Shandong
Happy Fan Shop	King of Power Saving	fantastic (idiom); awesome	Jingdong prefecture level city in Shandong
Happy Fan Shop	King of Power Saving	an excellent	Jingdong prefecture level city in Shandong
Happy Fan Shop	King of Power Saving	an excellent	Jingdong prefecture level city in Shandong
Happy Fan Shop	King of Power Saving	Want to buy! Recommended not to buy! No effect	Jingdong prefecture level city in Shandong
Happy Fan Shop	King of Power Saving	That's all there is to it. I can't figure out how to save electricity.	Jingdong prefecture level city in Shandong
Happy Fan Shop	King of Power Saving	It's no use.	Jingdong prefecture level city in Shandong
Happy Fan Shop	King of Power Saving	Things are more perfect than I imagined, very powerful, it is worth getting Packaging is very strong, things are well protected, it is worth the praise things are very satisfied Oh, beautiful and stylish atmosphere, customer service is super patient, service is meticulous and attentive, the big brand is not	Jingdong prefecture level city in Shandong
Happy Fan Shop	King of Power Saving	The effect is too good, the family has now several units, back to buy a number of times, the power saver received, power-saving effect is very good,, fine workmanship, long warranty, but also purify the air, before buying a used for a period of time, with the okay, and then bought a, and only now come to the	Jingdong prefecture level city in Shandong
Happy Fan Shop	King of Power Saving	Quite good, and imagine the same, casual ride are very good, satisfied! Baby received it 👌, really great ah! 🌈 quality is really nothing to say, very satisfied! 🆗 happy happy 😃 genuine, great, the price is very good, in short, quite good, will buy again a very good shopping experience, attentive service, fast logistics, distribution is also thoughtful, the attitude is also very good, value for money, ah,	Jingdong prefecture level city in Shandong
Happy Fan Shop	King of Power Saving	Power-saving effect: strong Cost-effective: quite good Appearance style: beautiful Workmanship material: meticulous Experience: looking forward to power-saving effect	Jingdong prefecture level city in Shandong
Happy Fan Shop	King of Power Saving	Cost-effective is okay, the appearance of the style of meticulous workmanship, looking forward to energy-saving effect.	Jingdong prefecture level city in Shandong
Happy Fan Shop	King of Power Saving	Very Good Very Power Saving This Power Saver is really power saving As soon as the power is turned on, the Power Saver starts the power saving function for you.	Jingdong prefecture level city in Shandong
Happy Fan Shop	King of Power Saving	Logistics is very fast, the price is favorable, friends introduced that it is very good to use, bought 2 at once, looking forward to the effect, used after the follow-up comment.	Jingdong prefecture level city in Shandong
Happy Fan Shop	King of Power Saving	It's all fake. It doesn't save any electricity.	Jingdong prefecture level city in Shandong
Happy Fan Shop	King of Power Saving	This Power Saver is so much better than the one I bought before, not only the effect is good, the quality is very good in all aspects, the customer service attitude is also very good, recommended to buy!	Jingdong prefecture level city in Shandong
Happy Fan Shop	King of Power Saving	Logistics is very fast, buy for parents, good quality, once bought 2, power-saving effect is good to buy 2 more, look forward to using the effect, satisfied will recommend to friends.	Jingdong prefecture level city in Shandong

Happy Fan Shop	King of Power Saving	With the description is the same, very high-grade, good value, to buy friends can order Oh!	Jingdong prefecture level city in Shandong
Happy Fan Shop	King of Power Saving	Not using much electricity in the winter saving over 100 per month! Ready to buy 2 more units in the summer ！！！！！ Good quality, good price, power saving effect is obvious, cost-effective, worth buying, can be recommended!	Jingdong prefecture level city in Shandong
Happy Fan Shop	King of Power Saving	Purchased a power saver received on the put into use, rent a room with many electrical appliances, power consumption is very large, we have a piece of electricity or 1.2 dollars a time, with this power saver is much better, every day can save a lot of money, very convenient and practical.	Jingdong prefecture level city in Shandong
Happy Fan Shop	King of Power Saving	The power saving effect is very good: my supermarket monthly electricity bill is more than 7000 yuan. On May 1st someone recommended buying a couple of power savers, and I bought 5 of them first. The electricity bill was about 2000 less in June. This time, I bought 5 more. The seller said 800 degrees of electricity with one. I feel it's basically enough. A total of 10 units	Jingdong prefecture level city in Shandong
Happy Fan Shop	King of Power Saving	I never thought I could get such a good baby at such an affordable price, it's very good value for money, I love it!	Jingdong prefecture level city in Shandong
Happy Fan Shop	King of Power Saving	Previously bought other brands of power savers, but found no effect at home, this time to see how the products on the Jingdong, inserted in the home socket, the LED display is blue, the night light is very nice to see, look at the instructions that have to be half a year in order to achieve the best results, try it, I believe that the quality of the Jingdong, it should not be wrong.	Jingdong prefecture level city in Shandong
Happy Fan Shop	King of Power Saving	Superb, inexpensive, saves a lot of points, been using it for a long time!	Jingdong prefecture level city in Shandong
Happy Fan Shop	King of Power Saving	There are a lot of appliances in the house, the summer air conditioning is very costly, I bought this in front of the air conditioning effect is very good, I explain that I bought three in one breath, I use more electricity a month down to 200 or so, very satisfied.	Jingdong prefecture level city in Shandong
Happy Fan Shop	King of Power Saving	A month can save more than 200, home monthly electricity bill of more than 600 dollars, inserted 3 or so, an amount of the balance of the average can save more than 200. Product is very good, has been buying this one, has given a lot of relatives recommended, the quality is also very good, before buying a look with this same, who knows to buy home has not been used for more than a week on the bad, the quality of this is really good with more than two years!	Jingdong prefecture level city in Shandong
Happy Fan Shop	King of Power Saving	Whole house power saving king, can really save electricity, friends recommended, high power saving appliances, only replacement, no repair, after-sales worry-free!	Jingdong prefecture level city in Shandong
Happy Fan Shop	King of Power Saving	Great item love it, great customer service, great shopping experience!	Jingdong prefecture level city in Shandong
Happy Fan Shop	King of Power Saving	Power saving, very good use, much cheaper than the physical store, satisfaction	Jingdong prefecture level city in Shandong
Happy Fan Shop	King of Power Saving	In use, very well packaged and fast delivery of credit!	Jingdong prefecture level city in Shandong
Happy Fan Shop	King of Power Saving	Product quality, customer service attitude is particularly good, like, praise!	Jingdong prefecture level city in Shandong
Happy Fan Shop	King of Power Saving	The logistics is very fast, the packaging is not broken, the quality is very good, I like it very much!	Jingdong prefecture level city in Shandong
Happy Fan Shop	King of Power Saving	The saver was well packaged and was as described when plugged in	Jingdong prefecture level city in Shandong
Happy Fan Shop	King of Power Saving	Energy efficient and very satisfied	Jingdong prefecture level city in Shandong
Happy Fan Shop	King of Power Saving	Great quality for the price and fast shipping.	Jingdong prefecture level city in Shandong
Happy Fan Shop	King of Power Saving	It's great, just as described. Perfect.	Jingdong prefecture level city in Shandong
Happy Fan Shop	King of Power Saving	There is no power saving effect, but rather ring up an increase in electricity costs, because this machine is on continuously all day long, uninterruptedly, its own electricity and increased costs, plus the cost of purchasing the machine, so I feel that the use of this machine instead of increasing the	Jingdong prefecture level city in Shandong
Happy Fan Shop	King of Power Saving	Recommended by the owner of the store next door, looked at his home does have to save electricity, decided to get. Indeed quite can, the voltage is much more stable, there is a reduction in electricity	Jingdong prefecture level city in Shandong
Happy Fan Shop	King of Power Saving	Goods have been received value for money, affordable and reasonable price, and the description of the same, power-saving safe and reliable!	Jingdong prefecture level city in Shandong
Happy Fan Shop	King of Power Saving	Listen to friends introduced to buy, tried to save a lot of electricity is good, quite good, worth buying.	Jingdong prefecture level city in Shandong
Happy Fan Shop	King of Power Saving	The product is easy to use, safe and environmentally friendly, only used for a short time, to the next month, after the electricity bill status display to know how the performance of the power saver efficacy.	Jingdong prefecture level city in Shandong
Happy Fan Shop	King of Power Saving	Last month's electricity bill is 178 yuan, with this power saver is 92 yuan, ready to buy two more back home, I was introduced to buy a friend, just started to not believe that I did not expect the effect!	Jingdong prefecture level city in Shandong
Happy Fan Shop	King of Power Saving	It looks nice, but after using it for a few days I can't see how it saves power?	Jingdong prefecture level city in Shandong
Happy Fan Shop	King of Power Saving	Recommended by a friend, it is genuine, as described by the merchant, cost-effective	Jingdong prefecture level city in Shandong
Happy Fan Shop	King of Power Saving	Works well, affordable and saves money.	Jingdong prefecture level city in Shandong
Happy Fan Shop	King of Power Saving	Feels good haven't compared it yet, the air conditioning has been on a lot in the past two months	Jingdong prefecture level city in Shandong
Happy Fan Shop	King of Power Saving	Saving about 5 kWh per day is a good thing	Jingdong prefecture level city in Shandong
Happy Fan Shop	King of Power Saving	Not bad, affordable, best value for money	Jingdong prefecture level city in Shandong
Happy Fan Shop	King of Power Saving	The goods are genuine and the logistics are faster.	Jingdong prefecture level city in Shandong
Happy Fan Shop	King of Power Saving	Goods have been received today, has been plugged into the outlet, looking forward to saving electricity, after a period of time to see how the effect in the follow-up comment!	Jingdong prefecture level city in Shandong
Happy Fan Shop	King of Power Saving	I just got it home and haven't used it for long, so I'll use it for a couple months and see what happens. Seeing that most people have good reviews, it should be fine.	Jingdong prefecture level city in Shandong
Happy Fan Shop	King of Power Saving	I'm looking forward to the results, so I'll give you a good review out of habit! Follow up review after a while	Jingdong prefecture level city in Shandong
Happy Fan Shop	King of Power Saving	Use a dish first to know if it will help save energy	Jingdong prefecture level city in Shandong
Happy Fan Shop	King of Power Saving	Whether it saves electricity or not, we don't know yet.	Jingdong prefecture level city in Shandong
Happy Fan Shop	King of Power Saving	Goods have been received, use a period of time to know how oh. The courier did not deliver to the door ah! Sent to the station ah! The heat is inconvenient ah!	Jingdong prefecture level city in Shandong
Happy Fan Shop	King of Power Saving	A little effect, before an hour a power, now an hour 0.76 degrees, a dollar a power = save 24 cents, although not a lot, first use it!	Jingdong prefecture level city in Shandong
Happy Fan Shop	King of Power Saving	Just received the goods, use a month to evaluate	Jingdong prefecture level city in Shandong
Happy Fan Shop	King of Power Saving	Home every month with a lot of electricity, inadvertently found this good thing online that can save the beginning of a skeptical attitude to consult customer service, customer service replied very professional, said 120 days can be refunded that first try it!	Jingdong prefecture level city in Shandong
Happy Fan Shop	King of Power Saving	After receiving the goods this afternoon, immediately installed in the bathroom Smith energy storage electric water heater on the power outlet (instead of connecting with a plug board), when used, found that the "Power Saving King" LCD display, is the value of the voltage, not the store's video said so	Jingdong prefecture level city in Shandong
Happy Fan Shop	King of Power Saving	Has made many days, did not feel the power saving, the effect is not obvious.	Jingdong prefecture level city in Shandong
Happy Fan Shop	King of Power Saving	Energy savers do not save energy at all, but rather consume a lot of electricity.	Jingdong prefecture level city in Shandong
Happy Fan Shop	King of Power Saving	It's useless. It's a waste of money.	Jingdong prefecture level city in Shandong

Happy Fan Shop	King of Power Saving	Last month saved more than 500, which came back to buy, the store bought three, and then buy 1 home use, my family is dry beauty salon, more instruments, especially electricity, a month almost 1000. or so, with this did not expect to save so much, great, five-star praise!	Jingdong prefecture level city in Shandong
Happy Fan Shop	King of Power Saving	It rained a couple days ago and I just went to get the item back today , not bad, hey I looked at the meter and that red display light takes several seconds to jump, did not make a mistake buying it. After a period of time to evaluate.	Jingdong prefecture level city in Shandong
Happy Fan Shop	King of Power Saving	Without further ado, the electricity bill came down, this month less 102 yuan, has been repurchased, I also recommended to my friends, all said that there are effects, the product is okay, the effect is quite	Jingdong prefecture level city in Shandong
Happy Fan Shop	King of Power Saving	After buying and using two months really see the effect, so only to evaluate! Originally held the mindset of trying, I did not expect really effective, worth having!	Jingdong prefecture level city in Shandong
Happy Fan Shop	King of Power Saving	In the use of this energy saver before the monthly electricity bill is more than 150 yuan or so, now save thirty percent, every day to work, six or seven o'clock in the afternoon to go home, I do not know why the electricity bill is so expensive, energy-saving appliances really have the effect of recommending	Jingdong prefecture level city in Shandong
Happy Fan Shop	King of Power Saving	It saves about 100 bucks a month. A friend recommended it to me, and he's been using it for a while, and the effect is so obvious that I bought one too.	Jingdong prefecture level city in Shandong
Happy Fan Shop	King of Power Saving	My dad asked me to buy one, but after I paid the bill, I asked and he said it was a lot less this month, so he asked me to buy another one.	Jingdong prefecture level city in Shandong
Happy Fan Shop	King of Power Saving	The specific effect will have to be tested for at least two months to compare whether there is a power saving effect or not.	Jingdong prefecture level city in Shandong
Happy Fan Shop	King of Power Saving	'The goods have been received, immediately go to use, please ask the boss you instructions do not allow to withdraw, there are two fuses installed there!	Jingdong prefecture level city in Shandong
Happy Fan Shop	King of Power Saving	First praise is using, after using if good then follow up, now because I have not yet seen the results	Jingdong prefecture level city in Shandong
Happy Fan Shop	King of Power Saving	I've just been using it, and I haven't seen if it has a power-saving effect yet, so I'll follow up after 10 days and half a month.	Jingdong prefecture level city in Shandong
Happy Fan Shop	King of Power Saving	Just received the intelligent power saving meter, immediately plugged in to use, see it is not bad, and order to sell the second time	Jingdong prefecture level city in Shandong
Happy Fan Shop	King of Power Saving	This power saver is a bit effective, used for half a month, the effect is still visible.	Jingdong prefecture level city in Shandong
Happy Fan Shop	King of Power Saving	I received the baby and it looks great, I'm trying it out now and will keep an eye on it afterward.	Jingdong prefecture level city in Shandong
Happy Fan Shop	King of Power Saving	I've just received it, but I'm still trying to see how it works, so I'll come back in a little while.	Jingdong prefecture level city in Shandong
Happy Fan Shop	King of Power Saving	Just used it wait to use it for a while and then comment thanks, all first five stars.	Jingdong prefecture level city in Shandong
Happy Fan Shop	King of Power Saving	Very trustworthy brand, quality is guaranteed, the price is still affordable, quite satisfied!	Jingdong prefecture level city in Shandong
Happy Fan Shop	King of Power Saving	Things received, I hope that the platform of Jingdong do not sell fake goods are available	Jingdong prefecture level city in Shandong
Happy Fan Shop	King of Power Saving	Energy saver received, the use of the effect is good and the business description of the same	Jingdong prefecture level city in Shandong
Happy Fan Shop	King of Power Saving	The surface looks beautiful, the actual effect is not known, after using additional evaluation.	Jingdong prefecture level city in Shandong
Happy Fan Shop	King of Power Saving	Tried it for a few months, it really saves electricity, and I bought it for a friend.	Jingdong prefecture level city in Shandong
Happy Fan Shop	King of Power Saving	It didn't work and wanted to return it, it really didn't work at all.	Jingdong prefecture level city in Shandong
Happy Fan Shop	King of Power Saving	Just used it, don't know how effective it is, looking forward to usefulness	Jingdong prefecture level city in Shandong
Happy Fan Shop	King of Power Saving	After two months before the effect I do not know if it is true!	Jingdong prefecture level city in Shandong
Happy Fan Shop	King of Power Saving	So fast! Ordered yesterday and received today 👍👍👍👍👍👍👍👍👍 🌹🌹🌹🌹🌹🌹🌹🌹🌹	Jingdong prefecture level city in Shandong
Happy Fan Shop	King of Power Saving	I've started using it. We'll see how it works.	Jingdong prefecture level city in Shandong
Happy Fan Shop	King of Power Saving	I just received the goods, I won't know the effect until after two.	Jingdong prefecture level city in Shandong
Happy Fan Shop	King of Power Saving	Just received the goods, the specific effect will be evaluated later	Jingdong prefecture level city in Shandong
Happy Fan Shop	King of Power Saving	I've received the goods, I don't know if it's any good yet	Jingdong prefecture level city in Shandong
Happy Fan Shop	King of Power Saving	Try it for a month and then come back with a concrete evaluation	Jingdong prefecture level city in Shandong
Happy Fan Shop	King of Power Saving	In use, after two months to see the effect of power saving	Jingdong prefecture level city in Shandong
Happy Fan Shop	King of Power Saving	Power Saver. It's authentic. It's black. It works.	Jingdong prefecture level city in Shandong
Happy Fan Shop	King of Power Saving	I've been using it for a week now and it's not working.	Jingdong prefecture level city in Shandong
Happy Fan Shop	King of Power Saving	I'm just using it. I don't know how it's going to work.	Jingdong prefecture level city in Shandong
Happy Fan Shop	King of Power Saving	Products are better looking out for power savings	Jingdong prefecture level city in Shandong
Happy Fan Shop	King of Power Saving	I received it and I've kept it on. I hope it works.	Jingdong prefecture level city in Shandong
Happy Fan Shop	King of Power Saving	It's really good! Worth recommending to buy!	Jingdong prefecture level city in Shandong
Happy Fan Shop	King of Power Saving	Try it for a few days and see if it doesn't work, then return it.	Jingdong prefecture level city in Shandong
Happy Fan Shop	King of Power Saving	It's in use and I hope to be pleasantly surprised.	Jingdong prefecture level city in Shandong
Happy Fan Shop	King of Power Saving	We'll see how it works when we use it	Jingdong prefecture level city in Shandong
Happy Fan Shop	King of Power Saving	Let's see if it works.	Jingdong prefecture level city in Shandong
Happy Fan Shop	King of Power Saving	I'm using it and will follow up with a review	Jingdong prefecture level city in Shandong
Happy Fan Shop	King of Power Saving	It really works. It saves energy.	Jingdong prefecture level city in Shandong
Happy Fan Shop	King of Power Saving	It does have some power savings!	Jingdong prefecture level city in Shandong
Happy Fan Shop	King of Power Saving	Jingdong shopping speed is convenient and timely	Jingdong prefecture level city in Shandong
Happy Fan Shop	King of Power Saving	Second time I've bought it. Everything's fine.	Jingdong prefecture level city in Shandong
Happy Fan Shop	King of Power Saving	I don't know if it works yet.	Jingdong prefecture level city in Shandong
Happy Fan Shop	King of Power Saving	Not used, don't know how well it works.	Jingdong prefecture level city in Shandong
Happy Fan Shop	King of Power Saving	No visible results so far	Jingdong prefecture level city in Shandong
Happy Fan Shop	King of Power Saving	It doesn't feel like it's working.	Jingdong prefecture level city in Shandong

Happy Fan Shop	King of Power Saving	Where's the effect? Where are the power savings?	Jingdong prefecture level city in Shandong
Happy Fan Shop	King of Power Saving	Let's see how it works in practice!	Jingdong prefecture level city in Shandong
Happy Fan Shop	King of Power Saving	I'll wait until I use it to give you a good review	Jingdong prefecture level city in Shandong
Happy Fan Shop	King of Power Saving	I'll follow up after I've used it	Jingdong prefecture level city in Shandong
Happy Fan Shop	King of Power Saving	Well, that's good for saving power.	Jingdong prefecture level city in Shandong
Happy Fan Shop	King of Power Saving	Received. Used in the know.	Jingdong prefecture level city in Shandong
Happy Fan Shop	King of Power Saving	It doesn't do much good.	Jingdong prefecture level city in Shandong
Happy Fan Shop	King of Power Saving	It's not working.	Jingdong prefecture level city in Shandong
Happy Fan Shop	King of Power Saving	I don't know. I just used it.	Jingdong prefecture level city in Shandong
Happy Fan Shop	King of Power Saving	Use it and come back to evaluate.	Jingdong prefecture level city in Shandong
Happy Fan Shop	King of Power Saving	Two words: useless.	Jingdong prefecture level city in Shandong
Happy Fan Shop	King of Power Saving	To be used and then evaluated	Jingdong prefecture level city in Shandong
Happy Fan Shop	King of Power Saving	No shipment.	Jingdong prefecture level city in Shandong
Happy Fan Shop	King of Power Saving	Fake. Speechless.	Jingdong prefecture level city in Shandong
Happy Fan Shop	King of Power Saving	fantastic (idiom); awesome	Jingdong prefecture level city in Shandong
Happy Fan Shop	King of Power Saving	an excellent	Jingdong prefecture level city in Shandong
Happy Fan Shop	King of Power Saving	an excellent	Jingdong prefecture level city in Shandong
Happy Fan Shop	King of Power Saving	Want to buy! Recommended not to buy! No effect	Jingdong prefecture level city in Shandong
Happy Fan Shop	King of Power Saving	That's all there is to it. I can't figure out how to save electricity.	Jingdong prefecture level city in Shandong
Happy Fan Shop	King of Power Saving	It's no use.	Jingdong prefecture level city in Shandong
Happy Fan Shop	King of Power Saving	Things are more perfect than I imagined, very powerful, it is worth getting Packaging is very strong, things are well protected, it is worth the praise things are very satisfied Oh, beautiful and stylish atmosphere, customer service is super patient, service is meticulous and attentive, the big brand is not	Jingdong prefecture level city in Shandong
Happy Fan Shop	King of Power Saving	The effect is too good, the family has now several units, back to buy a number of times, the power saver received, power-saving effect is very good,, fine workmanship, long warranty, but also purify the air, before buying a used for a period of time, with the okay, and then bought a, and only now come to the	Jingdong prefecture level city in Shandong
Happy Fan Shop	King of Power Saving	Quite good, and imagine the same, casual ride are very good, satisfied! Baby received it 👌, really great ah! 🌈 quality is really nothing to say, very satisfied! 🆗 happy happy 😃 genuine, great, the price is very good, in short, quite good, will buy again a very good shopping experience, attentive service, fast logistics, distribution is also thoughtful, the attitude is also very good, value for money, ah,	Jingdong prefecture level city in Shandong
Happy Fan Shop	King of Power Saving	Power-saving effect: strong Cost-effective: quite good Appearance style: beautiful Workmanship material: meticulous Experience: looking forward to power-saving effect	Jingdong prefecture level city in Shandong
Happy Fan Shop	King of Power Saving	Cost-effective is okay, the appearance of the style of meticulous workmanship, looking forward to energy-saving effect.	Jingdong prefecture level city in Shandong
Happy Fan Shop	King of Power Saving	Very Good Very Power Saving This Power Saver is really power saving As soon as the power is turned on, the Power Saver starts the power saving function for you.	Jingdong prefecture level city in Shandong
Happy Fan Shop	King of Power Saving	Logistics is very fast, the price is favorable, friends introduced that it is very good to use, bought 2 at once, looking forward to the effect, used after the follow-up comment.	Jingdong prefecture level city in Shandong
Happy Fan Shop	King of Power Saving	It's all fake. It doesn't save any electricity.	Jingdong prefecture level city in Shandong
Happy Fan Shop	King of Power Saving	This Power Saver is so much better than the one I bought before, not only the effect is good, the quality is very good in all aspects, the customer service attitude is also very good, recommended to buy!	Jingdong prefecture level city in Shandong
Happy Fan Shop	King of Power Saving	Logistics is very fast, buy for parents, good quality, once bought 2, power-saving effect is good to buy 2 more, look forward to using the effect, satisfied will recommend to friends.	Jingdong prefecture level city in Shandong
Happy Fan Shop	King of Power Saving	With the description is the same, very high-grade, good value, to buy friends can order Oh!	Jingdong prefecture level city in Shandong
Happy Fan Shop	King of Power Saving	Not using much electricity in the winter saving over 100 per month! Ready to buy 2 more units in the summer ！！！！！ Good quality, good price, power saving effect is obvious, cost-effective, worth buying, can be recommended!	Jingdong prefecture level city in Shandong
Happy Fan Shop	King of Power Saving	Purchased a power saver received on the put into use, rent a room with many electrical appliances, power consumption is very large, we have a piece of electricity or 1.2 dollars a time, with this power saver is much better, every day can save a lot of money, very convenient and practical.	Jingdong prefecture level city in Shandong
Happy Fan Shop	King of Power Saving	The power saving effect is very good: my supermarket monthly electricity bill is more than 7000 yuan. On May 1st someone recommended buying a couple of power savers, and I bought 5 of them first. The electricity bill was about 2000 less in June. This time, I bought 5 more. The seller said 800 degrees of electricity with one. I feel it's basically enough. A total of 10 units	Jingdong prefecture level city in Shandong
Happy Fan Shop	King of Power Saving	I never thought I could get such a good baby at such an affordable price, it's very good value for money, I love it!	Jingdong prefecture level city in Shandong
Happy Fan Shop	King of Power Saving	Previously bought other brands of power savers, but found no effect at home, this time to see how the products on the Jingdong, inserted in the home socket, the LED display is blue, the night light is very nice to see, look at the instructions that have to be half a year in order to achieve the best results, try it, I believe that the quality of the Jingdong, it should not be wrong.	Jingdong prefecture level city in Shandong
Happy Fan Shop	King of Power Saving	Superb, inexpensive, saves a lot of points, been using it for a long time!	Jingdong prefecture level city in Shandong
Happy Fan Shop	King of Power Saving	There are a lot of appliances in the house, the summer air conditioning is very costly, I bought this in front of the air conditioning effect is very good, I explain that I bought three in one breath, I use more electricity a month down to 200 or so, very satisfied.	Jingdong prefecture level city in Shandong
Happy Fan Shop	King of Power Saving	A month can save more than 200, home monthly electricity bill of more than 600 dollars, inserted 3 or so, an amount of the balance of the average can save more than 200. Product is very good, has been buying this one, has given a lot of relatives recommended, the quality is also very good, before buying a look with this same, who knows to buy home has not been used for more than a week on the bad, the quality of this is really good with more than two years!	Jingdong prefecture level city in Shandong
Happy Fan Shop	King of Power Saving	Whole house power saving king, can really save electricity, friends recommended, high power saving appliances, only replacement, no repair, after-sales worry-free!	Jingdong prefecture level city in Shandong
Happy Fan Shop	King of Power Saving	Great item love it, great customer service, great shopping experience!	Jingdong prefecture level city in Shandong
Happy Fan Shop	King of Power Saving	Power saving, very good use, much cheaper than the physical store, satisfaction	Jingdong prefecture level city in Shandong
Happy Fan Shop	King of Power Saving	In use, very well packaged and fast delivery of credit!	Jingdong prefecture level city in Shandong
Happy Fan Shop	King of Power Saving	Product quality, customer service attitude is particularly good, like, praise!	Jingdong prefecture level city in Shandong

Happy Fan Shop	King of Power Saving	The logistics is very fast, the packaging is not broken, the quality is very good, I like it very much!	Jingdong prefecture level city in Shandong
Happy Fan Shop	King of Power Saving	The saver was well packaged and was as described when plugged in	Jingdong prefecture level city in Shandong
Happy Fan Shop	King of Power Saving	Energy efficient and very satisfied	Jingdong prefecture level city in Shandong
Happy Fan Shop	King of Power Saving	Great quality for the price and fast shipping.	Jingdong prefecture level city in Shandong
Happy Fan Shop	King of Power Saving	It's great, just as described. Perfect.	Jingdong prefecture level city in Shandong
Happy Fan Shop	King of Power Saving	There is no power saving effect, but rather ring up an increase in electricity costs, because this machine is on continuously all day long, uninterruptedly, its own electricity and increased costs, plus the cost of purchasing the machine, so I feel that the use of this machine instead of increasing the	Jingdong prefecture level city in Shandong
Happy Fan Shop	King of Power Saving	Recommended by the owner of the store next door, looked at his home does have to save electricity, decided to get. Indeed quite can, the voltage is much more stable, there is a reduction in electricity	Jingdong prefecture level city in Shandong
Happy Fan Shop	King of Power Saving	Goods have been received value for money, affordable and reasonable price, and the description of the same, power-saving safe and reliable!	Jingdong prefecture level city in Shandong
Happy Fan Shop	King of Power Saving	Listen to friends introduced to buy, tried to save a lot of electricity is good, quite good, worth buying.	Jingdong prefecture level city in Shandong
Happy Fan Shop	King of Power Saving	The product is easy to use, safe and environmentally friendly, only used for a short time, to the next month, after the electricity bill status display to know how the performance of the power saver efficacy.	Jingdong prefecture level city in Shandong
Happy Fan Shop	King of Power Saving	Last month's electricity bill is 178 yuan, with this power saver is 92 yuan, ready to buy two more back home, I was introduced to buy a friend, just started to not believe that I did not expect the effect!	Jingdong prefecture level city in Shandong
Happy Fan Shop	King of Power Saving	It looks nice, but after using it for a few days I can't see how it saves power?	Jingdong prefecture level city in Shandong
Happy Fan Shop	King of Power Saving	Recommended by a friend, it is genuine, as described by the merchant, cost-effective	Jingdong prefecture level city in Shandong
Happy Fan Shop	King of Power Saving	Works well, affordable and saves money.	Jingdong prefecture level city in Shandong
Happy Fan Shop	King of Power Saving	Feels good haven't compared it yet, the air conditioning has been on a lot in the past two months	Jingdong prefecture level city in Shandong
Happy Fan Shop	King of Power Saving	Saving about 5 kWh per day is a good thing	Jingdong prefecture level city in Shandong
Happy Fan Shop	King of Power Saving	Not bad, affordable, best value for money	Jingdong prefecture level city in Shandong
Happy Fan Shop	King of Power Saving	The goods are genuine and the logistics are faster.	Jingdong prefecture level city in Shandong
Happy Fan Shop	King of Power Saving	Goods have been received today, has been plugged into the outlet, looking forward to saving electricity, after a period of time to see how the effect in the follow-up comment!	Jingdong prefecture level city in Shandong
Happy Fan Shop	King of Power Saving	I just got it home and haven't used it for long, so I'll use it for a couple months and see what happens. Seeing that most people have good reviews, it should be fine.	Jingdong prefecture level city in Shandong
Happy Fan Shop	King of Power Saving	I'm looking forward to the results, so I'll give you a good review out of habit! Follow up review after a while	Jingdong prefecture level city in Shandong
Happy Fan Shop	King of Power Saving	Use a dish first to know if it will help save energy	Jingdong prefecture level city in Shandong
Happy Fan Shop	King of Power Saving	Whether it saves electricity or not, we don't know yet.	Jingdong prefecture level city in Shandong
Happy Fan Shop	King of Power Saving	Goods have been received, use a period of time to know how oh. The courier did not deliver to the door ah! Sent to the station ah! The heat is inconvenient ah!	Jingdong prefecture level city in Shandong
Happy Fan Shop	King of Power Saving	A little effect, before an hour a power, now an hour 0.76 degrees, a dollar a power = save 24 cents, although not a lot, first use it!	Jingdong prefecture level city in Shandong
Happy Fan Shop	King of Power Saving	Just received the goods, use a month to evaluate	Jingdong prefecture level city in Shandong
Happy Fan Shop	King of Power Saving	Home every month with a lot of electricity, inadvertently found this good thing online that can save the beginning of a skeptical attitude to consult customer service, customer service replied very professional, said 120 days can be refunded that first try it!	Jingdong prefecture level city in Shandong
Happy Fan Shop	King of Power Saving	After receiving the goods this afternoon, immediately installed in the bathroom Smith energy storage electric water heater on the power outlet (instead of connecting with a plug board), when used, found that the "Power Saving King" LCD display, is the value of the voltage, not the store's video said so	Jingdong prefecture level city in Shandong
Happy Fan Shop	King of Power Saving	Has made many days, did not feel the power saving, the effect is not obvious.	Jingdong prefecture level city in Shandong
Happy Fan Shop	King of Power Saving	Energy savers do not save energy at all, but rather consume a lot of electricity.	Jingdong prefecture level city in Shandong
Happy Fan Shop	King of Power Saving	It's useless. It's a waste of money.	Jingdong prefecture level city in Shandong
Happy Fan Shop	King of Power Saving	Last month saved more than 500, which came back to buy, the store bought three, and then buy 1 home use, my family is dry beauty salon, more instruments, especially electricity, a month almost 1000. or so, with this did not expect to save so much, great, five-star praise!	Jingdong prefecture level city in Shandong
Happy Fan Shop	King of Power Saving	It rained a couple days ago and I just went to get the item back today , not bad, hey I looked at the meter and that red display light takes several seconds to jump, did not make a mistake buying it. After a period of time to evaluate.	Jingdong prefecture level city in Shandong
Happy Fan Shop	King of Power Saving	Without further ado, the electricity bill came down, this month less 102 yuan, has been repurchased, I also recommended to my friends, all said that there are effects, the product is okay, the effect is quite	Jingdong prefecture level city in Shandong
Happy Fan Shop	King of Power Saving	After buying and using two months really see the effect, so only to evaluate! Originally held the mindset of trying, I did not expect really effective, worth having!	Jingdong prefecture level city in Shandong
Happy Fan Shop	King of Power Saving	In the use of this energy saver before the monthly electricity bill is more than 150 yuan or so, now save thirty percent, every day to work, six or seven o'clock in the afternoon to go home, I do not know why the electricity bill is so expensive, energy-saving appliances really have the effect of recommending	Jingdong prefecture level city in Shandong
Happy Fan Shop	King of Power Saving	It saves about 100 bucks a month. A friend recommended it to me, and he's been using it for a while, and the effect is so obvious that I bought one too.	Jingdong prefecture level city in Shandong
Happy Fan Shop	King of Power Saving	My dad asked me to buy one, but after I paid the bill, I asked and he said it was a lot less this month, so he asked me to buy another one.	Jingdong prefecture level city in Shandong
Happy Fan Shop	King of Power Saving	The specific effect will have to be tested for at least two months to compare whether there is a power saving effect or not.	Jingdong prefecture level city in Shandong
Happy Fan Shop	King of Power Saving	'The goods have been received, immediately go to use, please ask the boss you instructions do not allow to withdraw, there are two fuses installed there!	Jingdong prefecture level city in Shandong
Happy Fan Shop	King of Power Saving	First praise is using, after using if good then follow up, now because I have not yet seen the results	Jingdong prefecture level city in Shandong
Happy Fan Shop	King of Power Saving	I've just been using it, and I haven't seen if it has a power-saving effect yet, so I'll follow up after 10 days and half a month.	Jingdong prefecture level city in Shandong
Happy Fan Shop	King of Power Saving	Just received the intelligent power saving meter, immediately plugged in to use, see it is not bad, and order to sell the second time	Jingdong prefecture level city in Shandong
Happy Fan Shop	King of Power Saving	This power saver is a bit effective, used for half a month, the effect is still visible.	Jingdong prefecture level city in Shandong
Happy Fan Shop	King of Power Saving	I received the baby and it looks great, I'm trying it out now and will keep an eye on it afterward.	Jingdong prefecture level city in Shandong
Happy Fan Shop	King of Power Saving	I've just received it, but I'm still trying to see how it works, so I'll come back in a little while.	Jingdong prefecture level city in Shandong
Happy Fan Shop	King of Power Saving	Just used it wait to use it for a while and then comment thanks, all first five stars.	Jingdong prefecture level city in Shandong
Happy Fan Shop	King of Power Saving	Very trustworthy brand, quality is guaranteed, the price is still affordable, quite satisfied!	Jingdong prefecture level city in Shandong

Happy Fan Shop	King of Power Saving	Things received, I hope that the platform of Jingdong do not sell fake goods are available	Jingdong prefecture level city in Shandong
Happy Fan Shop	King of Power Saving	Energy saver received, the use of the effect is good and the business description of the same	Jingdong prefecture level city in Shandong
Happy Fan Shop	King of Power Saving	The surface looks beautiful, the actual effect is not known, after using additional evaluation.	Jingdong prefecture level city in Shandong
Happy Fan Shop	King of Power Saving	Tried it for a few months, it really saves electricity, and I bought it for a friend.	Jingdong prefecture level city in Shandong
Happy Fan Shop	King of Power Saving	It didn't work and wanted to return it, it really didn't work at all.	Jingdong prefecture level city in Shandong
Happy Fan Shop	King of Power Saving	Just used it, don't know how effective it is, looking forward to usefulness	Jingdong prefecture level city in Shandong
Happy Fan Shop	King of Power Saving	After two months before the effect I do not know if it is true!	Jingdong prefecture level city in Shandong
Happy Fan Shop	King of Power Saving	So fast! Ordered yesterday and received today 👍👍👍👍👍👍👍👍👍 🌹🌹🌹🌹🌹🌹🌹🌹🌹	Jingdong prefecture level city in Shandong
Happy Fan Shop	King of Power Saving	I've started using it. We'll see how it works.	Jingdong prefecture level city in Shandong
Happy Fan Shop	King of Power Saving	I just received the goods, I won't know the effect until after two.	Jingdong prefecture level city in Shandong
Happy Fan Shop	King of Power Saving	Just received the goods, the specific effect will be evaluated later	Jingdong prefecture level city in Shandong
Happy Fan Shop	King of Power Saving	I've received the goods, I don't know if it's any good yet	Jingdong prefecture level city in Shandong
Happy Fan Shop	King of Power Saving	Try it for a month and then come back with a concrete evaluation	Jingdong prefecture level city in Shandong
Happy Fan Shop	King of Power Saving	In use, after two months to see the effect of power saving	Jingdong prefecture level city in Shandong
Happy Fan Shop	King of Power Saving	Power Saver. It's authentic. It's black. It works.	Jingdong prefecture level city in Shandong
Happy Fan Shop	King of Power Saving	I've been using it for a week now and it's not working.	Jingdong prefecture level city in Shandong
Happy Fan Shop	King of Power Saving	I'm just using it. I don't know how it's going to work.	Jingdong prefecture level city in Shandong
Happy Fan Shop	King of Power Saving	Products are better looking out for power savings	Jingdong prefecture level city in Shandong
Happy Fan Shop	King of Power Saving	I received it and I've kept it on. I hope it works.	Jingdong prefecture level city in Shandong
Happy Fan Shop	King of Power Saving	It's really good! Worth recommending to buy!	Jingdong prefecture level city in Shandong
Happy Fan Shop	King of Power Saving	Try it for a few days and see if it doesn't work, then return it.	Jingdong prefecture level city in Shandong
Happy Fan Shop	King of Power Saving	It's in use and I hope to be pleasantly surprised.	Jingdong prefecture level city in Shandong
Happy Fan Shop	King of Power Saving	We'll see how it works when we use it	Jingdong prefecture level city in Shandong
Happy Fan Shop	King of Power Saving	Let's see if it works.	Jingdong prefecture level city in Shandong
Happy Fan Shop	King of Power Saving	I'm using it and will follow up with a review	Jingdong prefecture level city in Shandong
Happy Fan Shop	King of Power Saving	It really works. It saves energy.	Jingdong prefecture level city in Shandong
Happy Fan Shop	King of Power Saving	It does have some power savings!	Jingdong prefecture level city in Shandong
Happy Fan Shop	King of Power Saving	Jingdong shopping speed is convenient and timely	Jingdong prefecture level city in Shandong
Happy Fan Shop	King of Power Saving	Second time I've bought it. Everything's fine.	Jingdong prefecture level city in Shandong
Happy Fan Shop	King of Power Saving	I don't know if it works yet.	Jingdong prefecture level city in Shandong
Happy Fan Shop	King of Power Saving	Not used, don't know how well it works.	Jingdong prefecture level city in Shandong
Happy Fan Shop	King of Power Saving	No visible results so far	Jingdong prefecture level city in Shandong
Happy Fan Shop	King of Power Saving	It doesn't feel like it's working.	Jingdong prefecture level city in Shandong
Happy Fan Shop	King of Power Saving	Where's the effect? Where are the power savings?	Jingdong prefecture level city in Shandong
Happy Fan Shop	King of Power Saving	Let's see how it works in practice!	Jingdong prefecture level city in Shandong
Happy Fan Shop	King of Power Saving	I'll wait until I use it to give you a good review	Jingdong prefecture level city in Shandong
Happy Fan Shop	King of Power Saving	I'll follow up after I've used it	Jingdong prefecture level city in Shandong
Happy Fan Shop	King of Power Saving	Well, that's good for saving power.	Jingdong prefecture level city in Shandong
Happy Fan Shop	King of Power Saving	Received. Used in the know.	Jingdong prefecture level city in Shandong
Happy Fan Shop	King of Power Saving	It doesn't do much good.	Jingdong prefecture level city in Shandong
Happy Fan Shop	King of Power Saving	It's not working.	Jingdong prefecture level city in Shandong
Happy Fan Shop	King of Power Saving	I don't know. I just used it.	Jingdong prefecture level city in Shandong
Happy Fan Shop	King of Power Saving	Use it and come back to evaluate.	Jingdong prefecture level city in Shandong
Happy Fan Shop	King of Power Saving	Two words: useless.	Jingdong prefecture level city in Shandong
Happy Fan Shop	King of Power Saving	To be used and then evaluated	Jingdong prefecture level city in Shandong
Happy Fan Shop	King of Power Saving	No shipment.	Jingdong prefecture level city in Shandong
Happy Fan Shop	King of Power Saving	Fake. Speechless.	Jingdong prefecture level city in Shandong
Happy Fan Shop	King of Power Saving	fantastic (idiom); awesome	Jingdong prefecture level city in Shandong
Happy Fan Shop	King of Power Saving	an excellent	Jingdong prefecture level city in Shandong
Happy Fan Shop	King of Power Saving	an excellent	Jingdong prefecture level city in Shandong
Happy Fan Shop	King of Power Saving	Want to buy! Recommended not to buy! No effect	Jingdong prefecture level city in Shandong

Happy Fan Shop	King of Power Saving	That's all there is to it. I can't figure out how to save electricity.	Jingdong prefecture level city in Shandong
Happy Fan Shop	King of Power Saving	It's no use.	Jingdong prefecture level city in Shandong
Happy Fan Shop	King of Power Saving	Things are more perfect than I imagined, very powerful, it is worth getting Packaging is very strong, things are well protected, it is worth the praise things are very satisfied Oh, beautiful and stylish atmosphere, customer service is super patient, service is meticulous and attentive, the big brand is not	Jingdong prefecture level city in Shandong
Happy Fan Shop	King of Power Saving	The effect is too good, the family has now several units, back to buy a number of times, the power saver received, power-saving effect is very good,, fine workmanship, long warranty, but also purify the air, before buying a used for a period of time, with the okay, and then bought a, and only now come to the	Jingdong prefecture level city in Shandong
Happy Fan Shop	King of Power Saving	Quite good, and imagine the same, casual ride are very good, satisfied! Baby received it 👌, really great ah! 🌈 quality is really nothing to say, very satisfied! 🆗 happy happy 😀 genuine, great, the price is very good, in short, quite good, will buy again a very good shopping experience, attentive service, fast logistics, distribution is also thoughtful, the attitude is also very good, value for money, ah,	Jingdong prefecture level city in Shandong
Happy Fan Shop	King of Power Saving	Power-saving effect: strong Cost-effective: quite good Appearance style: beautiful Workmanship material: meticulous Experience: looking forward to power-saving effect	Jingdong prefecture level city in Shandong
Happy Fan Shop	King of Power Saving	Cost-effective is okay, the appearance of the style of meticulous workmanship, looking forward to energy-saving effect.	Jingdong prefecture level city in Shandong
Happy Fan Shop	King of Power Saving	Very Good Very Power Saving This Power Saver is really power saving As soon as the power is turned on, the Power Saver starts the power saving function for you.	Jingdong prefecture level city in Shandong
Happy Fan Shop	King of Power Saving	Logistics is very fast, the price is favorable, friends introduced that it is very good to use, bought 2 at once, looking forward to the effect, used after the follow-up comment.	Jingdong prefecture level city in Shandong
Happy Fan Shop	King of Power Saving	It's all fake. It doesn't save any electricity.	Jingdong prefecture level city in Shandong
Happy Fan Shop	King of Power Saving	This Power Saver is so much better than the one I bought before, not only the effect is good, the quality is very good in all aspects, the customer service attitude is also very good, recommended to buy!	Jingdong prefecture level city in Shandong
Happy Fan Shop	King of Power Saving	Logistics is very fast, buy for parents, good quality, once bought 2, power-saving effect is good to buy 2 more, look forward to using the effect, satisfied will recommend to friends.	Jingdong prefecture level city in Shandong
Happy Fan Shop	King of Power Saving	With the description is the same, very high-grade, good value, to buy friends can order Oh!	Jingdong prefecture level city in Shandong
Happy Fan Shop	King of Power Saving	Not using much electricity in the winter saving over 100 per month! Ready to buy 2 more units in the summer ！！！！！ Good quality, good price, power saving effect is obvious, cost-effective, worth buying, can be recommended!	Jingdong prefecture level city in Shandong
Happy Fan Shop	King of Power Saving	Purchased a power saver received on the put into use, rent a room with many electrical appliances, power consumption is very large, we have a piece of electricity or 1.2 dollars a time, with this power saver is much better, every day can save a lot of money, very convenient and practical.	Jingdong prefecture level city in Shandong
Happy Fan Shop	King of Power Saving	The power saving effect is very good: my supermarket monthly electricity bill is more than 7000 yuan. On May 1st someone recommended buying a couple of power savers, and I bought 5 of them first. The electricity bill was about 2000 less in June. This time, I bought 5 more. The seller said 800 degrees of electricity with one. I feel it's basically enough. A total of 10 units	Jingdong prefecture level city in Shandong
Happy Fan Shop	King of Power Saving	I never thought I could get such a good baby at such an affordable price, it's very good value for money, I love it!	Jingdong prefecture level city in Shandong
Happy Fan Shop	King of Power Saving	Previously bought other brands of power savers, but found no effect at home, this time to see how the products on the Jingdong, inserted in the home socket, the LED display is blue, the night light is very nice to see, look at the instructions that have to be half a year in order to achieve the best results, try it, I believe that the quality of the Jingdong, it should not be wrong.	Jingdong prefecture level city in Shandong
Happy Fan Shop	King of Power Saving	Superb, inexpensive, saves a lot of points, been using it for a long time!	Jingdong prefecture level city in Shandong
Happy Fan Shop	King of Power Saving	There are a lot of appliances in the house, the summer air conditioning is very costly, I bought this in front of the air conditioning effect is very good, I explain that I bought three in one breath, I use more electricity a month down to 200 or so, very satisfied.	Jingdong prefecture level city in Shandong
Happy Fan Shop	King of Power Saving	A month can save more than 200, home monthly electricity bill of more than 600 dollars, inserted 3 or so, an amount of the balance of the average can save more than 200. Product is very good, has been buying this one, has given a lot of relatives recommended, the quality is also very good, before buying a look with this same, who knows to buy home has not been used for more than a week on the bad, the quality of this is really good with more than two years!	Jingdong prefecture level city in Shandong
Happy Fan Shop	King of Power Saving	Whole house power saving king, can really save electricity, friends recommended, high power saving appliances, only replacement, no repair, after-sales worry-free!	Jingdong prefecture level city in Shandong
Happy Fan Shop	King of Power Saving	Great item love it, great customer service, great shopping experience!	Jingdong prefecture level city in Shandong
Happy Fan Shop	King of Power Saving	Power saving, very good use, much cheaper than the physical store, satisfaction	Jingdong prefecture level city in Shandong
Happy Fan Shop	King of Power Saving	In use, very well packaged and fast delivery of credit!	Jingdong prefecture level city in Shandong
Happy Fan Shop	King of Power Saving	Product quality, customer service attitude is particularly good, like, praise!	Jingdong prefecture level city in Shandong
Happy Fan Shop	King of Power Saving	The logistics is very fast, the packaging is not broken, the quality is very good, I like it very much!	Jingdong prefecture level city in Shandong
Happy Fan Shop	King of Power Saving	The saver was well packaged and was as described when plugged in	Jingdong prefecture level city in Shandong
Happy Fan Shop	King of Power Saving	Energy efficient and very satisfied	Jingdong prefecture level city in Shandong
Happy Fan Shop	King of Power Saving	Great quality for the price and fast shipping.	Jingdong prefecture level city in Shandong
Happy Fan Shop	King of Power Saving	It's great, just as described. Perfect.	Jingdong prefecture level city in Shandong
Happy Fan Shop	King of Power Saving	There is no power saving effect, but rather ring up an increase in electricity costs, because this machine is on continuously all day long, uninterruptedly, its own electricity and increased costs, plus the cost of purchasing the machine, so I feel that the use of this machine instead of increasing the	Jingdong prefecture level city in Shandong
Happy Fan Shop	King of Power Saving	Recommended by the owner of the store next door, looked at his home does have to save electricity, decided to get. Indeed quite can, the voltage is much more stable, there is a reduction in electricity	Jingdong prefecture level city in Shandong
Happy Fan Shop	King of Power Saving	Goods have been received value for money, affordable and reasonable price, and the description of the same, power-saving safe and reliable!	Jingdong prefecture level city in Shandong
Happy Fan Shop	King of Power Saving	Listen to friends introduced to buy, tried to save a lot of electricity is good, quite good, worth buying.	Jingdong prefecture level city in Shandong
Happy Fan Shop	King of Power Saving	The product is easy to use, safe and environmentally friendly, only used for a short time, to the next month, after the electricity bill status display to know how the performance of the power saver efficacy.	Jingdong prefecture level city in Shandong
Happy Fan Shop	King of Power Saving	Last month's electricity bill is 178 yuan, with this power saver is 92 yuan, ready to buy two more back home, I was introduced to buy a friend, just started to not believe that I did not expect the effect!	Jingdong prefecture level city in Shandong
Happy Fan Shop	King of Power Saving	It looks nice, but after using it for a few days I can't see how it saves power?	Jingdong prefecture level city in Shandong
Happy Fan Shop	King of Power Saving	Recommended by a friend, it is genuine, as described by the merchant, cost-effective	Jingdong prefecture level city in Shandong
Happy Fan Shop	King of Power Saving	Works well, affordable and saves money.	Jingdong prefecture level city in Shandong
Happy Fan Shop	King of Power Saving	Feels good haven't compared it yet, the air conditioning has been on a lot in the past two months	Jingdong prefecture level city in Shandong
Happy Fan Shop	King of Power Saving	Saving about 5 kWh per day is a good thing	Jingdong prefecture level city in Shandong
Happy Fan Shop	King of Power Saving	Not bad, affordable, best value for money	Jingdong prefecture level city in Shandong
Happy Fan Shop	King of Power Saving	The goods are genuine and the logistics are faster.	Jingdong prefecture level city in Shandong

Happy Fan Shop	King of Power Saving	Goods have been received today, has been plugged into the outlet, looking forward to saving electricity, after a period of time to see how the effect in the follow-up comment!	Jingdong prefecture level city in Shandong
